# Supplementary material for: Discovery and characterization of Alu repeat sequences via precise local read assembly
Source: Nucleic Acids Res. 2015 Oct 25;43(21):10292–307. doi: 10.1093/nar/gkv1089 (PMC4666360; doi:10.1093/nar/gkv1089)
Supplement: SUPPLEMENTARY DATA [file supp_43_21_10292__index.html]

Discovery and characterization of Alu repeat sequences via precise local read assembly — SUPPLEMENTARY DATA 

# Discovery and characterization of *Alu* repeat sequences via precise local read assembly

## SUPPLEMENTARY DATA

- SUPPLEMENTARY DATA
- SUPPLEMENTARY DATA
- SUPPLEMENTARY DATA
- SUPPLEMENTARY DATA
- SUPPLEMENTARY DATA
- SUPPLEMENTARY DATA
- SUPPLEMENTARY DATA
- SUPPLEMENTARY DATA
- SUPPLEMENTARY DATA
- SUPPLEMENTARY DATA
- SUPPLEMENTARY DATA
