# Supplementary material for: Discovery and characterization of Alu repeat sequences via precise local read assembly
Source: Nucleic Acids Res. 2015 Oct 25;43(21):10292–307. doi: 10.1093/nar/gkv1089 (PMC4666360; doi:10.1093/nar/gkv1089)
Supplement: SUPPLEMENTARY DATA [file supp_gkv1089_nar-01397-h-2015-File005.docx]

Supplementary Figure Legends

Figure S1. Assembled *Alu* insertions. Summary of assembled insertions compared to the hg19 reference for all sites, depiction as in Figure 2. Information including the siteID, insertion coordinate, insertion size, predicted TSD, and genotyping are provided for each of 1,614 insertions.

Figure S2. Characteristics of regions of overlap at assembled insertions. A. Histogram of the length of overlapping sequence identified by comparing the assembled sequence with the hg19 genome reference. Negative values indicate deletions of sequence at the insertion site relative to the reference. B. Heatmap of the number of differences between the detected regions of overlap flanking each insertion. C. Number of mismatches between the 5’ overlapping segment, relative to the *Alu* orientation, and the hg19 reference. D. Number of mismatches between the 3’ overlapping segment, relative to the *Alu* orientation, and the hg19 reference.

Figure S3. Overlapping segments of identical sequence. A. A histogram of the length of identical sequence identified for the 1005 genotyped elements without a target site deletion. B. Density plot comparing the lengths of the region of overlap and the length of identical sequence identified for each site. The lengths are identical for most (864/1005) sites.

Figure S4. Trace alignments of 20 randomly selected validated insertions. Nucleotide alignments of Sanger sequencing results with the corresponding CAP3 assemblies for each validated site. A. Assembly strategy corresponding to the alignment. B. Trace alignments are shown both upstream and downstream the insertion for all validated sites. C. Information for the validated insertion and source sample for sequencing. A black line indicates TSDs flanking the insertion, when present; a red asterisk has been used to indicate the points of 5’ truncation for relevant insertions.

Figure S5. Trace alignments of selected validations biased to sites with unusual breakpoint characteristics. Alignments of Sanger sequencing traces with the CAP3 assembled sequence for each site as in Figure S2.

Figure S6. Trace alignments of validated *Alu*S and *Alu*J elements. Trace alignments with corresponding assembled *Alu*S and *Alu*J insertions are shown and labelled as in Figure S2. Where appropriate, a grey line has been used to indicate extended sequence associated with that element not present in the reference genome at that site.

Figure S7. *Alu*S and *Alu*J elements with high identity to reference elements. Alignments are depicted for four polymorphic elements having strong matches elsewhere in the reference genome. A. An *Alu*Jb element present at chr5:172054822 is also present at chr5:53789242. B. An *Alu*Jb element present at chr12:73056650 is also present at chr8:144374138. C. An *Alu*Sp element present at chr3:110413394 is also present at chr1: 109494853. D. An AluSx3 element present at chr17:46617220 is also present at chr17: 46615632.

Figure S8. Allele frequency distribution. A histogram of inferred allele frequencies across 53 individuals from 7 populations is plotted for 994 genotyped insertions located on the autosomes or pseudo-autosomal region of the X chromosome.

Figure S9. Comparison of recovered alleles at chr11:35425392. The sequence from the hg19 reference genome and the CAP3 assembled contig are compared for an insertion on chromosome 11. Extent of matching sequence between haplotypes is in yellow. The location of an indel coincident with the insertion site is depicted in blue; putative TSDs are underlined; the *Alu* insertion is in purple. Sequence present in the insertion haplotype but absent from the reference is in red. The green text corresponds to the extent of similar sequence (89.5% similarity) flanking the *Alu* insertion.
