# Supplementary figures and images for: Discovery and characterization of Alu repeat sequences via precise local read assembly
Source: Nucleic Acids Res. 2015 Oct 25;43(21):10292–307. doi: 10.1093/nar/gkv1089 (PMC4666360; doi:10.1093/nar/gkv1089)

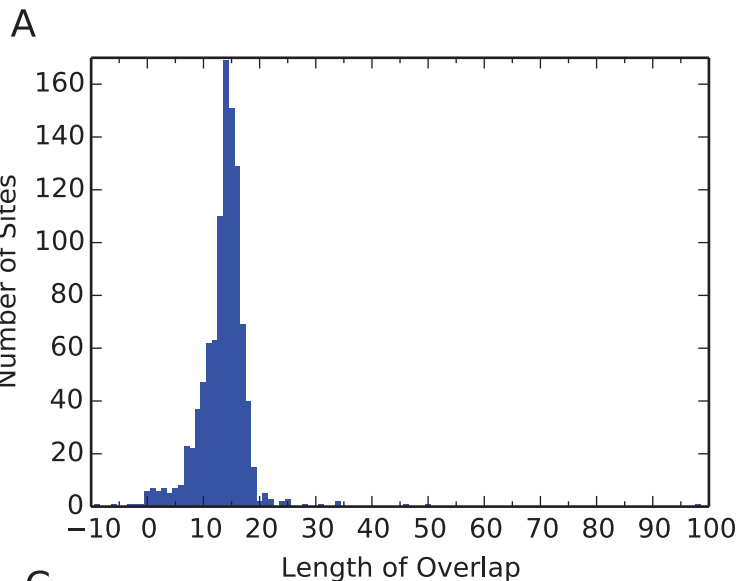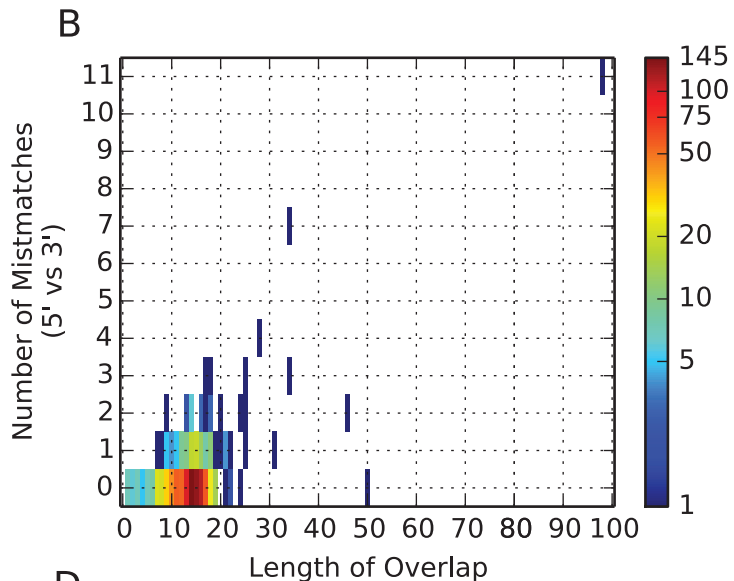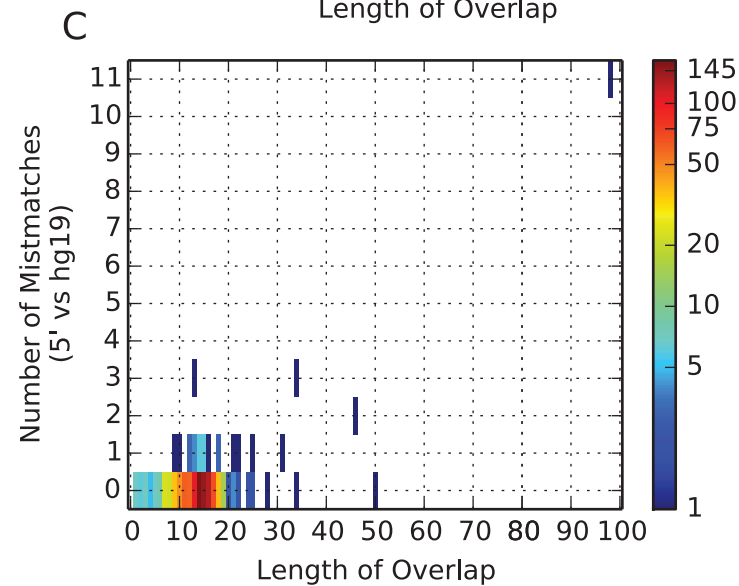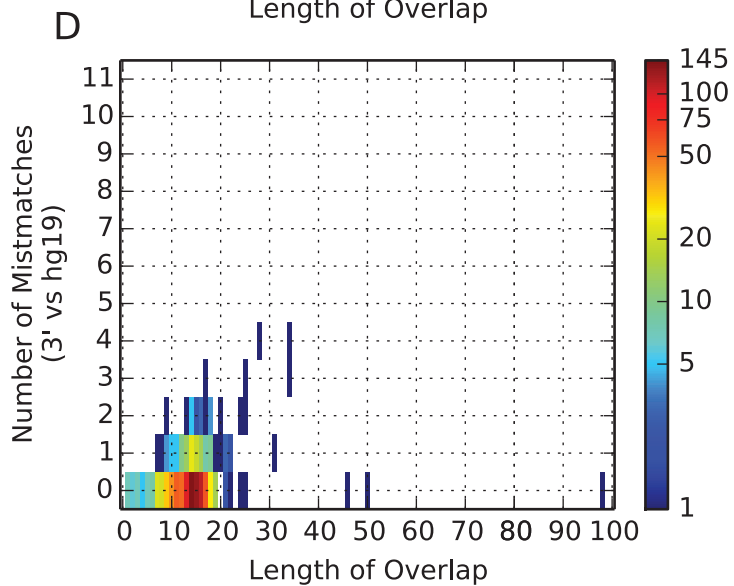

Supplement: SUPPLEMENTARY DATA [file supp_gkv1089_nar-01397-h-2015-File007.pdf]

A

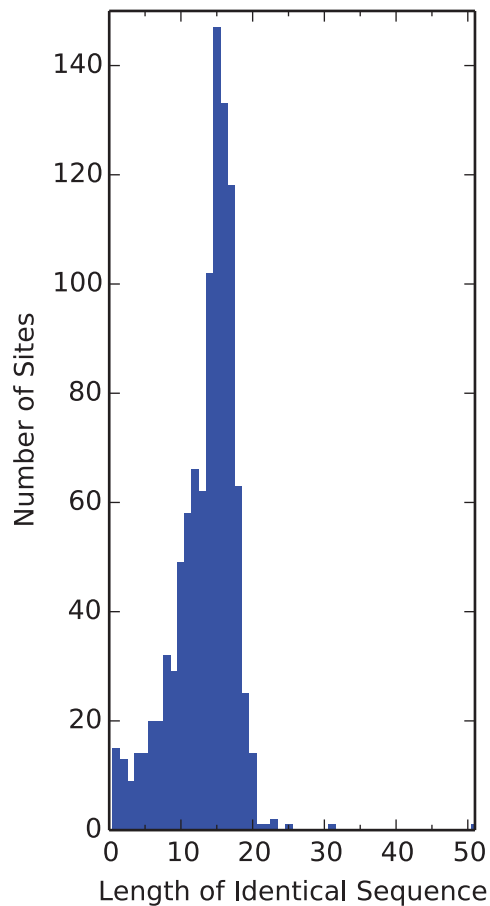

B

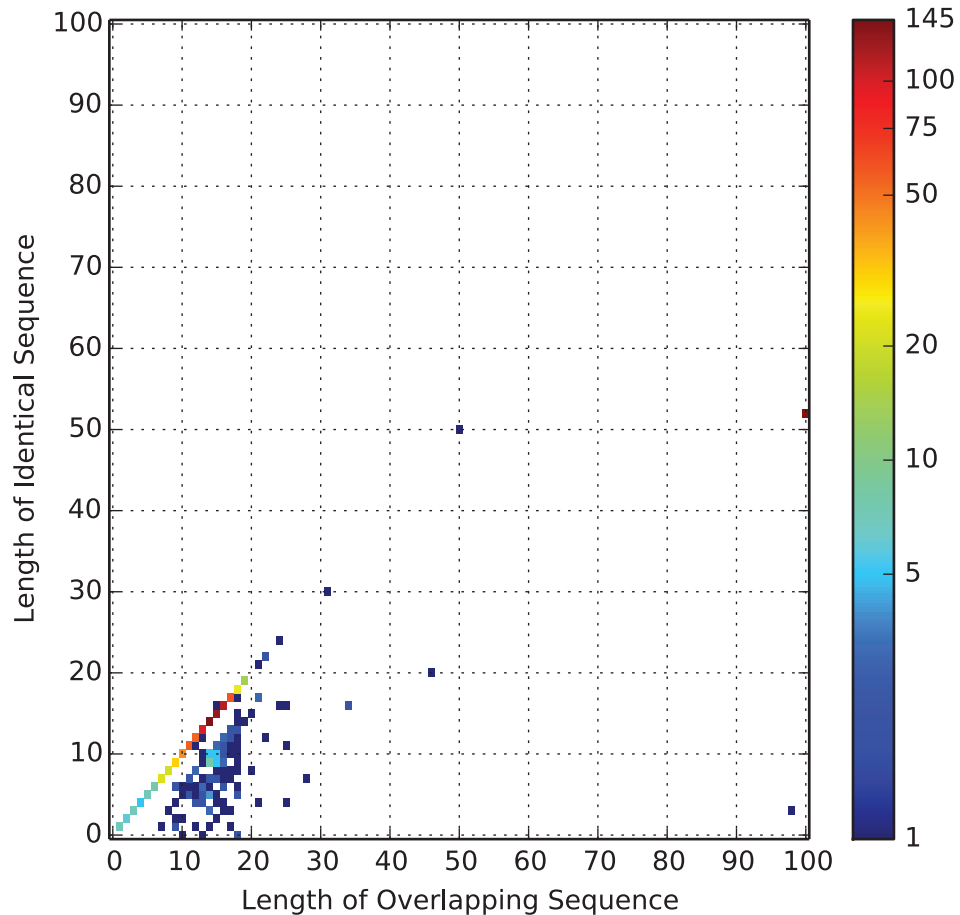

Supplement: SUPPLEMENTARY DATA [file supp_gkv1089_nar-01397-h-2015-File008.pdf]

**A**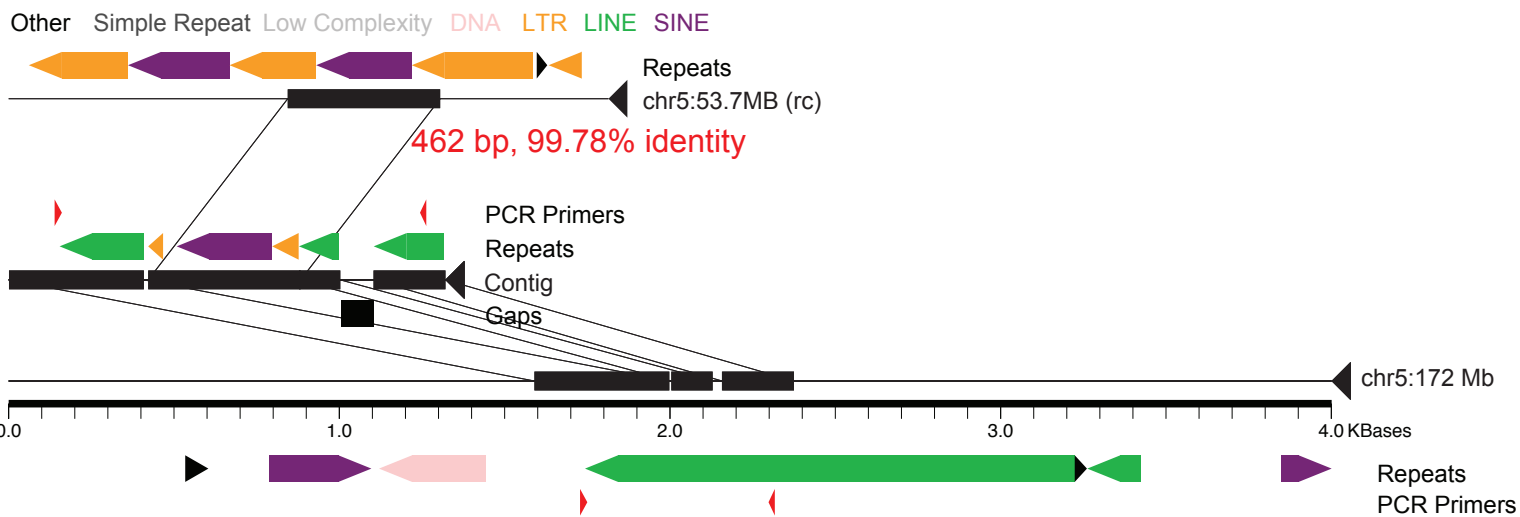**B**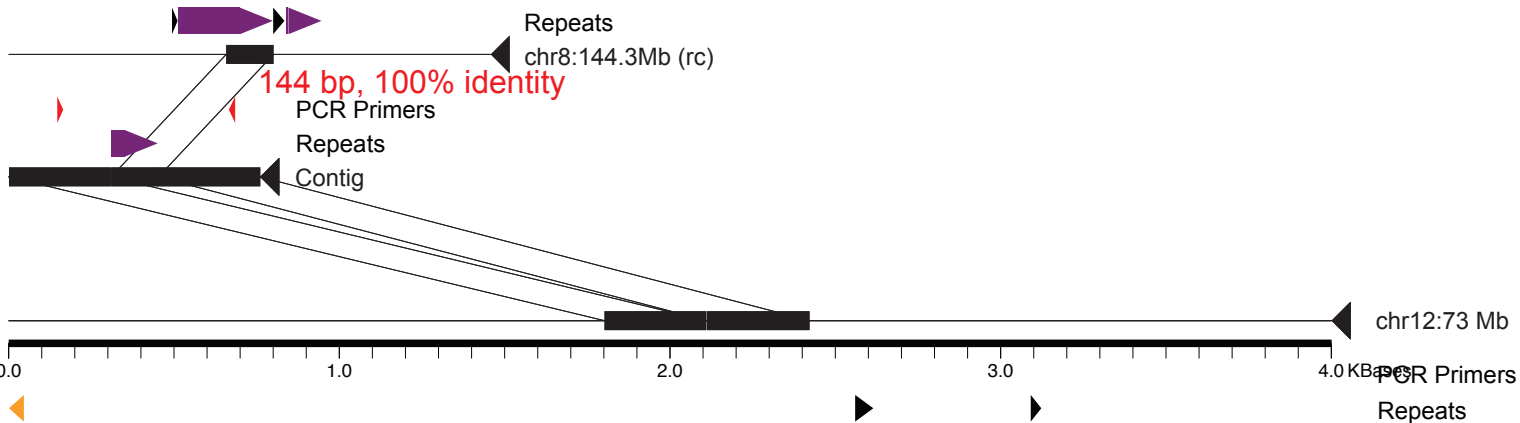**C**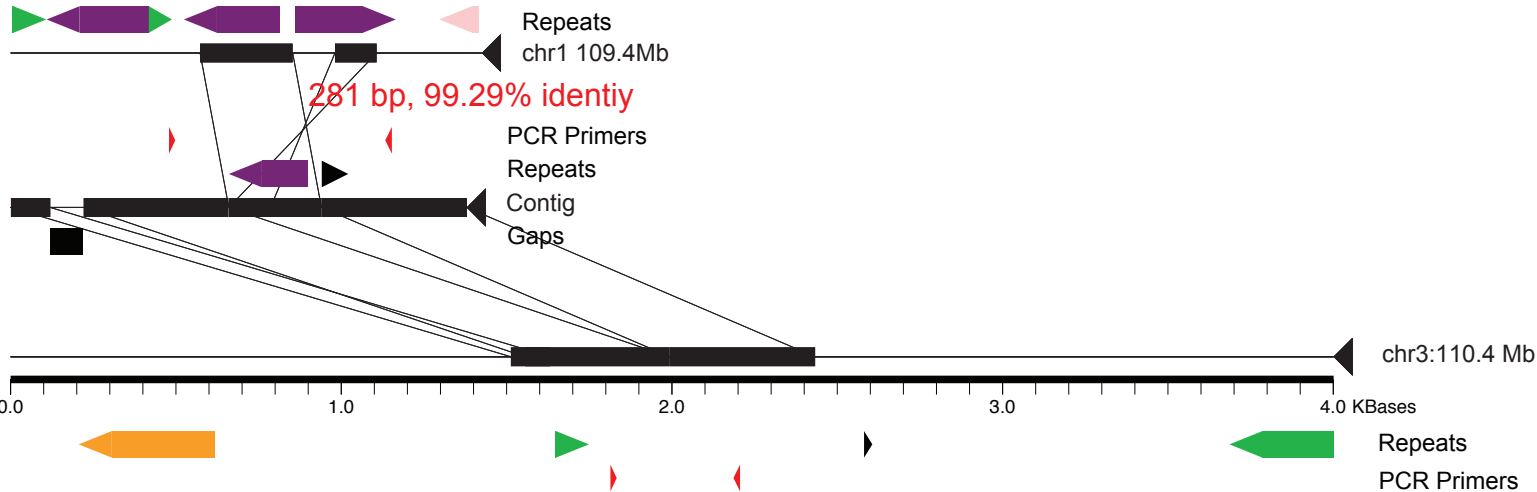**D**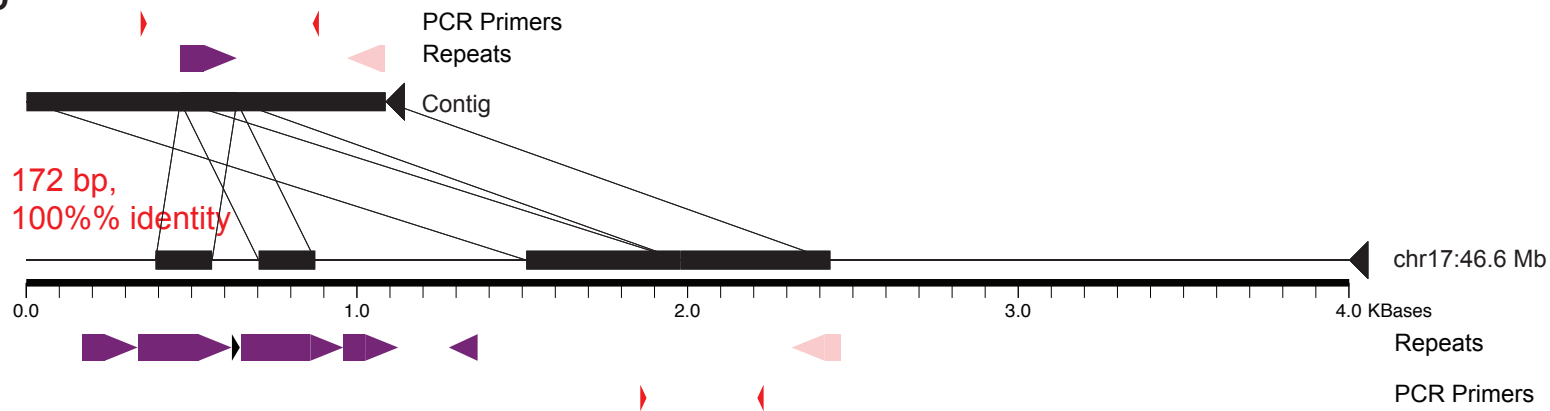

Supplement: SUPPLEMENTARY DATA [file supp_gkv1089_nar-01397-h-2015-File012.pdf]

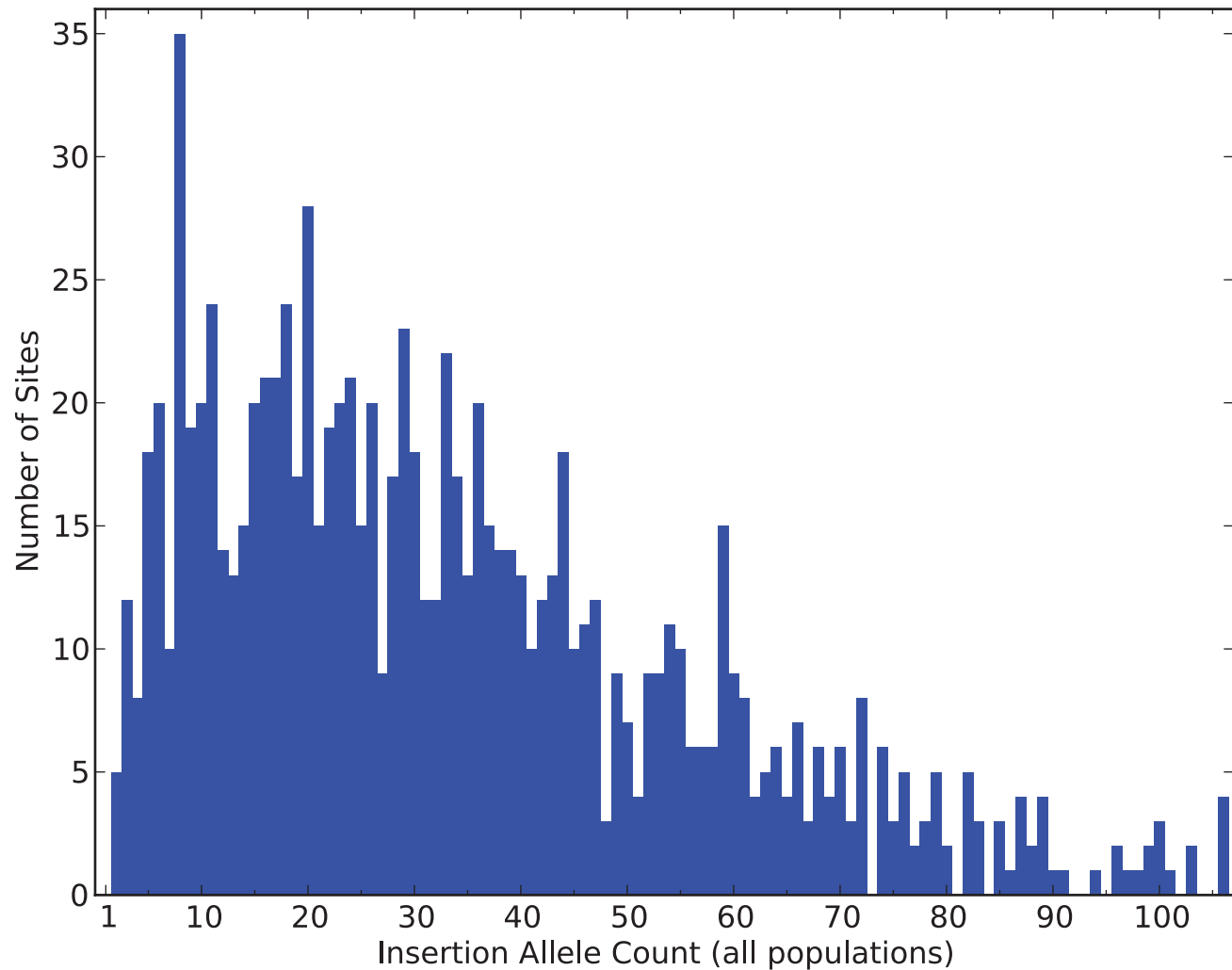

Supplement: SUPPLEMENTARY DATA [file supp_gkv1089_nar-01397-h-2015-File013.pdf]
