# Supplementary material for: Discovery and characterization of Alu repeat sequences via precise local read assembly
Source: Nucleic Acids Res. 2015 Oct 25;43(21):10292–307. doi: 10.1093/nar/gkv1089 (PMC4666360; doi:10.1093/nar/gkv1089)

siteID: chr1_104574495 Scafftig1 - Breakpoint: chr1:104574469
chr1:104574428-104574528
Overlap Length: 13 INS size: 289 Genotyped: Yes

```
left    CAAAGCTCCTATTA-----AAAGTAGTTTG-TTGCTTTT-TGATCC-----------CTTGTAATTTTTT
chrom   CAAAGCTCCTATTA-----AAAGTAGTTTG-TTGCTTTT-TGATCC-----------CTTGTAATTTTTT
right   CCGCCCGCCTCGGCCTCCCAAAGTGCTGGGATTACAGGCGTGAGCCACCGCGCCCGGCCTGTAATTTTTT
        *1111*1***111111111*****11*11*1**1*11111***1**11111111111*1***********

left    TTTTTTTTTTGAGACGGAGTCTCGCTCTGTCGCC-CAGGCTGGAGTGCAGTGGCGGGATCTC-GGCTCA
chrom   TTATTATT--------------AACTATAAAGCATCATG--GGTATAGAGTGTC----TCTCTGGGTTT
right   TTATTATT--------------AACTATAAAGCATCATG--GGTATAGAGTGTC----TCTCTGGGTTT
        ***2**2**2222222222222222**2*222**22**2*22**22*22****2*2222****2**2*22
```

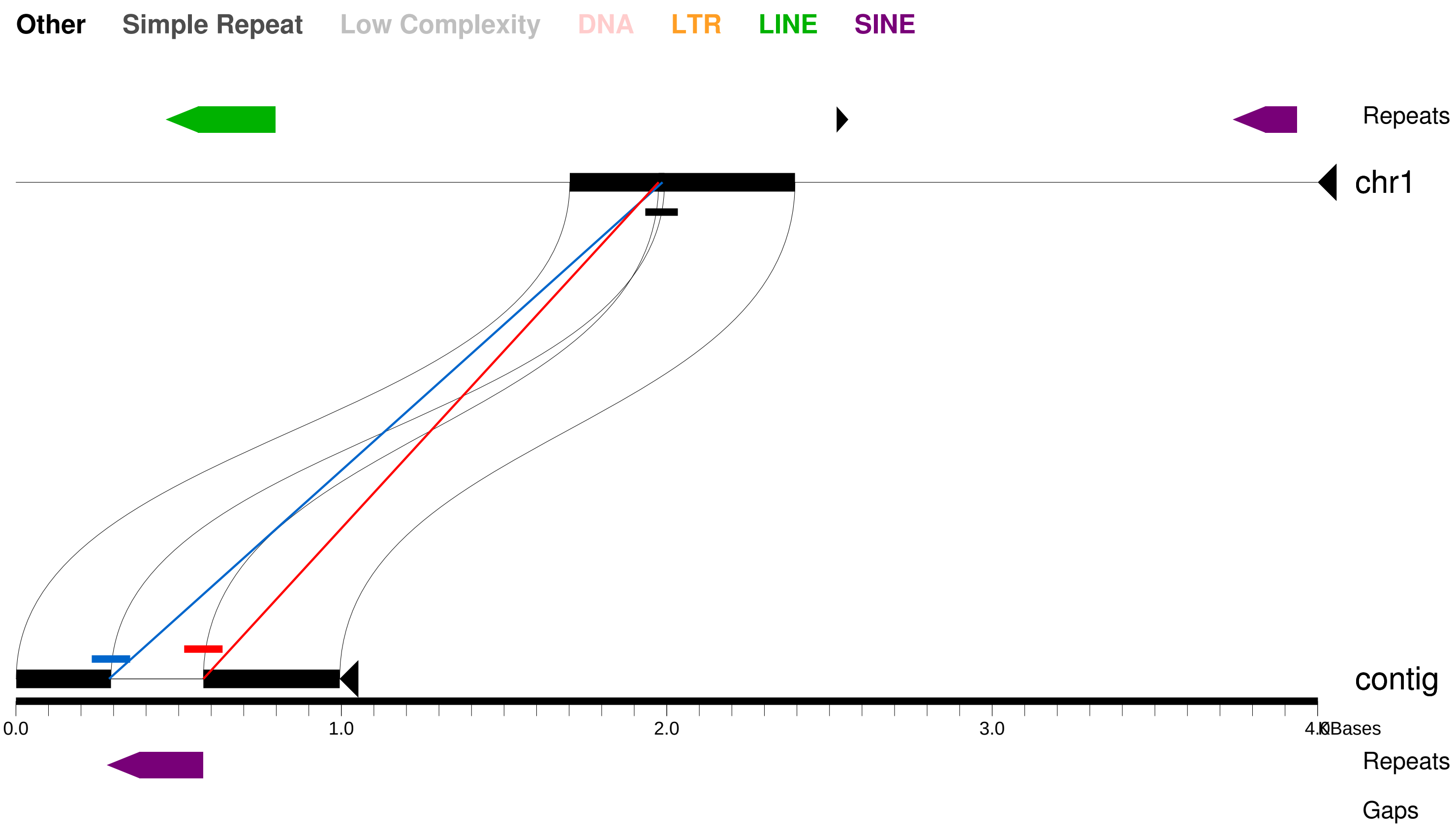

siteID: chr1_105054078 Scafftig11 + Breakpoint: chr1:105054075
chr1:105053914-105054243
Overlap Length: 9 INS size: 433 Genotyped: No

```
left   ----------ATCAGAATGAGAAAAGAGAAATTACTACAGATATTACAGATGTTAAGTGGATAATGAGAA
chrom  CATTTCCAATATCAGAATGAGAAAAGAGAAATTACTACAGATATTACAGATGTTAAGTGGATAATGAGAA
right  NNNNNNNNNNN--------AAAAAA---------------------------------------------
       NNNNNNNNNN111111111*1****111111111111111111111111111111111111111111111

left   ATATTATGGACAATTCTTTGCCAAAATTATTCAACTTACATATAATTTAAAAATTCTACACAAATTACCA
chrom  ATATTATGGACAATTCTTTGCCAAAATTATTCAACTTACATATAATTTAAAAATTCTACACAAATTACCA
right  ----------------------------------------------------------------------
       1111111111111111111111111111111111111111111111111111111111111111111111

left   AATTCATTTTTTAGTGATAAGAATAGCCCTGGCCGGGCGCGGTGGC----TCACGCCTGTAATCCCAGCA
chrom  AATTCATTTTTTAGTGATAATAATAGCCCTATTTTCAA-CTGTGTCACCTTCAGAAATGTA-TCCAAACT
right  ---------------------AATAGCCCTATTTTCAA-CTGTGTCACCTTCAGAAATGTA-TCCAAACT
       11111111111111111111N*********222222222*2***2*2222***2222****2***2*2*2

left   CTTTGGGAGGCC--------GAGGCGGGCGGATCACGAGGTCAGGAGATCGA-GACC--ATCCCGGCTAA
chrom  CGCCATGATTCACTTACTATGATACAGGAATATTAATAGTTCATACACTAGAAGATTGTATGAAGGTTAA
right  CGCCATGATTCACTTACTATGATACAGGAATATTAATAGTTCATACACTAGAAGATTGTATGAAGG----
       *22222**22*222222222**22*2**222**2*22**2***22222*2**2**2222**222**N111

left   A-----------ATGG--------TGAAACCCCGTCTCT-ACTAAA------
chrom  AGGAATTAACACATGGAAAGTCCTTGAAATAGTATCTATCACATAATATGAG
right  ----------------------------------------------------
       1           1111        11111NNNNN111N1 11NN11
```

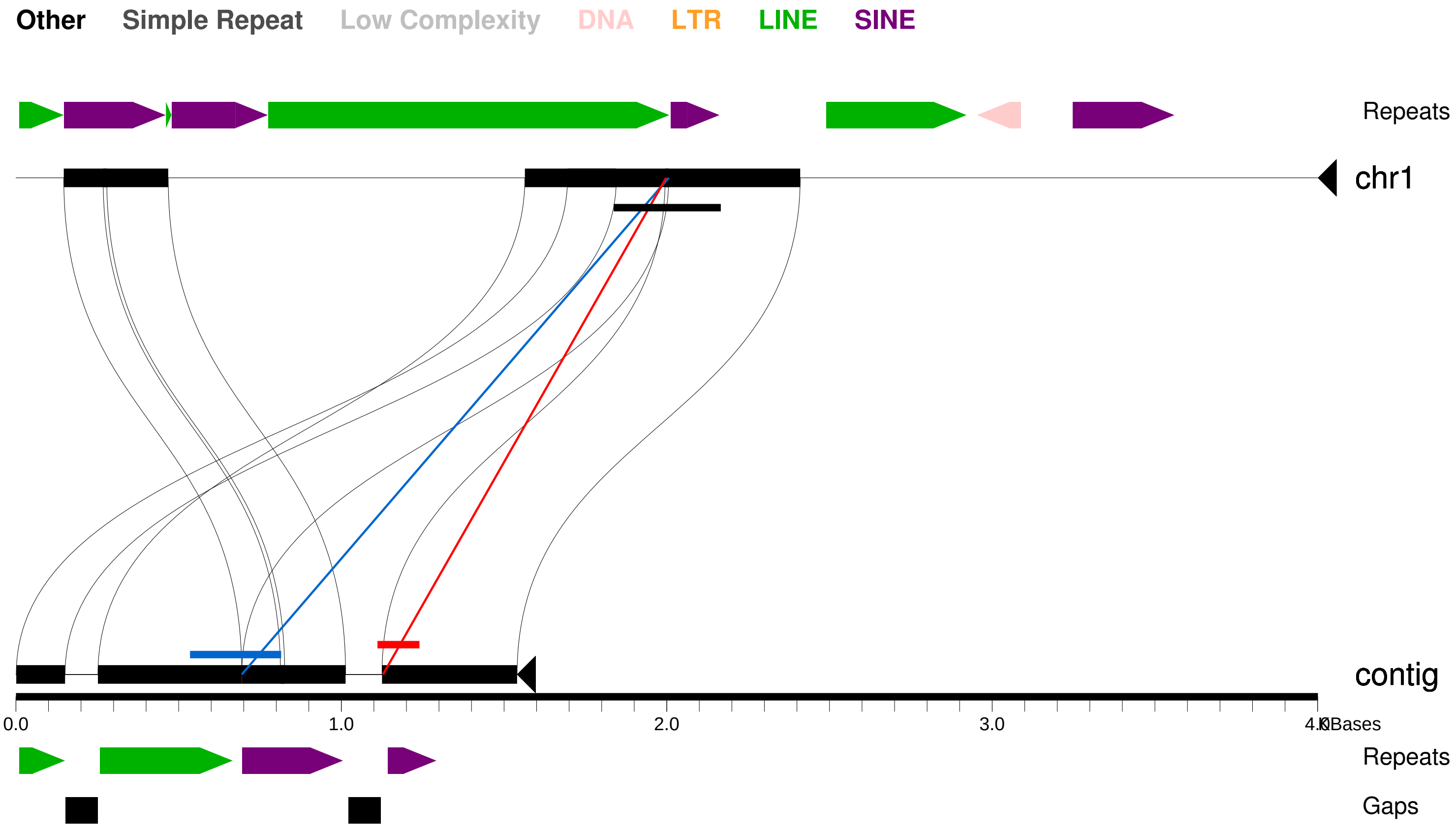

siteID: chr1_105070228 Scafftig2 + Breakpoint: chr1:105070232
chr1:105070072-105070404
Overlap Length: 13 INS size: 356 Genotyped: No

```
left   A-------------AATGATTAGAAAATCCTTTTGAACAGAACTACCATTTACACAATTTGCAAACCATA
chrom  ACTCTGATTGAAGAAATGATTAGAAAATCCTTTTGAACAGAACTACCATTTACACAATTTGCAAACCATA
right  NNNNNNNNNNNAAAAA-------AAAA-----------------------------------AAA-----
       1NNNNNNNNNN2N2**1111111****11111111111111111111111111111111111***11111

left   AATTTTACATTTGTGTTATATTAAATAAATGTTAACACATATATTATGTAAACCGCATTTTTCCCTGAAT
chrom  AATTTTACATTTGTGTTATATTAAATAAATGTTAACACATATATTATGTAAACCGCATTTTTCCCTGAAT
right  ----------------------AAAAAAA-----------------------------------------
       1111111111111111111111***1***11111111111111111111111111111111111111111

left   GGATTATAATGTGTGTTTATAAGAATTTATGGCG-----GCCGGGCG--------CGGTGGC--TCACGC
chrom  GGATTATAATGTGTGTTTATAAGAATTTATGGCTATACTGCAAAACGTATTTTAACAGTGTACTTTAAGG
right  --------------------AAGAATTTATGGCTATACTGCAAAACGTATTTTAACAGTGTACTTTAAGG
       11111111111111111111*************222222**2222**22222222*2***2222*2*2*2

left   CT----GTAA------------TCCCAGCACTTTG-----GGAGGCCGAGGCGGGCGG-ATCACGAGGTC
chrom  GTCGGTGTAAAAATGGGAAACATCAAAAAAGTATAAGTATGGATGCGAAAACGTGTATTAGAAAGAGAGC
right  GTCGGTGTAAAAATGGGAAACATCAAAAAAGTATAAGTATGGATGCGAAAACGTGTATTAGAAAGA----
       2*2222****222222222222**22*22*2*2*222222***2**22*22**2*2222*22*2**1NN1

left   AGGAG--ATCGAGACCATCC-TGGCT--AACACGGTGAAACCCCGTCTCTACTAAA
chrom  AGAAGTAATTTA-ACAATGCATGCCTTGATGAGAGTTAAATT--ATTAAAAATCAC
right  --------------------------------------------------------
       11N11  11NN1211N11N1 11N11  1NN1NN11N111NN22N1NNNN1N1N1N
```

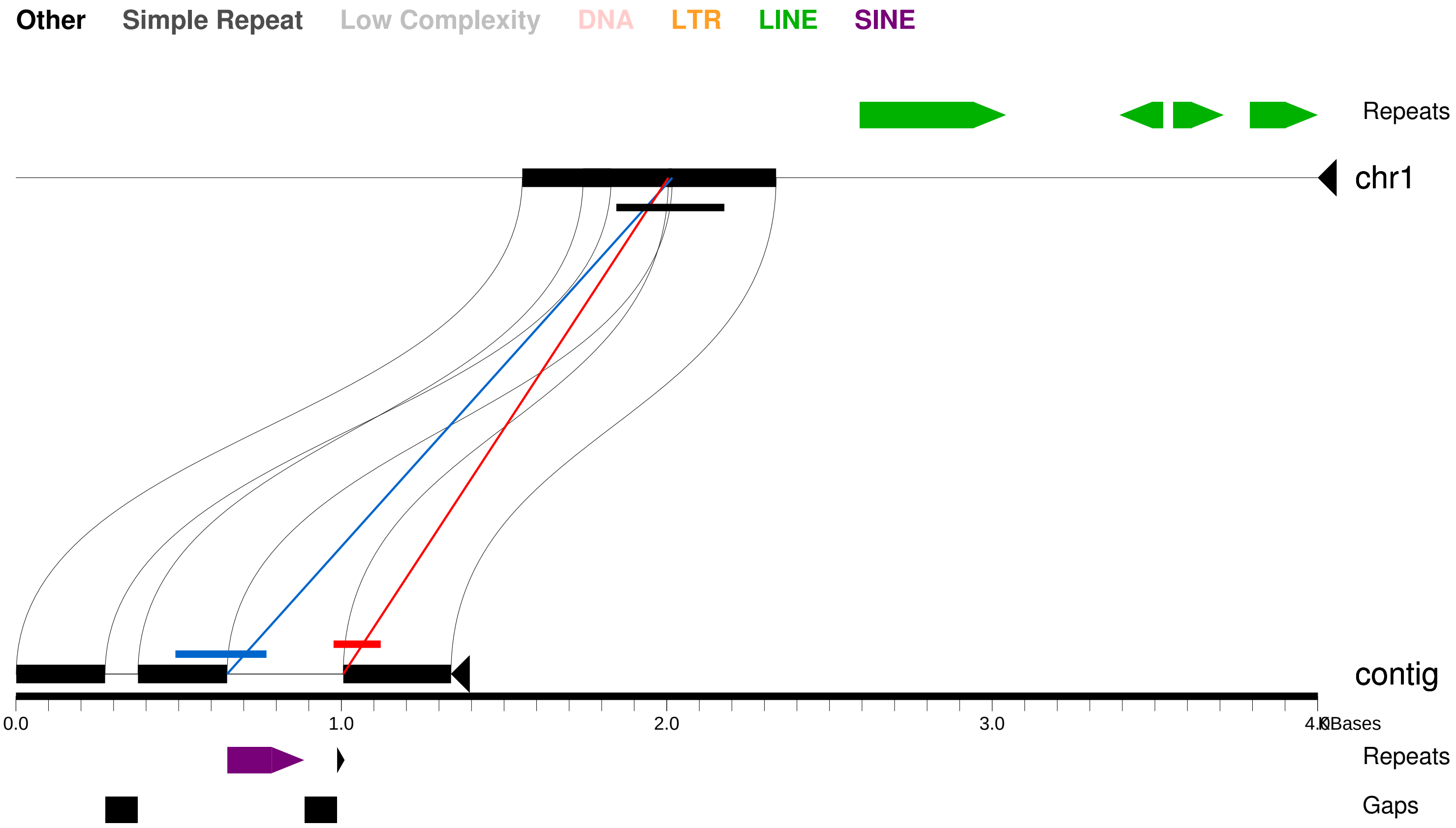

siteID: chr1_105138638 Scafftig2 + Breakpoint: chr1:105138642
chr1:105138602-105138701
Overlap Length: 9 INS size: 323 Genotyped: Yes

```
left     T-------------TATTTCCATTTAATTCTTTTTCATTTTTTTAGTTAT-------GTGCCATTTTTTT
chrom    T-------------TATTTCCATTTAATTCTTTTTCATTTTTTTAGTTAT-------GTGCCATTTTTTA
right    TCCACCCGCCTCGGCCTCCCAAAGTGCTGGGATTACAGGCGTG-AGCCACCGCGCCCGGCCCATTTTTTA
         *111111111111111*11*1*11*11*1111**1**1111*11**11*11111111*11*********2

left     TTTTTTTTTTTTTTTTTTTTTTTTTTTTTTTTTTTGAGACGGAGTCTCGCTCTGTTGCCCAGGCCGGAC
chrom    TCCTATTATTAATAACACCTTTTTAAAAGTCAAATGTGT--GATTA-----CTATTT------------
right    TCCTATTATTAATAACACCTTTTTAAAAGTCAAATGTGT--GATTA-----CTATTT------------
         *22*2**2**22*222222*****22222*2222**2*222**2*222222**2**2222222222222
```

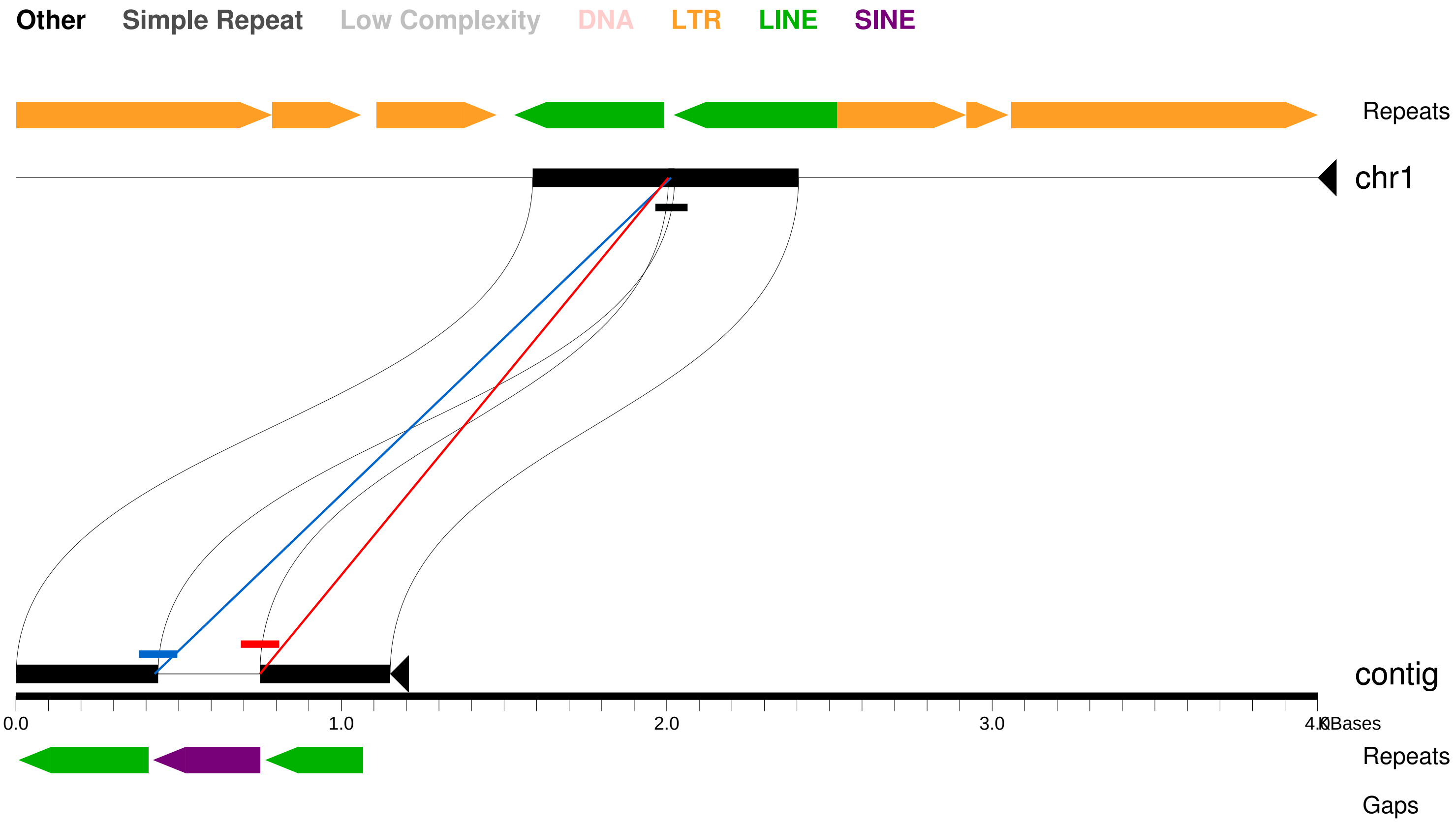

siteID: chr1_105231124 Scafftig2 + Breakpoint: chr1:105231104
chr1:105231052-105231163
Overlap Length: 8 INS size: 127 Genotyped: Yes

```
left    GGAAATGA-TGAAACTGAAGCAAAAAAAAAATTAAGGCA-----CTTTATCAAGGTAAC-AGTTTTTTTTTT
chrom   GGAAATGA-TGAAACTGAAGCAAAAAAAAAATTAAGGCA-----CTTTATCAAGGTAAC-AGTATTTTGAA
right   TCGGCTCACTGCAAGCTCCGCCTCCCGGGTTCACGCCATTCTCCTGCCTCAGCCTCCCGAGTATTTTGAA
        11111*1*1**1**11111**111111111*1*1*1**11111**111***111*11*1****2*****222

left    TTTTTTTTTTTTTTTTGAGACGGAGTCTCGCTCTGTCGCCCAGGCTGGAGTGCAGT-
chrom   TAATGTCACATGGTAGTA-ATAGAGGCAGG-------GCTATGATTTAAACCCAGTA
right   TAATGTCACATGGTAGTA-ATAGAGGCAGG-------GCTATGATTTAAACCCAGTA
        *22*2*2222*22*222*2*22***2*22*2222222**222*22*22*222****2
```

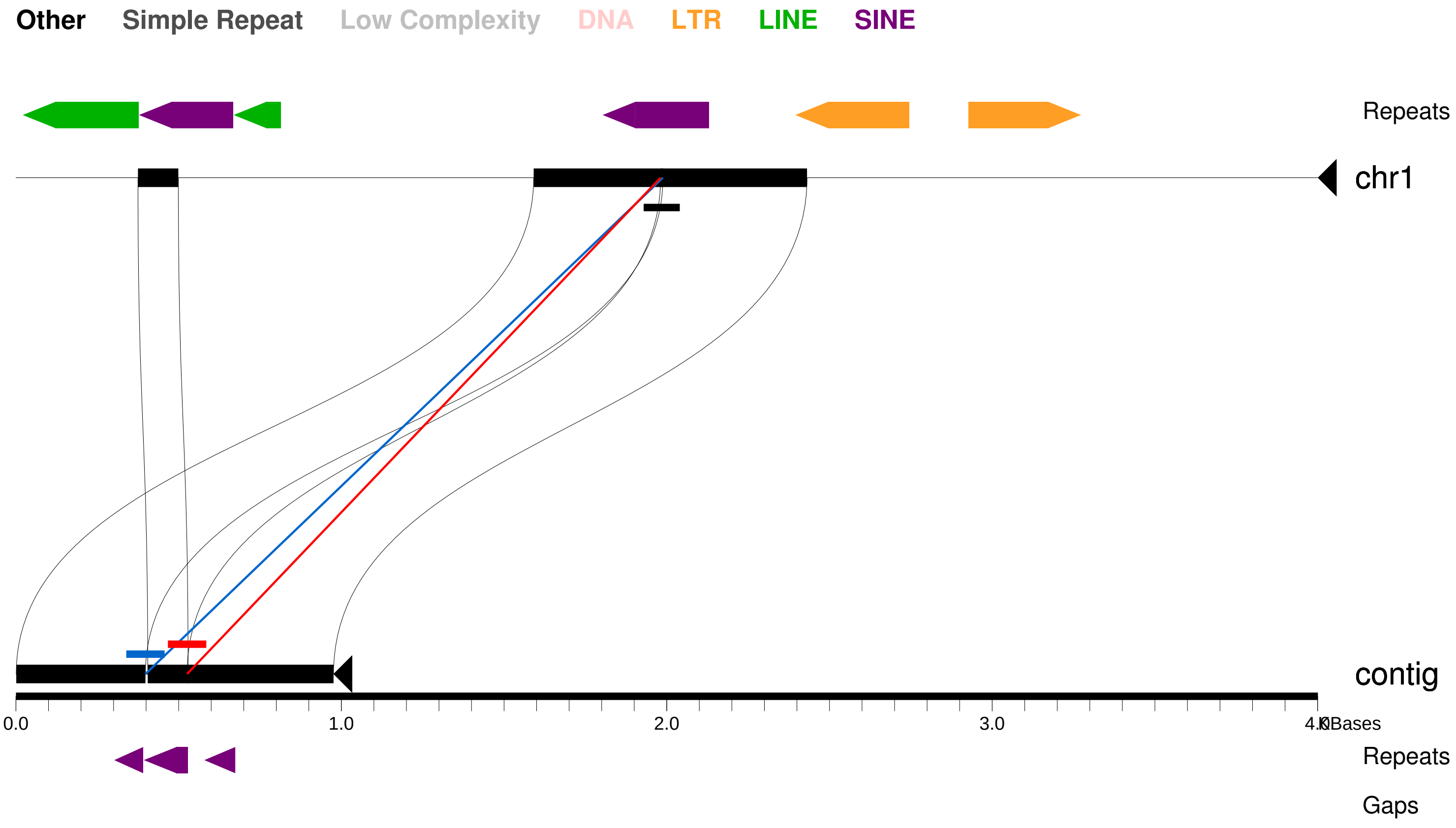

siteID: chr1_106326564 Scafftig3 + Breakpoint: chr1:106326575
chr1:106326415-106326761
Overlap Length: 27 INS size: 244 Genotyped: No

```
left    A------------------------------------------------------AATGTGAAAAATAAG
chrom   ACTACTAGGAATTG--ACTTCATATCTAAAGTAAGCAAAAATGCACTTCTCTAAAAATGTGAAAAATAAG
right   GCCACTACGCCCGGCTAATTTTT-TGTATTTTTAGTAGAGACGGGGTT-TC----ACCGTT-----TTAG
        12N2222N2NNNN2112N22NN2 2N22NNN2N22N2N2N2N2NNN22 22    *11**111111*1**

left    CTCAAACAATATGTGATTGGTTGATTTAATAATCTTAATTCCTCCATTAGTATTAGAAAATAATTTGGTT
chrom   CTCAAACAATATGTGATTGGTTGATTTAATAATCTTAATTCCTCCATTAGTATTAGAAAATAATTTGGTT
right   CTGGGATGGTCTC-GATCTCCTGACCTCGTGATCCGCCCGCCTC-----------------------GGC
        **111*111*1*11***1111***11*11*1***111111****11111111111111111111111*11

left    CAACCAACGA-CTATGTAACTTTGCTTCTTTTTAAAATATGAAACAATCCNNNNNNNNNNN---------
chrom   CAACCAACGA-CTATGTAACTTTGCTTCTTTTTAAAATATGAAACAATCCGCAGTGAAACATAAATGTAA
right   CTCCCAAAGTGCTGGGT------GCTTCTTTTTAAAATATGAAACAATCCGCAGTGAAACATAAATGTAA
        *11****1*11**11**111111***************************22222222222222222222

left    ----------------------------------------------------------------------
chrom   AACTTTAGATTGTTGTAATCCGAGGATCACGCAGAAAGAACGATGTAAAGATAGCTGATGCTTTGATT
right   AACTTTAGATTGTTGTAATCCGAGGATCACGCAGAAAGAACGATGTAAAGATANNNNNNNNNNNNNNN
        2222222222222222222222222222222222222222222222222222222NNNNNNNNNNNNNNN

left    ----------------------------------------------------------------------
chrom   AACCTAAAAGGAATATTTTTAGCTTTTATTTTAAGCTCTCAAAGATCTTTTACTTCCTTTGGTATTATCA
right   NNNNNNNNNNNNNNNNNNNNNNNNNNNNNNNNNNNNNNNNNN----------------------------
        NNNNNNNNNNNNNNNNNNNNNNNNNNNNNNNNNNNNNNNNNN
```

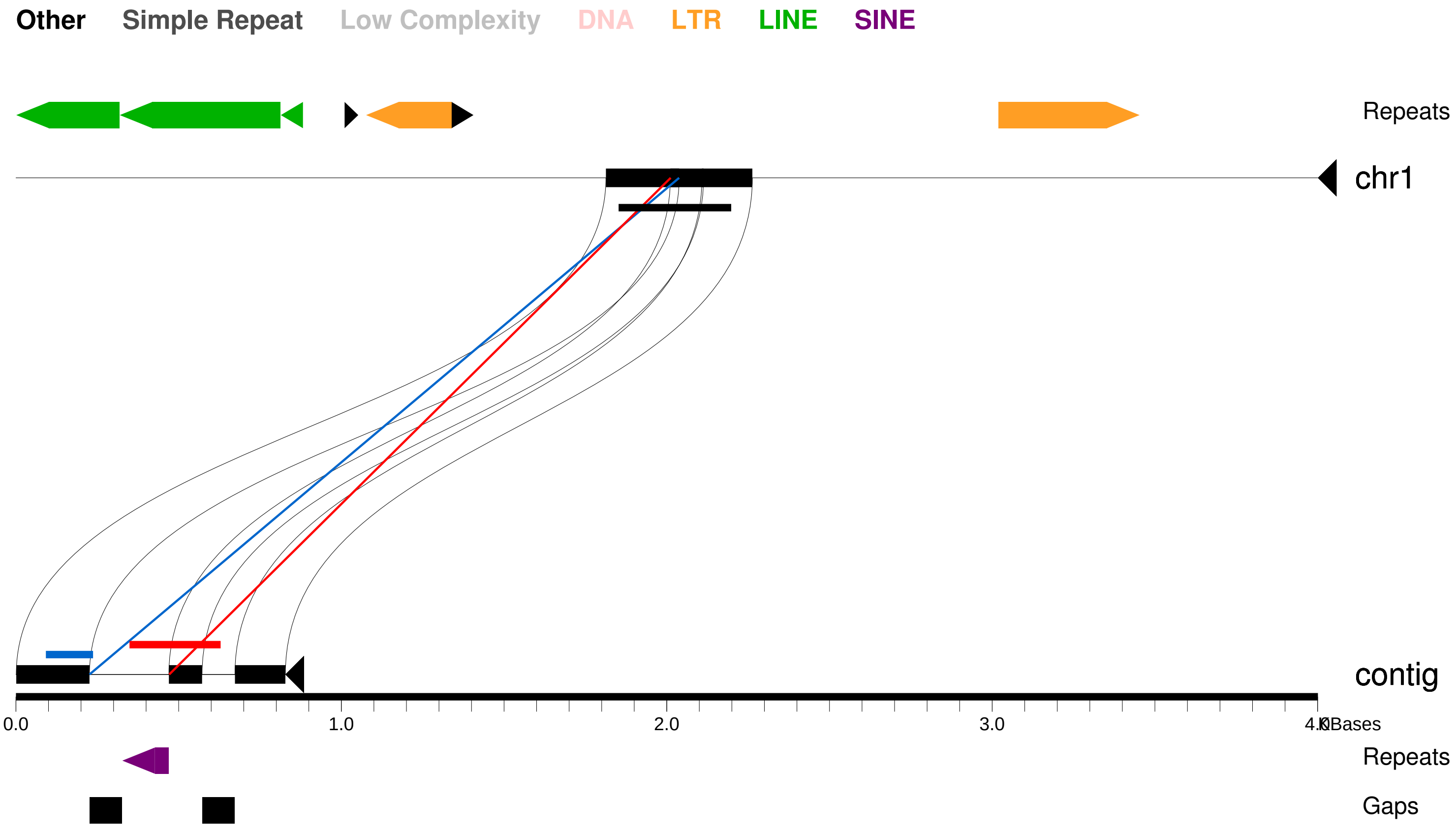

siteID: chr1_108142475 Scafftig2 + Breakpoint: chr1:108142467
chr1:108142420-108142526
Overlap Length: 7 INS size: 304 Genotyped: Yes

```
left    GTGTTTCACA-GATAGAAGAGTAAACTAATATCA----------ATAATAAGAG-GATACTGAACTTTTT
chrom   GTGTTTCACA-GATAGAAGAGTAAACTAATACCA----------ATAACAAGAG-GATACTGAACTATTC
right   CCTGACCTCATGATCCACCCGCCTCGGCCTCCCAAAGTGCTGGGATTACAGGCGTGAGCCTGAACTATTC
        111111*1**1***11*111*11111111*12**1111111111**1*2*1*1*1**11*******2**2

left    TTTTTTTTTTTTTTTTTTTTTTTTTTGAGACGGAGTCTCGCTCTGTCGCCCAGGTCGGACT
chrom   TTAAGAAACAACTCAAATCC---------ACAGA---TCACTCCTTACTTATAATAGACAA
right   TTAAGAAACAACTCAAATCC---------ACAGA---TCACTCCTTACTTATAATAGACAA
        **2222222222*2222*22222222222**2**222**2***22*22222222*2*2222
```

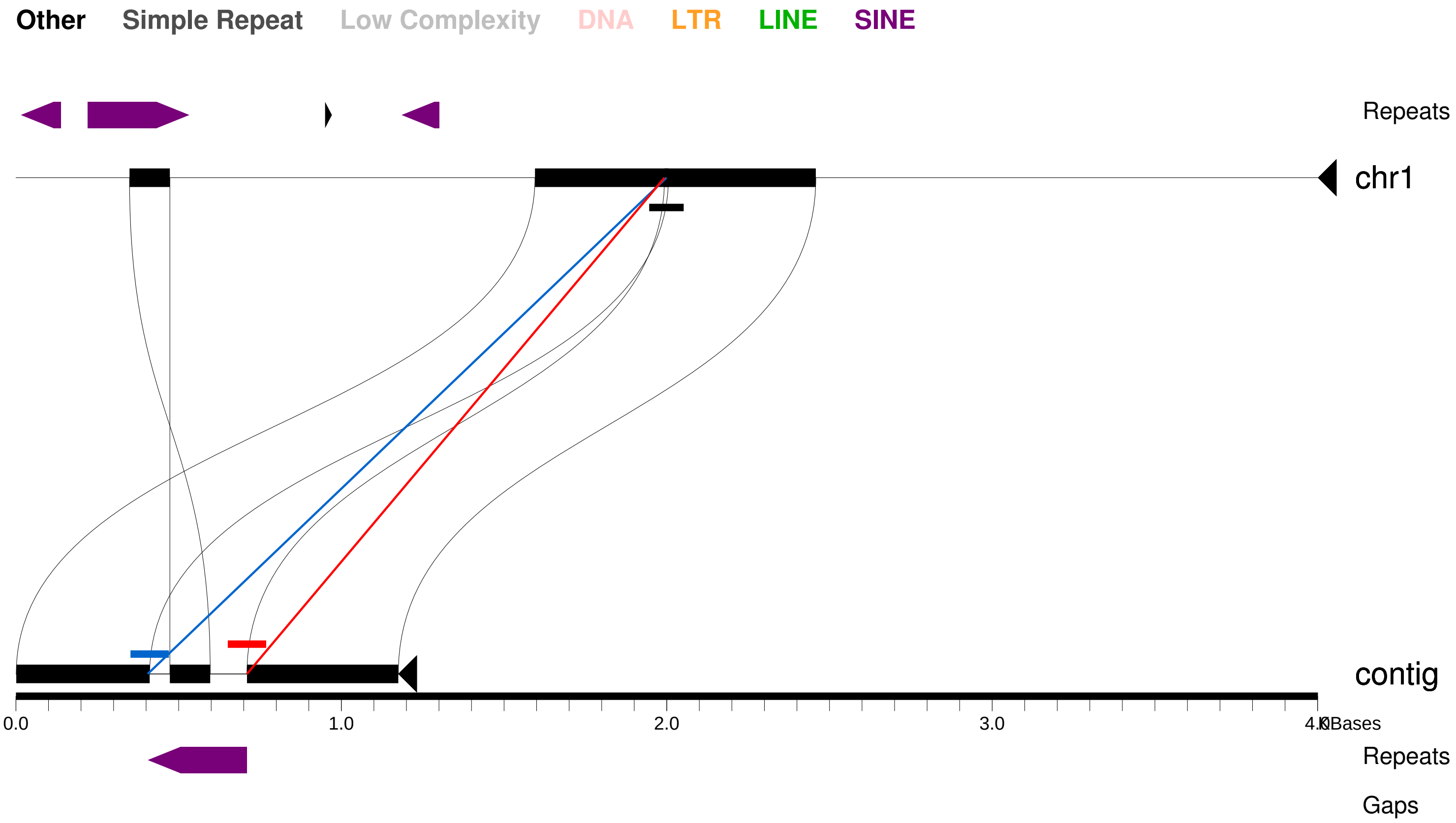

siteID: chr1_112032275 Scafftig3 + Breakpoint: chr1:112032269
chr1:112032225-112032328
Overlap Length: 16 INS size: 322 Genotyped: Yes

```
left    GGGTGA---------------AATATGGAGGCCTCCTACCATTGCTTGACGGAGGGCCTTGAAGAGCCTG
chrom   GGGTGA---------------AATATGGAGGCCTCCTACCATTGCTTGACGGAGGGCCTTGAAGAGCCTG
right   GAGCGAGACTCCGTCTCAAAAAAAAAAAAAAAATAAAAAAAAAATAAAATAAAATAAAATGAAGAGCCTG
        *1*1**111111111111111**1*111*1111*111*11*1111111*111*111111**********

left    AGGCTGCCGGGCGCGGTGGCTCACGCCTGTAATCCCAGCACTTTGGGAGGCCGAGGCGGGCGGA-
chrom   AGGCTTACCGTTA-GGAGGC-----CCTCTGTCCCTAGC----------TCCCACCCTACCTTAG
right   AGGCTTACCGTTA-GGAGGC-----CCTCTGTCCCTAGC----------TCCCACCCTACCTTAG
        *****22*2*2222**2***22222***2*222**2***22222222222**2*22*222*22*2
```

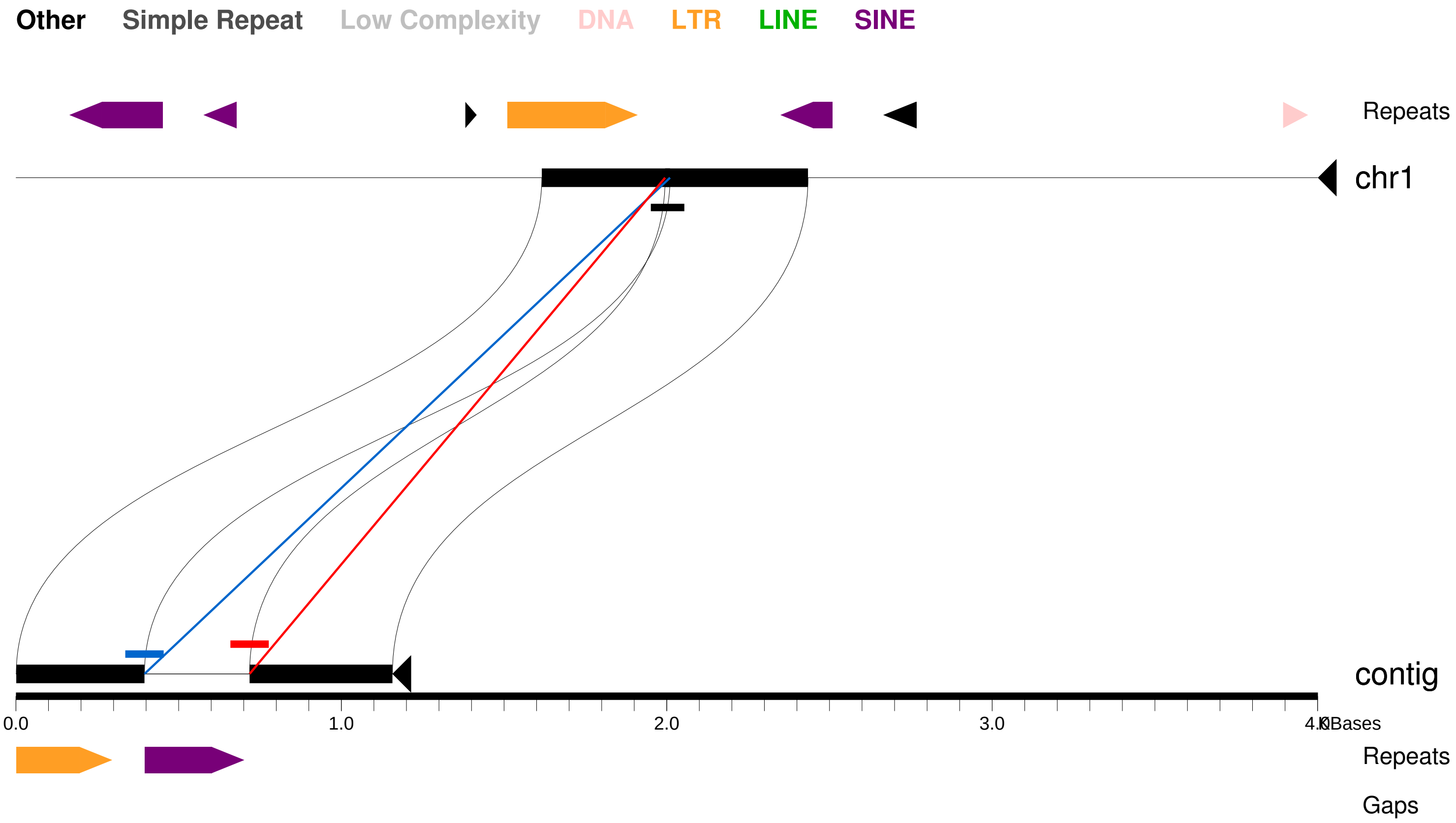

siteID: chr1_112736015 Scafftig1 - Breakpoint: chr1:112735999
chr1:112735948-112736058
Overlap Length: 9 INS size: 425 Genotyped: No

```
left   GTAAATGTGTATTGCAAACACTAGGACAATTACTAAACTAATTTTTGGAAA--------AGAAGTATAGG
chrom  GTAAATGTGTATTGCAAACACTAGGACAATTACTAAACTAATTTTTGGAAA--------AGAAGTATAA-
right  NNNNNNNNNNNNNNNNNNNNNNNNNNNNNNNNNNNNNNNNNNNNNNNNNNNNNNNNNNNAGAAGTATAA-
       11111111111111111111111111111111111111111111111111111111111*********22

left   CCGGGCGCGGTGGCTCACGCCTGTAATCCCAGCACTTTGGGAGGCCGAGGCGGGCGG
chrom  CTGATAAGAGAGGAGAGAAAATGAAATCATATAAAAT-----GTTCAATTAAGAT--
right  CTGATAAGAGAGGAGAGAAAATGAAATCATATAAAAT-----GTTCAATTAAGAT--
       *2*222222*2**22222222**2****22*22*22*22222*22*2*2222*2222
```

Other **Simple Repeat** **Low Complexity** **DNA** **LTR** **LINE** **SINE**

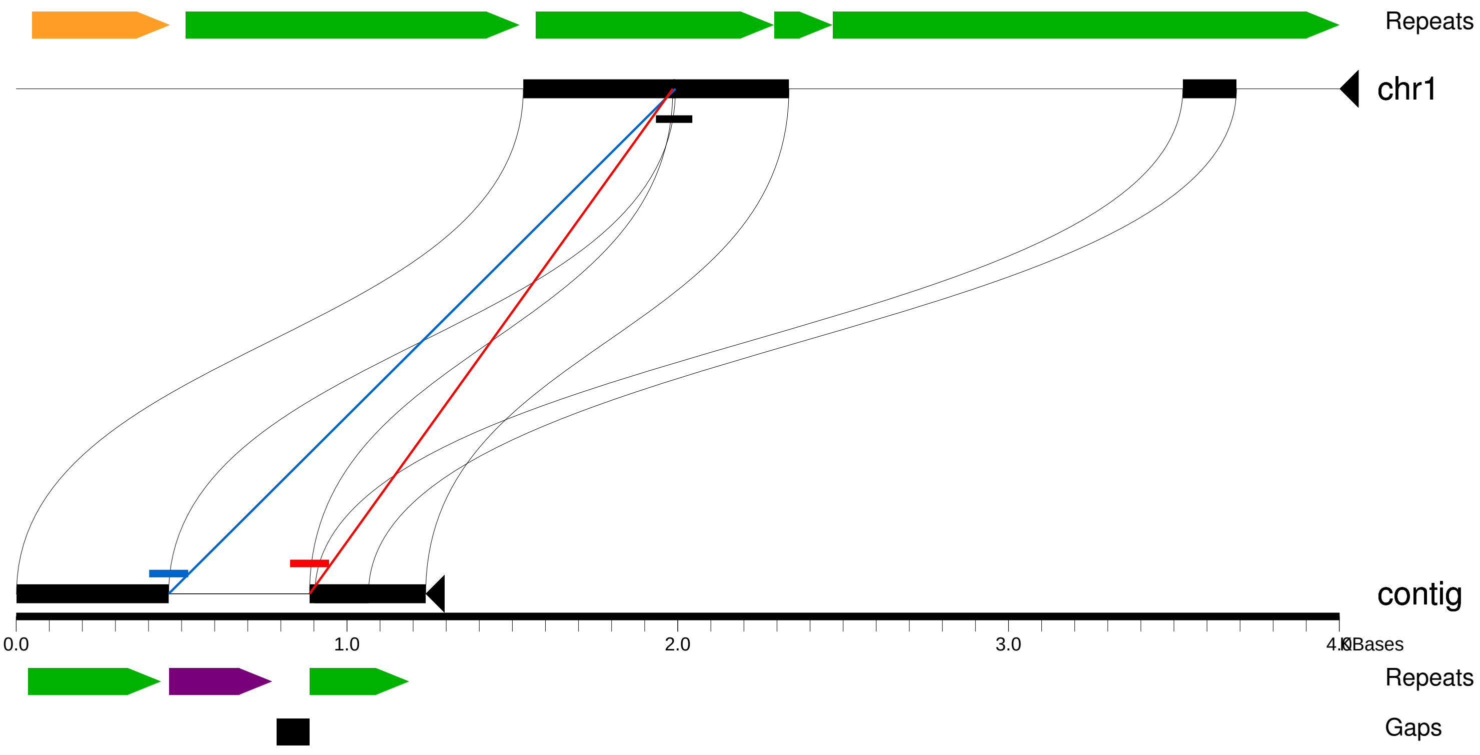

siteID: chr1_112992012 Scafftig9 - Breakpoint: chr1:112991995
chr1:112991835-112992169
Overlap Length: 15 INS size: 302 Genotyped: Yes

```
left   ------------GCCTGATACTTTCTTGTCAGCTGCCAGTTTTTCACAGTAATTCAATTGGGACATCTCA
chrom  TTTATAAATTATGCCTGATACTTTCTTGTCAGCTGCCAGTTTTTCACAGTAATTCAATTGGGACATCTCA
right  TTTAGTA---------GAGACGGGGTT-TCA----CCA--TTTTAGCCGGGATGGTCTCG----ATCTC-
       2222NN2     1111**1**1111**1***1111***11****11*1*11**1111*1*1111*****1

left   CGGGTATCAAAAAGCCCCTTAGTTTATGTAAGAGCCAGGAGATAAAAAAGAAAACACAGCT--TATTGCA
chrom  CGGGTATCAAAAAGCCCCTTAGTTTATGTAAGAGCCAGGAGATAAAAAAGAAAACACAGCT--TATTGCA
right  CTGACCTCGTGATCCGCCC--------------GCCTCGGCCTCCCAAAGT-------GCTGGGATTACA
       *1*111**111*11*1**111111111111111***11*111*111****11111111***111***1**

left   AATGTTAAGTTTAGAGAACGTGCCAGACACGTTATTT---------TTTTTTTTT---------------
chrom  AATGTTAAGTTTAGAGAACGTGCCAGACACGTTATTTAAGACAGCATTTCTTCTTGATATGTCACTCCAA
right  GGCGTGAGCCACCGCGCCCGGCCCAGACACGTTATTTAAGACAGCATTTCTTCTTGATATGTCACTCCAA
       111**1*111111*1*11**11**************222222222***2**2**222222222222222

left   ------TTTTTTTTTTTTTGAGACGGAG-----TCTCGCTCTG-----TCGCCCAGGCTGGAGTGCAGTG
chrom  AACACTTTGTATGTTTTTTCAGTTCTTGATCATTCAAACTTTGGGGTTTGGCCAAGCTTGTAGCTGACGG
right  AACACTTTGTATGTTTTTTCAGTTCTTGATCATTCAAACTTTGGGGTTTGGCCAAGCTTGTAGCTGACGG
       222222**2*2*2******2**22222*22222**222**2**22222*2***2**22**2**222*22*

left   GCAGGATCTCG----GCTCACTGCAAGCTCCGCCTCCCGGGTTCACGCCATTCTCCTGC
chrom  GAGTGTTCTCTATTTGCTTACTT-AAGAGATAAATAAAAAGATTAA-CAGGTATTTGAG
right  GAGTGTTCTCTATTTGCTTACTT-AAGAGATAAATAAAAAGATT---------------
       *222*2****22222***2***22***2222222*22222*2*21N21NNN1N1NNNNN
```

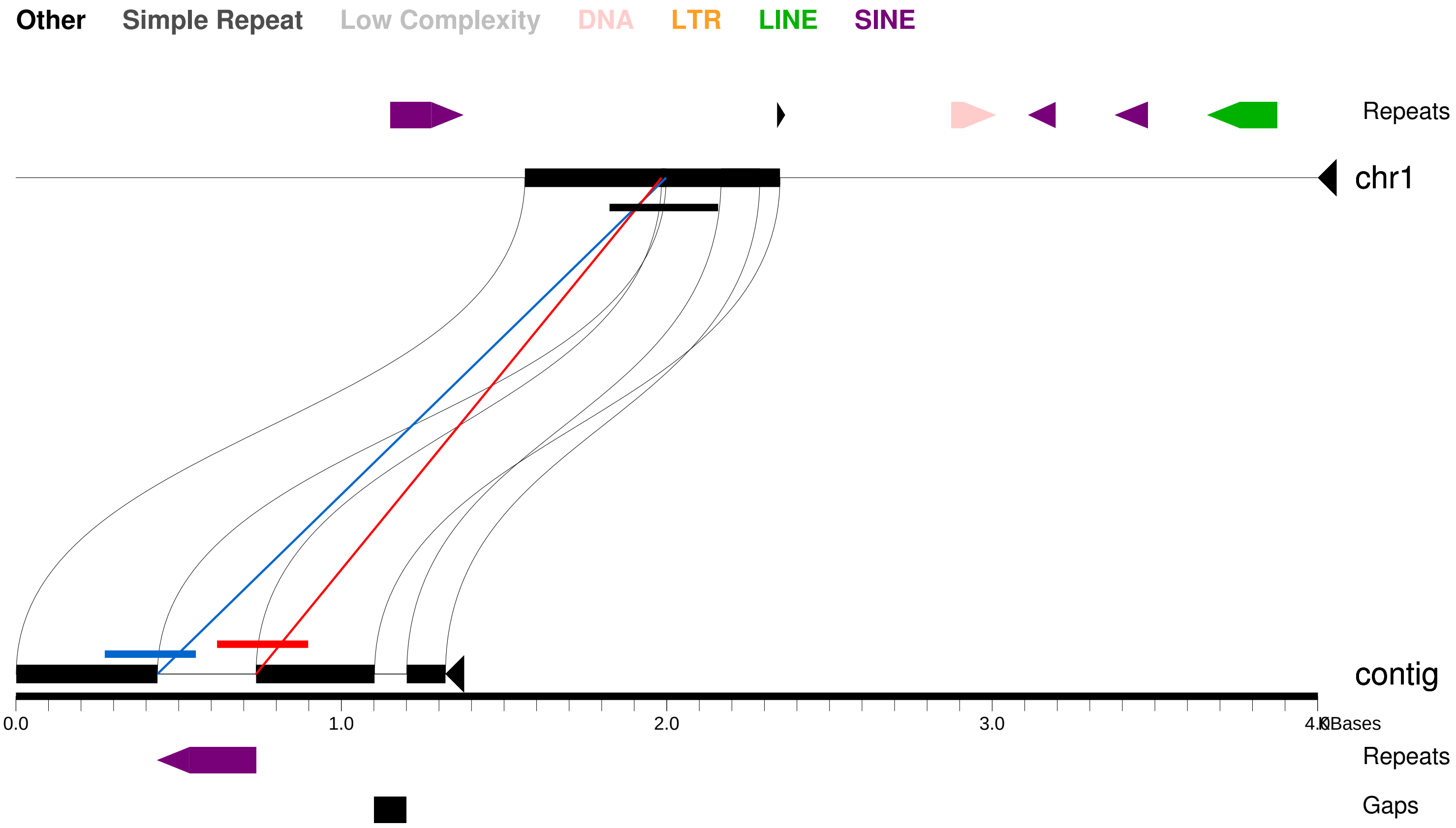

siteID: chr1_113517859 Scafftig1 - Breakpoint: chr1:113517774
chr1:113517753-113517833
Overlap Length: 60 INS size: 453 Genotyped: No

```
left   GTTTGGAAAACCATAGACCAT--------------------------------------AAACTTTTCTG
chrom  GTTTGGAAAACCATAGACCAT--------------------------------------AAACTTTTCTG
right  NNNNNNNNNNNNNNNNNNNNNNNNNNNNNNNNNNNNNNNNNNNNNNNNNNNNNNNNNNNAAACTTTTCTG
       11111111111111111111111111111111111111111111111111111111111***********

left   AACGTTCGATGCAGAGCTCCAACCGAAC-AGCAAAGAACATTTCCTCAAAGAAACGGAGTATTGGCCGGG
chrom  AACGTTCGATGCAGAGCTCCAACCGAACTAGCAAAGAACATTTCCTCAA---------------------
right  AACGTNCGATGCAGNGNNNCAACCNAACTAGCAAAGAACATTTCCTCAA---------------------
       *****1********1*111*****1***2********************222222222222222222222

left   CGCGGTGGCTCACGCCTG
chrom  ------------------
right  ------------------
       222222222222222222
```

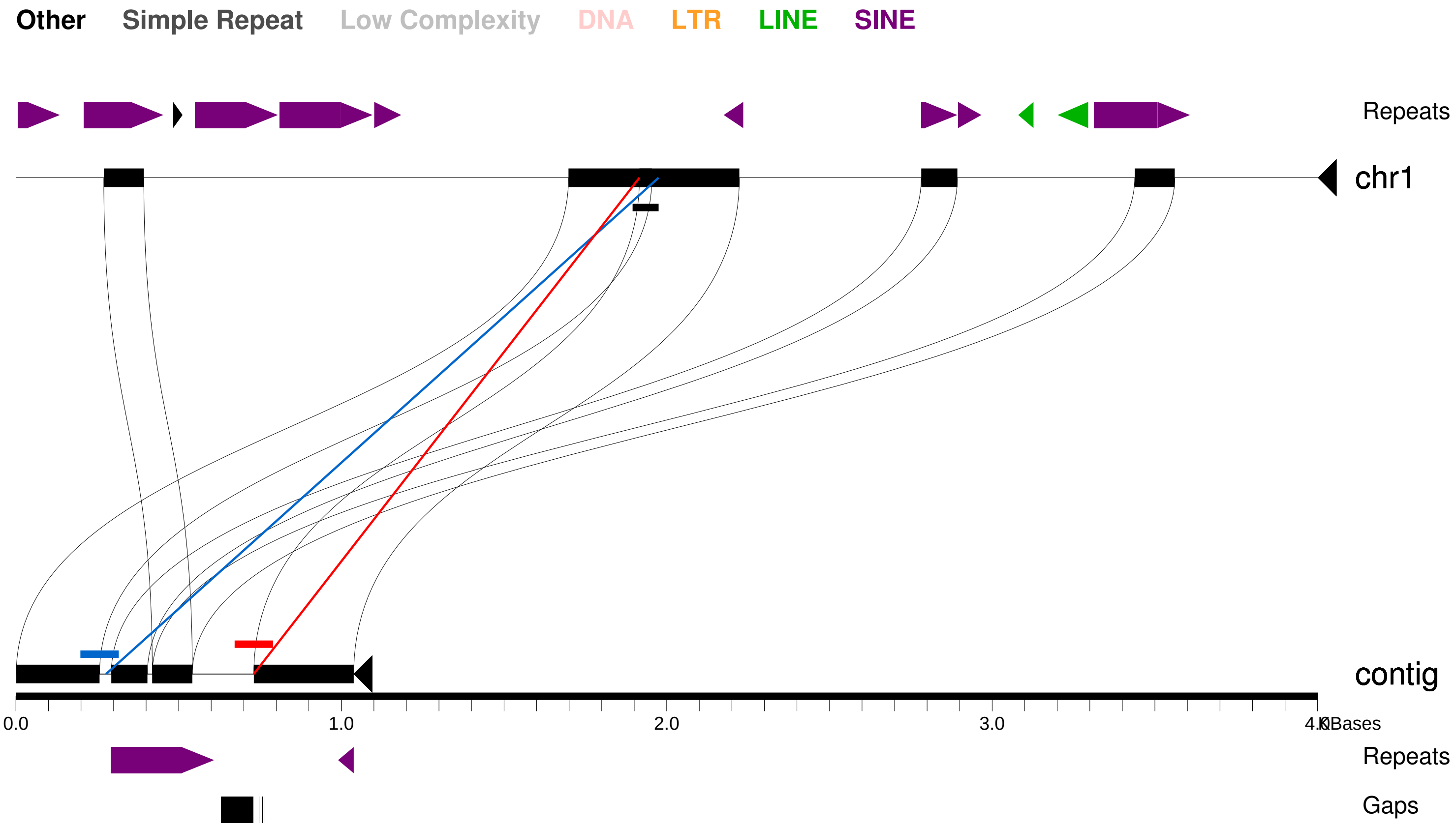

siteID: chr1_116983552 Scafftig2 + Breakpoint: chr1:116983560
chr1:116983503-116983619
Overlap Length: 3 INS size: 414 Genotyped: No

```
left     TGGGAGTTCTAAGTCTCC-ACTGTGCCTCCACT------GACAACCTCCTGGCTCGGTGAGGTAGGGNNN
chrom    TGGGAGTTCTAAGTCTCC-ACTGTGCCTCCACT------GACAACCTCCTGGCTCGGTGAGGTAGGGTTG
right    CCGC----CTCGGCCTCCCAAAGTGCTGGGATTACAGGCGTGAGCCACCGCGCCCGGCCTACC-GGGTTG
         11*11111**11*1****1*11****1111*1*111111*11*1**1**11**1***1111111***222

left     NNNNNNNNNNNNNNNNNNNNNNNNNNNNNNNNNNNNNNNNNNNNNNNNNNNNNNN
chrom    CTTCTTACTGATCTCCACATGGCCACCACAGGCACCATGGGGGTGAATGGCTCT--
right    CTTCTTACTGATCTCCACATGGCCACCACAGGCACCATGGGGGTGAATGGCTCT--
         2222222222222222222222222222222222222222222222222222222
```

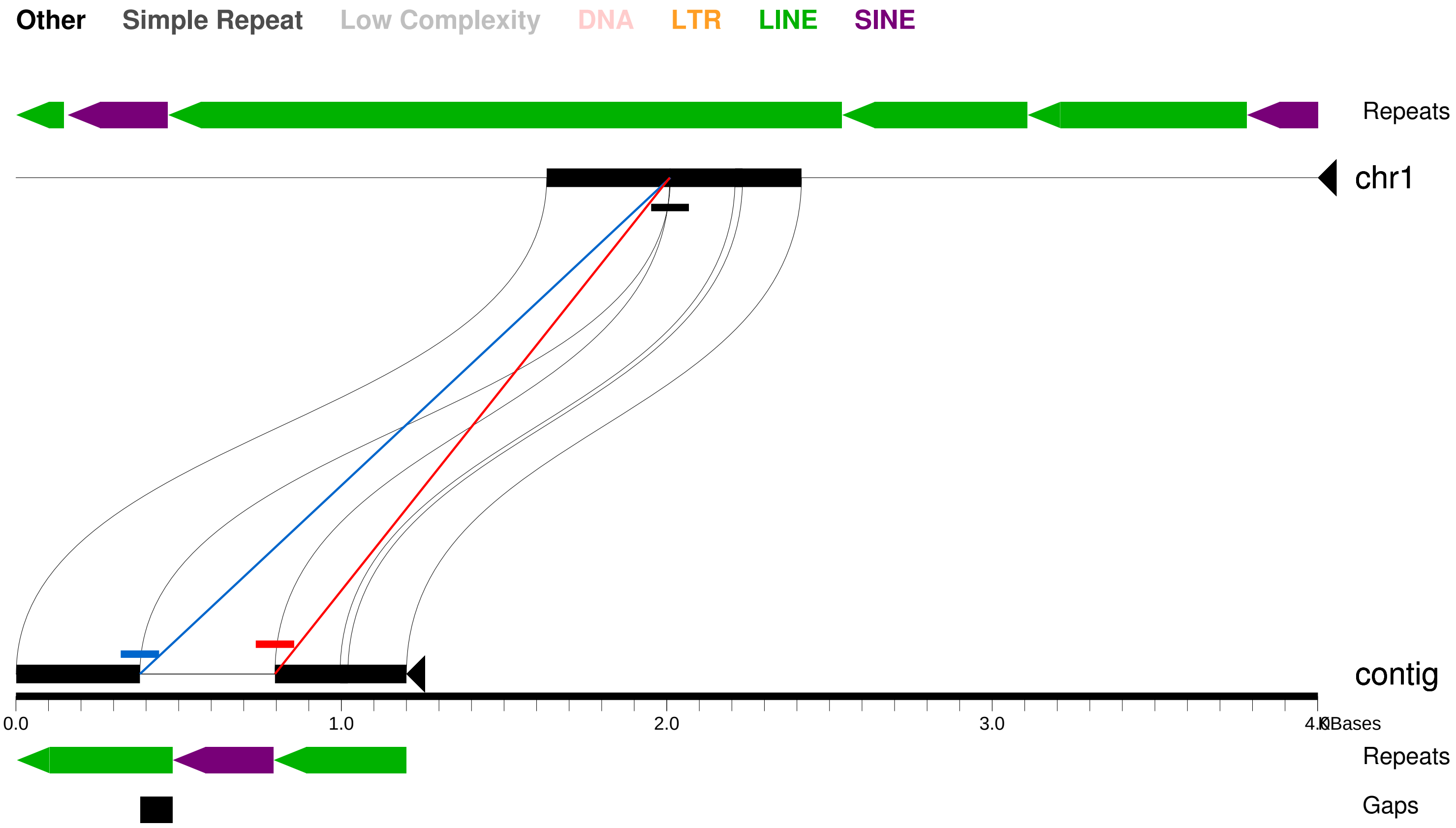

siteID: chr1_118379629 Scafftig1 + Breakpoint: chr1:118379680
chr1:118379630-118379739
Overlap Length: 10 INS size: 415 Genotyped: No

```
left   GACAGTTAAGAGTGATCAAATAGTGATGAGGGATAGCTTCTCTTTAAAGA---------ATTGTTCCAGG
chrom  GACAGTTAAGAGTGATCAAATAGTGATGAGGGATAGCTTCTCTTTAAAGA---------ATTGTTCCAGT
right  NNNNNNNNNNNNNNNNNNNNNNNNNNNNNNNNNNNNNNNNNNNNNNNNNNNNNNNNNNNNATTGTTCCAGT
       111111111111111111111111111111111111111111111111111111111111**********2

left   CCGGGCGCGGTGGCTCACGCCTGTAATCCCAGCACTTTGGGAGGCCGAGGCGGGCGGA
chrom  T---GATAAACAAATAAAGAATG--ATAAAATTAGTTTTTCAG-CATTTGCAACT---
right  T---GATAAACAAATAAAGAATG--ATAAAATTAGTTTTTCAG-CATTTGCAACT---
       2222*222222222*2*2*22**22**222*22*2***222**2*2222**2222222
```

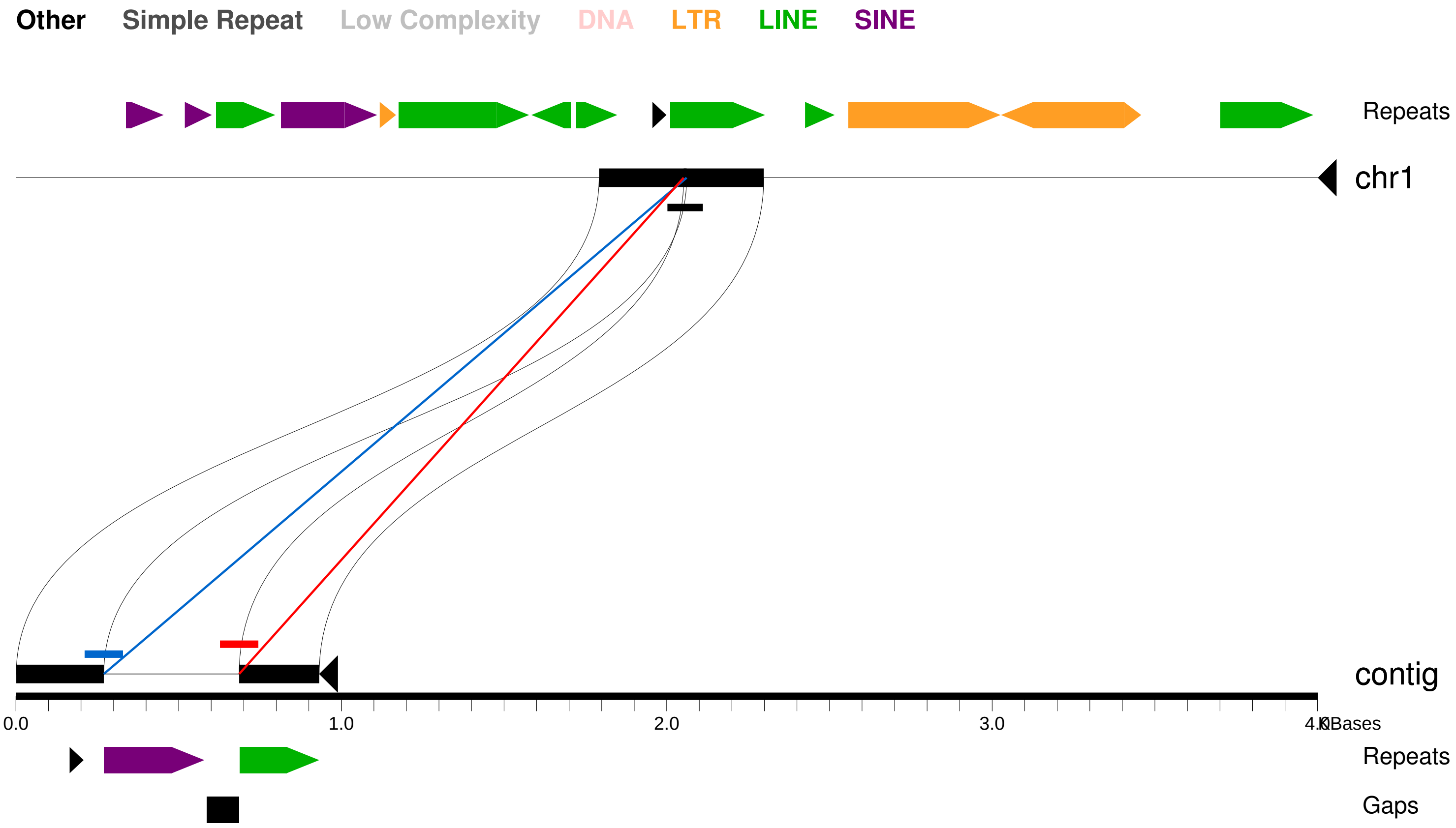

siteID: chr1_119080710 Scafftig2 + Breakpoint: chr1:119080692
chr1:119080645-119080751
Overlap Length: 13 INS size: 311 Genotyped: Yes

```
left     AAG-----------CTACCAAAG--ATTTAACGACCAGCTTAAAAGATTCTTTAATATTAGCAACCAGCT
chrom    AAG-----------CTACCAAAG--ATTTAACGACCAGCTTAAAAGATTCTTTAATATTAGCAACCAGCT
right    CCGCCCGCCTCGGCCTCCCAAAGTGCTGGGATTACAGGCGTGAGCCAC-CGCGCCCGGCCGCAACCAGCT
         11*11111111111**1******111*111*11**11**1*1*111*11*1111111111**********

left     TTTTTTTTTTTTTTTTTTTTTTTTTTTTTTTTTGAGACGGAGTCTCGCTCTGTCGCCCAGGC
chrom    TTTATAAGCTGGTGTGAGCC------------AGCTCCAGCATGCCACTGTGTAAATCCAAT
right    TTTATAAGCTGGTGTGAGCC------------AGCTCCAGCATGCCACTGTGTAAATCCAAT
         ***2*2222*22*2*222222222222222222*222*2*22*22*2**2***2222*2222
```

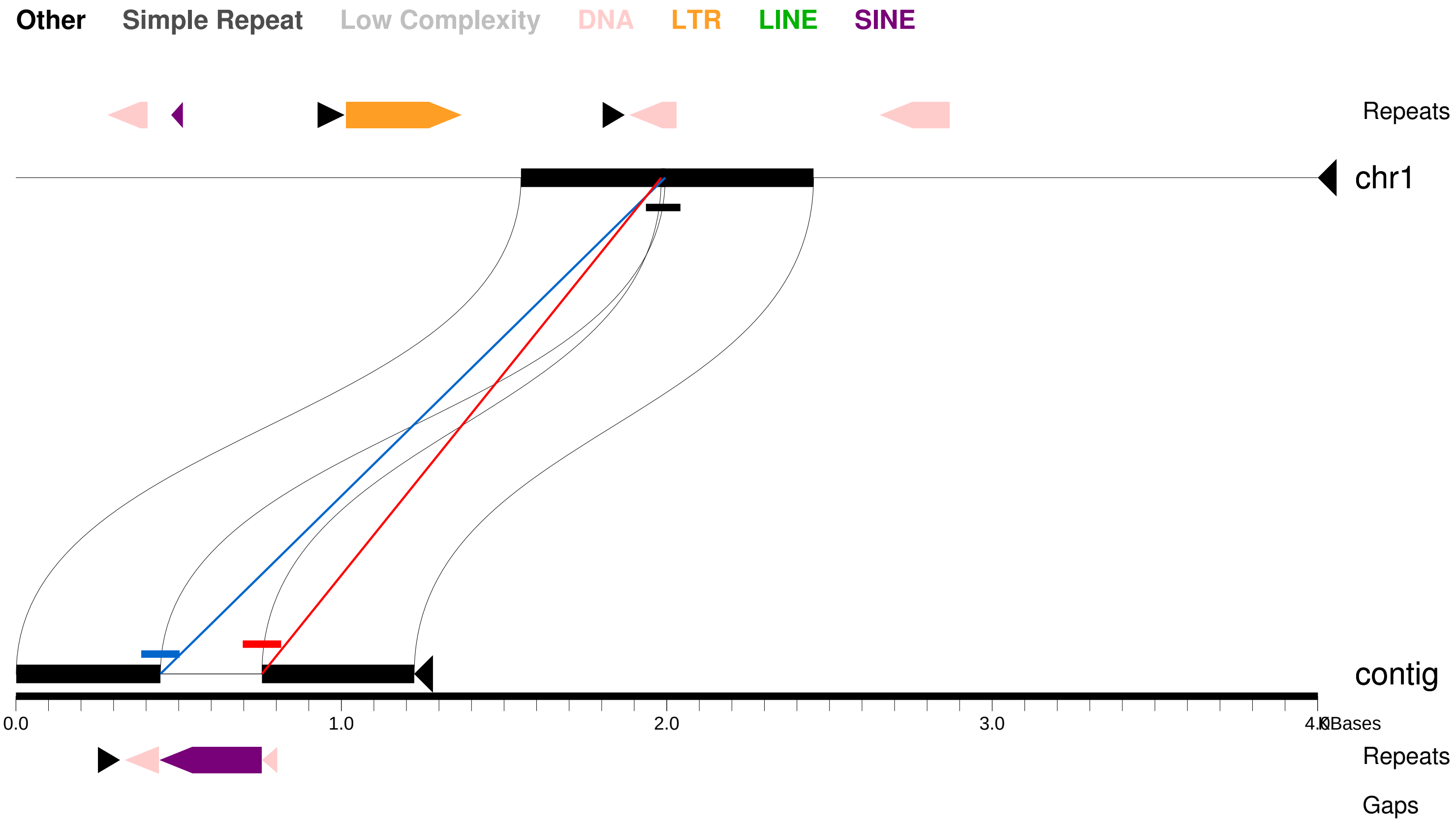

siteID: chr1_144950966 Scafftig1 - Breakpoint: chr1:144951011
chr1:144950524-144951173
Overlap Length: 16 INS size: 411 Genotyped: No

```
left   NNNNNNNNNNNNNNNNNNNNNNNNNNNNNNNNNNNNNNNNNNNNNNNNNNNNNNNNNNNNNNNNNNNNNN
chrom  AAAGTATAATAATTCATATCTCATGGGGCTGTTAAACAAGAACATAAAGTACTCAGCTGTATGGTAAGAG
right  NNNNNNNNNNNA-----------------------------------------------------------
       NNNNNNNNNNN2NNNNNNNNNNNNNNNNNNNNNNNNNNNNNNNNNNNNNNNNNNNNNNNNNNNNNNNNNN

left   NNNNNNNNNNNNNNNNNNNNNN------------------------------------------------
chrom  CTCAACAAAGCTTAGCCATCATTATTATTACTAGCCTGGCATGAATGAAGTTAAGGAATGGTGGTGGGTG
right  ----------------------------------------------------------------------
       NNNNNNNNNNNNNNNNNNNNNN

left   ----------------------------------------------------------------------
chrom  ATGAGTGTGGGAGAAATCAGAGGTTACATCATAAAAGGCTTTGATGGTCACATTAGGAGTATGGATCTTA
right  --------------------------------AAAA----------------------------------
                                       2222

left   ----------------------------------------------------------------------
chrom  TCTCCTAAAGGTATTGCAAAGCTTCTTAAATGGCTCTGCCATGGTGCGATTAAATTTATGTCTTGGAAAG
right  ----------------------------------------------------------------------

left   ----------------------------------------------------------------------
chrom  ATCACATTGGTCATGGTATGCACATGTGGTTTTTGCCTATTCAGTTAAAAGCTCCTTAAAGCTTTTTATA
right  ----------------------------------------------------------------------

left   ----------------------------------------------------------------------
chrom  CCACCACAGTCTTATGCACCTAAGAGGCTAAATTATTTGCCAATTTGTTGTTGTTGAATTATCTATTTCA
right  ----------------------------------------------------------------------

left   ---------------ATTAGGTTGAGATTGAATGAGAGGAGCAGGACTGGAAGTGGAGAGGACATTTAGA
chrom  GAACTACTCTGAATTATTAGGTTGAGATTGAATGAGAGGAGCAGGACTGGAAGTGGAGAGGACATTTAGA
right  -------------------------------------------------------------------AGA
                      1111111111111111111111111111111111111111111111111111***

left   AAGTTTCCTCGGTGGCCGAGCGCG------GTGGCTCACGCCTGTAAT--CCCAGC-ACTTTGGG-----
chrom  AAGTTTCCTCGGTAATCCAGACAGTAGATGGTGG-TGACCTCAATAATTGCCCAGTTACCTTCAGGACAA
right  AAGTTTCCTCGGTAATCCAGACAGTAGATGGTGG-TGACCTCAATAATTGCCCAGTTACCTTCAGGACAA
       *************222*2**222*222222****2*2**22*22****22*****22**2**22*22222

left   AGGCCGAGGCGGGT-----GGATCACGAGGTCAGGAGATCGAGACCATCCTGGCTAACACGGTGAAACCC
chrom  AGTCCAAGTTCCTTATAATGGTATACAAAGCTGGCACAATCAGACCCCTATTCTCAAAACAGCTTACCTC
right  AGTCCAAGTTCCTTATAATGGTATACAAAGCTGGCACAATCAGACCCCTATTCTCAAA------------
       **2**2**22222*22222**222**2*2*222*2*2*222*****2222*2222**211N1NNN1N1N1

left   ---CGTCTCTACTAAA-----
chrom  TCACTTGTCTTCTCACTTGCT
right  ---------------------
          1N1N111N11N1N
```

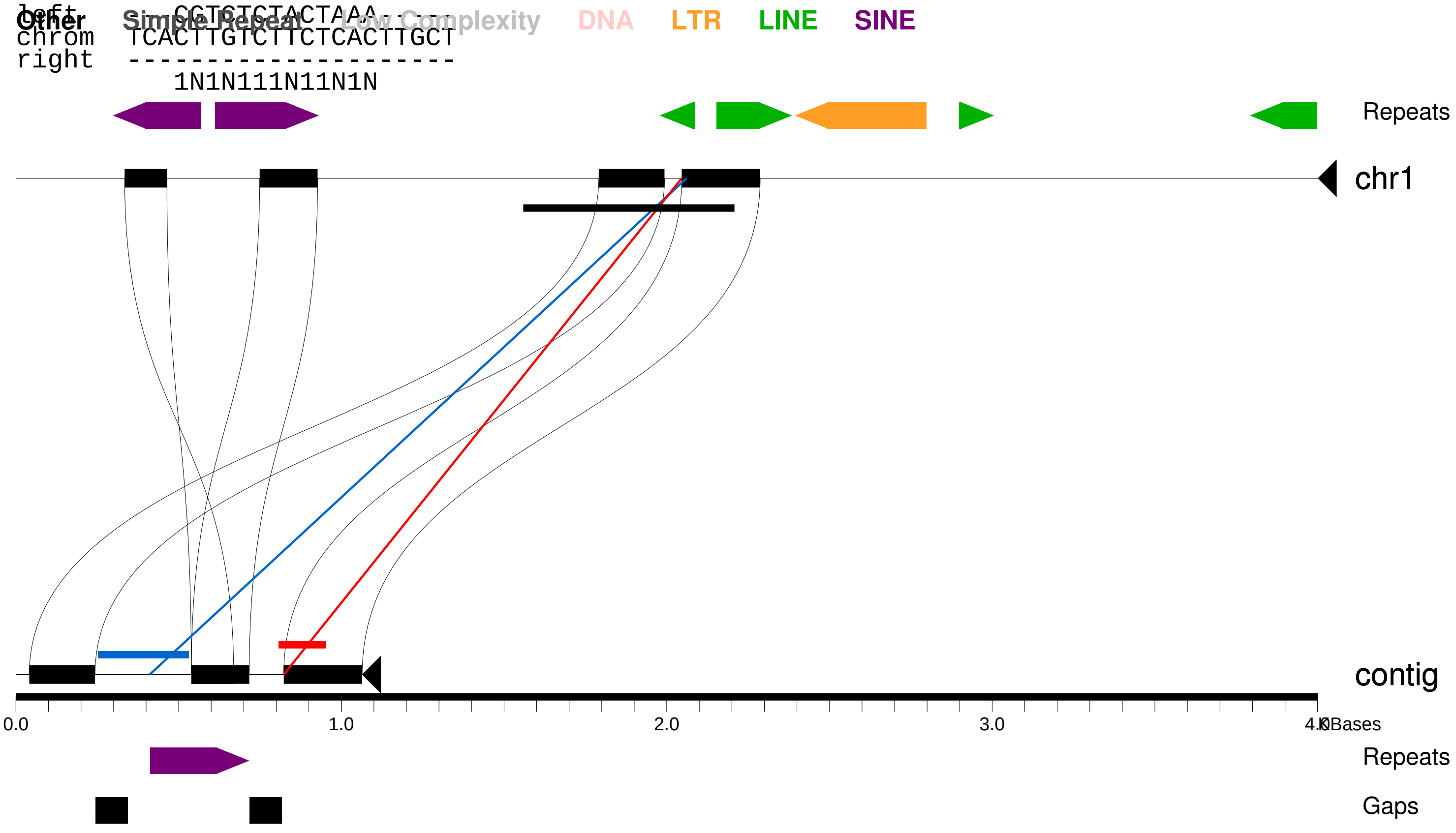

siteID: chr1_145098239 Scafftig1 - Breakpoint: chr1:145098242
chr1:145098198-145098301
Overlap Length: 16 INS size: 415 Genotyped: No

```
left    TTTTCCTACAGTGGGTGGCTGTGTGTTGGGGGT--AGGGGGGAT-----------GG---AATCTGGTCA
chrom   TTTTCCTACAGTGGGTGGCTGTGTGTTGGGGGT--AGGGGGGAT-----------GG---AATCTGGTCA
right   CAC-CCGCCTCGGCCTCCCAAAGTGCTGGGATTACAGGCGTGAGCCACCGCGCCCGGCCTAATCTGGTCA
        1111**11*111*11*11*111***1****11*11***1*1**111111111111**111**********

left    ATTTTTTNNNNNNNNNNNNNNNNNNNNNNNNNNNNNNNNNNNNNNNNNNNNNNNNNNNNNNNNNN
chrom   ATTTTT---------------AAATTATTGCATTGAATTCCAGTGTTAGCAAAGAATACAAAGTG
right   ATTTTT---------------AAATTATTGCATTGAATTCCAGTGTTAGCAAAGAATACAAAGTG
        ******22222222222222222222222222222222222222222222222222222222222
```

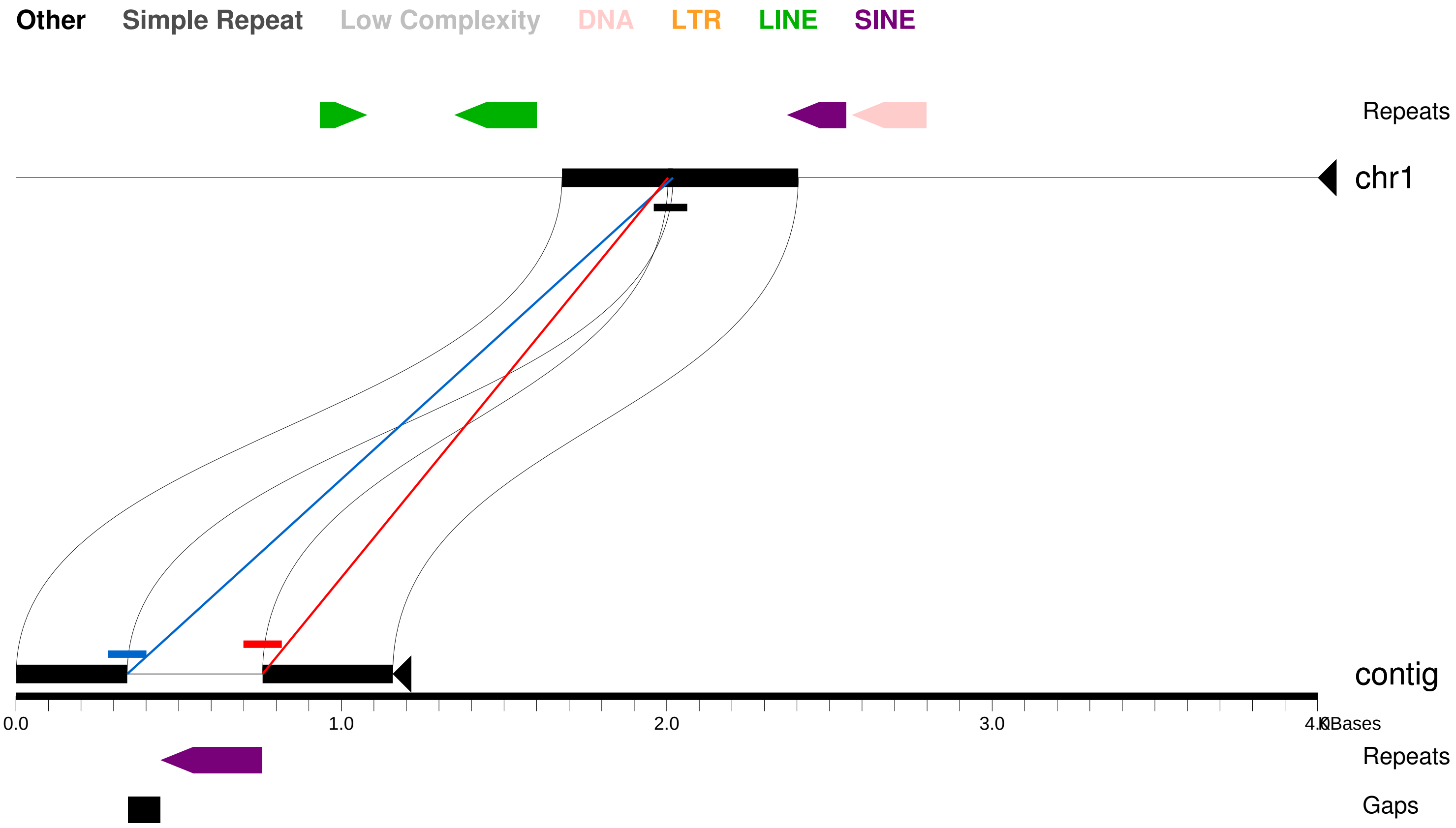

siteID: chr1_145115639 Scafftig1 + Breakpoint: chr1:145115546
chr1:145115402-145116066
Overlap Length: 87 INS size: 410 Genotyped: No

```
left   ------------------------------------------------------------------------
chrom  TCCCCAATTATAGATGAAGAAACTAGGTTTTAAATAGGTTACATGACTTGACCAATGTCACATAACTTGT
right  NNNNNNNNNNNA------------------------------------------------------------
       NNNNNNNNNNN2

left   ACAGGTGATATCTGAACCAAAGAGTCTGAGGCCAGAACCTGCACTTCTAACCACCTTACTATATAGCCTC
chrom  ACAGGTGATATCTGAACCAAAGAGTCTGAGGCCAGAACCTGCACTTCTAACCACCTTACTATATAGCCTC
right  ----------------------------------------------------------------------
       1111111111111111111111111111111111111111111111111111111111111111111111

left   AAAAATTCAGACATAATCTGATCAGCATCATCAGTTAATTAGTATATGCAATGTTAGCCTCAAAATAAAA
chrom  AAAAATTCAGACATAATCTGATCAGCATCATCAGTTAATTAGTATATGCAATGTTAGCCTCAAAATAAAA
right  ----ATTCAGACATAATCTGATCAGCATCATCAGTTAATTAGTATATGCAATGTTAGCCTCAAAATAAAA
       1111******************************************************************

left   ATGATGATTTAAGATTCATGG-----GCCGGGCG-----------------------------------C
chrom  ATGATGATTTAAGATTCATGGTTGTTGCAGTGCTGCTCATTTTGGTTATTTTTAATGCCTTAATACTTTC
right  ATGATGATTTAAGATTCATGGTTGTTGCAGTGCTGCTCATTTTGGTTATTTTTAATGCCTTAATACTT--
       *********************22222**2*2**222222222222222222222222222222222 1

left   GGT---------------------------------------------------------------GGCT
chrom  GGTAAAATACTTCCAGGGACTAATTAATGTTTTCCTTTTTTCCTCTTTGACAGCATTGGATTCAAAGGCT
right  ----------------------------------------------------------------------
       111                                                               1111

left   CAC----------GCCTGT------AATCCC------------AGCACTT--------------------
chrom  AACAATTTGTCCAGTCTGTCCAAGAAATACCGCCAGGATGCGAAGTACTTGAACATGCATTCCACTTATG
right  ----------------------------------------------------------------------
       N11          1N1111      111N11            11N1111

left   ------------------------------------------TGGGAGGCCGAGGCGGGCGGAT------
chrom  CCAAACTTGCAGCAGTAGCTGTATTTTTCATCATGTTAATAGTGTATGTCCGATTCTGGTGGCTGTGAAA
right  ----------------------------------------------------------------------
                                                 11NNN1N1111NN1N11N11N1

left   --------------------------------------CACGAGGT---------CAGGAGATCGAGACC
chrom  TAATGAATACAGTCACTGGTAAGGGAGAACCTAGAACCCAGTAGGTGTATATTTTCAGGAAACTGAGCTC
right  ----------------------------------------------------------------------
                                             11NN1111         11111N1NN111NN1

left   A-------------------TCCCGG--------------CTAAA-ACGGTGAAA----------------
chrom  ACAGAGATGTGTATTAGAATCCAAGTGGAACTTCTGCCTCTAAAGACCTTGCAAGAAAAGAGATGTCCTG
right  ----------------------------------------------------------------------
       1                   111NN1              11111 11NN11N11

left   --------------CCCCGTCTCTACTAAA----
chrom  AAAATGAAAGGTTGCACCTCATTTAATGAAGCTTA
right  ----------------------------------
                     1N11NNN1N11N1N11
```

Other Simple Repeat Low Complexity DNA LTR LINE SINE

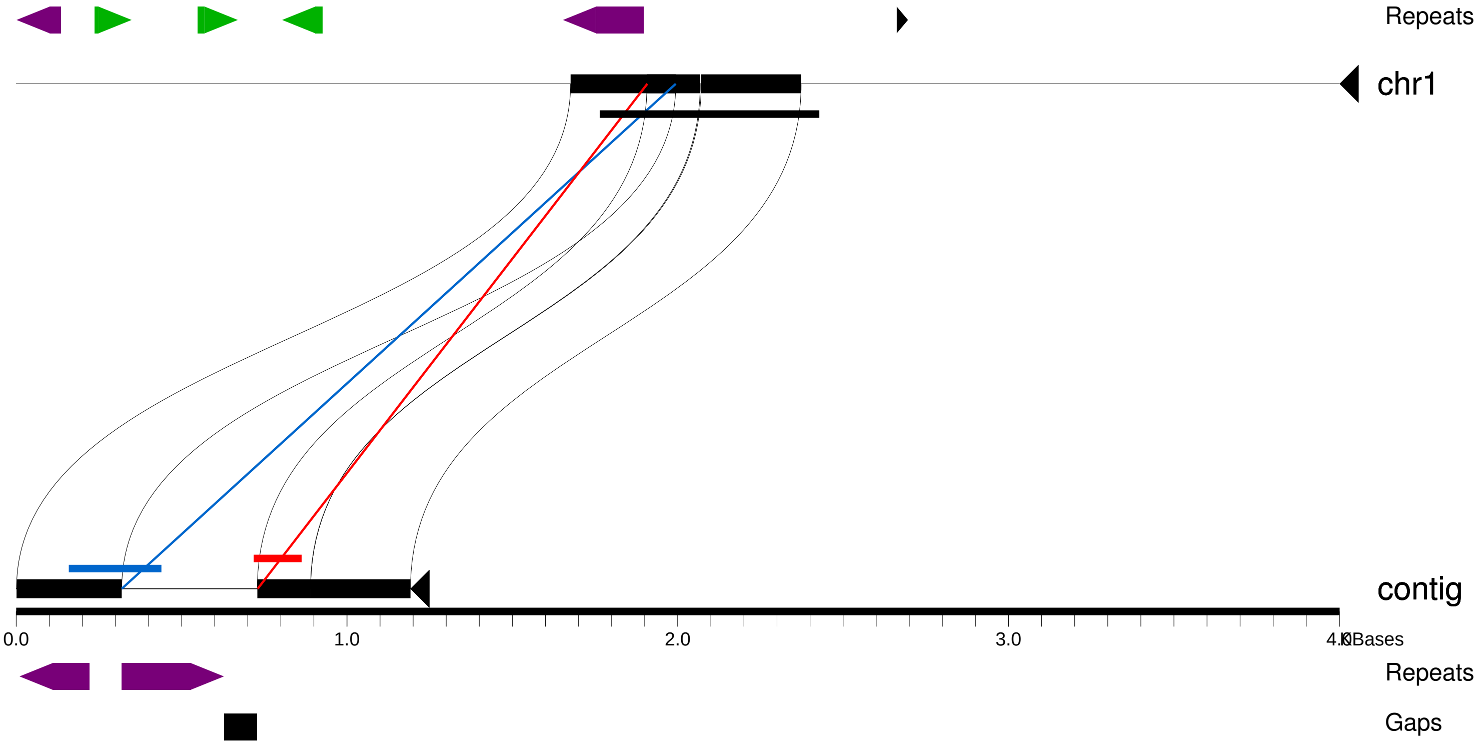

siteID: chr1_145211445 Scafftig2 + Breakpoint: chr1:145211317
chr1:145211157-145211483
Overlap Length: 7 INS size: 374 Genotyped: No

```
left   T------------------------------------TGGTAAATAACTTAATCTTCA-TGATATGTAGT
chrom  TGAGGGTAATAATCCTTGGTCTATCATTAACTTGTTTTGGTAAATAACTTAATCTTCA-TGATATGTAGT
right  GGC------TAAGGCGGAGAATGGCGTGAACCCGGGAGG--------CGGAGCTTGCAGTGAGCCGAGAT
       12N      222NN2NNN2NN2NN2N2N222NN2NNN1*11111111*11*111*1**1***111*111*

left   CTCTTCACCTATGAAGCAGGACTAAAGGAATAACCTGCTTTAGAGAATTGTAGGGAATAATAAATATGAT
chrom  CTCCTCACCTATGAAGCAGGACTAAAGGAATAACCTGCTTTAGAGAATTGTAGGGAATAATAAATATGAT
right  CCCGCCACT---------GCACTCCAG------CCTGGGCGACAGA-------GCGAGACTCCGTCTCAA
       *1*N1***1111111111*1***11**111111****1111*1***1111111*11*1*1*111*1*1*1

left   TGTAAAGTGATTTGAAAAATTAAGTATT------------------------------------------
chrom  TGTAAAGTGATTTGAAAAATTAAGTATTAAATAATATGTCAGAAATGGGATTACTCTGACACATAGGGCT
right  AAAAAAAA------AAAAAAAAAGTATTAAATAATATGTCAGAAATGGGATTACTCTGACACATAGGGCT
       111***11111111*****11******222222222222222222222222222222222222222222

left   ----------------------------------------------------------------------
chrom  TCTTTTATTATAGGATCTCAGTGCGTAAAACTATGCCCTCCACCTCTGCTCTGCTGAGGTGCTGCGATTA
right  TCTTTTATTATAGGATCTCAGTGCATAAAACTATGCCCTCCACCTCTGCTCTGCTGAGGTGCTNNNNNNN
       222222222222222222222222N222222222222222222222222222222222222222NNNNNNN

left   ----------------------------------GGCNNNNNNNNNNN
chrom  TTAGCTAGCTTTTAGGTAGTCAAGTTGAGTGTTAGTCAAGTTGACTCA
right  NNNNNNNNNNNNNNNNNNNNNNNNNNNNNNNNNNNNNNNNNNNNN-----
       NNNNNNNNNNNNNNNNNNNNNNNNNNNNNNNNNNN1N1NNNNNNNNNNN
```

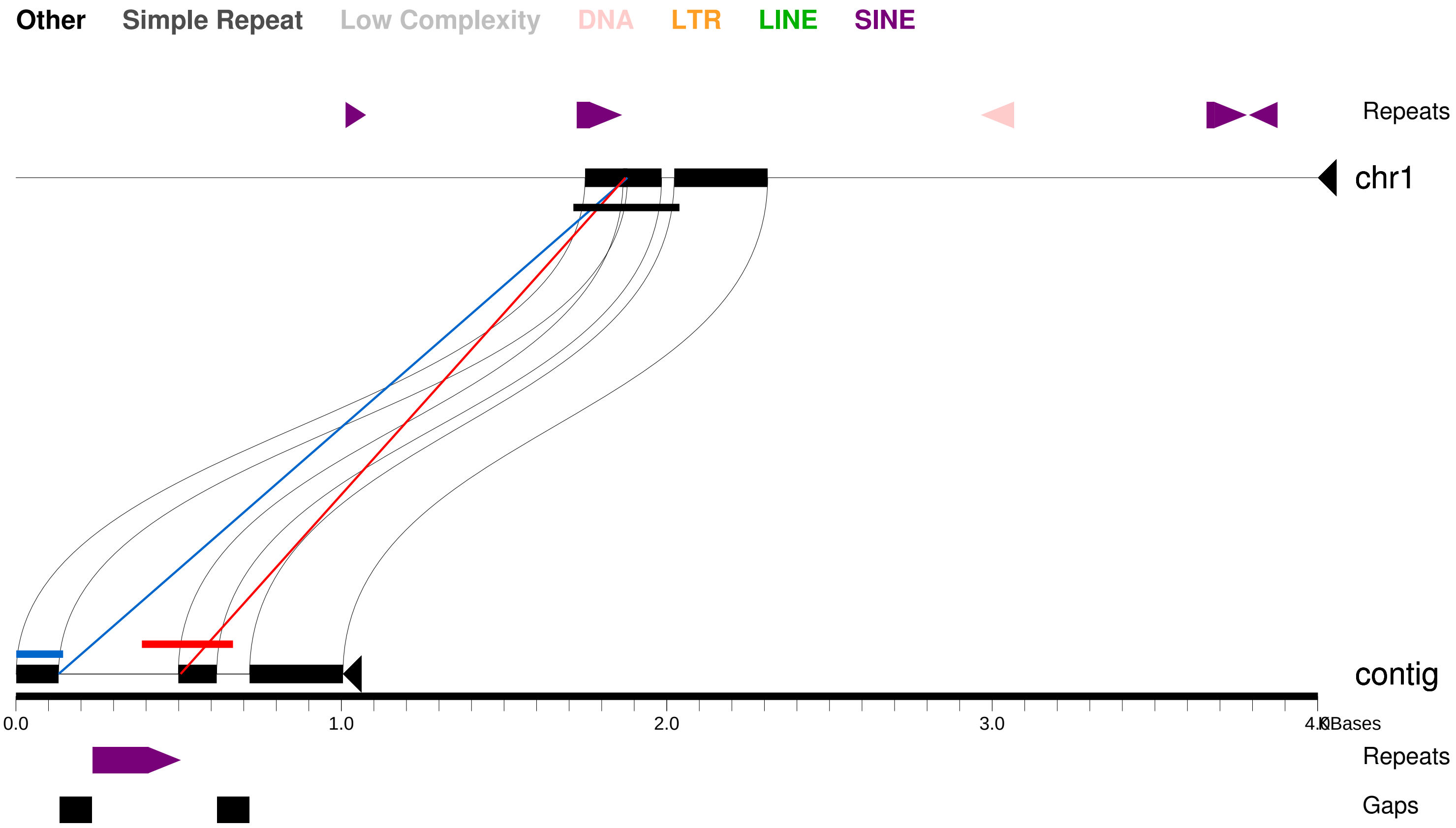

siteID: chr1_146658201 Scafftig2 + Breakpoint: chr1:146658206
chr1:146658160-146658265
Overlap Length: 14 INS size: 303 Genotyped: Yes

```
left     ACAA-CAAACATTTATTAAGCACCT--ACCACATGTC----------AAGAACTCTGCTAAAGACATACA
chrom    ACAA-CAAACATTTATTAAGCACCT--ACCACATGTC----------AAGAACTCTGCTAAAGACATACA
right    GCACTCCAGCCTGGGCGACAGAGCGAGACTCCGTCTCCAAAAAAAAAAAAAAAAAAAAAAAAAAACATACA
         1**11*1*1*1*11111*111*1*111**11*1*1**1111111111**1**1111111***1*******

left     AAGGGCCGGGCGCGGTGGCTCACGCCTGTAATCCCAGCACTTTGGGAGGCCGAGGCGGGCGG
chrom    AAGAT---GACTAAAAGAGTA---CCTGAGCTCCCAGTC--TAGGGATTACCACAAG-----
right    AAGAT---GACTAAAAGAGTA---CCTGAGCTCCCAGTC--TAGGGATTACCACAAG-----
         ***22222*2*22222*22*2222****222******2222*2****222*2*222*22222
```

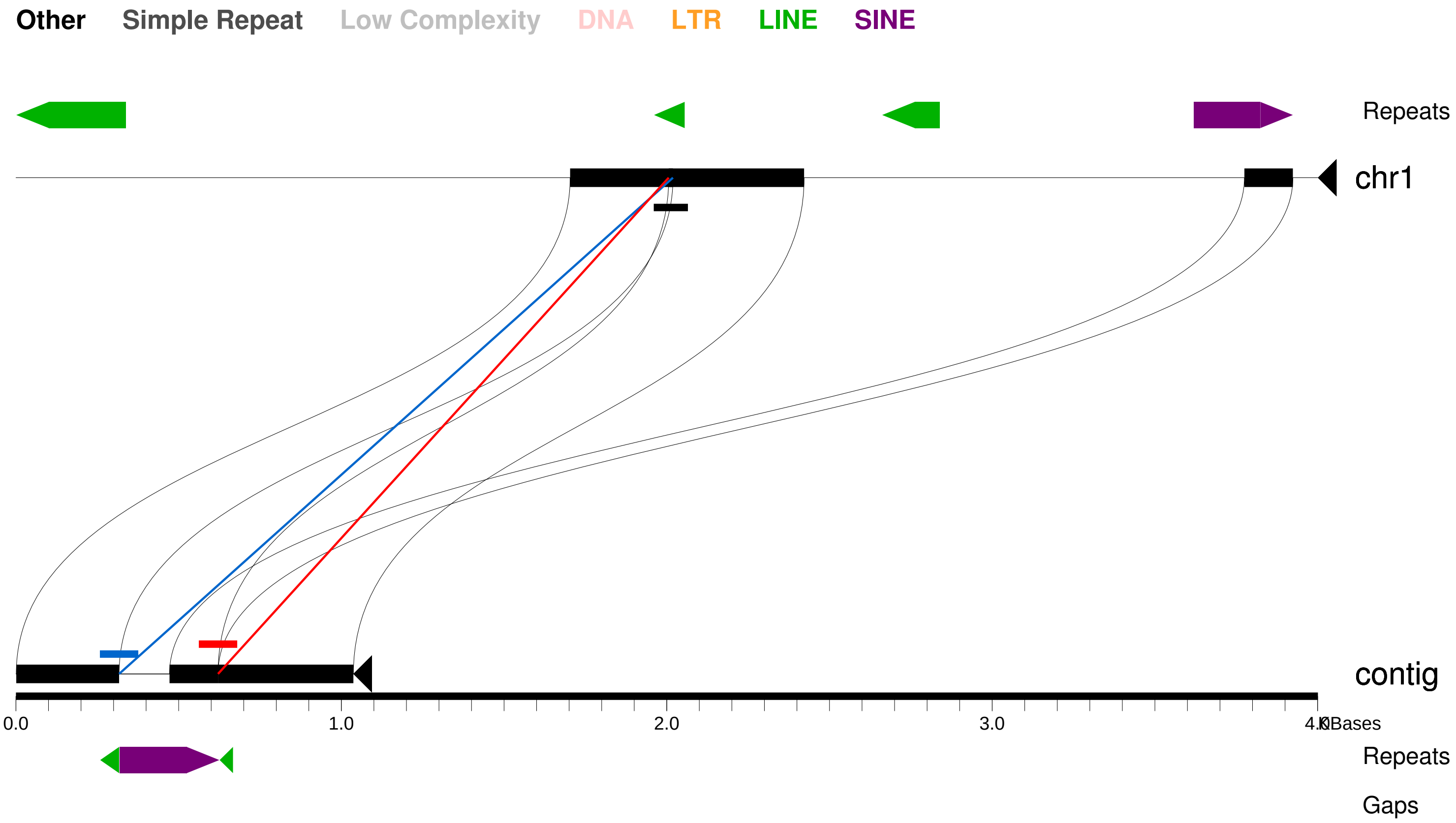

siteID: chr1_152748471 Scafftig1 + Breakpoint: chr1:152748416
chr1:152748256-152748588
Overlap Length: 13 INS size: 336 Genotyped: No

```
left   -------------------------------------GAAGTTACCTCTTTTGGCTTCAGTTTCTTTAT-
chrom  ACAA-ATGTGACTGTTAATGAATAACTTTGGGCCAGTGAAGTTACCTCTTTTGGCTTCAGTTTCTTTATT
right  ACTGCAAGCTCCGCTTCCCGGGT----TCACGCCATT--------CTCCT---GCCTCAGCCTCCC----
       22NN12N2NNN2NN22NNN2NN2    2NNN2222N211111111***1*111**1****11**11111

left   ---ATATTGAGTTGGTTGGAACAAATTGACTCCAACATCCTTTGCGGTTATAGTTTTCTCTATTTCTGCA
chrom  TATATATTGAGTTGGTTGGAACAAATTGACTCCAACATCCTTTGCGGTTATAGTTTTCTCTATTTCTGCA
right  --------GAGTAGCTGGGACTACAGGCGC-CCGCCACCGCGCCCGGCTAA--TTTTTTGTATTTTT---
          11111****1*1*1***11*1*1111*1**11**1*11111***1**111****1*1*****1*111

left   AACTGAAATATATCAGTTCTTCCAATAGAAATTTTNNNNNNNNNNNN-----------------------
chrom  AACTGAAATATATCAGTTCTTCCAATAGAAATTTATTTTGATTTTTCCTTAAAGAGATTTTCATCTCTAA
right  ------AGTAGAGACGGGGT-CCAATAGAAATTTATTTTGATTTTTCCTTAAAGAGATTTTCATCTCTAA
       111111*1**1*111*111*1*************2*2222222222222222222222222222222222

left   ----------------------------------------------------------------------
chrom  ACTGGTCTCAACATTGAACATTTTCAGAATTCTTGGTGATGTGATAGAATGAATGCTGGCCCGGAGCCAA
right  ACTGGTCTCAACATTGAACATTTTCAGAATTCTTGGTGATGTGATAGAATGAATGCTGGCCCGGAGCCAA
       2222222222222222222222222222222222222222222222222222222222222222222222

left   ------------------------------------------------------
chrom  TTTATTTCCTGGACTTCTCTCCCACACTTAGGAGCTTTTAATGAGCAGAAACTT
right  TTTATTTCCTGGACTTCTCTCCCACACTTAGGAGCTTTTAA-------------
       22222222222222222222222222222222222222222
```

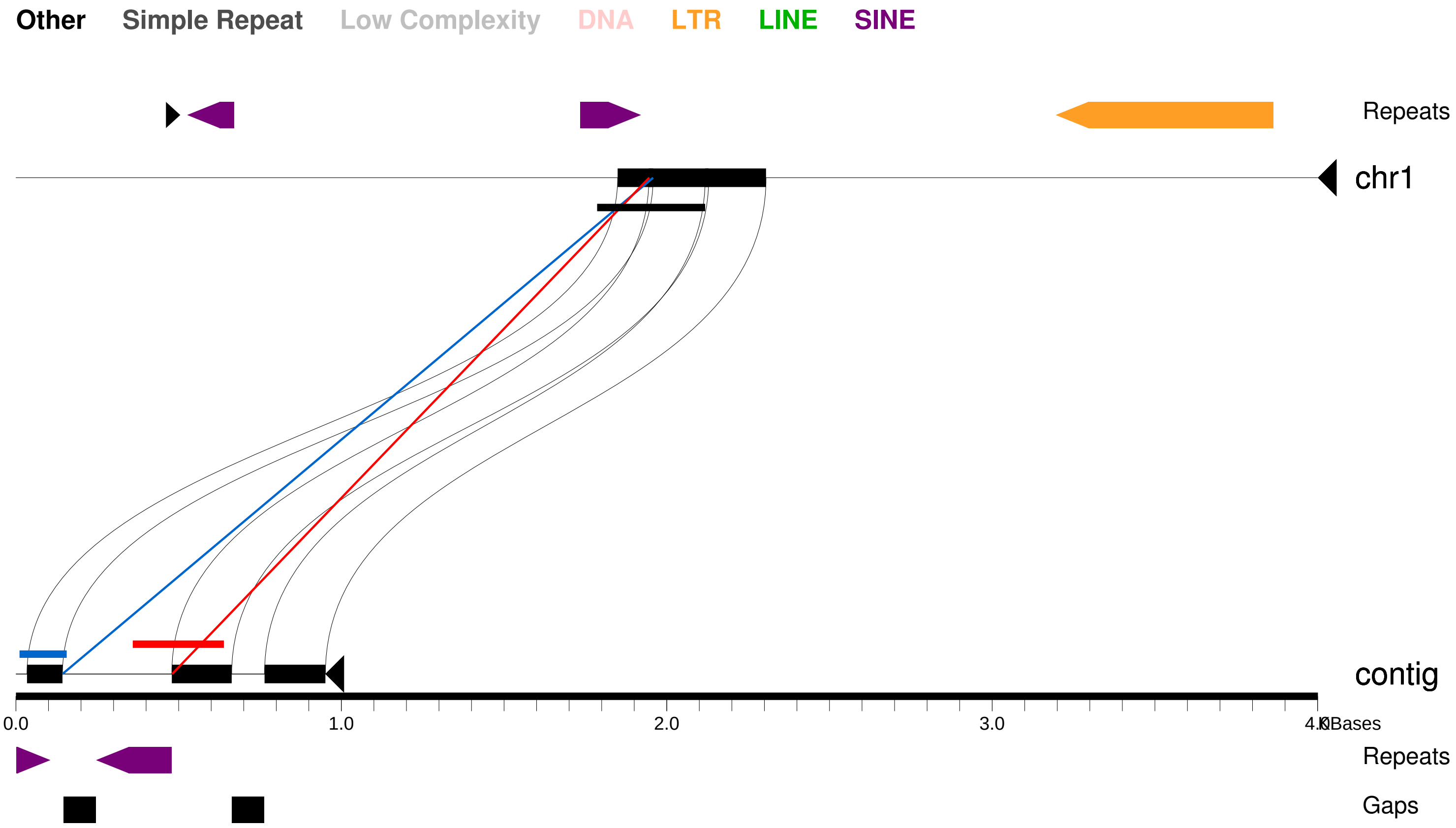

siteID: chr1_157235765 Scafftig3 + Breakpoint: chr1:157235732
chr1:157235683-157235791
Overlap Length: 11 INS size: 126 Genotyped: Yes

```
left   AACTTGTACATTTTTTAATAATAGCACTTTGTGGTT------TTTATTTCTTTTT-----GGGACTGTTT
chrom  AACTTGTACATTTTTTAATAATAGCACTTTGTGGTT------TTTATTTCTTTTT-----GGGACTGTTT
right  AG-TGGCGCAATCTCGGCTCACTGCAAGCTCCGCTTCCCGGGTTCACGCCATTCTCCTGCGGGACTGTTT
       *11*1*11**1*1*1111*1*11***111*11*1**111111**1*111*1**1*11111**********

left   TTTTTTTTTTTTTTTTTTTTTTTTTGAGACGGAGTCTCGCTCTGTCGCCCAGGCCGGACTG
chrom  TAATAGGTTATACTTTTC----TAGAAAATGATCCACTAAA-GTCTTCAAATA-----TA
right  TAATAGGTTATACTTTTC----TAGAAAATGATCCACTAAA-GTCTTCAAATA-----TA
       *22*222**2*22****22222*2**2*22**22*2*22222***22*2*22222222*2
```

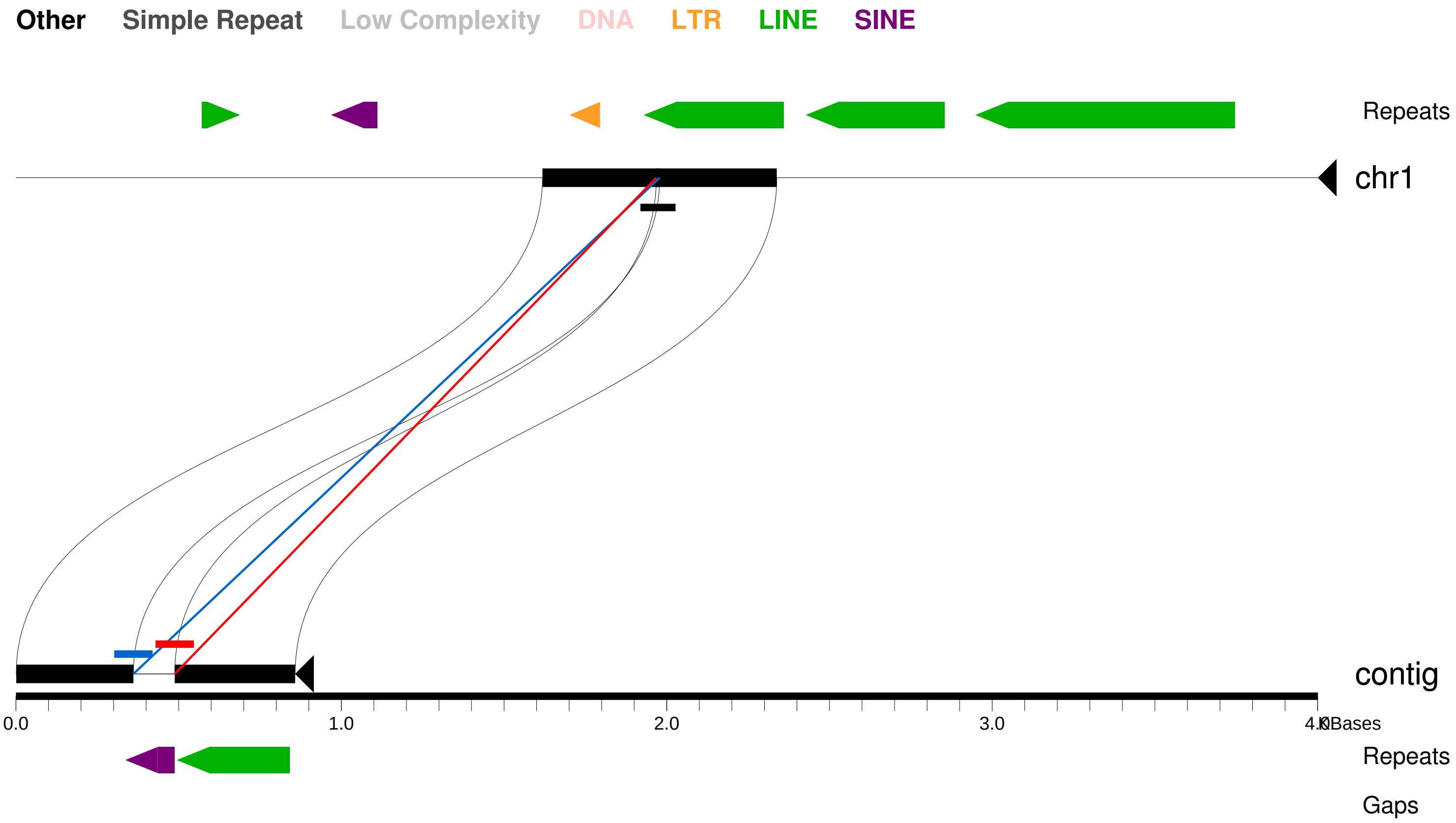

siteID: chr1_157363510 Scafftig7 + Breakpoint: chr1:157363488
chr1:157363443-157363547
Overlap Length: 15 INS size: 308 Genotyped: Yes

```
left    -----CAATTACAGCATTATAATATTCTGTGTTTTTCTAG---GAACTTACTA-------CCATGCATGA
chrom   -----CAATTACAGCATTATAATATTCTGTGTTTTTCTAG---GAACTTACTA-------CCATGCATGA
right   TCCACCCGCCTCGGCCTCCCAAAGTGCTGGGATTA-CAGGCGTGAGCCACCGCGCCCGGCCCATGCATGA
        11111*11111*1**1*111**11*1***1*1**11*11*111**1*111*111111111**********

left    GTTTTTTTTTTTTTTTTTTTTTTTTTGAGACGGAGTCTCGCTC-TGTCGCCCAGGCTGGACTGCG
chrom   GTTTTGTACCTGTAGATGATTTCTCATTG-------CTCACTAATGTC-------CTTTTCTTT-
right   GTTTTGTACCTGTAGATGATTTCTCATTG-------CTCACTAATGTC-------CTTTTCTTT-
        *****2*222*2*222*22***2*2222*2222222***2**22****2222222**222**222
```

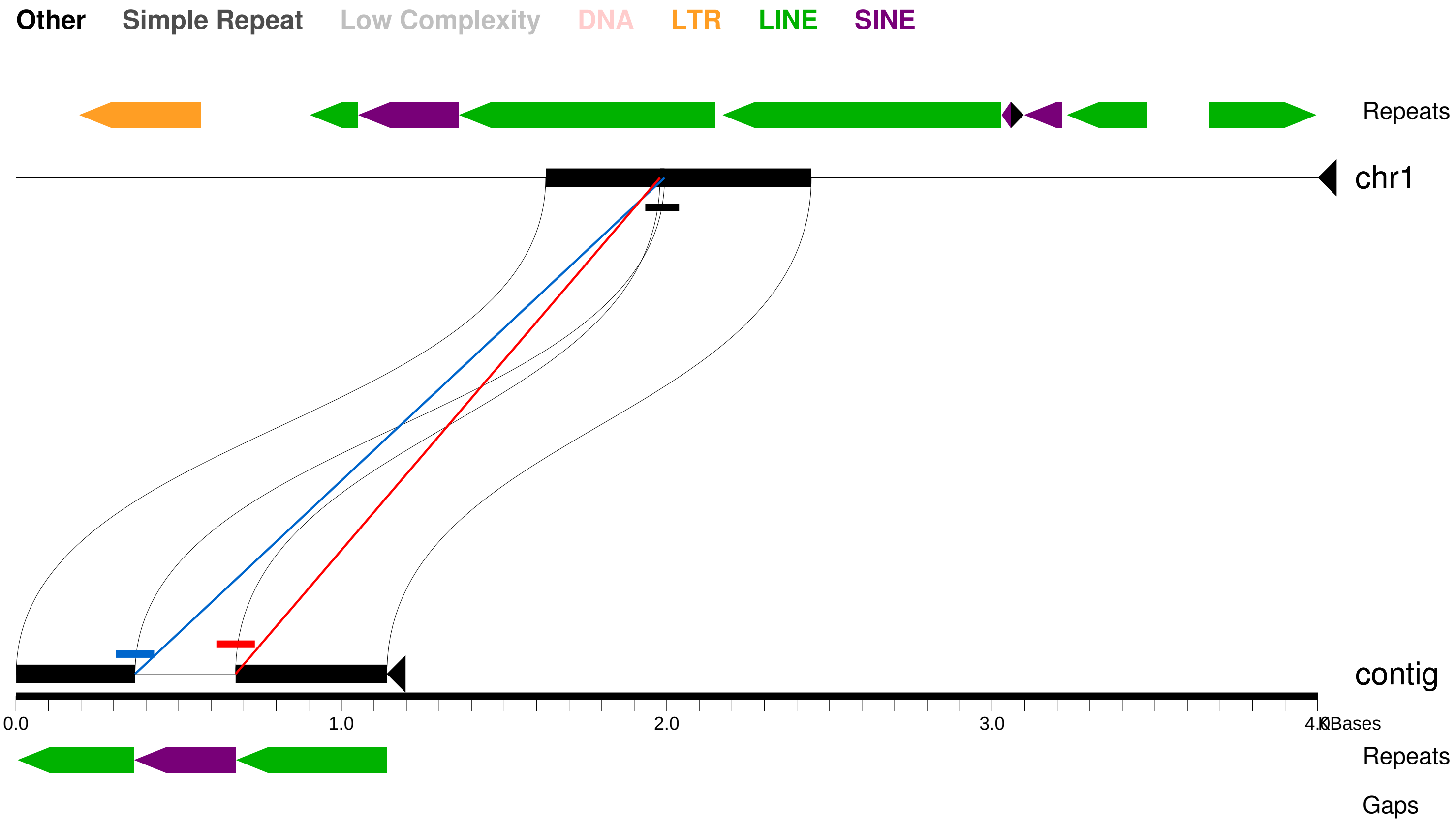

siteID: chr1_162080076 Scafftig2 + Breakpoint: chr1:162080083
chr1:162080040-162080142
Overlap Length: 17 INS size: 297 Genotyped: Yes

```
left    -TAAGTT---ATATATAGACCACTGTGC--------CTGTC-TGGGCACATGTTCA---CCAGCCAAATA
chrom   -TAAGTT---ATATGTAGACCACTGTGC--------CTGTC-TGGGCACATGTTCA---CCAGCCAAATA
right   ATCCGCCCGCCTCGGCCTCCCAAAGTGCTGGGATTACAGGCGTGAGCCACCGCGCCCGGCCAGCCAAATA
        1*11*111111*1121111***11****11111111*1*1*1**1**1111*11*1111***********

left    ACTTTTTTTTTTTTTTTTTTTTTTTGAGACGGAGTCTCGCTCTGTCGCCCAGGCTGGAGTGCAGTGG
chrom   ACTTTTAACATGCCCAAGGAAATGA-ACACAGGCTC-----------CAGGGTC---TG-AGGAA
right   ACTTTTAACATGCCCAAGGAAATGA-ACACAGGCTC-----------CAGGGTC---TG-AGGAA
        ******2222*22222222222***2**22**2***22222222222****2*2222**2**222
```

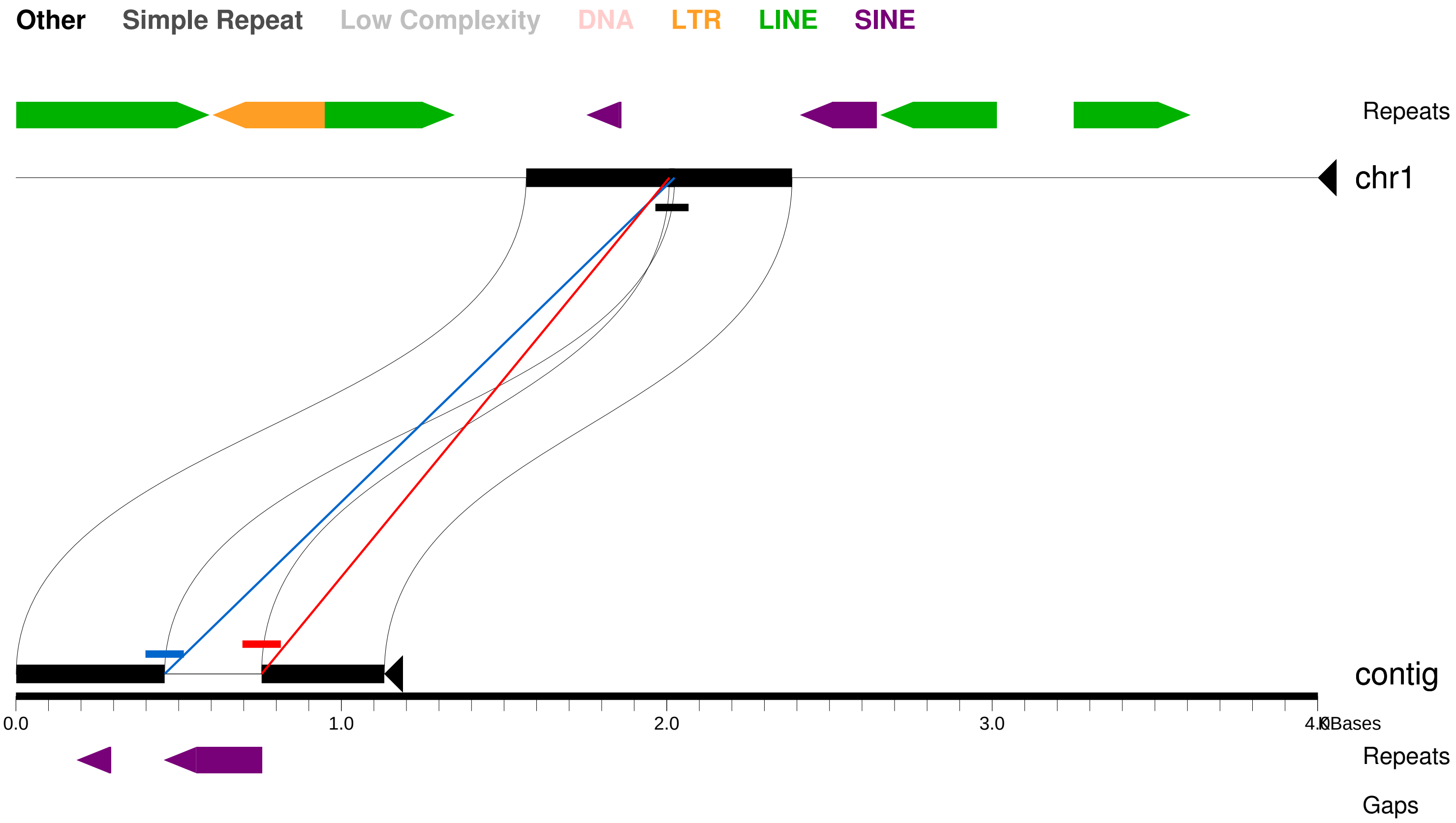

siteID: chr1_162761306 Scafftig3 + Breakpoint: chr1:162761309
chr1:162761079-162761483
Overlap Length: 14 INS size: 296 Genotyped: Yes

```
left    ----------------------------------------------------------------------
chrom   CGTTTCATTTAGCTCAGTGCTAGAGAGCTAACATACTCTTCAGAAACCCAGAGAAAATTAATCTTTTCCT
right   G------------------------------------CCTCGGCCTCCCAAAG-----------------
        N                                    2N22N2NNN2222N22

left    ----------------------------------------------------------------------
chrom   ATGAAGGCATGGTTGGGGTGCAGAATAACCAGCTCATAATAATTCCAGGGTTGCTCGTTTAGTCTCTCAG
right   -------------TGCGGGG---------------------ATTACAGGCGTG---------------AG
                     22N22N2                     222N2222NN22               22

left    ----------------TTTTACCTGTCATTTGCACTTCTGTGAAAAATGTGTGGAGGATGAAGCCAATCA
chrom   CCTCTTCTGTTGCTTGTTTTACCTGTCATTTGCACTTCTGTGAAAAATGTGTGGAGGATGAAGCCAATCA
right   CC------------------ACC--------GCGC--CCG----------------------GCCA----
        22              1111***11111111**1*11*1*1111111111111111111111****1111

left    AACCATTTTTCTACTAGTTGCATTTGGCATTTTTTTTTTTTT---------------TT-----TTGGAG
chrom   AACCATTTTTCTACTAGTTGCATTTGGCATTTTTAAATGATTAAGACAGAGGACATGTTAAACATTTAAG
right   --------------------CATTTGGCATTTTTAAATGATTAAGACAGAGGACATGTTAAACATTTAAG
        11111111111111111111**************222*22**222222222222222**22222**22**

left    ACGGAGTCTCGCTCTGTC------------GC------------CCAGGCTGG-AG-----TGCAGT-GG
chrom   ATTGATCCACTTTCAATTATTTAACCACATGCGTATTAACTAAGCCAGCTTGGTAGGTGTCTGCATTTGG
right   ATTGATCCACTTTCAATTATTTAACCACATGCATATTAACTAAGCTA-----------------------
        *22**22*2*22**22*2222222222222**N22222222222*1*1NN111 11     1111N1 11

left    C-----------------------GGGA------------------------TCT
chrom   CAACTTTTTAGCATACCCTCTCAAGTGACTTTAACCGTTGATTGGTGCTTCTTAT
right   -------------------------------------------------------
        1                       1N11                        1N1
```

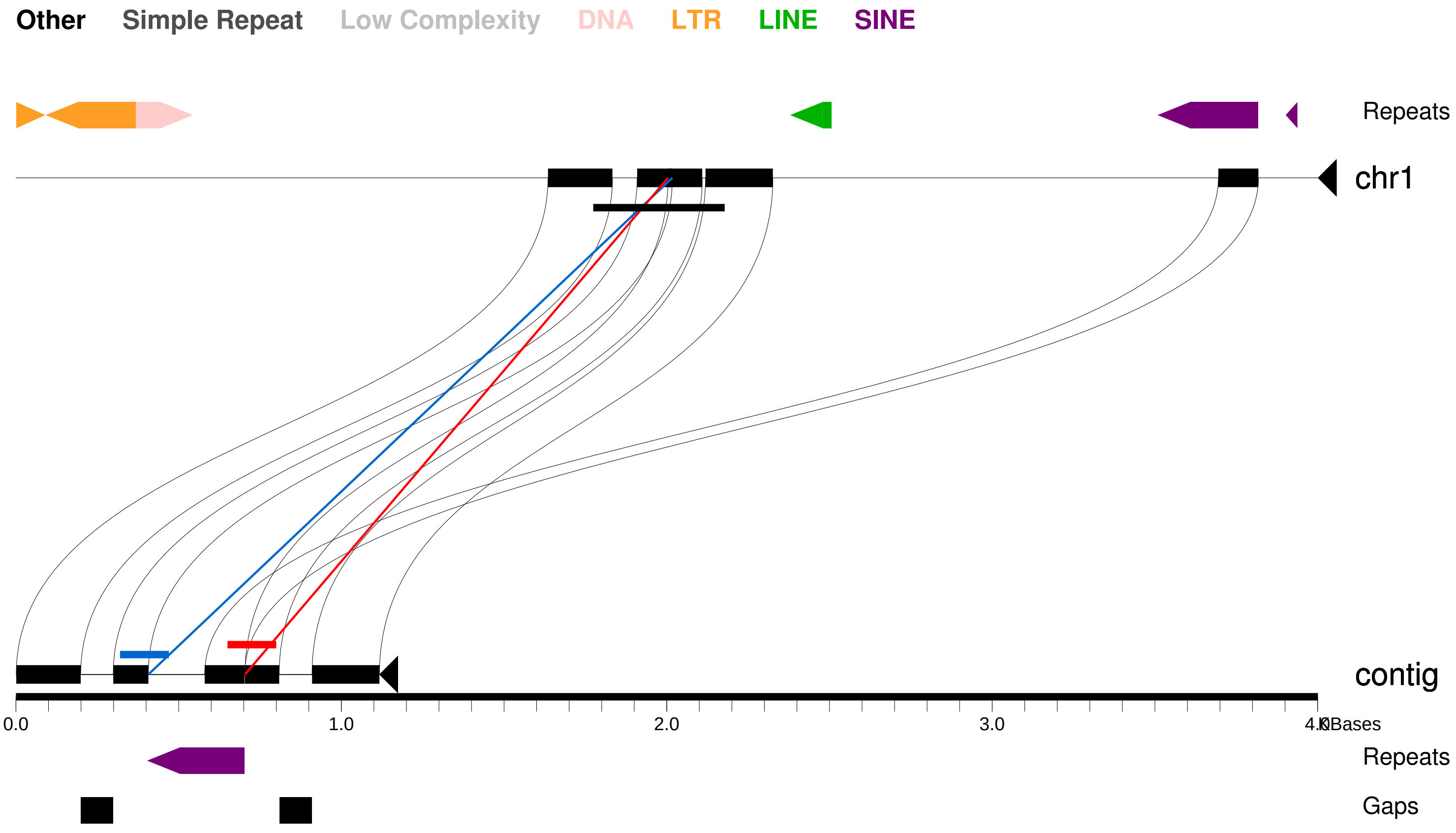

siteID: chr1_163314427 Scafftig5 - Breakpoint: chr1:163314433
chr1:163314384-163314480
Overlap Length: 11 INS size: 322 Genotyped: Yes

```
left   ATTATGGGTTACTGTGGGAGATGGATTG-----------ACGTGGTGCAATACAAACTTA----------
chrom  ATTATGGGTTACTGTGGGAGATGGATTG-----------ACGTGGTGCAATACAAACTTA----------
right  AGCCTGGGCGACAGAGCGAGACTCCGTCTCAAAAAAAAACAAAATAAAAAAAAAAAAAAAAAAAAAAAAA
       *111****11**1*1*1****11111*111111111111**1111*11**1*1***111*1111111111

left   -GAAGTGGGTAGGGCCGGGCGCGGTGGCTCACGCCTGTAATCCCAGCACTTTGGGAGGCCGAGGCGGGCG
chrom  -GAAGTGGGTAG------------------ACAATT----TACCATCT-TTTGTGAAGCAATGTTTTTAA
right  AGAAGTGGGTAG------------------ACAATT----TACCATCT-TTTGTGAAGCAATGTTTTTAA
       1***********222222222222222222**222*2222*2***2*22****2**2**222*2222222

left   G-
chrom  AC
right  AC
       22
```

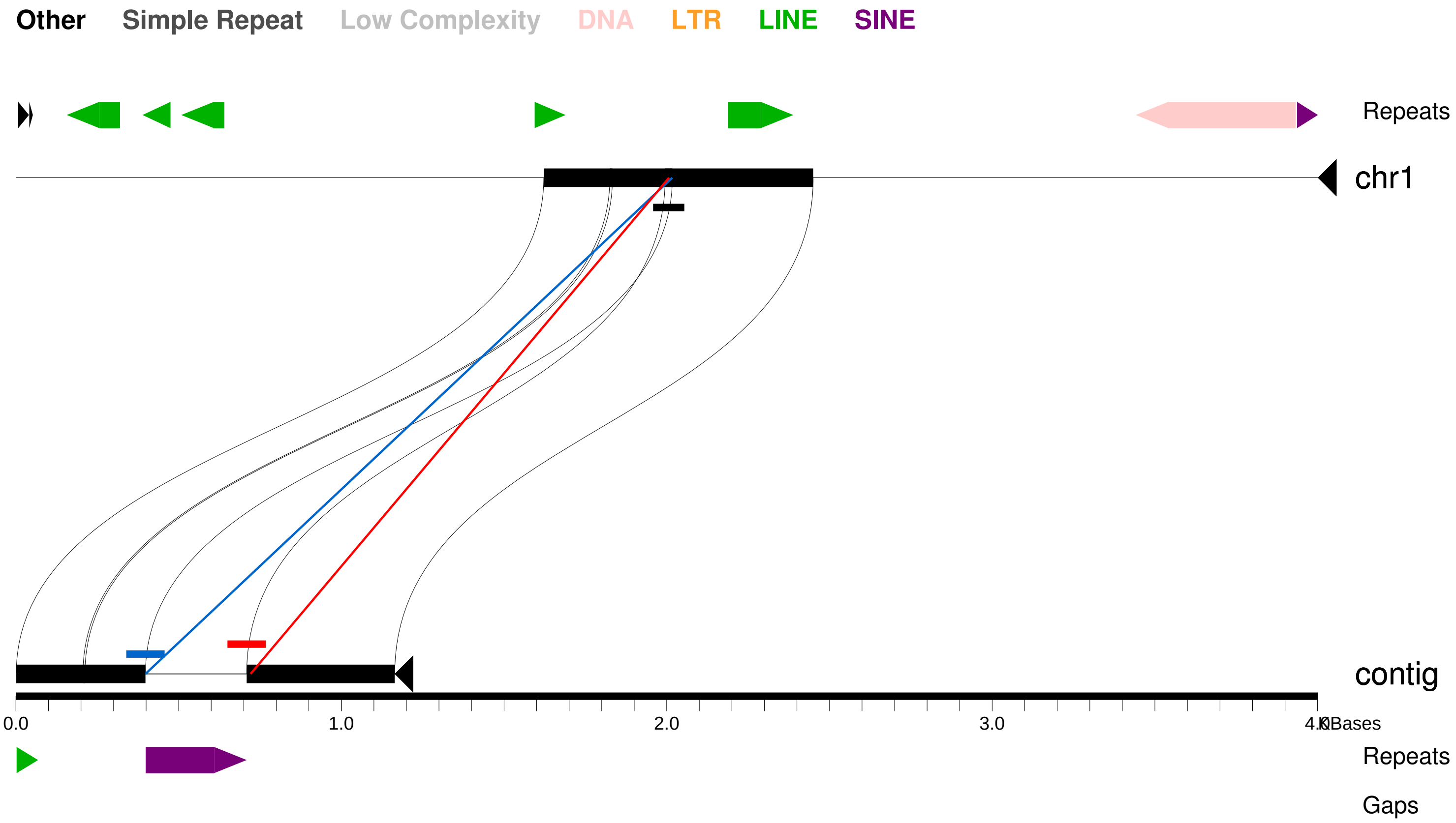

siteID: chr1_166189399 Scafftig2 + Breakpoint: chr1:166189433
chr1:166189386-166189492
Overlap Length: 13 INS size: 305 Genotyped: Yes

```
left     TAAAATTCTTTCTTGACTTTAGACATTCTGAATATAATATGCTGTGA----------------TGAAGTC
chrom    TAAAATTCTTTCTTGACTTTAGACATTCTGAATATAATATGCTGTGA----------------TGAAGTC
right    CCACCCGC---CTCGGCCTCCCAAAGTGCTGGGATTACAGGC-GTGAGCCACCGCGCCCGGCCTGAAGTC
         11*1111*111**1*1*1*111*1*1*111111**1*1*1**1****1111111111111111*******

left     CTTTTTTTTTTTTTTTTTTTTGAGACGGAGTCTCGCTCTGTCGCCCAGCAGGCCGGACTGCAGAC
chrom    CTTTTTACAATGTATTTTCCTG----GGGGTC---ATTGGGCCTCCTATAT-CTAGA----TGTT
right    CTTTTTACAATGTATTTTCCTG----GGGGTC---ATTGGGCCTCCTATAT-CTAGA----TGTT
         ******2222*2*2****22**2222**2***2222*22*2*22**222*22*22**22222*22
```

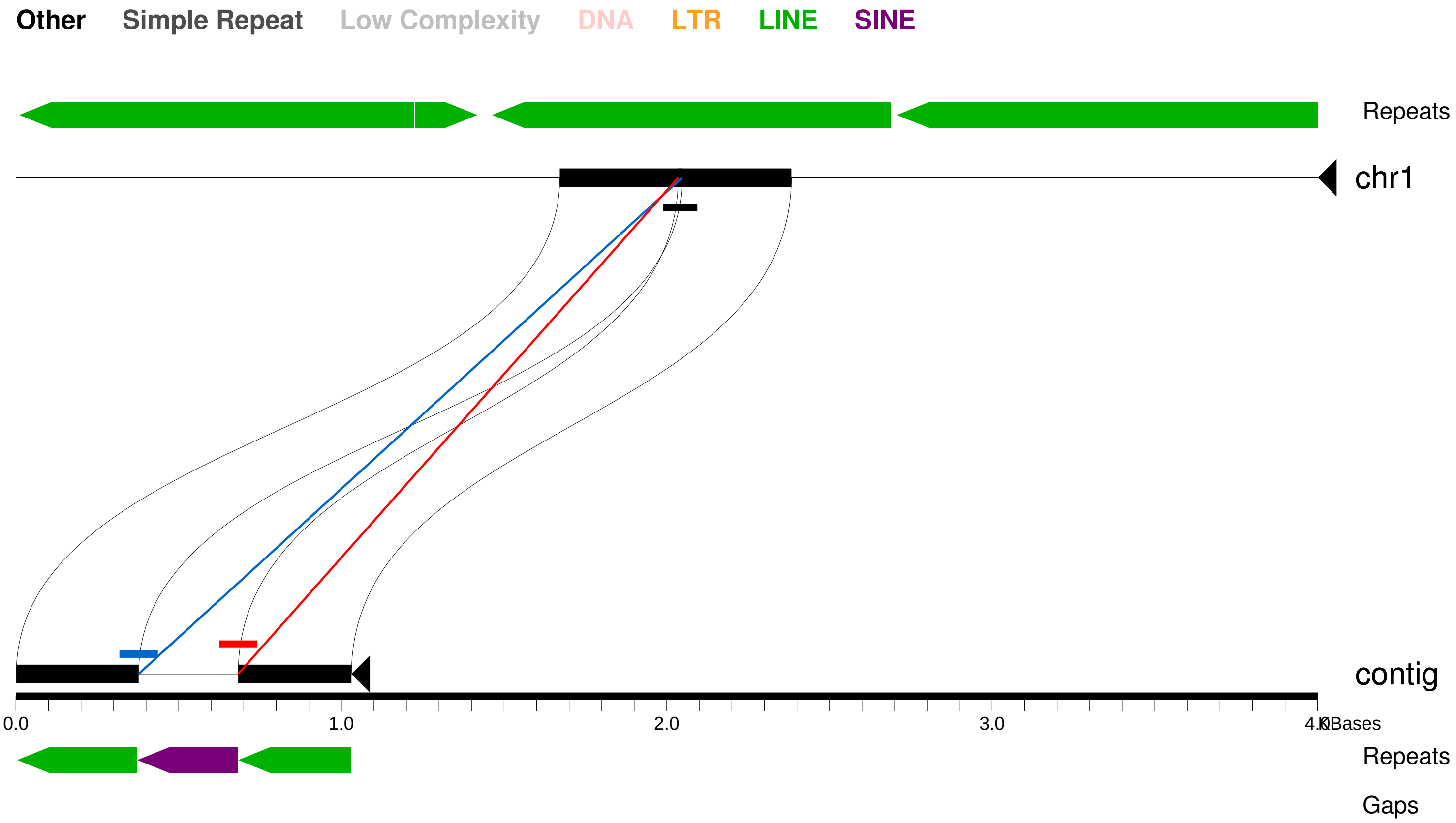

siteID: chr1_167506112 Scafftig1 - Breakpoint: chr1:167506095
chr1:167505962-167506154
Overlap Length: 16 INS size: 365 Genotyped: No

```
left    ----------------------------------------------------GTCACACAAGCAAGTTGG
chrom   CAAGTGCTTGGGATTAAATGTGCCTTCACTGAAACAAATCACTTCATTCTAAGTCACACA-GCAAGTTGG
right   NNNNNNNNNNNNNNNNNNNNNNNNNNNNNNNNNNNNNNNNNNNNNNNNNNNNNNNNNNAA----------
        NNNNNNNNNNNNNNNNNNNNNNNNNNNNNNNNNNNNNNNNNNNNNNNNNNNN1111111*2111111111

left    AGGTCCAACCTCCATCTTCCACTATATTCACTAAAGTACAAAGTCTGTAAGCAAAACGTTTGTTAAAATT
chrom   AGGTCCAACCTCCATCTTCCACTATATTCACTAAAGTACAAAGTCTGTAAGCAAAACGTTTGTTAAAATT
right   --------------------------------AAA-----------------------------AAAATT
        11111111111111111111111111111111***11111111111111111111111111111******

left    TCATTGAGGGTCCTGGCTAACACGGTGAAACCCCGTC-TCTACTAAAAATACAAAAAATTA----GCC--
chrom   TCATTGAGGG--------------GTTACTCCACGTAATGGAATATTGACACTTGGTGTTTTGGGGCT--
right   TCATTGAGGG--------------GTTACTCCACGTAATGGAATATTGACACTTGGTGTTTTGGGGCTAC
        **********22222222222222**2*22**2***22*22*2**222*2**222222**22222**211

left    --------------------------
chrom   --------------------------
right   AGTGATAAATATATCAGATGCAGACT
        11111111111111111111111111
```

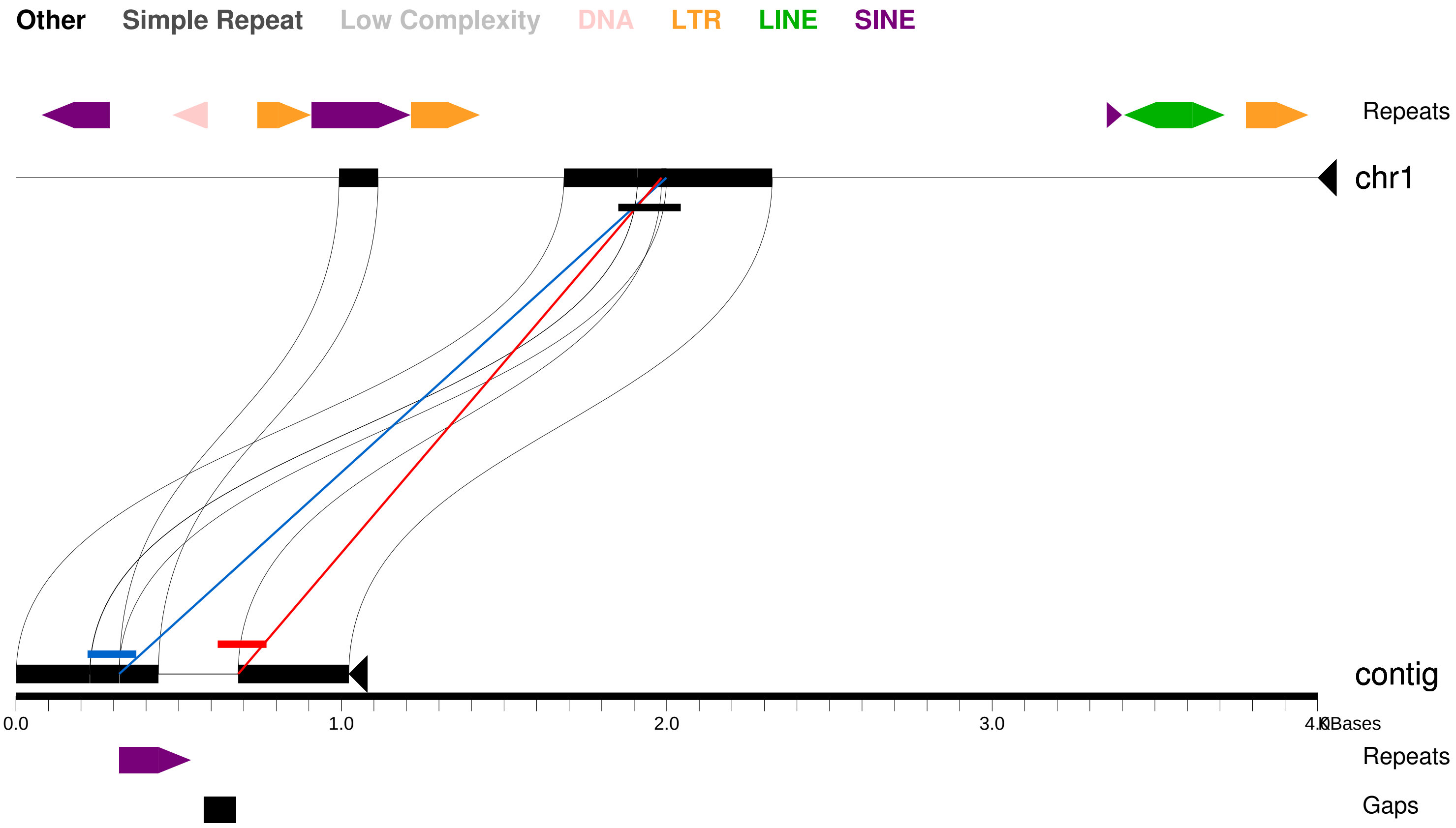

siteID: chr1_170651532 Scafftig1 - Breakpoint: chr1:170651485
chr1:170651433-170651544
Overlap Length: 3 INS size: 98 Genotyped: Yes

```
left    GTGCTCC-------CATGTTCTCAGTCACCCTTGATAGTATTATCACACTGCATGGCTTAAGCCTGC-AG
chrom   GTGCTCC-------CATGTTCTCAGTCACCCTTGATAGTATTATCACACTGCATGGCTTAAGAATGCCAG
right   GGGCGACAGCGAGACTCCGTCTCAAAAAAAAAAAAAAAAAAAAAAAAAAAAAAAAGAAAAAAGAATGCCAG
        *1**11*1111111*1111*****111*111111*1*11*11*11*1*1111*1*1111***22***2**

left    TGAGCCGAGATTGCGCCACTGCACTC-CAGCCTGGGCGA-CAGCGAGACTCCGTCTCAA
chrom   TAAG----GTTTTGGTGCCT-CAGTCACATCCTGCTCACTCCTCTAGAGT-----TCCC
right   TAAG----GTTTTGGTGCCT-CAGTCACATCCTGCTCACTCCTCTAGAGT-----TCCC
        *2**2222*2**22*222**2**2**2**2****22*222*22*2***2*22222**22
```

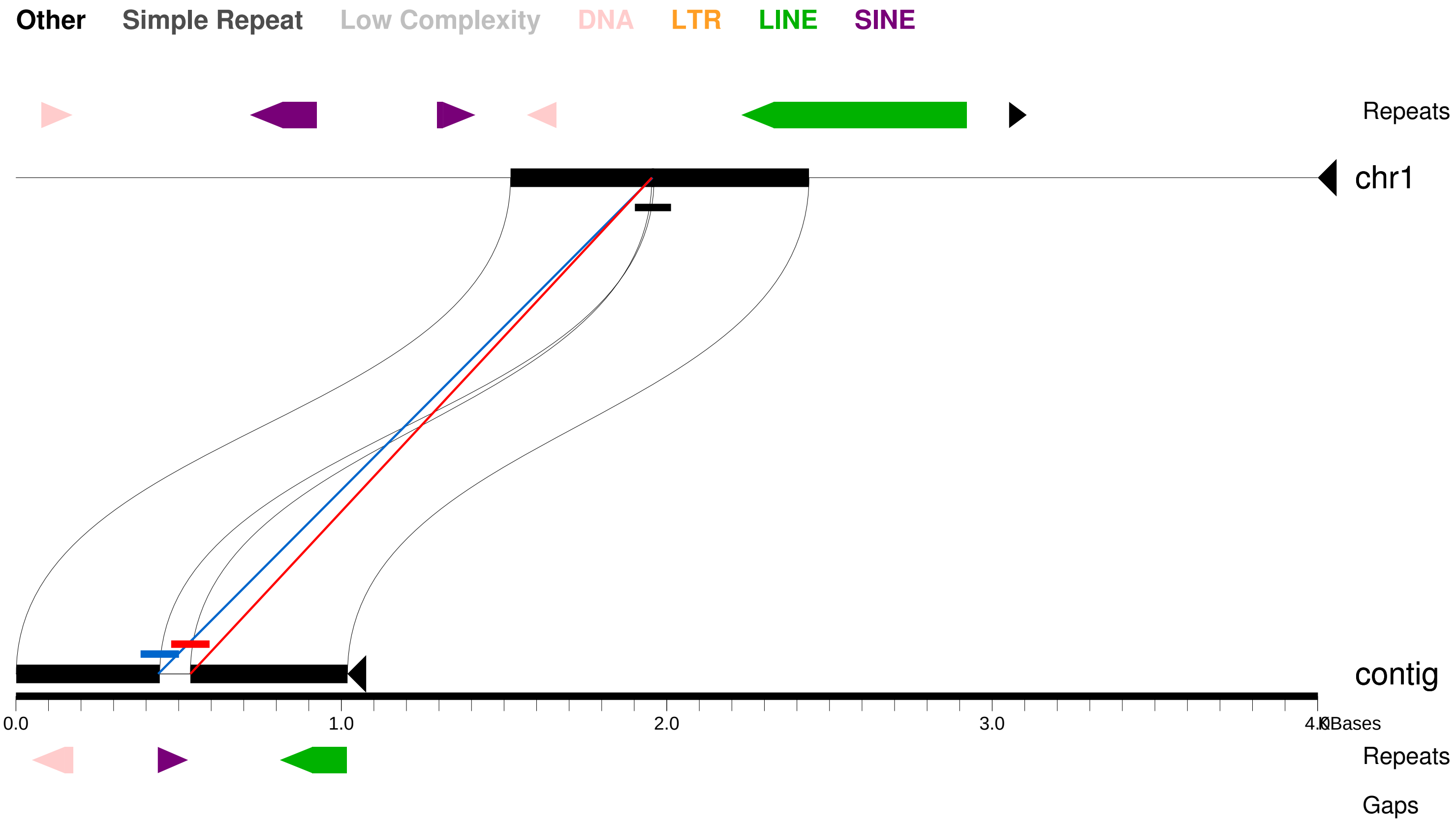

siteID: chr1_17234111 Scafftig9 - Breakpoint: chr1:17234101
chr1:17233941-17234277
Overlap Length: 17 INS size: 308 Genotyped: Yes

```
left   C------------------AGAAAATTATGTTTTTTCTTTGGTTAACTGCAGACTTTGGTCTGCACAATC
chrom  CCATA-CATCCTATTGGTCAGAAAATTATGTTTTTTCTTTGGTTAACTGCAGACTTTGGTCTGCACAGTC
right  GAATGGCATGAACCTGGGAGGCGGAGGTTGCAGTGAGCTGAGATCGCGCCAC-------TCTGCAC----
       1N22N1222NNNNN222NN1*111*111**111*1111*11*1*11*11**11111111*******1N11

left   TTTTTCTTTCTTGGTCTGAAAACCACCCCACAGTCCCATATGTAGCTTTTTTGGATAAACCTAGAAATGG
chrom  TTTTTCTTTCTTGGTCTGAAAACCACCCAGTAGTCCCATATGTAGCTTTTTTGGATAAACCTAGAAATTG
right  ----TCCAGCCTGGGCGACAGAGCA---AG-ACTCCG------------TCTCAAAAAAAAAAAAAAAAA
       1111**111*1***1*111*1*1**11122N*1***1111111111111*1*11*1***111*1***1N1

left   ACCCTTCTGGTCTTACAGCTTGAAACTTAATATTTGTT--GGCCGGGTGCGGT-----GGC--------T
chrom  ACCCTGCTGGTCTTAAAGCTTGAAACTTAATATTTGTTTCGTCTGAGTTCCTTCCTCAGGAAACAACCTT
right  A-------------AAAGAAAGAAACTTAGTATTTGTTTTATCTGAGTTCCTTCCTCAGGAAATGACCTT
       *1111N11111111*2**111********1********2N12*2*2**2*22*22222**222NN2222*

left   CACGCCTGT----AATCCCAGCA-------------CTTTGGGAGGCCGAGGCGGGTGGATCACGAGGTC
chrom  CAGGCCTCTCAAAAAAGCCATCAAAGAACTGAAATTCATCCAGACAATGAGATGCTGGGACCCCTCATTC
right  CAGGCCTGTCAAAAAAGCTATCAAAGTACTGAAATTCATCCAGAAAATGAGATGCTGGGACCCCTCATTC
       **2****N*2222**22*1*2**222N222222222*2*222**N222***22*222***2*2*2222**

left   AGGA-GATCGAGACCAT---CCTGGCTAACACGGTGAAGC---CCCGTCTCTACTAAA
chrom  ATCATGATTGCTTCCTTGCTCCTCCCTAGTTCCTGTTTTCTTACACATTGTTACATTT
right  ATCATGATTGCTTCCTTGCTCCTCCCTAGTTCCTGTTTTCTTAC-------------T
       *22*2***2*222**2*222***22***222*2222222*222*N1N1NNN111NNN2
```

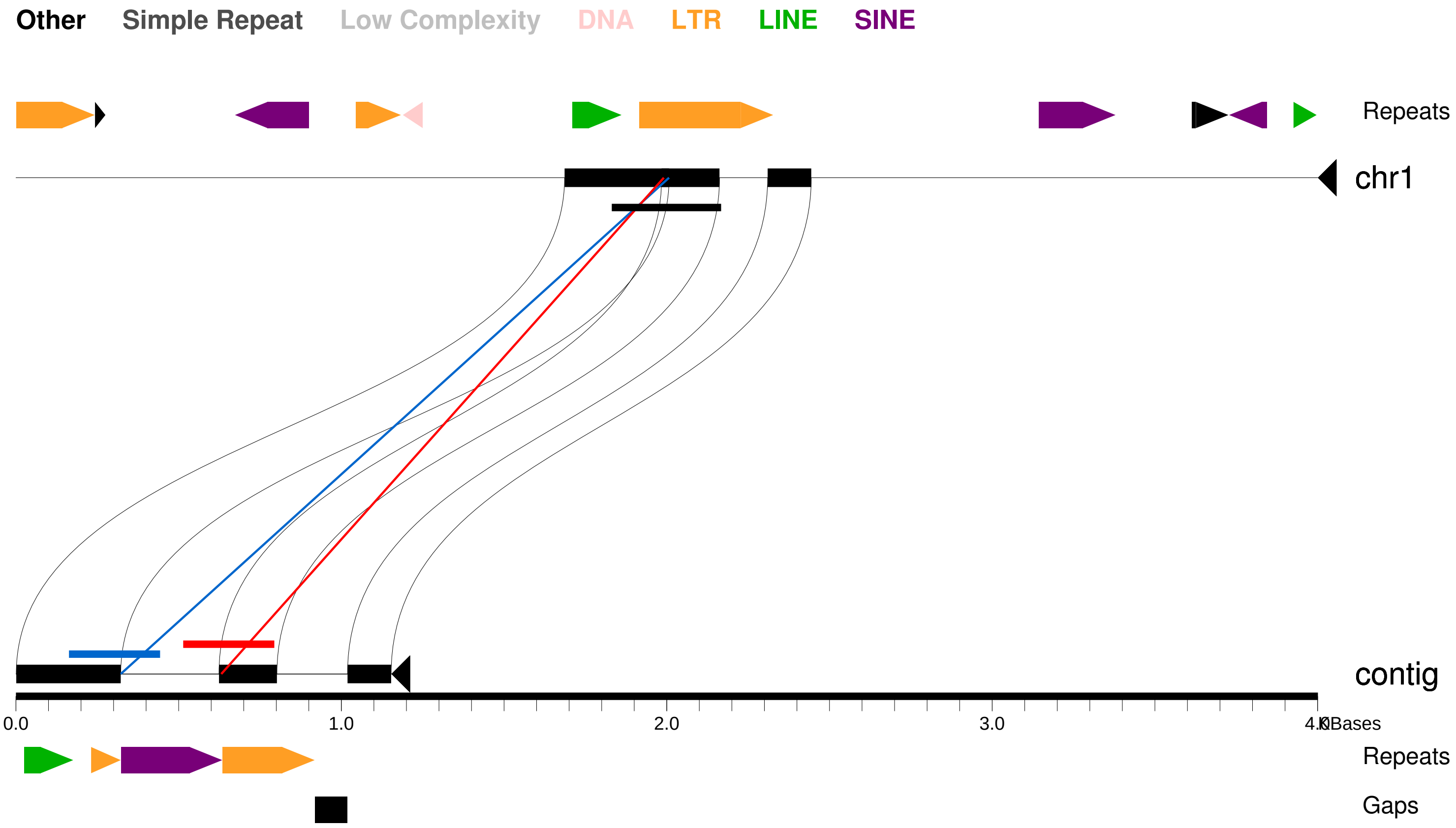

siteID: chr1_17441129 Scafftig1 - Breakpoint: chr1:17441131
chr1:17441075-17441190
Overlap Length: 4 INS size: 403 Genotyped: No

```
left    GAGAAGCCTGCACAAAATAAGTGTTCAATAAATGTCAACTAGTAACATTAAGAAAC---GATGGCTCACG
chrom   GAGAAGCCTGCACAAAATAAGTGTTCAATAAATGTCAACTAGTAACATTAAGAAAC---GATGAGA-AGT
right   NNNNNNNNNNNNNNNNNNNNNNNNNNNNNNNNNNNNNNNNNNNNNNNNNNNNNNNNNNNGATGAGA-AGT
        11111111111111111111111111111111111111111111111111111111111****2222*22

left    CCTGTAATCCCAGCACTTTGG-GAGGCCGAGGCGGGCGGATCACGAGGTCAGG
chrom   TGTGCAAAGAAAAAAAATGTGATGAAACAGAGTTCT-CGGGACTCAGGGT--AG
right   TGTGCAAAGAAAAAAAATGTGATGAAACAGAGTTCT-CGGGACTCAGGGT--AG
        22**2**2222*22*2*2**22**22*2***22222***22*2*22***222*
```

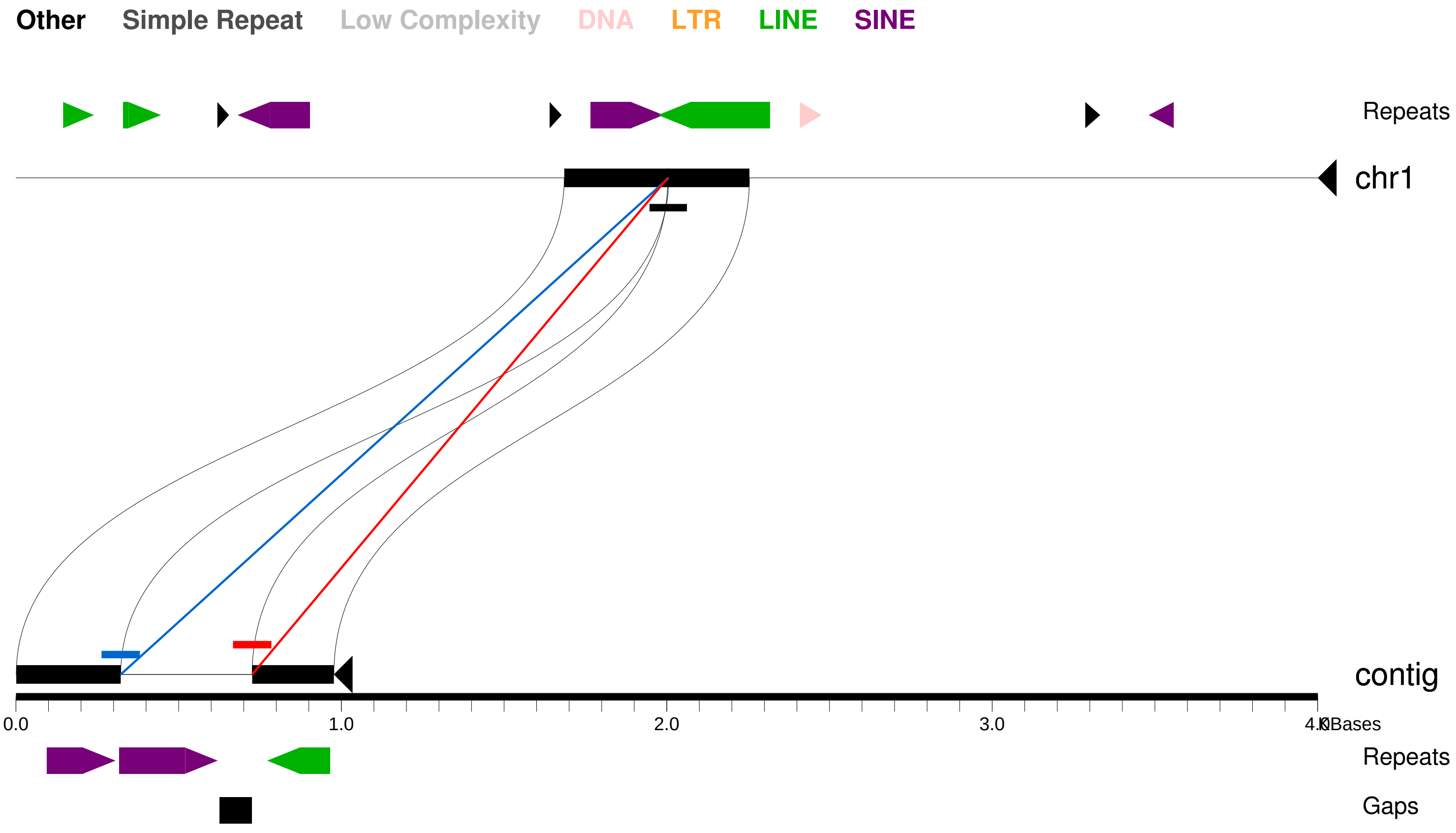

siteID: chr1_176232688 Scafftig1 + Breakpoint: chr1:176232695
chr1:176232636-176232755
Overlap Length: 1 INS size: 370 Genotyped: No

```
left    TCCTTCATTTCCCTCTCAATGCTGCCATCCCCCTGGTTTAGTCTTAAAATAGATAAAGGAGGCCGGGCGC
chrom   TCCTTCATTTCCCTCTCAATGCTGCCATCCCCCTGGTTTAGTCTTAAAATAGATAAAGGAATATCAGTAT
right   NNNNNNNNNNNNNNNNNNNNNNNNNNNNNNNNNNNNNNNNNNNNNNNNNNNNNNNNNNNA-TGTCAGTAT
        11111111111111111111111111111111111111111111111111111111111*N2N222*222

left    GGTGGCTCACGCCTGTAATCCCAGCACTTTGGGAGGCCG---AGGCGGGTGG
chrom   AGAGCCTCT--TCAAGCATCTGCTGACCTTCAAGGGCCATTAATTTAAAATA
right   AGAGCCTCT--TCAAGCATCTGCTGACCTTCAAGGGCCATTAATTTAAAATA
        2*2*2***2222*2222***22222**2**2222****2222*222222222
```

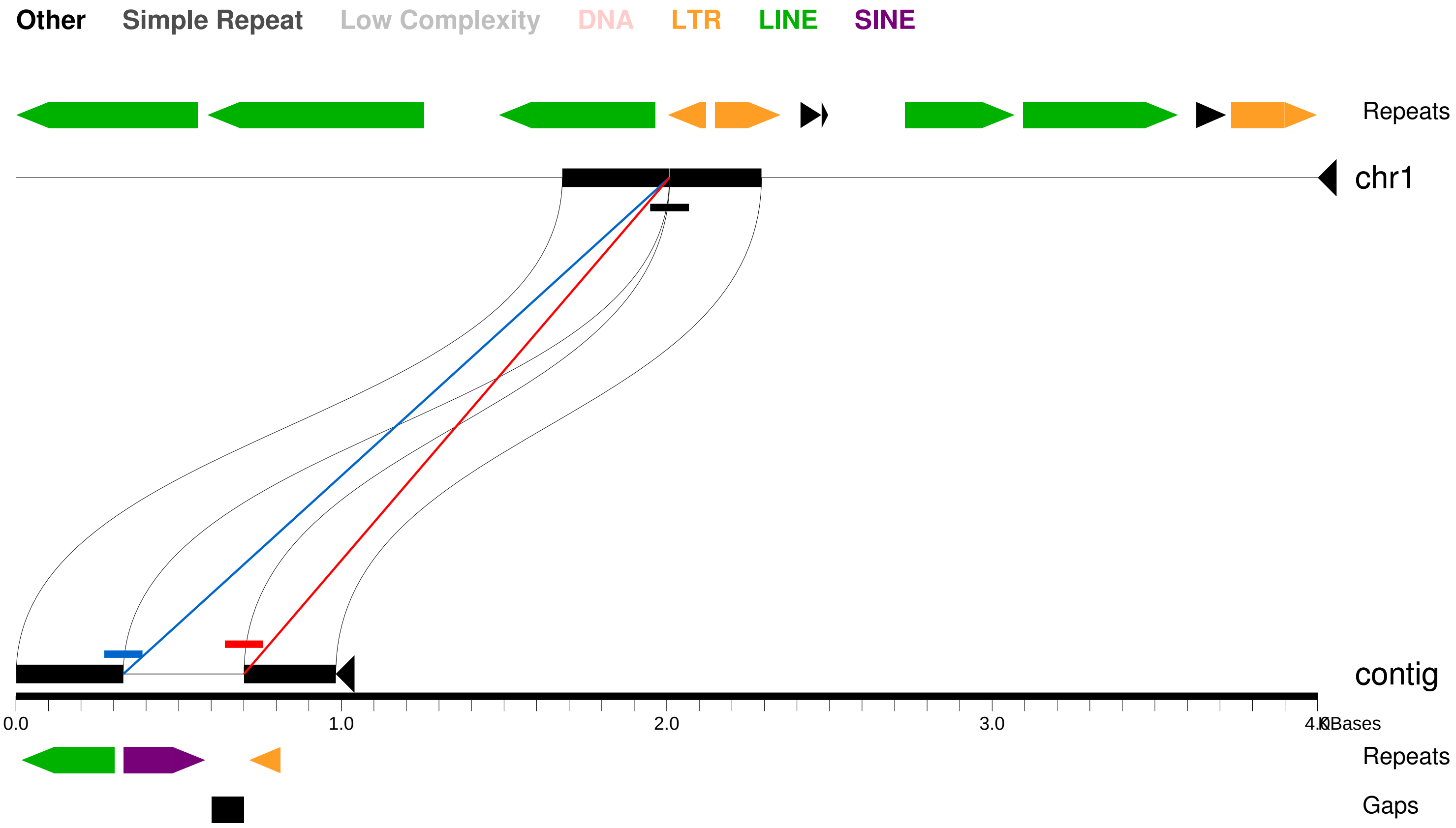

siteID: chr1_180857556 Scafftig1 - Breakpoint: chr1:180857550
chr1:180857390-180857724
Overlap Length: 15 INS size: 306 Genotyped: Yes

```
left   ---------------------TAACCCTATCTGTGGCATTGTTTTAAAATGTGGGATACAAATCTGG---
chrom  A-----AAATAATTAAGAAAATAACCCTATCTGTGGCATTGTTTTAAAATGTGGGATACAAATCTGG---
right  AGTCCCAGCTACTTGGGAGGCTGAGGCA----GGAGAATGGCGTGAACCCG--GGAGGCAGAGCTTGCAG
       2111112NN22N22NN22NNN*1*11*11111*11*1**1*11*1**111*11***11**1*1**1*111

left   TAAGCAGATTTCAGGTCATTATTGTTTTCTTTTAATGGTTTACAT-GAAATTTTCC-TCTCAAAAAACAA
chrom  TAAGCAGATTTCAGGTCATTATTGTTTTCTTTTAATGGTTTACAT-GAAATTTTCC-TCTCAAAAAACAA
right  TGAGCCGAGATCCCGCCACT---GCACTCCAGCC-TGGGCGACAGAGCGAGACTCCGTCTCAAAAAAAAA
       *1***1**11**11*1**1*111*111**111111***111***11*11*111***1**********1**

left   GTACCTCTTACTGTATAACATTTGAGTTTTTAAAATTGAGATAGC--------------AGGCCGGGCGC
chrom  GTACCTCTTACTGTATAACATTTGAGTTTTTAAAATTGAGATAGCTTCTGAGAGAAAAAAAGAAAGTTGA
right  TAAA----------ATAAAATAAAA-----TAAAATTGAGATAGCTTCTGAGAGAAAAAAAGAAAGTTGA
       11*1111111111****1**111*11111***************22222222222222*2*222*22*2

left   G---GTGGCTCACGCCTGTA----ATCCCAGCACTTT--------------GGGAGGCCGAGGC------
chrom  ATTTGTAGCTCTCTCAACTACAAAATCACCACACTTTTCTCAAATCTAATAGGAATTCAGTGTCATTACA
right  ATTTGTAGCTCTCTCAACTACAAAATCACCACACTTTTCTCAAATCTAATAGGAATTCAGTGTCATTACA
       2222**2****2*2*222**2222***2*22******22222222222222**2*22*2*2*2*222222

left   GGGCGGATCACGAGGTCAGGAGATCGAGACCATCCCGGCTAAAACGGTGAAACCCCGTCTCTACTAAA-
chrom  GATACGGTCTCTAG-TGACCAGGTGAAGACAATTGAAGCTTT---GGTAGAACTTCTGCAAAAGTTAGG
right  GATACGGTCTCTAG-TGACCAGGTGAA------------------------------------------
       *2222*2**2*2**2*2*22**2*22*111N11NNNN111NN222111NN111NN1NN1NNN1N1N1N
```

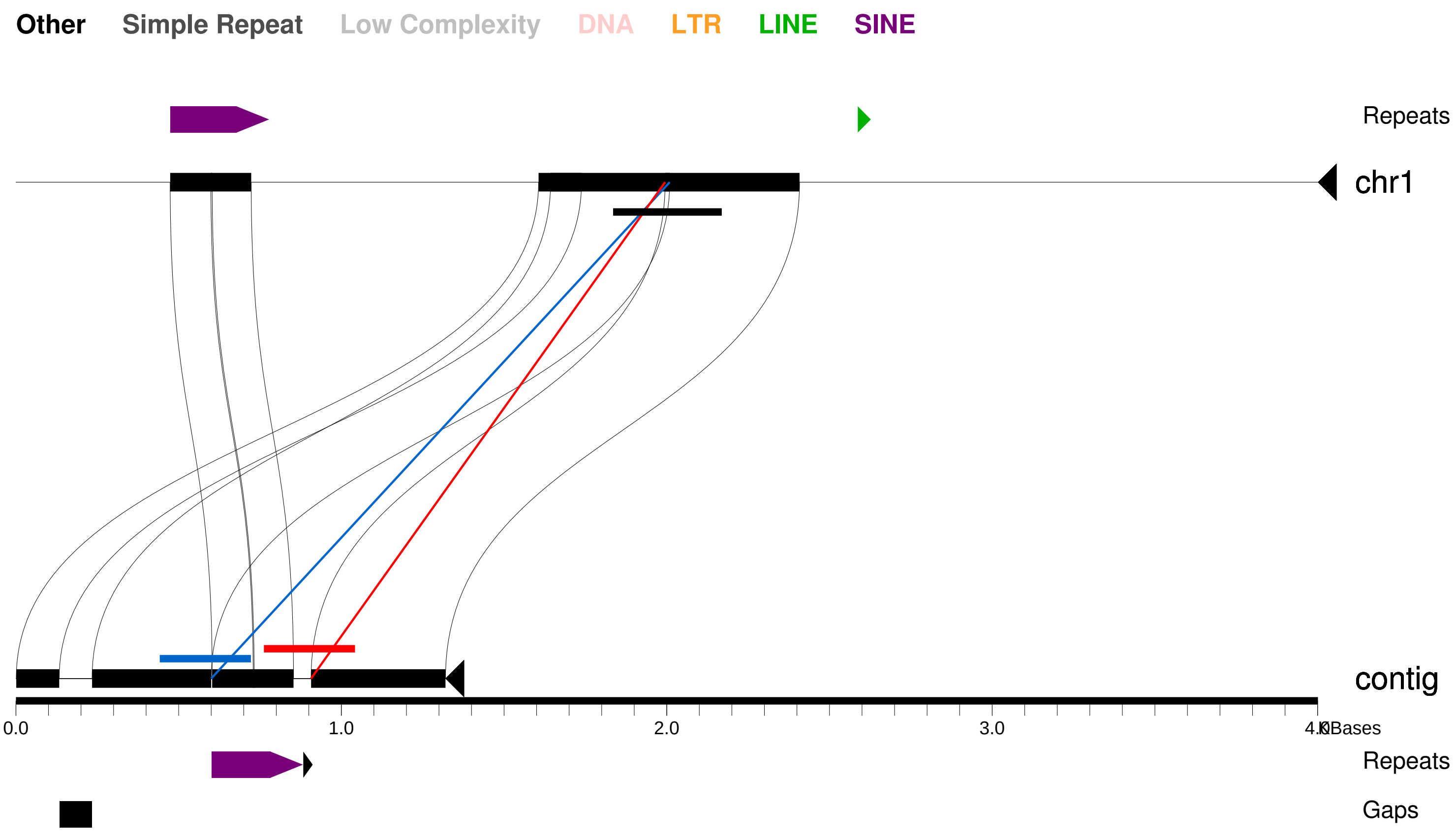

siteID: chr1_181588354 Scafftig1 - Breakpoint: chr1:181588347
chr1:181588301-181588406
Overlap Length: 6 INS size: 318 Genotyped: Yes

```
left    ------GGTGATACAGTACAAGGGGACGGGGTC---GGTTTTGGTAGGAGGGCCT----GATGGTTTTTT
chrom   ------GGTGATACAGTACAAGGGGACGGGGTC---GGTTTTGGTAGGAGGGCCT----GATGGTGTACT
right   CCACCCGCCTCGGCCTCCCAAAGTGCTGGGATTACAGGCGTGAGCCACCGCGCCCGGCCGATGGTGTACT
        111111*111111*1111***1*1*11***1*1111**11*11*11111*1***11111******2*22*

left    TTTTTTTTTTTTTTTTTTTTTTTTTGAGACGGAGTCTCGCTCTGTCGCCCAGGCCGGACTGC
chrom   TTTATATGGGGTAGTT----------AGGAAAGGCCTCACAGAGAAGTTGACTTTTGAA---
right   TTTATATGGGGTAGTT----------AGGAAAGGCCTCACAGAGAAGTTGACTTTTGAA---
        ***2*2*2222*22**2222222222**22222*2***2*222*22*222*22222**2222
```

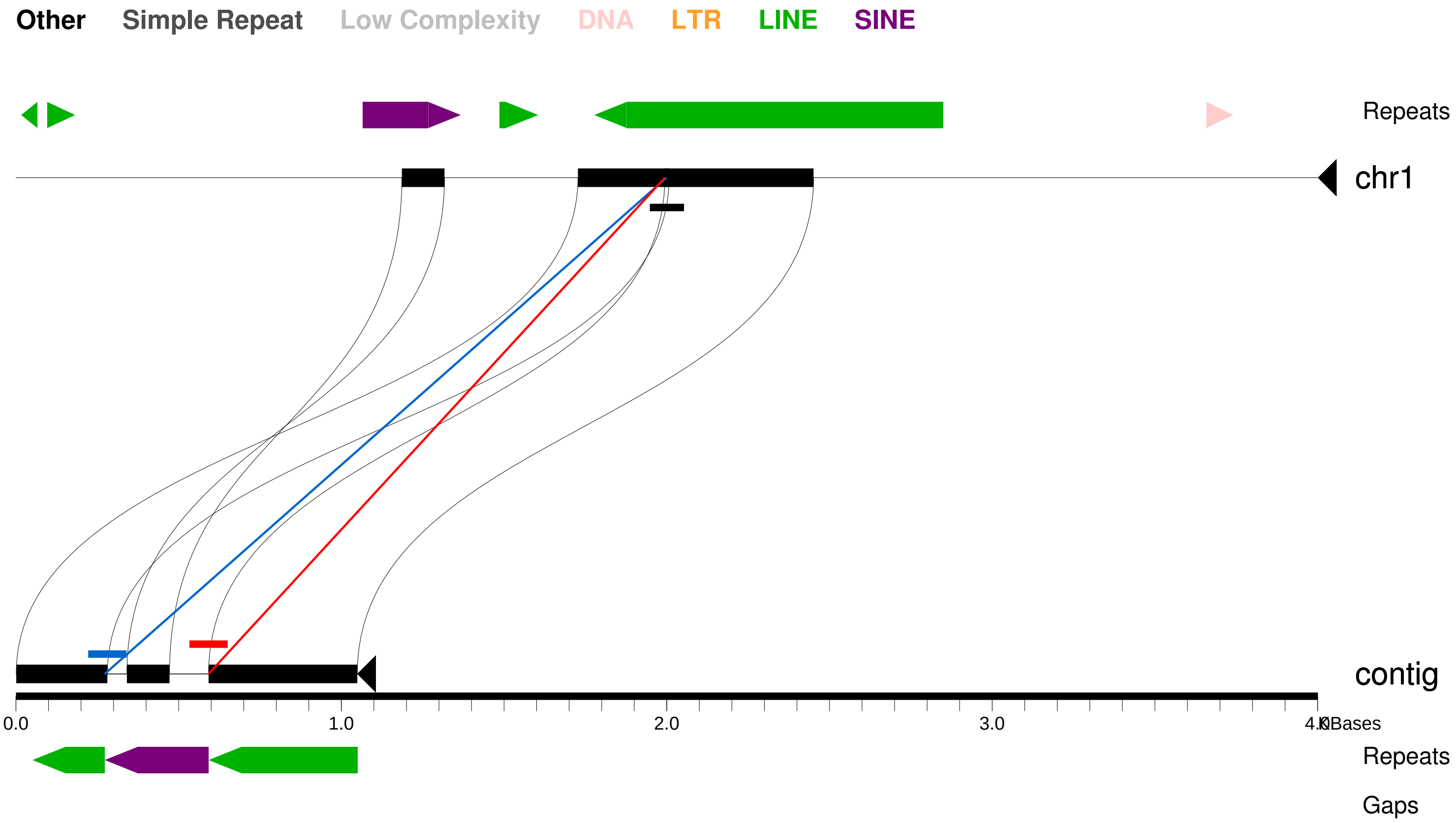

siteID: chr1_182420835 Scafftig1 + Breakpoint: chr1:182420841
chr1:182420232-182421001
Overlap Length: 17 INS size: 308 Genotyped: Yes

```
left   ------------------------------------------------------------------------
chrom  GATCTTGCCACTGCATTCCAGCCTGGGTGACGGAGTGAGACTTTGGCAAAAAAAAAAAAAAAAAAAAGATT
right  --TTTT--------AGTAGAGACGGGGT--------------------------------------------
         2N22        2N2NN22N2N2222

left   ------------------------------------------------------------------------
chrom  GAAGATTATCTTTTGAAAGAACAATTTATTCCACATTTACAGAACTTAGACATTGTGCAAACACTTTTAG
right  -----------------------------TTC-----------------------------ACCGTTTTAG
                                    222                             22NN222222

left   ------------------------------------------------------------------------
chrom  AGCCTAGAGGAAGAAAGGTGAAAAAACAAAGTCCTCCTCTCACACGCTCAGTCTGATGAGGAAGACAGA
right  --CCGGGATG-------GT-----------------------------CTCGATCTCCTGAC----------
         22NN22N2       22                             222NN222NN222N

left   ------------------------------------------------------------------------
chrom  TATATCGATGGATAAAAACAGTCAGACTTGTTAATTGTAATAATGGAGGATATACGTGATGGTATAGGGA
right  ---------------------------CT---------------------------CGTGAT----------
                                  22                           222222

left   ------------------------------------------------------------------------
chrom  GATAGTGATGAATTCTACCATTGCAGTAGATTCTACCTTTCTTGGCTCACCAAGATTATATGTAAACCAG
right  --------------------------------CCGCCCGCCTCGGCCTCCCAA---------------AG
                                       2NN22NNN22N222NNN2222               22

left   ------------------------------------------------------------------------
chrom  TTCTTAATGGATGACTATTAATTTGTGAGCTGACTGTGCTTAAGAAAACATTTGAAGCAATGGGAATATG
right  TGCT-----------------------------------------------------------GGGATTA--
       2N22                                                           2222N22

left   -----------------------------------------ATGCAGGAAACAGCAATGCCTTGGCTTAG
chrom  GGAACAAAGACATGGAGGTTTCAAGTGAGAGTACCTAGCACATGCAGGAAACAGCAATGCCTTGGCTTAG
right  ------------------------------------------------CAGG-------------------
                                                 111****1111111111111111111111

left   CTTGCAAACAGGAGGCAGAGTGCGCAGAGAGGCAGGAAATGAGACTGGGAGGAAACTCAAAAGCCAGATG
chrom  CTTGCAAACAGGAGGCAGAGTGCGCAGAGAGGCAGGAAATGAGACTGGGAGGAAACTCAAAAGCCAGATG
right  --------CGTGAGCCACCGCGCCC-----GGC---------------------------------------
       11111111*11***1**11*1**1*11111***1111111111111111111111111111111111111

left   GGGTAAAGCCTCAGATACTATACTAGAGAGTTTGGACTTTACCTTCTAGGACATCAAGGAGTTTTTTTTT
chrom  GGGTAAAGCCTCAGATACTATACTAGAGAGTTTGGACTTTACCTTCTAGGACATCAAGGAGATTTTGAAA
right  ------------------------------------------------GACATCAAGGAGATTTTGAAA
       1111111111111111111111111111111111111111111111111*************2****2222

left   [illegible]GAGACC[illegible]AGTCT[illegible]CT[illegible]T-[illegible]CAGGCTGG-----AGTGCA-
chrom  GACTTTAACAATAATAATATCA-----ACAGATTATGGTGGCAGTACGTGTAGGGTGGTTGGAAGGGCAA
right  GACTTTAACAATAATAATATCA-----ACAGATTATGGTGGCAGTACGTGTAGGGTGGTTGGAAGGGCAA
       222***22222*22*22*2*2222222**2**2*2*2*222*2**2**222***2***22222**2***2

left   GTGGC[illegible]CAATCTCGGCT[illegible]-TGCA[illegible]CTCC[illegible]------[illegible]-----ACC[illegible]-
chrom  GAGGTGGGAGCTAGGGAGACCATTTAAGAAGCTGATGCAGAAGTCTAGGTGAGAGAGCATAGAGGCCTGT
right  GAGGTGGGAGCTAGGGA[illegible]CTAGGT[illegible]AGAGCATAGAGGCCTGT
       *2**2*22*2**2**222**22*22**[illegible]*2*2*222**22222222222222222*2***222

left   -AT--
chrom  AATGG
right  AATG-
       2**2
```

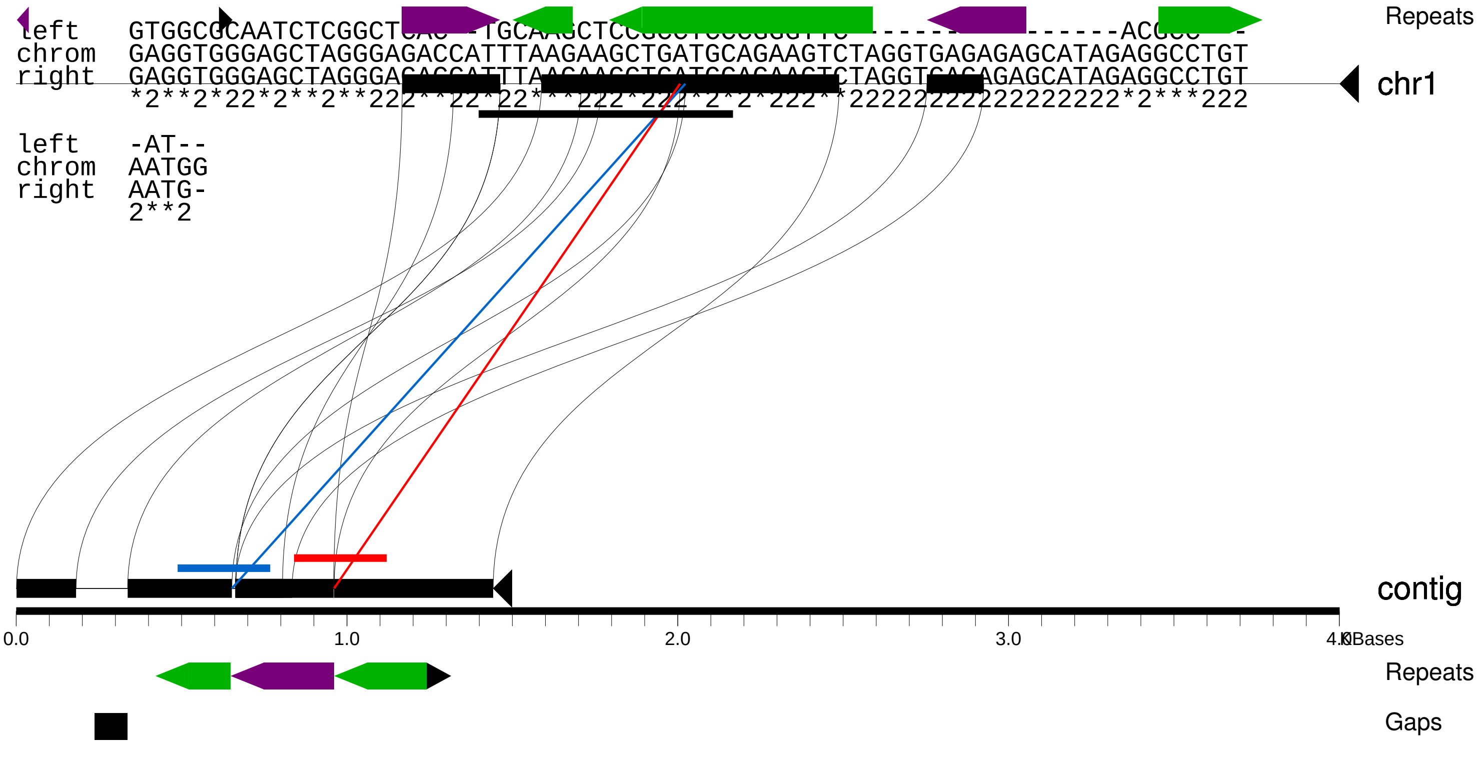

siteID: chr1_185504716 Scafftig1 - Breakpoint: chr1:185504703
chr1:185504658-185504736
Overlap Length: 15 INS size: 334 Genotyped: Yes

```
left   ------TTATGCATGG-----AGAGGAAGGC--------------------AAAAGATACAATA-AAATA
chrom  ------TTATGCATGG-----AGAGGAAGGC--------------------AAAAGATACAATA-AAATA
right  CCGCAGTCCGGCCTGGGCGACAGAGCGAGACTCCGTCTCAAAAAAAAAAAAAAAAAAAACAAAACAAAAA
       111111*111**1***11111****11**1*11111111111111111111****1*1****1*1***1*

left   AATGCTT--------CAAAGTGAATATTACGGCCGGGCGCGGTGGCTCACGCCTGTAGTCCCAGCACTTT
chrom  AATGCTT--------CAAAGTGAATATTACT----------------------TATAAGACCAGAGTAAT
right  AAAACAAAAAACAAACAAAGTGAATATTACT----------------------TATAAGACCAGAGTAAT
       **11*1111111111***************22222222222222222222222*2**222****22222*

left   GGGAGGCCGAGGCGGGTGG
chrom  G------------------
right  G------------------
       *222222222222222222
```

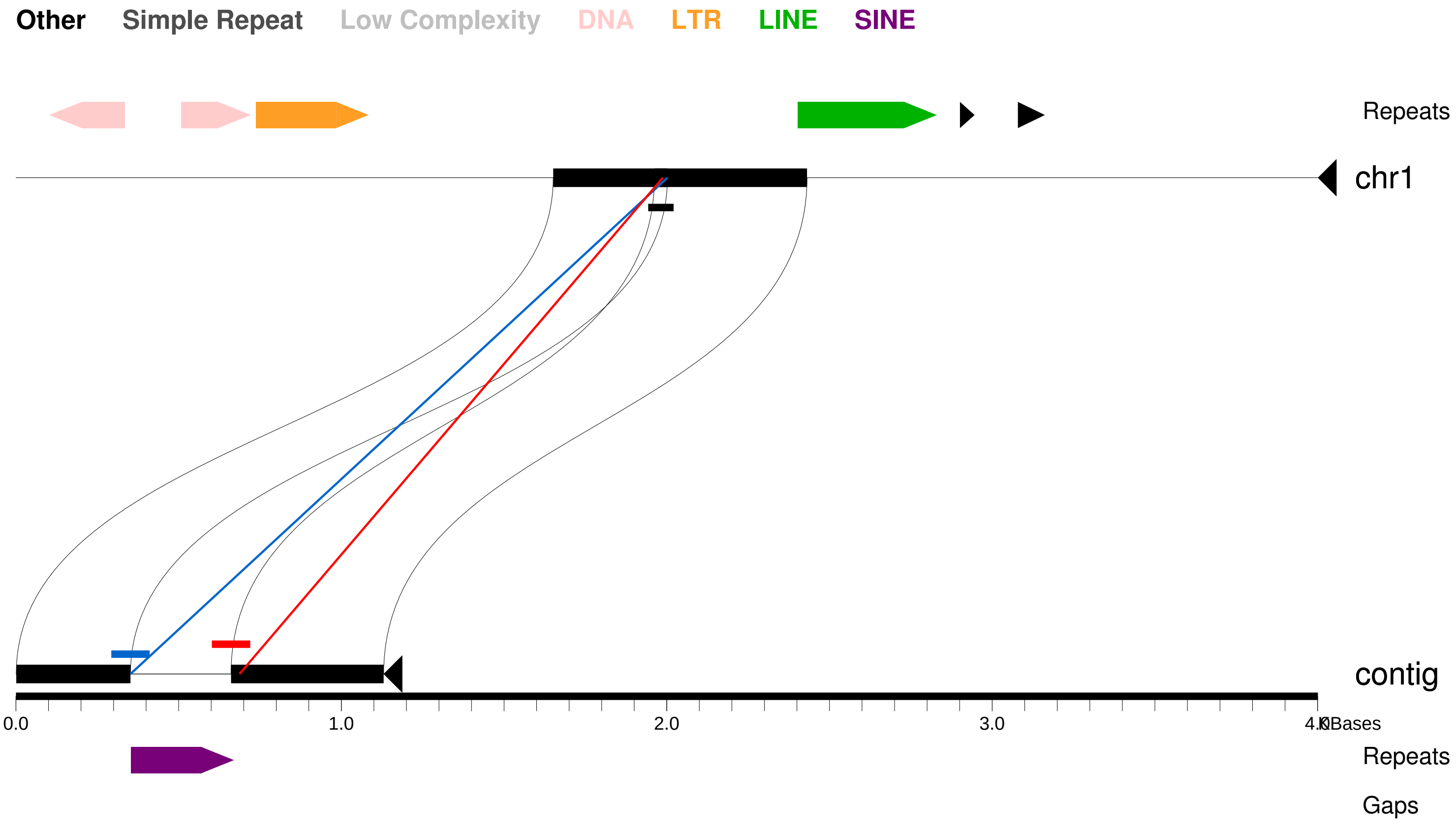

siteID: chr1_188174654 Scafftig1 + Breakpoint: chr1:188174642
chr1:188174598-188174701
Overlap Length: 16 INS size: 270 Genotyped: Yes

```
left    GTTAAATGTGCATT-TATAAAACACTTAACAT--TTATTT-------TAATATT-----TAAAAAATGCA
chrom   GTTAAATGTGCATT-TATAAAACACTTAACAT--TTATTT-------TAATATT-----TAAAAAATGCA
right   TTTTAGCCGGGATGGTCTCGATCTCCTGACCTCGTGATCCGCCCGCCTCGGCCTCCCAATTAAAAAATGCA
        1**1*1111*1**11*1*11*1*1*1*1**1*11*1**111111111*11111*11111***********

left    TTCTTTTTTTTTTTTTTTTTTTTTTTTTTTTTTGAGACGGAGTCTCGCTCTGTCGCCCAGGCTGG
chrom   TTCTTAAGGAAAGCTCTTTTCGAGTATCTTCTAAAATATACCCTCATT---------------G
right   TTCTTAAGGAAAGCTCTTTTCGAGTATCTTCTAAAATATACCCTCATT---------------G
        *****222222222*2****2222*2*2**2*2*2*222*22***22*222222222222222*
```

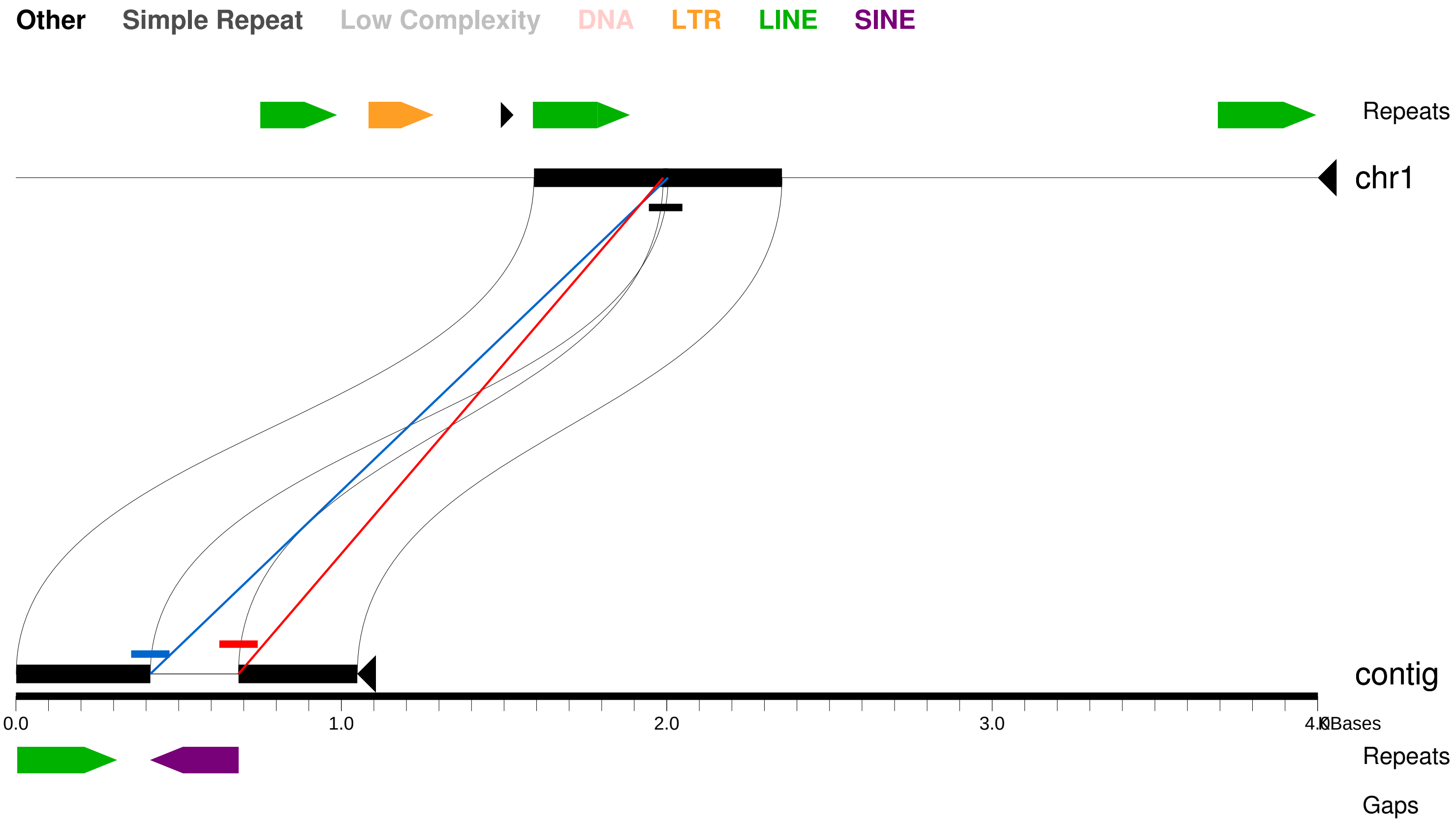

siteID: chr1_188438227 Scafftig3 + Breakpoint: chr1:188438202
chr1:188438042-188438373
Overlap Length: 12 INS size: 293 Genotyped: Yes

```
left    -----TGGT-GTGTTGTTATATACCAGTGTGCTTCAAATGGCTTACAGTTGTGAGATGCTGATTGCCTCT
chrom   CCCTCTGGT-GTGTTGTTATATACCAGTGTGCTTCAAATGGCTTACAGTTGTGAGATGCTGATTGCCTCT
right   ---TTTAGTAGAGACGGGGTTTCACCGTGTCAGCCA---GGATG---GTCTCGATCTCCTGA---CCTCG
           2N*1**1*1*11*111*1*11*1****1111**111**1*1111**111**11*1****111****1

left    TCCTATGTGTGCTATAGACAAAGATTGAAAGAAATTGCTTTACCAAATCAATTTATCTAAACATCTTTTC
chrom   TCCTATGTGTGCTATAGACAAAGATTGAAAGAAATTGCTTTACCAAATCAATTTATCTAAACATCTTTTC
right   TGATCCGCCCGCCTCGGCCTCCCA----AAGTGCTGGGATTAC--AGGCG--TGAGCCACCGCGCCCGGC
        *11*11*111**1111*1*1111*1111***111*1*11****11*11*111*1*1*1*11111*1111*

left    ATTTATTTATAAGAATTATTTCTATATTTTTTTTT----------TTTTTTTTGAGACGGAGTCTCGCTC
chrom   ATTTATTTATAAGAATTATTTCTATATTTTTTTATGACAACTTATTATTTATTATAACTGAGGCAAATAT
right   CT-------------------CTATATTTTTT-ATGACAACTTATTATTTATTATAACTGAGGCAAATAT
        1*1111111111111111111***********12*2222222222*2***2**222**2***2*222222

left    TGTCGCCCAG----GCTGGAGTGC----AG---TGGCGCGATCTCGGCTCACTGCAAGCTCCGCCTCCCG
chrom   TTCCTCCCCATATTGCTGATGACCCCTCAGGTTTGTTGCCTTTTACATTTGCTCCAAAGTGTGTTTGTTT
right   TTCCTCCCCATATTGCTGATGACCCCTCAGGTTTGTTGCCTTTTACATTTGCTCCAAAGTGTGTTTGTTT
        *22*2***222222****22*22*2222**222**22**22*2*2222*22**2***22*22*22*2222

left    GGTTCA------CGCCAT--TCTCCTGC-----------------CTCAGCCT
chrom   TGTTTTGTTGGTCACCATGATTTTATGATTATGGAGCACAAAACTCTTGGTTT
right   TGTTTTGTTGGTCACCATGATTTTATGATTATGGAGCACAA------------
        2***22222222*2****22*2*22**22222222222222    11NN1NN1
```

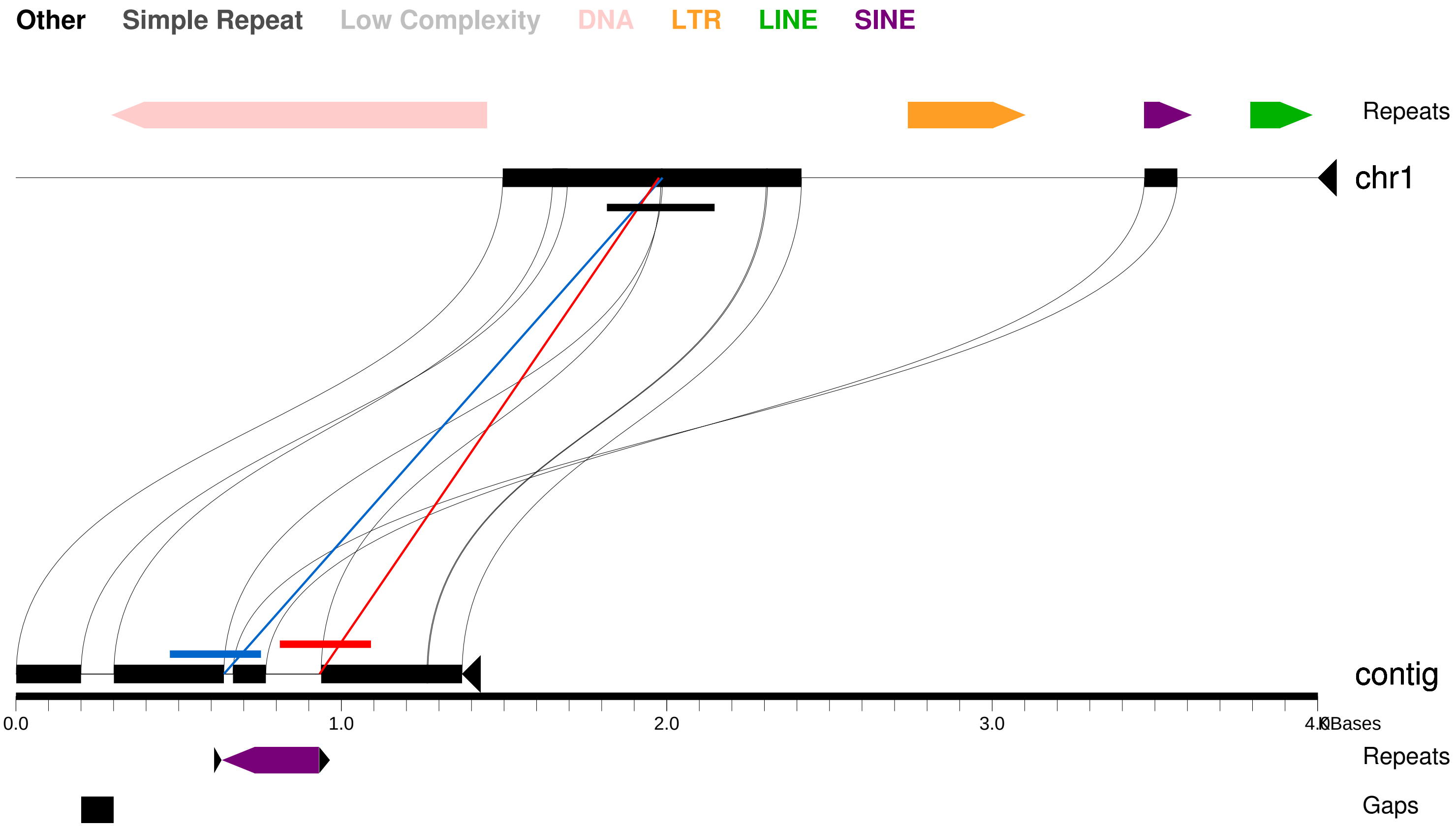

siteID: chr1_190874470 Scafftig2 + Breakpoint: chr1:190874445
chr1:190874410-190874501
Overlap Length: 13 INS size: 292 Genotyped: Yes

```
left    A-----------------CTTCCAGAGTGCT----TTA------TTAGAGTACGTTGCAGGGCTGAAAAC
chrom   A-----------------CTTCCAGAGTGCT----TTA------TTAGAGTACGTTGCAGGGCTGAAAAC
right   TGATCCGCCCGCCTCGGCCTCCCAAAGTGCTGGGATTACAGGCGTGAGCCACCGCGCCCGGCCTGAAAAC
        111111111111111111**1***1******1111***111111*1**1111**111*1**1********

left    TCTTTTTTTTTTTTTTTGAGACGGAGTCTCGCTCTGTCGCCCAGGCTGGAGTGCAGTGGCGCGATCTCGG
chrom   TCTTTATATTTGTGTTTCA-------TCTTGGT---------AAGC-------------CTCAACCTCCT
right   TCTTTATATTTGTGTTTCA-------TCTTGGT---------AAGC-------------CTCAACCTCCT
        *****2*2***2*2***2*2222222***2*2*222222222*2**2222222222222*2*2*2***22

left    CTCACT--
chrom   CTTCCTAG
right   CTTCCTAG
        **22**22
```

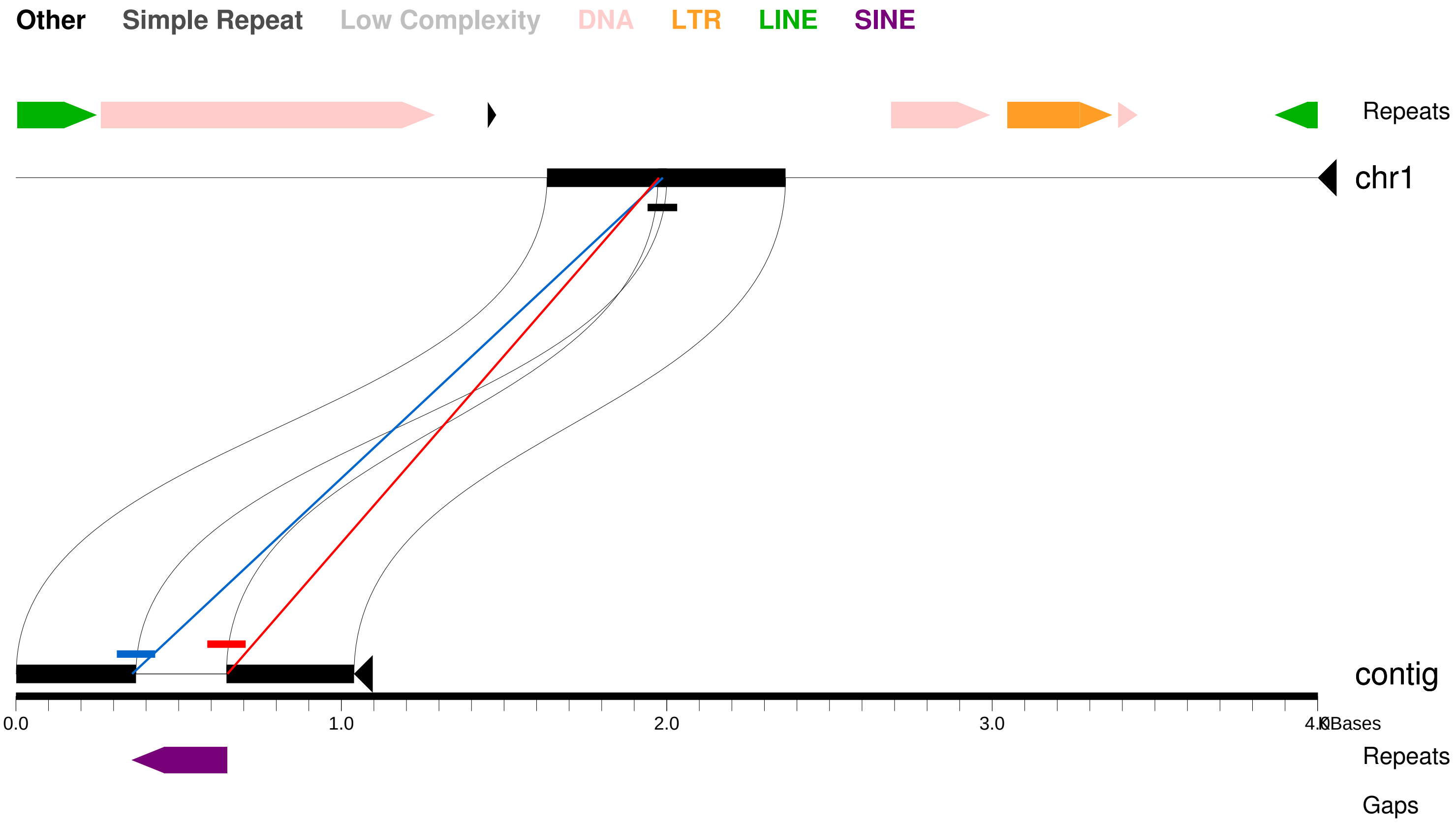

siteID: chr1_191477471 Scafftig4 + Breakpoint: chr1:191477451
chr1:191477419-191477510
Overlap Length: 15 INS size: 295 Genotyped: Yes

```
left    T------TGATTGATTTGCCA--GTTTTT---TTGTAAGCTTA----------------CACCTTTGCAG
chrom   T------TGATTGATTTGCCA--GTTTTT---TTGTAAGCTTA----------------CACCTTTGCAG
right   TCCGCCCGCCTCGGCCTCCCAAAGTGCTGGGATTACAGGCGTGAGCCACCGCGCCCGGCCACCTTTGCAG
        *111111111*1*111*1***11**11*1111**11*1**1*1111111111111111***********

left    TTTTTTTTTTTTTTTTTTGAGACGGAGTCTCGCTCTGTCGCCCAGGCTGGAGTGCAGTGGCGCGATCTCG
chrom   TTTTGATGGTTACTTTTAT-------GTCTCAATTTGA--------CTGG---------GCCCCA---CA
right   TTTTGATGGTTACTTTTAT-------GTCTCAATTTGA--------CTGG---------GCCCCA---CA
        ****22*22**22****222222222*****22*2**222222222****222222222**2*2*222*2

left    GCTCAC
chrom   GTACCC
right   GTACCC
        *22*2*
```

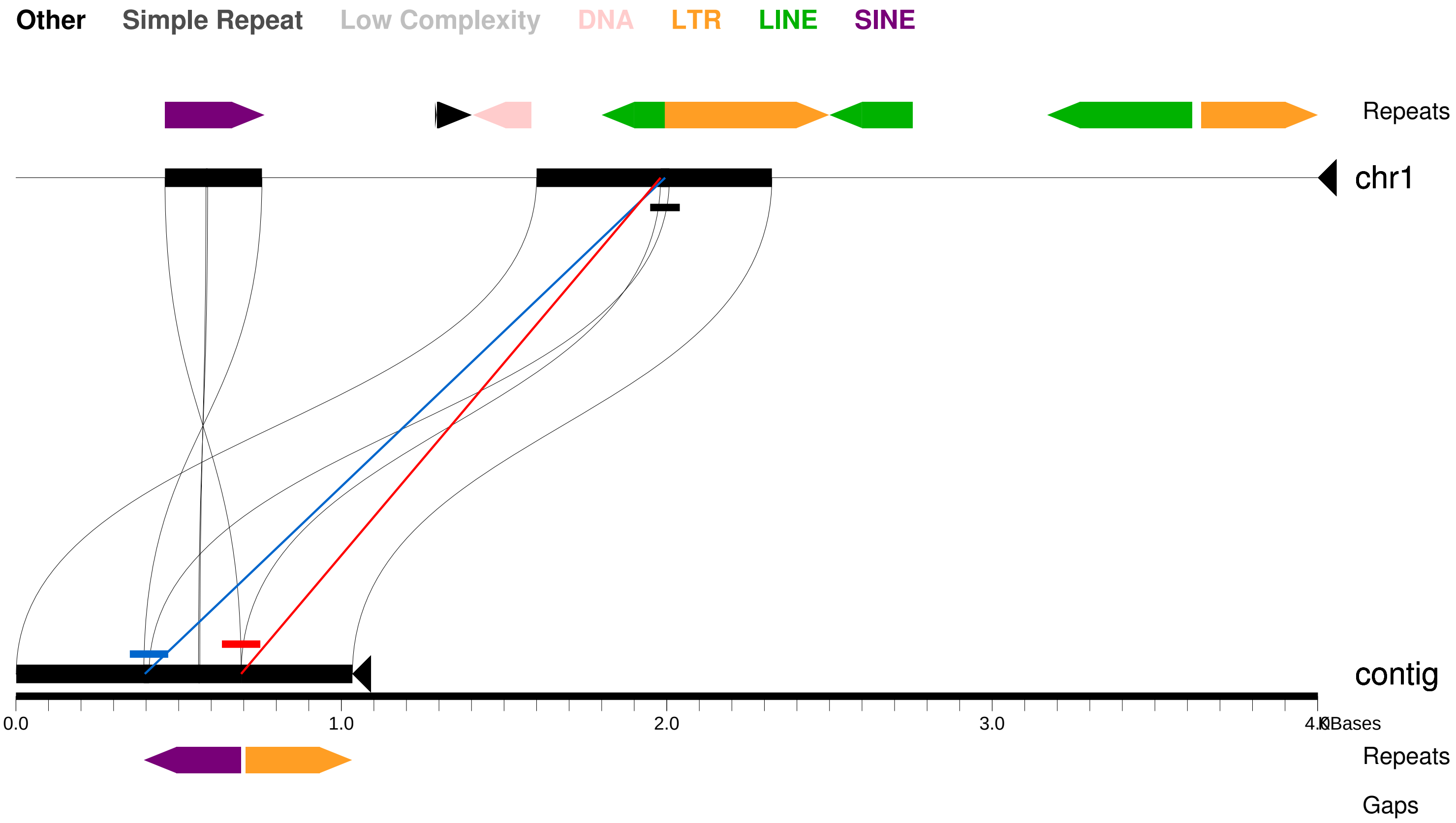

siteID: chr1_191580767 Scafftig1 + Breakpoint: chr1:191580769
chr1:191580722-191580828
Overlap Length: 13 INS size: 312 Genotyped: Yes

```
left     TTATGTAAAATTTACTGGAATCATTCT--------AATATTCTCTAGAATAAATT----TAAAATAAAAG
chrom    TTATGTAAAATTTACTGGAATCATTCT--------AATATTCTCTAGAATAAATT----TAAAATAAAAG
right    CCTGGGCGACAGAGCGAGACTCCGTCTCAAAAAAAAAAAAAATAAAAAATAAAATAAAATAAAATAAAAG
         1111*111*11111*11**1**11***11111111**1*111*11*1******1*1111***********

left     TAGGCCGGGCGCGGTGGCTCACGCCTGTAATCCCAGCACTTTGGGAGGCCGAGGCGGGCGG
chrom    TAAAATTTAT------GCTAAA-----TAATTA-ACTATTTAATGATTAACGTAAAAATGG
right    TAAAATTTAT------GCTAAA-----TAATTA-ACTATTTAATGATTAACGTAAAAATGG
         **22222222222222***2*222222****222*22*2**222**2222222222222**
```

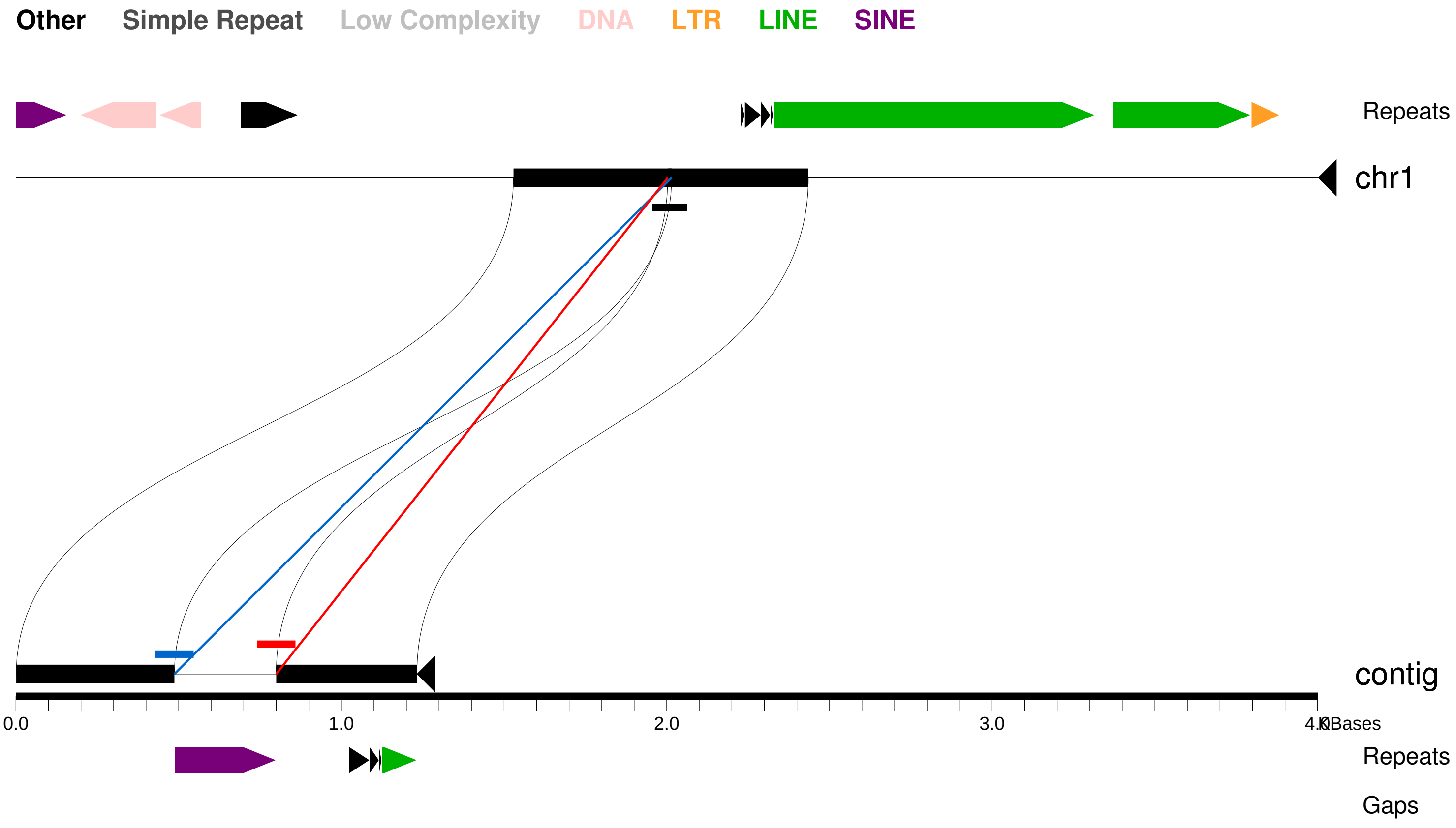

siteID: chr1_191817418 Scafftig2 - Breakpoint: chr1:191817426
chr1:191817378-191817485
Overlap Length: 12 INS size: 307 Genotyped: Yes

```
left    -------------ACAGATAAAAACCTATATATGTACATACATACACACACATACATATATATAAAATAT
chrom   -------------ACAGATAAAAACCTATATATGTACATACATACACACACATACATATATATAAAATAT
right   TCCAGCCTGGGCGACAGAGCGAGAC-TCCGTCTCAAAAAAAAAAAAAAAAAAAAATAAAAA-ATAAAATAT
        1111111111111*****111*1**1*111*1*11*1*1*1*1*1*1*1*1*1*1*1*1*1*********

left    CACGGCCGGGCGCGGTGGCTCACGCCTGTAATCCCAGCACTTTGGGAGGCCGAGGCGGGCGG
chrom   CACATTCA---------CATCACTCC--AAATATAAATTATAAGAGCTACACATATAAGAAC
right   CACATTCA---------CATCACTCC--AAATATAAATTATAAGAGCTACACATATAAGAAC
        ***222*222222222222****2**222***222*2222*22*2*222*22*22222*222
```

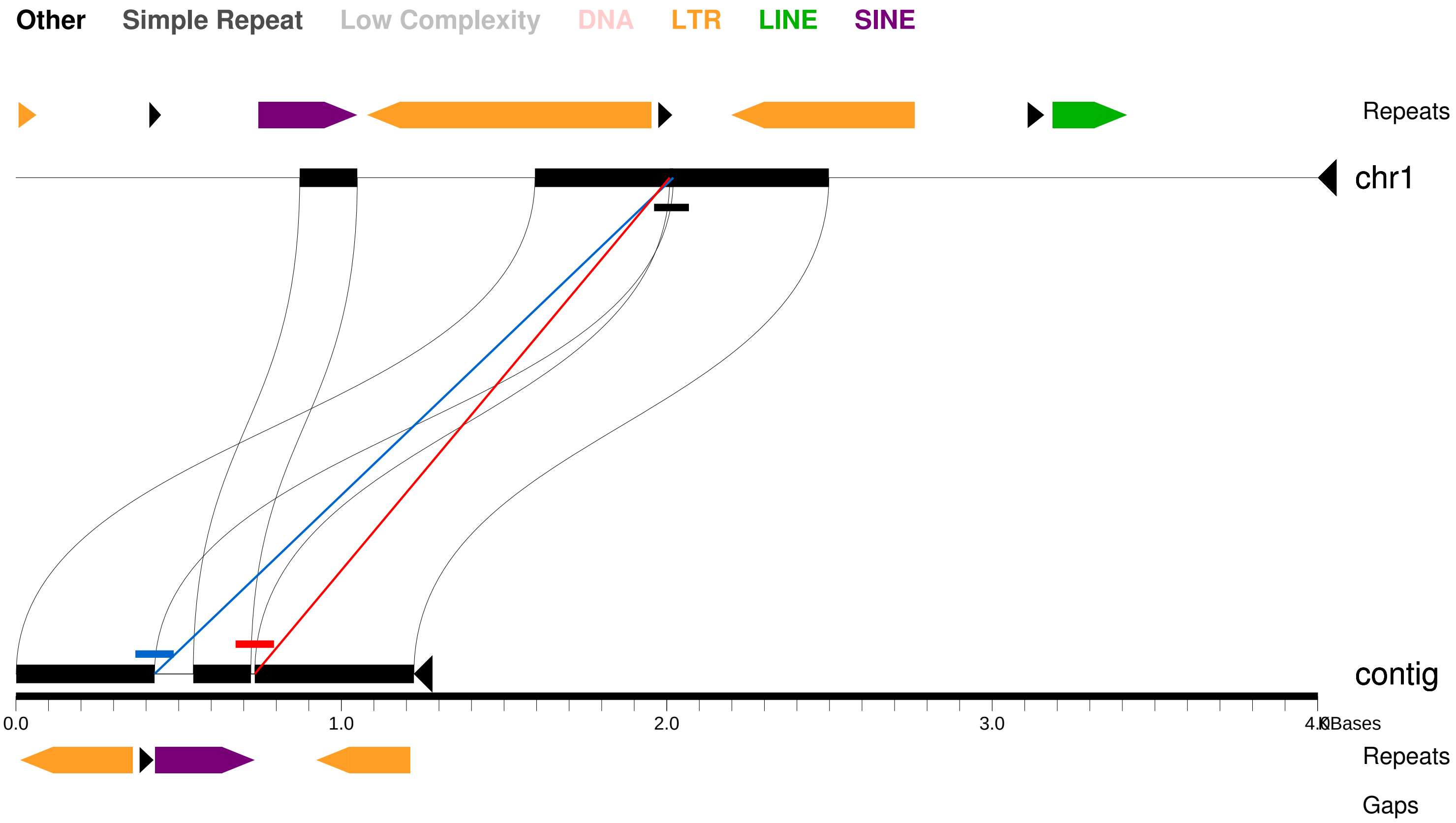

siteID: chr1_194595911 Scafftig1 - Breakpoint: chr1:194595948
chr1:194595901-194595995
Overlap Length: 13 INS size: 132 Genotyped: Yes

```
left    TGCAAT------ACT--AGTTCTG--------ACAAAAC-AGACTTC-------ACACCAACAAAGATTA
chrom   TGCAAT------ACT--AGTTCTG--------ACAAAAC-AGACTTC-------ACACCAACAAAGATTA
right   CGAGATCCCGCCACTGCACTCCAGCCTGGGCGACAGAGCGAGACTCCGTCTCAAAAAAAAAAAAAAAAAA
        1*11**111111***11*1*1*1*11111111***1*1*1*****1*1111111*1*11**1***1*11*

left    TAAAAGACAAAGAACTACTTGGGAGGCTGAGGCAGGAGAATGGCGTGAACCCGGGAGGCGGGGCTTGCAG
chrom   TAAAAGACAAAGAA--------GGGCATTA-----AATAATGG--TAAA----GGTTTCAG----TTCAA
right   AAAAAGACAAAGAA--------GGGCATTA-----AATAATGG--TAAA----GGTTTCAG----TTCAA
        1************22222222*2*22*2*222222*2*****22*2**2222**222*2*2222*2**2

left    TGA
chrom   C-A
right   C-A
        22*
```

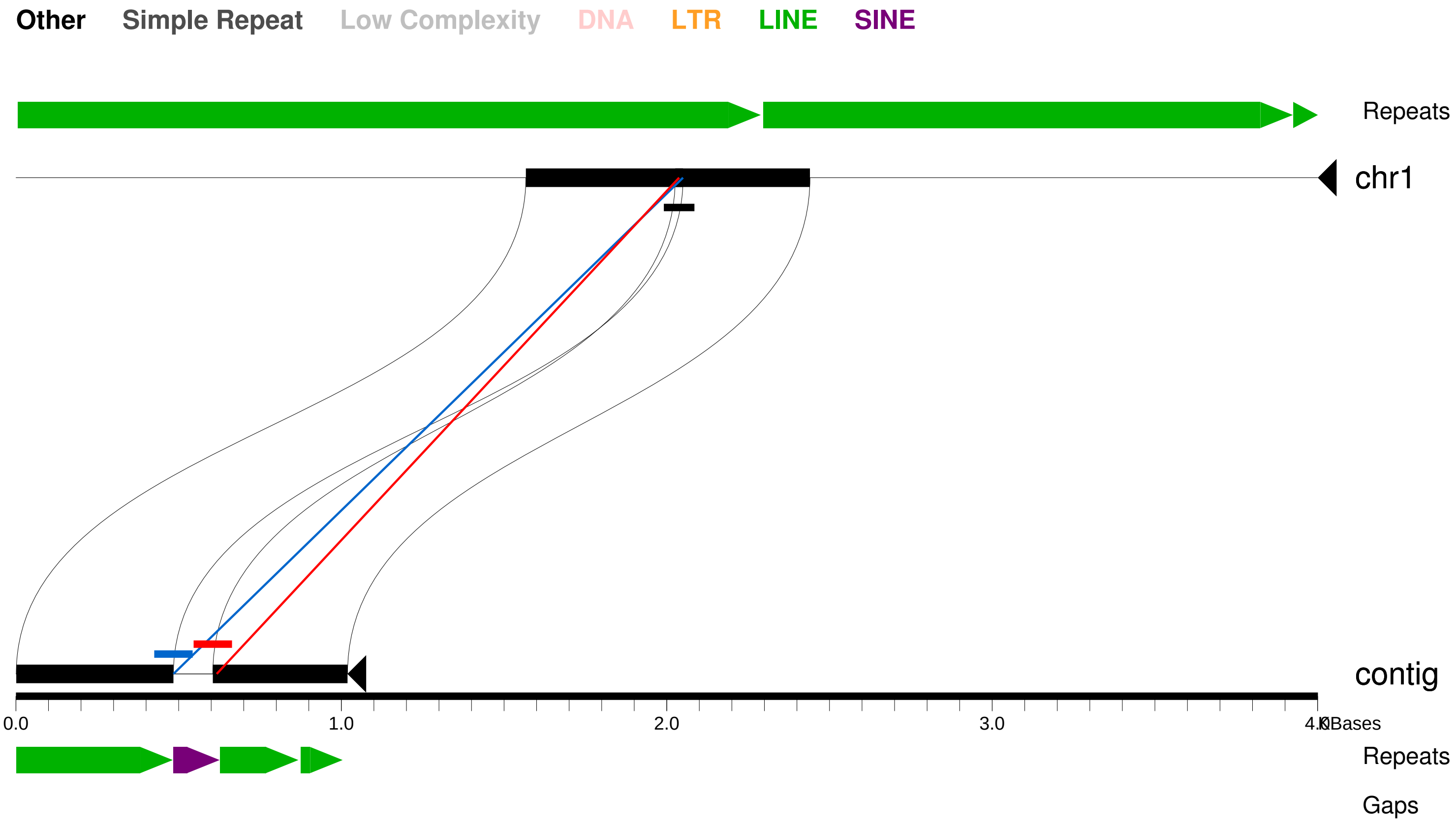

siteID: chr1_194675136 Scafftig3 - Breakpoint: chr1:194675126
chr1:194675081-194675185
Overlap Length: 15 INS size: 315 Genotyped: Yes

```
left     TAATCCACAGA---AGTCT------------AGAGAATATATACTTTCTGAATGTTGGTTAAAATTGAGT
chrom    TAATCCACAGA---AGTCT------------AGAGAATATATACTTTCTGAATGTTGGTTAAAATTGAGT
right    GGG-CGACAGAGCGAGACTCCGTCTCAAAAAAAAAAAAAAAAAAAAAAAAAAAAAAAAAAAAAAAATTGAGT
         1111*1*****111**1**111111111111*1*1**1*1*1*1111111**11111111**********

left     ATATCGGCCGGGCGCGGTGGCTCACGCCTGTAATCCCAGCACTTTGGGAGGCCGAGGCGGGCGG
chrom    ATATCATCAT----CAGTAAATAAA---TGTT----CATCTGAATGTCATTTTTTTTTCTTT---
right    ATATCATCAT----CAGTAAATAAA---TGTT----CATCTGAATGTCATTTTTTTTTCTTT---
         *****22*222222*2**222*2*2222***22222**2*2222**22*22222222*222222
```

Other Simple Repeat Low Complexity DNA LTR LINE SINE

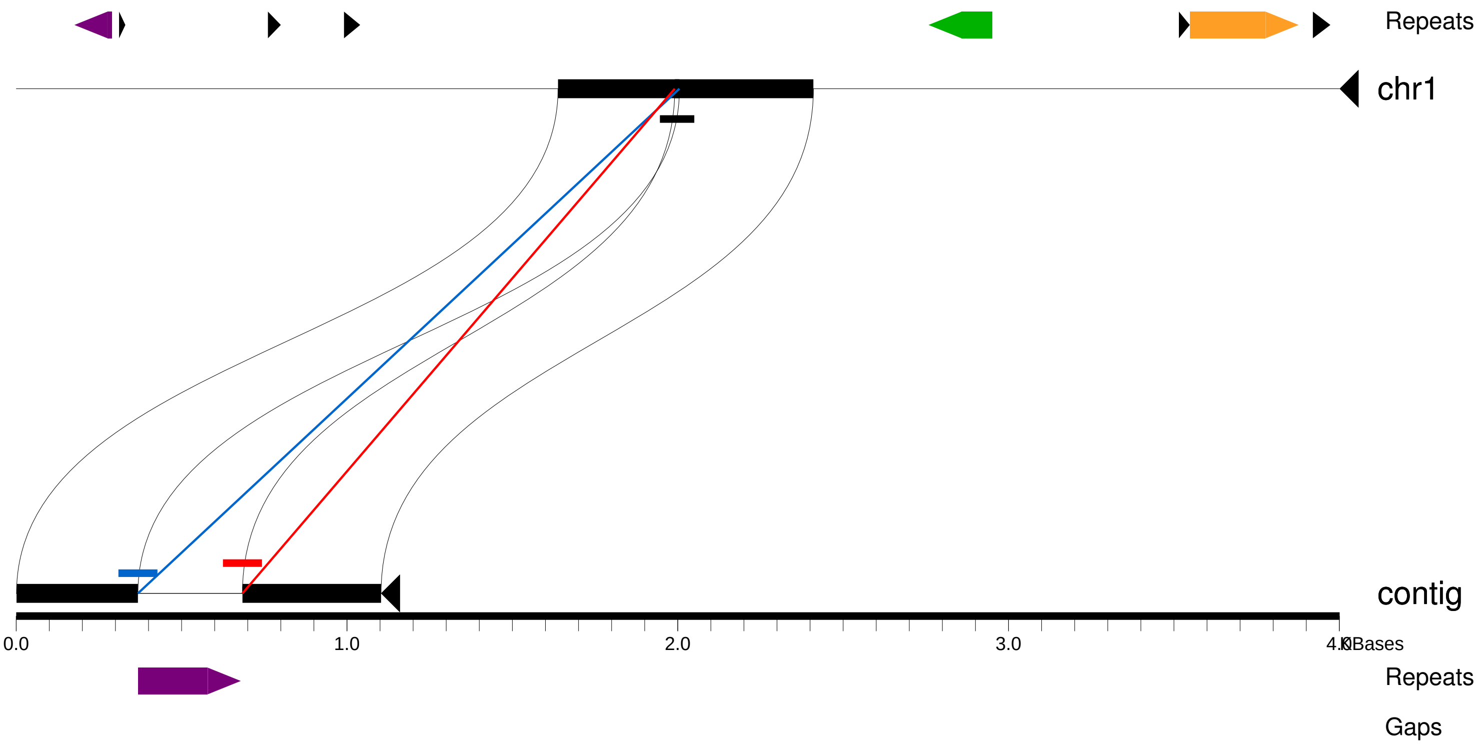

siteID: chr1_195530219 Scafftig3 + Breakpoint: chr1:195530245
chr1:195530201-195530304
Overlap Length: 16 INS size: 139 Genotyped: Yes

```
left    AAGAAAATG----------TGG----CACATATACACCATGG-AATACTATGCAGCCATAAAAAATGATG
chrom   AAGAAAATG----------TGG----CACATATACACCATGG-AATACTATGCAGCCATAAAAAATGATG
right   GCGCCACTGCACTCCAGCCTGGGCGACAGCAAGACTCCGTCTCAAAAAAAAAAAAAAAAAAAAAATGATG
        11*11*1**1111111111***1111**111*1**1**1*111**1*11*111*111*1***********

left    AGTTCTGTAGTCCCAGCTTCTCGGGAGGCTGAGGCAGGAGAATGGCGTGAACCCCAGGGGGCGG
chrom   AGTTCAT--GTCCT---TTGTAGGGA----CATGGATGAAATTGGAA--ATCATCATTCT----
right   AGTTCAT--GTCCT---TTGTAGGGA----CATGGATGAAATTGGAA--ATCATCATTCT----
        *****2222****2222**2*2****22222*2*2*2**2*2***2222*2*22**22222222
```

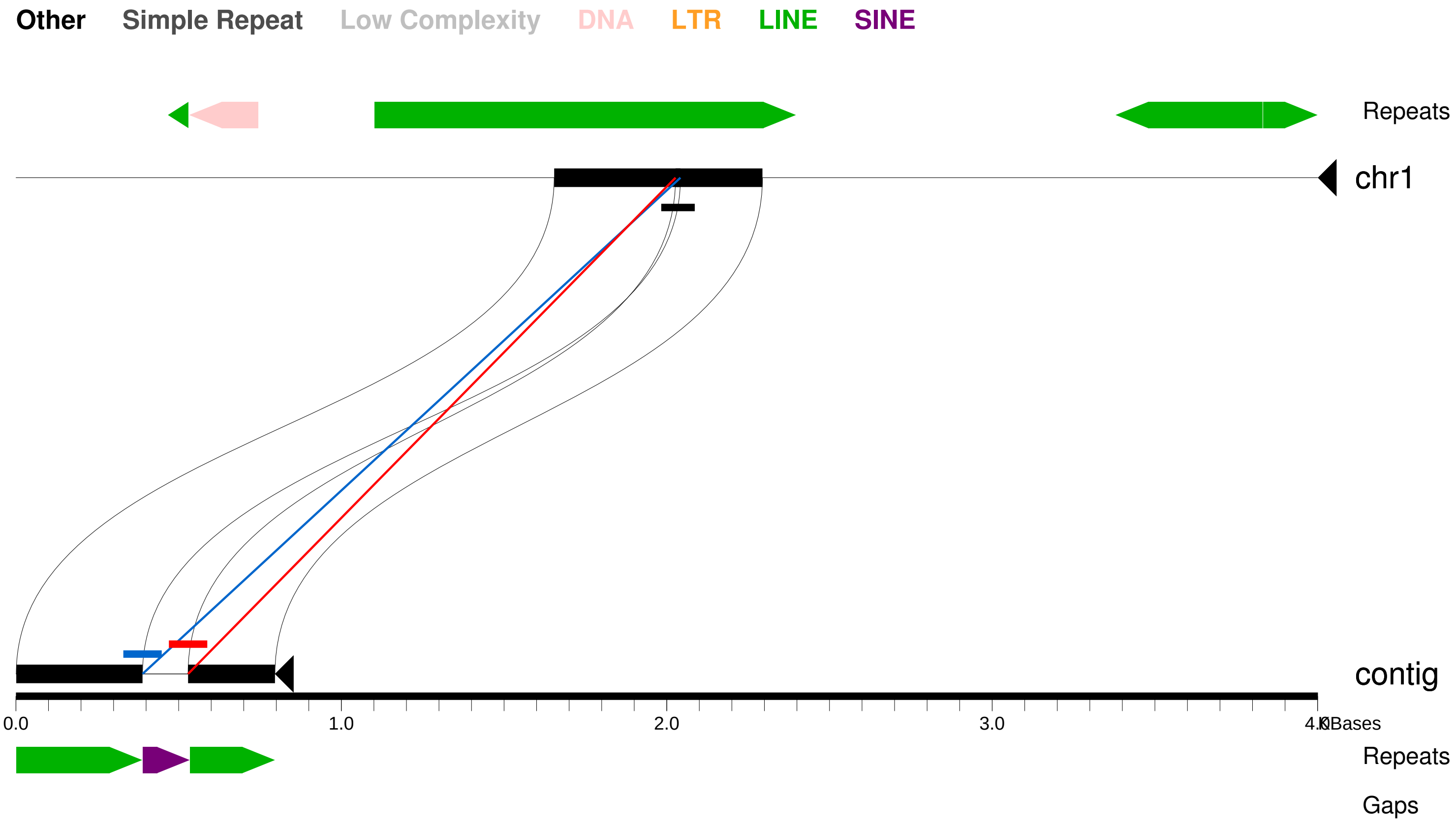

siteID: chr1_196779233 Scafftig7 - Breakpoint: chr1:196779192
chr1:196779144-196779248
Overlap Length: 12 INS size: 118 Genotyped: Yes

```
left    CCAGATGCATCACCTTTAAGAACTGTAACACTCACCGCGAGGGTC---------------TGCGGCTTCA
chrom   CCAGATGCATCACCTTTAAGAACTGTAACACTCACCGCGAGGGTC---------------CGCGGCTTCA
right   C-AGTGGCGGGATCTCGGCTCACTGCAAGCTCCGCCTCCCGGGTTCACGCCATTCTCCTGCGTGGCTTCA
        *1**11**111*1**111111****1**1111*1**1*11****11111111111111112*1*******

left    TTCTTTTTTTTTTTTTTTTTTTTTTTGAGACGGAGT-CTCGCTCTGTCGCCCAGGCTGGAGTGCAG
chrom   TTCTTGAA-----------GTCAGTGAGACCAAGAACCCACTAATTC---CAGACAC-ACTACCA
right   TTCTTGAA-----------GTCAGTGAGACCAAGAACCCACCAATTC---CAGACAC-ACTACCA
        *****222222222222222*222******22**22*2*2*1222**222***2*222*2*2*22
```

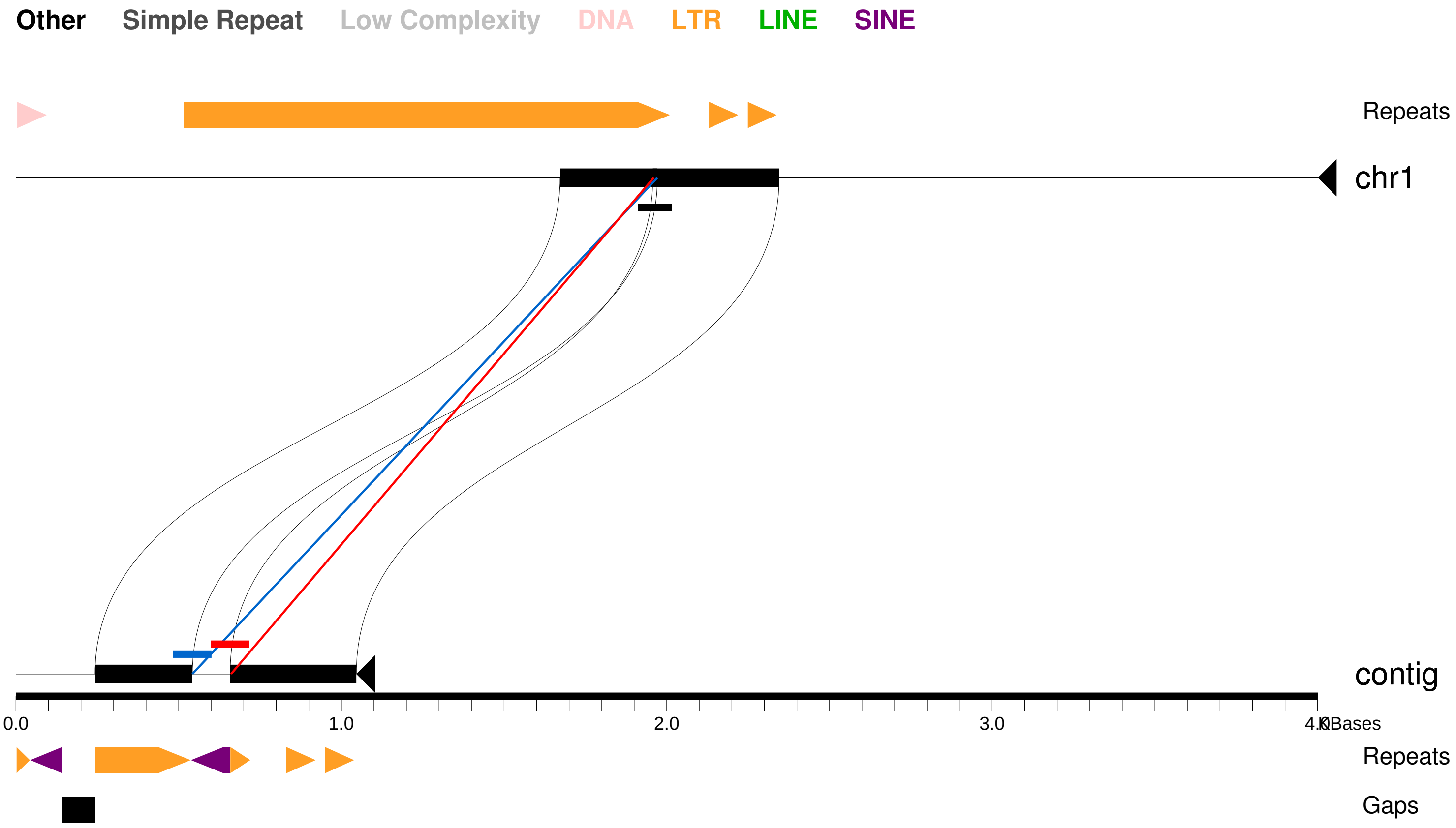

siteID: chr1_196780915 Scafftig6 + Breakpoint: chr1:196780912
chr1:196780869-196780971
Overlap Length: 17 INS size: 143 Genotyped: Yes

```
left     T-----------ACAGTTAAAG---------CAAAATAGTAAACAAGTAAATGGAAAGTGTTCAAAAAAT
chrom    T-----------ACAGTTAAAG---------CAAAATAGTAAACAAGTAAATGGAAAGTGTTCAAAAAAT
right    CCAGCCTGGGCGACAGAGCGAGACTCCGTCTCAAAAAAAAAAAAAAAAAAAAAAAAAAA----AAAAAAT
         111111111111****1111**111111111*****1*11***1**11***111***111111*******

left     GTAAATACTTTCAGCTACTTGGGAGGCTGAGGCAGGAGAATGGCGTGAACCCGGGAGGCGGAGCTTGCAG
chrom    GTAAATACTTT-------TTGTAAA----AAACAGTAGAATGTTTTATTAT----AAAATTAG-TAAGAT
right    GTAAATACTT-------TTGTAAA----AAACAGTAGAATGTTTTATTAT----AAAATTAG-TAAGAT
         **********2222222***22*22222*22***2******222*222222222*22222**2*222*2
```

Other **Simple Repeat** **Low Complexity** **DNA** **LTR** **LINE** **SINE**

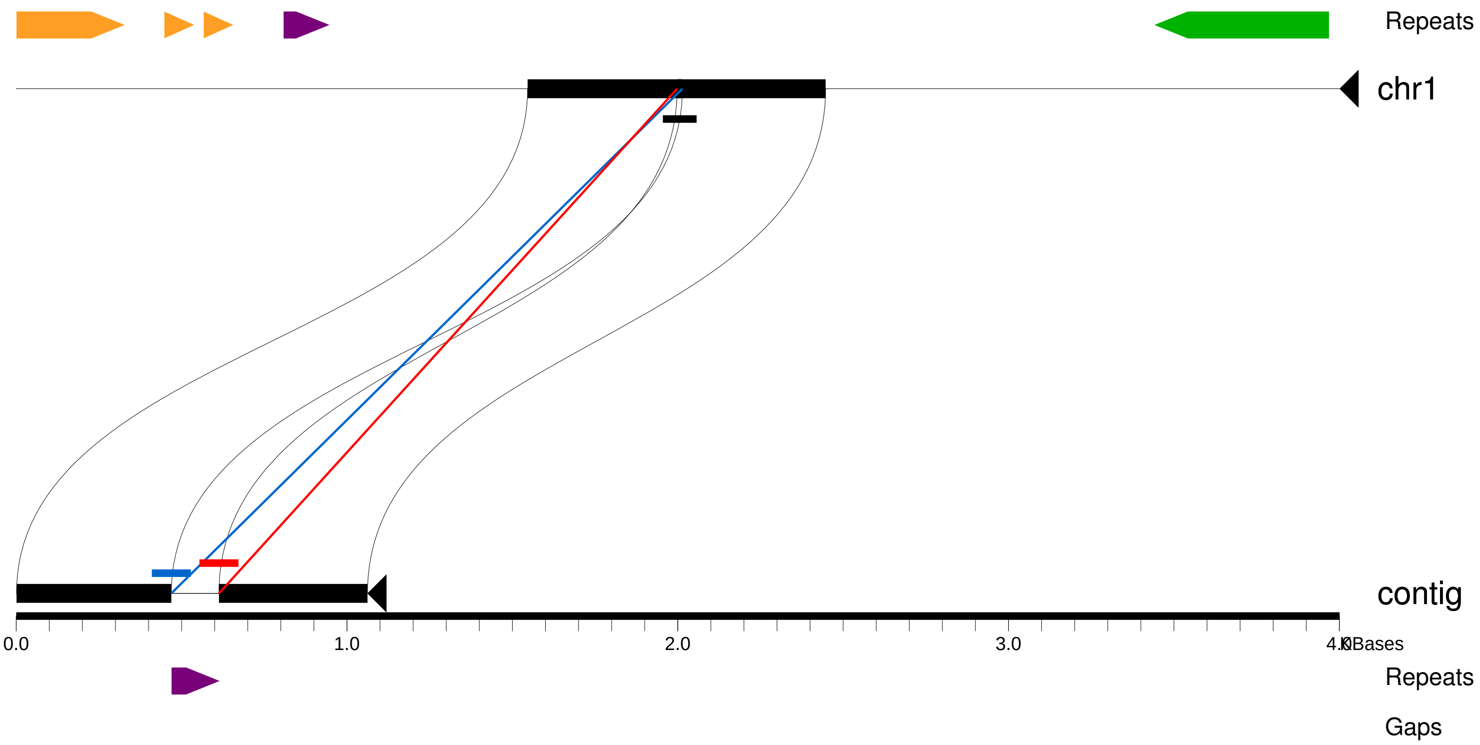

siteID: chr1_198243221 Scafftig3 + Breakpoint: chr1:198243289
chr1:198243256-198243348
Overlap Length: 12 INS size: 280 Genotyped: Yes

```
left     TGGAAGAAATGGAGAGCAGCTGGTG-CCT-GTAAT-------------------------TCTAGTTCTC
chrom    TGGAAGAAATGGAGAGCAGCTGGTG-CCT-GTAAT-------------------------TCTAGTTCTC
right    AGCCAGGA-TGGTCTCGATCTCCTGACCTCGTGATCCGCCCGCCTCGGCCTCCCAAAGTGTCTAGTTCTC
         1*11**1*1***11111*1**11**1***1**1**1111111111111111111111111**********

left     TTTTTTTTTTTTTTTTTTTTTTTTTTTTTTTTTTTGAGACGGAGTCTCGCTCTGTCGCCCAGGCTGGAGT
chrom    TTATGACTTCATTATTTCTCATCATTTAATTA------AC--ATTTTTCCT----------------AAT
right    TTATGACTTCATTATTTCTCATCATTTAATTA------AC--ATTTTTCCT----------------AAT
         **2*222**22**2***2*22*22***22**2222222**22*2*2*22**2222222222222222*2*

left     GCAGTG
chrom    CC--TC
right    CC--TC
         2*22*2
```

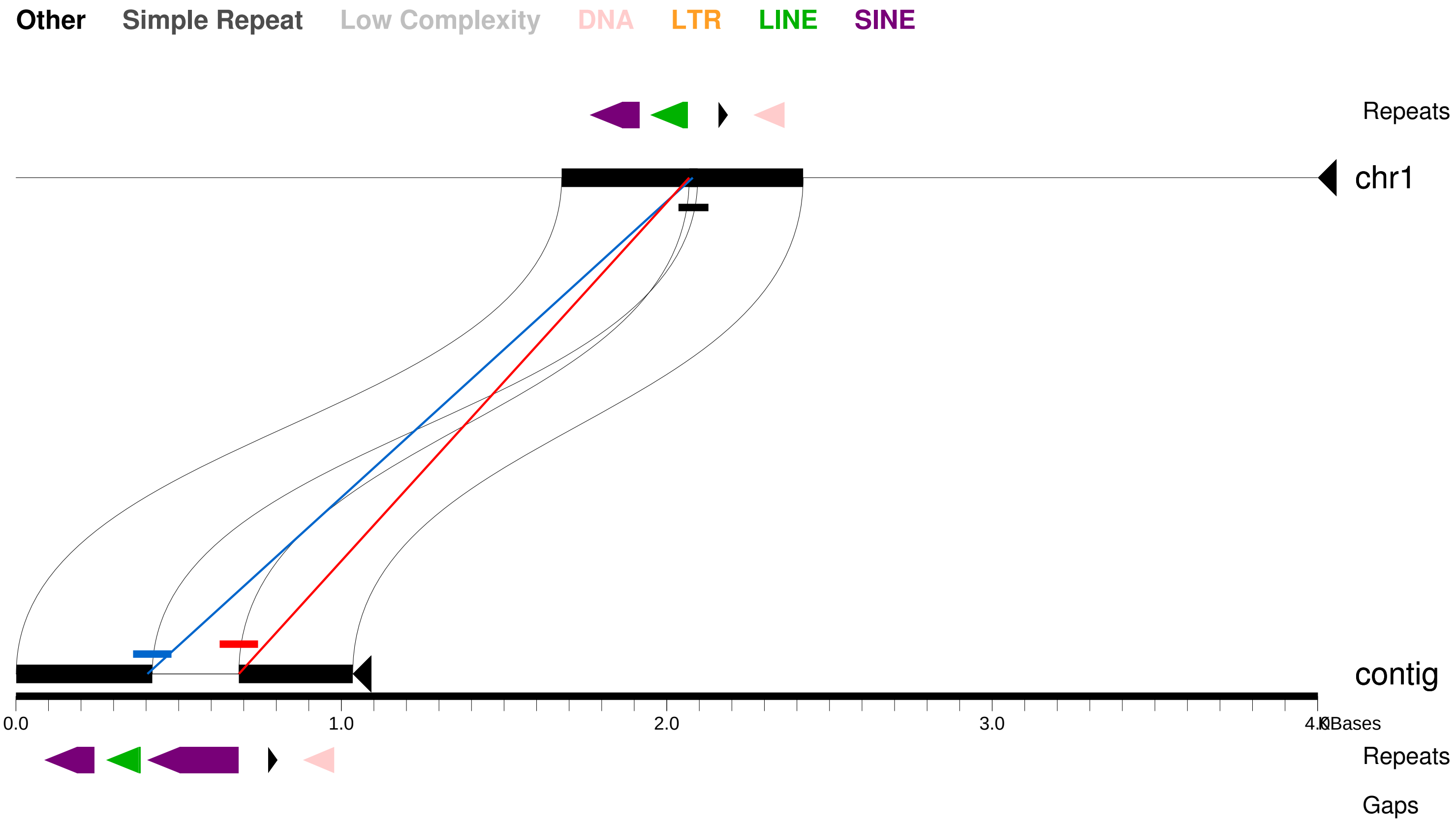

siteID: chr1_198456548 Scafftig1 + Breakpoint: chr1:198456456
chr1:198456331-198456665
Overlap Length: 85 INS size: 380 Genotyped: No

```
left   TTT-------------------------------------------------------------------
chrom  TTTCAACACCCTTTATTTAGAATTCAAGCTTCTAAAGATAAACTTTCTTAAATATTGTTCAAAATGTATA
right  NNNNNNNNNNNNNNNNNNNNNNNNNNNNNNNNNNNNNNNNNNNNNNNNNNNNNNNNNNN-----------
       111NNNNNNNNNNNNNNNNNNNNNNNNNNNNNNNNNNNNNNNNNNNNNNNNNNNNNNNN

left   ---------------------------------AATCTAACAATAGGAAATTACAAAATAATTGATCCAG
chrom  TTTTCCTACTAAAAATTACAGTTCTACTTATTTAATCTAACAATAGGAAATTACAAAATAATTGATCCAG
right  -------------------------------------------------------AAATAATTGATCCAG
                                        1111111111111111111111***************

left   TAGTCATATAGTGAAATACTCATAGTGATTAACAATGTGTTGATGTGCTAAATAAGAAAACAGGATATAA
chrom  TAGTCATATAGTGAAATACTCATAGTGATTAACAATGTGTTGATGTGCTAAATAAGAAAACAGGATATAA
right  TAGTCATATAGTGAAATACTCATAGTGATTAACAATGTGTTGATGTGCTAAATAAGAAAACAGGATATAA
       **********************************************************************

left   GGCCG--GGCGCGGTGGC-TCACGCCTGT--AATCCCAGCACTT------------------TGGG----
chrom  AACTGTAGAAAATATGACATCAATTTTGTTAAATACAAGTACTCACACTAAAGCTGATGAAATGTGTTCA
right  AACTGTAGAAAATATGACATCAATT---------------------------------------------
       22*2*22*222222**2*2***222N111  111N1N11N111N                  11N1

left   ---------AG---------------GCCGAGACGGGCGG---------------
chrom  TATGAAAACAGCAATCATATTTAAGTGGTGATATTGGAAGTAATTTTATTTTCCT
right  -------------------------------------------------------
                11               1NN11N1NN11NN1
```

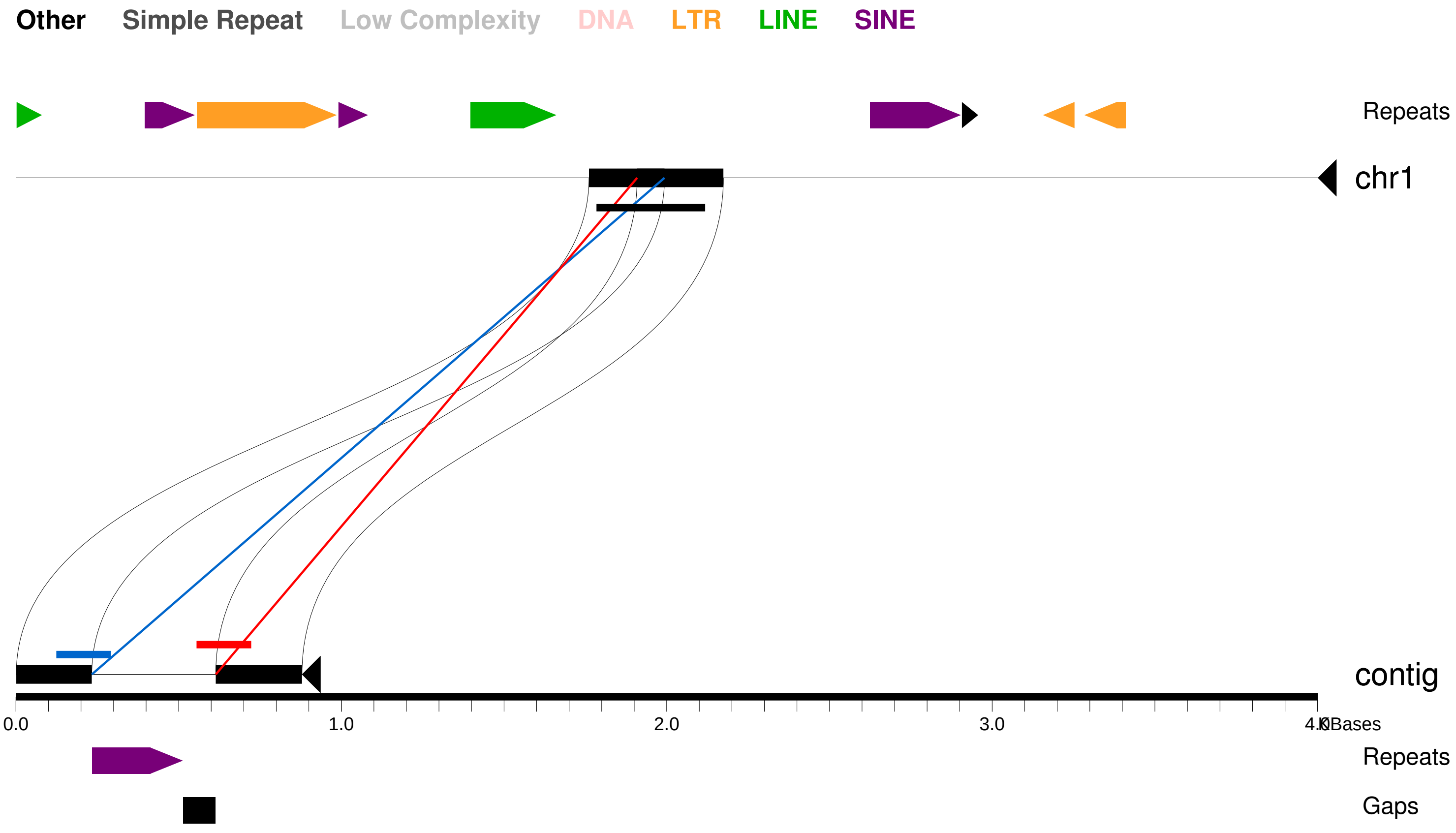

siteID: chr1_204238458 Scafftig6 - Breakpoint: chr1:204238456
chr1:204238415-204238515
Overlap Length: 19 INS size: 300 Genotyped: Yes

```
left     C------AAAGCCTGG-CCCCATGGTGGA-----GTAT------ATCAACCCGGCTCTCAGAAACCTCAT
chrom    C------AAAGCCTGG-CCCCATGGTGGA-----GTAT------ATCAACCCGGCTCTCAGAAACCTCAT
right    CTGCGCTCCAGCCTGGGCGACAGAGCGAAACTCCGTCTCAAAAAAAAAAAAAAAAAAAAAGAAACCTCAT
         *11111111*******1*11**11*1*1*11111**1*111111*11**1111111111***********

left     CTATTGTCGGCCGGGCGCGGTGGCTCACGC-CTGTAATCCCAGCACTTTGGGAGGCCGAGACGGGCGG
chrom    CTATTGTCTTCC--------TACCTCACCTTCTCTAAAACT--CAATACGGCACTCCAA---------
right    CTATTGTCTTCC--------TACCTCACCTTCTCTAAAACT--CAATACGGCACTCCAA---------
         ********22**22222222*22*****222**2***22*222**2*22**2*22**2*222222222
```

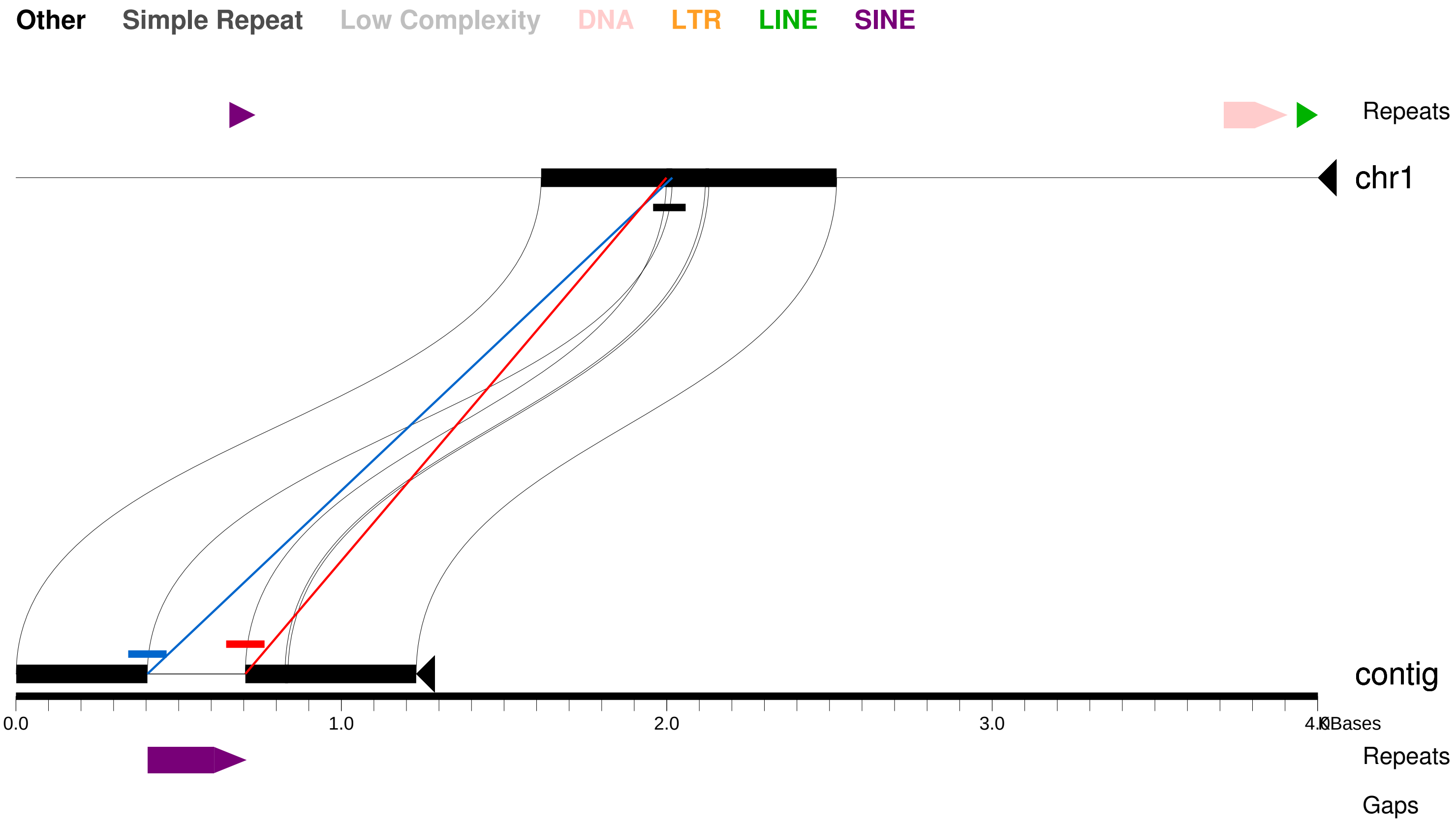

siteID: chr1_204345042 Scafftig2 + Breakpoint: chr1:204345041
chr1:204344987-204345100
Overlap Length: 6 INS size: 419 Genotyped: No

```
left    CCTGTTCCTCAGCCCCC---ACTGGTGCCTTTATTTCCCCAAGCCTCTACTTCCAAG--GCTTCTNNNNN
chrom   CCTGTTCCTCAGCCCCC---ACTGGTGCCTTTATTTCCCCAAGCCTCTACTTCCAAG--GCTTCTTTCAC
right   CGCCCGCCTCGGCCTCCCAAAGTGCTGGGATTACAGGCGTGAGCCACCGCGCCCGGCCCGCTTCTTTCAC
        *11111****1***1**111*1**1**111***1111*111****1*11*11**11111******22222

left    NNNNNNNNNNNNNNNNNNNNNNNNNNNNNNNNNNNNNNNNNNNNNNNNNNNNNN
chrom   TCTTGTCTCTCCTCCAGAATCAGCTCAAATGCTTCCTCCTCTGGGAAGC-----
right   TCTTGTCTCTCCTCCAGAATCAGCTCAAATGCTTCCTCCTCTGGGAAGC-----
        222222222222222222222222222222222222222222222222222222
```

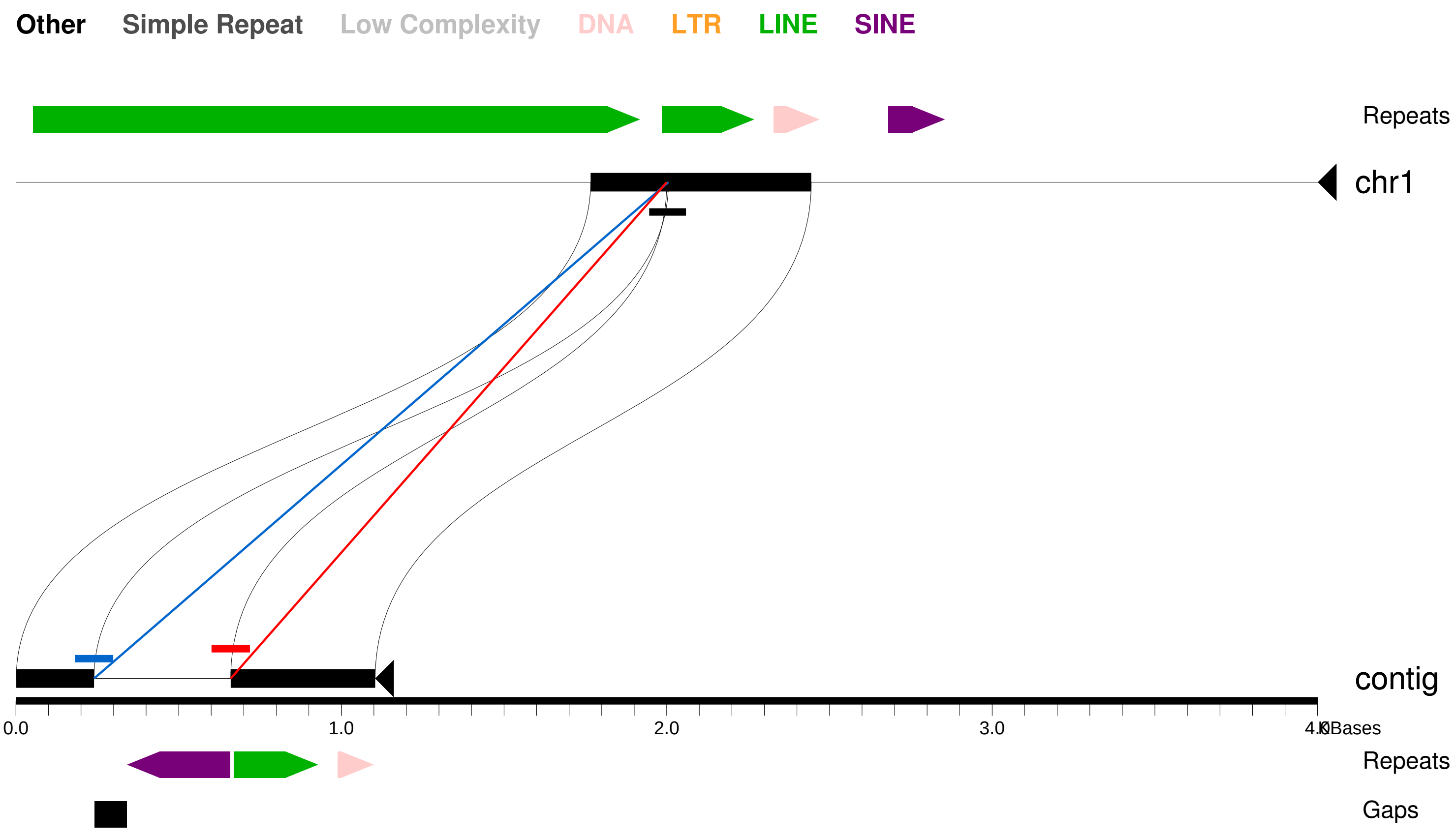

siteID: chr1_207624027 Scafftig1 + Breakpoint: chr1:207624030
chr1:207623870-207624205
Overlap Length: 16 INS size: 323 Genotyped: Yes

```
left   -----A----------------GGGCAGACAAGAATAAGCATGAAAA-GAATCATGAATCTAACTGT-AA
chrom  -----ATTTGGGAAATTTATGAGGGCAGACAAGAATAAGCATGAAAA-GAATCATGAATCTAACTGT-AA
right  CAGCTACTTGGGAGGCTGA----GGCAGG--AGAATG-GCGTGAACCCGGGAGGCGGAGCTTGCAGTGAG
       11111*N222222NNN2N2    1*****111*****11**1****111*111111*1*1**11*1**1*1

left   AATAAATGAAACAAT---ACACGTGCAAGAAGAAAACATGGATGAATTATTGTGTAACCTGGAGAATGGT
chrom  AATAAATGAAACAAT---ACACGTGCAAGAAGAAAACATGGATGAATTATTGTGTAACCTGGAGAATGGT
right  CCGAGATCCCACCACTGCACTCCAGCCTGGGCGACAGAGCGA-GACTCCGTCTCAAAAAAAATAAATAAA
       111*1**111**1*1111**1*11**11*1111*1*1*11**1**1*111*1*11**1111111***111

left   TTT-TAAAACCATGGCTCCTAATCCAGATGCAATAAAAGAAAAGATTGGCC-----GGGCGCGGTGGCTC
chrom  TTT-TAAAACCATGGCTCCTAATCCAGATGCAATAAAAGAAAAGATTTATCAACTTGACTGCATTAAAAC
right  TAAATAAATAAATAAATA--AATAAATA---AATAAAAGAAAAGATTTATCAACTTGACTGCATTAAAAC
       *111****111**111*111***11*1*111***************222*22222*222**22*2222*

left   A-----TGCCTGTAA----------TCCCAG---CACTTTGGGAGGCCGA--GGC-----GGGCGGAT-C
chrom  AGACAATGCTTACAAGGCAAAACGATGCCATAAACAAAGTCAAAAGACAACTGACAAACTGGGAGGAAAC
right  AGACAATGCTTACAAGGCAAAACGATGCCATAAACAAAGTCAAAAGACAACTGACAAACTGGGAGGAAAC
       *22222***2*22**2222222222*2***2222**222*222*2*2*2*22*2*22222***2***22*

left   ACGAGGTCAGGAGATCGAGACCATCCCGGCTAAAACGGTGAAACCCCGTCTCTACTAA---------A
chrom  ATTTGCAAAACACATCCAGAATAACAATCCTAATATA-TATAAATAAGTCTCAAACATTGAGAGAGGA
right  ATTTGCAAAACACA------------------------------------------------------
       *222*222*22*2*11N111NN1N1NNNN1111N1NN21NN11NNNN11111N1NN1N         1
```

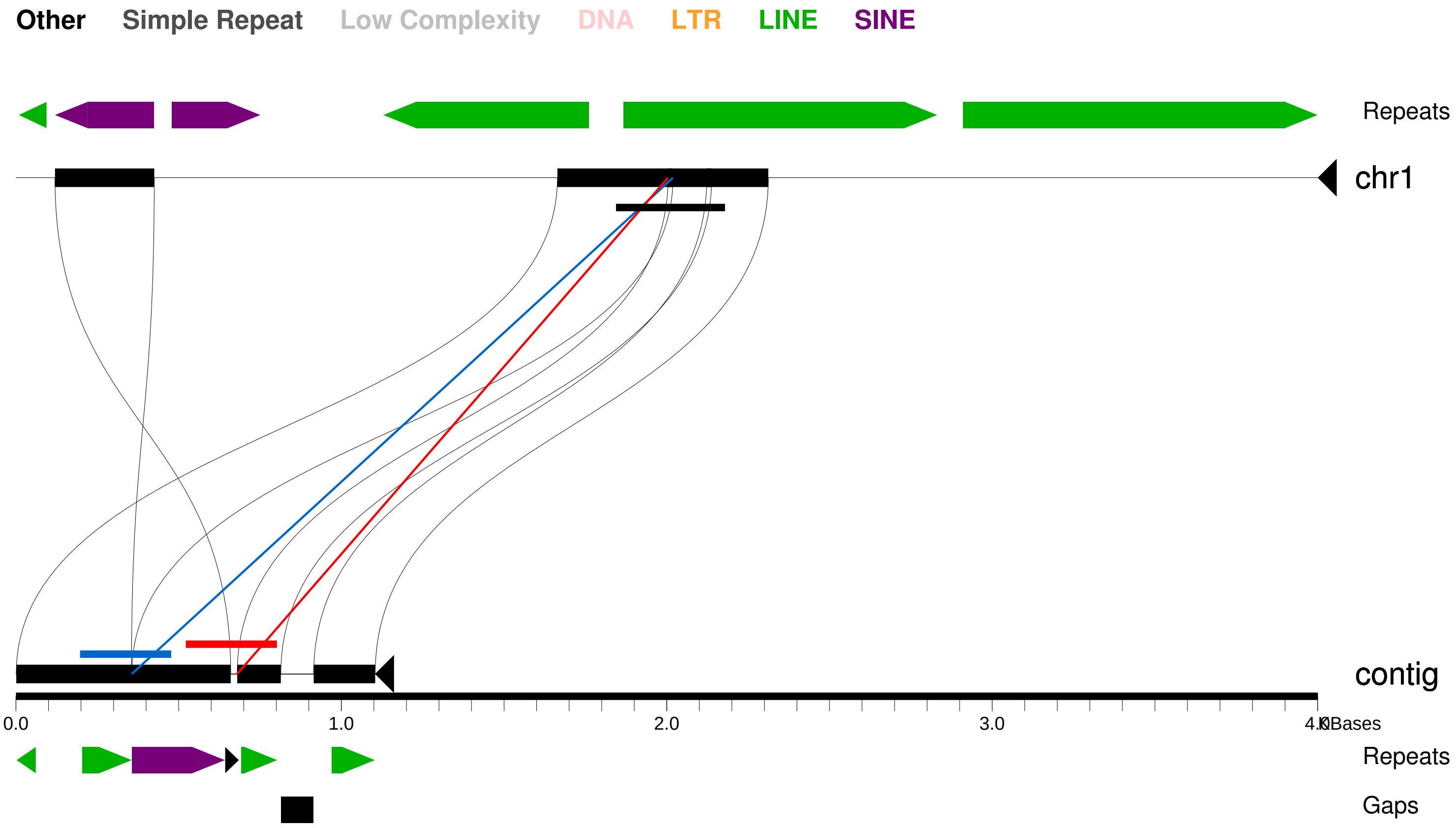

siteID: chr1_208691503 Scafftig2 + Breakpoint: chr1:208691481
chr1:208691439-208691540
Overlap Length: 14 INS size: 303 Genotyped: Yes

```
left     T-GAATGAAT--GAAT---AAACAAATGTTTTAAAAAATGTGGTCTTA-----------CAGATTTTACT
chrom    T-GAATGAAT--GAAT---AAACAAATGTTTTAAAAAATGTGGTCTTA-----------CAGATTTTACT
right    CCGCCCGCCTCGGCCTCCCAAAGTGCTGGGATTACAGGCGTGAGCCACCGCGCCCGGCCCAGATTTTACT
         11*111*11*11*11*111***1111**111*1*1*111***11*11111111111111***********

left     TTTTTTTTTTTTTTTTTTTTTTGAGACGGAGTCTCGCTCTGTTGCCCAGGCTGGAGTGCAGTGGCG-
chrom    TTTATTTG-------------CAAGTCTGATT-TAGTTTTTCTGCTAAGGCA--AATTC--TACCTT
right    TTTATTTG-------------CAAGTCTGATT-TAGTTTTTCTGCTAAGGCA--AATTC--TACCTT
         ***2***2222222222222222**2*2**2*2*2*2*2*22***22****222*2*2*22*22*22
```

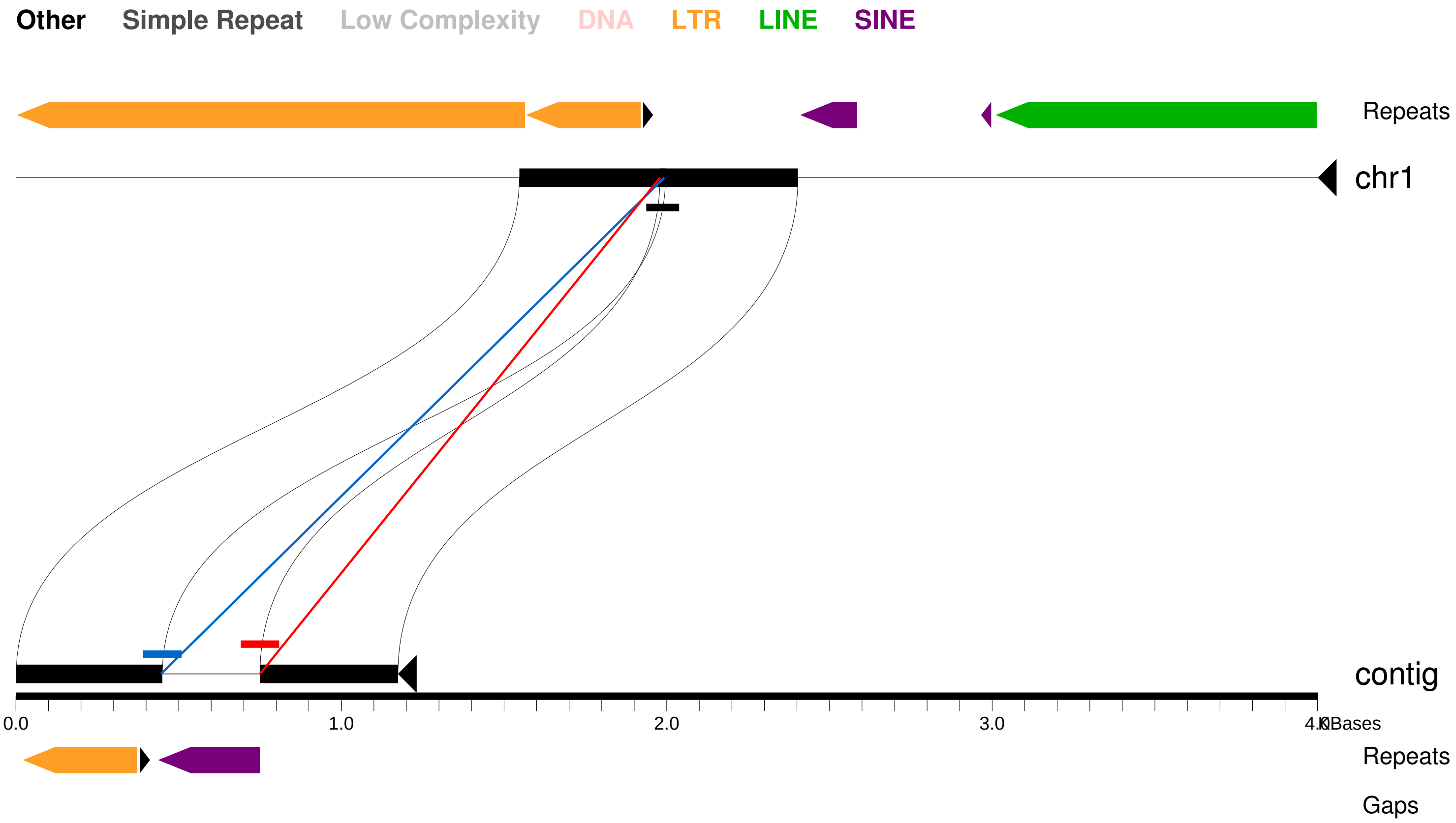

siteID: chr1_212765831 Scafftig8 + Breakpoint: chr1:212765835
chr1:212765800-212765894
Overlap Length: 14 INS size: 305 Genotyped: Yes

```
left    TCA---GGATC---CTAACAAGGTCCACACATTATATTTGG------------------TTGATATGTCT
chrom   TCA---GGATC---CTAACAAGGTCCACACATTATATTTGG------------------TTGATATGTCT
right   CCGCCCGCCTCGGCCTCCCAAAGTGCTGGGATTACAGGCGTGAGCCACCGCGCCCAGCCTTGATATGTCT
        1*1111*11**111**11***1**1*1111****1*111*1111111111111111111***********

left    CTTTTTTTTTTTTTTTTTTTTTTTTTTGAGACGGAGTCTCGCTCTGTCGCCCAGGCTGGAGTGCAGTGGC
chrom   CTTAAGTCTATTTTAACCTACCATATTC--------TTTCTCCTTATTTTTCATGCT-------------
right   CTTAAGTCTATTTTAACCTACCATATTC--------TTTCTCCTTATTTTTCATGCT-------------
        ***222*2*2****2222*2222*2**222222222*2**2*22*2*2222**2***2222222222222

left    GCG
chrom   ---
right   ---
        222
```

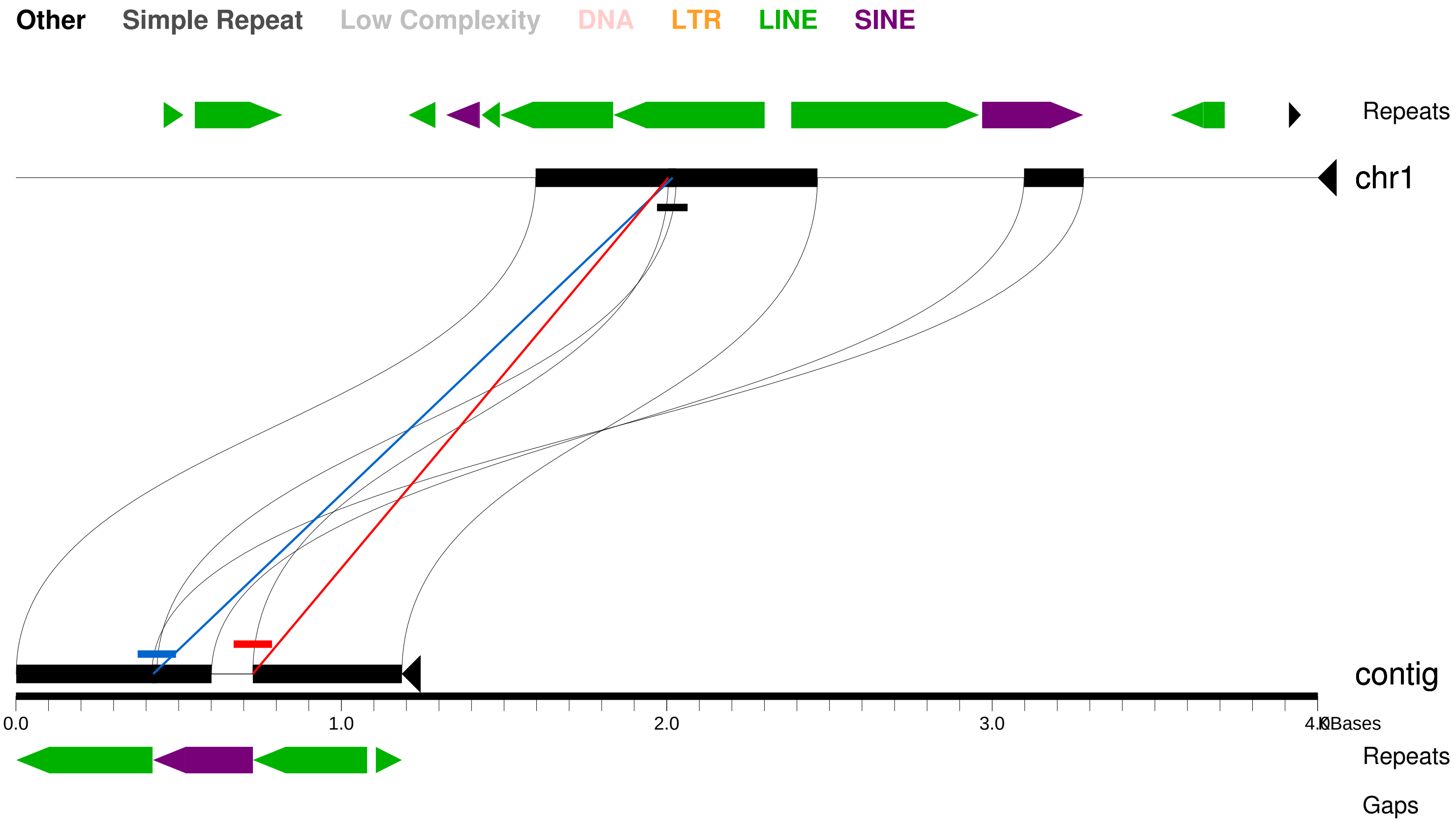

siteID: chr1_214911000 Scafftig2 - Breakpoint: chr1:214911000
chr1:214910954-214911048
Overlap Length: 14 INS size: 296 Genotyped: Yes

```
left    AGACATTA-------ACTT---TGCAGC-TG------AGGGTGAATGG-------AAGCAAAAATATTTT
chrom   AGACATTA-------ACTT---TGCAGC-TG------AGGGTGAATGG-------AAGCAAAAATATTTT
right   AGTCGAGATCGCGCCACTGCACTCCAGCCTGGGCGACAGAGCGAAACTCCGTCTCAAAAAAAAAAAAAAA
        **1*111*1111111***1111*1****1**111111**1*1***1111111111**11*****1*1111

left    GAAAAAACTTAAATTGGCCGGGTGCGGTGGCTCACGCCTGTAATCCCAGCACTTTGGGAGGCCGAGACGG
chrom   GAAAAAACTTAAATCA---------CTTTAGCA-ACTTCAACATTACAATTATTGT---------ATA---
right   GAAAAAACTTAAATCA---------CTTTAGCA-ACTTCAACATTACAATTATTGT---------ATA---
        *************2222222222*22*2**22**22*222*2*2*2*22*2*2*222222222*2*222

left    GCG
chrom   ---
right   ---
        222
```

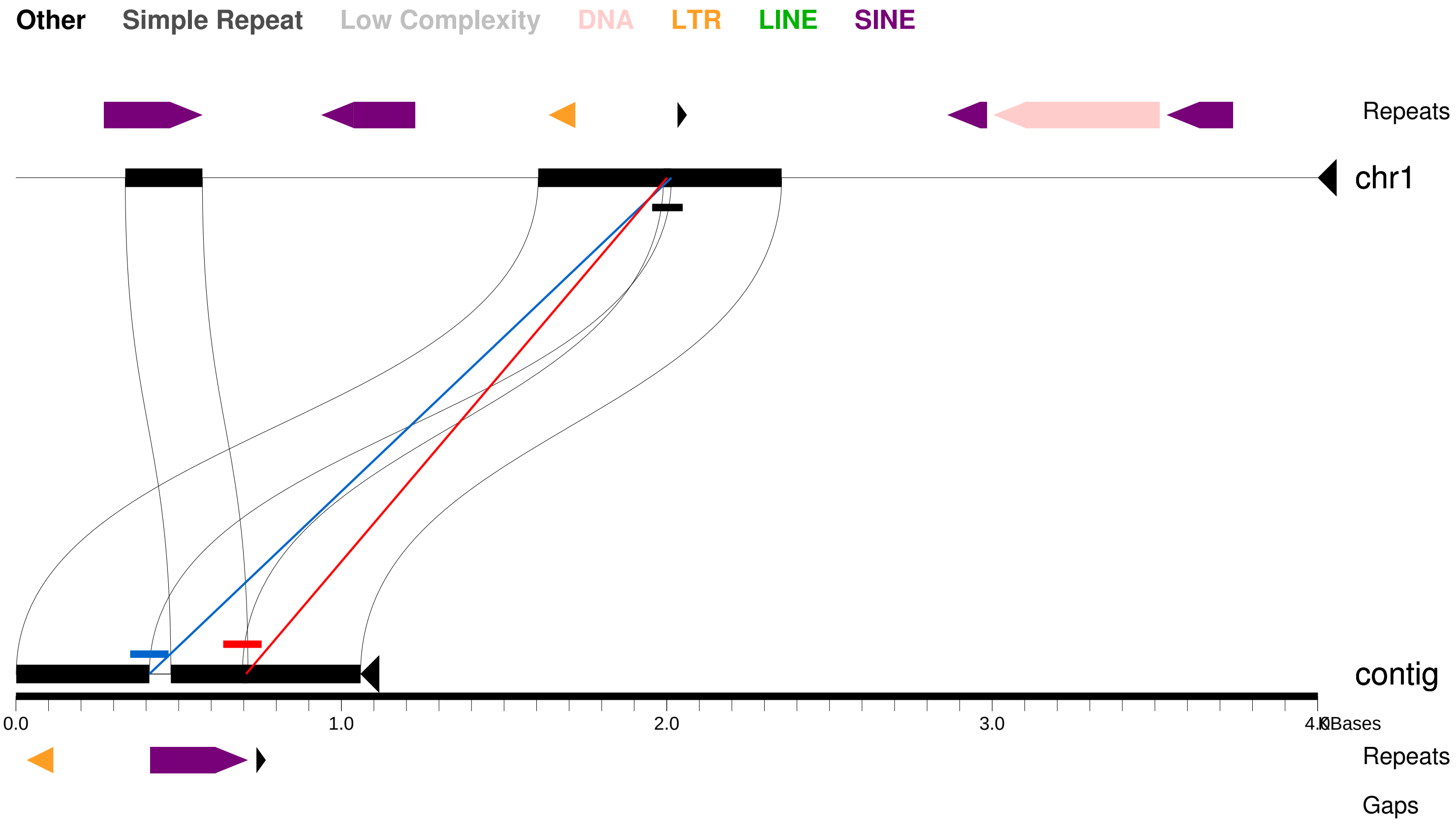

siteID: chr1_215285298 Scafftig1 + Breakpoint: chr1:215285275
chr1:215285135-215285484
Overlap Length: 70 INS size: 438 Genotyped: No

```
left    T---------------------------------------------------------------------
chrom   TTATGTGCCTTGTAACTTTTATGATTGGATCTACGCATGTGAAATAAAGAGTCACCTCTGTAAATCTCTA
right   TGATCTGCC-------------------------------------------CGCCTCGGCC--TCCCAA
        *N22N2222                                           2N2222N2NN  22N2N2

left    -------------------------------CACCAATCAGCCCTAATCAGCCTGCCTAGAGATTCTGTG
chrom   AGGACTGGATTCATGTGGGGAAATACCTAATCACCAATCAGCCCTAATCAGCCTGCCTAGAGATTCTGTG
right   AGTGCTGGGATTACAGGCG------------------TGAGCC------ACCGCGCCC------------
        22NN2222NN2N2NNN2N2            111111*1****111111*1*11***1111111111111

left    GGCCTCAAATCTTTTTTATAGTTGTGCGTTCTCTGGACTTGTGCCTGTAAGTTACCAAGTACAGGGATTT
chrom   GGCCTCAAATCTTTTTTATAGTTGTGCGTTCTCTGGACTTGTGCCTGTAAGTTACCAAGTACAGGGATTT
right   GGCCTCAAATCTTTTTTATAGTTGTGCGTTCTCTGGACTTGTGCCTGTAAGTTACCAAGTACAGGGATTT
        **********************************************************************

left    NNNNNNNNNNNNNNNNNNNNNNNNNNNNNNNNNNNNNNNNNNNNNNNNNNNNNNNNNNN-----------
chrom   ACTCTTTTCTTTTTCAGGGGCTTGTAATCTCTTGCTCTCTCTGGAGCTACCACATGCTGCTCAGCTCTTC
right   ACTCTTTTCTTTTTCAGGGGCTTGTAATCTCTTGCTCTCT------------------------------
        2222222222222222222222222222222222222222NNNNNNNNNNNNNNNNNNN

left    ----------------------------------------------------------------------
chrom   TTTGTTCTCAGTGGCTCCCAGACTTCTAGAGCGTGTTAGGTCCTGTTAATGCTGTTAGCTGGACAAGACA
right   ----------------------------------------------------------------------
```

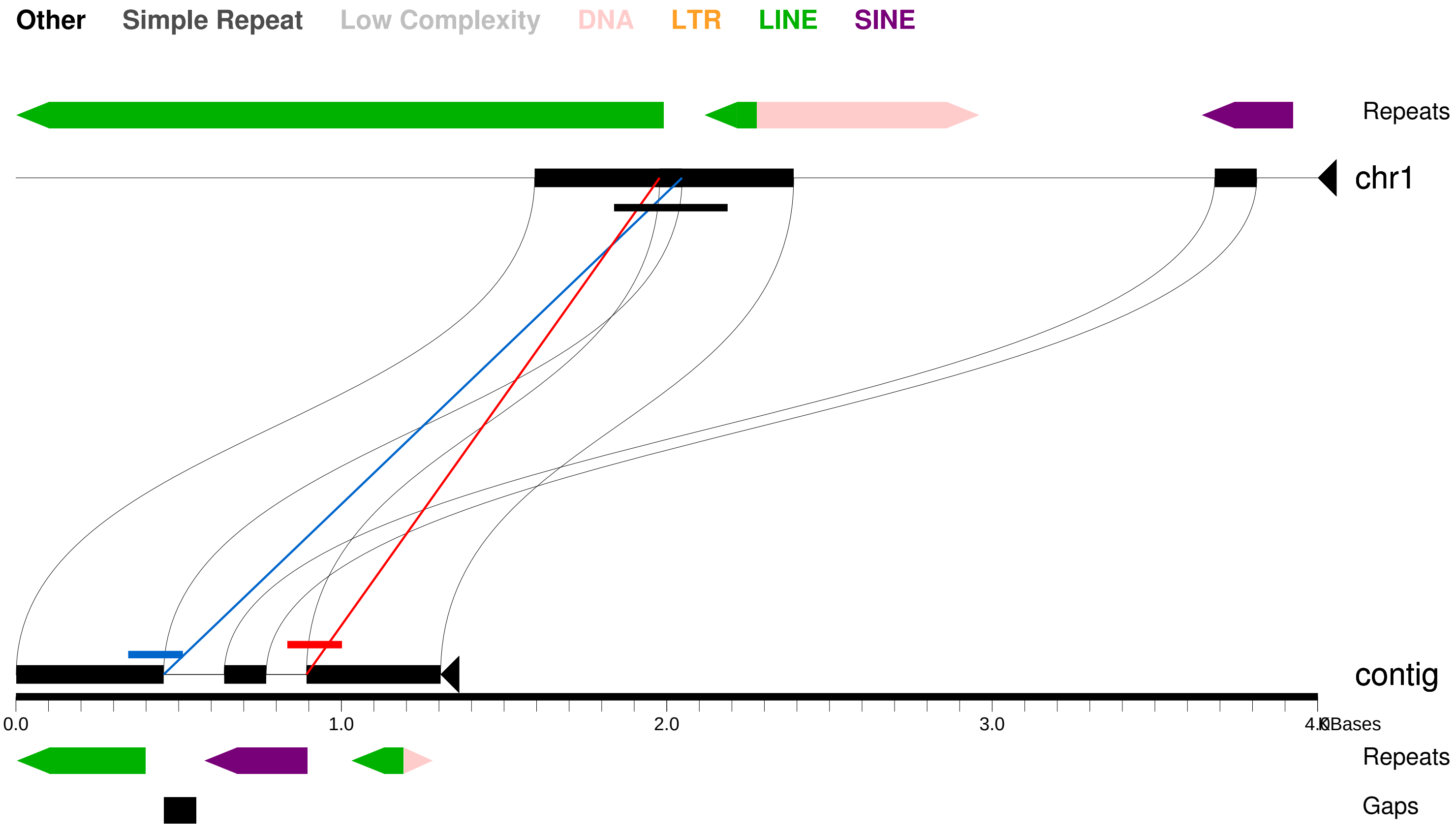

siteID: chr1_216438549 Scafftig1 + Breakpoint: chr1:216438530
chr1:216438472-216438588
Overlap Length: 2 INS size: 419 Genotyped: No

```
left     CTAGAATATATAAGAAAATCAACTCAATGGCCCCAAATAATCCAATTAAAACAGGTG--CAA--GGCCGG
chrom    CTAGAATATATAAGAAAATCAACTCAATGGCCCCAAATAATCCAATTAAAACAGGTG--CAAATGGTCTG
right    NNNNNNNNNNNNNNNNNNNNNNNNNNNNNNNNNNNNNNNNNNNNNNNNNNNNNNNNNNNNNNAAATGGTCTG
         1111111111111111111111111111111111111111111111111111111111**22**2*2*

left     GCGCGGTGGCTCACGCCTGTAATCCCAGCACTTTGGGAGGCCGAGACGGGC--GG
chrom    GACAGACATTTCTCAAAAGAAGACATACAAAT------AGCCAACAAGTACACGA
right    GACAGACATTTCTCAAAAGAAGACATACAAAT------AGCCAACAAGTACACGA
         *222*22222**2*2222*2*22*22*22*2*2222222***2*2*2*22*22*2
```

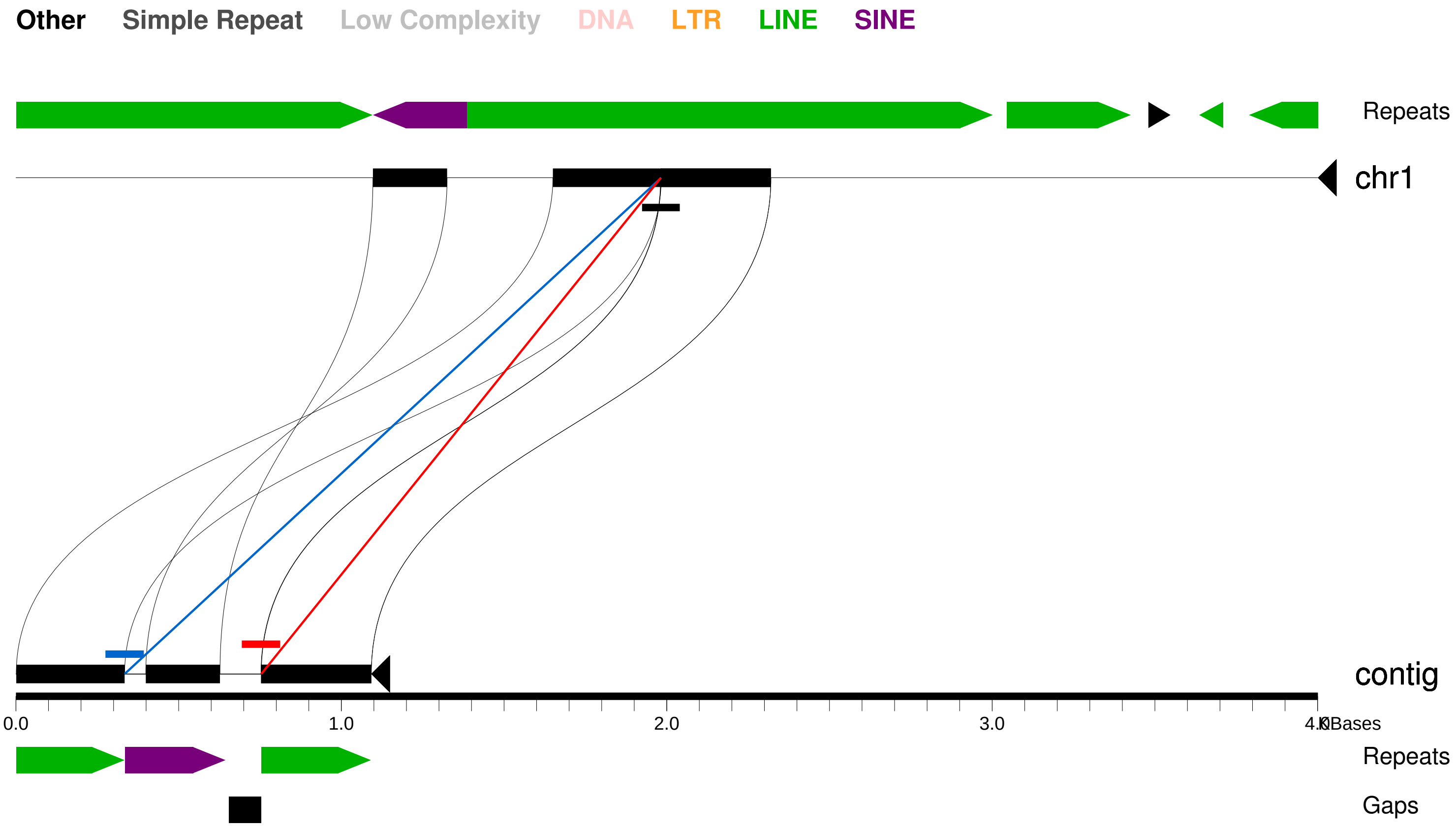

siteID: chr1_217913502 Scafftig1 - Breakpoint: chr1:217913502
chr1:217913400-217913723
Overlap Length: 62 INS size: 431 Genotyped: No

```
left    -----------------------------T-TTTGATTTTGTAT--CCAAGTTCTATGTACATCTT--TG
chrom   CTGTTTTTACTACTTAAAATAACTTACACT-TTTGATTTTGTAT--CCAAGTTCTATGTACATCTT--TG
right   -----TTTAGTAG--AGACGGGGTTTCACTGTGTTAGCCAGGATGGTCTCGATCTCCTGACCTCGTGATC
             2222N22N  2N2NNNNN22N222*1*1*1*1111*1**111*11*1***1111**1**1*11*1

left    TACCTTACCCG--TTCATATATGTTTTCAATTATA----TTAG----------------GCCTTTCTTCT
chrom   TACCTTACCCG--TTCATATATGTTTTCAATTATA----TTAG----------------GCCTTTCTTCT
right   CGCCCGCCTCGGCCTCCCAAAGTGCTGGGATTACAGGCGTGAGCCACCGCGCCCGGCCGGCCTTTCTTCT
        11**111*1**111**11*1*1111*111****1*1111*1**1111111111111111***********

left    TATATAAGACCTTTTCATATTTATTAAATTATTTATAGAGCAGAAACTTCTNNNNNNNNNNN--------
chrom   TATATAAGACCTTTTCATATTTATTAAATTATTTATAGAGCAGAAACTTCTTTATAGATTTTTATTTTT
right   TATATAAGACCTTTTCATATTTATTAAATTATTTATAGAGCAGAAACTTCTTTATAGATTTTTATTTTT
        ***************************************************2222222222222222222

left    ----------------------------------------------------------------------
chrom   ATGATTGCTTAGAAAATTACTGTACTTATATTTAGCATAATATGAAATTGGATACATGCATTTCTGAGTA
right   ATGATTGCTTAGAAAATTACTGTACTTATATTTAGCATAATATGAAATTGGATACATGCATTTCTGAGTA
        2222222222222222222222222222222222222222222222222222222222222222222222

left    ----------------------------------------------------------------------
chrom   ATTATCTTTATAGTGGTAGTTCTCTGGTTAATTTTTATTTTTTTCATAATATCAATAGTAAAGATTAAAA
right   ATTATCTT--------------------------------------------------------------
        22222222

left    -
chrom   T
right   -
```

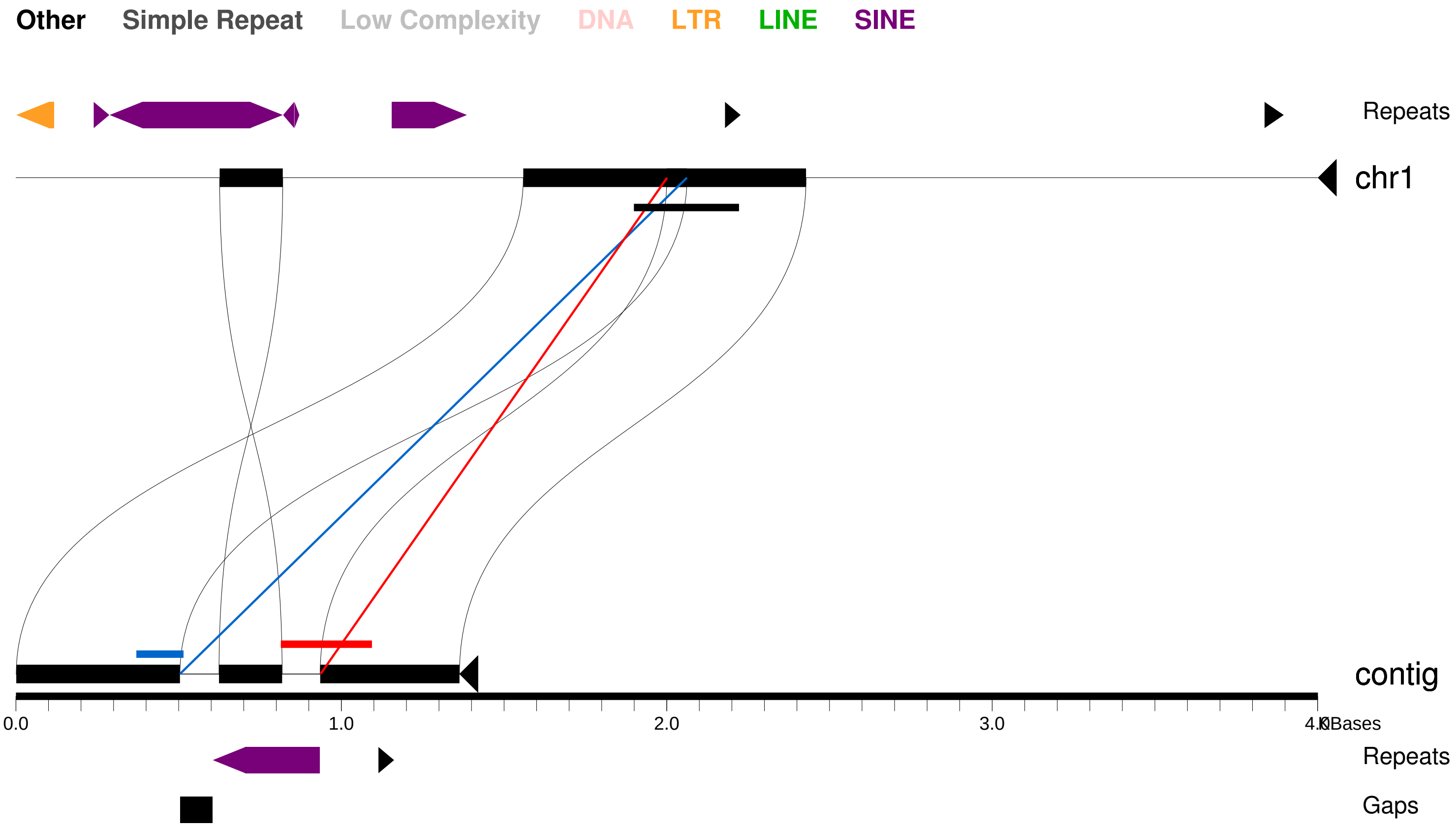

siteID: chr1_218001848 Scafftig3 + Breakpoint: chr1:218001826
chr1:218001666-218002003
Overlap Length: 18 INS size: 309 Genotyped: No

```
left    ----------------------------------------------CCAAATGCAAAAGAATGAAATTGG
chrom   GGGAACTGATAGTCTCTTTGACAAATGATGCAAGGAAACTGGATTTCCAAATGCAAAAGAATGAAATTGG
right   TTTAGTAGAGACGGGGTTTCACCGTTTTAGCCGGGA---TGG-TCTCGATCTCC-------TGACCTCG-
        NNN2NNN22N2NNNNN222N22NNN2NNN22NN222   222 2N2*1*11*1*1111111***11*1*1

left    ATGCTATTTTATACCATACATAAAAACCAACTTAAAATGGATCAAAGACCTAAATGTAACATCTGAATTT
chrom   ATGCTATTTTTTACCATACATAAAAACCAACTTAAAATGGATCAAAGACCTAAATGTAACATCTGAATTT
right   -TGAT--------CCGCCCGCCTCGGCCTCC-----------CAAAGTGCTGGGATTA----CAGGCGTG
        1**1*11111N11**111*1111111**11*11111111111*****11**11111**1111*1*111*1

left    ATACAAATCCTAGAAGAGGATATAGGTAGAACTTCTTT--------------------TTTNNNNNNNNN
chrom   ATACAAATCCTAGAAGAGGATATAGGTAGAACTTCTTTGATATTGACATTGGAAATGATTTTTTGAATAT
right   AGCCA---CCGCGCCCGGCCTATAGGTAGAACTTCTTTGATATTGACATTGGAAATGATTTTTTGAATAT
        *11**111**11*1111*11*****************22222222222222222222***222222222

left    NN--------------------------------------------------------------------
chrom   CACACCAAAAAAAAAA--GGCTACCAAAACAAAAATAAATAAAATGGGACTAAAAAGCTTCAAACTAAAC
right   CACACCAAAAAAAAAAAAGGCTACCAAAACAAAAATAAATAAAATGGGACTAAAAAGCTTCAAACTAAAC
        2222222222222222112222222222222222222222222222222222222222222222222222

left    ------------------------------------------------------------
chrom   TAAATCTAAACTAAAAAGCTTCTGCACAGCAAAGGAAACAATCAACAACATGAAAAGGCA
right   TAAATCTAAACTAAAAAGCTTCTGCACAGCAAAGGAAACA--------------------
        2222222222222222222222222222222222222222
```

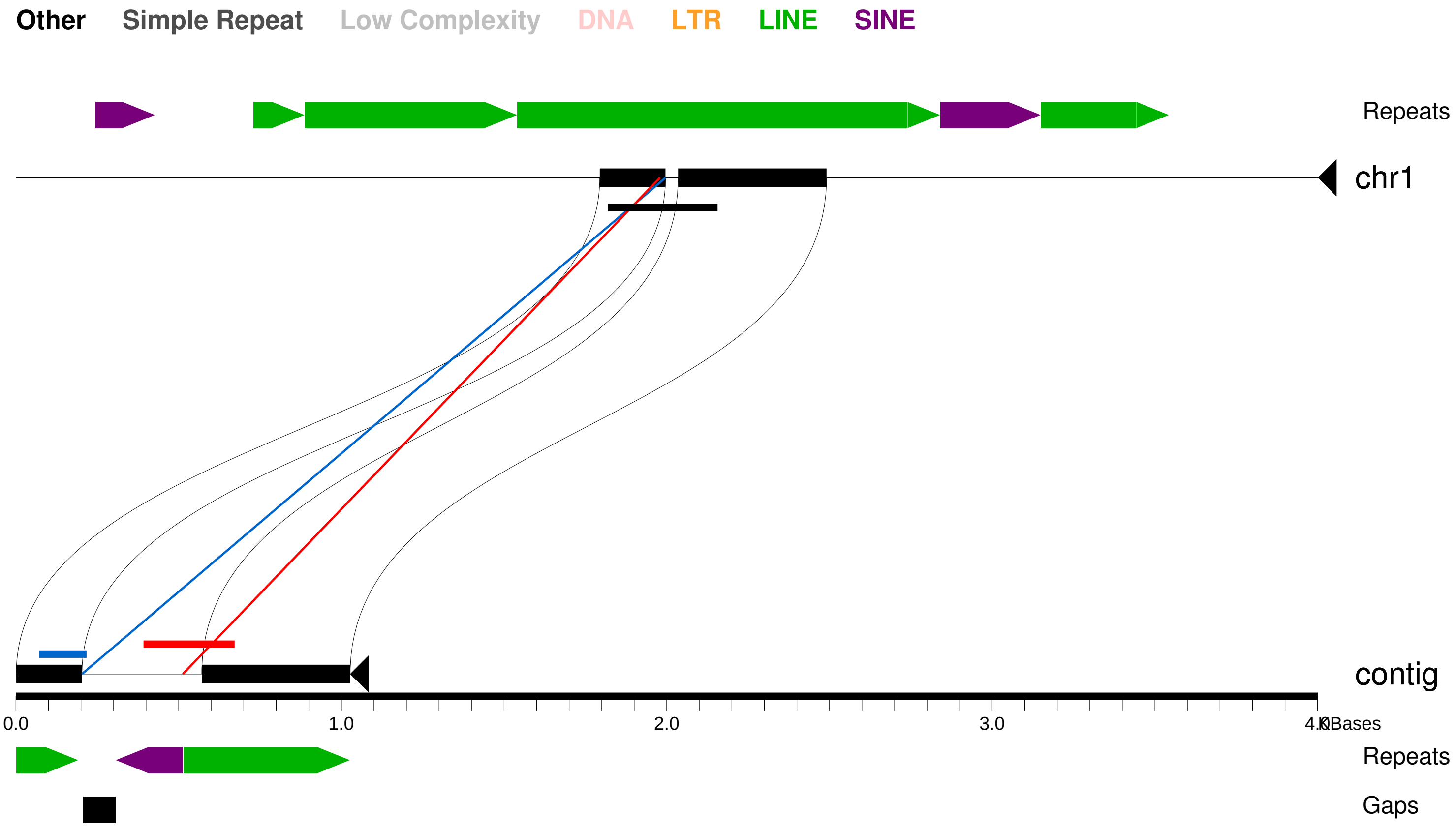

siteID: chr1_219574694 Scafftig1 + Breakpoint: chr1:219574680
chr1:219574633-219574737
Overlap Length: 13 INS size: 267 Genotyped: Yes

```
left    ------AT--TCTTAATAATGT----TTATAAGCCCCATGCTAGGTAGTAGCCTAATGC--CTTTTTTTT
chrom   ------AT--TCTTAATAATGT----TTATAAGCCCCATGCTAGGTAGTAGCCTAATGC--CTTTTCTTT
right   GCCGGGATGGTCTCGATCTCCTGACCTCGTGATCCGCCCGCCTCGGCCTCCCAAAGTGCGCCTTTTCTTT
        111111**11***11**1111*1111*11*1*1**1*11**111*111*11*11*1***11*****2***

left    TTTTTTTTTTTTTTTTTTTTTTGAGACGGAGTCTCGCTCTGTCGCCCAGGCTGGAGTGCAGTGG
chrom   TTTTAATTGCCACTTA------------ACCCTGCCTCCTTCTTGCAA--TAAAACATATTCA
right   TTTTAATTGCCACTTA------------ACCCTGCCTCCTTCTTGCAA--TAAAACATATTCA
        ****22**22222**2222222222222*22**22***22**222**222*22*2222*2*22
```

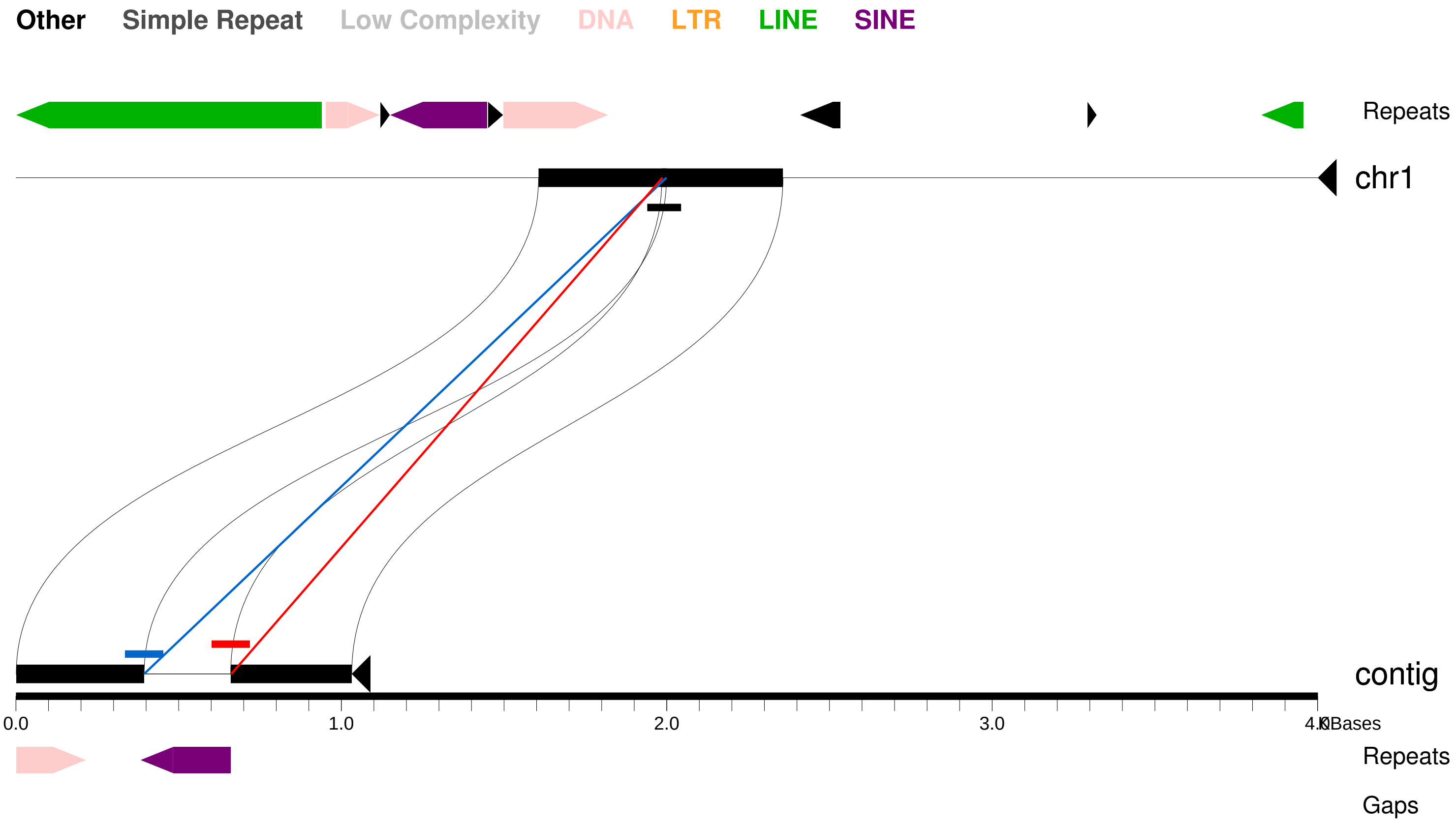

siteID: chr1_220119575 Scafftig1 + Breakpoint: chr1:220119531
chr1:220119378-220119703
Overlap Length: 10 INS size: 346 Genotyped: No

```
left   ----------------------------------------ATAAGTTCAAAGATATTTAATTTTGAAATT
chrom  --TAATAAACAGTGGTTTTGATTTCTTTGGCTATTAATACATAAGTTCAAAGATATTTAATTTTGAAATT
right  TTTAGTAGAGACGAGGTTTCACCGTTTTAGCCGGGA---------------------TGGTCTCGA----
       1122N22N2N2NNN2N222N2NNNN222N22NNNN2    11111111111111111*11*1*1**1111

left   TAATTTTCTGTTATAATTTGATAATAGGAAGGGATTCATATTGCTAAAATTTGGGGCATTTTTATATTGC
chrom  TAATTTTCTGTTATAATTTGATAATAGGAAGGGATTCATATTGCTAAAATTTGGGGCATTTTTATATTGC
right  ---TCTCCTG-----ACCTCGTGATCCGCCCGCCT-CGGCCTCCCAAAGTGCTGGGATTACAGGCGTGAG
       111*1*1***11111*11*11*1**11*111*11*1*1111*1*1***1*111***11*1111111*111

left   TGACATTGAATTGTTAGCATATCTTT----------TTT-------------------------------
chrom  TGACATTGAATTGTTAGCATATCTTGATACTATCACTTTGTGTCACTATATAATTGCCAATGAGCAATAT
right  CCACCGCGCCCGGCCAGCATATCTTGATACTATCACTTTGTGTCACTATATAATTGCCAATGAGCAATAT
       11**111*1111*11**********22222222222***2222222222222222222222222222222

left   -------------------------------------------------------TTTTNNNNNNNNNNN
chrom  TGAACATTTGGGGGCCTCAGAGTTAATTTAACTAGTATACTTACTCTTCTAACCCTTTTCAGTCATTGAG
right  TGAACATTTGGGGGCCTCAGAGTTAATTTAACTAGTATACTTACTCTTCTAACCCTTTTCAGTCATTGAG
       2222222222222222222222222222222222222222222222222222222****22222222222

left   ------------------------------------------------
chrom  GGTATCTAAATTGCCAAACCTGTTCCAAAGGATGAAATCCAAAGGAAG
right  GGTATCTAAATTGCCAAACCTGTTCCAAAGGATGA-------------
       22222222222222222222222222222222222
```

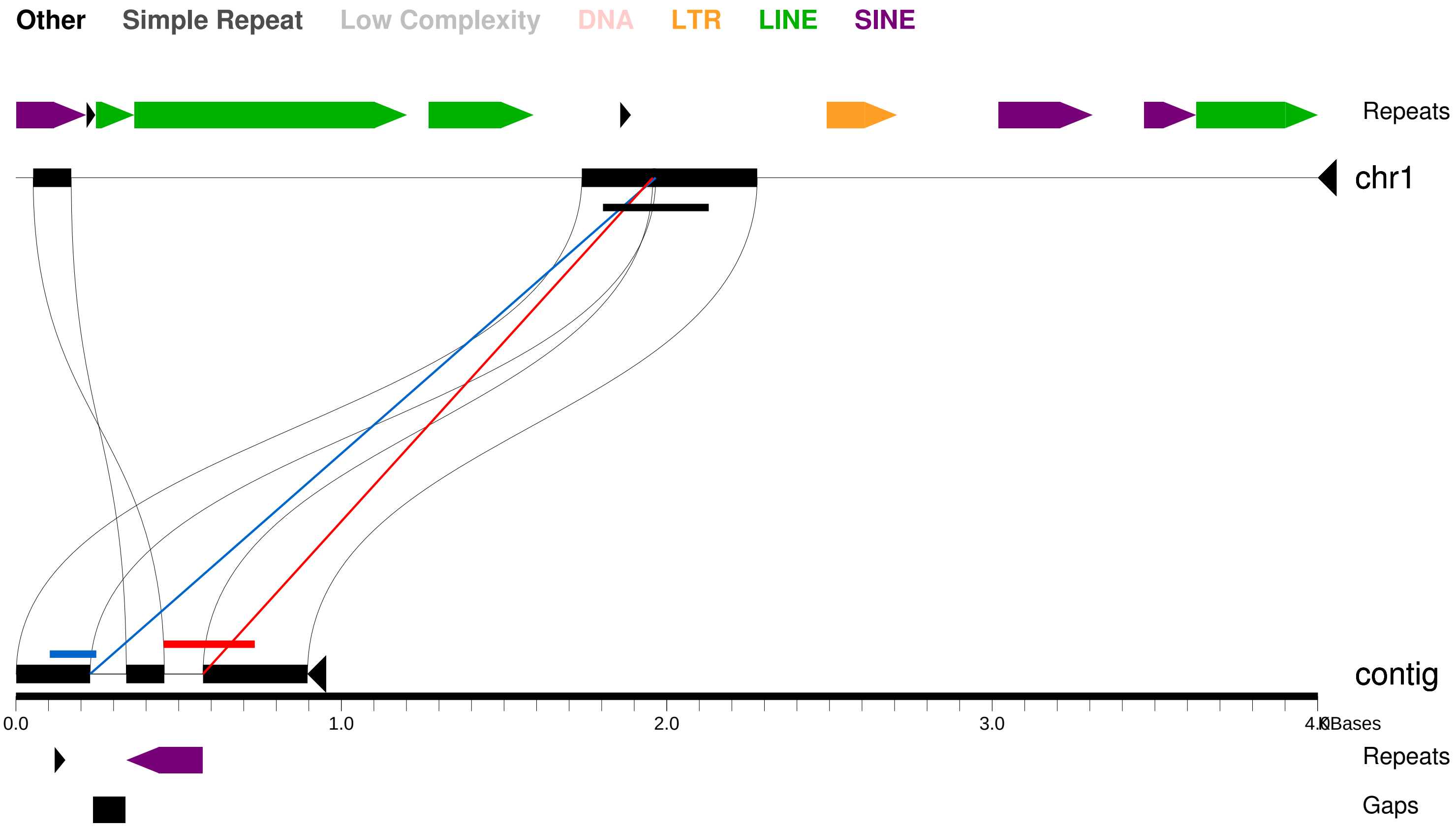

siteID: chr1_221969147 Scafftig1 + Breakpoint: chr1:221969156
chr1:221968996-221969331
Overlap Length: 16 INS size: 316 Genotyped: Yes

```
left   A----------GGCAGAGTCAGGACCCAAACCC--AAGCTCCCAGACCCCAAGCCCATATTCTCTGCATG
chrom  ACAGCTGGAAAGGCAGAGTCAGGACCCAAACCC--AAGCTCCCAGACCCCAAGCCCATATTCTCTGCATG
right  TTTAGTAGAGACGGGGTTTCACCGTGTTAGCCAGGATGGTCTC-GATCTCCTGACCTCGTGATCCGCCCG
       1NNNN2N22N21*11*11***1111111*1**111*1*1**1*1**1*1*11*1**111*11**1**11*

left   GCACAGTACTATCCTTCCCCTAACATATTGTCCCTGATGTAAGATGTCTGTCGTATCAAAACTACTGTCA
chrom  GCACAGTACTATCCTTCCCCTAACATATTGTCCCTGATGTAAGATGTCTGTCGTATCAAAACTACTGTCA
right  CCTCGG------------CCTCCCAAAGTG---CTGG-----GATTACAGGCGTG----AGCCACCGCGC
       1*1*1*11111111111***11**1*1**111***111111***11*1*1***11111*1*1**1*111

left   TTGTTATAGAAGTTAAAGTAGAAAGAAATTCATTTTTA--------TTTT--------TATTTT--TTTT
chrom  TTGTTATAGAAGTTAAAGTAGAAAGAAATTCATTTTTAGAATAAAGTTATACAAACTGTATTTTGGTTCT
right  CCGGCC----------------AAGAAATTCATTTTTAGAATAAAGTTATACAAACTGTATTTTGGTTCT
       11*1111111111111111111***************22222222**2*22222222******22**2*

left   TTTTATTTT-------------TTTTTTTTTTTTGAGACGGAGTCTCGCTCTGTCGCCCAGGCTGGAG--
chrom  TTGTACTTCCCGGAAAAAAAAATTATGTTTTATTAAGTC--ACCTTTGCCTTGTCAAAATATTAAGACAC
right  TTGTACTTCCCGGAAAAAAAAATTATGTTTTATTAAGTC--ACCTTTGCCTTGTCAAAATATTAAGACAC
       **2**2**22222222222222**2*2****2**2**2*22*222*2**22****2222222222**222

left   -TGCAGTGGCGCGATCTCG-----GCTCACTGCA--------AGCTCCGCCTCCCGGGTT
chrom  TTTAAACACCCCTCTCTCCCCCTTGCTCCCTTCCCTGCCACTAACTCTATGACCCCAACC
right  TTTAAACACCCCTCTCTCCCCCTTGCTCCCTTCCCTGCCNNNNNN---------------
       2*22*2222*2*22****222222****2**2*222222NNN1N111NNNNN111NNNNN
```

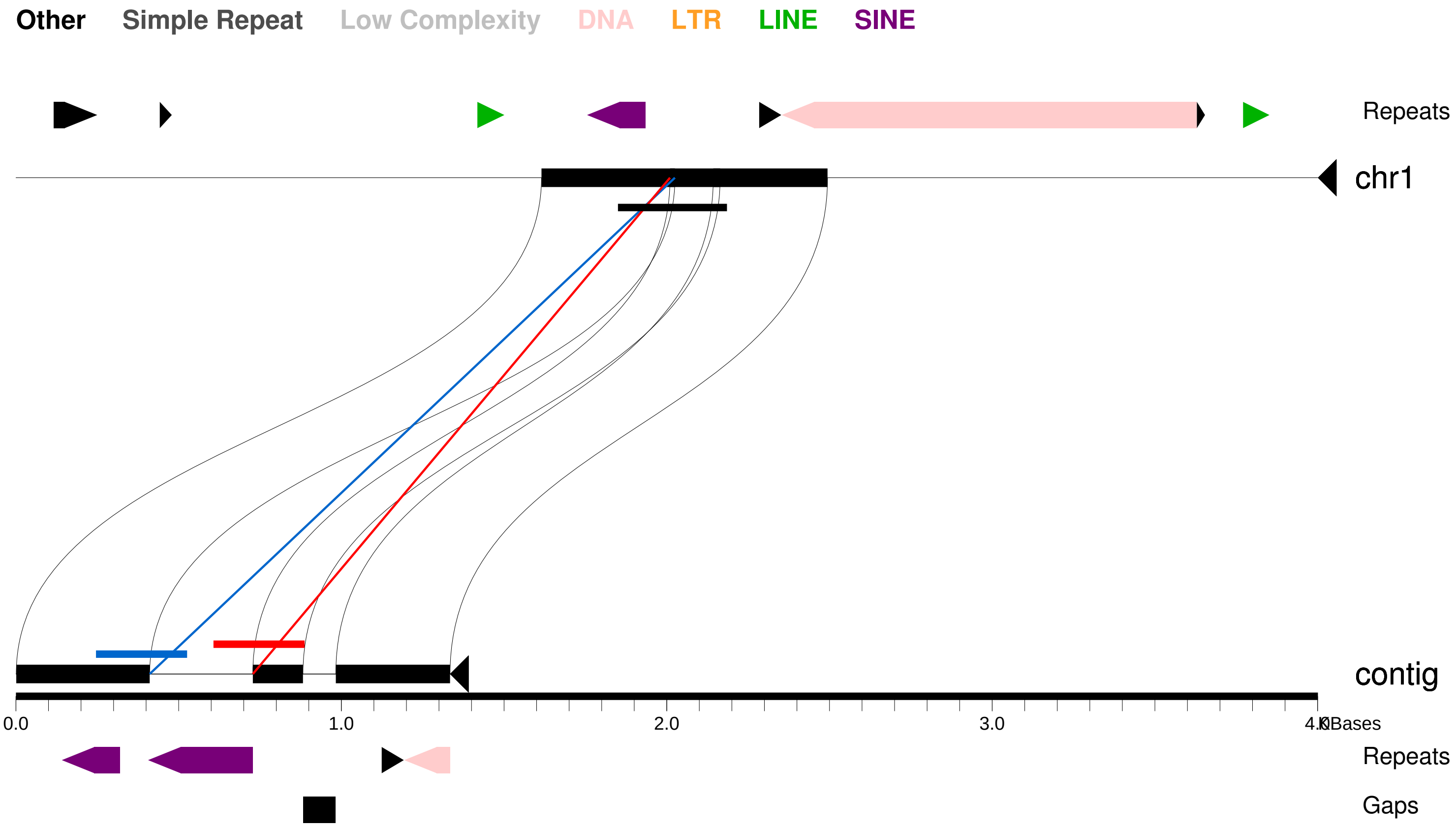

siteID: chr1_223911582 Scafftig3 + Breakpoint: chr1:223911480
chr1:223911414-223911882
Overlap Length: 95 INS size: 430 Genotyped: No

```
left     -TTGGAGGCCCCTGTCCCACCCTGGCTGCCTCAGCTTTGGCCATGGTACCCACGGTCTCCAGCTACATCA
chrom    CTTGGAGGCCCCTGTCCCACCCTGGCTGCCTCAGCTTTGGCCATGGTACCCACGGTCTCCAGCTACATCA
right    NNNNNNNNNNNC-------------------------------------------------------ATCA
         N11111111111*1111111111111111111111111111111111111111111111111111111****

left     GATGCCTGAAGAACAAATCTCTCCTTAGCTCTCCCTGCCCCCTCTCCAGGAATAGGAGCAGTGGGACCCC
chrom    GATGCCTGAAGAACAAATCTCTCCTTAGCTCTCCCTGCCCCCTCTCCAGGAATAGGAGCAGTGGGACCCC
right    GATGCCTGAAGAACAAATCTCTCCTTAGCTCTCCCTGCCCCCTCTCCAGGAATAGGAGCAGTGGGACCCC
         **********************************************************************

left     CTTCAAGAATCCCCCATTTCCG-----------------GCC---GGGCGCG----GTGG----------
chrom    CTTCAAGAATCCCCCATTTCCCATATACTTTCCTGCAAAGCCACAGGGCGTGCAAAGTGGGATTCCAGGG
right    CTTCAAGAATCCCCCATTTCCCATATACTTTCCTGCAAAGCCACAGGGC---------------------
         ********************2222222222222222222***222****1N1    1111

left     CTCACGCCTGTAATCCCAGCACTTTGGGAGGCCGAGGCG----------------GGTG--------GAT
chrom    CTCCAGCCTGCTCCCCATGGGCCTCTGGATCCAGGGGTGACTCTTTGCCTGTTTGGGTGCCTCTTTAGAT
right    ----------------------------------------------------------------------
         111NN11111NNNN11NN1NN1N1NN111NN1N1N11N1                1111        111

left     -------------CATGAGGTCAGGAGATCGAGACCA---------------------------TCCTGG
chrom    TTTCCACCAGCTTCCTGTGGCCAGGAG--CCAGACCACGCCCCTATACGGCCATAGTTGTCTTATCTTGT
right    ----------------------------------------------------------------------
                      1N11N11N111111221N111111                           11N11N

left     CTA-------ACAAGGT-GAAAC--------------CCCGTCT-CTACTAAA-----------------
chrom    CTCTGAGATTAGAAGGTTGGAACAGATTTTCCTGATGCCCCACTACTTCTGACATTCTGTGACTTTAGAA
right    ----------------------------------------------------------------------
         11N       1N11111 1N111              111NN11 11N11N1N

left     ---------------------------------------------------
chrom    TGTTACCTTCCTCGACAGCCCCCTGCACCCTCACACATCTGGCTTTAGCTG
right    ---------------------------------------------------
```

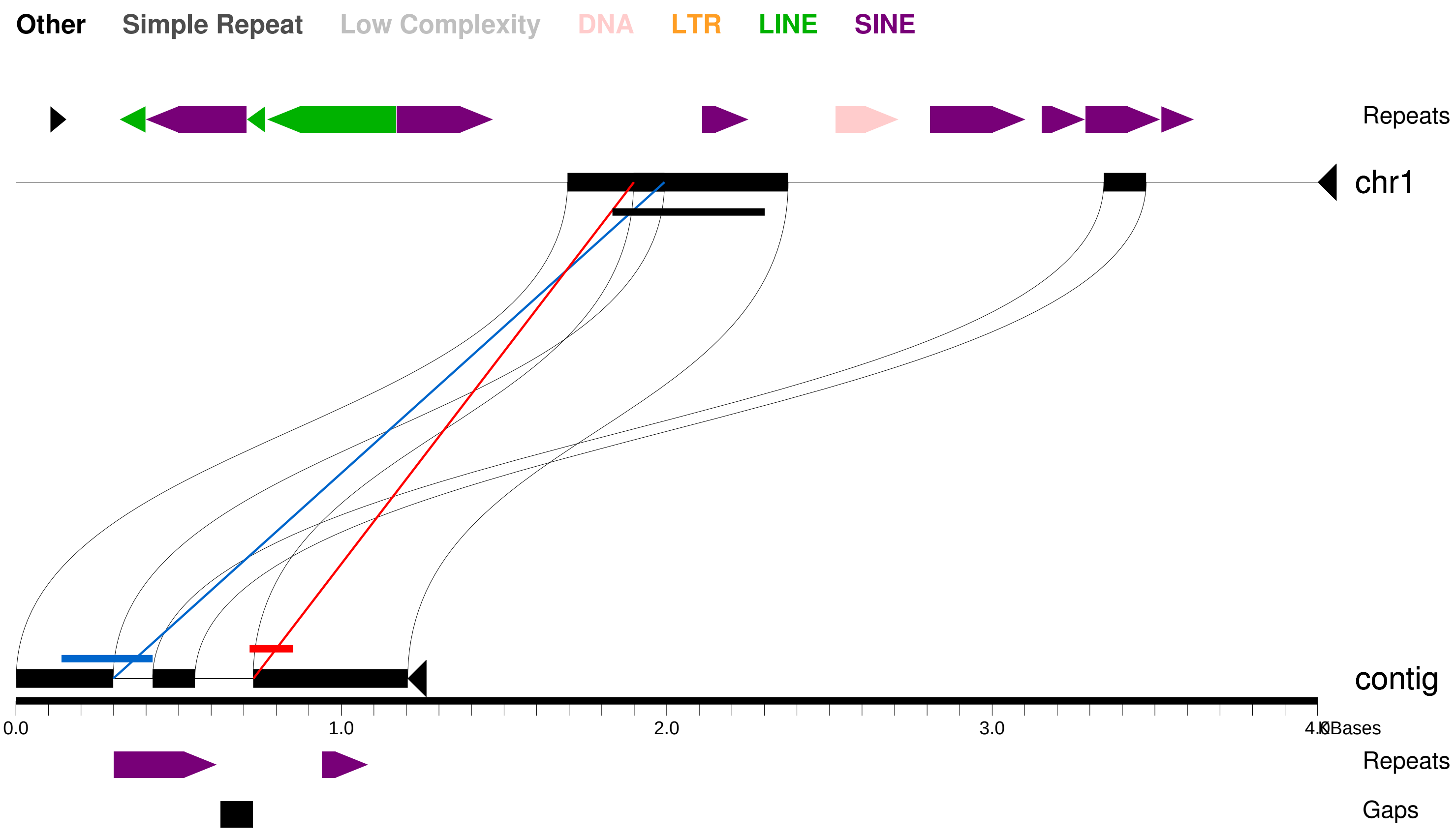

siteID: chr1_224241446 Scafftig5 - Breakpoint: chr1:224241448
chr1:224241402-224241507
Overlap Length: 14 INS size: 313 Genotyped: Yes

```
left    TCATCATGAGAAAACATGAAACAAACCCAAACGGAGAGACAGTCTC--------------TAAAACTACA
chrom   TCATCATGAGAAAACATGAAACAAACCCAAACGGAGAGACAGTCTC--------------TAAAACTACA
right   -CTGGGTGACAGAGCAAGACTCCATCTCAAAAAAAAAAAAAAATAAAAATAAAAAATAAAATAAAACTACA
        1*1111***1*1*1**1**11*1*1*1****111*1*1*1*1*11111111111111111**********

left    GGCCGGCCGGACGCGGTGGCTCACGCCTGTAATCCCA-GCACTTTGGGAGGCCGAAGCGGGTGG
chrom   GGCCTAC---ACAC-----CTAA---CAGCAAGGTCATGAAAAAT---AAACCAAAGCTGAGGA
right   GGCCTAC---ACAC-----CTAA---CAGCAAGGTCATGAAAAAT---AAACCAAAGCTGAGGA
        ****22*222**2*22222**2*222*2*2**222**2*2*222*222*22**2****2*22*2
```

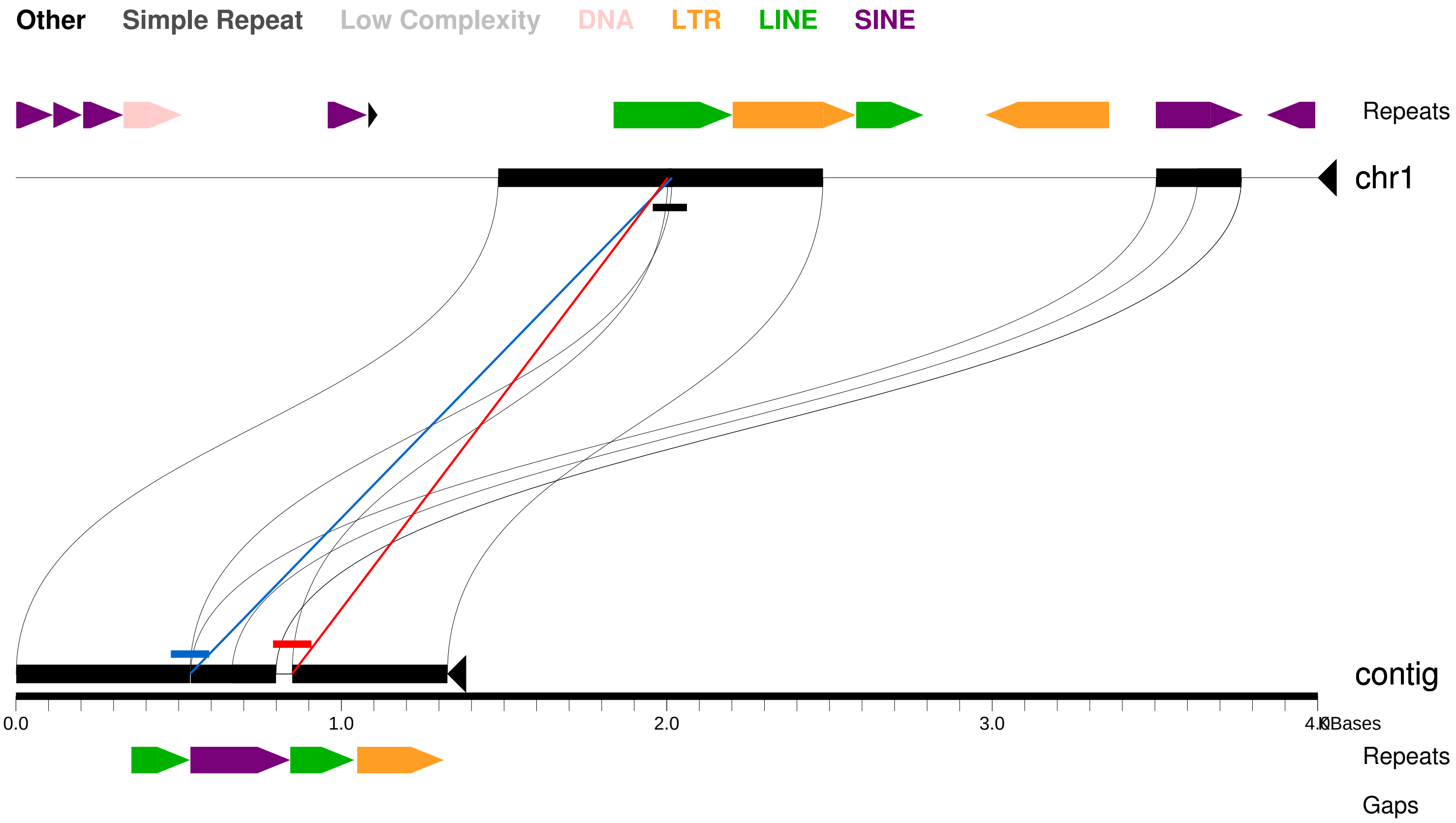

siteID: chr1_225996432 Scafftig2 - Breakpoint: chr1:225996421
chr1:225996374-225996480
Overlap Length: 13 INS size: 297 Genotyped: Yes

```
left   TGGCGTTAAGTCCTGACTC--CTAC-----------CTGTTTCACCATCACTGCAGTTTTAAGAGTAACG
chrom  TGGCGTTAAGTCCTGACTC--CTAC-----------CTGTTTCACCATCACTGCAGTTTTAAGAGTAACG
right  TCCACTGCACTCCAGCCTGGGCGACAGAGCGAGACTCCGTCTCAAAAAAAAAAAAAAAA-AAGAGTAACG
       *1111*11*1***1*1**111*1**11111111111*1**1***11*11*1111*11111**********

left   AATGGCCGGGCGCGGTGGCTCACGCCTGTAATCCCAGCACTT-TGGGAGGCCGAGGCGGGCGG
chrom  AATTT--GGGTCCCCTGG-TGAAGTGGCCAGAGGCAGAGCTTCTGTGTGAACT----------
right  AATTT--GGGTCCCCTGG-TGAAGTGGCCAGAGGCAGAGCTTCTGTGTGAACT----------
       ***2222***22*22***2*2*2*22222*2222***22***2**2*2*22*22222222222
```

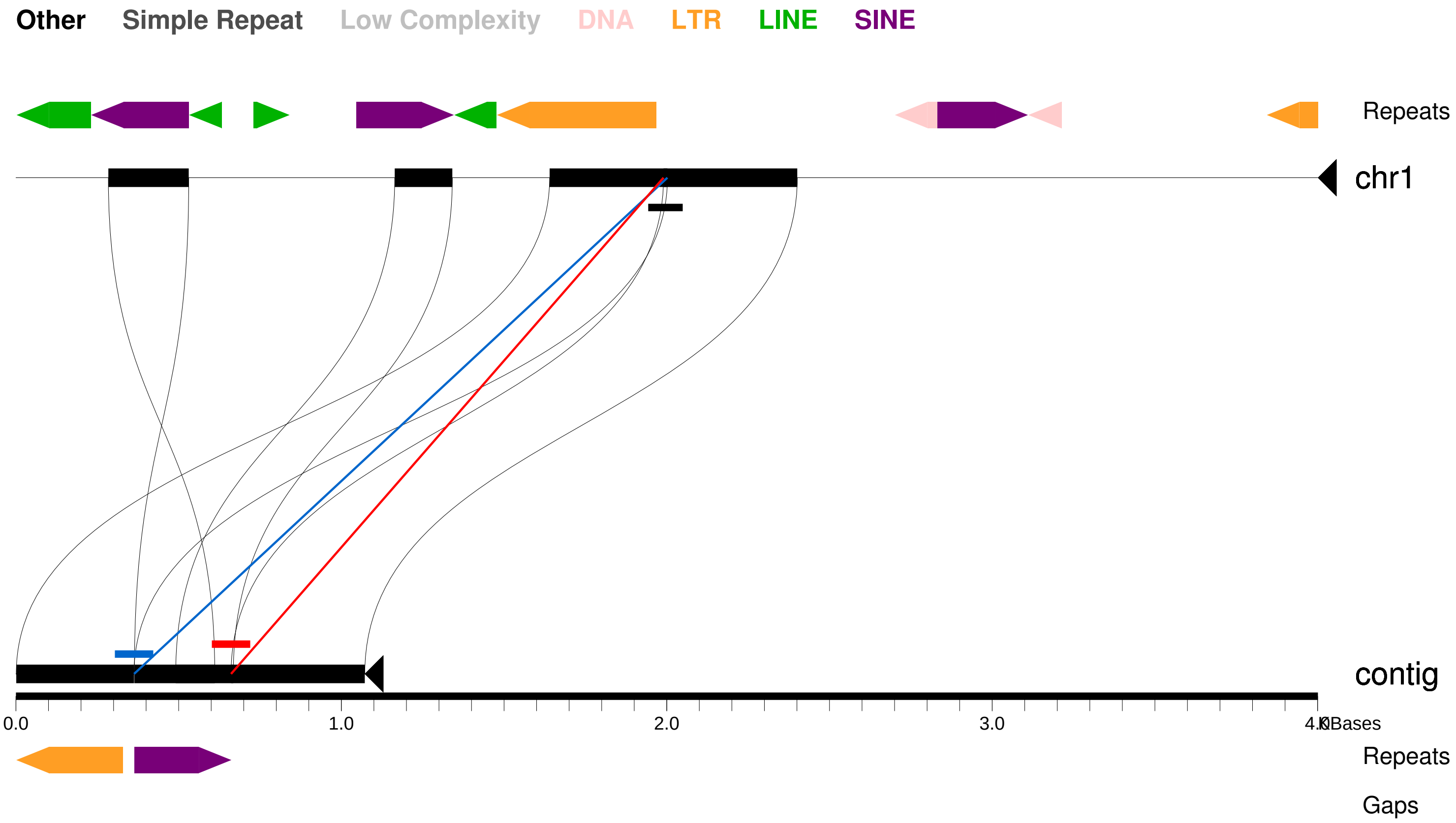

siteID: chr1_227462940 Scafftig2 - Breakpoint: chr1:227462943
chr1:227462897-227462987
Overlap Length: 14 INS size: 310 Genotyped: Yes

```
left   TTAAG----ACATAACTCTAAACAATTC-ACAG-----------GTC------------AAAAATAAAAA
chrom  TTAAG----ACATAACTCTAAACAATTC-ACAG-----------GTC------------AAAAATAAAAA
right  GATCGCGCCACTGCACTCCAGCCTGGGCGACAGAGCGAGACTCCGTCTCAAAAAAAAAAAAAAAAAAAAAA
       1111*1111**111****1*11*1111*1****11111111111***111111111111*****1*****

left   TTAGAAAGCAGAGGGCATGGCCGGGCGCGGTGGCTCACGCCTGTAATCCCAGCACTTTGGGAGGCCGAGG
chrom  TTAGAAAGCAGAGGGCAT----------------TTA-GAATGGAATAATAATAAA-----AAGACAACA
right  AAAAAAAGCAGAGGGCAT----------------TTA-GAATGGAATAATAATAAA-----AAGACAACA
       11*1*************2222222222222222*2*2*22**2***222*22*2222222*2*2*2*22

left   TGGGCGG
chrom  T------
right  T------
       *222222
```

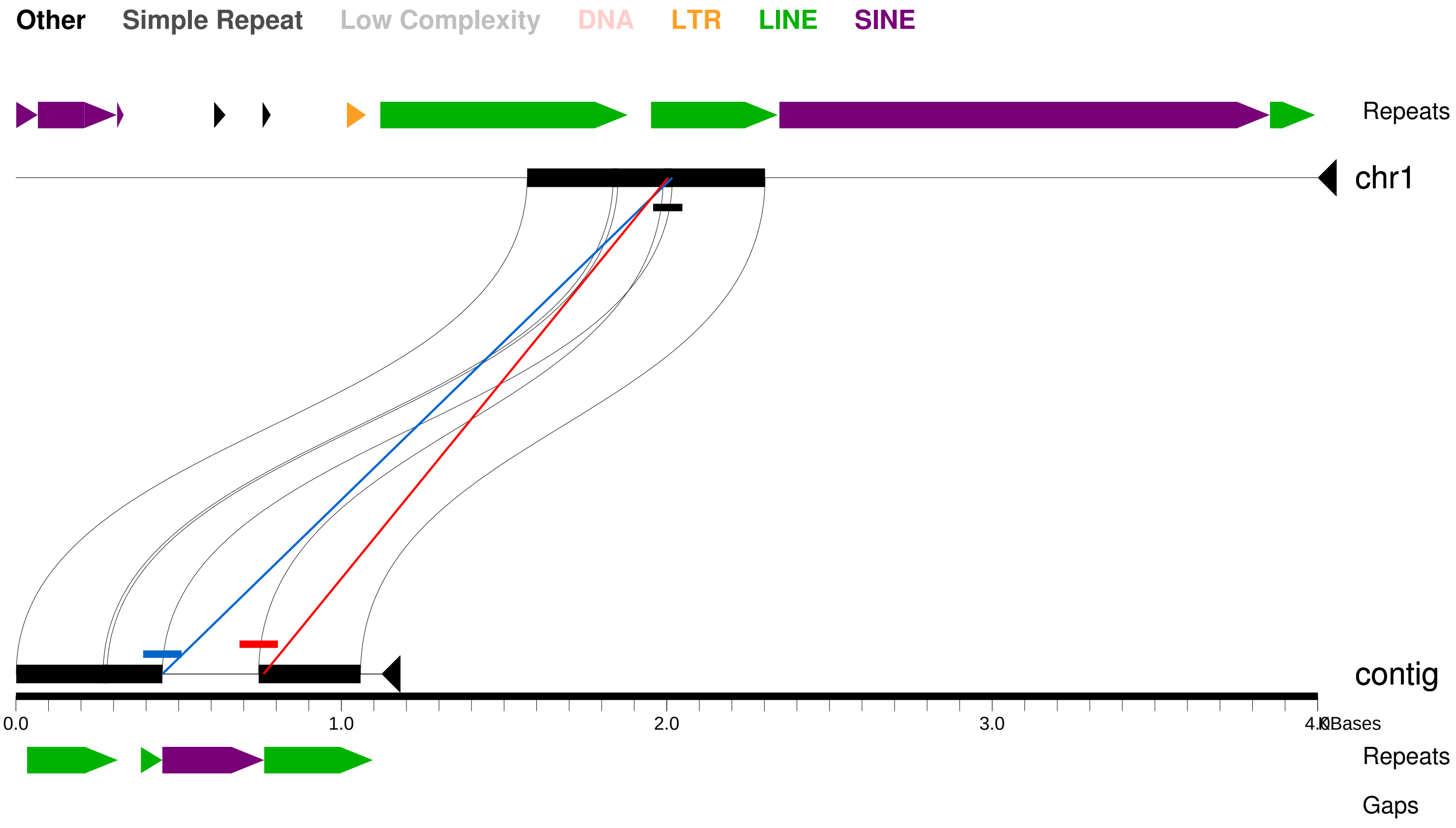

siteID: chr1_227602270 Scafftig6 + Breakpoint: chr1:227602266
chr1:227602106-227602438
Overlap Length: 13 INS size: 306 Genotyped: Yes

```
left    G------------A------------GAGAATCACTTGAGCCCAG-------GAGTTCAAGACAGTCTGG
chrom   GGAGGCCAAGGTGA------------GAGAATCACTTGAGCCCAG-------GAGTTCAAGACAGTCTGG
right   GGAGAATGGCGTGAACCCGGGAGGCGGAGCTTGCAGTGAGCCGAGATCCCGCCACTGCACTCCAGCCTGG
        *222NNNNNN222*111111111111***11*1111******1**11111111*1*1**111***1****

left    GCAACATAGTGACAGCCTCATCTCTTAAAAAAAAAAAAAAAAGTTAAACCAGGTATGCCTTTAATTGCAG
chrom   GCAACATAGTGACAGCCTCATCTCTTAAAAAAAAAAAAAAAAGTTAAACCAGGTATGCCTTTAATTGCAG
right   GCGACAGAGCGAGACTC-CGTCTCAAAAAAAAAAAAAAAAAA---AAA--------------AA------
        **1***1**1**1*11*1*1****11****************111***11111111111111**111111

left    AGCATTCCCAATCACTCAGCCCACCACTGCTCCTTTAAGAAGGCTCTTTGTGGCCGGGCGCGGTGGCTCA
chrom   AGCATTCCCAATCACTCAGCCCACCACTGCTCCTTTAAGAAGGCTCTTTGTGAT---GTTCTGTGGTTCT
right   ------------------------------------AAAAAGGCTCTTTGTGAT---GTTCTGTGGTTCT
        111111111111111111111111111111111111**1*************22222*22*2****2**2

left    CGCC-TGTAA--TCCCAGCA--CTTTGGGAGGC---------CGAGGCGGGCGGATCACGAGGT-CAGGA
chrom   AGCCCTGCACATTCCCAGTTTCCTCTTGCATGATTCTACCTTCCACTGAAACAGAGCAAGATCTGCTTAA
right   AGCCCTGCACATTCCCAGTTTCCTCTTGCATGATTCTACCTTCCACTGAAACAGAGCAAGATCTGCTTAA
        2***2**2*222******2222**2*2*2*2*2222222222*2*222222*2**2**2**22*2*222*

left    GATCG-----AGACCATCCCGGC---------TAAAATGGTGAAACC-----CCGTCTCTACTA------
chrom   GATCCTATAAAAATGACTTTGGCATGTGCCCTTTGCCTGGGAACAGCTTTTTCAGTTGCTGCTGCTGCTG
right   GATCCTATAAAAATGACTTTGGCATGTGCCCTTTGCCTGGGAACAGCTTTTTCAGTTGCTGCTG------
        ****222222*2*22*2222***222222222*2222***22*2*2*22222*2**22**2**2

left    ---AA
chrom   TGTAA
right   -----
          11
```

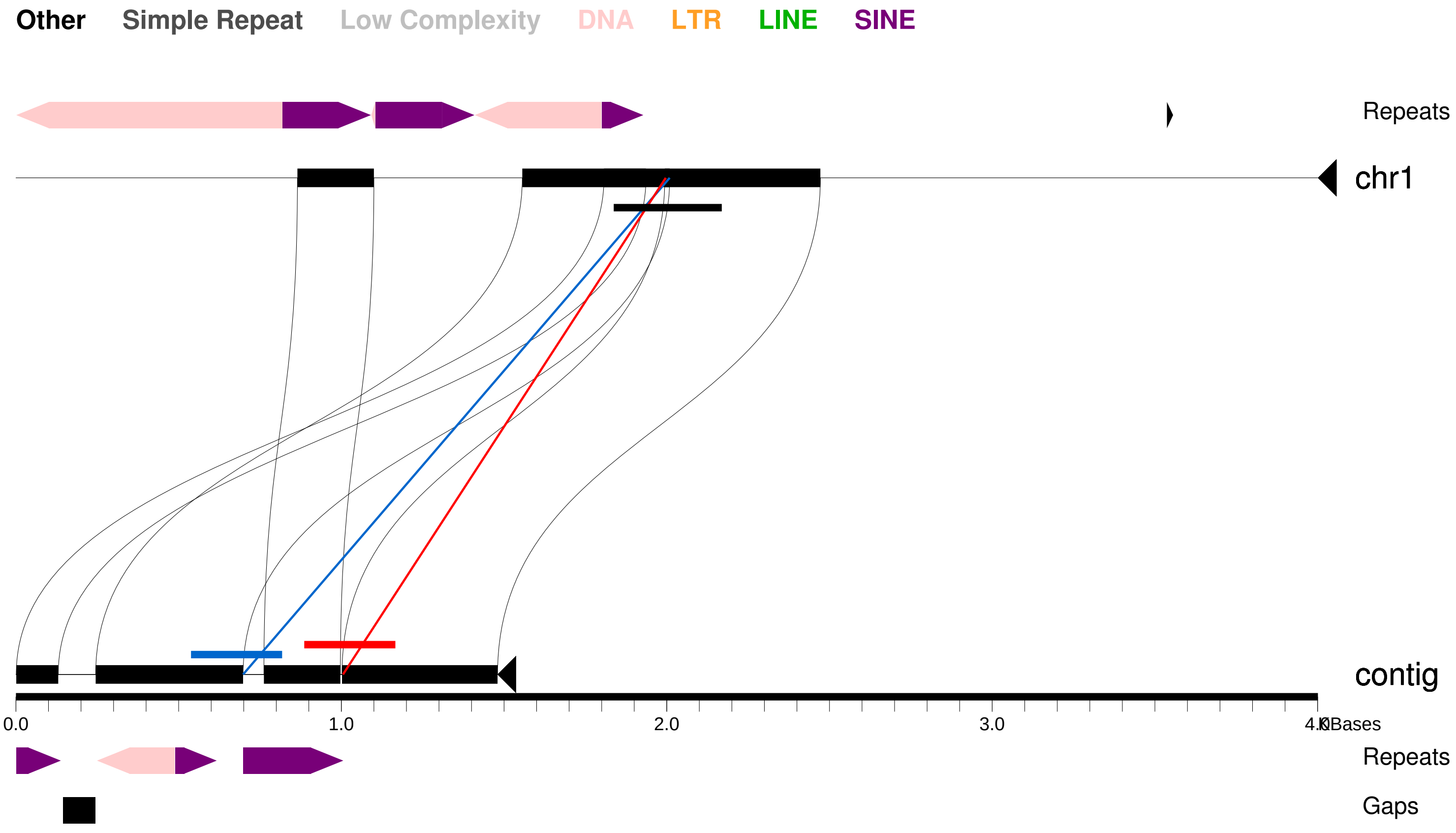

siteID: chr1_232587763 Scafftig7 - Breakpoint: chr1:232587761
chr1:232587715-232587820
Overlap Length: 14 INS size: 295 Genotyped: Yes

```
left    -------GCAAAACATTCTGT-----AGACCAAGAA--GGTCACTGAAAATGTGTTAGTTAAAAATGTGG
chrom   -------GCAAAACATTCTGT-----AGACCAAGAA--GGTCACTGAAAATGTGTTAGTTAAAAATGTGG
right   CGCCACTGCACTCCAGCCTGGGCGACAGAGCGAGACTCCGTCTCAAAAAAAAAAAAAAA-AAAAATGTGG
        1111111***111**11***111111***1*1***1111***1*11****111111*111**********

left    CATTGCCGGGCGCGGTGGCTCACGCCTGTAATCCCAGCACTTTGGGAGGCCGAGGCGGGCGGA
chrom   CATTATCAAACTGTAAGAATAACAA--GTAATTCCATTAGAT-----------GGCATCCTCA
right   CATTATCAAACTGTAAGAATAACAA--GTAATTCCATTAGAT-----------GGCATCCTCA
        ****22*222*22222*22*2**2222*****2***22*22*22222222222***222*22*
```

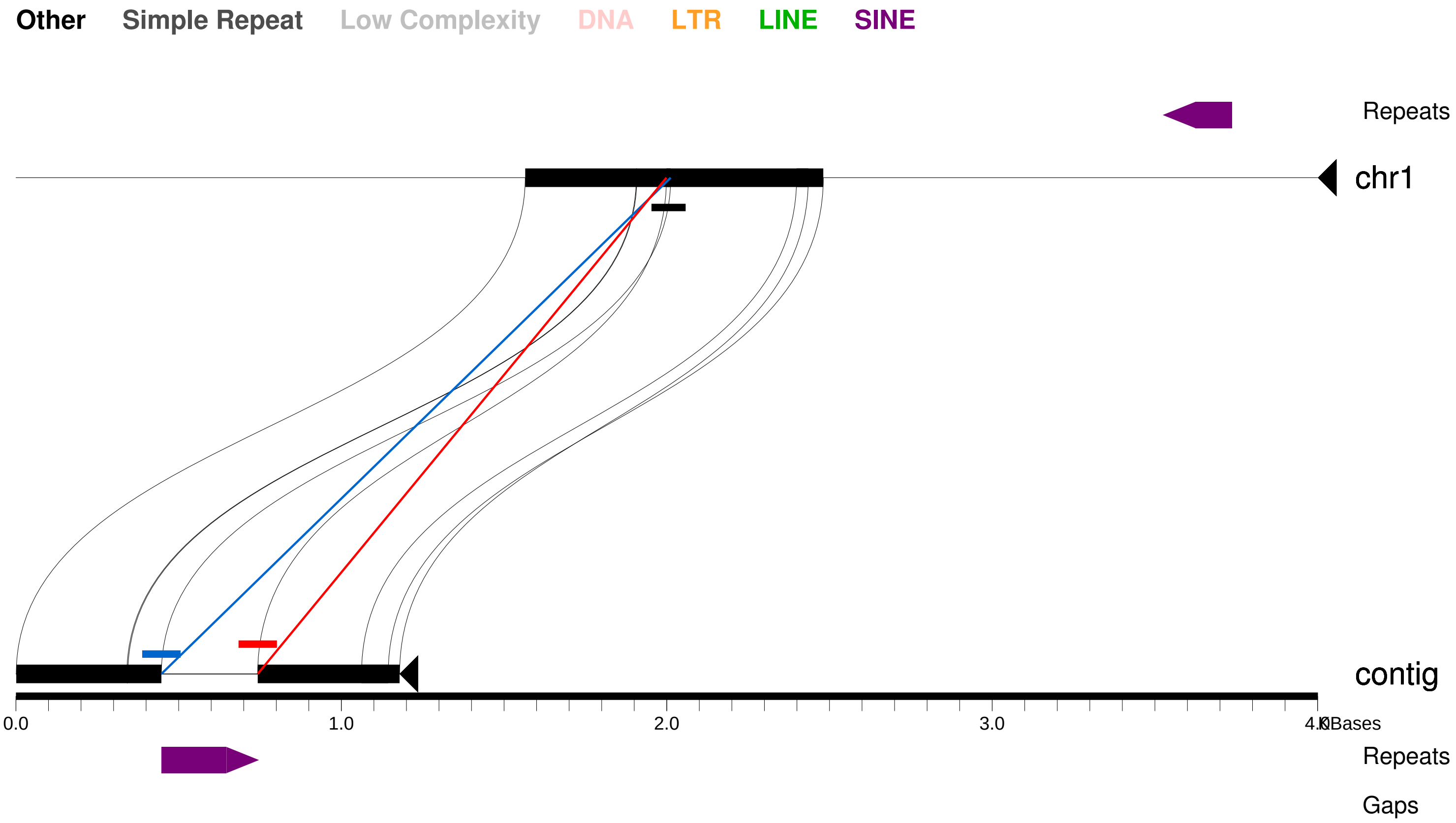

siteID: chr1_232748656 Scafftig1 + Breakpoint: chr1:232748628
chr1:232748468-232748804
Overlap Length: 17 INS size: 299 Genotyped: No

```
left   NNNNNNNNNNNNNNNNNNNNNNNNNNNNNNNNNNNNNNNNNNNNNNNNNNNNNNNNNNNNNNNNNNNN-N
chrom  AACTTGTATTGTCTCAGATGAGACTTTGGACTGTTAACTTTTGGGTTACTGCTGAAATGAGTAGACAT-T
right  T------------TTAGTAGAGAC---GGGGTTTCACCGTTT----TA--GCCGGGATG-GTCTCGATCT
       NNNNNNNNNNNNN2N22NN22222NNN22NN2N2N2N2N222NNNN22NN22N2NN222N22NNNN2212

left   NNNNNNNNNNNNNNNNNNNNNNNNNGG---------------ACATGAGGTTTGGAGAGGCCAGAGGTGC
chrom  GGGGGACTGTTGGGAAGGCATGATTGGTTTTGAAACGTCAGGACATGAGATTTGGAGAGGCCAGAGGTGC
right  CCTGACCTCGTGATCCGCCCGCCTCGGCCTCCCAAAGTGC-----TGGGATT---ACAGGCGTGAGCCAC
       NNN2NN22NN22NNNN2N2NNNN2N**NN2NNN22N22NN  111**1*2**111*1****11***111*

left   AATGATATGGTTTGGTTCTGTGTCCTCCCCCAAATCTTTTTTTTTTTTTTTT-----------------
chrom  AATGATATGGTTTAGTTCTGTGTCCTCACCCAAATCTTATCTTGAATTGTACTCCCATAATTCCTACACG
right  CGCGCCCGGCC----------GTCCTCACCCAAATCTTATCTTGAATTGTACTCCCATAATTCCTACACG
       111*1111*1111N1111111******2**********2*2**222**2*22*2222222222222222

left   TTTTGAGACGGAGTCTCGCTCTGTCGCCCAGGCTGGA------GTGCAGTGGCGG-------GATCTCGG
chrom  TTGTGGGAGGGAGGGACCTGGTGGAAGATAGTTTGAATCATGGGGGCAGTTTCCCCCATACTGTTCTCGT
right  TTGTGGGAGGGAGGGACCTGGTGGAAGATAGTTTGAATCATGGGGGCAGTTTCCCCCATACTGTTCTCGT
       **2**2**2****222*2222**222222**22**2*222222*2*****22*222222222*2*****2

left   CTCACTGCA--AG-CTC-CGCCTCCCG--GGTTCACGCCA-----TTCTCCTGCCTCA
chrom  GGTACTGAATAAGTCTCACGAGATCTGATGGTTTTCATCAAGTGTTTCCGCTTTTACA
right  GGTAGTGAATAAGTCTCACGAGATCTGATGGTTTTCATCAA-----------------
       222*1**2*22**2***2**2222*2*22****22*22**2    111NN11NNNN11
```

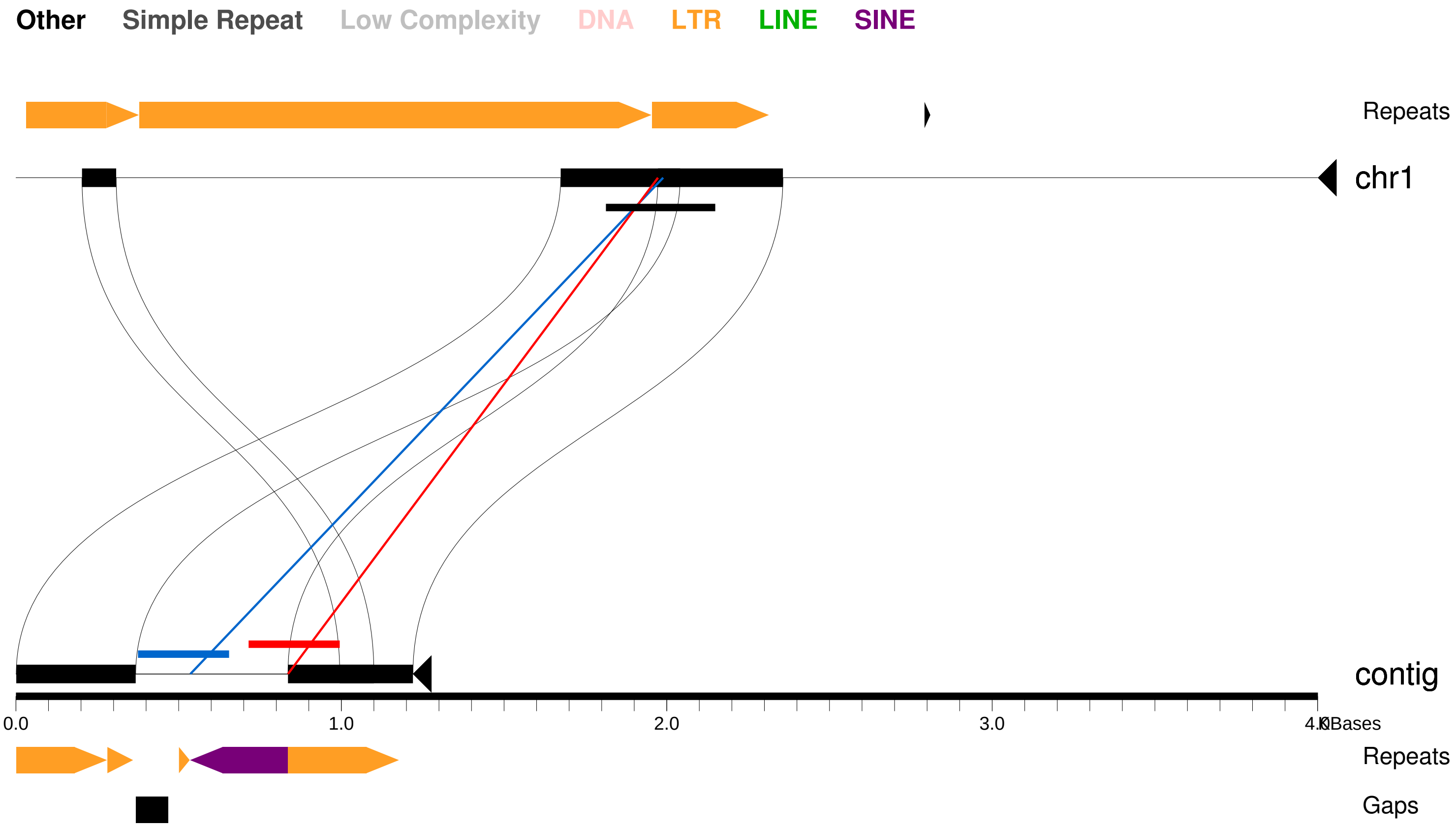

siteID: chr1_232869268 Scafftig5 - Breakpoint: chr1:232869263
chr1:232869103-232869388
Overlap Length: 1 INS size: 419 Genotyped: Yes

```
left   ACTTTTTGATTTTATATTTGTGACTCTAATCACTTATCCTGAAATTCTTGGTTCTGGCAAAGTTAACATA
chrom  ------------------TGTGACTCTAATCACTTATCCTGAAATTCTTGGTTCTGGCAAAGTTAACATA
right  ------------------C---AGCCTGGCGACAGAA--TGAGACTCCA---TCTCAAAAAAAAAAAAAA
       22222222222222222221111*11**1111**11*111***1*1**11111***111***111**1*1*

left   ATTA--------TTTGTTTTTACCTT----ACATGTCTTAGTT-------TCAGAAAAATGA-TGC---C
chrom  ATTA--------TTTGTTTTTACCTT----ACATGTCTTAGTT-------TCAGAAAAATGA-TGC---C
right  AATTCTCTAGAGTTTCTTTTTACCATTAGGATATATCCCACTTGGGACAGTCAGTCAAGTTACTGTATTT
       *1*111111111***1********1*1111*1**1**11*1**1111111****11**1*1*1**11111

left   CAAATCA-TTCAA----TATAAATATTGAATA---------CAGTTTAAAATT---CCTCCGCAGTAGGA
chrom  CAAATCA-TTCAA----TATAAATATTGAATA---------CAGTTTAAAATT---CCTCCGCAGTAGGA
right  TAAATCACTTGAAATACTTTAACCCTTGTGTGGTTAAGTGGCTATTTATTTTTAACCCTCTGAAAAAGAA
       1******1**1**1111*1***111***11*1111111111*11****111**111****1*1*11**1*

left   CA-TGCCTG-TAA--TACCAGCATTTTGGGAGGCCAAGGCAGGTGGACCAGCTGAGGTCAAAAGATCAAG
chrom  CA-TGCCTG-TTACGTTTTTCTGTTTTCAAATAACTACAAAGATTTTCT---TAACATTATAAGAGTAAT
right  TTGTTTTTGCTTACGTTTTTCTGTTTTCAAATAACTACAAAGATTTTCT---TAACATTATAAGAGTAAT
       111*111**1*2*22*2222222****222*222*2*222**2*222*2222*2*22*2*2****22**2

left   -TCCATCCTGG---------CCAACATGGTGAAGCCTGGTCTCTA---CTAAAAA--TAC---------
chrom  ATTCTTAGAGGAGTGTAGAGCTGAGAGGAAATACTTTTGTGTTTATAGCGAAAAAAGTATATTGAAGTG
right  ATTCTTAGAGGAGTGTAGAGCTGAGAGGAA---------------------------------------
       2*2*2*222**222222222*22*2*2*22NN1NNN1N11N1N11   1N11111  11N
```

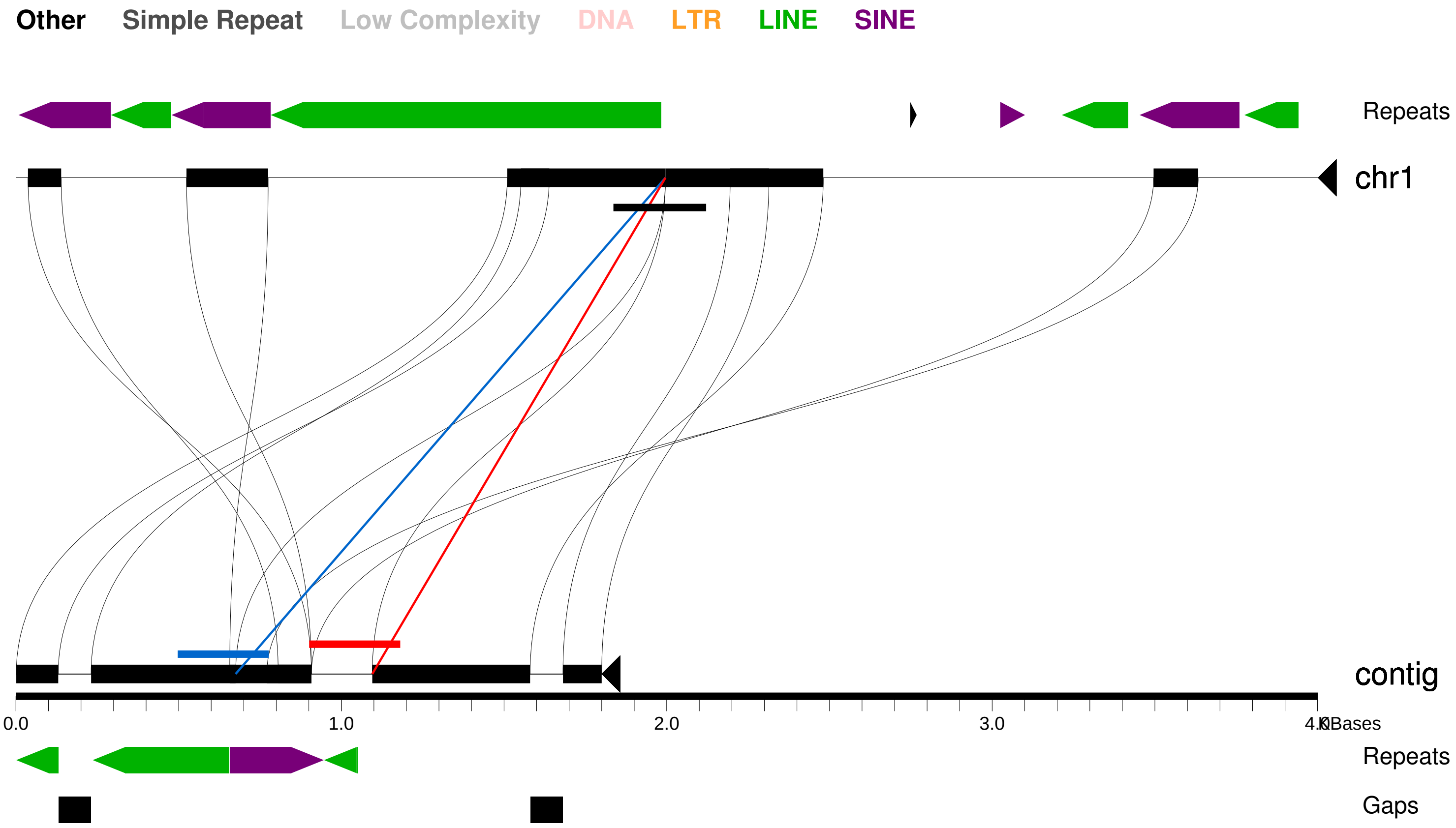

siteID: chr1_233519129 Scafftig2 - Breakpoint: chr1:233519131
chr1:233519085-233519190
Overlap Length: 14 INS size: 334 Genotyped: Yes

```
left   -TGC------------AATGATATTTCTCTCTGAGGAAATTATACGGAATGTAACTTATAAAAGCTTTAC
chrom  -TGC------------AATGATATTTCTCTCTGAGGAAATTATACGGAATGTAACTTATAAAAGCTTTAC
right  CTTCTCAAAAAAAAAAAAAAAAAAAAAAAAAAAAAAAAAAAAAAAAAAAAAAAAAAAAAAAAAAAAAGCTTTAC
       1*1*111111111111**11*1*1111111111*11***11*1*111**111**111*1***********

left   TGAGGCCGGGCGCGGTGGCTCACGCCTGTAATCCCAGCACTTTGGGAGGCCGAGGCGGGCGG
chrom  TGAATATAAG-------TTATAAGCATTTTATT------CATTAGAACTCCAAAATAGATGT
right  TGATTATAAG-------TTATAAGCATTTTATT------CATTAGAACTCCAAAATAGATGT
       ***N22222*22222222222*2**2*2*2**2222222*2**2*2*22**2*2222*22*2
```

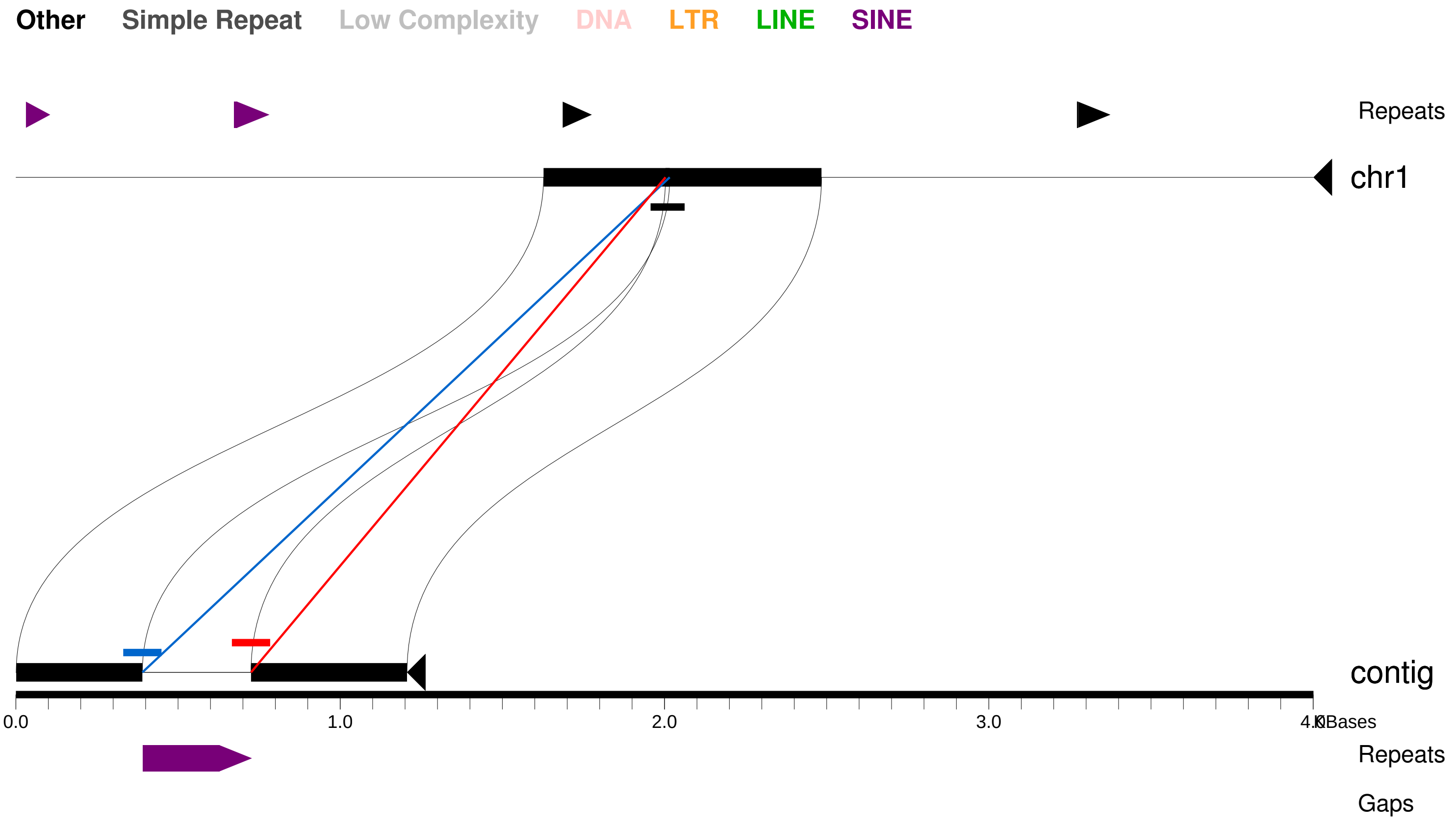

siteID: chr1_234070554 Scafftig1 - Breakpoint: chr1:234070553
chr1:234070505-234070612
Overlap Length: 12 INS size: 301 Genotyped: Yes

```
left    AAAGC-CACTGGCTTTCCCTCTGAAGCAAAGGTAGCTCCCTATGCAGAT----------AAACAATGTTT
chrom   AAAGC-CACTGGCTTTCCCTCTGAAGCAAAGGTAGCTCCCTATGCAGAT----------AAACAATGTTT
right   ACTGCACTCCAGCCTGGGCGACAGAGCGAGACTCCGTCTCAAAAAAAAAAAAAAAAAAAAAAAAAATGTTT
        *11**1*1*11**1*111*11111***1*111*111**1*1*111*1*11111111111***1*******

left    CG--GCCGGGCGCGGTGGCTCACGCCTGTAATCCCAGCACTTTGGGAGGCCGAGGCGGGCGG
chrom   CAATGAAAATCTAAGTGGATCCTGTCTTT--TACAAA--------GAAGCTGTGTCTAG---
right   CAATGAAAATCTAAGTGGATCCTGTCTTT--TACAAA--------GAAGCTGTGTCTAG---
        *222*22222*222****2**22*2**2*22*2*2*222222222**2**2*2*2*22*222
```

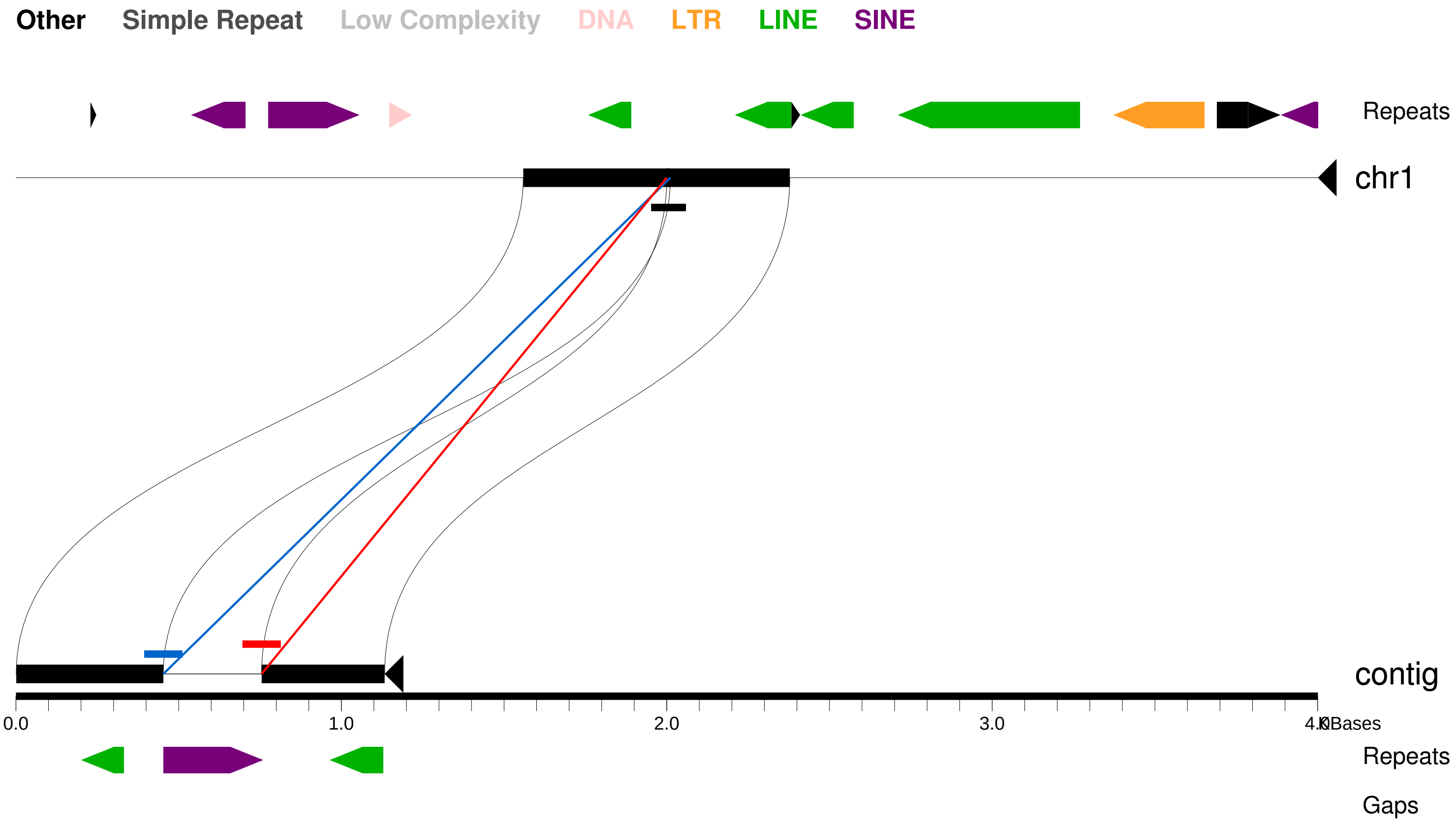

siteID: chr1_234805722 Scafftig1 - Breakpoint: chr1:234805713
chr1:234805553-234805886
Overlap Length: 14 INS size: 312 Genotyped: Yes

```
left     A--------GAA----TTGAAGAGTTTTGTTCTTGAT--TTAACATCAAATAAATTTTAGATACAGCCAT
chrom    ACAACAATAGAA----TTGAAGAGTTTTGTTCTTGAT--TTAACATCAAATAAATTTTAGATACAGCCAT
right    TTTAGTAGAGACGGGGTTTCACCGTTTTAGCCGGGATGGTCTCGATCTCCTGACCTCGTGATCCGCCC--
         1NN2NN2N2**11111**11*11*****111*11***11*1111***111*1*11*111***1*11**11

left     AATATGTCACAAAGGCAAAAACACAACAGTTTTAAACCTCTATAATCAATTACAAGAGTAAAATTAATGT
chrom    AATATGTCACAAAGGCAAAAACACAACAGTTTTAAACCTCTATAATCAATTACAAGAGTAAAATTAATGT
right    -----GCCTC---GGCCT---CCCAA-AGTGCTGGG-------------ATTACAGGCGTGAGCCACCGCG
         11111*1*1*111***11111*1***1***11*111111111111111******1*1**1*111111111

left     CCAAGCCATTTAAAATGGGGTTAAGTTGATTATATTTTTTTT------------TTTTTTTTTTTTTTTT
chrom    CCAAGCCATTTAAAATGGGGTTAAGTTGATTATATGTTTTCTAATAAAAAGGGCTCTGTGTCTATGTGTG
right    CCCGGCC-------------------TGATTATATGTTTTCTAATAAAAAGGGCTCTGTGTCTATGTGTG
         **11***1111111111111111111*********2****2*222222222222*2*2*2*2*2*2*2*2

left     TTTTTTTTTTTTTGAGGCGGAGTCTCGCTCTGT---CACC-CAGGCTGGA-----------GTGCAGTGG
chrom    TGTGTGTCTATAAAATACAAATTTTCCTTGTTTTTCCACCTCTTCCTTGACGGTCCATTCTGTACACTGG
right    TGTGTGTCTATAAAATACAAATTTTCCTTGTTTTTCCACCTCTTCCTTGACGGTCCATTCTGTACACTGG
         *2*2*2*2*2*222*22*22*2*2**22*2*2*222****2*222**2**22222222222**2**2***

left     CGGGA------TCT----CGGCTCA--CTGCAAGCTCCG----CCTCCCGGGTTCACG--
chrom    CACCAGATTAATCTTTTACAGGTGAGACTCCAAGTTCACAAACCTTCCCCAGCTCACCTT
right    CACCAGATTAATCTTTTACAGGTGAGACTCCAAGTTCACAAACCTT--------------
         *222*222222***2222*2*2*2*22**2****2**222222*2*111NN1N1111N
```

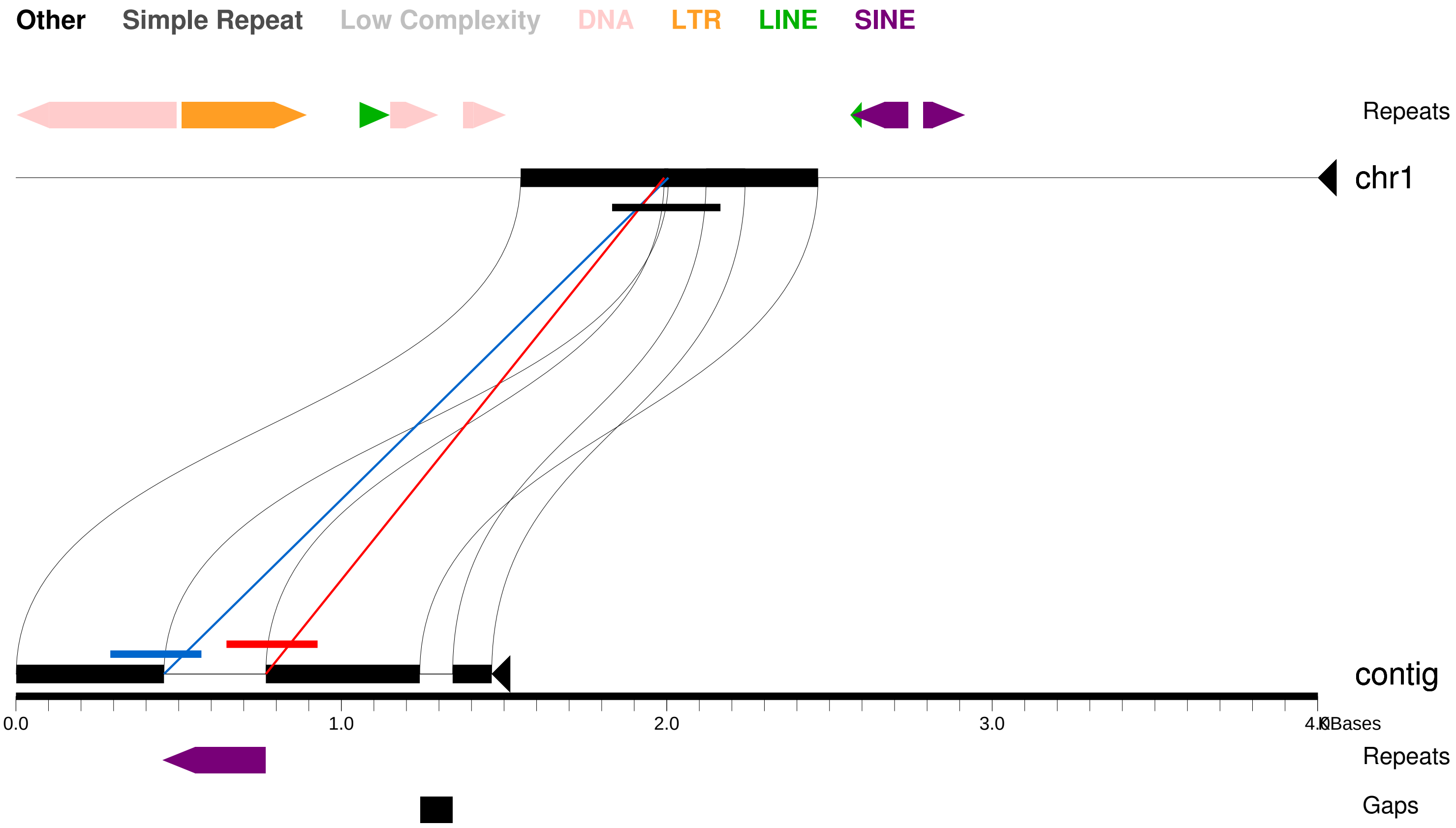

siteID: chr1_237569188 Scafftig4 + Breakpoint: chr1:237569195
chr1:237569162-237569254
Overlap Length: 14 INS size: 307 Genotyped: Yes

```
left    GTGGATTTGT-GACCACC------------ATT-CAGAAAAGTA------------GGTGAAAAAAACTTT
chrom   GTGGATTTGT-GACCACC------------ATT-CAGAAAAGTA------------GGTGAAAAAAACTTT
right   CCGCCCGCCTCGGCCTCCCAAAGTGCTGGGATTACAGGCGTGAGCCACCGCGCCCGGCCGAAAAAAACTTT
        11*111111*1*1**1**111111111111***1***1111*11111111111111*11***********

left    CTTTTTTTTTTTTTTTTTTTTTTTTTTTTTGAGACGGAGTCTCGCTCTGTCGCCCAGGCTGGAGTGCAGTG
chrom   CTTAACTTTATCTCTTCAT----------------------CCCACCATCAACCA--CTGAGGT--ATTC
right   CTTAACTTTATCTCTTCAT----------------------CCCACCATCAACCA--CTGAGGT--ATTC
        ***222***2*2*2**22*2222222222222222222222*2*2*22**22***22***22**22*2*2

left    GCGGG
chrom   TTGCA
right   TTGCA
        22*22
```

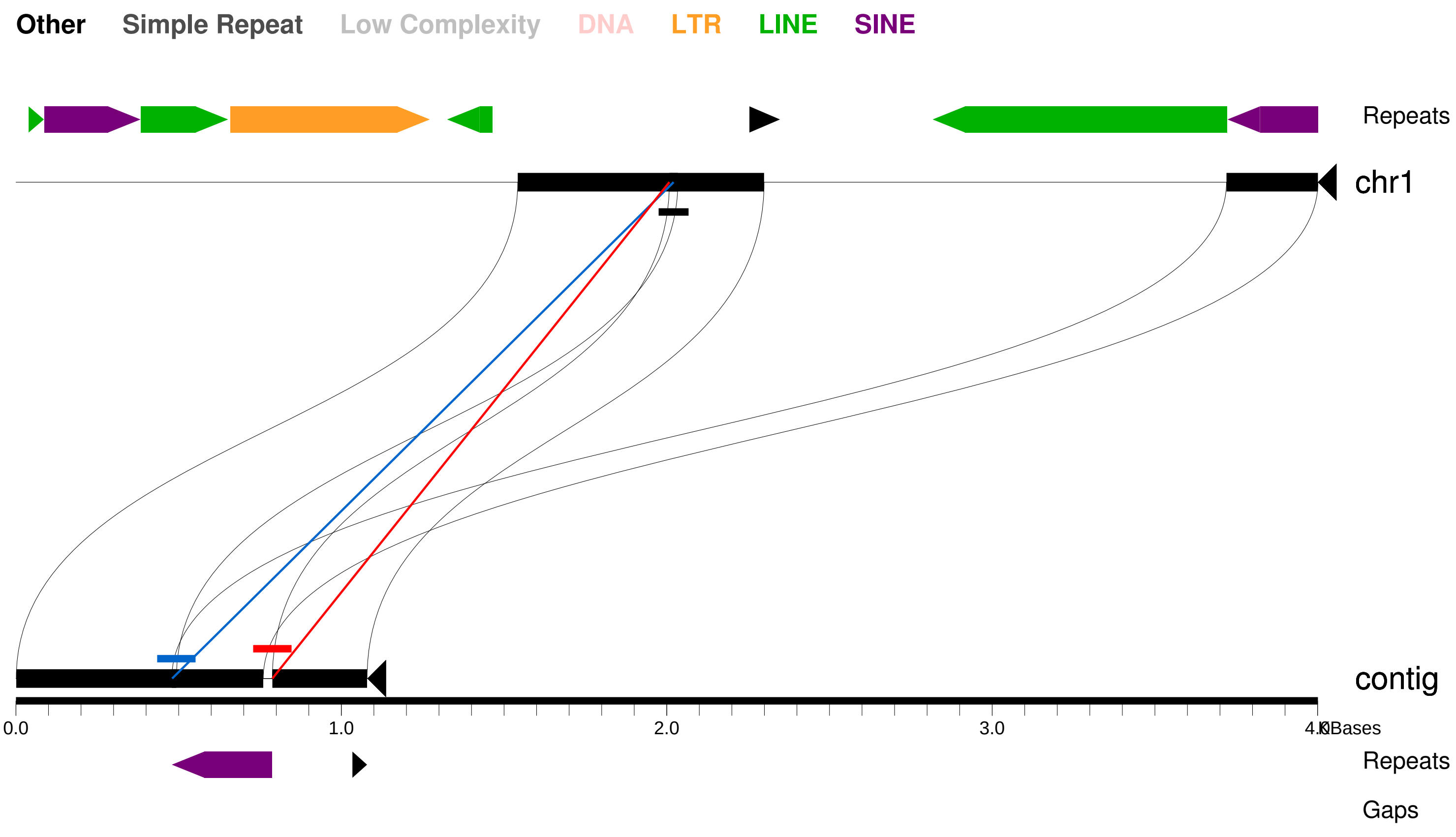

siteID: chr1_237967610 Scafftig5 + Breakpoint: chr1:237967612
chr1:237967452-237967787
Overlap Length: 16 INS size: 291 Genotyped: Yes

```
left    -----------CACTCAAGTTCCTGTGGGGTA-CACAAATATCCTTGAACT--TGCCCTCAAGCCTCTCC
chrom   TACTGCAGTGGCACTCAAGTTCCTGTGGGGTA-CACAAATGTCCTTGAACT--TGCCCTCAAGCCTCTCC
right   TTTAATAGAGACGGG---GTTTCACCGTTTTAGCCGGGATGGTCTCGATCTCCTGACCTCGTGATCCGCC
        2NNNNN22N2N*111111***1*111*111**1*1111**211**1**1**11**1****11*111*1**

left    AACACTGCCACACTTTTCCAACTTGGTCTTGCAAAACAGGAACCCACCACTGAAACCAAATTTTCCTTCC
chrom   AACACTGCCACACTTTTCCAACTTGGTCTTGCAAAACAGGAACCCACCACTCAAACCAAATTTTCCTTCC
right   C-----GCCTCGGCCTCCCAAAGTG--CTGGGATTACAGG---------CGTGAGCCA------CCGCGC
        111111***1*1111*1****11**11**1*1*11*****111111111*1N1*1***111111**111*

left    CAAGCAAACTCTGTTTAGTCTTACCTCAGTGTGCTTTTT----------TTTTTTTTTTTGAGAC--GGA
chrom   CAAGCAAACTCTGTTTAGTCTTACCTCAGTGTGCTTTTTAAAAATGCTGTTTTCTTTTCTGCCACTTGAC
right   CCGGC------------------CCTCAGTGTGCTTTTTAAAAATGCTGTTTTCTTTTCTGCCACTTGAC
        *11**111111111111111111***************2222222222****2****2**22**22*22

left    GTCTCGCT-CTGTCGCCCAGG---CTGGAGTGC-----AGTGGC---GCGATCTCGG------CTCACTG
chrom   CTCTAGCTTCAGTTCCACATGGTCCTTGAAATCCCTTTACTAGCTCAGCCCCCTCTTTTTATTCTCTGTG
right   CTCTAGCTTCACTTCCACATGGTCCTTGAAATCCCTTTACTAGCTCAGCCCCCTCTTTTTATTCTCTGTG
        2***2***2*21*22*2**2*222**2**222*22222*2*2**222**222***22222222***22**

left    CAAGCTCCG-----CCTCCCGGGTTCACGCCATTCTCCTGCCTCAGCCTC----------
chrom   AAAGATTAAGATATCTTACAGTCAAAATGAAGGTGTCC-GCCGCTTCCCCAAATGGGTGG
right   AAAGACTAAGATATCTTACAGTCAAAATGAAGGTGTCC-GCCGCT---------------
        2***2122222222*2*2*2*22222*2*2222*2***2***2*2N11N1
```

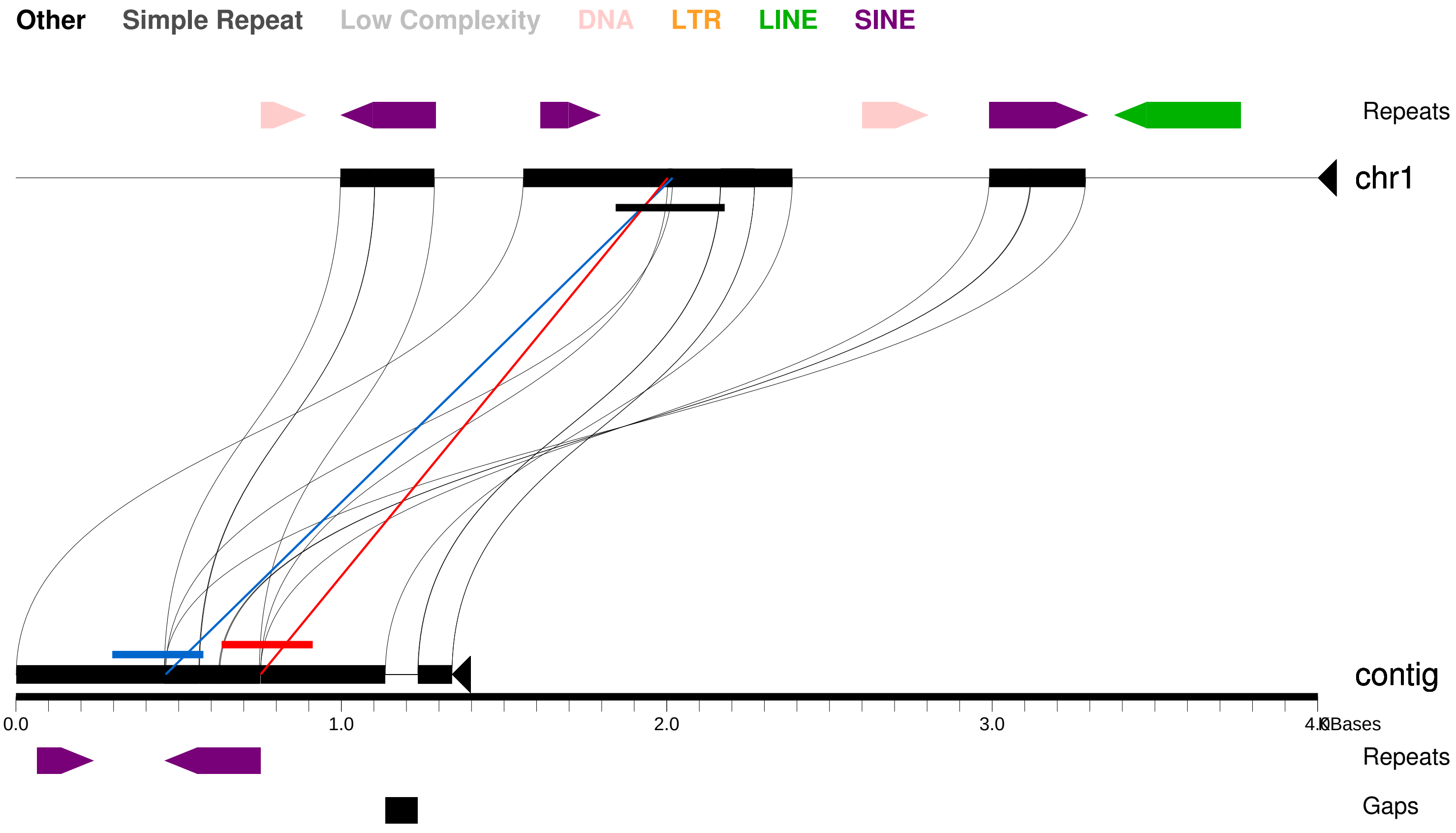

siteID: chr1_239426262 Scafftig4 + Breakpoint: chr1:239426255
chr1:239426095-239426427
Overlap Length: 13 INS size: 421 Genotyped: No

```
left    ------------CAACTCCTAGAAGCCAGCCTCAGGTCCTAACCACATTTTCTCCTCCTCTTGCAAAATG
chrom   GATCATCAACTTCAACTCCTAGAAGCCAGCCTCAGGTCCTAACCACATTTTCTCCTCCTCTTGCAAAATG
right   NNNNNNNNNNNNA---------------------------------------------------------
        NNNNNNNNNNNN111111111111111111111111111111111111111111111111111111111

left    GCAGCTTAGGTTTTCAAGGACAGTAAGAGAATACCTTTCATTTCGAATCTCTCTCTCTATCCAGTCTGCT
chrom   GCAGCTTAGGTTTTCAAGGACAGTAAGAGAATACCTTTCATTTCGAATCTCTCTCTCTATCCAGTCTGCT
right   ----------------------------------------------------------------------
        1111111111111111111111111111111111111111111111111111111111111111111111

left    AAGACAGTTTTGAATTACTTAAGAAGGAATTGGG---CCGGGCGCGGT-----GGCTCACGCCTGT----
chrom   AAGACAGTTTTGAATTACTTAAGAAGGAATTGGCTGTCCCATCATGTTCAAATGTCTCACCCATGTTGAA
right   --------------------AAGAAGGAATTGGCTGTCCCATCATGTTCAAATGTCTCACCCATGTTGAA
        11111111111111111111*************2222**222*22*2*22222*2*****2*2***2222

left    --------AATCCCA--------GCACTTTGGGAGGCC--GAGGCGGGCGGATCA-----------CGAG
chrom   GGAGAGGGAATAATACAGAGTGTGCACATCAGG-GGCCAGGAATCTTAGGGGTTATATTTTTCTGCCTAC
right   GGAGAGGGAATAATACAGAGTGAGCACATCAGG-GGCCAGGAATCTTAGGGGTTATATTTTTCTGCCTAC
        22222222***222*2222222N****2*22**2****22**22*2222**2*2*22222222222*2*2

left    GTCAGGAGATCGAGACCATC----CCGGCTAAAACGGTGAAACCCCGTCTCTACTAAA
chrom   TGTACCCGCTCACTAAAATCTTTTCTAGCGAAAAGGGAGAAT----GTTTTCACAAAC
right   TGTAC-----------------------------------------------------
        222*2NN1N11NNN1NN111    1NN11N1111N11N111N222211N1NN11N11N
```

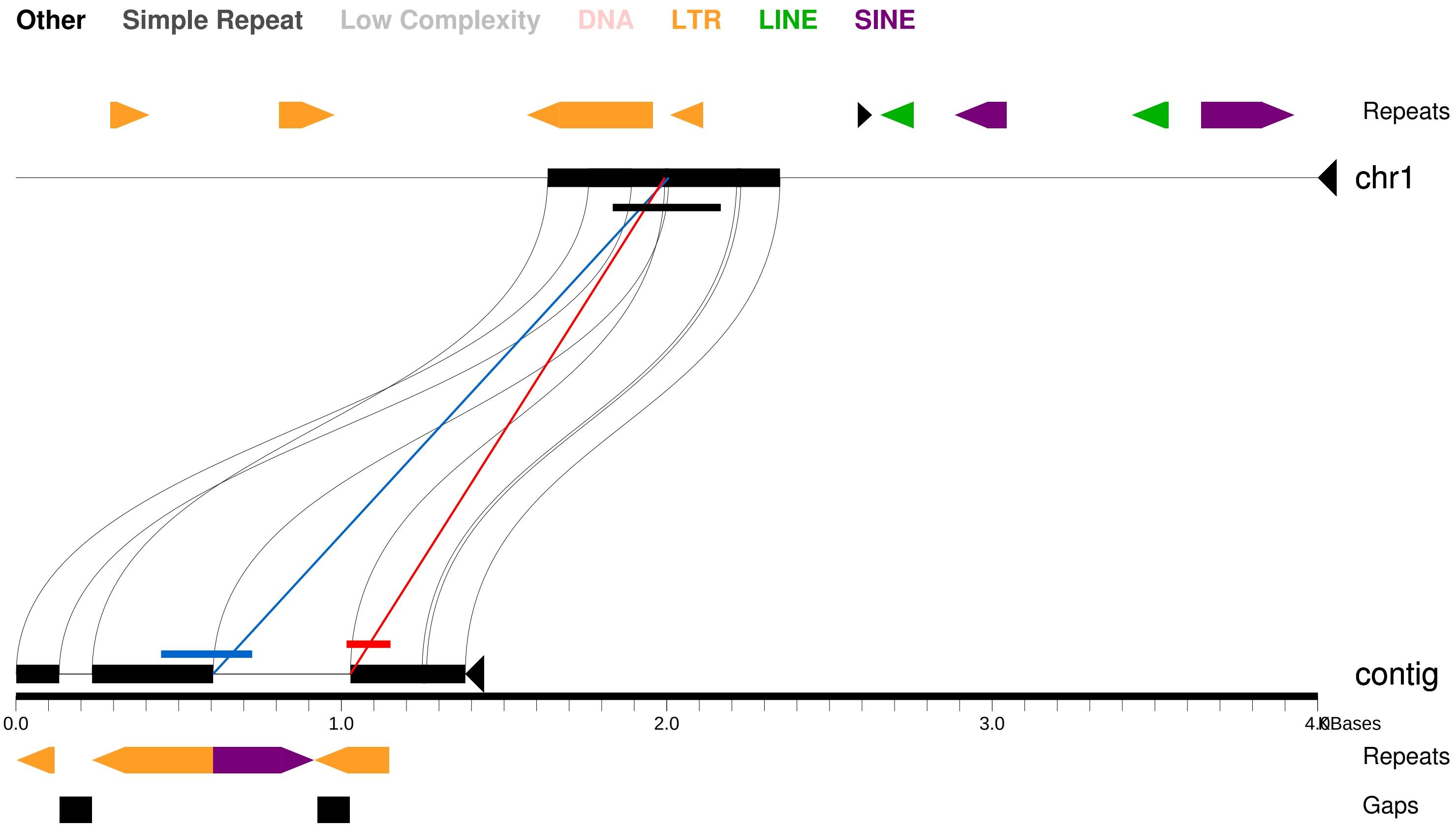

siteID: chr1_239780141 Scafftig1 - Breakpoint: chr1:239780149
chr1:239780098-239780205
Overlap Length: 9 INS size: 316 Genotyped: Yes

```
left   ATGTGGTATTTGAAGACAGGT---------GTTTCTAAATGATGTTATCAGATTTTTAAA---AATATTC
chrom  ATGTGGTATTTGAAGACAGGT---------GTTTCTAAATGATGTTATCAGATTTTTAAA---AATATTC
right  GTCCGGCCTGGGC-GACAGAGCGAGACTCCGTCTCAAAAAAAAAAAAAAAAAAAACAAAAAAAAATATTC
       1*11**11*11*11*****1111111111**1**1***11*1111*11*1*11111***111*******

left   CAGGCCGGGCGCGGTGGCTCACGCCTGTAATCCCA-GCACTTTGGGAGGCCGAGGCGGGTGG
chrom  CAAATC-----CATTAG-TCAT---TGTTGTGCAATGCAGAGTGC-AG--CATTCCTTTCTT
right  CAAATC-----CATTAG-TCAT---TGTTGTGCAATGCAGAGTGC-AG--CATTCCTTTCTT
       **222*22222*22*2*2***2222***22*2*2*2***222**22**22*2222*222222
```

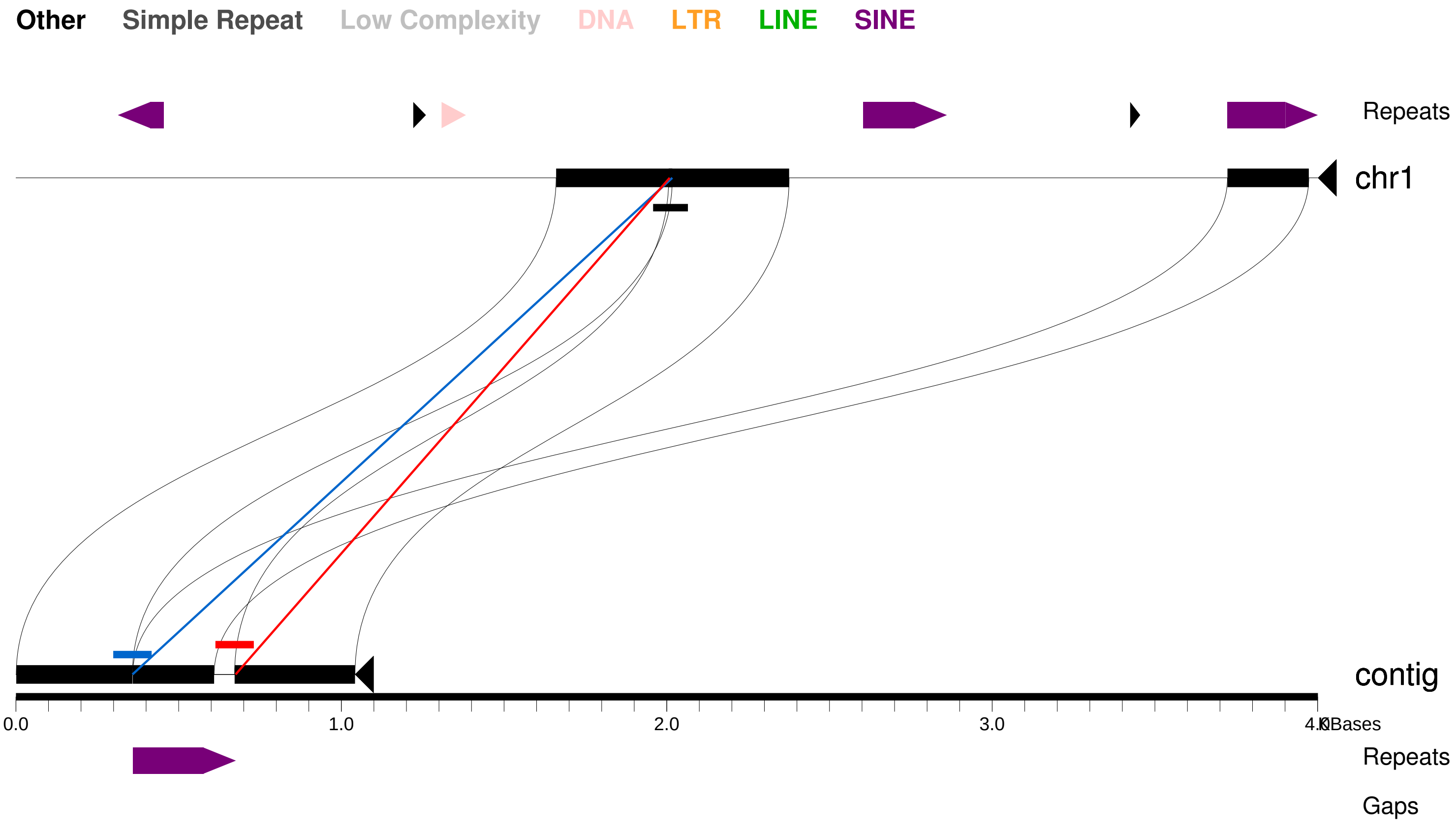

siteID: chr1_245989303 Scafftig10 + Breakpoint: chr1:245989305
chr1:245989288-245989372
Overlap Length: 15 INS size: 310 Genotyped: Yes

```
left   TG-----CTCCTACA--TCTAGTA--------------------------CCTAGCATGCTGCTTTTTTT
chrom  TG-----CTCCTACA--TCTAGTA--------------------------CCTAGCAT-CTGCTTTCTAT
right  CCTCGGCCTCCCAAAGTGCTGGGATTACAGGCGTGAGCCACCGCGCCCGGCCTAGCATGCTGCTTTCTAT
       1111111****1*1*111**1*1*111111111111111111111111111********N*******2*2*

left   TTTTTTTTTTTTTTTTTTTTTTTTTTTGAGACGGAGTCTCGCTCTGTCGCCCAGGCTGGAGTGCAGTGGC
chrom  GTTGTATTTAATTAAATATTTGTTAAATAAA---------------------------------AATGGA
right  GTTGTATTTAATTAAATATTTGTTAAATAAA---------------------------------AATGGA
       2**2*2***22**222*2***2**2222*2*222222222222222222222222222222222*2***2

left   GGGATCTCGGCT
chrom  AGAATATGCATC
right  AAAATATGCATC
       212**2*22222
```

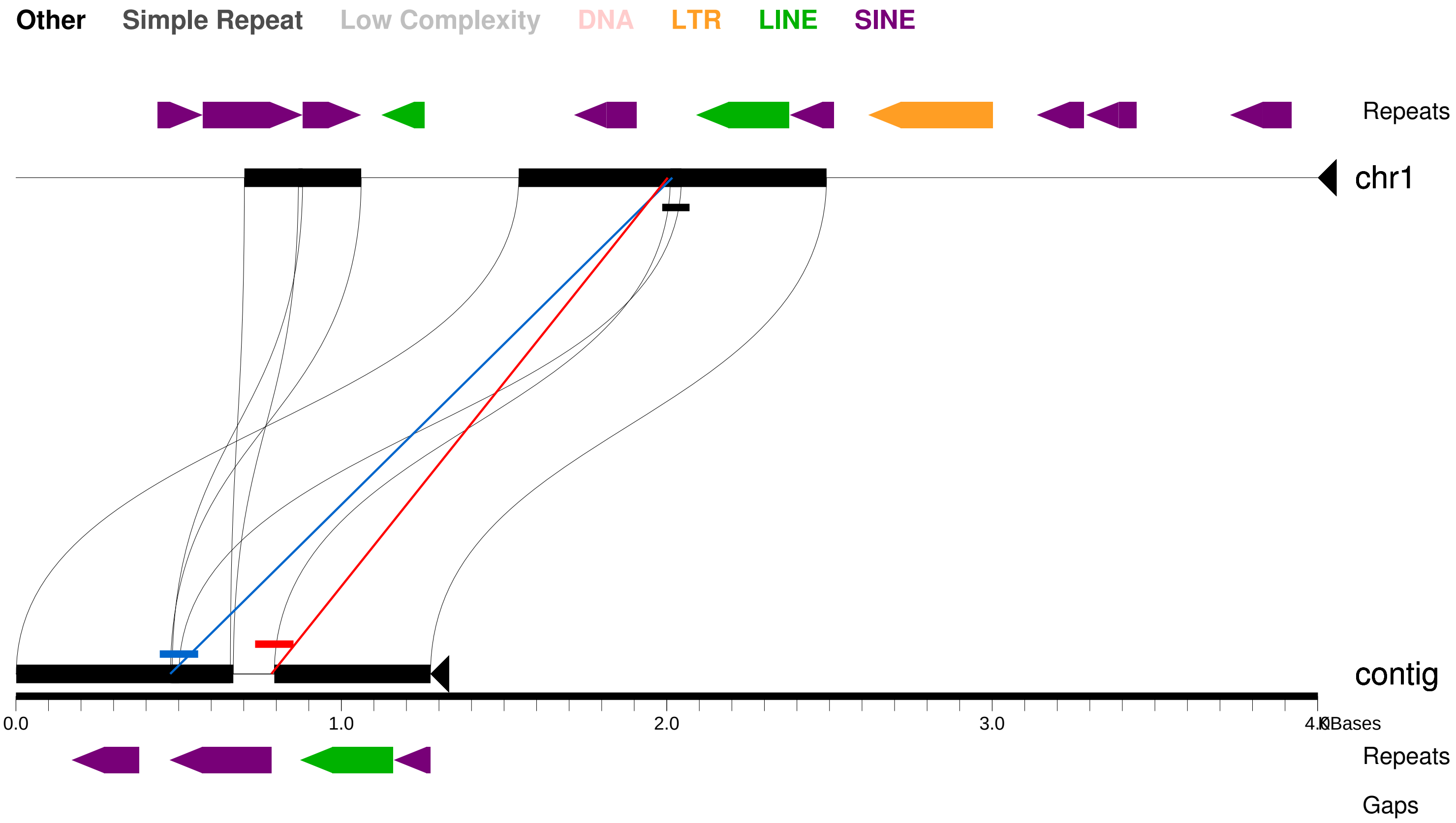

siteID: chr1_246592331 Scafftig1 - Breakpoint: chr1:246592331
chr1:246592171-246592497
Overlap Length: 7 INS size: 412 Genotyped: No

```
left    ---------------------------------AGAGCTAGCTTGCAGCTCAGTCTGGCTCCCCCTCAGGA
chrom   TCTCTCCAGTTCCCTCTGCTCTGCCCTCTTCCAGAGCTAGCTTGCAGCTCAGTCTGGCTCCCCGTCAGGA
right   TTT----AGTAGAGACGGGGTTTCAC-CTT-----GTTAGC---CAGGATGGTCTCGATCTCCTG-----
        2N2    222NNNNN2N2NNN2N2N2 222  111*1****111***1111****1*1**1**N111111

left    ATATTATAGATGCCAATGGGTCCTGGAACTACCCT-GTACTCATATAAAAGGGGAGAGCACAAGCATTCC
chrom   ATATTATAGATGCCAATGGGTCCTGGAACTACCCT-GTACTCATATAAAAGGGGAGAGCACAAGCATTCC
right   ACCTCGT-GATCCGCCCG---CCTCGGCCTCCCAAAGTGCTGGGATTACAGGCGTGAGC-----CACCGC
        *11*11*1***1*1111*111***1*11**1**111**1**111**1*1***1*1****11111**111*

left    TTCCTACCAAACAAAAGCTCTGAGTCTCNNNNNNNNNNN-------------------------------
chrom   TTCCTACCAAACAAAAGCTCTGAGTCTCACTTTAATAGGACAGATCAGGTATTGTTCCCACCACAGAACC
right   GCCCGGCC-------------GAGTCTCACTTTAATAGGACAGATCAGGTATTGTTCCCACCACAGAACC
        11**11**1111111111111*******222222222222222222222222222222222222222222

left    ----------------------------------------------------------------------
chrom   AACCACCGTAGACAGGGAATAAGGTTGCCCAGAGTTATCTATGCCAATGGGAACCCACCACAGGTACAGT
right   AACCACCGTAGACAGGGAATAAGGTTGCCCAGAGTTATCTATGCCAATGGGAACCCACCACAGGTACAGT
        2222222222222222222222222222222222222222222222222222222222222222222222

left    ----------------------------------------------
chrom   AATAAGGGAAGTCTACCCCACCAAACCTGCAAAACTGCCATGTAATGA
right   AATAAGGGAAGTCTACCCCACCAAACCTGCAAAACTGCCAT-------
        222222222222222222222222222222222222222222
```

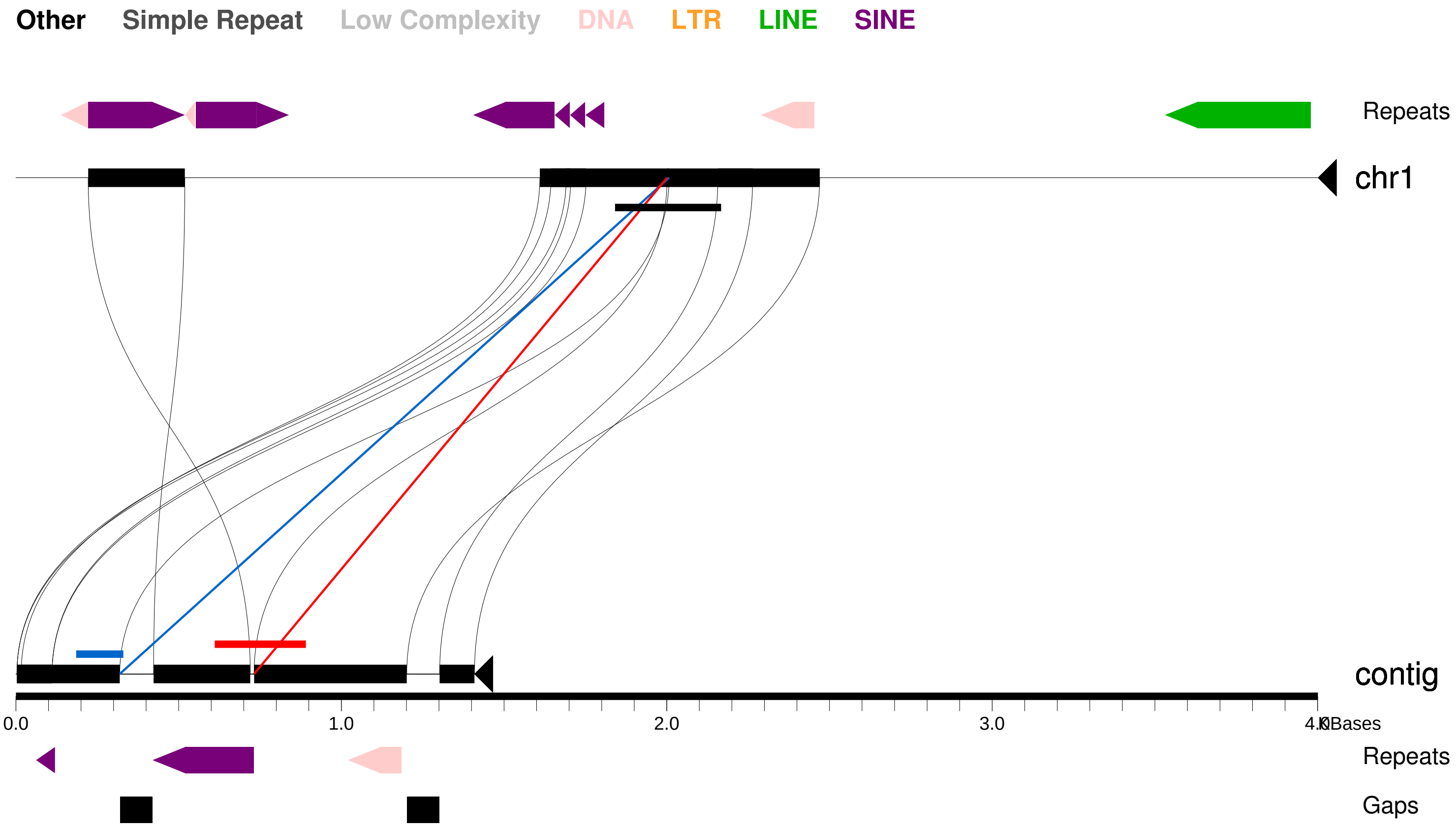

siteID: chr1_247208019 Scafftig1 + Breakpoint: chr1:247207923
chr1:247207889-247207982
Overlap Length: 23 INS size: 420 Genotyped: No

```
left   AAAAAGTGGTACATATACACCATGGAATACTATG-------------------------CAGCTATAAGA
chrom  AAAAAGTGGTACATATACACCATGGAATACTATG-------------------------CAGCTATAAGA
right  NNNNNNNNNNNNNNNNNNNNNNNNNNNNNNNNNNNNNNNNNNNNNNNNNNNNNNNNNNNCAGCTATAAGA
       11111111111111111111111111111111111111111111111111111111111***********

left   AATTAAATCATGGCCGGGCGCGGTGGCTCACGCCTGTAATCCCAGCACTTTGGGAGGCCGAGGCGGGCGG
chrom  AATTAAATCATGTCCTGT-------GCATAAACATG----GCCACAGCT---GGAGGCC-----------
right  AATTAAATCATGTCCTGT-------GCATAAACATG----GCCACAGCT---GGAGGCC-----------
       ************2**2*22222222**22*22*2**22222***222**222*******22222222222

left   ATCA
chrom  ATTA
right  ATTA
       **2*
```

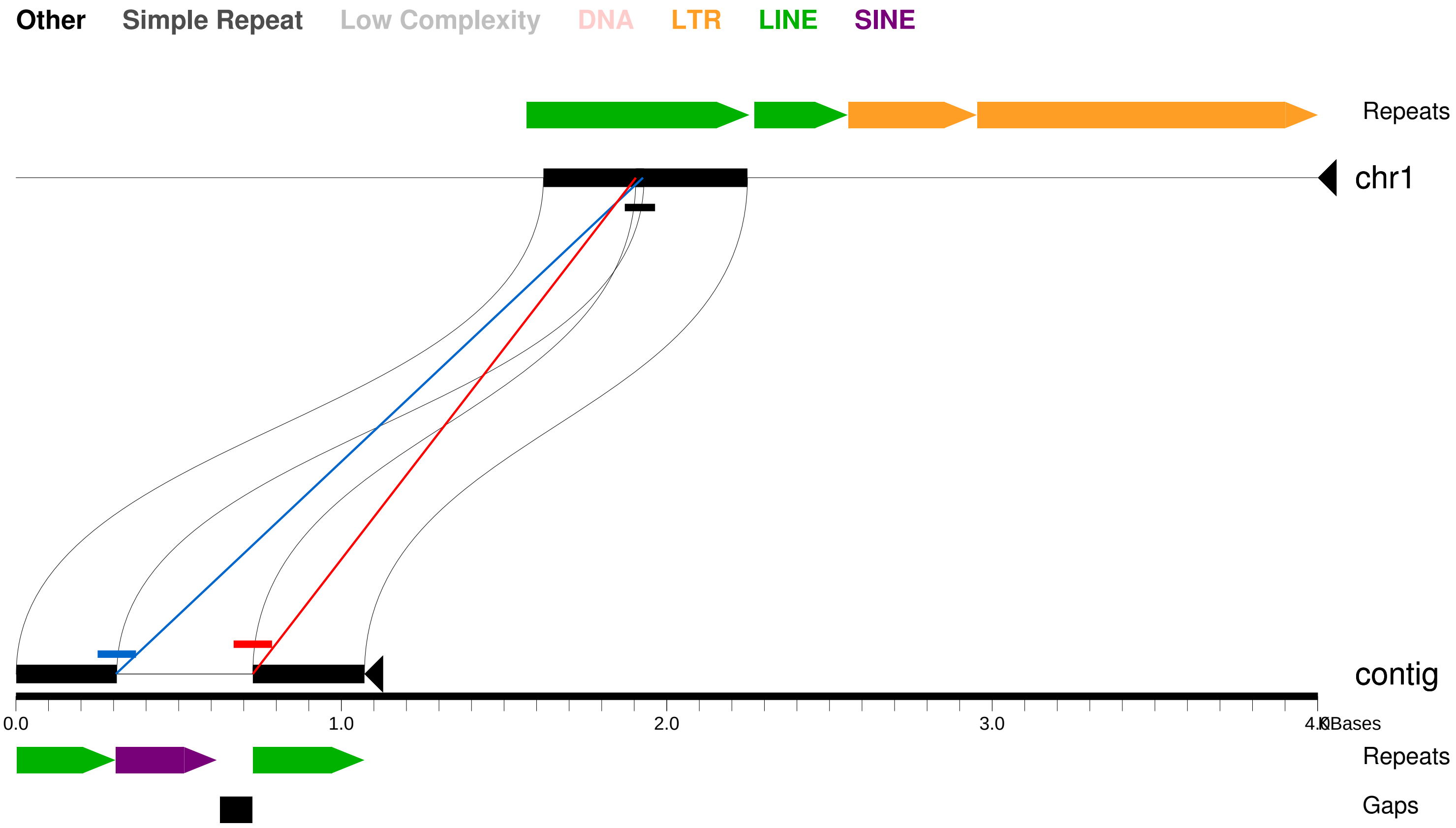

siteID: chr1_247865113 Scafftig1 - Breakpoint: chr1:247865123
chr1:247865075-247865175
Overlap Length: 12 INS size: 307 Genotyped: Yes

```
left     AA-----------CTAGTAAACTGTACCAAGAACC--TCTTAGTATTATTTCTTAAAACTA------CAT
chrom    AA-----------CTAGTAAACTGTACCAAGAACC--TCTTAGTATTATTTCTTAAAACTA------CAT
right    ACTGCACTCCAGCCTGGGCGACAG-AGCGAGACTCCGTCTCAAAAAAAAAAAAAAAAAAAAAAAAAACAT
         *111111111111**1*111**1*1*1*1***11*11***1*11*11*111111****11*111111***

left     GTGAAACTAGGCCGGGCGCGGTGGCTCACGCCTGTAATCCCAGCACTTTGGGAGGCCGAGGCGGGCGG
chrom    GTGAAACTAA-------------AATGAC-CTTATAAT---AACAAATTTTAATGGAAAAGTATACT-
right    GTGAAACTAA-------------AATGAC-CTTATAAT---AACAAATTTTAATGGAAAAGTATACT-
         *********2222222222222222*2**2*2*2****222*2**22**222*2*222*2*2222*22
```

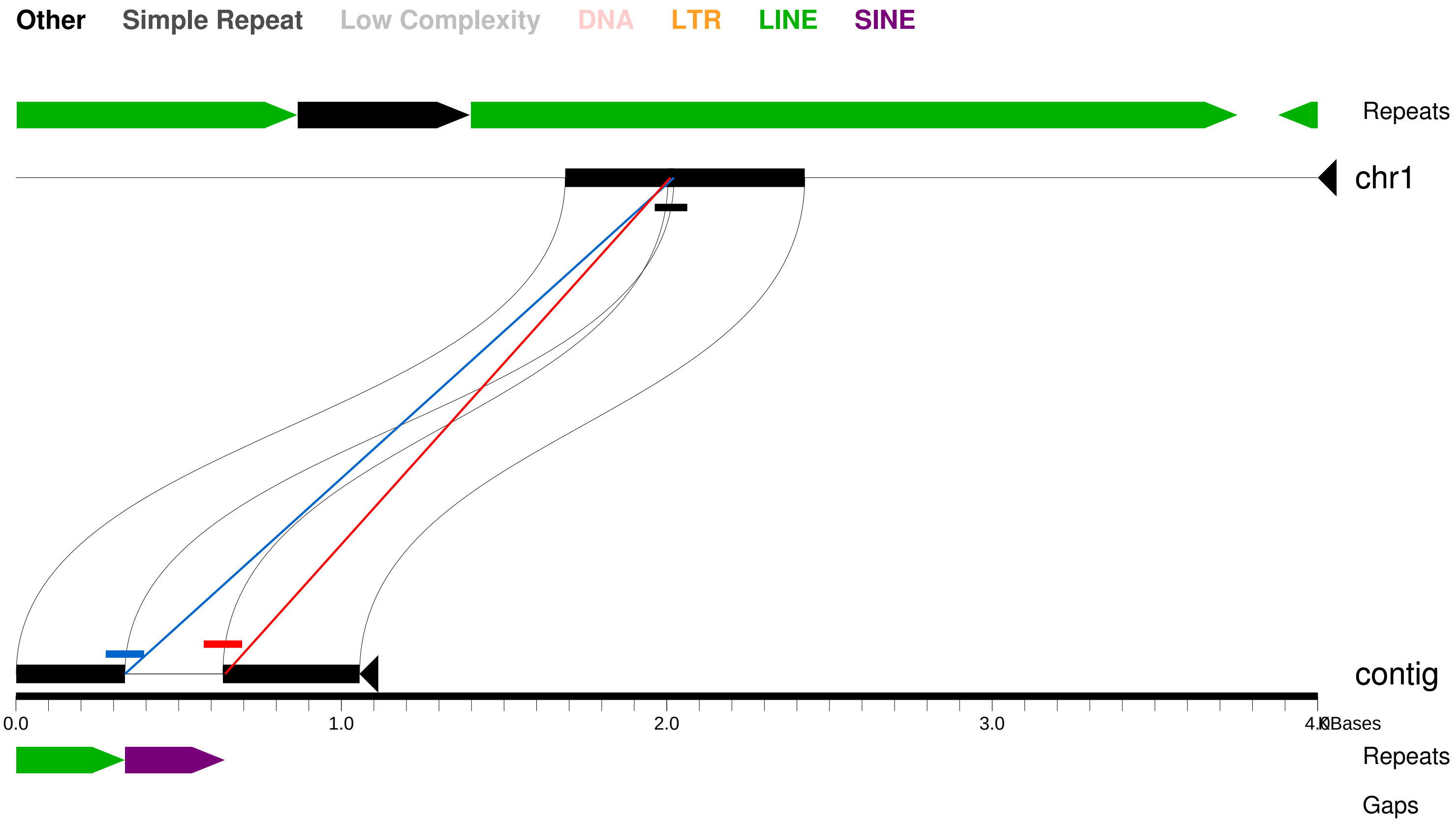

siteID: chr1_29862444 Scafftig1 - Breakpoint: chr1:29862408
chr1:29862248-29862580
Overlap Length: 13 INS size: 420 Genotyped: No

```
left    -----------------------------------------------ATTTGAATTAAAAAAATCCCATT
chrom   TAACACATTTTTAAAAAGATAATCTCAATAGCAATAATAAATCAGAAATTTGAATTAAAAAAATCCCATT
right   T------TTAGTAGAGACGGGGTTTCA----CCCTGTTAGC-CAGGA--TGGTCTCGATCTCCTGACCTC
        2      22NN22N2N2NNNNN2N222    2NN2NN22NN 222N211*1*11*11*11111*11*1*1

left    GAGAATGGCATAAAATGTGAAATACTCAGAAATACATCTAACAAAAGATTTATATAAATCTGACAATGGA
chrom   GAGAATGGCACAAAATGTGAAATACTCAGAAATACATCTAACAAAAGATTTATATAAATCTGACAATGGA
right   GTGATCCGCCCGC-----------CTCGG-----CCTC---CCAAAG--TGCTGGGATT---ACAGGCGT
        *1**111**121111111111111***1*11111*1**111*1****11*11*111*1*111***111*1

left    ATACTACTCAGCAATAAAAATGAATGAACTCTTT-----TTT----------------------------
chrom   ATACTACTCAGCAATAAAAATGAATGAACTCTTGATACATTTATCAACATGGCTAAATCATCAAATAATT
right   GAGCCACCGCGCCCGGCCC-TGAATGAACTCTTGATACATTTACCAACATGGCTAAATCATCAAATAATT
        111*1**111**11111111*************222222***2N2222222222222222222222222

left    ----------------------------------------------------------------------
chrom   ACACTGAGTGGAAGGAGCTGGATGAAAAAGCACACATACTGTGTGAGTTCATATATATTAAAATCTAGAA
right   ACACTGAGTGGAAGGAGCTGGATGAAAAAGCACACATACTGTGTGAGTTCATATATATTAAAATCTAGAA
        2222222222222222222222222222222222222222222222222222222222222222222222

left    -------------------------------------TTTTTNNNNNNNNNNN
chrom   CTGTAACCAATCTATAGTGACAGAAACAGATTAGTGCTTGTTTGGGGATGAAG
right   CTGTAACCAATCTATAGTGACAGAAACAGATTAGTGCTT--------------
        2222222222222222222222222222222222222**N11NNNNNNNNNNN
```

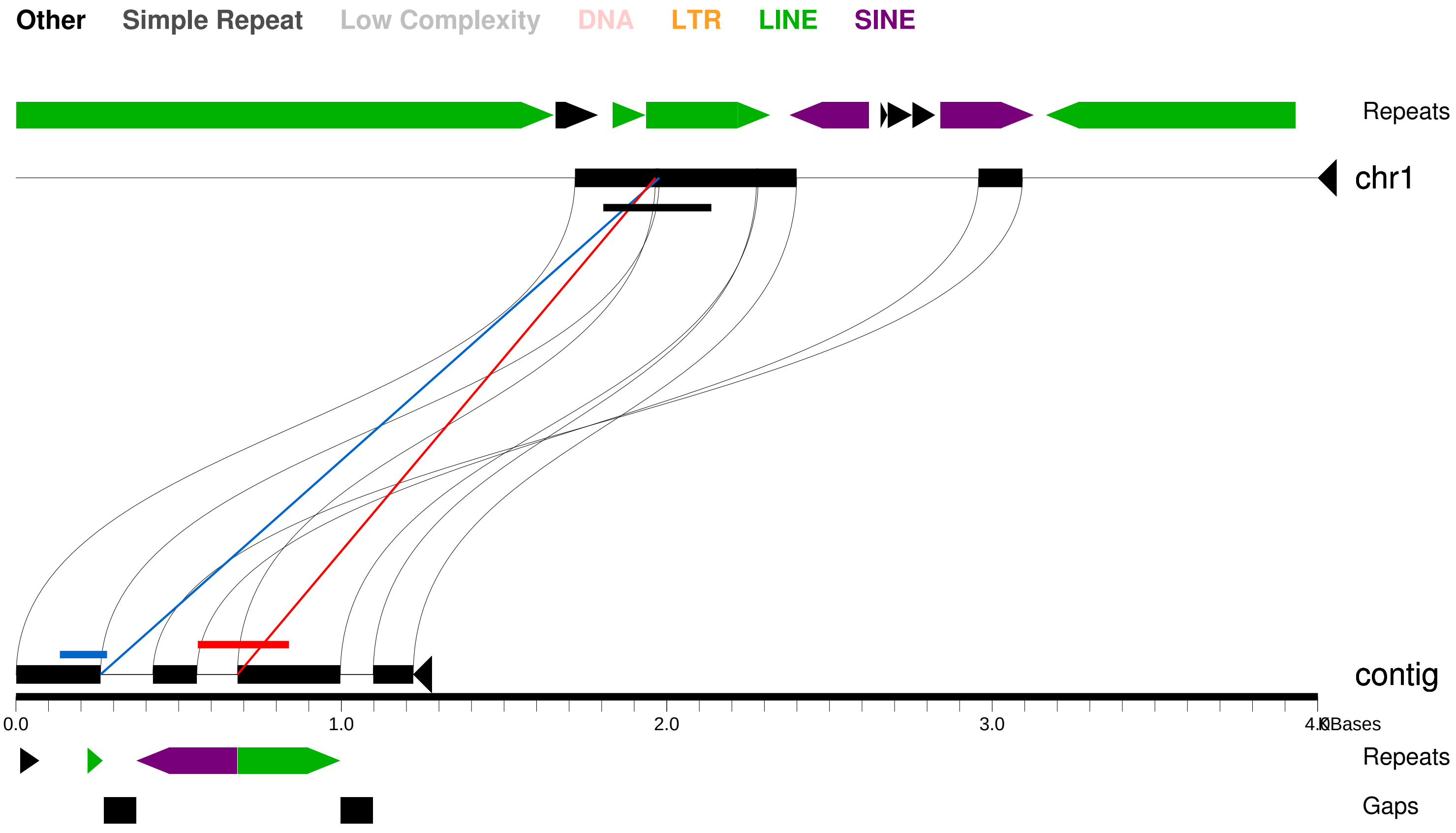

siteID: chr1_4288508 Scafftig1 - Breakpoint: chr1:4288451
chr1:4288306-4288629
Overlap Length: 15 INS size: 362 Genotyped: No

```
left   G-----AG--------------------------------GAGCCCAGTCCCTTGAGTTCCTGTGTGGCA
chrom  G-----AGCCACGGAAATTCGTGCCACGTGCAGAGGGGAGGAGCCCAGTCCCTTGAGTTCCTGTGTGGCA
right  TTTAGTAGAGACGGGGTTTCA---CCGTTTTAGCCGGGATGGTCTCGATCTCCTGACCTCGTGATCCGC-
       111111**NN2222NNN222N   2NNN2NN22NN2222N*11*1*11**1*1***11**1**1111**1

left   GGGTGGGAGCAGGTTAGCAGGCTTCCTTCTTACTCTGCTGAAAGCTTTCTGTTTTTCCTTTTTCGCCCAA
chrom  GGGTGGGAGCAGGTTAGCAGGCTTCCTTCTTACTCTGCTGAAAGCTTTCTGTTTTTCCTTTTTCGCCCAA
right  ---------CCGCCTCG--GCCTCCCAAAGTGCTGGGATTACAGGCGTGAG------CCACCGCGCCCGG
       111111111*1*11*1*11*1**1**1111*1**11*1*1*1**111*11*111111*11111*****11

left   TAAATTCCATTTTTCTCACCCTTCTTT-------------------------------------------
chrom  TAAATTCCATTTTTCTCACCCTTCTATGTGTCTGCGGGCCTAATGTTTCCTGGCTGTGTGACTAGAACCC
right  ------CC--TTTTCTCACCCTTCTATGTGTCTGCGGGCCTAATGTTTCCTGGTTGTGTGACTAGAACCC
       111111**11***************2*222222222222222222222222222N2222222222222222

left   -TTTTTNNNNNNNNNNN-----------------------------------------------------
chrom  ATTTTTTAGTTGAACTAAGGAGAAAGTCCTACAACAACACCACCTCCTTCTGCAGCGACACACCACTTCA
right  AGTTTTTAGCTGAACTAAGGAGAAAGTCCTACAACAACACCGCCTCCTTCTGCAGCGACACACCACTTCA
       21****222N222222222222222222222222222222N22222222222222222222222222222

left   ------------------------------------------------
chrom  TTGGTGATGAGGACAATGCAGTTTCTAGCAGCGGCGGATGTGCTTTTT
right  TTGGTGATGAGGACAATGCAGTTTCTAGCA------------------
       222222222222222222222222222222
```

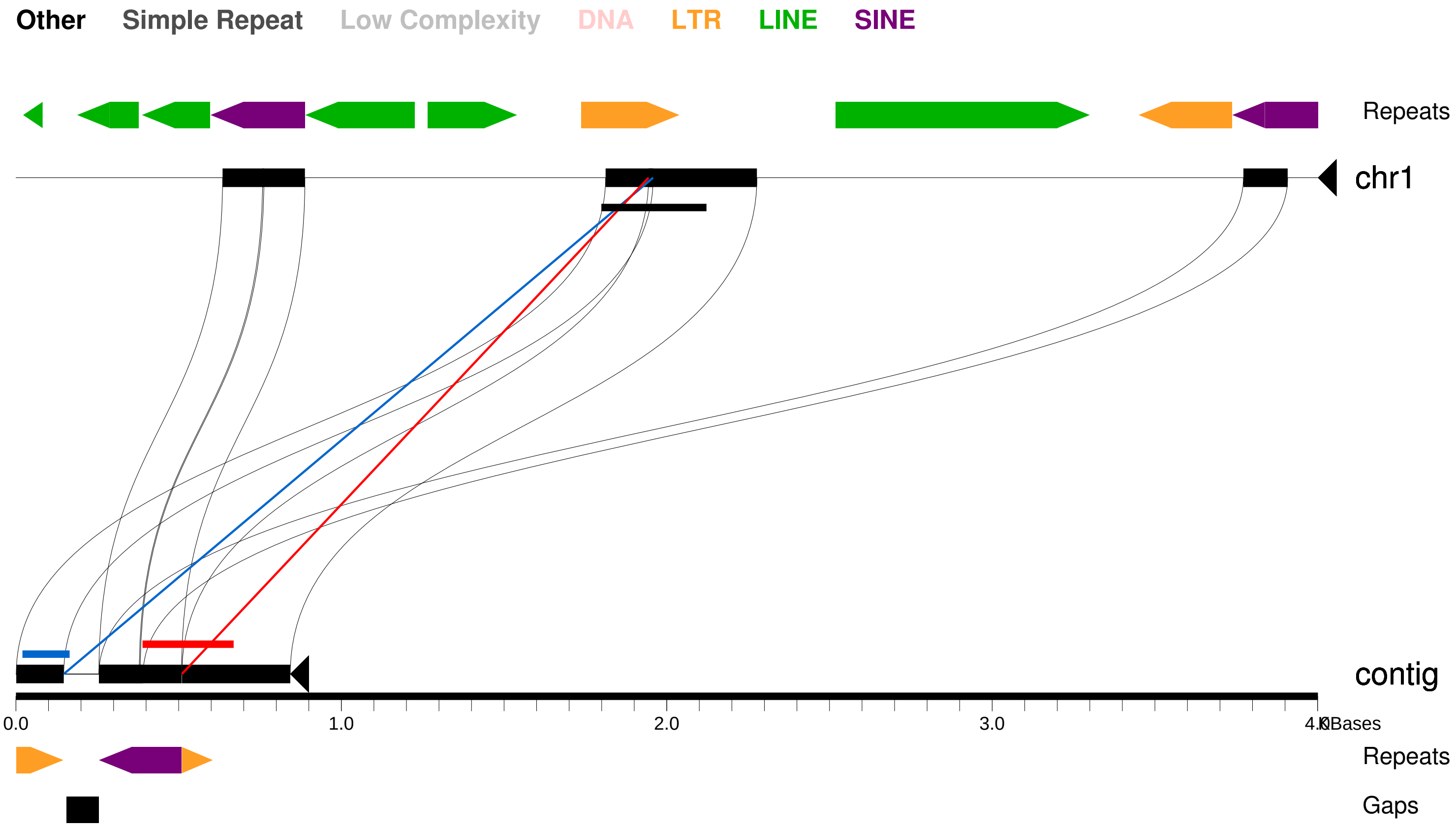

siteID: chr1_5934307 Scafftig2 - Breakpoint: chr1:5934289
chr1:5934242-5934348
Overlap Length: 13 INS size: 269 Genotyped: Yes

```
left    TCCATTAGATTCACTGGGCTTTA-----AGAAGCCCTTCCAACCAACTTAGT-------AAAGAAAATTA
chrom   TCCATTAGATTCACTGGGCTTTA-----AGAAGCCCTTCCAACCAACTTAGT-------AAAGAAAATTA
right   TCCGCAGTCCGGCCTGGGCGACAGAGCGAGACTCCGTCTAAAAAAAAAAAAAAAAAAAAAAAGAAAATTA
        ***1111111111******111*11111***11**1*11***11**111*111111111***********

left    GGTTGGGAGGCCGAGGCGGGTGGATCATGAGGTCAGGAGATCGAGACCATCCTGGCTAACA
chrom   GGAGGAGATGGTCCAGCTCGACACCCATCAGG-CTGGAAAA-GAGAA------GGGG----
right   GGAGGAGATGGTCCAGCTCGACACCCATCAGG-CTGGAAAA-GAGAA------GGGG----
        **22*2**2*22222**22*22222***2***2*2***2*22****2222222**222222
```

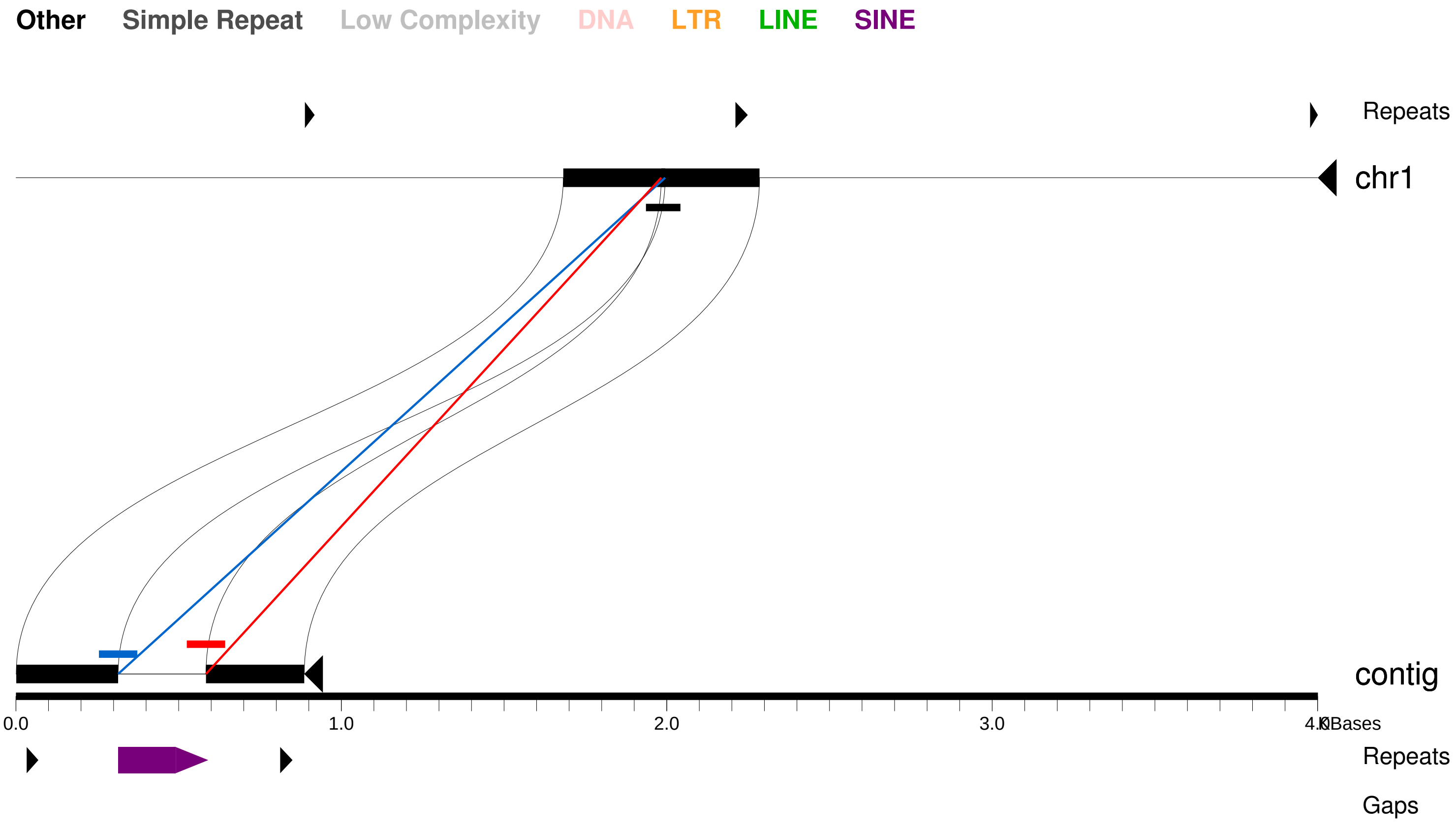

siteID: chr1_8446641 Scafftig2 - Breakpoint: chr1:8446628
chr1:8446468-8446795
Overlap Length: 8 INS size: 308 Genotyped: Yes

```
left   ------CCTGTCTCATAAAGACAGTAA-AGCCACACAAATAAGGGTTTCCACTCCCTGGGCCTGAGCTCT
chrom  TGTGGCCCTGTCTCATAAAGACAGTAA-AGCCACACAAATAAGGGTTTCCACTCCCTGGGCCTGAGCTCT
right  TTTAGTAGAGACGGGGTTTCACCGTTTTAGCCGGG------ATGGTCTCGATCTCCTGACCTCGTGATCC
       2N2N2N111*1*11111111**1**111****111111111*1***1**1*111****11*11*1*1**1

left   GAAACCCAGCCAAGACAGAATCCCAAACAGCCAGAGAGCTTCCTTTCTGAAGGCAGTGAACTGAACATAC
chrom  GAAACCCAGCCAAGACAGAATCCCAAACAGCCAGAGAGCTTCCTTTCTGAAGGCAGTGAACTGAACATAC
right  G----CCCGCCTCGGCC---TCCCAAAGTGCTGG---GATTAC-------AGGC-GTGAGCC--------
       *1111**1***11*1*1111******11**11*111*1**1*1111111****1****1*111111111

left   ATTATTTAAGATGAATATCTAGAACTCTTTTTTTTTTTTTTT----------TTTTTTTTTTTTTGAGAC
chrom  ATTATTTAAGATGAATATCTAGAACTCTTACAAATGAAAAAGAAAAGGACAATCCATTGAAAAATAAGCA
right  -------ACCGCGCCCGGCCCGAACTCTTACAAATGAAAAAGAAAAGGACAATCCATTGAAAAATAAGCA
       1111111*1111*11111*11********22222*22222222222222222*222**222222*2**22

left   GGAGTCTCGCTCTGTTGCCCAGGCTGGAGTG----CAGTGGCGGGATCTCGGCTCACTGCAAGCTCCGCC
chrom  AAAGACTTACACAG--GCCCTTCATGAAAAAGAAACACAAACAGCCAATAAACTCATTATAGGATGCCCA
right  AAAGACTTACACAG--GCCCTTCATGAAAAAGAAACACAAACAGCCAATAAACTCATTATAGGATGCCCA
       22**2**22*2*2*22****2222**2*2222222**2222*2*2222*222****2*22*2*2*2*2*2

left   T------------CCCGGG---TTCA--CGCC------------ATTCTCC
chrom  ATATGATTAGTAACCAGGGAAATACAAACTCCTCAAATGCCCAAATTCTTT
right  ATATGATTAGTAACCAGGGAAATACAAACTCCTCAAATGCCC---------
       2222222222222**2***222*2**22*2**2222222222  11111NN
```

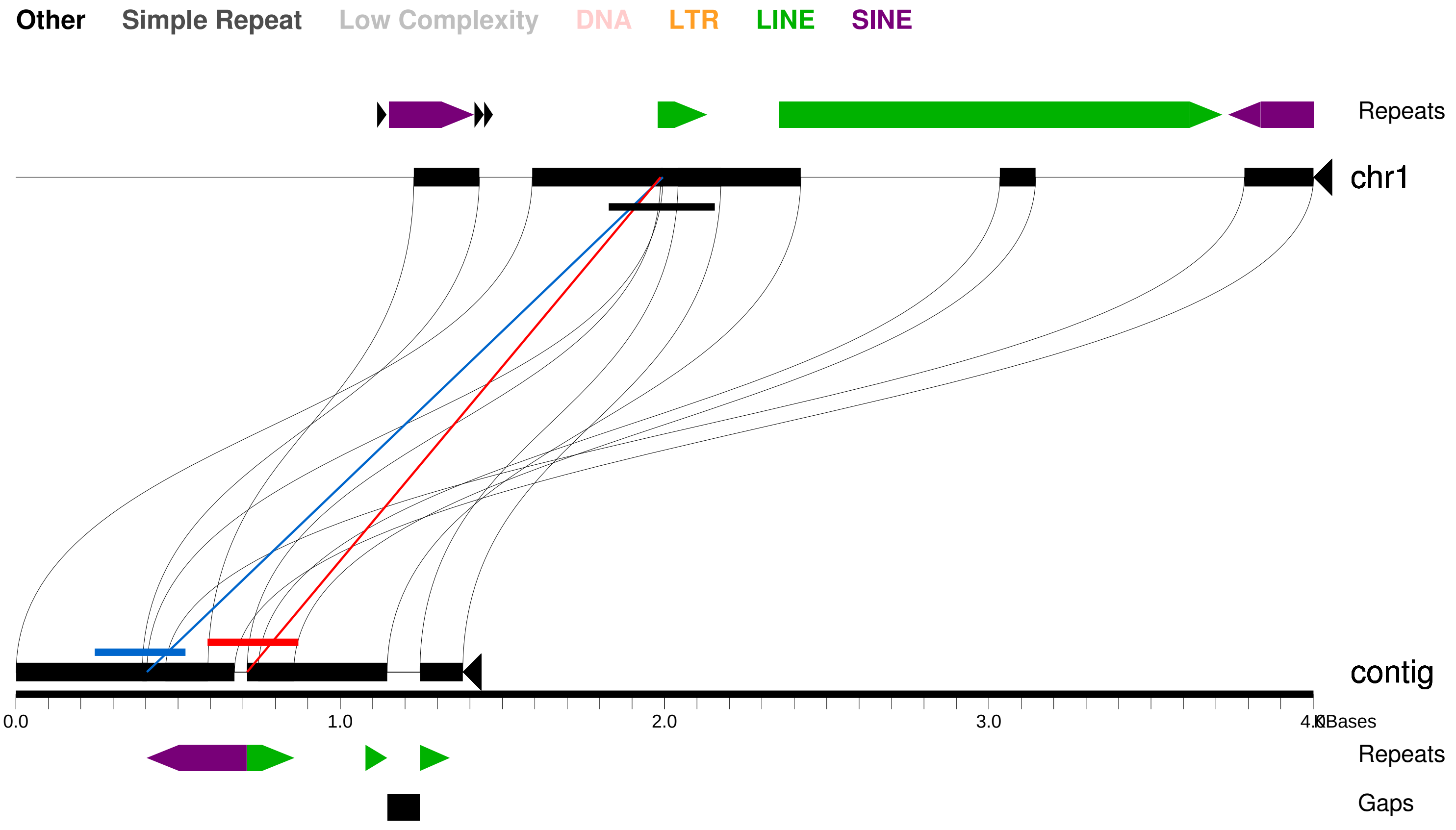

siteID: chr10_107285663 Scafftig1 + Breakpoint: chr10:107285490
chr10:107285447-107285549
Overlap Length: 17 INS size: 131 Genotyped: Yes

```
left    -----TGGTAATTGAAAATATAATA-ATTAT--TGATTAAAAATCAGAAAT--------GATTTTTGCTG
chrom   -----TGGTAATTGAAAATATAATA-ATTAT--TGATTAAAAATCAGAAAT--------GATTTTTGCTG
right   CAAGCTCCGCCTCCCGGGTTCACGCCATTCTCCTGCCTCAGCCTCCCAAGTAGCTGGGAGATTTTTGCTG
        11111*11111*111111*11*1111***1*11**11*1*111**11**1*11111111***********

left    ATTTTTTTTTTTTTTTTTGAGACGGAGTCTCGCTCTGTCGCCCAGGCTGGAGTGCAGTGGCGCGA
chrom   ATTTTTAAAAGATTTTATAAGTCAATGA---------------AAAATAAAATAAATTAGAAA-A
right   ATTTTTAAAAGATTTTATAAGTCAATGA---------------AAAATAAAATAAATTAGAAA-A
        ******222222****2*2**2*222*222222222222222*222*22*2*22*2*2*2222*
```

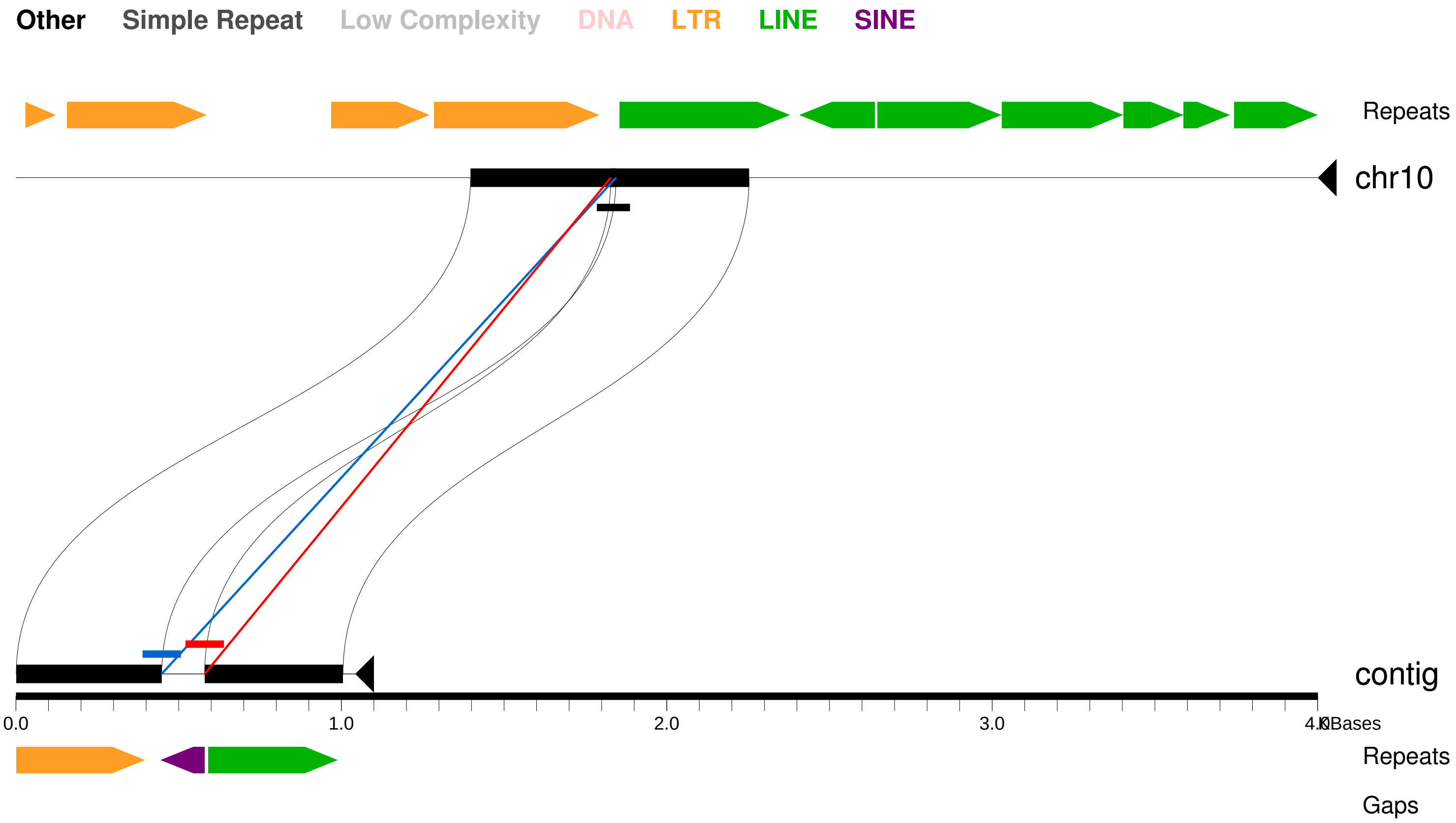

siteID: chr10_107891565 Scafftig12 + Breakpoint: chr10:107891547
chr10:107891502-107891606
Overlap Length: 15 INS size: 278 Genotyped: Yes

```
left    TCT-GTGGTCAACA-GAGAGG-------ACAGCGGTGCTGTTTC-----TCTGCTGATTGGAGCACACGT
chrom   TCT-GTGGTCAACA-GAGAGG-------ACAGGGGTGCTGTTTC-----TCTGCTGATTGGAGCACACGT
right   CCTCGTGATCCGCCCGCCTCGGCCTCCCAAAGTGCTGGGATTACAGGCGTGAGCCACCGGGAGCACACGT
        1**1***1**11*11*1111*1111111*1**N*1**111**1*11111*11**11111***********

left    TTTTTTTTTTGAGACGGAGTCTCGCTCTGTCGCCCAGGCTGGAGTGCAGTGGCGCGATCTCGG
chrom   TTTTAA-----------ATACT---TCTTTATGCCTTTATTGATCATGAATATGATATCTGGT
right   TTTTAA-----------ATACT---TCTTTATGCCTTTATTGATCATGAATATGATATTTGGT
        ****2222222222222*22**222***2*222**2222*2**2222222222*22**1*2*2
```

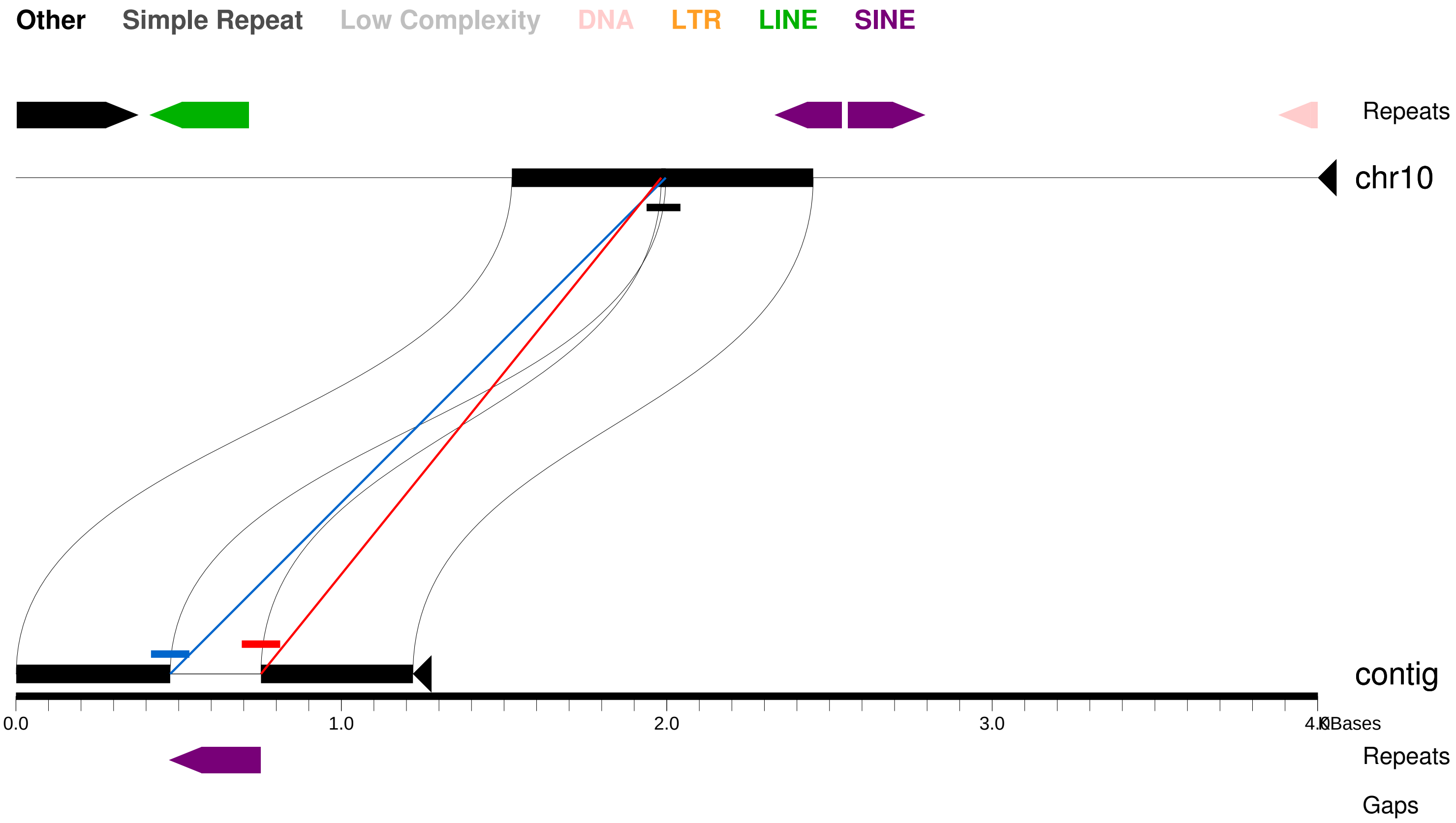

siteID: chr10_108991291 Scafftig4 + Breakpoint: chr10:108991269
chr10:108991225-108991328
Overlap Length: 16 INS size: 293 Genotyped: Yes

```
left    TAT----TTTCATTTTTTCACACCCCTTTGATTA----------ACACTGTGATAAGT-GCATATTACAC
chrom   TAT----TTTCATTTTTTCACACCCCTTTGATTA----------ACACTGTGATAAGT-GCATATTACAC
right   CCGCCCGCCTCGGCCTCCCAAAGTGCTGGGATTACAGGCGTGAGCCACCGCGCCCGGCCGCATATTACAC
        111111111**1111*11**1*111**11*****11111111111***1*1*1111*11***********

left    ATTTTTTTTTTTTTTTGAGACGGAGTCTCGCTCTGTCGCCCAGGCTGGAGTGCAGTGGCGGGAT
chrom   ATTTTAA---------GAACCTCAGTTTAGTTAAAAATAACTGA-TGAA----AGTCCCTCC-T
right   ATTTTAA---------GAACCTCAGTTTAGTTAAAAATAACTGA-TGAA----AGTCCCTCC-T
        *****22222222222**22*22***2*2*2*22222222*2*22**2*2222***22*2222*
```

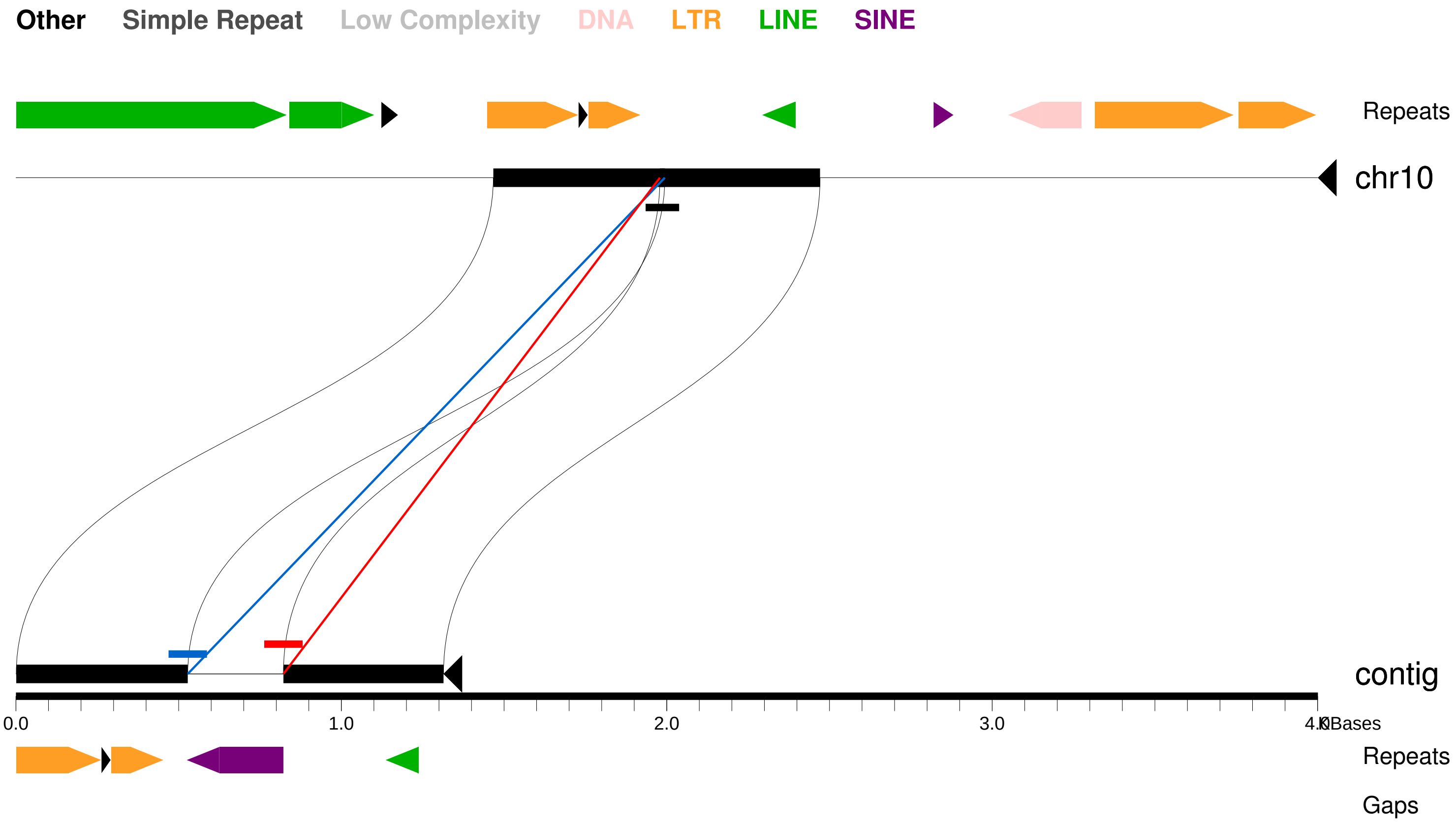

siteID: chr10_114073872 Scafftig8 - Breakpoint: chr10:114073854
chr10:114073815-114073907
Overlap Length: 21 INS size: 288 Genotyped: Yes

```
left     T---CTGTGA--------CT--AATCAAGACCTTTTCCA-AGA---AAAC----------AAACAGAAAT
chrom    T---CTGTGA--------CT--AATCAAGACCTTTTCCA-AGA---AAAC----------AAACAGAAAT
right    TGAGCCGAGATCGCGCCACTGCACTCCAG-CCTGGGCGACAGAGCGAGACTCCGTCTCAAAAAAAAAAAA
         *111*1*1**11111111**11*1**1**1***111*1*1***111*1**1111111111***1*1***1

left     AAAATATTGAGTTTCTGGCCGGGCGCGGTGGCTCACGCCTGTAATCCCAGCACTTTGGGAGGCCGAGGTG
chrom    AAAATATTGAGTTTCTGCC---GTGAAGTGACATA-GACTGAAATG---GAGCTT-------CC------
right    AAAATATTGAGTTTCTGCC---GTGAAGTGACATA-GACTGAAATG---GAGCTT-------CC------
         ****************2*222*2*22***2*22*2*2***2***2222*22***2222222**222222

left     GGCGGA
chrom    ------
right    ------
         222222
```

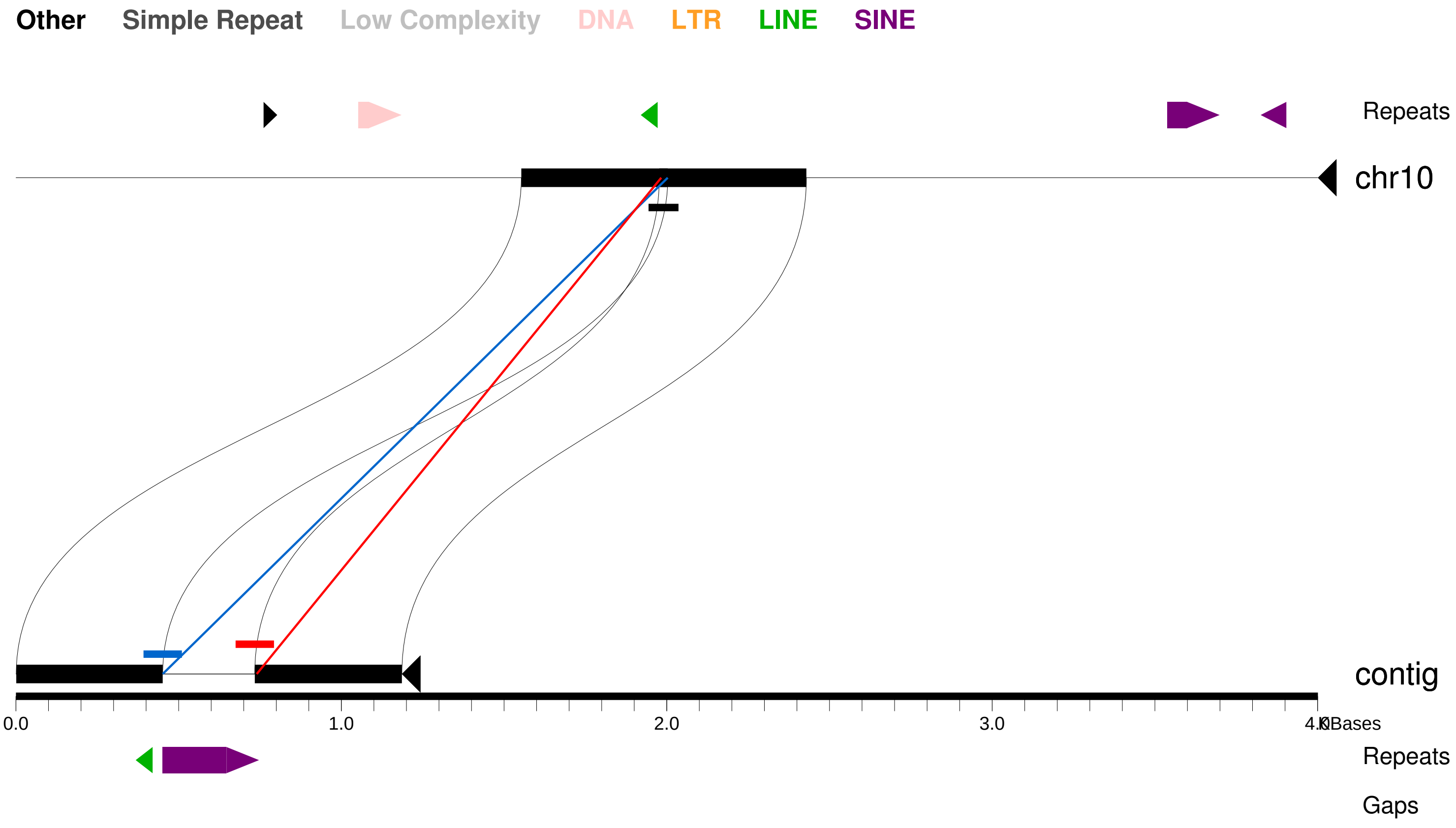

siteID: chr10_115048229 Scafftig3 - Breakpoint: chr10:115048251
chr10:115048206-115048310
Overlap Length: 15 INS size: 300 Genotyped: Yes

```
left   ----CAAACCTTGTTGTGC-----ATAGATAAT-----TTTAAAAACTTTTTTCTCTATAAAAATAATCA
chrom  ----CAAACCTTGTTGTGC-----ATAGATAAT-----TTTAAAAACTTTTTTCTCTATAAAAATAATCA
right  ACTGCACTCCAGCCTGGGCGACAGAGCGAGACTCCGTCTCAAAAAAAAAAAAAAAAAAAAAAAATAATCA
       1111**11**1111**1**11111*11**1*1*11111*11*****11111111111*1***********

left   TTGCGGCCGGGCGCGGTGGCTCACGCCTGTAATCCCAGCACTTTGGGAG-GCCGAGGCGGGCGG
chrom  TTGCT--------CATTGTAGAAAGTTTAGAA-------AATGTGGAAAAGCATAGAGAAGAAA
right  TTGCT--------CATTGTAGAAAGTTTAGAA-------AATGTGGAAAAGCATAGAGAAGAAA
       ****222222222*22**2222*2*22*22**2222222*2*2***2*22**22**2222*222
```

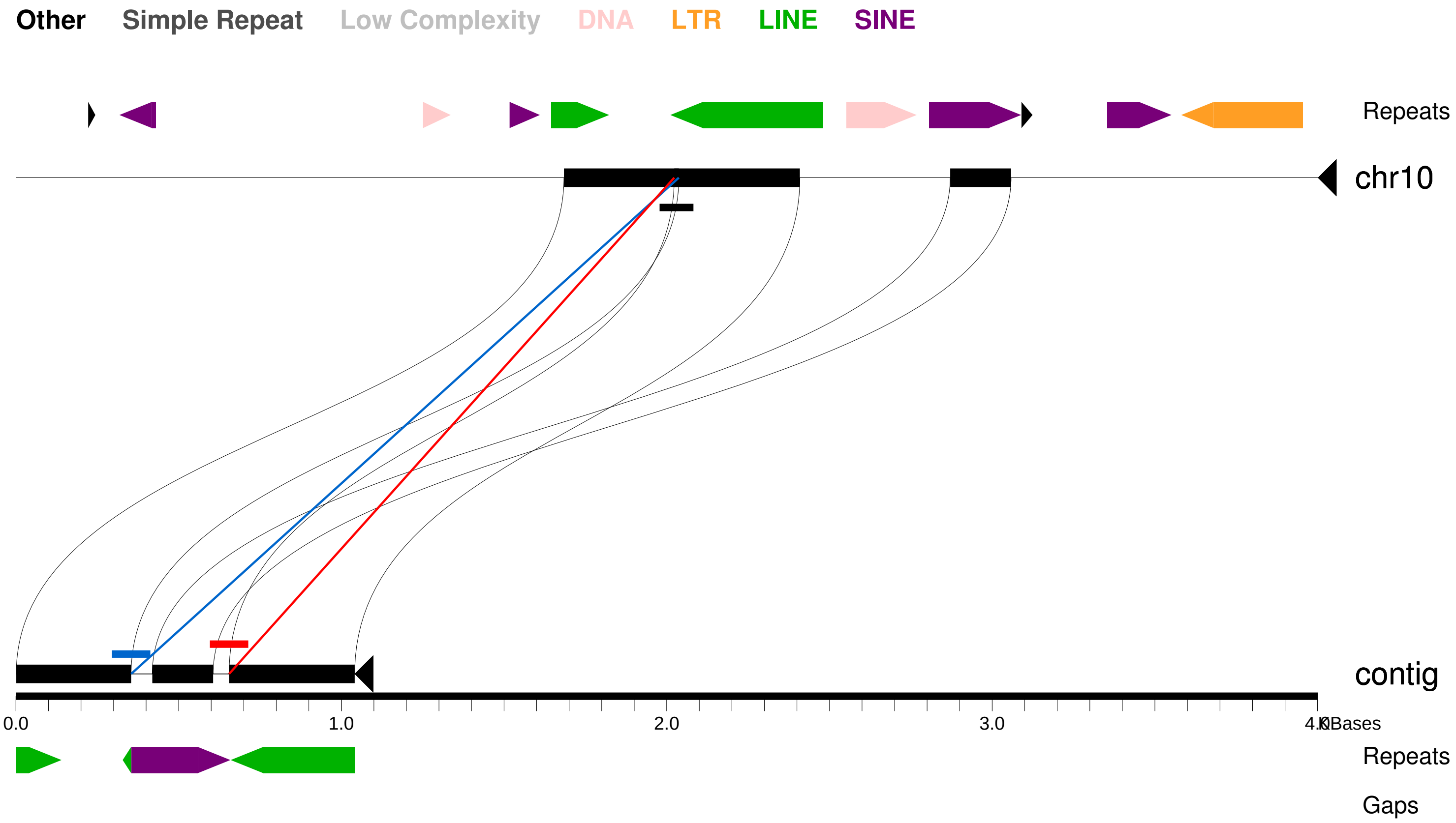

siteID: chr10_115250265 Scafftig3 + Breakpoint: chr10:115250270
chr10:115250227-115250329
Overlap Length: 17 INS size: 479 Genotyped: No

```
left    TGGAGGACATGACTGTGGGCTTTATATTTCTGTGAATTAATTT----------------AAGAAAAATAA
chrom   TGGAGGACATGACTGTGGGCTTTATATTTCTGTGAATTAATTT----------------AAGAAAAATAA
right   NNNNNNNNNNNNNNNNNNNNNNNNNNNNNNNNNNNNNNNNNNNNNNNNNNNNNNNNNNNNNAAAAGAAAAATAA
        11111111111111111111111111111111111111111111111111111111111***********

left    AAGGAAGGCCGGGCGCGGTGGCTCACGCCTGTAATCCCAGCACTTTGGGAGGC-CGAGGCGGGTGG--
chrom   AAGGAA------------TAGTTATTTCTTGTTCT-------CTTTGTGGTGCTCTAAGAATTTCACA
right   AAGGAA------------TAGTTATTTCTTGTTCT-------CTTTGTGGTGCTCTAAGAATTTCACA
        ******222222222222*2*2*2222*2***22*2222222*****2*22**2*2*2*2222*2222
```

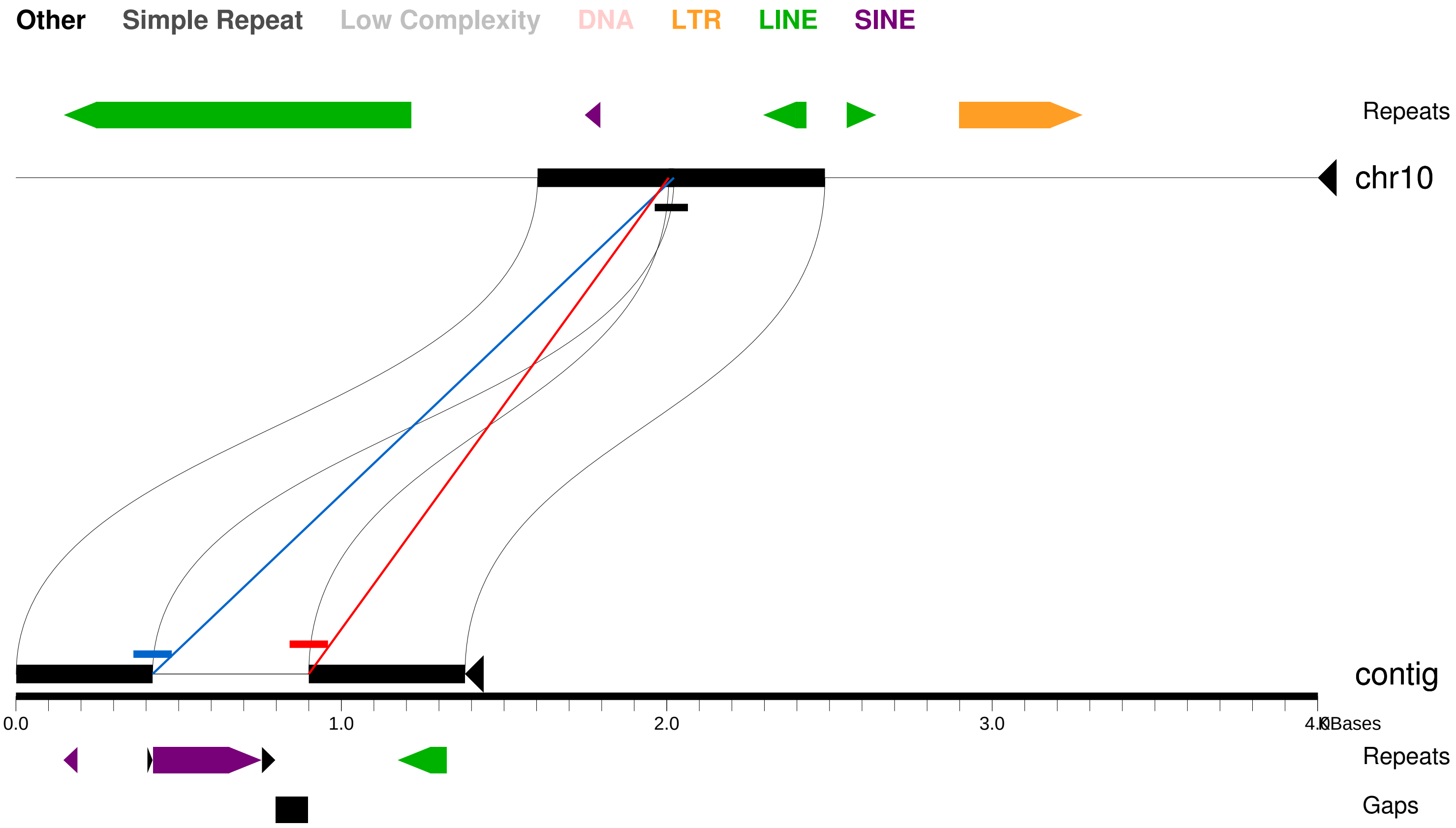

siteID: chr10_117006243 Scafftig8 + Breakpoint: chr10:117006328
chr10:117006283-117006387
Overlap Length: 15 INS size: 304 Genotyped: Yes

```
left    TCAAGA---TAG-------AAAATCCAGGAGAAAGAAAAGGATAATGGATAATGA------ATCTCTCTC
chrom   TCAAGA---TAG-------AAAATCCAGGAGAAAGAAAAGGATAATGGATAATGA------ATCTCTCTC
right   CCACCCGCCTCGGCCTCCCAAAGTGCTGGG--ATTACAGGCGTGAGCCACCGCGCCCGGCCATCTCTCTC
        1**111111*1*1111111***1*1*1**111*11*1*1*11*1*111*1111*1111111*********

left    ATTCTTTT----TTTTTTTTTTTTTTTGAGACGGAGTCTCGCTCTGTCGCCCAGGTCGGACTGCGGACTG
chrom   ATTCTTAACAAGTCTGTATCCATTTTGAGA--GA--------CTGT--------ATTGAATACAAAAAT
right   ATTCTTAACAAGTCTGTATCCATTTTGAGA--GA--------CTGT--------ATTGAATACAAAAAT
        ******222222*2*2*2*222********22**22222222****22222222222**2*2*22*222
```

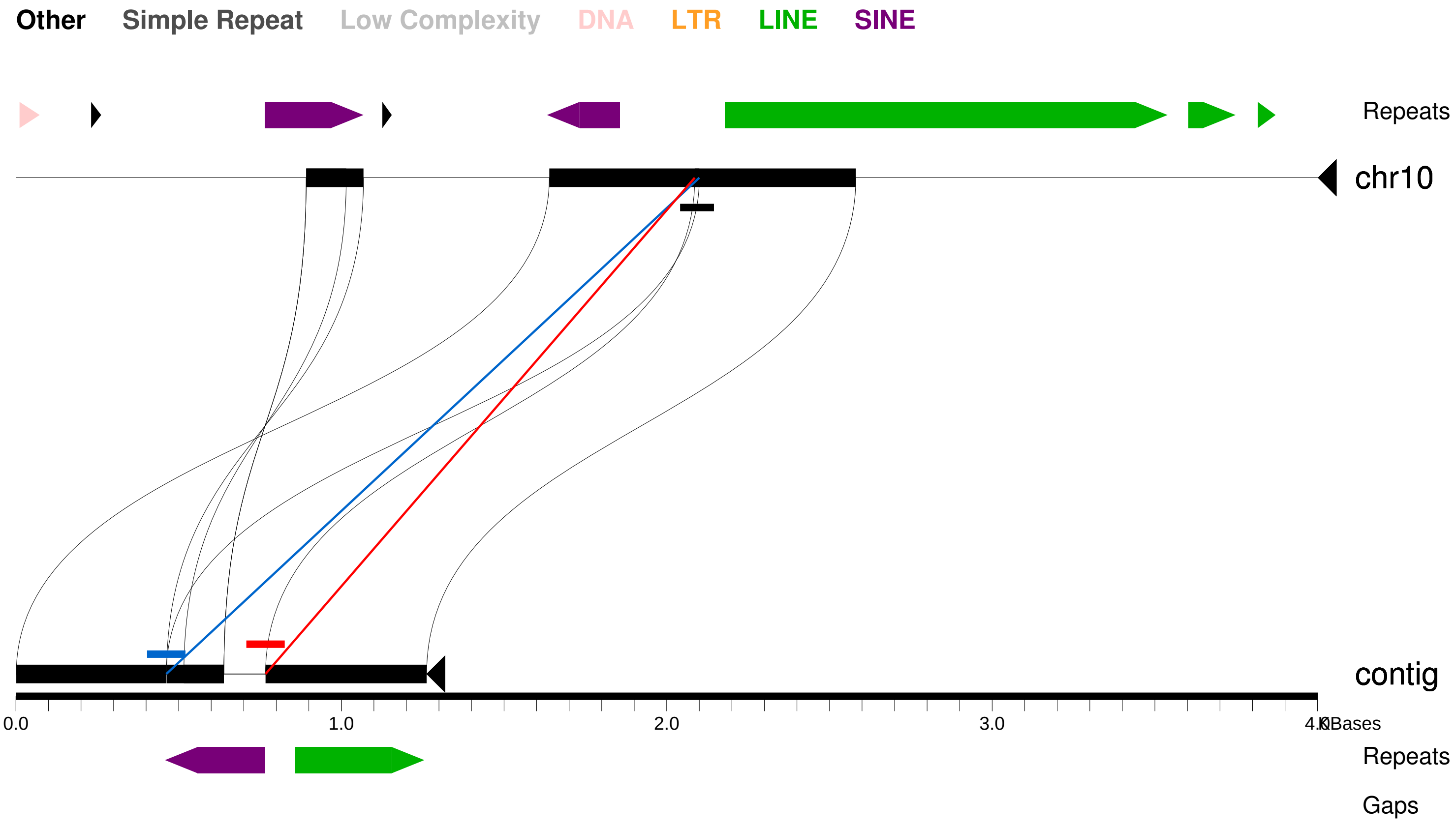

siteID: chr10_122140620 Scafftig1 - Breakpoint: chr10:122140590
chr10:122140464-122140750
Overlap Length: 14 INS size: 249 Genotyped: Yes

```
left   ---------------------------------------------------AGTTTCCAGTCAGCAAAAT
chrom  GCCCATTTGCAACTAACGAGTT---GCAATTC--GTTACAGGGCAATTTCTAGTTTCCAGTCAGCAAAAT
right  AGC----TCCGCCTCCCGGGTTCACGCCATTCTCCTGCCTCAGCC-TCCCAAGT----AGCTAGGACTAC
       NN2    2N2NN22NN22N22211122N222211N2NN2NNN22N 2NN2N***1111**11**1*11*1

left   TAGTTTCTTGCTTTACGTGGGGGAAAAGCAATCTAGATCTTTTTGGATAGATAG---TTCGGAAAACATC
chrom  TAGTTTCTTGCTTTACGTGGGGGAAAAGCAATCTAGATCTTTTTGGATAGATAG---TTCGGAAAACATC
right  AGGCGCCCGCCACTACGCCCGGCTATT----TTTTGTAGTTTTAGTAGAGACGGGGTTTCACCGAACATC
       11*111*111*11****111**11*111111*1*1*111****1*1*1***11*111***1111******

left   AAATTTTTTTTTTTTTTTTTTTTTTTTTTTTTTTTTTTTTTTTTTTTTTTTTTTTTTTTTGTTTTTTTTT
chrom  AAAATTTTATAGCAGCAATTGGTCTTGCTGAGAGTAAGAACAATTCTAGAACAGAAAGTGACAAAAATAC
right  AAAATTTTATAGCAGCAATTGGTCTTGCTGAGAGTAAGAACAATTCTAGAACAGAAAGTGACAAAAATAC
       ***2****2*22222222**22*2**22*22222*22222222**2*22222222222*22222222*22

left   TTTTGAGACGGAGTCTCGCTCTGTCGCCCAGGCTGGAGTGCAGTGGCGGGATCTCGGCTCACTGCAAGCT
chrom  TAAAAAGACGATGCA------------CCATGTTGTATAACT--------AATTCAAATCA-TTCTACCT
right  TAAAAAGACGATGCA------------CCATGTTGTATAACT--------AATTCAAATCA-TTCTACCT
       *2222*****22*22222222222222***2*2**2*222*222222222*22**222***2*2*2*2**

left   CCGCCTCCCGGGTTCACGCCATTCTCCTGCCTCAGCCTCCCAAGTAGCTAGGACT
chrom  CAGATT----GGATCTAATAATT--------TAAATATTTCAAATA-------CT
right  CAGATT----GGATCTAATAATT--------TAAATATTTCAAATA-------C-
       *2*22*2222**2**22222***22222222*2*222*22***2**2222222*1
```

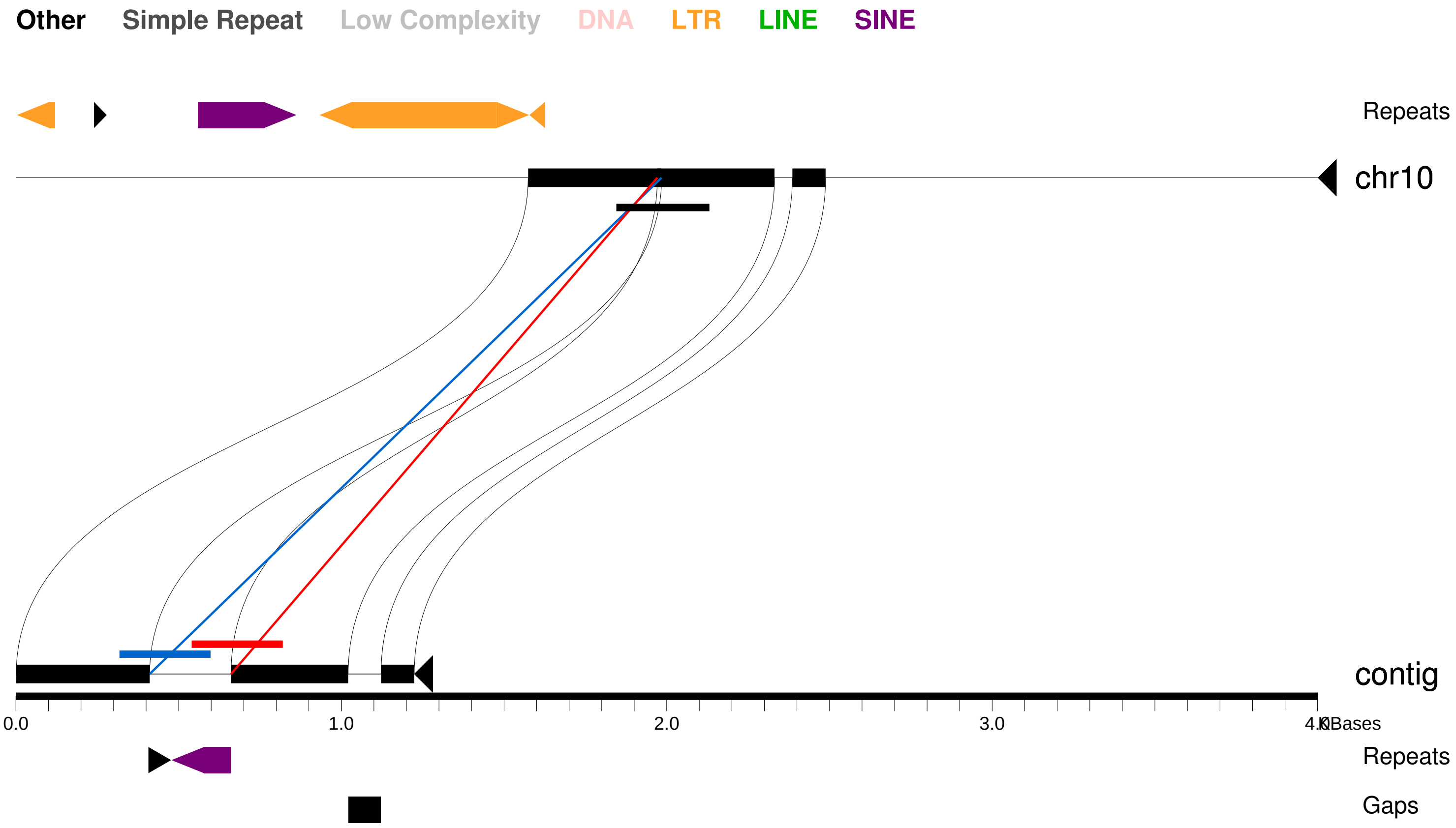

siteID: chr10_124736002 Scafftig10 + Breakpoint: chr10:124735991
chr10:124735831-124736164
Overlap Length: 14 INS size: 302 Genotyped: Yes

```
left   T----------------ATTGTCAAGATGTCATTTCTTCCCAACTTGATCTATAGATTCAACACAGTCTC
chrom  TCATGAATAGAAATAATATTGTCAAGATGTCATTTCTTCCCAACTTGATCTATAGATTCAACACAGTCTC
right  GCAGGA---GAATGGCGTGAACCCGGGAGGCGGAGCTTGCAG---TGAGCCG-AGATCGCGC-CA--CTA
       122N22   222NNNNN11111*11*11*1*1111***1*11111***1*111****1111*1**11**1

left   AACCCCAGCAAGCTGTTTTGTGGATATCAACAAAGTGATTCTAAAGTTTATGTGGCAAGGCAAAGGATCT
chrom  AACCCCAGCAAGCTGTTTTGTGGATATCAACAAAGTGATTCTAAAGTTTATGTGGCAAGGCAAAGGATCT
right  CACTCCAGC---CTG------GG----CGACAGAGCGAGACTCC-------GTCTCAAAAAAAAAAA---
       1**1*****111***111111**1111*1***1**1**11**111111111**11***111***11*111

left   AGGCTAGCCAGCACAATATTAAAGAAGGAGCAAAGGGGCCGGGCG--CGGTGGCTCAC-GC--------C
chrom  AGGCTAGCCAGCACAATATTAAAGAAGGAGCAAAACTAGAAGACTGACAGTAGCCAACTGCAAGACTTAC
right  ------------AAAAAAAAAAAAAAAGGAGCAAAACTAGAAGACTGACAGTAGCCAACTGCAAGACTTAC
       111111111111*1**1*11***1*********2222222*2*222*2**2**22**2**22222222*

left   TGTAATCCCAGCACTTT---GGGAGGCCGAGGCGGGCGGATCACGAGGTCAGGAGATCGA-GACCATCCT
chrom  TATAAAGCTATAATAATAAAGATAGTGTCATATTGGCAAAAGAATAGACAAATAGATCAATGAACAGAAT
right  TATAAAGCTATAATAATAAAGATAGTGTCATATTGGCAAAAGAATAGACAAATAGATCAATGAACAGAAT
       *2***22*2*22*222*222*22**2222*2222***22*22*22**222*22*****2*2**2**222*

left   GGCTAACACGGTGAAACCCCGTC------TCT---ACTAAA-------------
chrom  AGAGAGCCCACATAAATACAGTCAACTGATCTTTGACAAAGGAGCAAAGGCAAT
right  AGAGAGCCCACATAAATACAGTCAACTGATCTTTGACAAAGGAGCA--------
       2*22*2*2*2222***22*2***222222***222**2**222222
```

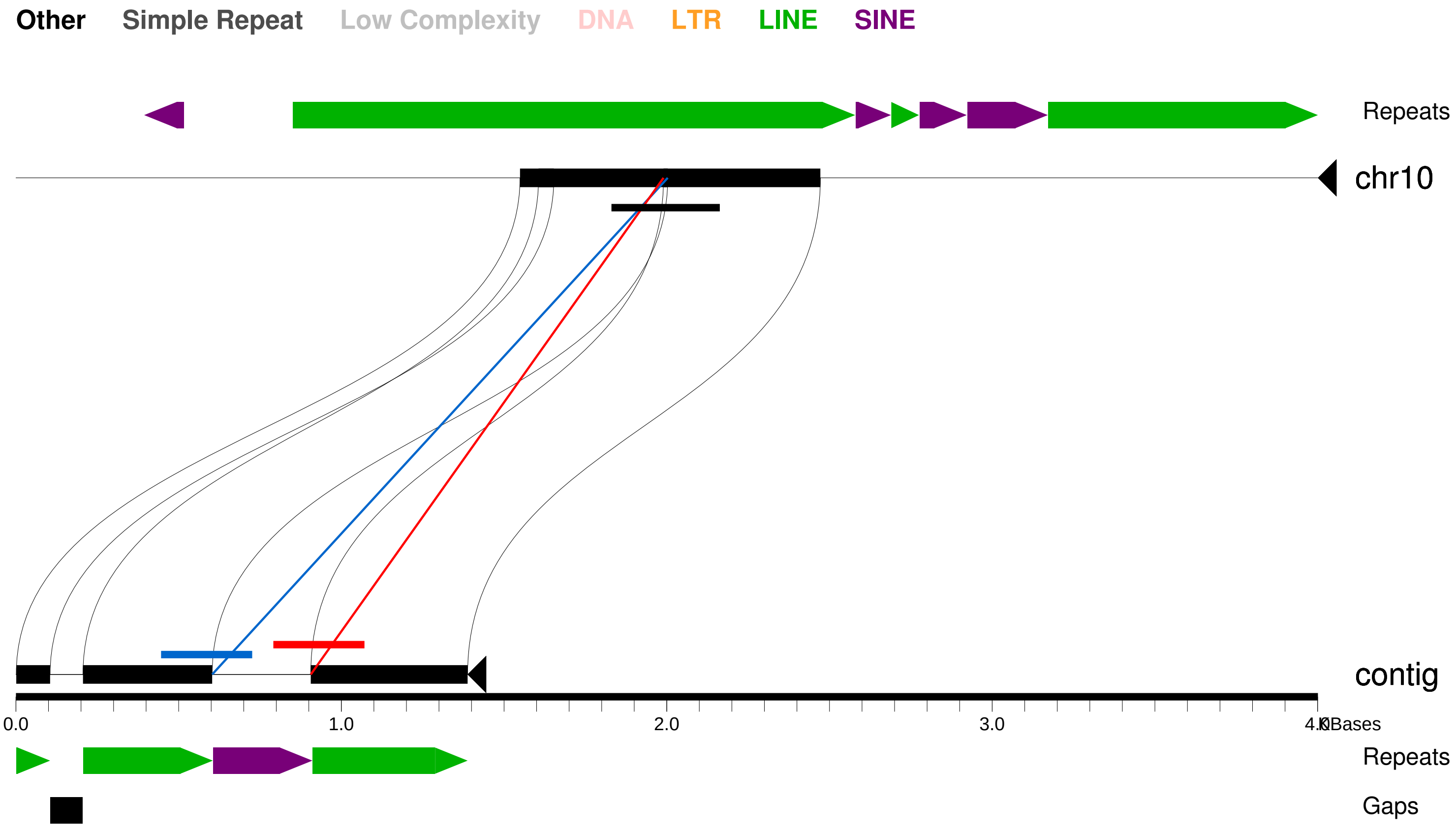

siteID: chr10_127299070 Scafftig2 + Breakpoint: chr10:127299077
chr10:127298915-127299556
Overlap Length: 11 INS size: 413 Genotyped: No

```
left   -------------CTGAACCCCAATTTGGGGGTTCTGTATATCCTGCCGTTTTCAAAGTGTGATCTTTGG
chrom  ATGGCCTCCCAGGCTGAACCCCAATTTGGGGGTTCTGTATATCCTGCCGTTTTCAAAGTGTGATCTTTGG
right  NNNNNNNNNNNG----------------------------------------------------------
       NNNNNNNNNNN2 111111111111111111111111111111111111111111111111111111111

left   AGCAGGGCATAAACTGCACCTGAAGTCTTGTTAGAAATGCAGACTCTCAGGCCCCACCCAAACCTGCTGA
chrom  AGCAGGGCATAAACTGCACCTGAAGTCTTGTTAGAAATGCAGACTCTCAGGCCCCACCCAAACCTGCTGA
right  ----------------------------------------------------------------------
       1111111111111111111111111111111111111111111111111111111111111111111111

left   ATCGGAACTTGTGTTTCAAAAGATCTCCTGGCG--GGCCGGGC-----GCGGTGGCTCACGC--------
chrom  ATCGGAACTTGTGTTTCAAAAGATCTCCTGGCGATGCCCATGCATATTGAAGTTGAAGAAGCTCGGTTTC
right  ----------------------ATCTCCTGGCGATGCCCATGCATATTGAAGTTGAAGAAGCTCGGCTTC
       1111111111111111111111***********22*2**22**22222*22**2*222*2**2222N222

left   -------CTGTAATC-------------------------------------CCA---------------
chrom  AAATGTTCTGAAATAATTCTTCCTCTTTTCTATGCGTTGAATTCATTTTCTGCCAATTTGTAGCAACTTT
right  AAATGTTCTGAAATAATTCTTCCTCTTTTCTATGCGTTGAATTCATTTTCTGCCAATTTGTAGCAACTTT
       2222222***2***22222222222222222222222222222222222222***222222222222222

left   -GCACTTTGGG-------------------AG-------------------------------GCCGA--
chrom  TGCATTTTGGGGATCTATCTTTTCATGTACAGATTTTTTTTAAATTTTAACCTTGTAGTATTTGCCAATA
right  TGCATTTTGGGGATCT------------------------------------------------------
       2***2******22222              11                               111N1

left   ------------GGCGGGCGGATCACGAGGTCAG------------------GAGATC-----GAGACCA
chrom  TTTTATGTTATGGTTTGTTTGCCCTCTTTGTCACTTAAATCCTTCTCTTCCTGAGATCACACAGATATGA
right  ----------------------------------------------------------------------
                   1NNN1NNN1NN1N1NNN1111N                  111111     11N1NN1

left   TCC--------CGGCTAAAA--------------------CGGT--------------GAAACCCC----
chrom  TGCTATATTTTCTTCTAAAAGTTTTTAGTTTTTCTTTTCACGATTGTTCTTTCATCTGGAATTTACATTT
right  ----------------------------------------------------------------------
       1N1        1NN111111                    11N1              111NNNN1

left   GTCTCTA-----CTAAA-----------------------------------------------------
chrom  GTCTCTAGTGTGCTATAATGCTCTCATTTTATTTTTTCATATTGATAATCAATTATCCCAGTGTGGTTCA
right  ----------------------------------------------------------------------
       1111111     111N1

left   ----------------------------------------------------------------------
chrom  TTGAATTATCCACCCATTGCCTCCTGACTGGTAGTGTCACTCGAGTCATAGGTGCTATCTTACGTTTCCG
right  ----------------------------------------------------------------------

left   ------------
chrom  TGGATGTGTGGT
right  ------------
```

Other Simple Repeat Low Complexity DNA LTR LINE SINE

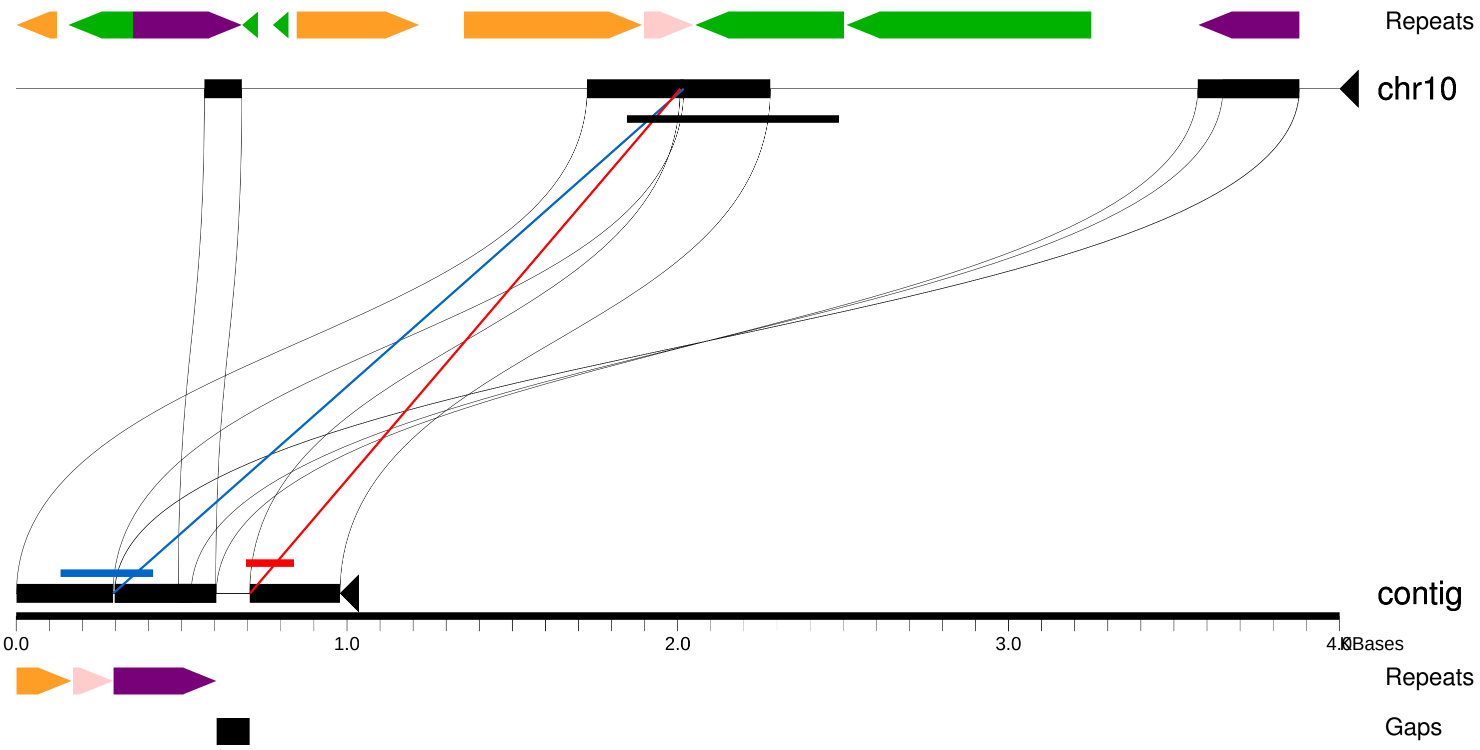

siteID: chr10_129190289 Scafftig1 - Breakpoint: chr10:129190293
chr10:129190133-129190466
Overlap Length: 14 INS size: 299 Genotyped: Yes

```
left    ----------GGCCTCTGGATCACAGCGTGAACTGCTTCAAAGATGCCCAAACCTCAGGGATTCTCCC-A
chrom   CTAATAAAGTGGCCTCTGGATCACAGCGTGAACTGCTTCAAAGATGCCCAAACCTCAGGGATTCTCCC-A
right   TTTA----GTAGAGACGGGGTTTCACCGT-------TTTA-----GCC---------GGGATGGTCTCGA
        N2N2    221*111*1**1*11**1***1111111**1*11111***111111111*****11**1*1*

left    TCTCCTCTGCAGGCGATCCAGGCCTCTCACCTGGCATGAGGCCCCTTAGAGTAGGAATCGAGCCTCCAGA
chrom   TCTCCTCTGCAGGCGATCCAGGCCTCTCACCTGGCATGAGGCCCCTTAGAGTAGGAATCGAGCCTCCAGA
right   TCTCCTGACCTCGTGATCCGCCCGCCTC----GGCCT-----CCCAAAGTGCTGGGAT------TACAGG
        ******111*11*1*****111*11***1111***1*11111***11**1*11**1**111111*1***1

left    TGTACCCTCTCATCACCCACAGCATCTGGCCTTCTTTTTTTTTTTTTTTTTTTG--AGAC---GGAGTCT
chrom   TGTACCCTCTCATCACCCACAGCATCTGGCCTTCTGAACATGCCCTCTGTTCCCCAAGCCCTTGCACCTT
right   CGTGAGCCACCGCGCCCGGCCGCATCTGGCCTTCTGAACATGCCCTCTGTTCCCCAAGCCCTTGCACCTT
        1**111*111*1111**11*1**************22222*2222*2*2**22222**2*222*2*222*

left    CGCTCTGTC-GCCCAGGC-TGGAG--------------TGCAGTGGCGGGATCTCGGCTCAC-TGCAAGC
chrom   CCCATGGTCTGCCCCGGCGTGGACCGGCTGCTTCTTGCTGCTGTCTCGTG-TGACGGCTGCCATGACAGC
right   CCCATGGTCTGCCCCGGCGTGGACCGGCTGCTTCTTGCTGCTGTCTCGTG-TGACGGCTGCCATGACAGC
        *2*222***2****2***2****222222222222222***2**22**2*2*22*****22*2**22***

left    TCC------GCCTCCCGGG-----TTCACGCCATTC------TCCTGCCTC-----
chrom   TGCCTTCCTGCCTTCTGGGCATATTTCACTGCTCTCAAACTTTCGTGGCTTTTGGA
right   TGCCTTCCTGCCTTCTGGGCATATTTCACTGCTCTCAAACTT--------------
        *2*222222****2*2***22222*****22*22**22222211N11N11N
```

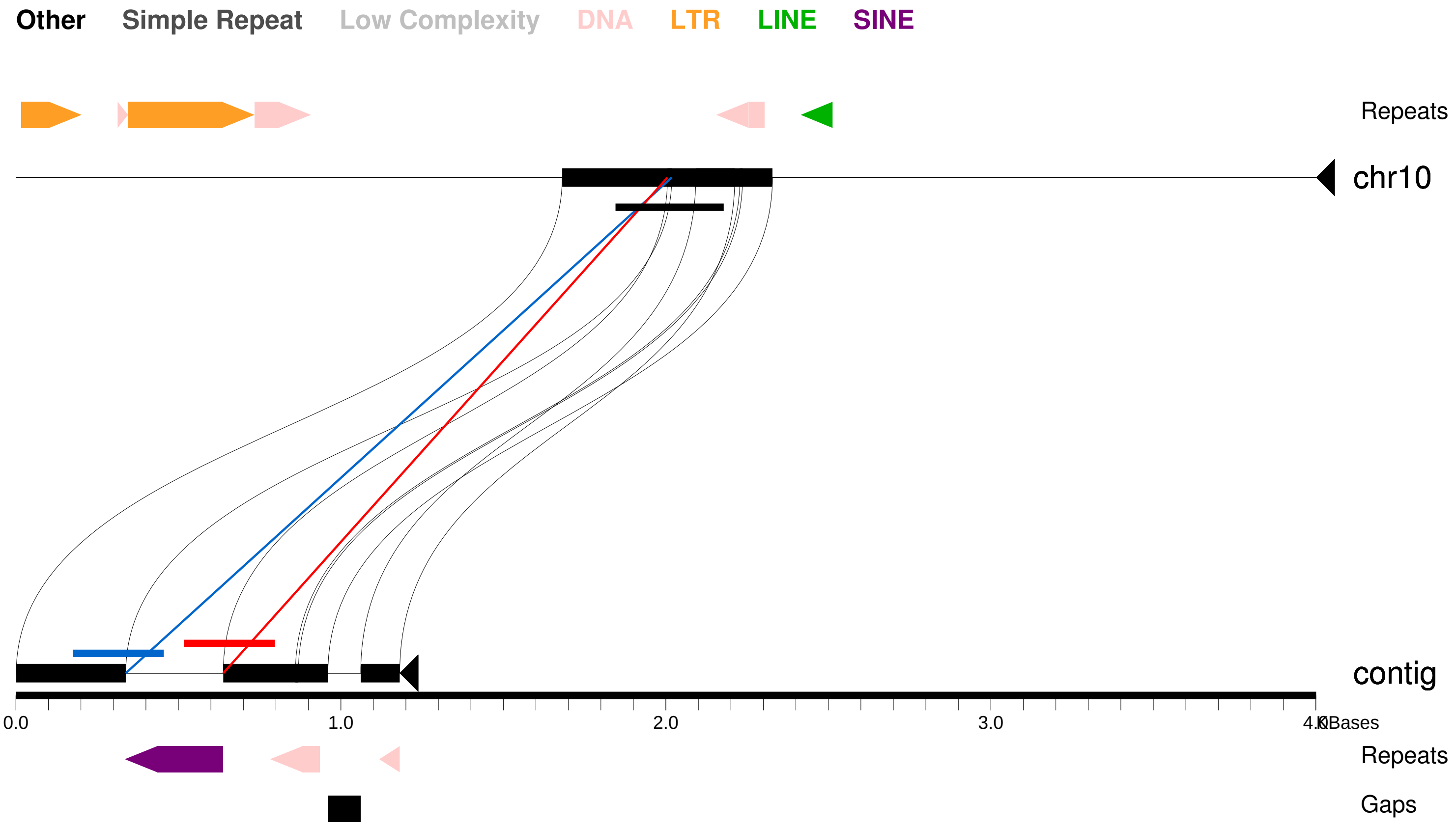

siteID: chr10_1292107 Scafftig1 + Breakpoint: chr10:1292091
chr10:1292048-1292150
Overlap Length: 17 INS size: 453 Genotyped: No

```
left    AAGTTTAACGAGAAATGACCAAGGACTATAGTGAATAAA----------------ACTTAAAAATCCTAT
chrom   AAGTTTAACGAGAAATGACCAAGGACTATAGTGAATAAA----------------ACTTAAAAATCCTAT
right   NNNNNNNNNNNNNNNNNNNNNNNNNNNNNNNNNNNNNNNNNNNNNNNNNNNNNNNAAAAAAAAATCCTAT
        1111111111111111111111111111111111111111111111111111111*111***********

left    TGAAGGCCGGGCGCGGTGGCTCACGCCTGTAATCCCAGCACTTTGGGAGGCCGAGGCGGGCGGAT
chrom   TGAAGGACA-----------CAACAAAATTTAAACAAACA-----GAAAGACTTGCCACATTTTT
right   TGAAGGACA-----------CAACAAAATTTAAACAAACA-----GAAAGACTTGCCACATTTTT
        ******2*22222222222222**22222*2*22*2*2**22222*2*2*2*22*2*2222222*
```

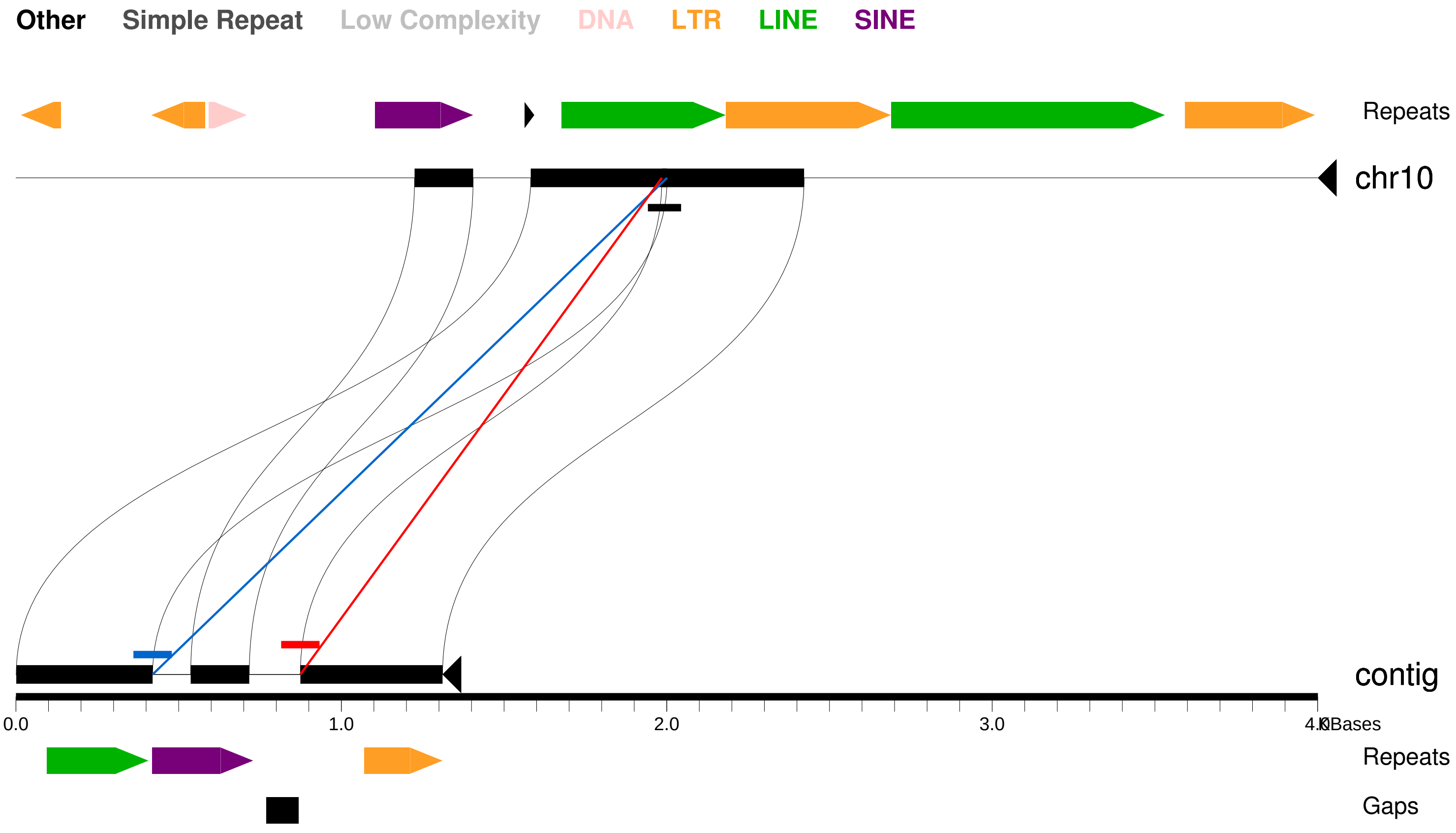

siteID: chr10_129373584 Scafftig7 + Breakpoint: chr10:129373582
chr10:129373536-129373641
Overlap Length: 14 INS size: 295 Genotyped: Yes

```
left   -------CATTTTCTTTCTATTCTGC----ATTGCAG---TGAACCCTATTG-GAATCACTGGGAATAAC
chrom  -------CATTTTCTTTCTATTCTGC----ATTGCAG---TGAACCCTATTG-GAATCACTGGGAATAAC
right  CCGCCCGCCTCGGCCTCCCAAAGTGCTGGGATTACAGGCGTGAGCC--ACCGCGCCCGGCCGGGAATAAC
       1111111*1*111*1*1*1*111***1111***1***111***1**11*11*1*11111*1*********

left   CTTTTTTTTTTTTTTTTTTTGAGACGGAGTCTCGCTCTGTCGCCCAGGCTGGAGTGCAGTGGCGG
chrom  CTTTTCCCTTGACT------AAGCGGGGTGTA--TGGGGGACCCAG-----AGAAGATAAGAGC
right  CTTTTCCCTTGACT------AAGCGGGGTGTA--TGGGGGACCCAG-----AGAAGATAAGAGC
       *****222**222*222222*22***2**2*222*22*222*****22222**222*222*2*2
```

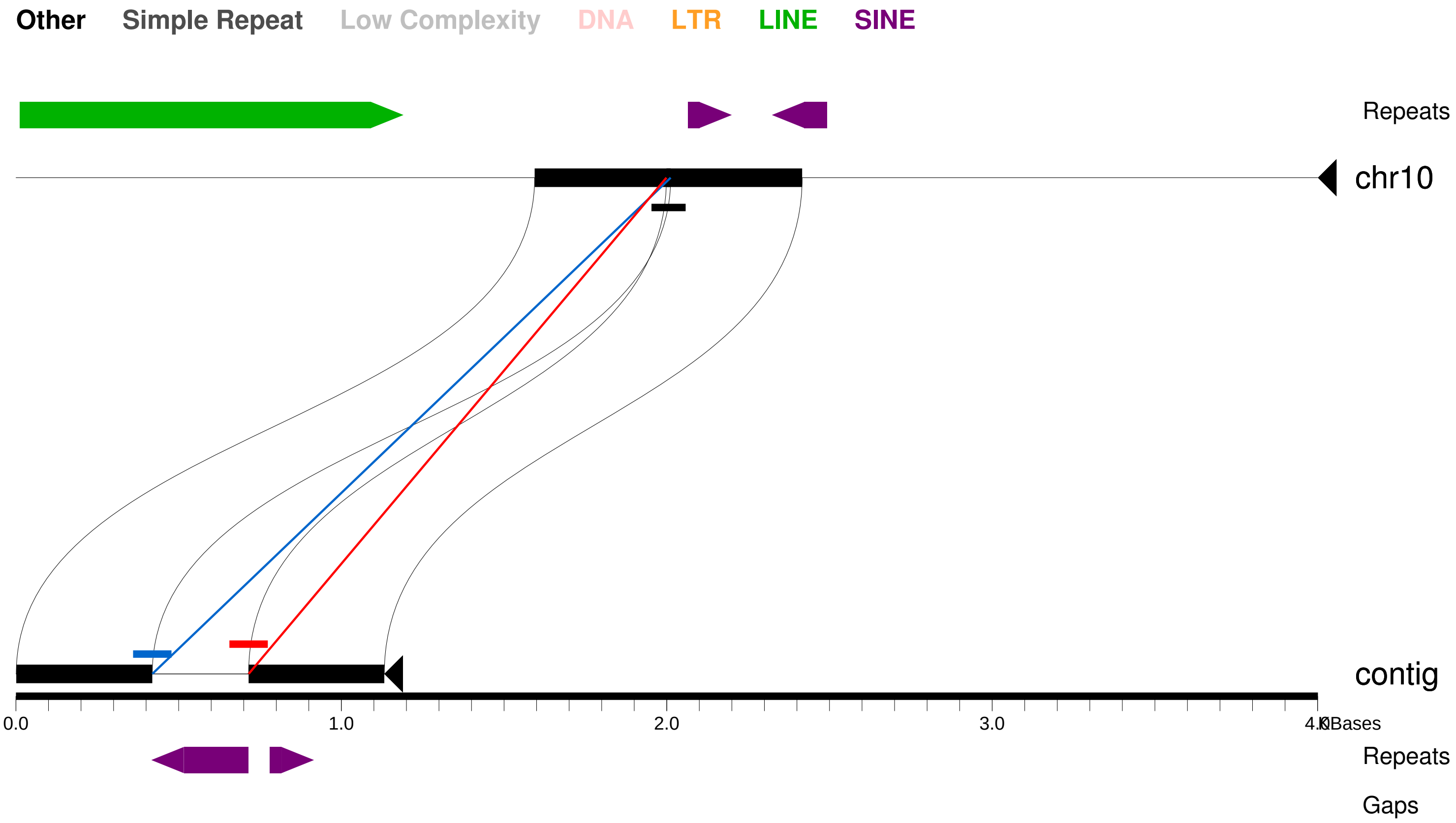

siteID: chr10_129553754 Scafftig1 - Breakpoint: chr10:129553743
chr10:129553701-129553802
Overlap Length: 18 INS size: 158 Genotyped: Yes

```
left     CAAAACAGTGAGTCTGT-----------------AAGCATTTTAGTCACCCTGGCCTTCAAGAACCATGT
chrom    CAAAACAGTGAGTCTGT-----------------AAGCATTTTAGTCACCCTGGCCTTCAAGAACCATGT
right    ACAGAGCGAGACTCCGTCTCAAAAAAAAAAAAAAAAAAAAAAAAAAAAAAAAAAAAAAAAAGAACCATGT
         11*1*11*1**1**1**11111111111111111**11*1111*111*11111111111***********

left     ATTTTTTTACTCGGGAGGCTGAGGCAGGAGAATGGCGTGAACCCGGGAAGCGGAGCTTGCAGTGAG
chrom    ATTTTTTAAT-----ATGCTT-GGTAGGAG-----CTACACACTTTGTAACAAATAGTTC------
right    ATTTTTTAAT-----ATGCTT-GGCAGGAG-----CTACACACTTTGTAACAAATAGTTC------
         *******2*222222*2***22**N*****22222*222*22*222*2*2*22*222*2*222222
```

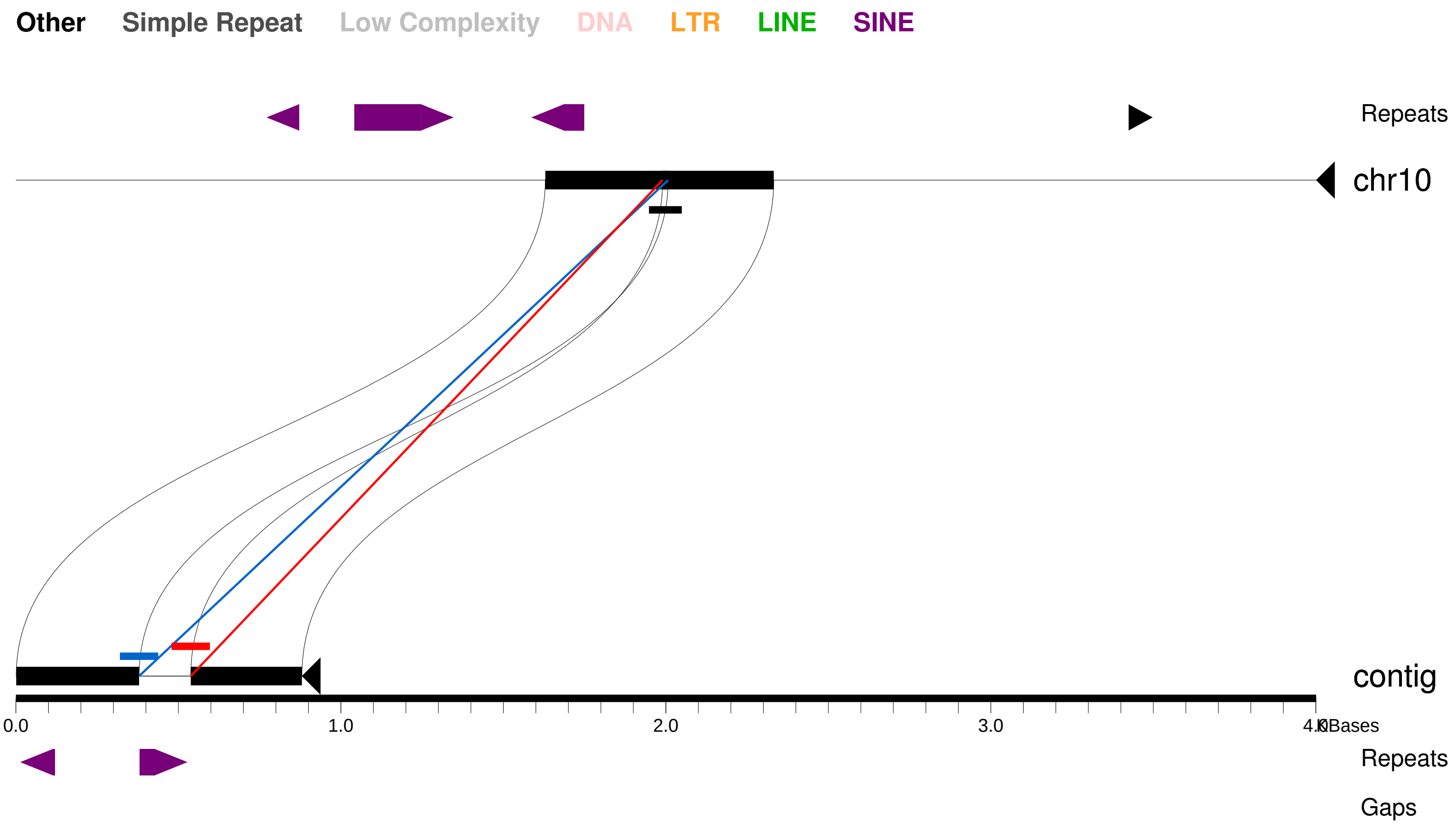

siteID: chr10_132145567 Scafftig2 + Breakpoint: chr10:132145572
chr10:132145527-132145631
Overlap Length: 15 INS size: 306 Genotyped: Yes

```
left   GTAAA--GCTAGACCCTTTTATTCATTTTGAGTAGAAGTAGAATCTT------------CTGTTAAAAAA
chrom  GTAAA--GCTAGGCCCTTTTATTCATTTTGAGTAGAAGTAGAATCTT------------CTGTTAAAGAA
right  CCACCCGCCTCGGCCTCCCAAAGTGCTGGGATTACAGGCGTGAGCCACCGCGCCCGGCCCTGTTAAAGAA
       11*11111**1*2**11111*11111*11**1**1*1*1111*1*111111111111111********2**

left   TTTTTTTTTTTTTTTTTTTTTTTGAGACGGAGTCTCGCTCTGTCGCCCAGGTCGGACTGCGGAC
chrom  TTTTGCATCATGTATCACAGGTGATA-GTTATCTGAATATTTTGTCTTT-------------C
right  TTTTGCATCATGTATCACAGGTGATA-GTTATCTGAATATTTTGTCTTT-------------C
       *****222*22*2*2*222222***2*2*222***222*2*2*2*2*2222222222222222*
```

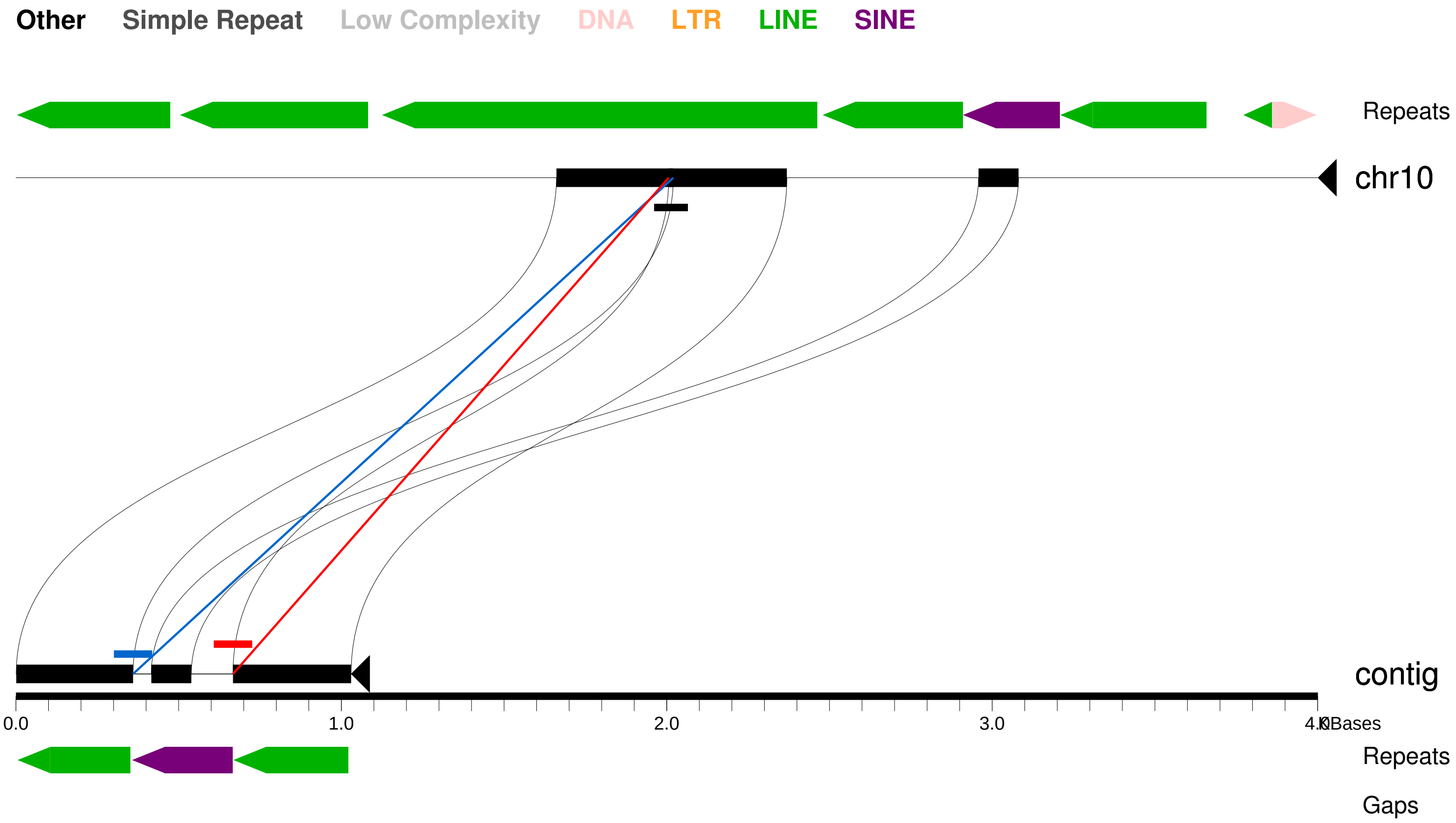

siteID: chr10_14396676 Scafftig1 - Breakpoint: chr10:14396684
chr10:14396524-14396858
Overlap Length: 15 INS size: 305 Genotyped: Yes

```
left   --------------TTATTCAGGGGAGGTAACAGCTTCATCTTATTGAAGAGGACTTCATTGCCTCTTGT
chrom  CTGACTGGGTTGGCTTATTCAGGGGAGGTAACAGCTTCATCTTATTGAAGAGGACTTCATTGCCTCTTGT
right  GAGAATGGCGTGAA-----CCCGGGAGGCGG-AGCTTGCAGTGAGCCGAGATCGCGCCACTGCA------
       NN22N222NN22NN11111*11******1111*****1111*1*1111***111*11**1***1111111

left   TGGTATCCCTAACCTCCCCACCATCCCCCCAGCCCCCTACTTTACAAAAAGTCCCTCTGGGAGTTTAACT
chrom  TGGTATCCCTAACCTCCCCACCATCCCCCCAGCCCCCTACTTTACAAAAAGTCCCTCTGGGAGTTTAACT
right  ------CTCCAGCCTGGGCGACAGAGCG--AGACTCCGTCTCAAAAAAAA-----------AAAAAAAAA
       111111*1*1*1***111*11**111*111**1*1**11**11*1*****11111111111*1111**11

left   TGGTCATACTCCCTTGCTACAAAACTCCAGTGCAG--GCCGGGCGCGGTGGCTC----------------
chrom  TGGTCATACTCCCTTGCTACAAAACTCCAGTGCAGTAGCCTCCCTTGAATAGTCTTCCTTTCCATATTGA
right  AAAAAAAA------------AAAACTCCAGTGCAGTAGCCTCCCTTGAATAGTCTTCCTTTCCATATTGA
       11111*1*111111111111***************22***222*22*22222**2222222222222222

left   ACGCCTGTAATCCCAG------------CACTTTGGGAGGC--CGAGGCGGGCGGA-TCACG---AGGTC
chrom  ACAGGTGTCATGAAAAGTTATTATTTAACAGTTACGGCACCTGCGATTTGGACAGAATCAGATTCATCGC
right  ACAGGTGTCATGAAAAGTTATTATTTAACAGTTACGGCACCTGCGATTTGGACAGAATCAGATTCATCGC
       **222***2**222*2222222222222**2**22**222*22***222**2*2**2***22222*222*

left   AGGAGATCGAGACCA---TCCCGG--CTAAAACGGTGAAACCCCGTCTCTACTAAA
chrom  CGGATTCCCAGACCCCTCTCTCAGAACTCCAAATATGCACCTTTGAAGCCTTTGT-
right  CGGATTCCCAGACCCCTCTCTCAGAACTCCAAATATGCACCTTT------------
       2***222*2*****2222**2*2*22**22**222**2*2*2221NNN1NNN1NN2
```

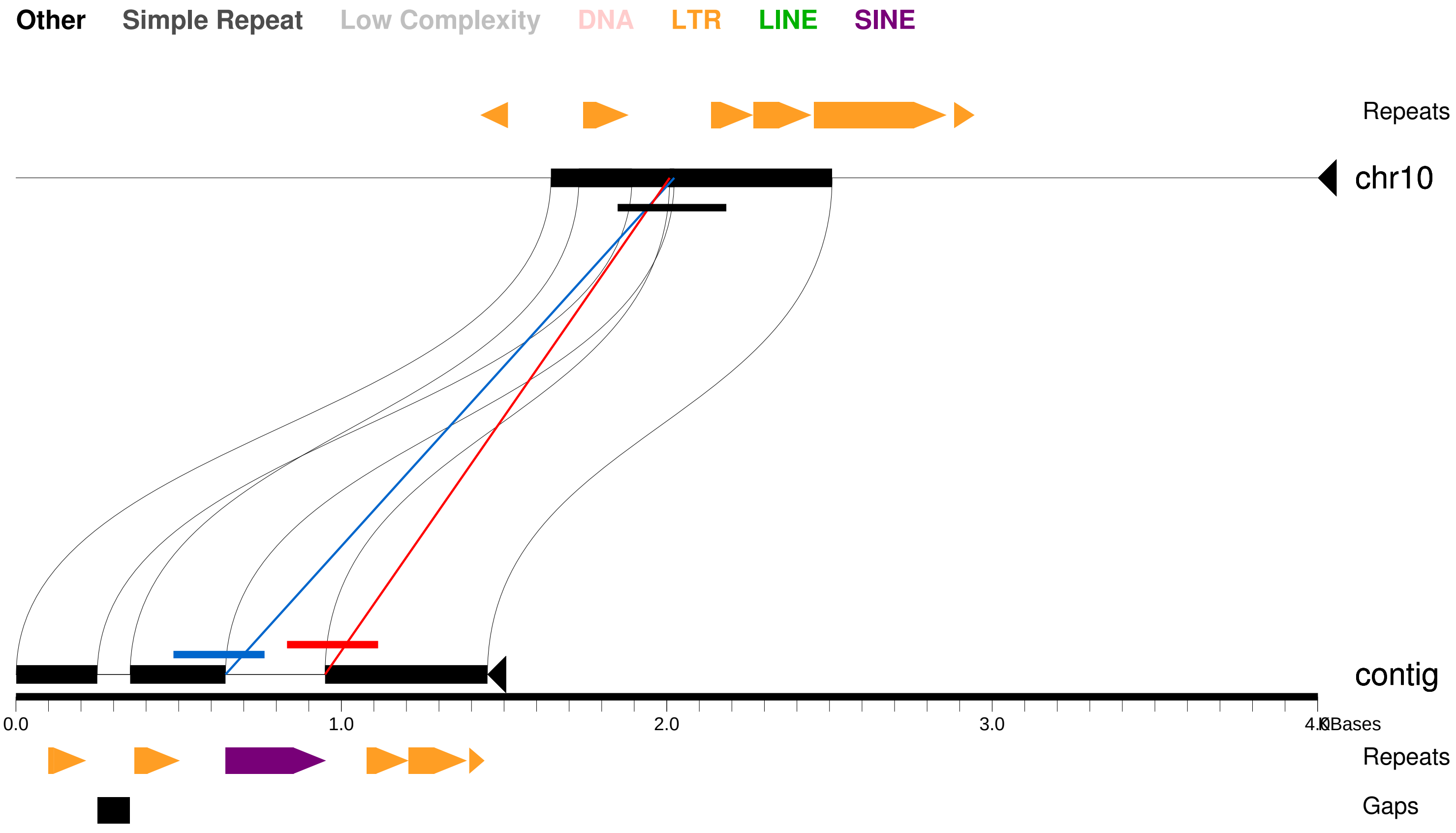

siteID: chr10_14484811 Scafftig9 - Breakpoint: chr10:14484814
chr10:14484755-14484883
Overlap Length: -9 INS size: 336 Genotyped: Yes

```
left   TAAAATATATCTACTAAAAATCTACAGCTTACATTATAACTCACTATAAAAGAAGGAATGGGCCGGGCGA
chrom  TAAAATATATCTACTAAAAATCTACAGCTTACATTATAACTCACTATAAAAGAAGGAATGTGTTTT---A
right  G------TCTCAAAAAAAAAAAAAAAAAAAAAAAAAGGAATGTGTTTTAGAAATAACATGTCTAT-----
       1111111*1**1*11*****111*1*1111*1*11*11*1*111*1*1*1*1*1111***212N2N2221

left   GGTGGCTCACGCCTGTAATC-CCAGCACTTTGGGA----GGCCGAGGCGGGCGG--------
chrom  GAAATAACATGTCTATACTTACCACCTCTATTCAATATTGTACCTAGCCAGTGTAACAAAAC
right  --AATAACATGTCTATACTTACCACCTCTATTCAATATTGTACCTAGCCAGTGTAACAAAAC
       1N22222**2*2**2**2*22***2*2**2*222*2222*22*222**22*2*222222222
```

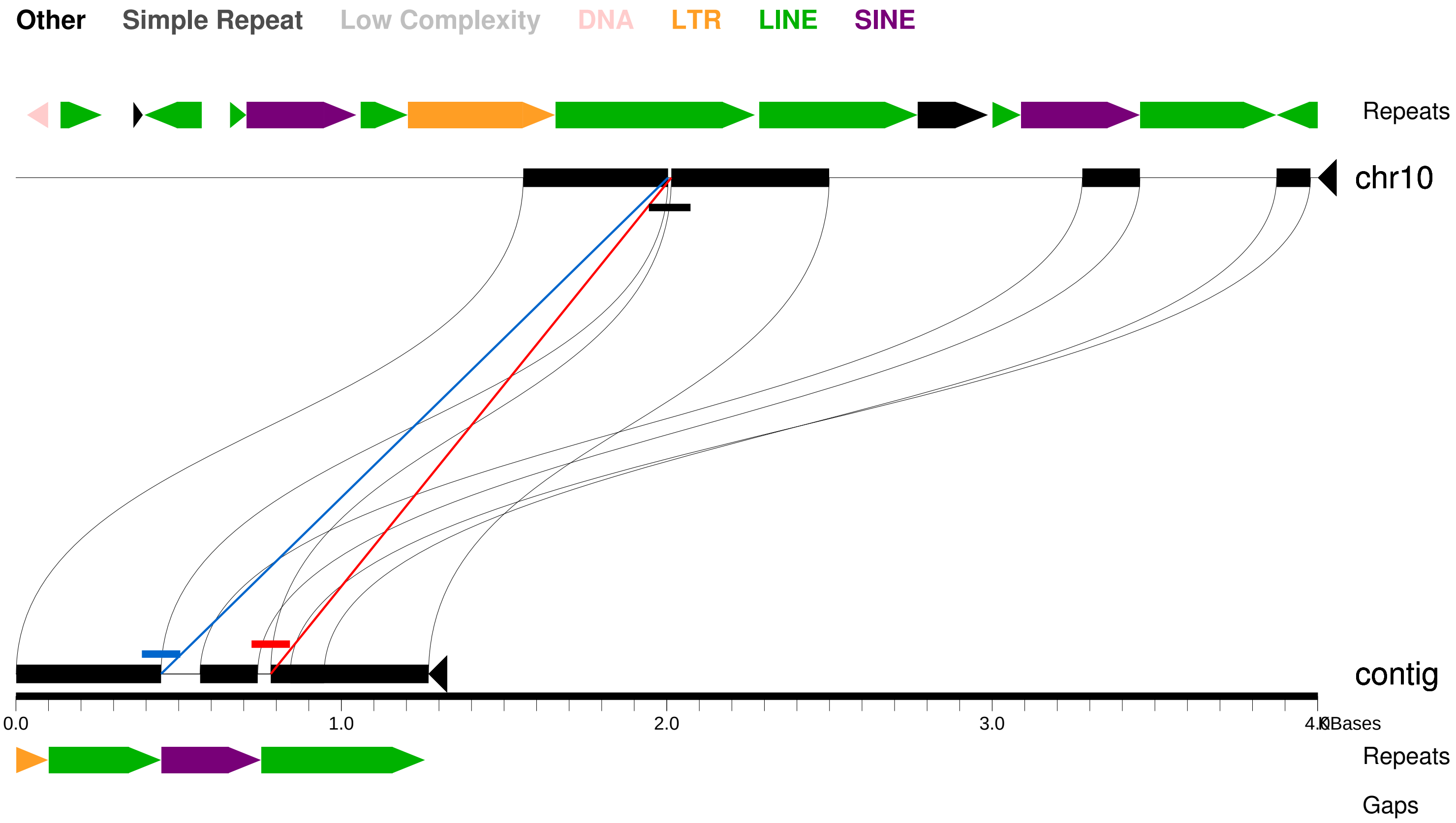

siteID: chr10_14537795 Scafftig4 - Breakpoint: chr10:14537776
chr10:14537616-14537948
Overlap Length: 13 INS size: 299 Genotyped: Yes

```
left    --------------GGTAGAACCTGCCTTTGAAACTACCTTTGCCTAGACTTTTAAGATTGTTTTTGACT
chrom   TTTCTGGACTTTCTGGTAGAACCTGCCTTTGAAACTACCTTTGCCTAGACTTTTAAGATTGTTTTTGACT
right   GG-CAGGAGAATGGCGTGAACCCGGGAGGCGGAGCTTGCAGTG---AGCCG----AGATCCCG-----CC
        NN 2N222NNN2NN1**11*1**1*11111*1*1**11*11**111**1*11111****111111111*1

left    TCTGTTCGATCTCTTTGAAGCATATATTATGTTTTTAAATTTTGGGTTTGTAATGTTATATTTACTCCCT
chrom   ACTGTTTGATCTCTTTGAAGCATATATTATGTTTTTAAATTTTGGGTTTGTAATGTTATATTTACTCCCT
right   ACTG--------CACTCCAGCCT---------------------GGGCGACAGAGCGAGA----CTCCGT
        2***11N11111*11*11***1*111111111111111111111**11111*11*11*1*1111****1*

left    GACTACAAATTATCTCATATAAAAAAAAATTTTCGG---------------------GCCGGGCGCGGTG
chrom   GACTACAAATTATCTCATATAAAAAAAAATTTTCATTAGCAGCTAAAAGAACACCACAGGAGGGCCTTAAG
right   CTCAAAAAAAAAAAAAAAAAAAAAAAAATTTTTATTTAGCAGCTAAAAAAACACCACAGGAGAGCCTTAAG
        11*1*1***11*1111*1*1*******1****1N2222222222222N222222222*22*1**22222*

left    GCTCACGCCTGTAATCCCAGCACTTTGG-------GAGGCCGAGGCGGGCGGATCACGAG--GTCAGGAG
chrom   CTTCAAGCTTTTGAATGTAGAATTTTTGTTCAGGAGAATCACAGGAGCGATGAGCTAGCTCTGTCCAGA-
right   CTTCAAGCTTTTGAATGTAGAATTTTTGTTCAGGAGAATCACAGGAGCGATGAGCTAGCTCTGTCCAGA-
        22***2**2*2*2*2222**2*2***2*2222222**22*22***2*2*22**2*22*2222***22**2

left    ATCGAGACCA-----TCCCGGCTAAAACGGTGAAACCCCGTCTCTACTAA---A
chrom   ATTGAAAGCAGGTGATGACCAAAAAAAGAAAGAAGCCAGAGAACAAGGAAGATA
right   ATTGAAAGCAGGTGATGACCAAAAAAAGAAAGAAGCCAGAGAACAAGG------
        **2**2*2**22222*22*2222****2222***2**222222*2*2211   1
```

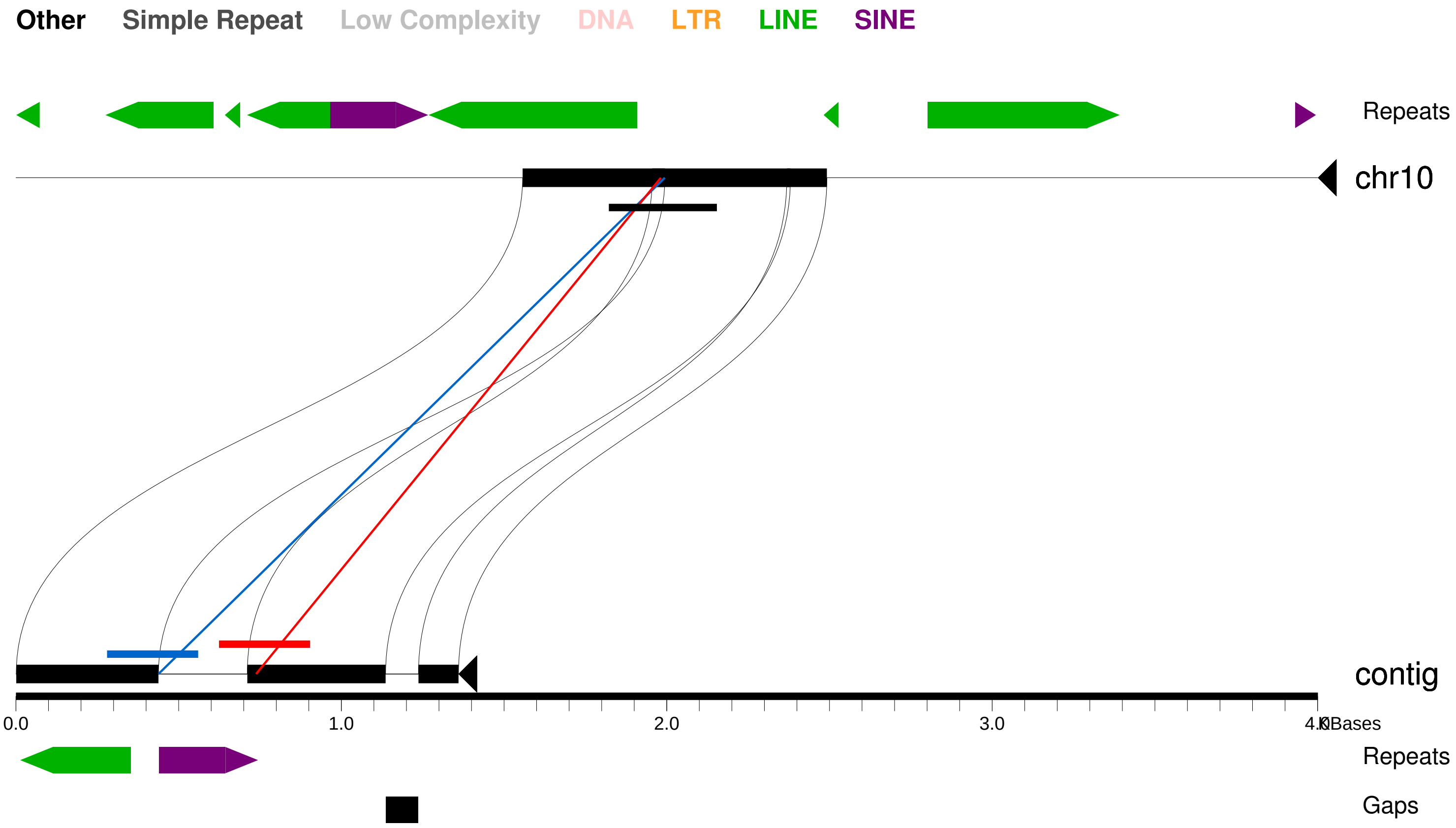

siteID: chr10_16000975 Scafftig4 - Breakpoint: chr10:16000950
chr10:16000908-16001009
Overlap Length: 18 INS size: 309 Genotyped: Yes

```
left    TCA-------CAAAATCAAGTTGCTT-GCTTCTTTGTATCATGCTTTTCT---------AGAAAAGTGAA
chrom   TCA-------CAAAATCAAGTTGCTT-GCTTCTTTGTATCATGCTTTTCT---------AGAAAAGTGAA
right   CCATCCTGGGCGACAGAGCGAGACTCCGCCTCAGAAAAAAAAAAAAAAAAAAAAAAAAAAGAAAAGTGAA
        1**1111111*1*1*1111*111**11**1**11111*11*111111111111111111**********

left    ACTTTGAGGCCGGGCGCGGTGGCTCACGCCTGTAATCCCAGCACTTTGGGAGGCCGAGGCGGGCGG
chrom   ACTTTGAAG-----------GAAATACCCAAGAAA--CAAGAATATT----GCCTCAATATTGTTT
right   ACTTTGGAG-----------GAAATACCCAAGAAA--CAAGAATATT----GCCTCAATATTGTTT
        ******12*22222222222*2222**2*22*2**22*2**2*22**2222*2*22*22222*222
```

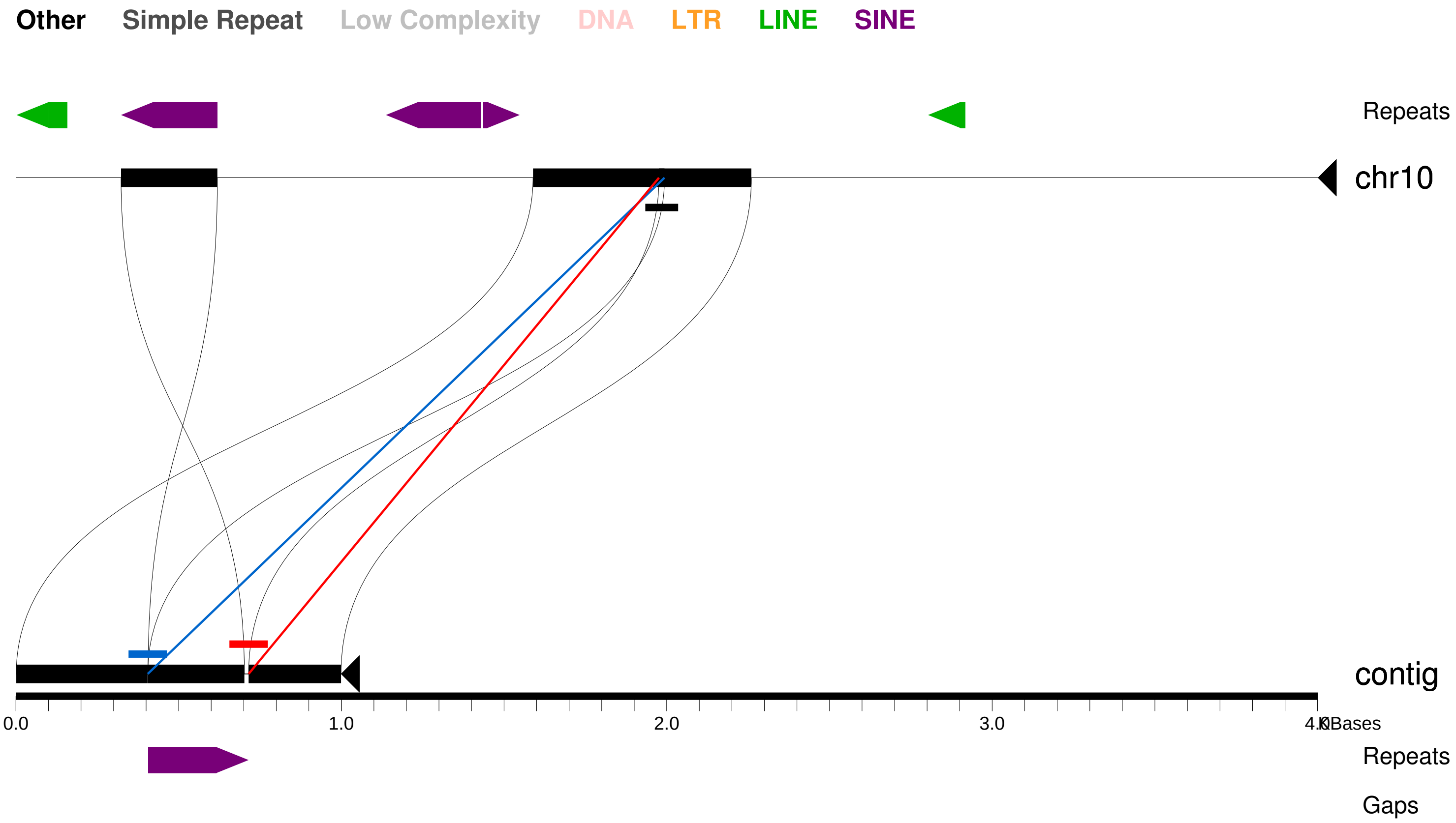

siteID: chr10_17751982 Scafftig1 - Breakpoint: chr10:17751963
chr10:17751917-17752022
Overlap Length: 14 INS size: 416 Genotyped: No

```
left   -------GGTTCTGTATCTTAGAAAAAAGTGTT---AAGTGT-AGCT--TGCACTCTTGCAGGATTTTTT
chrom  -------GGTTCTGTATCTTAGAAAAAAGTGTT---AAGTGT-AGCT--TGCACTCTTGCAGGATTTTTT
right  TCCGCCCGCCTCGGCCTCCCAAAGTGCTGGGATTACAGGCGTGAGCCACCGCGCCCGGCCAGGATTTTTT
       1111111*11**1*11**11*1*11111*1*1*111*1*1**1***1111**1*1*111***********

left   TTTTTTTNNNNNNNNNNNNNNNNNNNNNNNNNNNNNNNNNNNNNNNNNNNNNNNNNNNNNNN
chrom  TTTAAGTTGAAATCTGGTGGAAATGAATACCCTTCTGGAAAATAAACAG-------------
right  TTTAAGTTGAAATCTGGTGGAAATGAATACCCTTCTGGAAAATAAACAG-------------
       ***222**222222222222222222222222222222222222222222222222222222
```

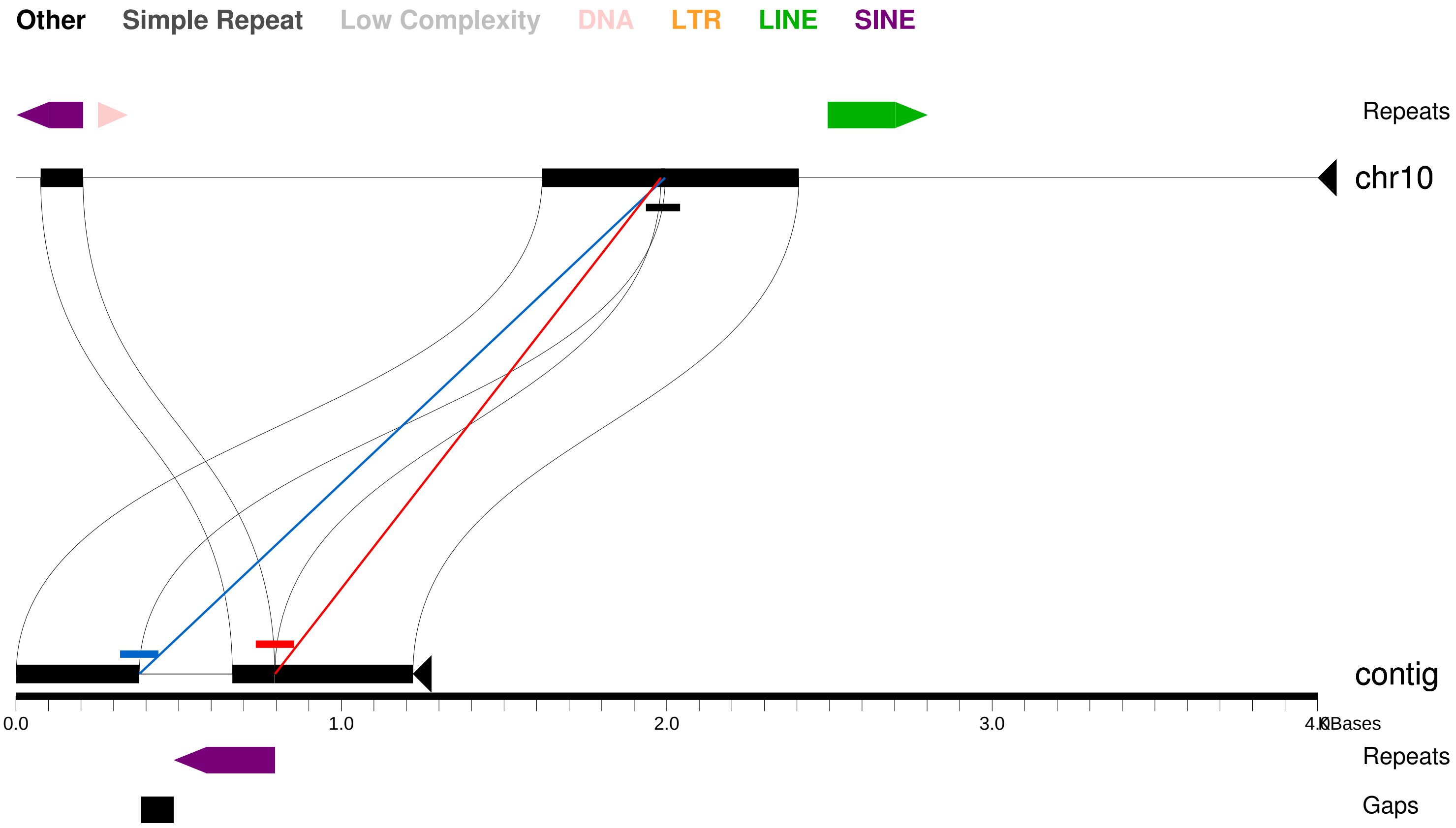

siteID: chr10_19550734 Scafftig4 - Breakpoint: chr10:19550721
chr10:19550674-19550780
Overlap Length: 13 INS size: 304 Genotyped: Yes

```
left    TTTACT--------TTTCTTTGGTACTTCAAAATCAACCTTTTCCCCTCGTGCCT----GCCTTGTAATT
chrom   TTTACT--------TTTCTTTGGTACTTCAAAATCAACCTTTTCCCCTCGTGCCT----GCCTTGTAATT
right   CCGCCCGCCTCGGCCTCCCAAAGTGCTGGGATTACAGGCGTGAGCCACCGCGCCCGGCCGCCTTGTAATT
        1111*1111111111*1*1111**1**111*111**11*1*111**11**1***11111***********

left    TTTTTTTTTTTTTTTTTTTTTTTTTGAGACGGAGTCTCGCTCTGTCGCCCAGGCTGGAGTG
chrom   TTAATGAAAGCAGTGTTCTC---CAGCAACCTGGGCTTGAATTTCCAC---------AGTC
right   TTAATGAAAGCAGTGTTCTC---CAGCAACCTGGGCTTGAATTTCCAC---------AGTC
        **22*22222222*2**2*222222*22**222*2**2*222*22*2*222222222***2
```

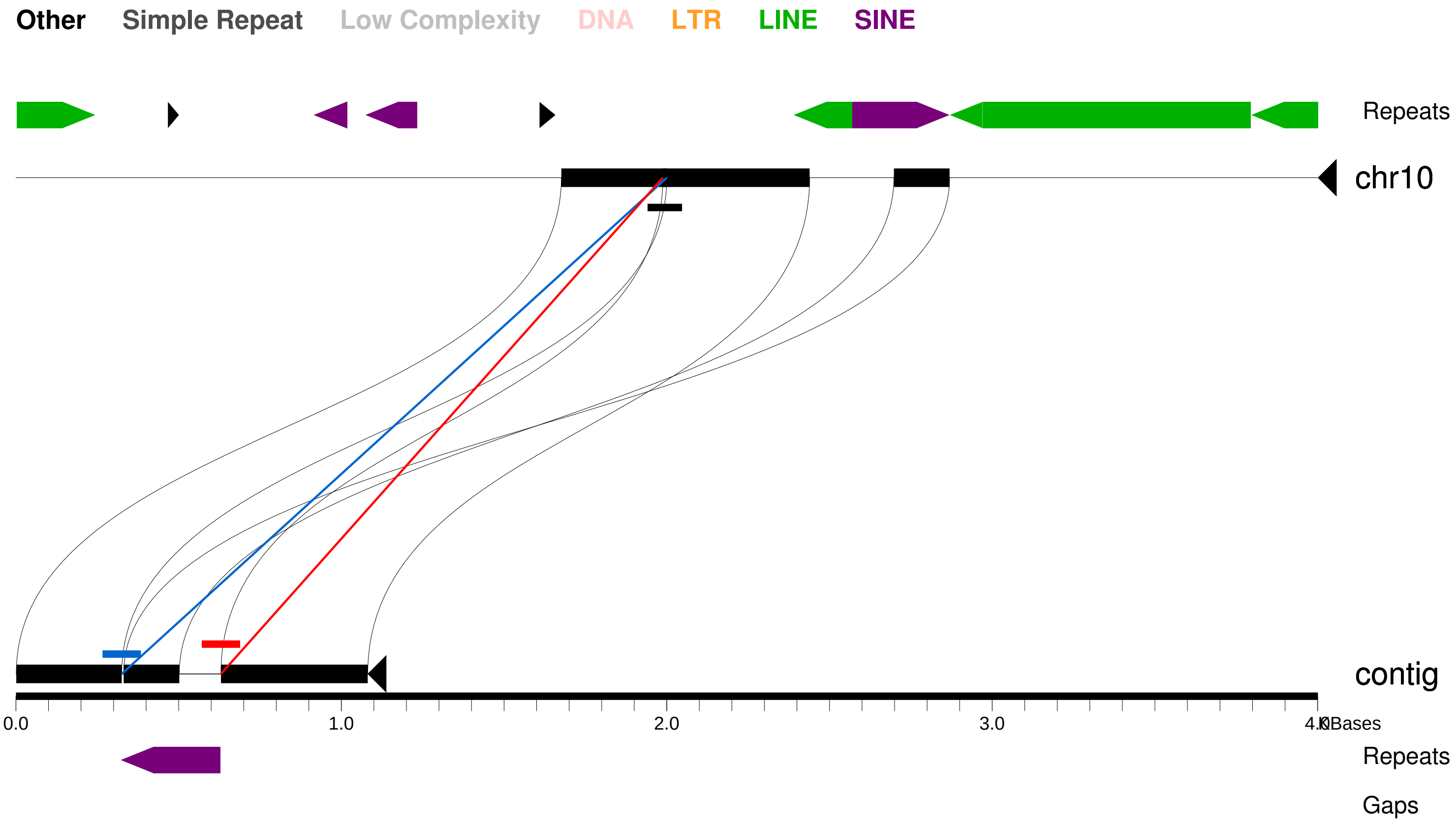

siteID: chr10_19786302 Scafftig3 - Breakpoint: chr10:19786296
chr10:19786250-19786343
Overlap Length: 14 INS size: 283 Genotyped: Yes

```
left    ------CATGACAAACA---------------CAAAAAAGGCACACAGAGCCC----ATAAATAAGCATA
chrom   ------CATGACAAACA---------------CAAAAAAGGCACACAGAGCCC----ATAAATAAGCATA
right   TTGCGCCACTGCAGTCCGCAGTCCGGCCTGGGCGACAAAGCGAGACTCCGTCTCAAAAAAAAAAAAAAAA
        111111**111**11*1111111111111111*1*1****11*1**111*1*11111*1***1**11*1*

left    CAAGAAGCAATAGATCTGTAATCCCAGCACTTTGGGAGGCCGAGGCGGGTGGATCATGAGGTCAGGAGAT
chrom   CAAGAAGCAATAGAT-TATA----------TAT---------AGTCATATAGA--ATGAACGAAAGGAAT
right   AAAGAAGCAATAGAT-TATA----------TAT---------AGTCATATAGA--ATGAACGAAAGGAAT
        1**************2*2**2222222222*2*222222222**2*222*2**22****2222*2*22**

left    CGAG
chrom   ---G
right   ---G
        222*
```

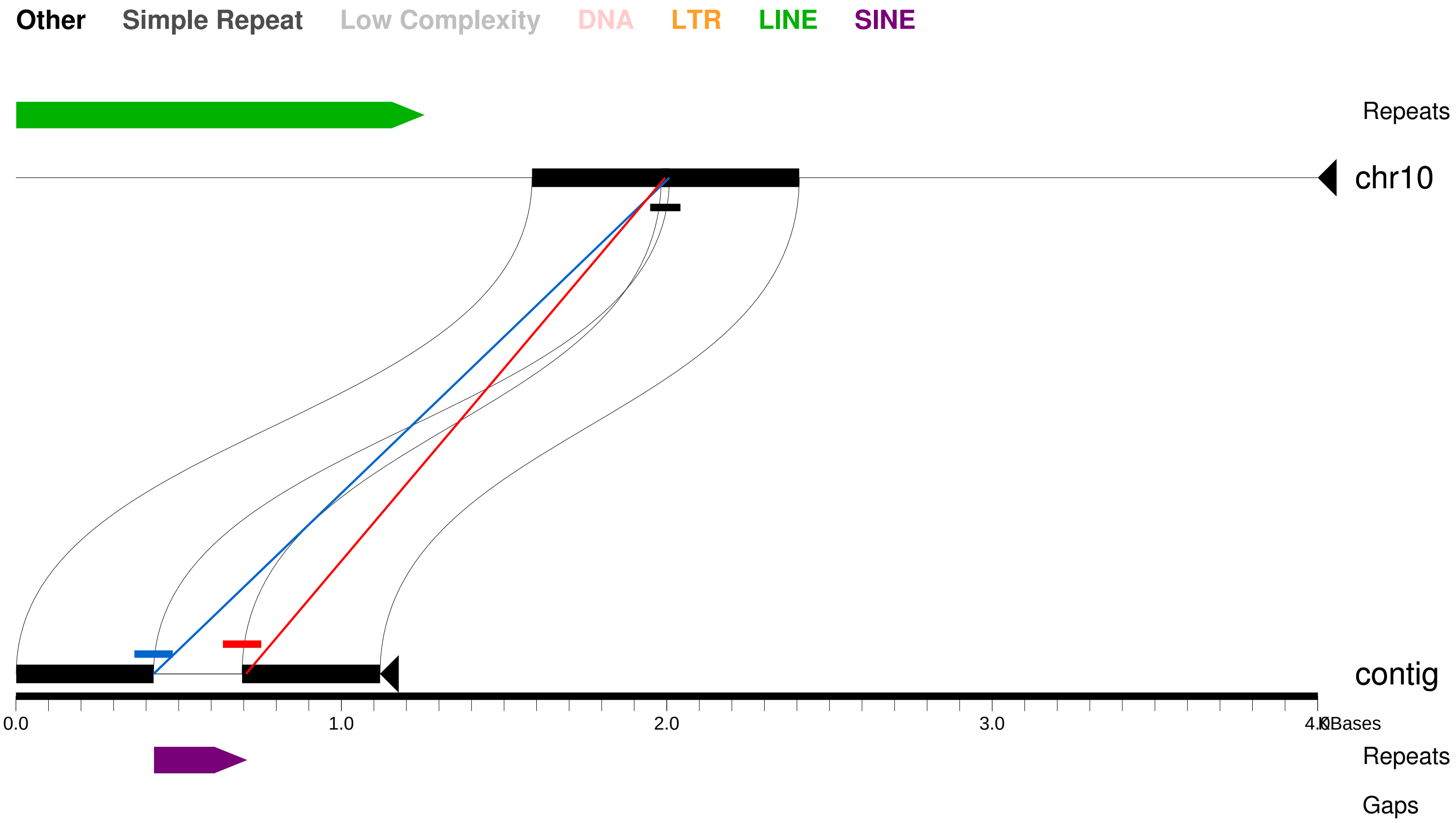

siteID: chr10_2077559 Scafftig2 - Breakpoint: chr10:2077543
chr10:2077501-2077602
Overlap Length: 18 INS size: 297 Genotyped: Yes

```
left     AC-ATTG---ACCATC-TGTTCT-CACATTGCAACTACTTTTTCCATT-----------AGAAATTTCAG
chrom    AC-ATTG---ACCATC-TGTTCT-CACATTGCGTCTACTTTTTCCATT-----------AGAAATTTCAG
right    ACCACTGCACTCCAGCCTGGGCGACAGAGCGAGACTCCGTCTCAAAAAAAAAAAAAAAAGAAATTTCAG
         **1*1**1111***1*1**11*11**1*11*12N**1*1*1*111*1111111111111**********

left     CATTTTAGGCCGGGCGCGGTGGCTCACGCCTGTAATCCCAGCACTTTGGGAGGCCGAGGCGGGCGG
chrom    CATTTTAATCATTTTAAATT--CCCAGTCTGATAATTCCACCATTCC----TGCC-----------
right    CATTTTAATCATTTTAAATT--CCCAGTCTGATAATTCCACCATTCC----TGCC-----------
         *******22*222222222*22*2**22*222****2***2**2*2222222***22222222222
```

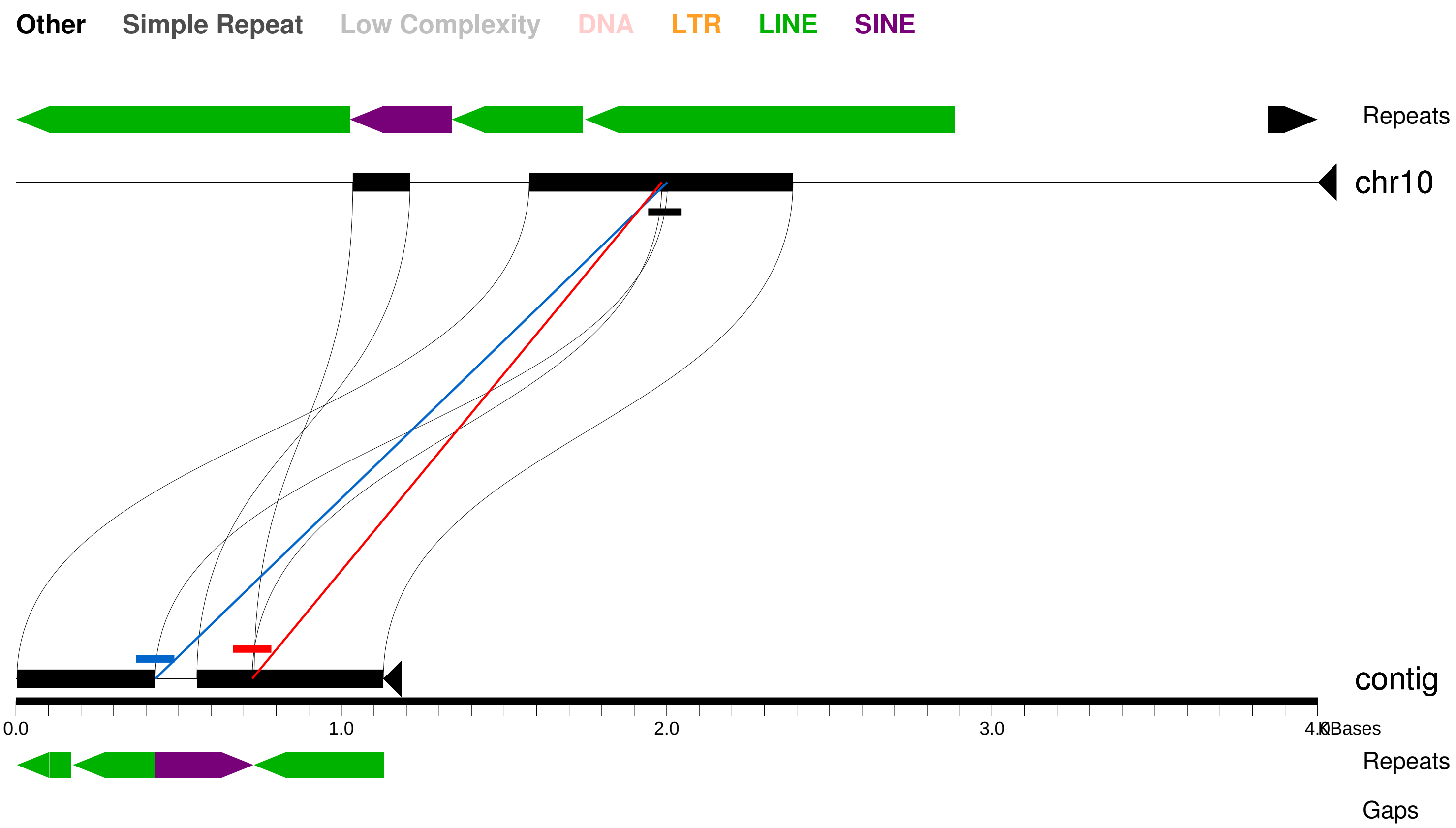

siteID: chr10_21192804 Scafftig4 - Breakpoint: chr10:21192818
chr10:21192774-21192877
Overlap Length: 16 INS size: 299 Genotyped: Yes

```
left   -TGTAACGGCCAATGGAGACT------CAGAAGGGAGA---------AGGGTAAGGGATTAAAATCTACG
chrom  -TGTAACGGCCAATGGAGACT------CAGAAGGGAGA---------AGGGTAAGGGATTAAAATCTACG
right  CTGCAGTCCGCAGTCCGGCCTGGGCGACAGA-GCGAGACTCCGTCTCAAAAAAAAAAAAAAAAATCTACG
       1**1*11111**1*111*1**111111****1*1****111111111*1111**111*11**********

left   GATTGGCCGGGCGCGGTGGCTC-ACGCCTGTAATCCCAGCACTTTGGGAGGCCGAGGCGGGTGGAT
chrom  GATTGG----GAACAATTACACTACTCAAGTGACAAGTGCACTA---AAATCTCAGA---------
right  GATTGG----GAACAATTACACTACTCAAGTGACAAGTGCACTA---AAATCTCAGA---------
       ******2222*22*22*22*2*2**2*22**2*22222*****22222*22*22**2222222222
```

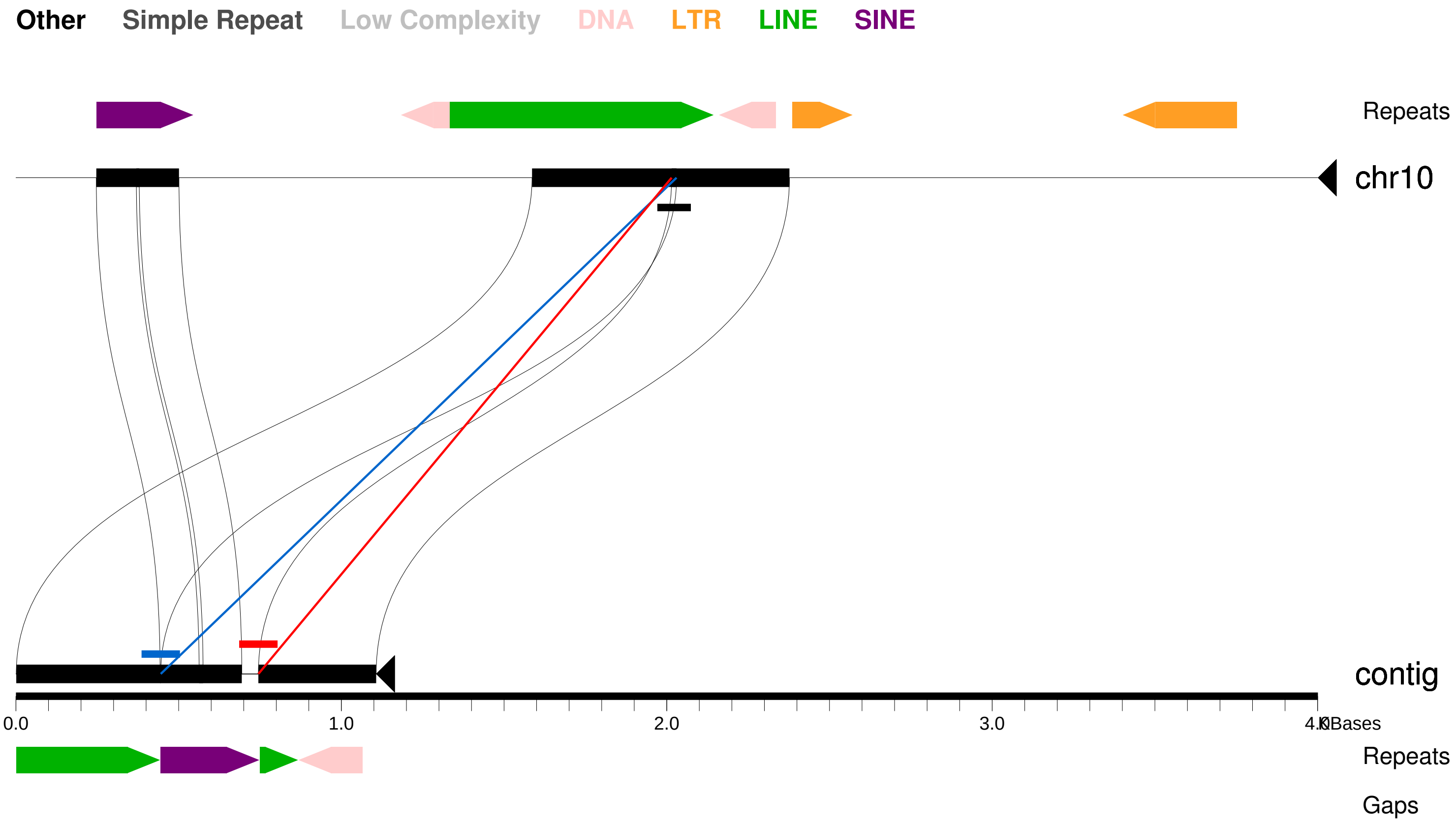

siteID: chr10_21688236 Scafftig2 + Breakpoint: chr10:21688225
chr10:21688180-21688284
Overlap Length: 11 INS size: 305 Genotyped: Yes

```
left   CT-CATTTATTACCCTTACCTCTTTATCAATTAAAAAACATGTTAT-------------AAAAGCACATT
chrom  CT-CATTTATTACCCTTACCTCTTTATCAATTAAAAAACATGTTAT-------------AAAAGCACATT
right  CCGCCCGCCTCGGCCTCCCAAAGTGCTGGGATTACAGGCGTGAGCCACCGCGCCCGGCCAAAAGCACATT
       *11*11111*111***11*1111*11*1111*1*1*11*1**11111111111111111**********

left   TTTTTTTTTTTTTTTTTTTTTTTTGAGACGGAGTCTCGCTCTGTCGCCCAGGCTGGAGTGCAG
chrom  CTTTATAAGAGGAAAGACAT-----AGA---AGTTATTCTCTTT--GCTATGCTC----ACAG
right  CTTTATAAGAGGAAAGACAT-----AGA---AGTTATTCTCTTT--GCTATGCTC----ACAG
       2***2*2222222222222*22222***222***2222****2*222*2*2***222222***
```

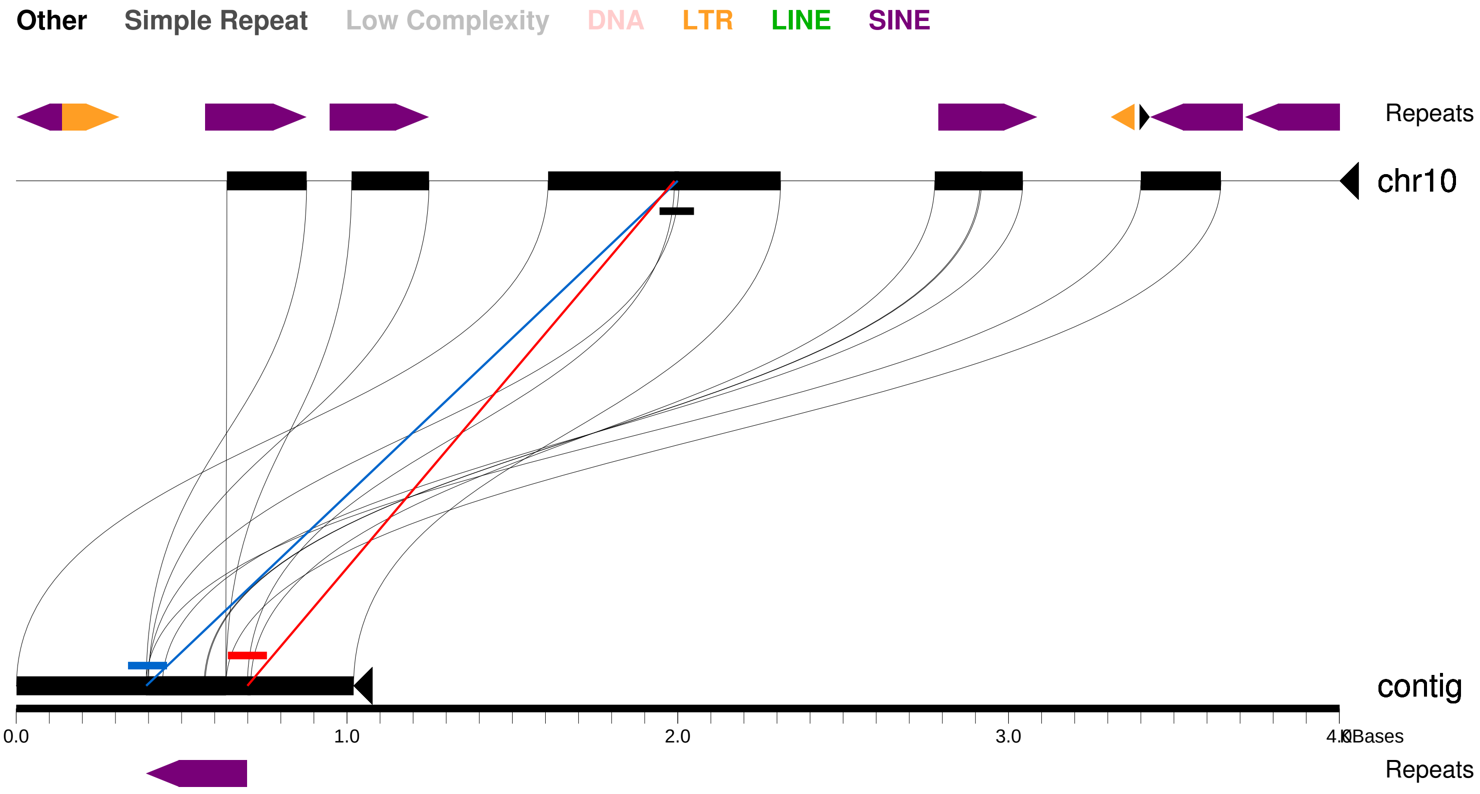

siteID: chr10_2241105 Scafftig1 + Breakpoint: chr10:2240998
chr10:2240952-2241057
Overlap Length: 14 INS size: 415 Genotyped: No

```
left    GAAAATTGAGGTACCCCAAAACCAAAAAAAAAAAGAAAACTTAAAA-------------AATAATAATTT
chrom   GAAAATTGAGGTACCCCAAAACCAAAAAAAAAAAGAAAACTTAAAA-------------AATAATAATTT
right   NNNNNNNNNNNNNNNNNNNNNNNNNNNNNNNNNNNNNNNNNNNNNNNNNNNNNNNNNNNNTAATAATAATTT
        11111111111111111111111111111111111111111111111111111111111***********

left    TGGGCCGGGCGCGGTGGCTCACGCCTGTAATCCCAGCACTTTGGGAGGCCGAGGCGGGCGGA
chrom   TGGA---------GAACTTCTAAGTTCTTATTTCAATACTT----ACTATGAAGATAAAGGT
right   TGGA---------GAACTTCTAAGTTCTTATTTCAATACTT----ACTATGAAGATAAAGGT
        ***2222222222*2222**22222*2*2**22**22****2222*2222**2*22222**2
```

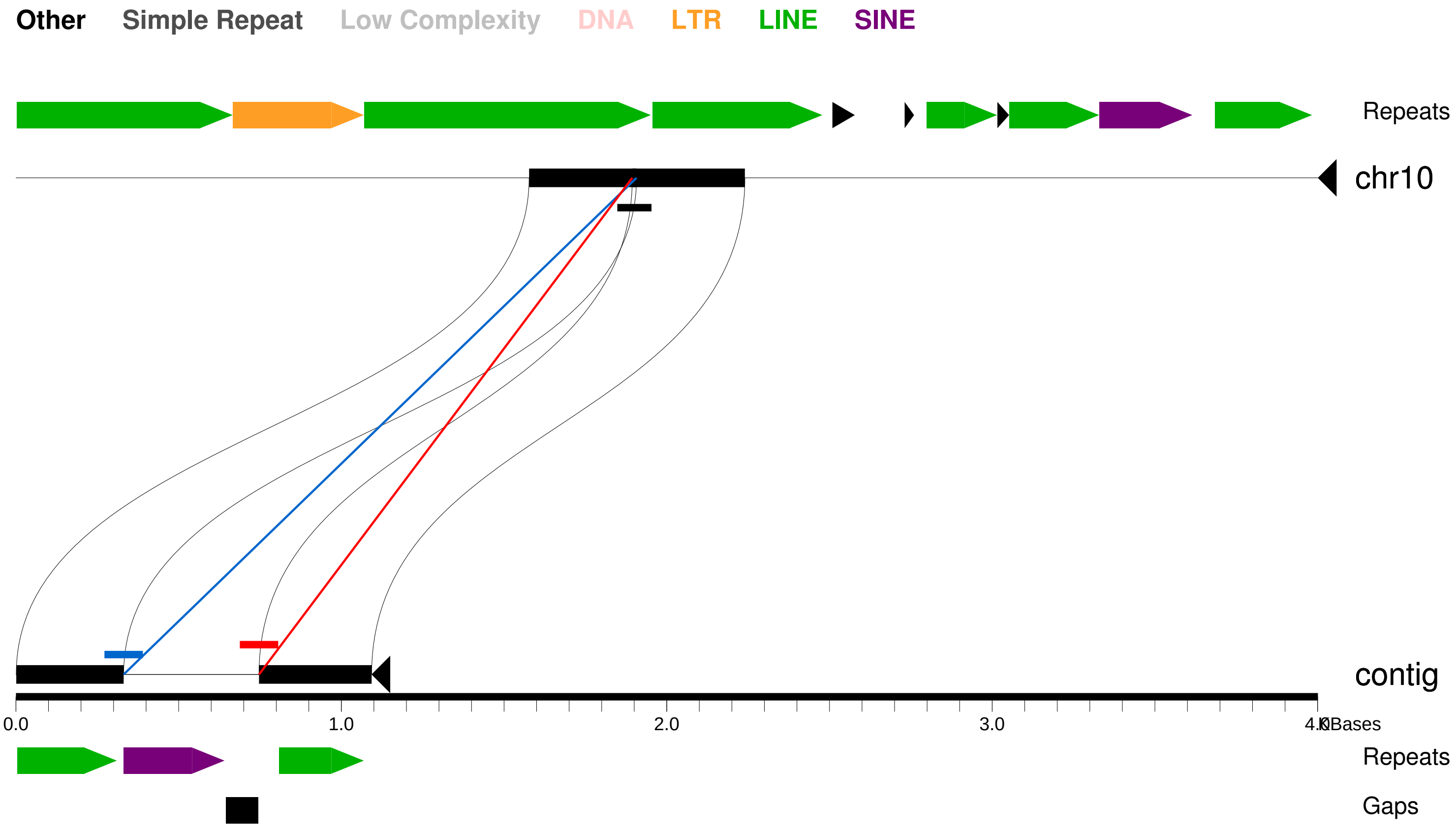

siteID: chr10_22867541 Scafftig2 - Breakpoint: chr10:22867575
chr10:22867256-22867735
Overlap Length: 8 INS size: 348 Genotyped: No

```
left     ----------------------------------------------------------------------
chrom    CTGGATTTTACTTTCCTTTCCCTTCAGAGCAACTGTTCCCCCTGAGATCTCAATGACCTTTGAGTGGTTA
right    ------TTTAGT-------------AGAG--ACGG----------GGTTTCA----CCTT------GTTA
               2222N2             2222  22N2          2N2N222    2222      2222

left     ----------------------------------------------------------------------
chrom    AATTTGACAGATGCTTCTCCGCCTTTCTCTCTCCTGAACCCCATGAGCAAACAGCGCTCTTGTCTGGGGC
right    -----GCCAG--GATGGTC------TCGATCTCCTGA-CCTCATGA------------------------
              2N222  2N2NN22      22NN22222222 22N22222

left     ----------------------------------------------------------------------
chrom    TTCCTCCCTCCCTTGGCTTCTGAGACGCTGTGTTCTCCTGGCAGTCCTCCTACTCCGTCCCATCTTTCCT
right    -TCCACCCGCC-TCGGCCTCCCAAA----GTG-----CTGGGA---------------------TTACAG
          222N222N22 2N222N22NN2N2    222     2222N2                     22N2NN

left     ----------------TTCCTCCTCTACATGCCCATTGTCAGGGCCCTGACCTAGATGTCCTCTCATCTT
chrom    GCTCTTTGGCCAATGCTTCCTCCTCTACATGCCCATTGTCAGGGCCCTGACCTAGATGTCCTCTCATCTT
right    GCG--TGAGCCACCGC--------------GCCC--------GGCC------------------------
         22N  2NN2222NN2211111111111111****11111111****111111111111111111111111

left     CTCTTCTTCACTGTGTACAGGCTACCTGATCCCCACCGTTTGTTTACNNNNNNNNNNN------------
chrom    CTCTTCTTCACTGTGTACAGGCTACCTGATCCACACCGTTTGTTTACATTTCTATCTGCGTCTCGATAAC
right    ---------------------------------------TTGTTTACATTTCTATCTGCGTCTCGATAAC
         111111111111111111111111111111111N111111*******22222222222222222222222

left     ----------------------------------------------------------------------
chrom    CCCCAAACTTATGATTCTCAGTCCACAACCCTTTCTTTTGAGTTGCACATCCACATAGGATTCATCTAGT
right    CCCCAAACTTATGATTCTCAGTCCACAACCCTTTCTTTTGAGTTGCACATCCACATAGGATTCATCTAGT
         2222222222222222222222222222222222222222222222222222222222222222222222

left     ------------------------------------------------------------
chrom    GTAATACAGCTTCCTGCCAAATGTAAGCTCCACGAAGGTGGCAAGCACTACCCTTAGCAG
right    GTAATACAGCTTCCTGCCAAATGTAAGCTCCACGAAGGTGGCAAGCACTACCCTTAGCA-
         22222222222222222222222222222222222222222222222222222222222
```

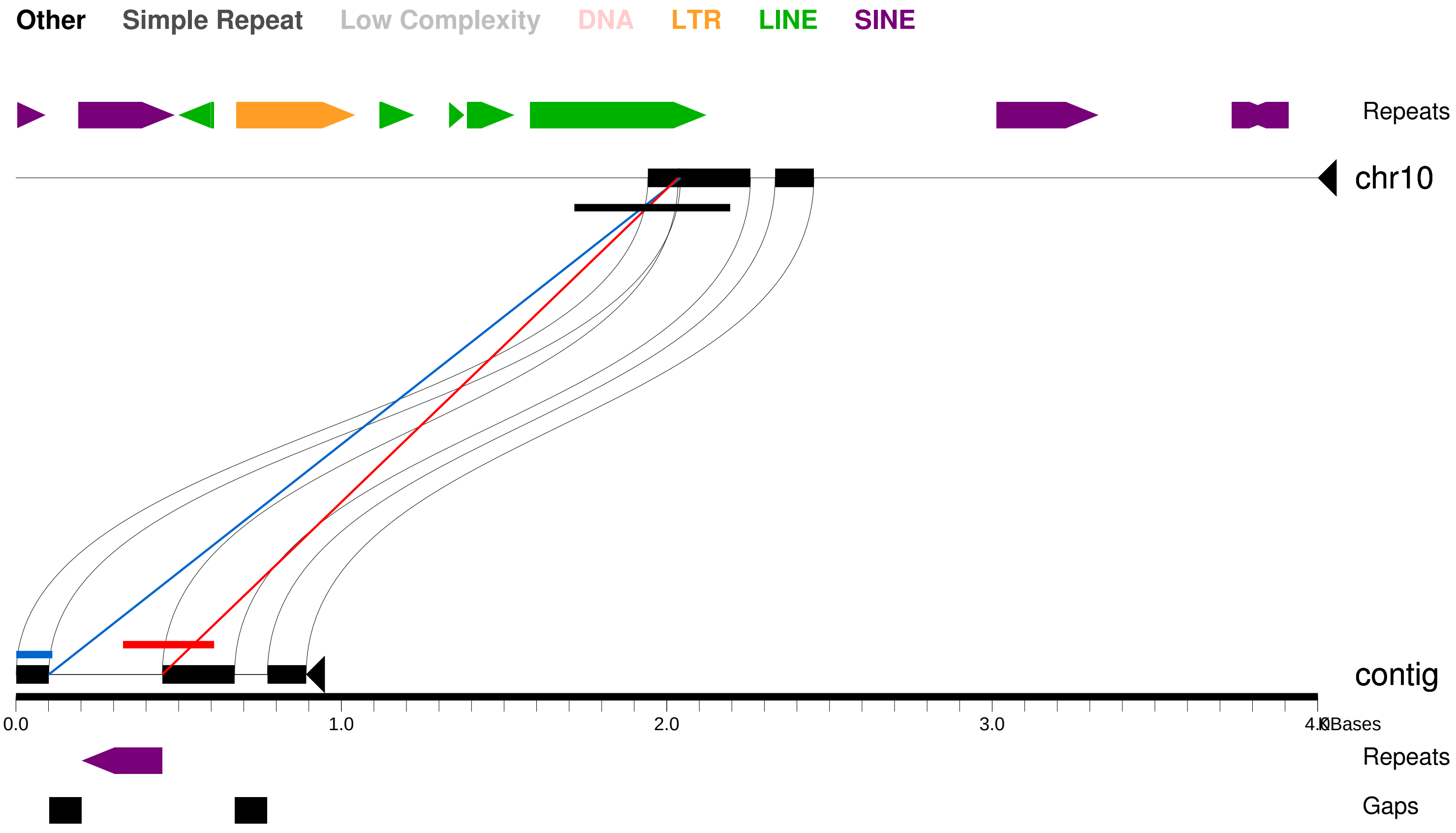

siteID: chr10_2388060 Scafftig2 + Breakpoint: chr10:2388048
chr10:2388022-2388107
Overlap Length: 12 INS size: 313 Genotyped: Yes

```
left    TTTCCTTAT---------------------TTAAATGCCTGTTCAGA------------TATTTTGCTCT
chrom   TTTCCTTAT---------------------TTAAATGCCTGTTCAGA------------TATTTTGCTCT
right   CACCCGCCTCGGCCTCCCAAAGTGCTGGGATTACAGGCGTGAGCCACCGCGCCCGGCCCTATTTTGCTCT
        111**111*111111111111111111111***1*1**1**11*111111111111111***********

left    TTTTTTTTTTTTTTTTTTTTTTTTTGAGACGGAGTCTCGCTCTGTCGCCCAGGCCGGACTGCGGACTGCA
chrom   TCTTAAACTGTGTTGTTGATTTT--GATAT-----------------------------TGTTGGCTTTA
right   TCTTAAACTGTGTTGTTGATTTT--GATAT-----------------------------TGTTGGCTTTA
        *2**2222*2*2**2**22****22**2*222222222222222222222222222222**22*2**22*

left    GTGGCGCAATCT
chrom   AAAGTTC--TTT
right   AAAGTTC--TTT
        222*22*22*2*
```

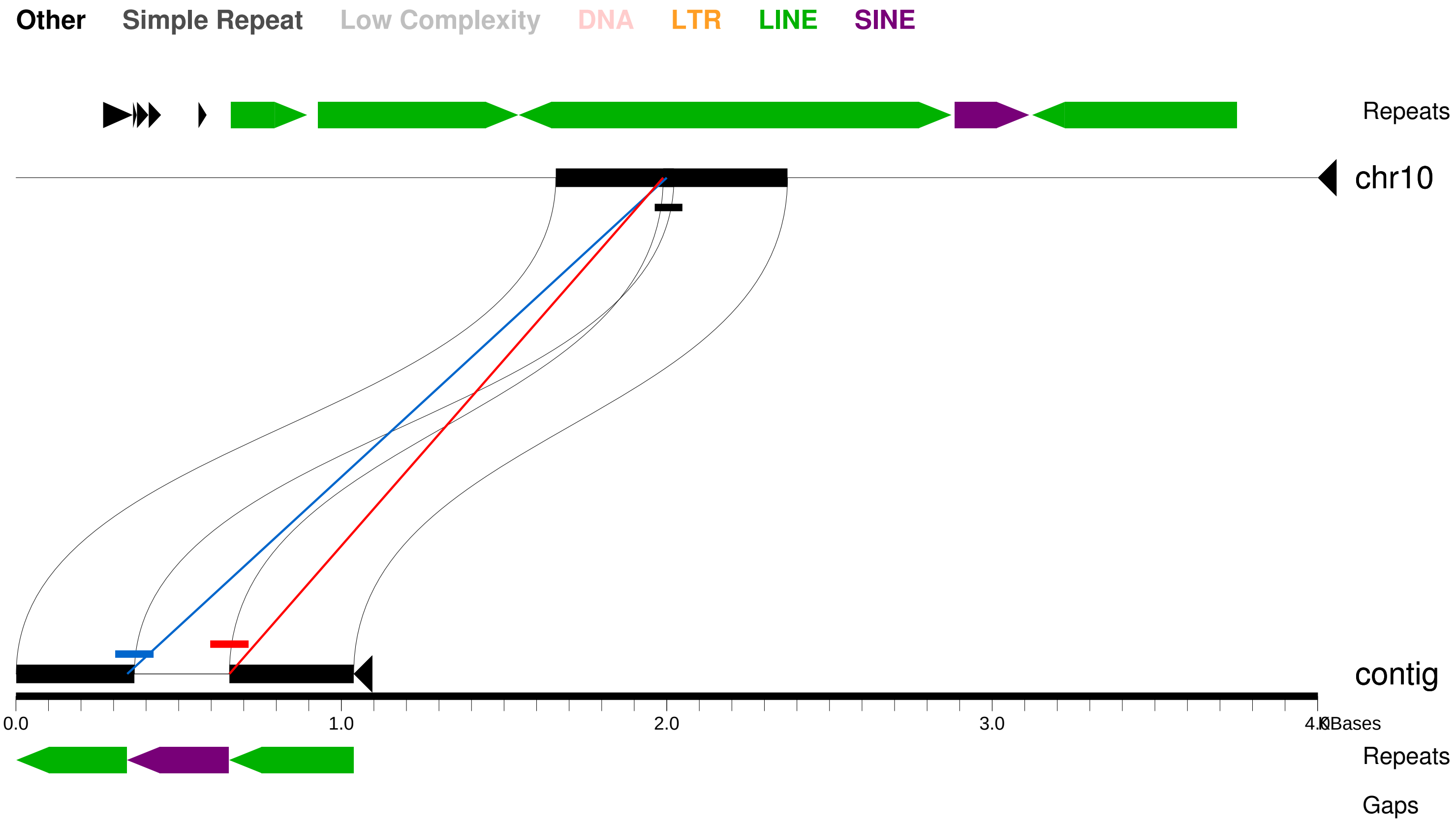

siteID: chr10_25487906 Scafftig4 + Breakpoint: chr10:25487915
chr10:25487866-25487974
Overlap Length: 11 INS size: 349 Genotyped: No

```
left   TATGCAACTGACATTTTCATCCTTGTTATTTGCTATAAGAGAGCATGTT----------AAAATATTTCT
chrom  TATGCAACTGACATTTTCATCCTTGTTATTTGCTATAAGAGAGCATGTT----------AAAATATTTCT
right  NNNNNNNNNNNNNNNNNNNNNNNNNNNNNNNNNNNNNNNNNNNNNNNNNNNNNNNNNNNNAAAAAAAATATTTCT
       111111111111111111111111111111111111111111111111111111111111***********

left   GGCCGGGCGCGGTGGCTCACGCCTGTAATCCCAGCACTTTGGGAGGCCGAGGCGGGTGG
chrom  AATCAGG-----TTGCCAATA--TTTAGAGAATTCGCTTT---AAGTTTTAGAAATAAT
right  AATCAGG-----TTGCCAATA--TTTAGAGAATTCGCTTT---AAGTTTTAGAAATAAT
       222*2**22222*2**22*2222*2**2222222*2****222*2*22222*2222222
```

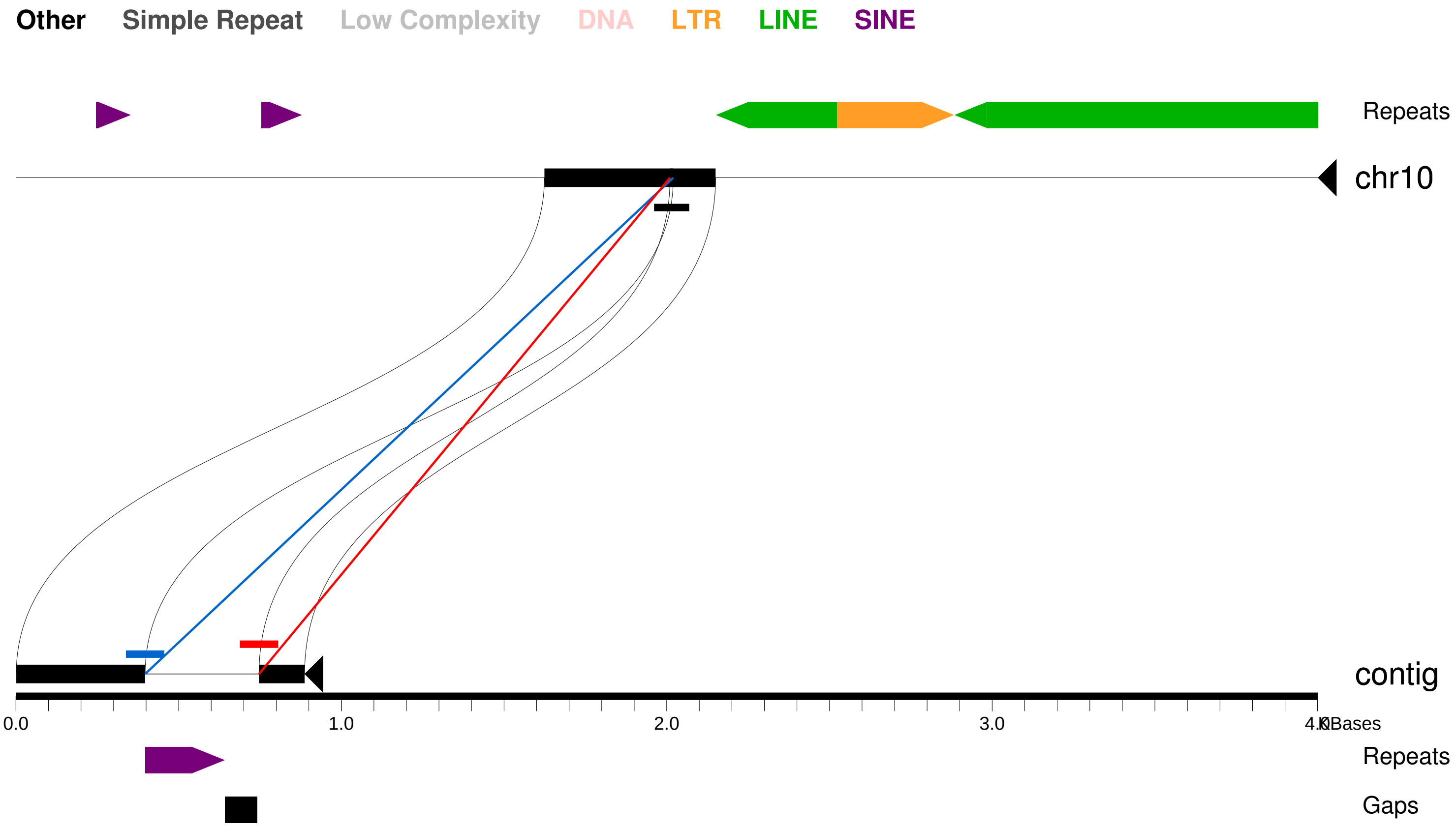

siteID: chr10_25957312 Scafftig2 - Breakpoint: chr10:25957316
chr10:25957267-25957368
Overlap Length: 11 INS size: 324 Genotyped: Yes

```
left    -----------AGA---ACATATAATCTCATTGGATAGCCATGATATTGCTACATTAACATAT---AAGA
chrom   -----------AGA---ACATATAATCTCATTGGATAGCCATGATATTGCTACATTAACATAT---AAGA
right   GCCTGGGCGACAGAGCGAGACTCCGTCTCAAAAAAAAAAAAAAAAAAAAAAAAAAAAAAAAAAAAAAAAGA
        11111111111***111*1*11111*****1111*1*111*11*1*11111*1*11**1*1*1111****

left    AGGGGAGGCCGGGCGCGGTGGCTCACGCCTGTAATCCCAGCACTTTGGGAGGCCGAGGCGGGTGGA
chrom   AGGGGAGATGAATTTTGGTGGA-CA-GCTAGCATTCTCCTCATTTAA---------------TTCT
right   AGGGGAGATGAATTTTGGTGGA-CA-GCTAGCATTCTCCTCATTTAA---------------TTCT
        *******222222222*****22**2**22*2*2**2*22**2**22222222222222222*222
```

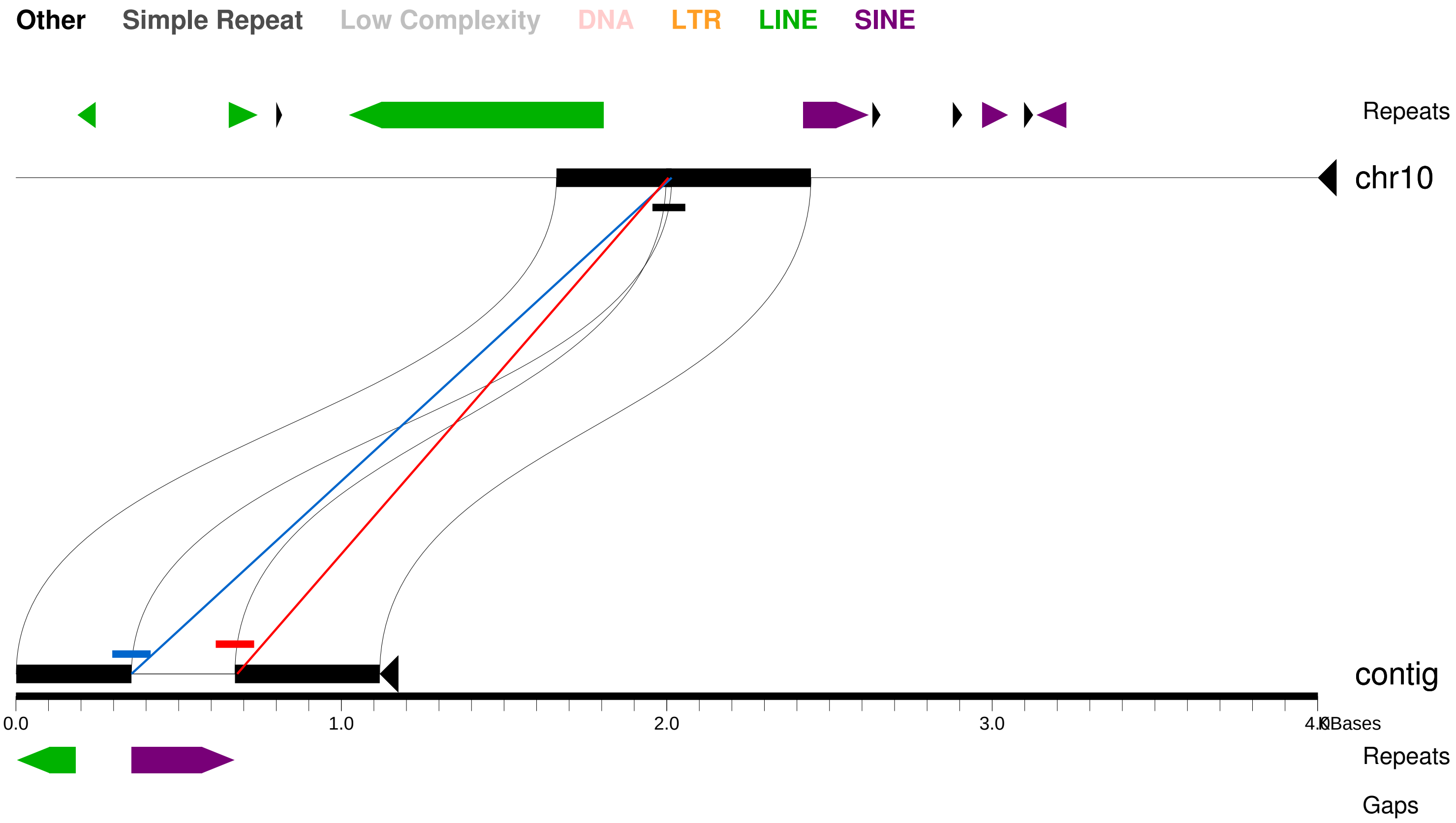

siteID: chr10_26252821 Scafftig1 - Breakpoint: chr10:26252893
chr10:26252850-26252952
Overlap Length: 17 INS size: 134 Genotyped: Yes

```
left    T-TCTTTGA-ATAGGCATGCACATTT--------------ATAGTGTTACTTCAGAGGTAAGAAAATATG
chrom   T-TCTTTGA-ATAGGCATGCACATTT--------------ATAGTGTTACTTCAGAGGTAAGAAAATATG
right   CCTGGGCGACAGAGCGAGACTCCGTCTCAAAAAAAAAAAAAAAAAAAAAAAAAAAAAAAAAGAAAATATG
        11*1111**1*1**11*11*1*11*111111111111111*1*11111*1111*1*111***********

left    AGGTGAGGCTGAGGCAGGAGAATGGCGTGAACCCGGGAGGCGGAGCTTGCAGTGAGCCGAGATCC
chrom   AGGTGA--------CCAGTGA-TGTCTTG--CTATTTAA---TAAATT--AATAGATTTTTATTG
right   AGGTGA--------CCAGTGA-TGTCTTG--CTATTTAA---TAAATT--AATAGATTTTTATTG
        ******22222222*22*2**2**2*2**22*22222*22222*22**22*2*22222222**22
```

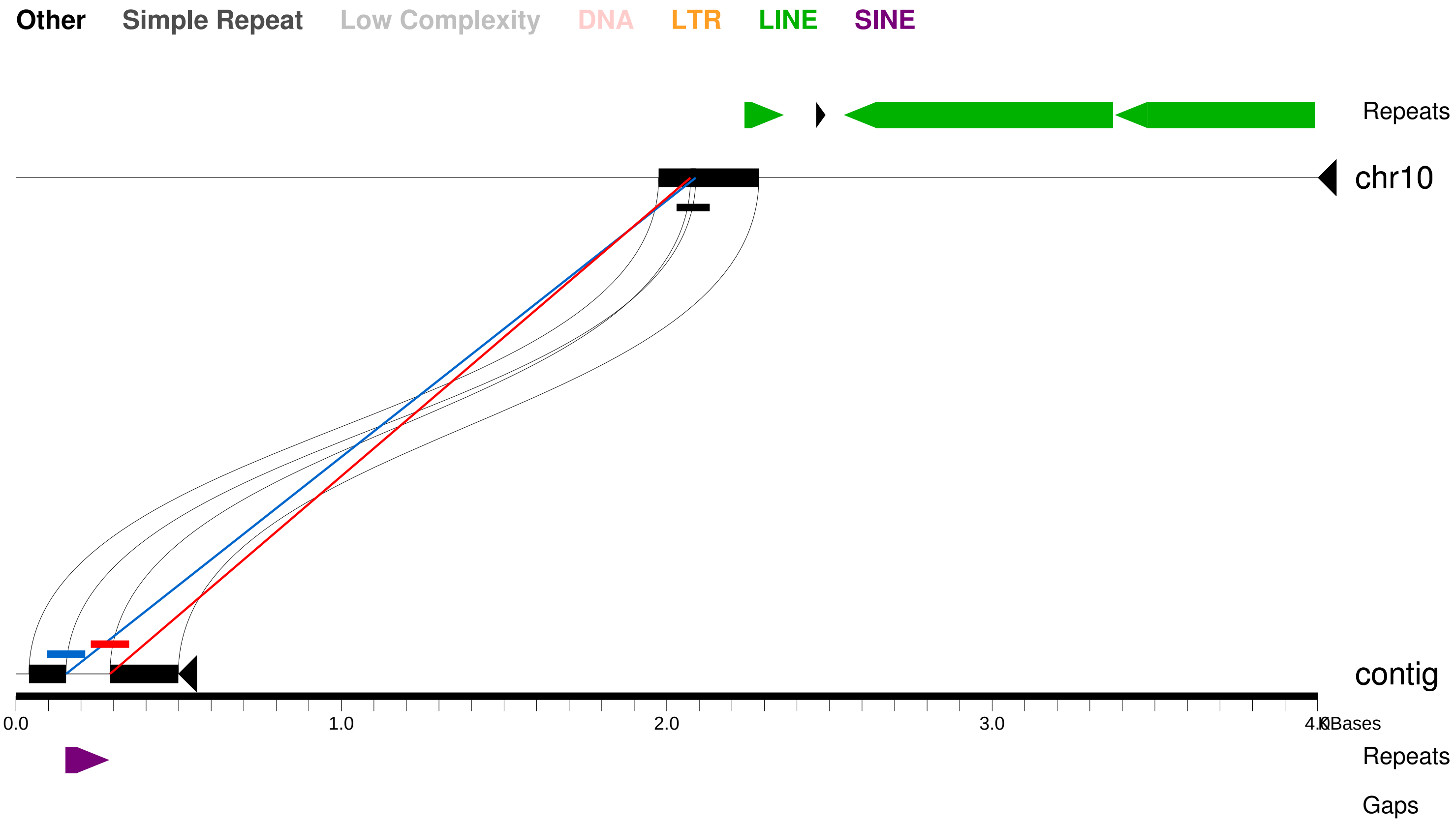

siteID: chr10_27929933 Scafftig4 + Breakpoint: chr10:27929919
chr10:27929759-27930088
Overlap Length: 10 INS size: 318 Genotyped: Yes

```
left   -----GTAGTATAACCAACAGAGAGCATTAAGTTCCCACAGTTCATGATAATGAATTAAAACAAAACCAA
chrom  AAAAAGTAGTATAACCAACAGAGAGCATTAAGTTCCCACAGTTCATGATAATGAATTAAAACAAAACCAA
right  TT----TAGTAGAG---ACGGGGT--------TTCACCTTGTTAGCCAGGATGGTCTCGATCT---CC--
       NN   1*****1*1111**1*1*111111111***1*111***1111*11***111*11*1*1111**11

left   TGAACATAGTTATGCTTTTTAAAAGTCCACCCCTAGATAACTGCACCAGTGAAGATGCCAAACAGCTACA
chrom  TGAACATAGTTATGCTTTTTAAAAGTCCACCCCTAGATAACTGCACCAGTGAAGATGCCAAACAGCTACA
right  TGACC----TCATGAT----------CCACCCGCCTCGGCCTCCCAAAGTGCTG--GGATTACAG-----
       ***1*1111*1***1*1111111111******11111111**1*111****11*11*1111****11111

left   GAGTTAGATTTAACTAGG-GTCAAGATTTTTTTTTTTTTTTTTTTTTTT------------------TTTTT
chrom  GAGTTAGATTTAACTAGG-GTCAAGATTTTTATCATGAGAGTATTCTGAAGCAAAAGAAGAGCAATTTTG
right  GCGTGAGCCACCGCGCCCAGCCAAGATTTTTATCATGAGAGTATTCTGAAGCAAAAGAAGAGCAATTTTG
       *1**1**111111*11111*1**********2*22*22222*2**2*222222222222222222****2

left   T---TTTTTTTTTGAGAC------GGAGTCTCGCTCT--GTCGCCCAGGCCGGACTG-CGGACTGCAGTG
chrom  TCAATTTTTCCTTCATATATTTTGGGAGTCT-GTTATTAGGTGTATAAGTATTTCTGTCTTTTTGCTATA
right  TCAATTTTTCCTTCATATATTTTGGGAGTCT-GTTATTAGGTGTATAAGTATTTCTGTCTTTTTGCTATA
       *222*****22**2*2*2222222*******2*2*2*22*22*222*2*22222***2*2222***22*2

left   GCGCAATCTCGGCTCAC-TGCAAGC----------TCCGCTTCCCGGG----
chrom  TTGAAATTTTTATTAATATATAATAGTAAAATTAATACACTTTAAATTTAAA
right  TTGAAATTTTTATTAATATATAATAGTAAAATTAATACACTTT---------
       22*2***2*2222*2*22*22**222222222222*2*2***2NNNNN
```

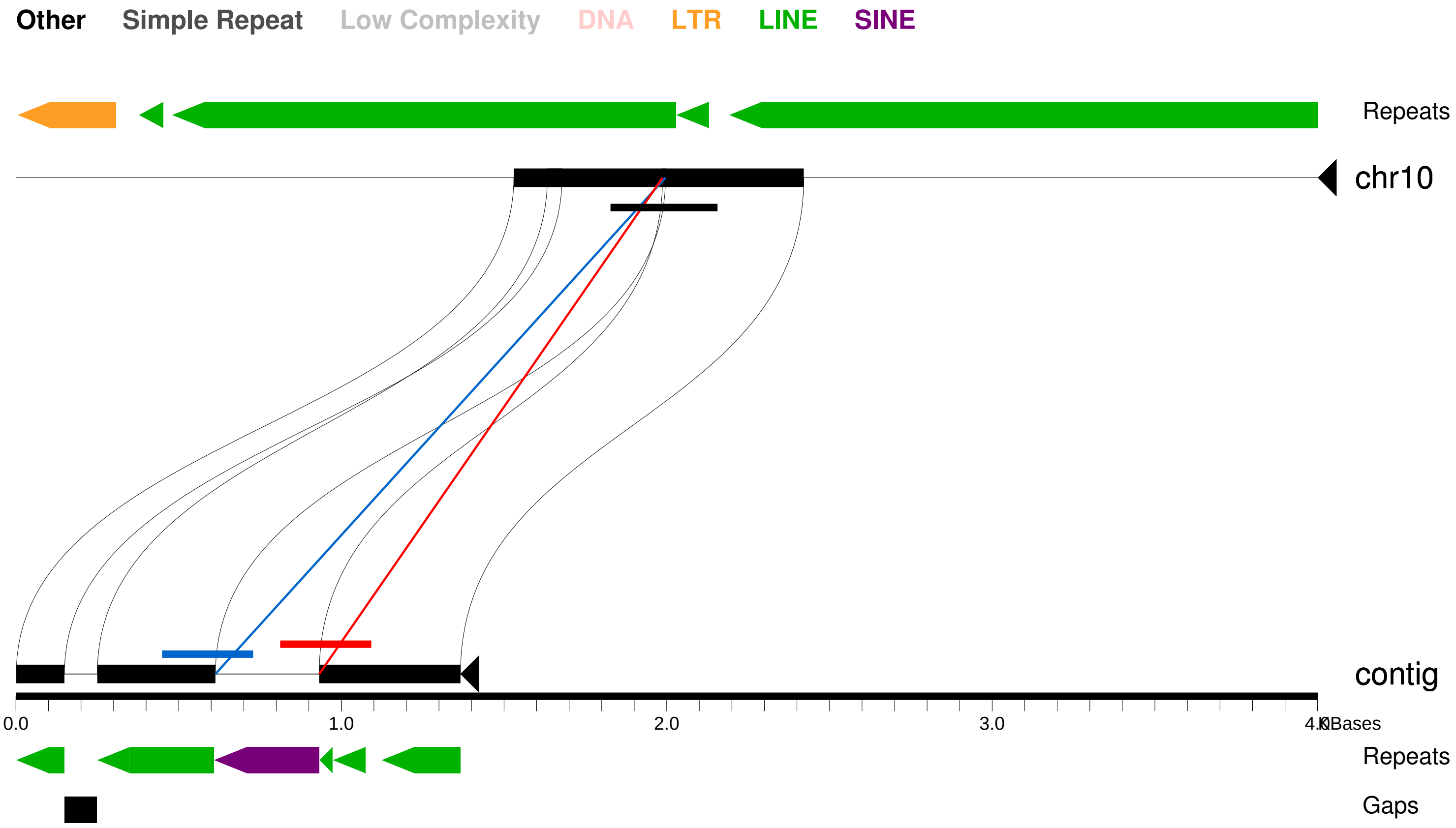

siteID: chr10_29102548 Scafftig10 + Breakpoint: chr10:29102528
chr10:29102482-29102587
Overlap Length: 14 INS size: 301 Genotyped: Yes

```
left   ------GTTTTTGGATGGTAA-------GAATAAAAATTGTGAAAGTGTCAATTTTTTTCTAAATCATCT
chrom  ------GTTTTTGGATGGTAA-------GAATAAAAATTGTGAAAGTGTCAATTTTTTTCTAAATCATCT
right  CCGCCCGCTTCGGCCTCCCAAAGTGCTGGGATTACAGGCGTGAGCCACCGCGCCCGGCCCTAAATCATCT
       111111*1**11*11*111**1111111*1**1*1*111****1111111111111111***********

left   ATTTTTTTTTTTTTTTTTTTTTTGAGACGGAGTCTTGCTCTGTCGCCCAGGCTGGAGTGCAG--
chrom  ATTAA------------TTCAATGCAACC---TCATGAAAAATTACAAAGGGATTGTTTTAGTC
right  ATTAA------------TTCAATGCAACC---TCATGAAAAATTACAAAGGGATTGTTTTAGTC
       ***22222222222222**222**22**2222**2**22222*22*22***222222*22**22
```

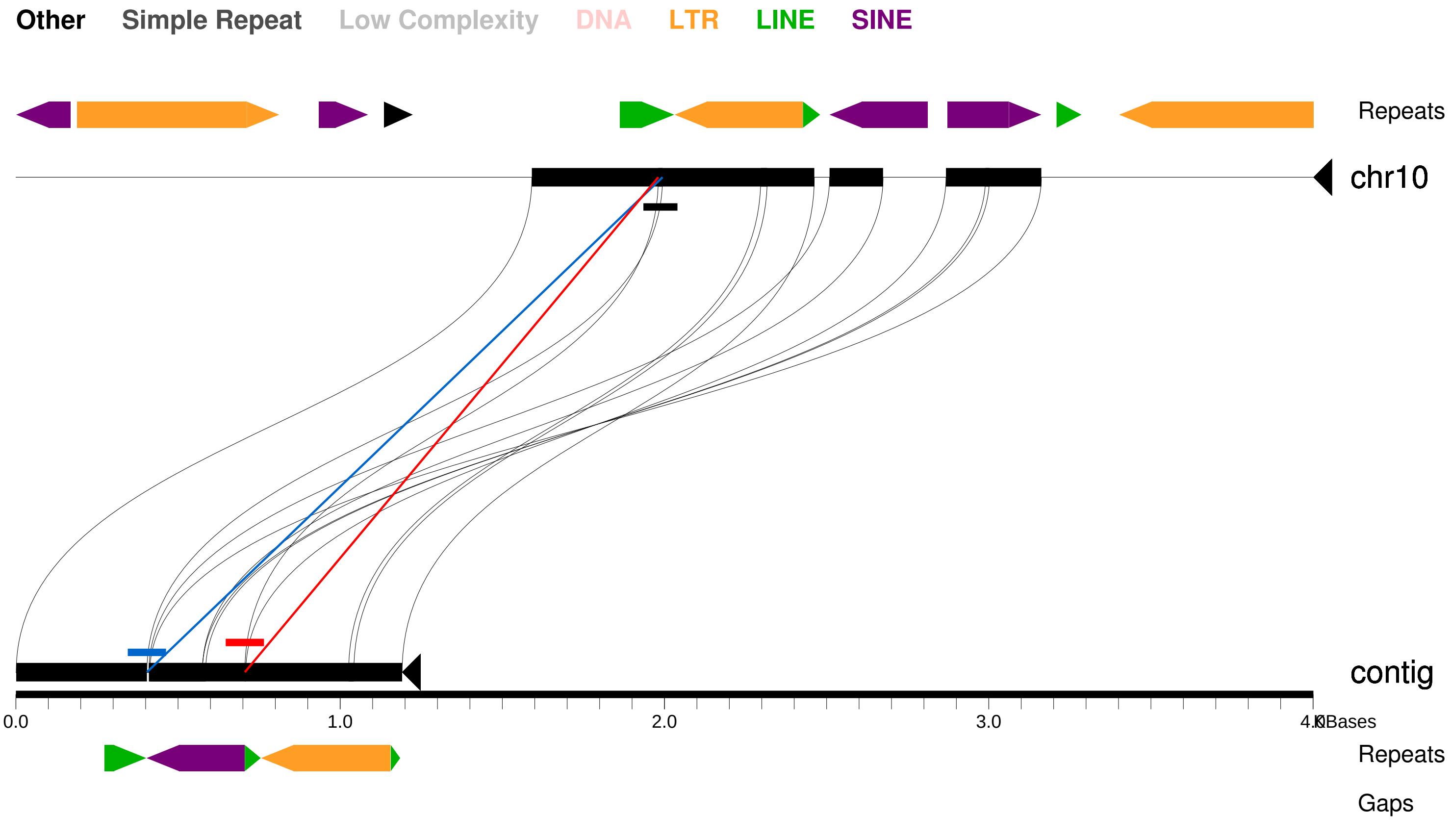

siteID: chr10_3569028 Scafftig4 - Breakpoint: chr10:3569009
chr10:3568382-3569169
Overlap Length: 17 INS size: 298 Genotyped: Yes

```
left   T-----------------------------------------------------------------------
chrom  TACTTTTTTAAATCTGTCCCATGGAAAATTGTACGAGTATGAGTGGAAAGCTGCAAGGTGACCGGATAGC
right  T----------------------------------GAG------------------GCAGG-------------
       *                                  222                  222N2

left   -----------------------------------------------------------------------
chrom  ACAGGGCTCAGGGAGCCACAGCCCAGCTTCAACCAAGAATGCATGATTCTTGTATGTGGCGAGGGCTTCC
right  -----------------------------------AGAATG-----------------GCGTG-------
                                          222222                  222N2

left   -----------------------------------------------------------------------
chrom  CCCACCCTCCGCAGCACCCTGGTTCCTTCACTTCATCTAGTGAGAAACAAGAGCAGCCCTGTTGCAAGGA
right  ---------------------------------------------AAC-----------------------
                                                    222

left   -----------------------------------------------------------------------
chrom  ATGTGCCTGGGGGCCCATCAGTGCTCTGGGCTGCCTGACATGAGGCCCTTGGGTCTCTGTGCCTCAGTGT
right  -----CCGGGAGGC-----------------------------GGAGCTTG-------------CAGTG-
            22N22N222                             22NN2222              22222

left   -----------------------------------------------------------------------
chrom  CCTCGAGTTCCAGTTGAGATGGGTGCCCATGGCTATGGATTGTTGATATCCTTCTGGCTCTGAAATTTGG
right  -----------AGTCGAGATCG-------------------------------------------------
                  222N22222N2

left   -----------------------------------------------------------------------
chrom  ATAGTTTCTTCTCCAAAGAACTAAGTGTTACTGGAGGCTCTTGAAAGAAATCTGCAGCGGCCTGGGATGT
right  ----------CGCCA------------------------------------CTGCAC-------------
                 2N222                                       22222N

left   -----------------------------------------------------------------GACCT
chrom  TACACATCCAGCCCCAGATTACCCCAAGGATGGGCTTGAACCTCACCCCATATCCAGCGTGAAATGACCT
right  ------TCCAGCC-----------------TGGGC--GA---------------CAGAGCGAAA----CT
             2222222                 22222  22               222N2N2222 111**

left   GCAGGCGTGTCTCGGGGTCTGGATATCAAGGCATCCAGTGACTATGGAGCAGGAACGAGGCCTTCAAGTC
chrom  GCAGGCGTGTCTCGGGGTCTGGATATCAAGGCATCCAGTGACTATGGAGCAGGAACGAGGCCTTCAAGTC
right  CC------GTCTC----------------------------------------------------------
       1*111111*****1111111111111111111111111111111111111111111111111111111111

left   CTGATGGCGAATGGCCACTTCTGCACATCCCCTAAAAACCTTATCTTCTAAAGACCTTCAACACATTAAA
chrom  CTGATGGCGAATGGCCACTTCTGCACATCCCCTAAAAACCTTATCTTCTAAAGACCTTCAACACATTAAA
right  ---------------------------------AAAAA-----------AAA-------AAAAAAAAAAA
       111111111111111111111111111111111*****11111111111***1111111**1*1*11***

left   AATTCTGTGAACTTGGCCCGGG[illegible]CGCGTGGCTCACGCCTGTAATCCCAGCACT-TTGGGA
chrom  AATTCTGTGAACTTTACTATTTCTAAAAATATCCTACCAGCTCCACCACTAAATATAACGTCTCTTCCAA
right  AATTCTGTGAACTTTACTATTTCTAAAAATATCCTACCAGCTCCACCACTAAATATAACGTCTCTTCCAA
       *************22*222222222222222*222222****222*2222***222*222**2**222*

left   [illegible]GGC-----GGATCACGAGGTCAGGAGA-TCGAGACCATCCTGGCTAACAC-GGTGAAA
chrom  CGATGAGTCAAATGCATTTTGCATCAATGGCTCTGCATACTTGAGA--AACGGAGTTGTTTGTGGTGAAG
right  CGATGAGTCAAATGCATTTTGCAT[illegible]A--AACGGAGTTGTTTGTGGTGAAG
       2*22***22*222**22222*2[illegible]*22*2*222*2*222222******2

left   CCCCGTCTCTA-----CTAAA---
chrom  ATATTTTTCCATGGTGCTGC----
right  ATATTTTTCCATGGTGCTGCTCCA
       22222*2**2*22222**22N111
```

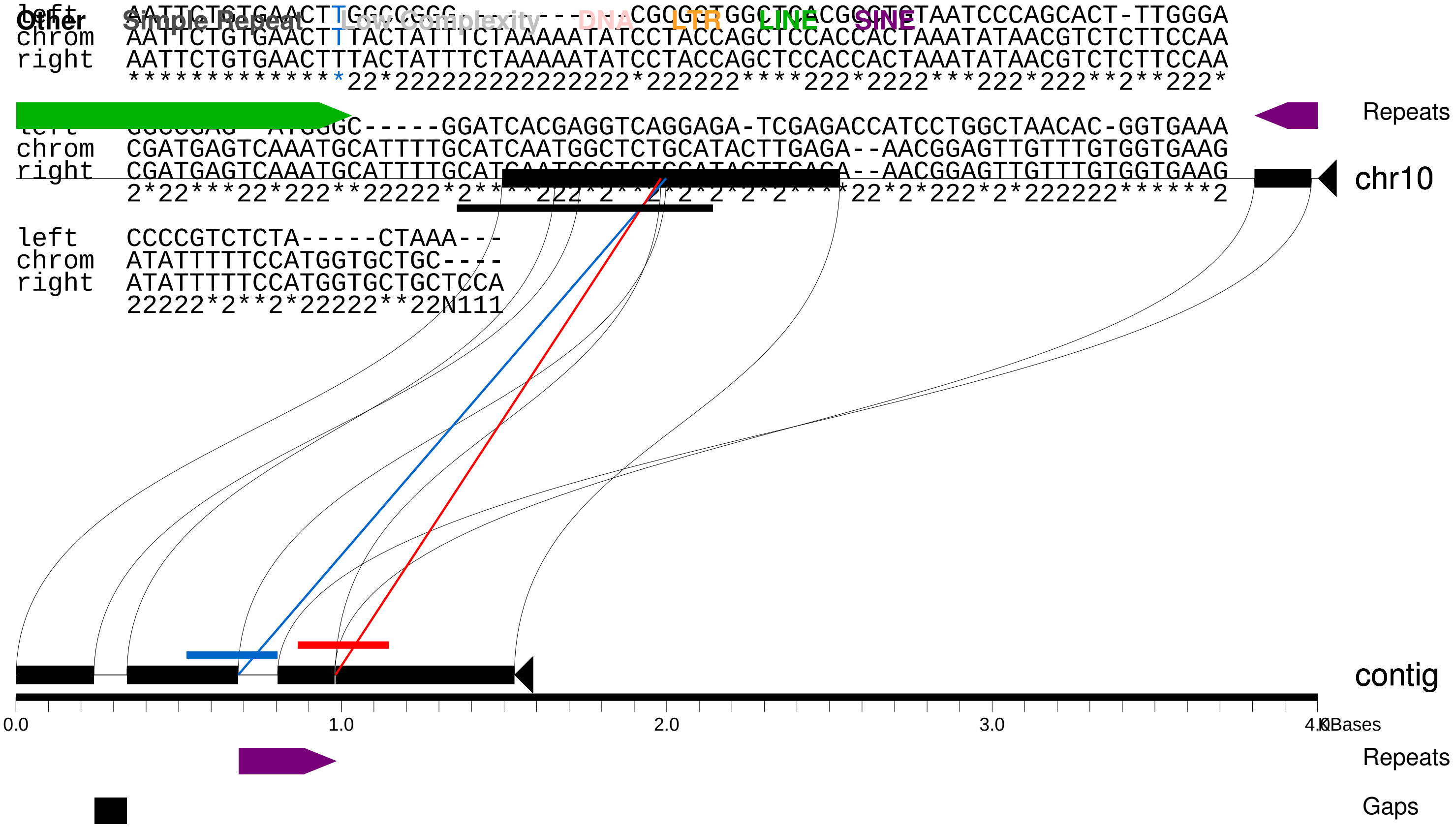

siteID: chr10_36467324 Scafftig1 + Breakpoint: chr10:36467292
chr10:36467247-36467351
Overlap Length: 15 INS size: 294 Genotyped: No

```
left    ------------GTAACCCAAAG-GAGACTATTCCAAGGATATGACAT-GTAAGCTAAATGATTAGACAT
chrom   ------------GTAACCCAAAG-GAGACTATTCCAAGGATATGACAT-GTAAGCTAAATGATTAGACAT
right   CCGCCCGCCTCGGCCTCCCAAAGTGCTGGGATTACAGGCGTGAGCCACCGCGCCCGGCCTGATTAGACAT
        111111111111*111*******1*11111***1**1*11*11*1**11*1111*1111***********

left    TTCTTTCTNNNNNNNNNNNNNNNNNNNNNNNNNNNNNNNNNNNNNNNNNNNNNNNNNNNNNN
chrom   TTCT--------------AAGGAAAGAAGGAAAATATACTGTAGGCAGGAGGAAGTGGATCTC
right   TTCT--------------AAGGAAAGAAGGAAAATATACTGTAGGCAGGAGGAAGTGGATCTC
        ****22222222222222222222222222222222222222222222222222222222222
```

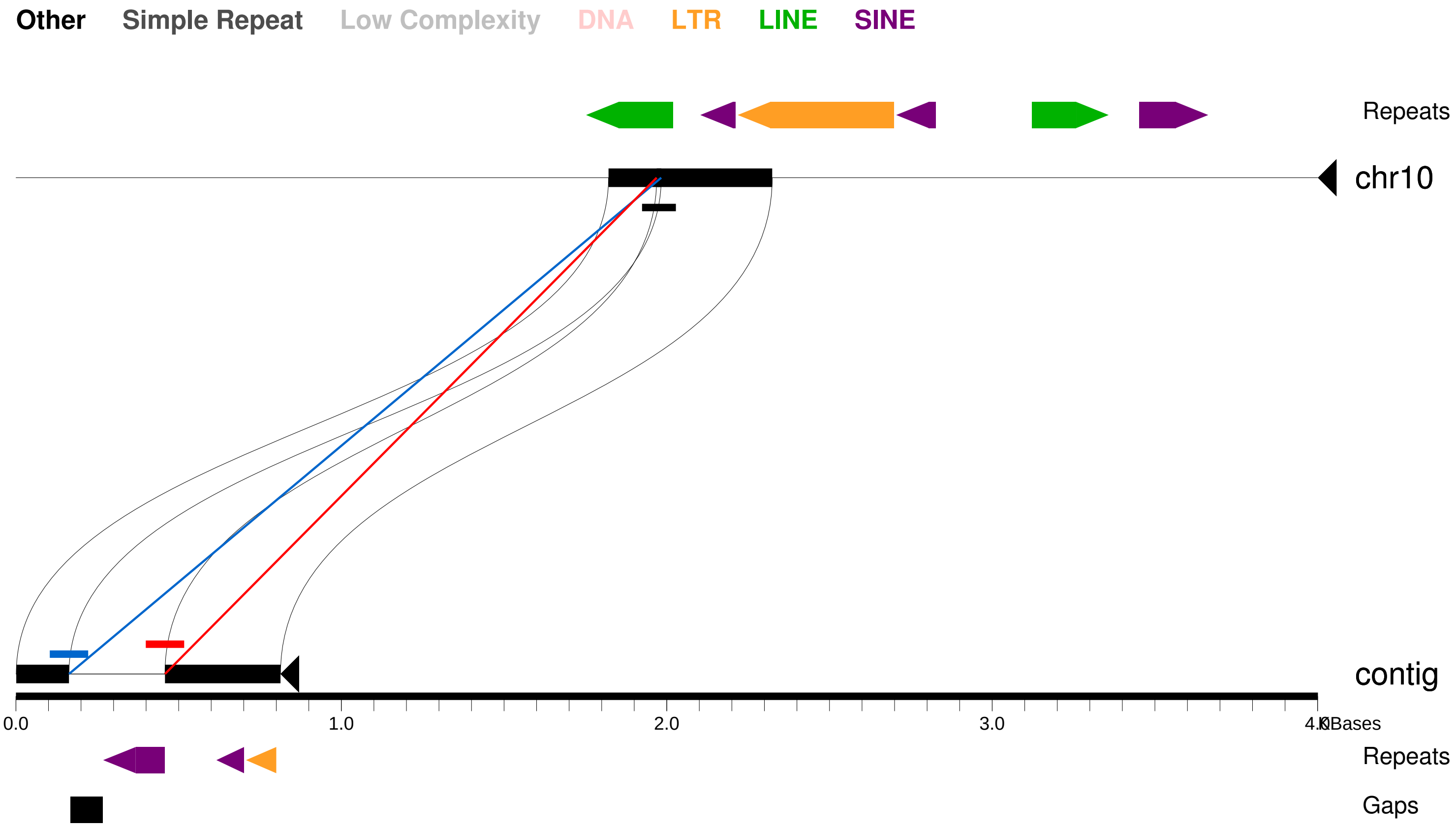

siteID: chr10_36593640 Scafftig3 + Breakpoint: chr10:36593622
chr10:36593462-36593787
Overlap Length: 6 INS size: 315 Genotyped: Yes

```
left   T---CTAAAATTATTTTAGCCAAATGAAACTAAATAATACTGCCTTTTAAATAGGTGGATAATTTGATGA
chrom  TATTCTAAAATTATTTTAGCCAAATGAAACTAAATAATACTGCCTTTTAAATAGGTGGATAATTTGATGA
right  TTTAGTA------------------GAGACGGGGTTTCAC--CTTGTTAACCAGGATGGTC--TCGAT--
       *N2N1**111111111111111111**1**1111*111**11*1*1****11***11*1*111*1***11

left   ATTCTTTGAAAAGAATTGTCCAAATAATATATTAAGTGTCTTTCCAATTGCTTATTTATGTCTACCTGTA
chrom  ATTCTTTGAAAAGAATTGTCCAAATAATATATTAAGTGTCTTTCCAATTGCTTATTTATGTCTACCTGTA
right  ---CTCCTGACCTCATGATCCACCCGCCTCG------GCCTCCCAAAGTGCTGGGAT-----TACAGGCG
       111**1111*1111**11****111111111111111*1**11*1**1****1111*11111***11*11

left   GTAATCTCTTATACTGACATGGATTTTTTTTTTTTT----TTTTTTTTTTTTTTTTTTGAGACGGGG----
chrom  GTAATCTCTTATACTGACATGGATTTCTTTATTGATAGGCTGTATGTATGTGGCTTTGGCTCCTACATAA
right  TGAGCCACCGCGCCCGGCC-GGATTTCTTTATTGATAGGCTGTATGTATGTGGCTTTGGCTCCTACATAA
       11*11*1*11111*1*1*11******2***2**22*2222*2*2*2*2*2*222****222*22222222

left   ---TCTCGCTCTGTCGCCCAGGCCGGACTGCGGACTGCAG----TGGCGCAATCTCG-GCTCA---CTGC
chrom  ATATCACCCCACTTCCTTTAAGAATAATTAATAAGTGAAAAGCTTGCTTCACTCTCTTGCTGAGCCCAGC
right  ATATCACCCCACTTCCTTTAAGAATAATTAATAAGTGAAAAGCTTGCTTCACTCTCTTGCTGAGCCCAGC
       222**2*2*2222**2222*2*2222*2*2222*2**2*22222**222**2****22***2*222*2**

left   ------AAGCTCCGC-------TTCCCGGG-----TTCACG-----
chrom  TTAATGAATATCGGTAAAATAATTGCATGACAACATTAATGATGAA
right  TTAATGAATATCGGTAAAATAATTGCATGACAACATTAAT------
       222222**22**2*22222222**2*22*222222**2*21
```

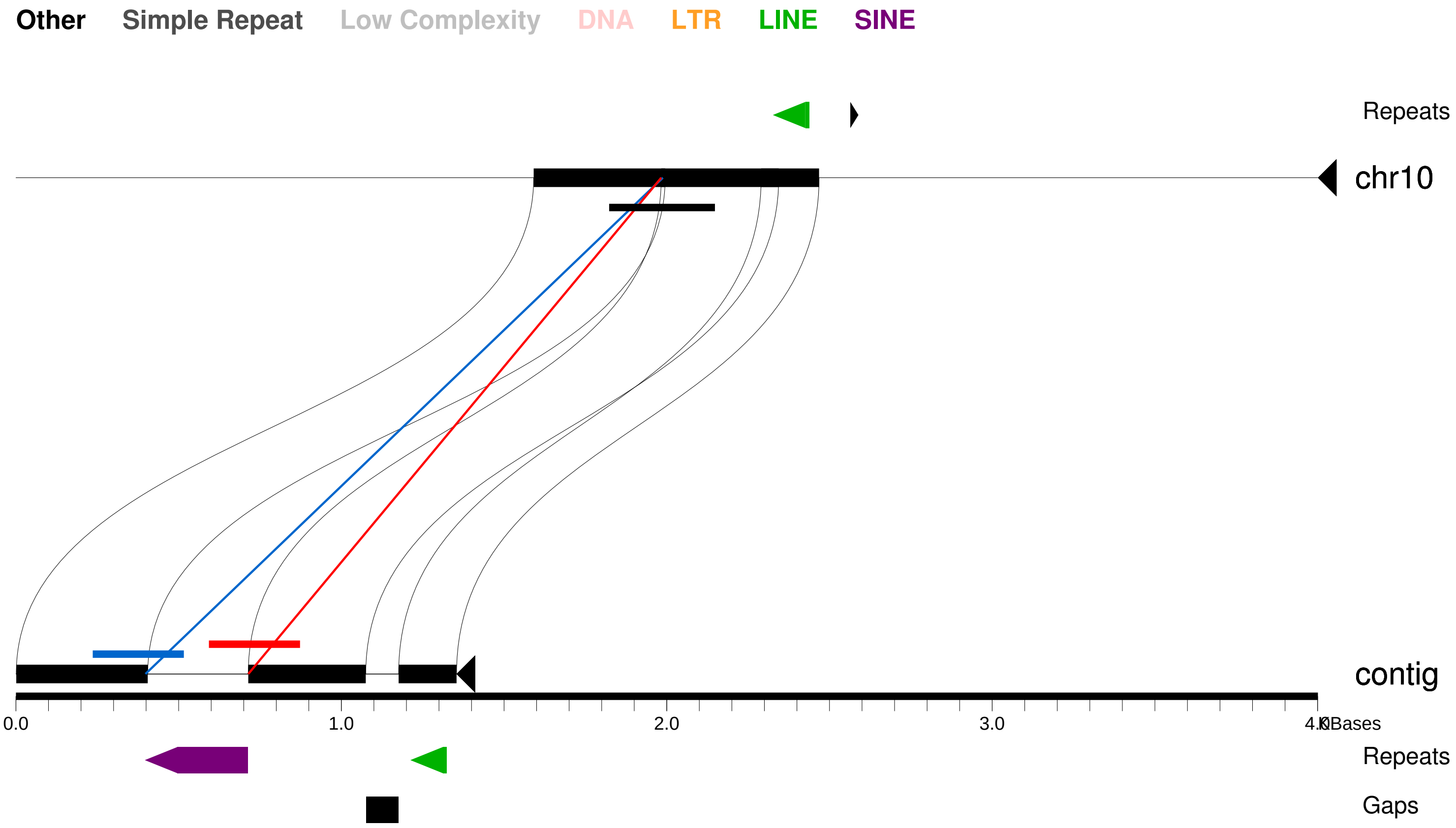

siteID: chr10_4317493 Scafftig2 - Breakpoint: chr10:4317420
chr10:4317260-4317591
Overlap Length: 12 INS size: 302 Genotyped: No

```
left     -----------TAATGCATGCTGGGCTCAATACCTAGGTAATGAGTTGATAGGTGCAGCAAACCACCATG
chrom    GGATAAACAGCTAATGCATGCTGGGCTCAATACCTAGGTAATGAGTTGATAGGTGCAGCAAACCACCATG
right    T--TCAGTAGAGACGG------GGTTTCA---CC----------GTTT-TAG----------CCGGGATG
         N  2N2NN22N1*11*111111**11***111**1111111111***11***1111111111**111***

left     GCACATGTTTGCCTACGTAATGAACCTGCACTTCCTGCACATGTACCCGAGAGCTTAAAATTTAAAAAAA
chrom    GCACATGTTTGCCTACGTAATGAACCTGCACTTCCTGCACATGTACCCGAGAGCTTAAAATTTAAAAAAA
right    GTCTCGATCTCCTGACCTCGTGATCC-GCCCGCCTCGGCC---TCCCAAAGTGCTGGGATT----ACAGG
         *111111*1*1*11**1*11***1**1**1*11*11*11*111*1**11**1***111*1*1111*1*11

left     ATTTAAAAAGTATAACTTGT-AGTCATGTATATTTTTTTTTTTTTTTTTTTTTTGAG-ACGGAGTCTCGC
chrom    ATTTAAAAAGTATAACTTGT-AGTCATGTATATCTTAAACAACTTTAAAAACTAATGTACTGTGTTCCCC
right    CGTGAGCCACCGCGCCCGGCCAGTCATGTATATCTTAAACAACTTTAAAAACTAATGTACTGTGTTCCCC
         11*1*111*111111*11*11***********2**2222222***222222*222*2**2*2**22*2*

left     TCTGTCGCCCAGGCTGGAGTGCAGTGGCGGGATCTCGGC-----TCA-------CT-----GCAAGCT--
chrom    CTTTTCCTTCAAGGAAAACCATTATAATATGATCTTGTCAATAATCAGCATTTTCTAGTTAGGAAACTTG
right    CTTTTCCTTCAAGGAAAACCATTATAATATGANNNNNNNNNNNNNNNNNNNNNNNNNNNNNNNNNNNNNN
         22*2**222**2*2222*222222*22222**111N1N1NNNNN111NNNNNNN11NNNNN1N11N11NN

left     ----CCGCCTCC---CGGGTTC---------ACGCCATTCTCC----TGCCTC
chrom    ATAACATTATCAAAACTGTTTTTTTGAGAGAATACAATTTTGACAATTTCTAC
right    NNNNNNNNNNNNNNNNNNNNNNNNNNNNNNNNNNNNNNNNN------------
         NNNN1NNNN11NNNN1N1N11NNNNNNNNNN1NN1N111N1NN    1N1NN1
```

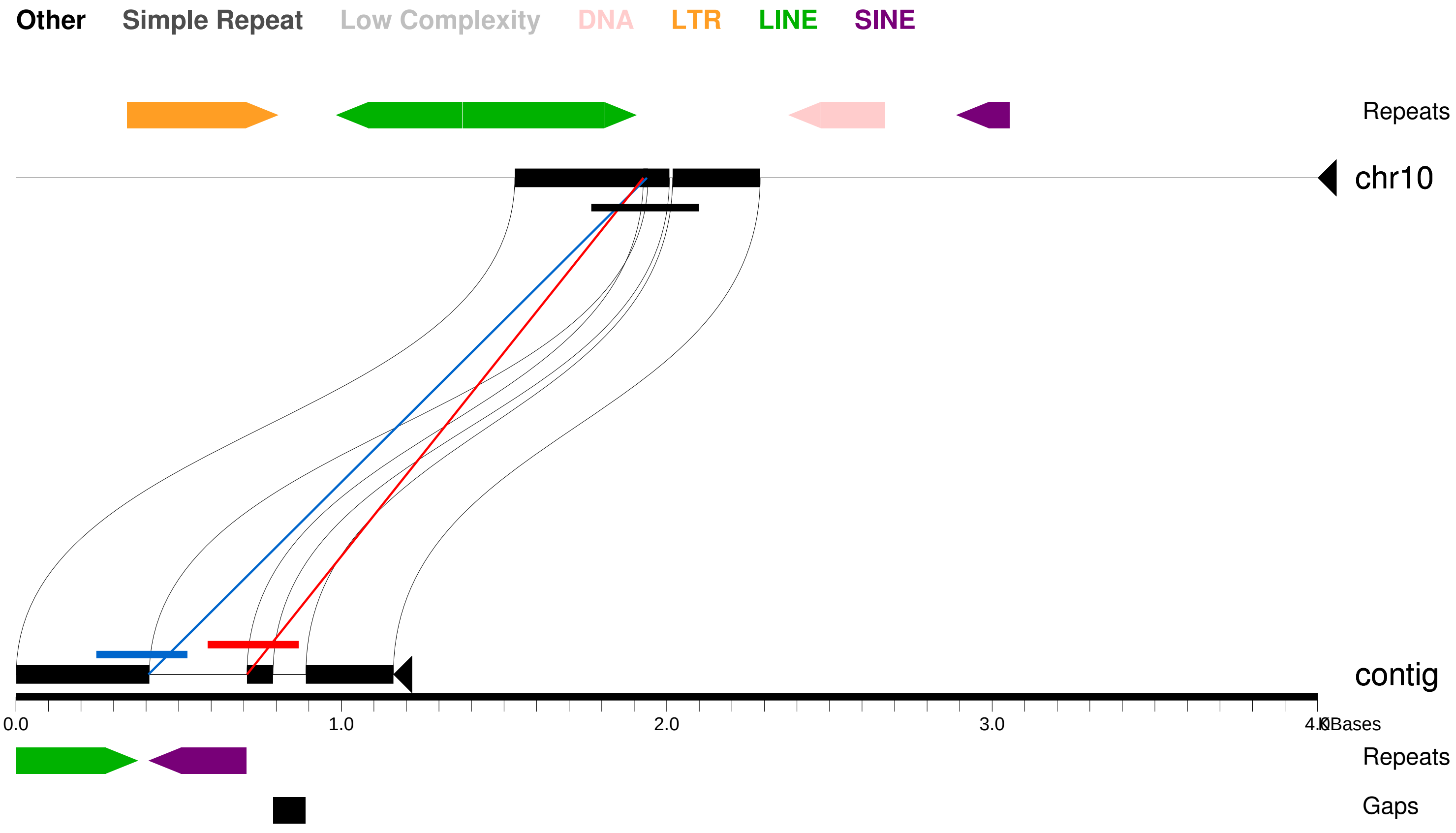

siteID: chr10_44068514 Scafftig1 - Breakpoint: chr10:44068501
chr10:44068341-44068671
Overlap Length: 11 INS size: 419 Genotyped: No

```
left    ------------------------------------CCTACCCTAGAGACACT-TATAGTGTATAGAGAG
chrom   ATCAACCTGAGTATAAAGGATGAATAACTCATTTTACCTACCCTAGAGACACT-TATAGTGTATAGAGAG
right   TTTA------GTAGAGACGGGGTTTCAC-CGTTTTAGC--CGGGATGGTCTCGATCTCCTG-ACCTCGTG
        N2N2      222N2N2N2NN2NN2N22 2N222221*11*111*11*1*1*11*1*11**1*1111*1*

left    ATATTTCACAATAAACCTTTGGATGTACAAAGATCAACAGAATAAAAAAGGAGTGAAACTCTCCTTTCAC
chrom   ATATTTCACAATAAACCTTTGGATGTACAAAGATCAACAGAATAAAAAAGGAGTGAAACTCTCCTTTCAC
right   ATCCGCCCGCCTCGGCCTCCCAAAGTGCTGGGATTAC-----------AGGCGTGAGCCACCGCGCCC--
        **1111*1111*111***1111*1**1*111***1*111111111111***1****11*1*11*111*11

left    TAGGTCTGGAAGAGAGAAGGAAGCTTAGTTCTNNNNNNNNNNN---------------------------
chrom   TAGGTCTGGAAGAGAGAAGGAAGCTTAGTTCTTAAAAAACACCACAGAGAGTTCTGATGCATAGCCTAGT
right   --GGCC---------------AGCTTAGTTCTTAAAAAACACCACAGAGAGTTCTGATGCATAGCCTAGT
        11**1*111111111111111************2222222222222222222222222222222222222

left    ----------------------------------------------------------------------
chrom   TGAAAAGCATTTCTAGAGTGATTTTGTAACCACTGTGCTGCCATTTAACGAATTTCCTCGTTTTTGACTC
right   TGAAAAGCATTTCTAGAGTGATTTTGTAACCACTGTGCTGCCATTTAACGAATTTCCTCGTTTTTGACTC
        2222222222222222222222222222222222222222222222222222222222222222222222

left    ----------------------------------------------------
chrom   ATAGTTAATAAATTATCACTATTAATATGCATTTTTTTGTGTACAGGTAATA
right   ATAGTTAATAAATTATCACTATTAATATGCATTTTTTTGTG-----------
        22222222222222222222222222222222222222222
```

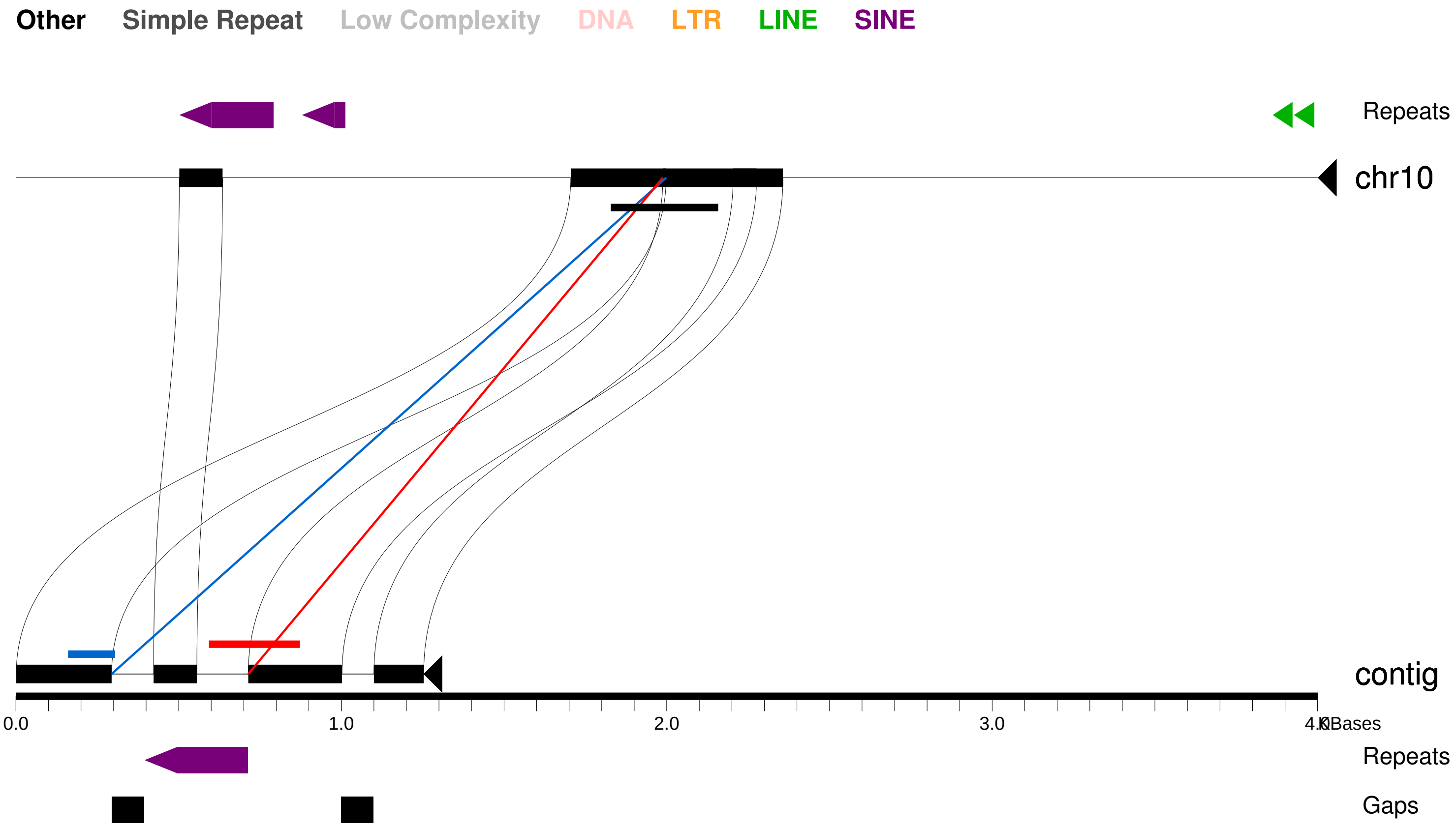

siteID: chr10_44192668 Scafftig1 - Breakpoint: chr10:44192642
chr10:44192599-44192701
Overlap Length: 17 INS size: 434 Genotyped: No

```
left    ------------GCATAACAAATTACCAC----ACAACGGGGAACCAACAGCTACTTATTGTCTTGCAGT
chrom   ------------GCATAACAAATTACCAC----ACAACGGGGAACCAACAGCTACTTATTGTCTTGCAGT
right   CCACCCGCCTCGGCCTCCCAAAGTGCTGGGATTACAGGCGTGAGCCACCGCGCCCGGCCTGTCTTGCAGT
        111111111111**1*11****1*1*1111111***111*1**1***1*11111*1111***********

left    TTCTTTTTTTTTTTTTTTTTTTGAGACGGAGNNNNNNNNNNNNNNNNNNNNNNNNNNNNNNNNNNNNNN
chrom   TTCTTT--------------AGGAC--AGAAGTTCAGGCCCGACGTGGCTAGCCTCTCCACGTAG
right   TTCTTT--------------AGGAC--AGAAGTTCAGGCCCGACGTGGCTGGCCTCTCCACGTAG
        ******2222222222222222***22**2222222222222222222222N22222222222222
```

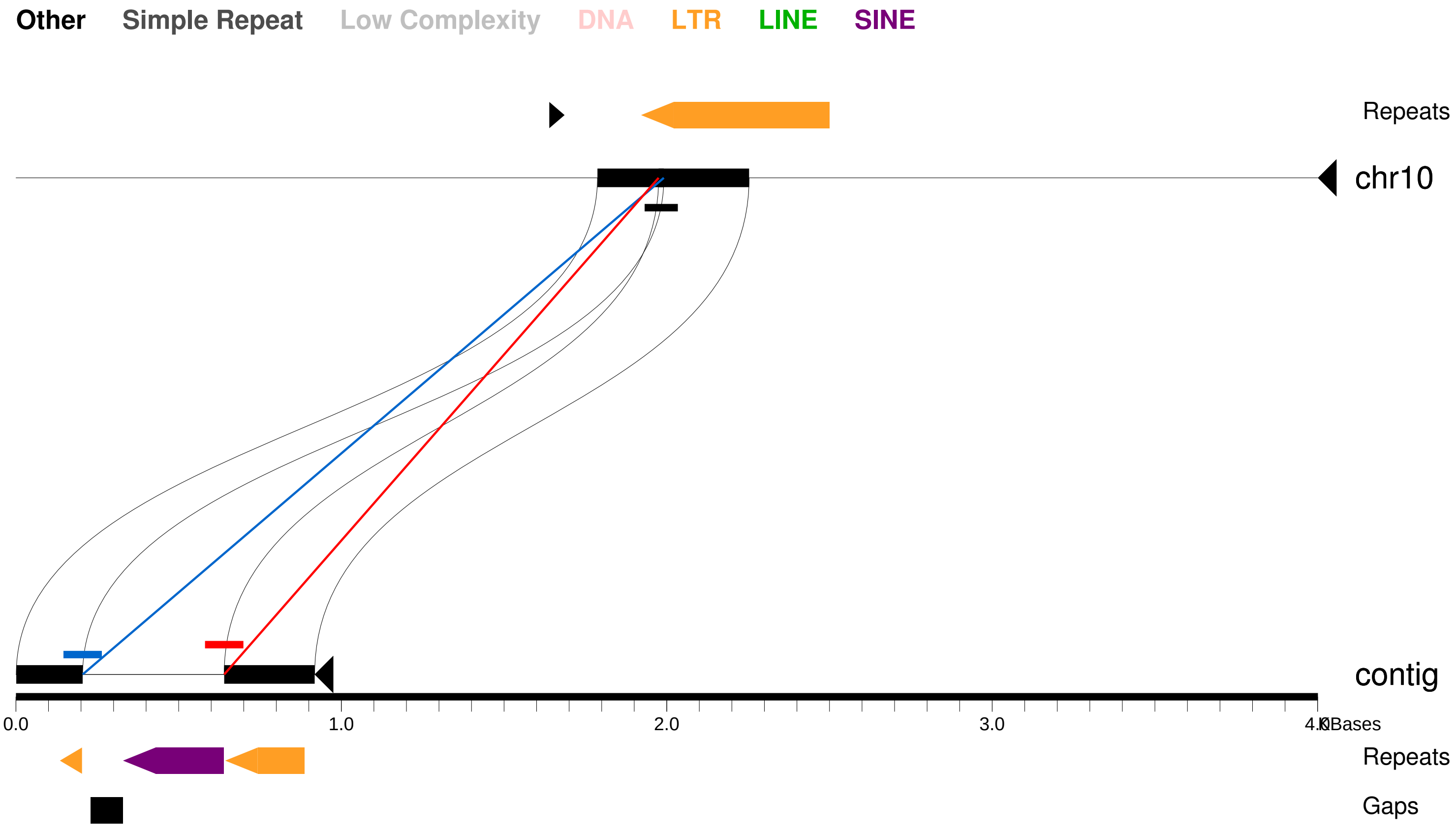

siteID: chr10_48419948 Scafftig13 + Breakpoint: chr10:48419944
chr10:48419893-48420003
Overlap Length: 9 INS size: 308 Genotyped: Yes

```
left   ----CTGAA-TTATGGTAGTCTTATGTGTCTATACCTAAATAAAGCAAAGACTGGC---ATAAAATTTTT
chrom  ----CTGAA-TTATGGTAGTCTTATGTGTCTATACCTAAATAAAGCAAAGACTGGC---ATAAAATTTAA
right  TCTCCTGACCTCGTGATCCGCCCGCCTCGGCCTCCCAAAGTGCTGGGATTACAGGCGTGATAAAATTTAA
       1111****11*11**1*111*11111*11111*1**1**1*111*11*11**1***111*********22

left   TTTTTTTTTTTTTTTTTTTTTTTTTTTTTTTTTTTTTTTTTTGAGACGGAGTCTCGC
chrom  GAAAATCTACACCAAAGTGTCTTCAGTCTCTGCCTTAGGTTT-------ACTTTCG-
right  GAAAATCTACACCAAAGTGTCTTCAGTCTCTGCCTTAGGTTT-------ACTTTCG-
       22222*2*222222222*2*2**222*2*2*222**222***2222222*2*2***2
```

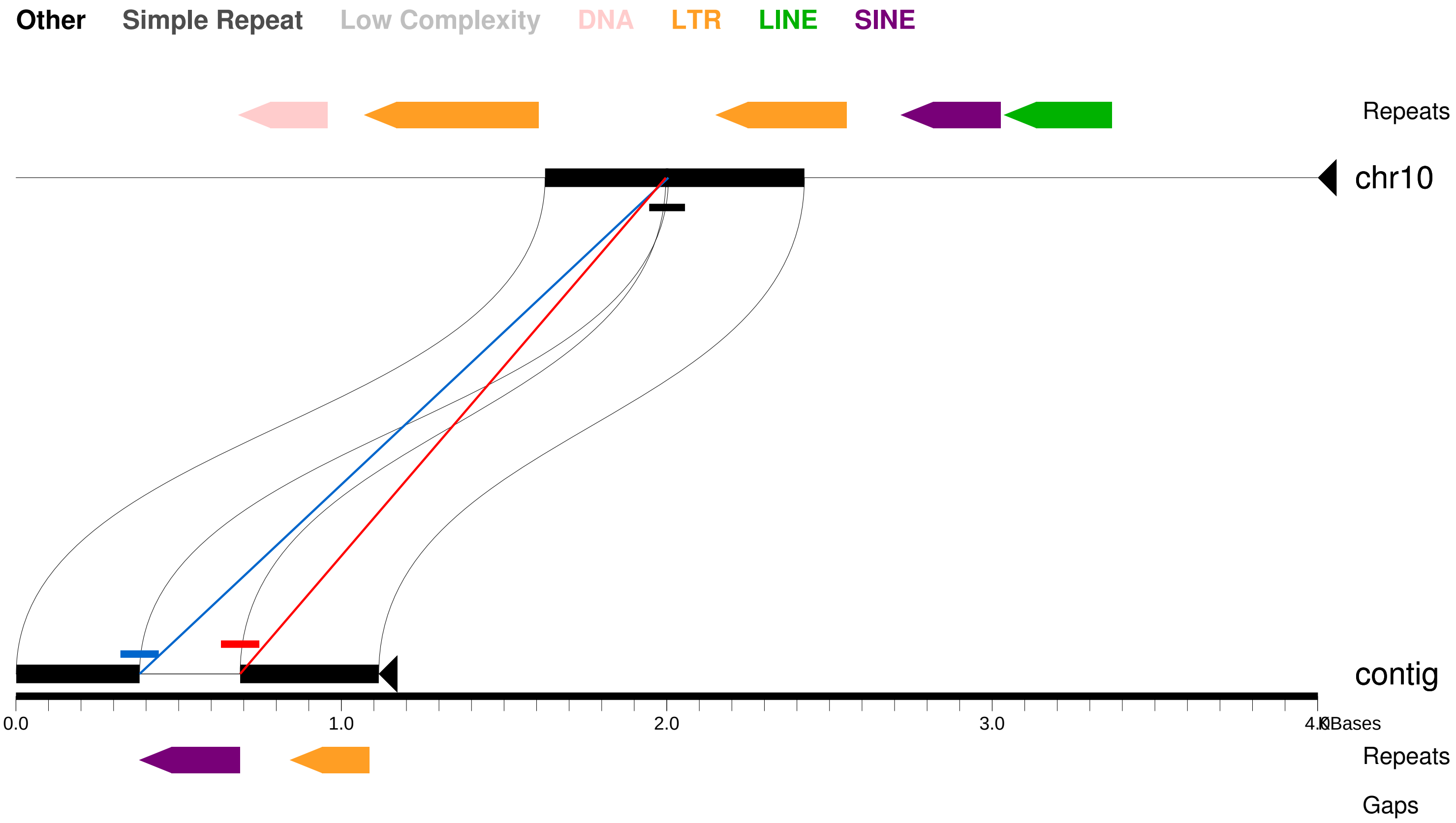

siteID: chr10_53862412 Scafftig3 - Breakpoint: chr10:53862415
chr10:53862047-53862575
Overlap Length: 15 INS size: 320 Genotyped: No

```
left   NNNNNNNNNNNNNNNNNNNNNNNNNNNNNNNNNNNNNNNNNNNNNNNNNNNNNNNNNNNNNNNNNNNNNN
chrom  ACATATTTAATTCAGCCTCTAGACCAGGAGTCACATGACGTTTAAGGCACAAACATCACAACCATATGCT
right  NNNNNNNNNNNA----------------------------------------------------------
       NNNNNNNNNNNNNNNNNNNNNNNNNNNNNNNNNNNNNNNNNNNNNNNNNNNNNNNNNNNNNNNNNNNNNN

left   NNNNNNNNNNNNNNNNNNNNNNNNNAA-------------------------------------------
chrom  GTGGAAAGAATTGACAATGCTCTGAAAGAGAATCTCTGCAGAGGGAACATCATAAGCATCTGGAAGGATT
right  ----------------------------------------------------------------------
       NNNNNNNNNNNNNNNNNNNNNNNNN11

left   ----------------------------------------------------------------------
chrom  TCACCAGTGAAAATGCCGTCATCCTTATAGAAAAATCTGTGAAAGCCATCAAGCCCAAAACAACAAATTT
right  ----------------------------------------------------------------------

left   ----------------------------------------------------------------------
chrom  CTGCTGGAGGAAACGGTCTAGATGTTGTGCATGAATTCATAGGATCTGTGACTGAGCCAATCACGAAAAT
right  ----------------------------------------------------------------------

left   ---------------------------------------AAATTGGTGGGAGGTAAGGGGTTTCAAGATA
chrom  CATGAAAGAGACTGTGGATATGGCAAAAAAAAGAAAAAAAAATTGGTGGGAGGTAAGGGGTTTCAAGATA
right  ----------------------------------------------------------------------
                                              1111111111111111111111111111111

left   GGGATCTTGGAGACATTCAAGAGCTAACAGACAGGCC----GAGGCGGG--CGGATCACGAGGTCA-GGA
chrom  GGGATCTTGGAGACATTCAAGAGCTAACAGACAACACACCAGAGGATTTAACAGAAGATGACTTGATGGA
right  ------------------AAGAGCTAACAGACAACACACCAGAGGATTTAACAGAAGATGACTTGATGGA
       111111111111111111***************222*2222****222222*2**22*2**22*2*2***

left   GATCGAGACCAT-----CCTGGCTAACACGGTGAA----ACCCCGTCTC--TACTAAAAATACAAA---A
chrom  GAT-GAGTACTTTGGAGCCAGTGTCAGACAATGAGGAAGACATAGACGCAGTGCCAGAAA-ACAAATTGA
right  GAT-GAGTACTTTGGAGCCAGTGTCAGACAATGAGGAAGACATAGACGCAGTGCCAGAAA-ACAAATTGA
       ***2***22*2*22222**2*22*2*2**22***22222**222*2*2*22*2*2*2***2*****222*

left   AATTAGCC-----GGGCGTAG-TGGCGGGCGCCTGTAGTC-
chrom  CATTAGACAATCTGGCAGAAGATTTCCACTTACTAAAGACT
right  CATTAGACAATCTG---------------------------
       2*****2*22222*1NN1N11 1NN1NNNNNN11NN11N1
```

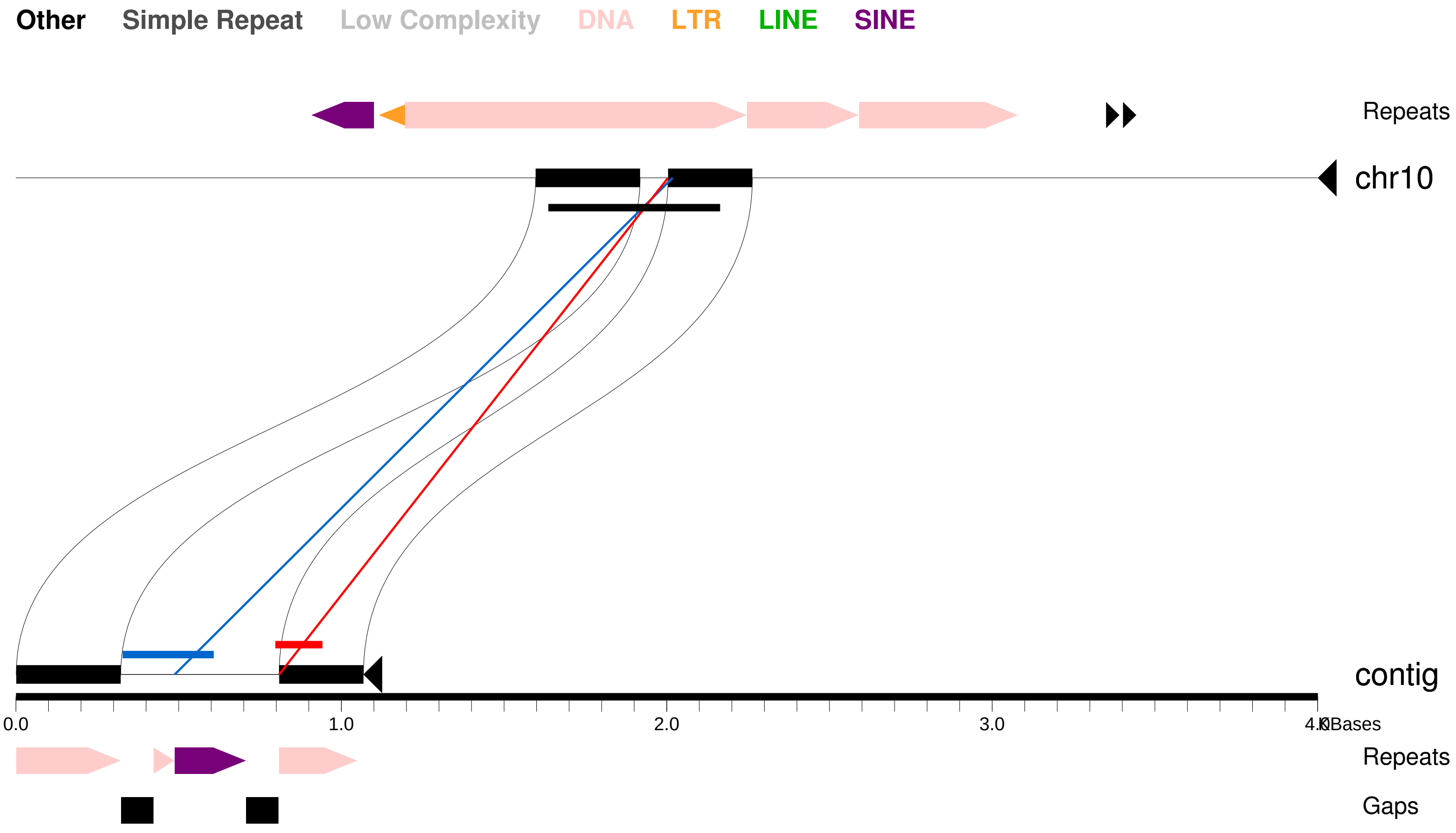

siteID: chr10_54008453 Scafftig1 - Breakpoint: chr10:54008436
chr10:54008425-54008495
Overlap Length: 49 INS size: 362 Genotyped: No

```
left    ATATTTAAGTG----------------------------------------------GATTTTGATGG
chrom   ATATTTAAGTG----------------------------------------------GATTTTGATGG
right   NNNNNNNNNNNNNNNNNNNNNNNNNNNNNNNNNNNNNNNNNNNNNNNNNNNNNNNNNNGATTTTGATGG
        11111111111111111111111111111111111111111111111111111111***********

left    TTAAAGATAAAATTTCTACCATTTAAGATGAAACTTAAGGCGGGCGGATCACGAGGTCAGGAGATCGAGA
chrom   TTAAAGAGAAAATTTCTACCATTTAAGATGAAACTTAAT--------AT-AC----TCAT----------
right   TTAAAGAGAAAATTTCTACCATTTAAGATGAAACTTAAT--------AT-AC----TCAT----------
        *******2*****************************222222222**2**2222***22222222222

left    CCATCCCGGCTAAAACGGTGAAACCCC
chrom   -------------------------CC
right   -------------------------CC
        2222222222222222222222222**
```

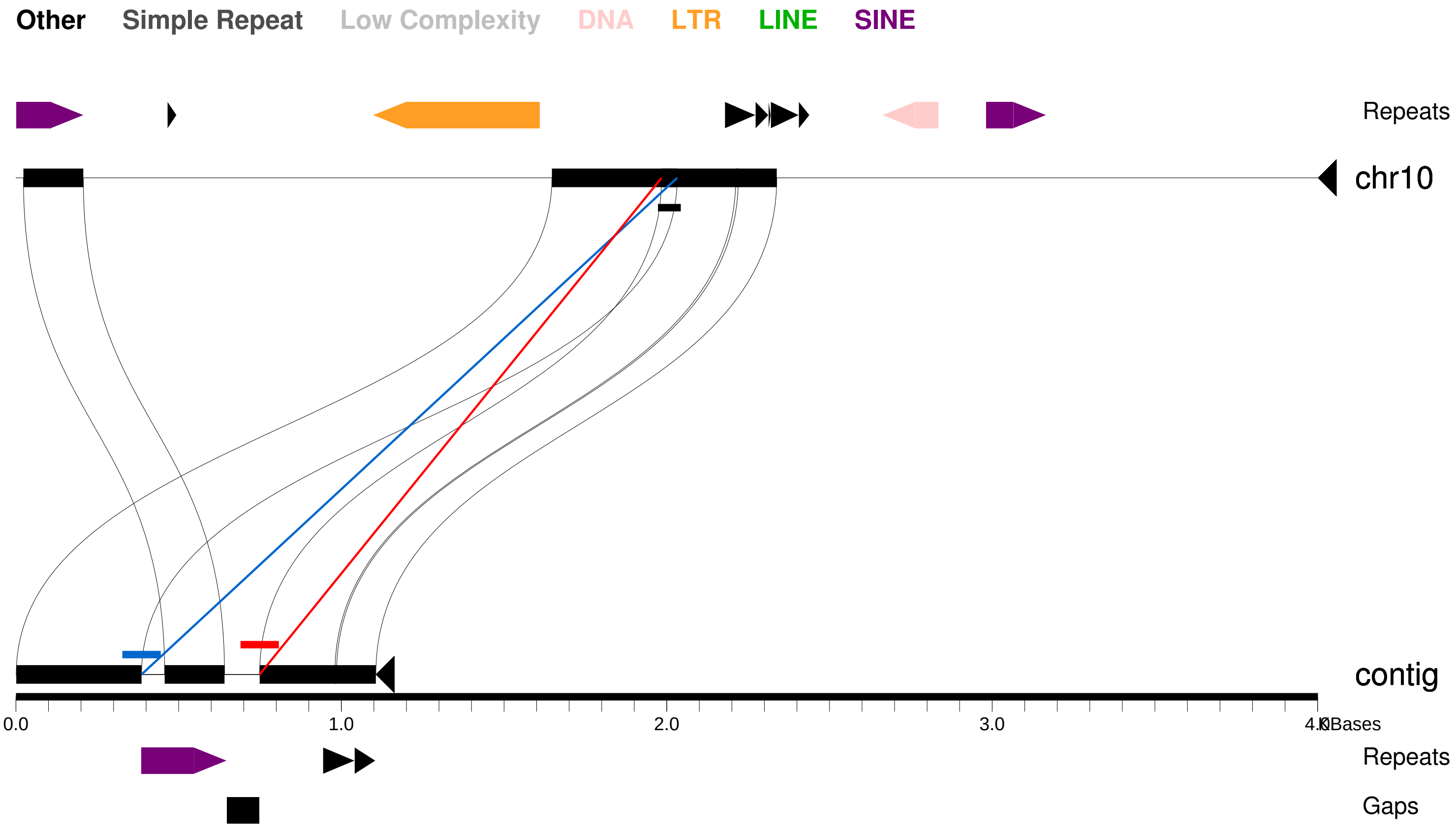

siteID: chr10_54395988 Scafftig1 + Breakpoint: chr10:54395991
chr10:54395970-54396050
Overlap Length: 39 INS size: 423 Genotyped: No

```
left    A----------------------TGTTTAGAT----------AGACACAAAGA------AAAACTGGATA
chrom   A----------------------TGTTTAGAT----------AGACACAAAGA------AAAACTGGATA
right   CCGCCCGTCTCGGCCTCCCAAAGTGCTGGGATTACAGGCGTGAGCCACCGCGCCCGGCCAAAACTGGATA
        11111111111111111111111**1*11***1111111111**1***111*1111111***********

left    TTTCATGTTGAGATGGATTTTTTCCTTTNNNNNNNNNNNNNNNNNNNNNNNNNNNNNNNNNNNNNNNNNN
chrom   TTTCATGTTGAGATGGATTTTTTCCTTTCTAAATAATTTCTAAATTTAT---------------------
right   TTTCATGTTGAGATGGATTTTTTCCTTTCTAAATAATTTCTAAATTTAT---------------------
        ****************************222222222222222222222222222222222222222222

left    NNNNNNNNNNNNNNNNN
chrom   -----------------
right   -----------------
        22222222222222222
```

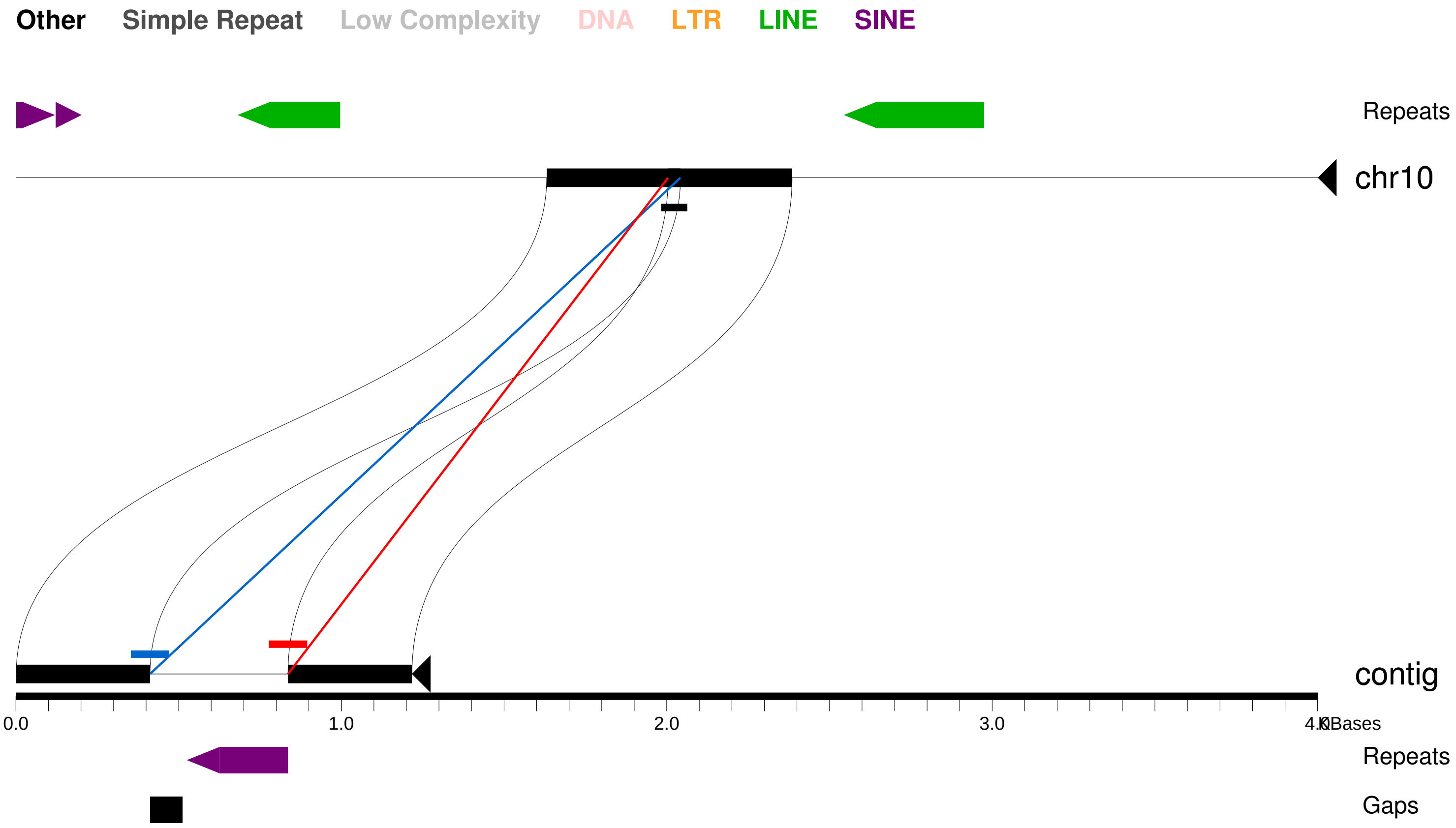

siteID: chr10_54466944 Scafftig5 - Breakpoint: chr10:54466933
chr10:54466883-54466988
Overlap Length: 9 INS size: 315 Genotyped: Yes

```
left   GATAA--------TGGAGAAGATGCAGGGAGTTTGT-TCTGCCCATTTTCTTCTTAAAA----GTGTCCT
chrom  GATAA--------TGGAGAAGATGCAGGGAGTTTGT-TCTGCCCATTTTCTTCTTAAAA----GTGTCCT
right  CTGCACTCCAGCCTGGGCGACAGAGCGAGACTCCGTCTCAAAAAAAAAAAAAAAAAAAAAAAAAGTGTCCT
       1111*11111111***111*1*1111*1**1*11**1**11111*1111111111****1111*******

left   TTCGGCCGGGCGCGGTGGCTCACG-CCTGTAATCCCAGCACTTTGGGAGGCCGAGGCGGGCGG
chrom  TT---CCTTCCTAAGTTAAGCAAGGCCTATAGT---AAGACTCT--CAGTGCTA------CTG
right  TT---CCTTCCTAAGTTAAGCAAGGCCTATAGT---AAGACTCT--CAGTGCTA------CTG
       **222**222*222**2222**2*2***2**2*222*22***2*222**22*2*222222*2*
```

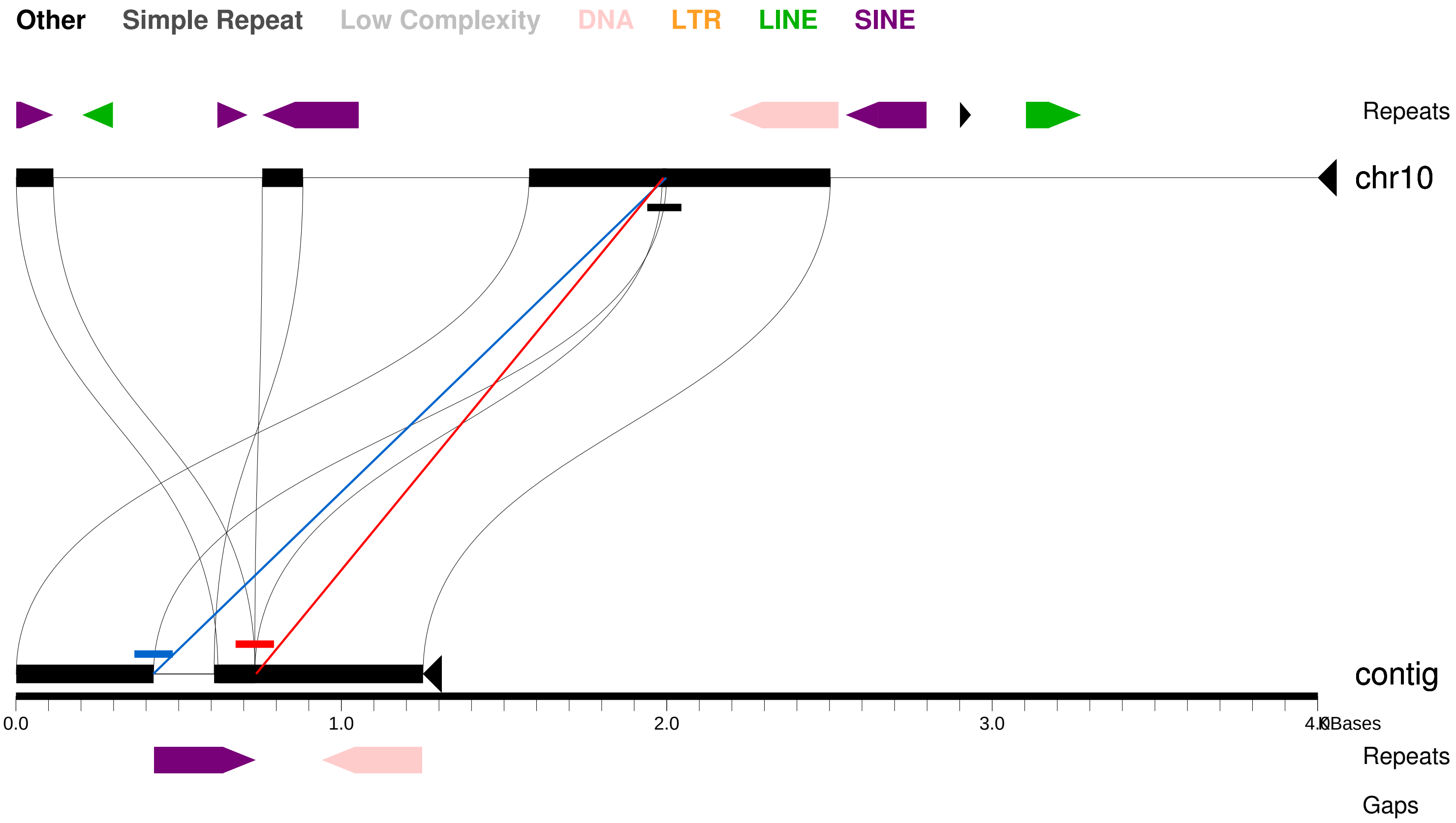

siteID: chr10_54682273 Scafftig2 - Breakpoint: chr10:54682254
chr10:54682239-54682313
Overlap Length: 45 INS size: 385 Genotyped: No

```
left    T--------TT--------AAAGC---------ACAA---TAA----------------TATTTTTTTTA
chrom   T--------TT--------AAAGC---------ACAA---TAA----------------TATTTTTTTTA
right   CCACCCGCCTTGGCCTCCCAAAGTGCTGGGATTACAAGCGTGAGCCACCGCGCCCGGCCTATTTTTTTTC
        111111111**11111111****1111111111****111*1*1111111111111111**********1

left    TTTTAACTACTTTGTTAAATGAGTTTTTATCAATNNNNNNNNNNNNNNNNNNNNNNNNNNNNNNNNNNNN
chrom   TTTTAACTACTTTGTTAAATGAGTTTTTATCAATAGATTCTGGTGAATC---------------------
right   TTTTAACTACTTTGTTAAATGAGTTTTTATCAATAGATTCTGGTGAATC---------------------
        **********************************222222222222222222222222222222222222

left    NNNNNNNNNNNNNNNNNNNNNNN
chrom   -----------------------
right   -----------------------
        22222222222222222222222
```

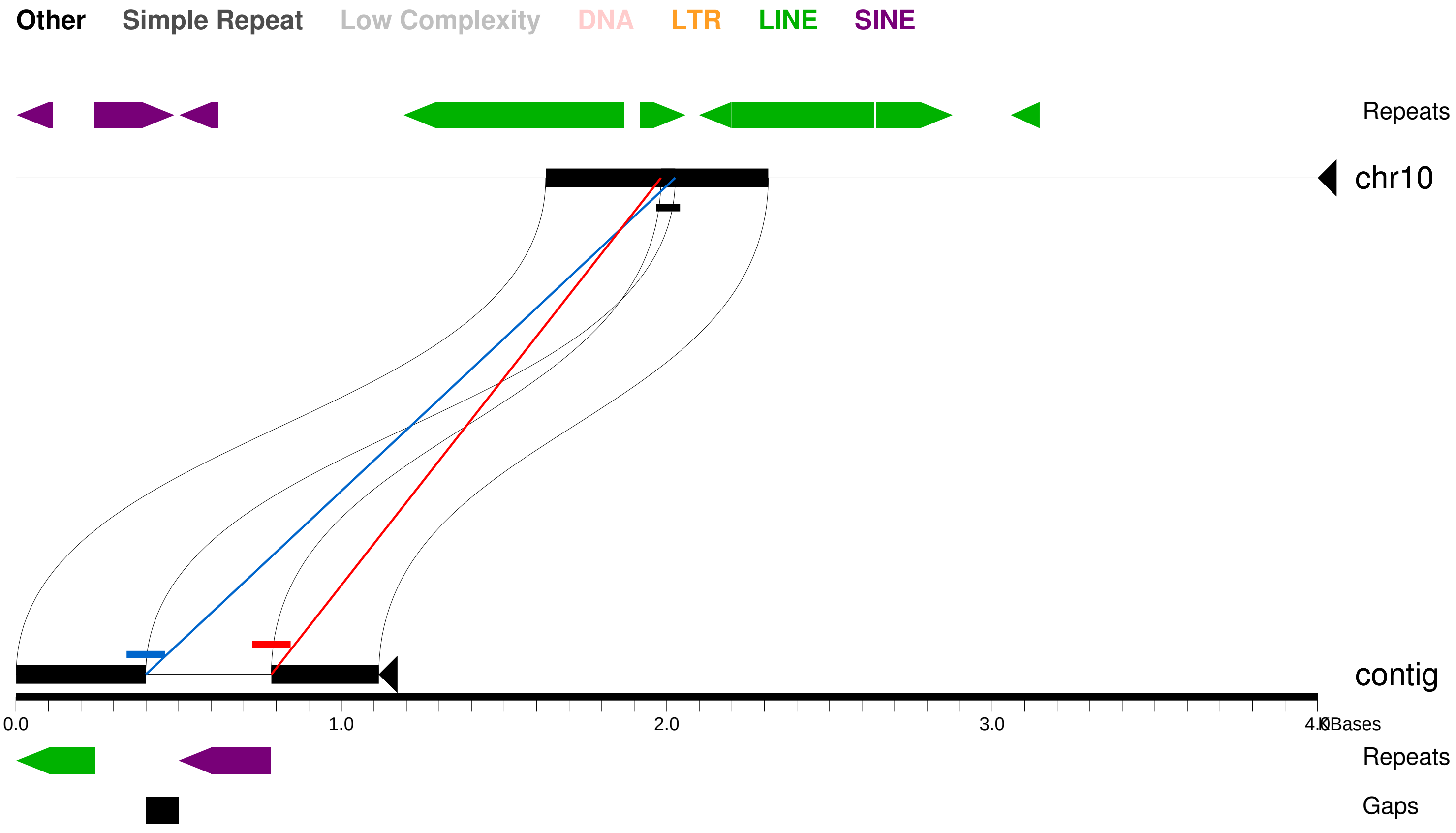

siteID: chr10_54805966 Scafftig2 + Breakpoint: chr10:54805956
chr10:54805911-54806015
Overlap Length: 15 INS size: 299 Genotyped: Yes

```
left   ---------------TCAAAAAAGG--GGATTTGGGGGCAATAGATATATTAAGGGATGTGACACTTTCA
chrom  ---------------TCAAAAAAGG--GGATTTGGGGGCAATAGATATATTAAGGGATGTGACACTTTCA
right  CCGCCCGCCTCGGCCTCCCAAAGTGCTGGGATTACAGGCGTGAGCCACCGC---GCCCGGCCCACTTTCA
       111111111111111**11***11*11**11**111***111**11*1111111*111*111********

left   AGAATTTTTTTTTTTTTTTTTTTTTTTTGAGACGGAGTCTCGCTGTCGCCCAGGCTGGAGTGCAGTGG--
chrom  AGAATTTAATACAATCATTTGTTCTTG----------------TCAGCAAGAAGAAAATAAAAGGCCA
right  AGAATTTAATACAATCATTTGTTCTTG----------------TCAGCAAGAAGAAAATAAAAGGCCA
       *******22*2222*22***2**2***2222222222222222**22*2**22222*2*22*22*222
```

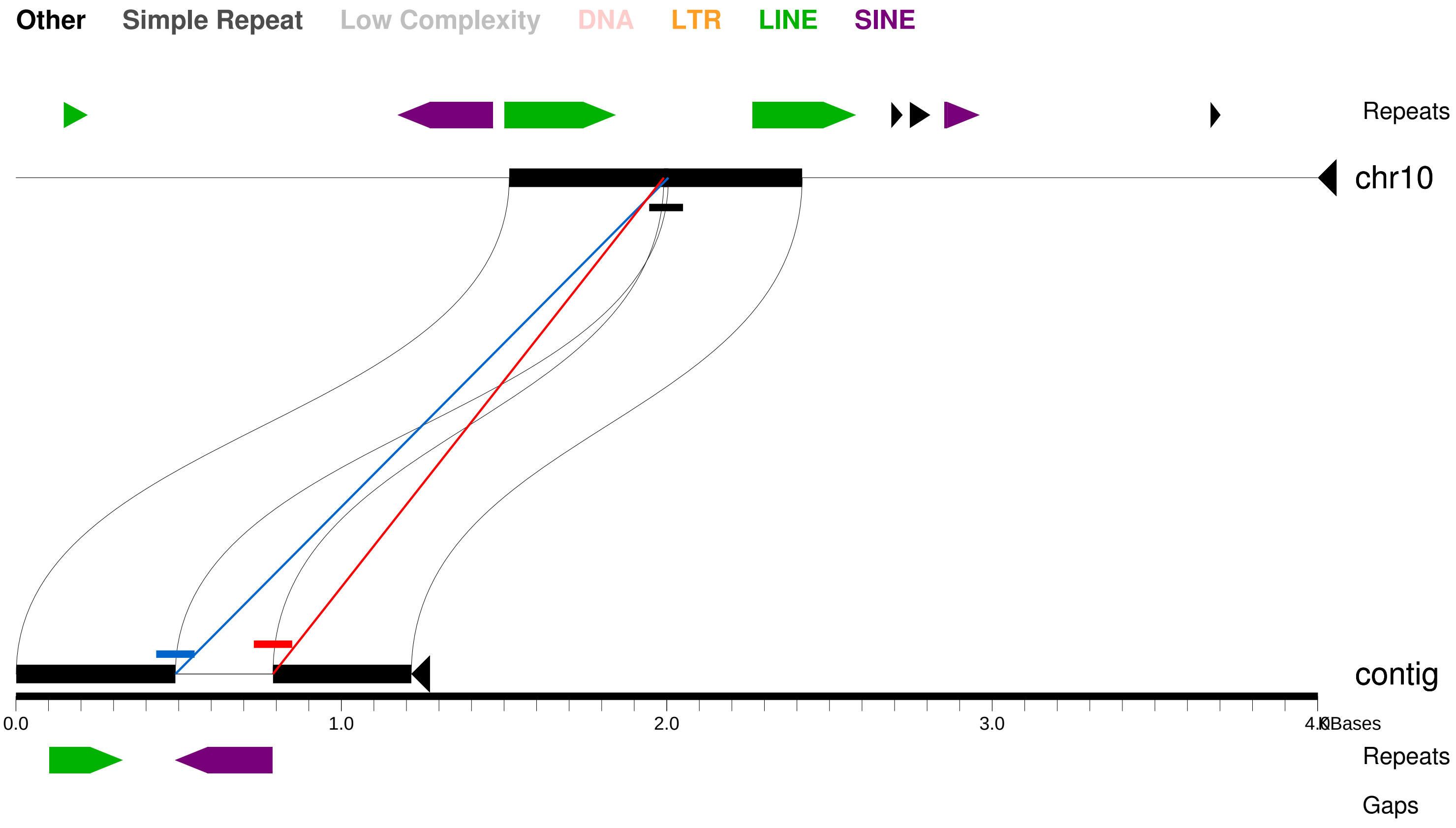

siteID: chr10_56140333 Scafftig2 + Breakpoint: chr10:56140319
chr10:56140264-56140378
Overlap Length: 5 INS size: 351 Genotyped: No

```
left    TGAGAAATTGAAAGGGAATTTATTTTCTCATCAAGAAAACTTATGTACAATAGGC----AGGTCGGCCGG
chrom   TGAGAAATTGAAAGGGAATTTATTTTCTCATCAAGAAAACTTATGTACAATAGGC----AGGTCAACTAA
right   NNNNNNNNNNNNNNNNNNNNNNNNNNNNNNNNNNNNNNNNNNNNNNNNNNNNNNNNNNNAGGTCAACTAA
        11111111111111111111111111111111111111111111111111111111111*****22*222

left    GCGCGGTGGCTCACGCCTGTAATCCCAGCACTTTGGGAGGCCGAGGCGGGTGG-
chrom   -CATTAACATTAATACCTTGACTCAATAGTGTTTAAGAGTA----GCAACTGCA
right   -CATTAACATTAATACCTTGACTCAATAGTGTTTAAGAGTA----GCAACTGCA
        2*22222222*2*22***22*2**2222222***22***222222**222**22
```

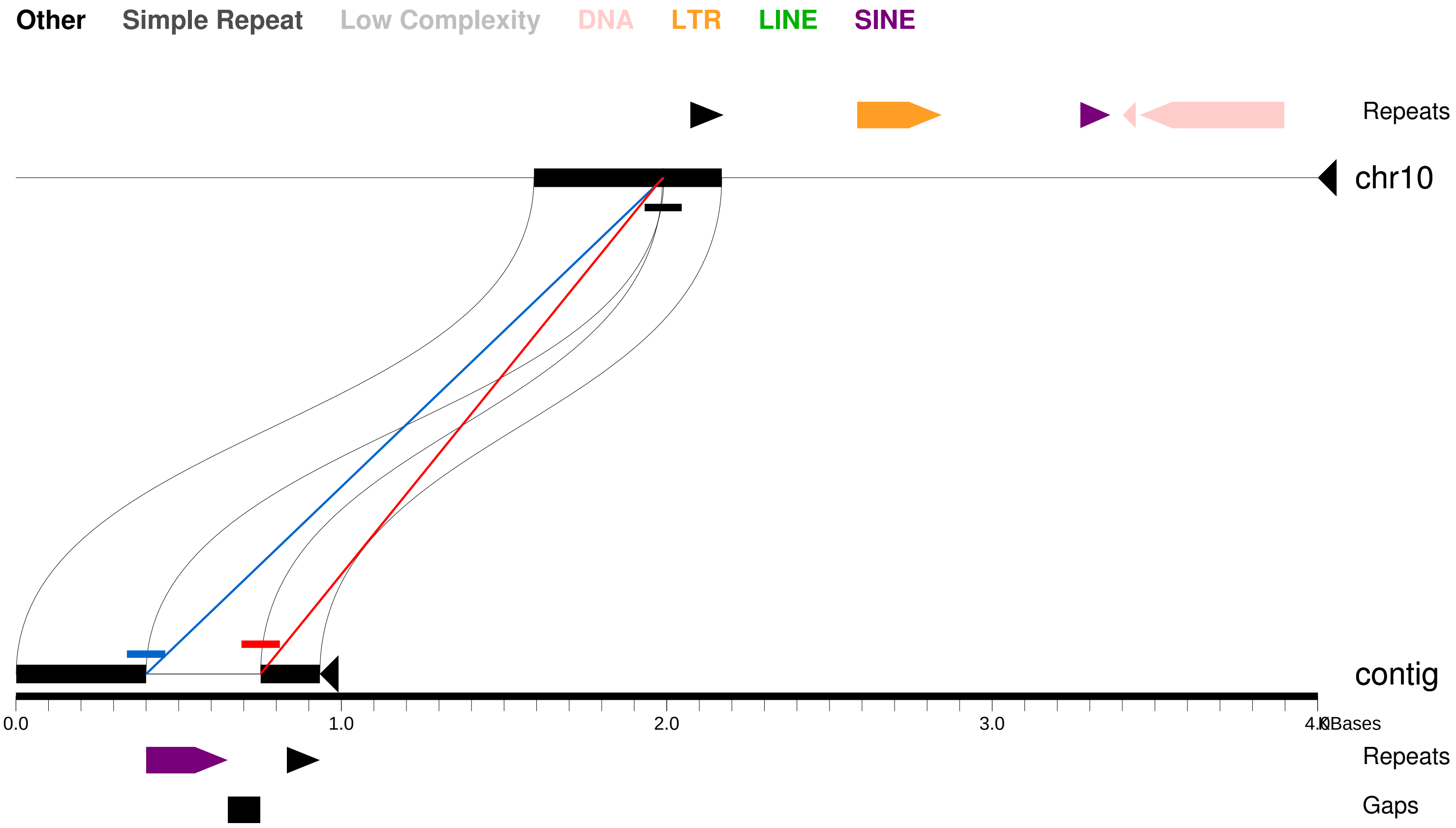

siteID: chr10_56664646 Scafftig2 - Breakpoint: chr10:56664597
chr10:56664450-56664806
Overlap Length: 63 INS size: 428 Genotyped: No

```
left   A---------------------------------------------------------------------
chrom  ACCAATCAGGCAAATGAACAAACAAAAACAGAATAATTTCTTTCCTAGCTGCCCCACACTTTATCACATC
right  NNNNNNNNNNNNNNNNNNNNNNNNNNNNNNNNNNNNNNNNNNNNNNNNNNNNNNNNNNN-----------
       1NNNNNNNNNNNNNNNNNNNNNNNNNNNNNNNNNNNNNNNNNNNNNNNNNNNNNNNNNN

left   -------------------------------TCAATTTCCATGTGCCATTTGAGGTACCACAGCTCACTC
chrom  ACTTCAGTATGCCCCACTTCCTTAACTCTAATCAATTTCCATGTGCCATTTGAGGTACCACAGCTCACTC
right  ----------------------------------------------------------------------
                                      111111111111111111111111111111111111111

left   TCAGAAGGTCAAGAGGTAAAGTGTGGAGAAAAGGGAAGAAGGTGGGTATAAATTAAAAAAAACAAAAAGG
chrom  TCAGAAGGTCAAGAGGTAAAGTGTGGAGAAAAGGGAAGAAGGTGGGTATAAATTAAAAAAAACAAAAAGG
right  -------GTCAAGAGGTAAAGTGTGGAGCAAAGGGAAGAAGGTGGGTATAAATTAAAAAAAACAAAAAGG
       1111111*********************1*****************************************

left   GCC-------------------------------------------------------GGGC----GCGG
chrom  ACAAAGATAAAATACAAGATAAATTAAAATTATATTTTTAAATTAGCTTTTGTTCCTTGAGCATCTGCTA
right  ACAAAGATAAAATACAAGATAAATTAAAATTATATTTTTAAATTAGC-----------------------
       2*222222222222222222222222222222222222222222222           1N11    11NN

left   TGGCTCACGC---CTGTAATCCC---------------------AGCACTTTGGGAGGCCGAGGCGGGCG
chrom  TGTATCAGGCATTCTCTATTACCTATTTTCGTTCTGGCCACACAACTACACTATGAAGCAGATATGACTG
right  ----------------------------------------------------------------------
       11NN111N11   11N11N1N11                     1NN11NN1NN11N11N11NNN1NNN1

left   GA-----
chrom  TACCACT
right  -------
       N1
```

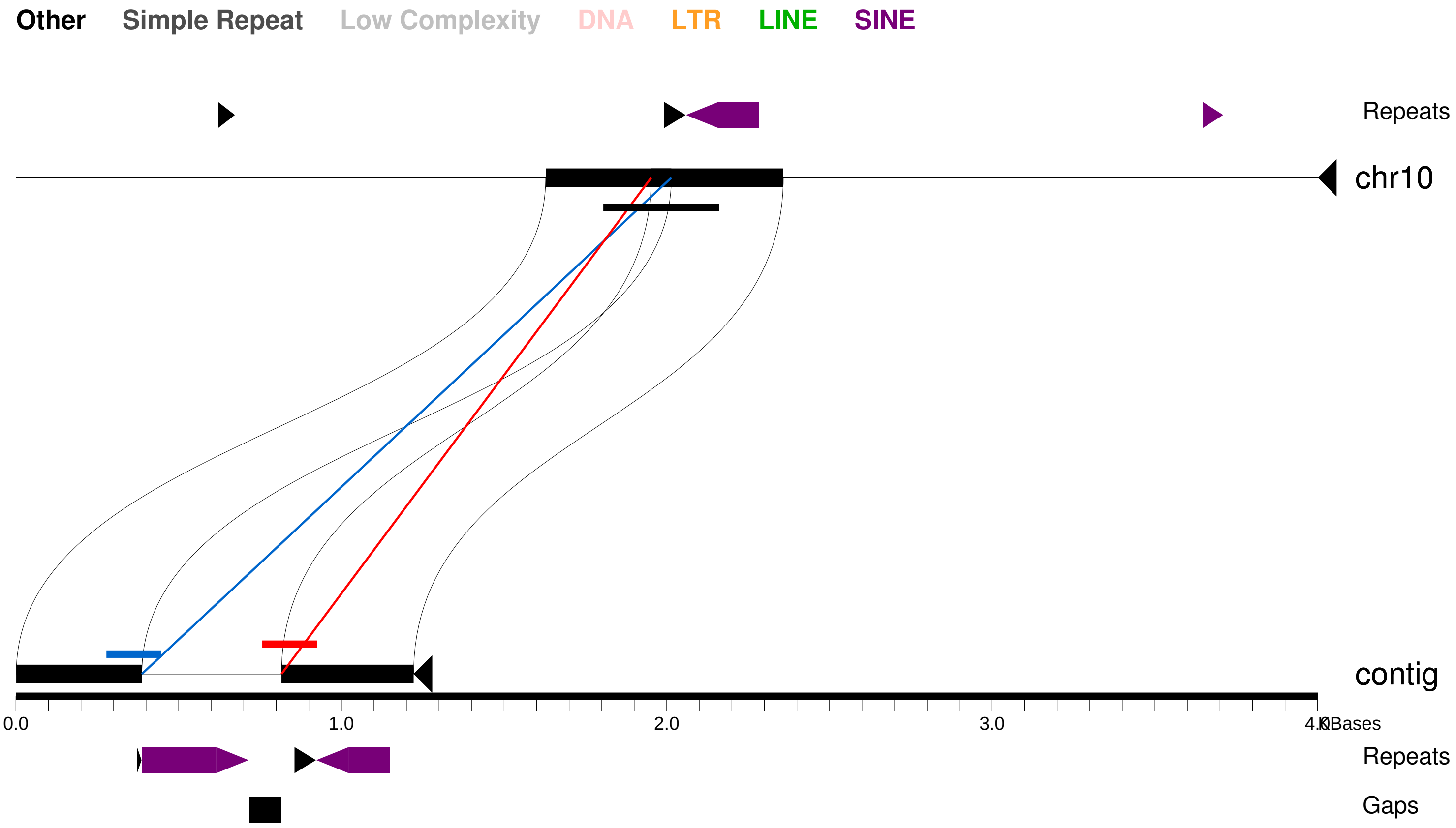

siteID: chr10_56902374 Scafftig6 + Breakpoint: chr10:56902354
chr10:56902319-56902413
Overlap Length: 25 INS size: 291 Genotyped: Yes

```
left    T-----ACATAG-------ATTGTG-TGTATCTATAGTTTTTTGCATA-----------TACTACAGTTA
chrom   T-----ACATAG-------ATTGTG-TGTATCTATAGTTTTTTGCATA-----------TACTACAGTTA
right   CCGCCCGCCTCGGCCTCCCAAAGTGCTGGGATTACAGGCGTGAGCCACCGCGCCCGGCCTACTACAGTTA
        1111111*1*1*1111111*11***1**1111**1**111*11**111111111111111***********

left    TTTTTTTATTTTTTTTTTTTTTTTGAGACGGAGTCTCGCTCTGTCGCCCAGGCTGGAGTGCAGTGGCGGGA
chrom   TTTTTATATATATTTTCATAAATAT-ATTTGTACTC---CTATGTAGATAT--------------------A
right   TTTTTATATATATTTTCATAAATAT-ATTTGTACTC---CTATGTAGATAT--------------------A
        *****2***2****22*222*2*2*222*2*2**222**2***2*222222222222222222222222*

left    TCT
chrom   TCT
right   TCT
        ***
```

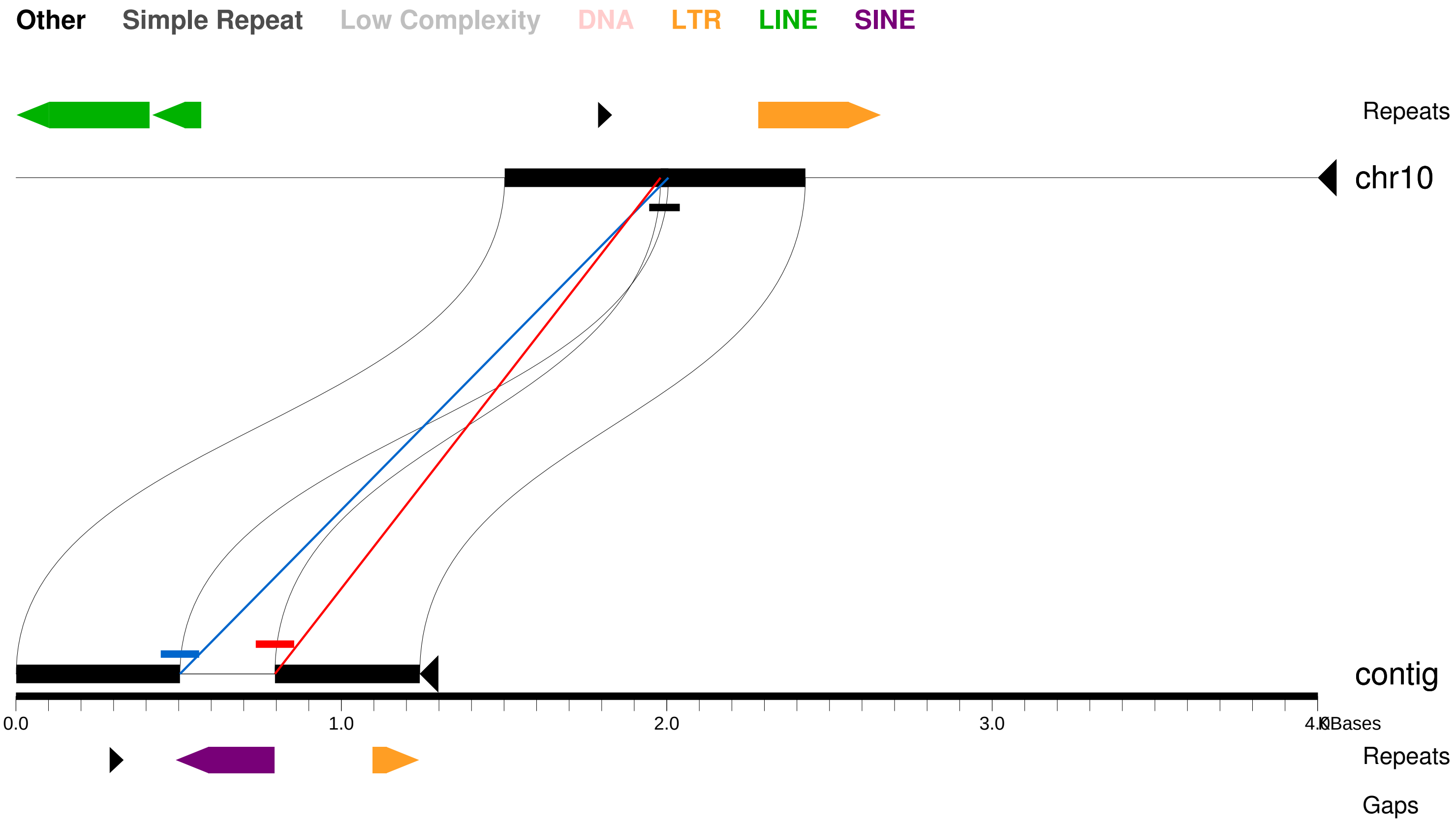

siteID: chr10_58327395 Scafftig1 - Breakpoint: chr10:58327355
chr10:58326724-58327515
Overlap Length: 15 INS size: 314 Genotyped: Yes

```
left   G-----------------------------------------------------------------------
chrom  GCATTGCAAGGTGACATGGGCTGGTGTCAACAGAAAGAACTACAGTAGCATGCTTCTTGACAATGTTTCA
right  C-------------------------GTGAAC----------------------------------------
       1                         22N222

left   ------------------------------------------------------------------------
chrom  GTCAATGAAGAACTGCTTGTATGACTGTGGTCTCATAAGGTTATAATGGACCTGAAAAATTACTATCACC
right  --CCAGGAAG------------------------------------TGGAGCT-----------------
         2N2N2222                                    2222N22

left   ------------------------------------------------------------------------
chrom  TAGTGATGATGTAGCTGCTGTTAGGCTGCAGCTCAATTACTTTATTTTAGAAAACAAATTTAGTGTACCC
right  ---------------TGCAGTGAG----------------------------------------------
                      222N22N22

left   ------------------------------------------------------------------------
chrom  TAAGTGTATAATGTTTGTAAAGTCTACAGTAGTGTACAGTAATTCTCTGGACCTTTACATTTACTCACCA
right  ------------------------------------------------------------------------

left   ------------------------------------------------------------------------
chrom  CTCACTCACTGACTCACCCAGAGCAACTTCCAGTTCTGCAAGCTTCATTCATGGAAGTACTGTATATAGG
right  ----------------CCGAGAT-----------------------------------------------
                       22N222N

left   ------------------------------------------------------------------------
chrom  TGTGCCATTTTTAGAATCTTTTATACTCACTTTTACAGTAAGCACACTGTAAAAAGATACTATACTTTTA
right  TGCGCCACTG-------------------------CAGTCCGCAGTCTGG--------------------
       22N2222N2N                          2222NN222NN222N

left   ---------------------------------------------------------------------TAT
chrom  CAGTATAAAAGATCCCATAAATGGCACAACTGTATACAGTATAAAGATTCTAAAAATGGCATACCTGTAT
right  ------------------------------------------------------------------------
                                                                            111

left   ATGGTACTTCCTGGGGTTATATTATAGCACCTTAGCACCTTATGGTATAGTTCAACAGTTACAAAATGAG
chrom  ATGGTACTTCCTGGGGTTATATTATAGCACCTTAGCACCTTATGGTATAGTTCAACAGTTACAAAATGAG
right  ---------CCTGGG-------------------------------------CGACAG------AGCGAG
       111111111*****111111111111111111111111111111111111*1****111111*11***

left   ACTTAGTTGGGTGGAATATAGAATCACCTTTGCCTAGAAGGCTAAGTTCCAAATAAGAATGAAAATTAAT
chrom  ACTTAGTTGGGTGGAATATAGAATCACCTTTGCCTAGAAGGCTAAGTTCCAAATAAGAATGAAAATTAAT
right  ACTCCGTC---------------TCA---------AAAA-----------AAAAAAAAAAAAAAAAAAAA
       ***11**111111111111111***111111111*1**1111111111***1**1**11****11**1

left   TAAAATCTATGTTTGCGGCCCGGGCGCG--[illegible]GTG[illegible]TCA[illegible]-C[illegible]GTAATCCCAGCACTT-TGGG
chrom  TAAAATCTATGTTTGCATACACTAATACTATTGTGTTACAATTACCTACAGTATTCAGTATACTAATGTG
right  AAAAATCTATGTTTGCATACACTAATACTATTGTGTTACAATTACCTACAGTATTCAGTATACTAATGTG
       1*************222*222222222222***222**22222*22*2***2**22222***22**2*

left   [illegible]--AGG[illegible]CCGAGG[illegible]-CGGGTG[illegible]CATGAGGTC[illegible]AGGAGATCG[illegible]AGACCATCCTG
chrom  CTATACAGGTTTGCAGCCTAGGAGCAATCGGCTATACTAT-AGGACCTAGGTGTTTGGTAGGCTATACCA
right  CTATACAGGTTTGCAGCCTAGGAG[illegible]ACGACCTAGGTGTTTGGTAGGCTATACCA
       222222***2222222**2***222222***2*22*22**2***2*22***2*2*2*22**2*2**2*22

left   GCTAACAAGGTGAAACCCCGTCTCTACTAAA---
chrom  TCTAGGTTTGTGTAAG--------TACACTA---
right  TCTAGGTTTGTGTAAG--------TACACTATTA
       2***22222***2**22222222***222*111
```

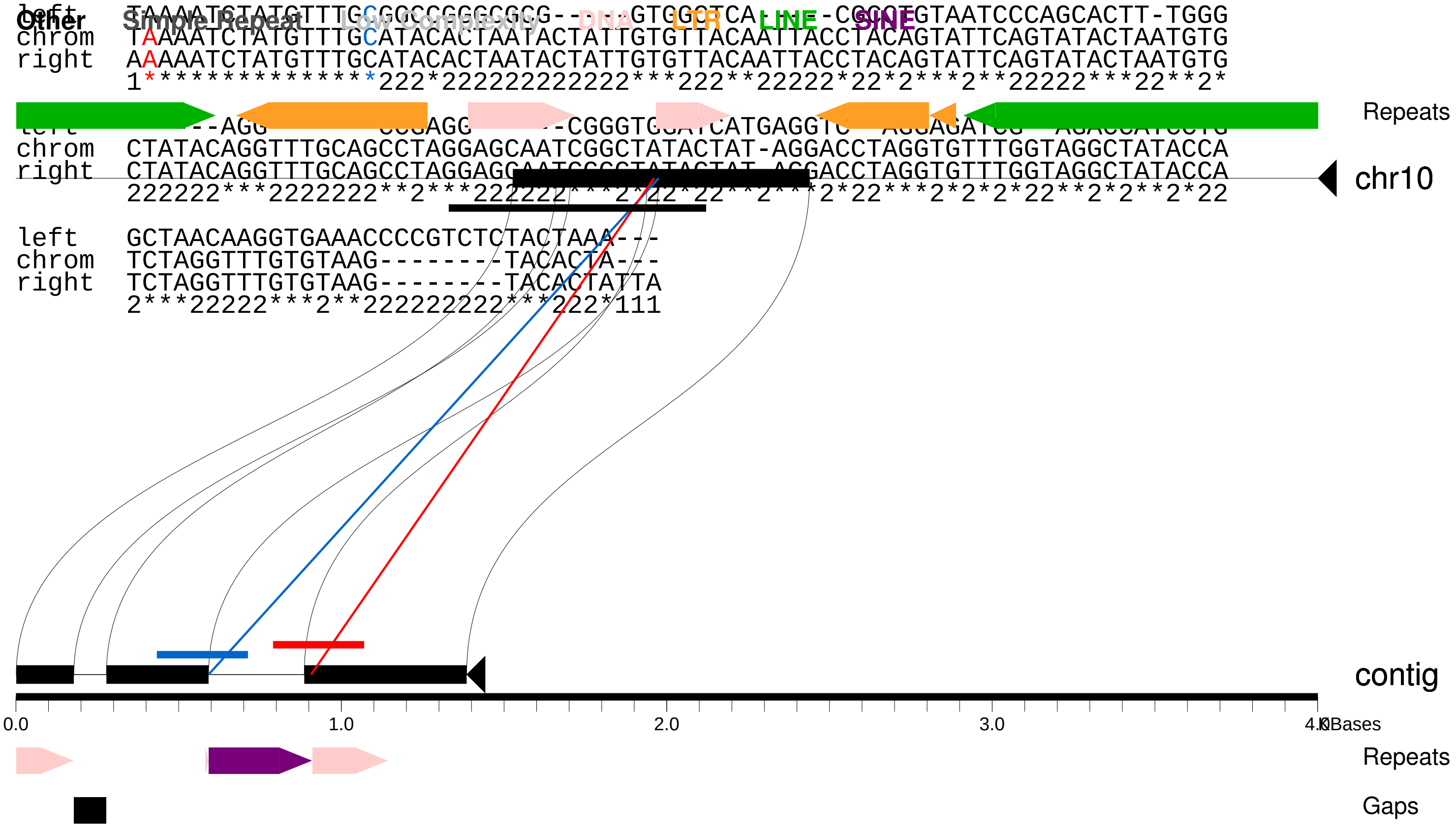

siteID: chr10_58370641 Scafftig4 + Breakpoint: chr10:58370653
chr10:58370611-58370712
Overlap Length: 18 INS size: 126 Genotyped: Yes

```
left     TACATG--TAACTGCAGTCTCAGA-----------AGGACAGAGTAGGG----AGAAGTTTGGACTGGAA
chrom    TACATG--TAACTGCAGTCTCAGA-----------AGGACAGAGTAGGG----AGAAGTTTGGACTGGAA
right    ATCTCGGCTCACTGCAAGCTCCGCTTCCCGGGTTCACGCCATTCTCCTGCCTCAGCCTCTTGGACTGGAA
         11*11*11*1******11***1*111111111111*1*1**111*111*1111**1111***********

left     AATTTTTTTTTTTTTTTTTTTGAGACGGAGTCTTGCTCTGTCGCCCAGGCCGGACTGCGGACAGCA
chrom    AAATTTTTAAAAATATTCTTCAATGAC------TTAAAATATTGATTAGA-----------AGAGTC
right    AAATTTTTAAAAATATTCTTCAATGAC------TTAAAATATTGATTAGA-----------AGAGTC
         **2*****22222*2**2**2222***222222**2222*2*2*222**222222222222*2**22
```

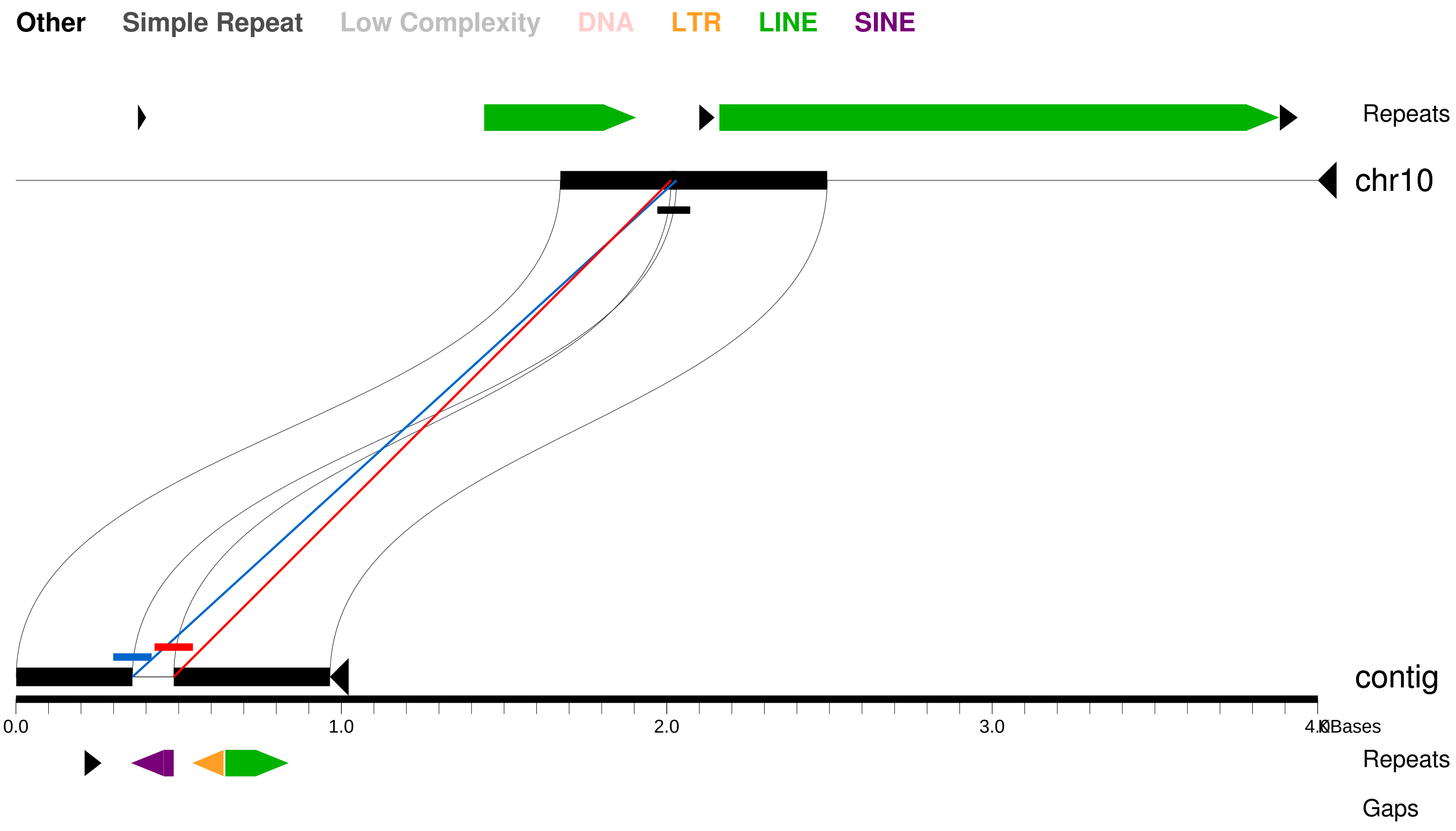

siteID: chr10_60984909 Scafftig1 - Breakpoint: chr10:60984904
chr10:60984862-60984954
Overlap Length: 18 INS size: 311 Genotyped: Yes

```
left    GCATCAAACTATCCCCTCCATA---AGACCTGGGCC-----------------------AAAATGAGTAA
chrom   GCATCAAACTATCCCCTCCATA---AGACCTGGGCC-----------------------AAAATGAGTAA
right   GCACTCCAGCCTGGGCGACAGAGCGAGACTCCGTCTCAAAAAAAAAAAAAAAAAAAAAAAAAAAAAAAAA
        ***1111*111*111*11**1*111****111*1*1111111111111111111111111****11*11**

left    GAAATGAAAATAGAGGGCCGGGCGCGGTGGCTCACGCCTGTAATCCCAGCACTTTGGGAGGCCGAGGCGG
chrom   GAAATGAAAATAGAGGAA------------AACTCTTCT-TAACAATAAAACTTATGTA-----AGG---
right   GAAATGAAAATAGAGGAA------------AACTCTTCT-TAACAATAAAACTTATGTA-----AGG---
        ***************2222222222222222*2*22**2***2222*22****22*2*22222***222

left    GCGGA
chrom   -----
right   -----
        22222
```

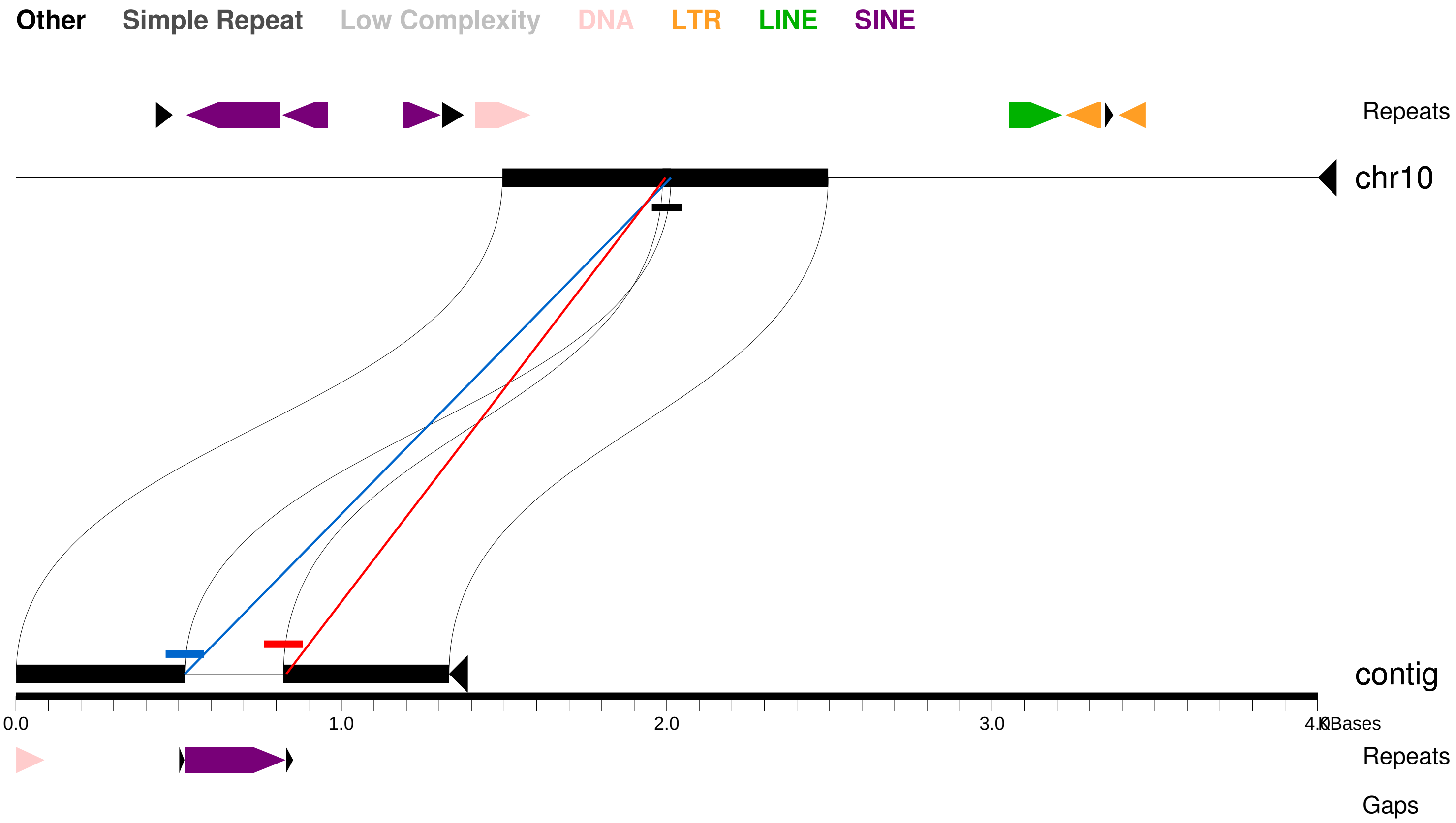

siteID: chr10_61077952 Scafftig1 - Breakpoint: chr10:61077953
chr10:61077952-61078012
Overlap Length: 59 INS size: 424 Genotyped: No

```
left    T----------------------------------------------------------CTAAGTTTCCC
chrom   T----------------------------------------------------------CTAAGTTTCCC
right   CCACCCGCCTCGGCCTCCCAAAGTGCTGGGATTACAGGCGTGAGCCACCGCGCCCGGCCCTAAGTTTCCC
        11111111111111111111111111111111111111111111111111111111111***********

left    TTTTATCCTTATGCCAAATTATCAGTGAAAGTGCTAACAATTTATCAANNNNNNNNNNNNNNNNNNNNNN
chrom   TTTTATCCTTATGCCAAATTATCAGTGAAAGTGCTAACAATTTATCAAA---------------------
right   TTTTATCCTTATGCCAAATTATCAGTGAAAGTGCTAACAATTTATCAAA---------------------
        ************************************************2222222222222222222222

left    NNNNNNNNNNNNNNNNNNNNNNNNNNNNNNNNNNNNN
chrom   -------------------------------------
right   -------------------------------------
        2222222222222222222222222222222222222
```

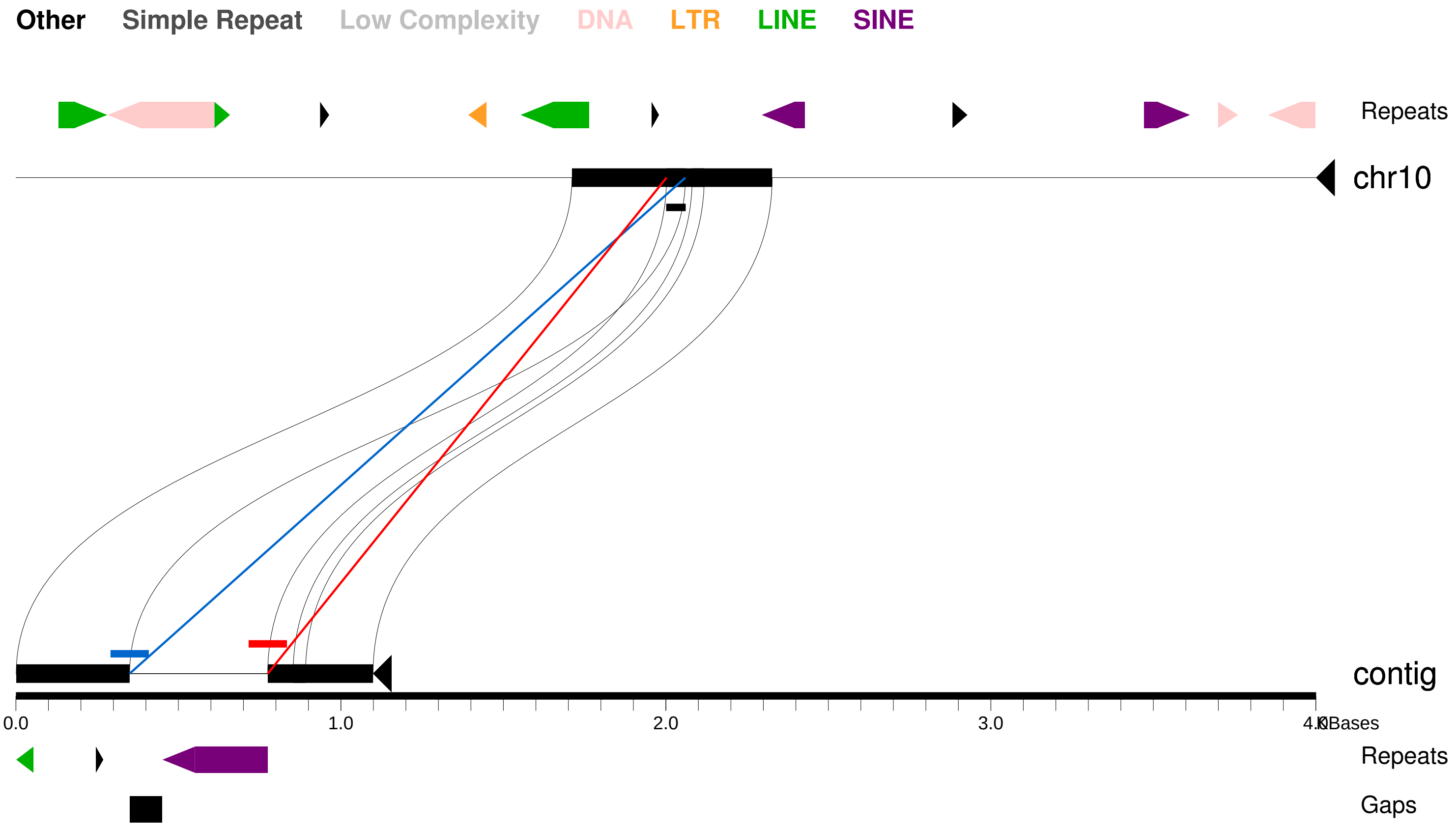

siteID: chr10_61628444 Scafftig4 - Breakpoint: chr10:61628672
chr10:61628493-61629204
Overlap Length: 2 INS size: 261 Genotyped: No

```
left    ----------------------------------------------------------------------
chrom   TGGTACATGCTAAGCAGAGCCAGCCCCCAGTAAAAACCCTCGGCTCCAATGAGCTTCCCTGGTAGATGAC
right   T---------TTAGTAGAGACGG------------------GGTTTCACCGTTTTAGCCGGGATGGTCTC
        2         2N22N2222N2N2                  22N2N22NN2NNN2NN22N22NN2N2NN2

left    ----------------------------------GAATTAAGCACATCCTGTGTGACTCTACTGGGAGAG
chrom   ACTTCACACGTGTGGTTACAATGCCACATTGGGGGAATTAAGCACATCCTGTGTGACTCTACTGGGAGAG
right   GATCTCCTGACCTCGTGATCCGCCCGCCTCGGC-----------CTCCCAAAGTG------CTGGGATTA
        NN2NNN2NNNNN2N22N2NNNNN22N2N2N22N 1111111111*11**111***111111******111

left    GACTCTTGGAAGCTATGCTTGGTTTCCTCTGGACTTTGTCC-----------------------------
chrom   GACTCTTGGAAGCTATGCTTGGTTTCCTCTGGACTTTGTCCCATGCACCTCTTTCTTATGCCAATTTTGC
right   CAGGCGTG--AGCCA----------CCGCG---------CCCGGCCACCTCTTTCTTATGCCAATTTTGC
        1*11*1**11***1*1111111111**1*1111111111**2NNN2222222222222222222222222

left    ----------------------------------------------------------------------
chrom   TTTGCGGCCTTTTGTTCTAATACATCTTAGCCATGAGTATTACTAGATACTGAGTCCTGAGTCTTTCTAG
right   TTTGCGGCCTTTTGTTCTAATACATCTTAGCCATGAGTATTACTAGATACTGAGTCCTGAGTCTTTCTAG
        2222222222222222222222222222222222222222222222222222222222222222222222

left    ----------------------------------------------------------------------
chrom   CAAATCACCAAACCTGAGGGTGGTCCTGGGGACTCCTGACACACCCTATATCTAAAAAGAATGCCTTTAA
right   CAAATCACCAAACCTGAGGGTGGTCCTGGGGACTCCTGACACACCCTATATCTAAAAAGAATGCCT----
        222222222222222222222222222222222222222222222222222222222222222222

left    ----------------------------------------------------------------------
chrom   CCTGTCTTTCAAAAGCCAATGGGAGCCATAGGAAAGAGCACACTTCATCCCTACCACGCCCTAATGATCT
right   ----------------------------------------------------------------------

left    ----------------------------------------------------------------------
chrom   CAGTGTAGTGCTATAAACTATAGCCCAAAGTCTTCCAGTTAAGTGCAACTTTACTAAGAGGGACATGAGA
right   ----------------------------------------------------------------------

left    ----------------------------------------------------------------------
chrom   GCACTTAGCCAGAAAAGCCTCCCTCAGATCTCCAGACTTAACCAACTGCCAACCTGTCGTCACCACCTGG
right   ----------------------------------------------------------------------

left    ----------------------------------------------------------------------
chrom   ACAGCATATAGGCACCTCCAACATAATCGACCCCAAACACCTCTCTGGATTCCTTCCCCTCTGTCCTAAG
right   ----------------------------------------------------------------------

left    ----------------------------------------------------------------------
chrom   CAGTGAGACATGGACTTGCTCAGGCAAAAAAAAAAAAAAAAAAAAAAAAAAAAACAGAAAAAAAGAAAAA
right   ----------------------------------------------------------------------

left    ANNNNNNNNNNN
chrom   AAAAAAACAGAA
right   ------------
        1NNNNNNNNNNN
```

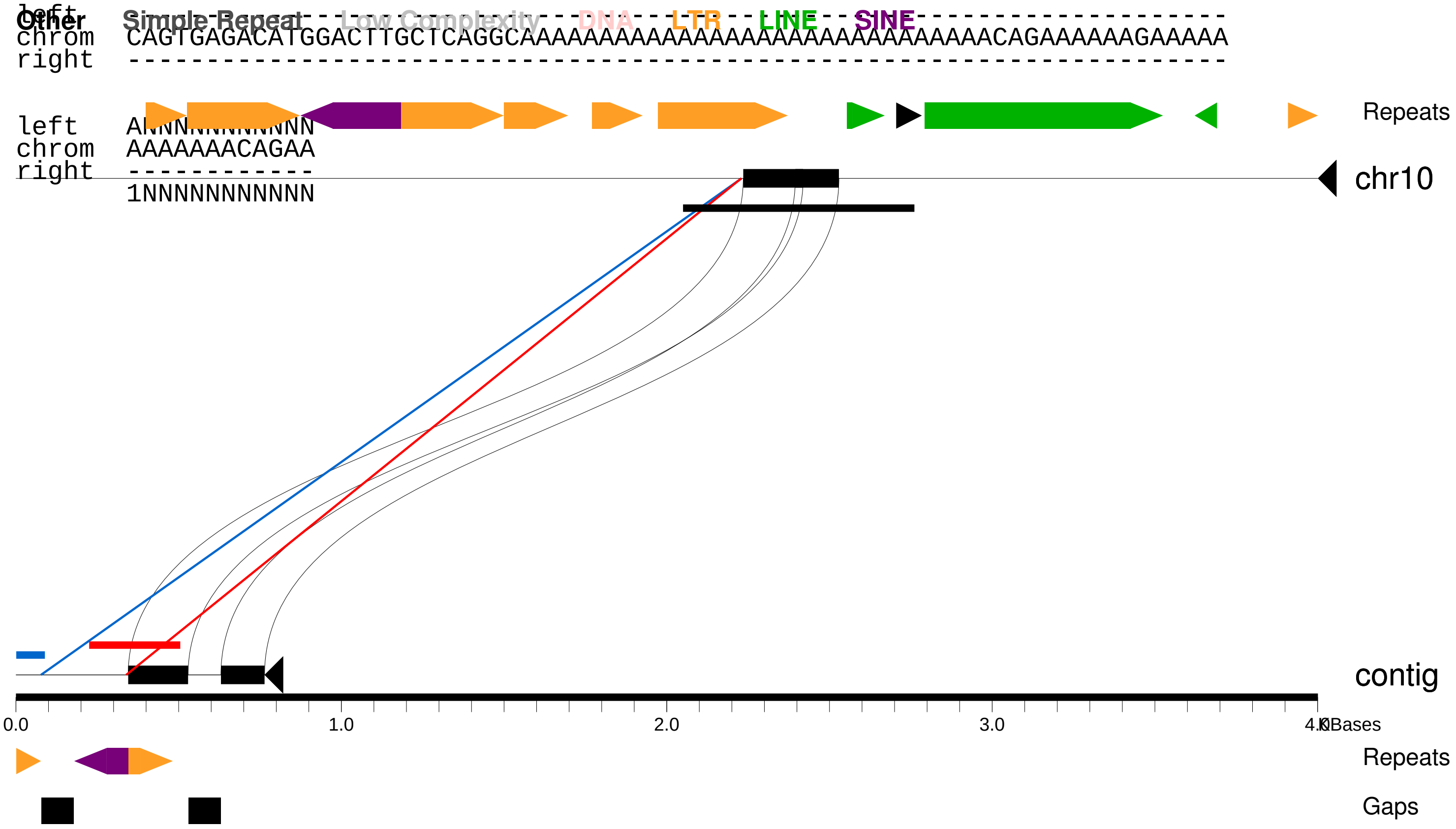

siteID: chr10_62124855 Scafftig2 - Breakpoint: chr10:62124845
chr10:62124799-62124904
Overlap Length: 14 INS size: 272 Genotyped: Yes

```
left   -----------------GGCTTTGGCTATTATTTGCCATTTTCCCTCATCTTGTATGGTGCCTAAAAAAT
chrom  -----------------GGCTTTGGCTATTATTTGCCATTTTCCCTCATCTTGTATGGTGCCTAAAAAAT
right  ACTGCAGTCCGCAGTCCGGCCTGGGCAACAGA--GCGAGACTCCGTC-TCAAAAAAAAAAAAAAAAAAAA-
       11111111111111111***1*1***1*111111**1*111***1**1**1111*11111111******1

left   AGATGCTCCCAGCACTTTGGGAGGCCGAGGCGGGTGGATCATGAGGTCAGGAGATCGAGACCATCC
chrom  AGATGCTAAAATATTTATTGAAAAAAGGAAAGAATAGAAAATGAGATGAGG-----GA--------
right  AGATGCTAAAATATTTATTGAAAAAAGGAAAGAATAGAAAATGAGATGAGG-----GA--------
       *******222*2222*2*2*2*2222*2222*22*2**22*****2*2***22222**22222222
```

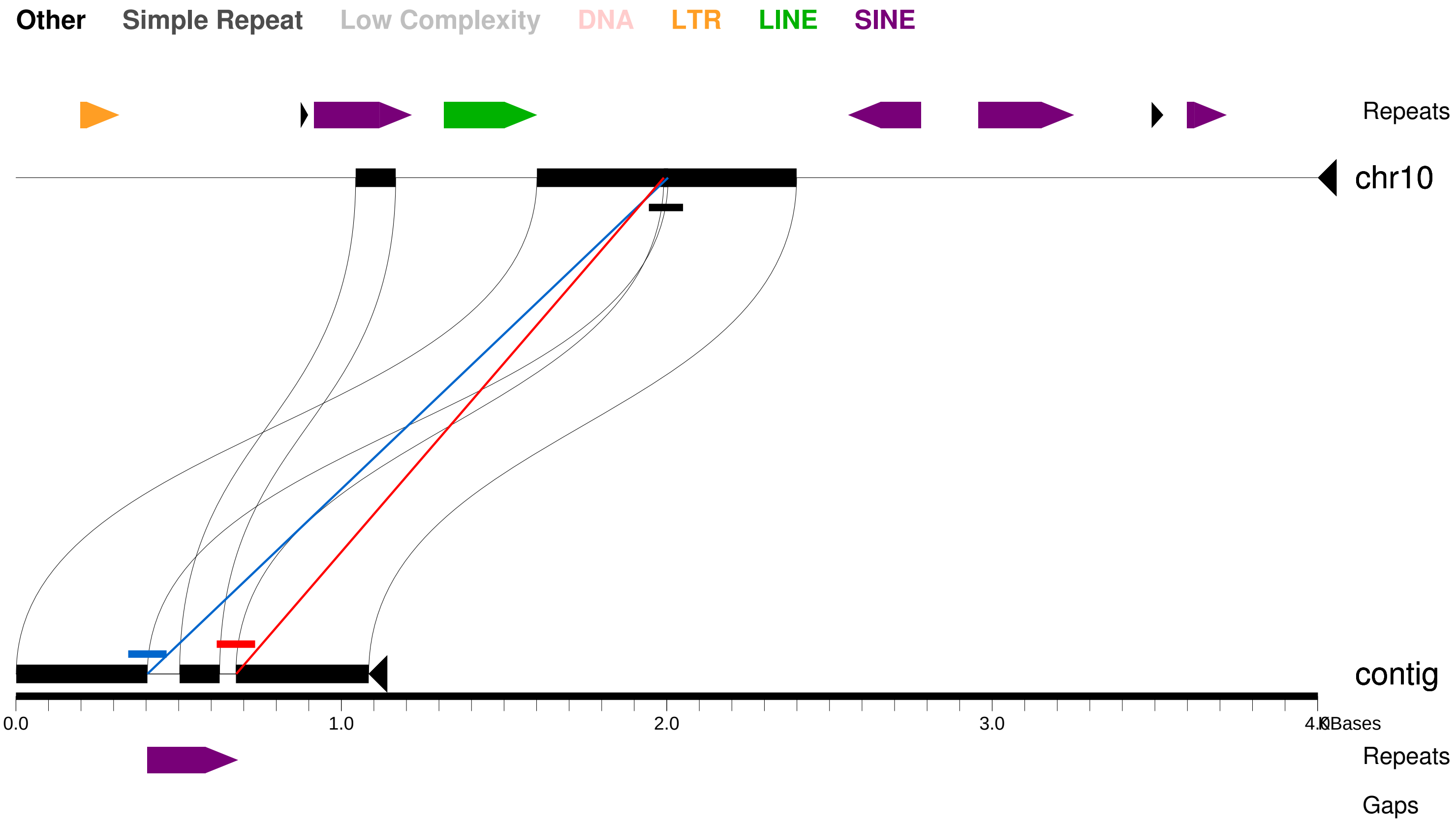

siteID: chr10_64233365 Scafftig1 - Breakpoint: chr10:64233370
chr10:64233210-64233538
Overlap Length: 9 INS size: 421 Genotyped: No

```
left    ----------------------------------------ATTTGGGGGCCAGACTTCCTGTGTTTGTAATA
chrom   TG----AGAGGCGCCTAGTATCCTGGTTATGAGTTTCGATTTGGGGGCCAGACTTCCTGTGTTTGTAATA
right   TTTAGTAGAGACGGG--GTTTCACCGTTTT-AGCTGGGAT----GGTCTCGATCTCCTGACCTCGTGATC
        2N11112222N22NN  22N22NNN222N2 22N2NN2**1111**1*11**11*****111*1**1**1

left    CTAGTTCTACTATTAGTTAGTTACGTGACATTGAACAAGTACTTCCACCTCTCTGTAATGAAGTTCTTCA
chrom   CTAGTTCTACTATTAGTTAGTTACGTGACATTGAACAAGTACTTCCACCTCTCTGTAATGAAGTTCTTCA
right   C--GCCCGCCTCGG---------CCTCCCA------AAGTGCTGGGA--TTACAGGCGTGAGCCACCGCG
        *11*11*11**111111111111*1*11**111111****1**111*11*11*1*111***1111*11*1

left    TCTGGGAAATGAAGAAGACAAATAGTGTCTTCTNNNNNNNNNNN-------------------------
chrom   TCTGGGAAATGAAGAAGACAAATAGTGTCTTCTTCTTATAGTTGTTATAAGGATTAAGTGGGCTAAAGCT
right   CCCGGCC------------------GTGTCTTCTTCTTATAGTTGTTATAAGGATTAAGTGGGCTAAAGCT
        1*1**1111111111111111111*********2222222222222222222222222222222222222

left    ----------------------------------------------------------------------
chrom   CTTAGAACCATGCCTGGCACATAGTAAATGCTGTGTTAGATGTGTTACTATTCACCTATAAATTGTCCCA
right   CTTAGAACCATGCCTGGCACATAGTAAATGCTGTGTTAGATGTGTTACTATTCACCTATAAATTGTCCCA
        2222222222222222222222222222222222222222222222222222222222222222222222

left    -----------------------------------------------------
chrom   CTGGGGGTTTGTTAGTGTATCGATTAGAATGCTTTTCTGGCAGCAAGTTGAAG
right   CTGGGGGTTTGTTAGTGTATCGATTAGAATGCTTTTCTGGCAGC---------
        22222222222222222222222222222222222222222222
```

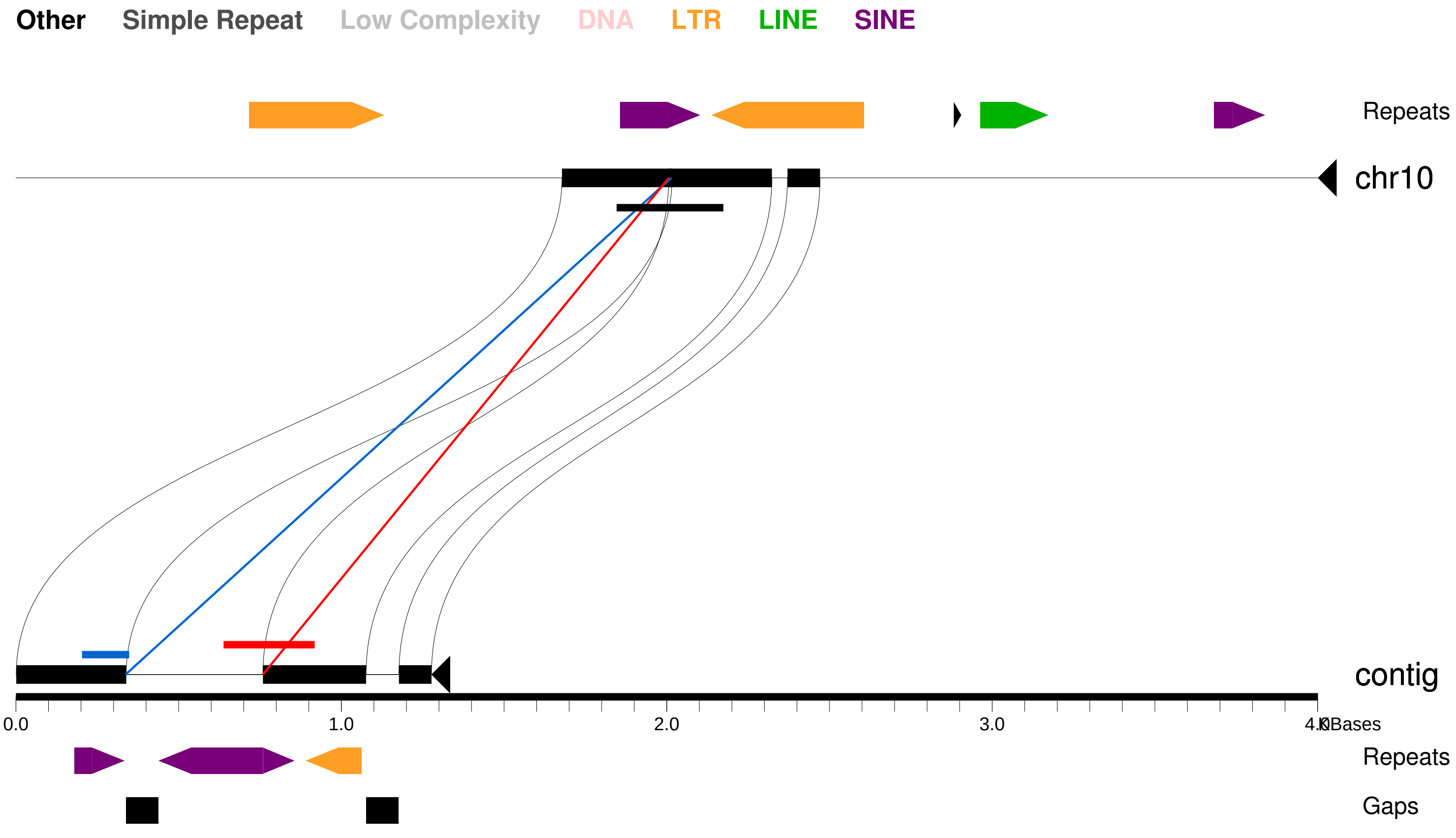

siteID: chr10_64459995 Scafftig3 + Breakpoint: chr10:64459994
chr10:64459950-64460053
Overlap Length: 16 INS size: 380 Genotyped: No

```
left    AGTAGCAG--TGCTAGATACAATGGAGCAGTGTGCCTATATTTCCC-------------CACCAAATGGC
chrom   AGTAGCAG--TGCTAGATACAATGGAGCAGTGTGCCTATATTTCCC-------------CACCAAATGGC
right   TACAGGCGCCCGCCACCGCGCCCGGCTAATTTTTTGTATTTTTAGTAGAGACGGGGTTTCACCAAATGGC
        111**11*111**1*11111111**111*1*1*111***1***1111111111111111***********

left    TTTTTTTTTNNNNNNNNNNNNNNNNNNNNNNNNNNNNNNNNNNNNNNNNNNNNNNNNNNNNNNN
chrom   TTTTTT--------------AAGAAAGAAAATAAATGAAAATATCCTCATATATTCAAGCCTCA
right   TTTTT---------------AAGAAAGAAAATAAATGAAAATATCCTCATATATTCAAGCCTCA
        ******2222222222222222222222222222222222222222222222222222222222
```

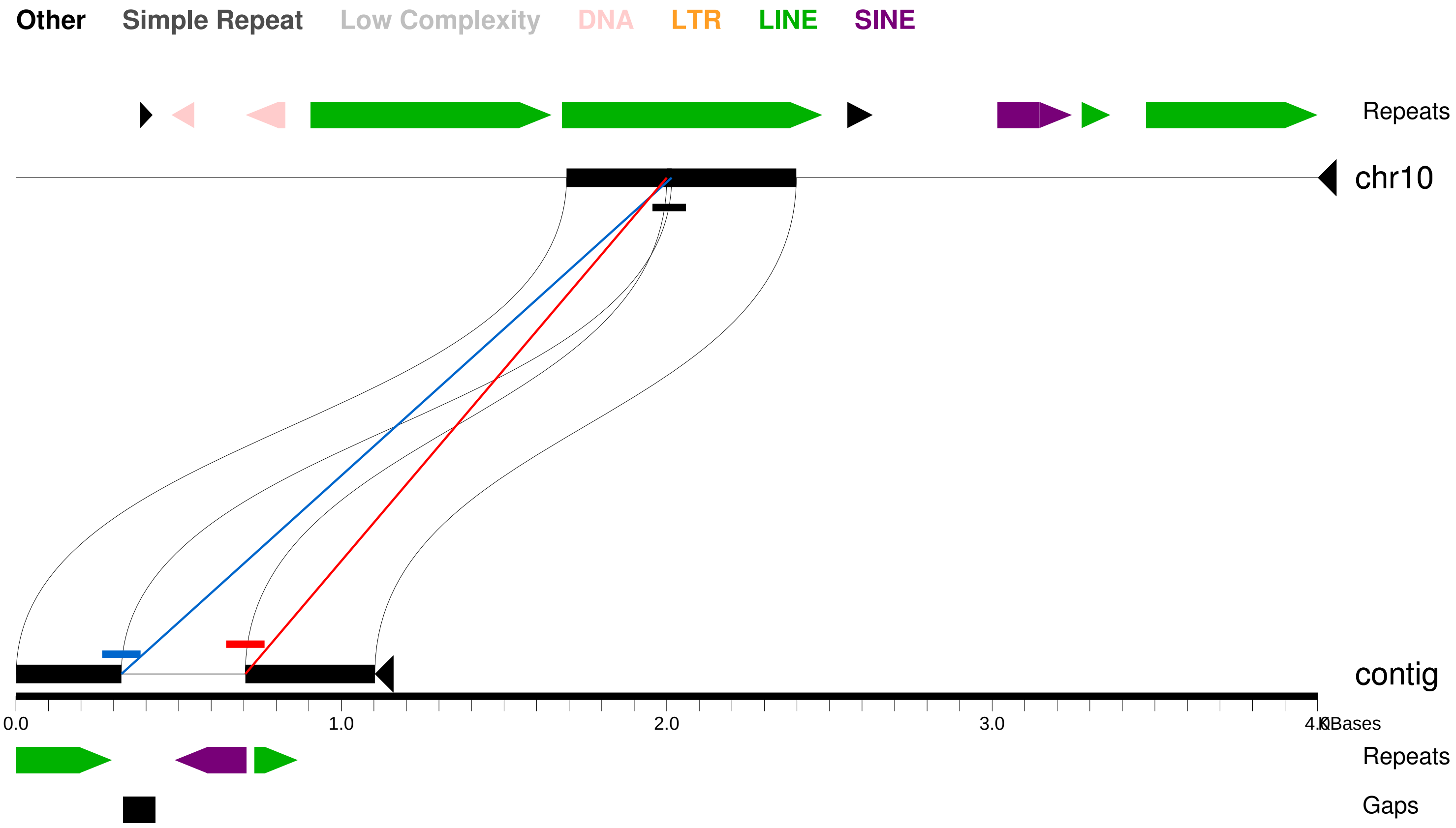

siteID: chr10_66837072 Scafftig3 + Breakpoint: chr10:66837055
chr10:66837010-66837114
Overlap Length: 15 INS size: 298 Genotyped: Yes

```
left    C------ATTTGATTTACA---TGTAGTTAATATAC-----AATCACTTTCATTAAATTTCAATGAGTTT
chrom   C------ATTTGATTTACA---TGTAGTTAATATAC-----AATCACTTTCATTAAATTTCAATGAGTTT
right   CGCCCGCCTCGGCCTCCCAAAGTGCTGGGATTACAGGCGTGAGCCACCGCGCCCGGCCCTCAATGAGTTT
        *1111111*11*11*11**111**11*11*1**1*111111*11***111111111111***********

left    TTTTTTTTTTTTTTTTTTTTGAGACGGAGTCTCGC-TCTATCGCCCAGGCTGGAGTGCAGTGGC
chrom   TTTTACACATATATA---CAGCCATGTAATCCATCATCTAT--------ATCAAGT----TATA
right   TTTTACACATATATA---CAGCCATGTAATCCATCATCTAT--------ATCAAGT----TATA
        ****22222*2*2*222222*22*2*2*2**222*2*****222222222*22***2222*222
```

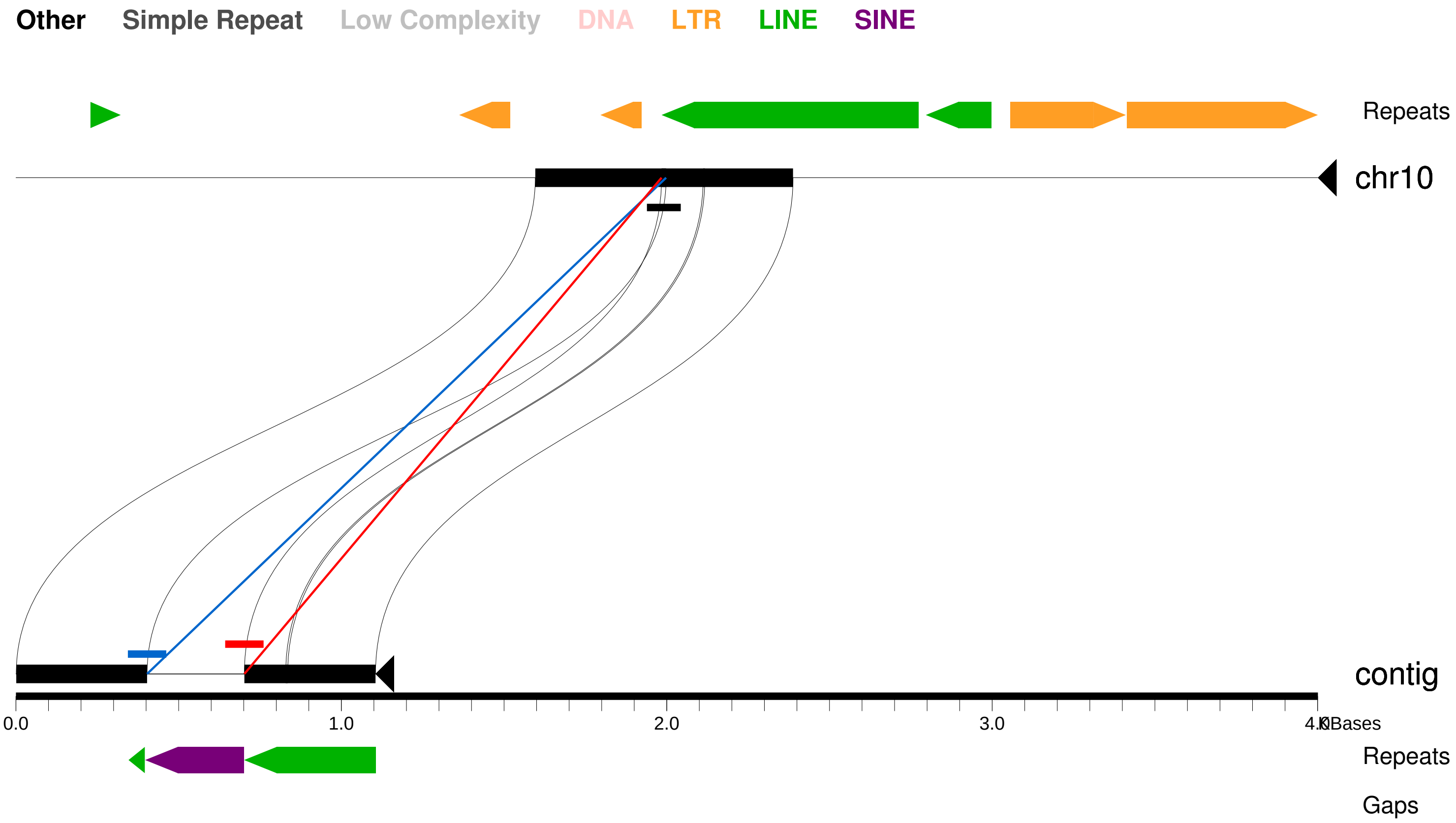

siteID: chr10_67196530 Scafftig3 + Breakpoint: chr10:67196551
chr10:67196493-67196610
Overlap Length: 2 INS size: 249 Genotyped: No

```
left    CTACATTTGAATTTTCAAATATTTATATAACTATTTTGGTATTATAAGAATTTTATTT-CTGGCCGGGCG
chrom   CTACATTTGAATTTTCAAATATTTATATAACTATTTTGGTATTATAAGAATTTTATTT-CTAA----GTG
right   NNNNNNNNNNNNNNNNNNNNNNNNNNNNNNNNNNNNNNNNNNNNNNNNNNNNNNNNNNNCTAA----GTG
        11111111111111111111111111111111111111111111111111111111111**222222*2*

left    CGGTGGCTCACGCCTGTAATCCCAGCACTTTGGGAGGC---CGAGGCGGGTGG
chrom   CATTTCTTTAAAAAAGTAGTAATGCATCTTTCAAAGTATTTCTATCAAATGAT
right   CATTTCTTTAAAAAAGTAGTAATGCATCTTTCAAAGTATTTCTATCAAATGAT
        *22*222*2*22222***2*2222222****222**22222*2*222222222
```

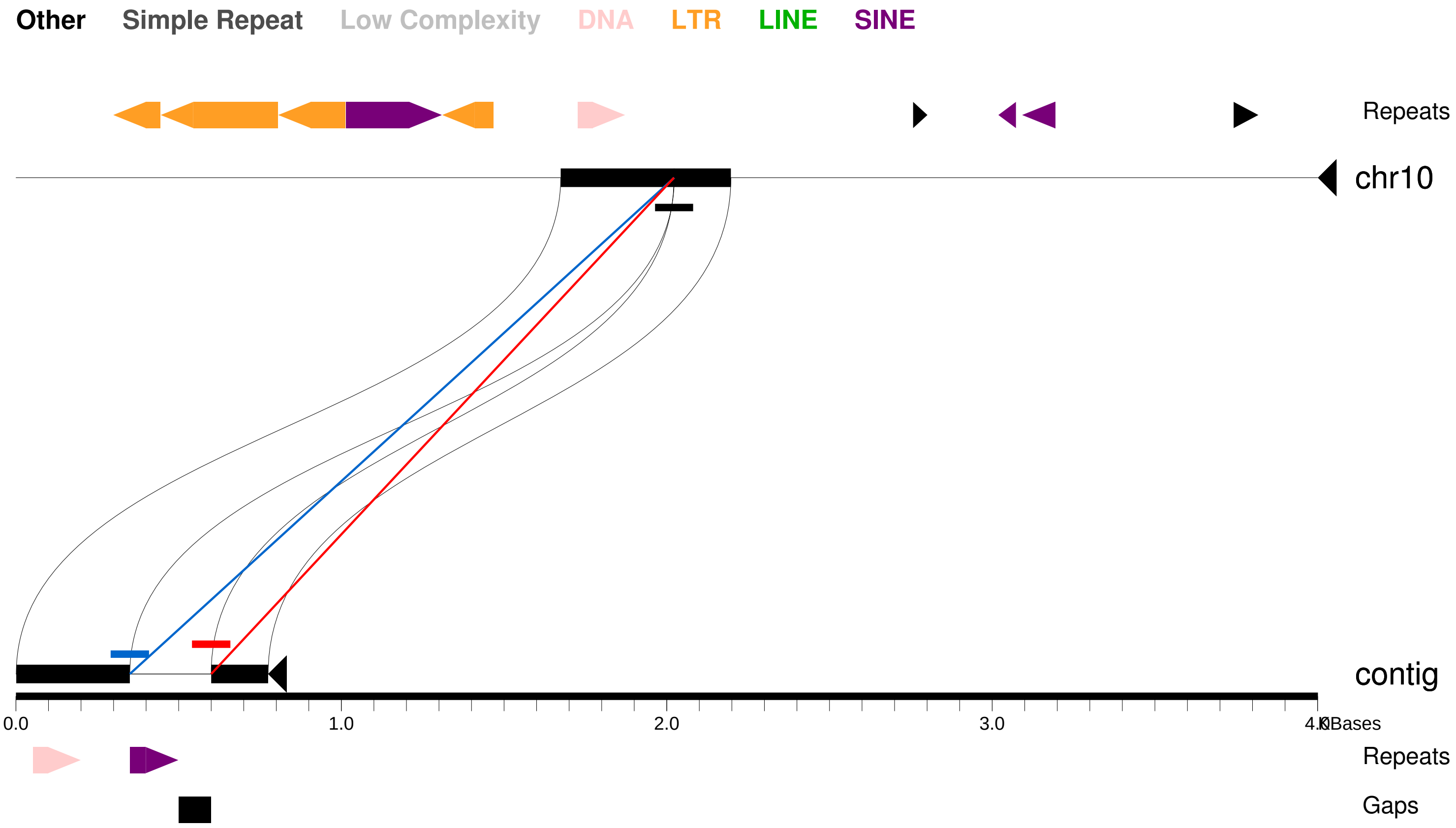

siteID: chr10_67431014 Scafftig1 - Breakpoint: chr10:67430984
chr10:67430944-67431043
Overlap Length: 20 INS size: 456 Genotyped: No

```
left    TCCTTGATTTTTGGAAAATCACATTTAAGTTTCCTCTGCA-------------------AAGAAAAGAAT
chrom   TCCTTGATTTTTGGAAAATCACATTTAAGTTTCCTCTGCA-------------------AAGAAAAGAAT
right   NNNNNNNNNNNNNNNNNNNNNNNNNNNNNNNNNNNNNNNNNNNNNNNNNNNNNNNNNNNAAGAAAAGAAT
        11111111111111111111111111111111111111111111111111111111111***********

left    AGGTAAATCGGCCGGGCGCGGTGGCTCACGCCTGTAATCCCAGCACTTTGGGAGGCCGAGGCGGGCGG
chrom   AGGTAAATCAA---------GTC-CTCTAGAAAAAAAGCATAAAAATAT---------AGTTTTGAAA
right   AGGTAAATCAA---------GTC-CTCTAGAAAAAAAGCATAAAAATAT---------AGTTTTGAAA
        *********22222222222**22***22*22222**2*22*22*2*2*222222222**2222*222
```

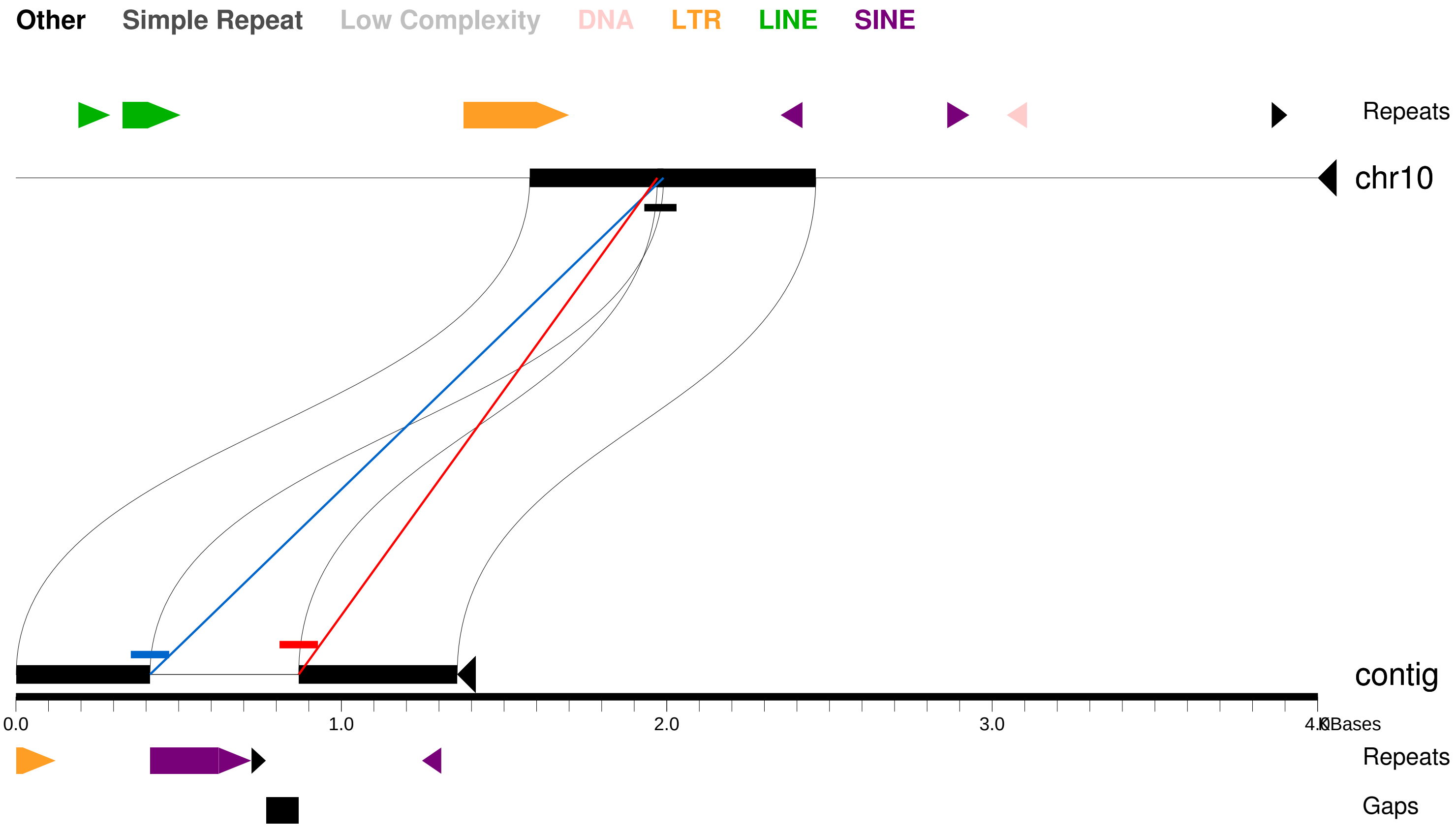

siteID: chr10_68049101 Scafftig3 - Breakpoint: chr10:68049106
chr10:68049047-68049166
Overlap Length: 0 INS size: 247 Genotyped: Yes

```
left    TAAGATTTTTAAAGAACAGGCCAGGCGTGGTG----GCTCATGCCTGTAATCCCAGCACTTTGGGAGACT
chrom   TAAGATTTTTAAAGAACAGGCCAGGCGTGGTG----GCTCATGCCTGTAATCCCAGCACTTTGGAA-ACA
right   CGACATCATGCCATTGCACTCCAGCCTGGGTGAAAAGAGAAAGACTCCA-TCTCAAAATA----AA-ACA
        11*1**11*111*111**11****1*11****1111*111*1*1**11*1**1**11*111112*2**2

left    GAGGAGGGCAGATCACCTGAGGTCTGGAGTT--TGAGGCTAGCCTGGCCAA-CACG-
chrom   AACAAGAA-AGATTTT-TAAAGAATAAATTTATTTTTCCTGTATTTGATATTCATTT
right   AACAAGAA-AGATTTT-TAAAGAATAAATTTATTTTTCCTGTATTTGATATTCATTT
        2*22**222****2222*2*2*22*22*2**22*2222**2222*2*22*22**222
```

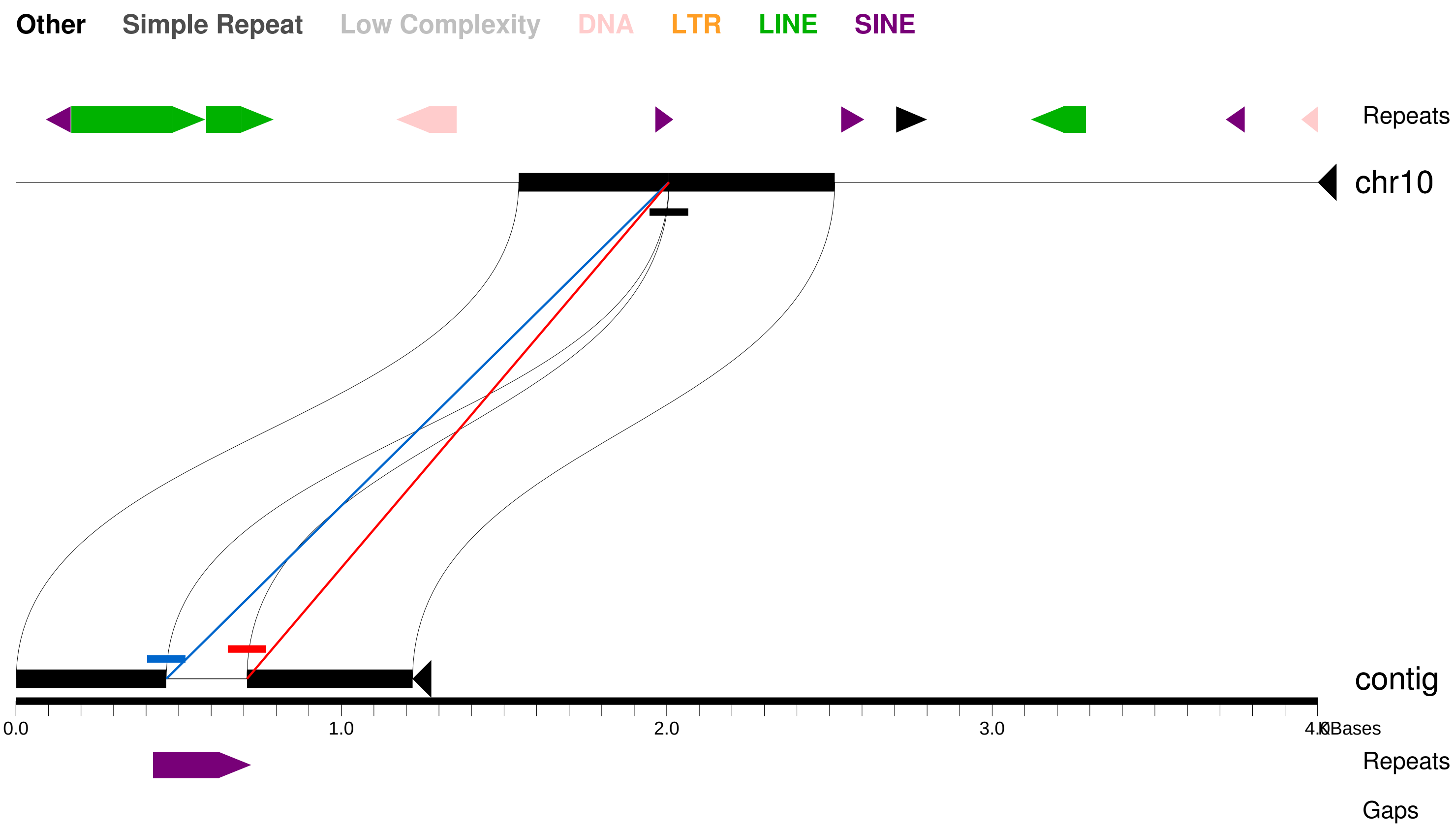

siteID: chr10_68244018 Scafftig1 + Breakpoint: chr10:68243986
chr10:68243376-68244146
Overlap Length: 16 INS size: 299 Genotyped: Yes

```
left   TT----------------------------------------------------------------------
chrom  TTCAGCTTTGAGAGATAGAGATGTAATGTACATGATATTGGAGACATGGACACTGGTTTATTGGAGGAAC
right  ----GCT----GAGACAG---------------GAGAATGGCG---TGAACCCGGGA-----GGCGGAGC
       11  222    2222N22               22N2N222N2   22N22N2N22N      22N222N2

left   ------------------------------------------------------------------------
chrom  TCTGCAGTTCACGGAATTCTTGCATATGTTATAGATCAGGAATATGTTAATTTGTCCCTGAAATTACTCT
right  T-TGCAGT-----GA---------------------------------------------------------
       2 222222     22

left   ------------------------------------------------------------------------
chrom  AACACCAATGGCAATTCTCATCCCCACTCACCCTTAGAAATCGTTAAGACTAAGCCAGATGGGGCTAATG
right  ------------------------------------------------------------------------

left   ------------------------------------------------------------------------
chrom  AACACTAGTTTCCATTGCCAAGAATTAGATTAACTTAAAAGAAGAGAAAAAAACTTAAAAAAATAAGATC
right  ----------------GCCGAGA-------------------------------------------------
                       222N222

left   ------------------------------------------------------------------------
chrom  TATCTGAAAAAGTCACTATGATATTAAAGACTACTCTAATTTCCTCGTCATACTGTACTACAATACTTTT
right  -----------------------------------------TCC-CGCCA--CTGCACTCCAG-------
                                                222 22N22  222N222N22N

left   ------------------------------------------------------------------------
chrom  TCCCCACTTTAGTTTATTAAATGATGAAAAAGGTAAAAAATGAGAACTACCTCTCCAAGGAAGTAATATT
right  ------------------------------------------------------------------------

left   -------------------------------------------GGAGACCAAGTTAAGCAAAATAAAATA
chrom  ACTCTATTTTCCTTGTGGTTGCTATAAGTATATTAGCACCCTTGGAGACCAAGTTAAGCAAAATAAAATA
right  ---------------------------------------CCTGGGCGAC---------------------
                                              222N**1***111111111111111111111

left   AACAAATAAACAAAAACCATGTTGCTAATGCAACCAAAGCTCCTGGAACTTA-----GAATCTGTGTCCA
chrom  AACAAATAAACAAAAACCATGTTGCTAATGCAACCAAAGCTCCTGGAACTTATAAAAGAATCTGTGTCCA
right  ------------------------------------AGAGCG---------------AGACTCCGTCTCAA
       111111111111111111111111111111111111*1***111111111111    2**1**1**1**1*

left   AAATTTTGTGTTCTTATACTTGGAAAGGAAAAGCATGAAGTATCATTTTAAAATATTTCAGTTTATGGCC
chrom  AAATTTTGTGTTCTTATACTTGGAAAGGAAAAGCATGAAGTATCATTTTAAAATATTTCAGTTTATTTTT
right  AAA--------------------AAAAAAAAA------------------AAAAATTTCAGTTTATTTTT
       ***11111111111111111111***11****111111111111111111***1***********2222

left   [illegible]CACTTT[illegible]AGGCCGAGGCGGGCGGATCA
chrom  CTTCTTCAAGAAGCAAAC-TACACTGCTAGACATTTTTTTTCAAAATGTTCAAACAATTAAAACTGTTCC
right  CTTCTTCAAGAAGCAAAC-TACACTGCTAGACATTTTTTTTCAAAATGTTCAAACAATTAAAACTGTTCC
       222*22222*222**22*2*22*2*2*2**2**2***2222222222*222222*2*222222*2*2**2

left   CGAGGTCAGGA[illegible]CGAGACCATCCCGGCTAAAACGG[illegible]GAAA-[illegible]
chrom  AAATTTTCTTTGAGGAAGTAATTTCAGTTTAAAGTTTTAAAAATAGCCAAAGAGGATAAGTGAATGCATT
right  AAATTTTCTTTGAGGAAGTAATTTCA[illegible]AATAGCCAAAGAGGATAAGTGAATGCATT
       22*22*22222**222**2222*2*2*22[illegible]2222*2***22222*222*2222**22*2**222222

left   ------
chrom  GG----
right  GGTTTA
       221111
```

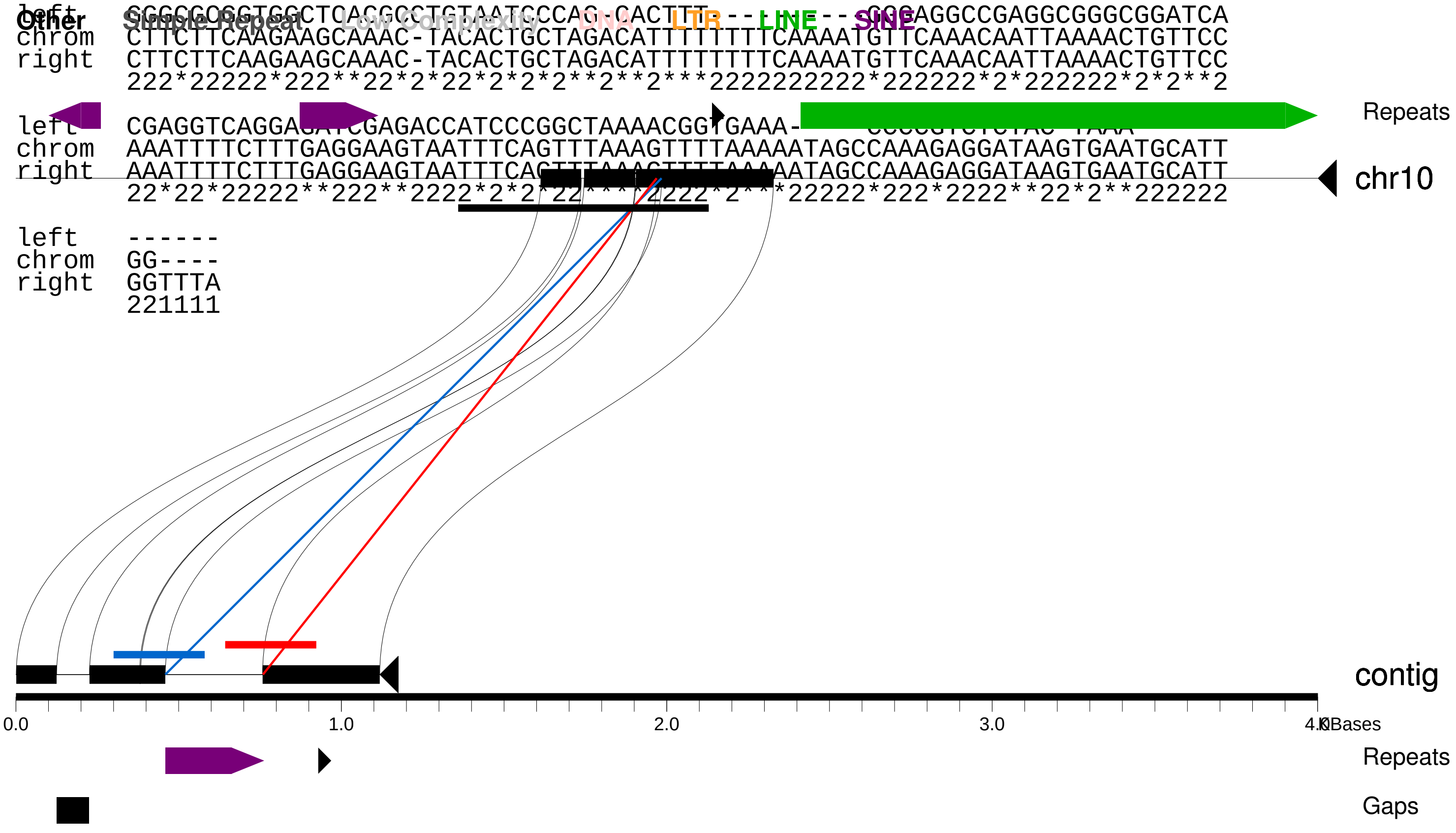

siteID: chr10_68336753 Scafftig6 + Breakpoint: chr10:68336759
chr10:68336710-68336818
Overlap Length: 11 INS size: 435 Genotyped: No

```
left    GTGTATA---AGCGTTCCCTTTTCTCTG------CAG-CTTAAACAGCATCTGTTGTTTCTTAACTTTTT
chrom   GTGTATA---AGCGTTCCCTTTTCTCTG------CAG-CTTAAACAGCATCTGTTGTTTCTTAACTTTTT
right   CCACCCACCTCGGCCTCCCAAAGTGCTGGGATTACAGGCGTGAGCCACCGCGCCCGGCCCTTAACTTTTT
        111111*1111*111****111111***111111***1*1*1*1*11*11*1111*111***********

left    TTNNNNNNNNNNNNNNNNNNNNNNNNNNNNNNNNNNNNNNNNNNNNNNNNNNNNNNNNNNNN
chrom   ----------AATAATAGCTATTCTGACTGATGTGAGATAGTATCTCATAGTTTGAAAT
right   ----------AATAATAGCTATTCTGACTGATGTGAGATAGTATCTCATAGTTTGAAAT
        2222222222222222222222222222222222222222222222222222222222222
```

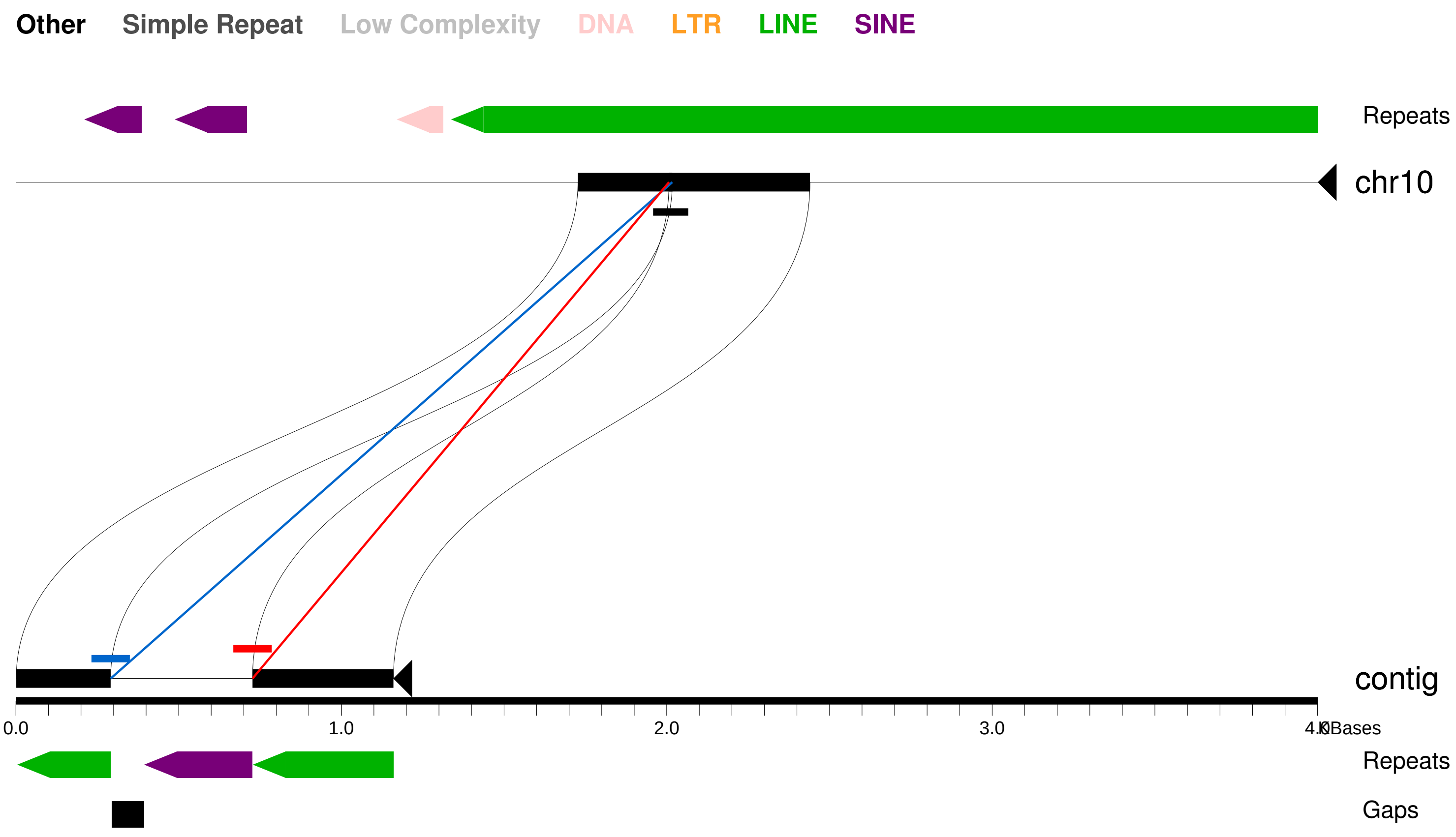

siteID: chr10_68977613 Scafftig1 + Breakpoint: chr10:68977598
chr10:68977546-68977657
Overlap Length: 8 INS size: 420 Genotyped: No

```
left   TAGTACTGTGAAAAATGTGCTATACAGGTTGAGCGTTGCTAATCTAAAAATC-------TGAAATCCGGC
chrom  TAGTACTGTGAAAAATGTGCTATACAGGTTGAGCGTTGCTAATCTAAAAATC-------TGAAATCCAAA
right  NNNNNNNNNNNNNNNNNNNNNNNNNNNNNNNNNNNNNNNNNNNNNNNNNNNNNNNNNNNTGAAATCCAAA
       11111111111111111111111111111111111111111111111111111111111********222

left   CGGGCGCGGTGGCTCACGC---CTGTAATCCCAGCACTTTGGGAGGCCGAGGCGGGCGG
chrom  ATGCTCCAAAATCTAAAACTTTCTG-AATGCCAATGTGATGGCA---CTAGG------G
right  ATGCTCCAAAATCTAAAACTTTCTG-AATGCCAATGTGATGGCA---CTAGG------G
       22*222*22222**2*22*222***2***2***222222***2*222*2***222222*
```

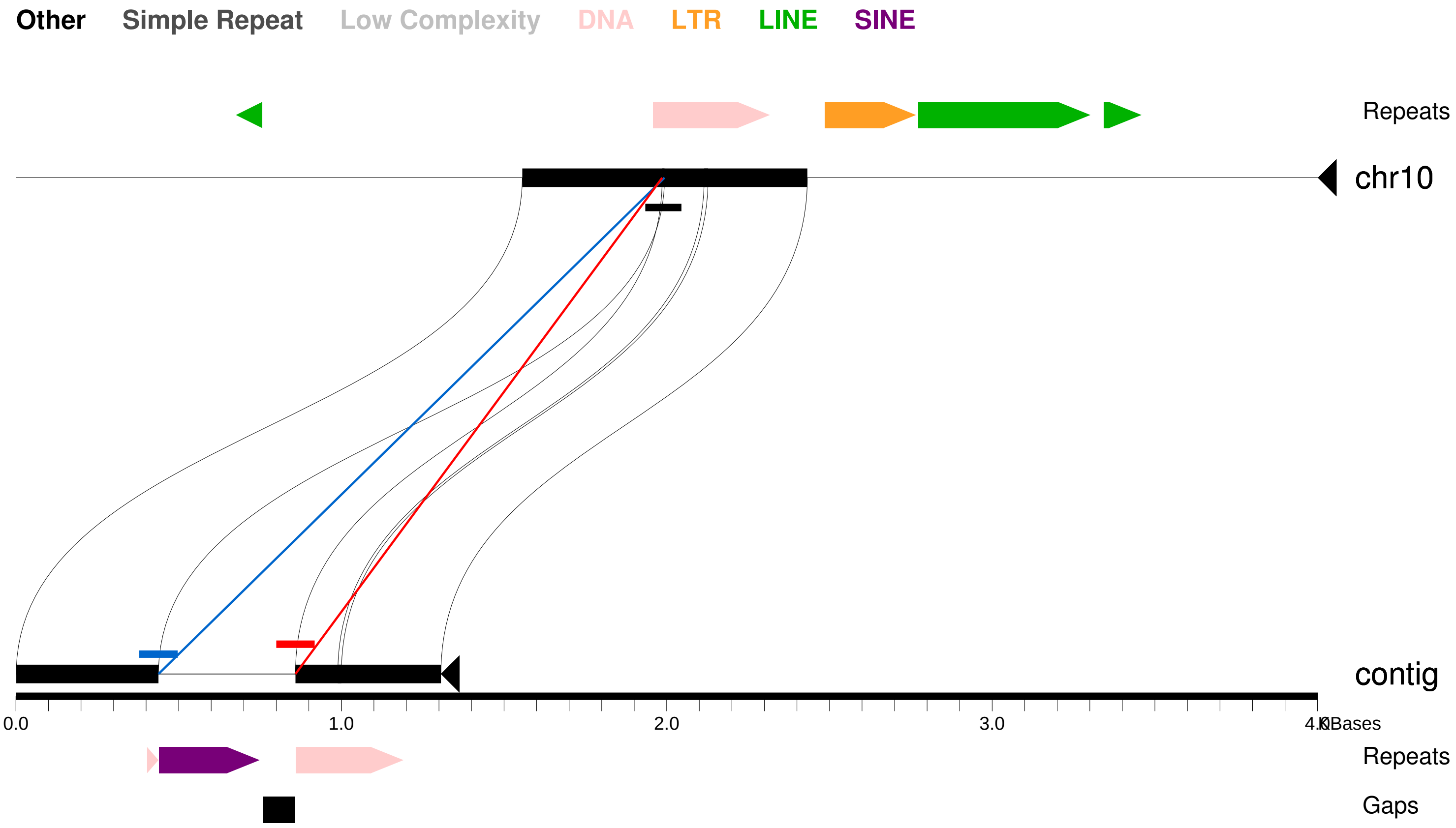

siteID: chr10_69138224 Scafftig4 + Breakpoint: chr10:69138283
chr10:69138237-69138342
Overlap Length: 14 INS size: 300 Genotyped: Yes

```
left     TTATTCTGTTTCAGGGTCCAATCAAAGATG---TTA------TGAAACATTTTGT------GGTCATCTT
chrom    TTATTCTGTTTCAGGGTCCAATCAAAGATG---TTA------TGAAACATTTTGT------GGTCATCTT
right    CCGCCC-GCCTCGGCCTCCCAA-AGTGCTGGGATTACAGGCGTGAGCCACCGCGCCCGGCCGGTCATCTT
         11111*1*11**1*11***1*11*11*1**111***111111***11**1111*1111111*********

left     CTTTTTTTTTTTTTTTTTTTTTTTGAGACGGAGTCTTGCTCTGTCGCCCAGGCTGGAGTGCAGT
chrom    CTTTTGTCTCCTCAGATCTGT---------GAGAATT---CTGT-GAGAAAGACAGTCTCCATC
right    CTTTTGTCTCCTCAGATCTGT---------GAGAATT---CTGT-GAGAAAGACAGTCTCCATC
         *****2*2*22*2222*2*2*222222222***22**222****2*222*2*222*22*2**22
```

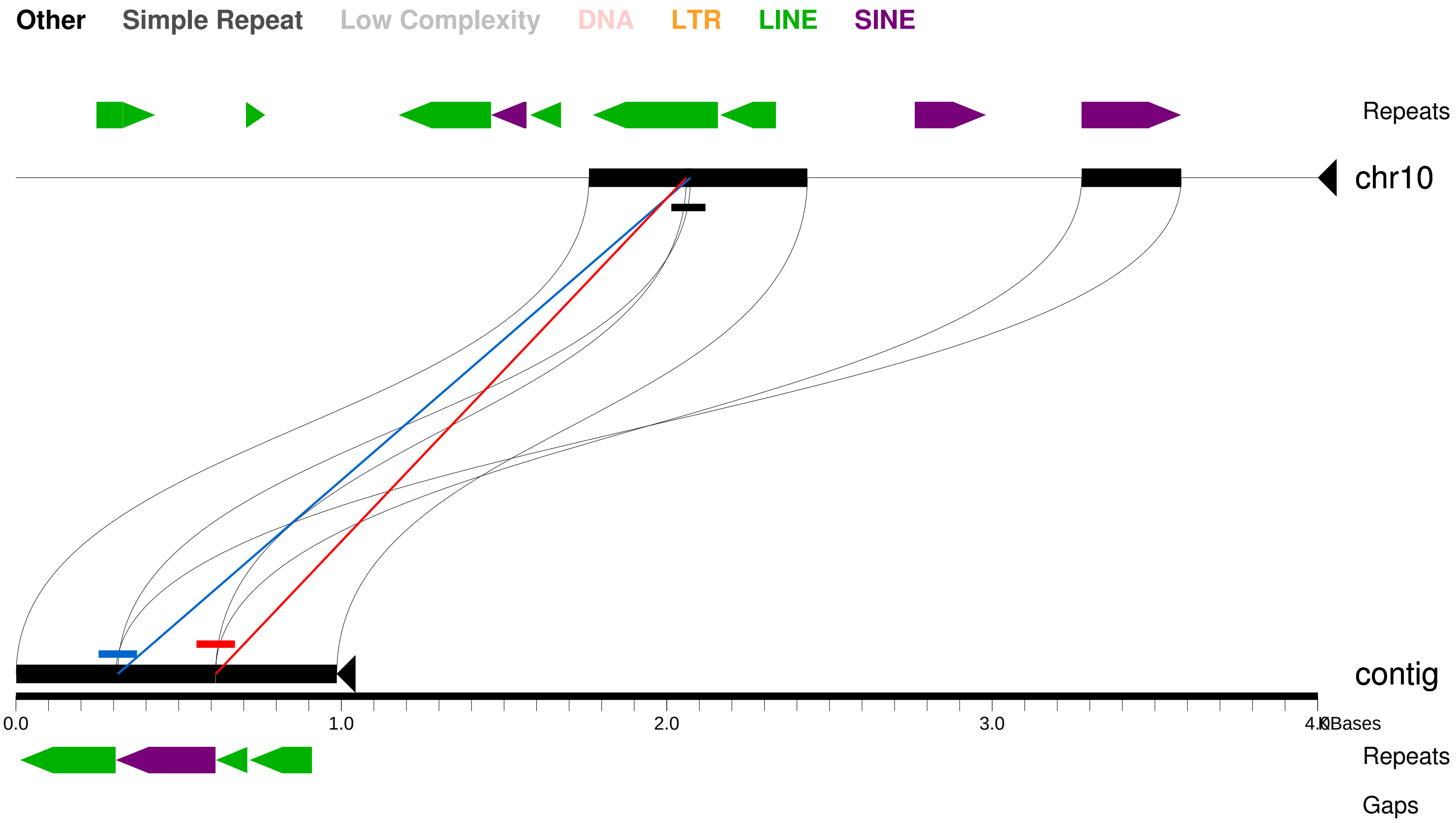

siteID: chr10_71308739 Scafftig10 - Breakpoint: chr10:71308735
chr10:71308689-71308794
Overlap Length: 14 INS size: 310 Genotyped: Yes

```
left     CTAGCTCTAGAGTGAAGGTTACTGT-AGTCCCATTTTTTAAGAGCTT--------------AAAAACAAG
chrom    CTAGCTCTAGAGTGAAGGTTACTGT-AGTCCCATTTTTTAAGAGCTT--------------AAAAACAAG
right    CA--CTCCAGCCTGGGCGACAGAGCGAGACTCCGTCTCAAAAAAAAAAAAAAAAAAAAAAAAAAAAACAAG
         *111***1**11**111*11*11*11**1*1*11*1*11**1*11111111111111111*********

left     CTTTAGGCCGGGCGCGGTGGCTCATGCCTGTAATCCCAGCACTTTGGGAGGCCGAGGCGGGCGG
chrom    CTTTAAAAAGATTATAGTTGT-----CTTTTAATGGC----CTCCAGGAC----AAAATGCAAC
right    CTTTAAAAAGATTATAGTTGT-----CTTTTAATGGC----CTCCAGGAC----AAAATGCAAC
         *****2222*222222**2*222222*2*2****22*2222**222***22222*2222*2222
```

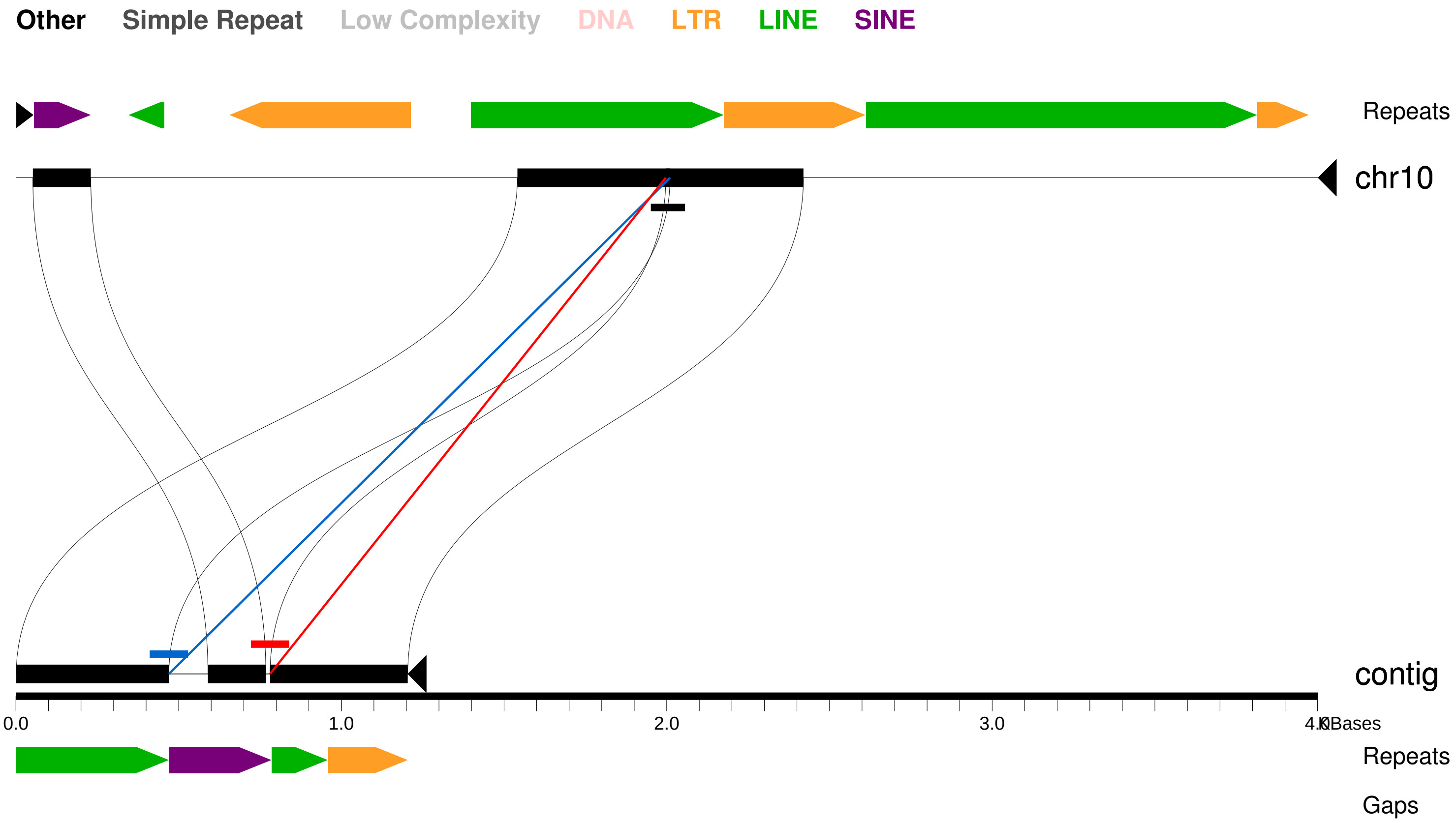

siteID: chr10_71610160 Scafftig7 - Breakpoint: chr10:71610164
chr10:71610004-71610335
Overlap Length: 12 INS size: 301 Genotyped: Yes

```
left   -----------ACAGTACGATACAGAGCAAAAGGCAGATTCTGGACTCCACAAGATCTGGGTTCAAATCC
chrom  GACTGTGCGACACAGTACGATACAGGGCAAAAGGCAGATTCTGGACTCCACAAGATCTGGGTTCAAATCC
right  AGGCAGGAGA-ATGGCATGAACCCGGG----AGGCGGAGCTTGCAGT------GAGCCGAG-----ATCC
       NNNNNN2N22 *11*1*1**11*1*2*1111****1**111**1*1*111111**1*1*1*11111****

left   CAGCTCTGGCATCAAGAAGCTTCAGGAAGTTGGGTTCTTTCAAAGCCTCGGCCCTGTCTCTAGTGGGATG
chrom  CAGCTCTGGCATCAAGAAGCTTCAGGAAGTTGGGTTCTTTCAAAGCCTCGGCCCTGTCTCTAGTGGGATG
right  CGCCACTG-CA--------CTCCAGCC---TGGGCGA---CAGAGCGA-GACTCCGTCTCAAAAAAAAAA
       *11*1***1**11111111**1***11111****111111**1***111*1*1*1*****1*11111*11

left   AGGATAACAATCCTCTTAAGAGACATCAGGAGCCGGGCGCGGTGGCTCACGCCTGTAATCCCAGCACTTT
chrom  AGGATAACAATCCTCTTAAGAGACATCAGGAGA-GGACCTGGAGCCACACGT-TAAACTGCCTGG-CATG
right  AAAAAAAAAA--------AAAGACATCAGGAGA-GGACCTGGAGCCACACGT-TAAACTGCCTGG-CATG
       *11*1**1**11111111*1***********22**2*22**2*2*2****22*22*2*2**2*22*2*2

left   GGGAGGC---CGAGGCGGGCGGAT-----CACGAGGTCAGGAG----ATCGAGA--CCATCC--CGGCTA
chrom  GGGTGACACTCGAGAAAGAGGGTTTCTTACCCCATGGCACATGTCTAATGCAGGGGCCTTCCAGCGGCCT
right  GGGTGACACTCGAGAAACAGGGTTTCTTACCCCATGGCGCATGTCTAATGCAGGGGCCTTCCAGCGGCCT
       ***2*2*222****222122**2*22222*2*2*2*2*1222*2222**22**222**2***22****22

left   AAACGGT------GAAACCCC----------GTCTCTACTAAA-----------A
chrom  CCTGCGTCTGGAGGAAGCCACCTACTGAGAGGTCTCTGCCAGCCTGCTCAGGGTA
right  CCTGCGTCTGGAGGAAGCCACCTACTGAGAGGTCTCTGCCAGCC-----------
       22222**222222***2**2*2222222222******2*2*222          1
```

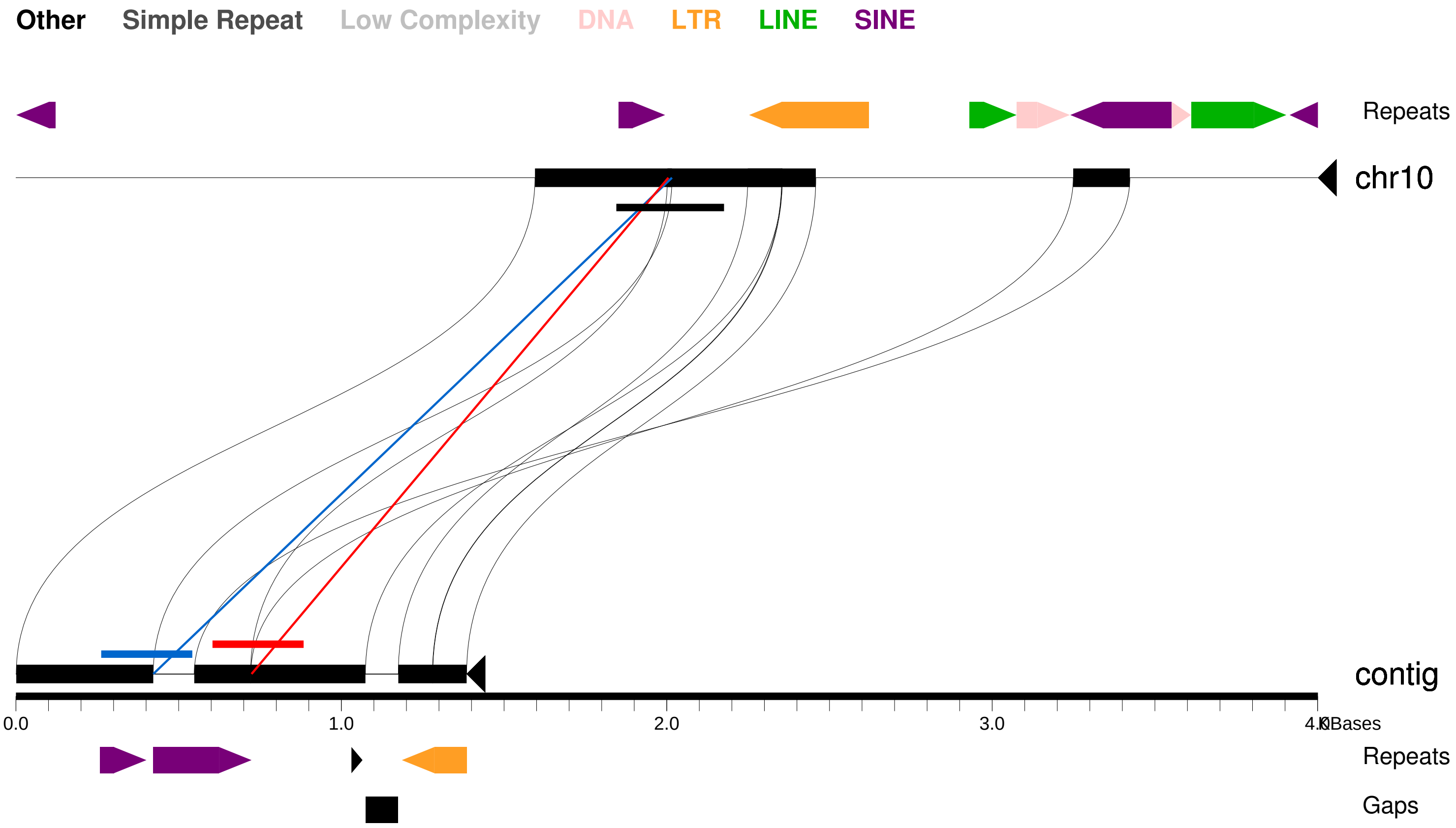

siteID: chr10_77229049 Scafftig1 - Breakpoint: chr10:77229053
chr10:77229010-77229112
Overlap Length: 11 INS size: 452 Genotyped: No

```
left     TGAGAGGAAAATGGCTTTTATAGAGAGCATGTTGCAATAAAGA----------------AACAGTGAGTC
chrom    TGAGAGGAAAATGGCTTTTATAGAGAGCATGTTGCAATAAAGA----------------AACAGTGAGTC
right    NNNNNNNNNNNNNNNNNNNNNNNNNNNNNNNNNNNNNNNNNNNNNNNNNNNNNNNNNNNAACAGTGAGTC
         11111111111111111111111111111111111111111111111111111111111***********

left     GCCGGGCGCGGTGGCTCACGCCT-GTAATCCCAGCACTTTGGGAGGCCGAGGCGGGTGGATCATGA
chrom    ACTGGG---GGAGCATCACCAATTGAAATGCCTTCAGTTATT--------------TGCCCTAACT
right    ACTGGG---GGAGCATCACCAATTGAAATGCCTTCAGTTATT--------------TGCCCTAACT
         2*2***222**2*22****222*2*2***2**22**2**22222222222222222**2222*222
```

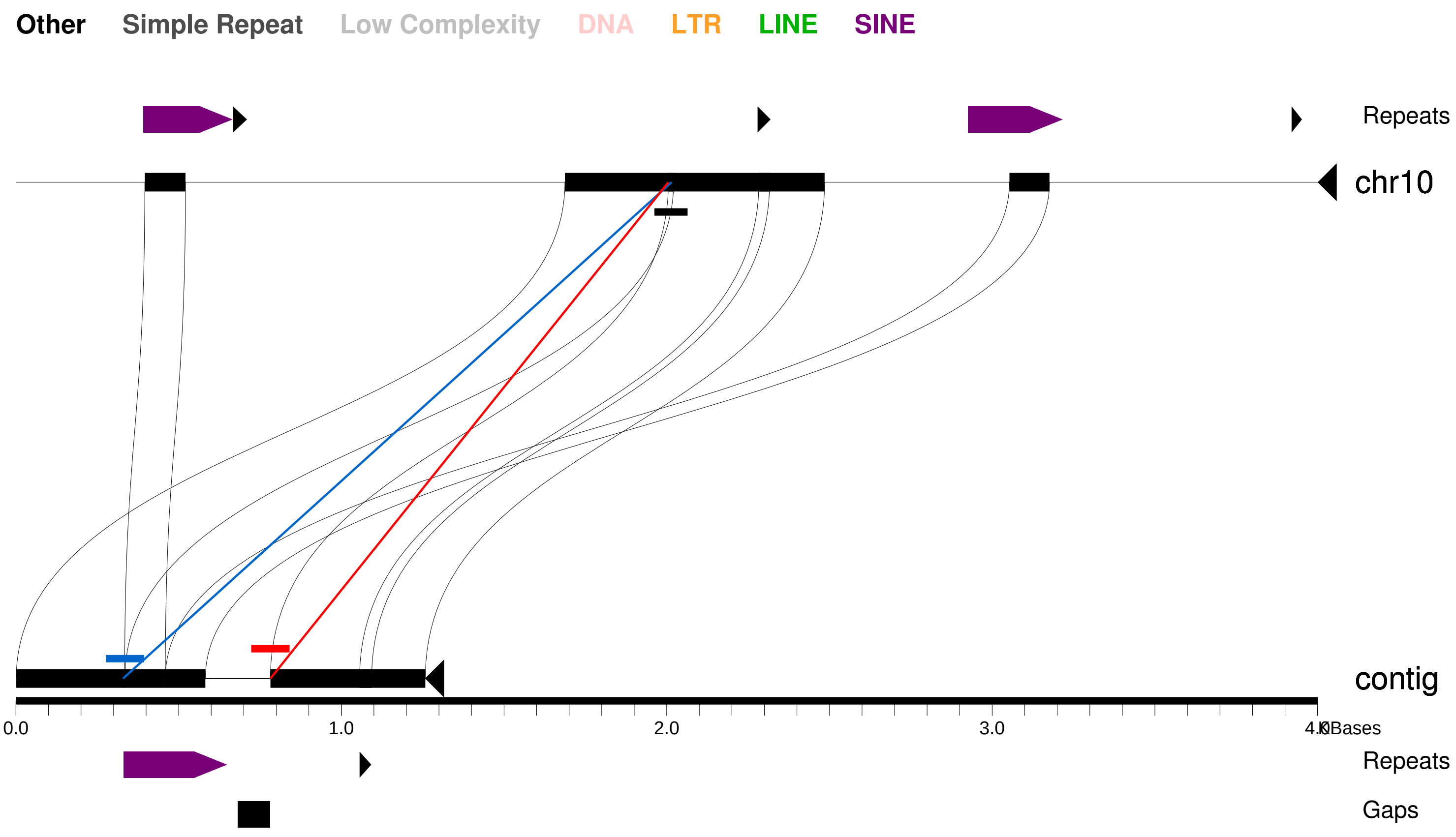

siteID: chr10_81651818 Scafftig1 - Breakpoint: chr10:81651943
chr10:81651892-81651999
Overlap Length: 9 INS size: 322 Genotyped: No

```
left   ATCTAAAGTCAGTCATGTAGAGATATGTACATTCCCTGAGCATTGTTT-----------AAGAGTCCTTG
chrom  ATCTAAAGTCAGTCATGTAGAGATATGTACATTCTCTGAGCATTGTTT-----------AAGAGTCCTTG
right  NNNNNNNNNNNNNNNNNNNNNNNNNNNNNNNNNNNNNNNNNNNNNNNNNNNNNNNNAAAAACAGTCCTTG
       111111111111111111111111111111111N1111111111111111111111111**1********

left   GAGGCCGAGGCGGGCGGATCACGAGGTCAGGAGATCGAGAC--CATCCCGGCTAAAACGGTG---
chrom  GTTTTCCAGCTT---TGAT----------GGAGATCTAGAAGGCGTTCAG---AAGTCTGTGTGC
right  GTTTTCCAGCTT---TGAT----------GGAGATCTAGAAGGCGTTCAG---AAGTCTGTGTGC
       *2222*2**2222222***2222222222*******2***222*2*2*2*222**22*2***222
```

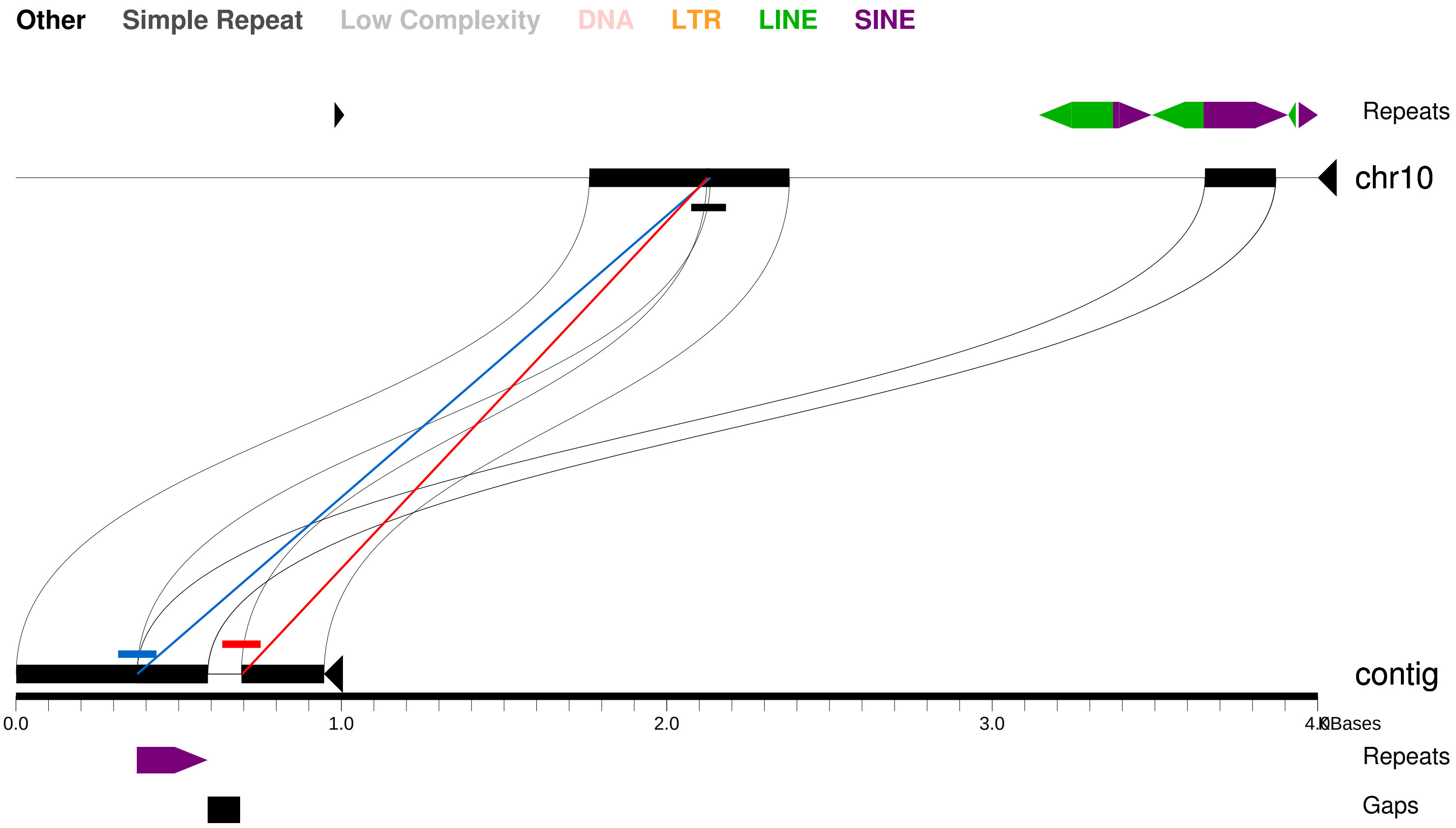

siteID: chr10_83333145 Scafftig1 - Breakpoint: chr10:83333091
chr10:83333044-83333150
Overlap Length: 13 INS size: 300 Genotyped: Yes

```
left   TCT----CATCATCAAGTCAAACAC----AAAAACAGCTTCTAATCAACATTTTA----GCAACTCACTC
chrom  TCT----CATCATCAAGTCAAACAC----AAAAACAGCTTCTAATCAACATTTTA----GCAACTCACTC
right  CCACCCGCCTCGGCCTCCCAAAGTGCTGGGATTACAGGCGTGAGCCACCGCGCCCGGCCGCAACTCACTC
       1*11111*1**11*1111****11111111*11****11111*11**1*1111111111***********

left   TTTTTTTTTTTTTTGAGACGGAGTCTCGCTCTGTCGCCCAGGCCGGACTGCGGACTGCAGT
chrom  TTAAAGCTGT-------ACGATTAAATGCAATGG----AATATCTGATGAAAGCCTACAA-
right  TTAAAGCTGT-------ACGATTAAATGCAATGG----AATATCTGATGAAAGCCTACAA-
       **22222*2*2222222***2222222**22**222222*222*2**22222*2**2**22
```

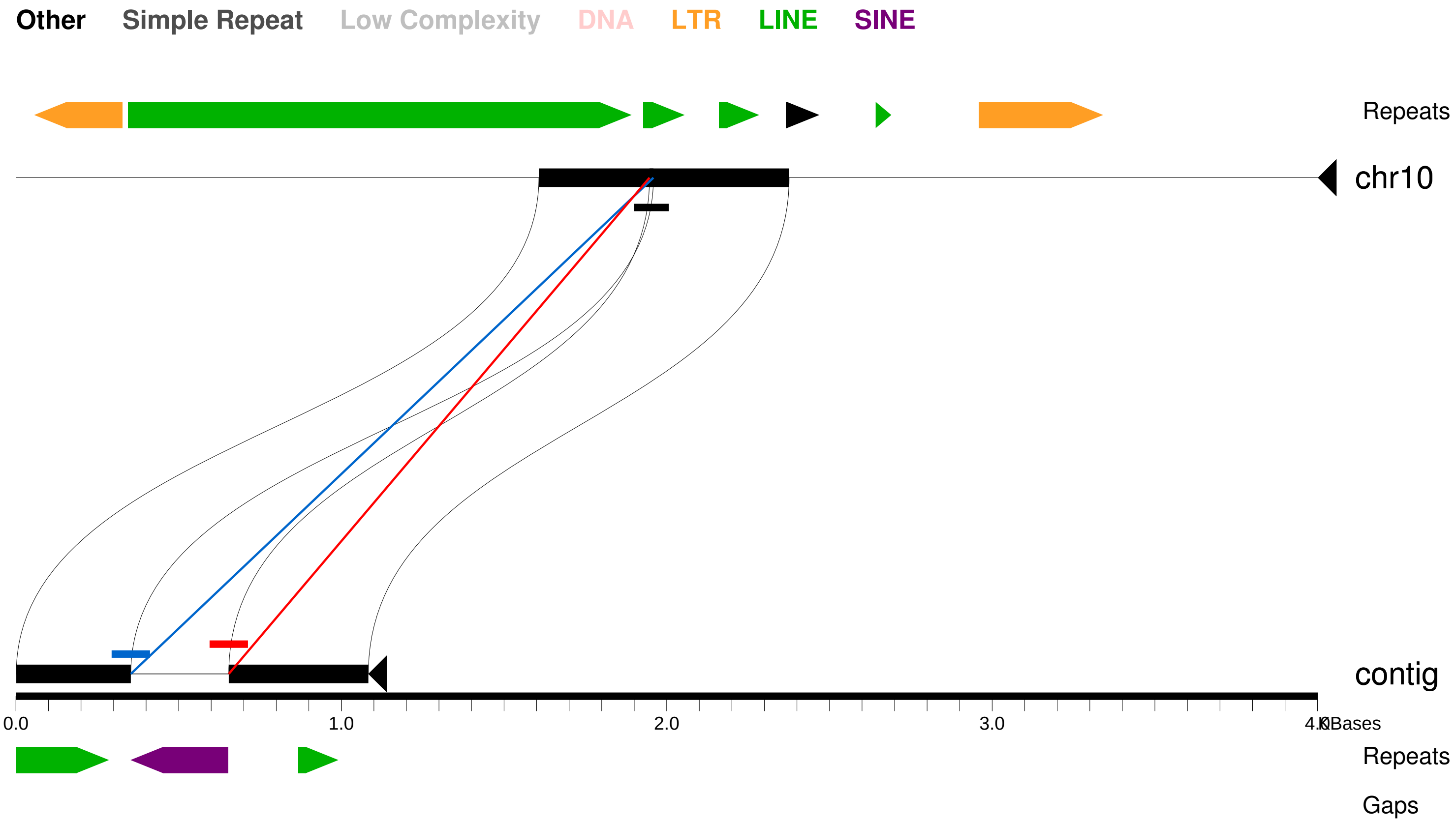

siteID: chr10_85983262 Scafftig3 + Breakpoint: chr10:85983244
chr10:85983200-85983303
Overlap Length: 15 INS size: 293 Genotyped: Yes

```
left    A--CCAATAAGGTATTATCAAAA------CAATACAGAA-TGTGAAAATTCAG------TTAATTGGGTA
chrom   A--CCAATAAGGTATTATCAAAA------CAATACAGAA-TGTGAAAATTCAG------TTAATTGGGTA
right   CCGCCCGCCTCGGCCTCCCAAAGTGCTGGGATTACAGGCGTGAGCCACCGCGCCCGGCCTTAATTGGGTA
        111**111111*111*11****11111111*1*****111**1*11*111*11111111***********

left    TTTTTTTTTTTTTTTTGAGACGGAGTCTCGCTCTGTCGCCCAGGCTGGAGTGCAG-TGGCGGGAT
chrom   TTTT-------TAAACCATCCAGAGTTT-----TGTC----ATAATGTATAACAAATACCTGGGC
right   TTTT-------TAAACCATCCAGAGTTT-----TGTC----ATAATGTATAACAAATACCTGGGC
        ****2222222*22222*22*2****2*22222****2222*222**2*222**22*22*2**22
```

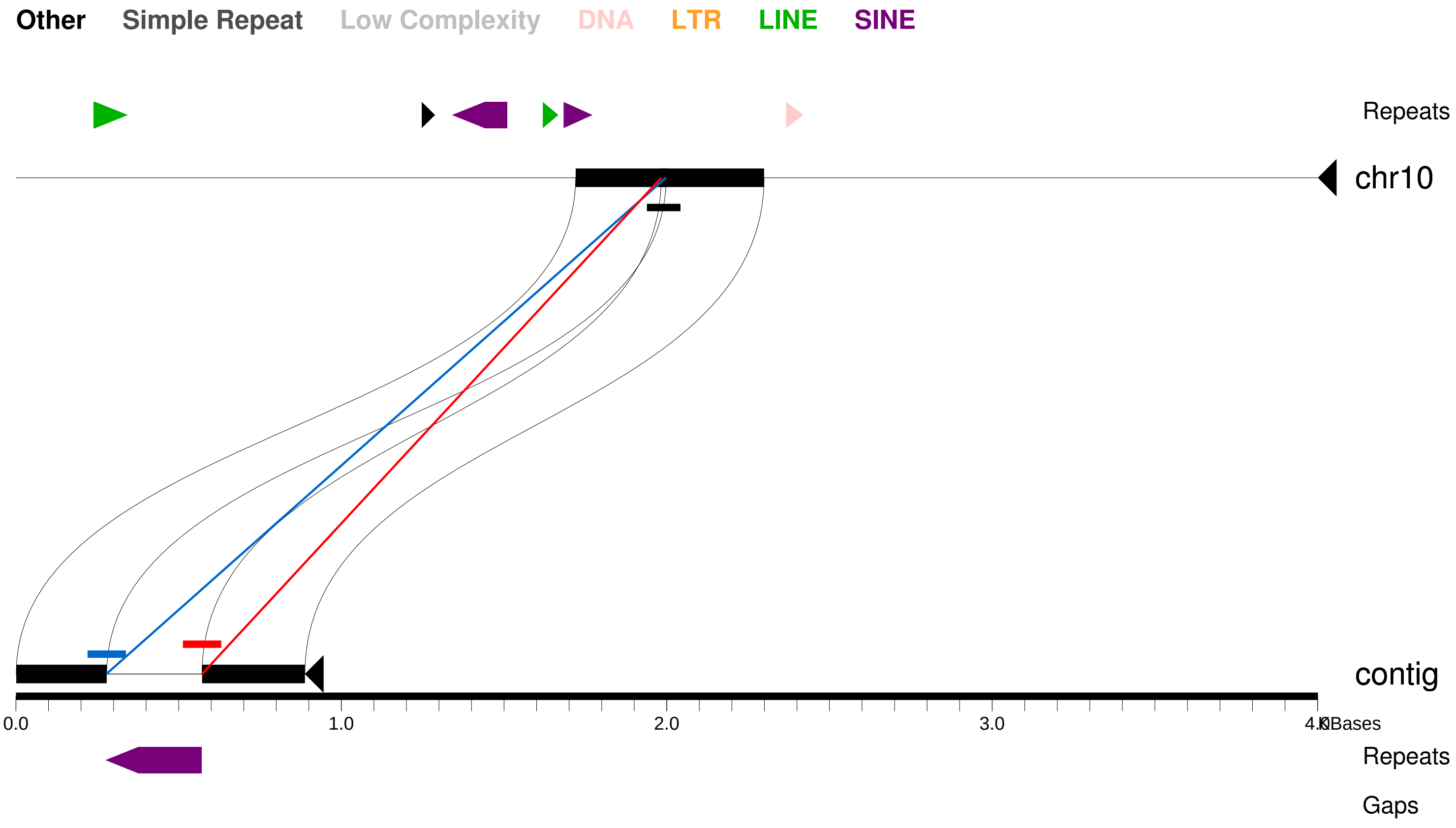

siteID: chr10_87963313 Scafftig3 + Breakpoint: chr10:87963292
chr10:87963247-87963351
Overlap Length: 15 INS size: 293 Genotyped: Yes

```
left    -------GCTGGGCATGAGGGGAAAGC-------CAGGACGAGCACTGGGCACCGGGTTAAAATTTTACA
chrom   -------GCTGGGCATGAGGGGAAAGC-------CAGGACGAGCACTGGGCACCGGGTTAAAATTTTACA
right   ACTCCAGCCTGGGCGACAGAGCGAGACTCCGTCTCAAAAAAAAAAAAAAAAAAAAAAAAAAAATTTTACA
        11111111*****111**1*11*11*1111111**11*11*11*111111*1111111***********

left    GAAACTCACGCCTGTAATCCCAGCACTTTGGGAGGCCGAGGCGGGTGGATCACGAGGTCAGGA
chrom   GAAAATG-----TGTAGAGGCAG---------AAACCAAGCCTCAATGCTTGTCATATCAGGA
right   GAAAATG-----TGTAGAGGCAG---------AAACCAAGCCTCAATGCTTGTCATATCAGGA
        ****2*222222****2222***222222222*22**2**2*22222*2*2222*22******
```

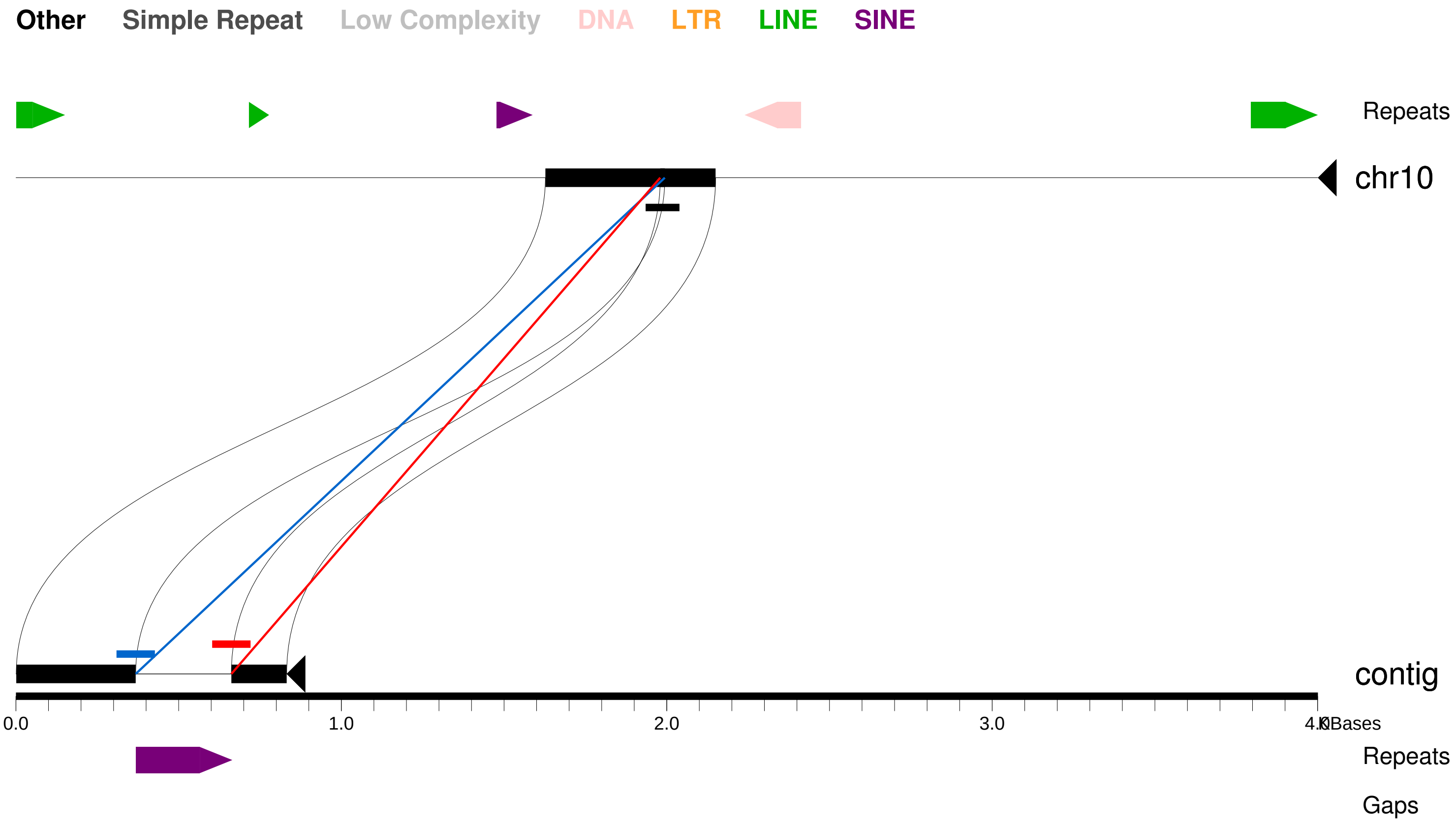

siteID: chr10_89814968 Scafftig16 - Breakpoint: chr10:89814961
chr10:89814384-89815121
Overlap Length: 16 INS size: 326 Genotyped: Yes

```
left   ---------------------------------------------------------------------
chrom  CAGTTTTCTAATCTGTAAAGTCAATGCTCGCTCCATTGGATCCATGTGAGAACTAAATAAGACAAAGCTT
right  -AG--------------------AATG-------------------GCGTGAAC-----------------
        22                    2222                   2N2N2222

left   ---------------------------------------------------------------------
chrom  TTATAATAGTATCTCCTGTAGAGCAAACCCTCAAAATAGTTGAGTTACAGTTAGTATTATGCTTTTTACT
right  --------------CCGGGAG-GCGGAGC---------------TTGCAGTGAG--------------CC
                     22N2N22 22NN2N2                22N2222N22              2N

left   ---------------------------------------------------------------------
chrom  GTTATTCATTTTATAGCATAGCCTCATGAATCTGATTACCTAGGTTCAAACCCAAGCTCTGGGTGAAATG
right  GAGATCCC------------GCCAC-------------------TGCACTCC--AGC-CTGGGCGACAGA
       2NN22N2N            222N2                   2N22NN22  222 22222N22N2NN

left   ---------------------------------------------------------------------
chrom  GAAAAAAGTTACCTAATGTCATTAAGCCTCAGTTTCTCATCTGTAAAATGGGGAAATAATAGACAATAAT
right  GCGAGA---------------------CTCCGT--CTCA-----AAAA-----AAAAAAAAAAAAAAAAA
       2NN2N2                     222N22  2222     2222     222N22N2N2N22N22N

left   ---------------------------------------------------------------------
chrom  AAAAAAAAAAAGGGCCTATTGCATAGGCTTGTGTGTAGACGGGATGAAGGAATGCATACCAAACATGTAG
right  AAAAAAAAAAA-------------------------------------------------AAA-------
       22222222222                                                 222

left   ---------------------------------------------------------------------
chrom  CTCAGATCCTGGCTCACAGTAAGAAGTCGGGAAATGTAAGCTACGGCTCTATTCCTTGTTGTGTGCTAGG
right  --------------------AAAAA------AAA------------------------------------
                           22N22      222

left   ---------------GATACAATTTCATTTCCCCTGCATCCAATAGAGTGGGTTATATGATCCCCATTTT
chrom  CACTTTTGTGTATGAGATACAATTTCATTTCCCCTGCATCCAATAGAGTGGGTTATATGATCCCCATTTT
right  ---------------------------------------------------------------------
                      1111111111111111111111111111111111111111111111111111111

left   ATAGATGAGGAGATTGAAGCATGGGGAGGTTAAGCAAATTTGTTCAAGGTCCACAGCCATAGATGATCTG
chrom  ATAGATGAGGAGATTGAAGCATGGGGAGGTTAAGCAA-TTTGTTCAAGGTCCGCAGCCATAGATGATCTG
right  ---------------------------------------------------------------------
       11111111111111111111111111111111111112111111111111111N1111111111111111

left   GTATAAGTTATTCCATTTAGAATGACTTGATACT---AGGCCGGGCACGGTGGCTC----ACGCCTGTAA
chrom  GTATAAGTTATTCCATTTAGAATGACTTGATACTTTTAGAATCTTTAGGAAAGTTCCTGTAGGCCCATAG
right  ------------------AGAATGACCTGATACTTTTAGAATCTTTAGGAAAGTTCCTGTAGGCCCATAG
       111111111111111111********1*******222**2222222*2*222*2**2222*2***22**2

left   TCCCAGGACTTT--GGCAGGCTGAGG-CGGCCGGATCACAAG--GTCAGGAGATCGAGACCATCCCG
chrom  GAAAAGGAGTTTATAGTTACACCTAGTTCATGACTATATTAATTTATGTCAGAAGGGAATTAGAATCATC
right  GAAAAGGAGTTTATAGTTACACCTAGTTCATGACTATATTAATTTATGTCAGAAGGGAATTAGAATCATC
       2222**2*2***222*22*22*22**22*22*222**222**22222*****2**222222*22***222

left   GCTAAAAC[illegible]-GGTGAAACCCCGTCTCT--ACTAAA-
chrom  ATCAAAAGTTAGAGTTGGAAGTAACTTTATTGAGTATTT
right  ATCAAAAGTTAGAGTTGGAAGTAA--
       222****222222*2**2**2222N1N1N1  1N11NN
```

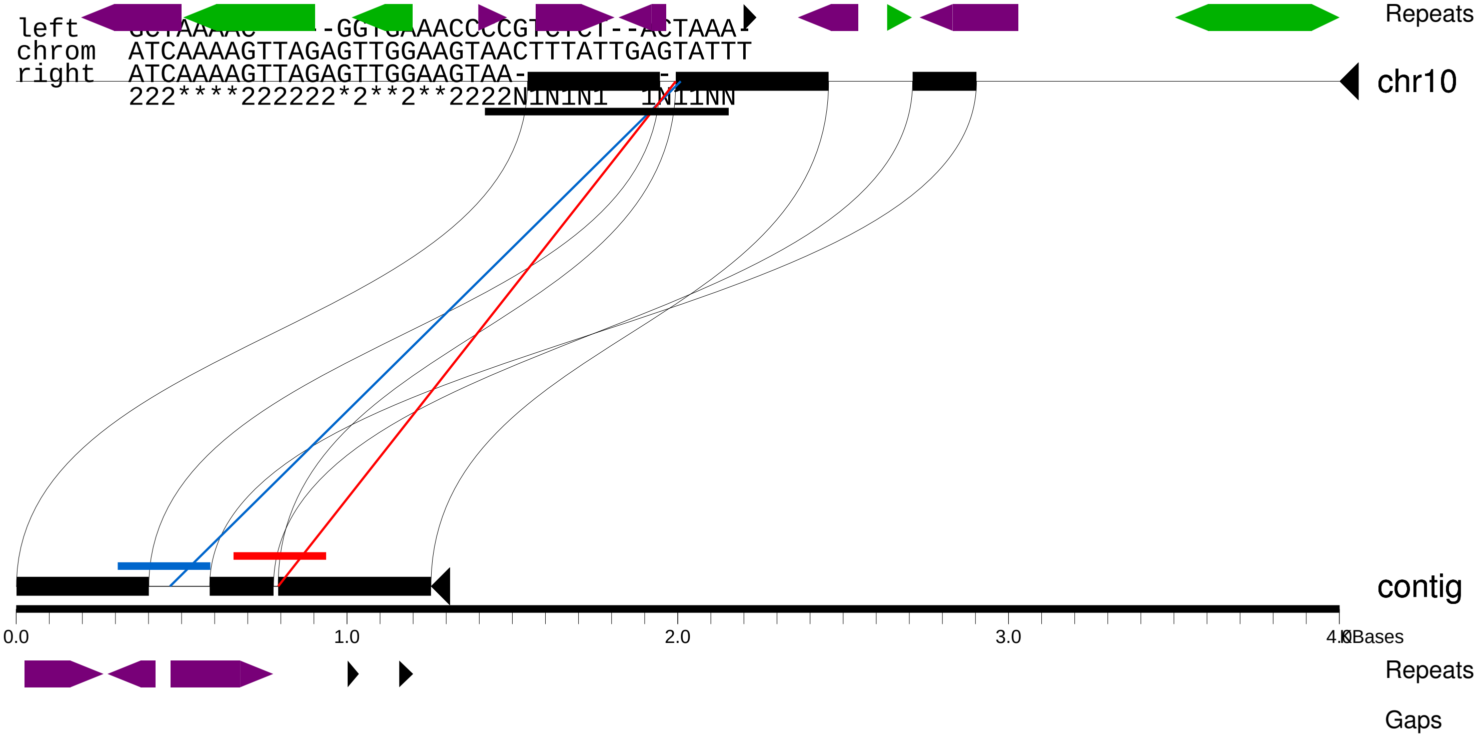

siteID: chr10_92215071 Scafftig6 + Breakpoint: chr10:92215057
chr10:92215013-92215116
Overlap Length: 16 INS size: 297 Genotyped: Yes

```
left    GTTGGCTT-----TGCGTATGTCTTCTTTTGAAAAGTGT------CTGTTCATGT----CCTTTGTCCAT
chrom   GTTGGCTT-----TGCGTATGTCTTCTTTTGAAAAGTGT------CTGTTCATGT----CCTTTGTCCAT
right   GCCCGCCTCGGCCTCCCAAAGTGCTGGGATTACAGGCGTGAGCCACCGCGCCCGGCCTACCTTTGTCCAT
        *111**1*11111*1*11*1**11*1111*1*1*1*1**111111*1*11*11*11111***********

left    TTTTTTTTTTTTTTTTTTTGAGACGGAGTCTCGCTCTGTCGCCCAGGCTGGAGTGCAGTGGCGC
chrom   TTTTTAATGGGATTGTTTGGT-------TTTTGCT-TGTT----AATCTG--TTGAAGTTTCA-
right   TTTTTAATGGGATTGTTTGGT-------TTTTGCT-TGTT----AATCTG--TTGAAGTTTCA-
        *****22*2222**2***2*22222222*2*2***2***22222*22***222**2***22*22
```

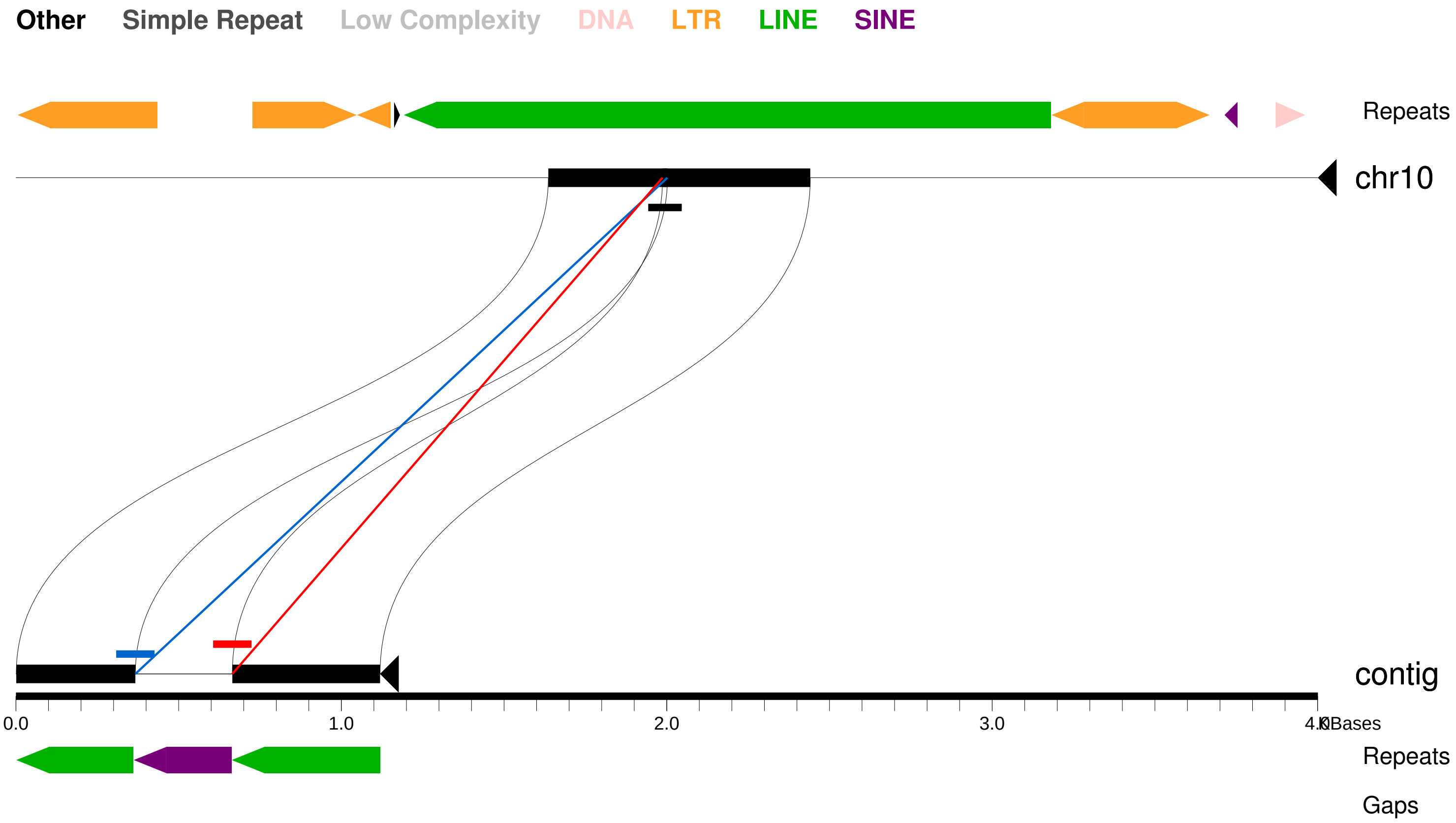

siteID: chr10_92227504 Scafftig1 + Breakpoint: chr10:92227498
chr10:92227372-92227660
Overlap Length: 3 INS size: 315 Genotyped: No

```
left   NNNNNNNNNNNNNNNNNNNN---------------------------------------------GGAT
chrom  ACAACTTTATTCAGATTTTATTCATTTTTACCTA-ATGCCCCCTTCCTATTCCAGGATCCCATTCAGGAT
right  TTTAGTAGAGACGGGGTTTCACCGTTTTAGCCGGGATGGTCTCGATCTCCT----GACCTCGT----GAT
       NNN2N2NN2NN2N2NN222NNN2N2222NN22NN1222NN2N2NNN22NN2     22N2N2N2    1***

left   ACTACATTAGCCTTTTT-TTTCATCCTCTTTAGTTTTCTCTTGGCTTGGACAATTTTC----CCCCTTTT
chrom  ACTACATTAGACTTAGT-TGTCATCCTCATTAGTTTACTCTTGGCTGTGACAATTTTC----CCCACTTT
right  CCGCCC---GCCTCGGCCTCCCAAAGTGCTGGGATTACAGGCGTGAGCCACCGCGCCCGGCCCCCACTTT
       1*11*1111*N**1N211*N1**111*1N*11*1**2*1111*1112N1**111111*1111***22***

left   TTTTTTTTTTTTTTTTTTTTTTTTTTTTTTGAGACGGAGTCTCGCTCTGTCGCCCAGGCTGGAGTGCAGTG
chrom  GTTTTTAATTGCATTGACAGTTTT-----GAGAAATACTA-------GTC----AGG-TATCTTGCAG--
right  GTTTTTAATTGCATTGACAGTTTT-----GAGAAATACTA-------GTC----AGG-TATCTTGCAG--
       2*****22**222**22222****22222****222*2*22222222***2222***2*2222*****22

left   GCGCAATCTCGGCTCACTGCAAGCTCCGCCTCCCGGGTTCACGCCATTCTCCTGCC--TCAGCCTCCCAA
chrom  ----AATGT---CTCCCTACC--CTCCCACTCCC----TCATTGAAATTGCCTGATATTTCTCTTATTAT
right  ----AATGT---CTCCCTACC--CTCCCACTCCC----TCATTGAAATTGCCTGATATTTCTCTTATTAT
       2222***2*222***2**2*222****22*****2222***2222*2*22****2222*222*2*222*2

left   GTAGCTGGGACTACAGGCGCCCGCCACTACGCCCGGCTAATTTTTTGTATTTTT
chrom  TCAAATAGGATTACCAGTTTTGGGGAGGAAGATCA-CAAA------GTGACATT
right  TCAAATAGGATTACCAGTTTTGGGGAGGAAGATCA-CAAA------GTGAC---
       22*22*2***2***22*22222*22*22*2*22*22*2**222222**222N11
```

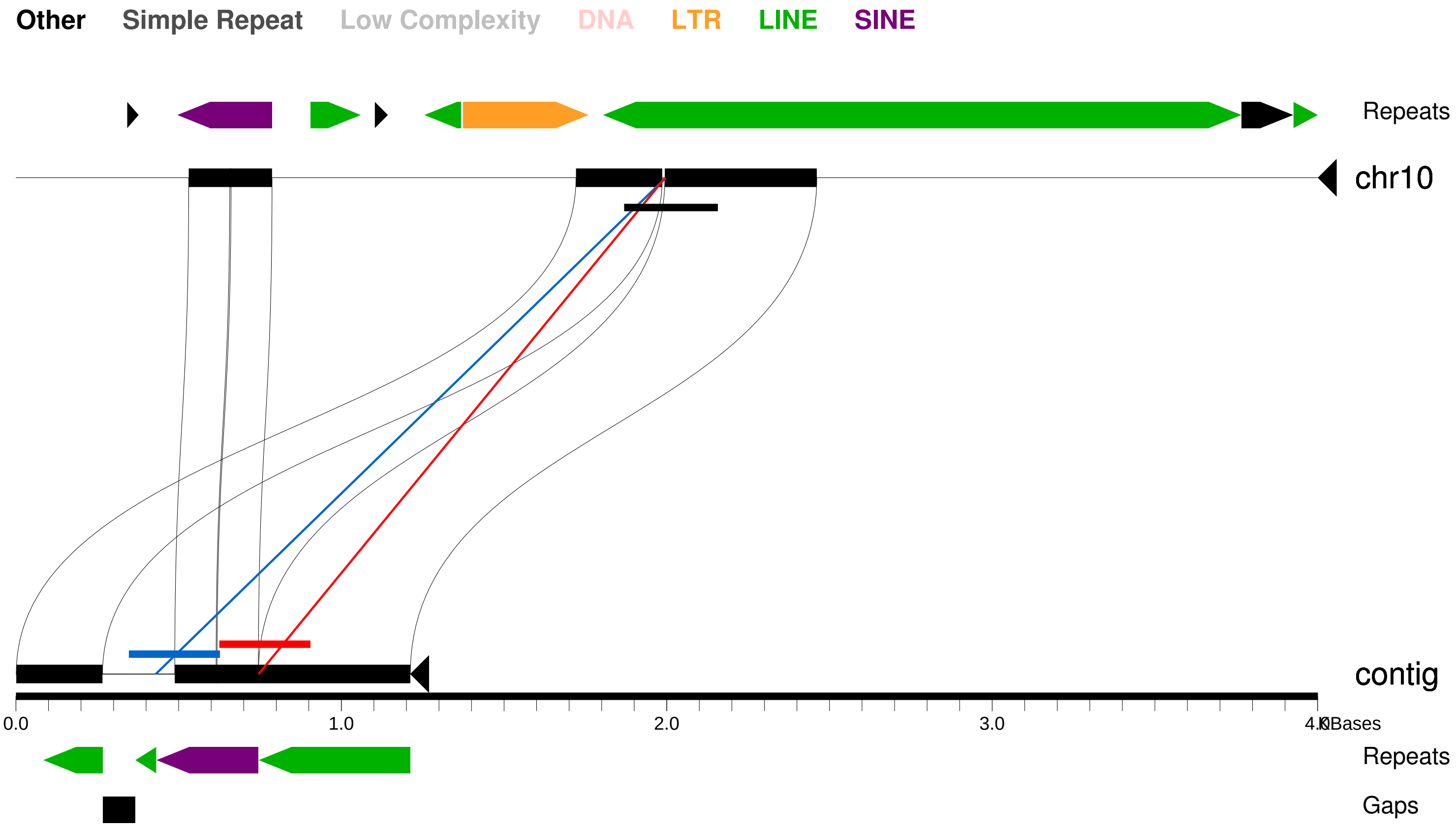

siteID: chr10_9474864 Scafftig5 + Breakpoint: chr10:9474847
chr10:9474825-9474906
Overlap Length: 9 INS size: 307 Genotyped: Yes

```
left    CTG-----------TGACAAATTAGGCAAATTT--------------------------TATTTTTTTTT
chrom   CTG-----------TGACAAATTAGGCAAATTT--------------------------TATTTTTTTAA
right   CGCCCGCCTCGGCCTCCCAAAGTGCTGGGATTACAGGCGTGAGCCACCGCGCCCGGCCCTATTTTTTTAA
        *1111111111111*11****1*111111***111111111111111111111111111*********22

left    TTTTTTATTTTTTTTTTTTTTTTGAGACGGAGTCTCGCTCTGTCGCCCAGGCTGGAGTGCAGTGGCGGGA
chrom   TTCTAATAATGGTTTTACTATTTTAGA-------TAACTTAATGGTTTAGTCT-----------------
right   TTCTAATAATGGTTTTACTATTTTAGA-------TAACTTAATGGTTTAGTCT-----------------
        **2*22222*22****22*2***2***2222222*22**222*2*222**2**22222222222222222

left    TCTCGGCTCACTGCAA
chrom   TTT-------------
right   TTT-------------
        *2*2222222222222
```

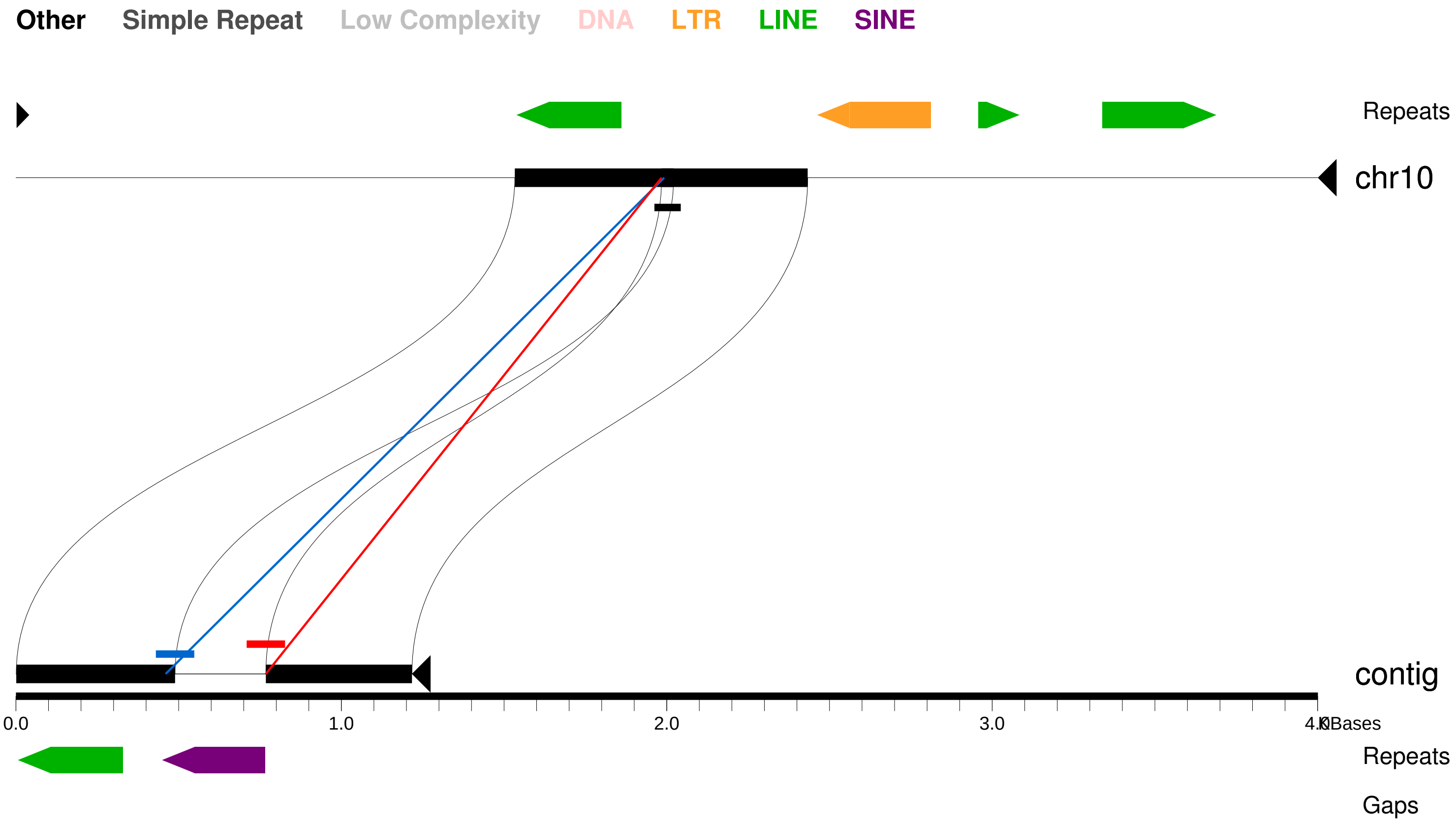

siteID: chr10_9510979 Scafftig4 + Breakpoint: chr10:9510982
chr10:9510822-9511154
Overlap Length: 13 INS size: 301 Genotyped: Yes

```
left   T------ATTCACACACTAAATATCTGTTTACAATTCACATGCAGTGTGTACTGACCACACATTGATTTT
chrom  TGACAATATTCACACACTAAATATCTGTTTACAATTCACATGCAGTGTGTACTGACCACACATTGATTTT
right  T-------TT-----AGTAGAGACGGGGTTTCA---CCGTTTTAGCCGGGATGGTC------TCGATCT-
       *      1**11111*1**1*1*111*1**1**111*111*11**111*1*11*1*111111*1***1*1

left   TCCTAATACATTTTGAACAAATATAAGTGAATTCCTATTTGAATATATACATATAAAATAAAATATGAAG
chrom  TCCTAATACATTTTGAACAAATATAAGTGAATTCCTATTTGAATATATACATATAAAATAAAATATGAAG
right  -CCTGA--CCTCGTGATCCGCCCGCCTCGGCCTCCCA-------AAGTGC---TGGGATTACAGGCGTGA
       1***1*11*1*11***1*1111111111*111***1*1111111*11*1*111*111**1*1*111*111

left   GCCCTTGATGGCATACGAGGTGCAAATTTTTTT--------TTTTTTTTTTTTTTTTTTTGA------GA
chrom  GCCCTTGATAGCATACGAGGTGCAAATTTTTTTAAGTGGAGTATTTTATGTTTGAATATAGAACTAAAGT
right  GCC----ACCGCGCCCGGCCTGCAAATTTTTTTAAGTGGAGTATTTTATGTTTGAATATAGAACTAAAGT
       ***1111*1N**111**111*************22222222*2****2*2***222*2*2**222222*2

left   CGGAGTCTC-------GCTCTGTCGCCCAGG----------CTGGAGTGCAGTGGCGGGATCTCGGCTCA
chrom  AGAAGTTTCAGGGTTAGTTTTTTAAAAAAGTAAAATTTTAACTGAAAGTCAGCCATTTTCTTTAAAAACA
right  AGAAGTTTCAGGGTTAGTTTTTTAAAAAAGTAAAATTTTAACTGAAAGTCAGCCATTTTCTTTAAAAACA
       2*2***2**2222222*2*2*2*22222**22222222222***2*222***22222222*2*22222**

left   CTGCAAGCTCT----GCCTCCCGGGTTCACGCCATT--CTCCTG----------
chrom  TTTCAGTTTTTAAGAGATTCAATTGTTTTTGA-ATTAGCTGATGAAAAATGCCA
right  TTTCAGTTTTTAAGAGATTCAATTGTTTTTGA-ATTAGCTG-------------
       2*2**222*2*2222*22**2222***222*22***22**2N11
```

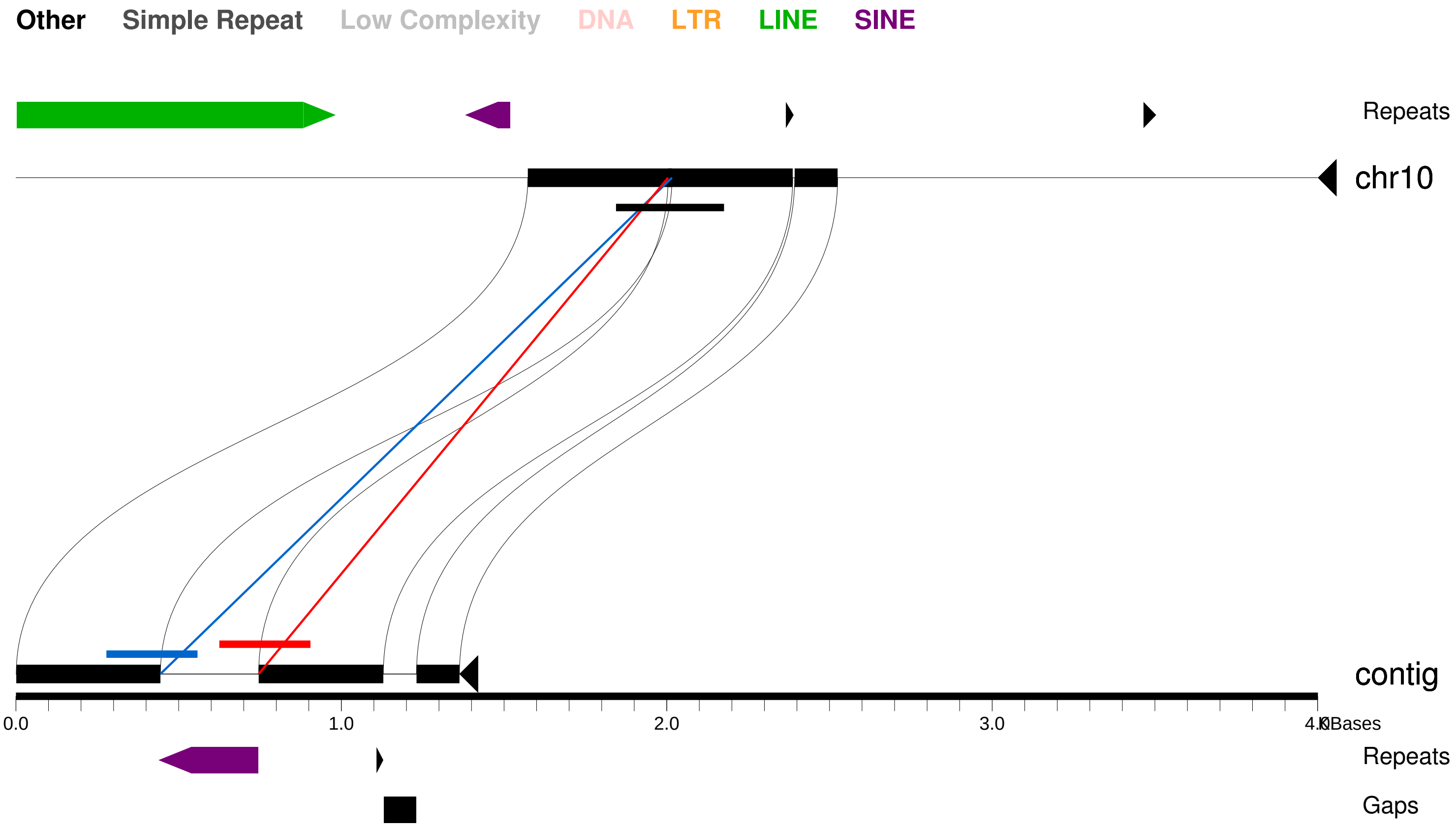

siteID: chr10_96763985 Scafftig3 + Breakpoint: chr10:96763973
chr10:96763928-96764032
Overlap Length: 15 INS size: 294 Genotyped: Yes

```
left     TATTTTTTTCCATCAGTTATTGGGGTAC--AAGTGGT---ATT-------TG-GTTAC--ATGAATAAGT
chrom    TATTTTTTTCCATCAGTTATTGGGGTAC--AAGTGGT---ATT-------TG-GTTAC--ATGAATAAGT
right    CCTCGTGATCCGCCCGCC-TCGGCCTCCCAAAGTGCTGGGATTACAGGCGTGAGCCACCGATGAATAAGT
         11*11*11***11*1*111*1**11*1*11*****1*111***1111111**1*11**11**********

left     TCTTTTTTTTTTTTTTTTTTTTTTTTTGAGACGGAGTCTCGCTCTGTCGCCCAGGCTGGAGTGC
chrom    TCTTTAGTGGTGATTTGTGAGATTTTGGTGCA----------TCCATCACCTGAGCAATA----
right    TCTTTAGTGGTGATTTGTGAGATTTTGGTGCA----------TCCATCACCTGAGCAATA----
         *****22*22*22***2*2222****2*2*222222222222**22**2**222**222*2222
```

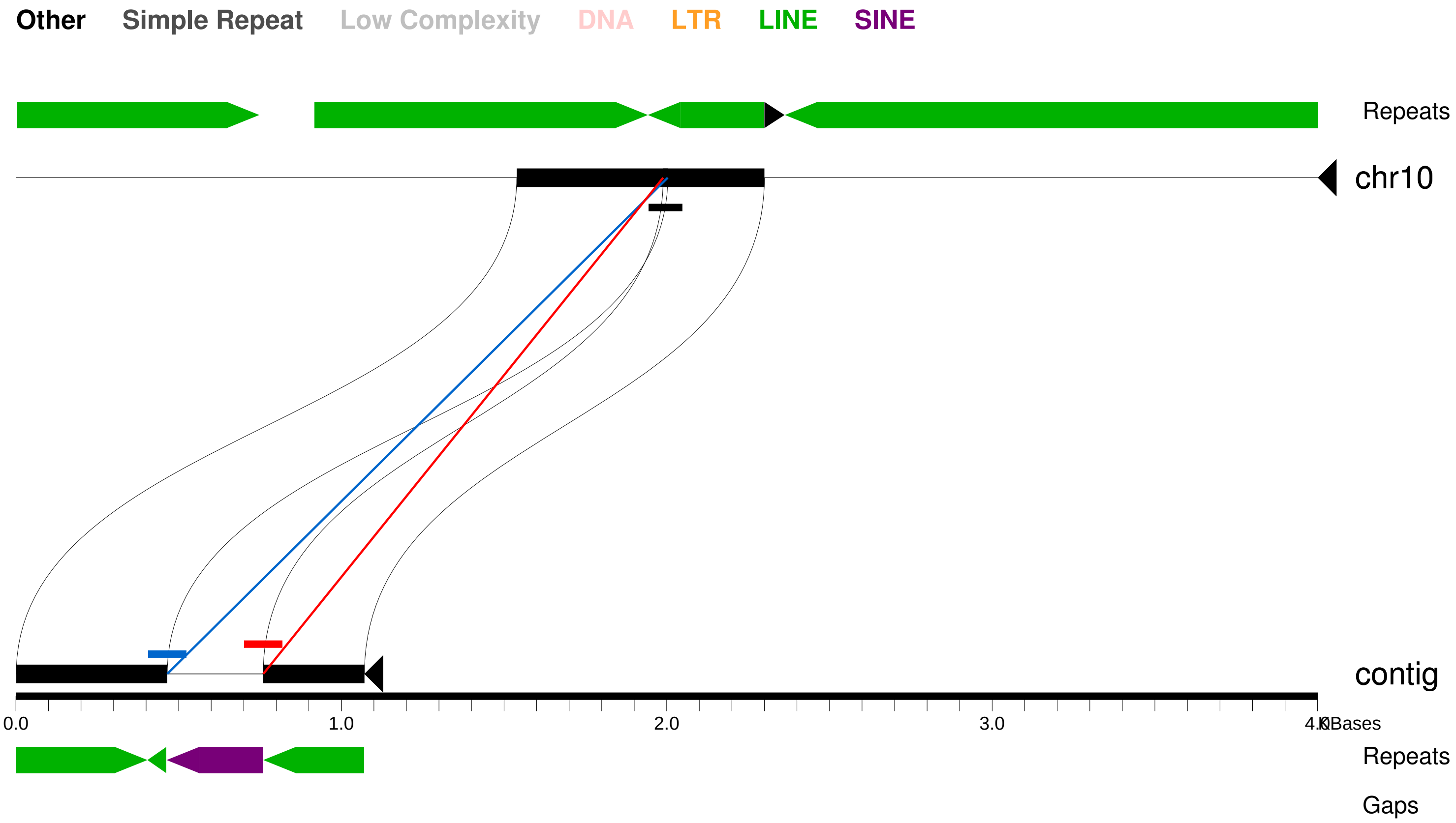

siteID: chr10_9904419 Scafftig1 + Breakpoint: chr10:9904393
chr10:9904348-9904452
Overlap Length: 15 INS size: 291 Genotyped: Yes

```
left     ATGTCTTTCAT-------CAAAGACATGTTATCTA--------ATCTAAGTACTTTTGTAGACTTTTAAC
chrom    ATGTCTTTCAT-------CAAAGACATGTTATCTA--------ATCTAAGTACTTTTGTAGACTTTTAAC
right    CCGCCCGCCTCGGCCTCCCAAAGTGCTGGGA-CTACAGGCGTGAGCCACCGCGCCCGGCCGACTTTTAAC
         11*1*111*111111111*****111**11*1***11111111*1*1*111111111*11**********

left     TTTTTTTTTTTTTTTGAGACGGAGTCTCGCTCTGTCGCCCAGGCTGGAGTGCAGTGGCGTGATC
chrom    TTTTTAACCTAGCAAAATA-GAAGAAAAGTTTTTTAATAAAAAAAGAA-------------AAT
right    TTTTTAACCTAGCAAAATA-GAAGAAAAGTTTTTTAATAAAAAAAGAA-------------AAT
         *****2222*222222*2*2*2**2222*2*2*2*22222*2222*2*2222222222222*22
```

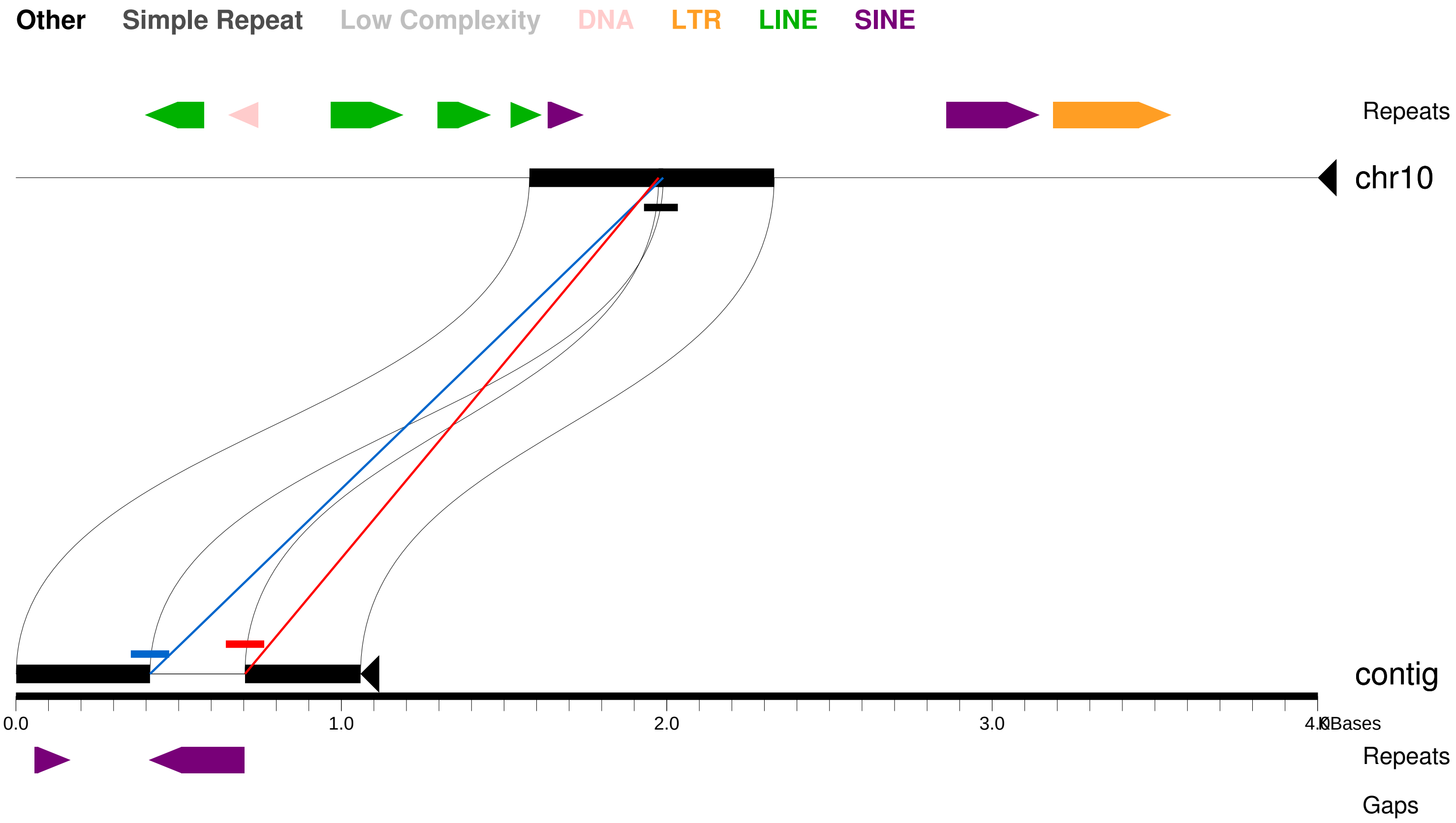

siteID: chr11_100781368 Scafftig1 + Breakpoint: chr11:100781380
chr11:100781334-100781439
Overlap Length: 14 INS size: 422 Genotyped: No

```
left     ACACAAGAGACAAGAGGTAGCTATATGGGGTTACATTACTAAGTCC-------------AAATAAGACTT
chrom    ACACAAGAGACAAGAGGTAGCTATATGGGGTTACATTACTAAGTCC-------------AAATAAGACTT
right    NNNNNNNNNNNNNNNNNNNNNNNNNNNNNNNNNNNNNNNNNNNNNNNNNNNNNNNNAAAAAAAAAAGACTT
         11111111111111111111111111111111111111111111111111111111111***1*******

left     GGCGGCCGGGCGCGGTGGCTCACGCCTGTAATCCCAGCACTTTGGGAGGCCGAGGCGGGTGG--
chrom    GGCTTACATGAGCAGTAAATCATGGCT--------------TTTGGAGGA-GATGTGAGTTGCA
right    GGCTTACATGAGCAGTAAATCATGGCT--------------TTTGGAGGA-GATGTGAGTTGCA
         ***222*22*2**2**222***2*2**22222222222222**2*****22**2*2*2**2*22
```

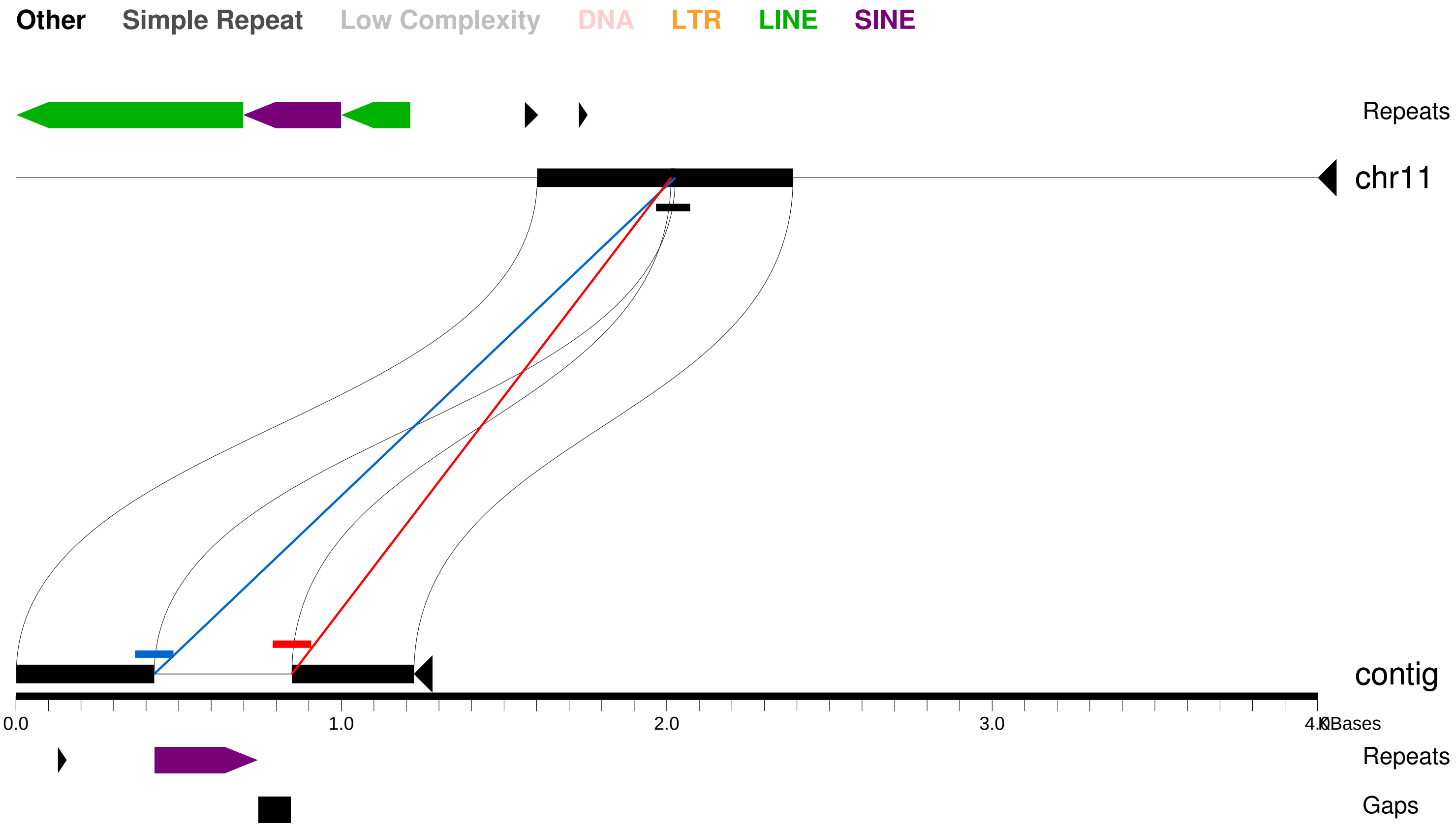

siteID: chr11_101303519 Scafftig1 - Breakpoint: chr11:101303558
chr11:101303499-101303617
Overlap Length: 1 INS size: 467 Genotyped: No

```
left    GAAGTCTGTGTTTAAAAATACATATGTA-TAGGAACACA----TAAAACATTTTGACTTTAAGACNNNNN
chrom   GAAGTCTGTGTTTAAAAATACATATGTA-TAGGAACACA----TAAAACATTTTGACTTTAAGACTTCTC
right   TCCACCCGCCTCGGCC--TCCCAAAGTGCTGGGATTACAGGCGTGAGCCACCGCGCCCG---GCCTTCTC
        11111*1*11*1111111*1*11*1**11*1***11***1111*1*11**1111*1*11111*1*22222

left    NNNNNNNNNNNNNNNNNNNNNNNNNNNNNNNNNNNNNNNNNNNNNNNNNNNNN
chrom   AGGGTCTTTAAGGTGATCAAGAGTGTGGTGAATCTTCAAGAGGGAGATAAAGTA
right   AGGGTCTTTAAGGTGATCAAGAGTGTGGTGAATCTTCAAGAGGGAGATAAAGTA
        22222222222222222222222222222222222222222222222222222222
```

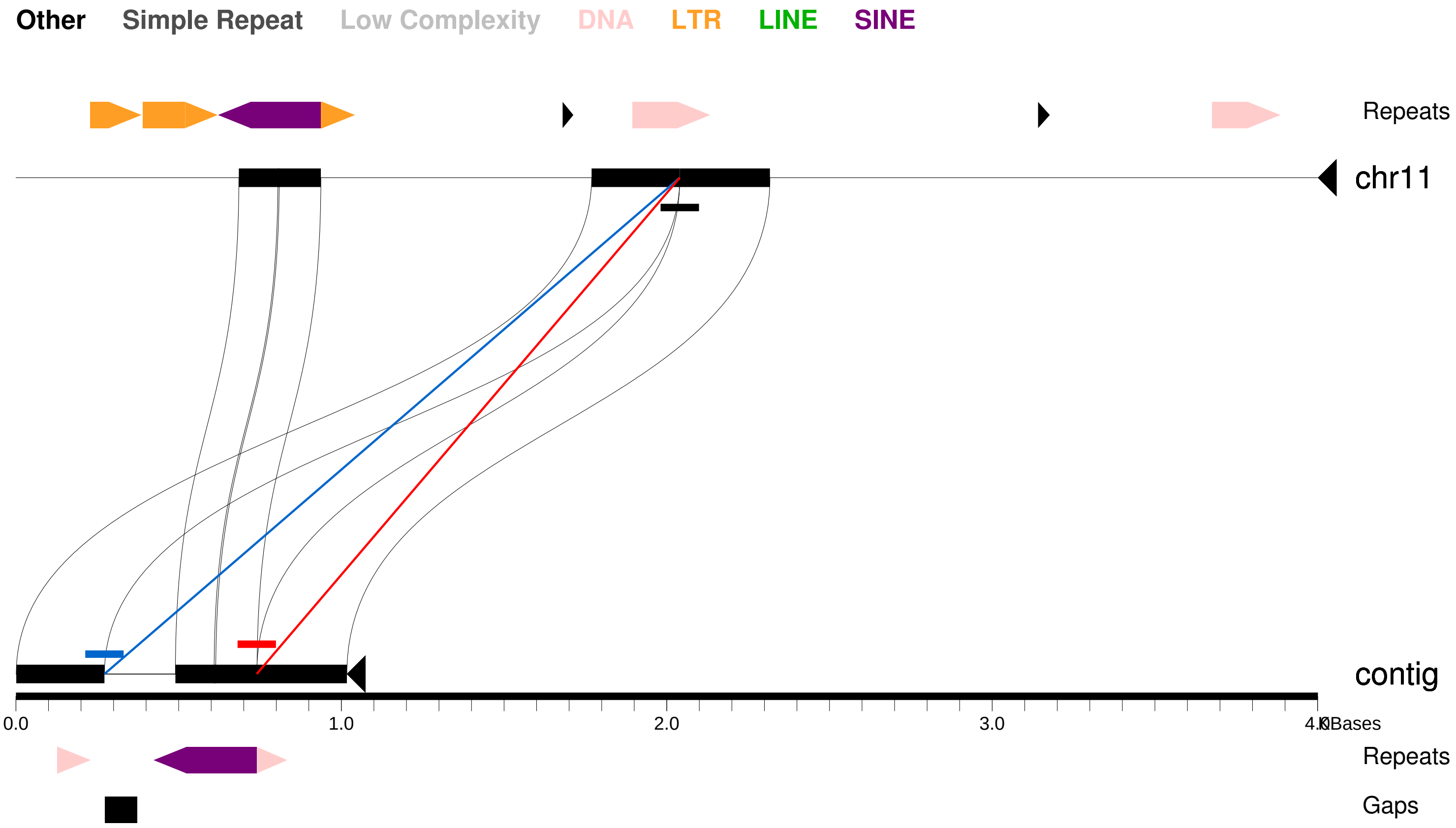

siteID: chr11_102169755 Scafftig1 + Breakpoint: chr11:102169754
chr11:102169695-102169813
Overlap Length: 1 INS size: 386 Genotyped: No

```
left    C--CCAGG--GATGAAGCTATCTTGA--TCGTGGTGGATGAGCTTTTTGATGTGCTGCTGGATTCANNNN
chrom   C--CCAGG--GATGAAGCTATCTTGA--TCGTGGTGGATGAGCTTTTTGATGTGCTGCTGGATTCAGTTT
right   TAGCCAGGATGGTCTCGATCTCCTGACCTCGTGATCCGCCCGCCTC-----GGCCTCCCAAAGT-AGTTT
        111*****11*1*111*1*1**1***11*****1*111111**1*111111*11**1*111*1*1*2222

left    NNNNNNNNNNNNNNNNNNNNNNNNNNNNNNNNNNNNNNNNNNNNNNNNNNNNNN
chrom   GCCAGTATTTTATTGAGGATTTTTGGATCAATGATCATCAGGGATATTGGTCTAC
right   GCCAGTATTTTATTGAGGATTTTTGGATCAATGATCATCAGGGATATTGGTCTAC
        2222222222222222222222222222222222222222222222222222222
```

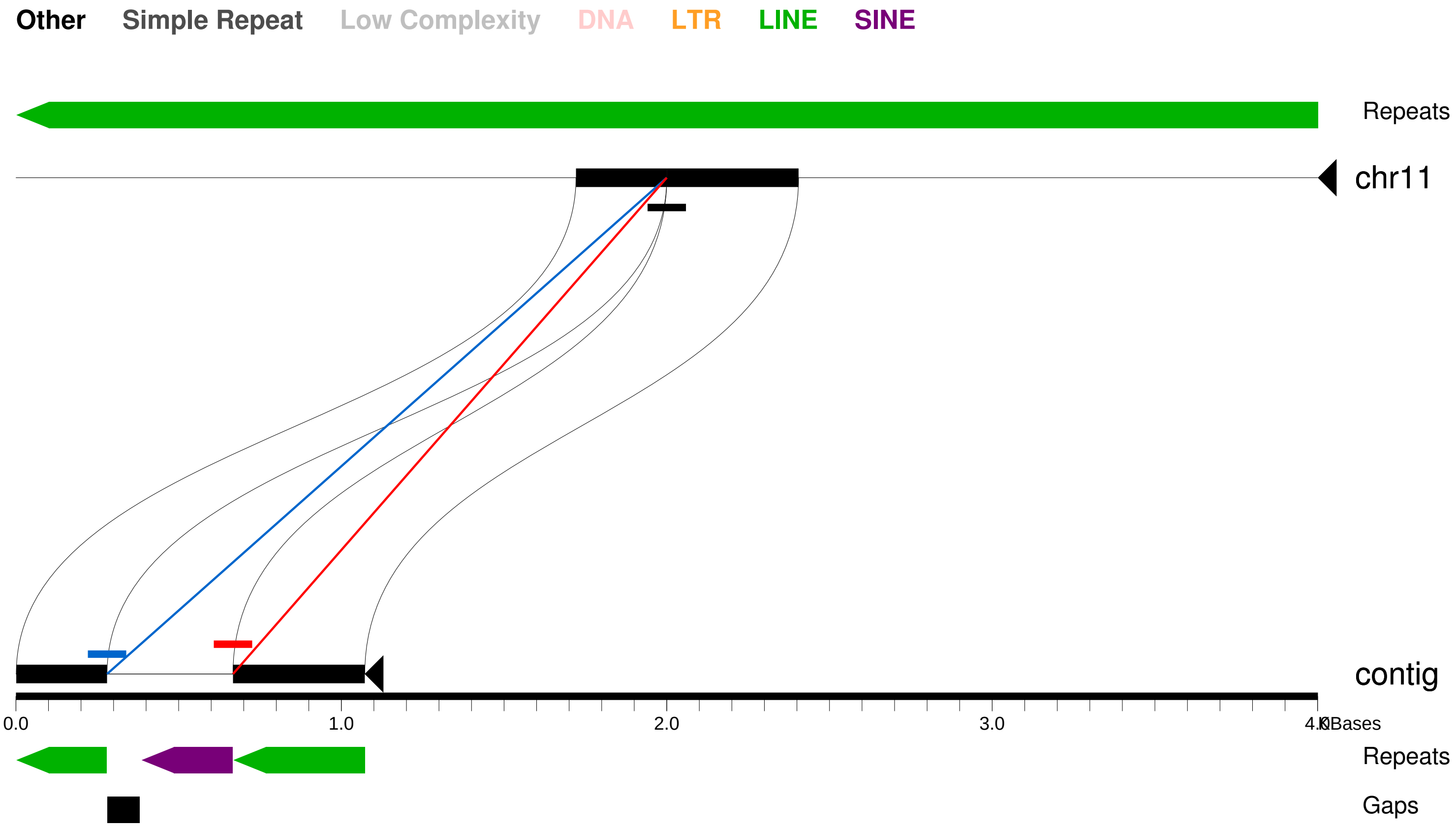

siteID: chr11_102348320 Scafftig4 + Breakpoint: chr11:102348313
chr11:102348267-102348372
Overlap Length: 14 INS size: 208 Genotyped: Yes

```
left    ACAAACTTTT--GAAAGGTTCACTGCAT-TAAATTCTGAAGCCCTAGGA----------CATGAACAATT
chrom   ACAAACTTTT--GAAAGGTTCACTGCAT-TAAATTCTGAAGCCCTAGGA----------CATGAACAATT
right   GCCCGCCACTACGCCCGGCTAATTTTTTGTATTTTTAGTAGAGACGGGGTTTCACCGTTCATGAACAATT
        1*111*111*11*111**1*1*1*111*1**11**11*1**11111**11111111111***********

left    TTTTTTTTTTTTTTTTTTTTTTTTTTTGAGACGGAGTCTCGCTCTGTCGCCCAGGCTGGAGTGC
chrom   TTTAAATTCTGCCTTGGTT-------AGA---AATCACCCTGATACAAAGAGGC---AAATC
right   TTTAAATTCTGCCTTGGTT-------AGA---AATCACCCTGATACAAAGAGGC---AAATC
        ***222**2*222**22**2222222***222*2**2*2**2222*2222****222*222*
```

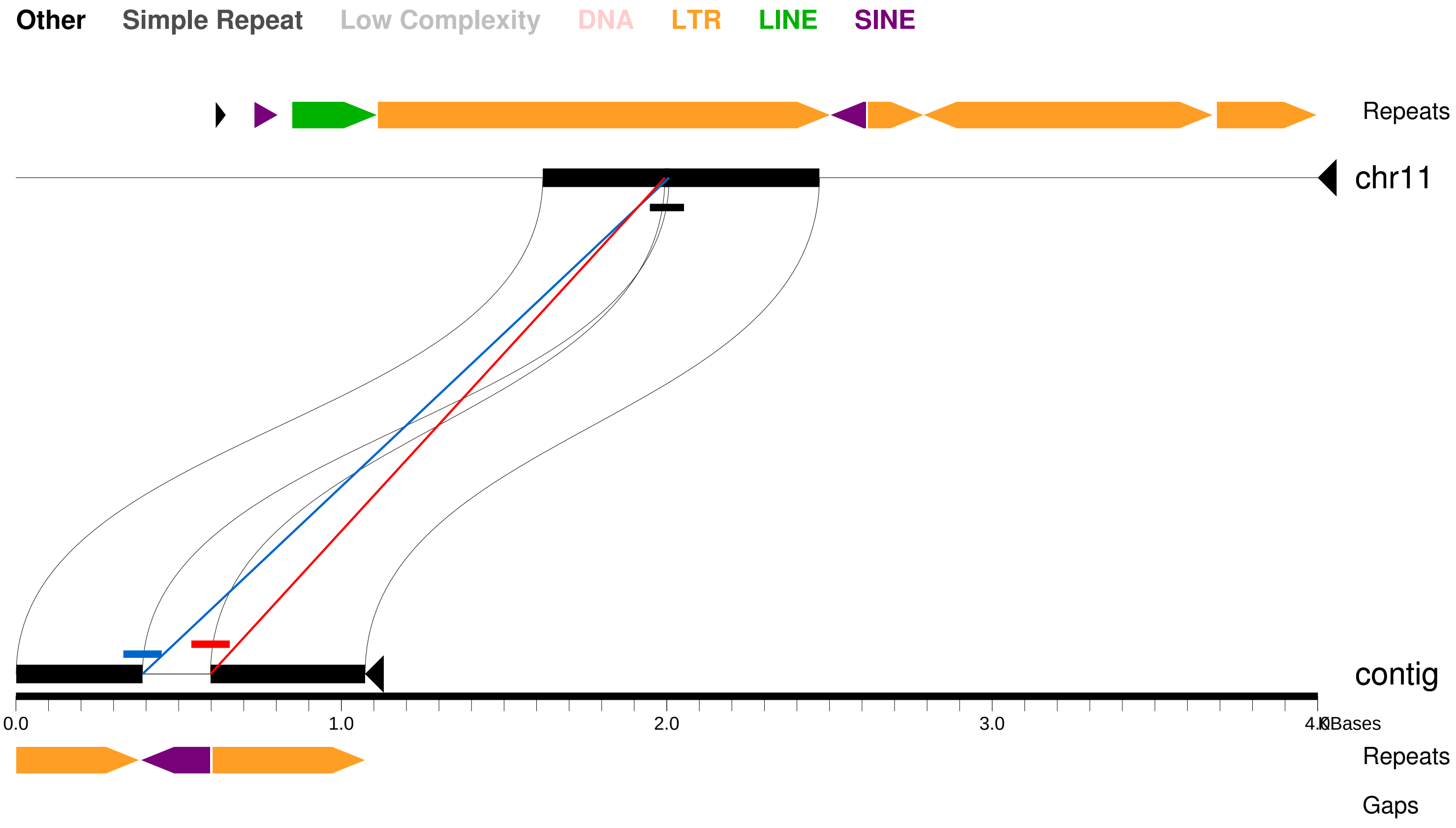

siteID: chr11_102525453 Scafftig1 - Breakpoint: chr11:102525467
chr11:102525307-102525636
Overlap Length: 10 INS size: 425 Genotyped: No

```
left   --------------------------------------GT---GCTCTGTAGTGCAC--GAGTTTACCTT
chrom  TGATATAAAGCTAGCATGGATATATGTGAATCTCCCTAGT---GCTCTGTAGTGCACATGAGTTTACCTT
right  TTTAGTAGAGACGGGGTTTCACCTTGTTAGCCAGGATGGTCTCGATCTCCTGACCTCGTGA-TCCACCCG
       2NNNN22N22NNN2NN2NNNNNNN222N2NN2NNNN2N**111*1***111*11*1*N2**1*11***11

left   CCTTTAAGCTTAAAAAAACAAATGTTGTGCTATAACTAATGTCTCAAAGGCTTATAGGAAGAAACACTTG
chrom  CCTTTAAGCTTAAAAAAACAAATGTTGTGCTATAACTAATGTCTCAAAGGCTTATAGGAAGAAACACTTG
right  CCTCG--GCCTCCCAAA---------GTGCTG----------------GGATTACAGG--------CGTG
       ***1111**1*111***111111111*****11111111111111111**1***1***11111111*1**

left   TGTTTCGCTACAACTGGACCCTTGCCTCGGGGGNNNNNNNNNNN--------------------------
chrom  TGTTTCGCTACAACTGGACCCTTGCCTCGGGGGTTCTTATTGACTCAGGCTTCCTAACATATTCCAAGGC
right  AG--------CCACCGCGCCCG-GCCGCGGGGGTTCTTATTGACTCAGGCTTCCTAACATATTCCAAGGC
       1*11111111*1**1*11***11****1*****2222222222222222222222222222222222222

left   ----------------------------------------------------------------------
chrom  ATCTGCAGAGGTCCTGCTGCCCAGACAGGTATGTTACATGTAGTTAAAGTTTGAACTGCTTTTGCCTATA
right  ATCTGCAGAGGTCCTGCTGCCCAGACAGGTATGTTACATGTAGTTAAAGTTTGAACTGCTTTTGCCTATA
       2222222222222222222222222222222222222222222222222222222222222222222222

left   -----------------------------------------------------
chrom  CTGTGAAGCTGCCTGTGCCTACTATGGGCCTGTAAGTTTGCTGAGGACAAAGC
right  CTGTGAAGCTGCCTGTGCCTACTATGGGCCTGTAAGTTTGCTGAGG-------
       2222222222222222222222222222222222222222222222
```

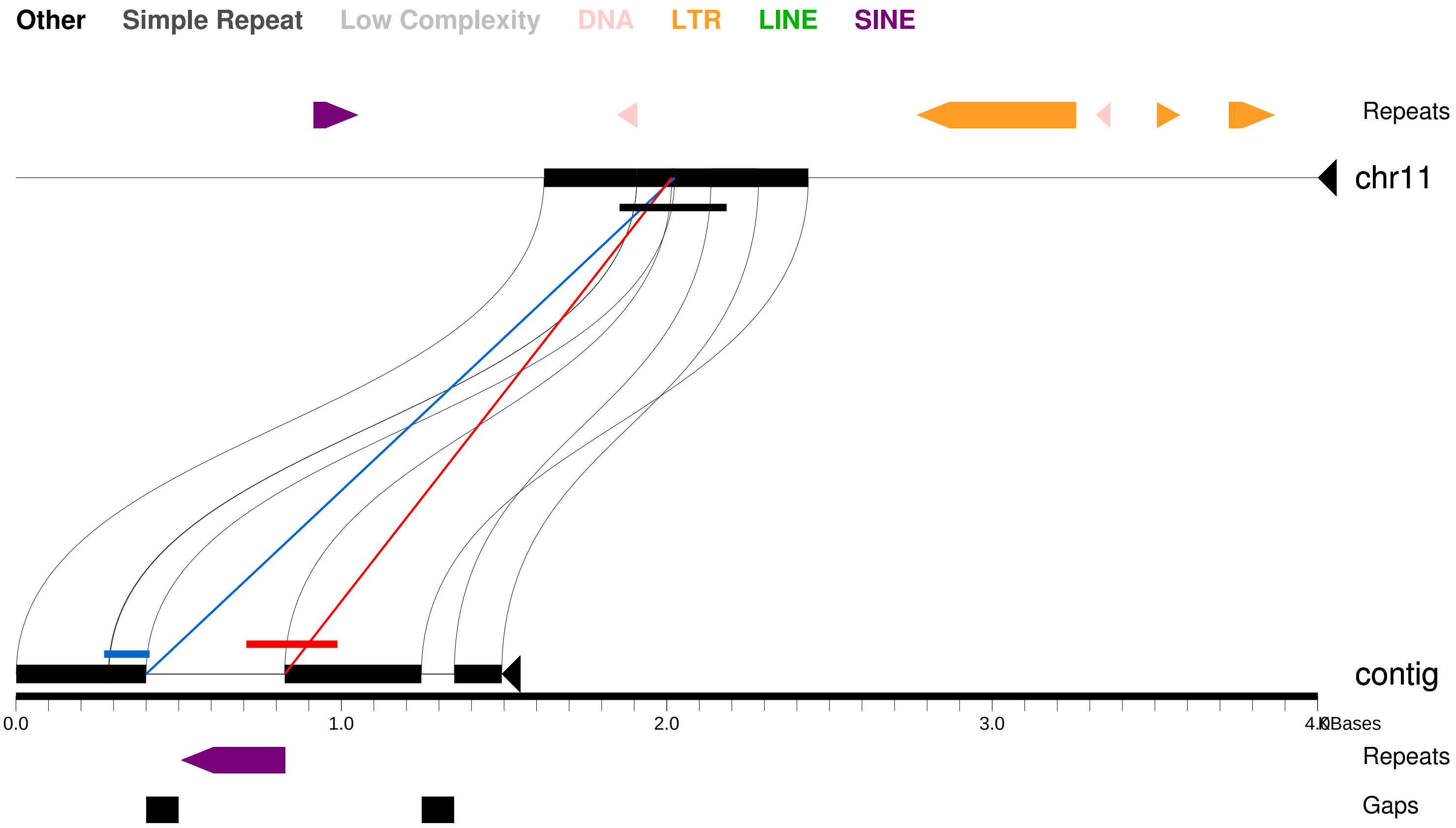

siteID: chr11_103201274 Scafftig1 - Breakpoint: chr11:103201267
chr11:103201221-103201326
Overlap Length: 10 INS size: 493 Genotyped: No

```
left     TTTT-GTTTGGTTAAAACAGACTTTTG---TTTCAGAAAATGAAAACTAA---------ACAATAAATTG
chrom    TTTT-GTTTGGTTAAAACAGACTTTTG---TTTCAGAAAATGAAAACTAA---------ACAATAAATTT
right    CACCCGCCTCGGCCTCCCAAAGTGCTGGGATTACAGGCGTGAGCCACCGCGCCCGGCCCACAATAAATTT
         11111*11*1*111111**1*1*11**111**1***111111111**111111111111**********2

left     TTTTTNNNNNNNNNNNNNNNNNNNNNNNNNNNNNNNNNNNNNNNNNNNNNNNNNNNNNNNNNNN
chrom    TTT-------------AAATAACTAAAGCTCTAACCTATTTTCATTAATATATATATCTTTATT
right    TTT-------------AAATAACTAAAGCTCTAACCTATTTTCATTAATATATATATCTTTATT
         ***2222222222222222222222222222222222222222222222222222222222222
```

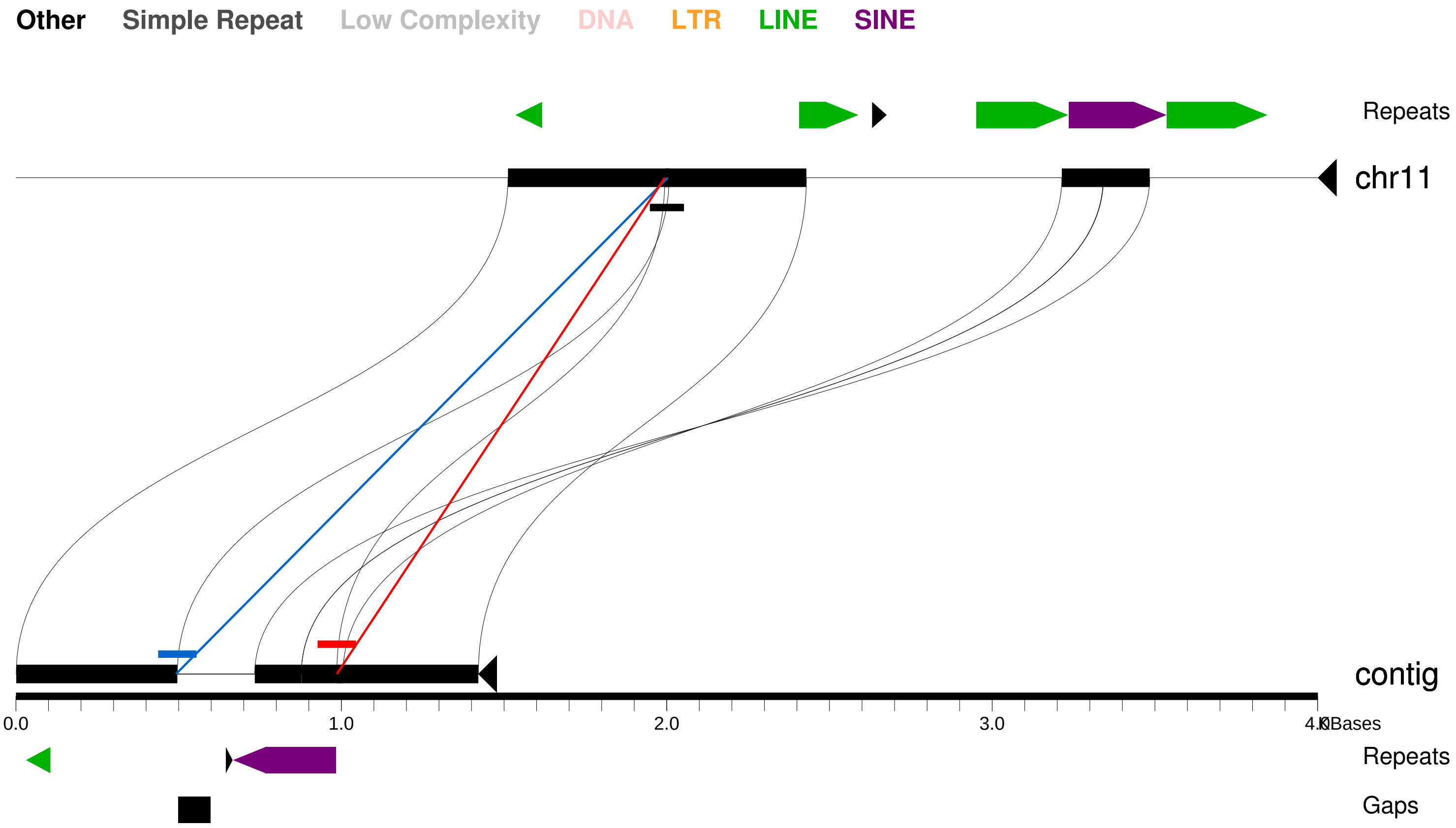

siteID: chr11_103792596 Scafftig7 - Breakpoint: chr11:103792589
chr11:103792545-103792648
Overlap Length: 16 INS size: 311 Genotyped: Yes

```
left    CT---TGTGCCATT--CAACA-AGGTAGTCATGGCCTTGTGTGGTAATTT---------AAATTTCAATT
chrom   CT---TGTGCCATT--CAACA-AGGTAGTCACGGCCTTGTGTGGTAATTT---------AAATTTCAATT
right   CGCAGTCCGGCCTGGGCAACAGAGCGAGACTCTGTCTCAAAAAAAAAAAAAAAAAAAAAAAATTTCAATT
        *1111*11*1*1*111*****1**11**1*121*1**11111111**111111111111***********

left    AGTTAGGCCGGGCGCGGTGGCTCACGCCTGTAATCCCAGCACTTTGGGAGGCCGAGGCGGGTGG
chrom   AGTTAAAACTAAATAA---AATTAAAAATTTAGTTTCT---ATTTGTGCTACACA---------
right   AGTTAAAACTAAATAA---AATTAAAAATTTAGTTTCT---ATTTGTGCTACACA---------
        *****222*222222222222*2*2222*2**2*22*22222****2*222*22*222222222
```

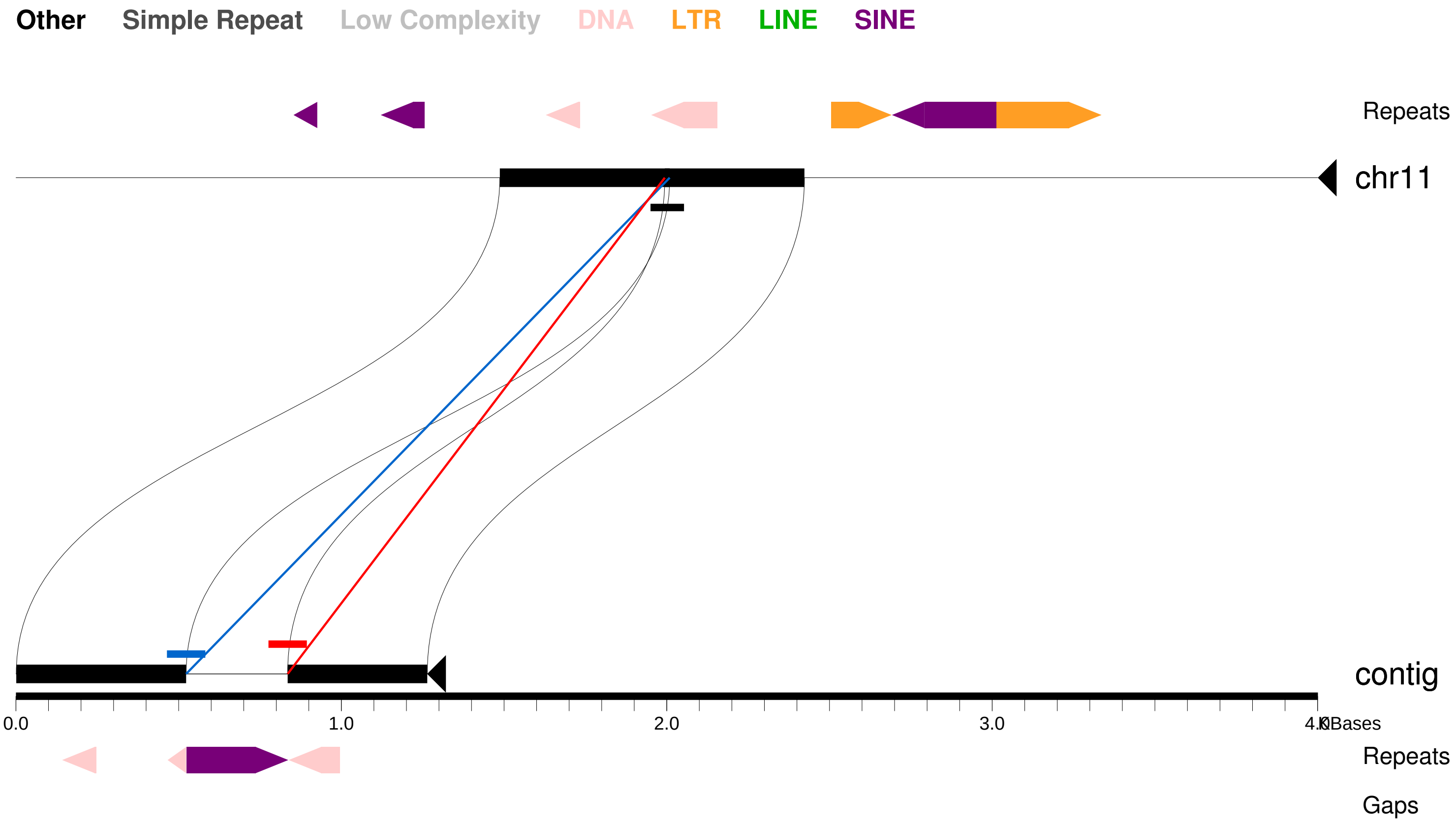

siteID: chr11_104144741 Scafftig2 - Breakpoint: chr11:104144735
chr11:104144689-104144794
Overlap Length: 14 INS size: 310 Genotyped: Yes

```
left    TG--TGCACCACATTATTGGCATTTTGCTGCCTTCCTACTGATGATTT------------AAAAATTTTT
chrom   TG--TGCACCACATTATTGGCATTTTGCTGCCTTCCTACTGATGATTT------------AAAAATTTTT
right   AGCCTGGGCGACAG-AGCGAGACTCCGTCTCAAAAAAAAAAAAAAAAAAAAAAAAAAAAAAAAAAATTTT
        1*11**11*1***11*11*11*1*11*111*111111*111*11*111111111111111*****1****

left    CCCCGGCCGGGCGCGGTGGCTCACGCCTGTAATCCCAGCACTTTGGGAGGCCGAGGCGGGCGG
chrom   CCCCAGTTTTACTAGTTG-CTCTC------AGTCAGAG---TTTAATA---CAAAGTGTCTAG
right   TCCCAGTTTTACTAGTTG-CTCTC------AGTCAGAG---TTTAATA---CAAAGTGTCTAG
        1***2*22222*22*2**2***2*222222*2**22**222***222*222*2*2*2*2222*
```

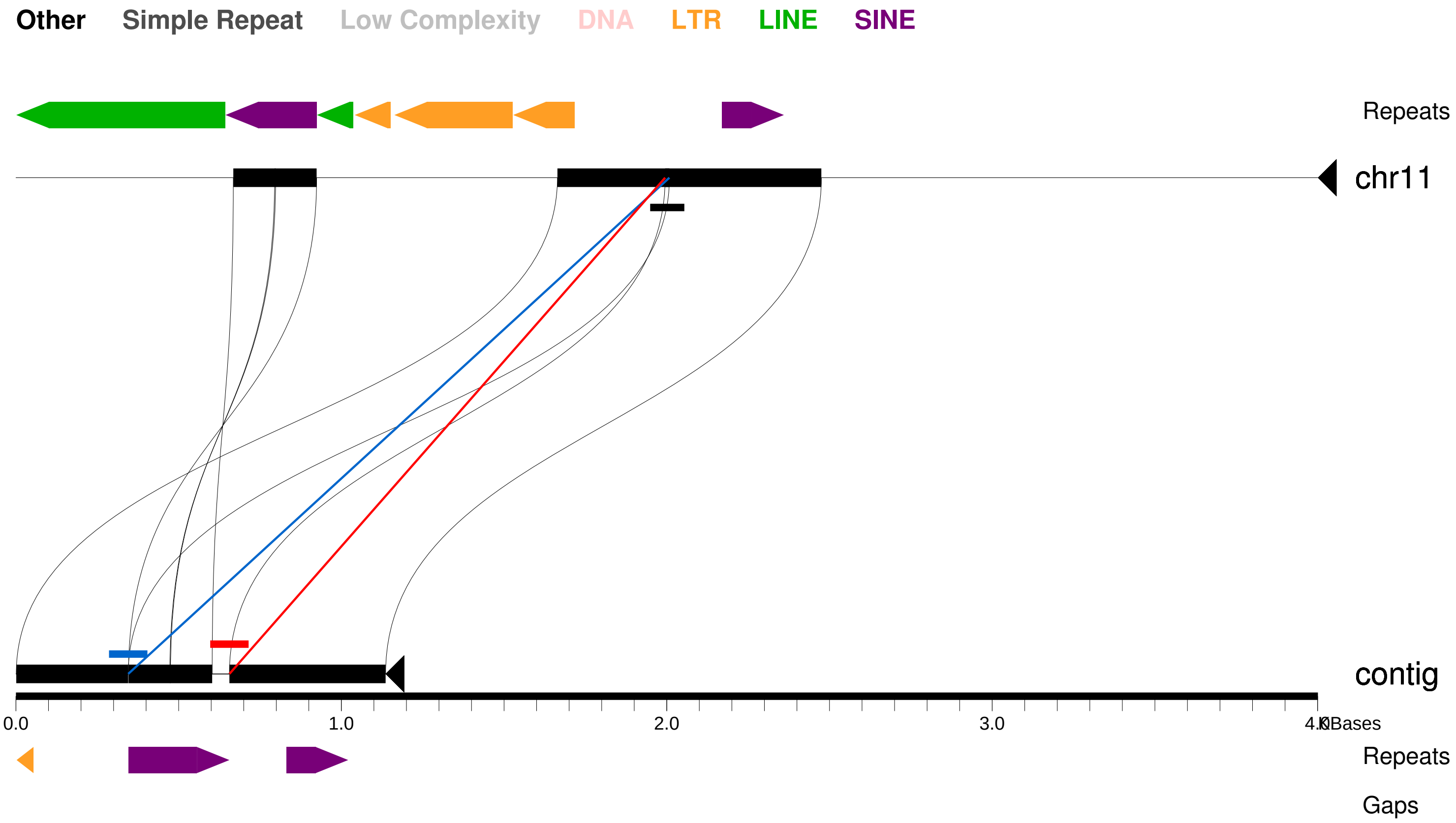

siteID: chr11_104786613 Scafftig7 - Breakpoint: chr11:104786599
chr11:104786549-104786650
Overlap Length: 10 INS size: 302 Genotyped: Yes

```
left    -----------AGGAAAGATT----GACA---TGCAAATAAGTATAATGAAACACCTTTTAAGAATTTAA
chrom   -----------AGGAAAGATT----GACA---TGCAAATAAGTATAATGAAACACCTTTTAAGAATTTAA
right   CCCGCCACTGCACTCCAGCCTGGGCGACAGAGCGAGACTCCGTCTCAAAAAAAAAAAAAAAAAAAAATAAAA
        11111111111*1111**11*1111****1111*11*1*11**1*1*11***1*111111**1***11**

left    ATATTGAGGCCGGGCGCGGTGGCTCACGCCTGTAATCCCAGCACTTTGGGAGGCCGAGGCGGGCGGA
chrom   ATATTGAG--------CAGATGCTCAAGAAAAGCATA--AGCAAAGTGTG--TCTCAGGTAG-----
right   A-ATTGAG--------CAGATGCTCAAGAAAAGCATA--AGCAAAGTGTG--TCTCAGGTAG-----
        *1******22222222*2*22*****2*222222**222****222**2*222*22***22*22222
```

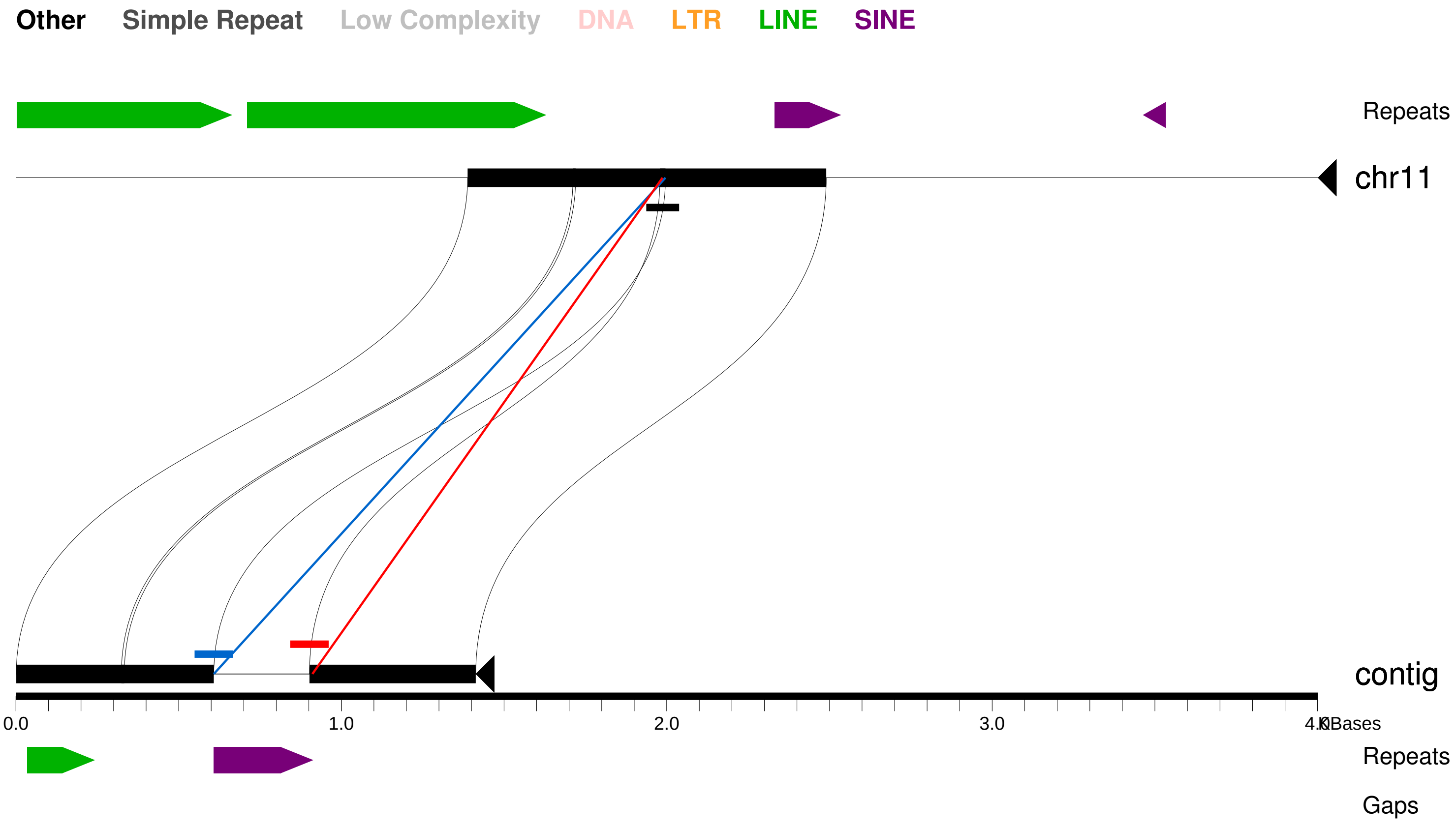

siteID: chr11_106044575 Scafftig13 - Breakpoint: chr11:106044565
chr11:106044522-106044624
Overlap Length: 17 INS size: 298 Genotyped: Yes

```
left    CCTCTGTA-TGCAGA-TGTTTGA------AATGCTTCTTCCCCAACGCCAT--------AAAGAAATAGC
chrom   CCTCTGTA-TGCAGA-TGTTTGA------AATGCTTCTTCCCCAACGCCAT--------AAAGAAATAGC
right   CCACTGCACTCCAGCCTGGGCGACAGAGCGAGACTCCGTCTCAAAAAAAAAAAAAAAAAAAAGAAATAGC
        **1***1*1*1***11**111**1111111*11**1*1**1*1**1111*111111111***********

left    ACTTGAGGCCGGGCGCGGTGGCTCACGCCTGTAATCCCAGCACTTTGGG-AGGCCGAGGCGGGCGG
chrom   ACTTGAA----------------CATAAATTTAATTTCCTCAGCAAGGCCATTTTTATACTTTCT-
right   ACGTGAA----------------CATAAATTTAATTTCCTCAGCAAGGCCATTTTTATACTTTCT-
        **1***2222222222222222**2222*2****22*22**2222**22*22222*22*222*22
```

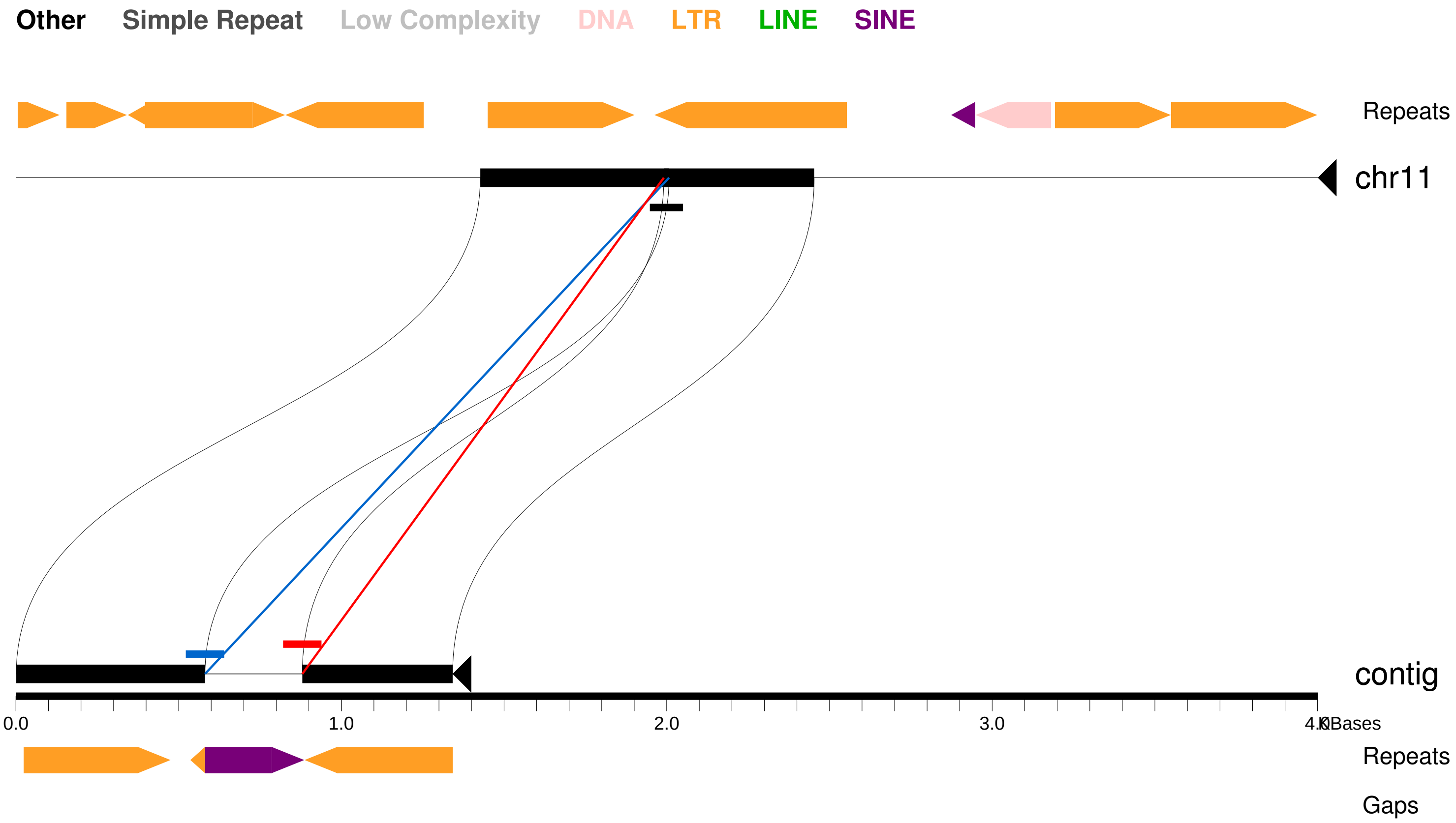

siteID: chr11_106170487 Scafftig9 - Breakpoint: chr11:106170468
chr11:106170309-106170641
Overlap Length: 14 INS size: 328 Genotyped: Yes

```
left    T-------------TCTCAGTCCAGTAAAATGTCAGTAGTGAGGAGGAGCCTCCTGTAATCCATTTCAAA
chrom   TGTTTTGACTCATTTCTCAGTCCAGTAAAATGTCAGTAGTGAGGAGGAGCCTCCTGTAATCCATTTCAAA
right   GG------CGGAGCTTGCAGT-----------------GAGCCGAGATCCCGCC---ACTGCACTCCA--
        12      2NN2NN*11****11111111111111111*1*11***111**1**111*1*1**1*1**11

left    TGGACTGGGTTTTAAGTTGCTCTTTGGGGCAGTGTGAATCTGAAAGAGTGCAATAGGAAATAGCCTTATC
chrom   TGGACTGGGTTTTAAGTTGCTCTTTGGGGCAGTGTGAATCTGAAAGAGTGCAATAGGAAATAGCCTTATC
right   --GCCTGGGCGACA-----------GAGCGAGACTCCGTCTCAAAAAAAAAAAAAAAAAAAAAAAAAAAA
        11*1*****1111*11111111111*1*11**11*111***1***1*1111**1*11***1*11111*11

left    CAGGTTTGCTATGTTTCTTGAAATAACTTTGCC---GGCCGGGCGCGG---TGGCTCACGCCTGTAA---
chrom   CAGGTTTGCTATGTTTCTTGAAATAACTTTGCCAAAGTTCTTTTAAGGGTATGATTCATCCTTTCTACCT
right   AAAAAAAAAAAAAAAAAAAGAAATAACTTTGCCAAAGTTCTTTTAAGGGTATGATTCATCCTTTCTACCT
        1*11111111*11111111**************222*22*222222**222**22***22*2*222*222

left    -TCC-CAGCACTTTGGGAGGCCGAGGCGGGCGGATC-ACGAGGT-----CAGGAGATCGAGACCATCCCG
chrom   TTCCTCAGGA-TTGGGGATGCCAACAGTCATGTATTTATAATCTATTCCCAGGACTTCCAAAAAGCTTTG
right   TTCCTCAGGA-TTGGGGATGCCAACAGTCATGTATTTATAATCTATTCCCAGGACTTCCAAAAAGCTTTG
        2***2***2*2**2****2***2*2222222*2**22*22*22*22222*****22**2*2*2222222*

left    GCTAA----AACGGTGAAACCCCGTCTCT---------------ACT----AAA
chrom   AGTGATTTTAGCTATGAAACAGGGTCTATTGTTTCCCTGGAGTGACTTTGGAAG
right   AGTGATTTTAGCTATGAAACAGGGTCTATTGTTTCCCTGGAGT-----------
        22*2*2222*2*22******222****2*22222222222222 111    11N
```

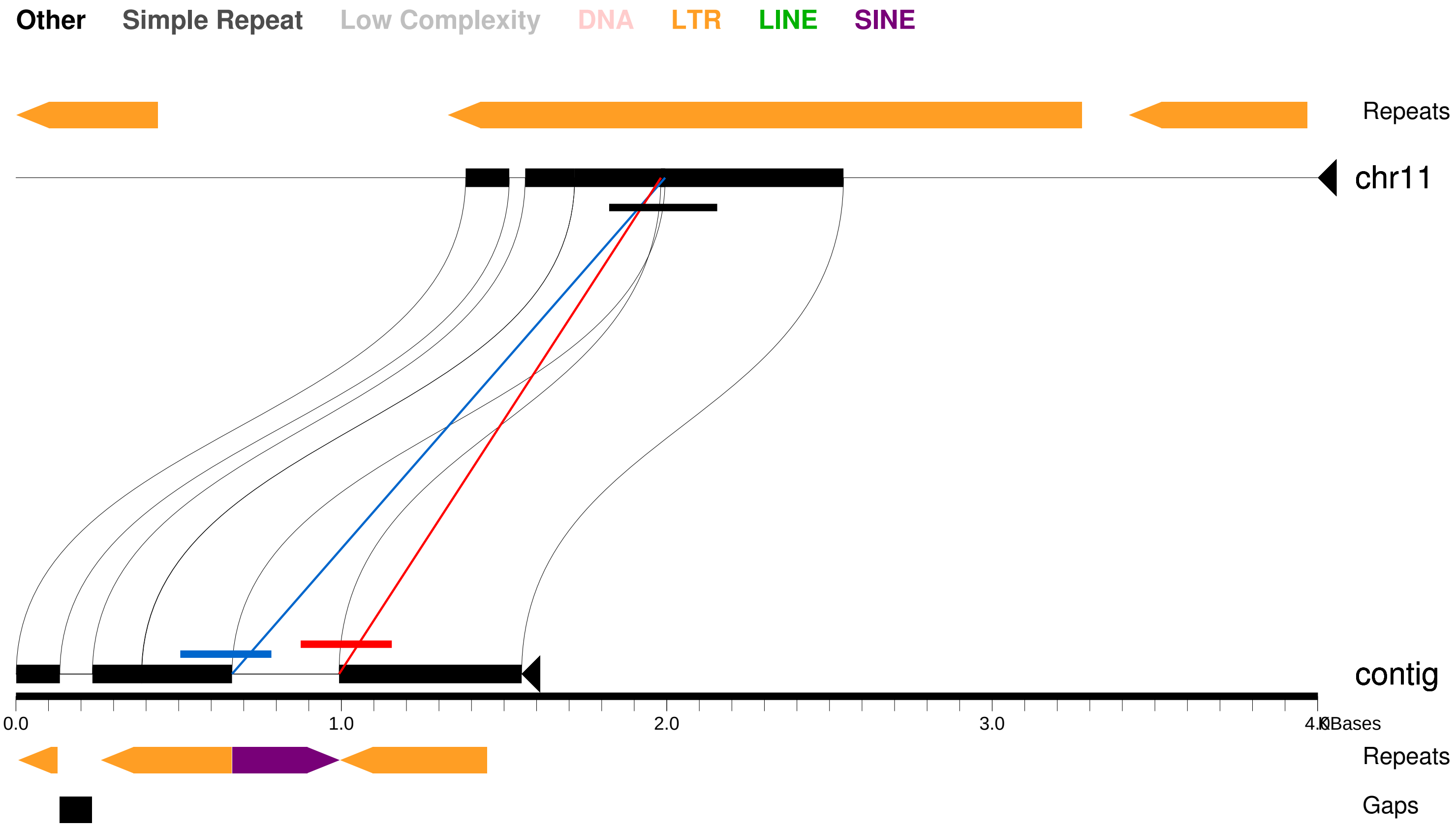

siteID: chr11_106893666 Scafftig1 - Breakpoint: chr11:106893657
chr11:106893612-106893716
Overlap Length: 15 INS size: 288 Genotyped: Yes

```
left    --------TTGTCCTCAAATGTCCTAAAAACAGATTTTAAAATATGCTGAATT------AAAATCACAAT
chrom   --------TTGTCCTCAAATGTCCTAAAAACAGATTTTAAAATATGCTGAATT------AAAATCACAAT
right   AGCGAGACTCCGTCTCAAAAAAAAAAAAAAAAAAAAAAAAAAAAAAAAAAAAAAAAAAAAAAATCACAAT
        11111111*1111******111111*****1*1*1111****1*11111**11111111***********

left    GTGACTTTGGGAGGCCGAGGCGGGCGGATCACGAGGTCAGGAGATCGAGACCATCCTGGCTAA
chrom   GTGA-----GTGGGTTATTGCATTAAAATAAATAT-----TAAAT--ACACAAT--TAACTTG
right   GTGA-----GTGGGTTATTGCATTAAAATAAATAT-----TAAAT--ACACAAT--TAACTTG
        ****22222*22**22222**222222**2*22*2222222*2**22*2**2**22*22**22
```

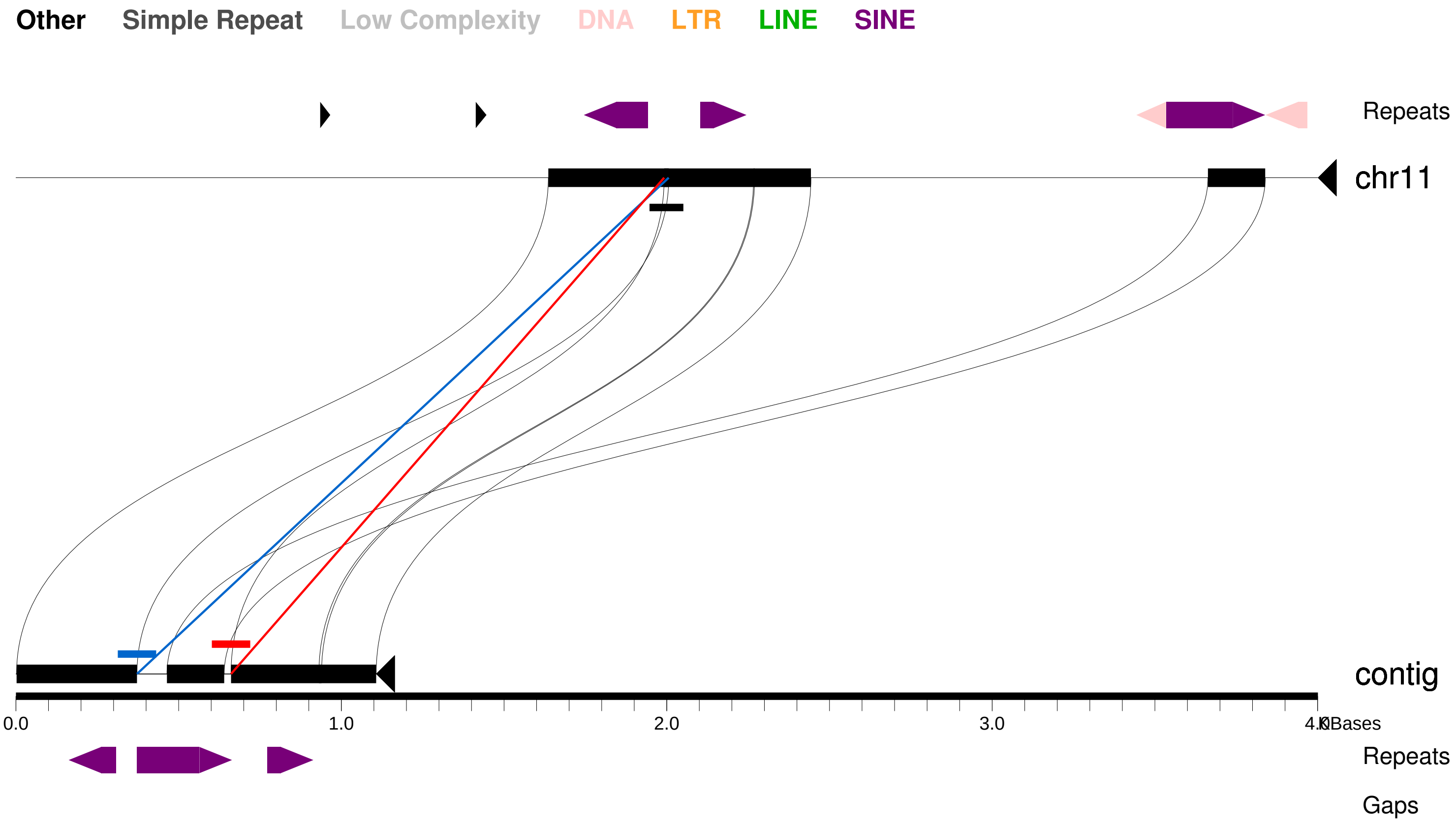

siteID: chr11_107354612 Scafftig2 - Breakpoint: chr11:107354618
chr11:107354458-107354795
Overlap Length: 18 INS size: 299 Genotyped: Yes

```
left    ------------TCTGATGGTTTTAATTAACTTTGCCCACACTTTAAAAATTTTTAAAATTTCAATAGCT
chrom   CTTCCACTTCCTTCTGATGGTTTTAATTAACTTTGCCCACACTTTAAAAATTTTTAAAATTTCAATAGCT
right   TTT--AGTAGAGACGG--GGTTTCACCTTG-TTAGCC----------AGGATGGTCTCGATCTCCTGACCT
        N22  2N2NNNN1*1*11*****1*11*111**1***111111111*11**11*1111**1**111*1**

left    TTATATGTACAACTGGTTTTTTGTTACATGGATGAATTGTATAGTGGTGAAGACTGCAGTGTACTCATCA
chrom   TTATATGTACAACTGGTTTTTTGTTACATGGATGAATTGTATAGTGGTGAAGATTTCAGTGTACTCATCA
right   C---ATGATCCACCCG----------CCTCG--GCCTCCCAAAGTGCTGG-GATTACAGG-----CGTGA
        1111***11*1**11*1111111111*1*1*11*11*111*1****1**11**2*N***111111*1*1*

left    CCCAAATAGTGTACATTGTACCCAACAGGTAATTTTTCTT---------------TTTTTTTTGAGACGG
chrom   CCCAAATAGTGTACATTGTACCCAACAGGTAATTTTTCATCCCTCACCCCCAACCTCCTCTTTGAGTTTC
right   GCCACCGCG----CCCGGCCCCCAACAGGTAATTTTTCATCCCTCACCCCCAACCTCCTCTTTGAGTTTC
        1***1111*1111*111*11*****************2*222222222222222*22*2******2222

left    -AGTCTCGCTCTGTCGCTCAGGCCAGACTGCGGACTGCAGTGGC---GCAATCTCGTCTCACTGCAAGC-
chrom   TAATGTCTATCATTCCATTCTGTAAGCCCCTGCATACCCATAGCTTAGCTCCTACTTATAAGTGAGAACA
right   TAATGTCTATCATTCCATTCTGTAAGCCCCTGCATACCCATAGCTTAGCTCCTACTTATAAGTGAGAACA
        2*2*2**22**22**22*222*22**2*222*2*222*22*2**222**22222*2*2*2*2**22*2*2

left    ---------------TCCGCTTCCCGGGTT----CAC----GCCATTCTCCTGCC--T
chrom   TAACGGTATTTGTTTTTCTGTTCCTGAGTTACTTCACTTAGGATAATAGCCTCCAGTT
right   TAACGGTATTTGTTTTTCTGTTCCTGAGTTACTTCACTTA------------------
        222222222222222*2*22****2*2***2222***222 1NN1N1NN111N1N  1
```

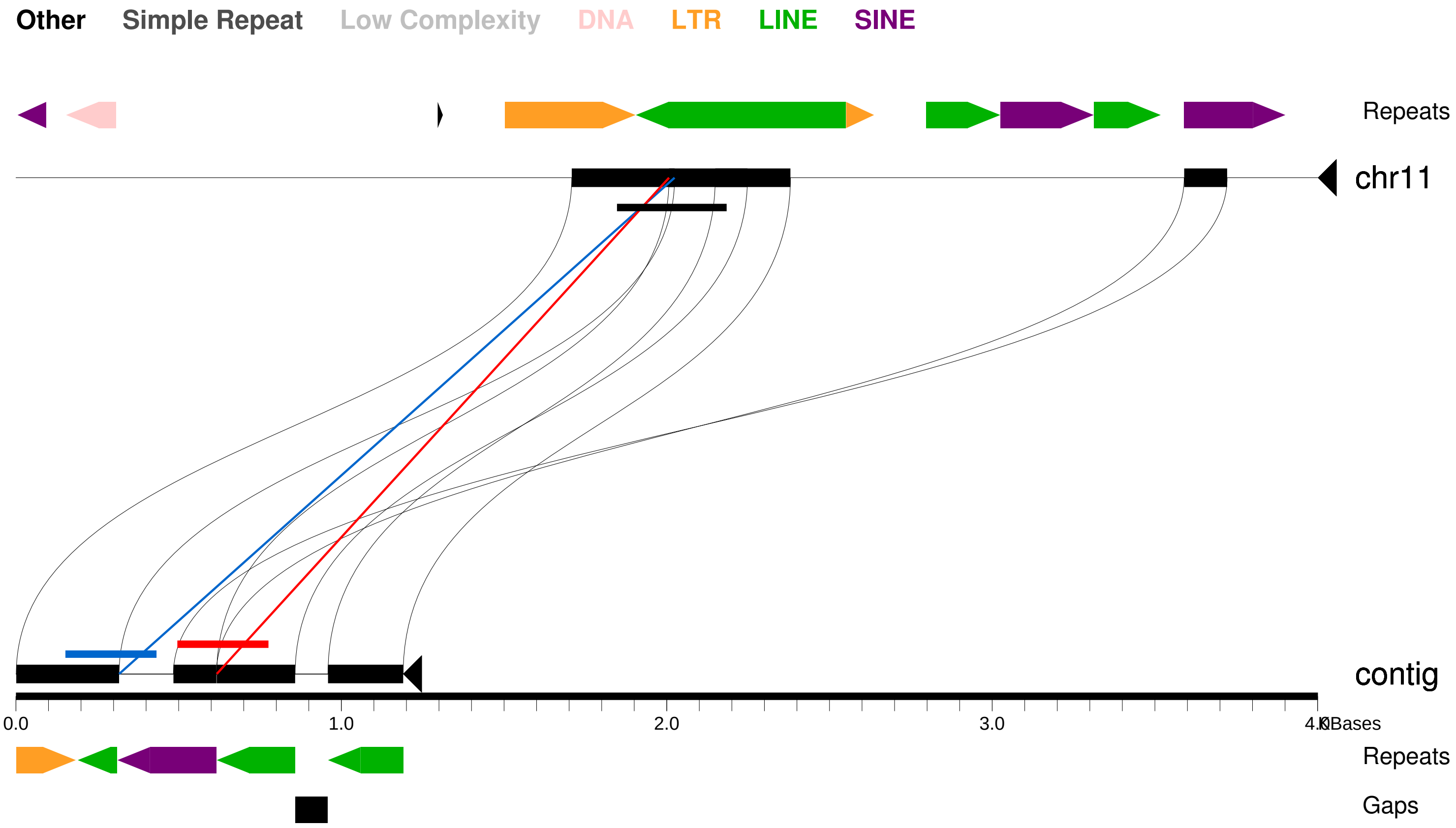

siteID: chr11_110276925 Scafftig1 + Breakpoint: chr11:110276923
chr11:110276864-110276982
Overlap Length: 0 INS size: 417 Genotyped: No

```
left    GCAACATGGCTTCTCCCCTTTCTAGGTCC---CATGGCAGCCATCTTGCTATTTCTCCCCTTC-NNNNNN
chrom   GCAACATGGCTTCTCCCCTTTCTAGGTCC---CATGGCAGCCATCTTGCTATTACTCACCTTC-GGGCCC
right   CCGCCC--GCCTCGGCC-TCCCAAAGTGCTGGGATTACAGGCGTGA-GCCACCGCGCCCGGCCCGGGCCC
        1*11*111**1**11**1*11*1*1**1*1111**11***1*1*111**1*11N*1*N*111*1222222

left    NNNNNNNNNNNNNNNNNNNNNNNNNNNNNNNNNNNNNNNNNNNNNNNNNNN
chrom   GGTATTTTTAACCTCCTTGTCAAATTTGTTTCCTCTAGGATCGAGGCTATCAA
right   GGTATTTTTAACCTCCTTGTCAAATTTGTTTCCTCTAGGATCGAGGCTATCAA
        22222222222222222222222222222222222222222222222222222
```

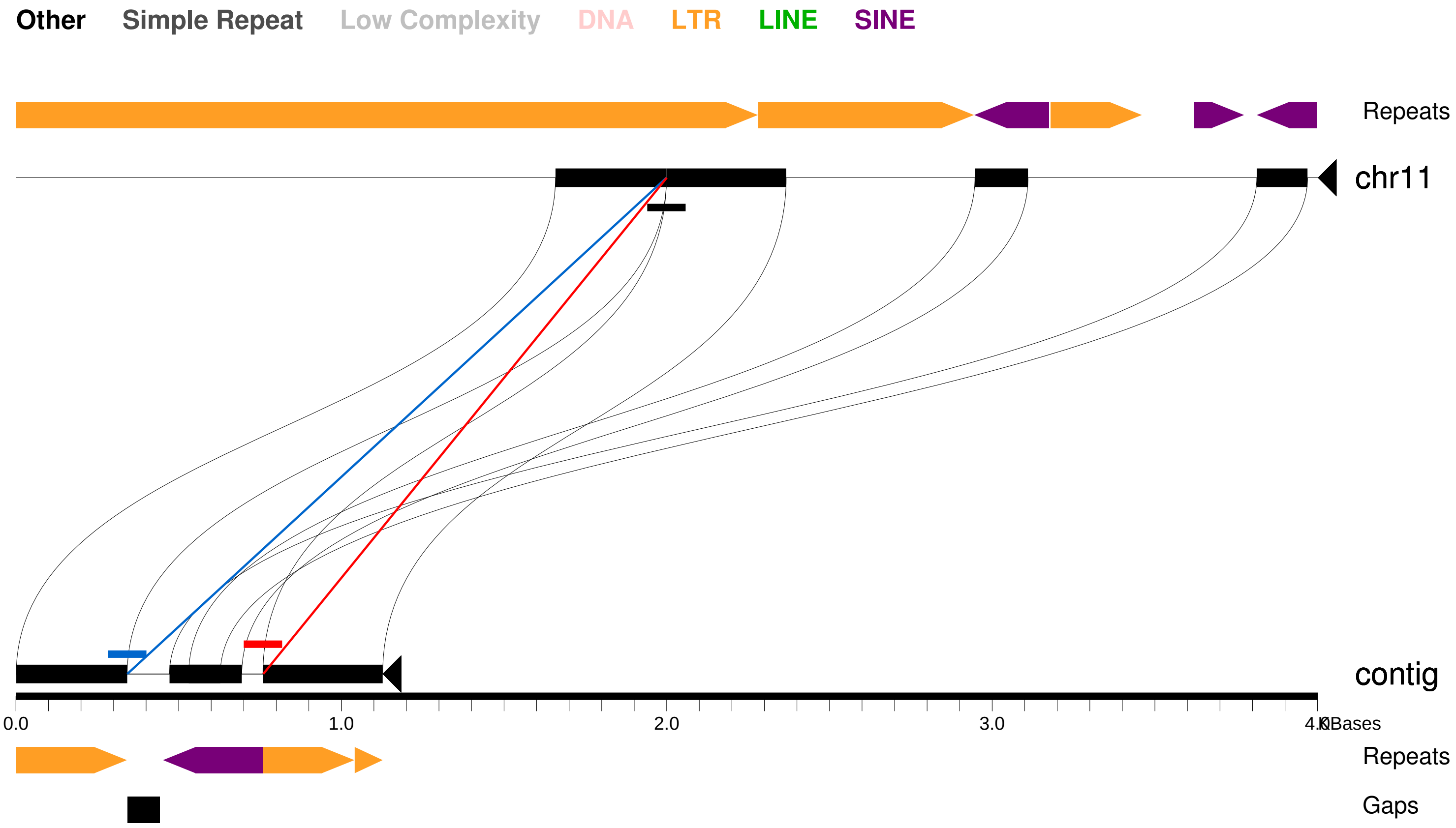

siteID: chr11_114686162 Scafftig3 - Breakpoint: chr11:114686163
chr11:114686118-114686222
Overlap Length: 15 INS size: 303 Genotyped: Yes

```
left    ----TAAAAAATGGG-GATAATAATAGAGCTGAACTCATAGGTTGTTGTG---------AAAATTTAAAA
chrom   ----TAAAAAATGGG-GATAATAATAGAGCTGAACTCATAGGTTGTTGTG---------AAAATTCAAAA
right   GCGCTCCAGCCTGGGCGACAGAGCGAGACTCCGTCTCAAAAAAAAAAAAAAAAAAAAAAAAAAATTTAAAA
        1111*11*111****1**1*11111***111111****1*1111111111111111111******N****

left    AGTTG--GCCGGGCGCGGTGGCTCACGCCTGTAGTCCCAGCACTTTGGGAGGCCGAGGCGGGCGG
chrom   AGTTAATGCATATAAAGAGGTTGGAATCATG----CCTAGCACCTAGAAAAGA------------
right   AGTTAATGCATATAAAGAGGTTGGAATCATG----CCTAGCACCTAGAAAAGA------------
        ****222**2222222*22*2222*22*2**2222**2*****2*2*22*2*222222222222
```

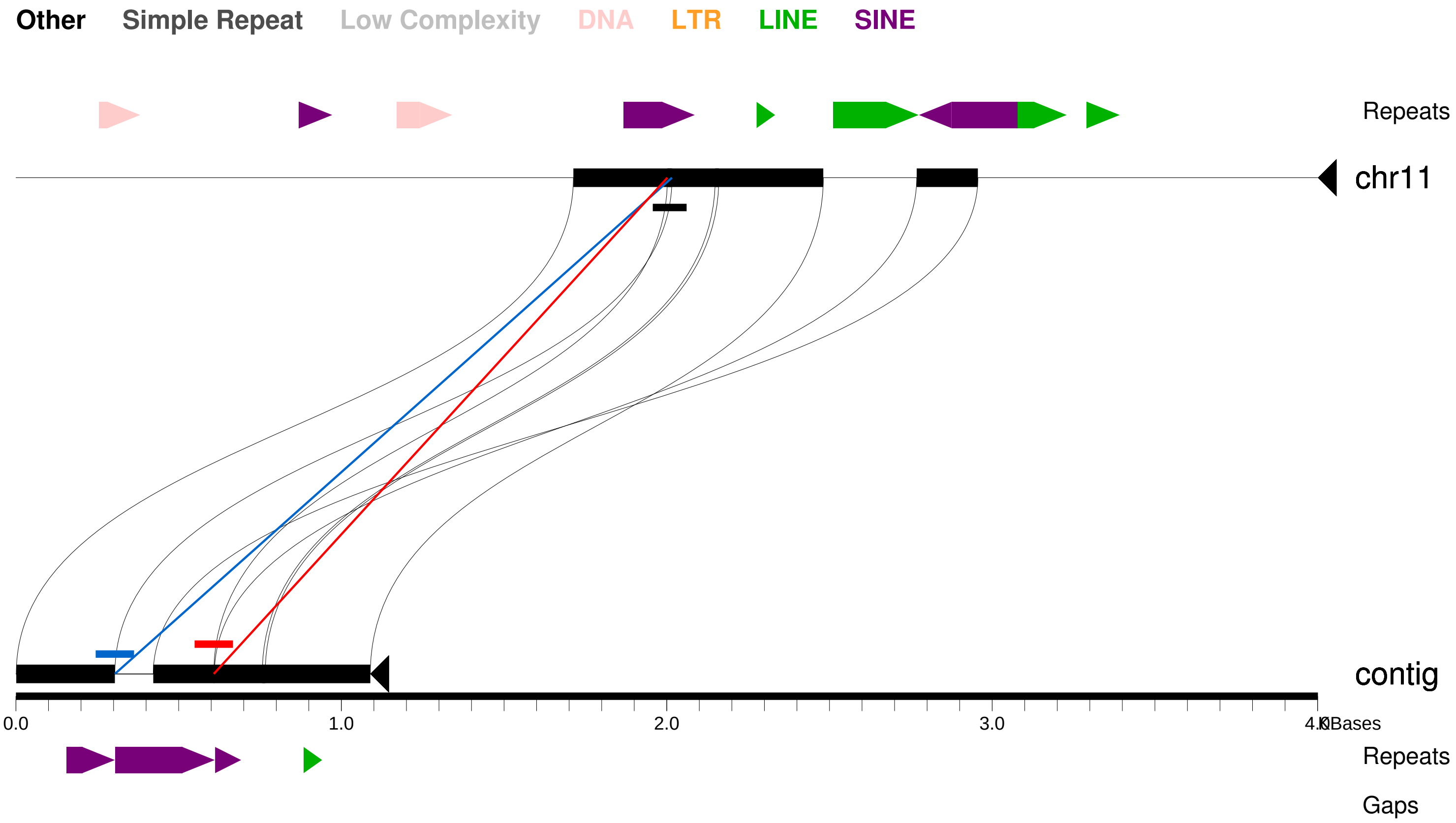

siteID: chr11_121957382 Scafftig2 + Breakpoint: chr11:121957374
chr11:121957220-121957575
Overlap Length: 56 INS size: 395 Genotyped: No

```
left   ----------------------------------------------------------------------------
chrom  GGAGTTTAATATATGTATATTGATTTTTGAATTTGTAAAAATGTGCTCAGGATTCCTGGAGAAATGAGCA
right  CCGCCCGCCTC-----------------GGCCTCCCAAA---GTGCTG-GGATTACAAGCG---TGAGCC
       NNNNNNNNN2N                 2NNN2NNN222    22222N 22222N2NN2N2    22222N

left   ------------------------------TGTGTATATATATGTATATGTATATGTATACATATATATA
chrom  TCCTCTGTACCCTCGTTATGGGCATATATATGTGTATATATATGTATATGTATATGTATACATATATATA
right  ACCGCG---CCC--------GGCCTATATATG--------------------------------------
       N22N2N   222        222N222222**11111111111111111111111111111111111111

left   TACATATATATATGGATATTTTTAAAGGAGCTGCATTTTCACGTTTGATTAGCAGATTAGCAGAATTATT
chrom  TACATATATATATGGATATTTTTAAAGGAGCTGCATTTTCACGTTTGATTAGCAGATTAGCAGAATTATT
right  --------------GATATTTTTAAAGGAGCTGCATTTTCACGTTTGATTAGCAGATTAGCAGAATTATT
       11111111111111********************************************************

left   NNNNNNNNNNNNNNNNNNNNNNNNNNNNNNNNNNNNNNNNNNNNNNNNNNNNNNNNNNNN-----------
chrom  TGAGTATATGGGATTCAAATTGGTCCAAATTCAATTTTGCTGTTAAAAATTATGACTACAGTGGATATTG
right  TGAGTATATGGGATTCAAATTGGTCCAAATTCAATTTTGCTGTTAA------------------------
       2222222222222222222222222222222222222222222222NNNNNNNNNNNNN

left   ----------------------------------------------------------------------
chrom  GTTGTCTCTTGATTTGAAAATAATTATATTTTCCATTTCCCTGAGATAGTTAGTGTAGCAGGAAGAGTGT
right  ----------------------------------------------------------------------

left   ------
chrom  TGTGTT
right  ------
```

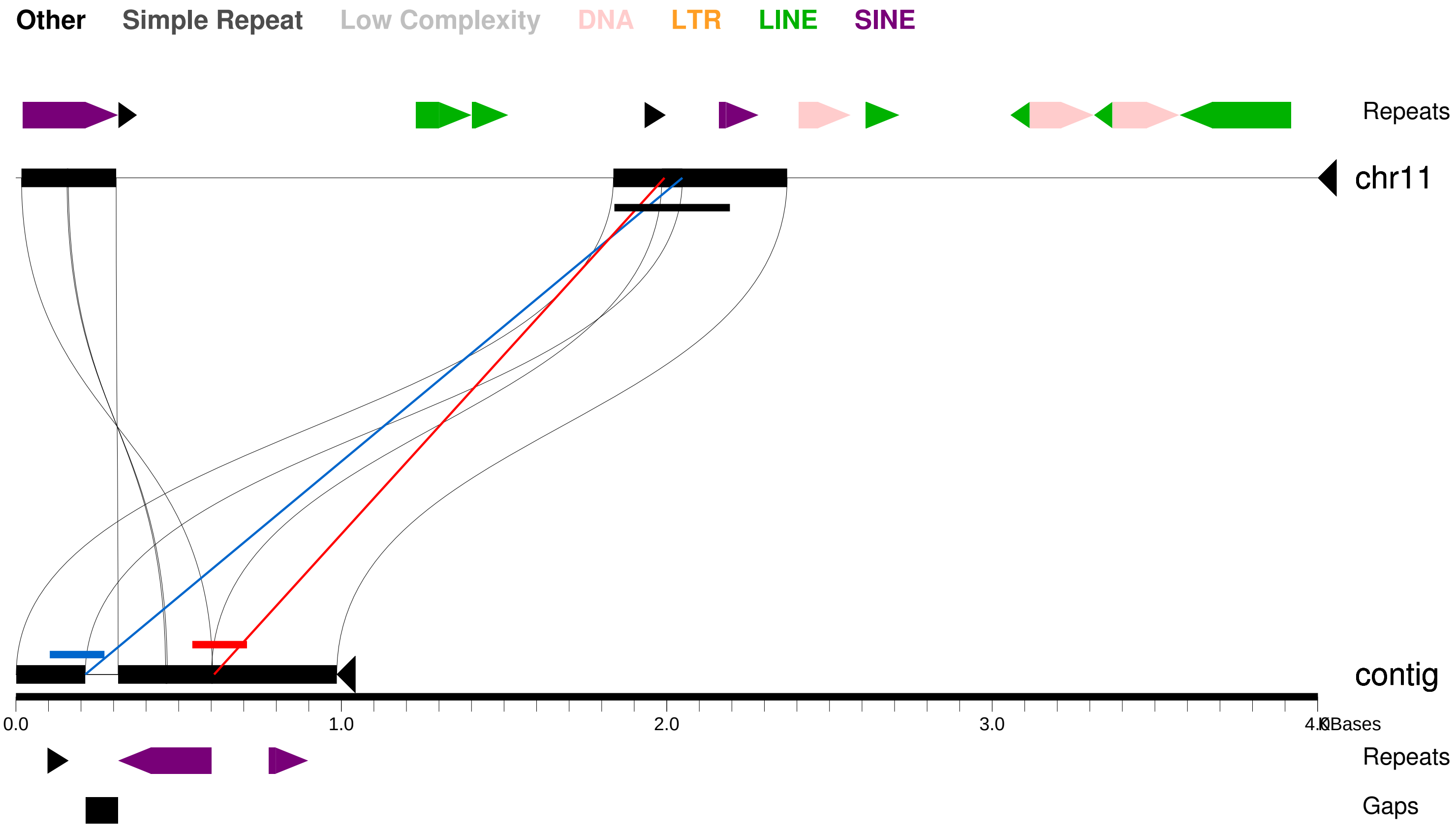

siteID: chr11_123866400 Scafftig1 + Breakpoint: chr11:123866400
chr11:123866348-123866459
Overlap Length: 19 INS size: 422 Genotyped: No

```
left     TGTGATTATATTTCCTATCTAAGTGTGCCCAGTGAAAAAATTTTCAGGTTTT-------AAAAAAAAATA
chrom    TGTGATTATATTTCCTATCTAAGTGTGCCCAGTGAAAAAATTTTCAGGTTTT-------AAAAAAAA-TA
right    NNNNNNNNNNNNNNNNNNNNNNNNNNNNNNNNNNNNNNNNNNNNNNNNNNNNNNNNNNNAAAAAAAA-TA
         11111111111111111111111111111111111111111111111111111111111********2**

left     TACTATCTGGGCCGGGCGCGGTGGCTCACGCCTGTAATCCCAGCACTTTGGGAGGC
chrom    TACTATCTGACCTGCACACTGAACCTAATACATT------CAGAACCTCTGAAGTT
right    TACTATCTGACCTGCACACTGAACCTAATACATT------CAGAACCTCTGAAGTT
         *********22*2*22*2*2*222**2*22*2*2222222***2**2*22*2**22
```

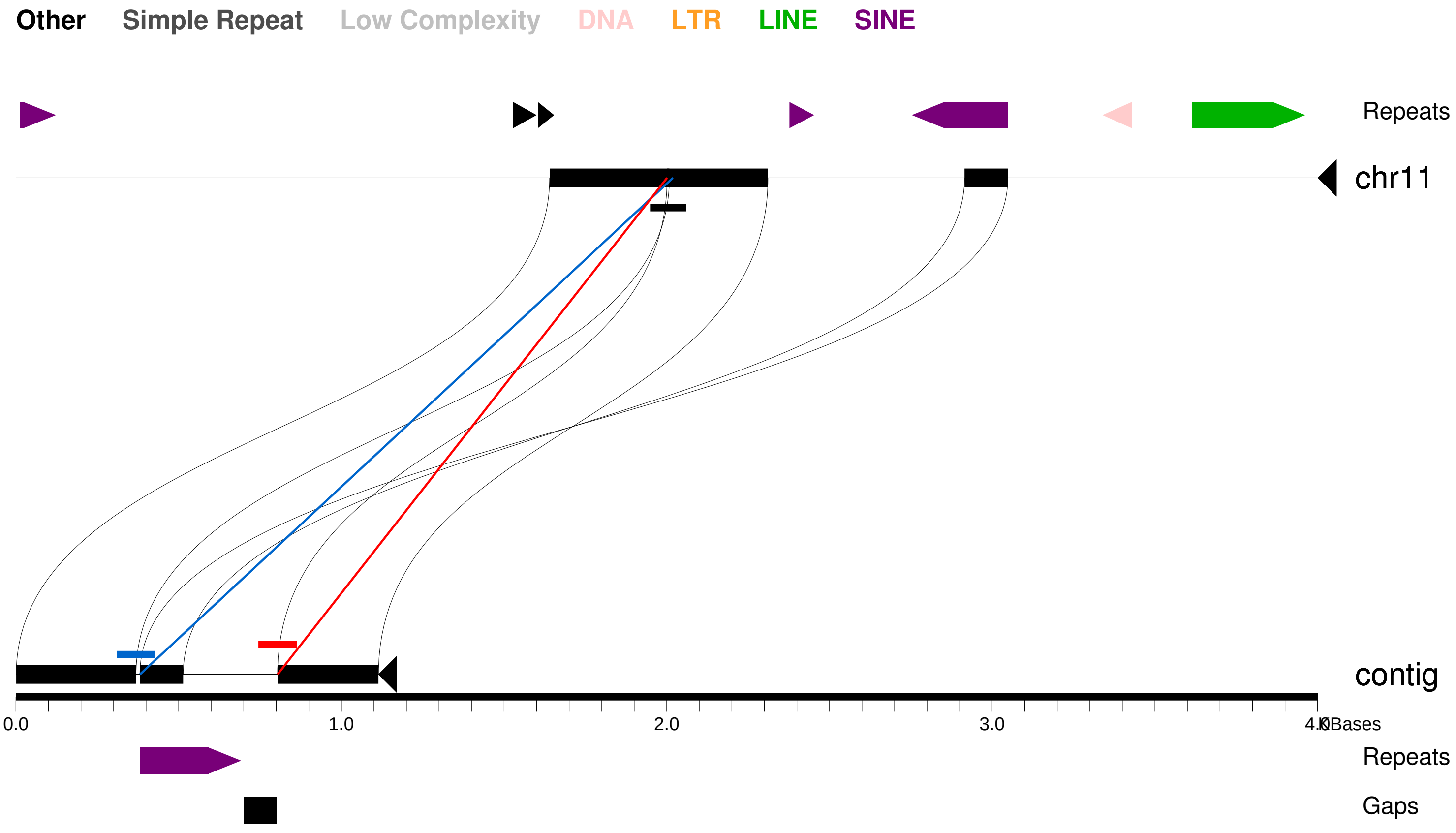

siteID: chr11_126903189 Scafftig1 - Breakpoint: chr11:126903173
chr11:126903013-126903347
Overlap Length: 15 INS size: 422 Genotyped: No

```
left   A----------------------------------------------TCAATCCAATTTTTTTTTTATAT
chrom  AGAAGTCTAAATTGGCACAGCTT-ATTTGGAGGGCAACTGGATGATATCAATCCAATTTTTTTTTTATAT
right  TTTAGTAGAGACGG----AGTTTCACCTTGTTAGCCA--GGATGGTCTCGATCTCCTGACCTCATGATC-
       1NN222NN2N2NN2    22N2212NN2N2NNN22N2  22222N2N**1***111*1111*11*1**11

left   CACATACTATTTGGTTCAGCAATGTTATTTCTGGGAGTTTGTCCTGCAGAAGTACTTATATAATGCACAA
chrom  CACATACTATTTGGTTCAGCAATGTTATTTCTGGGAGTTTGTCCTGCAGAAGTACTTATATAATGCACAA
right  --CACCCGCCTCGGCCTCCCAAAGTG----CTGGGATTA-------CAGGCGTG--------AGCCACCG
       11**11*111*1**11111***1**11111******1*11111111***11**111111111*11***11

left   CAATGTATGGAGAAGAATGTTCATTGCTGAATTATT----------------------------------
chrom  CAATGTATGGAGAAGAATGTTCATTGCTGAATTATTGATATTGATAATGGCACAAAACTGGAAGCTGCTA
right  C-------------GCCCGGCCATTGCTGAATTATTGATATTGATAATGGCACAAAACTGGAAGCTGCTA
       *1111111111111*111*11***************2222222222222222222222222222222222

left   ---TTTTTNNNNNNNNNNN---------------------------------------------------
chrom  TAGTATTTATCATTAGCAGAGTGTTTAGAAAGATTGTGGTGTATTTGTGTAATGGAATATGTTAACTAAG
right  TAGTATTTATCATTAGCAGAGTGTTTAGAAAGATTGTGGTGTATTTGTGTAATGGAATATGTTAACTAAG
       222*2***22222222222222222222222222222222222222222222222222222222222222

left   --------------------------------------------------------
chrom  AGAAGTAGCTATACAAGTACTGGGGTTGAAAGGCTAGGCCCAGCGTGCCAGAATGC
right  AGAAGTAGCTATACAAGTACTGGGGTTGAAAGGCTAGGCCCA--------------
       222222222222222222222222222222222222222222
```

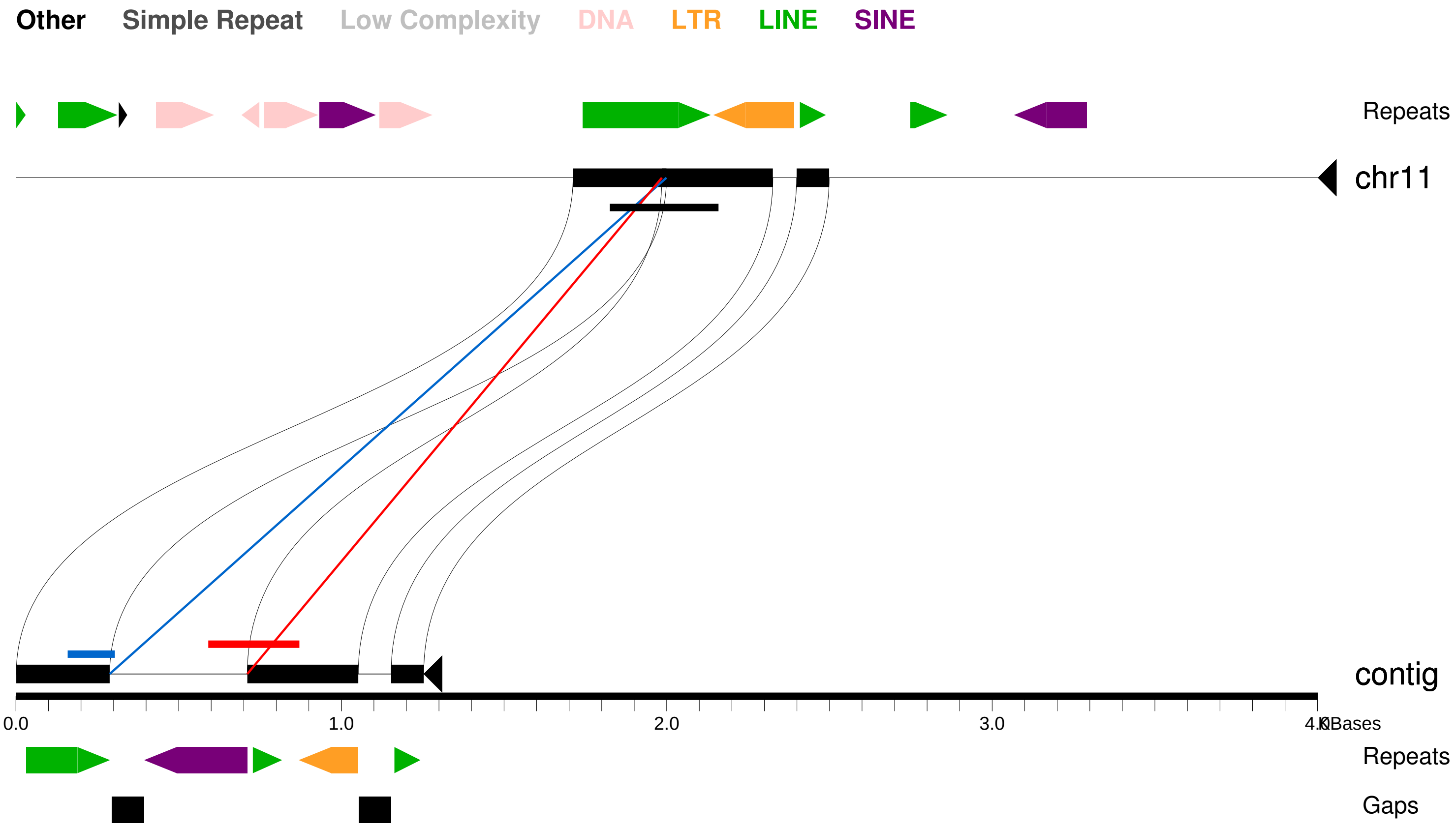

siteID: chr11_126941129 Scafftig8 - Breakpoint: chr11:126941118
chr11:126940958-126941291
Overlap Length: 14 INS size: 425 Genotyped: No

```
left    GCTC---------------------------------------TTCTCACTTCACAGGGCTGGTTTTTTA
chrom   GCTCACATCTCACCTCGGGAAGTAGGAAATTGAATGACTGCTCTTCTCACTTCACAGGGCAGGTTTTTTA
right   TTT-----------------AGTAGA---------GACGGGGTTTCAC-CTTGTTAGC-CAGGATGGTCT
        11*1                22222N         222N2NNN***1*1***111**11*2**1*11*11

left    TCCTCTGGGGAAAGTCTTTGTGAATGCTCACAGATTCTGCCCATAGAATCTAAGCTCCTTGAGGGCTAGG
chrom   TCCTCTGGGGAAAGTCTTTGTGAATGCTCACAGATTCTGCCCATAGAATCTAAGCTCCTTGAGGGCTAGG
right   CGATCTCCTGA----CCTCGTGA-TCCACCC-GCCTCGGCCTC------CCAAAGTGCTGGGATTACAGG
        111***111**1111*1*1****1*1*1*1*1*11**1***11111111*1**11*1**1*111111***

left    AGGAAGCAGGCTGCCATTGTCCACTGCTATATTTNNNNNNNNNNN-------------------------
chrom   AGGAAGCAGGCCGCCATTGTCCACTGCTATATTTTCAATGCCTGGTACACTTCCCAACACTTTGTAAGTG
right   CGTGAGCCACCGCGCCCGGCCCACTGCTATATTTTCAATGCCTGGTACACTTCCCAACACTTTGTAAGTG
        1*11***111*N11*111*1**************222222222222222222222222222222222222

left    ----------------------------------------------------------------------
chrom   CTCCCTACATATTCATTGCAGTGAATAAATAAAAATAACAACTAATACTTTTGTACCGCTTACTATGTAC
right   CTCCCTACATATTCATTGCAGTGAATAAATAAAAATAACAACTAATACTTTTGTACCGCTTACTATGTAC
        2222222222222222222222222222222222222222222222222222222222222222222222

left    ------------------------------------------------------
chrom   CAGACACTGTTGTAGGGGCTTTGCAGGTATTAATTTATTTCATCTCCACTACAA
right   CAGACACTGTTGTAGGGGCTTTGCAGGTATTAATTTATTTC-------------
        22222222222222222222222222222222222222222
```

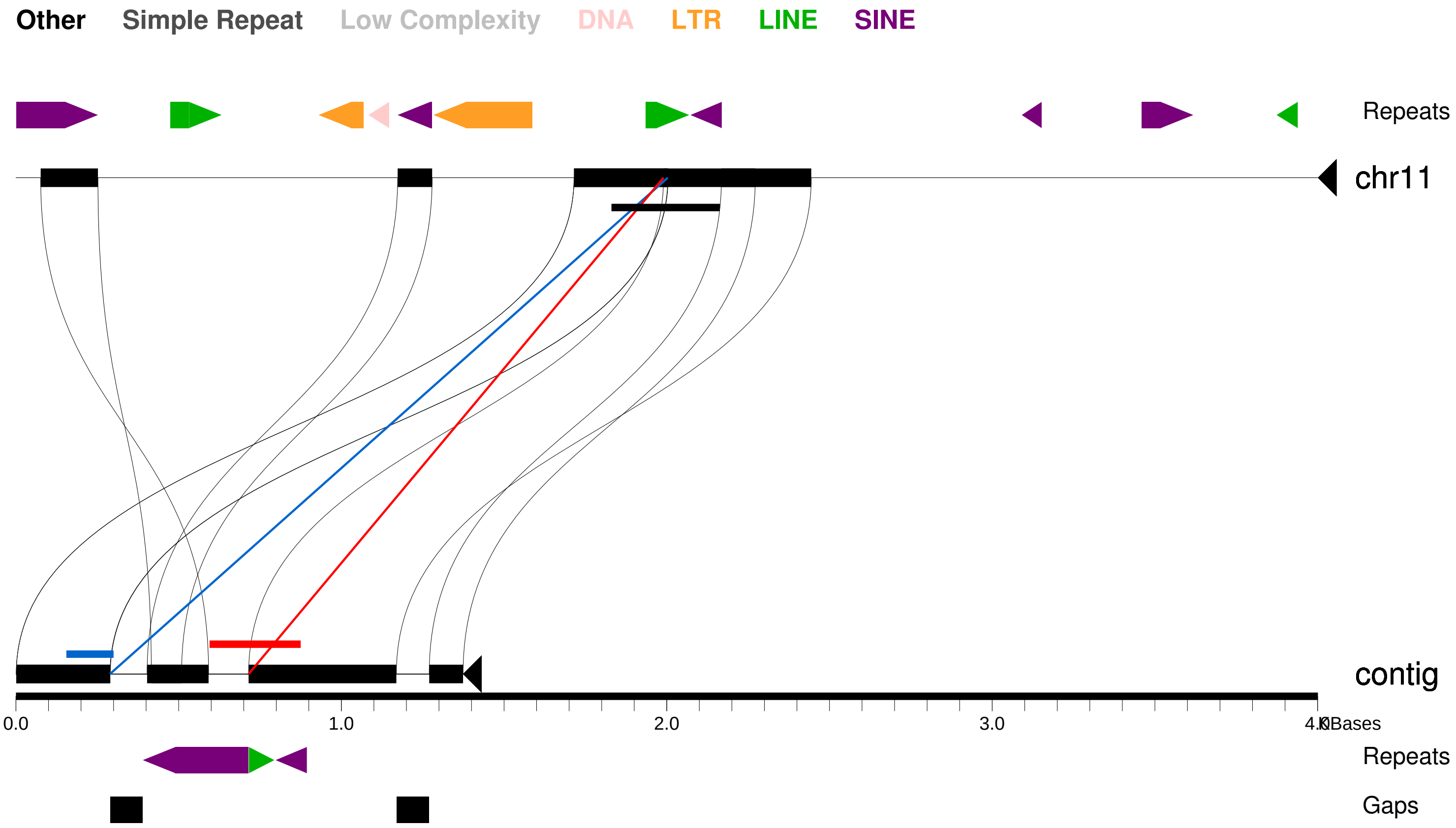

siteID: chr11_127997824 Scafftig1 + Breakpoint: chr11:127997827
chr11:127997774-127997886
Overlap Length: 7 INS size: 322 Genotyped: Yes

```
left   CATATATCTTGATCCCTACCTAACT-CTA----TACTGAAAT-ATCAATC-CAGATAGATAGCAGTTTTT
chrom  CATATATCTTGATCCCTACCTAACT-CTA----TACTGAAAT-ATCAATC-CAGATAGATAGCAGTTCTA
right  CCGCCCGCCTCGGCC-TCCCAAAGTGCTGGGATTACAGGCGTGAGCCACCGCGCCCGGCCAGCAGTTCTA
       *111111*1*111**1*1**1**1*1**11111***1*111*1*1*1*1*1*11111*11*******2*2

left   TTTTTTTTTTTTTTTTTTTTTTTTTTTTTTTTTTTTTGAGACGGAGTCTCGCTCTG
chrom  AATATAAGTAATATTACAATAAAGTTTTCAAAGAAAACATA-GAAGAC-----CTT
right  AATATAAGTAATATTACAATAAAGTTTTCAAAGAAAACATA-GAAGAC-----CTT
       22*2*222*22*2**2222*2222****2222222222*2*2*2**2*22222**2
```

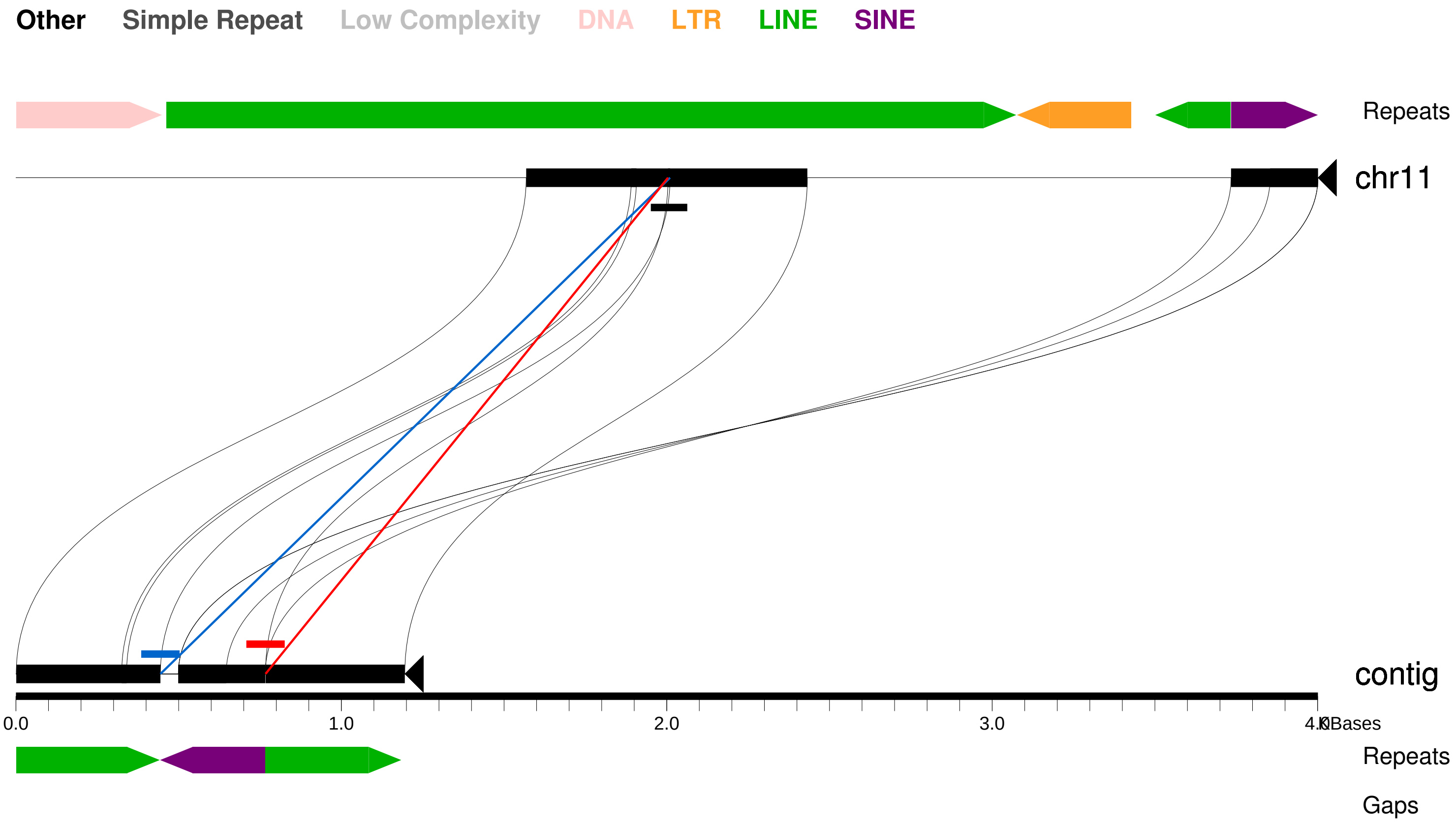

siteID: chr11_128997459 Scafftig3 + Breakpoint: chr11:128997467
chr11:128997307-128997640
Overlap Length: 14 INS size: 297 Genotyped: Yes

```
left    T-------------TCTGTCCAGCAATATTTTCCTAGAGCACAGAAAAGTTCCAGGAATATCCTAGCCTC
chrom   TGCCCATGTA-TGTTCTGTCCAGCAATATTTTCCTAGAGCACAGAAAAGTTCCAGGAATATCCTAGCCTC
right   AGA--ATGGCGTGAACCCGGGAGGCGGAGCTTGC-AGTGAGCCGAGA---TCCCGCCA----CTGCACTC
        12N  222NN122N1*11111**1111*11**1*1**1*11*1**1*111***1*11*1111**111***

left    TCAGTATATTTTTGTGAATGAACAAATACAATACACTTTAACATAACAAGGTTTTGTCATTTACTCTTTC
chrom   TCAGTATATTTTTGTGAATGAACAAATACAATACACTTTAACATAACAAGGTTTTGTCATTTACTCTTTC
right   -CAGCCTGG----GCGACAGAGCGAG---ACTCCGTCTCAAAAAAAAAA-------------------TA
        1***11*111111*1**11**1*1*1111*1*1*111*1**1*1**1**1111111111111111111*1

left    AAGATGAAGCCAGTAGAACTGAAAGGAAAATACTGGC-------TCA--CGCCTG------TAATC----
chrom   AAGATGAAGCCAGTAGAACTGAAAGGAAAATACTGTACTATTATTCAATCACCTGATGAACTAATATTTT
right   AAAAAAAAAAAAAAAAAA---AAAGGAAAATACTGTACTATTATTCAATCACCTGATGAACTAATATTTT
        **1*11**111*11*1**111**************222222222***22*2****222222****22222

left    --CCAGCACTTTGGGAGGCCGAGGCGGGCGG------ATCAC----GAGGTCAGG-AGATCGAG--ACC-
chrom   CACCTACATATTTTAAAAATAAAACAAATTATCTTAAATTTCTATTGAGAGCACATAGCTTGAGTTACTT
right   CACCTACATATTTTAAAAATAAAACAAATTATCTTAAATTTCTATTGAGAGCACATAGCTTGAGTTACTT
        22**22**22**222*22222*22*222222222222**22*2222***22**222**2*2***22**22

left    ATCCCG--GCTA-AAAC----GGTGAAACCCCGTCTCTACTAAAAATACAAAAAAT
chrom   ATTCTGTTGCTCCAAACCAAAGATTAAGAAAGTTCTTTTTTAGAAA-ATAGTTCAT
right   ATTCTGTTGCTCCAAACCAAAGATTAAGAAAGTTCTTTTTT---------------
        **2*2*22***22****2222*2*2**222222***2*22*1N11121N1NNNN11
```

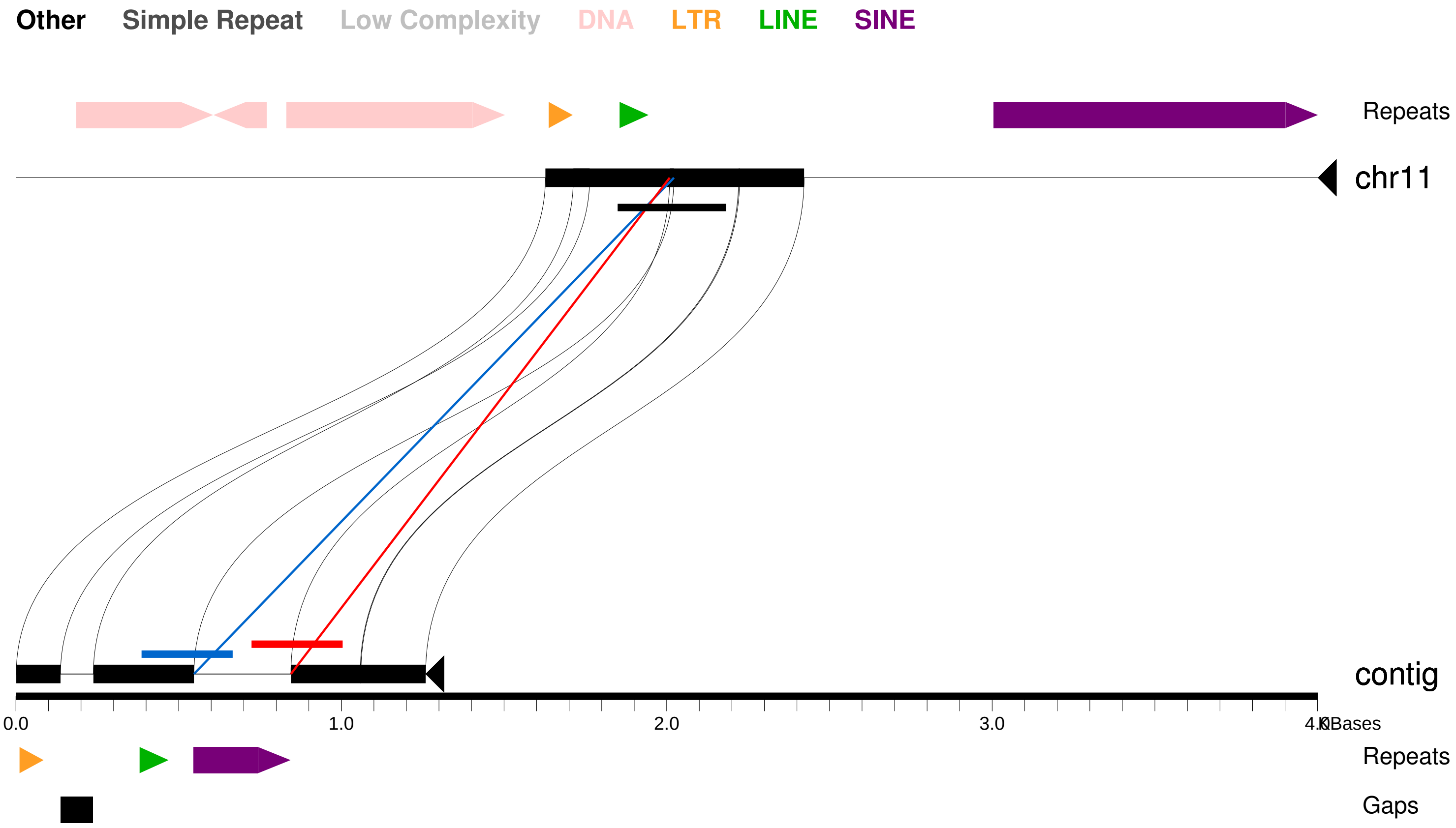

siteID: chr11_13092210 Scafftig1 + Breakpoint: chr11:13092186
chr11:13092141-13092245
Overlap Length: 15 INS size: 411 Genotyped: No

```
left     A-GTGCACCT-GGTCACACAA-GAGCTCTGCTCA-AGATGT----------ACAGGGAGATTAATGTAAT
chrom    A-GTGCACCT-GGTCACACAA-GAGCTCTGCTCA-AGATGT----------ACAGGGAGATTAATGTAAT
right    CCGCCCGCCTCGGCCTCCCAAAGTGCTGGGATTACAGGCGTGAGCCACCGCACCCGGCAATTAATGTAAT
         11*11*1***1**1*1*1***1*1***11*1*1*1**11**1111111111**11**11***********

left     TTTCNNNNNNNNNNNNNNNNNNNNNNNNNNNNNNNNNNNNNNNNNNNNNNNNNNNNNNNNNNN
chrom    TTTCATGCCTGCTGACAAAACATCCATTCTGTAGCCCATGGATCAAAGA--------------
right    TTTCATGCCTGCTGACAAAACATCCATTCTGTAGCCCATGGATCAAAGA--------------
         ****2222222222222222222222222222222222222222222222222222222222
```

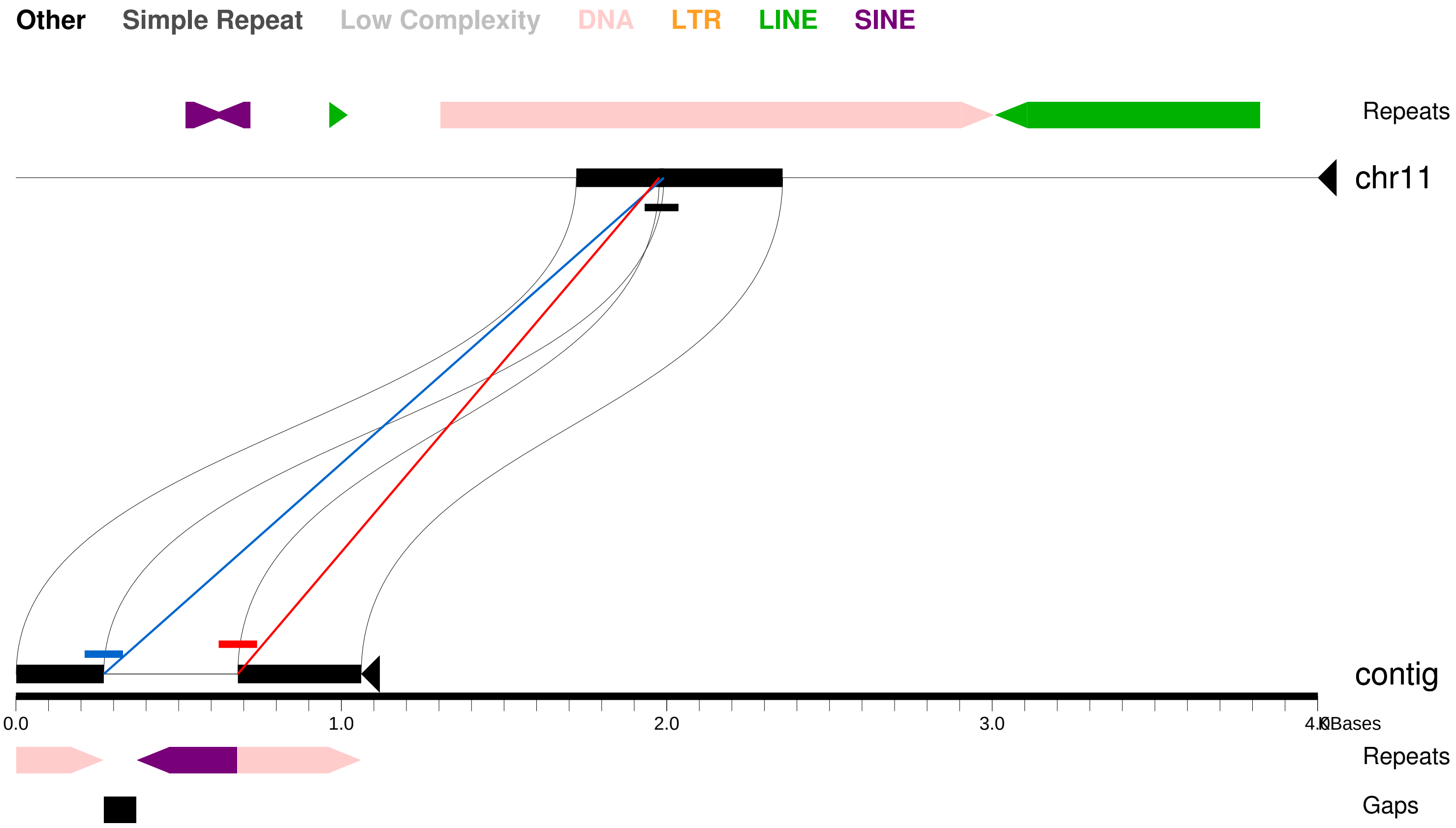

siteID: chr11_131361329 Scafftig4 - Breakpoint: chr11:131361321
chr11:131361279-131361380
Overlap Length: 18 INS size: 296 Genotyped: Yes

```
left    TTTCCTGGAGTTT-CTGATGTTTGTGGGTATATTTTAGCCTTT----------------AAAAAATTATT
chrom   TTTCCTGGAGTTT-CTGATGTTTGTGGGTATATTTTAGCCTTT----------------AAAAAATTATT
right   ATCGCGCCACTGCACTCCAGCCTGGGCGACAGAGCGAGACTCCGTCTCAAAAAAAAAAAAAAAAAAATATT
        1*11*111*1*111**111*11**1*1*11111111**1**111111111111111111******1****

left    ACTTGTGGCCGGGCGCGGTGGCTCACGCCTCTAATCCCAGC--ACTTTGGGAGGCCGAGGCGGGCGGA
chrom   ACTTGTGATTTCTCATTTTAATTAAATACTCACATCCATACCTAATTTT-------------------
right   ACTTGTGATTTCTCATTTTAATTAAATACTCACATCCATACCTAATTTT-------------------
        *******222222*2222*222*2*222***22****222*22*2***22222222222222222222
```

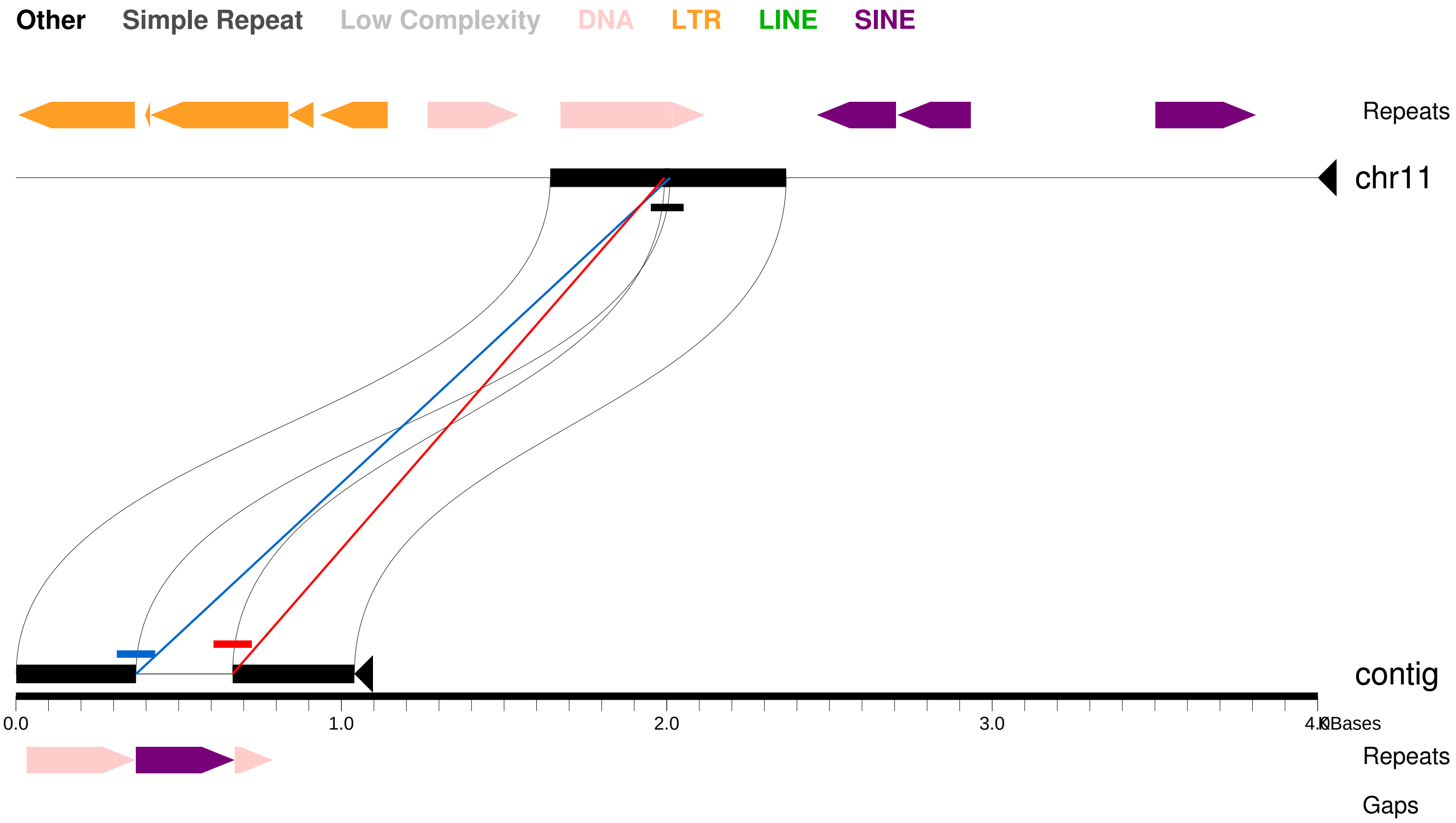

siteID: chr11_134659534 Scafftig1 + Breakpoint: chr11:134659542
chr11:134659495-134659601
Overlap Length: 13 INS size: 479 Genotyped: No

```
left     TTGTCAAT-TAAATTTGCCAAATT-------TAATTGACAT----CTCCACTTTTCTTTTTCTGCGGTAT
chrom    TTGTCAAT-TAAATTTGCCAAATT-------TAATTGACAT----CTCCACTTTTCTTTTTCTGCGGTAT
right    CCGCCCGCCTCGGCCTCCCAAAGTGCTGGGATTACAGGCGTGAGCCACCGCGCCCGGCCTTCTGCGGTAT
         11*1*1111*11111*1*****1*1111111*1*11*1*1*1111*1**1*11111111***********

left     TTNNNNNNNNNNNNNNNNNNNNNNNNNNNNNNNNNNNNNNNNNNNNNNNNNNNNNNNNNNNNN
chrom    TTGAAAGCAAATCCCCAAACATTCTATCATTTGTTCTGTAAGTACATCA------------
right    TTGAAAGCAAATCCCCAAACATTCTATCATTTGTTCTGTAAGTACATCA------------
         **2222222222222222222222222222222222222222222222222222222222222
```

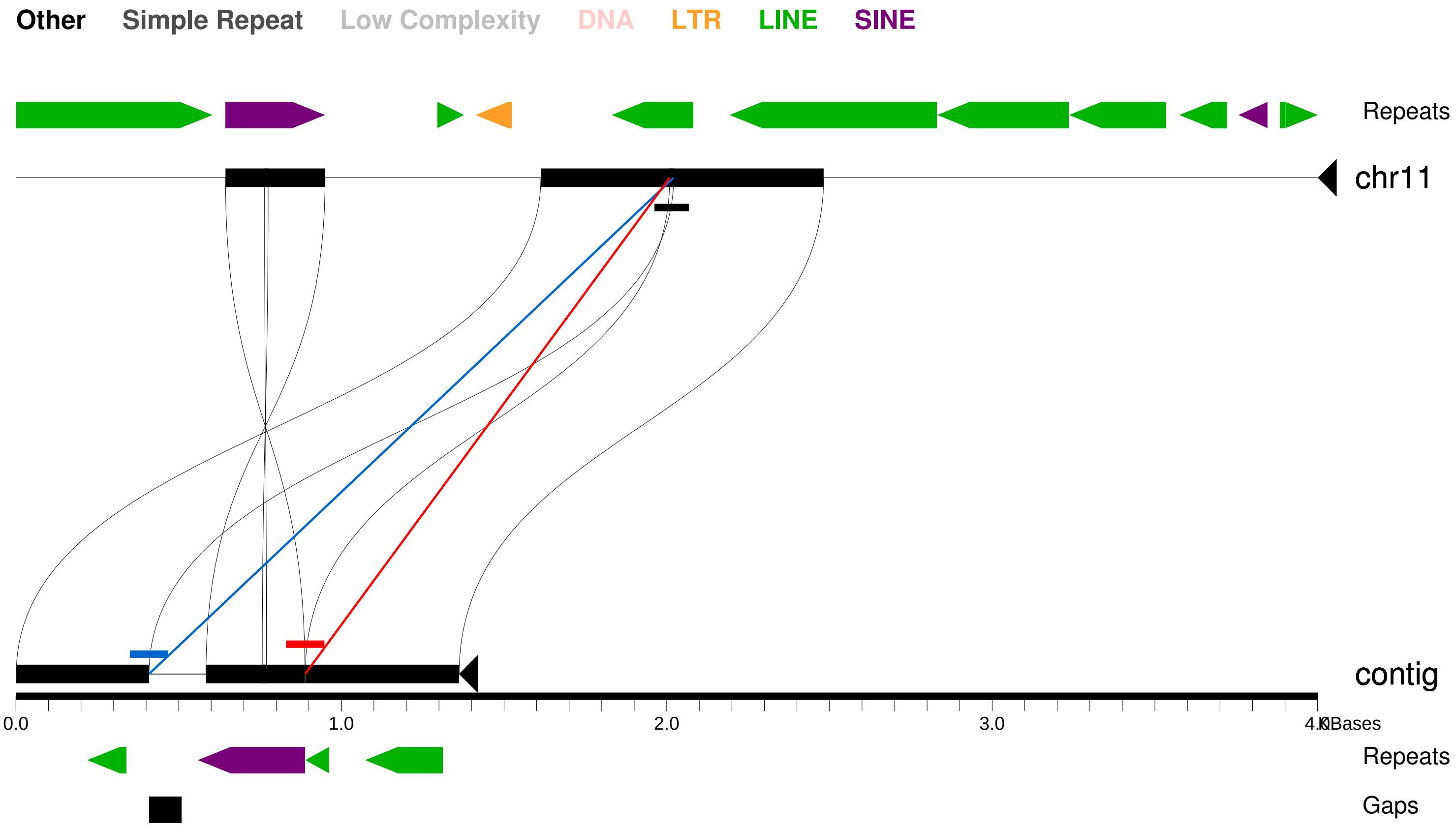

siteID: chr11_13813046 Scafftig4 + Breakpoint: chr11:13813020
chr11:13812975-13813079
Overlap Length: 15 INS size: 326 Genotyped: Yes

```
left    ATCATGAGGATGGAGCCCTC---------------ATGAATGGGAGTACTGCTTTTATAGAAAGAGGTCC
chrom   ATCATGAGGATGGAGCCCTC---------------ATGAATGGGAGTACTGCTTTTATAGAAAGAGGTCC
right   GACA-GAGCGAGACTCCGTCTCAAAAAAAAAAAAAAAAAAAAAAAAAAAAAAAAAAAAAAAAAAGAGGTCC
        11**1***111*111**1**111111111111111*11**1111*11*11111111*1*1**********

left    TGGGGGCCGGGCGCGGTGGCTCACGCCTGTAATCCCAGCACTTTGGGAGGCCGAGGCGGGTGGA
chrom   TGGGGAGCTGCCTTGTTCCTTCCAGCATGTGA-----GGATTCAGTAAG---AAGGC------A
right   TGGGGAGCTGCCTTGTTCCTTCCAGCATGTGA-----GGATTCAGTAAG---AAGGC------A
        *****22*2*2*22*2*222**22**2***2*22222*2*2*22*22**2222****222222*
```

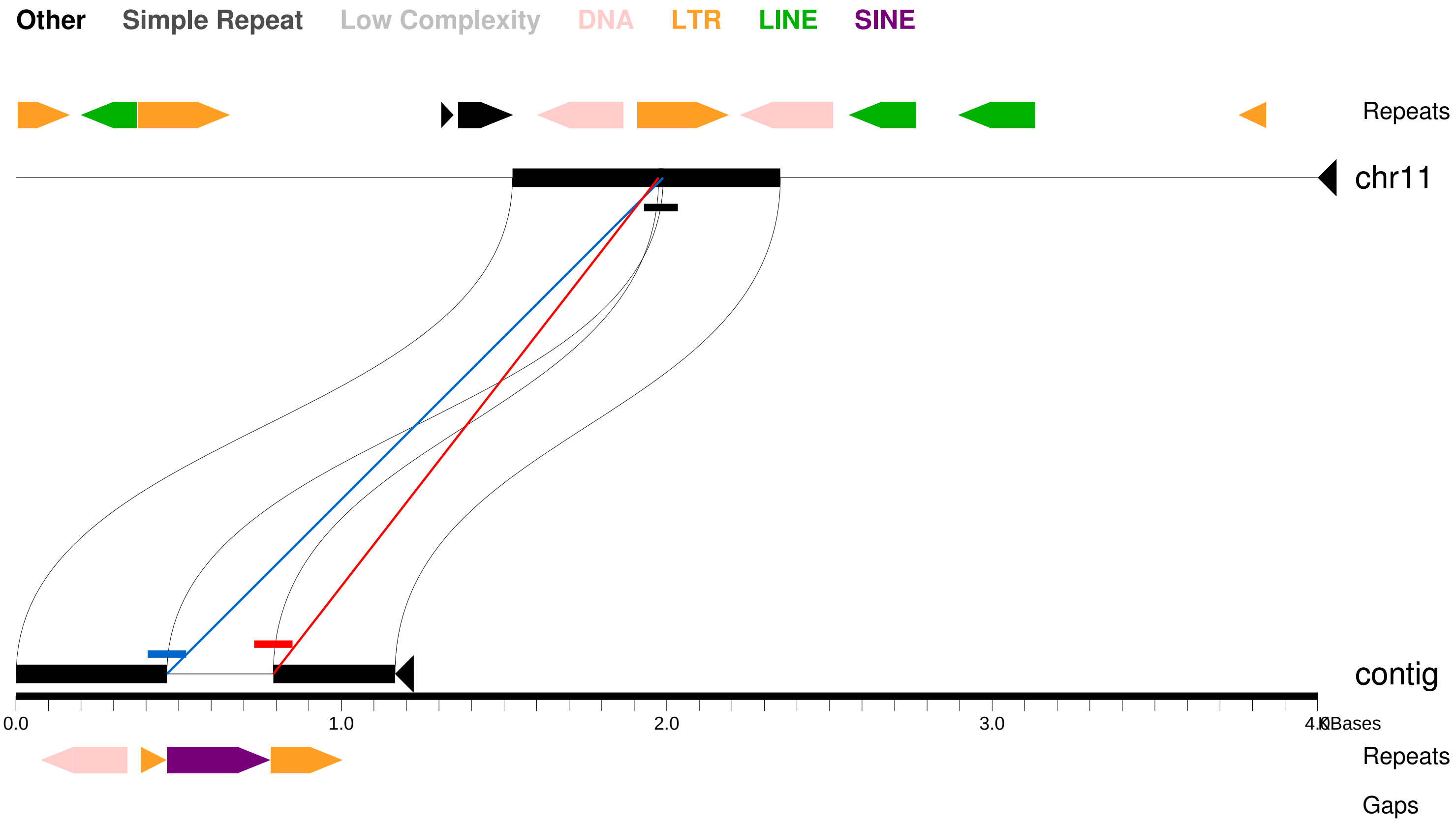

siteID: chr11_19382781 Scafftig1 + Breakpoint: chr11:19382761
chr11:19382614-19382969
Overlap Length: 63 INS size: 466 Genotyped: No

```
left   ----------------------------------------------------------------------
chrom  GAAAATTTTGTAACCTCGCTAAACTGGCTTAGATACTATAAAATGAGGATGGCAATAGTTTCTGCCTTGT
right  CA------------CCCGCC----------------------------------------TCGGCCTCCC
       N2            2N222N                                        22N2222NNN

left   ------------------------------AAGTACTTCGCACAACACCTGGTGTATTTTGTTGGTGCTG
chrom  AGGGTTGTATTAGGTTAGATAAAATGAGTAAAGTACTTCGCACAACACCTGGTGTATTTTGTTGGTGCTG
right  AAAGT-------GCTGGGATTACAGGCGTGAGCCACCGCGCCCGGC------------------------
       2NN22       2N2NN222N2N2N2N22N*111**11***1*11*111111111111111111111111

left   ACTAAAAGTGAGCTCTTTTTTTTTTCTCTCAGCCTCTGCTAAGCATTTTGTTATTGAGGTTTTAGTGGGT
chrom  ACTAAAAGTGAGCTCTTTTTTATGTCTCTCAGCCTCTGCTAAGCATTTTGTTATTGAGGTTTTAGTGGGT
right  -CTA---GTGAGCTCTTTTTTATGTCTCTCAGCCTCTGCTAAGCATTTTGTTATTGAGGTTTTAGTGGGT
       1***111**************2*2**********************************************

left   NNNNNNNNNNNNNNNNNNNNNNNNNNNNNNNNNNNNNNNNNNNNNNNNNNNNNNNNNNN-----------
chrom  CTACTTTGGGACTACCATTTCTTGTCCATAGAAATCTGAGAAAAAGTCACACTTTACAGTGGACTTGGGT
right  CTACTTTGGGACTACCATTTCTTGTCCATAAAAATCTGAGAAAAAG------------------------
       222222222222222222222222222222N222222222222222NNNNNNNNNNNNN

left   ----------------------------------------------------------------------
chrom  TCCAATCCCAGCATGTCACCTCTCGAGTGACAGGCATGACTGGGTGTCACATCTTATCCTTGGGTATCAG
right  ----------------------------------------------------------------------

left   ------
chrom  TTTGCT
right  ------
```

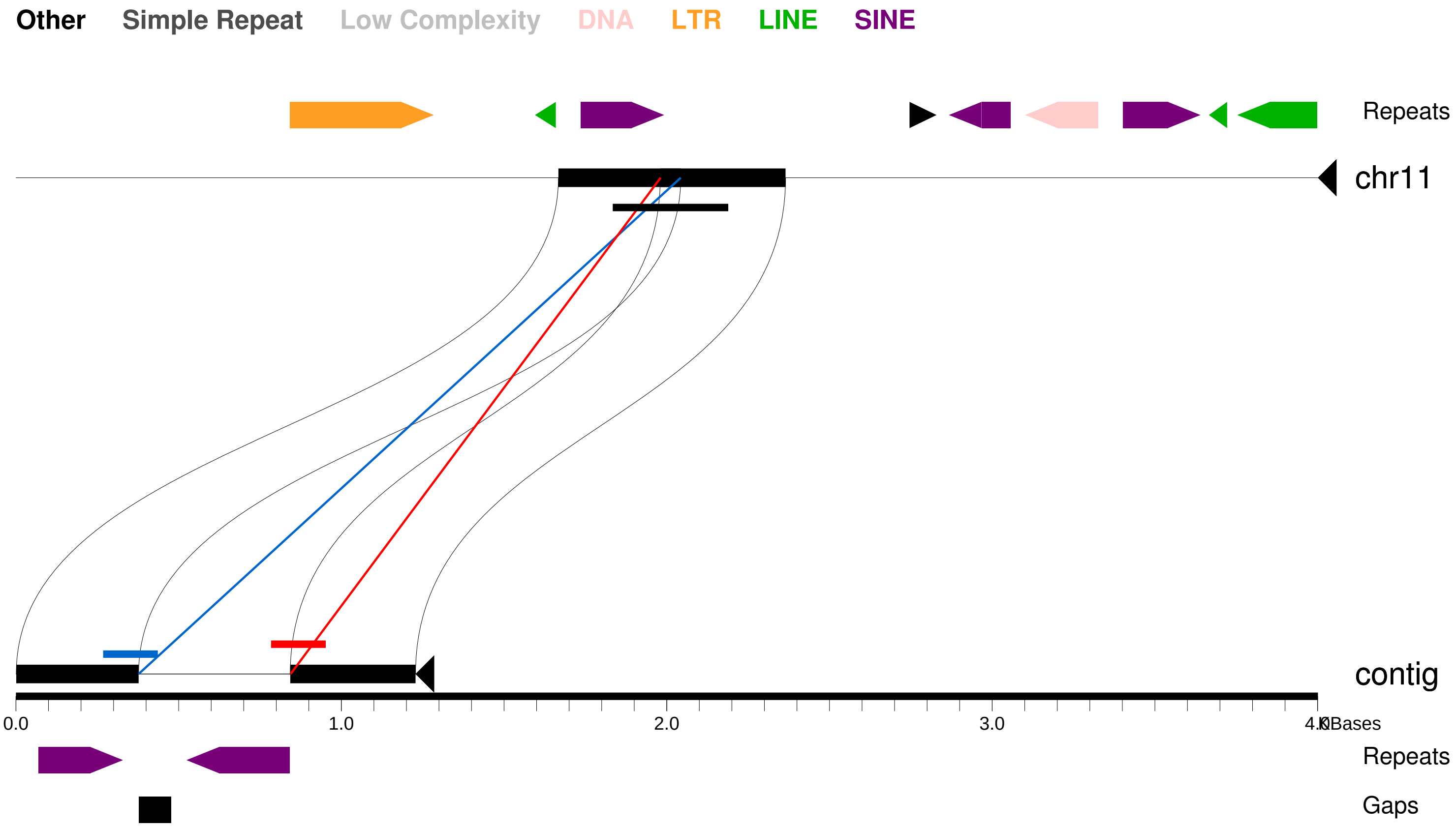

siteID: chr11_19963016 Scafftig1 + Breakpoint: chr11:19962994
chr11:19962834-19963172
Overlap Length: 19 INS size: 312 Genotyped: Yes

```
left    ----------------------GACTGTGCACTCTTTGGTGTCAAATCAAATGGGGTTGGTCGCTAACAA
chrom   CTGTGC--AGCCAGTAGTGCTTGACTGTGCACTCTTTGGTGTCAAATCAAATGGGGTTGGTCGCTAACAA
right   GGAGGCTGAGGCAGGAGAA--TGGC-GTGAACCCG--GG---------AAGCGGAGCTTGCAGTGAGCCG
        NNNN221122N222N22NN  2*1*1***1**1*111**111111111**11**1*1*1*11*11*1*11

left    AAATTCCTTATCAGGGTGACATGTCTTTATATTCAAGCAGTAGAAGTCACACATGTGACTATGTTGAATT
chrom   AAATTCCTTATCAGGGTGACATGTCTTTATATTCAAGCAGTAGAAGTCACACATGTGACTATGTTGAATT
right   AGATTGCGCCACTG-----CA-GTCCGCAG--TCCGGCC-TGGGCGACAGAGC-GAGACTCCGTCTCAAA
        *1***1*1111*1*11111**1***111*111**11**11*1*11*1**1*111*1****11**111*11

left    TTTTTTTTTATGGAGACACTGAATAATAAATAGCATGAGGTTGGC-----CGGGCGCGGTGGCTCACGCC
chrom   TTTTTT--TATGGAGACACTGAATAATAAATAGCATGAGGTTGAATTTAACTTACCCACAGCCAATCCTT
right   AAAAAA--AATAAATAAATAAATAAATAAATAGCATGAGGTTGAATTTAACTTACCCACAGCCAATCCTT
        11111221**11*1*1*111*11********************2222222*222*2*222*2*222*222

left    TGTAATCCCAGCACTTTGGGAGGCCGAGGCGGG--TGGATCATGAGGTCAGGA---GATCGA-------G
chrom   TGAAATGACAGTGCAT-GGTAGGACTATTTAGAAATGGACATTTACATAACTATTTGATCAATTGCTTAG
right   TGAAATGACAGTGCAT-GGTAGGACTATTTAGAAATGGACATTTACATAACTATTTGATCAATTGCTTAG
        **2***22***22*2*2**2***2*2*2222*222****222*2*22*2*22*222****2*2222222*

left    ACCATCCTGG--CTAA--CAA---GGTGAAAC--CCT----------GTC-TCTACTAAA----
chrom   TTCTTCCTCTTACTTATGCAAATGGGTGGGTTTTCCTATGGTGTGTGGTCATGTACAGAGCCTT
right   TTCTTCCTCTTACTTATGCAAATGGGTG------------------------------------
        22*2****2222**2*22***222****NNNN  111          111 1N111NN1N
```

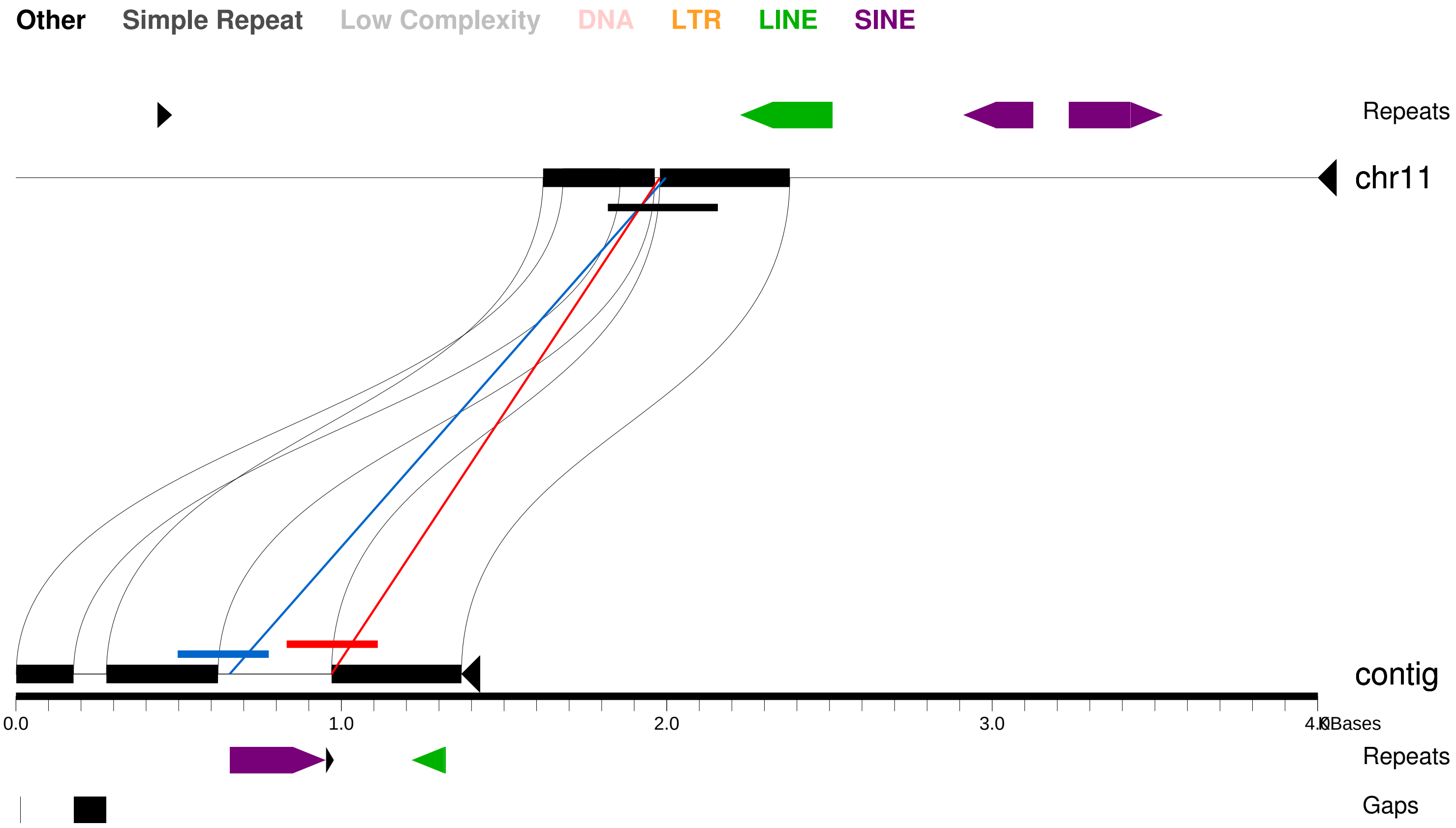

siteID: chr11_20375982 Scafftig1 + Breakpoint: chr11:20375997
chr11:20375837-20376170
Overlap Length: 14 INS size: 375 Genotyped: No

```
left   GAA--------------GTATGTCTCCCTGTCTGACTGTGTTATTTGATAAGATTGTTCTCATTGCATTC
chrom  GAAATGCGTTTTTGGAAGTATGTCTCCCTGTCTGACTGTGTTATTTGATAAGATTGTTCTCATTGCATTC
right  NNNNNNNNNNN-----------------------------------------------------------
       111NNNNNNNN      111111111111111111111111111111111111111111111111111111

left   TCTCTCTCTCTCTCTTTCCCTCTCCCTCCCCTCCTCCATCTACCCTCTTCCTCCTTTCTGAGGCCAAGGC
chrom  TCTCTCTCTCTCTCTTTCCCTCTCCCTCCCCTCCTCCATCTACCCTCTTCCTCCTTTCTGAGGCCAAGGC
right  ----------------------------------------------------------------------
       1111111111111111111111111111111111111111111111111111111111111111111111

left   TCTGAATTCTTTGAGGAGTTAAAACTGCAGGAAAGCCGGGCGCGGTGGCTCA----CGCCTGTAATCCCA
chrom  TCTGAATTCTTTGAGGAGTTAAAACTGCAGGAAATTCCTTTTGGAAGAATAAAATACAGCCATTCTCCTT
right  --------------------AAAACGGCAGGAATTTCCTTTTGGAAGAATAAAATACAGCCATTCTCCTT
       11111111111111111111*****1*******122*222222*22*22*2*2222*22*22*22***22

left   GCACTTT-------------GGGAGGC--CGAG----GCGGGTGGATCATGAG-GTCAGG-AGATCGA--
chrom  CCACTTTTCCTTTATTTAAAGGCAGCCTCCAAGCAGTGCTGCTTGTTCTTCATTGTCTTGCAGAGCTTTT
right  CCACTTTTCCTTTATTTAAAGGCAGCCTCCAAGCAGTGCTGCTTGTTCTTCAATGTCTTGCAGAGCTTTT
       2******2222222222222**2**2*22*2**2222**2*2*2*2**2*2*N2***22*2***2*2222

left   -GACCAT-CCTGGCTAA--CAAGG----TGAAACCCCGTCTCTACTAAAA----
chrom  TGTTCATTCCTTAGTAAAACAAGCGTTTTGGTTTTTCGGGTTCACTGTGTGTGC
right  TGTTCATTCCTTAGT---------------------------------------
       2*22***2***222*11  1111N    11NNNNNNN11NN1NN111NNNN
```

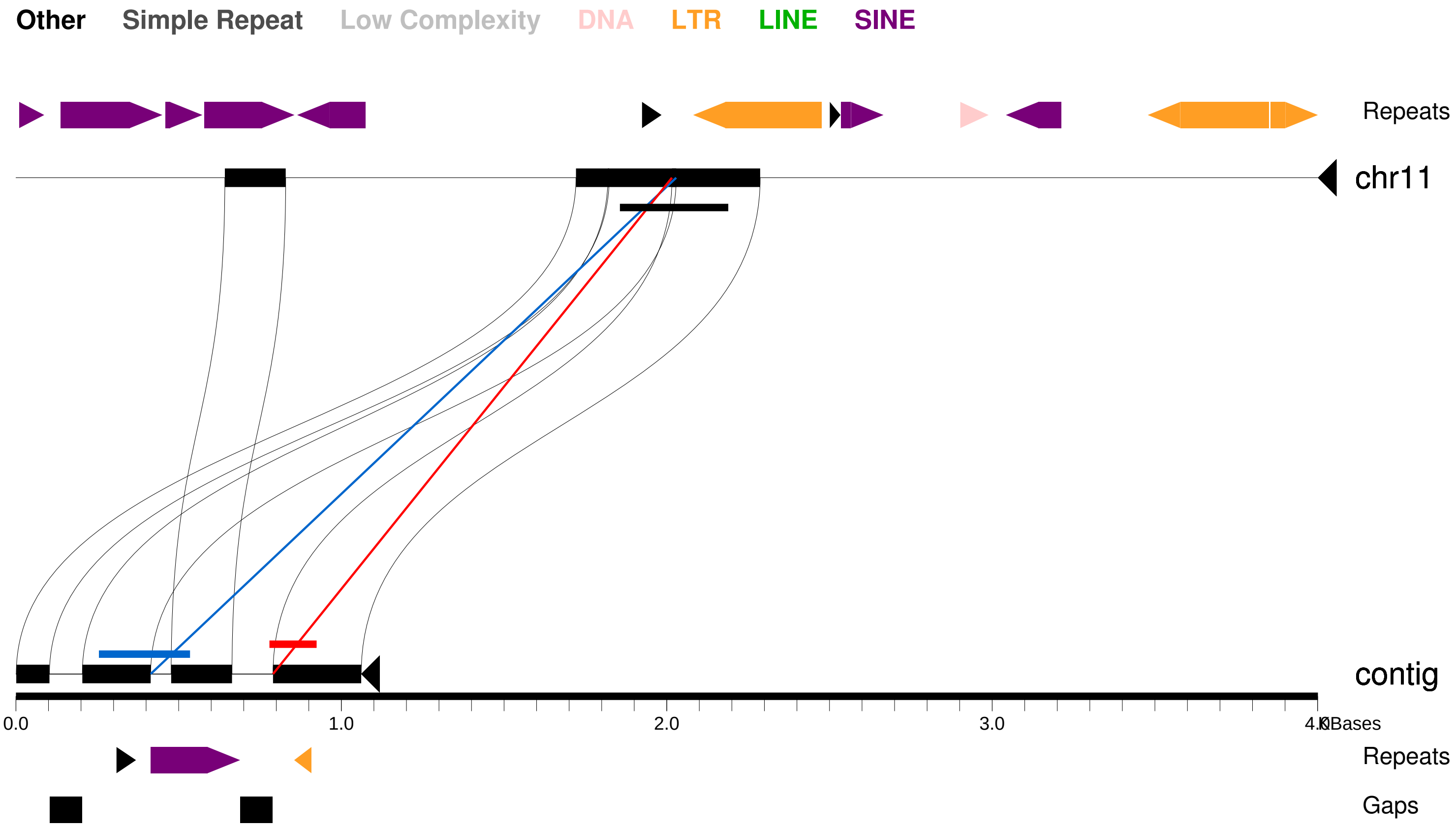

siteID: chr11_23270698 Scafftig5 - Breakpoint: chr11:23270707
chr11:23270547-23270876
Overlap Length: 10 INS size: 229 Genotyped: Yes

```
left   ------CTTTATT-TATTTTATCAGTGCTTTGCAGTTTGTGTTGTTGAGGTCTTTCACCTCATTGGTAAA
chrom  G-TCCTCTTTATT-TCTTTTATCAGTGCTTTGCAGTTTGTGTTGTTGAGGTCTTTCACCTCATTGGTAAA
right  GGTTCACGCCATTCTCCTGCCTCAGC-CTCCCGAGTAGCTGGGACTACAGGCGCCCGCCACCTTG-----
       212N2N*111***1*21*111****11**1111***111**1111*111*1*111*1**1*1***11111

left   ATGGAATCCTAGGTATGCTTTTTTTGCAGCTATTATAAACAAGATTGCTTTCTTATTTTTCAGCTAGTTT
chrom  ATGGAATCCTAGGTATGCTTTTTTTGCAGCTATTATAAACAAGATTGCTTTCTTATTTTTCAGCTAGTTT
right  -------CCCGGCTAA---TTTTTTGTATTTTTAGTAGAGACGGGG-----------TTTCACCGTGTT-
       1111111**11*1**1111*******1*11*1*11**1*1*1*11111111111111*****1*11***1

left   GTAATTGGTGTATAGAGTCACTATTGATTTTTTTTTTTTTTTTTTTTTTT-------TTTTTTTTTTTTTTTT
chrom  GTAATTGGTGTATAGAGTCACTATTGATGTTTGTATGTTGATTTTGTACCTGCATTTTTACTGAATTTTA
right  ---------------AGCCAGGATTGATTTTTGTATGTTGATTTTGTACCTGCATTTTTACTGAATTTTA
       111111111111111**1**11******N***2*2*2**22****2*2222222*****22*222****2

left   TTTTGAG----ACGGAGTCTCG--------CTGTCGCCCAGGCTGGAGTGCAG-TGGCGCAATCTC----
chrom  AAATCAGTTCTATGAATTTTCTGCTGCAGTCTGTAGCTTTTACTGTATATAAGATTACATCATCTACAAA
right  AAATCAGTTCTATGAATTTTCTGCTGCAGTCTGTAGCTTTTACTGTATATAAGATTACATCATCTACAAA
       222*2**2222*2*2*2*2**222222222****2**22222***2*2222**2*22*222****22222

left   --GGCTCACTGCAGGCTCCGC-----CCCCTGGG-------------GTTCA
chrom  AAGGGACAATTTGACTTCTTCTTTTTCAATTTGGATGCTGTTTATTTGTTTA
right  AAGGGACAATTTGACTTCTTCTTTTTCAATTTGGATGCCATTTA--------
       22**22**2*222222**22*22222*222*2**2222NN2222   111N1
```

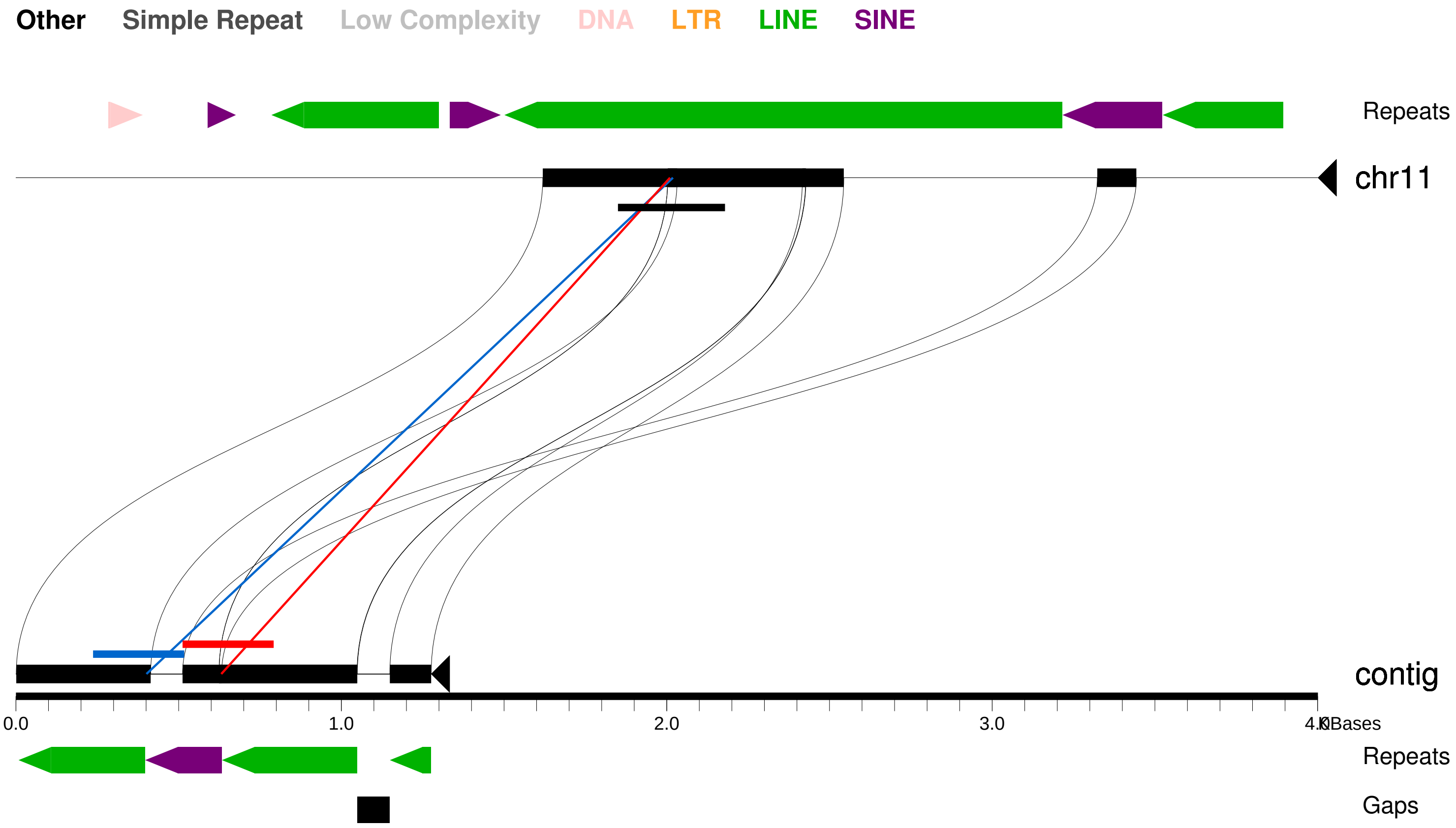

siteID: chr11_23358519 Scafftig1 - Breakpoint: chr11:23358504
chr11:23358458-23358553
Overlap Length: 14 INS size: 293 Genotyped: Yes

```
left    T-------------GCAAATAAAATTGTGTCAACATTAAAGAAAGAAAC----------AAAATGTAAGA
chrom   T-------------GCAAATAAAATTGTGTCAACATTAAAGAAAGAAAC----------AAAATGTAAGA
right   TGAGCCGAGATCCCGCCACTGCACTCCAGCCTGGGCGACAGAGCGAGACTCCGTCTCAAAAAAAAAAAAA
        *1111111111111**1*1*11*1*111*1*111111*1***11**1**1111111111****111**1*

left    AAATACTTTTTTCGGCCGGGCGCGGTGGCTCACGCCTGTAATCCCAGCACTTTGGGAGGCCGAGGCGGGT
chrom   AAATACTTTTTTCACA-----------GAACAA------AAGACTAGCAATTTGCCA--CCATAACCT--
right   AAATACTTTTTTCACA-----------GAACAA------AAGACTAGCAATTTGCCA--CCATAACCT--
        ************22222222222222*22**2222222**22*2****2****22*22**2222*2222

left    GG
chrom   --
right   --
        22
```

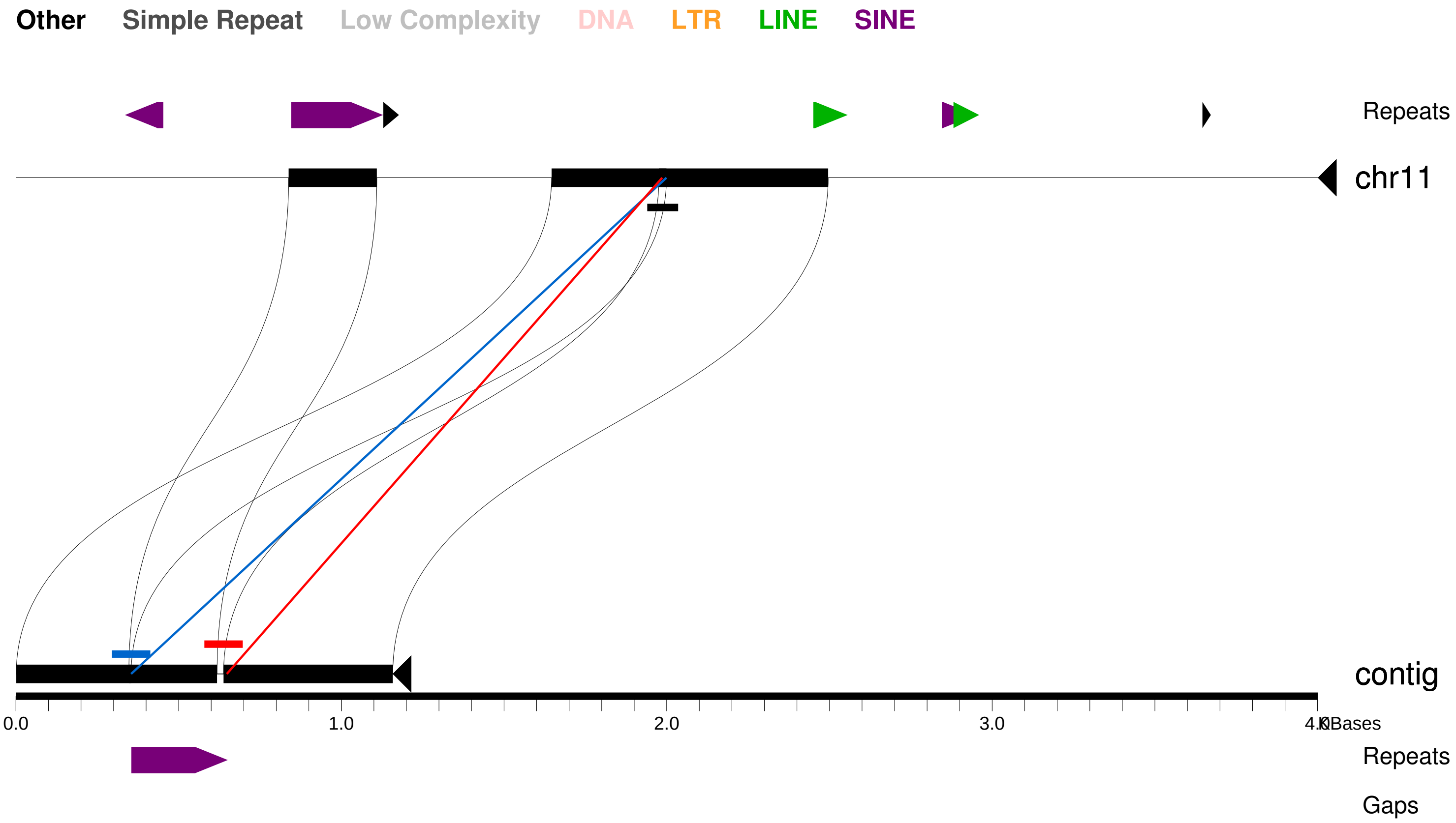

siteID: chr11_23522807 Scafftig5 - Breakpoint: chr11:23522791
chr11:23522745-23522850
Overlap Length: 14 INS size: 305 Genotyped: Yes

```
left    GGAAGTATTTATTGTCACTTTTTTGTCCATTTGATATTCTATTTTT----------------AATTGTAA
chrom   GGAAGTATTTATTGTCACTTTTTTGTCCATTTGATATTCTATTTTT----------------AATTGTAA
right   ---AGTCCACAGTCCGGCCTGGGTGACAGAGCGAGACTCCGTCTCAAAAAAAAAAAAAAAAAAATTGTAA
        111***1111*1*1111*1*111**1*11111**1*1**11*1*111111111111111111********

left    TTCATTGGCCGGGCGCGGTGGCTCACGCC-TGTAATCCCAGCACTTTGGGAGGCCGAGGCGGGTGG
chrom   TTCATTAT----ACTCTCTTTCT-ACGCTGTCTAA----AGCAAT-----AATCTTACTCACTAAA
right   TTAATTAT----ACTCTCTTTCT-ATGCTGTCTAA----GGCAAT-----AATCTTACTCACTAAA
        **1***2222222*2*22*22**2*1**22*2***22221***2*22222*22*22*22*222222
```

Other **Simple Repeat** **Low Complexity** **DNA** **LTR** **LINE** **SINE**

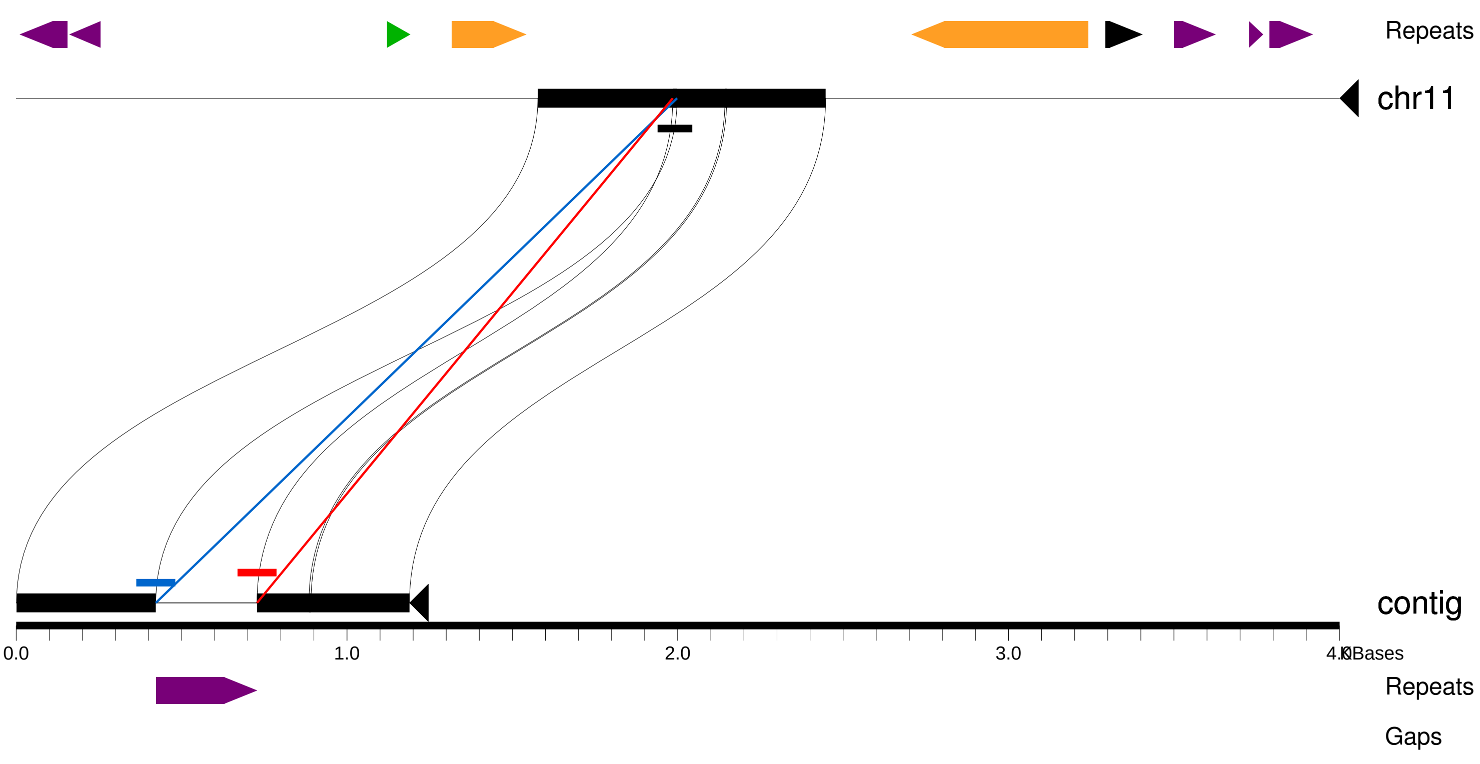

siteID: chr11_25140803 Scafftig3 + Breakpoint: chr11:25140802
chr11:25140756-25140861
Overlap Length: 14 INS size: 414 Genotyped: No

```
left     AAATTCT--TCCCCTTCCTTTAGTTTCTCGACTT-------TGACCT-----TCTCTGATACCATAGCCA
chrom    AAATTCT--TCCCCTTCCTTTAGTTTCTTGACTT-------TGACCT-----TCTCTGATACCATAGCCA
right    CCGCCCGCCTCGGCCTCCCAAAGTG-CTGGGATTACAGGCGTGAGCCACCGCGCCCGGCCACCATAGCCA
         11111*111**11*1***111***11**N*11**1111111***1*1111111*1*1*11**********

left     TACTNNNNNNNNNNNNNNNNNNNNNNNNNNNNNNNNNNNNNNNNNNNNNNNNNNNNNNNNNNN
chrom    TACTTTAAACACTGATCTCAAAATCTCTAATCCCAAATTGTAATCACACC-------------
right    TACTTTAAACACTGATCTCAAAATCTCTAATCCCAAATTGTAATCACACC-------------
         *****2222222222222222222222222222222222222222222222222222222222
```

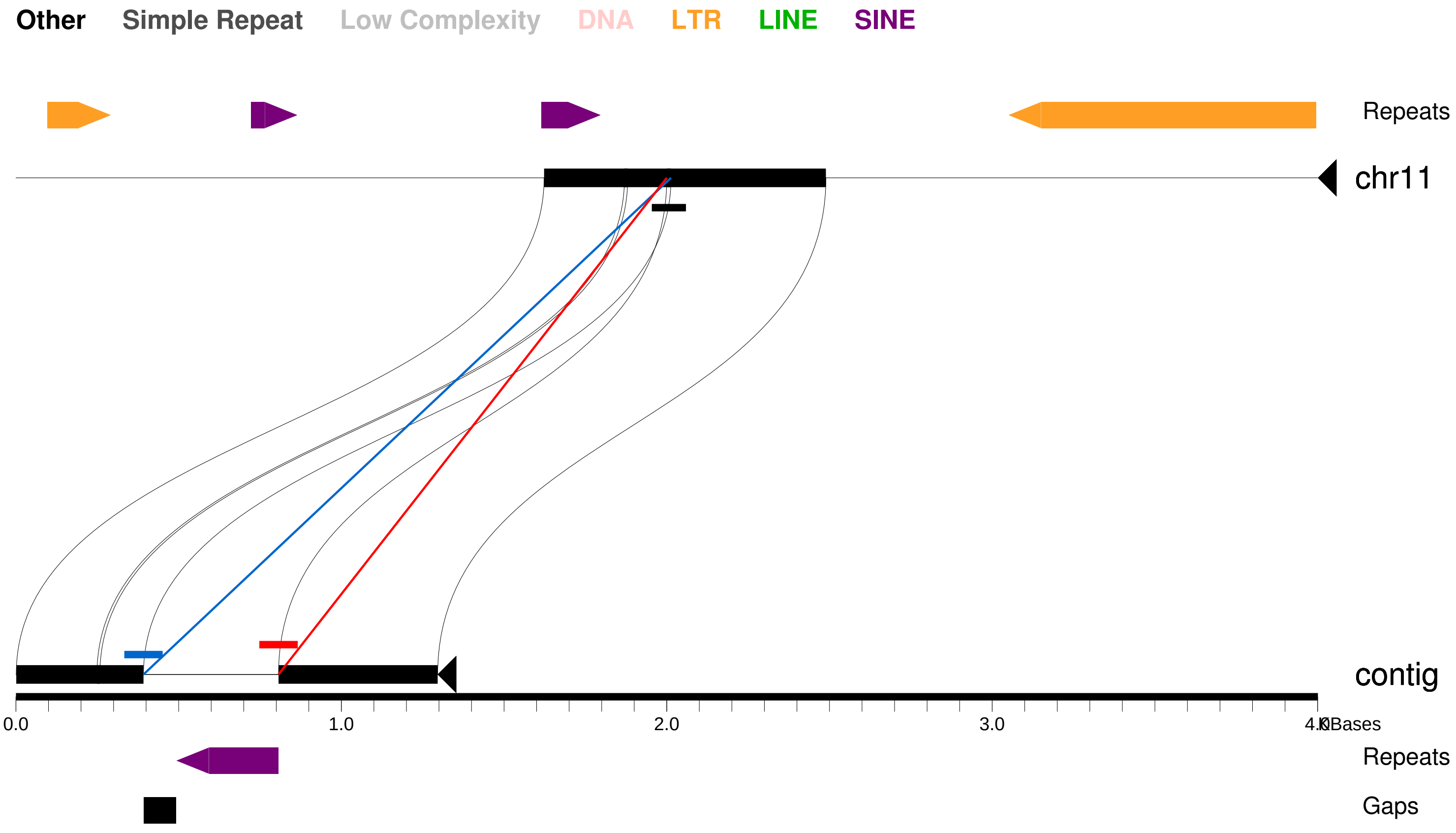

siteID: chr11_26128671 Scafftig2 + Breakpoint: chr11:26128652
chr11:26128626-26128711
Overlap Length: 14 INS size: 310 Genotyped: Yes

```
left   A--------TCTGTAT----ATCTTCTTGGGGAAATGT---------------------CAATTTCCCAT
chrom  A--------TCTGTAT----ATCTTCTTGGGGAAATGT---------------------CAATTTCCCAT
right  CCACCCGCCTCGGCCTCCCAAAGTGCTGGGATTACAGGCGTGAGCCACCGCGCCCGGCCCAATTTCCCAT
       111111111**1*11*1111*11*1**1**111*11*1111111111111111111111***********

left   TTTTTTTTTTTTTTTTTTTTTTTTTGAGACGGAGTCTCGCTCTGTTGCCCAGGCCGGACTGCGGACTGCA
chrom  TTTAATTGGATTTTAATCTTTTTA--------AGTTTTAA-----------------------GAGTTCA
right  TTTAATTGGATTTTAATCTTTTTA--------AGTTTTAA-----------------------GAGTTCA
       ***22**222****22*2*****222222222***2*22222222222222222222222222**2*2**

left   ATGGCGCAATCT
chrom  TTATAT--ATTA
right  TTATAT--ATTA
       2*222222**22
```

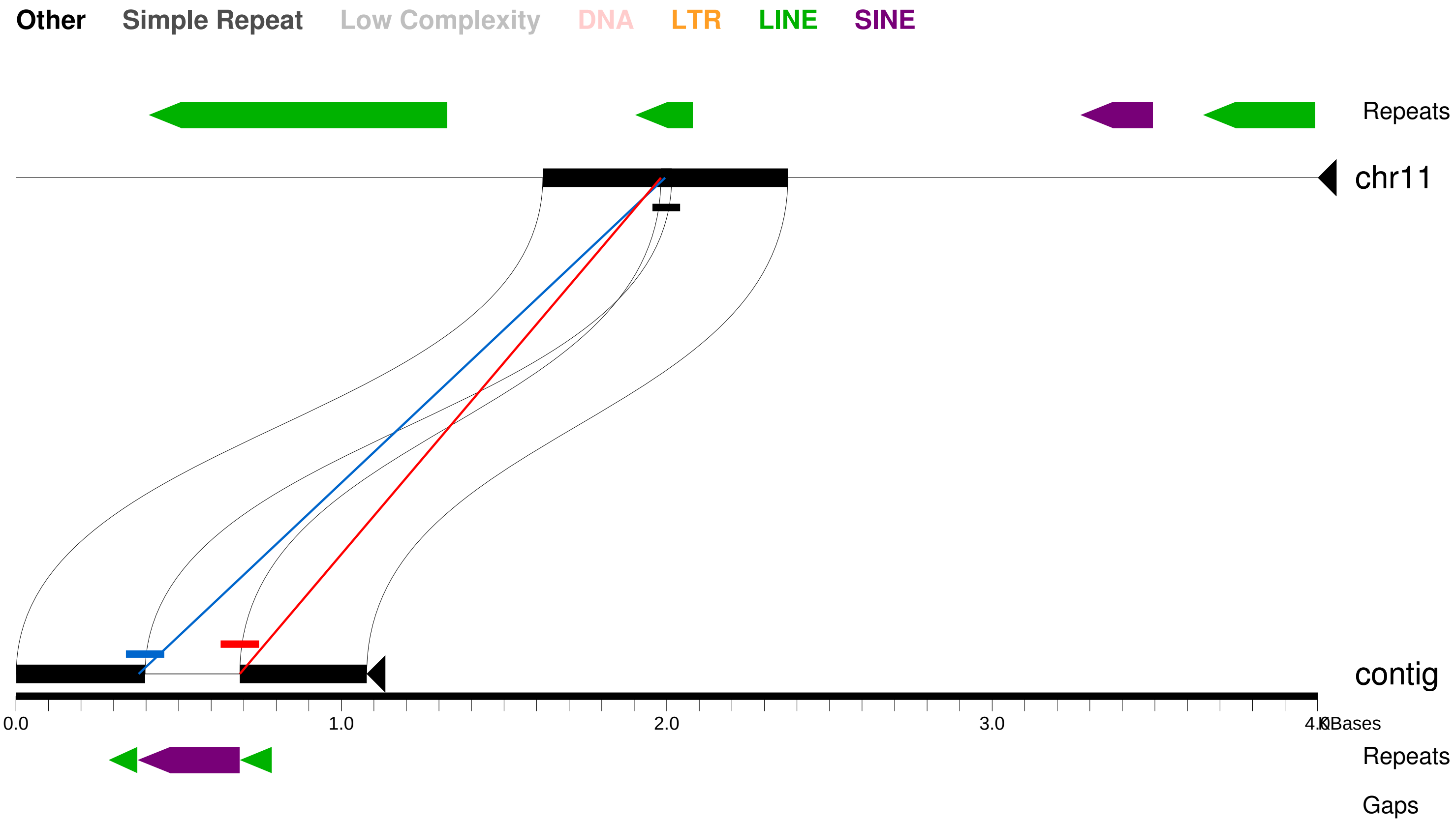

siteID: chr11_26601649 Scafftig3 + Breakpoint: chr11:26601646
chr11:26601587-26601709
Overlap Length: -3 INS size: 310 Genotyped: Yes

```
left   ATACAAAATGAGGTGCAAAAGTAATGAGAATGGAAAAAAATCACAAATTTATAAATTTTTTTTT---TTTT
chrom  ATACAAAATGAGGTGCAAAAGTAATGAGAATGGAAAAAAATCACAAATTTATAAATTTTTTAAA---ACTC
right  TCTCC---TGACCT-CATGATCCACCCGCCTCGGCC---TCCCAAAGTGCTGGGATTACAGGCGTGACTC
       111*1111***11*1**11*111*111*11*1*111111**1****1*11*1111**111NNN11122*2

left   T----TTTCTTTTTCTTTTTCTTTTTTTTTTTTTTTGAGACGGAGTCTCGCTCTGT
chrom  CAAAATAACATACACAAAAAGCAAAATCCAGTAGGTTAAAAATATTTTAATCAACT
right  CAAAATAACATACACAAAAAGCAAAATCCAGTAGGTTAAAAATATTTTAATCAACT
       22222*22*2*222*22222222222*2222*222*2*2*222*2*2*2222222*
```

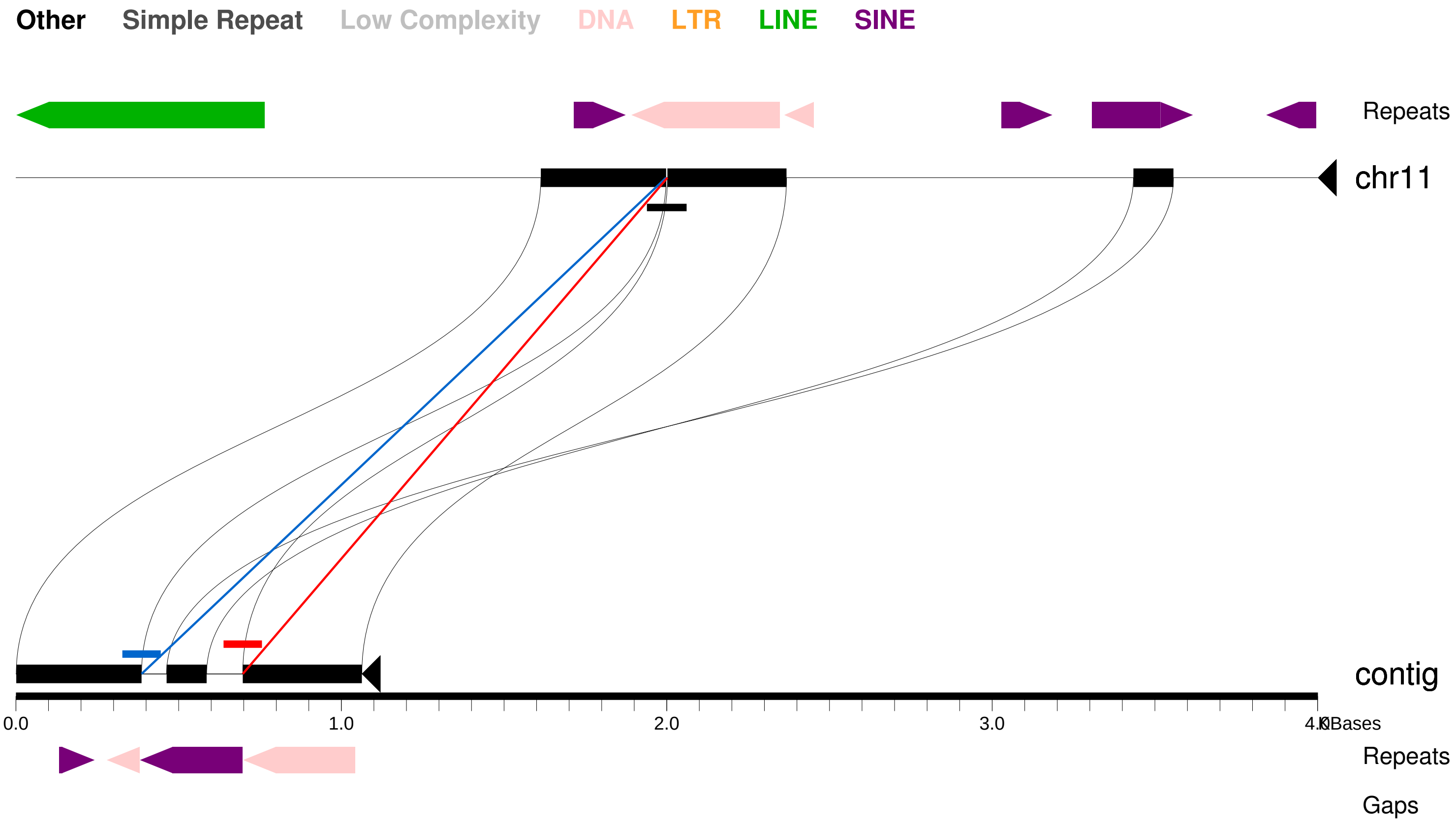

siteID: chr11_27025232 Scafftig2 - Breakpoint: chr11:27025232
chr11:27025189-27025288
Overlap Length: 17 INS size: 127 Genotyped: Yes

```
left    AT-----GTTCTTT---GGCTGAAA--AAGTTGTGTTTCA----AATACGCCTT-----TGGGATTCCAT
chrom   AT-----GTTCTTT---GGCTGAAA--AAGTTGTGTTTCA----AATACGCCTT-----TGGGATTCCAT
right   TGCAGTGGTGCGATCTAGGCTCACTGCAAGCTCCGCCTCCCGGGTTCACGCCATTCTCCTGCGATTCCAT
        1111111**1*11*111****1*1111***1*11*11**11111111*****1*11111**1********

left    GTATTTTCTTTTTTTTTTTTTTTTTTTTTTTTTTTTTTTTGAGACGGAGTCTCGCTCTGTCGCCCAGG
chrom   GTATTTTCTATGAGCCAGCCTTGACCTCCTCTTCCTG--------GCAGGCTC-CTCA----------
right   GTATTTTCTATGAGCCAGTCTTGACCTCCTCTTCCTG--------GCAGGCTC-CTCA----------
        *********2*2222222N2**2222*22*2**22*222222222*2**2***2***2222222222
```

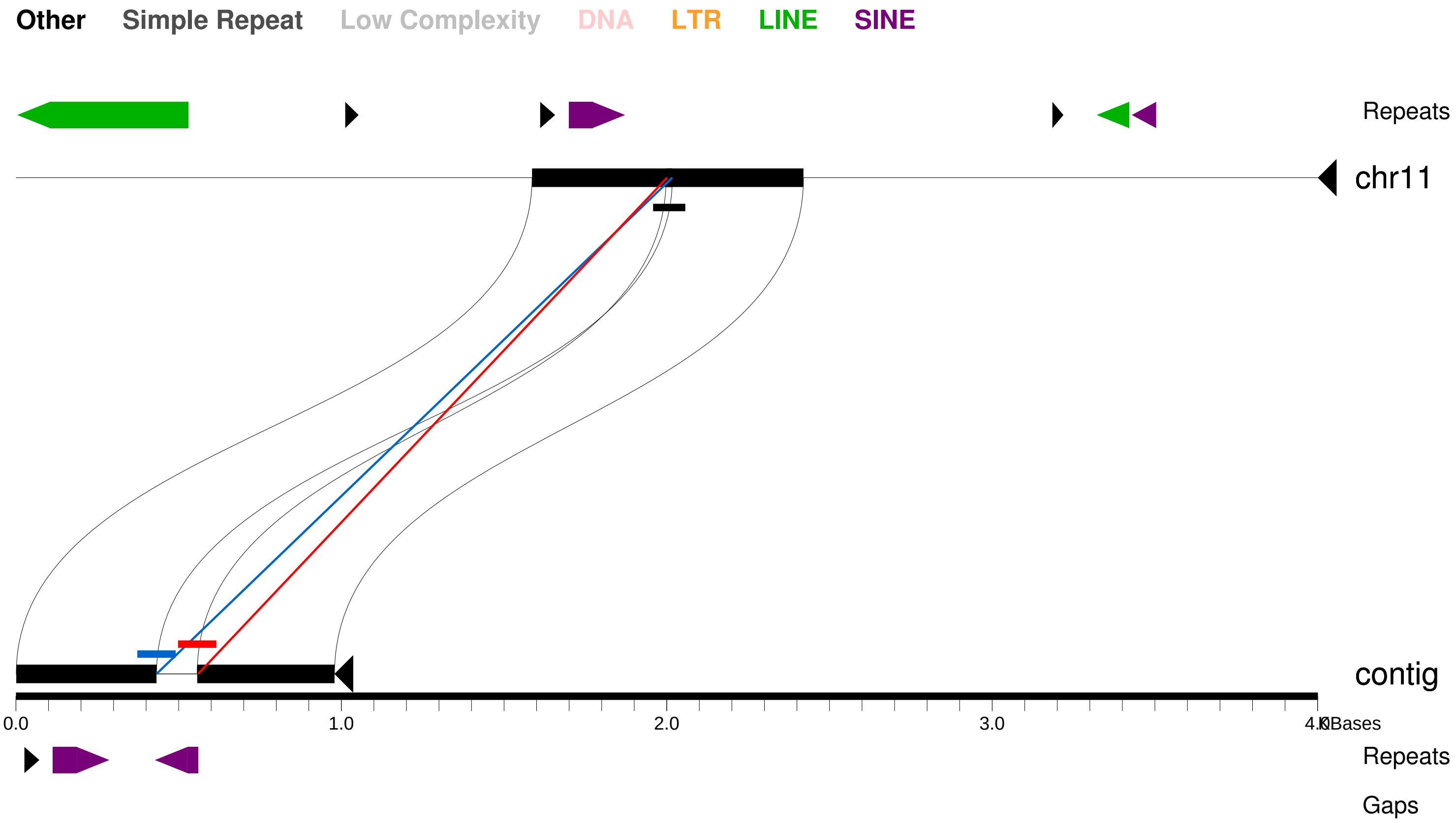

siteID: chr11_32434282 Scafftig13 - Breakpoint: chr11:32434264
chr11:32434219-32434323
Overlap Length: 15 INS size: 296 Genotyped: Yes

```
left     CTCAGAAAGATTCC--CC----------AGCA--ACGTTTTCTCCCAGGGACCCACTTTAAAAATCTAAT
chrom    CTCAGAAAGATTCC--CC----------AGCA--ACGTTTTCTCCCAGGGACCCACTTTAAAAATCTAAT
right    CGCCACTGCACTCCAGCCTGGGCGACAGAGCGAGACTCCGTCTCAAAAAAAAAAAAAAAAAAAATCTAAT
         *1*111111*1***11**1111111111***111**1111****11*111*111*1111**********

left     TTCAGGCCGGGCGCGGTGGCTCACGCCTGTAATCCCAGCACTTTGGGAGGCCGAGGCGGGCGG
chrom    TTCATTTGTTTGGGGGTG----AGG---GAGAAGAAAGTACTTT------CCGTAAAGTACC-
right    TTCATTTGTTTGGGGGTG----AGG---GAGAAGAAAGTACTTT------CCGTAAAGTACC-
         ****22222222*2****2222*2*222*22*2222**2*****222222***2222*22*22
```

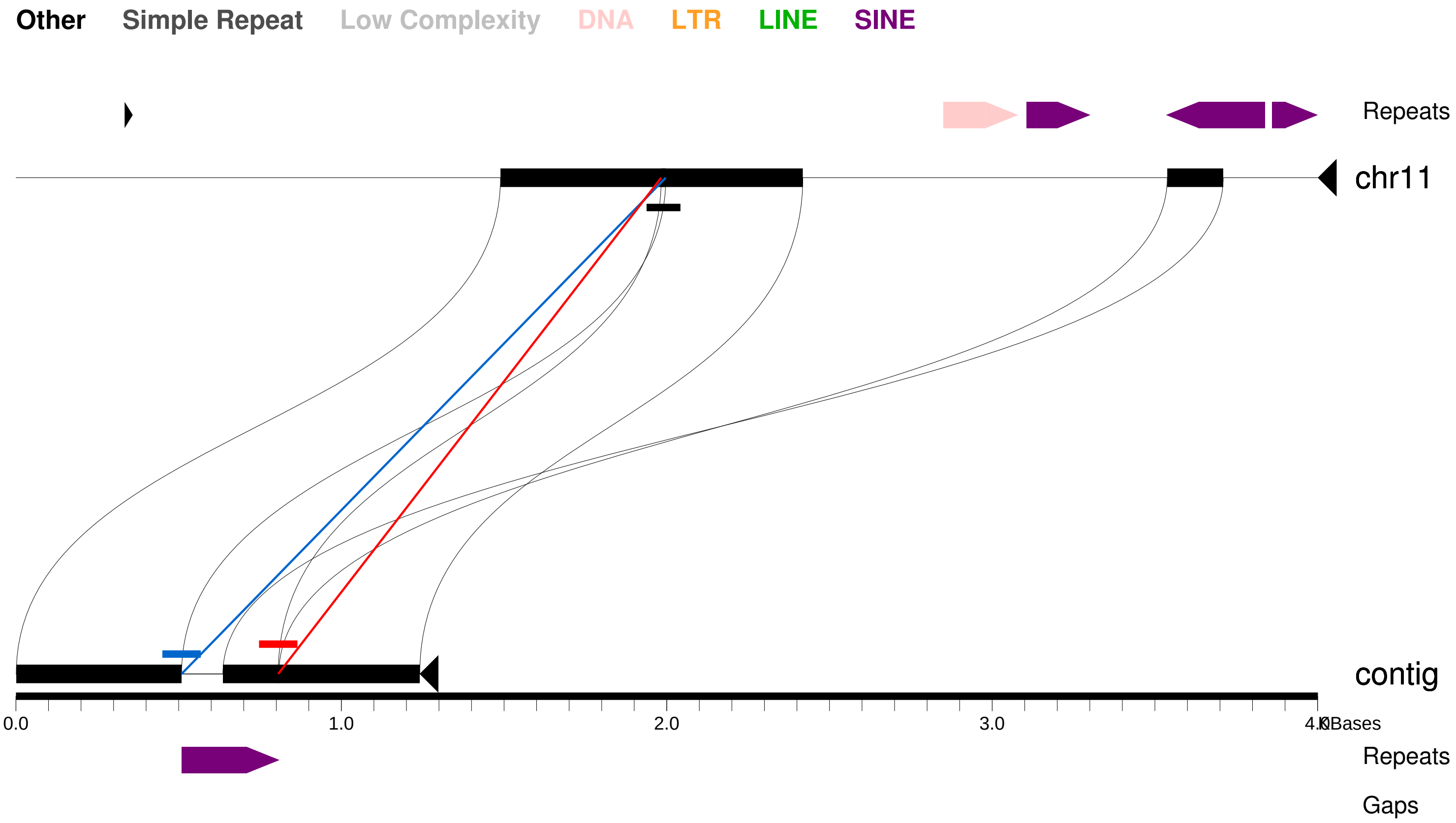

siteID: chr11_3267817 Scafftig10 + Breakpoint: chr11:3267797
chr11:3267750-3267856
Overlap Length: 13 INS size: 134 Genotyped: Yes

```
left    GAAAGGCAGGACAACTTGAAGAAGAGGCTTCCAGGT------CATAGG--TGGAT--------TCAAAGA
chrom   GAAAGGCAGGACAACTTGAAGAAGAGGCTTCCAGGT------CATAGG--TGGAT--------TCAAAGA
right   TCTCGGCTC----ACTGCAAGCTCCGCCTCCCGGGTTCACGCCATTCTCCTGCCTCAGCCTCCTCAAAGA
        1111***111111***11***1111*1**1**1***111111***11111**11*11111111*******

left    TTTTCTTTTTTTTTTTTTTTTTTTTTTTTTTTTTTGAGACGGAGTCTCGCTCTGTCGCCCAGG---CTG
chrom   TTTTCTGATTGGCAACTGGTTA---------------AAAGAGTTATTATCTAAAGACCTGGAATCAA
right   TTTTCTGATTGGCAATTGGTTA---------------AAAGAGTTATTATCTAAAGACCTGGAATCAA
        ******22**22222N*22**2222222222222222*22****22222***222*2**2**222*22
```

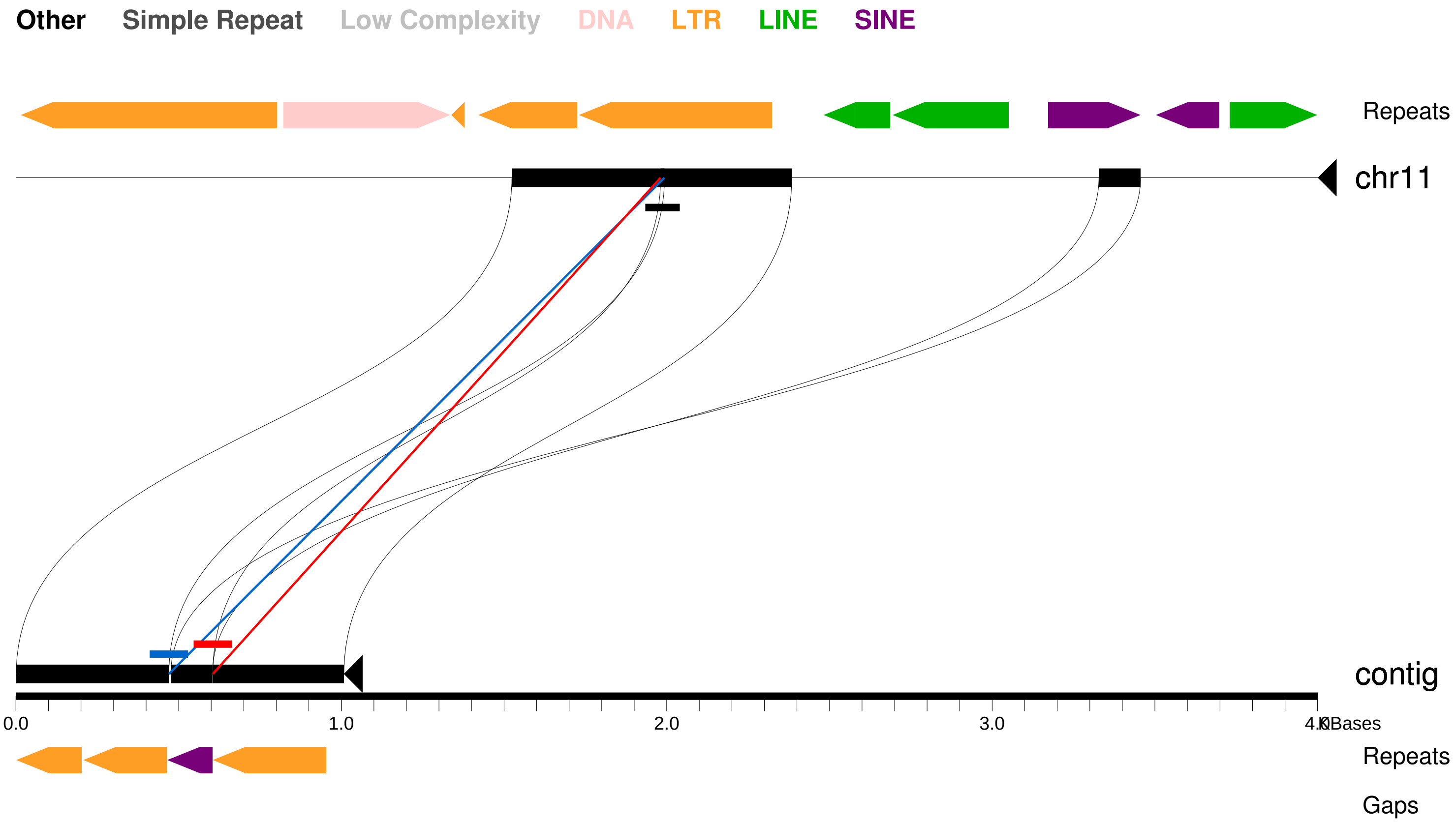

siteID: chr11_35148694 Scafftig1 - Breakpoint: chr11:35148514
chr11:35148455-35148574
Overlap Length: 10 INS size: 283 Genotyped: No

```
left    TGAGAAAGTGAGGCGGACAGAGAAAAAGAGAAGGGGAGAAAAAGTGGGAGAAAAACAGAAATTAAAAGAA
chrom   ----AAAGTGAGGAGGACAGAGAAAAAGAGAAGGGGAGAAAAAGTGGGAGAAAAACAGAAATTAAAAGAA
right   ----NNNNNNNNNNNNNNNNNNNNNNNNNNNNNNNNNNNNNNNNNNNNNNNNNNNNNNNNNNNNAAA-GAA
        2222111111111N1111111111111111111111111111111111111111111111111***1***

left    GAC-----GGCCGGGCGCGGT-------GGCTCAC-------------------G
chrom   GACAAAAAGGATGGG-GAGGTTGAGAAAGGTTAAAAATATCTGGGAAATTGGTAG
right   GACAAAAAGGATGGG-GAGGTTGAGAAAGGTTAAAAATATCTGGGAAATTGGTAG
        ***22222**22***2*2***2222222**2*2*2222222222222222222*
```

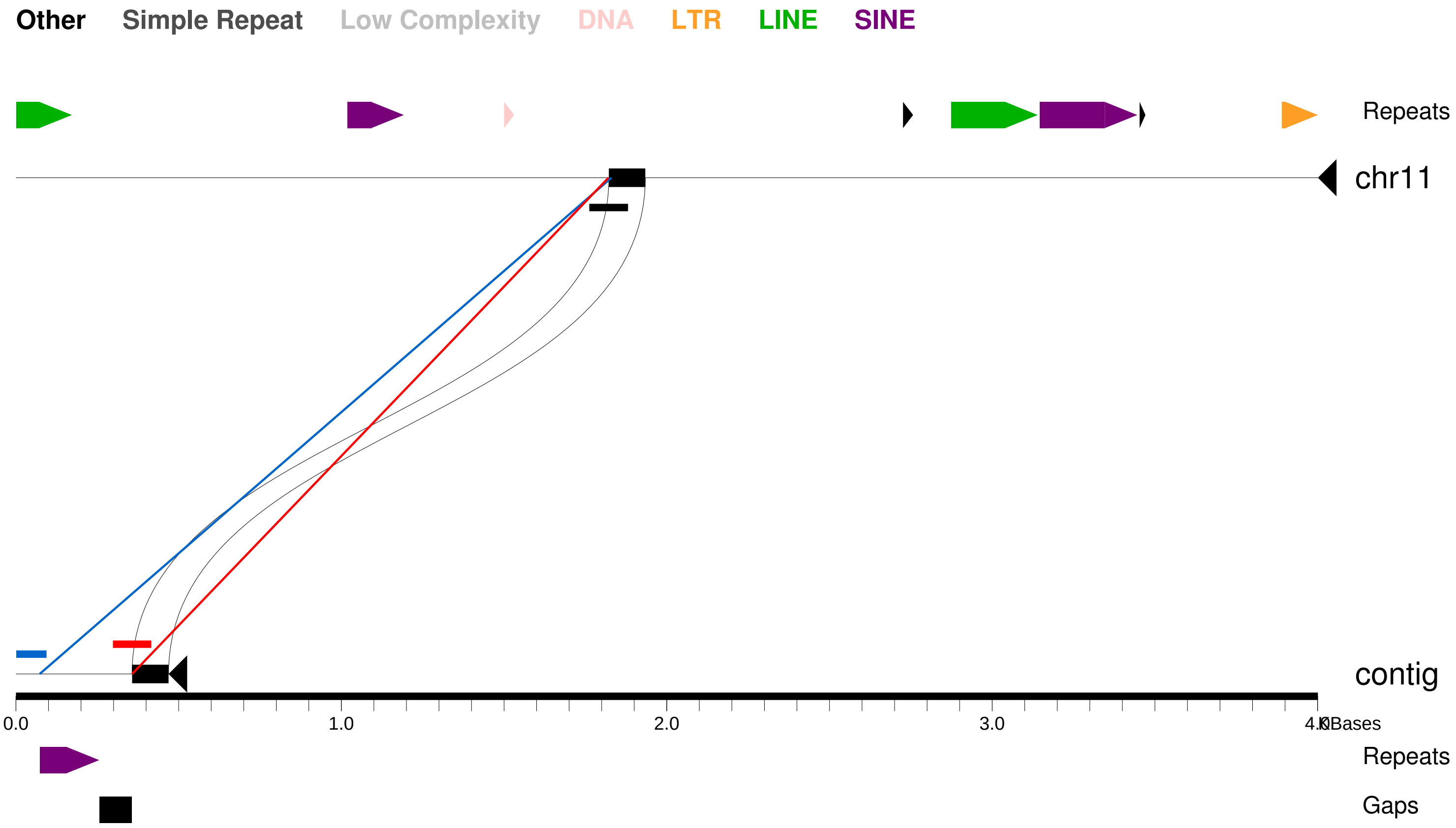

siteID: chr11_35425496 Scafftig1 + Breakpoint: chr11:35425392
chr11:35425173-35425621
Overlap Length: 98 INS size: 397 Genotyped: Yes

```
left   ----------------------------------------------------------------------
chrom  GTCGCATATCCATAAAG-CAGGTC---CTGCTTCCCAGG-GTTCCTATGTTTA-TGCCCAAATAATCTAA
right  AT----TTTTGGTAGAGACGGGTTTCACTGTTGGCCAGGCGGGCCTGGAACTCCTGACCTCATGATCC--
       N2    2N2NNN22N2212N222N111222N2NN2222212NN222NNNNN2N122N22NN22N222N

left   ----------------------------------------------------------------------
chrom  GATTGTCTTGAAGGAGAAACCAGAAATTCTTGAGGTAAAGAACTCATCCAGTGAGCTCTTCGGCTGCATG
right  GCCCACCTTG-----GCCTCCCAAAGTGCTGGGCTTACAGGCGTGAGCCATCGCGCCCGGCCG--GGAAA
       2NNNNN2222     2NNN22NN22N2N22N2NNN22N22NNN2N2N222NN2N22N2NN2N2  2N2NN

left   --------------------------------------------------------AATTTAATGCCCCC
chrom  CATTTATTTATTCAACAAATATCCTGTGATCTTATGACATGAGAAACTCAAGGATTAATTTAATGCCCCC
right  GTTCCCTTTA---AGCAAACAT-----------ATGG--------ACGCATGGATCAGTCCC-TGCTCCG
       NN2NNN2222   2N2222N22           222N        22N22N2222N*1*1111***1**1

left   TAACCAGAAACTAGAGCCACAGCTGGACAGAATCCTGATTTTCCTGTTCCCTAAAAGCAAGGGAACATCT
chrom  TAACCAGAAACTAGAGCCACAGCTGGACAGAATCCTGATTTTCCTGTTCCCTAAAAGCAAGGGAACATCT
right  TGATCACGGCC----GCCTCAGCTGCACAGAATCCTGATATTCCTGCTCCCGGCAAACAAGGGAACATCT
       *1*1**1111*1111***1******1*************1******1****111**1*************

left   TCATCTTGCTGGCTTGGCTCAGCAGCTTAGCTCAAAGGCAAAGGAGGAAA--CAGCTCAAAGGCAA-AGG
chrom  TCATCTTGCTGGCTTGGCTCAGCAGCTTAGCTCAAAGGCAAAGAGGGAAAGTCCTCTCTAAGCAAACATA
right  TCCTCTTGCTGGCTTGCCTCAGCAGCTTAGCTCAAAAGCAAAGAGGGAAAGTCCTCTCTAAGCAAACATA
       **1*************1*******************1******22*****22*22***2***22**2*22

left   GGGAAAGTCTTTTTTTTTTTTTTTTTTTTTTTGAGACAGAGTCTCACTCTGTGGCCCAGGCTGGAGTACA
chrom  TGGACA--CGTGGATCAGTCTCTGCTCCTC------CACGGGCGC-CTC-----CTAAGGC---AGT---
right  TGGACA--CGTGGATCAGTCTCT-----------------------------------------------
       2***2*22*2*222*222*2*2*NN1NN1N22222211NN1N1N12111222221NN1111222111222

left   GTGGTGCAATTTCAGCTCACTGCAACCTCCGCCTCCCGGGTTCAAGCGATTCTCTTGCCT
chrom  ---GTGCCCTTTACCAAAAACACAACGTTTTCACACCTATTTTAATTTTGTTGCTTGC--
right  ------------------------------------------------------------
       2221111NN111NNNNNN1NNN1111N1NNN1NNN11NNN11N11NNNNN1NN1111122
```

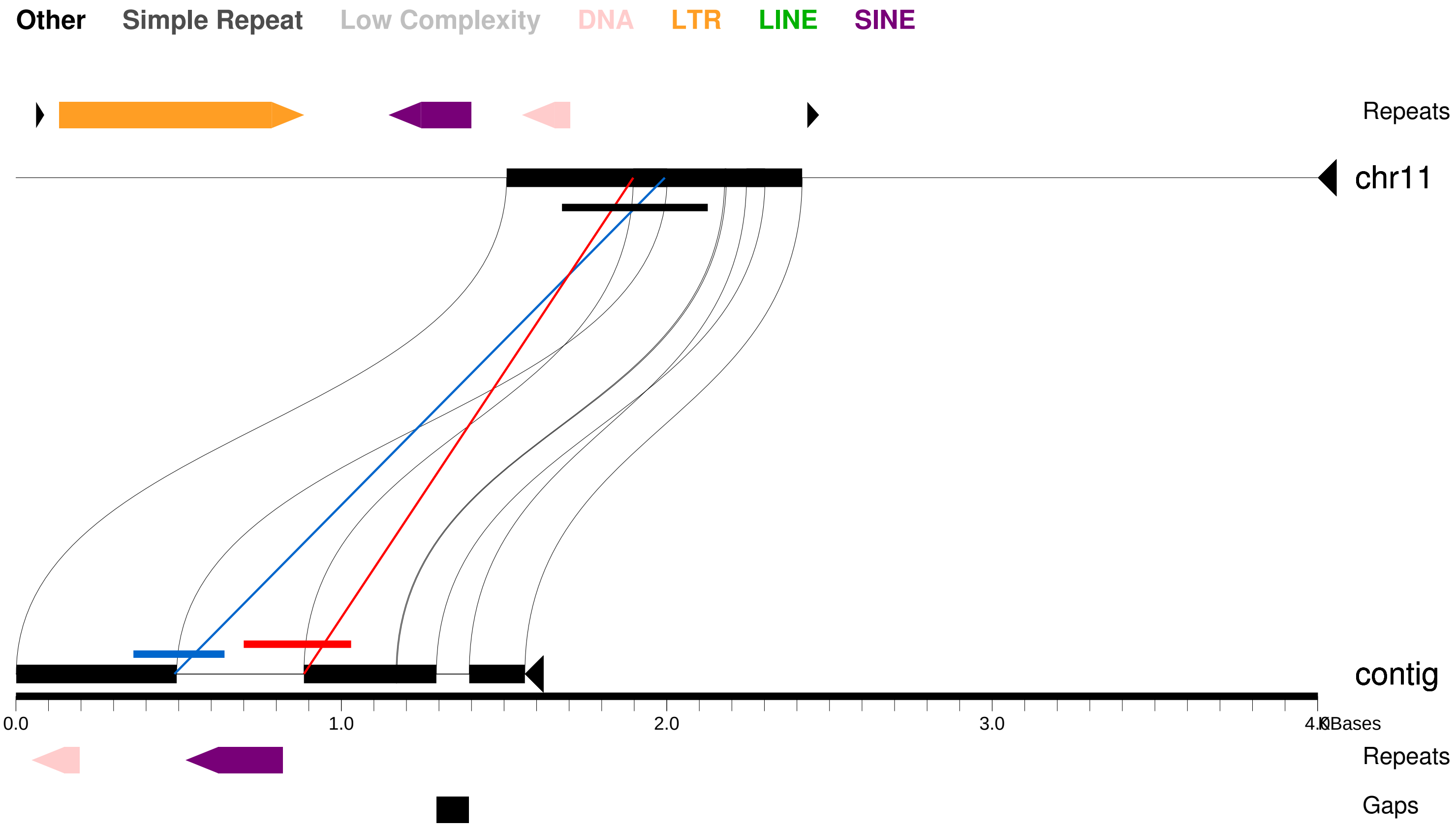

siteID: chr11_36277071 Scafftig1 - Breakpoint: chr11:36277077
chr11:36277031-36277133
Overlap Length: 14 INS size: 432 Genotyped: No

```
left     GAGC---AGGCTCTGG----------GGTGGGACTCCTTGGGCTAGAAGCCT-GCCA--CCACCCATTCT
chrom    GAGC---AGGCTCTGG----------GGTGGGACTCCTTGGGCTAGAAGCCT-GCCA--CCACCCATTCT
right    ATCCGCCCGCCTCGGCCTCCCAAAGTGCTGGGATTACAGGCGTGAGCCACCGCGCCCGGCCCCCCATTCT
         111*1111*1***1*11111111111*1*****1*1*11*1*11**111**11***111**1********

left     AGCTTTTTTTNNNNNNNNNNNNNNNNNNNNNNNNNNNNNNNNNNNNNNNNNNNNNNNNNNNNNNNNN
chrom    AGCTTTAT----------------GCTGGATAATTTAAGGATCTACTTTGTAAAGTTGTTGTGAT
right    AGCTTTAT----------------GCTGGATAATTTAAGGATCTACTTTGTAAAGTTGTTGTGAT
         ******2*22222222222222222222222222222222222222222222222222222222222
```

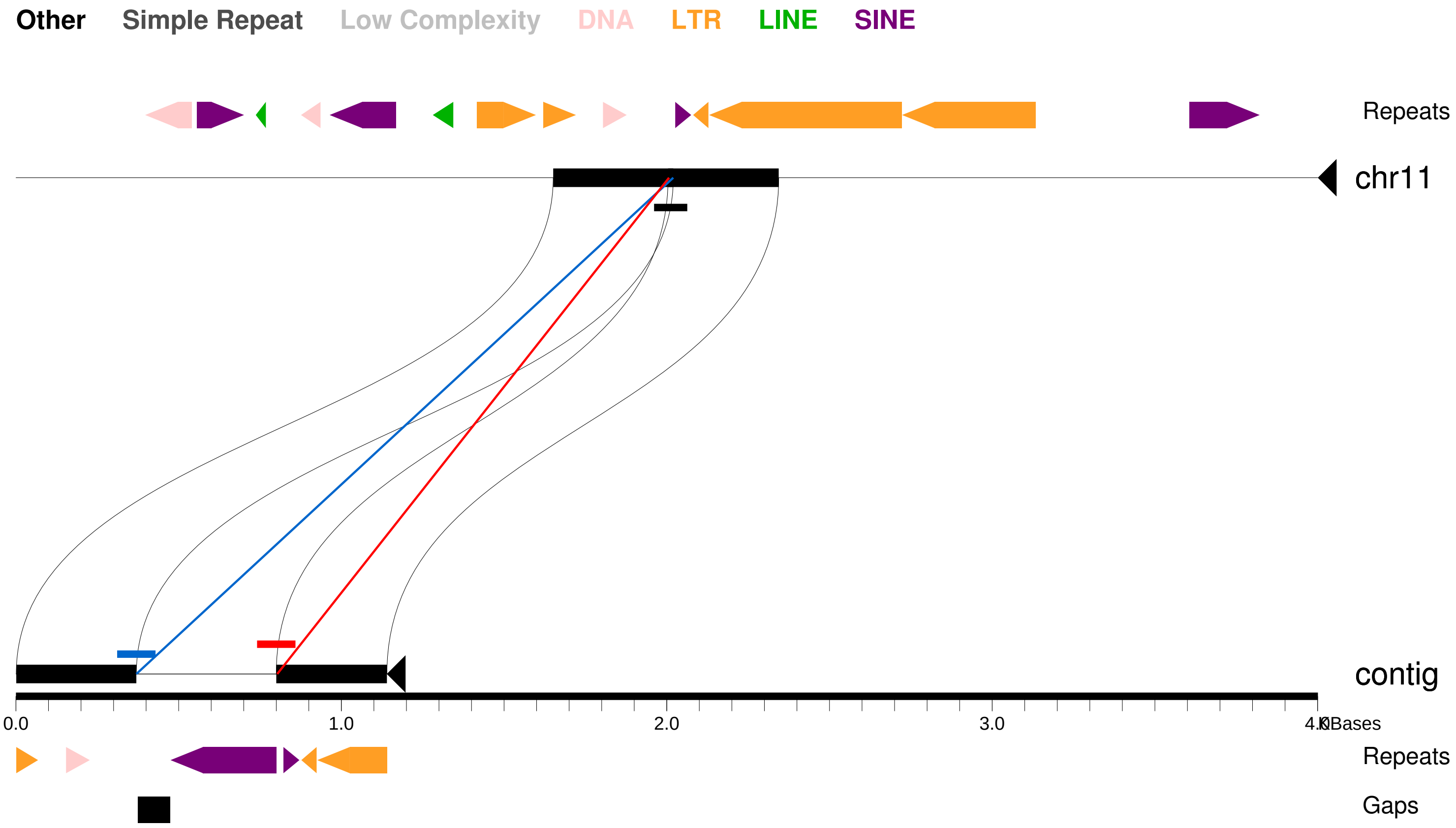

siteID: chr11_37544621 Scafftig4 - Breakpoint: chr11:37544621
chr11:37544578-37544680
Overlap Length: 17 INS size: 302 Genotyped: Yes

```
left    CTGCATTCCATGTTG--------------AAT---TTCCCCAAAAATTCTATAATTTTTTAAAAATTTTG
chrom   CTGCATTCCATGTTG--------------AAT---TTCCCCAAAAATTCTATAATTTTTTAAAAATTTTG
right   -TGCACTCCAGCCTGGGCGACAGAGCGAGACTCCGTCTCAAAAAAAAAAAAAAAAAAAAAAAAAATTTTG
        1****1****111**11111111111111*1*111*11*11*****1111*1**111111**********

left    CTAATTAGGCCGGGCGCGGTGGCTCACGCCTGTAATCCCAGCACTTTGGGAGGCCGAGGCGGGCGG
chrom   CTAATTAAG--------GATTGCTC---TTTGTACTGGGAGGTTCTGTGGATTTTGACAAA-----
right   CTAATTAAG--------GATTGCTC---TTTGTACTGGGAGGTTCTGTGGATTTTGACAAA-----
        *******2*22222222*2*2****22222****2*222**2222*22***2222**222222222
```

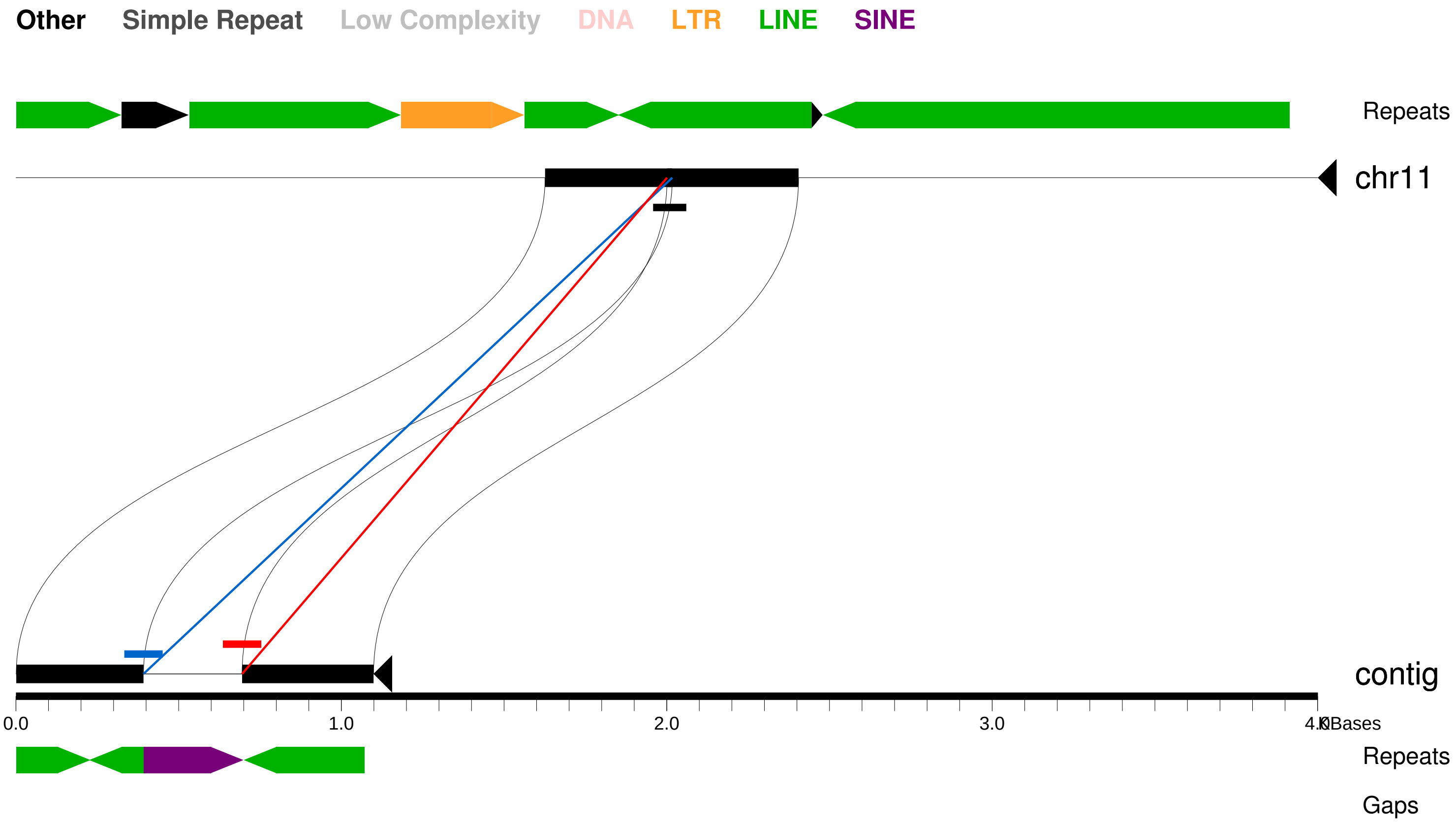

siteID: chr11_37706325 Scafftig6 + Breakpoint: chr11:37706271
chr11:37706236-37706396
Overlap Length: 25 INS size: 322 Genotyped: Yes

```
left    --------------------------AGTAAGTACTGGAAGCCCTGACCAATGCAATTAAGAAACAAAAA
chrom   --------------------------AGTAAGTACTGGAAGCCCTGACCAATGCAATTAAGAAACAAAAA
right   CAGAGCGAGACTCCGTCTCAAAAAAAAAAAAAAAAAAAAAAAAAAAAAAAAAAAAAAAAAAAAAAAAAAAAA
        11111111111111111111111111*11**11*1111**111111*11**111**11**1***1*****

left    GAAATGAAAGGCATCCGCCGGGCGCGG------------------TGGCTC-------AC-GCC------
chrom   GAAATGAAAGGCATCCAAATGGGAAAGAAAATGATAAACTTATCTTTGTTC-------ACAGACAACATA
right   AAAAAGAAAGGCATCCAAATGGGAAAGAAAATGATAAACTTATCTTTGTTTTATAATAATATAAAACATA
        1***1***********2222**2222*222222222222222222*2*2*11111111*12121222222

left    ----------TGTAA------TCCCAGCACTTTGGGAGACCGAG-GCGGGCGGA
chrom   ATATTATATTTATAAAAAATCTACATACACCACTAAAAACTGTTAGAACTCATA
right   ATATTATATTTATAAAAAATCTACATACACCACTAAAAACTGTTAGAACTCATA
        2222222222*2***222222*2*222***222222*2**2*222*2222*22*
```

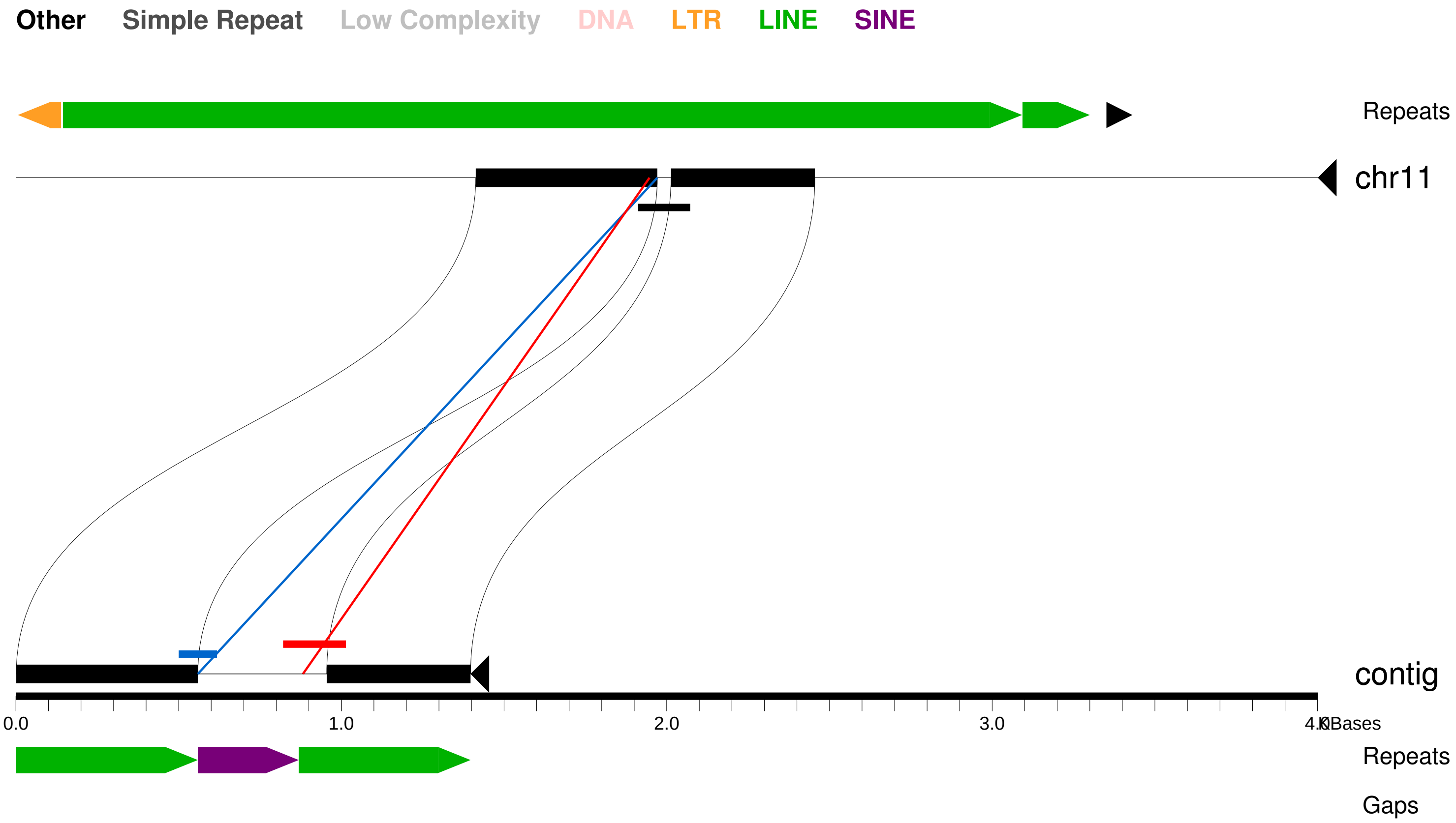

siteID: chr11_38047084 Scafftig1 + Breakpoint: chr11:38047094
chr11:38047040-38047147
Overlap Length: 6 INS size: 292 Genotyped: No

```
left    AACTAATGGGTACTAGGCTTAGTAAATGCATGATGAAATACTCTGT-----------ACAACAAACCCCC
chrom   AACTAATGGGTACTAGGCTTAGTAAATGCATGATGAAATACTCTGT-----------ACAACAAACCCCC
right   NNNNNNNNNNNNNNNNNNNNNNNNNNNNNNNNNNNNNNNNNNNNNNNNNNNNNNNNNAAAAAAAACCCCCC
        111111111111111111111111111111111111111111111111111111111*1**1**1******

left    AGGCCGGGCGCGGTGGCTCACGCCTGTAATCCCAGCACTTTGGGAGGCCGAGGCGGGCGG
chrom   AAGAC--ACACATTTACCTATA--TATCAAACCTGCACAT--GTATCTCTTAACT-----
right   AAGAC--ACACATTTACCTATA--TATCAAACCTGCACAT--GTATCTCTTAACT-----
        *2*2*222*2*22*22*22*2222*2*2*22**2****2*22*2*222*2222*222222
```

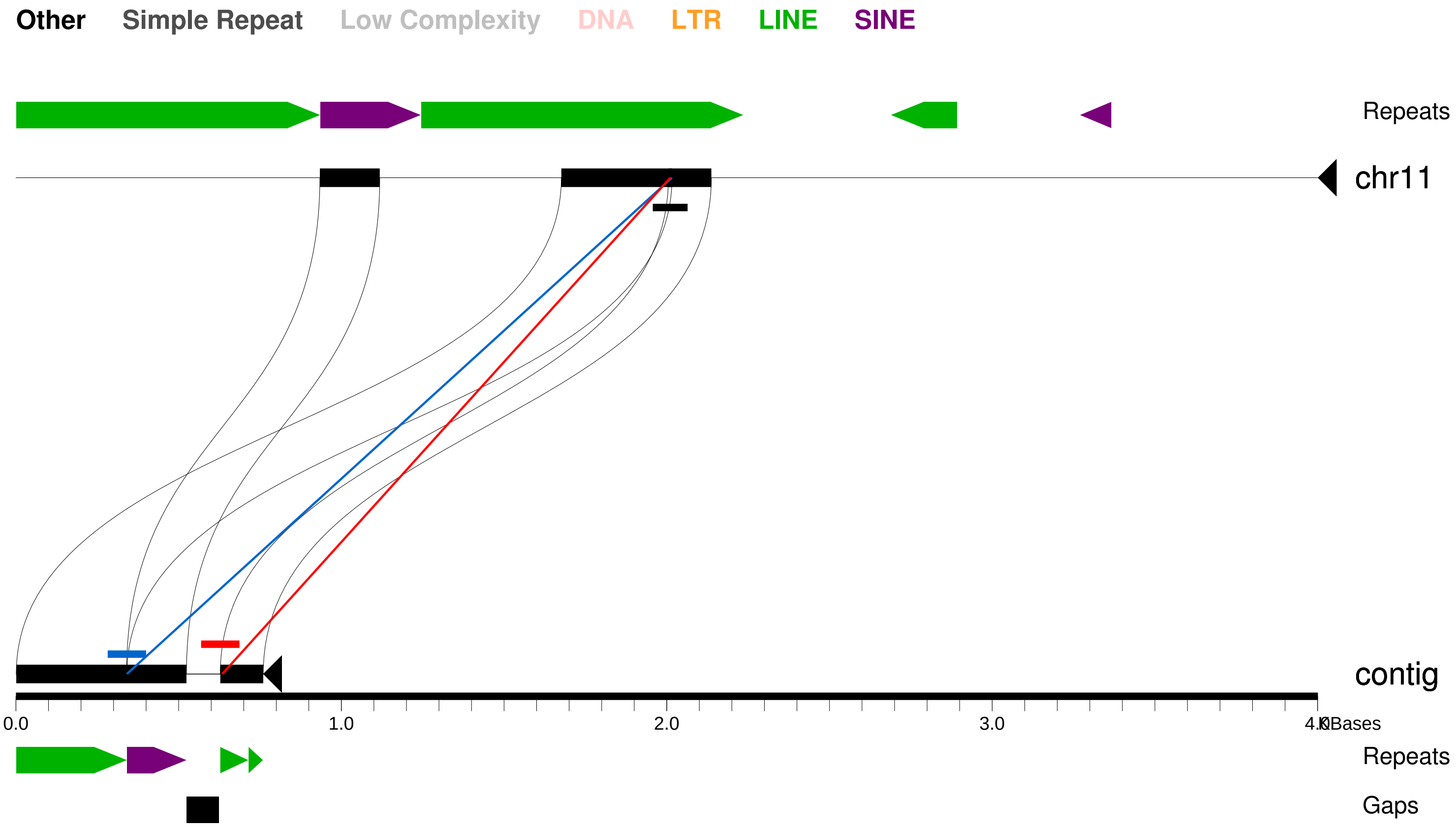

siteID: chr11_38801064 Scafftig1 + Breakpoint: chr11:38801063
chr11:38800903-38801235
Overlap Length: 13 INS size: 304 Genotyped: Yes

```
left   T-------------TACTGGCAGTTTCCAAATATTGACTGCAGACCCTCAGGAGAGCTTCCTGCAATATA
chrom  TCGTTGGTCTTCATTACTGGCAGTTTCCAAATATTGACTGCAGACCCTCAGGAGAGCTTCCTGCAATATA
right  CAGGAG---------AATGGCGTGAACC---------CGGGAGGCG-------GAGCTTGCAG-----TG
       1N2NN2        1*1****11111**111111111*1*1**1*11111111******1*1*11111*1

left   AATCTATAAGATATCCCTGTATTCCTGGTTTTTGTTTAGTTGTTTTCAAATGATTTTCTGTTAATTATAA
chrom  AATCTATAAGATATCCCTGTATTCCTGGTTTTTGTTTAGTTGTTTTCAAATGATTTTCTGTTAATTATAA
right  AGCCGAGATCCCGCCACTGCACTCCAGCCTGGGCGACAGAG-----CGA--GACTCCGTCTCAAAAAAAA
       *11*1*1*111111*1***1*1***1*11*1111111**1111111*1*11**1*111*1*1**11*1**

left   CCAAAATAATTTTGACTTAGAAAGTGGTTTATTGGCC--GGG--CGCGGTGGTTCAC------GCCT--G
chrom  CCAAAATAATTTTGACTTAGAAAGTGGTTTATTTAACATGGGAACACGCCAACTCAAAGTGTTGACTATG
right  AAAAAAAAA-----AAAAAAAAAGTGGTTTATTTAACATGGGAACACGCCAACTCAAAGTGTTGACTATG
       11****1**11111*111*1************222*22***22*2**22222***2222222*2**22*

left   TAATCCCAGCACTT---TGGGAGGC-----CGAGG----------CGGGCGGATCACGAG------GTCA
chrom  TGTTAACATTTTTTGACTAGCATGCAAAATCAAGGACAGGAGTTACGGGAATATGAATGGCATTTTGTCA
right  TGTTAACATTTTTTGACTAGCATGCAAAATCAAGGACAGGAGTTACGGGAATATGAATGGCATTTTGTCA
       *22*22**2222**222*2*2*2**22222*2***2222222222****222**2*222*222222****

left   GGAGAT---CGAGACCATCCCGGCTAAAACG-GTGAAACCCCGTCTCTACTAAA
chrom  A-AGATTTTCTCTAATATGCCTGCTTCATTTTGAGAAAATATGTATTTGGTAGA
right  A-AGATTTTCTCTAATATGCCTGCTTCATTTTGAGAAAATATGT----------
       22****222*222*22**2**2***22*2222*2****2222**N1N1NN11N1
```

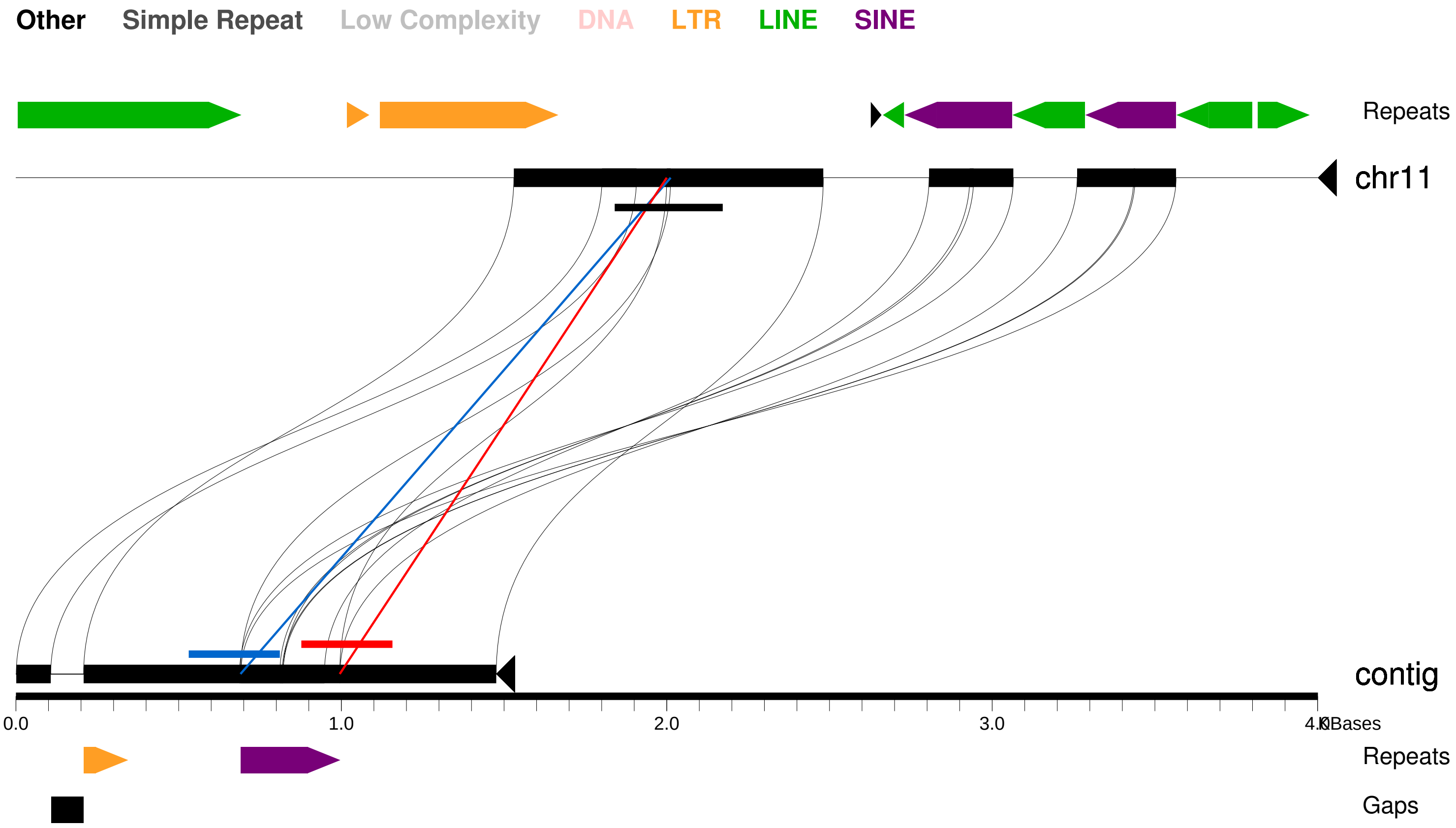

siteID: chr11_39567827 Scafftig1 - Breakpoint: chr11:39567813
chr11:39567782-39567872
Overlap Length: 29 INS size: 430 Genotyped: No

```
left    ATGTTGATGTCATTATCCTCAAACTTAACCA----------------------------ATAGGCTTACA
chrom   ATGTTGATGTCATTATCCTCAAACTTAACCA----------------------------ATAGGCTTACA
right   NNNNNNNNNNNNNNNNNNNNNNNNNNNNNNNNNNNNNNNNNNNNNNNNNNNNNNNNNNNATAGGCTTACA
        11111111111111111111111111111111111111111111111111111111111***********

left    TATCAAATGTGATATTATGGCCGGGCGCGGTGGCTCACGCCTGTAATCCCAGCA-CTTTGGGA-GGCCGA
chrom   TATCAAATGTGATATTATTCC-----------------------AATGTCAGCATCTCTTGTATGGTTGA
right   TATCAAATGTGATATTATTCC-----------------------AATGTCAGCATCTCTTGTATGGTTGA
        ******************22*22222222222222222222222***22*****2**2*2*2*2**22**

left    GGCGGGCGG
chrom   GT-------
right   GT-------
        *22222222
```

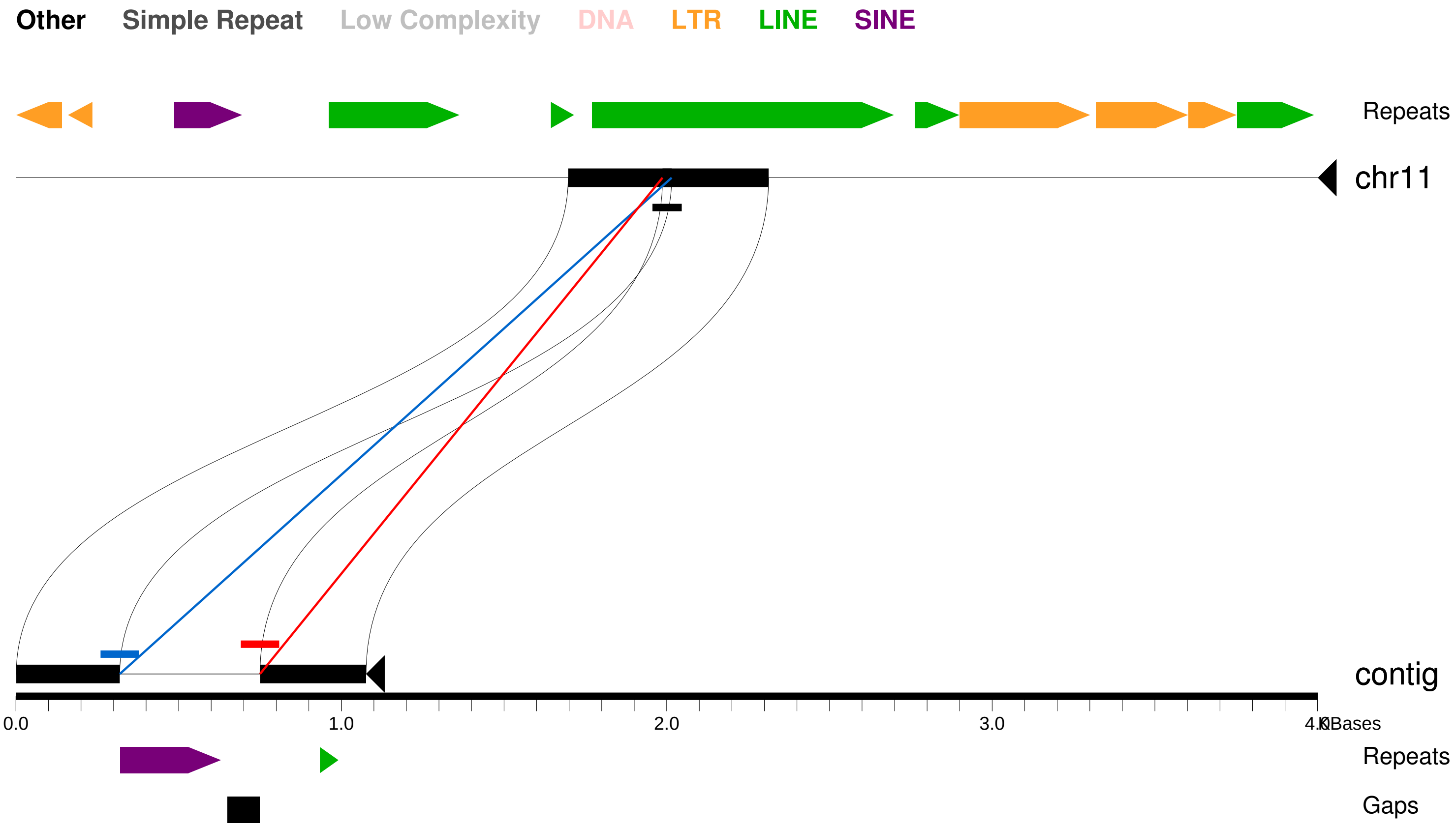

siteID: chr11_40727079 Scafftig1 - Breakpoint: chr11:40727082
chr11:40727038-40727141
Overlap Length: 16 INS size: 298 Genotyped: Yes

```
left    -------------------AGAATGATGG--TTTCCAGCTTCATCCACGTGGCTGCAAAGGACATGAACT
chrom   -------------------AGAATGATGG--TTTCCAGCTTCATCCACGTGGCTGCAAAGGACATGAACT
right   CCGCCCGCCTCGGCCTCCCAAAGTGCTGGGATTACAGGCGTGAGCCACCGCGCCC----GGCC--GAACT
        1111111111111111111*1*1**1***11**1*11**1*1*1****111**111111**1*11*****

left    CTTTTTTTTTTTTTTTTTTTTTTTTTTTTGAGACGGAGTCTCGCTCTGTCGCCCAGGCTGGAGTGCAGTGG
chrom   CATTCTTTTTTTATGCCTGCATAGTATTC----CGTGGTGTATATGTGCCACATTTTCTT-----------
right   CATTCTTTTTTTATGCCTGCATAGTATTC----CGTGGTGTATATGTGCCACATTTTCTT-----------
        *2**2******2*222*222*22*2**22222**22**2*222*2**2*2*22222**222222222222
```

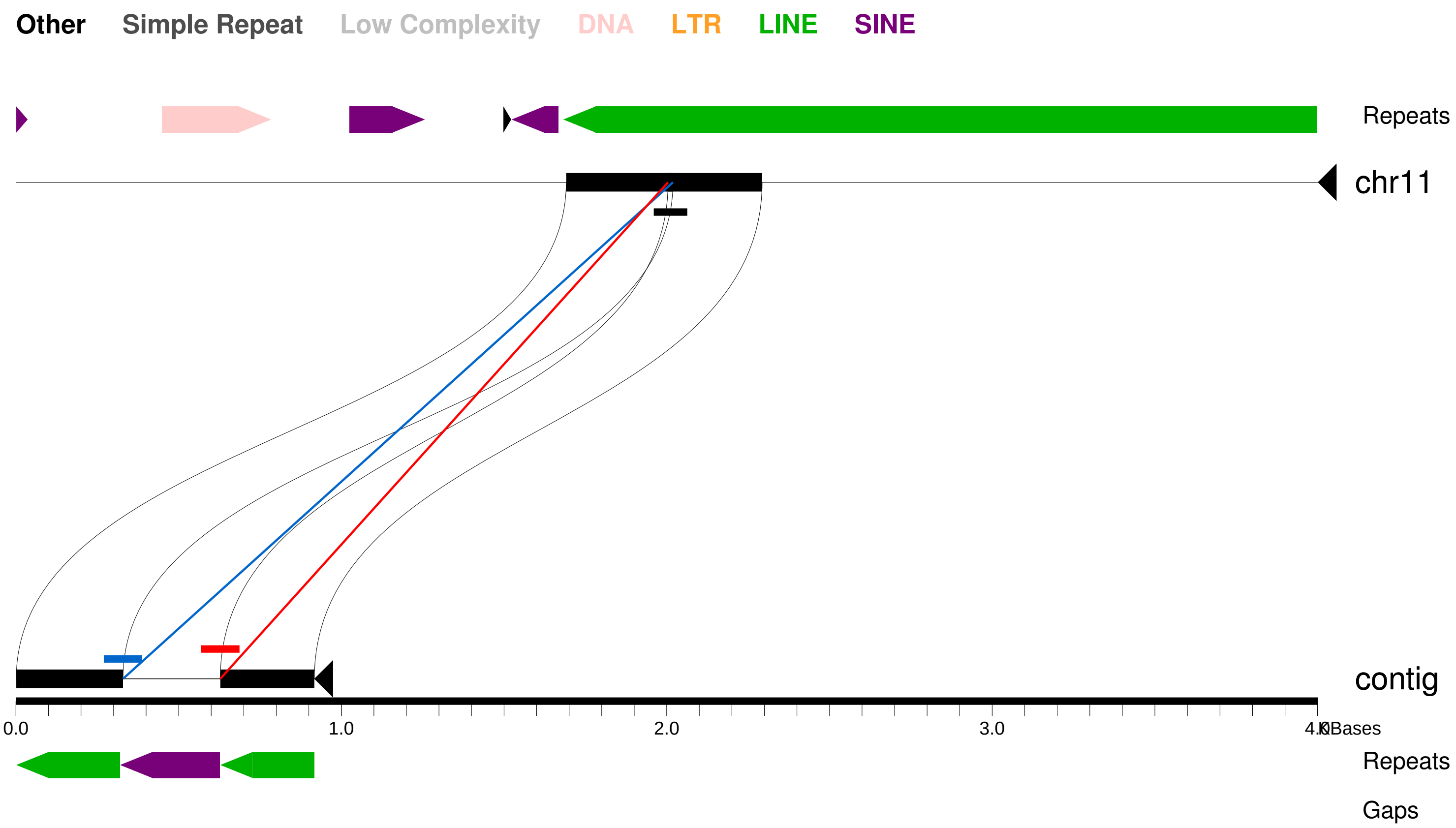

siteID: chr11_41530238 Scafftig1 - Breakpoint: chr11:41530235
chr11:41530075-41530408
Overlap Length: 14 INS size: 283 Genotyped: Yes

```
left    A--------------AAGAGACAGGGACCAGGAGAGAGAAAAAAATAGCTTTCAAAGGGGAAGTGCAACC
chrom   ATTCTAAGGATAGTAAAGAGACAGGGACCAGGAGAGAGAAAAAAATAGCTTTCAAAGGGGAAGTGCAACC
right   CGT--------------GAACCCGGGAAGCGGAG--------------CTTGCA--------GTG--AGC
        1N2            11**11*1****111****11111111111111***1**11111111***11*1*

left    CAAGAAAGATAATCTGTT-TCCCCTCTCCAAAGCTAAGAAATGGAG-GAGACCTTTAGGAGACAAATAAG
chrom   CAAGAAAGATAATCTGTT-TCCCCTCTCCAAAGCTAAGAAATGGAG-GAGACCTTTAGGAGACAAATAAG
right   CGAGATTGCGCCACTGCAGTCCGCAGTCCGGC-CTGGGCGACAGAGCGAGAC---TCCGTCTCAAAAAAA
        *1***11*11111***111***1*11***1111**11*11*11***1*****111*11*111****1**1

left    TAAAGTGAGATAAATCTTACGGAAAGAAAGACTAATCACTTTGGGAG---------GCCGAGGCGGTGG
chrom   TAAAGTGAGATAAATCTTACGGAAAGAAAGACTAATATTTTATTGACCAATATAATGTCCATGTTTGTGA
right   AAAAAAAAAAAAAAAAAAAAAAAAAAAAAGACTAATATTTTATTGACCAATATAATGTCCATGTTTGTGA
        1***111*1*1***1111*111***1**********222**222**2222222222*2*2*2*222***2

left    AT---------CATGA---------GGTCAGGAGATCGAGACCATCCTGGCTAACAAG---GT-------
chrom   ACATACCCAGACATGTCTGCAACAGGGAGAAGAGCACGAGAAAGGCATAGTAAAGATGAGAGTTTTAAAG
right   ACATACCCAGACATGTCTGCAACAGGGAGAAGAGCACGAGAAAGGCATAGTAAAGATGAGAGTTTTAAAG
        *2222222222****2222222222**22*2***22*****2222*2*2*22**2*2*222**2222222

left    ---GAAACCCCGTCTCTACTAAAAATACAAAAAATTAGCCGGGCGCGGTGGCGGGCG
chrom   AAGGAAACTACTTTTTCACCACTTGGCATGACAAGTTACCTAGATCTCCAGCTAGC-
right   AAGGAGCCTACTTTTTCACCACTTGGCATGACAAGTTACCTAG--------------
        222**11*22*2*2*22**2*222222222*2**2*22**22*NN1NNNN11NN112
```

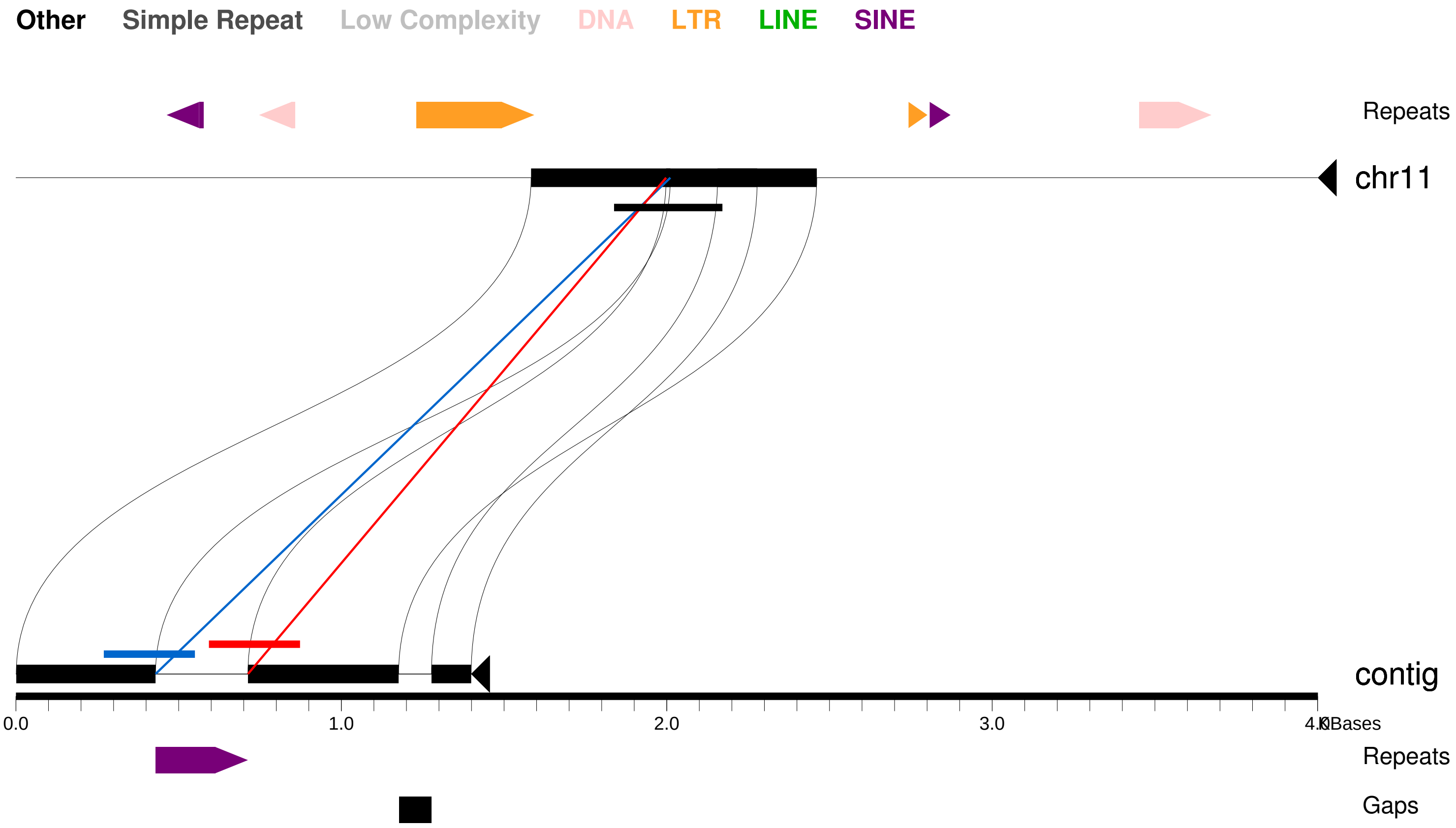

siteID: chr11_42663733 Scafftig2 + Breakpoint: chr11:42663965
chr11:42663923-42664024
Overlap Length: 18 INS size: 299 Genotyped: Yes

```
left   AGAGTGGGTGGTTGAGGACACCCAG---GATATGATAATA--------CCAAA-----------TAAACT
chrom  AGAGTGGGTGGTTGAGGACACCCAG---GATATGATAATA--------CCAAA-----------TAAACT
right  CGCCCGCCTCG-----GCCTCCCAAAGTGCTGGGATTACAGGCGTGAGCCACCGCGCCCGGCCCTAAACT
       1*111*11*1*11111*1*1****1111*1*11***1*1*11111111***11111111111111******

left   GAACCTTTTTTTTTTTTTTTTTTTTTTTTTGAGACGGAGTCTCGCTCTGTCGCCCAGGCTGGAGTGCAGTG
chrom  GAACCTATTTTTTATAT----------GCCAAGAGTGCTTGTCAC-CTGTCACA---TCTCTTTTTCCA--
right  GAACCTATTTTTTATAT----------GCCAAGAGTGCTTGTCAC-CTGTCACA---TCTCTTTTTCCA--
       ******2*****2*2*22222222222222***22*22*2**2*2*****2*22222**2222*2*2222

left   G
chrom  -
right  -
       2
```

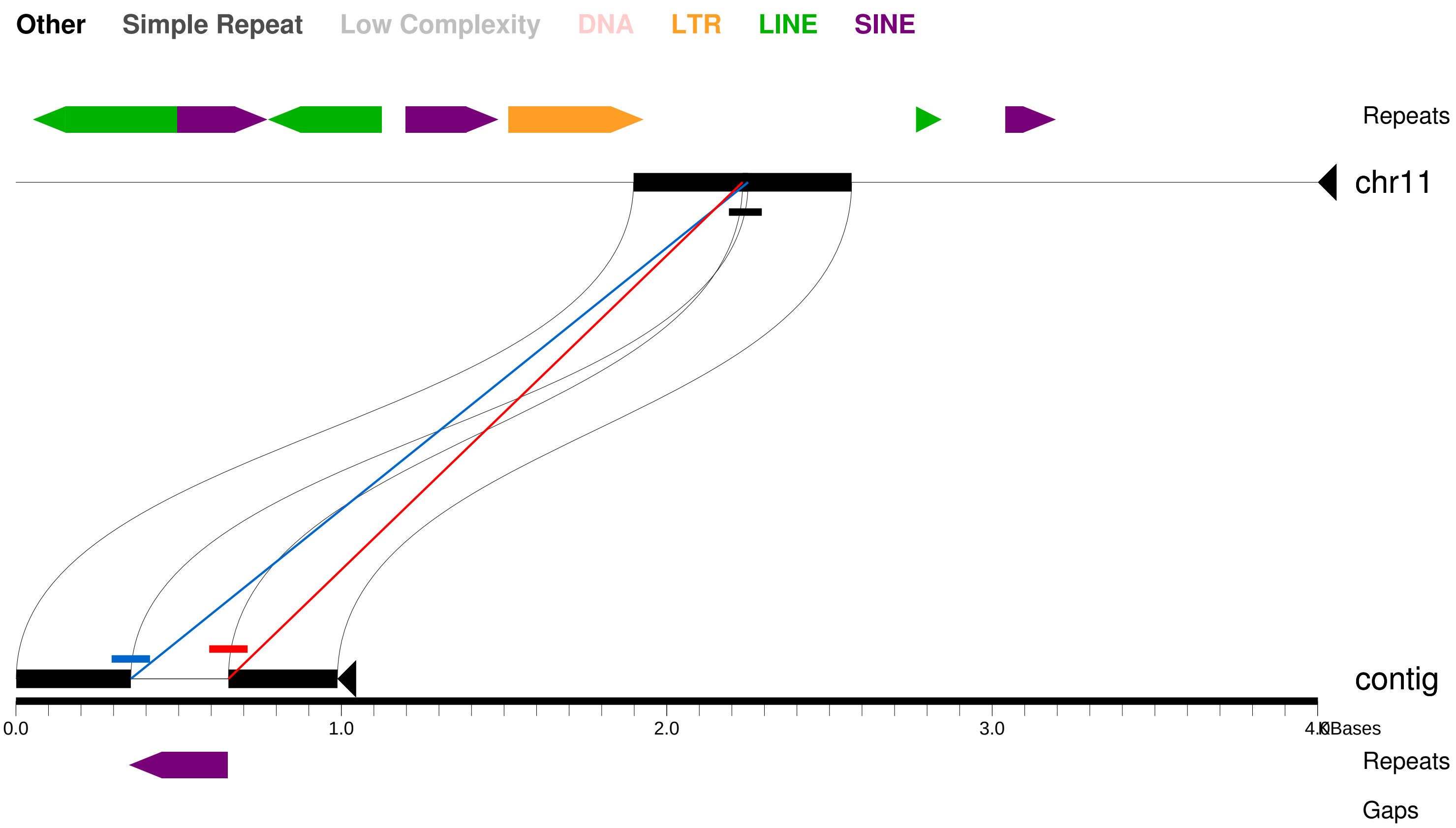

siteID: chr11_43191327 Scafftig2 - Breakpoint: chr11:43191331
chr11:43191284-43191390
Overlap Length: 13 INS size: 303 Genotyped: Yes

```
left    GGATAT------TAGGAACAATATCACAGGTGGGTGTACACCTTCTGCAGTAT--------AAAGAATAA
chrom   GGATAT------TAGGAACAATATCACAGGTGGGTGTACACCTTCTGCAGTAT--------AAAGAATAA
right   TGCAGTCCGCAGTCCGACCTGGGCGACAGA-GCGAG-ACTCCGTCTCAAAAAAAAAAAAAAAAAAAAATAA
        1*111*111111*11**1*111111****11*1*1*1**1**1***11*11*111111111***1*****

left    TGTTGGCCGGGCGCGGTGGCTCACGCCTGTAATCCCAGCACTTTGGGAGGCCGAGGCGGGTGG
chrom   TGTTATCTTCTC----TTCCTTTAGCTATTAAG---AACAATATCACATGGGAAG-----TAT
right   TGTTATCTTCTC----TTCCTTTAGCTATTAAG---AACAATATCACATGGGAAG-----TAT
        ****22*2222*2222*22**222**222***2222*2**2*2*222*2*222**22222*22
```

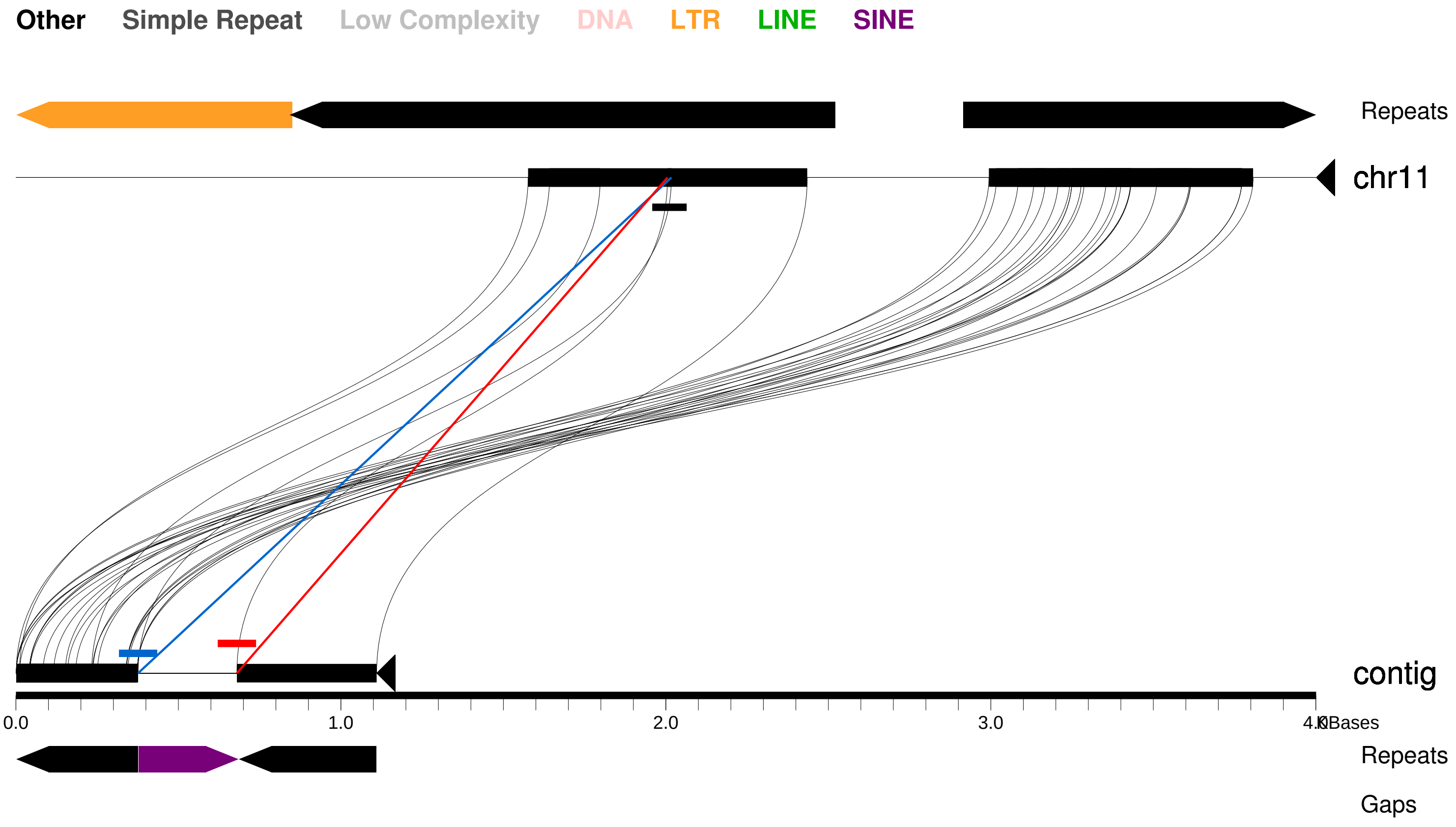

siteID: chr11_43877442 Scafftig1 + Breakpoint: chr11:43877440
chr11:43877389-43877499
Overlap Length: 9 INS size: 307 Genotyped: Yes

```
left   TCTGACACATGAAGCCCACTAAA---CTATGCTTTCTTA--TAATGCATATTTCTT----CTCAGTTTAT
chrom  TCTGACACATGAAGCCCACTAAA---CTATGCTTTCTTA--TAATGCATATTTCTT----CTCAGTTTAA
right  CCGCCCGCCTCG-GCCTCCCAAAGTGCTGGGATTACAGGCGTGAGCCACCGCGCCCGGCCCTCAGTTTAA
       1*111*1*1*111***11*1***111**11*1**1*11111*1*11**11111*111111*********2

left   TTTTTATTTTATTTTTTTTTTTTTTTGAGACGGAGTCTCGCTCTGTCGCCCAGGCTGGA
chrom  ATGTATGTAAATATCGAAGCTATATG-------GTAT-GATTTATAAAGATAAATGGG
right  ATGTATGTAAATATCGAAGCTATATG-------GTAT-GATTTATAAAGATAAATGGG
       2*2*222*22**2*222222*2*2**2222222**2*2*2*2*2*222222222***2
```

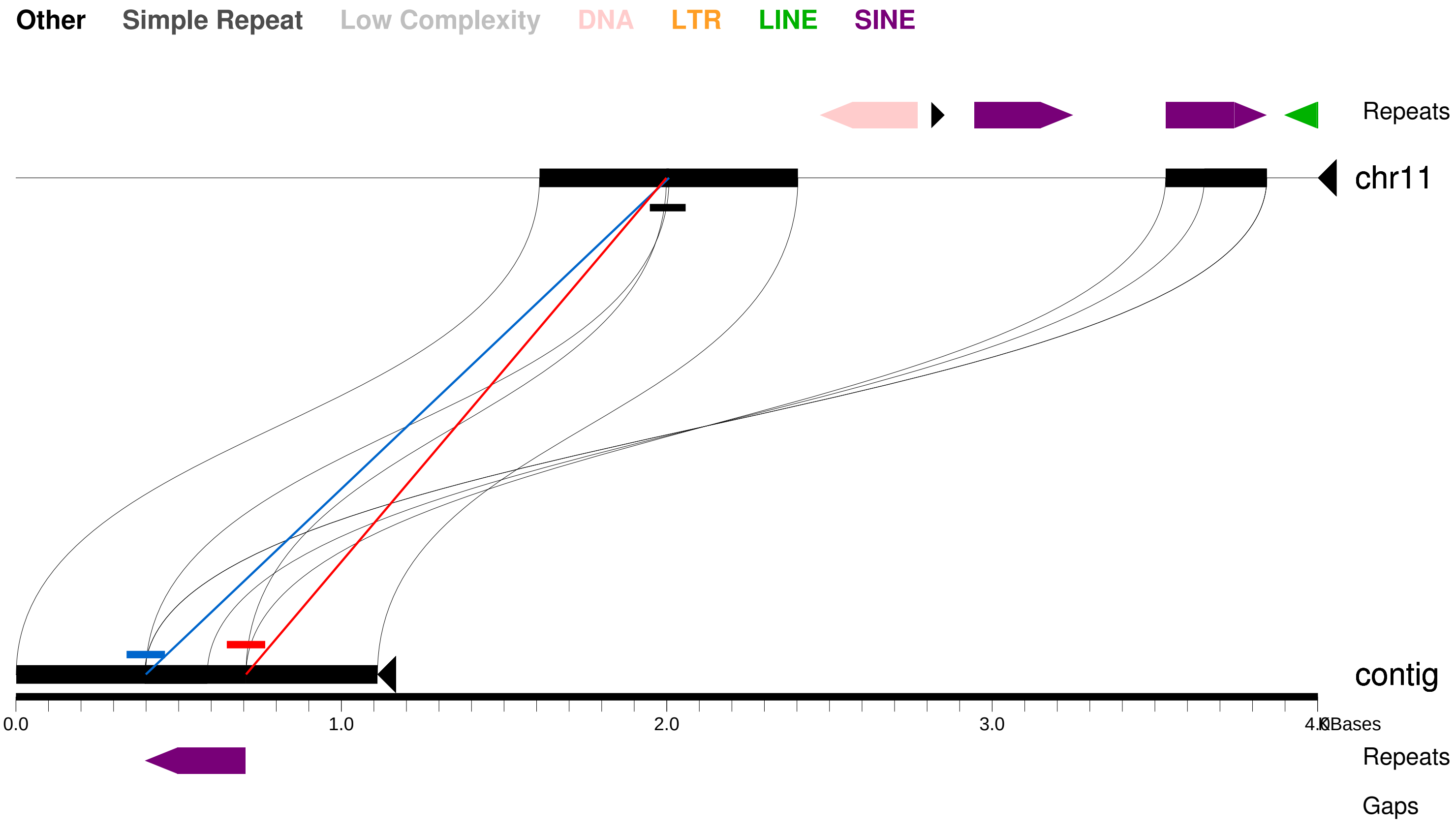

siteID: chr11_48933131 Scafftig15 + Breakpoint: chr11:48933110
chr11:48933066-48933169
Overlap Length: 16 INS size: 298 Genotyped: Yes

```
left    TC--------TGTGAGTTGAA---TGCACACATAACAAAG--AAGTTTCCGAGAATG--CTTCAGTCTAG
chrom   TC--------TGTGAGTTGAA---TGCACACATAACAAAG--AAGTTTCCGAGAATG--CTTCAGTCTAG
right   TCCACCCGCCTCGGCCTCCCAAAGTGCTGGGATTACAGGCGTGAGCCACCGCGCCCGGCCTTCAGTCTAG
        **11111111*11*11*111*111***1111**1***111111**111***1*111*11***********

left    ATTTTTTTTTTTTTTGAGACGGAGTCTCGCTCTGTCGCCCAGGTGGGACTGCGGACTGCAGTGG
chrom   ATTTTATGTGA-----AGAT--ATTCCCGT----TCGCAAAGGAGGC----CTCAAAGCAGTCC
right   ATTTTATGTGA-----AGAT--ATTCCCGT----TCGCAAAGGAGGC----CTCAAAGCAGTCC
        *****2*2*2222222***222*2**2**22222****22***2**22222*22*22*****22
```

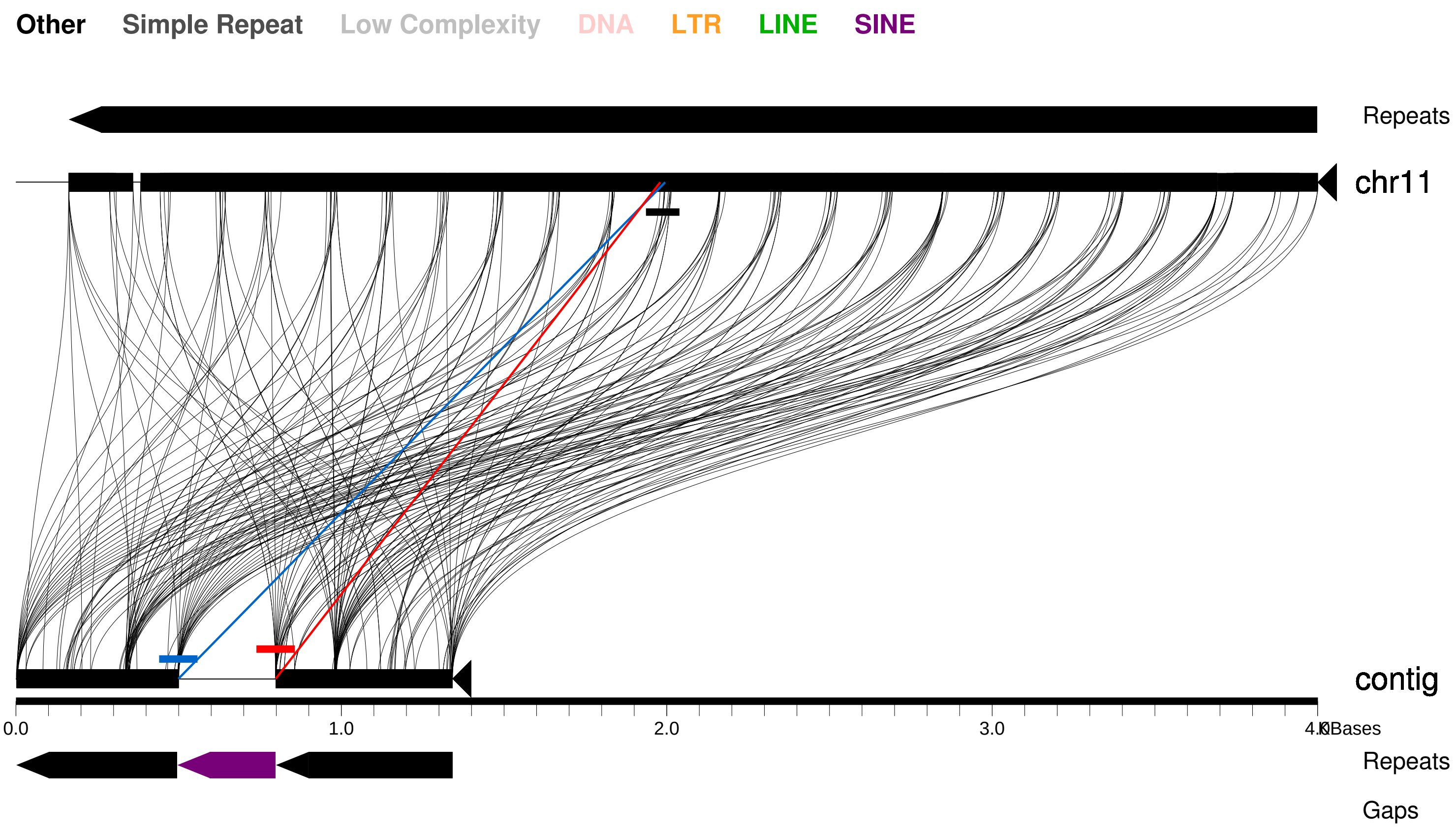

siteID: chr11_4907929 Scafftig1 + Breakpoint: chr11:4907948
chr11:4907904-4908007
Overlap Length: 16 INS size: 226 Genotyped: Yes

```
left     TATA----------TTTAGTTTCTATCATCTGACCCCAAACAAGGATTAAGGTC-----AAAAGACTTTA
chrom    TATA----------TTTAGTTTCTATCATCTGACCCCAAACAAGGATTAAGGTC-----AAAAGACTTTA
right    GCCACCGCGCCCGGCTAATTTTTTGTATTTTTAGTAGAGACGGGGTTTCACCTTGTTAGAAAAGACTTTA
         111*11111111111*1*1***1*1*11*1*1*1111*1**11**1**1*11*111111***********

left     TTTTTTTTTTTTTTTTTTTTTTTTTTTTTTTTTTGAGACGGAGTCTCGCTCTGTCGCCCAGGCC
chrom    TTTTTGACAGACAGCCTGTTGG------------CAAACTCAGAATAAAAC---CACAACCTTA
right    TTTTTGACAGACAGCCTGTTGG------------CAAACTCAGAATAAAAC---CACAACCTTA
         *****22222222222*2**222222222222222*2**22**22*2222*222*2*2222222
```

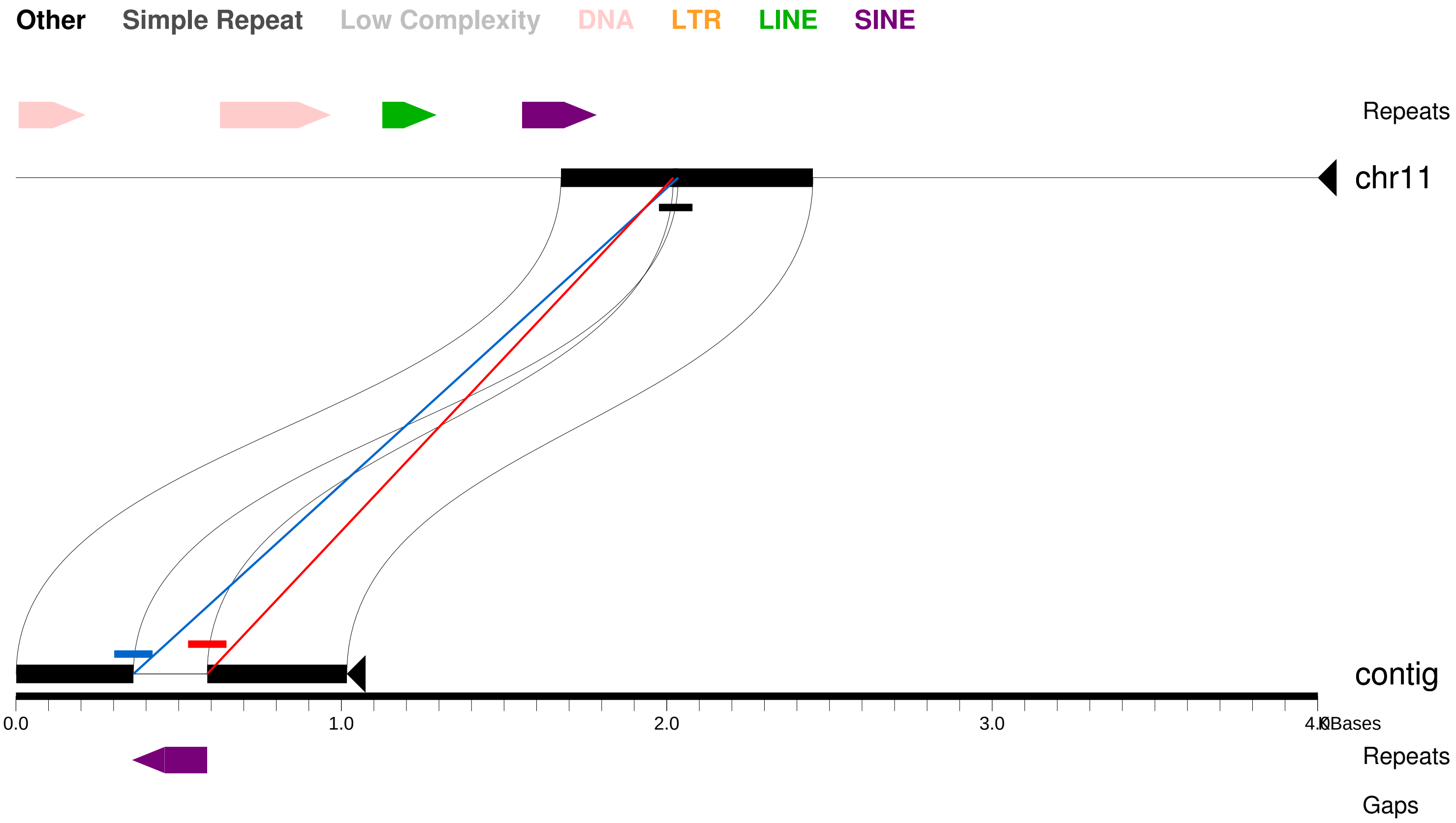

siteID: chr11_49655825 Scafftig5 - Breakpoint: chr11:49655810
chr11:49655765-49655869
Overlap Length: 15 INS size: 415 Genotyped: No

```
left    AAAA---ATATCATTGATAACAACATAAGATTTATTT----TCATAATTTGC--------CAGGTGTGTG
chrom   AAAA---ATATCATTGATAACAACATAAGATTTATTT----TCATAATTTGC--------CAGGTGTGTG
right   GGTCTCGATCTCCT-GACCTCGTGATCCGCCCGCCTCGGCCTCCCAAAGTGCTGGGATTACAGGCGTGTG
        1111111**1**1*1**111*111**11*111111*11111**11**11***11111111****1*****

left    TTCTTTTTTTTTTNNNNNNNNNNNNNNNNNNNNNNNNNNNNNNNNNNNNNNNNNNNNNNNNNN
chrom   TTCTTGAGATTTT--------------GAAGAAATTAGATGGAATGATTCTTTAGTATATGATGC
right   TTCTTGAGATTT--------------GAAGAAATTAGATGGAATGATTCTTTAGTATATGATGC
        *****2222***222222222222222222222222222222222222222222222222222
```

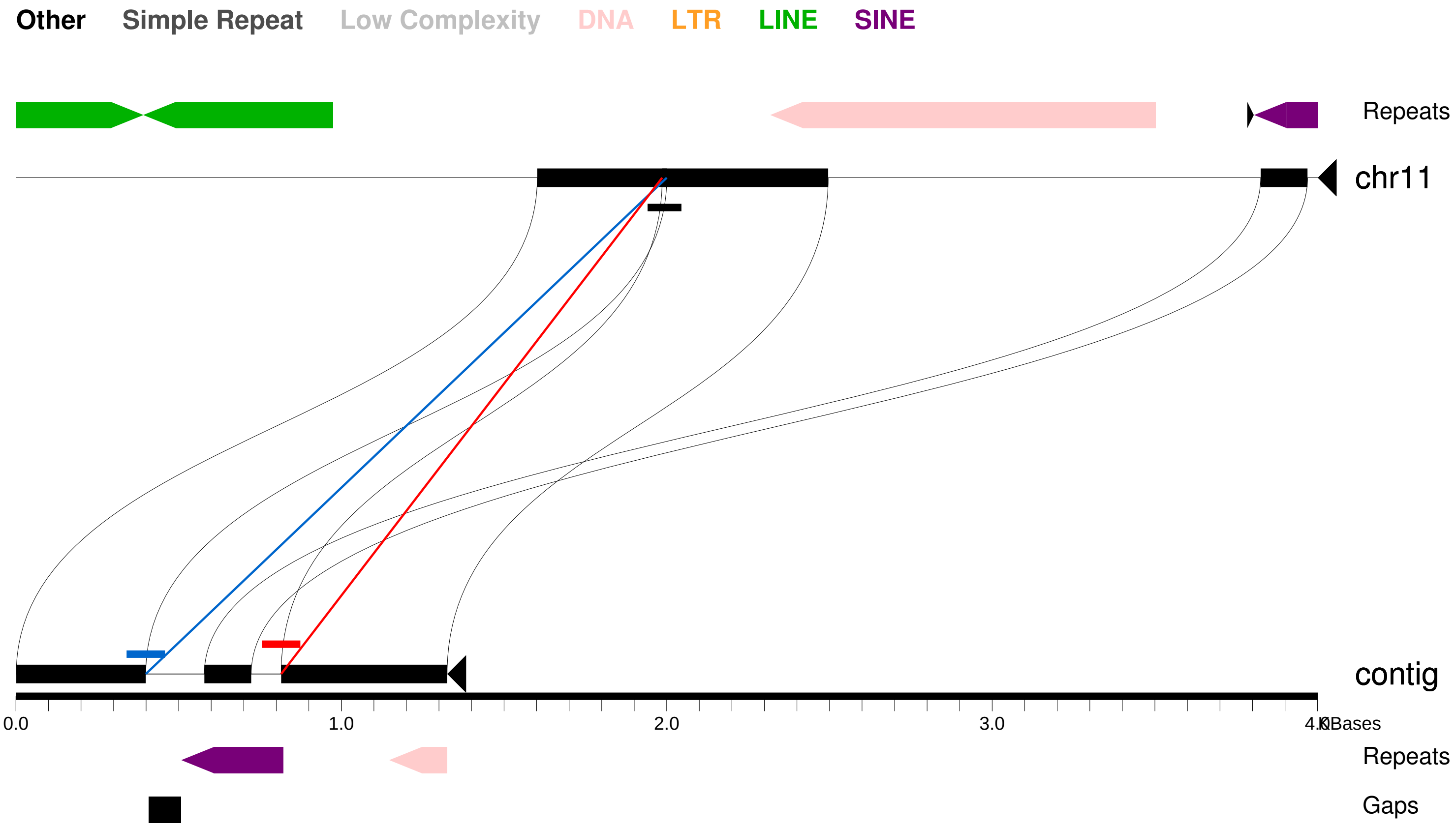

siteID: chr11_55180360 Scafftig2 - Breakpoint: chr11:55180299
chr11:55180253-55180358
Overlap Length: 14 INS size: 351 Genotyped: No

```
left     ACAA--------GACTCCACAGTTTTTGTGACTTAAAAC---AATCACCATTTTATT--TTAACTTTAAA
chrom    ACAA--------GACTCCACAGTTTTTGTGACTTAAAAC---AATCACCATTTTATT--TTAACTTTAAA
right    CCGCCTGCCTCGGCCTCCCAAAGTGCTGGGATTACAGGCTTGAGCCACCGCGCCCGGCCTTAACTTTAAA
         1*1111111111*1****11*11*11**1**1*11*11*111*11****1111111111***********

left     TTTNNNNNNNNNNNNNNNNNNNNNNNNNNNNNNNNNNNNNNNNNNNNNNNNNNNNNNNNNNN
chrom    TTTTATGGGCTAGGAATTCAGGCAGTTGCTGGCTGGGTATTTCTTCTGC-------------
right    TTTTATGGGCTAGGAATTCAGGCAGTTGCTGGCTGGGTATTTCTTCTGC-------------
         ***22222222222222222222222222222222222222222222222222222222222
```

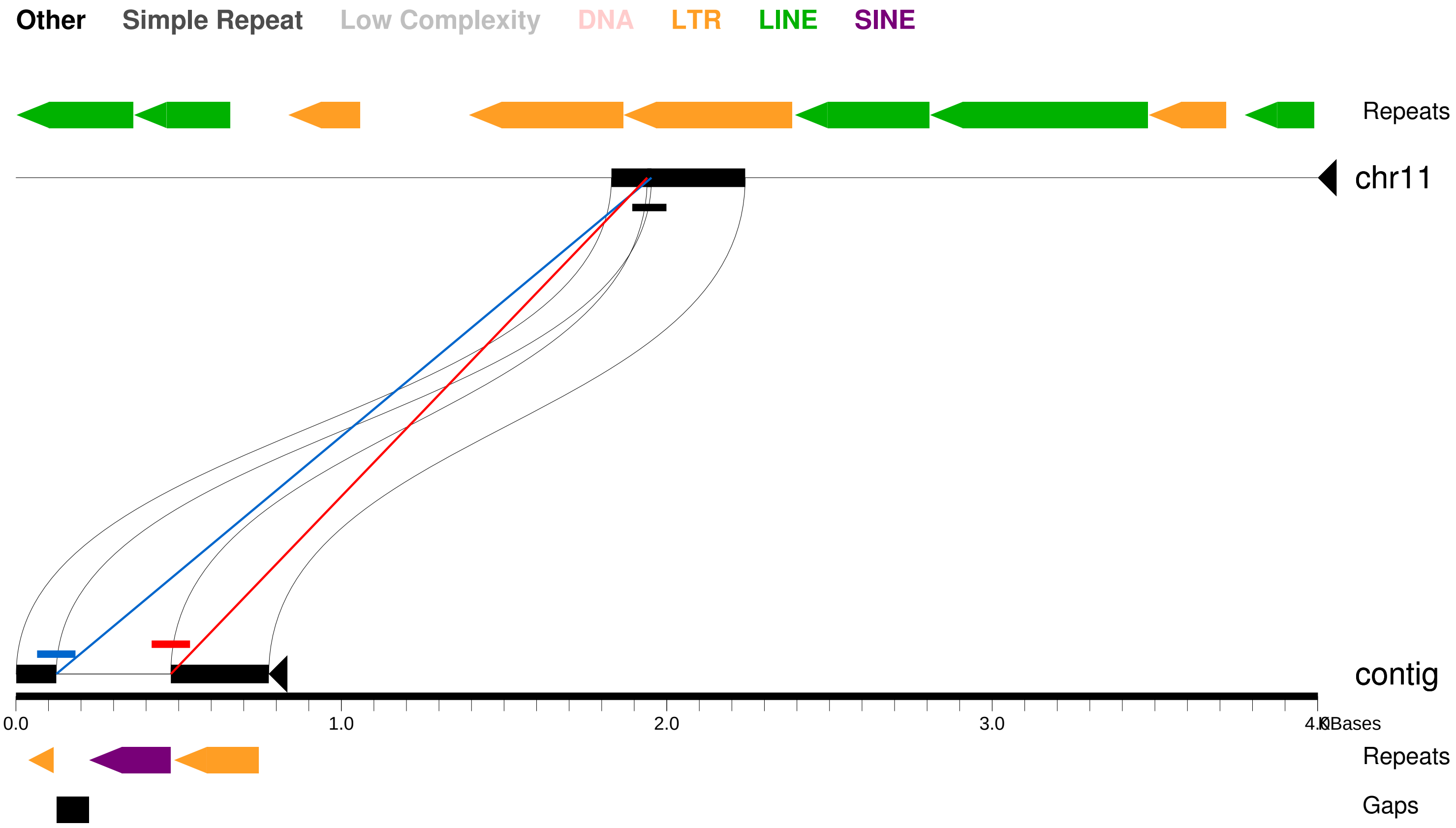

siteID: chr11_55771313 Scafftig1 - Breakpoint: chr11:55771276
chr11:55771273-55771335
Overlap Length: 57 INS size: 405 Genotyped: No

```
left   GTC--------------------------------------------------------TACCGATAAAA
chrom  GTC--------------------------------------------------------TACCAATAAAA
right  NNNNNNNNNNNNNNNNNNNNNNNNNNNNNNNNNNNNNNNNNNNNNNNNNNNNNNNNNNNTACCAATAAAA
       11111111111111111111111111111111111111111111111111111111111****2******

left   ATTCAACATCCGTTTATATTAAAAACCCTTAAAAAAGTAGGCATTGGGCCGGGCGCGGTGGCTCACGCCT
chrom  ATTCAACATCCGTTTACATTAAAAACCCTTAAAAAAGTAGGCATTG------------------------
right  ATTCAACATCCGTTTACATTAAAAACCCTTAAAAAAGTAGGCATTG------------------------
       ****************2*****************************222222222222222222222222

left   GTAATCCCAGCACTTTGGGAGGCCGAGGCGGGCGG
chrom  --AA------------------------------G
right  --AA------------------------------G
       22**222222222222222222222222222222*
```

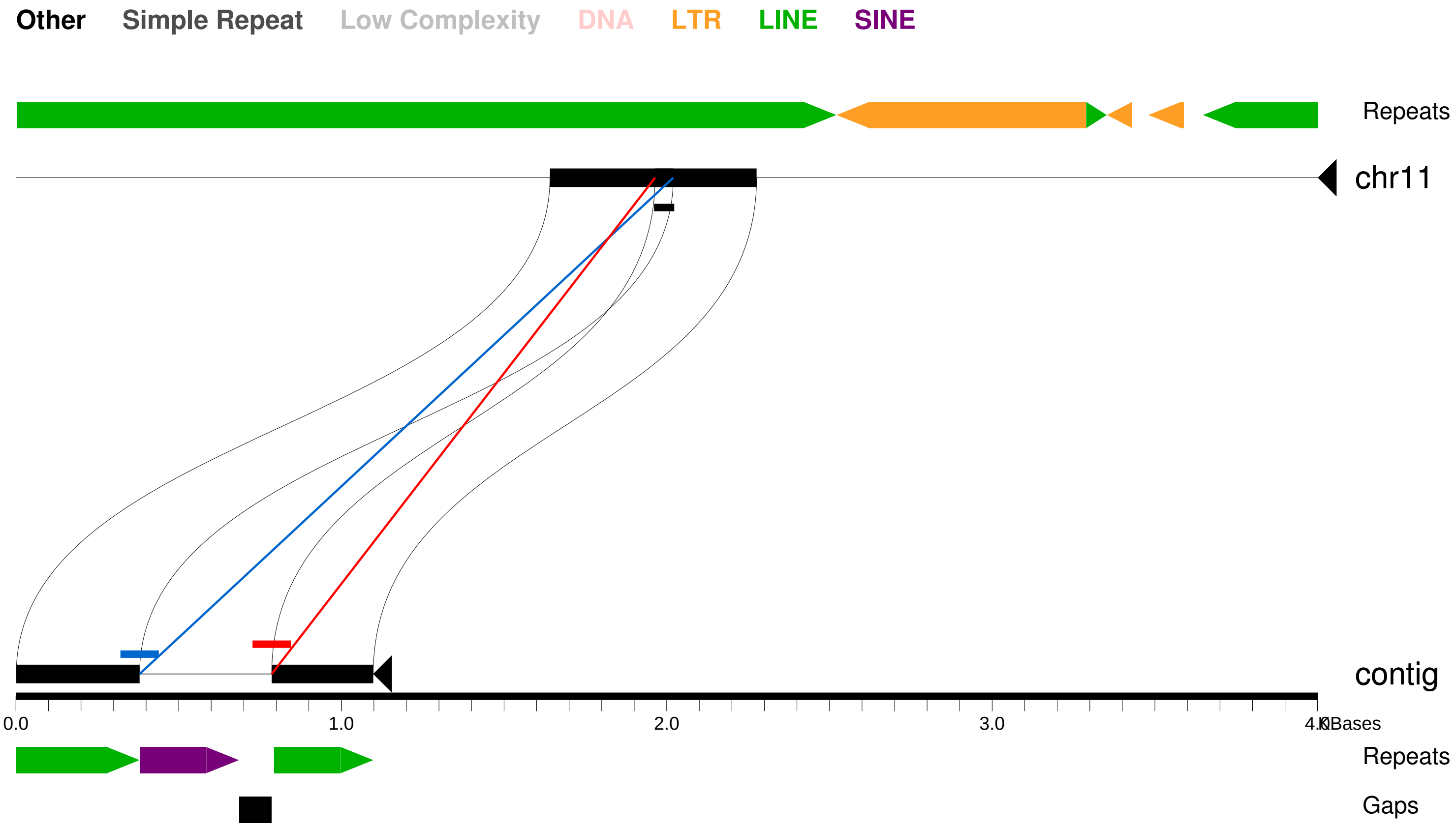

siteID: chr11_56303337 Scafftig4 - Breakpoint: chr11:56303343
chr11:56303304-56303402
Overlap Length: 21 INS size: 300 Genotyped: Yes

```
left     ---AATGCTTCATTGG-----AAGC-ACATTTTTCC-----------AATGATATACCGAAATAAAAACT
chrom    ---AATGCTTCATTGG-----AAGC-ACATTTTTCC-----------AATGATATACCGAAATAAAAACT
right    TGCACTCCAGCCTGGGCGACAGAGCGAAACTCCGCCTCAAAAAAAAAAAAAAAAAAAAAAAAAAAAAAACT
         111*1*1*11*1*1**111111***1*1*1*111**11111111111**11*1*1*111***1*******

left     TTTACTATAGGCCAGGCGCGGTGGCTCACGCCTGTAATCCCAGCACTTTGGGAGGCCGAGGCGGGCGGA
chrom    TTTACTATAGTCTA--------------CACCTA-AATC--AGCATTATCTGTCACTGTCTC---CCTC
right    TTTACTATAGTCTA--------------CACCTA-AATC--AGCATTATCTGTCACTGTCTC---CCTC
         *********2*2*22222222222222*2***22****22****2*2*22*222*2*222*222*222
```

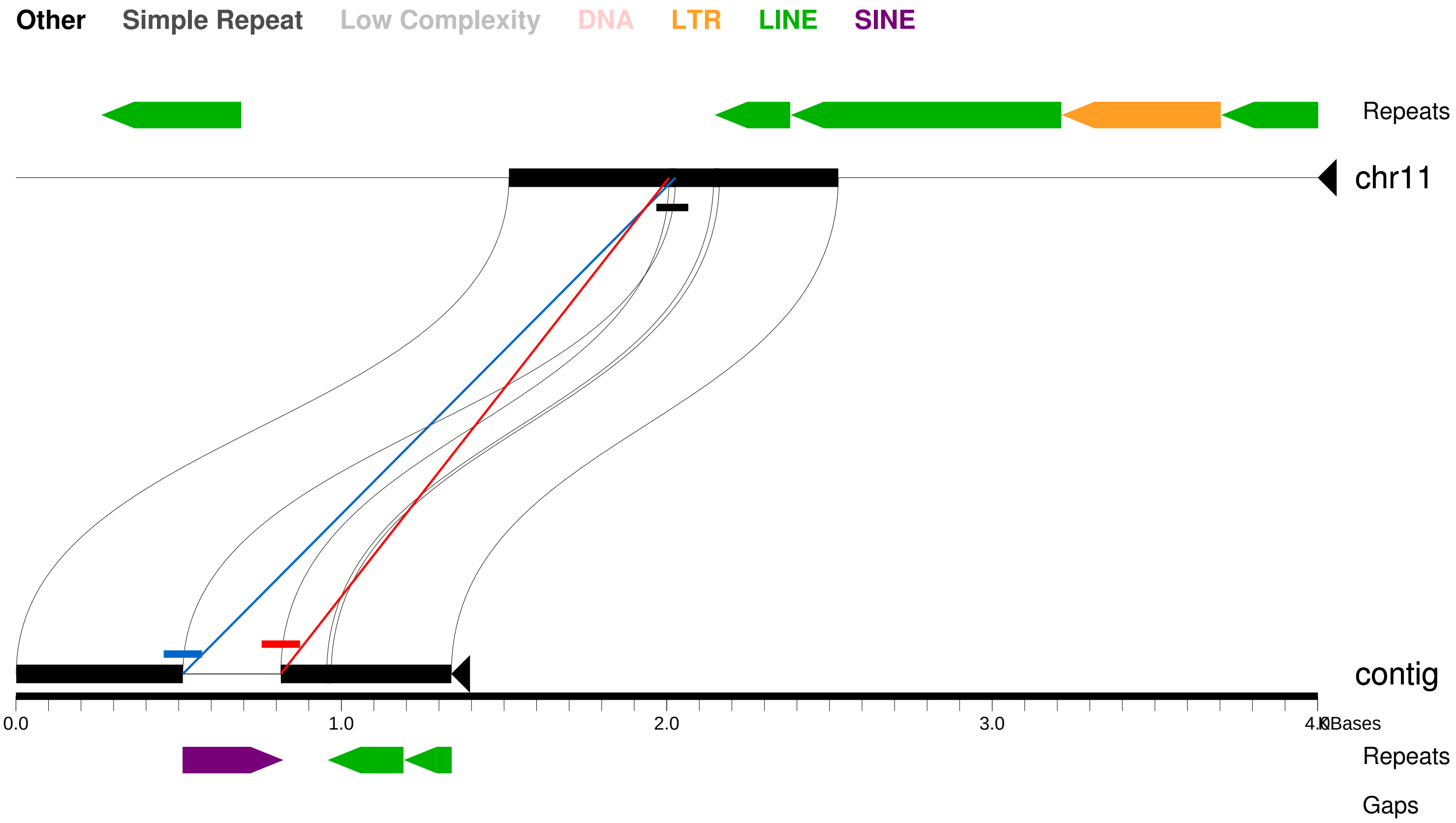

siteID: chr11_5885143 Scafftig11 - Breakpoint: chr11:5885137
chr11:5885090-5885196
Overlap Length: 13 INS size: 308 Genotyped: Yes

```
left    GATGCTATTTGT-CTATTCAG-GATTTTAA-TCTCTTACTGATTCATTTT---------AAGAGATTGTG
chrom   GATGCTATTTGT-CTATTCAG-GATTTTAA-TCTCTTACTGATTCATTTT---------AAGAGATTGTG
right   ACTCCAGCCTGGGCGACAGAGCGAGACTCCGTCTCAAAAAAAAAAAAAAAAAAAAAAAAAAGAGATTGTG
        11*1*1111**11*1*111**1**111*111****11*111*111*1111111111111***********

left    TGGCCGGGCGCGGTGGCTCACGCCTGTAATCCCAGCACTTTGGGAGGCCGAGGCGGGTGGA
chrom   TG-CTTCCCAAAAT---TTAAACATTTCCTCC-AG-ATTTT------CTCATTTGTGTGAA
right   TG-CTTCCCAAAAT---TTAAACATTTCCTCC-AG-ATTTT------CTCATTTGTGTGAA
        **2*2222*2222*222*2*22*2*2*22***2**2*2***222222*22*222*2***2*
```

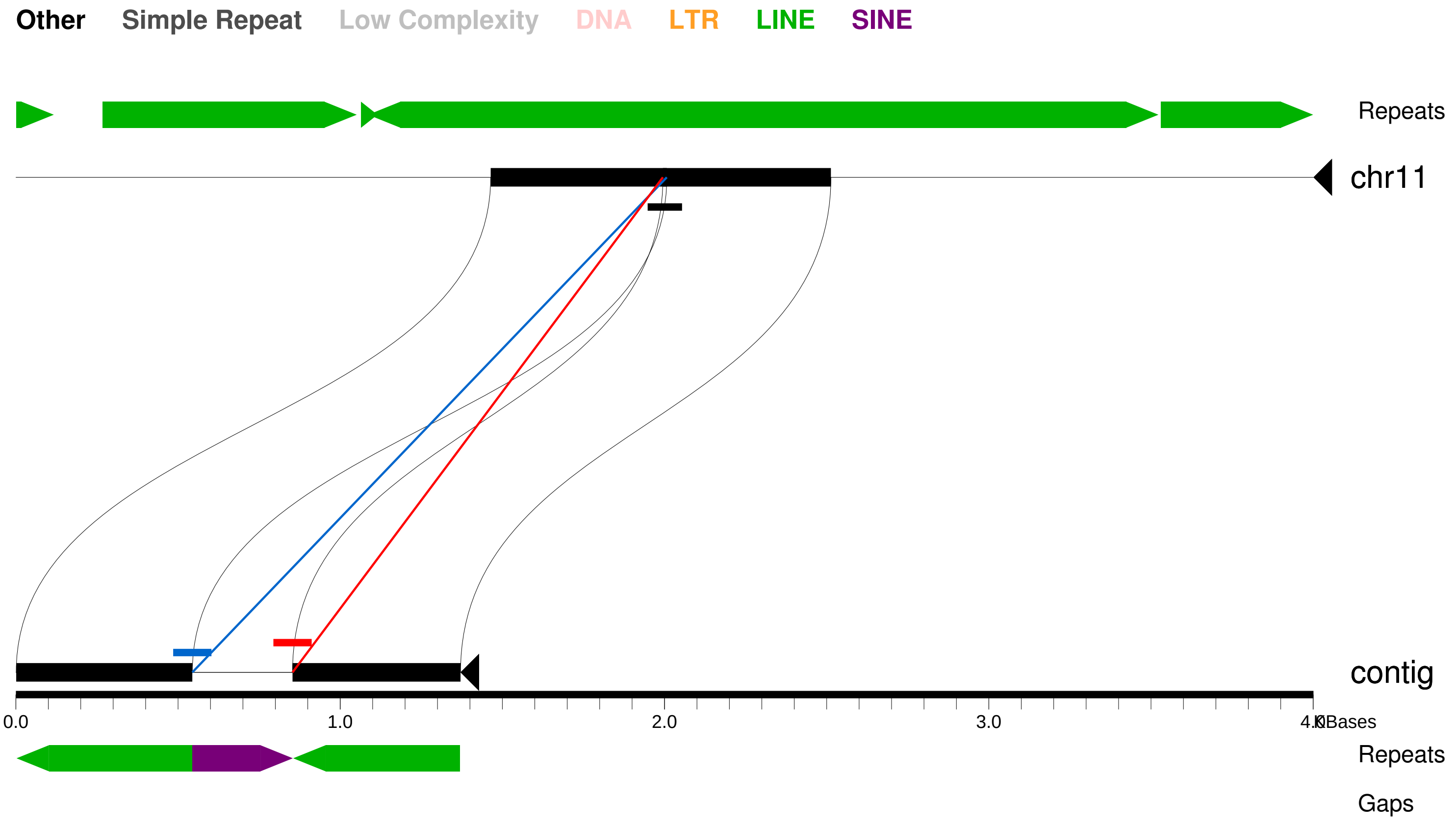

siteID: chr11_60293399 Scafftig2 - Breakpoint: chr11:60293384
chr11:60293341-60293443
Overlap Length: 17 INS size: 298 Genotyped: Yes

```
left     AATAGTTTTATTTTAATTA---TATTGGCTTTAAATTCAGAATC--------------ATAAAATATTAG
chrom    AATAGTTTTATTTTAATTA---TATTGGCTTTAAATTCAGAATC--------------ATAAAATATTAG
right    CC-ACTGCAGTCCGCAGTCCGGCCTGGGCGACAGAGCGAGACTCCGTCTCAAAAAAAAAAAAAATATTAG
         111*1*1111*1111*1*111111*1***111*1*111***1**11111111111111*1**********

left     TGGGGCAG--GCCGGGCGCGGTGGCTCACGCCTGTAATCCCAGCACTTTGGGAGGCCGAAGCGGGTGG
chrom    TGGGGCAAAAGACCTTAGCAAAG--TTAATACAA-AATCTCA-CATTTA---------AAGTG-----
right    TGGGGCAAAAGACCTTAGCAAAG--TTAATACAA-AATCTCA-CATTTA---------AAGTG-----
         *******222*2*2222**222*22*2*222*222****2**2**2**2222222222***2*22222
```

Other Simple Repeat Low Complexity DNA LTR LINE SINE

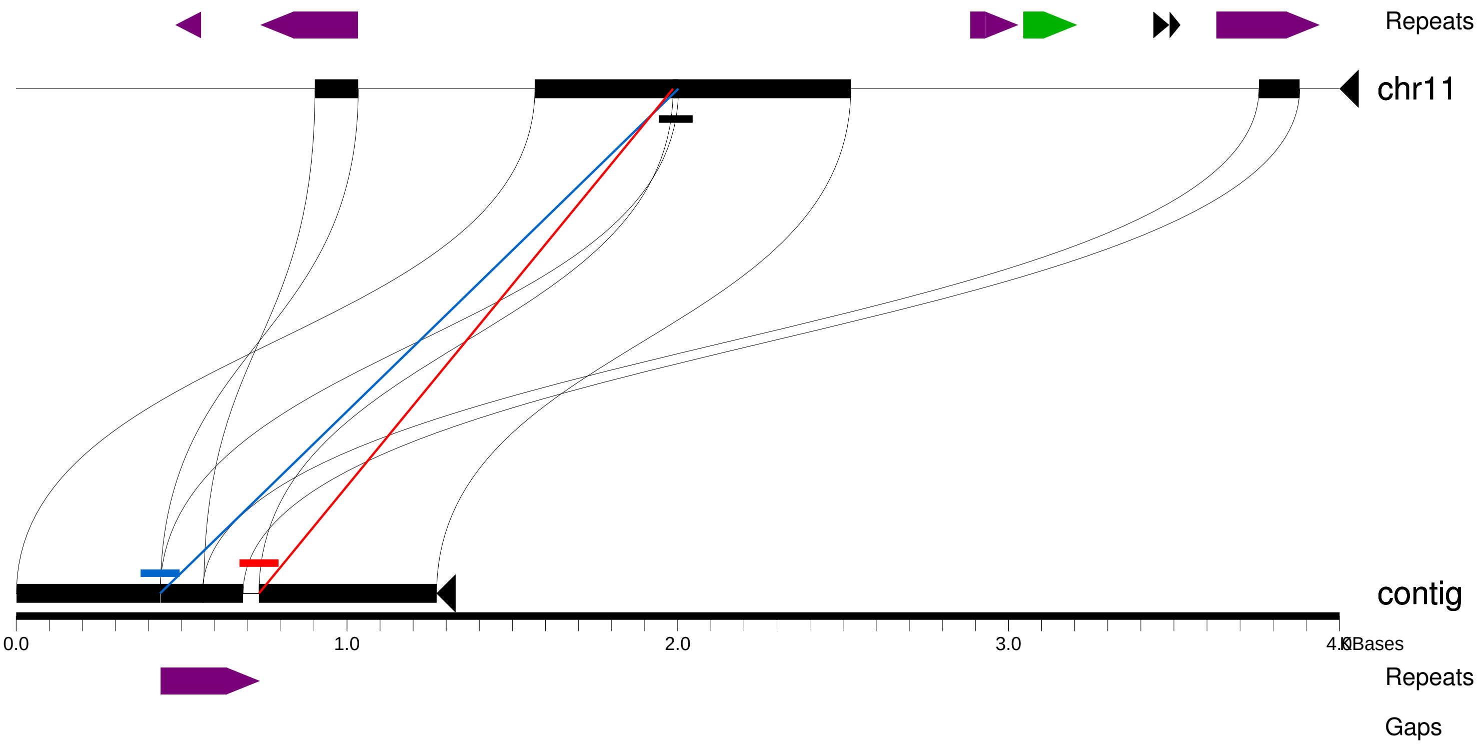

siteID: chr11_65503493 Scafftig4 + Breakpoint: chr11:65503476
chr11:65503430-65503535
Overlap Length: 14 INS size: 300 Genotyped: Yes

```
left     AATGCATCTATG----CCCA------TGGCATCATGTTTTTTATCTTT-GTGTTTGT--GAAACAACACC
chrom    AATGCATCTATG----CCCA------TGGCATCATGTTTTTTATCTTT-GTGTTTGT--GAAACAACAGC
right    CCGCCCGCCTCGGCCTCCCAAAGTGCTGGGATTACAGGCGTGAGCCACCGCGCCCGGCCGAAACAACACC
         1111*11*111*1111****111111***1**1*111111*1*1*1111*1*111*111*********N*

left     TTTTTTTTTTTTTTTTTTTTTTGAGACGGAGTCTCGCTC-TGTCGCCCAGGCTGGAGTGCAGT-
chrom    TTTAAGTCTTATA--------------GGAG-CTCACAGGTGTAAATCATGGTGTCCTCCTCTT
right    TTTAAGTCTTATA--------------GGAG-CTCACAGGTGTAAATCATGGTGTCCTCCTCTT
         ***222*2**2*222222222222222****2***2*222***2222**2*2**222*2*22*2
```

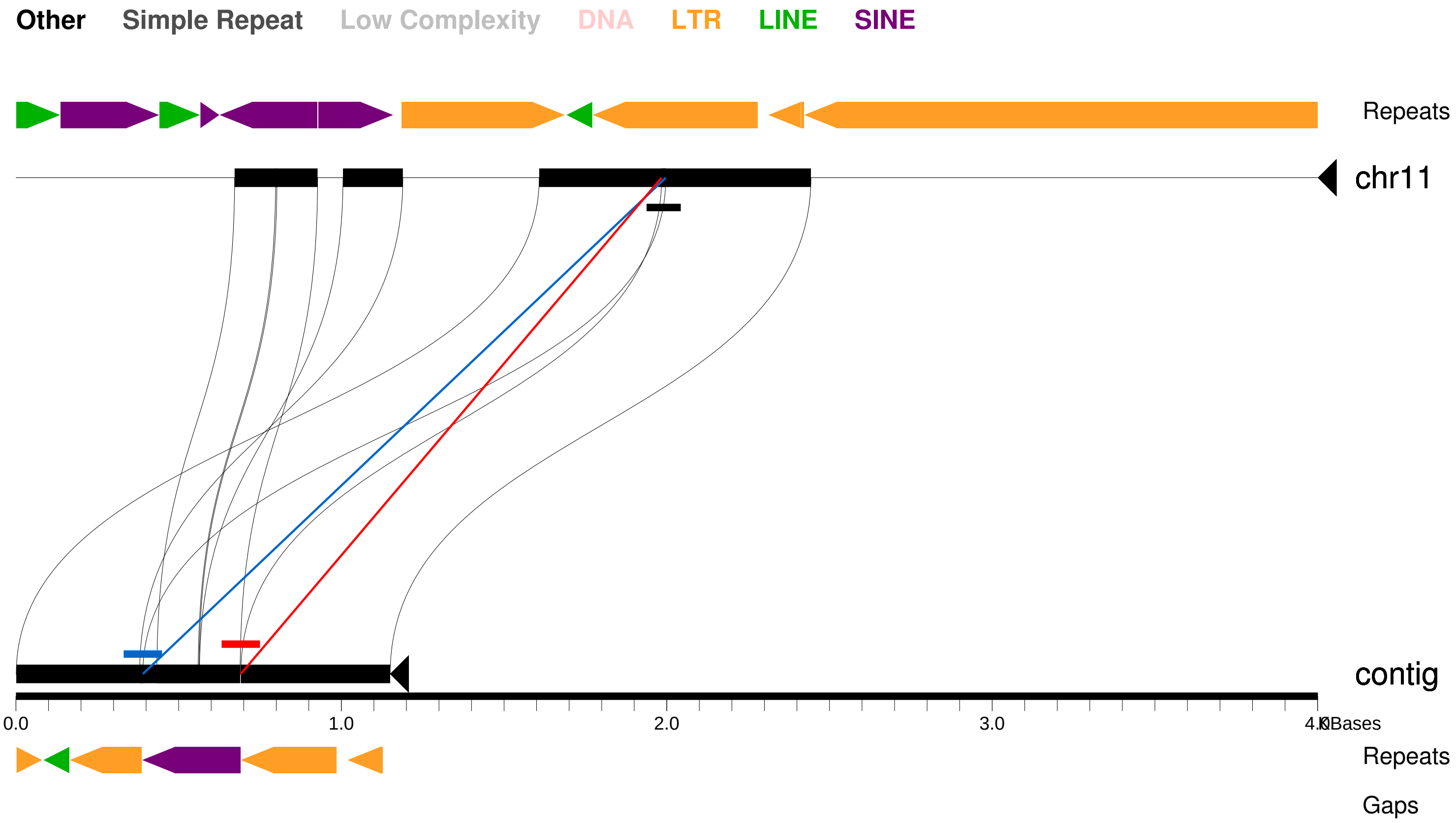

siteID: chr11_65984320 Scafftig1 + Breakpoint: chr11:65984326
chr11:65984269-65984385
Overlap Length: 3 INS size: 400 Genotyped: No

```
left    GTGGTTGGCTAAGATTTTC--AGT-CTGGGGGTAG-GTTGGCAGCAGCATATTCAGGCCTAGTGNNNNNN
chrom   GTGGTTGGCTAAGATTTTC--AGT-CTGGGGGTAG-GTTGGCAGCAGCATATTCAGGCCTAGTGGAGACT
right   CCGCCCACCTCGGCCTCCCAAAGTGCTGGGATTACAGGCGTGAGCCACCGCGCCCGGCC--GTGGAGACT
        11*11111**11*11*11*11***1*****11**11*11*11***11*11111*1****11***222222

left    NNNNNNNNNNNNNNNNNNNNNNNNNNNNNNNNNNNNNNNNNNNNNNNNNNNNN
chrom   TCTTAAAATCAACACAGGGTCATCCCTTCAACGATTCCAATGAACGCTCTA--
right   TCTTAAAATCAACACAGGGTCATCCCTTCAACGATTCCAATGAACGCTCTA--
        22222222222222222222222222222222222222222222222222222
```

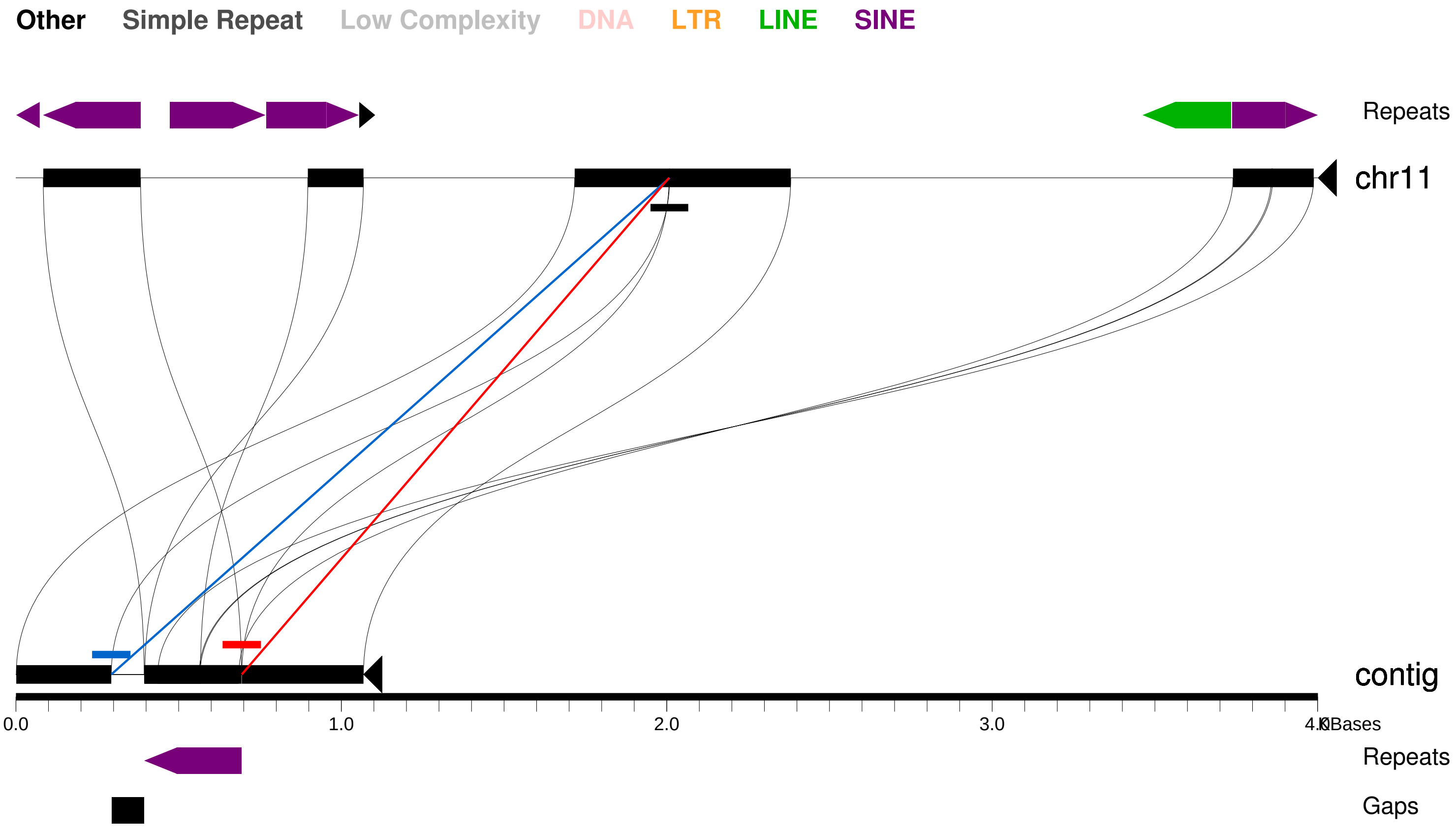

siteID: chr11_69548383 Scafftig2 + Breakpoint: chr11:69548376
chr11:69548333-69548425
Overlap Length: 17 INS size: 439 Genotyped: No

```
left   GAGAAAGAAAAGAAAGAAGAGAGAAAGGAAATA--------------------------AAGAAAAGATA
chrom  GAGAAAGAAAAGAAAGAAGAGAGAAAGGAAATA--------------------------AAGAAAAGATA
right  NNNNNNNNNNNNNNNNNNNNNNNNNNNNNNNNNNNNNNNNNNNNNNNNNNNNNNNNNNNNAAAAAAAAAAA
       111111111111111111111111111111111111111111111111111111111111**1****1*1*

left   GAAAAAAGAAGGAAGGGGGCCGGGCGCGGTGGCTCACGCCTGTAATCCCAGCACTTTGGGAGGCCGAGGC
chrom  GAAAAAAGAAGGAAGGAAGGAGG--------------------AAAAAAAGGAA----GGAAA--GAGAG
right  GAAAAAAGAAGGAAGGAAGGAGG--------------------AAAAAAAGGAA----GGAAA--GAGAG
       ****************22*22**2222222222222222222**2222**2*22222***2222***22

left   GGGCG
chrom  AAGAG
right  AAGAG
       22*2*
```

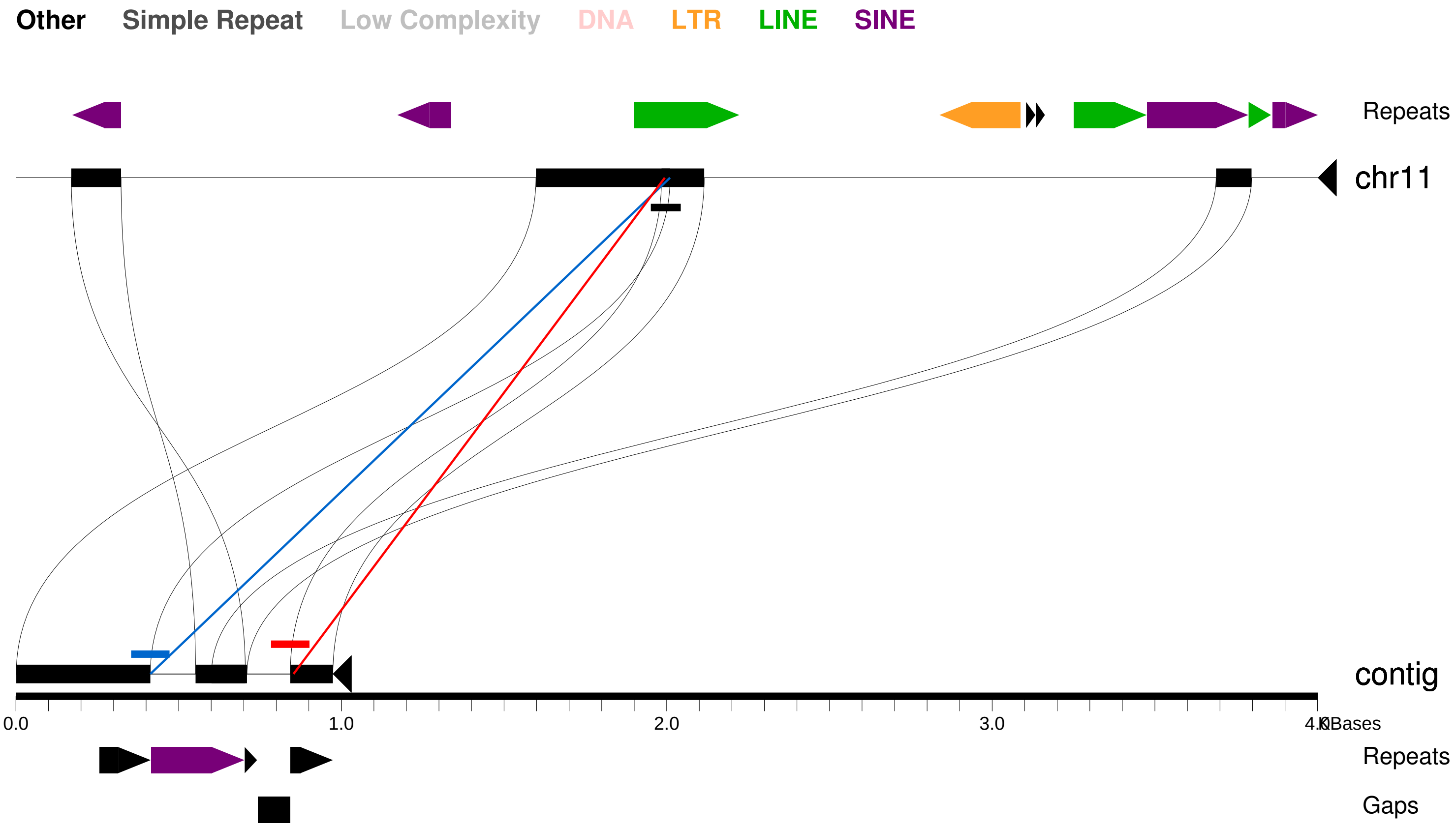

siteID: chr11_7092799 Scafftig13 - Breakpoint: chr11:7092789
chr11:7092741-7092848
Overlap Length: 12 INS size: 331 Genotyped: Yes

```
left    AAGCATAAAAC-TCTCTGAAAAGTATGTTTTTAATTGGATACAGAGCTT----------AAGAATGCAGG
chrom   AAGCATAAAAC-TCTCTGAAAAGTATGTTTTTAATTGGATACAGAGCTT----------AAGAATGCAGG
right   AGCGAGACTCCGTCTCAAAAAAAAAAAAAAAAAAAAAAAAAAAAAAAAAAAAAAAAAAGAAGAATGCAGG
        *111*1*111*1****11****11*1111111**1111*1*1*1*11111111111111***********

left    TGGCCGGGCGCGGTGGCTCACGCCTGTAATCCCAGCACTTTGGGAGGCCGAGGCGGGTG----G
chrom   TAA--GGG-----------AAGCCTGGA-TATGAGG-CTGGAGGATGCAGACAGGGAAGATCAG
right   TAA--GGG-----------AAGCCTGGA-TATGAGG-CTGGAGGATGCGGACAGGGAAGATCAG
        *2222***22222222222*2*****2*2*222**22**222***2**N**222**22*2222*
```

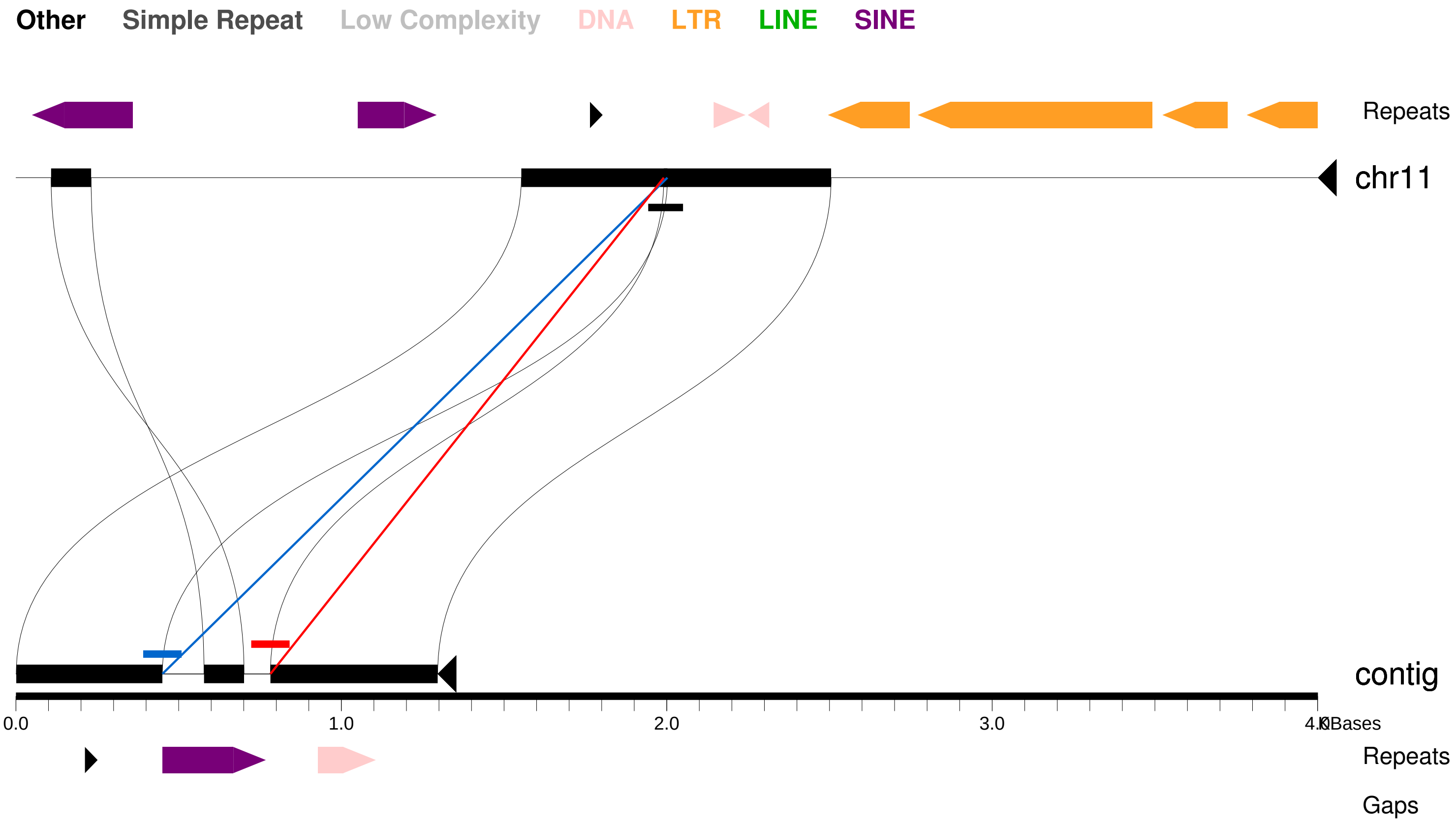

siteID: chr11_72320931 Scafftig2 + Breakpoint: chr11:72320909
chr11:72320749-72321082
Overlap Length: 14 INS size: 298 Genotyped: Yes

```
left   C---------------TCCCGCAAGCCCTTGGAAGTCCTTTCAGATGTCTTCCCAGTGGAAGGTTGGGTC
chrom  CCACCAGCCATGCCC-TCCCGCAAGCCCTTGGAAGTCCTTTCAGATGTCTTCCCAGTGGAAGGTTGGGTC
right  TGA---GGCAGGAGAATGGCGTGAACCC--GGGAGGC---------------------GGAGCTTG---C
       1N2   2N22N2NNN1*11**11*1***11**1**1*111111111111111111111*1**1***111*

left   TGTGTTCAGAAAGCAAAGTGATTAACTGCAAGATGAAGGAGACATGGCTGGGTCCCCATTCATGTGAACA
chrom  TGTGTTCAGAAAGCAAAGTGATTAACTGCAAGATGAAGGAGACATGGCTGGGTCCCCATTCATGTGAACA
right  AGTGAGCCGAGATTGCGCCACTGCACTCCAGCCTG--GGCGACAGAGCGAGACTCCGTCTCAAAAAAAAA
       1***11*1**1*111111111*11***1**111**11**1****11**11*111**111***1111**1*

left   TGATATGAGGTTGAGGGTCTTAAAAGACCACAGTTGGCCGGGCGCG----GTG---GC-TCAC--GCCTG
chrom  TGATATGAGGTTGAGGGTCTTAAAAGACCACAGTTCAGCATGAGCCCACAGTGTAAGCATGACTGGCAAG
right  AAAAAAAA-------------AAAAGACCACAGTTCAGCATGAGCCCACAGTGTAAGCATGACTGGCAAG
       11*1*11*1111111111111*************222*22*2**22222***222**2*2**22**22*

left   TAATCCCAGCACTT---TGGGAGGCCGAG------GCGGGCGGATCACGAGGTCAGGAGATCGAGACCAT
chrom  AAATAAAAATGCTCAAATTGCAGGCCATATTAATAGAGGTCTGTTAACCCAATTGGGAAGATGACAGTTC
right  AAATAAAAATGCTCAAACTGCAGGCCATATTAATAGAGGTCTGTTAACCCAATTGGGAAGATGACAGTTC
       2***222*222**222212*2*****222222222*2**2*2*2*2**2222*22***2222**2*2222

left   CCCGGCTAAAACAGT-------GA--AACCCCGTCTC------TACTAAA-----
chrom  CCCCACTTTTATAGTCTAGTTAGACCATCCCCTGTTCATTGTGTCCCAGGGGCTG
right  CCCCACTTTTATAGTCTAGTTAGACCATCCCCTGTTCATTGTGTC----------
       ***22**222*2***2222222**22*2****222**222222*21N1NN
```

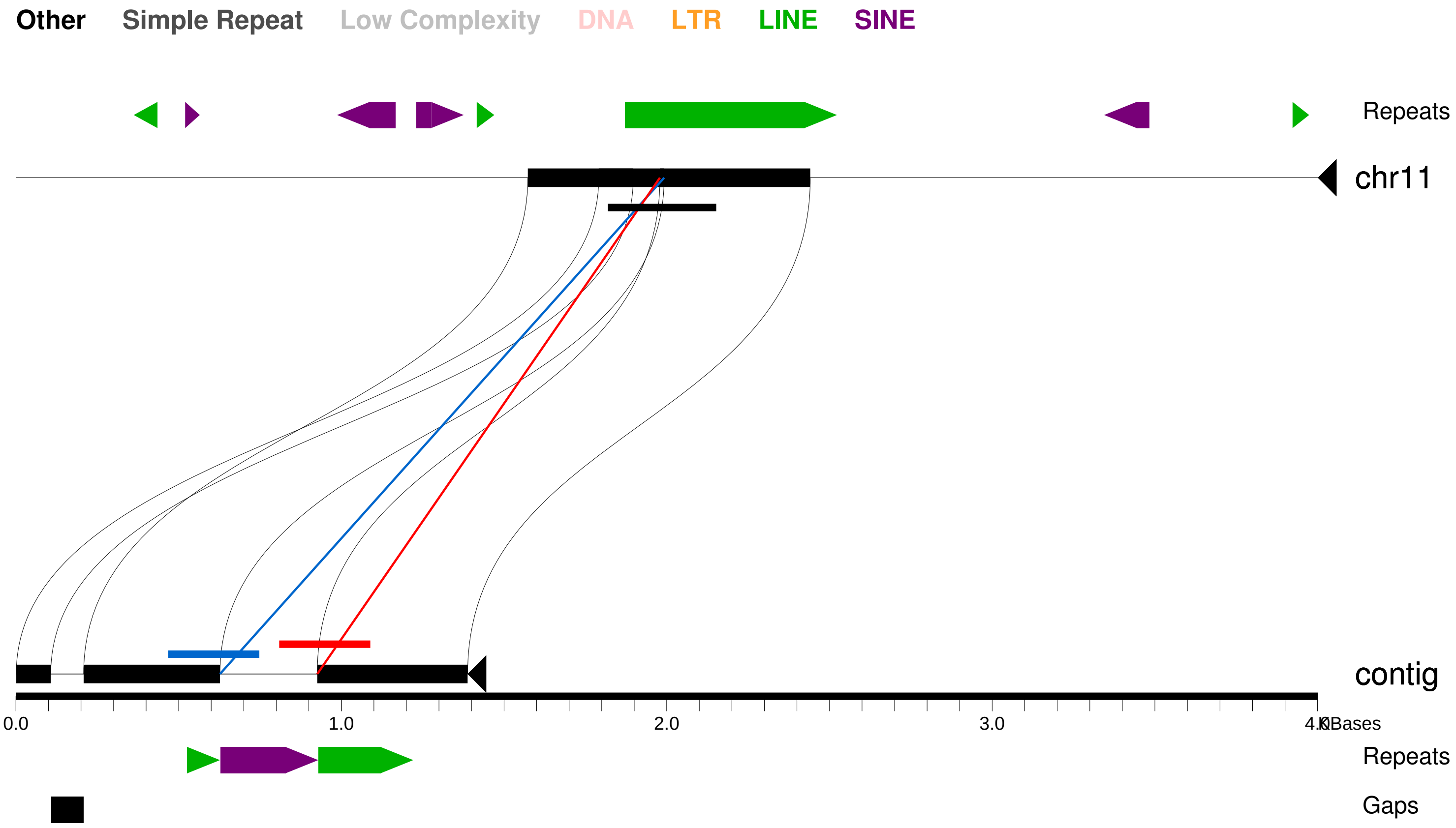

siteID: chr11_73562786 Scafftig1 - Breakpoint: chr11:73562777
chr11:73562741-73562836
Overlap Length: 17 INS size: 419 Genotyped: No

```
left   A----------GGCCTGCCATATGGGAGACATT-CAGTCATGATTGA------------AAGAATGACAG
chrom  A----------GGCCTGCCATATGGGAGACATT-CAGTCATGATTGA------------AAGAATGACAG
right  CCGCCCGCCTCGGCCTCCCAAAGTGCTGGGATTACAGGCGTGAGCCACCGCGCCCGGCCAAGAATGACAG
       11111111111*****1***1*11*11*11***1***1*1***111*111111111111***********

left   GTTCTTTTATATCNNNNNNNNNNNNNNNNNNNNNNNNNNNNNNNNNNNNNNNNNNNNNNNNNNNNNNNNN
chrom  GTTCTTAAATATCACTATACAATCCAGTTGTGTGGTATACAGTTGCTAA---------------------
right  GTTCTTAAATATCACTATACAATCCAGTTGTGTGGTATACAGTTGCTAA---------------------
       ******22*****2222222222222222222222222222222222222222222222222222222222

left   NN
chrom  --
right  --
       22
```

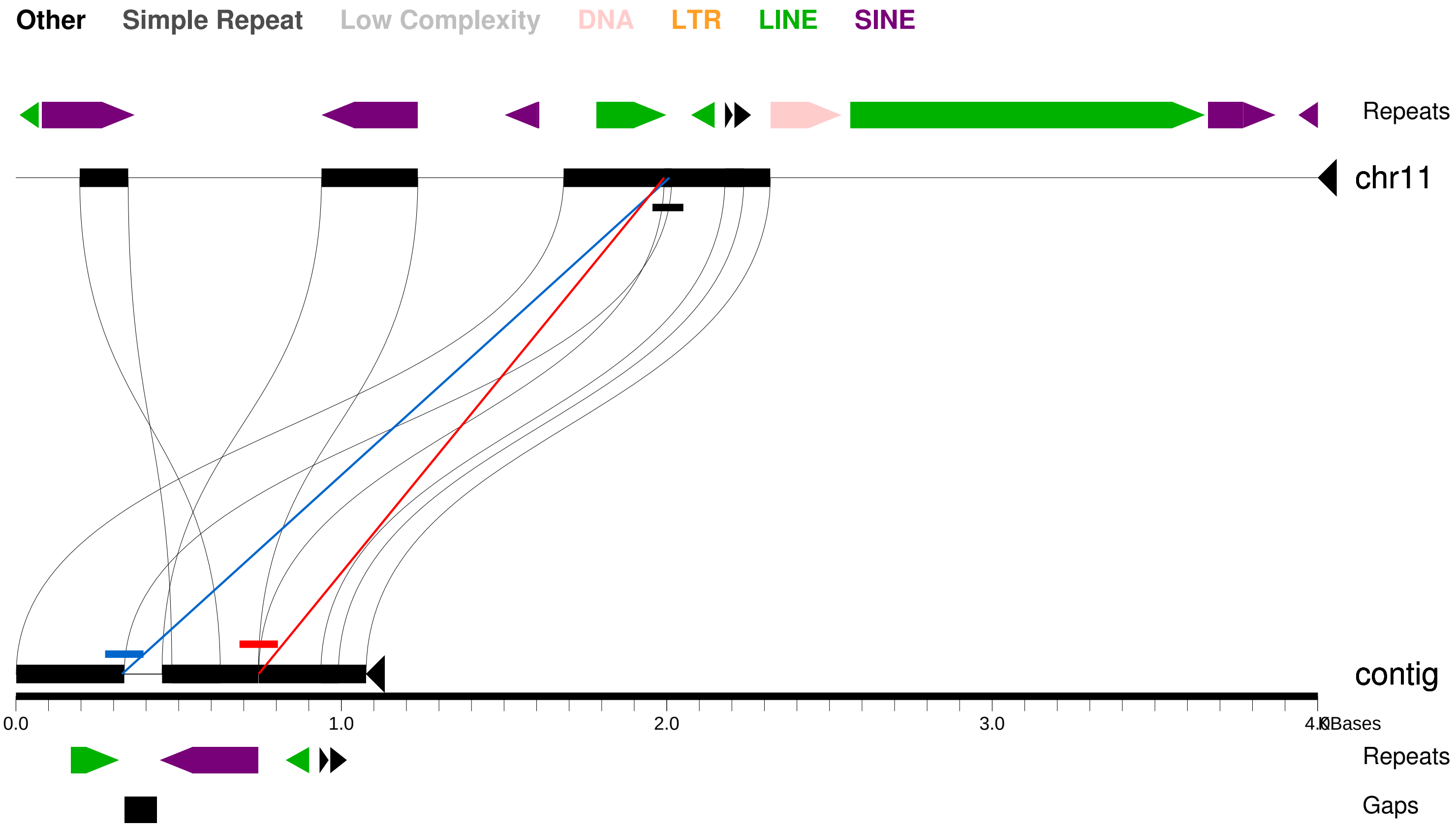

siteID: chr11_7612704 Scafftig9 + Breakpoint: chr11:7612688
chr11:7612528-7612857
Overlap Length: 10 INS size: 309 Genotyped: Yes

```
left    ----------CTAGGTGGCTCCGTGTCCATGTGGACTTCGGCACTGGGGTTCTACCTGCAGAGCTTCACT
chrom   GCTGCCAGTGCTAGGTGGCTCCGTGTCCATGTGGACTTCGGCACTGGGGTTCTACCTGCAGAGCTTCACT
right   ------AGAA-----TGGC-----------GTGAACCCAGGAAGCGGAGCT-----TGCAGTGAGCC---
              22NN11111****11111111111***1**111**1*11**1*1*11111*****1*111*111

left    TGGACACCTGGGTCCTGTTTAATTCTCTGTCC--ACTGGAAATCACCATCATGTGAGTTCTTTGACAGAG
chrom   TGGACACCTGGGTCCTGTTTAATTCTCTGTCC--ACTGGAAATCACCATCATGTGAGTTCTTTGACAGAG
right   --GAGA--TTGCGCCACTGCAGTCCGCAGTCCGGCCTGGG---CGACAGAGCGAGACTCCGTCTCAAAAA
        11**1*11*1*11**11*11*1*1*1*1****111****1111*11**1111*1**1*1*1*1111*1*1

left    AAGAAGTTGACCTGCTATTAAGAATTATTCTCGGCCGGGCGCGG---TGGCTC-----ACGCCTGTAATC
chrom   AAGAAGTTGACCTGCTATTAAGAATTATTCTCCTACCTTTTCTTCTCTGGTTCTGAGAAAATATAGATTA
right   AAAAAAAAAA------AAAAAAAATTATTCTCCTACCTTTTCTTCTCTGGTTCTGAGAAAATATAGATTA
        **1**1111*111111*11**1**********222*22222*22222***2**22222*2222*22*2*2

left    CCAGCACTTTGGGAGGCCGAGGCGGG----TGGATCATG--AGGTCAGGAG---------ATCGAGACCA
chrom   TAATATATTCTTGGATTCTAGTCAGAGAATTTGATCATGTGAGAACCGGAATATTAATTTAACAACAATA
right   TAATATATTCTTGGATTCTAGTCAGAGAATTTGATCATGTGAGAACTGGAATATTAATTTAACAACAATA
        22*2222**222*2222*2**2*2*22222*2*******22**22*N***2222222222*2*2*2*22*

left    TCCTGGC--TAACAAGG----------TGA-----AACCCCGTCTCTACTAAA
chrom   AACTGCCATTAACAAAGAAGTCAGTTGTGATTGTTAAGGTTCTTTGT-CTGTG
right   AACTGCCATTAACAAAGAAGTCAGTTGTGATTGTTAAGGTTCTT---------
        22***2*22******2*2222222222***22222**22222*21N1211NNN
```

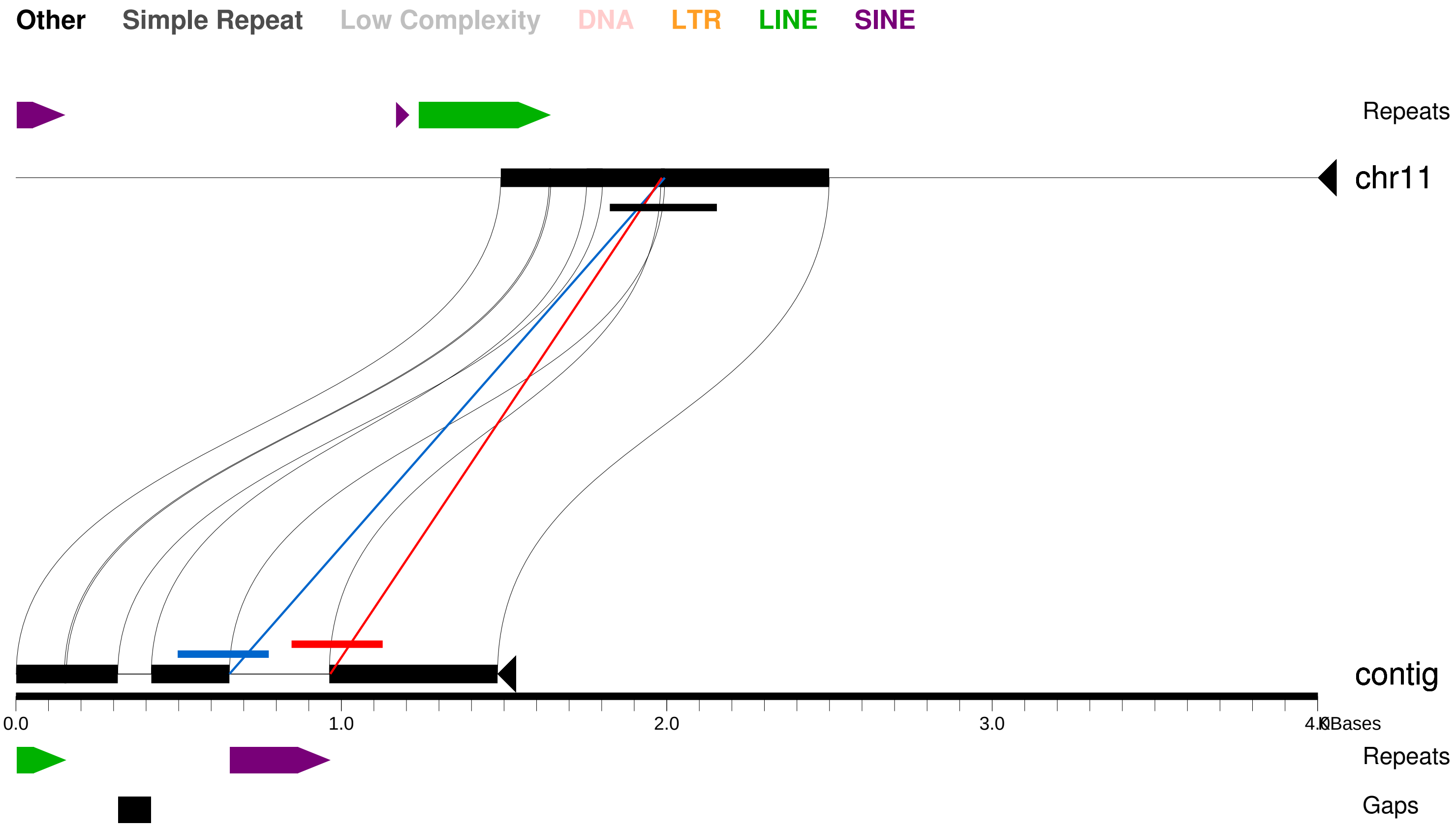

siteID: chr11_76990505 Scafftig1 - Breakpoint: chr11:76990571
chr11:76990526-76990630
Overlap Length: 15 INS size: 299 Genotyped: Yes

```
left     AGA----TAAGGCATAAGCAAATGTAGGGA----------GATAAAAAGAAGCAGTGTGTGAACAGCAGC
chrom    AGA----TAAGGCATAAGCAAATGTAGGGA----------GATAAAAAGAAGCAGTGTGTGAACAGCAGC
right    CGCCCGTCTCGGCCTCCCAAAGTGCTGGGATTACAGGCGTGAGCCACCGCGCCCGGCCCTGAACAGCAGC
         1*11111111***1*1111**1**11****1111111111**111*11*111*1*1111***********

left     TTTTTTTTTTTTTTTTTTTTTTTGAGACGGAGTTTCGCTCTGTTGCCCAGGCTGGAATGCAGTG
chrom    TTTTGCGGATAATTATAT----GAG---GAGTTTTAAAGTTGTGTTCTGTAGGCAA-------
right    TTTTGCGGATAATTATAT----GAG---GAGTTTTAAAGTTGTGTTCTGTAGGCAA-------
         ****22222*22**2*2*2222***222******22222*22**22*2*222*2**2222222
```

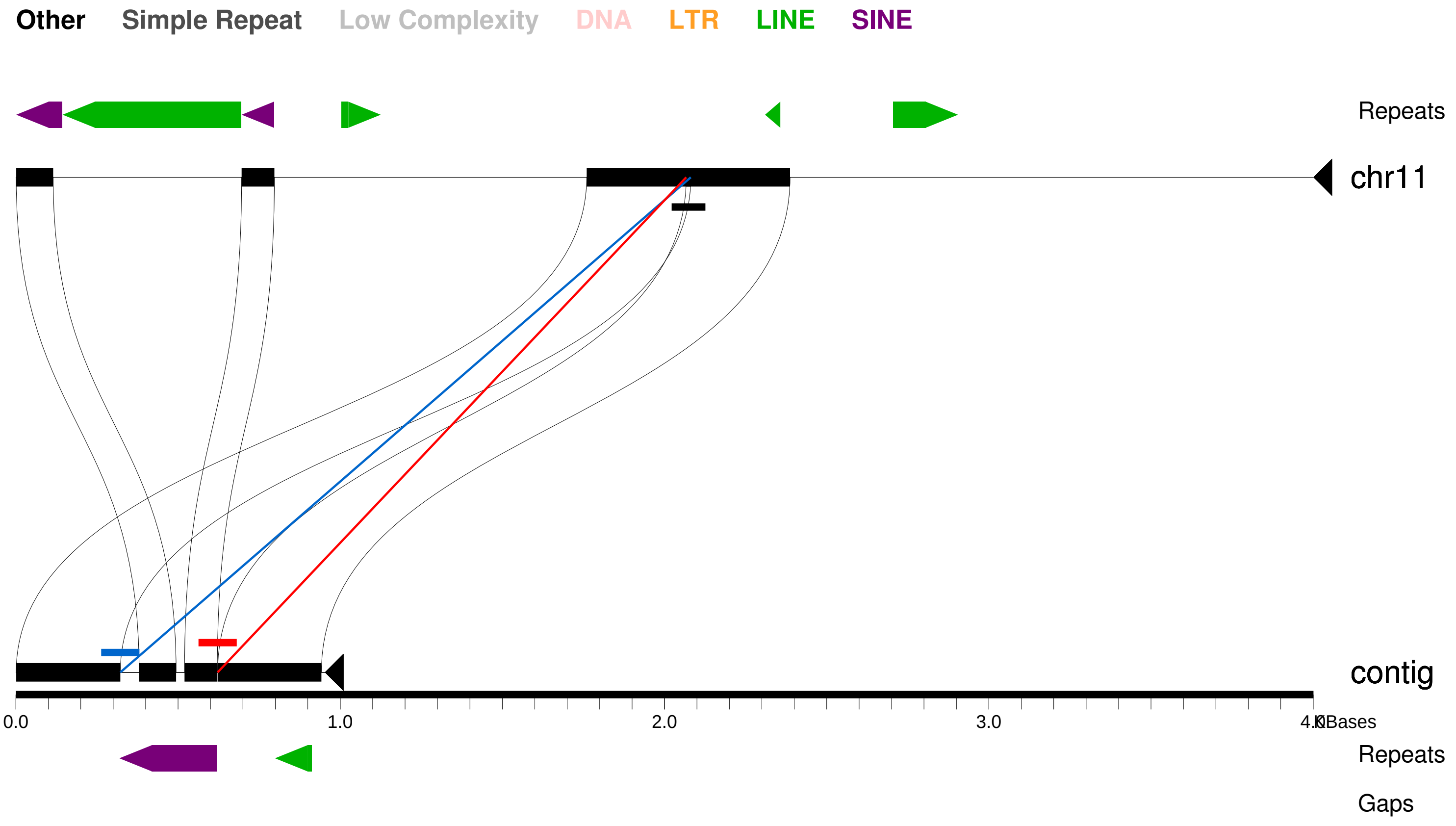

siteID: chr11_79191609 Scafftig1 + Breakpoint: chr11:79191615
chr11:79191568-79191666
Overlap Length: 13 INS size: 312 Genotyped: Yes

```
left    ----CCATGATTTT-------------TGATTAGGTTATAT---TTATTTATGATTTTTGGCCCATTAAA
chrom   ----CCATGATTTT-------------TGATTAGGTTATAT---TTATTTATGATTTTTGGCACATTAAA
right   TGATCCACCCGCCTCGGCCTCCCAAAGTGCTGGGATTACAGGCGTGAGCCACCGCGCCCGGCCCCT-AAA
        1111***111111*1111111111111**1*11*1***1*1111*1*111*11111111***N*1*1***

left    GGGTTTTTTTTTTTTTTTTTTTTTTTTTTTT-GAGACGGAGTCTCGCTCTGTCGCCCAGGCCGGACTGCG
chrom   GGGCTTTTTTAAAAA---------AAAAAAA-GAGAAGAAATC-----CTGAGAATCAG------CTTTG
right   GGGCTTTTTTAAAAA---------AAAAAAAAGAGAAGAAATC-----CTGAGAATCAG------CTTTG
        ***2******2222222222222222222221****2*2*2**22222***22222***222222**22*
```

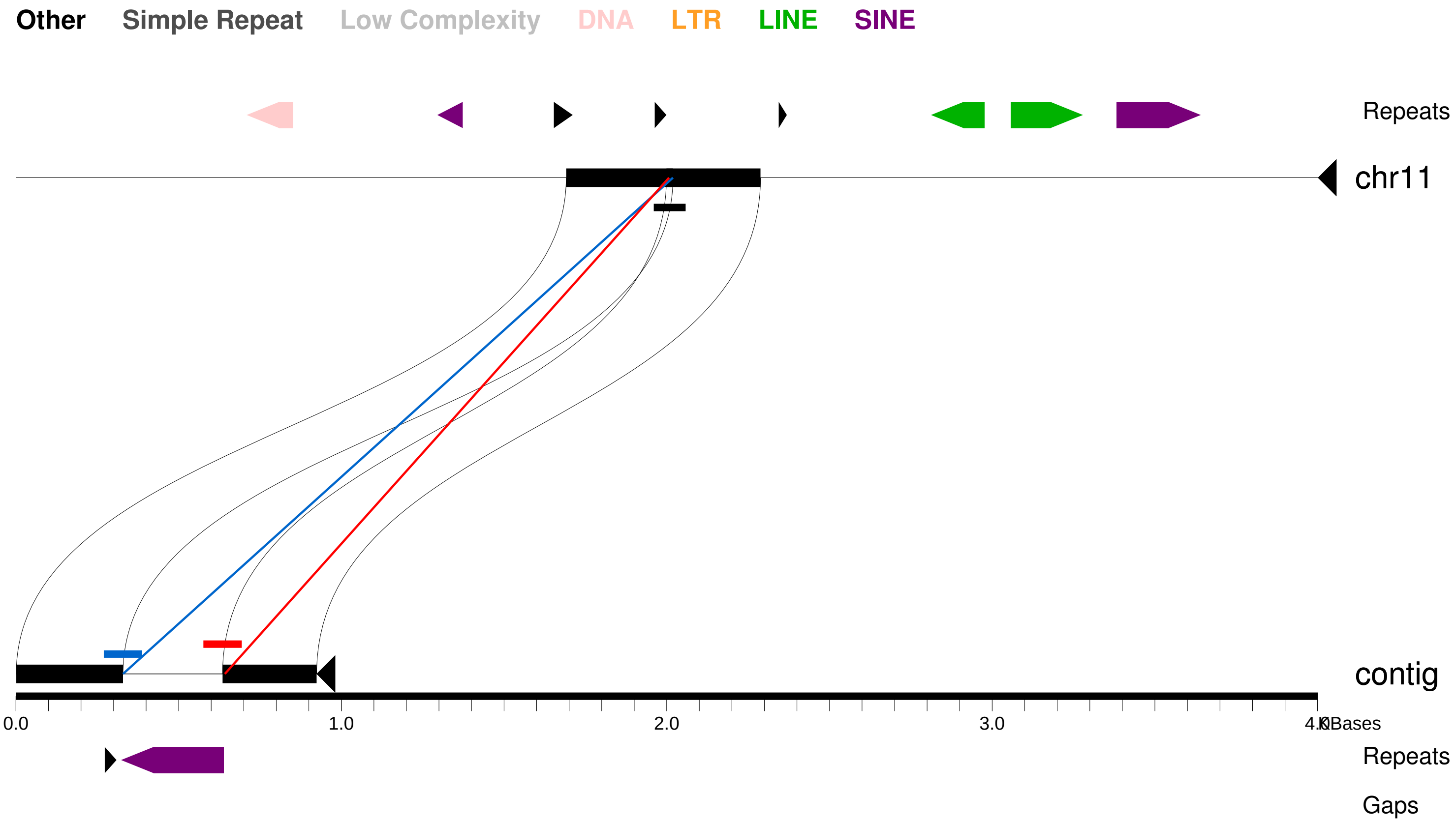

siteID: chr11_81283888 Scafftig4 + Breakpoint: chr11:81283868
chr11:81283825-81283927
Overlap Length: 17 INS size: 300 Genotyped: Yes

```
left    ATTCTAAATTC----------AGTGCA--AAATAATGTTATCAACTA--GTTCCC--CAATATTTCAACT
chrom   ATTCTAAATTC----------AGTGCA--AAATAATGTTATCAACTA--GTTCCC--CAATATTTCAACT
right   CCACCCGCCTCGGCCTCCCAAAGTGCTGGGACTACAGGCGTGAGCCACCGCGCCCGGCCATATTTCAACT
        111*11111**1111111111*****1111*1**11*111*1*1*1*11*11***11*1***********

left    TCTTTTTTTTTTTTTTTTGAGACGGAGTCTCGCTCTG-TCGCCCAGGCCGGACTGCGGACTGCAGT
chrom   TCTTTTAC-----------AGGCTGCATGGCTTTCTACTTGCTCTTGCTCGCTT--AGAC----GA
right   TCTTTTAC-----------AGGCTGCATGGCTTTCTACTTGCTCTTGCTCGCTT--AGAC----GA
        ******2222222222222**2*2*22*22*22***22*2**2*22**22*22*222***2222*2
```

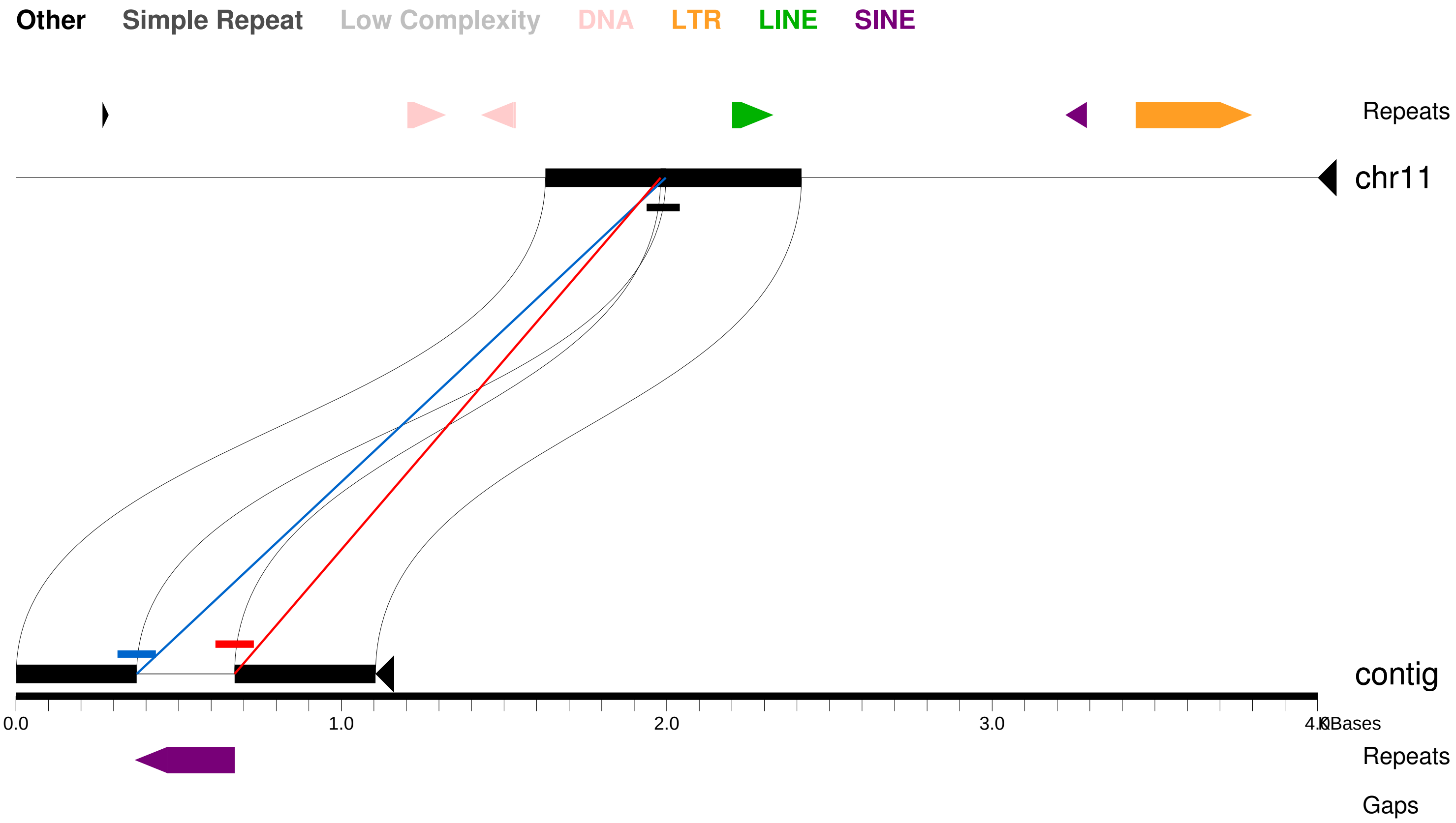

siteID: chr11_84701707 Scafftig4 - Breakpoint: chr11:84701717
chr11:84701672-84701776
Overlap Length: 15 INS size: 312 Genotyped: Yes

```
left    -------GAAA---------TGCAAATTAAAACCATGATGAGATACCACCACATACCTATTAAAATTCAT
chrom   -------GAAA---------TGCAAATTAAAACCATGATGAGATACCACCACATACCTATTAAAATTCAT
right   CCTGGGCGACAGAGCGAGACTCCGTCTCAAAAAAAAAAAAAAAAAAAAAAAAAAAAAAA--AAAATTCAT
        1111111**1*111111111*1*111*1****11*11*11*1*1*11*11*1*1*111*11*********

left    AAAATAGGCCGGGCGCGGTGGCTCACGCCTGTAATCCCAGCACTTTGGGAGGCCGAGGCGGGCGG
chrom   AAAATAAGAAG-------TAGCGACAGCACTAAATGCTGGAA----AGAATGCAGAGAAACT---
right   AAAATAAGAAG-------TAGCGACAGCACTAAATGCTGGAA----AGAATGCAGAGAAACT---
        ******2*22*2222222*2**2222**2222***2*22*2*22222*2*2**2***22222222
```

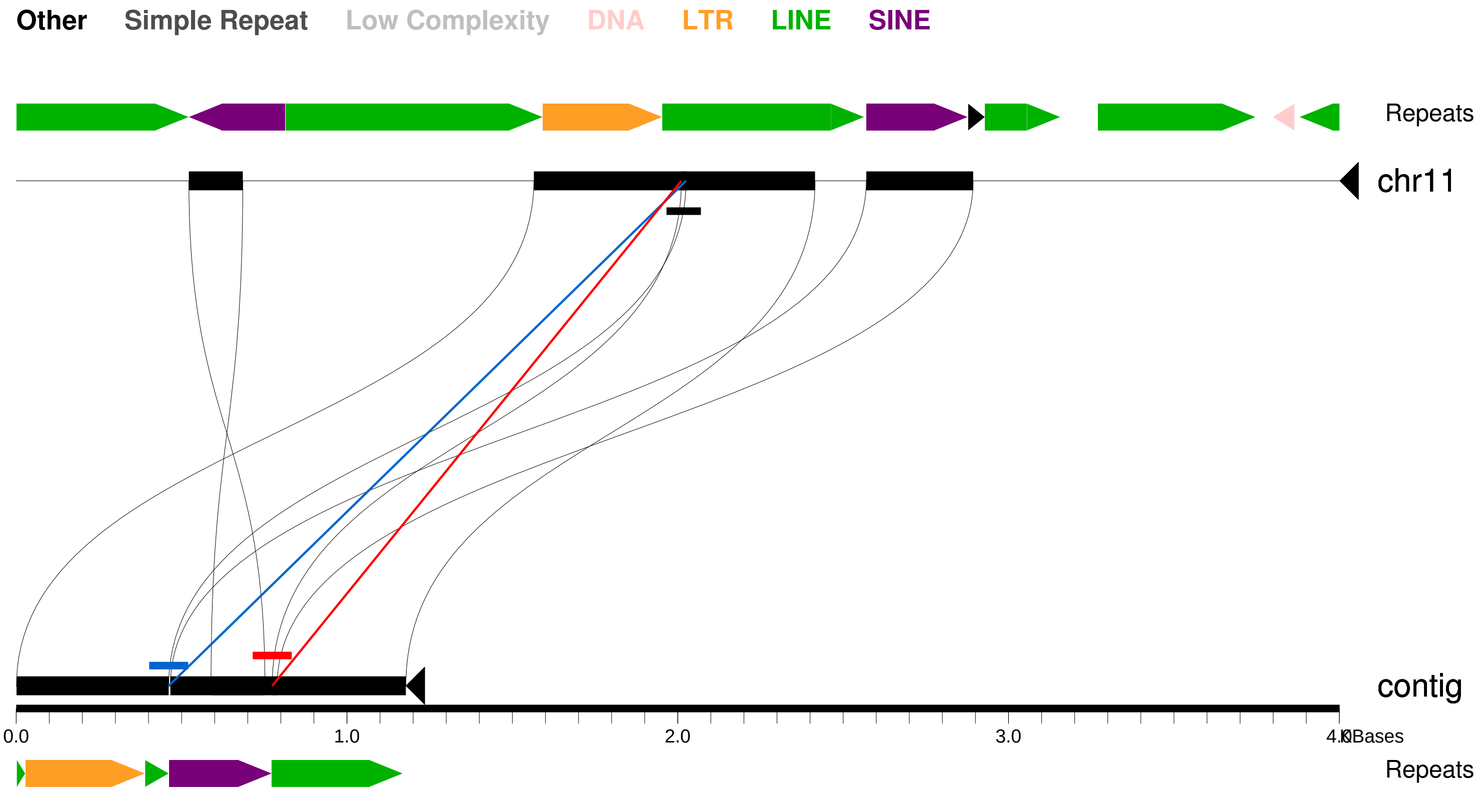

siteID: chr11_86886896 Scafftig1 + Breakpoint: chr11:86886785
chr11:86886611-86886945
Overlap Length: 94 INS size: 369 Genotyped: No

```
left   ----------------------------------------------------------------------
chrom  AAATGTGTCATAATTTTGTCCTTAAACAAAACAAACAACATCTGTTTAAGTATGGAACAATTAGGTTTTA
right  NNNNNNNNNNN-----------------------------------------------------------
       NNNNNNNNNNN

left   ----------------------------------------------------------------------
chrom  TTTTTCAAAAATTAGAAGGGTTCTTAAGATCATATCAATTTGTTTTGATTCTAATGGGAAGAGCTTTAGG
right  ----------------------------------------------------------------------

left   --------------------------GTAAGGCTGTACCTCCGCTACACCAACCACCCACTAAACTTAAA
chrom  TCAAGAAAGCAATTATTTCCACATGGGTAAGGCTGTACCTCCGCTACACCAACCACCCACTAAACTTAAA
right  ----------------------------------GTACCTCCGCTACACCAACCACCCACTAAACTTAAA
                                 11111111************************************

left   TTTGTCTACTCTGGTTGTATAGCTAGTTGTGGTTGAATGTATTAGAAGACACAGGCTTTGGCCG------
chrom  TTTGTCTACTCTGGTTGTATAGCTAGATGTGGTTGAATGTATTAGAAGACACAGGCTTTATTTAAGAAAC
right  TTTGTCTACTCTGTTTGTATAGCTAGATGTGGTTGAATGTATTAGAAGACACAGGCTTTATTTAAGAAAC
       *************1************2********************************22222222222

left   ------------------GGCGCGGTGGCT---------CACGCC---------T
chrom  AAAGATTTTAGATATATAGAAAATGTTTCTTTGTAGAAACATACCAAAAGAATTT
right  AAAGATTTTAGATATATAGAAAATGTTTC--------------------------
       222222222222222222*22222**22*1         11NN11         1
```

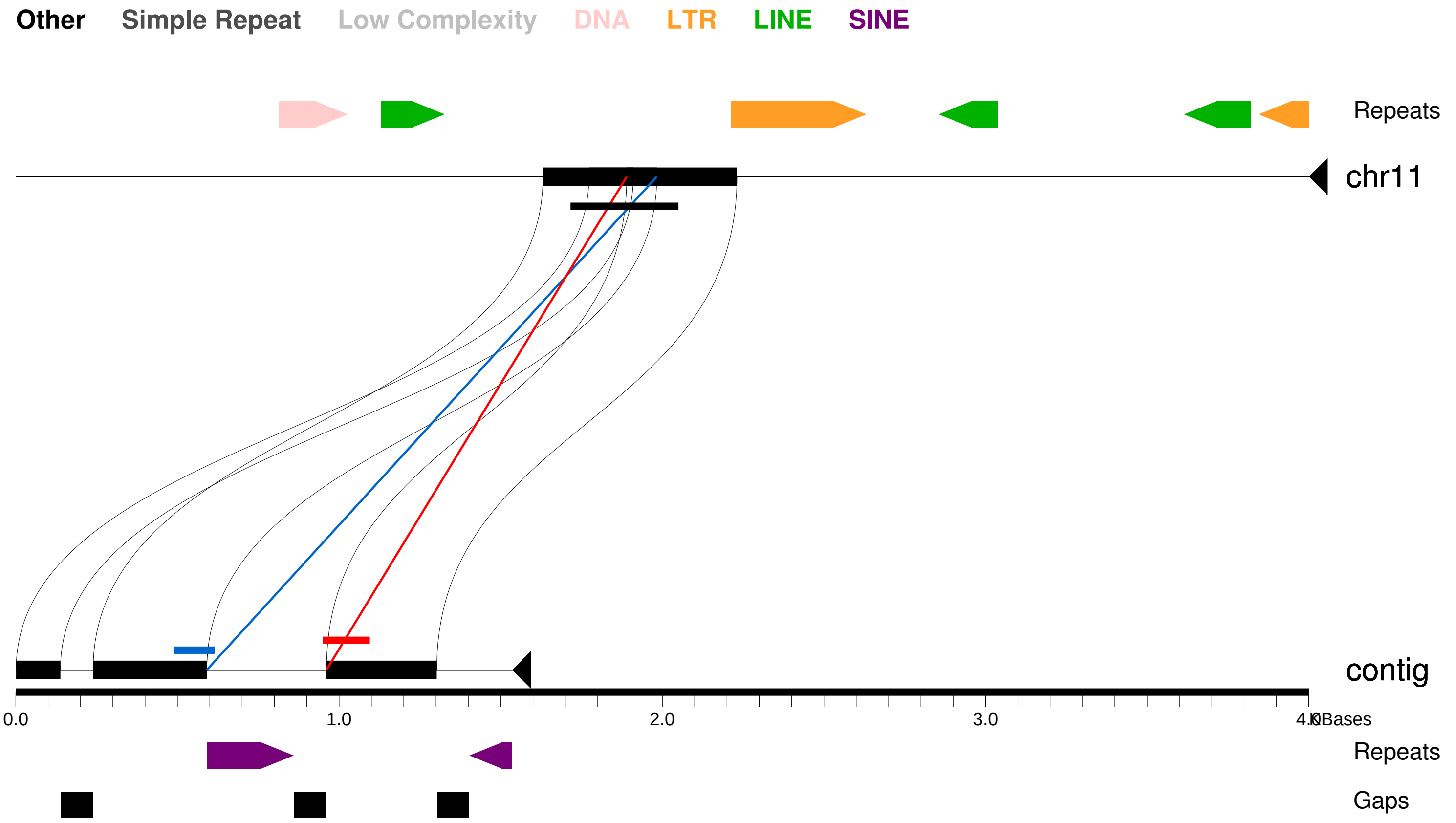

siteID: chr11_88908952 Scafftig3 + Breakpoint: chr11:88908938
chr11:88908902-88908994
Overlap Length: 13 INS size: 313 Genotyped: Yes

```
left    AGACGGGACAGC---AGACACA-AAAGCCCT-----TAAAGA------------------GCATGATGAA
chrom   AGACGGGACAGC---AGACACA-AAAGCCCT-----TAAAGA------------------GCATGATGAA
right   -GATCCGCCCGCCTCGGCCTCCCAAAGTGCTGGGATTACAGGCGTGAGCCACCGCGCCCGGCCTGATGAA
        1**111*1*1**1111*1*1*11****11**11111**1**1111111111111111111**1*******

left    GACTTTTTTTTTTTTTTTTTTTTTTTTTTTTTTTTGAGACGGAGTCTCGCTCTATCACCCAGGCTGGAGTG
chrom   GACTTTCTAAGTTATTTCACT------------------GGAAGCCTGAT-----AGTGGGGCA--AGTG
right   GACTTTCTAAGTTATTTCACT------------------GGAAGCCTGAT-----AGTGGGGCA--AGTG
        ******2*222**2***222*222222222222222222***22*22*2*22222*2222***222****

left    CAGTGG
chrom   TAA-GG
right   TAA-GG
        2*22**
```

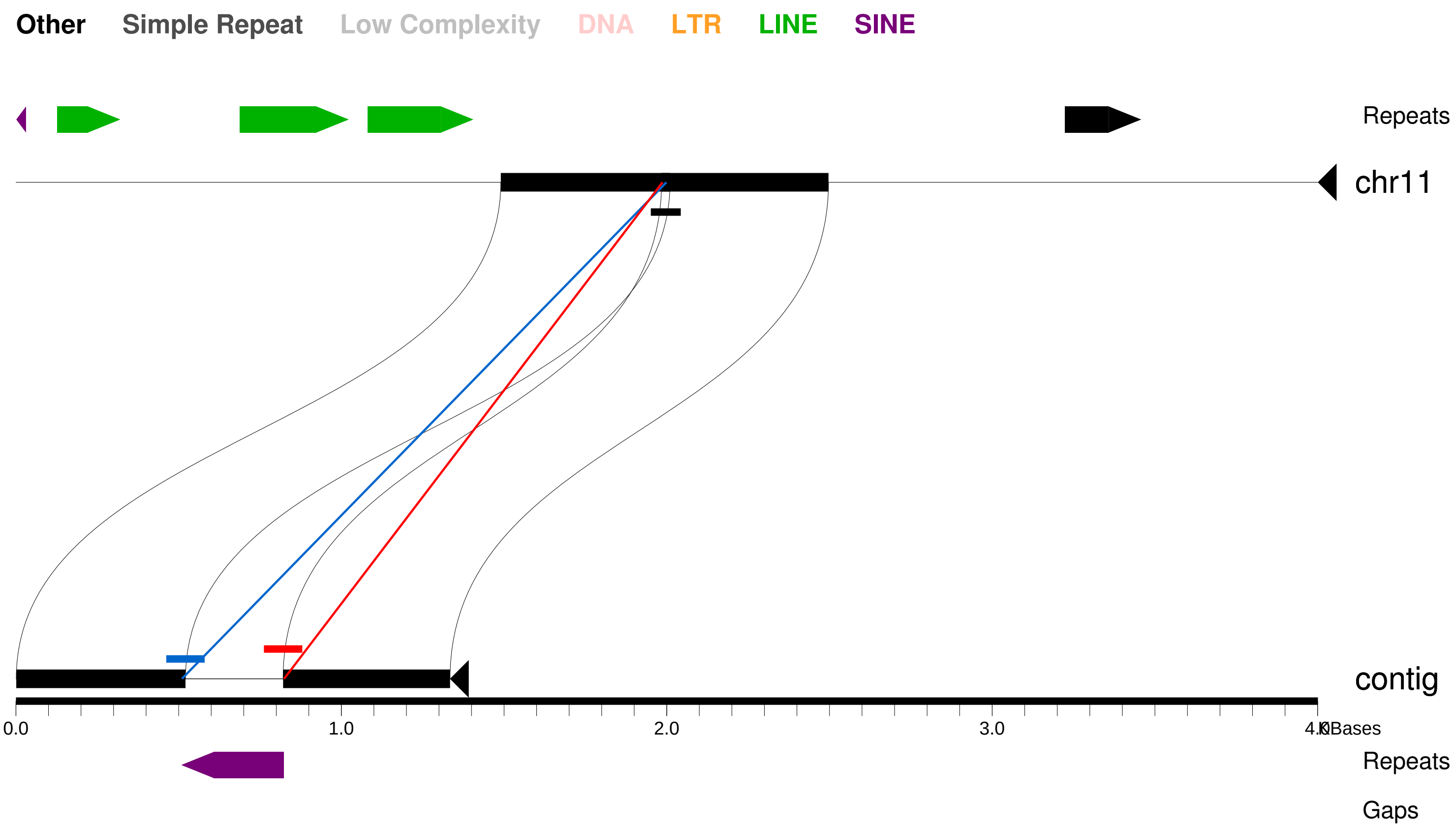

siteID: chr11_89196876 Scafftig1 - Breakpoint: chr11:89196877
chr11:89196831-89196936
Overlap Length: 14 INS size: 354 Genotyped: No

```
left     AAAGC--AGTAATTTTCATTGAC--------TGATATAAAAAGCAATTAATATGTC---CAGTTAGGTTA
chrom    AAAGC--AGTAATTTTCATTGAC--------TGATATAAAAAGCAATTAATATGTC---CAGTTAGGTTA
right    AATTTTTTGTATTTTTAGTAGAGACGGGGTTTCACCTTGTTAGCCAGGATGGTCTCGATCAGTTAGGTTA
         **111111***1****11*1**111111111*1*11*1111***1*11*111*1**111***********

left     TTTNNNNNNNNNNNNNNNNNNNNNNNNNNNNNNNNNNNNNNNNNNNNNNNNNNNNNNNNNN
chrom    TTTCTACATCTTAAGTTGGAACACCTTAGGGATACAGTTTAATGGATAT-------------
right    TTTCTACATCTTAAGTTGGAACACCTTAGGGATACAGTTTAATGGATAT-------------
         ***22222222222222222222222222222222222222222222222222222222222
```

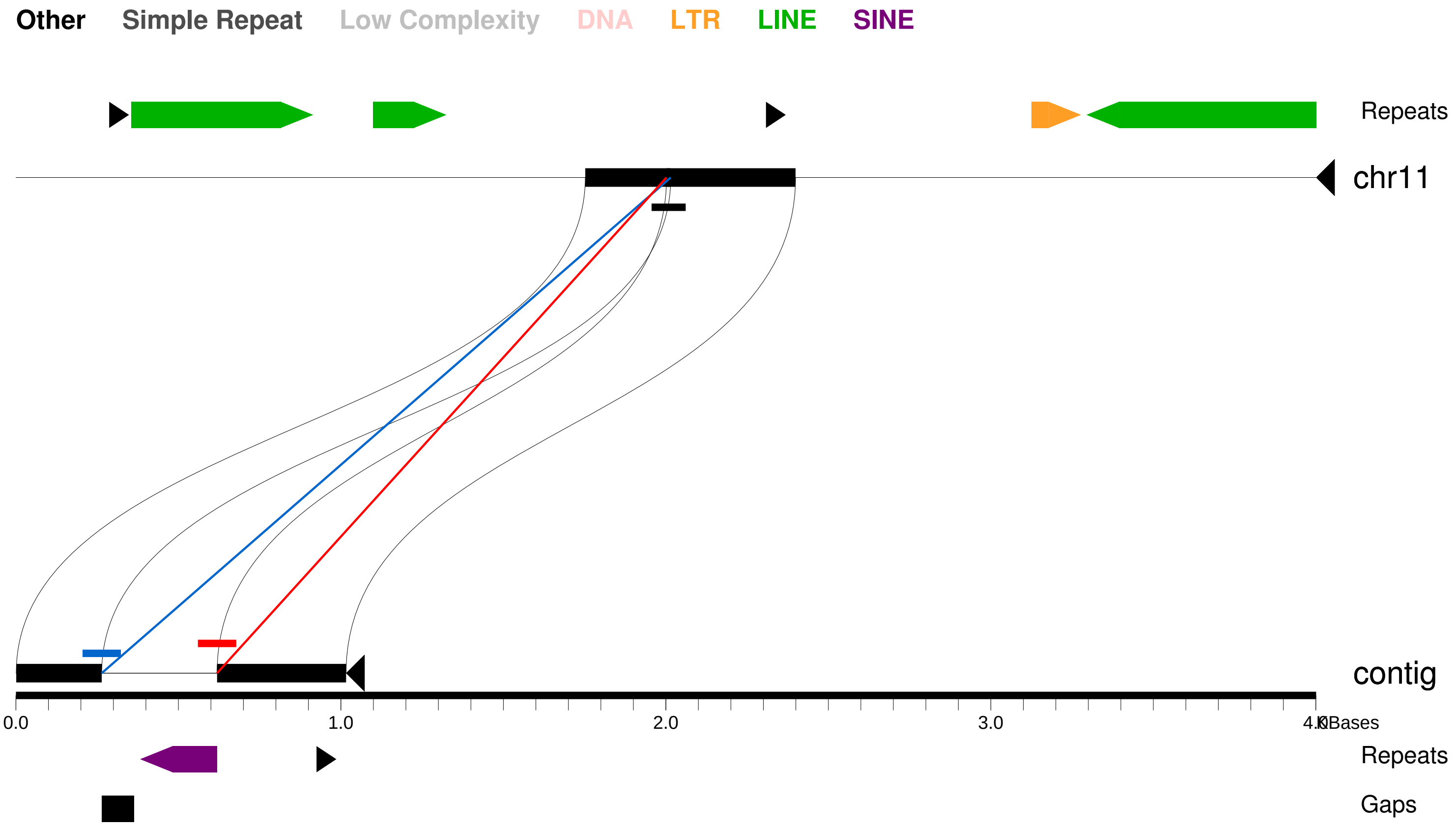

siteID: chr11_95371562 Scafftig1 + Breakpoint: chr11:95371574
chr11:95371414-95371747
Overlap Length: 14 INS size: 422 Genotyped: No

```
left    --------------TAGTGCCTATGCCCAGGTGATATGAAGTCATTTAAAATCTTCCCAGAAAAAAAATT
chrom   GTGGTCCTGGATTTTAGTGCCTATGCCCAGGTGATATGAAGTCATTTAAAATCTTCCCAGAAAAAAAATT
right   NNNNNNNNNNN-----------------------------------------------------------
        NNNNNNNNNNN   11111111111111111111111111111111111111111111111111111111

left    CAATTTCAACATGACCTAAAAGAATTCTATCAGGTAACAATCCAAGGGATATGAGCTAATAGCCAATAAT
chrom   CAATTTCAACATGACCTAAAAGAATTCTATCAGGTAACAATCCAAGGGATATGAGCTAATAGCCAATAAT
right   ----------------------------------------------------------------------
        1111111111111111111111111111111111111111111111111111111111111111111111

left    TGCAAATACACAGGCAAACAAAGAACCATGAATAGG--CCGGGCGCGGTG-----GCTCACGCCTGTAAT
chrom   TGCAAATACACAGGCAAACAAAGAACCATGAATAAGAACCAGCAGAAATAACATTGCAGAATCAGATAAA
right   --------------------AAGAACCATGAATAAGAACCAGCAGAAATAACATTGCAGAATCAGATAAA
        11111111111111111111**************2*22**2*22*222*222222**22*22*222***2

left    CCCAGCACT--------TTGGGAGG--CCGAGGC--------------GGGTGGATCATGAGGTC--AGG
chrom   CAAAAAACTCTTAAGTATTGGAATTATCAGATGCTAAACATAAACTAAGAATGTCTGATATGCTTGAAGG
right   CAAAAAACTCTTAAGTATTGGAATTATCAGATGCTAAACATAAACTAAGAATGTCTGATATGCTTGAAGG
        *22*22***22222222****2*2222*2**2**22222222222222*22**22*2**22*2*222***

left    AGATCGAG---ACCATCCTGGCT---AACAAGGTGAAACCCCGTCTCTCCTAAA
chrom   AGAGAGGTTTAAAAATATAAGCACGAAACAAGACTGTAAAAAGGATCTGAAGAG
right   AGAGAGGTTTA-------------------------------------------
        ***22*222221NN11NNNN11N   111111NNNNN1NNNN1NN111NNNN1N
```

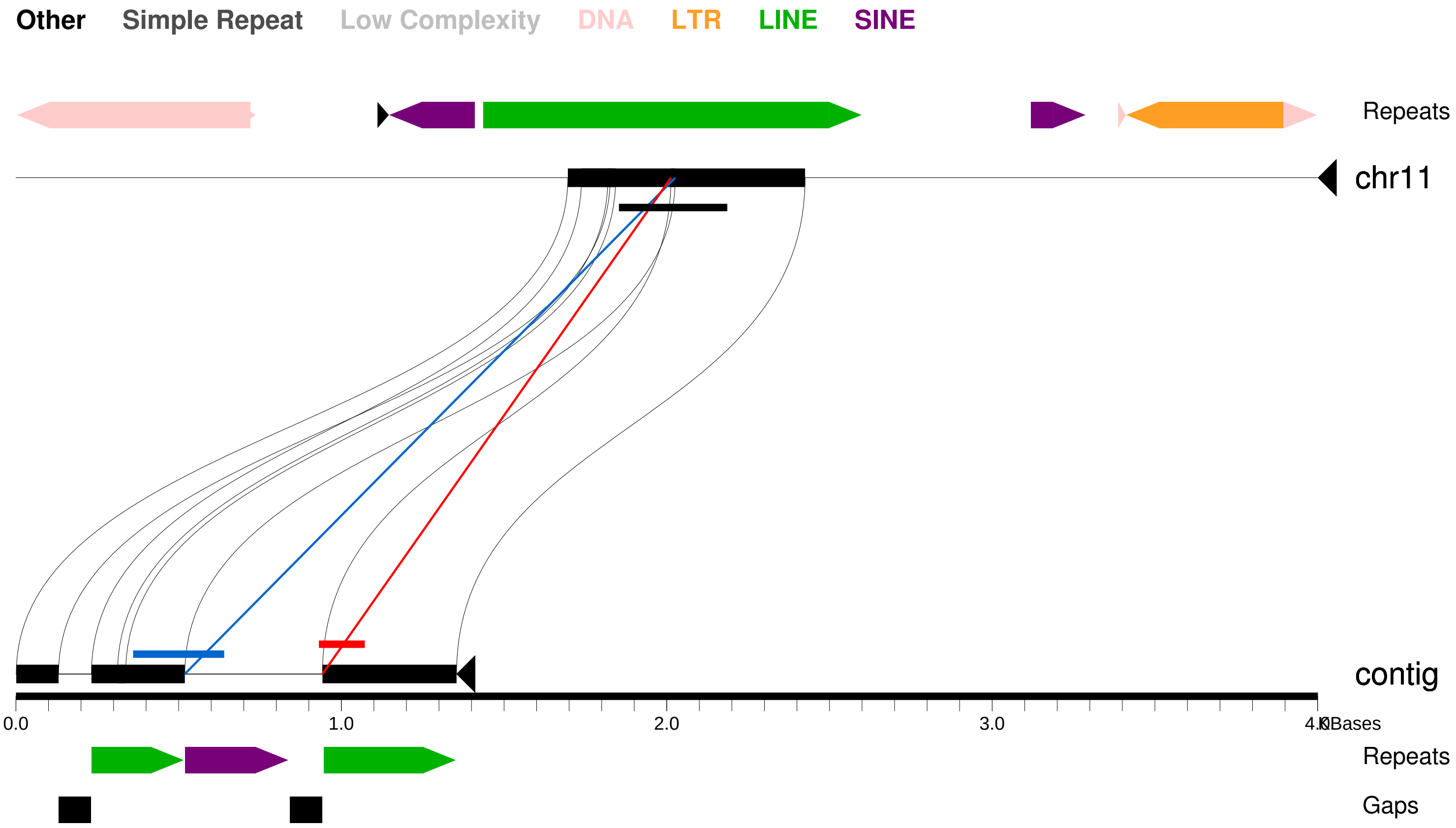

siteID: chr11_96620705 Scafftig1 + Breakpoint: chr11:96620697
chr11:96620645-96620756
Overlap Length: 8 INS size: 441 Genotyped: No

```
left   GGGGTATAATCAGGTGGTAAAAATACATTAGGGTTTAAAAAAGTTTTAAAAA-------GTATTGTCGGC
chrom  GGGGTATAATCAGGTGGTAAAAATACATTAGGGTTTAAAAAAGTTTTAAAAA-------GTATTGTCATA
right  NNNNNNNNNNNNNNNNNNNNNNNNNNNNNNNNNNNNNNNNNNNNNNNNNNNNNNNNNNNGTATTGTCATA
       11111111111111111111111111111111111111111111111111111111111********222

left   CGGGCGCGGTGGCTCACGCCTGTAATCCCAGCACTTTGGGAGGCCGAGGCGGGTGG-
chrom  AT-------TATAGCA-GCTTTACAACATAGAATATTTAAAATTAAACAATAGCCAA
right  AT-------TATAGCA-GCTTTACAACATAGAATATTTAAAATTAAACAATAGCCAA
       222222222*2222**2**2*222*2*22**2*22**222*22222*22222*2222
```

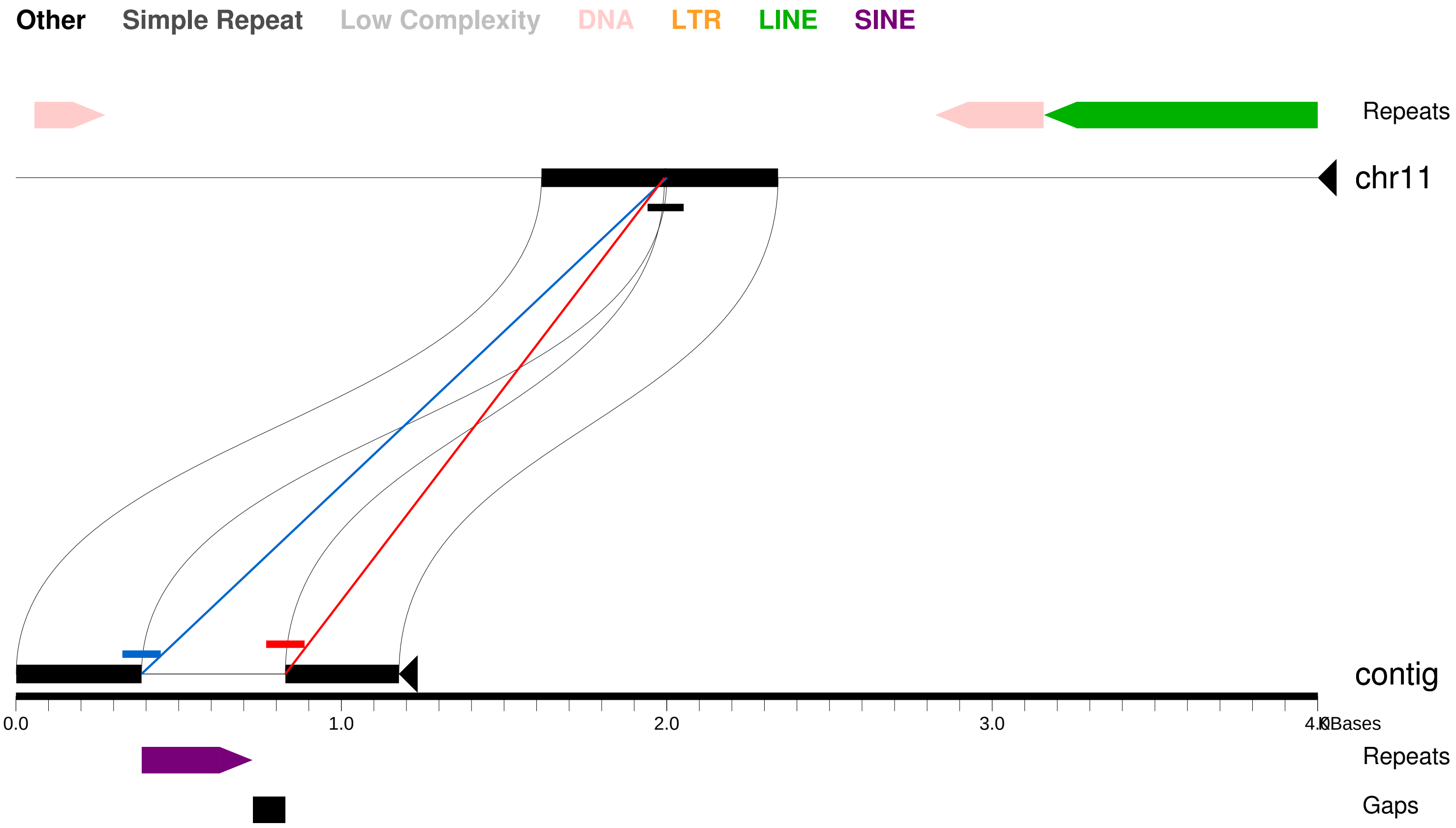

siteID: chr11_9757977 Scafftig4 - Breakpoint: chr11:9757986
chr11:9757826-9758161
Overlap Length: 16 INS size: 319 Genotyped: Yes

```
left   -----------------CATAAAATATTTATAACTGCTTTGAAGTCTTCATATGCTAAATCCACCATCTG
chrom  TCCTTTAGTTATTTGAGCATAAAATATTTATAACTGCTTTGAAGTCTTCATATGCTAAATCCACCATCTG
right  --CGTGA---ACCCGGGAAGCGGAGCTTGCAGTGAGCCGAGATTGCGCCAC-TGC--AGTCCGCAGTCCG
         2N2N2   2NNN2N21*1111*11**1111111**111**111*11**11***11*1***1*11**1*

left   GGCCT---CTTCAAAGGCAGTTTCCGTTGCCTGCTTTTTCCCTGTATATGGGCCATACTTTCCTTTTTCT
chrom  GGCCT---CTTCAAAGGCAGTTTCCGTTGCCTGCTTTTTCCCTGTATATGGGCCATACTTTCCTTTTTCT
right  G-CCTGGGCGACAGAGCGAGACTCCGT------CTCAAAACAAAAAAAAAAAACAAACAAAA--------
       *1***111*11**1**11**11*****111111**11111*1111*1*11111**1**111111111111

left   TTGCATGTCTCATAATTTCTTGTAGAAAACTAGACATTTGGGCCGG-GCGCGGTGGC---TCACGCCTGT
chrom  TTGCATGTCTCATAATTTCTTGTAGAAAACTAGACATTTTAGATAATGTGTGGTAGCAACTCTGGATTCT
right  -----------AAAA--------AGAAAACTAGACATTTTAGATAATGTGTGGTAGCAACTCTGGATTCT
       11111111111*1**11111111****************22*22222*2*2***2**222**22*22*2*

left   AATCCCAGCACTTTGG---GAGGCC-GAGGCGGGTGGATC----ATGAGGTCAG-GAGATCGA--GACCA
chrom  GATTCCACCCCTCTCCCCAGAGGCCTGCTGTTGTTTGCTCCTTTATTTGTTTAGTGACTTGGCTGGACTA
right  GATTCCACCCCTCTCCCCAGAGGCCTGCTGTTGTTTGCTCCTTTATTTGTTTAGTGACTTGGCTGGACTA
       2**2***2*2**2*22222******2*22*22*2*2*2**2222**22*2*2**2**22*2*222***2*

left   TCCTGG--------------CTAACAAGGTGAAACCCC-----GTCTCTACTAAA----
chrom  TTTCAGTGAAGTTTGTTTTCCTCACAGTGTGAAGCTTCTGACTGTTGCTCCTCAGAGGC
right  TTTCAGTGAAGTTTGTTTTCCTCACAGTGTGAAGCTTCTGACT----------------
       *2222*22222222222222**2***22*****2*22*2222211NN11N11N1N
```

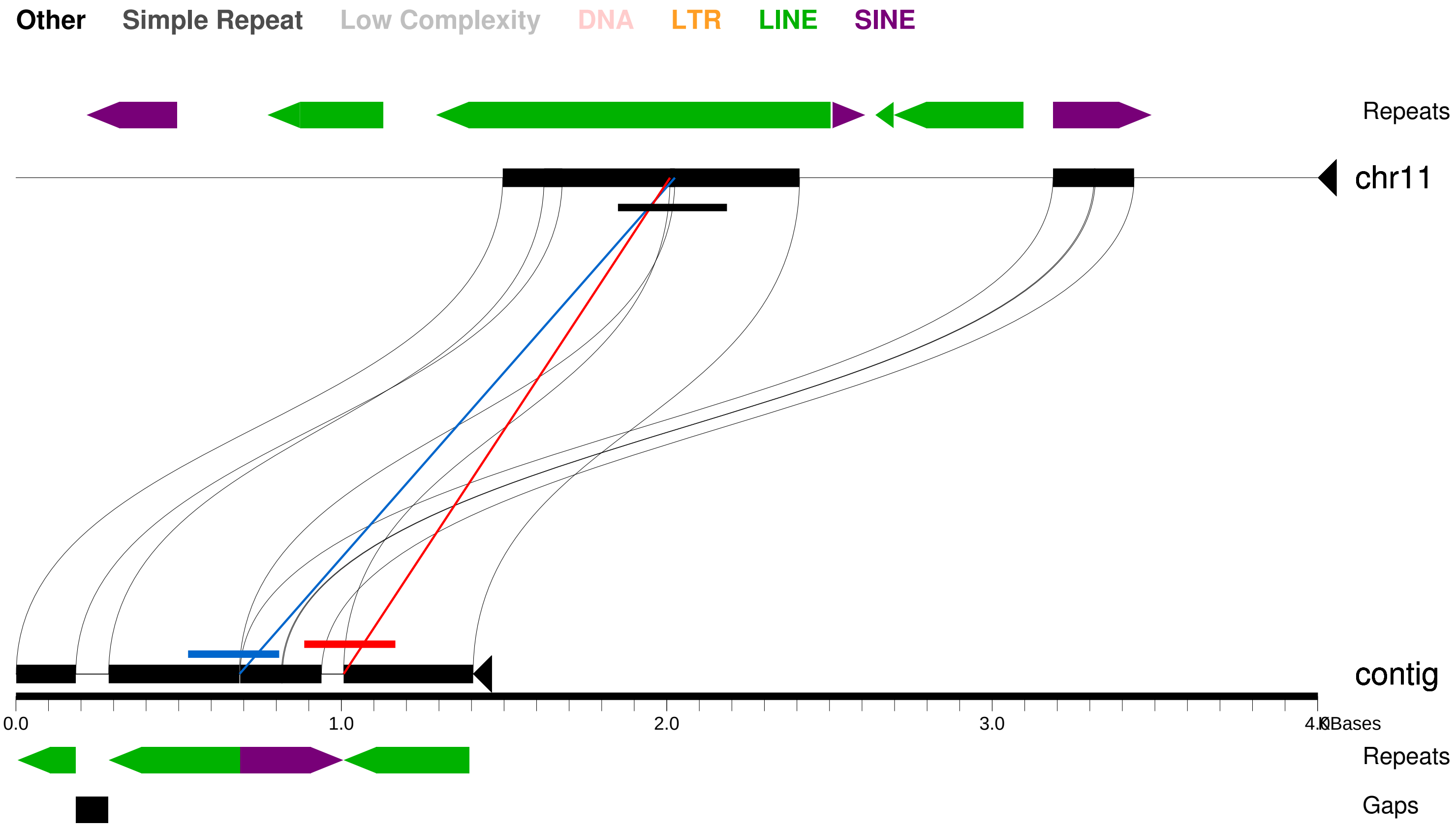

siteID: chr11_98774695 Scafftig3 - Breakpoint: chr11:98774691
chr11:98774643-98774790
Overlap Length: 12 INS size: 306 Genotyped: Yes

```
left    AAAGGAATATCAATTGGAGAAGTAATAAAAGTGTGTATTGTCATATTAAAATGTGATTGGGG-------C
chrom   AAAGGAATATCAATTGGAGAAGTAATAAAAGTGTGTATTGTCATATTAAAATGTGATTGGCTATTTTATC
right   AAAAAAA------------AAAAAAAAAAA------------------AAATGGGATTGGCTATTTTATC
        ***11**111111111111**11**1****111111111111111111*****1******222222222*

left    CGGG--------CGCGGTGGCTCA-CGCCTGTAATCCCAGCA---------CTTTGGGAGGCCGAGGCGG
chrom   CAAAAATATATTCAGATTAAAAAA-TAAATATTAACTAGACATATAGTGTTCTTTTTAAGTATAATTATT
right   CAAAAATATATTCAGATTAAAAAAATAAATATTAACTAGACATATAGTGTTCTTTTTAAGTATAATTATT
        *22222222222*2222*22222*12222*2*2*2*2222**222222222****222**2222*22222

left    GCGG-----
chrom   CCAGTTACA
right   CCAGTTACA
        2*2*22222
```

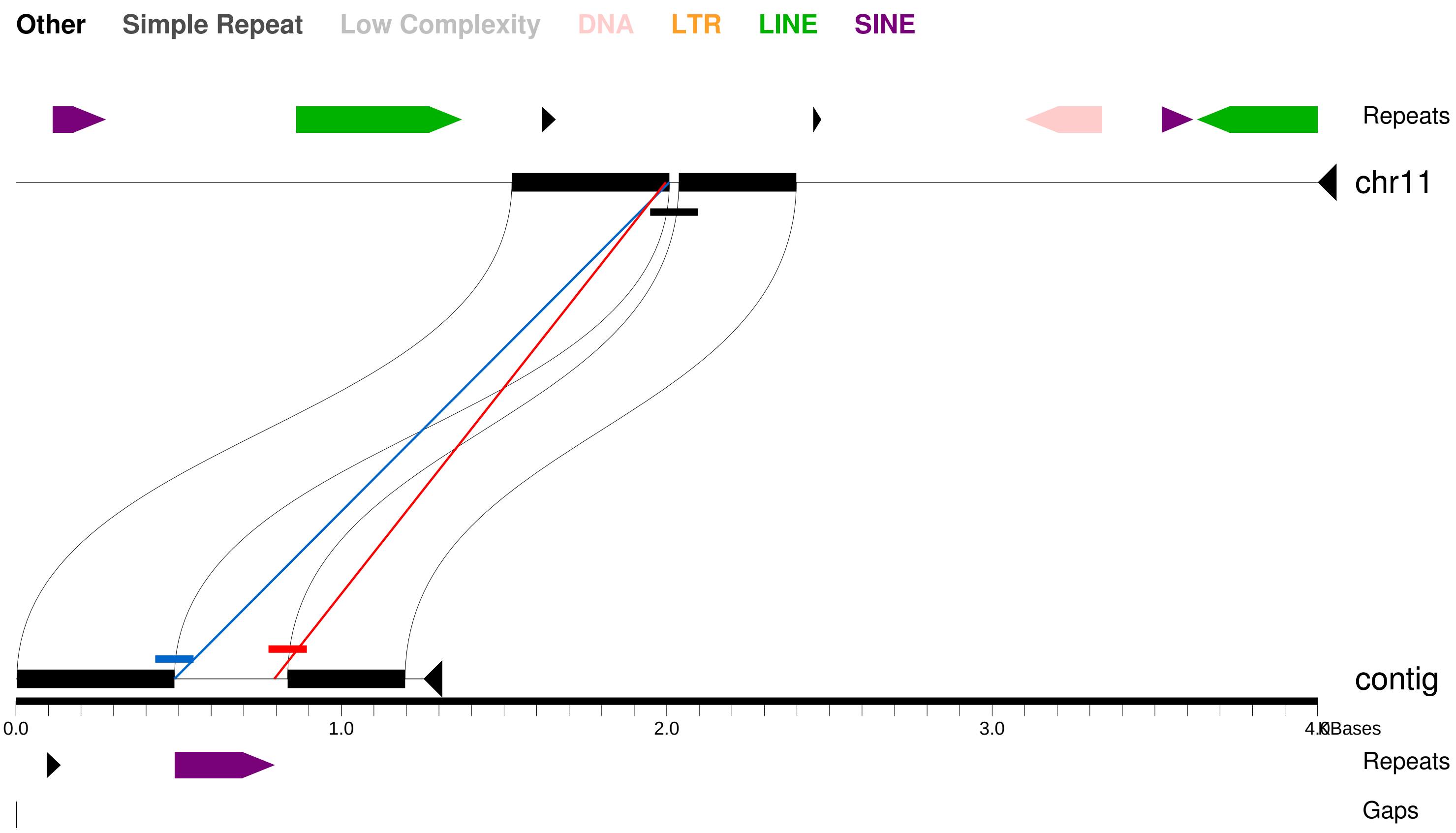

siteID: chr12_101450693 Scafftig11 + Breakpoint: chr12:101450676
chr12:101450634-101450735
Overlap Length: 15 INS size: 309 Genotyped: Yes

```
left    TTCTTTTATTTTTTTATACTTCAAGTTCTGGGAT-ACATGTGT---------------------GGAACG
chrom   TTCTTTTATTTTTTTATACTTCAAGTTCTGGGAT-ACATGTGT---------------------GGAACG
right   CCCGCCTCGGCCT-----CCCAAAGTGCTGGGATTACAGGCGTGAGCCACCGCGCCCGGCCGACGGAACG
        11*111*11111*11111*111****1*******1***1*1**111111111111111111111******

left    TGCAGGTTTTTTTTTTTTTTTTTTTTTTTTTTTTTTGAGACGGAGTCTCGCTCTGTCGCCCAGGCTGGAGTG
chrom   TGCAGGTTTCTTACATAGGTATACATGT-----------GCCATCGTGGTTTGCTGCAC--CCTTCA---
right   TGCAGGTTTCTTACATAGGTATACATGT-----------GCCATGGTGGTTTGCTGCAC--CCTTCA---
        *********2**222*222*2*222*2*22222222222*222*122*2*2**22**2*222**22*222

left    C
chrom   -
right   -
        2
```

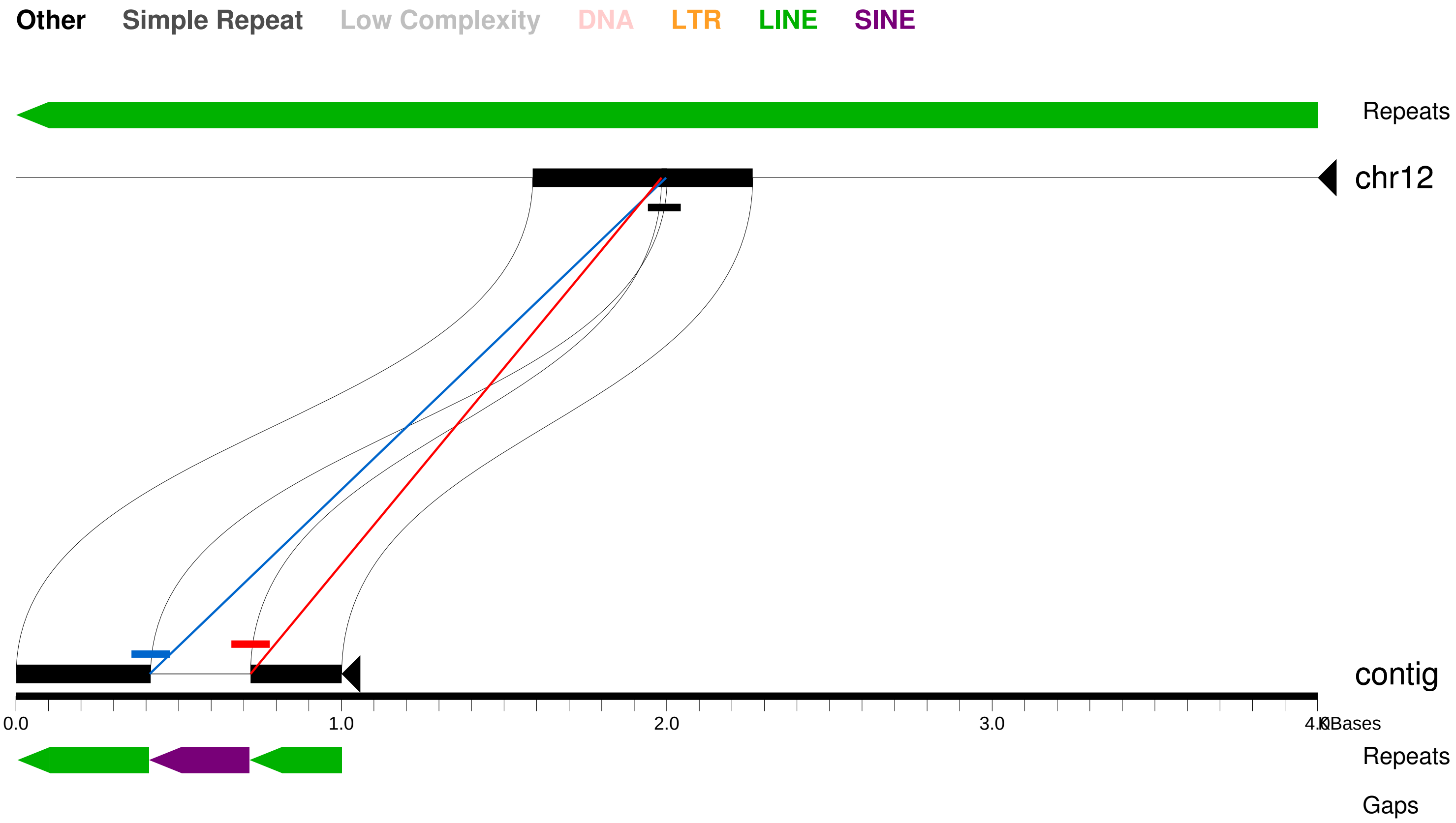

siteID: chr12_10234187 Scafftig16 + Breakpoint: chr12:10234188
chr12:10234143-10234244
Overlap Length: 15 INS size: 303 Genotyped: Yes

```
left    AAGT---------TTGGACTCT---AGTACATATATGAGAAGT-TGTATCACCTT----GGGCAAATTAA
chrom   AAGT---------TTGGACTCT---AGTACATATATGAGAAGT-TGTATCACCTT----GGGCAAATTAA
right   TGATCCGCCCGCCTCGGCCTCCCAAAGTGCTGGGATTACAGGCGTGAGCCACCGCGCCCGGCCAAATTAA
        111*111111111*1**1***1111***1*1111**1*1*1*11**111****111111**1********

left    TTTCTTTTTTTTTTTTTTTTTTTTTTTGAGACGGAGTCTCACTCTGTCGCCCAGGCTGGAGTG-CAG
chrom   TTTCTTTAAGTCTGTCTAAAAATGT-------GAAGTC-----ATG------AGGAGAAAATGACGG
right   TTTCTTTAAGTCTGTCTAAAAATGT-------GAAGTC-----ATG------AGGAGAAAATGACGG
        *******222*2*2*2*22222*2*2222222*2****222222**222222***2222*2**2*2*
```

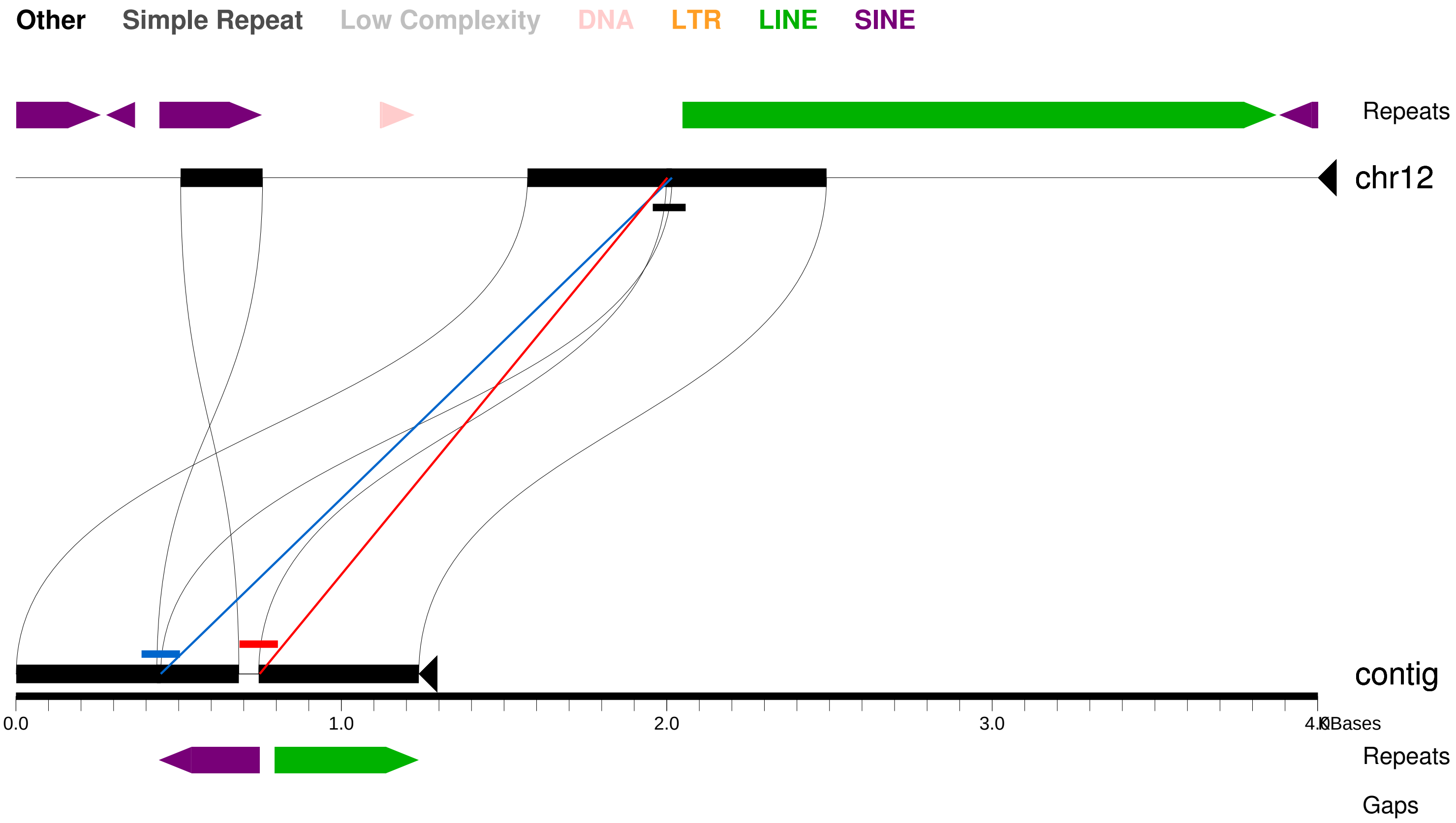

siteID: chr12_103089292 Scafftig1 - Breakpoint: chr12:103089283
chr12:103089236-103089342
Overlap Length: 13 INS size: 454 Genotyped: No

```
left    CATACGCTCATCTACAATGTCAAGGCTGCCCAAGAAAACATGACTTT------------AAGAAAGTAAC
chrom   CATACGCTCATCTACAATGTCAAGGCTGCCCAAGAAAACATGACTTT------------AAGAAAGTAAC
right   NNNNNNNNNNNNNNNNNNNNNNNNNNNNNNNNNNNNNNNNNNNNNNNNNNNNNNNNNAAAAGAAAGTAAC
        11111111111111111111111111111111111111111111111111111111111***********

left    TAGGCCGGGCGCGGTGGCTCACGCCTGTAATCCCAGCACTTTGGGAGGCCGAGGCGGGCGG
chrom   TAAATAAGGCACCAAGGATCAATTCTGGAGAAACAGAAGTAGGTGA------------CCT
right   TAAATAAGGCACCAAGGATCAATTCTGGAGAAACAGAAGTAGGTGA------------CCT
        **22222***2*222**2***222***2*2222***2*2*22*2**222222222222*22
```

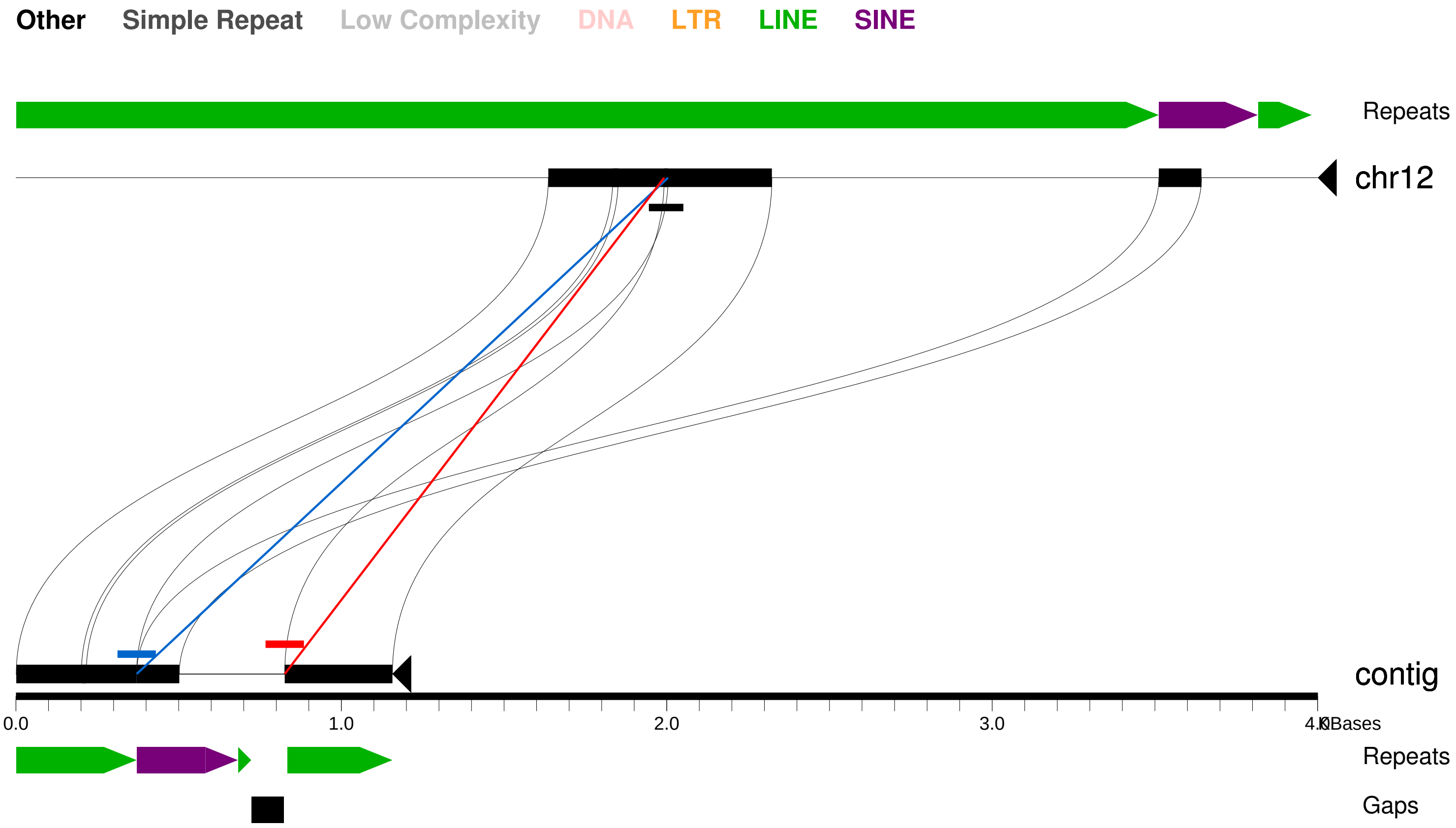

siteID: chr12_105830906 Scafftig9 - Breakpoint: chr12:105830890
chr12:105830846-105830949
Overlap Length: 16 INS size: 304 Genotyped: Yes

```
left     TGATTT---------------AAAAGGAACATGGCTTTTACAGAAAATCAGAGGGCCTTAGAAAAGCTGT
chrom    TGATTT---------------AAAAGGACCCTGGCTTTTACAGAAAATCAGAGGGCCTTAGAAAAGCTGT
right    CTCCGTCTCAAAAAAAAAAAAAAAAAAAAAAAAAAAAAAAAAAAAAAAAAAAAAAAAAGAAAAGCTGT
         11111*111111111111111****11*N1N11111111*1*1****11*1*1111111***********

left     AAATGATCCC--AGCACTTTGGGAGGCCGAGGCGGGCGGATCACGAGGTCAGGAGATCGAGACCAT
chrom    AAATGTTGGATAAGGTCTTTTGGAGTCTAA-----------CCCAAAGTCT-----TCTCTATGA-
right    AAATGTTGGATAAGGTCTTTTGGAGTCTAA-----------CCCAAAGTCT-----TCTCTATGA-
         *****2*22222**22****2****2*22*22222222222*2*2*2***222222**222*22*2
```

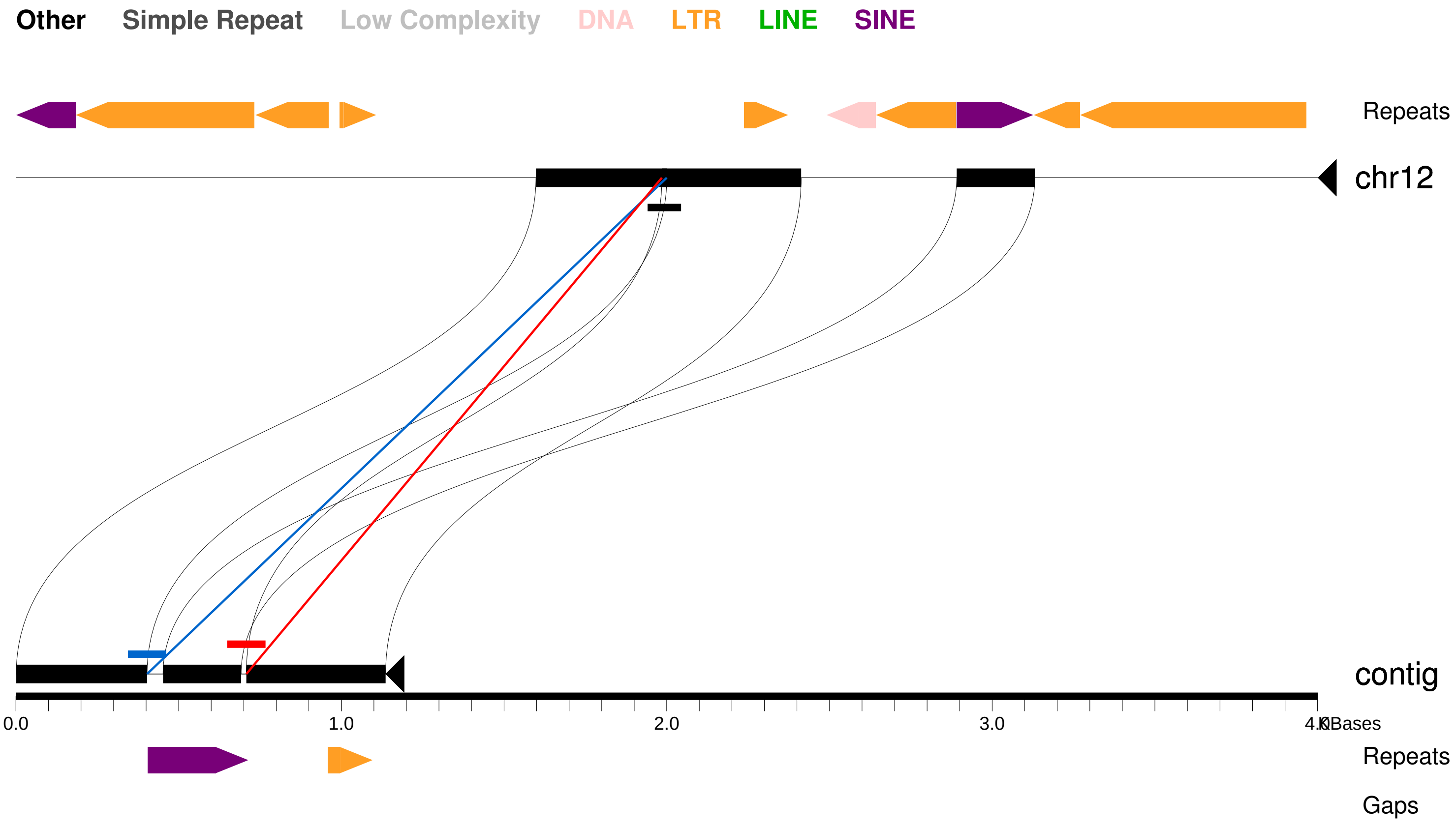

siteID: chr12_106551989 Scafftig2 - Breakpoint: chr12:106552012
chr12:106551962-106552065
Overlap Length: 10 INS size: 312 Genotyped: Yes

```
left    -----------------GGATGAGTTAAAGTTC-ACA-TCTTATTCAAGTCACCCCTATGCTTAAAACTC
chrom   -----------------GGATGAGTTAAAGTTC-ACA-TCTTATTCAAGTCACCCCTATGCTTAAAACTC
right   CTGCAGTCCGCAGTCCGGCCTGGGCGACAGAGCGAGACTCCGTCTCAAAAAAAAAAAAA----AAAAAAC
        11111111111111111*11**1*11*1**11*1*1*1**1111****111*11111*11111****11*

left    TTCAATGTCGGCCGGGCGCGGTGGCTCACGCCTGTAATCCCAGCACTTTGGGAGGCCGAGGCGGGTGG-
chrom   TTCAATGTCTTCCA-------TTTCTTTGG--TGTTA-------AAGTTCACAGGCCTTGACCACAGGA
right   TTCAATGTCTTCCA-------TTTCTTTGG--TGTTA-------AAGTTCACAGGCCTTGACCACAGGA
        *********22**22222222*22**222*22***2*2222222*22**222*****22*2*2222**2
```

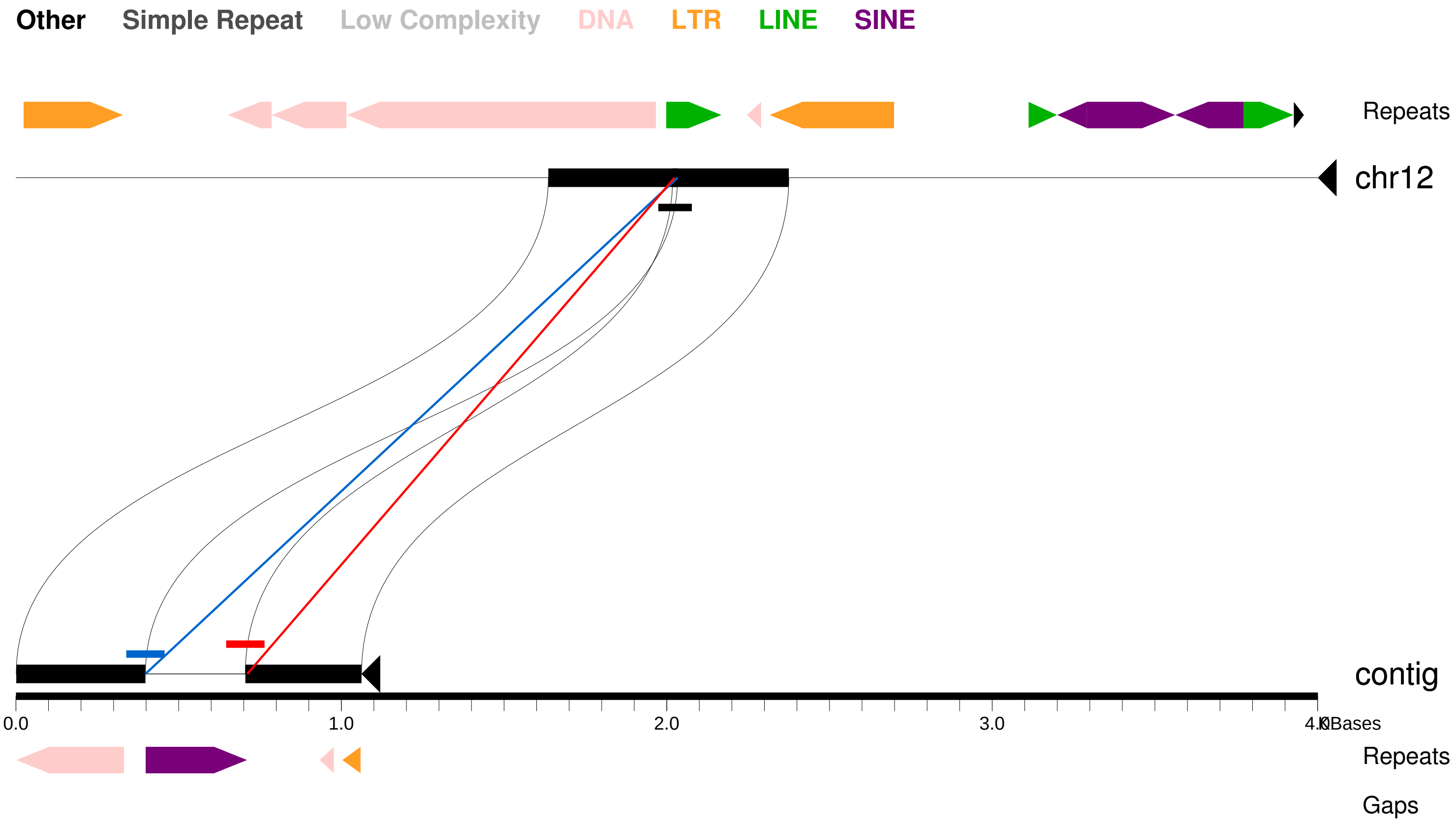

siteID: chr12_114131023 Scafftig3 - Breakpoint: chr12:114131014
chr12:114130964-114131073
Overlap Length: 10 INS size: 431 Genotyped: No

```
left    ---GTCCCCATTTTACAGACAAGGAAGCTGAGCCTAT----TTCAGG-ACAGGGTC--GTTGGATTTCTT
chrom   ---GTCCCCATTTTACAGACAAGGAAGCTGAGCCTAT----TTCAGG-ACAGGGTC--GTTGGATTTCTT
right   TCCGCCCGCCTCGGCCTCCCAAAGT-GCTGGGATTACAAGCGTGAGCCACCGCGCCCGGCTGGATTTCTT
        111*1**1*1*1111*111***1*11****1*11**111111*1**11**1*1*1*11*1**********

left    TTTTTTNNNNNNNNNNNNNNNNNNNNNNNNNNNNNNNNNNNNNNNNNNNNNNNNNN
chrom   ---------AACATAGGGGCAGGCAAACTTTTTTCTGAGGGGACGGATAGTAAATGTTT
right   ---------AACATAGAGGCAGGCAAACTTTTTTCTGAGGGGACGGATAGTAAATGTTT
        2222222222222222N22222222222222222222222222222222222222222
```

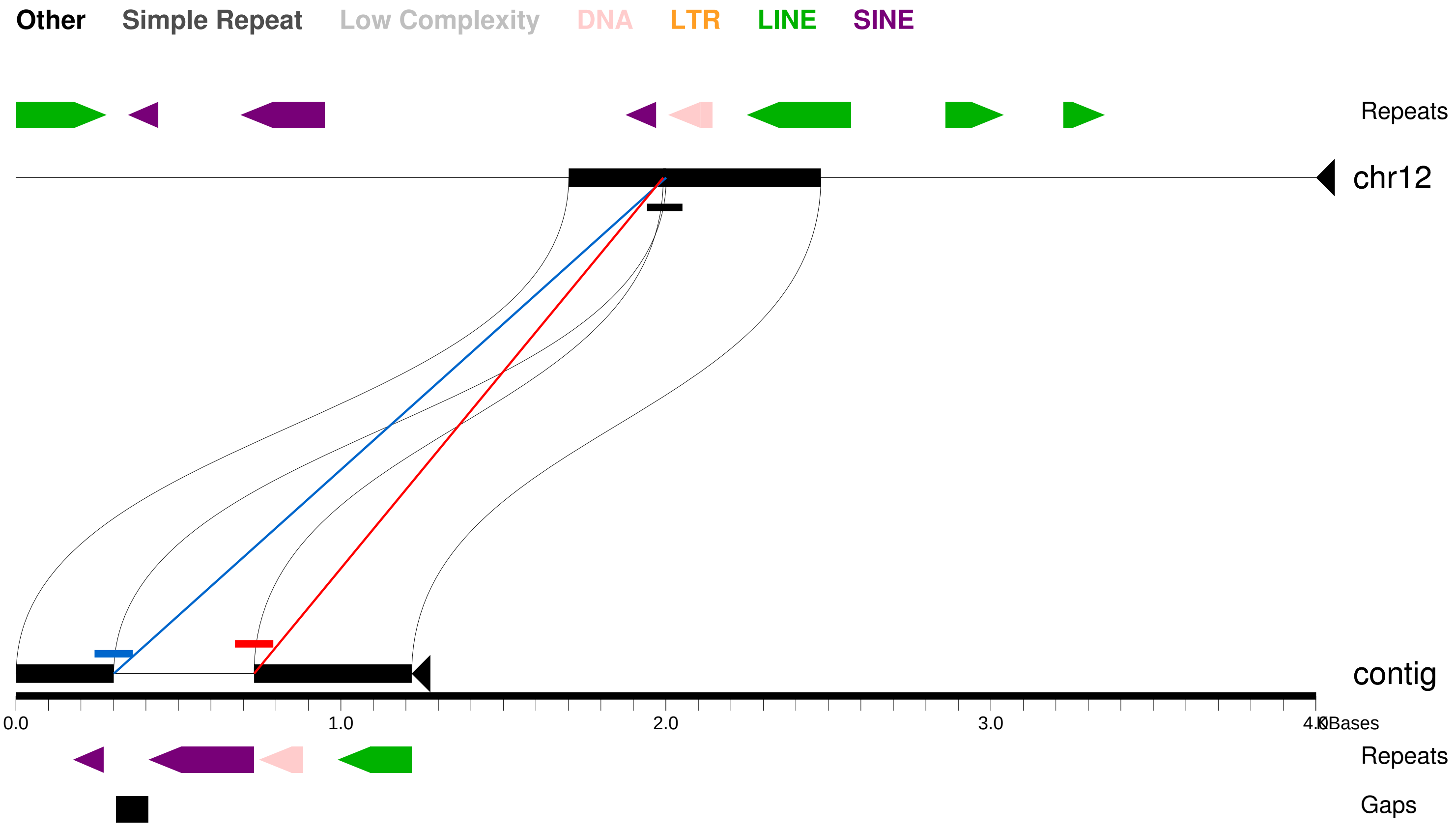

siteID: chr12_114545407 Scafftig1 + Breakpoint: chr12:114545416
chr12:114545256-114545589
Overlap Length: 14 INS size: 313 Genotyped: Yes

```
left   G--------------TTG-GCTATTTGGGT-CTGCTATTCCCTCATTACCACTTAAGGGTTTCCTGTGCC
chrom  GGTGGTCCTTAAAAGTTG-GCTATTTGGGT-CTGCTATTCCCTCATTACCACTTAAGGGTTTCCTGTGCC
right  CGTGAACCCGGGAAGCGGAGCTTGCAGTGAGCCGAGATTGCG------CCACTGCAG----TCCGCAGTC
       1222NN22NNNN22211*1***1111*1*11*1*11***1*1111111*****11**1111***111*1*

left   CTGCTTATTCTGGAGAGGTAGACACTGGTCATATCTATAGCAGCCACCTGAGCCCCATGGCTGTAGTGTA
chrom  CTGCTTATTCTGGAGAGGTAGACACTGGTCATATCTATAGCAGCCACCTGAGCCCCATGGCTGTAGTGTA
right  CGACCTGGGCGACAGAGCGAGACTCCG----TCTCAAAAAAAAAAA-----------------------A
       *11*1*111*111****11****1*1*1111*1**1*1*11*111*1111111111111111111111*

left   AAAGACACCTGTACTTGTATACAAAAAAAGTTTAGA------CG----------GTGGCTCAC--GCCTG
chrom  AAAGACACCTGTACTTGTATACAAAAAAAGTTTAGAAATGTTCGACTTGAACATGTTACTGACAAGGGAG
right  AAAAAAAAAAAAAAAAAAAAAAAAAAAAAGTTTAGAAATGTTCGACTTGAACATGTTACTGACAAGGGAG
       ***1*1*11111*11111*1*1**************222222**2222222222**22**2**22*222*

left   TAATCCCAGC--ACTTTGGGA-GGCCGAGGCGGGTGGATCA----TGAGGTCAGGA---GATCGAGACCA
chrom  TAAAGACTGGTTATTTTCTGATGGCACTGGTTATTAAAGCAGACCTGAAAAAAGTAAGTGATAGCAACCC
right  TAAAGACTGGTTATTTTCTGATGGCACTGGTTATTAAAGCAGACCTGAAAAAAGTAAGTGATAGCAACCC
       ***222*2*222*2***22**2***222**2222*22*2**2222***2222**2*222***2*22***2

left   TCCTG----------GCTAACAAGGTGAAACCCCGTCTCTACTAAAAATACAAAA--
chrom  TTCTATTGGCATATTGCTTTCTAGGATGCTACTTGAGT-TTCTTAATTTAGGAAAAC
right  TTCTATTGGCATATTGCTTTCTAGGATGCTANNNNNNN-------------------
       *2**22222222222***22*2***2222221NN1NN121N11N11NN11NN111
```

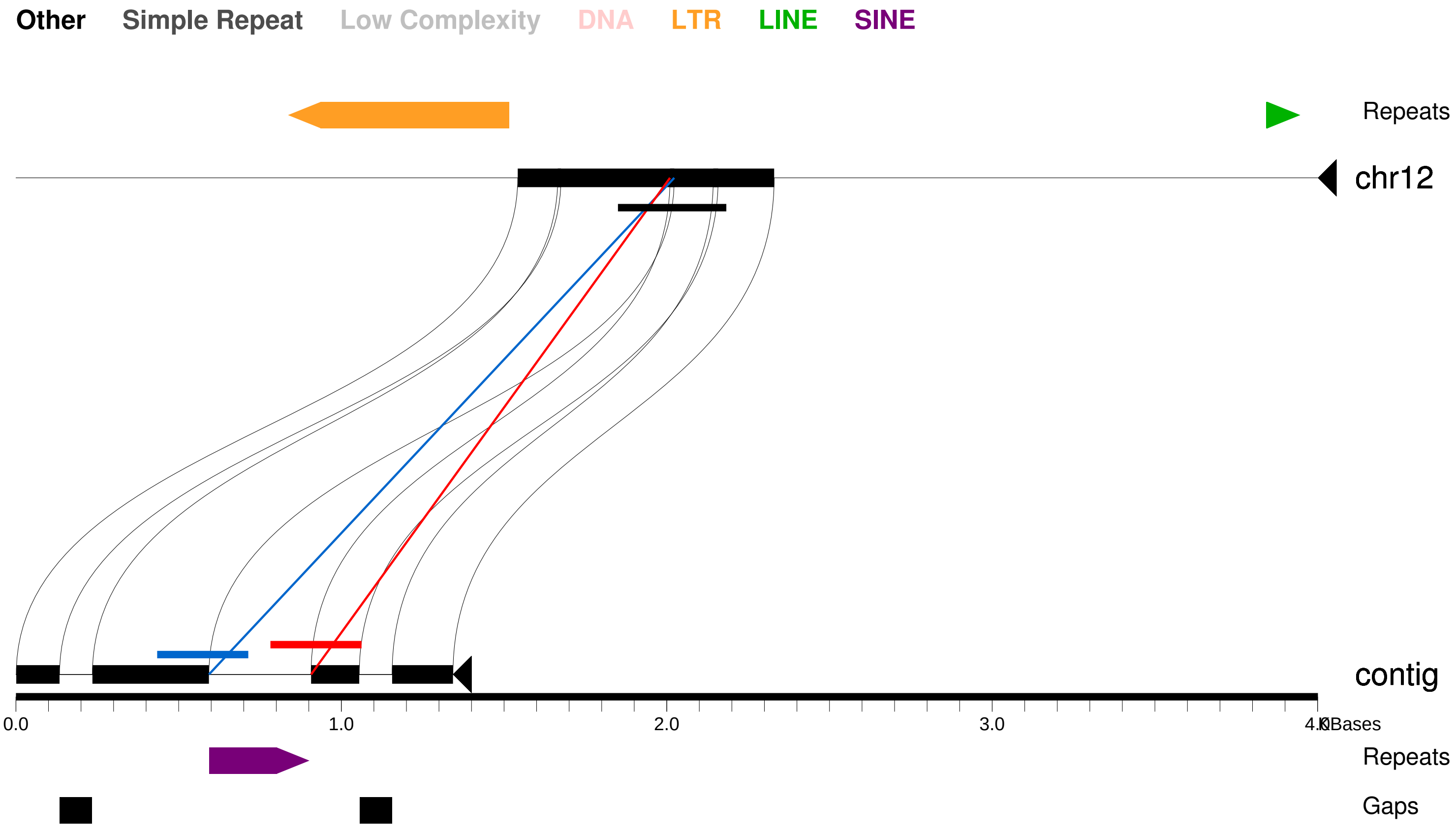

siteID: chr12_115731107 Scafftig2 + Breakpoint: chr12:115731078
chr12:115731034-115731137
Overlap Length: 16 INS size: 295 Genotyped: Yes

```
left     G---------TTGGCC------ATTTGTAGGTCTTCTTTTGAGAAATGTCTTTTCATGTCCTTTGCCTGT
chrom    G---------TTGGCC------ATTTGTAGGTCTTCTTTTGAGAAATGTCTTTTCATGTCCTTTGCCTGT
right    TCCGCCCGCCTCGGCCTCCCAAAGTGCTGGGATTACAGGCGTGAGCCACCGCGCCCGGCCCTTTGCCTGT
         1111111111*1****111111*1*11*1**11*1*1111*1**11111*1111*11*1***********

left     TTCTTTTTTTTTTTTTTTTTGAGACGGAGTCTCGCTCTGTCGCCCAGGCTGGAGTGCAGTGGCG-
chrom    TTCTTAATCAGGTTACTCATTTTCTTGAGT-----TCT-----------AGGAGTTTCTTATATA
right    TTCTTAATCAGGTTACTCATTTTCTTGAGT-----TCT-----------AGGAGTTTCTTATATA
         *****22*2222**22*22*222222****22222***222222222222*****2222*22222
```

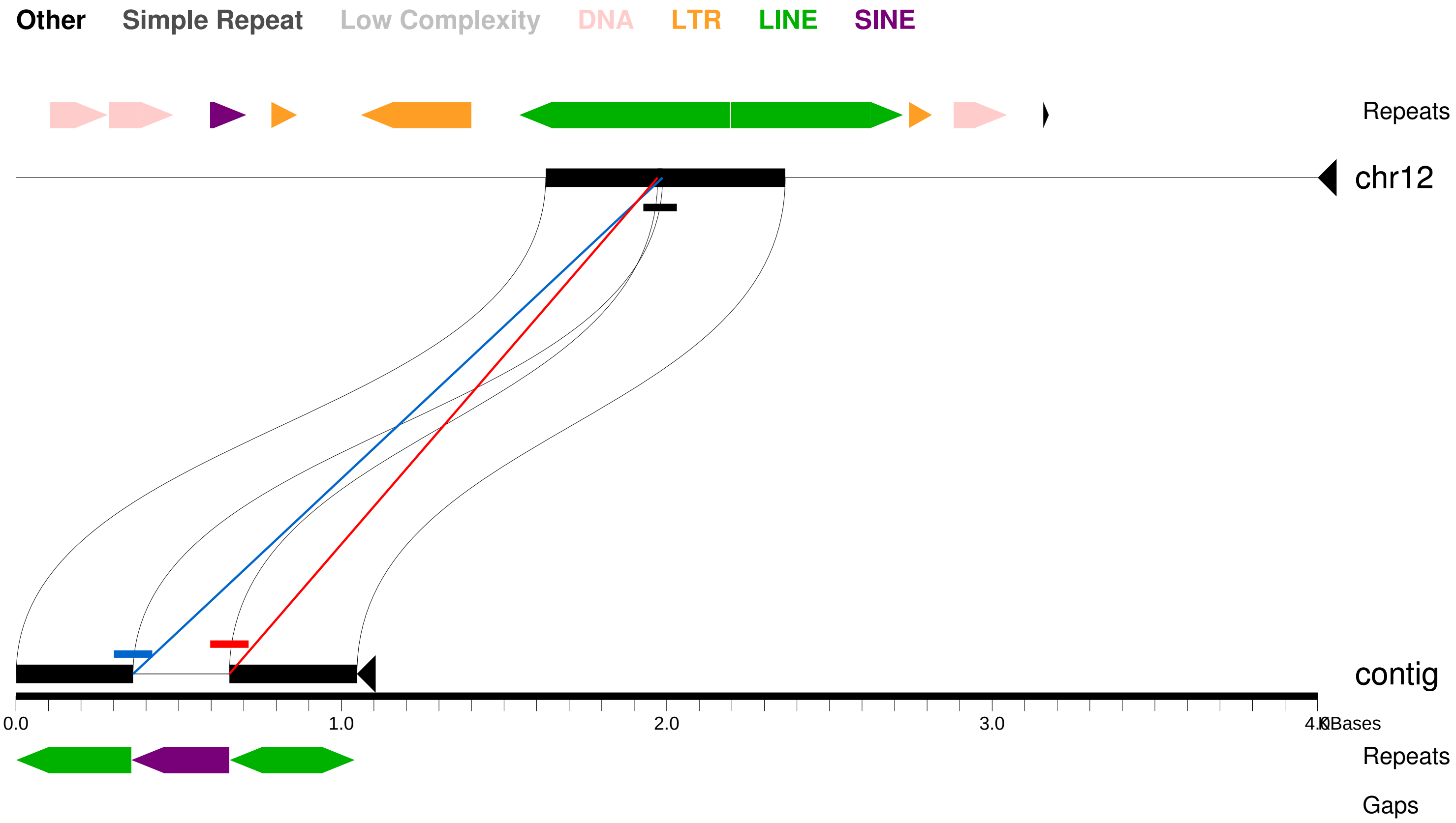

siteID: chr12_130455059 Scafftig2 + Breakpoint: chr12:130455046
chr12:130454886-130455215
Overlap Length: 10 INS size: 301 Genotyped: Yes

```
left   ----------CTGCATCTGACATGGAGTTAACATCCAGAATAAAAAAGGAATTCAAACAACTCAACCACA
chrom  TTTGCAGCCTCTGCATCTGACATGGAGTTAACATCCAGAATAAAAAAGGAATTCAAACAACTCAACCACA
right  CTG--AG-----GCAGGAGA-ATGGCGTGAAC--CCGGGAGGCGGA--GCTTGCAG-----TGAGCCG--
       N2N  22    11***111**1****1**1***11**1*1*11111*11*11*1**111111*1*1**111

left   ACAGAAACAAACACTCGAGTTAACAATGGCCAAAAGACCTGAACAGACATT-TCTCAAAAGAAGATTTAC
chrom  ACAGAAACAAACACTCGAGTTAACAATGGCCCAAAGACCTGAACAGACATT-TCTCAAAAGAAGATTTAC
right  --AGATCCCGCCACTGCACTCCAGCCTGGGCGACAGAGCG----AGACTCCGTCTCAAAAAAAAA-----
       11***11*111****11*1*11*111***1*N*1***1*11111****1111********1**1*11111

left   AAATGGCCAAAAAGTATCTTTAAAAAGGCTCGGCC-------------GGGCGCGGTGGCTCACGCC---
chrom  AAATGGCCAAACAGTATCTTTAAAAAGGCTCAACATCACTAGTCATCAGGGAAAGGTAAATCAAAACCAC
right  AAA-----AAA----------AAAAAGGCTCAACATCACTAGTCATCAGGGAAAGGTAAATCAAAACCAC
       ***11111***N111111111*********22*22222222222222***222***222***222*222

left   --TGTAATCCCAGCACTTTGGGAGGCCGAGGCGGGCGGATCACAAGGTCAGGAGATCGAGACCATCCCGG
chrom  AATGAAATGTCAACTCATCCC-AGTTAGAATAGGTATTATCCAGAAGAAAAAAAAAAATA-ACAAATGCTG
right  AATGAAATATCAACTCATCCC-AGTTAGAATAGGTATTATCCAGAAGAAAAAAAA--TA-ACAAATGCTG
       22**2***N2**2*2*2*2222**222**222**2222***222*2*22*22*2*NN2*2**2*222*2*

left   CTAAA-ACG--GTGAAAC-----CCCGTCTCTACT---------------AAA
chrom  CTAAGGACGTGGAGAAAGTGGAACTCTTCTGCACTGTTGATGGGAATGTAACT
right  CTAAGGACGTGGAGAAAGTGGAACTCTTCTGCACTGTTGATGGGAATGTA---
       ****22***22*2****222222*2*2***22***2222222222222221NN
```

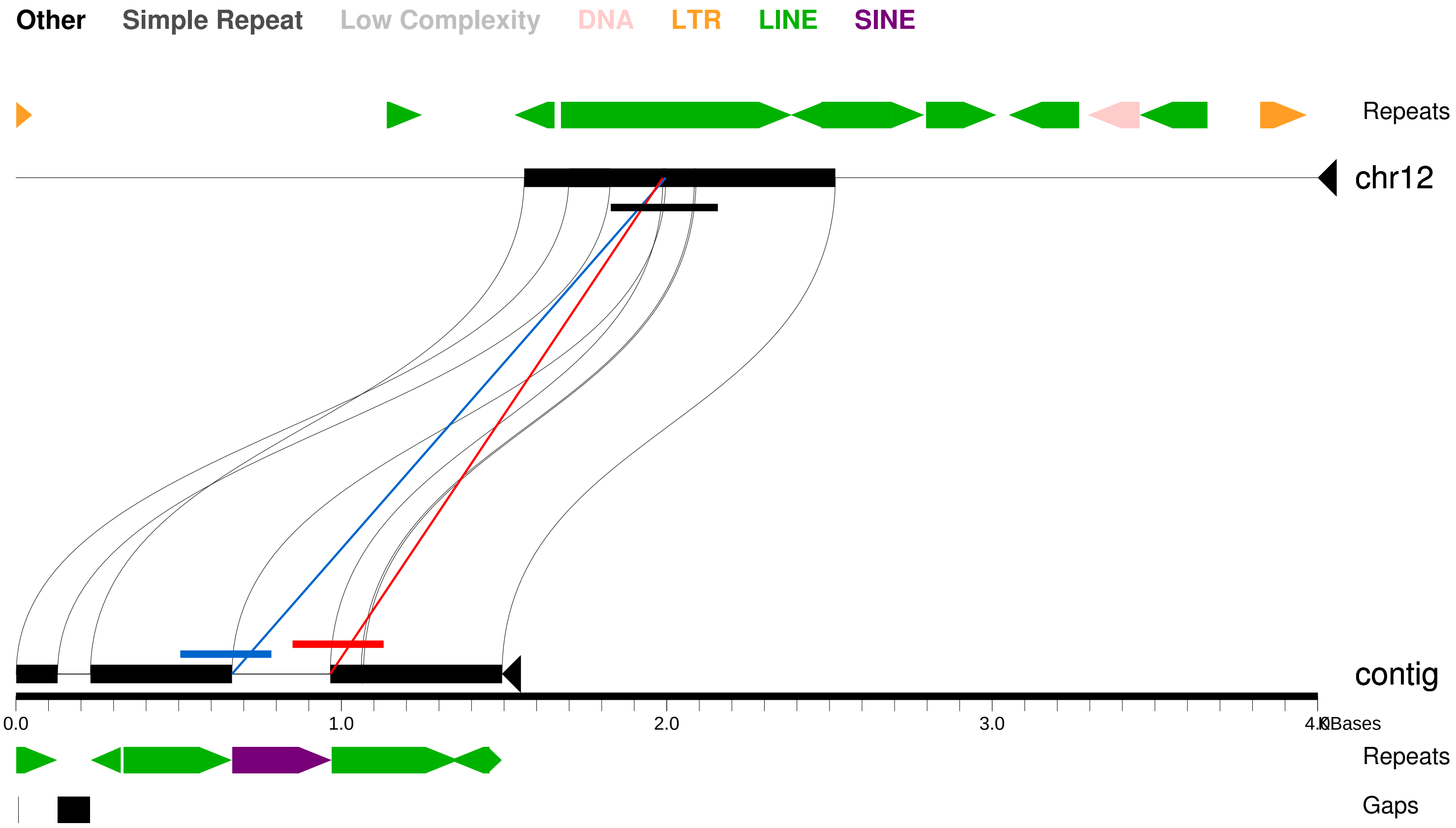

siteID: chr12_14173887 Scafftig1 + Breakpoint: chr12:14173870
chr12:14173820-14173929
Overlap Length: 10 INS size: 302 Genotyped: Yes

```
left   ATAAGCACTTAACATTAAGTTTGT-CTGTAAGCACTA-CTTTAGCTACCTCT-------CATAAATTTTT
chrom  ATAAGCACTTAACATTAAGTTTGT-CTGTAAGCACTA-CTTTAGCTACCTCT-------CATAAATTTTG
right  CCGCCCGCCTCGGCCTCCCAAAGTGCTGGGATTACAGGCGTGAGACACCGCGCCCGGCCCATAAATTTTG
       11111*1*1*11111*111111**1***11*11**111*1*1**11***1*11111111*********2

left   TTTTTTTTTTTTTTTTTTTTGAGACGGAGTCTCGCTCTGTCGCCCAGGCTGGAGTGCA
chrom  ATAGATTAGGTTTTAATCATTATTC--AGTTAAGCA-TATTTTCTA------ACTTAC
right  ATAGATTAGGTTTTAATCATTATTC--AGTTAAGCA-TATTTTCTA------ACTTAC
       2*222**222****22*22*2*22*22***222**22*2*222*2*222222*2*222
```

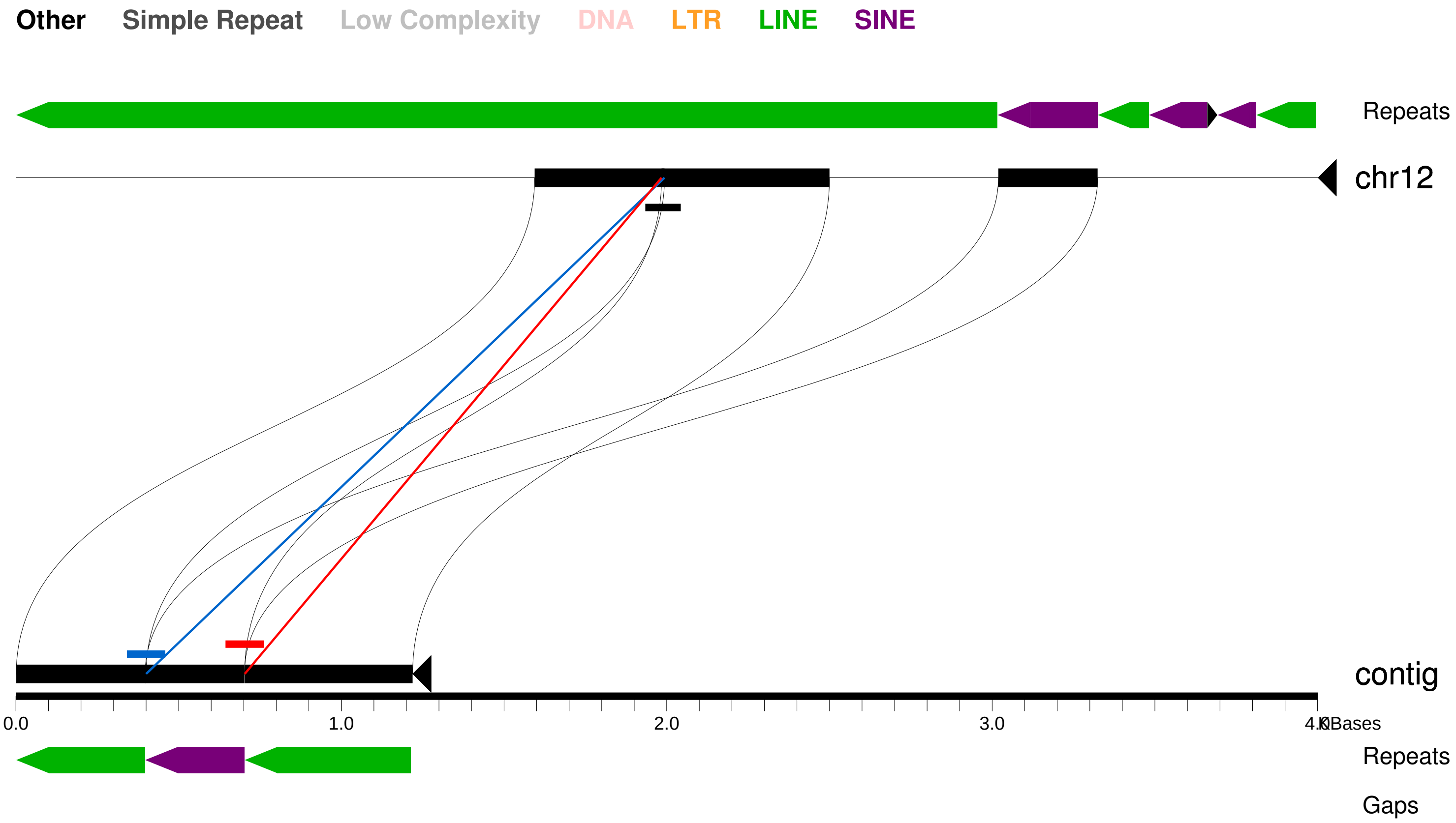

siteID: chr12_15104642 Scafftig3 - Breakpoint: chr12:15104633
chr12:15104588-15104692
Overlap Length: 15 INS size: 322 Genotyped: Yes

```
left    AGTCTTC--CTTCATCTCTTATTATCTACATTTACTTGTTTTGTGTT------------AAAACATTGAG
chrom   AGTCTTC--CTTCATCTCTTATTATCTACATTTACTTGTTTTGTGTT------------AAAACATTGAG
right   AGAGCGAGACTCCGTCTCAAAAAAAAAAAAAAAAAAAAAAAAAAAAAAAAAAAAAAAAAAAAAACATTGAG
        **1111111**1*1****11*11*111*1*111*1111111111111111111111111***********

left    GAGAGGCCGGGCGCGGTGGCTCACGCCTGTAATCCCAGCACTTTGGGAGGCCGAGGCGGGTGG
chrom   GAGAAATCTAGGCAAGTTATTTAC--CTGTTA----AGGG-TTAATGA--CTGAA-----TAG
right   GAGAAATCTAGGCAAGTTATTTAC--CTGTTA----AAGG-TTAATGA--CTGAA-----TAG
        *****222*22*2222**222*2**22****2*2222*1222**222**22*2**222222*2*
```

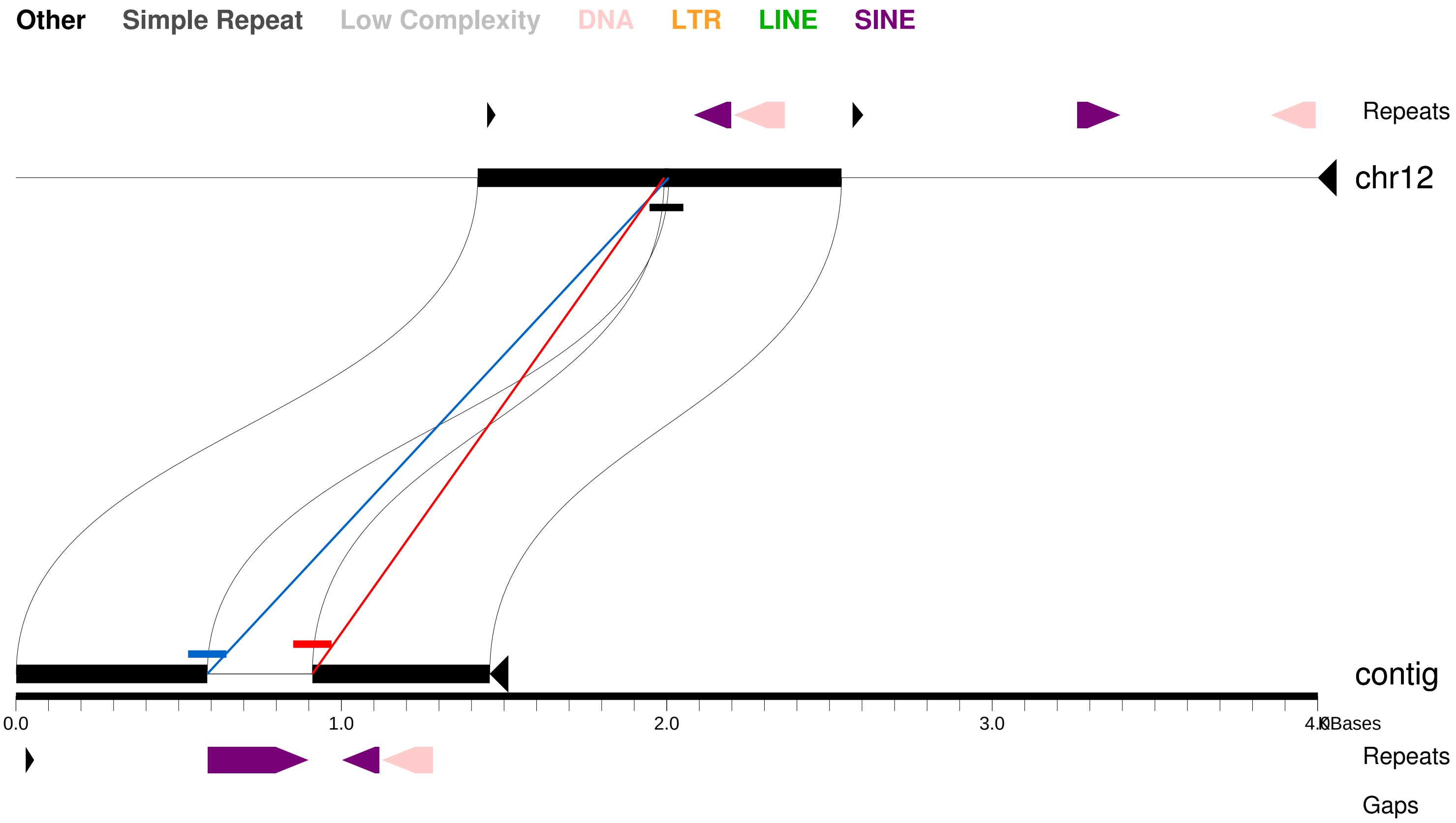

siteID: chr12_16207017 Scafftig1 + Breakpoint: chr12:16207040
chr12:16206880-16207210
Overlap Length: 11 INS size: 182 Genotyped: Yes

```
left   AAG----------AGTTTCTGCACAGCAAAAGAAACTATCAACAGAGTAAACAGACAA---CCTACAGAA
chrom  AAGCTAATTAAAGAGTTTCTGCACAGCAAAAGAAACTATCAACAGAGTAAACAGACAA---CCTACAGAA
right  AG----AATGGCGTGAACCCGGGAGGCGGAGCTTGCAGTGAGCCGAG-ATCCCGCCACTGCACTCCAGCC
       *11   2N2NNN21*111*1*1111**11*11111*11*1*1*1***1*11*1*1**11111**1***11

left   TCAGAGAAA-ATCTTCACAAACTATGCATCTGACAAAGGTCTAATATCCAGCAGCTACAAGAAACTTAAA
chrom  TCAGAGAAA-ATCTTCACAAACTATGCATCTGACAAAGGTCTAATATCCAGCAGCTACAAGAAACTTAAA
right  TGGGCGACAGAGCGAGAC------TCCGTCTCAAAAAAAAAAAAAAAAAAAAAAAAAAAAAAAAAAAAAA
       *11*1**1*1*1*111**111111*1*1***1*1***11111**1*111*11*111*1**1***111***

left   TTTACAAGAAAAAAAAACCCTATTAAAAAGTGGGCTG----TAGTCCCAGCTACTTGGGAGGCTGAGGCA
chrom  TTTACAAGAAAAAAAAACCCTATTAAAAAGTGGGCAAAGGATATGAACGGAGAGTTTTCAAAAGAAGACA
right  AAAAAAAAAAAAAAAAA----AAAAAAAAGTGGGCAAAGGATATGAACGGAGAGTTTTCAAAAGAAGACA
       111*1**1*********1111*11**********222222**2222*2*22*2**222*22222**2**

left   GGAGAATGGCGTGAACCCG-------GGAGGCGGAGCTTGC---------AGTGAGCCG-AGATC----C
chrom  TACATGTGGCCAACAAACATACAAAAGAAAGCTCAACATCTCTGATCATTAGAGAAACGCAAATCAAAAC
right  TACATGTGGCCAACAAACATACAAAAGAAAGCTCAACATCTCTGATCATTAGAGAAACGCAAATCAAAAC
       222222****2222*22*22222222*2*2**22*2*2*22222222222**2**22**2*2***2222*

left   CGC----------CACTGCACTCCAGCCTGGGCGAC----AGAGCGAGACTCCG-
chrom  CACAATAAGATACCACCTCACACCAGTCAGAATGACTATTATATTAAAAAAAAAA
right  CACAATAAGATACCAC---------------------------------------
       *2*2222222222***NN111N1111N1N1NNN111    1N1NNN1N1NNNNN
```

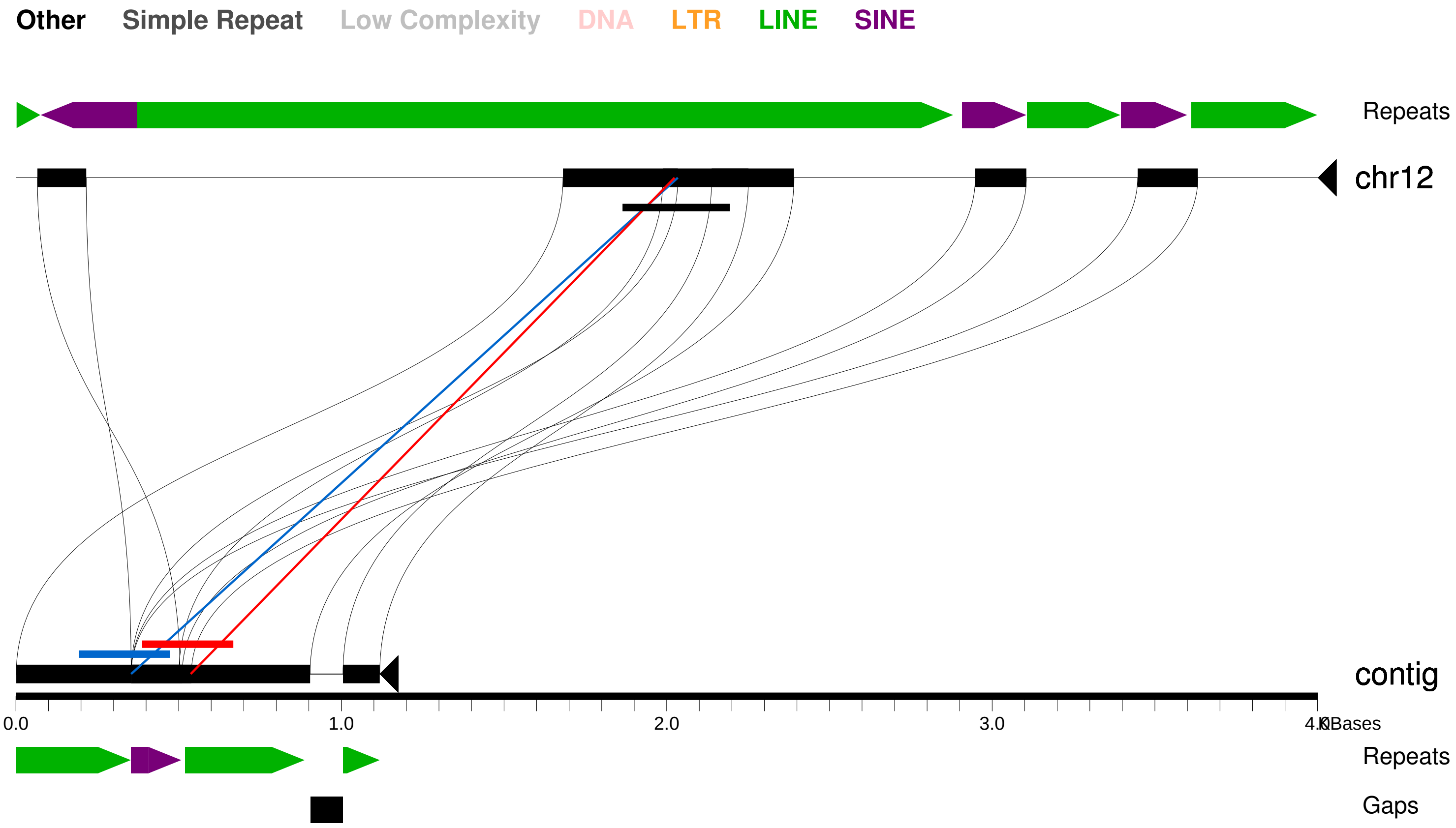

siteID: chr12_16450862 Scafftig1 + Breakpoint: chr12:16450839
chr12:16450679-16451009
Overlap Length: 11 INS size: 309 Genotyped: Yes

```
left     --GCATTAGGTCGTGGTCTGTTTGTCCAGTCATATTTCTACATGACTGTTCATACTTGGTTGAATCTAAG
chrom    TTGCATTAGGTCGTGGTCTGTTTGTCCAGTCATATTTCTACATGACTGTTCATACTTGGTTGAATCTAAG
right    TTTAGTAGAGACGGGGT----TTCACC-----TTGTTAGCCAGGA-TGGTCTCGATCTCCTGAC-CTCAT
         22111*111*1**1***1111**11**11111*11**111**1**1**1**1111*1111***11**1*1

left     CATAAAACTGGACAATTTCCTTTGTATCTTTGGGTCTTCATTCTGAAGGTTCCTGTGTCACATACAACTG
chrom    CATAAAACTGGACAATTTCCTTTGTATCTTTGGGTCTTCATTCTGAAGGTTCCTGTGTCACATACAACTG
right    GATCCACCCG--------CCTC----------GGCCTCCC-----AAAGTGCTGGGATTACAGGC----G
         1**11*1*1*11111111***11111111111**1**1*111111**1**1*11*11*1***11*1111*

left     TGATCAAATAAATGTGTATGCCTTTTCTCCTTTTTTTTTTTTTTTTTT-------TTTTGAG--------
chrom    TGATCAAATAAATGTGTATGCCTTTTCTCCTCTTAATCTGCCTTTTGTCAGCTGATCTTCAGCAACCCTT
right    TGAGCCACCGCGCCCGGC--CCTTTTCTCCTCTTAATCTGCCTTTTGTCAGCTGATCTTCAGCAACCCTT
         ***1*1*11111111*1111***********2**22*2*222****2*2222222*2**2**22222222

left     ---------ACGGAGTCTCGCTCTGTCGCCCAGGCC--AGACTGC--GGACTGC-----AGTGGCGCAAT
chrom    CAAAGGGCAAAGGGGAAGTGTTCCCTTGTCCCTTCCTTACACTACCCGTACTGTTCCTTATTCAGGCCAT
right    CAAAGGGCAAAGGGGAAGTGTTCCCTTGTCCCTTCCTTACACTACCCGTACTGTTCCTTATTCAGGCCAT
         222222222*2**2*2222*2**22*2*2**222**22*2***2*22*2****222222*2*222**2**

left     C------TCG-GCTC--ACTGCAAGCTCCGCTTCCCCGG-GTTCAC-----
chrom    CATTATTTCTTGCTATAATTATAATAAGAGATTCTCTACTGATCACCTTGC
right    CATTATTTCTTGCTATAATTATAATAAGAGATTCTCTACTG----------
         *222222**22***222*2*22**22222*2***2*2222*N1111
```

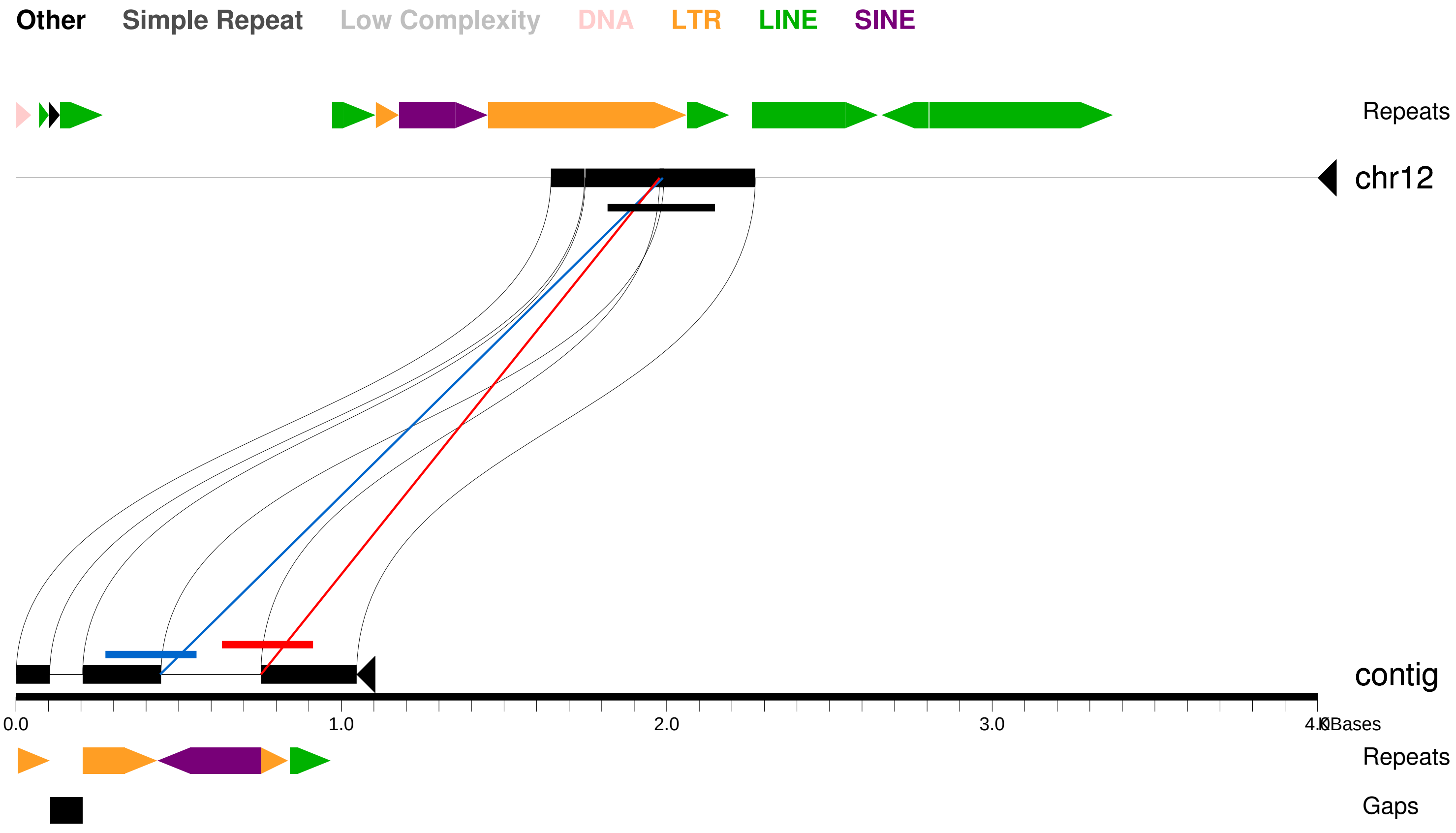

siteID: chr12_19806209 Scafftig7 - Breakpoint: chr12:19806210
chr12:19806163-19806267
Overlap Length: 13 INS size: 277 Genotyped: Yes

```
left    TTTTTTTCCACAG--------------TCAGAAACGTGACTGAAACATTTCTTCCTCTTAA-GAGGCATT
chrom   TTTTTTTCCACAG--------------TCAGAAACGTGACTGAAACATTTCTTCCTCTTAA-GAGGCATT
right   CCTGGG-CGACAGAGCGAGACTCCGTCTCAAAAAAAAAAAAAAAAAAAAAAAAAAAAAAAAAGAGGCATT
        11*1111*1****11111111111111***1***1111*111***1*111111111111**1********

left    AAGAGTTGGGAGGCCGAGGCGGGCGGATCACGAGGTCAAGAGATCGAGACCATCCCGGCTAAAA
chrom   AAGAG-----------AAAAGGATAGATTTCCTGTTTC---TTTTGCAATTTTCCTCTTTAAGA
right   AAGAG-----------AAAAGGATAGATTTCCTGTTTC---TTTTGCAATTTTCCTCTTTAAGA
        *****22222222222*222**222***22*22*2*2222222*2*22*222***2222***2*
```

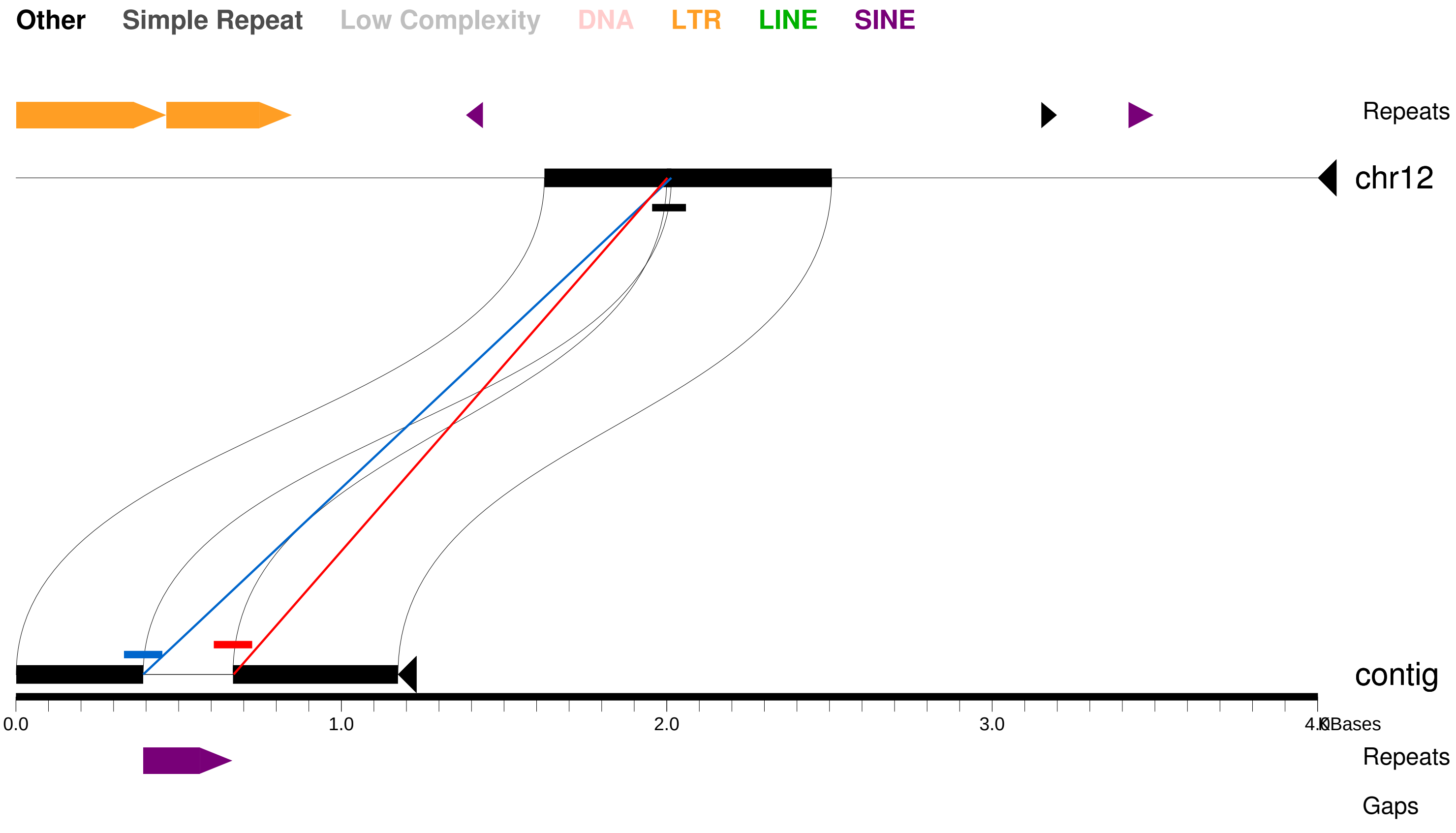

siteID: chr12_19846489 Scafftig1 - Breakpoint: chr12:19846477
chr12:19846436-19846535
Overlap Length: 16 INS size: 434 Genotyped: No

```
left     ATAAAAA---GGAAT---AAAACAATGGCATT------------CACCACAACCTGGAT-GGAATTGGTG
chrom    ATAAAAA---GGAAT---AAAACAATGGCATT------------CACCACAACCTGGAT-GGAATTGGTG
right    CACCCGCCTCGGCCTCCCAAAGTGCTGGGATTACAGGCGTGAGCCACCGCGCCCGGCCTTGGAATTGGTG
         1111111111**11*111***1111***1***111111111111****1*11**1*11*1**********

left     ACTCTTTTTNNNNNNNNNNNNNNNNNNNNNNNNNNNNNNNNNNNNNNNNNNNNNNNNNNNNNNNNNNN
chrom    ACTCTTATTGTAAGTGAAGTAACTCAGGAATGGAAAACCAAACATTGTA-------------------
right    ACTCTTATTGTAAGTGAAGTAACTCAGGAATGGAAAACCAAACATTGTA-------------------
         ******2**22222222222222222222222222222222222222222222222222222222222
```

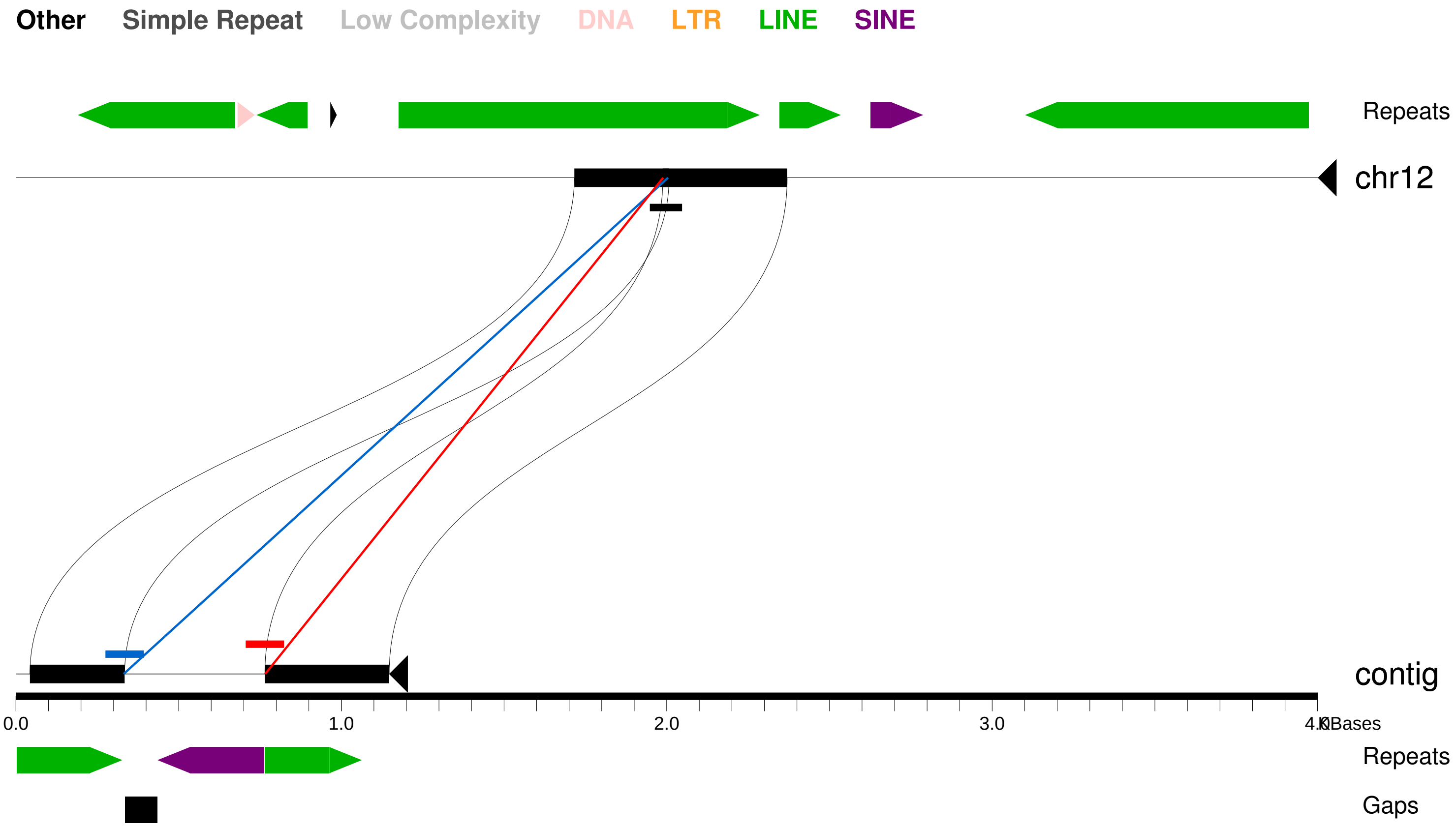

siteID: chr12_20473893 Scafftig7 - Breakpoint: chr12:20473879
chr12:20473719-20474053
Overlap Length: 15 INS size: 310 Genotyped: Yes

```
left    ---------------CTTGCTCTTGCTAAGAGAGACATCAGGTGT-CAGTGATTCCATCTCCTCGTCAGG
chrom   GTCCCTCTGATATTCCTTGCTCTTGCTAAGAGAGACATCCGGTGT-CAGTGATTCCATCTCCTCGTCAGG
right   A-------GA-ATGGCGTGAACCCGGGAGGCGGAGCTTGCAGTGAGCCGAGATTGCG---CCACTGCACT
        N       22 22NN*1**11*11*11*1*1*111*1*121***11*1*1****1*1111**1*11**11

left    TCAG--TTTTCCTGTGATCAAGAATAAGAAAGAAAGAAAAAAGACCTTTTATTCACTTGCTAACTGAAGC
chrom   TCAG--TTTTCCTGTGATCAAGAATAAGAAAGAAAGAAAAAAGACCTTTTATTCACTTGCTAACTGAAGC
right   CCAGCCTGGGCGACAGAGCGAGACTCCGTCTCAAAAAAAAAAAA-----------------AAAAAAA--
        1***11*111*1111**1*1***1*11*1111***1******1*11111111111111111**111**11

left    TGTATCTGTTTTTAACTAGCATTAAAATGTTCCATTCT--GGC-------CGGG-----CGCGGTGGCT-
chrom   TGTATCTGTTTTTAACTAGCATTAAAATGTTCCATTCTTTGTCTAACTTACATGAATTACTCTTAGGCTT
right   -------------AAAAAAAAAAAAAATGTTCCATTCTTTGTCTAACTTACATGAATTACTCTTAGGCTT
        1111111111111**11*11*11***************22*2*2222222*22*22222*2*222****2

left    ------CACGCCTGTAA-TCCCAGCACTTTGGGAGGCCGAGGCG----GGCGGAT--CACGAGGTCAGGA
chrom   TCCTTTCACTCTTTTAAATAACAGTTTTTTTCTAGCCATAGTGGATATGCCTGTTTTCAAAATATAACTG
right   TCCTTTCACTCTTTTAAATAACAGTTTTTTTCTAGCCATAGTGGATATGCCTGTTTTCAAAATATAACTG
        222222***2*2*2***2*22***222***222**2*22**22*2222*2*2*2*22**22*22*2*222

left    GATCGAGACCATCCCGGCTAAAACG---GTGAA--ACCCCGTCTCTACT-------AAA
chrom   CAAAGAG-CTATTGCTGCTTCAGTGATTGTGGATTACAAGCTATTTAGGTTGTCACAGC
right   CAAAGAG-CTATTGCTGCTTCAGTGATTGTGGATTACAAGCTATT--------------
        2*22***2*2**22*2***22*22*222***2*22**2222*2*211NN       1NN
```

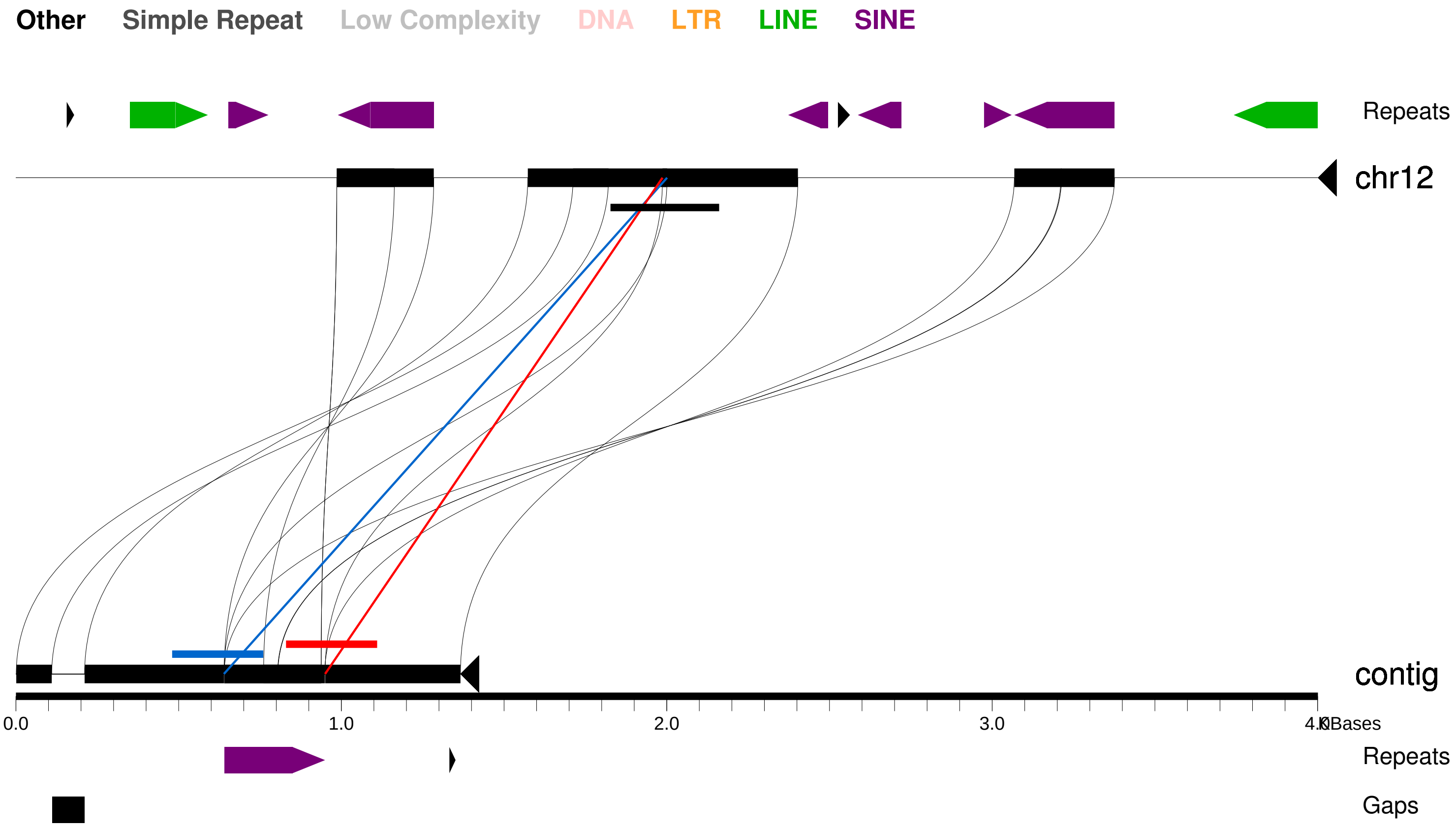

siteID: chr12_2089862 Scafftig1 + Breakpoint: chr12:2089842
chr12:2089797-2089901
Overlap Length: 15 INS size: 490 Genotyped: No

```
left    AAAT--------GCTTATAAGTGTCATTTTAAATCCAGTGTTTGGTAATACTA------CCTTTAACTAT
chrom   AAAT--------GCTTATAAGTGTCATTTTAAATCCAGTGTTTGGTAATACTA------CCTTTAACTAT
right   CCGCCCGCCTCGGCCTCCCAAAGTGCTGGGATTACAGGCGTGAGCCACCGCGCCCGGCCCCTTTAACTAT
        111111111111**1*111*11**11*111*111*11*1**11*11*111*11111111***********

left    TTTTTTTTTNNNNNNNNNNNNNNNNNNNNNNNNNNNNNNNNNNNNNNNNNNNNNNNNNNNNN
chrom   TTTTAAT--------------AAAGAGTTATTTATTTCGTTGCTTCTATAAAAATGAAAAAAA
right   TTTTAAT--------------AAAGAGTTATTTATTTCGTTGCTTCTATAAAAATGAAAAAAA
        ****22*222222222222222222222222222222222222222222222222222222222
```

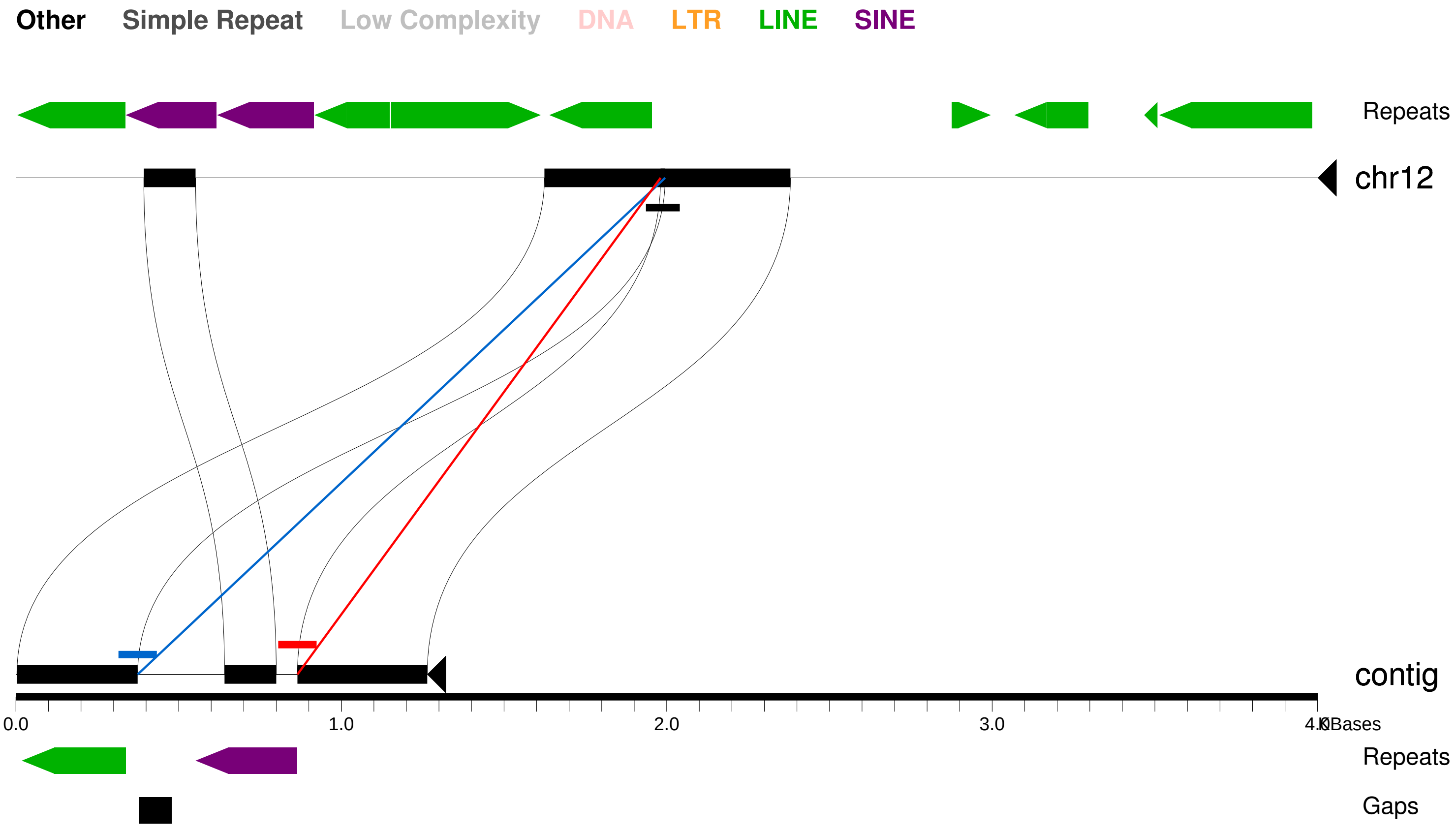

siteID: chr12_20908668 Scafftig10 + Breakpoint: chr12:20908671
chr12:20908626-20908730
Overlap Length: 15 INS size: 304 Genotyped: Yes

```
left     T--------TTGACAGAGGTAAG--CAGACTTTTTATGCCTCAGAAA----GCACAATTAGTTCTTTATT
chrom    T--------TTGACAGAGGTAAG--CAGACTTTTTATGCCTCAGAAA----GCACAATTAGTTCTTTATT
right    CCACCCGCCTCGGCCTCCCAAAGTGCTGGGATTACAGGCGTGAGCCACCGCGCCCGGCCAGTTCTTTATT
         111111111*1*1*111111***11*1*111**11*1**1*1**11*1111**1*1111***********

left     TTTTTTTTTTTTTTTTTTTTGAGACGGAGTCTCGCTCTGTCGCCCAGGCCGGACTGCGGACTGC
chrom    TTTTAAT-----------TAAACCAAACTCCTTTTCAGAAGTCAAGGA---AATTCCCTCAGT
right    TTTTAAT-----------TAAACCAAACTCCTTTTCAGAAGTCAAGGA---AATTCCCTCAGT
         ****22*22222222222*2*22*22*2**2222**2*22*2*2***2222*2*2*222*2*2
```

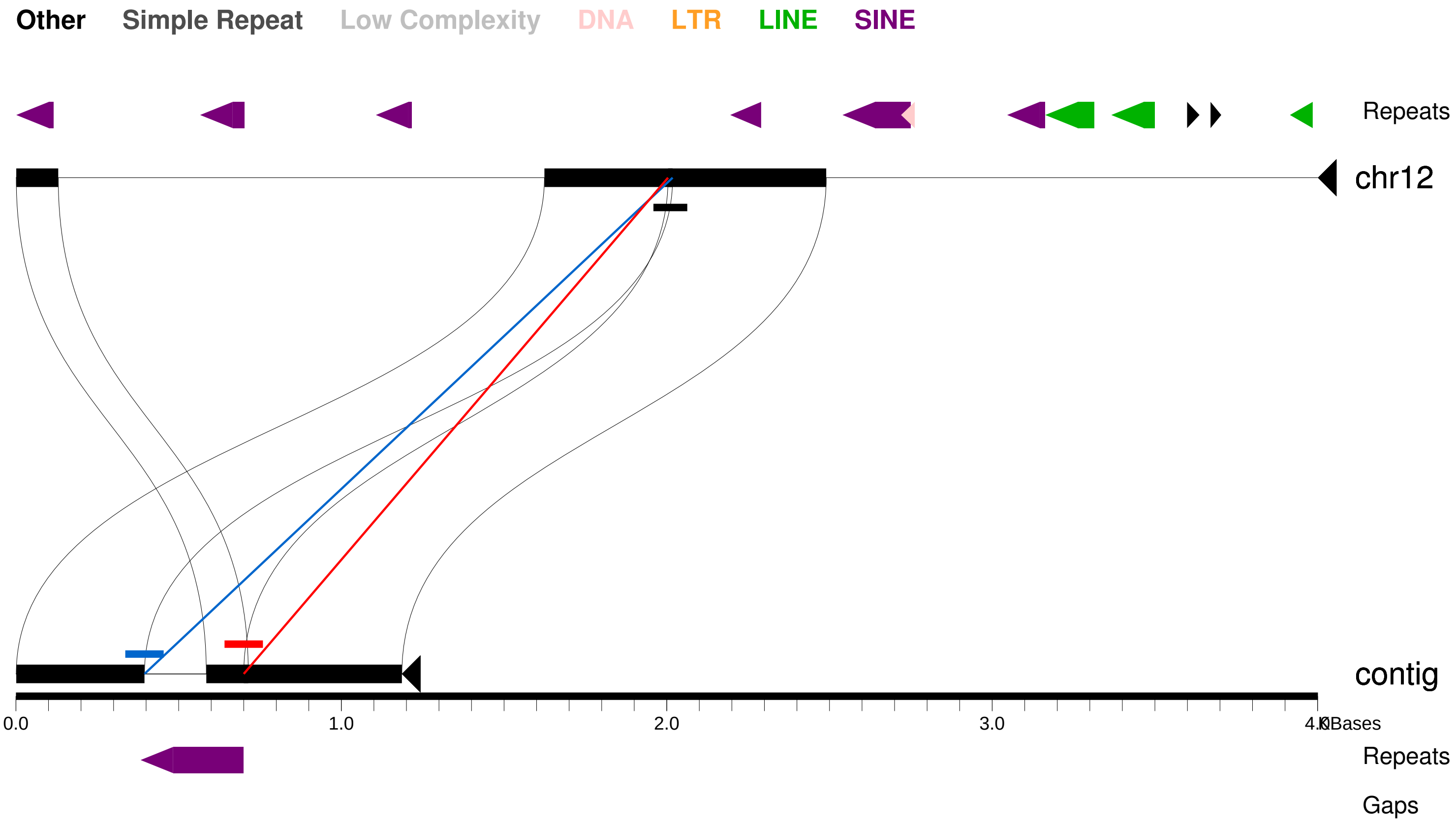

siteID: chr12_22085959 Scafftig2 - Breakpoint: chr12:22085944
chr12:22085896-22086003
Overlap Length: 12 INS size: 321 Genotyped: Yes

```
left    GCAAAGA----------CATGGAATCAACCTAAATGTCCATCAATGATAG-ACTGGATAAAGAAAATGTG
chrom   GCAAAGA----------CATGGAATCAACCTAAATGTCCATCAATGATAG-ACTGGATAAAGAAAATGTG
right   AGAGCGAGACTCCGTCTCAAAAAAAAAAAAAAAAAAACAAACAAAAAAAACAAACAAACAAGAAAATGTG
        11*11**1111111111**111**11**111***111*1*1***11*1*11*1111*11***********

left    GGCCGGGCGCGGTGGCTCGCGCCTGTAATCCCA-GCACTTTGGGAGGCCGAGGCGGGCGGA-
chrom   GTACATATACACTA---------TGGAATACTATGCA----GCCAGAAAAAAACAAGATAAT
right   GTACATATACACTA---------TGGAATACTATGCA----GCCAGAAAAAAACAAGATAAT
        *22*22222*22*2222222222**2***2*2*2***2222*22**2222*22*22*222*2
```

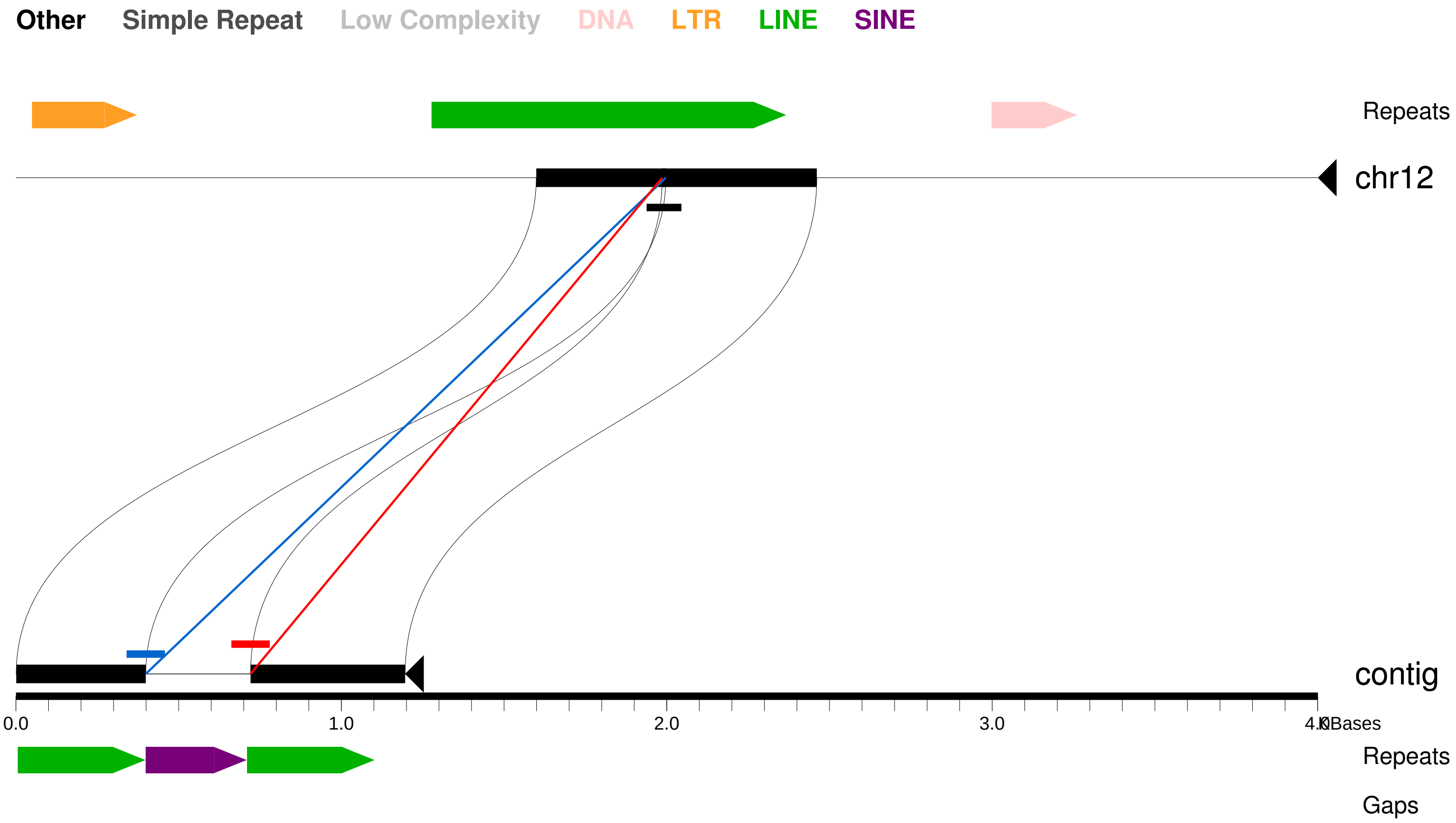

siteID: chr12_22123792 Scafftig11 + Breakpoint: chr12:22123783
chr12:22123737-22123842
Overlap Length: 14 INS size: 308 Genotyped: Yes

```
left    CCTT---GCTTGGAGTT----AGT-CTTGAATT-----CATCACTCACTGTCTCTCTGTTCCCTTCCATT
chrom   CCTT---GCTTGGAGTT----AGT-CTTGAATT-----CATCACTCACTGTCTCTCTGTTCCCTTCCATT
right   CCGCCCGCCTCGGCCTCCCAAAGTGCTGGGATTACAGGCGTGAGCCACCGCGCCCGGCCTCCCTTCCATT
        **111111**1**11*11111***1**1*1***11111*1*1*11***1*111*11111***********

left    CTTTTTTTTTTTTTTTTTTTTTTTTTTTTTTGAGACGGAGTCTCGCTCTGTCGCCCAGGCTGG
chrom   CTTATACAAATATTCAATCATTTAC-----GATAC--ACTCA-GCAATGA----CTGGCCT-
right   CTTATACAAATATTCAATCATTTAC-----GATAC--ACTCA-GCAATGA----CTGGCCT-
        ***2*22222*2**222*22***2222222**2**22*2**22**22**22222*2***222
```

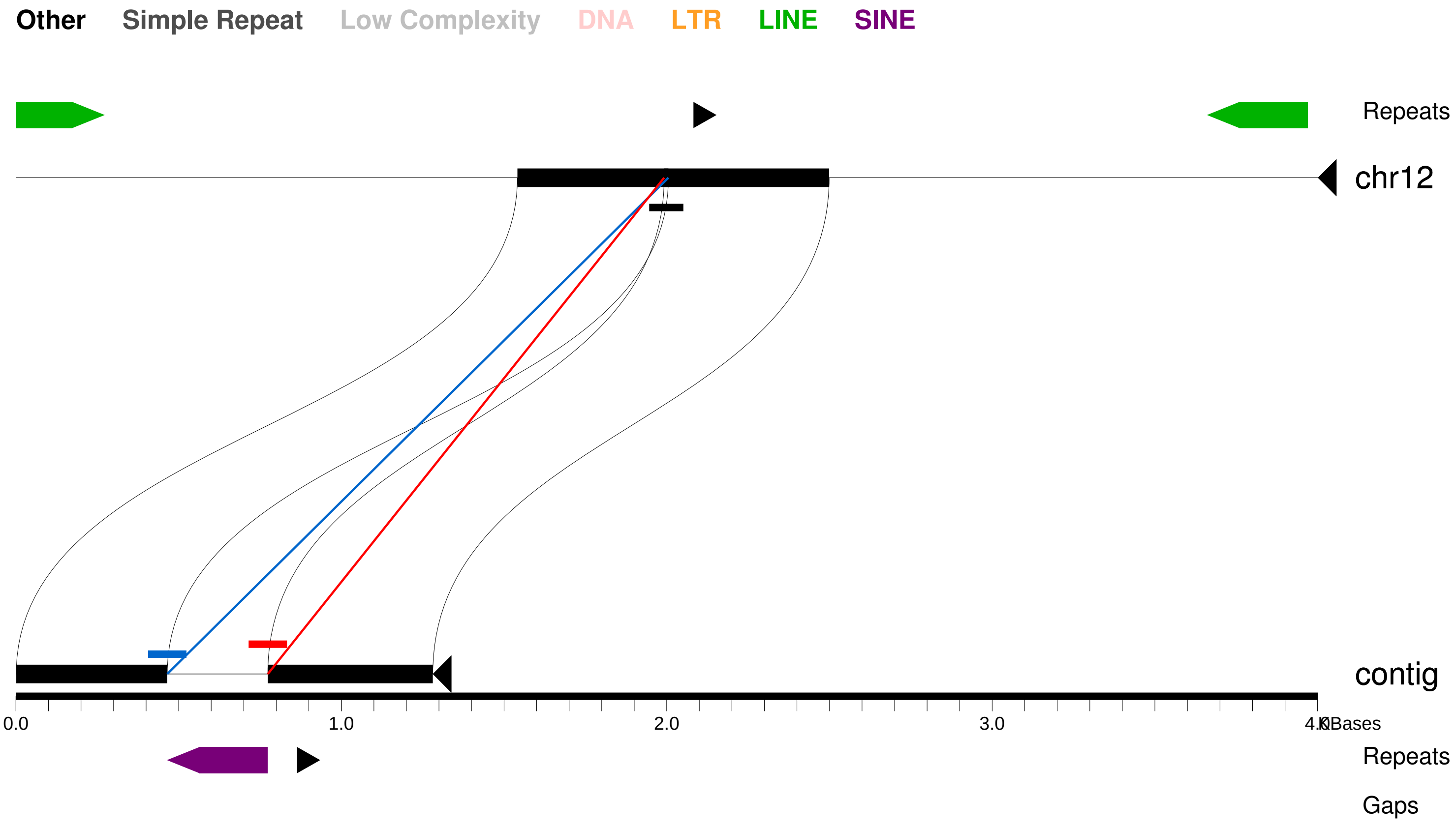

siteID: chr12_2307013 Scafftig1 - Breakpoint: chr12:2307017
chr12:2306962-2307076
Overlap Length: 5 INS size: 423 Genotyped: No

```
left     CAAAATCTGACAGTTTTGAGGACCCTTTAATAGAACCAGAGCTTCTGAAAATAAG----CACCTG--GCC
chrom    CAAAATCTGACAGTTTTGAGGACCCTTTAATAGAACCAGAGCTTCTGAAAATAAG----CACCTACTGAT
right    NNNNNNNNNNNNNNNNNNNNNNNNNNNNNNNNNNNNNNNNNNNNNNNNNNNNNNNNNNNNNCACCTACTGAT
         1111111111111111111111111111111111111111111111111111111111*****222*22

left     GGGCGCGGTGGCTCACGCCTGTAATCCCAGCACTTTGGGAGGCCGAGGCGGGCGG
chrom    GGGTTCAGTG---CAGAAATGAA--CCCAGGTCTA-GAACTGCAGTGTTAAGAGA
right    GGGTTCAGTG---CAGAAATGAA--CCCAGGTCTA-GAACTGCAGTGTTAAGAGA
         ***22*2***222**2222**2*22*****22**22*2222**2*2*2222*2*2
```

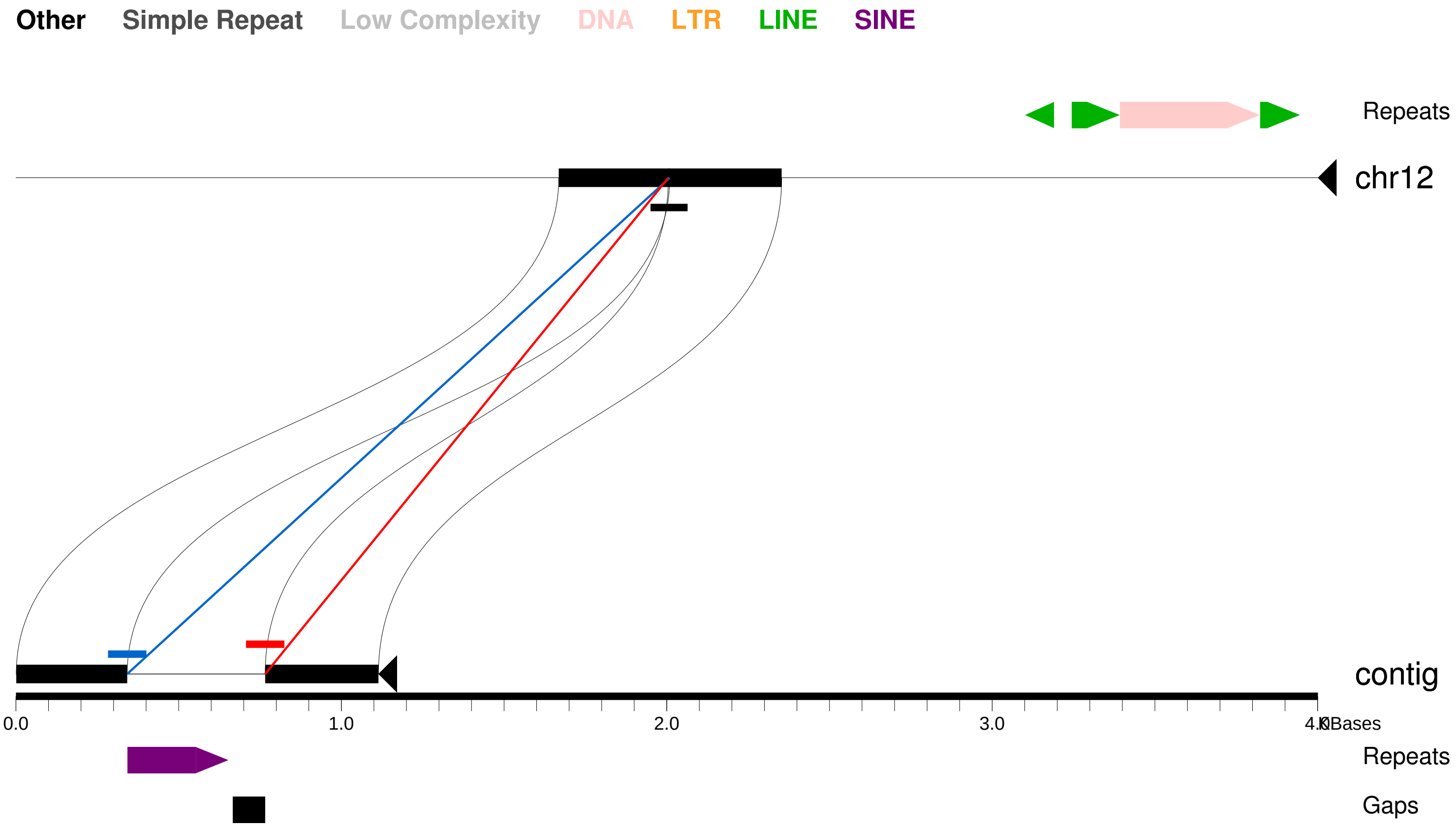

siteID: chr12_24518632 Scafftig1 + Breakpoint: chr12:24518612
chr12:24518568-24518671
Overlap Length: 16 INS size: 311 Genotyped: Yes

```
left     A--------TAATTATTGAAAATGATTTTCTTTACAAA--TTAATAAATA-----GACTTGATTAATACT
chrom    A--------TAATTATTGAAAATGATTTTCTTTACAAA--TTAATAAATA-----GACTTGATTAATACT
right    CCGCCCGCCTCGGCCTCCCAAAGTGCTGGGATTACAGGCGTGAGCCACCGCGCCCGGCCTGATTAATACT
         111111111*11111*111***1111*1111*****1111*1*111*11111111*1*1***********

left     TTTTTTTTTTTTTTTTTTTTTTTTTTTTTTTTTTTGAGACGGAGTCTCGCTCTGTCGCCCAGGC
chrom    TTTTTAACTGAAGTTGAAGAGATCTGGGATACATATAGACACAGGCTT---------------C
right    TTTTTAACTGAAGTTGAAGAGATCTGGGATACATATAGACACAGGCTT---------------C
         *****222*2222**2222222*2*2222*222*22****22**2**2222222222222222*
```

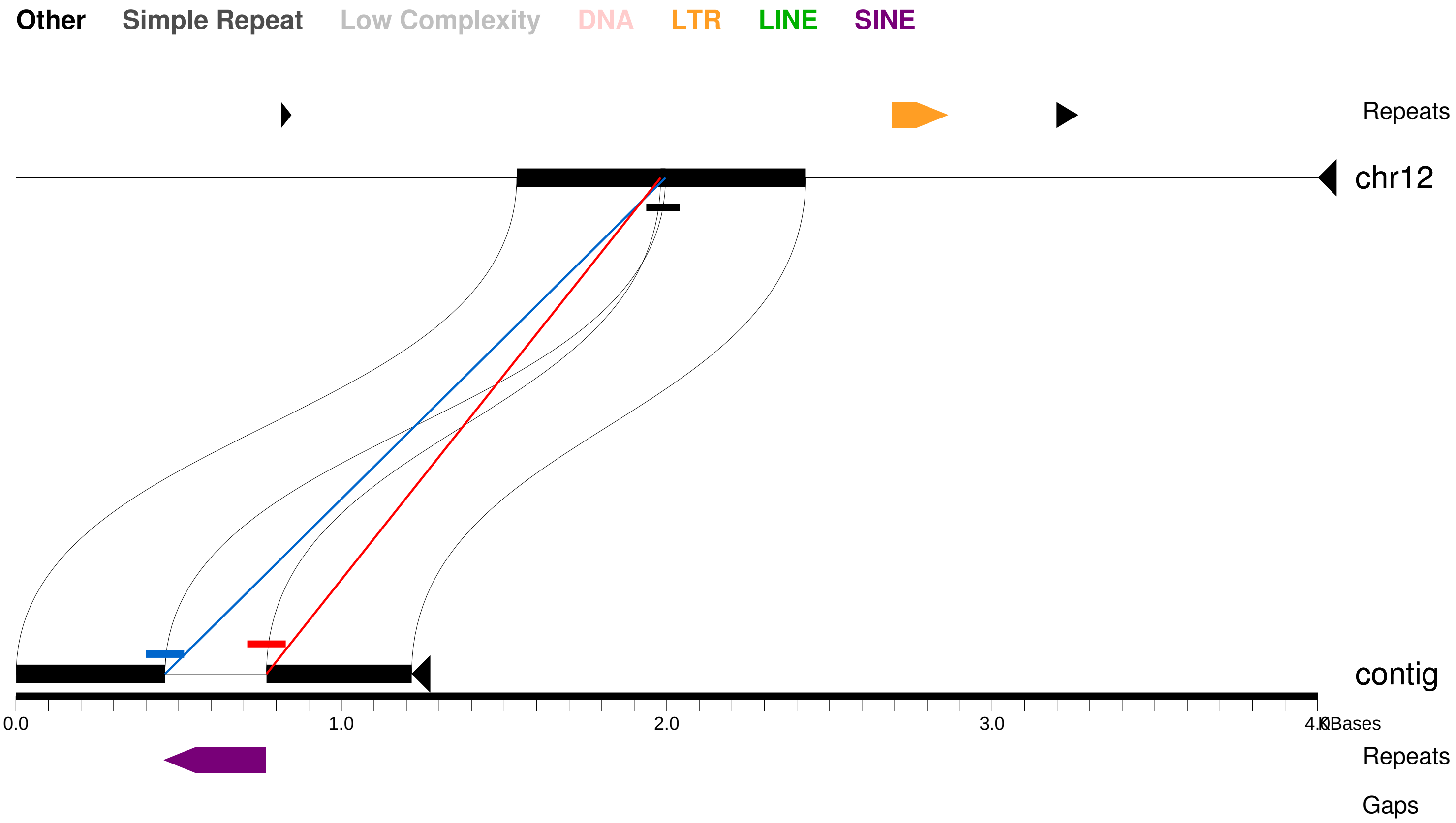

siteID: chr12_25462948 Scafftig1 - Breakpoint: chr12:25462934
chr12:25462774-25463109
Overlap Length: 16 INS size: 313 Genotyped: Yes

```
left   C---------------TGA--CTTGTAGGGTGAAGGGCTACTATGGTGAGAAAAGCCAAATGGAAGCCAT
chrom  CCCCATATTGGCTCCCTGA--CTTGTAGGGTGAAGGGCTACTATGGTGAGAAAAGCCAAATGGAAGCCAT
right  ----AGAATGGCG---TGAACCCGGGAGGCGGAGCTTGCAGTGAGCCGAGATCCCGCCACTGCACTCCAG
       1   2N2N2222N   ***11*11*1***11**111111*1*11*11****11111*1*1**1*11***1

left   TAGAGCTGCCTCTACCTAGAAAAATACTAAATGAAAAACAATATCTCATCCCTGGAGGGATTGTGGAGAT
chrom  TAGAGCTGCCTCTACCTAGAAAAATACTAAATGAAAAACAATATCTCATCCCTGGAGGGATTGTGGAGAT
right  CCTGGGTGACAG-AGCGAGACTCCGTCTCAAAAAAAAAAAAAA---------------------------
       1111*1**1*111*1*1***111111**1**11*****1**1*111111111111111111111111111

left   TAGTGCCACCATCAAGAACTTGAAAGATGCAGGGATGG-GCCGGGCGCGGTGGCTCACGCCTGTA-ATCC
chrom  TAGTGCCACCATCAAGAACTTGAAAGATGCAGGGATGGTGATTCCCAC-----CACATCCCTGTTCAACT
right  ----------AAAAAAAAAAAAAAAATGCAGGGATGGTGATTCCCAC-----CACATCCCTGTTCAACT
       1111111111*11**1**1111***1************2*22222*2*22222*2**22*****22*2*2

left   CAGCACTTTGG---GAGGCCGAGGCGGGCGGATCAC----GAGGTCAGGAGAT---------CGAGACCA
chrom  CGCCTATTTGGTCTGTGCAAAAGACAGATGGATCTCAGAGGATGACAGCAGATTATTGTAAGCTTAACCA
right  CGCCTATTTGGTCTGTGCAAAAGACAGATGGATCTCAGAGGATGACAGCAGATTATTGTAAGCTTAACCA
       *22*22*****222*2*2222**2*2*22*****2*2222**2*2***2****222222222*222****

left   ---------TCCCG--------GCTAAAACGG------TGAAACCCCGTCTC----TACTAAA
chrom  AGTGGTGACTCCAGTTGCAGCTGCTGTACCAGATGTGTTCATAGCTTGAAACAAATTAATACA
right  AGTGGTGACTCCAGTTGCAGCTGCTGTACCAGATGTGTTCATAGCTTGAAA------------
       222222222***2*22222222***22*2*2*222222*2*2*2*22*2221    11N11N1
```

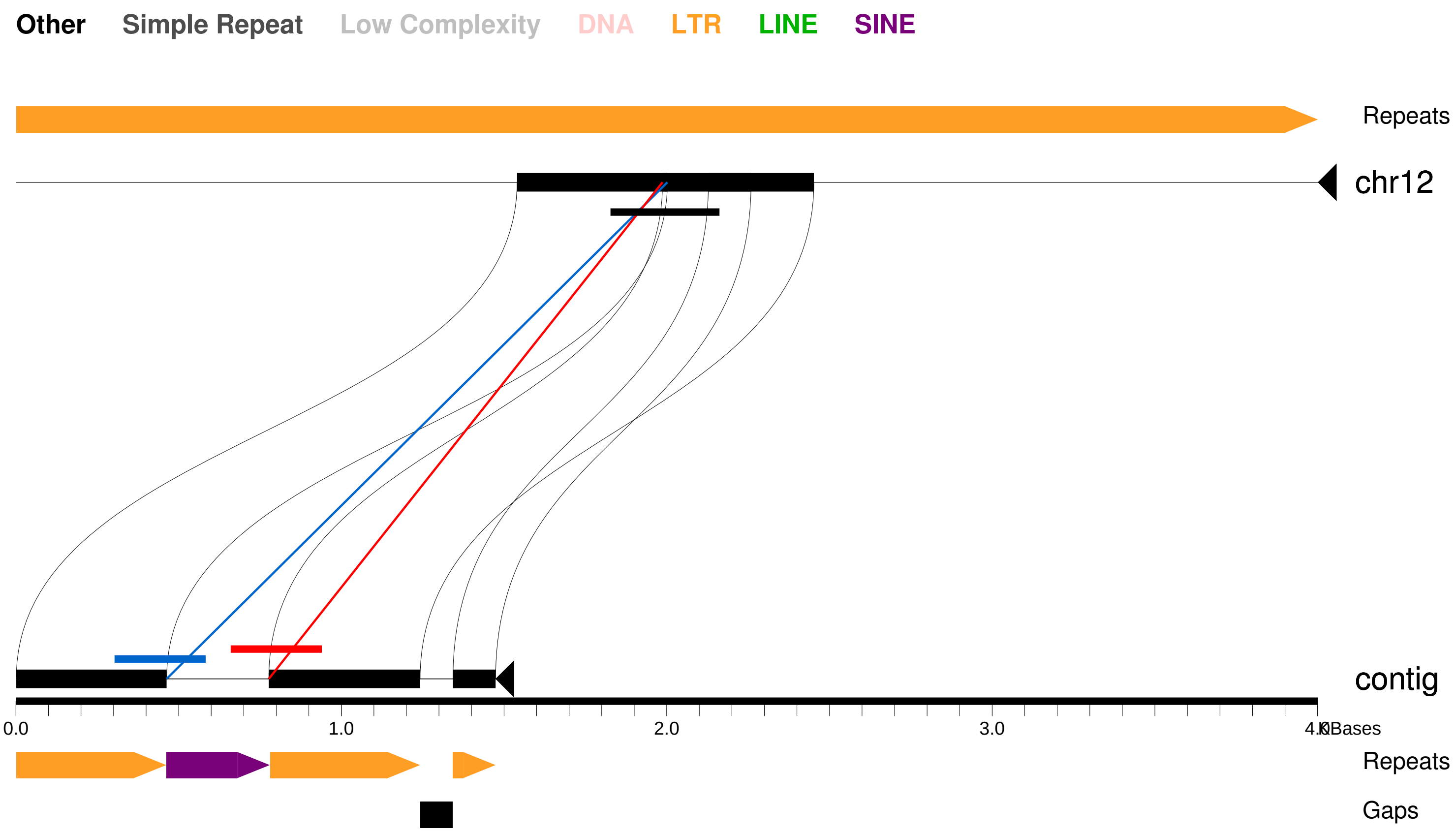

siteID: chr12_26958667 Scafftig14 + Breakpoint: chr12:26958660
chr12:26958601-26958716
Overlap Length: 0 INS size: 398 Genotyped: Yes

```
left    ATAAATT-ACATAG-CACGCTAAGGGATAAACACTATAGAAAAAAGGAA-ACATAAAACA-CGGTAAAAC
chrom   ATAAATT-ACATAG-CACGCTAAGGGATAAACACTATAGAAAAAAGGAA-ACATAAAACA-CGGCTCTAC
right   AGAAGGTGAGGGAGACATGC-AGGAGAACTGCATTCCAGGCAGAGGGAATAGCCAGGGCAACGACTCTAC
        *1**11*1*111**1**1**1*1*1**1111**1*11**11*1*1****1*111*111**1**12222**

left    TGGCCGGGCACCGTGGCTCACACCTGTAATCCCAGCACTTT--GGAAGGCCAA---GG
chrom   AGT---GGGACCGTGT-TCA---CTATGTTCCAAGACCTGCAAGGAAG-CTAATATGG
right   AGT---GGGACCGTGT-TCA---CTATGTTCCAAGACCTGCAAGGAAG-CTAATATGG
        2*2222**2*****22***222**2*22***2**22**2222*****2*2**222**
```

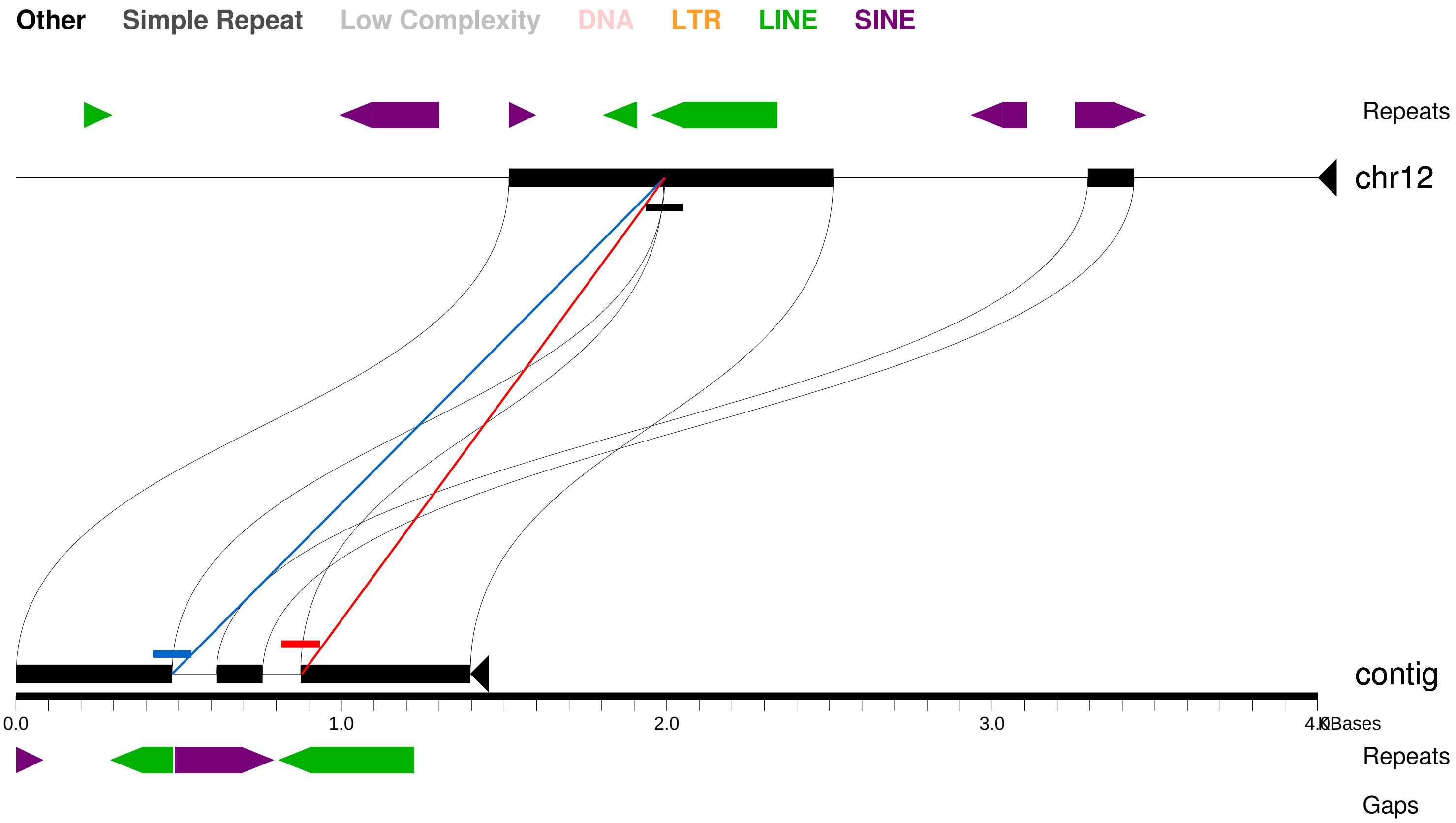

siteID: chr12_27864822 Scafftig2 + Breakpoint: chr12:27864796
chr12:27864766-27864855
Overlap Length: 16 INS size: 296 Genotyped: Yes

```
left     AATCCTTCATA--------------CTGGCTCTTCATAGGT---CTA------------ATAATTGTCTA
chrom    AATCCTTCATA--------------CTGGCTCTTCATAGGT---CTA------------ATAATTGTCTA
right    CCGCCCGCCTCGGCCTCCCAAAGTGCTGGGATTACAGGCGTGAGCCACCGCGCCCGGCCATAATTGTCTA
         111**11*1*1111111111111111****111*1**111**111*1*111111111111***********

left     TTTTTTTTTTTTTTTTTTTTGAGACGGAGTCTCGCTCTGTCGCCCAGGCTGGAGTGCAGTGGCGGGATCTC
chrom    TTTTTAAATTTTTCATTCTTAA-----ATTCT-----------------TGGAATAC-------TTAACTC
right    TTTTTAAATTTTTCATTCTTAA-----ATTCT-----------------TGGAATAC-------TTAACTC
         *****222****22**2**2*22222*2***22222222222222222****2*2*222222222*2***

left     GGCTCACT
chrom    CTATTGTA
right    CTATTGTA
         222*2222
```

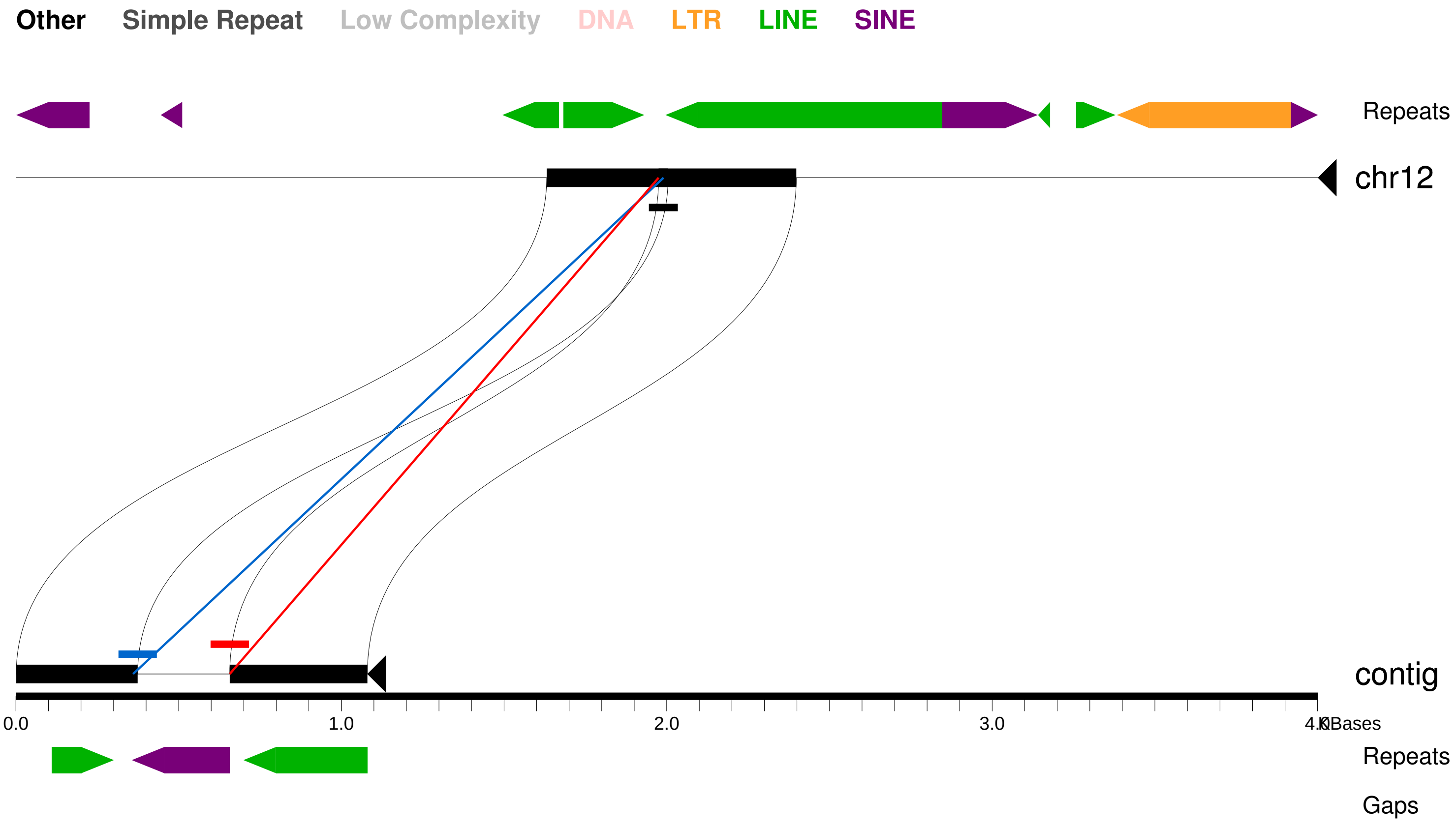

siteID: chr12_28163311 Scafftig1 + Breakpoint: chr12:28163320
chr12:28163272-28163379
Overlap Length: 12 INS size: 445 Genotyped: No

```
left    TGCTCTAGGAGTTATCAGAAAGAGAAGAGAGTAAAATCTAACCTGT-----------ATAAAAAGATGTG
chrom   TGCTCTAGGAGTTATCAGAAAGAGAAGAGAGTAAAATCTAACCTGT-----------ATAAAAAGATGTG
right   NNNNNNNNNNNNNNNNNNNNNNNNNNNNNNNNNNNNNNNNNNNNNNNNNNNNNNNNAAAAAAAAGATGTG
        111111111111111111111111111111111111111111111111111111111*1**********

left    GGCCGGGCGCGGTGGCTCACGCCTGTAATCCCAGCACTTTGGGAGGCCGAGGCGGGTGGA
chrom   GACTC-------TGGTAGATTTCTTAAAAGGAAGGAACTAGAGAT-CATAGG---GACTA
right   GACTC-------TGGTAGATTTCTTAAAAGGAAGGAACTAGAGAT-CATAGG---GACTA
        *2*222222222***222*222**22**2222**2*22*2*2**22*22***222*222*
```

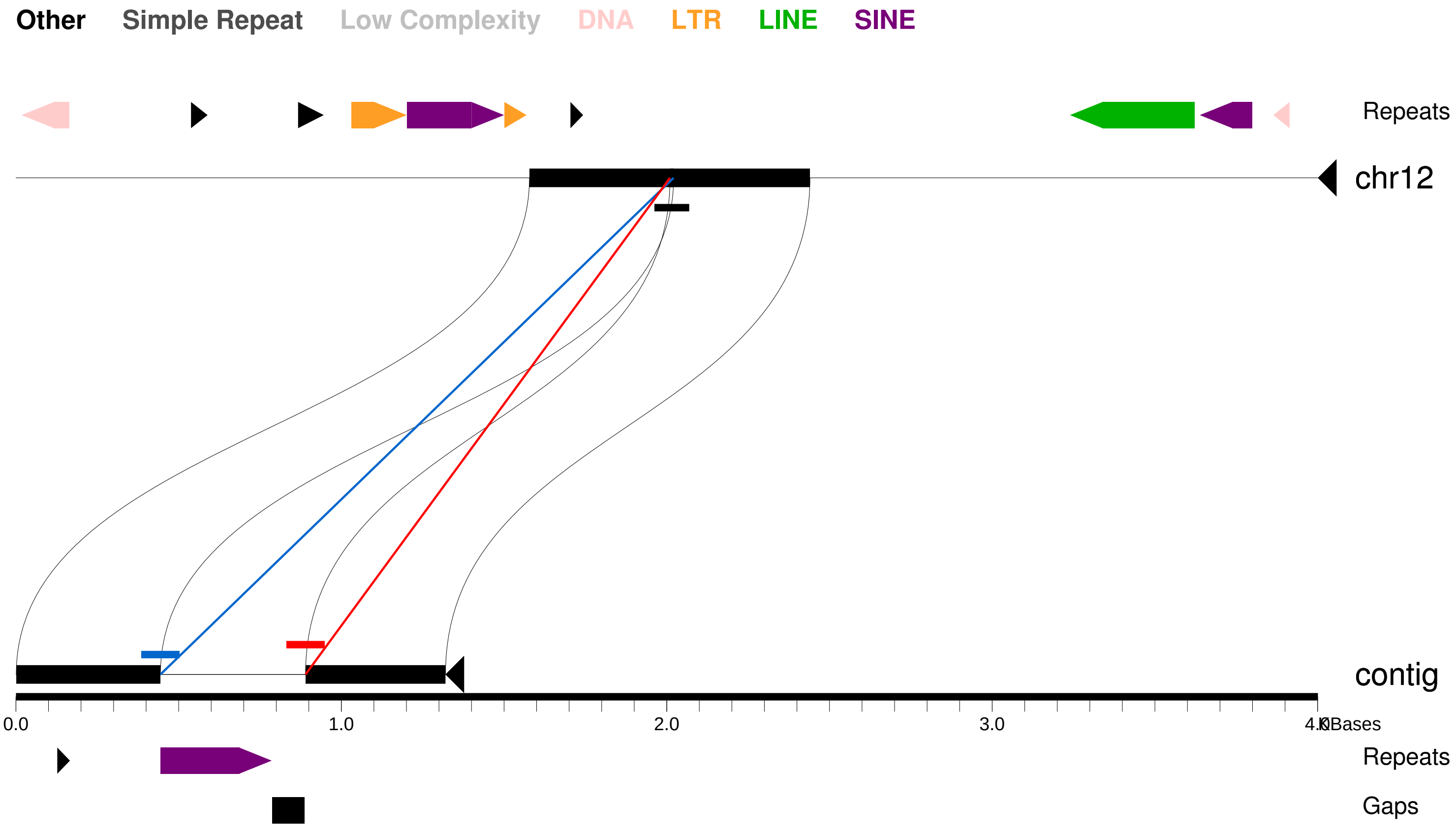

siteID: chr12_28438609 Scafftig6 + Breakpoint: chr12:28438597
chr12:28438563-28438656
Overlap Length: 8 INS size: 306 Genotyped: Yes

```
left   -----------GG------ATAATACTTAGATGA--------ACCCTGCCAGTGTTTATCTGTGTTTTTT
chrom  -----------GG------ATAATACTTAGATGA--------ACCCTGCCAGTGTTTATCTGTGTTTCTT
right  CCACCCGCCTCGGCCTCCCAAAGTGCTGGGATTACAGGCGTGAGCCACCGCGCCCGGCCCTGTGTTTCTT
       11111111111**111111*1*1*1**11***1*11111111*1**11*11*1111111********2**

left   TTTTTTTTTTTTTTTGAGACGGAGTCTCGCTCTGTCGCCCAGGTCGGACTGCGGACTGCAGTGGCGCAAT
chrom  TATTTAAATTTATTTC---------------CTTTATTTAAGTTTCTATGGCTGATTTCC------CAAT
right  TATTTAAATTTATTTC---------------CTTTATTTAAGTTTCTATGGCTGATTTCC------CAAT
       *2***222***2***2222222222222222**2*22222**2*222*22**2**2*2*2222222****

left   CTCG
chrom  ----
right  ----
       2222
```

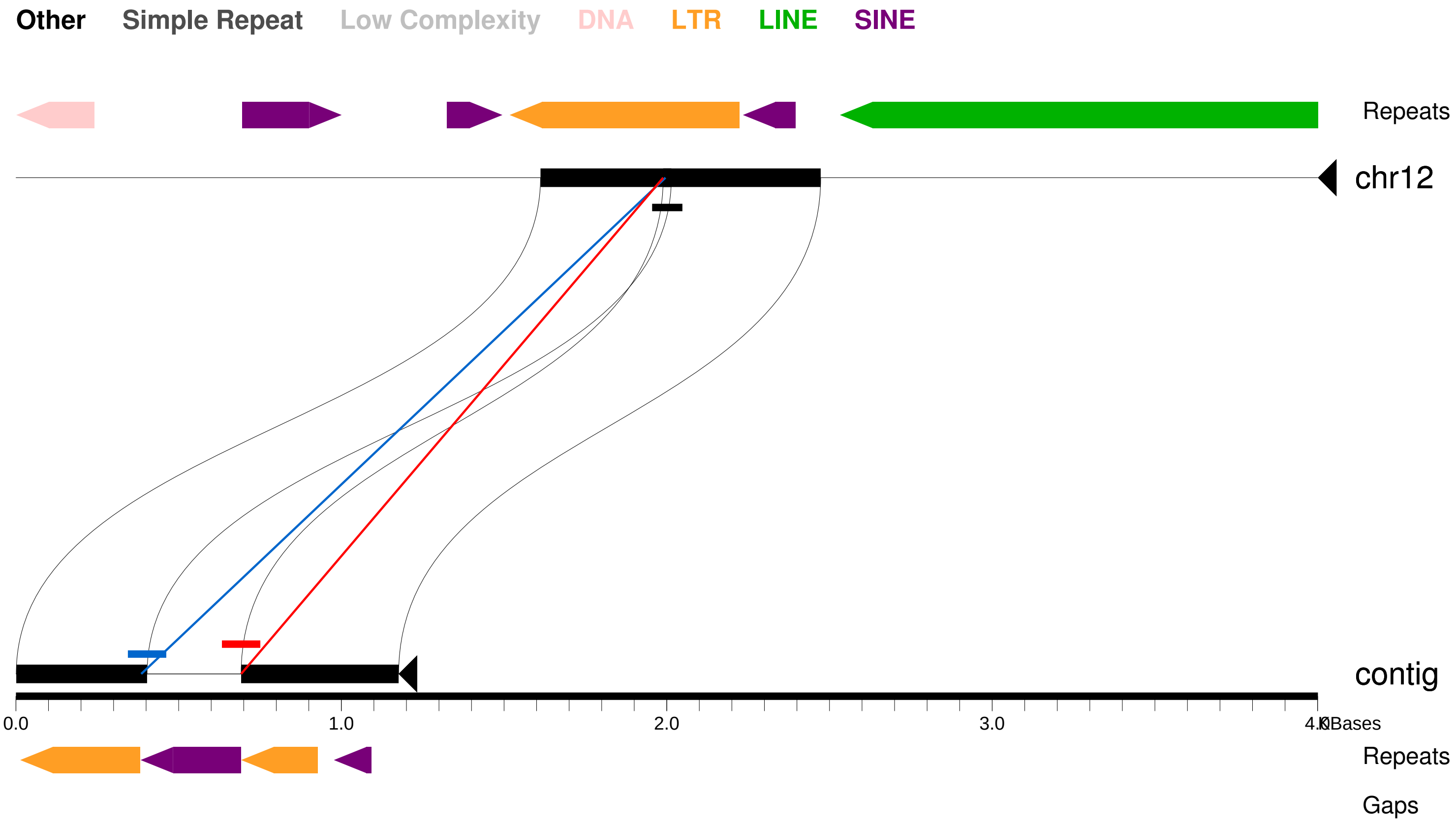

siteID: chr12_30420638 Scafftig5 + Breakpoint: chr12:30420646
chr12:30420611-30420705
Overlap Length: 12 INS size: 303 Genotyped: Yes

```
left    TAGTTT---------------AGAGCTGCTTGTAAAATTTT---------CTCACTTTTAAATTTCTTTT
chrom   TAGTTT---------------AGAGCTGCTTGTAAAATTTT---------CTCACTTTTAAATTTCTTTT
right   CCGCCCGCCTCGGCCTCCCAAAGTGCTGGGATTACAGGCGTGAGCCACCGCGCCCGGCCAAATTTCTTTT
        11*111111111111111111**1****1111**1*1111*111111111*1*1*1111***********

left    TTTTTTTTTTTTTTTTTTTTTTTGAGACGGAGTCTTGCTCTGTCACCCAGGCTGGAGTGTAGTGGCACGA
chrom   TAAATTATTGATTT--------------GTACTCTAGTTTTCTAACAC---------TGCAGTTCCATCA
right   TAAATTATTGATTT--------------GTACTCTAGTTTTCTAACAC---------TGCAGTTCCATCA
        *222**2**22***22222222222222*2*2***2*2*2*2*2**2*222222222**2***22**22*

left    TCT
chrom   GA-
right   GA-
        222
```

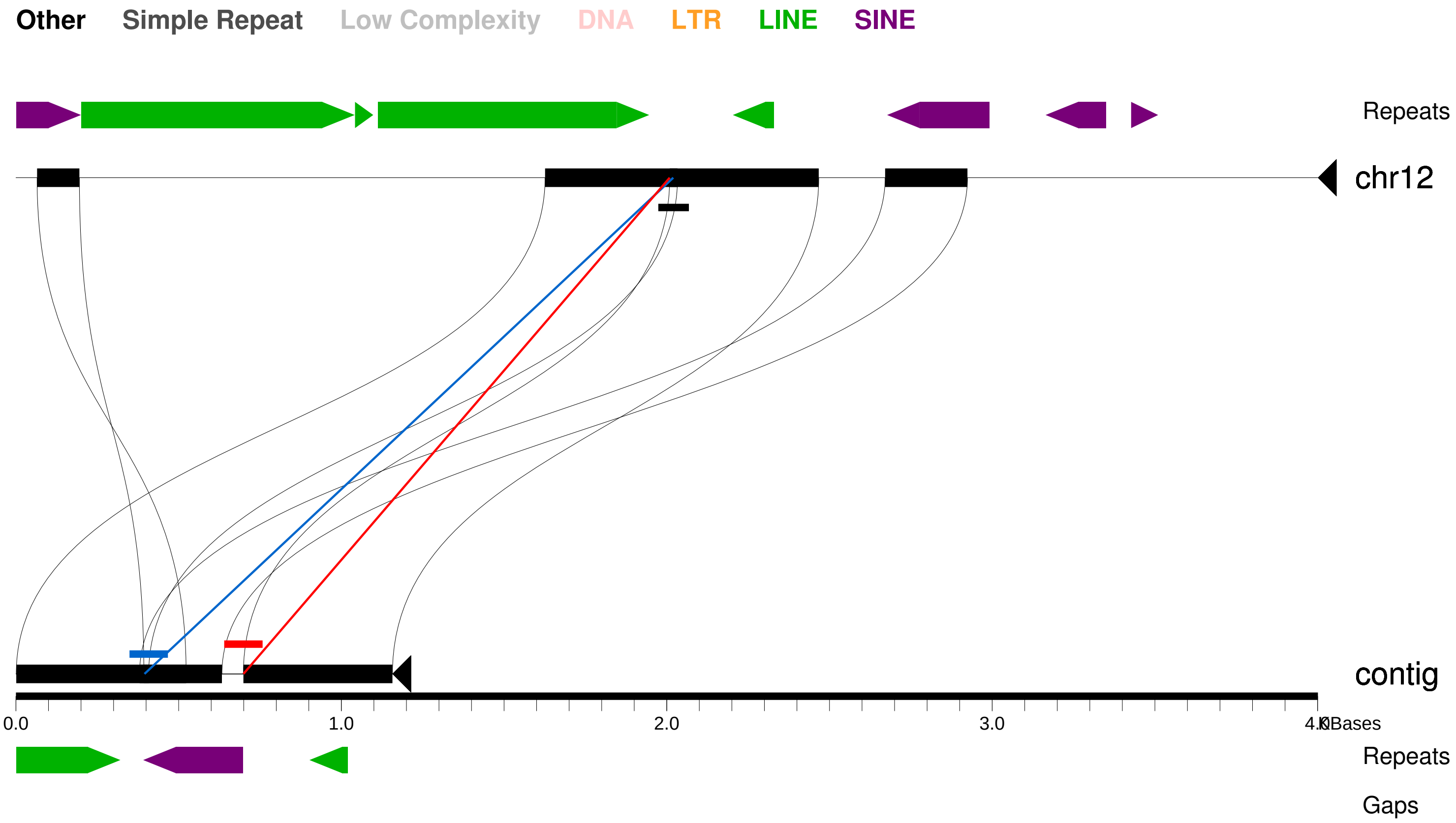

siteID: chr12_30471057 Scafftig4 - Breakpoint: chr12:30471069
chr12:30470909-30471229
Overlap Length: 1 INS size: 297 Genotyped: Yes

```
left   -ATTGTTTTAAAATCATTTTTAATATTTGTGCATGTATGTTCACAACAGGCTTAT-TTACAAT-AGCTAA
chrom  TATTGTTTTAAAATCATTTTTAATATTTGTGCATGTATGTTCACAACAGGCTTAT-TTACAAT-AGCTAA
right  AG--GCAGGAGAATG---------------GCGTGAA----CCCGGGAGGCGGAGCTTGCAGTGAGCCGA
       N111*1111*1***1111111111111111**1**1*1111*1*111****11*11**1**1*1***11*

left   AAGATGGAAACAACCCAAGAGCTCACTGATGGATGAATGGATAAACATAGTGTG-CTACGGATATAAGTG
chrom  AAGATGGAAACAACCCAAGAGCTCACTGATGGATGAATGGATAAACATAGTGTG-CTACGGATATAAGTG
right  --GAT--------CCCGCCACTGCACTCCAGCCTGGGCG-----ACAGAGCGAGACTCCGTCTCAAAAAA
       11***11111111***111*111****111*11**111*11111***1**1*1*1**1**11*11**111

left   AAAATTATTTAGCCTTAAAAAGGAGGCCGGGCGCGGTGGCTCACGCCTGTAA--TCCCAGCACTTTGGGA
chrom  AAAATTATTTAGCCTTAAAAAGGAAA---GAAACTCTAGCACACAC-TGCAACATAGAGGAACCTTGAGG
right  AAAA-------------AAAAAAAAA---AAAACTCTAGCACACAC-TGCAACATAGAGGAACCTTGAGG
       ****1111111111111****11*222221222*22*2**2***2*2**2**22*2222*2**2***2*2

left   GGCCGAGGCGG-GCGGATCACG--AGGTC----AGGAGATCGA--------------------GACCATC
chrom  ATATTATGCTAAGTGAATTAAGCCAGGCACTAAAGGAGAAACACTGTATAATTCCTTTTATATGAGCTTC
right  ATATTATGCTAAGTGAATTAAGCCAGGCACTAAAGGAGAAACACTGTATAATTCCTTTTATATGAGCTTC
       22222*2**222*2*2**2*2*22***222222*****222*22222222222222222222**2*2**

left   CTGG-CTAA-------CACGGTGAAACCCCGTCTCTACTAA-------A------
chrom  CTAGACTCATCAAATTCATGGAGATAGAGAGT-TAAACAATGTTTGCCA------
right  CTAGACTCATCAAATTCATGGAGATAGAGAGT-TAAACAATGTTTGCCAAAGGCT
       **2*2**2*2222222**2**2**2*2222**2*22**2*22222222*111111
```

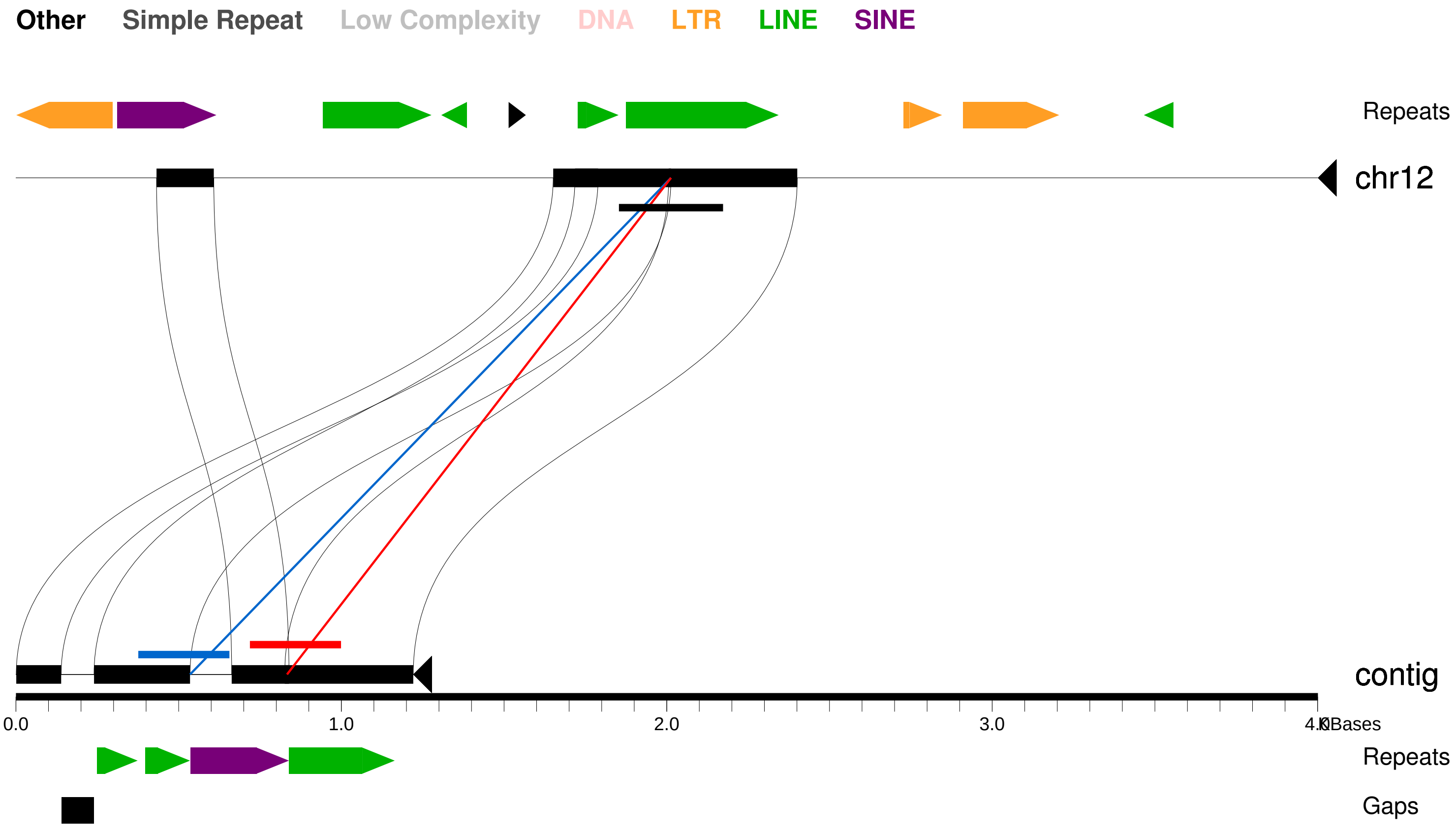

siteID: chr12_31120755 Scafftig3 + Breakpoint: chr12:31120731
chr12:31120697-31120790
Overlap Length: 13 INS size: 305 Genotyped: Yes

```
left    ------------CTCTGCC------AGGGC---GCTAGCAAAC---AGCAAACTC-CATGGCCTAGAATC
chrom   ------------CTCTGCC------AGGGC---GCTAGCAAAC---AGCAAACTC-CATGGCCTAGAATC
right   TGATCCGCCCGCCTCGGCCTCCCAAAGTGCTGGGATTACAGGCGTGAGCCACCGCGCCCGGCCTAGAATC
        111111111111***1***111111**1**111*1*11**11*111***1*1*1*1*11***********

left    TGTTTTTTTTTTTTTTTTTTTTTTTTTTTTGAGACGGAGTCTCGCTCTGTCGCCCAGGCTGGAGTGCAGT
chrom   TGCTTTCTTTGCTTTATCTGC---------------------CCCTCTGTGAAAGAACC---AATG-ACA
right   TGCTTTCTTTGCTTTATCTGC---------------------CCCTCTGTGAAAGAACC---AATG-ACA
        **2***2***22***2*2*22222222222222222222222*2******22222*22*222*2**2*22

left    GGCG
chrom   ACTG
right   ACTG
        222*
```

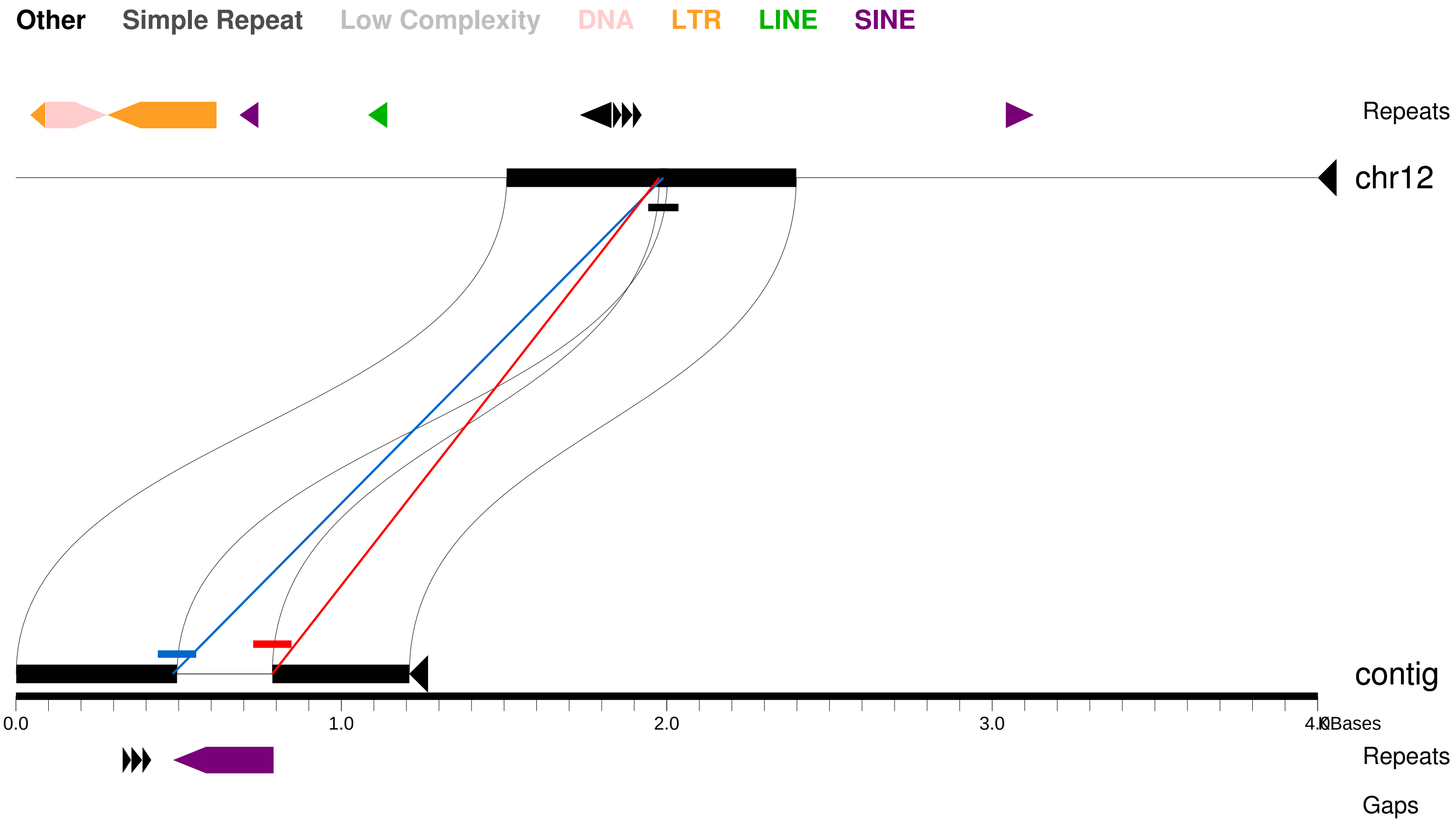

siteID: chr12_31406641 Scafftig4 + Breakpoint: chr12:31406636
chr12:31406583-31406695
Overlap Length: 7 INS size: 504 Genotyped: No

```
left    GAGGTGACCA--GATGTCAATAATGTTGGGAATTTAAAATGCATGTACCACACAT-------GAACACAN
chrom   GAGGTGACCA--GATGTCAATAATGTTGGGAATTTAAAATGCATGTACCACACAT-------GAACACAA
right   CCGCCCGCCTCGGCCTCCCAAAGTGCTGGGATT---ACAGGCGTGAGCCACCGCGCCCGGCCGAACACAA
        11*1111**111*1111*1*1*1**1*****1*111*1*1**1**11****11111111111*******2

left    NNNNNNNNNNNNNNNNNNNNNNNNNNNNNNNNNNNNNNNNNNNNNNNNNNNNNNNNNN
chrom   TTCTTTAAATGCAGCTGCAGGAAGTTCCGTTTGGGGGTCATCCTCTTTGAAA------
right   TTCTTTAAATGCAGCTGCAGGAAGTTCCGTTTGGGGGTCATCCTCTTTGAAA------
        2222222222222222222222222222222222222222222222222222222222
```

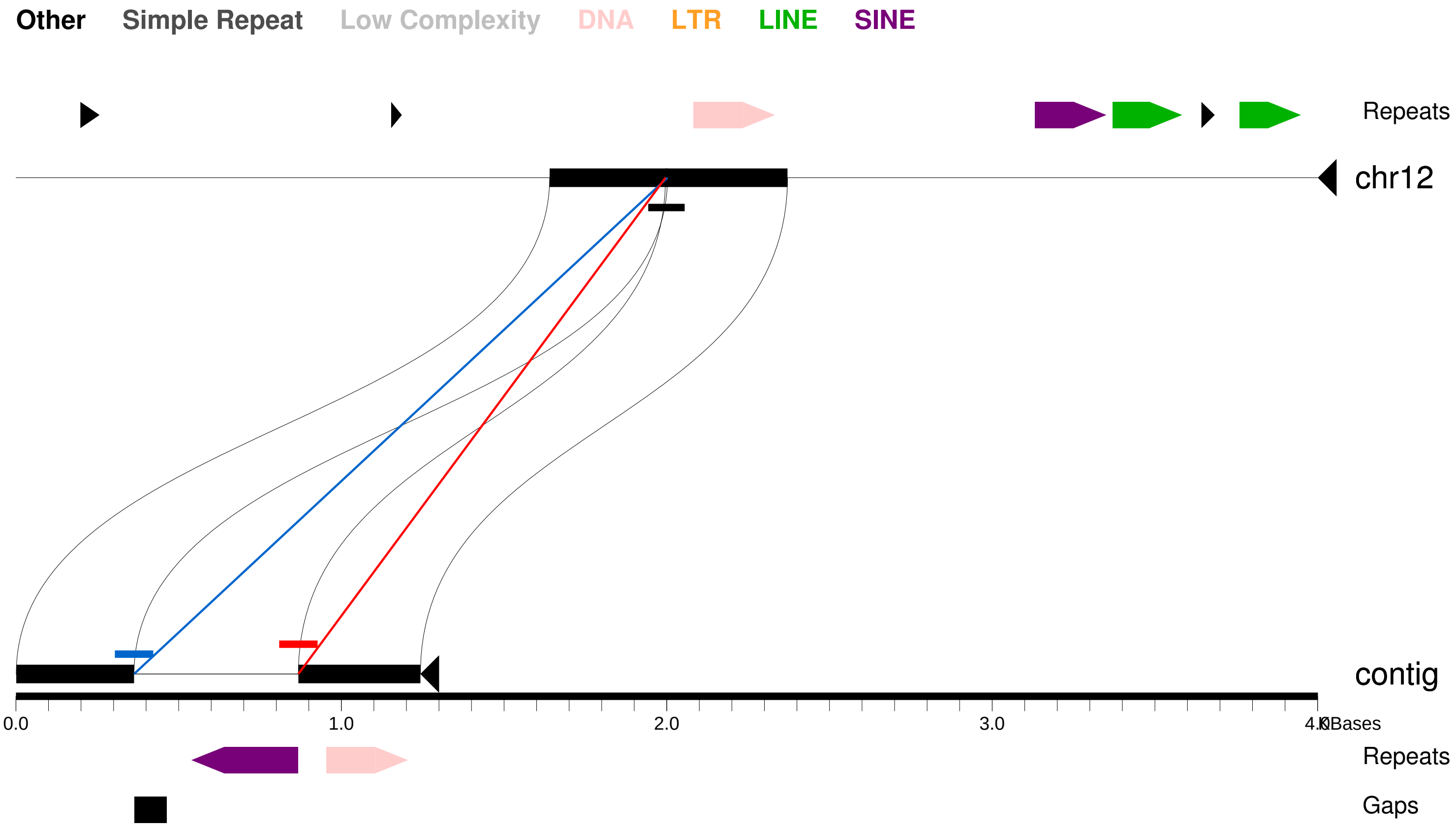

siteID: chr12_32076411 Scafftig4 - Breakpoint: chr12:32076414
chr12:32076368-32076473
Overlap Length: 14 INS size: 300 Genotyped: Yes

```
left    CATGACT---TCCTGGG--ACAAATCTAT--------TCCAGAACCAAAGGGCTTACTGAAATAATTGAT
chrom   CATGACT---TCCTGGG--ACAAATCTAT--------TCCAGAACCAAAGGGCTTACTGAAATAATTGAT
right   CTGCACTCCAGCCTGGGCGACAGAGCGAAACTCCGTCTCAAAAAAAAAAAAAAAAAAAAAAATAATTGAT
        *111***1111*****11***1*1*1*111111111**1*1**11***111111*111***********

left    CTCGGCCAGGCGCGGTGGCTCACGCCTGTAATCCCAGCACTTTGGGAGGCCGAGACGGGCGG
chrom   CTCAGT-------GGTTTCTAAGGAAA-TATTTAAATCAAAAGGGGA-----AAATCTGAAA
right   CTCAGT-------GGTTTCTAAGGAAA-TATTTAAATCAAAAGGGGG-----AAATCTGAAA
        ***2*22222222***22**2*2*2222**2*222*2**2222***122222*2*222*222
```

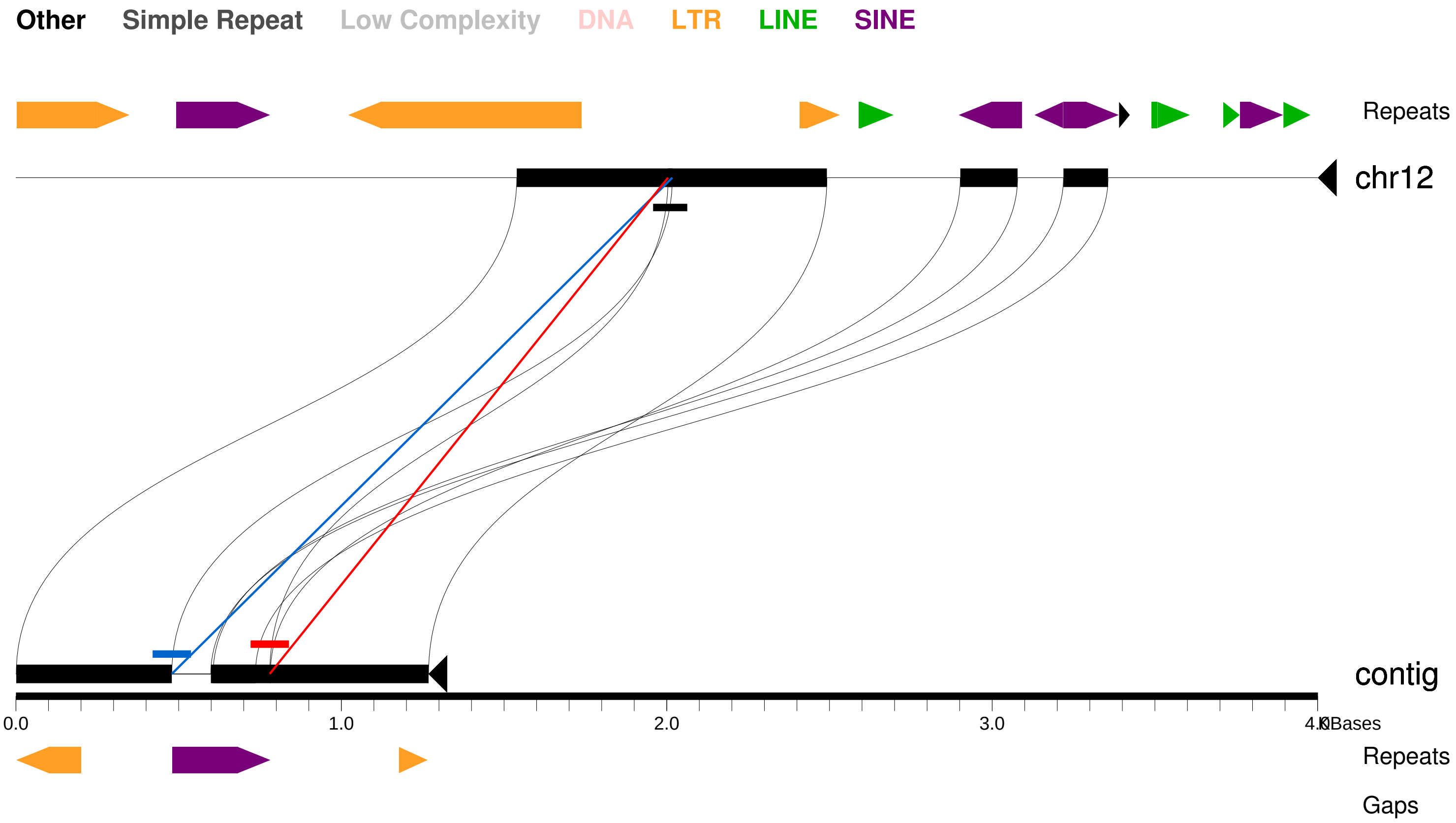

siteID: chr12_32358645 Scafftig2 + Breakpoint: chr12:32358650
chr12:32358603-32358709
Overlap Length: 13 INS size: 483 Genotyped: No

```
left   TG------------ATGTATAAGTGATCAATTTGCACTCTTTAAATATGTGCAATATATTATATGTCAAT
chrom  TG------------ATGTATAAGTGATCAATTTGCACTCTTTAAATATGTGCAATATATTATATGTCAAT
right  CCGCCCGCCTCGGCCTCCCAAAGTGCTGGGATTACAGGCGTGAGCCACCGCGCCCGGCCTATATGTCAAT
       111111111111111*1111*****1*1111**1**11*1*1*111*111111111111***********

left   TTGTTTTNNNNNNNNNNNNNNNNNNNNNNNNNNNNNNNNNNNNNNNNNNNNNNNNNNNNNN
chrom  TT------------TGACATATGTATATACTTGCGAAATCATCAACACAACCAAGATAATG
right  TT------------TGACATATGTATATACTTGCGAAATCATCAACACAACCAAGATAATG
       **22222222222222222222222222222222222222222222222222222222222
```

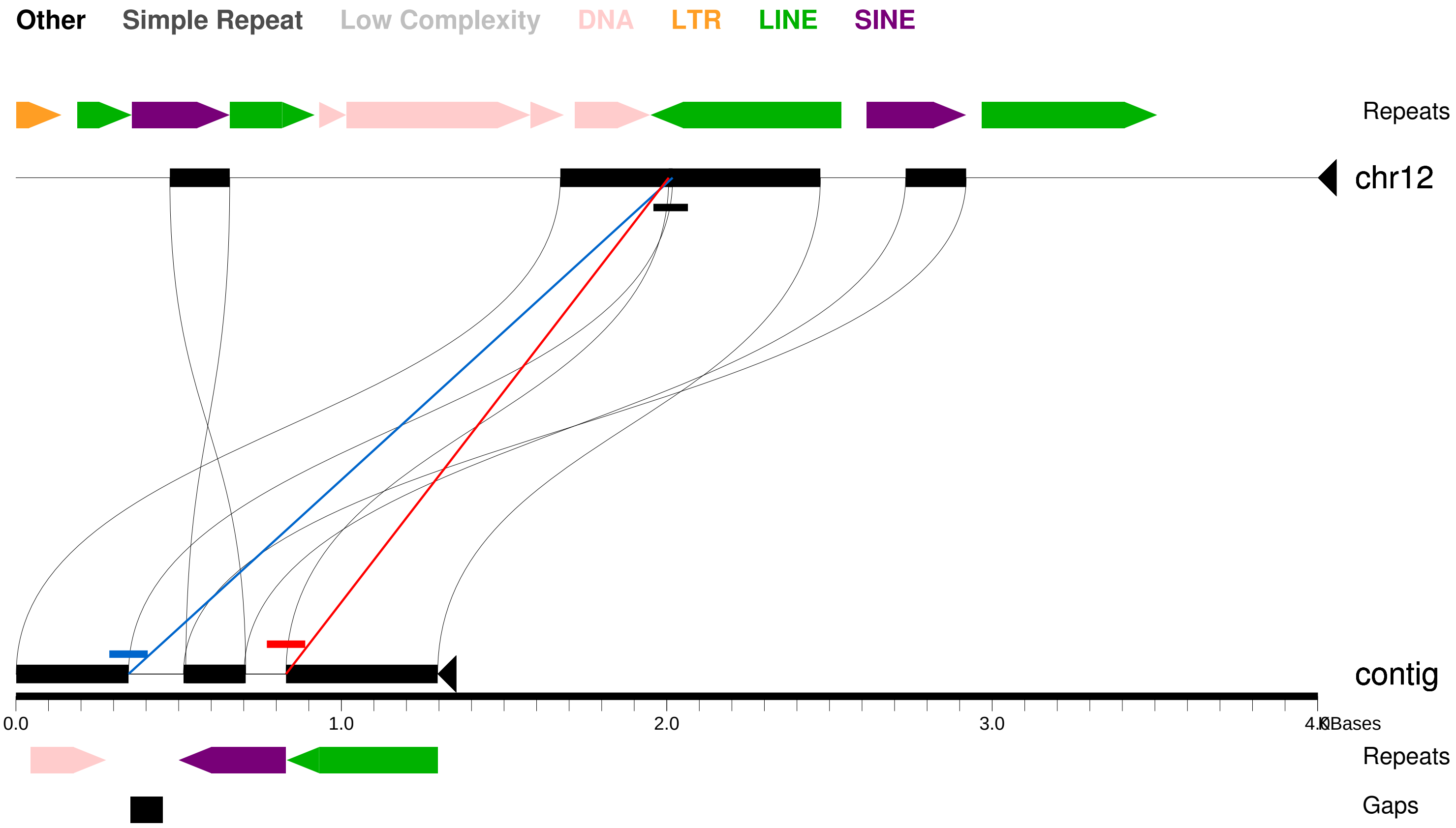

siteID: chr12_32658839 Scafftig3 + Breakpoint: chr12:32658848
chr12:32658823-32658907
Overlap Length: 35 INS size: 347 Genotyped: No

```
left   --------------TTCGCATAA-----GGCAAACAGAC-TGAAT--------------CTCTTGTATAT
chrom  --------------TTCGCATAA-----GGCAAACAGAC-TGAAT--------------CTCTTGTATAT
right  CCACCCGCCTCGGCCTCCCAAAGTGCTGGGATTACAGGCGTGAGCCACCGCGCCCGGCCCTCTTGTATAT
       111111111111111**1**1*111111**111****1*1***1111111111111111***********

left   TTTTAGATTCCTGTTATACATCCCNNNNNNNNNNNNNNNNNNNNNNNNNNNNNNNNNNNNNNNNNNNNNN
chrom  TTTTAGATTCCTGTTATACATCCCTAGATGTTTATTTTCACATAATTTA---------------------
right  TTTTAGATTCCTGTTATACATCCCTAGATGTTTATTTTCACATAATTTA---------------------
       ************************2222222222222222222222222222222222222222222222

left   NNNNNNNNNNNNN
chrom  -------------
right  -------------
       2222222222222
```

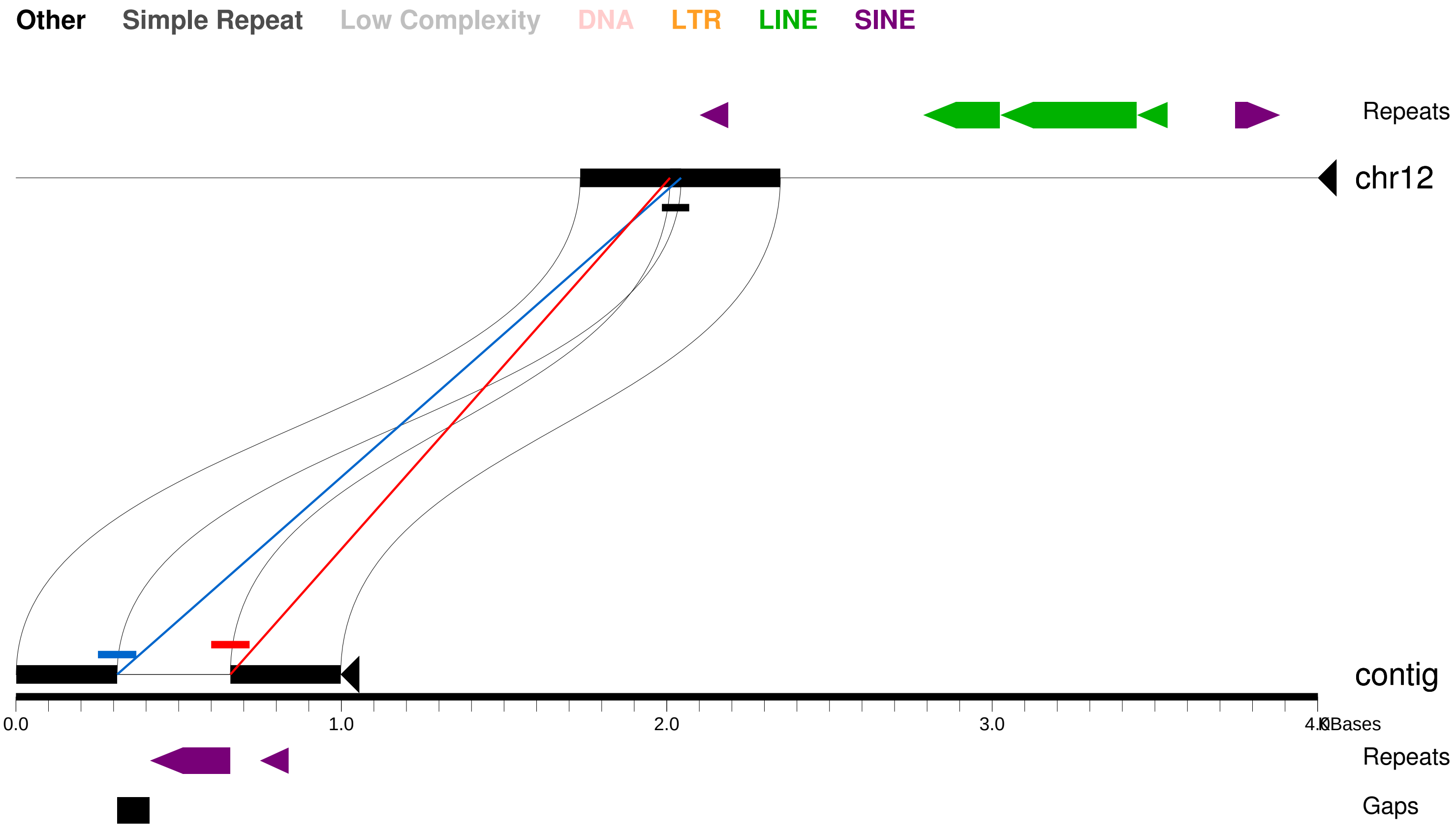

siteID: chr12_40210415 Scafftig2 - Breakpoint: chr12:40210386
chr12:40210348-40210435
Overlap Length: 22 INS size: 286 Genotyped: Yes

```
left     TTGTGCACA-----------TGTAC-----CCT-------AGAACTT-ACTT-------AAAGTATAATA
chrom    TTGTGCACA-----------TGTAC-----CCT-------AGAACTT-ACTT-------AAAGTATAATA
right    GTGAGCCGAGATCGCGCCACTGCACTCCAGCCTGGGCGACAGAGCGAGACTCCGTCTCAAAAAAAAAAAA
         1**1**11*11111111111**1**11111***1111111***1*111***11111111***11*1**1*

left     AAAAAAAAGAAAGAAAAATCAGCGCGGTGGCTCACGCCTGTAATCCCAGCACTTTGGGAGGCCGAGGCGG
chrom    AAAAAAAAGAAAGAAAAATCA------AGGGTAA-----GTAATACAAGAATTAT---------------
right    AAAAAAAAAAAAGAAAAATCA------AGGGTAA-----GTAATACAAGAATTAT---------------
         ********1************2222222**2*2*22222*****2*2**2*2*2*222222222222222

left     GCGGATCACG
chrom    ----ATTA-G
right    ----ATTA-G
         2222**2*2*
```

Other Simple Repeat Low Complexity DNA LTR LINE SINE

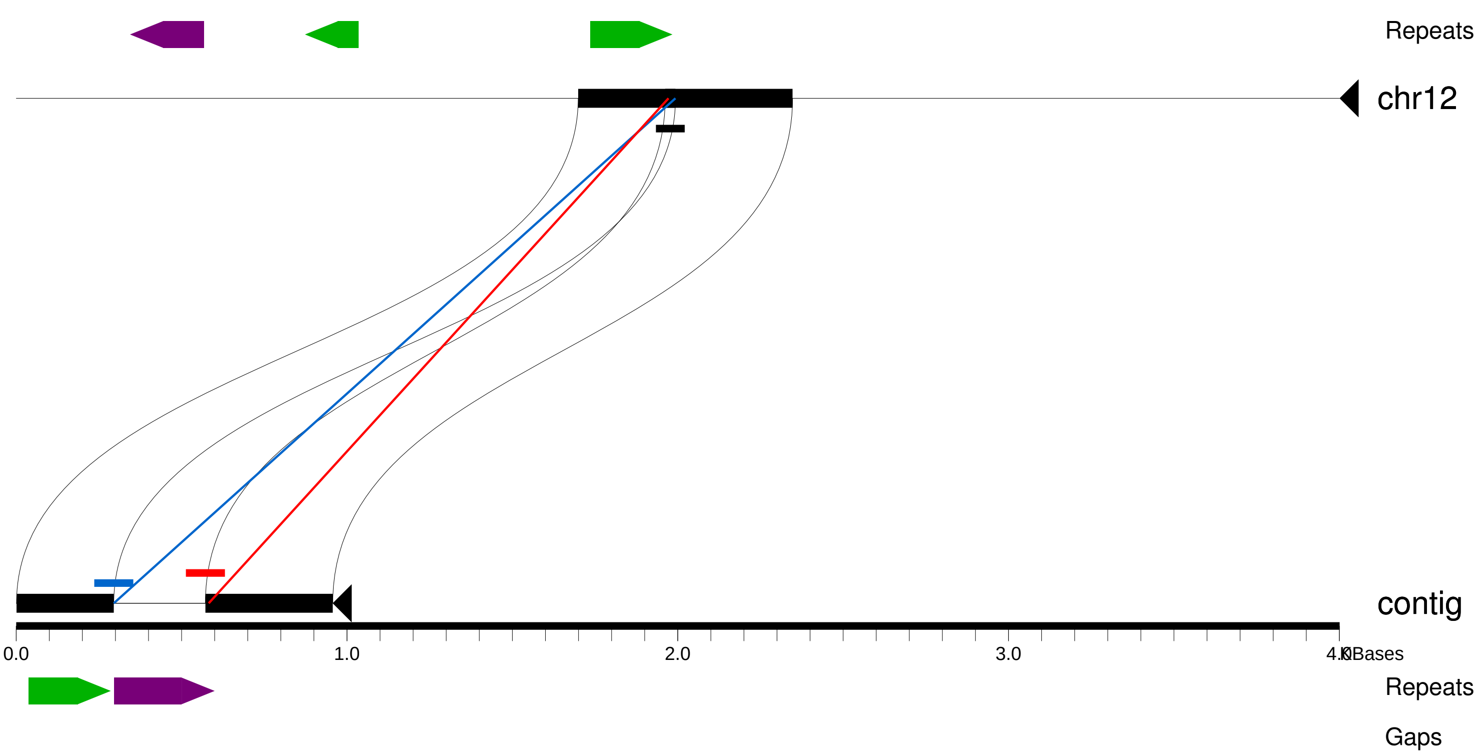

siteID: chr12_40673031 Scafftig1 + Breakpoint: chr12:40673010
chr12:40672968-40673069
Overlap Length: 18 INS size: 319 Genotyped: Yes

```
left     ATTTAATACACAT-----TATTAAA-TAAT--CAATAAAGTACTATGTTT---------GCCAATAGTTT
chrom    ATTTAATACACAT-----TATTAAA-TAAT--CAATAAAGTACTATGTTT---------GCCAATAGTTT
right    GATCCGCCCGCCTCGGCCTCCCAAAGTGCTGGGATTACAGGCGTGAGCCACCGCGCCCGGCCAATAGTTT
         11*11111*1*1*11111*111***1*11*111*1**1**111*11*111111111111***********

left     ACTTTTTTTTTTTTTTTTTTTTTTTTTTTTTTTTTTTTTTTTTTTTTTGAGACGGAGTCTCGCTCT
chrom    ACTTTTTAAACCTTACTGTATTTAATATCCCTACTGTATTT--------------AATATCCC---
right    ACTTTTTAAACCTTACTGTATTTAATATCCCTACTGTATTT--------------AATATCCC---
         ******22222**22*2*2***22*2*222*22*2*2***22222222222222*2*2**2*222
```

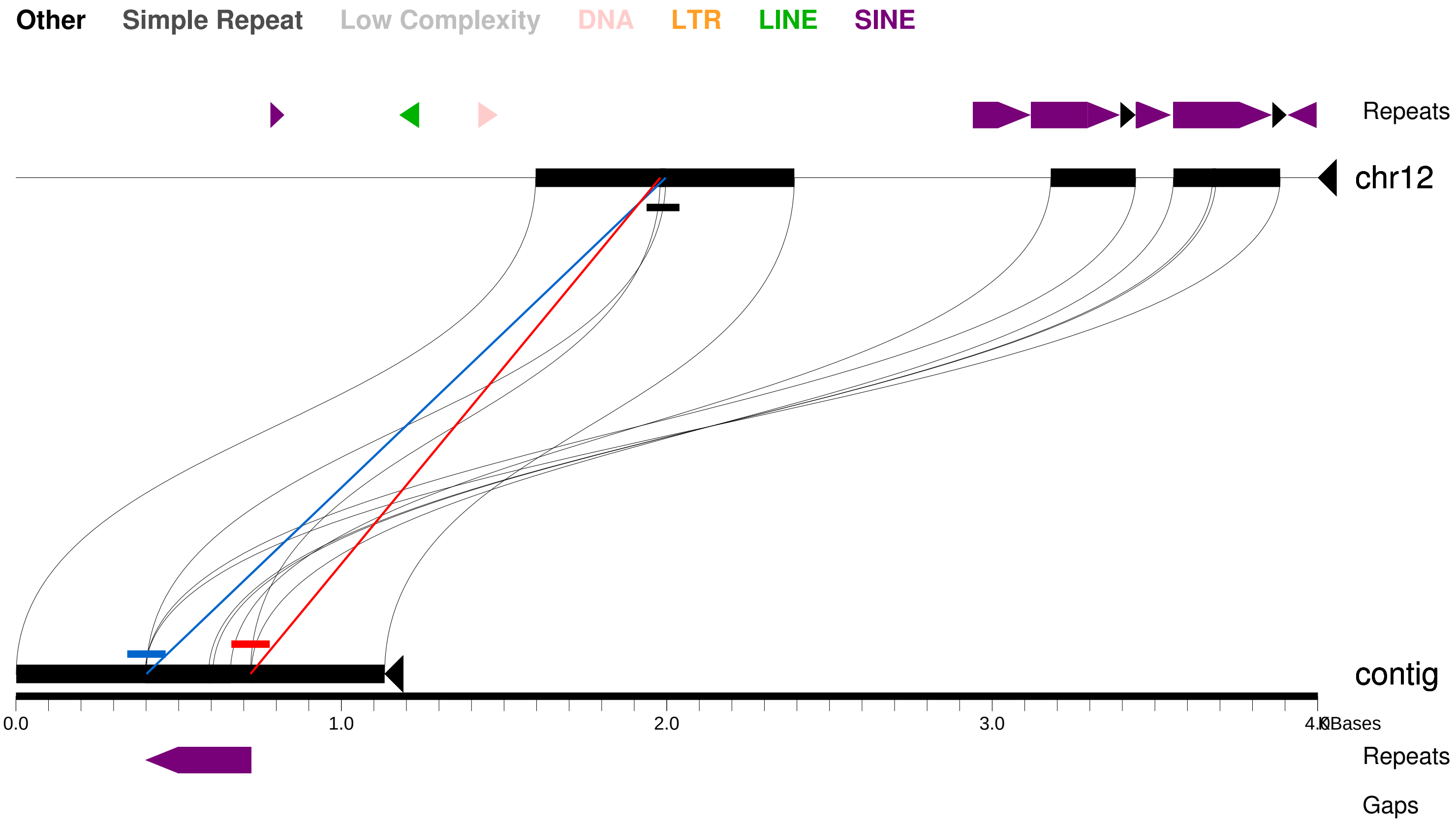

siteID: chr12_41847721 Scafftig3 - Breakpoint: chr12:41847712
chr12:41847664-41847768
Overlap Length: 12 INS size: 229 Genotyped: Yes

```
left    AAACAGAGTT-TCCTGTTT----------ATAAGGGTGCGTTTTTCAGCTCACTTTAAA---ATTTAATC
chrom   AAACAGAGTT-TCCTGTTT----------ATAAGGGTGCGTTTTTCAGCTCACTTTAAA---ATTTAATC
right   CGCCACTGCACTCCCGCCTGGGCCGCAGAGCAAGACTCCGTCTCAAAAAAAAAAAAAAAAAAATTTAATC
        111**11*111***1*11*111111111111***11*1***1*111*1111*1111***111********

left    TCCTGATCGAGACCATCCTGGCTAACACGGTGAAACCCCGTCTCTACTAAAAAAATACAAAAA
chrom   TCCTTCT-GAAATGTTTGATCTTTATAAGATGAA--------------TTTAATAGCTGTAACA
right   TCCTTCT-GAAATGTTTGATCTTTATAAGATGAA--------------TTTAATAGCTGTAACA
        ****22*2**2*222*222222*2*2*2*2****2222222222222*22**2*22222**2*
```

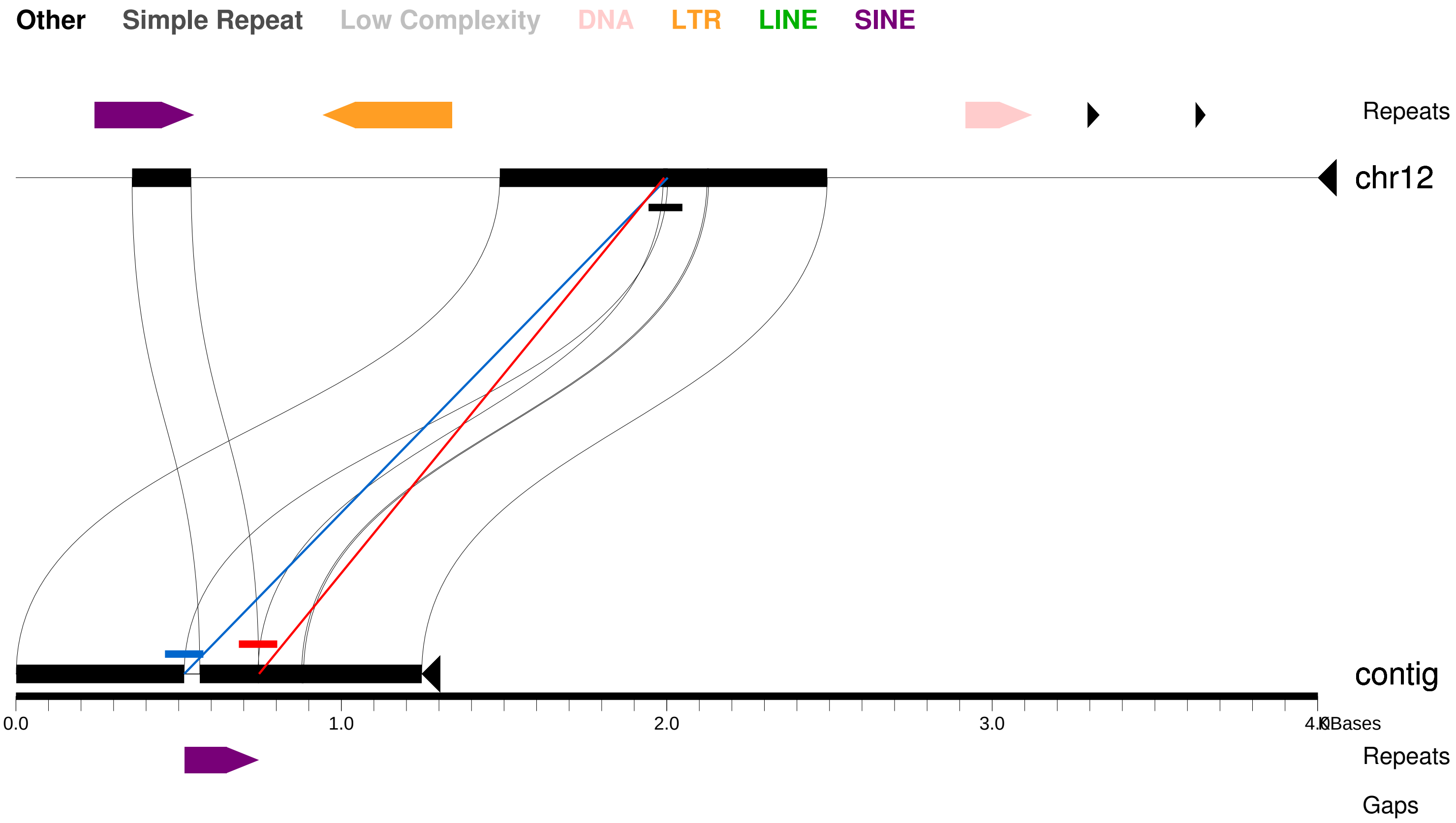

siteID: chr12_42482654 Scafftig7 - Breakpoint: chr12:42482686
chr12:42482639-42482740
Overlap Length: 13 INS size: 79 Genotyped: Yes

```
left     C--CCAGGTTATCC--CTGAGGTTCACTAGCACGCTGAGGGAGAGG-------------AAATGAAAGAA
chrom    C--CCAGGTTATCC--CTGAGGTTCACTAGCACGCTGAGGGAGAGG-------------AAATGAAAGAA
right    AGTCCGGCCTGGGCGACAGAGCGAGACTCCGTCTCAAAAAAAAAAAAAAAAAAAAAAAAAAAAAAAGAA
         111**1*11*111*11*1***1111***1111*1*11*111*1*1111111111111111***11******

left     CCACCCCGCCACTGCAGTCCGCAGTCCGGCCTGGGCGACAGAGCGAGACTCCGTCTCAAAAAAAAA
chrom    CCACCCCAA----GCACACTT-AGTGAGTC-TGCAGGA---AGCTGCAGTCAATCT--------AC
right    CCACCCCAA----GCACACTT-AGTGAGTC-TGCAGGA---AGCTGCAGTCAATCT--------AC
         *******222222***22*222***22*2*2**222**222***222*2**22***22222222*2
```

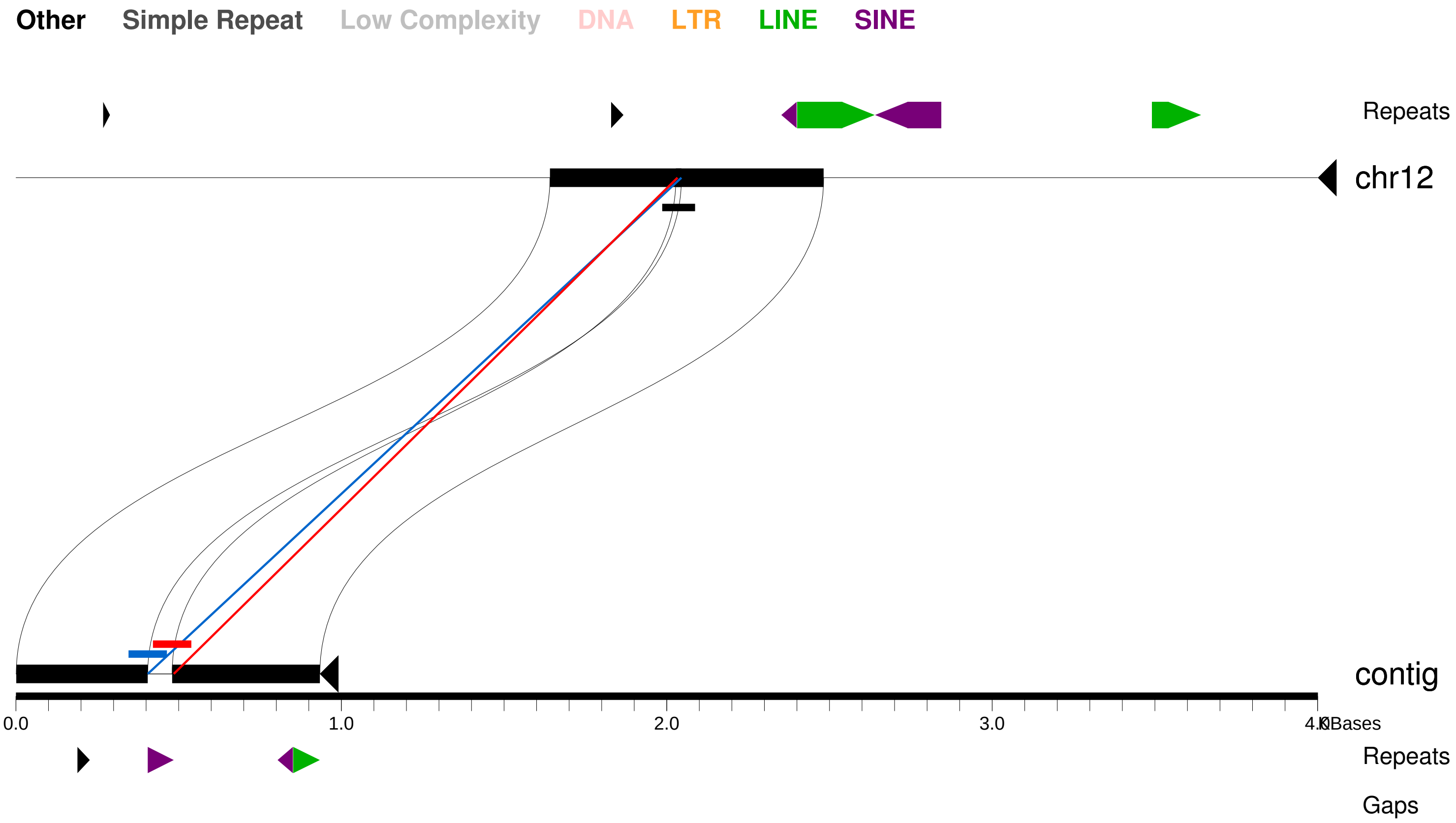

siteID: chr12_43717948 Scafftig4 + Breakpoint: chr12:43717929
chr12:43717894-43717988
Overlap Length: 12 INS size: 302 Genotyped: Yes

```
left   CCTTTTAA-TGTACCACCCA-----CCAAGACTCTAG--TTTA----------------CCAAATATTCT
chrom  CCTTTTAA-TGTACCACCCA-----CCAAGACTCTAG--TTTA----------------CCAAATATTCT
right  CCACCCGCCTCGGCCTCCCAAAGTGCTGGGATTACAGGCGTGAGCCACCGCGCCCGGCCCCAAATATTCT
       **1111111*111**1****11111*111**1*11**111*1*1111111111111111***********

left   TTTTTTTTTTTTTTTGAGACGGAGTCTTGCTCTGTCGCCCAGGTCGGACTGCGGACTGCAGTGGCGCAAT
chrom  TAATGTAACTTTTTA---ATTGAGCCATTCT-TGAATCCCTCTTT---------------------CATT
right  TAATGTAACTTTTTA---ATTGAGCCATTCT-TGAATCCCTCTTT---------------------CATT
       *22*2*222*****2222*22***2*2*2**2**222***222*222222222222222222222**2*

left   CTC-
chrom  CTCA
right  CTCA
       ***2
```

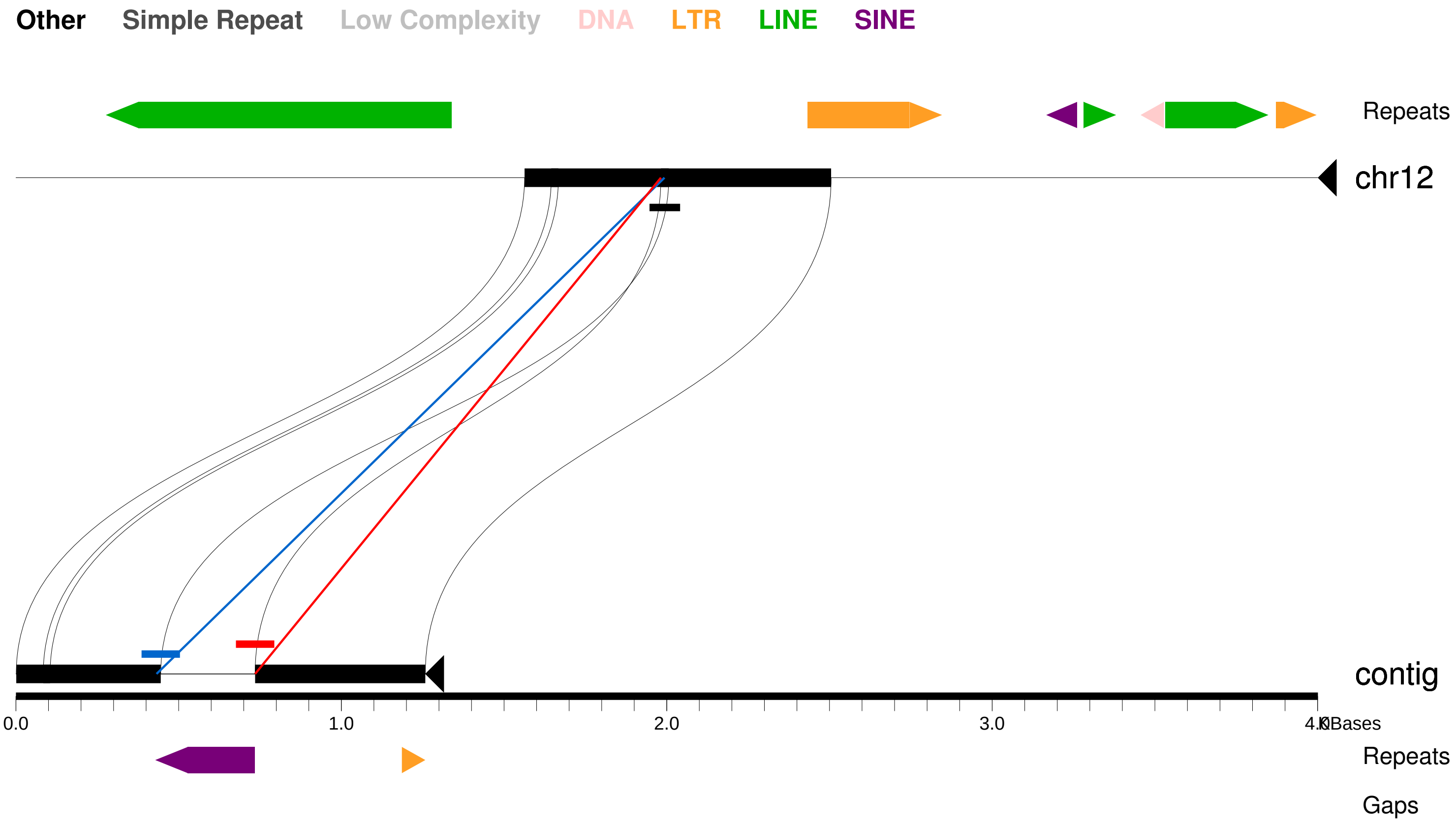

siteID: chr12_44139551 Scafftig3 - Breakpoint: chr12:44139556
chr12:44139512-44139615
Overlap Length: 16 INS size: 307 Genotyped: Yes

```
left     AATA---AATTCC-CCT----AACTAA---ATACTAC-TCT---ATATAGCAGATGCTTAAAAAAAATCT
chrom    AATA---AATTCC-CCT----GACTAA---ATACTAC-TCT---ATATAGCAGATGCTTAAAAAAAATCT
right    AGTCCGCAGTCCCACCTGGGCGACAGAGCGAGACTCCGTCTCAAAAAAAAAAAAAAAAAAAAAAAAATCT
         *1*1111*1*1**1***11112**11*111*1***1*1***111*1*1*11*1*11111**********

left     CTCTTGGCCGGGCGCGGTGGCTCACGCCTGTAATCCCAGCACTTTGGGAGGCCGAGGCGGGTGG
chrom    CTCTTA-----------TGAATGTAGATTGTCCCCTCTTCTCTGGAGGACACCATATCCT----
right    CTCTTA-----------TGAATGTAGATTGTCCCCTCTTCTCTGGAGGACACCATATCCT----
         *****222222222222**22*222*22***222*2*22*2**222***22**2222*222222
```

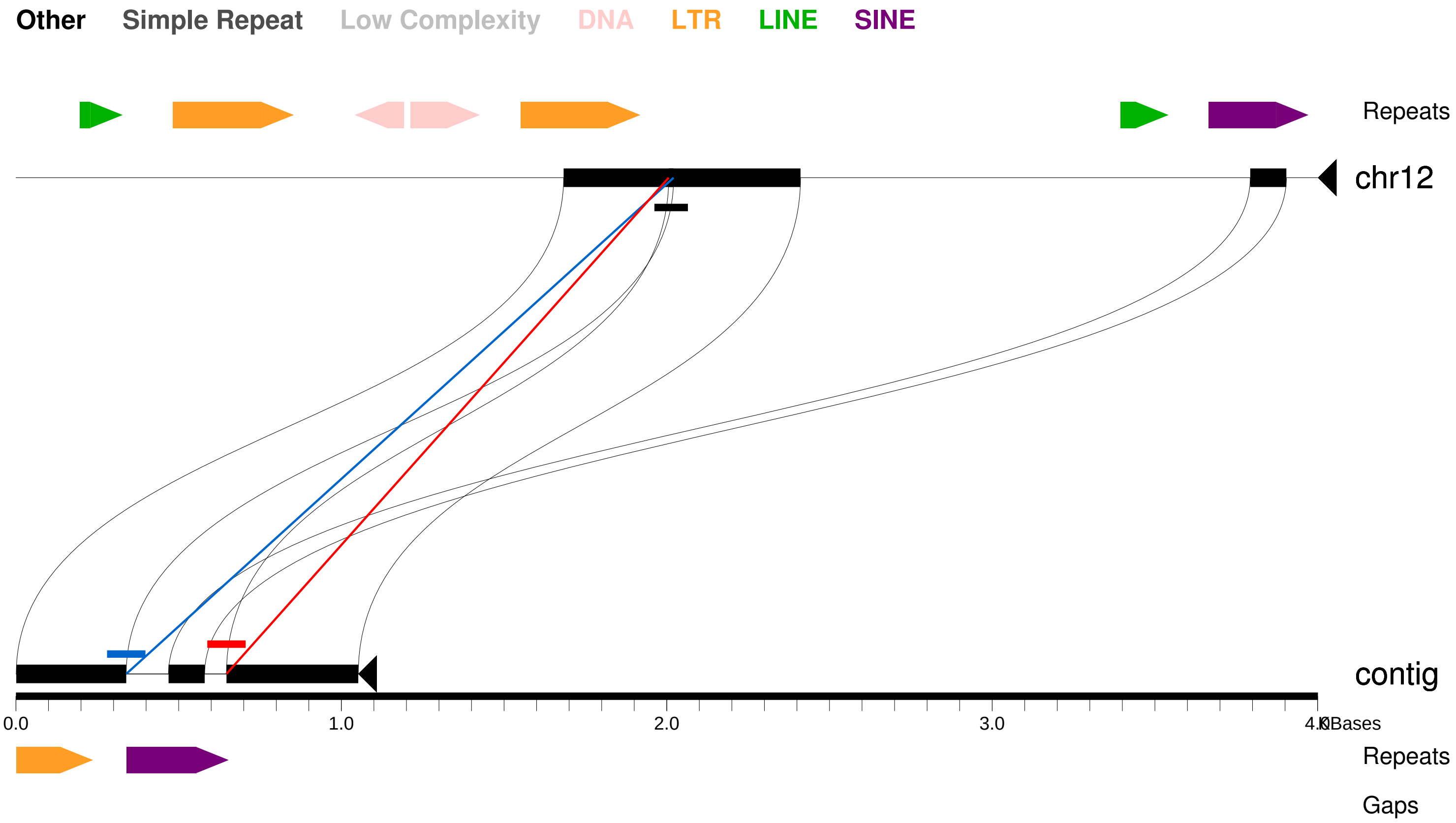

siteID: chr12_44150109 Scafftig1 + Breakpoint: chr12:44150142
chr12:44149982-44150310
Overlap Length: 9 INS size: 354 Genotyped: No

```
left   --------------------------------------AGGAATATATAAGATATACTTACTTTCCTTAATT
chrom  GAAATCACTACAAATTCTCCATATCCTAATTTCCTAAGGAATATATAAGATATACTTACTTTCCTTAATT
right  T---TTAGTAGAGACGGGGTTTCACCTTGTTAGCCA-GGATGGTCTC-GATCTCCTGAC-----------
       N   2N2N22N2N2NNNNNNN2NN222NN22NN2N21***111*1*11***1*1**1**11111111111

left   ACTTAATTTCCTGTACAGTTCCCTCACTTTCTTGCCCTAATTGAAATCTGGCTATTTCTTCAGGCTGCTT
chrom  ACTTAATTTCCTGTACAGTTCCCTCACTTTCTTGCCCTAATTGAAATCTGGCTATTTCTTCAGGCTGCTT
right  -CTCATGATCC---AC----CCGCCTCGGCCT--CCCAAAGTG----CTGGG-ATTACAGGCGTGAGCCA
       1**1*111***111**1111**11*1*111**11***1**1**1111****11***1*1111*111**11

left   CTTCTTCAGTCCTCTCAAGTAAAGGCTATNNNNNNNNNNN------------------------------
chrom  CTTCTTCAGTCCTCTCAAGTAAAGGCTATTTTTTCTTTCCCACCCATCTACCTTAGGACCTGGAGGCTGT
right  CCGCGCCCGGCCT-------AAAGGCTATTTTTTCTTTCCCACCCATCTACCTTAGGACCTGGAGGCTGT
       *11*11*1*1***1111111*********222222222222222222222222222222222222222222

left   ----------------------------------------------------------------------
chrom  AAATATTGGTCTATGTTTGTCACTGTTCGTTGCAAGCCATTTCTTCCTCTACTACCTCCAAAAACCCCAA
right  AAATATTGGTCTATGTTTGTCACTGTTCGTTGCAAGCCATTTCTTCCTNNNNNNNNNNNNNNNNNNNNNN
       222222222222222222222222222222222222222222222222NNNNNNNNNNNNNNNNNNNNNN

left   --------------------------------------------------
chrom  TTCTTTTGAAGAGCCTGCCATTGGGCTTTACCATCTGCTGTCATCCTCT
right  ****************************************----------
       ****************************************
```

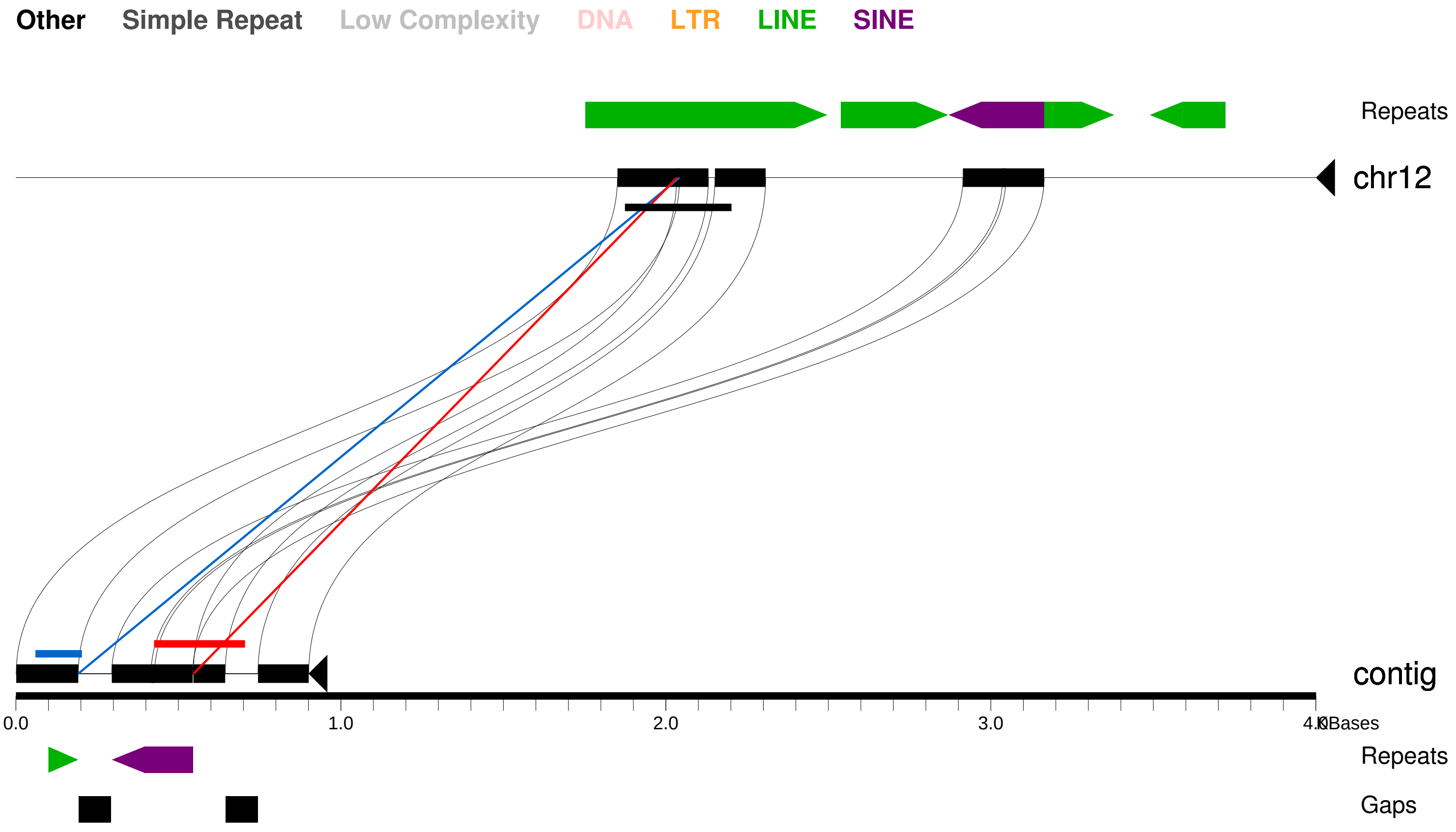

siteID: chr12_44487582 Scafftig2 - Breakpoint: chr12:44487571
chr12:44487564-44487630
Overlap Length: 53 INS size: 340 Genotyped: No

```
left    --------------------------------------------GGAATGT-------GAGAAAATTGA
chrom   --------------------------------------------GGAATGT-------GAGAAAATTGA
right   ATTTTTTGTATTTTTAGTAGAGACGGGGTTTCACCTTGTTAGCCAGGATGGTCTCGATCGAGAAAATTGA
        11111111111111111111111111111111111111111111***11**1111111***********

left    TCTTTCTTGGAGGAAGGCAGATAGACTGGCTGAACAATAAAGNNNNNNNNNNNNNNNNNNNNNNNNNNNN
chrom   TCTTTCTTGGAGGAAGGCAGATAGACTGGATGACCACTAAAGATTTCTT---------------------
right   TCTTTCTTGGAGGAAGGCAGATAGACTGGATGACCACTAAAGATTTCTT---------------------
        *****************************2***2**2*****2222222222222222222222222222

left    NNNNNNNNNNNNNNNNNNNNNNNNNNNNNNN
chrom   -------------------------------
right   -------------------------------
        2222222222222222222222222222222
```

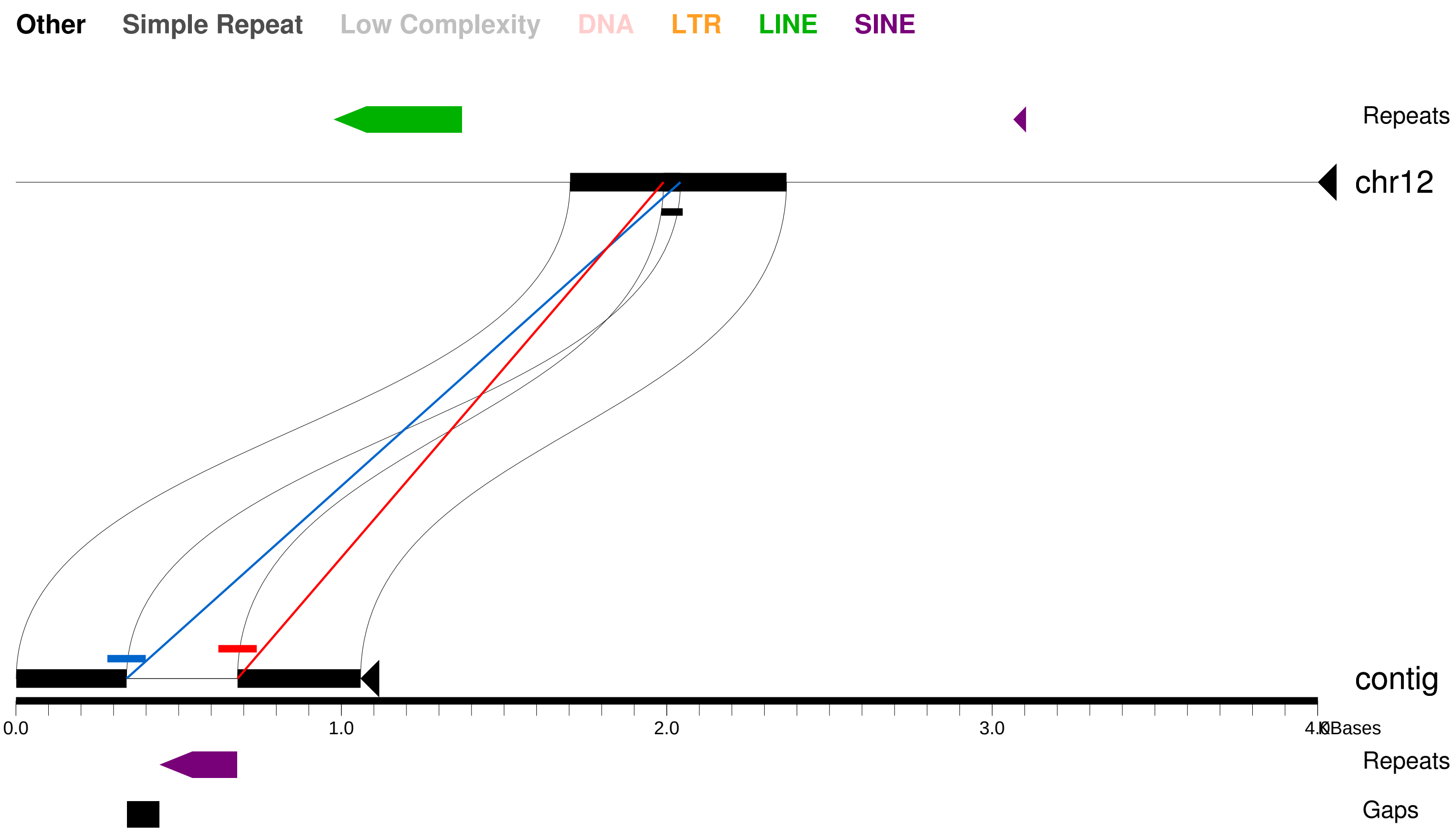

siteID: chr12_45064348 Scafftig1 - Breakpoint: chr12:45064360
chr12:45064318-45064419
Overlap Length: 18 INS size: 304 Genotyped: Yes

```
left    TTAA-------------AAAACAAGA-TGCAAGT---ACAAGAAACCTATTTCATCTATAAAGATACACA
chrom   TTAA-------------AAAACAAGA-TGCAAGT---ACAAGAAACCTATTTCATCTATAAAGATACACA
right   GCACTCCAGCCTGGGCGACAGCGAGACTCCGTCTCAAAAAAAAAAAAAAAAAAAAAAAAAAAGATACACA
        11*11111111111111*1*1*1***1*1*111*111*1**1***111*1111*111*1***********

left    TAGACTAGGCCGGGCGCGGTGGCTCACGCCTGTAATCCCAGCACTTTGGGAGGCCGAGGCGGGCAG
chrom   TAGACTAAAAGAGAAGGGACGAAAAAA------AATCCATGCAAAT---GAAAAC--------CAA
right   TAGACTAAAAGAGAAGGGACGAAAAAA------AATCCATGCAAAT---GAAAAC--------CAA
        *******22222*22*2*22*2222*2222222*****22***22*222**222*22222222**2
```

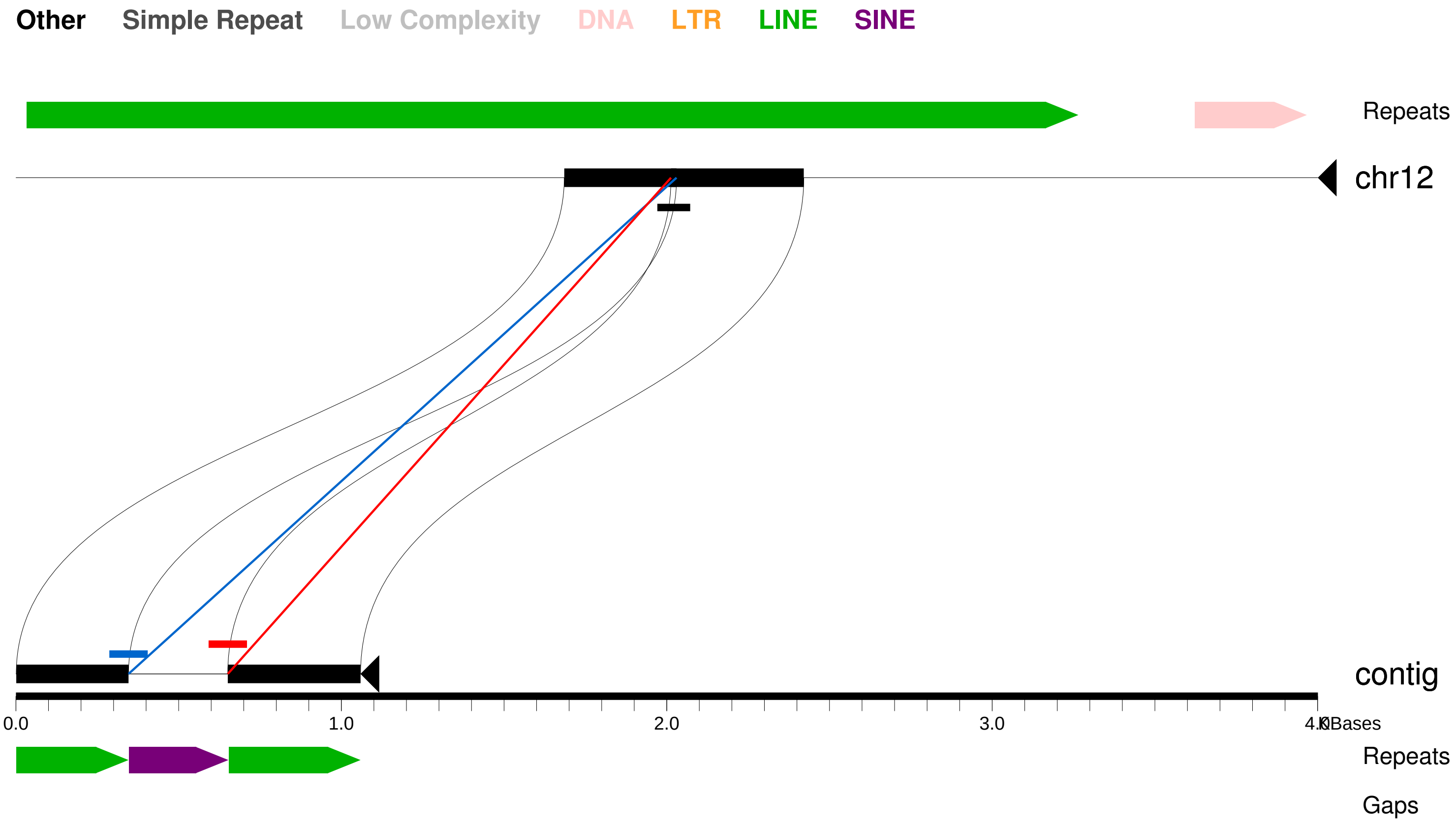

siteID: chr12_45131155 Scafftig2 + Breakpoint: chr12:45131135
chr12:45131115-45131194
Overlap Length: 40 INS size: 267 Genotyped: No

```
left    ---GCACACATTTAGTTCACAAT------------------------------------CAATAACTTTT
chrom   ---GCACACATTTAGTTCACAAT------------------------------------CAATAACTTTT
right   TCCGCCCGCCTCGGCCTCCCAAAGTGCTGGGATTACAGGCGTGAGCCACCGCGCCCGGCCAATAACTTTT
        111**1*1*1*11111**1***1111111111111111111111111111111111111***********

left    TTTTAATAAACAAAAAACTTGTTTGTTCTNNNNNNNNNNNNNNNNNNNNNNNNNNNNNNNNNNNNNNNNN
chrom   TTATAATAAACAAAAAACTTGTTTGTTCTTAGTTGATAAAATTATGCCT---------------------
right   TTATAATAAACAAAAAACTTGTTTGTTCTTAGTTGATAAAATTATGCCT---------------------
        **2**************************22222222222222222222222222222222222222222

left    NNNNNNNNNNNNNNNNNN
chrom   ------------------
right   ------------------
        222222222222222222
```

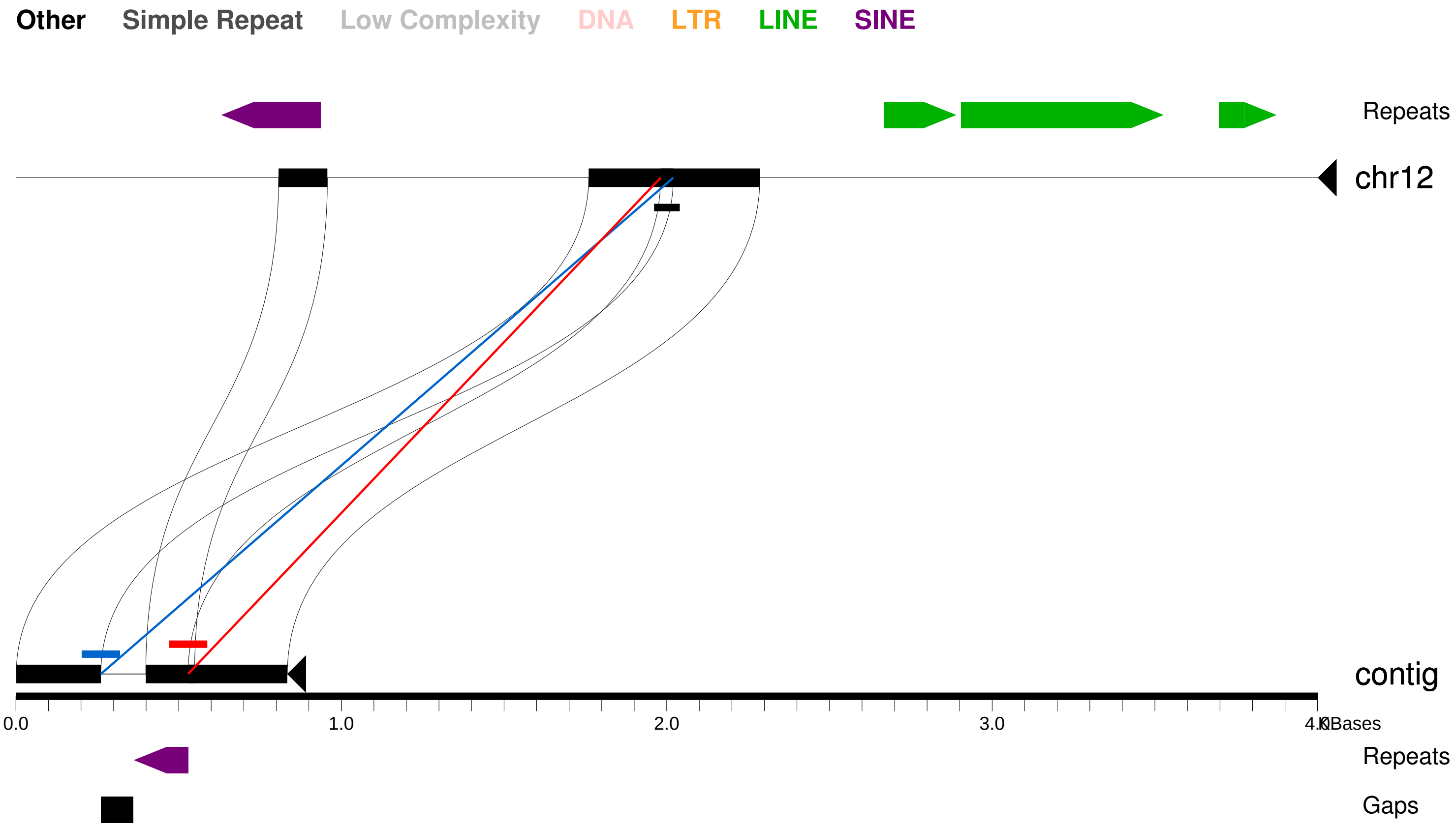

siteID: chr12_45419542 Scafftig4 - Breakpoint: chr12:45419545
chr12:45419496-45419568
Overlap Length: 10 INS size: 324 Genotyped: Yes

```
left   TAATTATTT----ACTG------------------------------------------AATAAACAAGT
chrom  TAATTATTT----ACTG------------------------------------------AATAAACAAGT
right  CCGAGATCCCGCCACTGCACTCCAGCCTGGGCGACAGAGCGAGACTCCGTCTCAAAAAAAAAAAAAAAAA
       11111**111111****111111111111111111111111111111111111111111**1***1**11

left   AAATGAATGTCATAAGTACTAGAATAGAAAGATCTGGGCCGGGCGCGGTGGCTCACGCCTGTAATCCCAG
chrom  AAATGAATGTCATAAGTACTAGAATAGAAAGATCT-------GCAAAGTG--------------------
right  AAAAAAAAAAAAAAAAAAAAAAAAAAGAAAGATCT-------GCAAAGTG--------------------
       ***11**1111*1**11*11*1**1*********2222222**222***22222222222222222222

left   CACTTTGGGAGGCCGAGGCGGGCGG
chrom  CACT-------------------GG
right  CACT-------------------GG
       ****2222222222222222222**
```

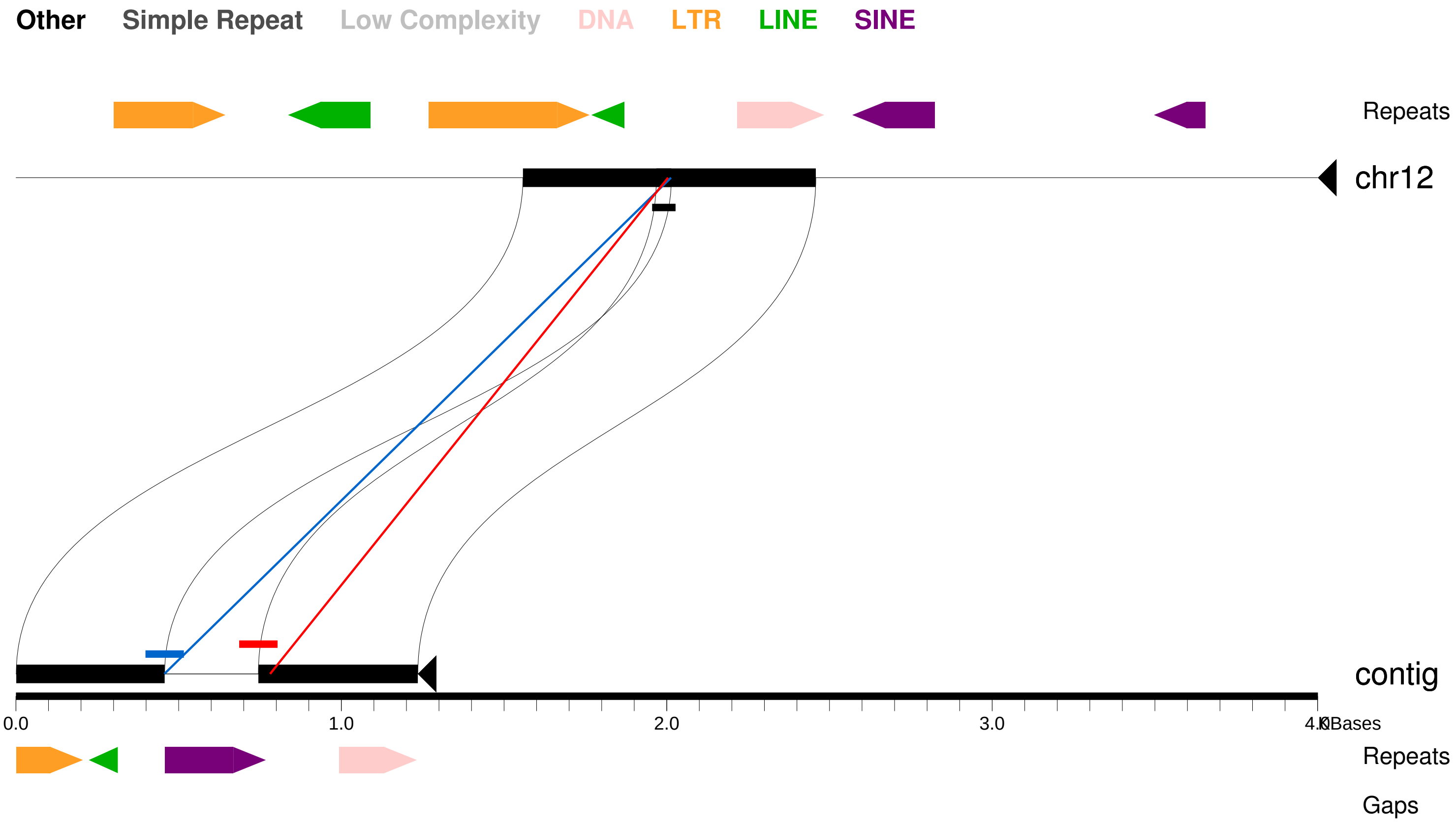

siteID: chr12_4770879 Scafftig4 - Breakpoint: chr12:4770875
chr12:4770826-4770934
Overlap Length: 11 INS size: 288 Genotyped: Yes

```
left     G--GACAGGAACAGTCGG---AAGAGTTG---ATACAGGG--GAAGCAGAATCAGTGCACTAGAGTTCTT
chrom    G--GACAGGAACAGTCGG---AAGAGTTG---ATACAGGG--GAAGCAGAATCAGTGCACTAGAGTTCTT
right    TCCGCCCGTCTCGGCCTCCCAAAGTGCTGGGATTACAGGCGTGAGCCACCGCGCCCGGCCTAGAGTTCTT
         111*1*1*111*1*1*11111***1*1**1111******111**11**11111111*11***********

left     TTTTTTTTTGAGACGGAGTTTTGCTCTGTCGCCCAGGCTGGAGCGCAGTGGCGCGATCT
chrom    GAGAAGGTTGAGAAACAG---------GTATCCCAGGA-GCAGATGAACAAAGAACAAG
right    GAGAAGGTTGAGAAACAG---------GTATCCCAGGA-GCAGATGAACAAAGAACAAG
         2222222******222**222222222**22******22*2**222*22222*222222
```

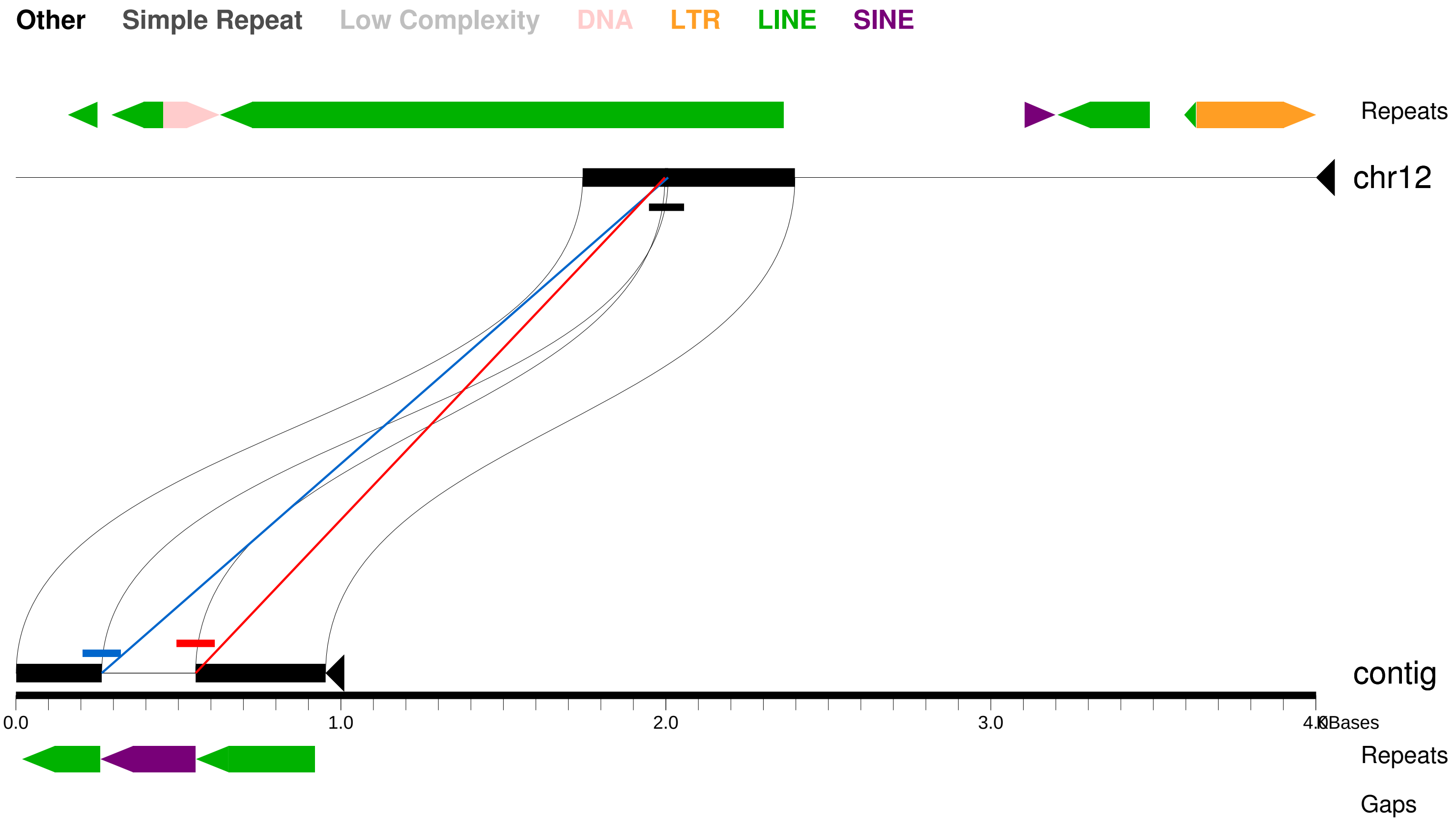

siteID: chr12_48312497 Scafftig1 - Breakpoint: chr12:48312476
chr12:48312431-48312535
Overlap Length: 15 INS size: 289 Genotyped: Yes

```
left     CTAT-GAAACAAAG-AGATCAGACAAACC--CAAGTCGAAGGACATTCT----------AAAAAATACCT
chrom    CTAT-GAAACAAAG-AGATCAGACAAACC--CAAGTCGAAGGACATTCT----------AAAAAATACCT
right    AGATCGCGCCACTGCACTCCAGCCTGGGCGACAGAGCGAGACTCCGTCTCAAAAAAAAAAAAAAATACCT
         11**1*111**11*1*111***1*1111*11**111***1111*11***1111111111***********

left     GACCGGCCGGGCGCGGTGGCTCACGCCTGT-AATCCCAGCACTTTGGGAGGCCGAGGCGGGCGG
chrom    GACCAGTC---CTC-----CTCAAAACTGTCAAGATCATCA-------AAAACTTGGGAAGTTT
right    GACCAGTC---CTC-----CTCAAAACTGTCAAGATCATCA-------AAAACTTGGGAAGTTT
         ****2*2*222*2*22222****222****2**222**2**2222222*222*22**222*222
```

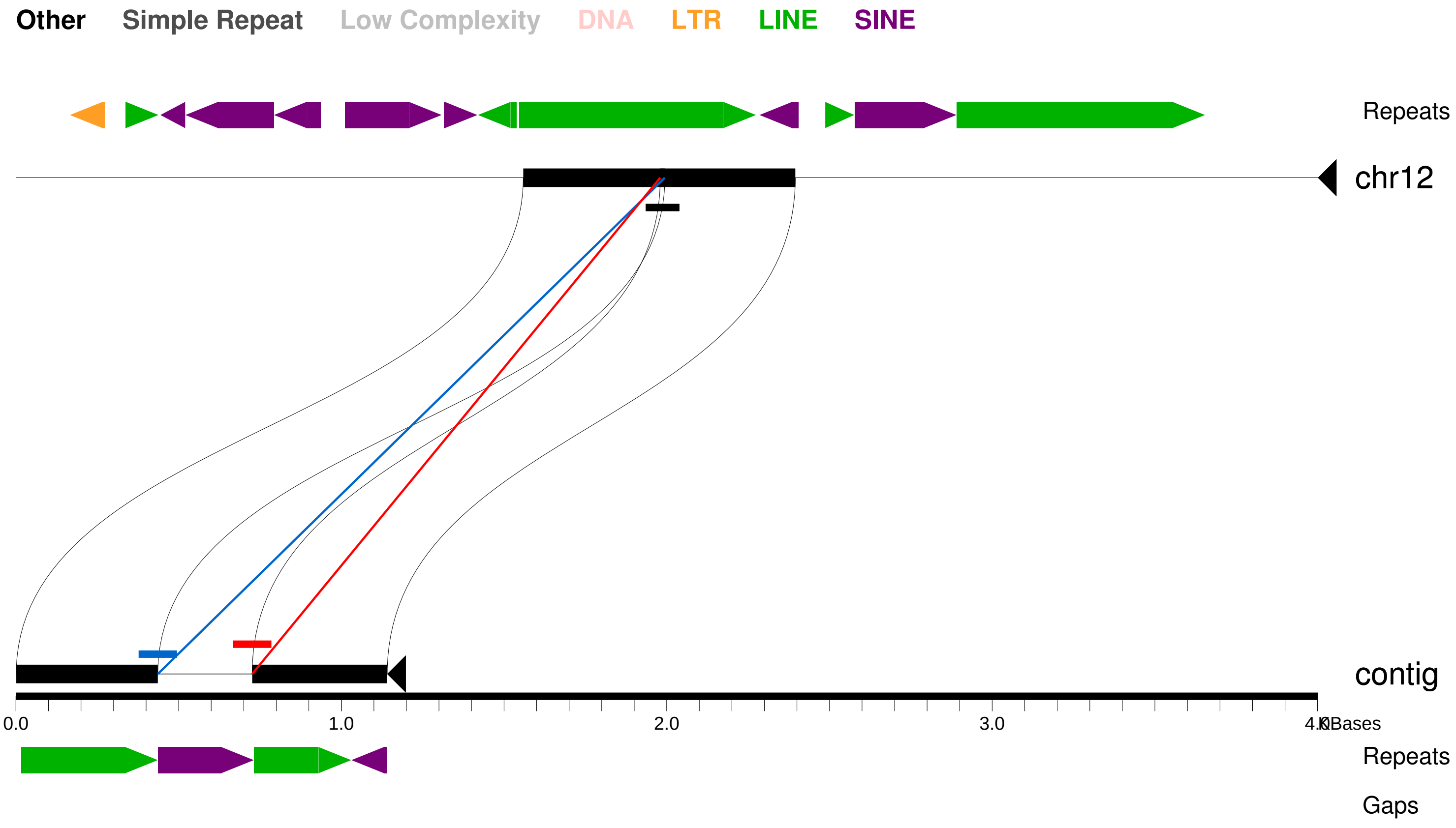

siteID: chr12_52813699 Scafftig3 - Breakpoint: chr12:52813681
chr12:52813636-52813740
Overlap Length: 15 INS size: 297 Genotyped: Yes

```
left   A-GCCATA--TGACCTCTCAGAG------GAT------CTTTCTCTACTATGTAATGGAAAGTTGTCATT
chrom  A-GCCATA--TGACCTCTCAGAG------GAT------CTTTCTCTACTATGTAATGGAAAGTTGTCATT
right  CCGCCCGCCTCGGCCTCCCAAAGTGCTGGGATTACAGGCGTGAGCCACCGCGCCC-GGCCAGTTGTCATT
       11***111111*1****1**1**111111***111111*1*111*1**111*1111**11**********

left   TTTTTTTTTTTTTTTTTTTTTGAGACGGAGTCTCGCTGTCGCCCAGG-CTGGAGTGCAGT-GGCGC
chrom  CTTTTA-------------AAACCACAGAGTCCC---ATCACACATGACTTACATGAAGCAGGATC
right  CTTTTA-------------AAACCACAGAGTCCC---ATCACACATGACTTACATGAAGCAGGATC
       2****2222222222222222222**2*****2*2222**2*2**2*2**2222**2**22**22*
```

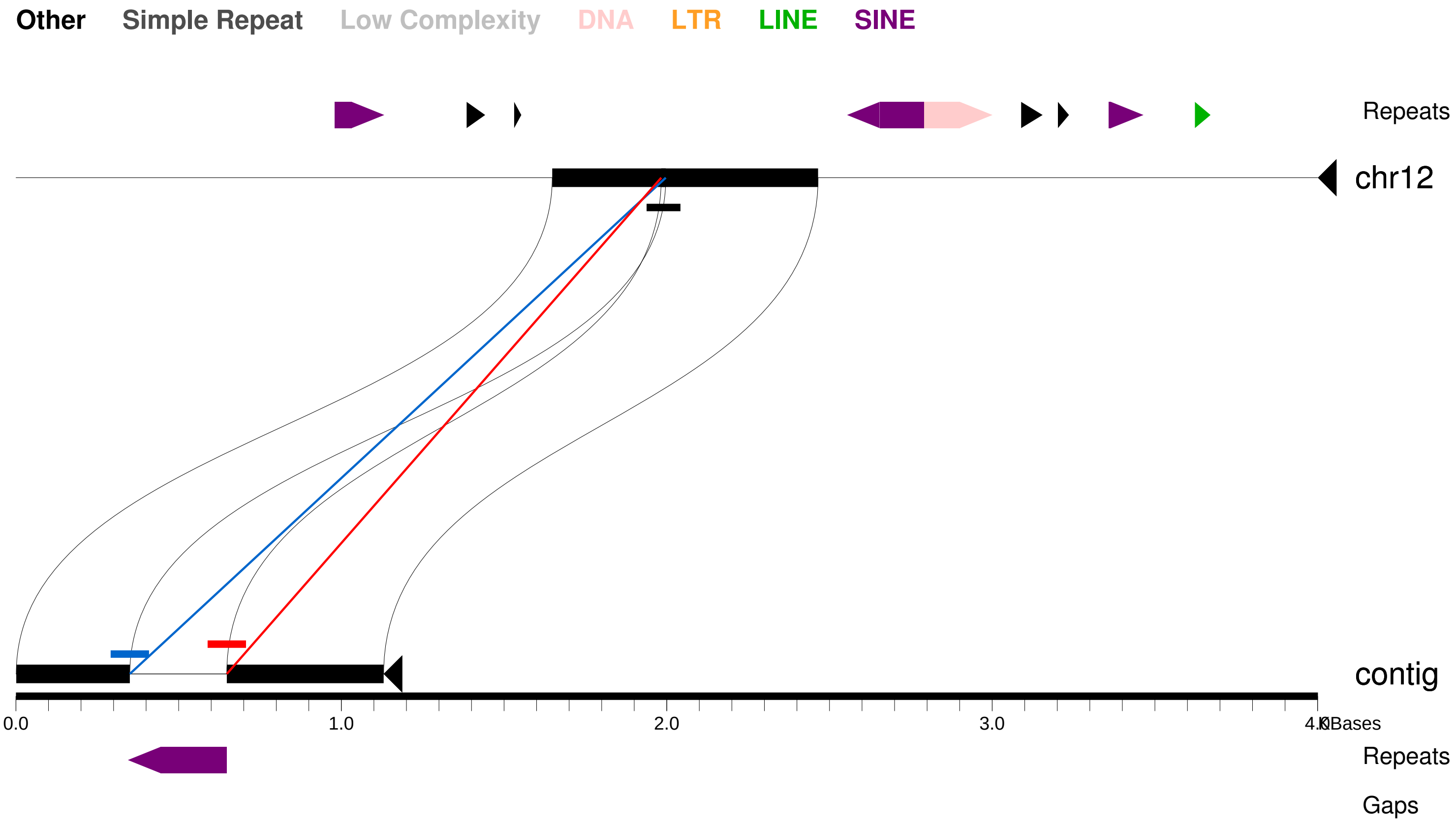

siteID: chr12_5769605 Scafftig7 - Breakpoint: chr12:5769595
chr12:5769543-5769654
Overlap Length: 8 INS size: 290 Genotyped: Yes

```
left    CAATCTTTTGTGTACTCCATCTCAGCCACACTGGCTTCCTTCCTTTTCTTAG--------AACACGCCG-
chrom   CAATCTTTTGTATACTCCATCTCAGCCACACTGGCTTCCTTCCTGTTCTTAG--------AACACGCCAA
right   AGCACTCCTGCCTGGGCGA-CAGAACGAGACTCCGTCTCAAAAAAAAAAAAAAAAAAAAAAACACGCCAA
        1111**11**1N*111*1*1*11*1*1*1***111*11*11111N11111*111111111********22

left    GCCGGGCGCGGTGGCTCACGCCTGTAATCCCAGCACTTTGGGAGGCCGAGGCGGGCGG
chrom   GCCGGCTTCCATAGCAAAACCTTAGCATGACTCCTCT--------CCTCACTCTCCAC
right   GCTGGCTTCCATAGCAAAACCTTAGCATGACTCCTCT--------CCTCACTCTCCAC
        **1**222*22*2**22*22*2*222**22*22*2**22222222**22222222*22
```

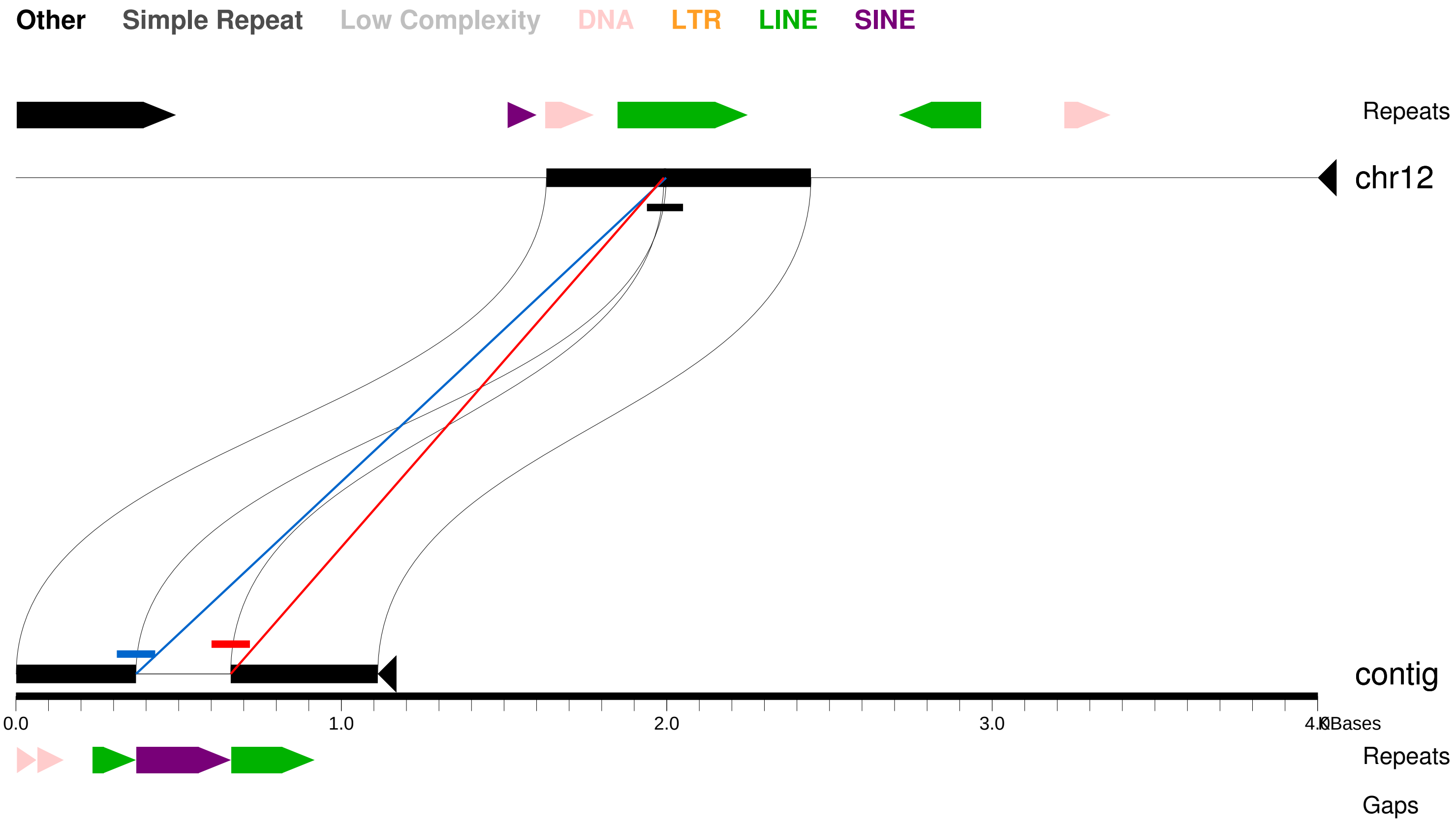

siteID: chr12_58359077 Scafftig1 - Breakpoint: chr12:58359058
chr12:58359012-58359117
Overlap Length: 14 INS size: 316 Genotyped: Yes

```
left    CTCAAAGACC-------------ACACGATCCAGCCATCTCACTCTTAAGTATATCCCCAAAAGAAAGGA
chrom   CTCAAAGACC-------------ACACGATCCAGCCATCTCACTCTTAAGTATATCCCCAAAAGAAAGGA
right   GCGACAGAGCGAAACTCCGCCTCAAAAAAAAAAAAAAAAAAAAAAAAAAAAAAAAAAAAAAAAGAAAGGA
        111*1***1*1111111111111*1*11*111*111*1111*11111**11*1*11111***********

left    AATGGCCGGGCGCGGTGGCTCACGCCTGTAATCCCAGCACTTTGGGAGGCCGAGGCGGGCGG
chrom   AATTAGTATATCAAGTG----ATATCTGTACGCTCACATTTATTGTAGCACTA---------
right   AATTAGTATATCAAGTG----ATATCTGTACGCTCACATTTATTGTAGCACTA---------
        ***2222222222***2222*222*****22*2**2222*2*2*2**22*2*222222222
```

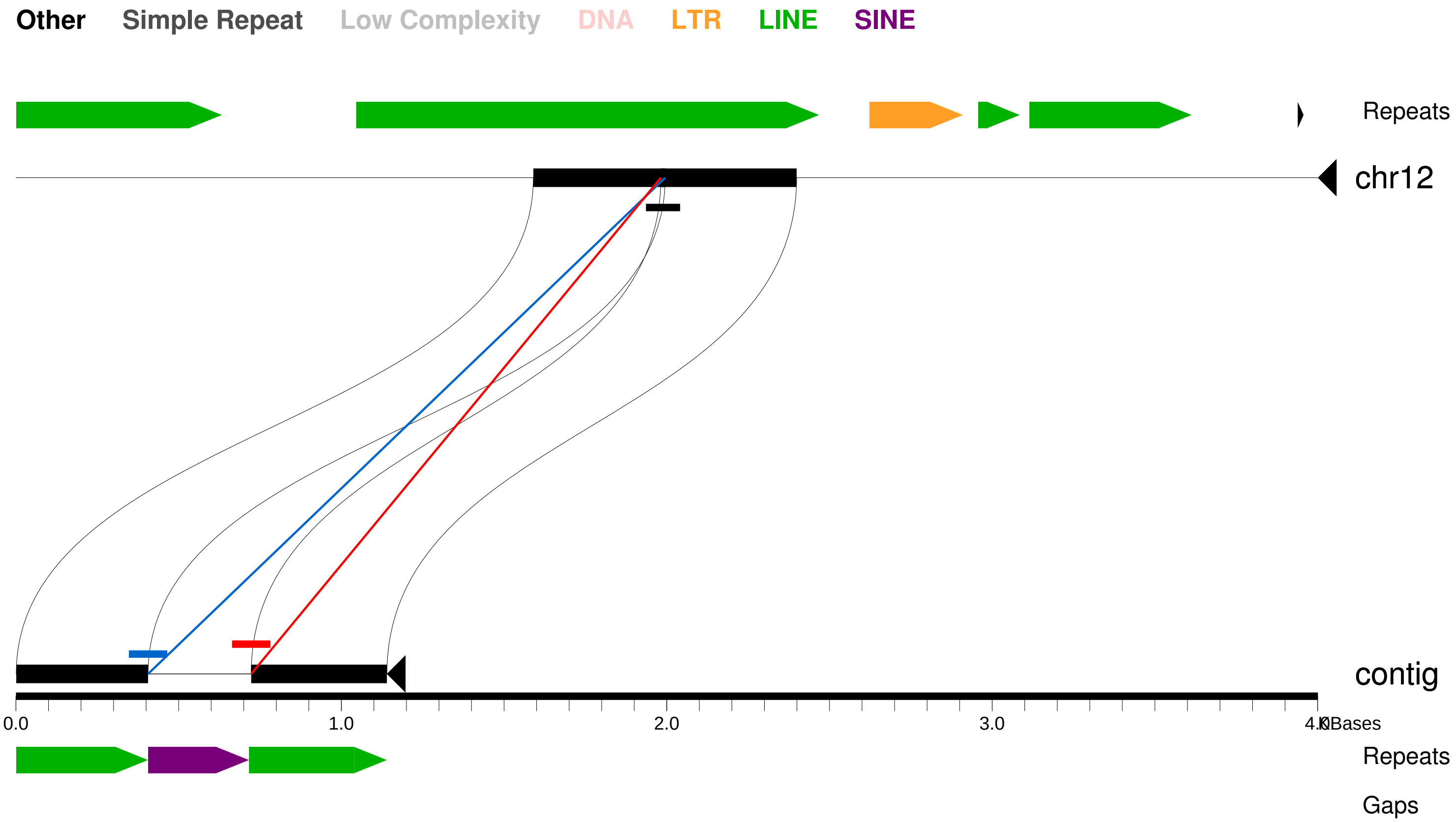

siteID: chr12_58458213 Scafftig12 - Breakpoint: chr12:58458207
chr12:58458164-58458263
Overlap Length: 17 INS size: 298 Genotyped: Yes

```
left    CTG-----GAAATCTAGTTTT--CGAAAGTTGAACACTGTGT------------AATTCAATAAAAATAT
chrom   CTG-----GAAATCTAGTTTT--CGAAAGTTGAACACTGTGT------------AATTCAATAAAAATAT
right   CCGCCACTGCACTCCAGCCTGGGCGACAGAGCGAGACTCCGTCTCAAAAAAAAAAAAAAAAAAAAAAAATAT
        *1*11111*1*1**1**11*111***1**1111*1***11**111111111111**111**1********

left    TCTAATGAGGGCCGGGCGCGGTGGCTCACGCCTGTAATCCCAGCACTTTGGGAGGCCGAGGCGGGCGG
chrom   TCTAATGAGAATAAAA-GCTA-GACAGAGGCA--TAAT---AATGCTTTT-----CCTAAT-------
right   TCTAATGAGAATAAAA-GCTA-GACAGAGGCA--TAAT---AATGCTTTT-----CCTAAT-------
        *********22222222**222*2*22*2**222****222*222****222222**2*222222222
```

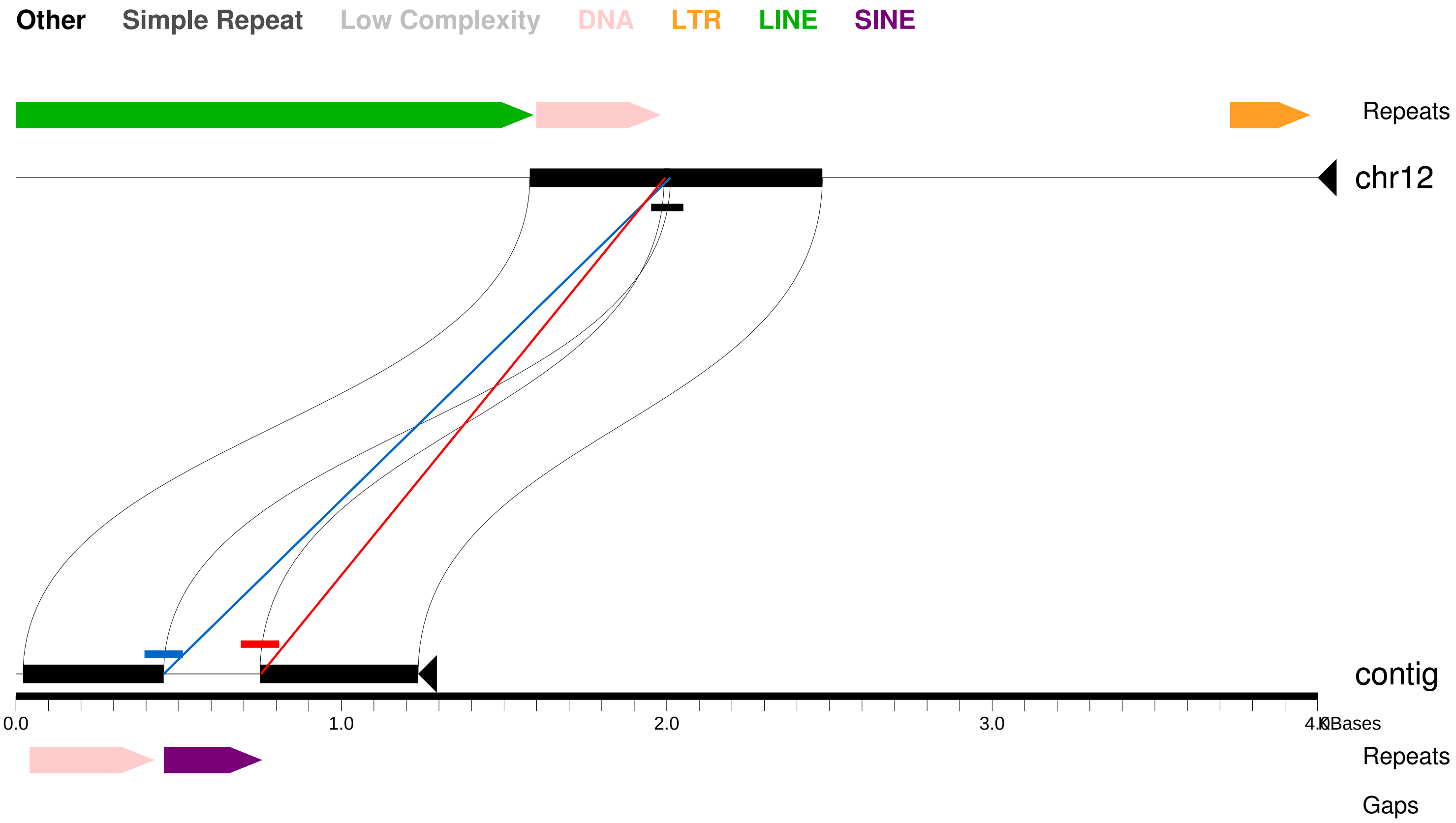

siteID: chr12_59219040 Scafftig1 - Breakpoint: chr12:59219048
chr12:59219003-59219104
Overlap Length: 15 INS size: 305 Genotyped: Yes

```
left     GATAAT--------------GGTCTAAATACCTAAAAA--GTAG-AATTTTTAACATTGAATAAGAAAGG
chrom    GATAAT--------------GGTCTAAATACCTAAAAA--GTAG-AATTTTTAACATTGAATAAGAAAGG
right    TGCAGTCCGCAGTCCGGCCTGGGCAACAGAGCGAGACTCCGTCTCAAAAAAAAAAAAAAAAAAAGAAAGG
         111*1*11111111111111**1*1*1*1*1*1*1*1111**111**11111**1*111**1********

left     AAGGCCCGGCCGGGCGCGGTGGCTCACGCCTGTAATCCCAGCACTTTGGGAGGCCGAGGCGGGTGG
chrom    AAGGCCCTACAACA-----TGCCACTTACAAGGAA-CTCAC--CTTAAGTATAAAGA---------
right    AAGGCCCTACAACA-----TGCCACTTACAAGGAA-CTCAC--CTTAAGTATAAAGA---------
         *******22*222222222**2*2*222*22*2**2*2**222***22*2*2222**222222222
```

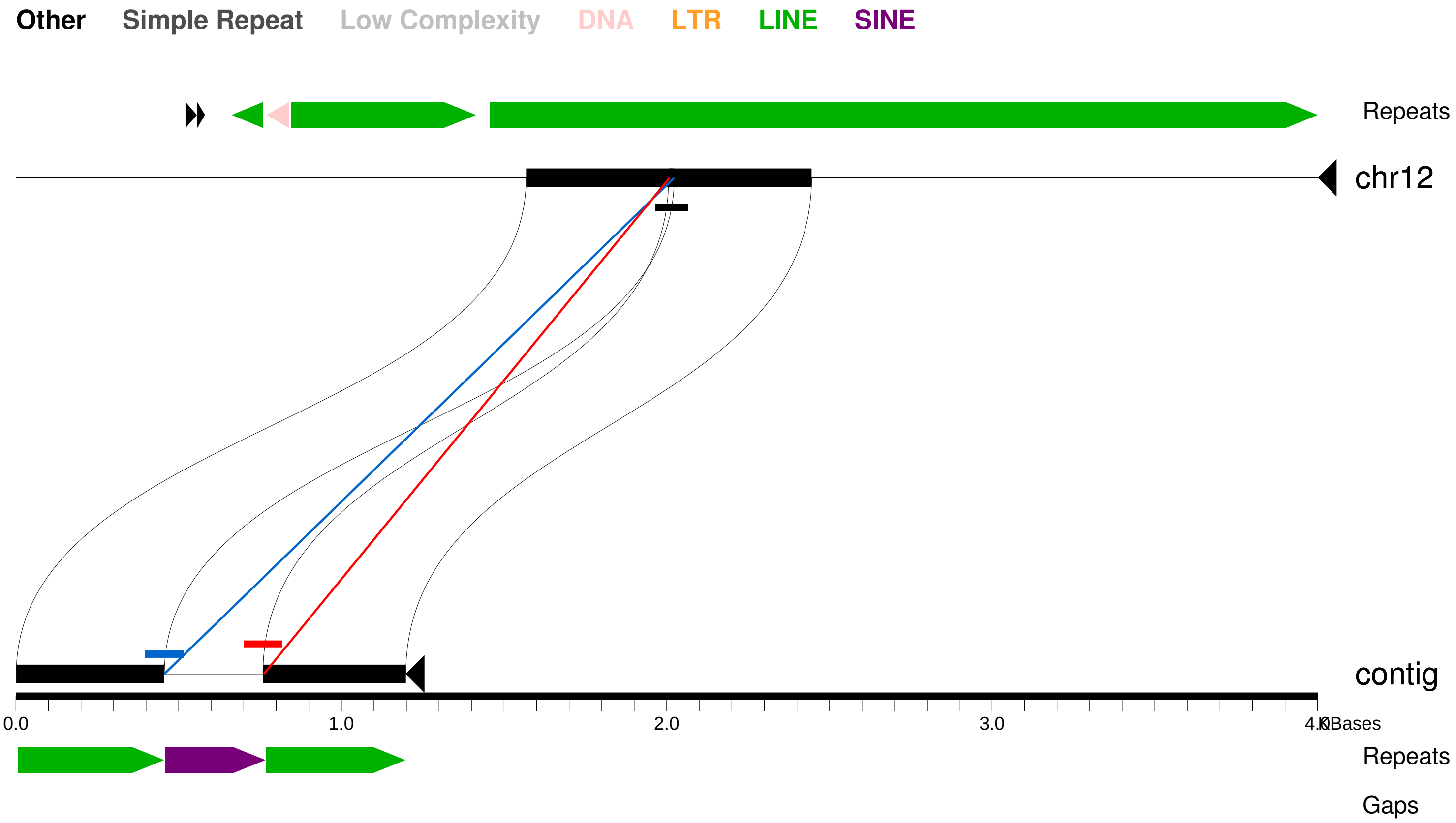

siteID: chr12_59381471 Scafftig1 - Breakpoint: chr12:59381457
chr12:59381409-59381516
Overlap Length: 12 INS size: 417 Genotyped: No

```
left    ATCTCATTATTGGATATACACCCAAAGGAATAT-----------AAATTGTTCTACTATAAAAACACATG
chrom   ATCTCATTATTGGATATACACCCAACGGAATAT-----------AAATTGTTCTACTATAAAAACACATG
right   NNNNNNNNNNNNNNNNNNNNNNNNNNNNNNNNNNNNNNNNNNNNNNNNNNNNNNAAAAAAAAAACACATG
        1111111111111111111111111N1111111111111111111111111111*11*1**********

left    CGGCCGGGCGCGGTGGCTCACGCCTGTAATCC---CAGCACT-TTGGGAGG--CCGAGGCG-GGTGG
chrom   C------------------ACGCTTATATTCATTGCAGCACTATTCATAATATCAAAGACGTGGAAT
right   C------------------ACGCTTATATTCATTGCAGCACTATTCATAATATCAAAGACGTGGAAT
        *222222222222222222****2*2**2**2222*******2**222*2222*22**2**2**222
```

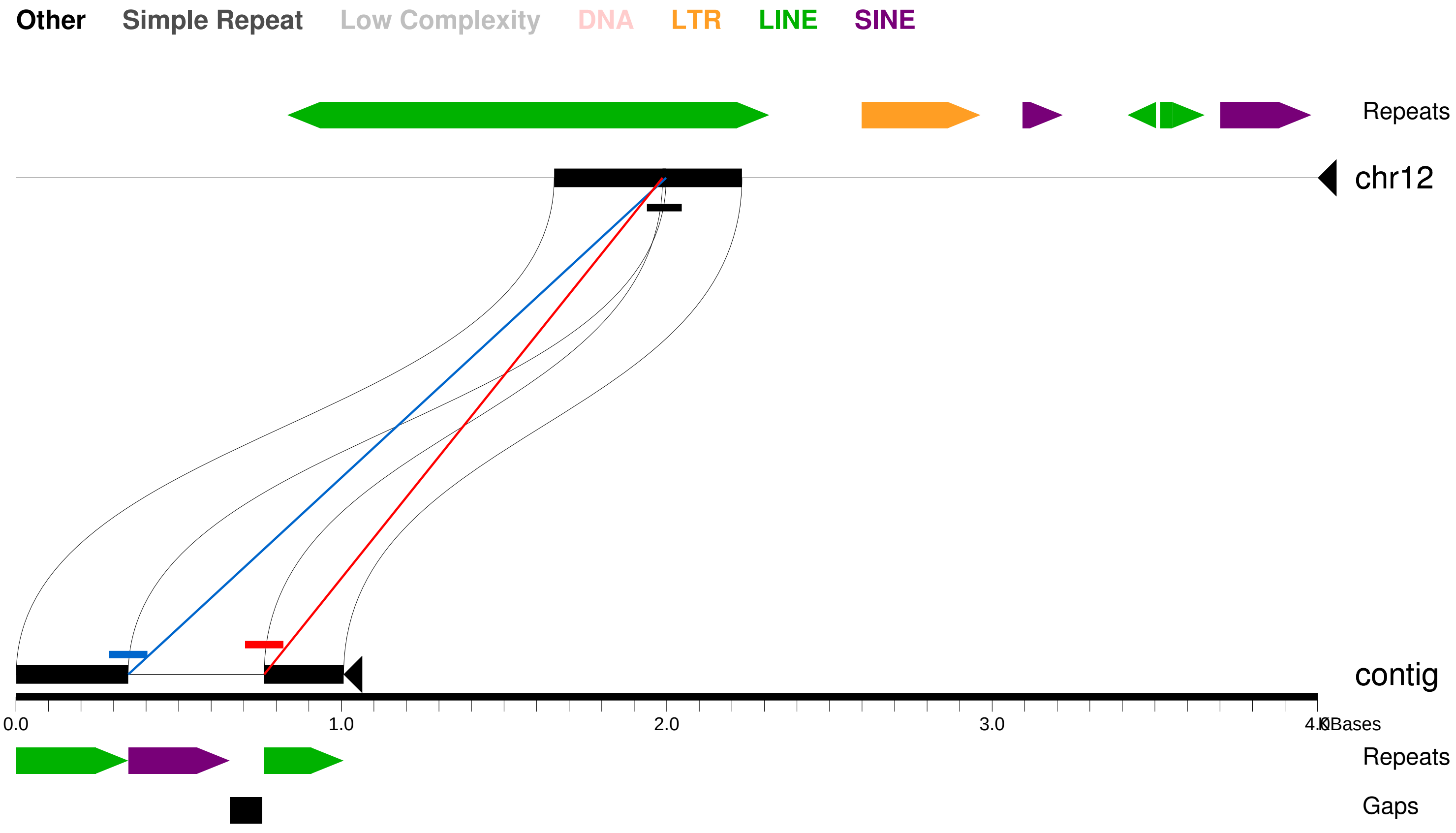

siteID: chr12_59491907 Scafftig7 + Breakpoint: chr12:59491937
chr12:59491777-59492098
Overlap Length: 0 INS size: 332 Genotyped: Yes

```
left   -GGGAAGTAGTTCTTAGAGACAAGGAGAGAATTGTTGAAACTGGTAGAACTGGCTCTGACTCCTCAATCT
chrom  CGGGAAGTAGTTCTTAGAGACAAGGAGAGAATTGTTGAAACTGGTAGAACTGGCTCTGACTCCTCAATCT
right  ---------------AGA---ATGGCGTGAACCCGGGA----GGCGGAGCTTGCAGTGAG-CCGAGATCG
        11111111111111***111*1**1*1***11111**1111**11**1**1**11***11**111***1

left   TGACTCCACATCCTATTCTTTTTAGTGACTAAAATGTCATTCATGTCTTTCTTTGGTTAAGACTGGCATC
chrom  TGACTCCACATCCTATTCTTTTTAGTGACTAAAATGTCATTCATGTCTTTCTTTGGTTAAGACTGGCATC
right  CGCCACTGCACTCCAGCCTGG---GCGACAGAGCGAGACTCCGTCTCAAAAAAAAAAAAAAAAAAAAAAA
       1*1*1*11**11*1*11**11111*1***11*1111111*1*1*1**11111111111**1*11111*11

left   CCCATAGAAAGAGCTGAGTGGGGCCGGGCGCG------GTGGCTCACGCCTGTAATCCCA---GCACTTT
chrom  CCCATAGAAAGAGCTGAGTGGCCATTGTCTAGAATACAGTGCCCTAGGTCAGAGGTCTGAAATGCTTTTT
right  AAAAAAAAAAAAAAAAAAAAACCATTCTCTAGAATACAGTGCCCTAGGTCAGAGGTCTGAAATGCTTTTT
       111*1*1***1*1111*11112222212*22*222222***2*22*2*2*2*222**22*222**22***

left   GG---GAGGCCGA--GGCGGGCGGATCACGAGGTCAGGAGATCGA----GACCATCC--TGGC--TAACA
chrom  GAAGAGAAGCAGAATGGTTGG-GGAACA-GAAGTCTCAAAATTGCCCTGGCTGATGAATTGGCAGTGAAA
right  GAAGAGAAGCAGAATGGTTGG-GGAACA-GAAGTCTCAAAATTGCCCTGGCTGATGAATTGGCAGTGAAA
       *2222**2**2**22**22**2***2**2**2***222*2**2*22222*222**2222****22*2*2*

left   CGGTGA------------AACCCCGTCTCTACTA--------AA
chrom  AGGTGTTTACAGCCAGGGAAGGCCATCAGTATGGTGTGGGGGAA
right  AGGTGTTTACAGCCAGGGAAGGCCATCAG---------------
       2****2222222222222**22**2**2211NNN        11
```

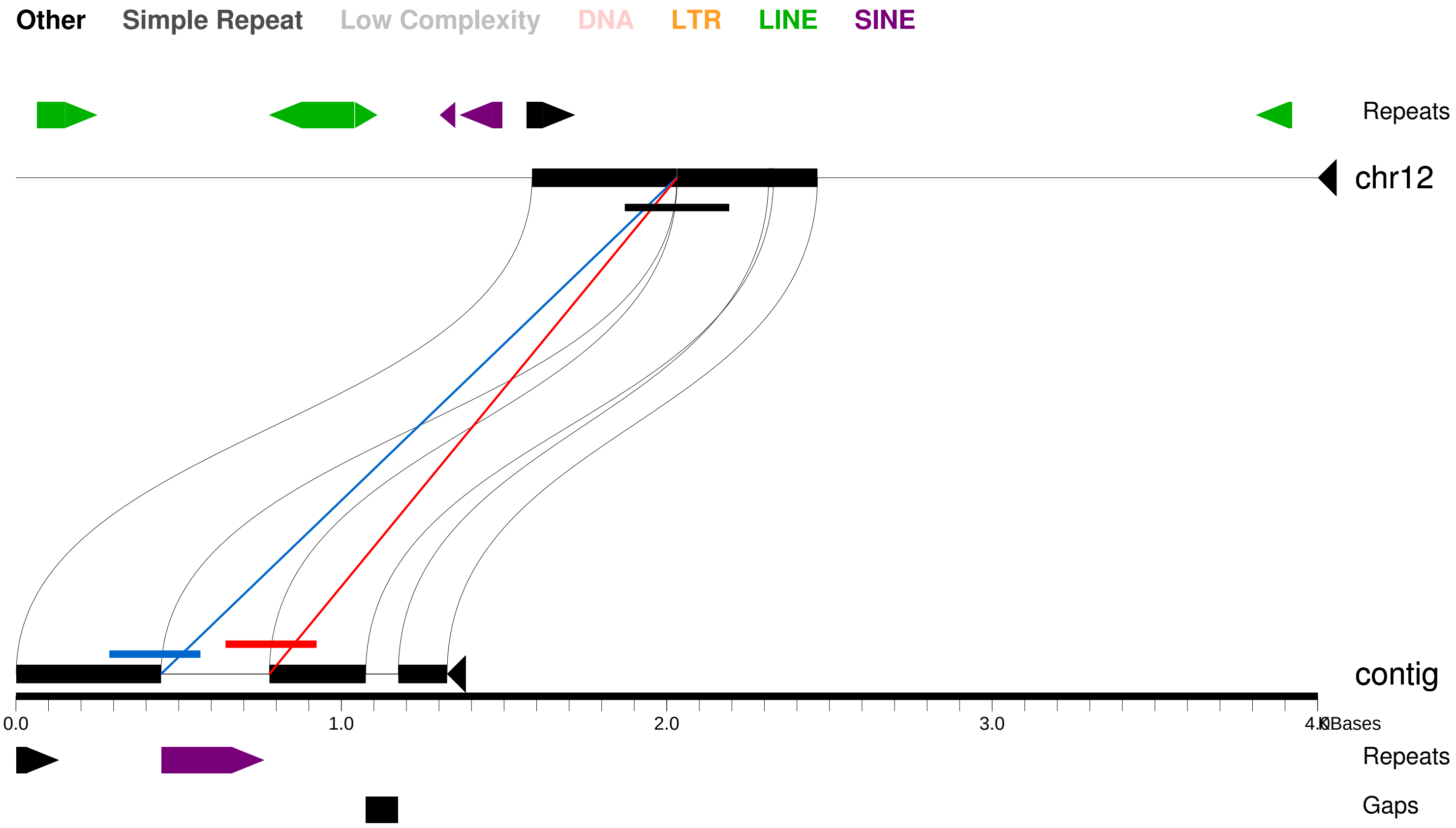

siteID: chr12_59848977 Scafftig1 + Breakpoint: chr12:59848971
chr12:59848968-59849030
Overlap Length: 57 INS size: 477 Genotyped: No

```
left   TCA--------------------------------------------------------CTCTGTCAGAT
chrom  TCA--------------------------------------------------------CTCTGTCAGAT
right  TCCACCCGCCTCGGCCTCCCAAAGTGCTGGGATTACAGGCGTGAGCCACCGCGCCCGGCCTCTGTCAGAT
       **111111111111111111111111111111111111111111111111111111111***********

left   ATTGAATGGAGATACTTTCTACAATGTGTTAACTTTTTCAGGTGCTNNNNNNNNNNNNNNNNNNNNNNNN
chrom  ATTGAATGGAGATACTTTCTACAATGTGTTAACTTTTTCAGGTGCTCAC---------------------
right  ATTGAATGGAGATACTTTCTACAATGTGTTAACTTTTTCAGGTGCTCAC---------------------
       **********************************************222222222222222222222222

left   NNNNNNNNNNNNNNNNNNNNNNNNNNNNNNNNNNN
chrom  -----------------------------------
right  -----------------------------------
       22222222222222222222222222222222222
```

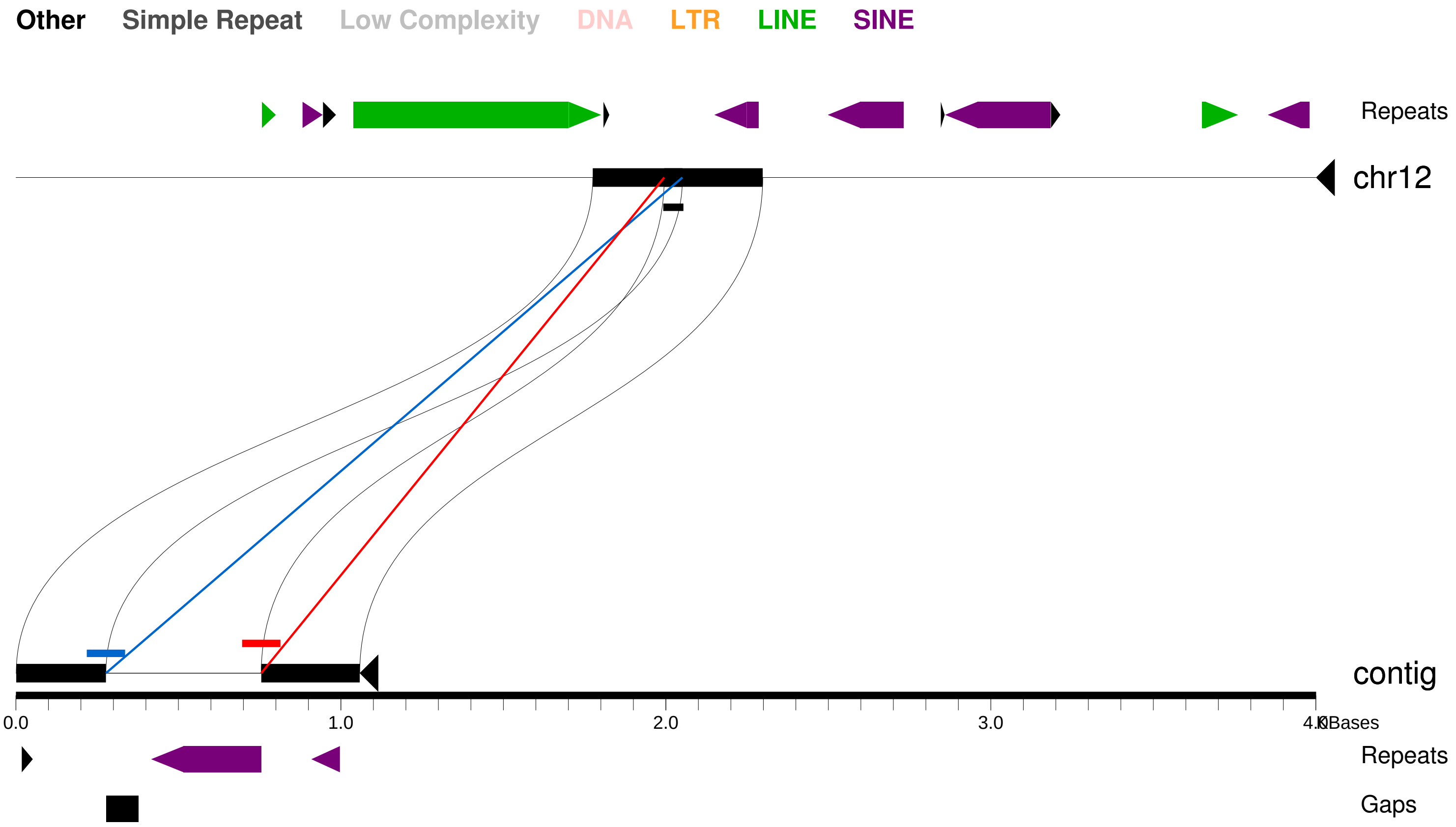

siteID: chr12_6066791 Scafftig2 + Breakpoint: chr12:6066792
chr12:6066632-6066963
Overlap Length: 12 INS size: 299 Genotyped: Yes

```
left    C------------TCAAGCTTCTGAAAACTAAAGAAAAACTGTTAAAGCATCCAATAGAAGGGGGGAAAT
chrom   CAAAAGCATATACTCAAGCTTCTGAAAACTAAAGAAAAACTGTTAAAGCATCCAATAGAAGGGGGGAAAT
right   -AATGGCGTGAAC--------CTGGGAGGCGGAG-----CTTGCAGTG-AGCCAAGATT---GTGCCACT
        122NN22N2NN2211111111***11*11111**11111**111*11*1*1****1*11111*1*11*1*

left    GCATACAGGAAAACAACAATAAAAATGGCGGCTGACTGGTCATCAGAAATAACACAAGCCAAAGACTAAG
chrom   GCATACAGGAAAACAACAATAAAAATGGCGGCTGACTGGTCATCAGAAATAACACAAGCCAAAGACTAAG
right   GCAATCCGGC---CTGGGCTAAAGA--GCGG--GACTCCGTCTCAAAAAAAAAAAAAA---AAAGA-----
        ***11*1**1111*11111****1*11****11****11111***1***1**1*1**111*****11111

left    AAACATACTTAAAGTGATGAAATTTAAAAACAGGCTGGGCGCGGTGG----CTCACGCCTGTAATCCCAG
chrom   AAACATACTTAAAGTGATGAAATTTAAAAACAAACCCGTCAACCTAGAATTCTATATTCAGTAAATGAAG
right   --------------------AATTTAAAAACAAACCCGTCAACCTAGAATTCTATATTCAGTAAATGAAG
        11111111111111111111***********22*22*2*2222*2*2222**22222*2****2222**

left    ------------CACTTTGGGAGG--------CCGAGGCGGGCGGA-TCA-CAAGGTCAGGAGATCGAGA
chrom   GCAGAATAGAGACATTTTCAGATAAATGAAATCTTAGAAATGTGTTGTCATCCAGCCCTGCACTAAAAGA
right   GCAGAATAGAGACATTTTCAGATAAATGAAATCTTAGAAATGTGTTGTCATCCAGCCCTGCACTAAAAGA
        222222222222**2***22**2222222222*22**2222*2*222***2*2**22*2*2*22222***

left    CCATCTT--GGCTAACACGG-------------TGAAACCCCGTCTCTACTAAA--
chrom   AGTTCTTTAGGCTGA-AGGGAAATGATATAATATGGAAATTCAGATATA-TAAG--
right   AGTTCTTTAGGCTGA-AGGGAAATGATATAATATGGAAATTCAGATATA-TAAGAA
        222****22****2*2*2**2222222222222**2**222*222*2**2***211
```

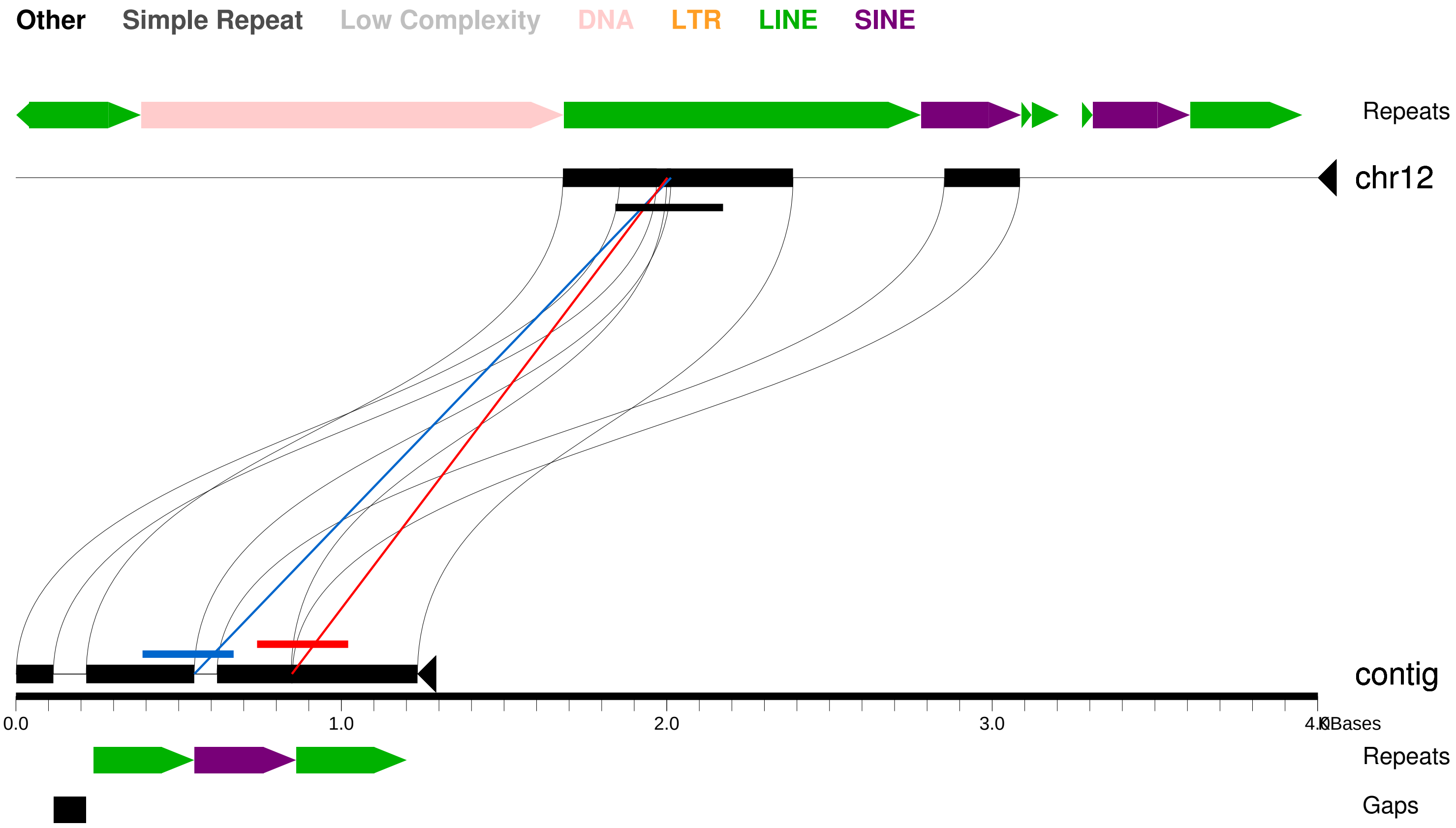

siteID: chr12_61053808 Scafftig5 - Breakpoint: chr12:61053816
chr12:61053766-61053858
Overlap Length: 10 INS size: 274 Genotyped: Yes

```
left    T-------------------------TGACCTTTAAAAATTACAGATAAT--TTATAGACAAAAAAATCTA
chrom   T-------------------------TGACCTTTAAAAATTACAGATAAT--TTATAGACAAAAAAATCTA
right   GATTGCGCCACTGCAGTCCGCAGTCTGGCCTGGGCGACAGAGCGAGACTCCGTCTCAAAAAAAAAAAAAA
        1111111111111111111111111**1***11111*111*11**1*1*111*1*11*1*******111*

left    GTGAATAAGAATATTTCACTTTGGGAGGCCGAGGCGGGTGGATCATGAGGTCAGGAGATCGAGACCATCC
chrom   GTGAATAAGAATATTTAAGAATGTGACTAC-------------CTTCATATCATGA----AAGAAC----
right   AAAAAAAAGAATATTTAAGAATGTGACTAC-------------CTTCATATCATGA----AAGAAC----
        111**1**********2*222**2**222*2222222222222*2*2*22***2**22222***2*2222

left    TGGCT
chrom   -----
right   -----
        22222
```

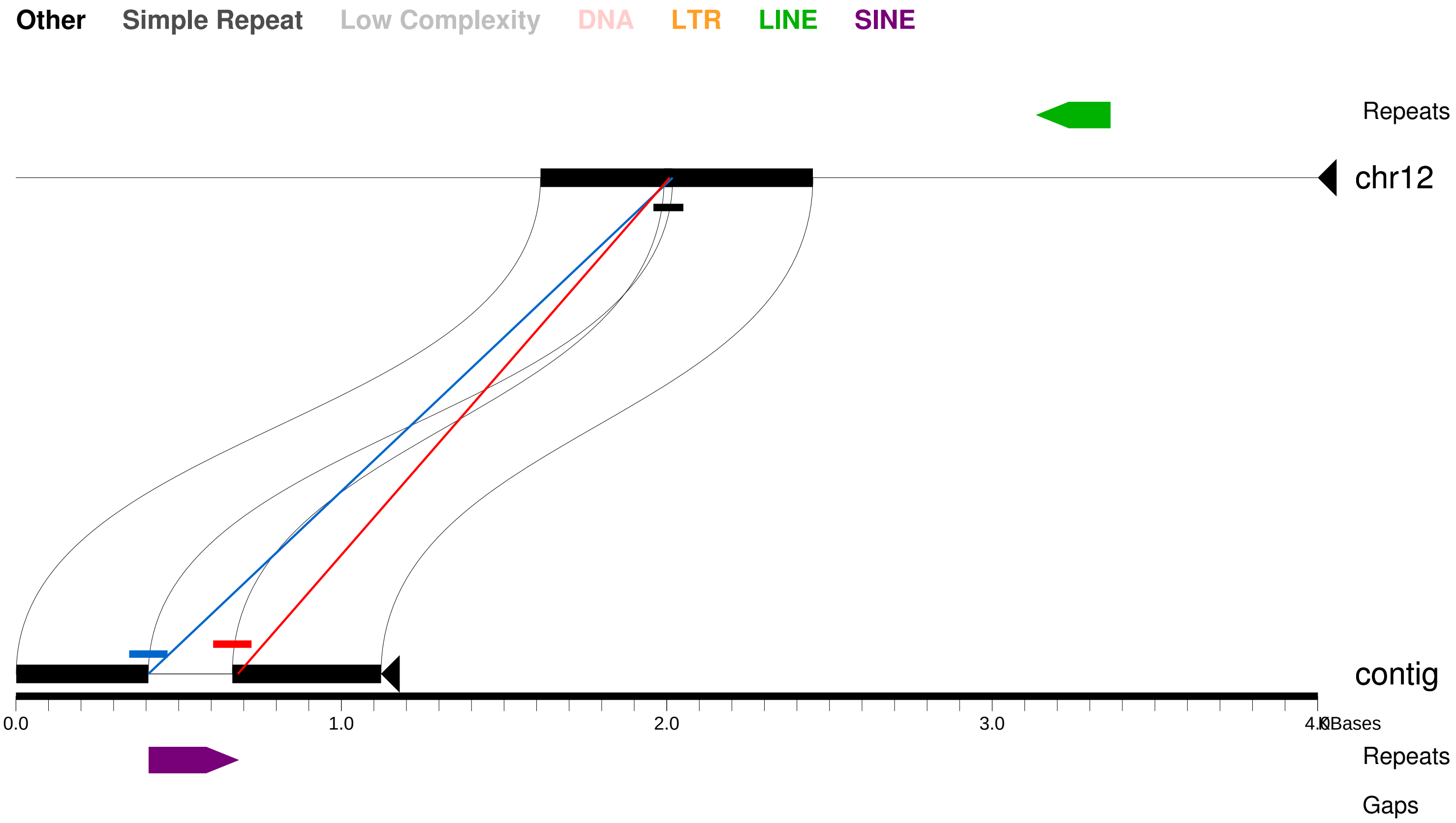

siteID: chr12_61247565 Scafftig1 + Breakpoint: chr12:61247547
chr12:61247510-61247606
Overlap Length: 13 INS size: 296 Genotyped: Yes

```
left    CAG-----------AATACAACTTGATGGGT---CAGAATAGAAATTCCAT--------AGTCTTTTTTT
chrom   CAG-----------AATACAACTTGATGGGT---CAGAATAGAAATTCCAT--------AGTCTTTTTTT
right   CCGCCCGCCTCGGCCTCCCAAAGTGCTGGGATTACAGGCGTGAGCCACCGCGCCCGGCCAGTCTTTTTTT
        *1*111111111111111***11**1****1111***1111**1111**1111111111***********

left    TTTTTTTTTTTTTT--TTTGAGACGGAGTCTCGCTCTGTCGCCCAGGCTGGAGTGCAGTGGCGCGATCTC
chrom   TTAATTATTATTATACTTTAAG-----TTCTAG------------GGTTTATGTGCAC-------AACGT
right   TTAATTATTATTATACTTTAAG-----TTCTAG------------GGTTTATGTGCAC-------AACGT
        **22**2**2**2*22***2**222222***2*222222222222**2*222*****22222222*2*22

left    GGC
chrom   GCA
right   GCA
        *22
```

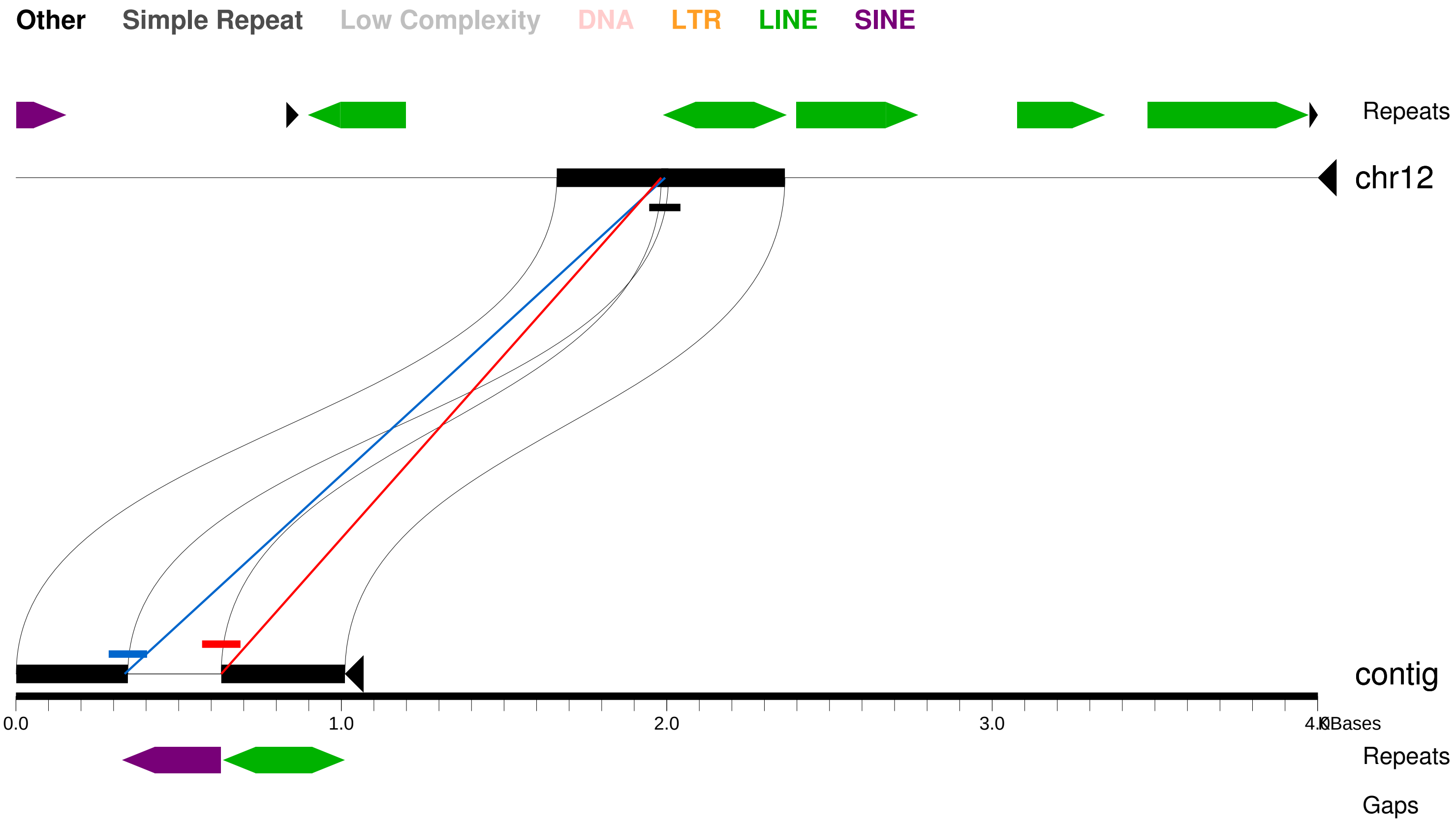

siteID: chr12_61537961 Scafftig7 + Breakpoint: chr12:61537939
chr12:61537894-61537998
Overlap Length: 15 INS size: 184 Genotyped: Yes

```
left     ATTTACATTTCATTT----AAAGTA-------TAAACTCTAGAATGAC---ACAAACTATGTCTCAGATT
chrom    ATTTACATTTCATTT----AAAGTA-------TAAACTCTAGAATGAC---ACAAACTATGTCTCAGATT
right    CCACCCGCCTCGGCCTCCCAAAGTGCTGGGATTACAGGCGTGAGCCACCGCGCCCGGCCTGTCTCAGATT
         11111*111**11111111*****11111111**1*11*11**111**1111*111111***********

left     TCTTTTTTTTTTTTTTTTTTTTTTTTTTTTTGAGACGGAGTCTCGCTCTGTCACCCAGGCCGGAG
chrom    TCTTAGGGGTTTCAATTATTCTTGGTAT--------AATAT---TATTTGA---ATTTATGTA
right    TCTTAGGGGTTTCAATTATTCTTGGTAT--------AATAT---TATTTGA---ATTTATGTA
         ****22222***222**2**2**22*2*22222222*2*2*222*2*2*2*222*22222*22
```

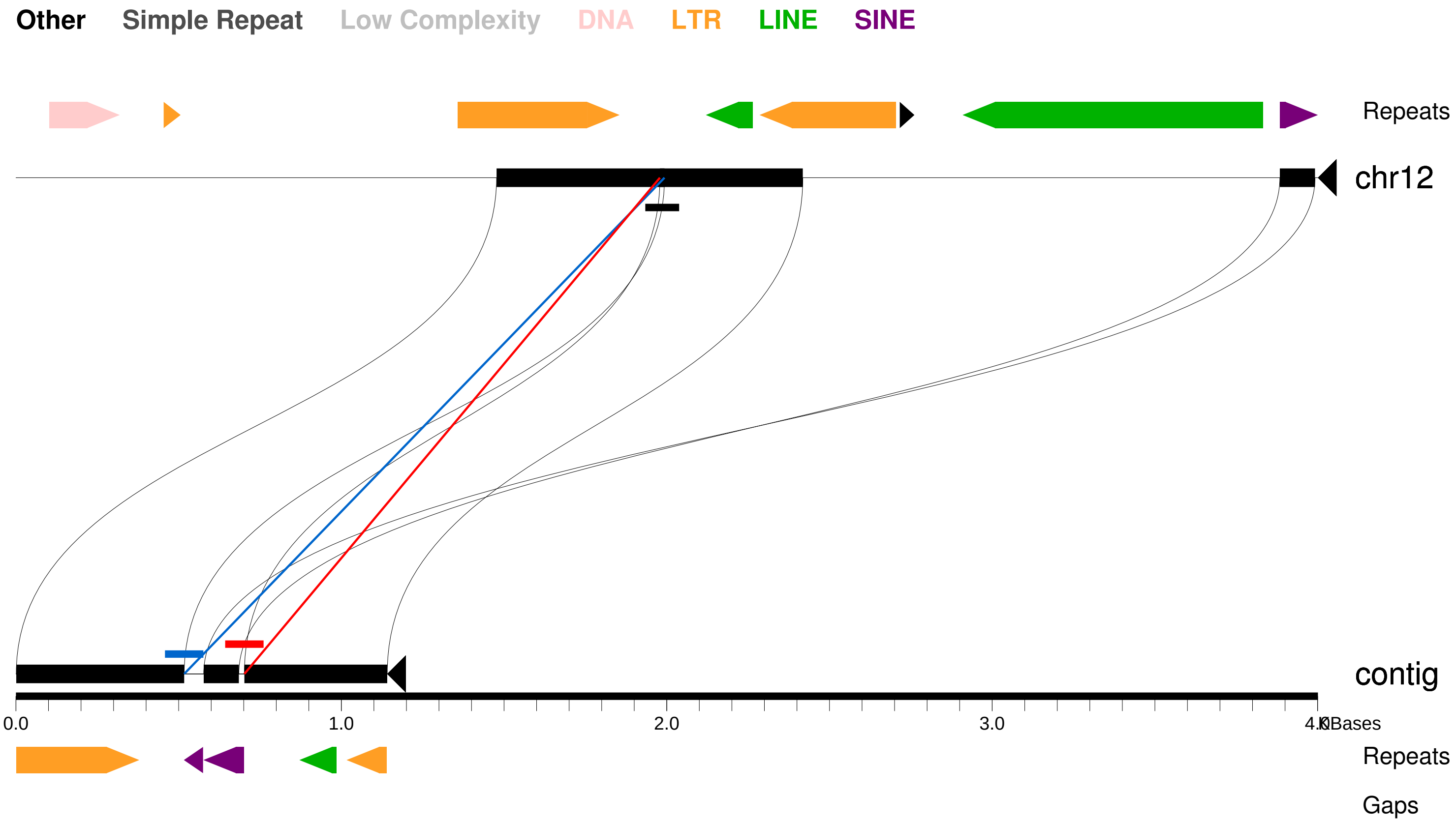

siteID: chr12_61704152 Scafftig2 - Breakpoint: chr12:61704136
chr12:61704088-61704195
Overlap Length: 12 INS size: 292 Genotyped: Yes

```
left   T-----CAAAAGGAAAACAC--------AAAAAGCATGCTTTCCTTCTTTTTATTTTGATTAAAAATTTT
chrom  T-----CAAAAGGAAAACAC--------AAAAAGCATGCTTTCCTTCTTTTTATTTTGATTAAAAATTTT
right  ATTGCGCCACTGCACTCCAGCCTGGGCGACAGAGCGAGACT-CCGTCTCAAAAAAAAAAA-AAAAATTTT
       111111*1*11*1*111**111111111*1*1***11*11*1**1***1111*11111*11*********

left   CTCGGCCGGGCGCGGTGGCTCACGCCTGTAATCCCAGCACTTTGGGAGGCCGAGGCGGGCGG
chrom  CTCAAT-GAAAAATTTTCATTACG--TGTATCTTGAACAAAAAAGTATTTCAAA--------
right  CTCAAT-GAAAAATTTTCATTACG--TGTATCTTGAACAAAAAAGTATTTCAAA--------
       ***2222*2222222*222*2***22****22222*2**22222*2*222*2*222222222
```

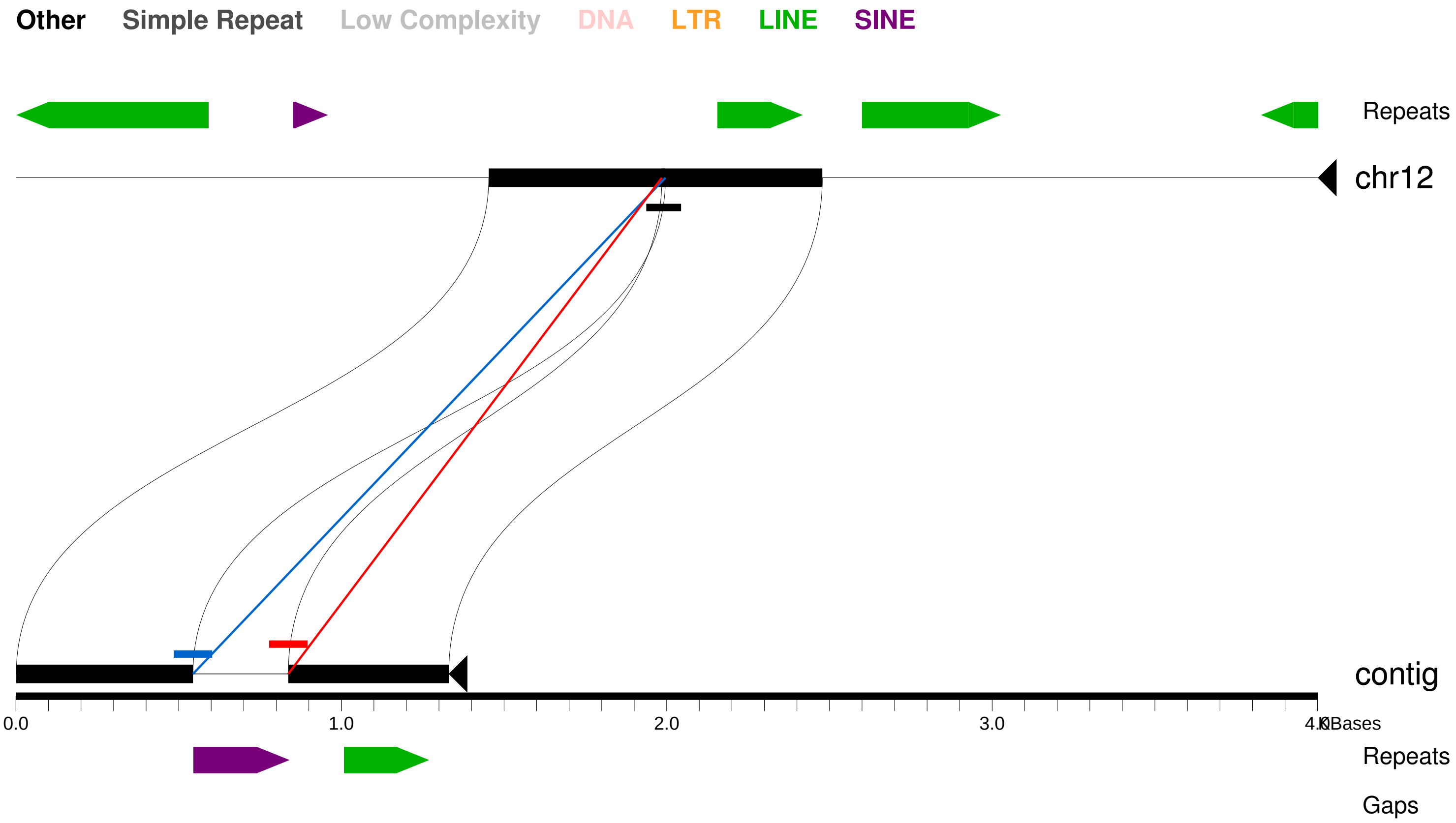

siteID: chr12_61838503 Scafftig5 - Breakpoint: chr12:61838506
chr12:61838451-61838565
Overlap Length: 5 INS size: 310 Genotyped: Yes

```
left    TCACCAGCATTAAAATTACCTTCTGTTTATTTCTTAGGACTCTGACATGACAATA----AATTTTTTTTT
chrom   TCACCAGCATTAAAATTACCTTCTGTTTATTTCTTAGGACTCTGACATGACAATA----AATTTCAAAAC
right   CCGCCCGCCTCGGCCTCCCAAAGTGCTGGGATTACAGGCGTGAGCCACCACGCCCGGCCAATTTCAAAAC
        1*1**1**1*11111*11*1111**1*1111*111***11*11*1**11**11111111*****222222

left    TTTTTTTTTTTTTTTTTTTTTTTGAGACGGAGTCTCGCTCTGTCGCCCAGGCT
chrom   AAGATTCAAGGAATCAGATATTC-AGAT--ATTATTGCTTAGGCTCT-ATTCA
right   AAGATTCAAGGAATCAGATATTC-AGAT--ATTATTGCTTAGGCTCT-ATTCA
        2222**2222222*2222*2**22***222*2*2*2***22*2*2*22*22*2
```

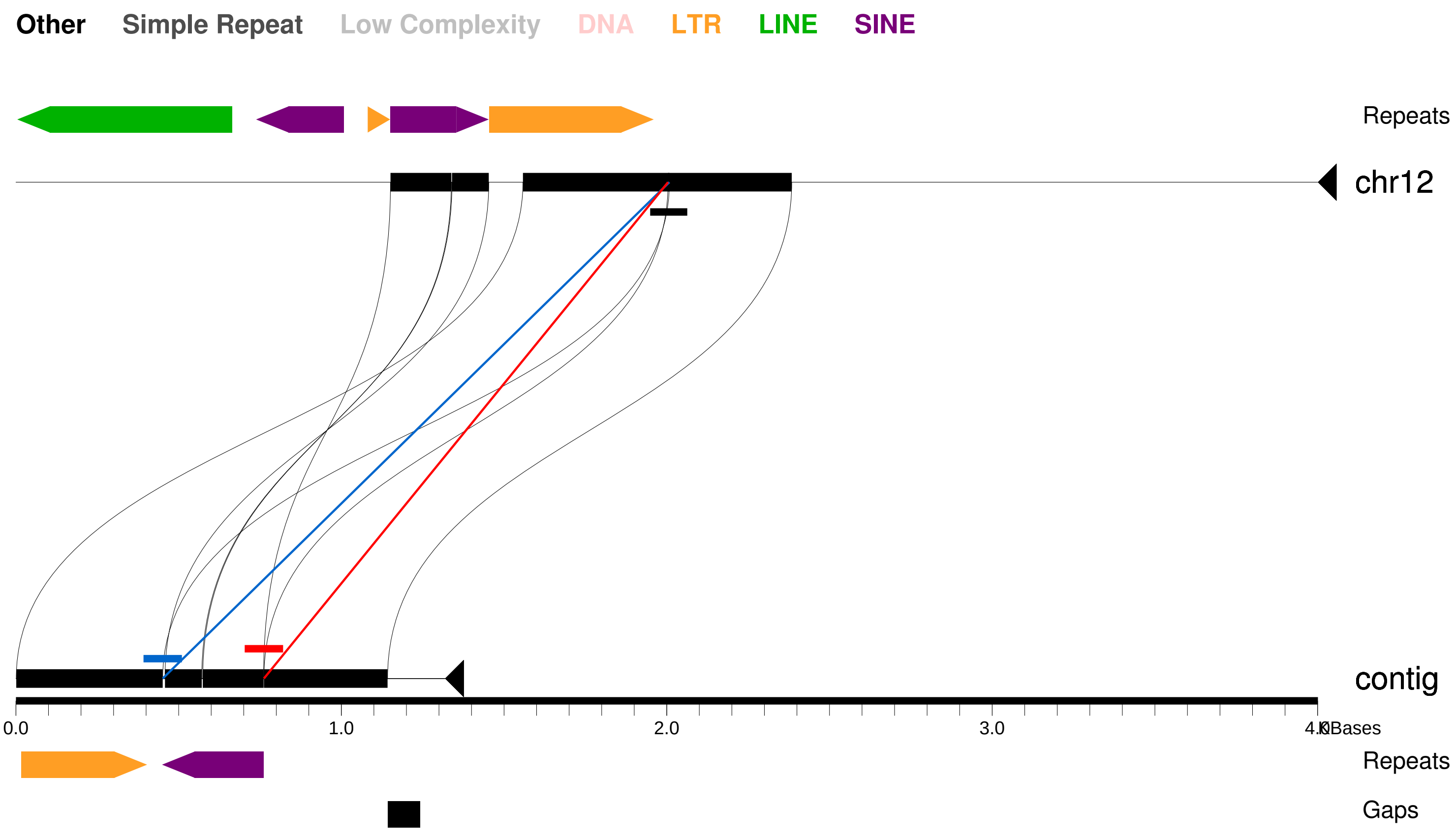

siteID: chr12_62417403 Scafftig1 + Breakpoint: chr12:62417375
chr12:62417333-62417434
Overlap Length: 18 INS size: 412 Genotyped: No

```
left     AAGACTTATGAACCATGAGCTTAAAATCATGATTTTGTAACT-----------------AAAAAGATACT
chrom    AAGACTTATGAACCATGAGCTTAAAATCATGATTTTGTAACT-----------------AAAAAGATACT
right    NNNNNNNNNNNNNNNNNNNNNNNNNNNNNNNNNNNNNNNNNNNNNNNNNNNNNNNNNNNAAAAAGATACT
         11111111111111111111111111111111111111111111111111111111111***********

left     GAAGTGTGGCCGGGCGCGGTGGCTCACGCCTGTAATCCCAGCACTTTGGGAGGCCGAGGCGGGCGG
chrom    GAAGTGTATCTGG--------AATTTCCTCTAAATTTCCAACA---------GCAGCAACATACCT
right    GAAGTGTATCTGG--------AATTTCCTCTAAATTTCCAACA---------GCAGCAACATACCT
         *******22*2**2222222222*22*22**22*2*2***2**222222222**2*222*222*22
```

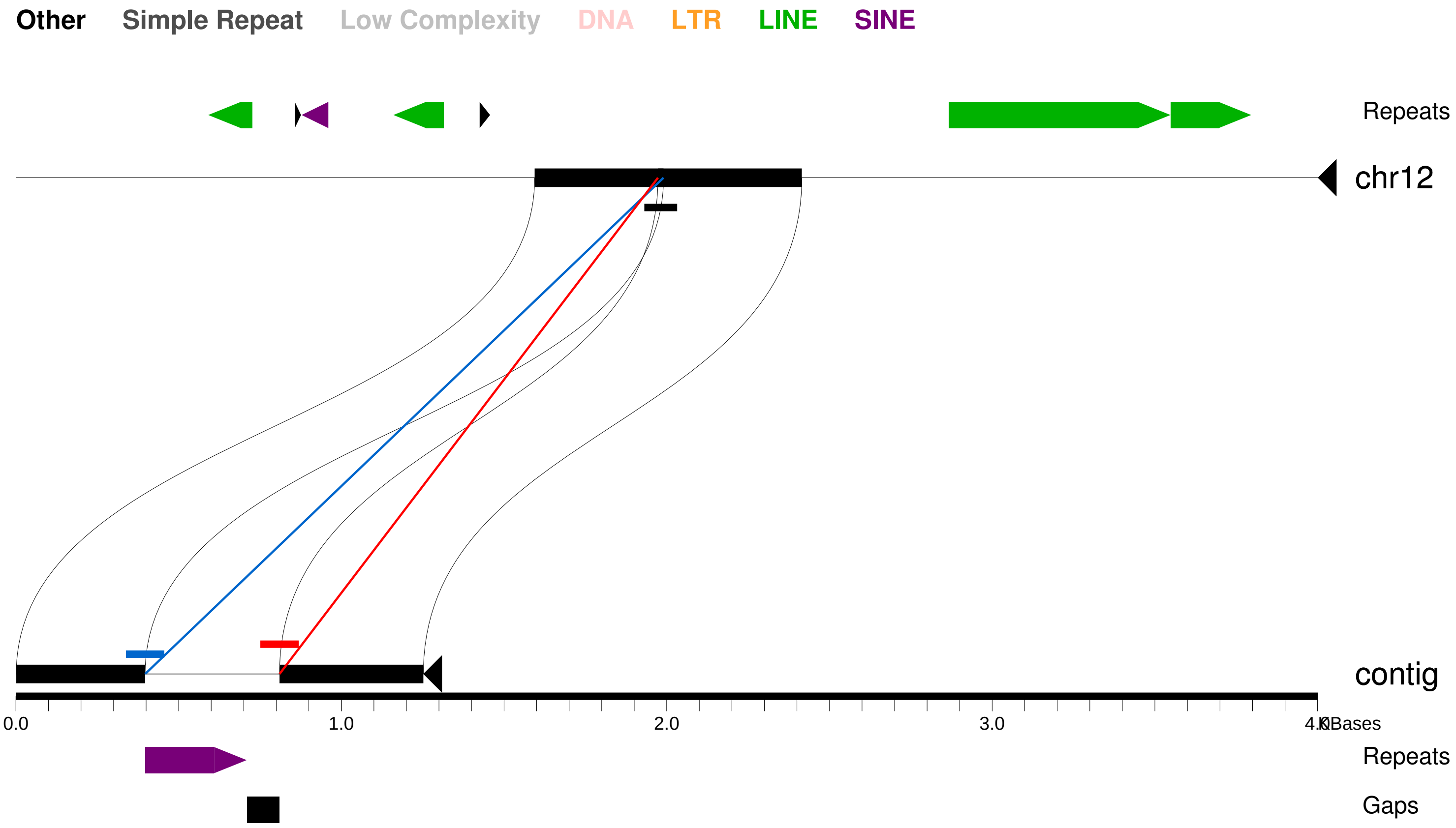

siteID: chr12_63630477 Scafftig1 + Breakpoint: chr12:63630478
chr12:63630424-63630537
Overlap Length: 6 INS size: 461 Genotyped: No

```
left    CACAGCCAATCCATCAACAATTCCTGTTACCTCTTGTTTGTGAAATAGATTCAA-----AACTGAGGCCG
chrom   CACAGCCAATCCATCAACAATTCCTGTTACCTCTTGTTTGTGAAATAGATTCAA-----AACTGAACCAC
right   NNNNNNNNNNNNNNNNNNNNNNNNNNNNNNNNNNNNNNNNNNNNNNNNNNNNNNNNNNNNAACTGAACCAC
        111111111111111111111111111111111111111111111111111111111111******22*22

left    GGCGCGGTGGCTCACGCCTGTAATCCCAGCACTTTGGGAGGCCGAGGCGGGTGG
chrom   TTCTTACAGCCTCTCCACTATCGACC-----TTGTCCAAGTCACTGTCATCTCT
right   TTCTTACAACCTCTCCACTATCGACC-----TTGTCCAAGTCACTGTCATCTCT
        22*2222212***2*22**2*222**222222*2*222**2*222*2*222*22
```

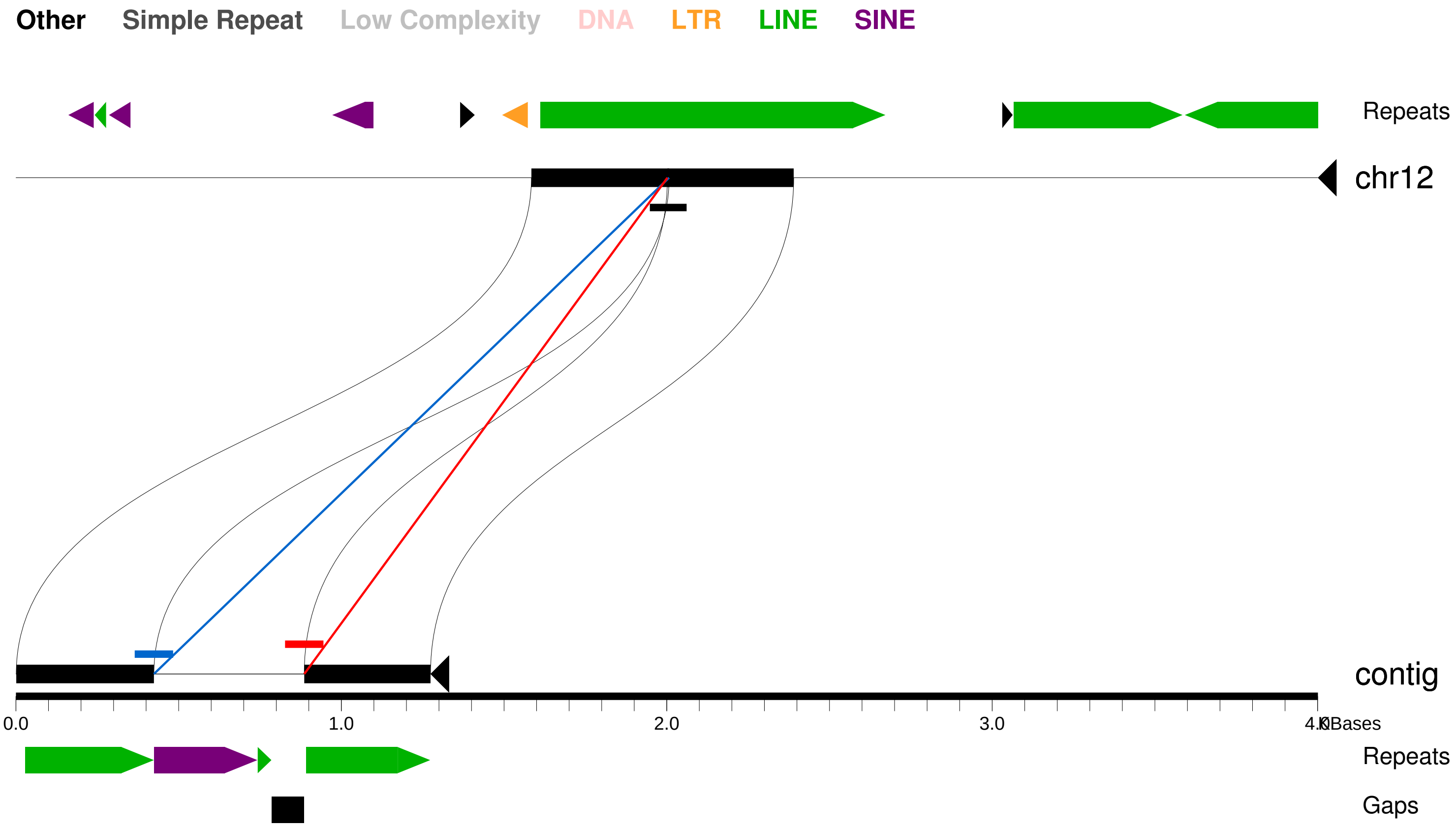

siteID: chr12_67011563 Scafftig1 - Breakpoint: chr12:67011569
chr12:67011529-67011630
Overlap Length: 20 INS size: 439 Genotyped: No

```
left    TACTACTGGCTCATGAAAGAAAAGATGTTCAACTTC-----------------ACTCAAAAGAAATGCAT
chrom   TACTACTGGCTCATGAAAGAAAAGATGTTCAACTTC-----------------ACTCATAAGAAATGCAT
right   NNNNNNNNNNNNNNNNNNNNNNNNNNNNNNNNNNNNNNNNNNNNNNNNNNNNAAAAAAAAGAAATGCAT
        11111111111111111111111111111111111111111111111111111*111*N***********

left    ATTAAAGGC-CGGGCGCGGTGGCTCACGCCTGTAATCCCAGCACTTTGGGAGGCCGAGGCGGGTGGA
chrom   ATTAAAGCCACAGGAAATAT-----AATCTTTT----TCACTTCTTAGATTAGCAAA---------A
right   ATTAAAGCCACAGGAAATAT-----AATCTTTT----TCACTTCTTAGATTAGCAAA---------A
        *******2*2*2**22222*22222*22*2*2*22222**222***2*2222**22*222222222*
```

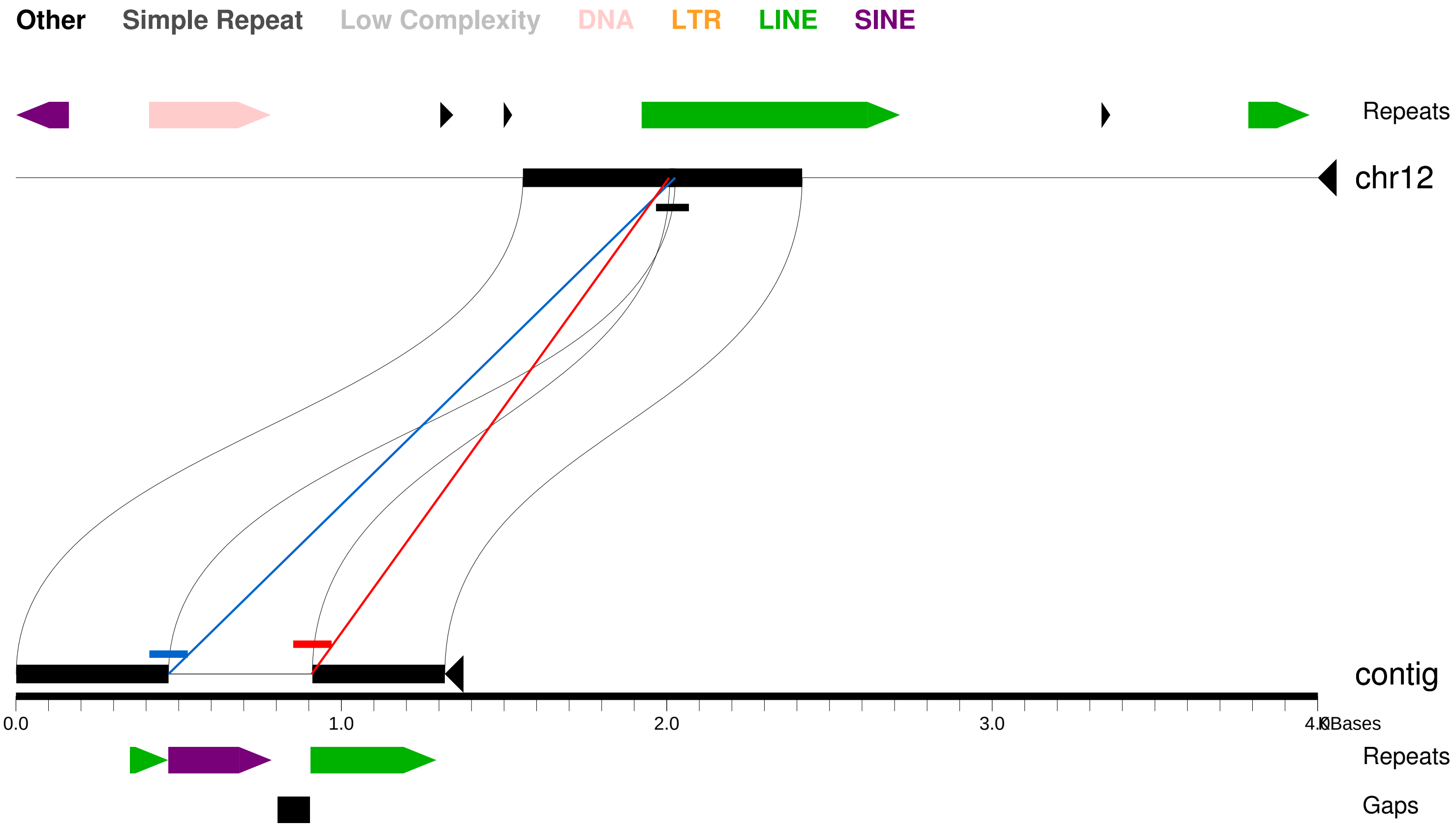

siteID: chr12_68229348 Scafftig1 + Breakpoint: chr12:68229323
chr12:68229286-68229382
Overlap Length: 16 INS size: 306 Genotyped: Yes

```
left    A------TCCTTTGCCCACTT--GTAATGGAATTATTGG-------------GCTCTT-TGCTATTGAGT
chrom   A------TCCTTTGCCCACTT--GTAATGGAATTATTGG-------------GCTCTT-TGCTATTGAGT
right   TCCGCCCGCCTCGGCCTCCCAAAGTGCTGGGATTACAGGCGTGAGCCACCGCGCCCGGCTGCTATTGAGT
        11111111***11***11*1111**11***1****11**1111111111111**1*111***********

left    TCCTTTTTTTTTTTTTTTTTTTTTTTTTTGAGACGGAGTCTCGCTCTGTCGCCCAGGCTGGAGTGCAGTG
chrom   TCCTTGTATGTTCTAGATATTAATCCCTTGTT--GGA---------TG-----CA---TAGTTTGCAA--
right   TCCTTGTATGTTCTAGATATTAATCCCTTGTT--GGA---------TG-----CA---TAGTTTGCAA--
        *****2*2*2**2*222*2**22*222***2222***222222222**22222**222*2*22****222

left    G
chrom   -
right   -
        2
```

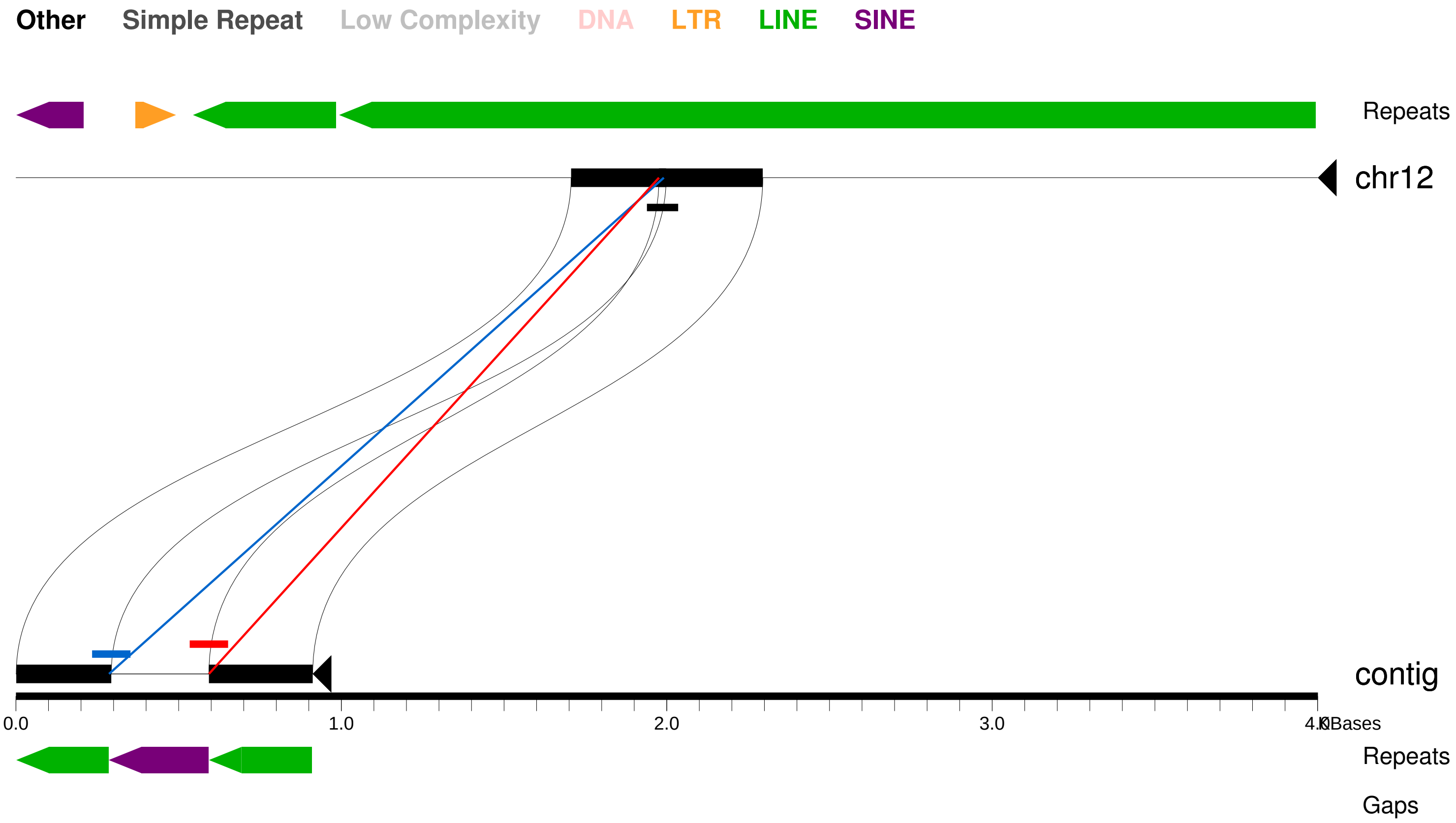

siteID: chr12_70679900 Scafftig2 + Breakpoint: chr12:70679887
chr12:70679875-70679946
Overlap Length: 48 INS size: 417 Genotyped: No

```
left     T------------------AAA------------------TAAGCGAT-----------CATATTTTCTA
chrom    T------------------AAA------------------TAAGCGAT-----------CATATTTTCAA
right    CCGCCCGTCTCGGCCTCCCAAAGTGCTGGGATTACAGGCGTGAGCCACCGCGCCCGGCCCATATTTTCAA
         1111111111111111111***111111111111111111*1***1*111111111111*********2*

left     ACACTATGTGAAAATAACAGAAGAGAGAACTTTATGAAGNNNNNNNNNNNNNNNNNNNNNNNNNNNNNNN
chrom    ACACTATGTGAAAATAACAGAAGAGAGAACTTTATGACGTTAGAGAAGT---------------------
right    ACACTATGTGAAAATAACAGAAGAGAGAACTTTATGAAGTTAGAGAAGT---------------------
         *************************************N*2222222222222222222222222222222

left     NNNNNNNNNNNNNNNNNNNNNNNNNN
chrom    --------------------------
right    --------------------------
         22222222222222222222222222
```

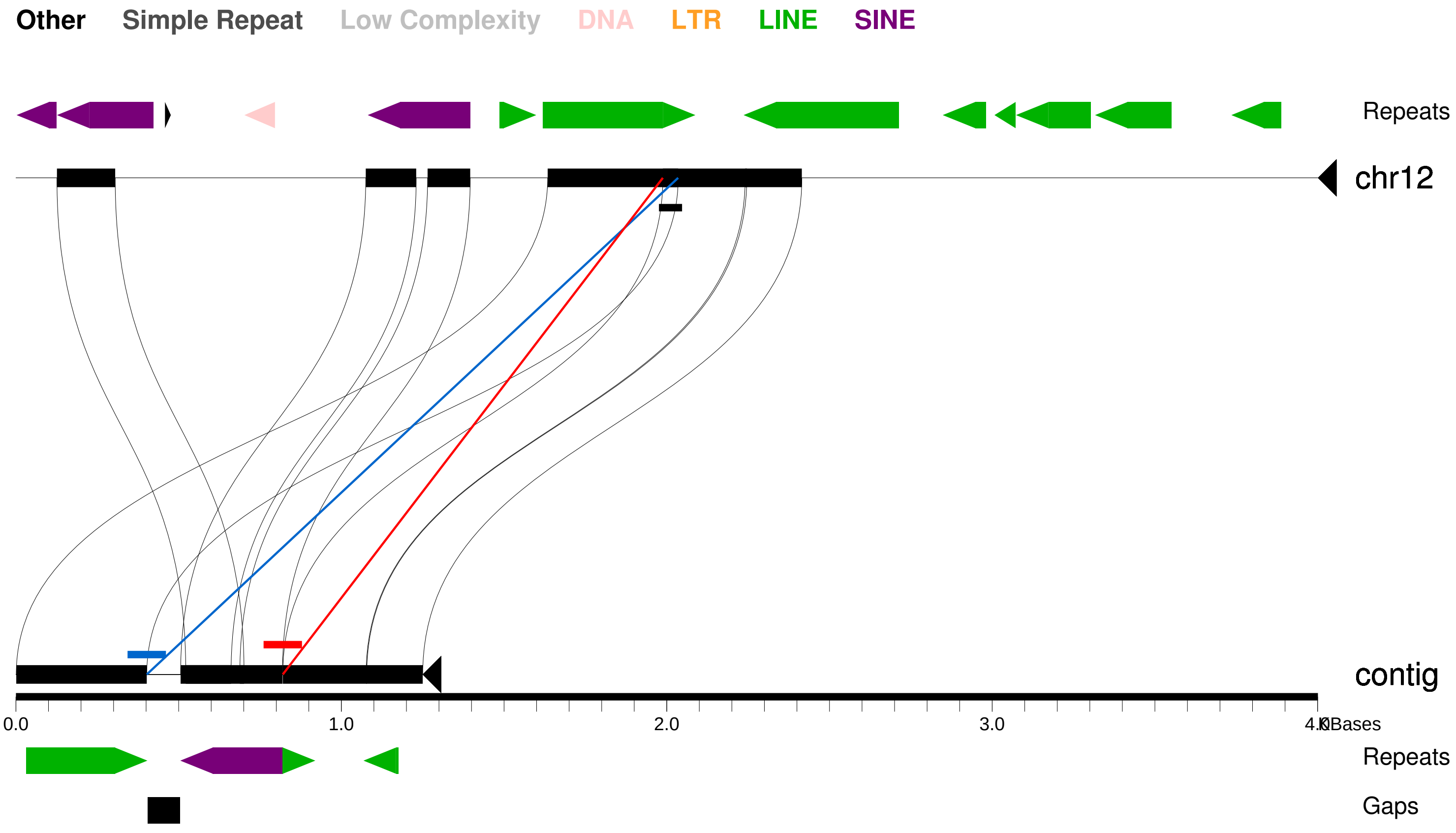

siteID: chr12_71525481 Scafftig1 + Breakpoint: chr12:71525468
chr12:71525420-71525527
Overlap Length: 12 INS size: 450 Genotyped: No

```
left   CATGTCTGATCCTAGCAAGGGTCTGTCTGACAT-----------GTAGTAGGCACTGAAGAATAACAGTA
chrom  CATGTCTGATCCTAGCAAGGGTCTGTCTGACAT-----------GTAGTAGGCACTGAAGAATAACAGTA
right  NNNNNNNNNNNNNNNNNNNNNNNNNNNNNNNNNNNNNNNNNNNNNNNNNNNNNAAAAAGGAATAACAGTA
       11111111111111111111111111111111111111111111111111111*111*1***********

left   AGGCCGGGCGCGGTGGCTCACGCCTGTAATCCCAGCACTTTGGGAGGCCGAGGCGGGTGG
chrom  AATAAATGAAATACTAATATCAATTGTAAT--------TTTTGTACACATTTC---TTCT
right  AATAAATGAAATACTAATATCAATTGTAAT--------TTTTGTACACATTTC---TTCT
       *222222*222222222*22*222******22222222***2*2*22*222222222*22
```

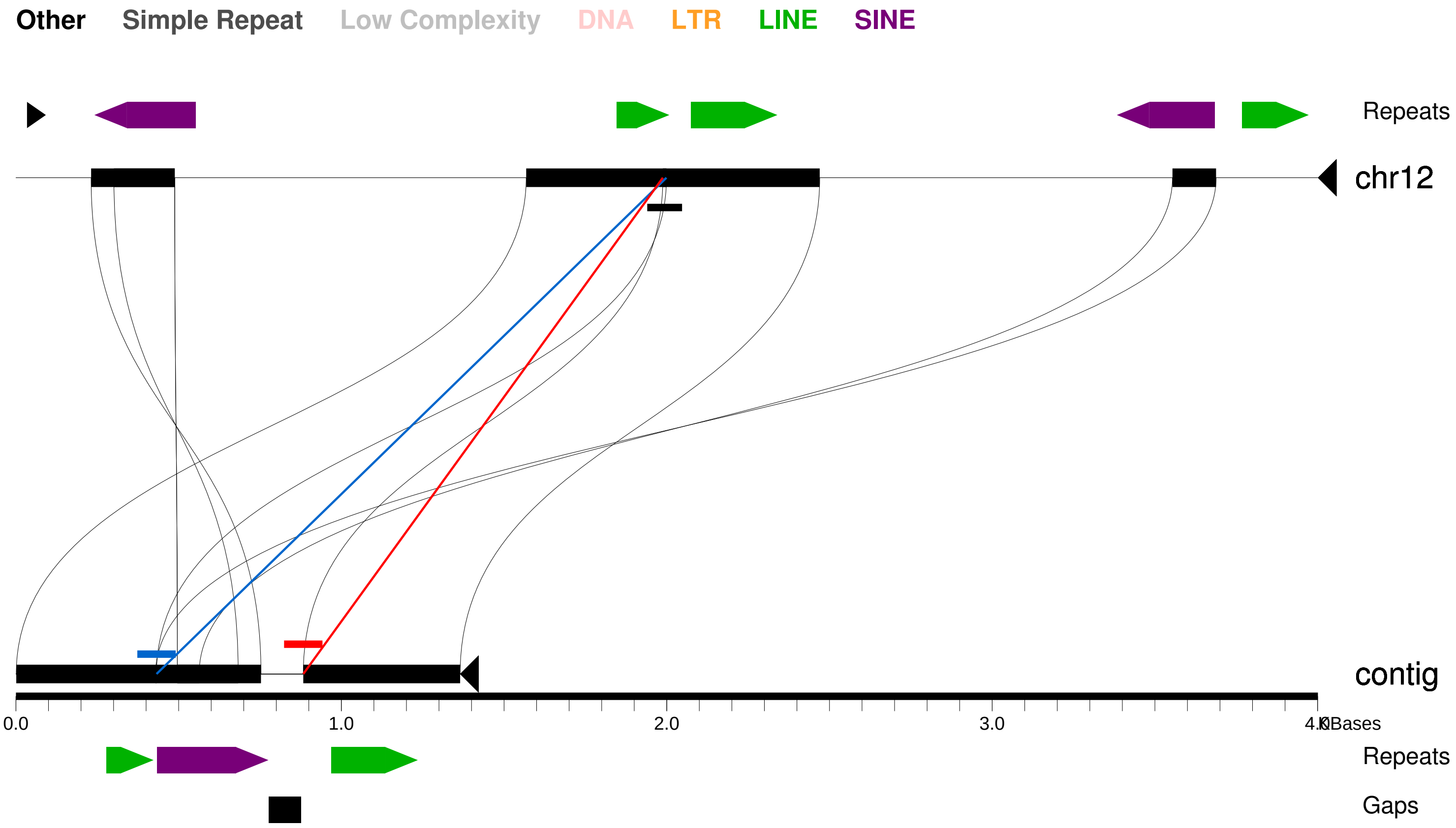

siteID: chr12_73056541 Scafftig1 - Breakpoint: chr12:73056650
chr12:73056591-73056711
Overlap Length: -1 INS size: 141 Genotyped: Yes

```
left    GTCCACTGTTTTTTTTTCTGT--TCCAAAAT--CAATTACATCACAACTAATTGACATAATCTGTATGGTG
chrom   GTCCACTGTTTTTTTTTCTGT--TCCAAAAT--CAATTACATCACAACTAATTGACATAATCTGTCATATT
right   ACCGAGA-TTTCGCCACTGCACTCCAGCTGGGCGTTGGAGTGAGACCC---TGTCTTAAAAAA--ATATT
        11*1*111***11111***111****111111*11*1111*1*1*1*1111**1*1***11111N222*2

left    GGCGTCTGTAGTCCCAGCT-ACTAGGGAGGCTGAGGTGGGAGGACTGCC-TGAGC-
chrom   TCATTCAGTTG-CCAAGCTCACTCCACAGTACTAAATATATCTTCTATAGTGGTCA
right   TCATTCAGTTG-CCAAGCTCACTCCACAGTACTAAATATATCTTCTATAGTGGTCA
        2222**2**2*2**2****2***2222**2222*22*2222222**2222**22*2
```

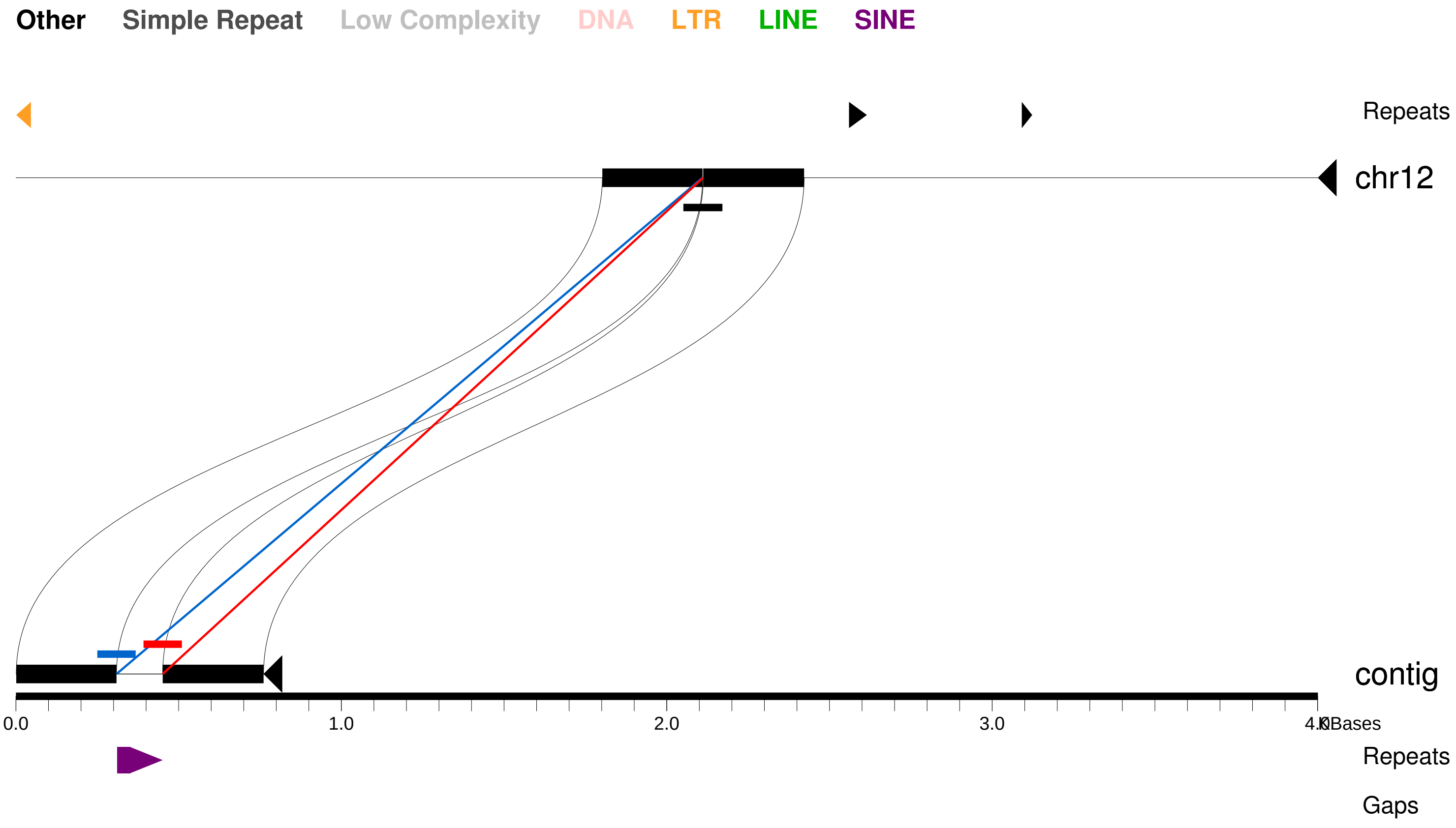

siteID: chr12_75320674 Scafftig5 + Breakpoint: chr12:75320677
chr12:75320632-75320736
Overlap Length: 15 INS size: 304 Genotyped: Yes

```
left     AAAAAGGATGAG--TTC--ATGTCCTTTGAATGGACATGTATGAA------------GCTGGAAACCATC
chrom    AAAAAGGATGAG--TTC--ATGTCCTTTGAATGGACATGTATGAA------------GCTGGAAACCATC
right    CACCCGCCTCGGCCTCCCAAAGTGCTGGGATT--ACAGGCGTGAGCCACCGCGCCCGGCCTGAAACCATC
         1*111*11*11*11*1*11*1**1**11**1*11***1*11***1111111111111**11*********

left     ATTCTTTTTTTTTTTTTTTTTGAGACGGAGTCTCGCTCTGTC-GCCCAGGTCGGACTGC--GGACTGC
chrom    ATTCTT-----------------GGCAAACTATCACAAAGACAGAAAACCAAACACTGCATGTTCTCA
right    ATTCTT-----------------GGCAAACTATCACAAAGACAGAAAACCAAACACTGCATGTTCTCA
         ******22222222222222222*2*22*2*2**2*222*2*2*222*222222*****22*22**22
```

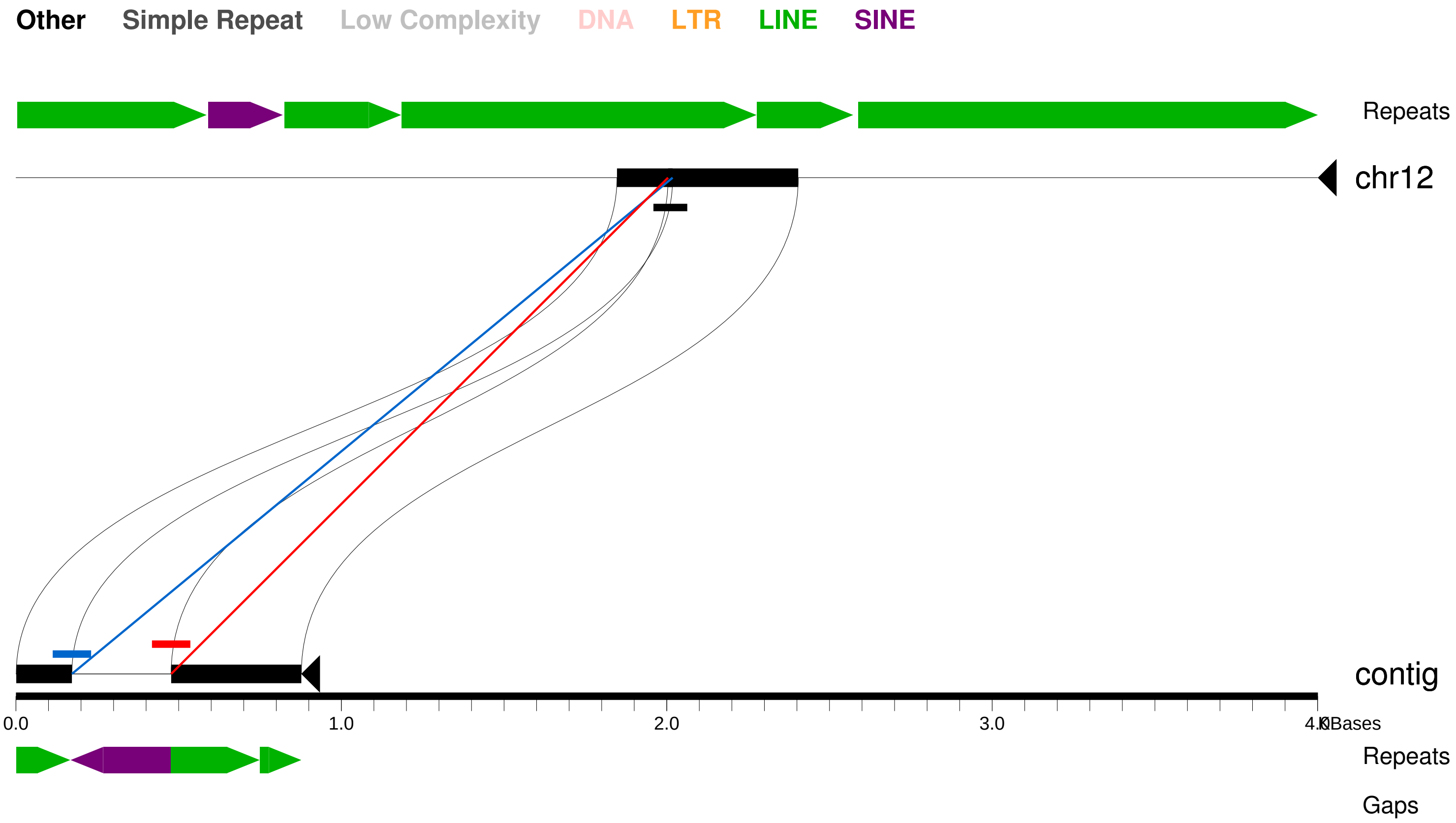

siteID: chr12_7603964 Scafftig2 + Breakpoint: chr12:7603969
chr12:7603950-7604028
Overlap Length: 41 INS size: 403 Genotyped: No

```
left    AA--------GT------------ACATCATTTTCC-----------------AAA---GAACGTGATTT
chrom   AA--------GT------------ACATCATTTTCC-----------------AAA---GAACGTGATTT
right   AGCCGGGATGGTCTCGATCTCTTGACCTCGTGATCCGCCCGCCTCGGCCTCCCAAAGTGGAACGTGATTT
        *111111111**111111111111**1**1*11***11111111111111111***111***********

left    TTTTAACTAGGTGACTGAAGTGACCAAGTGNNNNNNNNNNNNNNNNNNNNNNNNNNNNNNNNNNNNNNNN
chrom   TTTTAACTAGGTGACTGAAGTGACCAAGTGATATAGACATTAAAAATAA---------------------
right   TTTTAACTAGGTGACTGAAGTGACCAAGTGATATAGACATTAAAAATAA---------------------
        ******************************2222222222222222222222222222222222222222

left    NNNNNNNNNNNNNNNNNNN
chrom   -------------------
right   -------------------
        2222222222222222222
```

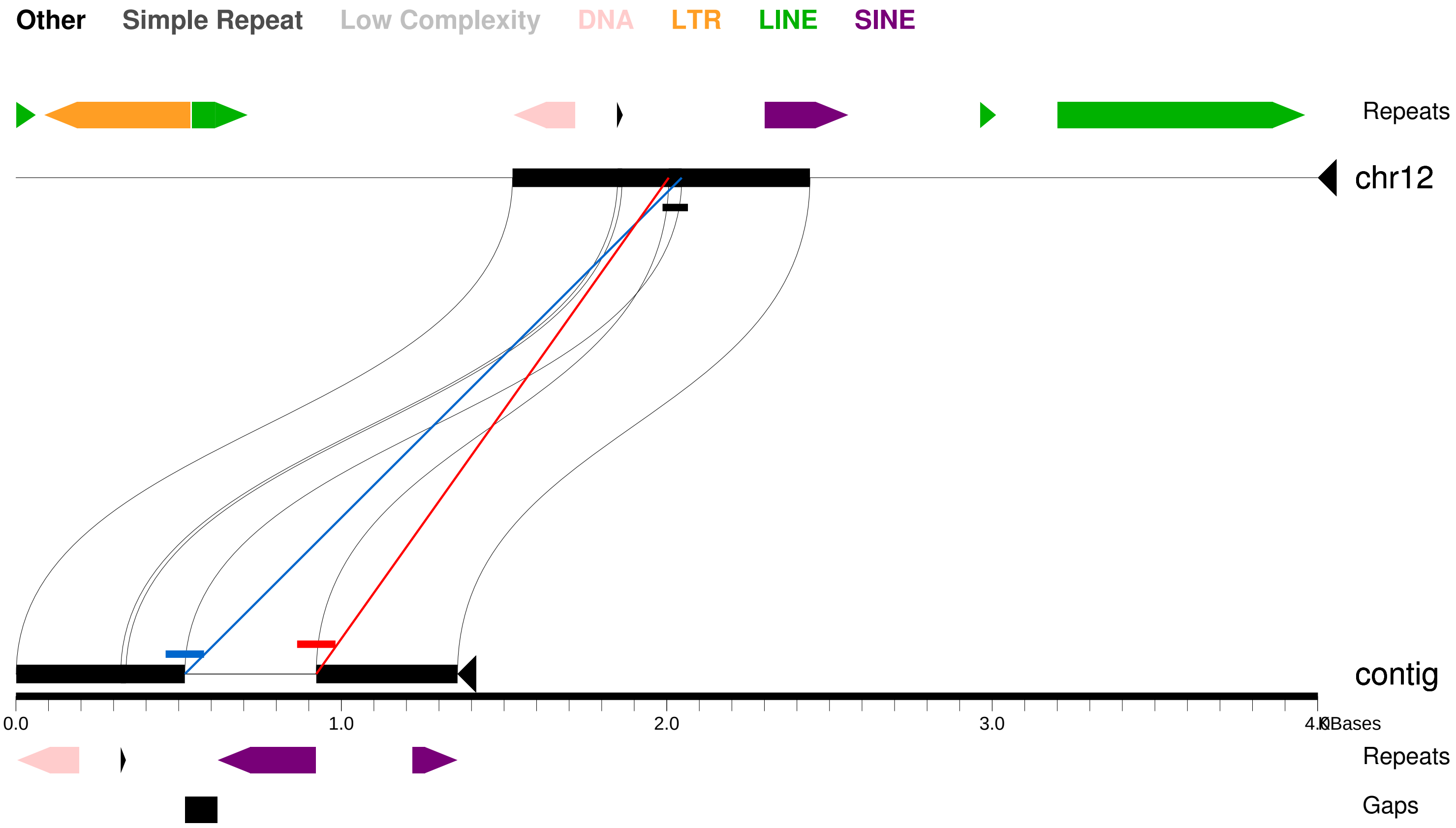

siteID: chr12_7624174 Scafftig2 - Breakpoint: chr12:7624181
chr12:7624137-7624231
Overlap Length: 16 INS size: 297 Genotyped: Yes

```
left   C------------------TTCAG--TGTGCATAAGAATTACTTTTATTCC----ATGGAAATACAGTAA
chrom  C------------------TTCAG--TGTGCATAAGAATTACTTTTATTCC----ATGGAAATACAGTAA
right  CGAGATCCCGCCACTGCACTCCAGCCTGGGCGACAGAGCGAGACTCCGTCTCAAAAAAAAAAAAAAAAAA
       *111111111111111111*1***11**1**111***111*111*111**11111*111***1*1*11**

left   GATTAAGAAATGCCGGCCGGGCGCGGTGGCTCACGCCTGTAATCCCAGCACTTTGGGAGGCCGAGGCGGG
chrom  GATTAAGAAATGCCATTTATAGGAGCTGGC----------AA-------------GATGGCTGAATAGGA
right  GATTAAGAAATGCCATTTATAGGAGCTGGC----------AA-------------GATGGCTGAATAGGA
       *************22222222*2*2****2222222222**2222222222222*22***2**222**2

left   CGG
chrom  AC-
right  AC-
       222
```

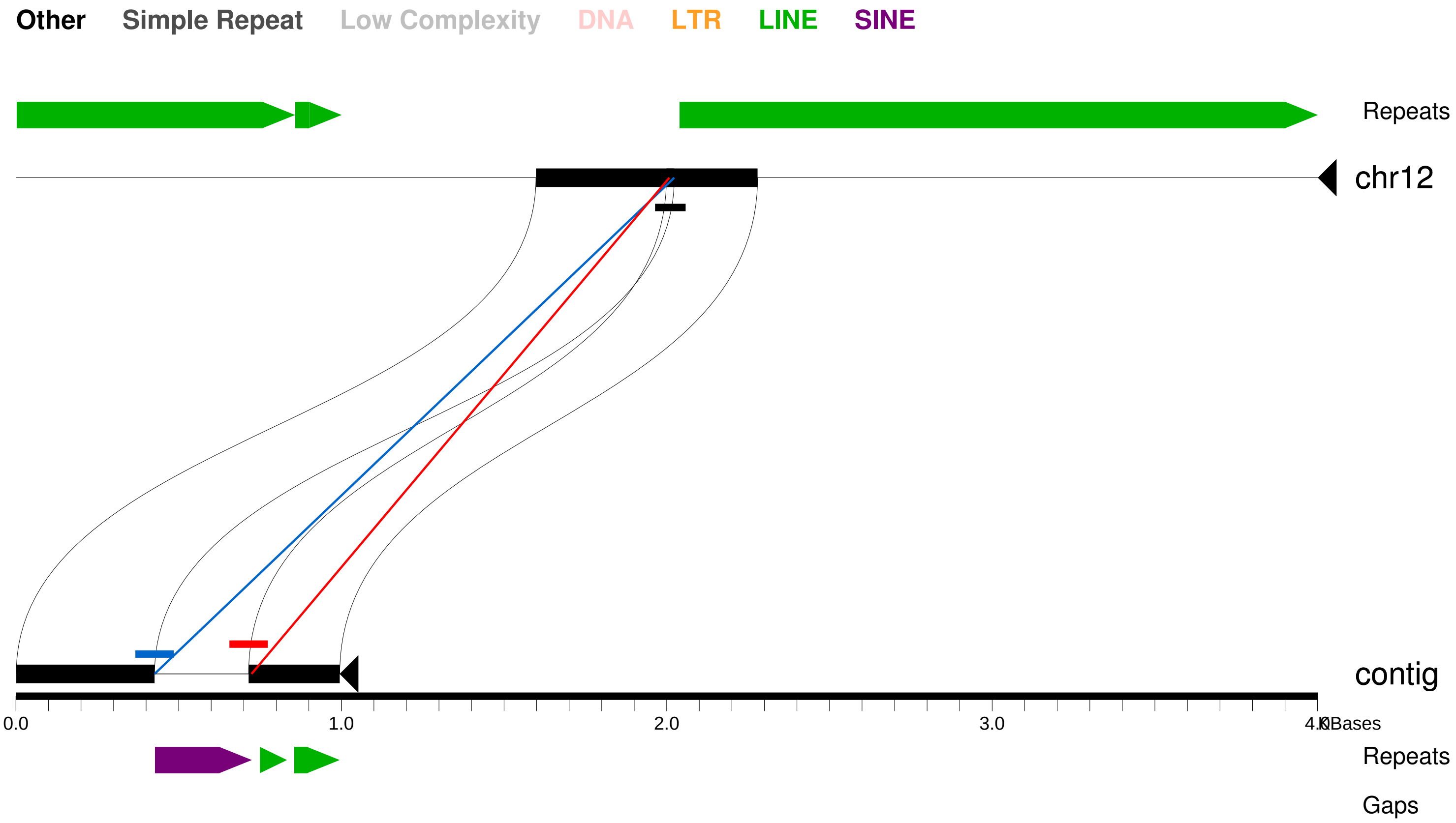

siteID: chr12_76823055 Scafftig1 - Breakpoint: chr12:76823044
chr12:76822913-76823113
Overlap Length: 15 INS size: 302 Genotyped: Yes

```
left   ATTAAATATGTCTCAACATGAAAGCACATGTAAATACACATACAAAAATAAAAGGATAGCTTAAAACAAT
chrom  A--------------ACATGAAAGCACATGTAAATACACATACAAAAATAAAAGGATAGCTTAAAACAAT
right  C--------------AGGAGAATGGC---GTGAACCCGGG-----------AGGCGGAGCTTGCAGTGAG
       122222222222222*111***1*11111**1**11*11111111111111*1*111*****11*111*1

left   TTTAGATTAG---ACAGCATTCTTATCCATGTACATATATTCATTGCAAGAAGTATACGACCGTACTTTC
chrom  TTTAGATTAG---ACAGCATTCTTATCCATGTACATATATTCATTGCAAGAAGTATACGACCGTACTTTC
right  CCGAGATTGCGCCACTGCAGTCCACAGTCCGGCCTGGGCGACAGAGCGAGAC---TCCGTCTCAAAAAAA
       111*****11111**1***1**11111111*11*1111111**11**1***1111*1**1*111*11111

left   CTCTGGATAAAAAGTTCTTTATAGGCCGGGCGCGGTGGCTCACGCCTGTAATCCCAGCACTTTGGGAGGC
chrom  CTCTGGATAAAAAGTTCTTTATAA-------------TTTCATATATATAATTAAA--------------
right  AAAAAAA-AAAAAGTTCTTTATAA-------------TTTCATATATATAATTAAA--------------
       111111*1***************2222222222222222***2222*2****222*22222222222222

left   CGAGGCGGGTGGATCATGAGGTCAGGAGATCGAGACCATTCTGGCTAACAAGGTGAAACCCCGTCTCTAC
chrom  ------------ATCATCCAATAAAAATTTAGTGAGCAC---------------------------CTAC
right  ------------ATCATCCAATAAAAATTTAGTGAGCAC---------------------------CTAC
       222222222222*****2222*2*22*22*2*2**2**2222222222222222222222222222****

left   TAAA------------------------------------------------------------------
chrom  TATG------------------------------------------------------------------
right  TATGTGTTGGGAACTATGTTAGGCACAGCTATGTTAGGCACCTTAGTTTCCGCTCTTACTGAGCTTTCAA
       **221111111111111111111111111111111111111111111111111111111111111111111

left   -----------------------------
chrom  -----------------------------
right  TATGAATGAGTACATGTTCATTATATGTT
       11111111111111111111111111111
```

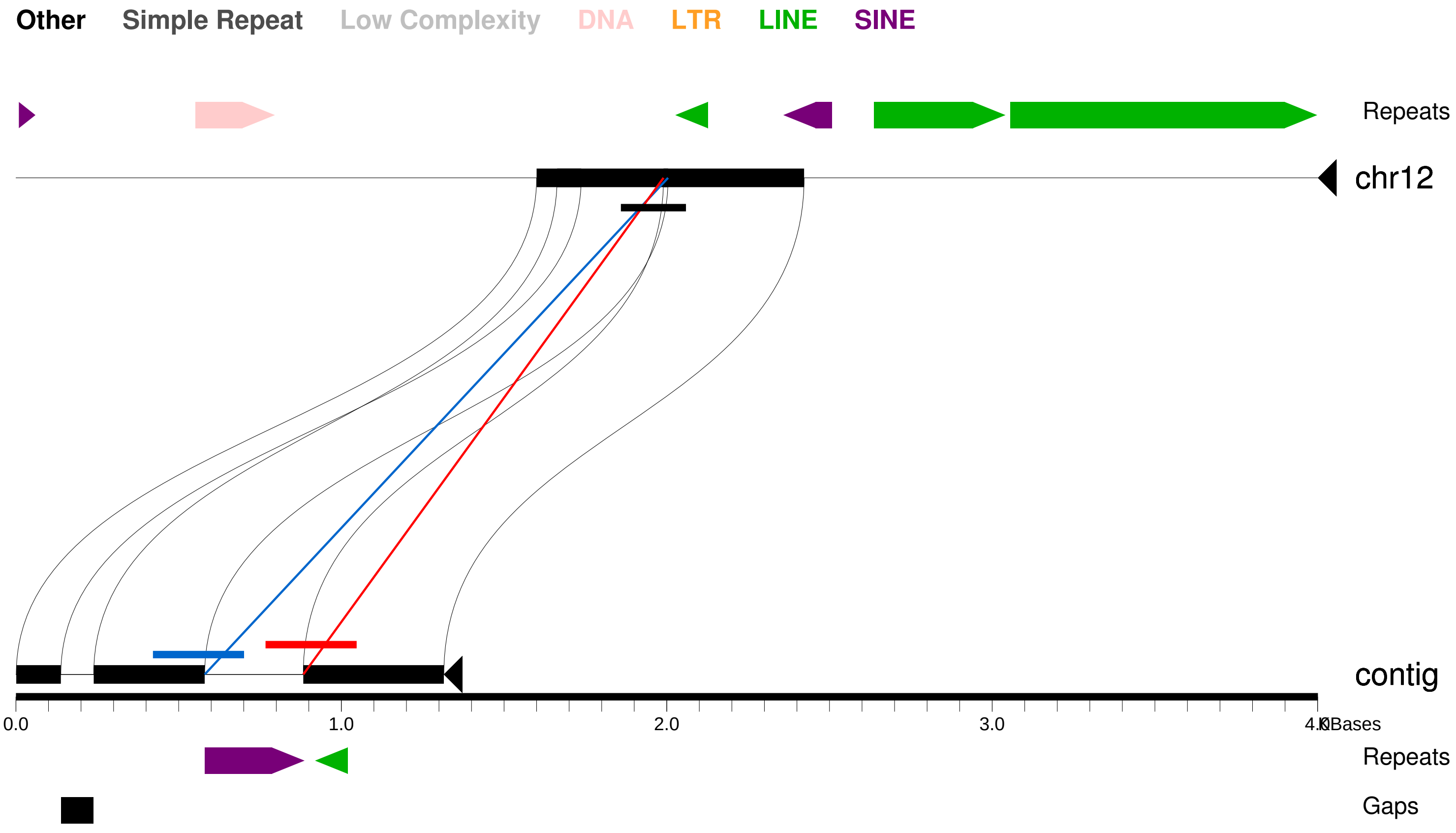

siteID: chr12_77965034 Scafftig1 - Breakpoint: chr12:77965041
chr12:77964997-77965100
Overlap Length: 16 INS size: 300 Genotyped: Yes

```
left     TTA---TCTAGC-TGGCCTTG-----GAAACCACTGG------ATTATATGATCCCTTTAAGAAGAAGTA
chrom    TTA---TCTAGC-TGGCCTTG-----GAAACCACTGG------ATTATATGATCCCTTTAAGAAGAAGTA
right    CTGCACTCCAGCCTGGGCGACAGAGCGAGACTCCGTCTCAAAAAAAAAAAAAAAAAAAAAAGAAGAAGTA
         1*1111**1***1***1*11111111**1**11*111111111*11*1*11*1111111***********

left     CTGTGGCCGGGCGCGGTGGCTCACGCCTGTAATCCCAGCACTTTGGGAGGCCGAGGCGGGCGGA
chrom    CTGTG------CATAGTGTAT-----CTGTAA-CCCAGTAAGGCACTTGACAGATTTGTTT---
right    CTGTG------CATAGTGTAT-----CTGTAA-CCCAGTAAGGCACTTGACAGATTTGTTT---
         *****222222*222***22*22222******2*****2*22222222*2*2**222*222222
```

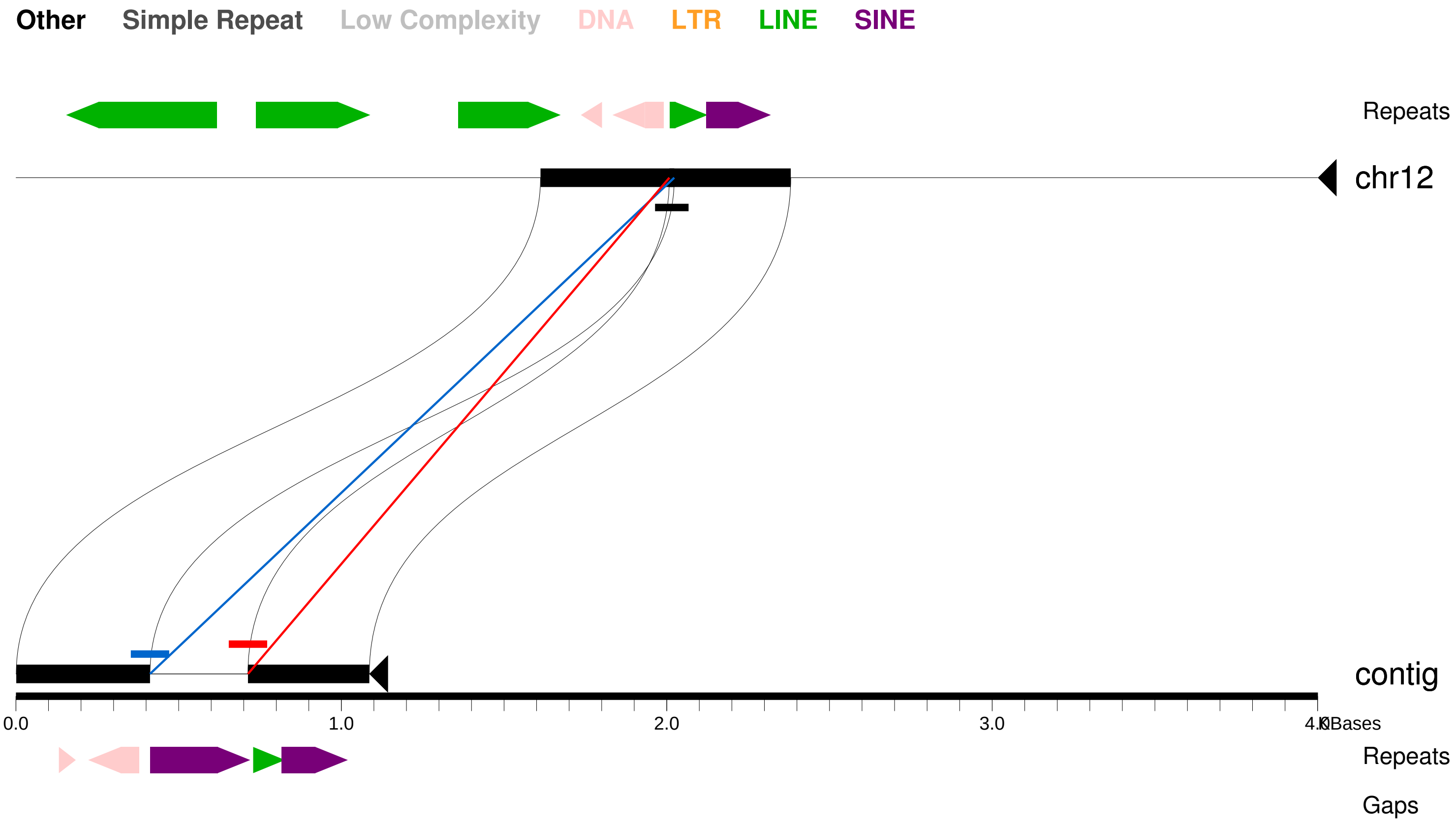

siteID: chr12_78396945 Scafftig1 + Breakpoint: chr12:78396934
chr12:78396774-78397108
Overlap Length: 15 INS size: 319 Genotyped: Yes

```
left    ------------AAATTGTACAACTCTCCACAGAGTGGCACTCTAATATTGCAAAGGTACAATATAAGCA
chrom   GTAAGCTTCTCTAAATTGTACAACTCTCCACAGAGTGGCACTCTAATATTGCAAAGGTACAATATAAGCA
right   TTTAG------TAGAG-------------ACGGGGTTTCACCTTGTTA--GCCAGGATGGTCTCGATCTC
        N2N22      2*1*11111111111111**1*1**11***11*11**11**1*1*1*1111*11*1111

left    TGTGCAGAATGAACAGCTCTTCTAGGATCCCTATAAAACTCCACCCCATGTTTCTGTTGAGTCACTATAA
chrom   TGTGCAGAATGAACAGCTCTTCTAGGATCCCTATAAAACTCCACCCCATGTTTCTTTTGAGTCACTATAA
right   CTGACCTCATGATCCACCCGCCTCGG---CCTCCCAAAGTGC---------------TGGG--ATTACAG
        1111*111****1*11*1*11**1**111***111***1*1*1111111111111N1**1*11*1**1*1

left    AATTGATG-ACTGAGGCAATACACGAGCAATTATTTTTTTTTTTTTTTTTTT------TTTTTTTTTTTTT
chrom   AATTGATG-ACTGAGGCAATACACGAGCAATTATTTATGCAAAGCAGTGTGAGGAGGTGCATGAGGATCC
right   GCGTGAGCCACCGCGCCCGGCCACGAGCAATTATTTATGCAAAGCAGTGTGAGGAGGTGCATGAGGATCC
        111***111**1*1*1*1111***************2*222222222*2*2222222*222*22222*22

left    TTTTGAGACGGAGTC-----TCGCTCTGTCGCCCAGGCCGG----ACT-------------GC----GGA
chrom   ACTTAATACCAAGACATATTTAGATTTGACTGCCACTTTTCTTCAACTTAATTTTTTTAAAGCATTTGGT
right   ACTTAATACCAAGACATATTTAGATTTGACTGCCACTTTTCTTCAACTTAATTTTTTTAAAGCATTTGGT
        22**2*2**22**2*22222*2*2*2**2*22***2222222222***2222222222222**2222**2

left    CTGCAGTGGCGCAATCTCGG--CTCACTG-----CAAGCTCCG---CTTCCCGGGT
chrom   ATGTTGTTGTGGAATTAAAGTCCTAATTATTTATCAAAATAAGTGGCATAGCCACA
right   ATGTTGTTGTGGAATTAAAGTCCTAATTATTTATCAAAATAA--------------
        2**22**2*2*2***2222*22**2*2*222222***22*221   1N1NN1NNNN
```

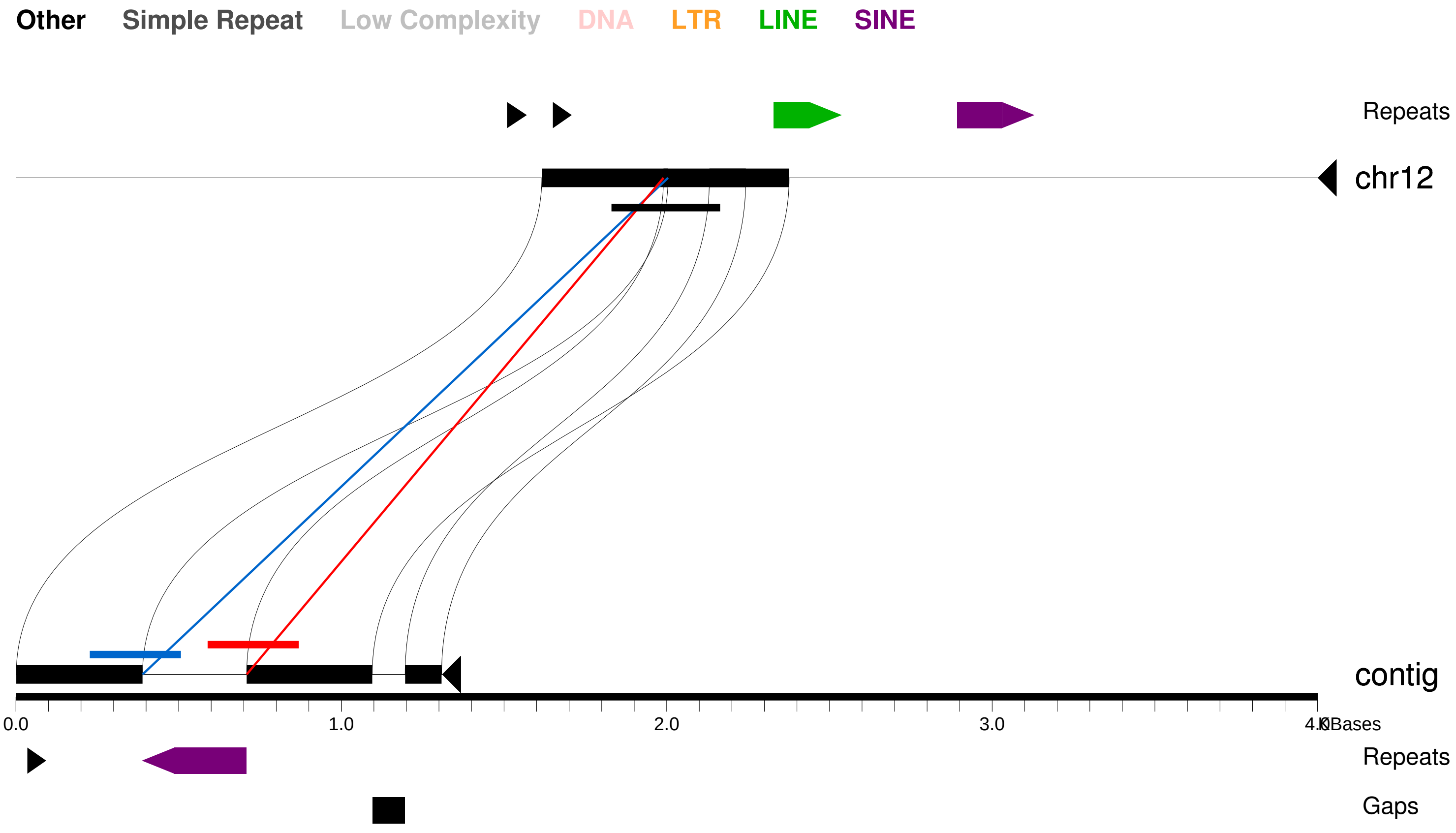

siteID: chr12_80189324 Scafftig1 + Breakpoint: chr12:80189273
chr12:80189137-80189482
Overlap Length: 74 INS size: 425 Genotyped: No

```
left   ----------------------------------------------------------------------
chrom  TTACCTATTATAAAATAATCAATACATGCACTCTGAGCTTCAGACATTATTATTTCTATTCCATAATTAG
right  NNNNNNNNNNNNNNNNNNNNNNNNNNNNNNNNNNNNNNNNNNNNNNNNNNNNNNNNNNNTC---------
       NNNNNNNNNNNNNNNNNNNNNNNNNNNNNNNNNNNNNNNNNNNNNNNNNNNNNNNNNNN22

left   ------------------------------CAATCTAAGTAGTTAACTATCCTTAAAAATAATTTTTCTT
chrom  GTCAGAACCACTCCTTTTAAGTAGTACCAACAATCTAAGTAGTTAACTATCCTTAAAAATAATTTTTCTT
right  --------------------------------------------------------------------TT
                                     11111111111111111111111111111111111111**

left   ATTTAACAACTCAGAAAACACAGTGATGATTTAAGGAATATGAAATCTAAGGCATATAAGAAATTATTAG
chrom  ATTTAACAACTCAGAAAACACAGTGATGATTTAAGGAATATGAAATCTAAGGCATATAAGAAATTATTAG
right  ATTTAACAACTCAGAAAGCACAGTGATGATTTAAGGAATATGAAATCTAAGGCATATAAGAAATTATTAG
       *****************1****************************************************

left   GCCG--------GGCGCGGT------------------------------------------GGCTC---
chrom  TATGTATTTTTGGACACATTAAGAATACTATCAAGAATACTATCAAATAATGTATTTTAATAGGTTCTCA
right  TATGTATTTTTGGACACATTAAGAATACTATCAAGA----------------------------------
       222*22222222*2*2*22*2222222222222222                          11N11

left   --------------------ACGCCTGTAA----TCCCAGCACTTTGGGAGGCCGAGGCGGGTGGA
chrom  CATCTTGTGTCCAAAATGAAACTCCTGTAGATCTTCTTTTCTCTCTTGGTCGTCGTCGTTCTCTGA
right  ------------------------------------------------------------------
                           11N111111N    11NNNN1N11N1N11NN1N11NN1NNNNNN11
```

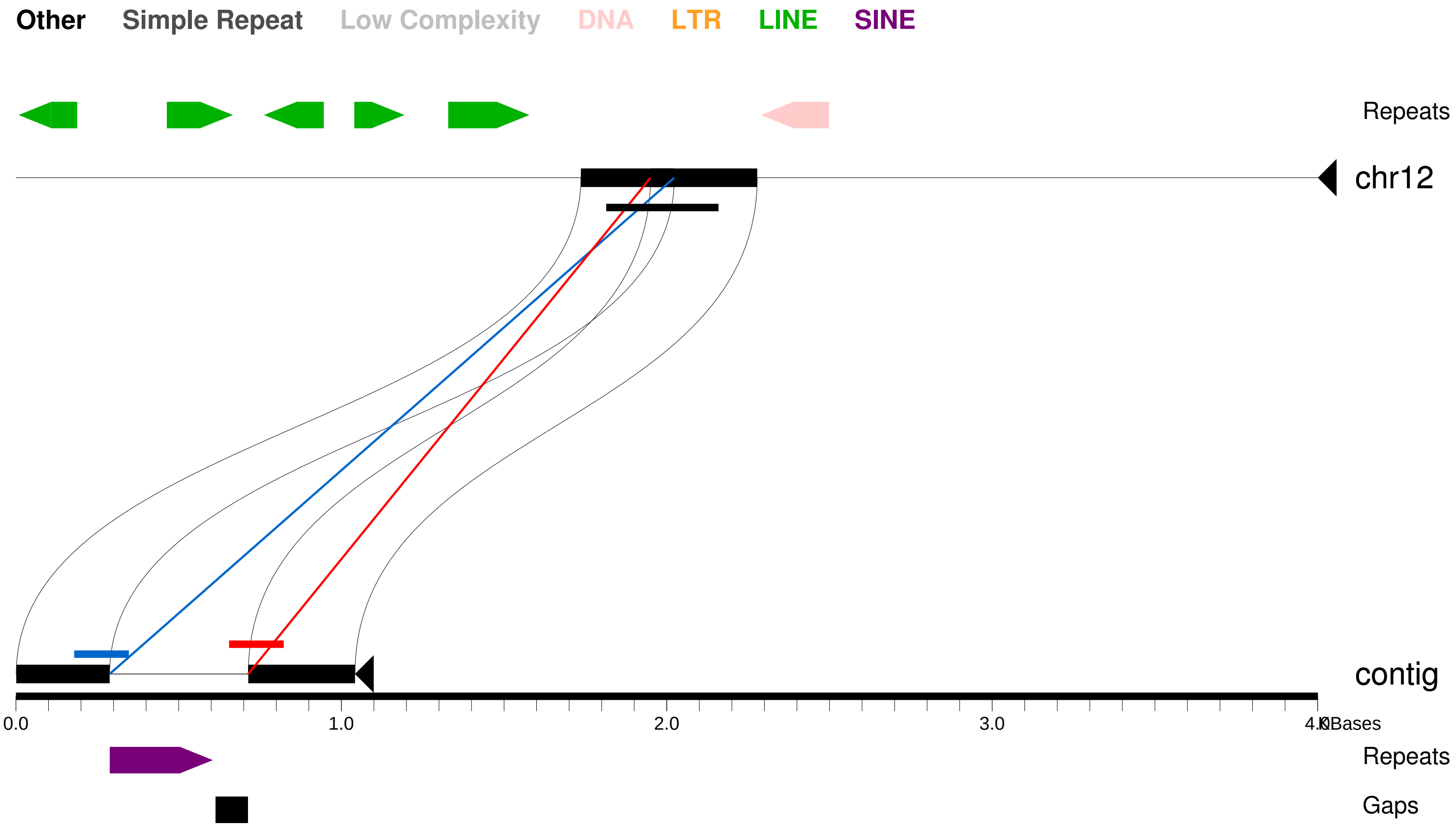

siteID: chr12_80480716 Scafftig1 - Breakpoint: chr12:80480718
chr12:80480667-80480777
Overlap Length: 9 INS size: 379 Genotyped: No

```
left    AGGAATGGAAGTAAAAAGTTGACGACACTTAGAATTTCAGTCTTAAAATTG--------GCCTTGGAGGC
chrom   AGGAATGGAAGTAAAAAGTTGACGACACTTAGAATTTCAGTCTTAAAATTG--------GCCTTGGAGA-
right   NNNNNNNNNNNNNNNNNNNNNNNNNNNNNNNNNNNNNNNNNNNNNNNNNNNNNNNNNNNGCCTTGGAGA-
        11111111111111111111111111111111111111111111111111111111111*********22

left    CGGGCGCGGTGGCTCACGCCTGTAATCCCAGCACTTTGGGAGGCCGAGGCGG----GCGG-A
chrom   ---GTGAAATGGTTCAAGG------TGCCAGGA---TGGGATTCAGGATCAGAAATGCAATA
right   ---GTGAAATGGTTCAAGG------TGCCAGGA---TGGGATTCAGGATCAGAAATGCAATA
        222*2*222***2***2*2222222*2****2*222*****22*2*222*2*2222**222*
```

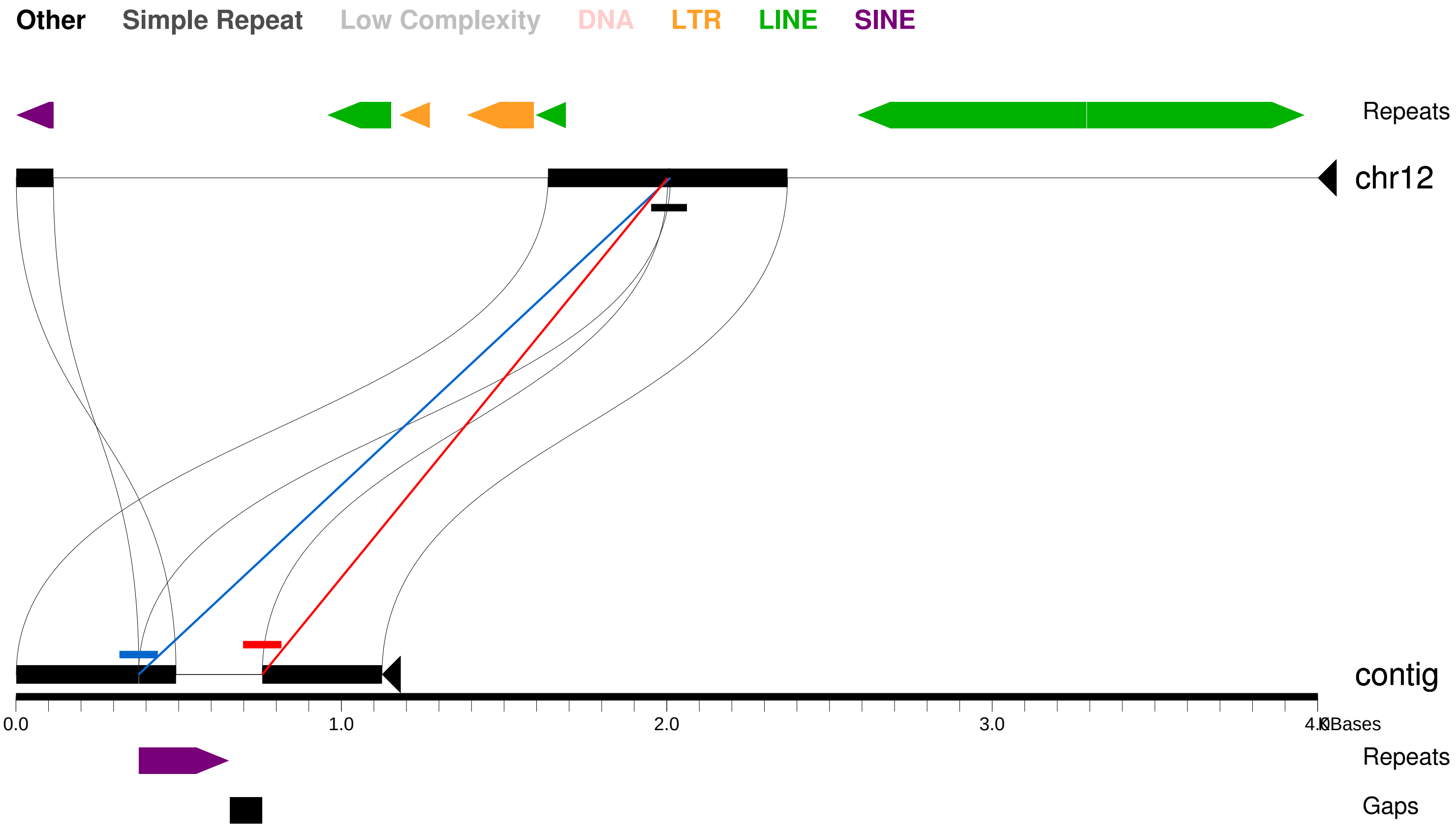

siteID: chr12_81315258 Scafftig3 - Breakpoint: chr12:81315220
chr12:81315060-81315393
Overlap Length: 14 INS size: 305 Genotyped: Yes

```
left   ------------GTTTATAAGTGACCTAGTCT-ATGGTATTTTATTATAGCAGCCCAAATAGACTAAAGC
chrom  TATATATCTGTAGTTTATAAGTGACCTAGTCT-ATGGTATTTTATTATAGCAGCCCAAATAGACTAAAGC
right  TA-------GTAGA---------GACGGGGTTTCACCGTTTTTA--------GCCGGGATGGTCTCGATC
       22       222*111111111***111**1*1*11**1*****11111111***111**1*1**11*1*

left   AGACTGCTGTAACAAAATGCCATACACTGAGTGGCTTAAGCAACTGATGTTTATTTCTCATAGTTTTGGA
chrom  AGACTGCTGTAACAAAATGCCATACACTGAGTGGCTTAAGCAACTGATGTTTATTTCTCATAGTTTTGGA
right  TC-CTGACCTCGTGATCCGCCCGCCTC-----GGCCTCCCAAAGTGCTGGG-ATTACA---------GGC
       111***111*1111*111***111*1*11111***1*1111**1**1**111***1*1111111111**1

left   GGCTGGCACTTCCAAGATCAGAGTGCCAGCAGATTTTTTTT----------TTTTTTTTTTTTTGAGACG
chrom  GGCTGGCACTTCCAAGATCAGAGTGCCAGCAGATTCTACCTGACGAGGGTCTTCTTCTGGTTCTGAACAG
right  GTGAGCCACCGCGCCCGGCCCAGTGCCAGCAGATTCTACCTGACGAGGGCCTTCTTCTGGTTCTGAACAG
       *111*1***11*111111*11*************2*222*22222222N2**2**2*22**2***222*

left   GAG---TCTCGCTCTGTCGCCC--AGGCTGGAGTGCAGTGGCGGGATCTCG-GCTCACTA----------
chrom  CAGCCTTCTGGATCTGTCTTCACAAGTCAGAAGTG-AGAGG-GAGATCAAGTGAGCACAAAGGCGTTGGG
right  CAGCCTTCTGGATCTGTCTTCACAAGTCAGAAGTG-AGAGG-GAGATCAAGTGAGCACGAAGGCGTTGGG
       2**222***2*2******22*222**2*2*2****2**2**2*2****22*2*22***N*2222222222

left   ---------------CAAGCTCCGC-CTCC-CGGGTTCACGCCATTCTCCTGCCTCA
chrom  GAGGGTGGGAGGGAGCATGCTCTGCTCTCCTCTTCTTAGAAGGCTAATAATCCCATC
right  GAGGGTGGGAGGGAGCATGCTCTGCTCTCCTCTTCTTAGAAG---------------
       222222222222222**2****2**2****2*222**22222NN1NN1NN1N11NNN
```

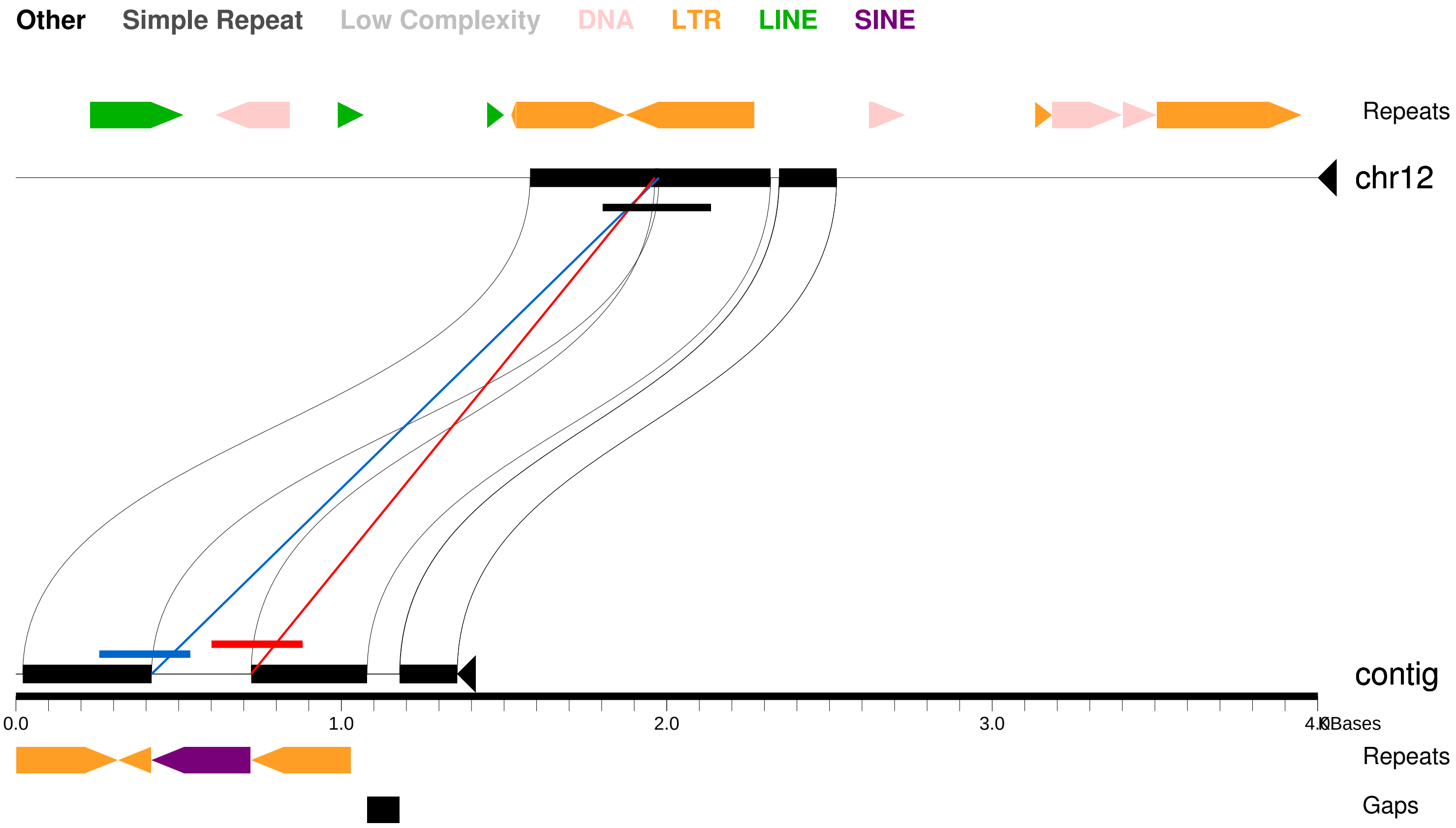

siteID: chr12_84086116 Scafftig3 - Breakpoint: chr12:84086120
chr12:84086065-84086236
Overlap Length: 5 INS size: 449 Genotyped: No

```
left     TGTTGTTAGTTTTCTCTTTGCTTGTTTATGCAATCAGCAGTAAGCTGTCACCAGTTTTTT----------
chrom    TGTTGTTAGTTTTCTCTTTGCTTGTTTATGCAATCAGCAGTAAGCTGTCACCAGTTTTTTAAAAAAATAA
right    T------------------------------------------------------TTTTTAAAAAAATAA
         *111111111111111111111111111111111111111111111111111111*****2222222222

left     -TTNNNNNNNNNNNNNNNNNNNNNNNNNNNNNNNNNNNNNNNNNNNN-NNNNNNNNNNNNN---------
chrom    GTTGAAAGATATTATTTGCAAGCCTCATGGTAAACTTAAATCAAAAA-CTTATAACAGATACACAATAAA
right    GTTGAAAGATATTATTTGCAAGCCTCATGGTAAACTTAAATCAAAAAACTTATAACAGATACACAATAAA
         2**222222222222222222222222222222222222222222221222222222222222222222222

left     ---------------------------------
chrom    ACAAAAGCAAGAAATTAAAACATACCACCTGAA
right    ACAAAAGCAAGAAATTAAAACATACCACCTGAA
         222222222222222222222222222222222
```

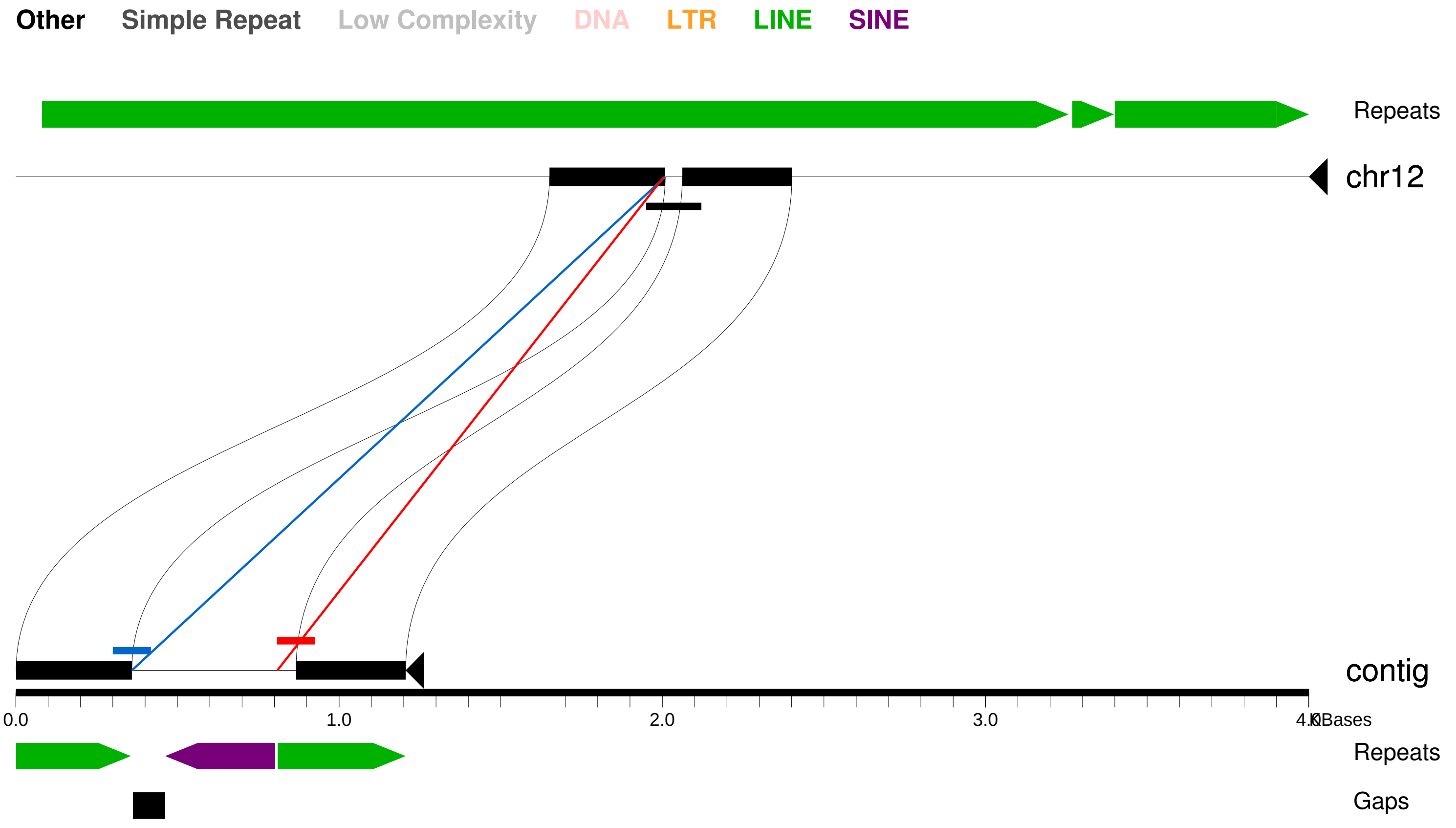

siteID: chr12_87870833 Scafftig4 + Breakpoint: chr12:87870815
chr12:87870793-87870874
Overlap Length: 9 INS size: 309 Genotyped: Yes

```
left   TTAT----------------AGGTGC----ATT-------TGTTCAATA----------CTGTTTCTTTT
chrom  TTAT----------------AGGTGC----ATT-------TGTTCAATA----------CTGTTTCTTAT
right  CCACCCGCCTCGGCCTCCCAAAGTGCTGGGATTACAGGCGTGAGCCACCGCGCCCGGCCCTGTTTCTTAT
       11*11111111111111111*1****1111***1111111**11*1*111111111111*********2*

left   TTTTTTTTTTTTTTTTTTTTTTTTTTGAGACGGAGTCTCGCTCTGTCGCCCAGGCTGGAGTGCAGTGGCG
chrom  ATTCTACAAATTTCTTTGCATTTTCTG----------------TCTAGCATATACT--------------
right  ATTCTACAAATTTCTTTGCATTTTCTG----------------TCTAGCATATACT--------------
       2**2*22222***2***222****2**2222222222222222*2*2**22*22**22222222222222

left   CGATCTCGGCTCACTG
chrom  ----CTCATCT---TT
right  ----CTCATCT---TT
       2222***22**222*2
```

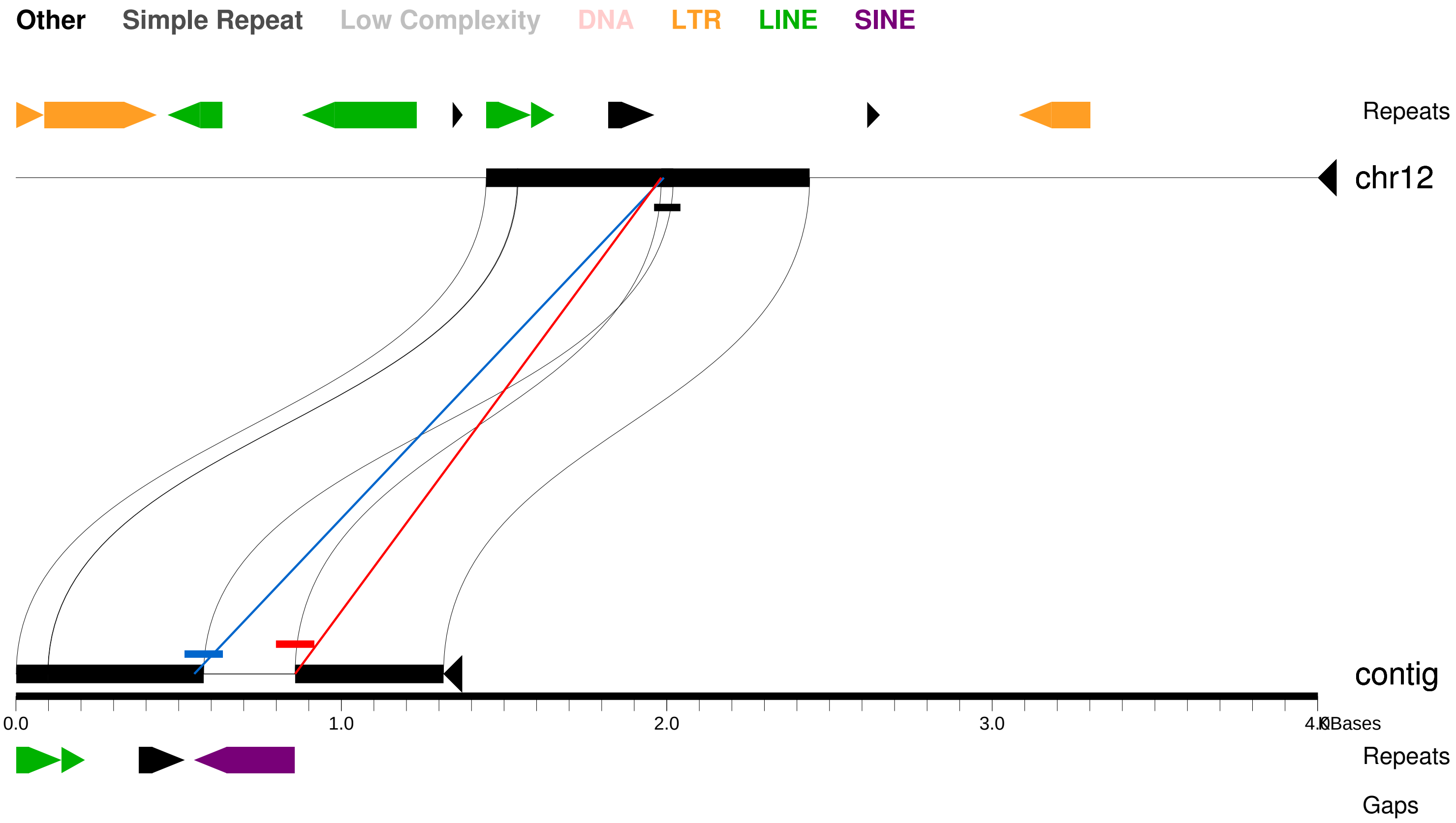

siteID: chr12_88338175 Scafftig12 - Breakpoint: chr12:88338160
chr12:88338111-88338219
Overlap Length: 11 INS size: 297 Genotyped: Yes

```
left    ---------CTAGAAAATAG----CAAAGGTAAATTCTGACTAGAGGGAGATATAGAAATTTAAAAGAAA
chrom   ---------CTAGAAAATAG----CAAAGGTAAATTCTGACTAGAGGGAGATATAGAAATTTAAAAGAAA
right   GCCACTGCACTCCAGCCTGGGCGACAGAGCGAGACTCCGTCTCAAAAAAAAAAAAAAAA---AAAAGAAA
        111111111**11*111*1*1111**1**11*1*1**1*1**11*111*1*1*1*1***111********

left    ATGGGCCGGGCGCGGTGGCTCACGCCTGTAATCCCAGCACTTTGGGAGGCCGAGGCGGGCGG----
chrom   ATG--------------ACTCATTTCTTTTATCCTTTTGTTGTAACATGTACAAGTTATTTTTTAA
right   ATG--------------ACTCATTTCTTTTATCCTTTTGTTGTAACATGTACAAGTTATTTTTTAA
        ***222222222222222****222**2*2****222222*2*222*2*222*2*22222222222
```

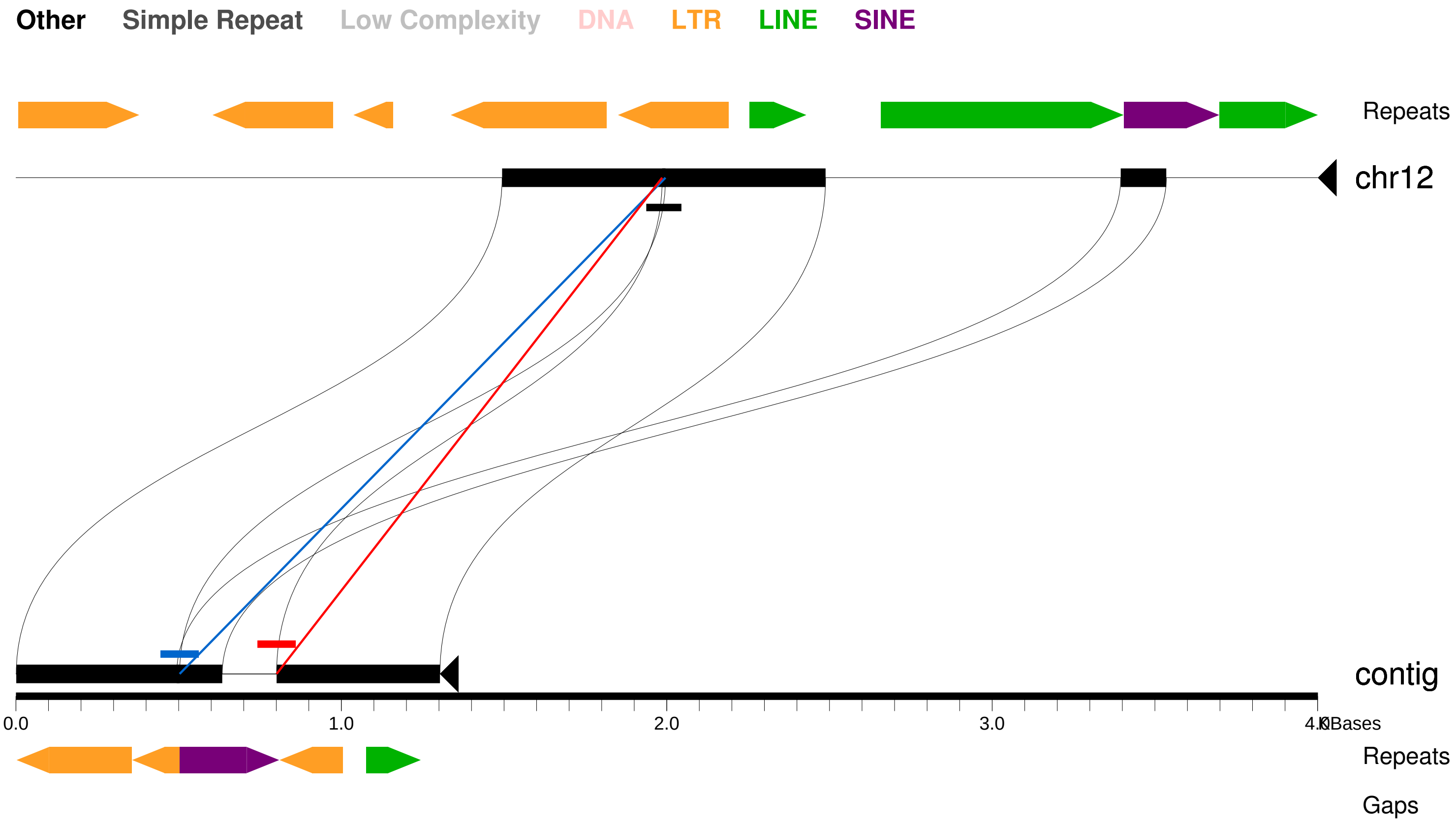

siteID: chr12_89383745 Scafftig2 - Breakpoint: chr12:89383752
chr12:89383705-89383811
Overlap Length: 13 INS size: 335 Genotyped: Yes

```
left    -------------ATAATTCAATTTAGTTGAACACATAATTTTACATTTTACTTAATTTTAAACATTGTG
chrom   -------------ATAATTCAATTTAGTTGAACACATAATTTTACATTTTACTTAATTTTAAACATTGTG
right   GACTCCGTCTCAAAAAAACAAAAAAAAAAAAAAACAAAAAAACAAAAAAAAAAAAAAAA-AAACATTCTG
        1111111111111*1**111**111*1111**1***1**1111*1*1111*111**1111*******1**

left    TAGGCCGGGCGCGGTGGCTCACGCCTG-TAATCCCAGCACTTTGGGAGGCCGAGGCGGGTGGA
chrom   TAGAA--GTAATGTTGGCTTAT--CTGATTGGTAAAATAGTTTAAGAATATTA---------A
right   TAGAA--GTAATGTTGGCTTAT--CTGATTGGTAAAATAGTTTAAGAATATTA---------A
        ***2222*2222*2*****2*222***2*222222*22*2***22**22222*222222222*
```

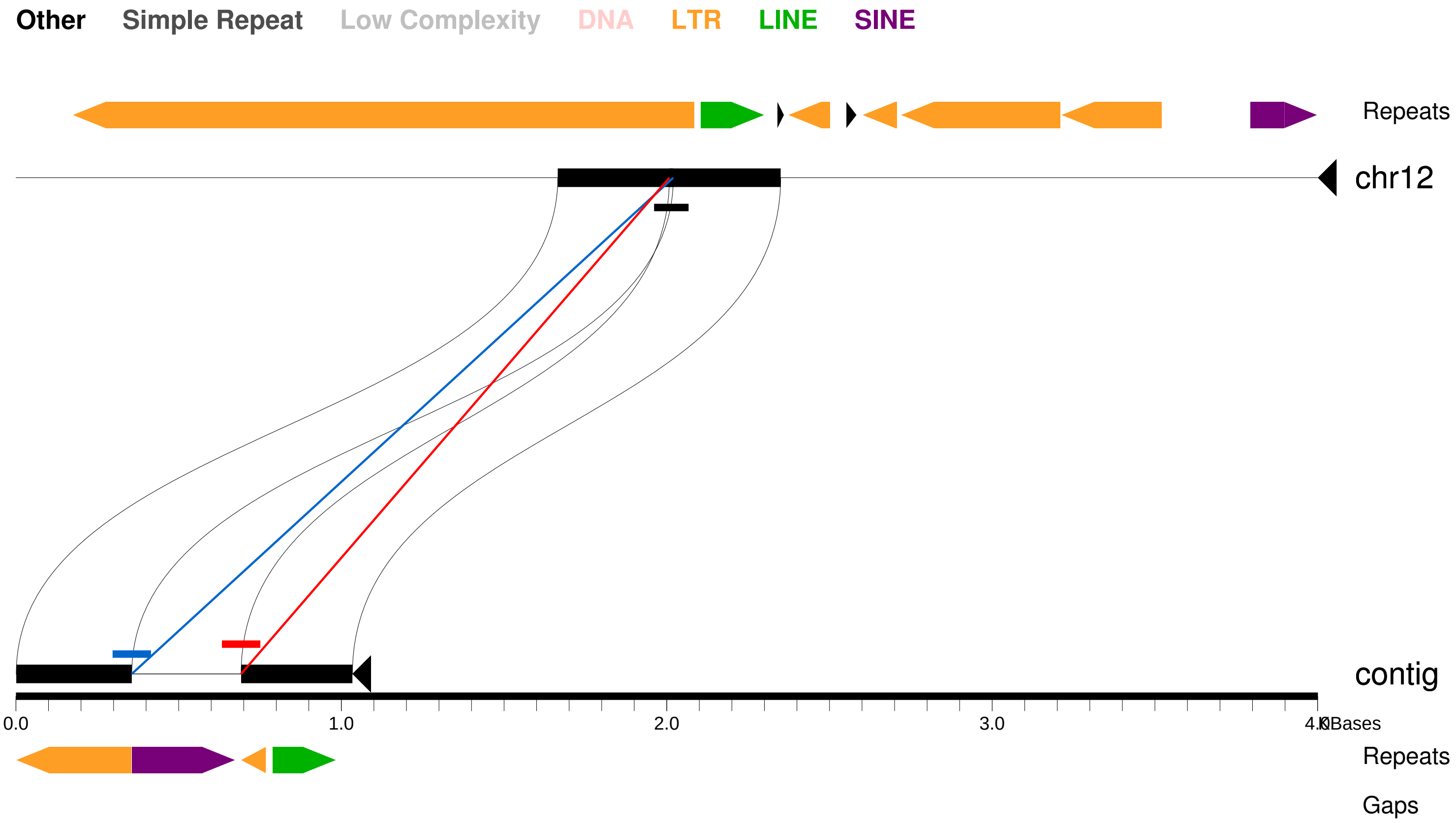

siteID: chr12_89531649 Scafftig1 - Breakpoint: chr12:89531600
chr12:89531463-89531809
Overlap Length: 73 INS size: 329 Genotyped: No

```
left   AA--------------------------------------------------------------------
chrom  AATGGACTTTGGGGACTGAGAAGAAAAGGTGGGAGGGGGGTGAGGGATAAAAGGCTACAAATTGAGTACA
right  NNNNNNNNNNNNNNNNNNNNNNNNNNNNNNNNNNNNNNNNNNNNNNNNNNNNNNNNNNN-----------
       11NNNNNNNNNNNNNNNNNNNNNNNNNNNNNNNNNNNNNNNNNNNNNNNNNNNNNNNNN

left   --------------------------------ATCTCAGAAATCACCACTAAATAACTCATTTGTAAGAA
chrom  GTGTATACTGCTCAGGTGATGGGTGCACCAAAATCTCAGAAATCACCACTAAATAACTCATTTGTAAGAA
right  -------------------------------------------------------------------GAA
                                       11111111111111111111111111111111111***

left   ACCAAACACCACTTGTTCCCCATAAACCTATTGAAATTTTTTTAAACTTTTTTTTTAAAAAGAATGAGCC
chrom  ACCAAACACCACTTGTTCCCCATAAACCTATTGAAATTTTTTTAAACTTTTTTTTTAAAAAGAATGAGCC
right  ACCAAACACCACTTGTTCCCCATAAACCTATTGAAATTTTTTTAAACTTTTTTTTTAAAAAGAATGAGCC
       **********************************************************************

left   TCC--TGGCTAACAA--------------GGTGAA-----------------ACCCCGTC--TCTACTAA
chrom  ACAATTTGGTAATGATAATTGTGGCAGATGCTGAAGACTGGTTCATTTAACAACCATTTCAATCTCCTAG
right  ACAATTTGGTAATGATAATTGTGGCAGATGCTGAAGA---------------------------------
       2*222*2*2***22*22222222222222*2****22               111NNN11  111N111N

left   AA-----------------ATACAA-----AAAATTAG------------CCGG--------GC-GC
chrom  CATGCCCTCCTGCTGTGAGATACAGGCTGGAAAGTTAAATCTCACATTCTCCAGAATGTTCTGCAGG
right  -------------------------------------------------------------------
       N1                 11111N     111N111N            11N1        11 1N
```

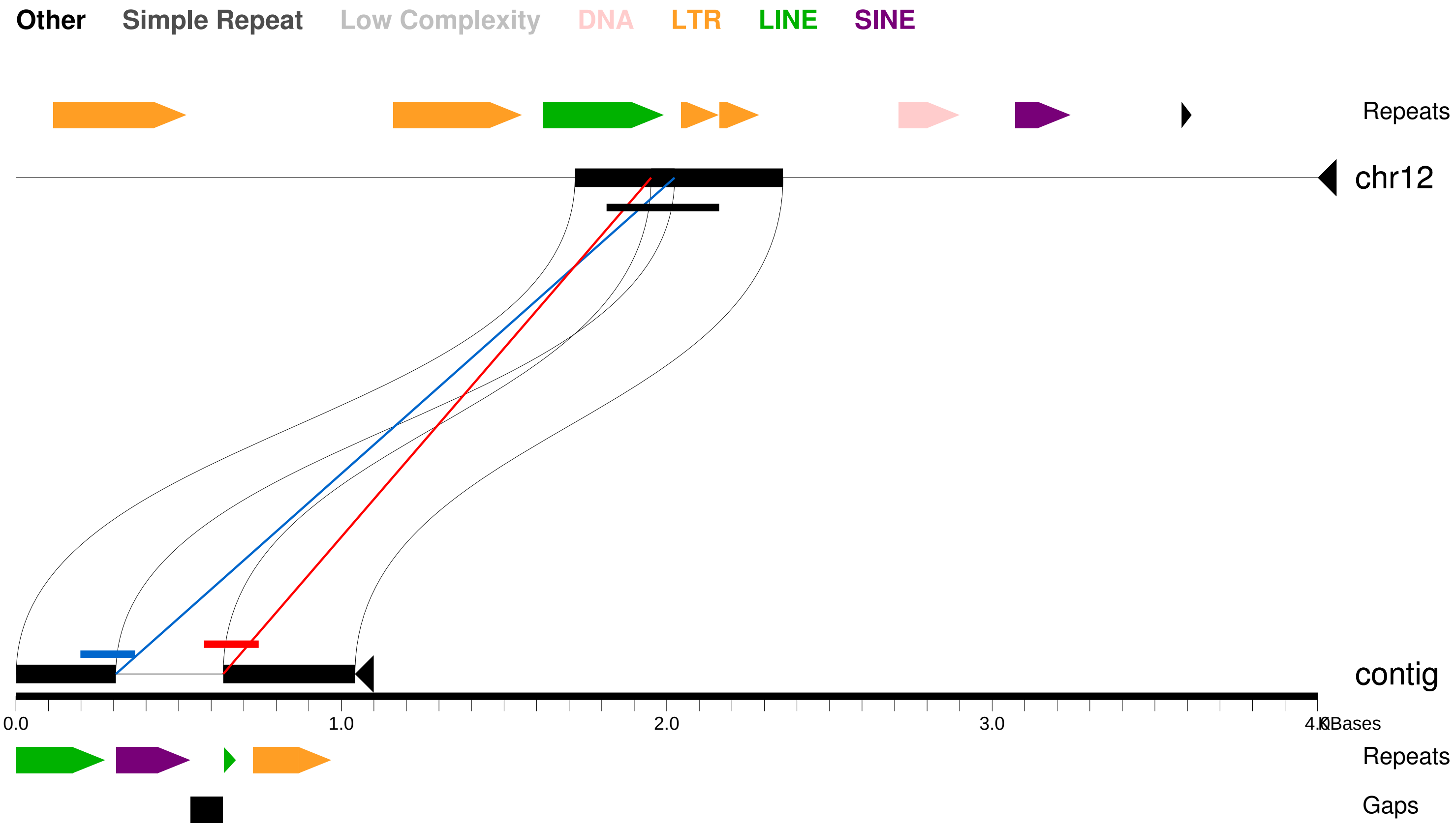

siteID: chr12_9369225 Scafftig8 - Breakpoint: chr12:9369226
chr12:9369180-9369282
Overlap Length: 14 INS size: 299 Genotyped: Yes

```
left     TTTTTGAAAGGA-TAGATAA-GGTTGATAGAAC---ACT-----------AGATAGACTAATAAAGAAAA
chrom    TTTTTGAAAGGA-TAGATAA-GGTTGATAGAAC---ACT-----------AGATAGACTAATAAAGAAAA
right    CGCGCCACTGCACTCCAGCCTGGGCGACAGAGCGAGACTCCGTCTCAAAAAAAAAAAAAAAAAAAAAAAA
         111111*11*1*1*11*1111**11**1***1*111***1111111111*1*1*1*11**1***1****

left     AAAGAGGCCGGGCGCGGTGGCTCACGCCTGTAATCCCAGCACTTTGGGAGGCCGAGGCGGGCGGA
chrom    AAAGAGA--------GAAGATCCAAATAAGTACAATCAGAAAT----GAAAATGAGAATTT----
right    AAAGAGA--------GAAGATCCAAATAAGTACAATCAGAAAT----GAAAATGAGAATTT----
         ******222222222*22*222**22222***2222***2*2*2222**2222***222222222
```

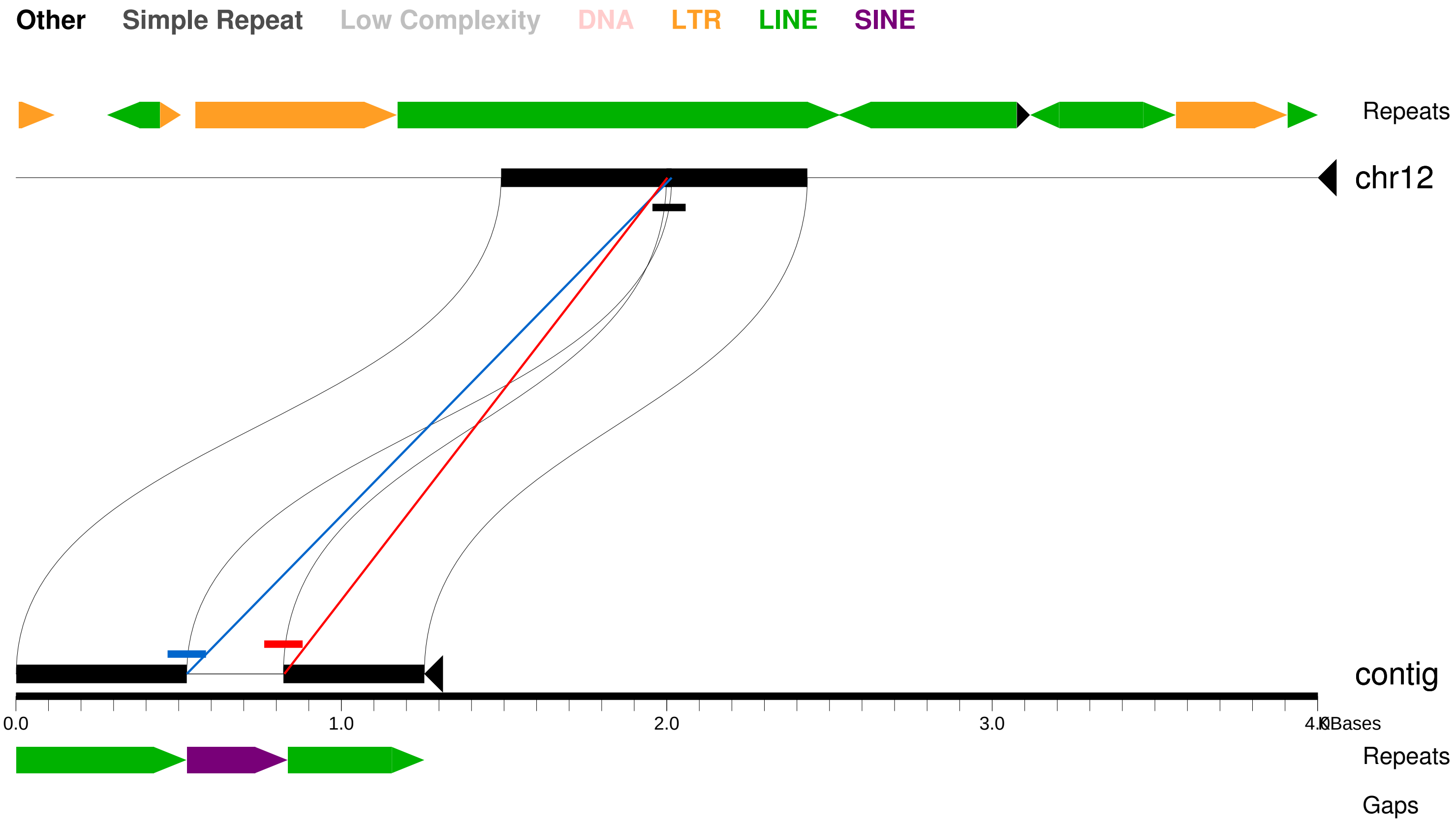

siteID: chr12_97420077 Scafftig2 + Breakpoint: chr12:97420075
chr12:97419925-97420509
Overlap Length: 11 INS size: 290 Genotyped: Yes

```
left   GATCCTTCATAATTTGAAAGGTGGTTAAACGTGTGGATTCC-AGAGATGAAGATGTTGCTTCAAAGCCAT
chrom  ---CCTTCATAATTTGAAAGGTGGTTAAACGTGTGGATTCC-AGAGATGAAGATGTTGCTTCAAAGCCAT
right  ---CCGCGCCC-----------GGCTAATTTTTTGTATTTTTAGCCAGGATGGTCTCGAT------CTCT
       222**1111111111111111**1***111*1**1***111**11*1**1*1*1*1*1*111111*11*

left   TGAGTAGCT-ATGAGACTTTGAACTAGTAACTTGACTTCTTT--GTGCCTCACTTTCCTCATCTGTGAAA
chrom  TGAGTAGCT-ATGAGACTTTGAACTAGTAACTTGACTTCTTT--GTGCCTCACTTTCCTCATCTGTGAAA
right  TGAC---CTCATGATCC-----ACCCGC--CTCGGCCTCCCAAAGTGC-TGGGATTACAGGCGTGAGCCA
       ***1111**1****11*11111**11*111**1*1*1**11111****1*1111**1*11111**1*11*

left   TTGGGGTGATAATAATAGTACTTATTTT------TTTTTT------------------------------
chrom  TTGGGGTGATAATAATAGTACTTATTTTAAAAGGTTTTTTGGGAGATCAAATAATCTGAGTGAAATACCT
right  CCGCGCCCGGCCTA---GTACTTATTTTAAAAGGTTTTTTGGGAGATCAAATAATCTGAGTGAAATACCT
       11*1*1111111**111**********222222******222222222222222222222222222222

left   ------------------------------------------TTTTTTTTTTTTTT-----------TTT
chrom  CTCATTGTACCTGGTACATTAAAAGTGCTCAATGATGAATCATTATTTTAATGTGTCCTCTGCTCACTTA
right  CTCATTGTACCTGGTACATTAAAAGTGCTCAATGATGAATCATTATTTTAATGTGTCCTCTGCTCACTTA
       222222222222222222222222222222222222222222**2****22*2*2*22222222222**2

left   TTGAGAC-----------GGAGTCTC------------------------------GCTCTGTCGCCCAG
chrom  TCGAGCCCCATGTTCCATGGAATCCCCCATATTGTAGGGTCTACTCCCAGCTAATTGCTCTTTGACCCTG
right  TCGAGCCCCATGTTCCATGGAATCCCCCATATTGT-----------------------------------
       *2***2*22222222222***2**2*222222222                     11111N1NN111N1

left   GCC-----------------------------------------------GGACTGCG------------
chrom  ACCCATTTTTTCAGGTCGTTGTATCTTTCATCCACACTAGTCTTTCTGTTGGAATGCCTTTGCCAATTTT
right  ----------------------------------------------------------------------
       N11                                               111N111N

left   GACTGCAGTGGCG-----------------CAAT-CTCGGCTCA-CTGCAAGCTCC--------------
chrom  GACTGCC-TGGCAAACTCTTATTCATCCTTCAATTCTCAGGTAAACTGCTTGGTCCTTGGTGGACTTATG
right  ----------------------------------------------------------------------
       111111N21111N                 1111 111N1N1N1 1111NN1N111

left   ----GCTTCCC--GGGTTCAC---------GC--------------------------------------
chrom  TGTTGATTCTCTTGGGCTCTCATTAATAAAGCTAATACCCCTGTTCTCCTAAGACTGTGCACTTCTGAGG
right  ----------------------------------------------------------------------
           1N111N1  111N11N1         11

left   ---------------------------------
chrom  CCAAGGATTGGGTCTTTCTGTCTATGTTGCTAT
right  ---------------------------------
```

Other **Simple Repeat** **Low Complexity** **DNA** **LTR** **LINE** **SINE**

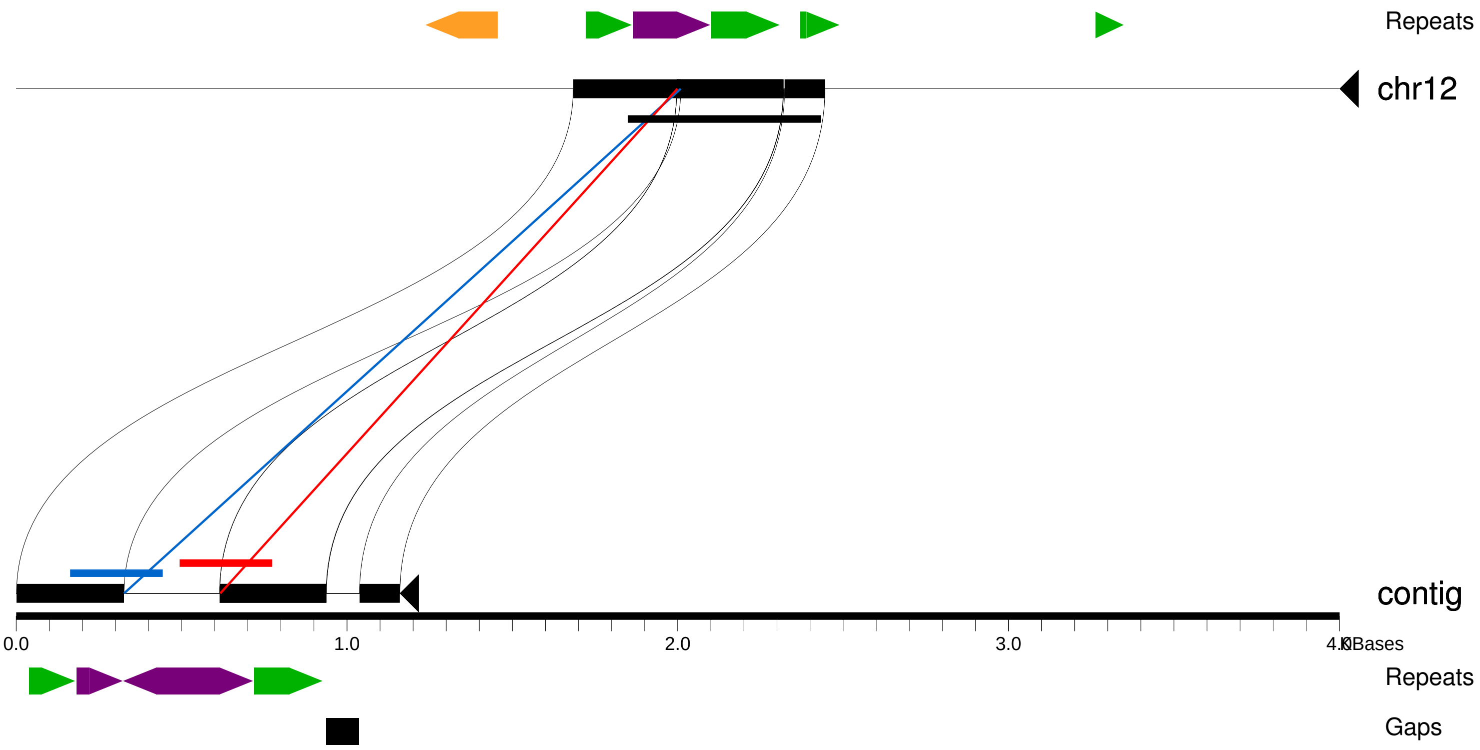

siteID: chr12_97495339 Scafftig1 - Breakpoint: chr12:97495339
chr12:97495299-97495398
Overlap Length: 20 INS size: 491 Genotyped: No

```
left     TATCTGAAGGTAGGTGAATCTAAATTGATAAAAATTCTAA-------------------CTTTTTAAAAA
chrom    TATCTGAAGGTAGGTGAATCTAAATTGATAAAAATTCTAA-------------------CTTTTTAAAAA
right    NNNNNNNNNNNNNNNNNNNNNNNNNNNNNNNNNNNNNNNNNNNNNNNNNNNNNNNNNNNCTTTTTAAAAA
         11111111111111111111111111111111111111111111111111111111111***********

left     CAAATGTAGGCCGGGCGCGGTGGCTCACGCCTGTAATCCCAGCACTTTGGGAGGCCGAGGCGGGTGGA
chrom    CAAATGTAGAGGCAATACAATGGATAA--------AAGATAGTTTTTT-------CAAGAATGG----
right    CAAATGTAGAGGCAATACAATGGATAA--------AAGATAGTTTTTT-------CAAGAATGG----
         *********22222222*22***2*2*22222222*2222**222***2222222*2**222**2222
```

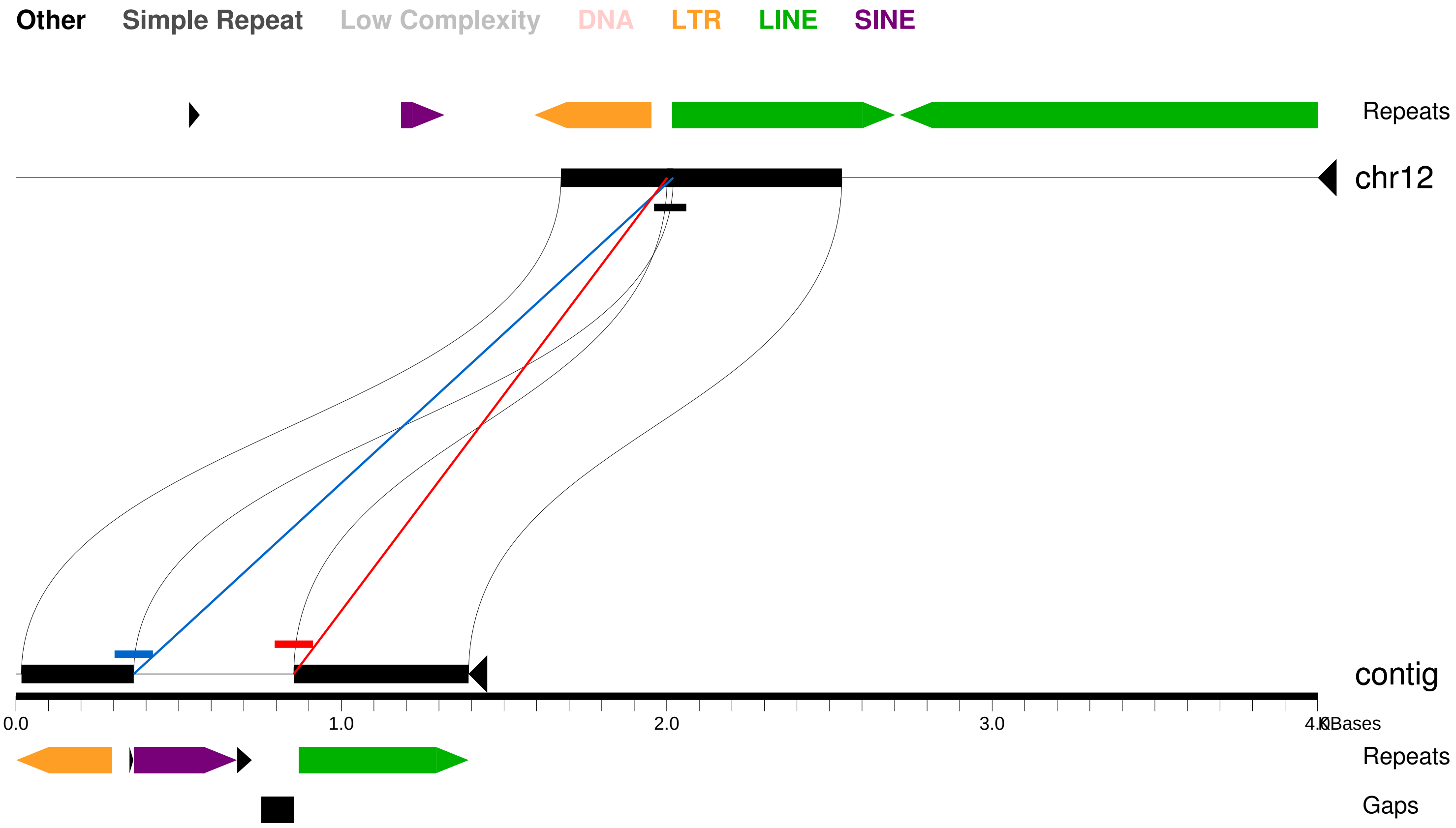

siteID: chr12_97550757 Scafftig1 + Breakpoint: chr12:97550759
chr12:97550599-97550932
Overlap Length: 14 INS size: 309 Genotyped: Yes

```
left   TGT----------AAGAATAAGCAGTGGAAGTACACTGACTGTGGTCATTTACACTTGGCTTCAACATCA
chrom  TGTTCTCTACTGTAAGAATAAGCAGTGGAAGTACACTGACTGTGGTCATTTACACTTGGCTTCAACATCA
right  TTT--------------------AGTAGA--------GACGG-GGT--TTCAC-CTTG---TTAGC--CA
       *1*          1111111111***1**11111111***1*1***11**1**1****111*1*1*11**

left   GTGACAAACTAGAAATGATGGAGACTATGAAAATTGTTACTAATGCCTCTCA---TTCAGGGGTTCATTC
chrom  GTGACAAACTAGAAATGATGGAGACTATGAAAATTGTTACTAATGCCTCTCA---TTCAGGGGTTCATTC
right  G-GATGGTCTCGATCTCCTGACCTC-ATGATCCACCCGCCTCG-GCCTCCCAAAGTGCTGGGATTACAGG
       *1**1111**1**11*11**1111*1****111111111**111*****1**111*1*1***1**11111

left   AATAATCTACTTTGGCTGAAAATGTAGGACCAGTTTTTTTTTTTTTTTTTTTTTTTTTGAGACGGA----
chrom  AATAATCTACTTTGGCTGAAAATGTAGGACCAGTTTTTGCCAGATCTAATTTTTCAGT-GAAAGGGAACAT
right  CGTGAGCCACCGCGCCCGGCC--GTAGGACCAGTTTTTGCCAGATCTAATTTTTCAGT-GAAAGGGAACAT
       11*1*1*1**111*1*1*11111**************222222*2*22*****222*2**2*2***2222

left   ----GTCTCGCTCTG---TCGCC-CAGGCCAGACTGCGGACTGCAGTGGCGCAATCT-------------
chrom  TTAGGTTTCTATGGGAAATCTCTTCATTTTAAACTTTGGATCTAATTAAAACAAACAATCAAATAAAATA
right  TTAGGTTTCTATGGGAAATCTCTTCATTTTAAACTTTGGATCAAATTGAAACAAACAATCAAATAAAATA
       2222**2**22*22*222**2*22**2222*2***22***22N2*2*N222***2*22222222222222

left   CGGCT--------CACTGCA--AGCTCCACT---TCCTGGGTTCACGCCATT------
chrom  CAGCTGTGCTAAACATAGCAGAAGTTTAGCTCTGTCCAAGGCTCATGAGTTTGCAGCC
right  CAGCTGTGCTAAACATAGCAGAAGTTTAGCTCTGTCCAAGGCTC--------------
       *2***22222222**22***22**2*222**222***22**2**1N1NNN11
```

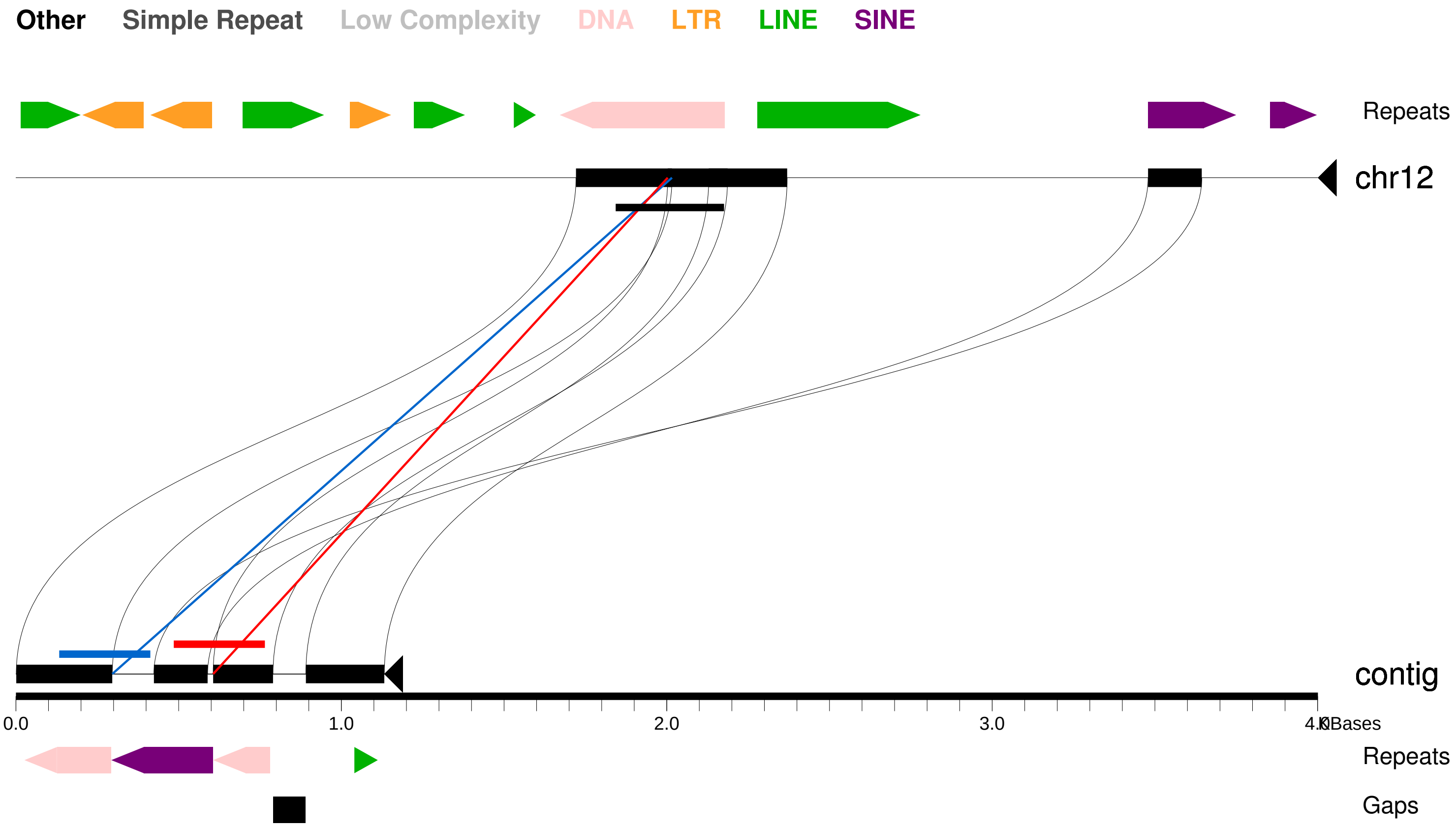

siteID: chr12_98340778 Scafftig6 - Breakpoint: chr12:98340924
chr12:98340309-98341084
Overlap Length: 15 INS size: 142 Genotyped: Yes

```
left   ------------------------------------------------------------------------
chrom  ACCTAGTATATTTATATTTGATATTGGTGATAAACTTTTTCTAGTAGACAGAGACAGACTGTAATGATCA
right  A----------------------------------------------------------GAATGGCGTGAAC-
       2                                                          22N22NNN222N2

left   ------------------------------------------------------------------------
chrom  ATTCTAAAATTGTGTTCAGGAGCAAGTGTACCTATCTGAGGTTTTCTGAATCTATAATTTACCCTGCTTT
right  ----------------CCGG---------------------GAGG----CGGA----------------GCTT-
                       2N22                     2222    2N22                2222

left   ------------------------------------------------------------------------
chrom  TTAAATCAGAATGACTTCTTTAAAATGTCCATACTGTTCTAGAACATCTTTTCTTCTCTTTGATTCCTCA
right  ------------------------------------------------------------------------

left   ------------------------------------------------------------------------
chrom  ACAATATGCAATAAAAGTTTGGTGAGCTGACTTACAAATTATCAACAACCATGAGAAATAATCTGCATTC
right  -------GCA-----------GTGAGCCGAG---------ATC--CCGCCA-----------CTGCACTC
              222           222222N22N         222  2NN222            22222N22

left   ------------------------------------------------------------------------
chrom  AAGTGCAAAAAAAAAAAAGTGTCATTTGTGGCACCTCAGTTGTCTGCCTTTCAAACTCTTGTCCAGTATC
right  CAG------------------------------CCT-----------------------------------
       N22                              222

left   ------------------------------------------------------------------------
chrom  CGGCTGAGGTTTCAGTTCCTTTATACCAGAGTTTATTTATTTCTTACCTCATTCATTCATTCAATACATA
right  -GGGCGA-------------------CAGAGC---------------------------------------
        22NN22                   22222N

left   -------------------------------------------------TCATCAGAAAACAGGAAGAAA
chrom  TGTACTGAGAGCCTCCTATGTACCAAGTGTTGATGGAAATGTTGAAGATTCATCAGAAAACAGGAAGAAA
right  --------GAGACTCCG--------------------------------TC-TCAAAAAAAA--AAAAAA
               222N2222N                                **1***1****1*11**1***

left   TCCTTCAAGGAGTTTACATTTAATCAAATAAAAATGTAGCATTATAGAGTGCCAGAGAGAGATAAGTTGT
chrom  TCCTTCAAGGAGTTTACATTTAATCAAATAAAAATGTAGCATTATAGAGTGCCAGAGAGTGATAAGTTGT
right  -------------------------AAAAAAAAA-------------------------------------
       1111111111111111111111111***1*****1111111111111111111111111N1111111111

left   ATGGAGGTAAATATTTTAGGGAAGAAAGAAAAAGAGGCTGGTGTGAGTGTGGATTAAAATTTTAAAAAGA
chrom  ATGGAGGTAAATATTTTAGGGAAGAAAGAAAAAGAGGCTGGTGTGAGTGTGGATTAAAATTTTAAAAAGA
right  ------------------------AAAAAAAAA----------------------AAAATTTTAAAAAGA
       111111111111111111111111***1*****1111111111111111111111***************

left   [illegible]CTTG[illegible]GGAG[illegible]GCTGAGGCA--[illegible]GCAGA[illegible]GAGGCGGAGCTTGCAGTGAG
chrom  AAGGTCATGAAGGAATTTGCTGATTCATTTGAACAAAGGATTGAAGGAGGT-GAAGGAGCAAGCCATGCA
right  AAGGTCATGAAGGAATTTGCTGATTCATTTGAACAAAGGATTGAAGGAGGT-GAAGGAGCAAGCCATGCA
       22222*2**22***2222*****22**222*2*2**2**22****222**22*22*****22**22**22

left   [illegible]GATCCCGCCA[illegible]GCGAC[illegible]TCCG[illegible]AAAA
chrom  GGTATTTATGGTAAGGAGGTTCCAAGCAAAGGGAGCAATAAGTACTAAATTCCCAAGACAGGAATGTGCC
right  GGTATTTATGGTAAGGAGGTTCCAAGCAA[illegible]AATTCCCAAGACAGGAATGTGCC
       222*22*222*22*222222****22*22[illegible]222*2*2***2*2*22**222222

left   -------
chrom  TGACATG
right  TGAC---
       2222
```

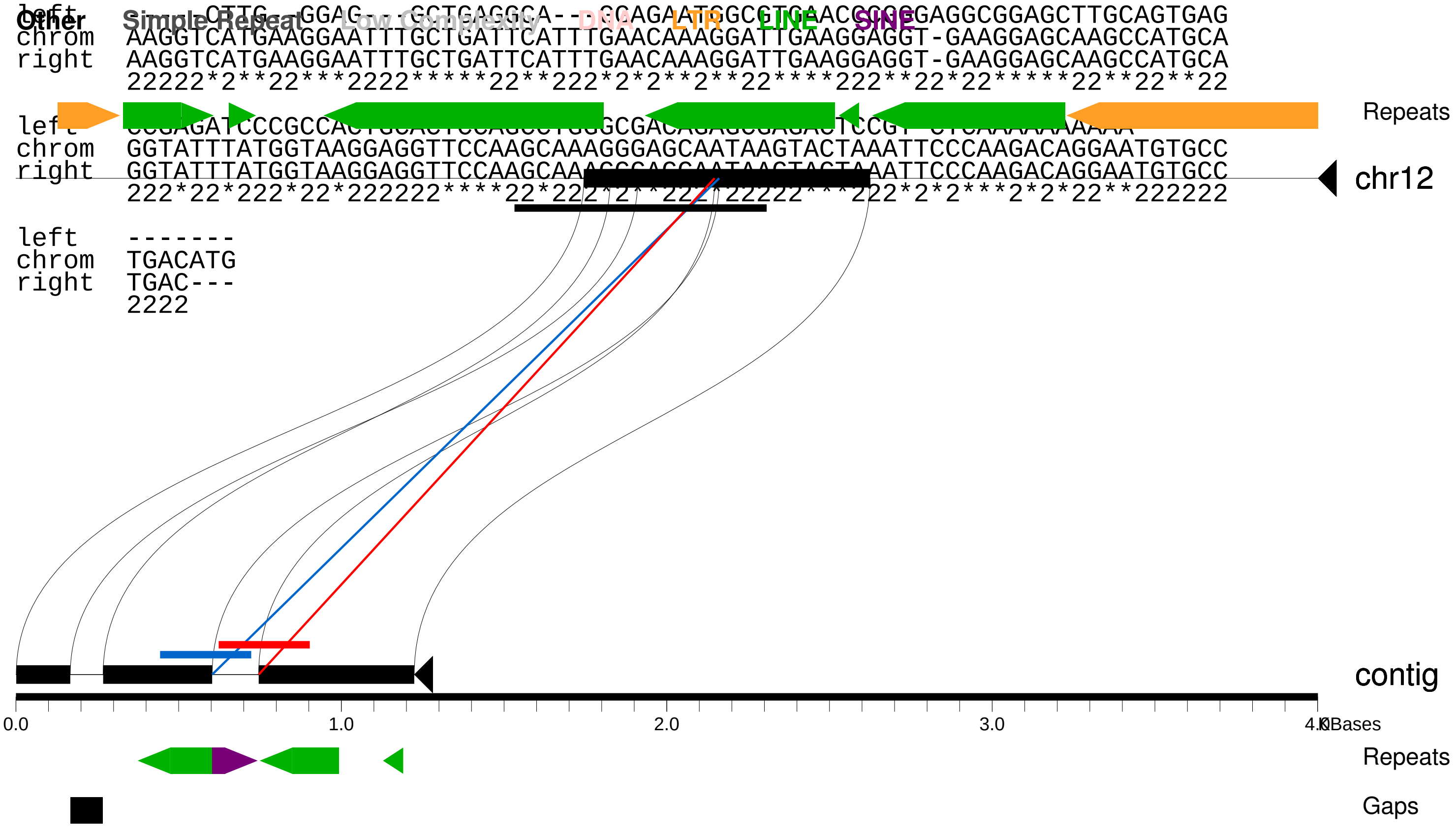

siteID: chr12_987941 Scafftig1 + Breakpoint: chr12:987954
chr12:987794-988120
Overlap Length: 7 INS size: 300 Genotyped: Yes

```
left   ATTGATGTA---ACAGGTATTAGAAATAACAGGTTAA--ATAAAGGTGCTCTGGTAACTGAACCTATGAC
chrom  --TGATGTA---ACAGGTATTAGAAATAACAGGTTAA--ATAAAGGTGCTCTGGTAACTGAACCTATGAC
right  --TTTAGTAGAGACGGGGTTTC-----ACCTTGTTAGCCAGGATGGT-CTCGATCTCCTGACCTCATGAT
       22*111***111**1**11**111111*1*11****111*11*1***1***111111****1*11****1

left   ATTCCCTAGAATTGTGTCAGCTATGT-CTGATATTCTAGGTTTGAAACTATGCTTTATTTTCATGTAGAT
chrom  ATTCCCTAGAATTGTGTCAGCTATGT-CTGATATTCTAGGTTTGAAACTATGCTTTATTTTCATGTAGAT
right  CCACCC--GCCTCGGCCTCCCAAAGTGCTGGGATTACAGGCGTGAGCCACCGC-----------------
       111***11*11*1*111111*1*1**1***11***11***11***11*111**11111111111111111

left   TTCTGCACTGTTTAATGTGTGGAATGTTTTTTTTTTTTTTTTTTT------------TGAGACGGAGTC
chrom  TTCTGCACTGTTTACTGTGTGGAATGTTTTTTTTTCTTAAGTAGTCCTCCTAATCACTTGAGAAGTACCC
right  ----GCCCGGCCT---------------TTTTTTTTCTTAAGTAGTCCTCCTAATCACTTGAGAAGTACCC
       1111**1*1*11*1N1111111111111*******2**222*22*2222222222222*****2*2*22*

left   TC----------GCT---CTG--TCGCCCAGGTCGGACTGCG-----GACTGCAGTGGCGCAA-TCTCGG
chrom  TGTAGAAGGTATGCTTTTCTGAATGACCTTTTTTCAACTTTATTTTTGAGTACTGTTGTGCATGTATGTG
right  TGTAGAAGGTATGCTTTTCTGAATGACCTTTTTTCAACTTTATTTTTGAGTACTGTTGTGCATGTATGTG
       *22222222222***222***22*22**2222*222***22222222**2*2*2**2*2***22*2*22*

left   CTCACTGCAA-GCTCCGCT------TCCCGGGTTC----ACGCCATTCTCCT---
chrom  CACAGTGTATTGTTACCCTAATATTTCATCACATCTGAGATGCTATCAACTTTAA
right  CACAGTGTATTGTTACCCTAATATTTCATCACATCTGAGATGCTATC--------
       *2**2**2*22*2*2*2**222222**222222**2222*2**2**2NN1N1
```

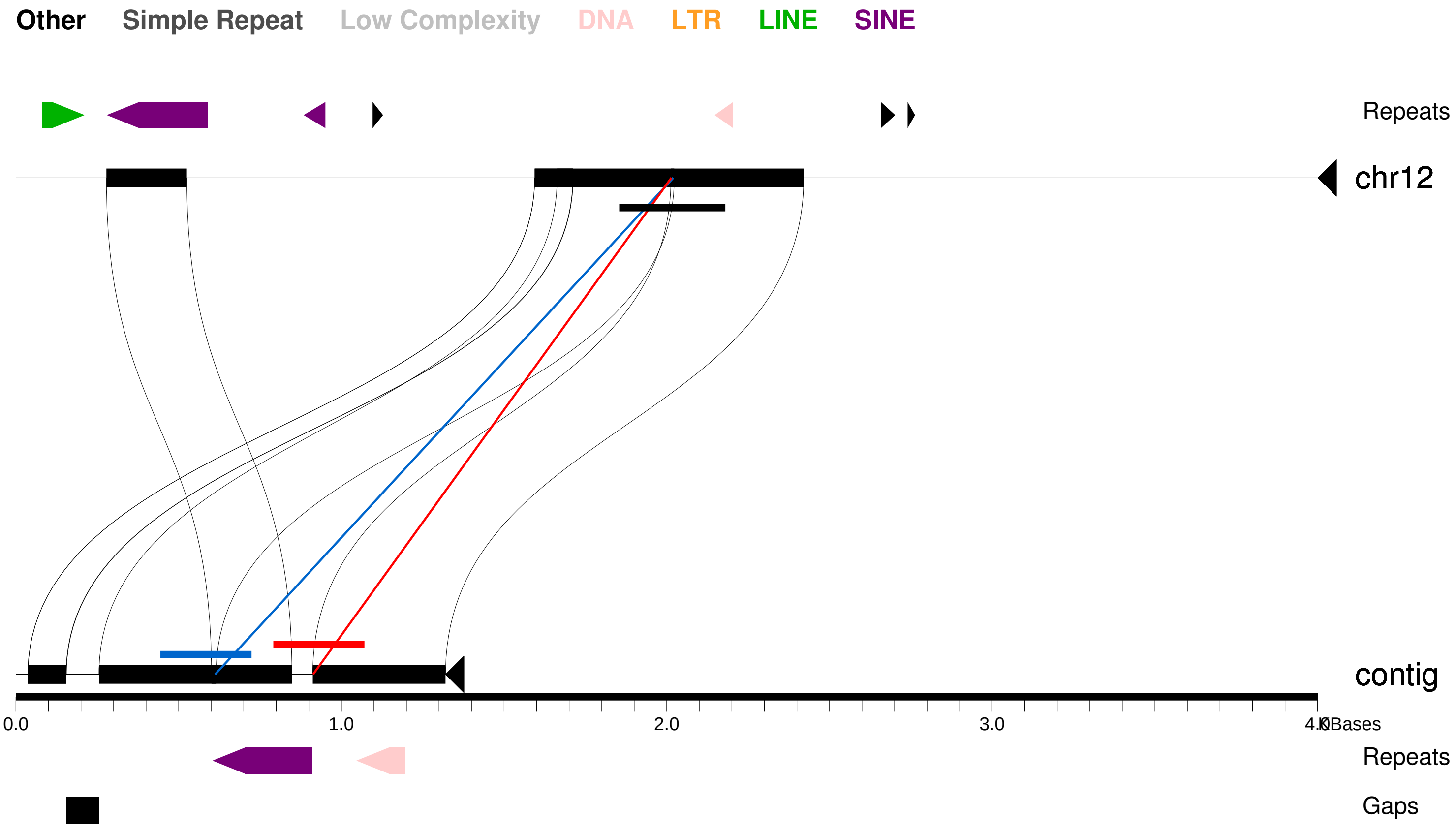

siteID: chr12_9903071 Scafftig1 - Breakpoint: chr12:9903052
chr12:9903007-9903111
Overlap Length: 15 INS size: 375 Genotyped: No

```
left     TTACAAATAGACCACGAATTCCACAAG---------GCCAG--TTGCCATGCGGAA---TTTGCTGAGTA
chrom    TTACAAATAGACCACGAATTCCACAAG---------GCCAG--TTGCCATGCGGAA---TTTGCTGAGTA
right    AGCCGGGATGGTCTTGATCTCCTGACCTCGTGATCCGCCCGCCTCGGCCTCCCAAAGTGTTTGCTGAGTA
         111*11111*11*11**11***11*11111111111***1*11*1*1*1*1*11**111***********

left     TTTTTTNNNNNNNNNNNNNNNNNNNNNNNNNNNNNNNNNNNNNNNNNNNNNNNNNNNNNNN
chrom    TTTT--------------AACTCTGGTGTCCACCATAGTAGTAGGTGCTTACTTATAACTTGT
right    TTTT--------------AACTCTGGTGTCCACCATAGTAGTAGGTGCTTACTTATAACTTGT
         ****22222222222222222222222222222222222222222222222222222222222
```

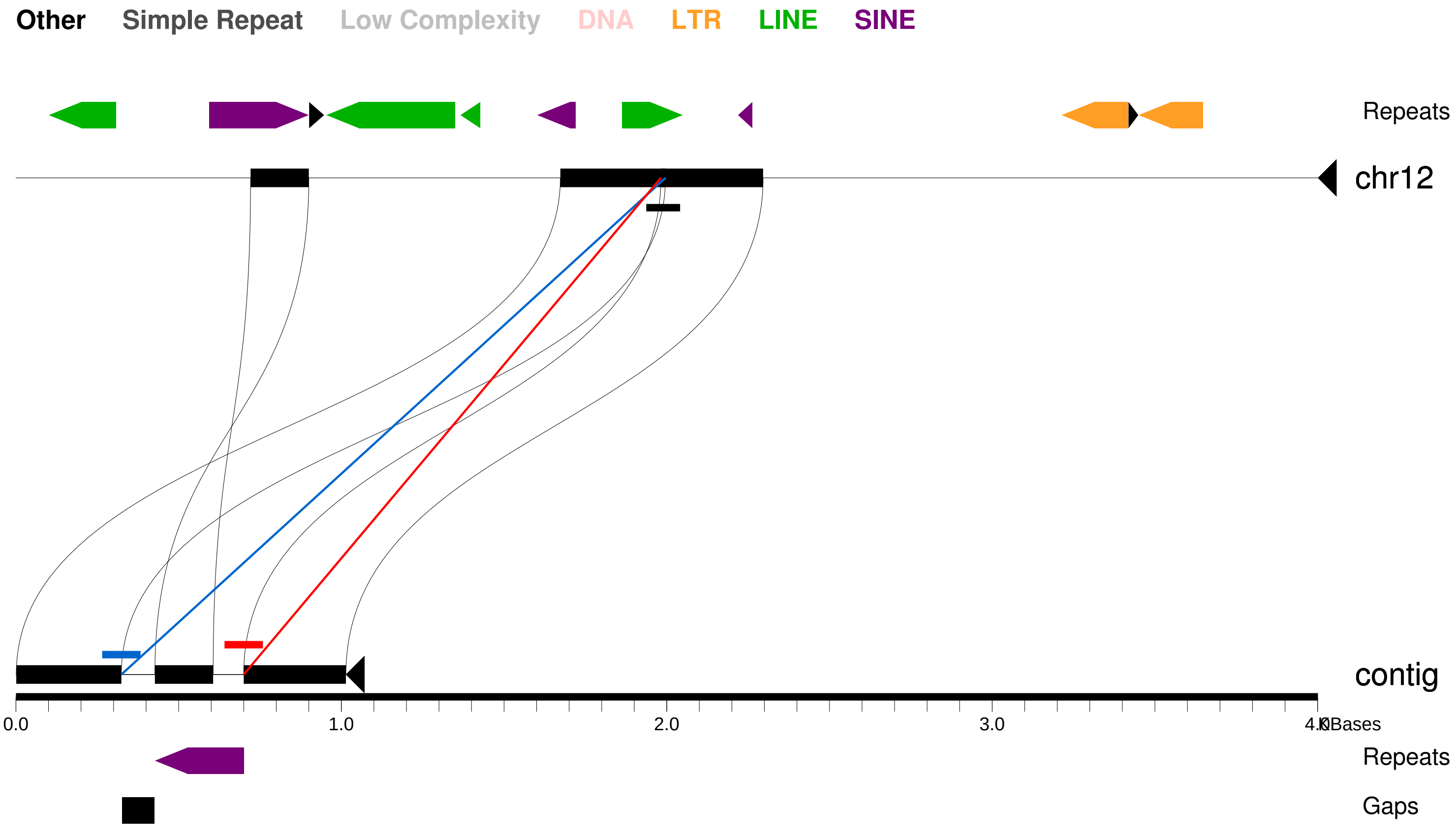

siteID: chr12_99227709 Scafftig1 - Breakpoint: chr12:99227704
chr12:99227656-99227764
Overlap Length: 0 INS size: 306 Genotyped: Yes

```
left    AGG---GTAATGGCCTTCA-----GCTGCA--TCCATGC-TGCTGCAAAGGACATGATTTTTTTTTTTTT
chrom   AGG---GTAATGGCCTTCA-----GCTGCA--TCCATGC-TGCTGCAAAGGACATGATTTCATTCTTTTT
right   CCGCCCGTCTCGGCCTCCCAAAGTGCTGGGATTACAGGCGTGAGCCACCGCACCCGGCC-CATTCTTTTT
        11*111**111*****1*111111****1111*1**1**1**111**11*1**11*111122**2*****

left    TTTTTTTTTTTTTTTTGAGACGGAGTTTCGCTCTGTCGCCCAGGCTGGAGTGCAGTGGCG
chrom   TATGTCTGTGTAGTATTTCATCTACCTTGACTTTTTAT-------TTGCCT--ATTTGT-
right   TATGTCTGTGTAGTATTTCATCTACCTTGACTTTTTAT-------TTGCCT--ATTTGT-
        *2*2*2*2*2*22*2*222*222*22**22**2*2*222222222*2*22*22*2*2*22
```

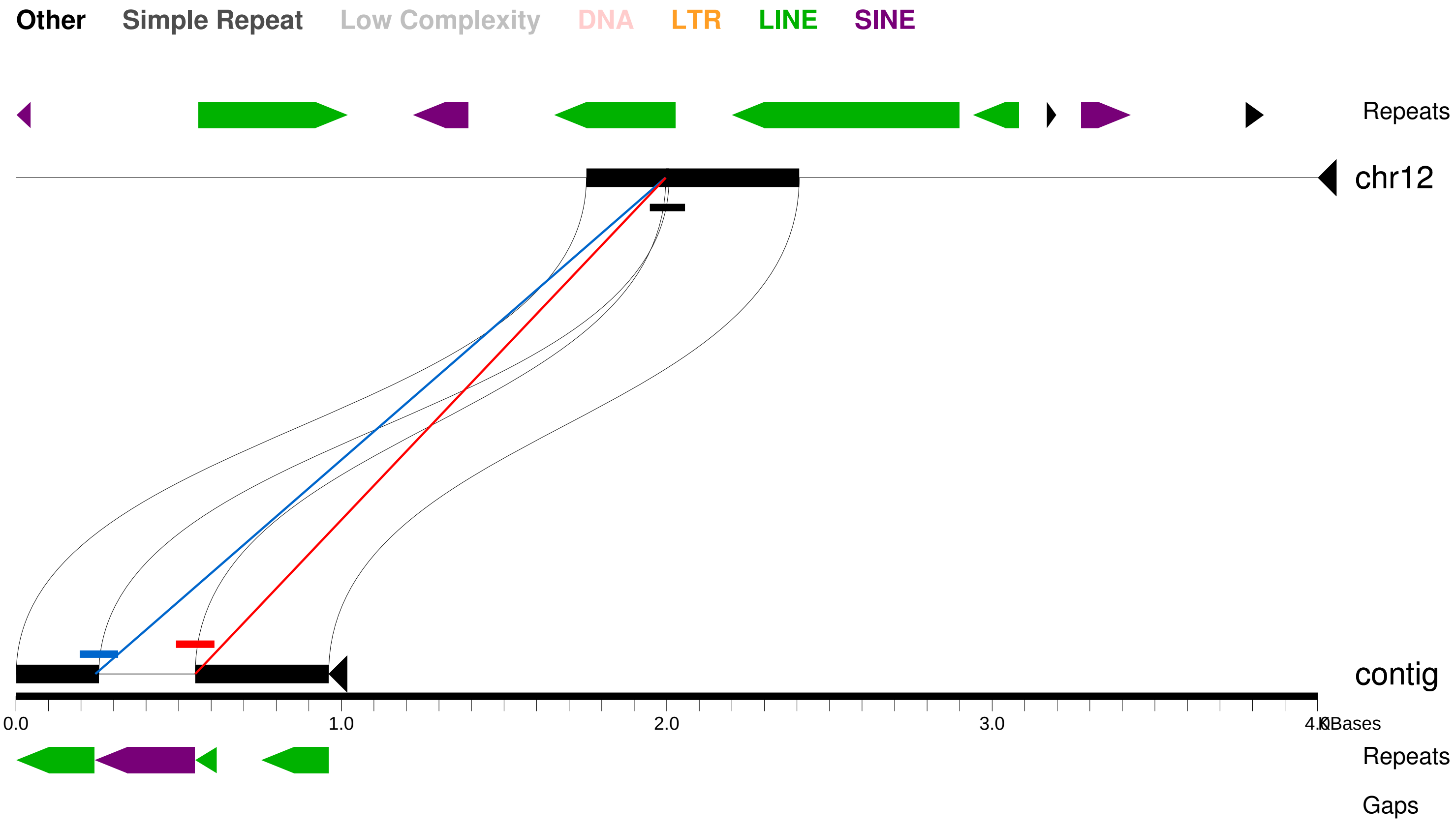

siteID: chr13_101577225 Scafftig1 + Breakpoint: chr13:101577234
chr13:101577074-101577404
Overlap Length: 11 INS size: 301 Genotyped: Yes

```
left   A----GGAATAGTGGGAGTTAAGACATTTTTA---ATAATGG-CTTTATCTCTTC-CATCCCCCTCCTTT
chrom  ATATAGGAATAGTGGGAGTTAAGACATTTTTA---ATAATGG-CTTTATCTCTTC-CATCCCCCTCCTTT
right  TT-TAGTAG-AGACGGGGTTTC-ACCGTGTTAGCCAGGATGGTCTCGATCTCCTGACCTCGTGATCCGCC
       12 22*1*11**11**1***111**11*1***111*11****1**11*****1*11*1**1111***111

left   TGCTAAAAAATGTAAAATTCTGTGAGAGCAAAGCAAAGTGCACTTTAAGAAATATTGAGTGCTAAAAATG
chrom  TGCCAAAAAATGTAAAATTCTGTGAGAGCAAAGCAAAGTGCACTTTAAGAAATATTGAGTGCTAAAAATG
right  CGCC--------------TCGG------CCTCCCAAAGTGC----TGGGATTACAGGCGTG-----AGCC
       1**21111111111111**1*111111*1111********1111*11**111111*1***11111*111

left   ACCTTTTTCATTTATTTAACCATAAGCAATTTTTTT----TTTTTTT--------TTTTT-------TTT
chrom  ACCTTTTTCATTTATTTAACCATAAGCAATTCTTTTCAGCTATTCTTAGGACTCATTTTTAGTCTGCTTT
right  ACCGCGCCCGG--------CCA---GCAATTCTTTTCAGCTATTCTTAGGACTCATTTTTAGTCTGCTTT
       ***11111*1111111111***111******2****2222*2**2**22222222*****2222222***

left   TTTTG-----AGACGGAGTCTCGCTCTGTCGCCCAGGCT----GGAGTGCAGTGGCGCG--ATCTCGGC-
chrom  ATTAGCTGGAAAGCAGAGACT-GCT-TGTTGAAGAAGCTAACAGTAGGTTAGTATTGCAGAATCTTTTAA
right  ATTAGCTGGAAAGCAGAGACT-GCT-TGTTGAAGAAGCTAACAGTAGGTTAGTATTGCAGAATCTTTTAA
       2**2*22222*22*2***2**2***2***2*222*2***2222*2**222***222**222****22222

left   ---TCACTGCAAG----CTCCGCCTCCC----------GGGTTCACGCCATTCTCCTG
chrom  ATGTCACTGGAAGGCATCTAAAACTACAAAAAATTTTAGGATACATTATATAAAACCC
right  ATGTCACTGGAAGGCATCTAAAACTACAAAAAATTTTAGGATACAT------------
       222******2***2222**2222**2*22222222222**2*2**2NNN11NNNN1NN
```

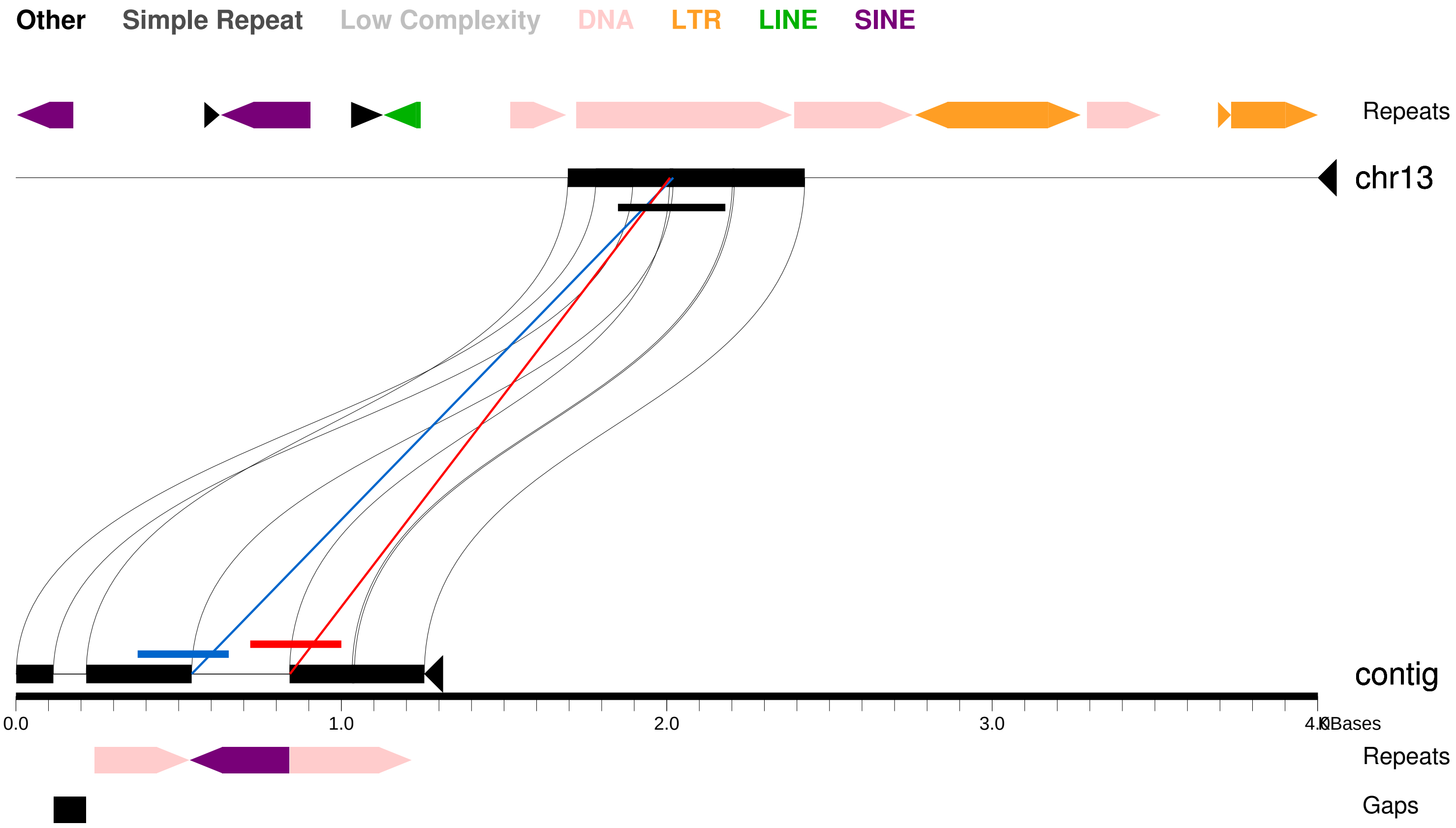

siteID: chr13_102389383 Scafftig2 + Breakpoint: chr13:102389367
chr13:102389349-102389426
Overlap Length: 16 INS size: 326 Genotyped: Yes

```
left    -----------------------------AT--CAGTCTT-AGCAACTAT---------TCTATTTTTTA
chrom   -----------------------------AT--CAGTCTT-AGCAACTAT---------TCTATTTTTTA
right   CACCCGCCTCGGCCTCCCAAAGTGCTGGGATTACAGGCGTGAGCCACCGCGCCCGGCCCTCTATTTTTTA
        11111111111111111111111111111**11***1*1*1***1**111111111111***********

left    ATCTTTTTTTTTTTTTTTTTTTTTTTTTTTTTTTTTTTTTTTGAGACGGAGTCTCGCTCTGTCGCCCAGGC
chrom   ATCTTATTTCATTATTTTAGCCTTATGTTTTATAA-------------------GCATTG-------GGT
right   ATCTTATTTCATTATTTTAGCCTTATGTTTTATAA-------------------GCATTG-------GGT
        *****2***22**2****2222**2*2****2*222222222222222222222**22**2222222**2

left    TGGACTGCGGACTGCAGTGG
chrom   TGG---------------GA
right   TGG---------------GA
        ***222222222222222*2
```

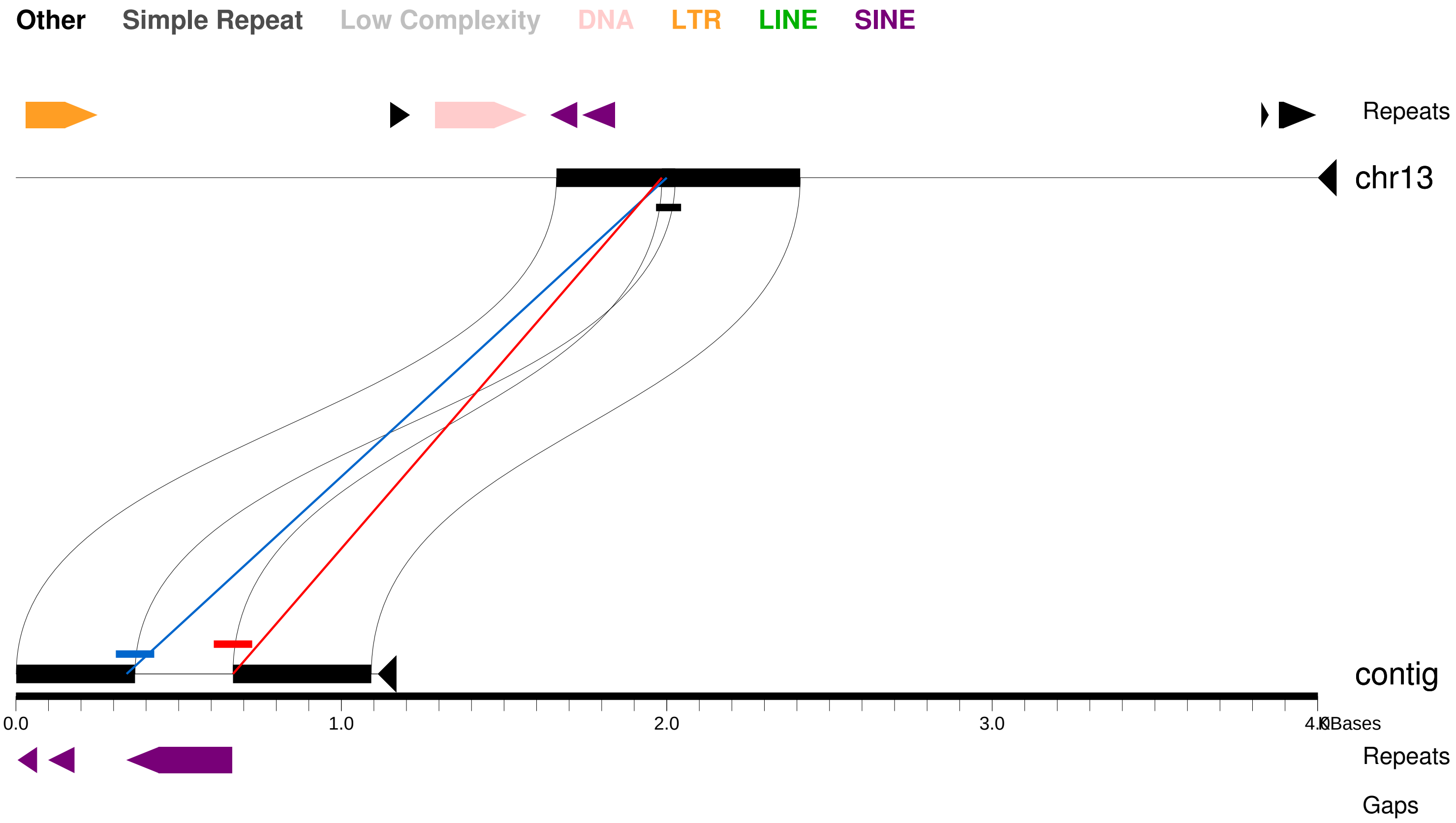

siteID: chr13_105714939 Scafftig13 + Breakpoint: chr13:105714921
chr13:105714878-105714980
Overlap Length: 17 INS size: 271 Genotyped: Yes

```
left     ACTTGCTAAA----TAAAAAGCTGAAGGCTTCCT-ATCTGACAAATTT-----------GACTTTGCTGC
chrom    ACTTGCTAAA----TAAAAAGCTGAAGACTTCCT-ATCTGACAAATTT-----------GACTTTGCTGC
right    GTTAGCCAGGATGGTCTCGATCTCCTGACCTCATGATCTACCCGCCTCGGCCTCCCAAAGACTTTGCTGC
         11*1**1*111111*1111*1**111*2*1**1*1****11*1111*111111111111***********

left     TGTTTTTTTTTTTTTTTTT---TTTTTTTTGAGACGGAGTCTCGCTCTGTCGCCCAGGCCGGACTGCGG
chrom    TGTTTTATATGCTAGCATAAATTTATTGTTAAAAACA-TTTCAATATT------------------GT
right    TGTTTTATATGCTAGCATAAATTTATTGTTAAAAACA-TTTCAATATT------------------GT
         ******2*2*22*2222*222***2**2*2*2*222*2*2**22*2*222222222222222222*2
```

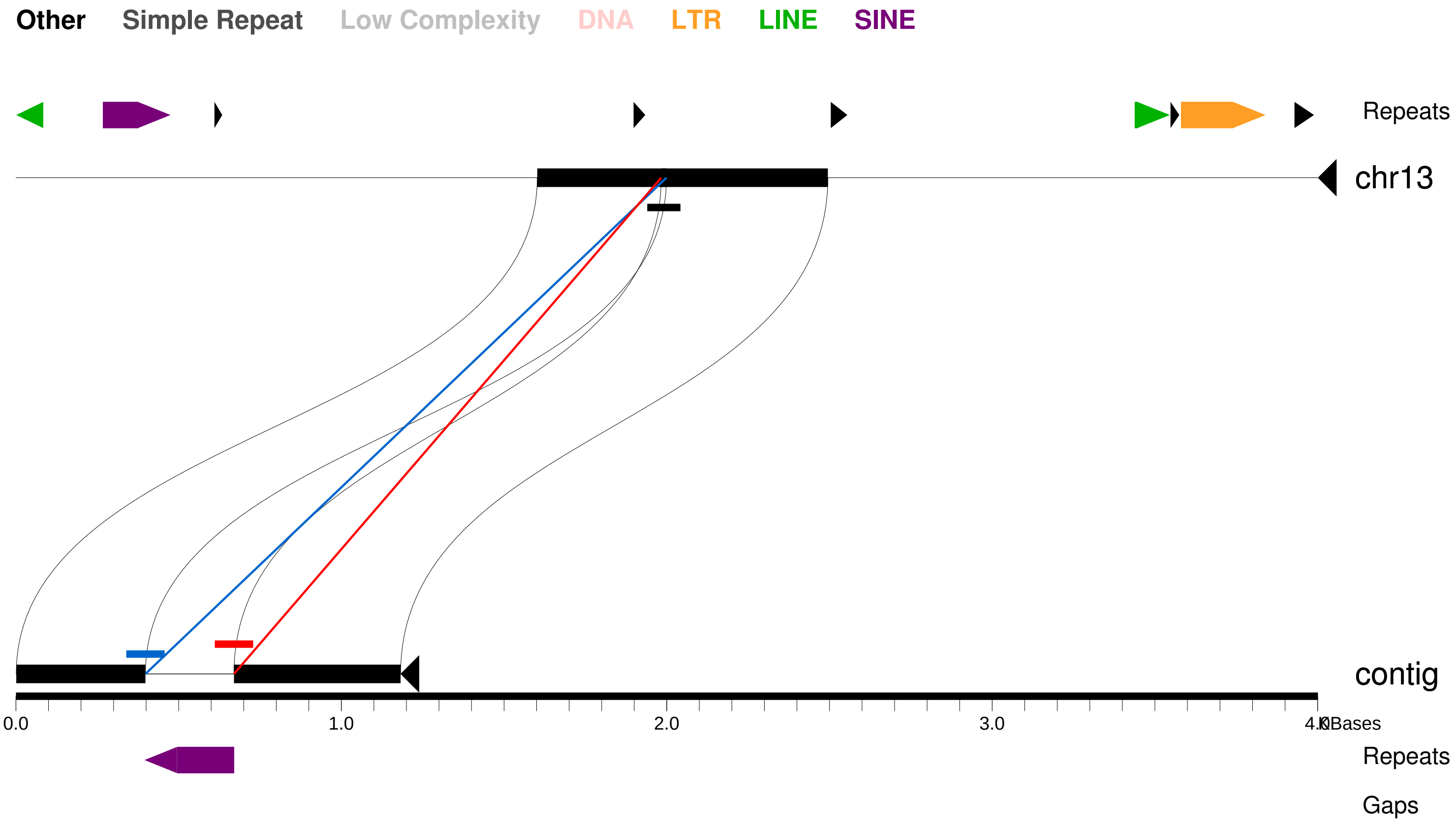

siteID: chr13_108669033 Scafftig4 - Breakpoint: chr13:108669013
chr13:108668971-108669072
Overlap Length: 18 INS size: 304 Genotyped: Yes

```
left    C------TTCTGAG---CAG-GCAAAACA-------TAATTAAAGGTCAAACCTGGTTTAAAGTATGTTC
chrom   C------TTCTGAG---CAG-GCAAAACA-------TAATTAAAGGTCAAACCTGGTTTAAAGTATGTTC
right   CACTCCAGCCTGGGCGACAGAGCGAGACTCCGTCTCAAAAAAAAAAAAAAAAAAAAAAAAAAGTATGTTC
        *11111111***1*111***1**1*1**111111111**11***1111***11111111***********

left    TAATCTGGGCCGGGCGCGGTGGCTCACGCCTGTAATCCCAGCACTTTGGGAGGCCGAGGCGGGCGG
chrom   TAATCTGATCGTTAGGCAGTTTGTCTCCAGTGGATG---AGCATTCTTGCAC--------------
right   TAATCTGATCGTTAGGCAGTTTGTCTCCAGTGGATG---AGCATTCTTGCAC--------------
        *******22*22222**2**222**2*222**2*22222****2*2*2*2*222222222222222
```

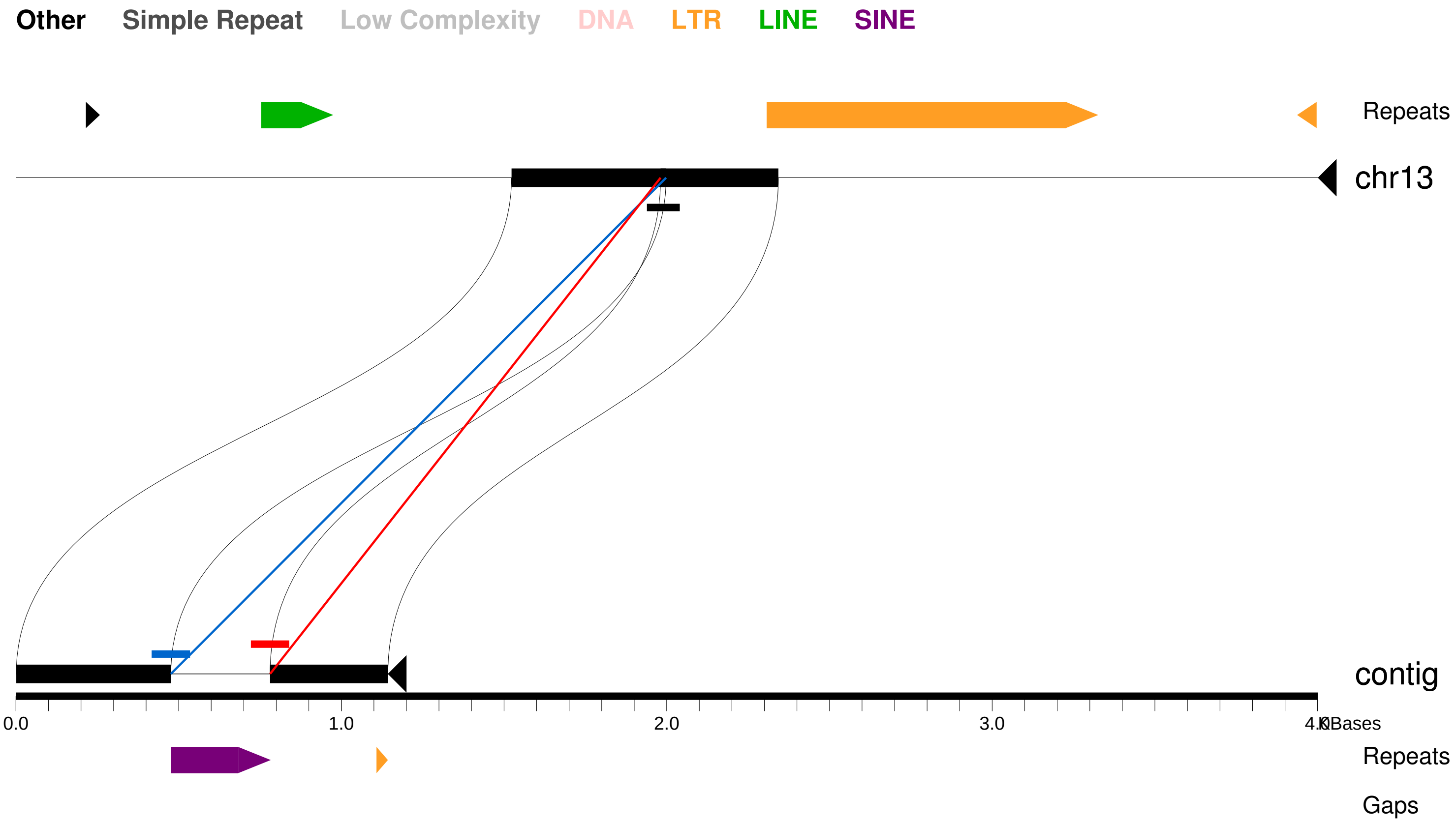

siteID: chr13_109990725 Scafftig1 + Breakpoint: chr13:109990807
chr13:109990647-109990979
Overlap Length: 13 INS size: 323 Genotyped: No

```
left    NNNNNNNNNNNNNNNNNNNNNNNNNNNNNNNNNNNNNNNNNNNNNNNNNNNNNNNNNNNNNNNNNNNNN-
chrom   AGTACATAAGGAAATGTAGTATATTACAAAGATGGCATCTCAAATCACTGAAATAATGGTGGACGTTTTA
right   NNNNNNNNNNNAAA--------------------------------------------------------
        NNNNNNNNNNN222NNNNNNNNNNNNNNNNNNNNNNNNNNNNNNNNNNNNNNNNNNNNNNNNNNNNNNN

left    --------------GACAAATGAATAGCCATTTGGAAACAGGTAAAATTAGATTCATGTCTTGTATCTAA
chrom   ATAAGTTTGGCTAGGACAAATGAATAGCCATTTGGAAACAGGTAAAATTAGATTCATGTCTTGTATCTAA
right   ----------------------------------------------------------------------
                      11111111111111111111111111111111111111111111111111111111

left    GAATATACTCTAAATTGATTAGAAATAAATATAGTGG----------CCGGGCGCGGTGGCTCACGCCTG
chrom   GAATATACTCTAAATTGATTAGAAATAAATATATTAAAGAAATCAAACAATTAGTAGAAGAAAATGTATA
right   --------------------AGAAATAAATATATTAAAGAAATCAAACAATTAGTAGAAGAAAATGTATA
        11111111111111111111*************2*222222222222*22222*22*22*222*2*22*2

left    TAATC-CCAGCACTTTGGG--AGGCCGAGGCGGGCGGA-------TCA-----CG-AGGT-------CAG
chrom   AATTCTCCCTAACATTGGTGTAGAGAAAAGCTTTCTAACTATGACTCAAACTTCGTAGGCAATAAAACAA
right   AATTCTCCCTAACATTGGTGTAGAGAAAAGCTTTCTAACTATGACTCAAACTTCGTAGGCAATAAAACAA
        2*2**2**222**2****222**2222*2**222*22*2222222***22222**2***22222222**2

left    GAGATCGAGACCATT--CTGGCTAACACGGTGAAACCCCGTC----TCTACTAAA
chrom   AAGATTAATACATTTTACTAAATAAAAAAT--ATACCTCTTCAAATTTTACATGA
right   AAGATTAATACA-------------------------------------------
        2****22*2**2N11  11NNN111N1NNN221N111N1N11    1N111NNN1
```

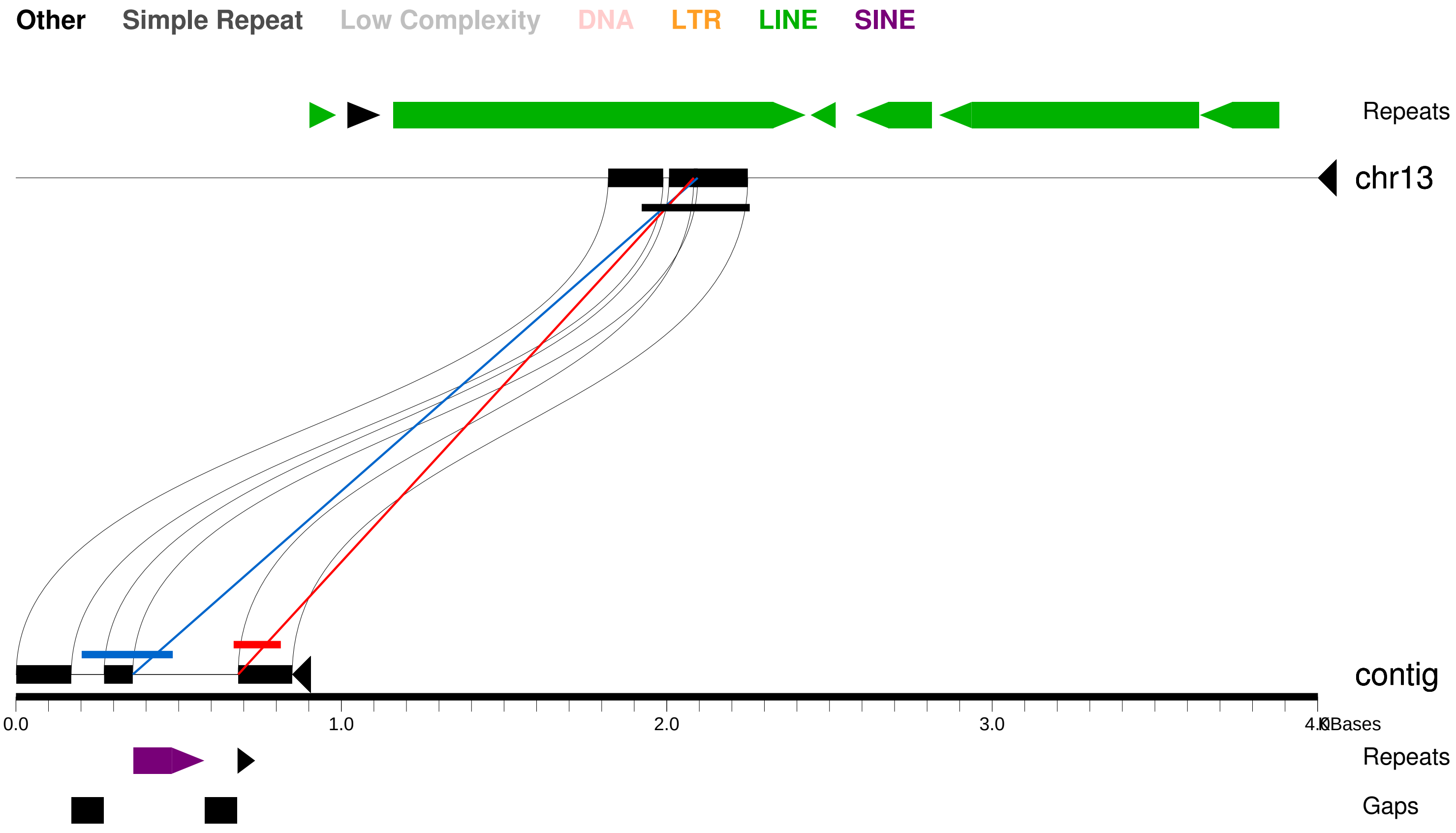

siteID: chr13_111076815 Scafftig11 + Breakpoint: chr13:111076796
chr13:111076752-111076855
Overlap Length: 16 INS size: 305 Genotyped: Yes

```
left    -------ATTTGGA-----AAATTTTTTA---TACTGAAATGACCTTAAAAAGAACCCAAATAAGTTAAT
chrom   -------ATTTGGA-----AAATTTTTTA---TACTGAAATGACCTTAAAAAGAACCCAAATAAGTTAAT
right   CCGCCCGCCTCGGCCTCCCAAAGTGCTGGGATTACAGGCGTGAGCCACCGCGCCCGGCCAATAAGTTAAT
        111111111*1**111111***1*11*11111***1*111***1*111111111111*1***********

left    TCTTTTTTTTTTTTTTTTTTTTTTTTTTTTTGAGACGGAGTCTCGCTCTGTCGCCCAGGCTGGAGT
chrom   TCTTTCATCCCAACCCAGTATTATCATTTCACAATGA---------------CATGACTTTAGT
right   TCTTTCATCCCAACCCAGTATTATCATTTCACAATGA---------------CATGACTTTAGT
        *****22*2222222222*2**2*22***2*2*22**222222222222222*22*2**22***
```

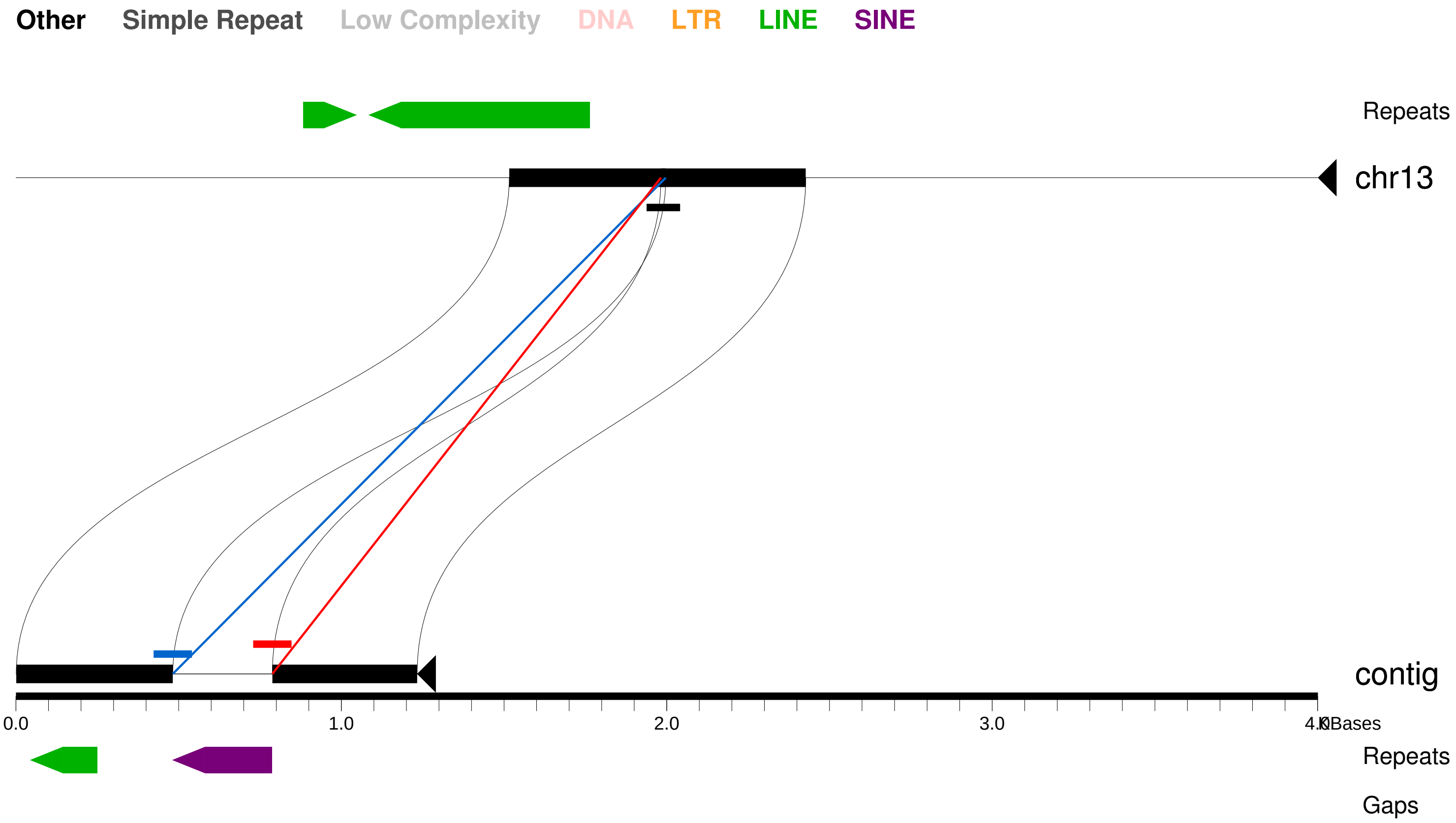

siteID: chr13_113436786 Scafftig1 - Breakpoint: chr13:113436762
chr13:113436718-113436821
Overlap Length: 13 INS size: 339 Genotyped: No

```
left    ---------TTGTATTAAAAGATATCTGT------ATGCTTTTCTCTCTTTTTCTGGTTCACTTATATTC
chrom   ---------TTGTATTAAAAGATATCTGT------ATGCTTTTCTCTCTTTTTCTGGTTCACTTATATTC
right   CCGCCCGCCTCGGCCTCCCAAAGTGCTGGGATTACAGGCGTGAGCCACCGCGCCCGGCCCACTTATATTC
        111111111*1*111*111*1*111***1111111*1**1*1111*1*11111*1**11***********

left    TTTTTTTTTNNNNNNNNNNNNNNNNNNNNNNNNNNNNNNNNNNNNNNNNNNNNNNNNNNNNNNN
chrom   TTATTCAGT---------------GCAATTTCCTGATTTCACGAGAATGAGAAGCATCTATTTG
right   TTATTCAGT---------------GCAATTTCCTGATTTCACGAGAATGAGAAGCATCTATTTG
        **2**222*2222222222222222222222222222222222222222222222222222222
```

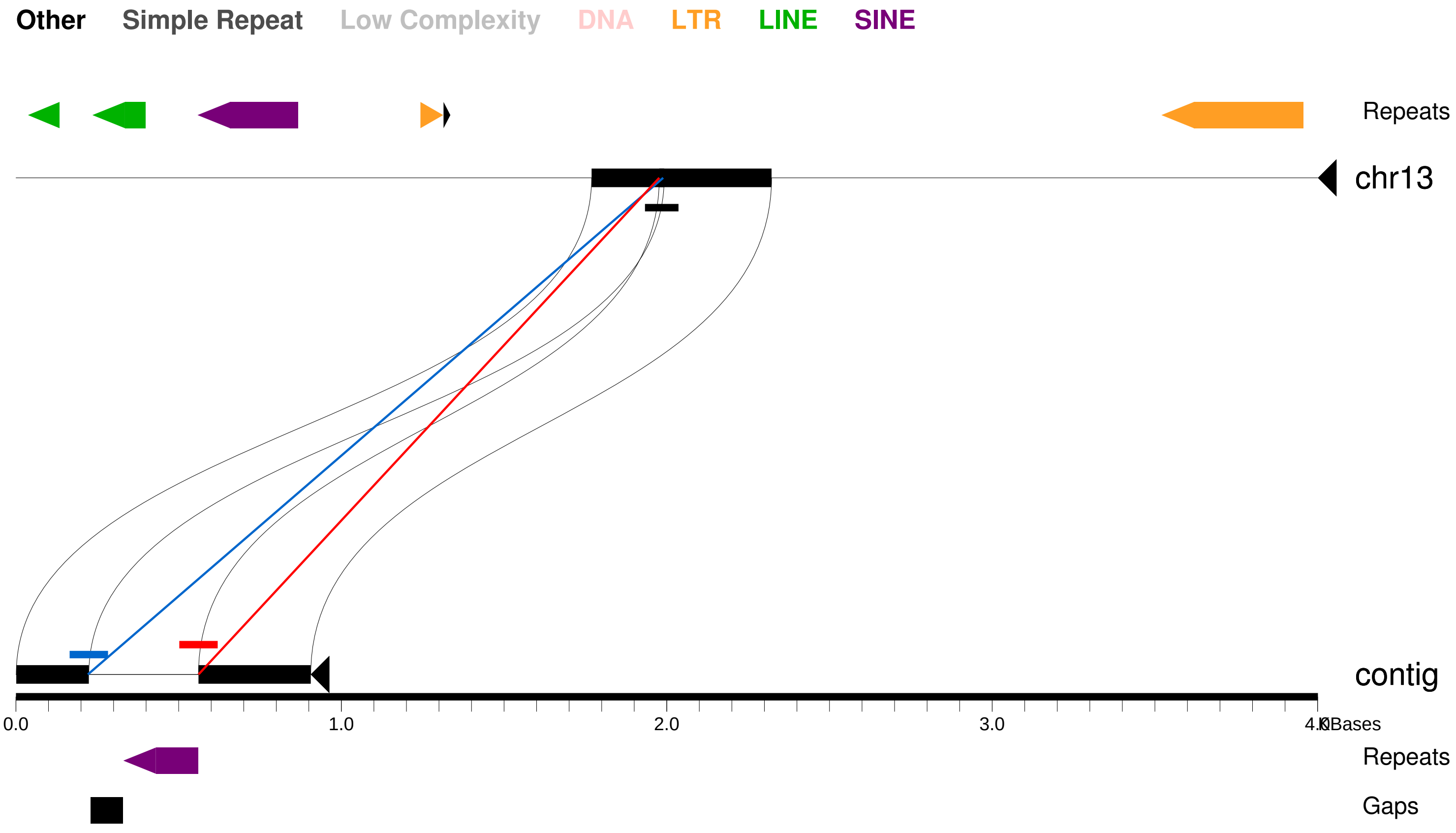

siteID: chr13_113520966 Scafftig2 + Breakpoint: chr13:113520954
chr13:113520794-113521127
Overlap Length: 14 INS size: 300 Genotyped: Yes

```
left    ----------CCGGTGGGGCCCTGTGCTTCCTGAGAGAGCCACACCAGGCATTCGGTAGATGTCAGAGAC
chrom   TGTGGGAGGCCCGGTGGGGCCCTGTGCTTCCTGAGAGAGCCACACCAGGCATTCGGTAGATGTCAGAGAC
right   TTTAGTAGAGACGAGGTTTCACCGTTTTAGCCGGGATGGTCTCGA-------TCTCCTGACCTC-GTGAT
        2N2N2N22NN1**11*111*1*1**11*11*1*1**11*1*1*111111111**1111**11**1*1**1

left    CATCCCTGCTTAAATGCCTCAGGTATTTAACTAGCCGTACAAGCAGACACCAGCGCTGACCTCGGAGAAA
chrom   CATCCCTGCTTAAATGCCTCAGGTATTTAACTAGCCGTACAAGCAGACACCAGCGCTGACCTCGGAGAAA
right   CCGCCCGCCTCG---GCCTCCCAAAGTG--CTGGGATTACAGGCGTGAGCCACCGCG---CCCGG-----
        *11***11**11111*****1111*1*111**1*111****1**11111***1***1111*1***11111

left    GTCCCTCTTCAGCAGCCCCTTCTGGGTGACTTCTT--------TTTTTTTTTTTTTTTTTTGAGACGGAG
chrom   GTCCCTCTTCAGCAGCCCCTTCTGGGTGACTTCTAAACAGAAATCGGCTTCGTTCCTTGTTTAGCCTGAC
right   ------------------CCTCTGGGTGACTTCTAAACAGAAATCGGCTTCGTTCCTTGTTTAGCCTGAC
        111111111111111111*1*************222222222*2222**22**22**2**2**2*2**2

left    -TCTCGCTC----------TGTCGCCCAGGCTG--GAGTGCAGTGGCGGGATCTCGGCT-------CACT
chrom   CTCTCTCTCCCTGTGTGTTTGTCCCTCAGACTGCTGGGTTCAAACACAAAAGTCCTGCTATGTGTCCTGT
right   CTCTCTCTCCCTGTGTGTTTGTCCCTCAGACTGCTGGGTTCAAACACAAAAGTCCTGCTATGTGTCCTGT
        2****2***2222222222****2*2***2***22*2**2**2222*222*222*2***2222222*22*

left    GCAAGCTC---CGCCTCC------CGGGTTCACGCCATTCTCCT-------GCC--T
chrom   GCACCATT---TACCTGCTTTAGTCGTTTTCATTCCTCTTTACTAAACACTGCTGTT
right   GCACCATTATTTACCTGCTTTAGTCGTTTTCATTCCTCTT-----------------
        ***222*211122***2*222222**22****22**22*21N11       11N  1
```

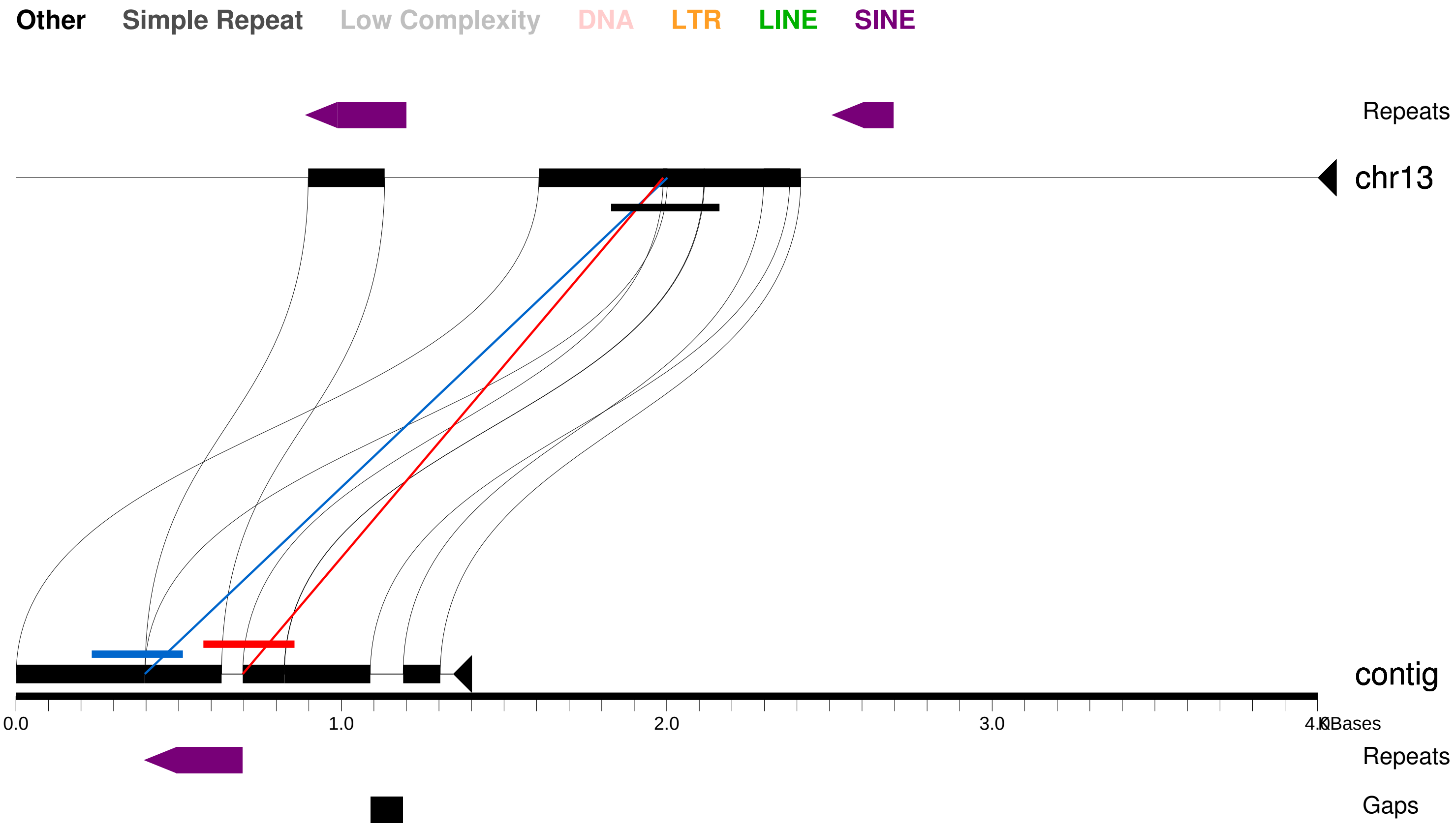

siteID: chr13_114881995 Scafftig1 - Breakpoint: chr13:114882006
chr13:114881846-114882181
Overlap Length: 16 INS size: 307 Genotyped: Yes

```
left    ----------------GCATTCTGAGGAATCCGAAAGACTCCAGCGTAGGACAGACCGCTGTTCCAAGCC
chrom   TTTTCATAAACATGCCGCATTCTGAGGAATCCGAAAGACTCCAGCGTAGGACAGACCGCTGTTCCAAGCC
right   --------AGAATGGCGTGAACCCGGGAAGCGGA--GCTTGCAGTG-AGCCGAGATTGCG---CCACTGC
                2NN222N2*1111*111****1*1**11*11*1***1*1**111***11**1111***111*

left    ACTGGGAAAGGGGAAACTGGTGACTGAGCG-GCGCCCGTCAGAGCCATTAAAACGTGAGGGAAAACTGCA
chrom   ACTGGGAAAGGGGAAACTGGTGACTGAGCG-GCGCCCGTCAGAGCCATTAAAACGTGAGGGAAAACTGCA
right   AGTCCGCAGTCCGGCCTGGGCGACAGAGCGAGACTCCGTCTCA---AAAAAAA--------AAAA-----
        *1*11*1*1111*11111**1***1*****1*111*****11*111*11****11111111****11111

left    CCTTACGGCACACGAACCCTTAGAAGAGGCAAAACACGGCCGGGCGCGGTGGCTCACGCCT--GTAATCC
chrom   CCTTACGGCACACGAACCCTTAGAAGAGGCAAAACACTACTGAGTTAA-TGCTTAATTTATTTGTAATGA
right   ---------AAAAAAA-----AGAAGAGGCAAAACACTACTGAGTTAA-TGCTTAATTTATTTGTAATGA
        111111111*1*11**11111***************22*2*2*22222**22*2*2222*22*****22

left    CAGCACTTTGGGAGGCCGAGGCGGGTGGATCATGAGGTCAGGAGATCGAGACCATCCTG--------GCT
chrom   TAAAAGTTTTTATCACTTTAAATAAAGTTATTTGAAAAAAGTTATTCAATGGCAGCCTAATAATTTTGCT
right   TAAAAGTTTTTATCACTTTAAATAAAGTTATTTGAAAAAAGTTATTCAATGGCAGCCTAATAATTTTGCT
        2*22*2***222222*2222222222*22222***2222**2222**2*222**2***222222222***

left    -----AACAAGG-----TG-AAACCCCGTCTCTACTAAA-------------------
chrom   TAATGAAAAATTAACTCTGCAAATACAGTTTCCACTATTCACATGGAGCCTGCAGGTT
right   TAATGAAAAATTAACTCTGCAAATACAGTTTCCACTATTCACATGG------------
        22222**2**2222222**2***22*2**2**2****222222222
```

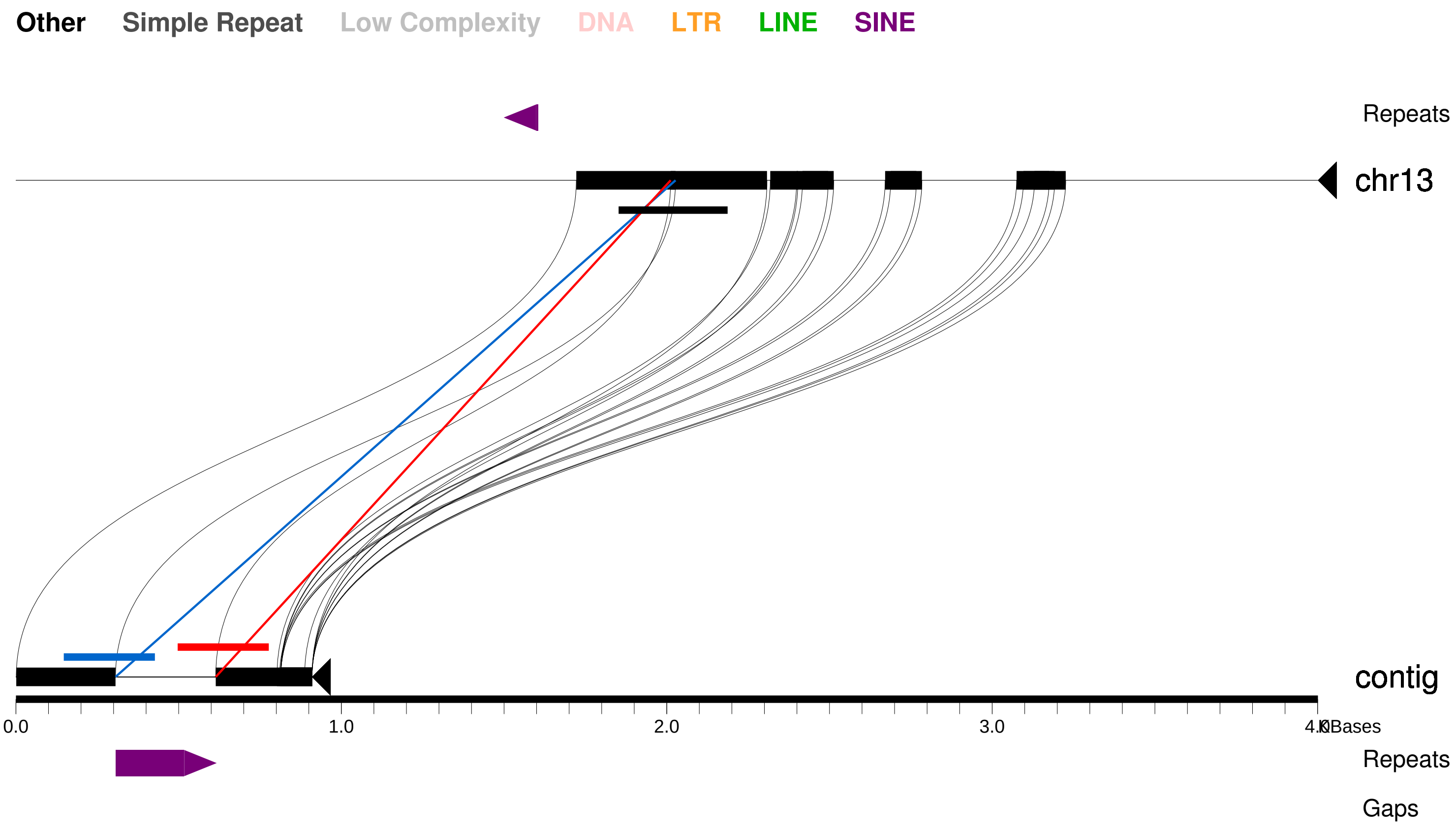

siteID: chr13_21894779 Scafftig1 + Breakpoint: chr13:21894765
chr13:21894605-21894938
Overlap Length: 14 INS size: 271 Genotyped: Yes

```
left    --------------GCACCCCCTCTTGAAGAAGCTGAACTTCAAGCGGGTGGGAGCCGGCATGCTGGAGC
chrom   CGAAGTGAAGG--AGCACCCCCTGTTGAAGAAGCTGAACTTCAAGCGGGTGGGAGCCGGCATGCTGGAGC
right   C-AGGCGCCCGCCACCGCGCCCGGCT-AATTTTTTGTATTTTTAGTAG-----AGACGGGGT--------
        2 2N2N2NNN21121*1*1***121*1**11111**1*1**11**11*11111**1***11*11111111

left    CGCCCTTCAAGCCTGACCCTCGGGCCATTTACTGCAAGGATATTCTGGACATTAAACAGTTCTCTAGGGT
chrom   CGCCCTTCAAGCCTGACCCTCGGGCCATTTACTGCAAGGATATTCTGGACATTAAACAGTTCTCTAGGGT
right   -----TTCA--------CCTTG------TTA--GCCAGGATGGTCTCGATCTC---CTGACCTCATGATC
        11111****11111111***1*111111***11**1*****11***1**11*1111*1*11***11*111

left    CAAGGGTGTGGAGCTGGAGGCTACCGACCAAGACTTTTTT-------TTT-----------TTTTTTTTTT
chrom   CAAGGGTGTGGAGCTGGAGGCTACCGACCAAGACTTCTACCAGAAGTTTGCCACAGGCAGTGTGCCCATC
right   CACCCGCCTCGGCCTCC---CAACCGACCAAGACTTCTACCAGAAGTTTGCCACAGGCAGCGTGCCCATC
        **111*11*1*11**11111*1**************2*22222222***2222222222212*22222*2

left    TTTTTTGAGACGGAG-TCTCG----CTCTGT-CGCCCAGGC-CAGACTGC----GGACTGCAGTGGCGCA
chrom   CCCTGGCAGAAGGGGATGGTGGAGACTGAGTGCTTCCAGGAGCTGAATGTCTTTGGGCTGGA-TGGCTCA
right   CCCTGGCAGAACGGGATGGTGGAGACTGAGTGCTTCCAGGAGCTGAATGTCTTTGGGCTGGA-TGGCTCA
        222*222***21*2*2*222*2222**22**2*22*****22*2**2**22222**2***2*2****2**

left    AT---CTCGGCTC---ACTGCAAGCTCCG-----CTTCCCGGGTTCACGCCATT--C
chrom   GTTCCCCCAGACCTGGACTGGAAGGGCCAGCCACCTGCAAGGGACTGCTGCAGAGAC
right   GTTCCCCCAGACCTGGACTGGAAGGGCCAGCCACCTGCAAGGGA-------------
        2*222*2*2*22*222****2***22**222222**2*22***2NNN1NN11NN  1
```

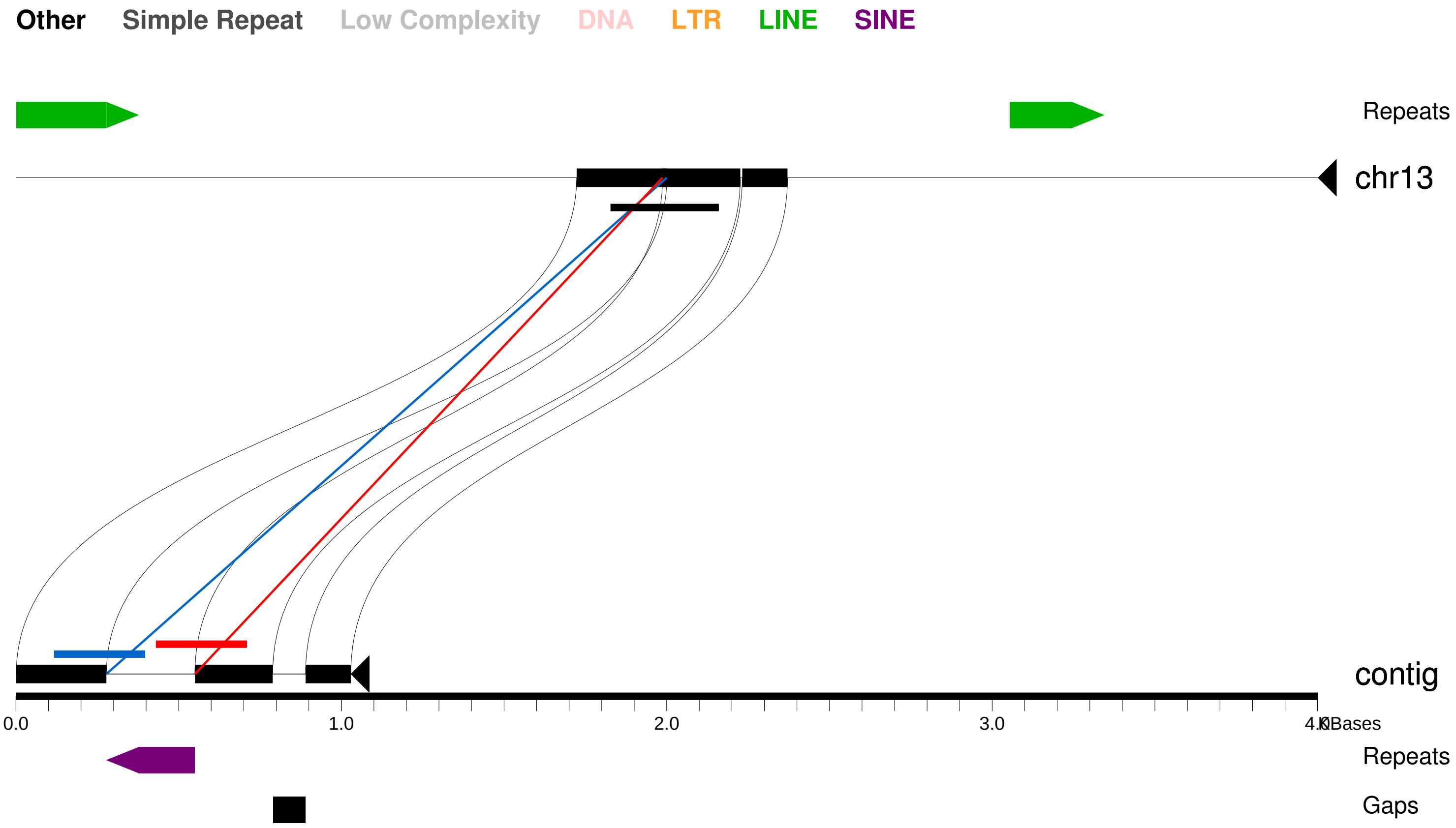

siteID: chr13_22556635 Scafftig3 - Breakpoint: chr13:22556640
chr13:22556593-22556699
Overlap Length: 13 INS size: 228 Genotyped: Yes

```
left    GGGGCGACT----TGGGGCATACTCTCCGTACTGGGCATTTCTCTCGAGTT---------AAAAAGATCT
chrom   GGGGCGACT----TGGGGCATACTCTCCGTACTGGGCATTTCTCTCAAGTT---------AAAAAGATCT
right   GCGCC-ACTGCGGTCCGGCCTGGGCGACAGAGCGAGACTCCGTCTCAAAAAAAAAAAAAAAAAAAGATCT
        *1*1*1***1111*11***1*111*11*11*11*1*11*111****2*111111111111**********

left    GCGGTCAGGAGATCGAGACCATCCTGGCTAA-CAAGGTGAAACCCCGTCTCTACTAAAAAT-AC
chrom   GCGTA----AGTTAAAGAGATTCATTGCACAGCATGGTGA----------CTACAGTTAATGAC
right   GCGTA----AGTTAAAGAGATTCATTGCACAGCATGGTGA----------CTACAGTTAATGAC
        ***222222**2*22***222**2*2**22*2**2*****2222222222****2222***2**
```

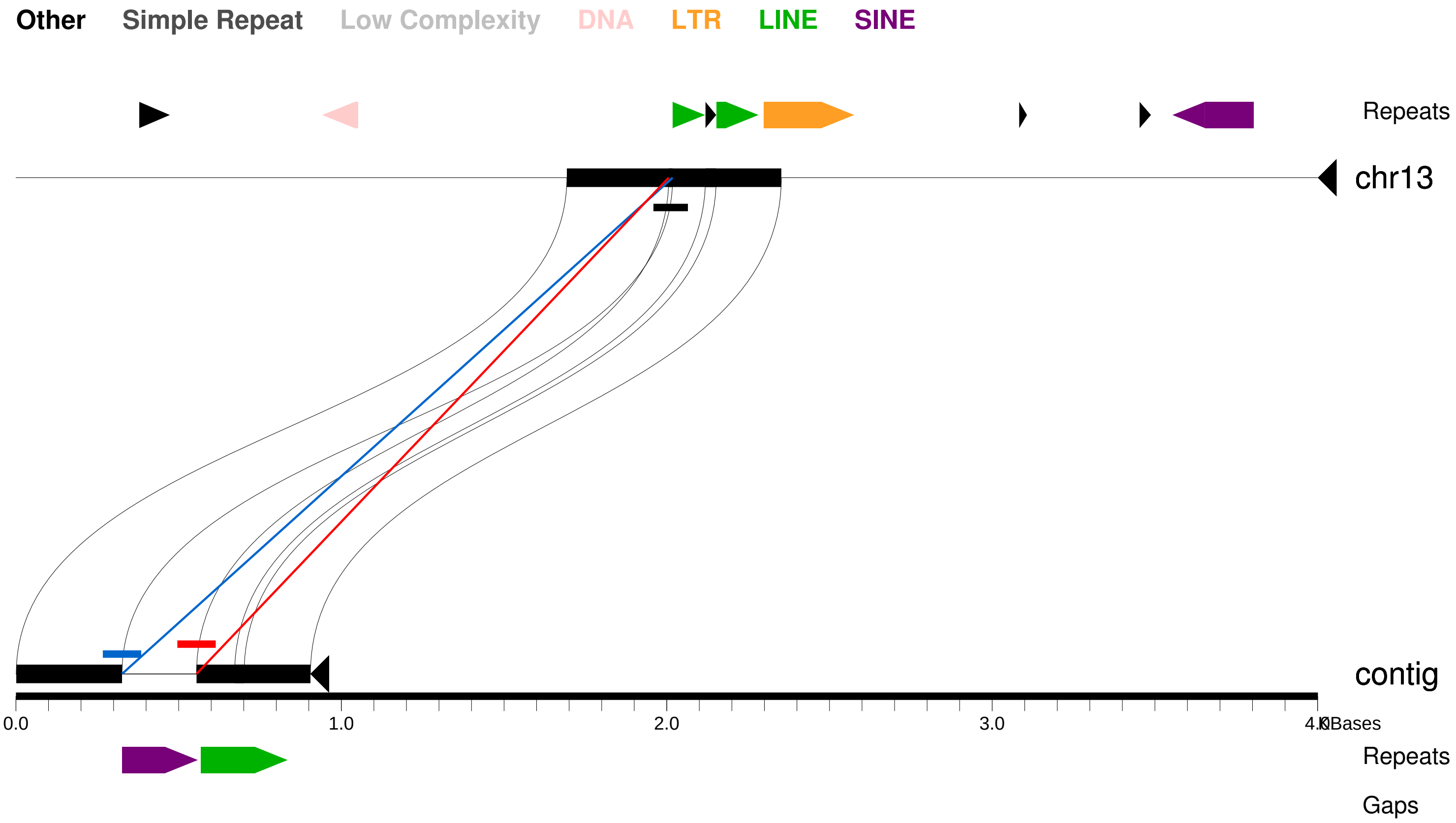

siteID: chr13_22975697 Scafftig1 - Breakpoint: chr13:22975700
chr13:22975656-22975759
Overlap Length: 16 INS size: 469 Genotyped: No

```
left    AATTTTCAGAATCACATCTCGACTTTTAAAAATCACATTTGTTT---------------AAAAATACCTG
chrom   AATTTTCAGAATCACATCTCGACTTTTAAAAATCACATTTGTTT---------------AAAAATACCTG
right   NNNNNNNNNNNNNNNNNNNNNNNNNNNNNNNNNNNNNNNNNNNNNNNNNNNNNNNNNNNAAAAAATACCTG
        11111111111111111111111111111111111111111111111111111111111***********

left    TGATGGGCCGGGCGCGGTGGCTCACGCCTGTAATCCCAGCACTTTGGGAGGCCGAGGCGGGCGG
chrom   TGATG------------TATCTAAAGCCTTTTTCTTGTTAGCTGTGTGAAGTTTAAG---GTGT
right   TGATG------------TATCTAAAGCCTTTTTCTTGTTAGCTGTGTGAAGTTTAAG---GTGT
        *****222222222222*22**2*2****2*2222222222**2**2**2*222*2*222*2*2
```

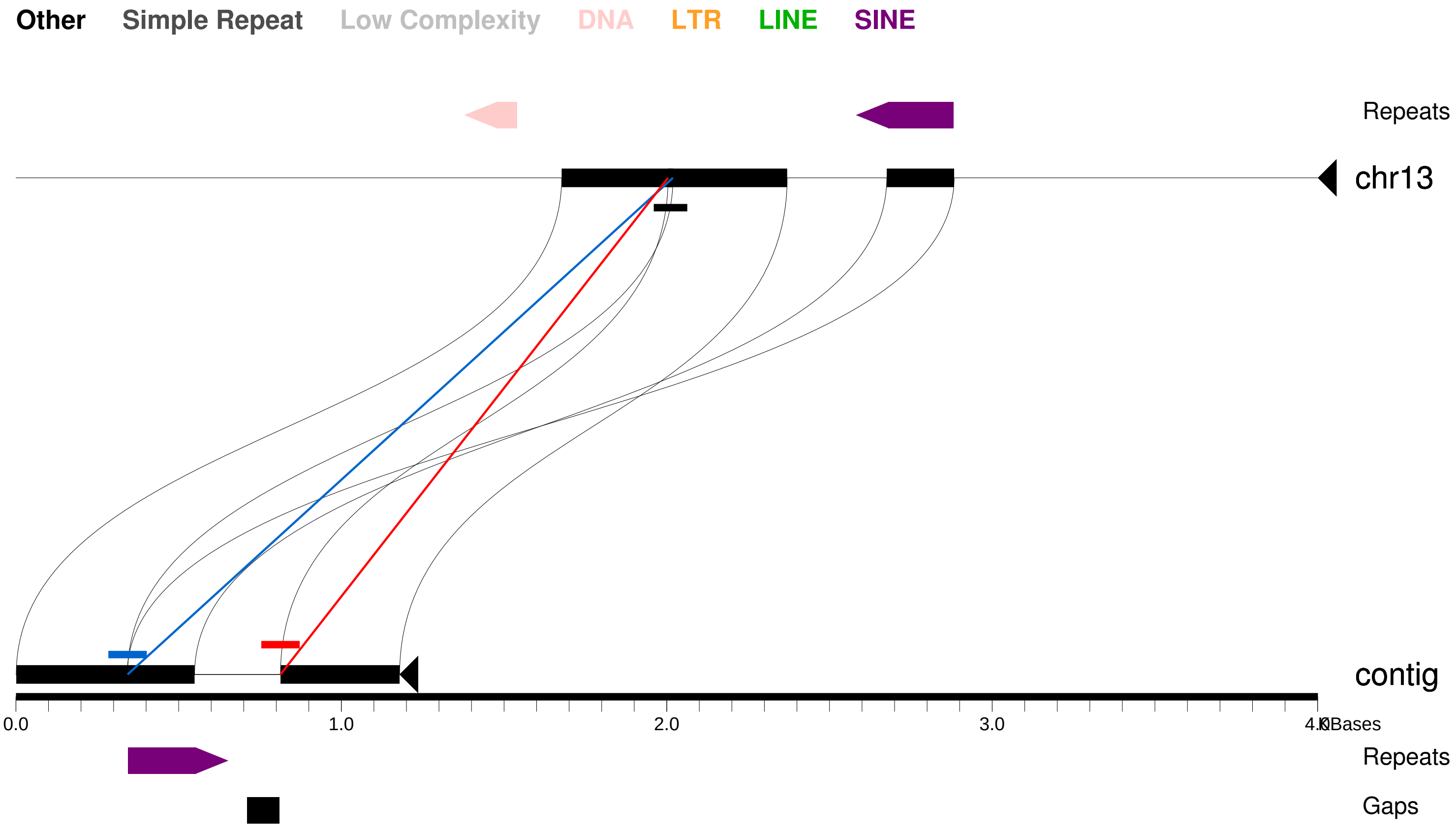

siteID: chr13_23662812 Scafftig5 + Breakpoint: chr13:23662917
chr13:23662867-23663053
Overlap Length: 10 INS size: 291 Genotyped: Yes

```
left    GAACTTTGGGCTTCCTTTGATAGCTAAAGAGAA---AATAGAGGCAAAAAT-----------GGCAGATG
chrom   GAACTTTGGGCTTCCTTTGATAGCTAAAGAGAA---AATAGAGGCAAAAAT-----------GGCAGATG
right   GATCC---GCCCGCCTCGGCCTCCCAAAGTGCTGGGATTACAGGCGTGAGCCACCGCGCCCGGCCAGATG
        **1*1111*1*11***11*1111*1****1*11111*1**1****111*1111111111111*1******

left    TTTTTTTTTTTTTTTG---------------------------------AGACGGAGTCTCGC------T
chrom   TTTTAAGATTCTTGAGCATAATTAAATACTAAAGTGACAAGGGTGATAAAGAGGGAGAGGACAAAATAAT
right   TTTTAAGATTCTTGAGCATAATTAAATACTAAAGTGACAAGGGTGATAAAGAGGGAGAGGACAAAATAAT
        ****2222**2**22*222222222222222222222222222222222***2****222222222222*

left    CTGTCGCCCAGGCT------GGAGTGC------AGTGGCGCGAT-----------------
chrom   CAATGCCACAGGCTCTAAAGGAACTGAGGACATAGGGACAGGACAGGAAGAGACAAACCTC
right   ----GCCACAGGCTCTAAAGGAACTGAGGACATAGGGACAGGACAGGAAGAGACAAACCTC
        1NN122*2******222222*2*2**2222222**2*2*22**222222222222222222
```

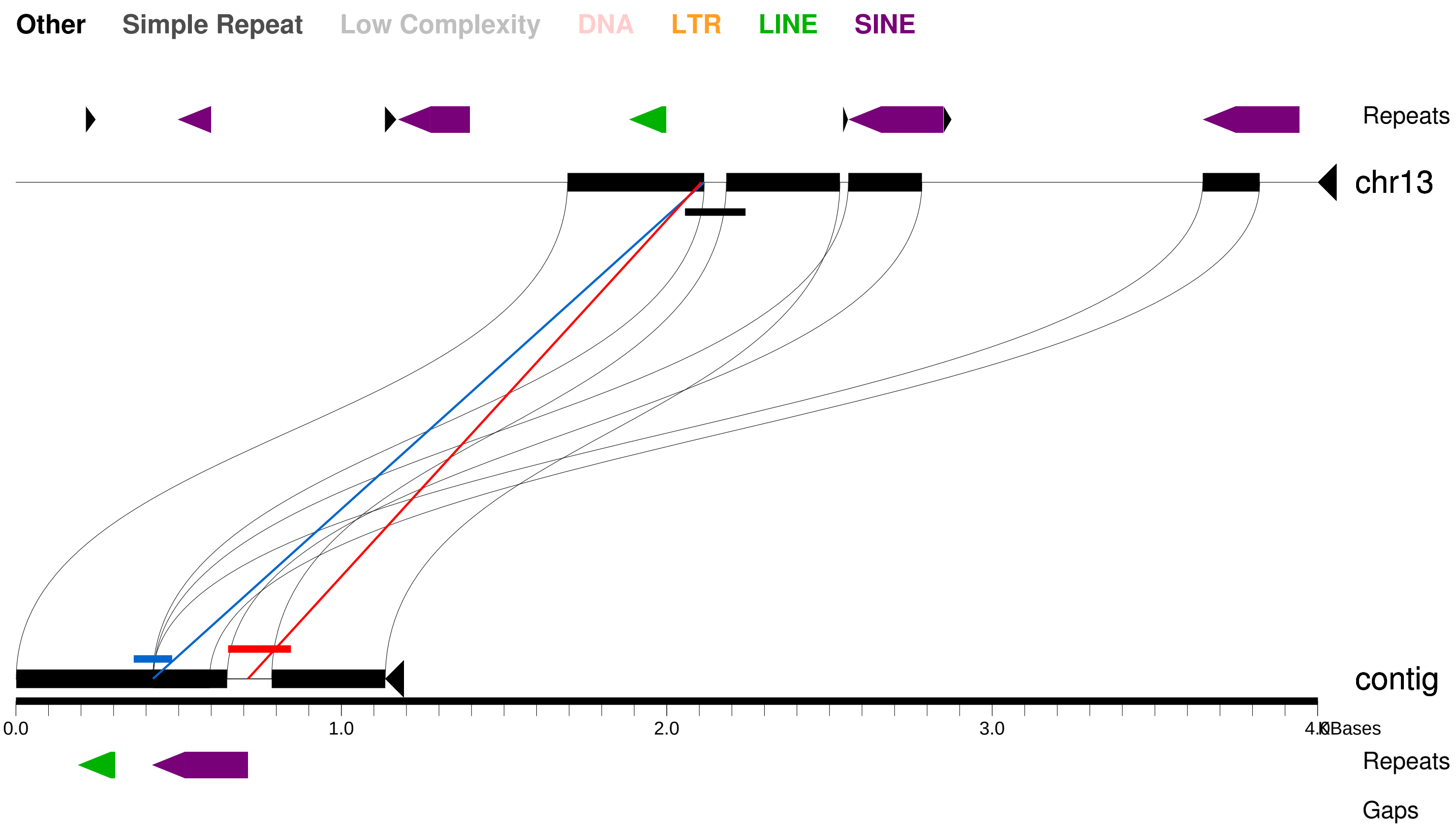

siteID: chr13_27856030 Scafftig1 + Breakpoint: chr13:27855984
chr13:27855937-27856043
Overlap Length: 13 INS size: 410 Genotyped: No

```
left   TTGACAAAATATCTGGGCACTCCATGGCTCAGTCAAGTTGACACATA------------AAATGAATAAT
chrom  TTGACAAAATATCTGGGCACTCCATGGCTCAGTCAAGTTGACACATA------------AAATGAATAAT
right  NNNNNNNNNNNNNNNNNNNNNNNNNNNNNNNNNNNNNNNNNNNNNNNNNNNNNNNNNNNNAAAGAATAAT
       111111111111111111111111111111111111111111111111111111111111***1*******

left   CAGGCCGGGCGCGGTGGCTCACGCCTGTAATCCCAGCACTTTGGGAGGCCGAGGCGGGCGG
chrom  CATAT---GCAGTATGGGTTTTAGCTGCATT---ATCTCGTTGAATTCCCCCAAC------
right  CATAT---GCAGTATGGGTTTTAGCTGCATT---ATCTCGTTGAATTCCCCCAAC------
       **222222**2222***2*22222***2*2*222*2*2*2***22222**2222*222222
```

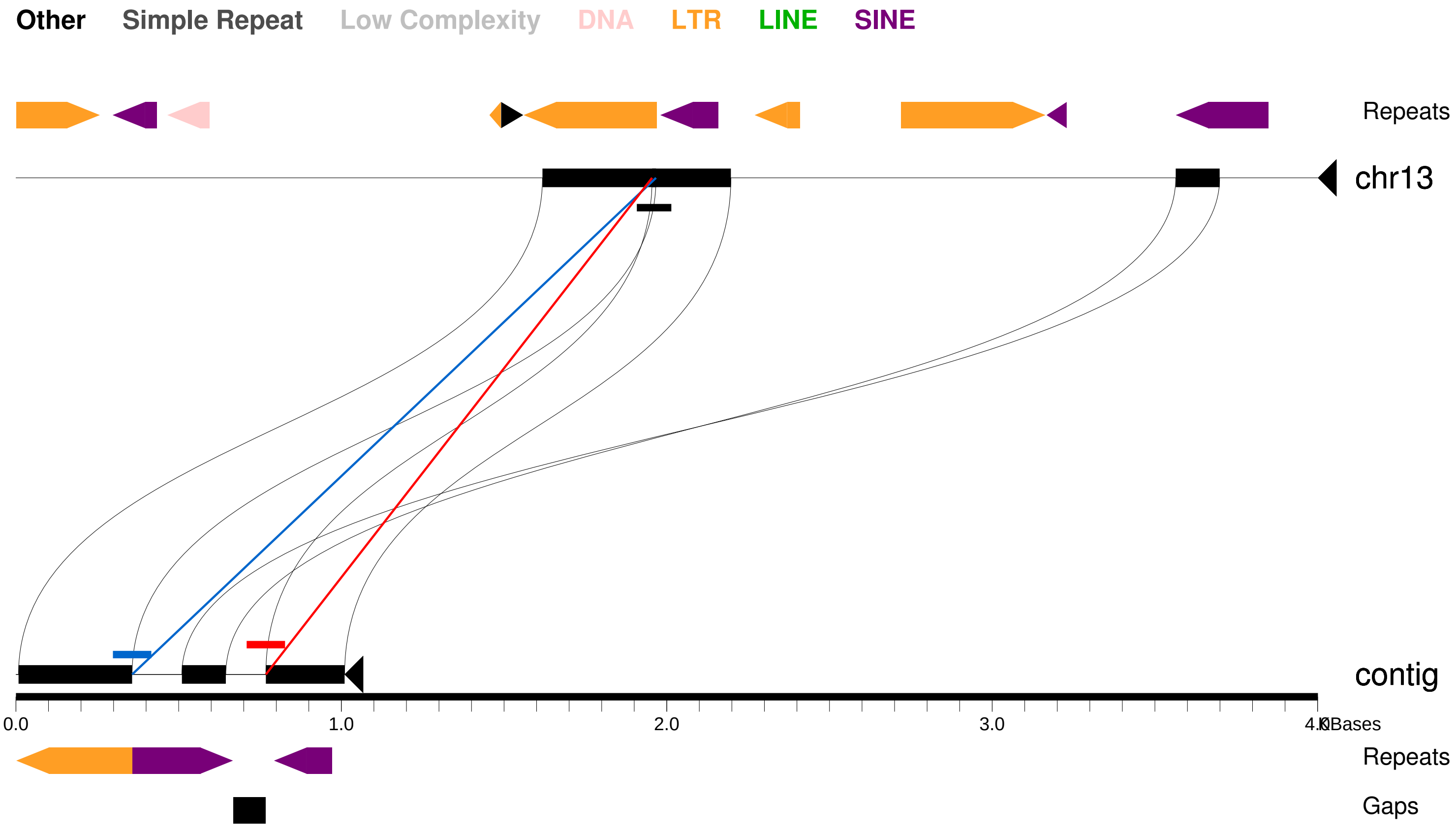

siteID: chr13_29538897 Scafftig10 + Breakpoint: chr13:29538876
chr13:29538831-29538935
Overlap Length: 15 INS size: 305 Genotyped: Yes

```
left     GGGTCAGTGTTTCAGTCT------GT-CTGAGCTTT-AGTCATGTACAA--------GAAGCTTTTGTGT
chrom    GGGTCAGTGTTTCAGTCT------GT-CTGAGCTTT-AGTCATGTACAA--------GAAGCTTTTTTGT
right    CCGCCCGC--TTCGGCCTCCCAAAGTGCTGGGATTACAGGCGTGAGCCACCGCGCCCGGCCCTTTTTTGT
         11*1*1*111***1*1**111111**1***1*1**11**1*1**11*1*11111111*111*****2***

left     GTTTTTTTTTTTTTTTTTTTTTTTTTTTTGAGACGGAGTCTCGCTCTGTCGCCCAGGCTGGAGTGC-
chrom    GTTTTTAATGCCTGGGTCTTGGGTT----AGAAGAA------CCCTAATGCCCAA-----AGTCCC
right    GTTTTTAATGCCTGGGTCTTGGGTT----AGAAGAA------CCCTAATGCCCAA-----AGTCCC
         ******22*222*222*2**222**2222***2*2*222222*2**222*****222222***2*2
```

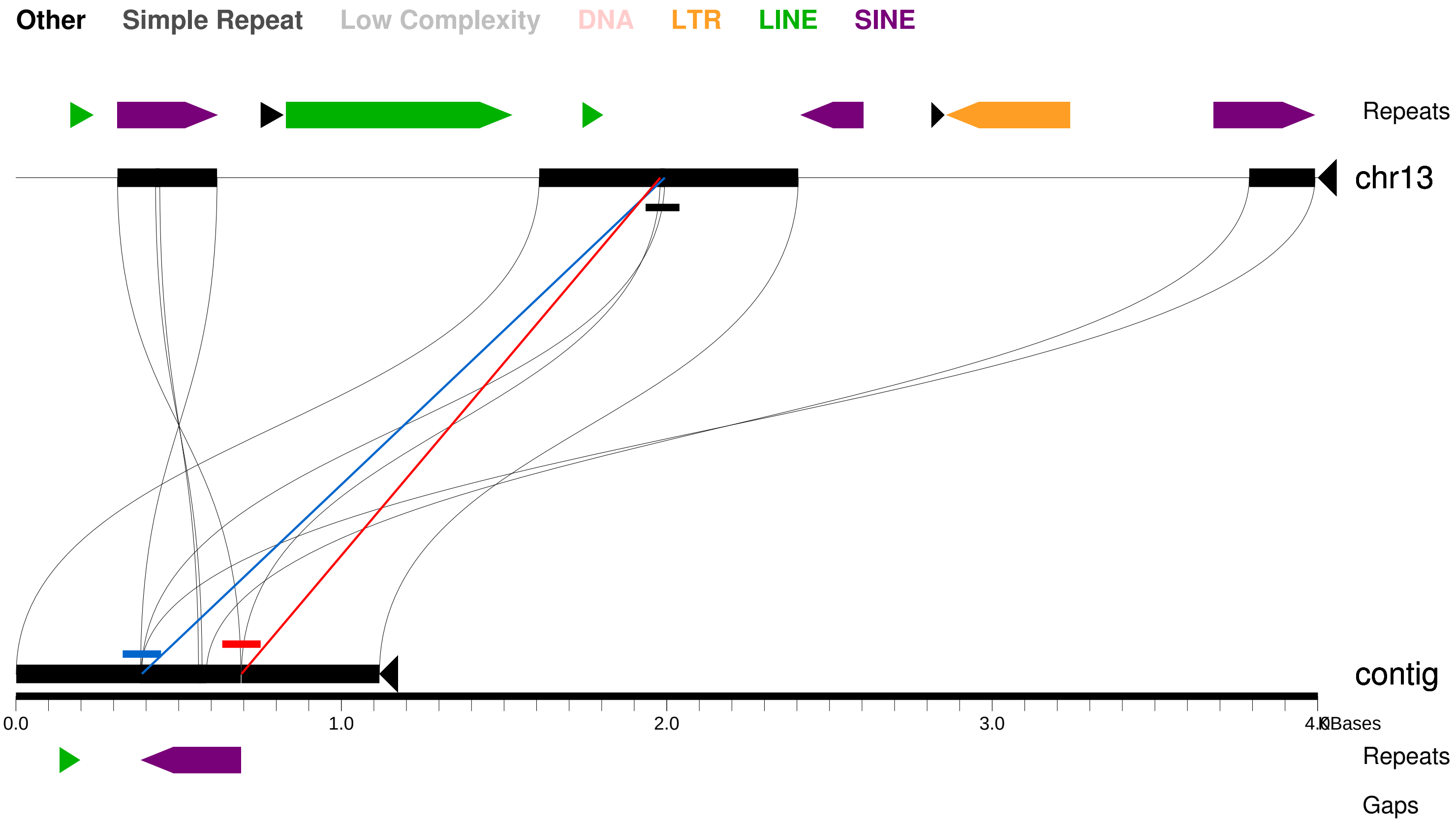

siteID: chr13_31891942 Scafftig1 + Breakpoint: chr13:31892033
chr13:31891986-31892077
Overlap Length: 13 INS size: 129 Genotyped: Yes

```
left   AGAAC--------ACTC-AGT-------AAAGA---ATATTTTAGCCCTTGAAGAAATGATATAAA----
chrom  AGAAC--------ACTC-AGT-------AAAGA---ATATTTTAGCCCTTGAAGAAATGATATAAA----
right  ATCCCGCCACTGCACTCCAGCCTGGGCGACAGAGCGAGACTCCGTCTCAAAAAAAAAAAAAAAAAAAAAA
       *111*11111111****1**11111111*1***111*1*1*1111*1*111**1***11*1*1***1111

left   ----AAAGTATGTCATAGGCTGAGGCAGGAGAATGGCGTGAACCCGGGAGGCGGAGCTTGCAGTGAGCCG
chrom  ----AAAAGTATGTCATA--CT--TGCT--AGAATGTCCCTAACAATGG---------TTGC---------
right  AAAAAAAGTATGTCATA--CT--TGCT--AGAATGTCCCTAACAATGG---------TTGC---------
       1111*************22**222**222******2*222***222**222222222****222222222

left   AGATCC
chrom  ---TTC
right  ---TTC
       222*2*
```

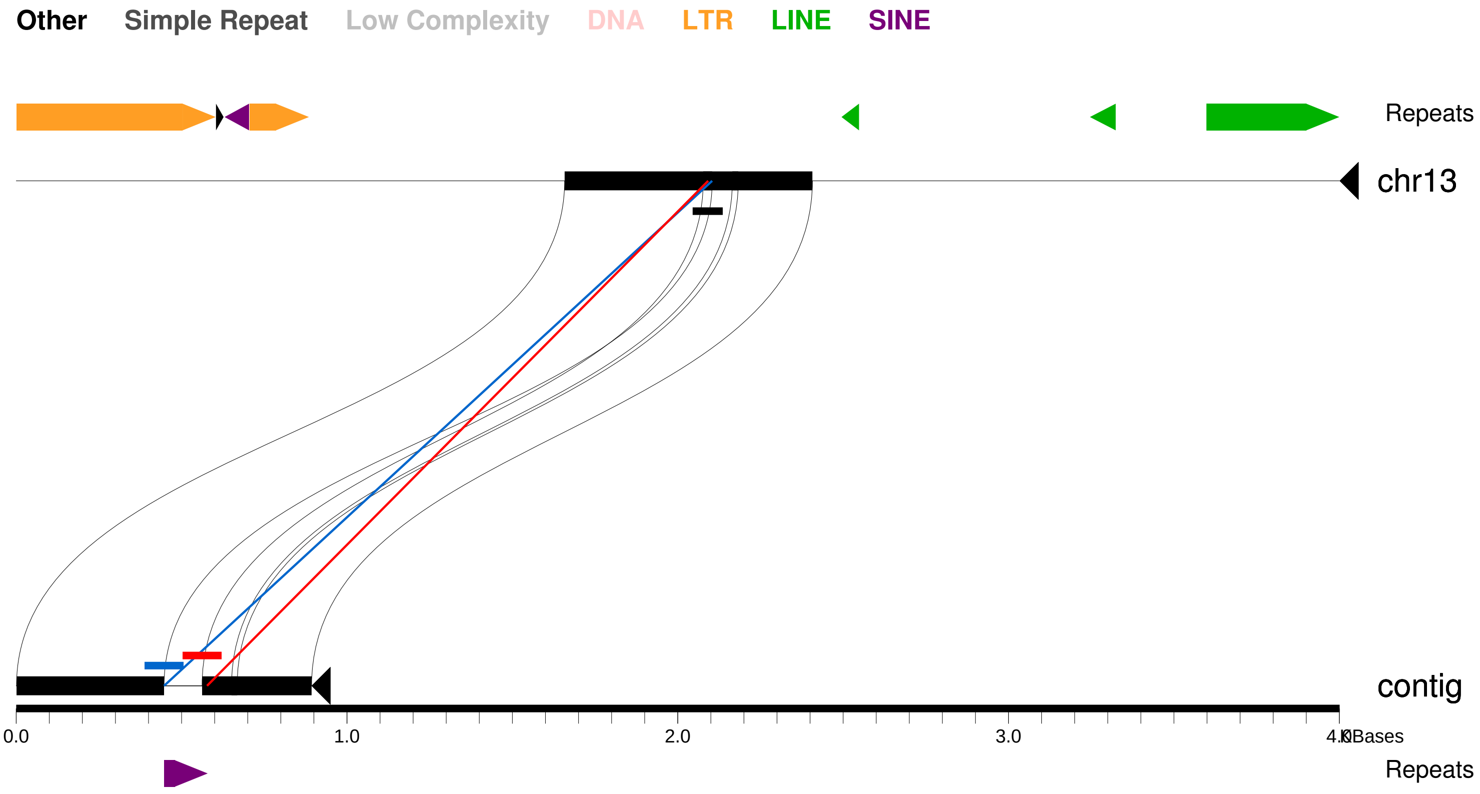

siteID: chr13_32706403 Scafftig2 - Breakpoint: chr13:32706388
chr13:32706341-32706447
Overlap Length: 13 INS size: 311 Genotyped: Yes

```
left     T--------AAAGA-----AATCTGTTTTATTCTTACTATAAAAAGGAATCTTAATTTTTAAAAACTCAG
chrom    T--------AAAGA-----AATCTGTTTTATTCTTACTATAAAAAGGAATCTTAATTTTTAAAAACTCAG
right    GCCTGGGCGACAGAGTGAGACTCCGTCTCAAAAAAAAAAAAAAAAAAAAAAAAAAAAAA-AAAAACTCAG
         111111111*1***11111*1**1**1*1*11111*11*1*****11**1111**11111**********

left     CTAGGCCGGGCGCGGTGGCTCACGCCTGTAATCCCAGCACTTTGGGAGGCCGAGGCGGG--CGG
chrom    CTAAACAATACAGGTTTGATA-----TTTATTGCC--CAGT-------GCCCATCATGGTACAT
right    CTAAACAATACAGGTTTGATA-----TTTATTGCC--CAGT-------GCCCATCATGGTACAT
         ***22*2222*22*2*2*2*222222*2**2*2**22**2*2222222***2*2222**22*22
```

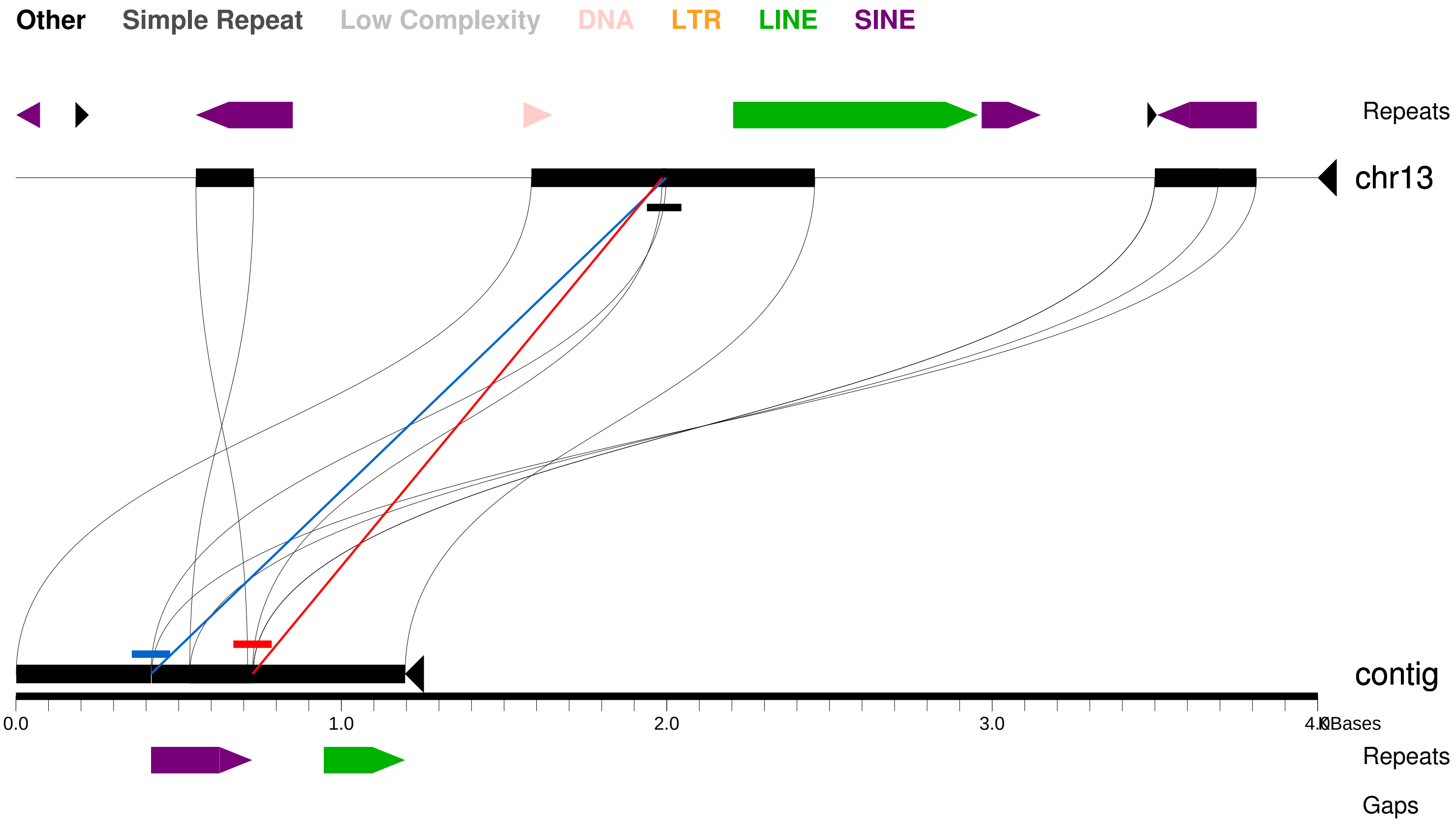

siteID: chr13_32922420 Scafftig2 - Breakpoint: chr13:32922439
chr13:32922279-32922601
Overlap Length: 1 INS size: 316 Genotyped: Yes

```
left   ---AAGAATCATGAAAGTTCCCAGAATACAGAATAAAATGAAAAAAGGTATGAAAAGTCAATTCTGTGGA
chrom  TGGAAGAATCATGAAAGTTCCCAGAATACAGAATAAAATGAAAAAAGGTATGAAAAGTCAATTCTGTGGA
right  ----AGAATGGTGTGAA--CCCGGGAGGCGGAGCT---TGCA-----GTG-----AGCCGA-------GA
          1*****11**11*111***1*1*11*1**111111**1*11111**111111**1*1*1111111**

left   TCTATCATCTGAAAATACAGAGTTTGAGAAGGAAGGCACAGAAGAGAAATGAAGAAAGAAATTTTAAAAT
chrom  TCTATCATCTGAAAATACAGAGTTTGAGAAGGAAGGCACAGAAGAGAAATGAAGAAAGAAATTTTAAAAT
right  TCCCGCCACTGCAC-TCCAGCCTGGGCGACAGAGCG---AGACTCCGTCTCAAAAAAAAAAA---AAAAA
       **111*11***1*11*1***11*11*1**11**11*111***1111111*1**1***1***1111****1

left   AAATACATAATTTTAAAAGTTTT------------GGCC--------------------GGGCGCGGTGG
chrom  AAATACATAATTTTAAAAGTTCTACTAGTACTGAAGGACATGAGTTTCCTTAATTAAAAGGGCCCACTGA
right  AAAAAAAAAA----AAAAAATTTACTAGTACTGAAGGACATGAGTTTCCTTAATTAAAAGGGCCCACTGA
       ***1*1*1**1111****11*N*222222222222**2*22222222222222222222****2*22**2

left   CT---CACGCCTGTAATCCCAGCACTTTGGGAGGCCGAGGCGGGCGGATCACGAGGTCAGGAGATCGAGA
chrom  GTGAGCACACAAGTAAAAATGACCCACAGTAAGGCACATCCTTGTGAATTTTTAGAATAATAGAGGCAGA
right  GTGAGCACACAAGTAAAAATGACCCACAGTAAGGCACATCCTTGTGAATTTTTAGAATAATAGAGGCAGA
       2*222***2*22****222222*2*222*22****22*22*22*2*2**2222**222*22***222***

left   CCATCCCGGCTAAAACGGTGAA--ACCCCGTCTCTAC--TAAA
chrom  CAGGAACCTTAAATTCATTAGAGGACCAAGAAGTTAGGTTTCA
right  CAGGAACCTTAAATTCATTAGAGGACCAAGAAGTTAGG-----
       *22222*2222**22*22*22*22***22*2222**22 1NN1
```

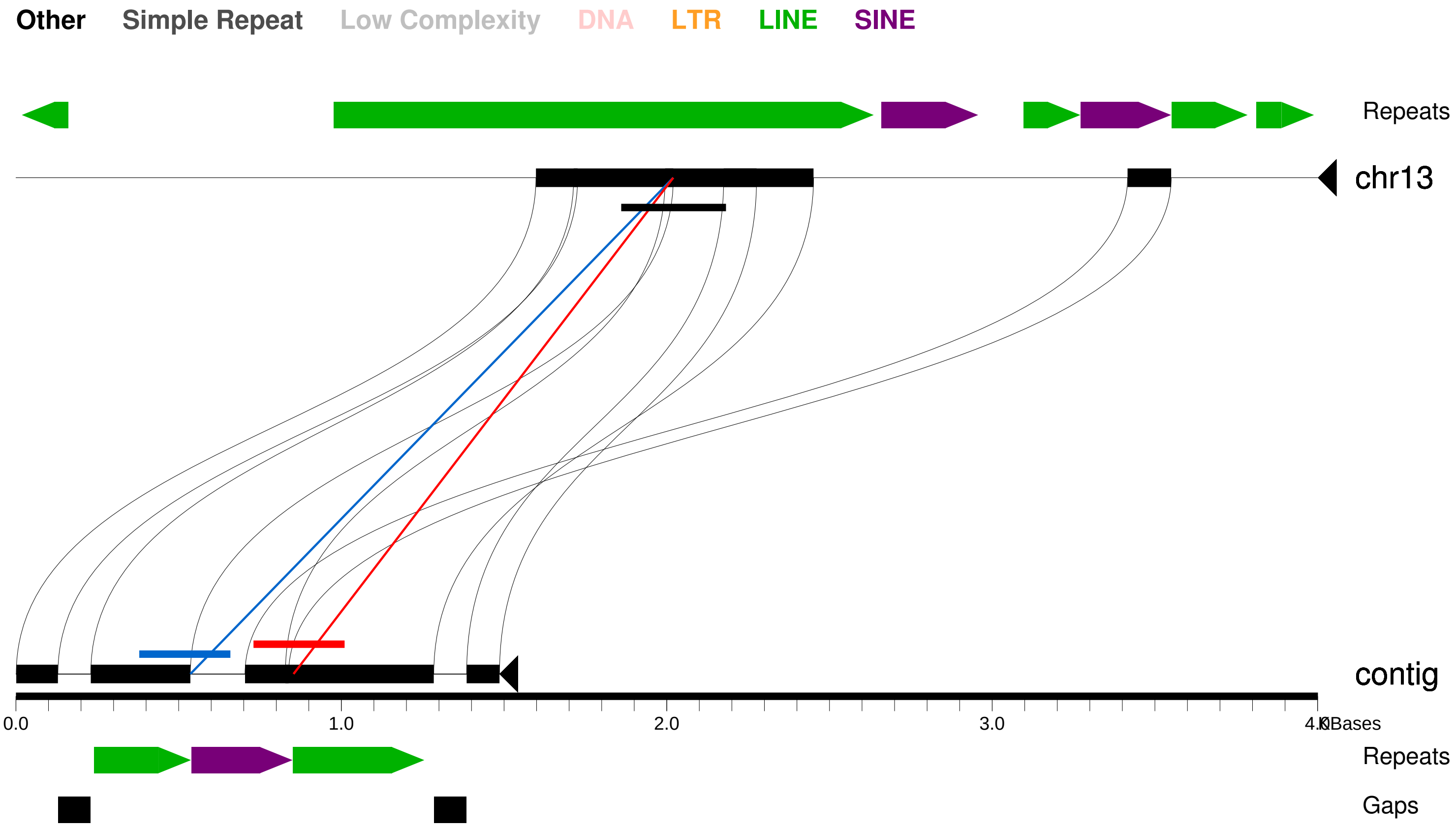

siteID: chr13_34738976 Scafftig1 + Breakpoint: chr13:34738956
chr13:34738796-34739126
Overlap Length: 11 INS size: 280 Genotyped: Yes

```
left   ----------ACAGACACTAACCTGGCGGTAAGTCAGGGCAATTTCATTACAAGTGCACGCTTGCATTTT
chrom  TATGGCAATCACAGACACTAACCTGGCGGTAAGTCAGGGCAATTTCATTACAAGTGCACGCTTGCATTTT
right  CGTG--AACCCGGGAAGCGGAGCTTGCAGTGAGCCGAG--------------ATTGCGCCACTGCAGT--
       NN22  22N2111**11*11*1**1**1**1**1*11*11111111111111*1***1*111****1*11

left   ATGCCCCTTAATCACTGTTACTGCTCTCCCAAGCAGCACATTCTTTCAGCTTGGATTTTGACAATACGTT
chrom  ATGCCCCTTAATCACTGTTACTGCTCTCCCAAGCAGCACATTCTTTCAGCTTGGATTTTGACAATACGTT
right  -----CCGCAGTC-CGGC--CTGGGCGACAGAGCGAGACTCCGTCTCAAAAAAAAAAAAAAAAAAAAA--
       11111**11*1**1*1*111***11*11*11***111**1111*1***111111*11111*1**1*1111

left   CTTTGTCAGAGGAATGCTGCAAAGAGGGTGA---------CTTTGGGAGGCCGAGGCGGGTGGATCATGA
chrom  CTTTGTCAGAGGAATGCTGCAAAGAGGGTGAAAGGGGCTGCTATTGCCAGAACAGTTCCCTGGCACCTGA
right  -------AAAAAAA------AAAGAGGGTGAAAGGGGCTGCTATTGCCAGAACAGTTCCCTGGCACCTGA
       1111111*1*11**111111***********222222222**2*2*222*222**22222***22*2***

left   GG---------TCAGGAGATCGA----GACCATC----CTGGCTAACAAGGTGAAACCCCGTCTCTACTA
chrom  GGCGGAGGTACTTAAAAAAGCCACTGTGTGCATTTACACTTGCTCCATTGGTGCA-CACAGCCT-TACTC
right  GGCGGAGGTACTTAAAAAAGCCACTGTGTGCATTTACACTTGCTCCATTGGTGCA-CACAGCCT-TACTC
       **222222222*2*22*2*2*2*2222*22***22222**2***22222****2*2*2*2*2**2****2

left   AAAATACAAAAAATTA-GCCGGGCGCGGT-------GGCGG-GCG-------C
chrom  AAAAGAATAATGATAATGCAGGTAACAGTCTTATCTGGCTCAGCGGTTTTACC
right  AAAAGAATAATGATAATGCAGGTAACAGTCTTATCTGGCTCAGC---------
       ****2*22**22**2*2**2**222*2**2222222***222**1       1
```

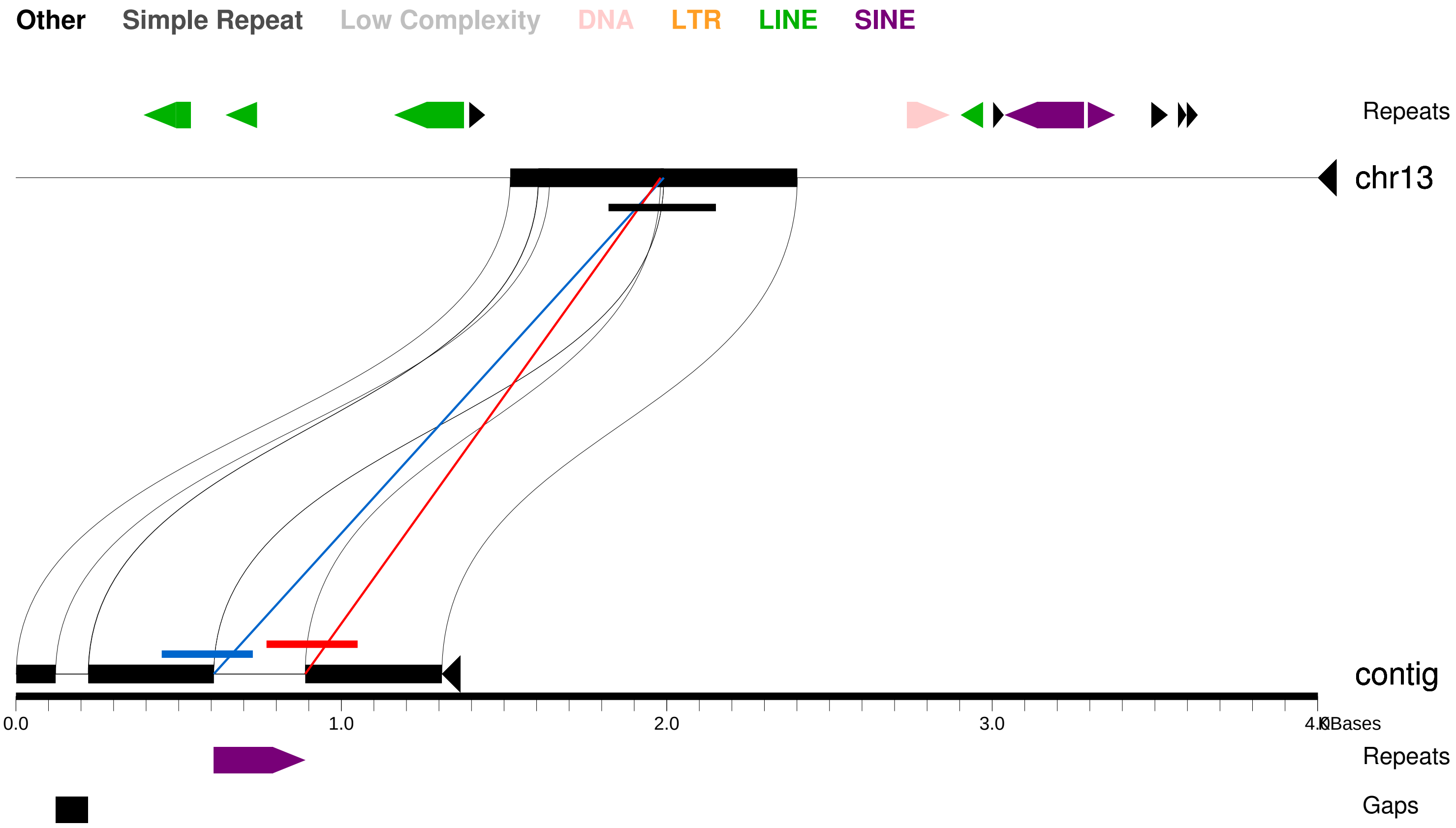

siteID: chr13_37568751 Scafftig2 - Breakpoint: chr13:37568741
chr13:37568695-37568792
Overlap Length: 14 INS size: 410 Genotyped: No

```
left     TATTCAAACCCAGTTAACCAAAAAATAAAAAAAAGAG--------------------AGAAGAGAATAAA
chrom    TATTCAAACCCAGTTAACCAAAAAATAAAAAAAAGAG--------------------AGAAGAGAATAAA
right    NNNNNNNNNNNNNNNNNNNNNNNNNNNNNNNNNNNNNNNNNNNNNNNNNNNNNNNNNAAAAAAAAAAAAA
         111111111111111111111111111111111111111111111111111111111*1**1*1**1***

left     AGTTAACAGCATGTAATCCCAGCACTTTGGGAGGCCGAGGCGGGCGGATCATGAGGTCAGGAGATCGAGA
chrom    AGTTAACAGCAAA-AACAGCAACAAT-----ACACCTTTTCTTAC--------AAGTCACAA-------A
right    AGTTAACAGCAAA-AACAGCAACAAT-----ACACCTTTTCTTAC--------AAGTCACAA-------A
         ***********222**222**2**2*22222*22**2222*222*22222222*2****22*2222222*
```

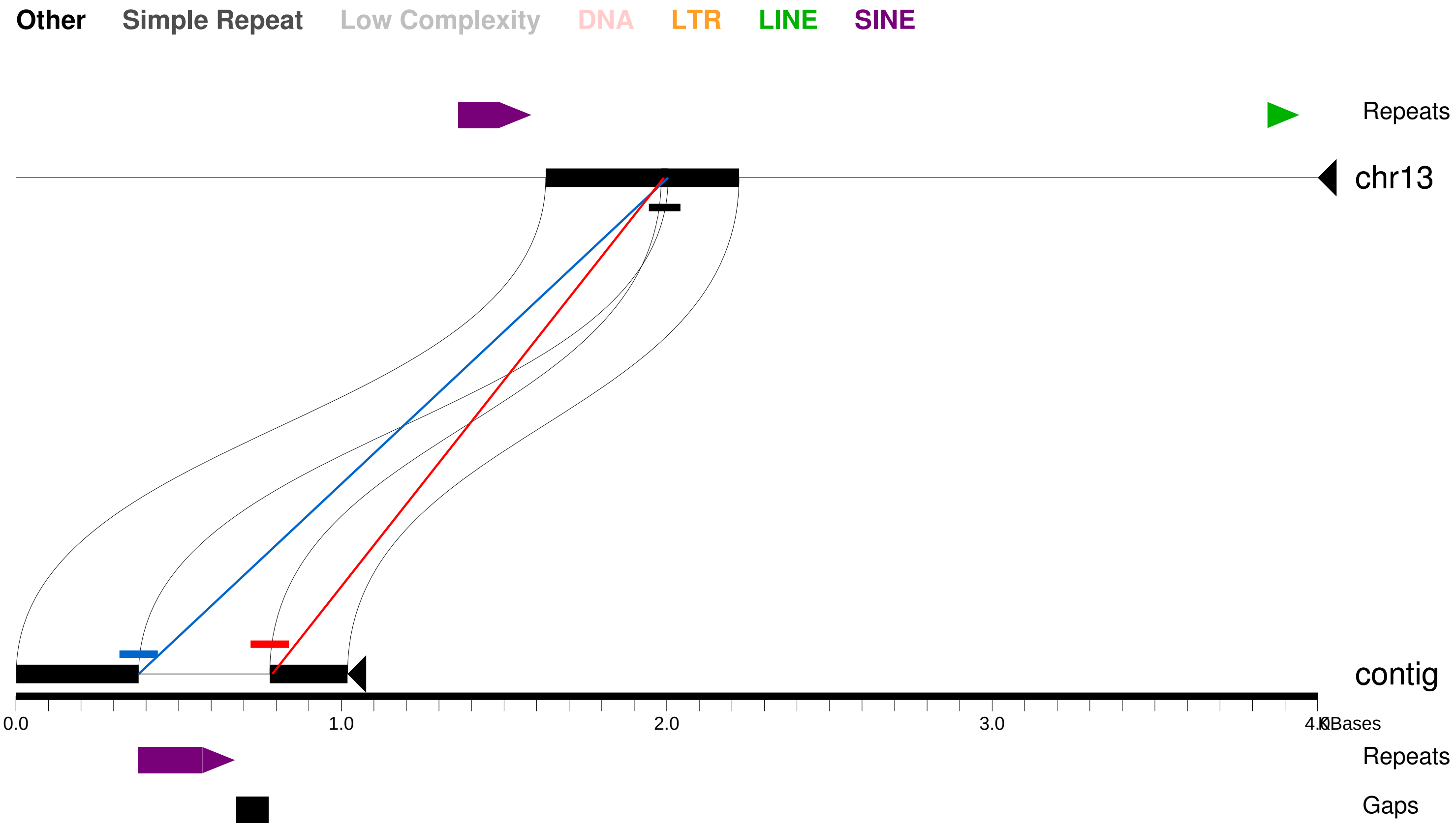

siteID: chr13_38181980 Scafftig2 - Breakpoint: chr13:38181978
chr13:38181932-38182037
Overlap Length: 14 INS size: 439 Genotyped: No

```
left    CCACAATGAGATACCATCTCAGGCCAGTTAGAATGGCGATTGTTAA-------------AAAGTCAGGAA
chrom   CCACAATGAGATACCATCTCAGGCCAGTTAGAATGGCGATTGTTAA-------------AAAGTCAGGAA
right   NNNNNNNNNNNNNNNNNNNNNNNNNNNNNNNNNNNNNNNNNNNNNNNNNNNNNNNNNNNNAAAGTCAGGAA
        111111111111111111111111111111111111111111111111111111111111***********

left    ACAGGCCGGGCGCGGTGGCTCACGCCTGTAATCCCAGCACTTTGGGAGGCCGAGGCGGGTGG
chrom   ACA--ACAGGTGCTGGAGAGGATG--TGGAG-------ACATAGGAACGCTTTTACACTT--
right   ACA--ACAGGTGCTGGAGAGGATG--TGGAG-------ACATAGGAACGCTTTTACACTT--
        ***222*2**2**2*22*222*2*22**2*22222222**2*2**2*2**22222*222*22
```

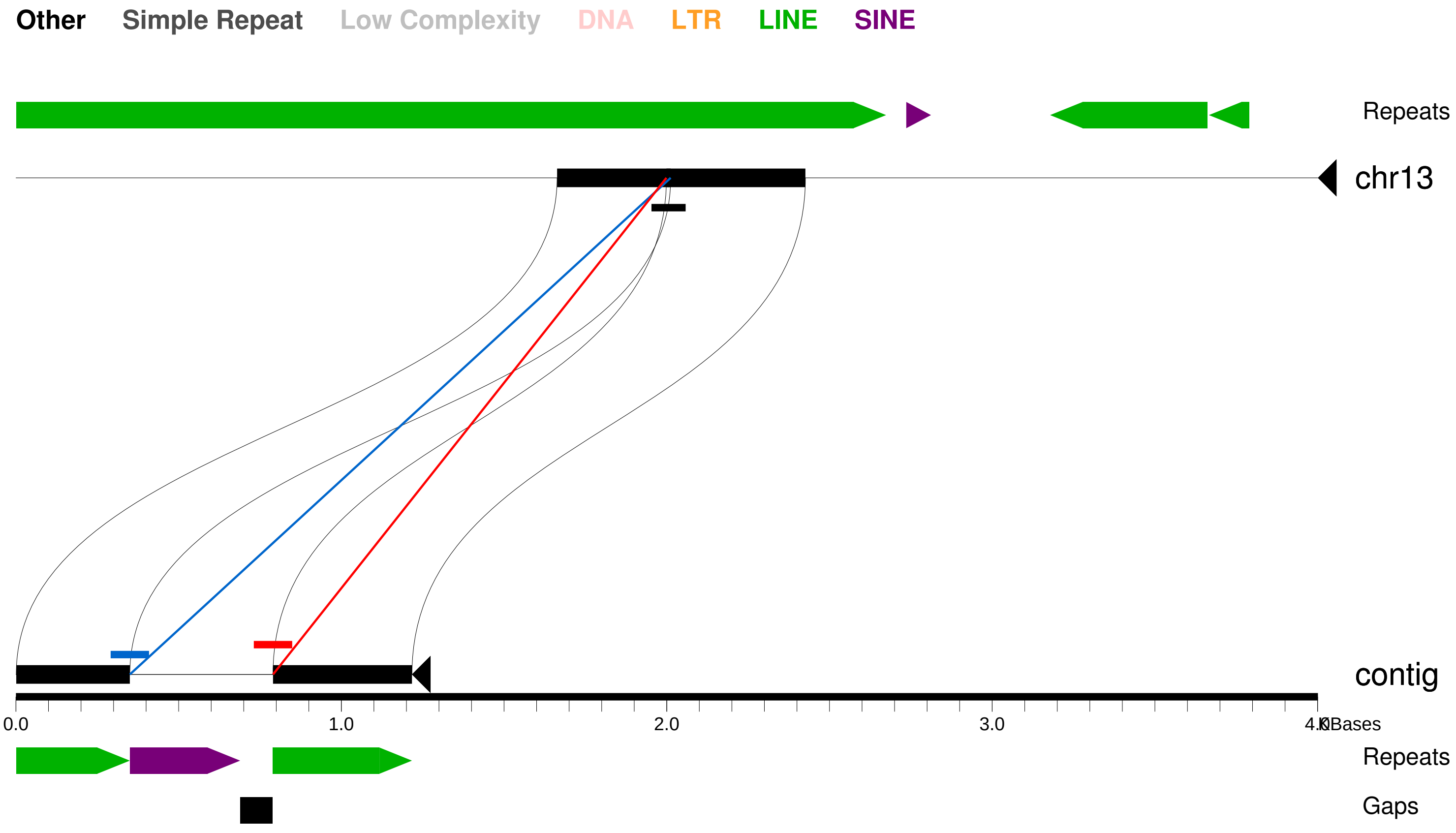

siteID: chr13_39430882 Scafftig2 + Breakpoint: chr13:39430876
chr13:39430716-39431042
Overlap Length: 7 INS size: 314 Genotyped: Yes

```
left   C------TCAGAGTAAAGAGGGAACTATTAATTCAGTGCCACAACTCATAAAACATATTCGAACCCACAA
chrom  CAACATCTCAGAGTAAAGAGGGAACTATTAATTCAGTGCCACAACTCATAAAACATATTCGAACCCACAA
right  -----TTT---AGTAGAGACGGGGTTTCACCGTGTTAGCCAGGA-TGGTCTCG-ATCTCCTGACCTCGTG
       1    2N*111****1***1**111*111111*1111****11*1*11*11111**1*1*11***11111

left   AGCAGTTAGC-TAACCCTCCAAATCAAAGTCTGTTTCTCCTGGCTTTATCAGAATTTGATGTCCACAGCC
chrom  AGCAGTTAGC-TAACCCTCCAAATCAAAGTCTGTTTCTCCTGGCTTTATCAGAATTTGATGTCCACAGCC
right  ATCCGCCCGCCTCGGCCTCC----CAAAGTG--------CTGGGATTA-CAGGCGT-GAG--CCACCGCG
       *1*1*111**1*111*****1111******111111111****11***1***111*1**111****1**1

left   TCGTGATCAAAATGTTGCAGTCCTAAATTTTTTTTTTTTTTTTTTTTTTTTTTTTTTTGAG------ACG
chrom  TCATGATCAAAATGTTGCAGTCCTAAATCTTGTTTTCTATACTTACTTAATTTATATTGTCTCACTAACC
right  CC---------------CGGCCCTAAATCTTGTTTTCTATACTTACTTAATTTATATTGTCTCACTAACC
       1*N11111111111111*1*1*******2**2****2*2*22**22**22***2*2***22222222**2

left   GAGTCTCGCTC----TG-------TCGCCCAGGCTGGAGTGC-----AGTGGCGCGATCTCGGCTCACTG
chrom  AGGTTTTCCTCAAAATGAACATAATTCCACAGAATGTTGACCTTTATATTGGAAATAATTTTTAAAACTA
right  AGGTTTTCCTCAAAATGAACATAATTCCACAGAATGTTGACCTTTATATTGGAAATAATTTTTAAAACTA
       22**2*22***2222**2222222*22*2***22**22*22*22222*2***2222*22*222222***2

left   CAAG-CTCC-------GCCTCCCGGGTTCACGCCATTC----------
chrom  CGTGTCTTTTTGGAATGTATTAATTTTTTATGTCTTTGCTTACATTTA
right  CGTGTCTTTTTGGAATGTATTAATTTTTTATGTCTTTGCTTA------
       *22*2**222222222*22*222222**2*2*2*2**22222
```

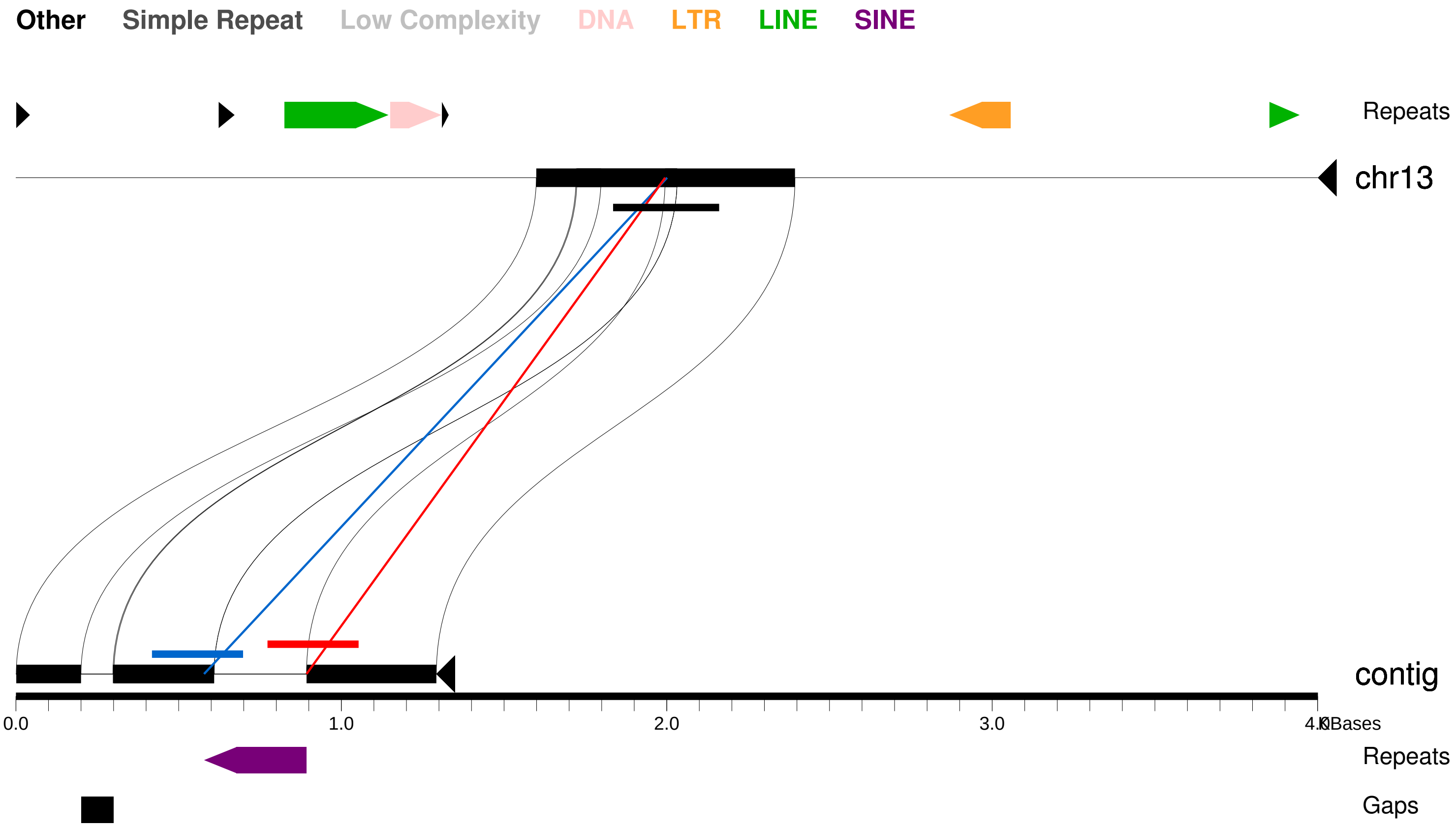

siteID: chr13_39499612 Scafftig2 - Breakpoint: chr13:39499622
chr13:39499578-39499663
Overlap Length: 16 INS size: 336 Genotyped: Yes

```
left   GTGT-----------------TCTCTCTCATATGTGGGAGCTTAAAAAAAAATTAGCTCAT---------
chrom  GTGT-----------------TCTCTCTCATATGTGGGAGCTTAAAAAAAAATTAGCTCAT---------
right  GCCTGGGCGACAGAGCGAGACTCCGTCTCAAAAAAAAAAAAAAAAAAAAAAAAAAAAAAAAAAAAAAAAA
       *11*11111111111111111**11*****1*111111*1111*********11*1111*1111111111

left   -------GAAAGTAGAGAGTTGAGGCCGGGCGCGGTGGCTCACGCCTGTAATCCCAGCACTTTGGGAGGC
chrom  -------GAAAGTAGAGAGTTGAAT---------------------TGTAGG--------TATTAGAGGC
right  AAAAAAAGAAAGTAGAGAGTTGAAT---------------------TGTAGG--------TATTAGAGGC
       1111111****************22222222222222222222222****2222222222*2*22*****

left   CGAGGCGGGCGG
chrom  TG----GGAAAG
right  TG----GGAAAG
       2*2222**222*
```

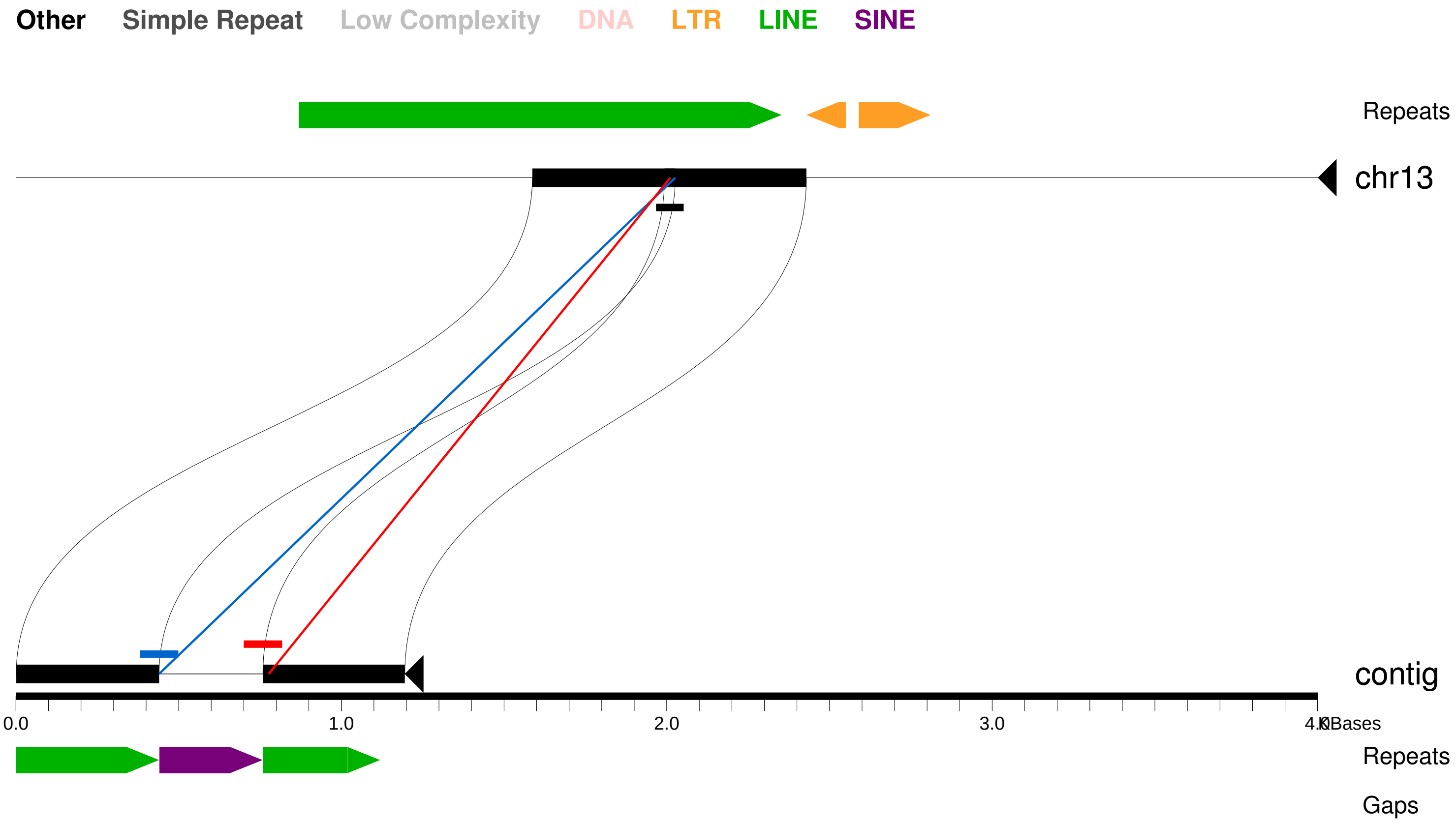

siteID: chr13_42571554 Scafftig12 - Breakpoint: chr13:42571548
chr13:42571501-42571607
Overlap Length: 13 INS size: 300 Genotyped: Yes

```
left    CTTACTAGGGAACTCTCATACCTTTAC-ATTGATACAATCTCCCTTTT------------AAAAATCGTT
chrom   CTTACTAGGGAACTCTCATACCTTTAC-ATTGATACAATCTCCCTTTT------------AAAAATCGTT
right   ACTGC-AGTCCGCAGTCCGGCCTGGGCGACAGAGCGAGACTCCGTCTCAAAAAAAAAAAAAAAAATCGTT
        11*1*1**1111*11**111***111*1*11**111*11****1*1*111111111111**********

left    TTAGGCCGGGCGCGGTGGCTCACGCCTGTAATCCCAGCGCTTTGGGAGGCCGAGGCGGGTGG
chrom   TTAATTGTGGTAAGAT---TCAC----TTAA---CATAAAATTGACCATCTTAACC--ATGT
right   TTAATTGTGGTAAGAT---TCAC----TTAA---CATAAAATTGACCATCTTAACC--ATGT
        ***22222**222*2*222****22222***222**22222***22222*22*22*222**2
```

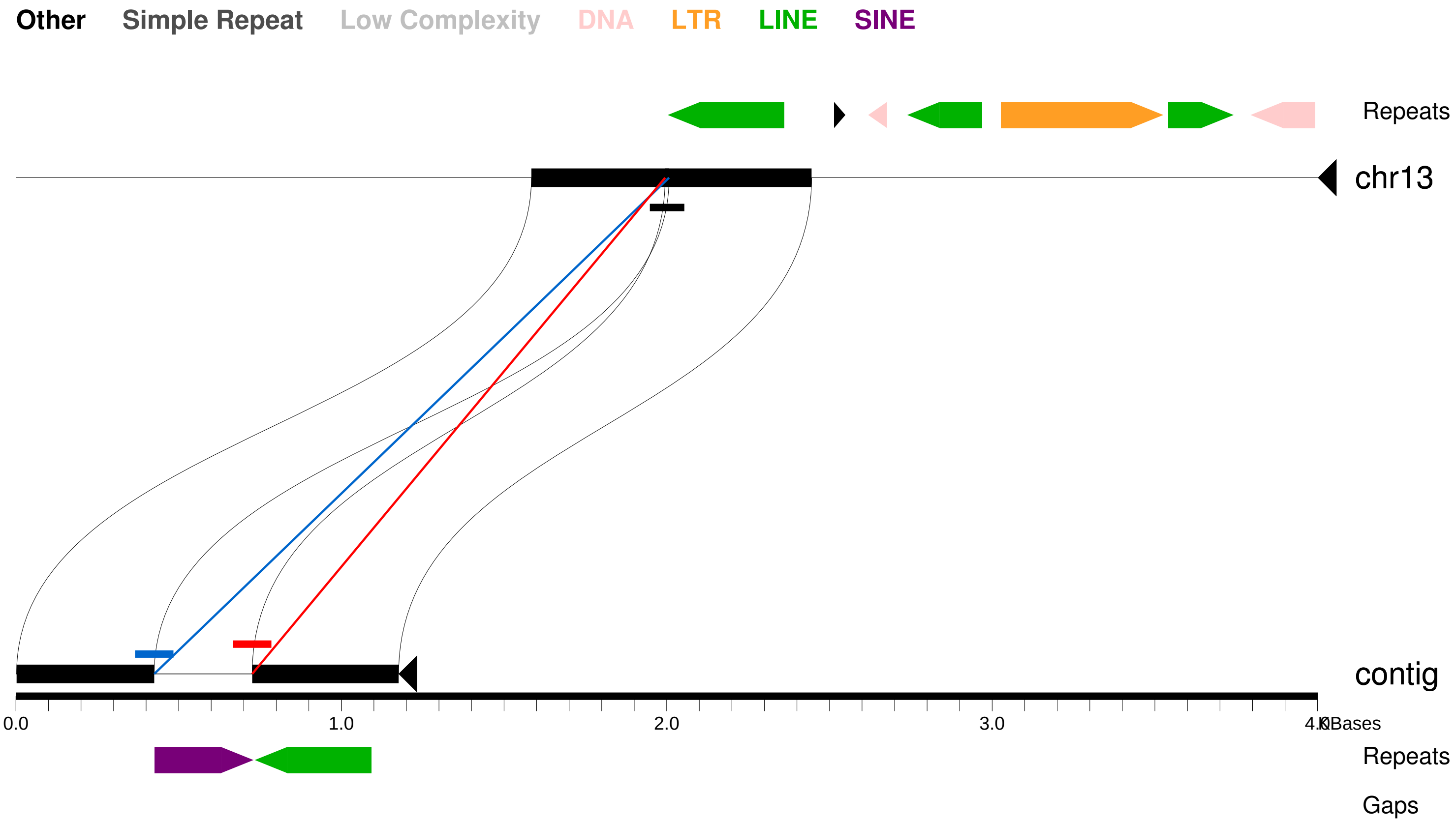

siteID: chr13_42726773 Scafftig1 + Breakpoint: chr13:42726787
chr13:42726742-42726846
Overlap Length: 15 INS size: 417 Genotyped: No

```
left    TCCTTTTCCCATGG------AAG-GCAGGG----CAGGAG---GGCACAGCTGCACAGGGACCTCTTTAT
chrom   TCATTTTCCCATGG------AAG-GCAGGG----CAGAAG---GGCACAGCTGCACAGGGACCTCTTTAT
right   CCACCCGCCTCGGCCTCCCAAAGTGCTGGGATTACAGGCGTGAGCCACCGCGCCCGGCCGACCTCTTTAT
        1*21111**111*1111111***1**1***1111***N1*111*1***1**11*11111***********

left    TCTTTNNNNNNNNNNNNNNNNNNNNNNNNNNNNNNNNNNNNNNNNNNNNNNNNNNNNNNNNNN
chrom   TCTT--------------GATGGTTTCACTATCACCTTCTATTTTTGATGCTCTGGTGAACCA
right   TCTT--------------GATGGTTTCACTATCACCTTCTATTTTTGATGCTCTGGTGAACCA
        *****2222222222222222222222222222222222222222222222222222222222
```

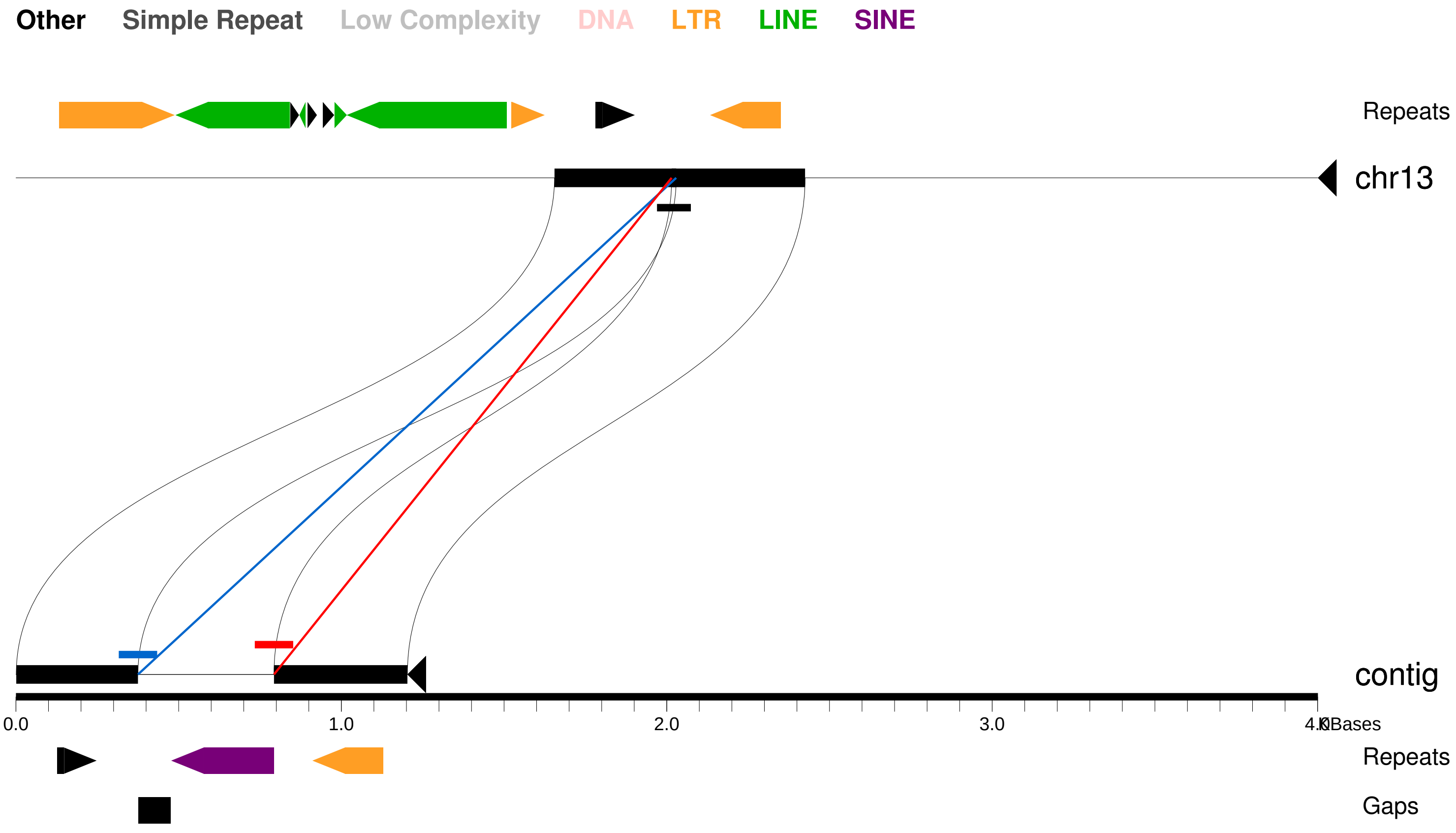

siteID: chr13_42940095 Scafftig8 - Breakpoint: chr13:42940073
chr13:42940029-42940132
Overlap Length: 16 INS size: 467 Genotyped: No

```
left    CTTATCCACTTTCAGGGTGTAAATCTTTACCTGTGTTTTC---------------AATTAAAAAAAATAGT
chrom   CTTATCCACTTTCAGGGTGTAAATCTTTACCTGTGTTTTC---------------AATTAAAAAAAATAGT
right   NNNNNNNNNNNNNNNNNNNNNNNNNNNNNNNNNNNNNNNNNNNNNNNNNNNNNNNAAAAAAAAAAAAAATAGT
        1111111111111111111111111111111111111111111111111111111**11***********

left    TTTTCGGGCCGGGAGCGGTGGCTCACGCCTGTAATCCCAGCACTTTGGGAGGCCGAGGCGGGTG
chrom   TTTTCT-------ATCTCTT-CTTGAGCA---AATTCTGTTAATTTTAATTTCCTACAAT----
right   TTTTCT-------ATCTCTT-CTTGAGCA---AATTCTGTTAATTTTAATTTCCTACAAT----
        *****22222222*2*22*22**222**2222***2*2222*2***222222**2*22222222
```

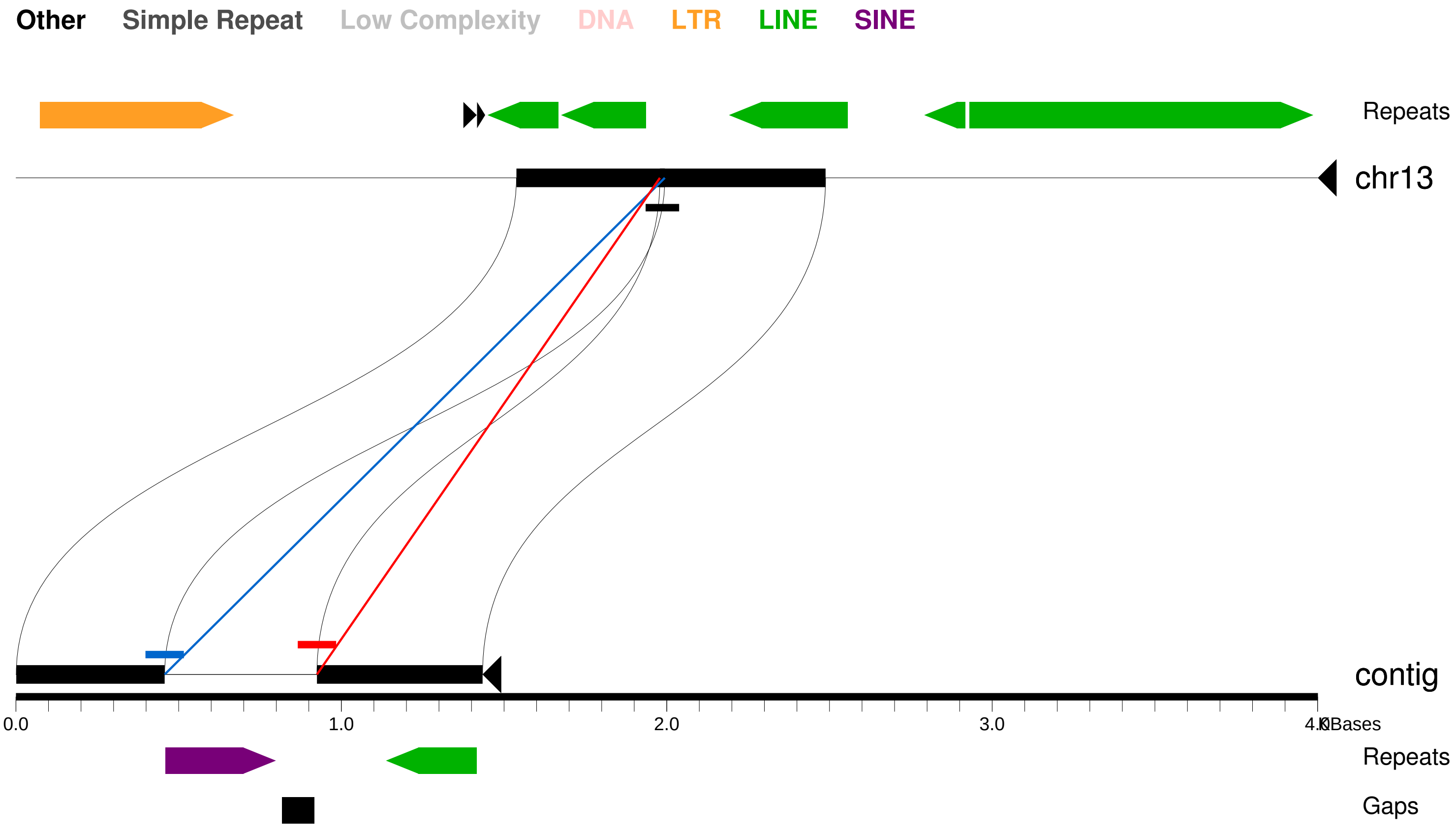

siteID: chr13_46268080 Scafftig9 - Breakpoint: chr13:46268066
chr13:46267906-46268239
Overlap Length: 14 INS size: 300 Genotyped: Yes

```
left   --------------GGTTGAATTATGTCTCCCCAAAAAGATGTA-TTGAAGTCCTACCAGGAGACCTAAT
chrom  TAGCTTCTATTATGGGTTGAATTATGTCTCCCCAAAAAGATGTA-TTGAAGTCCTACCAGGAGACCTAAT
right  --GGCAGGAGAATGGCGTGAA---------CCCGGGAAGCGGAGCTTGCAGT--------GAGCC-----
         2NNNNN2NN222*11****111111111***111***11*111***1***11111111***1*11111

left   TTGGAAATAGGATCATTTCAGATGTAACTAGTTAAGATGAGGTCCAACTACAGTCTAGTGGCCCTCAATC
chrom  TTGGAAATAGGATCATTTCAGATGTAACTAGTTAAGATGAGGTCCAACTACAGTCTAGTGGCCCTCAATC
right  ---GAGATTGCGCCACTGCAG---TCCGCAGTCC-GACCTGGGCGAC--AGAG-CGAGACTCCGTC--TC
       111**1**1*111**1*1***111*1111***111**111**1*1*111*1**1*1**111**1**11**

left   CAATATGACTGGTGTCCTTATAAGAAGAGGATACCGGCCGGGCGCGGTGGCTCACGCCTGTAATCCCAG-
chrom  CAATATGACTGGTGTCCTTATAAGAAGAGGATACCAG----ATACAGACACACGAGGGTAGAATACTATT
right  AAAAAAAAAA---------AAAAGAAGAGGATACCAG----ATACAGACACACGAAGGTAGAATACTATT
       1**1*11*11111111111*1**************2*2222222*2*222*2*22122*22***2*2*22

left   CACTTTGGGAGGCCGAGGCGG--GTGGATCATGAG--GTCAG-GA----GATCGA----GACCATC----
chrom  CAATAAGAGAGGCAGAGATTGAAGTGCTGCAGCACAAGTCAGAGAATAGGATTGCTGATGACCACCAGAA
right  CAATAAGAGAGGCAGAGATTGAAGTGCTGCAGCACAAGTCAGAGAATAGGATTGCTGATGACCACCAGAA
       **2*22*2*****2***222*22***222**22*222*****2**2222***2*22222*****2*2222

left   -CTGGCTAACA---AGGTGA--AACCCCGTCTCTACT---AAA----------------
chrom  GCTGGGAGAGAGGCAGGCAAGGACCCTCCCCTCTGATGTCAGAGGGAGCGTGGCTCTGC
right  GCTGGGAGAGAGGCAGGGAAGGACCCTCCCCTCTGATGTCAGAGGGAGCG---------
       2****222*2*222***N2*22*2**2*22****22*222*2*2222222
```

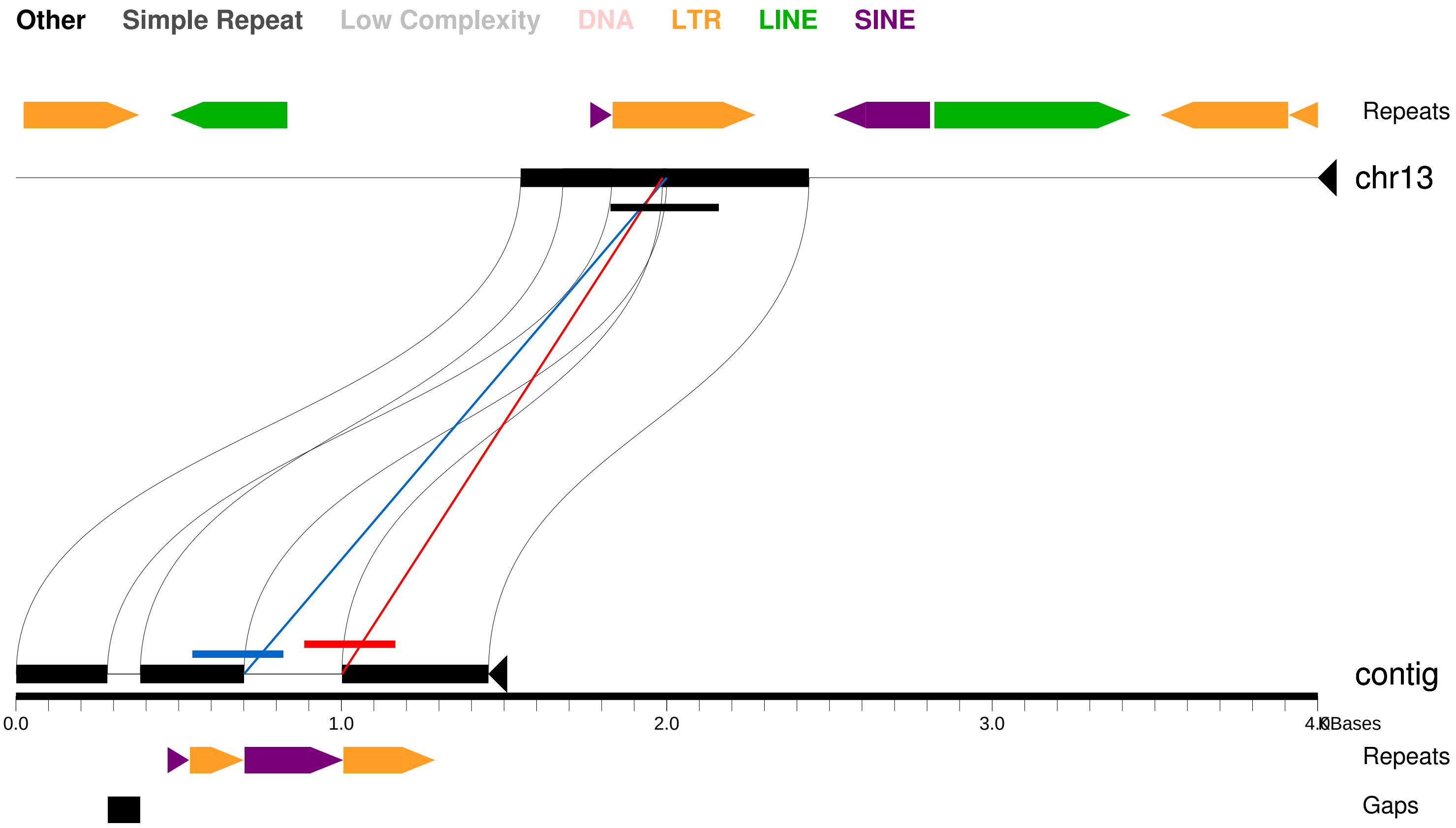

siteID: chr13_46647648 Scafftig4 - Breakpoint: chr13:46647733
chr13:46647689-46647792
Overlap Length: 16 INS size: 472 Genotyped: No

```
left     TAA-----------CTCCTTCAGTCCT----TTCCTCTACTTAGTAAACATATTATAATCAATGTACTAT
chrom    TAA-----------CTCCTTCAGTCCT----TTCCTCTACTTAGTAAACATATTATAATCAATGTACTAT
right    CCGCCCGCCTCGGCCTCCCAAAGTGCTGGGATTACAGGCGTGAGCCACCGCGCCCGGCCCAATGTACTAT
         11111111111111****111***1**1111**1*11111*1**11*1*1111111111***********

left     TTCTTTTTTNNNNNNNNNNNNNNNNNNNNNNNNNNNNNNNNNNNNNNNNNNNNNNNNNNNNNN
chrom    TTCTTAAT---------------CATTTTCCACTGTTTAGCTCCAGGCAGAAATGTAGAAAAGA
right    TTCTTAAT---------------CATTTTCCACTGTTTAGCTCCAGGCAGAAATGTAGAAAAGA
         ******22*2222222222222222222222222222222222222222222222222222222
```

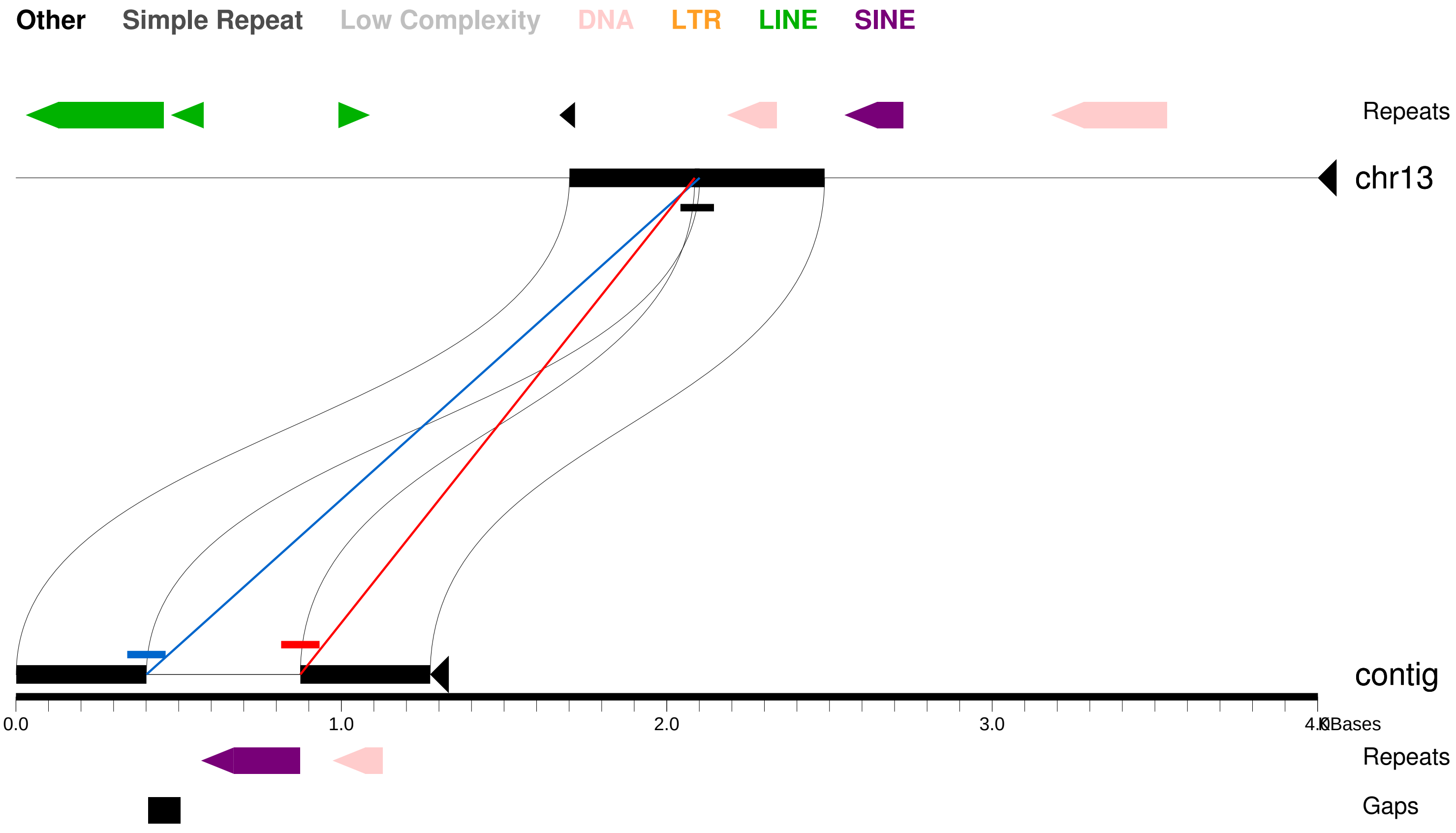

siteID: chr13_48475976 Scafftig1 - Breakpoint: chr13:48475971
chr13:48475920-48476030
Overlap Length: 9 INS size: 298 Genotyped: No

```
left    ACAGTGAAAACCTAATTTGAACCAGGTTAACTGAACAGAGGAATTCACTGG--------AAAGATCCATG
chrom   ACAGTGAAAACCTAATTTGAACCAGGTTAACTGAACAGAGGAATTCACTGG--------AAAGATCCAGC
right   NNNNNNNNNNNNNNNNNNNNNNNNNNNNNNNNNNNNNNNNNNNNNNNNNNNNNNNAAAAAAAAGACCCAGC
        11111111111111111111111111111111111111111111111111111111111*****1***22

left    TAATCCCAGCACTTTGGGAGGCCGAGGCGGGCGGATCACGAGG-TCAGGAGATCGAGA
chrom   GTTTCTCACAAACTTCAAAGAC--AGGGATGCAG----CCAGCCTCAGGA-ATGGA--
right   GTTTCTCACAAACTTCAAAGAC--AGGGATGCAG----CCAGCCTCAGGA-ATGGA--
        222**2**22*22**222**2*22***222**2*2222*2**22******2**2**22
```

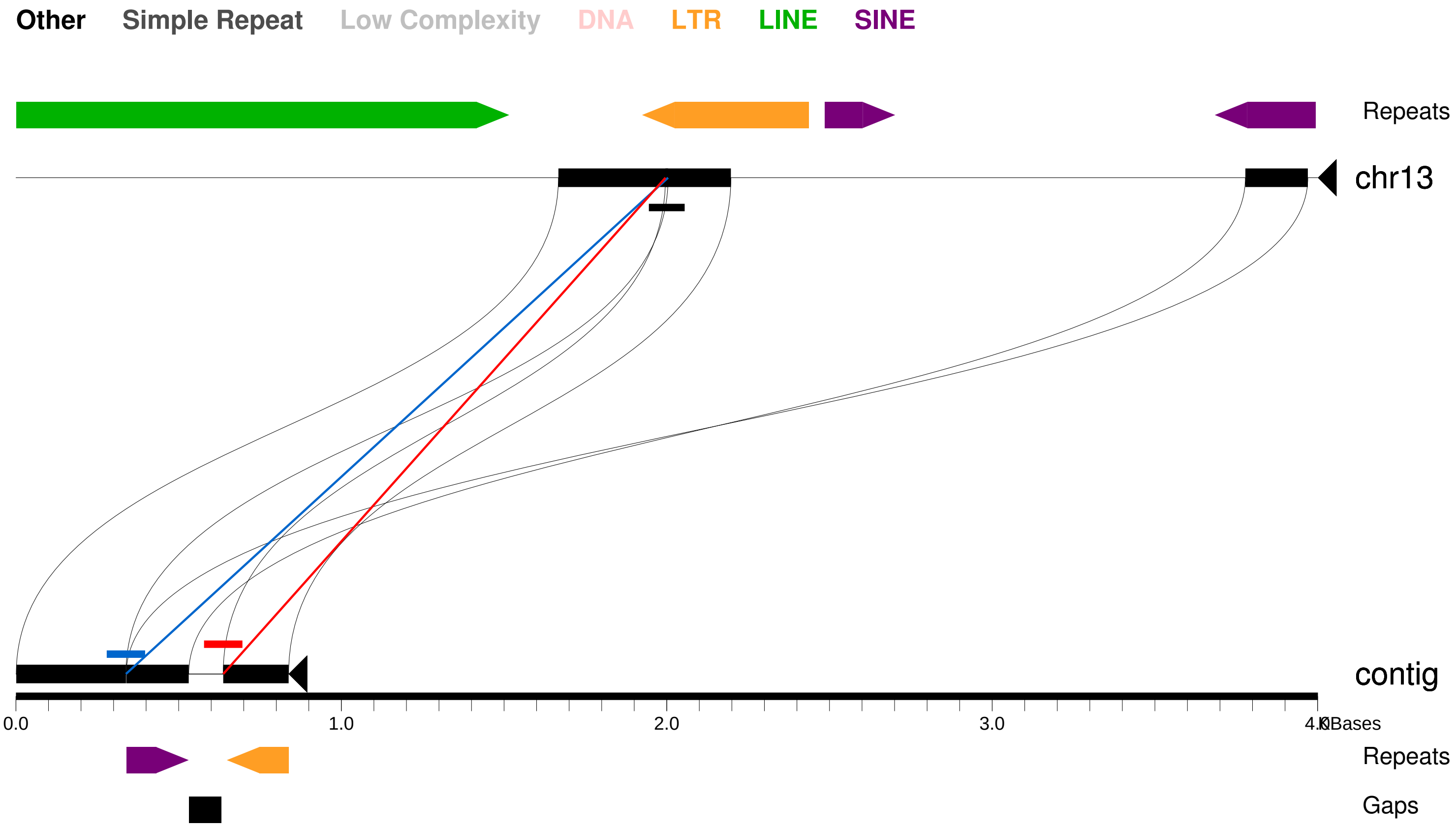

siteID: chr13_49336679 Scafftig1 + Breakpoint: chr13:49336663
chr13:49336503-49336833
Overlap Length: 11 INS size: 332 Genotyped: Yes

```
left   -----------CTCATATCGGGAGATGAGAGCCCTGGACATAAT-GTTATTGTGTTTATTGTTCTCTTGA
chrom  TCCTCTGACTTCTCATATCGGGAGATGAGAGCCCTGGACATAAT-GTTATTGTGTTTATTGTTCTCTTGA
right  AGAA-TGGCGTG--AACCCGGGAGGCG-GAGCT-TGCAGTGAGCCGAGATCGCGCC-ACTGCACTCCAGC
       NNNN 22N2N2111*111*****11*1****11**1*111*111*11**1*1*111*1**11***11*1

left   ---GGTCACTTAGCCATGGCCTAGCTTTCTCACTAGACTAAAAACCCCTTAAGGTGAGACTGTCTCTCTT
chrom  ---GGTCACTTAGCCATGGCCTAGCTTTCTCACTAGACTAAAAACCCCTTAAGGTGAGACTGTCTCTCTT
right  CTGGGCGACAGAGCGA--GACT--CCGTCTCAAAAAA-TAAAAAAAAAAAAAAATAAATAAAAATAAAAA
       111**11**11***1*11*1**11*11*****11*1*1******111111**11*1*1111111*11111

left   ATTTGTCCCTCTCTCACAGCCTATAGAAGTTCCTG--GGCCGGGCGCGGTGGCTCACGCC--------TG
chrom  ATTTGTCCCTCTCTCACAGCCTATAGAAGTTCCTGATGACACATAGCTTGTACTCAATCAATAGATGGTG
right  AAATA------------AAAATAAAGAAGTTCCTGATGGCACATAGCTTGTACTCAATCAATAGATGGTG
       *11*1111111111111*111**1**********22*N*22222**22222****22*222222222**

left   TAATCCCAGCACTTTGGGAGGCCGAGGCGGGCGGATCACGAGG--TCAGGAGATCGAGACCATCC-----
chrom  AATTCAGAAGCCTATGTAAATAACACT-GGGGGGATAGACAGGAATCAGCATTTCTTAAGGATGCCCCAT
right  AATTCAGAAGCCTATGTAAATAACACT-GGGGGGATAGACAGGAATCAGCATTTCTTAAGGATGCCCCAT
       2*2**22*222**2**22*22222*222***2****2222***22****2*22**222*22**2*22222

left   -TGGCTAACACGGTGAAA------CCC-------CGTCTCTACTA---------AA
chrom  GTGTCCAGCACTTGCACATATTATCTCATTAAATCTTCTCAACAACCTTCCAAGAT
right  GTGTCCAGCACTTGCACATATTATC-------------------------------
       2**2*2*2***2222*2*222222*N1       1N1111N11N1         1N
```

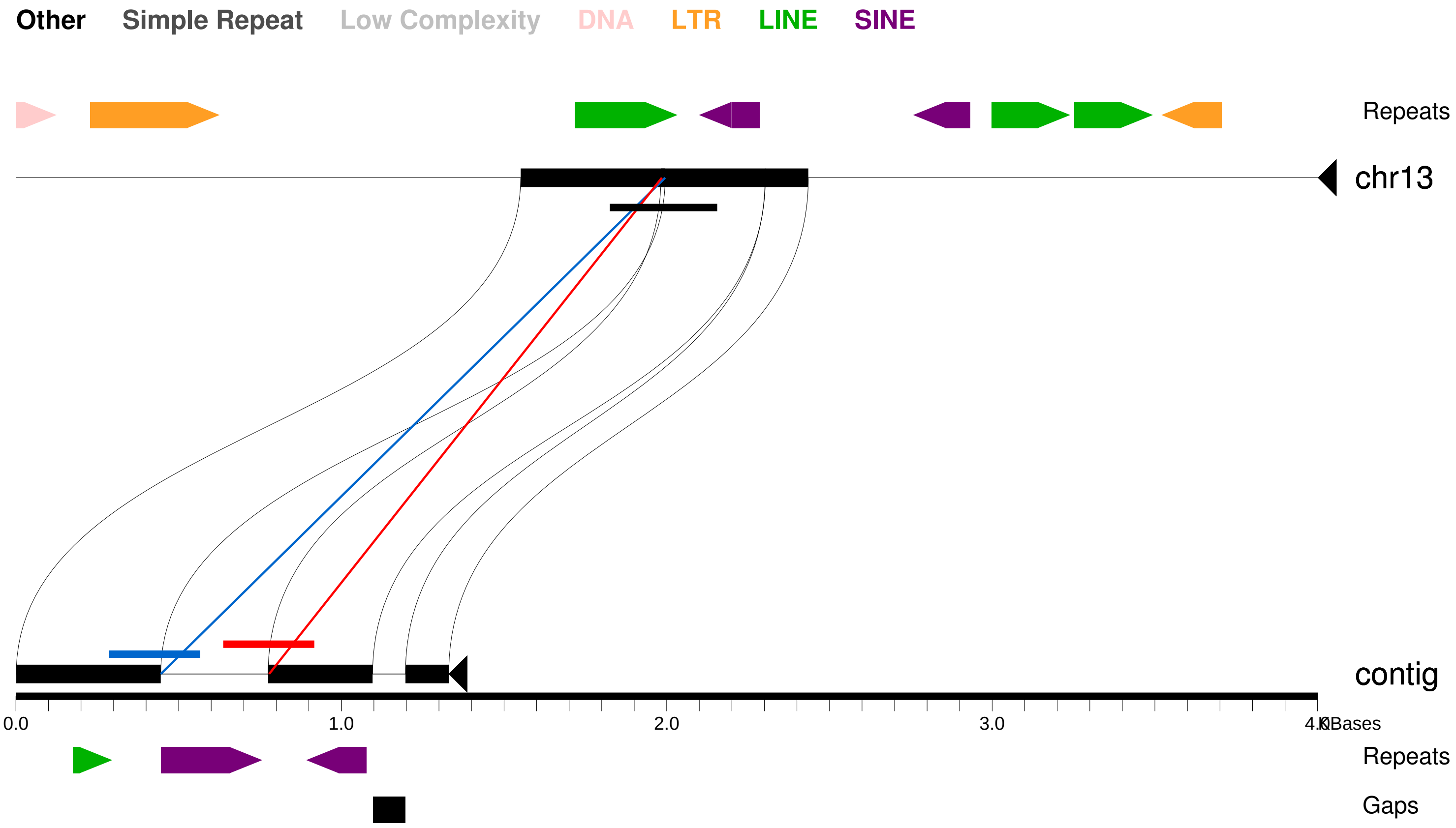

siteID: chr13_50042762 Scafftig1 + Breakpoint: chr13:50042751
chr13:50042716-50042810
Overlap Length: 14 INS size: 463 Genotyped: No

```
left    AAAAC-------------AAAGAAGTATCTTTAGAA-----AGAAACTTCTTT------GTTACCCTAAT
chrom   AAAAC-------------AAAGAAGTATCTTTAGAA-----AGAAACTTCTTT------GTTACCCTAAT
right   CACCCGCCTCGGCCTCCCAAAGTGCTGGGATTACAGGCGTGAGCCACCGCGCCCGGCCCGTTACCCTAAT
        1*11*1111111111111****111*1111***1*111111**11**11*111111111***********

left    TTCTTTTTTTTTTTTNNNNNNNNNNNNNNNNNNNNNNNNNNNNNNNNNNNNNNNNNNNNNNNNNNNNNNN
chrom   TTCATTTGTTCTTT------------------------CAGCTTAATATTAAATTGACTAAGAAAAACTA
right   TTCATTTGTTCTTT------------------------CAGCTTAATATTAAATTGACTAAGAAAAACTA
        ***2***2**2***22222222222222222222222222222222222222222222222222222222

left    NNN
chrom   TAT
right   TAT
        222
```

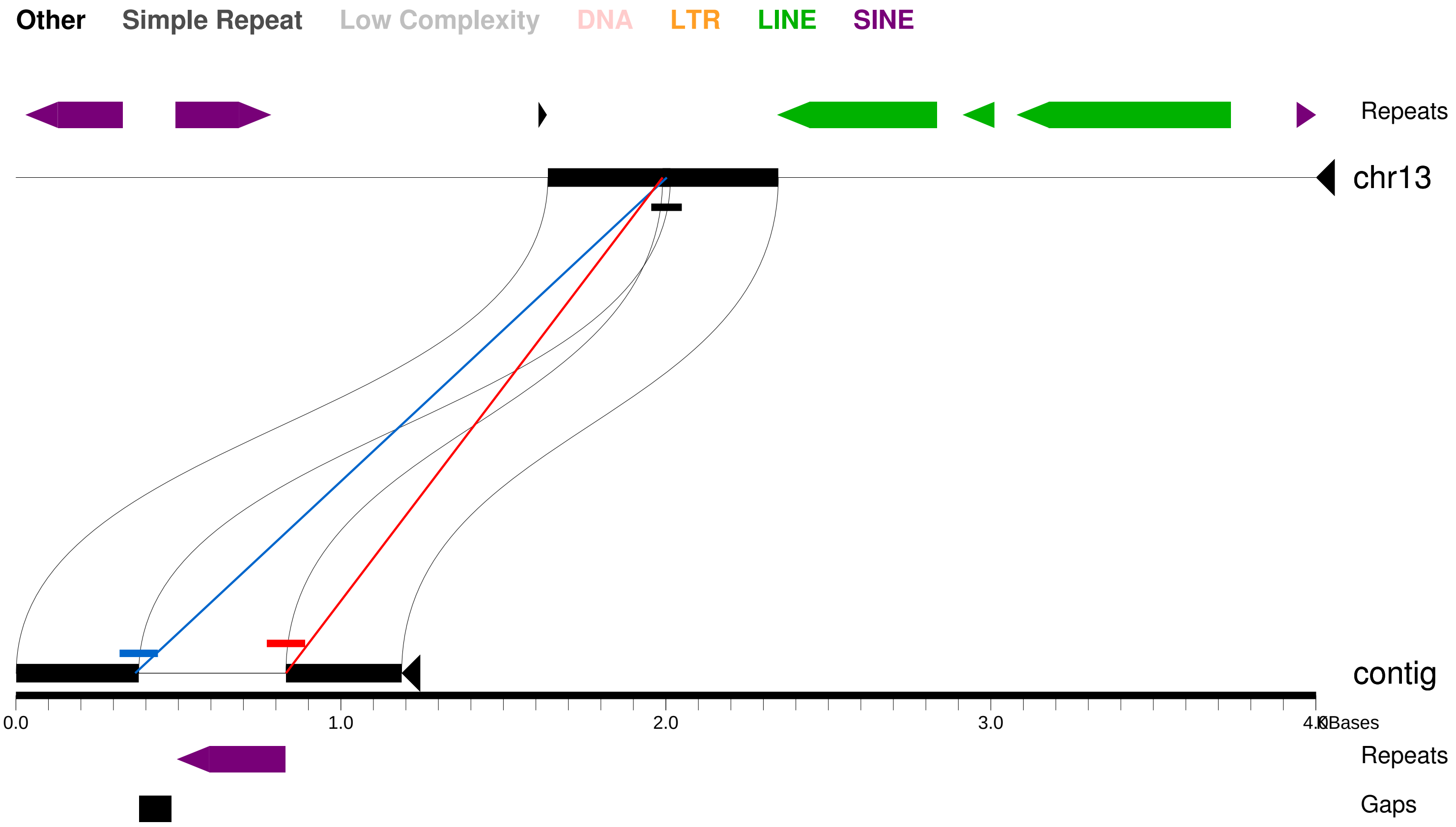

siteID: chr13_50173368 Scafftig2 - Breakpoint: chr13:50173359
chr13:50173199-50173528
Overlap Length: 10 INS size: 303 Genotyped: Yes

```
left   -------AGAAGTAGACATAGGAGACTCCATTTTGTTCTGTACTAAGACAAATTCTTCTGCCTTGAGATT
chrom  TGTAGAAAGAAGTAGACATAGGAGACTCCATTTTGTTCTGTACTAAGACAAATTCTTCTGCCTTGAGATT
right  TTTAG-------TAGAGACGGGGT--TTCACCGTGTT--------AGCCAGGATGGTCTC----GATCTC
       2N222  11111****1*11**1111*1**111****11111111**1**111*11***11111**11*1

left   CTGTTAATCTATGACCTTACTCCCAACCCTGTGCTCTCTGAAACATGTGCTGTGTCAAACTCAGGGTTAA
chrom  CTGTTAATCTATGACCTTACTCCCAACCCTGTGCTCTCTGAAACATGTGCTGTGTCAAACTCAGGGTTAA
right  CTG---ACCT----CGTGATCCGCCCGCCTCGGC-CTCCCAAA---GTGCTGGGATTA---CAGGAGTGA
       ***111*1**1111*1*1*11*1*111***11**1***11***111******1*111*111****11*1*

left   ATGGATTAAGGGCGGTGCAAGATGTGCTTTTTTTTTTTTTTTTTTTT--------TTTTTGAGACGGAGT
chrom  ATGGATTAAGGGCGGTGCAAGATGTGCTTTGTTAAACAGATGCTTGAAGGCAGCATGCTCGTTAAG-AGT
right  ---GCCACCGCGCCCGGCC-GATGTGCTTTGTTAAACAGATGCTTGAAGGCAGCATGCTCGTTAAG-AGT
       111*11111*1**111**11**********2**2222222*22**2222222222*22*2*22*2*2***

left   C-TCACTCTTTCGCCCAAGCTGGACTG--CAGTGGGGCTATCCCGGCT------CACTGCAAGCTCCGCC
chrom  CATCAC-CACTC-CCTAATCTCAAGTATCCAGGGACACAAACACTGCGGAAGGCCACAGGGACCTCTGCC
right  CATCAC-CACTC-CCTAATCTCAAGTATCCAGGGACACAAACACTGCGGAAGGCCACAGGGACCTCTGCC
       *2****2*22**2**2**2**22*2*222***2*222*2*2*2*2**2222222***2*22*2***2***

left   TC-------------TTG-----GGTT------CATGCCATTCTCC----TGC
chrom  TAGGAAAGCCAGGTATTGTCCAAGGTTTCTCCCCATGTGATAGTCTGAAATAT
right  TAGGAAAGCCAGGTATTGTCCAAGGTTTCTCCCCATGTGATAG----------
       *22222222222222***22222****222222****22**2211N    1NN
```

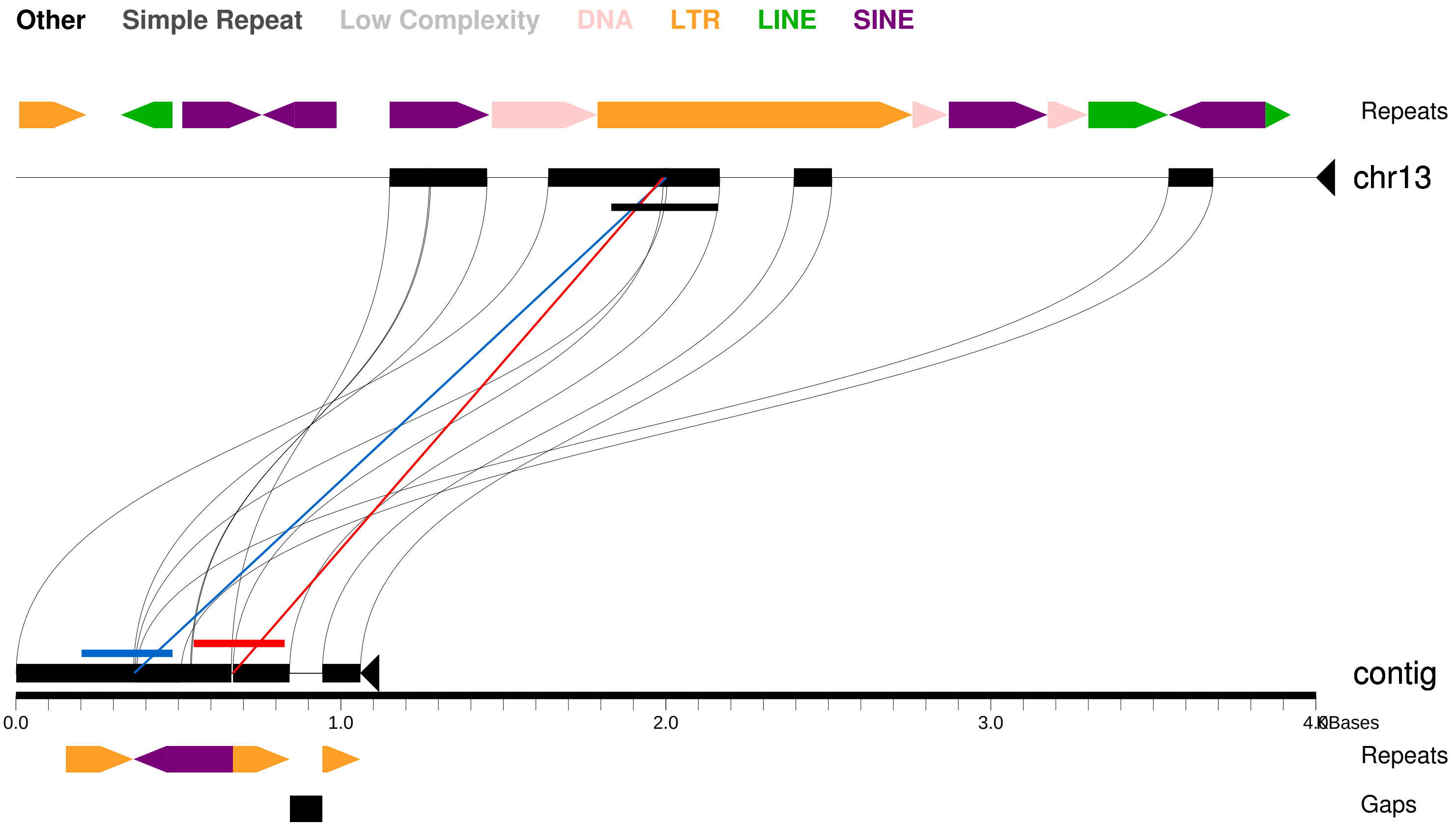

siteID: chr13_54606509 Scafftig2 + Breakpoint: chr13:54606515
chr13:54606355-54606689
Overlap Length: 15 INS size: 318 Genotyped: Yes

```
left   -------------GATTTCTCAAAAACTAAAAACAAAGCTACCATTCAACCCAGCAATCCATTACTGGGT
chrom  AGAGCAGTTTGGAGATTTCTCAAAAACTAAAAACAAAGCTACCATTCAACCCAGCAATCCATTACTGGGT
right  -------TTTAG---------------TAGAGACGGGGTTTCC------CCGTTTTAGCCAGGATGGTCT
              222N2 11111111111111**1*1**111*1*1**111111**11111*1***11*11*11*

left   AAATACCCAAAGGAAAAGAGATGATTATGCCAAAAAGACACATTAGTTCATATGTTCTTTGCCACACTAT
chrom  AAATACCCAAAGGAAAAGAGATGATTATGCCAAAAAGACACATTAGTTCATATGTTCTTTGCCACACTAT
right  CGATCTCCTGACCTC--GTGATCCGCCCGCCTCGGCCTCCCAA-AGTGC---TGGGATTACAGGCGTGAG
       11**11**11*111111*1***111111***1111111*1**11***1*111**111**11111*111*1

left   TCACAACAGCAAAAACATTTTATCAGTCCCTTCTTTTTTTTTTTTTTTTTTTTTT--------TTTTTTT
chrom  TCACAACAGCAAAAACATTTTATCAGTCCCTTCTTATGCTGCTAATAAAGACATACCCAAGACTGGGTAG
right  CCACCGCGCC-----CGGCCTATCAGTCCCTTCTTATGCTGCTAATAAAGACATACCCAAGACTGGGTAG
       1***11*11*11111*1111***************2*22*22*22*2222222*222222222*222*22

left   TTT--------------TTTTTTTGAGACGGAGTCTCGCTCTGTC--GCC-CAGG---CTGG--AGTGCA
chrom  CTTATAAAGGAAACAGGTTTTATTGACTCACAGTTCAGCATGGGGAGGCCTCAGGAAACTTACAATTATG
right  CTTATAAAGGAAACAGGTTTTATTGACTCACAGTTCAGCATGGGGAGGCCTCAGGAAACTTACAATTATG
       2**22222222222222****2****22*22***222**222*2222***2****222**2222*2*222

left   GTGGCGGG-------ATCTCGGCTCACTGCA-AGCTCCGCCTCCCGG-GTTCA--
chrom  GTGGAAGGGGAATCAAACACATCTTTCTTCACATGGCAGGAGCAAGAAGTTCAGA
right  GTGGAAGGGGAATCAAACACATCTTTCTTCACATGGCAGG---------------
       ****22**2222222*2*2*22**22**2**2*222*2*2NN1NN1N 11111
```

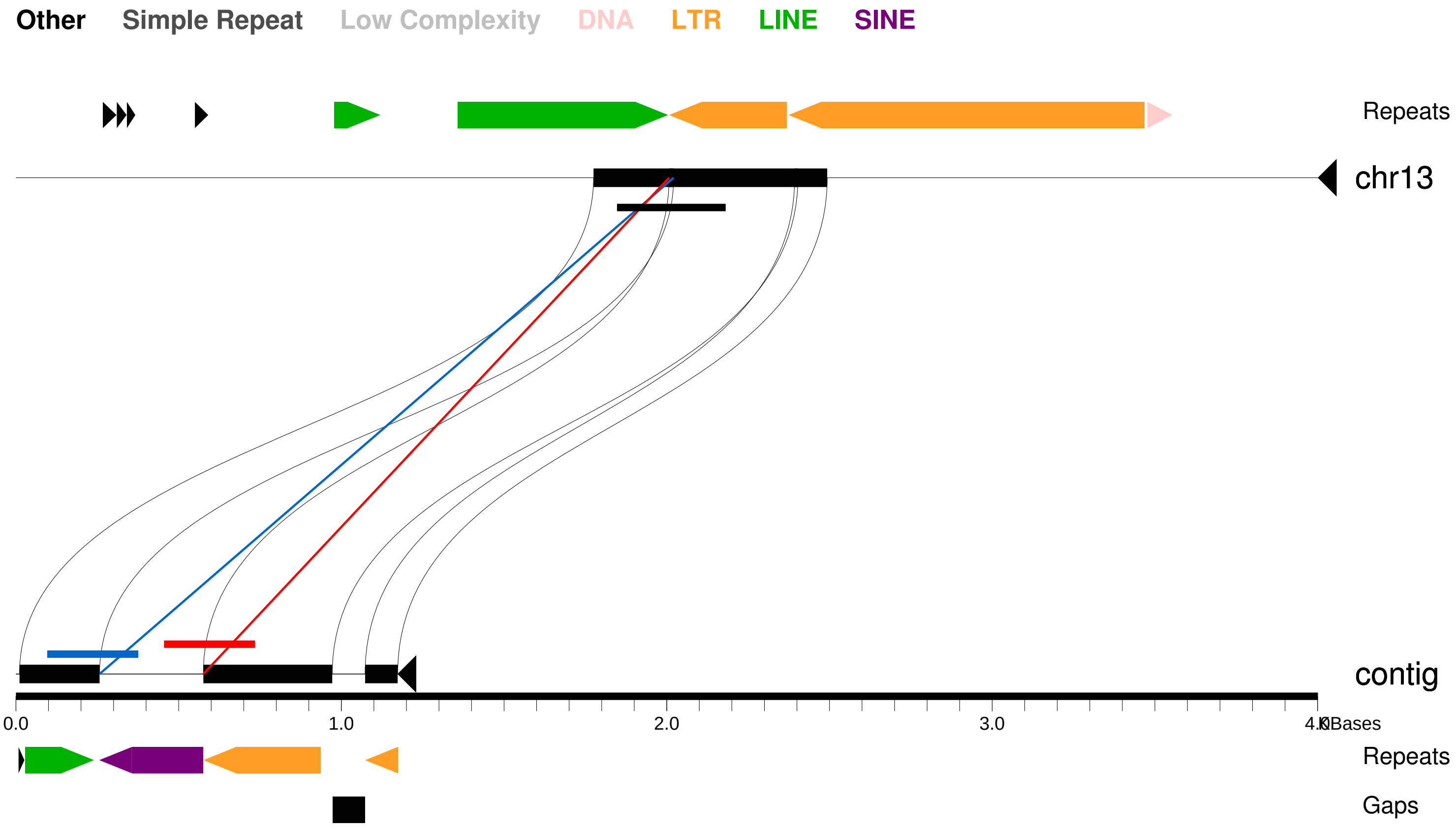

siteID: chr13_55438080 Scafftig7 - Breakpoint: chr13:55438076
chr13:55438028-55438131
Overlap Length: 12 INS size: 310 Genotyped: Yes

```
left     TGTA----------AATATTTTAACAATTGGT-TCAATGTAATTTTGCAACATTTAAAA----GTTCACC
chrom    TGTA----------AATATTTTAACAATTGGT-TCAATGTAATTTTGCAACATTTAAAA----GTTCACC
right    ACTCCAGCCTGGGCGACAGAGCGAGACTCCGTCTCAAAAAAAAAAAAAAAAAAAAAAAAAAAAAGTTAACC
         11*111111111111*1*1*11111*1*1*11**1****111**111111**1*111****1111***1***

left     TTTTTGGCCGGGCGCGGTGGCTCACGCCTGTAATCCCAGCACTTTGGGAGGCCGAGGCGGGCGG-
chrom    TTTTTAT----------------ACATATGTATACATATACTTTTTATATATAAATGTATAAATA
right    TTTTTTT----------------ACATATGTATACATATACTTTTTATATATAAATGTATAAATA
         *****N22222222222222222**222****22*22*2222***222*22222*2*22222222
```

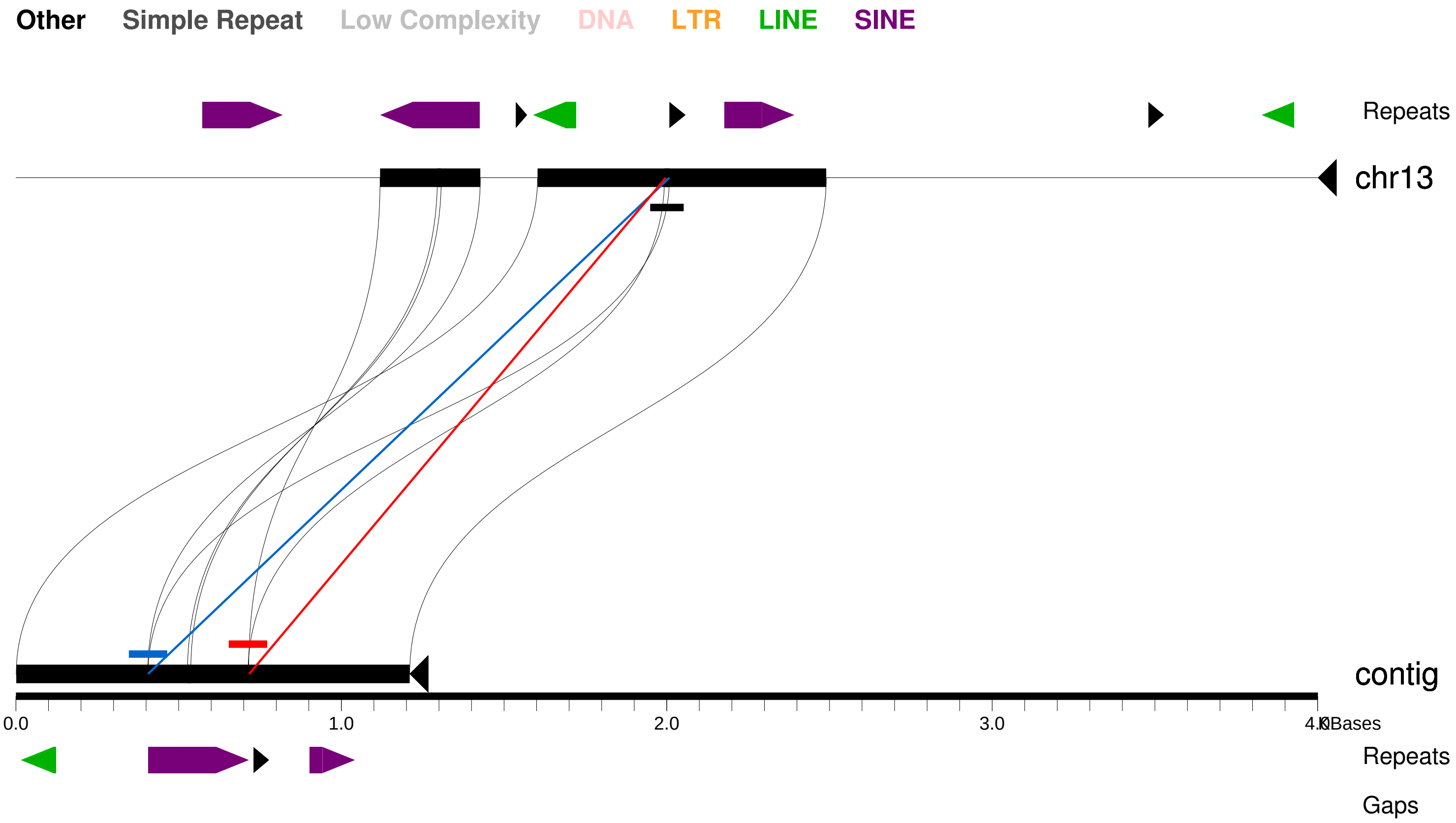

siteID: chr13_55849712 Scafftig1 - Breakpoint: chr13:55849658
chr13:55849612-55849711
Overlap Length: 14 INS size: 363 Genotyped: No

```
left    --------TGGTCCATCAATTT--TGTTGATTTTTTCCAAAAACC-----------AGCTCCTGGATTCA
chrom   --------TGGTCCATCAATTT--TGTTGATTTTTTCCAAAAACC-----------AGCTCCTGGATTCA
right   AGCCAGGATGGTC--TCGATCTCCTGACCTCGTGATCCGCCCGCCTCGGCCTCCCAAAGTGCTGGGATCA
        11111111*****11**1**1*11**111111*11***11111**11111111111*11*1****11***

left    TTGATTTTTTTTTNNNNNNNNNNNNNNNNNNNNNNNNNNNNNNNNNNNNNNNNNNNNNNNNNNNNNNNNN
chrom   TTGATTTTTTT-------------------GAAGGGTTATTTGTGTCTCTATTTCCTTCAGTTATGCTCT
right   TTGATTTTTTT-------------------GAAGGGTTATTTGTGTCTCTATTTCCTTCAGTTATGCTCT
        ***********2222222222222222222222222222222222222222222222222222222222
```

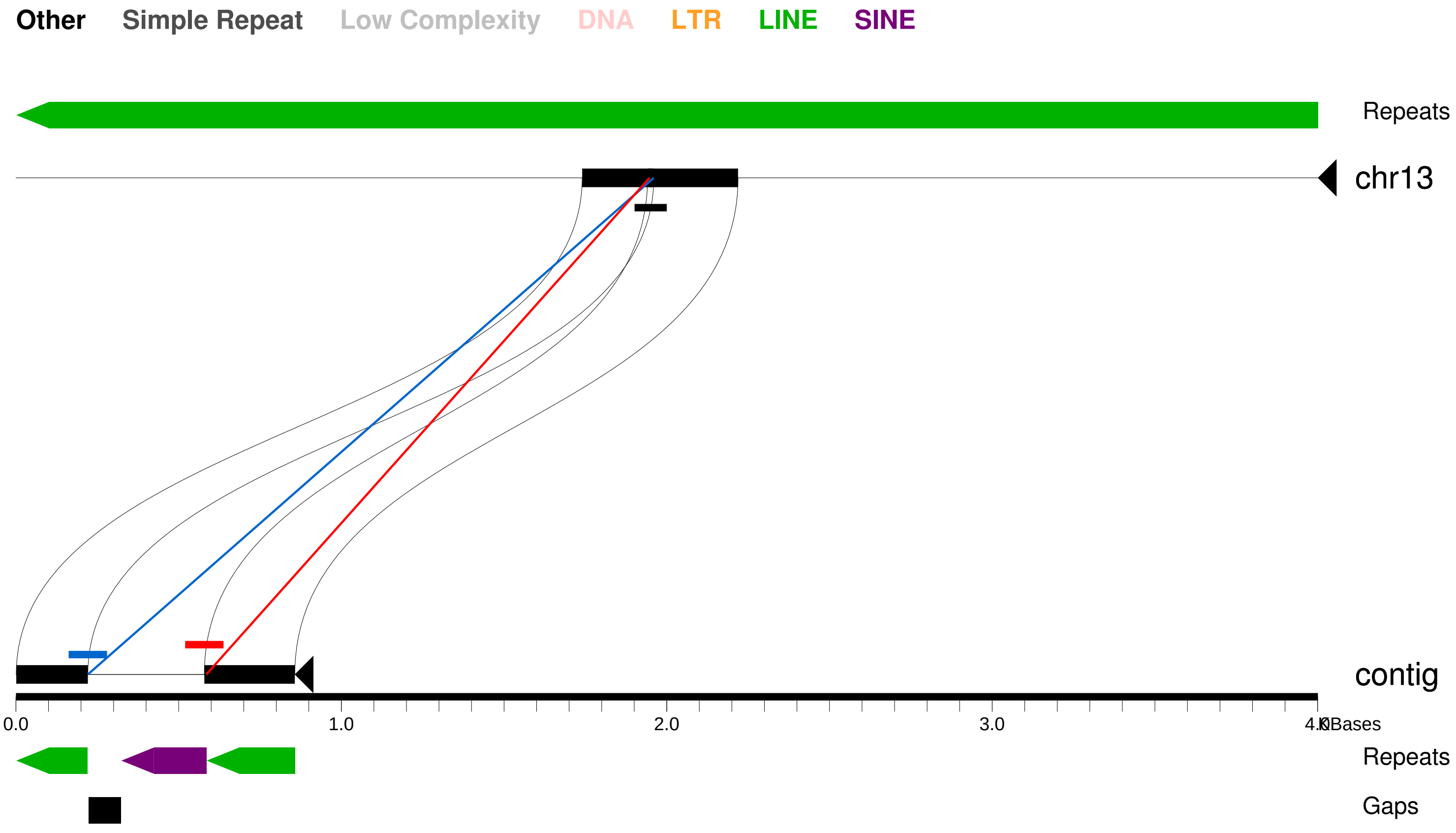

siteID: chr13_56186580 Scafftig10 + Breakpoint: chr13:56186560
chr13:56186517-56186619
Overlap Length: 17 INS size: 300 Genotyped: Yes

```
left    GATATAAACTT---ATTTAAAAGTTTTGAAAAAAATTAAAATTTTT-----------------TAAAAGT
chrom   GATATAAACTT---ATTTAAAAGTTTTGAAAAAAATTAAAATTTTT-----------------TAAAAGT
right   CCACCCGCCTCGGCCTCCCAAAGTGCTGGGA----TTACAGGCGTGAGCCACCACGCCCGGCCTAAAAGT
        11111111**11111*111*****11**11*1111***1*1111*111111111111111111*******

left    TTTTTTTTTTTTTTTTTTTTTTTTTTTTTGAGACAGCGTCTGGCTCTGTCGCCCAGGCTGGAGTGCAGT
chrom   TTTTTTTTTTGCATAAGTAATTTGA-----AGAC----TCCAATAATTTATCCAATCATGAA-------
right   TTTTTTTTTTGCATAAGTAATTTGA-----AGAC----TCCAATAATTTATCCAATCATGAA-------
        **********222*222*22***2222222****2222**222222*2*22**2*222**2*2222222
```

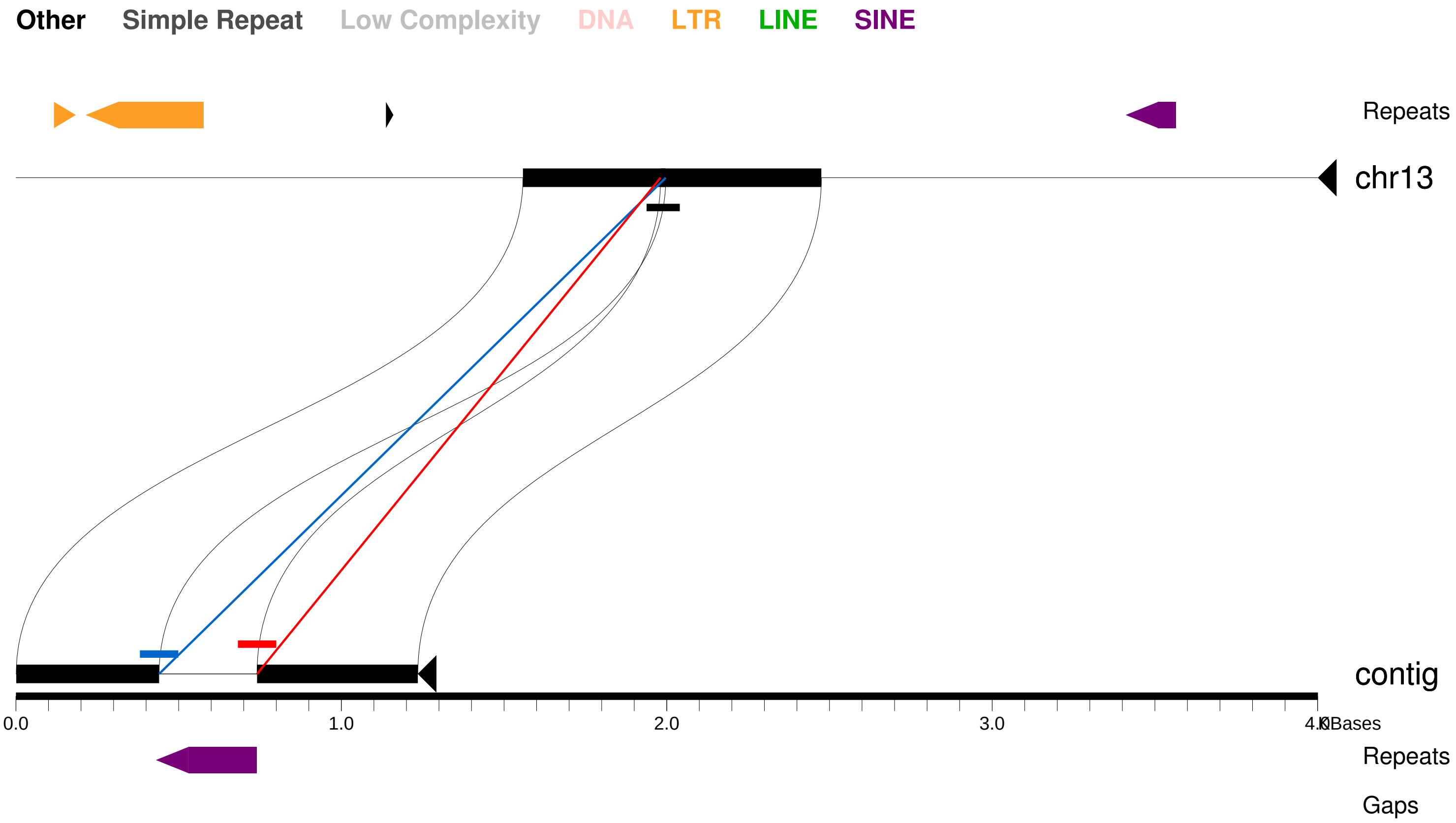

siteID: chr13_57589043 Scafftig3 + Breakpoint: chr13:57589015
chr13:57588970-57589074
Overlap Length: 15 INS size: 116 Genotyped: Yes

```
left     CACTCAGTTAAGCCTGAACTTTC--AATCCACAAAAACTGTGAGATA------------TATACGTGATT
chrom    CACTCAGTTAAGCCTGAACTTTC--AATCCACAAAAACTGTGAGATA------------TATACGTGATT
right    CGCCCAGGCTGGAGTGCAGTGGCGCAATCTCGGCTCACTGCAAGCTCCGCCTCCCGGGTTATACGTGATT
         *1*1***1111*11**1*1*11*11****1111111****11**1*1111111111111***********

left     TTTTTTTTTTTTTTTTTTTTTTTTTTTTTTTTTTTTTTTTTGAGACGGAGTCTCGCTCTGTCG
chrom    TTTTAGACTATTAGGTTTATGTAATTTGTTACCT------AGAAACCAA---TAACTA-----
right    TTTTAGACTATTAGGTTTATGTAATTTGTTACCT------AGAAACCAA---TAACTA-----
         ****2222*2**222***2*2*22***2**222*2222222**2**22*222*22**222222
```

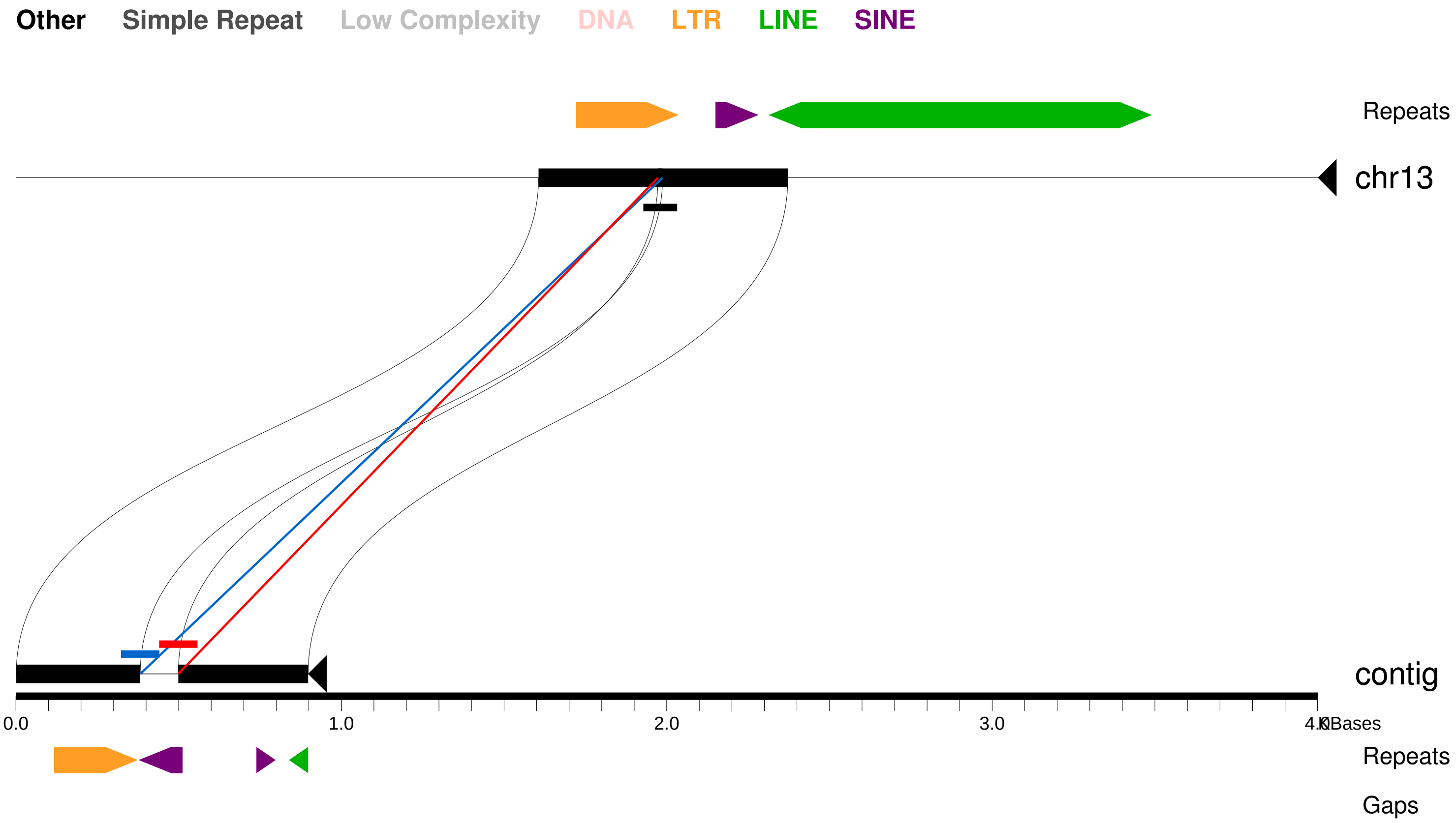

siteID: chr13_58621074 Scafftig14 + Breakpoint: chr13:58621052
chr13:58621007-58621111
Overlap Length: 15 INS size: 277 Genotyped: Yes

```
left    ---CTGTATAATACAAAAATAA-------ATTTTTT----GTTTTAAAATTAAAAACACAAAATGAATAA
chrom   ---CTGTATAATACAAAAATAA-------ATGTTTT----GTTTTAAAATTAAAAACACAAAATGAATAA
right   AGCCAGGATGGTCTCGATCTCCTGACCTCATGATCCACCCGCCTCGGCCTCCCAAAGTGAAAATGAATAA
        111*1*1**11*1111*11*111111111**21*111111*11*11111*111***111***********

left    TATTTTTTTTTTTTTTTTTTTTTTTTGAGACGGAGTCTCGCTCTGTCGCCCAGGCCGGACTGC
chrom   TATTAATAAAATCAATCAAAATT----AGTTGAATT-----TCTATGGTACA--CTAGAT---
right   TATTAATAAAATCAATCAAAATT----AGTTGAATT-----TCTATGGTACA--CTAGAT---
        ****22*2222*222*22222**2222**22*2*2*22222***2*2*22**22*22**2222
```

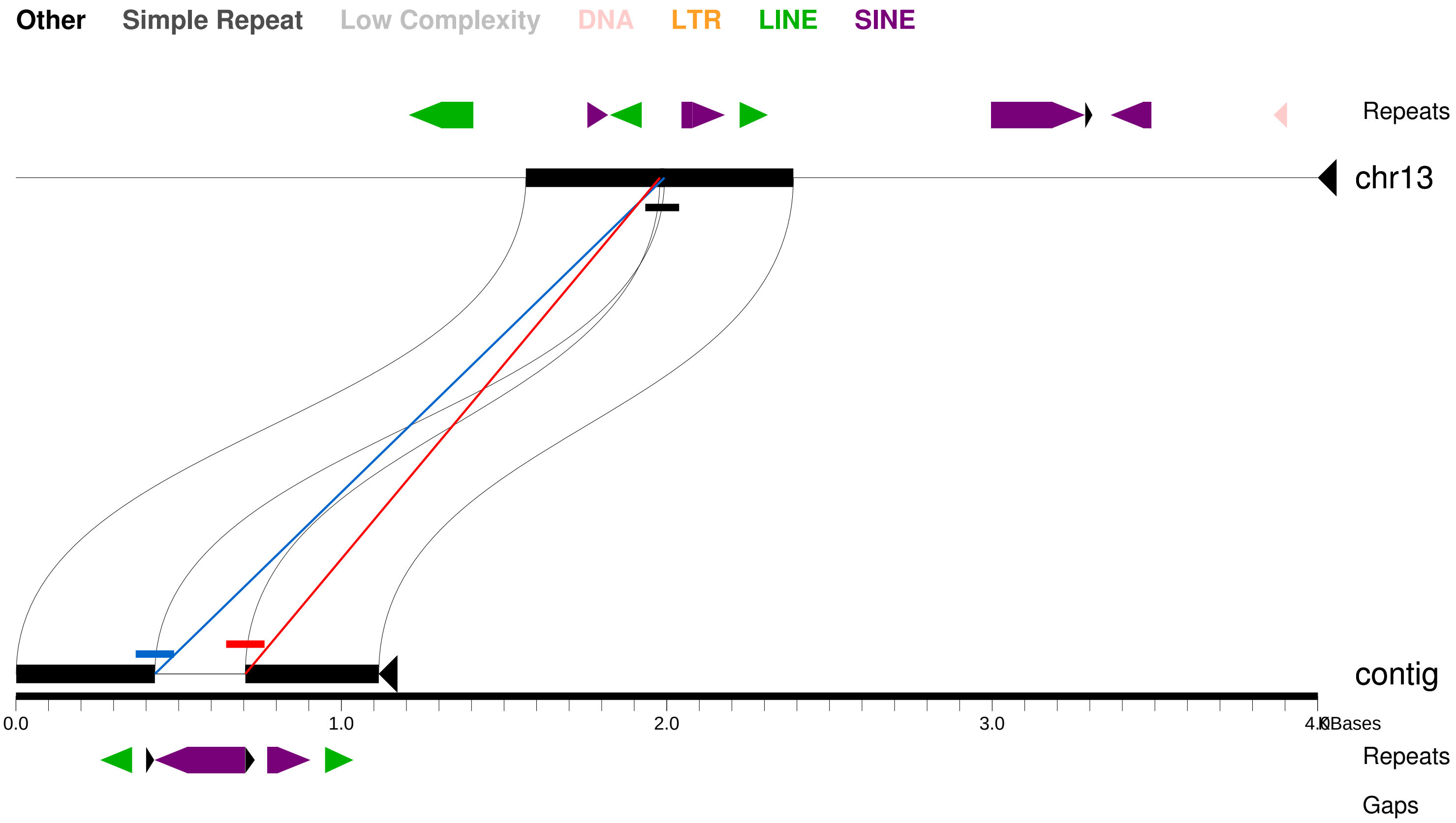

siteID: chr13_58761410 Scafftig1 + Breakpoint: chr13:58761416
chr13:58761366-58761475
Overlap Length: 10 INS size: 422 Genotyped: No

```
left   AGGACTGTATTTTCATCCCATTGACTGTAGGCTTTTCTCTTGTCAAAATA---------AAAACGTCCGG
chrom  AGGACTGTATTTTCATCCCATTGACTGTAGGCTTTTCTCTTGTCAAAATA---------AAAACGTCCGT
right  NNNNNNNNNNNNNNNNNNNNNNNNNNNNNNNNNNNNNNNNNNNNNNNNNNNNNNNNNNNNAAAACGTCCGT
       111111111111111111111111111111111111111111111111111111111111**********2

left   CCGGGCGCGGTGGCTCACGCCTGTAATCCCAGCACTT--TGGGAGG-----CCGAGGCGGGCGGA
chrom  ----------------ATGACTCTAATATTTGCCTTTGCTGGTATGAATACCATAGCAGGTATAA
right  ----------------ATGACTCTAATATTTGCCTTTGCTGGTATGAATACCATAGCAGGTATAA
       2222222222222222*2*2**2****2222**22**22***2*2*22222*22**22**2222*
```

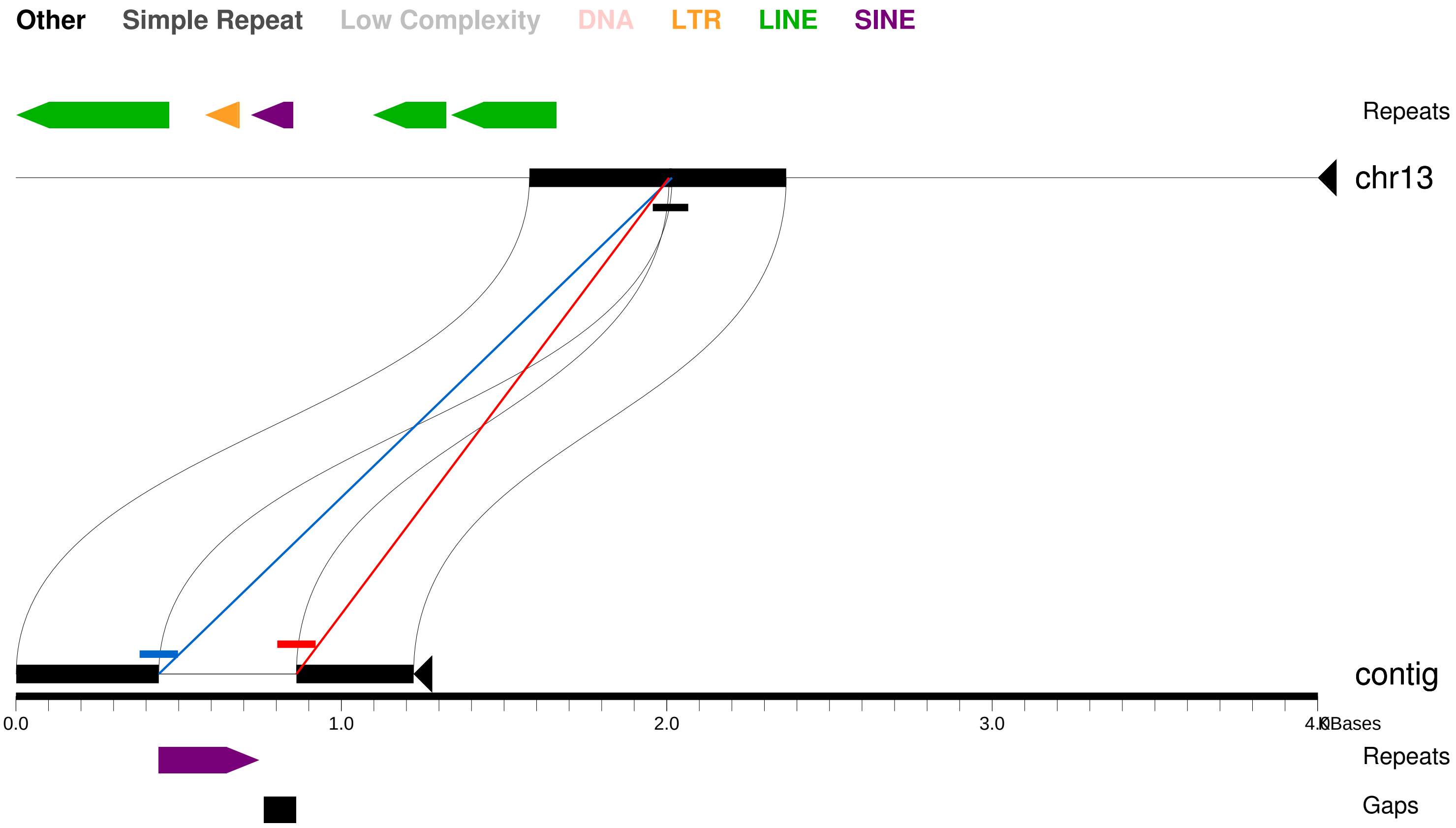

siteID: chr13_59377694 Scafftig2 + Breakpoint: chr13:59377671
chr13:59377625-59377730
Overlap Length: 14 INS size: 304 Genotyped: Yes

```
left    GTATTCTTCCTGTGTTCTCA-----CTTGGA-----GGAG-GAGGCAAGGCTGCTTT--CTGGGGTCTCT
chrom   GTATTCTTCCTGTGTTCTCA-----CTTGGA-----GGAG-GAGGCAAGGCTGCTTT--CTGGGGTCTCT
right   CCGCCCGCCTCGGCCTCCCAAAGTGCTGGGATTACAGGCGTGAGCCACCGCGCCCGGCCCTGGGGTCTCT
        11111*11*11*111**1**11111**1***11111**1*1***1**11**11*11111***********

left    TTTTTTTTTTTTTTTTTTTTTTTT-TGAGA-CGGAGTCTCGCTCTGTCGCCCAGGCTGGAGTGC
chrom   TTTATAAGGGCTCTAAACCTGTTTATGAGGTCTGAGTC---CTAATT------------ACTAA
right   TTTATAAGGGCTCTAAACCTGTTTATGAGGTCTGAGTC---CTAATT------------ACTAA
        ***2*222222*2*22222*2***2****22*2*****222**222*222222222222*2*22
```

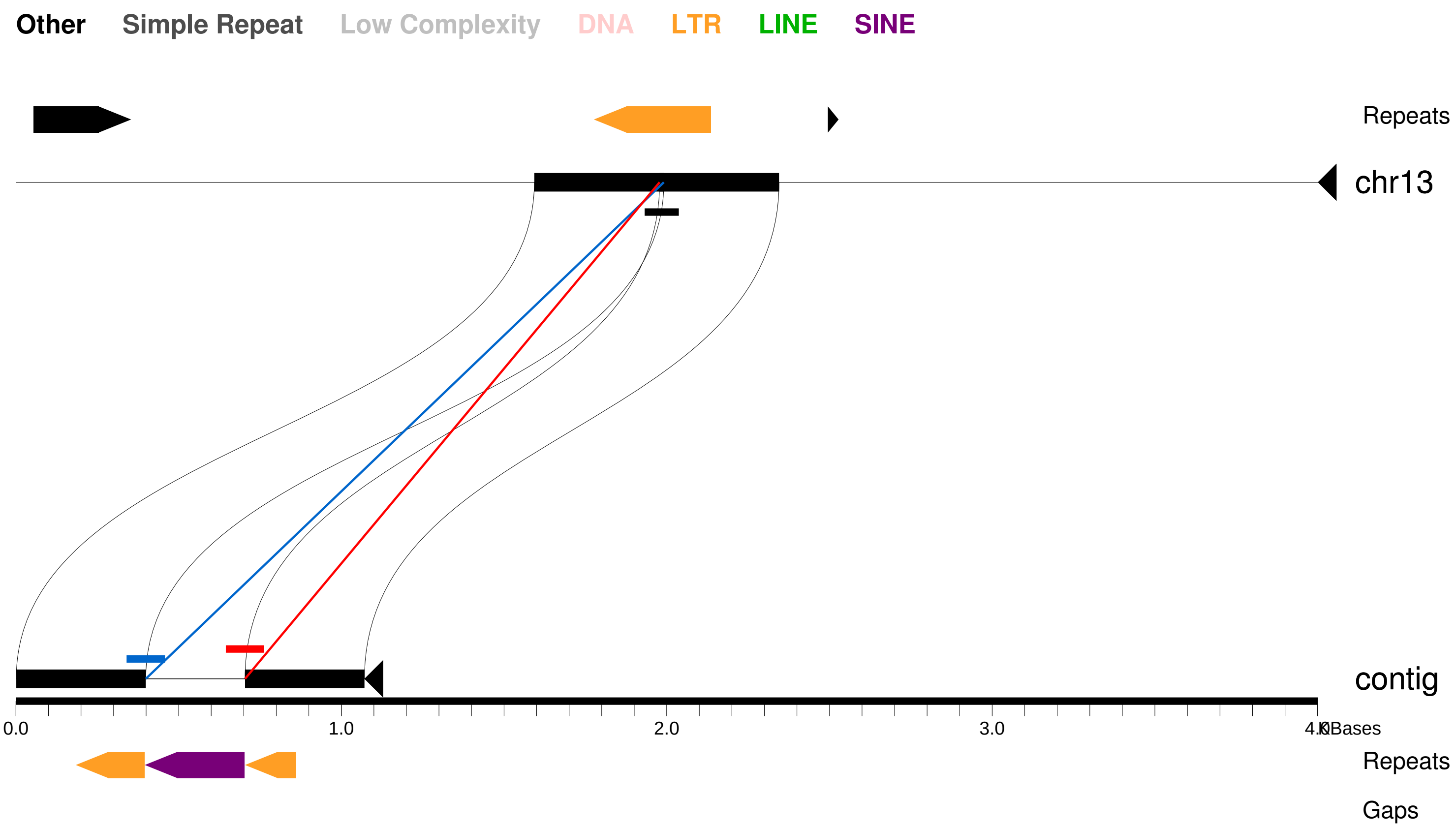

siteID: chr13_59396866 Scafftig14 + Breakpoint: chr13:59396880
chr13:59396836-59396939
Overlap Length: 16 INS size: 300 Genotyped: Yes

```
left    GGGAA-------GTGTACTATTGTGTCCAGAATTGGTGCGG-----ACCTTGCAGT---GAGTGTTACAG
chrom   GGGAA-------GTGTACTATTGTGTCCAGAATTGGTGCGG-----ACCTTGCAGT---GAGTGTTACAG
right   CCGCCCGTCTCGGCCTCCCAAAGTGCTGGGATTACAGGCGTGAGCCACCGCGCCCGGCCGAGTGTTACAG
        11*111111111*11*1*1*11***1111**1*1111***111111***11**111111***********

left    TTCTTTTTTTTTTTTTTTTTTTTTTTGAGACGGAGTTTCGCTCTGTCGCCCAGGCTGGAGTGCAG
chrom   TTCTTAAAGATGGTATCTGGAGTTTG---------TTCCTTGTGATGTTCGGAC--GTGT----
right   TTCTTAAAGATGGTATCTGGAGTTTG---------TTCCTTGTGATGTTCGGAC--GTGT----
        *****22222*22*2*2*2222****222222222***22*2**22*22*2*2*22*2**2222
```

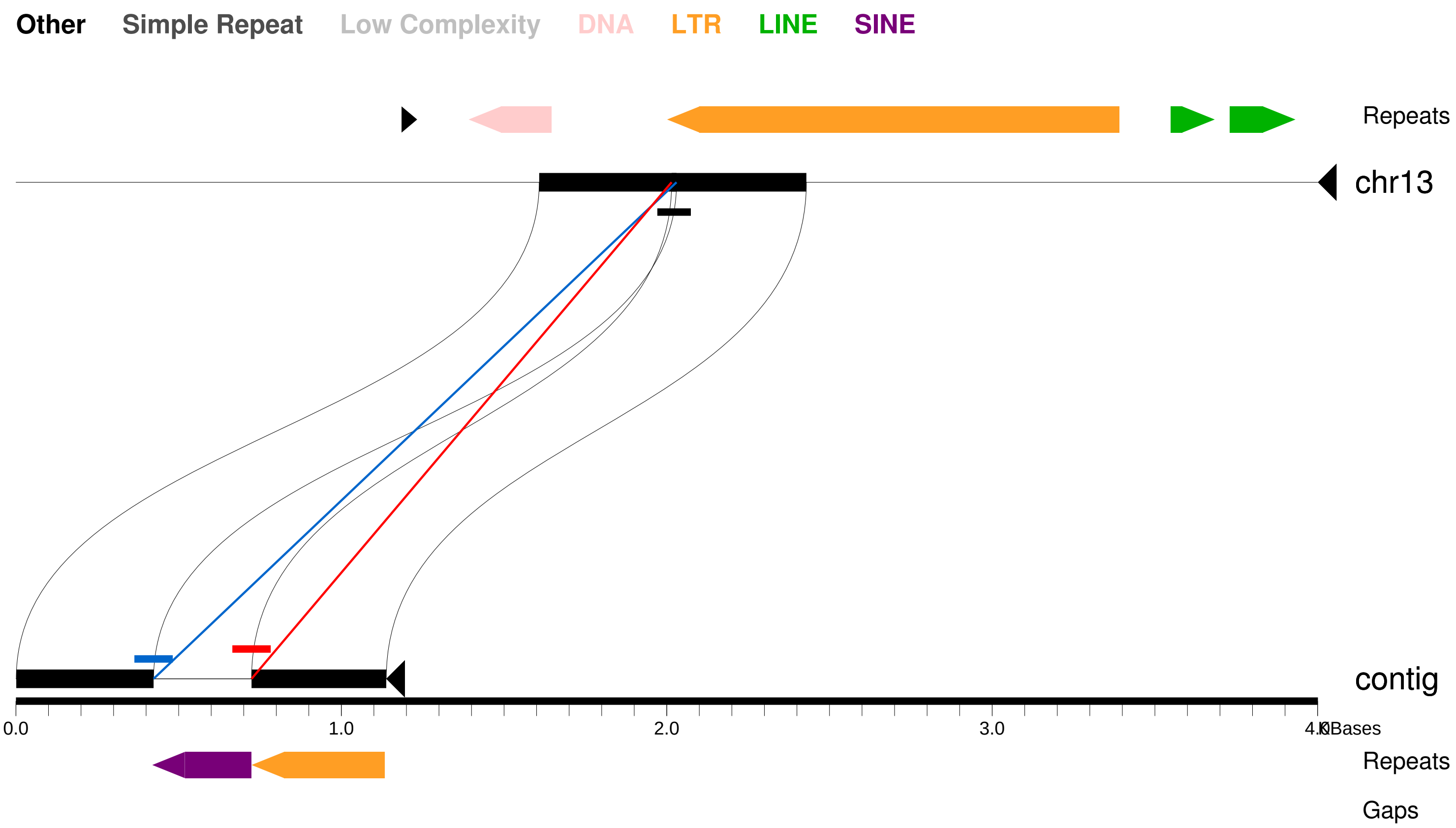

siteID: chr13_60032504 Scafftig3 - Breakpoint: chr13:60032546
chr13:60032502-60032605
Overlap Length: 16 INS size: 99 Genotyped: Yes

```
left    AAATA-ATTATGTG------ATTTGGACTGTGGATTATAATAAAGATTCTT--------AAAAGATGCTG
chrom   AAATA-ATTATGTG------ATTTGGACTGTGGATTATAATAAAGATTCTT--------AAAAGATGCTG
right   CACTCCAGCCTGGGCGACAGAGCGAGACTCCGTCTCAAAAAAAAAAAAAAAAAAAAAAAAAAAAGATGCTG
        1*1*11*111**1*111111*1111****11*11*1*1**1***1*1111111111111***********

left    GGAACCGGGAGGCGGAGCCTCCAGTGAGCCGA-GATCCCGCCACTGCACTCCAGCCTGGGCGACA
chrom   GGAACACAGTAGAAAAAGTTCCGTTTCTCTGAAGATG------------TCAA---TGATAGA-A
right   GGAACACAGTAGAAAAAGTTCCGTTTCTCTGAAGATG------------TCAA---TGATAGA-A
        *****222*22*222*222***22*222*2**2***2222222222222**2*222**222**2*
```

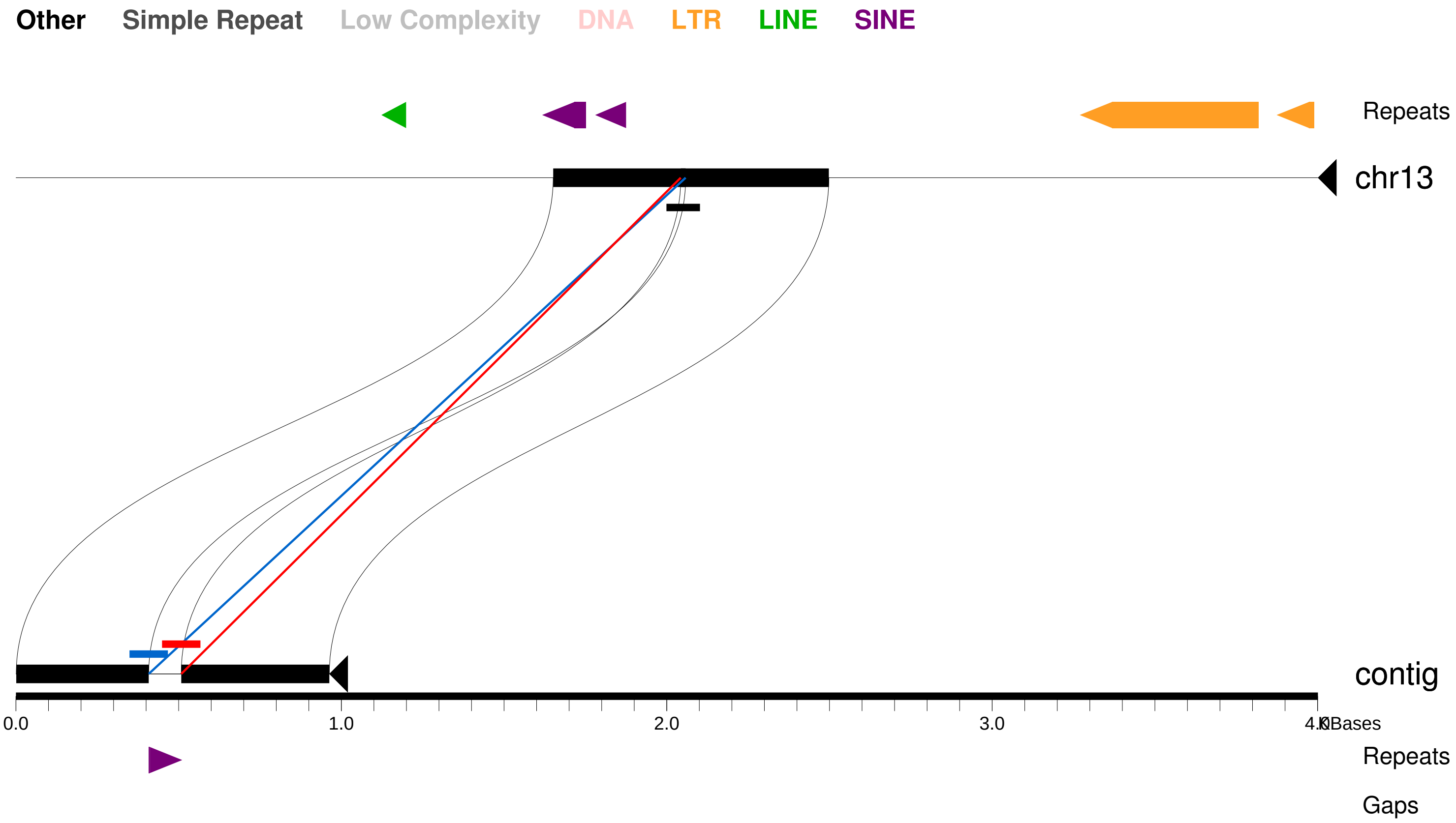

siteID: chr13_60465048 Scafftig1 + Breakpoint: chr13:60465043
chr13:60464882-60465213
Overlap Length: 11 INS size: 416 Genotyped: No

```
left   T-----------CCTTTATAATTAATAGTTTATGGGGAGCTGTTTCGAGGCTATGTAAATAGTATGTATA
chrom  TACTGTTTCTTTCCTTTATAATTAATAGTTTATGGGGAGCTGTTTCGAGGCTATGTAAATAGTATGTATA
right  NNNNNNNNNNNT----------------------------------------------------------
       1NNNNNNNNNN21111111111111111111111111111111111111111111111111111111111

left   TAACCTATTTTCACCAAATTTTCACATTTGTATCAACAGAGATAGCACACTAACTAGAAAAATACAGAAT
chrom  TAACCTATTTTCACCAAATTTTCACATTTGTATCAACAGAGATAGCACACTAACTAGAAAAATACAGAAT
right  ----------------------------------------------------------------------
       1111111111111111111111111111111111111111111111111111111111111111111111

left   CTACTCAACTTTATTAAAACTGAACTTTACGGGCCGGGCGCGGTGGCTCACG-CCTGTAATCCCAGCACT
chrom  CTACTCAACTTTATTAAAACTGAACTTTACGGACCCTTTCAGAAACAGCAGTACCTTCAAGAGCATGACA
right  ---------------------GAACTTTACGGACCCTTTCAGAAACAGCAGTACCTTCAAGAGCATGACA
       111111111111111111111**********2**222222*222222**222***22**222**22**2

left   TTGGGAGGCCGAGGC----GGGTGGATC----ATGAGGTCAGG-------AGATC-GAGACCATCCTGGC
chrom  TAAAAGTGCAAATGAACCAGAATATATCTACAATAATTTCACTTTAAGCCACAACTGCAACAAAATTGTC
right  TAAAAGTGCAAATGAACCAGAATATATCTACAATAATTTCACTTTAAGCCACAACTGCAACAAAATTGTC
       *222222**22*2*22222*22*22***2222**2*22***222222222*2*2*2*22**2*222**2*

left   TAACA------------------AGGTGAAACCCCGTCTCTACTAAA-----
chrom  TAACTTCAGTGATTTAGTTCCATAAGTCCAGCCTCCTCTTTTCCATTATGTT
right  TAACTTCAGTGATTT-------------------------------------
       ****22222222222        1N11NN1N11N1N111N1N1N1NN
```

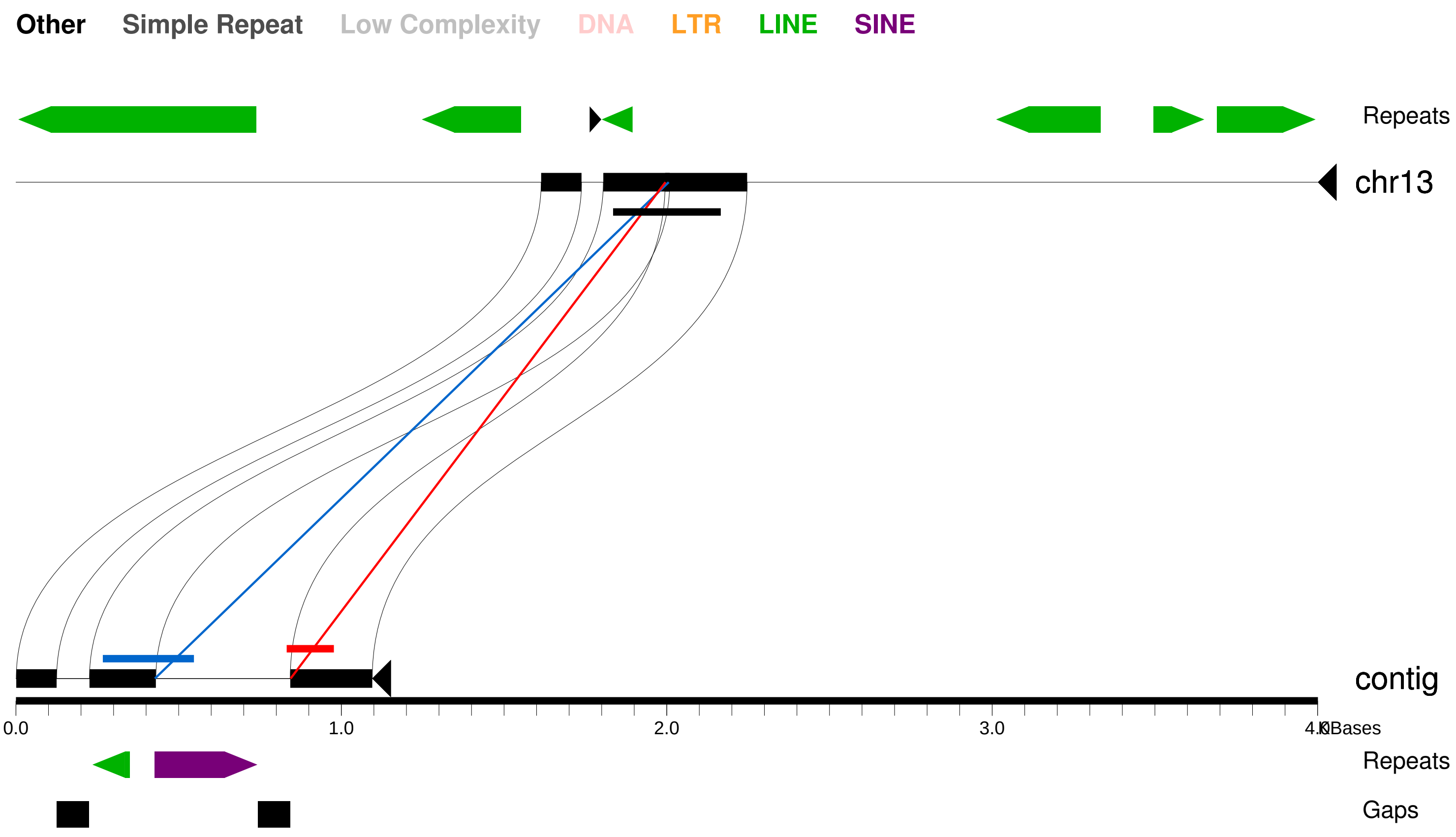

siteID: chr13_61162460 Scafftig1 + Breakpoint: chr13:61162457
chr13:61162413-61162516
Overlap Length: 16 INS size: 300 Genotyped: Yes

```
left    TTATTATTAAACTCAG--TCACAATTTCAT----TTAAAATCATGA--CATT----------TTAAATGA
chrom   TTATTATTAAACTCAG--TCACAATTTCAT----TTAAAATCATGA--CATT----------TTAAATGA
right   CCACCCGC---CTCGGCCTCCCAAAGTGCTGGGATTACAGGCGTGAGCCACCGCGCCCGGCCTTAAATGA
        11*11111111***1*11**1***11*11*1111***1*11*1***11**111111111111********

left    ACTTTTTTTTTTTTTTTTTTTGAGACGGAGTCTCGCTCTGTCGCCCAGGCCGGACTGCGGACTGCAGT
chrom   ACTGTTTTAAAGT---------GGCCAAATGT-GCAATTTA---AATTTCTTA-TAAACTCT-CTCT
right   ACTGTTTTAAAGT---------GGCCAAATGT-GCAATTTA---AATTTCTTA-TAAACTCT-CTCT
        ***2****-*2222*222222222*2*22*2*2*2**22*2*22222*222*22*2*22222**2*22*
```

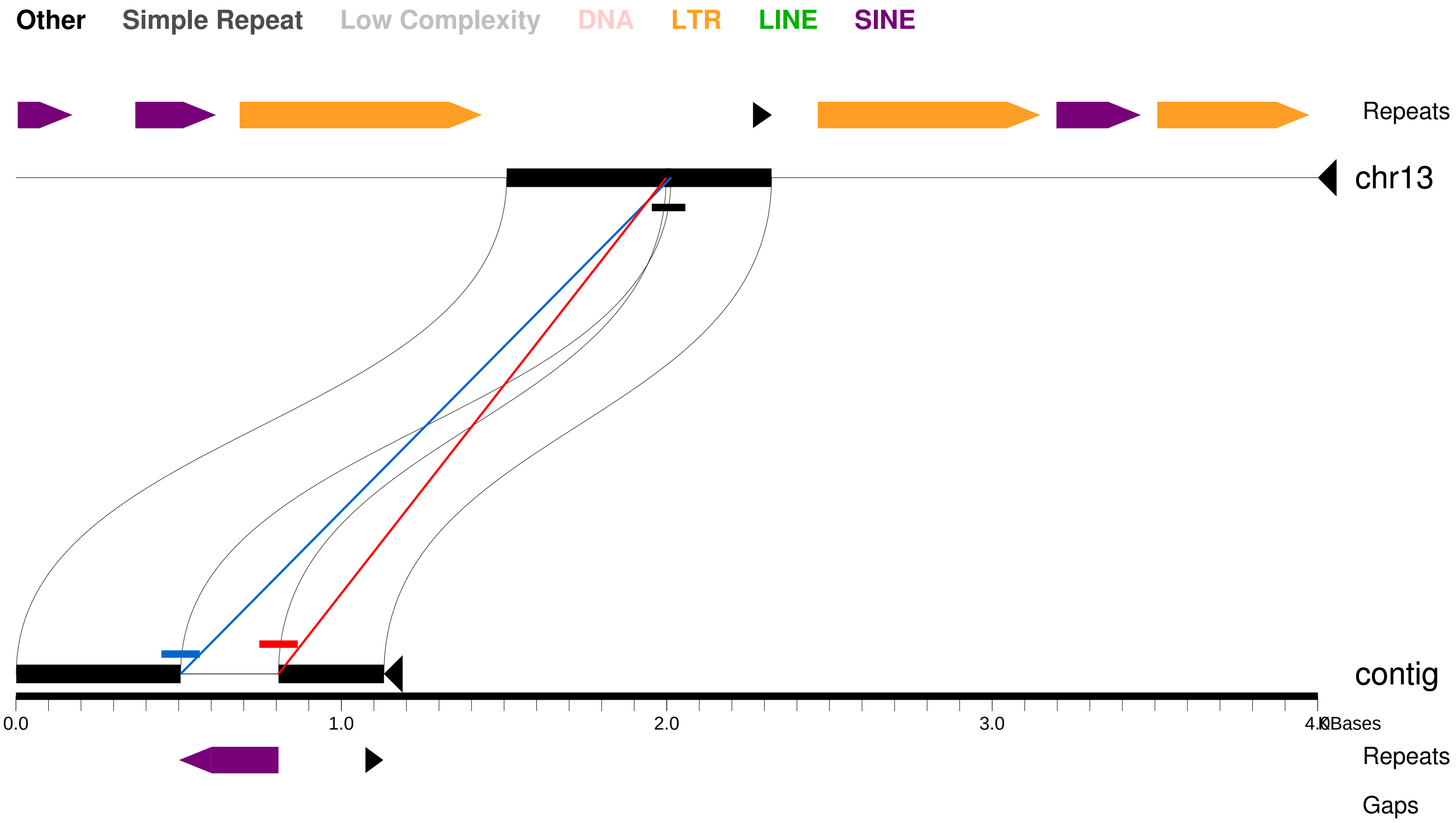

siteID: chr13_61332418 Scafftig2 + Breakpoint: chr13:61332424
chr13:61332385-61332483
Overlap Length: 12 INS size: 434 Genotyped: No

```
left   ACATGGCTTCTTTCGGATCTTGTAGGAGACTTCCATAGG--------------------AAAAAAAGAAC
chrom  ACATGGCTTCTTTCGGATCTTGTAGGAGACTTCCATAGG--------------------AAAAAAAGAAC
right  NNNNNNNNNNNNNNNNNNNNNNNNNNNNNNNNNNNNNNNNNNNNNNNNNNNNNNNNNNNNAAAAAAAGAAC
       111111111111111111111111111111111111111111111111111111111111***********

left   CTGGCCGGGCGCGGTGGCTCACGCCTGTAATCCCAGCACTTTGGGAGGCCGAGGCGGGTGGATCATGAG
chrom  CCTGCA--------TGGCTGAGAAATTTATTTTC----CCT---GAGTTCCA--CTTATCAATTGT---
right  CCTGCA--------TGGCTGAGAAATTTATTTTC----CCT---GAGTTCCA--CTTATCAATTGT---
       *22**222222222*****2*2222*2**2*22*2222*2*222***22*2*22*222*22**22*222
```

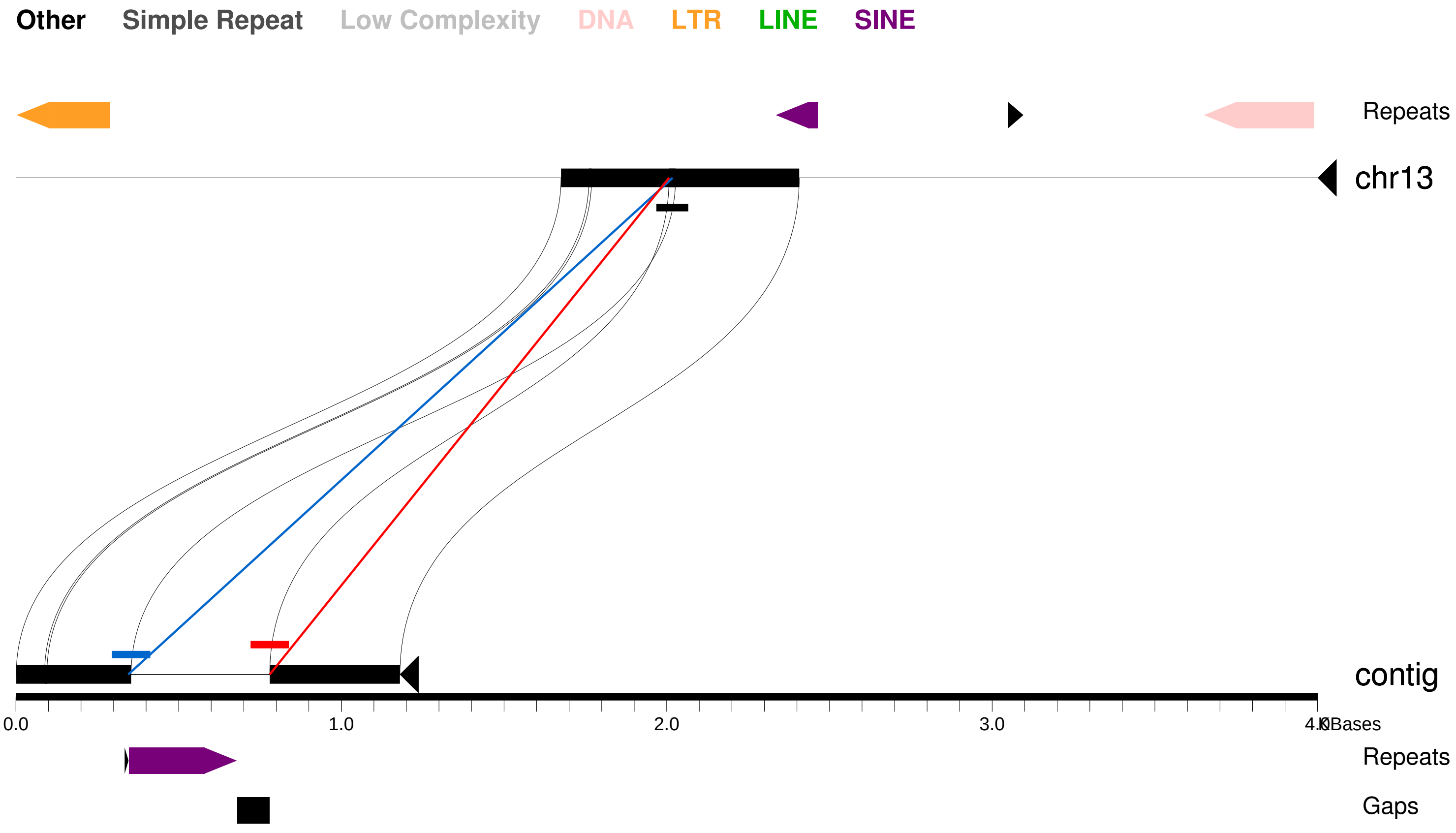

siteID: chr13_61367403 Scafftig1 - Breakpoint: chr13:61367395
chr13:61367349-61367454
Overlap Length: 14 INS size: 293 Genotyped: Yes

```
left     GGTGAAGATGCTTATTTTC--CATATCTTCCAGA---AGTAACTGTC---------AGTCAAAAAGCATT
chrom    GGTGAAGATGCTTATTTTC--CATATCTTCCAGA---AGTAACTGTC---------AGTCAAAAAGCATT
right    A-TCCCGCCACTGCACTCCAGCCTGGGCAACAGAGCGAGACTCTGTCTCAAAAAAAAAAAAAAAAGCATT
         11*111*111**1111*1*11*1*111111****111**111*****111111111*111**********

left     TGTGGGCCGGGCGCGGTGGCTCACGCCTGTAATCCCAGCACTTTGGGAGGCCGAGGCGGGCGG
chrom    TGTGAACACTT-GCTGTGGAT-ACTGCTGGGAT------AAT---GGAGGAAGTGAAGATC--
right    TGTGAACACTT-GCTGTGGAT-ACTGCTGGGAT------AAT---GGAGGAAGTGAAGATC--
         ****22*22222**2****2*2**22***22**222222*2*222*****22*2*22*22*22
```

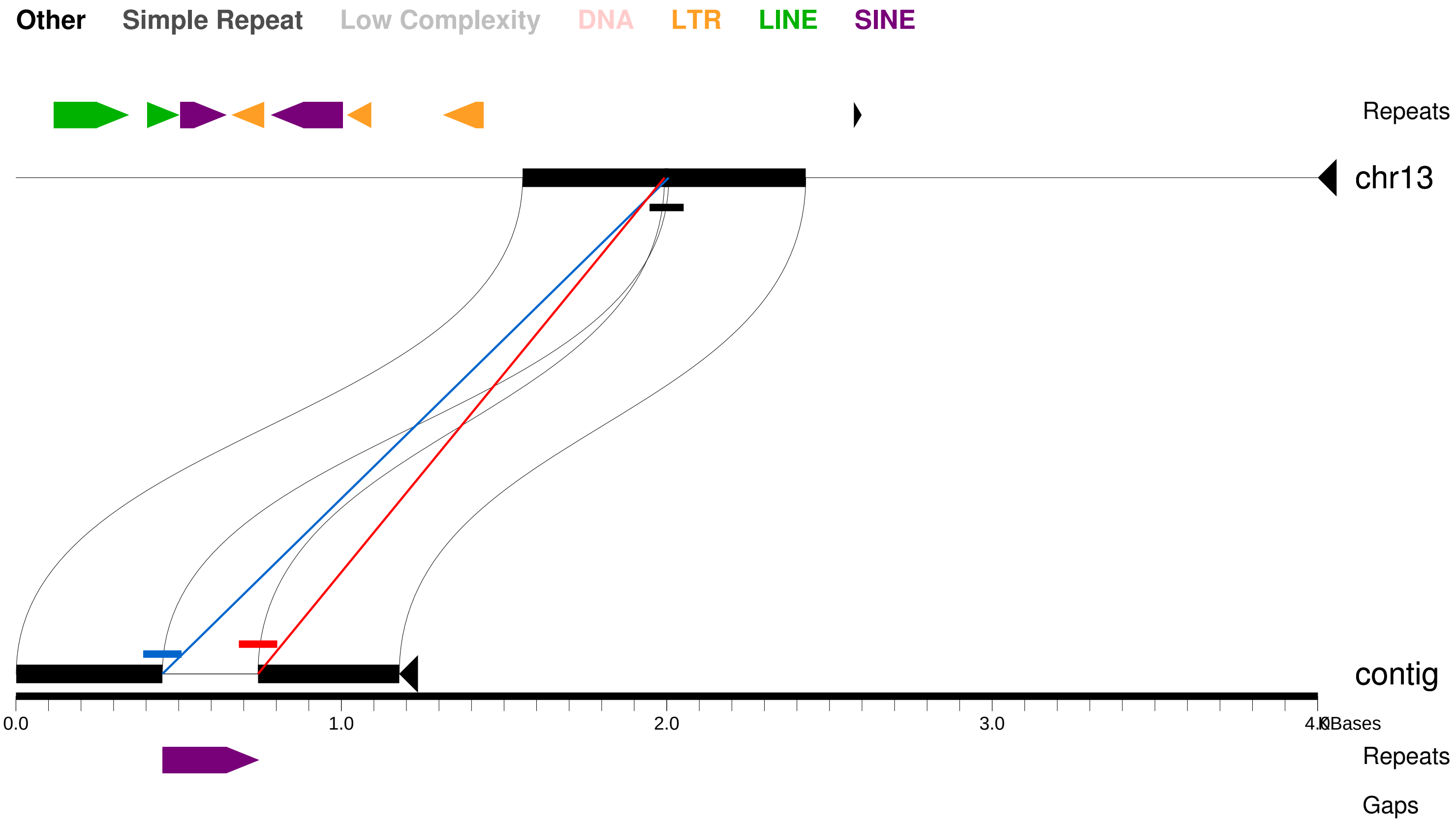

siteID: chr13_61676098 Scafftig1 - Breakpoint: chr13:61676093
chr13:61676047-61676152
Overlap Length: 14 INS size: 141 Genotyped: Yes

```
left    -------CAAAAATGTG----TATGTCT--AATTCACTGCATATATGTAGAAGTTTTATAAAAGTGGTTA
chrom   -------CAAAAATGTG----TATGTCT--AATTCACTGCATATATGTAGAAGTTTTATAAAAGTGGTTA
right   GCCTGGGCGACAGAGCGAGACTCCGTCTCAAAAAAAAAAAAAAAAAAAAAAAAAAAAAAAAAAAGTGGTTA
        1111111*1*1*11*1*1111*11****11**111*1111*1*1*111*1**11111*1***********

left    GCTACTTGGGAGGCTGAGGCAGGAGAATGGCGTGAACCCGGGAGGCAGAGCTTGCAGTGAGC
chrom   GCT--TTCAGAAATCCATCCC------TCGATTCTACTATGAATGCAAAGCTTCTC-----C
right   GCT--TTCAGAAATCCATCCC------TCGATTCCACTATGAATGCAAAGCTTCTC-----C
        ***22**22**22222*22*2222222*2*22*2N**222*2*2***2*****22222222*
```

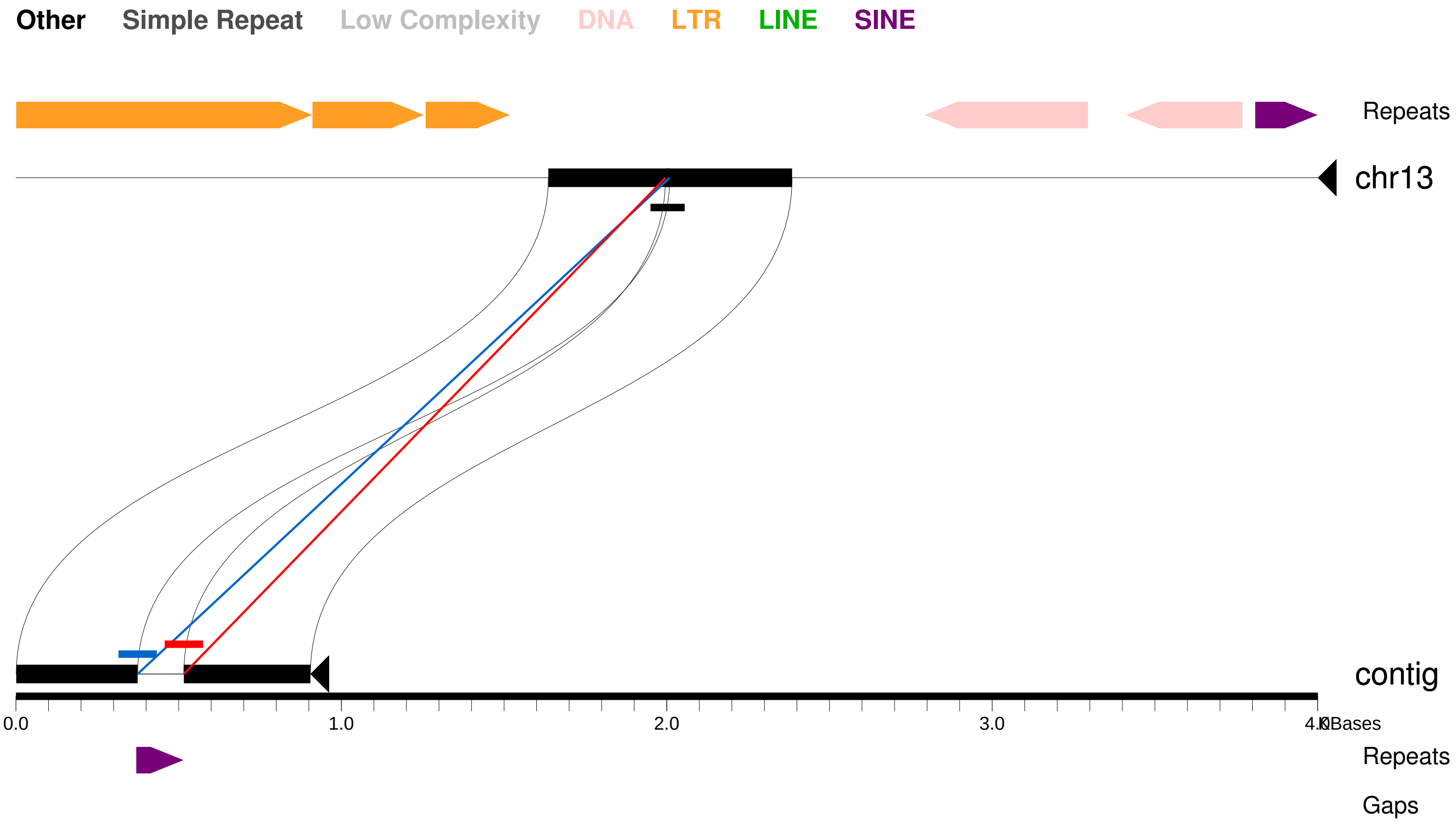

siteID: chr13_61787416 Scafftig1 + Breakpoint: chr13:61787402
chr13:61787243-61787578
Overlap Length: 17 INS size: 306 Genotyped: Yes

```
left   -A----------------CTTTAATGCTTTGAAAGCATTGAAAATAAACTTATTCCTTCAAAAACTCTAT
chrom  -AGTTCACTGTAACTTAACTTTAATGCTTTGAAAGCATTGAAAATAAACTTATTCCTTCAAAAACTCTAT
right  GAGAATGGCGTGA---ACCCGGGAGGCGGAGCTTGCAGTGAGCCGAGA-----TCCCGCCACTGCACT--
       1*2NNNNNN22N2   2N*1111*1**111*111***1***1111*1*11111***11*1*111*1**11

left   TAACCACCAAAAACATTAATATGCTTCAAATTCCCATGAAGTAAATGCATTAAACTTCAGGAAAACATGA
chrom  TAACCACCAAAAACATTAATATGCTTCAAATTCCCATGAAATAAATGTATTAAACTTCAGGAAAACATGA
right  ---CCAGCCTGGGCGACAGAGCGAGACTCCCTCTCAGAAAAAAAAAAAAAAAAAA-------AAAAAA---
       111***1*11111*111*1111*111*1111**1**11**21***11N*11***1111111****1*111

left   GGCTTATTAAGTTAGTGTGTAAAATTTAATTATTTTTGGCCGGGCGCGG---------TGGCTCACGCCT
chrom  GGCTTATTAAGTTAGTGTGTAAAATTTAATTATTTTTATTATAGAGTATAAAACATACTGTATAAATTTT
right  --------------------AAAATTTAATTATTTTTATTATAGAGTATAAAACATACTGTATAAATTTT
       11111111111111111111****************222222*2*222222222222**22*2*2222*

left   GT----AATCCCAGCACTTT----------GGGAGGCCGAGGCGGGCGGATCACGAG--------GTCAG
chrom  TTTTAAAAGATGATAACTTTACAGTCAAATGGTAGGTTCTGAGAGGAGTTTAAATAGTACTTTGTGTACT
right  TTTTAAAAGATGATAACTTTACATTCAAATGGTAGGTTCTGAGAGGAGTTTAAATAGTACTTTGTGTACT
       2*2222**2222*22*****222N222222**2***2222*222**2*22*2*22**22222222**222

left   GAGATCGAGACCATCCC--GGCTAAAATGGTGAAACCCCGTCTCTACTAAA--------
chrom  TAGGTAAATGGTCTCCTCTGGAAAAAAACGAT--ATCAGAGTTGAAAGAAAAAAGTACAT
right  TAGGTAAATGGTCTCCTCTGGAAAAAAACGAT--ATCAGAGTTGAAA-------------
       2**2*22*22222***222**22****2*2*22*2*222**2222*NN111
```

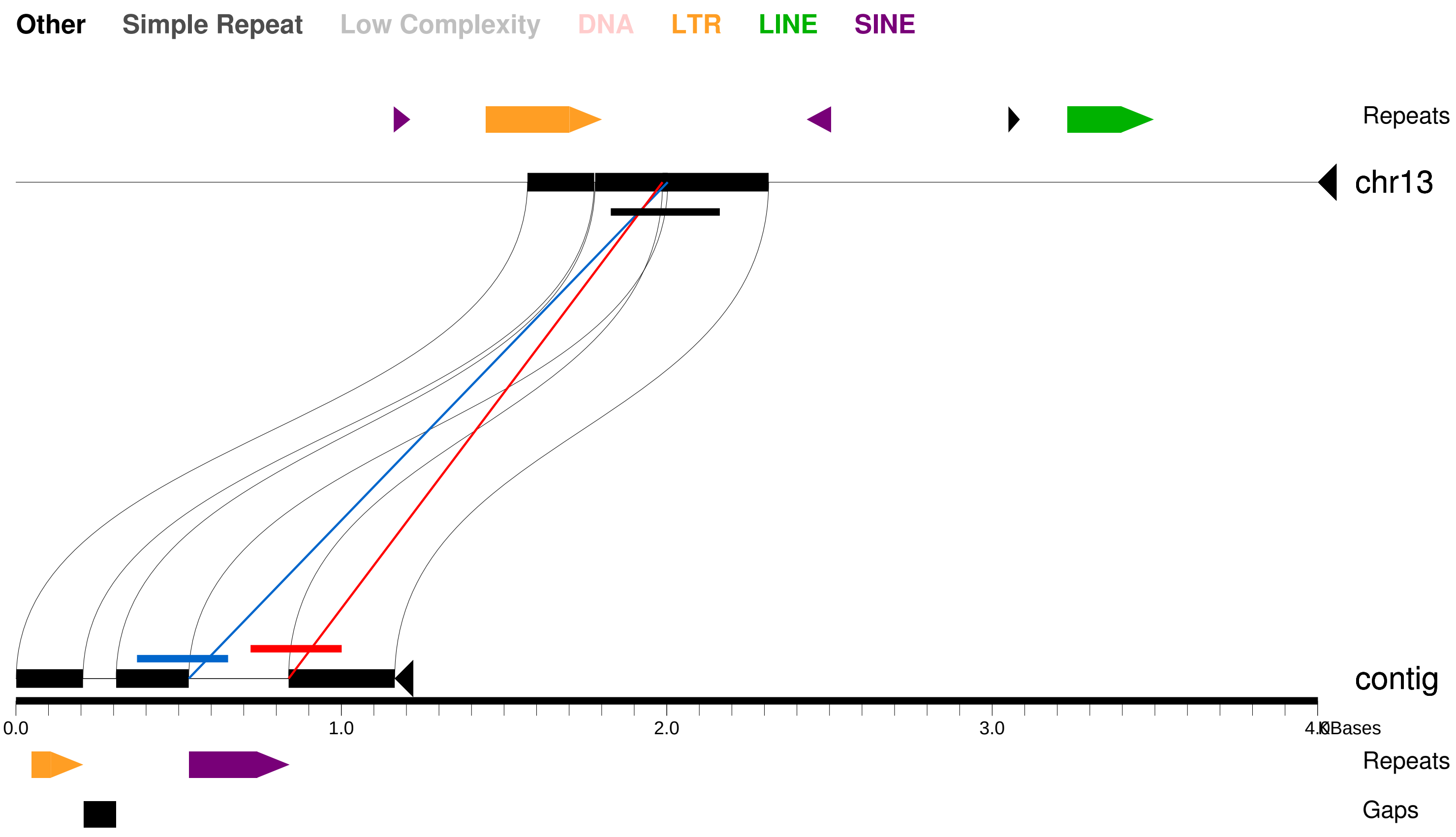

siteID: chr13_62588978 Scafftig3 + Breakpoint: chr13:62588959
chr13:62588913-62589018
Overlap Length: 14 INS size: 292 Genotyped: Yes

```
left    TACACACATTATTTTTATTCTCTCAAAAAAACAAGTTTT--TGTATGA----------------CCGTTC
chrom   TACACACATTATTTTTATTCTCTCAAAAAAACAAGTTTT--TGTATGA----------------CCGTTC
right   C-CGCCCGCC----TCGGCCTCCCAAAGTGCTGGGATTACAGGCGTGAGCCACCGCGCCCGGCCCCGTTC
        11*1*1*1111111*1111***1****1111111*1**1111*11***1111111111111111******

left    ATGTTTTTTTTTTTTTTTTGAGACGGAGTCTCGCTCTGTCGCCCAGGCTGGAGTGCAGTGGCGGGATC
chrom   ATGTTTTTACATGGCAT---GATGACCACT-GAATTGTAA--CATTCTGTTCT----TGGTGGA---
right   ATGTTTTTACGTGGCAT---GATGACCACT-GAATTGTAA--CATTCTGTTCT----TGGTGGA---
        ********22N*2222*222**2*2222**2*222***2222**22***222*2222***2**2222
```

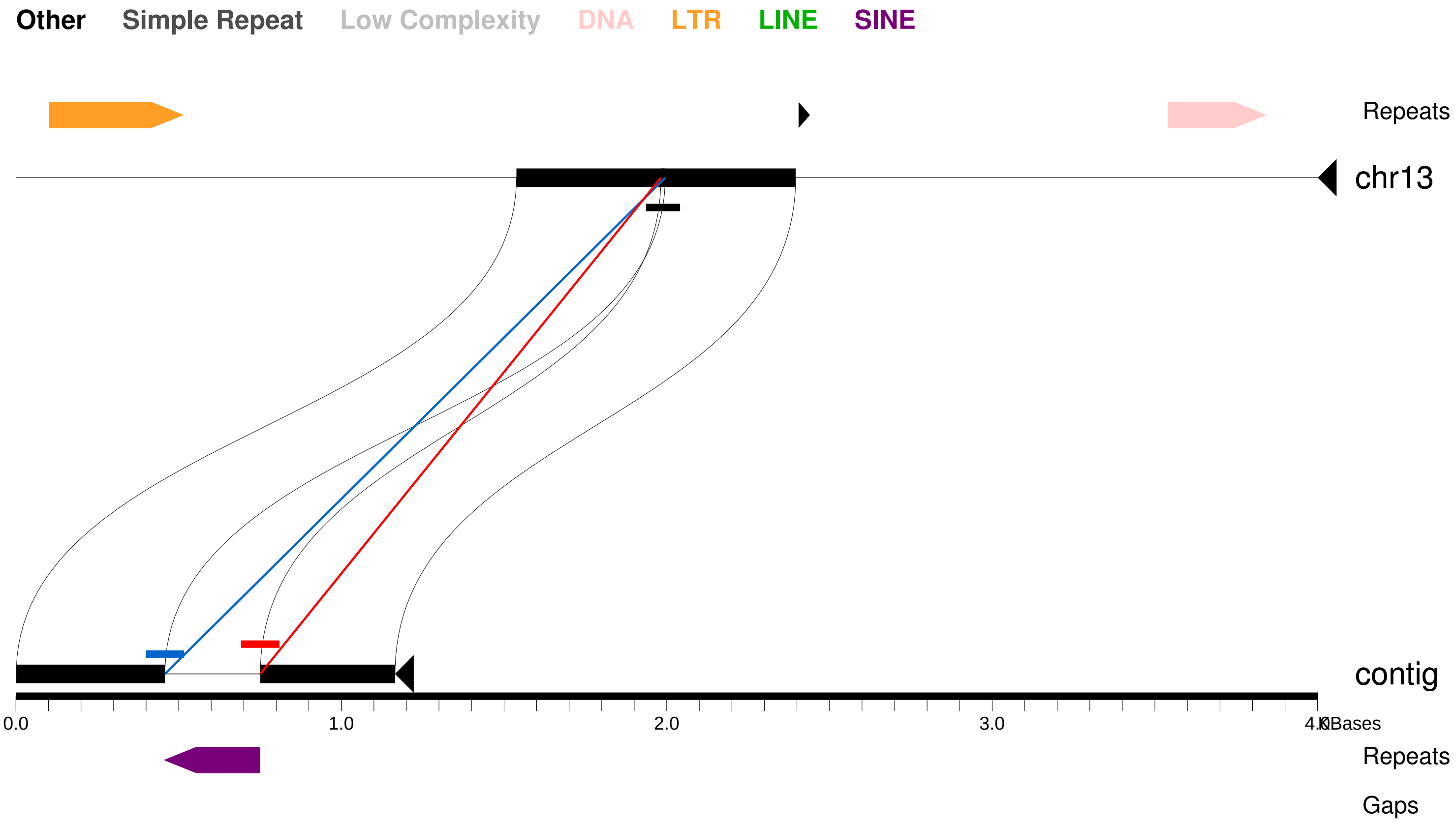

siteID: chr13_62721066 Scafftig2 - Breakpoint: chr13:62721066
chr13:62721015-62721125
Overlap Length: 9 INS size: 300 Genotyped: Yes

```
left   CCCT-ACTCC--TCAGAACCAATTTTCTGTGATAGGAC-----ATTTTTGTATTGTTATAAAGAAATAGG
chrom  CCCT-ACTCC--TCAGAACCAATTTTCTGTGATAGGTC-----ATTTTTGTATTGTTATAAAGAAATA--
right  ACTGCACTCCAGCCTGGGCGACAGAGCGAGACTCCGTCTCAAAAAAAAAAAAAAAAAAAAAAGAAATA--
       1*111*****111*1*11*1*11111*11111*11*2*11111*1111111*11111*1*********22

left   CCGGGCGCGGTGGCTCACGCCTGTAATCCCAGCACTTTGGGAGGC---CGAGGCGGGCGG
chrom  -CCTGAGTGATTTATAAAG-----AAA---AGGTATTTAATTGACTCACTATTCCGTAGG
right  -CCTGAGTGATTTATAAAG-----AAA---AGGTATTTAATTGACTCACTATTCCGTAGG
       2*22*2*2*2*222*2*2*22222**2222**222***2222*2*222*2*22*2*22**
```

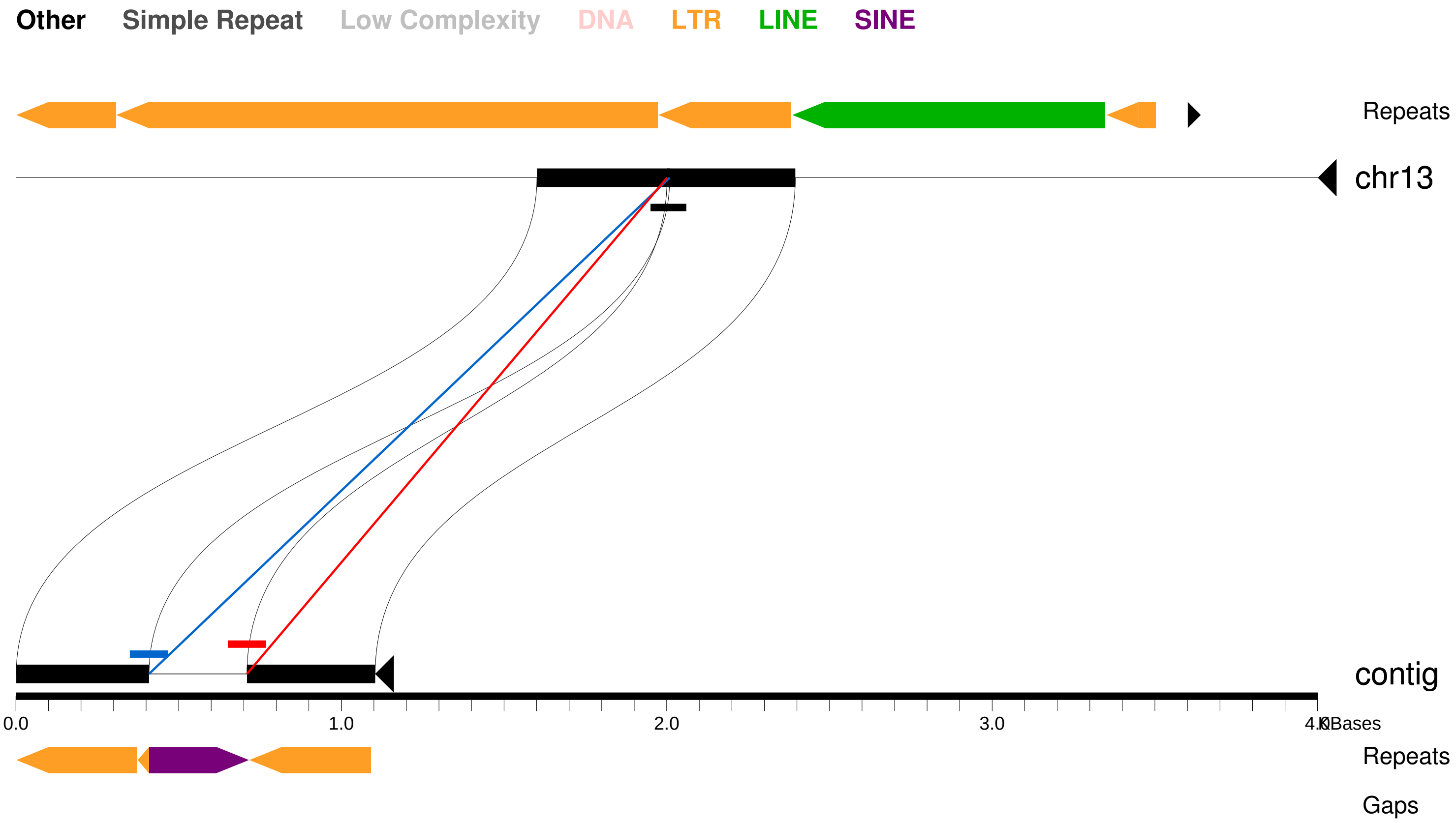

siteID: chr13_63488242 Scafftig3 - Breakpoint: chr13:63488266
chr13:63488219-63488325
Overlap Length: 13 INS size: 444 Genotyped: No

```
left    ATATATCTTCTGCAGTTTCACAAGCTAAATTGTCAACTTCAGGTC------------ATAAAAATCACTG
chrom   ATATATCTTCTGCAGTTTCACAAGCTAAATTGTCAACTTCAGGTC------------ATAAAAATCACTG
right   NNNNNNNNNNNNNNNNNNNNNNNNNNNNNNNNNNNNNNNNNNNNNNNNNNNNNNAAAAAAAAAATCACTG
        111111111111111111111111111111111111111111111111111111111*1***********

left    CTGGCCGGGC-GCGGTGGCTCACGCCTGTAATCCCAGCACTTTGGGAGGCCGAGGCGGGCGG
chrom   CTATTTTGAAAGTGGATGCTGAC------ATGGTCAGCGGTTA-------CCAGTTCCTCAT
right   CTATTTTGAAAGTGGATGCTGAC------ATGGTCAGCGGTTA-------CCAGTTCCTCAT
        **22222*222*2**22***2**222222*2222****22**22222222*2**22222*22
```

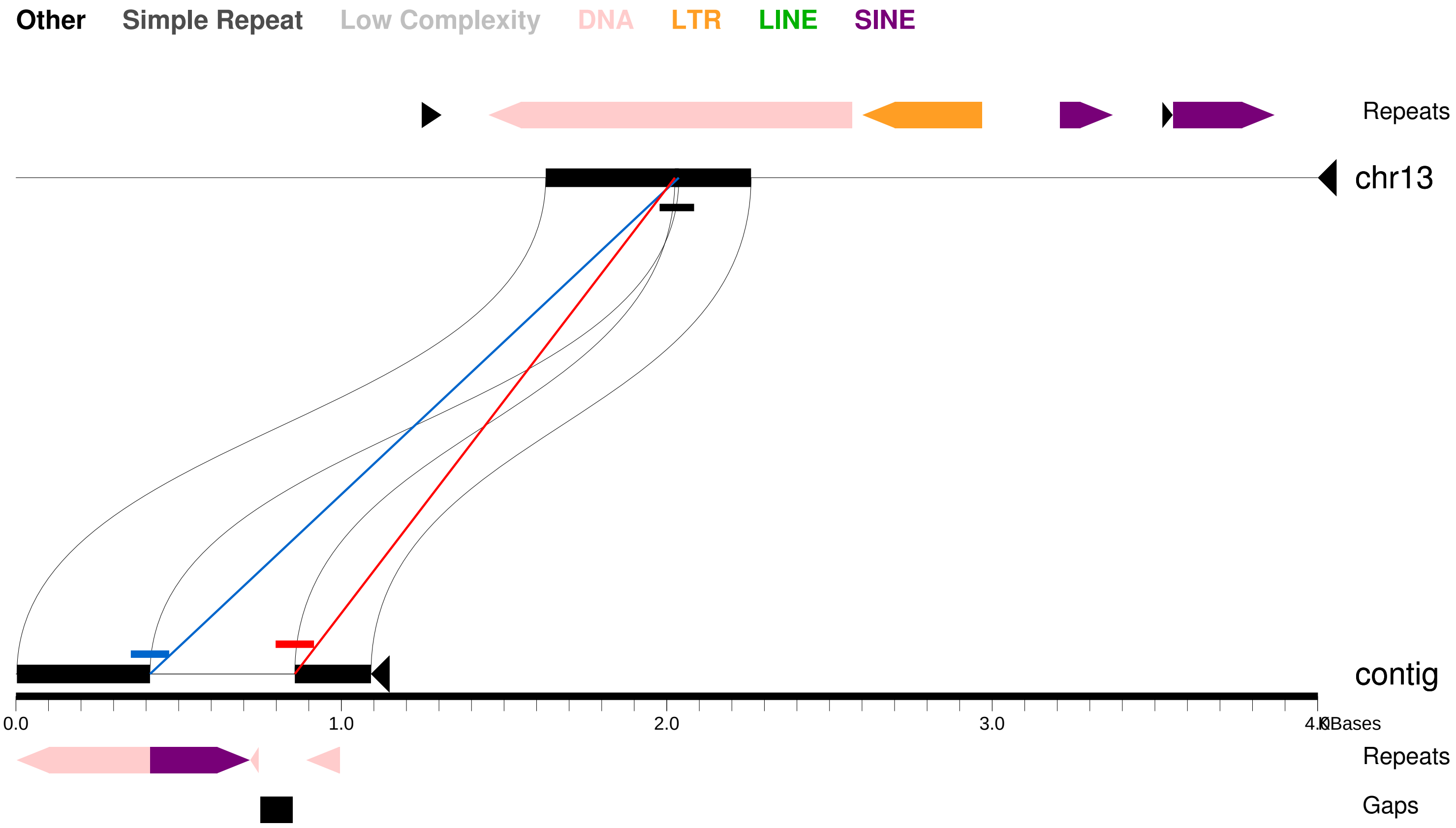

siteID: chr13_64964519 Scafftig3 - Breakpoint: chr13:64964520
chr13:64964476-64964579
Overlap Length: 16 INS size: 439 Genotyped: No

```
left     TTAATGTAATATTTCCTGTTTGATGTTGCCCTACTAGAGCCTAT---------------AAGAACTGTGG
chrom    TTAATGTAATATTTCCTGTTTGATGTTGCCCTACTAGAGCCTAT---------------AAGAACCGTGG
right    NNNNNNNNNNNNNNNNNNNNNNNNNNNNNNNNNNNNNNNNNNNNNNNNNNNNNNNNNNNAAGAACTGTGG
         1111111111111111111111111111111111111111111111111111111111******N****

left     TACTGGCCGGGCGCGGTGGCTCACGCCTGTAATCCCAGCACTTTGGGAGGCCGAGGCGGGCGGA
chrom    TACTGATGTTTTAATAGGTCTCAC------ATTCCCAGTGCCTA---ATACAGTGCCT------
right    TACTGATGTTTTAATAGGTCTCAC------ATTCCCATTGCCTA---ATACAGTGCCT------
         *****222222222222*2*****222222*2*****122*2*2222*22*2*2*2*2222222
```

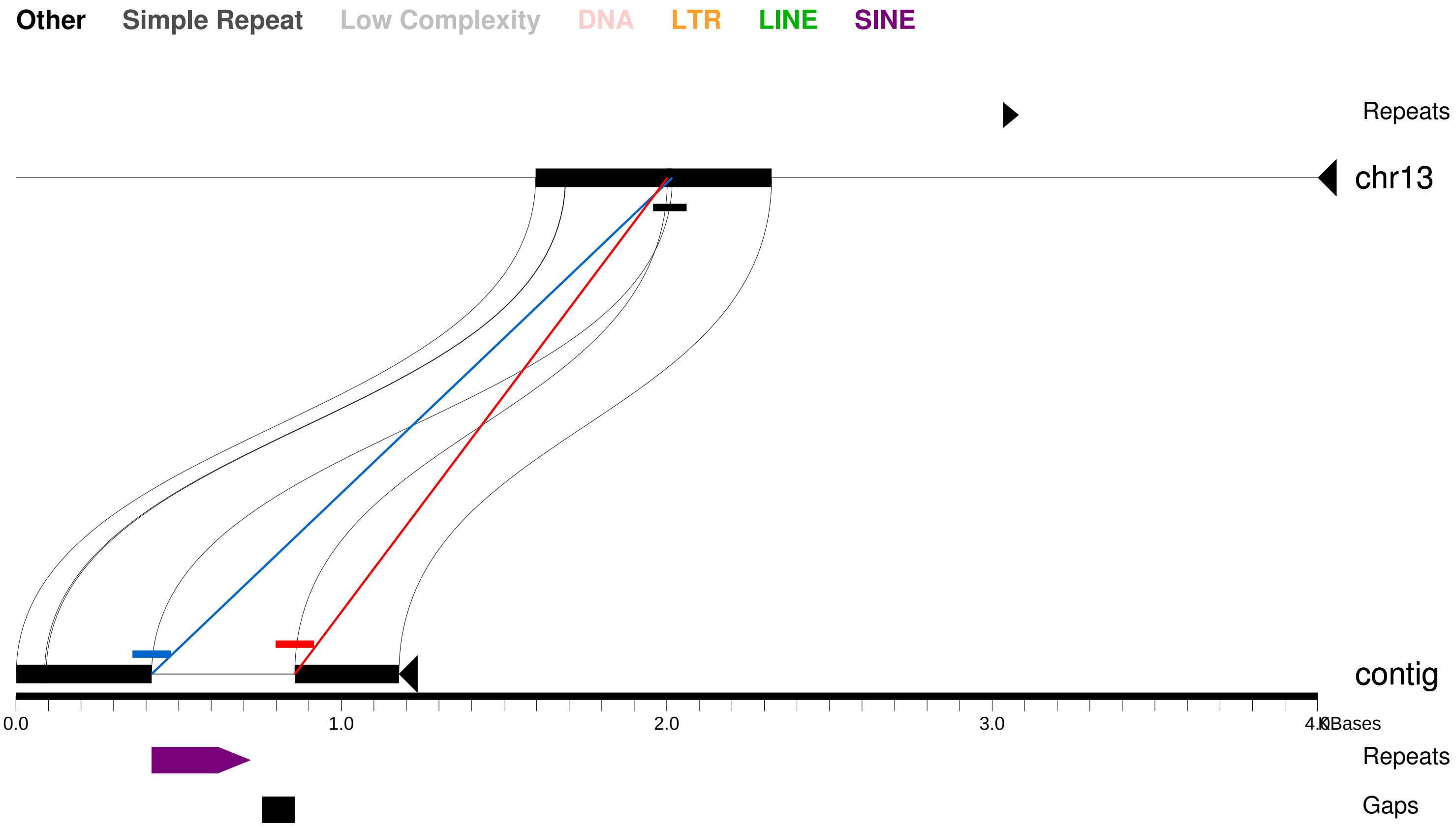

siteID: chr13_65892986 Scafftig1 + Breakpoint: chr13:65892950
chr13:65892891-65893009
Overlap Length: 1 INS size: 419 Genotyped: No

```
left    GAAGACACTCTTTTGCAGTTATATATTAGAAAAGATTAAATGCTTTTTTTCAGTCGTCA---CNNNNNNN
chrom   GAAGACACTCTTTTGCAGTTATATATTAGAAAAGATTAAATGCTTTTTTTCAGTCGTCA---CTCAGACA
right   TCC-ACCCGCCTCGGCC-TCCCAAAGT-GCTGGGACTACAGGCGTGAGCCACCGCGCCCGGCCTCAGACA
        1111**1*1*1*11**11*111*1*1*1*1111**1**1*1**1*111111111**1*1111*2222222

left    NNNNNNNNNNNNNNNNNNNNNNNNNNNNNNNNNNNNNNNNNNNNNNNNNNNN
chrom   CTTCTTAAACTAGAAAGCCTGGGCTCATTATAGTTGAAGATAATGATTCTCT
right   CTTCTTAAACTAGAAAGCCTGGGCTCATTATAGTTGAAGATAATGATTCTCT
        2222222222222222222222222222222222222222222222222222
```

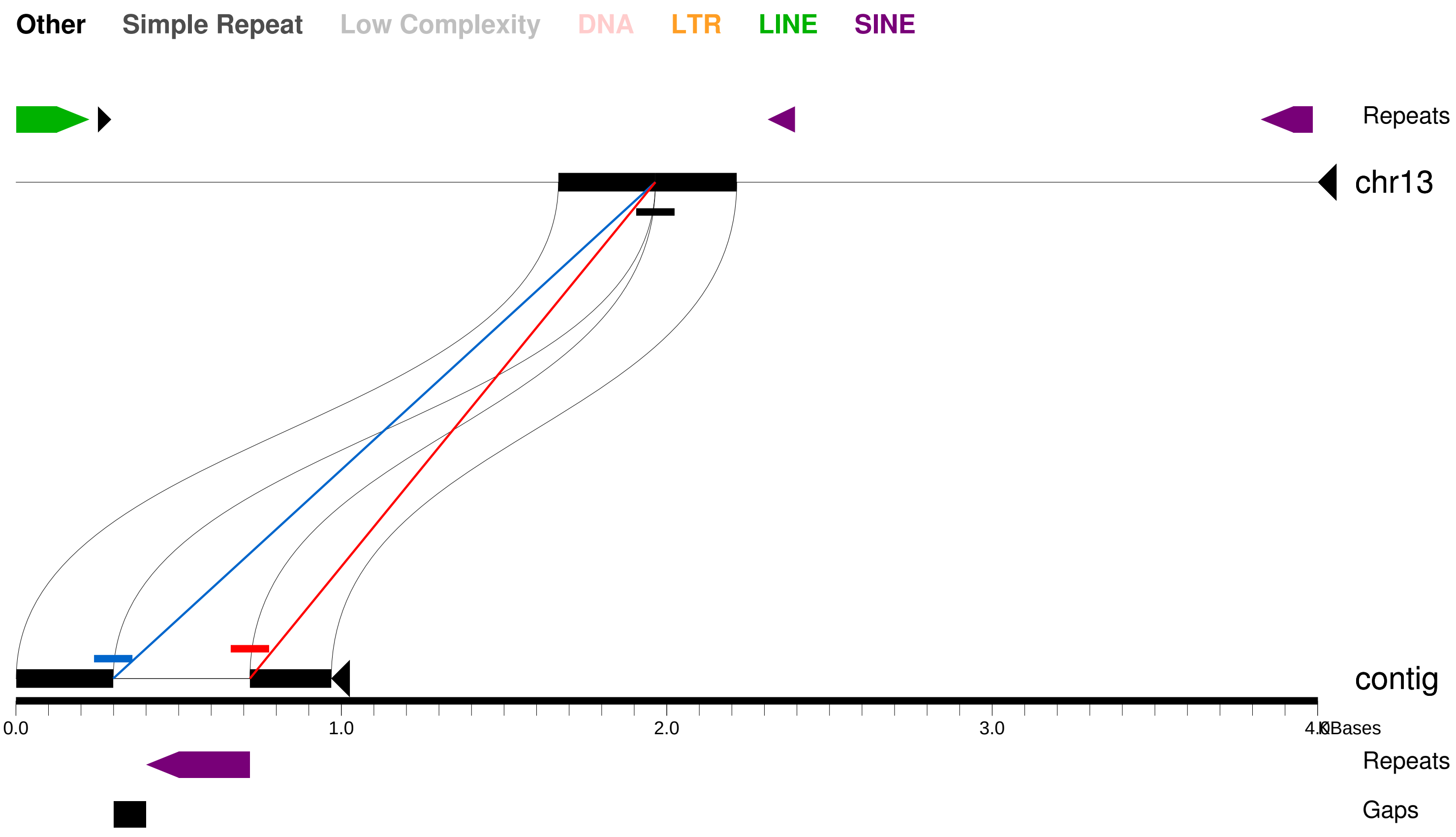

siteID: chr13_66475610 Scafftig1 - Breakpoint: chr13:66475596
chr13:66475436-66475756
Overlap Length: 1 INS size: 419 Genotyped: No

```
left   ---------------------------CTACTAGATGATTTTGCTTTGTGGCTGTCGTAAGGCTGACAAAA
chrom  TTCCCTTCCTTTTATTTTTTGTGCTTCTACTAGATGATTTTGCTTTGTGGCTGTCGTAAGGCTGACAAAA
right  TT-------TAGTAGAGACGGGGTTTCACCGTG-----TTAGCCAGGATGGTCTCGATCTCCTGACC---
       22       2NN22NNNNNN2N2N22*11*11*11111**1**111*11*1*1***11111*****1111

left   GTTTTTATAGTTACAACTGATTATTTTAAACTAAAAACAACTTAACTCTAATGGCAAATAAAATAAAACA
chrom  GTTTTTATAGTTACAACTGATTATTTTAAACTAAAAACAACTTAACTCTAATGGCAAATAAAATAAAACA
right  -TCGTGATCCGCCCGCCTCGGCCTCCCAAAGTG-------CTGGGAT-TACAGGCG-------TGAGCCA
       1*11*1**11111*11**11111*111***1*11111111**1111*1**11***11111111*1*11**

left   CTTTCCATTTTTACTCCATTCNNNNNNNNNNN--------------------------------------
chrom  CTTTCCATTTTTACTCCATTCCCTACATACATATTTTAAATTTCTGATGTCACAATAAACAAAAATAAAA
right  CCGCGC---------CCGGCCCCTACATACATATTTTAAATTTCTGATGTCACAATAAACAAAAATAAAA
       *1111*111111111**111*22222222222222222222222222222222222222222222222222

left   ----------------------------------------------------------------------
chrom  ATAAATTAATATTCCATATTTTTAAATAATAATTTTGTCTTGAATCCTTTACACAAAAGATACAAGTGAT
right  ATAAATTAATATTCCATATTTTTAAATAATAATTTTGTCTTGAATCCTTTACACAAAAGATACAAGTGAT
       2222222222222222222222222222222222222222222222222222222222222222222222

left   -----------------------------------------
chrom  TTATACACCACCATTACAGTAATAGAGTATTCTCAATTTGA
right  TTATACACCACCATTACAGTAATAGAGTATTCTCAATTTGA
       22222222222222222222222222222222222222222
```

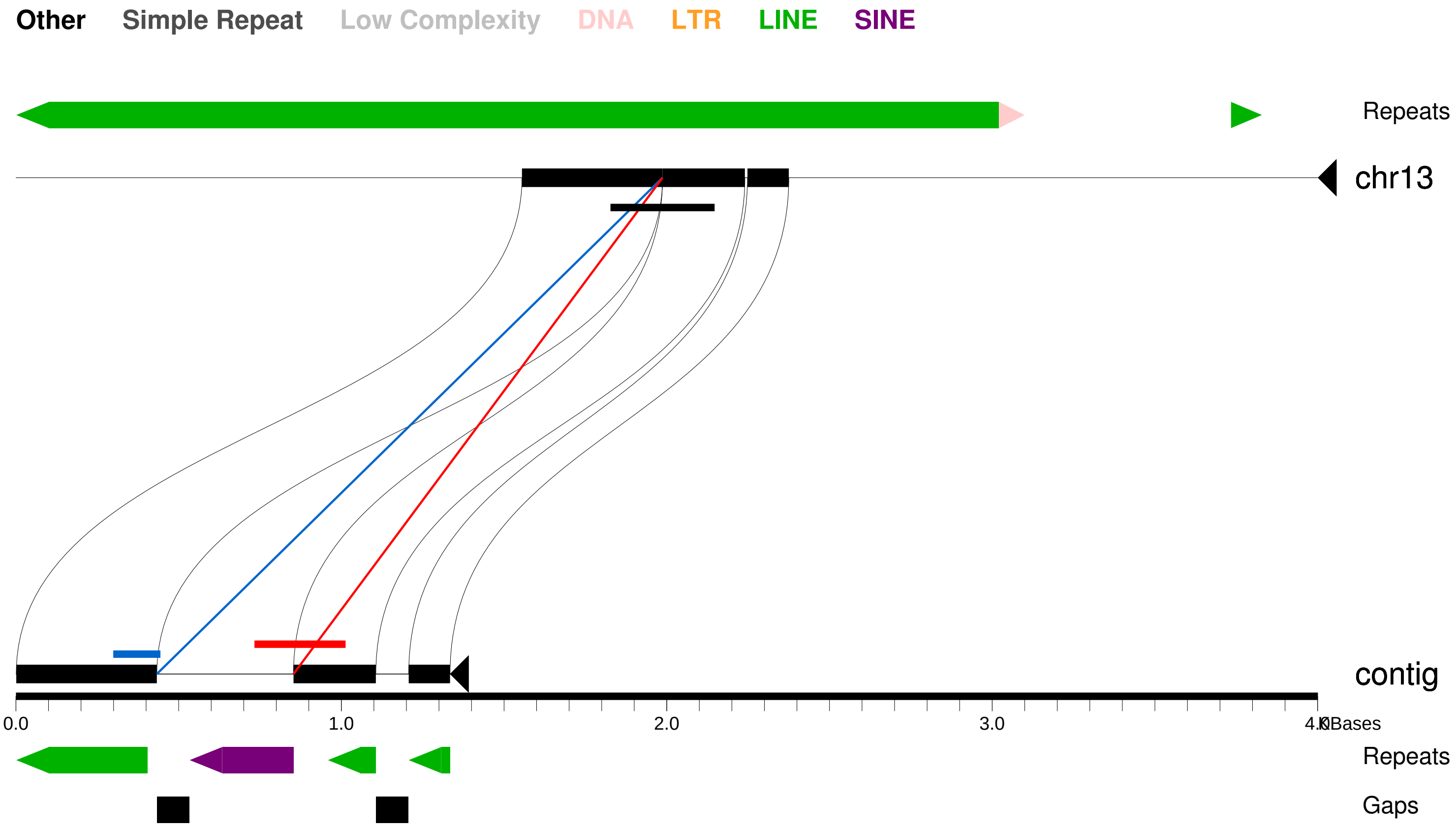

siteID: chr13_67903458 Scafftig9 - Breakpoint: chr13:67903455
chr13:67903412-67903514
Overlap Length: 7 INS size: 227 Genotyped: Yes

```
left    ATTCTGGCCTGTTTTTCCATGGTATTCTCTAACACCT--TTTC------TT--------TATCTATTTTT
chrom   ATTCTGGCCTGTTTTTCCATGGTATTCTCTAACACCT--TTTC------TT--------TATCTATCTGT
right   CGCCCGGCTAATTTTTTTGTATTTTTAGTAGAGACGGGGTTTCACCGTGTTAGCCAGGATATCTATCTGT
        111*1***111*****111*11*1**11111*1**1111****111111**11111111*******2*2*

left    TTTTTTTTTTTTTTTTTTTTTTTTTTTGAGACGGAGTCTCGCTGTCGCCCAGGCTGGAGTGCAGT
chrom   ATTCTTAACAT---------CCTTTATGAACCAGA---TCAACTTCAATCTT-CTGCAG---AAA
right   ATTCTTAACAT---------CCTTTATGAACCAGA---TCAACTTCAATCTT-CTGCAG---AAA
        2**2**2222*22222222222***2***22*2**222**2222**222*222***2**222*22
```

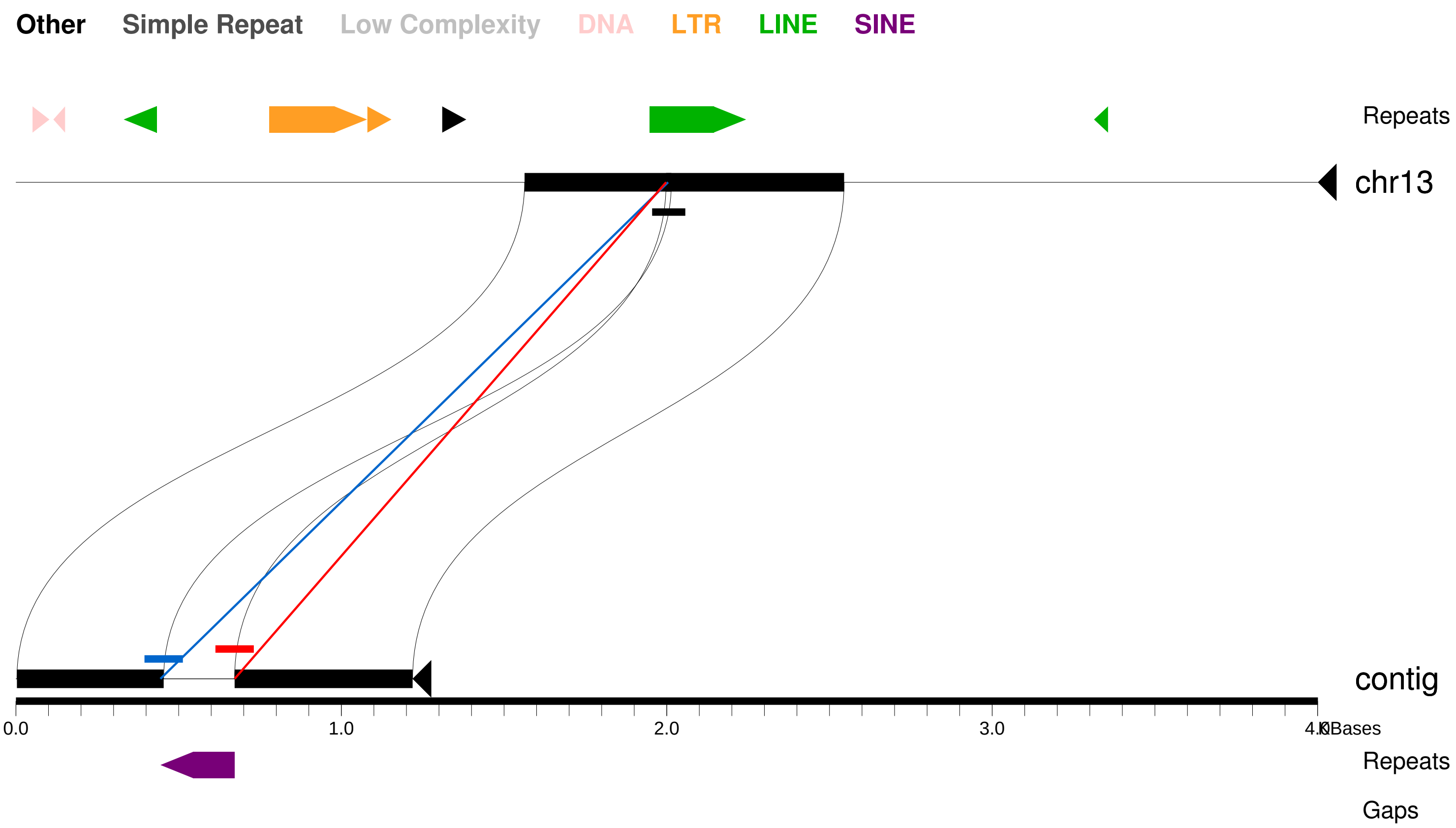

siteID: chr13_67967172 Scafftig2 + Breakpoint: chr13:67967152
chr13:67967108-67967211
Overlap Length: 16 INS size: 314 Genotyped: Yes

```
left   -------TCTGTGGAACCCAACCATTTCGC-TTATTTTCATAAATGCCACCA---------ACATTCAGG
chrom  -------TCTGTGGAACCCAACCATTTCGC-TTATTTTCATAAATGCCACCA---------ACATTCAGG
right  CCACCCGCCTCGGCCTCCCAAAGTGCTGGGATTACAGGCGTGA--GCCACCGCGCCCGGCCACATTCAGG
       11111111**11*111*****11111*1*11***1111*1*1*11******1111111111*********

left   CTTTATTTTTTTTTTTTTATTTTATTTTTTTTTTTGAGACGGAGTCTCGCTCTGTCGCCCAGGCCGGA
chrom  CTTTATTACCTCTTACATTATAACTGGTTTACTTCCCCAAACTCTAGC-CTC--------------
right  CTTTATTACCTCTTACATTATAACTGGTTTACTTCCCCAAACTCTAGC-CTC--------------
       *******222*2**2222*2*2*2*22***22*2222*22*2***2**2**222222222222222
```

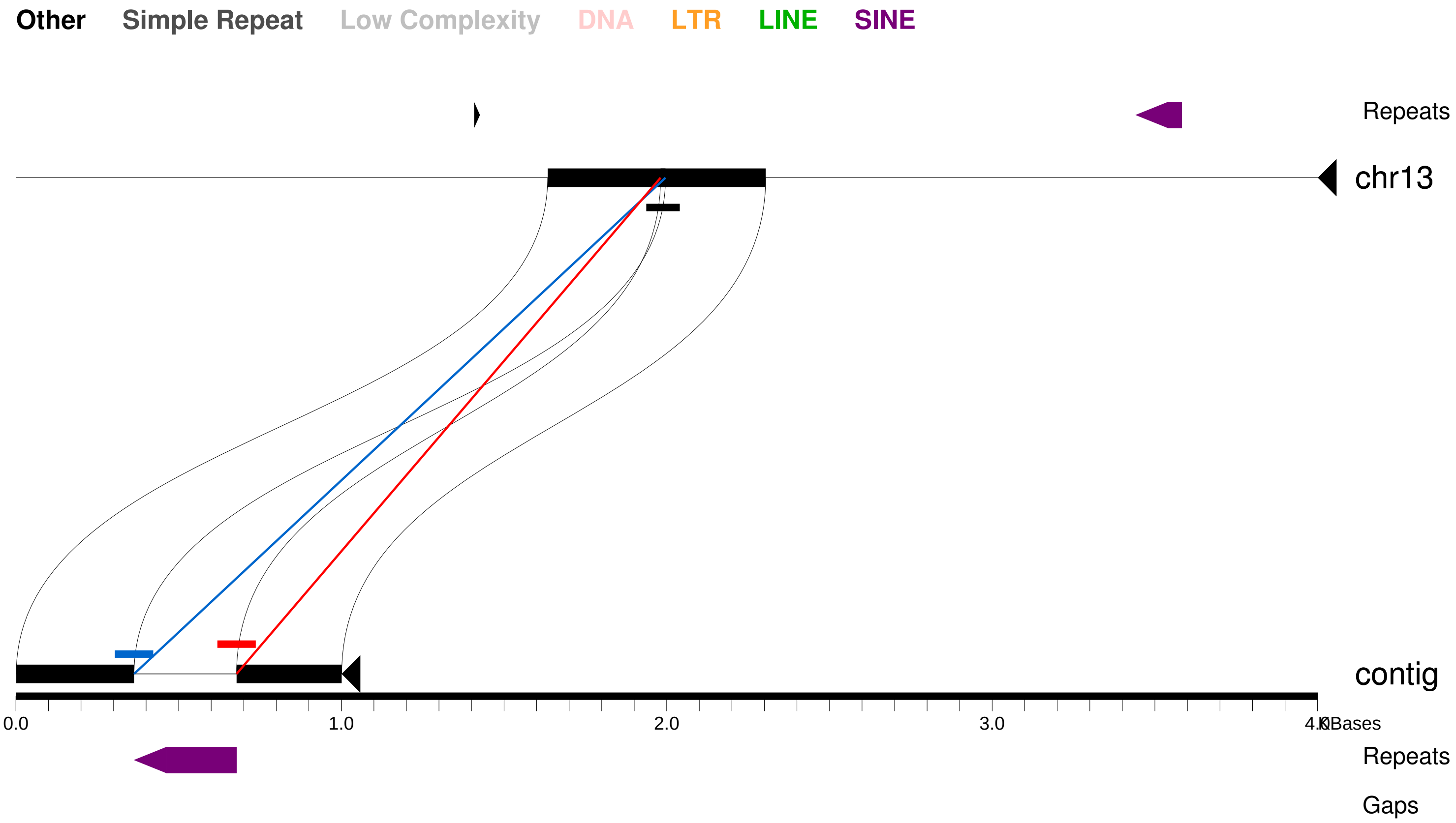

siteID: chr13_68097899 Scafftig2 - Breakpoint: chr13:68097897
chr13:68097864-68097956
Overlap Length: 27 INS size: 312 Genotyped: No

```
left    CT-----CATCTATCTACCACAGATATTCATATT----GCTT------------------TCAAACTTTT
chrom   CT-----CATCTATCTACCACAGATATTCATATT----GCTT------------------TCAAACTTTT
right   CCACCCGCCTCGGCCTCCCAAAG-TGCTGGGATTACAGGCGTGAGCCACCGCGCCCGGCCTCAAACTTTT
        *111111*1**111**1***1**1*11*111***1111**1*111111111111111111**********

left    TTAAAATGTAATAATTGNNNNNNNNNNNNNNNNNNNNNNNNNNNNNNNNNNNNNNNNNNNNNNNNNNNN
chrom   TTAAAATGTAATAATTGCTATACACCCTTGACCATATGCCTGCTATTTTA--------------------
right   TTAAAATGTAATAATTGCTATACACCCTTGACCATATGCCTGCTATTTTA--------------------
        ****************2222222222222222222222222222222222222222222222222222222

left    NNNNNN
chrom   ------
right   ------
        222222
```

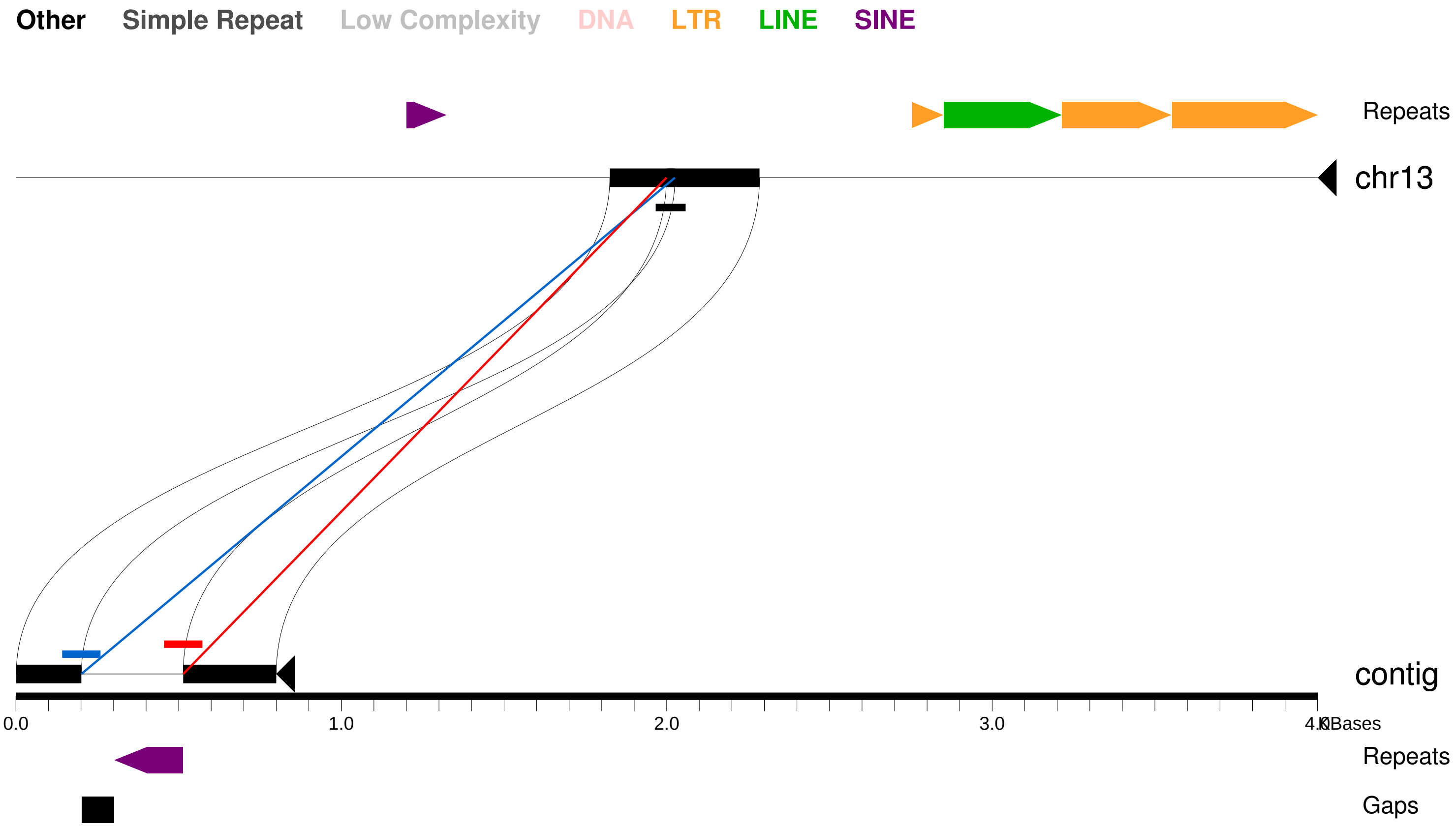

siteID: chr13_69670749 Scafftig6 - Breakpoint: chr13:69670752
chr13:69670592-69670924
Overlap Length: 13 INS size: 145 Genotyped: Yes

```
left   ---------ATGCTAATAACTGGCAATAAATTTAAAAATAGGTTAAATGGTGAAATCATTTATTAAACAA
chrom  TTCTGGTGGATGCTAATAACTGGCAATAAATTTAAAAATAGGTTAAATGGTGAAATCATTTATTAAACAA
right  TTTTTTTTTTT-------------------TTTGAGACGGAGTC-----------TCGCTCTGTCGCCCA
       22N2NN2NN1*1111111111111111111***1*1*1111**111111111111**11*111*111*1*

left   TGTTGAACAAATTTATCTACTGA-AGTACTTTTCAGA-CATTTTAAGATGTGACTGTGTTGTATATTTTA
chrom  TGTTGAACAAATTTATCTACTGA-AGTACTTTTCAGA-CATTTTAAGATGTGACTGTGTTGTATATTTTA
right  GGCTGGAG-------TGCAGTGGCAGGA--TCTCGGCTCACTGCAAGCTCCGCCTCCCGGGTTCACGCCA
       1*1**1*11111111*11*1**11**1*11*1**1*11**1*11***1*11*1**11111**11*1111*

left   AGTGACTACAGTCAGTATTTGTCATTCATTCTTTTTT--------TTT-------TTTTTTTTTTTTTTT
chrom  AGTGACTACAGTCAGTATTTGTCATTCATTCTTTTACAGAGAGAATGTGCAAGCATTTTTCTTCTTTTAA
right  TTCTCCTGCC-TCAGCCTCCCACATTCATTCTTTTACAGAGAGAATGTGCAAGCATTTTTCTTCTTTTAA
       11111**1*11****11*1111*************2222222222*2*2222222*****2**2****22

left   TTTTTTTTTTTTTTTTTGAGACGGAGTCTCGCTCT------GTCGCCCAGG---------CTGGAGTGCA
chrom  TCAAGTACATGTATTTAGCACAGCAATGAAGCTATAATATAGTCAAAAAGAGAATTTTCTCTAAAATATT
right  TCAAGTACACGTATTTAGCACAGCAATGAAGCTATAATATAGTCAAAAAGAGAATTTTCTCTAAAATATT
       *2222*22212*2***2*2222*2*2*222***2*222222***2222**2222222222**22*2*222

left   GTGGCAGG-ATCTCGGCT-CACT--GCAA--GCTCCGCCTCCCGGGTT-------
chrom  CTTCCAAATAACTCACCTACATTAAGAAAATGCTGAGCACCAATTGAGACACCAA
right  CTTCCAAATAACTCACCTACATTAAGAAAATGCTGAGCACC--------------
       2*22**222*2***22**2**2*22*2**22***22**22*NNNN1NN
```

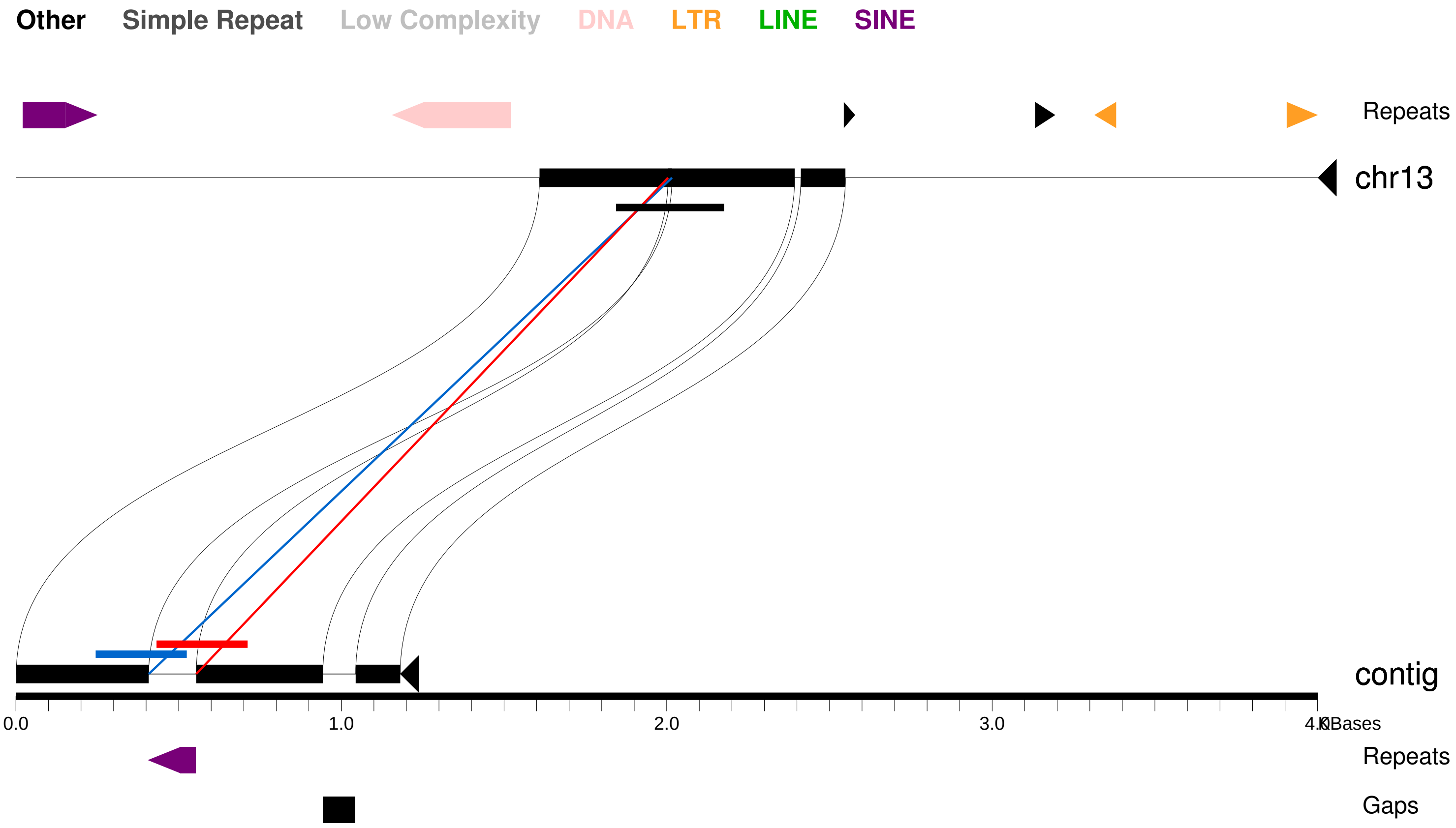

siteID: chr13_70056940 Scafftig1 - Breakpoint: chr13:70056950
chr13:70056790-70057121
Overlap Length: 12 INS size: 315 Genotyped: Yes

```
left   ------TCATGCGTCACGTAGTTCTCGTGCCATGGTTTTCAGCTCCATCAAGTCATTTGAGGTCTTCTCT
chrom  GCTTGTTCATGCATCACGTAGTTCTCGTGCCATGGTTTTCAGCTCCATCAAGTCATTTGAGGTCTTCTCT
right  TTTAGTAGAG-----ACGGGGTT-TCA--CCGTGTTAGCCAGG---ATG--GTC--TCGA-----TCTCC
       NN2N2211*111N11***11***1**111**1**1*111***1111**111***11*1**11111****1

left   ACACTGTTTATTCTAGTTAGCCATTCATCTCATCTTTTTTCAAGGTTTTTAGCTTCTTTGTGATGGGTTC
chrom  ACACTGTTTATTCTAGTTAGCCATTCATCTCATCTTTTTTCAAGGTTTTTAGCTTCTTTGTGATGGGTTC
right  TGAC--------CTCGTGATCCGCCCACCTCGGCCTCC--CAAAGTGCTGGGATTACAAGCG-TGAG---
       11**11111111**1**1*1**111**1***11*1*1111***1**11*11*1**1111*1*1**1*111

left   AAACATCCTCCTTTAGCTTGGAGAAGTTTATT--TATTTAT-----------TTATTTATTTATTTAT--
chrom  AAACATCCTCCTTTAGCTTGGAGAAGTTTATTATTACCGATCGTCTGAAGCCTTCTCTCTCAACTCTTCA
right  ---CCACCGCGCCCGGCC--GAGAAGTTTATTATTACCGATCGTCTGAAGCCTTCTCTCTCAACTCTTCA
       111*11**1*11111**111************22**222**22222222222**2*2*2*22*2*22*22

left   ---TTATTTTTTGAGACGGAGTCTCGCTCTGTGGCCC---AGGCG------------GGAGT-GCAGTGG
chrom  AAGTCATTCTCTGACA---AGCTTTGTTCTGTTGCTGGTGAGGAGCTGCATTCCTTTGGAGGAGAAGAGG
right  AAGTCATTCTCTGACA---AGCTTTGTTCTGTTGCTGGTGAGGAGCTGCATTCCTTTGGAGGAGAAGAGG
       222*2***2*2***2*222**22*2*2*****2**22222***2*222222222222****22*2**2**

left   TGC-------------AATCTCGGCT------CACTGCAAGCTCCGCCTCCAGGGTTCAC
chrom  TGCTCTGATTTTTAGAATTTTCAGCTTTTCTGCTCTGGTTTCTCCCCATC-----TTTGT
right  TGCTCTGATTTTTAGAATTTTCAGCTTTTCTGCTCTGGTTTCT-----------------
       ***2222222222222*2*2**2***222222*2***2222**11N1N112222211NNN
```

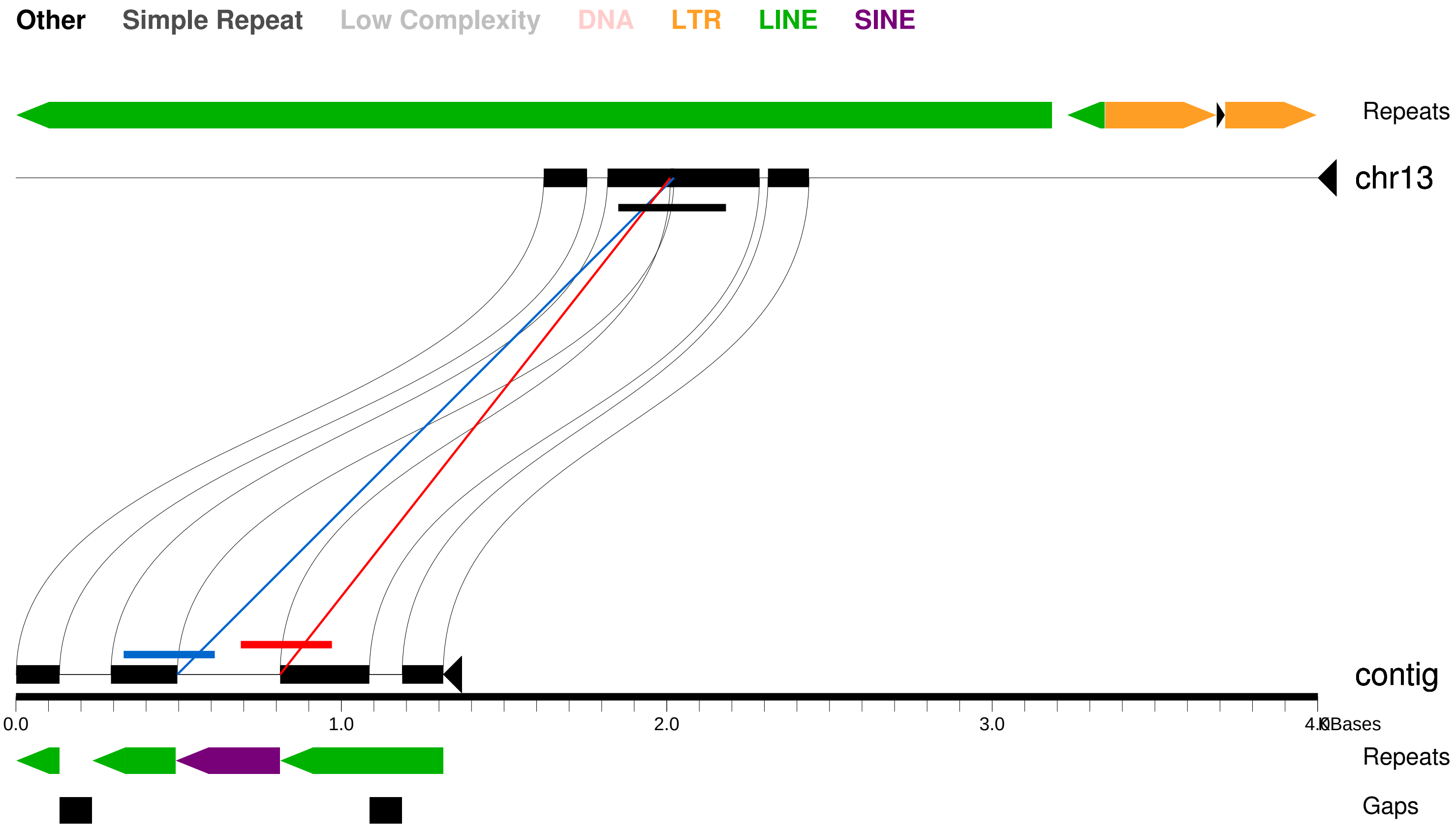

siteID: chr13_70264426 Scafftig1 - Breakpoint: chr13:70264397
chr13:70264342-70264456
Overlap Length: 5 INS size: 409 Genotyped: No

```
left    TTGGTGTTTCACAAAGTTTAATAAGCCACATTAGGGTATCTGTTAAAAAAACAAG----ATCTCGGCCGG
chrom   TTGGTGTTTCACAAAGTTTAATAAGCCACATTAGGGTATCTGTTAAAAAAACAAG----ATCTCATTTAG
right   NNNNNNNNNNNNNNNNNNNNNNNNNNNNNNNNNNNNNNNNNNNNNNNNNNNNNNNNNNNNATCTCATTTAG
        111111111111111111111111111111111111111111111111111111111111*****22222*

left    GCGCGGTGGCTCACGCCTGTAATCCCAGCACTT-TGGGAGGCCGAGGCGGGTGG
chrom   TAGCTCTGATTGGGCCCTCAGATAC-AGCATTTCTAATAAGC----TCCTAGGA
right   TAGCTCTGATTGGGCTCTCAGATAC-AGCATTTCTAATAAGC----TCCTAGGA
        22**22**22*22221**222**2*2****2**2*222*2**22222*2222*2
```

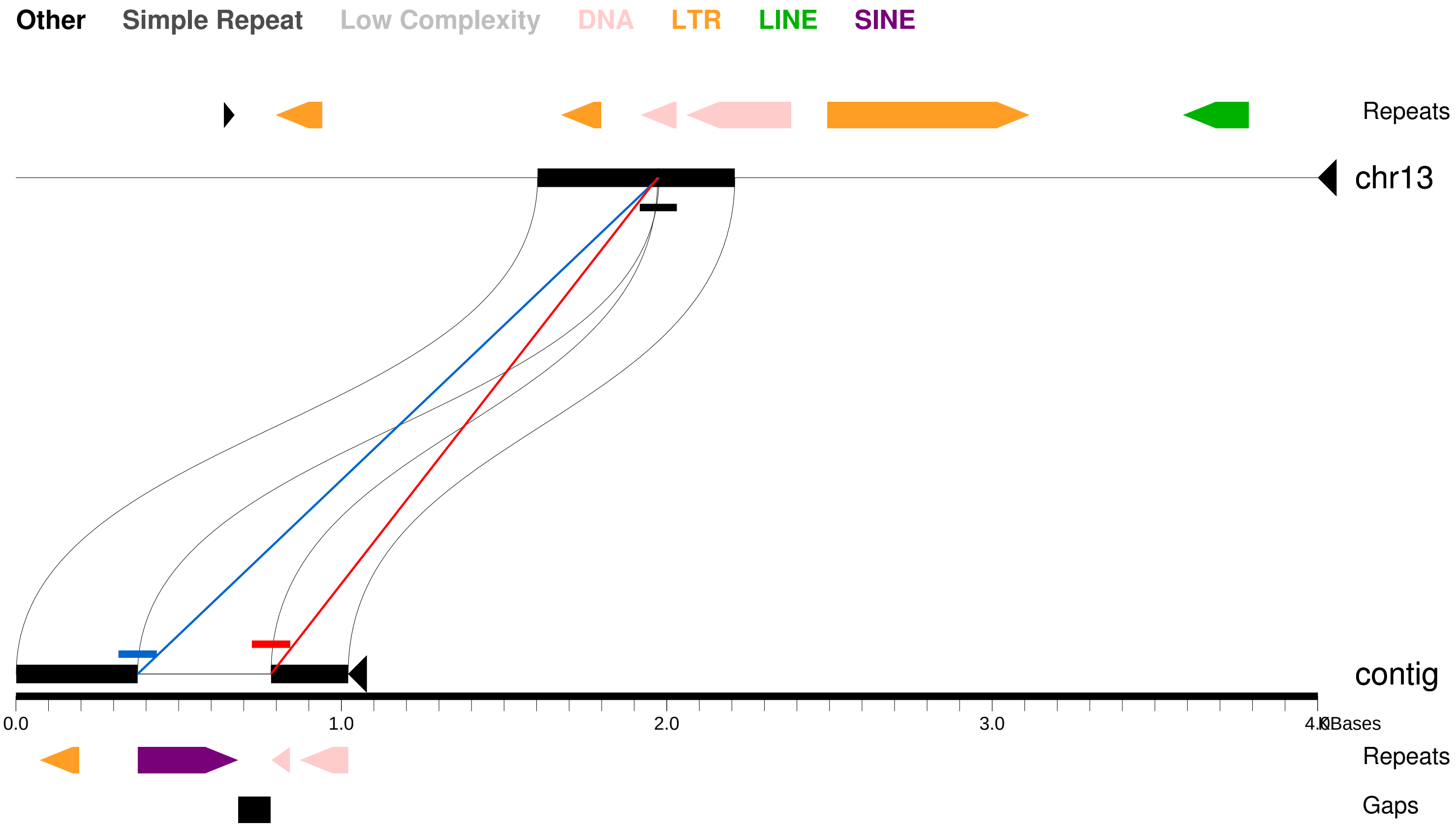

siteID: chr13_71041852 Scafftig2 + Breakpoint: chr13:71041864
chr13:71041818-71041923
Overlap Length: 14 INS size: 468 Genotyped: No

```
left   CTGGAATGAATGTATATGTCAAAATCAACTAATAGTAACTCTGC-------------ATATAAGTCATCC
chrom  CTGGAATGAATGTATATGTCAAAATCAACTAATAGTAACTCTGC-------------ATATAAGTCATCC
right  NNNNNNNNNNNNNNNNNNNNNNNNNNNNNNNNNNNNNNNNNNNNNNNNNNNNNNAAAAAATAAGTCATCC
       111111111111111111111111111111111111111111111111111111111*1***********

left   ATAGCCGGGCGCGGTGGCTCACGCCTGTAATCCCAGC--ACTTTGGGAGGCCGAGGCGGGCGGA
chrom  ATATATAGT---GGTT-CTGACTTTTATGATCTATTTCTACTTTT----TCCAAAAT-------
right  ATATATAGT---GGTT-CTGACTTTTATGATCTATTTCTACTTTT----TCCAAAAT-------
       ***2222*2222***22**2**222*2*2***2222222*****222222**2*2222222222
```

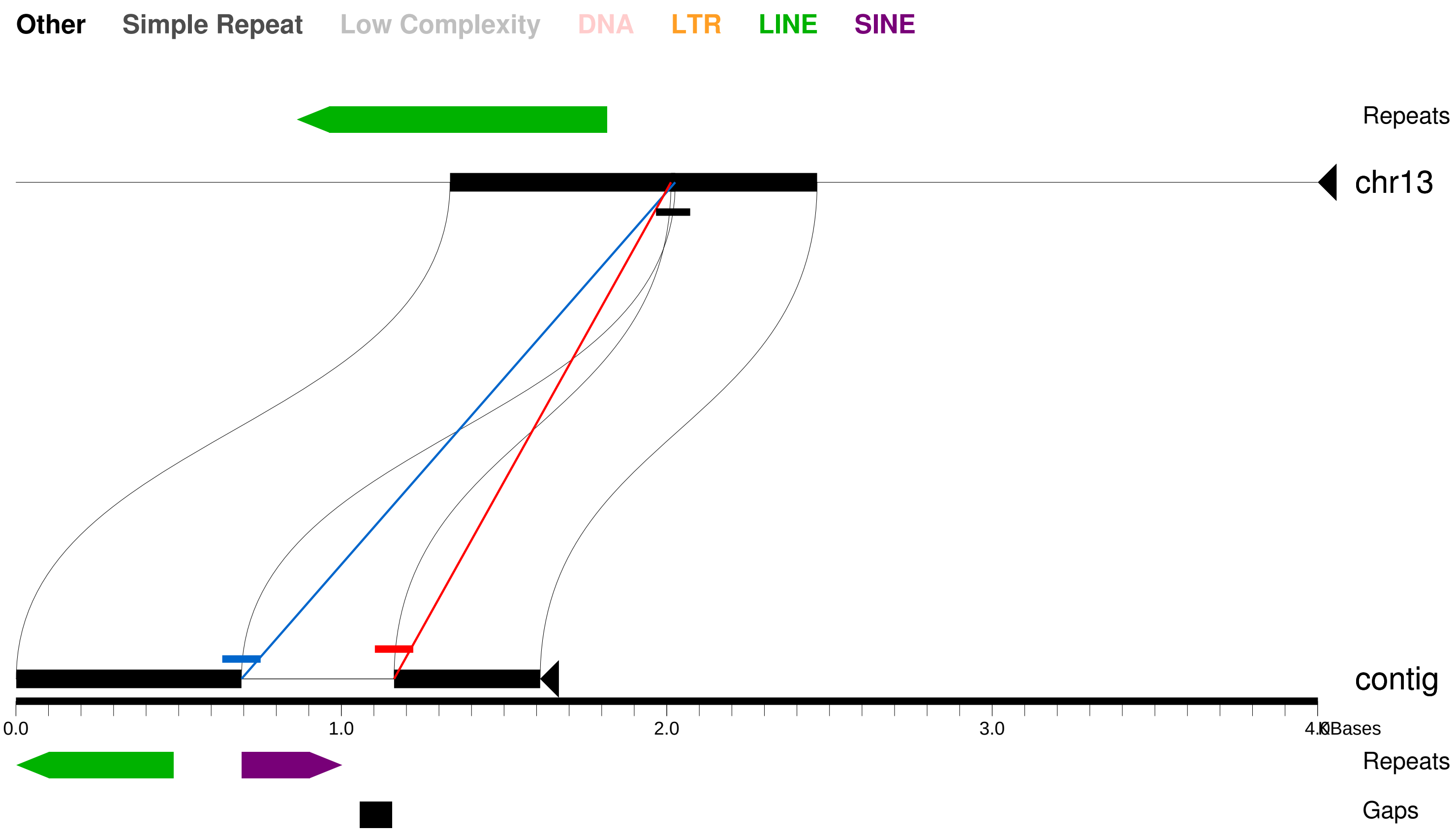

siteID: chr13_71142396 Scafftig2 - Breakpoint: chr13:71142394
chr13:71142234-71142564
Overlap Length: 11 INS size: 163 Genotyped: Yes

```
left   T---------AGCGTGTCTTGCCTGTGTACCTACAAAGCCACACTTGTGTGTTGTGTGCAATACGAAAAT
chrom  TCTTTTCCATAGCGTGTCTTGCCTGTGTACCTACAAAGCCACACTTGTGTGTTGTGTGCAATACGAAAAT
right  GC----CCAGGTCG------GACTGCGGAC-TGCAGTGGCGCAATCTCG-GCTCACTGCAAG-CTCCGCT
       12    222N11**111111*1***1*1**1*1**11*1*1**1*111*1*1*111*****11*11111*

left   GCCACAAGGTCAATCATATTCTT-TGAAAGTAAGACTCCAATATTTTTGTGGTGTTGGATATTGAAACAA
chrom  GCCACAAGGTCAATCATATTCTT-TGAAAGTAAGACTCCAATATTTTTGTGGTGTTGGATATTGAAACAA
right  TCC-CGGGTTCACGCC-ATTCTCCTGCC--TCAGCCTCCC--------GAGTAGCTGGG-------ACTA
       1**1*11*1***11*11*****11**1111*1**1****111111111*1*11*1***11111111**1*

left   TAATAGAACATTAAAAGAATTCCAAACTTCTTTT-----------------------TTT----------
chrom  TAATAGAACATTAAAAGAATTCCAAACTTCTTATCATGTACATGCTGAAGTAGTAGGTTTCACACAGCAG
right  CAGGCGCCCG-----------CCAAACTTCTTATCATGTACATGCTGAAGTAGTAGGTTTCACACAGCAG
       1*111*11*111111111111**********2*22222222222222222222222***2222222222

left   -TTTTTTTTTTTTTTTTTTTGAGACGGAGTCTCGCTCTGTCGCCCAGGTCGGAC-TGCGGACTGCAGTGG
chrom  ATGTTTTTCTTTCATTTTATGAATAATTGTATTATACATGGTACAATATAATACATGTACATTATACATG
right  ATGTTTTTCTTTCATTTTATGAATAATTGTATTATACATGGTACAATATAATACATGTACATTATACATG
       2*2*****2***22****2***222222**2*2222*222222*2*22*222**2**222*2*22*222*

left   CGCA----ATCTCGGCTCACTGCAAGCTCCGCTTCCCGGGTTCACGCCAT---
chrom  TACATAGTATAAAGAATATATAGAATATGTAAATATAGTATTTACAT-ATAGA
right  TACATAGTATAAAGAATATATAGAATATGTAAATATAGTATTTA---------
       22**2222**222*22*222*22**22*22222*222*22**2*1NN211
```

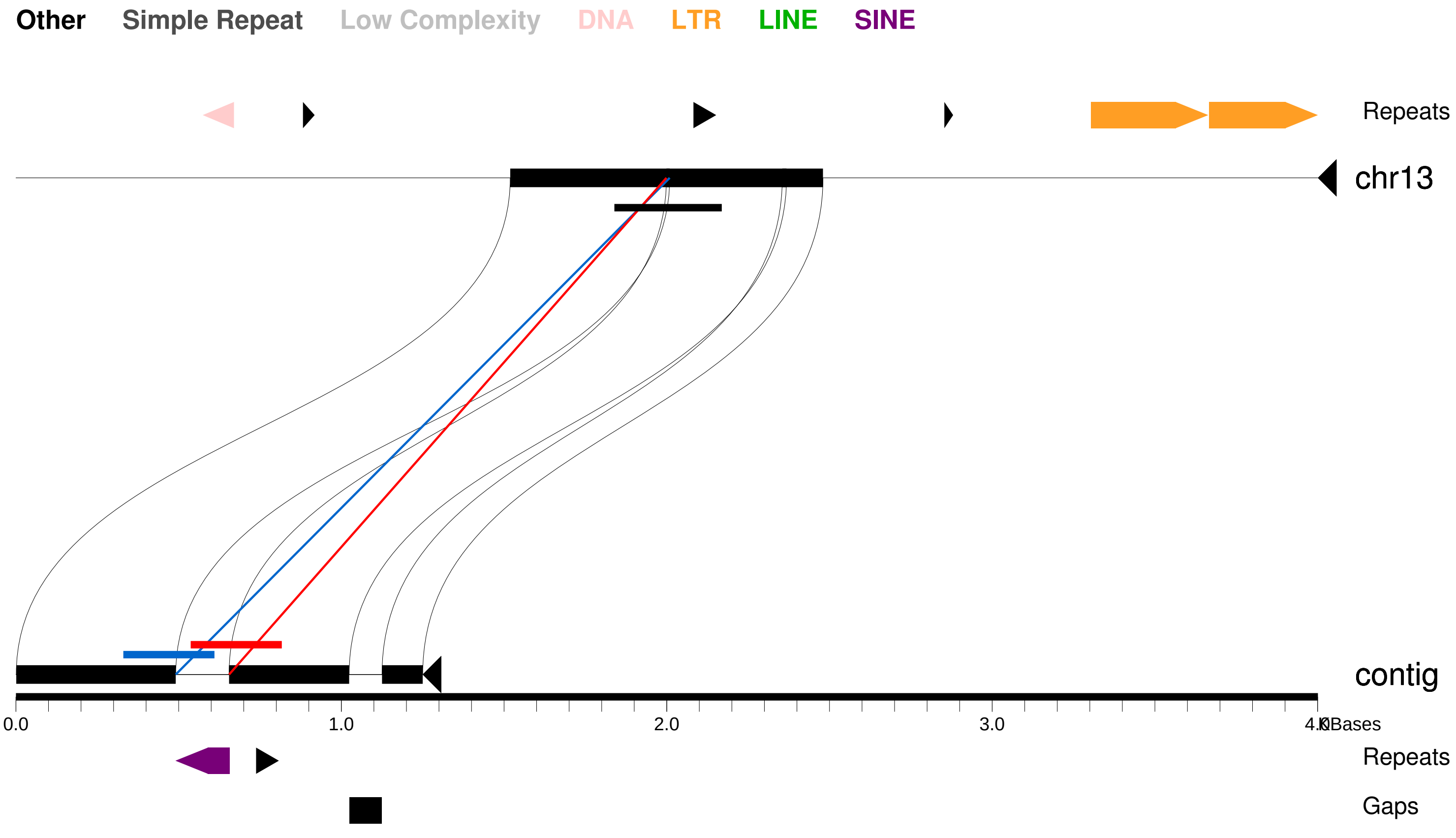

siteID: chr13_74058503 Scafftig1 + Breakpoint: chr13:74058486
chr13:74058430-74058545
Overlap Length: 4 INS size: 414 Genotyped: No

```
left    TCAAGCCACATTCAGAGTGTTAAAATTATTTGAACTAATTTTATTATAAAAGAAAT---ATAGGGCCGGG
chrom   TCAAGCCACATTCAGAGTGTTAAAATTATTTGAACTAATTTTATTATAAAAGAAAT---ATAGAACAGTT
right   NNNNNNNNNNNNNNNNNNNNNNNNNNNNNNNNNNNNNNNNNNNNNNNNNNNNNNNNNNNNATAGAACAGTT
        111111111111111111111111111111111111111111111111111111111111****22*2*22

left    CGCGGTGGCTCACGCCTGTAATCCCAGCACTTTGG-GAGGCCG--AGGCGGGTGG
chrom   AAATATGCTACAA------AAACCAATTACAATGAAGAGACTTGAAAATTTTTCA
right   AAATATGCTACAA------AAACCAATTACAATGAAGAGACTTGAAAATTTTTCA
        22222**222**2222222**2**2*22**22**22***2*2222*222222*22
```

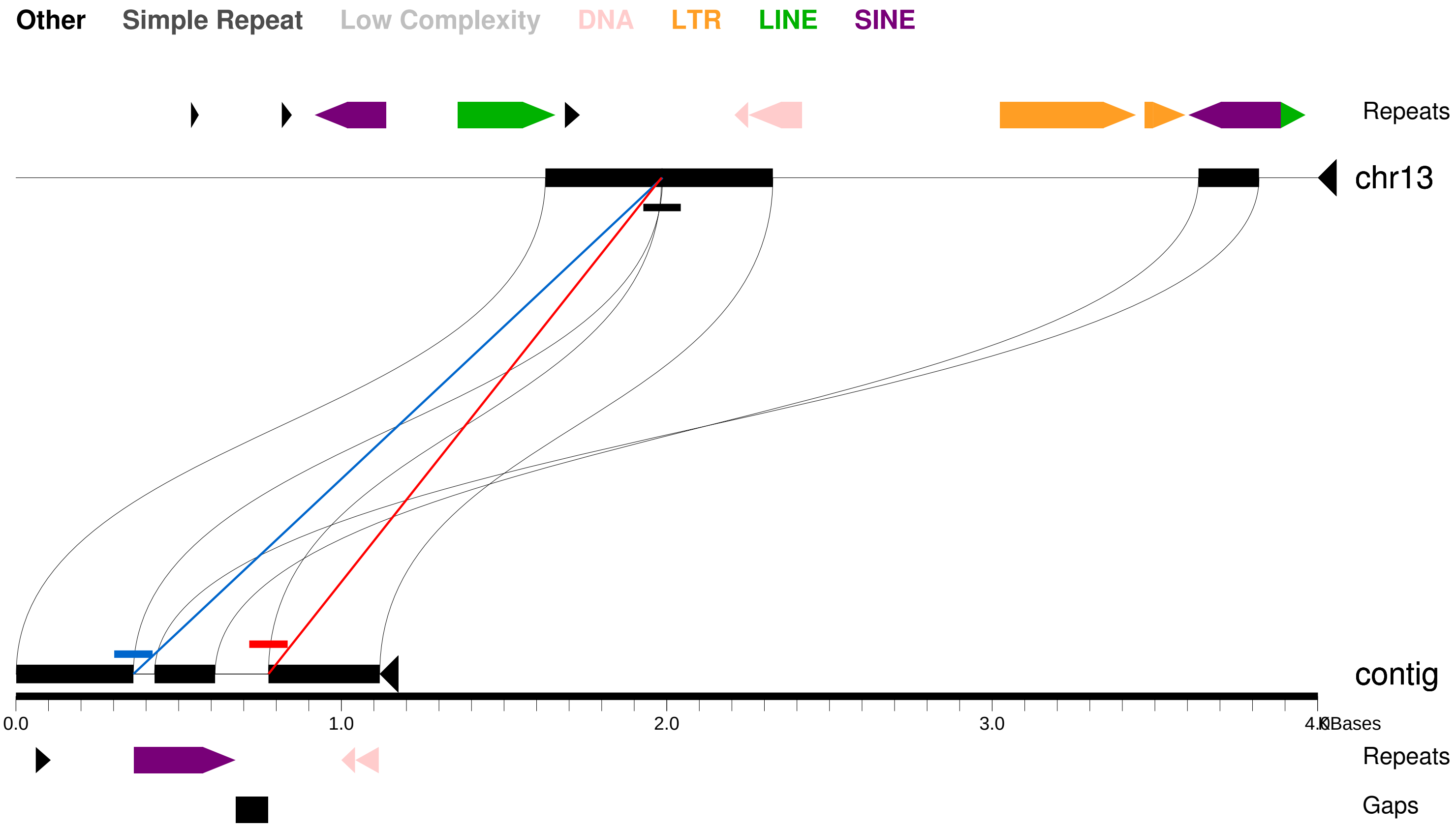

siteID: chr13_78584671 Scafftig1 - Breakpoint: chr13:78584664
chr13:78584612-78584723
Overlap Length: 8 INS size: 444 Genotyped: No

```
left     TAGTGACTTATATGATTACCATTATTCC----TTAAGGAACTTACACTCAGTG---GAGCAAATCTTTTT
chrom    TAGTGACTTATATGATTACCATTATTCC----TTAAGGAACTTACACTCAGTG---GAGCAAATCTT---
right    TCCGCCCGCCTCGGCCTCCCAAAGTGCTGGGATTACAGGCGTGAGCCACCGCGCCCGGCCAAATCTT---
         *11111*111*11*11*1***111*1*11111***11*111*1*11*1*1*1*111*11********222

left     NNNNNNNNNNNNNNNNNNNNNNNNNNNNNNNNNNNNNNNNNNNNNNNNNNNNNNNN
chrom    ----AAAAGCTAATTCAGAGGTCGGATAAATTACATATGCAAAAATTATAAAAAGA
right    ----AAAAGCTAATTCAGAGGTCGGATAAATTACATATGCAAAAATTATAAAAAGA
         22222222222222222222222222222222222222222222222222222222
```

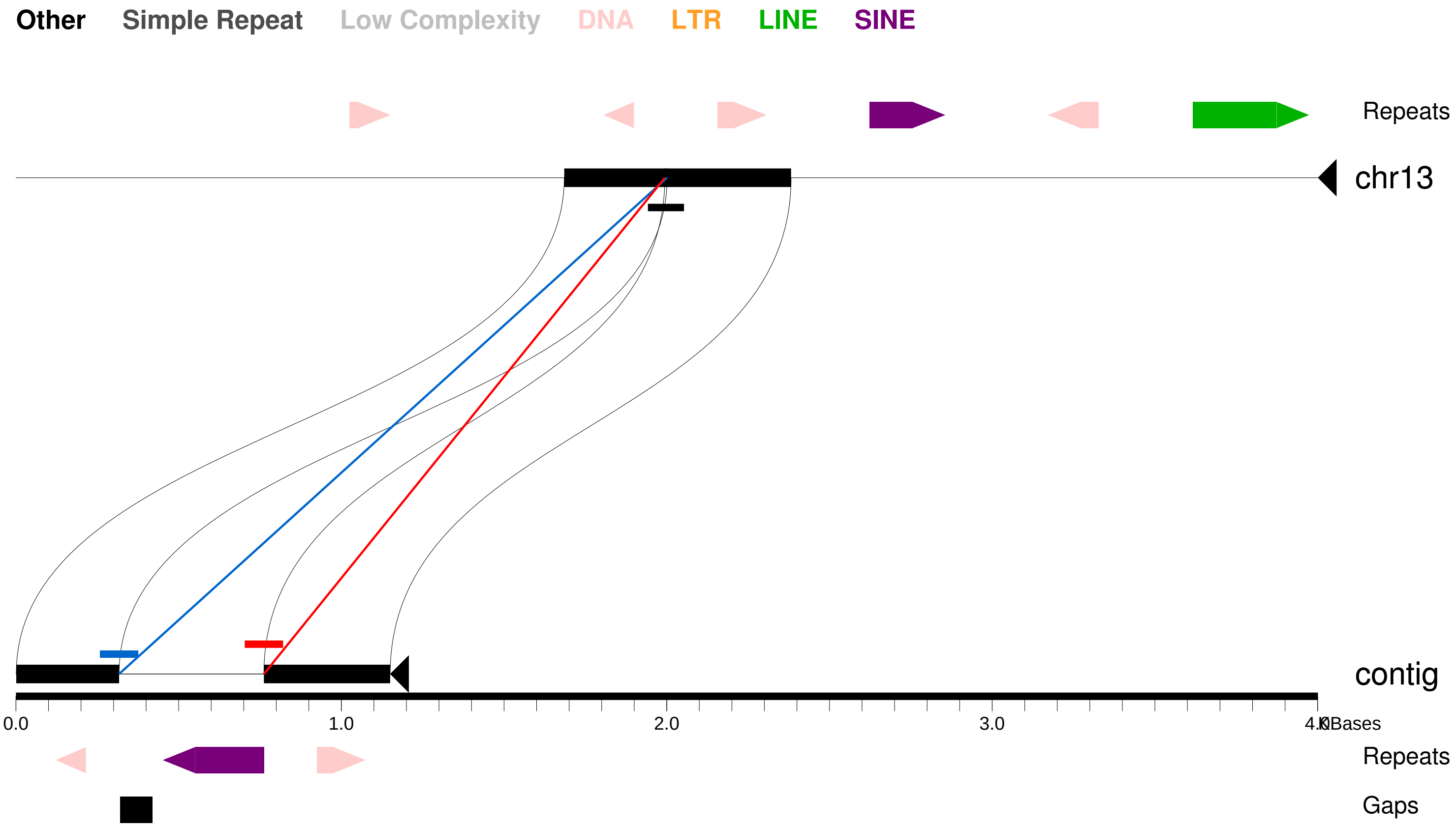

siteID: chr13_78651700 Scafftig10 - Breakpoint: chr13:78651672
chr13:78651628-78651731
Overlap Length: 16 INS size: 299 Genotyped: Yes

```
left    ---TGCAGTCTTGGCTTTAAAAAAAAATAGAGA------------AACTTTAATTGCCATAAAAATGGGAA
chrom   ---TGCAGTCTTGGCTTTAAAAAAAAATAGAGA------------AACTTTAATTGCTATAAAAATGGGAA
right   CACTGCACTCCAGCCTGGGCGACAGAGCGAGACTCCGTCTCAAAAAAAAAAAAAAAAAAAAAAATGGGAA
        111****1**11*1**11111*1*1*11****111111111111**1111**1111N*1***********

left    TTGTTGGCCGGGCGCGGTGGCTCACGCCTGTAATCCCAGCACTTTGGGAGGCCGAGGCGGGCGG
chrom   TTGTTAAACAAGT------TTTGACATAT-TAATGCT-GCAGAATGCACTGCAG-------CAC
right   TTGTTAAACAAGT------TTTGACATAT-TAATGCT-GCAGAATGCACTGCAG-------CAC
        *****222*22*222222222*2**222*2****2*22***222**2222**2*2222222*22
```

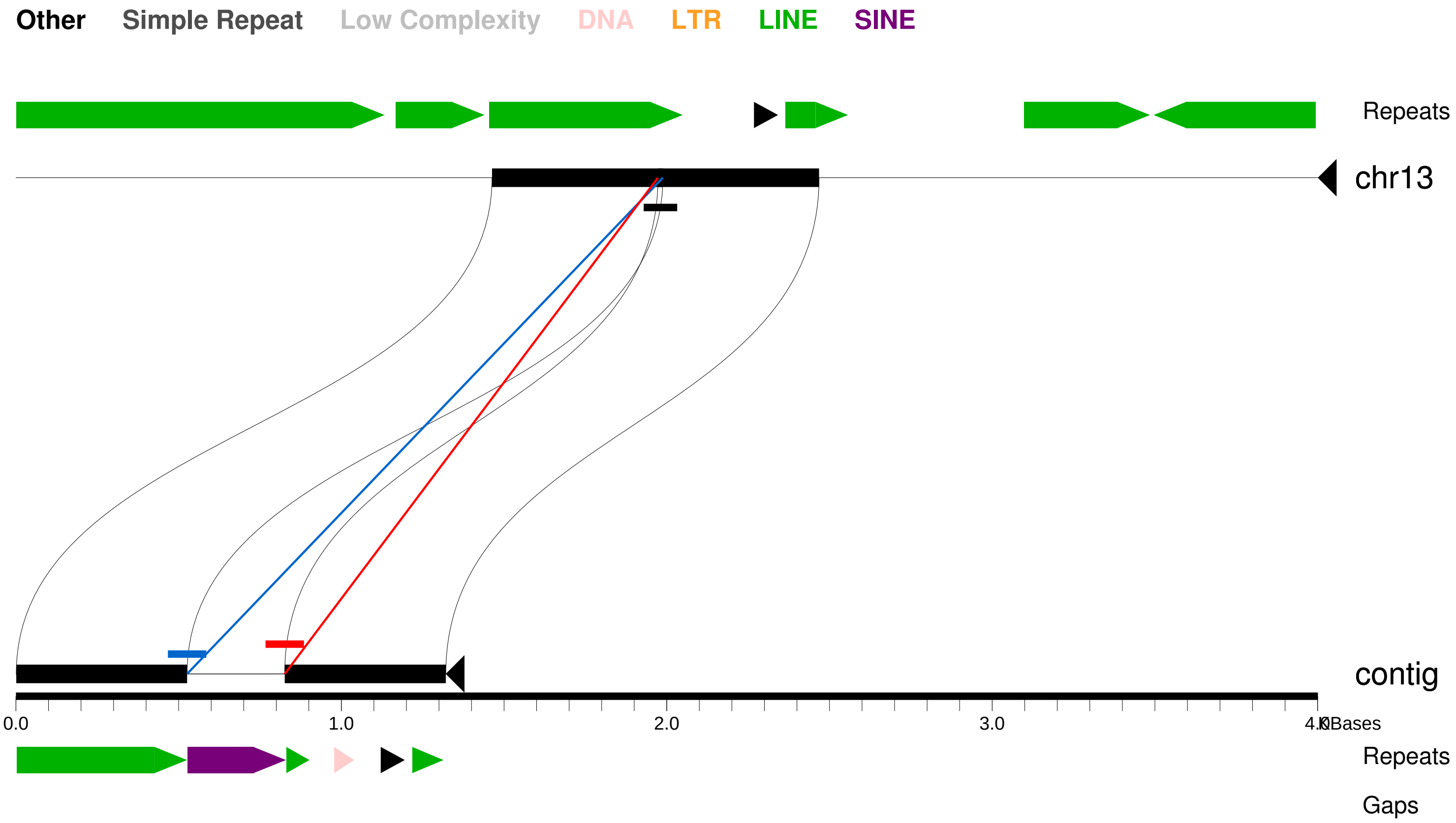

siteID: chr13_79271591 Scafftig5 - Breakpoint: chr13:79271588
chr13:79271542-79271647
Overlap Length: 14 INS size: 304 Genotyped: Yes

```
left     AGACACATTTAATAACTGGAGGAAGTTTTGGATTGTCTGTCTTGAG------------------AAATGG
chrom    AGACACATTTAATAACTGGAGGAAGTTTTGGATTGTCTGTCTTGAG------------------AAATGG
right    -----CACTCCAGCCTGGGCGACAGAGCGAGACTCCGTCTCAAAAAAAAAAAAAAAAAAAAAAAAAAATGG
         11111**1*11*11111**1*11**11111**1*111*1**111*1111111111111111111******

left     GCATATTCGGCCGGGCGCGGTGGCTCAAGCCTGTAATCCCAGCACTTTGGGAGGCCGAGGCGGGCGG
chrom    GCATATTCTACATAT-GTGATGAC---AG---GTGCACATGGATCTTTCGGTG------TCGTGTCC
right    GCATATTCTACATAT-GTGATGAC---AG---GTGCACATGGATCTTTCGGTG------TCGTGTCC
         ********22*22222*2*2**2*222**222**222*222*22****2**2*2222222**2*222
```

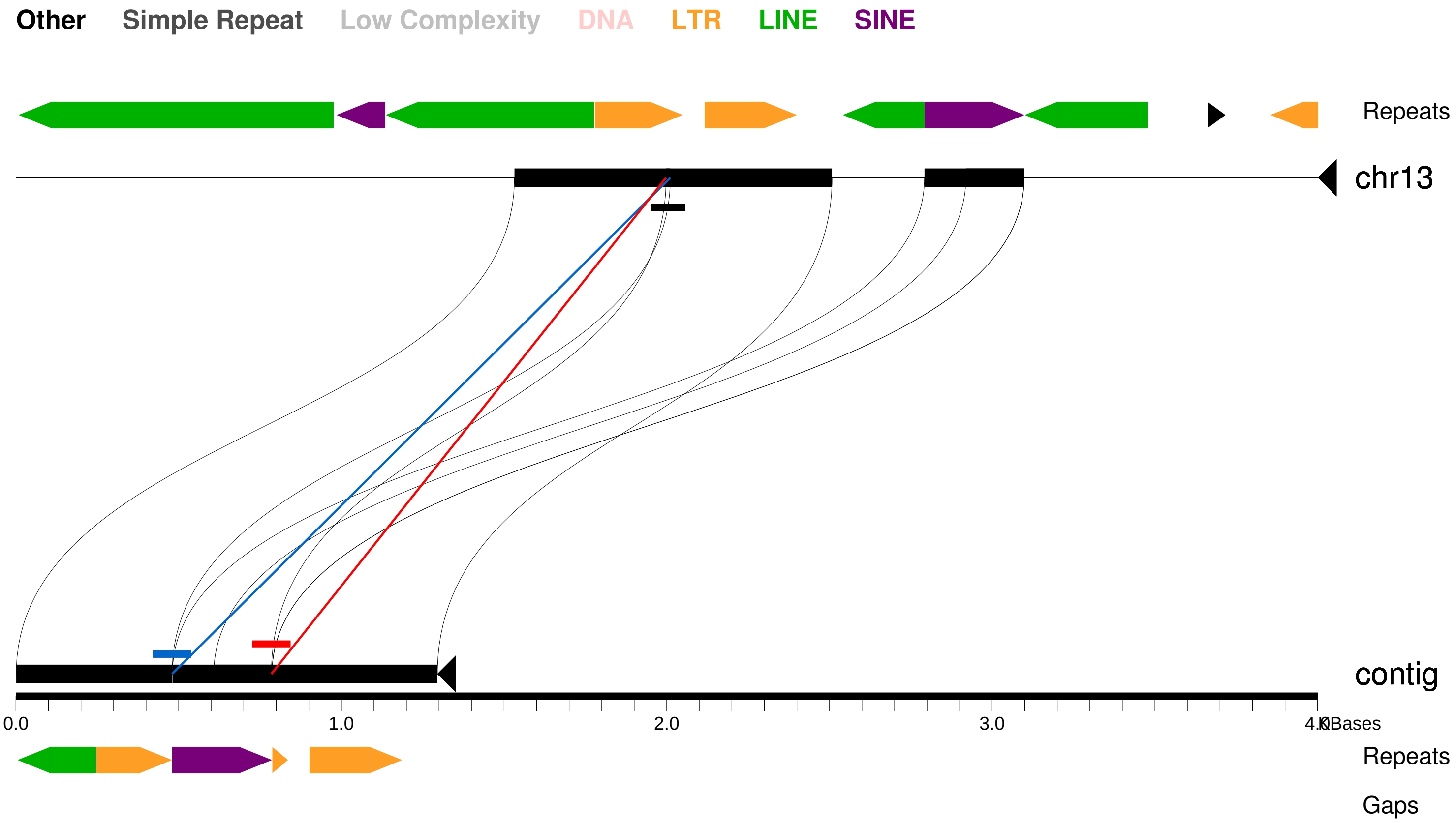

siteID: chr13_79774254 Scafftig1 + Breakpoint: chr13:79774274
chr13:79774229-79774333
Overlap Length: 15 INS size: 380 Genotyped: No

```
left    CTCAAGTAGAACCTTATCAAGAGTACTCCGAGTAAAGCAAAGG--------------ACAAAAAGTCTTC
chrom   CTCAAGTAGAACCTTATCAAGAGTACTCCGAGTAAAGCAAAGG--------------ACAAAAAGTCTTC
right   NNNNNNNNNNNNNNNNNNNNNNNNNNNNNNNNNNNNNNNNNNNNNNNNNNNNNNNNAAAAAAAAGTCTTC
        111111111111111111111111111111111111111111111111111111111*1***********

left    AATATTGGGAGGCCGAGGCGGGCGGATCACGAGGTCAGGAGATCGAGACCATCCCGGCTAAAA
chrom   AATAACAGTATTGCTATCATTGGTGGT-----GGT--GGTGGTGGTGCTTTTCACT-------
right   AATAACAGTATTGCTATCATTGGTGGT-----GGT--GGTGGTGGTGCTTTTCCCT-------
        ****222*2*222*2*22222*22*2*22222***22**2*2*2*2*2222**N*22222222
```

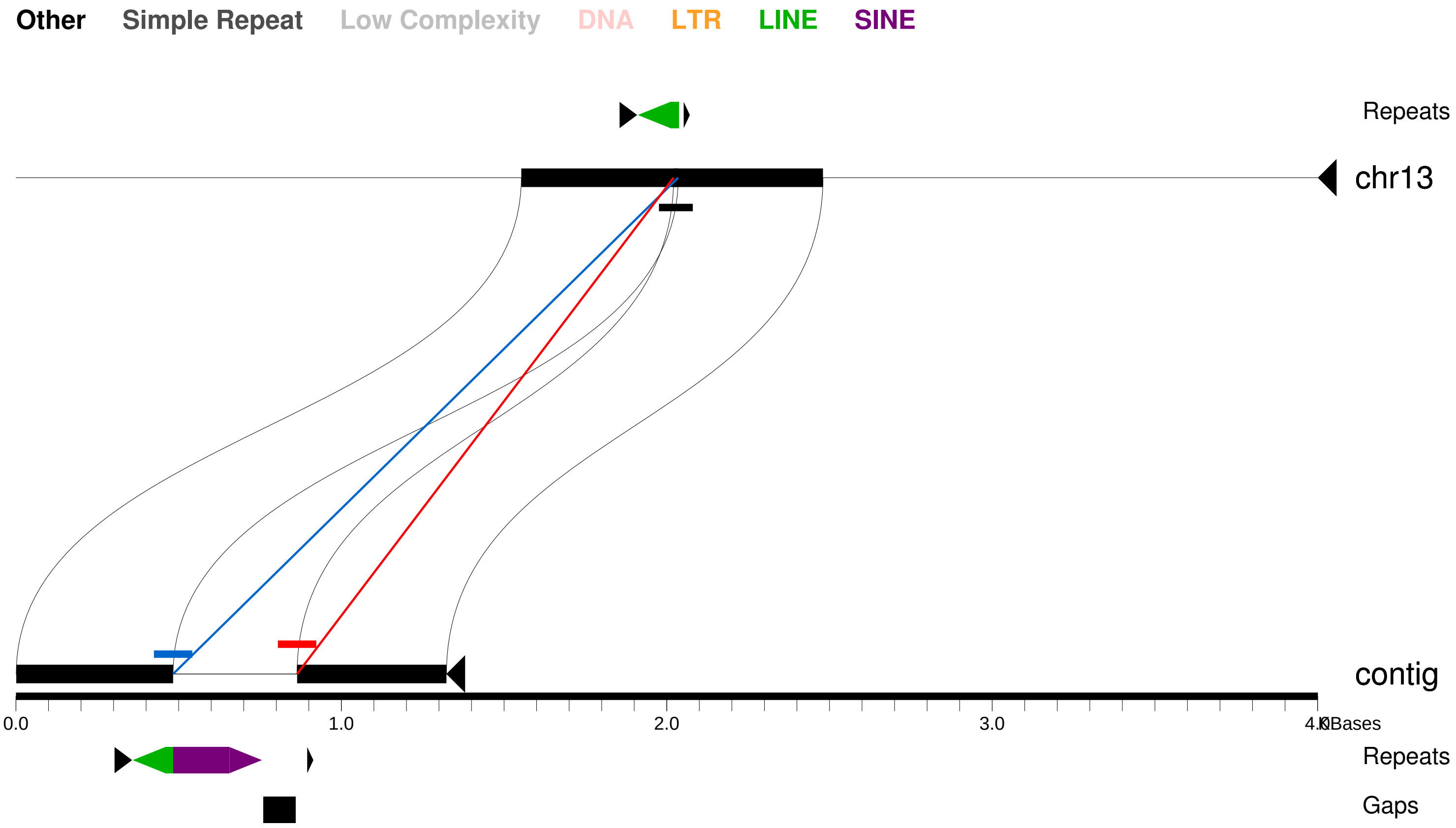

siteID: chr13_79861017 Scafftig1 + Breakpoint: chr13:79860990
chr13:79860678-79861150
Overlap Length: 8 INS size: 83 Genotyped: Yes

```
left     NNNNNNNNNNNNNNNNNNNNNNNNNNNNNNNNNNNNNNNNNNNNNNNNNNNNNNNNNNNNNNNNNNNNNNNN
chrom    GGAAACCAAAAAATGTAAAAGTGTGATATTCACTTTATTCTAGTGGTCTGGAACCAAACTTGCAATATCT
right    ----------------------------TTTAATTTCT------------------------CAGCATTT
         NNNNNNNNNNNNNNNNNNNNNNNNNNNN22N2N222N2NNNNNNNNNNNNNNNNNNNNNNNN22NN22N2

left     NNN-------------------------------------------------------------------
chrom    CTGAGGTGTGCCTGTAGAAAAAGCCTACACAAAATGTACCAGGAGTGAAATATTTCCCTAGCTCTGTAAA
right    CGGAGGT-----TGTAGCAAA------CA-----------------GAAAGACATCCCG-----------
         2N22222     22222N222      22                 2222N2NN2222N

left     -------------------------------------------------------------------TCTT
chrom    AAAAAAAAAATTCACCTCTTAAATGTATCATTTGTGTTTCAAAGTAGTAAGCATTTCATACTATAATCTT
right    --------------CCACTGCACTCCAGCCTGGGAGA--CAGAG----------------CGAGACTCCG
                       22N22NN2N2NN2N2N2NN2N2N  22N22                2N2N2N**11

left     TTTTTAAATAAAATGGGATGAAACATTGTAAAAAGATTTGTGCACACTCAAATTTTCAAATTGGATTTAA
chrom    TTTTTAAATAAAATGGGATGAAACATTGTAAAAAGATTTGTGCACACTCAAATTTTCAAATTGGATTTAA
right    TCTCAAAAAAAAAA-------AAAAAAAAAAAAAAAA-------------AAA-----AAA----------
         *1*11***1****1111111***1*1111*****1*1111111111111***11111***1111111111

left     TTTCTCAGCATTTCGGAGGTTGTAGCAAACAGAAAGACATCCCGC-CACT---GCACTCCAGCCTGGGA-
chrom    TTTCTCAGCATTTCGGAGGTTGTAGCAAACAGAAAGACATATTGTTCTCTTTAGGATGCGACTGTGTAAA
right    --------------------------AAAAAAAAAGACATATTGTTCTCTTTAGGATGCGACTGTGTAAA
         11111111111111111111111111***1*1********222*22*2**222*2*22*2*222**22*2

left     -GACAGAGCGA-GACTCCGTCTCAAAAAAAAAAAAAAAAAAAAAAAAAAAA--------------------
chrom    TGCAAGAGTTATGTATGTGTTTGTATATATATATATACATACACACTAAATGCTGTATATACATGTGTAT
right    TGCAAGAGTTATGTATGTGTTTGTATATATATATATACATACACACTAAATGCTGTATATACATGTGTAT
         2*22****22*2*22*22**2*22*2*2*2*2*2*2*2*2*2*22***2222222222222222222

left     ----AAAAAAAAA--------------------AAAAGACATATT--------
chrom    ATCTATTTAAACATTTCTTCCATTAATCATTTCATATGACCTACTAAAAATAA
right    ATCTATTTAAACATTTCTTCCATTAATCATTTCATATGACCTACT--------
         2222*222***2*2222222222222222222*2*2***2**2*
```

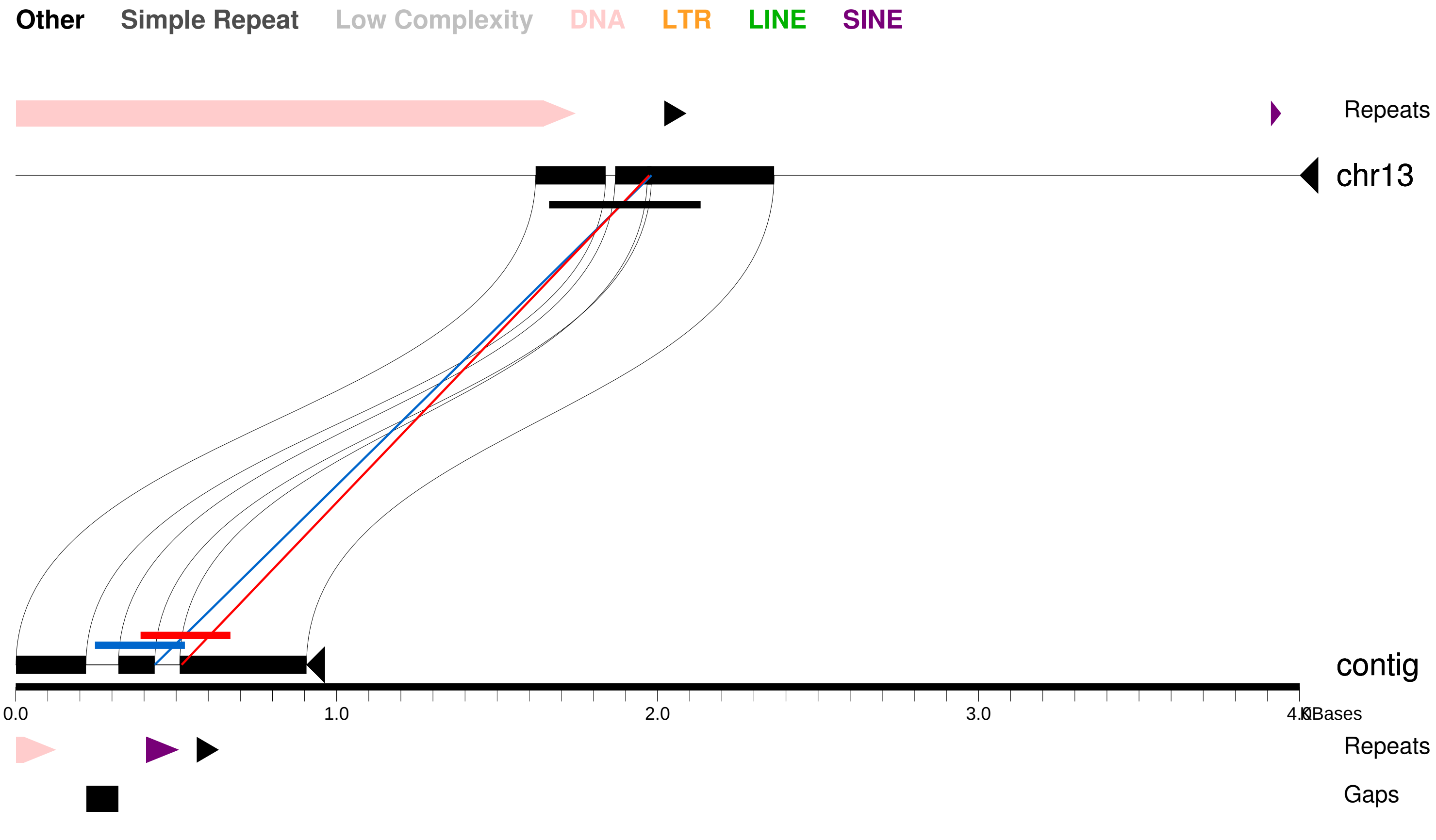

siteID: chr13_80296998 Scafftig4 + Breakpoint: chr13:80296968
chr13:80296924-80297024
Overlap Length: 13 INS size: 315 Genotyped: Yes

```
left     TAGTAAACCTTC--AGTTCTTCC----TCTTGCTAACATAGTTGGAA------------GGAGGGTTCTT
chrom    TAGTAAACCTTC--AGTTCTTCC----TCTTGCTAACATAGTTGGAA------------GGAGGGTTCTT
right    TGATCCGCCCGCCTCGGCCTCCCAAAGTGCTGGGATTACAGGCGTGAGCCACCGCGCCCGGCGGGTTCTT
         *11*111**11*111*11**1**1111*11**11*11*1**11*11*111111111111**1********

left     TTATTTTTTTTTTTTTATTTATTTTTTTTTTTTTTTTTGAGACGGAGTCTCGCTCTGTCGCCCAGGC
chrom    TTATTATTGAAATAACCA---AAGTCCTTCACTCCATTG---------CTTGCAA---CTCCAA---
right    TTATTATTGAAATAACCA---AAGTCCTTCACTCCATTG---------CTTGCAA---CTCCAA---
         *****2**2222*2222*222*22*22**222*222***222222222**2**22222*2**2*222
```

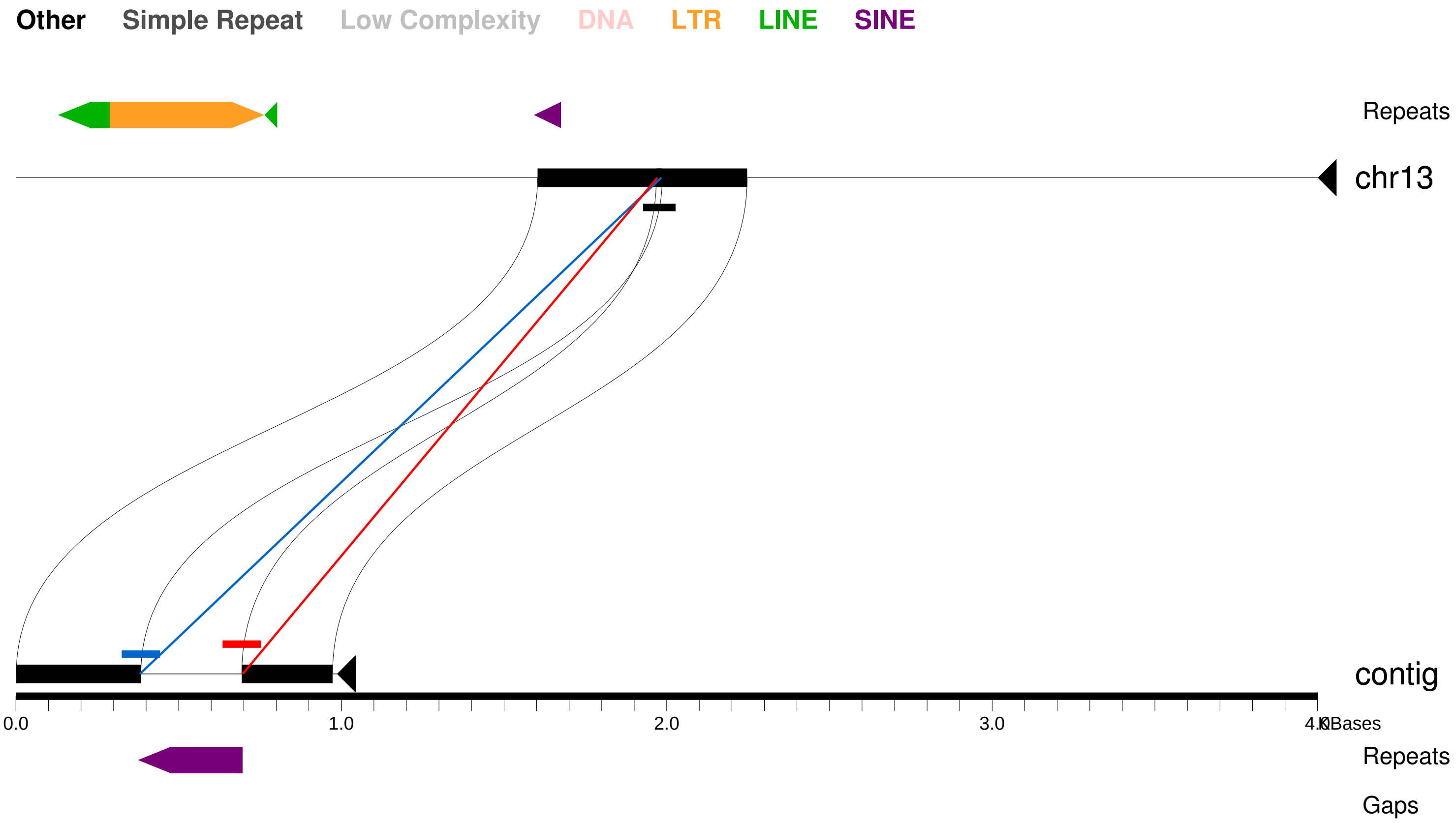

siteID: chr13_81373646 Scafftig1 - Breakpoint: chr13:81373725
chr13:81373681-81373784
Overlap Length: 16 INS size: 417 Genotyped: No

```
left     TGTTAAAAAAGCGAAGGCAATCAATTGGA---------------TGACATTAAAAATATAGAATGAACTG
chrom    TGTTAAAAAAGCGAAGGCAATCAATTGGA---------------TGACATTAAAAATATAGAATGAACTG
right    NNNNNNNNNNNNNNNNNNNNNNNNNNNNNNNNNNNNNNNNNNNNNNNNNNNNNNNNNNNNAAAGAATGAACTG
         111111111111111111111111111111111111111111111111111111111*1***********

left     AGAAAGGCCGGGCGCGGTGGCTCACGCCTGTAATCCCAGCACTTTGGGAGGCCG-AGGCGGGTGG-
chrom    AGAAAAAA---------TAGTTTAA------AATAC--GTATGTGTAGAGGGTGTATACTTCTGGC
right    AGAAAAAA---------TAGTTTAA------AATAC--GTATGTGTAGAGGGTGTATACTTCTGGC
         *****222222222222*2*2*2*2222222***2*22*2*22*222****22*2*22*222***2
```

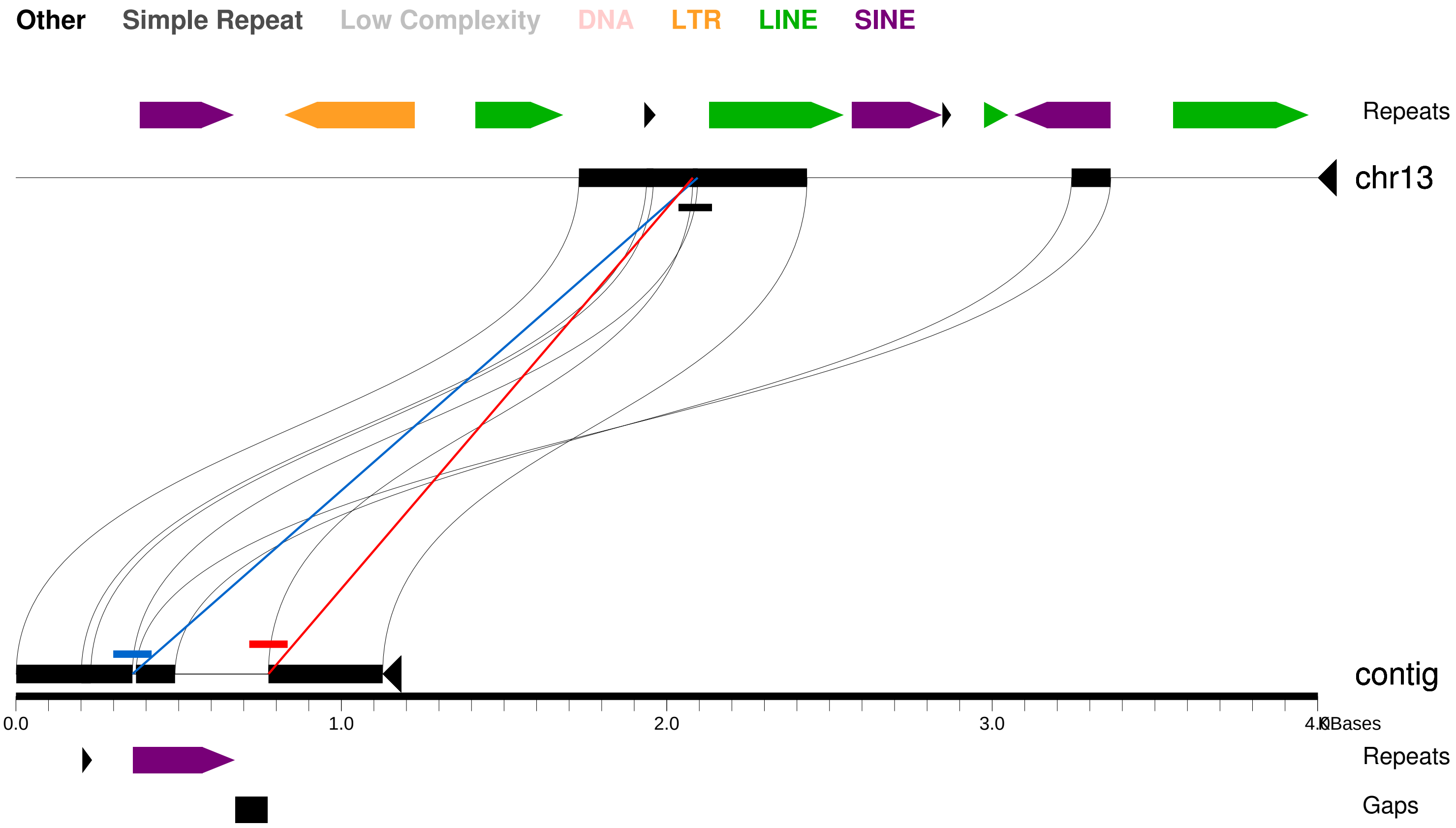

siteID: chr13_81523087 Scafftig7 + Breakpoint: chr13:81523071
chr13:81523030-81523130
Overlap Length: 19 INS size: 312 Genotyped: Yes

```
left    C------ACAAATATTGTTGAAGTGTAGTGTTTAAATA--TTGGTAATT----------GTAAAATGATA
chrom   C------ACAAATATTGTTGAAGTGTAGTGTTTAAATA--TTGGTAATT----------GTAAAATGATA
right   CCGCCCGCCTCGGCCTCCCAAAGTGCTGGGATTACAGGCGTGAGCCACCGCGCCCGGCCGTAAAATGATA
        *1111111*111111*1111*****11*1*1***1*1111*11*11*111111111111***********

left    ACGTTTCATTTCTTTTTTTTTTTTTTTTTTTTTTTTTTTTGAGACGGAGTCTCGCTCTGTCGCCCAGG
chrom   ACGTTTCAATGACAAGTGTGACTTTTGGAATTCTTAATAAAAA-----------TTTGAA-------
right   ACGTTTCAATGACAAGTGTGACTTTTGGAATTCTTAATAAAAA-----------TTTGAA-------
        ********2*222222*2*222****2222**2**22*22*2*22222222222*2**222222222
```

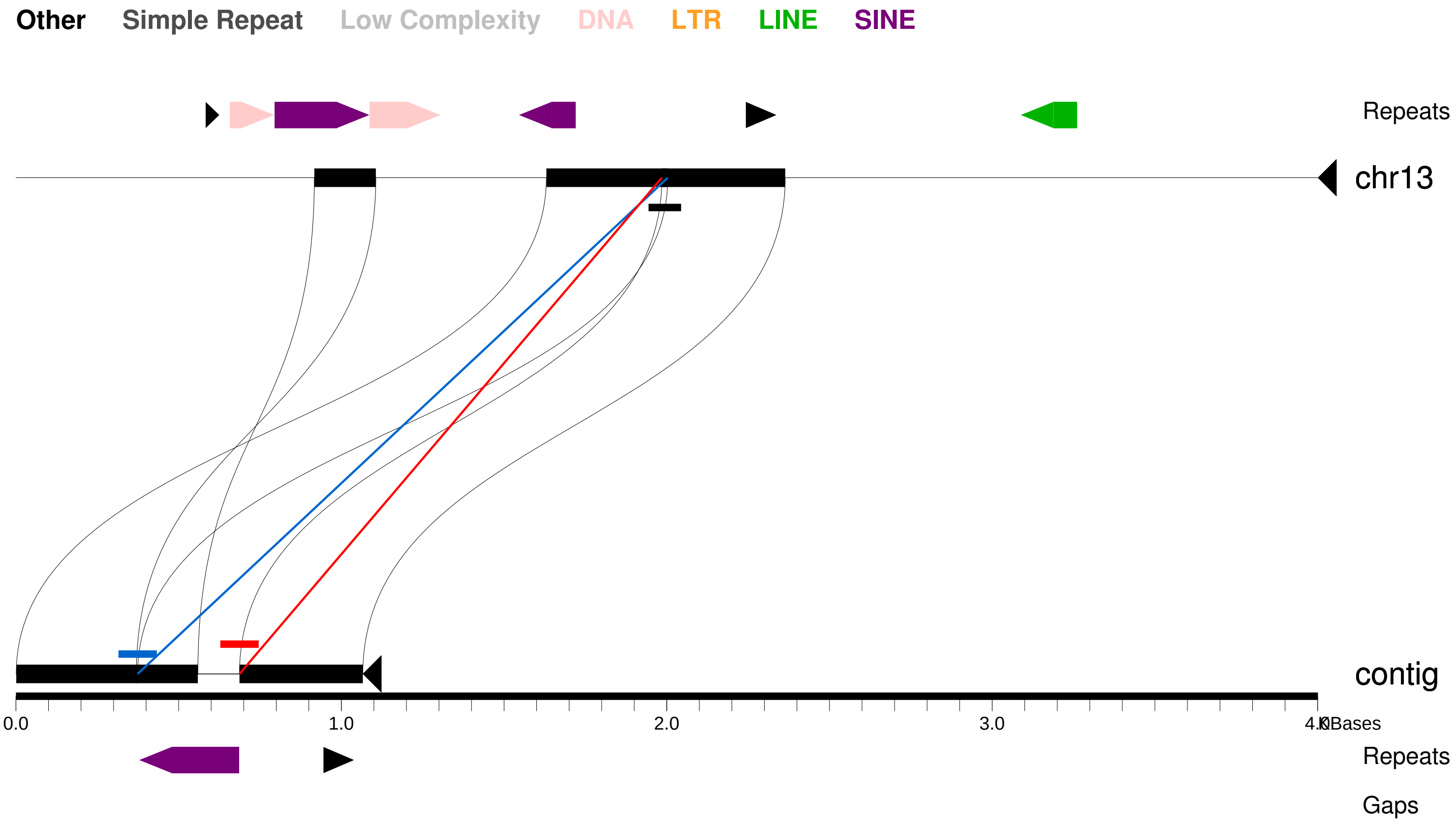

siteID: chr13_86166454 Scafftig1 - Breakpoint: chr13:86166445
chr13:86166405-86166504
Overlap Length: 2 INS size: 173 Genotyped: Yes

```
left     GCAG--ATTATGGGAAAGTCAGACTG---AGTAG-----AAAACAAACTC----------TTTTTTTTTT
chrom    GCAG--ATTATGGGAAAGTCAGACTG---AGTAG-----AAAACAAACTC----------TTGATTCATT
right    CACGCCATTCTCCTGCC-TCAGCCTCCCAAGTAGCTGGGACTACAGGCGCCCGCCACTATTTGTTTCATT
         111*11***1*1111111****1**1111*****11111*11***11*1*1111111111**2N**22**

left     TTTTTTTTTTTTTTTTTTTTTTTTTTGAGACGGAGTCTCGCTCTGTCGCCCAGGCTGGAGTGCAGTGGC
chrom    TAGTTATCTTA----------------AGCCAGAGTGGAAAGGTTGCGATTAGATTC--TTAGAATG-C
right    TAGTTATCTTA----------------AGCCAGAGTGGAAAGGTTGCGATTAGATTC--TTAGAATG-C
         *22**2*2**2222222222222222**2*2****2222222*22**222**22*2222*22*2**2*
```

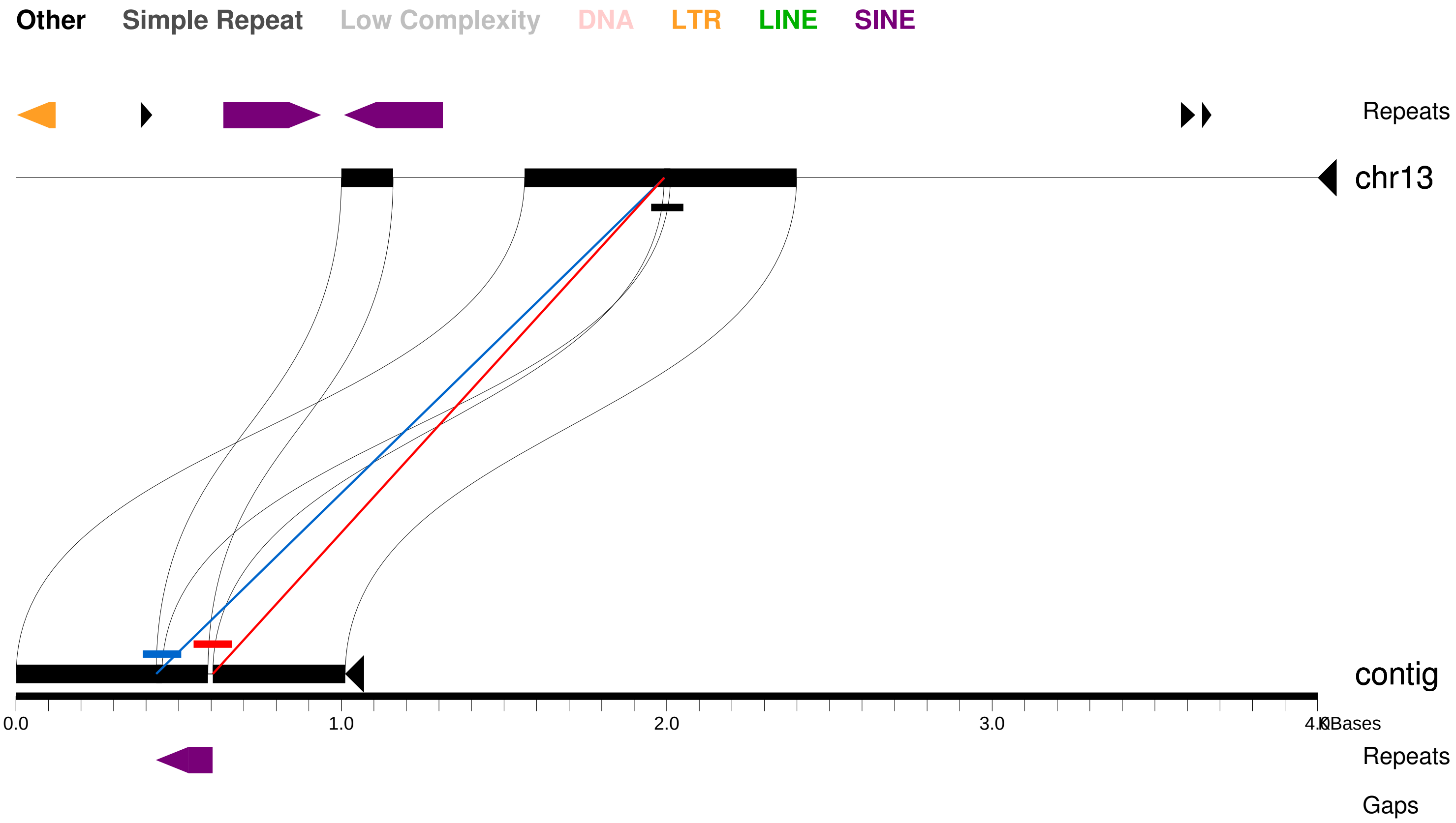

siteID: chr13_86361633 Scafftig2 - Breakpoint: chr13:86361565
chr13:86361405-86361736
Overlap Length: 12 INS size: 99 Genotyped: Yes

```
left   -----------ACTCTTAAAATTCAACAAAAAGAAAATGAACATTTGGATAAAAAAGAGGAAAAGACCTA
chrom  TTATACAAAGAACTCTTAAAATTCAACAAAAAGAAAATGAACATTTGGATAAAAAAGAGGGAAAGACCTA
right  A-ATGTAAA------TTAAGA--CAACAATGCG-----GAGC--TTGCAGTGAGCCG--GGATAGCGCCA
       N 22NN222  1111****1*11******111*11111**1*11***1*111*111*11*2*1**11*1*

left   AA-CAGATAACCTCAGCAAAGAAGGTATATGTATGGCCATAAATATATGGAAAGATGATCCACATTACAT
chrom  AA-CAGATAACCTCAGCAAAGAAGGTATATGTATGGCCATAAATATATGGAAAGATGATCCACATTACAT
right  CTGCAGTCCAGCTTGGGCGAAAGAGTGAGACTCCGTCTCAAAAAACA---AAAAACAAACAA----ACA-
       111***111*1**11*111*1*11**11111*11*1*111***1*1*111***1*11*1*1*1111***1

left   GTCATCAGGGAAATGTAAATTAAGACAACAATGCGG--AGCTTGCAGTGAGCCGGGATAGCGCCACTGCA
chrom  GTCATCAGGGAAATGTAAATTAAGACAACAATGAGATAAAATTAGAATGGTCAAAAGTCAGACAACTG-A
right  ---AACA---AAA--------AAGACAACAATGAGATAAAATTAGAATGGTCAAAAGTCAGACAACTG-A
       111*1**111***11111111***********2*222*22**22*2**22*22222*2222*2****2*

left   GTCCAGCTTGGGCGAAAGAG--TGAG-------ACTCCGTC--TCAA----------AAAACAAAAAA--
chrom  CAACACCAAGAGCTGATGAGGATGTGGAACAACAATTCTTCATTCATTGTTGGTGGGAATGCAAAATTGT
right  AAACACCAAGAGCTGATGAGGATGTGGAACAACAATTCTTCATTCATTGTTGGTGGGAATGCAAAATTGT
       N22**2*22*2**22*2***22**2*2222222*2*2*2**22***22222222222**22*****2222

left   -CAAACA----AACAAAC------------AAAAAAGACAACAATGAGATAAAAT
chrom  GCAGCCACTTTAGTAGACGGTTAGTTGCTTAGAAAGCAAAACACAGACTTATTA-
right  ACAGCCACTTTAGTAGACGGTTAGTTGCTTAGAAAGCAAAAC-------------
       N**22**2222*22*2**222222222222*2***22*2***1NN11NN11NN12
```

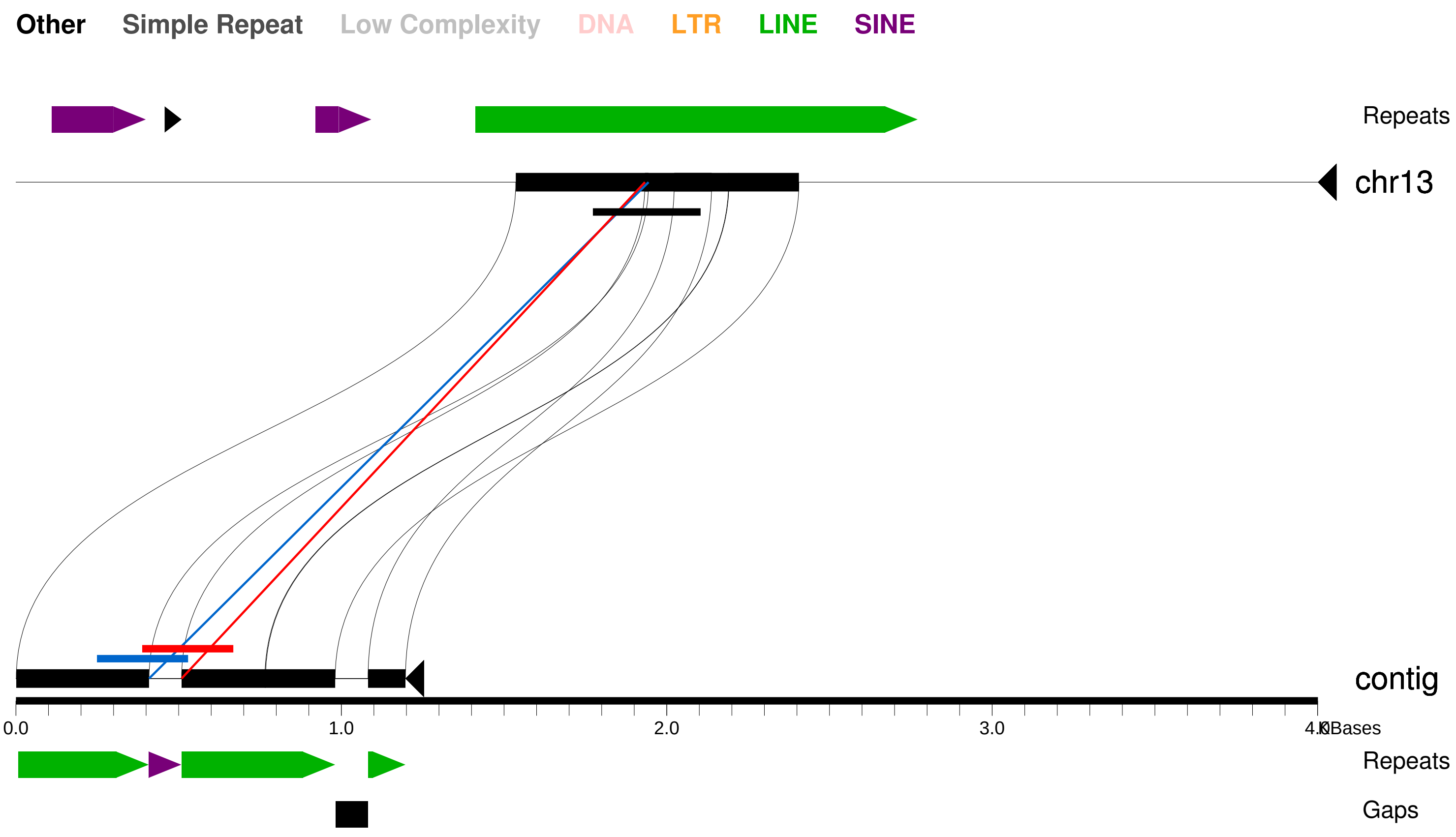

siteID: chr13_87392969 Scafftig2 + Breakpoint: chr13:87392948
chr13:87392910-87393004
Overlap Length: 16 INS size: 279 Genotyped: Yes

```
left    T------AAGCAATTGCTATCA-GAATTTTAAAT-------------------AAAGGTTACTTTTAACT
chrom   T------AAGCAATTGCTATCA-GAATTTTAAAT-------------------AAAGGTTACTTTTAACT
right   AGCCGGGATGGTCTCGATCTCCTGACCTCGTGATCCGCCCGCCTCGGCCTCCCAAAG--TGCTGTTAACT
        1111111*1*111*1*1*1**11**11*1111**1111111111111111111****11*1**1******

left    GAACATTTCTTTTTTTTTTTTTTTTTTTTTTTTTTTTTGAGACGGAGTCTCGCTCTGTCGCCCAGGCTG
chrom   GAACATTTCTAATTTTAGTGTATAAATGTATATTT----------GTGTCTGTATTAGT-----------
right   GAACATTTCTAATTTTAGTGTATAAATGTATATTT----------GTGTCTGTATTAGT-----------
        *********22****22*2*2*222*2*2*2***2222222222*2****222*22**22222222222

left    GAGTG
chrom   ---TT
right   ---TT
        222*2
```

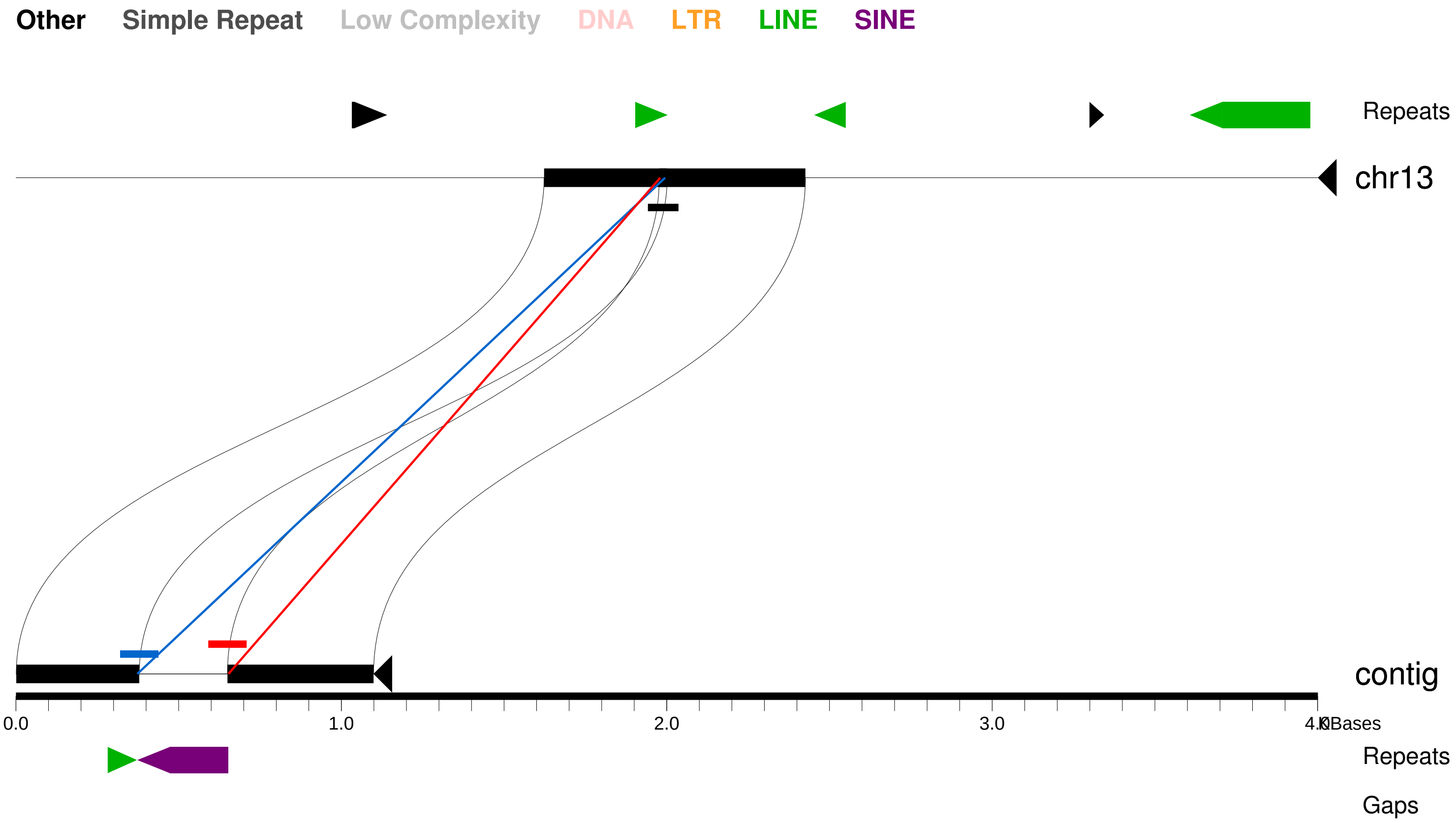

siteID: chr13_87696248 Scafftig1 - Breakpoint: chr13:87696240
chr13:87696198-87696299
Overlap Length: 18 INS size: 243 Genotyped: Yes

```
left    --ACAT-GA-ACTTAG-CTCTGGACCAAGCAGACCTAATAGACATCT------------AAAGAACTCTC
chrom   --ACAT-GA-ACTTAG-CTCTGGACCAAGCAGACCTAATAGACATCT------------AAAGAACTCTC
right   CGACAGCGAGACTCCGTCTCAAAAAAAAAAAAAAAAAAAAAAAAAAAAAAAAAAAAAACAAAGAACTCTC
        11***11**1***11*1***111*11**11*1*111**1*1*1*111111111111111***********

left    CACCCCATCGAGACCATCCTGGCTAACACGGTGAAACCCCGTCTCTACTAAAAATACAAAAAATTA
chrom   CACCCCAA-------ATC------AACAGAATATACCTTCTTCTC----AACACCACATCGCATTT
right   CACCCCAA-------ATC------AACAGAATATACCTTCTTCTC----AACACCACATCGCATTT
        *******22222222***222222****222*22*2*22*2****2222**2*22***2222***2
```

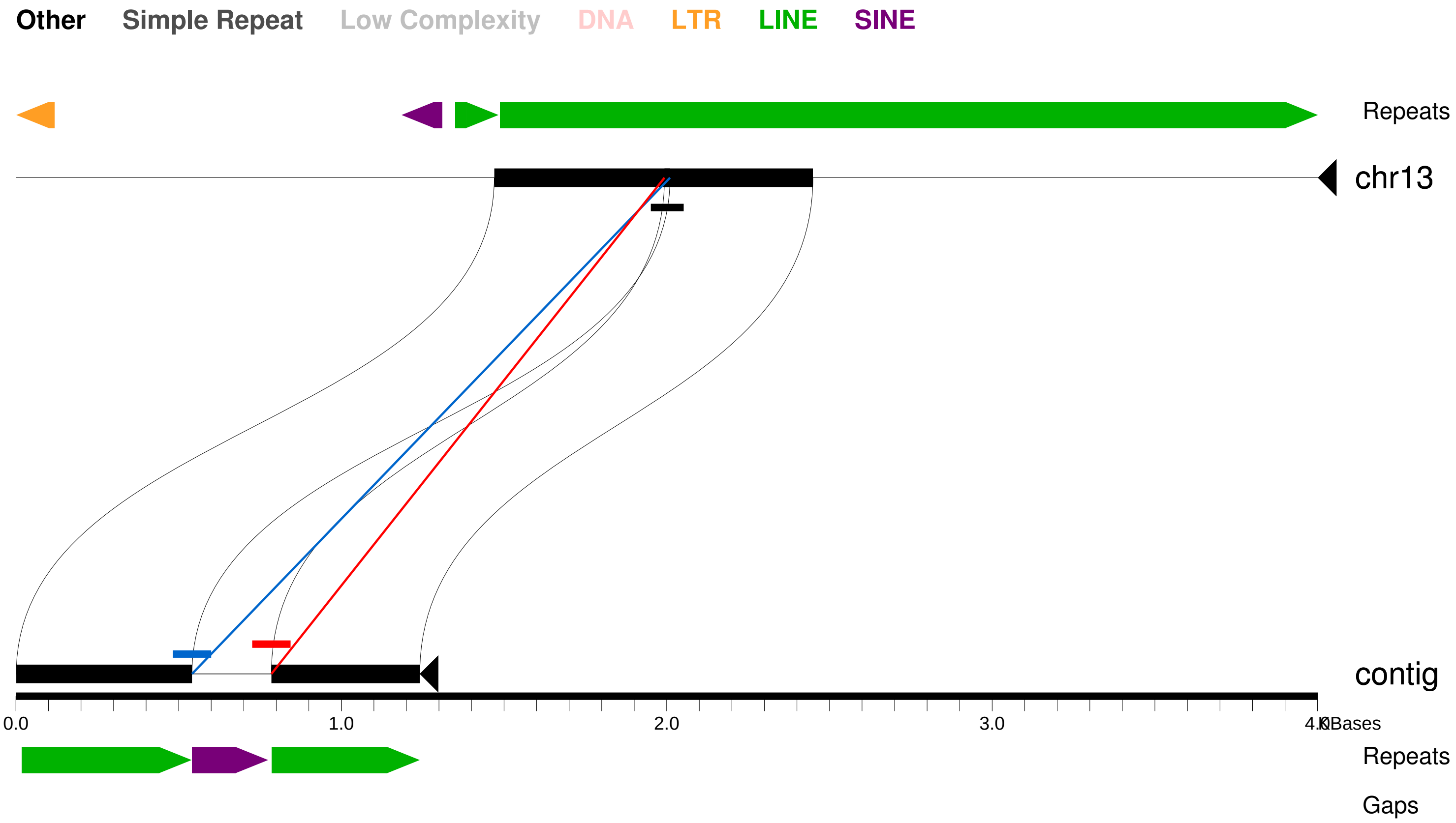

siteID: chr13_87869619 Scafftig7 - Breakpoint: chr13:87869616
chr13:87869567-87869672
Overlap Length: 11 INS size: 297 Genotyped: Yes

```
left     CATC--TACTG-------ACATTG---AGAGTGCAGGAAGGC-TGTGAATCTTGAGGACAATAAAGAAAG
chrom    CATC--TACTG-------ACATTG---AGAGTGCAGGAAGGC-TGTGAATCTTGAGGACAATAAAGAAAG
right    TTGCGCCACTGCACTCCCACCTGGGCCACAGAGCGAGACTCCGTCTCAAAAAAAAAAAAAAAAAAAAAAG
         111*111****1111111**1*1*111*1**1**11**111*1*1*1**11111*11*1**1***1****

left     TTTGGCCGGGCGCGGTGGCTCACGCTTGTAATCCCAGC---ACTTTGGGA--GGCCGAGGCGGGCGG
chrom    TTTAACA------------------TTGTAATTGCAGGGAGAGGTAGAGTATGGCTAAAAATTGAAG
right    TTTAACA------------------TTGTAATTGCAGGGAGAGGTAGAGTATGGCTAAAAATTGAAG
         ***22*222222222222222222*******22***2222*22*2*2*222***22*22222*22*
```

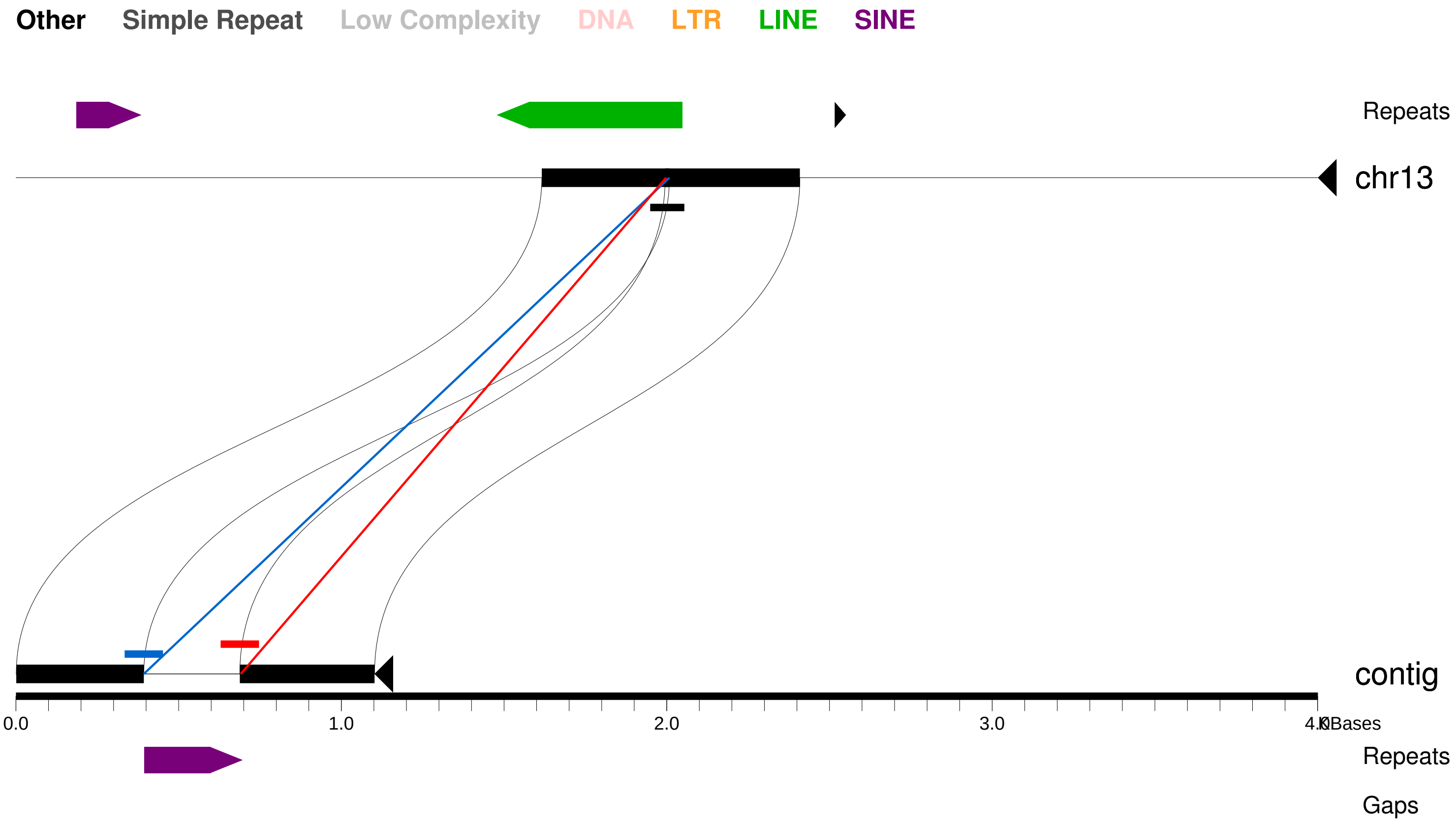

siteID: chr13_90401823 Scafftig3 - Breakpoint: chr13:90401833
chr13:90401787-90401892
Overlap Length: 14 INS size: 315 Genotyped: Yes

```
left     TAGAATTT----ACA-ATCGA--------TGCACACCCAAAAATATTGAAGGCATGTAGAAATGAGTTTT
chrom    TAGAATTT----ACA-ATCGA--------TGCACACCCAAAAATATTGAAGGCATGTAGAAATGAGTTTT
right    CCGGCCTGGGCGACAGAGCGAGACTCCGTCTCAAAAAAAAAAAAAAAAAAAAAAAAAAAAAAAATGAGTTTT
         11*111*11111***1*1***1111111111**1*111*****1*111**111*111*1***********

left     ATAGGCCGGGCGCGGTGGCTCACGCCTGTAATCCCAGCACTTTGGGAGGCCGAGGCGGGTGG
chrom    ATAAACAATGAATAGTT----ATTTCTGGAG----AGAAATATAAAATGTATTAATT-----
right    ATAAACAATGAATAGTT----ATTTCTGGAG----AGAAATATAAAATGTATTAATT-----
         ***22*222*2222**22222*222***2*22222**2*2*2*222*2*2222222222222
```

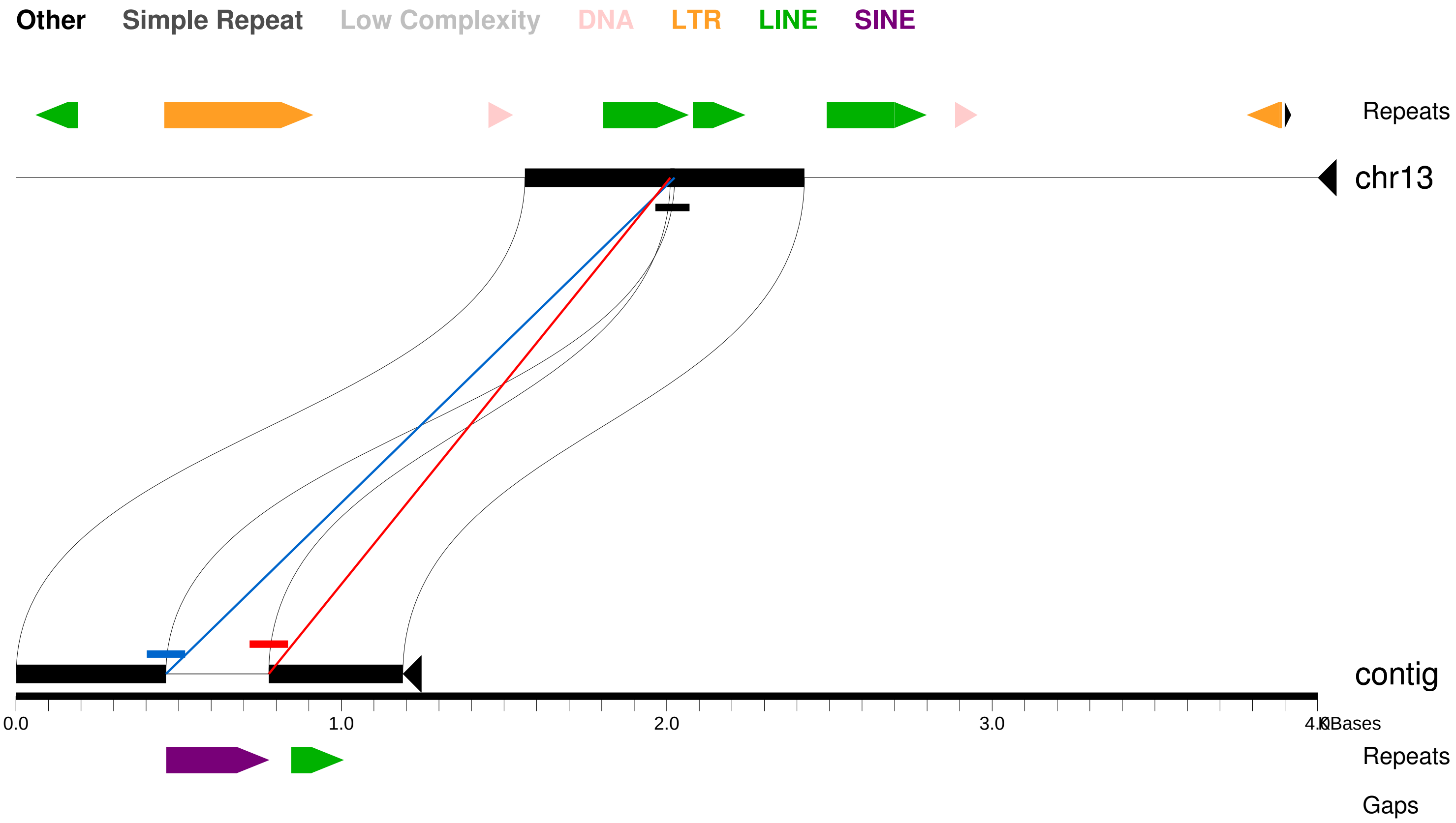

siteID: chr13_90510384 Scafftig1 + Breakpoint: chr13:90510394
chr13:90510234-90510565
Overlap Length: 12 INS size: 346 Genotyped: No

```
left   ------------AAATTGTAATCTTTTCTCCACAGCTTTGAAATGTAAATCTTTTTAGGAGCCTTTACTA
chrom  TGGAAAATATATAAATTGTAATCTTTTCTCCACAGCTTTGAAATGTAAATCTTTTTAGGAGCCTTTACTA
right  NNNNNNNNNNN-----------------------------------------------------------
       NNNNNNNNNNN 111111111111111111111111111111111111111111111111111111111

left   GTCTTTCTCAAGGACCTAGGAATCATCATTTTGAACTGTAATCATTGAGGAAGACAGGATCTCTATCTTC
chrom  GTCTTTCTCAAGGACCTAGGAATCATCATTTTGAACTGTAATCATTGAGGAAGACAGGATCTCTATCTTC
right  ----------------------------------------------------------------------
       1111111111111111111111111111111111111111111111111111111111111111111111

left   TGTTTTCTGCGGGAGCTTAAGAATGGGTTCCTG-GCCGGGCGCGGTGG--CTCACGCCTGTAATC-----
chrom  TGTTTTCTGCGGGAGCTTAAGAATGGGTTCCTTTGCCTCAAGTTGTGAAACTGCTTCCTGTCATGAAGAT
right  --------------------GAATGGGTTCCTTTGCCTCAAGTTGTGAAACTGCTTCCTGTCATGAAGAT
       11111111111111111111***********22***2222*22***222**2222*****2**222222

left   -CCAG------CACTTTGGG---AGGCTGAGGCGGGCGG--ATCACGAGGTCAGGAGATCGAGAC-CATC
chrom  ACCAGAAAATTCACTTTTCCTTTAAGTCAAGCCAATCAGTAAACAC-AGATGGCCAGTAAGCCACTCACC
right  ACCAGAAAATTCACTTTTCCTTTAAGTCAAGCCAATCAGTAAACAC-AGATGGCCAGTAAGCCACTCACC
       2****222222*****222222*2*222**2*222*2*22*2***2**2*2222**222*22**2**2*

left   CCGGCT---AAAACGGTGAAACC--------CCGTC-TCTA---CTAA----A
chrom  CCAGCTTTTAAAAATTTTCATCTTTTTTTTTCAGTGATGTAGAGCTCAGACTA
right  CCAGCTTTTAAAAATT-------------------------------------
       **2***222****2221NN1N1N        1N11N 1N11   11N1    1
```

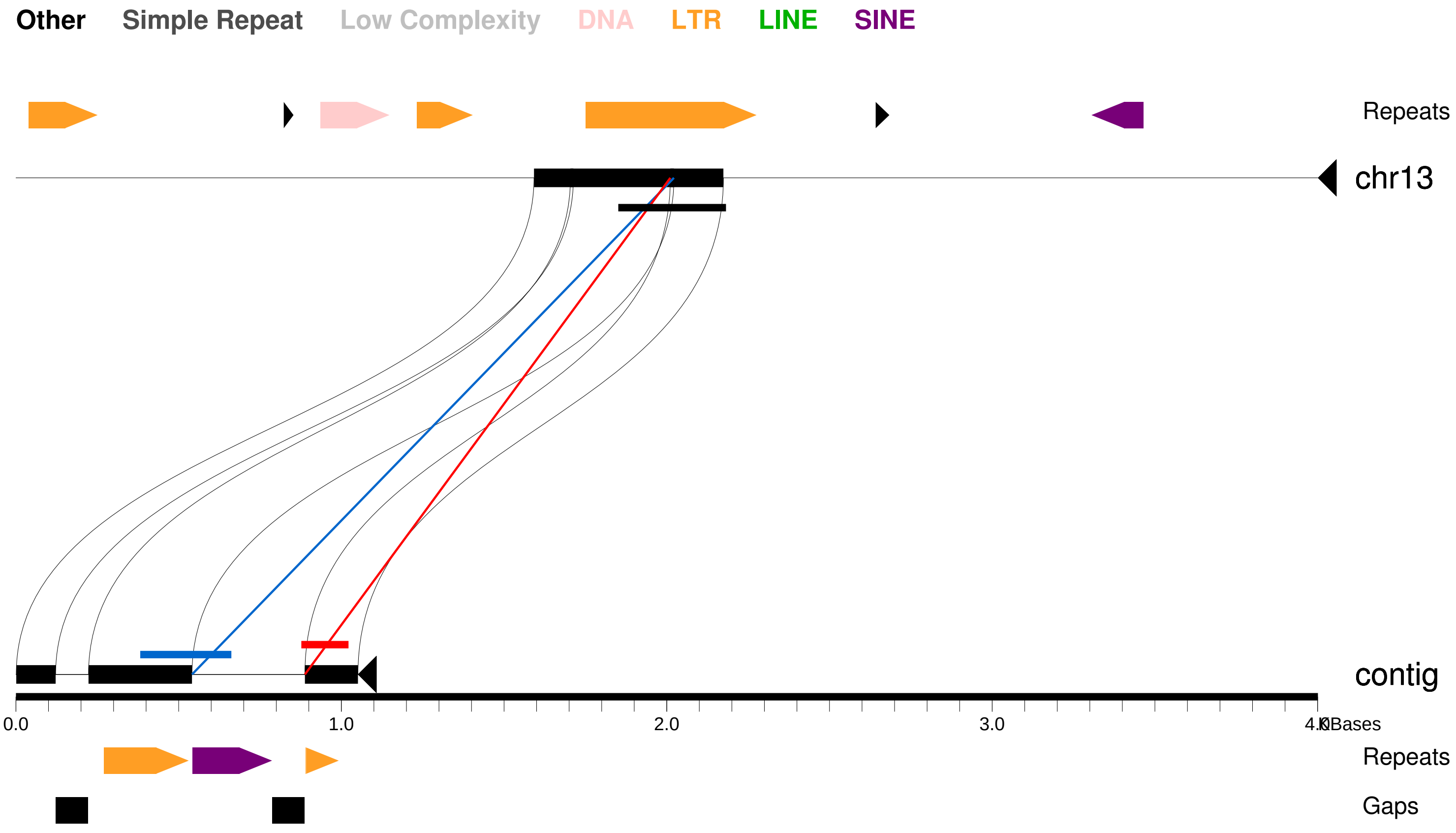

siteID: chr13_90550562 Scafftig5 - Breakpoint: chr13:90550561
chr13:90550516-90550620
Overlap Length: 15 INS size: 308 Genotyped: Yes

```
left     ATACCTAAGG-----------GAATGTGTATCAGGAGAGAG---GTGATGGTGGTGGTTAACAGGAGACA
chrom    ATACCTAAGG-----------GAATGTGTATCAGGAGAGAG---GTGATGGTGGTGGTTAACAGGAGACA
right    CCAGCCTGGGCGACAGAGCGAGACTCCGTCTCAAAAAAAAATAAATAAATAAATAAATAAACAGGAGACA
         11*1*111**11111111111**1*11**1***11*1*1*11111*1*111111111*1***********

left     GATGGCCGGGC--ACGGTGGCTCACGCCTGTAATCCCAGCACTTTGGGAGGCCGAGGCGGGCGGA
chrom    GATG-CTGAGATTAAAGCAGTTTTC-CTTGAAATCC------ATTATCAG-CAGATAC-------
right    GATG-CTGAGATTAAAGCAGTTTTC-CTTGAAATCC------ATTATCAG-CAGATAC-------
         ****2*2*2*222*22*22*2*22*2*2**2*****2222222**222**2*2**22*2222222
```

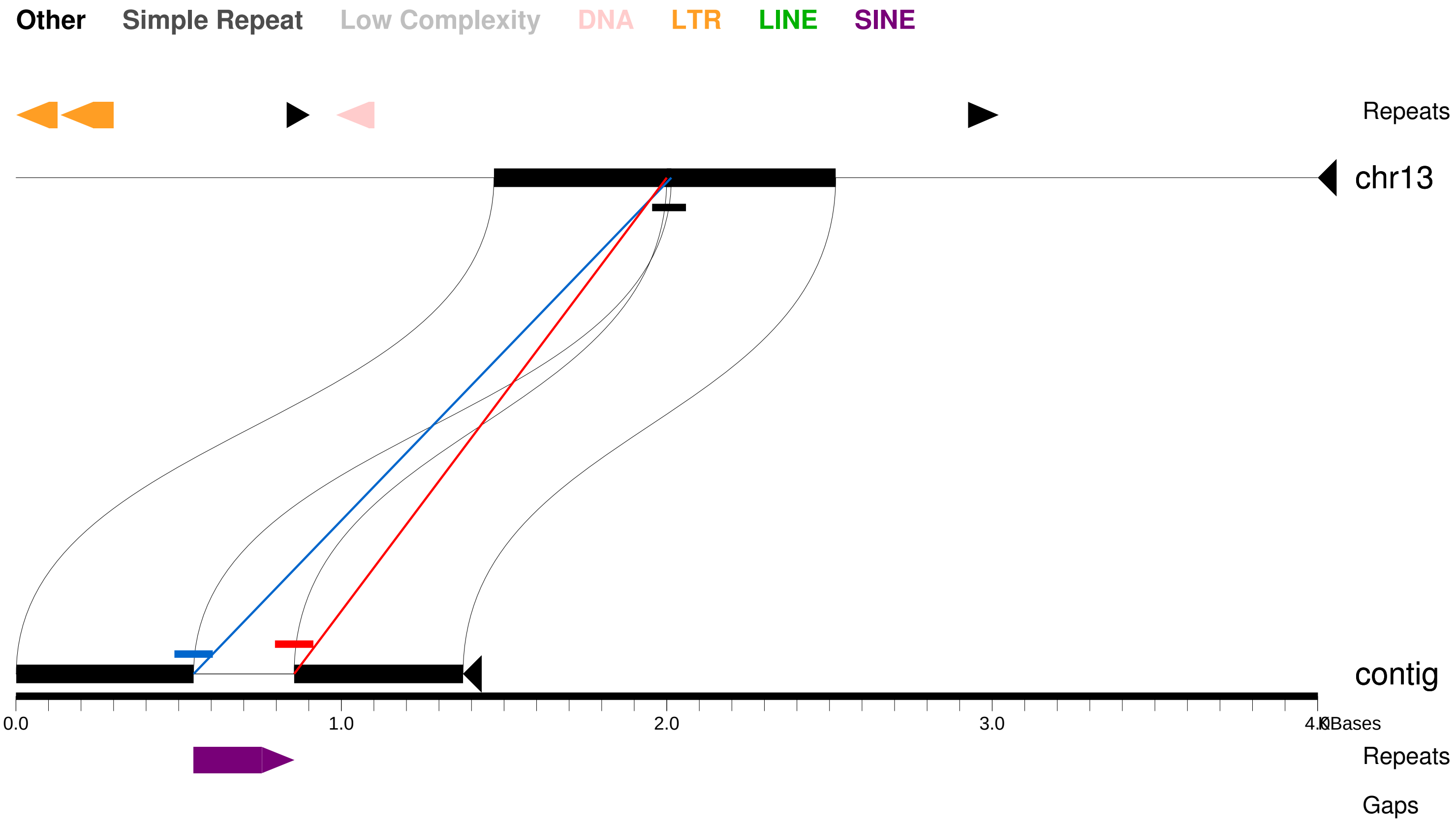

siteID: chr13_90947659 Scafftig10 + Breakpoint: chr13:90947643
chr13:90947595-90947702
Overlap Length: 9 INS size: 313 Genotyped: Yes

```
left     AAA------TATTTAAAACTAACTTATGT-ATTAAT--CATAAAATAAGGA--CAGAGGTGCAGTTTCTT
chrom    AAA------TATTTAAAACTAACTTATGT-ATTAAT--CATAAAATAAGGA--CAGAGGTGCAGTTTCAT
right    CCACCCGCCTCGGCCTCCCAAAGTGCTGGGATTACAGGCGTGAGCCACCGCGCCCGGCCTGCAGTTTCAT
         11*111111*11111111*1**1*11**11****1111*1*1*111*11*111*1*111*********2*

left     TTTTTTTTTTTTTTTTTTTTTGAGACGGAGTCTCGCTCTGTCGCCCAGGCGGGACTGCGG
chrom    TACATGCTTGAAAA-------AACACTGA---TCATTTAGTTCAAGAAAATTTACTTTT-
right    TACATGCTTGAAAA-------AACACTGA---TCATTTAGTTCAAGAAAATTTACTTTT-
         *222*22**2222222222222*2**2**222**22*22**22222*222222***2222
```

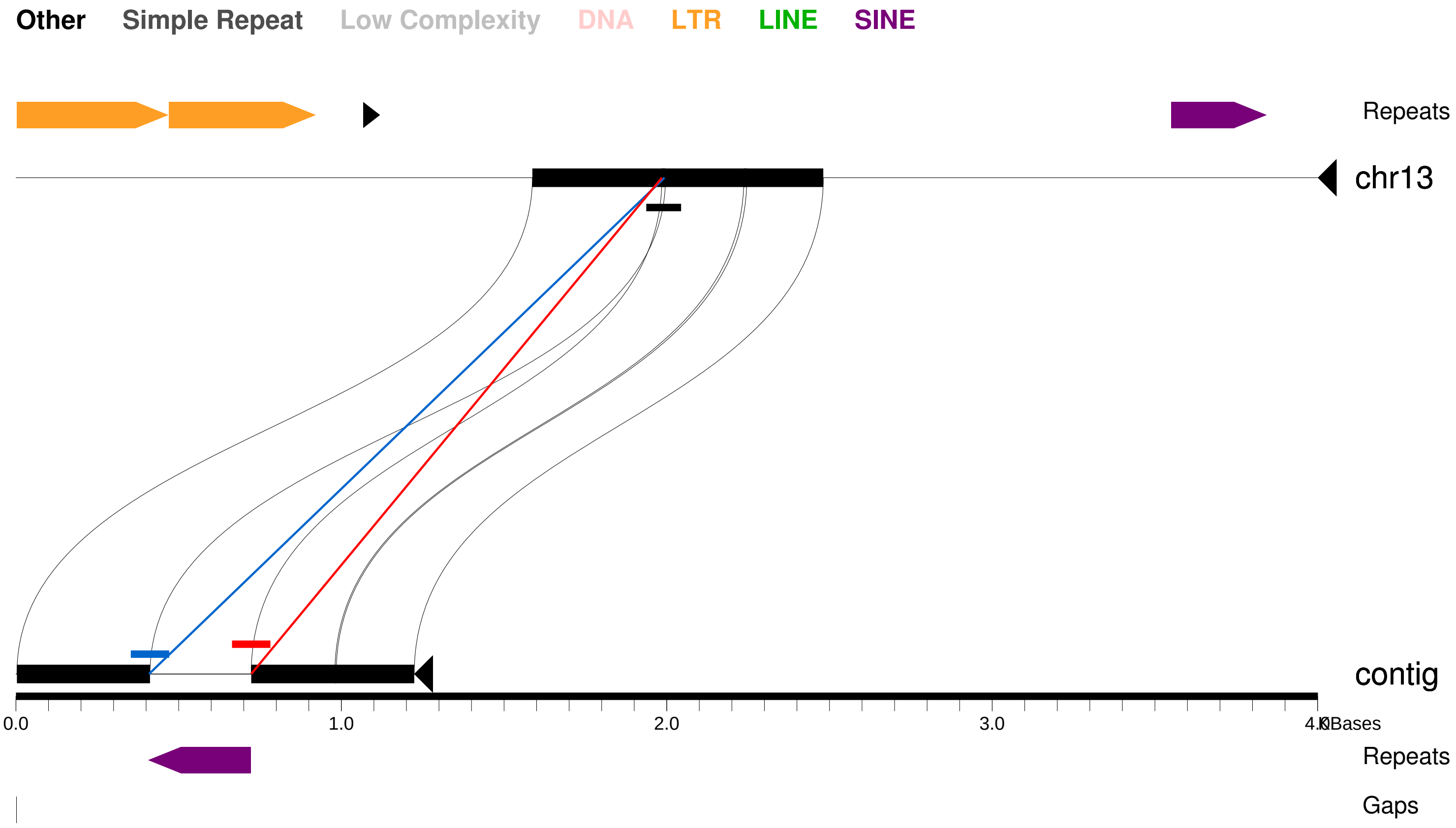

siteID: chr13_92161687 Scafftig9 + Breakpoint: chr13:92161659
chr13:92161615-92161718
Overlap Length: 16 INS size: 309 Genotyped: Yes

```
left    AGTTACTTCAATTAGGTCTTTAAAAGTGCTCATAAAGAAGGCTT------------------AACATATT
chrom   AGTTACTTCAATTAGGTCTTTAAAAGTGCTCATAAAGAAGGCTT------------------AACATATT
right   CCACCCGCC---TCGGCCTCCCAAAGTGCTGGGATTACAGGCGTGAGCCACCGCGCCCGGCCAACATATT
        11111*11*111*1**1**111********111*1111****1*111111111111111111********

left    CATATTTTTTTTTTTTTTTTTTTTTTTTGAGACGGAGTCTCGCTCTGTCGCCCAGGTGGGACTGCGGA
chrom   CATATTTTAAGGATCAAATAGT-----GAGA--TAGTCA---TATTTG---CA--TAGGCATTCTTA
right   CATATTTTAAGGATCAAATAGT-----GAGA--TAGTCA---TATTTG---CA--TAGGCATTCTTA
        ********22222*2222*22*22222****222****2222*2*2*2222**22*2**22*2*22*
```

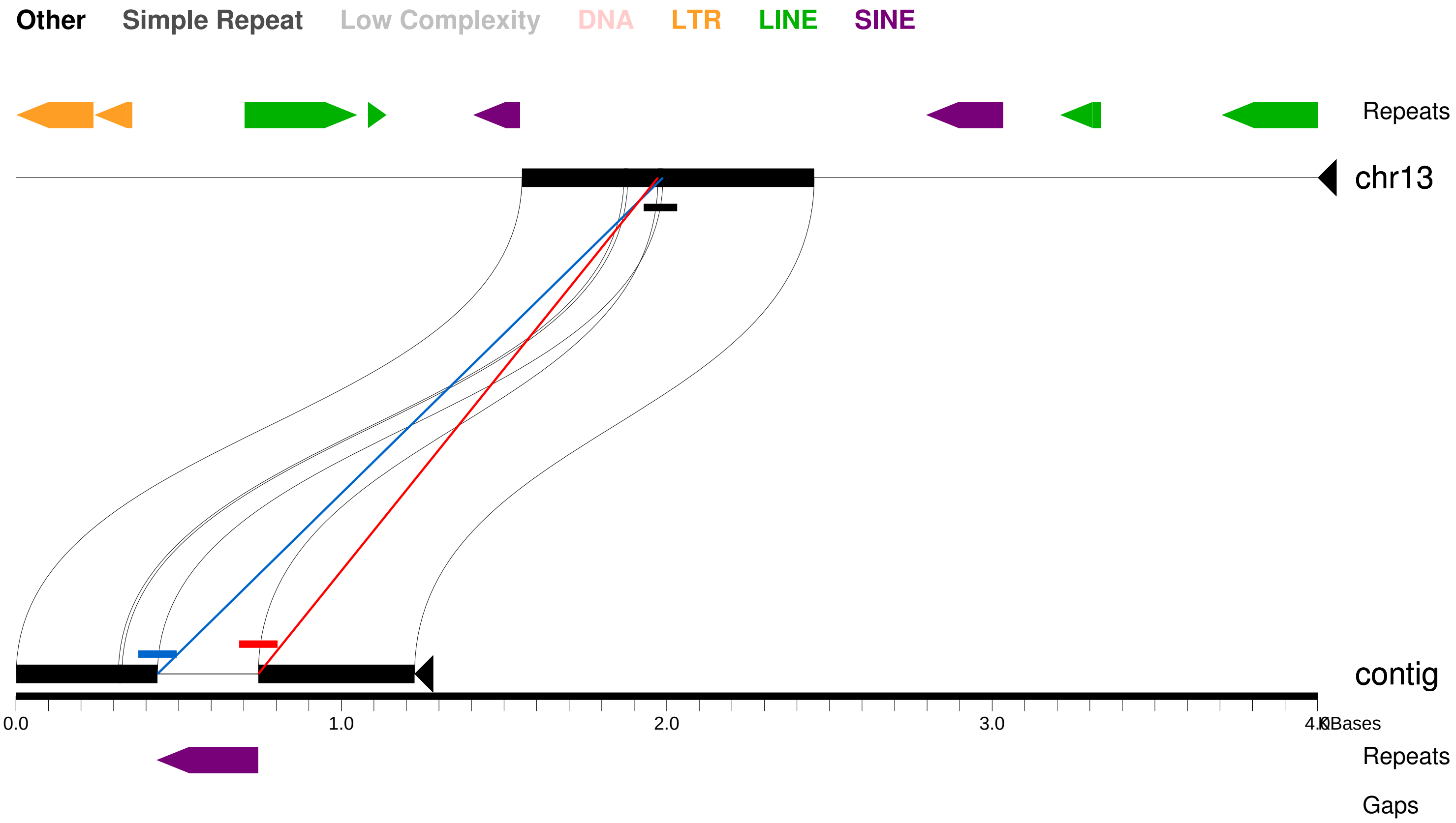

siteID: chr13_92261273 Scafftig4 - Breakpoint: chr13:92261274
chr13:92261227-92261333
Overlap Length: 13 INS size: 310 Genotyped: Yes

```
left     TCAAATTCAGTGTGACAACATTGTAGGTGTGTGCCATCAAGGCATAA-----------------AATTTA
chrom    TCAAATTCAGTGTGACAACATTGTAGGTGTGTGCCATCAAGGCATAA-----------------AATTTA
right    ----AGCCTGGGCGACAG-AGCGAAACTCCGTCTCAAAAAAAAAAAAAAAAAAAAAAAAAAAAAGAATTTA
         1111*11*1*1*1****11*11*1*11*11**11**11**111*1**11111111111111111******

left     GATTTGAGGGCCGGGCGCGGTGGCTCACGCCTGTAATCCCAGCACTTTGGGA----GGCCGAGACGGGTG
chrom    GATTTGA---CCAG---CAATCTCTAATG-------ACACAGGAGTTGGTGATAGTGTCCTTGTCAG---
right    GATTTGA---CCAG---CAATCTCTAATG-------ACACAGGAGTTGGTGATAGTGTCCTTGTCAG---
         *******222**2*222*22*22**2*2*22222222*2***2*2**2*2**2222*2**22*2*2*222
```

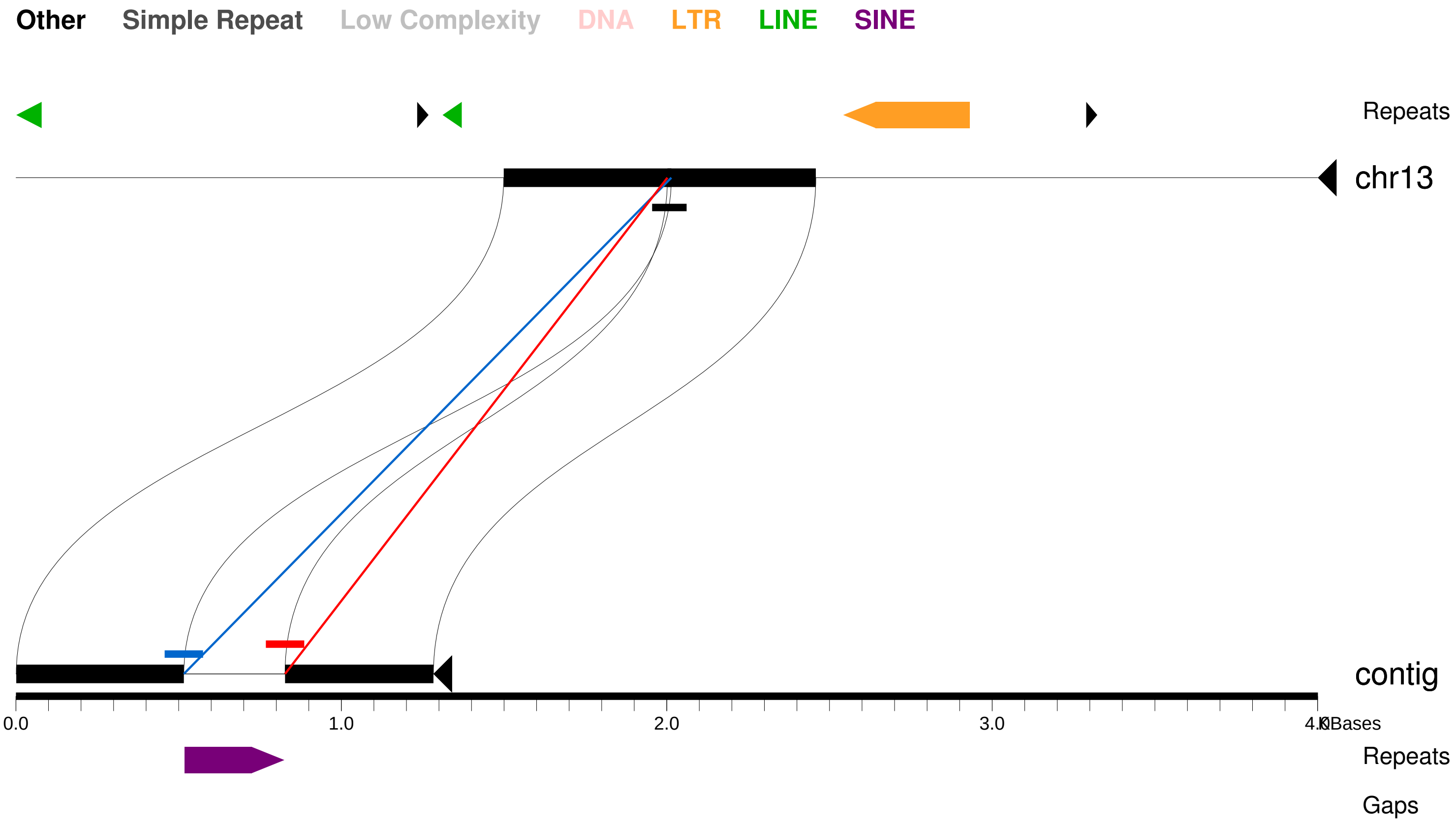

siteID: chr13_92513972 Scafftig7 - Breakpoint: chr13:92513963
chr13:92513921-92514022
Overlap Length: 18 INS size: 263 Genotyped: Yes

```
left   -ATCTCTACCTTACAAT------TGGATAATTTAG------TTCAT----AAATATGTTAAATAAAACAT
chrom  -ATCTCTACCTTACAAT------TGGATAATTTAG------TTCAT----AAATATGTTAAATAAAACAT
right  GATCCCGCCACTGCACTCCAGCCTGGACGACAGAGCGAGACTCCGTCTCAAAAAAAAAAAAAAAAAAACAT
       1***1*11*11*1**1*111111****11*111**111111*1*1*1111***1*1111***1*******

left   GTTGCTATCCCAGCACTTTGGGAGGCCGAGGCGGGCGGATCACGAGGTCAGGAGATCGAGACCATC
chrom  GTTGCTA--------CTTTTTAAAACTGTA-CAATCTGTT-------TCTTGTT-TCTCTTCTCTT
right  GTTGCTA--------CTTTTTAAAACTGTA-CAATCTGTT-------TCTTGTT-TCTCTTCTCTT
       *******22222222****222*22*2*222*222*2*2*2222222**22*222**2222*22*2
```

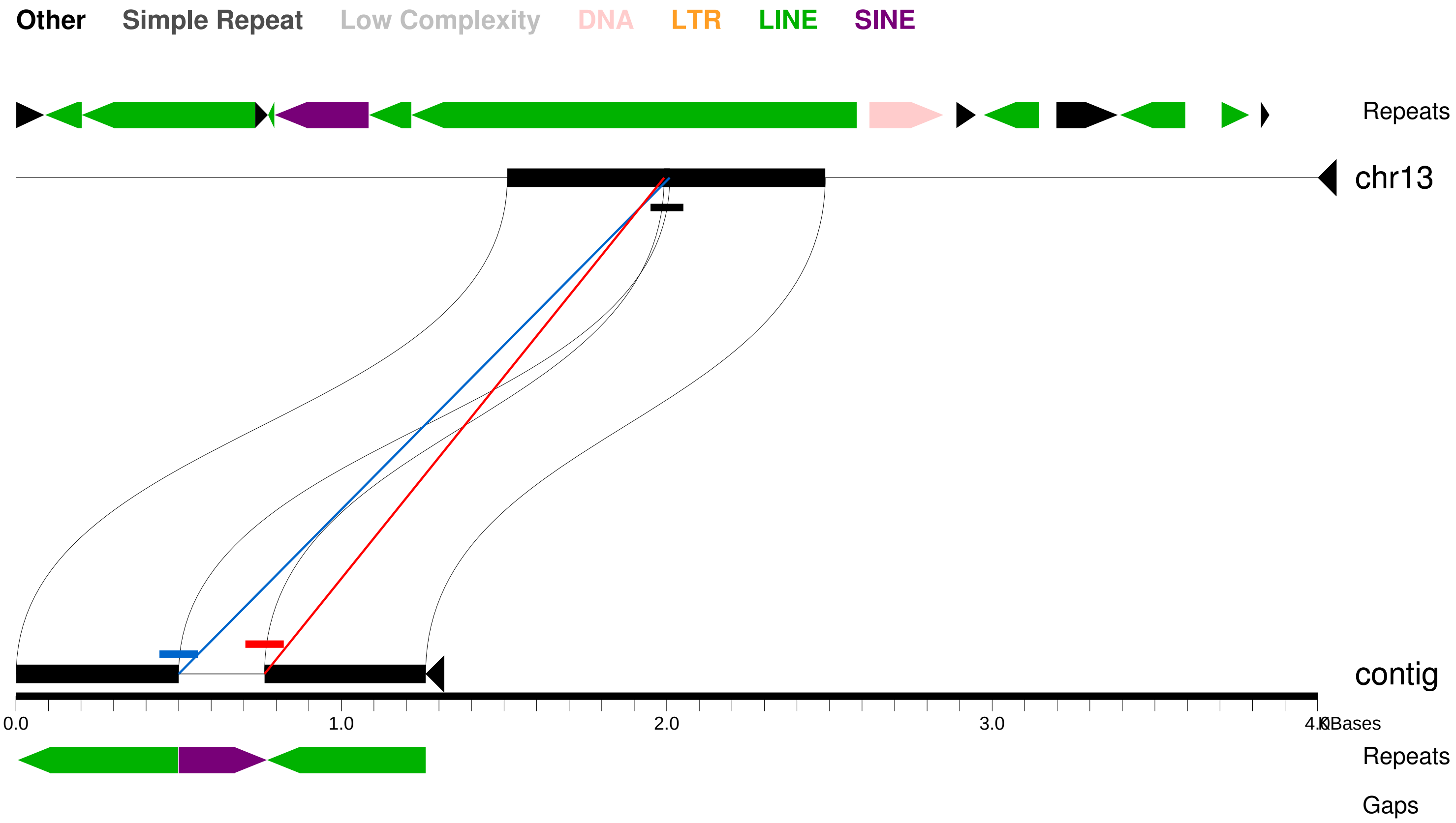

siteID: chr13_92825228 Scafftig1 - Breakpoint: chr13:92825227
chr13:92825179-92825278
Overlap Length: 12 INS size: 331 Genotyped: Yes

```
left    A-------------------AAAAATTTAGGCAATTATTTTAATTTGACTATACCTGTTAACAATATAAG
chrom   A-------------------AAAAATTTAGGCAATTATTTTAATTTGACTATACCTGTTAACAATATAAG
right   AGAGCGAGACTCCGCCTCAAAAAAAAAAAAAAAAAAAAAAAAAAAAAAAAAAAAAAAAAAAAAAAAAAAG
        *1111111111111111111*****111*111**11*1111**1111*11*1*111111**1**1*1***

left    AAACGTTGCGGCCGGGCGCGGTGGCTCACGCCTGTAATCCCAGCACTTTGGGAGGCCGAGGCGGGCGG
chrom   AAACGTTGCAACAGTCAAACGAG---CAACTCTTAAGTCTTATTACAT----------------GTTA
right   AAACGTTGCAACAGTCAAACGAG---CAACTCTTAAGTCTTATTACAT----------------GTTA
        *********22*2*222222*2*222**222**22*2**22*22**2*2222222222222222*222
```

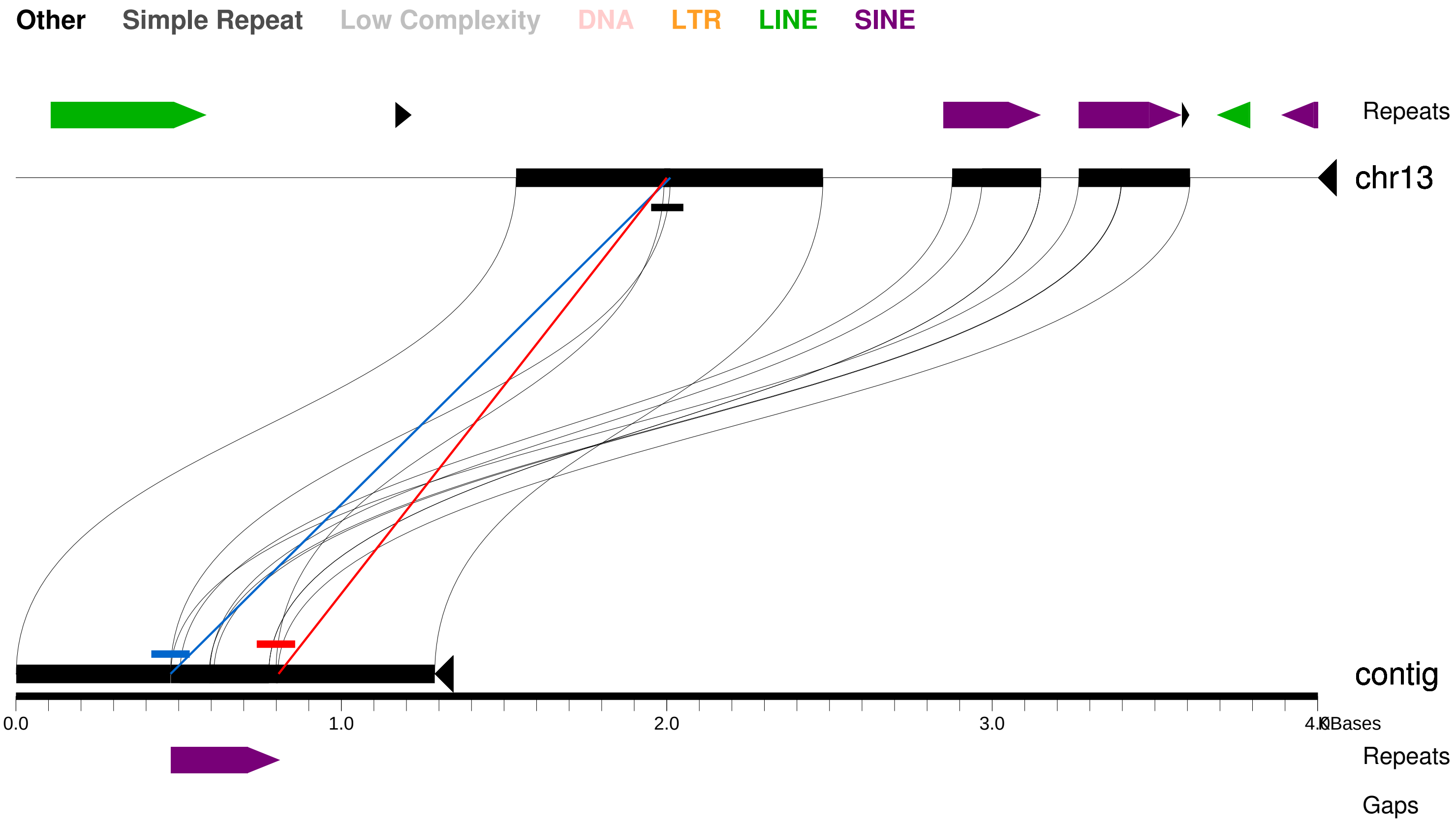

siteID: chr13_93101143 Scafftig8 - Breakpoint: chr13:93101135
chr13:93101091-93101194
Overlap Length: 16 INS size: 249 Genotyped: Yes

```
left    CTTTGAG----------------AGATTCCTGCTTAATGGTATAAGAGAAATTCTTCATTAAAGAAGCCC
chrom   CTTTGAG----------------AGATTCCTGCTTAATGGTATAAGAGAAATTCTTCATTAAAGAAGCCC
right   ACTCCAGCCTGGGCGACAGAGCGAGACTCCGTCTCAAAAAAAAAAAAAAAAAAAAAAAA-AAAGAAGCCC
        11*11**1111111111111111***1***11**1**1111*1**1*1***111111*11**********

left    ACACAGCGGATCACGAGGTCAGGAGATCGAGACCATCCCGGCTAAAACGGTGAAACCCCGTCTCT
chrom   ACACAGTTATACCCCA---CAA-AAAGCTAATGCTATTGGGC----ACTGTAGAA----GTG---
right   ACACAGTTATACCCCA---CAA-AAAGCTAATGCTATTGGGC----ACTGTAGAA----GTG---
        ******22222*2*2*222**22*2*2*2*222*22222***2222**2**22**2222**2222
```

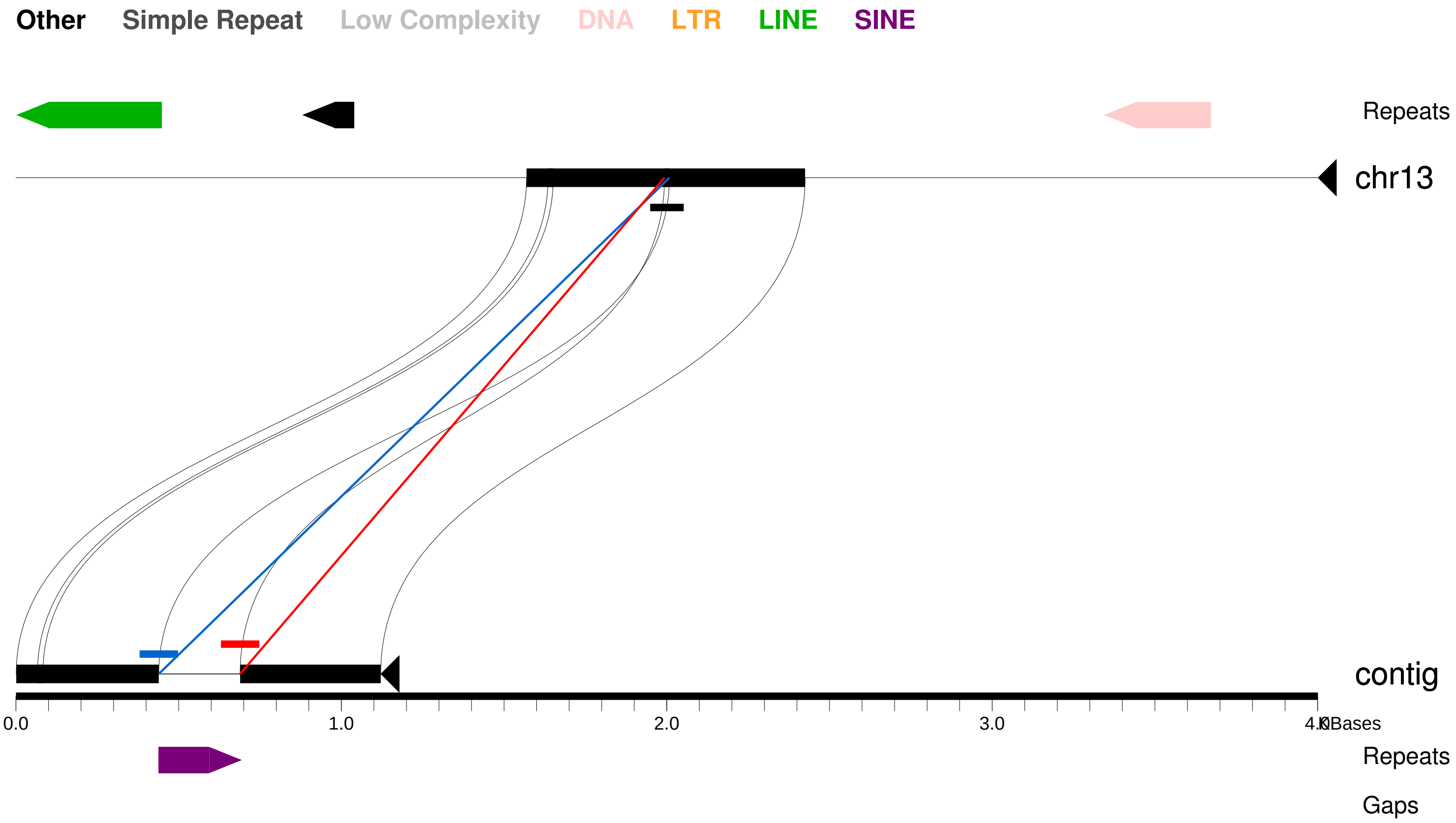

siteID: chr13_93828423 Scafftig3 + Breakpoint: chr13:93828407
chr13:93828247-93828581
Overlap Length: 15 INS size: 288 Genotyped: Yes

```
left   A----------TCCAACCACATGTAAAAAACTTAATTCACCATAATCAAGTGGGCTTTATTCCTTGGATG
chrom  AGCAACCCAAATCCAACCACATGTAAAAAACTTAATTCACCATAATCAAGTGGGCTTTATTCCTTGGATG
right  TT---------TTGTATTTTTAGTAGAGACGGGGTTTCACCAT------------TTTTTAGCCGGGATG
       1N         *111*111111***1*1*111111********111111111111***1*11*11*****

left   CAAAGTTGGTTCAACACACACAGATCAATAAATGTGATTCATCATA-TAAGCAGAACTAAAAACAAAAAC
chrom  CAAAGTTGGTTCAACACACACAGATCAATAAATGTGATTCATCATA-TAAGCAGAACTAAAAACAAAAAC
right  -------GTCTCGATCTCCTGACCTC-------GTGATCCGCCCGCCTCGGCCTCCCAAAGTGCTGGGAT
       1111111*11**1*1111*11*11**1111111*****1*11*1111*11**1111*1**111*1111*1

left   CCCATGATCATCTCAATACATGCAGAAAAGGTTTTTTTTTTTTTTT---------TT--TTTTGAGACG--
chrom  CCCATGATCATCTCAATACATGCAGAAAAGGTTTTTGATAAAATTCAGCACAGCTTCATGTTAAAAAGCT
right  TACAGG-----CGTGAGCCACGCAGAAAAGGTTTTTGATAAAATTCAGCACAGCTTCATGTTAAAAAGCT
       11**1*11111*111*11**1**************22*2222**222222222**22*2**2*2*2*22

left   -GAGTCTCG-CTCTGTCGCCCAGGCTGGAG---TGC-------AGTG-GCGGGATCTCGG-----CTCAC
chrom  AGAGGCTCTACAAAGTAGGTCTTGAAGAAACATTTCTCAAAATAGTAAGAGCCATCTATGACAAGCCCAC
right  AGAGGCTCTACAAAGTAGGTCTTGAAGAAACATTTCTCAAAATAGTAAGAGCCATCTATGACAAGCCCAC
       2***2***22*222**2*22*22*22*2*2222*2*2222222***22*2*22****22*22222*2***

left   TGCAAGCTCCG--CCTCCCGGGTTCACGC------CATTCTCCTG-----CCTCAG
chrom  AGCCAACATCATACAGAATGGGAAAAAGCTGGAAGCATTTCCCTTGAGAACCAGAA
right  AGCCAACATCATACAGAATGGGAAAAAGCTGGAAGCATTTC---------------
       2**2*2*22*222*22222***222*2**222222****22111N     11NN1N
```

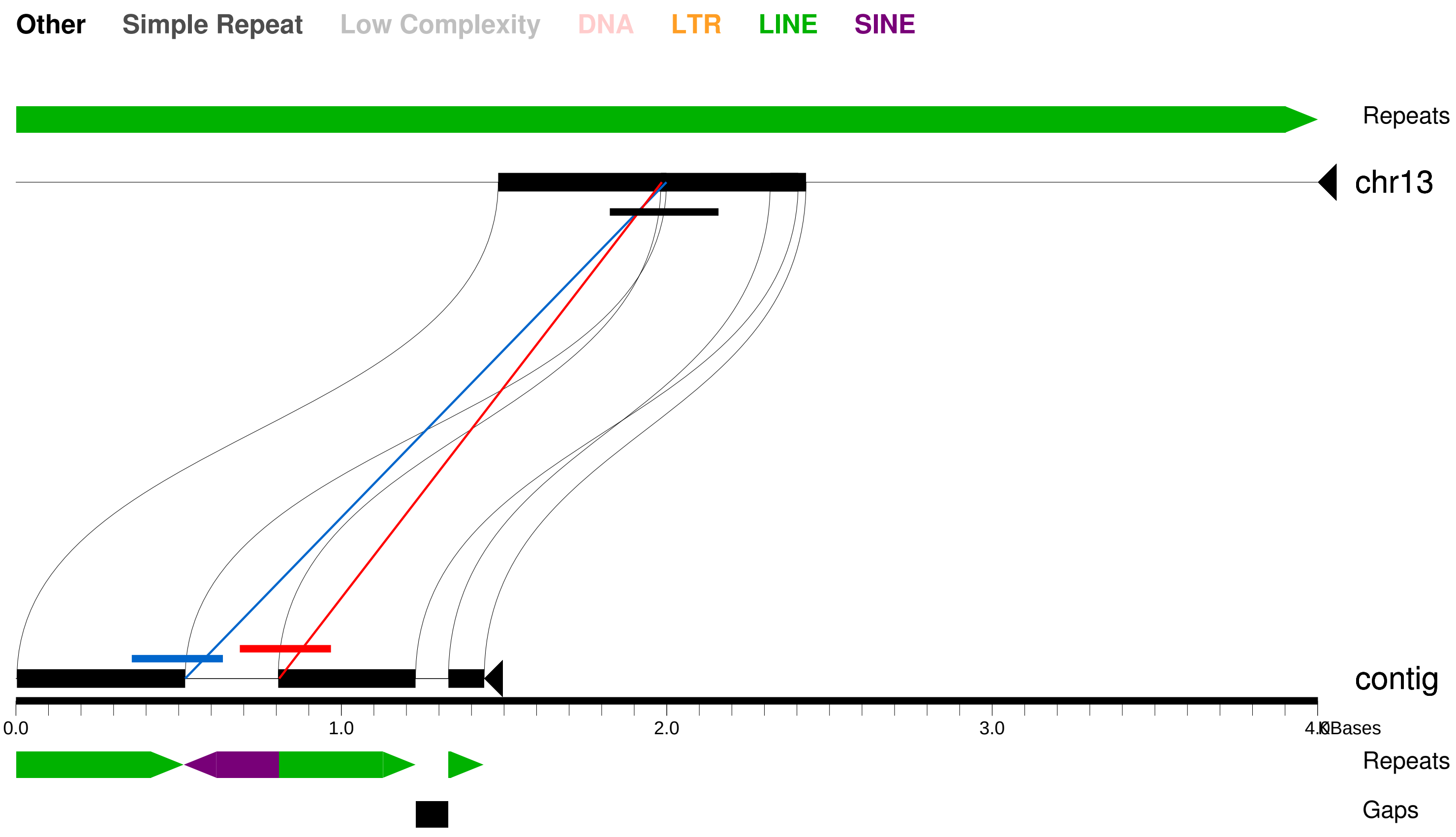

siteID: chr13_94105819 Scafftig3 - Breakpoint: chr13:94105804
chr13:94105751-94105863
Overlap Length: 7 INS size: 302 Genotyped: Yes

```
left    GGTGGGTTCTTGGTCTCACTGACTTCAAGAATGA-AGCCGCGGACC----CTCGCAGT-GAGTGTTTTTT
chrom   GGTGGGTTCTTGGTCTCACTGACTTCAAGAATGA-AGCCGCGGACC----CTCGCAGT-GAGTGTTACAA
right   CCACCCGCCTCGGCCTCCCAAAGTGCTGGGATTACAGGCGTGAGCCACCACGCCCGGCCGAGTGTTACAA
        1111111**1**1***1*11*1*1*11*1**1*1**1**1*11**1111*1*1*1*11********2222

left    TTTTTTTTTTTTTTTTTGAGACAGCGTCTGGCTCTGTCGCCCAGGCTGGAGTGCA
chrom   CTCTTAAGGTGGCACATCTG---GAGTTTGTTCCTT---CTGATGTTCGGATGTG
right   CTCTTAAGGTGGCACATCTG---GAGTTTGTTCCTT---CTGATGTTCGGATGTG
        2*2**2222*222222*22*222*2**2**222**2222*22*2*2*2*22**22
```

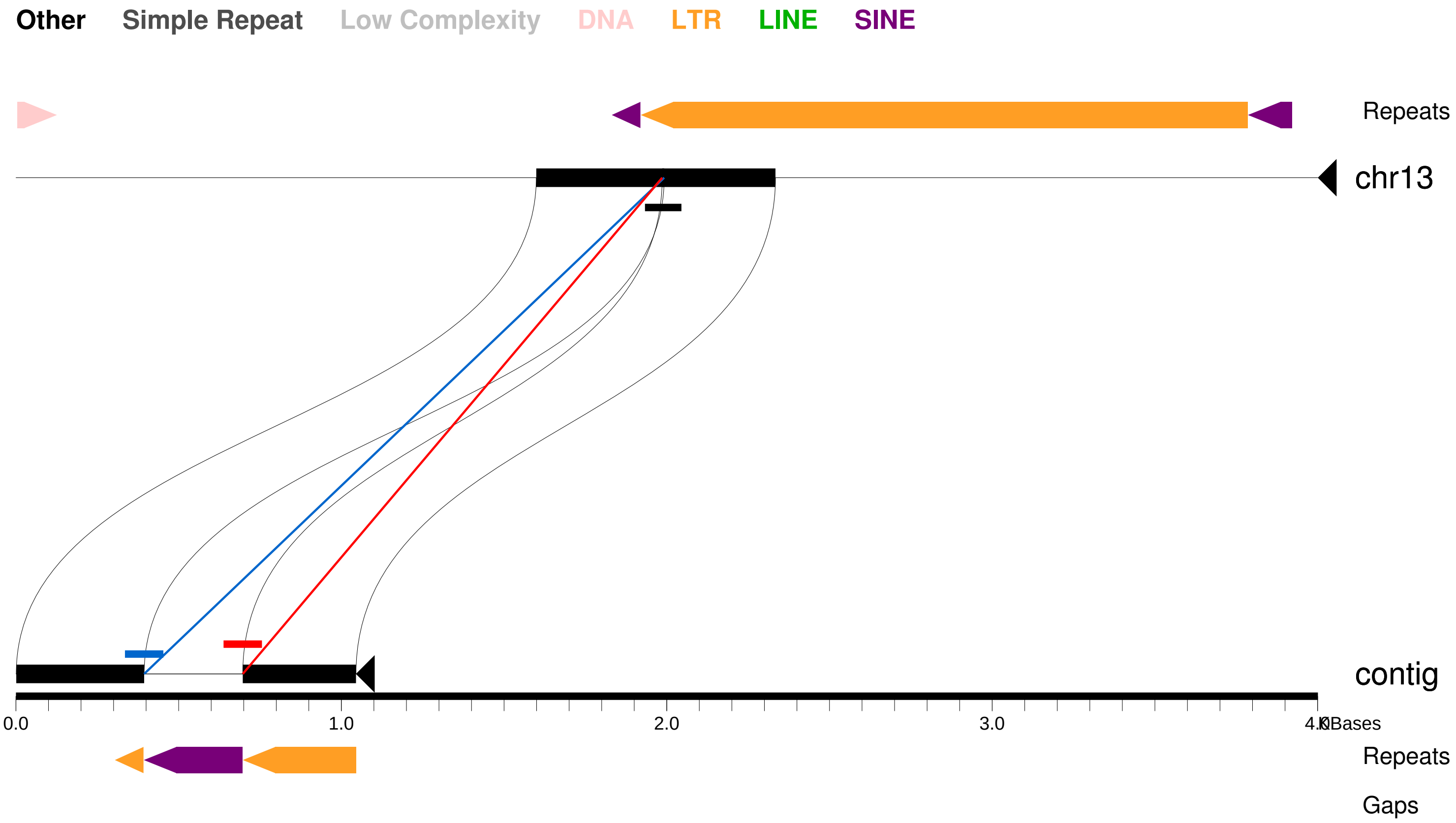

siteID: chr13_98523883 Scafftig1 + Breakpoint: chr13:98523770
chr13:98523723-98523829
Overlap Length: 13 INS size: 330 Genotyped: No

```
left    GA-GCCAGA-TGAAATGAAGACCATTAAGTTTTTACAG--CTAAAAAAACT--------CTCATTTCATC
chrom   GA-GCCAGA-TGAAATGAAGACCATTAAGTTTTTACAG--CTAAAAAAACT--------CTCATTTCATC
right   TCCGCCCGCCTCGGCCTCCCAAAGTGCTGGGATTACAGGCGTGAGCCACCGCGCCCGGCCTCATTTCATC
        111***1*11*111111111*111*111*111******111*1*111*1*111111111***********

left    TTNNNNNNNNNNNNNNNNNNNNNNNNNNNNNNNNNNNNNNNNNNNNNNNNNNNNNNNNNNN
chrom   TTTATATTGTATAGGTTCATTAATATTTCAATCAATTACATAATTACAA------------
right   TTTATATTGTATAGGTTCATTAATATTTCAATCAATTACATAATTACAA------------
        **22222222222222222222222222222222222222222222222222222222222
```

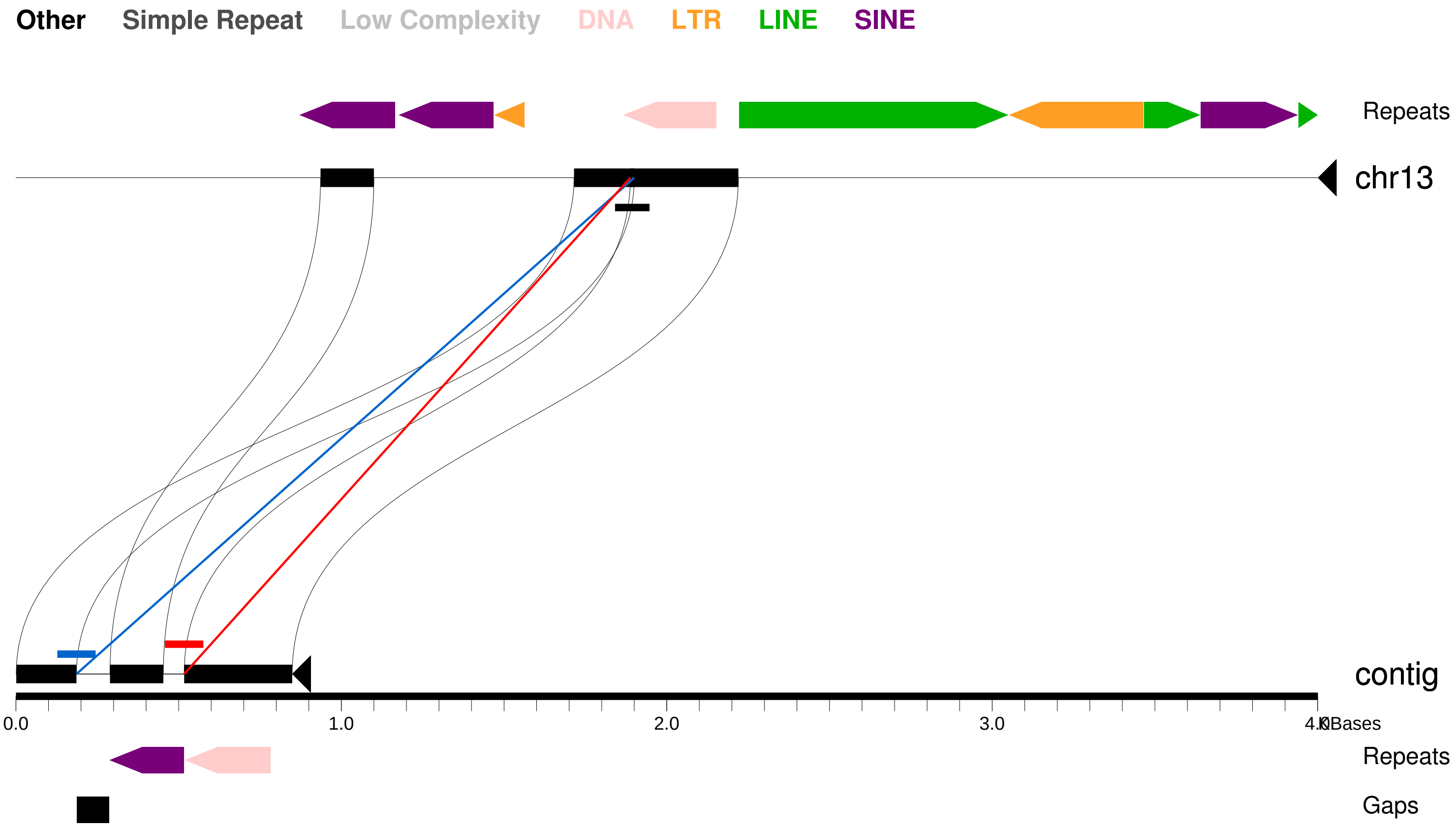

siteID: chr13_98734903 Scafftig1 + Breakpoint: chr13:98734895
chr13:98734854-98734954
Overlap Length: 19 INS size: 406 Genotyped: No

```
left    TTTCTCTTTTCTTGTCC-CTCACTTT-------TTAC---------CTAAAACTCCTA---GACAGTTTG
chrom   TTTCTCTTTTCTTGGCC-CTCACTTT-------TTAC---------CTAAAACTCCTA---GACAGTTTG
right   CCACCCGCCTC--GGCCTCCCAAAGTGCTGGGATTACAGGCGTGAGCCACCGCGCCCGGCCGACAGTTTG
        111*1*111**11*2**1*1**111*1111111****111111111*1*111*1**11111*********

left    CCATTAACCTNNNNNNNNNNNNNNNNNNNNNNNNNNNNNNNNNNNNNNNNNNNNNNNNNNNNNNNNNNNNN
chrom   CCATTAACCTTAAATCCTAATATAGATTGAACATTTGTGTCCCTCCCAGAT------------------
right   CCATTAACCTTAAATCCTAATATAGATTGAACATTTGTGTCCCTCCCAAAT------------------
        **********22222222222222222222222222222222222222N22222222222222222222
```

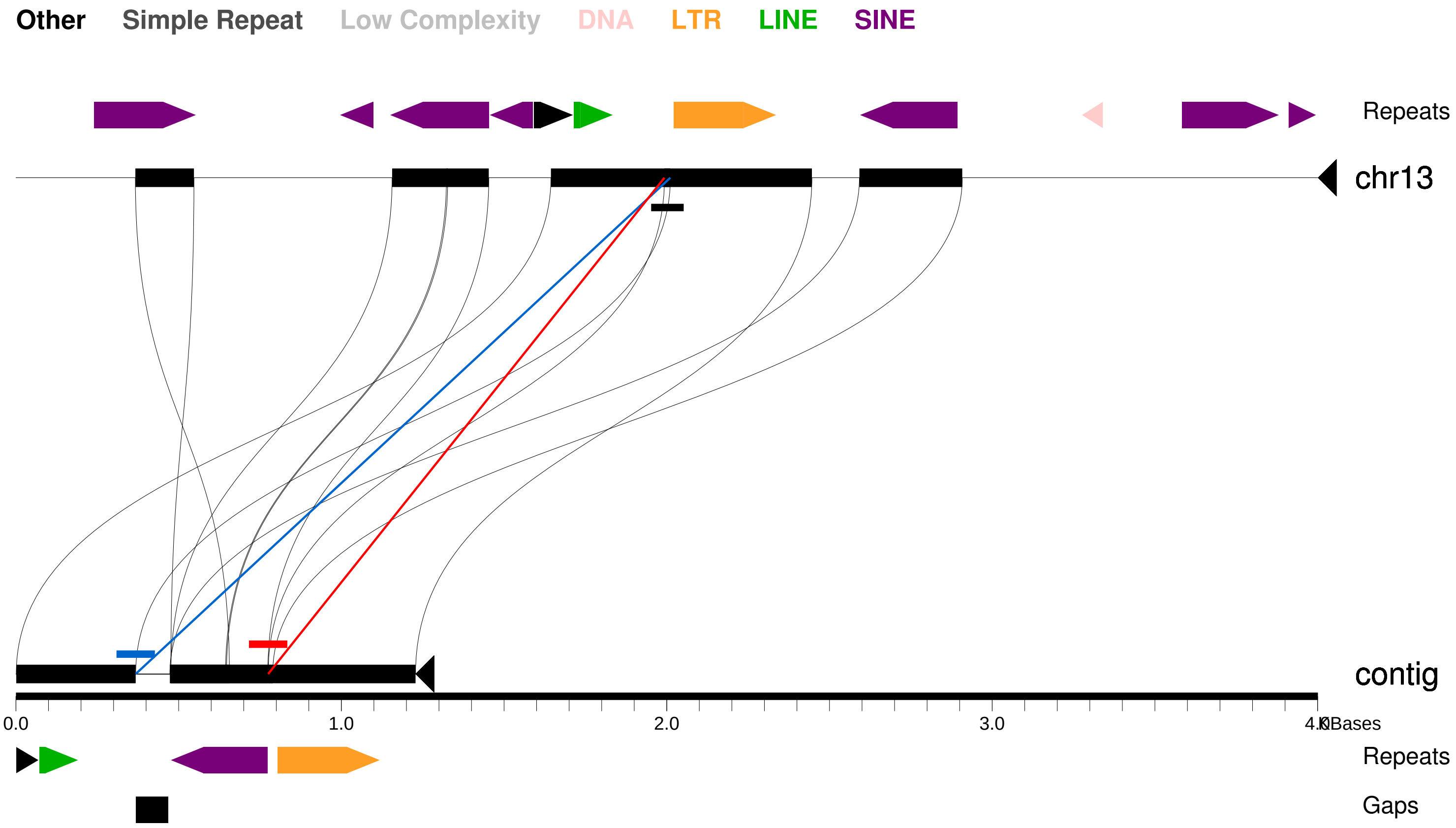

siteID: chr13_98803291 Scafftig1 + Breakpoint: chr13:98803298
chr13:98803255-98803357
Overlap Length: 14 INS size: 307 Genotyped: Yes

```
left     TTTCTGCAGTTTCAGTTACCCACGGT----CAACCACAGTC-TGAATA------------AACAATTCAC
chrom    TTTCTGCAGTTTCAGTTACCCACGGT----CAACCACAGTC-TGAATA------------AACAATTCAC
right    TC-CGCCCGTCTCGGCCTCCCAAAGTGCTGGGATTACAGGCGTGAGCCACCGCGCCCGGCAACAATTCAC
         *11*11*1**1**1*111****11**111111*11****1*1***1111111111111111*********

left     AGGTTTTTTTTTTTTTTTTTTTTTTTTTTTTTGAGACGGAGTCTCGCTCTGTCGCCCAGGCTGGAG
chrom    AGGTCTTAAACGGTGTGCTGTTACGAGTAGCTT-GATGAAATCTCT---------------TAGCG
right    AGGTCTTAAACGGTGTGCTGTTACGAGTAGCTT-GATGAAATCTCT---------------TAGCG
         ****2**222222*2*22*2**22222*222*22**2*2*2****2222222222222222*2*2*
```

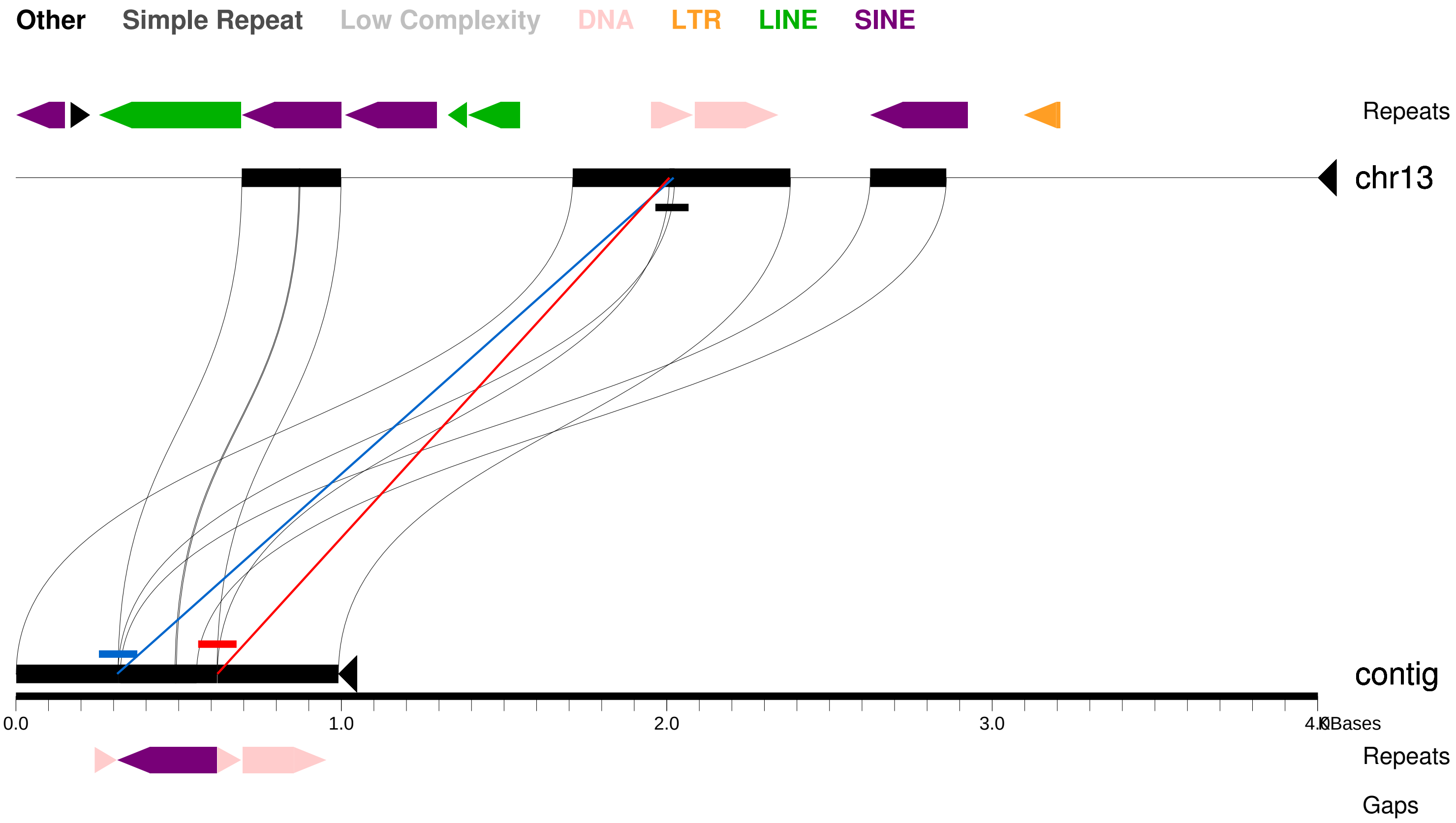

siteID: chr13_99765010 Scafftig3 - Breakpoint: chr13:99764995
chr13:99764949-99765054
Overlap Length: 14 INS size: 443 Genotyped: No

```
left    --GATGGTCTTGTCTTTTTGAGAATATTGTATATATTGAAATCATACA------------GTATGTAGCC
chrom   --GATGGTCTTGTCTTTTTGAGAATATTGTATATATTGAAATCATACA------------GTATGTAGCC
right   CCGCCCGCCTCGGCCTCCCAAAGTGCTGGGAT-TACAGGCGTGAGCCACCGCGCCCGGCCGTATGTAGCC
        11*111*1**1*1*1*1111*11111*1*1**1**11*111*1*11**111111111111**********

left    TTTTNNNNNNNNNNNNNNNNNNNNNNNNNNNNNNNNNNNNNNNNNNNNNNNNNNNNNNNNNNN
chrom   TTTTCAAAGCAGCTTCTTTTACTCAGCATAATGCCTCTGAGTTTCACACA-------------
right   TTTTCAAAGCAGCTTCTTTTACTCAGCATAATGCCTCTGAGTTTCACACA-------------
        ****222222222222222222222222222222222222222222222222222222222222
```

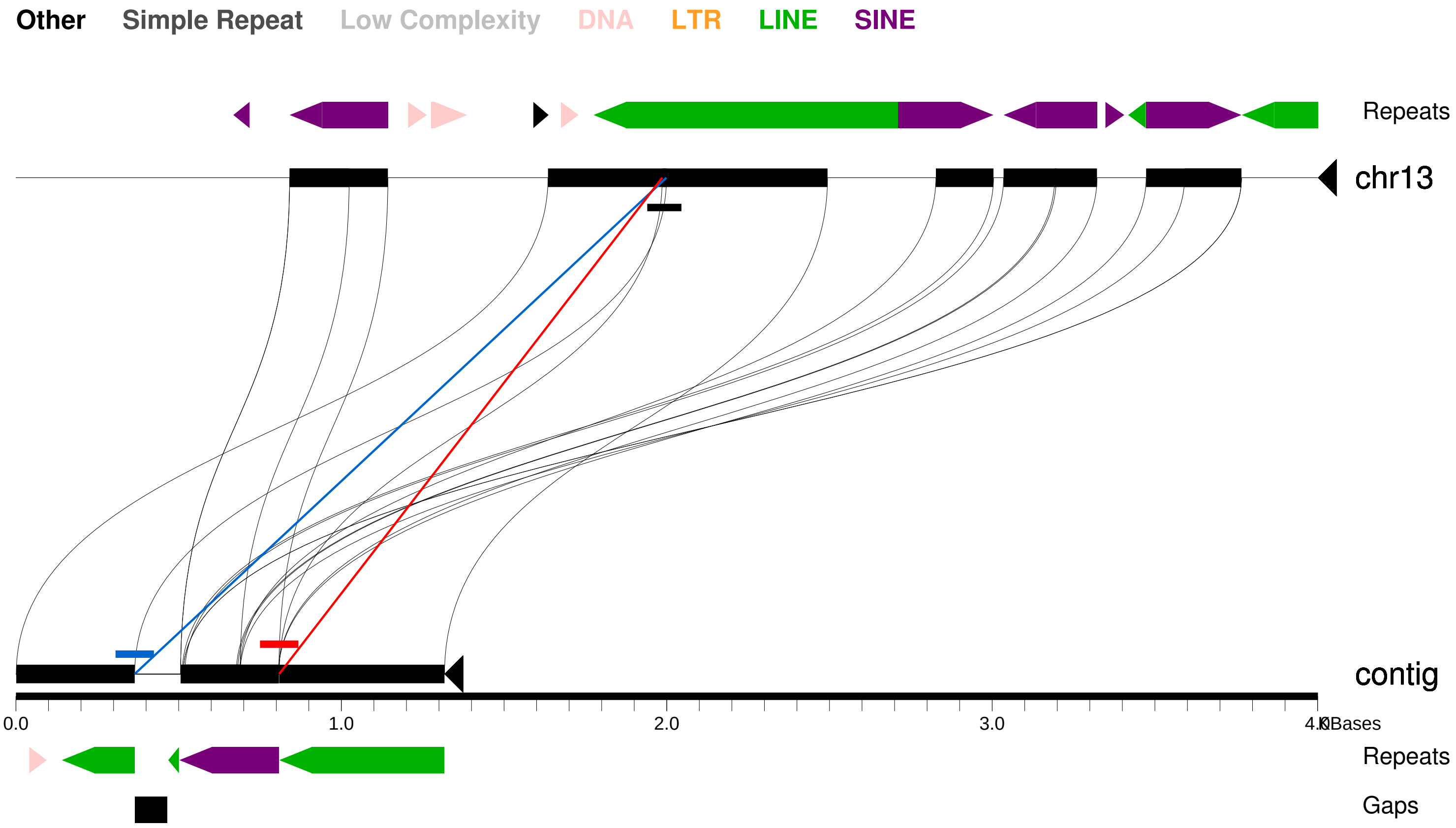

siteID: chr14_20282011 Scafftig3 + Breakpoint: chr14:20282004
chr14:20281955-20282063
Overlap Length: 11 INS size: 297 Genotyped: Yes

```
left    ATGG-TCTTGCTCTCCTTGTG----GACTA-----GTATCTCTCTGTTTCTTCACCTTTTTTTTTTTTTT
chrom   ATGG-TCTTGCTCTCCTTGTG----GACTA-----GTATCTCTCTGTTTCTTCACCTTTTTTTTTTTTTT
right   CCGCCTCGGCCTCCCAAAGTGCTGGGATTACAGGCGTGAGCCACCGCGCCCGGCCCACCTTTTTTTTTTT
        11*11**111***1*111***1111**1**11111**1111*1*1*111*1111**111***********

left    TTTTTTTTGAGACGGAGTCTCGCTCTGTCGCCCAGGCTGGAGTGCAGTGGCGCGATCTC
chrom   AAGTCATACA-ACTATCTTTCCATTTAT-GTCTCTGCTAGTCTTCTTT-------TC-C
right   AAGTCATACA-ACTATCTTTCCATTTAT-GTCTCTGCTAGTCTTCTTT-------TC-C
        222*22*22*2**2222*2**22*2*2*2*2*222***2*22*2*22*2222222**2*
```

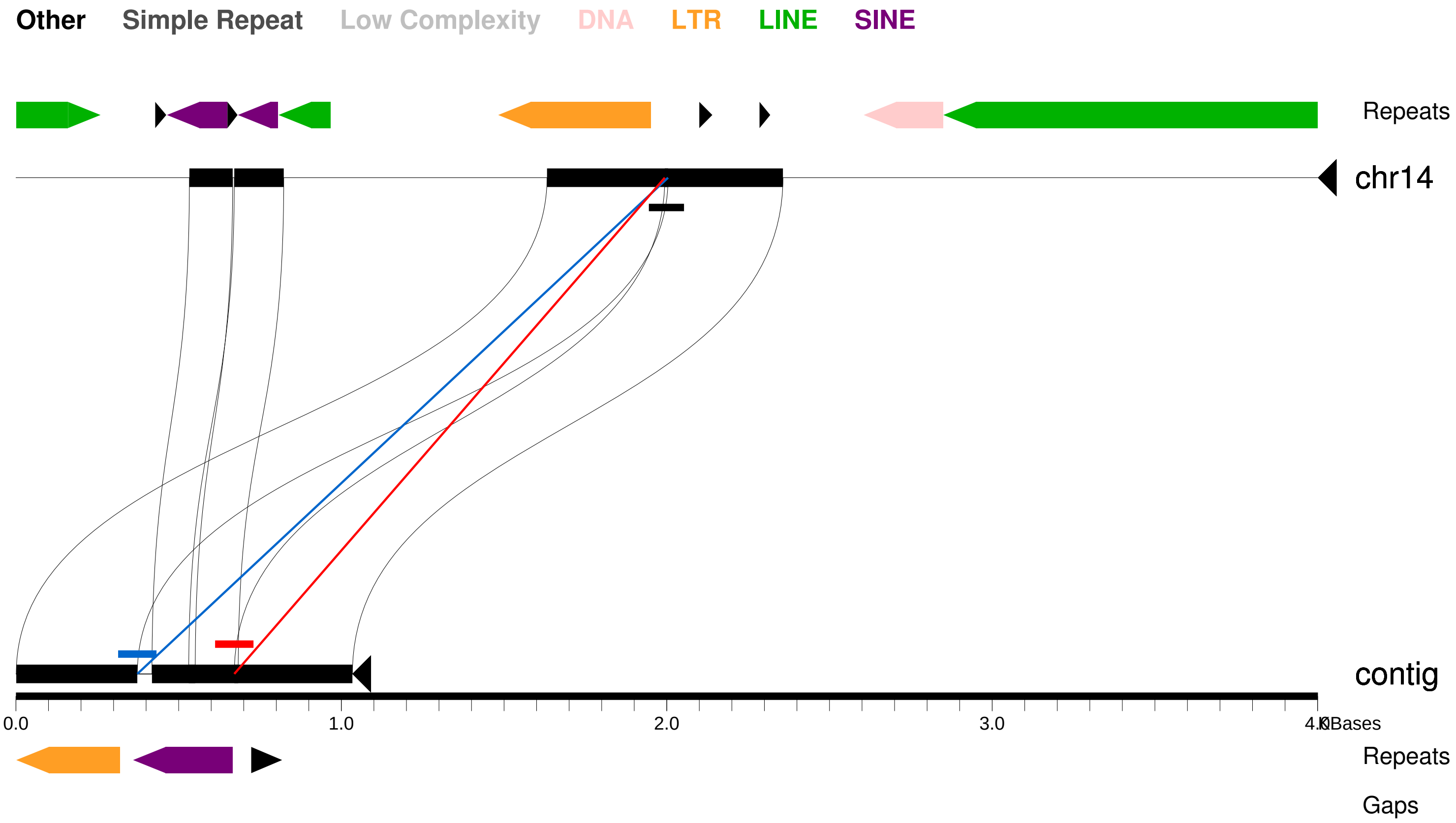

siteID: chr14_20484295 Scafftig6 - Breakpoint: chr14:20484296
chr14:20484253-20484355
Overlap Length: 17 INS size: 352 Genotyped: Yes

```
left     AA-----GCTTC--ACTTA-AAAATAATGTCA--ACA--CGTAAGACATGGGAAA----CTGAATGTACA
chrom    AA-----GCTTC--ACTTA-AAAATAATGTCA--ACA--CGTAAGACATGGGAAA----CTGAATGTACA
right    TCCACCCGCCTCGGCCTCCCAAAGTGCTGGGATTACAGGCGTGAGCCACCGCGCCCGGCCTGAATGTACA
         1111111**1**111**111***1*11**11*11***11***1**1**11*11111111***********

left     TTATTATTATTATTATTATTATTATTTTTTTTTTTTTTTTTTTTTTTTTTTTTTTTTTTTTTTTTT
chrom    TTATTAAG----------------TGTCTCTTCCTCTTCCTCTCTCTCCATTTACACACACAAAC
right    TTATTAAG----------------TGTCTCTTCCTCTTCCTCTCTCTCCATTTACACACACAAAC
         ******2222222222222222*2*2*2**22*2**22*2*2*2*222***222222222222
```

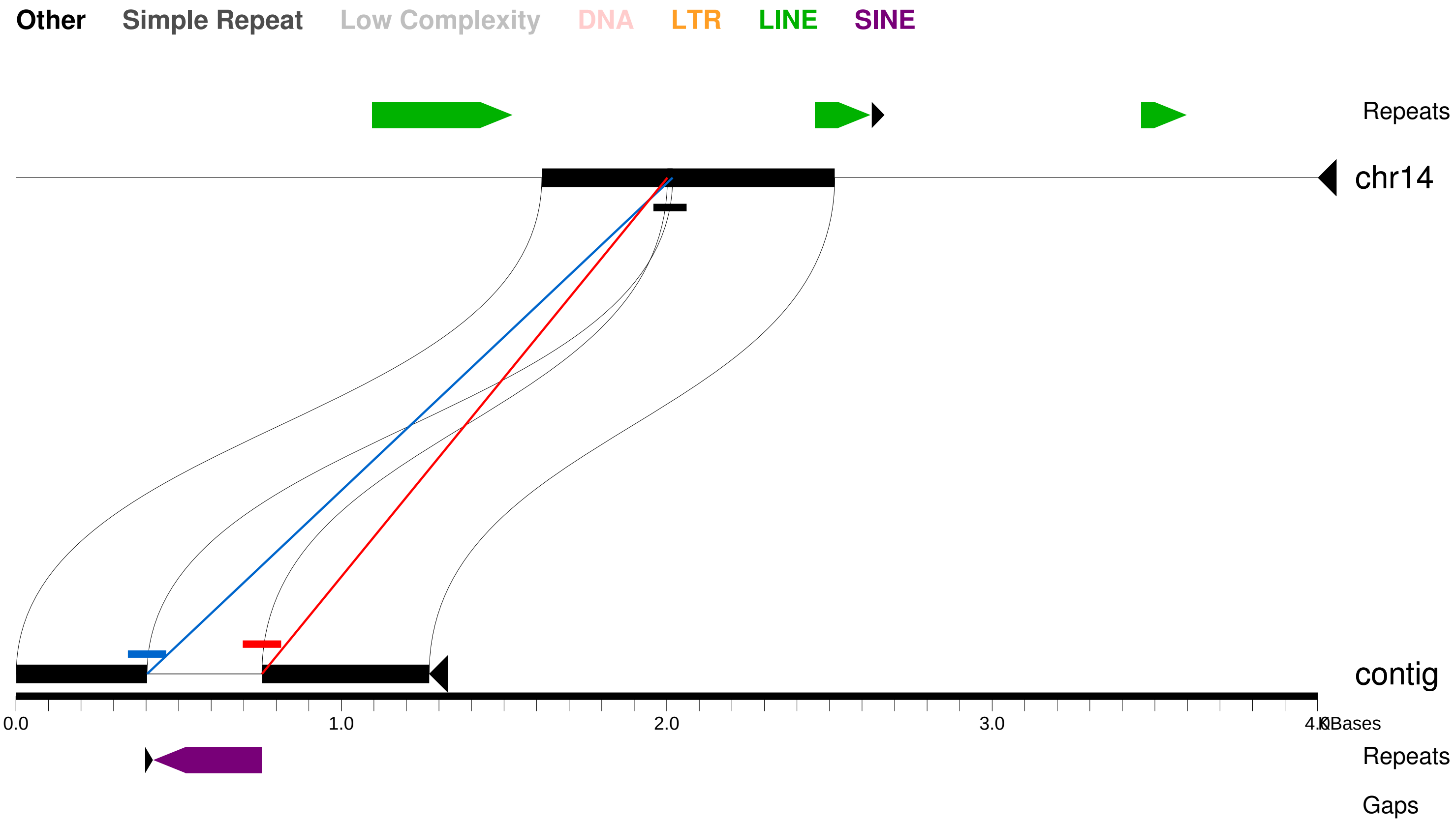

siteID: chr14_20698626 Scafftig1 - Breakpoint: chr14:20698576
chr14:20698416-20698742
Overlap Length: 7 INS size: 361 Genotyped: No

```
left    -------------------------------CATGAAATATGCCCTCCATCATGTCT-TCTGTGGAATG
chrom   AACCCCCTCATCTACAGCCTACGCAACAAGGACATGAAATATGCCCTCCATCATGTCT-TCTGTGGAATG
right   -------TTTAGTAGAGACGGGGTTTCACCGT-------TTTAGCCGGGATGGTCTCGATCTCCTGACCT
               2NNNN22N22N2NNN2NNN22NN2N1111111*1*11**111**11*1**11***111**111

left    AGAATTATCCAGAGATCATGAATAGGGTTTTTTATAACCCAATGACTCTTAATGCGATTGGAAGAATAAT
chrom   AGAATTATCCAGAGATCATGAATAGGGTTTTTTATAACCCAATGACTCTTAATGCGATTGGAAGAATAAT
right   CG--TGATCCG-----CCCGCCTCGGCCT--------CCCAAAG-----TGCTGGGATTACAGGCGTGAG
        1*11*1****111111*11*11*1**11*11111111*****1*11111*11**1****11*1*11*1*1

left    CCTCTTCTTTTAACACTAACTGACTTGANNNNNNNNNNN-------------------------------
chrom   CCTCTTCTTTTAACACTAACTGACTTGATTTTATTCATGGTCATCGCCATCCTTTTGTACAAGCAAAACT
right   CCACCGCGCCCGGCCCT----GACTTGATTTTATTCATGGTCATCGCCATCCTTTTGTACAAGCAAAACT
        **1*11*111111*1**1111*******222222222222222222222222222222222222222222

left    ----------------------------------------------------------------------
chrom   CTTCATGTATCTGATCATATTTTGACGAGCTTCTAGTTTCAGAAATGTGCTCCATGGGAGCAGGTTGGCC
right   CTTCATGTATCTGATCATATTTTGACGAGCTTCTAGTTTCAGAAATGTGCTCCATGGGAGCAGGTTGGCC
        2222222222222222222222222222222222222222222222222222222222222222222222

left    ---------------------------------------------------
chrom   CCAATATATGGTAAAAATAACACATAAAAATCTTAATATCTTCATCCA
right   CCAATATATGGTAAAAATAACACATAAAAATCTTAATAT---------
        222222222222222222222222222222222222222
```

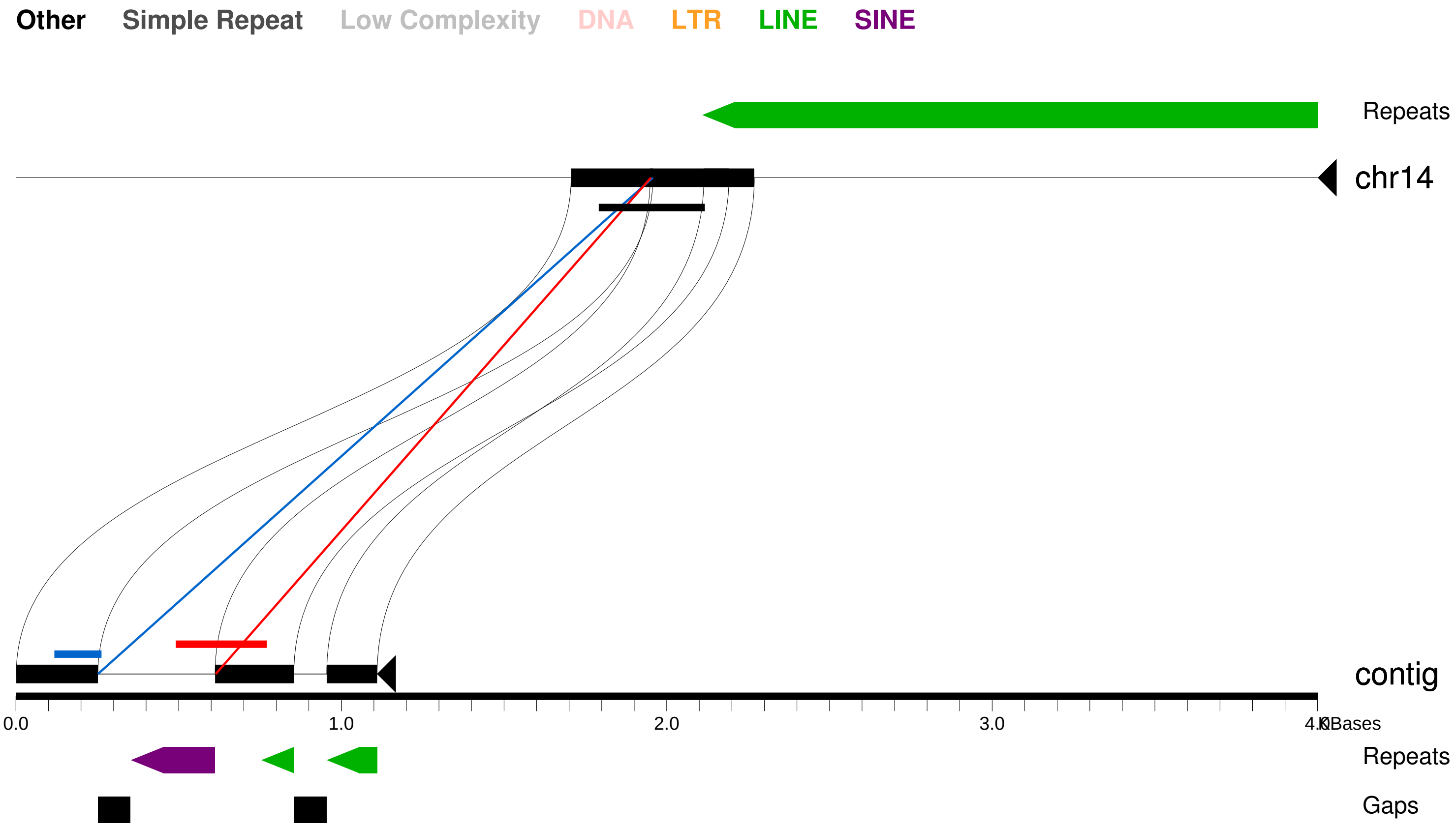

siteID: chr14_20950437 Scafftig2 - Breakpoint: chr14:20950441
chr14:20950392-20950500
Overlap Length: 11 INS size: 317 Genotyped: Yes

```
left    TTCCT---CTTTATTTTGA-ACTGTTTCT---ATCTGAGCTCCTGTTGTAATTTAT---AATAAAAAGTT
chrom   TTCCT---CTTTATTTTGA-ACTGTTTCT---ATCTGAGCTCCTGTTGTAATTTAT---AATAAAAAGTT
right   GGCCTGGGCGACAGAGCGAGACTCCGTCTCAAAAAAAAAAAAATAAAAAAAAATAAAAAAATAAAAAGTT
        11***111*111*1111**1***111***111*1111*11111*11111**11**1111***********

left    GGCCGGGCGCGGTGGCTCACGCCTGTAATCCCAGCACTTTGGGAGGCCGAGGCGGGTGG
chrom   AGTAGAATATAGTACTTCTCTTGTGTA--GCCTGACCTGATCTAGTTCTAT--------
right   AGTAGAATATAGTACTTCTCTTGTGTA--GCCTGACCTGATCTAGTTCTAT--------
        2*22*222222**222**2*222****222**2*22**22222**22*2*222222222
```

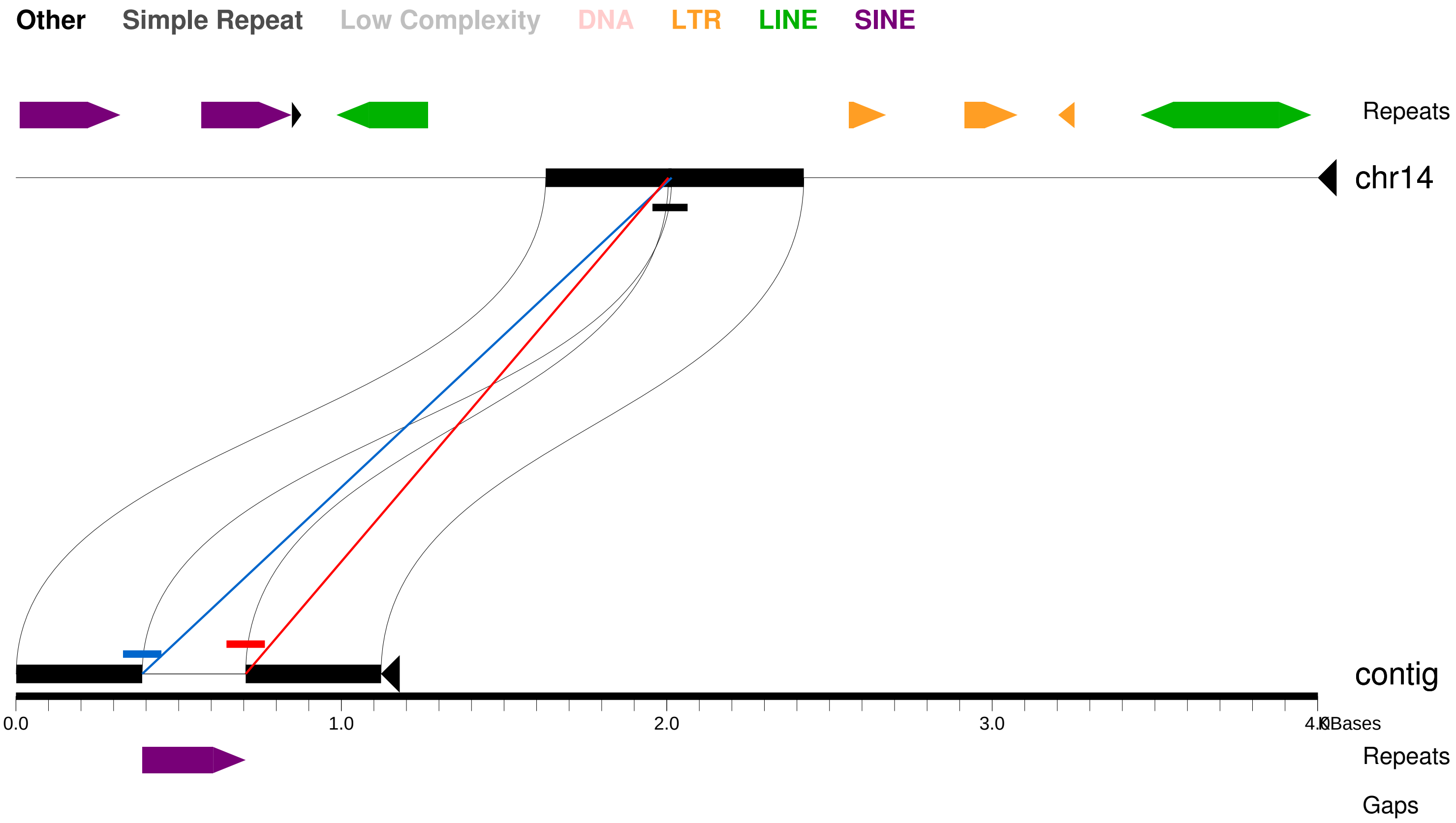

siteID: chr14_22032387 Scafftig2 - Breakpoint: chr14:22032347
chr14:22032299-22032406
Overlap Length: 12 INS size: 199 Genotyped: Yes

```
left   AAA--TCAAGATCAACCATGAA-GGAGAAGTAAATAGGGCCCGT--TACATGC------CGCAGAACTCT
chrom  AAA--TCAAGATCAACCATGAA-GGAGAAGTAAATAGGGCCCGT--TACATGC------CGCAGAACTCT
right  TACAGGCGCCCGCCACTACGCCCGGCTAATTTTTTTGTATTTTTAGTAGAGACGGGGTTCGCAGAACTCT
       1*1111*11111*1**1*1*111**11**1*111*1*111111*11**1*11*111111***********

left   TTTTTTTTTTTTTTTTTTTTTGAGACGGAGTCTCGCTCTGTCGCCCAGGCTGGAGTGCAG
chrom  TGTATCATCAAAACAA------AGACTCCTTCCAGTGATGT--TCTTGTCTT---TGACT
right  TGTATCATCAAAACAA------AGACTCCTTCCAGTGATGT--TCTTGTCTT---TGACT
       *2*2*22*22222222222222****2222**22*222***222*22*2**2222**222
```

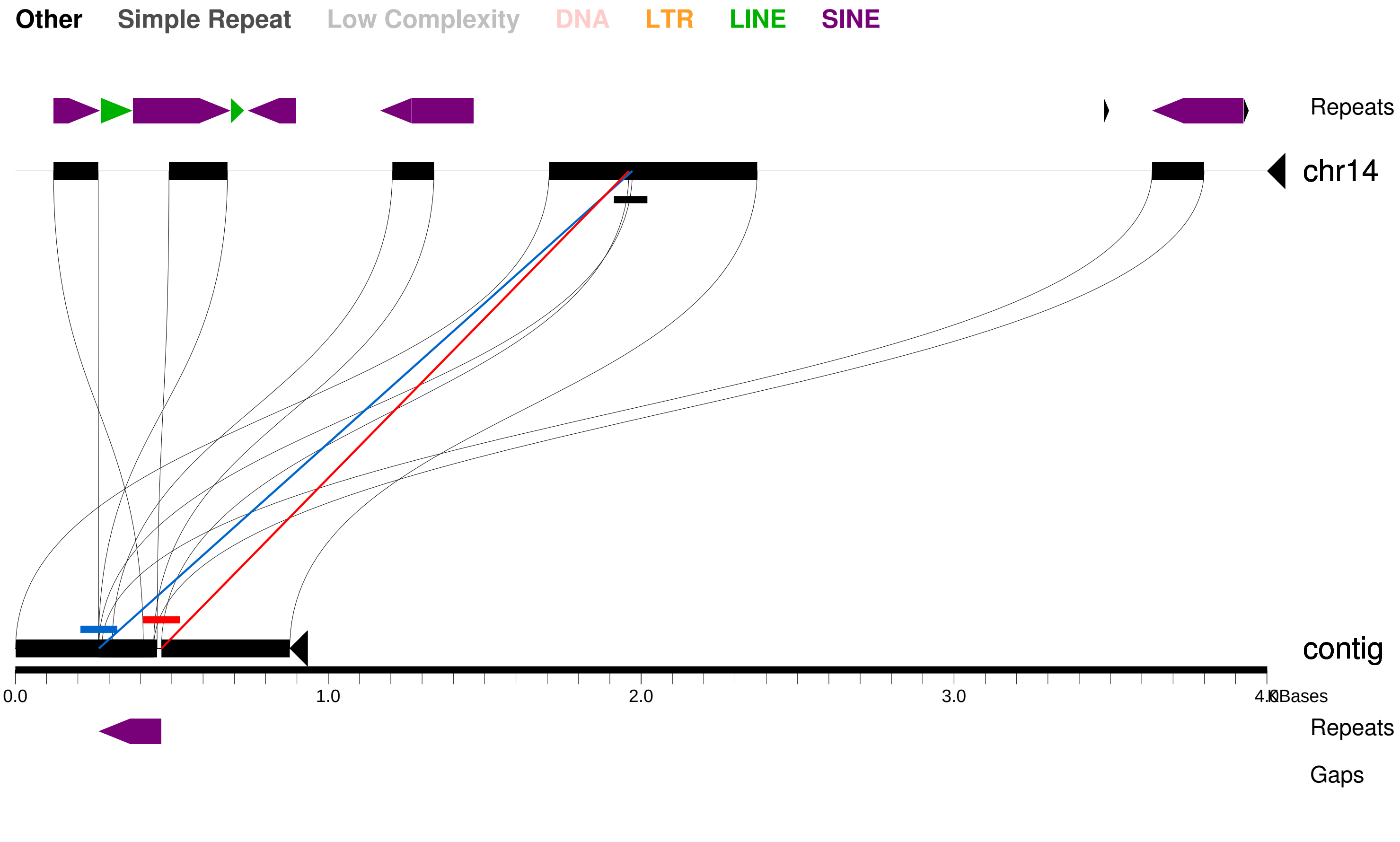

siteID: chr14_22326221 Scafftig2 + Breakpoint: chr14:22326228
chr14:22326182-22326287
Overlap Length: 14 INS size: 425 Genotyped: No

```
left    TTGATGATGAATTTTCCCTGCTTTCTTTTGTGATTCTAGAGC-------------AATTAGAAATGTGCA
chrom   TTGATGATGAATTTTCCCTGCTTTCTTTTGTGATTCTAGAGC-------------AATTAGAAATGTGCA
right   NNNNNNNNNNNNNNNNNNNNNNNNNNNNNNNNNNNNNNNNNNNNNNNNNNNNNAAAGAAAGAAATGTGCA
        1111111111111111111111111111111111111111111111111111111*111***********

left    ATAGGCCGGGCGCGGTGGCTCACGCCTGTAATCCCAGCACTTTGGGAGGCCGAGATGGGCAG
chrom   ATA--ACAGATGCAGATACTTCC-----TAAATATTTCAAATTGTAAAG--GAA----GTAG
right   ATA--ACAGATGCAGATACTTCC-----TAAATATTTCAAATTGTAAAG--GAA----GTAG
        ***222*2*22**2*222**22*22222***222222**22***22*2*22**22222*2**
```

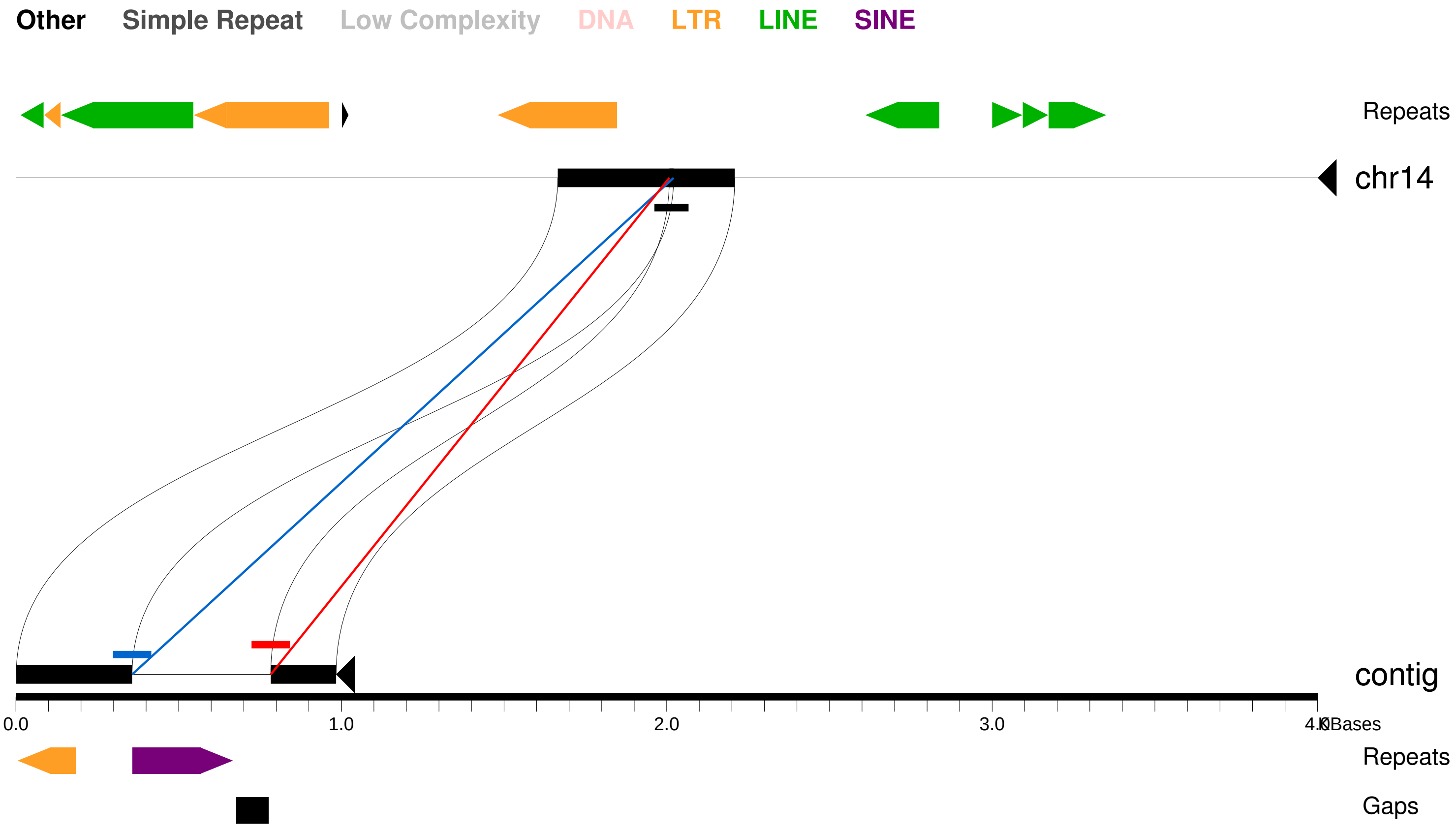

siteID: chr14_25581770 Scafftig3 + Breakpoint: chr14:25581752
chr14:25581709-25581811
Overlap Length: 12 INS size: 305 Genotyped: Yes

```
left     TGATGT--------TCATGAAGATTT---ATTAACAGCATGGGGAAATGATTAT-----TAAAATATATA
chrom    TGATGT--------TCATGAAGATTT---ATTAACAGCATGGGGAAATGATTAT-----TAAAATATATA
right    CGCCCGCCTCGGCCTCCCAAAGTGCTGGGATTACAGGCGTGAGCCACCGCGCCCGGCCCTAAAATATATA
         1*111111111111**111***111*111****111**1**1*11*11*1111111111***********

left     TATATATATTTTTTTTTTTTTTTTTGAGACGGAGTCTCGCTCTGTCGCCCAGGCTGGAGTGCAGTG-
chrom    T-------------TTTTAAGTAGGATA-GAAGT-TGTATATGTGG--CACGGTGAAGTTTGGTAA
right    T-------------TTTTAAGTAGGATA-GAAGT-TGTATATGTGG--CACGGTGAAGTTTGGTAA
         *2222222222222****222*22**2*2*2***2*222*2***2*22**2*2**2***222**22
```

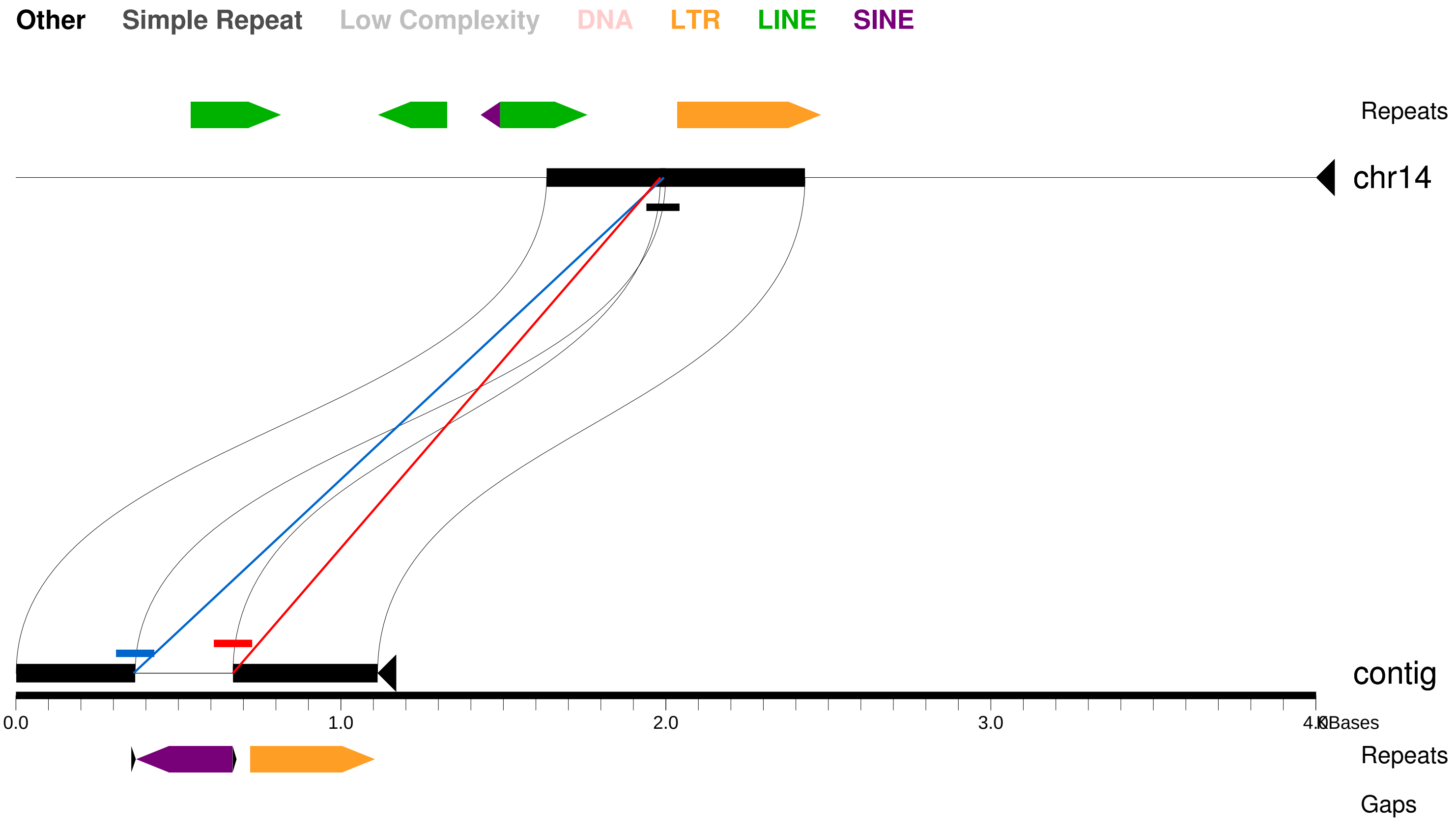

siteID: chr14_26046853 Scafftig1 + Breakpoint: chr14:26046845
chr14:26046797-26046904
Overlap Length: 12 INS size: 327 Genotyped: Yes

```
left    ATA----GAGAATATATCACT-------TACACACACATACAATATGATGAACATACTTAAAAAAAATTC
chrom   ATA----GAGAATATATCACT-------TACACACACATACAATATGATGAACATACTCAAAAAAAATTC
right   ACAGAGCGAGACTCCATCTCAAAAAAAATAAATAAATAAATAAAAAAAAAAAAAAAAAAAAAAAAAATTC
        *1*1111****1*11***1*11111111**1*1*1*1*1*1**1*11*11**1*1*11N***********

left    TGGCCGGGCGCGGTGGCTCACGCCTGTAATCCCAGCACTTTGGGAGGCCGAGGCGGGTGG
chrom   TAAATAAG-----TAACACATGACTCAAATCTGACTCCTCAAAATTGGAAAAGA------
right   TAAAAAAG-----AAACACATGACTCAAATCTGACTCCTCAAAATTGGAAAAGA------
        *222N22*22222122*2**2*2**22****22*222**2222222*222*2*2222222
```

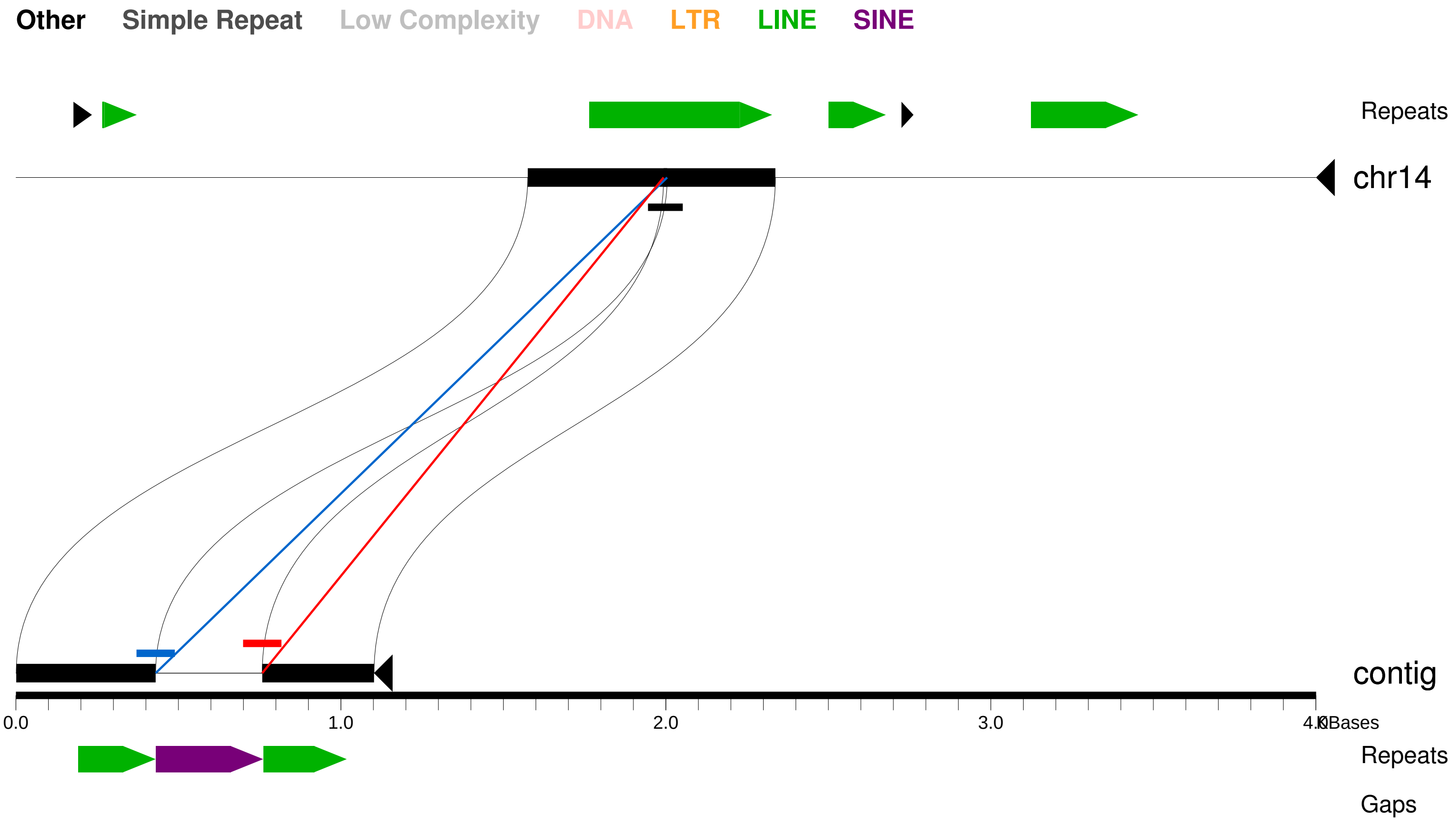

siteID: chr14_26524462 Scafftig3 - Breakpoint: chr14:26524434
chr14:26524392-26524493
Overlap Length: 18 INS size: 302 Genotyped: Yes

```
left     TAT--------CTGGATCTCCCACCAAAA-TCAGT--------AGATAATCAAACTGCTAAAAATAAATG
chrom    TAT--------CTGGATCTCCCACCAAAA-TCAGT--------AGATAATCAAACTGCTAAAAATAAATG
right    TGCACTCCAGCCTGGGCGACAGAGCGAGACTCCGTCTCAAAAAAAAAAATAAATAAAAAAAAAATAAATG
         *1111111111****1111*11*1*1*1*1**1**11111111*1*1***1**111111***********

left     GAGCCGTGGCCGGGCGCGGTGGCTCACGCCTGTAATCCCAGCACTTTGGGAGGCCGAGGCGGGCGG
chrom    GAGCCGTATC---------TTATACAAAA-TGTTATC--TGCATCTGCTGAGGAAGAATAC-----
right    GAGCCGTATC---------TTATACAAAA-TGTTATC--TGCATCTGCTGAGGAAGAATAC-----
         *******22*222222222*2222**2222***2***222***22*222****22**222222222
```

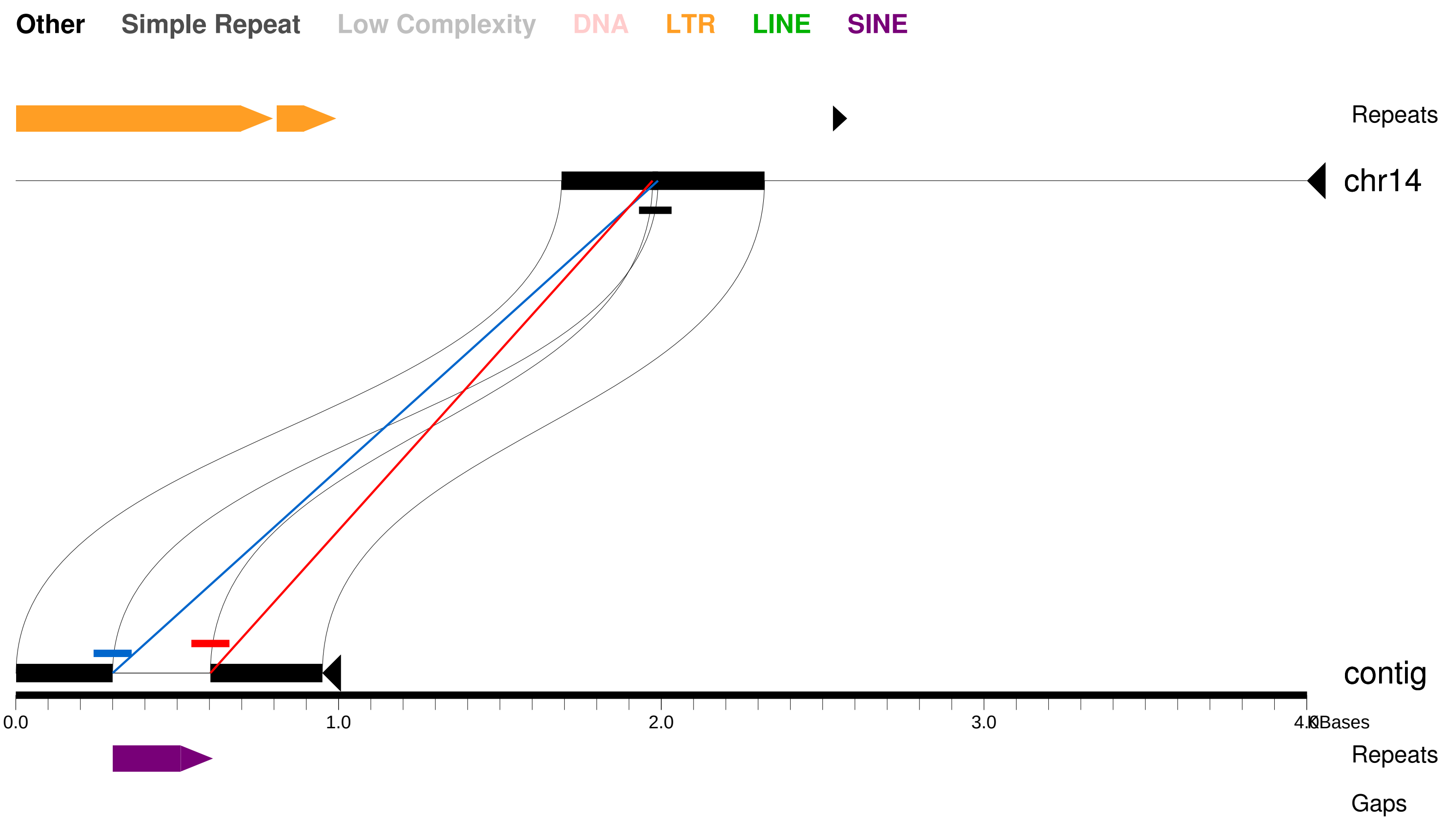

siteID: chr14_27526134 Scafftig3 - Breakpoint: chr14:27526146
chr14:27526098-27526205
Overlap Length: 12 INS size: 308 Genotyped: Yes

```
left   AGCATT--GGCTTTGG---------AAAAGTTGGCATCATTTAACTATTTGCCCATGACAAAA-GTCTTA
chrom  AGCATT--GGCTTTGG---------AAAAGTTGGCATCATTTAACTATTTGCCCATGACAAAA-GTCTTA
right  -GCAGTCCGGCCTGGGCGACAGAGCAAGACTCCGTCTCAAAAAAAAAAAAAAAAAAAAAAAAAAGTCTTA
       1***1*11***1*1**111111111**1*1*11*11***111**11*1111111*11*1****1******

left   CCGCCGGGCGCGGTGGCTCACGCCTGTAATCCCAGCACTTTGGGAGGCCGAGGCGGGTGGA
chrom  CCTATGAGAAGTTTTGTTAAA-----TATTTCCTAAAGGATCTAAGATAGTGATG------
right  CCTATGAGAAGTTTTGTTAAA-----TATTTCCTAAAGGATCTAAGATAGTGATG------
       **222*2*22222*2*2*2*222222**2*2**222*222*222**222*2*22*222222
```

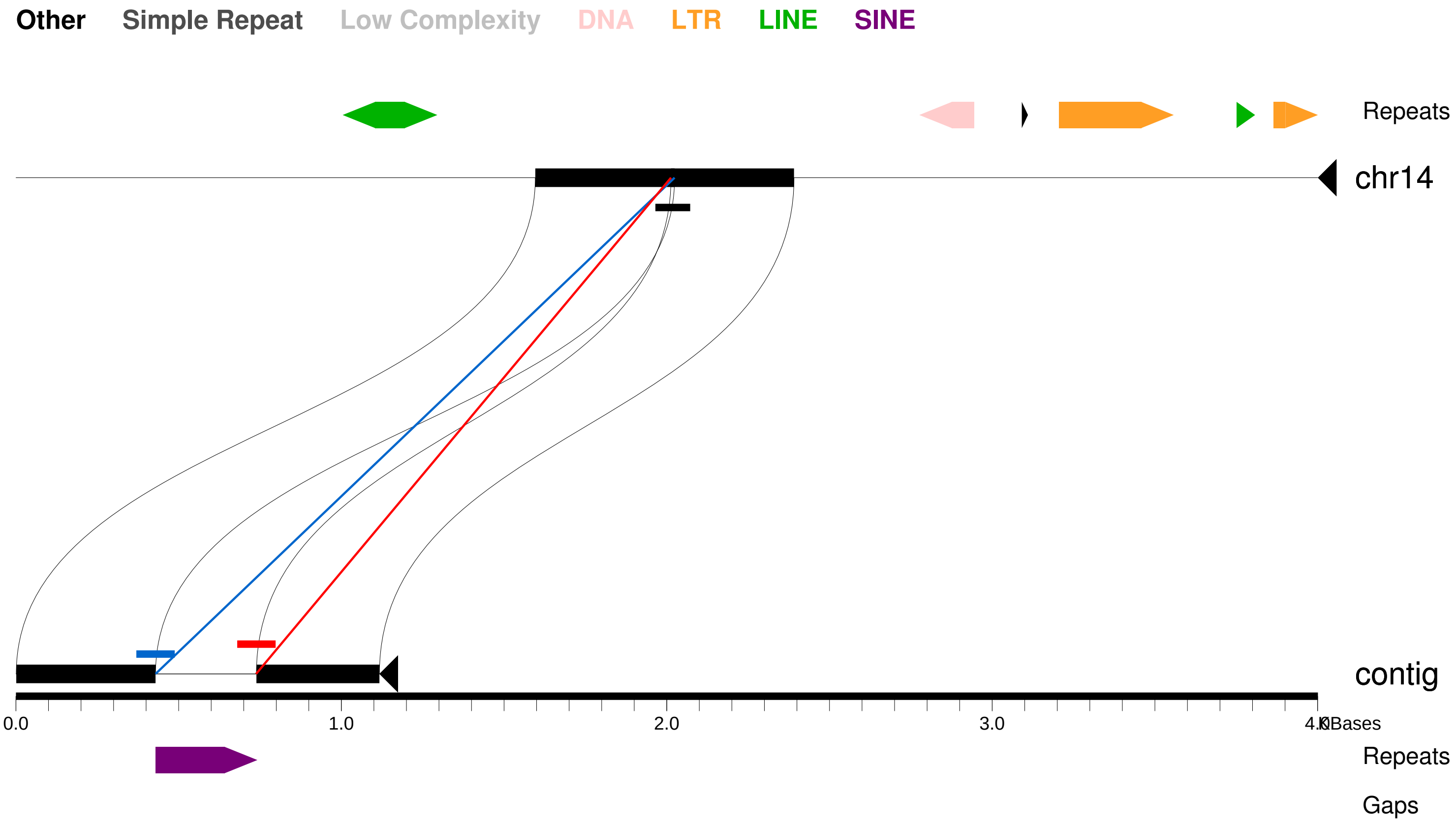

siteID: chr14_28538404 Scafftig2 - Breakpoint: chr14:28538381
chr14:28538337-28538440
Overlap Length: 16 INS size: 288 Genotyped: Yes

```
left    AAAAATT---GAAAATG----ATAGTTTTTTCATAATAAAG--------TATTATATTTAAAAATAGGAC
chrom   AAAAATT---GAAAATG----ATAGTTTTTTCATAATAAAG--------TATTATATTTAAAAATAGGAC
right   CCGAGATCCCGCCACTGCACTCCAGCCTGGGCGACAGAGCGAGACTCCGTCTCAAAAAAAAAAATAGGAC
        111*11*111*11*1**111111**11*111*111*1*11*11111111*1*1*1*111***********

left    AGGTTGGGCCGGGCGCGGTGGCTCACGCCTGTAATCCCAGCACTTTGGGAGGCCGAGGCGGGCG
chrom   AGGTT--------CCCTCTTCTTCA------TAAGCAAATCAAAATTTTCGTTC-ATCCTATCA
right   AGGTT--------CCCTCTTCTTCA------TAAGCAAATCAAAATTTTCGTTC-ATCCTATCA
        *****22222222*2*22*222***222222***2*22*2**222*2222*22*2*22*222*2
```

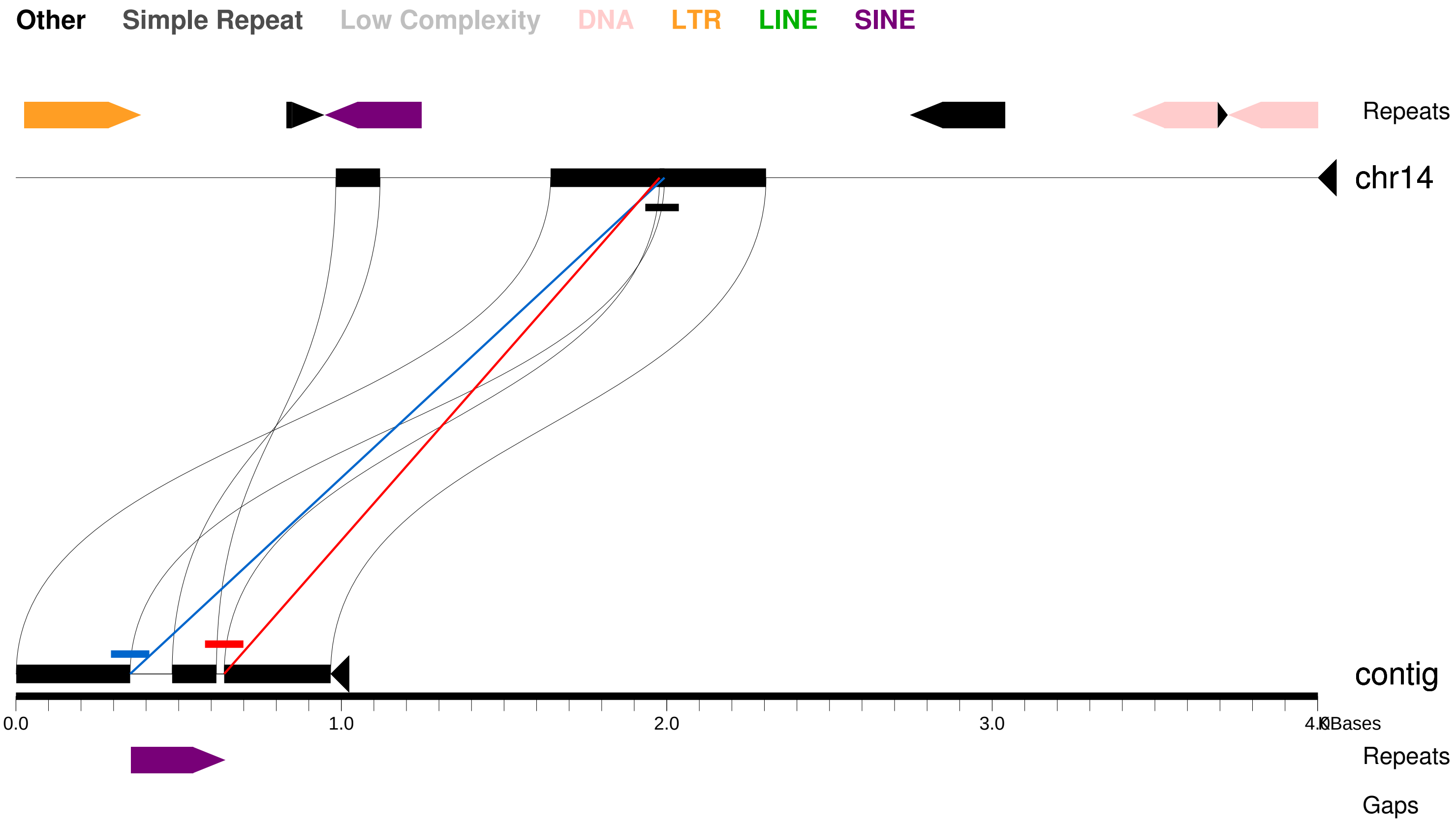

siteID: chr14_30044250 Scafftig2 - Breakpoint: chr14:30044303
chr14:30044250-30044362
Overlap Length: 7 INS size: 264 Genotyped: No

```
left    ATTAACTGTTCTCCTTGATGCTTTATACCTTTACATCTTATTTTCTAAAAGCA--------CCAAATTNN
chrom   ATTAACTGTTCTCCTTGATGCTTTATACCTTTACATCTTATTTTCTAAAAGCA--------CCAAATTTG
right   ACCG--TGTTAGCCAGGATGGTCTCGATCTCCTGACCTCGTGATCCGCCCGCCTCGGCCTCCCAAAGTTG
        *11111****11**11****1*1*11*1**1111*1**11*11**11111**111111111*****1*22

left    NNNNNNNNNNNNNNNNNNNNNNNNNNNNNNNNNNNNNNNNNNNNNNNNNNNNNNNNN
chrom   TTTATTCACTTCTATATAATAAATTTATTTTGTGCAGGGACCATGTCTTAT------
right   TTTATTCACTTCTATATAATAAATTTATTTTGTGCAGGGACCATGTCTTAT------
        222222222222222222222222222222222222222222222222222222222
```

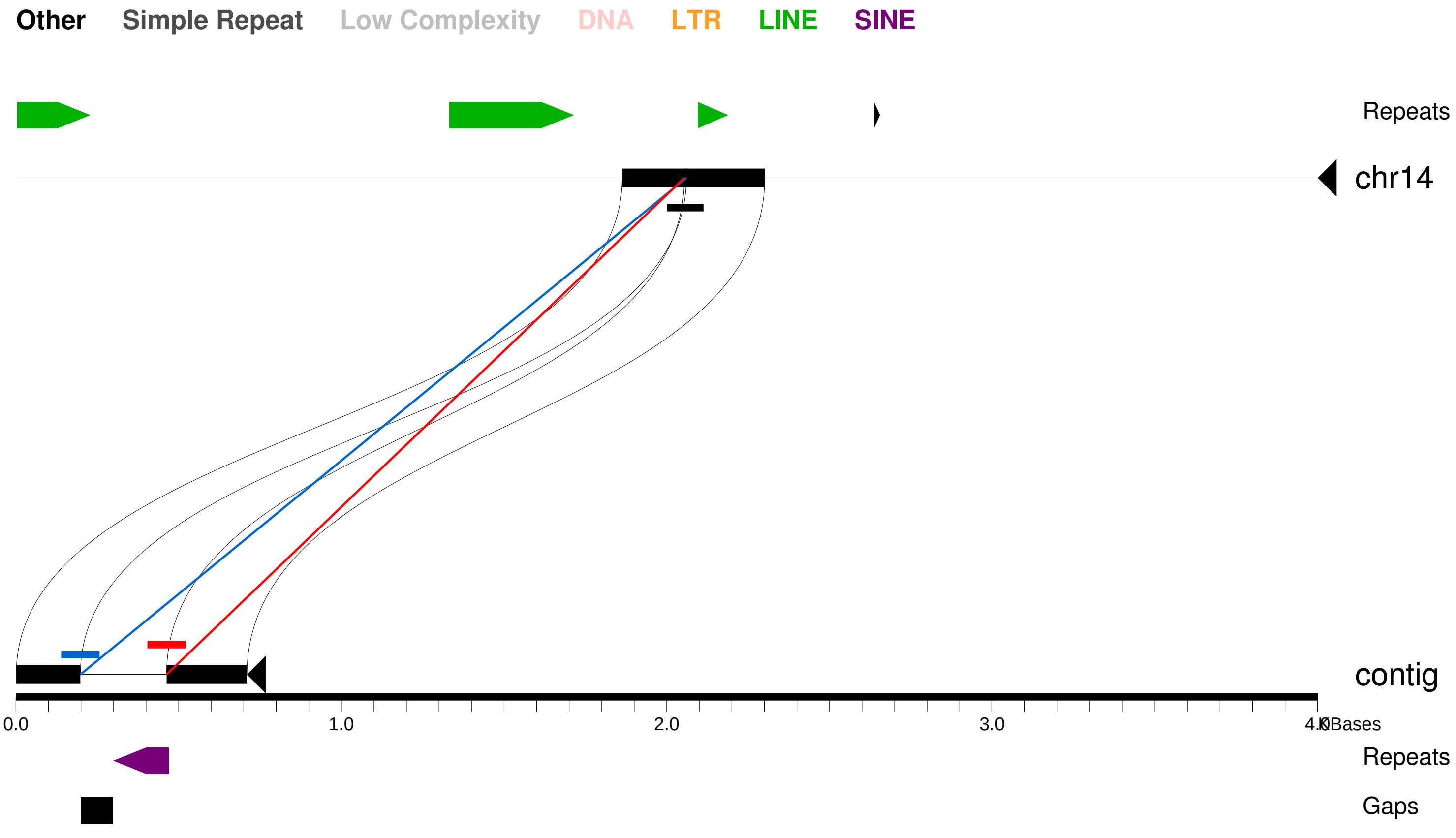

siteID: chr14_31181269 Scafftig1 - Breakpoint: chr14:31181265
chr14:31181105-31181428
Overlap Length: 4 INS size: 427 Genotyped: No

```
left    ------------------------------CTGGGTGAGAGAGGGATAGATGTGTCTTGGTGAGTTCACAT
chrom   GATTTGCCACGAGTTAATACTTGTGGAAGCTGGGTGAGAGAGGGATAGATGTGTCTTGGTGAGTTCACAT
right   --TTT------AGTAGAGAC--GGGGTTTCACCGTTTTAGCCGG---GATG-GTCTCGAT-----CTCCT
          222      222NN2N22  2N22NNN*111**111**11**111****1****1*1*11111*1*1*

left    AATCTCATTTTCTTTATTGCCATGAACACGAGATGATTTTTAAACTCATCTGTAAGATAAGGGTATTAGA
chrom   AATCTCATTTTCTTTATTGCCATGAACACGAGATGATTTTTAAACTCATCTGTAAGATAAGGGTATTAGA
right   GACCTCGTGATCC-----GCCCGC--CTCG----GCCTCCCAAAGTG--CTGGGAT-TACAGGCGTGAGC
        1*1***1*11**111111***11111*1**1111*11*111***1*111***11*11**11**11*1**1

left    TACACTCTGTAGATCACAGTCTGGNNNNNNNNNNN-----------------------------------
chrom   TACACTCTGTAGATCACAGTCTGGTTTATAATTTTAAATTTTGGAAAGAGACAGCCCATCTTATTCCCTC
right   CACC-------GCGCCCGGCCTGGTTTATAATTTTAAATTTTGGAAAGAGACAGCCCATCTTATTCCCTC
        1**11111111*11*1*1*1****222222222222222222222222222222222222222222222

left    ----------------------------------------------------------------------
chrom   TCCTCGTAGCTTCACTTCACTGGCAACTTTCCACCCTTCTATCATTGATACATCTCTCATCTCCAGTGTT
right   TCCTCGTAGCTTCACTTCACTGGCAACTTTCCACCCTTCTATCATTGATACATCTCTCATCTCCAGTGTT
        2222222222222222222222222222222222222222222222222222222222222222222222

left    ---------------------------------------------
chrom   TCCAGTGTCCACACACGCCATCTAATTCTTTACTTGGCTGATAA
right   TCCAGTGTCCACACACGCCATCTAATTCTTTACTTGGCTGA---
        2222222222222222222222222222222222222222222
```

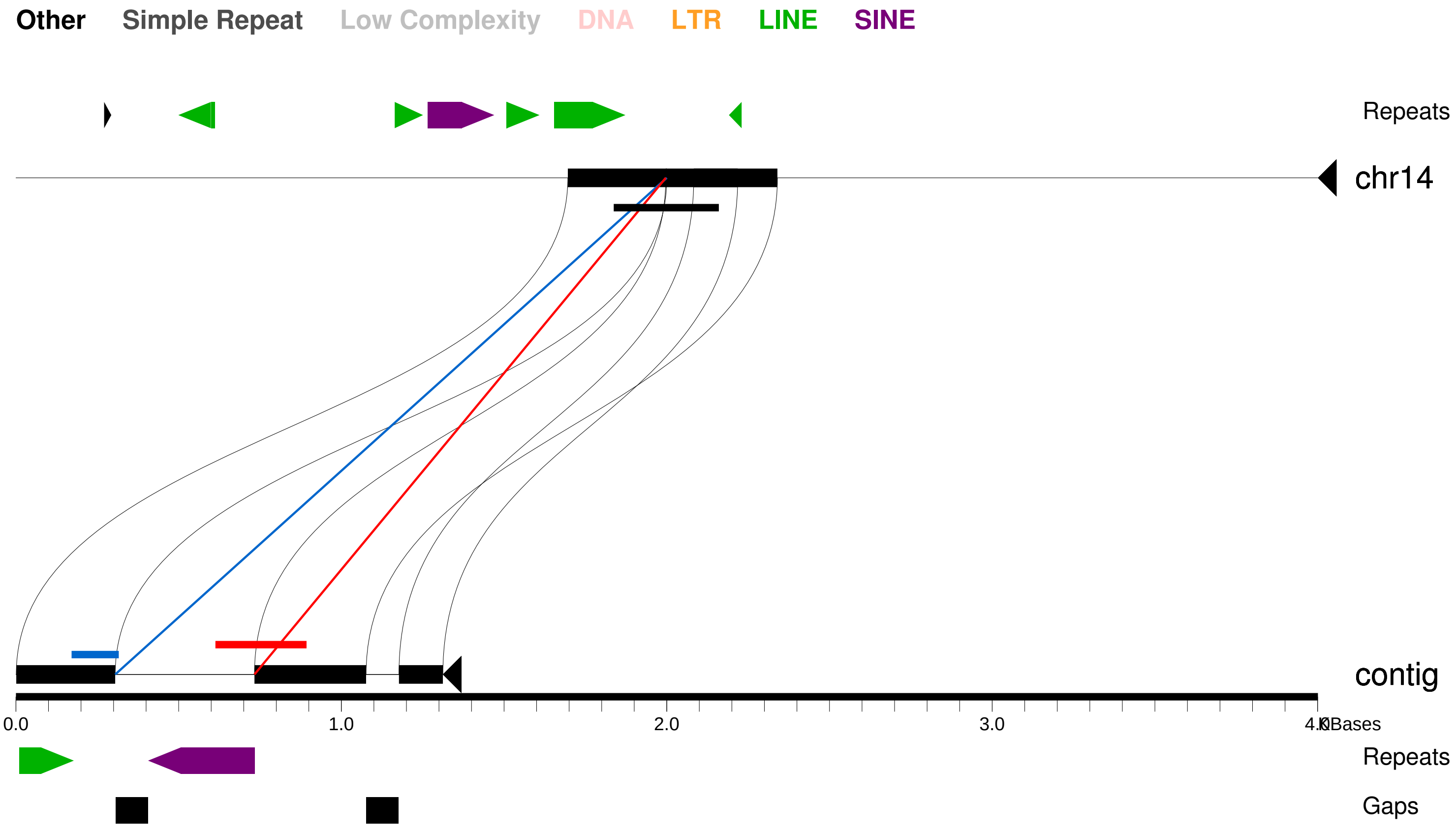

siteID: chr14_32669235 Scafftig4 - Breakpoint: chr14:32669218
chr14:32669174-32669277
Overlap Length: 16 INS size: 235 Genotyped: Yes

```
left     TTTTGAACACT------TGGTCTCAAGGG---------GTCTTCCCACCTTGGCCTCACAAAACTCTGGG
chrom    TTTTGAACACT------TGGTCTCAAGGG---------GTCTTCCCACCTTGGCCTCACAAAACTCTGGG
right    CAGTCCGCAGTCCAGCCTGGGCGACAGAGCGAGACTCCGTCTCAAAAAAAAAAAAAAAAAAAACTCTGGG
         111*111**1*111111***1*111**1*111111111****1111*1111111111*1***********

left     ACCTCCAGGAGATCGA-GACCATCCTGGCTAACAAGGTGAAACCCCGTCTCTACTAAAAATACAA
chrom    ACCTC-ACCAGGCCTACGACCATT-----TATAAAGATTTT------TCTCCCCTAA----TGGA
right    ACCTC-ACCAGGCCTACGACCATT-----TATAAAGATTTT------TCTCCCCTAA----TGGA
         *****2*22**22*2*2******222222**22***2*222222222****22****2222222*
```

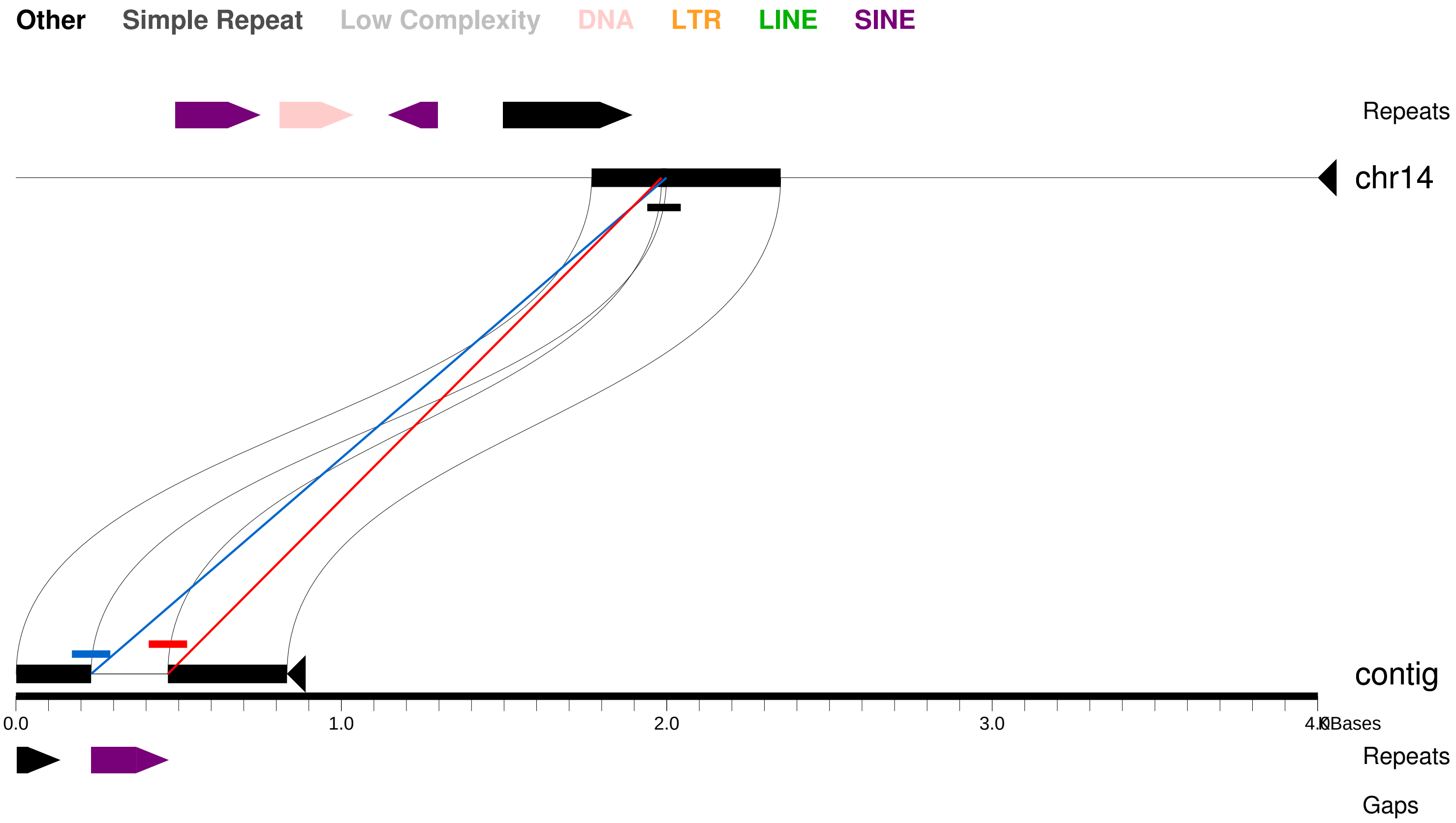

siteID: chr14_33254190 Scafftig1 + Breakpoint: chr14:33254187
chr14:33254142-33254246
Overlap Length: 15 INS size: 427 Genotyped: No

```
left    ACTGCCTGGAATTCCTTTTTGAATTGAATACAAGAAGAATCATAT--------------AAATGATTGAT
chrom   ACTGCCTGGAATTCCTTTTTGAATTGAATACAAGAAGAATCATAT--------------AAATGATTGAT
right   NNNNNNNNNNNNNNNNNNNNNNNNNNNNNNNNNNNNNNNNNNNNNNNNNNNNNNNNNNNAAATGATTGAT
        11111111111111111111111111111111111111111111111111111111111***********

left    AGCAGGCCGGGCGCGGTGGCTCACGC-CTGTAATCCCAGCACTTTGGGAGGCCGAGGCGGGTGG
chrom   AGCA---CAGTAATTTTTGTTAATATACTGTTTTCTATTCA-TTAGTCAGTTC-----------
right   AGCA---CAGTAATTTTTGTTAATATACTGTTTTCTATTCA-TTAGTCAGTTC-----------
        ****222*2*222222*2*2*2*2222****22**2222**2**2*22**22*22222222222
```

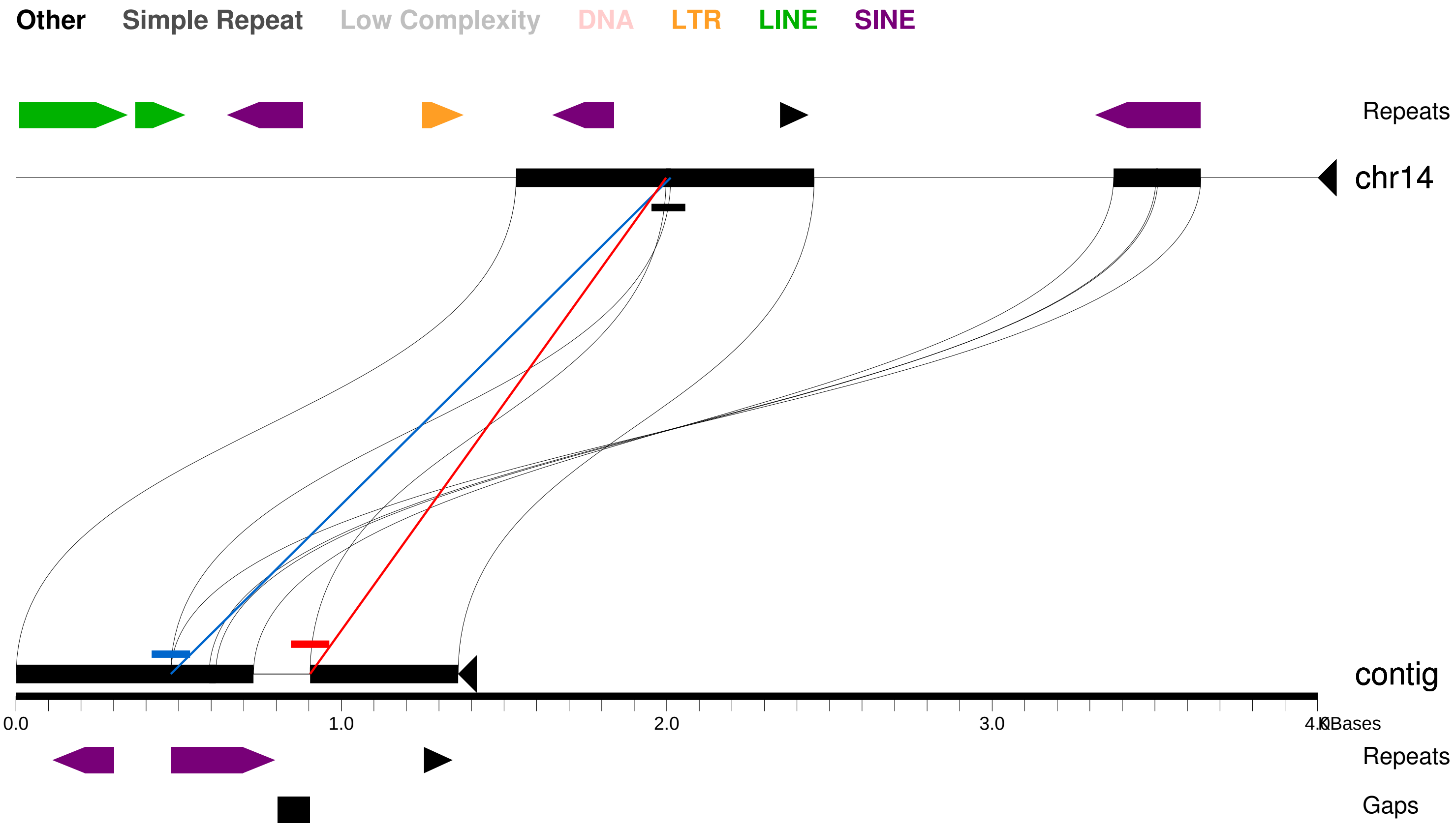

siteID: chr14_34055459 Scafftig1 - Breakpoint: chr14:34055441
chr14:34055395-34055500
Overlap Length: 11 INS size: 469 Genotyped: No

```
left   ------CTATTTTAATAAAACCAAATAACA------ACAATCTTTACTTTCCAAGCAA-TCAGATGTTTT
chrom  ------CTATTTTAATAAAACCAAATAACA------ACAATCTTTACTTTCCAAGCAA-TCAGATGTTTT
right  GATCCGCCCGCCTCGGCCTCCCAAAGTGCTGGGATTACAGGCGTGAGCCACCGCGCCCGTCAGATGTTTT
       111111*11111*1111111*****111*1111111***11*1*1*1111**11**111***********

left   TTTTTTNNNNNNNNNNNNNNNNNNNNNNNNNNNNNNNNNNNNNNNNNNNNNNNNNNNNNNNNN
chrom  ATT-------------GACTGTTTCATAGCATAATAATTCACTTTCCAAAAGGATTTTGAGT
right  ATT-------------GACTGTTTCATAGCATAATAATTCACTTTCCAAAAGGATTTTGAGT
       2**222222222222222222222222222222222222222222222222222222222222
```

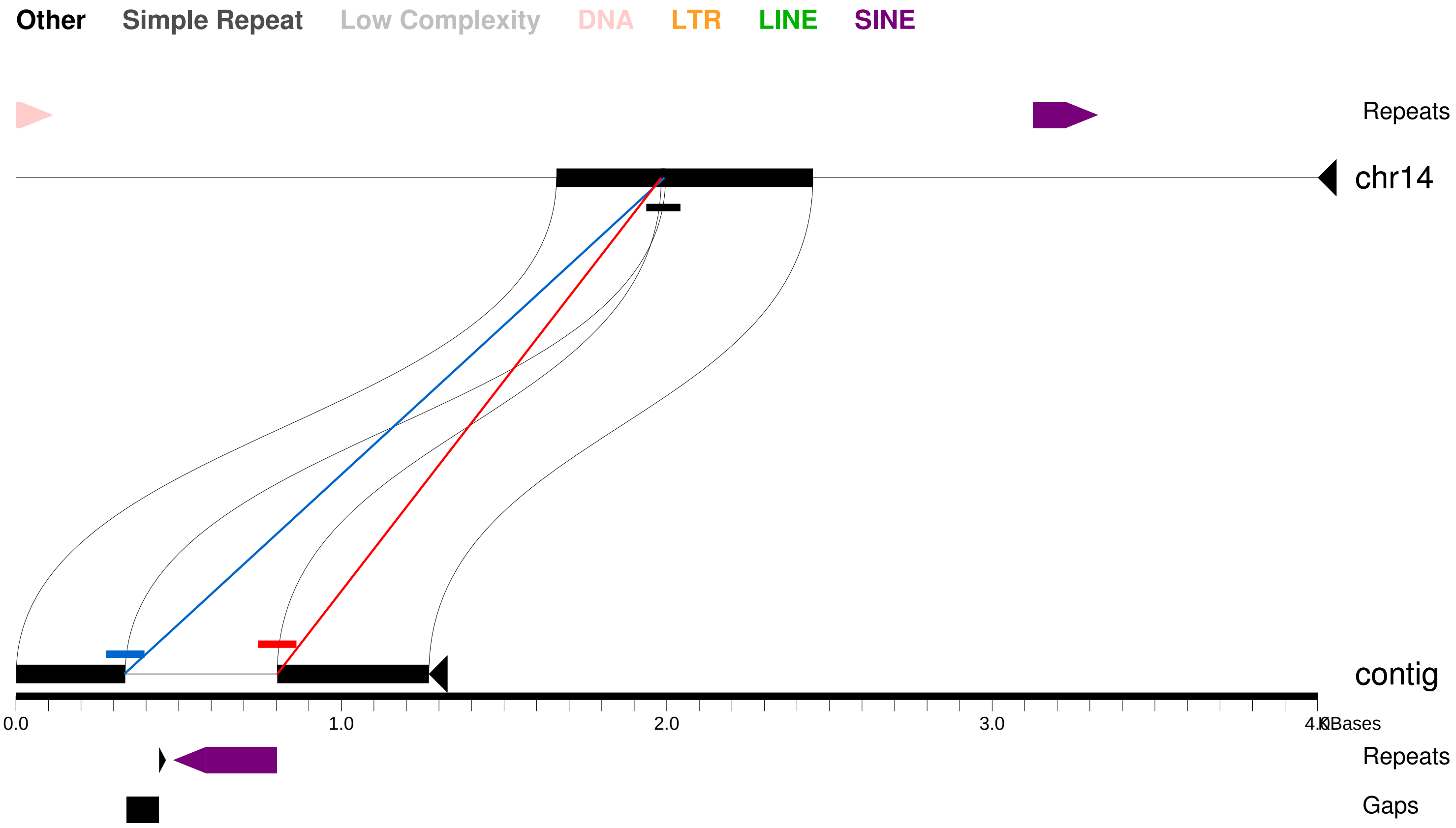

siteID: chr14_34493356 Scafftig4 + Breakpoint: chr14:34493339
chr14:34493295-34493398
Overlap Length: 16 INS size: 297 Genotyped: Yes

```
left     ATTAGAGAAAGCACCTCCT------CTCAGAATA-ATACTTTGTTGAAAAA--------TAAAGGCGGAC
chrom    ATTAGAGAAAGCACCTCCT------CTCAGAATA-AGACTTTGTTGAAAAA--------TAAAGGCGGAC
right    CCGCCCGCCTCGGCCTCCCAAAGTGCTGGGATTACAGGCGTGAGCCACCGCGCCCGGCCTAAAGGCGGAC
         111111*111111*****1111111**11**1**1*21*1*11111*111111111111***********

left     TTTCTTTTTTTTTTTTTTTTGAGACGGAGTCTCGCTCTGTCGCCCAGGCTGGAGTGCAGTGGC-
chrom    TTTCT--------------AAGAAGAGCAGT-TCCATAAAT-GCAGAGAATAATAAATATTTTAA
right    TTTCT--------------AAGAAGAGCAGT-TCCATAAAT-GCAGAGAATAATAAATATTTTAA
         *****2222222222222222**222*2***2**22*222*2**22**22*2222222*2*2222
```

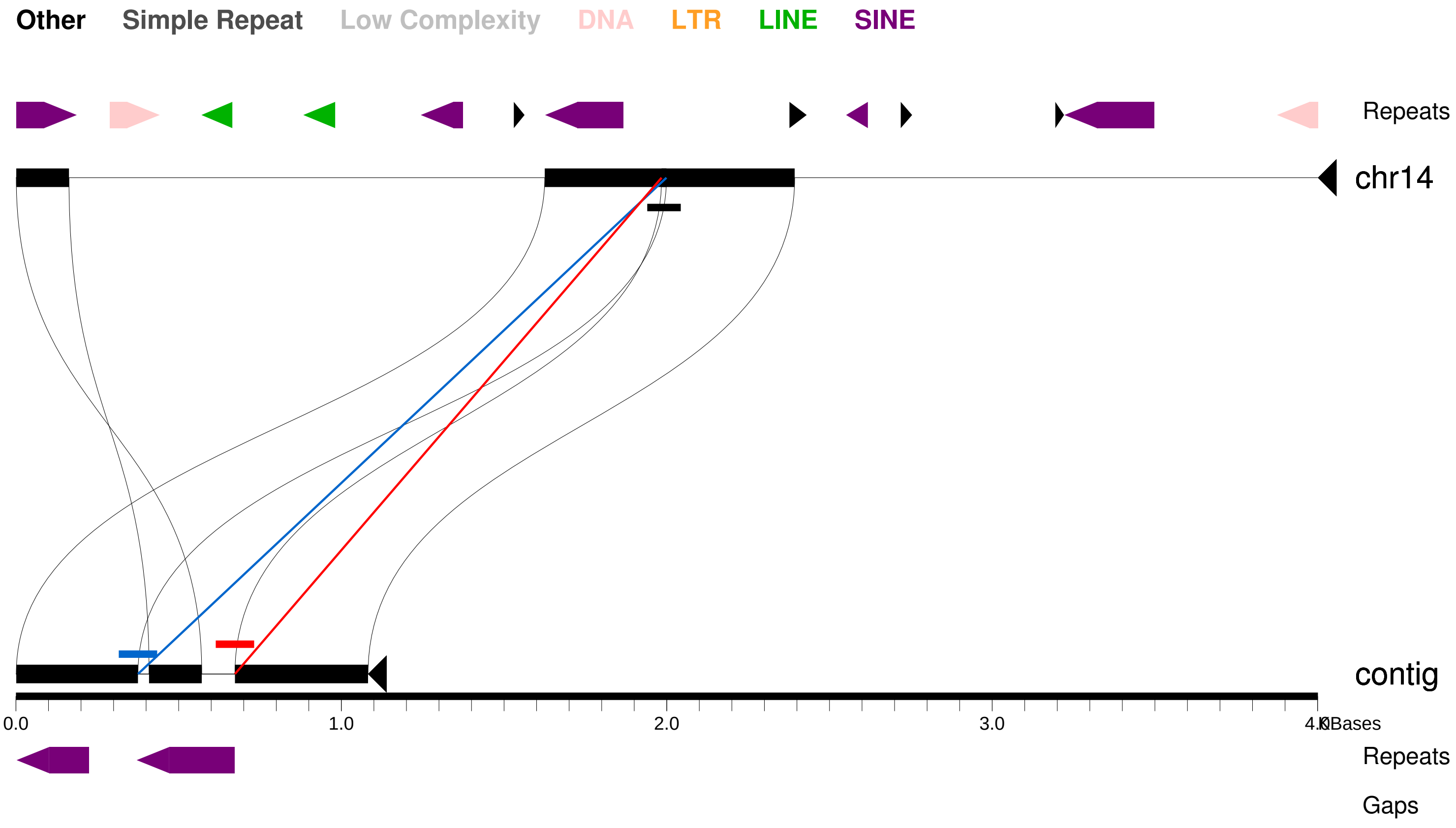

siteID: chr14_34847471 Scafftig1 + Breakpoint: chr14:34847639
chr14:34847595-34847698
Overlap Length: 16 INS size: 112 Genotyped: Yes

```
left     T----------TCCCTGCTGACTATGAGATTC----------TTAATGACATGGACCTTGTTTTTAATTT
chrom    T----------TCCCTGCTGACTATGAGATTC----------TTAATGACATGGACCTTGTTTTTAATTT
right    CAGTGGCGCGATCTCGGCTCACTGCAAGCTCCGCCTCCCGGGTTCACGCCATTCTCCTG-----TAATTT
         11111111111**1*1***1***111**1*1*1111111111**1*1*1***111***111111******

left     GGCTTTTTTTTTTTTTTTTTTTTTTTTTGAGACGGAGTCTCGCTCTGTCGCCCAGGCTGGAGTGCAGTGG
chrom    GGCTTTTTTTTAAGTGCCTAGTCCATT-----CTGAGTTTATAGTAGGCACTCAA---------CAATT-
right    GGCTTTTTTTTAAGTGCCTAGTCCATT-----CTGAGTTTATAGTAGGCACTCAA---------CAATT-
         *********222*222*22*222**22222*2****2*222222*2*2*2**2222222222**2*22
```

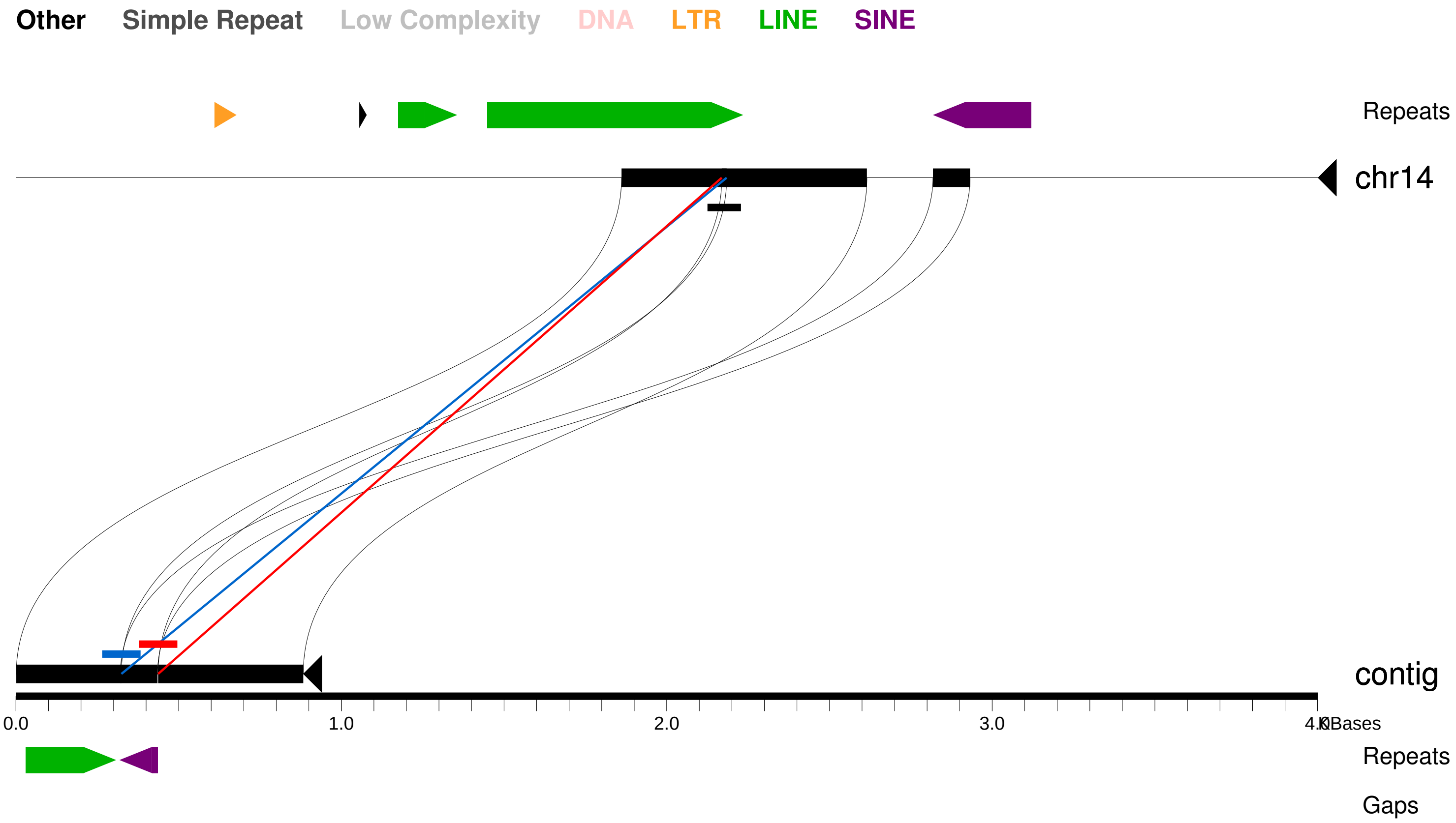

siteID: chr14_36120185 Scafftig3 + Breakpoint: chr14:36120148
chr14:36120102-36120207
Overlap Length: 14 INS size: 300 Genotyped: Yes

```
left     CTA----TATTTGC---CCATATGGCT---ATTGAGCACTTGATATTT-GCAAACGA--GGAATTACATT
chrom    CTA----TATTTGC---CCATATGGCT---ATTGAGCACTTGATATTT-GCAAACGA--GGAATTACATT
right    CCGCCCGCCTCGGCCTCCCAAAGTGCTGGGATTACAGGCGTGAGCCACCGCGCCCGGCCGGAATTACATT
         *11111111*11**111***1*11***111***11111*1***111111**111**111***********

left     TTTTTTTTTTTTTTTTTTTTGAGACGGAGTCTCGCTCTGTCGCCCAGGCTGGAGTGCAGTGG
chrom    TTTCATGTGAACTTT------AAACTAAATTTAG---TGTCAACT-GAAAGCAATACAC---
right    TTTCATGTGAACTTT------AAACTAAATTTAG---TGTCAACT-GAAAGCAATACAC---
         ***22*2*2222***222222*2**22*2*2*2*222****22*22*222*2*2*2**2222
```

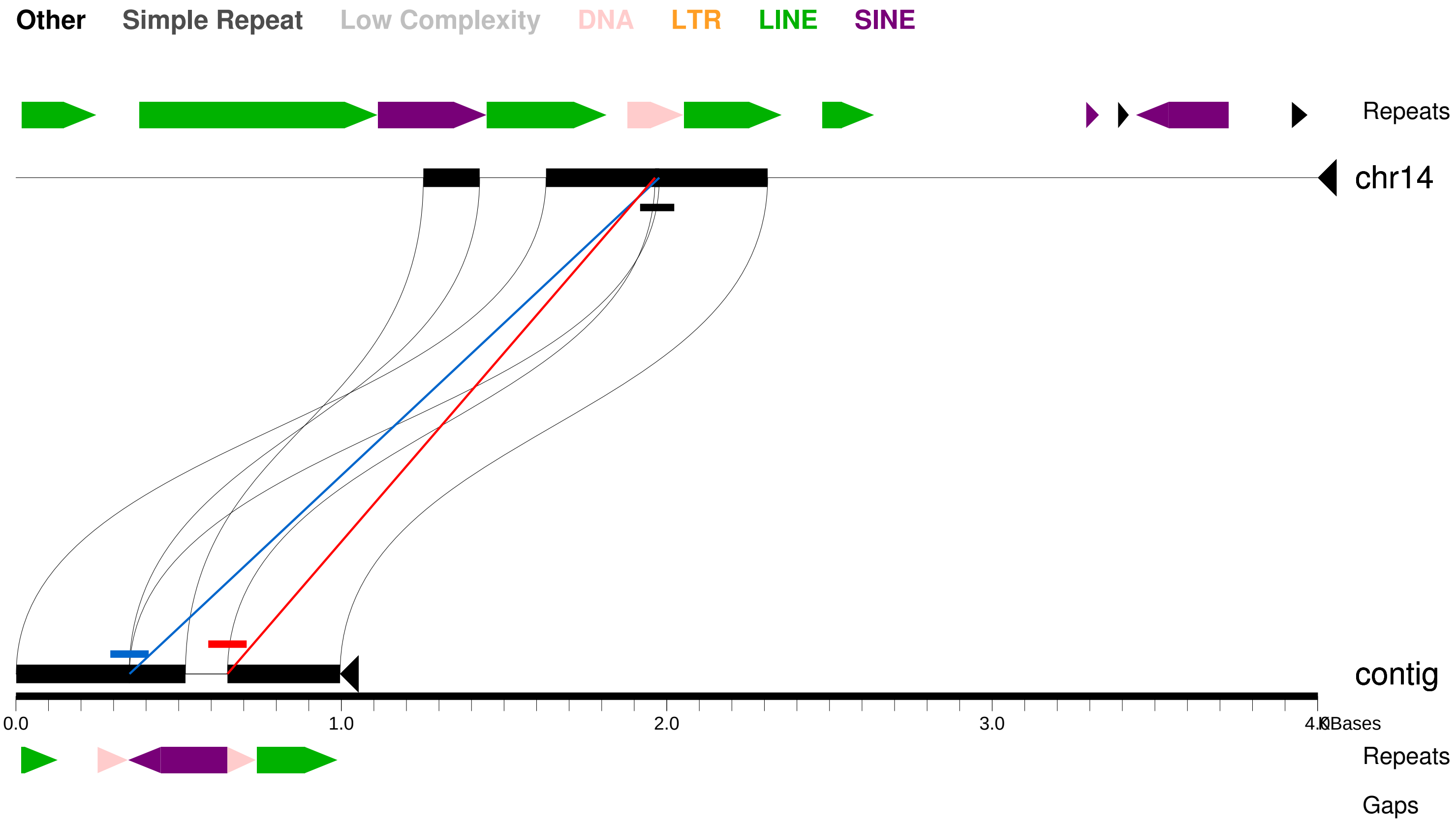

siteID: chr14_38359649 Scafftig7 - Breakpoint: chr14:38359623
chr14:38359584-38359682
Overlap Length: 21 INS size: 317 Genotyped: Yes

```
left    AGT----------GACACC----------AAATTTAAAAGAAATTTCTTATGTGGGTTTAATAATGTATA
chrom   AGT----------GACACC----------AAATTTAAAAGAAATTTCTTATGTGGGTTTAATAATGTATA
right   GGCCACAGAGCGAGACTCCGTCTCAAAAAAAAAAAAAAAAAAAAGAAAAAAAAAAAAGAAATAATGTATA
        1*11111111111***1**1111111111***111****1***111111*111111111***********

left    GAAAATTCTTGGCCGGGCGCGGTGGCTCACGCTTGTAATCCCAGCACTTTGGGAGGCCGAGGCGGGCGG
chrom   GAAAATTCTTTAA---TCATATAGATTTA---TTTAAATGTC----CTTTTATAAAATA----------
right   GAAAATTCTTTAA---TCATATAGATTTA---TTTAAATGTC----CTTTTATAAAATA----------
        **********2222222*22222*22*2*222**22***22*2222****222*222222222222222
```

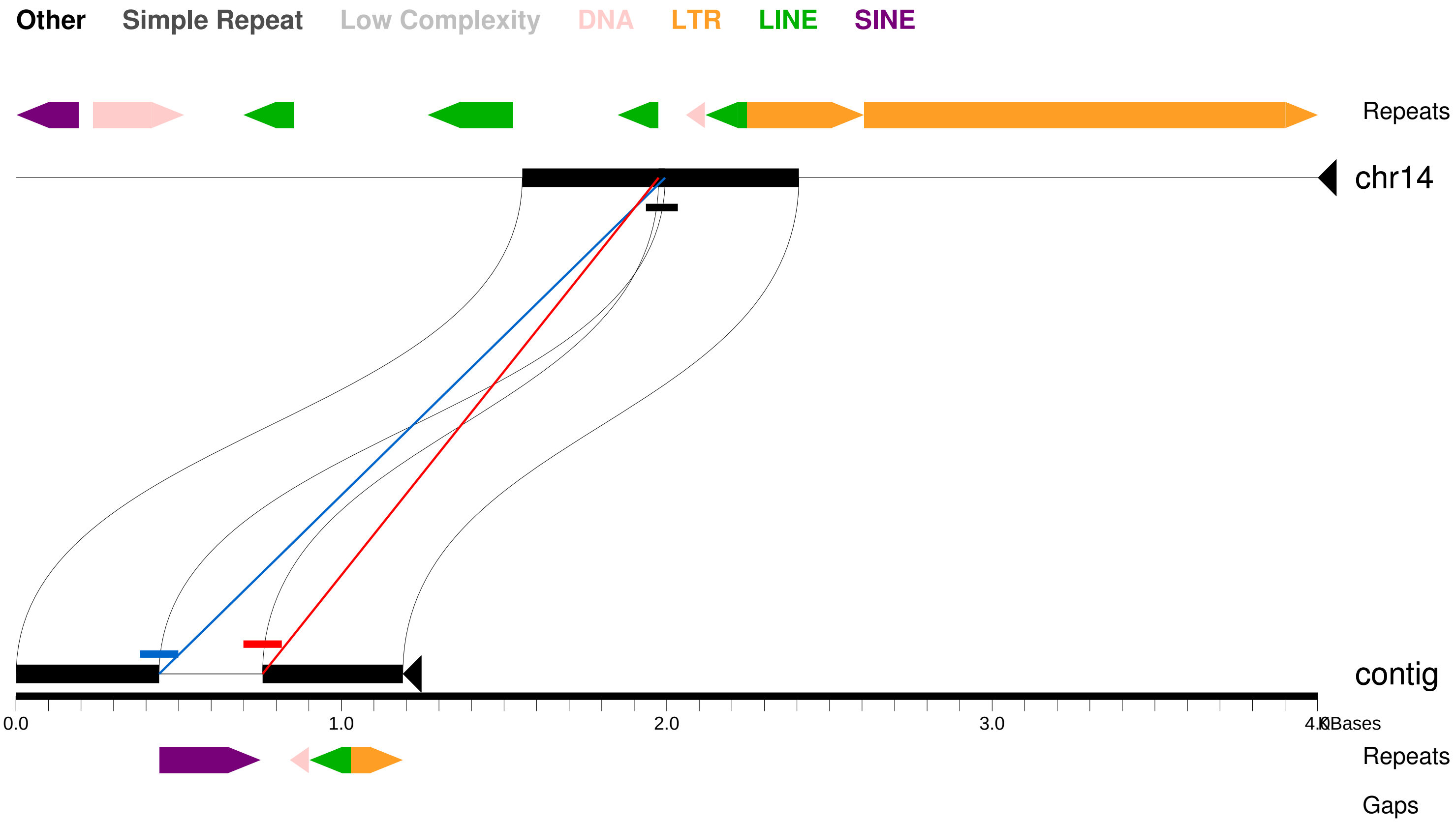

siteID: chr14_39508542 Scafftig1 - Breakpoint: chr14:39508524
chr14:39508378-39508732
Overlap Length: 64 INS size: 427 Genotyped: No

```
left   AAAT------------------------------------------------------------------
chrom  AAATCAATTTTATTTAGTATGTGTTGGTAAAATTAATTTTATTAACTTAATTTTTAAAAGATTAAACTTT
right  CCACCC--------------GCCTCGGCC-----------------------------------------
       11*12N              2NN2N22NN

left   ----------------------------------AATAAATGCACCTCTTCTCTCCAAATCATACATCCA
chrom  GAAATGTATAGGTCACTAAGTGTTACAAATAAATAATAAATGCACCTCTTCTCTCCAAATCATACATCCA
right  ------------TCCCAAAGTGCTGGGATTACAGGCGTTAGCCACCGCGCCCGGCCA-------------
                   22N2N22222N2NNN2N22N2N11111*11****1*11*111***1111111111111

left   AAATAACAAATACATTGTTTGGTCCAAAACTCAGAATATAAAAATTCTGAGTGCAAAAATTATGACAAGG
chrom  AAATAACAAATACATTGTTGAGTCCAAAACTCAGCATATAAAAATTATGTGTGCAACAATTATGACAAGG
right  ------CAAATACATTGTTGAGTCCAAAACTCAGCATATAAAAATTATGTGTGCAACAATTATGACAAGG
       111111*************22*************2***********2**2******2*************

left   NNNNNNNNNNNNNNNNNNNNNNNNNNNNNNNNNNNNNNNNNNNNNNNNNNNNNNNNNNN-----------
chrom  TTGGTGTGAAATTACTAAACACTTGGTCAGAAGTTCAAAATCCCAAATAATGATAAAAACAAAAGCCATG
right  TTGGTGTGAAATTACTAAACACTTGGTCAGAAGTTCAAAATCCCA-------------------------
       222222222222222222222222222222222222222222222NNNNNNNNNNNNNN

left   ----------------------------------------------------------------------
chrom  AAAATCTGTATGTTCTTTTGCTTAAAATAATGACCCTTAAGCTTTCTTAAACCACAGGTGATTCTATGAG
right  ----------------------------------------------------------------------

left   -----
chrom  ACAAG
right  -----
```

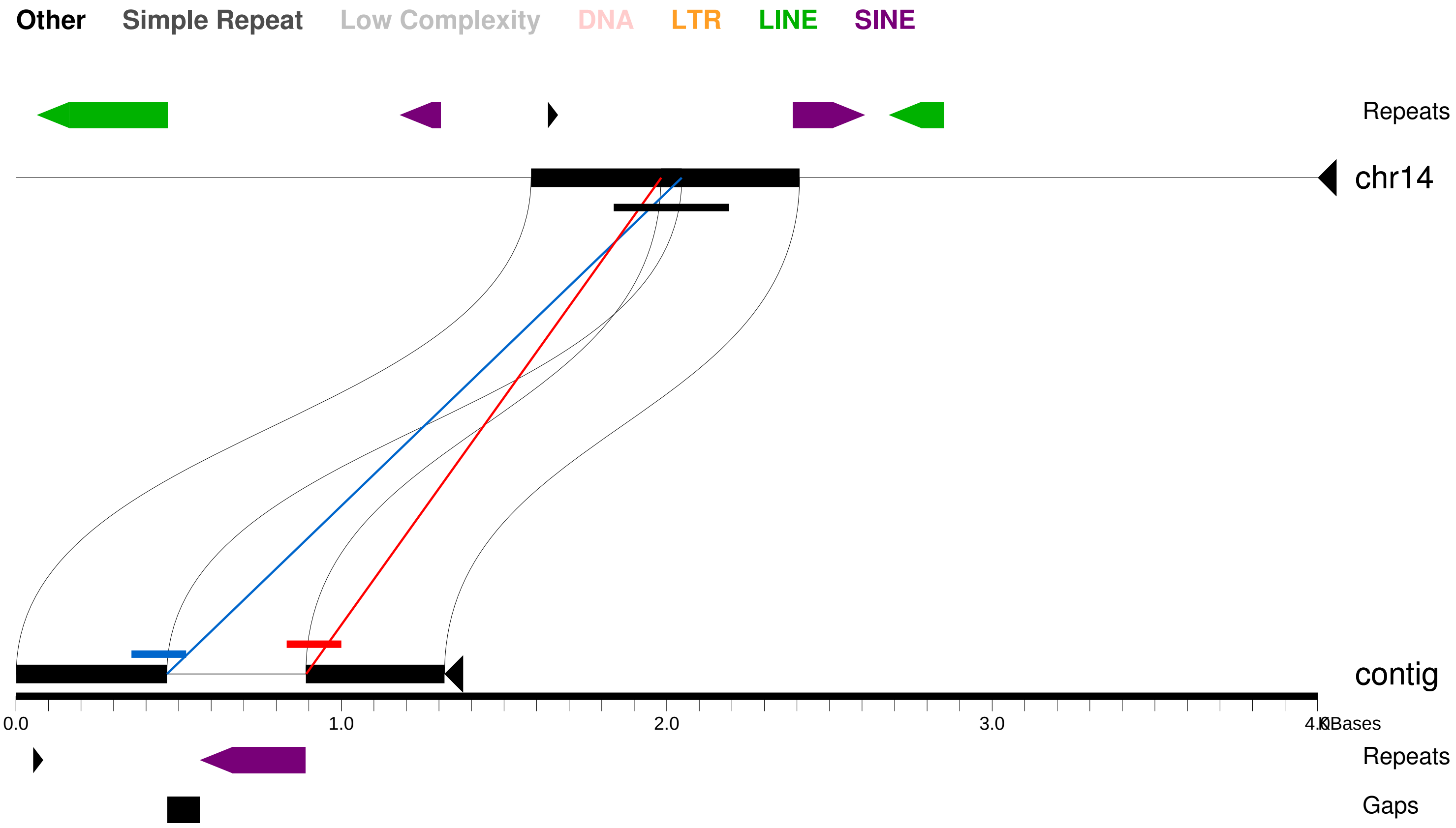

siteID: chr14_39804826 Scafftig1 - Breakpoint: chr14:39804796
chr14:39804745-39804855
Overlap Length: 9 INS size: 416 Genotyped: No

```
left    --AACTGCT-----CTAACTTATCTCTTTTCTCATATGCCCTATTC-CAGACATTCTTTTAAAAAATANN
chrom   --AACTGCT-----CTAACTTATCTCTTTTCTCATATGCCCTATTC-CAGACATTCTTTTAAAAAATATT
right   CCACCCGCCTCGGCCTCCCAAAGTGCTGGGATTACAGGCGTGAGCCACCGCGCCCGGCCTAAAAAATATT
        11*1*1**111111**11*11*111**1111*1*1*1**111*11*1*1*111111111*********22

left    NNNNNNNNNNNNNNNNNNNNNNNNNNNNNNNNNNNNNNNNNNNNNNNNNNNNNNNNN
chrom   TTAATAATGTTATCTTTTATTTGAGATTCACAGGGTACATGTGCAGGTT--------
right   TTAATAATGTTATCTTTTATTTGAGATTCACAGGGTACATGTGCAGGTT--------
        222222222222222222222222222222222222222222222222222222222
```

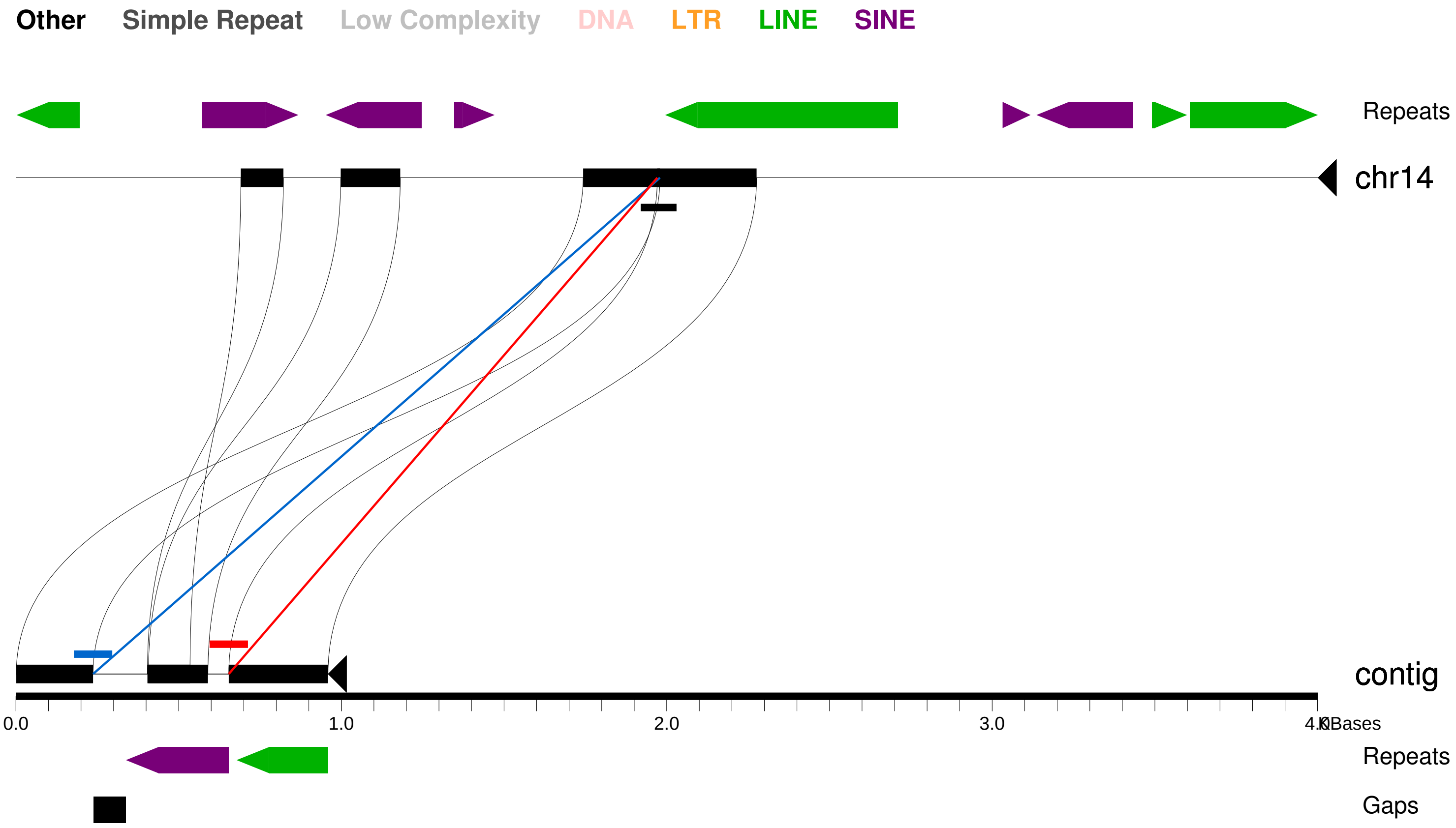

siteID: chr14_40312975 Scafftig15 + Breakpoint: chr14:40312948
chr14:40312907-40313007
Overlap Length: 19 INS size: 303 Genotyped: Yes

```
left    -------TTTCTACCAATACAAACTCATTGAAAA-------TGATCTA----GAAAAGA-GATTCTTATC
chrom   -------TTTCTACCAATACAAACTCATTGAAAA-------TGATCTA----GAAAAGA-GATTCTTATC
right   CCGCCCGCCTCGGCCTCC-CAAAGTGCTGGGATTACAGGCGTGAGCCACCGCGCCCGGCCGATTCTTATC
        111111111**11**1111****1*11*1*1*111111111***1*1*1111*1111*11**********

left    AACTTTTTTTTTTTTTTTTTTTTTTTTTTTGAGACGGAGTCTCGCTCTGTCGCCCAGGCTGGAGTGCAG
chrom   AACTCTTTTGGGAAAAGCTGAGTGTTGG-GAGAAG----------CTGAGGCAG-GGCT----TGC--
right   AACTCTTTTGGGAAAAGCTGAGTGTTGG-GAGAAG----------CTGAGGCAG-GGCT----TGC--
        ****2****222222222*222*2**222****2*2222222222***22**222****2222***22
```

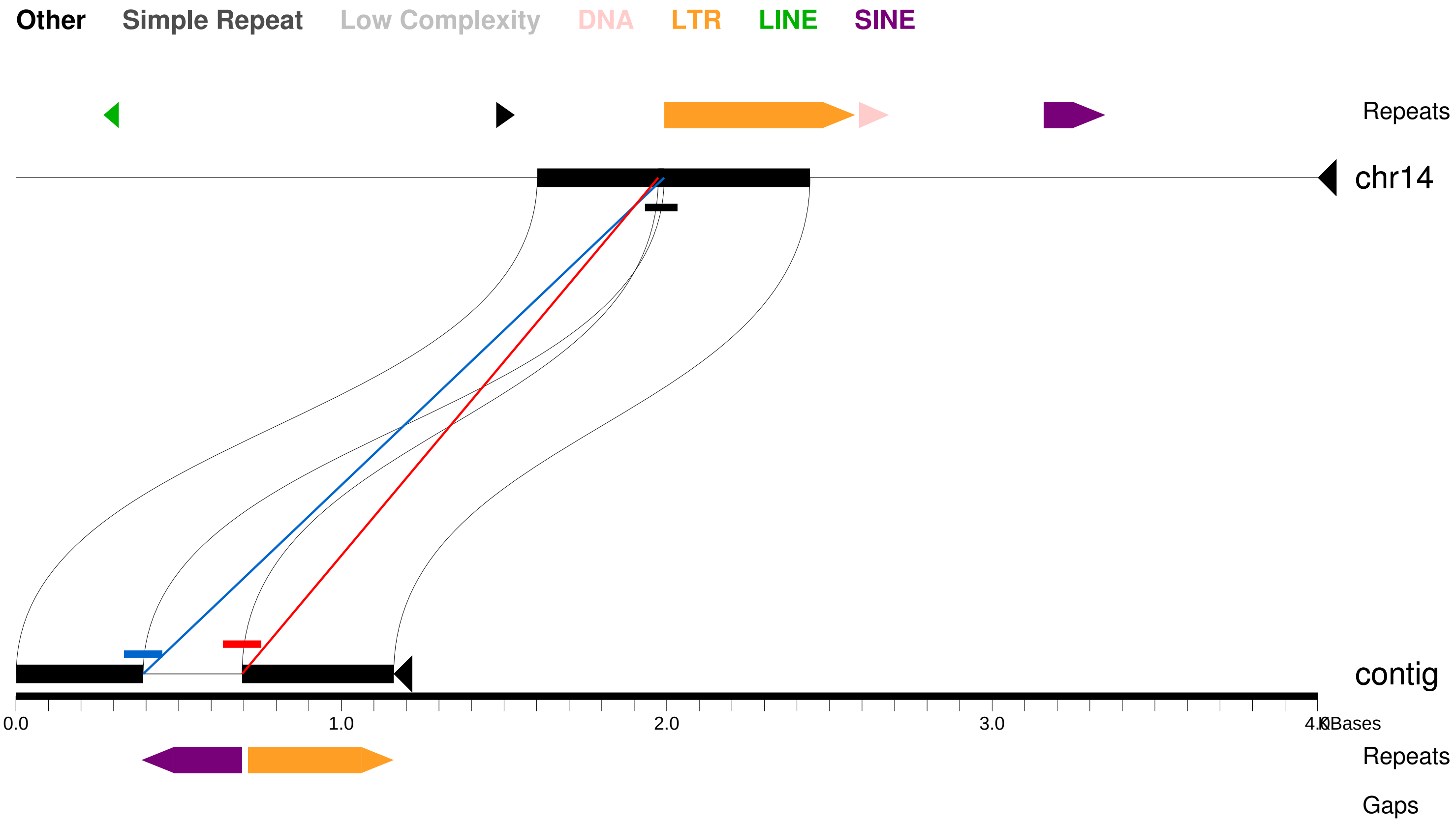

siteID: chr14_40579680 Scafftig2 - Breakpoint: chr14:40579675
chr14:40579626-40579734
Overlap Length: 11 INS size: 429 Genotyped: No

```
left    ATTTGCATGGTGAAGCTAGTTTAAAGTTATGACTGTAAAGGCAGTCAAA----------ACGGGCCATAT
chrom   ATTTGCATGGTGAAGCTAGTTTAAAGTTATGACTGTAAAGGCAGTCAAA----------ACGGGCCATAT
right   NNNNNNNNNNNNNNNNNNNNNNNNNNNNNNNNNNNNNNNNNNNNNNNNNNNNNNNNNNNACGGGCCATAT
        11111111111111111111111111111111111111111111111111111111111***********

left    GGCCGGGCGCGGTGGCTCACGCCTGTAATCCCAGCACTTTGGGAGGCCGAGGCGGGCGG
chrom   A-CTGTGCAATCTCAATGAAAA-TAAATGTCCAGAA--TAGGAAAATCTATA------G
right   A-CTGTGCAAACTCAATGAAAA-TAAATGTCCAGAA--TAGGAAAATCTATA------G
        22*2*2**22N2*222*2*2222*22*222****2*22*2**2*222*2*22222222*
```

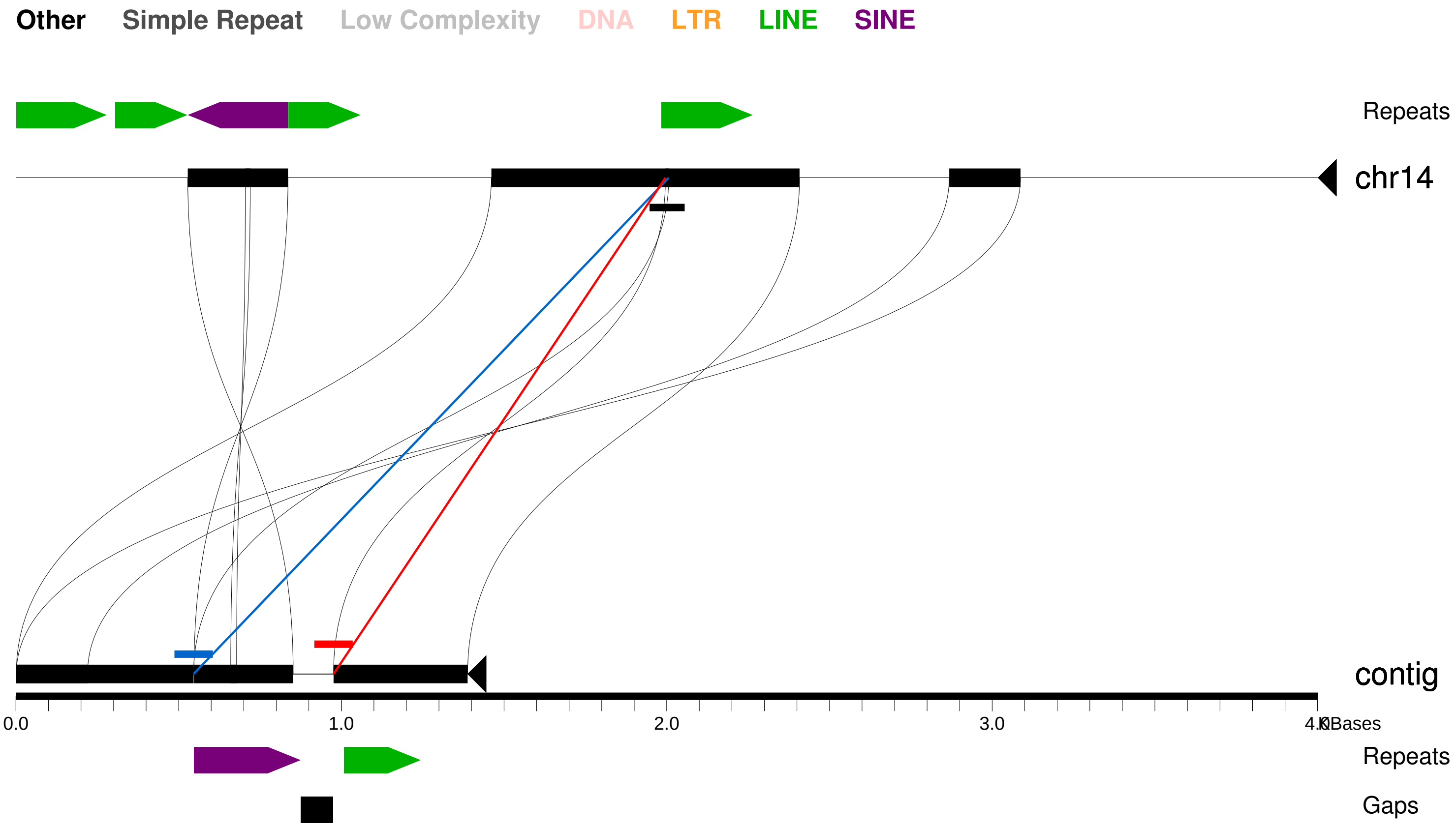

siteID: chr14_40592352 Scafftig5 + Breakpoint: chr14:40592430
chr14:40592384-40592488
Overlap Length: 14 INS size: 118 Genotyped: Yes

```
left     ACATA---------TTCATGCTTCACTGAGTAATGTTCT-GTGATTCA---TTCTCATC-GTTTCTTTCT
chrom    ACATA---------TTCATGCTTCACTGAGTAATGTTCT-GTGATTCA---TTCTCATC-GTTTCTTTCT
right    CAATCTCGGCTCACTGCAGGCTCCGCCCCCTGGGGTTCACGCCATTCTCCTGCCTCAGCCGTTTCTTTCT
         11**1111111111*1**1***1*1*1111*111****11*11****111111****1*1**********

left     TTCTTTTTTTTTTTTTTTTTGAGACGGAGTCTCGCTGTCGCCCAGGCTGGAGTGCAGTGGCGC
chrom    TTCTAAAGCTGATTAATTCTTTAAAG----CTAGCTCAAGTTCA----------CATTTACCT
right    TTCTAAAGCTGATTAATTCTTTAAAG----CTAGCTCAAGTTCA----------CATTTACCT
         ****22222*22**22**2*222*2*2222**2***222*22**2222222222**2*22*22
```

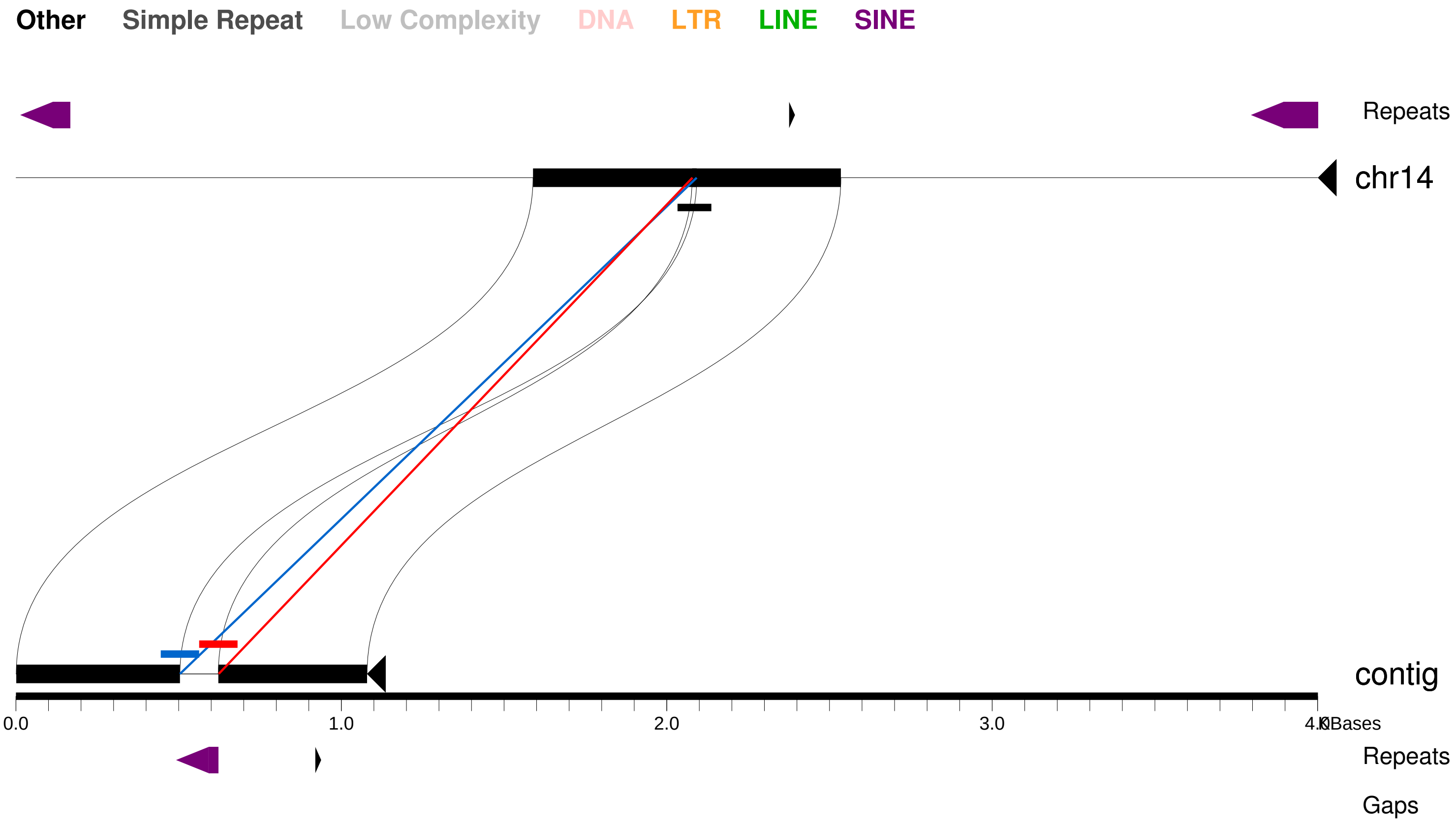

siteID: chr14_40882890 Scafftig4 - Breakpoint: chr14:40882887
chr14:40882841-40882946
Overlap Length: 14 INS size: 318 Genotyped: Yes

```
left     T------TTCTTGCTTACATTGTTCCAGCTTT--GGCCATTGGTTACAGTT---GGT--TCCTTTGTCCT
chrom    T------TTCTTGCTTACATTGTTCCAGCTTT--GGCCATTGGTTACAGTT---GGT--TCCTTTGTCCT
right    CGCCCGCCTCGGCCTCCCAAAGTGCTGGGATTACAGGCGTGAGCCACCGCGCCCGGCCCTCCTTTGTCCT
         11111111**111**11**11**1*11*11**111*1*1*11*11**1*11111**111***********

left     CTATTTTTTTTTATTTTTTTATTTTATTTTTTTTTTTTTGAGACGGAGTCTCGCTCTGTCGC
chrom    CTA---------ACGTGTCCTCATCACTTTGTATTTTCAGA-ACTTCCTTACCTTCTGG---
right    CTA---------ACGTGTCCTCATCACTTTGTATTTTCAGA-ACTTCCTTACCTTCTGG---
         ***222222222*22*2*22222*2*2***2*2****22**2**2222*22*22****2222
```

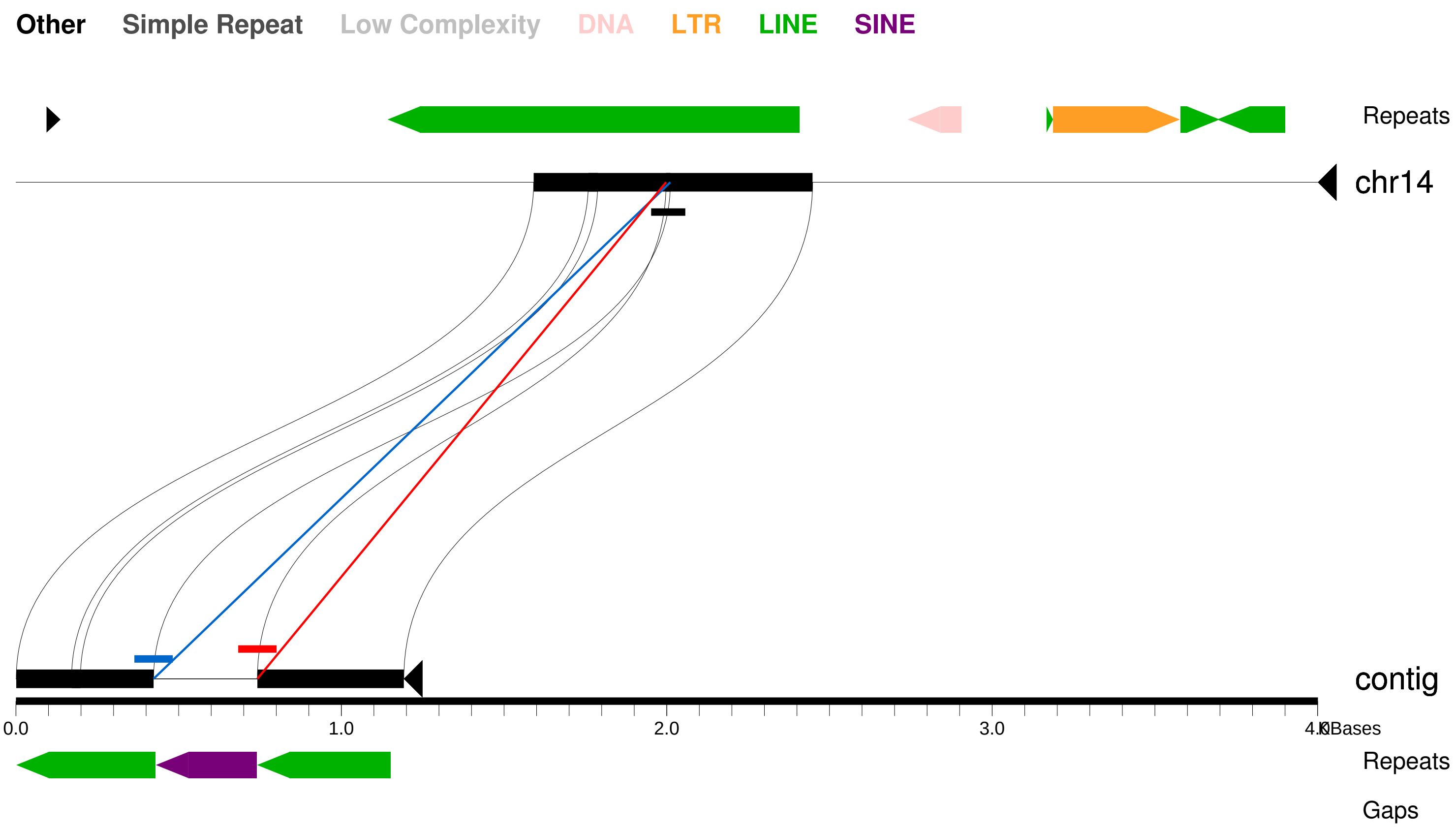

siteID: chr14_41455946 Scafftig1 - Breakpoint: chr14:41455965
chr14:41455907-41456024
Overlap Length: 2 INS size: 411 Genotyped: No

```
left    GCTAGCCAGCATTTCTCTATGGATGACATGGTCTGTAAACTCACATTAAGAAGCAATC-TTGGCCGGGCG
chrom   GCTAGCCAGCATTTCTCTATGGATGACATGGTCTGTAAACTCACATTAAGAAGCAATC-TTTT--TGACC
right   NNNNNNNNNNNNNNNNNNNNNNNNNNNNNNNNNNNNNNNNNNNNNNNNNNNNNNNNNNNTTTT--TGACC
        11111111111111111111111111111111111111111111111111111111111**22222*2*2

left    CGGTGGCTCACGCCTGTAATCCCAGCACTTTGGGAGGCCG--AGGCGGGCGG
chrom   CAACTAGAGAAACCAGAAAGCTGTCCAATCTTGGCAGCTGCTAGGTCTGTG-
right   CAACTAGAGAAACCAGAAAGCTGTCCAATCTTGGCAGCTGCTAGGTCTGTG-
        *22222222*22**2*2**2*2222**2*2*2**22**2*22***222*2*2
```

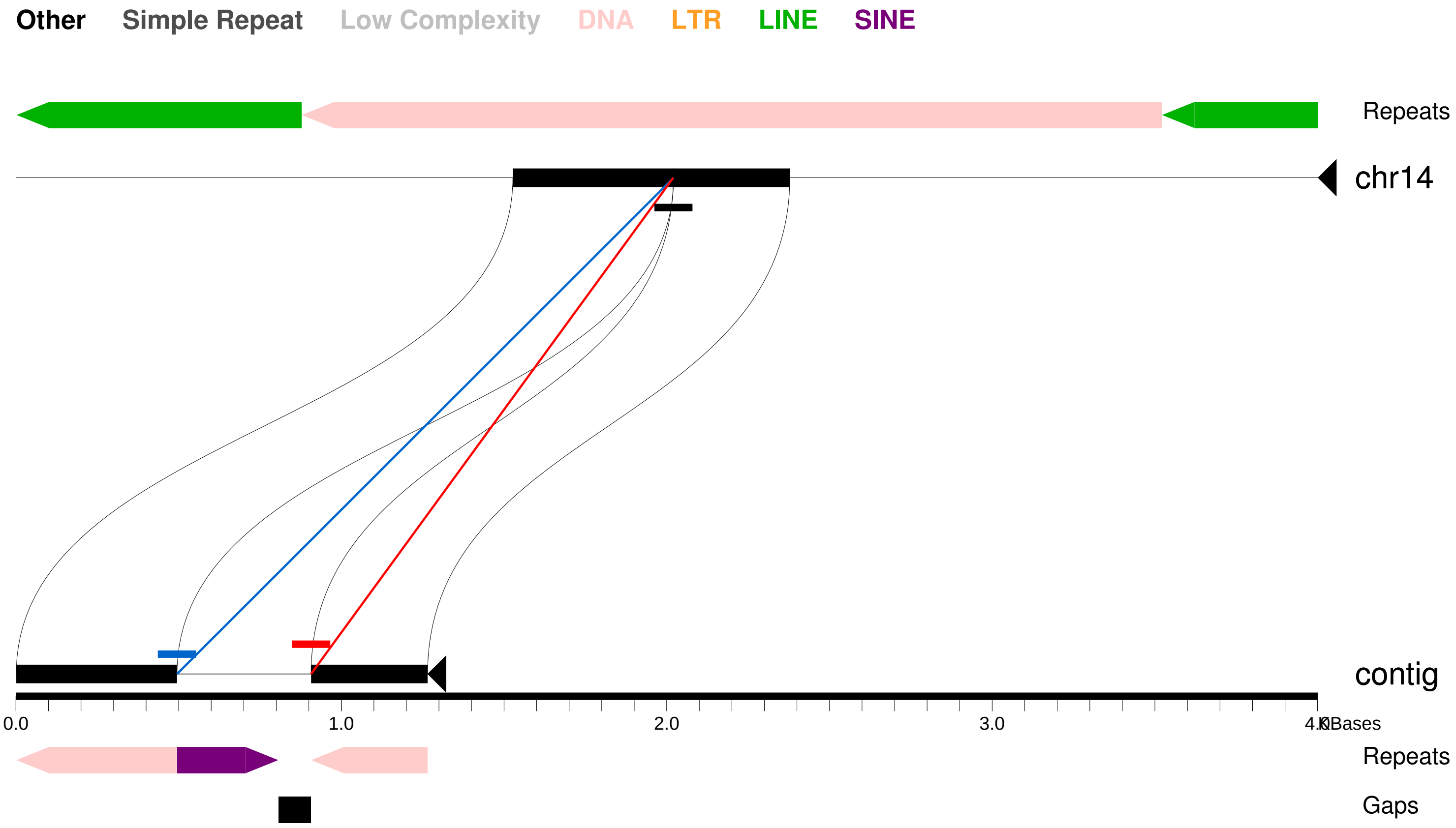

siteID: chr14_41840256 Scafftig6 + Breakpoint: chr14:41840258
chr14:41840213-41840317
Overlap Length: 15 INS size: 448 Genotyped: No

```
left    ACATTTCTGTTGAATAGCAAAAGAGT--------------AAGTTAAGGAGATCTATATAAAACAGGGTG
chrom   ACATTTCTGTTGAATAGCAAAAGAGT--------------AAGTTAAGGAGATCTATATAAAACAGGGTG
right   NNNNNNNNNNNNNNNNNNNNNNNNNNNNNNNNNNNNNNNNCAAAAAAAAAAAAAAAAAAAAAAAACAGGGTG
        1111111111111111111111111111111111111111**111**11*1*111*1*1***********

left    ATTAGGCCGGGCGCGGTGGCTCACGCCTGTAATCCCAGCACTTTGGG-AGGCCGAGGCGGGCGG
chrom   ATTA------------TAGTTAATAC-TATATTA--AATTCTTAGAATATGCTTAGAGAGTGAA
right   ATTA------------TAGTTAATAC-TATATTA--AATTCTTAGAATATGCTTAGAGAGTGAA
        ****222222222222*2*2*2*22*2*2**2*222*222***2*222*2**22**222*2222
```

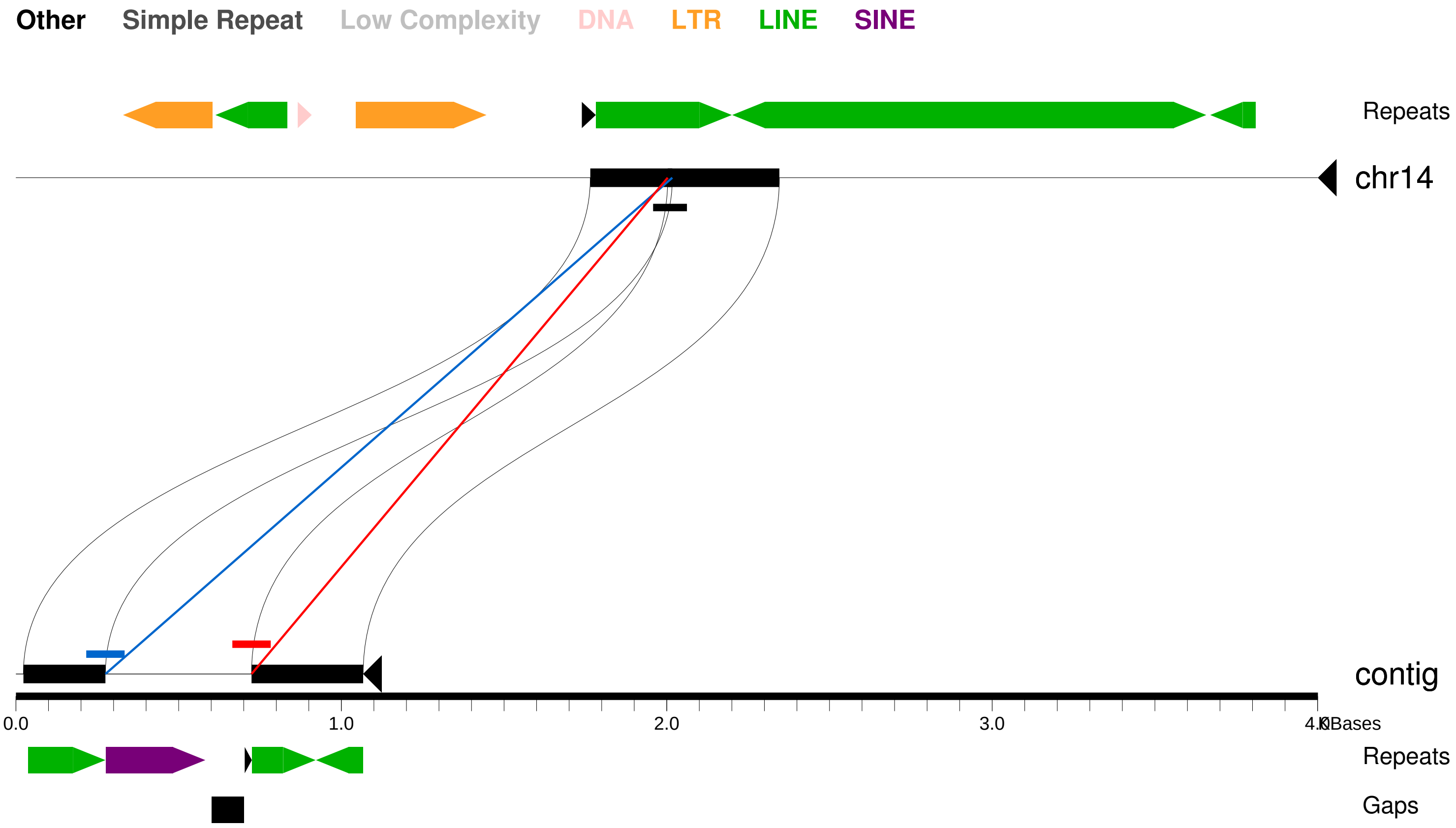

siteID: chr14_41926827 Scafftig1 - Breakpoint: chr14:41926821
chr14:41926763-41926880
Overlap Length: 2 INS size: 420 Genotyped: No

```
left    TTGTTTCTTCTGACCTATTTTACTATTGTTGGTTATGGC-TTATATTTTCCTTCTTTTTGTNNNNNNNNN
chrom   TTGTTTCTTCTGACCTATTTTACTATTGTTGGTTATGGC-TTATATTTTCCTTCTTTTTGTGAGTGCTAA
right   CCGCCCGCCTCGGCCTCCCAAAGTGCTGGGATTACAGGCGTGAGCCACCGCGCCCGGCCGTGAGTGCTAA
        11*11111111*1***11111*1*11**1111*111***1*1*1111111*11*11111**222222222

left    NNNNNNNNNNNNNNNNNNNNNNNNNNNNNNNNNNNNNNNNNNNNNNNNNN
chrom   TTTTTTATTGTATGCTTTATATTGTAACTAATGTACTTTAGAGAGCCCA-
right   TTTTTTATTGTATGCTTTATATTGTAACTAATGTACTTTAGAGAGCCCA-
        22222222222222222222222222222222222222222222222222
```

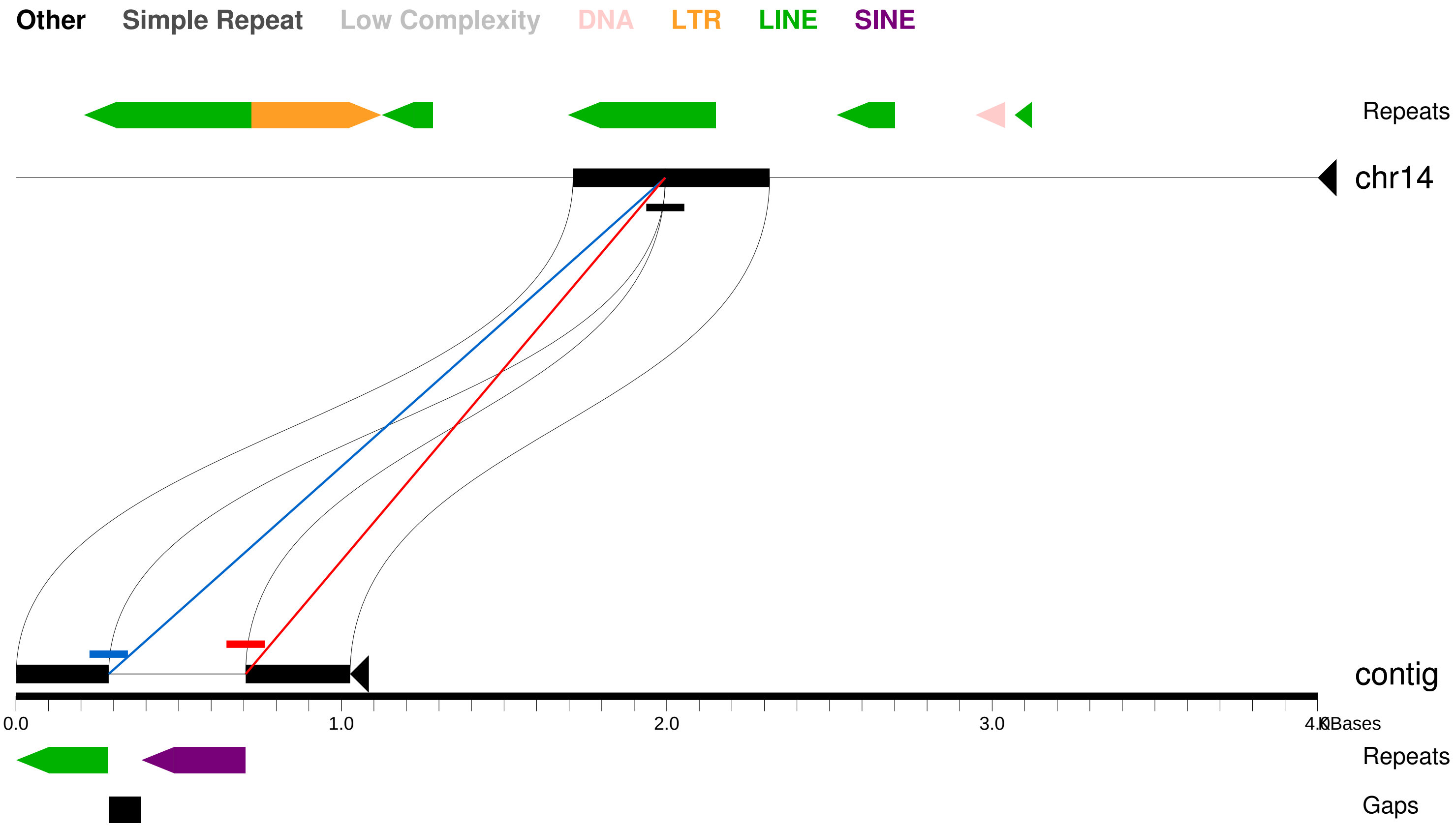

siteID: chr14_42511221 Scafftig2 + Breakpoint: chr14:42511224
chr14:42511064-42511411
Overlap Length: 28 INS size: 293 Genotyped: Yes

```
left     T------------------------TTTTATTTCTGAGGCATCTGTTGTAATGTCCCCTTTTTCATTTAT
chrom    TCATAAGATAGTCTCGTATGATCCTTTTTATTTCTGAGGCATCTGTTGTAATGTCCCCTTTTTCATTTAT
right    C--TAATTTTTT---GTAT------TTTTAGTAGAGACGGG---GTT------TCACCGTTTTA------
         1  222NN2NN2   2222      *****1*111**1*11111***111111**1**1****1111111

left     GATTTTGTGTGCTTGTAGCTTCTCTTTTTTCTTACTTAGTCTAGCAAGGGTTTCTTTATCTTCTAAATAA
chrom    GATTTTGTGTGCTTGTAGCTTCTCTTTTTTCTTACTTAGTCTAGCAAGGGTTTCTTTATCTTCTAAATAA
right    ----------GCCGGGATGGTCTCGATCTCCTGACCTCGTGATCCGCCCGCCTCGGCCTC--CCAAAGTG
         1111111111**11*1*111****11*1*1**1**1*1**1111*1111*11**1111**11*1***111

left     AACCAACTCTCAGTTTAGTTGATTTTTTATTTTT--TATT-TTTTTAT------TTT-------------
chrom    AACCAACTCTCAGTTTAGTTGATTTTTAATTTTTAATATTATTTTTATGATGTATTTGTAGTATTAAGTT
right    CTGGGACTA-CAGGCATGA-GATTTTTAATTTTTAATATTATTTTTATGATGTATTTGTAGTATTAAGTT
         11111***11***1111*11*******2******22****2*******222222***2222222222222

left     ------TTTTTTTTATTTTTTTTT-ATTTTTTGAGACGGAGTC--TCGCTCTGTCGCCCAGGCT----GG
chrom    AGGTGGTTTATTTTATATATTTTT-CTTTTTTTA-ATGTAGGCAATTACTACTACAACTTCTCTTTTAGT
right    AGGTGGTTTATTTTATATATTTTTTCTTTTTTTA-ATGTAGGCAATTACTGCTACAACTTCTCTTTTAGT
         222222***2******2*2*****12******2*2*2*2**2*22*22**N222*22*2222**2222*2

left     AGTGCAGTGGCGGGATCTCGGCTC-------ACTGCAAGCTCCGCCTCCCGGGT-TAACGCCATTCTC--
chrom    ACTGTTTTTGCAACATTTTGTTTGTTTTGGTATTGTGTTTTCATTTTCTTTTGTCTTTAGGTATTTAAAA
right    ACTGTTTTTGCAACATTTTGTTTGTTTTGGTATTGTGTTTTC----------------------------
         *2**222*2**222**2*2*22*22222222*2**22222**NNNN11NNNN11 1NNN1NN111NNN
```

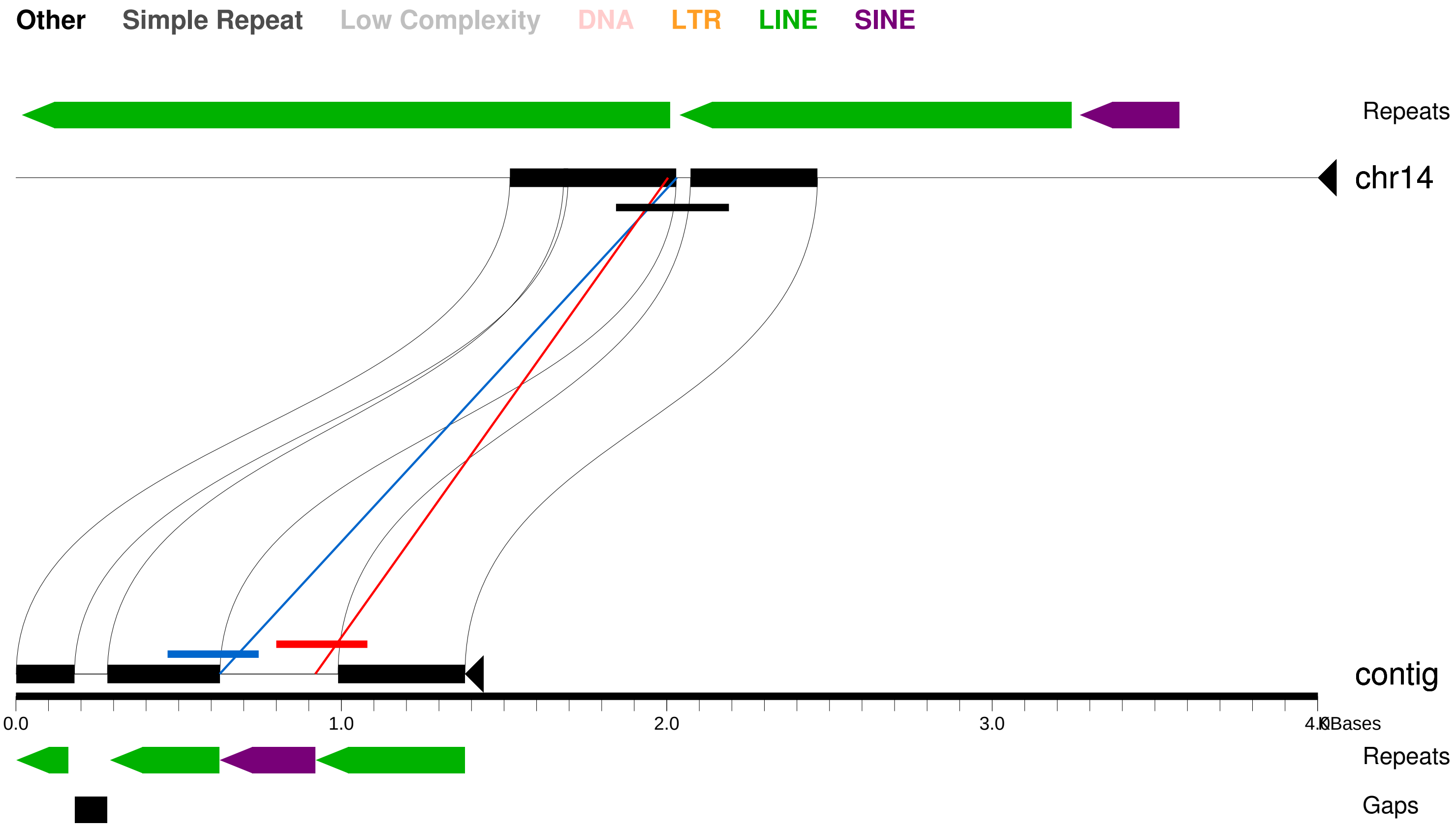

siteID: chr14_43478798 Scafftig2 - Breakpoint: chr14:43478793
chr14:43478746-43478852
Overlap Length: 13 INS size: 300 Genotyped: Yes

```
left    TT----TACA---TTGA--AAATTACTAAATT--GTCACATTACTTAATAATCCTTAGAAA-GATGCAAA
chrom   TT----TACA---TTGA--AAATTACTAAATT--GTCACATTACTTAATAATCCTTAGAAA-GATGCAAA
right   CTGCACTCCAGCCTGGGCGACAGAGCGAGACTCCGTCTCAAAAAAAAAAAAAAAAAAAAAAAGATGCAAA
        1*1111*1**111*1*111*1*111*1*1*1*11***1**11*111**1**11111*1***1********

left    TTGGCCGGGCGTGGTGGCTCACACCTGTAATCCCAGCACTTTGGGAGGCCGAGACGGGCGG
chrom   TTAAAACA--ATCCACACTTTCAGGTGT--TGCAAGGA---TGGGCATTGTAGTCT-----
right   TTAAAACA--ATCCACACTTTCAGGTGT--TGCAAGGA---TGGGCATTGTAGTCT-----
        **222222222*22222**22**22***22*2*2**2*222****222222**2*222222
```

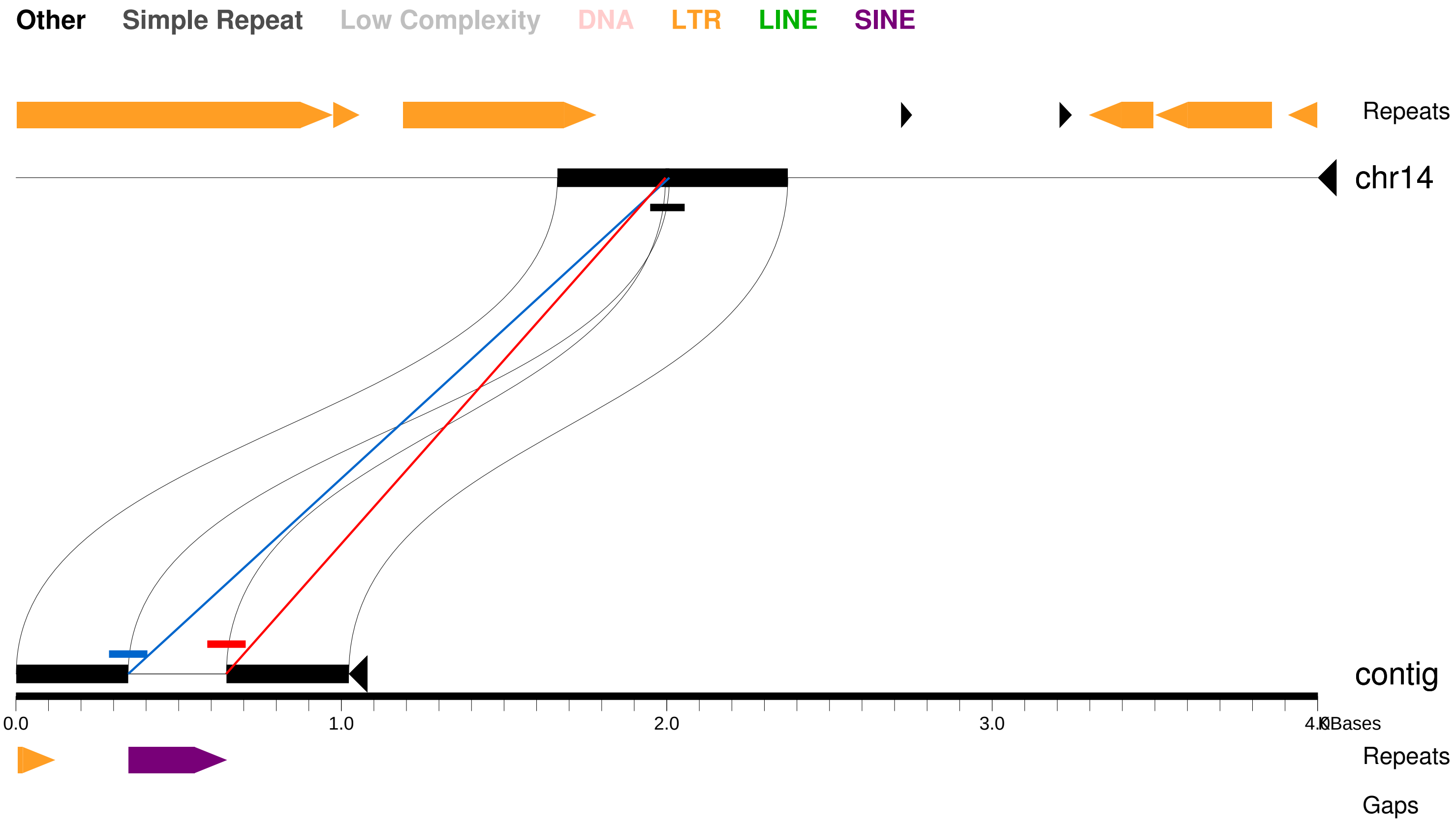

siteID: chr14_43740604 Scafftig1 + Breakpoint: chr14:43740621
chr14:43740578-43740680
Overlap Length: 17 INS size: 478 Genotyped: No

```
left    GGTGATTTTGGTCACATAATAAGGATAATTGTGCAGTAAGCTG----------------AAAGAAGCCTG
chrom   GGTGATTTTGGTCACATAATAAGGATAATTGTGCAGTAAGCTG----------------AAAGAAGCCTG
right   NNNNNNNNNNNNNNNNNNNNNNNNNNNNNNNNNNNNNNNNNNNNNNNNNNNNNNAAAAAAAAGAAGCCTG
        11111111111111111111111111111111111111111111111111111111111***********

left    GATCCCGGCCGGGCGCGGTGGCTCACGCCTGTAATCCCAGCACTTTGGGAGGCCGAGGCGGGCGG
chrom   GATCCCAAA-GAACTTTGAGAAACATCATGATAGTATCATCCTTGTATTA---------------
right   GATCCCAAA-GAACTTTGAGAAACATCATGATAGTATCATCCTTGTATTA---------------
        ******2222*22*222*2*222**222222**2*22**2*22*2*222*222222222222222
```

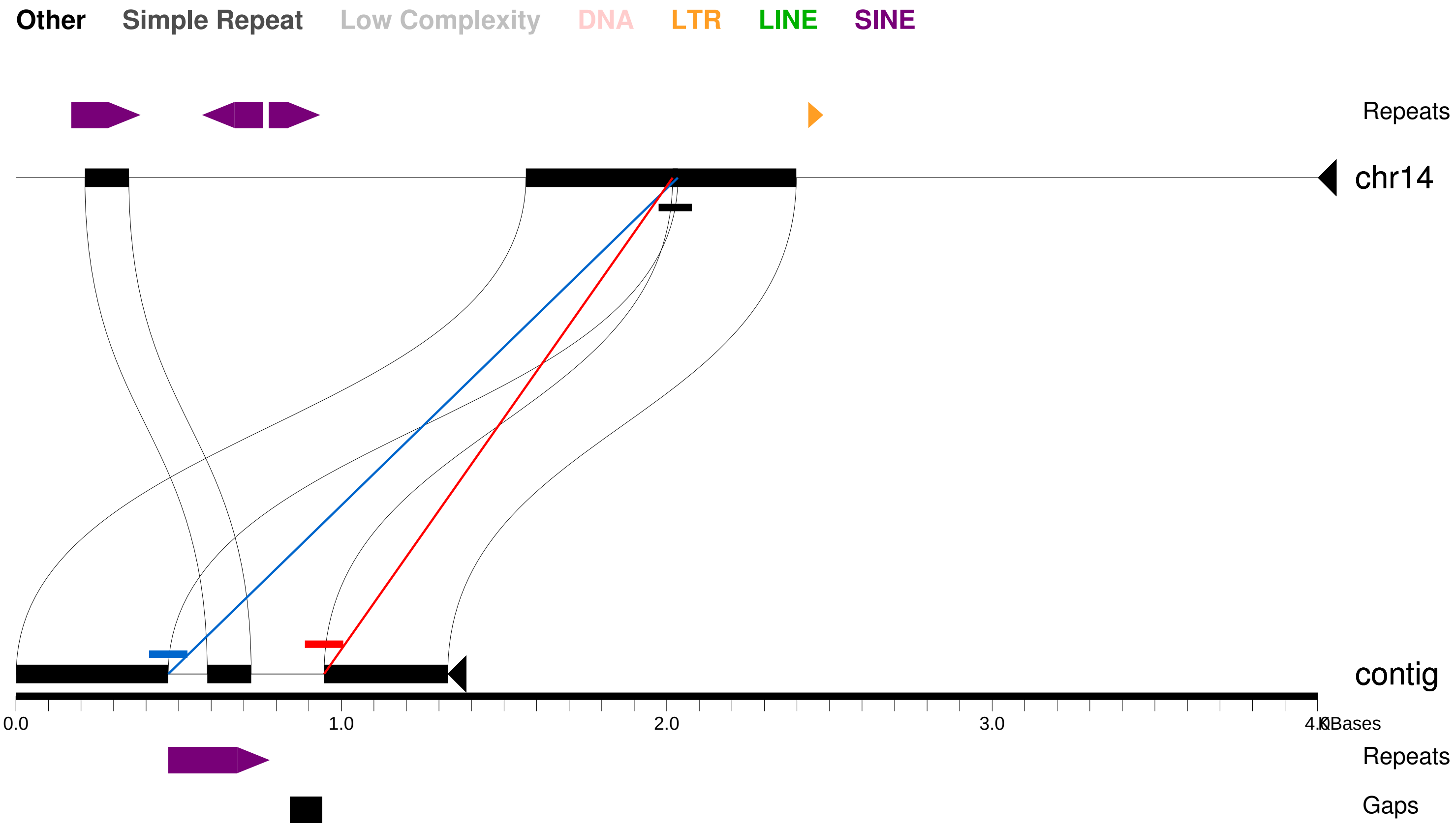

siteID: chr14_43791746 Scafftig1 - Breakpoint: chr14:43791753
chr14:43791746-43791812
Overlap Length: 53 INS size: 434 Genotyped: No

```
left    ------------------AATATGT----------------------------------ACAACTTTTTA
chrom   ------------------AATATGT----------------------------------ACAACTTTTTA
right   CGCCCGCCTCGGCCTCCCAAAGTGCTGGGATTACAGGCGTGAGCCACCGCGCCCGGCCCACAACTTTTTA
        111111111111111111**11**11111111111111111111111111111111111***********

left    TAAAATCATTAATACCTCAACAAAATAGTCTCAAATAAAAGGNNNNNNNNNNNNNNNNNNNNNNNNNNNN
chrom   AAAAATCATTAATACCTCAACAAAATAGTCTCAAATAAAAGGCTACAAA---------------------
right   AAAAATCATTAATACCTCAACAAAATAGTCTCAAATAAAAGGCTACAAA---------------------
        2*****************************************2222222222222222222222222222

left    NNNNNNNNNNNNNNNNNNNNNNNNNNNNNNN
chrom   -------------------------------
right   -------------------------------
        2222222222222222222222222222222
```

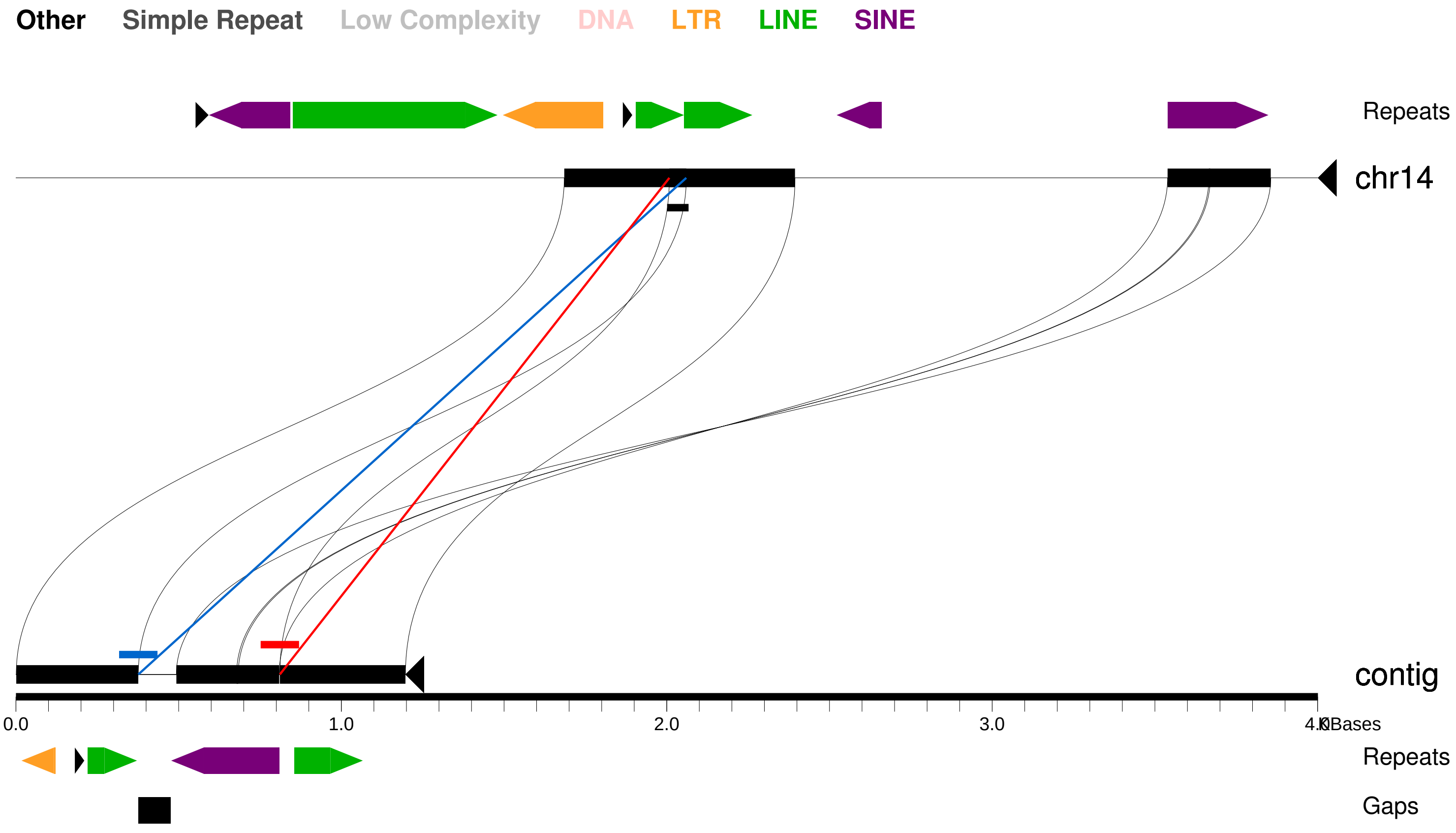

siteID: chr14_44522310 Scafftig1 + Breakpoint: chr14:44522303
chr14:44522254-44522362
Overlap Length: 11 INS size: 294 Genotyped: Yes

```
left    A--CCTGCTATGAATTGTTAAACTG------TACCACTGAAGATTCAACAGAATTGT--GAGAATTTCTT
chrom   A--CCTGCTATGAATTGTTAAACTG------TACCACTGAAGATTCAACAGAATTGT--GAGAATTTCTT
right   CCGCCCGCCTCGGCCTCCCAAAGTGCTGGGATTACAGGCGTGAGCCACCGCGCCCGGCCGAGAATTTCTT
        111**1**111*111*111***1**111111*11**11111**11**1*111111*111***********

left    TTTTTTTTTTTTTGAGACGGAGTCTCGCTCTGTCGCCCAGGCTGGAG-TGCAGTGGCGCA
chrom   AAGTA---------AGATAGAATATGCAGGAGTCAA--ATGAAAAATATATAGTCATGCA
right   AAGTA---------AGATAGAGTATGCAGGAGTCAA--ATGAAAAATATATAGTCATGCA
        222*2222222222***22**N*2*222222***2222*2*2222*22*22***222***
```

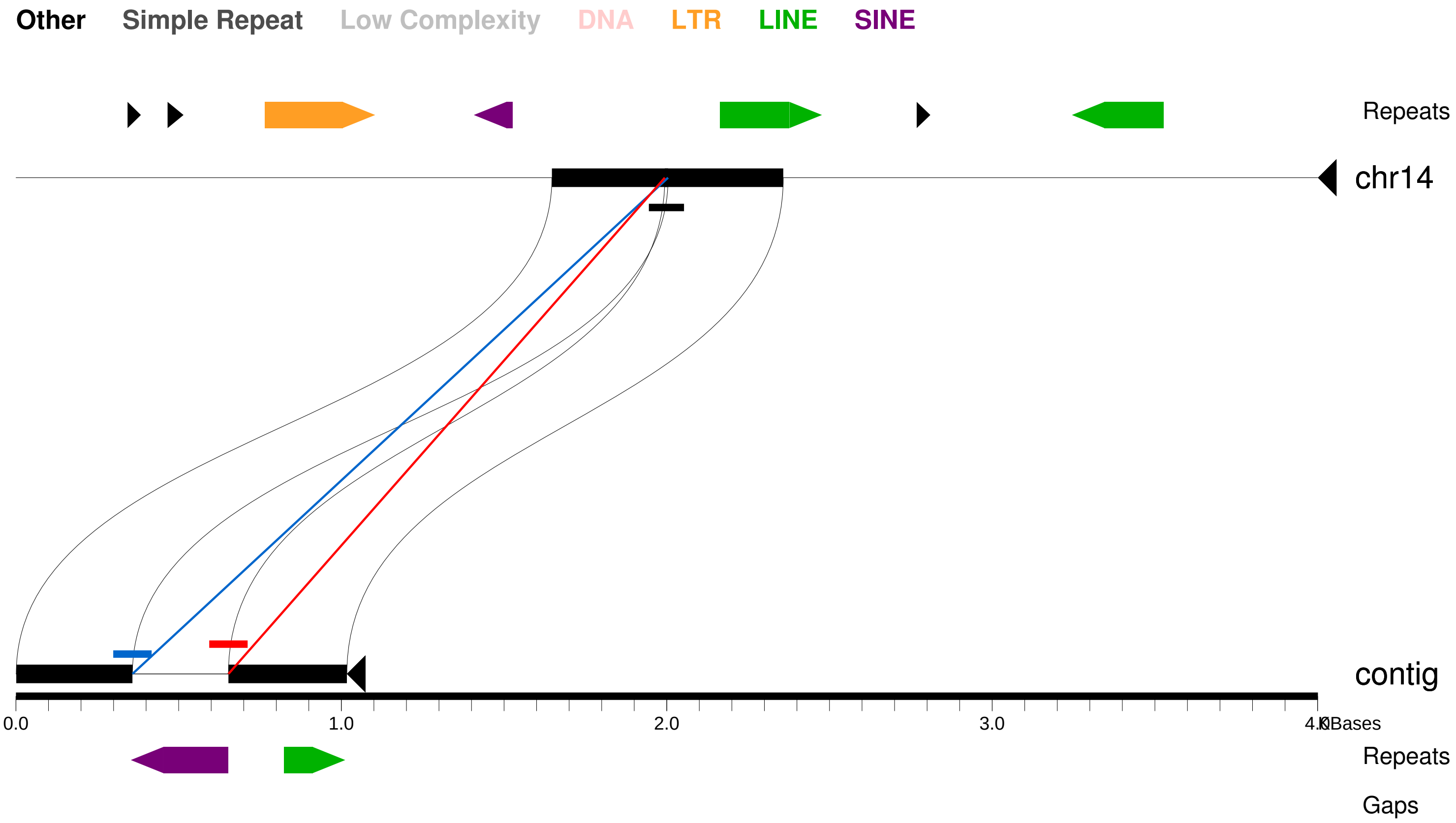

siteID: chr14_47435982 Scafftig1 + Breakpoint: chr14:47435959
chr14:47435913-47436018
Overlap Length: 14 INS size: 307 Genotyped: Yes

```
left     TTG------------TAATAAAGTCATTAAGTAACAAGTGC---CCAAGATTAATGATTTTAGCATTTTC
chrom    TTG------------TAATAAAGTCATTAAGTAACAAGTGC---CCAAGATTAATGATTTTAGCATTTTC
right    CCGCCCGCCTCGGCCTCCCAAAGTGCTGGGATTACAGGCGTGAGCCACCGCGCCCGGCC--AGCATTTTC
         11*111111111111*111*****11*1111*1***1*1*1111***11111111*11111*********

left     TTTTTTTTTTTTTTTTTTTTTTTTTTTTTTTGAGACGGAGTCTCGCTCTGTCGCCCAGGC--TGGA
chrom    TTTTTGTATCTACTGGCTC---------------AAGATGTCCAGCAAGGTTTCTAAATACTTAGT
right    TTTTTGTATCTACTGGCTC---------------AAGATGTCCAGCAAGGTTTCTAAATACTTAGT
         *****2*2*2*22*222*2222222222222222*2*22***22**222**22*22*22222*2*2
```

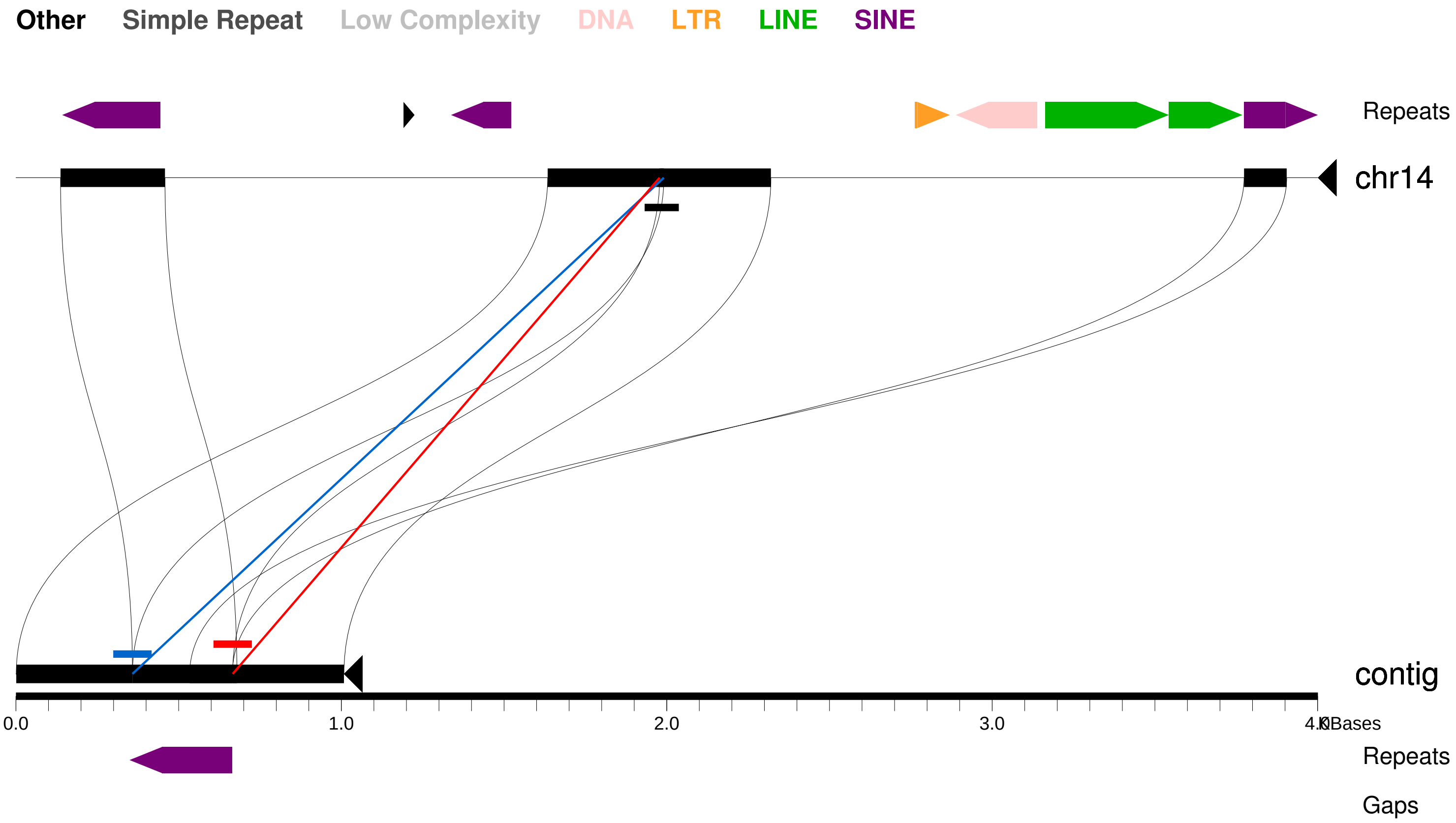

siteID: chr14_47836123 Scafftig1 - Breakpoint: chr14:47836089
chr14:47836072-47836148
Overlap Length: 43 INS size: 421 Genotyped: No

```
left   ACTA---------------AAAGT--TAGATATA-------------------------ATGAAATTACT
chrom  ACTA---------------AAAGT--TAGATATA-------------------------ATGAAATTACT
right  CCACCCGCCTCGGCCTCCCAAAGTGCTGGGATTACAGGCGTGAGCCACCGCGCCCGGCCATGAAATTACT
       1*1111111111111111*****11*1*111**11111111111111111111111111***********

left   TTTATTTTTTCTTTGGCCTACTAGTTTTGCATNNNNNNNNNNNNNNNNNNNNNNNNNNNNNNNNNNNNNN
chrom  TTTAGTTTGACTTTGGCCTACTAGTTTTGCATACAACAAAAAAAATAGAA--------------------
right  TTTAGTTTGACTTTGGCCTACTAGTTTTGCATACAACAAAAAAAATAGAA--------------------
       ****2***22**********************22222222222222222222222222222222222222

left   NNNNNNNNNNNNNNNNNNNNN
chrom  ---------------------
right  ---------------------
       222222222222222222222
```

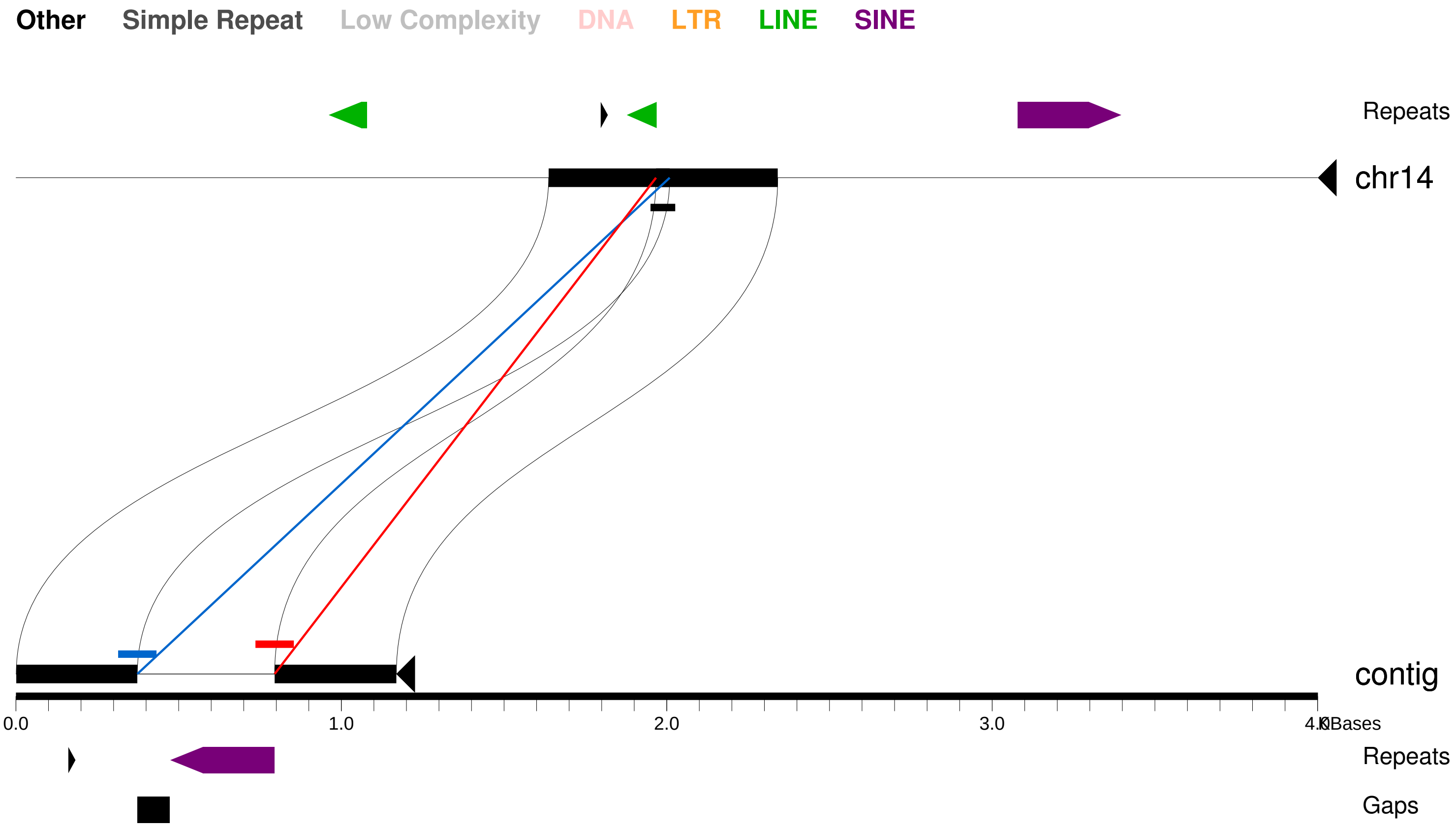

siteID: chr14_51432080 Scafftig4 - Breakpoint: chr14:51432080
chr14:51432035-51432139
Overlap Length: 15 INS size: 326 Genotyped: Yes

```
left     CTAAGAGG--TAGA---CCCA--------GGAT-ACAAGCAAAACCAGACACCATCTTTCTGGAATTAAA
chrom    CTAAGAGG--TAGA---CCCA--------GGAT-ACAAGCAAAACCAGACACCATCTTTCTGGAATTAAA
right    TCCACCCGCCTCGGCCTCCCAAAGTGCTGGGATTACAGGCGTGAGCCACCGCGCCCGGCCTGGAATTAAA
         111*111*11*1*1111****11111111****1***1**111*1*111*1*111*111***********

left     TCTTTTTTTTTTTTTTTTTTTTTTTTTTTTTTTTTTTTTTTTTTTTTGAGACGGAG-TCTCGCTCTGT
chrom    TCTTGAGCAGTGAGACAATA-------------AAATGAGGATGAGCTGGAGCTTTTGCGCC--
right    TCTTGAGCAGTGAGACAATA-------------AAATGAGGATGAGCTGGAGCTTTTGCGCC--
         ****222222*2222222*2222222222222222*22222****22****2*2*2**2*222
```

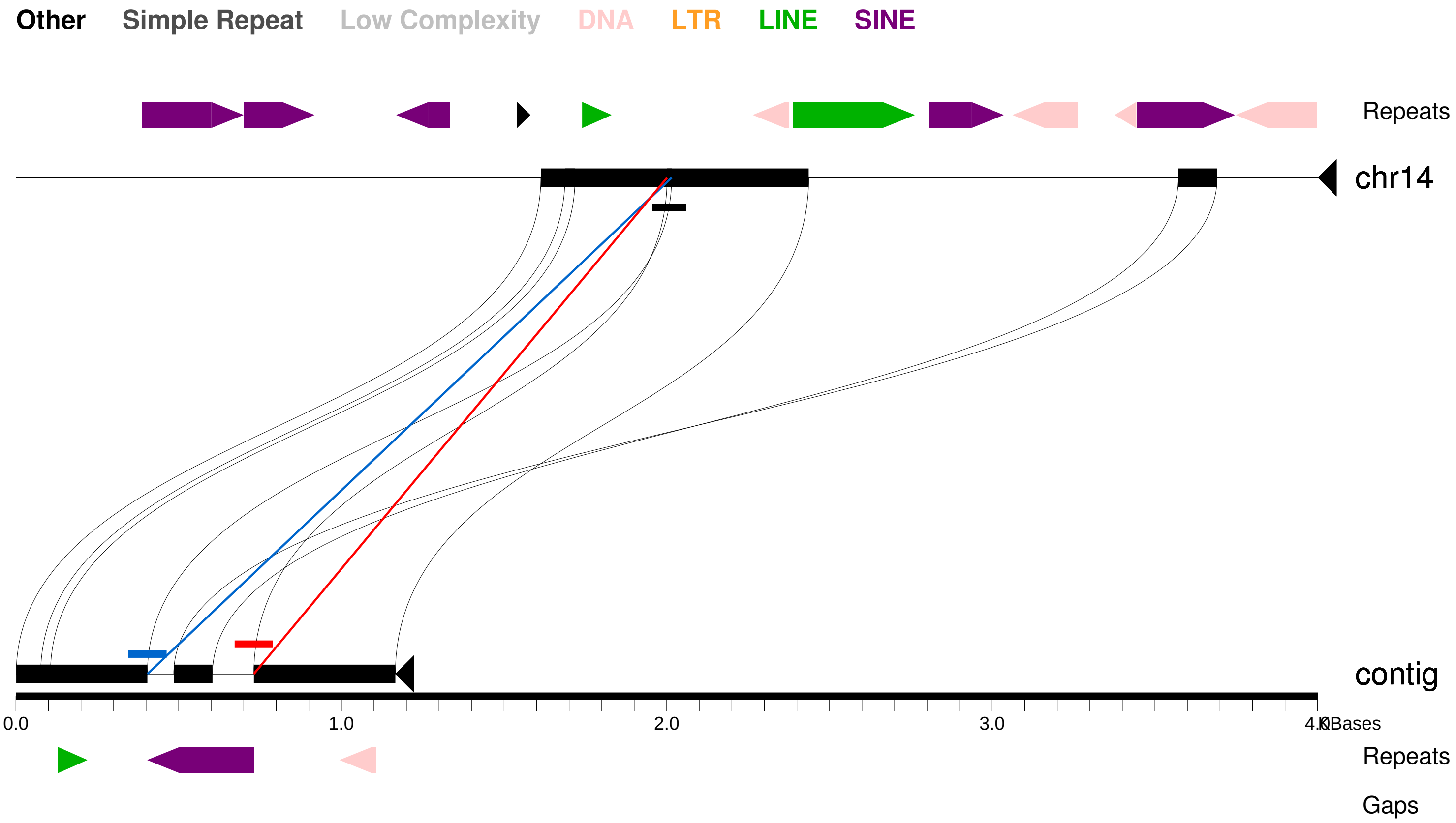

siteID: chr14_51718983 Scafftig8 - Breakpoint: chr14:51718987
chr14:51718941-51719046
Overlap Length: 14 INS size: 297 Genotyped: Yes

```
left   --------ACTTAAGTTTTTCTGTC----CTCTATTATGA-TCCACAGTAAAAGTCACTAAAGAAATCTT
chrom  --------ACTTAAGTTTTTCTGTC----CTCTATTATGA-TCCACAGTAAAAGTCACTAAAGAAATCTT
right  GCCACTGCACTCCAGCCTGGGCGACAGAGCGAGACTCCGTCTCAAAAAAAAAAAAAAAAAAAGAAATCTT
       11111111***11**11*1111*1*1111*111*1*11*11**1*1*11****111*11***********

left   TCCGGCCGGGCGCGGTGGCTCACGCCTGT-AATCCCAGCACTTTGGGAGGCCGAGGCGGGCGG
chrom  TCC---------CTTTAAATA---TCTGTTAATTTGGAGGGATTGAAAGGTATAA--AGTAAA
right  TCC---------CTTTAAATA---TCTGTTAATTTGGAGGGATTGAAAGGTATAA--AGTAAA
       ***222222222*22*222*22222****2***222222222***22***222*2222*2222
```

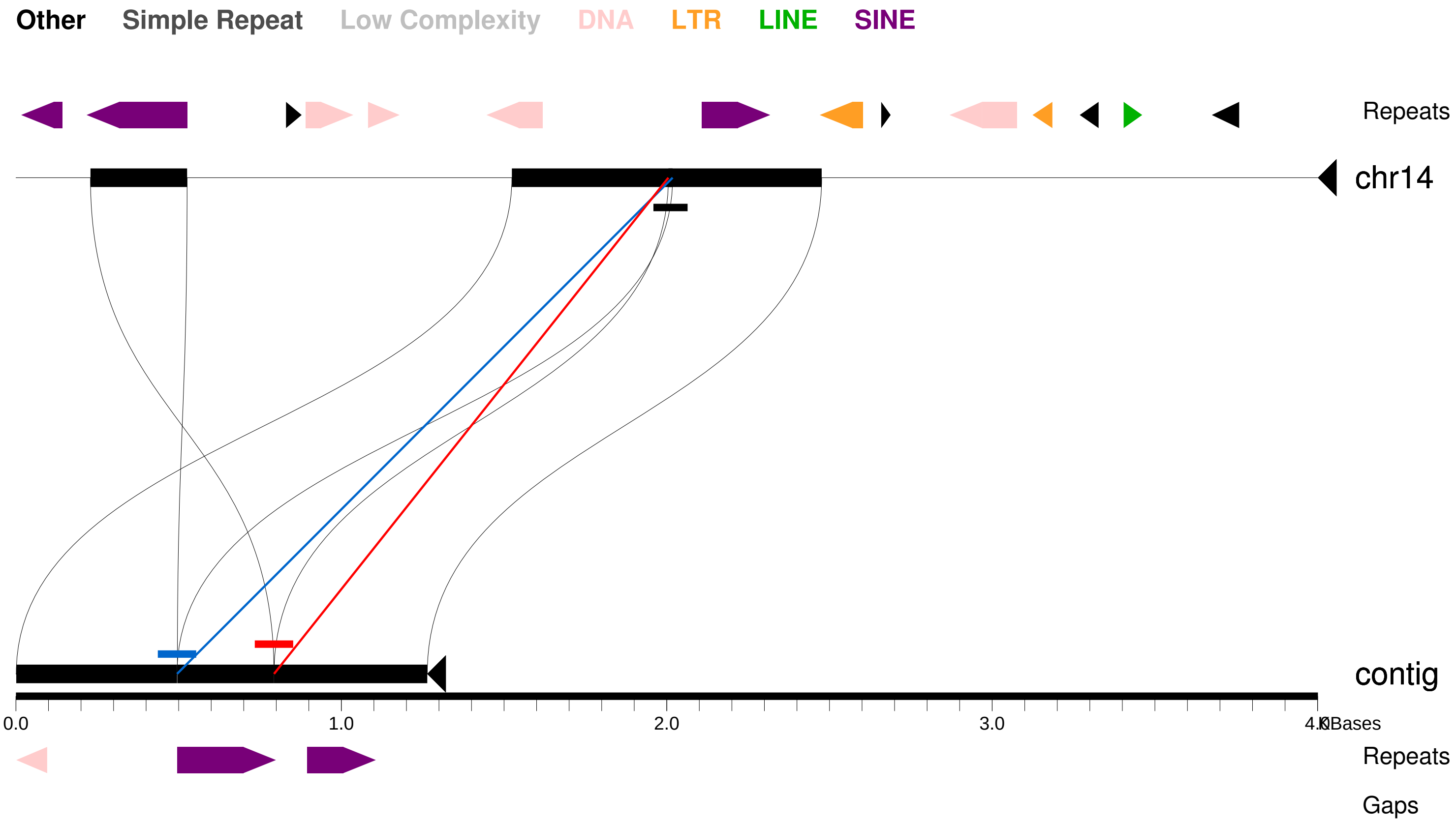

siteID: chr14_52561815 Scafftig1 - Breakpoint: chr14:52561822
chr14:52561777-52561881
Overlap Length: 15 INS size: 454 Genotyped: No

```
left     TGGCAGTCCTCCATGGGTTGTGCTTCTGTG--------------TCTGCACAGGTCATTAAGAAGTCTAA
chrom    TGGCAGTCCTCCATGGGTTGTGCTTCTGTG--------------TCTGCACAGGTCATTAAGAAGTCTAA
right    NNNNNNNNNNNNNNNNNNNNNNNNNNNNNNNNNNNNNNNNNNNNNNNNNNNNNNNNAAAAAGAAGTCTAA
         11111111111111111111111111111111111111111111111111111111*11***********

left     AGCCGGCCGGGCGCGGTGGCTCACGCCTGTAATCCCAGCACTTTGGGAGGCCGAGGCGGGCGG
chrom    AGCCAA------------ATTTACACCA-TAACTCCAGTAACATGC-ATATAGAATCTACAGT
right    AGCCAA------------ATTTACACCA-TAACTCCAGTAACATGC-ATATAGAATCTACAGT
         ****2222222222222222*2**2**22***22****2*222**22*2222**22*2222*2
```

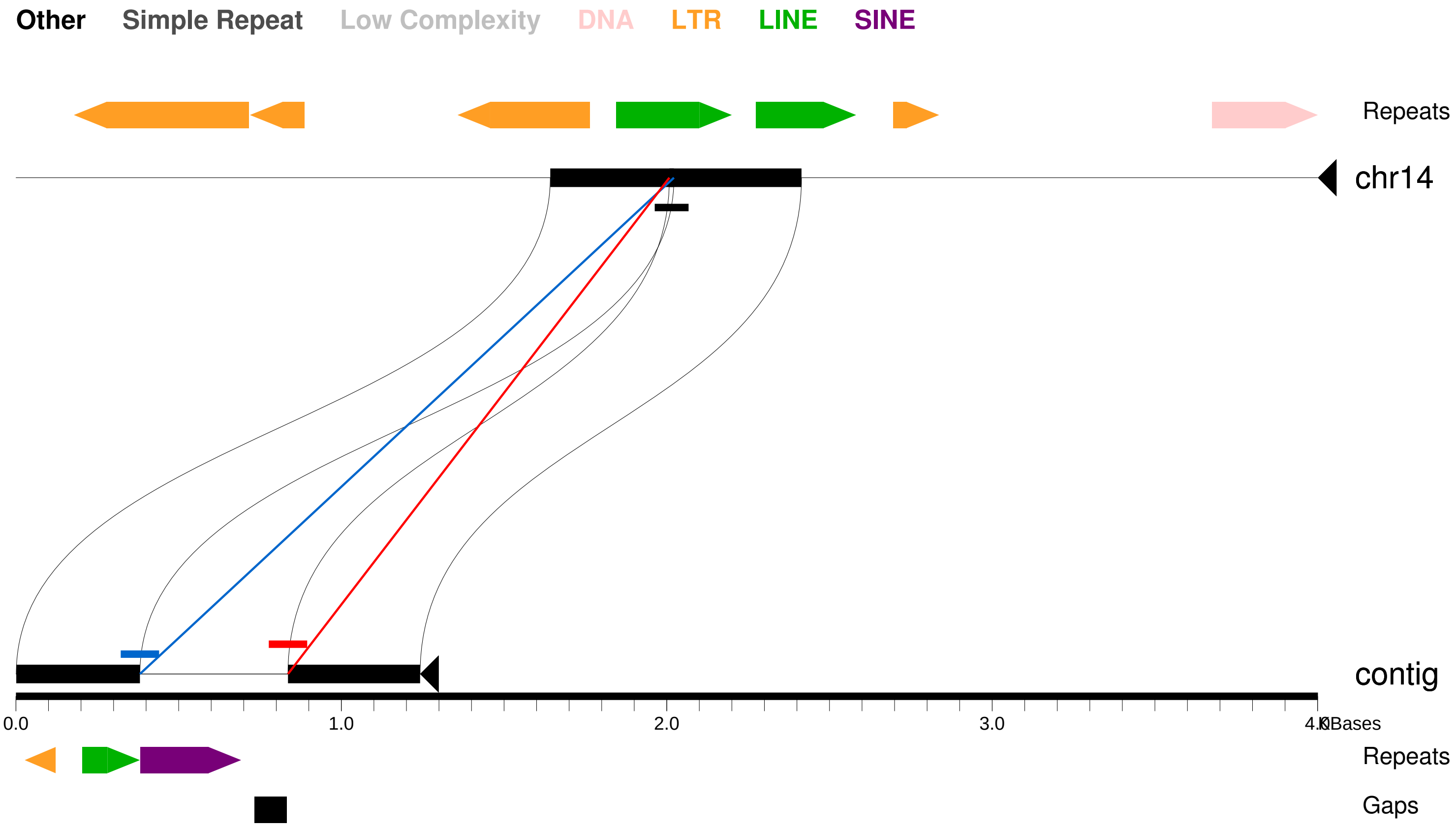

siteID: chr14_52643753 Scafftig1 + Breakpoint: chr14:52643754
chr14:52643708-52643813
Overlap Length: 14 INS size: 314 Genotyped: Yes

```
left   GG----CATAGTTCTTGGTGTACAGTCTCCATCACTGTT----TCTCTGGCT-----CTCCTGGAGTTTC
chrom  GG----CATAGTTCTTGGTGTACAGTCTCCATCACTGTT----TCTCTGGCT-----CTCCTGGAGTTTC
right  TGACCTCATGATCCACCCGCCTCGGCCTCCCAAAGTGCTGGGATTACAGGCGTGAGCCACCTGGAGTTTC
       1*1111***11*1*11111111*1*1****111*1**1*1111*11*1***111111*1***********

left   TTTTTTTTTTTTTTTTTTTTTTTTTTTTTTTTTTTTTTTTTGAGACGGAGTCTCGCTCTGTC
chrom  TTTAAGATACCAAATATCCTTTAGTAAACATTCTTTCTGCTCTAA-------------TTAG
right  TTTAAGATGCCAAATATCCTTTAGTAAACATTCTTTCTGCTCTAA-------------TTAG
       ***2222*N22222*2*22***22*22222**2***2*22*222*2222222222222*222
```

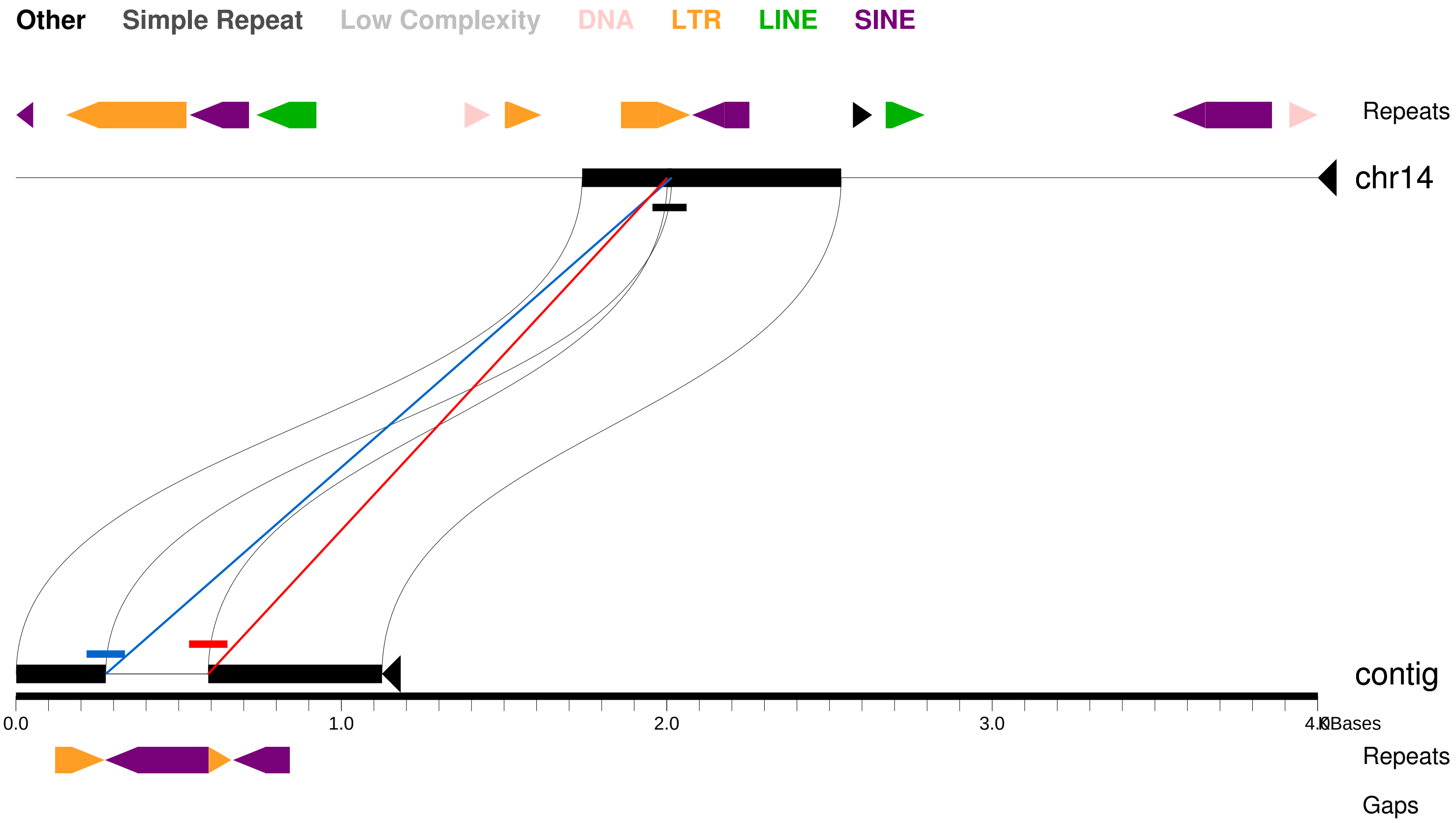

siteID: chr14_53778098 Scafftig16 - Breakpoint: chr14:53778106
chr14:53777454-53778266
Overlap Length: 22 INS size: 298 Genotyped: Yes

```
left   ----------------------------------------------------------------------
chrom  GAATCAAATCAAGAACTCAACCCCTTTTACAACAGCTGTGAAGAAAAATAAAGTACTTAGGAATATACCT
right  T---------------------------------------------------------------------
       N

left   ----------------------------------------------------------------------
chrom  AACCAAAGAGGCAAAAGATGTCTACAAGGAAACCTACAAAACACTGCTGAAAGAAATCATAGATGACACA
right  -------GAGGCAGGAGA----------------------------------------------------
              222222NN222

left   ----------------------------------------------------------------------
chrom  AACAAATGGAAACACATTCTCTCATGGATGGGTAGAATCAATATTGTGAAAATGGCCATACTGCCAAAAG
right  -----ATGG-----------------------------------TGTGAA-----CC-------------
            2222                                   222222     22

left   ----------------------------------------------------------------------
chrom  CAATCTACAGATTCAATGCAATTTACATCAAAATGCCATCATCATTCTTCACAGAACTAGAAAAAACAAT
right  ----------------------------------------------------------------------

left   ----------------------------------------------------------------------
chrom  CCTAAAATTCATATGGAACCAAAAAAAAAAAAAAAAAAAAAGACTGCACATGGCCAAAGCAAGACTAAGCA
right  ----------------------------------------------------------------------

left   ----------------------------------------------------------------------
chrom  AAAAGAACAAATCTGGAGGCATCACATTAGCCAAATTCAAACTATACTACAAGGATATAGTTACCAAAAC
right  ------------CGGGAGGC--------------------------------GGA---------------
                   2N222222                                222

left   ----------------------------------------------------------------------
chrom  AGCATGGTACTGGTATAAAAGTAGGCATGTAGACCAATGGAACAGAATAGAAAACCCAGAAATAAAGCCA
right  ------------------------GCTTGCAGT-----------GAGCCGAGATCCCA------------
                               22N22N22N           22NNN22N2N2222

left   -----------------------CAACAAAGCAAACAAAAACAAAGTGGGGAAAGTACACTGTTTTCAAC
chrom  AATATTTATAGCCAACTGATCTTCAACAAAGCAAACAAAAACAAAGTGGGGAAAGTACACTGTATTCAAC
right  ------------CCACTGCACTCCAGC-------------------------------------------
                   2N2222NN22N**1*1111111111111111111111111111111111111N111111

left   AAATGATGCTGGAATCATTGGCAAGCCACGTGTAGAAGAATGAAACTAGATCTTCACCTCTCACCTTATA
chrom  AAATGATGCTGGAATCATTGGCAAGCCACGTGTAGAAGAATGAAACTAGATCTTCACCTCTCACCTTATA
right  --------CTGG------------GCGAC-------AGAGCGAGACTC--------CGTCTCA----AAA
       11111111****111111111111**1**1111111***11**1***111111111*1*****1111*1*

left   GAAAAATCAAGTCAAGATGATCAAAGAGTTAAATCTAAGACCTGG-GCGGGCGCGGTG-----GCTCAC
chrom  CAAAAATCAACTCAAGATGATCAAAGAGTTAAATCTAAGACCTGATACCATAAAAATTCTACAAGATAAC
right  GAAAAAAAAA---AAAA-----AAAGAGTTAAATCTAAGACCTGATACCATAAAAATTCTACAAGATAAC
       1*****11**111**1*11111********************222**22222222*222222*2*2**

left   [illegible]
chrom  ATCAGAAAAACTCTTCTAG-ACATTGGCTTAGGCAAAGAGTTCATGACCAAGAACCCAAAAGCAAACAAA
right  ATCAGAAAAACTCTTCTAG-ACATTGGCTTAGGCAAAGAGTTCATGACCAAGAACCCAAAAGCAAACAAA
       22*2*2**22222*2*2**2**2**[illegible]222*[illegible]22*2222222**2**2**222**22**22*2*2*2

left   ACCATCCCG--GCTAAAACGGTGAAACCCCGTCTCTACTAAA-----
chrom  ACAAAAACAAAGATAAATAGACAGGACCTAAT-TAAACTAAAAGT--
right  ACAAAAACAAAGATAAATAGACAGGACCTAAT-TAAACTAAAAGTCT
       **2*222*222*2****22*22222***222*2*22*****22211
```

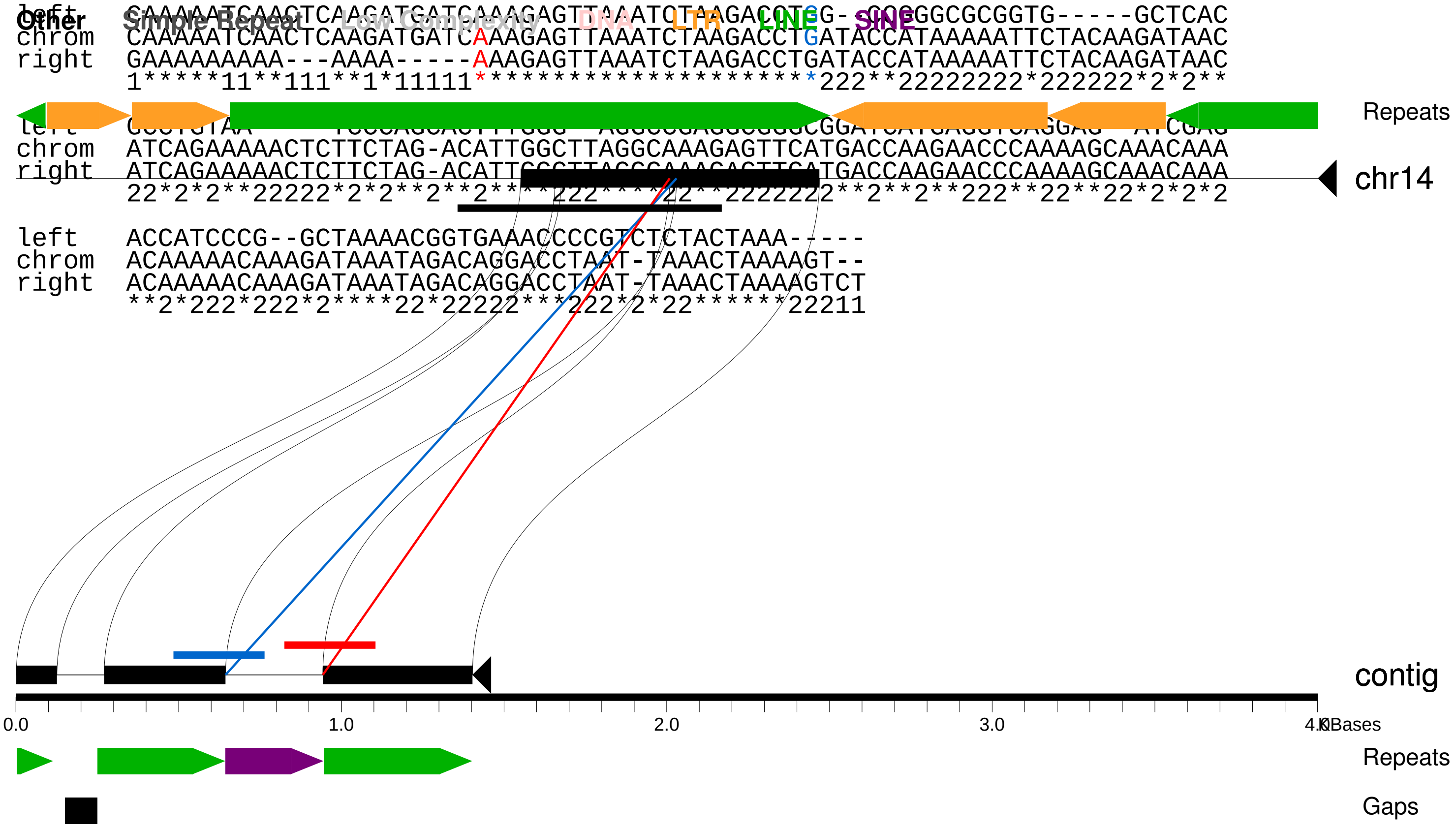

siteID: chr14_53800200 Scafftig5 + Breakpoint: chr14:53800171
chr14:53800156-53800230
Overlap Length: 42 INS size: 427 Genotyped: No

```
left    AT-----------------AAA----TGG--TTTC---CTT------------------TCTACCTCATT
chrom   AT-----------------AAA----TGG--TTTC---CTT------------------TCTACCTCATT
right   CCGCCCGTCTCGGCCTCCCAAAGTGCTGGGATTACAGGCGTGAGCCACCGCGCCCGGCCTCTACCTCATT
        1111111111111111111***1111***11**1*111*1*1111111111111111111***********

left    TCTTTTTTTTTTTTTTTATTTCTATCAGTGCCATNNNNNNNNNNNNNNNNNNNNNNNNNNNNNNNNNNNN
chrom   TCTTTATTTACTCATTTATTTCTATCAGTGCAATGTTATGGTTTTCTAT---------------------
right   TCTTTATTTACTCATTTATTTCTATCAGTGCAATGTTATGGTTTTCTAT---------------------
        *****2***22*22*****************2**222222222222222222222222222222222222

left    NNNNNNNNNNNNNNNNNNNNNNN
chrom   -----------------------
right   -----------------------
        22222222222222222222222
```

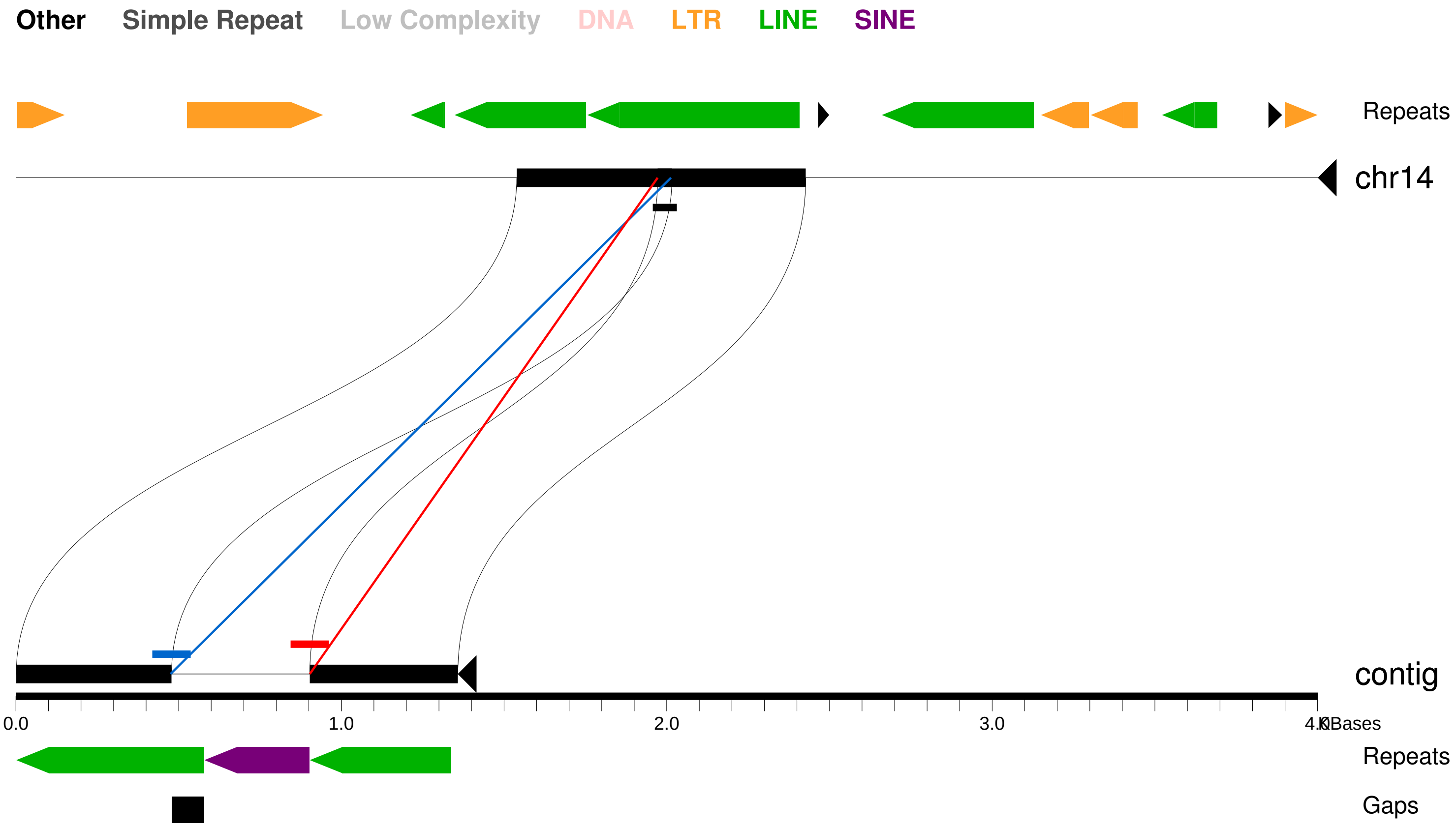

siteID: chr14_54815727 Scafftig3 + Breakpoint: chr14:54815728
chr14:54815568-54815903
Overlap Length: 16 INS size: 304 Genotyped: Yes

```
left   -----------CAGAGAGAGGCGCCTGTGAGCAGCTGGAGAGGACAGGGAGGACTATTTGTCTAGGAAAT
chrom  AAAAGAATACCCAGAGAGAGGCGCCTGTGAGCAGCTGGAGAGGACAGGGAGGACTATTTGTCTAGGAAAT
right  TTTAGTA--------GAGACGGGGTTT----CACCTTGTTAGC-CAGGATGGTCTCGATCTCCTGACCTC
       NNN22N2    1111****1*1*11*11111**1**1*11**11****11**1**111*1**11*11111

left   TAGAGCTGTTAGGCAGAGCTGAACTGCCTAGAGAATGGGAAATCATAATTTGTGAAACGCTTTTATTGCA
chrom  TAGAGCTGTTAGGCAGAGCTGAACTGCCTAGAGAATGGGAAATCATAATTTGTGAAACGCTTTTATTGCA
right  ATGATCCACCCGCCTCGGC----CTCCCAAAGTGCTGGGATTACAGGC---GTGA---GC--------CA
       11**1*11111*1*111**1111**1**1*11111*****111**111111****111**11111111**

left   CCCCGCCATGGTGGATTCCTTGTCCCAAATATTCTTTTTTTTTTTTTTTTTGAGACGGAG----------
chrom  CCCCGCCATGGTGGATTCCTTGTCCCAAATATTCTTACGCAAAATTTATTCGGGAAGGAGAAAGAAAGGG
right  CCGCGCCCGG--------CCTGTCCCAAATATTCTTACGCAAAATTTATTCGGGAAGGAGAAAGAAAGGG
       **1****11*11111111*1***************22222222***2**2*2**2****2222222222

left   -------------------TCTCGC---TCTGTTGC--CCAGGCCGGACTGCGGACT--GCAGTGGCGCA
chrom  CATGCGAGTTCAGAACCTGTCTCACGCCTCTAAAACTTCCAGGCCACCTTCAGTACAAAGAATGGGGGGA
right  CATGCAAGTTCAGAACCTGTCTCACGCCTCTAAAACTTCCAGGCCACCTTCAGTACAAAGAATGGGGGGA
       22222N2222222222222****2*222***2222*22*******2222*22*2**222*2*22**2*2*

left   ATCTC--GGCT---CACTGCAAGCTCCGCTTCCCGGGTTCACGCCATTC--TCCT-
chrom  ATGTCCGGGCGAGCCACGGGAGGCGTTGGGGAGAGGGGAAAACGGATTCCTTCCTG
right  ATGTCCGGGCGAGCCACGGGAGGCGTTGGGGAGAGGGGAA----------------
       **2**22***2222***2*2*2**222*222222***2221NNNN1111  1111
```

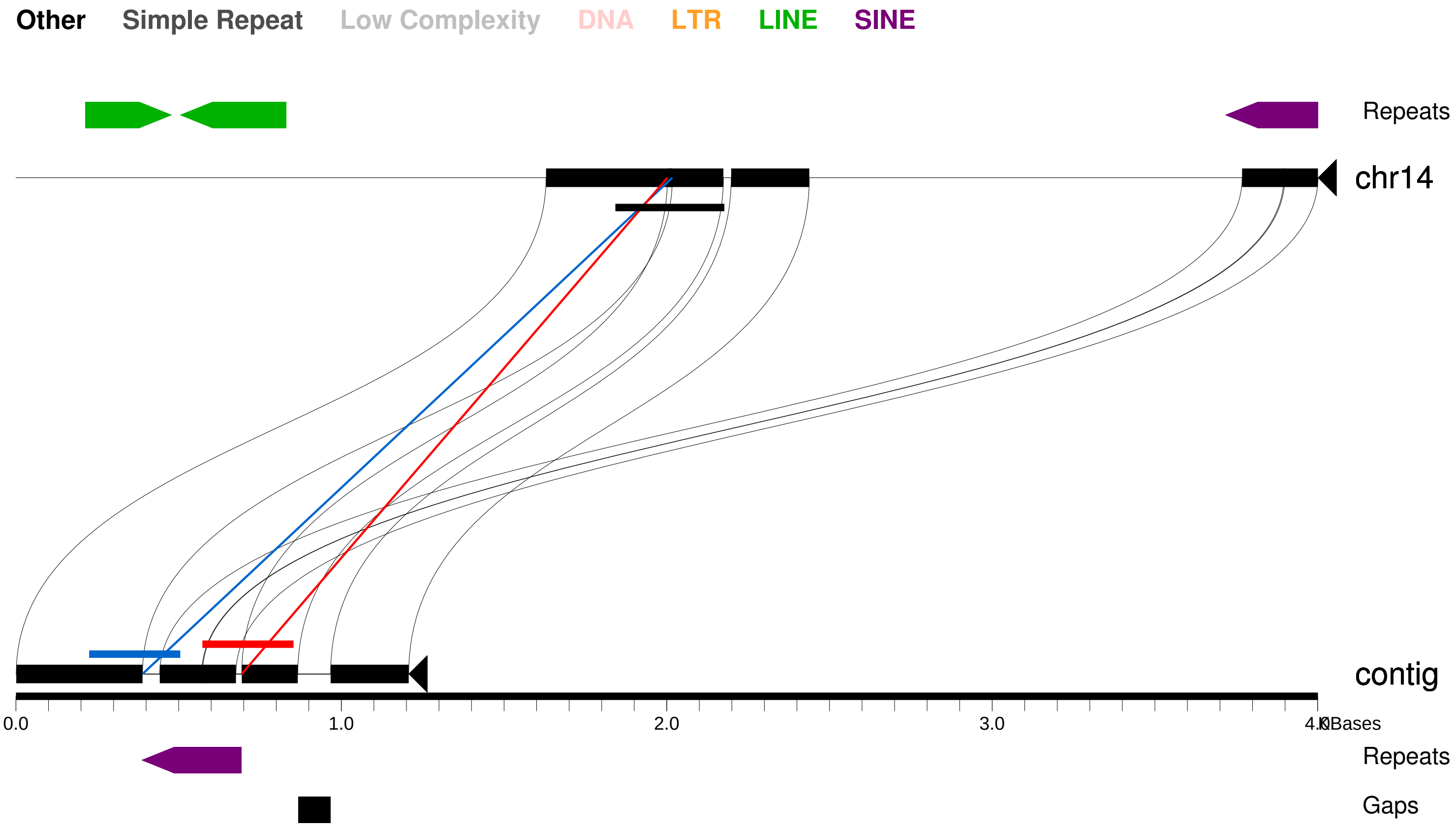

siteID: chr14_55795850 Scafftig5 + Breakpoint: chr14:55795853
chr14:55795812-55795912
Overlap Length: 19 INS size: 291 Genotyped: Yes

```
left   -----------GGCCCAGGAAAGGGCTGAGGG-AGATTTGTGAAAAATGTCTT------TAAAACGTGGT
chrom  -----------GGCCCAGGAAAGGGCTGAGGG-AGATTTGTGAAAAATGTCTT------TAAAACGTGGT
right  CCGCCCGCCTCGGCCTCCCAAAGTGCTGGGATTACAGGCGTGAGCCACCGCGCCCGGCCTAAAACGTGGT
       11111111111****1111****1****1*111*1*111****111*111*11111111***********

left   TTATTTCTTTTTTTTTTTGAGACGGAGTCTCGCTCTGTCGCCCAGGCTGGAGTGCAGTGGCGCGATC
chrom  TTATTTCTAAAATTCATGCAG-CTGAGT-----TCT-TTTCTCA--------TATATTGTCCCA---
right  TTATTTCTAAAATTCATGCAG-CTGAGT-----TCT-TTTCTCA--------TATATTGTCCCA---
       ********2222**22*22**2*2****22222***2*22*2**22222222*22*2**2*2*2222
```

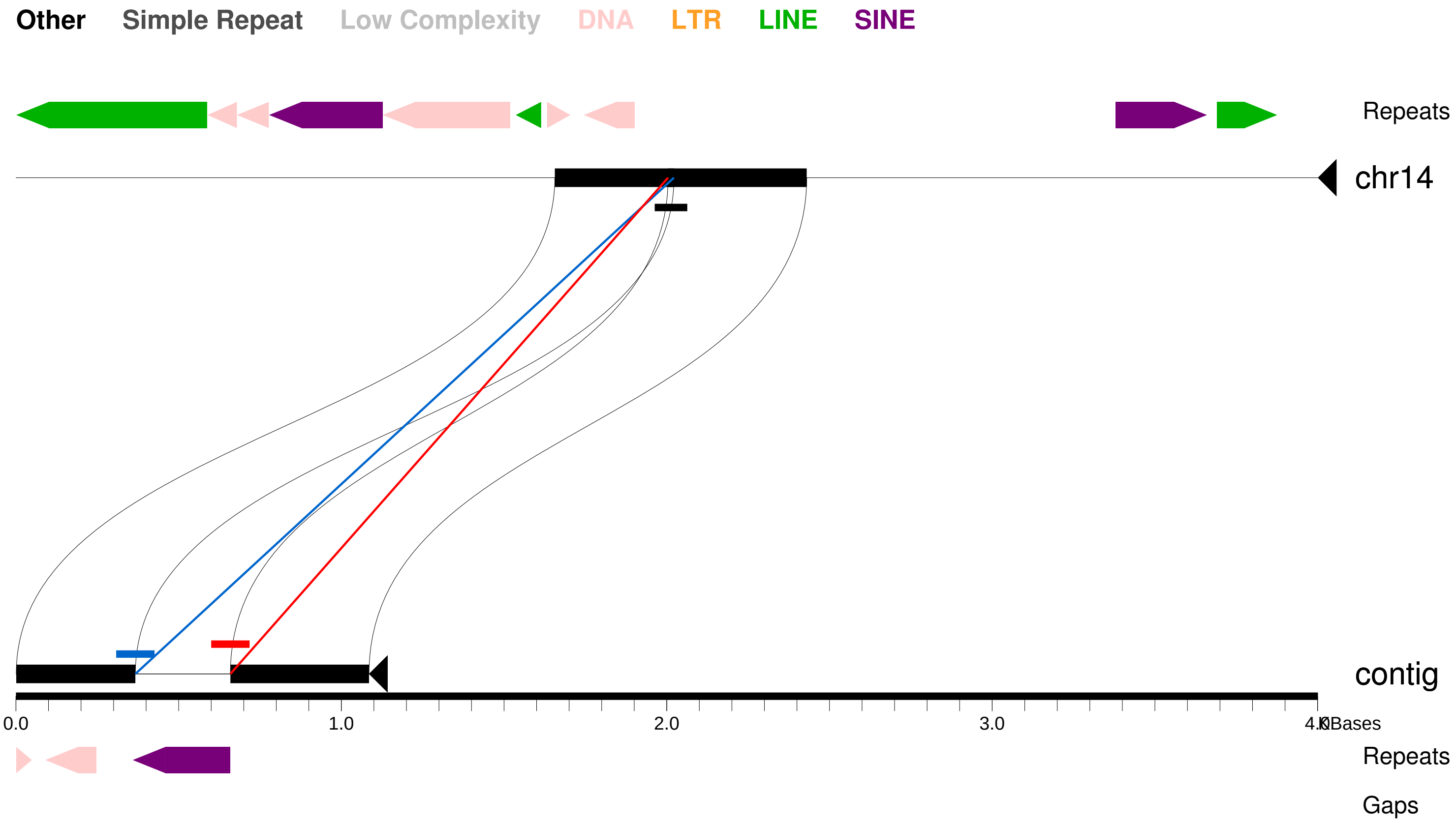

siteID: chr14_56430912 Scafftig4 + Breakpoint: chr14:56430899
chr14:56430853-56430958
Overlap Length: 14 INS size: 308 Genotyped: Yes

```
left    CTTTTTCATGCTTGATAT-GC---AC-CTGGCCCAGGAGTCAGAGAAGCTG----------AAAAACGAT
chrom   CTTTTTCATGCTTGATAT-GC---AC-CTGGCCCAGGAGTCAGAGAAGCTG----------AAAAACGAT
right   CCAG--CCTGGGCGACAGAGCGAGACTCCGTCTCAAAAAAAAAAAAAAAAAAAAAAAAAAAAAAAAAAACGAT
        *11111*1**111**1*11**111**1*1*1*1**11*111*1*1**11111111111111*********

left    CCAGCGGCCGGGCGCGGTGGCTCACGCCT-GTAATCCCAGCACTTTGGGAGGCCGAGGCGGGCGG
chrom   CCAGCAGGAGCTGGAGTCTGTGTGGGCCTTGTTGTTGCACCACC------GTCCGCC--------
right   CCAGCAGGAACTGGAGTCTGTGTGGGCCTTGTTGTGGCACCACC------GTCCGCC--------
        *****2*221222*2*222*22222****2**22*N2**2***2222222*2***2222222222
```

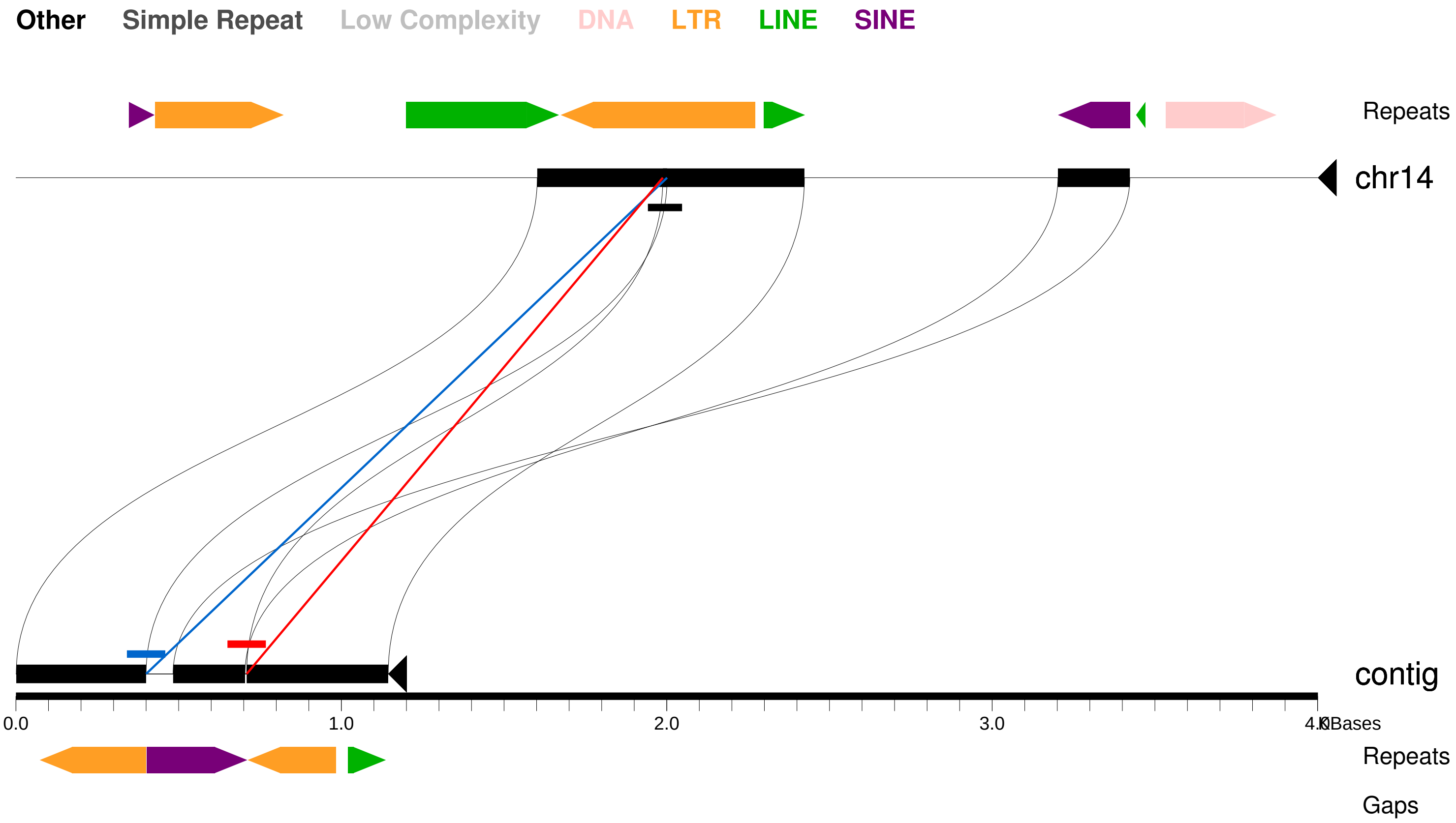

siteID: chr14_56437930 Scafftig3 + Breakpoint: chr14:56437912
chr14:56437866-56437971
Overlap Length: 14 INS size: 403 Genotyped: No

```
left   -----TCTTCT-GATTA--AGACCCTGTGATTGCTCTTCATTTCCCTTCAAA-----TAAAGTCCAAATT
chrom  -----TCTTCT-GATTA--AGACCCTGTGATTGCTCTTCATTTCCCTTCAAA-----TAAAGTCCAAATT
right  CGGGATGGTCTCGATCTCCTGACCTCGTGATCCGCCCGCCTCGGCCTCCCAAAGTGCTGAAGTCCAAATT
       11111*11***1***11111****11*****1111*11*1*111***1*1**11111*1***********

left   CTTTTNNNNNNNNNNNNNNNNNNNNNNNNNNNNNNNNNNNNNNNNNNNNNNNNNNNNNNNNN
chrom  CTT-------------GAACAAAGCTTAAAAAGCCTTCATAATCCAACCTCTGCTTCCTTCT
right  CTT-------------GAACAAAGCTTAAAAAGCCTTCATAATCCAACCTCTGCTTCCTTCT
       ***22222222222222222222222222222222222222222222222222222222222
```

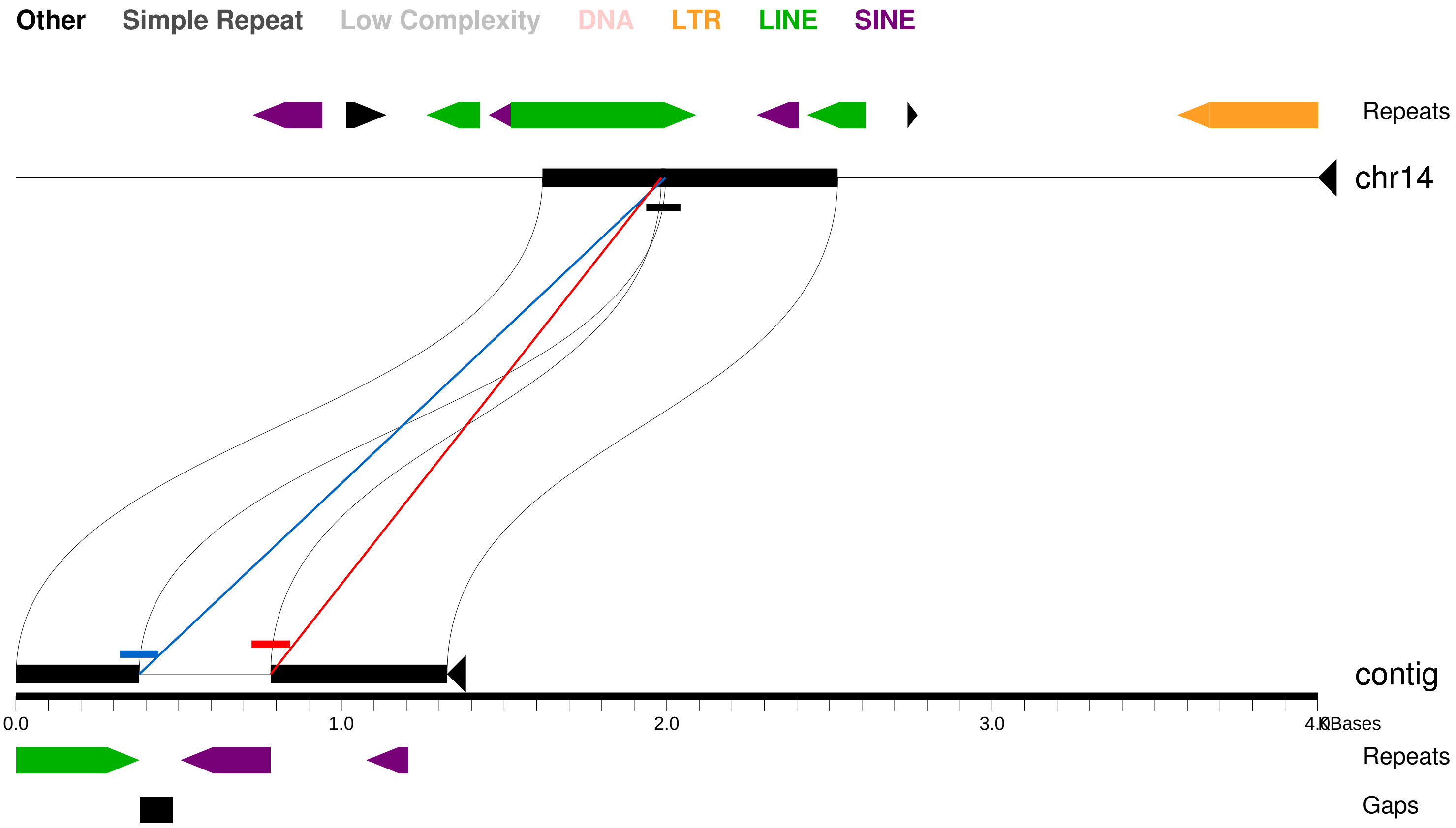

siteID: chr14_56606687 Scafftig9 - Breakpoint: chr14:56606669
chr14:56606625-56606728
Overlap Length: 16 INS size: 312 Genotyped: Yes

```
left   ---TTAGTATTTGA---AGTTCGTC---------ATTTGTAGGGCAGCTCATTGCTGTTAAAATTTCTGG
chrom  ---TTAGTATTTGA---AGTTCGTC---------ATTTGTAGGGCAGCTCATTGCTGTTAAAATTTCTGG
right  GCCTGGGCGACAGCGAGACTCCGTCTCAAAAAAAAAAAAAAAAAAAAAAAAAAAAAAAAAAATTTTCTGG
       111*11*11111*1111*1*1****111111111*11111*1111*1111*11111111***1*******

left   CTTTTGGCCGGGCGCGGTGGCTCACGCCTGTAATCCCAGCACTTTGGGAGGCCGAGGCGGGCGG
chrom  CTTTT---CTGGCT---TTCATCAGAAATG-AATG--ATTAATTT---ATTCTGAAAATGG---
right  TTTTT---CTGGCT---TTCATCAGAAATG-AATG--ATTAATTT---ATTCTGAAAATGG---
       1****222*2***2222*222***2222**2***222*22*2***222*22*2**2222**222
```

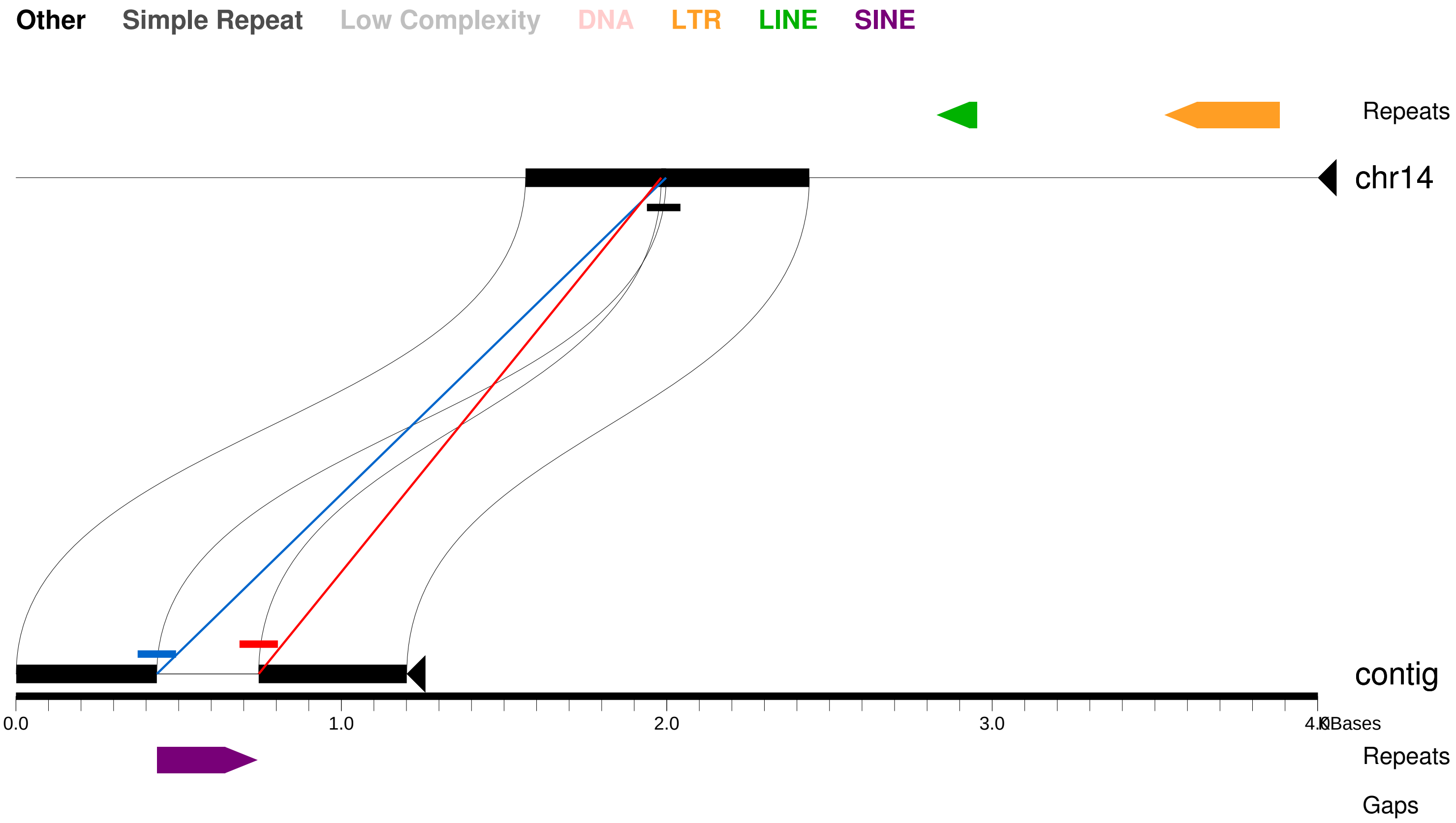

siteID: chr14_57744449 Scafftig6 - Breakpoint: chr14:57744434
chr14:57744391-57744485
Overlap Length: 17 INS size: 257 Genotyped: Yes

```
left   ACAT---AATGAATTCC-----------CACA---------CATGTCAAATAGTAGGTAAAACATTAAAA
chrom  ACAT---AATGAATTCC-----------CACA---------CATGTCAAATAGTAGGTAAAACATTAAAA
right  TCCCGCCACTGCACTCCAGCCTGGGCGACAGAGCGAGACTCCGTCTCAAAAAAAAAAAAAAAAAAAAAAA
       1*11111*1**1*1***11111111111**1*111111111*1*1*****1*11*111****1*11****

left   -GTGAAACATTTCAGGCCGAGGCGGGCGGATCACGAGGTCAAGAGATCGAGACCATCCCGGCTAAAACGG
chrom  -GTGAAACATTTCA-----------------CATTAGAAATTGAAAACAAAAGTA---GGTTTATAATT-
right  AGTGAAACATTTCA-----------------CATTAGAAATTGAAAACAAAAGTA---GGTTTATAATT-
       1************22222222222222222**22**22222**2*2*2*2*22*2222*22**2**222

left   TGA
chrom  ---
right  ---
       222
```

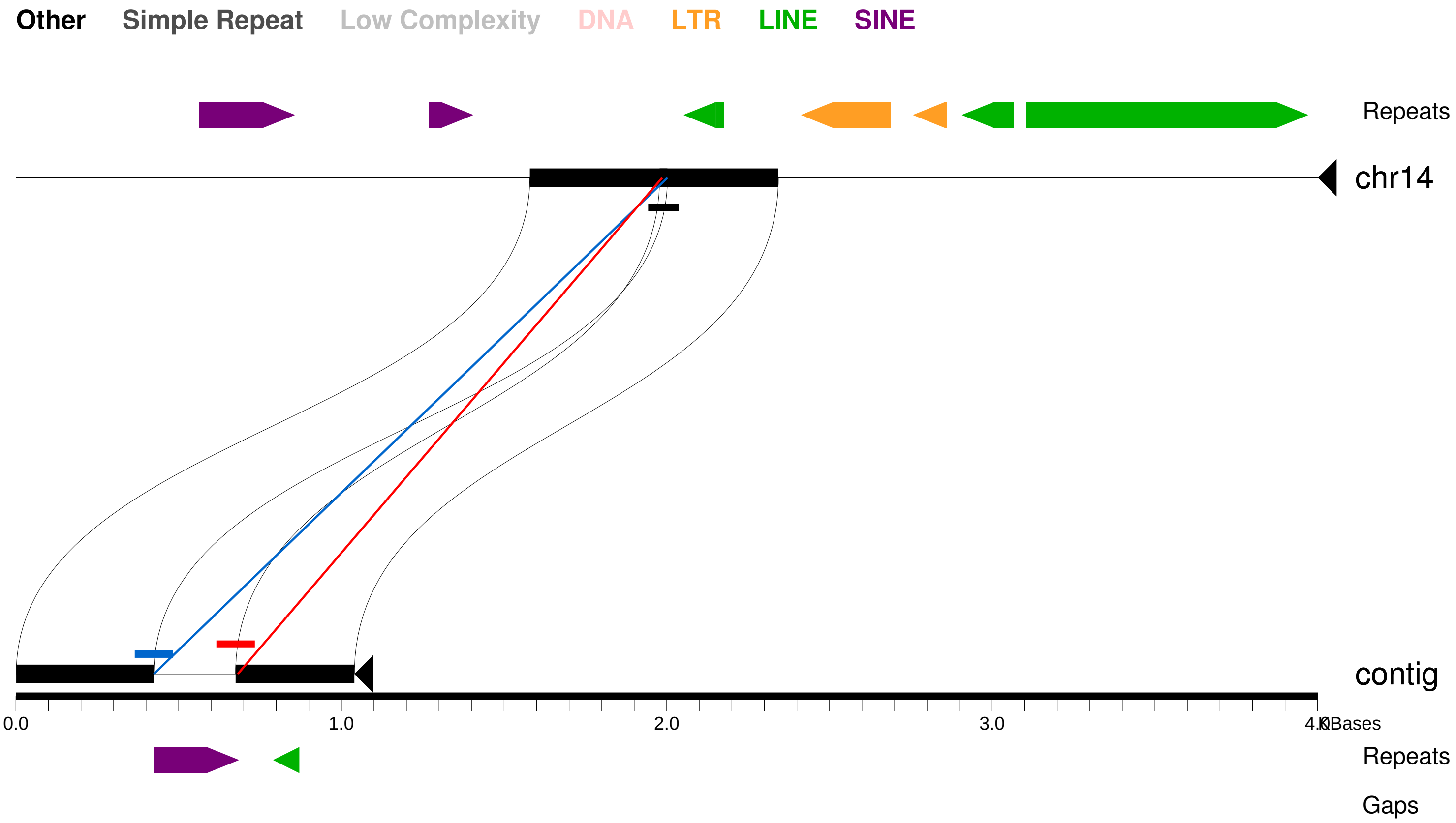

siteID: chr14_60454173 Scafftig7 + Breakpoint: chr14:60454156
chr14:60453996-60454331
Overlap Length: 16 INS size: 312 Genotyped: Yes

```
left    -------------AAAAATGTAACTGCGTTTCTT-TTGTTGCTTTTGGCAACCAGTTACATTATTTGTAA
chrom   TTTGCCATTTTTAAAAAATGTAACTGCGTTTCTT-TTGTTGCTTTTGGCAACCAGTTACATTATTTGTAA
right   TTTAGTA------------GAGACGGGGTTTCACCTTGTT---------AGCCAGG---ATGGTCTCGAT
        222NNN2      111111*11**1*1*****111*****111111111*1****1111**11*1*11*1

left    CAACTTGTTATCATTACAGAGGAAGTTTATATGCATTGTATAAAAACAGAAAATGTAGGCCAACAACAAA
chrom   CAACTTGTTGTCATTACAGAGGAAGTTTATATGCATTGTATAAAAACAGAAAATGTAGGCCAACAACAAA
right   CTCCT-GACCTCATGA---------TCCACCCGCCTCGGCCT----CCCAAAGTGCTGGG--ATTACAGG
        *11**1*11N****1*111111111*11*111**1*1*11111111*11***1**11**111*11***11

left    ACATTCTCACCACCCAGAGATCAACCTTAACTTCTTTTTTTTTTTTTTTTTTTTTTTTTTTTTGA--GACG
chrom   ACATTCTCACCACCCAGAGATCAACCTTAACTTCTTTGTACTCTGTCTTTTAAATCTCTTTTAATTGATA
right   CGTGAGCCACCGCGCCCGGC-CAACCTTAACTTCTTTGTACTCTGTCTTTTAAATCTCTTTTAATTGATA
        1111111****1*1*111*11***************2*22*2*2*2****222*2*2****2*22**22

left    GAGTCTCGCTCTGTCGCCCAG------------GCCGGACTGCGGAC-TGC---------AGTGGC--GC
chrom   GATTTCCCTTCCATTTCCCATAGTTTATCTCTAGAAATACTTGAGCCATTCGACCCAAAGAGTTTCCAGC
right   GATTTCCCTTCCATTTCCCATAGTTTATCTCTAGAAATACTTGAGCCATTCGACCCAAAGAGTTTCCAGC
        **2*22*22**22*22****2222222222222*2222***222*2*2*2*222222222***22*22**

left    AATCTCGG-----CTCACTGCAAGCTCC----------GCCTCCCGGGTTCACGCC--
chrom   AGCCTGGGATTTGCTTACTT-AAGATACATCGCTATATGCCTCCCATCTTCTATATTT
right   AGCCTGGGATTTGCTTACTT-AAGATACATCGCTATATGCCTC---------------
        *22**2**22222**2***22***2*2*2222222222*****11NNN111NNNNN
```

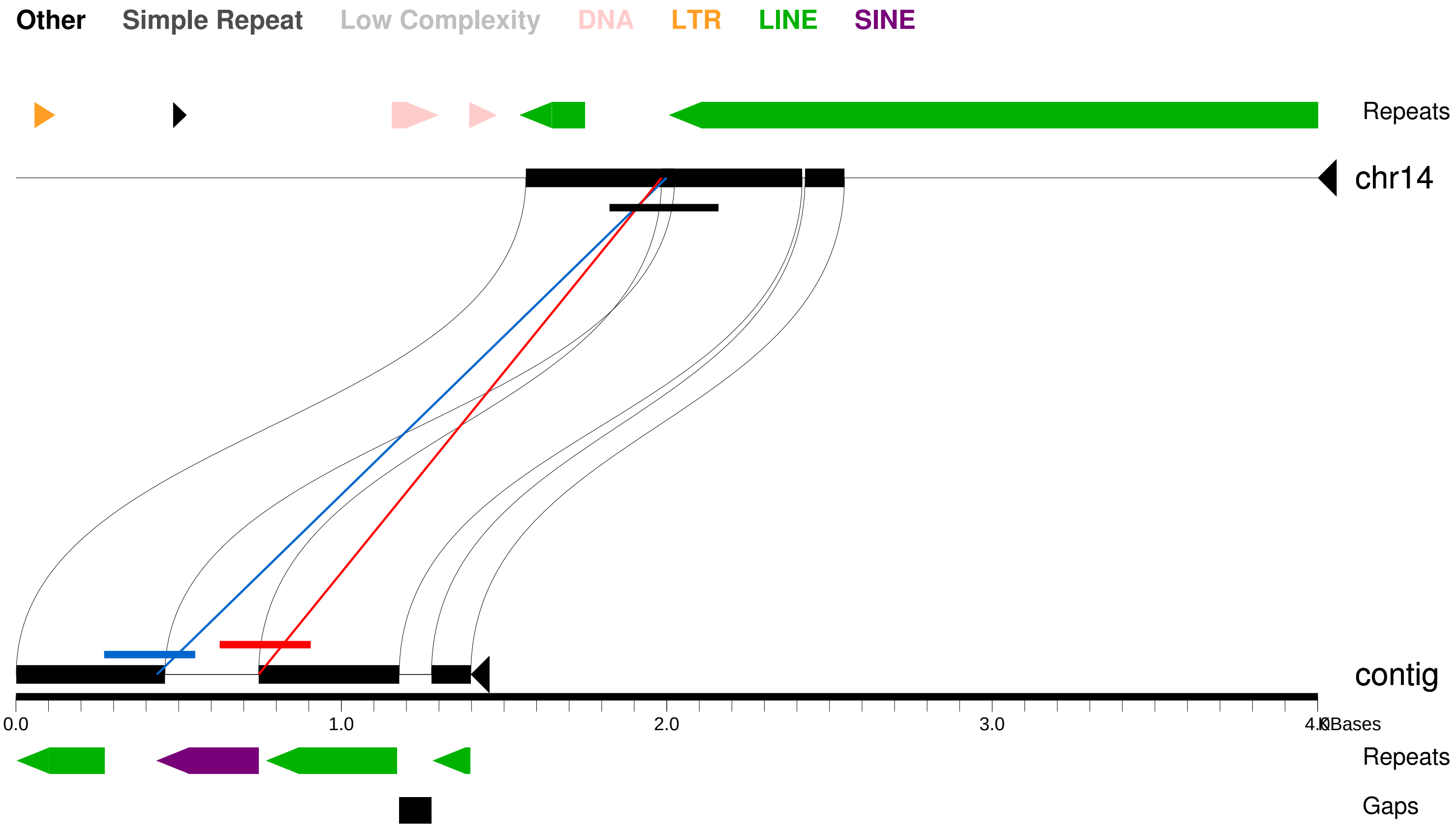

siteID: chr14_60575466 Scafftig4 + Breakpoint: chr14:60575487
chr14:60575440-60575546
Overlap Length: 13 INS size: 302 Genotyped: Yes

```
left    --------------ATACAAAATTTATTTCTAATTTTATGATAGCAACAATAGTAGAAGTAATATTGATT
chrom   --------------ATACAAAATTTATTTCTAATTTTATGATAGCAACAATAGTAGAAGTAATATTGATT
right   CCGCCCGCCTCGGCCTCCCAAAGTGCTGGG--ATTACAGGCGTGAGCCACCGCGCCCGGCCATATTGATT
        111111111111111*1*1***1*11*11111***11*1*111*111**111111111*11*********

left    TTTTTTTTTTTTTTTTTTTTTTTTTTGAGACGGAGTC--TCGCTCTGTCGCCCAGGCTGGAGTGCA
chrom   TTTTAAAAACCAACTTGTTACAGAAGAAAAGTAGAAAATAGTTTTTTTAAC--------------
right   TTTTAAAAACCAACTTGTTACAGAAGAAAAGTAGAAAATAGTTTTTTTAAC--------------
        ****2222222222**2**222222**2*2*2**2222*2*2*2*222*22222222222222
```

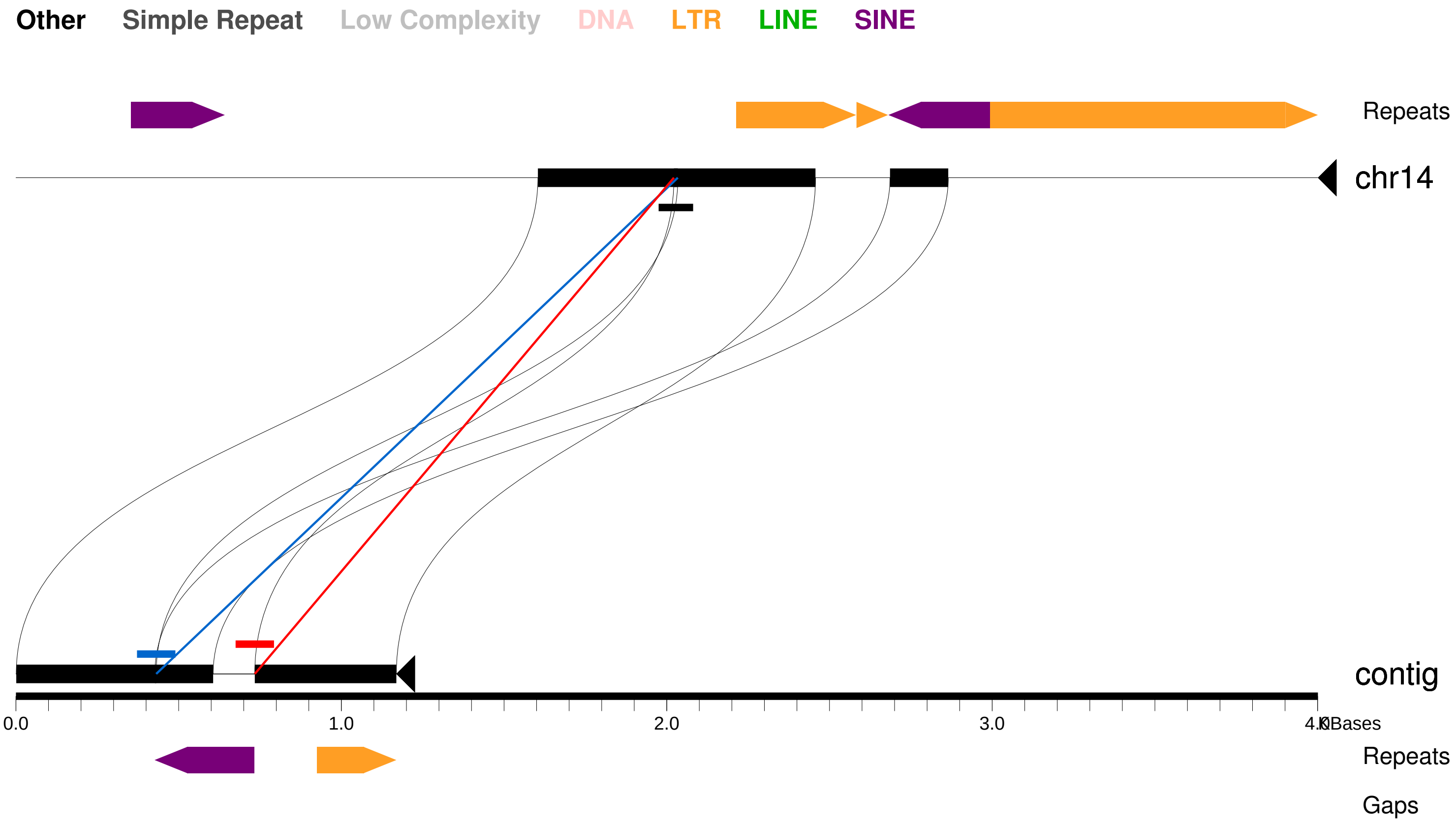

siteID: chr14_60741397 Scafftig12 - Breakpoint: chr14:60741385
chr14:60741341-60741444
Overlap Length: 16 INS size: 310 Genotyped: Yes

```
left     TTACAGCAGAAAAGTAGGAGGAGAGGGTT---------------ATGAAAGAATTCATTGAAATGGTTCA
chrom    TTACAGCAGAAAAGTAGGAGGGGAGGGTT---------------ATGAAAGAATTCATTGAAATGGTTCA
right    AGCCTGGGCCACAGAGCGAGACTCCGTCTCAAAAAAAAAAAAAAAAAAAAAAAAAAAAAGAAATGGTTCA
         111*1*1111*1**111***1N111*11*111111111111111*11***1**111*11***********

left     CTCATGGCCGGGCGCGGTGGCTCACGCTTGTAATCCCAGCACTTTGGGAGGCCGAGGCGGGCGG
chrom    CTCATTTATTGATCTTTTTTTTTC-----TGTAGTATC--CAATCTGCCATTCATTG--------
right    CTCATTTATTGATCTTTTTTTTTC-----TGTAGTATC--CAATCTGCCATTCATTG--------
         *****22222*222222*222**22222****2*22*22**2*2**22*22*222*22222222
```

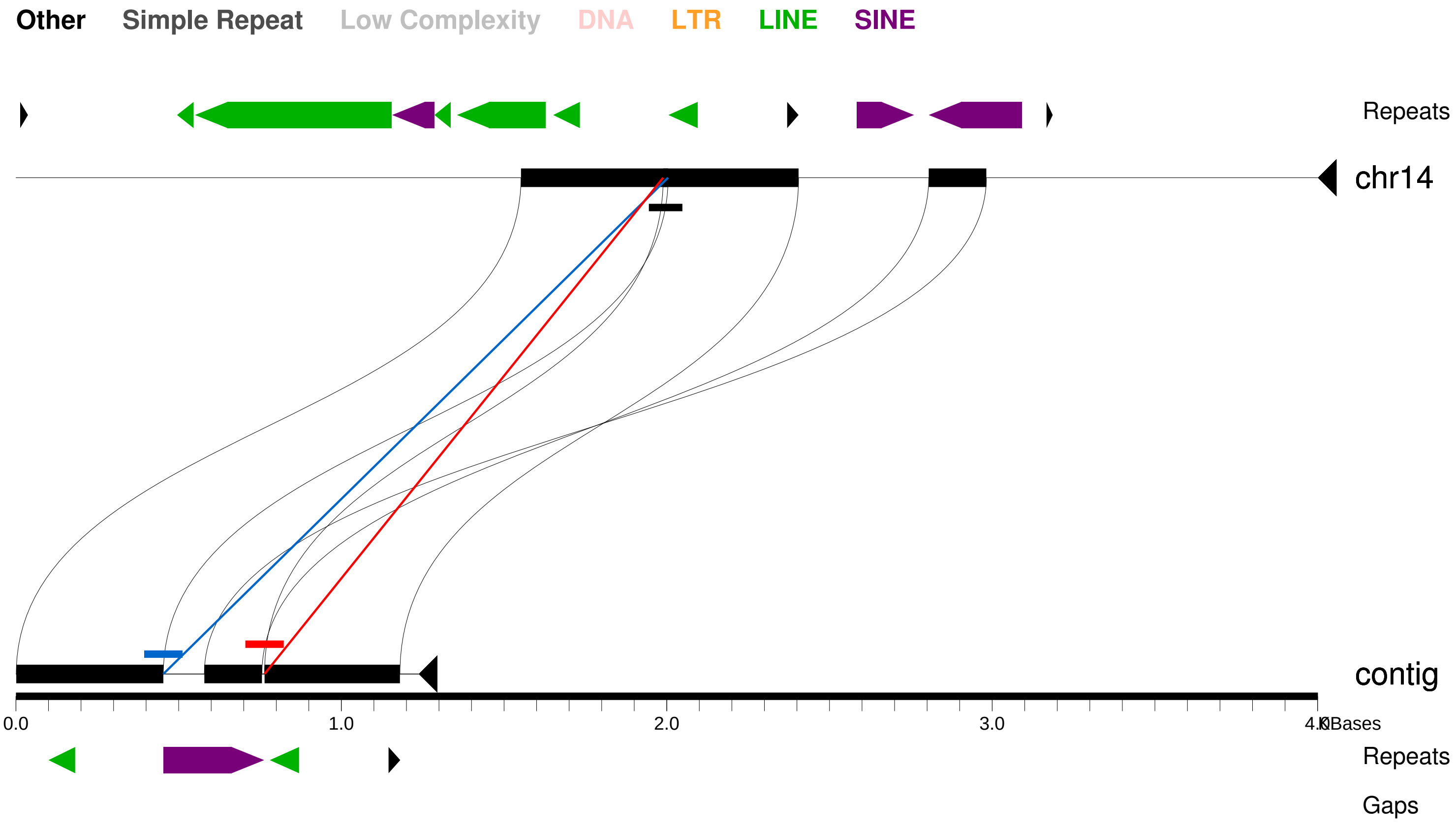

siteID: chr14_63226270 Scafftig3 + Breakpoint: chr14:63226284
chr14:63225919-63226444
Overlap Length: 9 INS size: 497 Genotyped: No

```
left   ----------------------------------------------------------------------
chrom  TAAATCTATCTCTGGTCTTCTTTCATCAAAATTAGTCAGCTGTTTTTGACAAGCTGAAGCACACCTGAGG
right  T------------------------------TTAGT-AGA-------GAC----------------GGGG
       2                              22222 22N       222                2N22

left   ----------------------------------------------------------------------
chrom  ATTGAATAGTCCCACTGTCTGAAATCCAGACTAAAGTAGCAGTTTTCTAAATTAGGCATATTTTTTCTGA
right  TTT---------CAC-----------------------------------------CATATT--------
       N22         222                                         222222

left   ----------------------------------------------------------------------
chrom  AGAGCCTTTAAGAAAATGAGAGAATGCTTAGATAATTTGAAATCCTGTGGCATCAATTATGAATTTATTT
right  --AGCC--------------AGGATGGTC-------TTGATCTCCTG----ACC--TTGTGA-------T
         2222              22N222N2N       2222NN22222    2N2  22N222       2

left   ----------------------------------------------------------------------
chrom  CTGGGGCATGGCTATTTGATTATGGAAAATACTGAGGTGCCTGTGCTTACAGAAGGATATTGGCACATTT
right  CCG---CCCGCCTC---------GGCC--TCCCAAAGTGC-TGGGATTACAGGCG---------------
       2N2   2NN2N22N         22NN  2N2NN2N2222 22N2N222222NN2

left   --------------------AAGCTGAAAATTCAAATCCTTTATATTCCATTTGATATCATAAAGCTGGT
chrom  GTTTTATAACATTTGCTCAAAAGCTGAAAATTCAAATCCTTTATATTCCATTTGATATCATAAAGCTGGT
right  ------------------------TGAG-------------------CCAC-------------------
                           1111***11111111111111111111***11111111111111111111

left   TTACCCTCCAGGCGTAGGGTATAT----------------------------------------------
chrom  TTACCCTCCAGGCGTAGGGTATATTTTTTAATAATTACATTTGTTTAAGTCTTTTCTGCTGACAAACTTG
right  ---CGCGCCCGGCC-AGGGTATATTTTTTAATAATTACATTTGTTTAAGTCTTTTCTGCTGACAAACTTG
       111*1*1**1***11*********2222222222222222222222222222222222222222222222

left   ----------------------------------------------------------------------
chrom  TCCTGATTGAATTTTTGAATTTTTACAGTCCTTTCTTTCTCTCTCTCTTTCAATATTATCTGATCATCAC
right  TCCTGATTGAATTTTTGAATTTTTACAGTCCTTTCTTTCTCTCTCTCTTTCAATATTATCTGATCATCAC
       2222222222222222222222222222222222222222222222222222222222222222222222

left   -----------------ATATATTTNNNNNNNNNNN
chrom  CACTGTTACCAACCTCCATAAAATTGCTGGTTCAAA
right  CACTGTTACCAACCTCCATAAAATTGCTGGTTCAA-
       22222222222222222***2*2**2222222222N
```

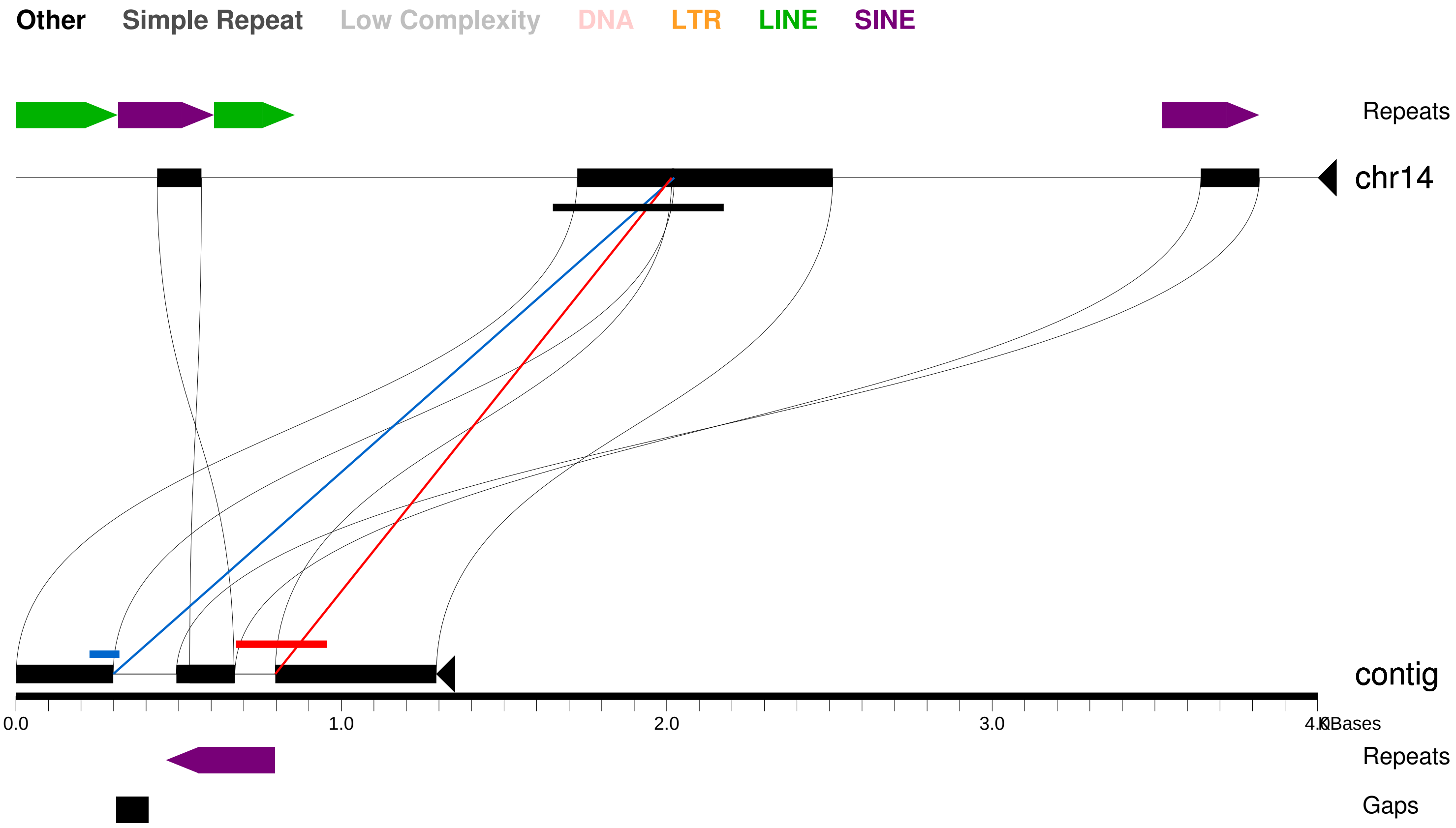

siteID: chr14_66934443 Scafftig6 - Breakpoint: chr14:66934447
chr14:66934402-66934506
Overlap Length: 15 INS size: 299 Genotyped: Yes

```
left    AA-----GCTGTTTAGC-----TGACA----ACTGCCTAGAGTCGTGAAACAGGTTATCAAGAATCTGAA
chrom   AA-----GCTGTTTAGC-----TGACA----ACTGCCTAGAGTCGTGAAACAGGTTATCAAGAATCTGAA
right   CGCCACTGCACTCCAGCCTGGGTGACAGAGCAAGACTCCGTCTCAAAAAAAAAAAAAAAAAGAATCTGAA
        1111111**11*11***11111*****1111*111*111*11**111***1*1111*11***********

left    AGTC-AGGCCGGGCGCGGTGGCTCACGCCTGTAATCCCAGCACTTTGGGAGGCCGAGGCGGGCG
chrom   AGTCTAAGATAAGAAAAAAAG---AGTTTTATAA----ATCTCTAAG--------ATGTATTTC
right   AGTCTAAGATAAGAAAAAAAG---AGTTTTATAA----ATCTCTAAG--------ATGTATTTC
        ****2*2*2222*2222222*222*2222*2***2222*2*2**22*22222222*2*222222
```

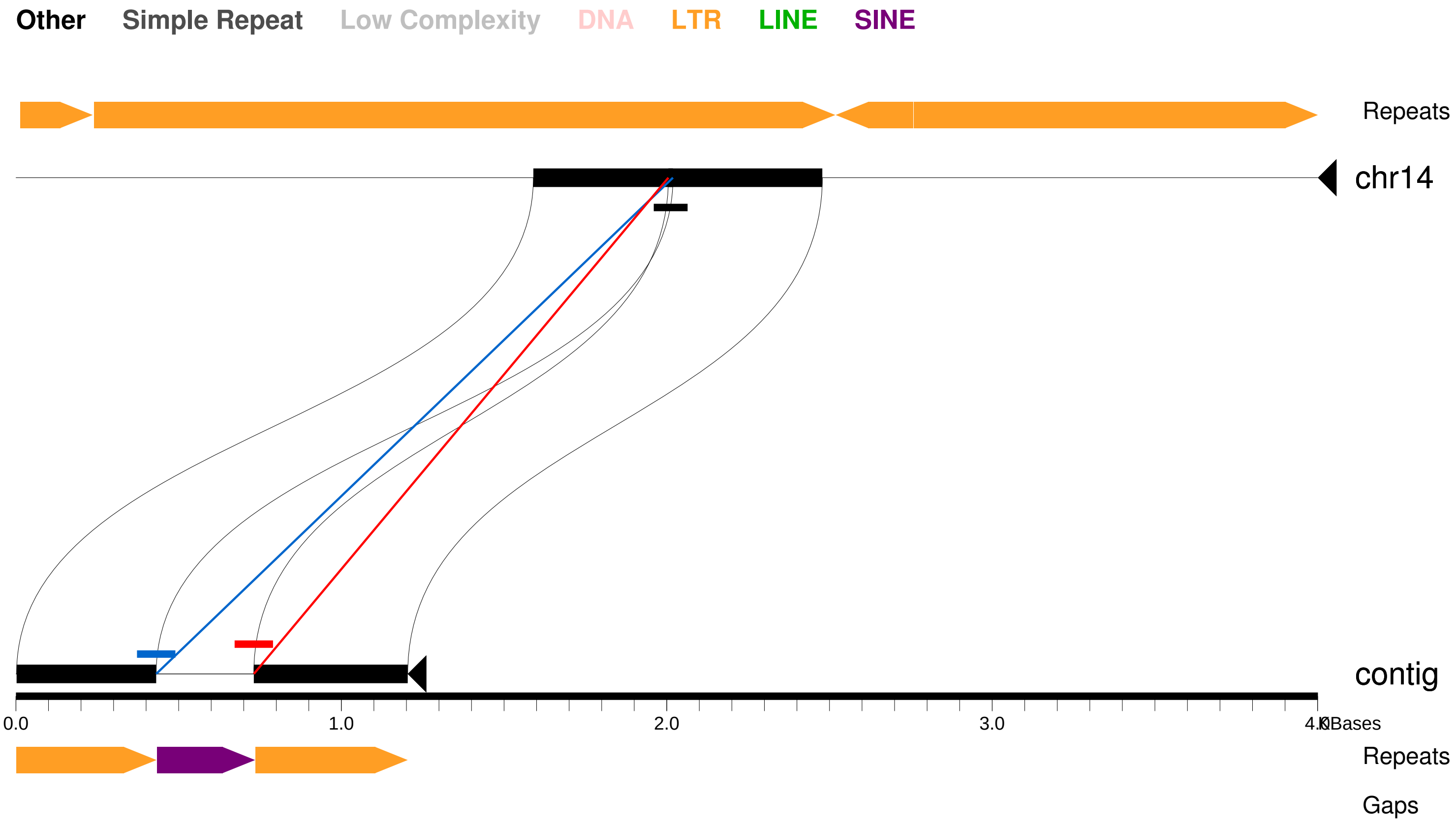

siteID: chr14_71480274 Scafftig2 - Breakpoint: chr14:71480268
chr14:71480225-71480327
Overlap Length: 17 INS size: 433 Genotyped: No

```
left    AATA----------TGATGCAAAGTTCTAT-ATAACAGTTATAAGA-----GGAAACTTCTGTTTCCCTG
chrom   AATA----------TGATGCAAAGTTCTAT-ATAACAGTTATAAGA-----GGAAACTTCTGTTTCCCTG
right   TCCACCCGCCTCGGCCTCCCAAAGTGCTGGGATTACAGGCGTGAGCCACCGCGCCCGGCCTGTTTCCCTG
        111*111111111111111******1**111**1****111*1**1111111*111111***********

left    GTTCTTTTTTTNNNNNNNNNNNNNNNNNNNNNNNNNNNNNNNNNNNNNNNNNNNNNNNNNNNNNN
chrom   GTTCTT----------------AACACTACTCTCTTAATGGCAAACACAAACTTGGTGACCAACA
right   GTTCTT----------------AACACTACTCTCTTAATGGCAAACACAAACTTGGTGACCAACA
        ******22222222222222222222222222222222222222222222222222222222222
```

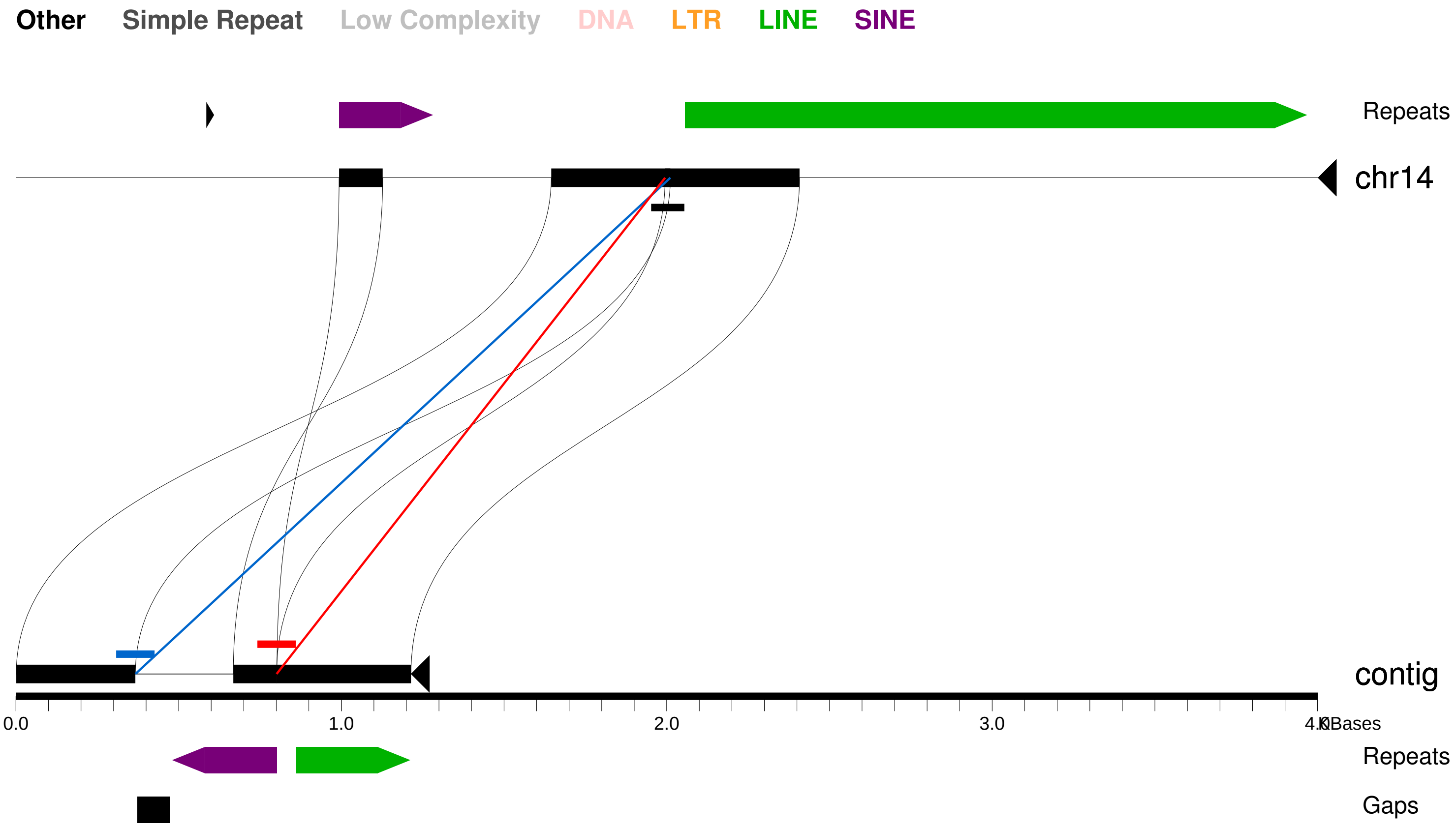

siteID: chr14_74942135 Scafftig4 + Breakpoint: chr14:74942145
chr14:74942099-74942204
Overlap Length: 14 INS size: 306 Genotyped: Yes

```
left     AGTCAAGCAGCTGG-----AAAAAGTTGG------AGG--TGAGATATAAACCCAGCTTGTATTTAACAT
chrom    AGTCAAGCAGCTGG-----AAAAAGTTGG------AGG--TGAGATATAAACCCAGCTTGTATTTAACAT
right    CCGCCCGCCTCGGCCTCCCAAAGTGCTGGGATTACAGGCGTGAGCCACCGCGCCCGGCCGTATTTAACAT
         111*11**11*1*111111***11*1***111111***11****11*11111**1*111***********

left     TTTTTTTTTTTTTTTTTTTTTTTTTTTTGAGACGGAGTCTCGCTCTGTCGCCCAGGCTGGAG
chrom    TTTAAATGTTACAGAAATCTGT-------AAACA-ACTA-----CTGCTTCCAAAAGTGAAA
right    TTTAAATGTTACAGAAATCTGT-------AAACA-ACTA-----CTGCTTCCAAAAGTGAAA
         ***222*2**2222222*2*2*2222222*2**22*2*222222***222**2*222**2*2
```

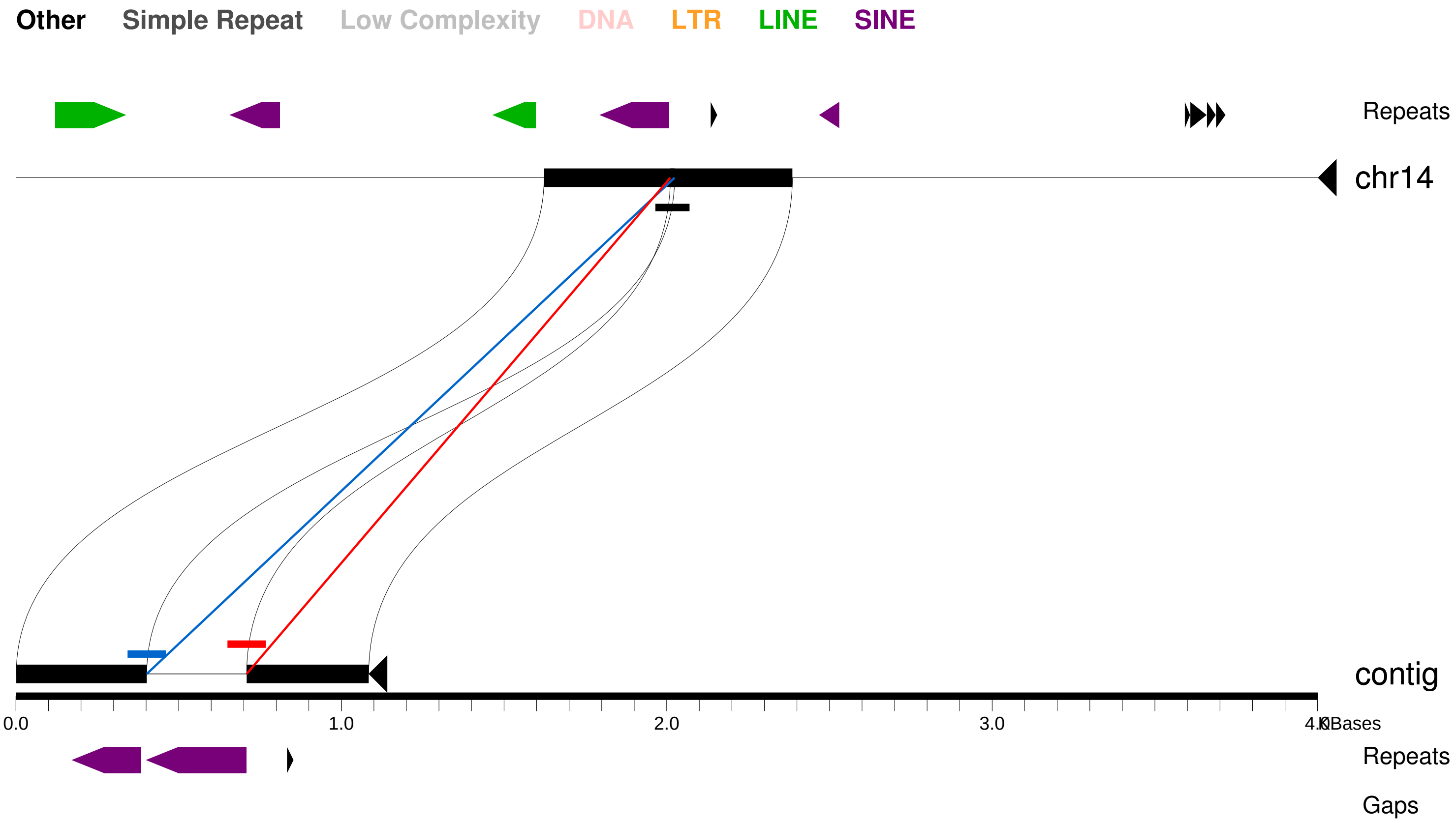

siteID: chr14_75499560 Scafftig7 - Breakpoint: chr14:75499568
chr14:75499408-75499737
Overlap Length: 10 INS size: 315 Genotyped: Yes

```
left    ----------CACTCCTTAGGTCCTTGCCTAACTCTTTGACATTACTCATTGGCCACAGGAGTAAAGACT
chrom   GTCCATGAAGCACTCCTTAGGTCCTTGCCTAACTCTTTGACATTACTCATTGGCCACAGGAGTAAAGACT
right   TTTAGTAGAG--------ACGGGGTTTC---ACCGTTTTA------------GCCGGGATGGTCTCGA-T
        N2NNN2NN2211111111*1*111**1*111**11***1*111111111111***111111**111**1*

left    TTCCTGGTATTTTACCACTTGGTGAACACACTGCCTCATGATGAGGAAAAGGGTAGCAAAAAAAAACATT
chrom   TTCCTGGTATTTTACCACTTGGTGAACACACTGCCTCATGATGAGGAAAAGGGTAGCAAAAAAAAACATT
right   CTCCTG----------ACCTCGTGATCCACCCGCCTCGGCCTCCC--AAAGTGCTGGGATTACAGGCGTG
        1*****1111111111**1*1****1*111*1*****1111*11111****1*11*11*11*1*11*1*1

left    TAAAAAGCATATATTGCTTTGGAAAGATAGTTTTTTTTTTTTTTTTTTTTTTTTTTTTTTTTTTGAGACGG
chrom   TAAAAAGCATATATTGCTTTGGAAAGATAGATTTAAAAACCAGAGATATTGGTTGTCTCTGGGGAGAGAA
right   -AGCCACCGCGCCCGGCCT-GGAAAGATAGATTTAAAAACCAGAGATATTGGTTGTCTCTGGGGAGAGAA
        1*111*1*1111111**1*1**********2***222222222222*2**22**2*2*2*222****222

left    AGTCTC-GCTCTGTCGCCCAGGCTGGAGTGCAG------------------TGGCG------------GG
chrom   ACTAGGAGGGGTGGGGACAAGGATAGAAAGGAGACTTTATTATATTCCCTTTTGTGCCTTTCAGAATTGG
right   ACTAGGAGGGGTGGGGACAAGGATAGAAAGGAGACTTTATTATATTCCCTTTTGTGCCTTTCAGAATTGG
        *2*2222*222**22*2*2***2*2**22*2**222222222222222222*2*2*222222222222**

left    ATCTCG--GCTCA--CTGCAAGCTCC---GCCTCCCGGGTTCATGCCAT--
chrom   ATCTATTTGATTATACTGCATATTCAAAAGCAAACTAAATTT-TAAAATCA
right   ATCTATTTGATTATACTGCATATTCAAAAGCAAACTAAA------------
        ****2222*2*2*22*****222**2222**222*222211N21NNN11
```

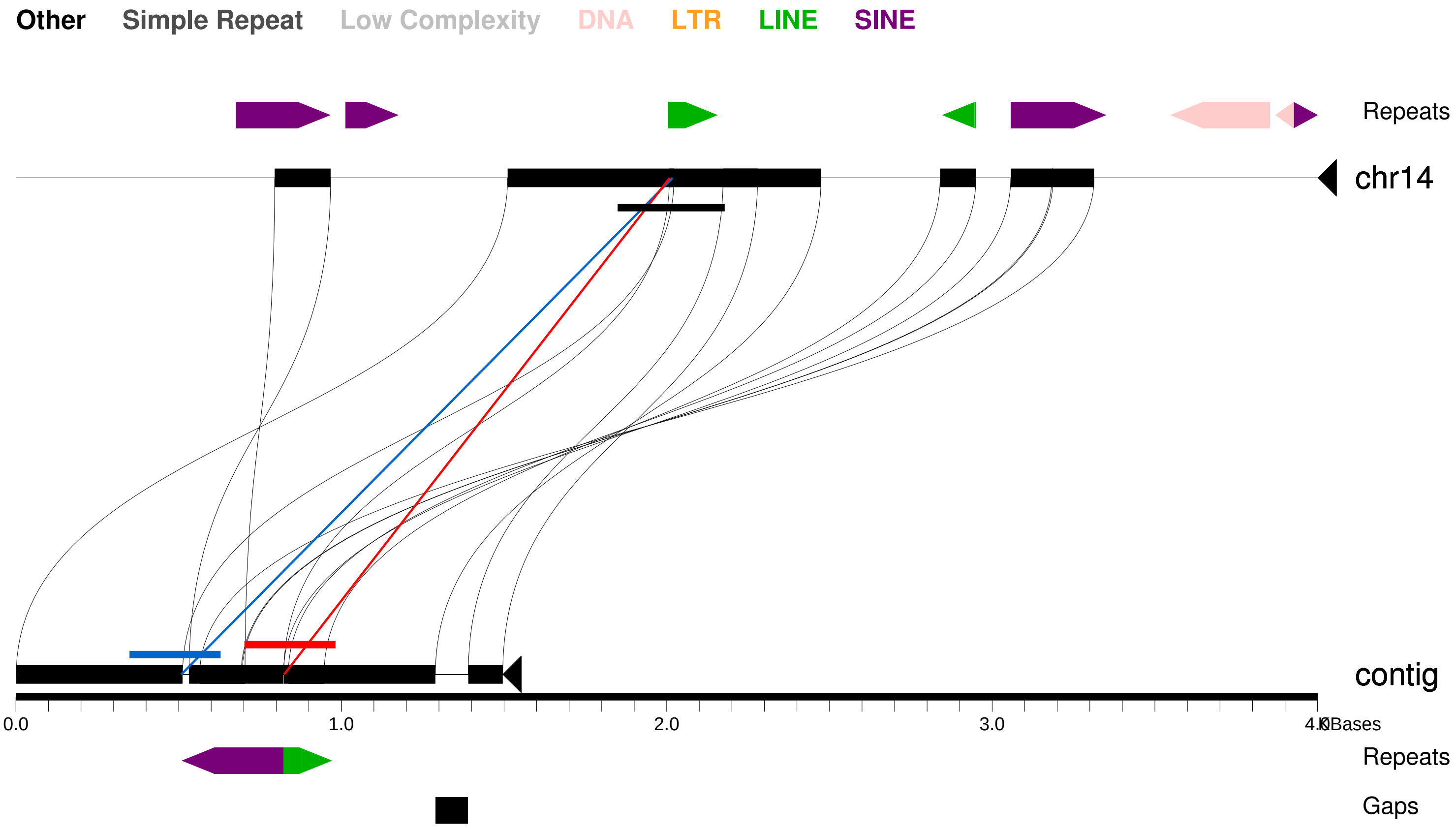

siteID: chr14_77116242 Scafftig4 + Breakpoint: chr14:77116229
chr14:77116184-77116288
Overlap Length: 15 INS size: 302 Genotyped: Yes

```
left    CT---------TATTTTTATGATCTAACTGTATTTCCAAAAGATGATTCTAATC------CCTGCTGTGT
chrom   CT---------TATTTTTATGATCTAACTGTATTTCCAAAAGATGATTCTAATC------CCTGCTGTGT
right   ATCCACCCGCCTCGGCCTCCCAAAGTGCTGGGATTACAGGCG-TGAGCCACCGCGCCCGGCCTGCTGTGT
        1*111111111*11111*111*11111***111**1**111*1***11*1111*111111**********

left    TTCTTTTTTTTTTTTTT---TTTTGAGACGGAGTCTCGCTCTGTCGCCCAGGCCGGACTGCGGACTG
chrom   TTCTTAATAGTTCTGTGGCCTTATCAGCCAAATCCTTGATTTC---------CCTGAGT--------
right   TTCTTAATAGTTCTGTGGCCTTATCAGCCAAATCCTTGATTTC---------CCTGAGT--------
        *****22*22**2*2*2222**2*2**2*22*22**2*2*2*2222222222**2**2*22222222
```

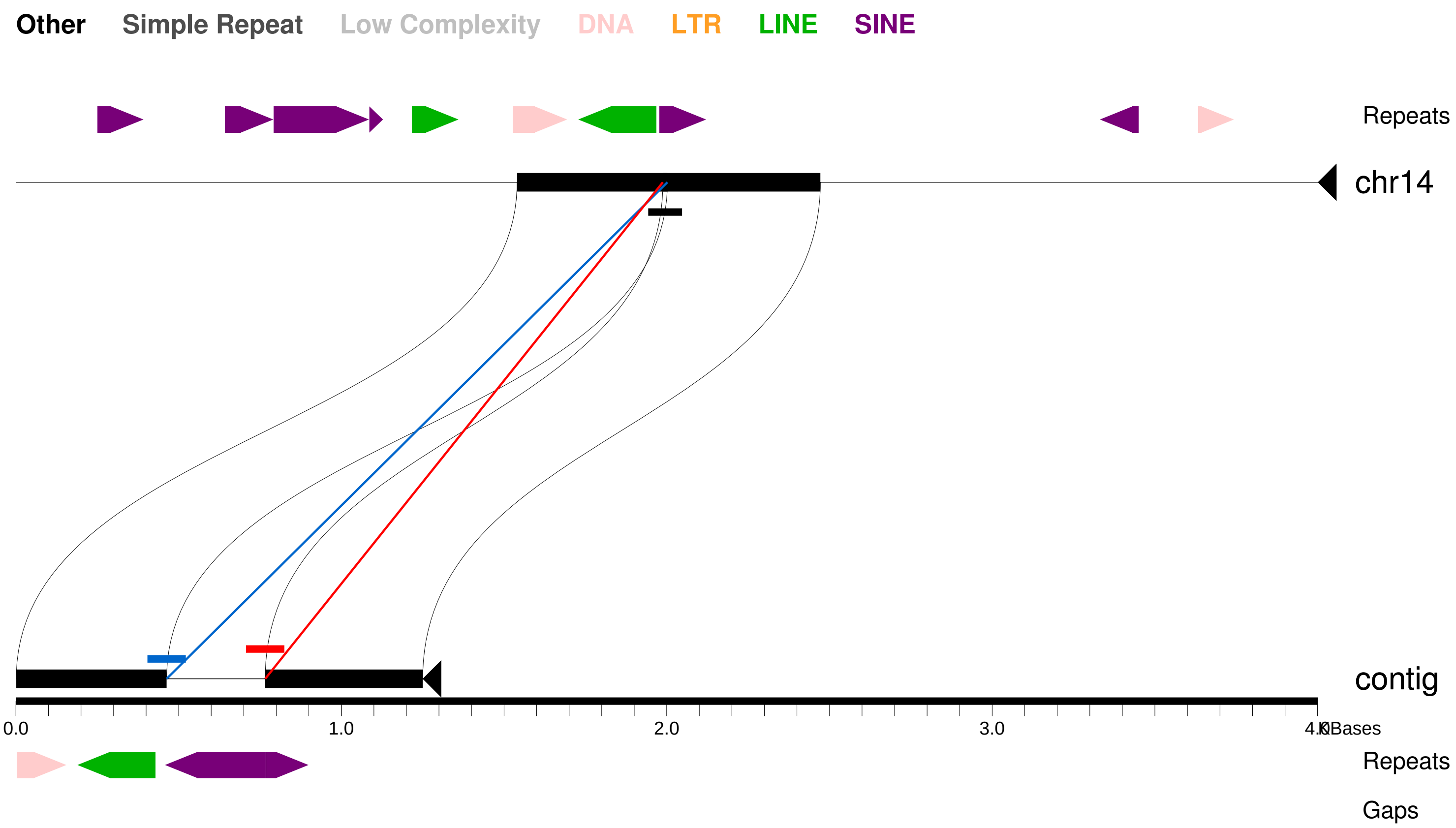

siteID: chr14_78982699 Scafftig4 - Breakpoint: chr14:78982701
chr14:78982650-78982760
Overlap Length: 9 INS size: 446 Genotyped: No

```
left     GTCACTGGCTTCTCTCTAATAGTTGTTATCCCTTTTAATTCTATAGAATAG--------AAAAGAAGGGG
chrom    GTCACTGGCTTCTCTCTAATAGTTGTTATCCCTTTTAATTCTATAGAATAG--------AAAAGAAGGAA
right    NNNNNNNNNNNNNNNNNNNNNNNNNNNNNNNNNNNNNNNNNNNNNNNNNNNNNNNNNNNAAAAGAAGGAA
         11111111111111111111111111111111111111111111111111111111111*********22

left     CCGGGCGCGGTGGCTCACGCCTGTAATCCCAGCACTTTGGGAGGCCGAGGCGGGTG--G
chrom    TTGGG-GCTA-GGCTGA-GC-----AGCAGAGGAGA--GATTGGCTGTGGAGAGTATAG
right    TTGGG-GCTA-GGCTGA-GC-----AGCAGAGGAGA--GATTGGCTGTGGAGAGTATAG
         22***2**222****2*2**22222*2*22**2*2222*222***2*2**2*2**222*
```

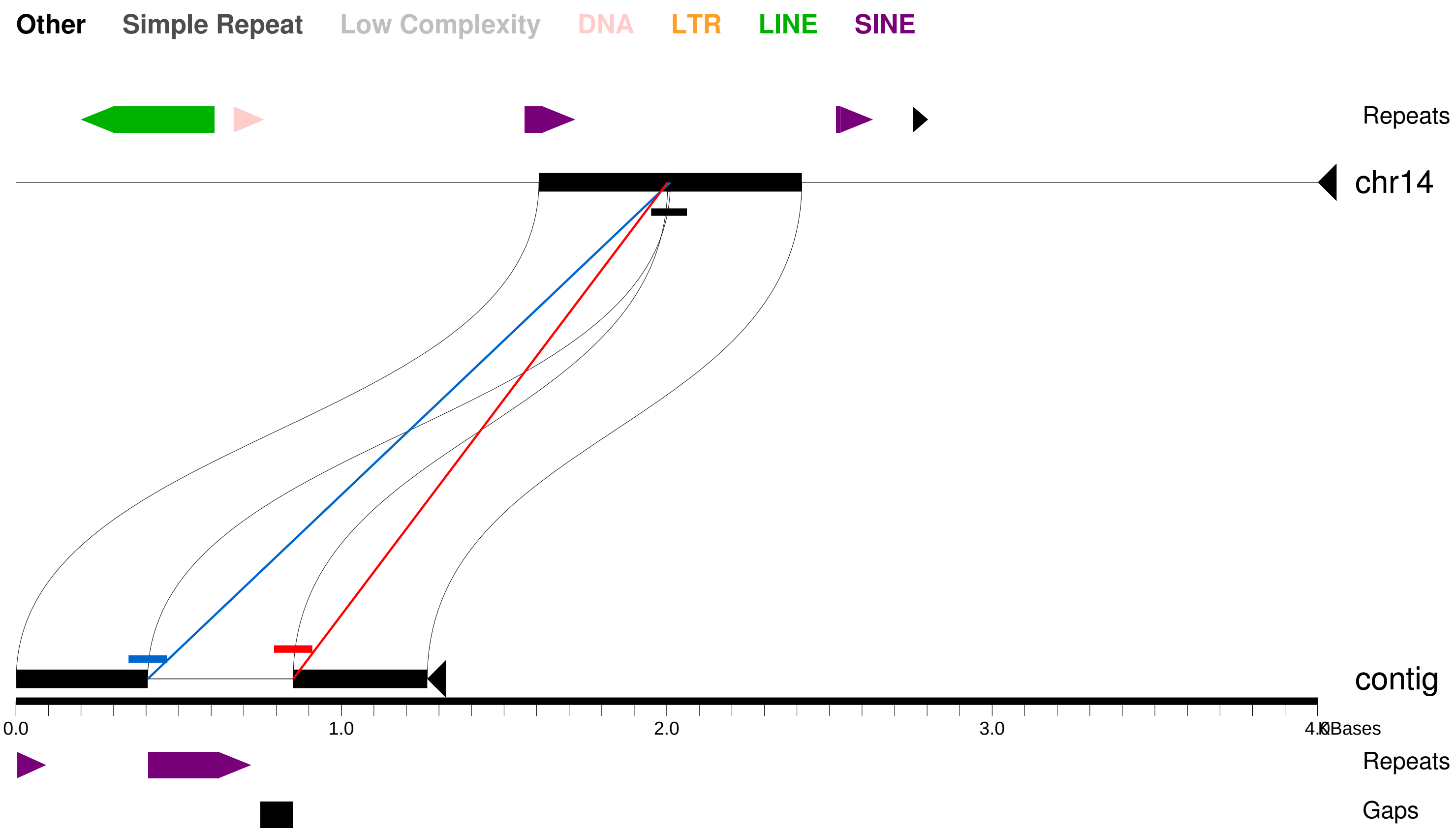

siteID: chr14_86630692 Scafftig2 - Breakpoint: chr14:86630694
chr14:86630643-86630748
Overlap Length: 9 INS size: 297 Genotyped: Yes

```
left     TGAAAACA----ACTCTGCTCAGGTGGAATGACAGA-------TGTCAAGAGCTTACAAAAA--ACTACC
chrom    TGAAAACA----ACTCTGCTCAGGTGGAATGACAGA-------TGTCAAGAGCTTACAAAAA--ACTACC
right    TCGCGCCACTGCACTCCAGCCTGGGCAACAGAGCGAGACTCCATCTCAAAAAAAAAAAAAAAAAACTACC
         *11111**1111****1111*1**111*11**11**1111111*1****1*1111*1*****11******

left     TTGCCGGGCGCGGTGGCTCACGCCTGTAATCCCAGCACTTTGGGAGGCCGAGGCGGGCGGAT
chrom    TTGGCCTGTTTAAAGG---ACATAAAGAAAACCAGCATCTTAAGCTGCAGTG----------
right    TTGGCCTGTTTAAAGG---ACATAAAGAAAACCAGCATCTTAAGCTGCAGTG----------
         ***2*22*222222**222**222222**22******22**22*22**2*2*2222222222
```

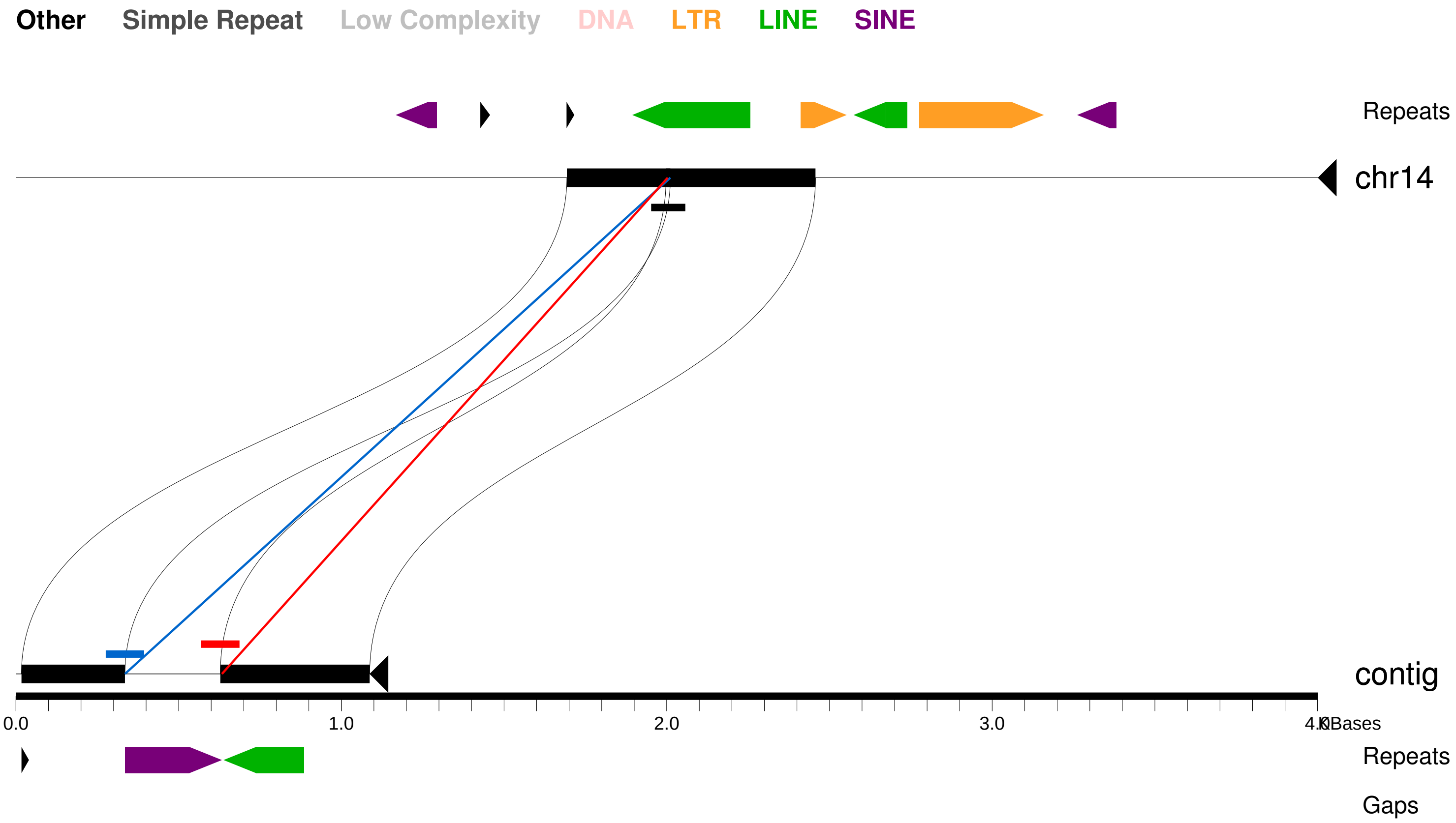

siteID: chr14_87375554 Scafftig1 - Breakpoint: chr14:87375554
chr14:87375508-87375613
Overlap Length: 14 INS size: 445 Genotyped: No

```
left    ACTCACACACAGAGAACACCACCAGAAACTGAGTGGGAGAAGGCTT-------------GAAATGTTATC
chrom   ACTCACACACAGAGAACACCACCAGAAACTGAGTGGGAGAAGGCTT-------------GAAATGTTATC
right   NNNNNNNNNNNNNNNNNNNNNNNNNNNNNNNNNNNNNNNNNNNNNNNNNNNNAAAAAAGAAATGTTATC
        11111111111111111111111111111111111111111111111111111111111***********

left    CAAGGCCGGGCGCGTGGCTCAC-GCCTGTAATCCCAGCACTTTGGGAGGCCGAGGCGGGCGGA
chrom   CAAAACAGT-----TTTCTGAGAGACTATAAGAT-ATCACAT-----GGCAGAAAATTAT---
right   CAAAACAGT-----TTTCTGAGAGACTATAAGAT-ATCACAT-----GGCAGAAAATTAT---
        ***22*2*222222*22**2*22*2**2***2222*2***2*22222***2**2222222222
```

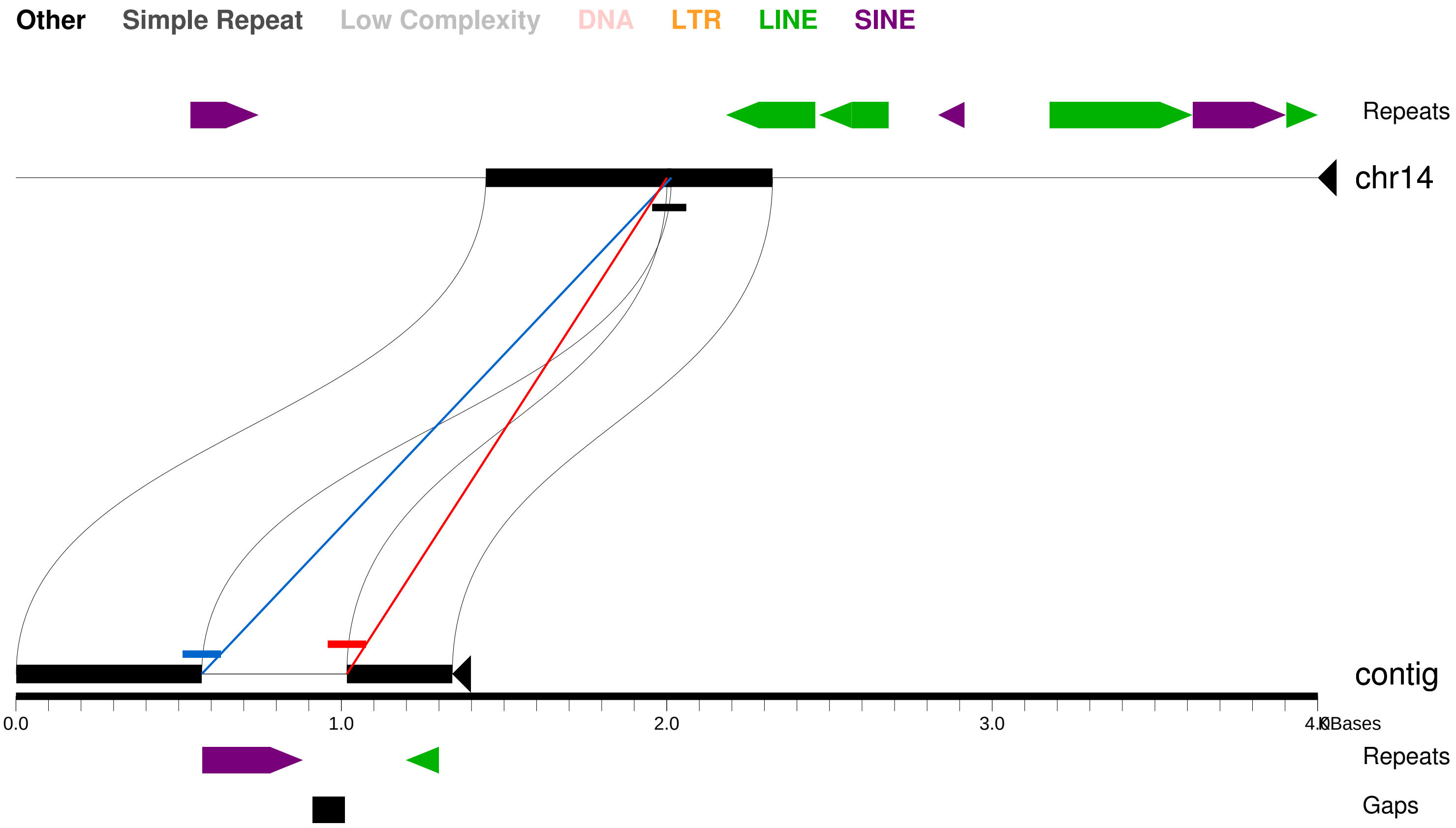

siteID: chr14_88853731 Scafftig1 - Breakpoint: chr14:88853738
chr14:88853689-88853797
Overlap Length: 11 INS size: 322 Genotyped: Yes

```
left   TA---GCTTCATTTTTAGGAAATAATAT-ATGATAAT-GTCAAATACTGTTATT-----TTGAATTTTTA
chrom  TA---GCTTCATTTTTAGGAAATAATAT-ATGATAAT-GTCAAATACTGTTATT-----TTGAATTTTTA
right  CACCCGCCTCGGCCTCCCAAAGTGCTGGGATTACAGGCGTGAGCCACCGCGCCCGGCCCTTGAATTTTTA
       1*111**1**1111*1111**1*11*111**1*1*111**1*111**1*1111111111***********

left   TTTTTTTTTTATTTTATTTTATTTTTTTTTTTTTGAGACGGAGTGTCGCTCTGTCGCCCA
chrom  A---------AATAAATGTAATCACCAAATAACCAGG-GCAGTCAGCTTTCATGTACTG
right  A---------AATAAATGTAATCACCAAATAACTGGG-GCAGTCAGCTTTCATGTACTG
       2222222222*2*22**2*2**2222222*222N1*22*2***22222*222*222*22
```

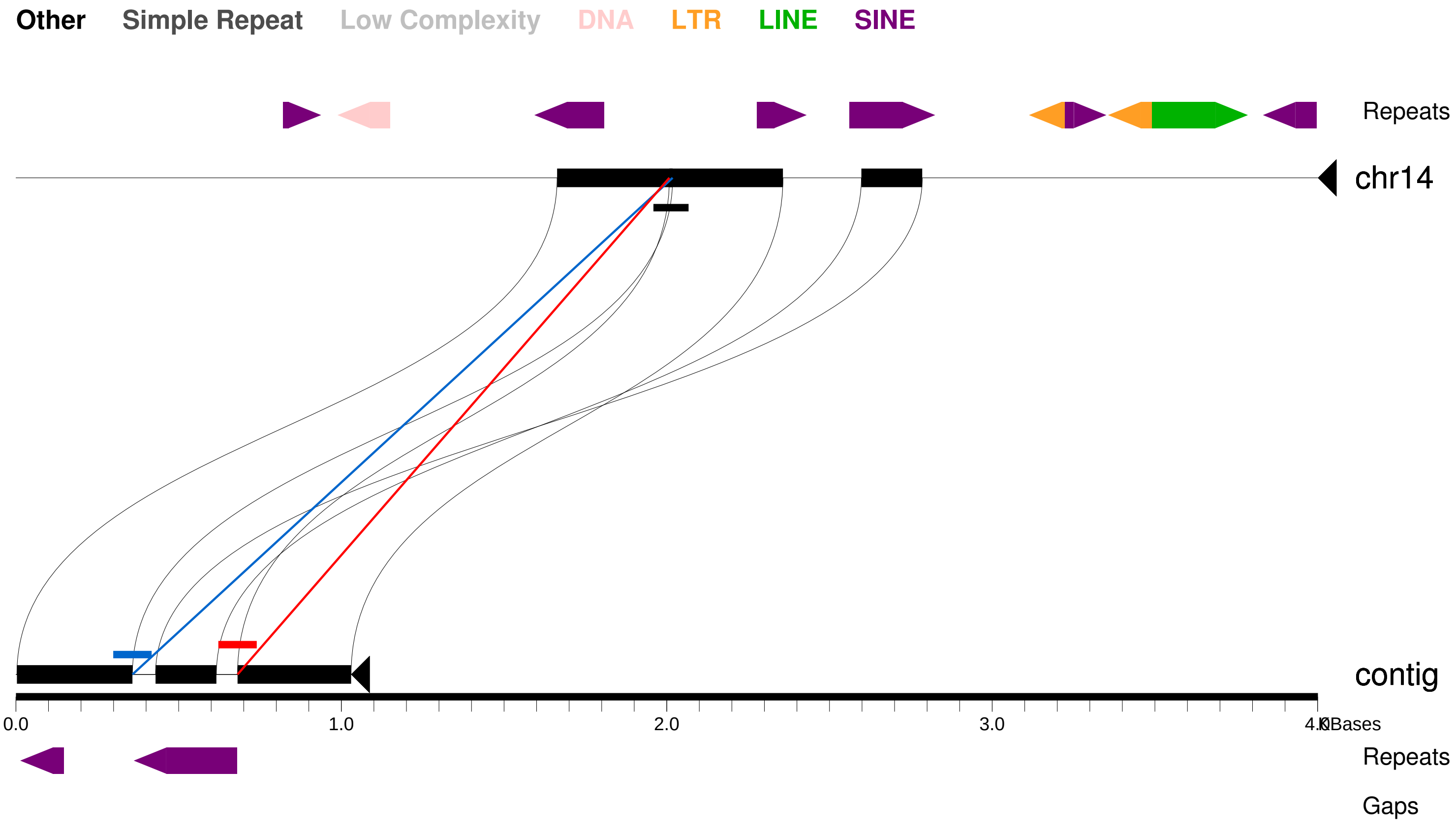

siteID: chr14_92125054 Scafftig5 - Breakpoint: chr14:92125054
chr14:92124894-92125228
Overlap Length: 15 INS size: 296 Genotyped: Yes

```
left   ---------------AAAGGA-TAAATTCTTGATGTGATGGATACCCCATTTACCCCGATATGATTATTA
chrom  GAATGTTTGTAACATAAAGGA-TAAATTCTTGATGTGATGGATACCCCATTTACCCCGATATGATTATTA
right  GAG-----GCAGGAGAATGGCGTGAACCCGGGAGGCGGAGCTTGC---AGTGAGCC-GA---GATCCCGC
       22N     2N2NN2N**1**11*1**11*11**1*1*11*11*1*111*1*1*1**1**111***11111

left   CACAGTGTATGCCTGTATCAAAATATCTCATGTATCCCATAAATTTATACACTTACTATGTACCCACAAA
chrom  CACAGTGTATGCCTGTATCAAAATATCTCATGTATCCCATAAATTTATACACCTACTATGTACCCACAAA
right  CAC--TGCACTCCAG-------------CCTGGG--CAACAGAGCGAGACTCCGTCT---------CAAA
       ***11**1*11**1*1111111111111*1**1111*1*1*1*111*1**1*211**111111111****

left   AATTAAGAATAAAATATTTTTAAAAAAAGGAAGGGAGGCCGGGCGCGGTGGCTCAT-GCCTGTAATCCCA
chrom  AATTAAGAATAAAATATTTTTAAAAAAAGGAAGGGAAGA-GAAGGAAGAAACTAACAGCTTGTAAAAGAT
right  AA--AAAAAAAAAA-------AAAAAAAGGAAGGGAAGA-GAAGGAAGAAACTAACAGCTTGTAAAAGAT
       **11**1**1****1111111**************2*22*222*22*222**2*22**2*****22222

left   GCACTTTGGGAGGCCGAGGCGG-GCGGATCACGAGGTCAGGAG----ATTGAGACC---ATCCCGGCTAA
chrom  AAAAAGAATCAAGTTGAGACAAAGCACATCCCAAGAT-AGGAGGTAAATTTACTTCCATATTCTGCCTCA
right  AAAAAGAATCAAGTTGAGACAAAGCACACCCCAAGAT-AGGAGGTAAATTTACTTCCATATTCTGCCTCA
       22*2222222*2*22***2*222**22*1*2*2**2*2*****2222***2*222*222**2*2*2**2*

left   AACGGTG--------------------AAACCCCGTCTCTA---CT---------AAA
chrom  AAAAATCTTAATAAATTAAATTCAACAAAACAAGATCTCAAAGACTCAATAATAGAAA
right  AAAAATCTTAATAAATTAAATTCAACAAAACAAGATCTCAAAGACTCAAT--------
       **222*22222222222222222222****2222****2*222**2222     111
```

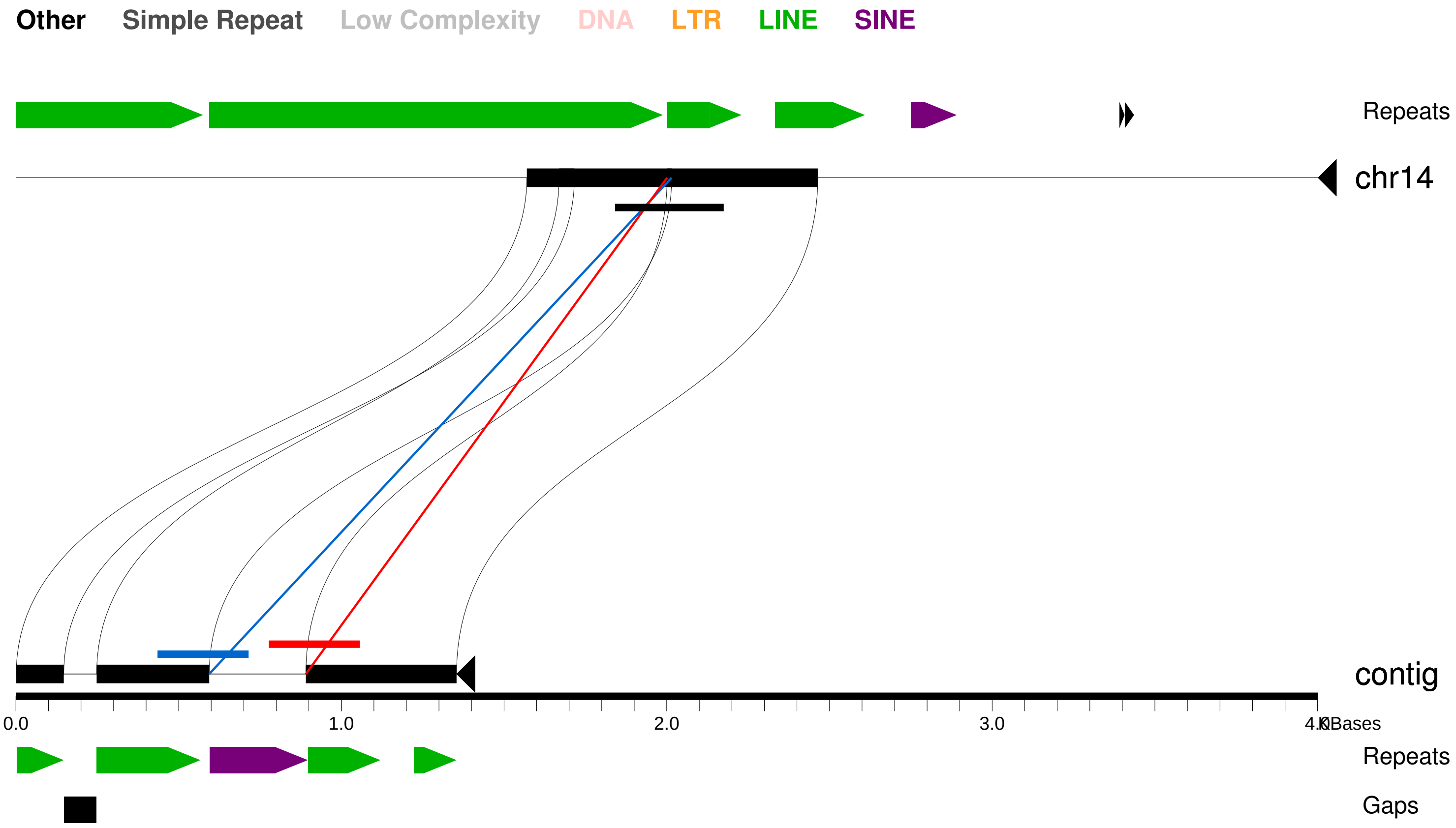

siteID: chr14_95024636 Scafftig2 - Breakpoint: chr14:95024606
chr14:95024446-95024781
Overlap Length: 16 INS size: 294 Genotyped: Yes

```
left    A-----------TTTCTTACAAGACTGGAAGGAAACAAATTATATCTAGTCATAAGCTGAACACCAGGTT
chrom   AGCACATACCCATTTCTTACAAGACTGGAAGGAAACCAATTATATCTAGTCATAAGCTGAACACCAGGTT
right   ------------TTTAGTAGA-GACGGGGTTTCACCG--TTTTAGCCGG------GATGGTCTCGA--TC
        1           ***11**1*1***1**11111*1*N11**1**1*11*111111*1**11*1*1*11*1

left    TGCAAATGATTTTATTTTCTTCTCCAGGTTTTTTTTACTTTCCCATATTTTTCATGATGACCATTATTGC
chrom   TGCAAATGATTTTATTTTCTTCTCCAGGTTTTTTTTACTTTCCCATATTTTTCATGATGACCATTATTGC
right   TCC---TGACCTCGTGATC--CGCCCGCCTCGGCCT-----CCCAAAGTGCTGG-GATTACAG-----GC
        *1*111***11*11*11**11*1**1*11*11111*11111****1*1*11*111***1**1111111**

left    ATTGCCAATCAAATTCAAAACAAGGACAGCCTTTTTTTTT-----TTTTTTTTTTGAGA------GGAGT
chrom   ATTGCCAATCAAATTCAAAACAAGGACAGCCTTTTTAAGGGCAGATTAATTTCCTGGCTCCATTTGGAAT
right   GTGAGCCACCGCGCCCGGCCCAAGGACAGCCTTTTTAAGGGCAGATTAATTTCCTGGCTCCATTTGGAAT
        1*111*1*1*11111*1111***************222222222**22***22**222222222***2*

left    CTCGCTCTGTCGCCCAGGCTGGAGTGCAGTG-GCGGGATCTCGGC-TCACTGCAAGCTCCGCCTCCCGGG
chrom   CCAGGGAATGGGAAGGGGATTGGGAGCAGTTAGCAGGAGGGCAGCATAAGTGGAGCCTGTGCTGGAATGG
right   CCAGGGAATGGGAAGGGGATTGGGAGCAGTTAGCAGGAGGGCAGCATAAGTGGAGCCTGTGCTGGAATGG
        *22*2222222*2222**2*2*2*2*****22**2***222*2**2*2*2**2*22**22**222222**

left    TTCAC-------GCCATTCTC------CTGCCT--------------CAGC----C
chrom   GAGACCAAGACAGCCATTGAGAAAGGGCTGGGTTGCAATTTAGGATGCAGTGGAGC
right   GAGACCAAGACAGCCATTGAGAAAGGGCTGGGTTGCAATT----------------
        222**2222222*****222222222***22*2222222       111N    1
```

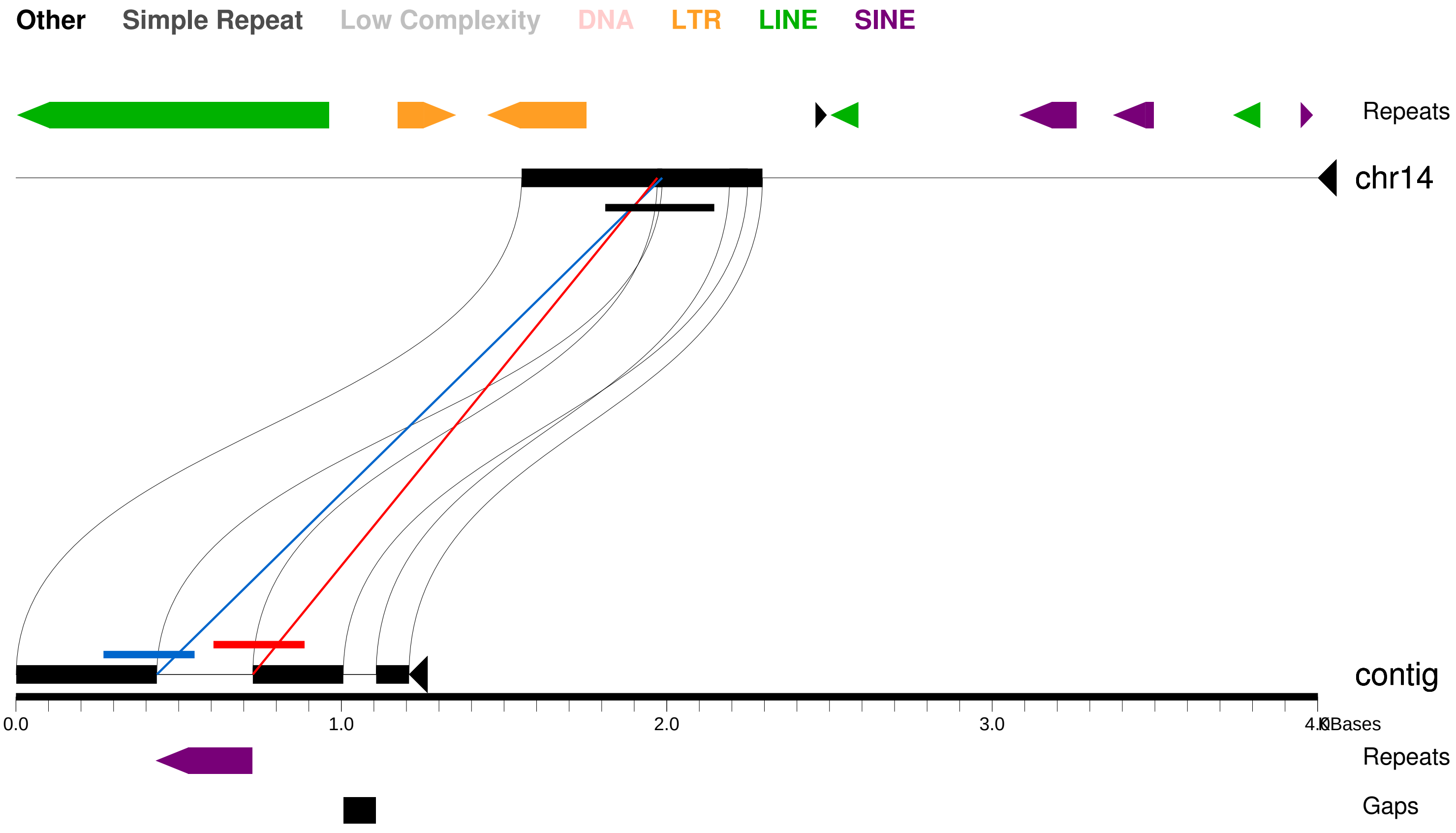

siteID: chr14_98494406 Scafftig3 + Breakpoint: chr14:98494377
chr14:98494334-98494436
Overlap Length: 17 INS size: 313 Genotyped: Yes

```
left     GTCAT--------GGGGTCTCCAT---CTGGGGAAACCCATATCATAAAAC-----CATCCAACTCAAAG
chrom    GTCAT--------GGGGTCTCCAT---CTGGGGAAACCCATATCATAAAAC-----CATCCAACTCAAAG
right    ATCCACCCGCCTCGGCCTCCCAAAGTGCTGGGATTACAGGTGTGAGCCACCGCGCCCGGCCAACTCAAAG
         1**1111111111**11**1*1*1111*****111**111*1*1*111*1*11111*11***********

left     ACAGTTTTGTTTTGTTTTGTTTTTTTTTTTTTTGAGACGGAGTCTCGCT-CTGTCGCCCAGGCCGG
chrom    ACAGTTGCGTAG-GAACTGA--------------AAAGGAACACTTGGAACATTTG--GAATCCAC
right    ACAGTTGCGTAG-GAACTGA--------------AAAGGAACACTTGGAACATTTG--GAATCCAC
         ******22**222*222**222222222222222*2*2*2*22**2*222*22*2*222*22**22
```

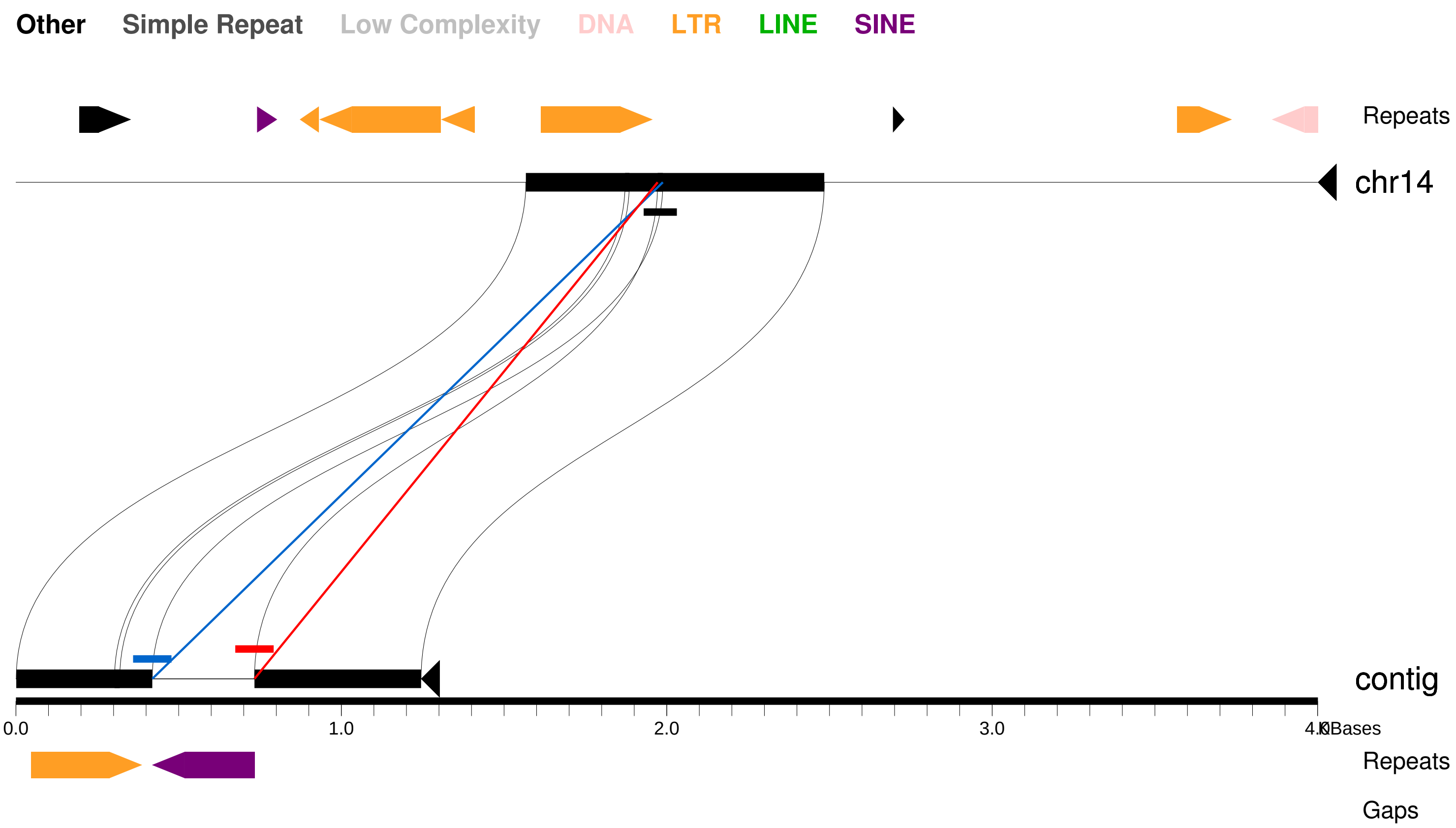

siteID: chr15_100812812 Scafftig11 - Breakpoint: chr15:100812804
chr15:100812758-100812860
Overlap Length: 14 INS size: 319 Genotyped: Yes

```
left    AAGAATTTATTAGCATTCTCTCTGTCTCTCTCTCTGTGTGTGT----------------AAGATTTAGGG
chrom   AAGAATTTATTAGCATTCTCTCTGTCTCTCTCTCTGTGTGTGT----------------AAGATTTAGGG
right   GGCGACAGAGCGAGACTCCATCTCAAAAAAAAAAAAAAAAAAAAAAAAAAAAAAAAAAAAAAATTTAGGG
        1111*111*11111*1**11***111111111111111111111111111111111111**1********

left    TTTTCTGGCCAGGCGCGGTGGCTCACGCCTGTAATCCCAGCACTTTGGGAGGCCGAGGCGGGCGG
chrom   TTTTCT--ACATGTAATAAATATGTCATCTGTGAACAGAGATCATTTTACT--------------
right   TTTTCT--ACATGTAATAAATATGTCATCTGTGAACAGAGATCATTTTACT--------------
        ******222**2*222222222*22*22****2*2*22**22*2**2222222222222222222
```

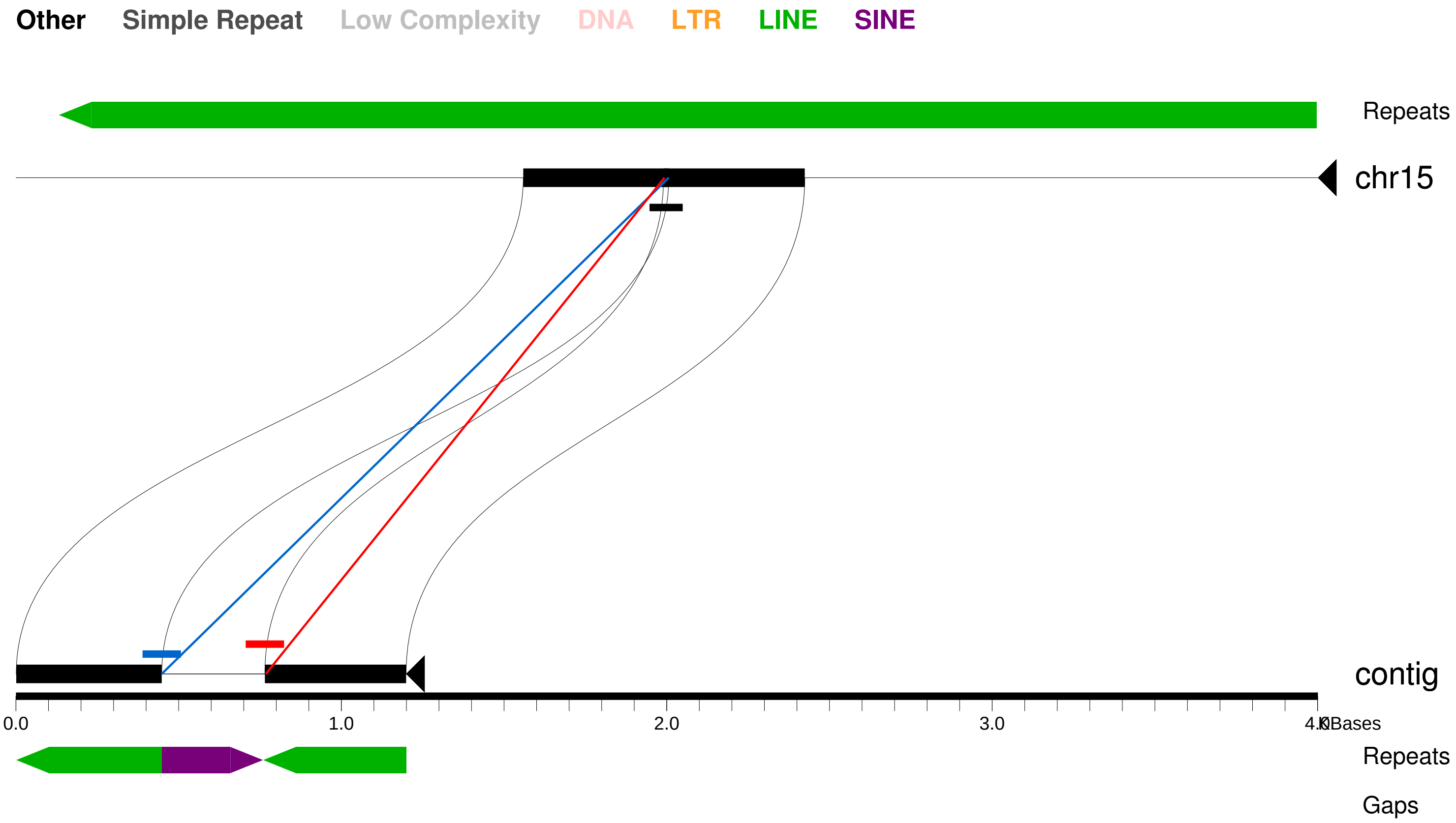

siteID: chr15_20158920 Scafftig2 + Breakpoint: chr15:20158908
chr15:20158748-20159074
Overlap Length: 7 INS size: 422 Genotyped: No

```
left    -------CAAGAAAAAAACAAACAACCCCATCAACAAGTGGGTGAAGGATATGAACAGACACTTCTCAAA
chrom   AAATTTACAAGAAAAAAACAAACAACCCCATCAACAAGTGGGTGAAGGATATGAACAGACACTTCTCAAA
right   NNNNNNNNNNN-----------------------------------------------------------
        NNNNNNN111111111111111111111111111111111111111111111111111111111111111

left    AGAAAACATTTGTGCAGCCAAAAGACACATGAAAAAATGCTCATCTTCACTGGCCATCAGAGAAATGCAA
chrom   AGAAAACATTTGTGCAGCCAAAAGACACATGAAAAAATGCTCATCTTCACTGGCCATCAGAGAAATGCAA
right   ----------------------------------------------------------------------
        1111111111111111111111111111111111111111111111111111111111111111111111

left    ATCAAAACCACGATGAGATACCATCTCGGCCGGGCGCG---GTGGCTCAC------GCCTGTAATCC-CA
chrom   ATCAAAACCACGATGAGATACCATCTCACACCAGTTAGAATGACGATCATTAAAAAGTCAGGAAACAACA
right   --------------------CCATCTCACACCAGTTAGAATGACGATCATTAAAAAGTCAGGAAACAACA
        11111111111111111111*******222*22*222*222*22*2***2222222*2*2*2**2*22**

left    GCACTTTGGGAGGCCGAGGCGGGC--GGA------TCACGAGGTCAGGAG-------ATCGAGACCATCC
chrom   GGTGCTGGAGAGGATGTGGAGAAATAGGAATACTTTCACACTGTTGGTGGGACTGTAAACTAGTTCAACC
right   GGTGCTGGAGAGGATGTGGAGAAATAGGAATACTTTCACACTGTTGGTGGGACTGTAAACTAGTTCAACC
        *2222*2*2****22*2**2*22222***222222****222**22*22*2222222*2*2**22**2**

left    CGGCTAAAA--CGGTGA---AACCCCGTC----TCTA---CTA--AA
chrom   ATTGTGGAAGTCAGTGTGGCAATTCCTTAGGGATCTAGAACTAGAAA
right   ATTG-------------------------------------------
        22221NN11  1N111N   11NN11N1N    1111   111  11
```

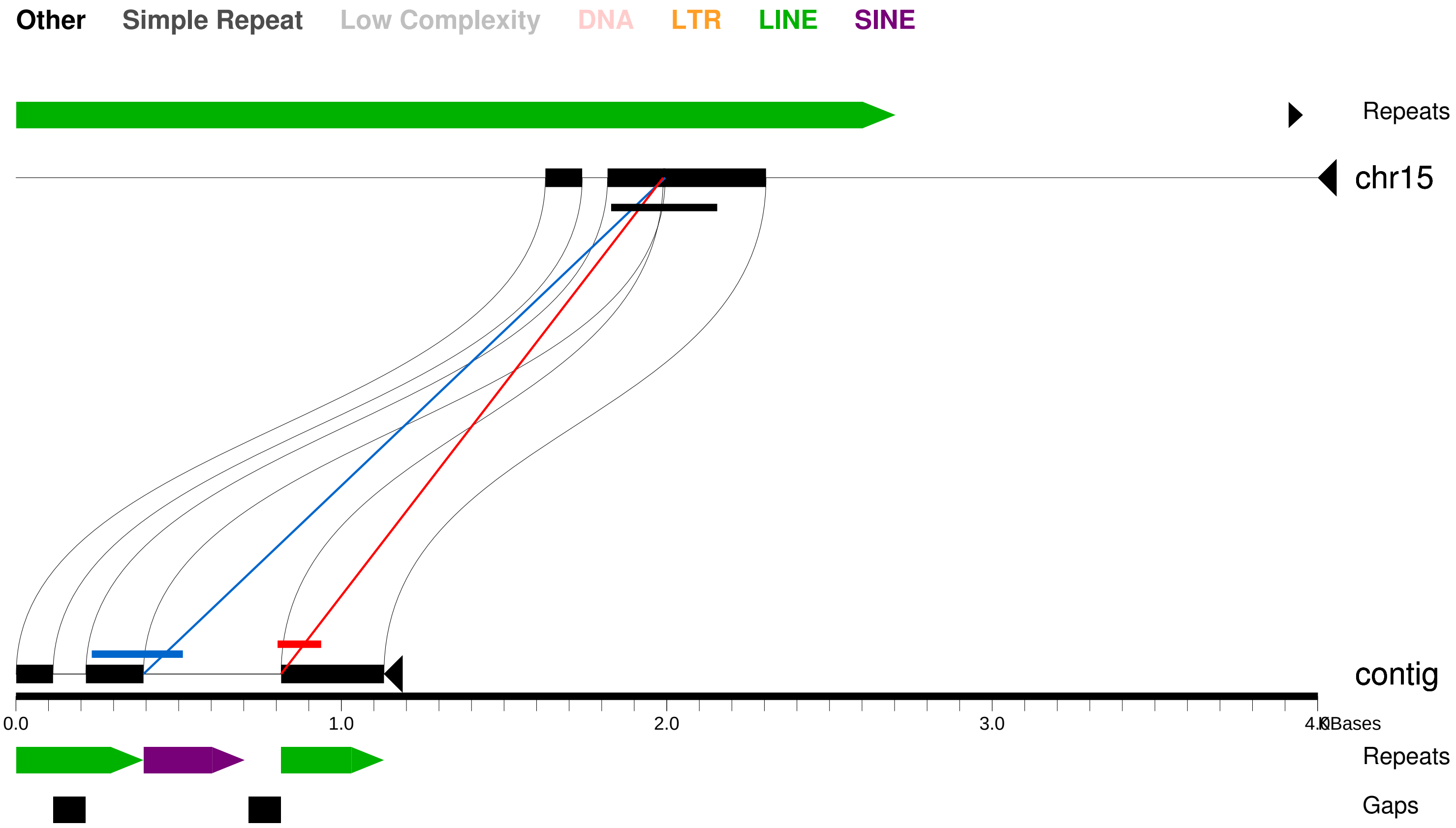

siteID: chr15_20586592 Scafftig1 - Breakpoint: chr15:20586688
chr15:20586639-20586747
Overlap Length: 11 INS size: 66 Genotyped: Yes

```
left     CTATTTTACCTTCATGACTACT---------ACAGGCTCTGAAACATATTGG--TGCTTAGAGACATTCT
chrom    CTATTTTACCTTCATGACTACT---------ACAGGCTCTGAAACATATTGG--TGCTTAGAGACATTCT
right    TTTTTTTTTTTTTTTTTTTTTTTTTTTTTTGAGACAGAGTCTCGCTCTT-TTGCCCAGGCTGGAGACATTCT
         1*1****111**11*111*11*111111111****11***1111*1*1***1111*11*1**********

left     TTTTTTTTTTTTTTTTTTTTTTTTTTTTTTTTTTTTGAGACAGAGTCTCGCTCTTTTGCC
chrom    TAAAAACATTAATGAATGATGAAAACATTTAAAATTGTGTCACAAACAAG----------
right    TAAAAACATTAATGAATGATGAAAACATTTAAAATTGTGTCACAAACAAG----------
         *2222222**22*222*22*2222222***2222***2*2**2*22*22*2222222222
```

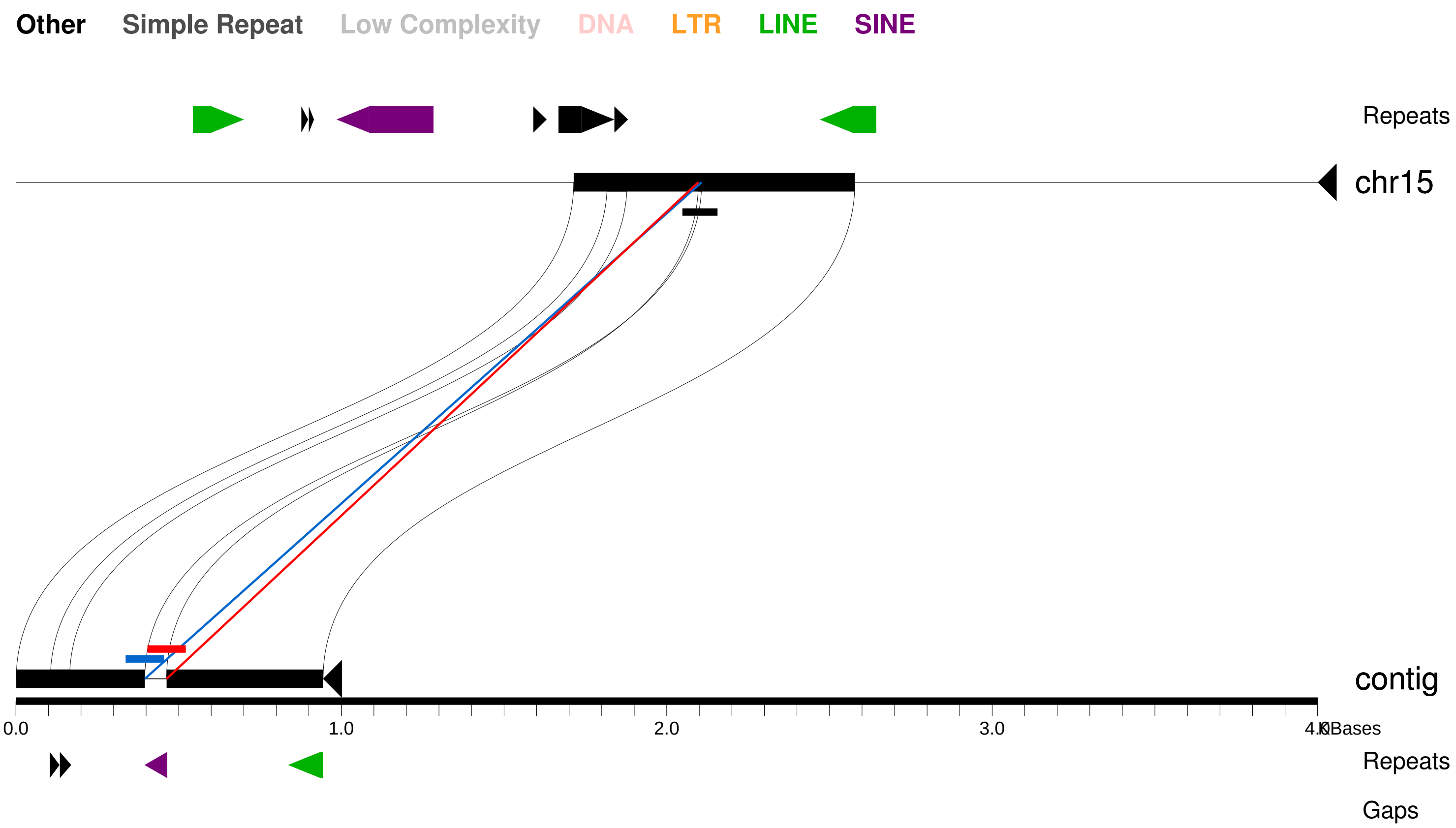

siteID: chr15_21941237 Scafftig2 - Breakpoint: chr15:21941241
chr15:21941081-21941410
Overlap Length: 10 INS size: 180 Genotyped: Yes

```
left    ---------GAGTAACTTCAATTCATATGGTCCAAGTCTGACAAAAATGCATTCTAACACCTGCATCTAA
chrom   CAGATGATAGAGTAACTTCAATTCATATGGTCCAAGTCTGACAAAAATGCATTCTAACACCTGCATCTAA
right   -----------GGAGCTTGCAGTGAG-----CCGAG--------------ATCCCGCCAC-TGCA-----
                 11*1*1***11*1*1*111111**1**11111111111111**1*111***1****11111

left    CACCAGCTTTATTGA-ATAATTTCATCACGTTTCAAAATCTGTGTCTCCTAGACCAGTTGAGCTCACAAC
chrom   CACCAGCTTTATTGA-ATAATTTCATCACGTTTCAAAATCTGTGTCTCCTAGACCAGTTGAGCTCACAAC
right   CTCCAGCCTGGGCGACAGAGCGAGACTCCGTCTCAAAAA-----------AAAAAAAAAAAAAAAAAAAA
        *1*****1*1111**1*1*11111*111***1******111111111111*1*11*1111*1111*1**1

left    AAAAAGCAATTGTAGAGAGTTAGAAAAATATTGGC------GGGCGC----CTGTAG----TCCCAGCTA-
chrom   AAAAAGCAATTGTAGAGAGTTAGAAAAATATATGAAATTAGTGAACAAAACAGTGAAACCTTCCAACCAG
right   AAAAAAAAAAAAAAAAAAAAAAGAAAAATATATGAAATTAGTGAACAAAACAGTGAAACCTTCCAACCAG
        *****11**1111*1*1*111*********222222222*2*22*2222*2**222222*2***2*2*2

left    ------TTTGGGAGG---CTGAGGCAGGAGAATGAC---ATGAACCCGGGAGG--------CGGAGCTTG
chrom   GAATGTTTTTGGAACTTTCTTTGTTAGTAATAGTTCCTTATGGTTCCTAAATATTCAACCTCCCAGCATG
right   GAATGTTTTTGGAACTTTCTTTGTTAGTAATAGTTCCTTATGGTTCCTAAATATTCAACCTCCCAGCATG
        222222***2***22222**22*22**2*22*222*222***222**222*2222222222*22***2**

left    CA-GTGAGCCG-------AGATCCCGCCACTGCACTCCAGCCTGGGCGACAGAG
chrom   AATGTGTCTCCCATCAAAAGAGCCACAGAATAAACACAACCTCTTTTTTCA---
right   AATGTGTCTCCCATCAAAAGAGCCACAGAATAAACACAACCTCTTTTT------
        2*2***222*22222222***2**2222*2*22**2*2*2*2222222N11222
```

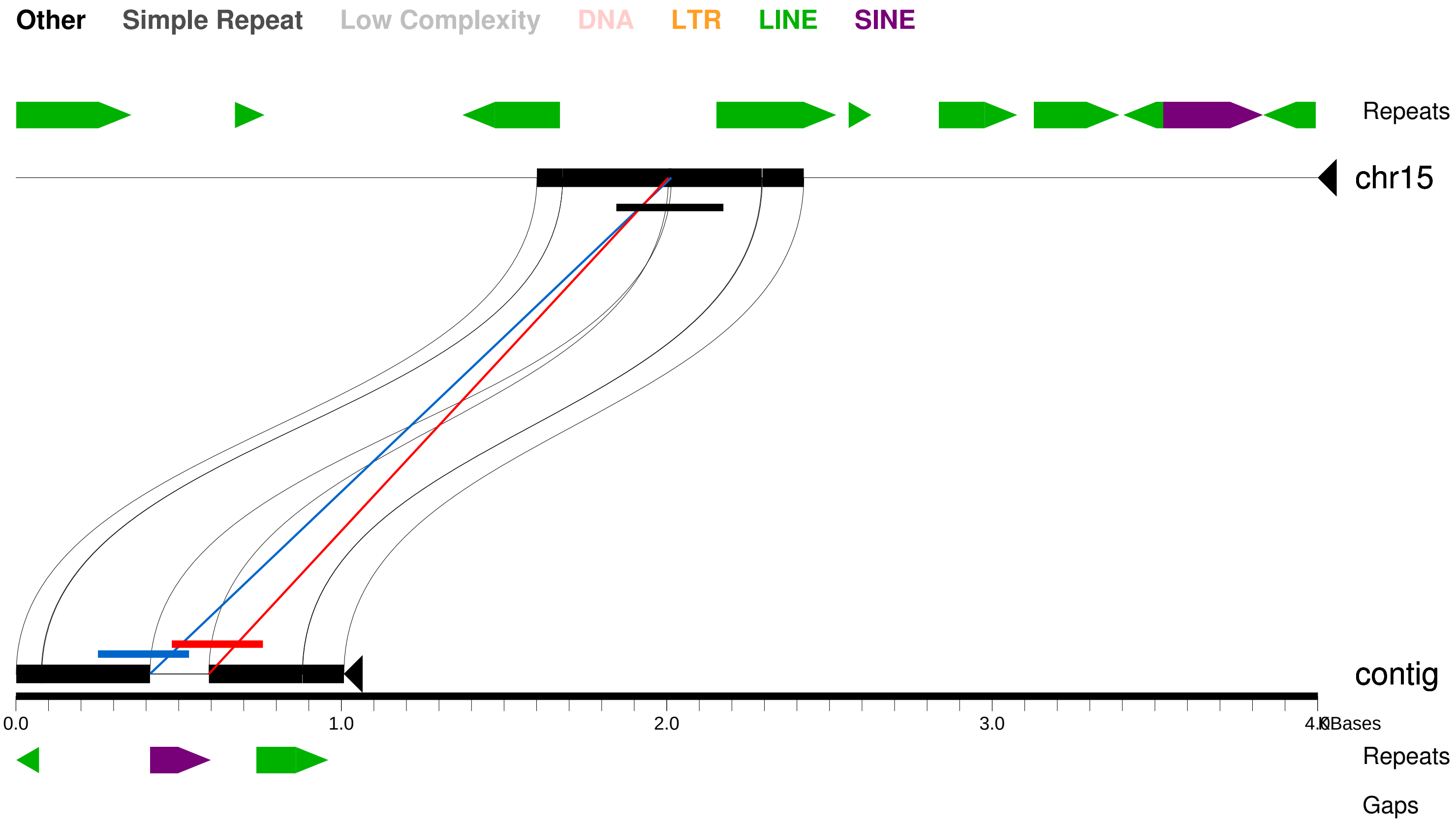

siteID: chr15_22769064 Scafftig1 - Breakpoint: chr15:22769065
chr15:22769015-22769107
Overlap Length: 10 INS size: 307 Genotyped: Yes

```
left   --ATAT------AAAGAACTCTCAAAACTCA----------AAATTAAGAA--------AAAAAATTTTA
chrom  --ATAT------AAAGAACTCTCAAAACTCA----------AAATTAAGAA--------AAAAAATTTTA
right  CGAGATTGCGCCACTGCAGTCCGCAGTCCCACCTGGGCGACAGAGCGAGACTCCGTCTCAAAAAAAAAAA
       11*1**111111*11*1*1**111*11*1**1111111111*1*111***111111111******1111*

left   ATGAACAAAATATTTAGGCCGGGCGCGGTGGCTCACGCCTGTAATCCCAGCACTTTGGGAGGCCGAGGCG
chrom  ATGAACAAAATATTTAAACAGA---CATT---TCACC-----AAAAAAAGTA-TCTGTCAA---------
right  AAAAAAAAAAAATTTAAACAGA---CATT---TCACC-----AAAAAAAGTA-TCTGTCAA---------
       *11**1*****1*****22*2*2222*22*222****222222**2222**2*2*2**22*2222222222

left   GGTGG
chrom  -----
right  -----
       22222
```

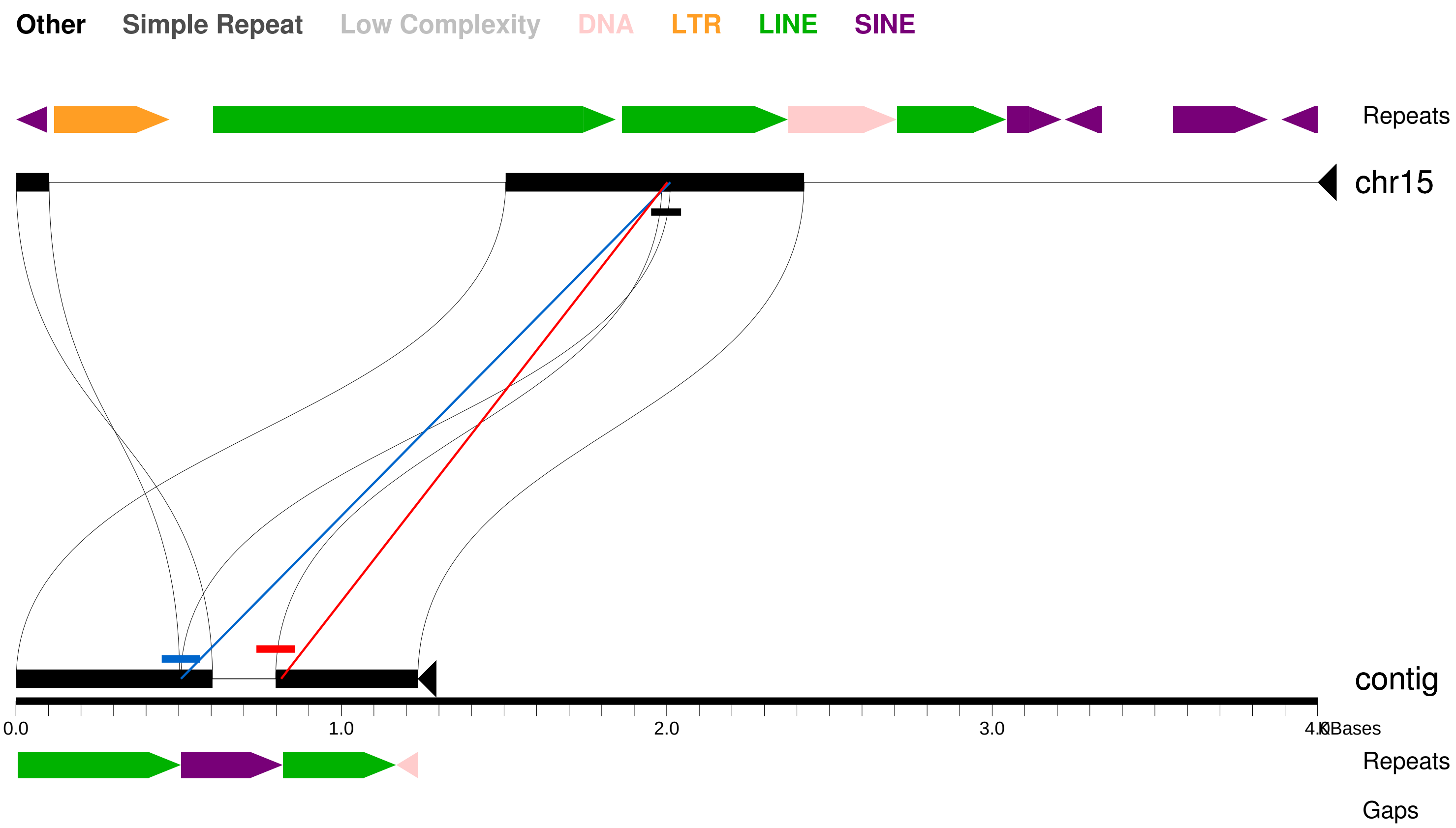

siteID: chr15_23214991 Scafftig5 - Breakpoint: chr15:23214969
chr15:23214923-23215028
Overlap Length: 14 INS size: 302 Genotyped: Yes

```
left     TA---TCA--AGTT---TCTCAATGTCAGTTCTAGAGGA-----AAAAAAATTCCTTATAAAAATTTAAC
chrom    TA---TCA--AGTT---TCTCAATGTCAGTTCTAGAGGA-----AAAAAAATTCCTTATAAAAATTTAAC
right    TGCAGTCCGCAGTCCGGCCTGGGCAACAGAGCGAGACTCCGTCTCAAAAAAAAAAAAAAAAAAAATTTAAC
         *1111**111***11111**111111***11*1***111111111******111111*1***********

left     CTCGGCCGGGCGCGGTGGCTCACGCCTGTAATCCCAGCACTTTGGGAGGCCGAGGCGGGTGG
chrom    CTCAT---------GTGTAACA-GTTTCCATTCCCATAGCAATGACATTT-GATATACAT--
right    CTCAT---------GTGTAACA-GTTTCCATTCCCATAGCAATGACATTT-GATATACAT--
         ***22222222222***222**2*22*22*2*****222*22**22*2222**222222*22
```

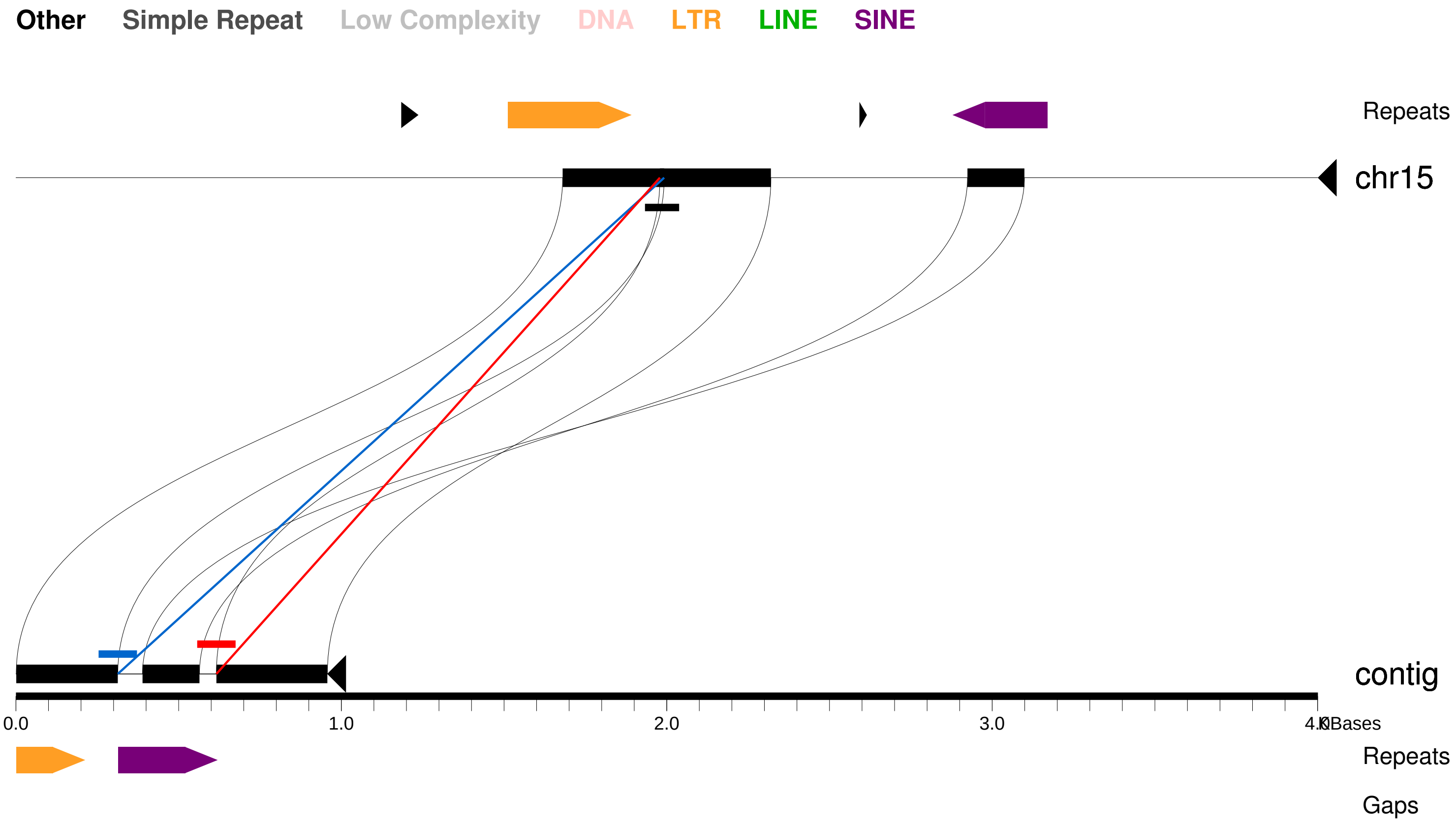

siteID: chr15_24591148 Scafftig2 - Breakpoint: chr15:24591229
chr15:24591069-24591396
Overlap Length: 8 INS size: 295 Genotyped: Yes

```
left    GGCATCCAGAGAGCGGTCCCCCCAGCAGTGTCATTCGGGTAGTAATGCG----GGAAGGCCCCGTCCGGT
chrom   C-CTTTCAGATTACT-TATGATGATATGTGAGCTTTTCATAG--ATGCCCTTTGAAATGAAGAAA---GT
right   ----TTTAG--TA---------GAGACGGGG---TTTCACCG----------TGTTAGCCAGGAT---GG
        N21N*21**1N221N21NNNNN2*N2N*1*NNN1*222211*221111N   2*N1*N1N22N2N222*1

left    TCGTGTTCAGTGCCTAATTTGCTGCATGTTTTTATAATACTGAATCTGGGAATTTTCAAGTGCTTGTTGC
chrom   TC-TCTTATCTGCCTAATTTGCTGCATGTTTTTATAATACTGAATCTGGGAATTTTCAAGTGCTTGTTGC
right   TC-TCG-ATCT-CCTGACCTCGTG---------ATCCGCCCGCCTC--GGCCTCCCAAAGTGCTGGGATT
        **2*211222*1***1*11*11**111111111**1111*1*11**11**11*1111*******1*1111

left    ACACCTTCTGAGATGATTGTGTAGTTTTTATTCTTTTTTTTTTT--TTTTTGAGACGGAGTCTCGC-TCT
chrom   ACACCTTCTGAGATGATTGTGTAGTTTTTATTCTTTATTAATATAGTGTATGACATTAATTGTATCATAT
right   ACAGGCG-TGAGCC-ACCGCGCCCGGCCTATTCTTTATTAATATAGTGTATGACATTAATTGTATCATAT
        ***11111****111*11*1*1111111********2**22*2*22*2*2***2*222*2*2*22*2*2*

left    GTCG--CCCAGGCTGGAGTGCAGTGGC-----------GCGAT-----------CTCG---------GCT
chrom   GTTGAACCAAACTTGCATTCCTGAGGTAAATACCTTTTGCCATAGTTTATAATACTTTCTACATGTTGCT
right   GTTGAACCAAACTTGCATTCCTGAGGTAAATACCTTTTGCCATAGTTTATAATACTTTCTACATGTTGCT
        **2*22**2*222**2*2*2*2*2**222222222222**2**22222222222**22222222222***

left    CACTGCAA--GCTTCCGCCTCCCGGGT--------TCACGCCATTCT-----CCTG
chrom   GATTTCATTTGATAATGTCTCCCTGGTGATTTTTGTCTCTGTATTTATAAGGCATA
right   GATTTCATTTGATAATGTCTCCCTGGTGATTTTTGTCTCTGTATTTAT--------
        2*2*2**222*2*222*2*****2***22222222**2*222***222    1N1N
```

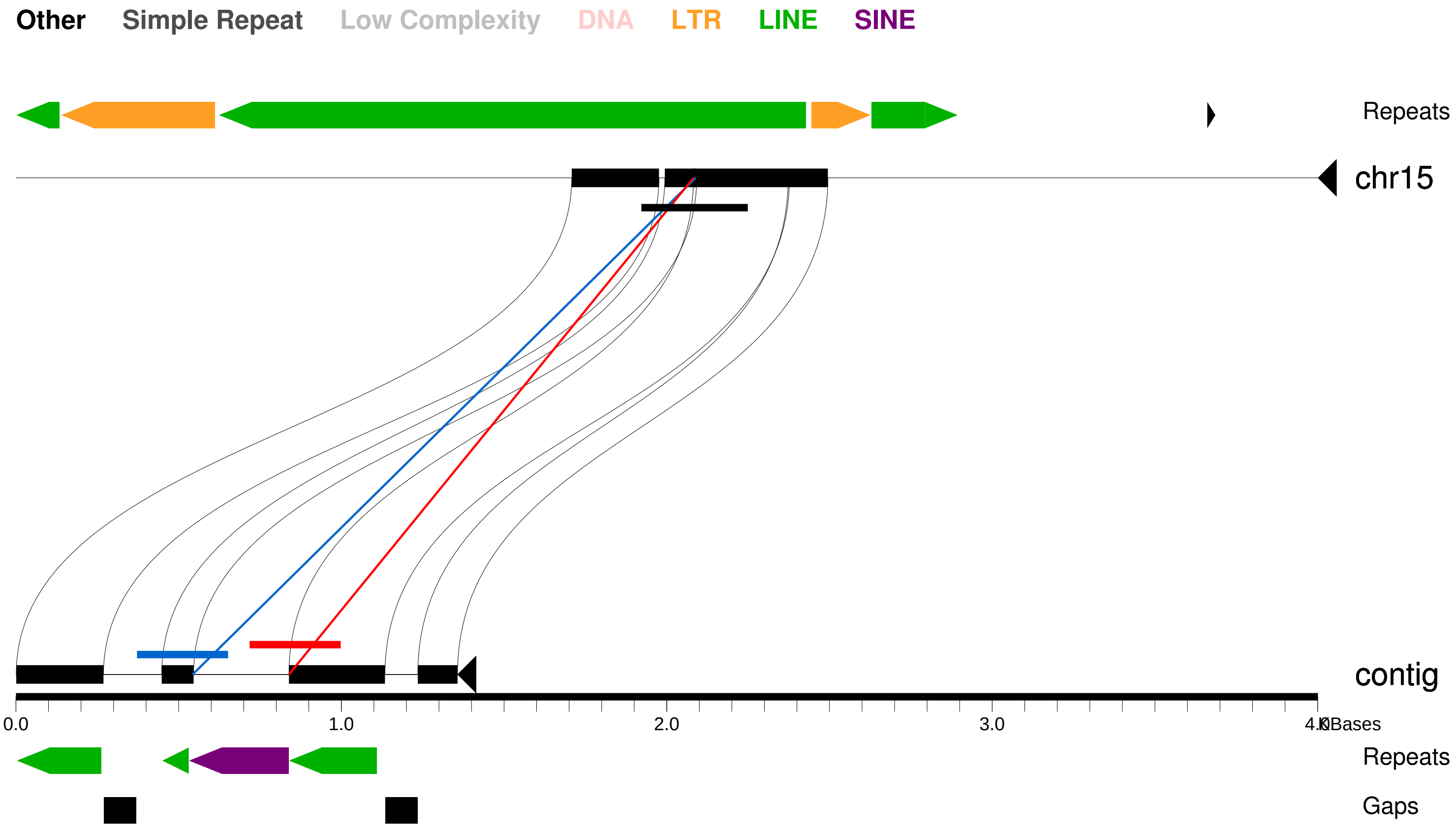

siteID: chr15_25735654 Scafftig15 - Breakpoint: chr15:25735654
chr15:25735610-25735713
Overlap Length: 16 INS size: 308 Genotyped: Yes

```
left    T----GACTGGCAGA---AGAAATAAT--------ATAATCCAATTCTATTCTGTCTATAAGAGATACAG
chrom   T----GACTGGCAGA---AGAAATAAT--------ATAATCCAATTCTATTCTGTCTATAAGAGATACAG
right   CTCCAGCCTGGGCGACAGAGTGAGACTCCGTCTCAAAAAAAAAAAAAAAAAAAAAAAAAAAGAGATACAG
        11111*1****11**111**11*1*1*11111111*1**111**1111*11111111*1***********

left    TTTAGGCTGGGCGCGGTGGCTCACGCCTGTAATCCCAGCACTTTGGGAGGCCGAGGTGGGC----GGA
chrom   TTTAGATT-------------------TGAAAACCAAATACATTAAAAGTAAAATGATGGAAAAAGGG
right   TTTAGATT-------------------TGAAAACCAAATACATTAAAAGTAAAATGATGGAAAAAGGG
        *****22*2222222222222222222**2**2**2*22**2**222**2222*2*22**22222**2
```

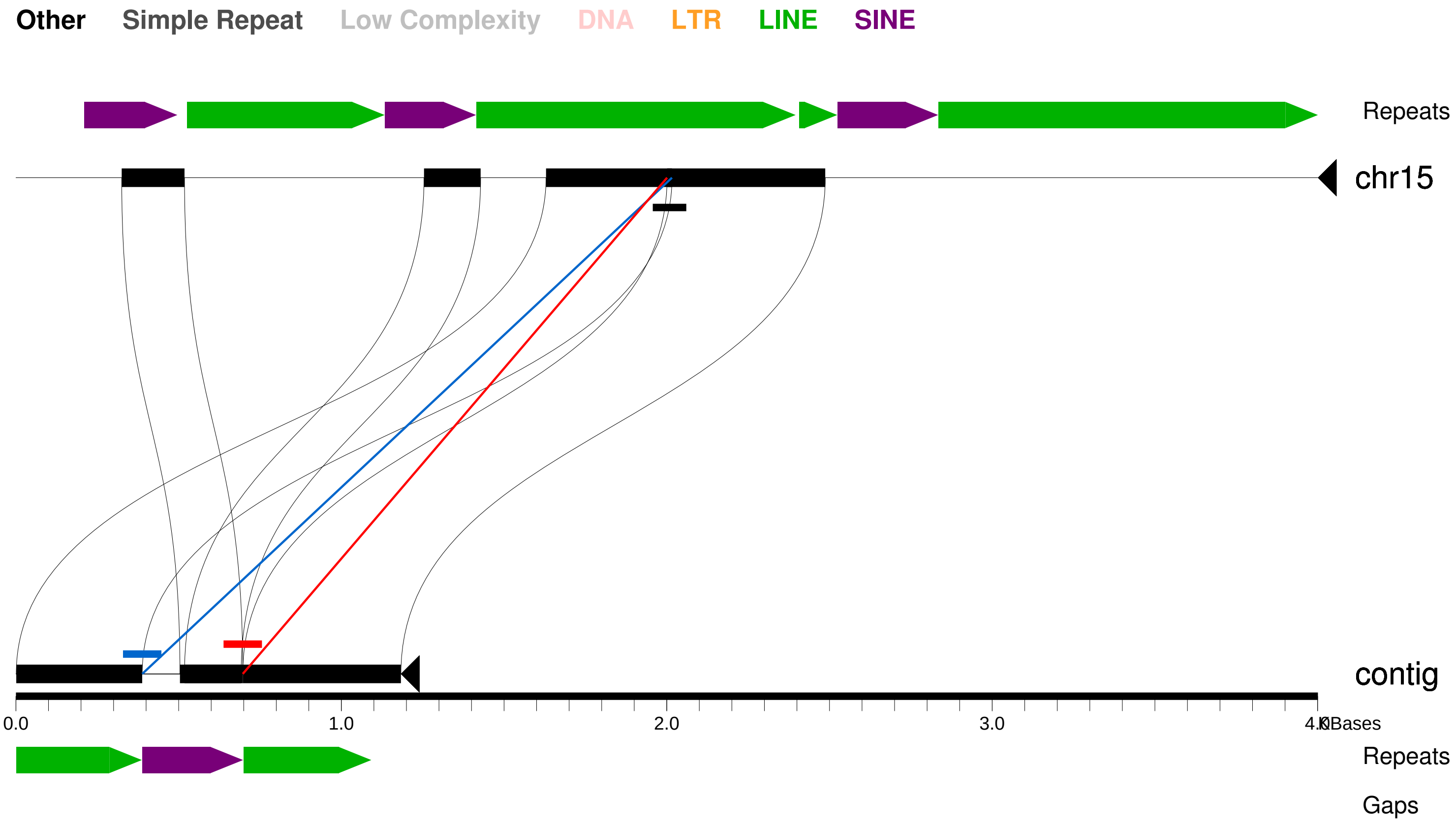

siteID: chr15_26141229 Scafftig2 + Breakpoint: chr15:26141233
chr15:26141205-26141292
Overlap Length: 15 INS size: 301 Genotyped: Yes

```
left    AAG-----------------ATGAATTGGAAACAAAT---TGTTCCAT-----------AGTAATCTAAT
chrom   AAG-----------------ATGAATTGGAAACAAAT---TGTTCCAT-----------AGTAATCTAAT
right   CCGCCCGCCTCGGCCTCCCAAAGTGCTGGGATTACAGGCGTGAGCCACCGCGCCCGGCCAGTAATCTAAT
        11*11111111111111111*1*111***1*11*1*1111**11***111111111111***********

left    TTTTTTTTTTTTTTTTTTTTTTTGAGACGGAGTCTCGCTCTGTCACCCAGGCTGGAGTGCAGTGGCGGGA
chrom   TTTTAATAATCTCATGTTTTT-------GCATT-----TGTGCCA-------------------GAAGGA
right   TTTTAATAATCTCATGTTTTT-------GCATT-----TGTGCCA-------------------GAAGGA
        ****22*22*2*22*2*****2222222*2*2*22222*2**2**2222222222222222222*22***

left    TCTCGGCTCA
chrom   AATAATCTCC
right   AATAATCTCC
        22*222***2
```

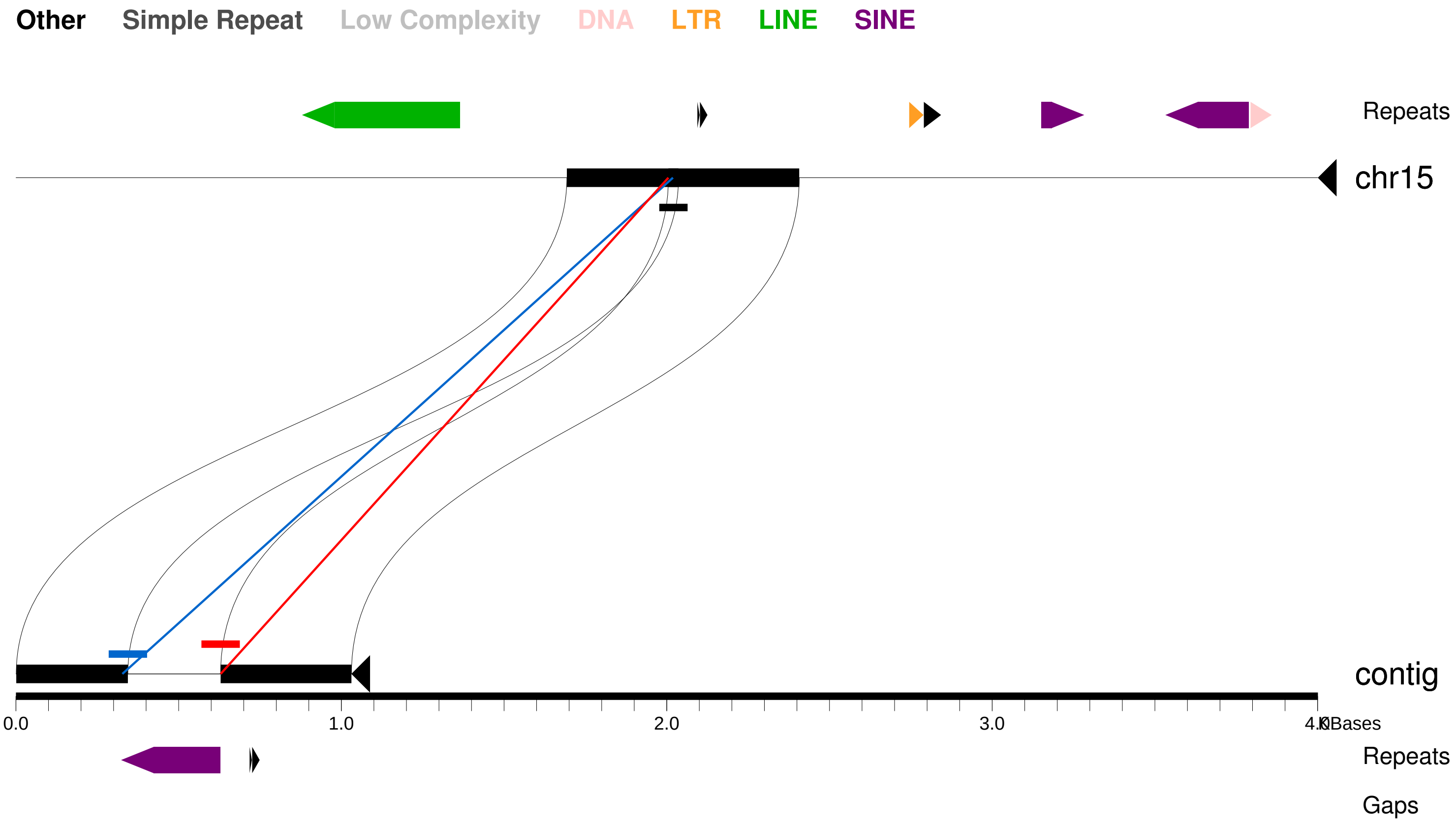

siteID: chr15_26872530 Scafftig5 + Breakpoint: chr15:26872513
chr15:26872355-26872683
Overlap Length: 11 INS size: 319 Genotyped: Yes

```
left   ----GAAAAAACATTCCTTTCAAAATGTTA--CTGCTCATTTTCAATGTACCTGGTCAC-TCAAGAGCTC
chrom  TC-AGAAAAAACATTCCTTTCAAAATGTTA--CTGCTCATTTTCAATGTACCTGGTCAC-TCAAGAGCTC
right  TTTAGTAGAGACGGGG-TTTCACCGTGTTAGCCAGGATGGTCTCGATCT-CCTGACCTCGTGATCCGCCC
       2N12*1*1*1**11111*****111*****11*1*11111*1**1**1*1****11*1*1*1*111**1*

left   TAATGGATATGTACAAAGAGATGAATGTTGTTTTCATGTCTGCTAACACAACATCCATTCTACAGCCCAT
chrom  TAATGGATATGTACAAAGAGATGAATGTTGTTTTCATGTCTGCTAACACAACATCCATTCTACAGCCCAT
right  GCCTCGGCCTCC-CAAAGTGCTGG-----GATTACAGGCGTG----------AGCCA-----CCGCGCC-
       111*1*111*111*****1*1**111111*1**1**1*11**1111111111*1***11111*1**1*11

left   AGATCAAGGAGAAACTGACTTTCAAGTTTTATTTATTTATTTATTTATTT--------------------
chrom  AGATCAAGGAGAAACTGACTTTCAAGTTTTATTATTTAAGAAATACATTTCACAAGGCTGTAGCTGCCAT
right  --------------CGGCC---CAAGTTTTATTATTTAAGAAATACATTTCACAAGGCTGTAGCTGCCAT
       11111111111111*1*1*111***********22**2*222**22****22222222222222222222

left   ----ATTTATTTTTTTTTTTTTTTTGAG--ACGGAGTCT-----CGCTCTGTC---GCCCAGG-------
chrom  AGACAGTGATTATTTTTACGGATCTGGGCAAAGTAAACTGAAAACATTCTGGAAAGGACTAGATGTCATT
right  AGACAGTGATTGTTTTTACGGATCTGGGCAAAGTAAACTGAAAACATTCTGGAAAGGACTAGATGTCATT
       2222*2*2***N*****22222*2**2*22*2*2*22**22222*22****22222*2*2**22222222

left   CTGGAGTGCAGTG-GCGCGATCTCGGCTCACTGCA--AGCTCCGCCTCCCGG-
chrom  CTAGATGTCAGTAAGAACATTCATGATTTATAGGAGGAGGTCAAAATATCAAC
right  CTAGATGTCAGTAAGAACATTCATGATTTATAGGAGGAGGTC-----------
       **2**222****22*22*22**22*22*2*22*2*22**2**NNNN1NN1NN
```

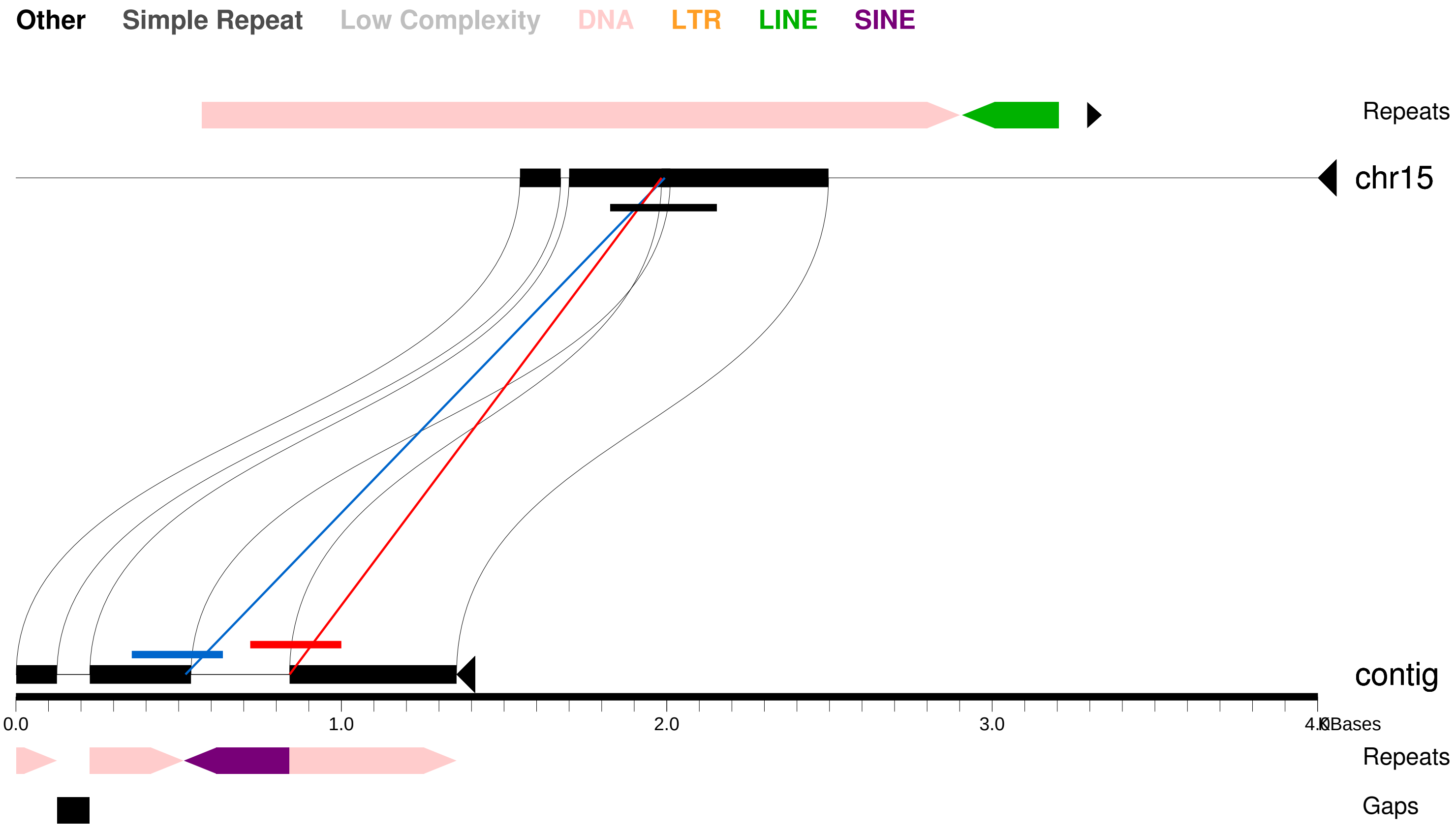

siteID: chr15_27765800 Scafftig7 + Breakpoint: chr15:27765781
chr15:27765735-27765840
Overlap Length: 11 INS size: 316 Genotyped: Yes

```
left    TGAGCACACA--------CTAAAACTCAGTCTTTTCATGAATTTTTCTCTG-----GTTCCCAAAATCTTT
chrom   TGAGCACACA--------CTAAAACTCAGTCTTTTCATGAATTTTTCTCTG-----GTTCCCAAAATCTTT
right   TCCACCCGCCTCGGCCTCCCAAAGTGCTGGGATTACAGGCGTGAGCCACCGCGCCCGGCCCCAAAATCTT
        *111*1*1*111111111*1***111*1*111**1**1*11*1111*1*1*11111*11************

left    TTTTTTTTTTTTTTTTTTTTTTTTTTTGAGACGGAGTCTCGCTCTGTCGCCCAGGCCGGACT
chrom   CTTAATAAAACACTAACTTTCAGTAGT---------TTTAGAACTGT-GCAACGT---GTAT
right   CTTAATAAAACACTAACTTTCAGTAGT---------TTTAGAACTGT-GCAACAT---GTAT
        2**22*2222222*222***222*22*222222222*2*2*22****2**22212222*22*
```

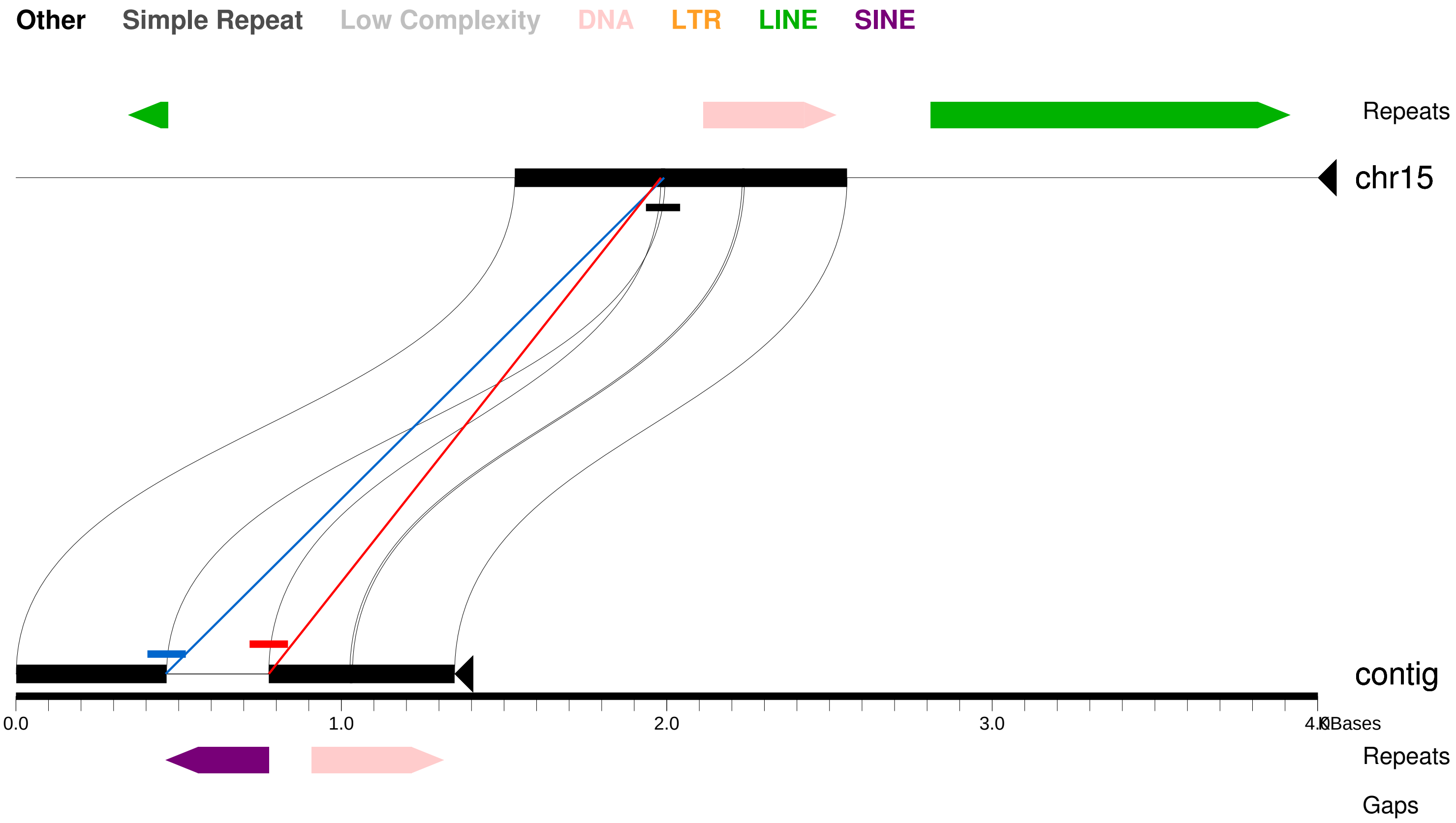

siteID: chr15_28174925 Scafftig8 + Breakpoint: chr15:28174961
chr15:28174918-28175019
Overlap Length: 17 INS size: 106 Genotyped: Yes

```
left   ATAAATTTTTAATTCTCAAATTCAAC---AAGTAAACA---------------ACACAATTCAAATGGGA
chrom  ATAAATTTTTAATTCTCAAATTCAAC---AAGTAAACA---------------ACACAATTCAAATGGGA
right  CTGGAGTGCAGTGGCGCGATCTCGGCTCACTGCAAGCTCCGCCTCCCGGGTTCACGCCAT-CAAATGGGA
       1*11*1*1111111*1*1*11**11*11111*1**1*1111111111111111**1*1**1*********

left   AAAATTTTTTTTTTTTTTTTTTTTTTTTTGAGACAGAGTCTCGCTCT--GTCGCCC-AGGCTGGAGTGCAGTG
chrom  AATATTTT-------------------AAACAGACACTTCAACAAAGAAGACATAAGATTGACA--AATG
right  AATATTTT-------------------AAACAGACACTTCAACAAAGAAGACATAAGATTGACA--AATG
       **2*****2222222222222222222*2*****22**2222*222*22*2*22*2*2*2**2222*2**

left   -
chrom  A
right  A
       2
```

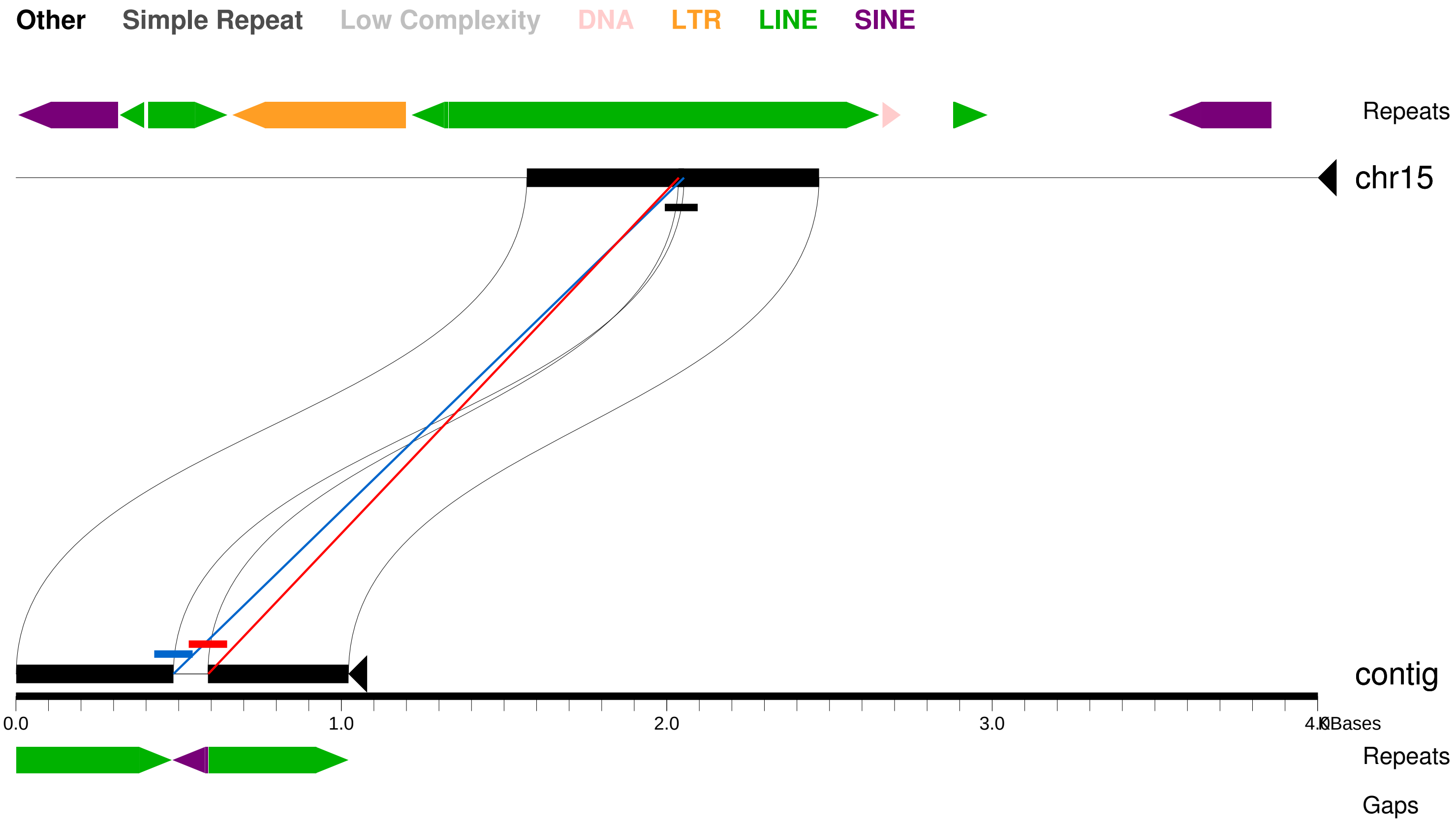

siteID: chr15_34207226 Scafftig3 + Breakpoint: chr15:34207228
chr15:34207184-34207287
Overlap Length: 16 INS size: 383 Genotyped: No

```
left    GGAAACAGCAGGGA---------------CAAGATGCATACCAGAAAGAAAGTACCCTCAAAAAATGATG
chrom   GGAAACAGCAGGGA---------------CAAGATGCATACCAGAAAGAAAGTACCCTCAAAAAATGATG
right   NNNNNNNNNNNNNNNNNNNNNNNNNNNNNNNNNNNNNAAAAAAAAAAAAAAAAAAAAAAAAAAAAATGATG
        1111111111111111111111111111111111111*1*11*1***1***11*11111***********

left    GGGAAGGCCGGGCGCGGTGGCTCACGCCTGTAATCCCAGCACTTTGGGAGGCCGAGGCGGGCGG
chrom   GGGAACA----------TG--TCACAAATATACAGC-AGTAGTTAAAAAGGGCT--TCCAACTG
right   GGGAACA----------TG--TCACAAATATACAGC-AGTAGTTAAAAAGGGCT--TCCAACTG
        *****222222222222**22****222*2**222*2**2*2**2222***2*2222*222*2*
```

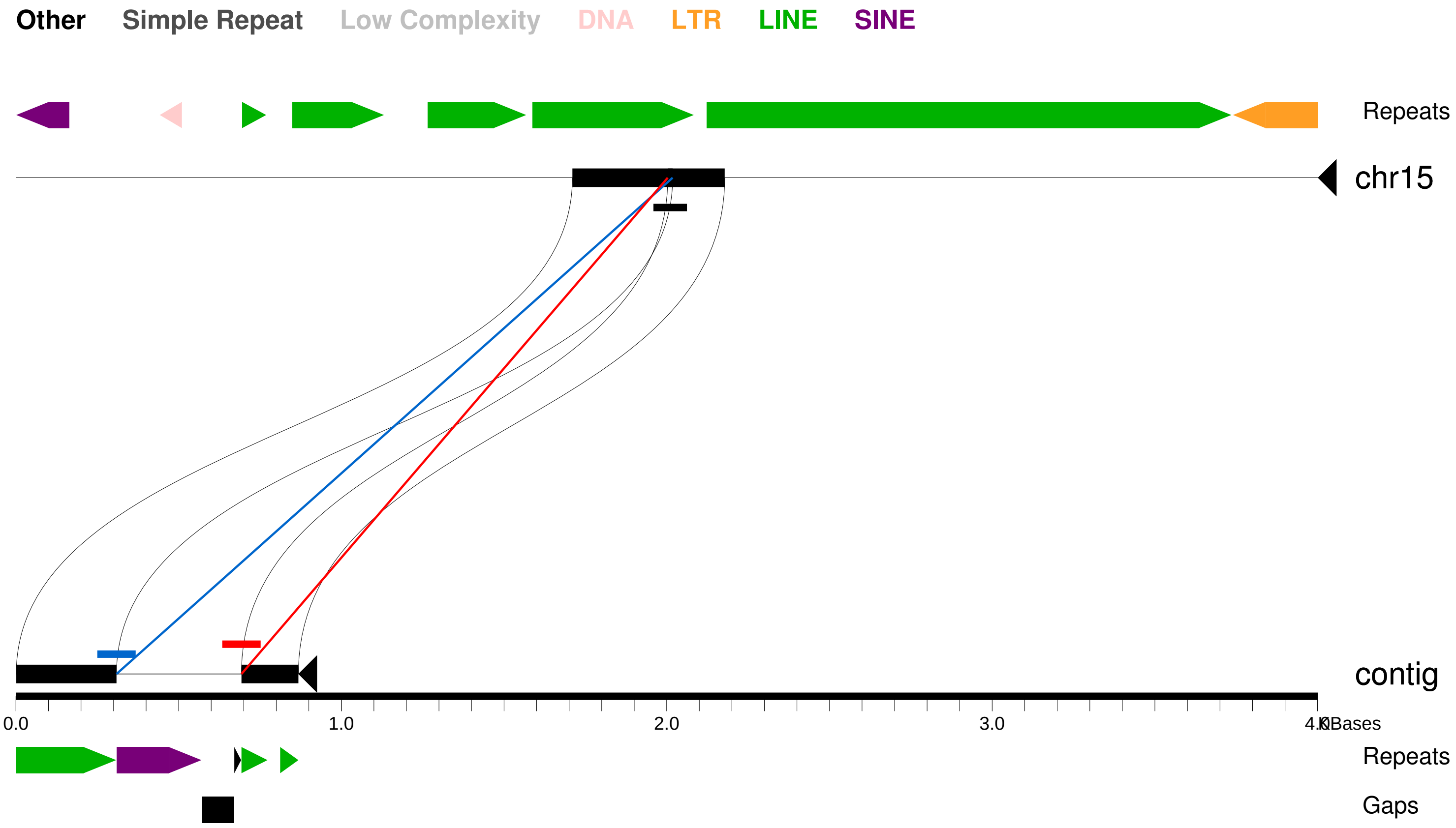

siteID: chr15_35473608 Scafftig1 + Breakpoint: chr15:35473615
chr15:35473574-35473674
Overlap Length: 19 INS size: 286 Genotyped: Yes

```
left    ------------CAACATATGTAAACGCAGGCACTAAATATAGCAGCCATCCT------CCCTGTGTTCA
chrom   ------------CAACATATGTAAACGCAGGCACTAAATATAGCAGCCATCCT------CCCTGTGTTCA
right   ATCCGCCCGCCTCGGCCTCCCAAAGTGCTGGGATTACAGGCGTGAGCCACCGCGCCCGGCCCTGTGTTCA
        111111111111*11*1*1111**11**1**1*1**1*111111*****1*11111111***********

left    CTATTCTTTTTTTTGAGACGGAGTCTCGCTCTGTCGCCCAGGCTGG-AGTGCAGTGGCGGGATCTCGG
chrom   CTATTCTTGCCCT-----CAGATTCTCT---TGTCAGTTATAAAAA-AAAAC----------TCTGCT
right   CTATTCTTGCCCT-----CAGATTCTCT---TGTCAGTTATAAAAACAAAAC----------TCTGC-
        ********2222*22222*2**2****2222****2222*2222221*222*2222222222***22N
```

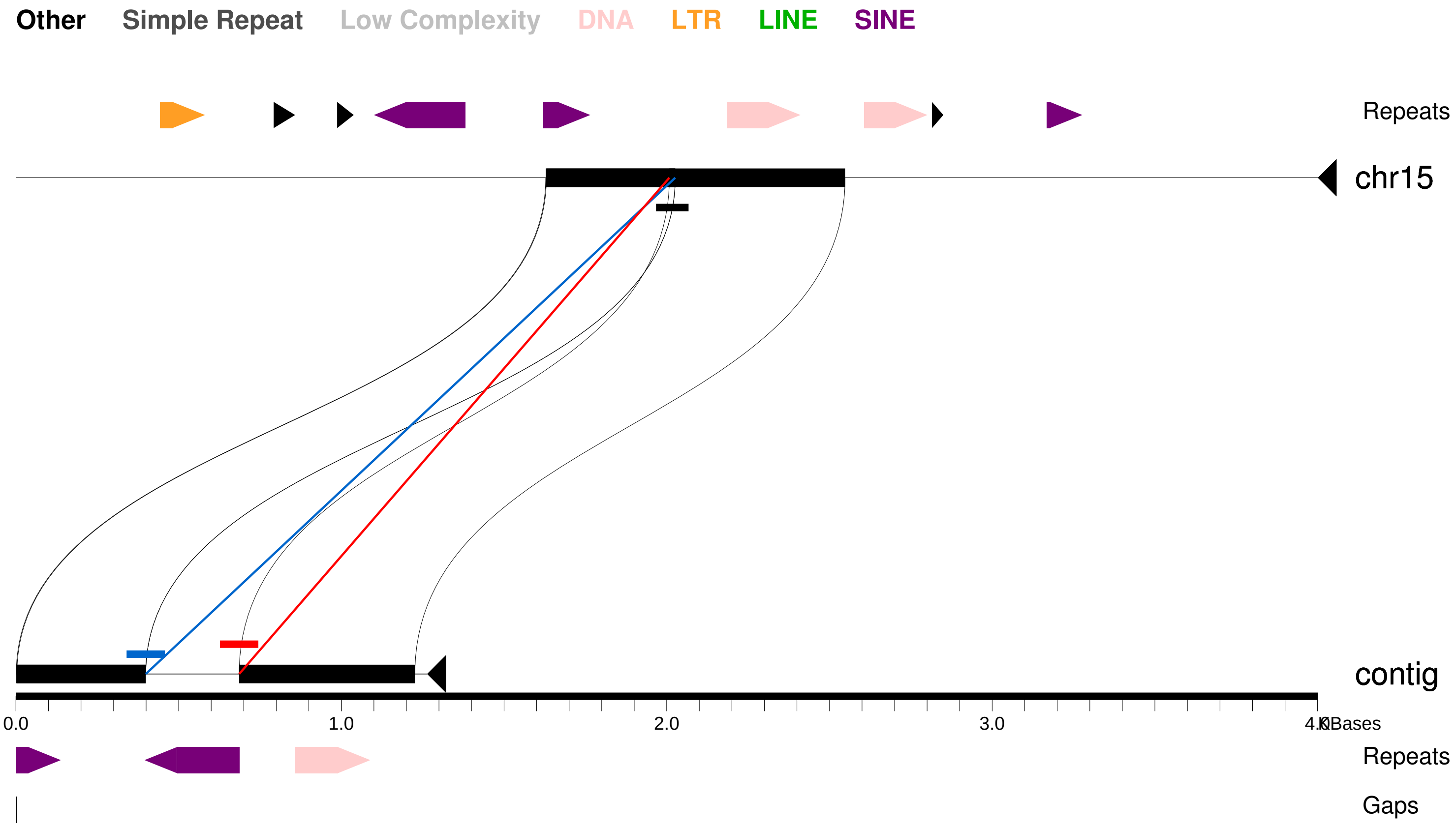

siteID: chr15_36096323 Scafftig4 + Breakpoint: chr15:36096309
chr15:36096265-36096368
Overlap Length: 16 INS size: 294 Genotyped: Yes

```
left    -TAATCTCTTTTCTGTCTCAAAA-----TGTGATTTCTGTATTTATAT---------GTCAGTTTCAGTA
chrom   -TAATCTCTTTTCTGTCTCAAAA-----TGTGATTTCTGTATTTATAT---------GTCAGTTTCAGTA
right   ATCCGCCCGCCTCGGCCTCCCAAAGTGCTGGGATTACAGGCGTGAGCCACCGCGCCCGGCAGTTTCAGTA
        1*111*1*111**1*1***11**11111**1****1*1*111*1*111111111111*1***********

left    TTTTTTTTTTTTTTTTTTTTGAGACGGAGTCTCGCTCTGTCGCCCAGGCTGGAGTGCAGTGGCG
chrom   TTTTTAATGAACATTT---CAAAACT---TTTCAATTTAAAA---AGGC---AACAAAATG---
right   TTTTTAATGAACATTT---CAAAACT---TTTCAATTTAAAA---AGGC---AACAAAATG---
        *****22*22222***22222*2**2222*2**22*2*2222222****222*2222*2**222
```

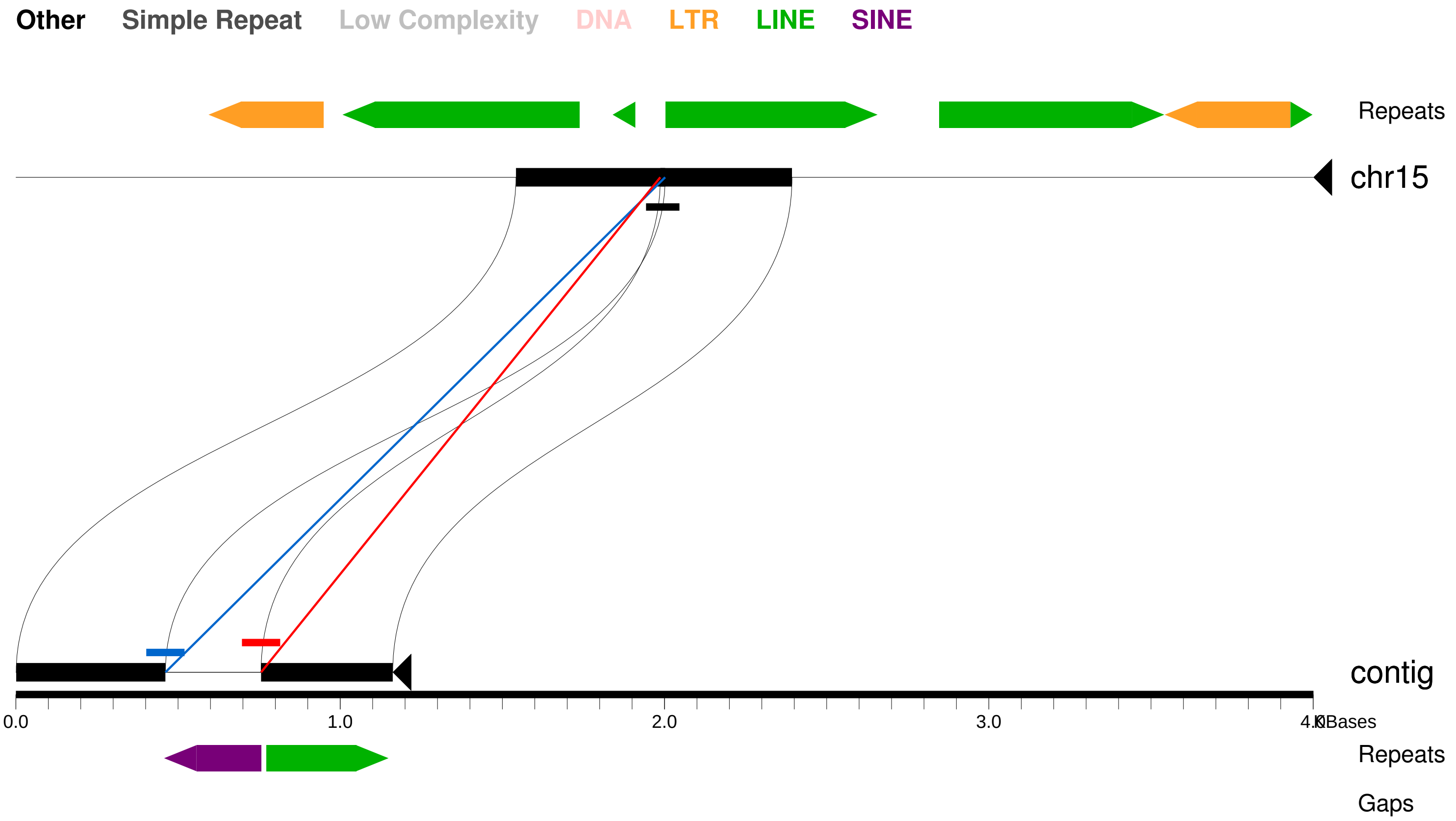

siteID: chr15_36640196 Scafftig4 - Breakpoint: chr15:36640171
chr15:36640126-36640230
Overlap Length: 15 INS size: 226 Genotyped: Yes

```
left    AGTT-ATAGATGCCTAG---AAAGATGCAGGTTTTTT----------AAACCAATTCTTAAAAATCAGCC
chrom   AGTT-ATAGATGCCTAG---AAAGATGCAGGTTTTTT----------AAACCAATTCTTAAAAATCAGCC
right   ACTGCACTCCAGCCTGGGCGACAGAGCGAGACTCCGTCTCAAAAAAAAAAAAAAAAAAAAAAAAACAGCC
        *1*11*11111****1*111*1***111**11*111*1111111111***11**11111*****1*****

left    TCTTGA--TC-GAGACCATCCCGGCTAAAACGGTGAAACCCCGTCTCTACTAAAAATACAAAAAAT
chrom   TCTTTACTTCAGAGTTTTTTCCTACACATACAT---------GTTTCTC--------ACTATGAAT
right   TCTTTACTTCAGAGTTTTTTCCTACACATACAT---------GTTTCTC--------ACTATGAAT
        ****2*22**2***2222*2**22*22*2**22222222222**2***222222222**2*22***
```

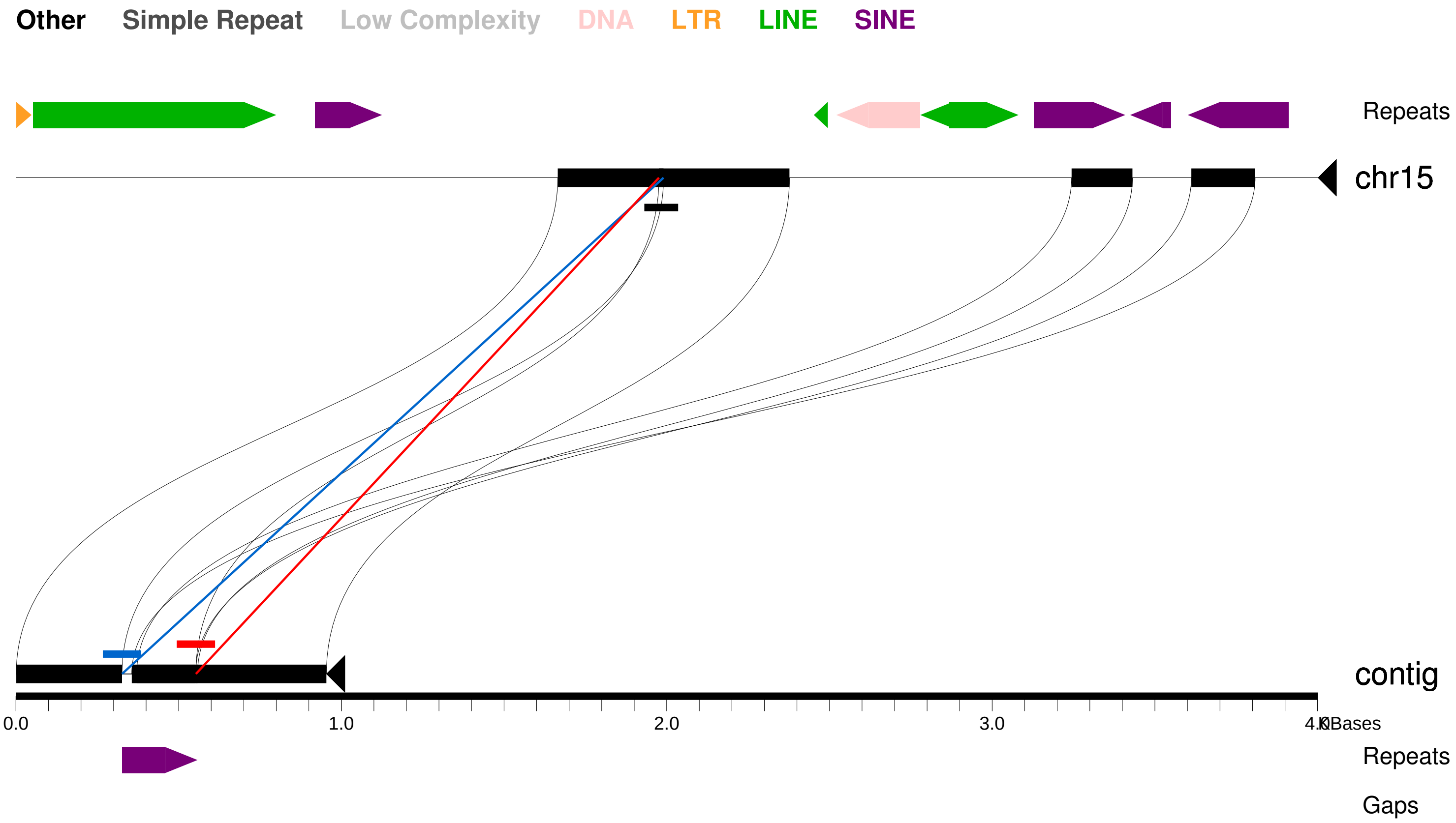

siteID: chr15_36672695 Scafftig2 - Breakpoint: chr15:36672679
chr15:36672640-36672738
Overlap Length: 18 INS size: 290 Genotyped: Yes

```
left   --------------------ATTGTGATGTCATTATAATATATTTGAAATTATTTTTATTAAATTTTTTC
chrom  --------------------ATTGTGATGTCATTATAATATATTTGAAATTATTTTTATCAAATTTTTTC
right  TCCGCCCGCCTCGGCCTCCCAAAGTGCTGGGATTACAGGCGTGAGCCACCGCGCCCGGCCAAATTTTTTC
       11111111111111111111*11***1**11****1*1111111111*111111111112**********

left   TATTTTTTTTTTTTTTTCAGACGGAGTCTCGCTCTGTCGCCCAGGCTGGAGTGCAGTGGCGGGATCTCGG
chrom  TATTTTTATTACAATTCTCCCTGCGAGT-----TTTTGA----GCTATATTTCA-----------TATT
right  TATTTTTATTACAATTCTCCCTGCGAGT-----TTTTGA----GCTATATTTCA-----------TATT
       *******2**2222***222*2*2*22*22222*2*2*22222***22*2*2**22222222222*222
```

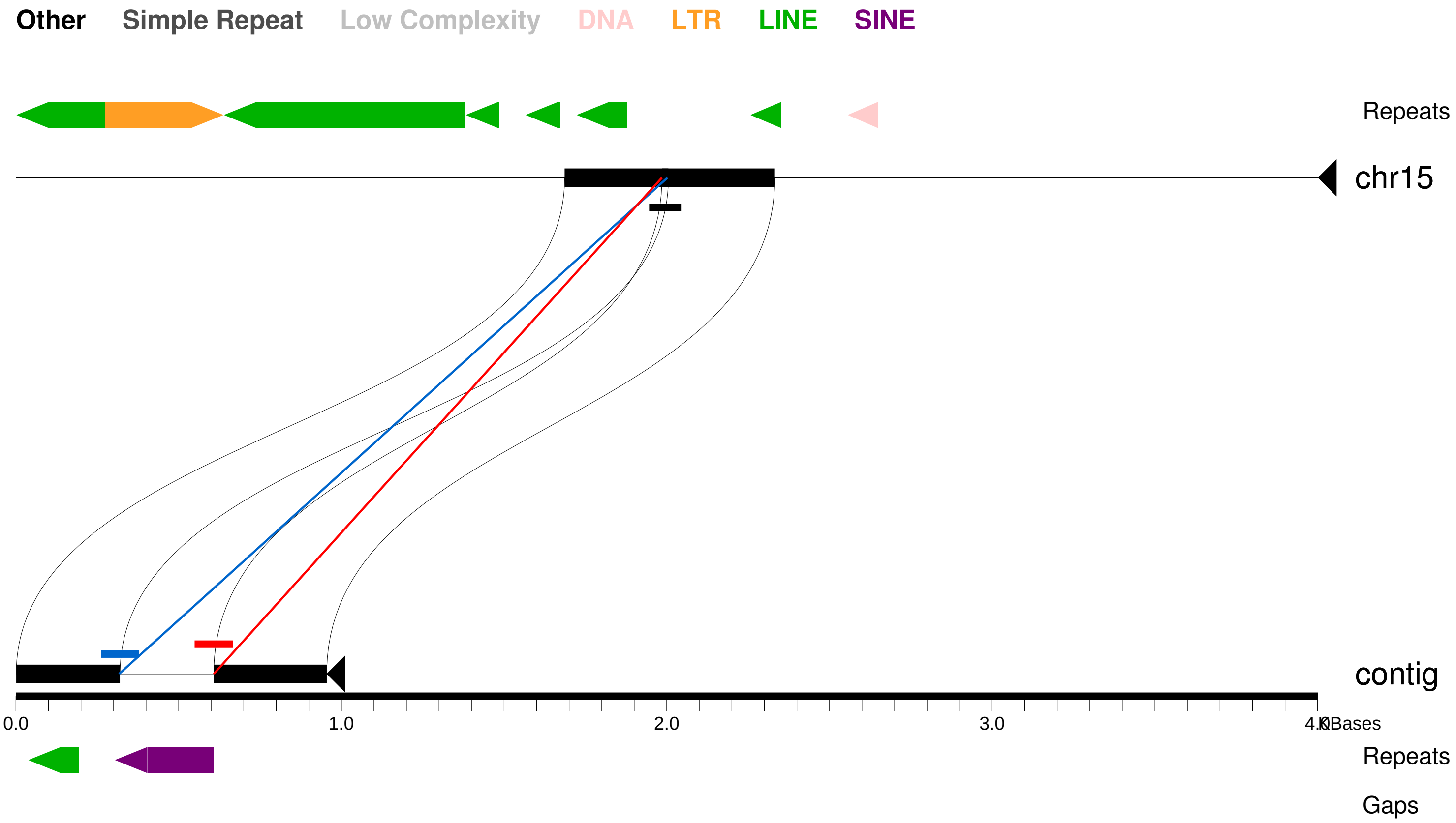

siteID: chr15_39691605 Scafftig4 - Breakpoint: chr15:39691591
chr15:39691546-39691650
Overlap Length: 15 INS size: 292 Genotyped: Yes

```
left   TCACTCCCTCAGGACCCTCCTCCCAAG--------------TGGAAACTAACTTATTTTTAAATGTCTGT
chrom  TCACTCCCTCAGGACCCTCCTCCCAAG--------------TGGAAACTAACTTATTTTTAAATGTCTGT
right  TCTCAAAAAAAAAAAAAAAAATAAAAATAAAAATAAAAAAAATAAAAAAAAAAAAAAAAATAAATGTCTGT
       **1*111111*11*111111*111**111111111111111*11***11**111*1111***********

left   GTTCG--GCCGAGGCGGCGGATCACGAGGTCAGGAGATCGAGACCATCCTGGCTAACACGGTGA
chrom  GTTCCCTGTCAATGCAGGCAGA---CATTTTAAGGAGA-------CTT--TAG---ACACTATG-
right  GTTCCCTGTCAATGCAGGCAGA---CATTTTAAGGAGA-------CTT--TAG---ACACTATG-
       *****222*2*2*2**2***2**222*2222*2******2222222*2*22*2*222****22**2
```

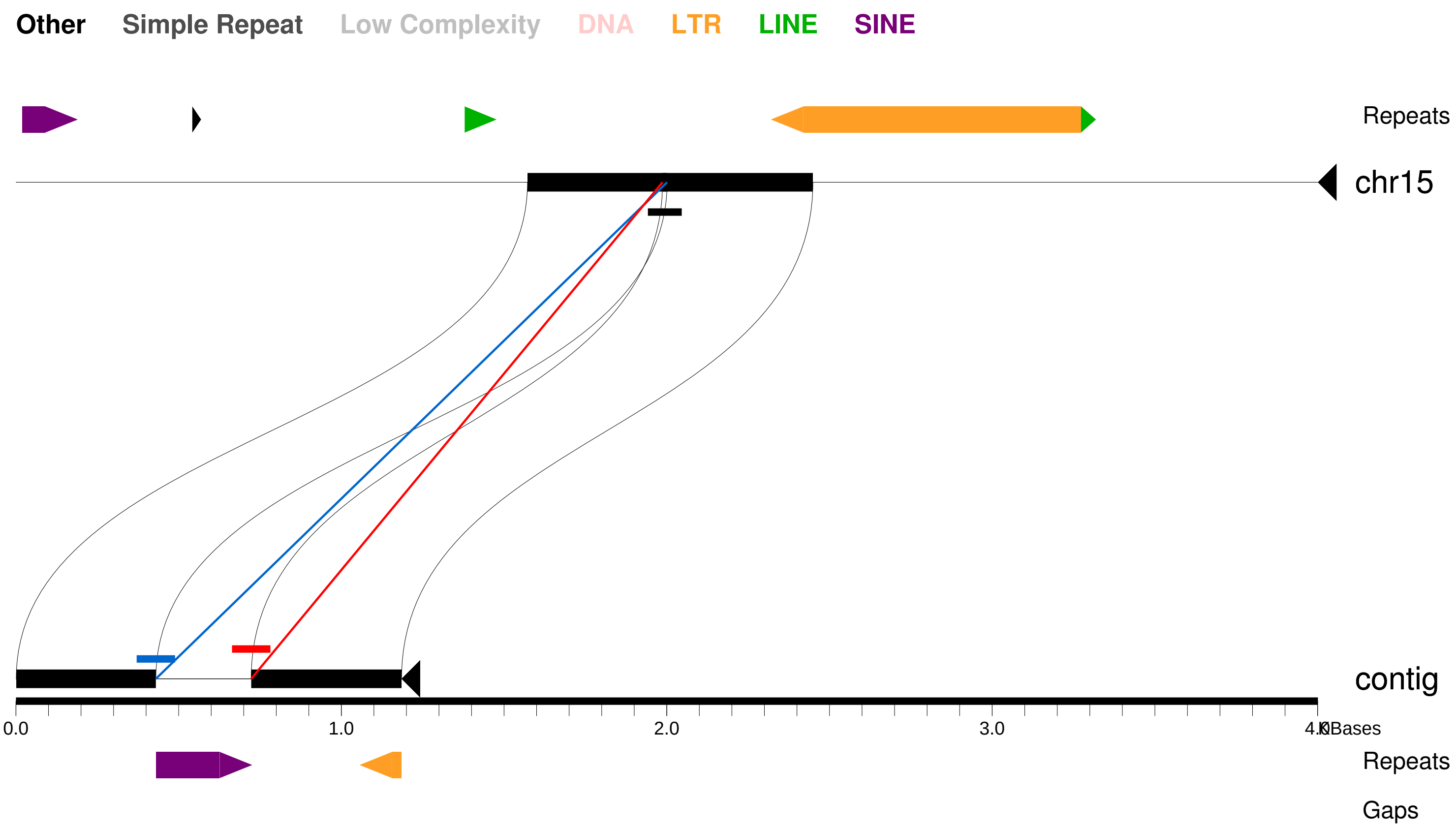

siteID: chr15_41039888 Scafftig1 + Breakpoint: chr15:41039811
chr15:41039262-41039970
Overlap Length: 75 INS size: 388 Genotyped: No

```
left   AA--------------------------------------------------------------------
chrom  AAAATGGGTAGATGGTTTATCTGGAACGGGAAGCTGGAATTGCATCATCTCCCTTTGCTAGAAGGAGGGG
right  NNNNNNNNNNNA----------------------------------------------------------
       11NNNNNNNNN2

left   ----------------------------------------------------------------------
chrom  GAGACCCTATTTCTTGCTGAGTTGAGGGCTGCCTCAGCCAATCAGGAGTCCAAAGAGGAGTCTCTCTGCC
right  ----------------------------------------------------------------------

left   ----------------------------------------------------------------------
chrom  TCCAGCACTCCCAGGGAGGGACCTAGCCCTGCTGGAGCCCAGGCATCAGCACAGAATCCCCTGGGACCTG
right  ----------------------------------------------------------------------

left   ----------------------------------------------------------------------
chrom  AGCTGCGATGCTGCCATCCCTCTGGATCCTAAAGGCCCAGCTCACAAAGCCAAGAAGAGAGGTCAGGGAG
right  ----------------------------------------------------------------------

left   ----------------------------------------------------------------------
chrom  CCTTGCTGCCACAAACAGAGTGAAAGATGGTAGAGCCCTGTGAGGCAGGGAGGCTCCAGGGAAGCTGGCA
right  ----------------------------------------------------------------------

left   ----------------------------------------------------------------------
chrom  TAGAAAGTAGACTTTAATTGCATCTCAGCCCCACTTTAAGGAACCCTCAACAATCTGAAACCCTACTGCC
right  ----------------------------------------------------------------------

left   --------------------------------------------GGGCTACCACATTTACACCATTCTCT
chrom  TGAGGCAGCTTCTGGGGCCTCAGAAGAGATTAATCACATGCCAAGGGCTACCACATTTACACCATTCTCT
right  ----------------------------------------------------------------------
                                                   11111111111111111111111111

left   CTCACTTTCCACCTCCCAGACTAACCCAGCCAGCCTTTGGAGAAATCTACCAGAGAGGAGTGACCCTTTT
chrom  CTCACTTTCCACCTCCCAGACTAACCCAGCCAGCCTTTGGAGAAATCTACCAGAGAGGAGTGACCCTTTT
right  -----------------------------------------------------------GTGACCCTTTT
       111111111111111111111111111111111111111111111111111111111111***********

left   AACATACCCTGAGGCTTTTGAGATCTGCCAGAGACGAGCAGGGACCTTTGAAAAGGCCCTTTGGCGCGGT
chrom  AACATACCCTGAGGCTTTTGAGATCTGCCAGAGACGAGCAGGGACCTTTGAAAAGGCCCTTTGGTCCTGT
right  AACATACCCTGAGGCTTTTGAGATCTGCCAGAGACGAGCAGGGACCTTTGAAAAGGCCCTTTGGTCCTGT
       ***************************************************************22*2**

left   GGCTCAGGCCTGTAATCCCAGCACTTTGGGAGGCCGAGGCGGGCGGATCACGAGGTCAGGAGATCGAGAC
chrom  G---CACTCC------------------GGAGGAC-AGGAGTTCCCA-CAGTGGGCCAG-AGACCCAAGG
right  G---CACTCC------------------GGAGGAC-AGGAGTTCCCA-CAGTGGGCCAG-AGACCCAAGG
       *222***2**222222222222222222*****2*2***2*22*22*2**222**2***2***2*2*222

left   CATCCTGGCTAACACGGTGAAACCCCGTCTCTACTAAAAATAC
chrom  CTCCCT--CTGA---GCAGAGACTGCCACTCTACTCTT-----
right  CTCCCT--C----------------------------------
       *22***22*1N12221NN11N11NN1NN111111NNN22222
```

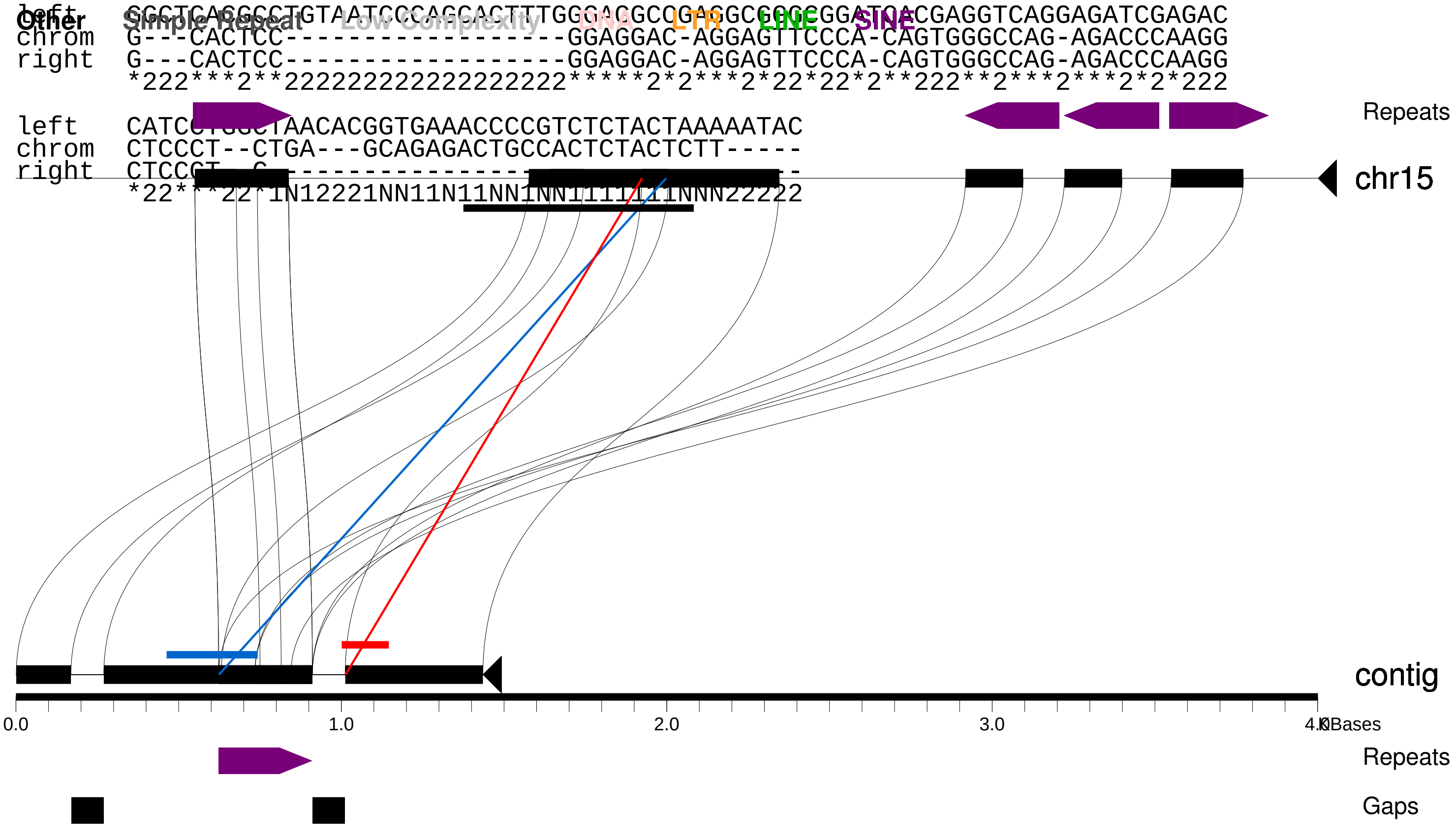

siteID: chr15_41100317 Scafftig1 + Breakpoint: chr15:41100319
chr15:41100159-41100491
Overlap Length: 13 INS size: 305 Genotyped: Yes

```
left   TTT----------------GATGAAGTCATTGATCCTCAGTTTCCATTTCTGTAAAATGGGGCTAACATT
chrom  TTTCCAGCTGTGTGGTTTTGATGAAGTCATTGATCCTCAGTTTCCATTTCTGTAAAATGGGGCTAACATT
right  TTTAGTAGAGACGGGGTTTCACCGTGTTAGCCAGGATG-GTCTCGATCTC------------CTGAC---
       ***NNNNNN2NNN22N2221*1111**1*111*111*11**1**1**1**111111111111**1**111

left   CTCTCGCTTTCAGGGTTTATGTGAGGGTCCACTGAAGTGCTTTAGGTGAGAGTTCCCTATAAACTGTAAA
chrom  CTCTCGCTTTCAGGGTTTATGTGAGGGTCCACTGAAGTGCTTTAGGTGAGAGTTCCCTATAAACTGTAAA
right  --CTCGTGATCCGCCCGTCTC----GGCCTCCCAAAGTGCTGG-GATTACAGG-CGTGAGCCAC------
       11****111**1*1111*1*11111**1*11*11*******111*1*1*1**11*111*111**111111

left   GCGCGCTGCCGTCGTTTTTGCAAAGCTACTCTTTAATTTTTTTTTTTTTTTTTTTTTTTGAGACGGAGTT
chrom  GCGCGCTGCCGTCGTTTTTGCAAAGCTACTCTT----TTCTGTCGGCCCAGTGTAGCATCTGGCGG--TT
right  -CGCGC--CCGGC-------CAAAGCTACTCTT----TTCTGTCGGCCCAGTGTAGCATCTGGCGG--TT
       1*****11***1*1111111*************2222**2*2*22222222*2*2222*22*2***22**

left   TCGCTCTGTCGCCCAGGCT-----GGAGTGCAGTGGCG-------CAATCTCG--ACTCACT-------G
chrom  TTCTTCTCTCCACCCTGCTCCTCCGAAGCGCGGTGTCCATCACACCAAGCCCGTTACTCCCTCTGCTCTG
right  TTCTTCTCTCCACCCTGCTCCTCCGAAGCGCGGTGTCCATCACACCAAGCCCGTTACTCCCTCTGCTCTG
       *222***2**22**22***22222*2**2**2***2*22222222***2*2**22****2**2222222*

left   CAAGCTCCGCCTCCCGGGTTCATGCCATTCTCCTG--------------------CCT
chrom  AAATCTTCCCCATCCCGCAGCCTGCCCTTCTCCAGGTCGCTGGAGTTAGATTACCACAC
right  AAATCTTCCCCATCCCGCAGCCTGCCCTTCTCCAGGTCGCTGGAGTT------------
       2**2**2*2**22**2*222*2****2******2*222222222222         1NN
```

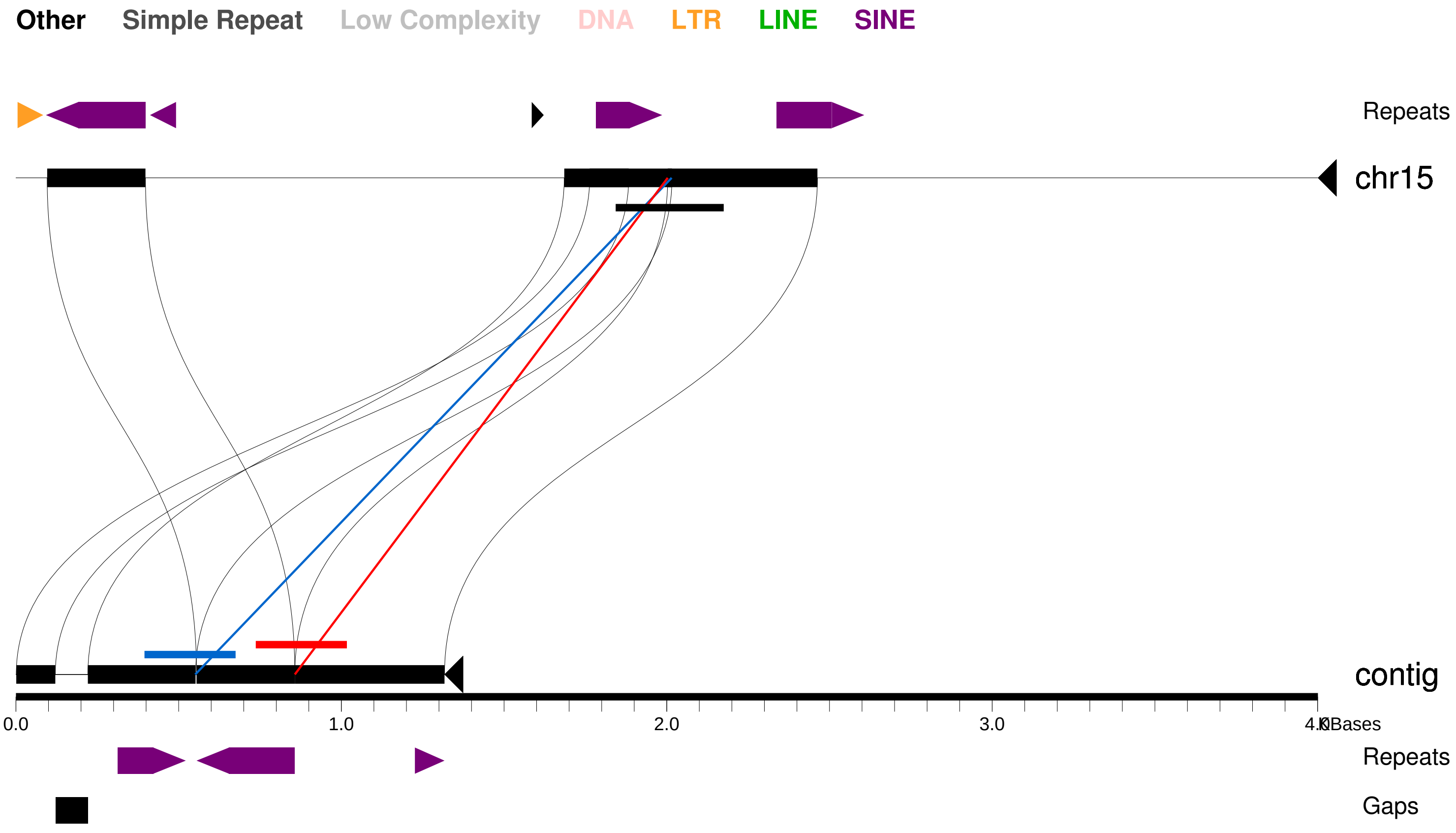

siteID: chr15_48318839 Scafftig1 - Breakpoint: chr15:48318845
chr15:48318731-48319053
Overlap Length: 96 INS size: 369 Genotyped: No

```
left   ----------------------------------------------------------------------
chrom  AATGAAGTCCAAACCACCCCAAGCTCATAGAGATTTGGCATATTCCTAGATATCCCCAAGGAAGATGATA
right  TTTTA-GTAGAGAC---------------GGGGTTT--CA----CCTTGTTAGCC------AGGATGGTC
       NN2N2 22NN2N22               2N2N222  22    222N2N22N22      2N2222N2N

left   -------------------------------TTAAATACACCATAGTATCATGGTCTTTCTGATACATGA
chrom  T-GATCATTCCACAACCCTTGGTTCCAGACTTTAAATACACCATAGTATCATGGTCTTTCTGATACATGA
right  TCGATC-----------------TCCTGACCTCAA----------GTATCATGGTCTTTCTGATACATGA
       212222                 222N222N*1**1111111111*************************

left   TCAATATCTACAACCCGATGAAACATACACACACACTTTTTCTCTTGTCTTCTTGCAAAAAGAAGAACAG
chrom  TCAATATCTACAACCCGATGAAACATACACACACACTTTTTCTCTTGTCTTCTTGCAAAAAGAAGAACAG
right  TCAATATCTACAACCCGATGAAACATACACACACACTTTTTCTCTTGTCTTCTTGCAAAAAGAAGAACAG
       **********************************************************************

left   GNNNNNNNNNNNNNNNNNNNNNNNNNNNNNNNNNNNNNNNNNNNNNNNNNNNNNNNNNNN----------
chrom  GACCACGCAGGTTATGAAACTAATGAGAGTTTCTCCCATTCCCAGGATCCCCATTTTTAATCTGAGCATC
right  GACCACGCAGGTTA--------------------------------------------------------
       *2222222222222NNNNNNNNNNNNNNNNNNNNNNNNNNNNNNNNNNNNNNNNNNNNNN

left   --------------------------------------------
chrom  CCATGAACAGACGGGATCCTCTACATACCAGTATGTGACACCCA
right  --------------------------------------------
```

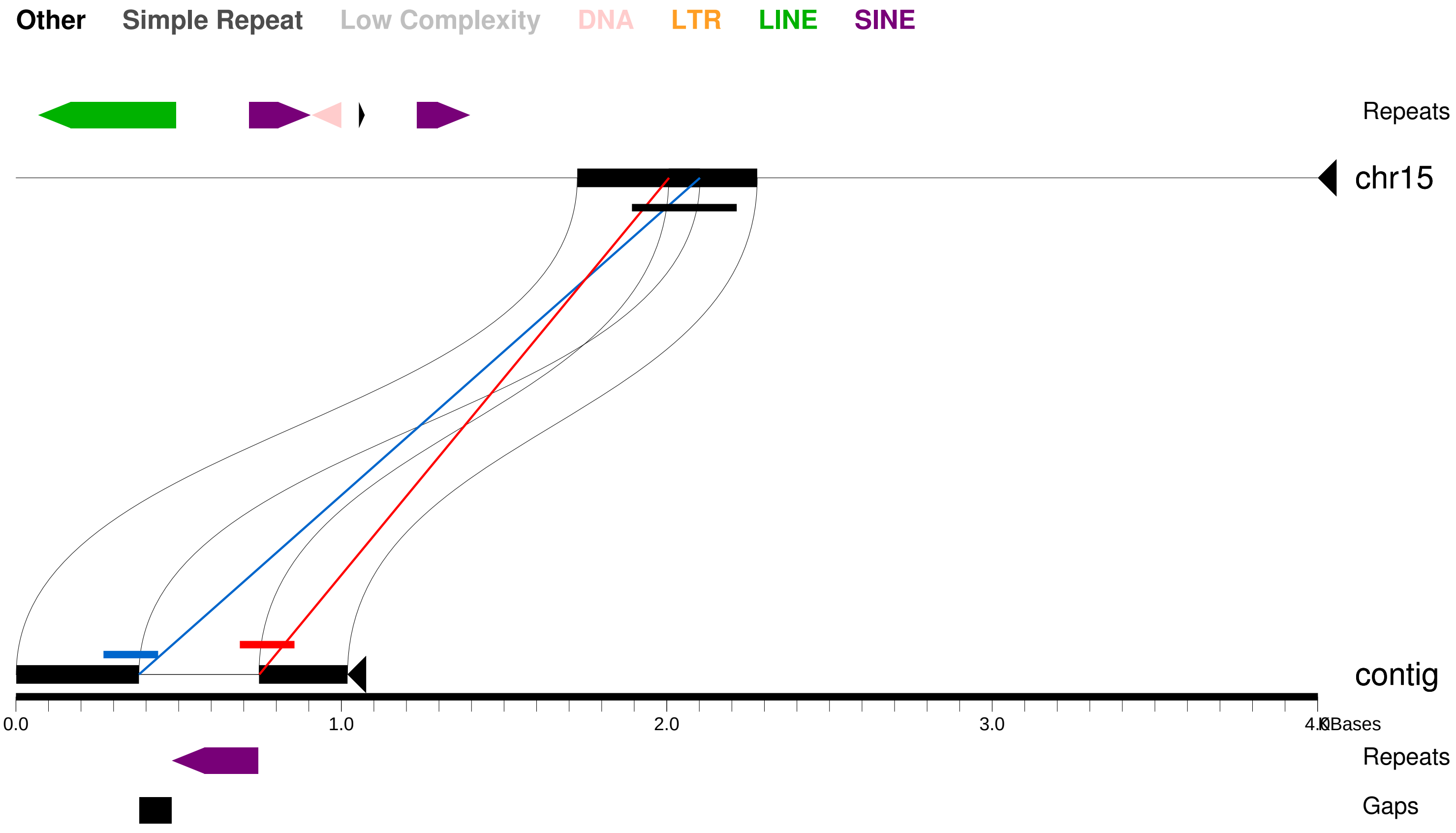

siteID: chr15_48642084 Scafftig1 - Breakpoint: chr15:48642048
chr15:48641888-48642264
Overlap Length: 57 INS size: 359 Genotyped: No

```
left   G---------------------------------------------------------CAGGGCTCCCTG
chrom  GTCCCCAGACCACCTATGTCTTACTTGCCCTTCTTTCCCCCTGCGTCTTCACCTCCAGCAGGGCTCCCTG
right  NNNNNNNNNNN-----------------------------------------------------------
       1NNNNNNNNNN                                               111111111111

left   GTAGGACGGTACCAATAGTTCATACTATCTATGCCATCCAGCTTCCTTTCTCTCCCTACTGTTTATTTCT
chrom  GTAGGACGGTACCAATAGTTCATACTATCTATGCCATCCAGCTTCCTTTCTCTCCCTACTGTTTATTTCT
right  ----------------------------------------------------------------------
       1111111111111111111111111111111111111111111111111111111111111111111111

left   ACTACATACCCTAAGGTAGTGGGGATCGCCTATCAGTTACCAGAACTGCAGATTACATAGGATAAAAGTA
chrom  ACTACATACCCTAAGGTAGTGGGGATCGCCTATCAGTTACCAGAACTGCAGATTACATAGGATAAAAGTA
right  --------------------GGGGTTCGCCTATCAGTTACCAGAACTGCAGATTACATAGGATAAAAGTA
       11111111111111111111****1*********************************************

left   ACTCACGGGCCGGGCGCGGTGGCTCACGCCTGTAATCCCAGCACTTTGGGAGGCCGAGGCGGGCGGATCA
chrom  ACTCACGAATGGAAAACCAAACAT--CGTATGTTCTCACTGATATGTGGGAG-CTAAGCTATGAGGA-CG
right  ACTCACGAATGGAAAACCAAACAT--CGTATGTTCTCACTGATATGTGGGAG-CTAAGCTATGAGGA-CG
       *******2222*2222*222222*22**22***22**2*2*222*2******2*22**2222*2***2*2

left   CGAGGTCAGGAGATCGAGACCAT------------CCCGGCTAAAACGGTGAAACCCCG-----------
chrom  CAAAAGCAGTAGAATGATACAATGGACTTTGGGGACTCGGGGGGAAGAGTGGGAGCAGGGAGAGGGATAA
right  CAAAAGCAGTAGAATGATA---------------------------------------------------
       *2*222***2***22**2*1N11            1N111NNNN11NN111NN1N1NN1

left   -----TCTCTACTAAA---------------
chrom  AAGACTCTACACTACAGAGGGTGCAGCGTAT
right  -------------------------------
            111NN1111N1
```

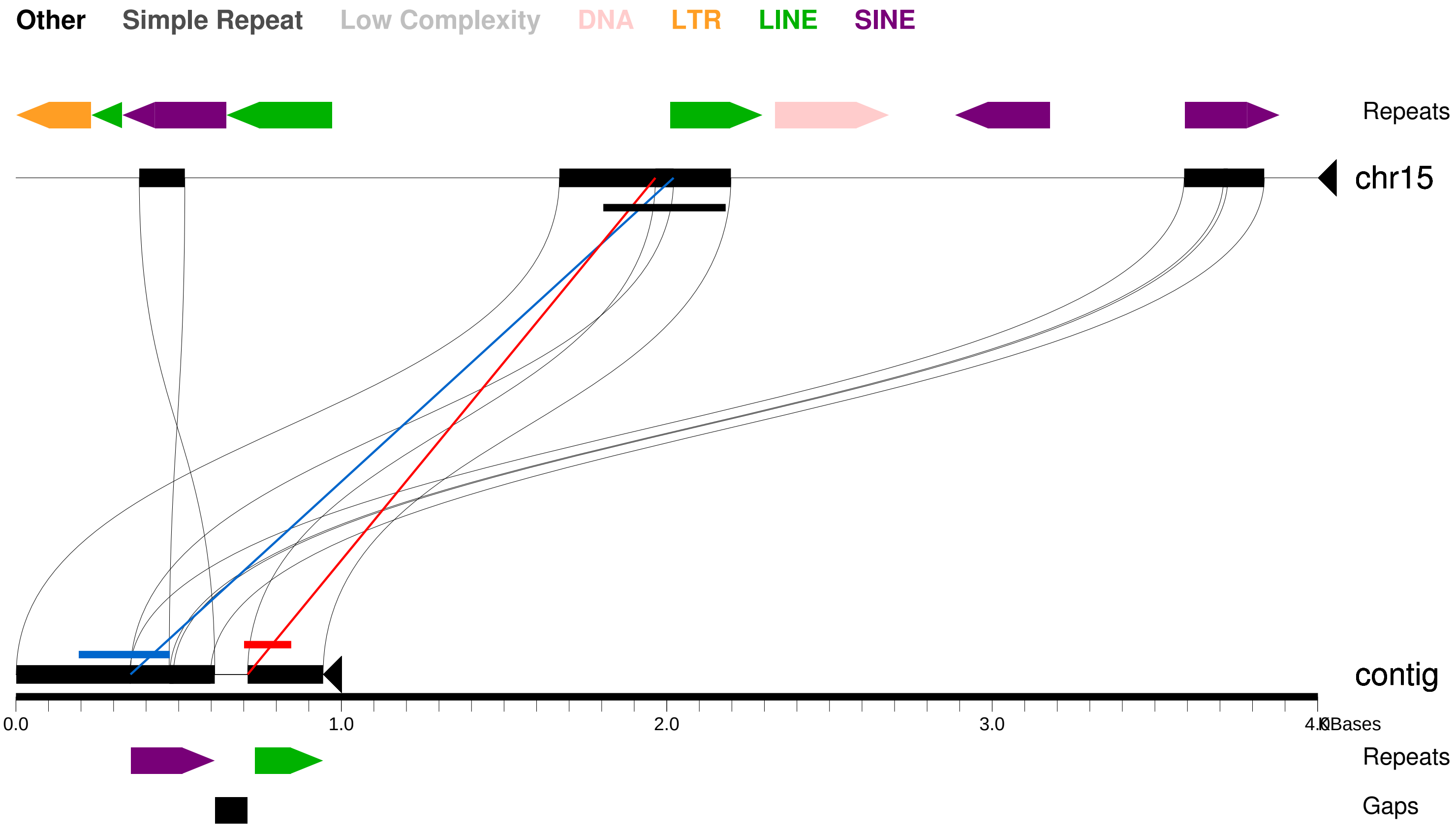

siteID: chr15_49609595 Scafftig1 + Breakpoint: chr15:49609591
chr15:49609545-49609650
Overlap Length: 14 INS size: 415 Genotyped: No

```
left    ACCACAATGAGATACCATCTCACACCAGTTAGAAAGGCGGTTATTA-------------AAAAGTCAGGA
chrom   ACCACAATGAGATACCATCTCACACCAGTTAGAAAGGCGGTTATTA-------------AAAAGTCAGGA
right   NNNNNNNNNNNNNNNNNNNNNNNNNNNNNNNNNNNNNNNNNNNNNNNNNNNNNNNNNNNAAAAGTCAGGA
        11111111111111111111111111111111111111111111111111111111111***********

left    AGCGGCCG--GGCGCGGTGGCTCACGCCTGTAATCCCAGCACTTTGGGAGGCCGAGGCGGGCGG
chrom   AGCAATAGATGCTGGAGAGGATGTGG--AGAAATAGGAATGCTTTTACACT-------------
right   AGCAATAGATGCTGGAGAGGATGTGG--AGAAATAGGAATGCTTTTACACT-------------
        ***2222*22*22*22*2**2*222*222*2***222*222****222*222222222222222
```

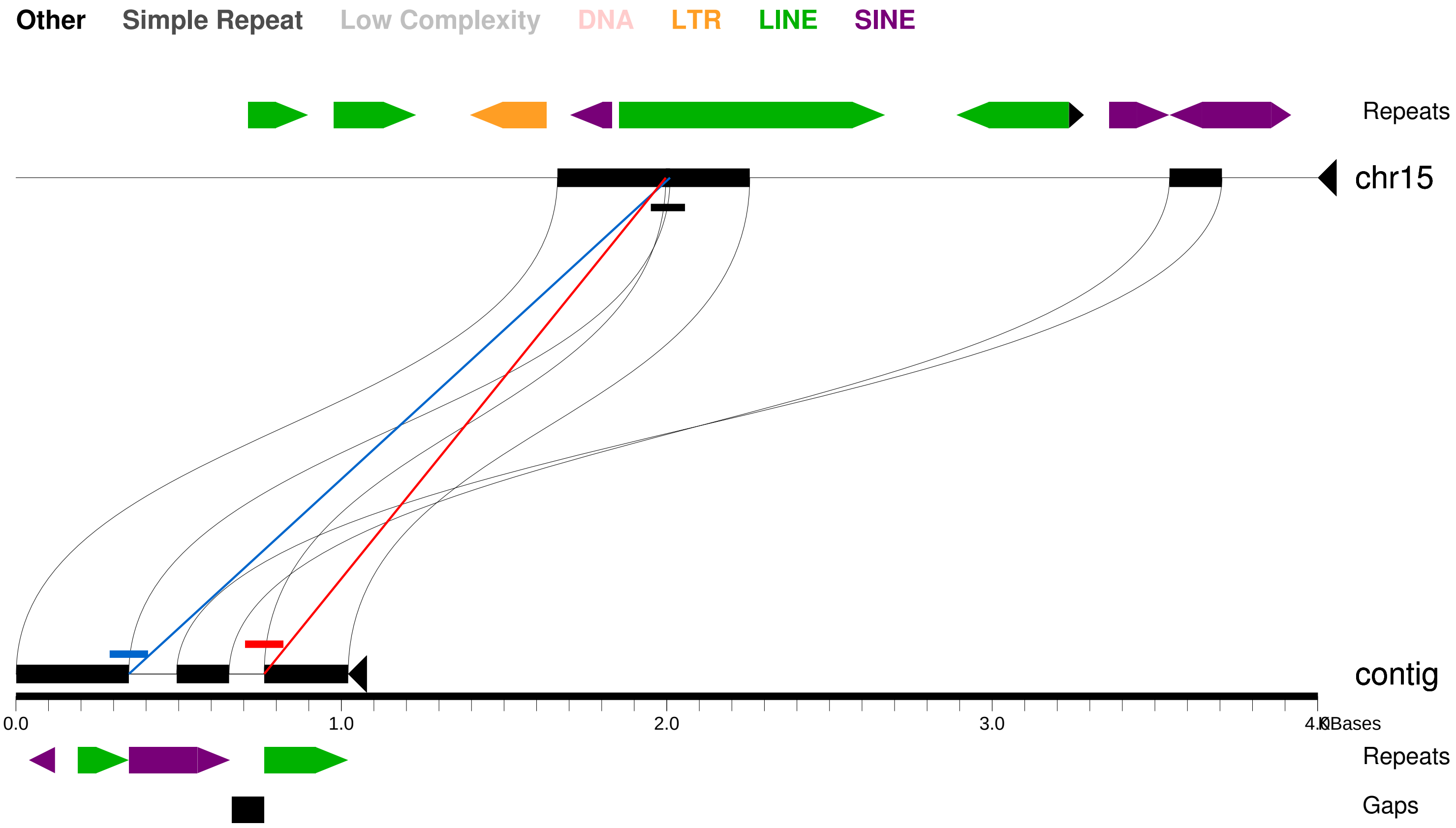

siteID: chr15_52649008 Scafftig1 - Breakpoint: chr15:52648982
chr15:52648925-52649070
Overlap Length: 3 INS size: 436 Genotyped: No

```
left    AAAAAAAAAAAATTAGCTCAGTTAATAGACCAAAAGTGGCACTCCCCTGTTGATTTAATTTNNNNNNNNNN
chrom   AAAAAAAAAAAATTAGCTCAGTTAATAGACCAAAAGTGGCACTTCGCTGTTGATTTAATTTGCATTTCTTT
right   GGA------------------TTACAGGC---ATGAGCCACTGCGCC--TGGCCTTCTTTGACTTTTGAT
        11*1111111111111111111*1*1**1*111*1*1*1****N*2*111**111*11***2NN222NNN2

left    NNNNNNNNNNNNNNNNNNNNNNNNNNNNNNNNNNNNNNNNNNNNNNNNNN---------------------
chrom   GACTTTTTTTTTTTTTTTTTTTTTATTTTCAGGAATTGTATATTAATTAGCTACCTTTGGTCTTTCATGAATT
right   GAGATTGAATAATTTTT----ATTTTCAGGAATTGTATATTAATTAGCTACCTTTGGTCTTTCATGAATT
        22NN22NNN2NN22222NNNN2222222222222222222222222222222222222222222222222

left    ------
chrom   CAGAGT
right   CAGAGT
        222222
```

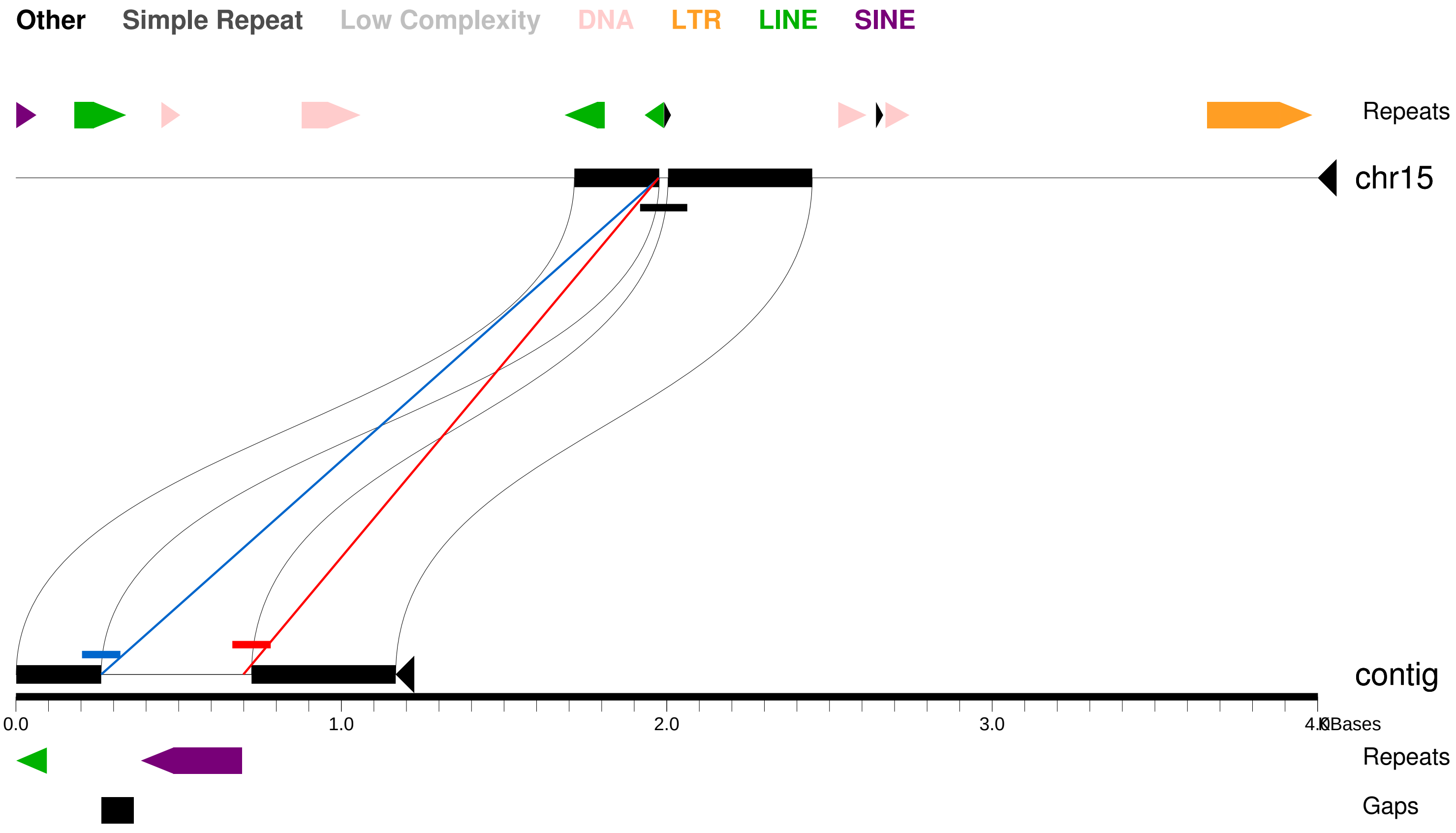

siteID: chr15_53453641 Scafftig2 - Breakpoint: chr15:53453612
chr15:53453567-53453671
Overlap Length: 15 INS size: 323 Genotyped: Yes

```
left     TCTCTTTC-TCTTTCTTTCT-----CTG------CATGTGTGTGTGTTTTAGA--GGAAAAGAGCTGGCC
chrom    TCTCTTTC-TCTTTCTTTCT-----CTG------CATGTGTGTGTGTTTTAGA--GGAAAAGAGCTGGCC
right    CCGCCCGCCTCGGCCTTCCAAAGTGCTGGGATTACAGGCGTGAGCCACCGCGCCCGGCCAAGAGCTGGCC
         1*1*111*1**111***1*111111***111111**1*1***1*1111111*111**11***********

left     TCTTTTTTTTTTTTTTTTTTTTTTTTTTTTTTTTTTTTTTTTTTTTTGAGACGGAGTCTCGCTC
chrom    TCTTAAGGCTAGGCTACAGGAACTGACTCATGGCCATTTTGGTTT-----------CTTG---
right    TCTTAAGGCTAGGCTACAGGAACTGACTCATGGCCATTTTGGTTT-----------CTTG---
         ****22222*2222*22222222*222*22*22222****22***22222222222**2*222
```

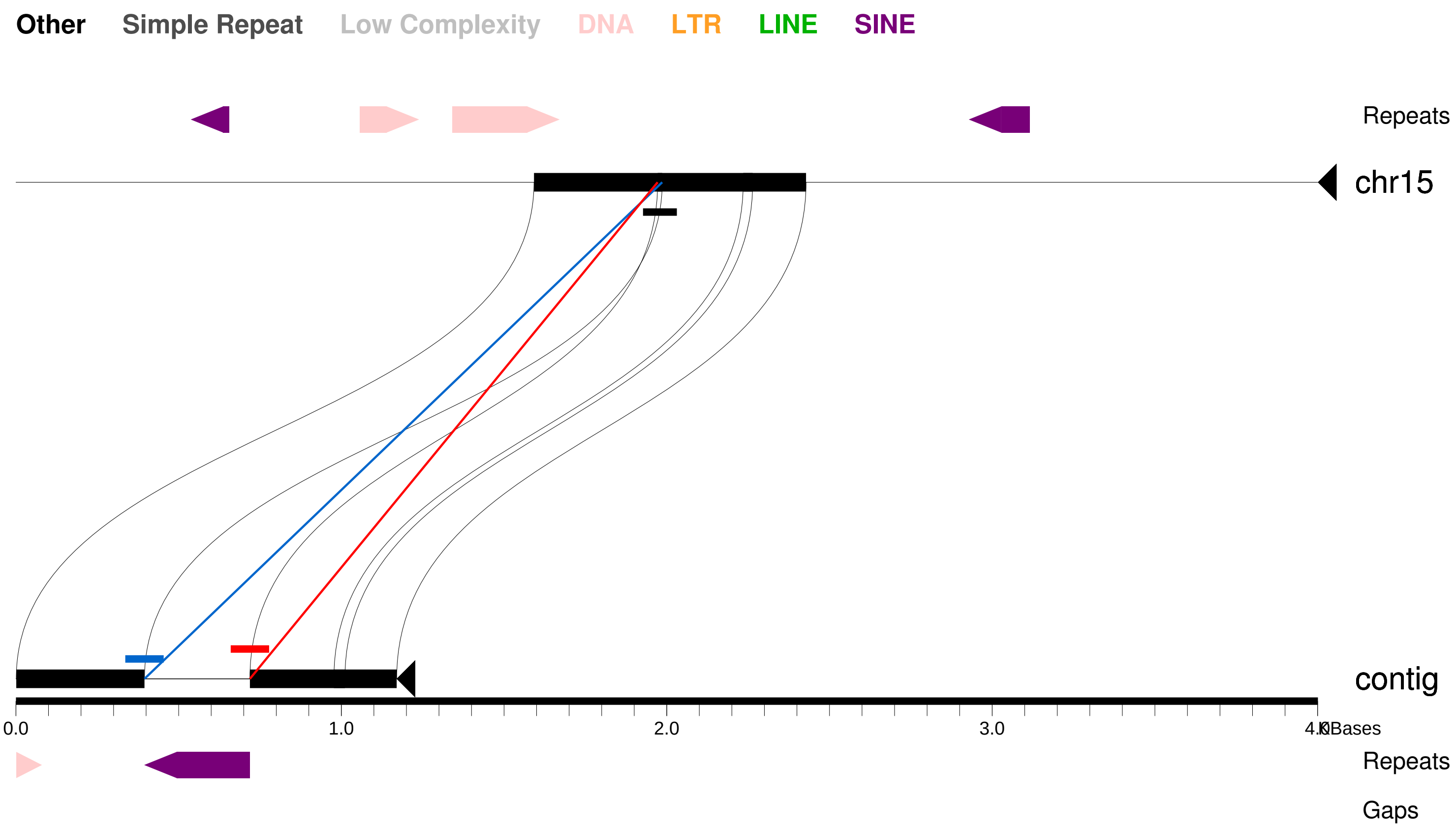

siteID: chr15_54188207 Scafftig3 - Breakpoint: chr15:54188208
chr15:54188161-54188267
Overlap Length: 13 INS size: 334 Genotyped: Yes

```
left    ATTCTCTTTCTTCAGAGCTCTGCCAACATTTTATTTGATTTT-------------TACTTTAAAATGCATC
chrom   ATTCTCTTTCTTCAGAGCTCTGCCAACATTTTATTTGATTTT-------------TACTTTAAAATGCATC
right   CGTCTCAAAAAAAAAAAAAAAAAAAAAAAAAAAAATAAAATAAAATAAAATAAAATAAAATAAAATGCATC
        11****1111111*1*11111111**1*1111*1*11*1*11111111111111**111************

left    TCGGCCGGGCGCGGTGGCTCACGCCTGTAATCCCAGCACTTTGGGAGGCCGAGGCGGGCGG
chrom   TCAAACAAA-----------ATGATAATAAAAACAACACATGAATTTAAATATGCTAATG-
right   TCAAACAAA-----------ATGATAATAAAAACAACACATGAATTTAAATATGCTAATG-
        **222*22222222222222*2*2222***222**2***2*2222222222*2**2222*2
```

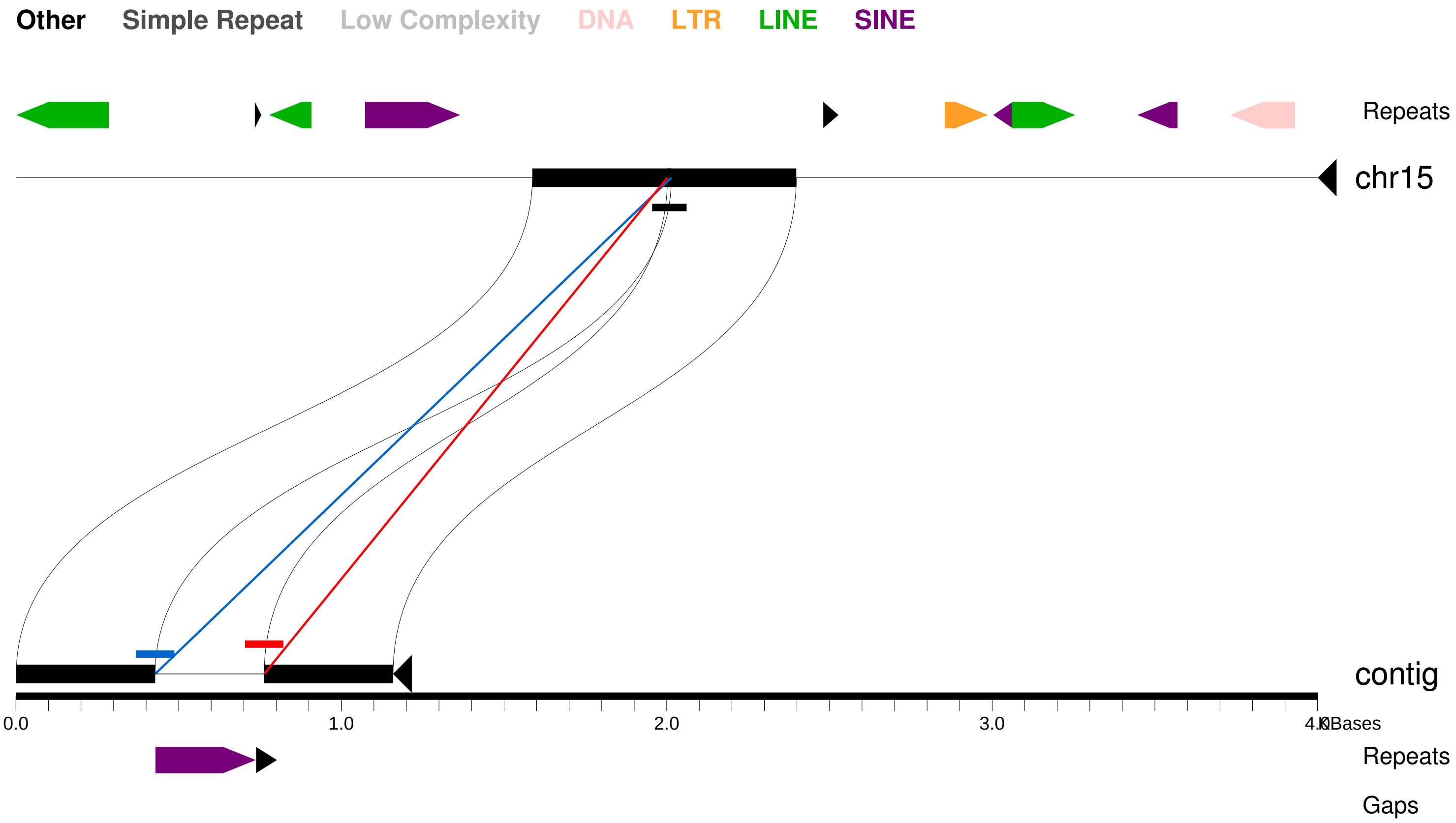

siteID: chr15_54248784 Scafftig5 - Breakpoint: chr15:54248771
chr15:54248725-54248830
Overlap Length: 14 INS size: 314 Genotyped: Yes

```
left   AAA----------ACAGAGAGGTAAAGAGGTTTAGGGTATAGCAAGAGTGGTACTT---AGAACTGAAGC
chrom  AAA----------ACAGAGAGGTAAAGAGGTTTAGGGTATAGCAAGAGTGTTACTT---AGAACAGAAGC
right  TCCGGCCTGGGCGACAGAGCGAGACTCCGTCTCAAAAAAAAAAAAAAAAAAAAAAAAAAGAACTGAAGC
       1111111111111******1*11*1111*11*1*1111*1*11**1*111N1*111111*****N*****

left   TTCGGCCGGGCGCG-GTGGCTCACGCCTGTAATCCCAGCACTTTGGGAGGCCGAGGCGGGTGG
chrom  TTCATAAGTTAGCAAGTGAAAAAGG---GAGAAGCTTGATGATTGGAATAAA-----------
right  TTCATAAGTTAGCAAGTGAAAAAGG---GAGAAGCTTGATGATTGGAATAAA-----------
       ***2222*222**22***2222*2*222*22*22*22*2222****2*22222222222222
```

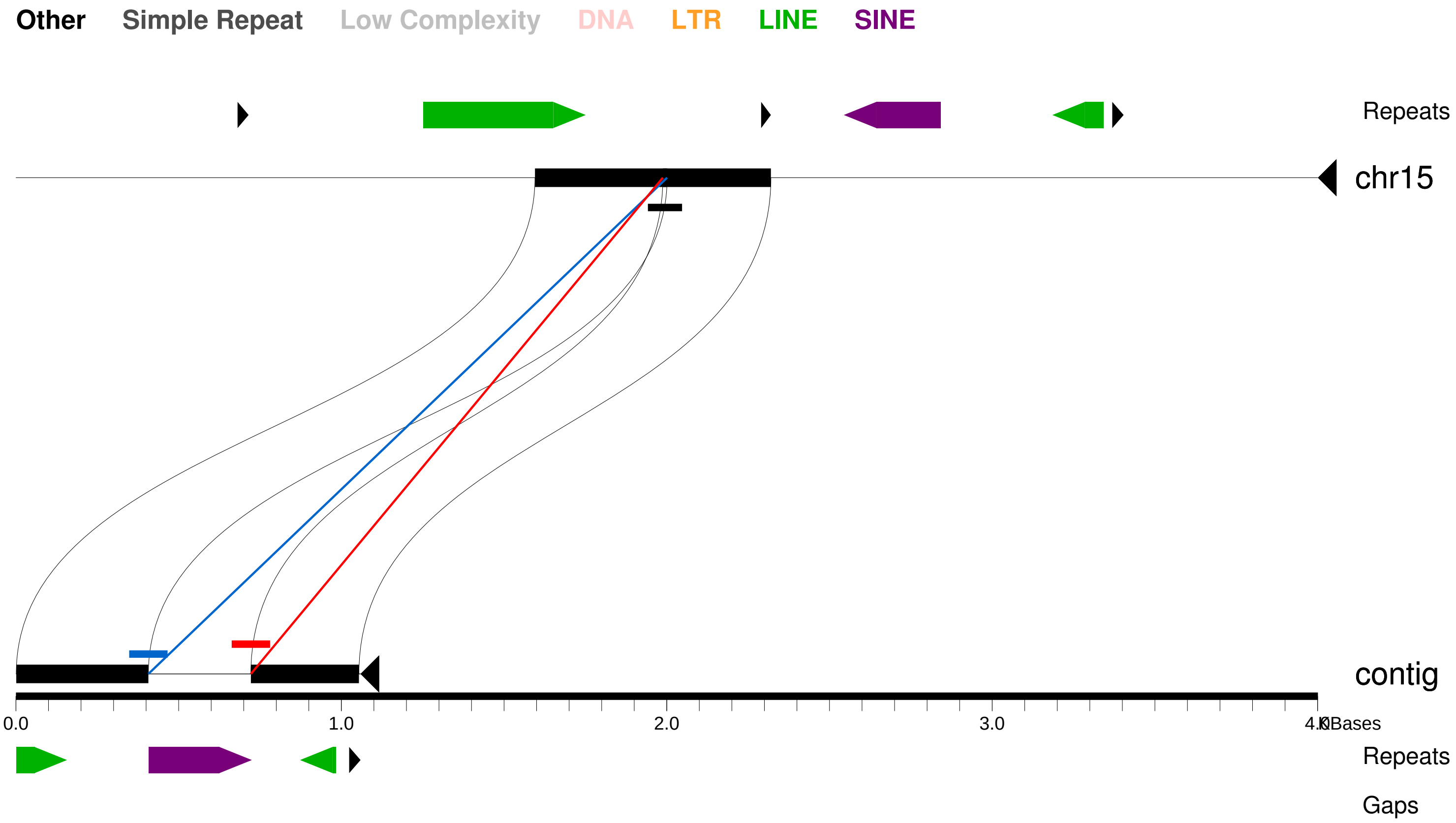

siteID: chr15_54376450 Scafftig1 + Breakpoint: chr15:54376442
chr15:54376387-54376501
Overlap Length: 5 INS size: 342 Genotyped: No

```
left    TCTCTTGAGAGCCCGTCCATTCACAGTAACTGTGATC-CA--CGT----CCCCTTTTTTCTTCAGTTNNN
chrom   TCTCTTGAGAGCCCGTCCATTCACAGTAACTGTGATC-CA--CGT----CCCCTTTTTTCTTCAGTTCTG
right   TC-CACCCGC-CTCGGCCTCCCAAAGTG-CTGGGATTACAGGCGTGAGCCACCGCGCCCGGCCAGTTCTG
        **1*1111*11*1**1**111**1***11***1***11**11***1111*1**111111111*****222

left    NNNNNNNNNNNNNNNNNNNNNNNNNNNNNNNNNNNNNNNNNNNNNNNNNNNNNNNN
chrom   TACTCTTTCTACTCTCTTTCTATTGGTCAGTTGGATTTAAGTTCCTCGTGTA----
right   TACTCTTTCTACTCTCTTTCTATTGGTCAGTTGGATTTAAGTTCCTCGTGTA----
        22222222222222222222222222222222222222222222222222222222
```

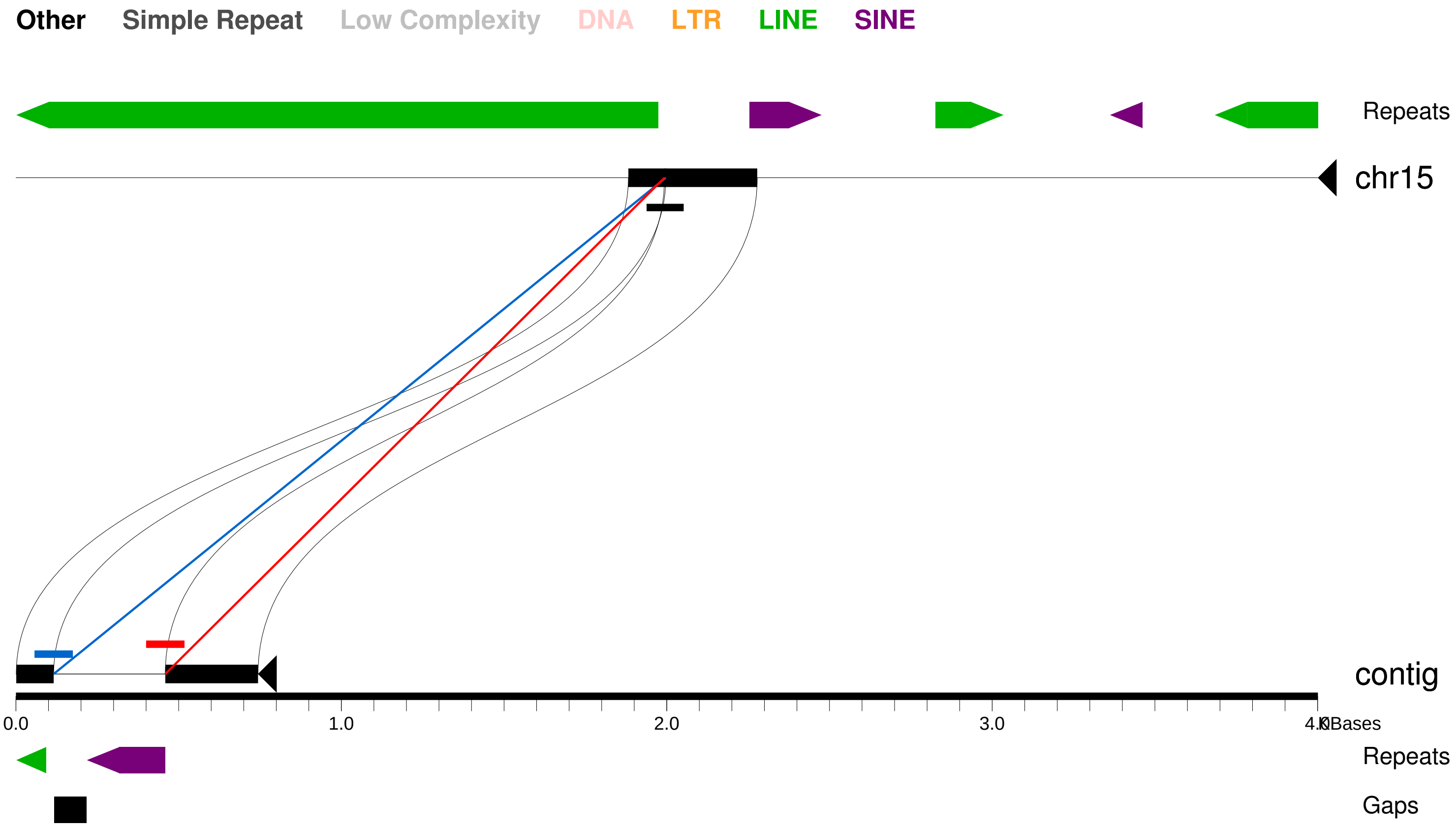

siteID: chr15_54448974 Scafftig7 - Breakpoint: chr15:54448975
chr15:54448932-54449034
Overlap Length: 17 INS size: 295 Genotyped: Yes

```
left     GTCTGAG-AATAGAGAAGCTG-GACGGTGCCTGCAACTGTGGTTC----------------AAAACTAGA
chrom    GTCTGAG-AGTAGAGAAGCTG-GACGGTGCCTGCAACTGTGGTTC----------------AAAACTAGA
right    GCCACTGCACTCCAGCCTGGGCGACAGAGC--GAAACTCCGTCTCAAAAAAAAAAAAAAAAAAAAACTAGA
         *1*111*1*N*11**11111*1***1*1**11*1****11*11**1111111111111111*********

left     CATATTTGGCCGGGCGCGGTGGCTCACGCCTGTAATCCCAGCACTTTGGGAGGCCGAGACGGGCGGA
chrom    CATATTTGATGAAAGACTGTG--TCA-----GTA---GCAGTGAAATTGATGGGCAACAT------A
right    CATATTTGATGAAAGACTGTG--TCA-----GTA---GCAGTAAAATTGATGGGCAACAT------A
         ********22222222*2***22***22222***2222***2N222*2*22**2*2*2*2222222*
```

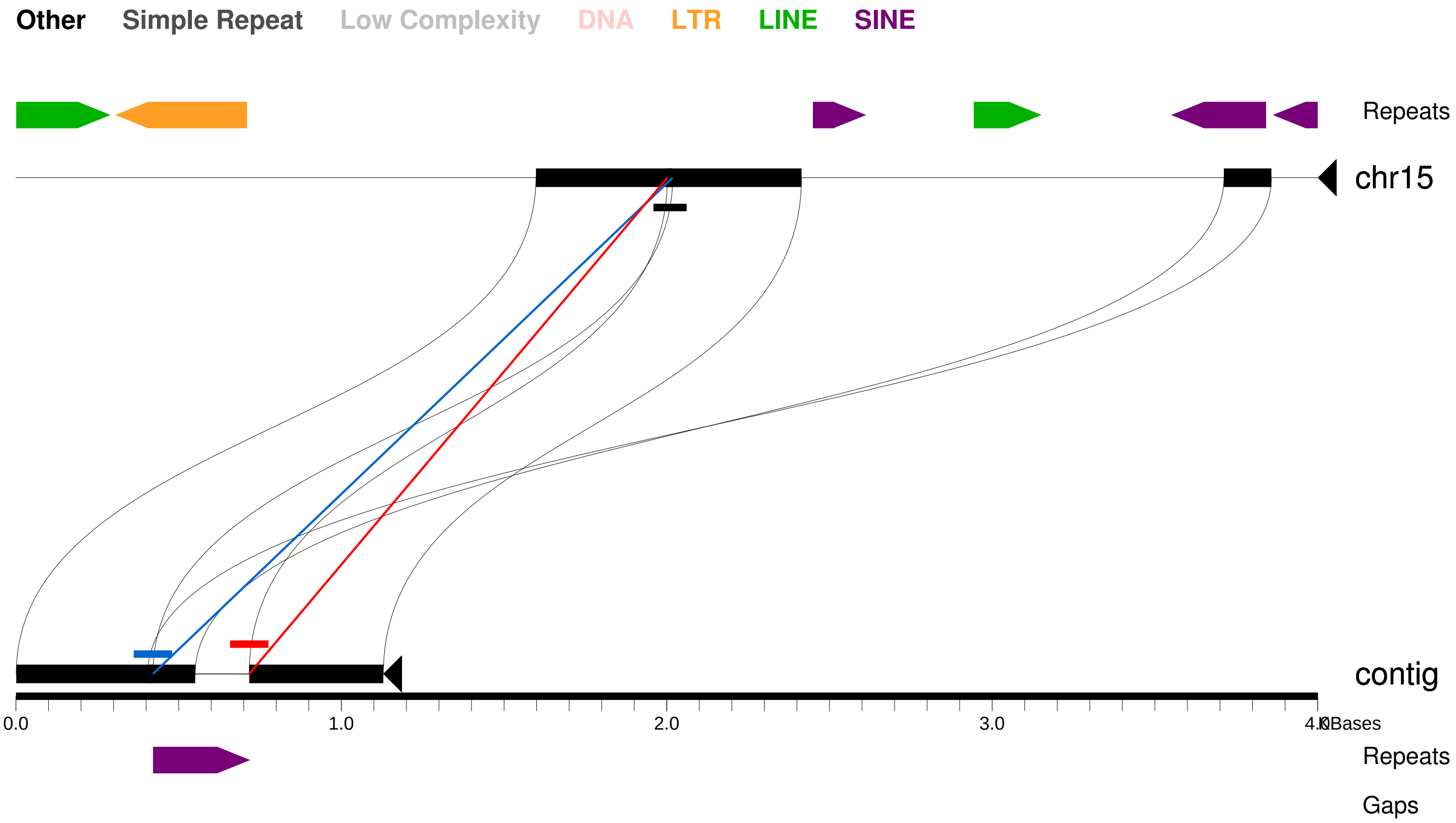

siteID: chr15_54800415 Scafftig1 - Breakpoint: chr15:54800310
chr15:54800152-54800551
Overlap Length: 82 INS size: 432 Genotyped: No

```
left   ----------------------------------------------------------------------
chrom  CTAAATTATTCTAAAGAAAGCTACTAGTTTTCTCCCTGAAACCTCATTGACCTCTTGCTGACAGCTCATT
right  NNNNNNNNNNN-----------------------------------------------------------
       NNNNNNNNNNN

left   ----------AGTCCTAATGGTTCTTGAGAATTCTATAAGATGCTTCTTTTCCTTAAATTAATATGCTGC
chrom  AAGATAAGTCAGTCCTAATGGTTCTTGAGAATTCTATAAGATGCTTCTTTTCCTTAAATTAATATGCTGC
right  ----------------------------------------------------------------------
                 111111111111111111111111111111111111111111111111111111111111

left   CCAGAAATTTTATTAGAACTTTTTGACAACTCAAACAATTATTTCTTATTGCTATTGATTATACTCAAAA
chrom  CCAGAAATTTTATTAGAACTTTTTGACAACTCAAACAATTATTTCTTATTGCTATTGATTATACTCAAAA
right  ------------------CTTTTTGACAACTCAAACAATTATTTCTTATTGCTATTGATTATACTCAAAA
       111111111111111111****************************************************

left   AGCAAGATATTTATTTATTGAAAGTATATTGGCCGGG----CGCGGTGGCTCACGCCT---GT-------
chrom  AGCAAGATATTTATTTATTGAAAGTATATTATTTCTGATTACAAGATAATACATGTCTAAAGTGTTACAT
right  AGCAAGATATTTATTTATTGAAAGTATATTATTTCTGATTACAAGATAATACATGTCTAAAGTGTTACAT
       ******************************222222*2222*22*2*2222**2*2**222**2222222

left   ----AATCCCAGCACTTTGGGAGGCCGAGGCGGGCGGATC--------ACGAGGTCAGG-AGATCGA---
chrom  TGAAAATCAAAACAGTATCTGATAATTTCACAAAAGGATCCTGTTTATACTAGGACAGCCAAATTGAAGA
right  TGAAAATCAAAAC---------------------------------------------------------
       2222****22*2*1N1N1NN11NNNNNNNN1NNNN11111        11N111N111N 1N11N11

left   -----------GACCATCCTGGCTAAAATGGTGAAACCCCGTCTCTACTAAA
chrom  TTTTGAAAGGTGACCATG--AGCTCCTAAGGCTAATTCCTGCCATTCAGAAA
right  ----------------------------------------------------
                  111111N22N111NNN1N11NN11NN11N1N1NN1NNN111
```

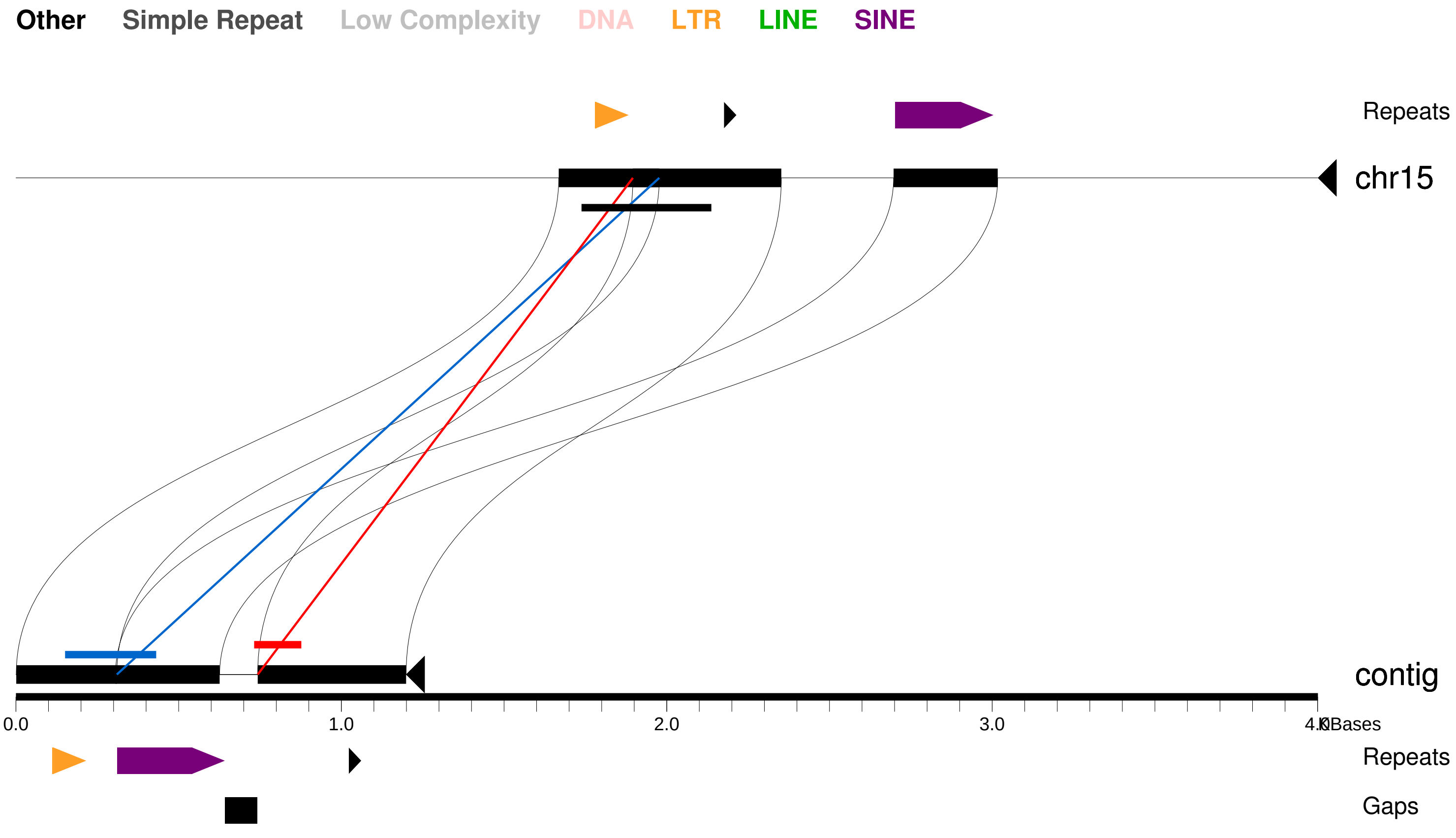

siteID: chr15_55213469 Scafftig1 + Breakpoint: chr15:55213439
chr15:55213386-55213498
Overlap Length: 7 INS size: 407 Genotyped: No

```
left    CTTGTTTCTCTAGTTCTT----TGAGGTGTGATCTTAGACTGTCTATTTCTGTTCTT----TCAGACTNN
chrom   CTTGTTTCTCTAGTTCTT----TGAGGTGTGATCTTAGACTGTCTATTTCTGTTCTT----TCAGACTTT
right   CCGCCCAC-CTCGGCCTCCCAAAGTGCTGGGATTACAGGC-GTGAGCCACCGCGCCCGGCCTCAGACTTT
        *111111*1**1*11**111111*1*1**1***111**1*1**111111*1*11*111111*******22

left    NNNNNNNNNNNNNNNNNNNNNNNNNNNNNNNNNNNNNNNNNNNNNNNNNNNNNNNNN
chrom   TTGATGTAGGCATTTAATGCTATGAGCTTTCCTCTTAGCACTGCTTTTGCT------
right   TTGATGTAGGCATTTAATGCTATGAGCTTTCCTCTTAGCACTGCCTTTGCT------
        22222222222222222222222222222222222222222222N222222222222
```

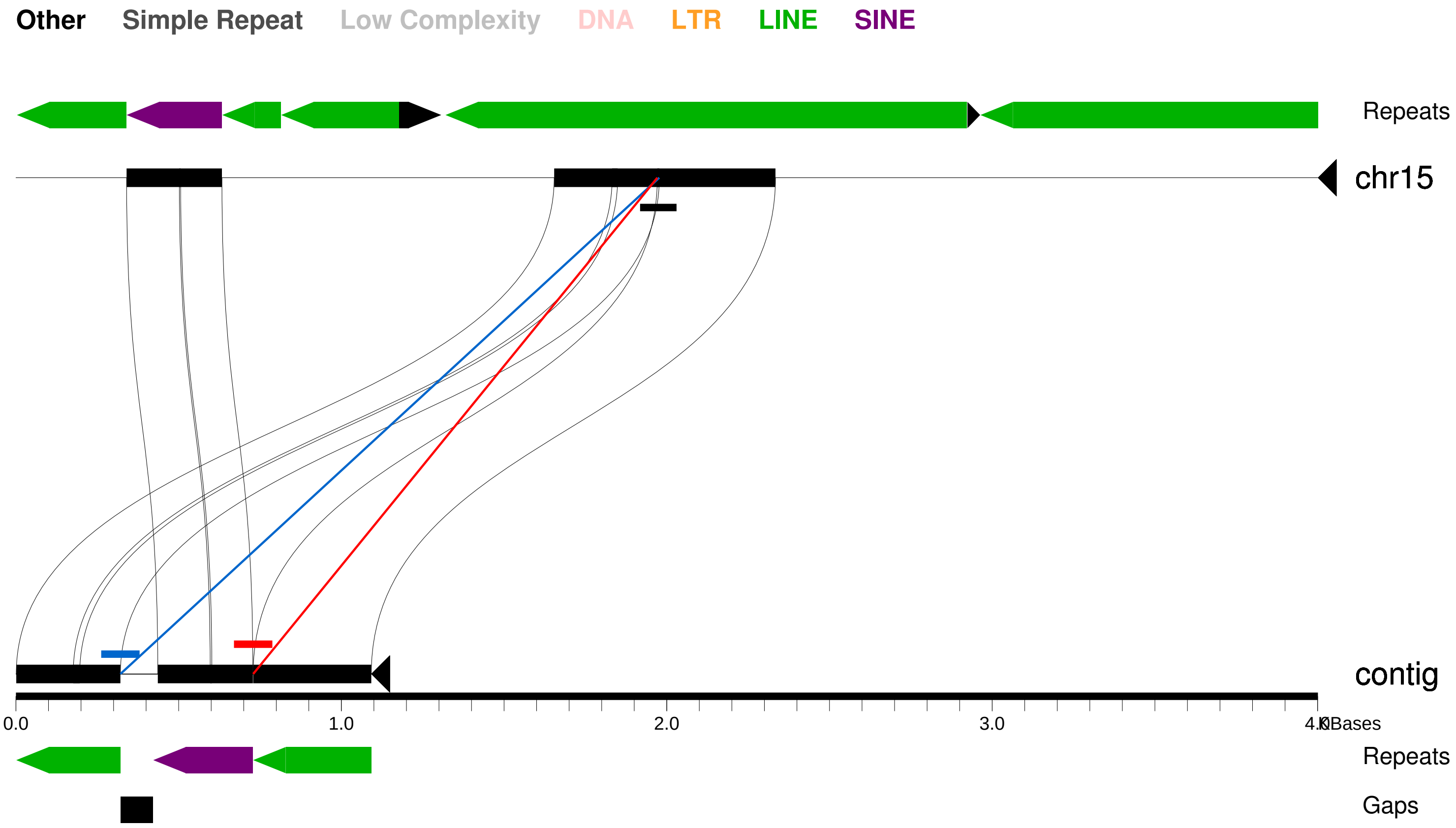

siteID: chr15_55228586 Scafftig1 - Breakpoint: chr15:55228567
chr15:55228518-55228622
Overlap Length: 11 INS size: 302 Genotyped: Yes

```
left    CA-------TCCAT-----GTAACCAAACACCATTTTTGCCCCTAAAGCCATTGATTAAAA--CACTAAT
chrom   CA-------TCCAT-----GTAACCAAACACCATTTTTGCCCCTAAAGCCATTGATTAAAA--CACTAAT
right   CCACTGCACTCCAGCCTGGGCGACAGAGCGAGACTCCGTCTCAAAAAAAAAAAAAAAAAAATCACTAAT
        *11111111****111111*11**11*1*111*1*1111*1*11***111*111*11****11*******

left    TTTTGGCCGGGCGCGGTGGCTCACGCCTGTAATCCCAGCACTTTGGGAGGCCGAGGCGGGCGG
chrom   TTTTAA---GTCAAGCTGGATTAT-------ATAGAAGAACTA---GAAATAAAAACACAAC-
right   TTTTAA---GTCAAGCTGGATTAT-------ATAGAAGAACTA---GAAATAAAAACACAAC-
        ****22222*2*22*2***2*2*22222222**222**2***2222**22222*22*222222
```

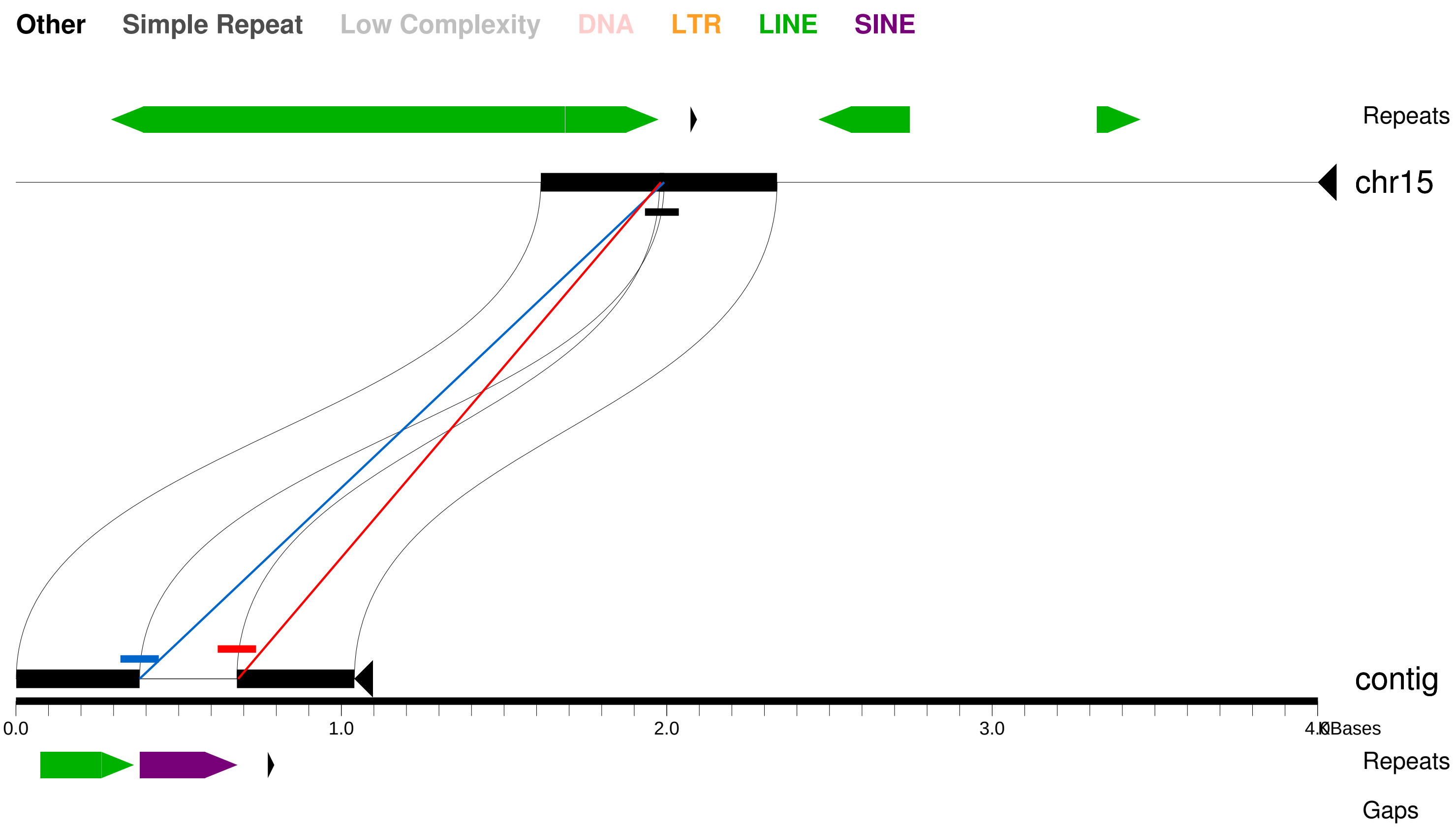

siteID: chr15_57380187 Scafftig1 - Breakpoint: chr15:57380190
chr15:57380029-57380363
Overlap Length: 14 INS size: 314 Genotyped: Yes

```
left   GG------------TGTAATAGATAATTCTGATAGTATTGCTCTGTCCTTCATAATGTATGTCATCTCAT
chrom  GGCTAAAAGGCTGGTGTAATAGATAATTCTGATAGTATTGCTCTGTCCTTCATAATGTATGTCATCTCAT
right  TTTAGTAGAGACGGGGTTTCACCTTGTTA-GCCAGGATGG---TCTCGATC-TCCTG------ACCTCAT
       11NNNN2NN2NN221**111*11*11**11*11**1**1*111*1**11**1*11**111111*1*****

left   GTATTAAAAAATCTCGTTTATCTTAGTTGCTATTAACAGAATTCAAGATCTTAATTAGGAGTCCTGGGAG
chrom  GTATTAAAAAATCTCGTTTATCTTAGTTGCTATTAACAGAATTCAAGATCTTAATTAGGAGTCCTGGGAG
right  G-ATCCACCCGCCTCGGCCTCCCAAAGTGCTG------GGATTACAGGCGT-------GAG-CCACCGCG
       *1**11*11111****11111*11*11****1111111*1***11**111*1111111***1**111*1*

left   CCATGTTTGACCCCTTTTATTAGCATAGTAACTTTTTTTTTTTT----------------TTTTTTTTT
chrom  CCATGTTTGACCCCTTTTATTAGCATAGTAACTTTCTATCAATTTGAAATATGCAGCATGATCATTGTAT
right  CCCGG-------CCT------AGCATAGTAACTTTCTATCAATTTGAAATATGCAGCATGATCATTGTAT
       **11*1111111***111111**************2*2*222***2222222222222222*22**2*2*

left   TTTT-----TGAGACGGAGT--CTCGCTCTGTCGCCCAGGCCGGACTGCGGA--------CTG----CAG
chrom  ATTTAATGATGTCACTGATTTACTTCCTTTATATTTTAAAATTGAAATAGCATTCATTTACTGGTCCCAT
right  ATTTAATGATGTCACTGATTTACTTCCTTTATATTTTAAAATTGAAATAGCATTCATTTACTGGTCCCAT
       2***22222**22**2**2*22**22**2*2*22222*22222**2222*2*22222222***2222**2

left   TGGTGCAATCTC---GGCTC--ACTGCAAGCTCCGCTTCCCGGGTTCACGCCA---
chrom  TGGTTGATCCACTCGGGCTCCAACTT-AACCATTGTTTGCTTGGTTTTTGTTCTTT
right  TGGTTGATCCACTCGGGCTCCAACTT-AACCATTGTTTGCT---------------
       ****22*22*2*222*****22***22**2*222*2**2*2N1111NNN1NNN
```

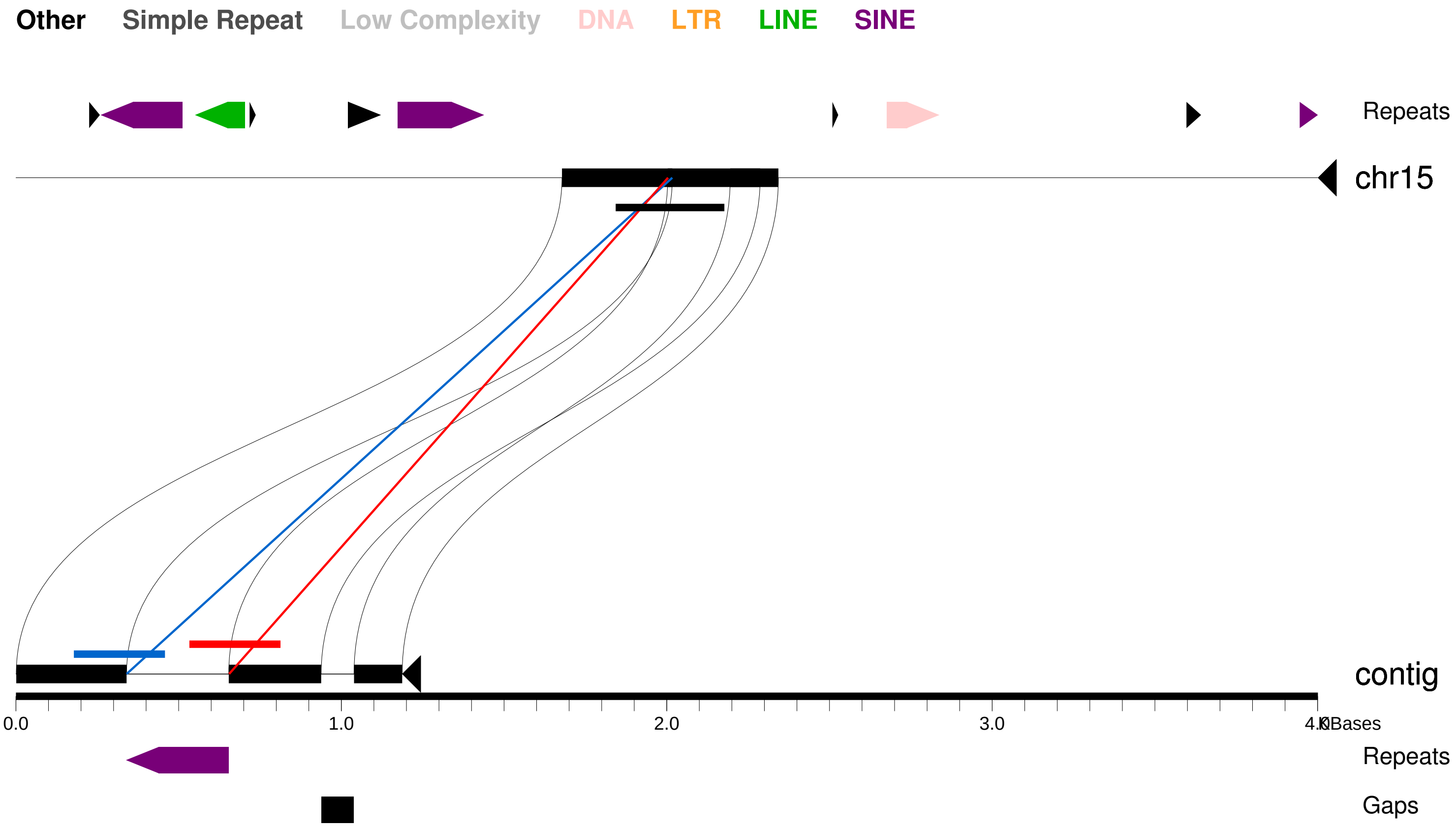

siteID: chr15_60657255 Scafftig1 + Breakpoint: chr15:60657244
chr15:60657200-60657303
Overlap Length: 16 INS size: 436 Genotyped: No

```
left    ---------CTATAATTATAAAA--ACTGTCATGACAA--ATGAGTCA--GATTTAATATGGCAGGTGTA
chrom   ---------CTATAATTATAAAA--ACTGTCATGACAA--ATGAGTCA--GATTTAACACGGCAGTTGTA
right   TCCGCCCGCCTCGGCCTCCCAAAGTGCTGGGATTACAGGCGTGAGCCACCGCGCCCGGCCGGCAGTTGTA
        111111111**11111*111***111***11**1***1111****1**11*111111N12*****2****

left    TTTTTNNNNNNNNNNNNNNNNNNNNNNNNNNNNNNNNNNNNNNNNNNNNNNNNNNNNNNNNNNN
chrom   TTTTTAATAGCATAATTGATTAACACAATCACACTTATTGAAGACAAAA---------------
right   TTTTTAATAGCATAATTGATTAACACAATCACACTTATTGAAGACAAAA---------------
        *****2222222222222222222222222222222222222222222222222222222222
```

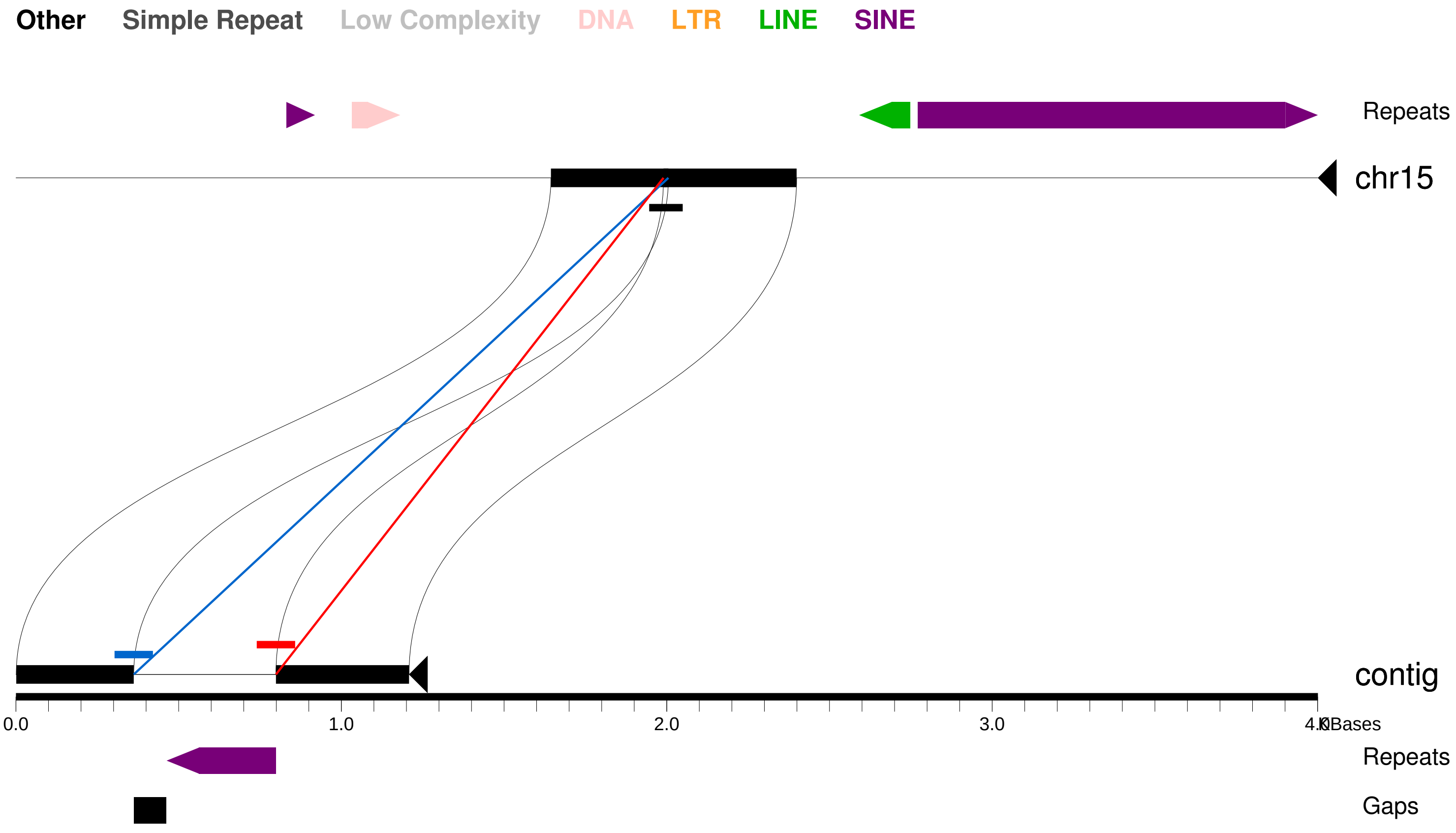

siteID: chr15_62318266 Scafftig5 - Breakpoint: chr15:62318266
chr15:62318221-62318325
Overlap Length: 15 INS size: 313 Genotyped: Yes

```
left    AGAAGACAGTTGGATTAGCATA----TCTGCTT-CTGCCTTCAACCT---------GTTGAGATATGTTG
chrom   AGAAGACAGTTGGATTAGCATA----TCTGCTT-CTGCCTTCAACCT---------GTTGAGATATGTTG
right   CCGCCCGCCTCGGCCTCCCAAAGTGCTGTGATTACAGGCGTGAGCCACCGCGCCCGGCCGAGATATGTTG
        111111111*1**11*11**1*1111*1**1**1*1*1*1*1*1**1111111111*11***********

left    TTTTTTTTTGTTTTTTTTGTTTTTTTTTTTTTTGAGACGGAGTCTCGCTCTGTCGCCCAGGCT
chrom   TTTTAATT-GAAGTACATGAT------------GAAAATCTGA-TCTCACTATAGATATATAA
right   TTTTAATT-GAAGTACATGAT------------GAAAATCTGA-TCTCACTATAGATATATAA
        *****22**2*222*222**2*222222222222**2*2222*22**2*2**2*2*22222222
```

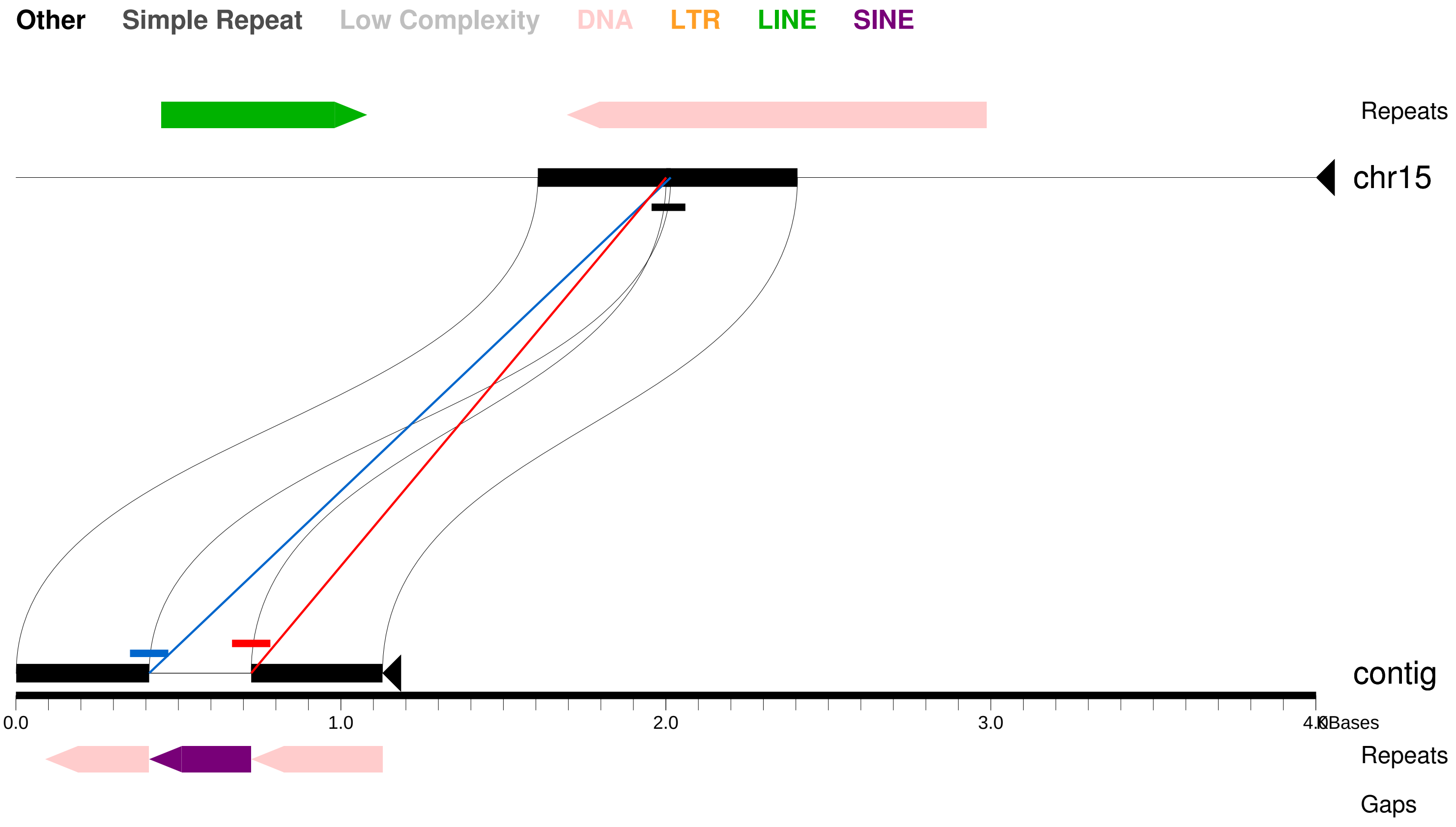

siteID: chr15_62606057 Scafftig2 - Breakpoint: chr15:62606047
chr15:62605996-62606106
Overlap Length: 9 INS size: 352 Genotyped: No

```
left    T------TCATGTATTGCTAGTGGGAACGCA--AAATGGTACAGCCACTGAAAAAAATAGTTTAGCAGNN
chrom   T------TCATGTATTGCTAGTGGGAACGCA--AAATGGTACAGCCACTGAAAAAAATAGTTTAGCAGAT
right   CCGCCCGCCTCGGCCTCCCAAAGTGCTGGGATTACAGGCGTGAGCCACCGCGCCCGGCCGTTTAGCAGAT
        11111111*11*111*1*1*11*1*111*1*11*1*1*1111******1*111111111*********22

left    NNNNNNNNNNNNNNNNNNNNNNNNNNNNNNNNNNNNNNNNNNNNNNNNNNNNNNNNN
chrom   TCTTATAAAGTTAAGAACACATTTACTATACAACCCAGTAGTCCCATTC--------
right   TCTTATAAAGTTAAGAACACATTTACTATACAACCCAGTAGTCCCATTC--------
        222222222222222222222222222222222222222222222222222222222
```

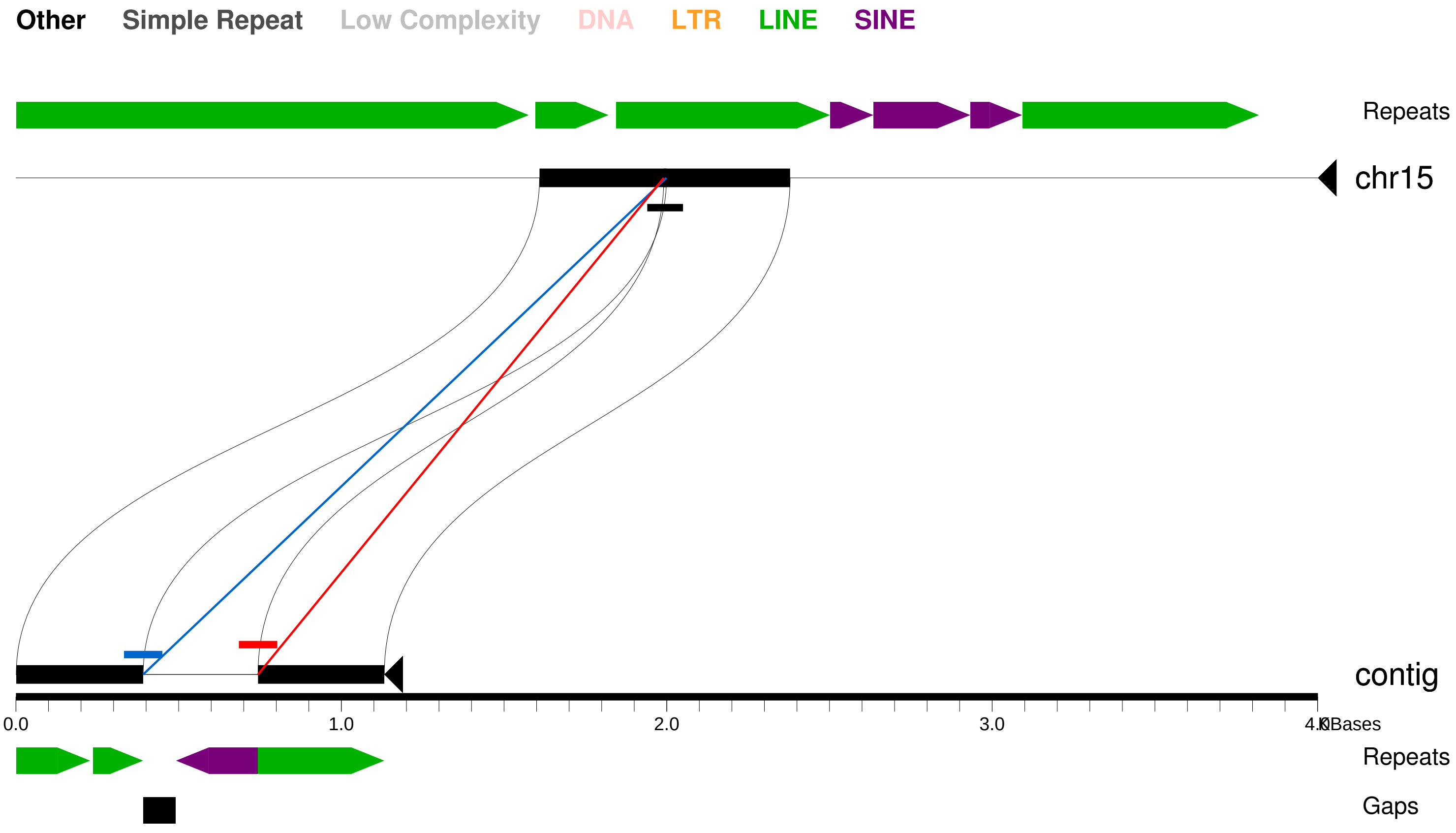

siteID: chr15_63287064 Scafftig2 - Breakpoint: chr15:63287072
chr15:63287027-63287131
Overlap Length: 15 INS size: 298 Genotyped: Yes

```
left    TTTCTCAGCAATTCAGGGAAGGAACAGCAGTGCCTGAAGTCCTTC--------------------AAAAG
chrom   TTTCTCAGCAATTCAGGGAAGGAACAGCAGTGCCTGAAGTCCTTC--------------------AAAAG
right   ---CACTGCACTCCAGCCTGGG--CGACAGAGCGAGAC-TCCATCTCAAAAAAAAAAAAAAAAAAAAAAG
        111*1*1***1*1***1111**11*11***1**11**11***1**11111111111111111111*****

left    TCTACCCGGGGCCGGGCGCGGTGGCTCACGCCTGTAATCCCAGCACTTTGGGAGGCCGAGGCGGGCGGA
chrom   TCTACCCGGGCAAATG----GTGT-TAAGGGGTTT---CCCTTCCCTTTGTGGAGTTCAGCCT------
right   TCTACTCGGGCAAATG----GTGT-TAAGGGGTTT---CCCTTCCCTTTGTGGAGTTCAGCCT------
        *****1****22222*2222***22*2*2*22*2*222***22*2*****2*22*222**2*2222222
```

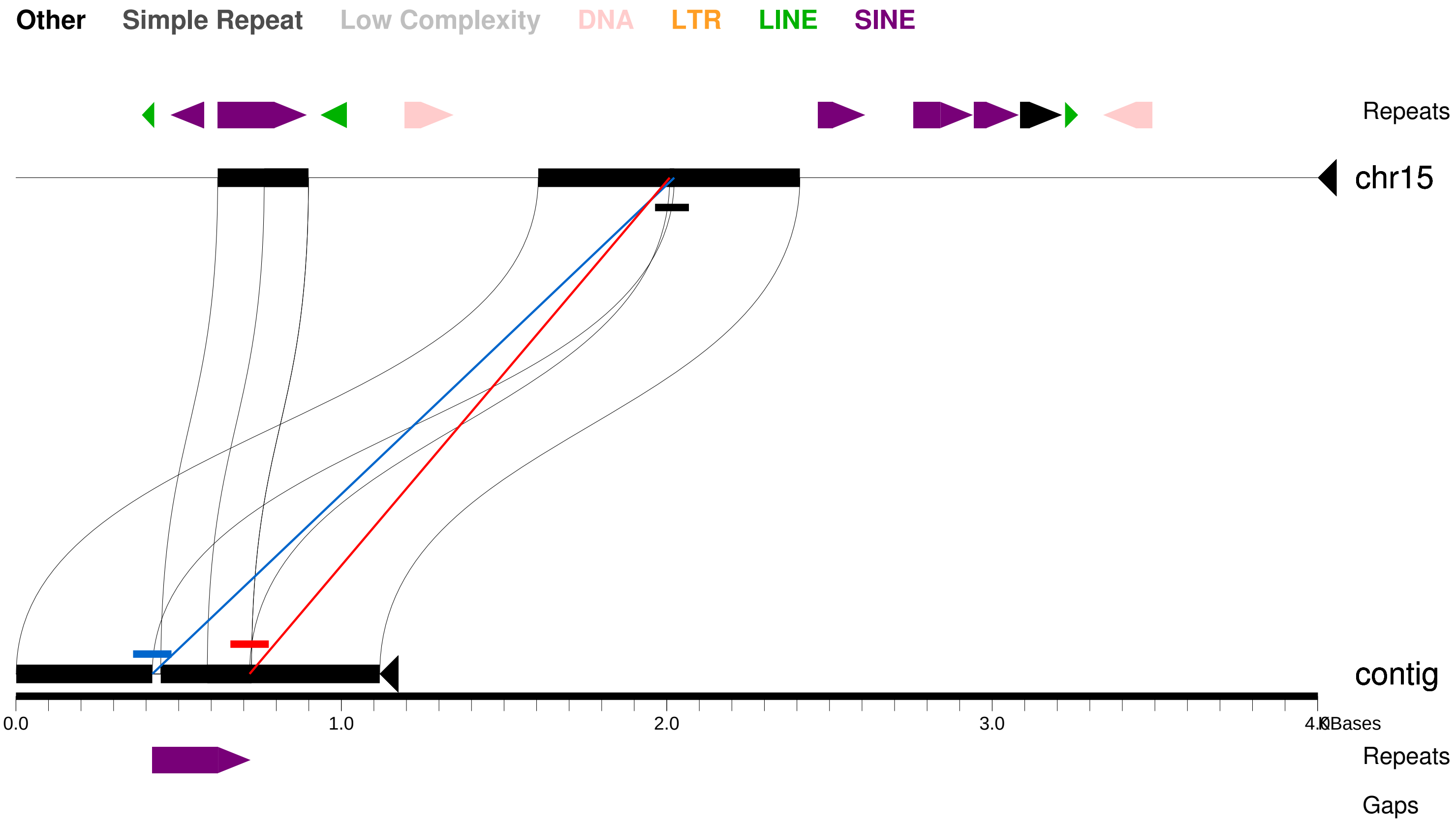

siteID: chr15_64085914 Scafftig1 + Breakpoint: chr15:64085838
chr15:64085823-64085897
Overlap Length: 45 INS size: 270 Genotyped: No

```
left    -----------------------GCAGACATT-----CTTTAT----------------TCCTTTACTTT
chrom   -----------------------GCAGACATT-----CTTTAT----------------TCCTTTACTTT
right   CACCCGCCTCGGCCTCCCAAAGTGCTGGGATTACAGGCGTGAGCCACCGCGCCCGGCCCTCCTTTACTTT
        11111111111111111111111**1*11***11111*1*1*1111111111111111***********

left    CTTAATAAACTCACTTTCACTTTACTCTGTGGACNNNNNNNNNNNNNNNNNNNNNNNNNNNNNNNNNNNN
chrom   CTTAATAAACTCACTTTCACTTTACTCTGTGGACTCGCCCTGAATTCTT---------------------
right   CTTAATAAACTCACTTTCACTTTACTCTGTGGACTCGCCCTGAATTCTT---------------------
        **********************************222222222222222222222222222222222222

left    NNNNNNNNNNNNNNNNNNNNNNN
chrom   -----------------------
right   -----------------------
        22222222222222222222222
```

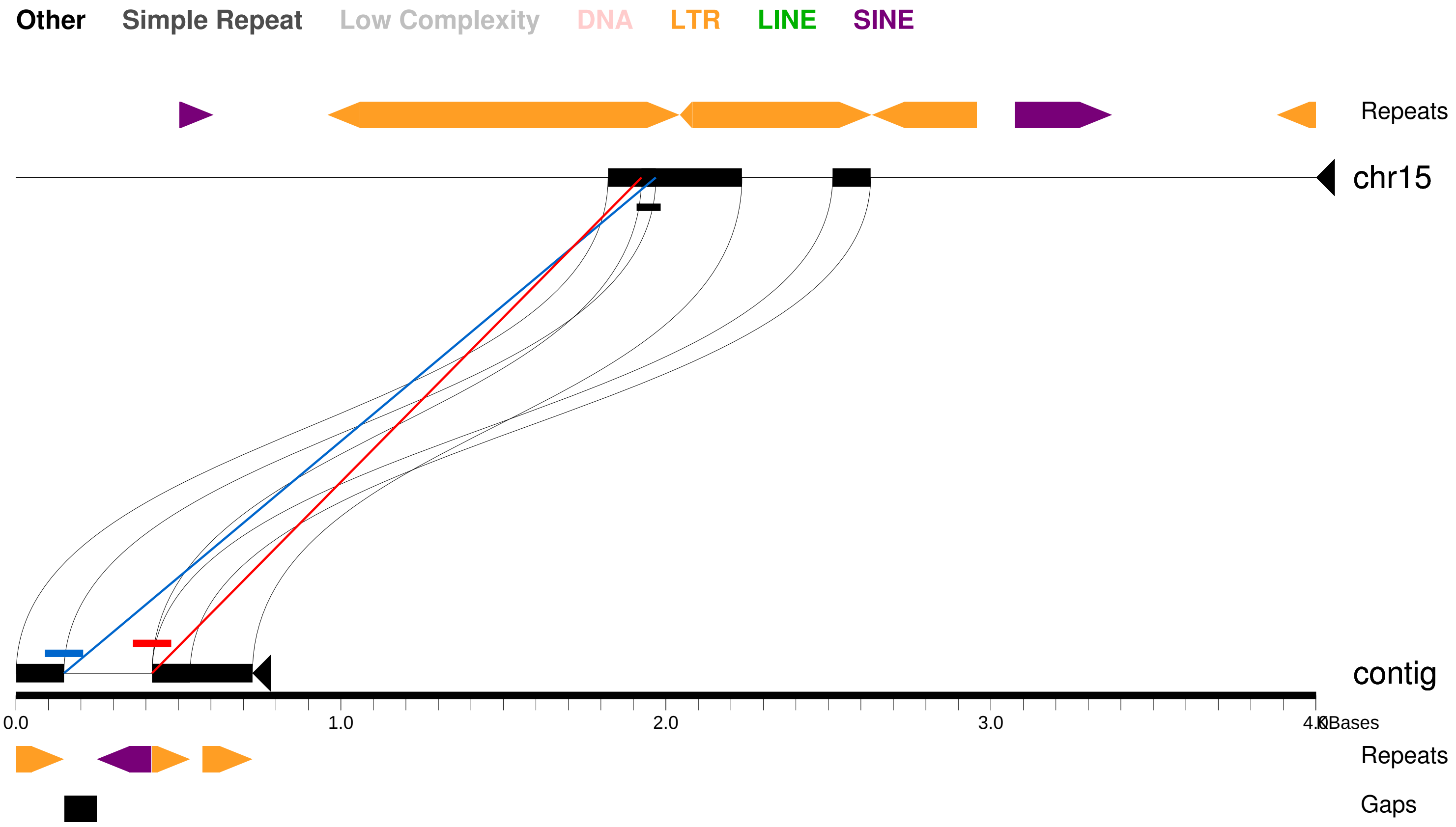

siteID: chr15_70408646 Scafftig2 - Breakpoint: chr15:70408626
chr15:70408571-70408685
Overlap Length: 12 INS size: 434 Genotyped: No

```
left    TTTCTGCTTGATTTTTCTGTAAACCTAAAACTGATTTTTTTTAAAGTCCTTTAAA----AAAAAAGAGCG
chrom   TTTCTGCTTGATTTTTCTGTAAACCTAAAACTGATTTTTTTTAAAGTCCTTTAAA----AAAAA-GAGCG
right   NNNNNNNNNNNNNNNNNNNNNNNNNNNNNNNNNNNNNNNNNNNNNNNNNNNNNNNNNNNNAAAAA-GAGCG
        111111111111111111111111111111111111111111111111111111111111*****2*****

left    TCGGCCGGGCGCGGTGGC-TCACGCCTGTAATCCCAGCACTT-TGGGAGGCCGA-G
chrom   TC------ATGAAGTGTAATCACGGAGAAATGCATAGGTGTTATGGGAGCAATCTG
right   TC------ATGAAGTGTAATCACGGAGAAATGCATAGGTGTTATGGGAGCAATCTG
        **22222222*22***222*****22222*22*22**222**2******222222*
```

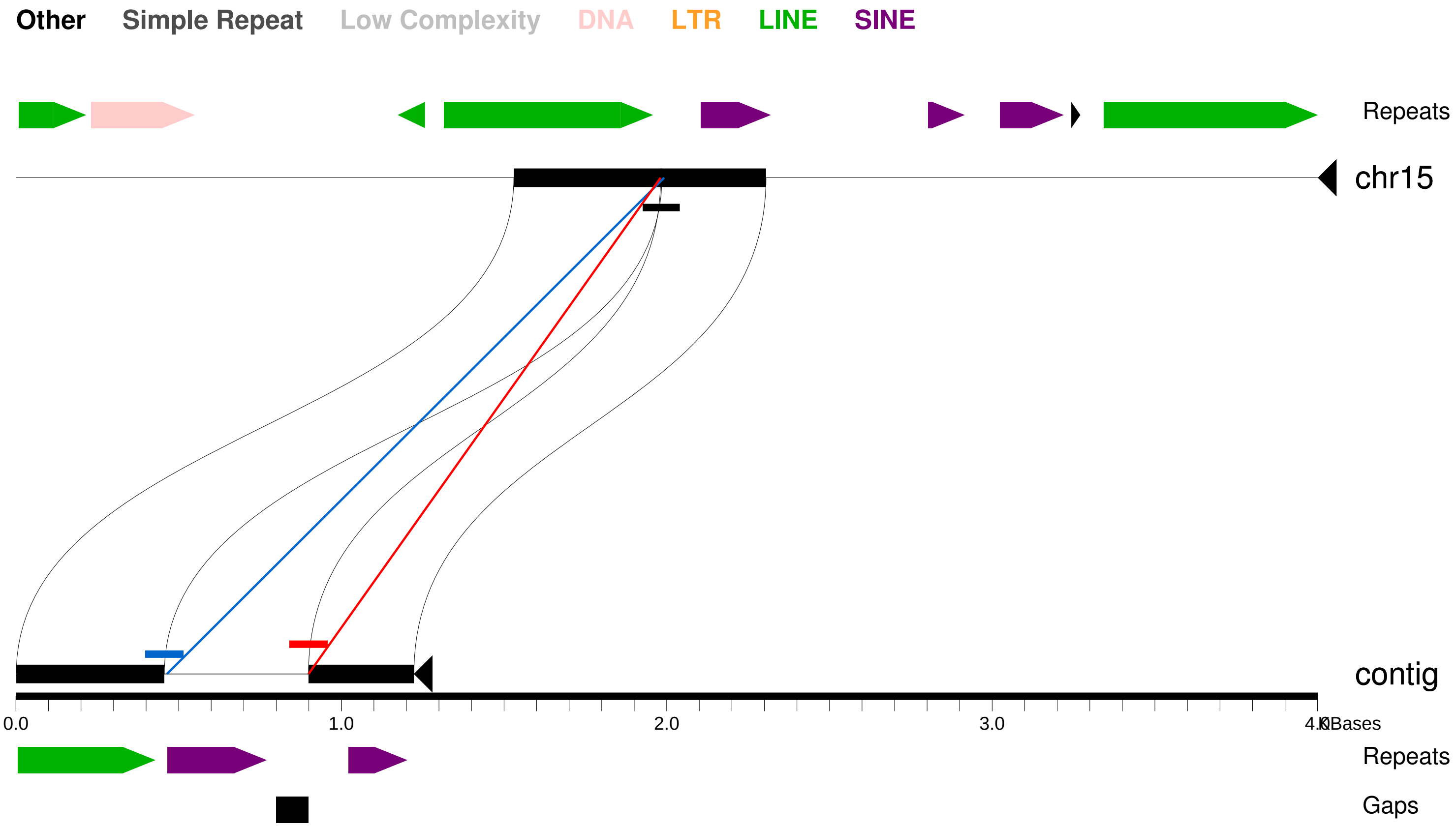

siteID: chr15_73983323 Scafftig2 - Breakpoint: chr15:73983311
chr15:73983151-73983479
Overlap Length: 9 INS size: 313 Genotyped: Yes

```
left   -----GCCCACTGTGGTGAGGGGCAAGTAGGAAGAGGTGAGGCTGGGCAGGTGGGCAGGGCCAGGGCAGA
chrom  AAGCAGCCCACTGTGGTGAGGGGCAAGTAGGAAGAGGTGAGGCTGGGCAGGTGGGCAGGGCCAGGGCAGA
right  -----------TTTAGT---------------AGAGACGGGGTTTCACCGTTTTA----GCCAGG----A
            111111*1*1**111111111111111****11*1**1*111*1*1*1111111******1111*

left   TGGGGCTCTGTAGGCTATGGAGGAGCT-GCGATCTTATCACAAGGAAAGTGAACAGCCCTATGATTAACT
chrom  TGGGGCTCTGTAGGCTATGGAGGAGCT-GCGATCTTATCACAAGGAAAGTGAACAGCCCTATGATTAACT
right  TGGT-CTCGATCTCCT------GACCTCGTGATCCGCCCGCCTCGGCCTCCCAAAGTGCTGGGATTACAG
       ***11***11*111**111111**1**1*1****1111*1*111*1111111*1**11**11*****111

left   ATTGAAGGCAGTGATATAATT-TATGCTTTTTTTTTTTTTTTTAATTTTTTTT----TTTTTTTGAG----
chrom  ATTGAAGGCAGTGATATAATT-TATGCTTTTATATTAATAGCAATTATTCTGGCTGTTGTATAGAGTATA
right  GCGTGAGCCACCGCGCCCGGCCTATGCTTTTATATTAATAGCAATTATTCTGGCTGTTGTATAGAGTATA
       11111**1**11*111111111*********2*2**22*222****2**2*22222**2*2*2***2222

left   ---------ACGGAGT------------CTCGCTCTGTCGCCCAGGCTGGAGTGCAGTGGC-------GG
chrom  GATTGGAGAACAGAATTGGGTGGGGGAACTAGAGAAGGTGAACAGACTGG--TGAAGAGGCTTTTATAGT
right  GATTGGAGAACAGAATTGGGTGGGGGAACTAGAGAAGGTGAACAGACTGG--TGAAGAGGCTTTTATAGT
       222222222**2**2*222222222222**2*2222*22*22***2****22**2**2***2222222*2

left   GATCTCGGC------TCACTGCAAGCTCCGCCTCCCGGGTTCA----CGCCATT
chrom  TATCCAGGCAAGAGATGA-TGGTGACTTGGAAGACCAGGAAGAGAAGCAGGGGT
right  TATCCAGGCAAGAGATGA-TGGTGACTTGGAAGACCAGGAAGAGA---------
       2***22***222222*2*2**2222**22*2222**2**222*22  1NNNNN1
```

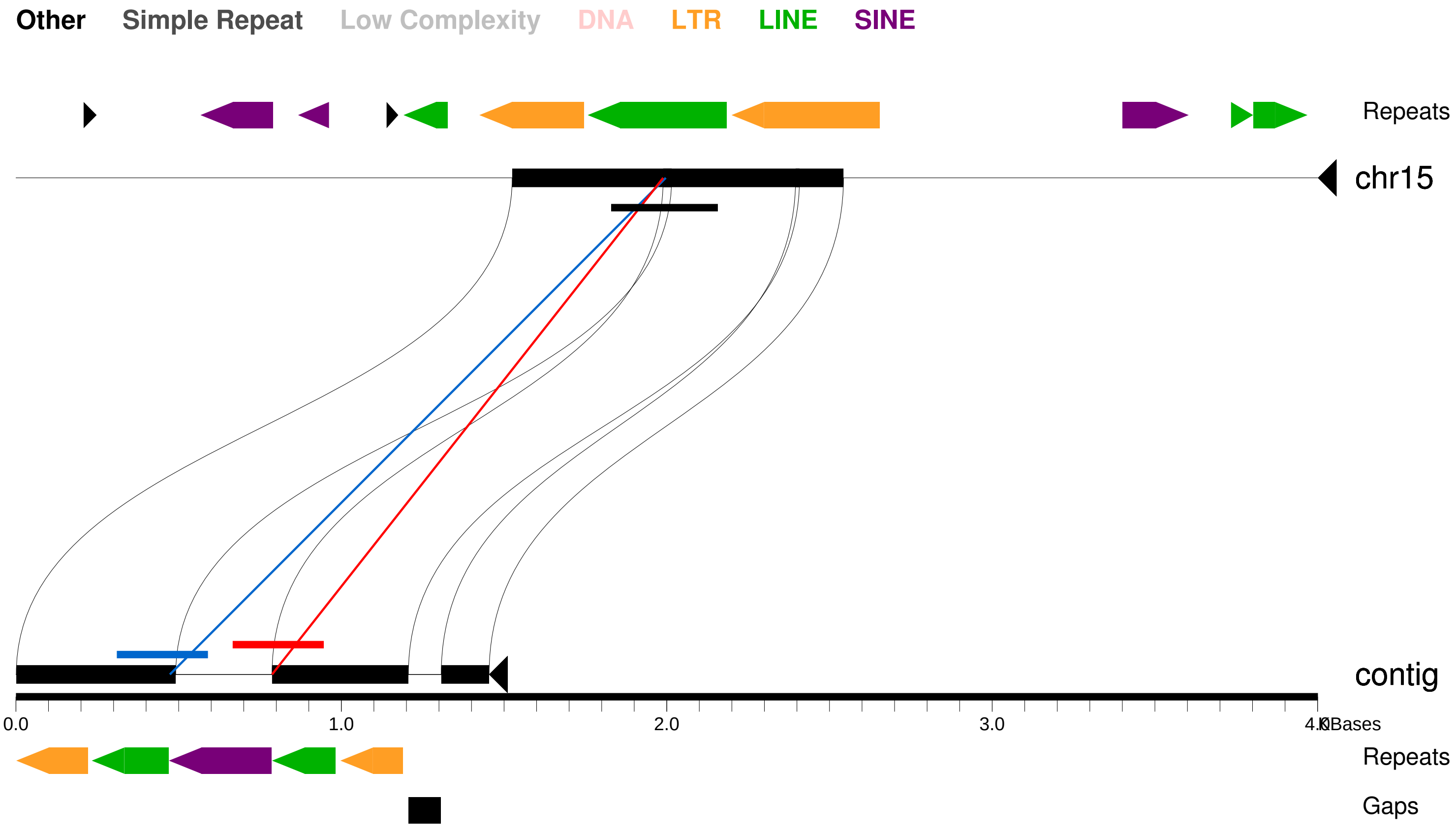

siteID: chr15_76826034 Scafftig3 - Breakpoint: chr15:76825980
chr15:76825960-76826037
Overlap Length: 40 INS size: 343 Genotyped: No

```
left   ATGAGTGTACATATGTAT-----------------------------------------GATCTATGGAC
chrom  ATGAGTGTACATATGTAT-----------------------------------------GATCTATGGAC
right  NNNNNNNNNNNNNNNNNNNNNNNNNNNNNNNNNNNNNNNNNNNNNNNNNNNNNNNNNNNNNTCTATGGAC
       1111111111111111111111111111111111111111111111111111111111111*********

left   ATATCTTAAGCAATGTTAAAATTATTACACAGCACTTTGGGAGGCCGAGGCGGGCGGATCACGAGGTCAG
chrom  ATATCTTAAGCAATGTTAAAATTATTACACA----TTT------CCT-----------TCAC--------
right  ATATCTTAAGCAATGTTAAAATTATTACACA----TTT------CCT-----------TCAC--------
       *******************************2222***222222**222222222222****22222222

left   GAGATCGAGACCATCCCGGC
chrom  ----TCTTGCTC--------
right  ----TCTTGCTC--------
       2222**22*22*22222222
```

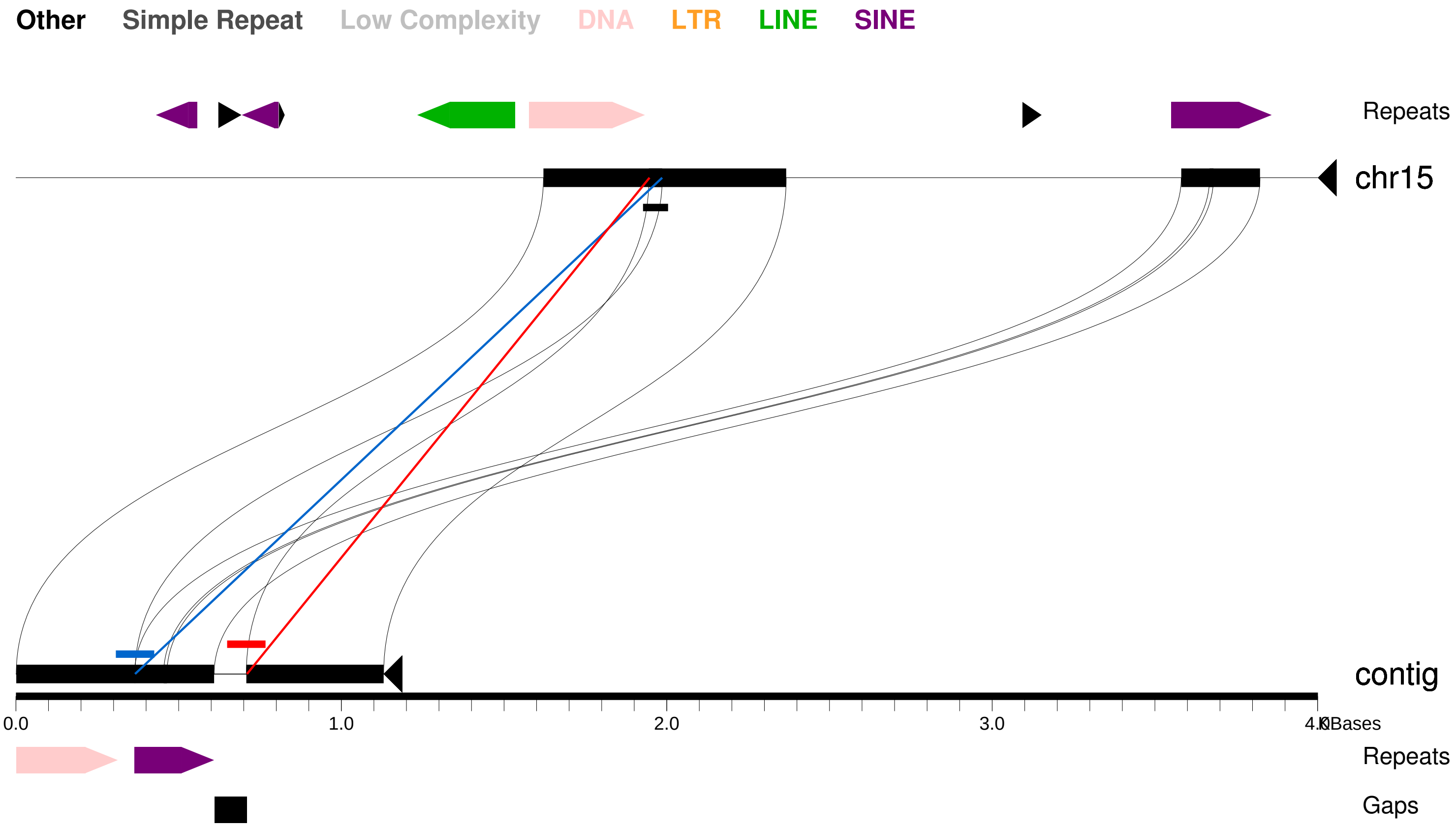

siteID: chr15_77092474 Scafftig3 + Breakpoint: chr15:77092489
chr15:77092442-77092545
Overlap Length: 13 INS size: 272 Genotyped: Yes

```
left    -TCCC---CTCCTG------TG-TCAGAAC----CTGTATAC-AGTATTCTGC-ATAACATATACATGGA
chrom   -TCCC---CTCCTG------TG-TCAGAAC----CTGTATAC-AGTATTCTGC-ATAACATATACATGGA
right   GTCTCGATCTCCTGACCTCGTGATCCGCCCGCCTCGGCCTCCCAAAGTGCTGGGATTACATA--CATGGA
        1**1*111******111111**1**1*11*1111*1*11*1*1*111*1***11**1*****11******

left    TATTTTTTTTTTTTTTTTTTTTTTTTTTTTGAGACGGAGTC-TCGTTCTGTCGCCCAGGCGGGAGTG
chrom   TATTTTTATAGTAGAATAT---------------CAGAATCATAATACTATAATA-ACTAAGAAAAT
right   TATTTTTATAGTAGAATAT---------------CAGAATCATAATACTATAATA-ACTAAGAAAAT
        *******2*22*2222*2*222222222222222*2**2**2*22*2**2*22222*2222*2*222
```

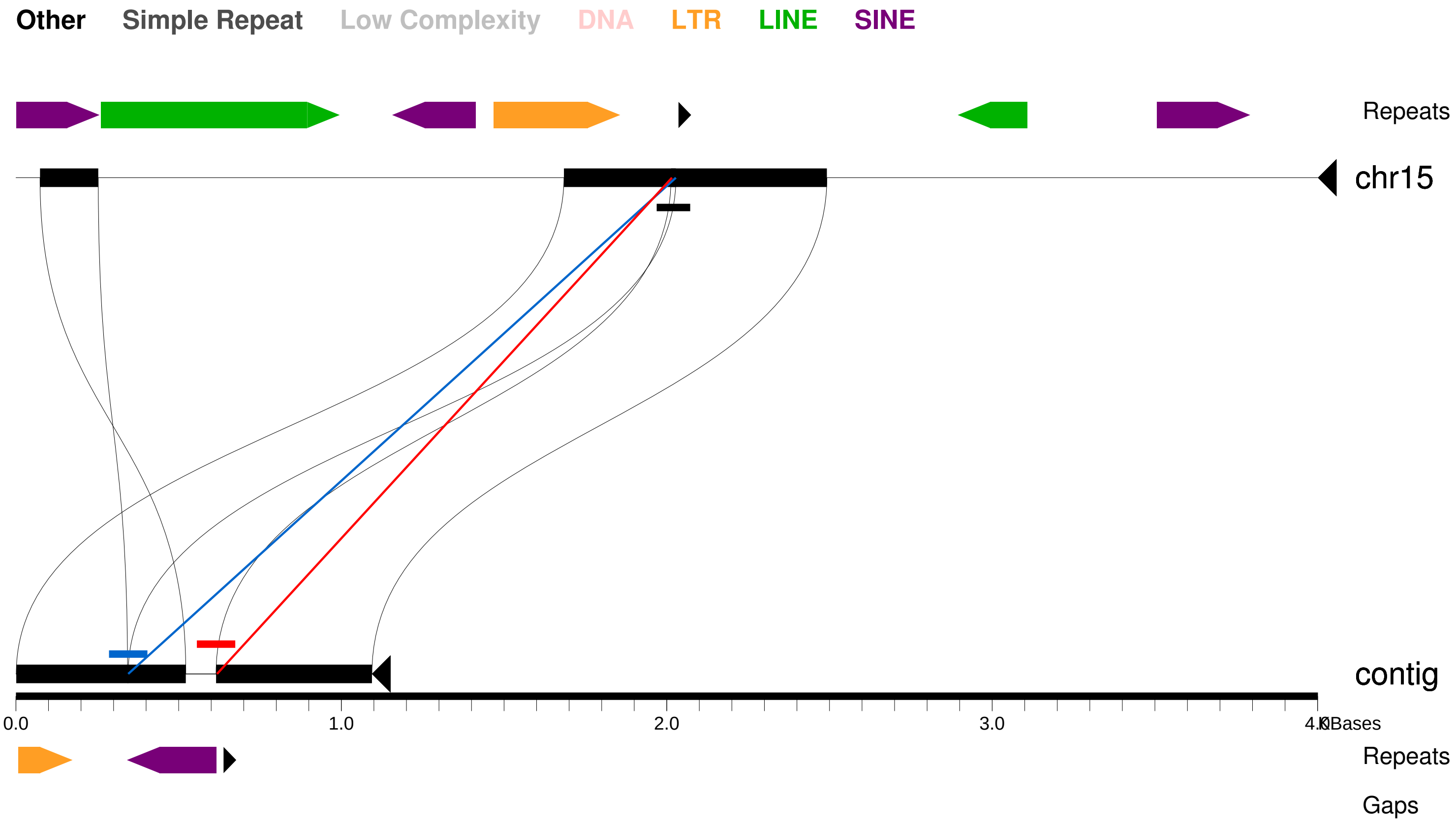

siteID: chr15_79795856 Scafftig5 - Breakpoint: chr15:79795873
chr15:79795713-79796045
Overlap Length: 13 INS size: 302 Genotyped: Yes

```
left   N---NNNNNN-NNNNNNNNNNNNNNNNNNNNNNNNNNNNNNNNNNNNNNNT-----------GAGATTGA
chrom  T---GTGGAC-CTCATTTTCTCCATTAAAAACAACATCAACAACAAACTCTTCAAAGTGACTGAACTTGA
right  TTTAGTAGAGACGGGGTTTCACCGTTTTAGCTGGGATGGTCT-CGATCTC----------CTGACCTCGT
       211122N22N12NNNN2222N22N22NN2NNNNNN22NNN2NN2N2N2221         22**N2*1*1

left   GAATGAGTATGCTCTCTTTAAATTTTCATACTCC-ACAGTGCTATAATAGAATTTTTAGGCATATTCTAA
chrom  GAATGAGTATGCTCTCTTTAAATTTTCATACTCC-ACAGTGCTATAATAGAATTTTTAGGCATATTCTAA
right  GA-----TCCGCCCGCCT--------CGGCCTCCCAAAGTGCTGGGAT------TACAGGCGTG-----A
       **11111*11**1*1*1*11111111*111****1*1******111**111111*11****1*111111*

left   GTCCATTAAGAATTATACAGGGCATCACATACATTCTTTTTTTTTTTTTTTTTTTTTTTTTTTGAGACAGAG
chrom  GTCCATTAAGAATTATACAGGGCATCACATACATTCTTAAAGAGACTTGGTGGTTGTATGTGAGATTCAA
right  G-CCACCGCG-------CCCGGC--CACATACATTCTTAAAGAGACTTGGTGGTTGTATGTGAGATTCAA
       *1***1111*1111111*11***11*************22222222**22*22**2*2*2*****222*2

left   TCTCGCTCTGTCGCCAGGCTGGAGTGCAG-----------TGGCGGGATCTCGGCTCACTGCA-AGCT-C
chrom  ATTTTCTA-GTCATCAGGCTTGGGAAGAGAAATATCCTAATTTCACAATATCCCCCAAGAACAGAGCTGC
right  ATTTTCTA-ATCATCAGGCTTGGGAAGAGAAATATCCTAATTTCACAATATCCCCCAAGAACAGAGCTGC
       22*22**221**22******2*2*222**22222222222*22*222**2**22*22*222**2****2*

left   CGCC-----------TCCCGGGTTCAC--GCCATTCTC-----------CTGCC-----
chrom  CGACATATATAATTATGCAGACTCCACCAGTGATTCCCTGATGTTGTTTCTCACTAATT
right  CGACATATATAATTATGCAGACTCCACCAGTGATTCCCTGATGTTGT------------
       **2*22222222222*2*2*22*2***22*22****2*222222222  11NN1
```

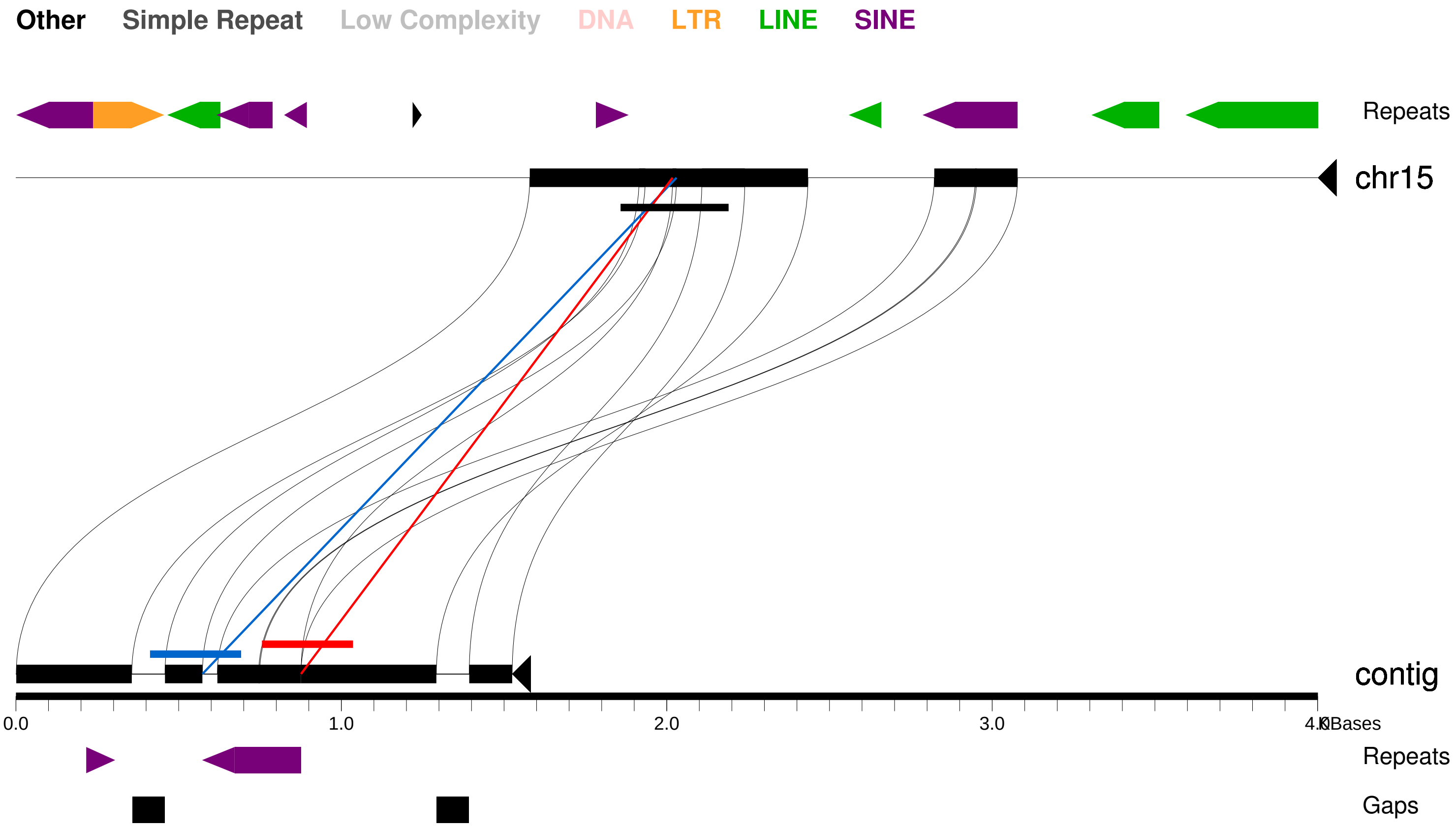

siteID: chr15_81392579 Scafftig1 + Breakpoint: chr15:81392586
chr15:81392543-81392645
Overlap Length: 12 INS size: 375 Genotyped: No

```
left    CAA--------GAATTATTTAACATCTGC-ATTAT-----TTTGAAACTGTATTCAT--AATACTTATTT
chrom   CAA--------GAATTATTTAACATCTGC-ATTAT-----TTTGAAACTGTATTCAT--AATACTTATTT
right   CCGCCCGCCTCGGCCTCCCAAAGTGCTGGGATTACAGGCGTGAGCCACCGCGCCCGGCCAATACTTATTT
        *1111111111*111*1111**111***11****111111*11*11**1*1111*1111***********

left    CTTTTTTTTNNNNNNNNNNNNNNNNNNNNNNNNNNNNNNNNNNNNNNNNNNNNNNNNNNNNNNNN
chrom   CATCTTAGTTCTAAGTTTAAATTATTTCAGTGCTGATTATCTGTCCTAC----------------
right   CATCTTAGTTCTAAGTTTAAATTATTTCAGTGCTGATTATCTGTCCTAC----------------
        *2*2**22*22222222222222222222222222222222222222222222222222222222
```

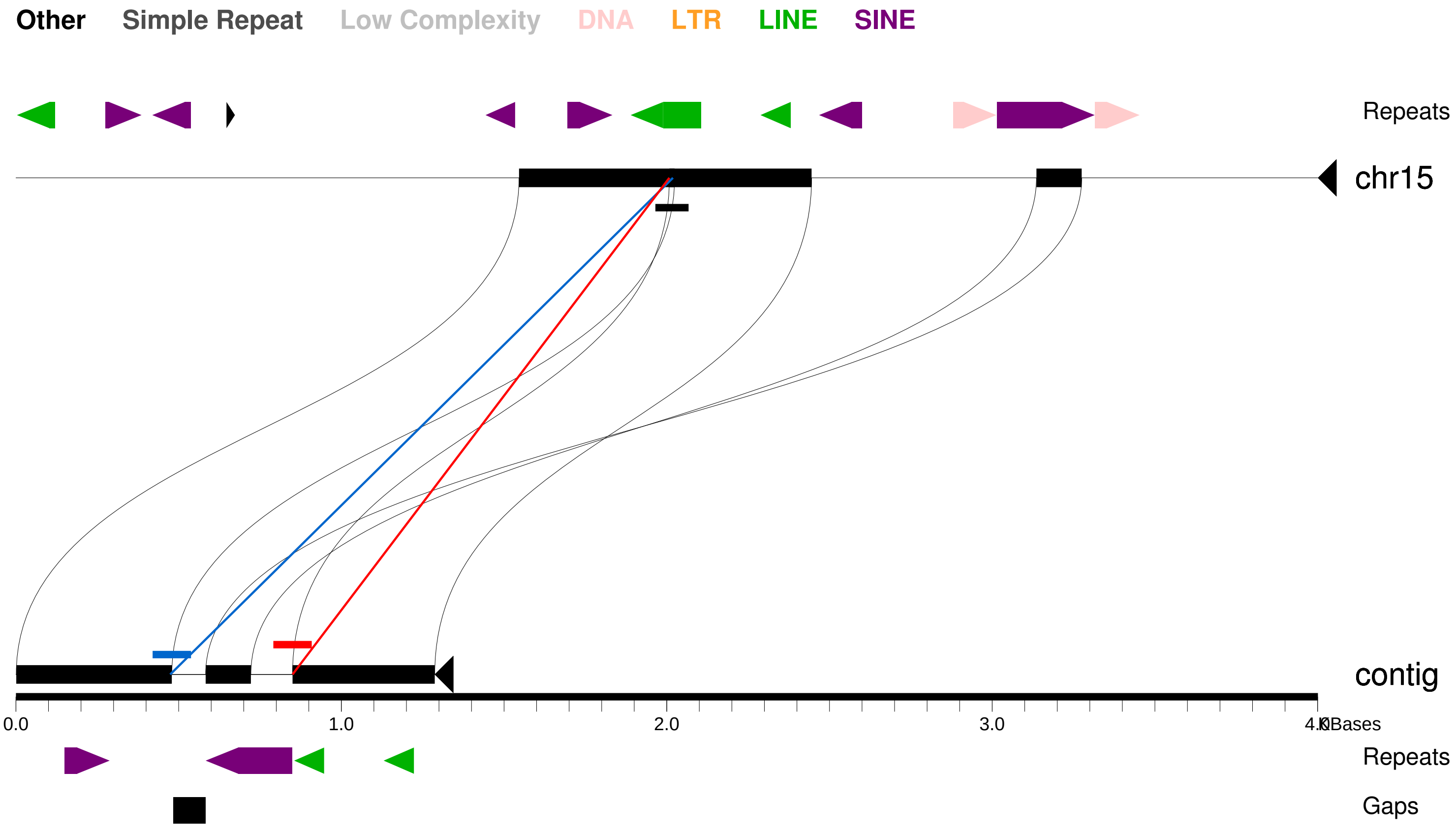

siteID: chr15_82035645 Scafftig3 - Breakpoint: chr15:82035657
chr15:82035610-82035716
Overlap Length: 13 INS size: 316 Genotyped: Yes

```
left     TAGGTTTCCTCTGGTT------GTACTCTT-TGATAGAAATTGGCTTCAAAGTT-----AAGCTTATTTT
chrom    TAGGTTTCCTCTGGTT------GTACTCTT-TGATAGAAATTGGCTTCAAAGTT-----AAGCTTATTTT
right    CCGCCCGCCTCGGCCTCCCAAAGTGCTGGGATTACAGGCGTGAGCCACCGCGCCCGGCCAAGCTTATTTT
         11*1111****1*11*111111**1**1111*1*1**111*11**11*111*1111111***********

left     CTTTCTTTTTTTTTTTTTTTTTTTTTTTTTTTTTTTGAGACGGAGTCTCGCTCTGTCGCCCAG
chrom    CTAACTGGGCAGATGTCCATATTTTTTAATACTCTGGA-GGAGT-TAATTC----------
right    CTAACTGGGCAGATGTCCATATTTTTTAATATTCTGGA-GGAGT-TAATTC----------
         **22**2222222*2*222*2******22*2N*222**2*****2*222**2222222222
```

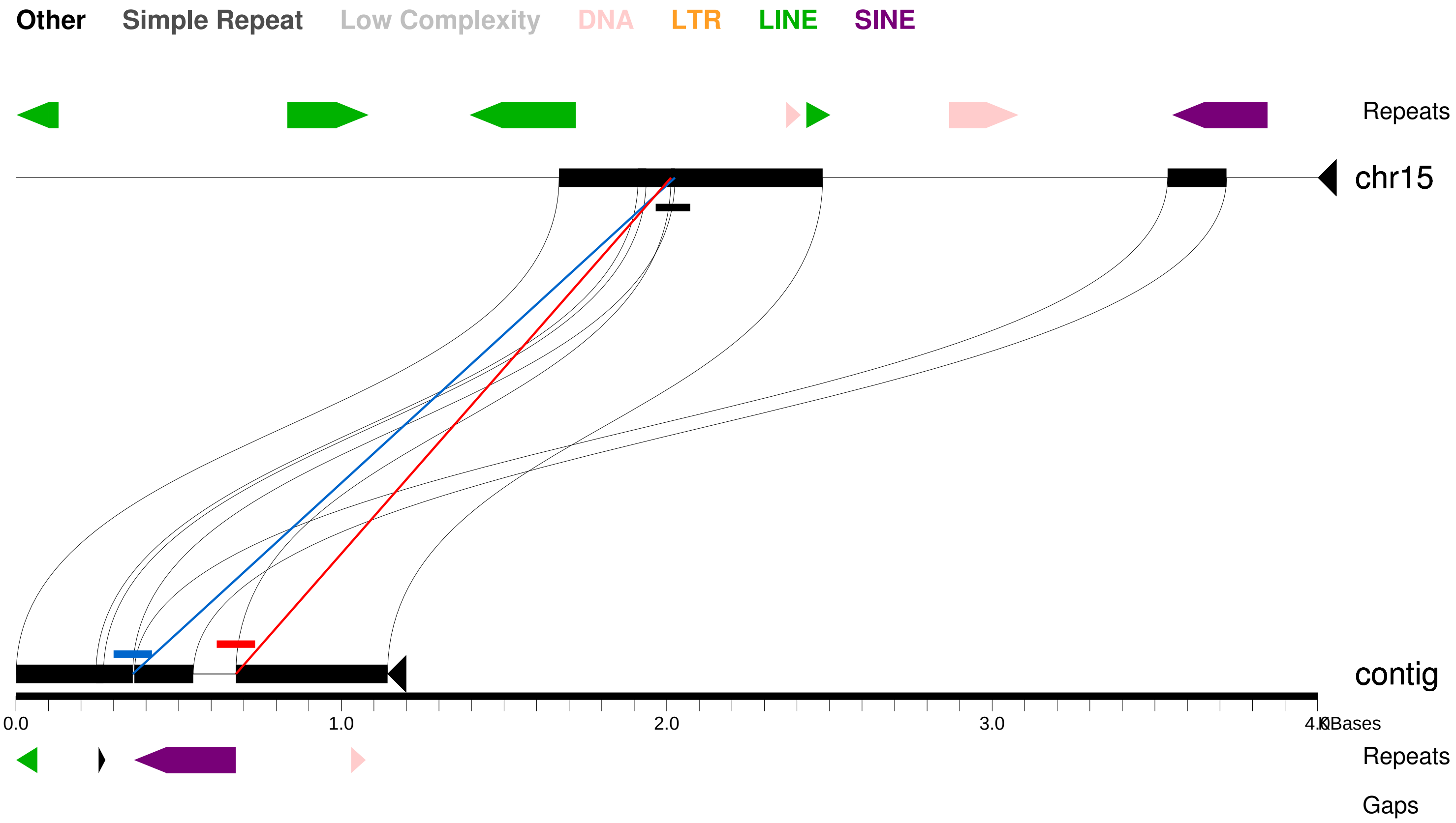

siteID: chr15_86224926 Scafftig4 + Breakpoint: chr15:86224951
chr15:86224904-86225010
Overlap Length: 13 INS size: 299 Genotyped: Yes

```
left    CCTGTTTGCTAATCT------ATTTCTAGCAATTCT--CATGAAAATATTAGAGA----TGAGCTAGATC
chrom   CCTGTTTGCTAATCT------ATTTCTAGCAATTCT--CATGAAAATATTAGAGA----TGAGCTAGATC
right   CCACCCACCTCGGCCTCCCAAAGTGCTGGGATTACAGGCGTGAGCCACTGCGCCCGGCCTGAGCTAGATC
        **111111**111*1111111*1*1**1*1*1*1*111*1***11111*11*1111111***********

left    TTTTTTTTTTTTTTTTTTTTGAGACGGAGTCTCGCTCTGTCACCCAGGCTGGAGTGCAGTG--
chrom   TTATAGAAACATAT-------AAAAGCAGTAT-------TAACGGATAAAATATGACATTTAA
right   TTATAGAAACATAT-------AAAAGCAGTAT-------TAACGGATAAAATATGACATTTAA
        **2*2222222*2*2222222*2*2*2***2*2222222*2**22*222222*222**2*222
```

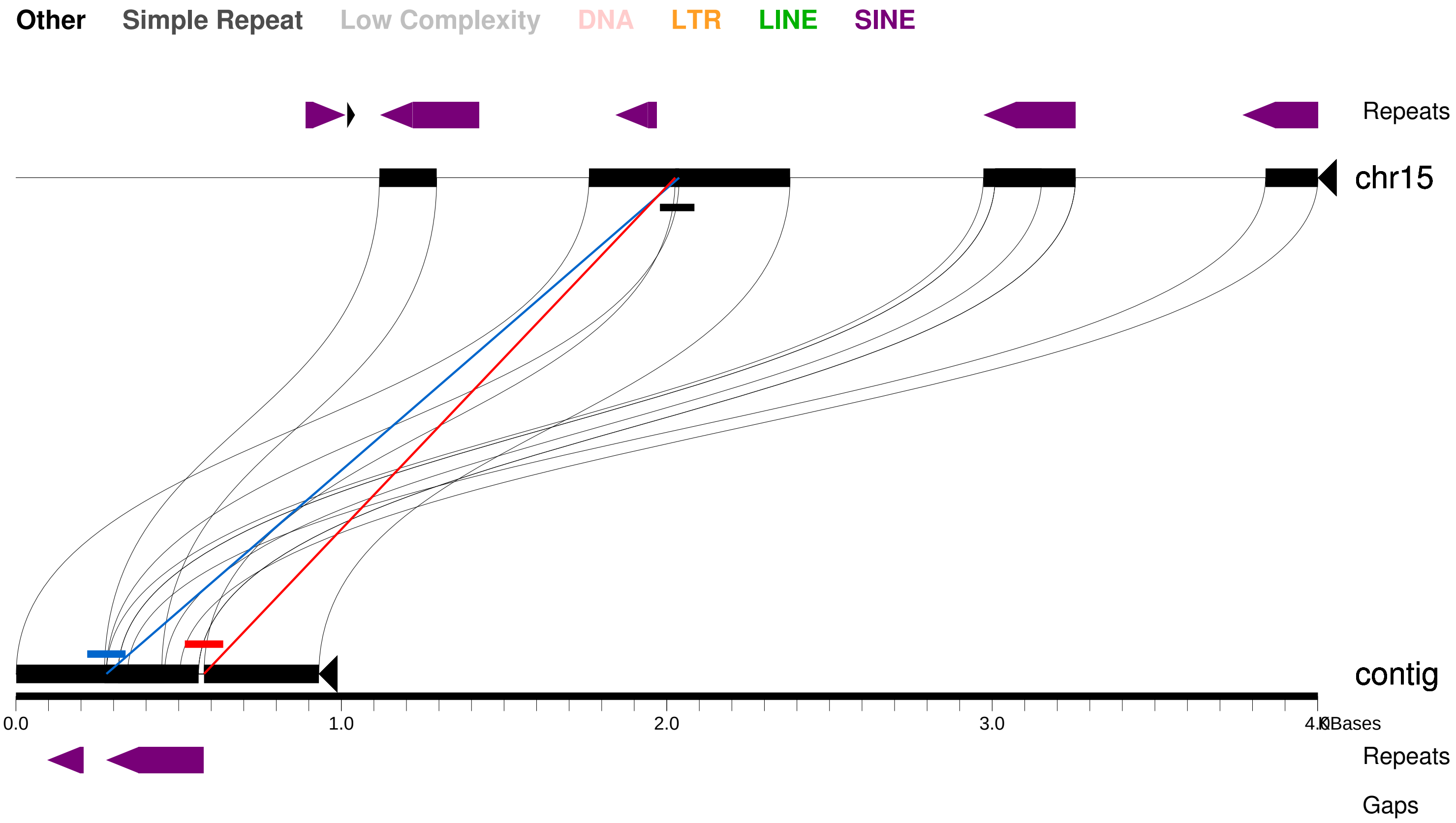

siteID: chr15_88629748 Scafftig2 - Breakpoint: chr15:88629728
chr15:88629690-88629798
Overlap Length: 22 INS size: 447 Genotyped: No

```
left   TAGATATATTGGGCTAAAAT-ACATAATTAGAATTA---------ATTTCACCTGTTTCTTTTTTTTTTT
chrom  TAGATATATTGGGCTAAAAT-ACATAATTAGAATTA---------ATTTCACCTGTTTATTTTATTTTTT
right  GCCTCCCAAAGTGCTGGGATTACAGGCGT-GAGCCACCGCGCCCGGCCTCACCTGTTTCTTTTATTTTTT
       1111111*11*1***111**1***1111*1**111*111111111111**********N****2******

left   TTTTTTTTTTTTAGA-CGGNNNNNNNNNNNNNNNNNNNNNNNNNNNNNNNNNNNNNNNNN
chrom  AA----------AAA-CATGGCTACTAGAAAACATCAAAGTATGTATGGGGCTCTCTCGT
right  AA----------AAAACATGGCTACTAGAAAACATCAAAGTATGTATGGGGCTCTCTCGT
       222222222222*2*1*2222222222222222222222222222222222222222222
```

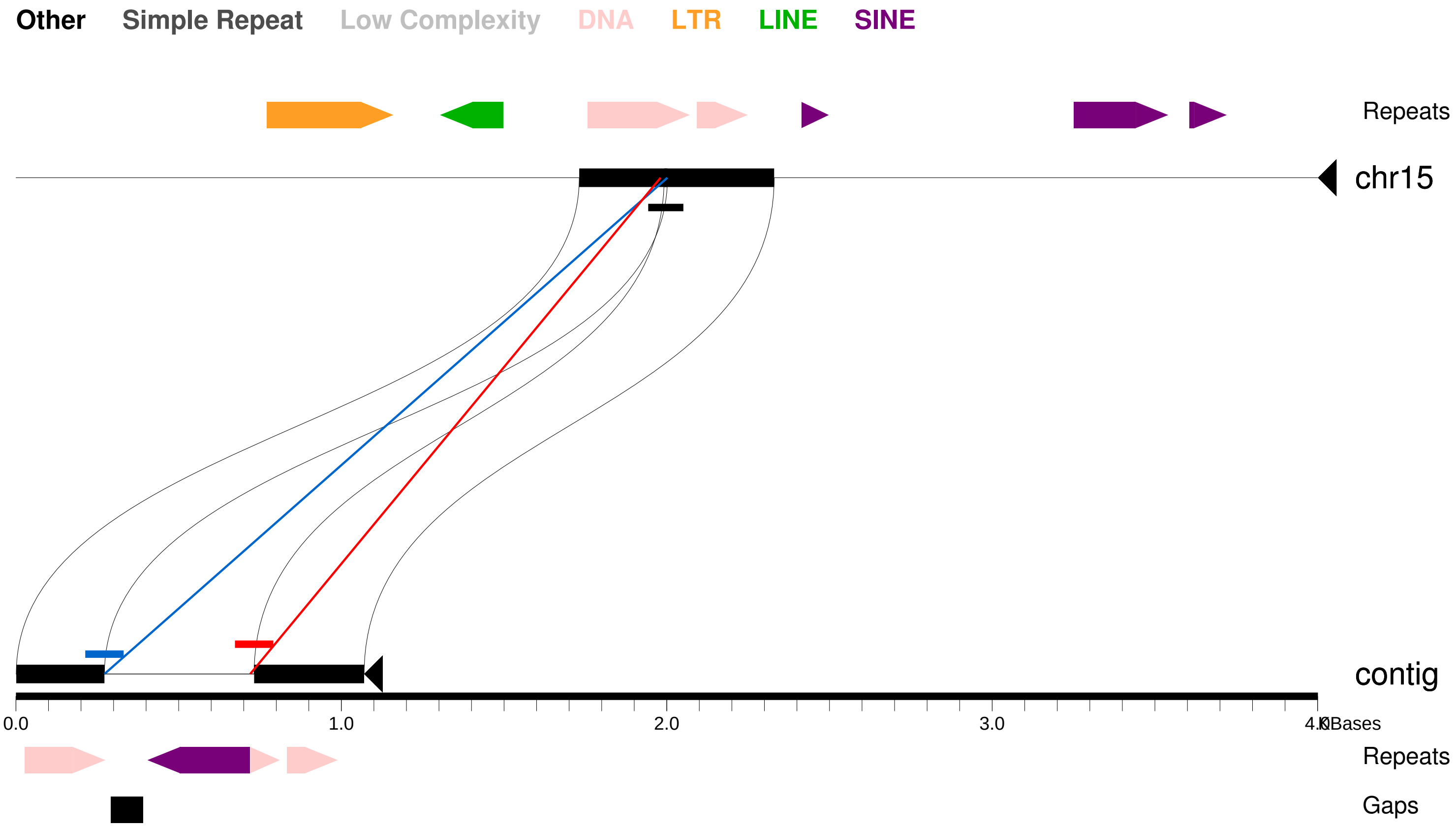

siteID: chr15_92573047 Scafftig4 + Breakpoint: chr15:92573044
chr15:92572995-92573103
Overlap Length: 11 INS size: 297 Genotyped: Yes

```
left    GCCCCAGGATCTGGGCTCCC--AGTGCAGCTG----AGGCCTG------CTCACTTGGCTTCATACATTT
chrom   GCCCCAGGATCTGGGCTCCC--AGTGCAGCTG----AGGCCTG------CTCACTTGGCTTCATACATTT
right   CCGCCCGCCTC-GGCCTCCCAAAGTGCTGGGATTACAGGCGTGAGCCACCGCGCCCGGCC-CATACATTT
        1*1**1*11**1**1*****11*****1*1111111****1**111111*1*1*11***11*********

left    TTTTTTTTTTTTTTTTTTGAGACGGAGTCTCGCTCTGTCGCCCAGGCTGGAGTGCAGTGG--C
chrom   TTAATAGAACTCTT--------CCGTCTCACGTTATGA----GAGATAAAAGATCTATGATTC
right   TTAATAGAACTCTT--------CCGTCTCATGTTATGA----GAGATAAAAGATCTATGATTC
        **22*22222*2**22222222*2*22**21*2*2**222222**22222**22*22**222*
```

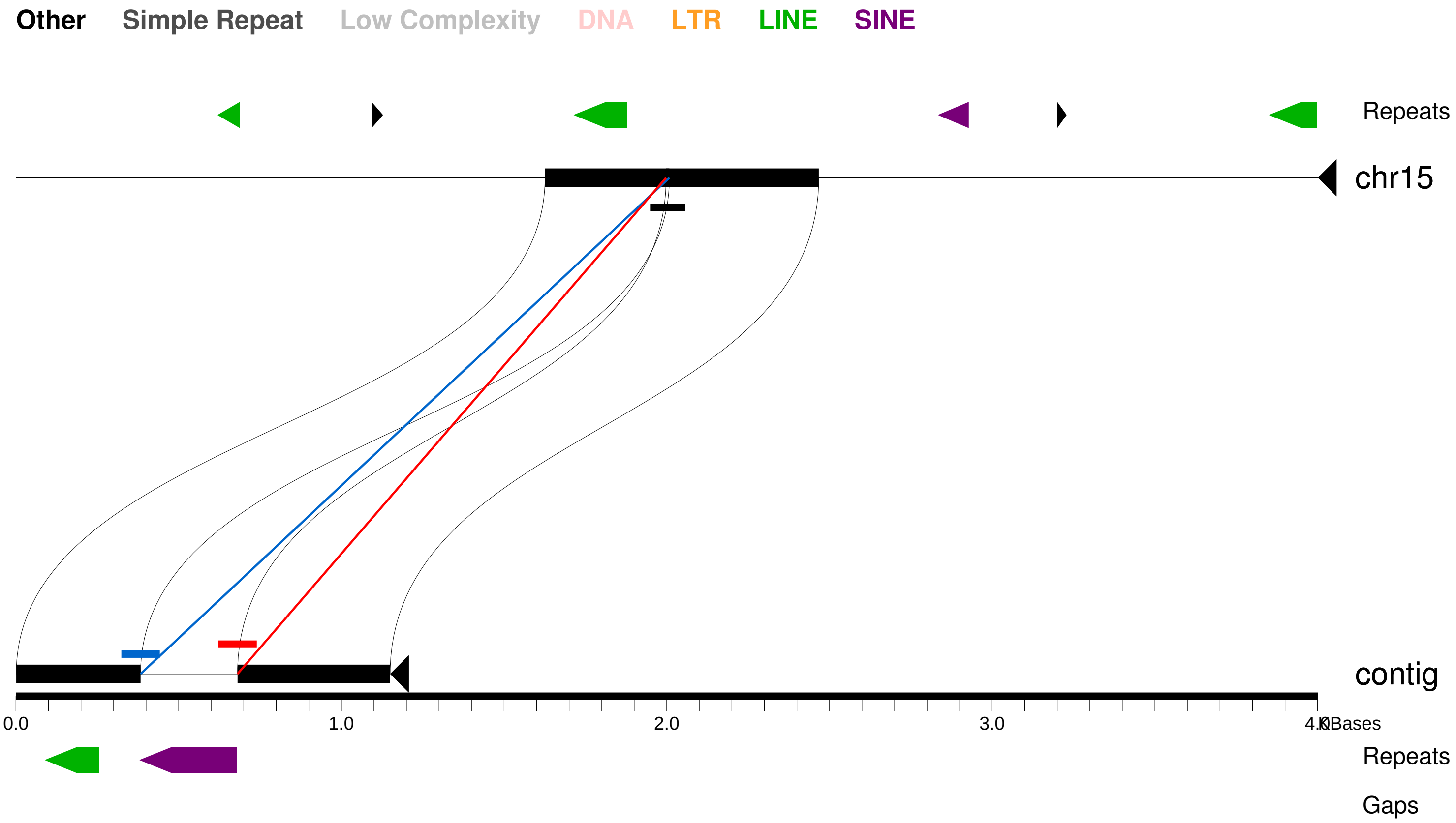

siteID: chr15_94715723 Scafftig4 - Breakpoint: chr15:94715731
chr15:94715684-94715790
Overlap Length: 13 INS size: 298 Genotyped: Yes

```
left    AACCACAG----------TGAGATACC-ATCTC-ACACCCATTAGGATGACTATGATTTAAAAAACAAAA
chrom   AACCACAG----------TGAGATACC-ATCTC-ACACCCATTAGGATGACTATGATTTAAAAAACAAAA
right   CGCCACTGCACTCCAGCCTGGGCGACAGAGCGAGACTCCGTCTCAAAAAAAAAAAAAAAAAAAAAAAAAA
        11****1*1111111111**1*11**11*1*111**1**111*111*11*11*11*111******1****

left    ACGGCCGG-GCGCGGTGGCTCACGCCTGTAATCCCAGCACTTTGGGAGGCCGAGGCGGGCGG
chrom   ACAAAAGCAGAAAACAAGTACTGGCAAGGATGCAAAGAAATT--GGAGACC-----------
right   ACAAAAGCAGAAAACAAGTACTGGCAAGGATGCAAAGAAATT--GGAGACC-----------
        **2222*22*2222222*22*22**22*2*22*22**2*2**22****2**22222222222
```

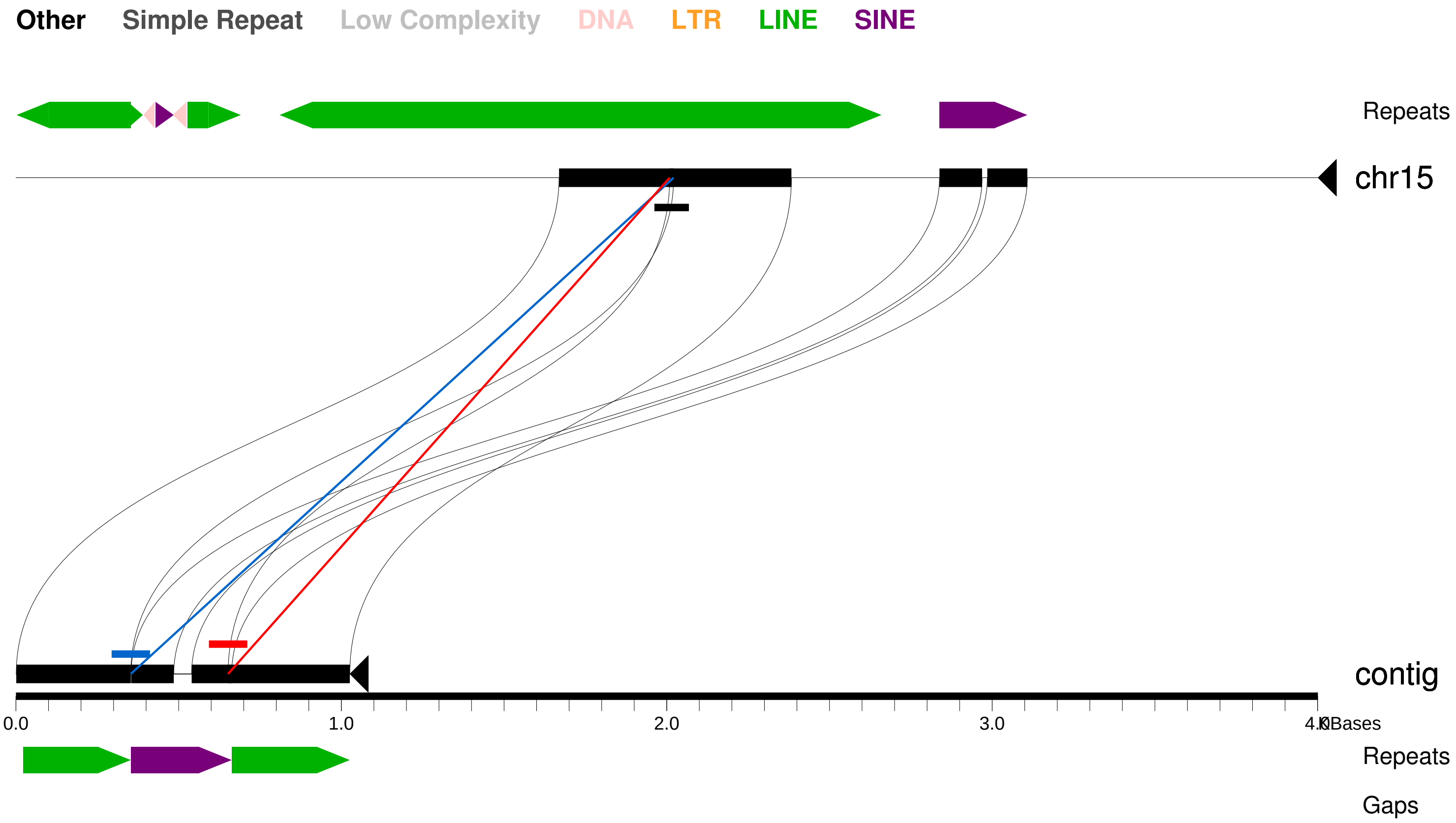

siteID: chr15_94819888 Scafftig2 + Breakpoint: chr15:94819891
chr15:94819731-94820063
Overlap Length: 13 INS size: 293 Genotyped: Yes

```
left   -----------CTGAAAGTAGTATAACACATGACTCAACCTAAACAAAGAACAAAGGAAAAATTACAGGT
chrom  GTCTTTCTTTGCTGAAAGTAGTATAACACATGACTCAACCTAAACAAAGAACAAAGGAAAAATTACAGGT
right  -----------CTGAG-GCAGGAGAA----TGGCGT------------GAACCCGGGA---------GGC
                  ****11*1**1*1**1111**1*11111111111111****111***111111111**1

left   TGATATTACATGTAAACTAG-TATGGACAAATAATAATATGTTCGTAATCTGAATTCAGCAATGGTATTT
chrom  TGATATTACATGTAAACTAG-TATGGACAAATAATAATATGTTCGTAATCTGAATTCAGCAATGGTATTT
right  GGAGCTTGCAGTGAGCCGAGATCCCGCCACTGCACTCCAGCCTGGGCGACAGA-----GCGAGACTCCGT
       1**11**1**111*11*1**1*111*1**1111*1111*111*1*1111*1**11111**1*111*111*

left   TTAAAATATTACAATGCAAATAAGAAATATTTGG--------CCGGGCGC----GGTGGCTCACGCCTGT
chrom  TTAAAATATTACAATGCAAATAAGAAATATTTGGTTATATCTCAGAATGAAAAAGATAGTTTAACATTAG
right  CTCAAAAAAAAAAAA----AAAAAGAAATATTTGGTTATATCTCAGAATGAAAAAGATAGTTTAACATTAG
       1*1***1*11*1**1111**1*************22222222*2*222*22222*2*2*2*2*2222*22

left   AATCCCA--GCACTTTGGGAGG----CCGA-----GGCGGGCGGA----TCACGAGGTCAGGAGATCGAG
chrom  AATATCAATGTAATTTACTACATTAACAGATAAAAGGAGAAAAGAAACATGATTACTTAAACAGATGCAG
right  AATATCAATGTAATTTACTACATTAACAGATAAAAGGAGAAAAGAAACATGATTACTTAAACAGATGCAG
       ***22**22*2*2***222*222222*2**22222**2*2222**2222*2*22*22*2*22****22**

left   ----ACCATCCC--GGCTAAAA-CGGTGAAACCCCGTCT------CTACTAA-A
chrom  GAAAAAAATCTTTTGGCAAAATTCAGCACTTGTCCATCTTAAGAGCTGTTAGTA
right  GAAAAAAATCTTTTGGCAAAATTCAGCACTTGTCCATCTTAAGAGCT-------
       2222*22***2222***2***22*2*2222222**2***222222**NN11N 1
```

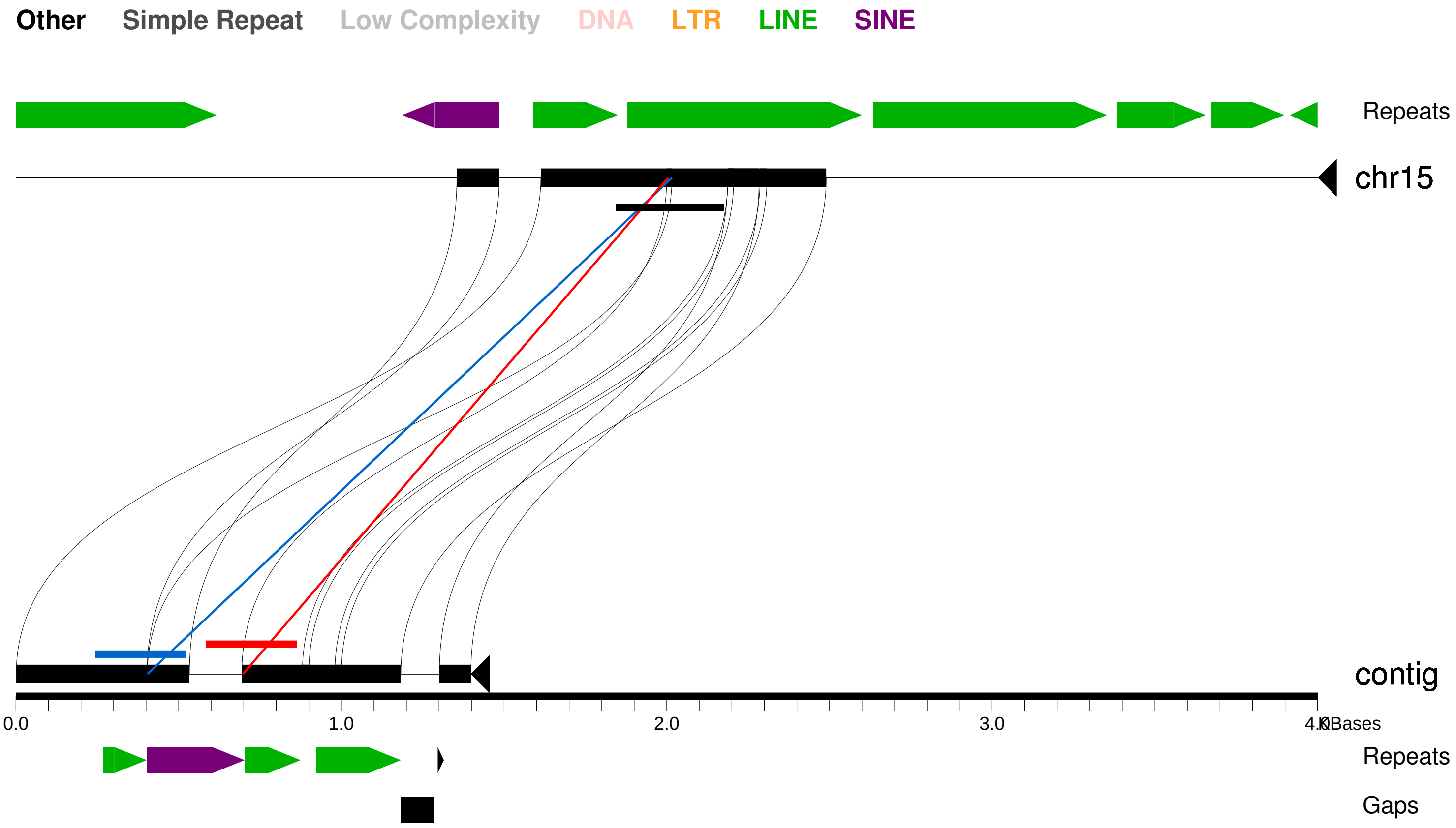

siteID: chr15_95164998 Scafftig2 - Breakpoint: chr15:95165007
chr15:95164971-95165066
Overlap Length: 24 INS size: 282 Genotyped: Yes

```
left    ATAT-------TTGGTAAACT--ACTAAAACAT-----------TGAATTT---TATAGAAAAATATAAA
chrom   ATAT-------TTGGTAAACT--ACTAAAACAT-----------TGAATTT---TATAGAAAAATATAAA
right   GTGAGCCGAGATCGCGCCACTGCACTCCAGCCTGGGCGACAGAGCGAGACTCCGTCTCAAAAAAAAAAAA
        1*111111111*1*1111***11***11*1*1*111111111111**111*111*1*11*****1*1***

left    ATAACATCATGAGGGCCGGGCGCGGTGGCTCACGCCTGTAATCCCAGCACTTTGGGAGGCCGAGGCGGGC
chrom   ATAACATCATGAGAT--------GTTATTTCAAGAAAGATGT--------TTTTTAATGACATAT-----
right   ATAACATCATGAGAT--------GTTATTTCAAGAAAGATGC--------TTTTTAATGACATAT-----
        *************2222222222*2*222***2*222*222122222222***222*2*2*222222222

left    GG
chrom   --
right   --
        22
```

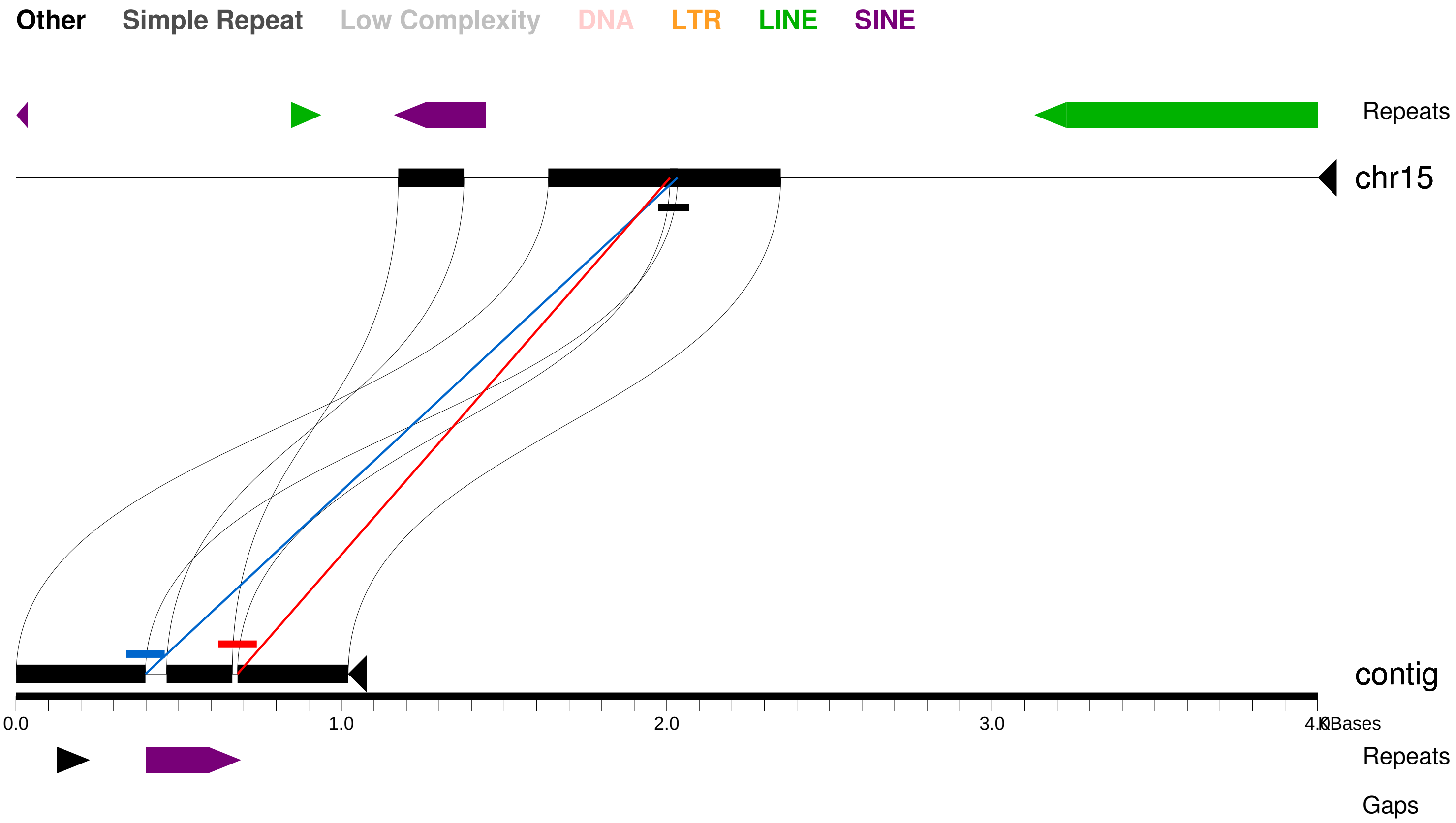

siteID: chr15_95775878 Scafftig1 - Breakpoint: chr15:95775881
chr15:95775822-95775940
Overlap Length: 1 INS size: 418 Genotyped: No

```
left    GGATAAGGAGGAAGAGGAGGAGGGGGAGGAGAAAGAGGAGGAGAAATGAAAGGAAAAGAAGGCCGGGCGC
chrom   GGATAAGGAGGAAGAGGAGGAGGGGGAGGAGAAAGAGGAGGAGAAATGAAAGGAAAAGAAATAATAATTA
right   NNNNNNNNNNNNNNNNNNNNNNNNNNNNNNNNNNNNNNNNNNNNNNNNNNNNNNNNNNNNAATAATAATTA
        11111111111111111111111111111111111111111111111111111111111*2222222222

left    GGTGGCTCACGCCTGTAATCCCAGCACTTTGGGAGGCCGAGGCGGGTGG---
chrom   --TTATTATGAAATGAAAAATCATG-CTGACAACAGTAGAAGCAAATAAATT
right   --TTATTATGAAATGAAAAATCATG-CTGACAACAGTAGAAGCAAATAAATT
        22*222*222222**2**222**222**2222222*22**2**222*22222
```

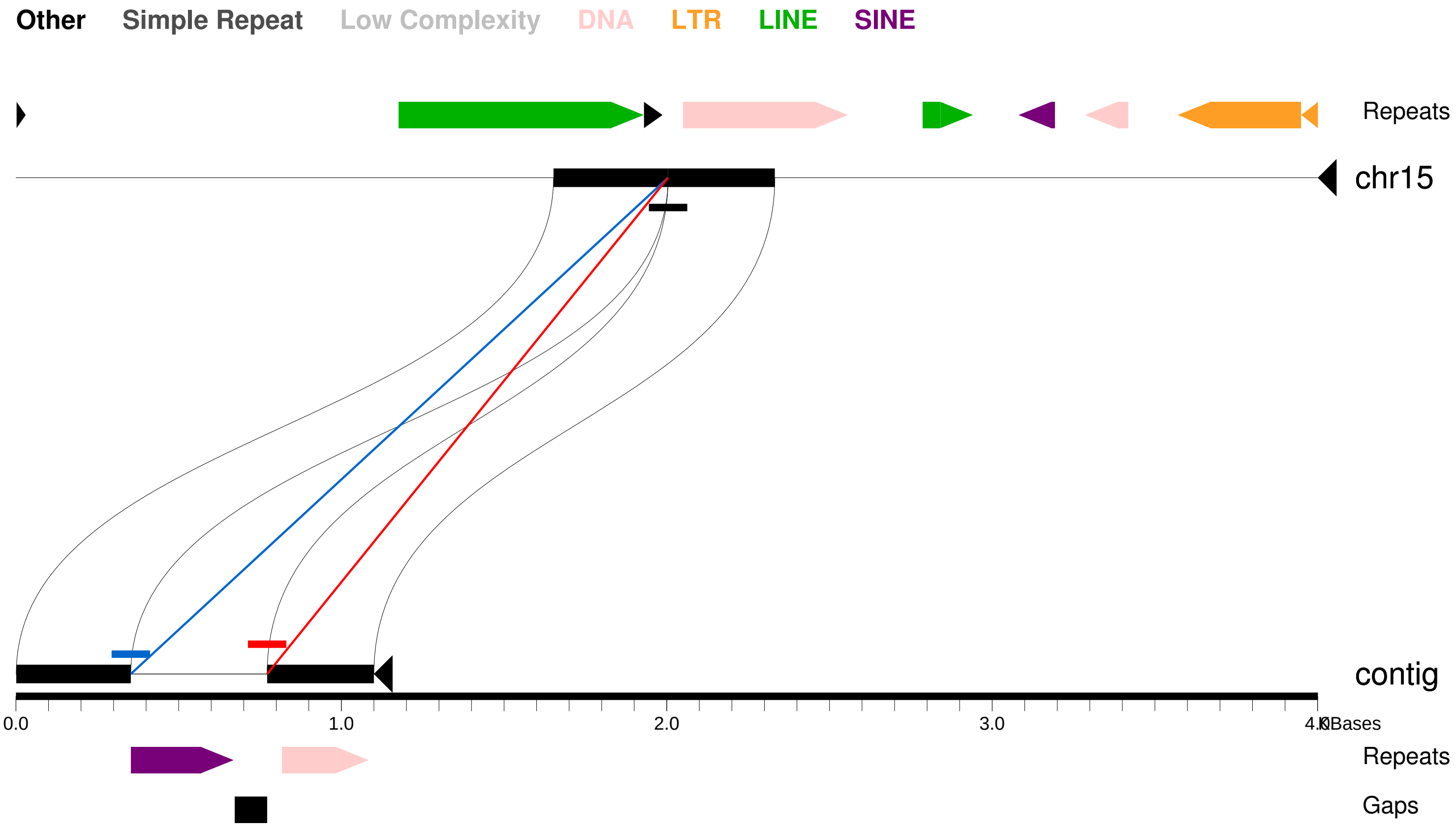

siteID: chr15_96127788 Scafftig4 - Breakpoint: chr15:96127767
chr15:96127720-96127826
Overlap Length: 13 INS size: 275 Genotyped: Yes

```
left    ---GCTAGTC--CTAATCCAAAGCTCTTTGT--------ATAGCATCTAGTCTGTATTTTAAGATGAAAT
chrom   ---GCTAGTC--CTAATCCAAAGCTCTTTGT--------ATAGCATCTAGTCTGTATTTTAAGATGAAAT
right   CAGCCTGGGCGACAGAGCGAGA-CTCCGTCTCAAAAAAAAAAAAAAAAAAAAAAAAAAAAAAGATGAAAT
        1111**1*1*11*11*1*1*1*1***11*1*11111111*1*11*111*111111*1111**********

left    TGACACTTTGGGAGGCCGAGGCGGGCGGATCACGAGGTCAGGAGATCGAGACCATCCCGGCT
chrom   TGAAATAGT----TGCTGTTTCC-----ATCCTTGGGTAATCTTATCCCTTCCATTAT---T
right   TGAAATAGT----TGCTGTTTCC-----ATCCTTGGGTAATCTTATCCCTTCCATTAT---T
        ***2*222*22222**2*222*222222***2222***2*2222***2222****222222*
```

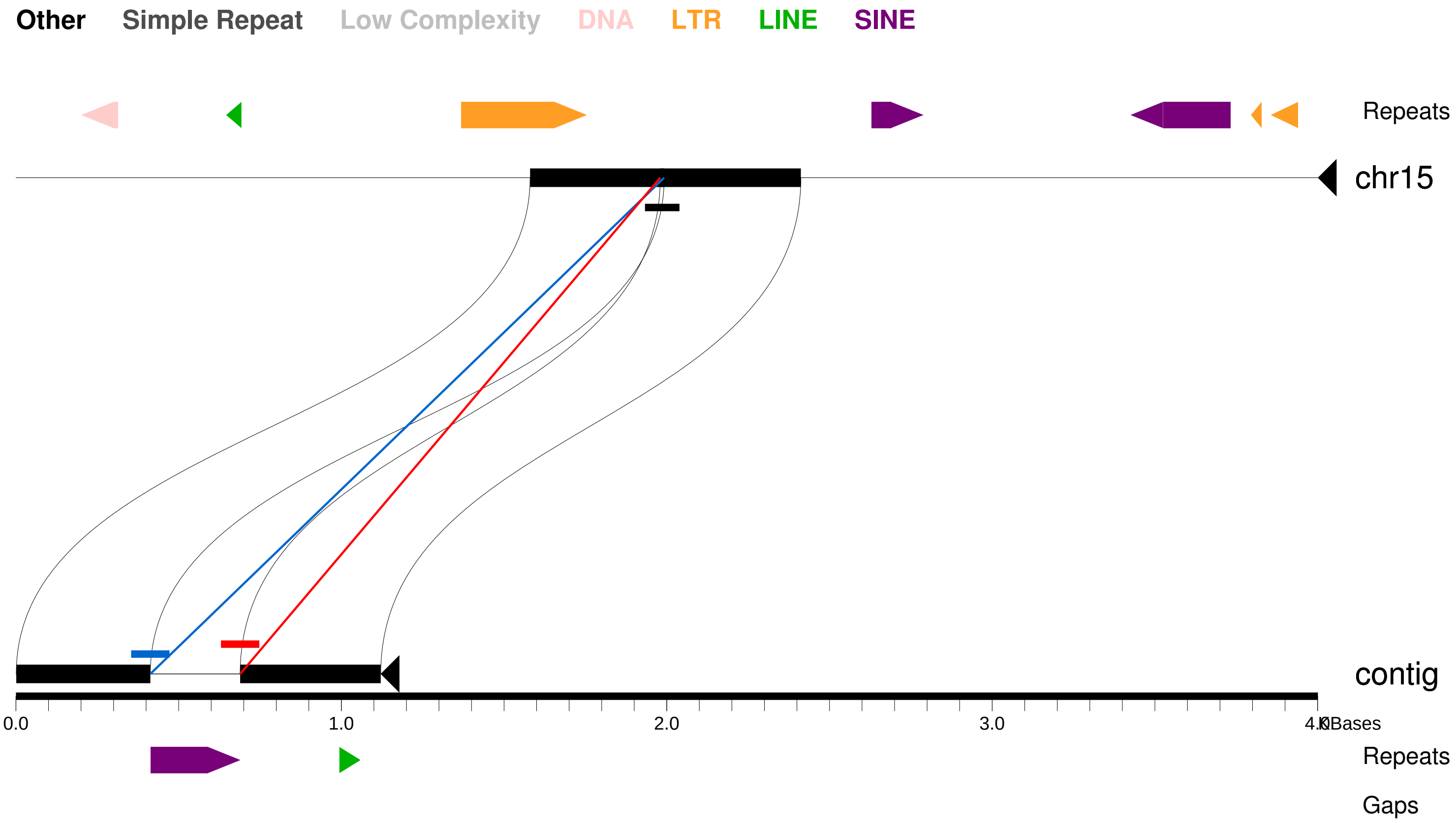

siteID: chr15_97321248 Scafftig2 - Breakpoint: chr15:97321186
chr15:97321184-97321245
Overlap Length: 58 INS size: 472 Genotyped: No

```
left   CC---------------------------------------------------------TTTGGCATTCC
chrom  CC---------------------------------------------------------TTTGGCATTCC
right  NNNNNNNNNNNNNNNNNNNNNNNNNNNNNNNNNNNNNNNNNNNNNNNNNNNNNNNNNNNTTTGGCATTCC
       11111111111111111111111111111111111111111111111111111111111***********

left   TTCTTTGTAAAGAATGTGTTGGGGCTCCACAAAGAGTGACAGCAGAGGGCCGGGCGCGGTGGCTCACGCC
chrom  TTCTTTGTAAAGAATGTGTTGGGGCTCCACAAAGAGTGACAGCAGAGAG---------------------
right  TTCTTTGTAAAGAATGTGTTGGGGCTCCACAAAGAGTGACAGCAGAGAG---------------------
       ***********************************************2*222222222222222222222

left   TGTAATCCCAGCACTTTGGGAGGCCGAGGCGGGTGG
chrom  ------------------------------------
right  ------------------------------------
       222222222222222222222222222222222222
```

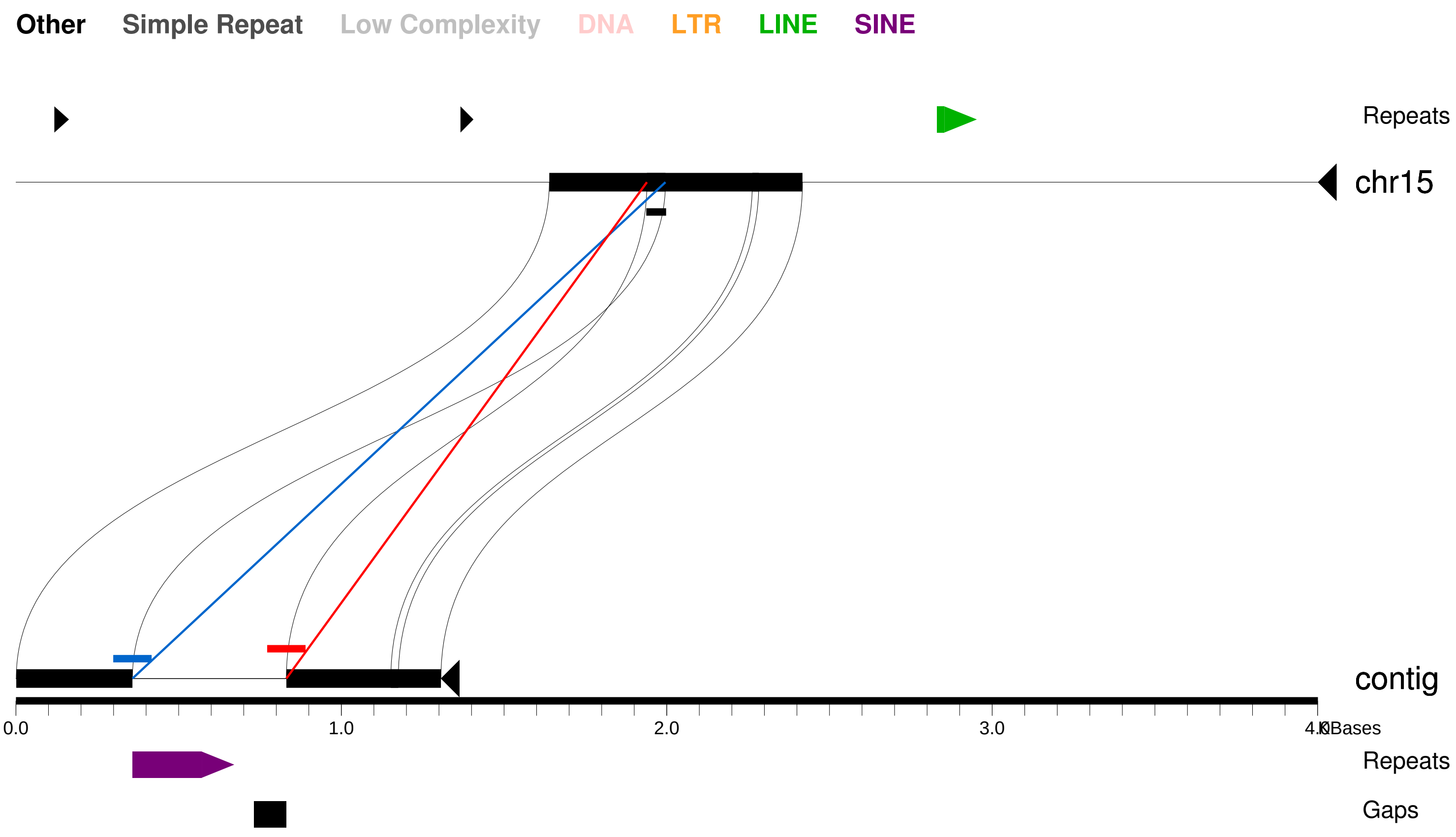

siteID: chr15_97585929 Scafftig1 + Breakpoint: chr15:97585929
chr15:97585876-97585988
Overlap Length: 7 INS size: 416 Genotyped: No

```
left     TAT--TAATAGTCAGATAATGAAACTTTTTCC-AGAA------GATAAAAGCTTGTTTGGGGCAGAATAN
chrom    TAT--TAATAGTCAGATAATGAAACTTTTTCC-AGAA------GATAAAAGCTTGTTTGGGGCAGAATAC
right    TGACCTCATGATCCACTC--GCCTCGGCCTCCCAGAGTGCTGGGATTACAGGC-GTGAGCCACAGAATAC
         *1111*1**11**111*111*111*1111***1***1111111***1*1**111**11*111*******2

left     NNNNNNNNNNNNNNNNNNNNNNNNNNNNNNNNNNNNNNNNNNNNNNNNNNNNNNNNNN
chrom    TTACTTTTTATTAGTATTCTCTTTGAACAGTTGGAGGGCTTTCTCTTTTCCT------
right    TTACTTTTTATTAGTATTCTCTTTGAACAGTTGGAGGGCTTTCTCTTTTCCT------
         2222222222222222222222222222222222222222222222222222222222
```

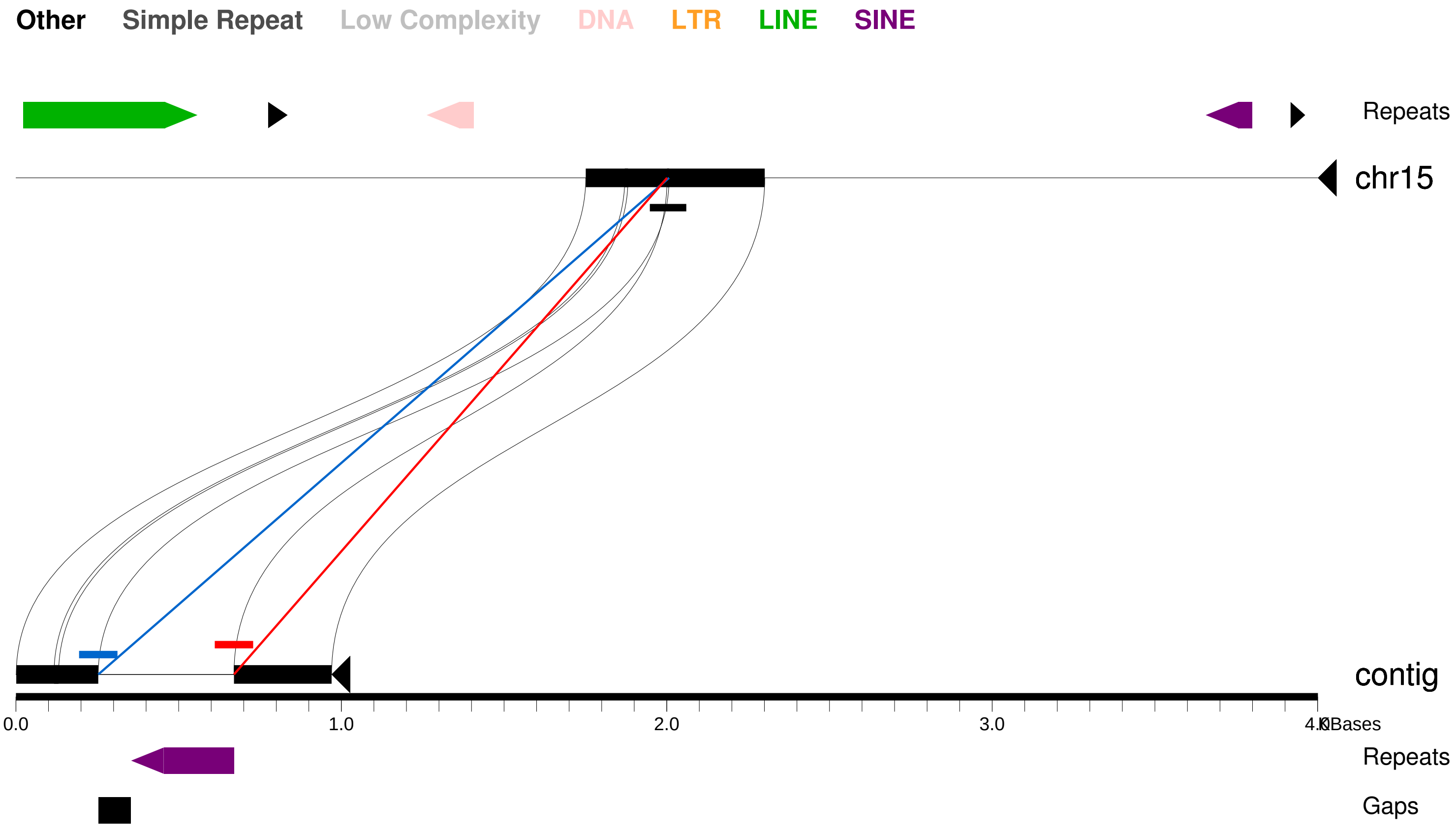

siteID: chr15_97770218 Scafftig5 - Breakpoint: chr15:97770219
chr15:97770059-97770394
Overlap Length: 16 INS size: 311 Genotyped: Yes

```
left   -----------------AGGAGAGATAAAATGGCACGTGCCTTAAGAGACGCAAATTACCAAAATTGACT
chrom  TGATATGTCAGAAAATTAGGAGAGATAAAATGGCACGTGCCTTAAGAGACGCAAATTACCAAAATTGACT
right  AGA-ATGGCGTGAACCCGGGAGGC-------GGAGCTTGCAGT--GAGCCG-AGATCCCGCCACTGCACT
       N22 222N2NNN22NNN1****111111111**11*1***11*11***1**1*1**11*111*1*11***

left   TATGAA---GAAATAGAACATTTGAATATACTGATAACAAGCATAGAAAGTATGTAATAACAAATATCCC
chrom  TATGAA---GAAATAGAACATTTGAATATACTGATAACAAGCATAGAAAGTATGTAATAACAAATATCCC
right  CCAGCCTGGGCGACAGAGC----GAGACTCCGTCTCAAAAAAAAAAAAA------AAAAAAAAAAA----
       111*11111*11*1***1*1111**111*1*111*1*1**11*1*1***111111**1**1***1*1111

left   TCTCTCCCAAAAATTTCAGGCTCAGAAGATTTCTTTGGTAGGCCGGGCGCGGTGGCT----CACGCC--T
chrom  TCTCTCCCAAAAATTTCAGGCTCAGAAGATTTCTTTGGTGAATACAATAAAATATTTAAACCAAACCAAT
right  --------AAAAA----------AGAAGATTTCTTTGGTGAATACAATAAAATATTTAAACAAAACCAAT
       11111111*****1111111111****************2222222222222*222*22221*22**22*

left   GTAATCCCAGCACTTTGGGAGGCC-GAGG----------CGGGC--------------GGA-TCACGAGG
chrom  CTTTTACAAGCTCTTTCAGATGATAGAGGTAGAGAAAACCTGTCCCAACTCATTCTAAGGACTCACGATT
right  CTTTTACAAGCTCTTTCAGATGATAGAGGTAGAGAAAACCTGTCCCAACTCATTCTAAGGACTCACGATT
       2*22*2*2***2****22**2*222****2222222222*2*2*22222222222222***2******22

left   TCAG-GAGATCGAGACC--ATCCCGGCT----AAAACGGTGAAACCCCGTCTCTACTAAA
chrom  ACTCTGAAACTGAGGCCTGATGAAGACACCACAATAAAATAAAACTGCAGACC-AGGAAC
right  ACTCTGAAACTGAGGCCTGATGAAGACACCACAATAAAATAAAA----------------
       2*222**2*22***2**22**222*2*22222**2*222*2***1NN1NNNN121NN11N
```

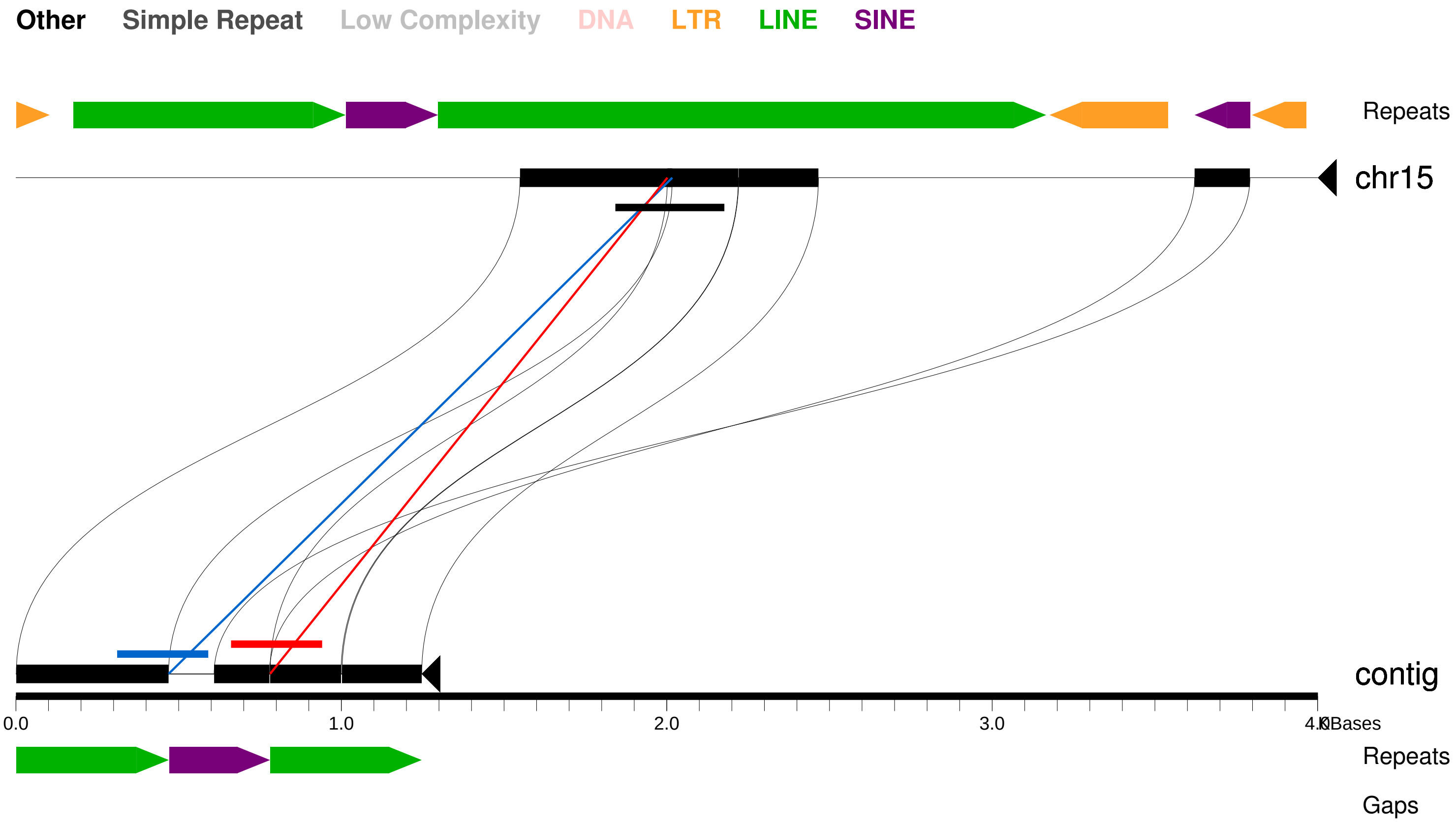

siteID: chr15_98140576 Scafftig1 - Breakpoint: chr15:98140608
chr15:98140553-98140667
Overlap Length: 5 INS size: 365 Genotyped: No

```
left    ATAAAATACAAACCAATTGCTCAGCAGAAACTGTATCCCCAGTTAAAAAAAAAAT----TATTGGCCGAG
chrom   ATAAAATACAAACCAATTGCTCAGCAGAAACTGTATCCCCAGTTAAAAAAAAAAT----TATTGTCACCG
right   NNNNNNNNNNNNNNNNNNNNNNNNNNNNNNNNNNNNNNNNNNNNNNNNNNNNNNNNNNNNTATTGTCACCG
        111111111111111111111111111111111111111111111111111111111111*****2*222*

left    GCGGGCGGATCACGAGGTCAGGAGATCGAGACCATCCTGGCTAACACGGTGAA
chrom   TCTTGGTCCCAACCAGGACTCCTCACACAGGGAATTCTGG-TAATA---TAAT
right   TCTTGGTCCCAACCAGGACTCCTCACACAGGGAATTCTGG-TAATA---TAAT
        2*22*222222**2***2*22222*222**222**2****2***2*222*2*2
```

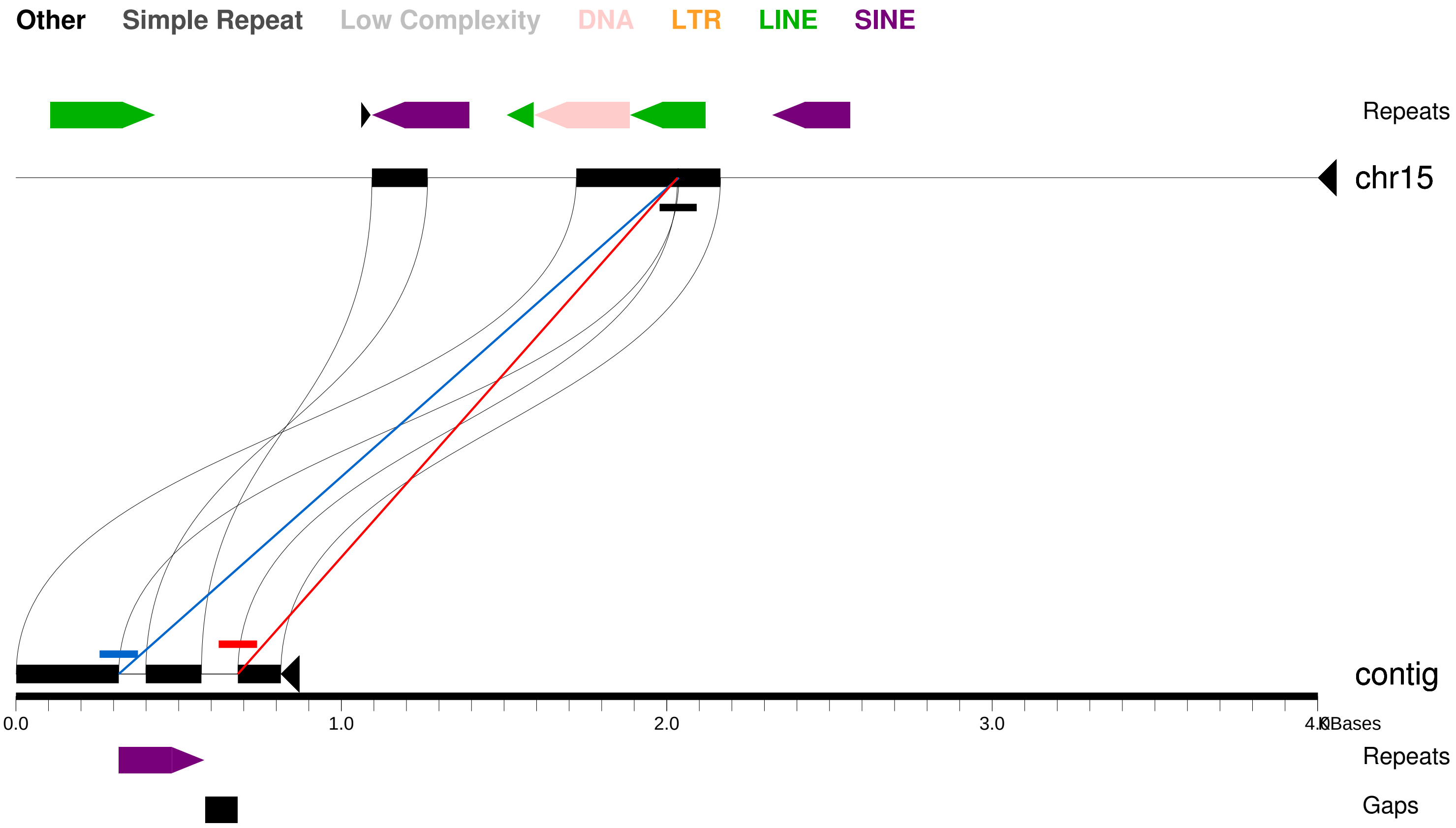

siteID: chr15_98553616 Scafftig2 + Breakpoint: chr15:98553591
chr15:98553543-98553647
Overlap Length: 12 INS size: 427 Genotyped: No

```
left    AT----GTTAG-------AGGTTCAGGGAATGCTA-----AACGAATGCTGCTTTTTAATTTTCCTTTCT
chrom   AT----GTTAG-------AGGTTCAGGGAATGCTA-----AACGAATGCTGCTTTTTAATTTTCCTTTCT
right   GCCCGCCTCGGCCTCCCAAAGTGCTGGGATTACAAGCATGAGCCACCGCGCCCGGCTCCTTT--CTTTCT
        1111111*11*1111111*1**1*1****1*1*1*11111*1*1*11**11*1111*11***11******

left    TTTTTTTNNNNNNNNNNNNNNNNNNNNNNNNNNNNNNNNNNNNNNNNNNNNNNNNNNNNNNNNNN
chrom   TGTTTT--------------AAAAAGAATGCCTGCATTCAGCTCTCCCTCCAAGCATTTGTTAAA
right   TGTTTT--------------AAAAAGAATGCCTGCATTCAGCAATCCCTCCAAGCATTTGTTAAA
        *2****222222222222222222222222222222222222NN222222222222222222222
```

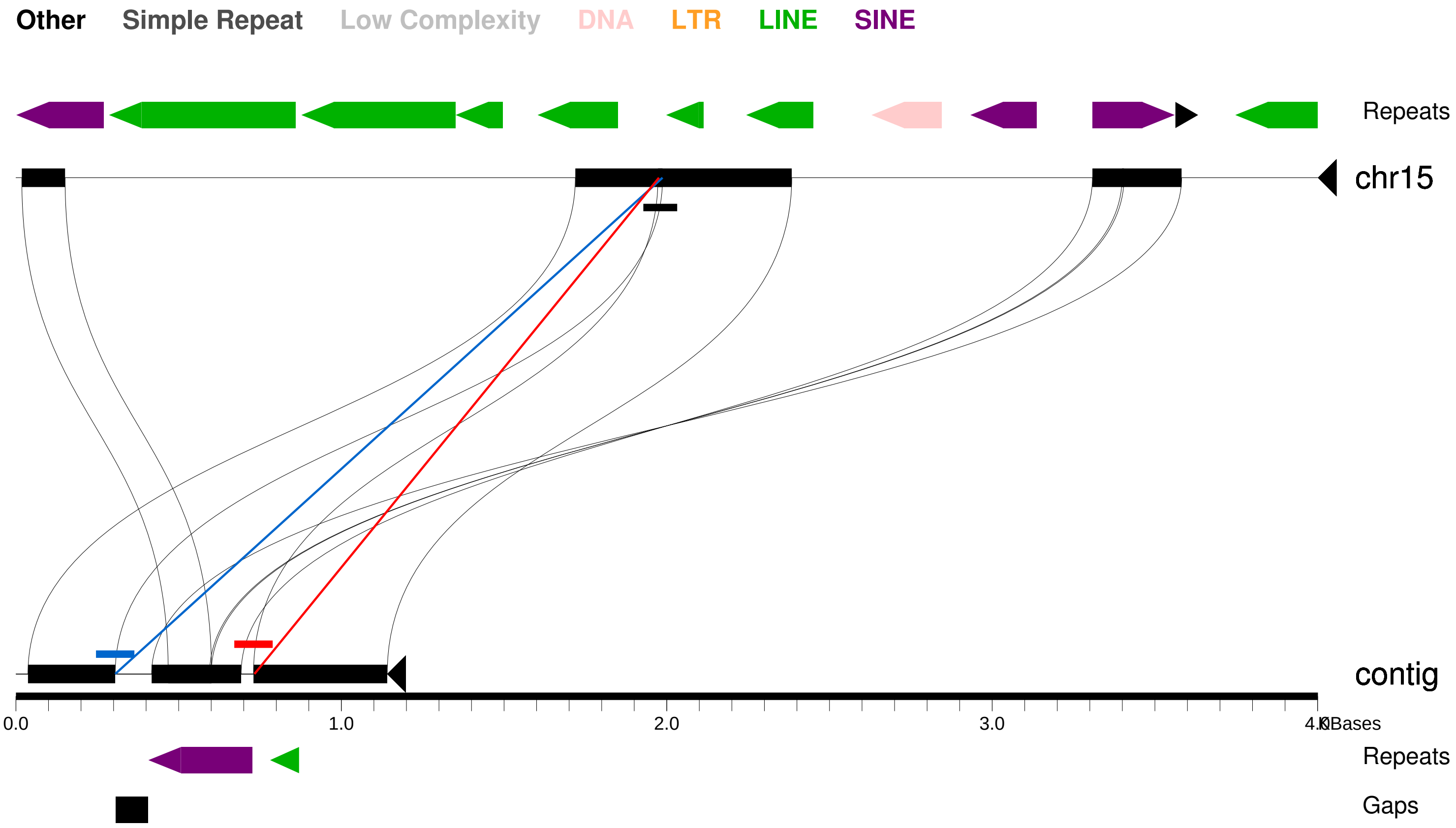

siteID: chr16_11086397 Scafftig3 - Breakpoint: chr16:11086393
chr16:11086349-11086449
Overlap Length: 16 INS size: 299 Genotyped: Yes

```
left    TCAAATTGTACCCCATA-----------------ACCATGTA-CAATTATAATGCATCCATTAAAAAATA
chrom   TCAAATTGTACCCCATA-----------------ACCATGTA-CAATTATAATGCATCCATTAAAAAATA
right   CGCCACTGCACTCCAGCCTGGGCGACAGAGCGAGACTCCGTCTCAAAAAAAAAAAAAAAATAAAAAAATA
        1111*1**1**1***1111111111111111111**111**11***11*1**111*111**1********

left    AAAAAGAAGGCCGGGCGCGGTGGCTCACGCCTGTAATCCCAGCACTTTGGGAGGCCGAGGCGGGCGG
chrom   AAAAAGAAAAA-GATAACATTAAAT---GGTTTTATTC-----ACT---------CAAATAATATTG
right   AAAAAGAAAAA-GATAGCATTAAAT---GGTTTTATTC-----ACT---------CAAATAATATTG
        ********2222*222N*22*222*222*22*2**2**22222***222222222*2*22222222*
```

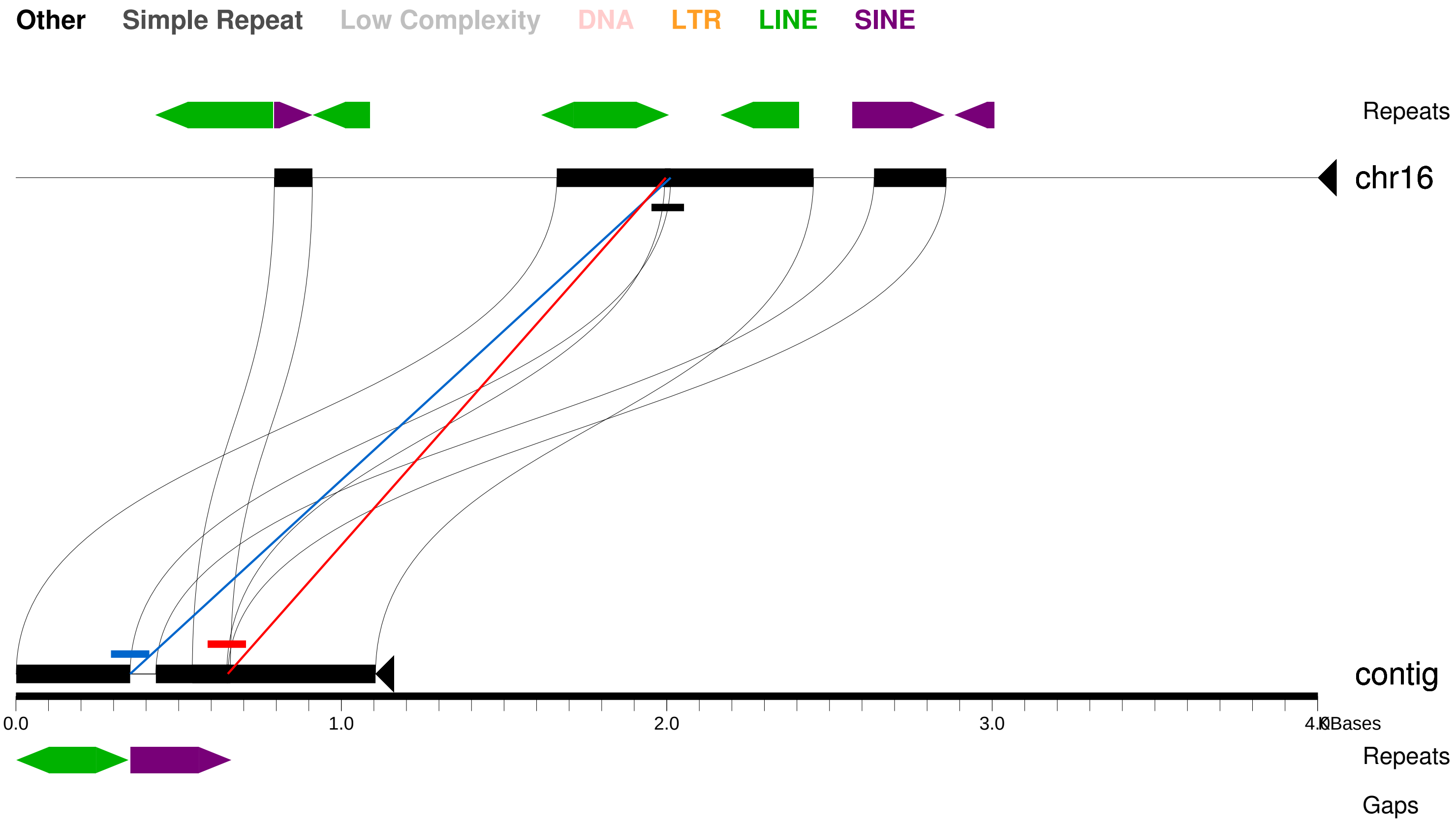

siteID: chr16_18117815 Scafftig1 + Breakpoint: chr16:18117795
chr16:18117744-18117854
Overlap Length: 9 INS size: 421 Genotyped: No

```
left     AGTACCACTCATTGCCTGG---ACTGCTATGATT-------TGACCCTTCCTCACCTCACTGATCCTCTT
chrom    AGTACCACTCATTGCCTGG---ACTGCTATGATT-------TGACCCTTCCTCACCTCACTGATCCTCTT
right    CCACCCGC-CTCGGCCTCCCAAAGTGCTGGGATTACAGGCGTGAGCCA-CCGCGCCCGGCCGATCCTCTT
         1111**1*1*111****11111*1****11****1111111***1**11**1*1**111*1*********

left     NNNNNNNNNNNNNNNNNNNNNNNNNNNNNNNNNNNNNNNNNNNNNNNNNNNNNNNNNNN
chrom    TCAAATGTCCCTTTCACATAGGCTTCAGGCACCTTTGCTTTTATACTGGTT--------
right    TCAAATGTCCCTTTCACATAGGCTTCAGGCACCTTTGCTTTTATACTGGTT--------
         22222222222222222222222222222222222222222222222222222222222
```

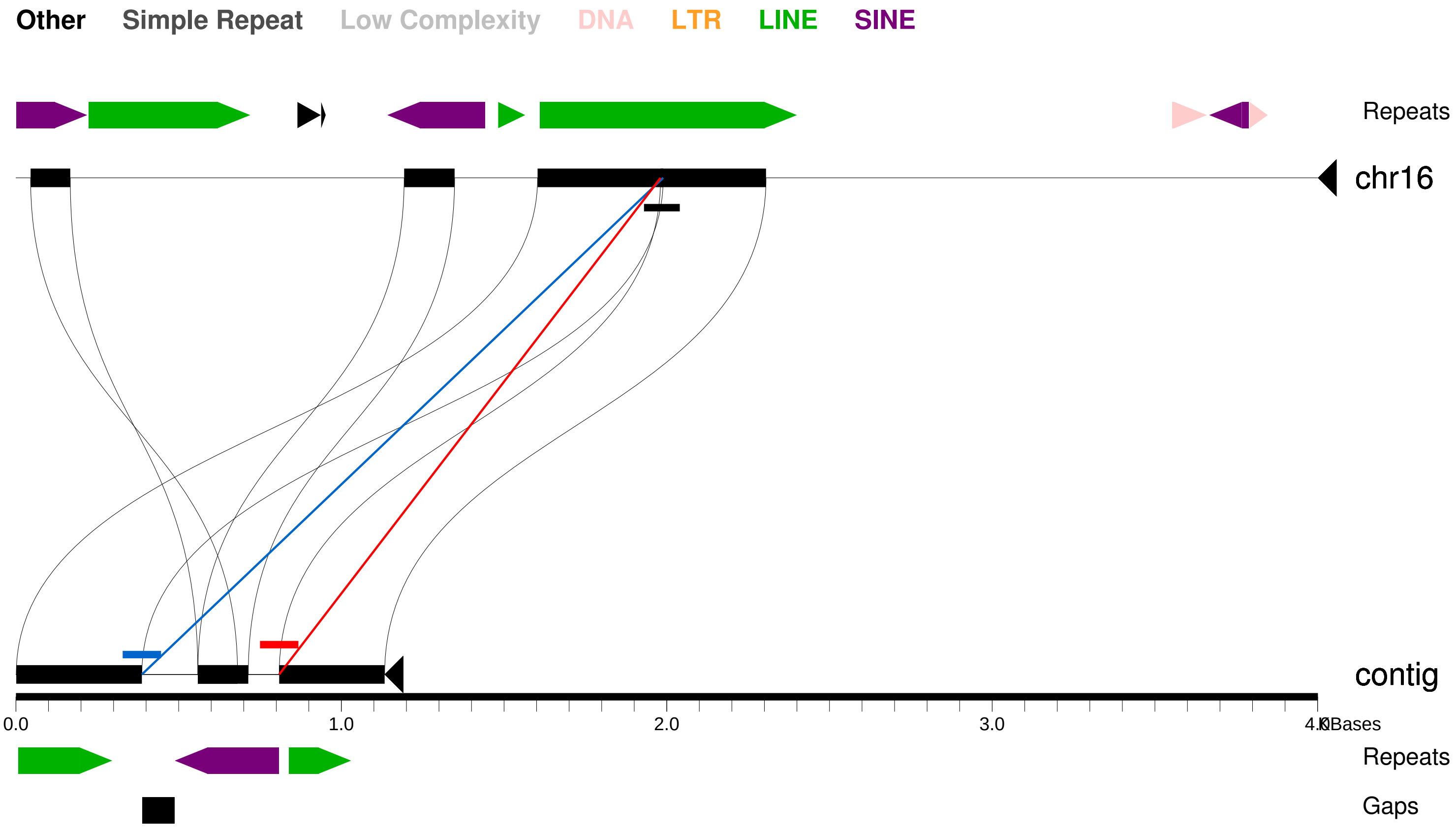

siteID: chr16_22845998 Scafftig5 - Breakpoint: chr16:22845999
chr16:22845958-22846058
Overlap Length: 19 INS size: 295 Genotyped: Yes

```
left    -------TATTTCAGAGGAATTTTGGGGTTTGTGGGTAACCATGCTTT-----------AAAAAGTATCC
chrom   -------TATTTCAGAGGAATTTTGGGGTTTGTGGGTAACCATGCTTT-----------AAAAAGTATCC
right   ATCCCGCCACTGCACTCCAGCCTGGGCGACAGAGCGAGACTCCGTCTCAAAAAAAAAAAAAAAAGTATCC
        11111111*1*1**1111*111*1**1*111*1*1*11**111*11*111111111111***********

left    ATTTTTTGGGCCGGGCGCGGTGGCTCACGCCTGTAATCCCAGCACTTTGGGAGGCCGAGGCGGGCGG
chrom   ATTTTTTGATACA---------AATTAC-CAAGTTTTCCCCACCCCTATGGCAATAGAG--------
right   ATTTTTTGATACA---------AATTAC-CAAGTTTTCCCCACCCCTATGGCAATAGAG--------
        ********222*222222222222*2**2*22**22****22*2*2*22**22222***22222222
```

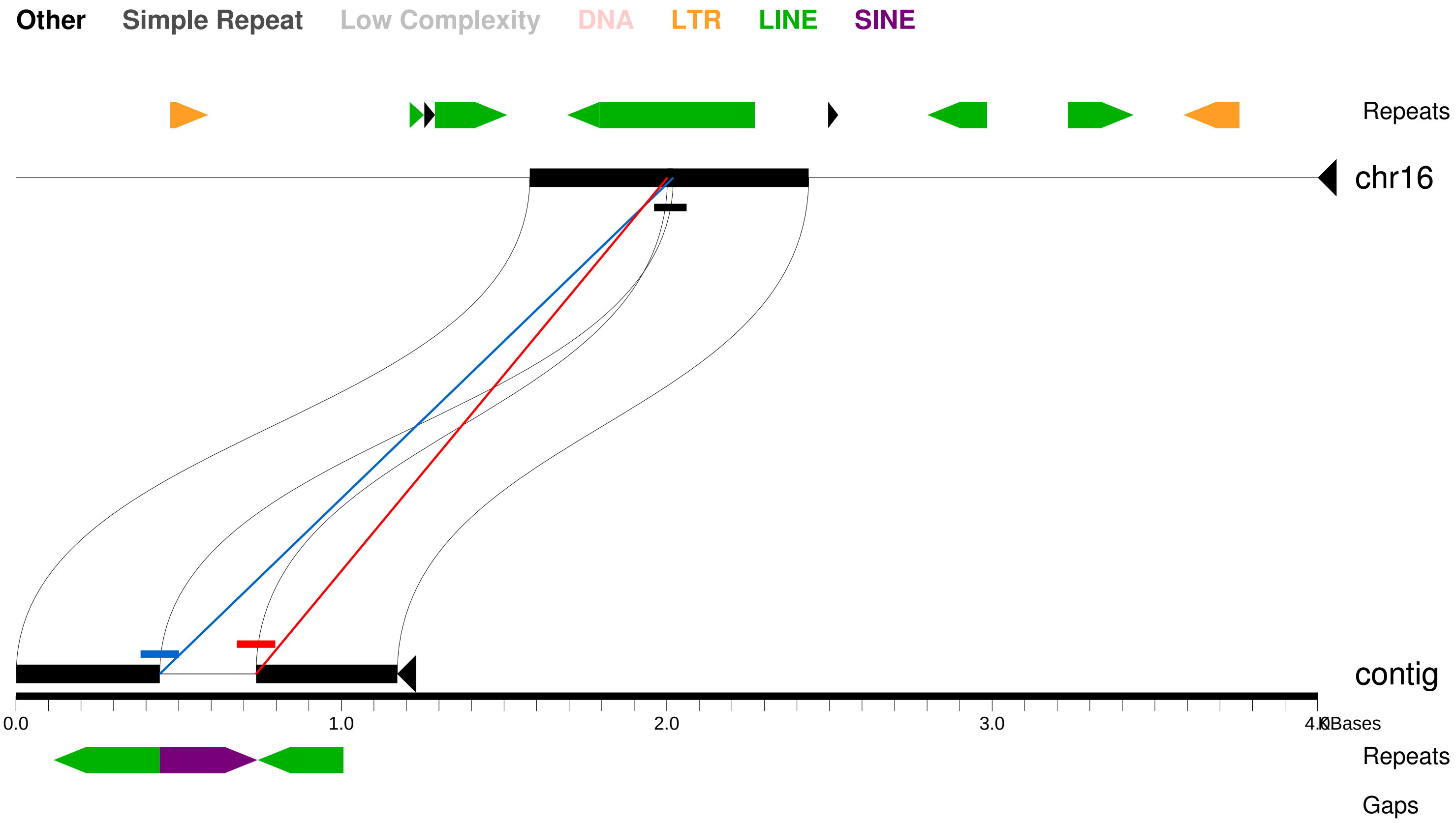

siteID: chr16_23865299 Scafftig5 + Breakpoint: chr16:23865288
chr16:23865236-23865344
Overlap Length: 8 INS size: 421 Genotyped: No

```
left    AT-TCAGCATGGCTGGGG-------AGGCCTCACAATCATGGCAGAAGGCAAAGGAA--GGTCAAGGCAC
chrom   AT-TCAGCATGGCTGGGG-------AGGCCTCACAATCATGGCAGAAGGCAAAGGAA--GGTCAAGGCAC
right   TGATCCGCCCGCCTCGGCCTCCCAAAGTGCTGGGATTACAGGCGTGAGCCACCGCGCCCGGCCAAGGCAC
        111**1**11*1**1**11111111**11**111*1*111***111**1**11*11111**1********

left    NNNNNNNNNNNNNNNNNNNNNNNNNNNNNNNNNNNNNNNNNNNNNNNNNNNNNNNNNN
chrom   TTCTTACATGGCAGCAGGCAAGAGAGCGTGCACAGGGGAACTGCCCTTT----------
right   TTCTTACATGGCAGCAGGCAAGAGAGCGTGCACAGGGGAACTGCCCTTT----------
        2222222222222222222222222222222222222222222222222222222222
```

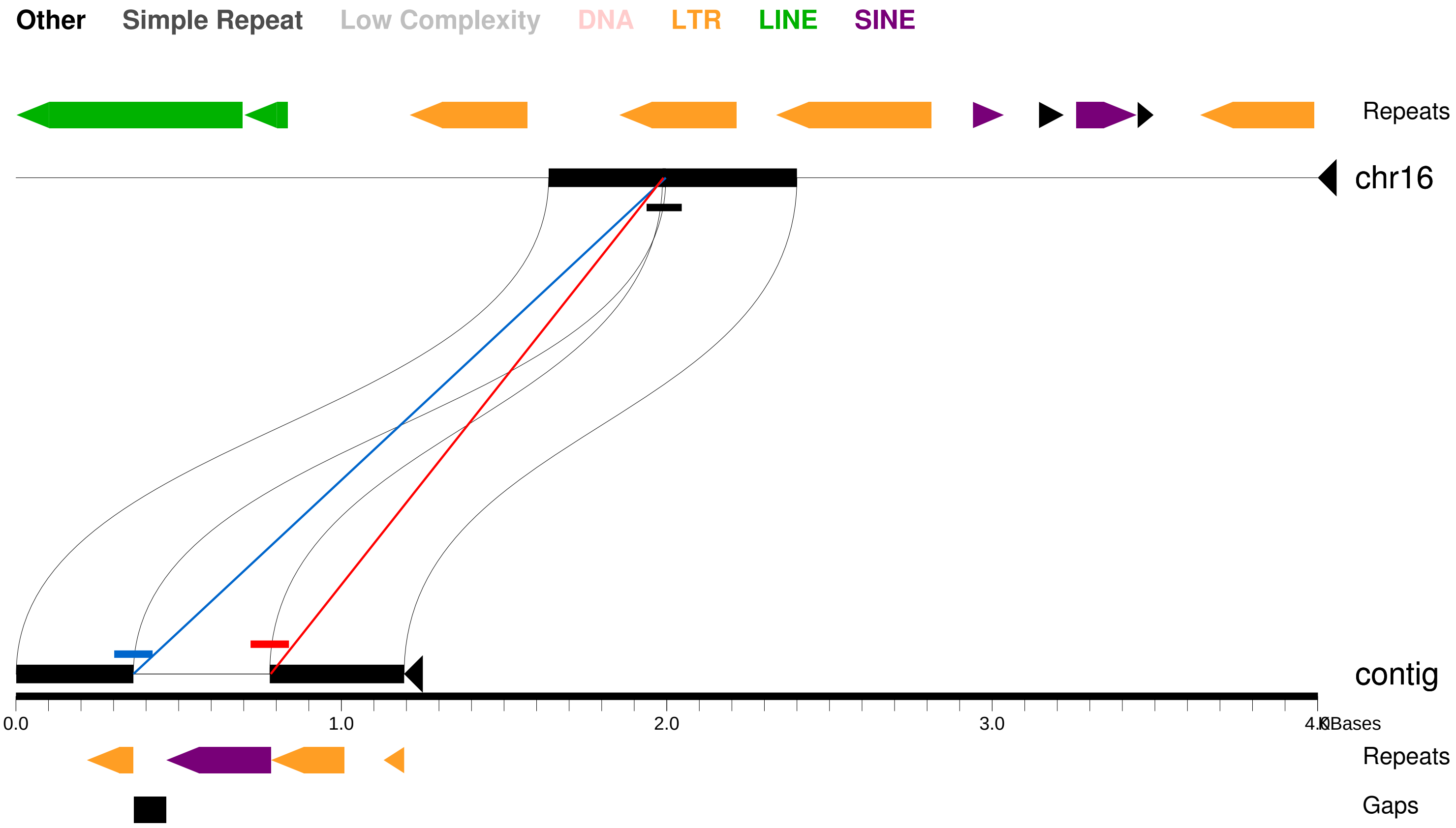

siteID: chr16_53874978 Scafftig5 - Breakpoint: chr16:53874970
chr16:53874810-53875141
Overlap Length: 12 INS size: 299 Genotyped: Yes

```
left    T-----------CCTTTTATCAGTTGGCAACTCATTTAGATTTGTTAAGGAAATATGGTTGAGGTCAAGC
chrom   TTTAGGCAAAGTCCTTTTATCAGTTGGCAACTCATTTAGATTTGTTAAGGAAATATGGTTGAGGTCAAGC
right   G--AGGCA-----------------GGAGAATGGCGTGAACCCGGGAAGCGGA---GCTTGCAGTGAGCC
        1  22222    1111111111111**11*1*1111*11*111*11***111*111*1***11**1*11*

left    TACACACCTTCGTGAGTTCTTTTCTGGTCCATGAACTCTAGTTTAGCAAATTCTGGTATTTTCAAGTGTA
chrom   TACACACCTTCGTGAGTTCTTTTCTGGTCCATGAACTCCAGTTTAGCAAATTCTGGTATTTTCAAGTGTA
right   GAGAT---TGCGCCACTGCA------GTCC--GCAGTCCCGCCTGGGAGA--CAG---------AGCGAG
        1*1*1111*1**11*1*1*1111111****11*1*1**21*11*1*1*1*11*1*111111111**1*11

left    ACAGTGTATTATGTATGAATAAGAAGTGCCTGG----------CCGGGCGCGGTGGC-------TCACGC
chrom   ACAGTATATTATGTATGAATAAGAAGTGCCTGTAGAATGTCATCAGACATCGATAGCAACTATCTAATGC
right   ACTCCGTCTCAAAAAAAAAAAAGAAGTGCCTGTAGAATGTCATCAGACATCGATAGCAACTATCTAGTGC
        **111N*1*1*111*11**1***********22222222222*2*2222**2*2**2222222*212**

left    CTGTAATCCCAG-CACT-----TTGGGAGGCCGAGGCGGGTGGATCATGAGGTCAGGAGATCGAGACCAT
chrom   TGGTGCTGCCACTCACTAACCCTTGTAGTGCCCATGACAGAC-ATCAGTAATTGATCATAAC-ACTCTTT
right   TGGTGCTGCCACTCACTAACCCTTGTAGTGCCCATGACAGAC-ATCAGTAATTGATCATAAC-ACTCTTT
        22**22*2***22****22222***2222***2*2*222*222****22*22*2*22*2*2*2*22*22*

left    CCTGGCTAACAAGGTGAAA--------------CCCC-----GTCTCTACTAAA
chrom   CCTGCTGAGCTAGGAGAAAGTTTCAGATTTCTTCTCCACTAGGGCTCTAAAAAG
right   CCTGCTGAGCTAGGAGAAAGTTTCAGATTTCTTCTCCACTAGGGCTC-------
        ****222*2*2***2****22222222222222*2**22222*2***11NN11N
```

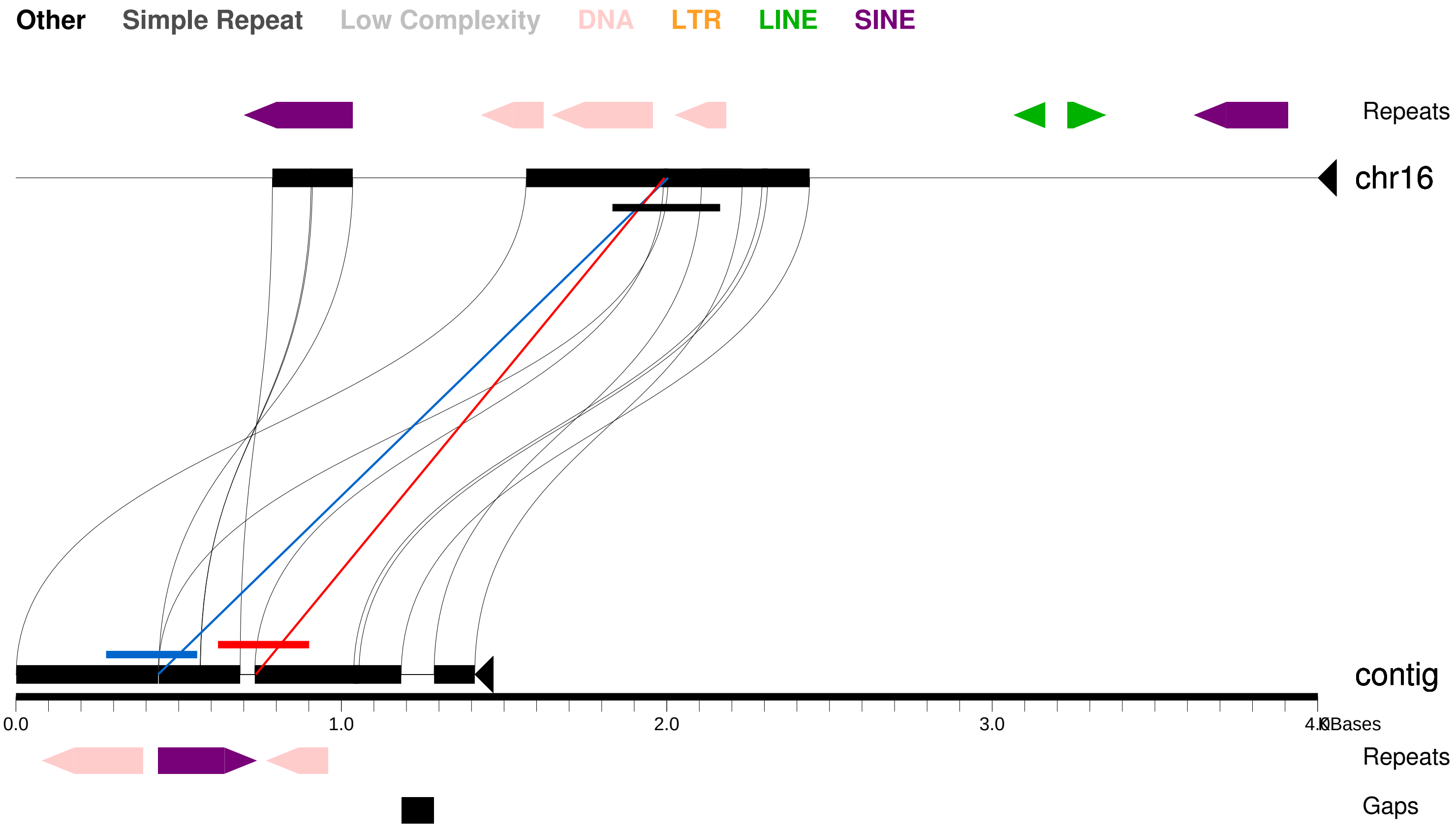

siteID: chr16_61092186 Scafftig1 + Breakpoint: chr16:61092191
chr16:61092139-61092250
Overlap Length: 8 INS size: 425 Genotyped: No

```
left     GGAGAACA--CTTGAACAACAAAAGCCAT---CTTATATATTTGTCCTCACAGATAC---CCTACAACNN
chrom    GGAGAACA--CTTGAACAACAAAAGCCAT---CTTATATATTTGTCCTCACAGATAC---CCTACAACTA
right    ATCCACCCGCCTCGGCCTCCCAAAGTGCTGGGATTACAGGCGTGAGC-CACCGCGCCCGGCCTACAACTA
         1111*1*111**1*11*11*1****111*1111***1*1111**11*1***1*111*111********22

left     NNNNNNNNNNNNNNNNNNNNNNNNNNNNNNNNNNNNNNNNNNNNNNNNNNNNNNNNN
chrom    TCTTAAGTCAAGATGAAAAAGAGAAAAAAGTTGTGATGATGACACACGCA-------
right    TCTTAAGTCAAGATGAAAAAGAGAAAAAAGTTGTGATGATGACACACGCA-------
         222222222222222222222222222222222222222222222222222222222
```

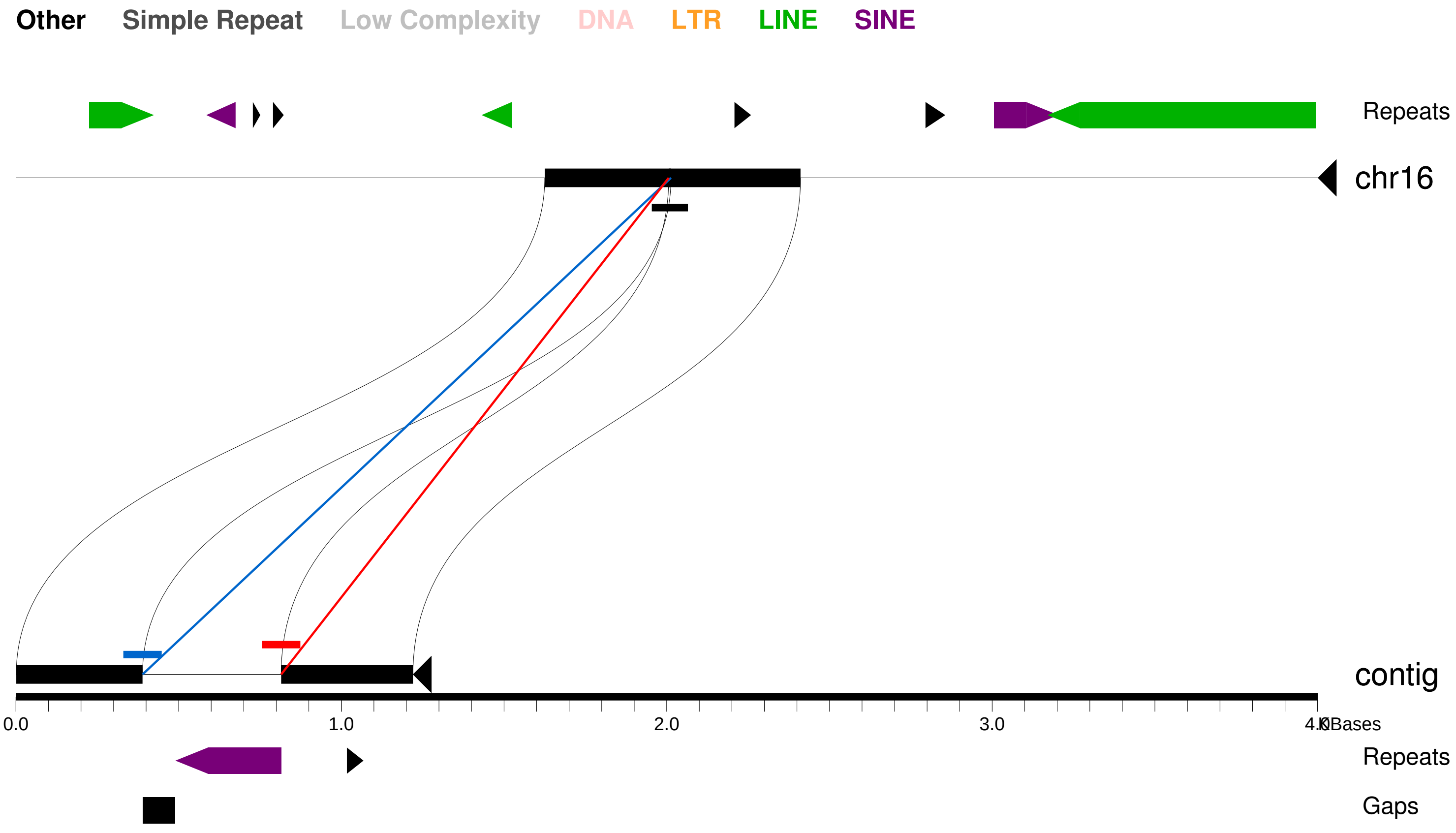

siteID: chr16_65482207 Scafftig2 + Breakpoint: chr16:65482188
chr16:65482145-65482247
Overlap Length: 17 INS size: 298 Genotyped: Yes

```
left     T-------------ATCAGAAACTGGTGAGTTTCTAACAAGAATTTACC---CTAAACACAGAAAACAAT
chrom    T-------------ATCAGAAACTGGTGAGTTTCTAACAAGAATTTACC---CTAAACACAGAAAACAAT
right    CCGCCCGCCTCGGCCTCCCAAAGTGCTGGGATTAAAGGCGTGAGCCACCGCGCCCGGCCCAGAAAACAAT
         111111111111111**11***1**1**1*1**11*111111*111***111*1111*1***********

left     TTTTTTTTTTTTTTTTTTTTTTTGAGACGGAGTCTCGCTCTGTCGCCCAGGCTGGAGTGCAGTGG
chrom    TTTTTTAA----------------AAATAGGGCTTACTTCTGGAAGGCAGGATAATGCACAGAAC
right    TTTTTTAA----------------AAATGGGGCTTACTTCTGGAAGGCAGGATAATGCACAGAAC
         ******222222222222222222*2*2N*2*22*222****22222****2*222*22***222
```

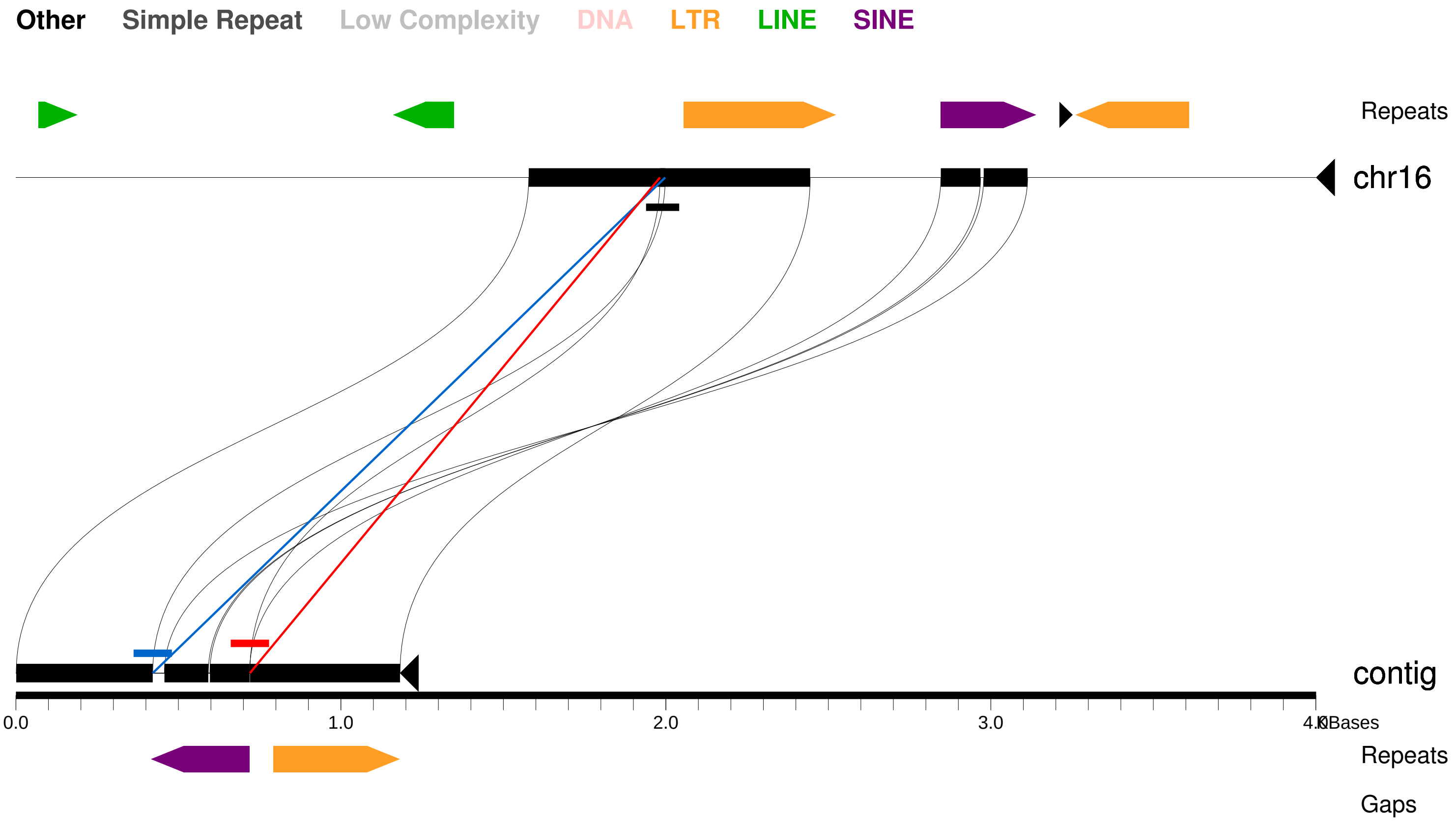

siteID: chr16_7226227 Scafftig3 + Breakpoint: chr16:7226224
chr16:7226178-7226283
Overlap Length: 14 INS size: 300 Genotyped: Yes

```
left    ACTGTGTTAGCTTTGGTTAATGAAACTG-TAAAATGAGATTAATAAT---------------AATACTTG
chrom   ACTGTGTTAGCTTTGGTTAATGAAACTG-TAAAATGAGATTAATAAT---------------AATTCTTG
right   CC---GCCCGCCTCGGCCTCCCAAAGTGCTGGGATTACAGGCGTGAGCCACCGCGCCCGGCCAATACTTG
        1*111*111**1*1**111111***1**1*111**1*1*1111*1*1111111111111111***N****

left    GTTCTTTTTTTTTTTTTTTTTTTTTGAGACGGAGTCTCGCTCTGTCGCCCAGGCTGGAGTGC--AGT
chrom   GTTCTTAAAATCATGGATAGGATT--ACATGAGATAATGCA-TGTA-----------AGTGCTTAG-
right   GTTCTTAAAATCATGGATAGGATT--ACATGAGATAATGCA-TGTA-----------AGTGCTTAG-
        ******2222*22*222*2222**22*2*2*222*222**22***222222222222*****22**2
```

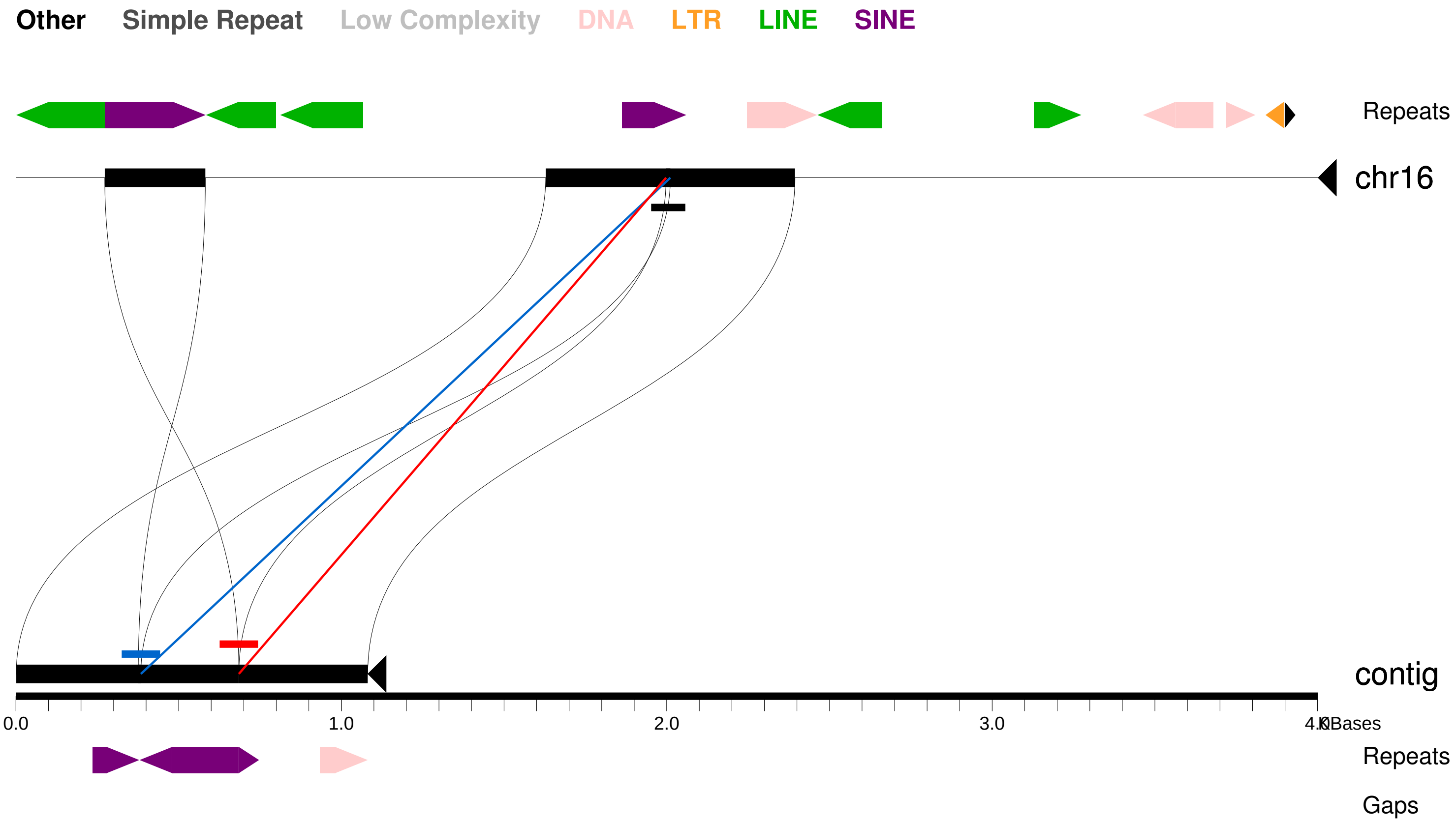

siteID: chr16_75655160 Scafftig8 + Breakpoint: chr16:75655155
chr16:75655117-75655214
Overlap Length: 16 INS size: 305 Genotyped: Yes

```
left    C-------------TTTCTGGACAGATGTTCTTA------TCTGTCTTCTTTTTC--CAGGCATTTTATT
chrom   C-------------TTTCTGGACAGATGTTCTTA------TCTGTCTTCTTTTTC--CAGGCATTTTATT
right   CCGCCCGCCTCGGCCTCCCAAAGTGCTGGGATTACAGGCGTGAGCCACCGCGCCCGGCCGGCATTTTATT
        *11111111111111*1*111*11*1**111***111111*11*1*11*11111*11*1***********

left    TTTCTTTTTTCTTTTTTTTTTTTTTTTTTTGAGACGGAGTCTCGCTCTGTCGCCCAGGCTGGAGTGCAGTG
chrom   TTTCTCCTTTCC-------------CCTACAG-CAGAGTGTTTCTCTACCGCCC----TCTAGTAAA---
right   TTTCTCCTTTCC-------------CCTACAG-CAGAGTGTTTCTCTACCGCCC----TCTAGTAAA---
        ******22****222222222222222*22**2*2****2*22****22*****2222*22***22*222
```

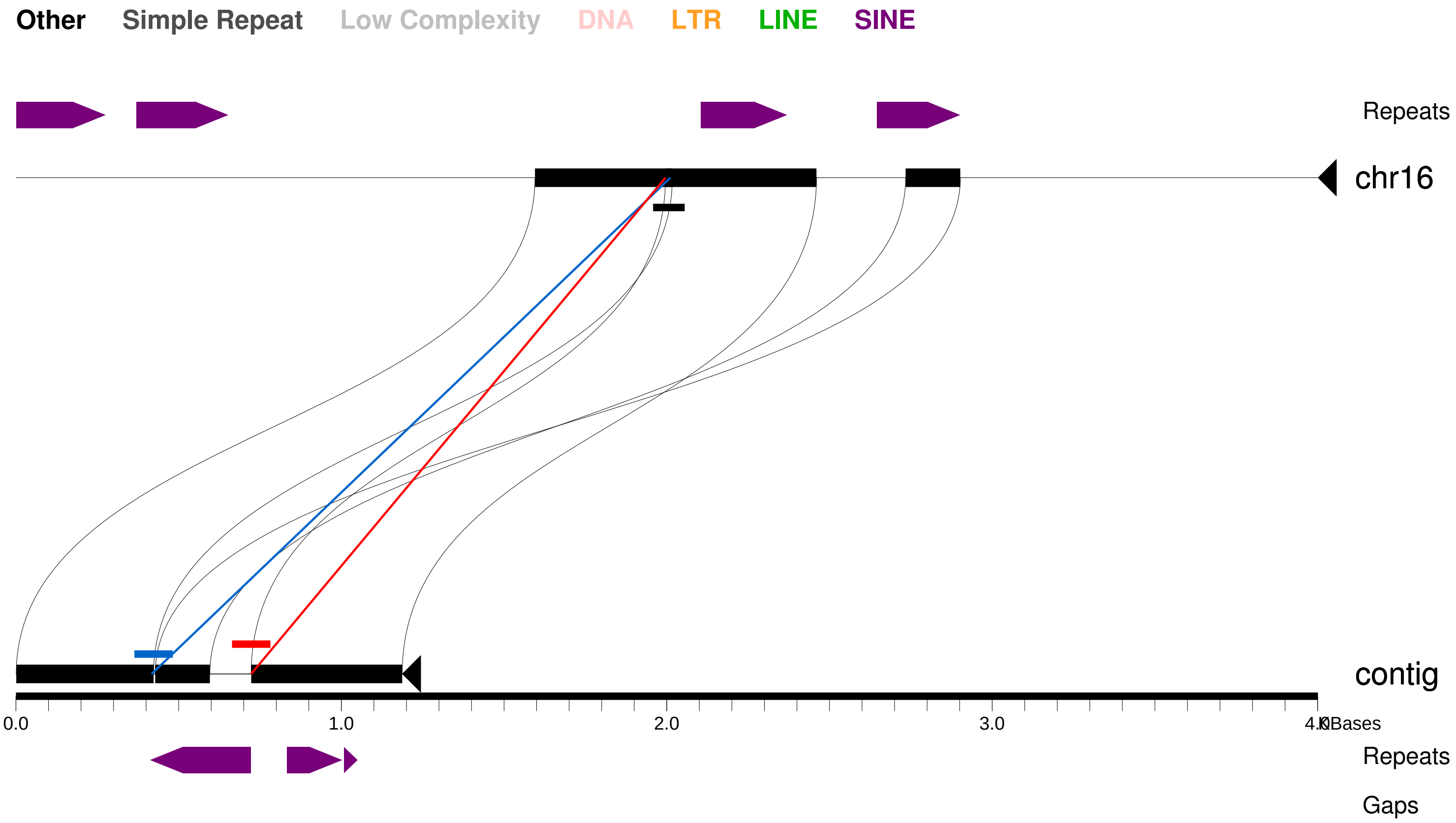

siteID: chr16_7688970 Scafftig1 - Breakpoint: chr16:7688965
chr16:7688909-7689024
Overlap Length: 4 INS size: 405 Genotyped: No

```
left   GGAAATAAGGAAAATTCTTTTAAGTG-TGCACCCTCATGTATTTTTTGCATTT--TGTACCATNNNNNNN
chrom  GGAAATAAGGAAAATTCTTTTAAGTG-TGCACCCTCATGTATTTTTTGCATTT--TGTACCATTTTATCA
right  TCCGCCCGCCTCGGCCTCCCAAAGTGCTGGGATTACAGGCGTGAGCCACCGCGCCCGGCCCATTTTATCA
       111111111111111111111*****1**111111**1*11*111111*1111111*11****2222222

left   NNNNNNNNNNNNNNNNNNNNNNNNNNNNNNNNNNNNNNNNNNNNNNNNN
chrom  CATTTTAAATGACGTGAAGTAGTACATAAAGATAGGTACCCTTCTCCTG---
right  CATTTTAAATGACGTGAAGTAGTACATAAAGATAGGTACCCTTCTCCTG---
       2222222222222222222222222222222222222222222222222222
```

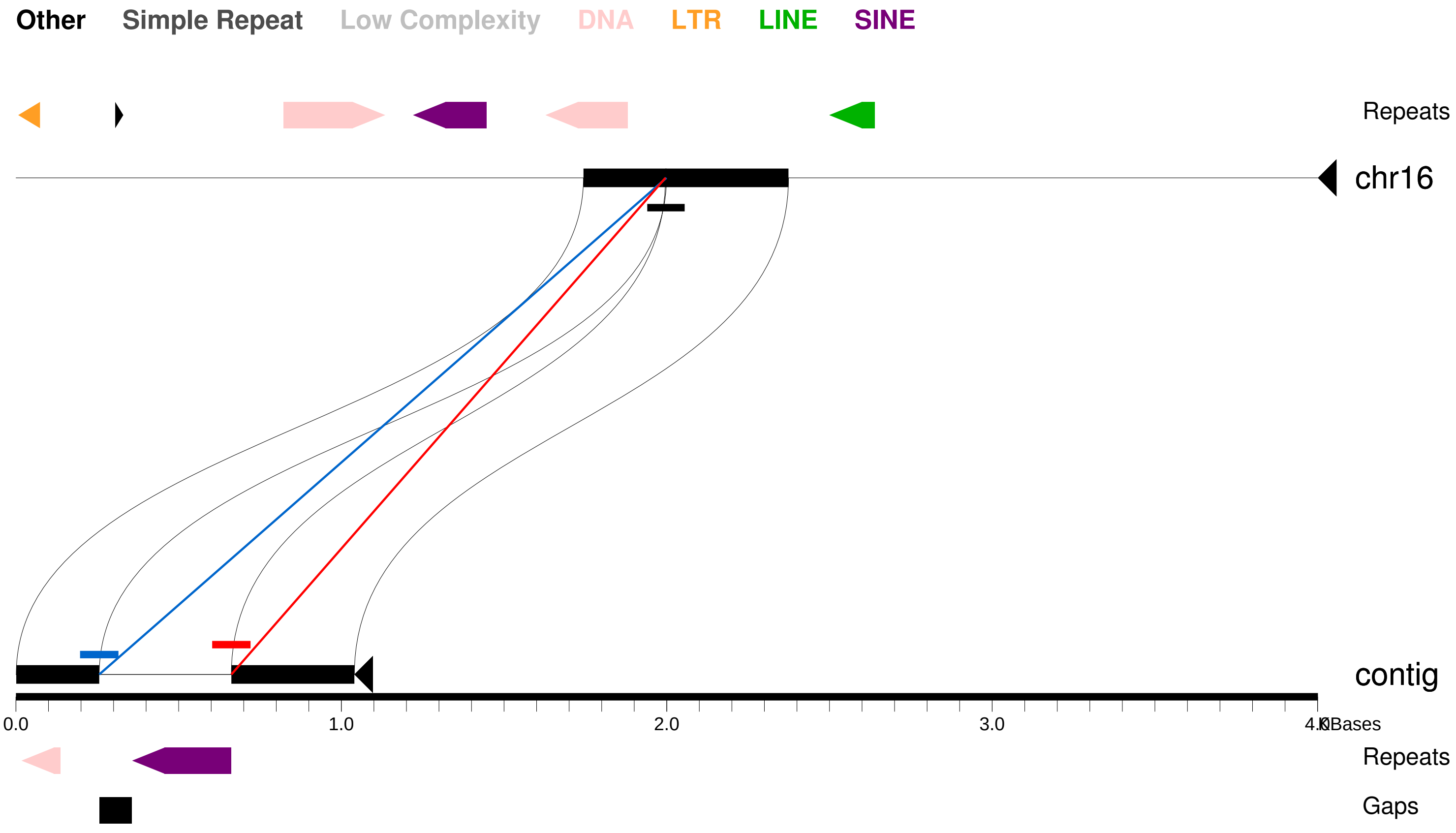

siteID: chr16_80217433 Scafftig6 - Breakpoint: chr16:80217431
chr16:80217380-80217484
Overlap Length: 9 INS size: 312 Genotyped: Yes

```
left    CACA--------------AAAGACAAATGCTGCGTGATCTCACTTATAGGTGGTATCTTAAGAAGTCAAA
chrom   CACA--------------AAAGACAAATGCTGCGTGATCTCACTTATAGGTGGTATCTTAAGAAGTCAAA
right   TGCACTCCAGCCTGGGCGACAGACCGAGACTCCGTCTCAAAAAAAAAAAAAAAAAAAAAAAAAAATCAAA
        11**11111111111111*1****11*11**1***111111*111*1*111111*1111**1**1*****

left    TTCAGGCCGGGCGCGGTGGCTCACGCCTGTAATCCCAGCACTTTGGGAGGCCGAGGCGGGCGG-
chrom   TTCA---CAGAAGCAGAG---AAC------AAT---AGAATGATGGTTGCCTGGGACTGGGGGC
right   TTCA---CAGAAGCAGAG---AAC------AAC---AGAATGATGGTTGCCTGGGACTGGGGGC
        *****222*2*22**2*2*2222**222222**1222**2*222***22*2*2*2*2*2**2**2
```

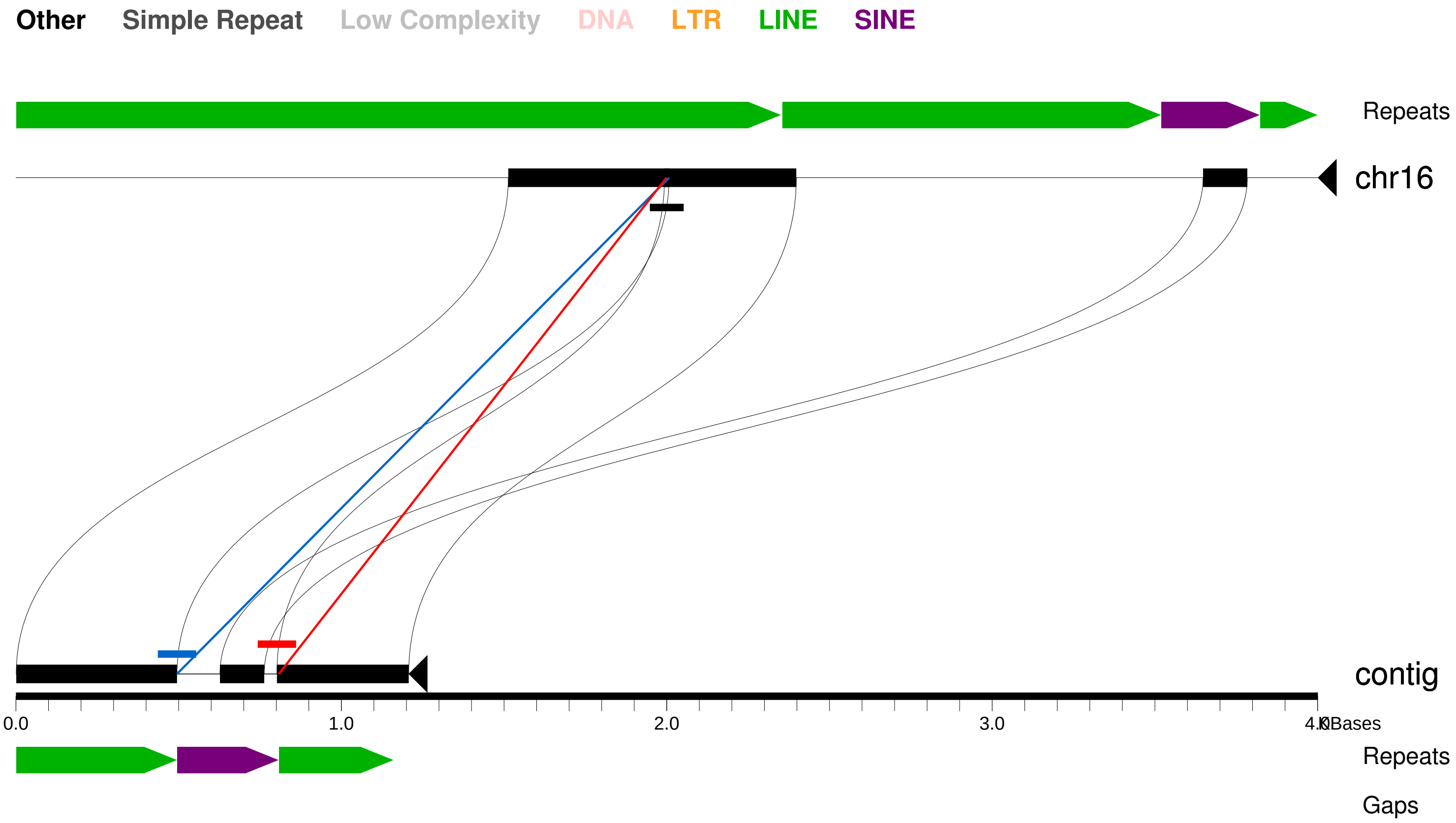

siteID: chr16_80848071 Scafftig3 - Breakpoint: chr16:80848060
chr16:80848018-80848119
Overlap Length: 18 INS size: 307 Genotyped: Yes

```
left     A----------GACATT---ATA-TCAT-CTCATCTGAAAGTGTCAATA--TGTATCTCTAAATAAGAAT
chrom    A----------GACATT---ATA-TCAT-CTCATCTGAAAGTGTCAATA--TGTATCTCTAAATAAGAAT
right    CCAACCTGGGCGACAGAGCGAGACTCTTTCTCAAAAAAATAAATAAATAAATAAATAAATAAATAAGAAT
         11111111111****11111*1*1**1*1****1111**1111*1****11*11**111***********

left     TGTTCTCGCCGGGCGCGGTGGCTCACGCCTGTAATCCCAGCACTTTGGGAGGCCGAGGTGGGCGGA
chrom    TGTTCTCTTAAAT----GTAAC-CACGACT------CCATCATT-----ACACCTAAATTCACAC-
right    TGTTCTCTTAAAT----GTAAC-CACGACT------CCATCATT-----ACACCTAAATTCACAC-
         *******2222222222**22*2****2**222222***2**2*22222*22**2*22*222*222
```

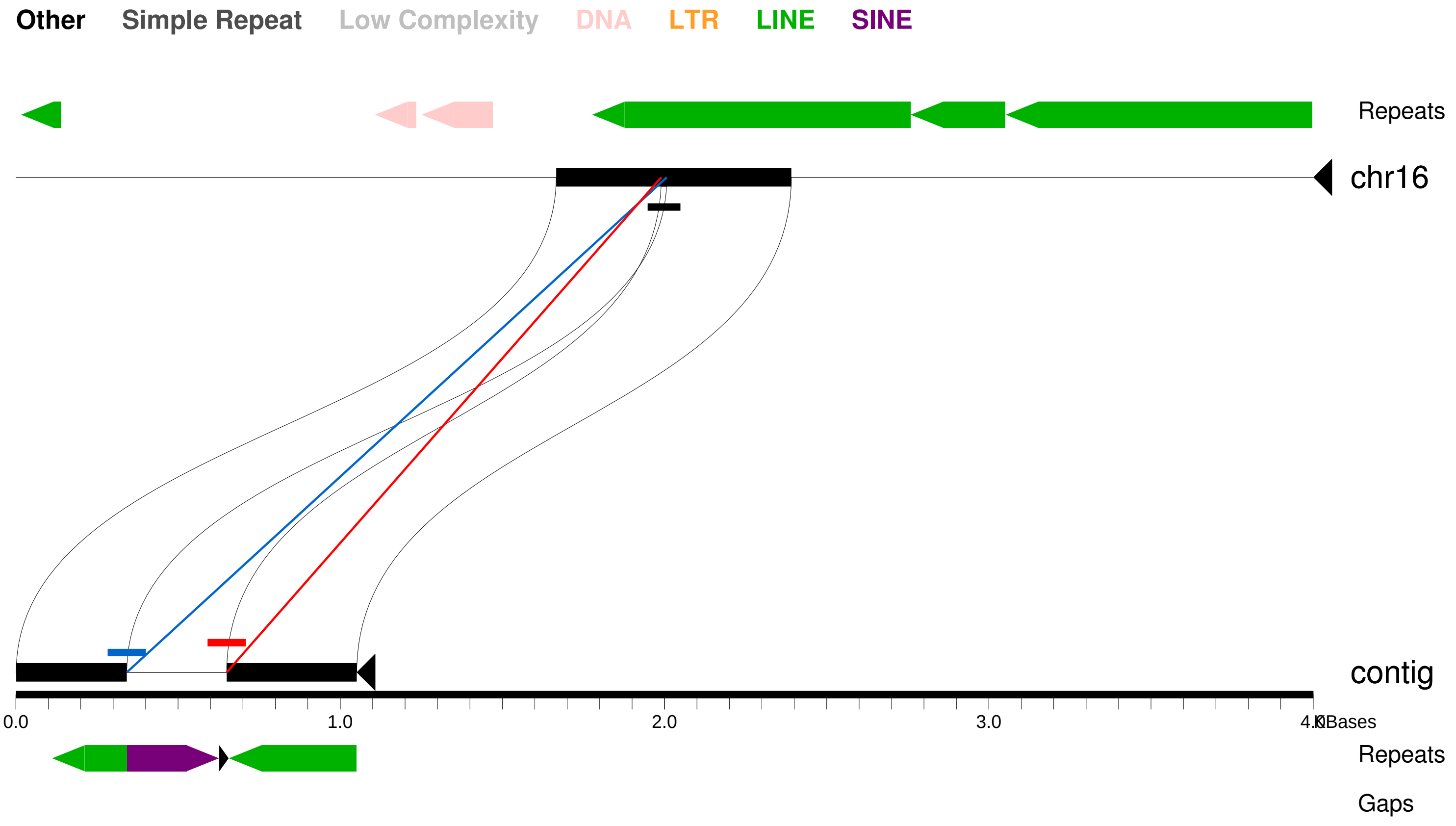

siteID: chr16_82164694 Scafftig9 - Breakpoint: chr16:82164679
chr16:82164519-82164850
Overlap Length: 12 INS size: 291 Genotyped: Yes

```
left    ------------ATACACACACACATATATGAAATTGTAAATTACCTAGAGCACTCAAAACAATTTTGAA
chrom   GCGTATATACACATACACACACACATATATGAAATTGTAAATTACCTAGAGCACTCAAAACAATTTTGAA
right   GG--AGAATGGCGTGAACCCGGG-AGGCA-GAGCTTGCAGTGAGCCGAGATCGCGCCACTGCACTCC---
        2N  2N2NNNN21*11**1*1111*111*1**11***1*11111**1***1*1*1*1*1111*1*11111

left    AAGAAAGGACAAAGTTGGATAAATACACTACTGTAGTTCAAGACTTAATATAAAGCTGCAGTCATCAAGA
chrom   AAGAAAGGACAAAGTTGGATAAATACACTACTGTAGTTCAAGACTTAATATAAAGCTGCAGTCATCAAGA
right   -AGCCTGGGC-------GACAGA-ACAAGACTCCGTCTCAAAA---AAAAAAAA-----------AAAA
        1**111**1*1111111**1*1*1***11***11111****1*111**1*1***111111111111**1*

left    TAGGTAAATATTAGTGTAAGAATGGACACATGT----------CACACC----TGT---------AATCC
chrom   TAGGTAAATATTAGTGTAAGAATGGACATATGGATAAATGGAGCAGAACAGAGTGTCCAGAAATAAATCC
right   AA---AAAAA-------AAAAATGGACACATGGATAAATGGAGCAGAATAGAGTGTCCAGAAATAAATCC
        1*111***1*1111111**1********N***22222222222**2*212222***222222222*****

left    -CAGCA-CTTTGGGAGG-CCGAGG-----CGGGCGGATCACGAG--GT--------CAGGAGATCGAGAC
chrom   ACAGCAGCGTAATCAGTACCAAGGATAGTCTGTAGGGAAAAGTGTGGTGTTTTAACCAGTAGCACTAGAG
right   ACAGCAGCGTAATCAGTACCAAGGATAATCTGTAGGGAAAAGTGTGGTGTTTTAACCAGTAGCACCAGAG
        2*****2*2*2222**22**2***222N2*2*22**222*2*2*22**22222222***2**22*N***2

left    CATCCTGGCTAACACGGTGAAACCCCGTCTCTACTAAAAATACAAAAAATTAGC-
chrom   CAAC-TGAATATTTGTATGAAAACAAAACAAAAC--AAAACCAGGAATCTCAGCC
right   CAAC-TGAACATTTGTATGAAAACAAAACAAAAC--CAAACCAGG----------
        **2*2**221*222222*****2*2222*222**221***2222211NN1N111
```

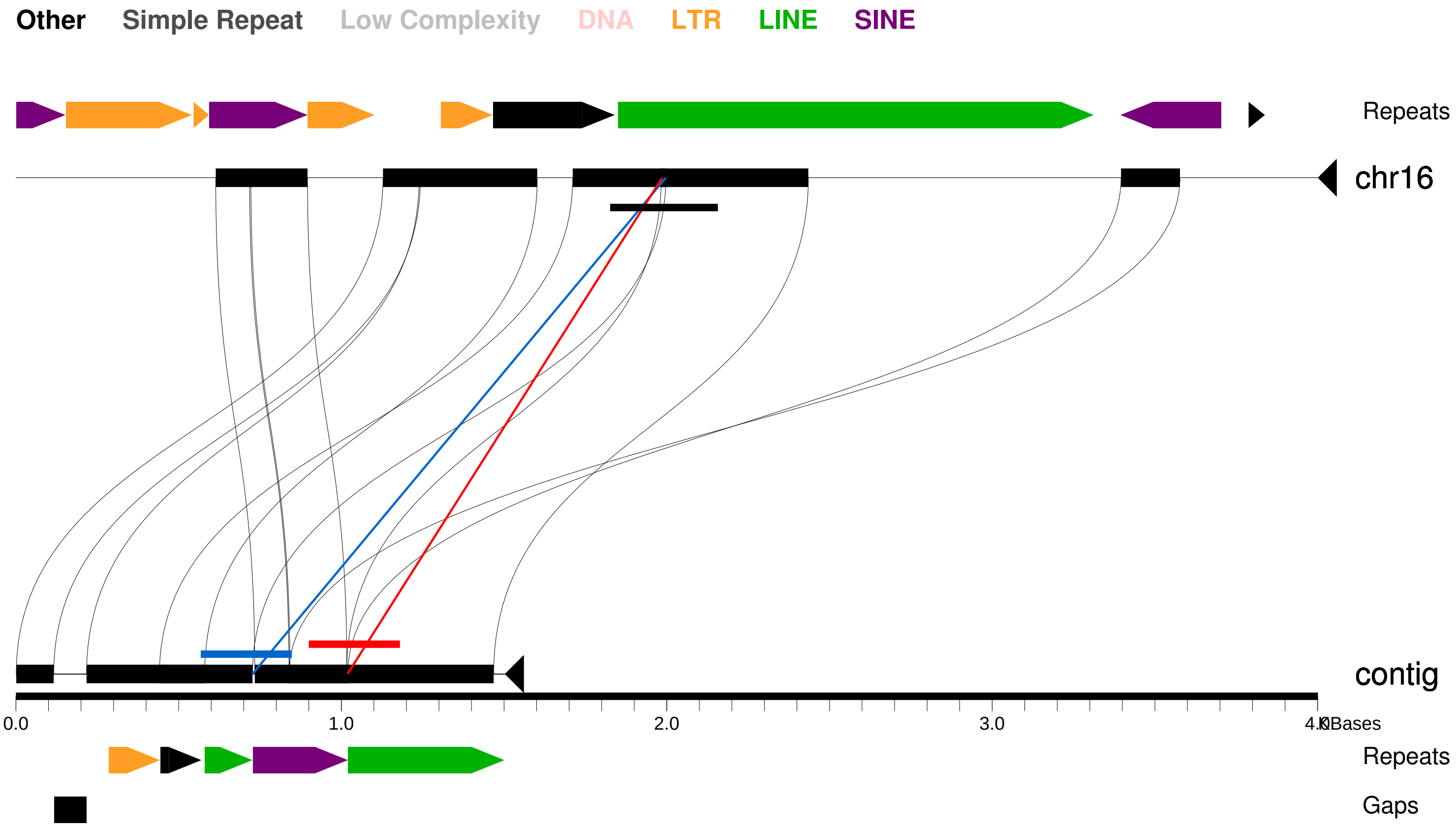

siteID: chr16_82827962 Scafftig4 + Breakpoint: chr16:82827959
chr16:82827800-82828127
Overlap Length: 9 INS size: 418 Genotyped: No

```
left   AG--------CTGTCGTTAGTGTTATTTTATGTGTTGCCAAAGATCATTCTTCTTCTTCCACTGTGGCCC
chrom  AGTTCATCAGCTGTCGTTAGTGTTATTTTATGTGTTGCCAAAGATCATTCTTCTTCTTCCACTGTGGCCC
right  NNNNNNNNNNN-----------------------------------------------------------
       11NNNNNNNN111111111111111111111111111111111111111111111111111111111111

left   AGGGAAGCCAAAAATTTGAAAACCCCTGGTTTAAATCCTCTCTACAAACTCGCCAGCTCTGCAAAATAAT
chrom  AGGGAAGCCAAAAATTTGAAAACCCCTGGTTTAAATCCTCTCTACAAACTCGCCAGCTCTGCAAAATAAT
right  ----------------------------------------------------------------------
       1111111111111111111111111111111111111111111111111111111111111111111111

left   CATCTTTGTTGGTCTTAAGAACACTTTA---CACTTTGG--------GAGGCCGAGGCGGGTGGATCATG
chrom  CATCTTTGTTGGTCTTAAGAACACTTTAAATCAGGTTTAAATCATCTGAACATCAGAAAGGAATTTTTTT
right  -------------------AACACTTTAAATCAGGTTTAAATCATCTGAACATCAGAAAGGAATTTTTTT
       1111111111111111111*********222**22**2222222222**22222**222**2222*22*2

left   AGGTCAGGAGATCGAGACCATCCTGGCTAAC---AGGGTGAAACCCCGTC-TCTACTAAAAATACA----
chrom  TTCTTTTCTTTTCTTTTGTTTTTTTTCTCCCTTGAGAGGGAAATCTAGGGGTGGAGTAGAGAATCATTTA
right  TTCTTTTCTTTTCTTTTGTTTTTTTTCTCCCTTGAGAGGGAAATCTAGGG--------------------
       222*2222222**2222222*22*22**22*222**2*2****2*22*22 1NN1N11N1N1NN11

left   AAAAATTAGCCG------------GGCGCGG--TGGCGGGCG------
chrom  AAATATTTCCCTTCAGAGACCAACGGCTAGGAATAGCTGATAAATAAC
right  ------------------------------------------------
       111N111NN11N            111NN11  1N11N1NNN
```

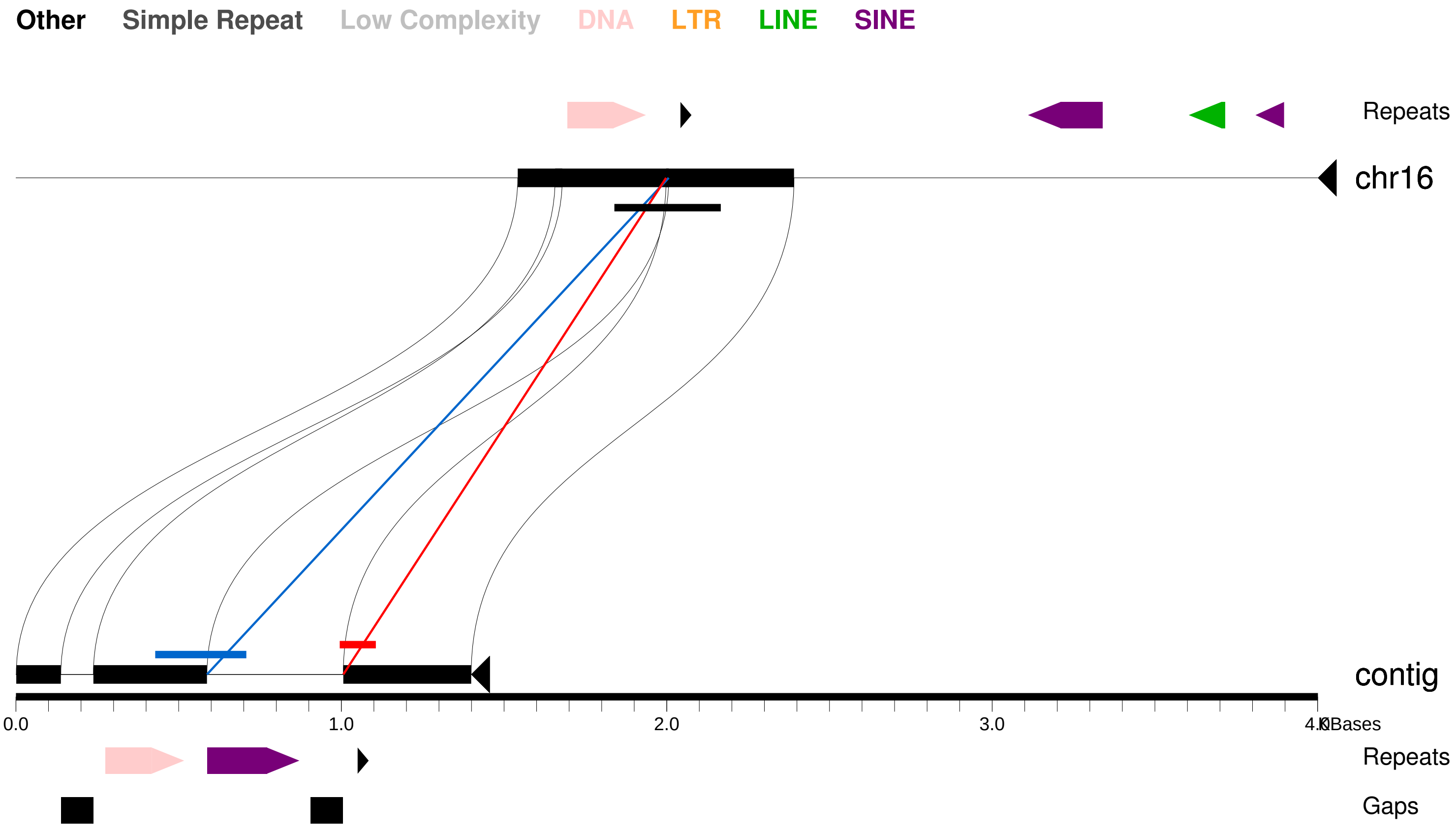

siteID: chr16_82912119 Scafftig2 - Breakpoint: chr16:82912113
chr16:82912056-82912162
Overlap Length: 3 INS size: 311 Genotyped: Yes

```
left    CA-CACAGATACATTTCAGTAAGGTTG--TCTCTGTTACTTGAGGCTTACAAGAAGGAAA---------G
chrom   CA-CACAGATACATTTCAGTAAGGTTG--TCTCTGTTACTTGAGGCTTACAAGAAGGAAA---------G
right   CTGCACTCCAGCGTGGGCGACAGAGCGAGACTCCGTCTCAAAAAAAAAAAAAAAAAAAAAAAAAAAAAAG
        *11***11111*1*1111*11**111*111***1**11*111*11111*1**1**11***111111111*

left    AAGGCCGGGCGCGGTGGCTCACGCCTGTAATCCCAGCACTTTGGGAGGCCGAGGCGGGCGG
chrom   AATTCC---CTCTCTAAA-CACAC-TCTAAACACA-CA-----GGAGTT-GAGAACGGGGA
right   AATTCC---CTCTCTAAA-CACAC-TCTAAACACA-CA-----GGAGTT-GAGAACGGGGA
        **22**222*2*22*2222***2*2*2***2*2**2**22222****222***222**2*2
```

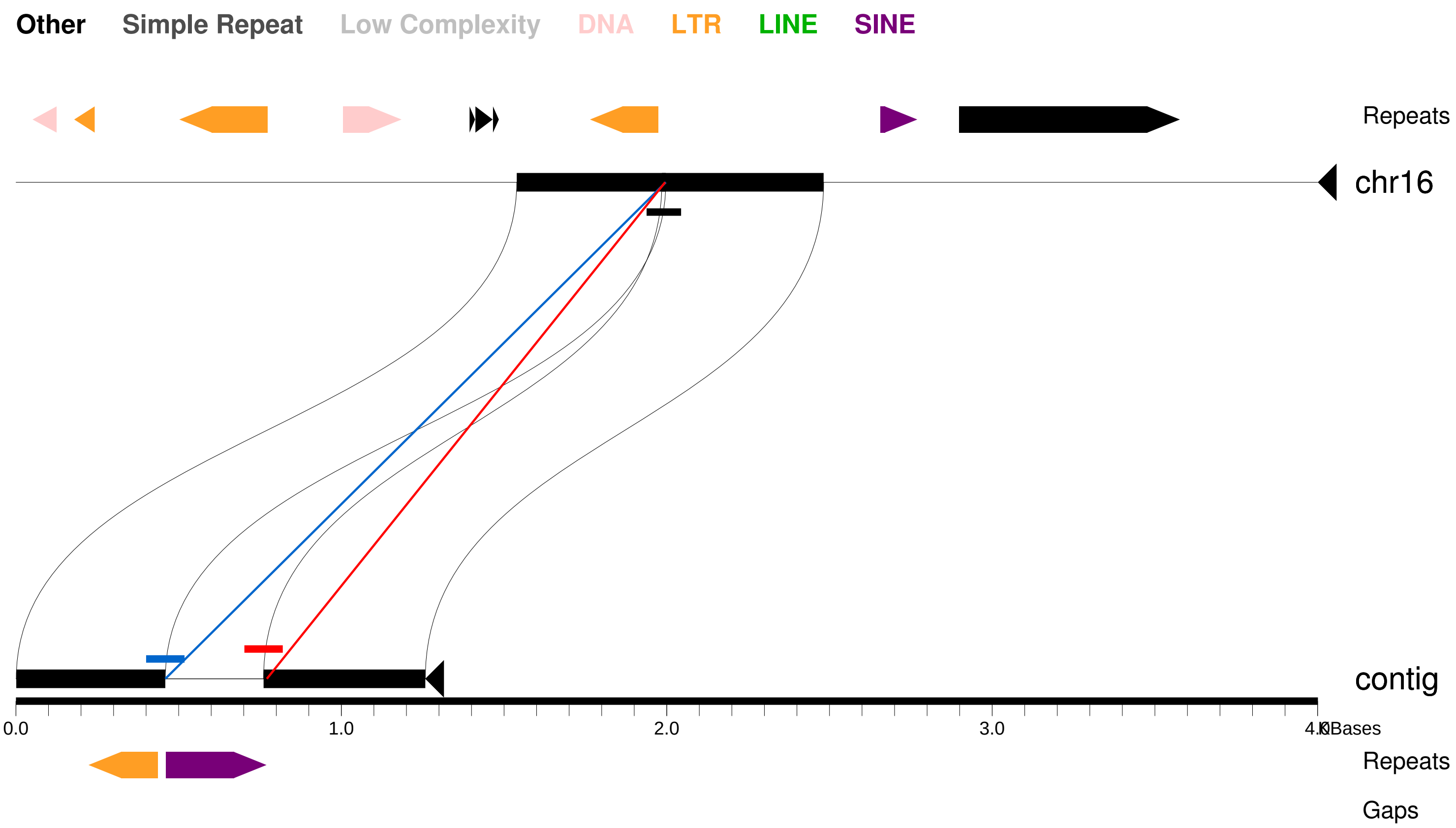

siteID: chr16_83666364 Scafftig4 - Breakpoint: chr16:83666361
chr16:83666315-83666420
Overlap Length: 14 INS size: 319 Genotyped: Yes

```
left     A--AGAATTCAGGGCGAG--------TCCATAGAGTAAAGTGAA---AGCAAGTTTATTAAGAAAGTAAA
chrom    A--AGAATTCAGGGCGAG--------TCCATAGAGTAAAGTGAA---AGCAAGTTTATTAAGAAAGTAAA
right    CCTGGGCGACAGAGCGAGACTCCGTCTCAAAAAAAAAAAATAAATAAAAAAAAAAAAAAAAGAAAGTAAA
         1111*1111***1*****11111111**1*1*1*11***1*1**111*11**1111*11***********

left     GGAGGCCGGGCGCGGTGGCTCACGCCTGTAATCCCAGCACTTTGGGAGGCCGAGGCGGGTGG
chrom    GGAATAAAAGAATGGCTGCTC-----TATAGGCAGAGCAAC-----AGCATGAG-CTGCT--
right    GGAATAAAAGAATGGCTGCTC-----TATAGGCAGAGCAAC-----AGCATGAG-CTGCT--
         ***222222*222**22****22222*2**22*22****2222222**222***2*2*2*22
```

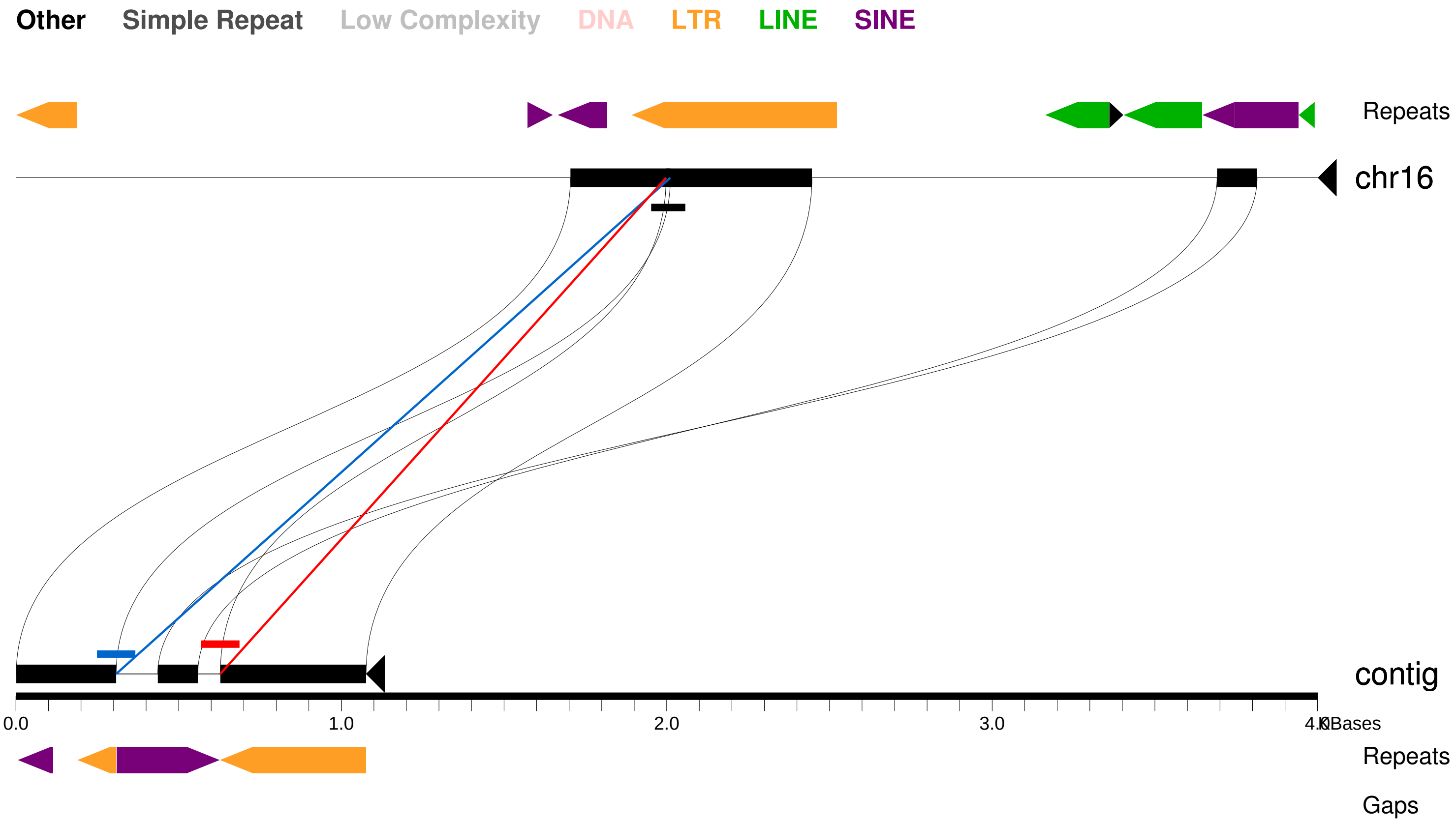

siteID: chr16_8418198 Scafftig3 + Breakpoint: chr16:8418180
chr16:8418020-8418356
Overlap Length: 17 INS size: 295 Genotyped: Yes

```
left   AA---------------CTGCATGGGTTCTTTTTGTATAGGTTGCCCCAAAATTTCCACAAGGGTATGTT
chrom  AAGATGCCGAAGGAAAACTGCATGGGTACTTTTTGTATAGGTTGCCCCAAAATTTCCACAAGGGTATGTT
right  ----TGAGGCAGGAGAATGGCGTGAACCCGGGAGGCGGAGCTTGC------AGTGAGCCGAGA-------
       11  22NN2N2222N2211**1**111N*11111*111**1****111111*1*1111*1**11111111

left   TTACTTCAAAGCTTCCTGCAAAGCAAGAAGGGCCAAATGGTTGATGACCCTCTTGAATGAGGCAGATGAA
chrom  TTACTTCAAAGCTTCCTGCAAAGCAAGAAGGGCCAAATGGTTGATGACCCTCTTGAATGAGGCAGATGAA
right  TCGCGCCA-------CTGGACCCCAGCCTGGGCGACAGAGCGAGACTCCGTCT---------CAAAAAAA
       *11*11**1111111***1*111**1111****1*1*11*1111111**1***111111111**1*11**

left   CAAAACAGATTCTTGACTACAAGAAGCTAACATAAGGCCGGGCGCGGTGGCTCACGCCTGTAATCCCA--
chrom  CAAAACAGATTCTTGACTACAAGAAGCTAACATAAGGTAGGTATTA-TTACTAACTCCATCAGACAGATG
right  AAAAAAAAA-----------AAGAAGCTAACATAAGGTAGGTGTTA-TTACTAACTCCATCAGACAGATG
       1****1*1*11111111111****************22**2N2222*22**2**2**222*22*22*22

left   --GCACT----TT--GGGAGGCCGAGGC-----GGGCGGATCAC-GAG---GTCAGGAGATCGAGACCAT
chrom  AAGAACTGGGGTTCAGGGAGGTTAAGTAACTAAGTCAAGGTCACAGAGCATGTATCTATATATGGAAAAC
right  AAGAACTGGGGTTCAGGGAGGTTAAGTAACTAAGTCAAGGTCACAGAGCATGTATCTATATATGGAAAAC
       22*2***2222**22*****222**2222222*2222*2****2***222**2222*2**222**22*2

left   CCCGGCTAAAA-CGGTGAAACC---CCGTCT-------CTACTAAA-------------
chrom  CCAGGCTTCTGGCTTTCAAAACTACCCTTCTAGTTTAGCTA-TAAATGTGTGATTGAGT
right  CCAGGCTTCTGGCTTTCAAAACTACCCTTCTAGTTTAGCTA-TAAAT------------
       **2****22222*22*2***2*222**2***2222222***2****2
```

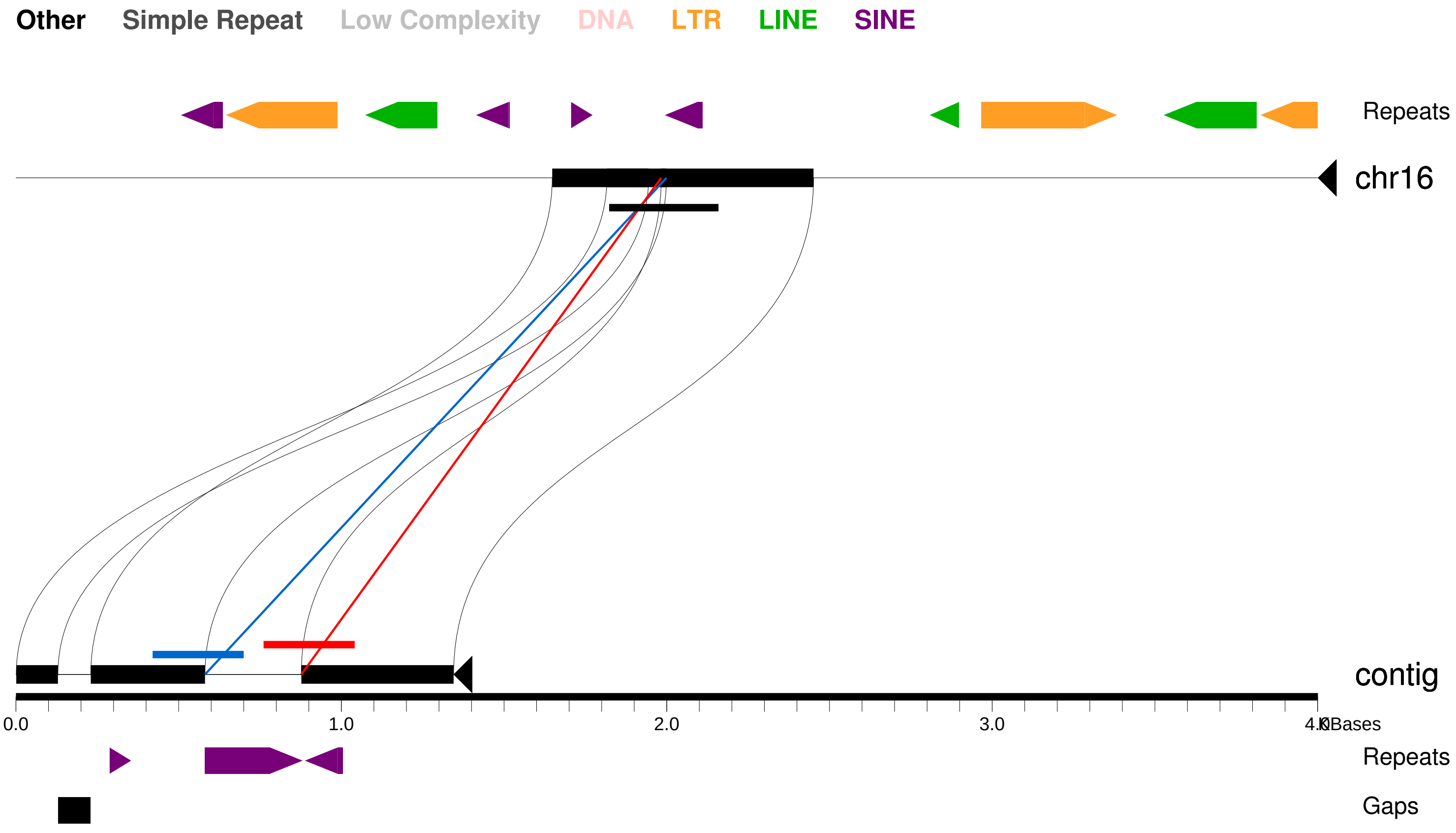

siteID: chr16_85108692 Scafftig2 + Breakpoint: chr16:85108693
chr16:85108533-85108868
Overlap Length: 16 INS size: 315 Genotyped: Yes

```
left    CT----TATATAAGTAACGTTGGGGAGGGAGGGGATTAAAGTCCCAACCCTGCCAAAACACAAAAATTCT
chrom   TTGTCCTATATAAGTAACGTTGGGGAGGGAGGGGATTAAAGTCCCAACCCTGCCAAAACACAAAAATTCT
right   TTT---------AGTAGAGACGGGGTTTCACCGTGTTA--GCCAGGATGGTCTCGAT-CTCCTGACCTCG
        2*N   111111****11*11****1111*11*11***11*1*111*111*11*1*11*1*111*11**1

left    TGCCATCACTTGGTCCCTGAGCATATATATGTTTGTATAGTTGCAAAAACAAA-GTGAAACAAAAACCCT
chrom   TGCCATCACTTGGTCCCTGAGCATATATATGTTTGTATAGTTGCAAAAACAAA-GTGAAACAAAAACCCT
right   TG--ATCCGCCCG--CCTCGGCCTCCCAAAGT-------GCTGGGATTACAGGCGTGAGCCACCGCGCCC
        **11***11111*11***11**1*1111*1**1111111*1**11*11***111****11**11111**1

left    TTTACTATTTAGACAATATGGGATTTTTTAGTTTTAT----TTTATTTTATT------------------
chrom   TTTACTATTTAGACAATATGGGATTTTTTAGTTTTATAATCTTTAGTTTGTTAAACATCTAAAGTAAAAA
right   G----------GCC-------GATTTTTTAGTTTTATAATCTTTAGTTTGTTAAACATCTAAAGTAAAAA
        11111111111*1*1111111****************2222****2***2**222222222222222222

left    -TTA--TTTTATTTTTATTTTTTTTTGAGACGGAGT-CTCGCTCTGTCGCCCAGGCTG-----------G
chrom   GTTAATTTTTACTTTTAGTCTTTTAT-AGTTTTATTACTAGTTTAGTTGATTAGTTTTTTATATAAAAAG
right   GTTAATTTTTACTTTTAGTCTTTTAT-AGTTTTATTACTAGTTTAGTTGATTAGTGTTTTATATAAAAAG
        2***22*****2*****2*2****2*2**2222*2*2**2*2*22**2*222**2N*222222222222*

left    AGTGCAGTGGCGCGATCTCGGCTCACTG-CAAGCTC----CGCCTCCC-------------
chrom   ATAAAAATGGCC---TTTAATCCCACTGTCTAGATAATTTCTTTTGACTTGTAAAGTAATA
right   ATAAAAATGGCC---TTTAATCCCACTGTCTAGATAATTTCTTTT----------------
        *2222*2****2222*2*222*2*****2*2**2*22222*222*NN1
```

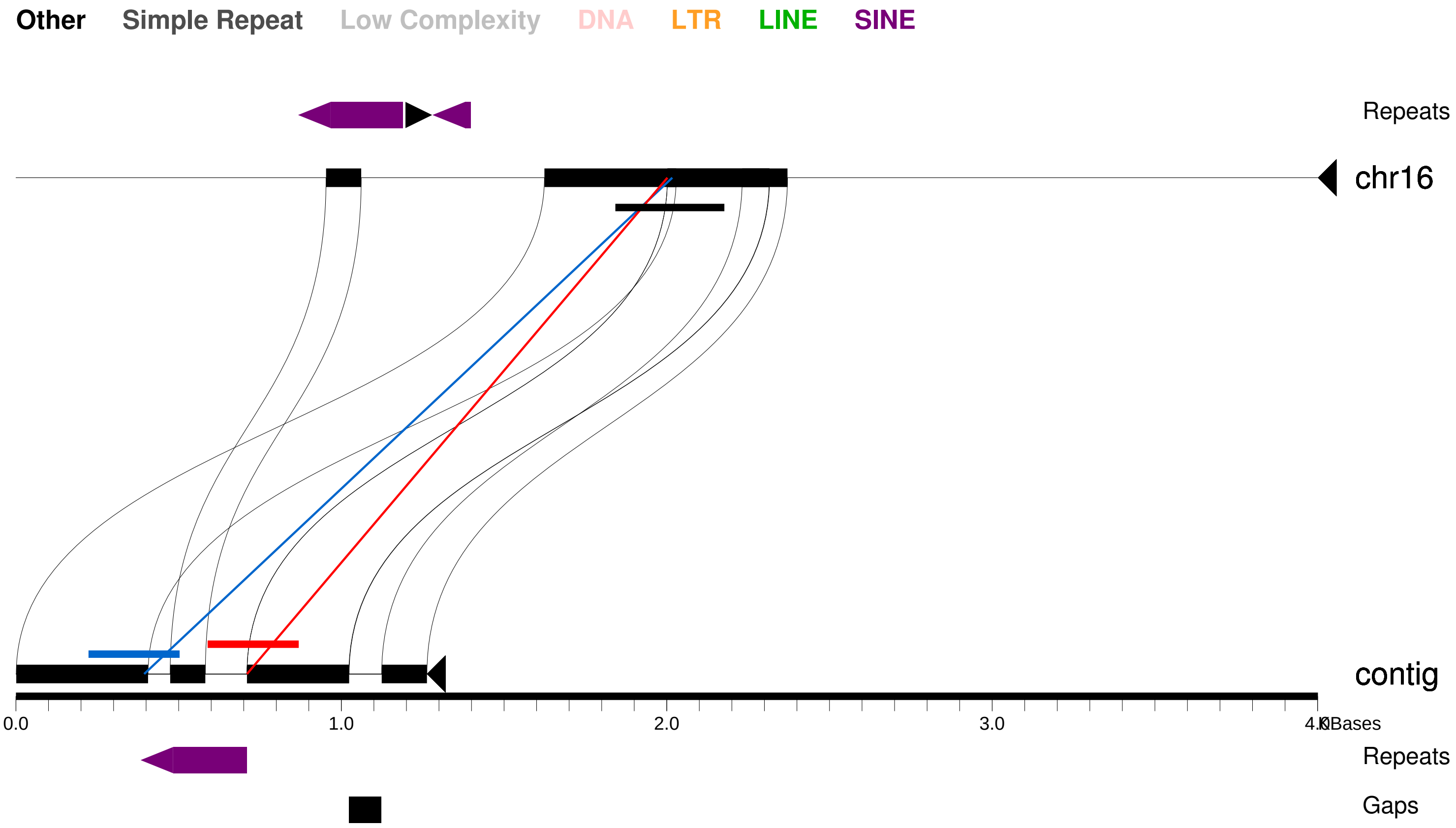

siteID: chr16_87191672 Scafftig3 + Breakpoint: chr16:87191662
chr16:87191613-87191721
Overlap Length: 11 INS size: 312 Genotyped: Yes

```
left    CAATACCCCACAAGCAC--AA---GCAACCAAAGCAAAAATGGACAAATG-----GGATCACCTCAAGTT
chrom   CAATACCCCACAAGCAC--AA---GCAACCAAAGCAAAAATGGACAAATG-----GGATCACCTCAAGTT
right   CCGCCCGCCTCGGCCTCCCAAAGTGCTGGGATTACAGGCGTGAGCCACCGCGCCCGGCCCACCTCAAGTT
        *1111*1**1*111*1*11**111**1111*111**1111**11*1*11*11111**11***********

left    TTTTTTTTTTTTTTTTTTTTTTTTTTTTTTTGAGACGGAGTCT-CGCTCTGTCGCCCAGG
chrom   AAAAAGCTTCTGCACAGCAA---------AGGAAAC--AATCAACAAAGTGATGAGACAA
right   AAAAAGCTTCTGCACAGCAA---------AGGAAAC--AATCAACAAAGTGATGAGACAA
        2222222**2*2222222222222222222**2**22*2**22*2222**22*222222
```

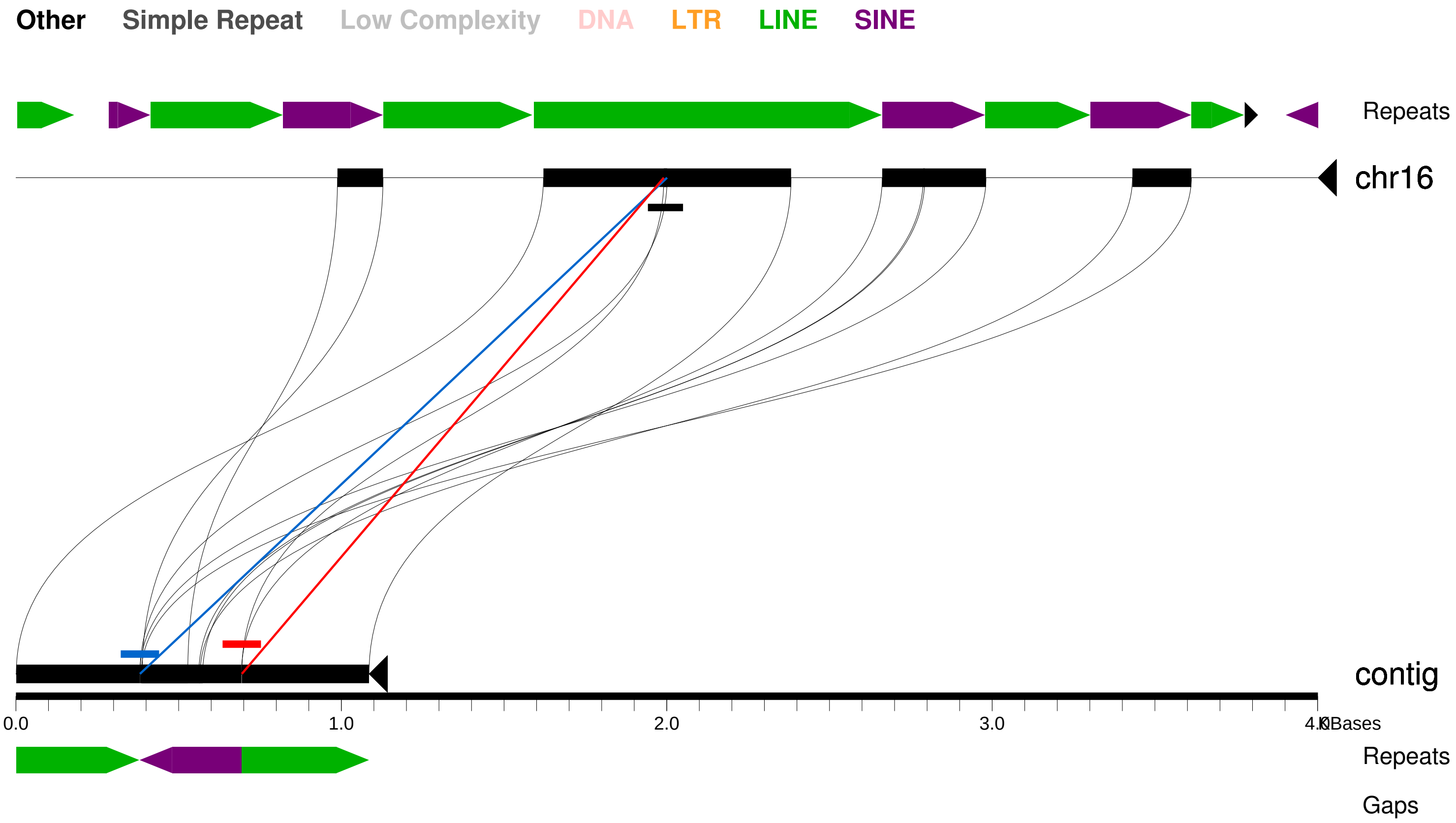

siteID: chr17_13045148 Scafftig3 + Breakpoint: chr17:13045143
chr17:13044983-13045315
Overlap Length: 13 INS size: 308 Genotyped: Yes

```
left   A-------------ATGGACTTTCTCTAGGAAAGTTAAAGGAGAACTTCATTTATTGTACC-ATGTTATG
chrom  ATGGCTTGGCCACAATGGACTTTCTCTAGGAAAGTTAAAGGAGAACTTCATTTATTGTACC-ATGTTATG
right  TTCAGTAGAG----ACGGGGTTTCACC------GTTTTAGCCGGG--------ATGGTCTCGATCTCCTG
       12NNN2N2NN    *1**11****1*1111111***11**11*1111111111**1**11*1**1*11**

left   AT-TAGTGTTGCTCCA-CTACACCCTCAAATAGTATTTTTAAGGCTCAAATTTTTATTGTAGACATAACT
chrom  AT-TAGTGTTGCTCCA-CTACACCCTCAAATAGTATTTTTAAGGCTCAAATTTTTATTGTAGACATAACT
right  ACCTCGTGATCCGCCCGCCTCGGCCTCCCAAAGTGCTG----GG-----------ATTACAGGCGTGA--
       *11*1***1*1*1**11*11*11****11*1***11*11111**1111111111***11**1*1*1*11

left   TTTAGATTTTGGACTATTAGAAGGGCCGAATCTTTCTT-----TTTTTTTTTTTTTTTTT---------T
chrom  TTTAGATTTTGGACTATTAGAAGGGCCGAATCTTTCAAACAACTCTGGCTGCATTTGGATAGCAAAGACT
right  -----------GCCACCGCGCCCGGCCAAATCTTTCAAACAACTCTGGCTGCATTTGGATAGCAAAGACT
       11111111111*1*11111*111****1********2222222*2*222*222***222*222222222*

left   TTTTTTTTTTTGAG--ACGGAGTCTCGCTCTGTCGCCCAGGCTGGAGTG-----CAGTGGCGG-------
chrom  TCTATGTCATTGAGTAATTAAGCCATGCATTCTTGTGCAGGATTATATGATAACCATTCATAACTTCTTA
right  TCTATGTCATTGAGTAATTAAGCCATGCATTCTTGTGCAGGATTATATGATAACCATTCATAACTTCTTA
       *2*2*2*22*****22*222**2*22**22*2*2*22****2*2222**22222**2*222222222222

left   -GATC------TCGGCTCACTGCAAG--CTCCGCCTCCCGGGTTCACG--CCATTC
chrom  AGATCCTTTCCTCCTTTTGTTGCCATACCTTTACATCAGTGTTTCCCAGACTCTAA
right  AGATCCTTTCCTCCTTTTGTTGCCATACCTTTACATCAGTGTTTCCC---------
       2****222222**222*222***2*222**222*2**222*2***2*N  1NN1NN
```

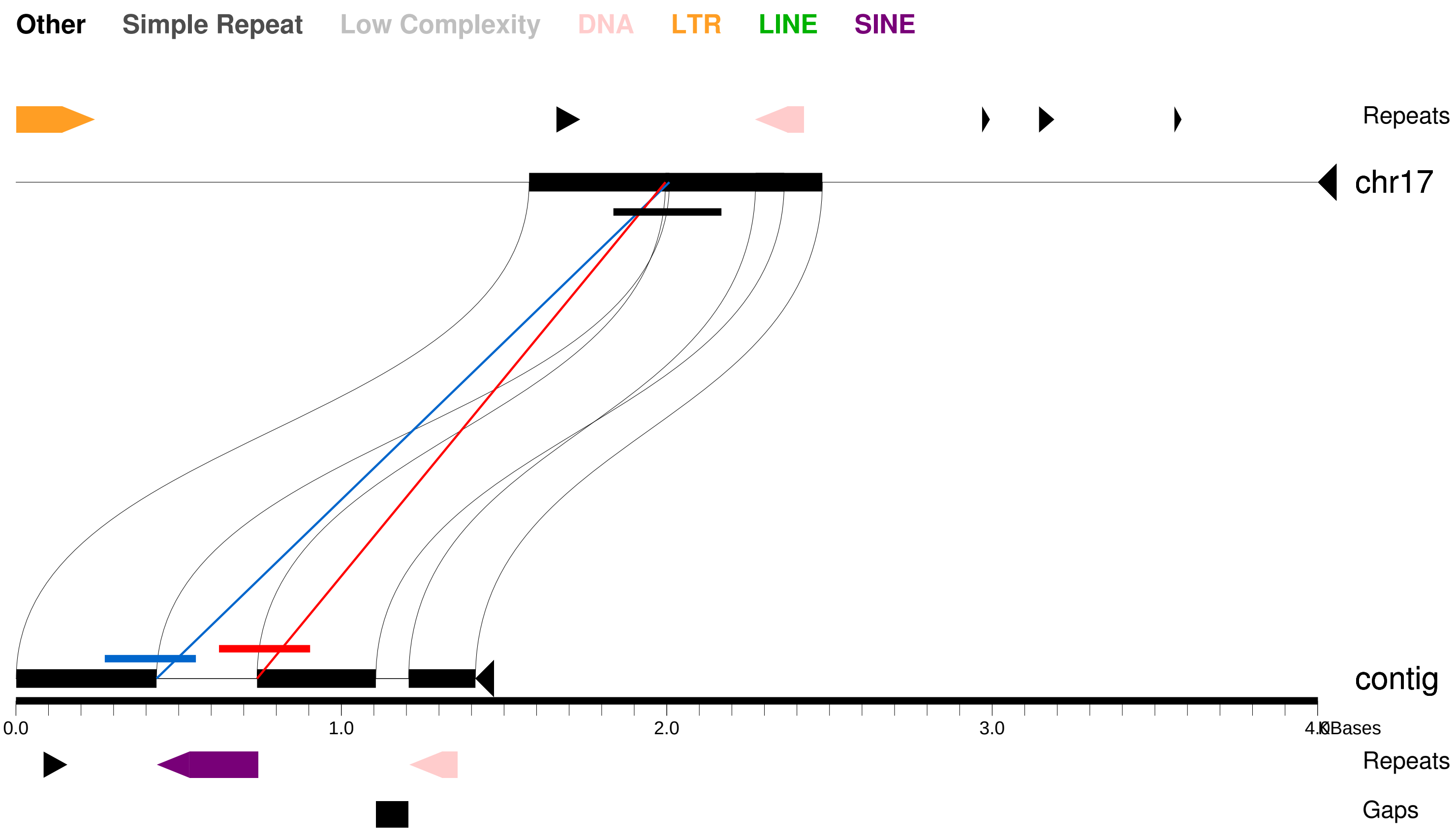

siteID: chr17_13436850 Scafftig4 + Breakpoint: chr17:13436832
chr17:13436788-13436891
Overlap Length: 16 INS size: 279 Genotyped: Yes

```
left     TAA----GAAAAATAATAAT--GAAAATATA-------CAAAGAATAC---TTATAAACTGAAGTTATTA
chrom    TAA----GAAAAATAATAAT--GAAAATATA-------CAAAGAATAC---TTATAAACTGAAGTTACTA
right    CGATCTCTTGACCTCGTGATCCGCCCGCCTCGGCCTCCCAAAGTGCTGGGATTACAGGC-GAAGTTATTA
         11*1111111*11*11*1**11*111111*11111111*****11111111***1*11*1*******N**

left     ATTTTTTTTTTTTTTTTTTTTTTGAGACGGAGTCTCGCTCTGTCGCCCAGGCTGGAGTGCAGTG---G
chrom    ATTTTTA-----------------AGGCA-ACTCTGTATCTGTCATTTGAGATTGGTTCCATTCATTG
right    ATTTTTA-----------------AGGCA-ACTCTGTATCTGTCATTTGAGATTGGTTCCATTCATTG
         ******222222222222222222**2*22*2***222******222222*2*2*22*2**2*2222*
```

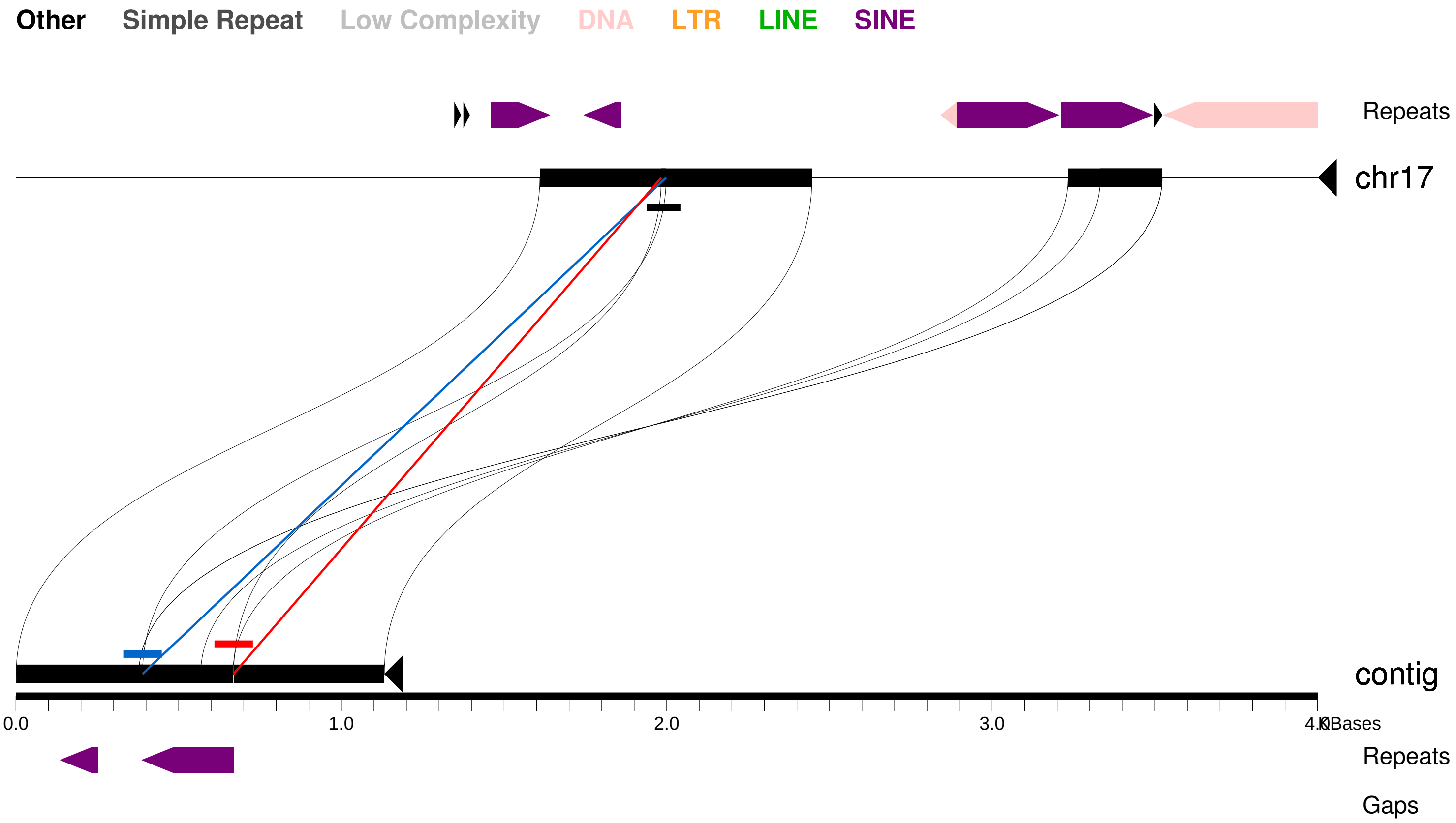

siteID: chr17_13799652 Scafftig14 + Breakpoint: chr17:13799643
chr17:13799483-13799816
Overlap Length: 14 INS size: 299 Genotyped: Yes

```
left    --------------AACAGAAACTGAGTGACTTGGTGAAAGGGGTGGGACAGAAATTCATTAACACCATC
chrom   TGTCTGCCACTGAAAACAGAAACTGAGTGACTTGGTGAAAGGGGTGGGACAGAAATTCATTAACACCATC
right   AG---GAGAATGGCGT--GAACCCGGGAGGC--GGAGCTTGCAGTGAGCC-GAGATCCCGCCACTGCA-C
        N2   2NN2N22NN1111***1*1*1*1*1*11**1*111*11***1*1*1**1**1*1111**11**1*

left    TATTAACTAGTCAAAAAATTAATTAACTACTTAGATATTCCTTACCTGTATTAAAGACACAGAAAAAGAA
chrom   TATTAACTAGTCAAAAAATTAATTAACTACTTAGATATTCCTTACCTGTATTAAAGACACAGAAAAAGAA
right   TCCAGCCTGGGCGACAGAGC-------------GAGACTCC------GTCTCAAAAAAAAAAAAAAAAAA
        *11111**1*1*1*1*1*1*111111111111111**1*1***111111**1*1***1*1*1*1*****1**

left    GGTTCTGTAGGAACATACTCAAAACAATAGTAAGGGC--------CGGGCGCGGTGGC----TCACGCCT
chrom   GGTTCTGTAGGAACATACTCAAAACAATAGTAAGAACATATATGACATACGTATTTGAGTTATCCAGGCA
right   --------------------AAAAAAAAAGTAAGAACATATATGACATACGTATTTGAGTTATCCAGGCA
        11111111111111111111****1**1******22*22222222*222**222*2*22222**22*2*2

left    GTAATCCCAGC--ACTTTGG-GAGGCCGAGGCGGGCGGATCACG-AGGTCAGGAGATC---GA-------
chrom   AGAGCCACAGCTGACGGTGGAGAAGCTAAGTTAGA--AATCAAGGACTTCAGAATATCTTTGAATACTCT
right   AGAGCCACAGCTGACAGTGGAGAAGCTAAGTTAGA--AATCAAGGACTTCAGAATATCTTTGAATACTCT
        22*22*2****22**N2***2**2**22**222*2222****2*2*22****2*2***222**2222222

left    GACCATCCCGGC-TAAAACGGTGAAACCC----CGTCTCT-ACTAAA---------
chrom   GAAGTTACAGCCCTAAAATGAAGCAGCAAGATGCATCTGTTAATAGAATTCCTGTC
right   GAAGTTACAGCCCTAAAATGAAGCAACAAGATGCATCTGTTAATAGAATTC-----
        **222*2*2*2*2*****2*22*2*N*222222*2***2*2*2**2*2222
```

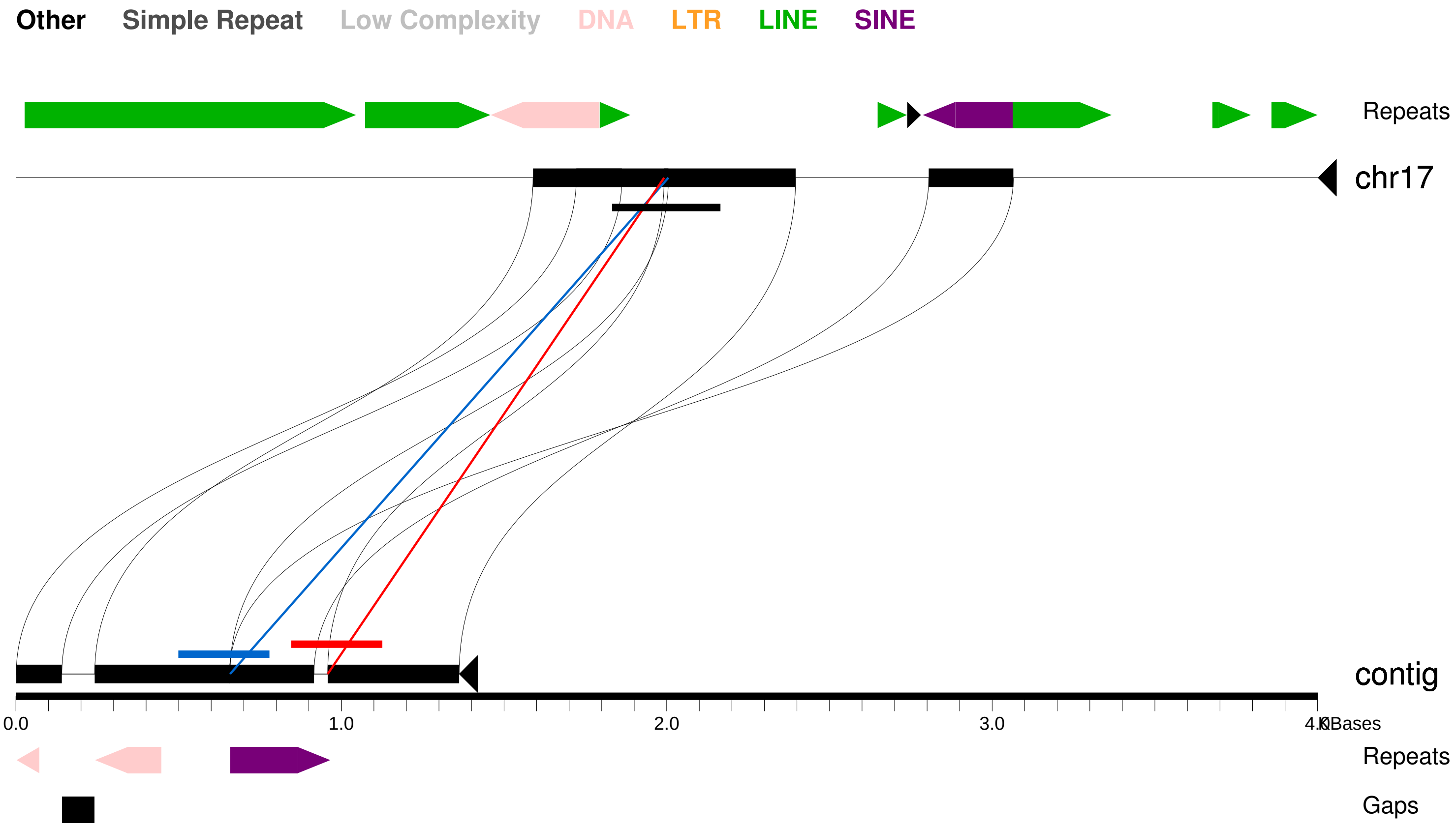

siteID: chr17_13884342 Scafftig2 + Breakpoint: chr17:13884330
chr17:13884295-13884389
Overlap Length: 15 INS size: 310 Genotyped: Yes

```
left    CA-------TTAGAATAATAAAG-GCTCTGAGAA-------GACTTACAA---------TAAAGAAACCT
chrom   CA-------TTAGAATAATAAAG-GCTCTGAGAA-------GACTTACAA---------TAAAGAAACCT
right   CCGCCCGTCTCGGCCTCCCAAAGTGCTGGGATTACAGGCGTGAGCCACCGCGCCCGGCCTAAAGAAACCT
        *11111111*11*11*111****1***11**11*1111111**111**11111111111***********

left    TTTTTTTTTTTTTTTTTTTTTTTTTTTTTTTTTTGAGACGGAGTCTCGCTCTGTCGCCCAGTCTGGAGTG
chrom   TTTTAATGTTGTTTAATCCAGTGTGT-----------------TCAAACTTTAT---------TTGACTG
right   TTTTAATGTTGTTTAATCCAGTGTGT-----------------TCAAACTTTAT---------TTGACTG
        ****22*2**2***22*2222*2*2*22222222222222222**222**2*2*222222222*2**2**

left    CAG--
chrom   CAGGA
right   CAGGA
        ***22
```

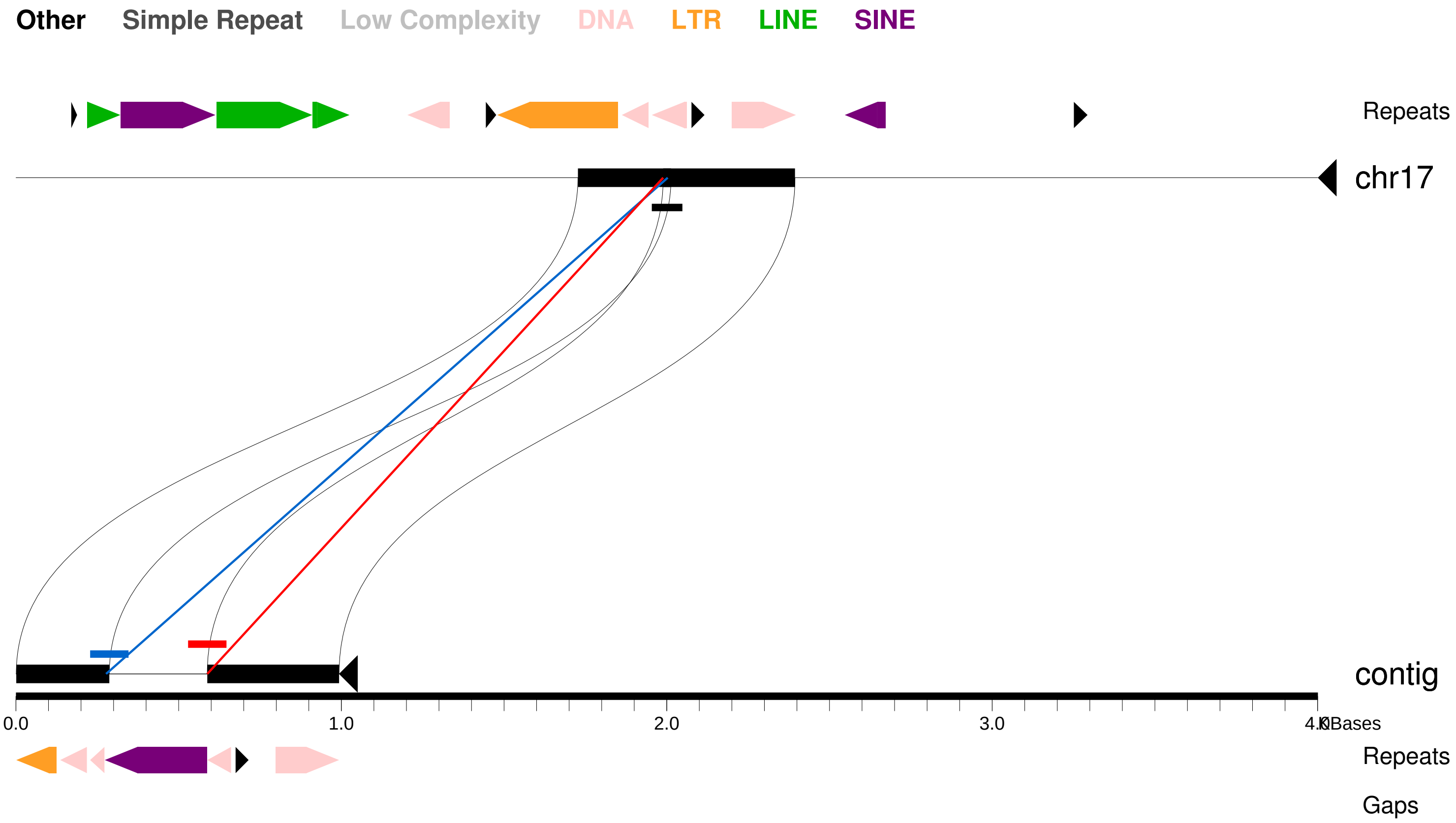

siteID: chr17_14527641 Scafftig2 - Breakpoint: chr17:14527640
chr17:14527595-14527699
Overlap Length: 15 INS size: 301 Genotyped: Yes

```
left     TATTATTC-AGTCTCCT-GTTGTAGCAATTGTCACTATCTTCCTCTT------------AAAGCTTCCAT
chrom    TATTATTC-AGTCTCCT-GTTGTAGCAATTGTCACTATCTTCCTCTT------------AAAGCTTCCAT
right    CTGCACTCCAGCCTGGGCGACAGAGCGAGACTCCGTCTCAAAAAAAAAAAAAAAAAAAAAAAACTTCCAT
         1111*1**1**1**1111*1111***1*111**11*1**1111111111111111111***1*******

left     TTGCGGCCGGGCGCGGTGGCTCACGCCTGTAATCCCAGCACTTTGGGAGGCCGAGGCGGGCGG
chrom    TTGC------TTGTGCTGACCATTTCCTATCTTCTCTCCAATT------GCTAAAGC--TCTT
right    TTGC------TTGTGCTGACCGTTTCCTATCTTCTCTCCAATT------GCTAAAGC--TCTT
         *****22222222*2*2**2*2N222***2*22**2*22**2**222222**22*2**222*22
```

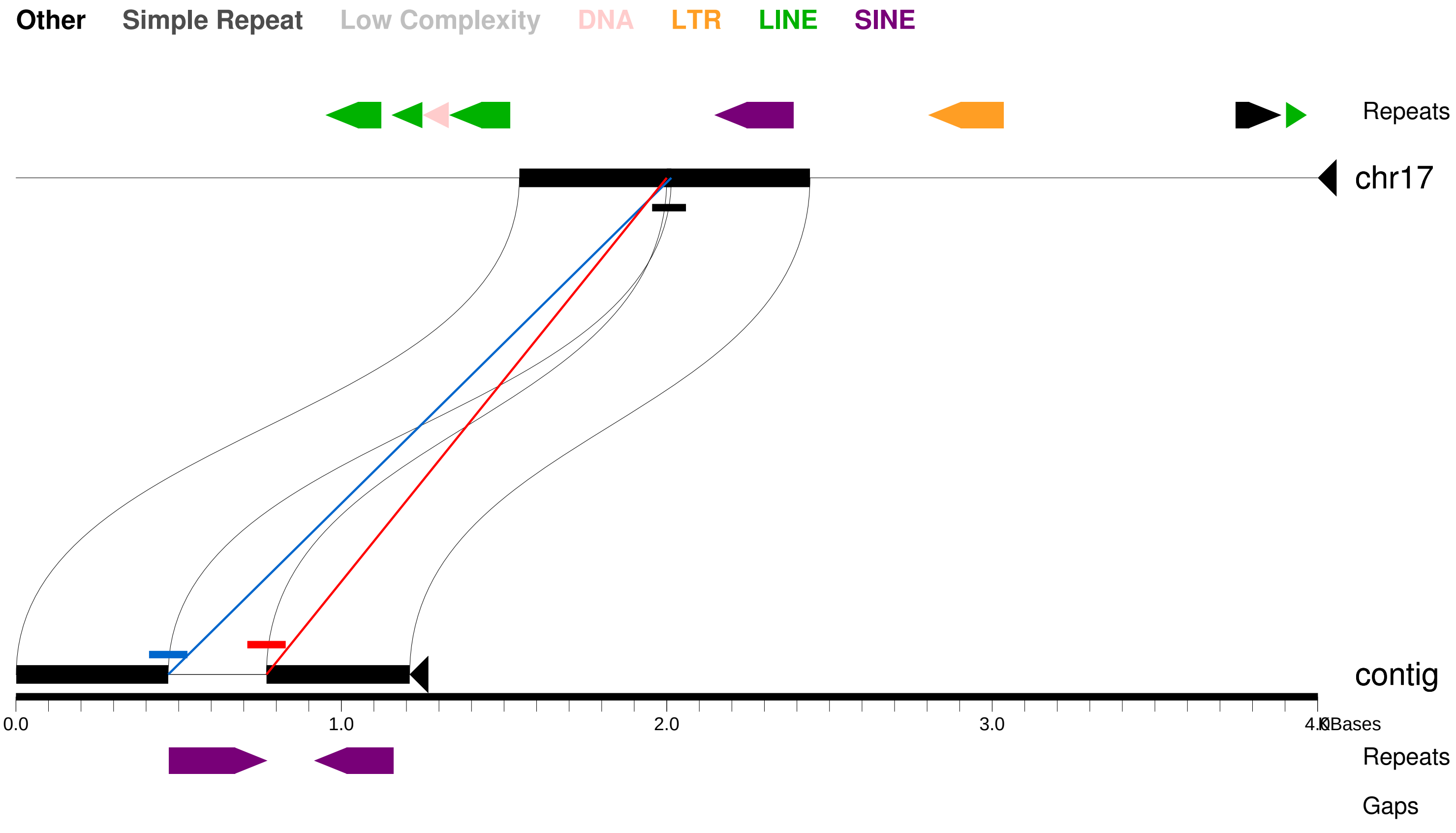

siteID: chr17_21668228 Scafftig3 + Breakpoint: chr17:21668213
chr17:21668053-21668384
Overlap Length: 12 INS size: 155 Genotyped: Yes

```
left   -------------TTCCTCAAAAGTATATATAAAAAAAGAAACATGTAGCAACATCACTAAAGTGACTAT
chrom  AAGTGTACAGATTTTACTCAAAAGTATAAA-AAAAAAAGAAACATGTAGTAACATCACCAAAGTGACTAT
right  A---GAATGGCGTGAACCCGGGAGGC------------GGAGCTTGCAGTGAG-----CCGAGATCCCGC
       2   2N2NN2NN2112*1*111**1111N121111111*1*1*1**1**21*111111211**111*111

left   AAATAAAAGAGTTTGAAATTTGGGACACTTTTTAAGATGTTACAGAGAGGAGTAGAATTAGAAATAAAGT
chrom  AAATAGAAGAGTTTGAAACTTGGGACACTTTTTAAGATGTTACAGAGAGGAGTAGAATTAGAAATAAAGT
right  CACTGCAC-----TCCAGCCTGGGCGAC-------------AGAGCGAGACTCCGTCTCAAAAAAAAAAA
       1*1*1N*111111*11*121****11**1111111111111*1**1***11111*11*1*1***1***11

left   AGAAGGAAAATCTGAAGGTAGAAACATGAAGAT---TGTAGTCC----------CAGCTACTTGGGAGGC
chrom  AGAAGGAAAATCTGAAGGTAGAAACATGAAGATAATTGGAGACCAGGACAAGAGCAGTTTCTCTGGGGTA
right  AAAAAAAAAAAAAAAAAAAA-AAACATGAAGATAATTGGAGACCAGGACAAGAGCAGTTTCTCTGGGGTA
       *1**11****1111**111*1***********222**2**2**2222222222***2*2**22**2*22

left   TGAG--GCAGGAGAATGGCGTGAA------CCCGGGAGGCGGAGCT-----------TGCAGTGAGCCGA
chrom  GTAGCAGCAAAAAAATGGTGGGAGTGGATTCAATGAAGTCATATATGAAAAATTAGATGCAGATAAACTT
right  GTAGCAGCAAAAAAATGGTGGGAGTGGATTCAGTGAAGTCATATATGAAAAATTAGATGCAAATAAACTT
       22**22***22*2*****2*2**2222222*2N2*2**2*22*22*22222222222****122*22*22

left   GATCCC--GCC-----ACTGCACTCCAGCCTGGGCGACAGAGCGAGACTCCGT
chrom  TATGTTTTGCATAAATATTGCTGTAAATGAAAAAAGAGAAAAGGGTTGTGAGC
right  TATGTTTTGCATAAATATTGCTGTAAATGAAAAAAGAGAA-------------
       2**22222**222222*2***22*22*22222222**2*21NN1NNNN1NN1N
```

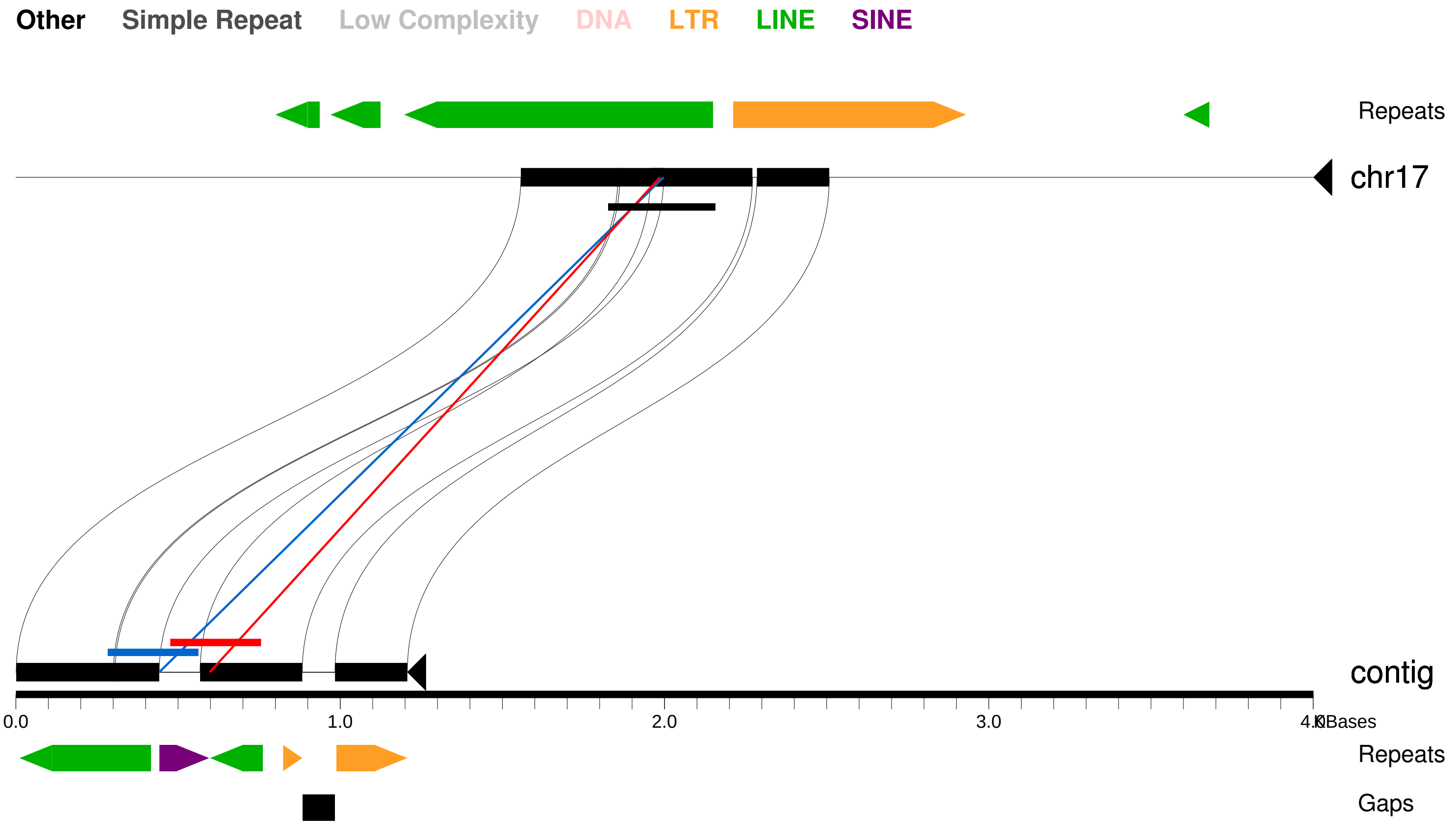

siteID: chr17_35922005 Scafftig1 - Breakpoint: chr17:35922016
chr17:35921969-35922075
Overlap Length: 13 INS size: 304 Genotyped: Yes

```
left    ---TCTTTAAGCCGTAATA----ACA-ACTGGAATGGCGTTGGAAGTAGATTATT----AAAGAATGATG
chrom   ---TCTTTAAGCCGTAATA----ACA-ACTGGAATGGCGTTGGAAGTAGATTATT----AAAGAATGATG
right   CAGTCCGCAGTCCGGCCTGGGCGACAGAGCGAGACTCCGTCTCAAAAAAAAAAAAAAAAAAAGAATGATG
        111**111*11***111*11111***1*11*11*111***111**11*1*11*111111***********

left    ACGGCCGGGCGCGGTGGCTCACGCCTGTAATCCCAGCACTTTGGGAGGCCGAGGCGGGTGG
chrom   ACTTCCTGCAATTTTAACTTAAG---GTC--CATAAAACT-------GCAAAAGTGAACTG
right   ACTTCCTGCAATTTTAACTTAAG---GTC--CATAAAACT-------GCAAAAGTGAACTG
        **22**2*222222*22**2*2*222**222*22*22***2222222**22*2*2*2222*
```

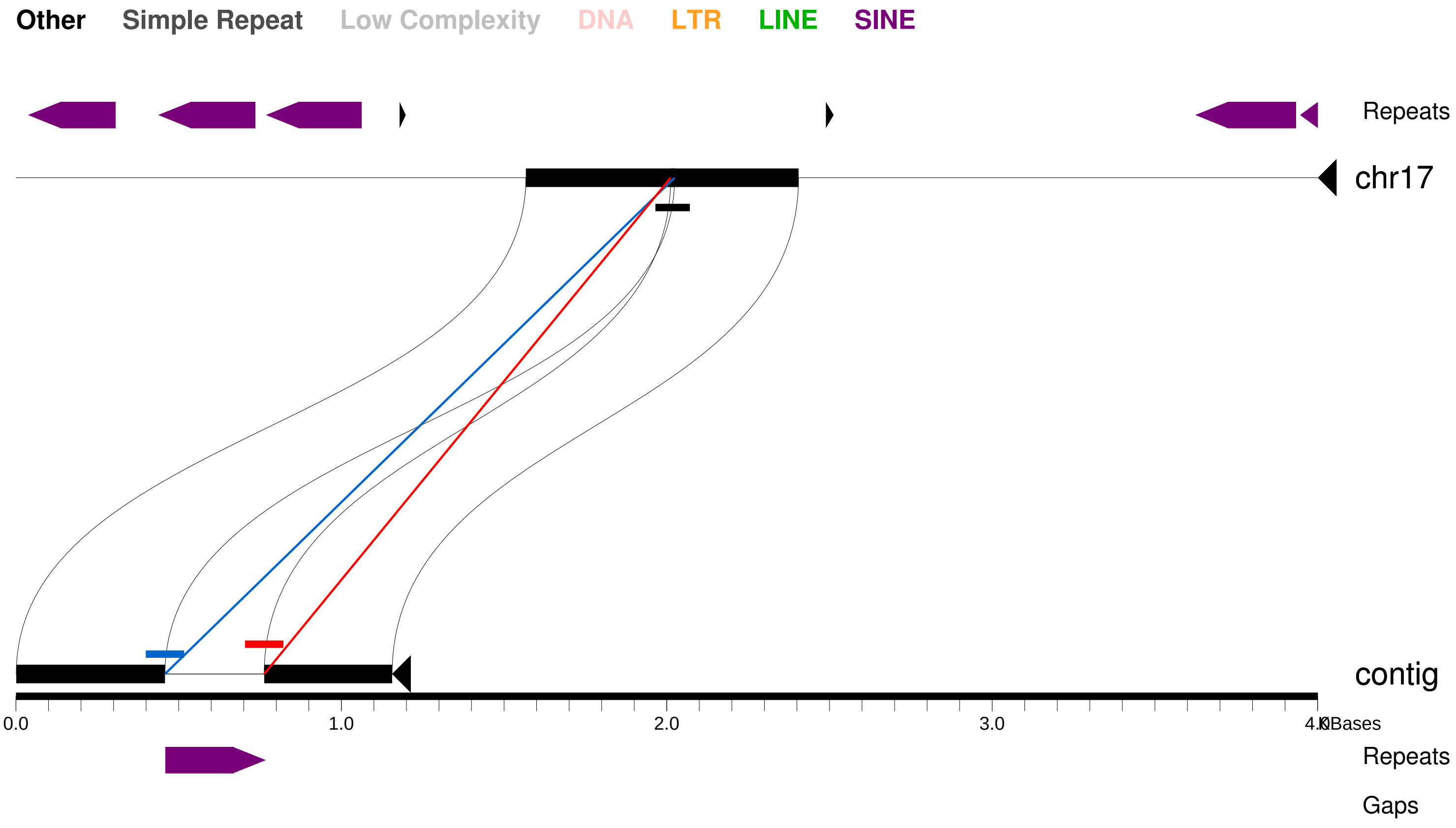

siteID: chr17_36025919 Scafftig3 - Breakpoint: chr17:36025927
chr17:36025882-36025986
Overlap Length: 15 INS size: 266 Genotyped: Yes

```
left    --GGATTATAGGTATGAGCTACCACGCCTGG--CCAT-----------AGAATGCATTTTAAAAGAGATA
chrom   --GGATTATAGGTATGAGCTACCACGCCTGG--CCGT-----------AGAATGCATTTTAAAAGAGATA
right   CTGCACTCCAGCC-TGGGCGACAGAGCGAGACTCCGTCTCAAAAAAAAAAAAAAAAAAAAAAAAAGAGATA
        11*1*1*11**111**1**1**111**11*111**2*11111111111*1**111*1111**********

left    CTTCACTTTGGGAGGCCGAGGCGGGCGGATCACGAGGTCAGGAGATCGAGGCCATCCCGGCTAA
chrom   CTTCA--------GAACAAACAATGCTTTTACCCATTTAA----AT-GAGTTTTTAACAG-TGG
right   CTTCA--------GAACAAACAATGCTTTTACCCATTTAA----AT-GAGTTTTTAACAG-TGG
        *****22222222*22*2*22222**222*22*2*22*2*2222**2***2222*22*2*2*22
```

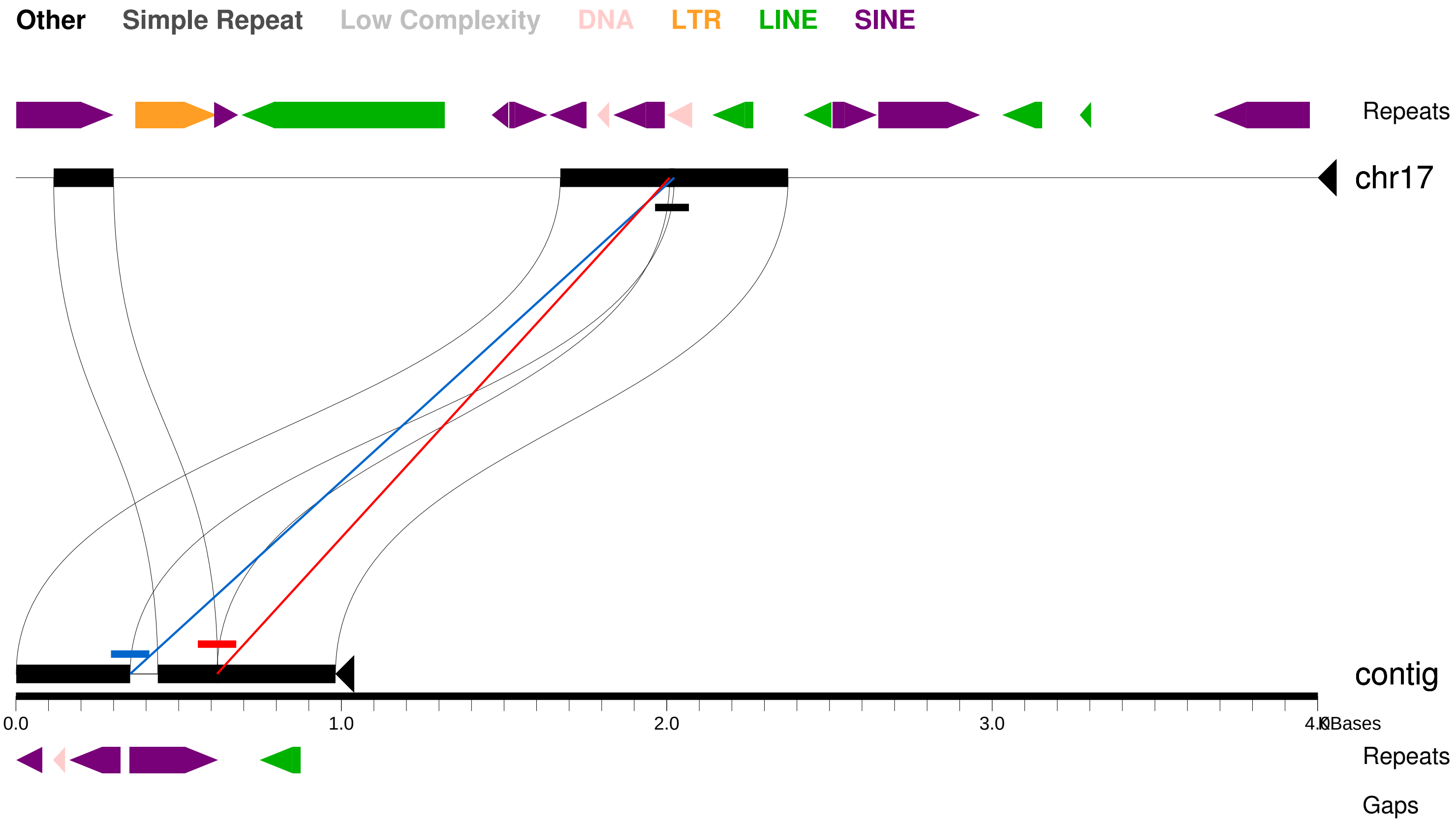

siteID: chr17_39177017 Scafftig1 + Breakpoint: chr17:39177019
chr17:39176972-39177076
Overlap Length: 13 INS size: 417 Genotyped: No

```
left    TTTGGGACATCATCTCCAGTTGAGGTCATCACCTGAATTCATTTA--------------AAATAATAATA
chrom   TTTGGGACATCATCTCCAGTTGAGGTCATCACCTGAATTCATTTA--------------AAATAATAATA
right   NNNNNNNNNNNNNNNNNNNNNNNNNNNNNNNNNNNNNNNNNNNNNNNNNNNNNNNNNNNNNATAATAATA
        1111111111111111111111111111111111111111111111111111111111111*********

left    CATAGGCCGGGCGCGGTGGCTCACGCCTGTAATCCCAGCACTTTGGGAGGCCGAGGCGGGCGG
chrom   CATAA-----------TTGTTCTGT--TATACTCCAGGGTTTATCTGCTTTGTCTACGGGAT-
right   CATAA-----------TTGTTCTGT--TATACTCCAGGGTTTATCTGCTTTGTCTACGGGAT-
        ****222222222222*2*2**22222*2**2***22*222*2*22*222222222****222
```

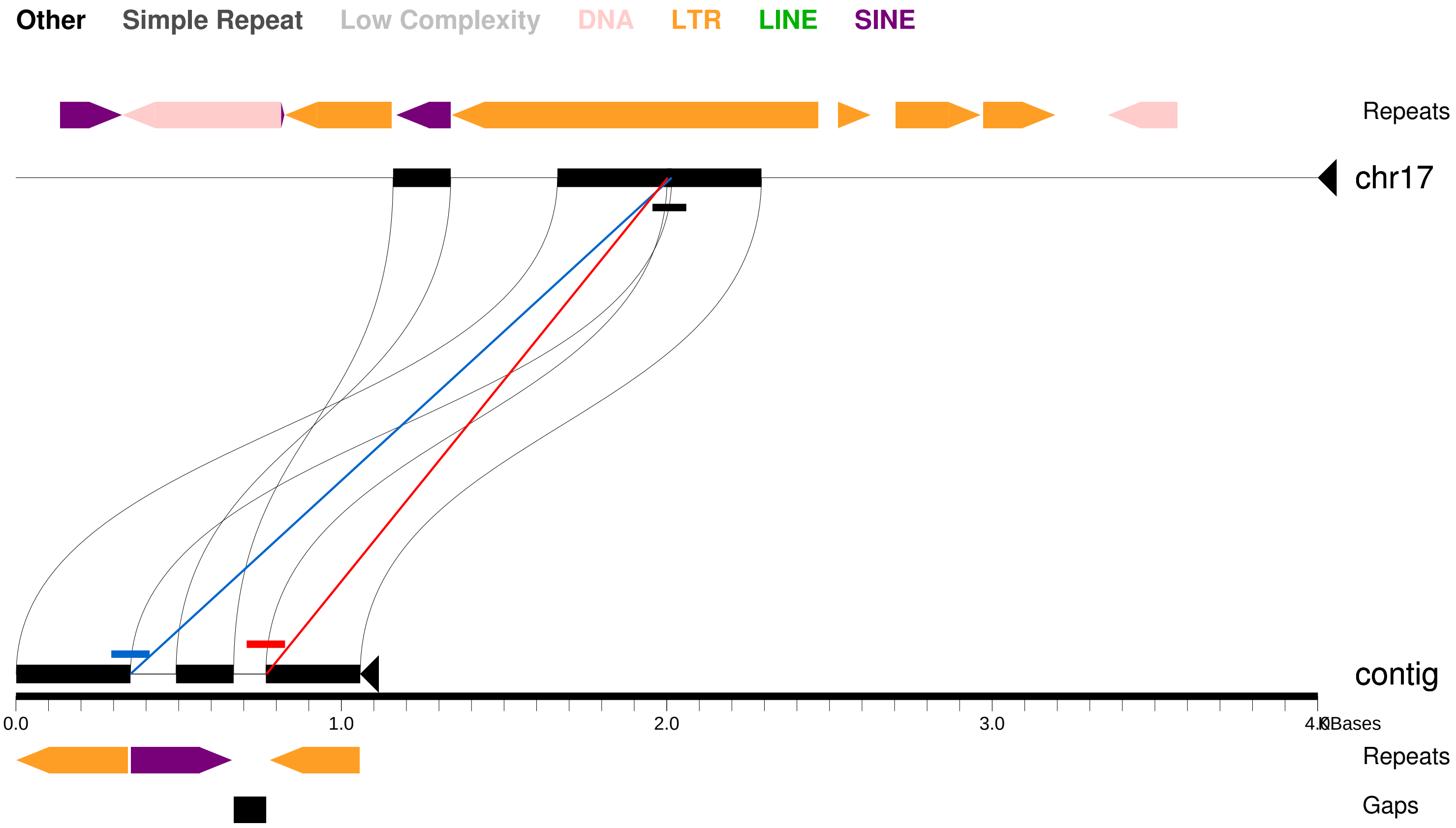

siteID: chr17_46504985 Scafftig5 + Breakpoint: chr17:46505001
chr17:46504842-46505357
Overlap Length: 2 INS size: 320 Genotyped: No

```
left   -GAGGTTTCTAGGTCGAGTGAAACATTTCCAAGTGTCTCAAATTTTATATTTAAACAAAATTAATTTTAG
chrom  TGAGGTTTCTAGGTCGAGTGAAACATTTCCAAGTGTCTCAAATTTTATATTTAAACAAAATTAATTTTAG
right  AGAA---------TGGCGTGAACCC-------GGGAGGCGGAGCTTGCAGTGAGCCGAGATCCC------
       N**1111111111*1*1*****1*11111111*1*111*11*11**11*1*1*11*1*1**111111111

left   GCCTTGGGAATTTCTGTTCTCTTCATTATGCAAAATTTTGTTATCTACCTTTCACAATGCTTTACATATT
chrom  GCCTTGGGAATTTCTGTTCTCTTCATTATGCAAAATTTTGTTATCTACCTTTCACAATGCTTTACATATT
right  GCCA---------CTGCACTCCAGCCTGGGCGACAGAGCGAGA-CTCCGTCTCAAAAAAAAAAAAAAAAA
       ***1111111111***11***11111*11**1*1*1111*11*1**1*1*1***1**111111*1*1*11

left   TTATTCTTAAAAGCACACA---CA-----------------------------------------------
chrom  TTATTCTTAAAAGCACACA---CATTATGTCAAAAAATGCCCCTGTCATTTATGCTTGTGTAGTTATAAA
right  AAAAAAAAAAAAAAAAAAAAAACATTATGACAAAAAATGCCCCTGTCATTTATGCTTGTGAATTTAAAAA
       11*11111****11*1*1*111**22222N22222222222222222222222222222222N2N222N222

left   --------------------GGCCGGG----------------CGCGGTG-----GCTCACGTCT-----
chrom  TAAATAAATGCTTAAGTTATGGCTGTGATGTCCAGTGTTAAATCACTGTGCAAATGCTTATGCCTTGGTT
right  AAAATAAATGCTTANNNNNNNNNNNNNNNNNNNNNNNNNNNNNNNNNNNNNNNNNNNNNNNNNNNNNNNN
       N2222222222222NNNNNN111N1N1NNNNNNNNNNNNNNNN1N1N111NNNNN111N1N1N11NNNNN

left   ----------GTAATCC--------------------CAGCACTTTGGGAGGCCGAGGCGGGCGG----
chrom  TATGAAAGTAGTAATTCATTGTGGAAACCCCACCCCCACAGCCATGTGGCAGCACTAGAGTTGCAGCCCT
right  NNNNNNNNNNNNNNNNNNNNNNNNNNNNNNNNN-------------------------------------
       NNNNNNNNNN11111N1NNNNNNNNNNNNNNNN     1111NN1N111N11NN1N11NNNN11N1

left   ---ATCATGAG---------------GTCAGGAGATCGAGACCA-----TCC------------------
chrom  CACATCCTGTGACTCCCTCCCACAGAGTGACTATATAGAGATCAGGACCTCCATTTTTTCTAATTGTCAA
right  ----------------------------------------------------------------------
          111N11N1               11N1NN1N11N1111N11     111

left   CGGCTAA-----AACGGTG-------------------------AAACCCC-------------------
chrom  CAGTTAACCTTAAAAAGTGTGTGGTGGGGGAGACAAATAGCAACAAACAACAAATTGATGGCCATTTTGG
right  ----------------------------------------------------------------------
       1N1N111     11NN111                              1111NN1

left   GTCTCTACTA-----------------AA
chrom  GTTTCTAATATGGACAGATTATTTGTAAC
right  -----------------------------
       11N1111N11                 1N
```

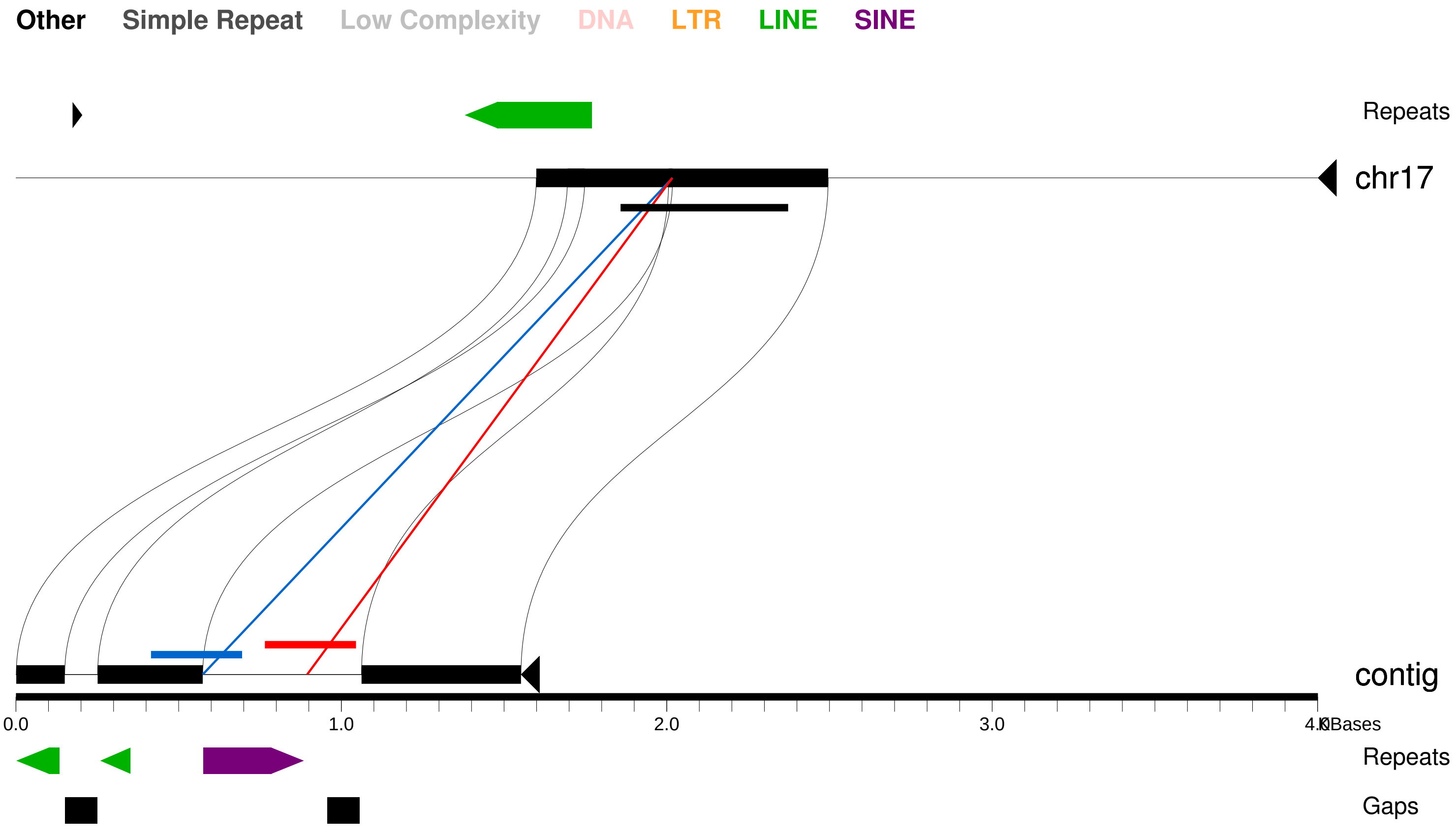

siteID: chr17_46617242 Scafftig2 + Breakpoint: chr17:46617220
chr17:46617161-46617280
Overlap Length: 0 INS size: 166 Genotyped: Yes

```
left     TTCATTGCCTCTTTGT--GCTG---CCGTGGTATCATCAGAGACGGTGAGTTTCATAAATTTGGACGGAT
chrom    TTCATTGCCTCTTTGT--GCTG---CCGTGGTATCATCAGAGACGGTGAGTTTCATAAATTTGGAAAGAA
right    TCCCA-GCTACTTGGGAGGCTGAGACAGGAGAATCACTTGAACCCAGAAGGTGGAGG---TTG--AAGAA
         *1*111**11***1*111****111*1*11*1****111**11*1111**1*11*11111***1122**2

left     CACTTGAGGTCAGGAGTTCGAGGCCAGCCTGGCCAACATGGTGAAACCTCATCT----
chrom    GACAGCCAGCAACGCTTTTGTGTTT--CCTGTA-AAAAGAGTTTCAACTCCTCTCCAA
right    GACAGCCAGCAACGCTTTTGTGTTT--CCTGTA-AAAAGAGTTTCAACTCCTCTCCAA
         2**22222*22*2*22**2*2*22222****222**2*22**222*2***2***2222
```

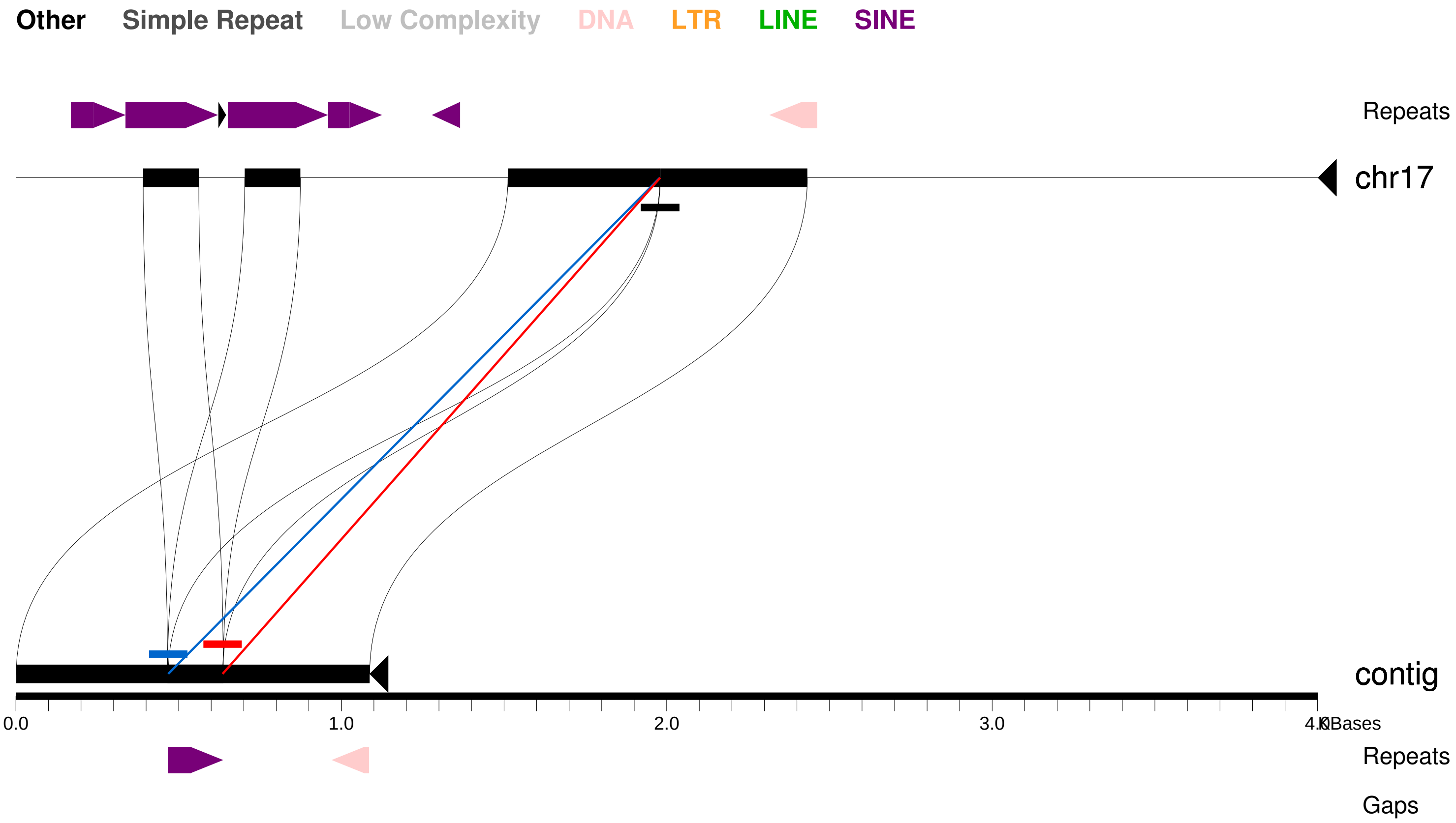

siteID: chr17_49529393 Scafftig2 - Breakpoint: chr17:49529391
chr17:49529341-49529431
Overlap Length: 10 INS size: 292 Genotyped: Yes

```
left    TT----AT------ACAAAAATTAAGA-TGGATTAAAGA-----------TTT------AAAGATAAGAC
chrom   TT----AT------ACAAAAATTAAGA-TGGATTAAAGA-----------TTT------AAAGATAAGAC
right   GCGGAGATCGCGCCACGGCACTCCCGCCTGGGCGACAGAGCGAGACTCCGTCTCAAAAAAAAAAAAAAAA
        111111**111111**111*1*111*11***111*1***11111111111*1*111111***1*1**1*1

left    TTAAAACTATAAAAACCCGCCGGGCGCGGTGGCTCACGCCTGTAATCCCAGCACTTTGGGAGGCCGAGGC
chrom   TTAAAACTATAAAAACCC----------------------TAGAAG---AAAACCTAGGCAGTACCATTC
right   AAAAAAAAATAAAAACCC----------------------TAGAAG---AAAACCTAGGCAGTACCATTC
        11****11**********2222222222222222222222*22**2222*22**2*2**2**22*2*22*

left    GGGCGGA
chrom   AGG---A
right   AGG---A
        2**222*
```

Other Simple Repeat Low Complexity DNA LTR LINE SINE

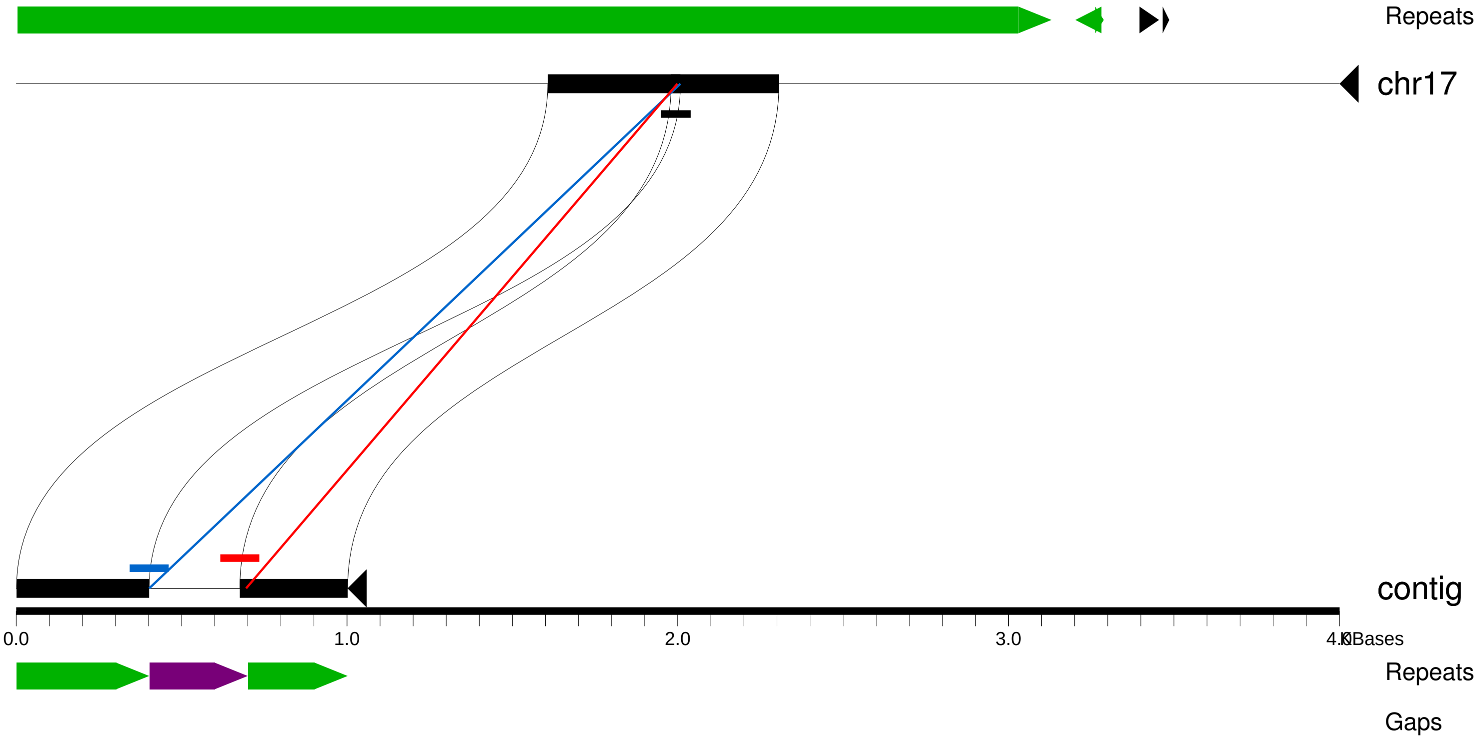

siteID: chr17_50049746 Scafftig1 - Breakpoint: chr17:50049731
chr17:50049682-50049786
Overlap Length: 11 INS size: 303 Genotyped: Yes

```
left    GTC----CTCAATCTTT------ATTTTTAGATTCCATATCTAAAGTTAATACTCAAGA----TTATCGA
chrom   GTC----CTCAATCTTT------ATTTTTAGATTCCATATCTAAAGTTAATACTCAAGA----TTATCGA
right   CACTGCACTCCAGCCTGGGCGACAGAGCGAGACTCCGTCTCAAAAAAAAAAAAAAAAAAAAAATTATCGA
        11*1111***1*1*1*1111111*11111***1***1*1**1***111**1*111**1*1111*******

left    TGGTGGCCGGGCGCGGTGGCTCACGCCTGTAATCCCAGCACTTTGGGAGGCCGAGGCGGGCGG
chrom   TGGTATTCTAA-------ACTAAGTTTTGTA--CAATGCACTTTT-----CTGAACATCTAAG
right   TGGTATTCTAA-------ACTAAGTTTTGTA--CAATGCACTTTT-----CTGAACATCTAAG
        ****222*22222222222**2*2222****22*222*******222222*2**22222222*
```

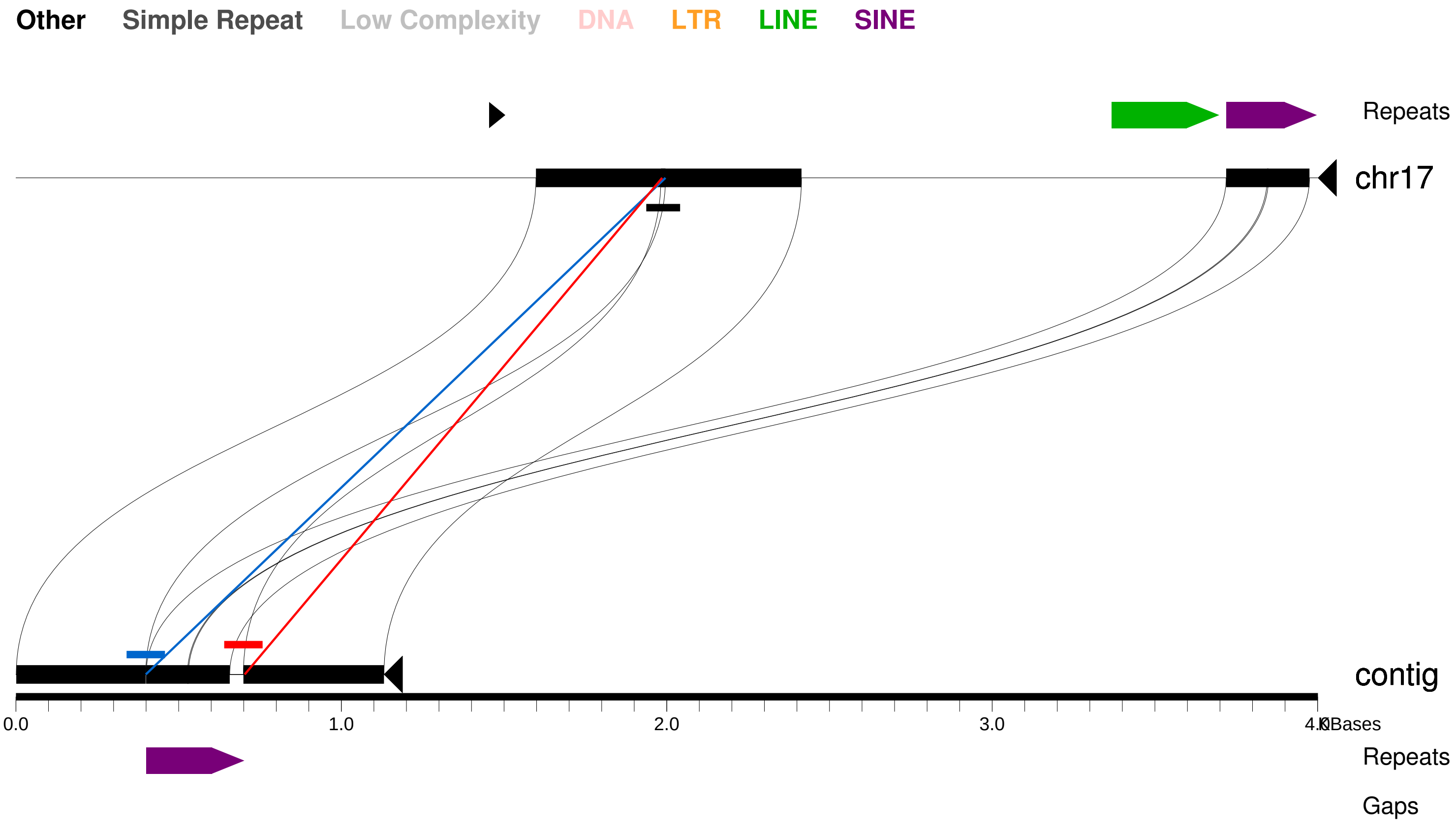

siteID: chr17_50398494 Scafftig4 - Breakpoint: chr17:50398490
chr17:50398442-50398549
Overlap Length: 12 INS size: 420 Genotyped: No

```
left   --CATCTTTATTAGCCTACTGCAGTGGTTT--TCAAAGGCATGGTCTGGAGA-------CCCCAAATTCT
chrom  --CATCTTTATTAGCCTACTGCAGTGGTTT--TCAAAGGCATGGTCTGGAGA-------CCCCAAATTCT
right  TCCGCCCGCCTCGGCCTCCCAAAGTGCTGGGATTACAGGCGTGAGCCACCGCGCCCGGCCCCCAAATTCT
       11*11*1111*11****1*111****1*1111*1*1****1**11*1111*11111111***********

left   TNNNNNNNNNNNNNNNNNNNNNNNNNNNNNNNNNNNNNNNNNNNNNNNNNNNNNNNNNNNNN
chrom  TTATCAAGGTATATGAAGCCAAATTATTTCCATAATAATTCTAACATGA-----------
right  TTATCAAGGTATATGAAGCCAAATTATTTCCATAATAATTCTAACATGA-----------
       *2222222222222222222222222222222222222222222222222222222222222
```

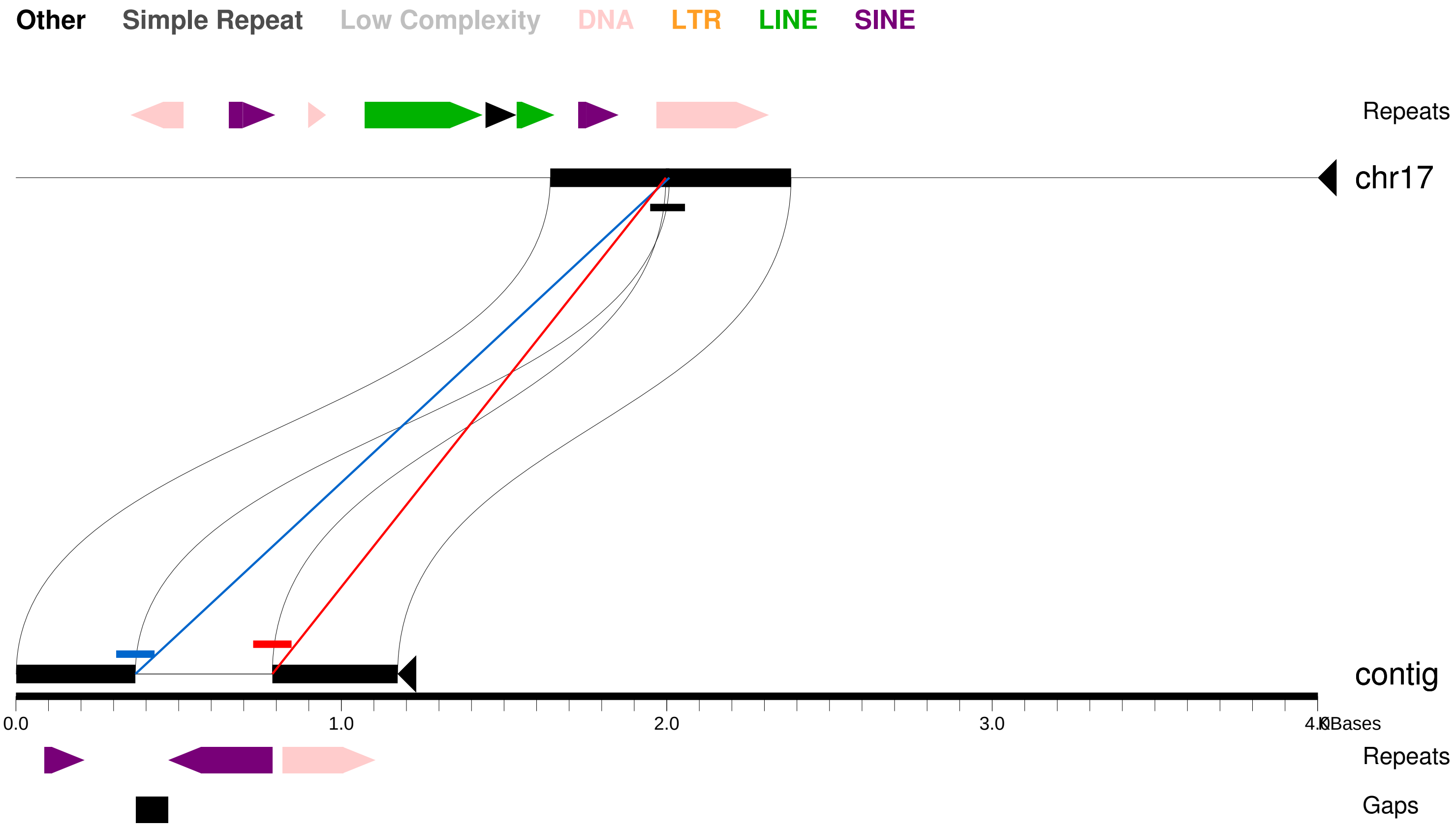

siteID: chr17_51427902 Scafftig3 - Breakpoint: chr17:51427857
chr17:51427811-51427916
Overlap Length: 14 INS size: 67 Genotyped: Yes

```
left    TGT--TGT-AGGTAT---------AGAGAGGTTGGGGGTGAATGTACCAACTGGGGATAAAA-CTGAGAA
chrom   TGT--TGT-AGGTAT---------AGAGAGGTTGGGGGTGAATGTACCAACTGGGGATAAAA-CTGAAAA
right   TCTGCAGTCAGGCCTGGGCGACAGAGCGAGACTCCGTCTCAAAAAAAAAAAAAAAAAAAAAAACTGAAAA
        *1*111**1***11*111111111**1***11*11*11*1**111*11**111111*1****1****2**

left    GTTCC-ACTGCAGTCTGCAGTCAGGCCTGGGCGACAGAGCGAGACTCCGTCTCAAAAAAAAAA
chrom   GTTAAGACAGGATTGTCCAC-CATGTTTGTGA---ATATCATGTTAGCCTCTG----------
right   GTTAAGACAGGATTGTCCAC-CATGTTTGTGA---ATATCATGTTAGCCTCTG----------
        ***222**2*2*2*2*2**22**2*22**2*2222*2*2*22*2222*2***22222222222
```

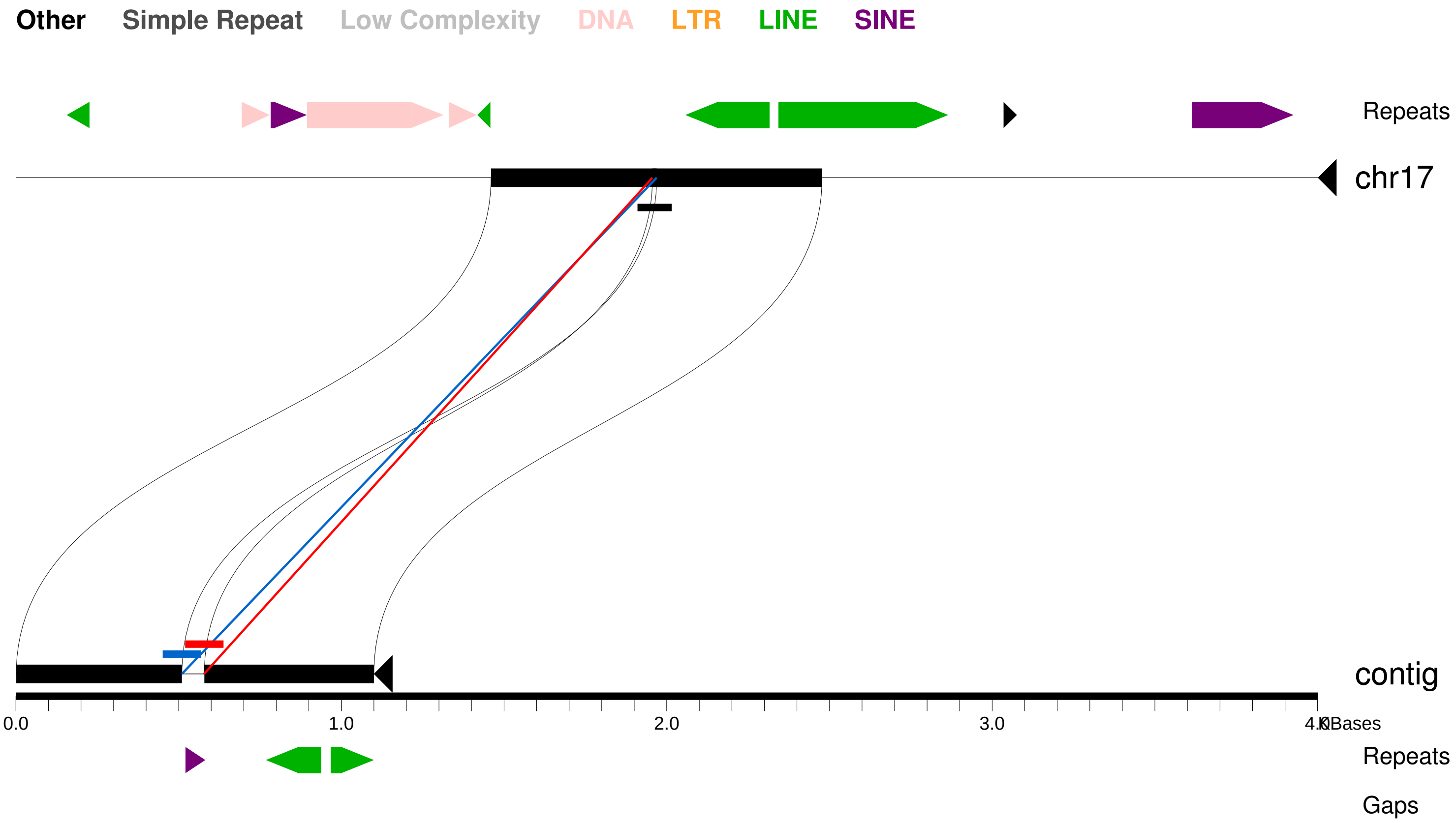

siteID: chr17_51457985 Scafftig1 - Breakpoint: chr17:51457993
chr17:51457948-51458052
Overlap Length: 15 INS size: 307 Genotyped: Yes

```
left     ATCTTT--------------ACATAACTCTA-TATTTTTCAAAAAATGTGTTCATTTTATAAAATTATTT
chrom    ATCTTT--------------ACATAACTCTA-TATTTTTCAAAAAATGTGTTCATTTTATAAAATTATTT
right    TCCGCAGTCCGGCCTGGGCGACAGAGCGAGACTCCGTCTCAAAAAAAAAAAAAAAAAAA-AAAATTATTT
         11*11111111111111111***1*1*111*1*111*1********1111111*1111*1**********

left     TTTCTGGCCGGGCGCGGTGGCTCACGCCTGTAATCCCAGCACTTTGGGAGGCCGAGGCGGGTGG
chrom    TTTCTTTATTTT-----TGTCT---GGCTGGATT------ACTTCAAAAGACCAGTCTTTAAGC
right    TTTCTTTATTTT-----TGTCT---GGCTGGATT------ACTTCAAAAGACCAGTCTTTAAGC
         *****222222222222**2**222*2***2*2*222222****2222**2**222222222*2
```

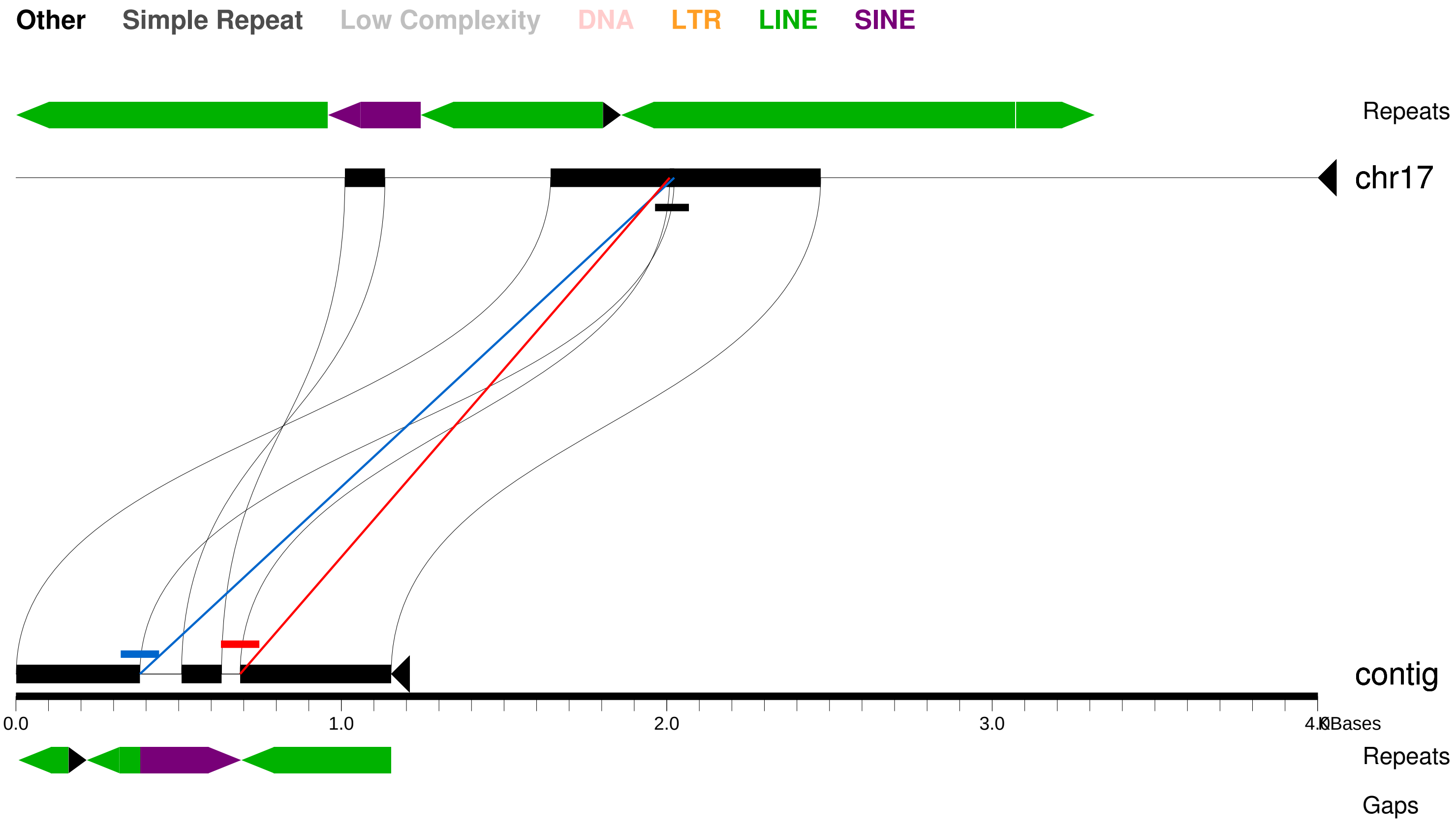

siteID: chr17_52456347 Scafftig1 - Breakpoint: chr17:52456342
chr17:52456182-52456518
Overlap Length: 17 INS size: 159 Genotyped: Yes

```
left   ----------------TTTTATTAGATACGTTATAATATTTTATAAATGAATATTTGCCAATTATAGGAA
chrom  GTGGCATGAGAATAATTTTTATTAGATACGTTATAATATTTTATAAATGAATATTTGCCAATTATAGGAA
right  TTG-----AGACGGAGTCT---------CGCTCTG-----TCGCCCAGGCCGGACTGCGGACTGCAG---
       N22     222NNN2N*1*111111111**1*1*111111*11111*1*111111***11*1*11**111

left   ACTTGTCTACCTATATCCTCTTCAAGTAAAATTTCATGTTCCCTGAAATGACCCTAATAACTTCTAGAAT
chrom  ACTTGTCTACCCATATCCTCTTCAAGTAAAATTTCATGTTCCCTGAAATGACCCTAATAACTTCTAGAAT
right  ---TGGCG--CAATCTCGGCTC--ACTGCAAGCTCCGCTTCCCGGGT-TCACGCCATT--CTCCT-GCCT
       111**1*111*N**1**11**111*1*11**11**111*****1*111*1**1*1*1*11**1**1*11*

left   AAGTTGTGATATACAAGAACACGTAGCTACTATTTCTTTTTTTTTTTTTTTTTTTTTTTTTTTTTTTTTT
chrom  AAGTTGTGATATACAAGAACACGTAGCTACTATTTCTAATGGTGATCACTCATCAGTTTCTTACCCACTG
right  CAGCCT-------CCCGAGTACGTAGCTACTATTTCTAATGGTGATCACTCATCAGTTTCTTACCCACTG
       1**1111111111*11**11****************22*22*22*222*22*222***2**222222*2

left   TT--------------TTTTTTGAGACGGAGTCTCGCTCT----GTCGCCCAGGCCGGACTGCGGACTG-
chrom  ATAAAGGAACAGGAAATGTGATCATTTCATGTCTCAGTCATAAAGGTGCTTATATTGTTTTAATTACTTG
right  ATAAAGGAACAGGAAATGTGATCATTTCATGTCTCAGTCATAAAGGTGCTTATATTGTNNNNNNNNNNNN
       2*22222222222222*2*22*2*222222*****22**22222*22**22*2222*2NN1NNNN111NN

left   ----CAGTGG----CGCAATCTCGGCTC--ACTGCAAGC------TCCG-----CTT
chrom  TTAACATTTTTTATCAAGATTTCAATTTTTAATAGAAACAATTTTTCTGGTTTACTT
right  NNNNNNNNNNNNNNNNNNNNNNNNNNNNNNNNNNNNNNNNNN---------------
       NNNN11N1NNNNNN1NNN11N11NNN1NNN1N1NN11N1NN    11N1     111
```

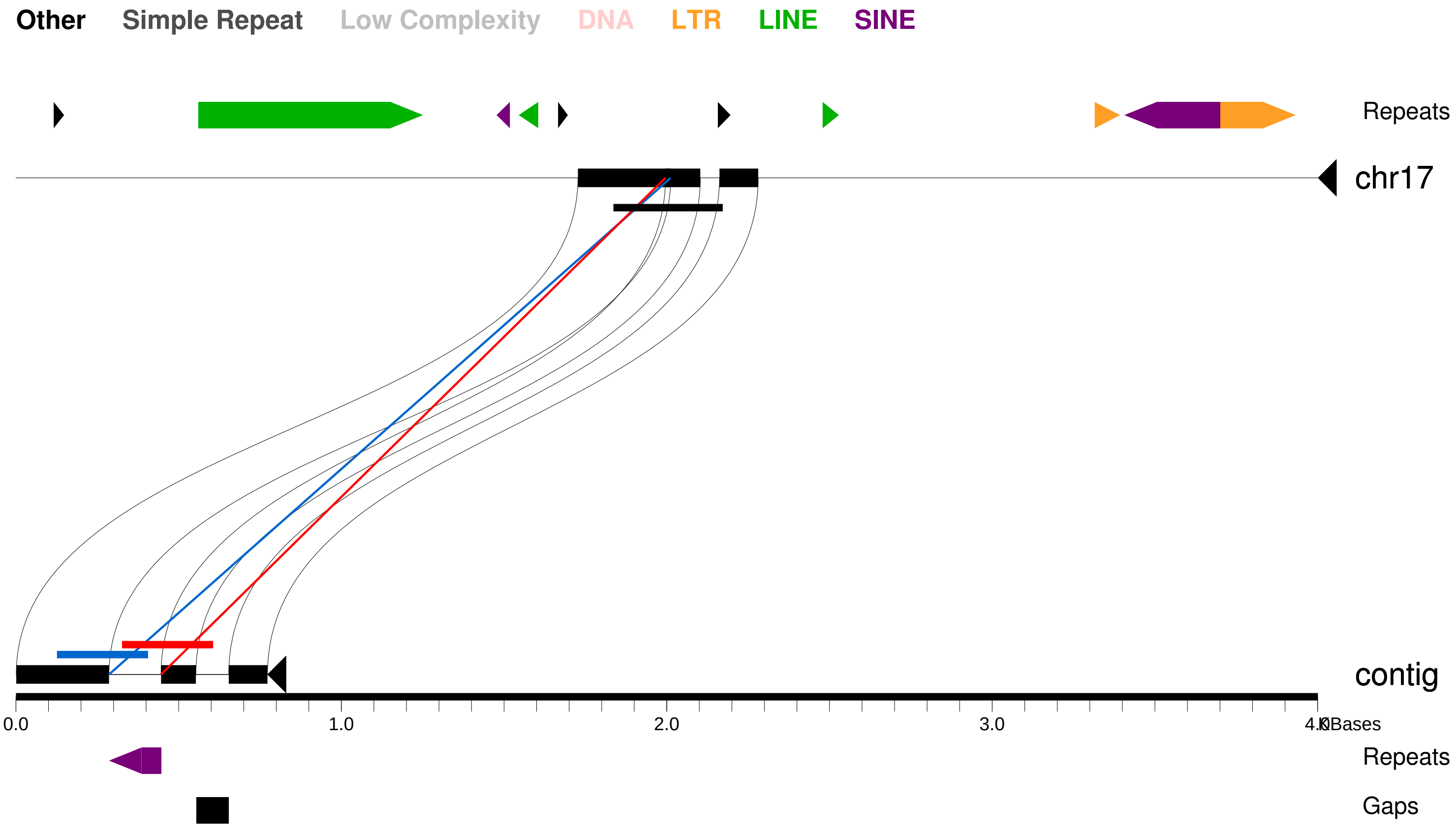

siteID: chr17_53308230 Scafftig1 + Breakpoint: chr17:53308238
chr17:53308187-53308291
Overlap Length: 9 INS size: 321 Genotyped: Yes

```
left     T---------------CTCATTTTACAGTGTAACCATTTAACATTTTCTCAGGCCTATAGAAATATCCCC
chrom    T---------------CTCATTTTACAGTGTAACCATTTAACATTTTCTCAGGCCTATAGAAATATCCCC
right    GGGCGACAGAGCGAGACTCCGTCT-CAAAAAAAAAAAAAAAAAAAAAAAAAAAAAAAAAAAAAAAAACCCC
         1111111111111111***11*1*1**1111**11*111**1*1111111*11111*1*1***1*1****

left     TTTGTGGCCGGGCGCGGTGGCTCACGCCTGTAATCCCAGCACTTTGGGAGGCCGAGGCGGGCGG
chrom    TTTGTT-CATTCATCAAGCACTTAT-CATGTGTACC----ACTTGGTCAG--CTAGGT------
right    TTTTTT-AATTCATCAAGCACTTAT-CATGTGTACC----ACTTGGTCAG--CTAGGT------
         ***1*221222222*22222**2*22*2***222**2222****2*22**22*2***2222222
```

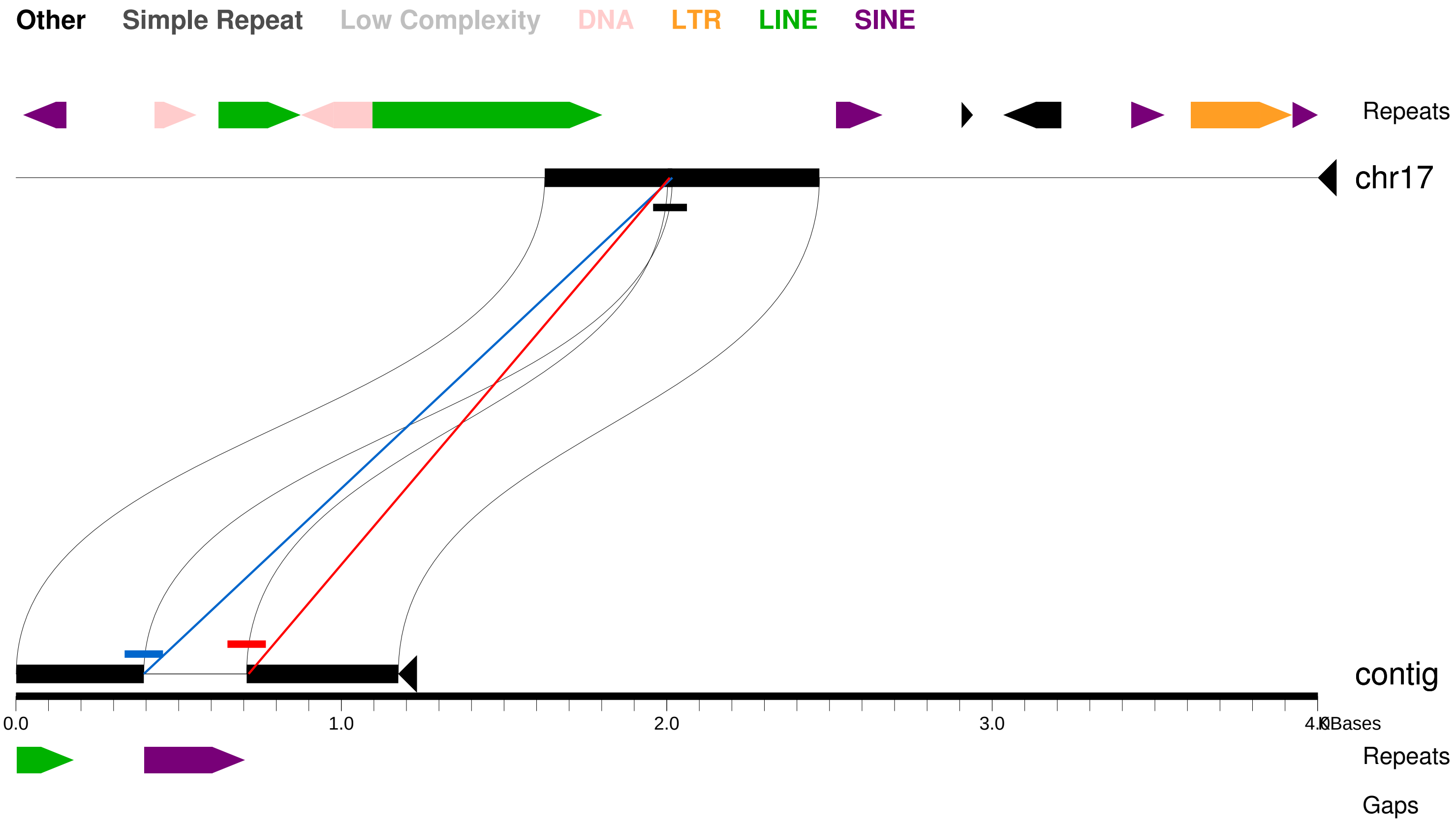

siteID: chr17_54594352 Scafftig1 - Breakpoint: chr17:54594334
chr17:54594286-54594393
Overlap Length: 12 INS size: 414 Genotyped: No

```
left   AGATTTTTTGCTGTTTAAGGGACAGTTTCCAAGCCCATTTGTCTTAAG-----------TGTGGAAAAAT
chrom  AGATTTTTTGCTGTTTAAGGGACAGTTTCCAAGCCCATTTGTCTTAAG-----------TGTGGAAAAAT
right  NNNNNNNNNNNNNNNNNNNNNNNNNNNNNNNNNNNNNNNNNNNNNNNNNNNNNNNNNNNNTGTGGAAAAAT
       111111111111111111111111111111111111111111111111111111111111***********

left   GGGCCGGGCGCGGTGGCTCACGCCTGTAATCCCAGCACTTTGGGAGGCCGAGGCGGGCGG
chrom  GTT---GATTCAATAATAAACAAATAGAATTCTTAATACTTGGCTTACTTAA--------
right  GTT---GATTCAATAATAAACAAATAGAATTCTTAATACTTGGCTTACTTAA--------
       *22222*222*22*22222**222*22***2*2222222****2222*22*222222222
```

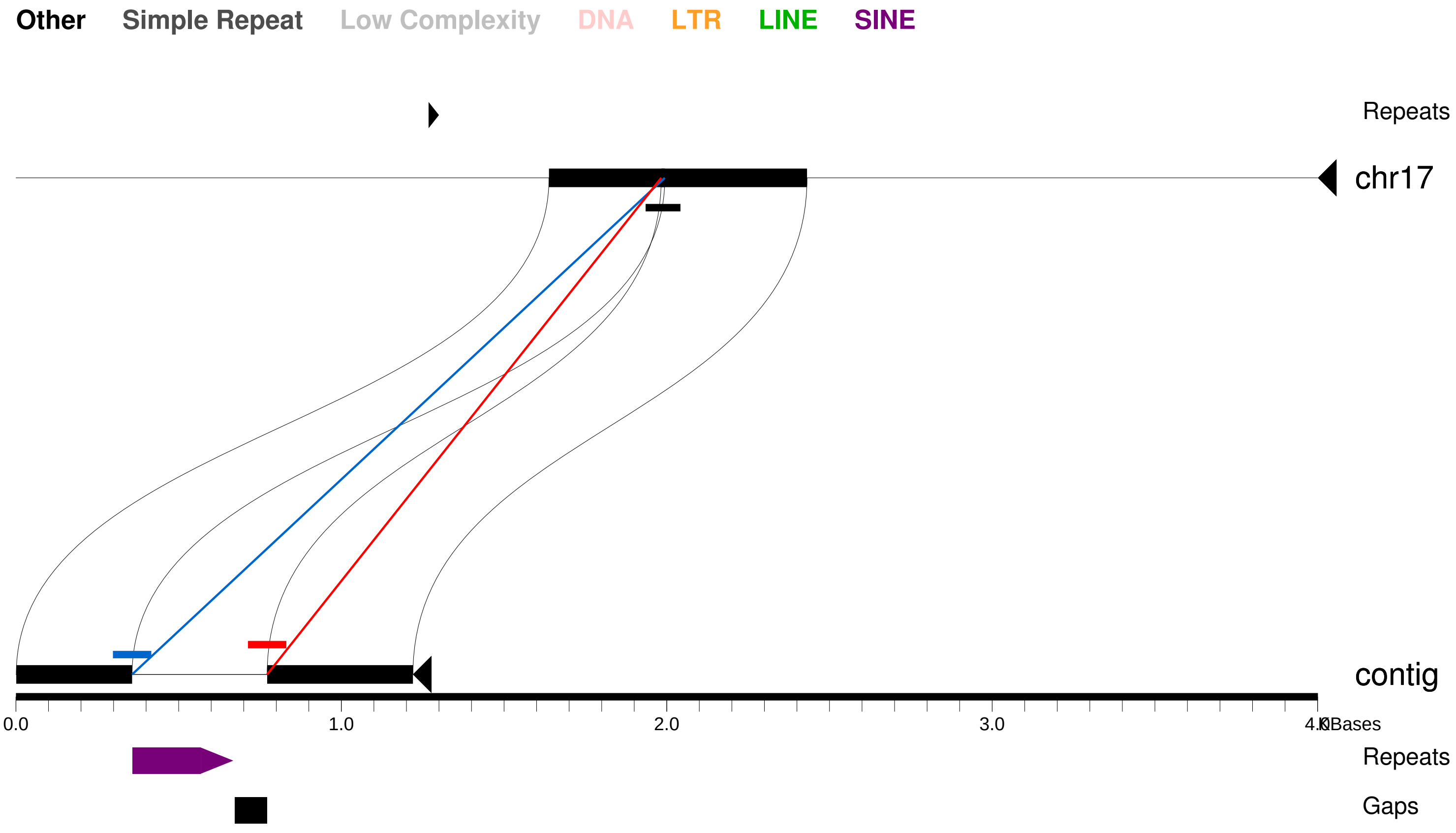

siteID: chr17_60434048 Scafftig2 + Breakpoint: chr17:60434032
chr17:60433989-60434091
Overlap Length: 17 INS size: 452 Genotyped: No

```
left     TCTTCTCTTT----TCCC----TGGTG--AATCCAG-------CCTACGTTCCTCATGCAGTATACATTA
chrom    TCTTCTCTTT----TCCC----TGGTG--AATCCAG-------CCTACGTTCCTCACGCAGTATACATTA
right    CACCCGCCTCGGCCTCCCAAAGTGCTGGGATTACAGGCGTGAGCCACCGTGCCCGGC-CGGTATACATTA
         1111*1*1*11111****1111**1**11*1*1***1111111**11***1**11121*1**********

left     TTTCTTTTTTTNNNNNNNNNNNNNNNNNNNNNNNNNNNNNNNNNNNNNNNNNNNNNNNNNNNNNNNNNNNN
chrom    TTTCTTTTGT----------------GCTGGGTCCTGGTCCCTGTTTTGCCACAGACTTGCTGACAA
right    TTTCTTTGT----------------GCTGGGTCCTGGTCCCTGTTTTGCCACAGACTTGCTGACAA
         *******2*22222222222222222222222222222222222222222222222222222222
```

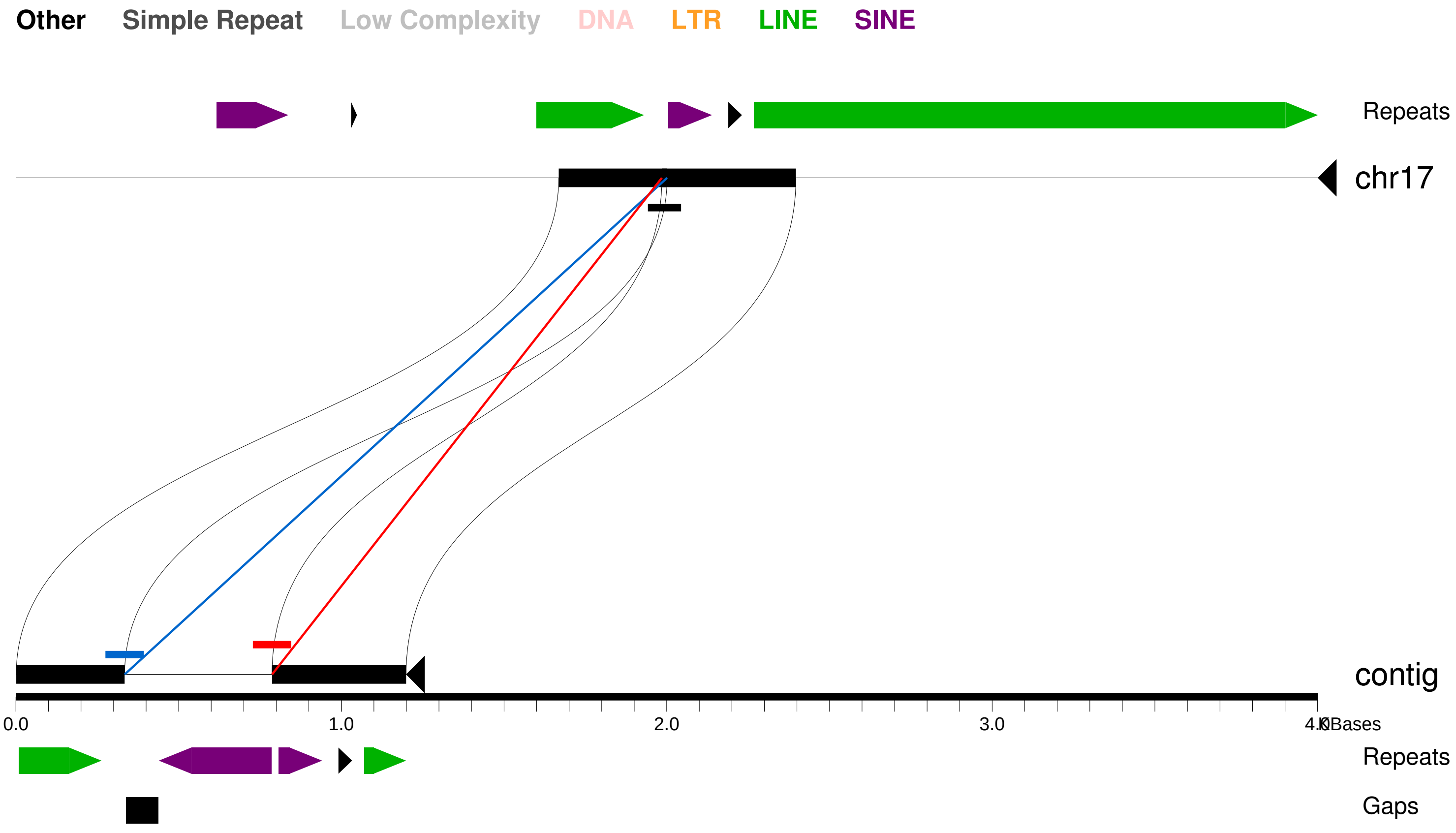

siteID: chr17_61745469 Scafftig3 + Breakpoint: chr17:61745366
chr17:61745206-61745541
Overlap Length: 16 INS size: 328 Genotyped: No

```
left    GA---------------------------------------------AAGCTCTGTGGCTGCTACCAGTC
chrom   GATATGGAGGGCCAACTATATATACAACCCTCTGCTAACTAGTCAGAAAGCTCTGTGGCTGCTACCAGTC
right   TTTA-GTAGAG---ACGGGGTTTC--ACCTTGT------TAGCCAGGATGGTCTCGATCTCCT----GAC
        1122 2N22N2   22NNNN2N2N  222N2N2      222N222N*1*1***1111**1**1111*1*

left    CTTCCCAGGACAGGAATCTAAATCAGGCCTGTGCATTATTACTTGTTAACCCAGGATTCCTGTGGATT-C
chrom   CTTCCCAGGACAGGAATCTAAATCAGGCCTGTGCATTATTACTTGTTAACCCAGGATTCCTGTGGATT-C
right   CT---CATGA------TCCACCT---GCCTCGGCCTCCCAAAGTGCTG-----GGATTACAGGCGTGAGC
        **111**1**111111**1*11*111****11**1*1111*11**1*111111*****1*1*11*1111*

left    CACTTTTGGGAGTCTGGGGAGAAAGCCCCCATTTCTT---------------------------------
chrom   CACTTTTGGGAGTCTGGGGAGAAAGCCCCCATTTCTTGAATTGTGGAACTTGTATCAAAAGGGAAATCTG
right   CACC-----GCGCCCGGCCA-AAAGCCCCCATTTCTTGAATTGTGGAACTTGTATCAAAAGGGAAATCTG
        ***111111*1*1*1**11*1****************222222222222222222222222222222222

left    ----------------------------------------------------------------------
chrom   GTCTTATCAAACATCGTGTTGAGCCTGCTGCCTTAATGTTGTGGAGACCCCTGCTGCTGCTAGAAAGCCA
right   GTCTTATCAAACATCGTGTTGAGCCTGCTGCCTTAATGTTGTGGAGACCCCTGCTGCTGCTAGAAANNNN
        222222222222222222222222222222222222222222222222222222222222222222NNNN

left    --TTT----------------------------------------TNNNNNNNNNNN
chrom   GCTTTGCCCTTATCCATATAGGTAGAATTGCTTCAAATTGAAGTGTCCTCTGAAGAT
right   NNNNNNNNNNNNNNNNNNNNNNNNNNNNNNNNNNNNNNNN-----------------
        NN111NNNNNNNNNNNNNNNNNNNNNNNNNNNNNNNNNNN     1NNNNNNNNNNN
```

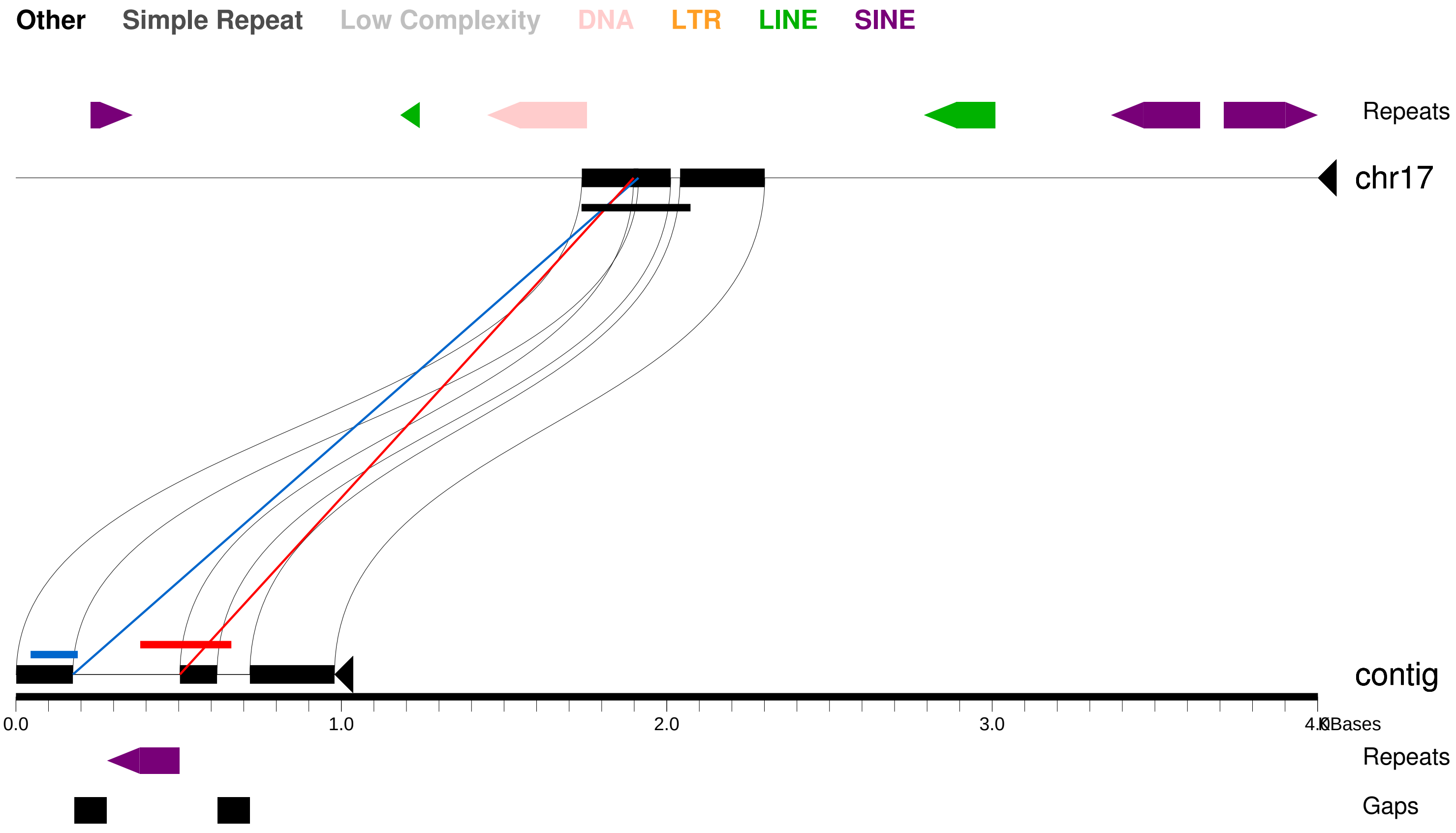

siteID: chr17_6538654 Scafftig1 - Breakpoint: chr17:6538658
chr17:6538498-6538831
Overlap Length: 14 INS size: 302 Genotyped: Yes

```
left   --------------TTCAGACCAACAGCGTTGAACAAGGGAAAAGAACAGATAAAACACAGTGCCATGTG
chrom  CGAAACAAAGCTACTTCAGACCAACAGCGTTGAACAAGGGAAAAGAACAGATAAAACACAGTGCCATGTG
right  -GAGA------------------ATGGCGT-GAACCCGGGAGGCG----GAGCTTGCAGTGAGCCGAGAT
        22N2         111111111*11****1****11****111*1111**11111**11*1***11*11

left   CATGTAGCAATATACTAGAAGTAGCCAATAGTAACCTCATACTGAGAGTGACTGCTTAATGACAGGTAAA
chrom  CATGTAGCAATATACTAGAAGTAGCCAATAGTAACCTCATACTGAGAGTGACTGCTTAATGACAGGTAAA
right  CCCGC--CACTGCACTCCA----GCC--TGGG--CGACAGAGCGAGACTCCGT-CTCAAAAAAAAAAAAA
       *11*111**1*11***11*1111***11*1*111*11**1*11****1*111*1**1**11*1*111***

left   TGATAGTTTCTTCCATTTATAAAAGCAACCACAC---GGCCGGGCGCGGTGGCTCACGCCTGTAATCCCA
chrom  TGATAGTTTCTTCCATTTATAAAAGCAACCACACTTTGGAAGTTCCCAATTTCAAAAACAAAAAAAAATA
right  AAAAA-------------AAAAAAACAAACACACTTTGGAAGTTCCCAATTTCAAAAACAAAAAAAAATA
       11*1*1111111111111*1****1***1*****222**22*22*2*22*22*22*22*2222**2222*

left   G-----CACTT----------TGGGAGGCC---GAGGCGGGCGGATCACGAGGTCAGGAGATCGAGA---
chrom  GGGGAACACTTACTATTTTAATAATAATCTCTTGAGCCCTTTCCATTCCAAATTTCAAATCTCAAAGGTC
right  GGGGAACACTTACTATTTTAATAATAATCTCTTGAGCCCTTTCCATTCCAAATTTCAAATCTCAAAGGTC
       *22222*****2222222222*222*22*2222***2*222222**22*2*22*2222*22**2*22222

left   -CCATCCCGGCT--------AAAACGGTGAAACCCCGTC-TCTACTAAA-----
chrom  TCCAGCAAGGCGTTATTCCCAAAACAGAGCTTTGCCATGGTAAACTAGCATGTT
right  TCCAGCAAGGCGTTATTCCCAAAACAGAGCTTTGCCATGGTAAACTAGC-----
       2***2*22***222222222*****2*2*22222**2*22*22****22
```

Other **Simple Repeat** **Low Complexity** **DNA** **LTR** **LINE** **SINE**

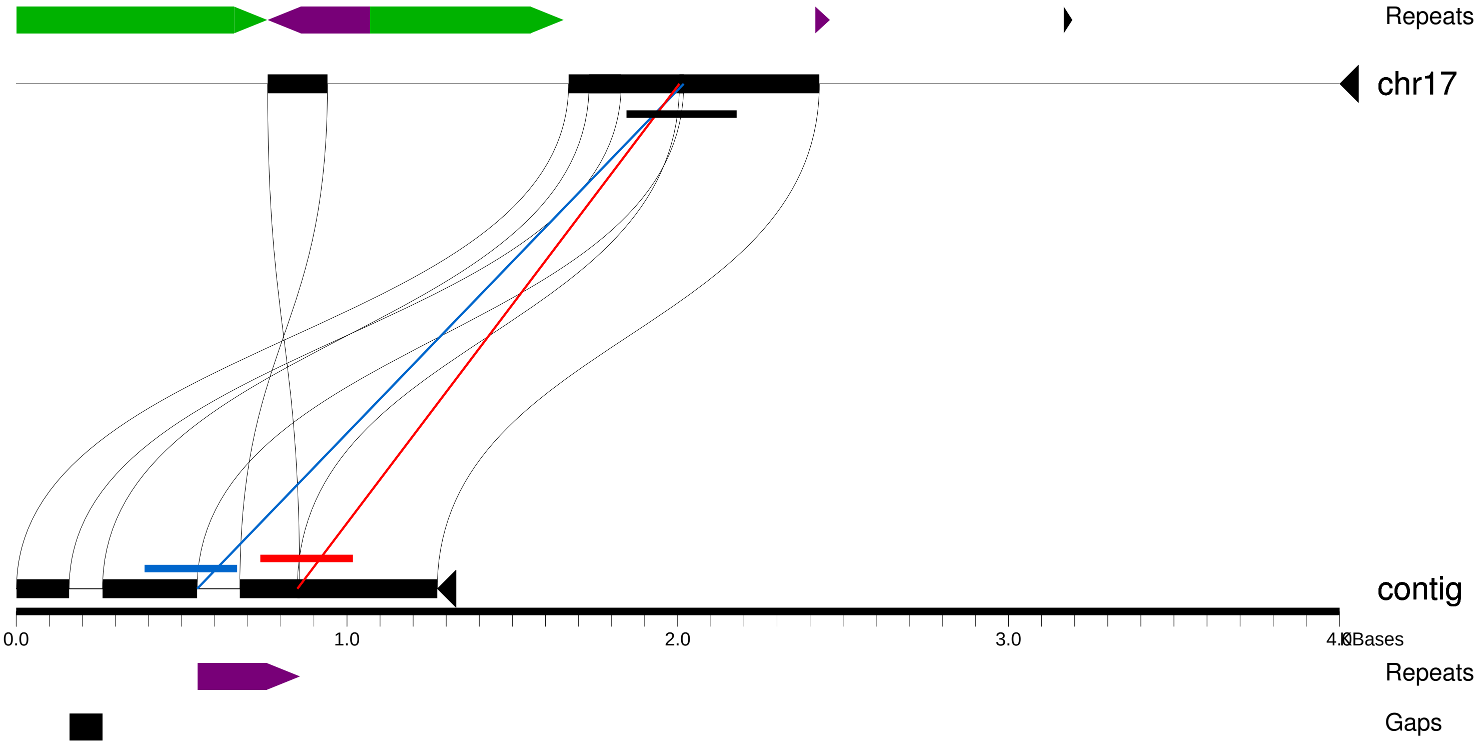

siteID: chr17_67875366 Scafftig3 + Breakpoint: chr17:67875350
chr17:67875303-67875409
Overlap Length: 13 INS size: 349 Genotyped: No

```
left    TTACCAGCA-----ATATTTA--TTTTTGGA--ACCAGAAATATATAGGTTGACAT---GTAATGCCATC
chrom   TTACCAGCA-----ATATTTA--TTTTTGGA--ACCAGAAATATATAGGTTGACAT---GTAATGCCATC
right   CCGCCTGCCTCGGCCTCCCAAAGTGCTGGGATTACAGGCGTGAGCCACCGTGCCCGGCCGTAATGCCATC
        111**1**1111111*1111*11*11*1***11**11*1111*111*111**1*11111***********

left    TTTTTNNNNNNNNNNNNNNNNNNNNNNNNNNNNNNNNNNNNNNNNNNNNNNNNNNNNNNNNNN
chrom   TT------------AAAGATCATGATGAATGATGAGCTACTGGGAAAAGAAGGCTTTGGTG
right   TT------------AAAGATCATGATGAATGATGAGCTACTGGGAAAAGAAGGCTTTGGTG
        **2222222222222222222222222222222222222222222222222222222222222
```

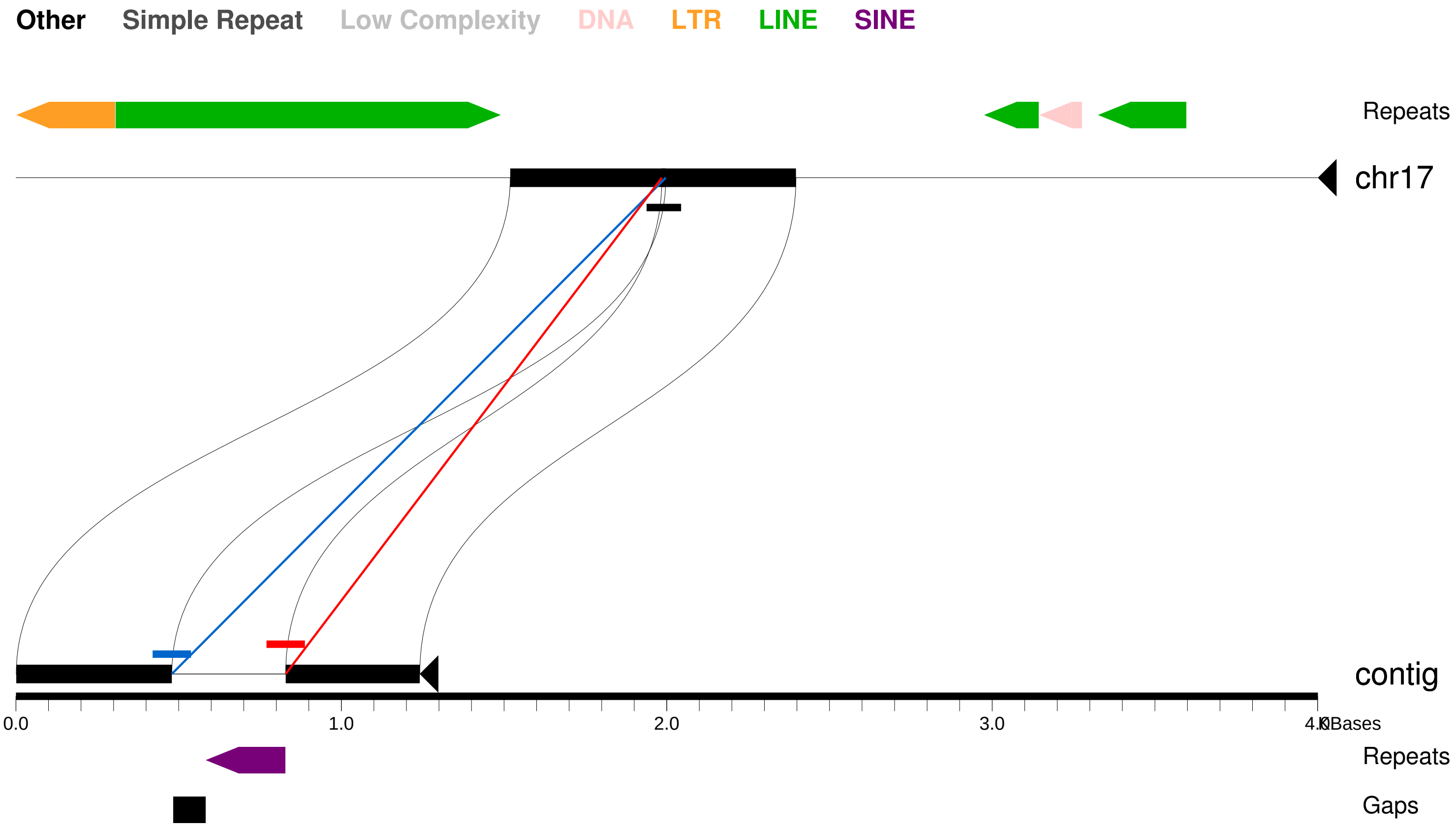

siteID: chr17_68309143 Scafftig1 + Breakpoint: chr17:68309121
chr17:68309075-68309180
Overlap Length: 14 INS size: 411 Genotyped: No

```
left    GAGCCGCATTCTTTCTACTTCACTCAAATTAATTTTACCATTAAAA-------------ATTAATTCAAT
chrom   GAGCCGCATTCTTTCTACTTCACTCAAATTAATTTTACCATTAAAA-------------ATTAATTCAAT
right   NNNNNNNNNNNNNNNNNNNNNNNNNNNNNNNNNNNNNNNNNNNNNNNNNNNNNNNNNNNATTAATTCAAT
        11111111111111111111111111111111111111111111111111111111111***********

left    TTCCCTGTAATCCCAGCACTTTGGGAGGCAGAGGCGGGCGGATCATGAGGTCAGGAGATCGA
chrom   TTCAATG-------ATCAGTTT---ATTTTAATTC--TCTAAAAATGTG-TCATCGTTTTTT
right   TTCAATG-------ATCAGTTT---ATTTTAATTC--TTTAAAAATGTG-TCATCGTTTTTT
        ***22**2222222*2**2***222*22222*22*222122*22***2*2***22222*222
```

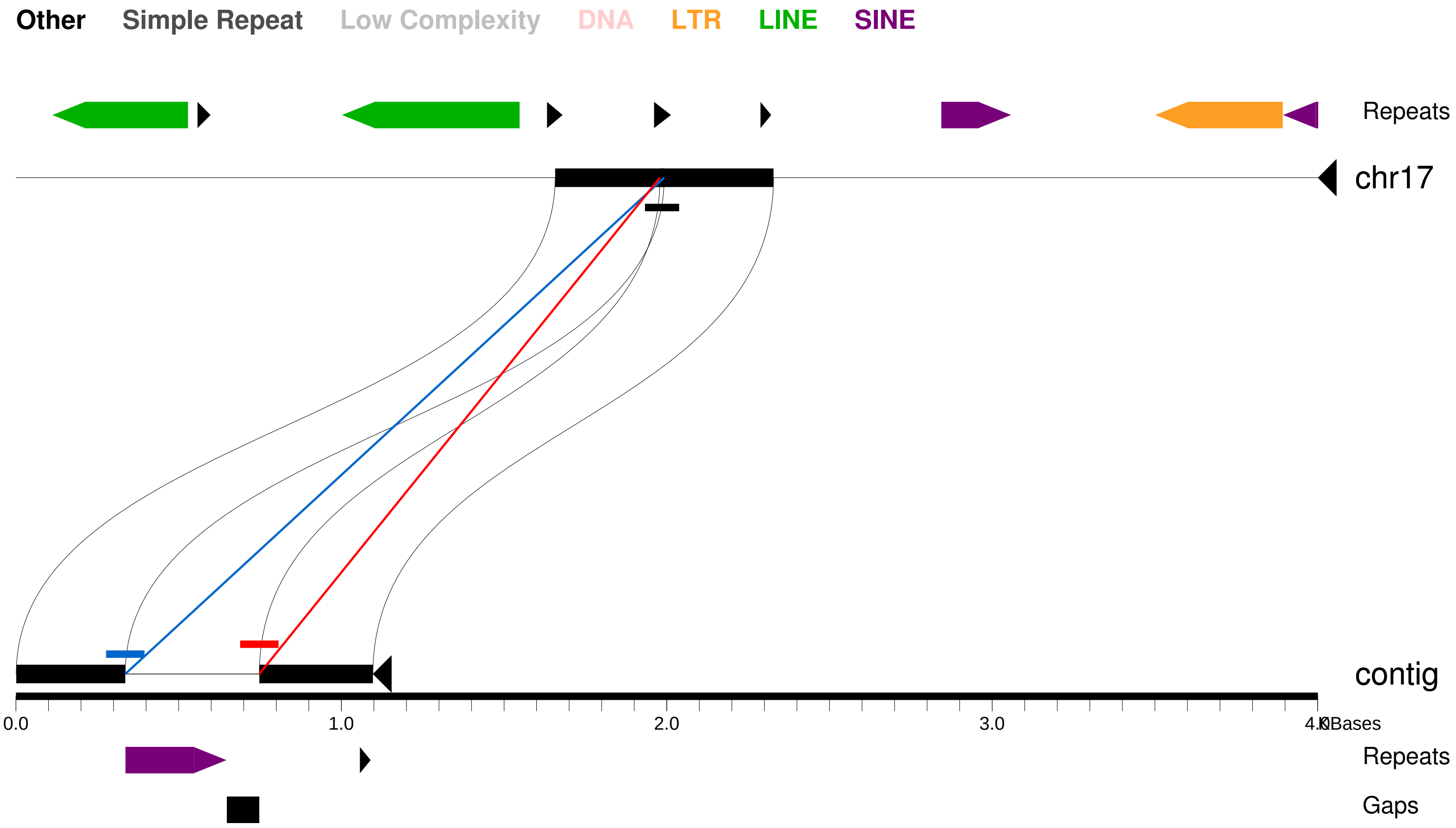

siteID: chr17_68327417 Scafftig1 + Breakpoint: chr17:68327425
chr17:68327380-68327484
Overlap Length: 15 INS size: 305 Genotyped: Yes

```
left     ATAAAAAAGGATGAGTTC---ATGTCC----ATTGCAGG-----GACATGGATGATG--CTGGAAACCAT
chrom    ATAAAAAAGGATGAGTTC---ATGTCC----ATTGCAGG-----GACATGGATGATG--CTGGAAACCAT
right    TCCGCCCGCCTCGGCCTCCCAAAGTGCTGGGATTACAGGCGTGAGCCACCGCGCCCGGCCTGGAAACCAT
         111111111111*111**111*1**1*1111***1****11111*1**11*11111*11***********

left     CATTTTTTTTTTTTTTTTTTTTTTTTTTTGAGACGGAGTCTCGCTCTGTCGCCCAGGCTGGAG
chrom    CATTCTAAGCAAACTATTG------CAAGGACAAAAAAACCAAACACTA-----CATGTTC---
right    CATTCTAAGCAAACTATTG------CAAGGACAAAAAAACCAAACACTA-----CATGTTC---
         ****2*22222222*2**22222222222**2*222*22*222*2**222222**2*2*2222
```

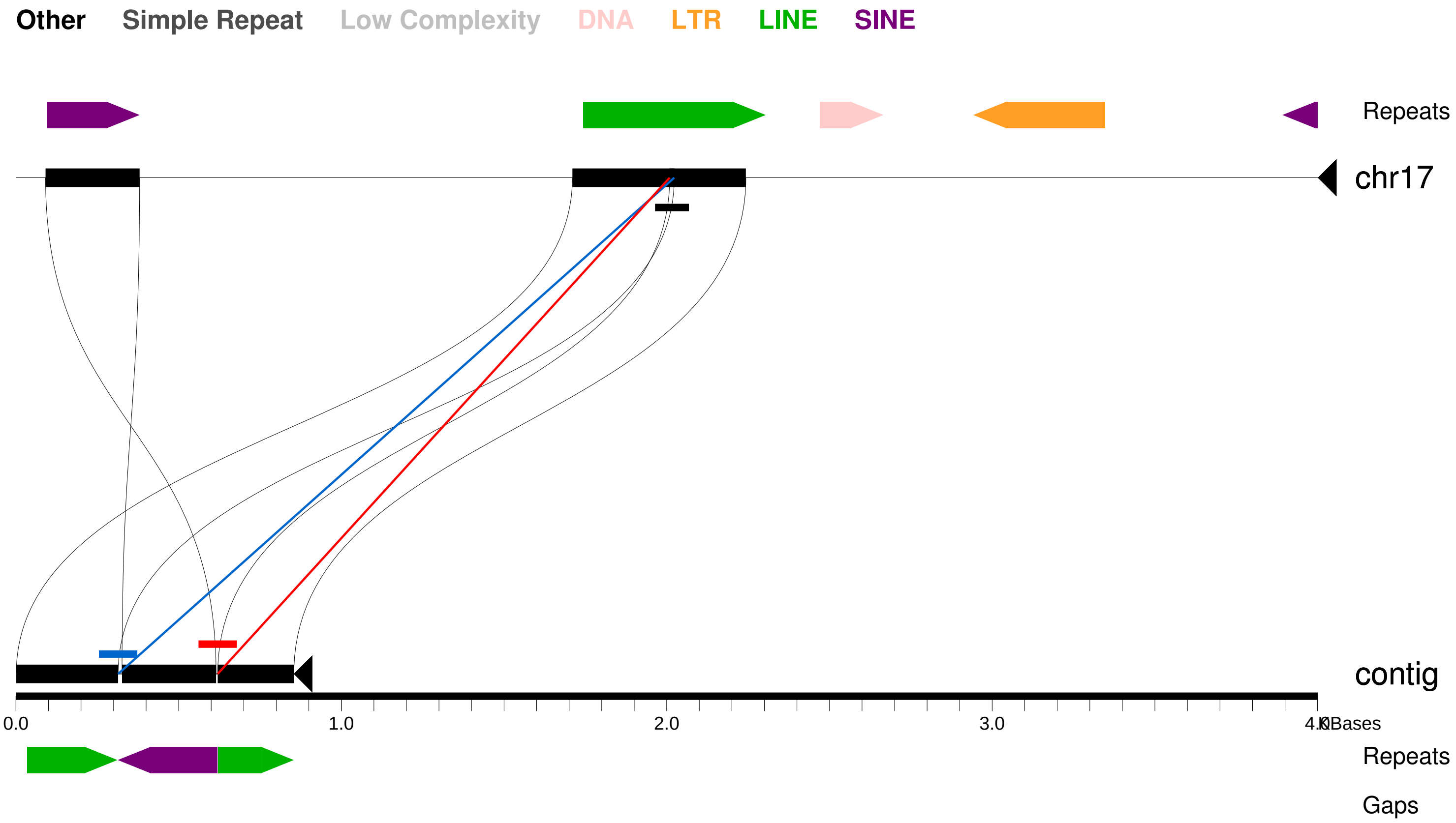

siteID: chr17_72620197 Scafftig10 - Breakpoint: chr17:72620207
chr17:72620158-72620266
Overlap Length: 11 INS size: 296 Genotyped: Yes

```
left    A---------TCGATCTAAGGAG-CTTCCCTTCTCTATGGCTGCCTACTTTTGAATCATAAAACACCCTC
chrom   A---------TCGATCTAAGGAG-CTTCCCTTCTCTATGGCTGCCTACTTTTGAATCATAAAACACCCTC
right   GCCACTGCACTCCAGCCTGGGCGACAGAGCGAAACTCCGCCTCAAAAAAAAAAAAAAAAAAAACACCCTC
        1111111111**1*1*111**1*1*1111*1111**11*1**1111*111111**11*1***********

left    GGCCG--GGCGCGGTGGC--TCACGCCTGTAATCCCAGCACTTTGGGAGGCCGAGACGGGCGG
chrom   TACTTAAGGAGCTGTGGTGATCACTC-------CCCATGTCTTTCTCA-------ACCGTTAT
right   TACTTAAGGAGCTGTGGTGATCACTC-------CCCATGTCTTTCTCA-------ACCGTTAT
        22*2222**2**2****222****2*2222222****222****222*2222222**2*2222
```

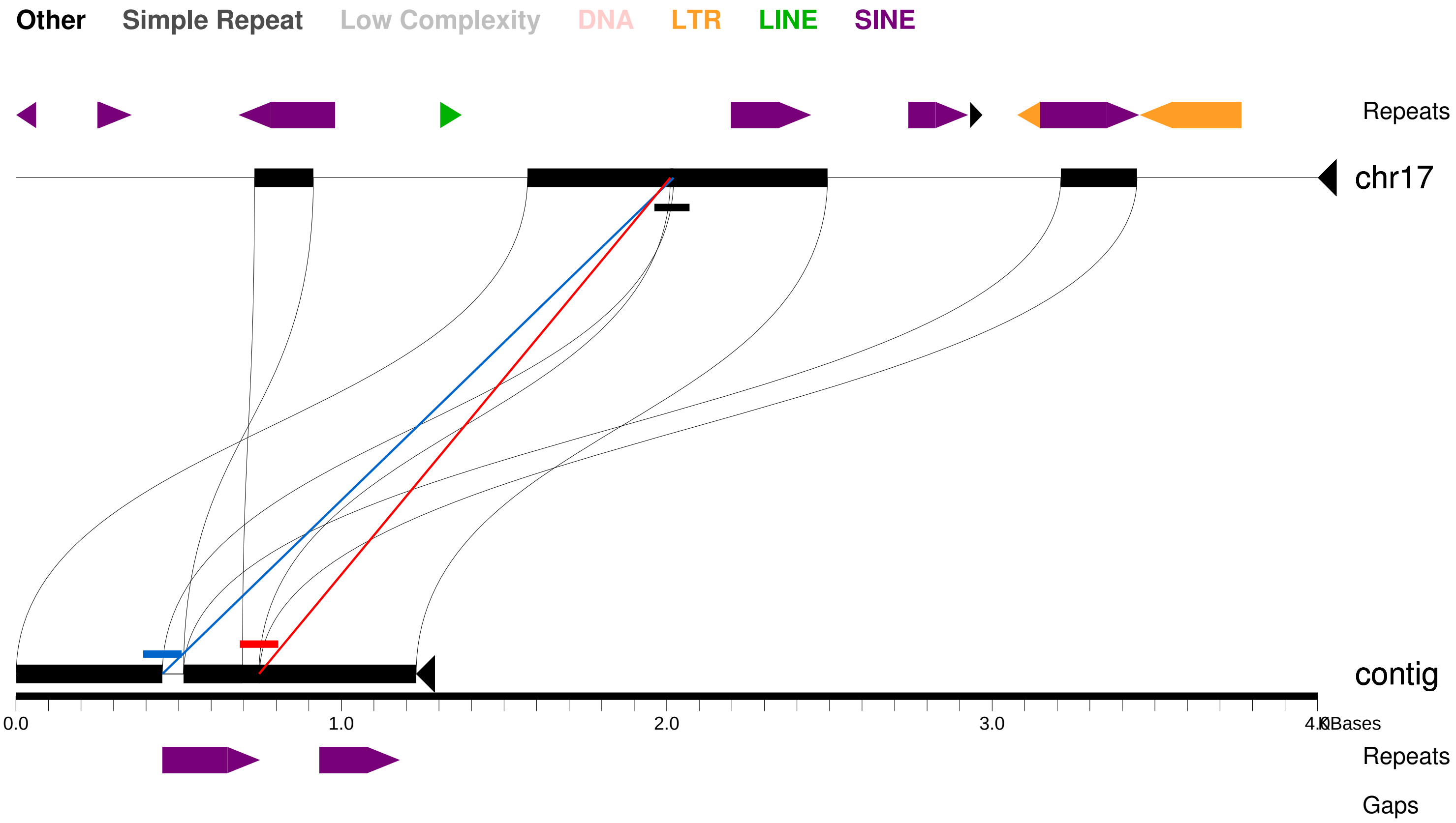

siteID: chr17_74046828 Scafftig1 - Breakpoint: chr17:74046810
chr17:74046765-74046869
Overlap Length: 15 INS size: 460 Genotyped: No

```
left    -------TAATGGTTTCTCATAGCCTTAGTCATTCA----TTATTCATATGGAAAG---GGGAGTTCTAT
chrom   -------TAATGGTTTCTCATAGCCTTAGTCATTCA----TTATTCATATGGAAAG---GGGAGTTCTAT
right   CCGCCCGCCTCGGCCTCCCAAAGTGCTGGGATTACAGGCGTGAGCCACCGCGCCCGGCCGGGAGTTCTAT
        11111111111**11**1**1**111*1*111*1**1111*1*11**1111*111*111***********

left    TTTTNNNNNNNNNNNNNNNNNNNNNNNNNNNNNNNNNNNNNNNNNNNNNNNNNNNNNNNNNNN
chrom   TTTTAAAAGCAAACATGCTGAAAAAGCTGTCTGCATAACTGATCTGAGA--------------
right   TTTTAAAAGCAAACATGCTGAAAAAGCTGTCTGCATAACTGATCTGAGA--------------
        ****22222222222222222222222222222222222222222222222222222222222
```

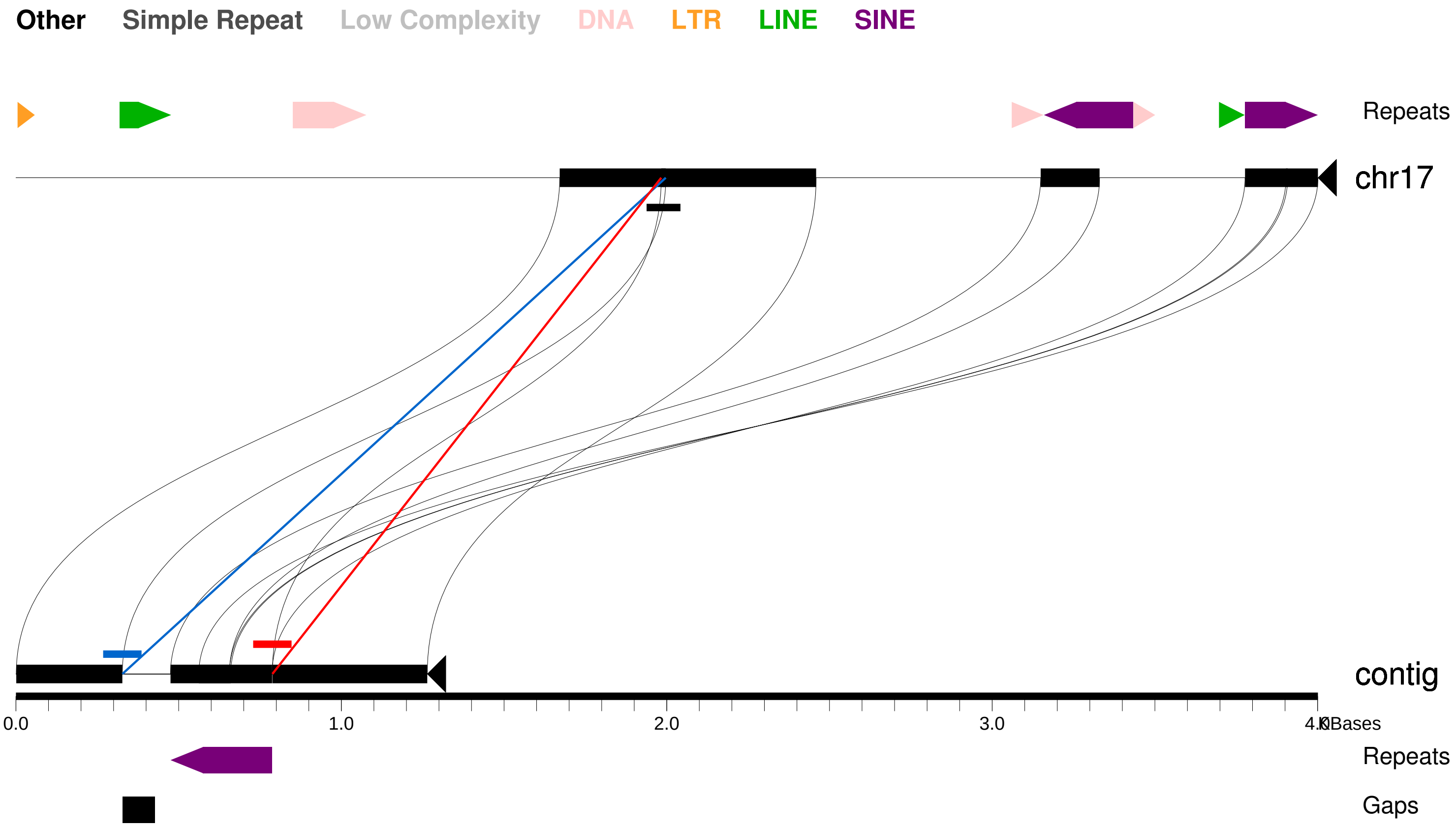

siteID: chr17_78356489 Scafftig3 - Breakpoint: chr17:78356522
chr17:78356362-78356695
Overlap Length: 14 INS size: 417 Genotyped: No

```
left     C------------------------------------------------------------CACATC-TG
chrom    CAGAGTCACACTGATGAAATGACAGAATCCTGGAAGCAGGTGGACAGTTCCCGCAGCACTCCACATC-TG
right    TTTAGT---AGAGACGGGGTTTCAC---CTTGTTAGCCAG--GATAGT-CTCG-ATCTCCTGACCTCATG
         1NN222   2NN22N2NNN2NN22N   2N22NN222NN2  22N222 2N22 2N2N2NN1**1**1**

left     TGTGGTCTGCCTCAGGGTTTGGGTGGCAGCAGCTGAGACGGCTGGCGGTAGCCTGGGCCACTCTTACTAT
chrom    TGTGGTCTGCCTCAGGGTTTGGGTGGCAGCAGCTGAGACGGCTGGCGGTAGCCTGGGCCACTCTTACTAT
right    ATCCACCCGCCTCGGCCTCCCAAAGT-----GCTGGGATTACAGGCG------TGAGCCACC--------
         111111*1*****1*11*111111*111111****1**111*1****111111**1*****111111111

left     TGAGCTCAAGTTCTGTTATTTCAGTAATTTTTTTC-----------------------------------
chrom    TGAGCTCAAGTTCTGTTCTTTCAGTAATTTTTTTCAAAATGGGTCTAAGATTACAATTATGTAGGATAGA
right    -GCGCCCGGC-----------CAGTAATTTTTTTCAAAATGGGTCTAAGATTACAATTATGTAGGATAGA
         1*1**1*1111111111N111**************22222222222222222222222222222222222

left     ----TTTTT-----TTTTTTTTTTTTT-------------------------------------------
chrom    GGAATTTTTAGAGATTGATGTTATCTTCAAAAGGTCATTAAGTTGGTGGTAACAACAGGAACTACTTCAA
right    GGAATTTTTAGAGATTGATGTTATCTTCAAAAGGTCATTAAGTTGGTGGTAACAACAGGAACTACTTCAA
         2222*****22222**22*2**2*2**2222222222222222222222222222222222222222222

left     -----------------------------------------TTTNNNNNNNNNNN
chrom    AATACTTAGATTAATTTAGAAAGCTATTACCTCAAAACGAGTGTTATTTCTATTA
right    AATACTTAGATTAATTTAGAAAGCTATTACNNNNNNNNNNNN-------------
         222222222222222222222222222222NNNNNNNNNNN1N1NNNNNNNNNNN
```

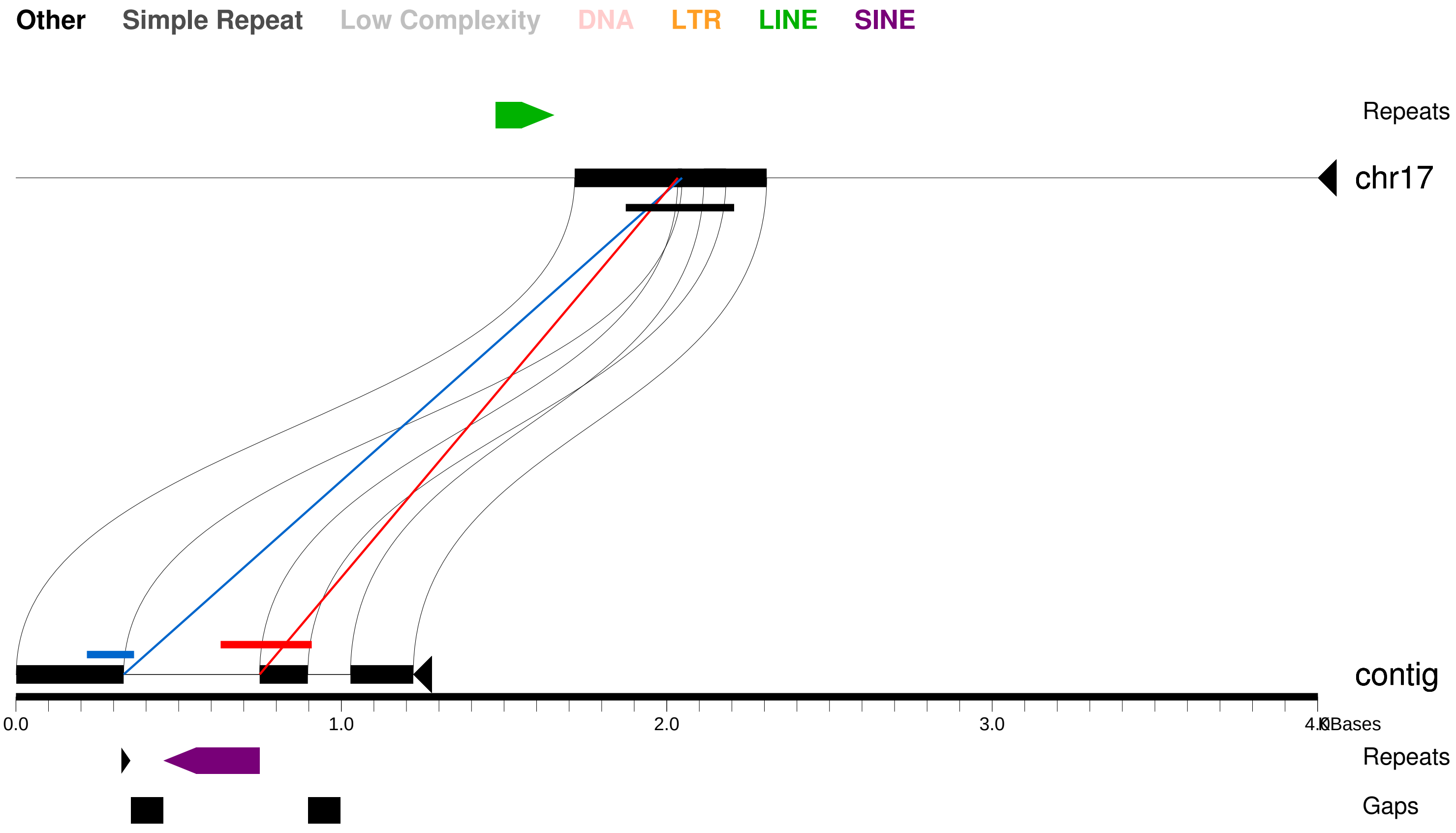

siteID: chr18_11069069 Scafftig1 - Breakpoint: chr18:11069073
chr18:11068913-11069248
Overlap Length: 16 INS size: 298 Genotyped: Yes

```
left   A------------TGAATAAATTCCTAGCAATATACAACCTACCATGATGGAATCATGAAGAAATAAAAA
chrom  AACCTAGAGGAAATGAATAAATTCCTAGCAATATACAACCTACCATGATGGAATCATGAAGAAATAAAAA
right  TT--TAGTAGAGACGGGGT--TTC--ACCGTTTTAG------CCGGGATGGTCTC-----GATCTC----
       1N  222NN22N21*111111***11*1*11*1**1111111**11*****11**11111**11*11111

left   ATCTGAGCAGACTAGTAAAAAGTAAGGAGATTGGAATCGGTGATAAAAAGTCTCCCCTCAAAAATAAATG
chrom  ATCTGAGCAGACTAGTAAAAAGTAAGGAGATTGGAATCGGTGATAAAAAGTCTCCCCTCAAAAATAAATG
right  --CTGACC----TCGTGATCCGCCCGCC--------TCGGCCTCCCAAAGTG----CTGGGAT-TACAGG
       11****1*1111*1**1*111*111*1111111111****111111*****11111**111*11**1*1*

left   AATAAATAAATAAAAGCC-CAGGACCTCATGAATTCTT-------TTT------------------TTTT
chrom  AATAAATAAATAAAAGCC-CAGGACCTCATGAATTCTACCAAACATTTAAGGAGAAATAATATCAATTCT
right  CGTGAGCCACCGCGCCCGGCCGGACCTCATGAATTCTACCAAACATTTAAGGAGAAATAATATCAATTCT
       11*1*111*1111111*11*1***************22222222***2222222222222222222**2*

left   TTT----TTTTTG---------------AGACGGAGT-CTCGCTCTGTCGCCCAGGCTGGAGTGCAGTGG
chrom  TCTCAAATTCTTCCAAGAAAATTAAAGAAGAGGGAATACTTGCAAAGTCATCCTATGTGGTCATCACTAC
right  TCTCAAATTCTTCCAAGAAAATTAAAGAAGAGGGAATACTTGCAAAGTCATCCTATGTGGTCATCACTAC
       *2*2222**2**2222222222222222***2***2*2**2**222***22**2222***2222**2*22

left   CGGGATCTCGGCTCACTGCAAGCTCCGCCTCCCGGGTTCACGCCATTCTCCTGCCTCAG--
chrom  CCTGATACCAAAACCAGACAAGGATAA--TACAAGG--AAAGAAAATTACAGGCCAGTGTC
right  CCTGATACCCACAC-AGACT---------TACA--------GCAAATTT--GGTCTTACNN
       *22***22*N2N2*N222*111NNNNN22*2*2N1122N1N*N2*2*2N1N2*1*NNN1NN
```

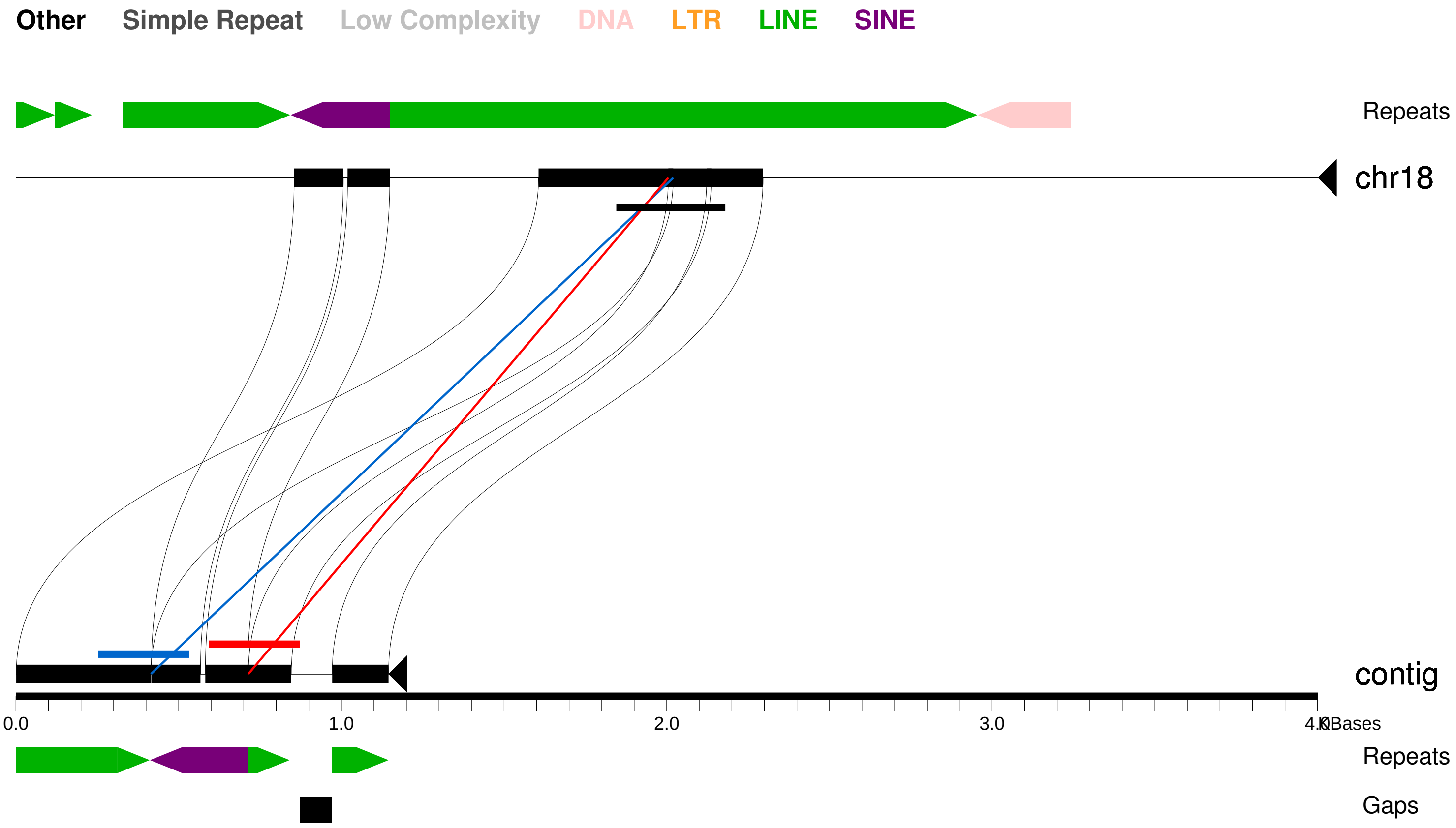

siteID: chr18_1235779 Scafftig17 - Breakpoint: chr18:1235778
chr18:1235731-1235837
Overlap Length: 13 INS size: 311 Genotyped: Yes

```
left    ATATGATTGCTTTTAAG--------TCAGTT----AAGAAATTTGCAAATATAGTTTTTAAAAATAATTA
chrom   ATATGATTGCTTTTAAG--------TCAGTT----AAGAAATTTGCAAATATAGTTTTTAAAAATAATTA
right   TCCAGCCTGGGCGACAGAGCGAGACTCCGTCTCAAAAAAAAAAAAAAAAAAAAAAAAAAAAAAAATAATTA
        1111*11**111111**11111111**1**11111**1***11111***1*1*111111***********

left    CAGGCCGGGCGCGGTGGCTCACGCCTGTAATCCCAGCACTTTGGGAGGCCGAGGCGGGCGG
chrom   CATAA-----------ACAAGTACTTTTACTGGCA-CTCTATTTTATCATGTGGACTTGAA
right   CATAA-----------ACAAGTACTTTTACTGGCA-CTCTATTTTATCATGTGGACTTGAA
        **222222222222222*22222*2*2**2*22**2*2**2*222*2222*2**2222222
```

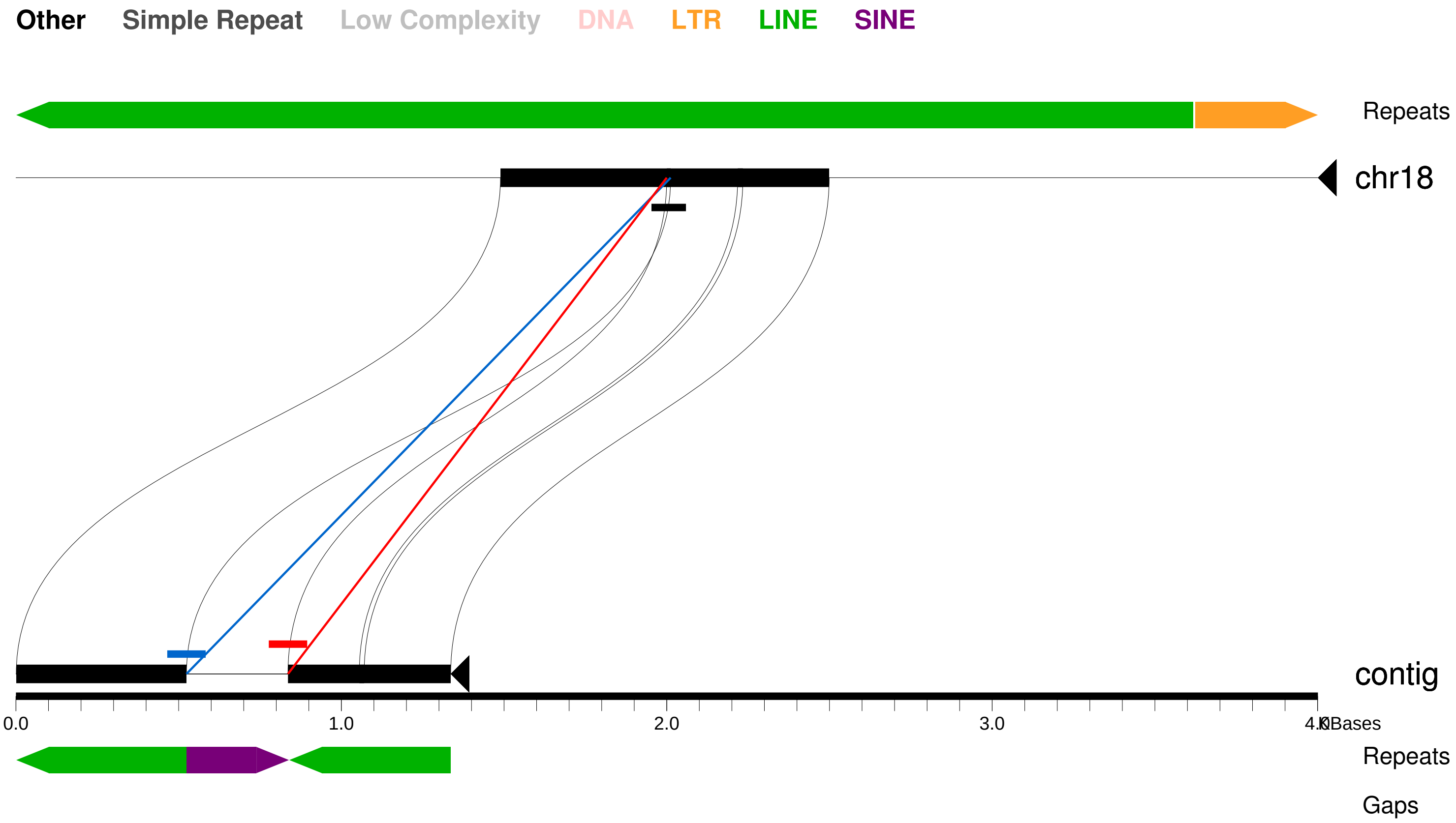

siteID: chr18_1830649 Scafftig2 + Breakpoint: chr18:1830654
chr18:1830607-1830713
Overlap Length: 13 INS size: 295 Genotyped: Yes

```
left    AACAC--------GCCTACTATA-TGTTAGTCTCA-----GTTACTCATAAAAACATAGTACTGGACAGA
chrom   AACAC--------GCCTACTATA-TGTTAGTCTCA-----GTTACTCATAAAAACATAGTACTGGACAGA
right   TCCGCCCGCCTCGGCCTCCCAAAGTGCTGGGATTACAGGCGTGAGCCACCGCGCCC--GGCCTGGACAGA
        11*1*11111111****1*1*1*1**1*1*11*1*11111**1*11**111111*111*11*********

left    TCTTTTTTTTTTTTTTTTTTGAGACGGAGTCTCGCTGTCGCCCAGGCAGGAGTGCAGTGGCGC
chrom   TCTTATAAACAGAAATTTTTA----GTAGACTAGAA-TCTCCCCTCTAGGCTTGA-------C
right   TCTTATAAACAGAAATTTTTA----GTAGACTAGAA-TCTCCCCTCTAGGCTTGA-------C
        ****2*222222222*****22222*2**2**2*222**2***2222***22**22222222*
```

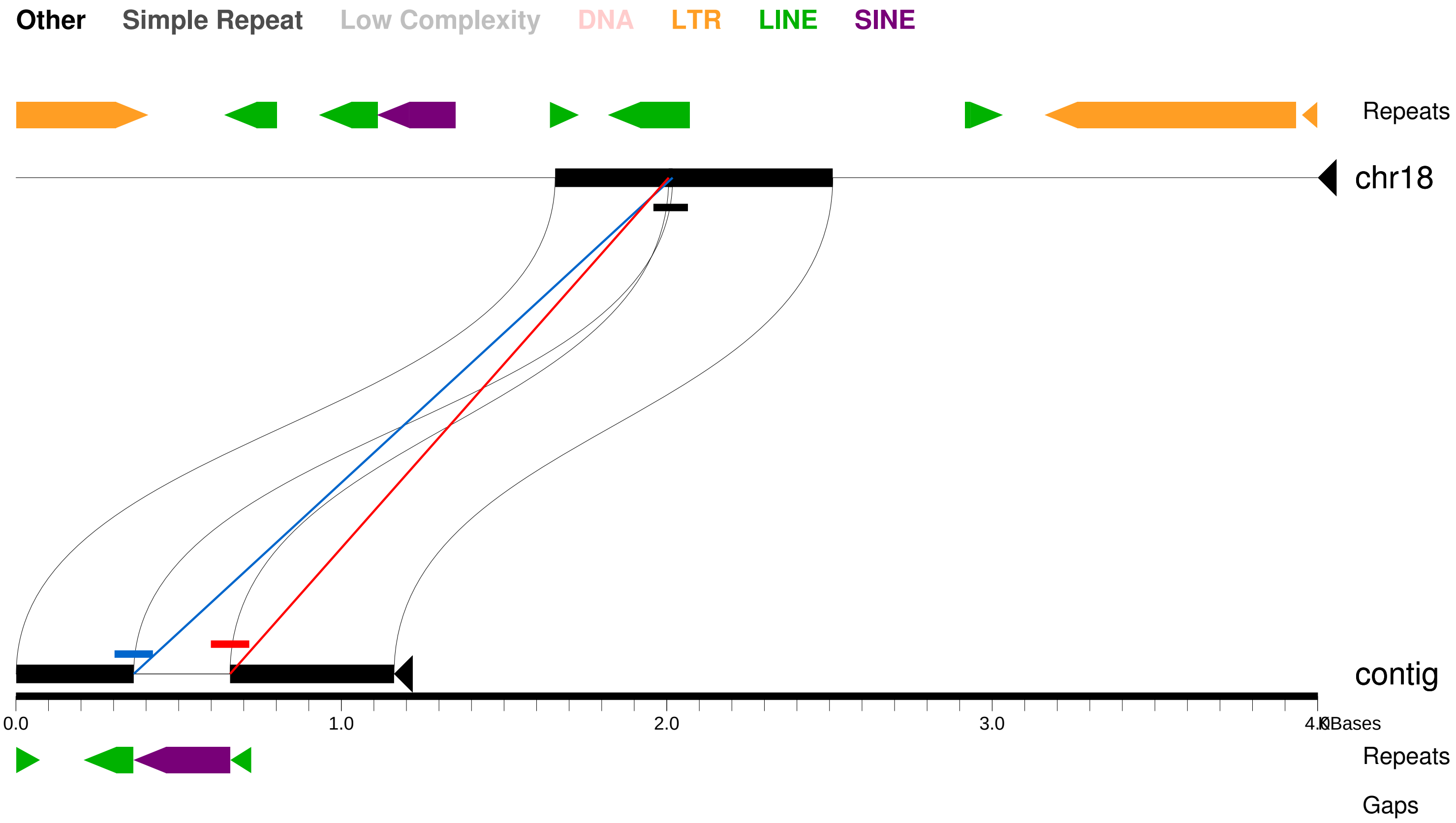

siteID: chr18_19705164 Scafftig2 - Breakpoint: chr18:19705151
chr18:19705102-19705210
Overlap Length: 11 INS size: 482 Genotyped: No

```
left    --------TATAGTTACAAACATTTTACAAATATTG-CATCA-CCTTTTATTTAACATAAAAAACATTTT
chrom   --------TATAGTTACAAACATTTTACAAATATTG-CATCA-CCTTTTATTTAACATAAAAAACATTTT
right   CGCCCGCCTCGGCCTCCCAAAGTGCTGGGATTACAGGCGTGAGCCACCGCGCCCGGCCCAAAAACATTTT
        11111111*11111*1*1**11*11*111*1**11*1*1*1*1**111111111111111**********

left    TTTTTTTNNNNNNNNNNNNNNNNNNNNNNNNNNNNNNNNNNNNNNNNNNNNNNNNNNNNN
chrom   ACATT----------ACTAAAAATCATTATAAATACAATTTCTGATGTGTAATGTAGAT
right   ACATT----------ACTAAAAATCATTATAAATACAATTTCTGATGTGTAATGTAGAT
        222**222222222222222222222222222222222222222222222222222222
```

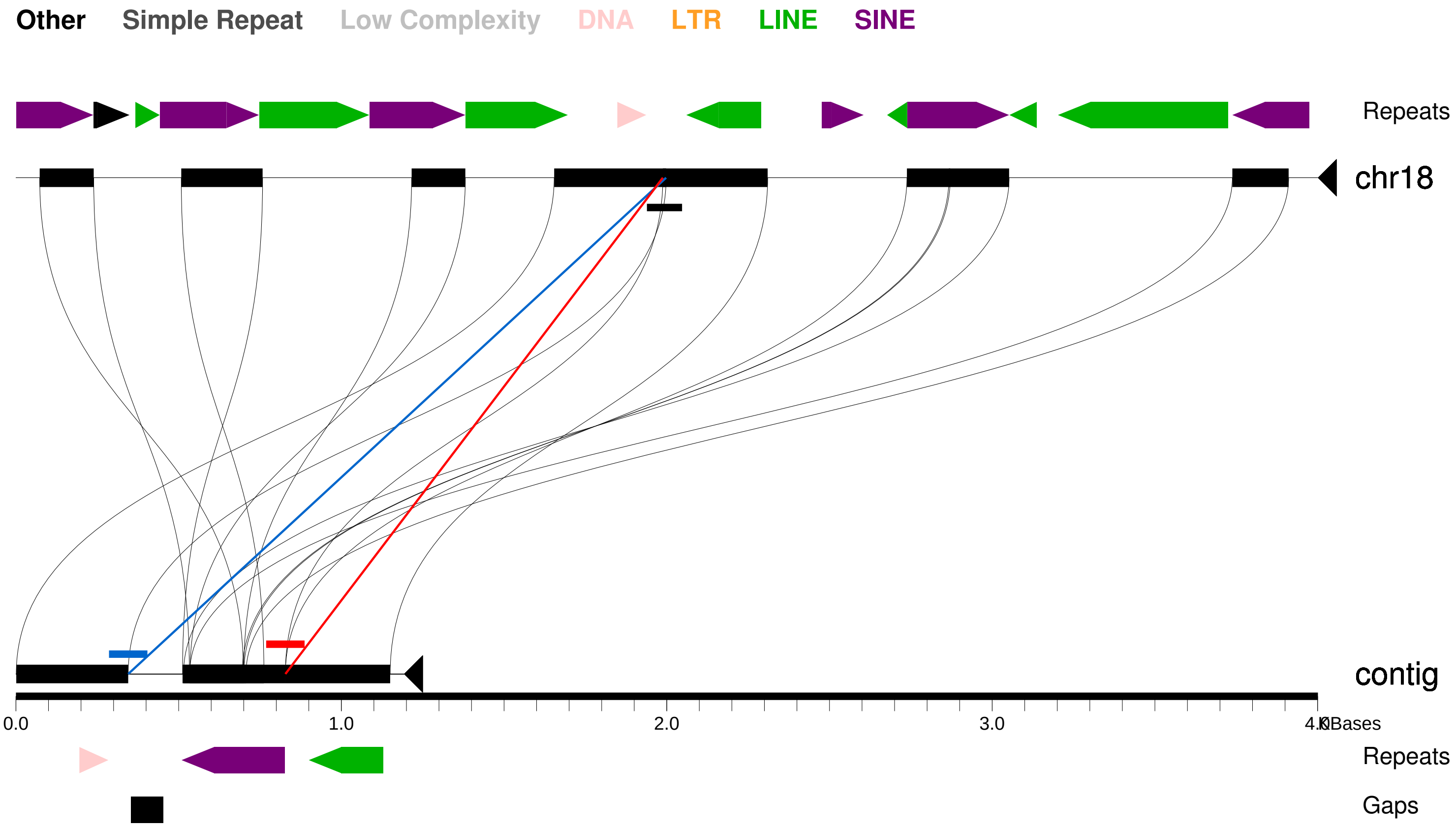

siteID: chr18_22390137 Scafftig3 - Breakpoint: chr18:22390149
chr18:22390103-22390208
Overlap Length: 14 INS size: 300 Genotyped: Yes

```
left    ---GAAAA--AGCCTGTAGTTCCTA-CAATAC--AGT-----AGAATTAAAATTTGTTTAAAAGAGTTTT
chrom   ---GAAAA--AGCCTGTAGTTCCTA-CAATAC--AGT-----AGAATTAAAATTTGTTTAAAAGAGTTTT
right   ACTGCACTCCAGCCTGGGCGACAGAGCGAGACTCCGTCTCAAAAAAAAAAAAAAAAAAAAAAAGAGTTTT
        111*1*1111******11111*11*1*1*1**111**11111*1**11****1111111***********

left    ATGGGCCGGGCGCGGTGGCTCACGCCTGTAATCCCAGCACTTTGGGAGGCCGAGGCGGGCGG
chrom   ATGAAAAGTAAACAAAAACTAA----------ACCAACACATA---AGATTATAGTAAATAA
right   ATGAAAAGTAAACAAAAACTAA----------ACCAACACATA---AGATTATAGTACATAA
        ***2222*2222*22222**2*22222222222***2***2*2222**222222*22N2222
```

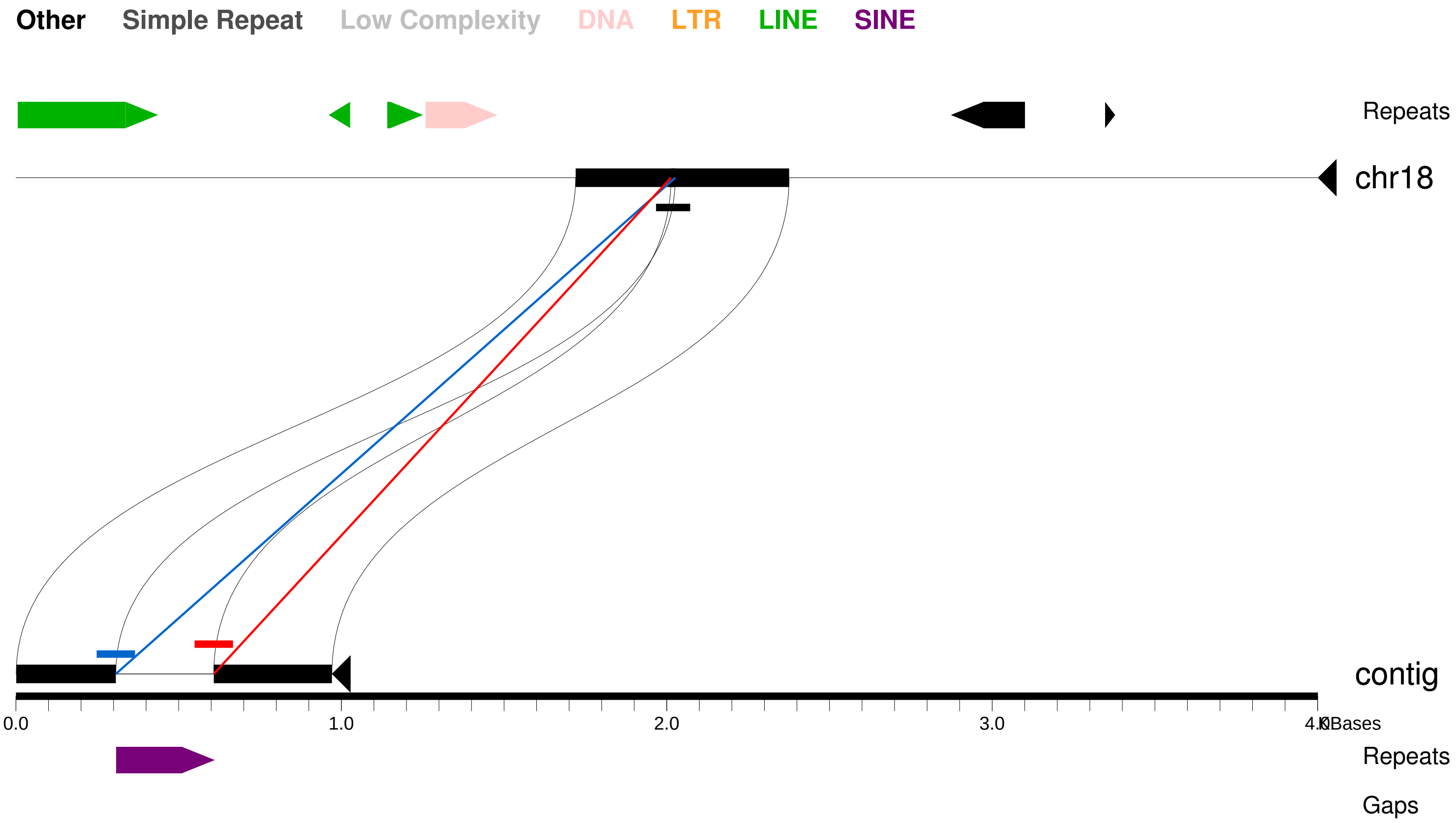

siteID: chr18_2304388 Scafftig2 + Breakpoint: chr18:2304393
chr18:2304348-2304452
Overlap Length: 15 INS size: 257 Genotyped: Yes

```
left     ATTATGTAA--AT----TCCAAGAGGTGTAAAAG-GTTCTGAAACTTTTG--CT-----TCTACTGGGCT
chrom    ATTATGTAA--AT----TCCAAGAGGTGTAAAAG-GTTCTGAAACTTTTG--CT-----TCTACTGGGCT
right    TGTTAGCCAGGATGGTCTCGATCTCCTGACCTCGTGATCCGCCCGTCTCGGCCTCCCAATTCTACTGGGCT
         11*11*11*11**1111**1*11111**11111*1*1**1*1111*1*1*11**11111***********

left     TTTTTTTTTTTTTTTTTTTTGAGACGGAGTCTCGCTCTGTCGCCCAGGCCGGAGTG-CAGTGGCG
chrom    TTTTAACCCAA-------AGAATCAAAGGAAAGTTTTGTGACACA------AATGACAATGA--
right    TTTTAACCCAA-------AGAATCAAAGGAAAGTTTTGTGACACA------AATGACAATGA--
         ****222222222222222**22*22**2222*2*2***22*2**222222*2**2**2**222
```

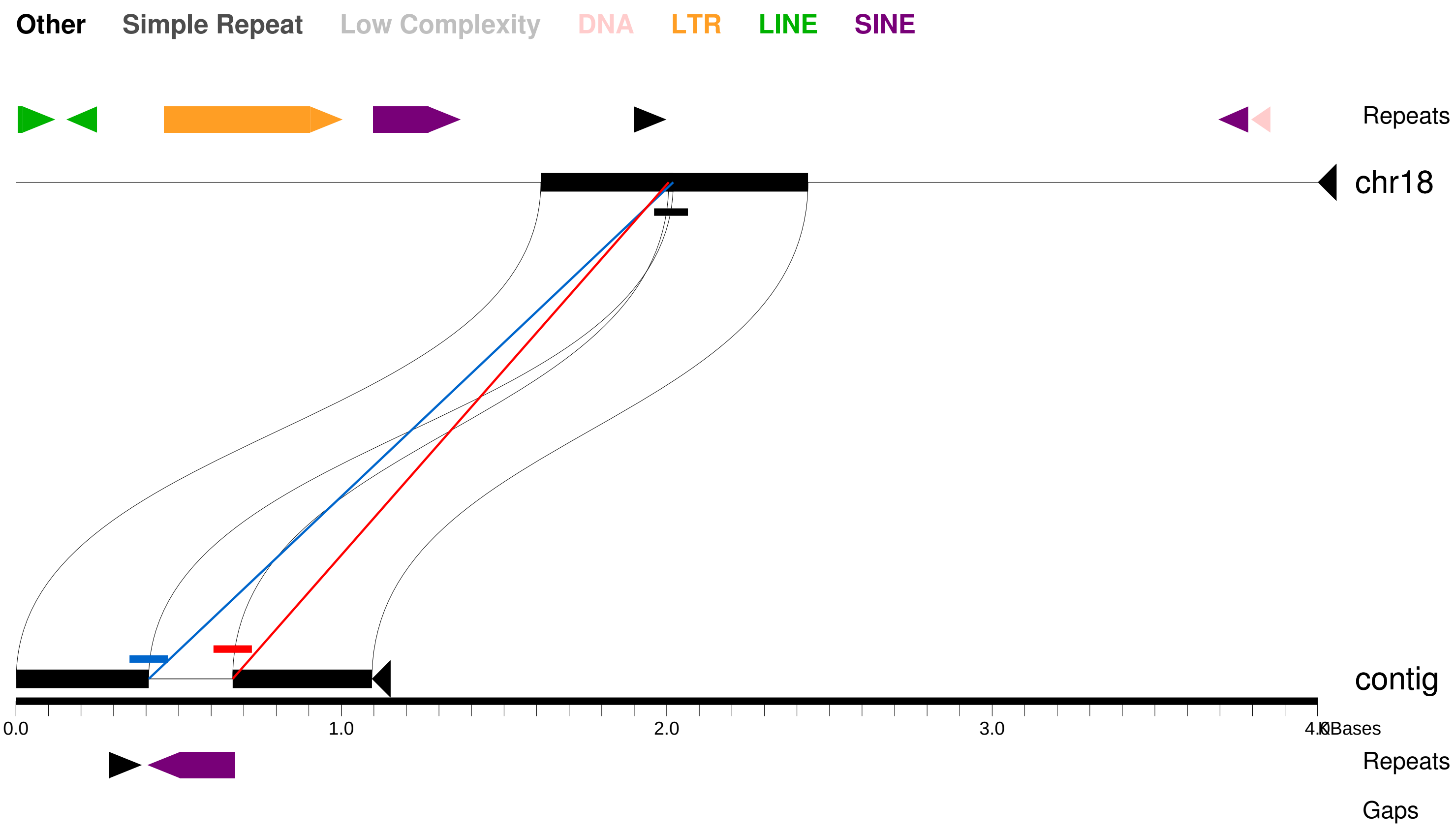

siteID: chr18_25851192 Scafftig1 + Breakpoint: chr18:25851172
chr18:25851012-25851347
Overlap Length: 16 INS size: 336 Genotyped: Yes

```
left   AT--------TTATGTCTATTTTTATGTGATTACCATGCCATTTTGATTACTGTAACTTTGAAATATATT
chrom  CTATTTGTCTTTATGTCTATTTTTATGTGATTACCATGCCATTTTGATTACTGTAACTTTGAAATATATT
right  TT--TAGTAGAGACGGGG--TTTCACCTTGTTA----GCCAGGATGGTCTCG-------------ATCTC
       N*  2N22NN11*1*11111***1*11*11***1111****111**1*11*11111111111111**1*1

left   TTGAAATCAGGGAGTGAGGCTTCCCATTTTGATCTTTTCACTCAAGATTGCTTTGGCTATTTAAGATCAT
chrom  TTGAAATCAGGGAGTGAGGCTTCCCATTTTGATCTTTTTACTCAAGATTGCTTTGGCTATTTAAGATCAT
right  CTGACCTCA-----TGATCCA-CCCGCCTCGGCCT-----CCCAA-AGTGCTGGGATTA--------CAG
       1***11***11111***11*11***111*1*11**111N1*1***1*1****11*11**11111111**1

left   TTGTGGTCCAATATGAATTT--TAGGATTGTTTTTTCTTTTTTTTT------TTTTTTTTTTTTTTTGAG
chrom  TTGTGGTCCAATATGAATTT--TAGGATTGTTTTTTCTAATTGTGTAAAGAATGCCATTGGGATTTAGGT
right  GCGTGAGCCACCGCGCCCGGCCTAGGATTGTTTTTTCTAATTGTGTAAAGAATGCCATTGGGATTTAGGT
       11***11***1111*1111111****************22**2*2*222222*2222**2222***2*22

left   ACGGAGTC---TCGCTCTGTCGCCCAGGCTGG-----------------ACTCGCTCTGTCGCCCAGGCC
chrom  AGGGATTGCATTGACTCTGTAGATCTCTTTGGGTAGTATAAACATTTTAACAATTTAATTCTTCCAATTC
right  AGGGATTGCATTGACTCTGTAGATCTCTTTGGGTAGTATAAACATTTTAACAATTTAATTCTTCCAATTC
       *2***2*2222*22******2*22*2222***22222222222222222**2222*222**22***222*

left   -----GGACTGCGGACTG-----CAGTGGCG-CAATCTCGGC-----TCACTG-----
chrom  AAAAGGGATGTCATTCTATTTATCTGTGCCTTCTTTCATTTCATTCATCAATGTCTAA
right  AAAAGGGATGTCATTCTATTTATCTGTGCCTTCTTTCATTTC----------------
       22222***222*222**222222*2***2*22*22**2222*     111N11
```

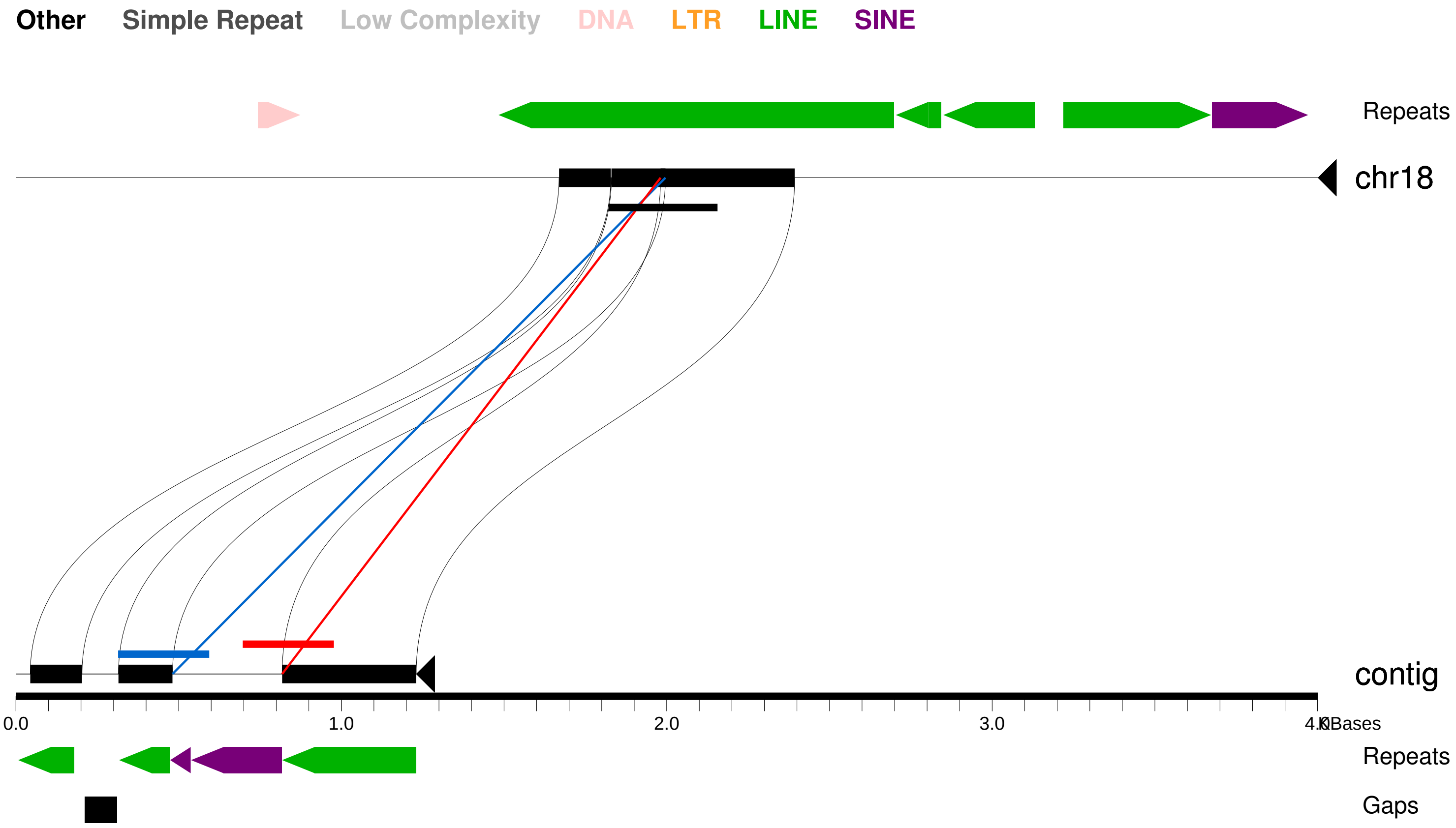

siteID: chr18_25871810 Scafftig1 - Breakpoint: chr18:25871819
chr18:25871770-25871875
Overlap Length: 11 INS size: 297 Genotyped: Yes

```
left     A--GTCATTTCTCTT-----TGTGTG---TAGGTTGA-------TAAAAATAACAGCTTATATAATAATC
chrom    A--GTCATTTCTCTT-----TGTGTG---TAGGTTGA-------TAAAAATAACAGCTTATATAATAATC
right    CCCGCCACTGCACTCCAGCCTGGGCGACAGAGCGAGACTCTGTCTCAAAAAAAAA----AAAAAAAAATC
         111*1**1*1*1**111111**1*1*1111**111**1111111*1****1**1*1111*1*1**1****

left     ACATTTTGGCCGGGCGCGGTGGCTCACGCCTGTAATCCCAGCACTTTGGGAGGCCGAGGCGGGCGG
chrom    ACATTTTAT------------AATCTT-CAAATTTTCTGTTCAGCTAAATATAGTTAAGAATACAA
right    ACATTTTAT------------AATCTT-CAAATTTTCTGTTCAGCTAAATATAGTTAAGAATACAA
         *******2222222222222222**222*222*22**2222**22*2222*22222*2*2222*22
```

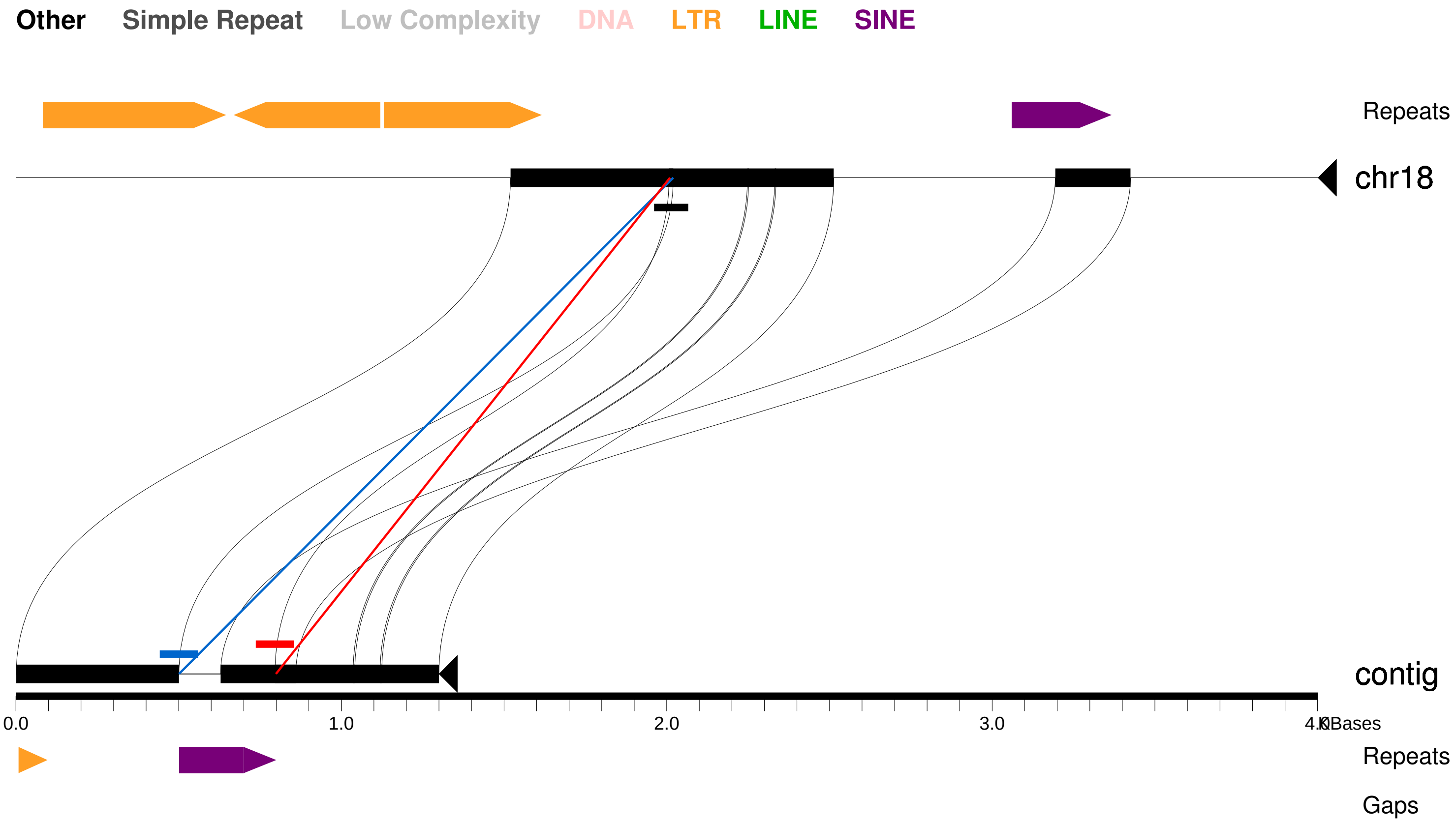

siteID: chr18_27156352 Scafftig2 + Breakpoint: chr18:27156356
chr18:27156311-27156415
Overlap Length: 15 INS size: 305 Genotyped: Yes

```
left    --------------TTTTCAAAAC-CAGTAATAGCACAAGTCCTCTCAAGCTTTCTATCTCTCTTGTCTC
chrom   --------------TTTTCAAAAC-CAGTAATAGCACAAGTCCTCTCAAGCTTTCTATCTCTCTTGTCTC
right   CCACCCGCCTCGGCCTCCCAAAGTGCTGGGATTACAGGCGTGAGCCACCGCGCCCGGCC-CTCTTGTCTC
        111111111111111*11****111*1*11**11**111**111*1111**111*111*1**********

left    TTCTTTTTTTTTTTTTTTTTTTGAGACGGAGTCTCGCTCTGTCGCCCAGGTCGGACTGCGGACT
chrom   TTCTTCCACAATCACATCT-----------GTCTGTC-CTACCATTCTGCTTTTCCTTTT--CC
right   TTCTTCCACAATCACATCT-----------GTCTGTC-CTACCATTCTGCTTTTCCTTTT--CC
        *****222222*2222*2*22222222222****22*2**22*222*2*2*2222**22222*2
```

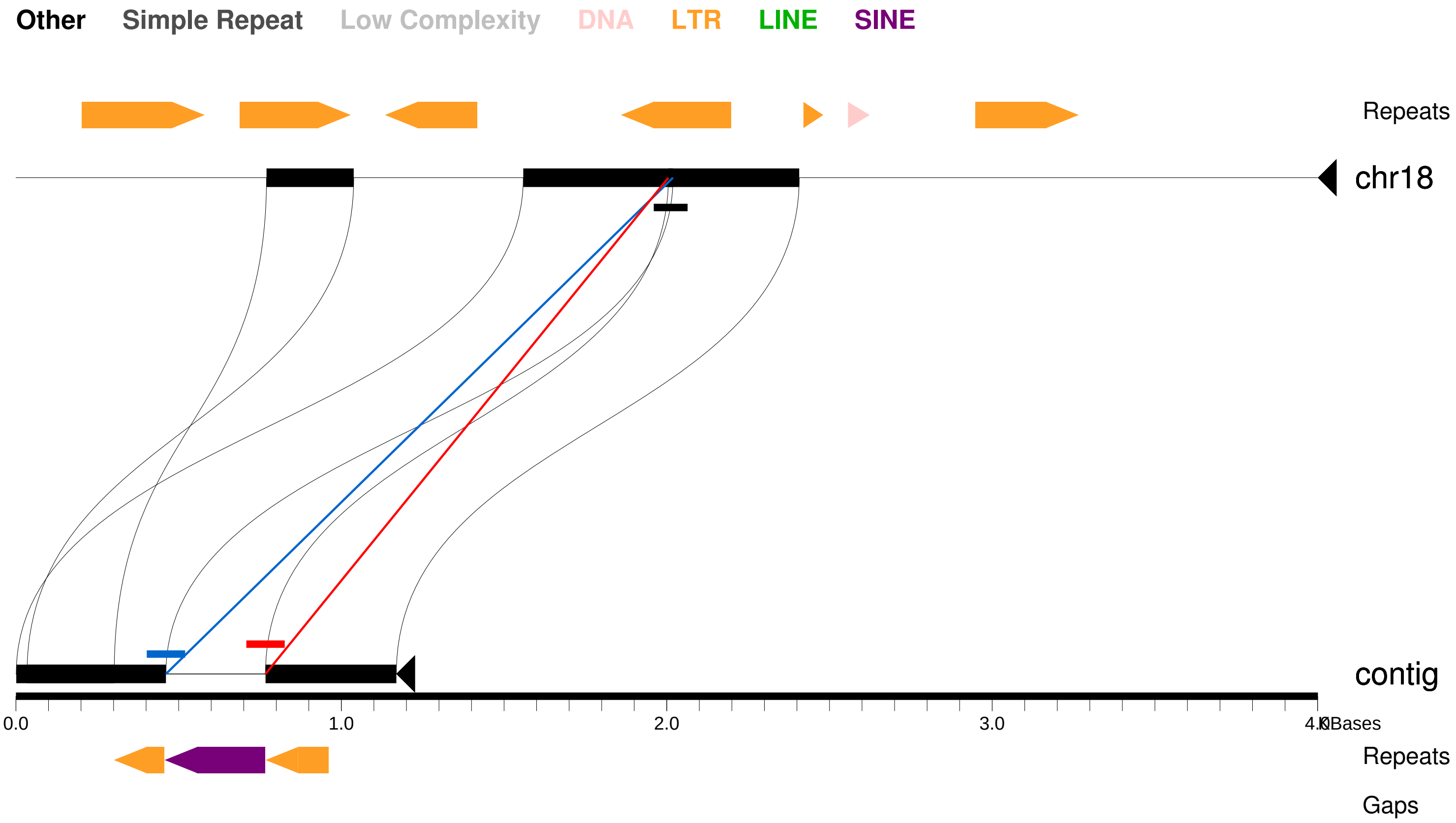

siteID: chr18_27182106 Scafftig1 - Breakpoint: chr18:27182090
chr18:27182045-27182149
Overlap Length: 15 INS size: 425 Genotyped: No

```
left    AGACAACCAAACCAACAATGGACAAATGAGATTACGTTGAGATAA--------------AAAGCTTCTAC
chrom   AGACAACCAAACCAACAATGGACAAATGAGATTACGTTGAGATAA--------------AAAGCTTCTAC
right   NNNNNNNNNNNNNNNNNNNNNNNNNNNNNNNNNNNNNNNNNNNNNNNNNNNNNNNNNNNAAAGCTTCTAC
        11111111111111111111111111111111111111111111111111111111111***********

left    ACAGGCCGGGCGCGGTGGCTCACGCCTGTAATCCCAGCACTTTGGGAGGCCGAGGCGGGCGGA
chrom   ACAGCAAGGGAA--ATGATTAACG-----AAGTGGAGA-------GATAACCCACCTAATGGG
right   ACAGCAAGGGAA--ATGATTAACG-----AAGTGGAGA-------GATAACCCACCTAATGGG
        ****222***22222**22*2***22222**2222**22222222**222*2222*2222**2
```

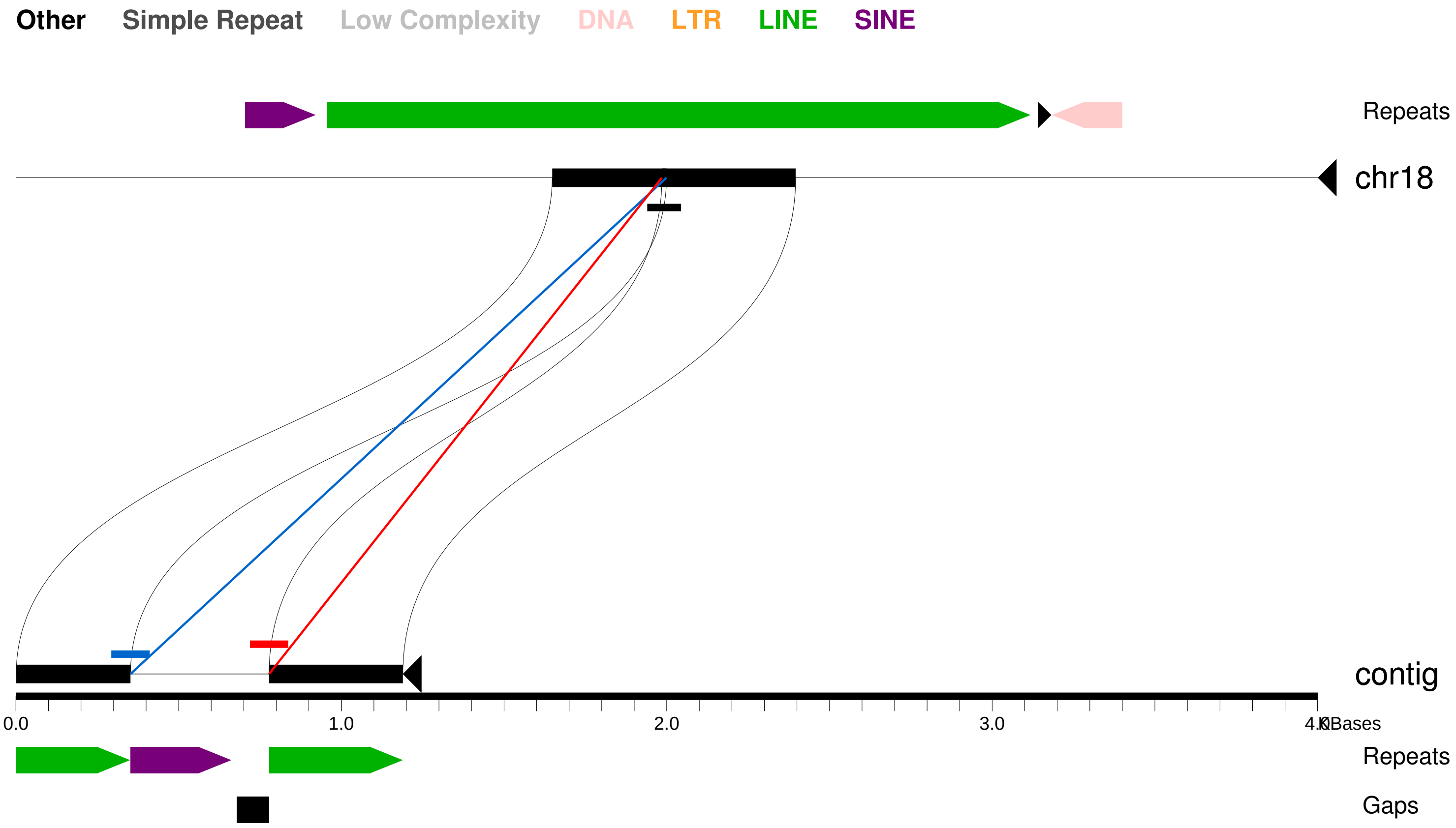

siteID: chr18_29003599 Scafftig4 - Breakpoint: chr18:29003588
chr18:29003552-29003651
Overlap Length: 34 INS size: 156 Genotyped: Yes

```
left   AT---GTAGAGAACATGAAACT---------------AGATAG-----GAATTATTAAAAAAAAAAAAAA
chrom  AT---GTAGAGAACATGAAACT---------------AGATAG-----GAATTATTAAAAAAAAAAAAAA
right  AGTGAGCCGAGATCGCGCCACTGCACTCCAGCCTGGGAGACAGAGCGAGACTCCGTCTCAAAAAAAAAAA
       *1111*11****1*11*11***111111111111111***1**11111**1*111*111***********

left   AAAGAGTAAAAAACAAGTATTTACTACAAAAAATTAGCCGGGCGTGGTAGCGGGCGCCTGTAGTCCCAGC
chrom  ---GAGTAAAAAACAAGTATTTACTAGTA----TGTGTCTAGCAC--TAG-----GCTAATTGTTTC---
right  -------AAAAAACAAGTATTTACTAGTA----TGTGTCTAGCAC--TAG-----GCTAATTGTTTC---
       2221111*******************22*2222*22*2*22**2222***22222**222*2**22*222

left   TA
chrom  --
right  --
       22
```

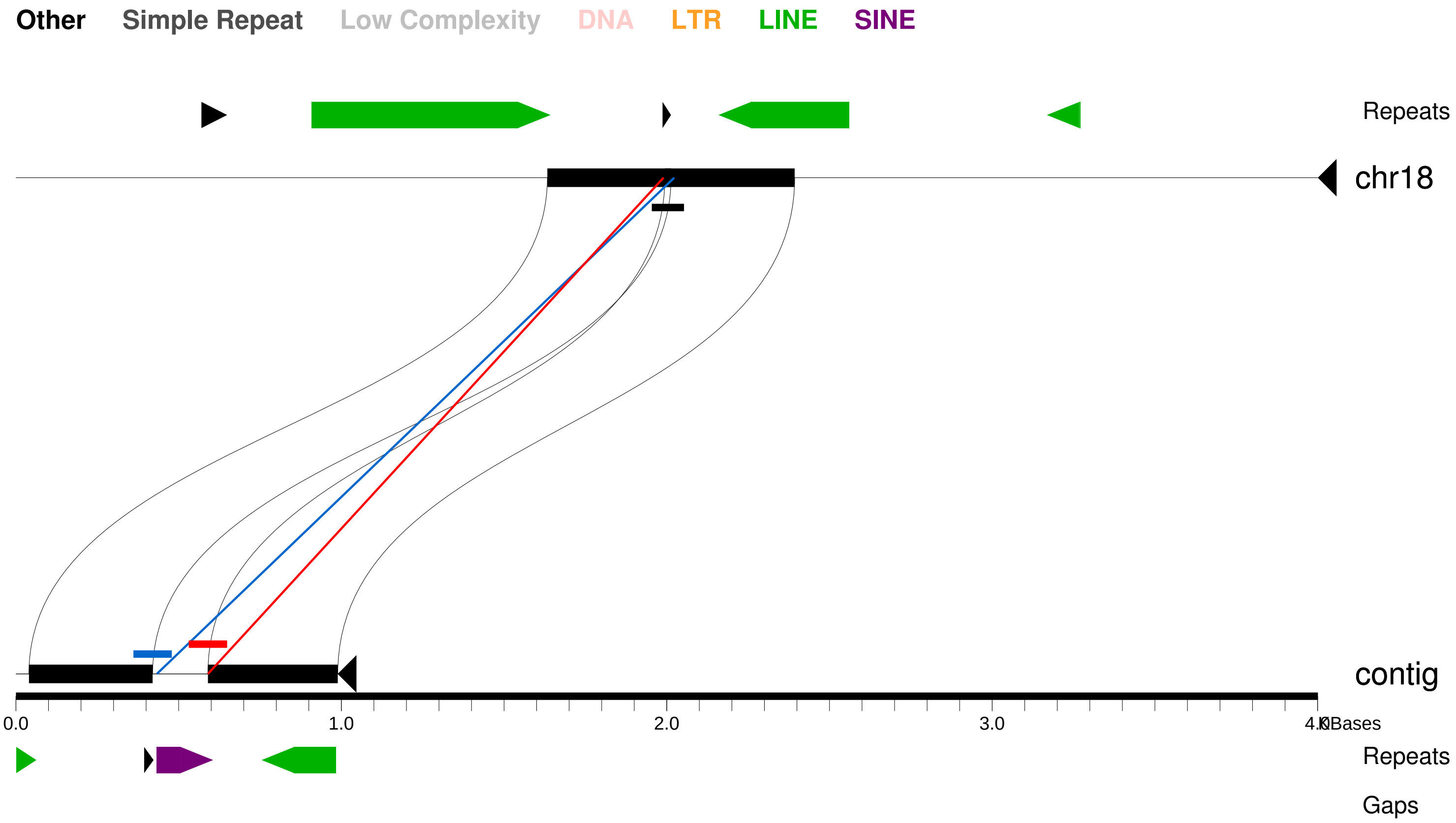

siteID: chr18_29398919 Scafftig1 - Breakpoint: chr18:29398920
chr18:29398865-29398966
Overlap Length: 5 INS size: 311 Genotyped: Yes

```
left    -----GATATCGATTTTGAAATTTAAAG---------GTACCAGAATATGCCCCACAAAAAATATA---C
chrom   -----GATATCGATTTTGAAATTTAAAG---------GTACCAGAATATGCCCCACAAAAAATATA---C
right   CCACTGCACTCCAGCCTGGGCGACAGAGCGAGACTCCGTCTCAAAAAAAAAAAAAAAAAAAAAAAAAACC
        11111*111**1*111**111111*1**111111111**11**1**1*111111*1******1*1*111*

left    CACTTTGGCCGGGCGCGGTGGCTCACGCCTGTAATCCCAGCACTTTGGGAGGCCGAGGCGGGCGGA
chrom   CACTTTGACAAGAATTATT--TTGA-----GTTATAG--GCAACT---GAGAACCAGGAG-----A
right   CCCTTTGACAAGAATTATT--TTGA-----GTTATAG--GCAACT---GAGAACCAGGAG-----A
        *1*****2*22*222222*222*2*22222**2**2222***22*222***22*2***2*22222*
```

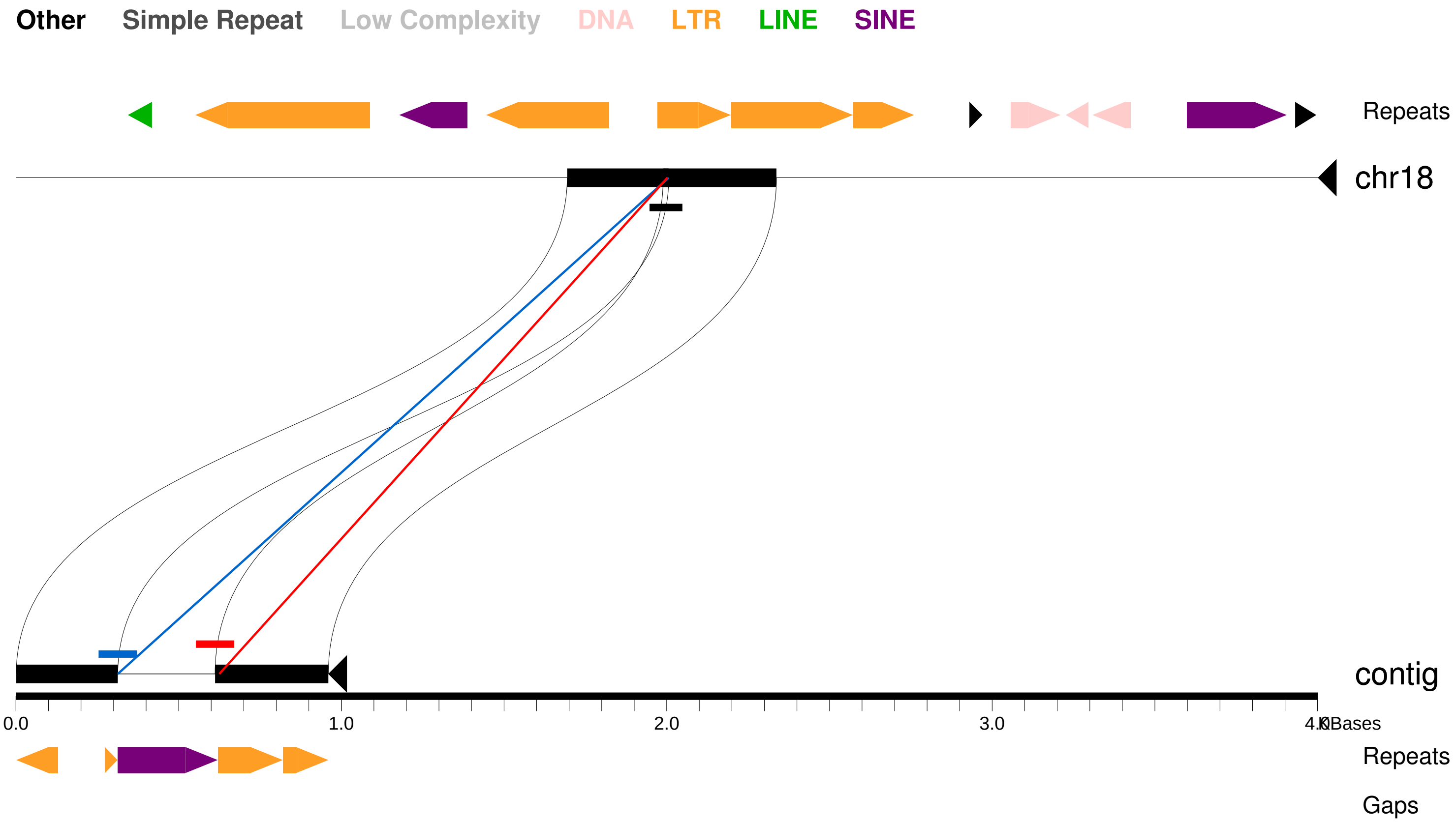

siteID: chr18_30047878 Scafftig3 - Breakpoint: chr18:30047887
chr18:30047842-30047946
Overlap Length: 15 INS size: 299 Genotyped: Yes

```
left     TAC--CAGCATGTCCTCAAC--CTGTCCTAAACTGTGTG----------AAACATGTTTAAAAACATGCA
chrom    TAC--CAGCATGTCCTCAAC--CTGTCCTAAACTGTGTG----------AAACATGTTTAAAAACATGCA
right    CACTGCACTCCAGCCTGGGCGACAGAGCGAGACTCCGTCTCAAAAAAAAAAAAAAAAAAAAAAACATGCA
         1**11**111111***111*11*1*11*1*1***11**11111111111***1*11111***********

left     AAGAGGCCGGGCGCGGTGGCTCACGCCTGTAA--TCCCAGCACTTTGGGAGGCCGAGGCGGGCGG
chrom    AAGAATCTA-------TGATTGACTCTTTTAAAGTCTACCCAAATT---------AAACTGCTAT
right    AAGAATCTA-------TGATTGACTCTTTTAAAGTCTACCCAAATT---------AAACTGCTAT
         ****22*222222222**22*2**2*2*2***22**2222**22**222222222*22*2*2222
```

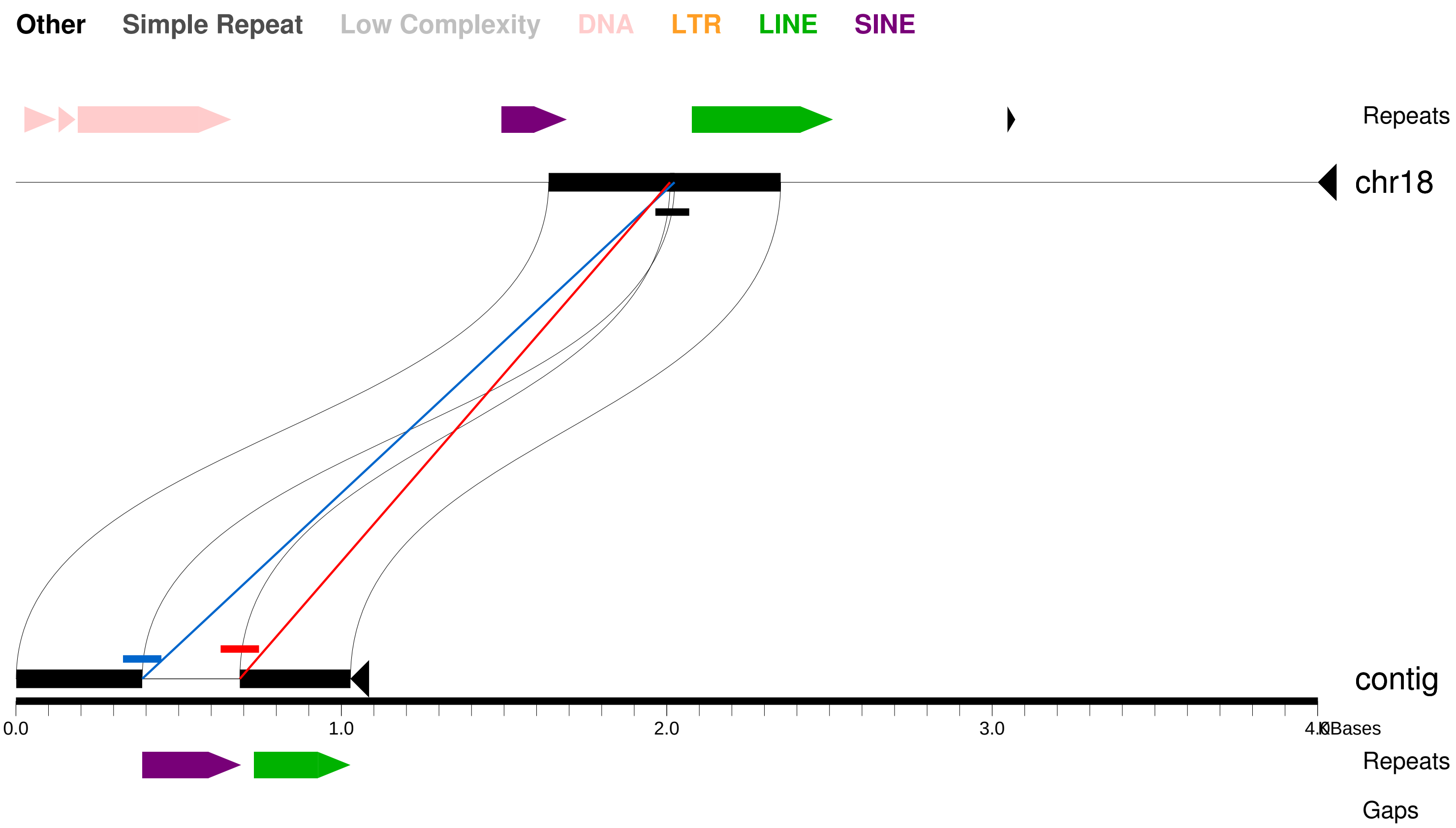

siteID: chr18_30189794 Scafftig2 - Breakpoint: chr18:30189808
chr18:30189795-30189849
Overlap Length: 42 INS size: 422 Genotyped: No

```
left    AG-----------------------------------------------------------------AAAA
chrom   AG-----------------------------------------------------------------AAAA
right   ATCCCGCCACTGCACTCCAGCCTGGGCGACAGAGCGAGACTCCGTCTCAAAAAAAAAAAAAAAAAAAAAAA
        *111111111111111111111111111111111111111111111111111111111111111111****

left    TATTAAGAGAGAGGCTGCATGGTTTTGGTCAGACTGTATAAAATATTTCCCGCTNNNNNNNNNNNNNNNNN
chrom   TATTAAGAGAGAGGCTGCATGGTTTTGGTCAGACTGTATAAAATATTTC----------------------
right   AAAAAAAAGAGAGGCTGCATGGTTTTGGTCAGACTGTATAAAATATTTC----------------------
        1*11**1*****************************************2222222222222222222222

left    NNNNNNNNNNNNNNNNNNNNNNNNNNNNNNNNNNNNNNNNNNN
chrom   -------------------------------------------
right   -------------------------------------------
        2222222222222222222222222222222222222222222
```

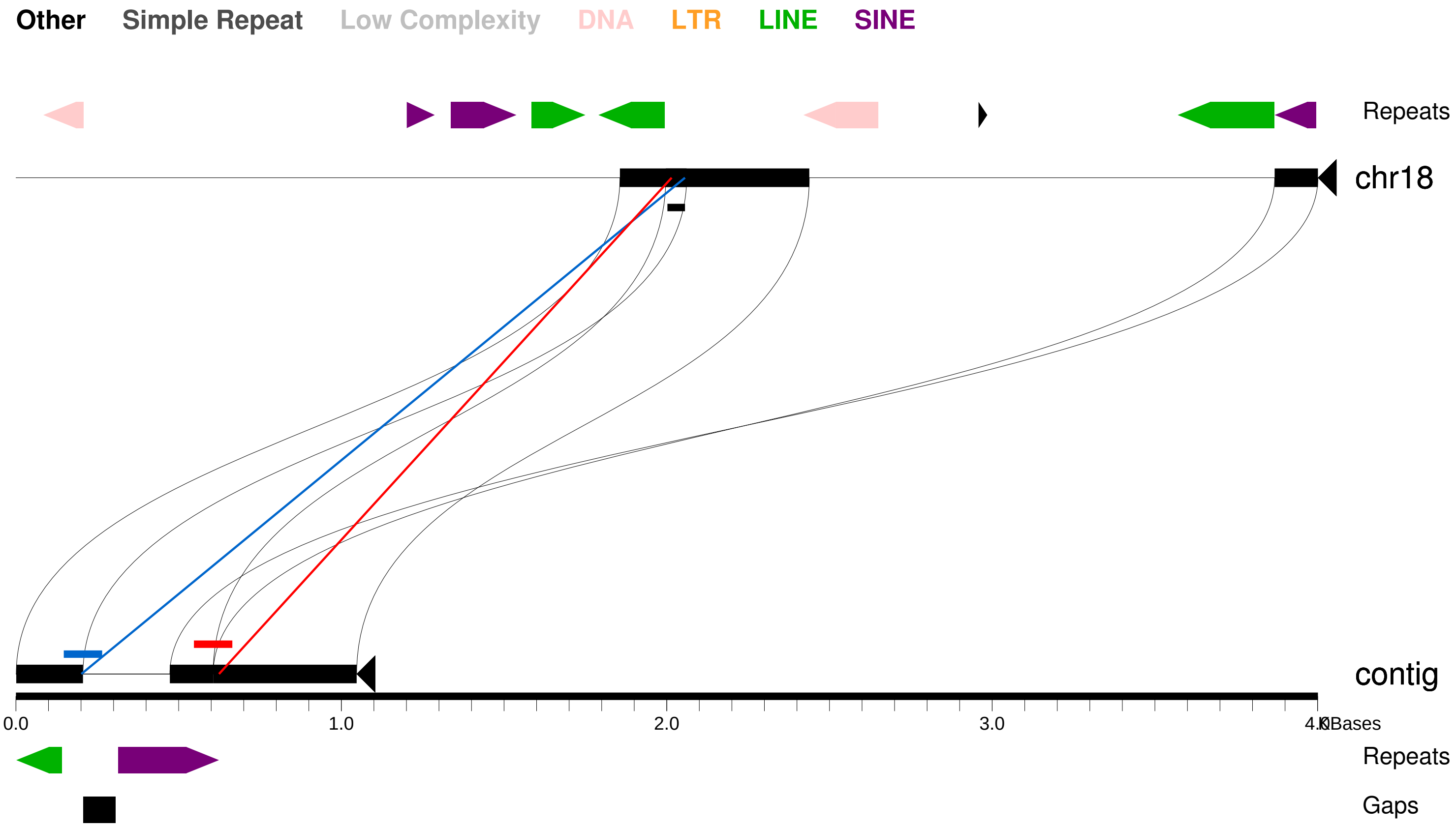

siteID: chr18_36512550 Scafftig7 - Breakpoint: chr18:36512533
chr18:36512487-36512592
Overlap Length: 14 INS size: 304 Genotyped: Yes

```
left   ------ATTATAAGTGATTGTTCAACTATTTACCTCAGTTTCAAGAAATCTC-------AAAAGCAAATT
chrom  ------ATTATAAGTGATTGTTCAACTATTCACCTCAGTTTCAAGAAATCTC-------AAAAGCAAATT
right  CACTCCAGCCTGGGCGACAGAGCGAGACTCCGTCTCAAAAAAAAAAAAAAAAAAAAAAAAAAAAGCAAATT
       111111*111*11*1**11*11*1*111*1211****11111**1***11111111111***********

left   TGAGGCCGGGCGCGGTGGCTCACGCCTGTAATCCCAGCACTTTGGGAGGCCGAGGCGGGCGG
chrom  TGAAAC---TCAAG-----TCATGGCAACTAGATAGACAAGTTTTAATTCATATTTT-----
right  TGAAAC---TCAAG-----TCATGGCAACTAGATAGACAAGTTTTAATTCATATTTT-----
       ***22*2222*22*22222***2*2*2222*222222**22**222*22*22*222222222
```

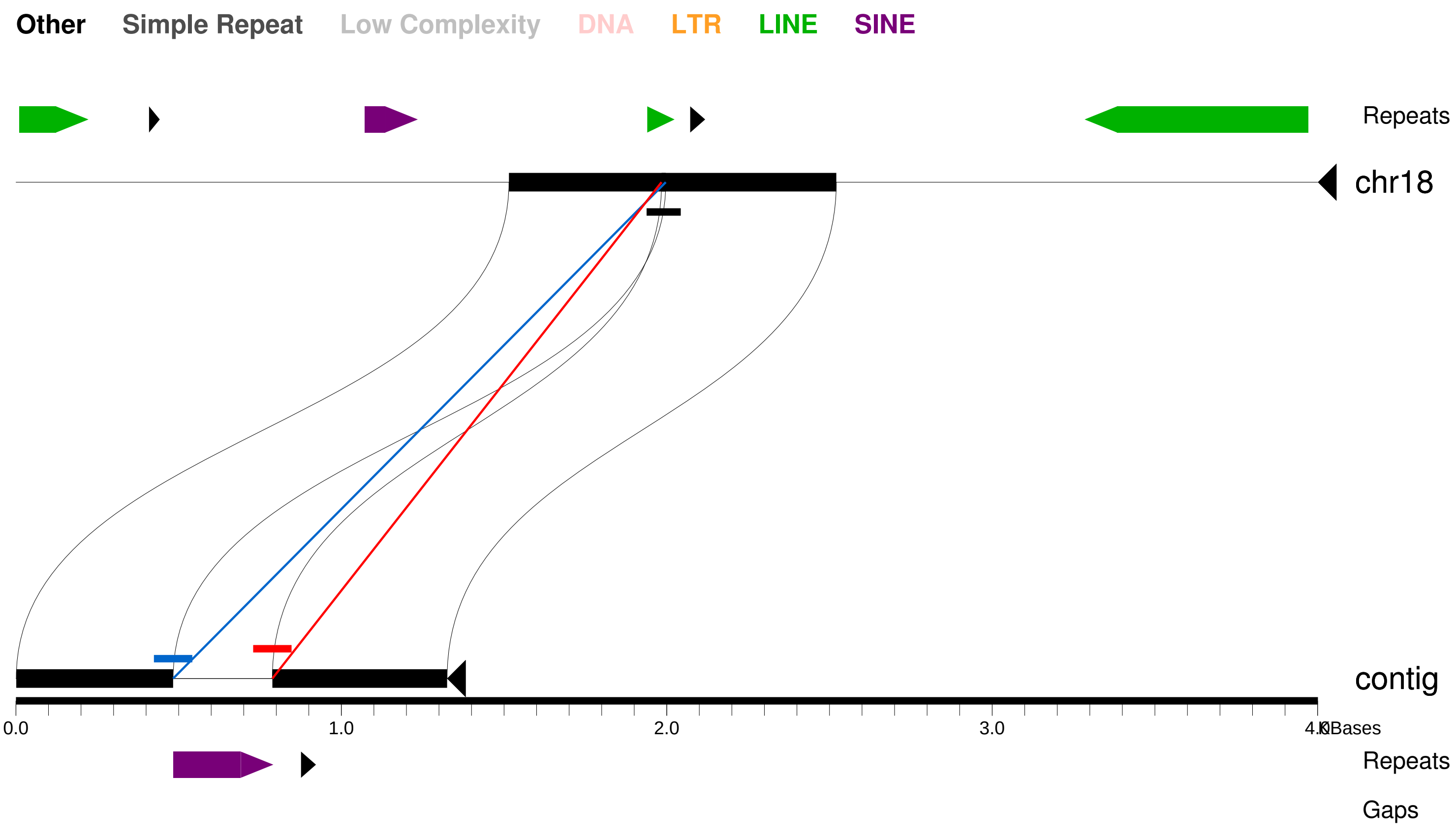

siteID: chr18_4355875 Scafftig1 + Breakpoint: chr18:4355868
chr18:4355822-4355927
Overlap Length: 14 INS size: 294 Genotyped: Yes

```
left     TTC--TCTTGAGTCTCCCATACTAGACTAGCTGCTGC-TCTC----TGCT-----TCC--TCTTCTGCTT
chrom    TTC--TCTTGAGTCTCCCATACTAGACTAGCTGCTGC-TCTC----TGCT-----TCC--TCTTCTGCTT
right    CTCGATCTCCTGAC-CTCATGATCCACCCGCCTCGGCCTCCCAAAGTGCTGGGATTACAGTCTTCTGCTT
         1**11***111*1*1*1***11*11**11**11*1**1**1*1111****11111*1*11**********

left     TTTTTTTTTTTTTTTTTTTTTTTTTTTTTTTTGAGACGGAGTCTCGCTCTGTCGCCCAGGCCG
chrom    TTTTAATAGAAAACTTCTCAAAATTTATCT-----ACT---TGTTGC-CTTTA----ATACCT
right    TTTTAATAGAAAACTTCTCAAAATTTATCT-----ACT---TGTTGC-CTTTA----ATACCT
         ****22*2222222**2*22222***2*2*22222**2222*2*2**2**2*22222*22**2
```

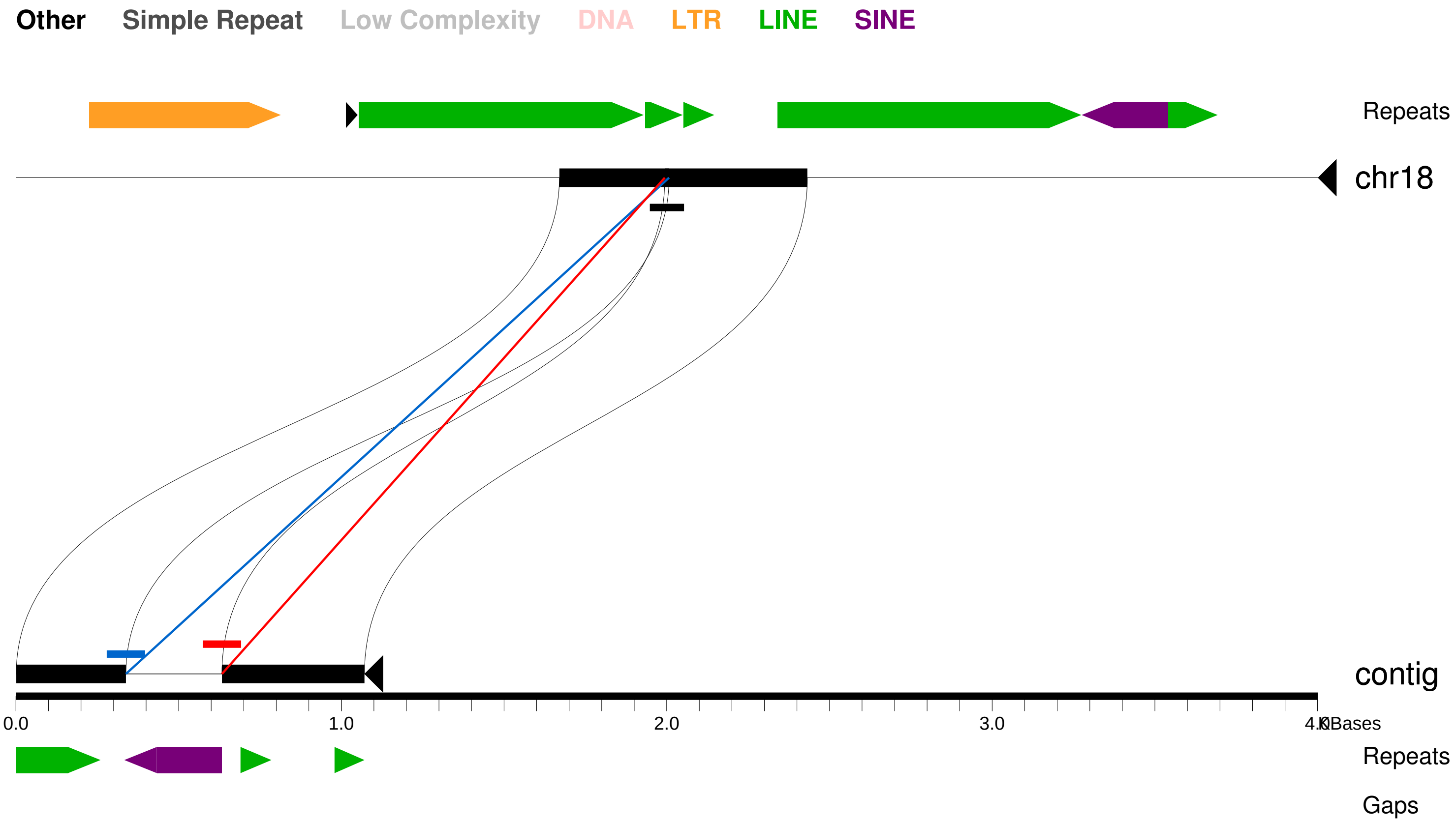

siteID: chr18_44641860 Scafftig4 + Breakpoint: chr18:44641881
chr18:44641835-44641940
Overlap Length: 14 INS size: 351 Genotyped: No

```
left    CTTATTAACTTTTAATGACTTGCTGTCTTCTAATGAATTCACAAAG-------------AACTGCTGGGA
chrom   CTTATTAACTTTTAATGACTTGCTGTCTTCTAATGAATTCACAAAG-------------AACTGCTGGGA
right   NNNNNNNNNNNNNNNNNNNNNNNNNNNNNNNNNNNNNNNNNNNNNNNNNNNNNNNNNNNAACTGCTGGGA
        11111111111111111111111111111111111111111111111111111111111***********

left    ATTGGCCGGGCGCGGTGGCTCACGCCTGTAATCCCAGCACTTTGGGAGGCCGAGGCGGGCGG
chrom   ATT------ACACTCAGATACAC-----TAAAGAAAG--GTGATAGATTATTAAATTTGGGA
right   ATT------ACACTCAGATACAC-----TAAAGAAAG--GTGATAGATTATTAAATTTGGGA
        ***2222222*2*222*222***22222***2222**222*2222**22222*22222*2*2
```

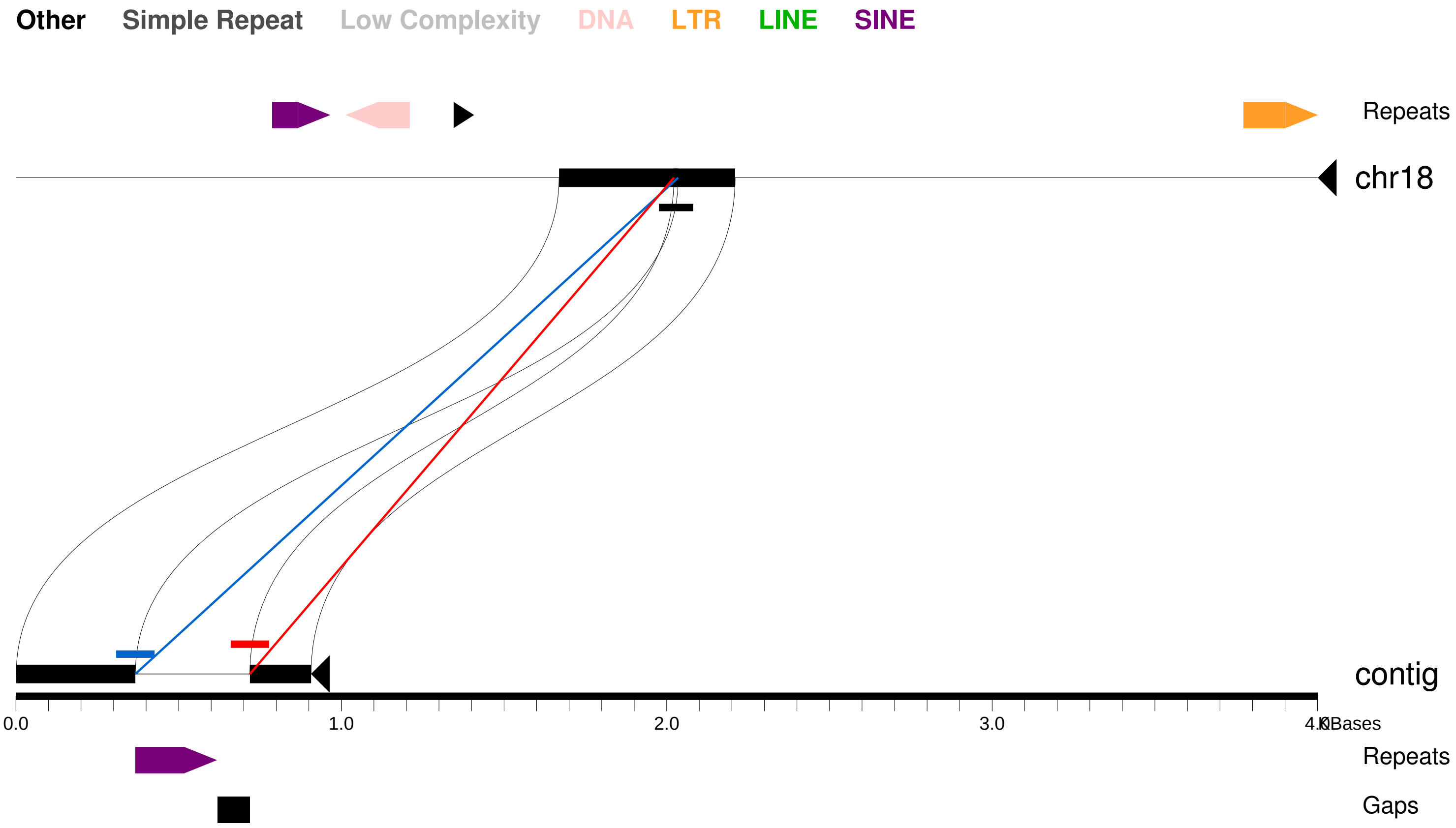

siteID: chr18_45837926 Scafftig1 + Breakpoint: chr18:45837929 chr18:45837928-45837988
Overlap Length: 59 INS size: 402 Genotyped: No

```
left   G----------------------------------------------------------GACAGGACAGT
chrom  G----------------------------------------------------------GACAGGACAGT
right  CCGCCCGCCTCGGCCTCCCAAAGTGCTGGGATTACAGGCGTGAGCCACCGCGCCCGGCCGACAGGACAGT
       11111111111111111111111111111111111111111111111111111111111***********

left   CTTGATACAACAGATGAGAAAACTGAGGTTCAGAGAGGTAAAGCAATGNNNNNNNNNNNNNNNNNNNNNN
chrom  CTTGATACAACAGATGAGAAAACTGAGGTTCAGAGAGGTAAAGCAATGT---------------------
right  CTTGATACAACAGATGAGAAAACTGAGGTTCAGAGAGGTAAAGCAATGT---------------------
       ************************************************2222222222222222222222

left   NNNNNNNNNNNNNNNNNNNNNNNNNNNNNNNNNNNNN
chrom  -------------------------------------
right  -------------------------------------
       2222222222222222222222222222222222222
```

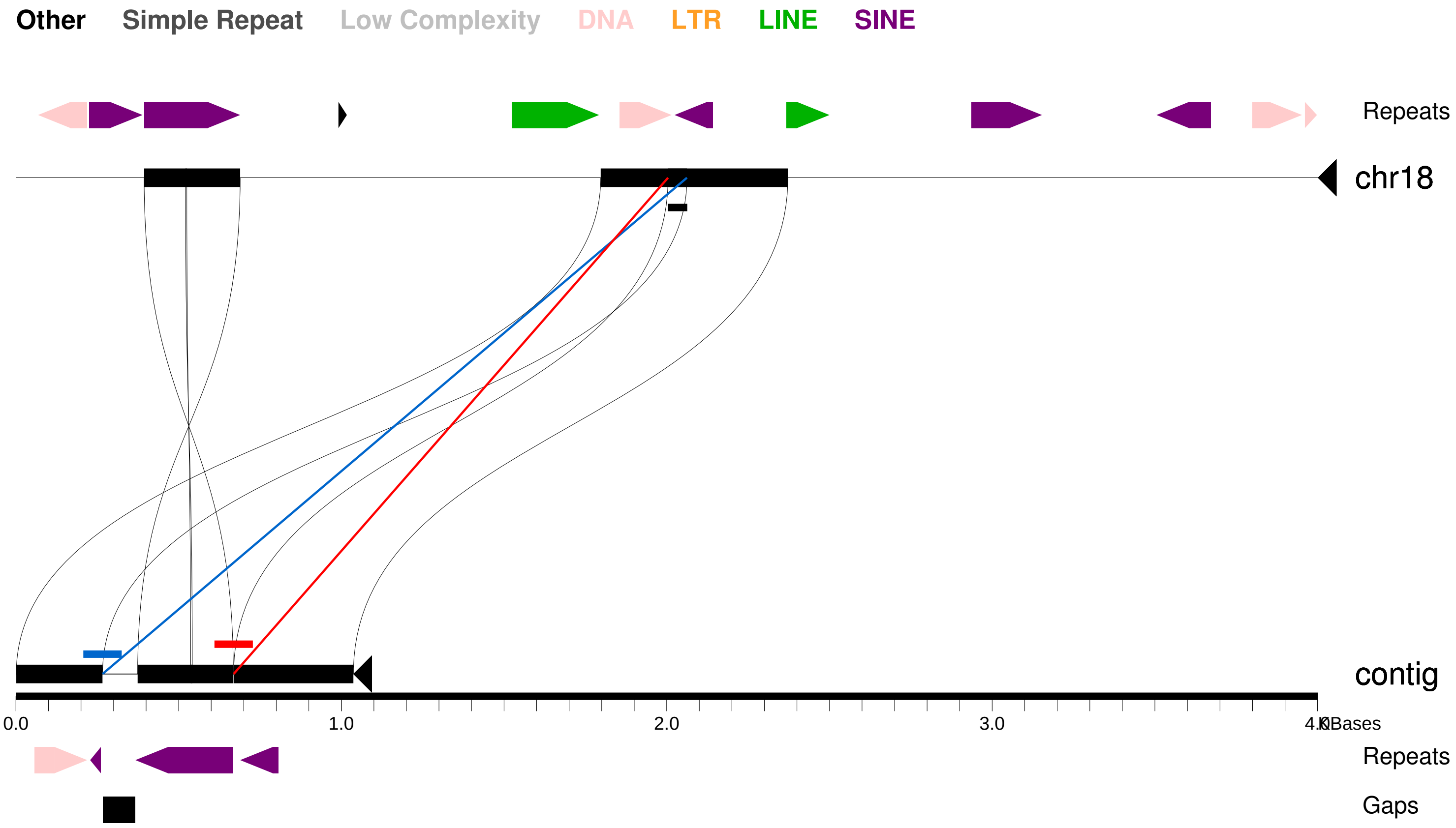

siteID: chr18_46523831 Scafftig3 + Breakpoint: chr18:46523840
chr18:46523680-46524006
Overlap Length: 7 INS size: 108 Genotyped: Yes

```
left    T-----TCACCTCCCAGGCACGTTTTCAAAACTAATCATAGGTCAATTCCCCTTCTTGCCAGCTCCAGGG
chrom   TGTCTTTCACCTCCCAGGCACGTTTTCAAAACTAATCATAGGTCAATTCCCCTTCTTGCCAGCTCCAGGG
right   AGAAA----------AGGGACG-----------AAGCGGAG-------------CTTGC-------AGTG
        12NNN 111111111***1***11111111111**1*11**1111111111111*****1111111**1*

left    ACCTGCAGAGATGGGAGGCGCTGCTGAAAACCCTCTAA---GGGGGGCAGCGCCGCCTTACCTCTCAGAG
chrom   ACCTGCAGAGATGGGAGGCGCTGCTGAAAACCCTCTAA---GGGGGGCAGCGCCGCCTTACCTCTCAGAG
right   A---GCCGAGATTGC-GCCACTGCAGTCCGCAGTCCGACCTGGGCGACAGAGCGAGACTCCATCTCAAAA
        *111**1*****1*11*1*1****1*1111*11**11*111***1*1***1**11111*1*1*****1*1

left    CTGCATTTCCCTCTCTTAGAAAA---GGGACGAA----GCGGAGCTTGCAGTGAGCCGAGATTGCGCCAC
chrom   CTGCATTTCCCTCTCTTAGAAAA---GGGACGAGCTAGGTGGATTTTAAAGTTC-CTGCCAGTTC-CCAA
right   AAAAAAAAAAAAAAAAAAAAAAAAAAGGGACGAGCTAGGTGGATTTTAAAGTTC-CTGCCAGTTC-CCAA
        1111*111111111111*1****111*******22222*2***22**22***222*2*22*2*2*2***2

left    TGCAGTCCGCAGTCCG------ACCTGGGCGA-----CAGAGC----------GAGACT-CCATCTCAAA
chrom   TTATTTACCCAGAACTGATTTTAACTGAGTGTTTGACCAAAGCTTTGGTCCATGAGCCTTCCATTGCCGG
right   TTATTTACCCAGAACTGATTTTAACTGAGTGTTTGACCAAAGCTTTGGTCCATGAGCCTTCCATTGCCGG
        *2222*2*2***22*2222222*2***2*2*222222**2***2222222222***2**2****22*222

left    A-----------AAAAAAAAAAAAAAAAAAAAAAAAAAGGGACGAGCTA------
chrom   GGTGTTTATTTTACATAAAATTTATCTGGATACTCACACGTAGAAGTTTTTCTTT
right   GGTGTTTATTTTACATAAAATTTATCTGGATACTCACACGTAGAAGTT-------
        222222222222*2*2****222*22222*2*222*2*2*2*22**2*N
```

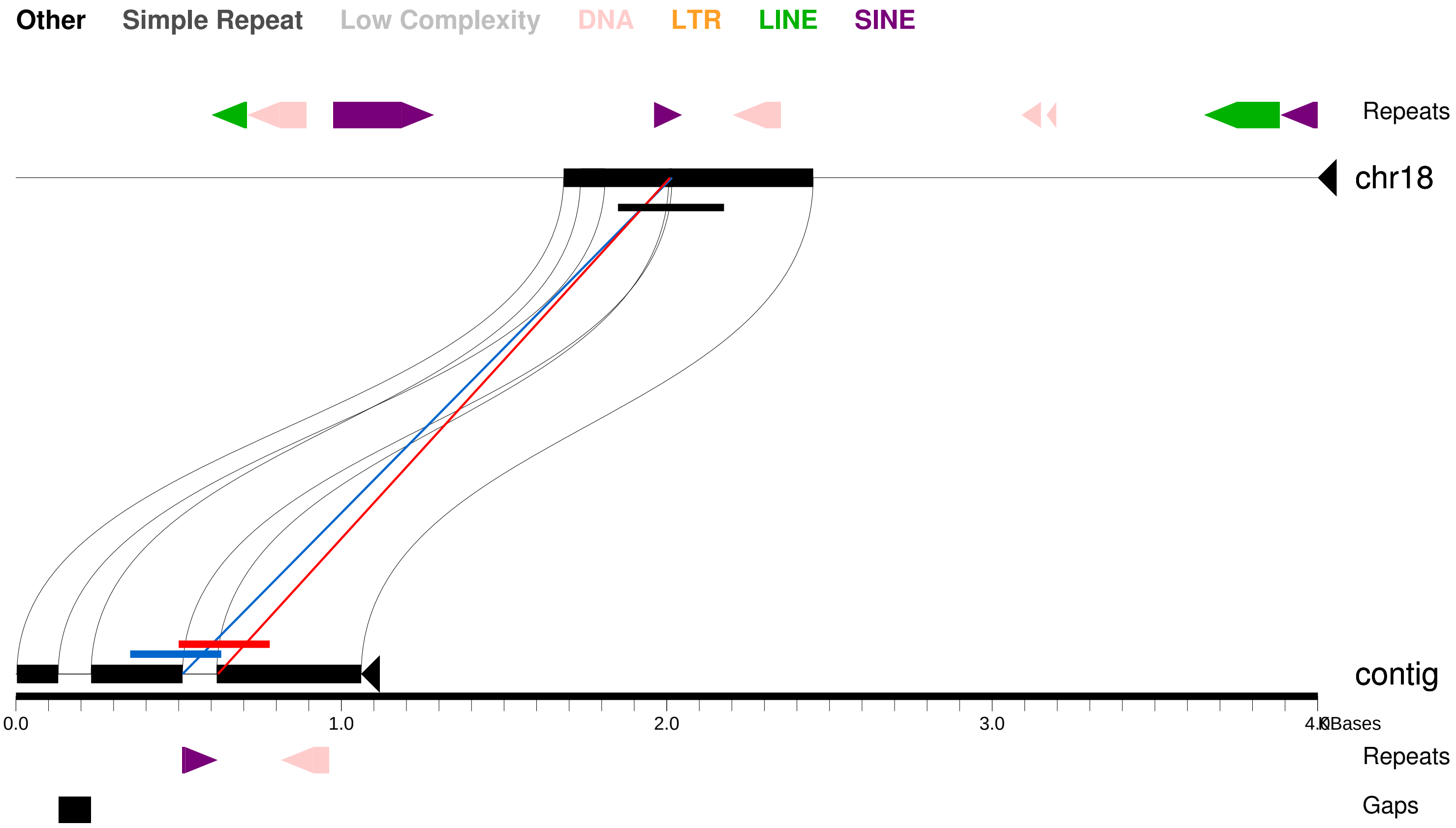

siteID: chr18_49795421 Scafftig7 - Breakpoint: chr18:49795408
chr18:49795355-49795467
Overlap Length: 7 INS size: 288 Genotyped: Yes

```
left   CATGAGATAAAGTTAG-----CAGTGCTGTTATTA---TTCACAATGTCATAGCTCCTTAGAACTTGTGG
chrom  CATGAGATAAAGTTAG-----CAGTGCTGTTATTA---TTCACAATGTCATAGCTCCTTAGAACTTGTTT
right  C-TGCACTCCAGCCTGGGCGACAGAGC-GAGACTCCGTCTCAAAAAAAAAAAAAAAAAAAAACTTGTTT
       *1**111*11**111*11111***1**1*11*1*11111***1**1111*1*1111111*1*******22

left   CTCACGCCTGTAATCCCAGCACTTTGGGAGGCCGAGGCGGGCGGATCACGAGGTCAG
chrom  TATTGAAACAAAATACATGTTCTTATTTTG--CATTTC---CCTACTACCAT-TCAC
right  TATTGAAACAAAATACATGTTCTTATTTTG--CATTTC---CCTACTACCAT-TCAC
       22222222222***2*22*22***22222*22*2222*222*22*22**2*22***2
```

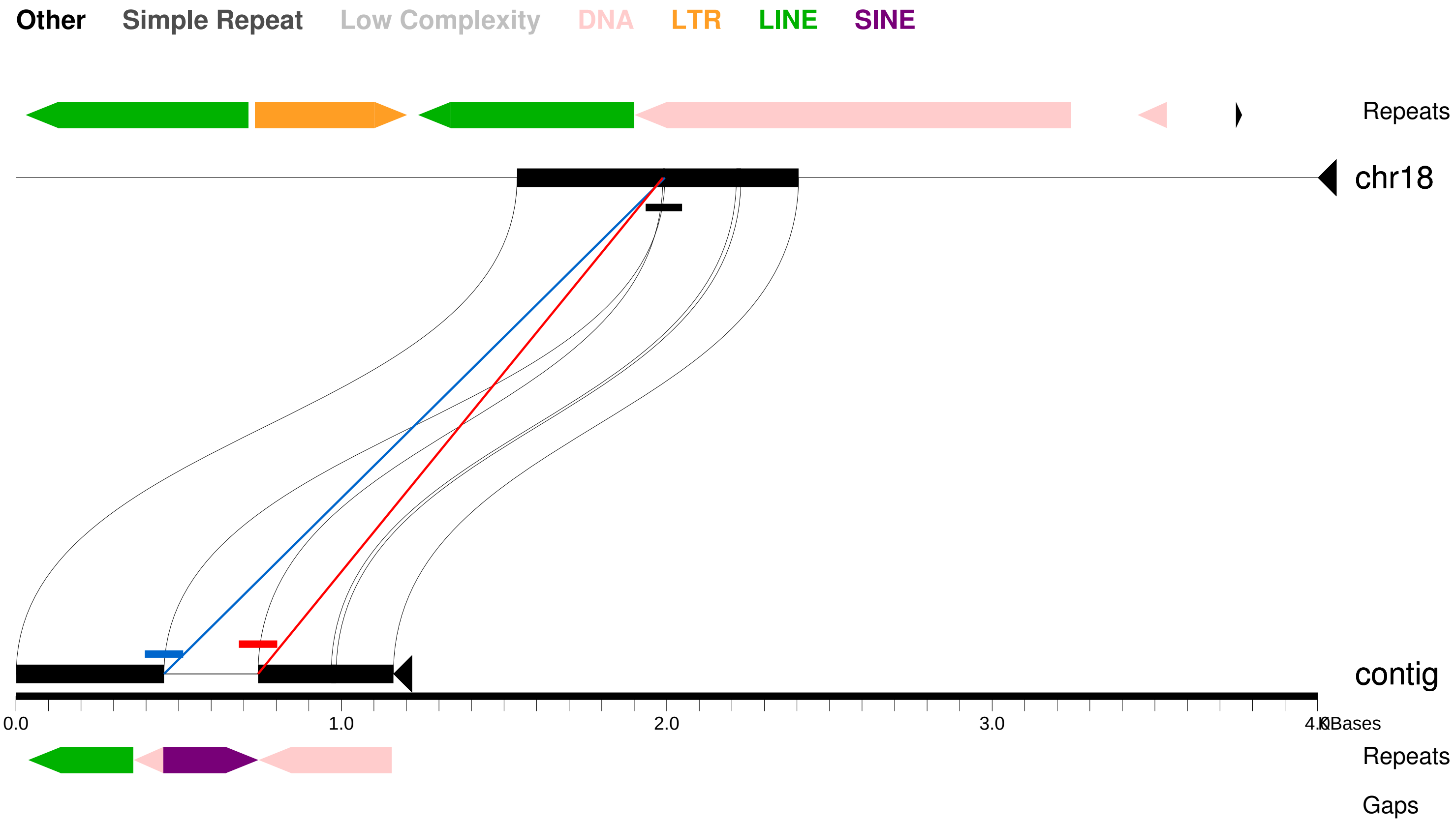

siteID: chr18_50452272 Scafftig1 - Breakpoint: chr18:50452229
chr18:50452070-50452428
Overlap Length: 40 INS size: 252 Genotyped: No

```
left   AT--------------------------------------GTTATTTGAGATGACAGGGTTTCTGACATT
chrom  ATTGTACAAAATATTTATCCTTGAGGGGAAAAAGGCAGATGTTATTTGAGATGACAGGGTTTCTGACATT
right  NNNNNNNNNNN-----------------------------------------------------------
       11NNNNNNNNN                            111111111111111111111111111111

left   AAATTCAGTATGTTGGTAAGATTTTTTTATTAACATGGGAATACTGTGAGTGTTGAAAGTGAACCATTAA
chrom  AAATTCAGTATGTTGGTAAGATTTTTTTATTAACATGGGAATACTGTGAGTGTTGAAAGTGAACCATTAA
right  ----------------------------------------------------------------------
       1111111111111111111111111111111111111111111111111111111111111111111111

left   AATAAAGTATATGTAAGGAAAAGTTGCATAGCAAGAATATAATTTAAAGAGATTTAAGG-GCCGGGCGCG
chrom  AATAAAGTATATGTAAGGAAAAGTTGCATAGCAAGAATATAATTTAAACAGATTTAAGGAGGATTGAGGG
right  -------------------AAAGTTGCATAGCAAGAATATAATTTAAAGAGATTTAAGGAGGATTGAGGG
       1111111111111111111*****************************N**********2*2222*2*2*

left   GT--------------------GGCTCACGCCTGT---AATCCCAGCA-------CTTTGGGAGGCCGAG
chrom  GTTCATTATATTCCAAAAGGCAGGGTTACCCATGTGTCATTGCAAACAAATAATTCTTCTACATGGC-AG
right  GTGCATTATATTCCAAAAGGCAGGGTTCCCCATGTGTCATTGCAAACAAATAATTCTTCTACATGGC-AG
       **N2222222222222222222**2*21*2*2***222*2*2*2*2**2222222***2222*2*2*2**

left   GCGGGCGGATCACGAGGTCAGGAGATCGAGACCATCCCGGCTAA-----AACGGTGAAACCCCGATTCT-
chrom  TTATTCAAACAAACACATATTTTTATC-ACTTACTCCAGATTTATTTTAACCAGTTTTATTTGAATTATG
right  TTATTCAAACAAACA-------------------------------------------------------
       22222*22*22*22*NN1NNNNNN11121NNNNN111N1NN1N1     1N1N11NNN1NNNNN111N1

left   -ACT----AAA
chrom  TAGTTTGGAGA
right  -----------
        1N1    1N1
```

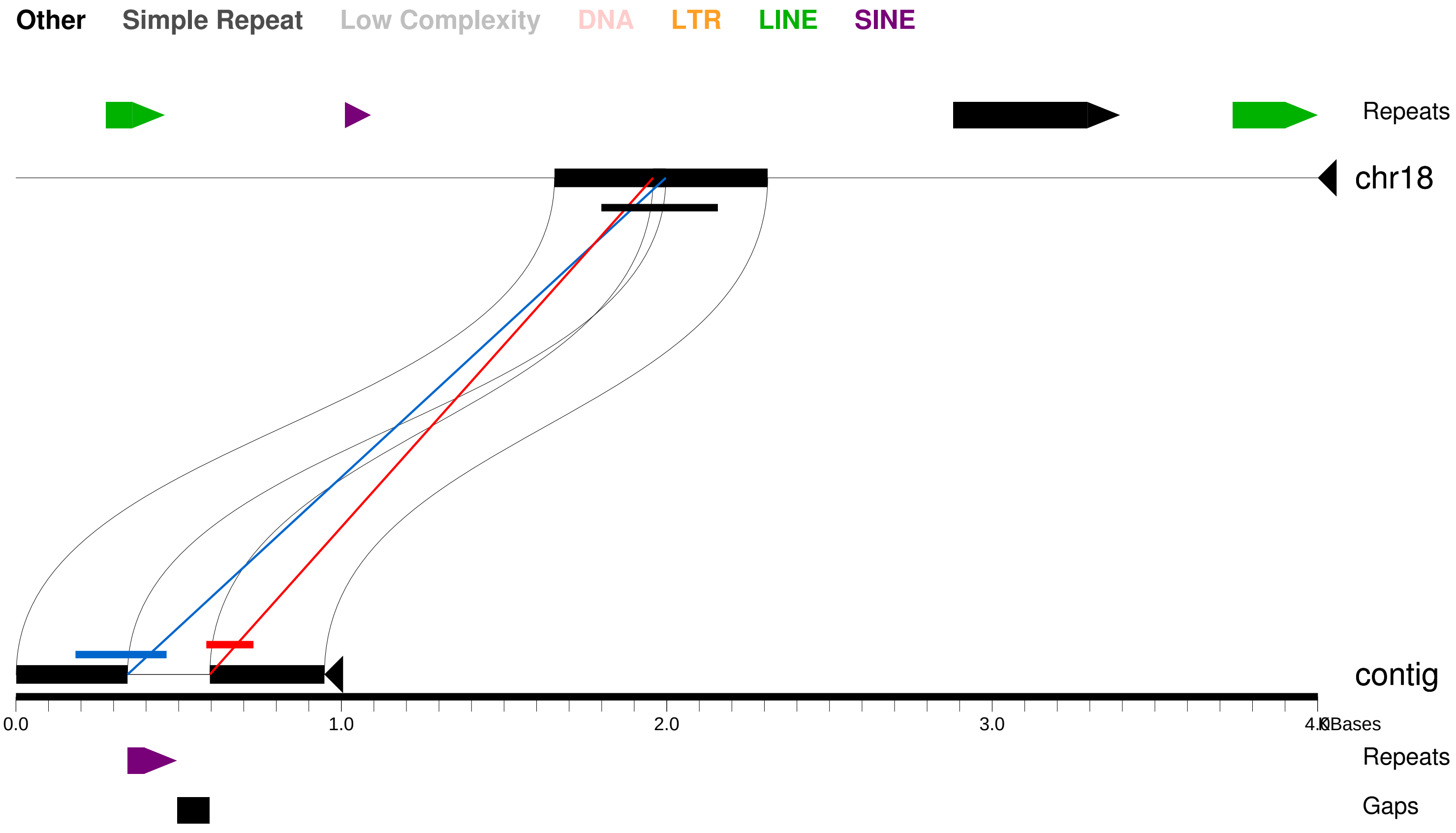

siteID: chr18_51584000 Scafftig3 + Breakpoint: chr18:51584008
chr18:51583954-51584064
Overlap Length: 6 INS size: 295 Genotyped: No

```
left   AAAGC--AAGATGTGTAAGGAA-TGCTCTGATTTCA------ATATTTCATTCCATGCATTTGCTCCTCN
chrom  AAAGC--ACGATGTGTAAGGAA-TGCTCTGATTTCA------AGATTTCATTCCATGCATTTTCTCCTCC
right  CCACCCGCCTCGGCCTCCCAAAGTGCTGGGATTACAGGCGTGAGCCACCGCGCCCGGCCTTG-CTCCTCC
       11*1*1112111*11*1111**1****11****1**111111*21111*111**11**1**1N******2

left   NNNNNNNNNNNNNNNNNNNNNNNNNNNNNNNNNNNNNNNNNNNNNNNNNNNNNNNNNN
chrom  TTCTATTCCTTGTTCCCTCTTTCATGACACCTAGATCTCCTTTGGTATTT--------
right  TTCTATTCCTTGTTCCCTCTTTCATGACACCTAGATCTCCTTTGGTATTT--------
       2222222222222222222222222222222222222222222222222222222222
```

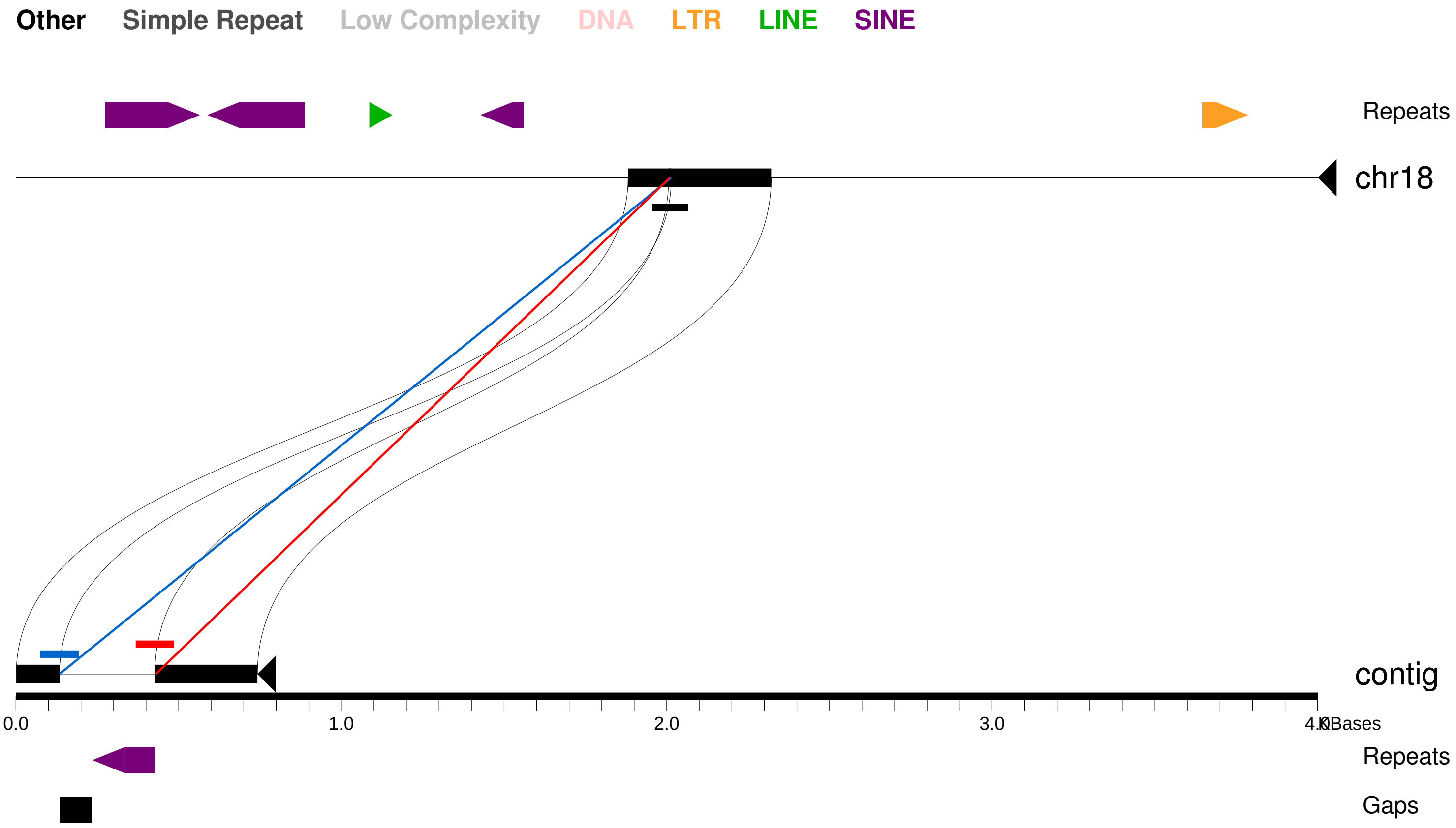

siteID: chr18_53146062 Scafftig1 - Breakpoint: chr18:53146062
chr18:53146016-53146121
Overlap Length: 14 INS size: 322 Genotyped: Yes

```
left     AAC-----CAGAGAAAGTCCACAGTTCTGTGGCATAAGGAGATTTAAACTG--------CAACAAACAGT
chrom    AAC-----CAGAGAAAGTCCACAGTTCTGTGGCATAAGGAGATTTAAACTG--------CAACAAACAGT
right    TCCGCCCGCCTCGGCCTCCCAAAGTGCTGGGATTACAGGCGTGAGCCACCGCGCCCGGCCAACAAACAGT
         11*11111*111*11111***1***1***1*11111***1*111111**1*11111111***********

left     TTTTTTTTTTTTTTTTTTTTTTTTTTTTTTTTTTTTTTTTTTTTTTTTGAGACGGAGTCTCGCTC
chrom    TTTAAAATTAAAAATGTTCAAACTTTAAGGTGAAAATTATAAATGTTAA-------------
right    TTTAAAATTAAAAATGTTCAAACTTTAAGGTGAAAATTATAAATGTTAA-------------
         ***2222**22222*2**22222***2222*22222**2*222*2222*2222222222222
```

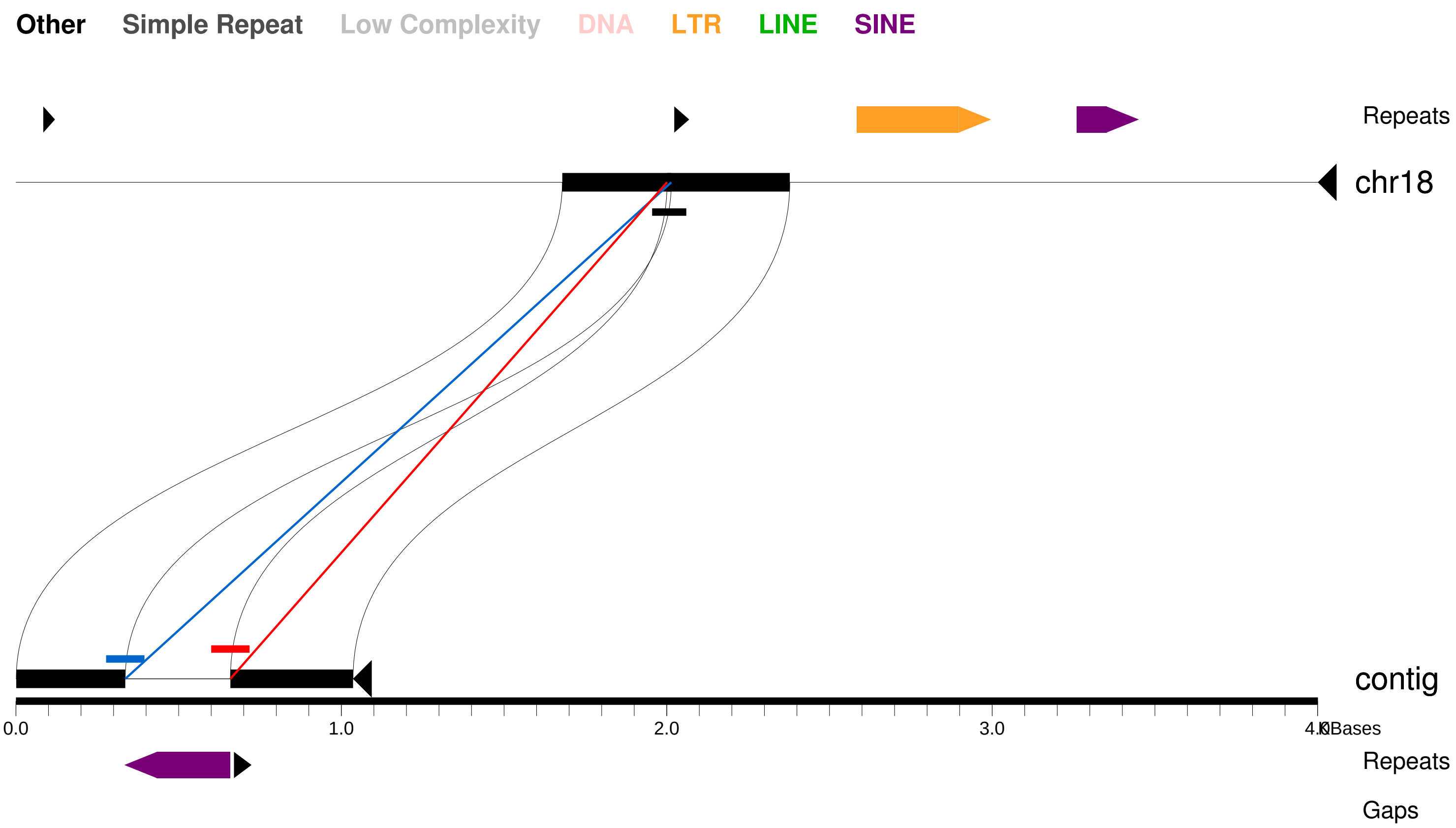

siteID: chr18_5411684 Scafftig1 + Breakpoint: chr18:5411652
chr18:5411606-5411711
Overlap Length: 14 INS size: 296 Genotyped: Yes

```
left     ACA--------GATATC---AACTAAT--CATTAATAAAGTTCCCTTGATCAAATATCTGTGATTAATAT
chrom    ACA--------GATATC---AACTAAT--CATTAATAAAGTTCCCTTGATCAAATATCTGTGATTAATAT
right    CCGCCCGCCTCGGCCTCCCAAAGTGCTGGGATTACAGGCGTGAGCCACCGCGCCCGGCCGTGATTAATAT
         1*111111111*111**111**1*11*111****11111**111*11111*111111*1***********

left     TTTTTTTTTTTTTTTTTTGAGACGGAGTCTCGCTCTGTCGCCCAGGCTGGAGTGCAGTGGCG
chrom    TTTGAAAACAAAAA---TGAAAATGGGAGGCGA------GTCTATGATGG--TGAATCTG--
right    TTTGAAAACAAAAA---TGAAAATGGGAGGCGA------GTCTATGATGG--TGAATCTG--
         ***22222222222222***2*22*2*222**2222222*2*2*2*2***22**2*222*22
```

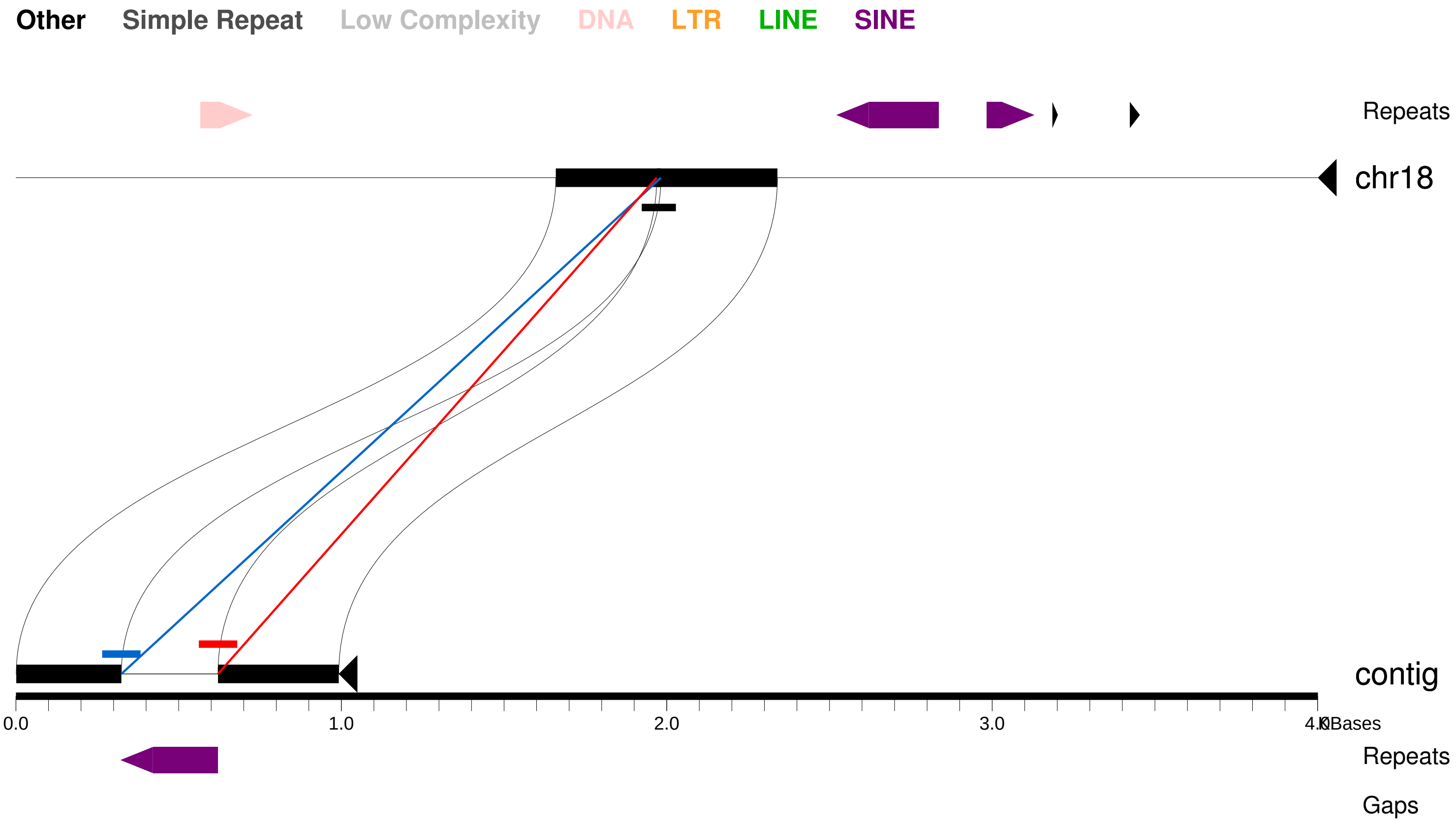

siteID: chr18_54706177 Scafftig11 + Breakpoint: chr18:54706181
chr18:54706021-54706353
Overlap Length: 13 INS size: 306 Genotyped: Yes

```
left     T-------------CCAGCTCTAGTCCACAGCCTTCCTCCCCAGGACATGTGATTCAAGCCTGAAGCATA
chrom    TTTTTTTTCCTCTTCCAGCTCTAGTCCACAGCCTTCCTCCCCAGGACATGTGATTCAAGCCTGAAGCATA
right    -----------------------AGGAGAATGGCGTGAACCCGGGAAGCGGAGCTTGCAG--TGA-GCCGA
         1             11111111**111*11*1*1*111***11*1*111*1*1**11**11***1**11*

left     CAATGGGAGCACAAAGGAGGAGGGGACAGAGAAAAGAAATGAGCATCAGCCTTGGGAATGTCTCC-TCTG
chrom    CAATGGGAGCACAAAGGAGGAGGGGACAGAGAAAAGAAATGAGCATCAGCCTTGGGAATGTCTCC-TCTG
right    GATTGCGC---CACTGCAGTCCG---CAGTCC---GGCCTGGGCGACAGA---GCGA--GACTCCGTCTC
         1*1**1*1111**11*1**111*111***111111*111**1**11***1111*1**11*1****1***1

left     ACTGTAGAGTCAGGAGACATTAAGATGAACATTCGGCCGGGCGC---------GGTGGCTCACGCCTG--
chrom    ACTGTAGAGTCAGGAGACATTAAGATAAACATTCACTGGAACATATACATTCTGGTAGATTCCGA-TGAG
right    AAAAAAAA---AAAAAAAAAAAAGATGAACATTCACTGGAACATATACATTCTGGTAGATTCCGA-TGAG
         *1111*1*111*11*1*1*11*****N*******2222*22*22222222222***2*2*22**22**22

left     ---TAATCCCAGCACTT----TGGGAGGC--CGAG-GCGGGTGGATCATGAGGTCAGG---AGATCGAG-
chrom    AATTAAGCCCATTCATTCATCTGGTAAATATCTAGTGAACATCTACCATGCA-TCAGGCAGAGAACTAGG
right    AATTAAGCCCATTCATTCATCTGGTAAATATCTAGTGAACATCTACCATGCA-TCAGGCAGAGAACTAGG
         222***2****2222**2222***2*22222*2**2*2222*22*2****222*****222***2*2**2

left     -------ACCATCCTGGCTAA--CAAG----GTGAAACC---CCGTCTCTACTAAA
chrom    TGCTGGGATTGTAGTGGGGAAGGCAACAGTTGTGACACCTGCCCTTATGGCTCAC
right    TGCTGGGATTGTAGTGGGGAAGGCAACAGTTGTGACACCTGCCCT-----------
         2222222*222*22***22**22***22222****2***222**21N1NNN11N1N
```

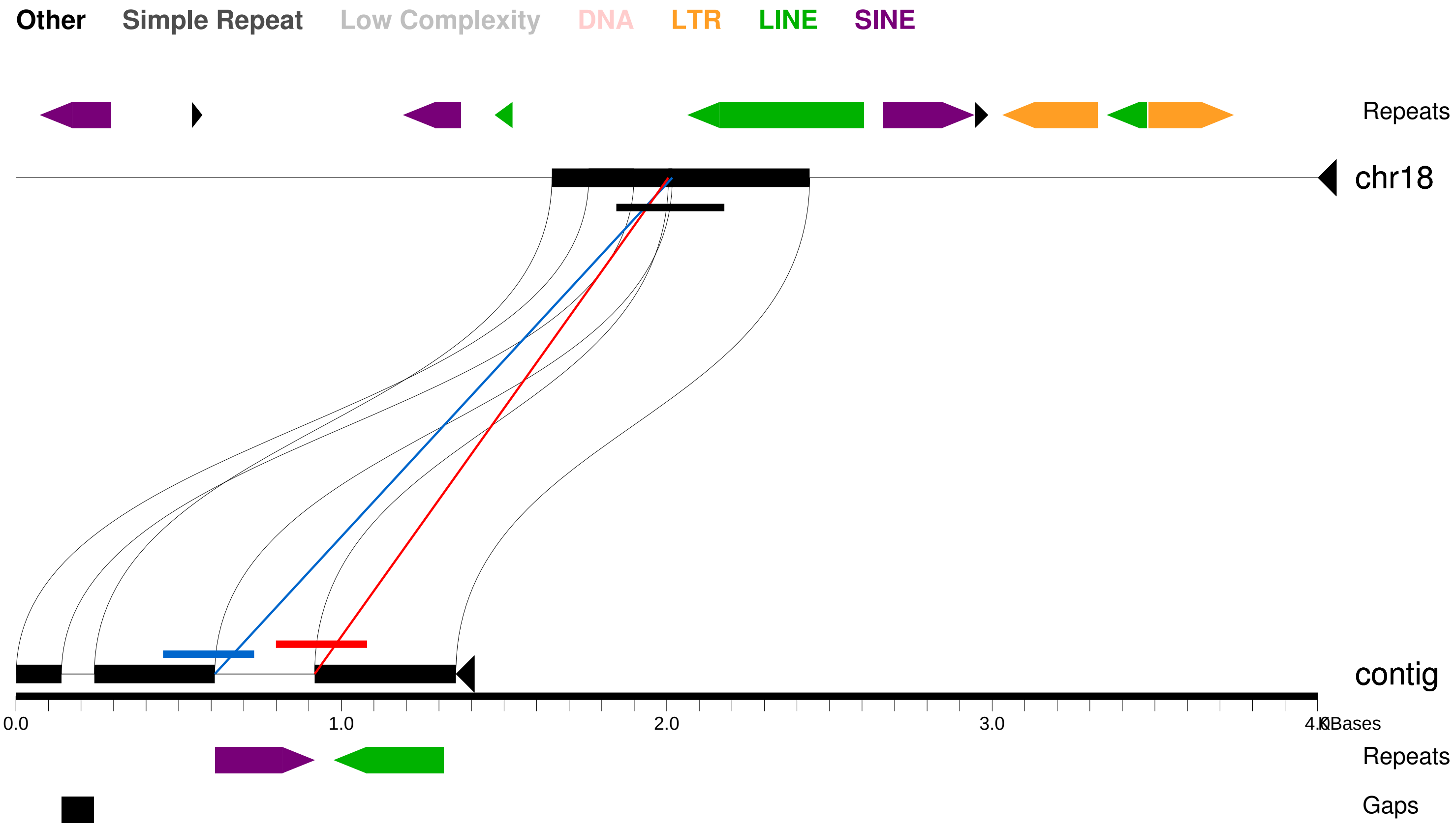

siteID: chr18_57150109 Scafftig1 + Breakpoint: chr18:57150108
chr18:57150079-57150167
Overlap Length: 14 INS size: 301 Genotyped: Yes

```
left    T-------------ACCCCAAGATATTTTGACTATAAAGGTT-----------------AAATTACCGTT
chrom   T-------------ACCCCAAGATATTTTGACTATAAAGGTT-----------------AAATTACCGTT
right   CCGCCCGCCTCGGCCTCCCAAAGTGCTGGGATTACAGGCGTGAGCCACCGCGCCCGGCCAAATTACCGTT
        1111111111111111*****11*11*11**1**1*111**1111111111111111111***********

left    TTTTTTTTTTTTTTTTTTTTTGAGACGGAGTCTCGCTCTGTCGCCCAGGCTGGAGTGCAGTGGCGGGATC
chrom   TTTAAGAAGTATTTTTTTTTTGTAA------------------------CT--ATTAAAGACATAAGATT
right   TTTAAGAAGTATTTTTTTTTTGTAA------------------------CT--ATTAAAGACATAAGATT
        ***222222*2*********22*22222222222222222222222222**22*2*22**222222***2

left    TCGGCTCAC
chrom   CAAGGT---
right   CAAGGT---
        222*2*222
```

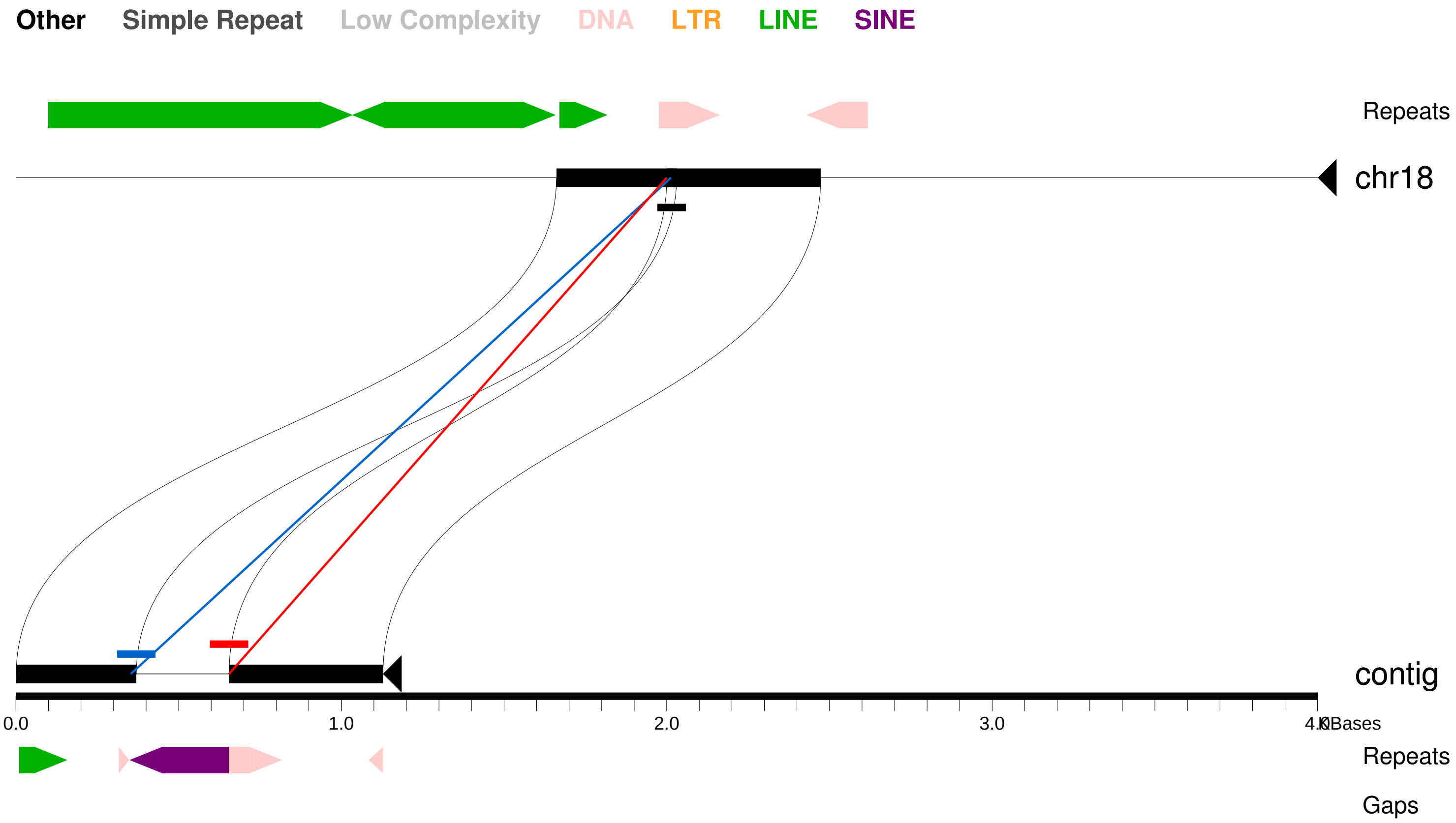

siteID: chr18_57372774 Scafftig11 + Breakpoint: chr18:57372759
chr18:57372712-57372818
Overlap Length: 13 INS size: 302 Genotyped: Yes

```
left   ------GACTA---CTCACAGAGAGC----ATTTCTGCAATCA-CCACAAGGAAATACCTAGGCAAAATG
chrom  ------GACTA---CTCACAGAGAGC----ATTTCTGCAATCA-CCACAAGGAAATACCTAGGCAAAATG
right  CCGCCCGCCTCGGCCTCCCAAAGTGCTGGGATTACAGGCGTGAGCCACCGCGCCCGTCC--GGCAAAATG
       111111*1**1111***1**1**1**1111***1*1*111*1*1****111*11111**11*********

left   TTTTTTTTTTTTTTTTTTTTTTTTTGAGACGGAGTCTCGCTCTGTCGCCCAGGCTGGAGTGCA
chrom  TTTTAAAAACTTCAGAGTA------CAG-CATAGTC---CTGTGGAACCTACTTCTAATTG--
right  TTTTAAAAACTTCAGAGTA------CAG-CATAGTC---CTGTGGAACCTACTTCTAATTG--
       ****222222**22222*22222222**2*22****222**2**222**2*222222*2**22
```

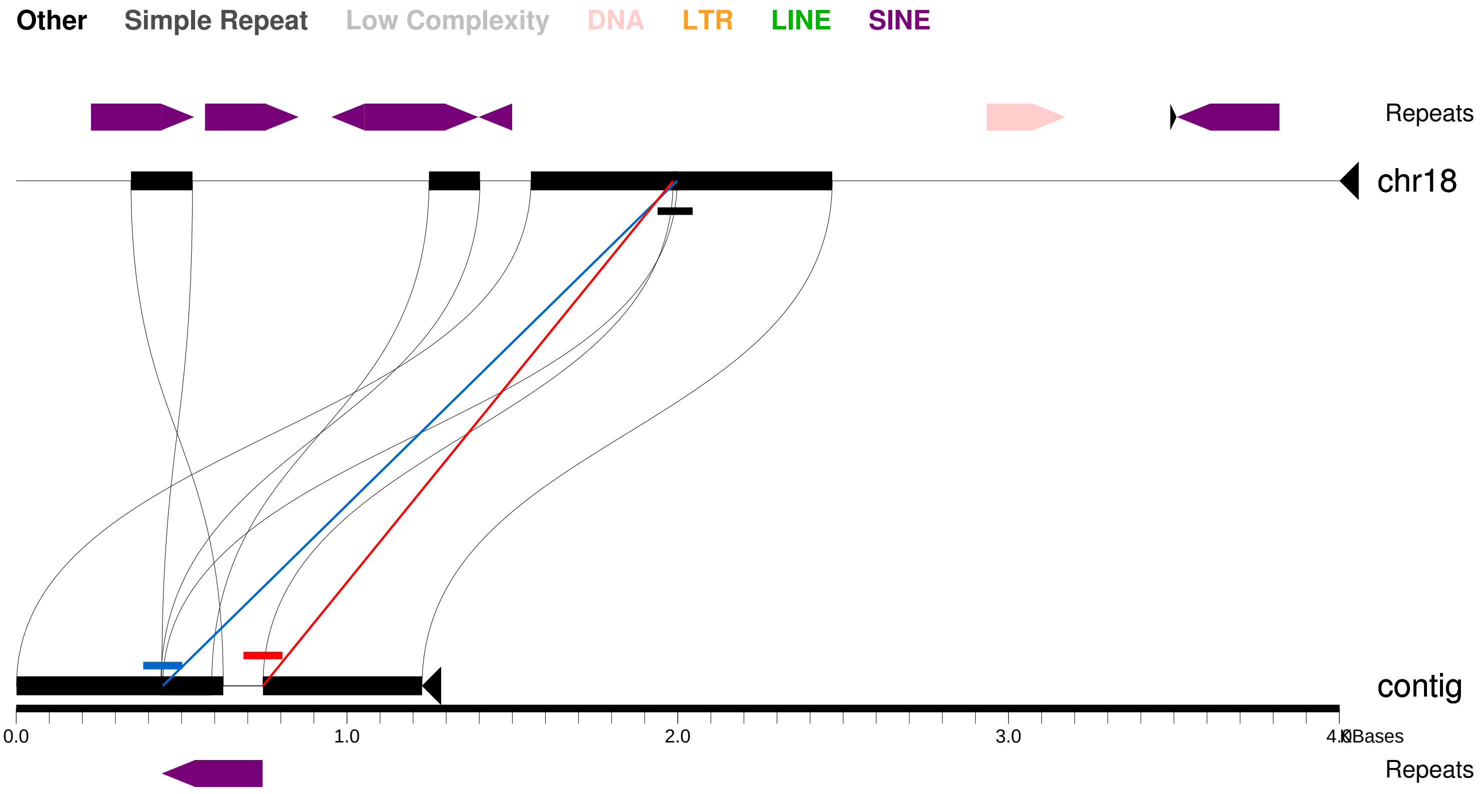

siteID: chr18_58264581 Scafftig1 - Breakpoint: chr18:58264751
chr18:58264721-58264810
Overlap Length: 30 INS size: 195 Genotyped: No

```
left   AGAAACATTTATTAATAGTTTAAAAAATAC-----------------------------ATATGGTTTGA
chrom  AGAAACATTTATTAATAGTTTAAAAAATAC-----------------------------ATATGGTTTGA
right  NNNNNNNNNNNNNNNNNNNNNNNNNNNNNNNNNNNNNNNNNNNNNNNNNNNNNNNNNNNATATGGTTTGA
       11111111111111111111111111111111111111111111111111111111111***********

left   TAGAAATAAGACCTAGTGTGACCATCCTG-GCTAACAAGGTGAAACCCCGTCTCTACTAAAAATACAAAA
chrom  TAGAAATAAGACCTAGTGTT-------TGAGTTTACAA--TAA---------TCTATTTTATATC-----
right  TAGAAATAAGACCTAGTGTT-------TGAGTTTACAA--TAA---------TCTATTTTATATC-----
       *******************22222222**2*2*2****22*2*222222222****2*22*2**222222

left   AAAAAATTA
chrom  -------TC
right  -------TC
       2222222*2
```

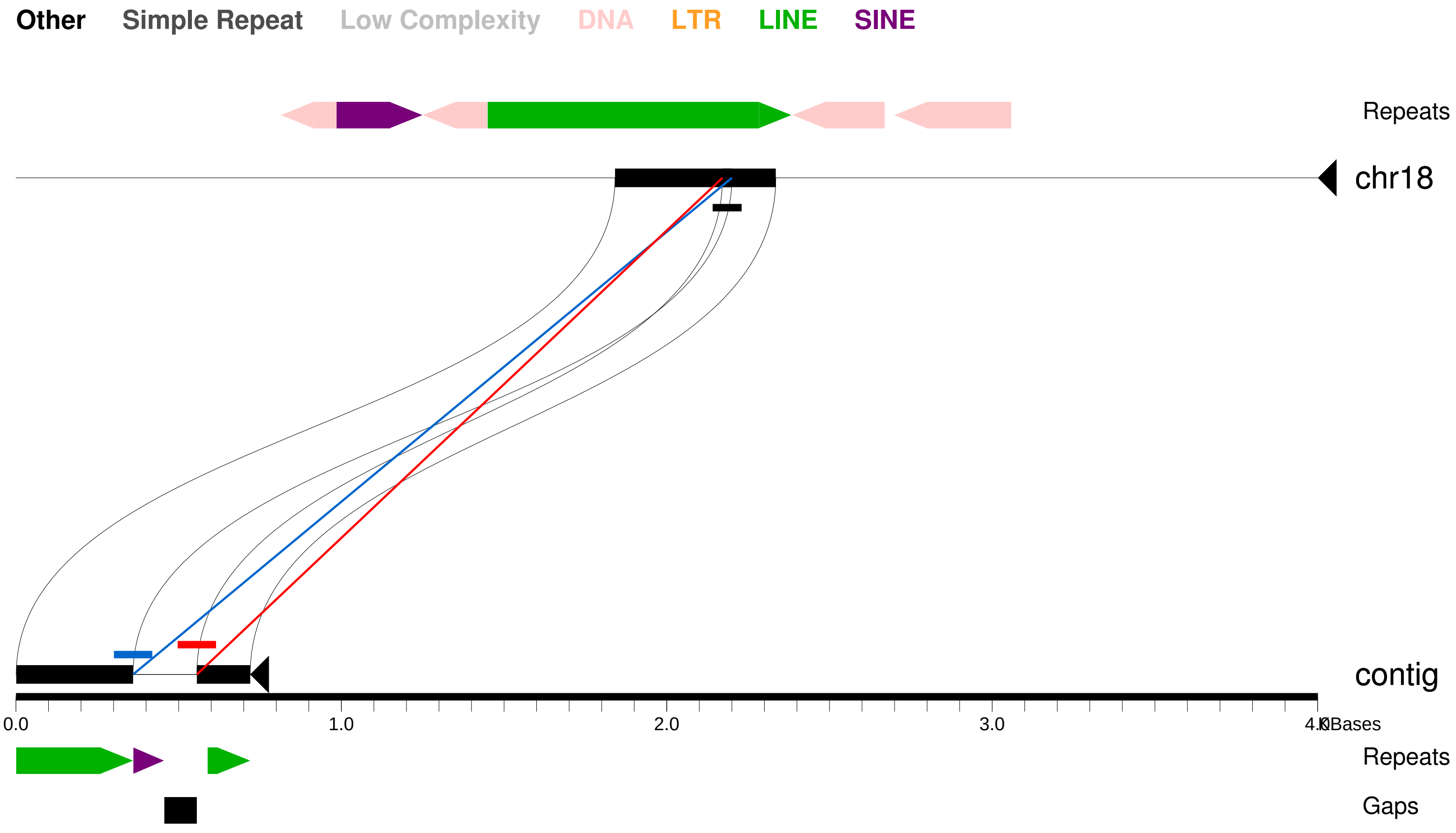

siteID: chr18_58454019 Scafftig2 + Breakpoint: chr18:58453995
chr18:58453951-58454054
Overlap Length: 16 INS size: 465 Genotyped: No

```
left     TACCGTACTTTTTTTATCTACTATGTTCCAAACAACTGCTGATT---------------AAAAGAATAAA
chrom    TACCGTACTTTTTTTATCTACTATGTTCCAAACAACTGCTGATT---------------AAAAGAATAAA
right    NNNNNNNNNNNNNNNNNNNNNNNNNNNNNNNNNNNNNNNNNNNNNNNNNNNNNNNNNAAAAAAGAATAAA
         11111111111111111111111111111111111111111111111111111111111***********

left     ATCTCGGCCGGGCGCGGTGGCTCACGCCTGTAATCCCAGCACTTTGGGAGGCCGAGGCGGGCGG
chrom    ATCTCATCATA----AGAGGGAAA----TTTAATG--AGTTCTCATGAACAATTAAAAA-----
right    ATCTCATCATA----AGAGGGAAA----TTTAATG--AGTTCTCATGAACAATTAAAAA-----
         *****22*22222222*2**222*2222*2****222**22**222*2*22222*222222222
```

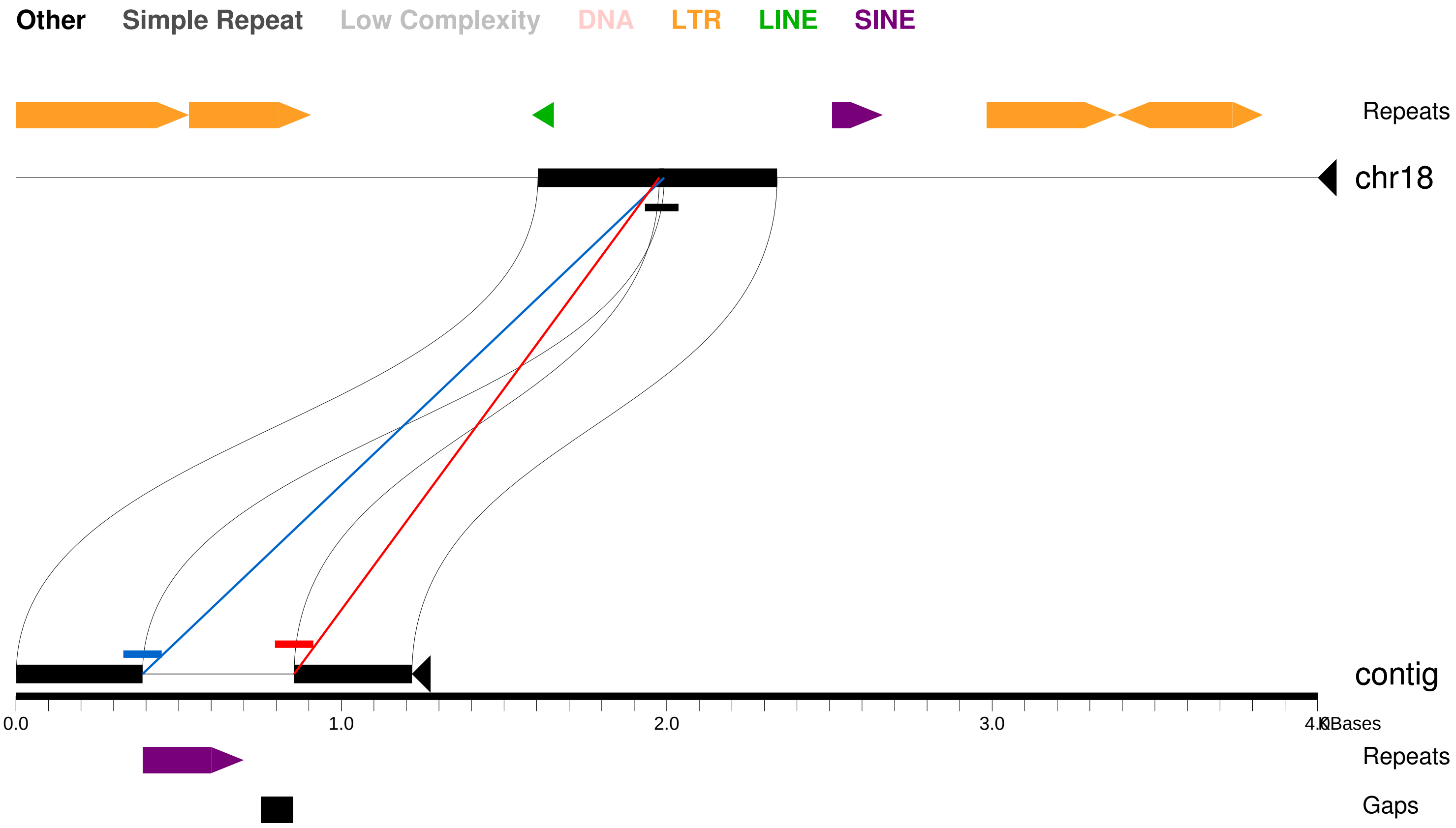

siteID: chr18_58775004 Scafftig4 - Breakpoint: chr18:58774981
chr18:58774937-58775040
Overlap Length: 16 INS size: 322 Genotyped: Yes

```
left    --------AATGAATATAATATTGATAATAAAAGCCA-------ACAAATATTTCATTTAAAAAGACAAC
chrom   --------AATGAATATAATATTGATAATAAAAGCCA-------ACAAATATTTCATTTAAAAAGACAAC
right   GGCGACAGAGCGAGACTCCGTCTCAAAAAAACACACACACAAAAAAAAAAAAAAAAAAAAAAAAAGACAAC
        11111111*11**111*11111*1*1**1**1*11**1111111*1***1*1111*111***********

left    TATAGGCCGGGCGCGGTGGCTCACGCCTGTAATCCCAGCACTTTGGGAGGCCGAGGCGGGTGGA
chrom   TATAGATTAACCTCA----CTTACG-----AATATTAACATTAAAAAAATCCT------GAGTA
right   TATAGATTAACCTCA----CTTACG-----AATATTAACATTAAAAAAATCCT------GAGTA
        *****222222*2*22222**2***22222***222*2**2*22222*22**2222222*2*2*
```

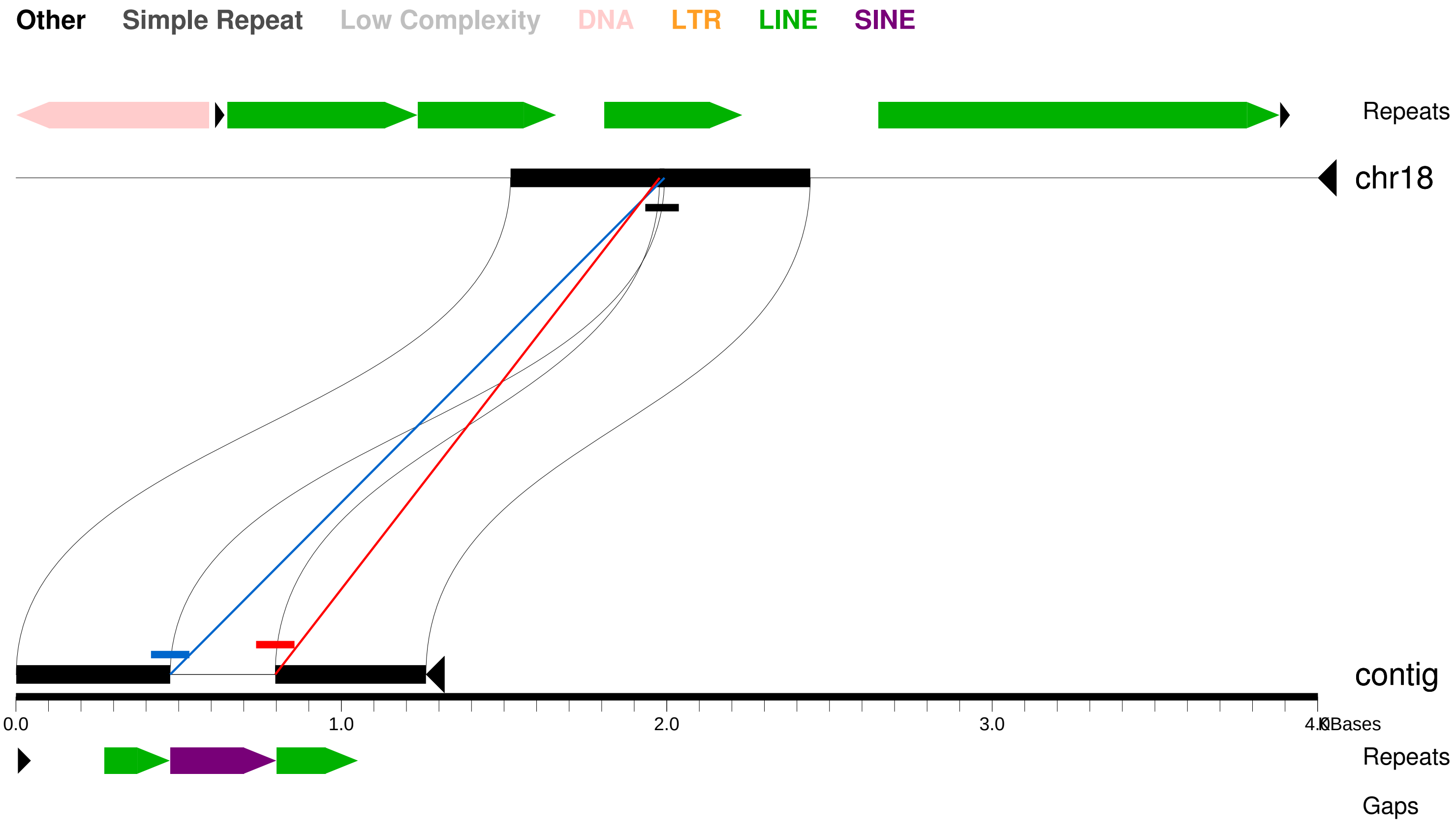

siteID: chr18_59713719 Scafftig8 + Breakpoint: chr18:59713720
chr18:59713675-59713779
Overlap Length: 15 INS size: 298 Genotyped: Yes

```
left     AGAGGAGCA-CGAAC-CCTGTCGTGAACTGTGCTTGCAAGGG--ATCTA------------GGTTGTGCA
chrom    AGAGGAGCA-CGAAC-CCTGTCGTGAACTGTGCTTGCAAGGG--ATCTA------------GGTTGTGCA
right    CCGCCCGCCTCGGCCTCCCAAAGTG--CTGGGATTACAGGCGTCAGCCACCGCGCCCGGCCGGTTGTGCA
         111111**11**11*1**1111***11***1*1**1**1*1*11*1*1*111111111111*********

left     CTTCTTTTTTTTTTTTTTTTTTTGAGACGGAGTCTCGCTCTGTCGCCCAGGCTGGAGTGCAGTGG-
chrom    CTTCTTATGAGAAT------CTAACGAATGCCTGATGATCTG------AGG-TGGAA--CAGTTTC
right    CTTCTTATGAGAAT------CTAACGAATGCCTGATGATCTG------AGG-TGGAA--CAGTTTC
         ******2*22222*2222222*222**22*22*222*2****222222***2****222****222
```

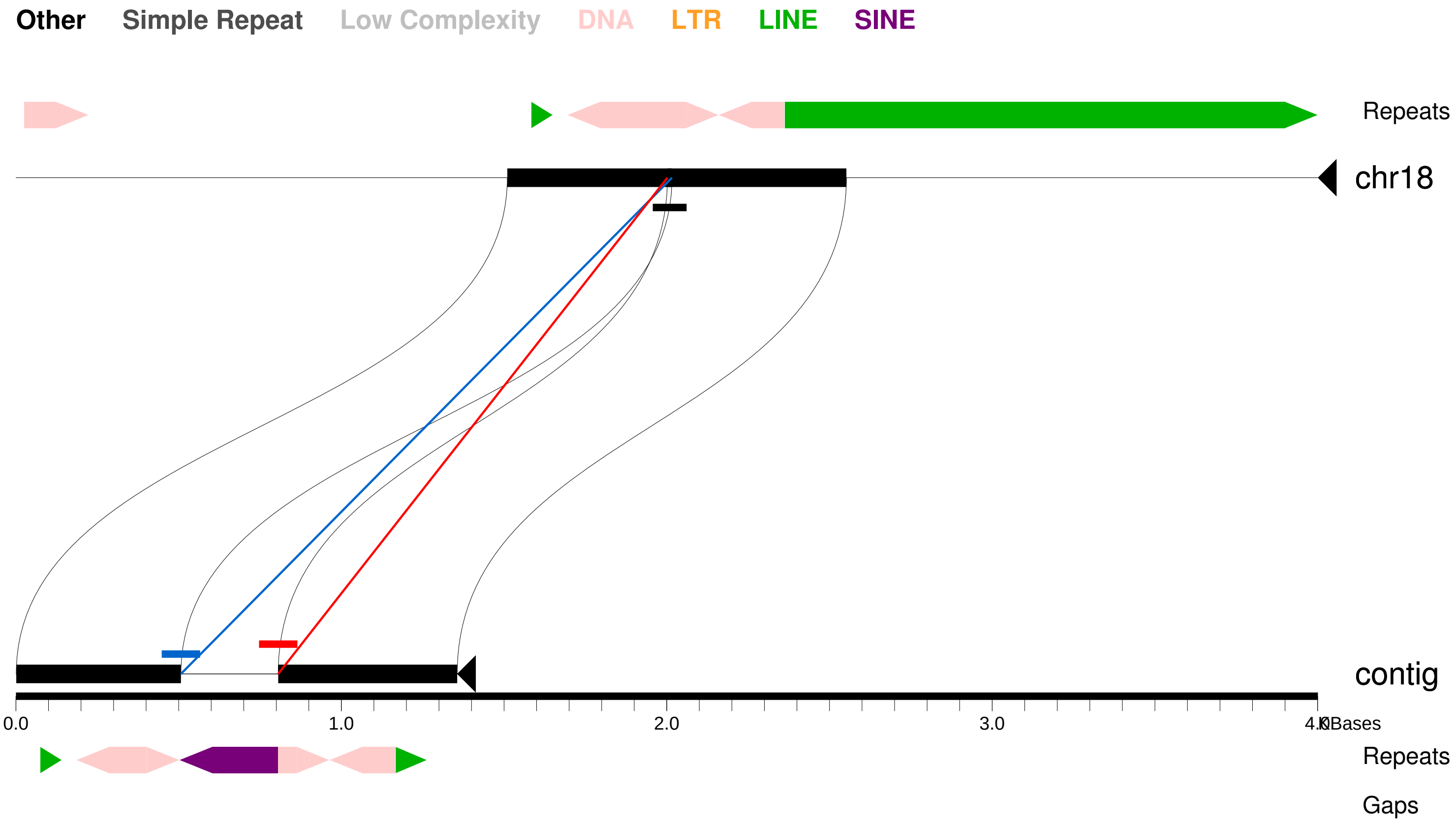

siteID: chr18_62173801 Scafftig6 + Breakpoint: chr18:62173782
chr18:62173622-62173958
Overlap Length: 17 INS size: 304 Genotyped: Yes

```
left    -------------CACTGTACTTACAATTTTTTCCATTTTTATATTTGCCTTATCATTTTTTATTTTGTG
chrom   AAGACATTATTTCCACTGTACTTACAATTTTTTCCATTTTTATATTTGCCTTATCATTTTTTATTTTGTG
right   T-----TTAGTAGAGGCGGGGTTTCA-------CCACGTTA------GCCAGGATGGTCTCGATCTCCTG
        N     222N2NN1111*111**1**1111111***11**1111111***1111111*1*11**1*11**

left    CAAATTCAAGTCTATACATTTTTCCATTTTTATTACTTCTAATATGTTTG--TTTCAGTATAATTTAACT
chrom   CAAATTCAAGTCTATACATTTTTCCATTTTTATTACTTCTAATATGTTTG--TTTCAGTATAATTTAACT
right   --ACCTCATG---ATCCGCCCGCC------TCTGCCTCCCAAAGTGCTGGGATTACAGGCGTGAGCCACC
        11*11***1*111**1*111111*111111*1*11**1*1**11**1*1*11**1***111111111**1

left    ATGCACTTTTTAAAAATTTTTACAAAAGGAATTGCTTTTTTTTTTTTTTT----------TTTTTTTTTG
chrom   ATGCACTTTTTAAAAATTTTTACAAAAGGAATTGCTTTTAATCTTCATATAATAAATAGATGTCTTTATA
right   GCGCCCGGC-------------CAAAAGGAATTGCTTTTAATCTTCATATAATAAATAGATGTCTTTATA
        11**1*1111111111111111****************22*2**22*2*2222222222*2*2***2*2

left    AGAC-GAAGTCTCGCTCTG----------TCGCCCAGGCTGG----AGTGCAGTGGCGCGACCTCGGC--
chrom   ATTCAGAAGCAGAGCATTGCAGACAAAAATCACTCAAAATCGTACAACTACAAAATGATGACCATGAAGA
right   ATTCAGAAGCAGAGCATTGCAGACAAAAATCACTCAAAATCGTACAACTACAAAATGATGACCATGAAGA
        *22*2****2222**22**2222222222**2*2**222*2*2222*2*2**2222222****22*2222

left    --TCACTGCAAGCTCC--------------GCCTCCCGGGTTCACGCCATTCTCCTGCC
chrom   TATTAATACATAATCTTTTTGACAGATATTGCTTCTAATCTTAAAATGATTTATCAAAT
right   TATTAATACATAATCTTTTTGACAGATATTGCTTCTAATCTTA----------------
        22*2*2*2**222**222222222222222**2**22222**21NNNN111NNN1NNNN
```

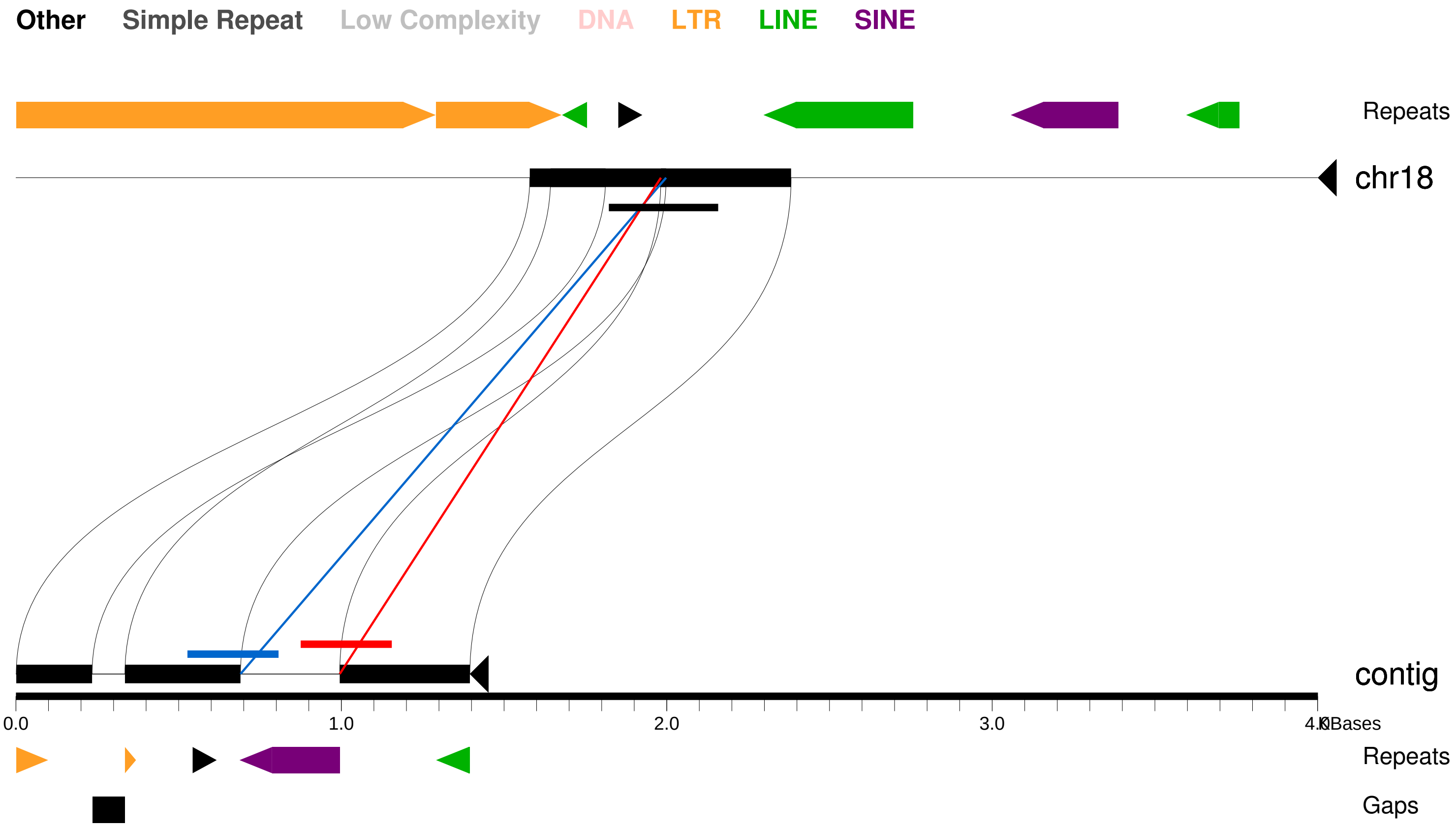

siteID: chr18_62566665 Scafftig1 - Breakpoint: chr18:62566673
chr18:62566513-62566847
Overlap Length: 15 INS size: 309 Genotyped: Yes

```
left   ---------------TTATAAAATGTTACTTGTCATTTAATATTTTTTATTTTACTGCTATTTAATTAAT
chrom  CTAAAATGTAACTTCTTATAAAATGTTACTTGTCATTTAATATTTTTTATTTTACTGCTATTTAATTAAT
right  ATGGCGTGAACCCGGGAGGCGGAGCTTGCA-GTGAGCTGAGATCGCGCC----ACTGCAGTCCGC--AGT
       N2NNNN22N2N2NNN1111111*11**1*11**1*11*1*1**1111111111*****11*111111*1*

left   GTGAAAATGACAAATAAGAAAAAGAGATCATTCTTTGATGTATCATTTTTCTACAATCTCAATTATGTAG
chrom  GTGAAAATGACAAATAAGAAAAAGAGATCATTCTTTGATGTATCATTTTTCTACAATCTCAATTATGTAG
right  CCGGCCTGGGC------GACAGAGCGA---------GA------------CTCCG-TCTCAAAAAAAAAA
       11*11111*1*111111**1*1**1**111111111**111111111111**1*11******11*111*1

left   TTAGAGTCTTGTTCTATGATAAAAAGTCTTTTCTT-----------------GGCCGG------------
chrom  TTAGAGTCTTGTTCTATGATAAAAAGTCTTTTCTTAAAATAAGTTTTTTTAAGTCCTTTTTTTAAAAAAT
right  AAAAAA---------AAAAAAAAAAGTTTTTTCTTAAAATAAGTTTTTTTAAGTCCTTTTTTTAAAAAAT
       11*1*1111111111*11*1*******1*******22222222222222222*2**22222222222222

left   ----GCG---CGGTGGCTCACGCCTGTAATCC---CAGCACTTTGGGAGGCCGAGGCGGGTGGATCATGA
chrom  TTTTGCCTTTCGGACAGTTGCTTCTGCAATTCTCACAGCCCTTATAATTTCCATCTTTTCACTTTCTTTC
right  TTTTGCCTTTCGGACAGTTGCTTCTGCAATTCTCACAGCCCTTATAATTTCCATCTTTTCACTTTCTTTC
       2222**2222***2222*22*22***2***2*222****2***2222222**222222222222**2*22

left   GGTCAGGAGATCGAGACCATCCTGGCTAACA------AGGTGAAACCCCGTCTCTACTAAA
chrom  ATTCAAGTGAGAAAATCCGTCAAGGTGATCAGAAAAAATGTCAAACATGGTC------AGA
right  ATTCAAGTGAGAAAATCCGTCAAGGTGATCAGAAAAAATGTCAAA----------------
       22***2*2**222*22**2**22**22*2**222222*2**2***1NNN1112222221N1
```

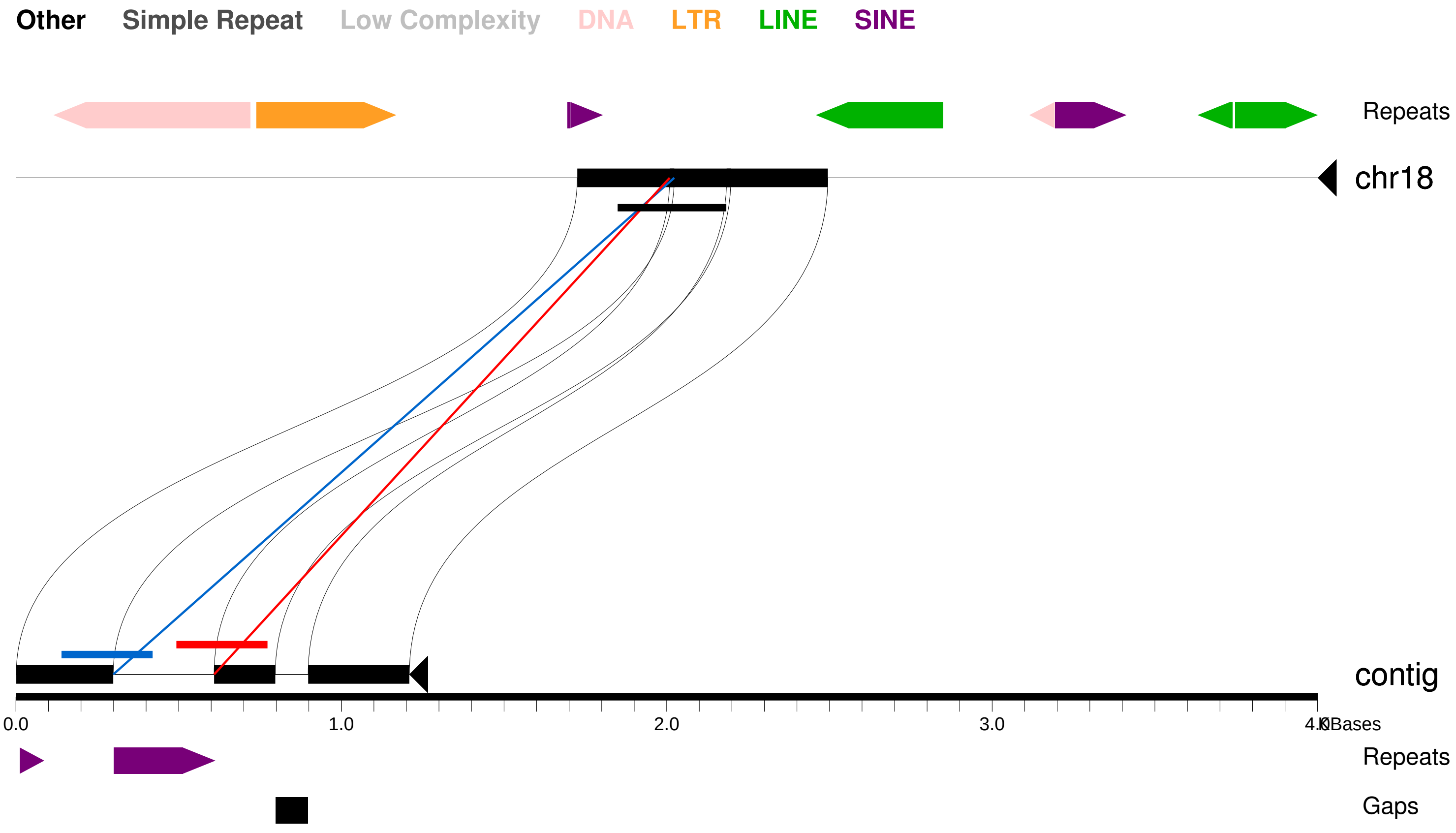

siteID: chr18_63481595 Scafftig1 - Breakpoint: chr18:63481602
chr18:63481557-63481661
Overlap Length: 15 INS size: 297 Genotyped: Yes

```
left    C-----------------TGGTTTACTTAAAAAAATACTGTATCCATTTTTTTATGTGTCTTGAAAAAGA
chrom   C-----------------TGGTTTACTTAAAAAAATACTGTATCCATTTTTTTATGTGTCTTGAAAAAGA
right   CGCCACTGCACTCCAGCCTGGGCGACAGAGCGAGACTCCGTCTCAAAAAAAAAAAAAAA---GAAAAAGA
        *11111111111111111***111**11*111*1*11*1**1**1*1111111*11111111*******

left    TAAATGATGGCCGGGCGCGGTGGCTCACGCCTGTAATCCC-AGCACTTTGGGAGGCCGAGGCGGGCG
chrom   TAAATGAAAATTTTCAAATAAGCTTAATG--TGGAATCAGTAACATTTTGTA-------------CG
right   TAAATGAAAATTTTCAAATAAGCTTAATG--TGGAATCAGTAACATTTTGTA-------------CG
        *******22222222222222*22*2*2*22**2****222*2**2****222222222222222**
```

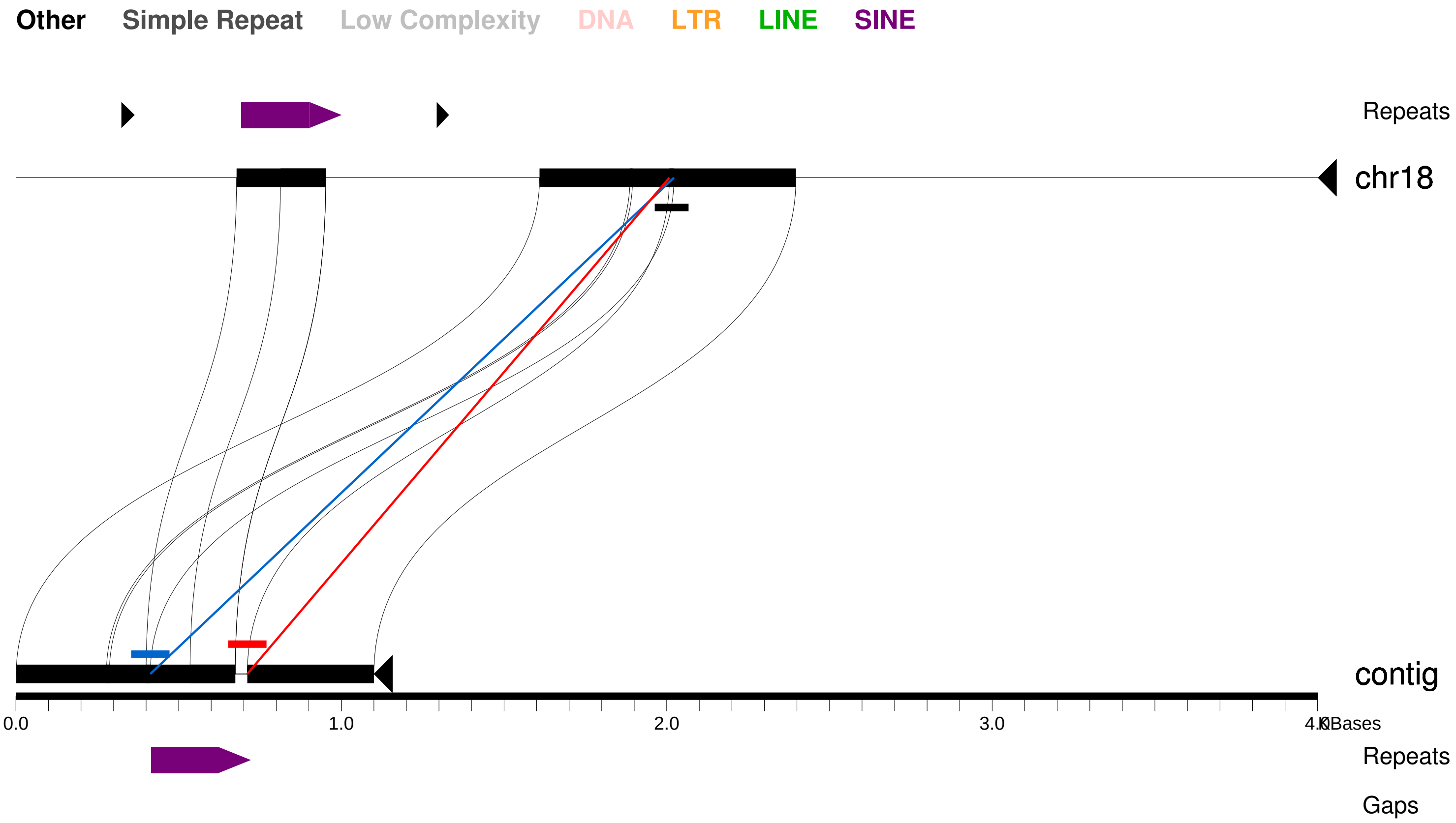

siteID: chr18_63487290 Scafftig11 + Breakpoint: chr18:63487270
chr18:63487110-63487447
Overlap Length: 18 INS size: 432 Genotyped: No

```
left   G---------------AGGGTAATTCGAAGCCTGAGTATGCAGAACCCGTGTTCCTTTGCTTCCCTAAAG
chrom  GTTGTCTAGGCTCCTGAGGGTAATTCGAAGCCTGAATATGCAGAACCCGTGTTCCTTTGCTTCCCTAAAG
right  NNNNNNNNNNN-------------------------------------------------------AAA-
       1NNNNNNNNNN     111111111111111111N11111111111111111111111111111***1

left   TGCGAAAGATTTCAGAAATGGCTATCGTCTGTTACATGAGGTACATATACCAAATTTTTAAGAAGATTCA
chrom  TGCGAAAGATTTCAGAAATGGCTATCGTCTGTTACATGAGGTACATATACCAAATTTTTAAGAAGATTCA
right  ----AAAAA-------------------------------------------------------------
       1111***1*1111111111111111111111111111111111111111111111111111111111111

left   ATAGTAGCAGAATATGCTTTAAAAAAATCATCTGAGGCCGGGCG--CGGTGGCTCA-CGCCTGTAATCCC
chrom  ATAGTAGCAGAATATGCTTTAAAAAAATCATCTGAGGCTGATAGGACATTAGATCATCAGATTTTTTTCC
right  --------------------AAAAAAATCATCTGAGGCTGATAGGATATTAGATCATCAGATTTTTTTCC
       11111111111111111111*****************2*222*22122*2*2***2*222*2*22*2**

left   AGCACTTTGGGAGGCCGAGGCGGGCGGATCACGAGGTCAGGAGAT------CGAG-ACCATCCTGGCTA-
chrom  TATACTTAA--AAACAGCTTTTGAACAATAGCAATATAAGTTTATATTTTACATGCACAATGCTTTTTAT
right  TATACTTAA--AAACAGCTTTTGAACAATAGCAATATAAGTTTATATTTTACATGCACAATGC-------
       222****2222*22*2*22222*2222**22*2*22*2**222**222222*22*2**2**2*1NNN11

left   --ACACGG------------TGAAA----CCCCGTC--------------TCTA-CTAAA
chrom  GAACATGGATTATAAATAAATGAAAAGTTCTGAGTTATGGTATCAGATGATATAGCTATA
right  ------------------------------------------------------------
         111N11            11111    1NNN11N              1N11 111N1
```

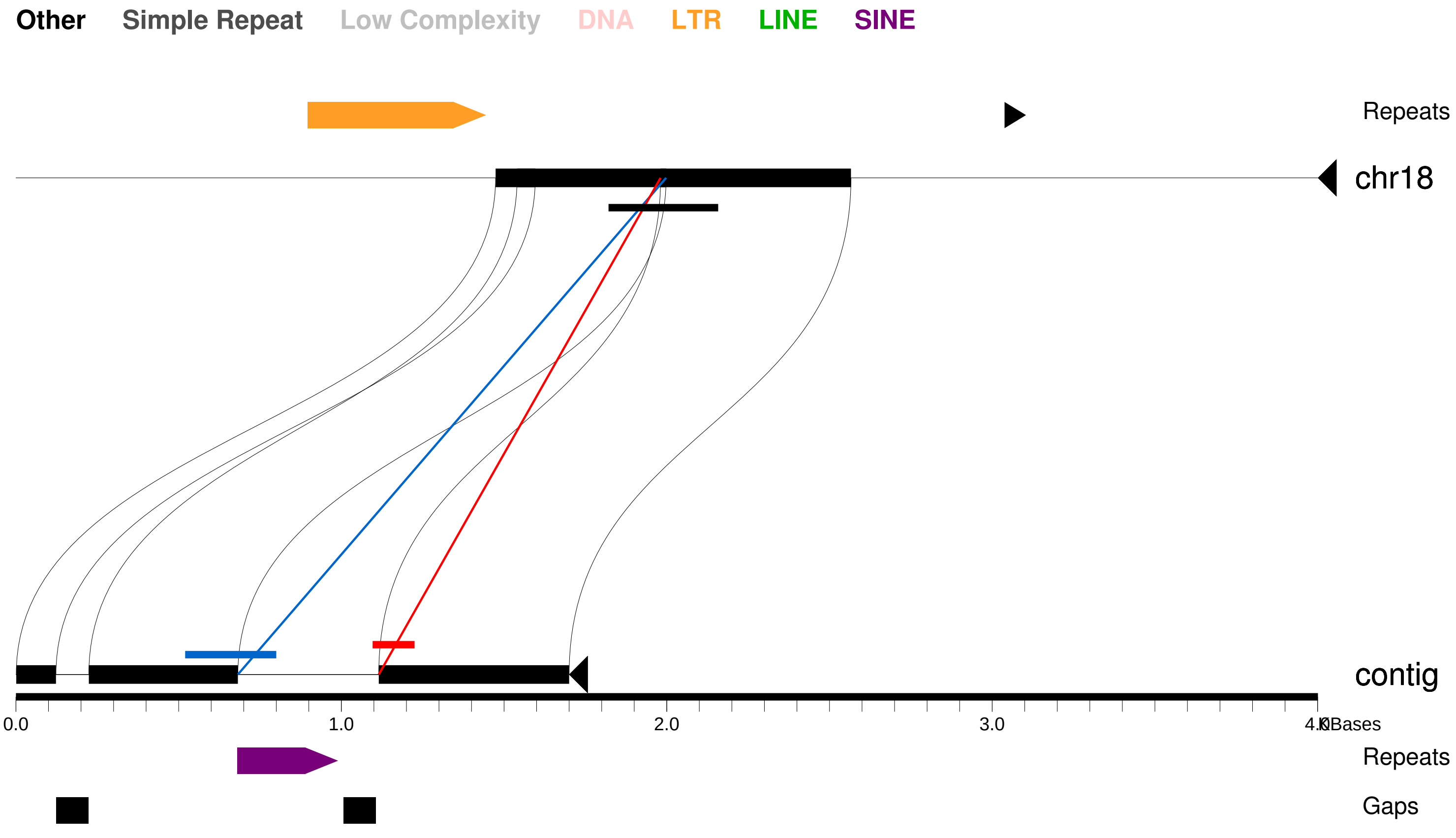

siteID: chr18_63661983 Scafftig1 - Breakpoint: chr18:63661987
chr18:63661827-63662169
Overlap Length: 23 INS size: 455 Genotyped: No

```
left   ----------------------------------------------------------------------
chrom  TGACATAATTTACATTTCTATCCACAGCATATAAGGAGTCCCTTTCTCAACACTCTCGCTGACTACATAT
right  TT---TAGTAGAGACGGGGTTTCAC--CTTGTTAGCCAGGATGGTCTCGA---TCTC-CTGACCTCATGA
       2N   22N2NN2N2NNNNNN2N222  2N2N2N22NNNNNNNNN2222N2   2222 22222NN222NN

left   ------------------------CCATTCTAATAGATGTGATGTGATATTTCATGGTGGTTATGATTTG
chrom  TATCTCTTGTCTTTTTGATAACAGCCATTCTAATAGATGTGATGTGATATTTCATGGTGGTTATGATTTG
right  TC-CACCCGCCT---------CGGCCTCCCAAA-----GTGCTGGGAT--TACA-GGCG----TGAGCCA
       2N 2N2NN2N22         2N2**111*1**11111***1**1***11*1**1**1*1111***1111

left   TATTTTTCTGATGATTGATAAAGTTGAGCATTTTTTAAACATANNNNNNNNNNN----------------
chrom  TATTTTTCTGATGATTGATAAAGTTGAGCATTTTTTAAACATACCTGTTGGTCATTTTTATGACATCTTT
right  -------CCGC-GCCCGGCCAAGTTGAGCATTTTTTAAACATACCTGTTGGTCATTTTTATGACATCTTT
       1111111*1*11*111*111***********************222222222222222222222222222

left   ----------------------------------------------------------------------
chrom  GGAAATATGTCTATTCAGATCTTTGCCCATTTTTGAATGTTTCTTTGCTTGTTTTTCCTTTTTGCTATTG
right  GGAAATATGTCTATTCAGATCTTTGCCCATTTTTGAATGTTTCTTTGCTTGTTTTTCCTTTTTGCTATTG
       2222222222222222222222222222222222222222222222222222222222222222222222

left   ---------------------------------------------------------------
chrom  AGTTGTGTGAGTTCCTTATATATTTTTGTATTTAGTCCCTTGATTAATGAGATATTTGGTTCA
right  AGTTGTGTGAGTTCCTTATATATTTTTGTATTTAGTCCCT-----------------------
       2222222222222222222222222222222222222222
```

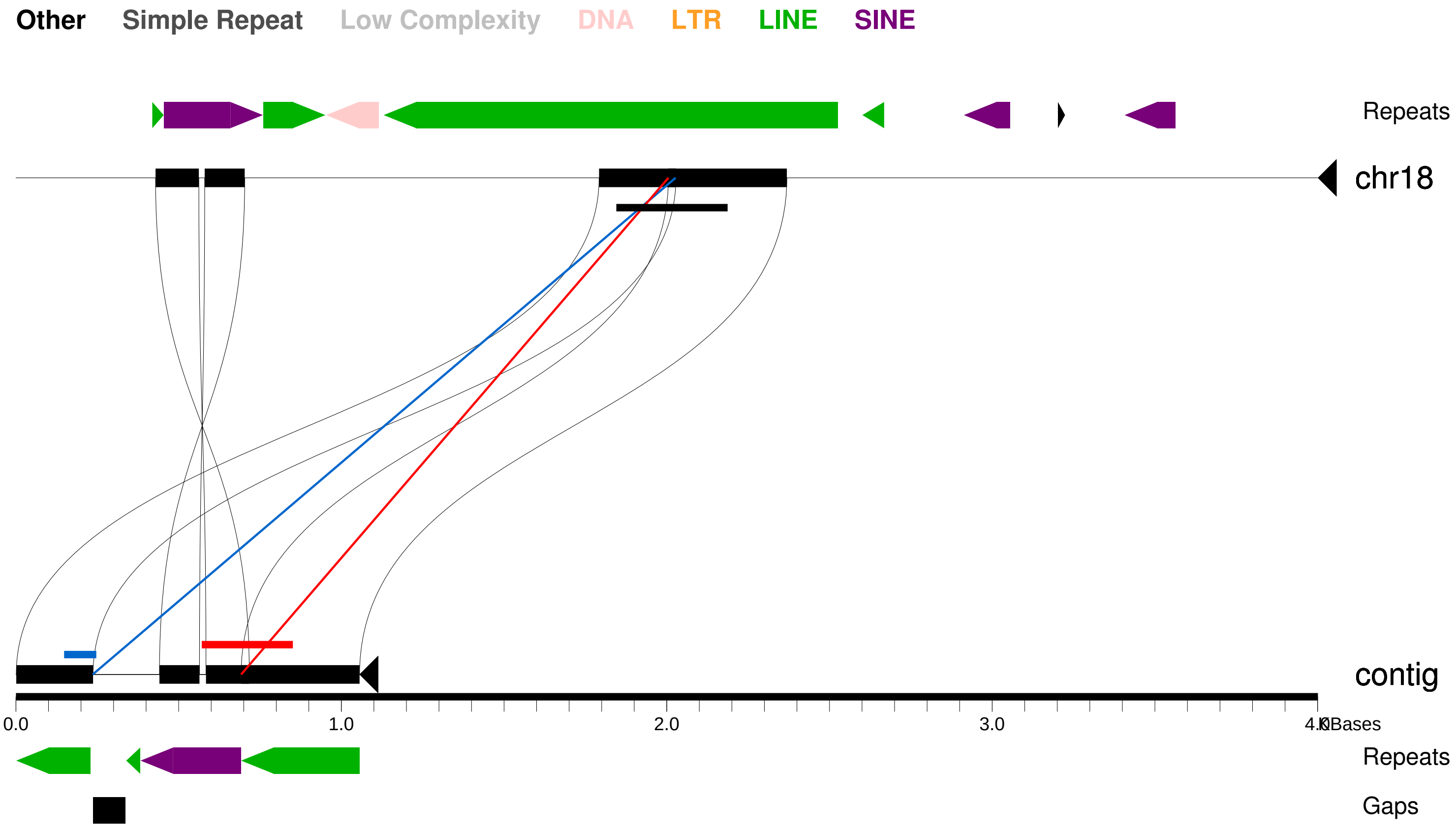

siteID: chr18_66572512 Scafftig1 - Breakpoint: chr18:66572495
chr18:66572335-66572661
Overlap Length: 7 INS size: 326 Genotyped: Yes

```
left    ----------------TAAAATCTCCTTCATGTGTATCAAACAGGTGATATGTTAACCCATATCCAATTA
chrom   AGTTTATATTATTCTTTAAAATCTCCTTCATGTGTATCAAACAGGTGATATGTTAACCCATATCCAATTA
right   ----TTTAGTAG-----AGACGGGGTTTCACC-GTTTTAGCCAGG----ATGGTCTCG-ATCTCC---TG
            2N22N22N    1*1*111111****111**1*1*11****1111***1*11*11**1***111*1

left    AAACAAAGATGCAAAATTTGACAATTTAAATCACTTATATTTAATAAAATACCGTAGCAAAAATTATGAC
chrom   AAACAAAGATGCAAAATTTGACAATTTAAATCACTTATATTTAATAAAATACCATAGCAAAAATTATGAC
right   ACCTCATGATCCACC----------------CGCCTCGGCCTCCCAAAGTGC--TGGGATTACAGGCGTG
        *1111*1***1**11111111111111111*1*1*11111*111***1*1*1N*1*1*11*11111*11

left    ATTTACGAATGTCAGCTGTGAAATTATTTTTTTTT--ATTTTTATTTTTATT----TATT----------
chrom   ATTTACGAATGTCAGCTGTGAAATTATATATTTTTTAATGTTTGATGTAACTAGCATACTACTAATAATC
right   AGCCACCGCGCCCGGCCG--AAATTATATATTTTTTAATGTTTGATGTAACTAGCATACTACTAATAATC
        *111**111111*1**1*11******2*2*****22**2***22*2*2*2*2222**2*2222222222

left    ---TATTTTTTT---------TGAGACGGAG-----TCTCGCT--CTGTCGCCCAGGTCGGACTGCGGAC
chrom   CACTATATTATTACAAAATAATGAGATAAATAATTATTTACCTTCCTATTTAAAAGGACATAAAAC--AC
right   CACTATATTATTACAAAATAATGAGATAAATAATTATTTACCTTCCTATTTAAAAGGACATAAAAC--AC
        222***2**2**222222222*****222*222222*2*22**22**2*22222***2*22*222*22**

left    TGCAGTGACGCAATCTCGGCTCACTGCAAGCTCCGCTTCCCGGGTTCACGCC
chrom   TATTGTTACAAAGATTATTTAAAATTAAAACTAAAATGCAAGT-TTGAGT--
right   TATTGTTACAAAGATTATTTAAAATTAAAACTAAAATGCAA-----------
        *222**2**22*222*222222*2*22**2**2222*2*221N211N1NN22
```

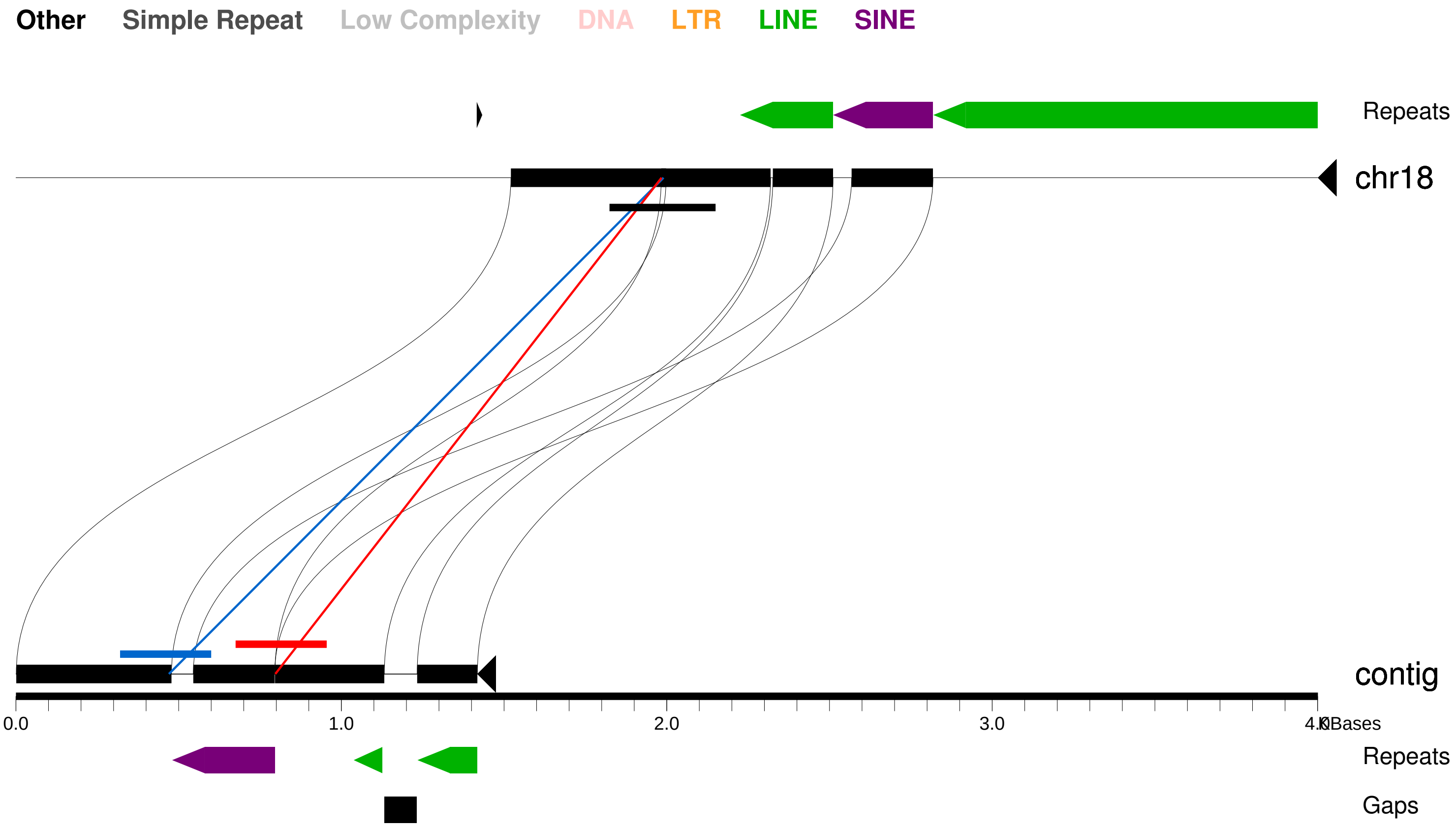

siteID: chr18_67160053 Scafftig4 + Breakpoint: chr18:67160054
chr18:67159894-67160226
Overlap Length: 13 INS size: 300 Genotyped: Yes

```
left    A--------ATTTTGTTTTCTGTTCATGAATTAATTCACTTAGACTA-ATGACCTCCAACTGCATTCATG
chrom   AT--GCAGAATTTTGTTTTCTGTTCATGAATTAATTCACTTAGACTA-ATGACCTCCAACTGCATTCATG
right   TTTAGTAGAGACGGGGTTTC-------------ACCGTTTTAGCCGGGATGGTCTCGATCTCCTGACCTC
        12112N22211111*1****1111111111111*11111****1*111***11***1*1**1*111*1*1

left    TTGCTGCAAAGGACATGGTTTCATTCTTTTTCTATGGCTGCATAATATTCCATGGTGTATATGTACCAAA
chrom   TTGCTGCAAAGGACATGGTTTCATTCTTTTTCTATGGCTGCATAATATTCCATGGTGTATATGTACCAAA
right   ATGATCCGCCCGCCTCGGCCTCC--------CAAAG--TGC-TGGGATTACA-GGTGT----GAGCCA--
        1**1*1*1111*1*11**11**111111111*1*1*11***1*111***1**1*****1111*11***11

left    TTTTCTTTACCCAGTCCACCATTGGTGGGTTTTTTTTTTTTTTTTTTTTTTTTTTGAGACGGAGT------
chrom   TTTTCTTTACCCAGTCCACCATTGGTGGGCTTTTTTATAGCTTTGGATTTTTAAG-GATGGTGTAAGAAA
right   ---------CCGCGCCCGGCCT-GGTGGGCTTTTTTATAGCTTTGGATTTTTAAG-GATGGTGTAAGAAA
        111111111**11*1**11*1*1******2******2*222***222*****22*2**2**2**222222

left    --CTC----GCTCTGTCGCCCAGGCTGGAGTGCAGTGGCGGGATCTCAGCTCACTG-----CAAGCTCCG
chrom   TACTCAAAAGGAACGTTTTCTTTTCTTGAGTATTTAGCTTCAAACCCAGCTCTGTCATTTACTACCTGTG
right   TACTCAAAAGGAACGTTTTCTTTTCTTGAGTATTTAGCTTCAAACCCAGCTCTGTCATTTACTACCTGTG
        22***2222*2222**222*2222**2****22222*22222*2*2******22*222222*2*2**22*

left    CCTCC-CGGGTTCAC---------GCCATTCT-CCTGC-------------------
chrom   CTTTTTCCTTTTCAAATGAGAATAGTAATACTACCTGCCTTATATTTAAGAATCTTA
right   CTTTTTCCTTTTCAAATGAGAATAGTAATACTACCTGCCTTAT--------------
        *2*222*222****2222222222*22**2**2*****22222
```

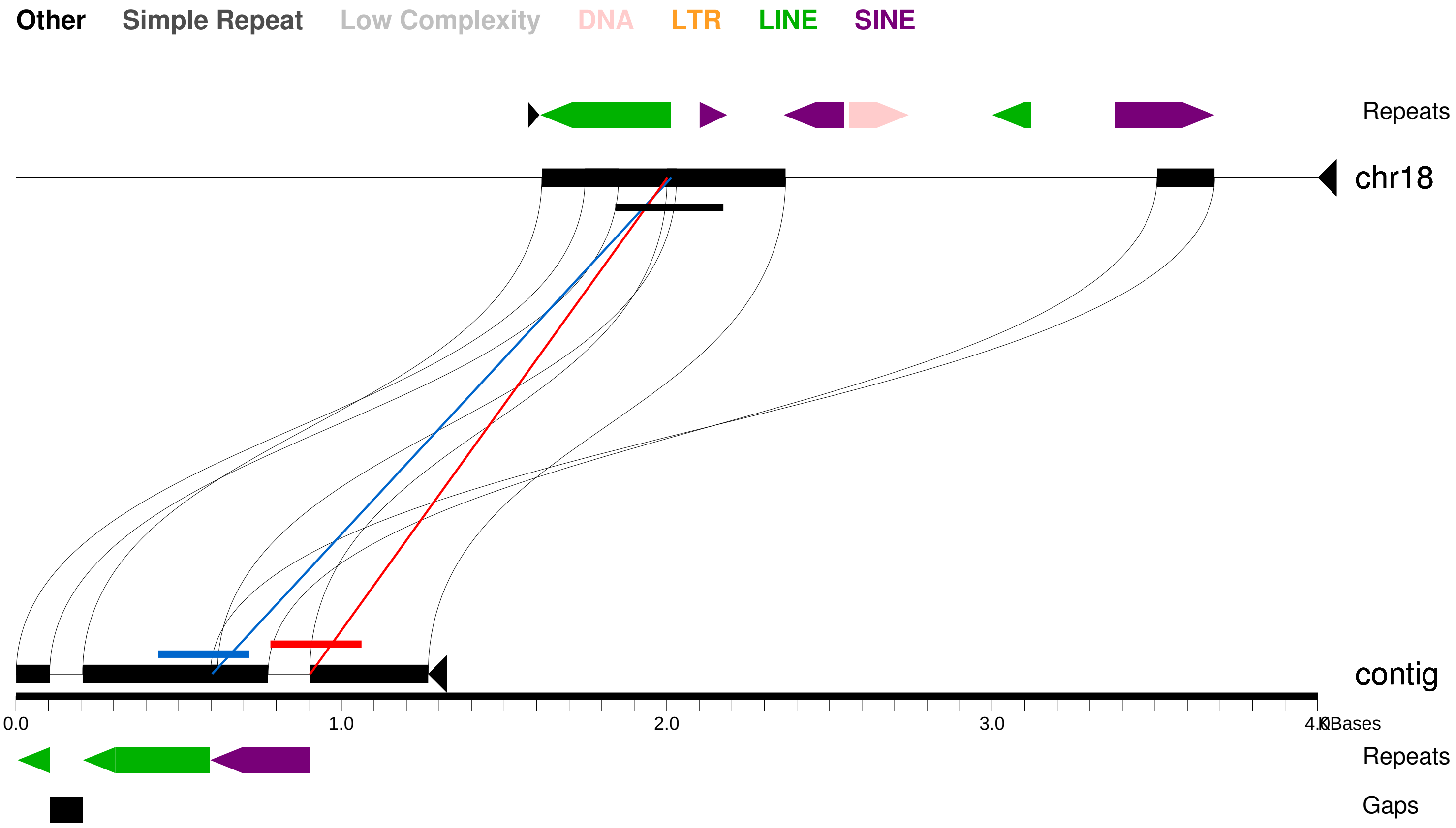

siteID: chr18_68097009 Scafftig2 - Breakpoint: chr18:68097052
chr18:68096999-68097084
Overlap Length: 7 INS size: 309 Genotyped: Yes

```
left   ---------------CCATT---------TTTAGATGAATAAATGTGA---------AGAAAGAGACGAA
chrom  ---------------CCATT---------TTTAGATGAATAAATGTGA---------AGAAAGAGACGAA
right  TGAGTCGAGATCGCGCCACTGCACTCCAGCCTGGGCGACAGAGCGAGACTCCGTCTCAAAAAAAAAAAAA
       111111111111111***1*11111111111*1*11**111*11*1**111111111*1***1*1*11**

left   AGAACATAGAATTTAGAAAAATGGGCCGGGCGCGGTGGCTCACGCCTGTAATCCCAGCACTTTGGGAGGC
chrom  AGAACATAGAATTTAGAAAAATGAT---------GTGGAT-------GTATTA-----ACT-----ATGC
right  AAAAAAAAAAAAAAAAAAAAATGAT---------GTGGAT-------GTATTA-----ACT-----ATGC
       *1**1*1*1**111*1*******22222222222****2*2222222***2*222222***22222*2**

left   CGAGACGGGCGG
chrom  CTAA-------G
right  CTAA-------G
       *2*22222222*
```

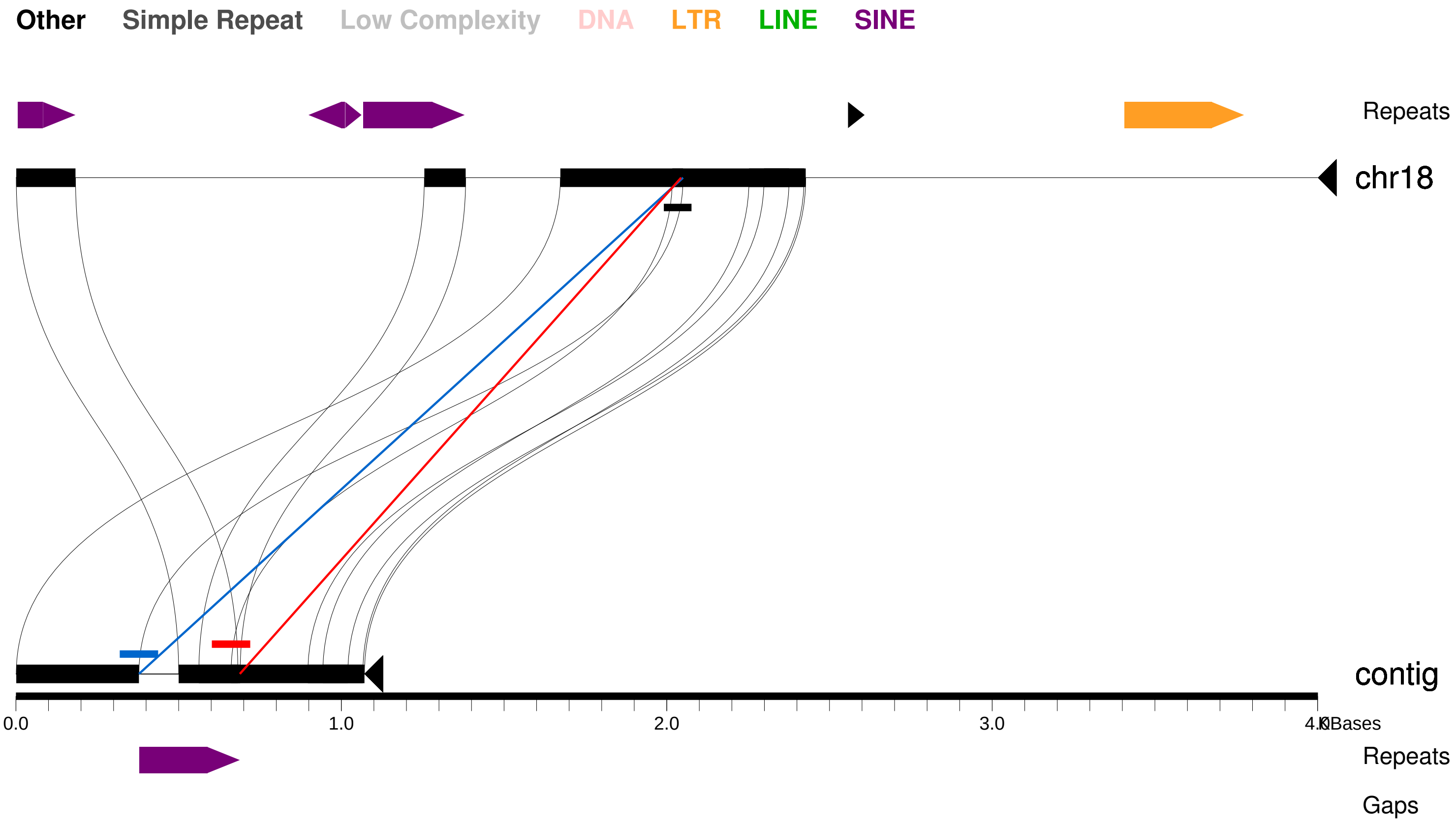

siteID: chr18_68438293 Scafftig3 - Breakpoint: chr18:68438274
chr18:68438114-68438441
Overlap Length: 8 INS size: 397 Genotyped: No

```
left   GAA----------------------------------ATGAAGAAATTTCAAGAAAACTGCAACTTACTA
chrom  GAAGTCTATGATAACAAATTTT-TAATTTTAGATGAAATGAAGAAATTTCAAGAAAACTGCAACTTACTA
right  G--------GCTAATTTTTTTGTATTTTTAGTAGAGACGGGG---TTTCAC--------CGTTTTTTTTT
       *11      2N222NNNN2222122N222222NN22N*1*11*111*****111111111*111**11*1

left   AAACTGACACAAGAAGAAAGTCTAAAATTTTTCA--TATTTATGTAATTGAATCTGT-TACAAATGAAAG
chrom  AAACTGACACAAGAAGAAAGTCTAAAATTTTTCA--TATTTATGTAATTGAATCTGT-TACAAATGAAAG
right  AGCCGG---------GATGGTCTCGATCTCCTGACCTCGTGATCCGCCCGCCTCGGCCTCCCAAAGTGCT
       *11*1*111111111**11****11*11*11*1*11*11*1**111111*11**1*11*1*1**1*1111

left   GAAGGATTTCTTTATATATTCTGGATAGTAATNNNNNNNNNNN---------------------------
chrom  GAAGGATTTCTTTATATATTCTGGATAGTAATCACTTTTTATAATACATGTTTGAAATACCTTTCACCCA
right  G--GGATTAC--------------ATAGTAATCACTTTTTATAATACATGTTTGAAATACCTTTCACCCA
       *11*****1*11111111111111********22222222222222222222222222222222222222

left   ----------------------------------------------------------------------
chrom  CTTGTCACTTGACTTTTAATACCCTCTATGGTGCTCTCTGACACATAGAAGTTTTAAAAATGTAAGTAGA
right  CTTGTCACTTGACTTTTAATACCCTCTATGGTGCTCTCTGACACATAGAAGTTTTAAAAATGTAAGTAGA
       2222222222222222222222222222222222222222222222222222222222222222222222

left   ----------------------------------------------------
chrom  ATGTCCAAGATATTACTAGTGTTTCCTGATGTGCCTTGTTCATCTTACCTTT
right  ATGTCCAAGATATTACTAGTGTTTCCTGATGTGCCTTGTTCATCT-------
       222222222222222222222222222222222222222222222
```

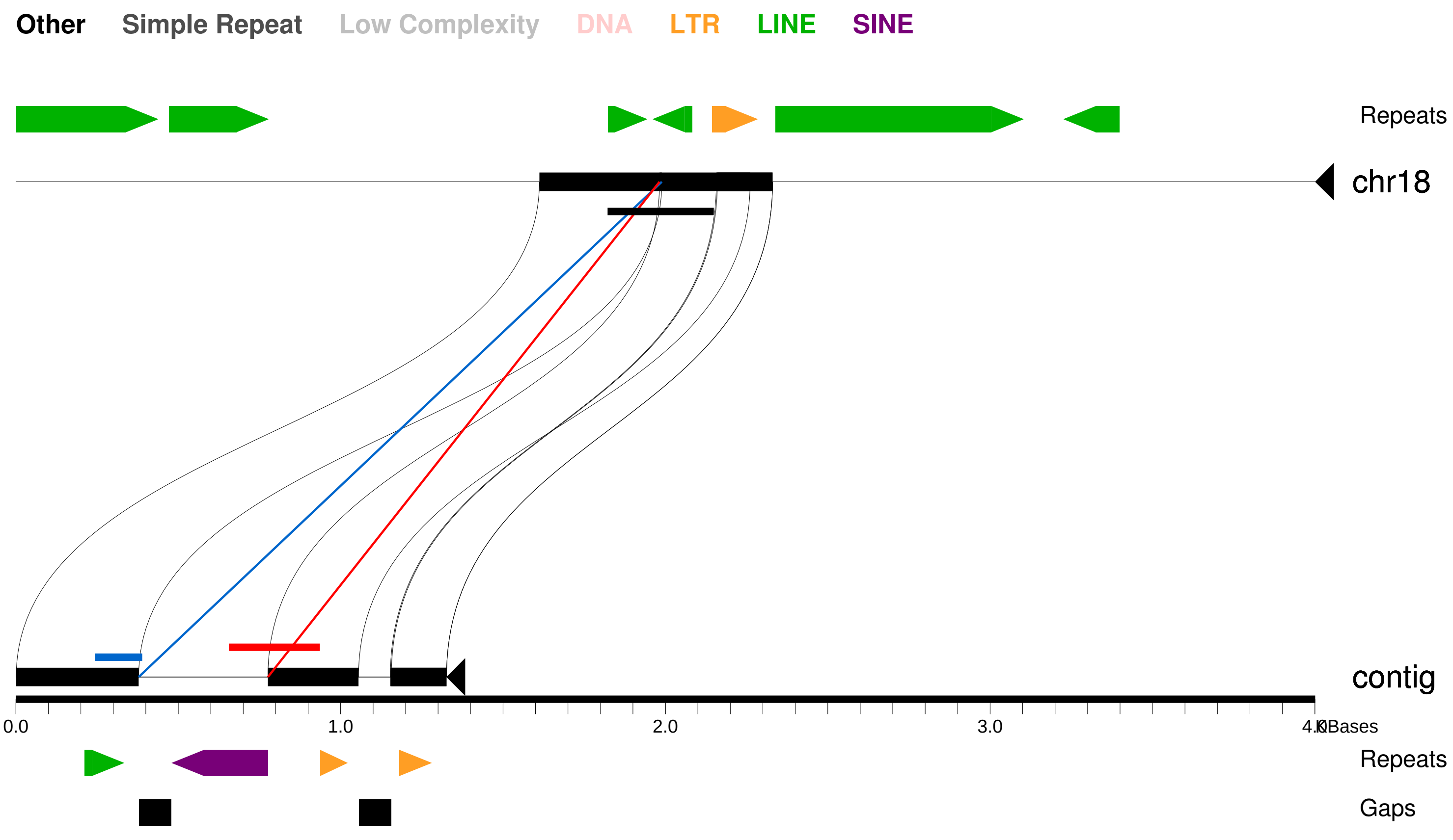

siteID: chr18_68572084 Scafftig1 - Breakpoint: chr18:68572142
chr18:68572098-68572201
Overlap Length: 16 INS size: 183 Genotyped: Yes

```
left     TAAG---ATAGAAAT---AAATTTCT---ATCTCTTTTAAGATTA------CTCAACTTGGAAGCTTCCT
chrom    TAAG---ATAGAAAT---AAATTTCT---ATCTCTTTTAAGATTA------CTCAACTTGGAAGCTTCCT
right    CACCCGCCTCGGCCTCCCAAAGTGCTGGGATTACAGGCGTGAGCCACCGCGCCCGGCCAGGAAGCTTCCT
         1*111111*1*111*111***1*1**111**11*111111**111111111*1*11*11***********

left     GTTTTTGTTTTTTTTTTTTTTTTTTTTTTTTTTTTTTTTTTGAGACGGAGTCTCGCTCTGTCGC
chrom    GTTTTTA-------------TAAAATAAAACCTATCCTAGTTAATACCAACCACAGGCC--ACCA
right    GTTTTA-------------TAAAATAAAACCTATCCTAGTTAATACCAACCACAGGCC--ACCA
         *****22222222222222*2222*222222*2*22*22**2*2**22*22222*22*222*22
```

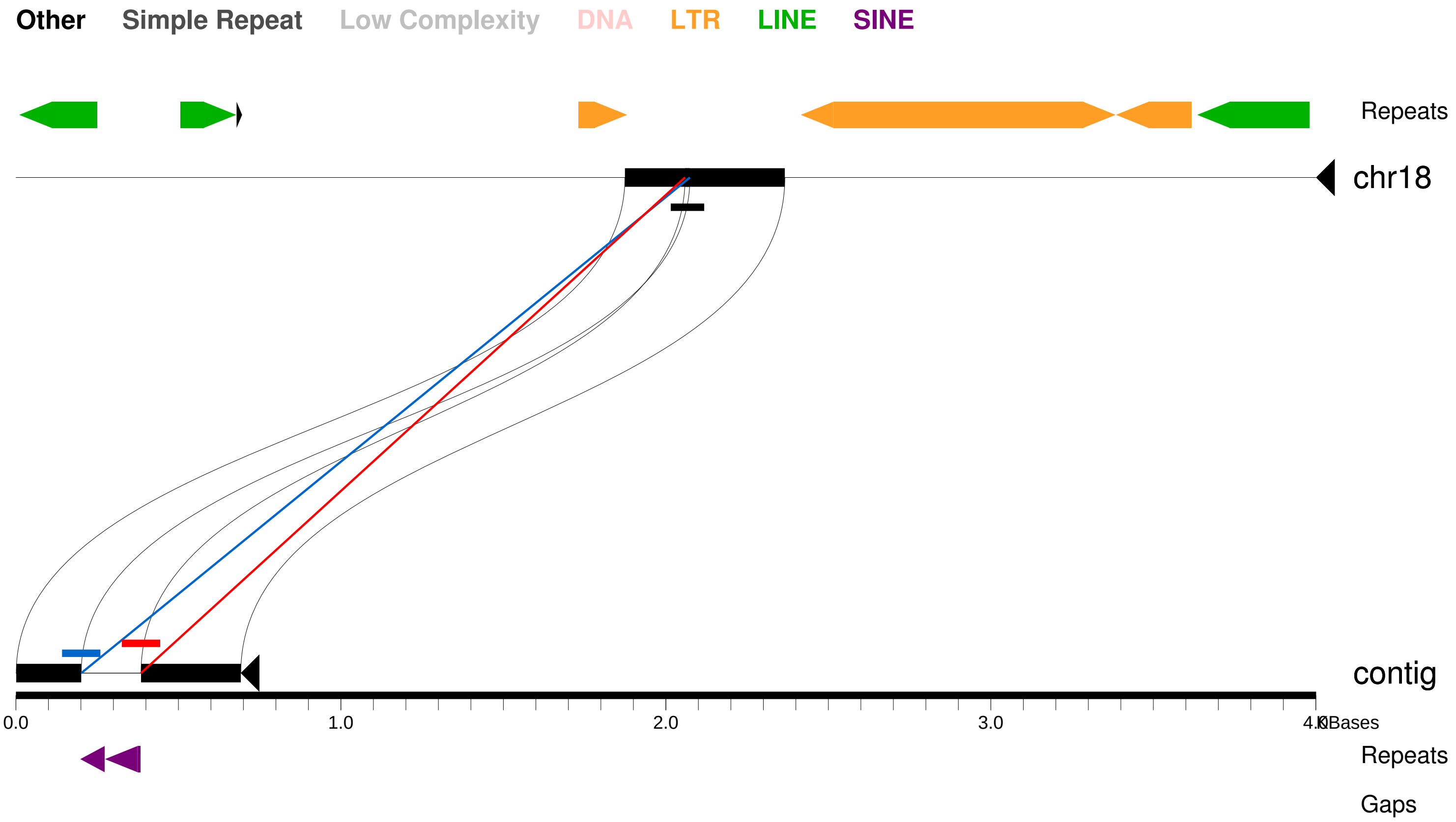

siteID: chr18_69600911 Scafftig1 - Breakpoint: chr18:69600984
chr18:69600928-69601043
Overlap Length: 4 INS size: 360 Genotyped: No

```
left    TATGAAGACCTTATAATTAAATAAAACATATAAAGATCCATTAATAAAAATAATCT---AATTCACGAGG
chrom   TATGAAGACCTTATAATTAAATAAAACATATAAAGATCCATTAATAAAAATAATCT---AATTATCAAGA
right   NNNNNNNNNNNNNNNNNNNNNNNNNNNNNNNNNNNNNNNNNNNNNNNNNNNNNNNNNNNAATTATCAAGA
        11111111111111111111111111111111111111111111111111111111111****22*2**2

left    TCAGGAGATCGAGACCATCCCGGCTAAAACGGTGAAACCCCGTCTCTACTAA
chrom   CTTAAAAATGCATAGCATTCTGCATTAGCAACTAATATTCC--CAAAAC-AG
right   CTTAAAAATGCATAGCATTCTGCATTAGCAACTAATATTCC--CAAAAC-AG
        22222*2**22*2*2***2*2*22*2*22222*2*2*22**22*222**2*2
```

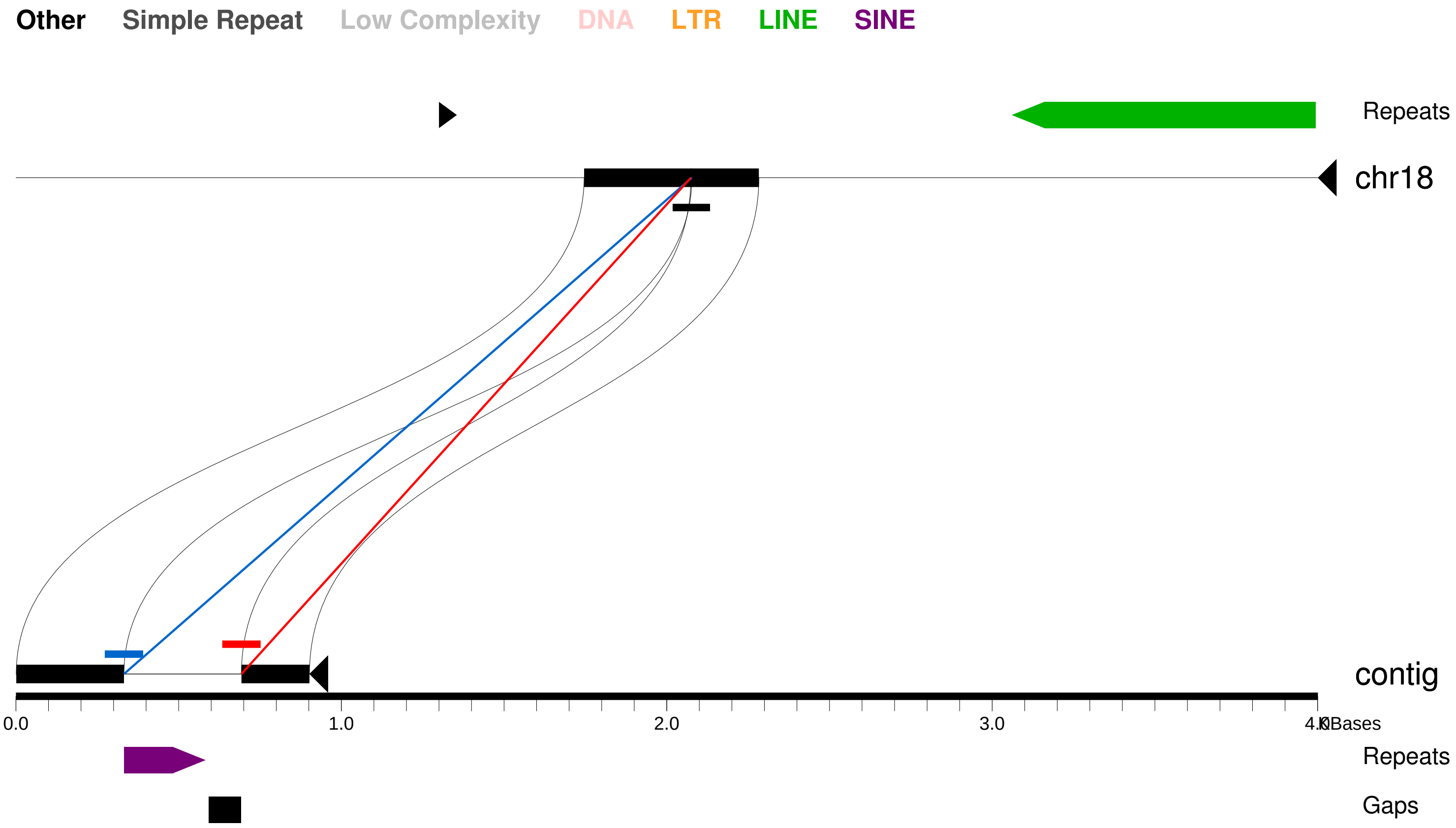

siteID: chr18_71324643 Scafftig1 + Breakpoint: chr18:71324647
chr18:71324599-71324706
Overlap Length: 12 INS size: 355 Genotyped: No

```
left   ATCCTTATACAGTAACAGATA-----CGTGTTTGTA--GATACATGAGAGA----AATAAAGGCTTTTCT
chrom  ATCCTTATACAGTAACAGATA-----CGTGTTTGTA--GATACATGAGAGA----AATAAAGGCTTTTCT
right  TTTTTTTAGTAGAGACGGGGTTTCACCGTGTTAGCCAGGATGGTCTCGATCTCCTGACCAAGGCTTTTCT
       1*11**1111**11**1*11111111******1*1111***111111**1111111*11***********

left   TTTTTNNNNNNNNNNNNNNNNNNNNNNNNNNNNNNNNNNNNNNNNNNNNNNNNNNNNNN
chrom  TGACTGCTGAATACCGCATGCCTTTGAGTCTGCGCAGCTGTTTAAGGTG-----------
right  TGACTGCTGAATACCGCATGCCGTTGAGTCTGCGCAGCTGTTTAAGGTG-----------
       *222*222222222222222222N222222222222222222222222222222222222
```

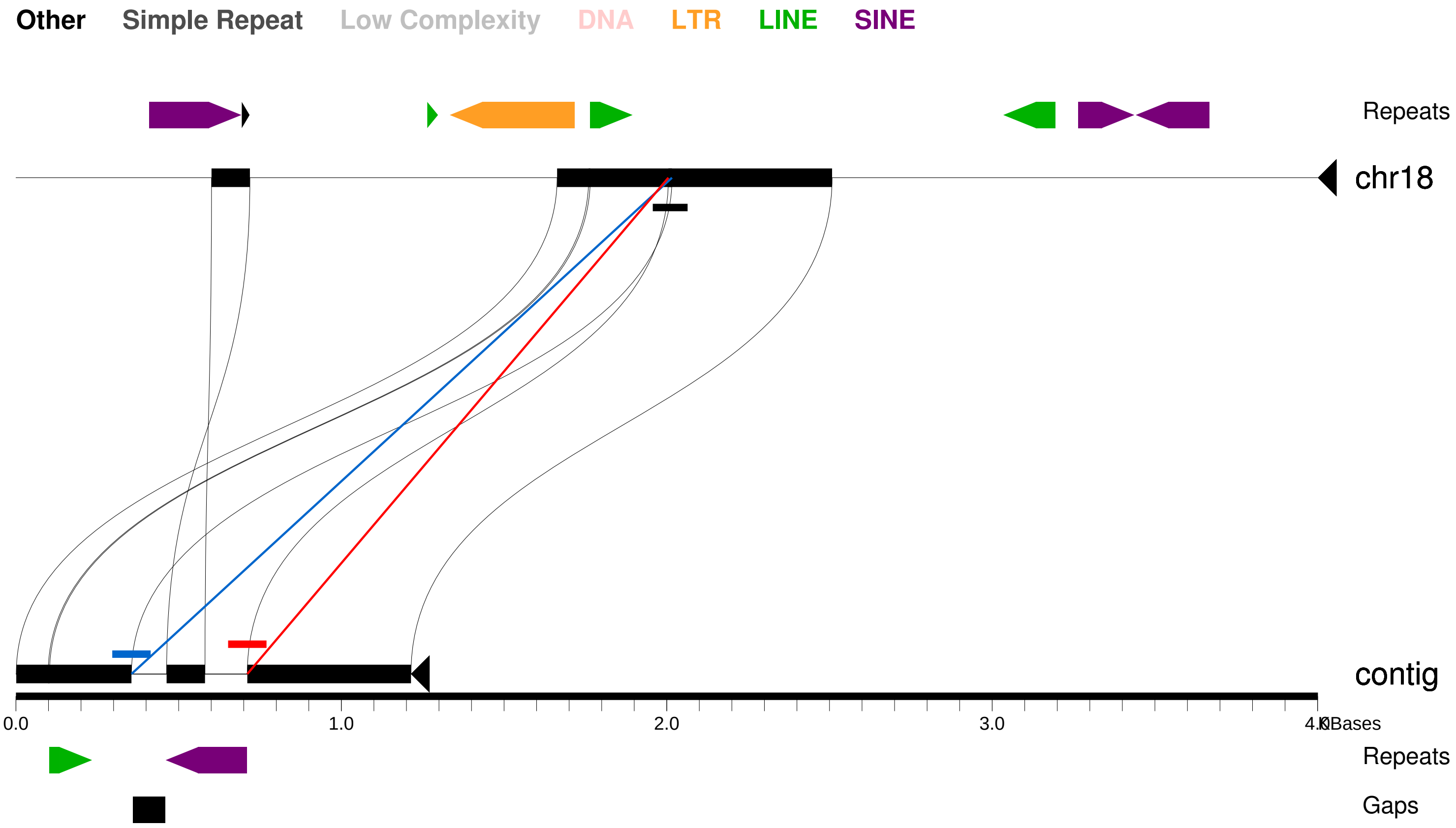

siteID: chr18_73953944 Scafftig7 + Breakpoint: chr18:73953925
chr18:73953765-73954099
Overlap Length: 15 INS size: 308 Genotyped: Yes

```
left     -------------ATCTAAAATATGTCTGTGTGTAATGATCATGAGAAGTTTATTCCAGGAGTTCCAGGA
chrom    GTAAAAAAAAAAAAATCTAAAATATGTCTGTGTGTAATGATCATGAGAAGTTTATTCCAGGAGTTCCAGGA
right    TTTAGTAGAGACGGGGTTTCA-----CTGTGT-TAGCCAGGATG----GTCTC------GATCTCCTGAC
         N2N2NN2N2N2NN111*111*11111******1**111*11***1111**1*1111111**11***1*11

left     GTTTAGTTCCAGAAGTTTATTCTAGTTTAATATTTGAAAAT-CAATCAATATAATTGTTATAGACTGAAT
chrom    GTTTAGTTCCAGAAGTTTATTCTAGTTTAATATTTGAAAAT-CAATCAATATAATTGTTATAGACTGAAT
right    CTCGTGATCCGCCCGCCTCGGCC---------TCCCAAAGTGCTGGGATTACAAGCGTGAGCCACCG---
         1*111*1***1111*11*111*1111111111*111***1*1*1111*1**1**11**1*111**1*111

left     GTTTATATCTCCCTCAAAAATCATAAGCTAAATCTTT----------TTTTTTTTTTTTTTTTTTTTTTT
chrom    GTTTATATCTCCCTCAAAAATCATAAGCTAAATCTTAACACTCAAAGTGATGGTATTTGGAGGTGTGCCT
right    --------CGCCCGGCC----CATAAGCTAAATCTTAACACTCAAAGTGATGGTATTTGGAGGTGTGCCT
         11111111*1***11111111**************2222222222*22*22*2***22222*2*222*

left     TGAGACGG-----AGTCTCGCT--CTGTGGCC-CA----GGTGGGAGT-GCAGTGGCGC-----AA---T
chrom    TTAGAAGTTATTAAGTCATGAAAGCAGAGCCCTCAAAGTGATGGTATTTGGAGATGGGCCTTTGAAAGTT
right    TTAGAAGTTATTAAGTCATGAAAGCAGAGCCCTCAAAGTGATGGTATTTGGAGATGGGCCTTTGAAAGTT
         *2***2*222222****22*2222*2*2*2**2**2222*2***2*2*2*2**22*2**22222**222*

left     CTCGGCTCACTGCAAGCTCCGCC----TCCCAGGTTCACGCCATTC--------TCCT
chrom    ATTAGGTCA-TGAGGGTACAGCCCTTGTGAATGATACTAGAGATTCCAGAGAACTCC-
right    ATTAGGTCA-TGAGGGTACAGCCCTTGTGAATGATACTAGAG----------------
         2*22*2***2**222*22*2***2222*2222*2*2*22*221111        1112
```

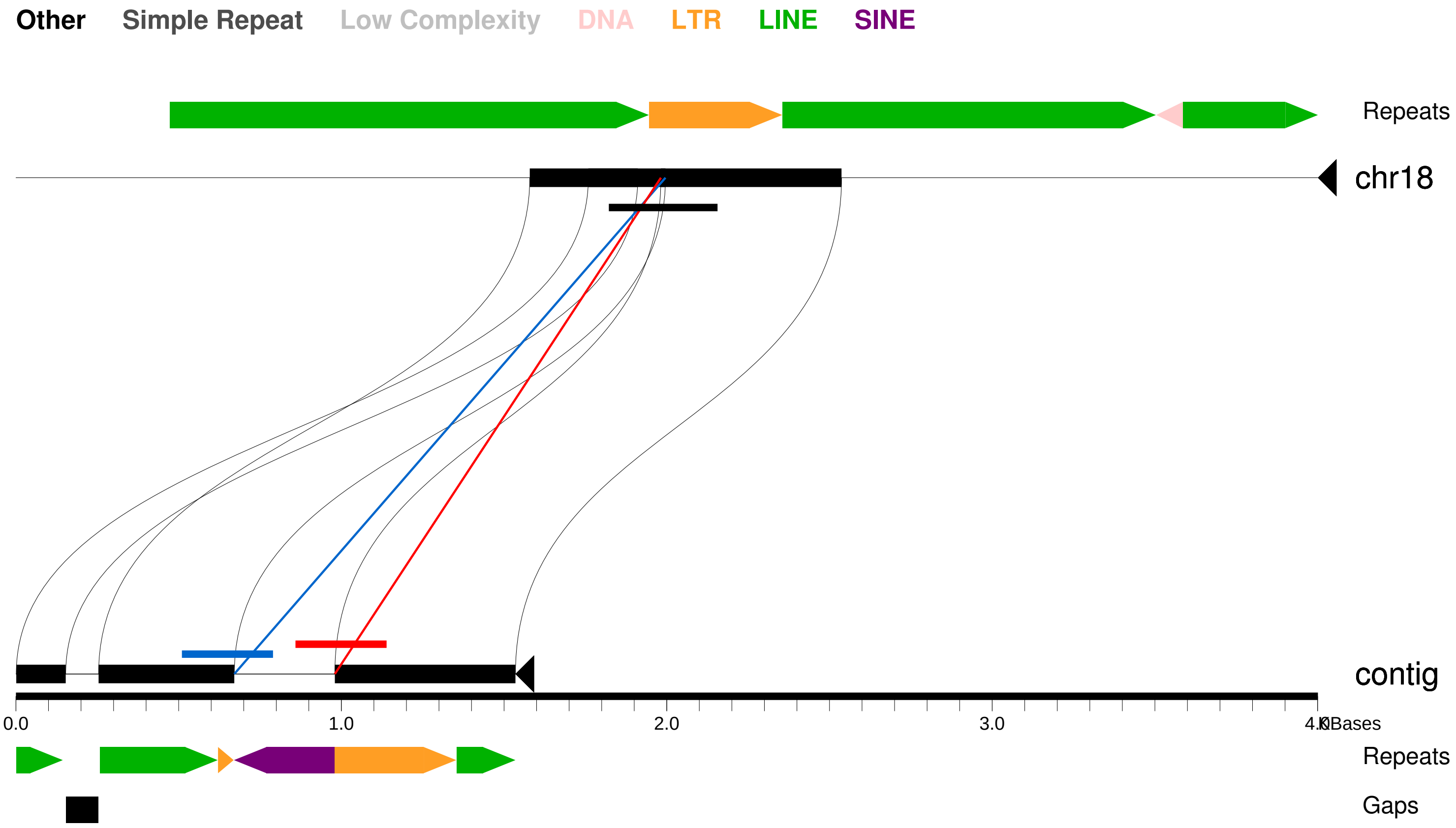

siteID: chr18_74414054 Scafftig1 - Breakpoint: chr18:74414034
chr18:74413991-74414093
Overlap Length: 17 INS size: 425 Genotyped: No

```
left    TAAGTCATGGTACAAAAGAATTTAATAAGTGACACATCGGTAT----------------AAAAAACATGT
chrom   TAAGTCATGGTACAAAAGAATTTAATAAGTGACACATCGGTAT----------------AAAAAACATGT
right   NNNNNNNNNNNNNNNNNNNNNNNNNNNNNNNNNNNNNNNNNNNNNNNNNNNNNNNNNNNAAAAAACATGT
        11111111111111111111111111111111111111111111111111111111111***********

left    CACTTGGCCGGGCGCGGTGGCTCACGCCTGTAATCCCAGCACTTTGGGAGGCCGAGGCGGGCGGA
chrom   CACTTG-----------TAGCTCAT-CCTTTAAAACCAGAATAGCCAAATGAAAAATCAGT----
right   CACTTG-----------TAGCTCAT-CCTTTAAAACCAGAATAGCCAAATGAAAAATCAGT----
        ******22222222222*2*****22***2***22****2*2222222*2*222*22*2*22222
```

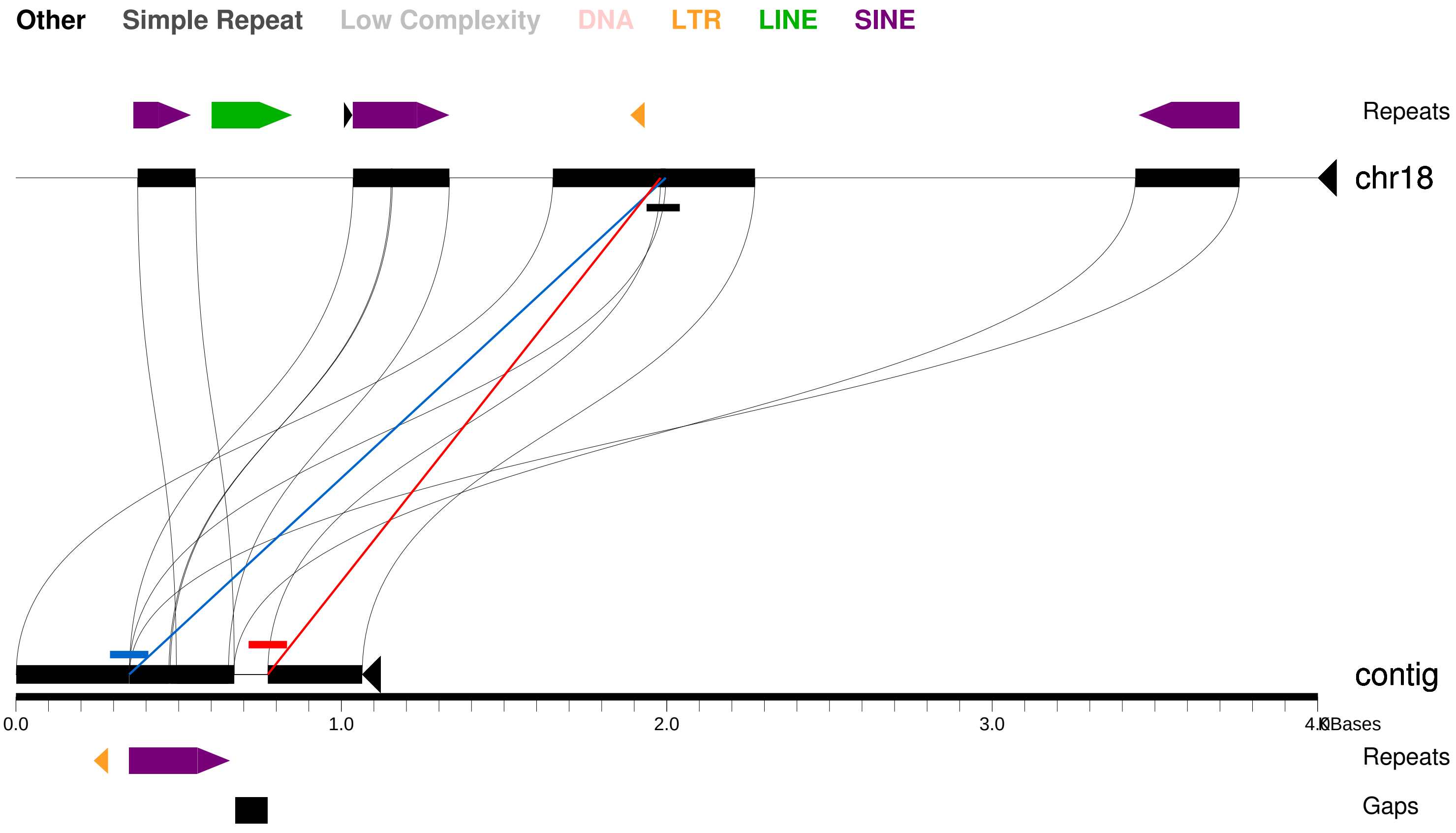

siteID: chr18_74638697 Scafftig12 + Breakpoint: chr18:74638702
chr18:74638605-74638861
Overlap Length: 46 INS size: 294 Genotyped: Yes

```
left   AA-----------------------------------------------------------------GGG
chrom  AAAGGGTGATTAGACTGTGTGTGTATATATGGTATAAAGAGTGATTAGACTGTGTGTATGGTATAAAGGG
right  GATGGCACC----ACTGCAC-TCTAG-TCTGGGCGACTGAGCGAG-AGACTGTCTCAAA---AAAAA---
       1*N22NNNN    2222NNN 2N22N 2N222NNN2NN222N22N 2222222N2NN2N    2N222111

left   TGATTAGACCATGTGTGTGTATGGTATAAAGGGTGATTAGACAGTGTGTGTGTGTATGGTATAAAGGGTG
chrom  TGATTAGACCATGTGTGTGTATGGTATAAAGGGTGATTAGACAGTGTGTGTG--TATGGTATAAAGGGTG
right  -------------------------AAAAAGGGTGATTAGACAGTGTGTGTG--TATGGTATAAAGGGTG
       1111111111111111111111111*1*************************22****************

left   ATTAG---GGCCGGGTGT----GGT---------GGCTCAT--------GCCTGTA-ATCC---------
chrom  ATTAGACAGTGTGTGTGTATATGGTATAAATAAGGGGTGATTAGACAGTGTGTGTATATGGTATAAAGGG
right  ATTAGACAGTATGTGTGTG---------------------------------------------------
       *****222*2N2*2****N   111         11N1N11        1NN1111 11NN

left   --------CAGCACTT-TGGG---------------AGG----------
chrom  TGATTAAACAGCATATATGTGTATATGGTATAAAGTAGGTGTTCGTATT
right  -------------------------------------------------
               11111NN1 11N1               111
```

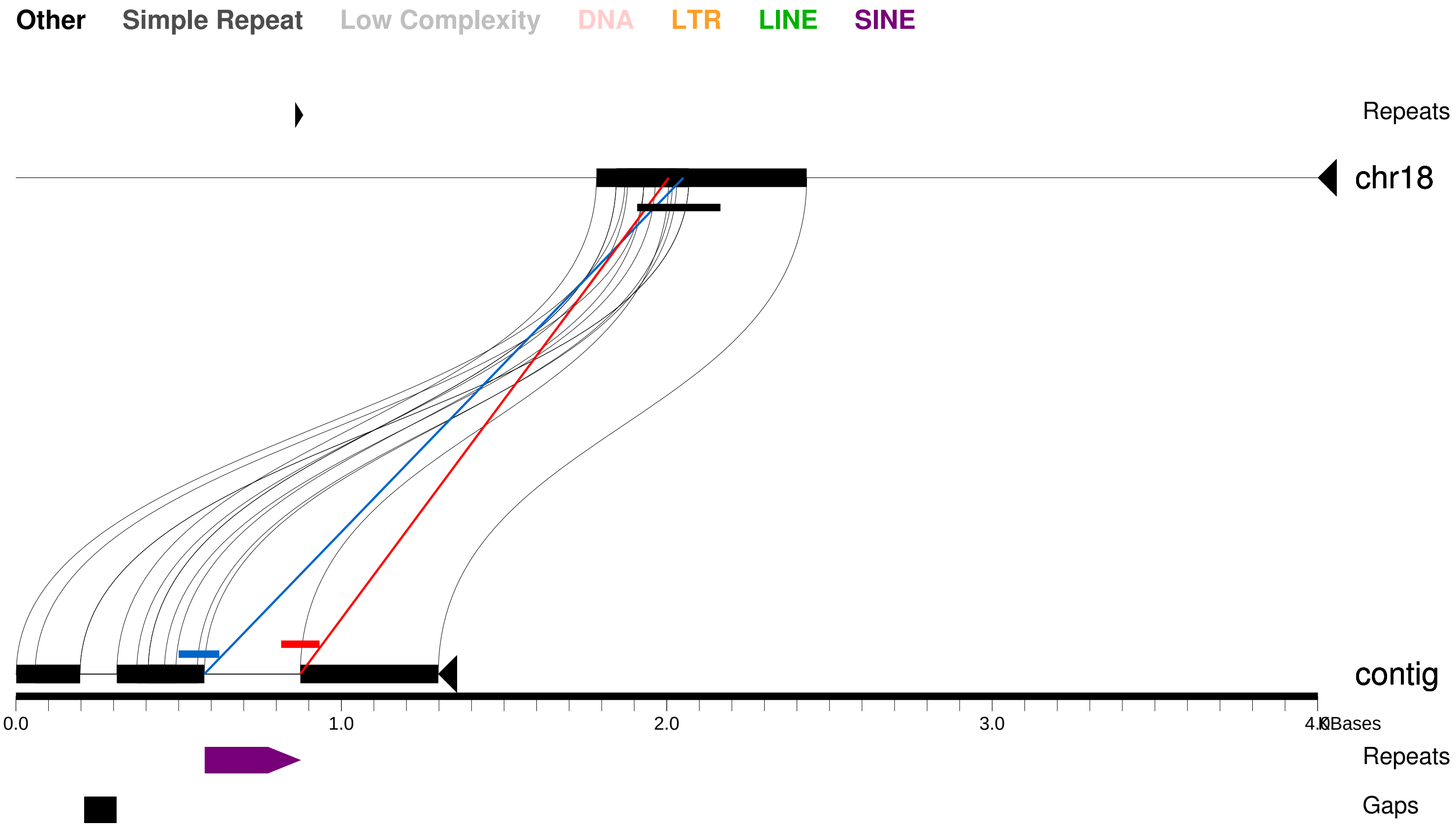

siteID: chr18_74756896 Scafftig3 - Breakpoint: chr18:74756872
chr18:74756826-74756977
Overlap Length: 14 INS size: 326 Genotyped: Yes

```
left    TGTTGTATCCTGTGTGTAAGTGTGTGTGACTGTGTGAGTGTGTAAGAAAATGTGAGTCTGGGCCGG----
chrom   TGTTGTATCCTGTGTGTAAGTGTGTGTGACTGTGTGAGTGTGTAAGAAAATGTGAGTCTGTGAGAGAACA
right   AAAAAAAAAAAAAAAAAAAAAAA--------------------AAAAAAATGTGAGTCTGTGAGAGAACA
        111111*1111111111**111111111111111111111111**1*************2*222*2222

left    -GCGCG-GTGGCTC-ACGCCTGTAATCCCAGC----ACTTTGGGAG-GCCGAGG-------------CGG
chrom   TGAGTGTGTGTGTGTAAGTGTGGTGTGTGAGTGTGGAGTGTGTGAGTGTTCAAGATTCCCTACATAACGG
right   TGAGTGTGTGTGTGT-------------GAGTGTGGAGTGTGTGAGTGTTCAAGATTCCCTACATAACGG
        2*2*2*2***22*221N1NN11NNN1NN2**22222*2*2**2***2*222*2*2222222222222***

left    GT--------GG
chrom   GATACACACAGA
right   GATACACACAGA
        *222222222*2
```

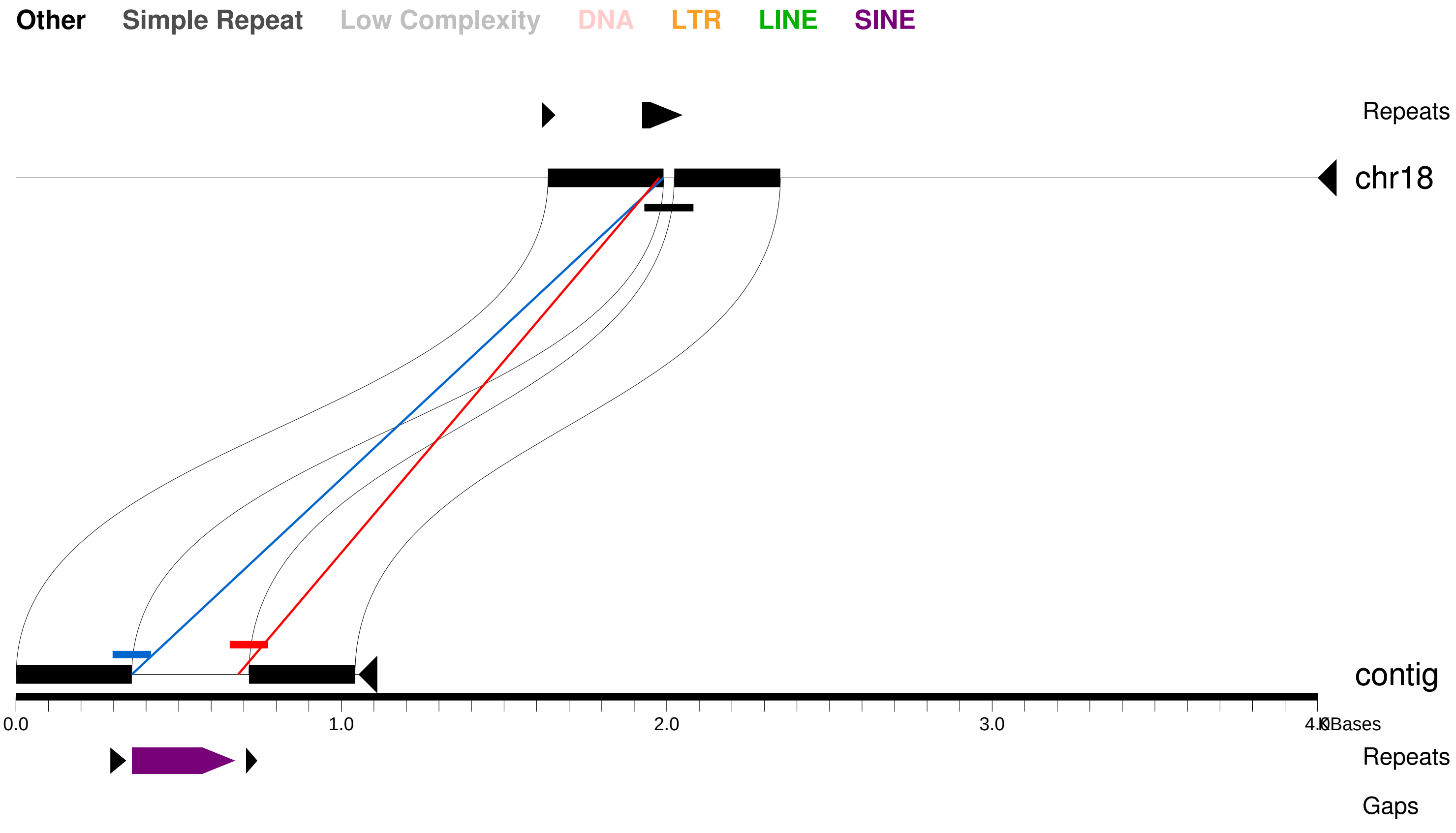

siteID: chr19_30448192 Scafftig5 + Breakpoint: chr19:30448199
chr19:30448153-30448258
Overlap Length: 14 INS size: 290 Genotyped: Yes

```
left    TC-----AAATGAAGGTC------TGCTTTTATTCCTTCCCCTTGGTTTCTTTATAG--CCGCTTTTGTT
chrom   TC-----AAATGAAGGTC------TGCTTTTATTCCTTCCCCTTGGTTTCTTTATAG--CCGCTTTTGTT
right   TCCGCCCGCCTCGGCCTCCCAAAGTGCTGGGATTACAGGCGTGAGCCACCGCGCCCGGCCCGCTTTTGTT
        **11111111*11111**111111****111***1*111*1111*1111*111111*11***********

left    TTTTTTTTTTTTTGAGACGGAGTCTCGCTCTGTCGCCCAGGCTGGAGTGCAGTGGCGGGATC
chrom   TTTAT---------AGATTGCCTTGTACCTTGTA----ATTTTGAACTGGATGCCAGATATT
right   TTTAT---------AGATTGCCTTGTACCTTGTA----ATTTTGAACTGGATGCCAGATATT
        ***2*222222222***22*22*2222*22***22222*222**2*2**2*22222*22**2
```

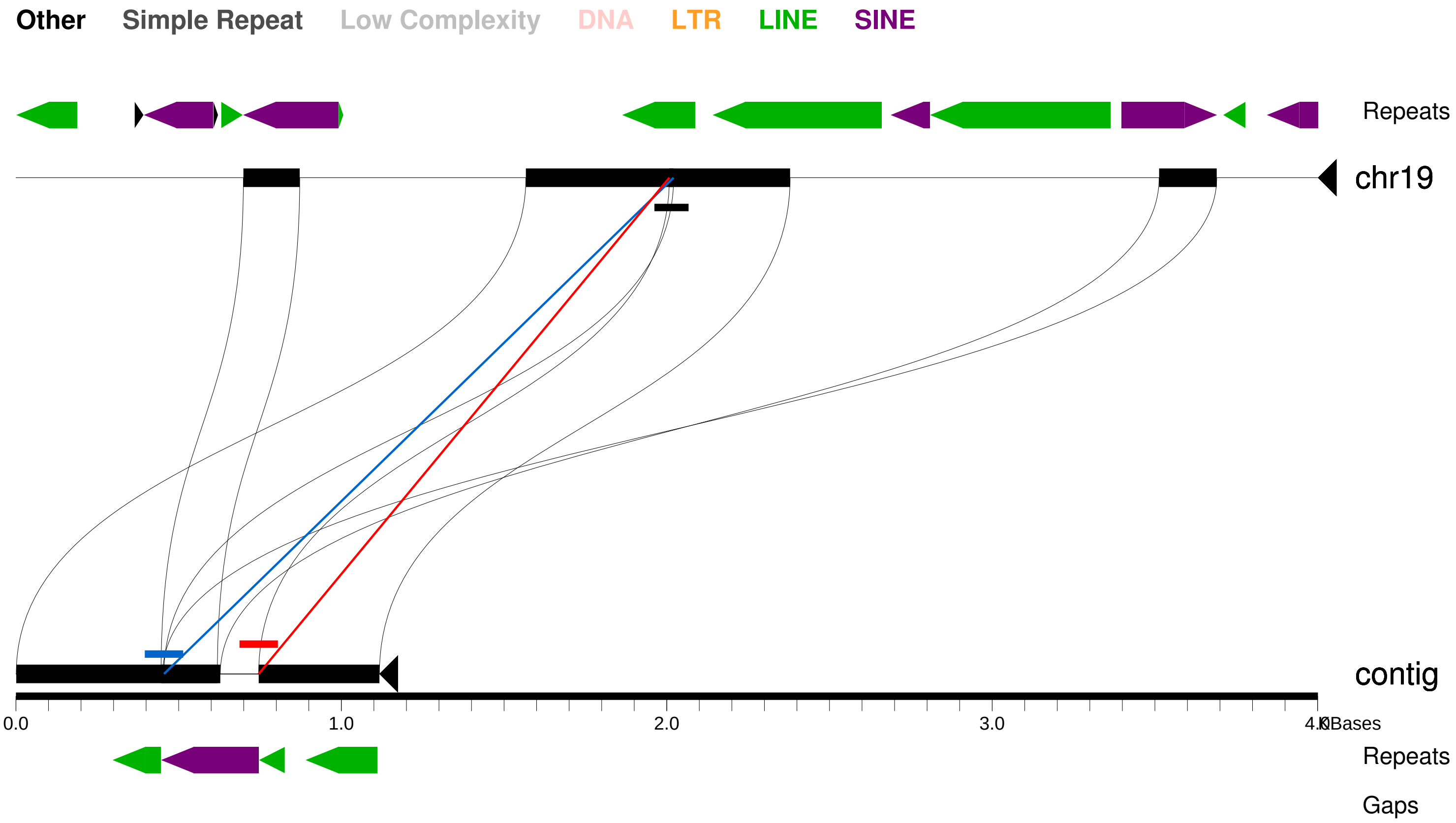

siteID: chr19_35073561 Scafftig4 - Breakpoint: chr19:35073547
chr19:35073493-35073603
Overlap Length: 6 INS size: 311 Genotyped: Yes

```
left    ATA--CCTAATGAACT-AAAAAAACT-----TAAAATCGCAAAAATCTCATAATGTTTTAAGAAAGTTGG
chrom   ATA--CCTAATGAACT-AAAAAAACT-----TAAAATCGCAAAAATCTCATAATGTTTTAAGAAAGTTTA
right   TCCAGCCTGGGGGACAGAGCGAGACTCCGTCTCAAAAAAAAAAAAAAAAAAAAAAAAAAAAAAAAGTTTA
        11111***111*1**11*111*1***11111*1***1111*****1111*1**111111**1******22

left    CCGGGCGCGGTGGCTCACGCCTGTAATCCCAGCACTTTGGGAGGCCGAGGCGGGCGG
chrom   CAAATTTGTGTTGGGCCGCACTGAAAGCC----ACCCTGGGCCGC--AAGCATCC--
right   CAAATTTGTGTTGGGCCGCATTGAAAGCC----ACCCTGGGCCGC--AAGCATCC--
        *22222222**2*22*22221**2**2**2222**22****22**22*2**222*22
```

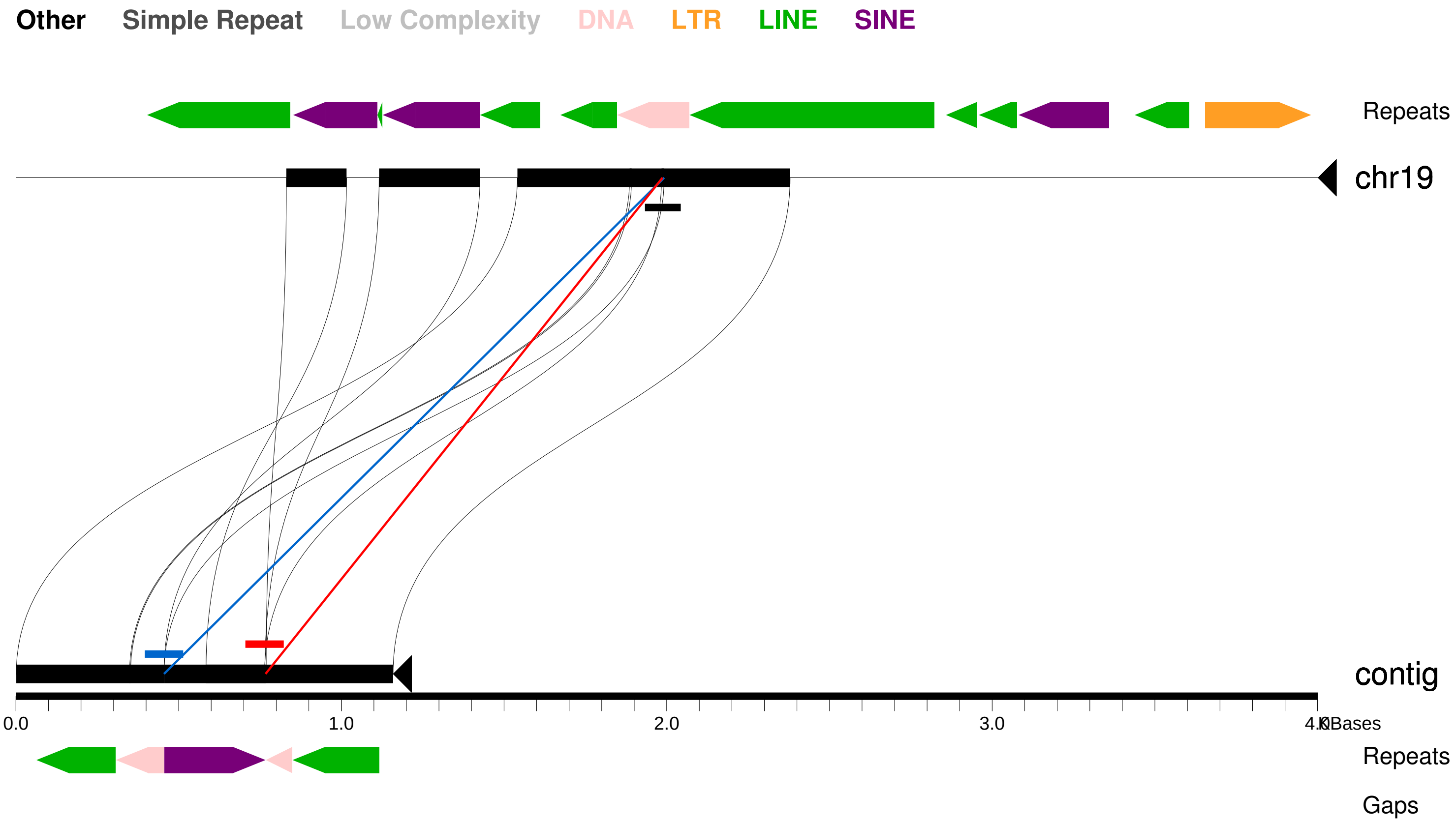

siteID: chr19_53814370 Scafftig2 + Breakpoint: chr19:53814557
chr19:53814397-53814730
Overlap Length: 14 INS size: 72 Genotyped: No

```
left   NNNNNNNNNNNNNNNNNNNNNNNNNNNNNNNNNNNNNNNNNNNNNNNNNNNNNNNNNNNNNNNNNNNNNN
chrom  TGCTTCCACGATCATTTAGGCGGCTGACAAATCTCCTTTTCTTCTCATGATTTCTACCTGCCTATCCTCT
right  --CTGC------------GGTGTCT--CTAACTTCTTTGTCTGCT-----TTAAGAGCT-------CACT
       NN22N2NNNNNNNNNNNN22N2N22NN2N22NN22N22N222N22NNNNN22NNN2N22NNNNNNN2N22

left   NNNNNNNN-NNNNNN-----------TCCCCCTCTTCCCTCTAAGCCGTGACTTCCTCTTCTGCGGTGTC
chrom  TCAGAGCC-AGCAGCAGCCTGTGAAATCCCCCTCTTCCCTCTAAGCCGTGACTTCCTCTTCTGCGGTGTC
right  T--GAGCCGAGATCCCGCCACTGCACTCCAGCCTGGGCGACAGAGC-GAGACTCC------------GTC
       2NN22222122NNN2N222NN22N2N***11*11111*11*11***1*1****1*111111111111***

left   TCTAACTTCTTTGTCTGCTTTAAGAGCTCACTTGAG-----------CCGAGATCC-------------C
chrom  TCTAACTTCTTTGTCTGCTTTAAGAGCTCACTTGATTACGTTAGACTCCTGGATCATCCAGGATAATCTC
right  TCAAAAAAAAAAAAAAAAAAA-AAGAGCTCACTTGATTATGTTAGACTCCTGGATCATCCAGGATAATCTC
       **1**1111111111111111**************222N22222222**22****22222222222222*

left   GCCACTGCACTCCAGCCTGGGCG------ACAGAGCGAGACTCCGTCTCAAAAAAAAAAAAAAAAAAAAAG
chrom  CCGAGTTCATTTCAGC-TGATTGTTAATTACATCCACAAAGTCTCTTTTGCCTACGTGGAACTTAACATA
right  CCGAGTTCATTTCAGC-TGATTGTTAATTACATCCACAAAGTCTCTTTTGCCTACGTGGAACTTAACATA
       2*2*2*2**2*2****2**222*222222***22222*2*2**22*2*22222*22222**222**2*22

left   AGCTCACTTGATTATGTTAGACTCCTGGATCATCCAG-------------GATAAT
chrom  ACCACACGATAACATCAGGGATTGGAGAACCAACCAATGCATTTTTTTATGGTAAA
right  ACCACACGATAACATCAGGGATTGGAGAACCAACCAATGCATTTTT----------
       *2*2***222*22**2222**2*222*2*2**2***2222222222    1N111N
```

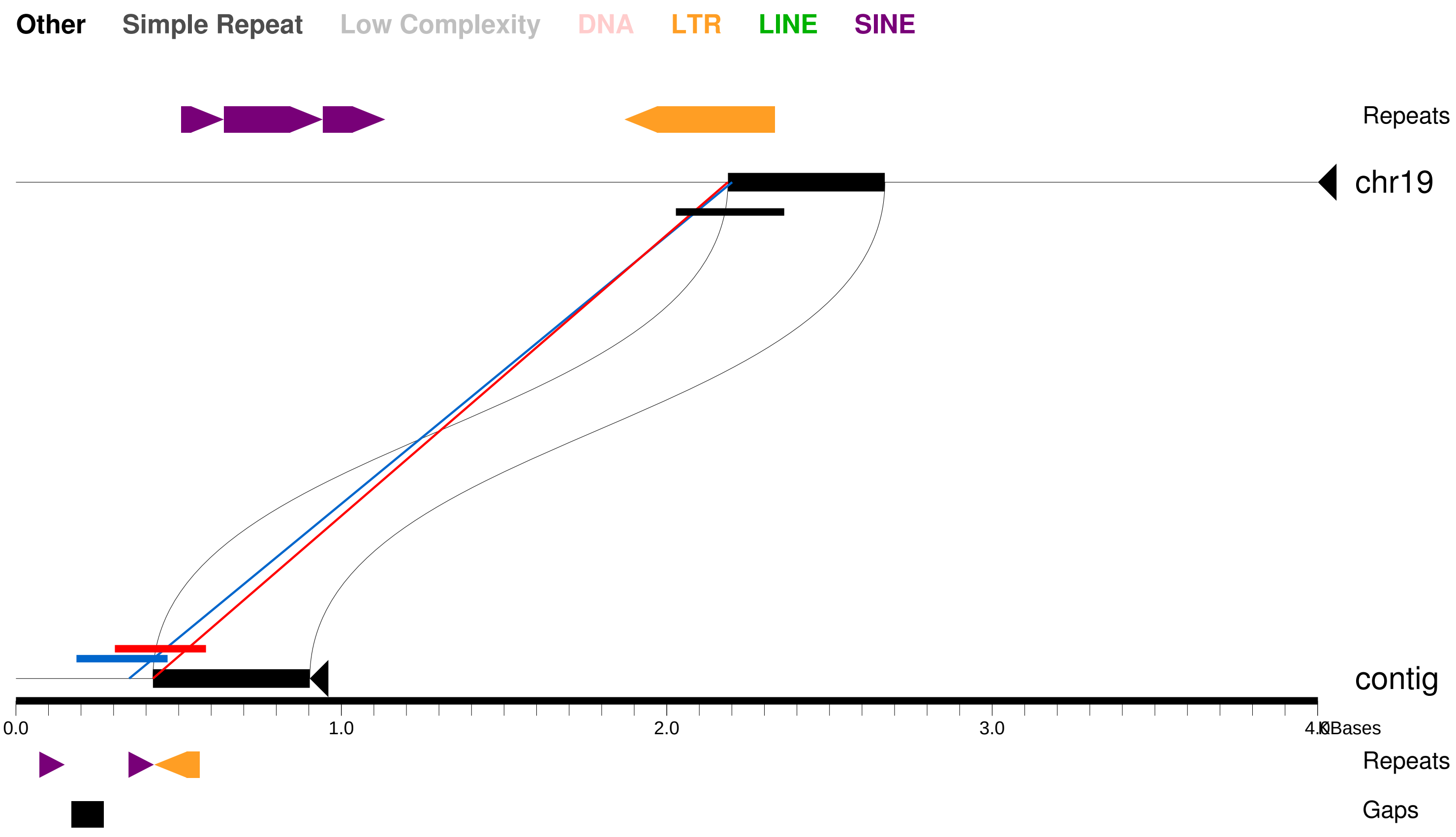

siteID: chr19_56966041 Scafftig1 - Breakpoint: chr19:56966041
chr19:56966002-56966093
Overlap Length: 21 INS size: 294 Genotyped: Yes

```
left    ---AAGA------CACTAGACTCCAATCT------------------CTTGCCATATGCAAAAATCAAAT
chrom   ---AAGA------CACTAGACTCCAATCT------------------CTTGCCATATGCAAAAATCAAAT
right   GCCGAGATCCCGCCACTGCACTCCAGCCTGGGCGACAGAGCGAGACTCTGTCTCAAAAAAAAAAAAAAAA
        1111***111111****11******11**111111111111111111**11*111*111*****11***1

left    AAAAATGGATTAAAGACGGCCGGGCGCGGTGGCTCACGCCTGTAATCCCAGCACTTTGGGAGGCCGAGGC
chrom   AAAAATGGATTAAAGACAAATCTACGA----CCTAAAAC----AATCAAACTACTAA-------------
right   AAAAATGGATTAAAGACAAATCTACGA----CCTAAAAC----AATCAAACTACTAA-------------
        *****************2222222**222222**2*22*2222****22*22***222222222222222

left    GGGCGG
chrom   ------
right   ------
        222222
```

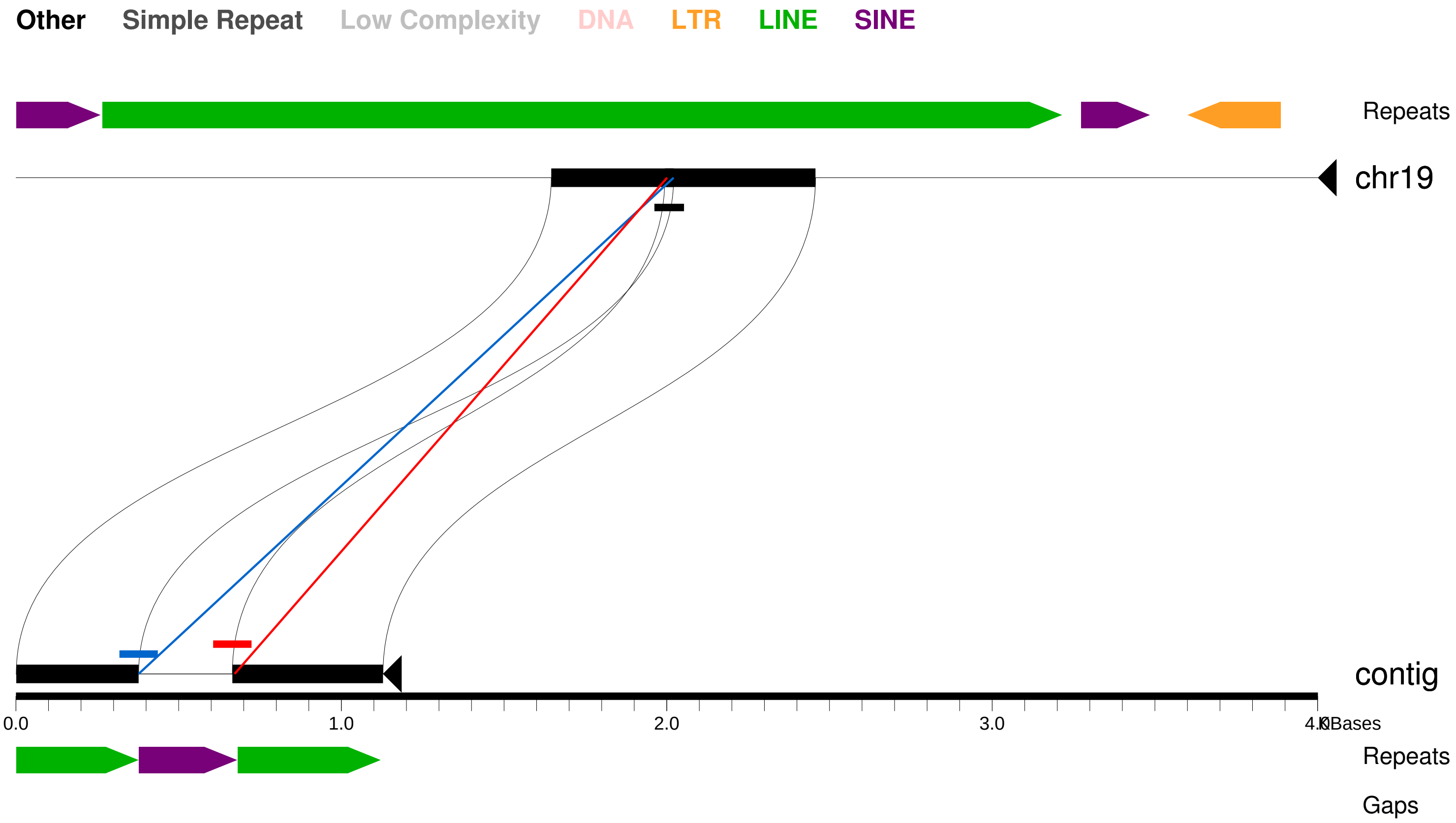

siteID: chr19_57637008 Scafftig1 + Breakpoint: chr19:57637011
chr19:57636969-57637070
Overlap Length: 18 INS size: 380 Genotyped: No

```
left    --------------CTTCCCATTTGCTTGGCAAATATTCCT---CCATCCCTTTATTTTGAGCCTATGTA
chrom   --------------CTTTCCATTTGCTTGGCAAATATTCCT---CCATCCCTTTATTTTGAGCCTATGTA
right   CCGCCCGCCTCGGCCTCCCAAAGTGCTGGGATTACAGGCGTGAGCCACCGCGCCCGGCCGAGCCTATGTA
        11111111111111**1N*1*11****1**111*1*11*1*111***1*1*11111111***********

left    TGTCTTTTTTNNNNNNNNNNNNNNNNNNNNNNNNNNNNNNNNNNNNNNNNNNNNNNNNNNNNNNNN
chrom   TGTCTTT-----------------GCACATGAGATAGGTCTCCTGAATATAGCACATCAATGGGTC
right   TGTCTTT-----------------GCACATGAGATAGGTCTCCTGAATATAGCACATCAATGGGTC
        *******22222222222222222222222222222222222222222222222222222222222
```

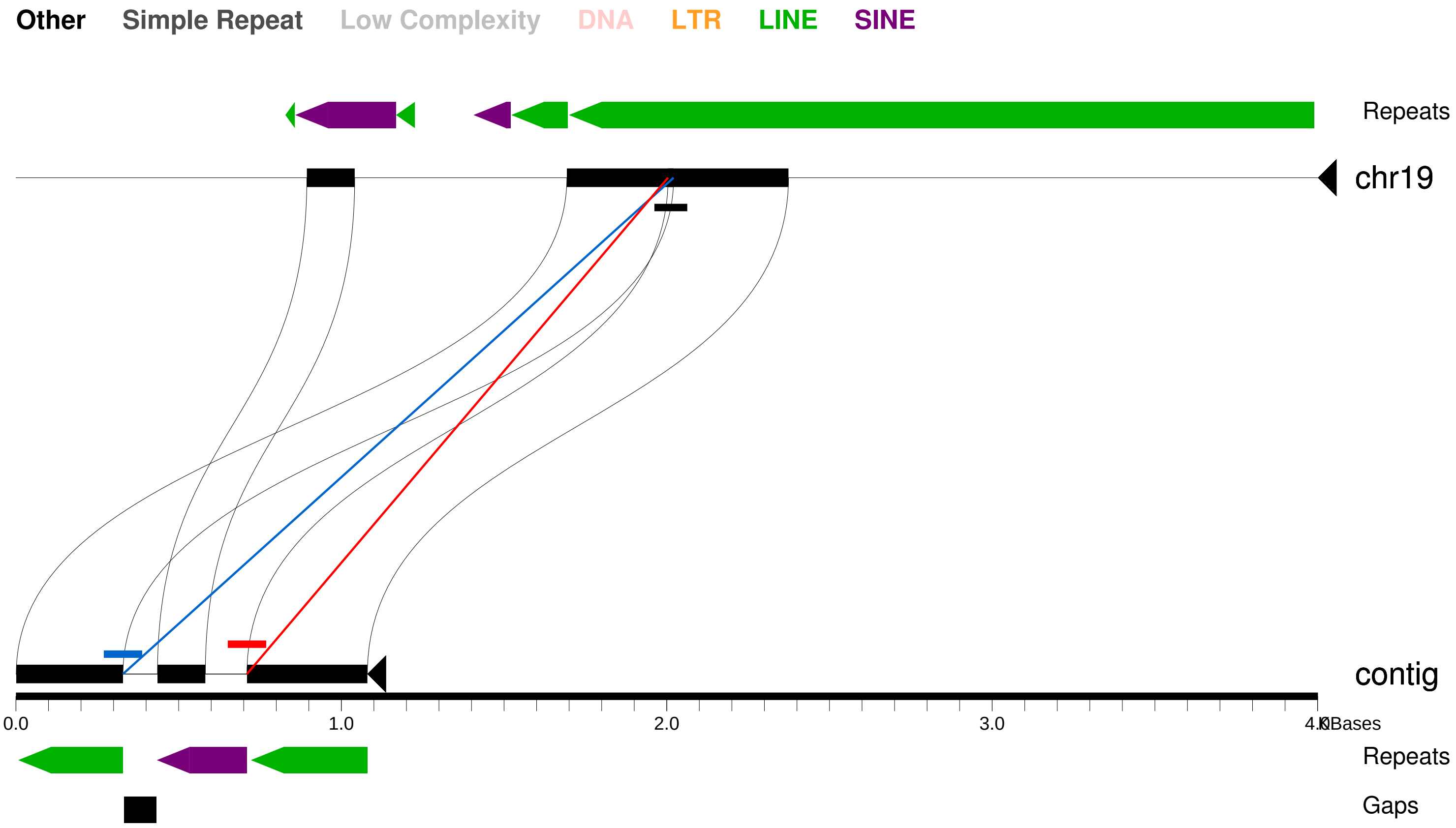

siteID: chr2_100785851 Scafftig1 - Breakpoint: chr2:100785854
chr2:100785808-100785913
Overlap Length: 14 INS size: 480 Genotyped: No

```
left     AACTGCCAAACTAAATTAACACAAAATGAATCATACACCTACATGT-------------AAAACCTAAAA
chrom    AACTGCCAAACTAAATTAACACAAAATGAATCATACACCTACATGT-------------AAAACCTAAAA
right    NNNNNNNNNNNNNNNNNNNNNNNNNNNNNNNNNNNNNNNNNNNNNNNNNNNNNNNNNNNAAAAACCTAAAA
         11111111111111111111111111111111111111111111111111111111111***********

left     CCCGGCTGGGCGCGGTGGCTCACGCCTGTAATCCCAGCACTTTGGGAGGCCGAGGCGGGCGG
chrom    CCCA-----------TACATTTCTAGAGAAAAACAA--AATAAAGATATTCATGAACTTAGG
right    CCCA-----------TACATTTCTAGAGAAAAACAA--AATAAAGATATTCATGAACTTAGG
         ***222222222222*222*22*2222*2**22*2*22*2*222*22222*22*222222**
```

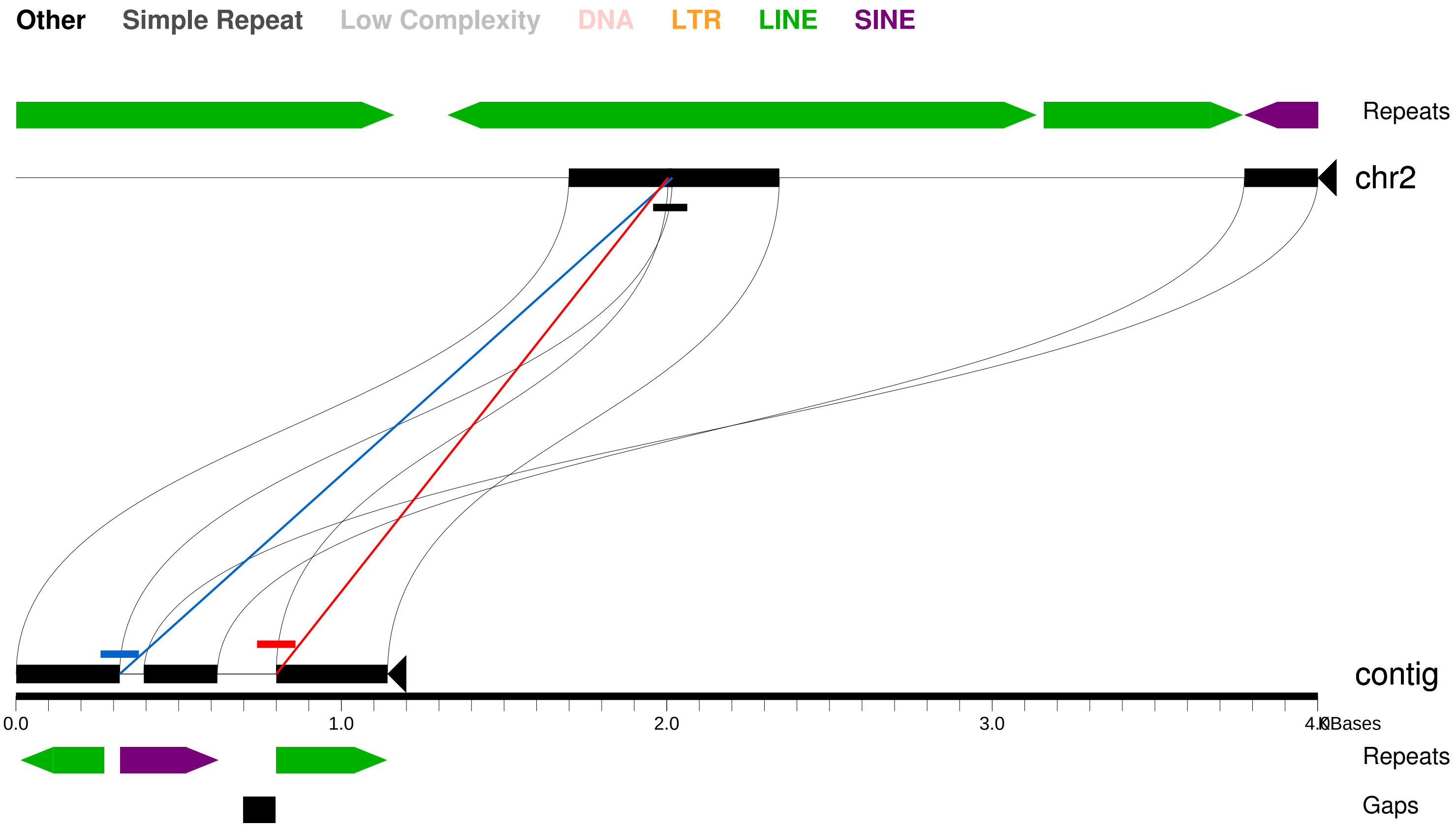

siteID: chr2_106452573 Scafftig9 + Breakpoint: chr2:106452567
chr2:106452534-106452626
Overlap Length: 12 INS size: 314 Genotyped: Yes

```
left    ---------TCATTATTTTAGATTCTT----TTTTAGGCATTTTAT-------------GGATTTCCTTT
chrom   ---------TCATTATTTTAGATTCTT----TTTTAGGCATTTTAT-------------GGATTTCCTTT
right   CCGCCCGCCTCGGCCTCCCAAAGTGCTGGGATTACAGGCGTGAGCCACCGCGCCCGGCCGGATTTCCTTT
        111111111**1111*111*1*1*11*1111**11****1*111111111111111111***********

left    TTTTTTTTTTTTTTTTTTTTTTTTTTTTTTTTTTTGAGACGGAGTCTCGCTGTCGCCCAGGCTGGAGTGC
chrom   TCTTTGTTCTGTTATTAGAGAATT--------------ACTGTGTTTCTTTGG-----AGGTTTTATT--
right   TCTTTGTTCTGTTATTAGAGAATT--------------ACTGTGTTTCTTTGG-----AGGTTTTATT--
        *2***2**2*2**2**222222**22222222222222**2*2**2**22**222222***2*22*2*22

left    AGTGG
chrom   -----
right   -----
        22222
```

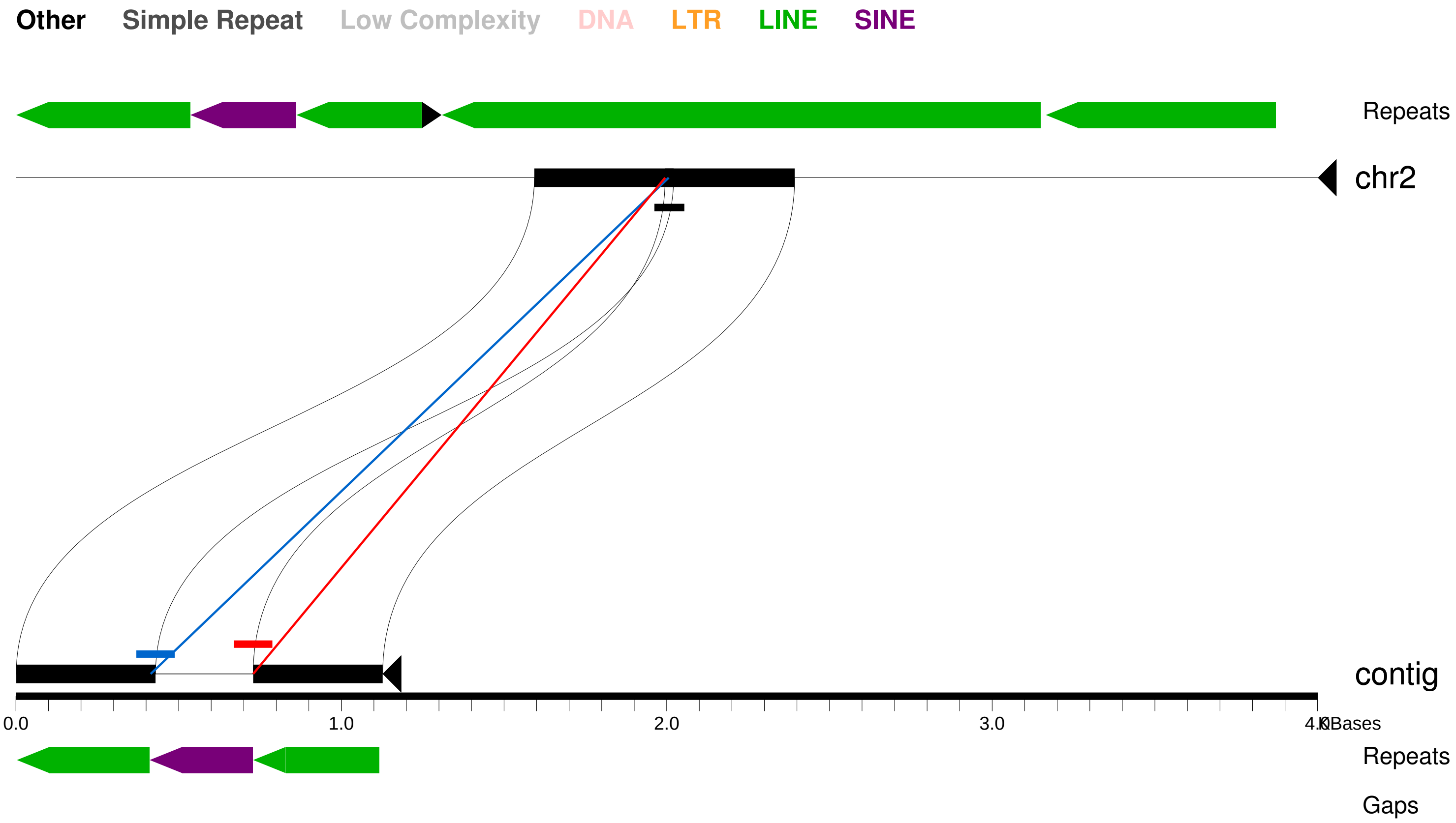

siteID: chr2_106585635 Scafftig4 - Breakpoint: chr2:106585611
chr2:106585451-106585787
Overlap Length: 17 INS size: 311 Genotyped: Yes

```
left    TTTT----------------ATTATCAATGTTTTAATGGCTATTATCAATGTTTTAATGGCTATTATTTA
chrom   TTATCAATGTTTTAATGGCTATTATCAATGTTTTAATGGCTATTATCAATGTTTTAATGGCTATTATTTA
right   AGA-----------ATGGCGTGAACCCGGG----AGGCGGAGCTTGCAGTG----AGCCGAGATCCCGCC
        1121          22222N111*1*111*1111*111*1111*11**1**1111*111*11**111111

left    ATTATTATCAATGTTTTAATTATCAATGGGATAACACAG-GAGAAACAGGAGGAGCCCAATGATGAAGAT
chrom   ATTATTATCAATGTTTTAATTATCAATGGGATAACACAG-GAGAAACAGGAGGAGCCCAATGATGAAGAT
right   ACTGCACTCCAGCCT-------------GGGCGACAGAGCGAGACTCCGTCTCAAAAAAAAAAAAAAAAA
        *1*1111**1*111*1111111111111**111***1**1****11*1*1111*1111**11*11**1*1

left    TACTGTCCCTACCCATGTTTTAAATCCTCCTAAATTAGCCGGGCGCGGTGGCTCACGCCT-GTAA--TCC
chrom   TACTGTCCCTACCCATGTTTTAAATCCTCCTAAATTAGAGAACCACCCTCCCAGAAGGTTTGCCAGGTCC
right   AAAAAAAAAAAAAAA------AAATCCTCCTAAATTAGAGAACCACCCTCCCAGAAGGTTTGCCAGGTCC
        1*11111111*111*111111*****************22222*2*22*22*22*2*22*2*22*22***

left    CAGCACTT-----TGGGAGGCCG-AGGCGGGCGGATCAC--GAGGTCAGGAGA----TCGAGACC-----
chrom   CATCCCTTCCAGGTTTGAGTTGGTACTTGGGCTACTTTTCTGATGTTTGAAGCAATTTCTAGAACTGCTT
right   CATCCCTTCCAGGTTTGAGTTGGTACTTGGGCTACTTTTCTGATGTTTGAAGCAATTTCTAGAACTGCTT
        **2*2***22222*22***222*2*222****222*22222**2**22*2**22222**2***2*22222

left    -----------ATCCCGGCTAAAACGG---TGAAACCCCGT---CT--CTACTGAAA-
chrom   TTTCCATTATCATCTATGTTAAAACAGCAATTAGAGACATTAAACTTACCACAGACCC
right   TTTCCATTATCATCTATGTTAAAACAGCAATTAGAGACA-------------------
        22222222222***222*2******2*222*2*2*22*2N1   11  1N11N11NN
```

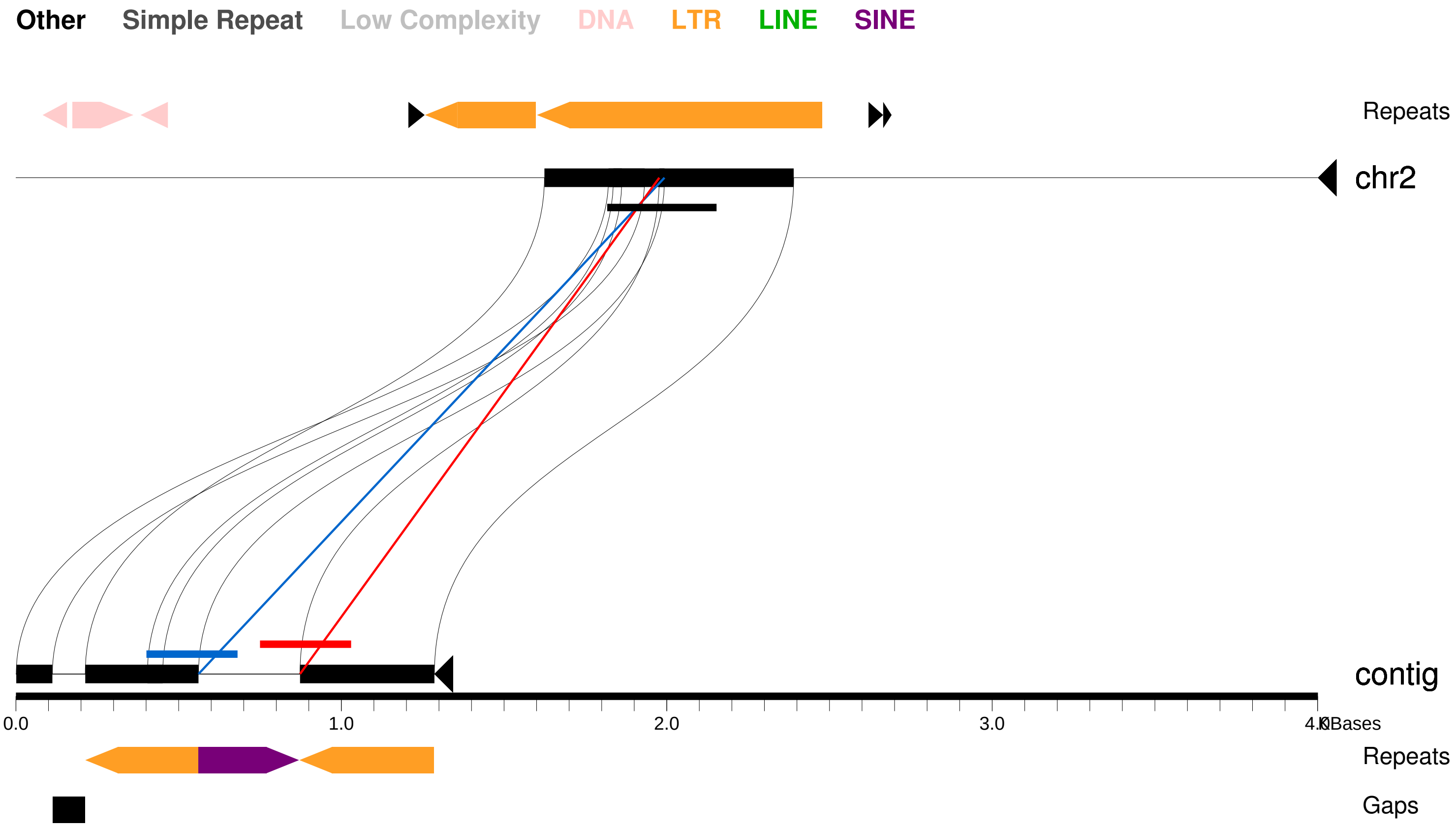

siteID: chr2_107924640 Scafftig1 + Breakpoint: chr2:107924608
chr2:107924448-107924784
Overlap Length: 17 INS size: 255 Genotyped: Yes

```
left    -----------TAATTTTCTTTCTCACCAATAGTGTATAAGAGTTCCCTTTTCTCTGGATCCTCAAAGCA
chrom   AATTGCTGTACTAATTTTCTTTCTCACCAATAGTGTATAAGAGTTCCCTTTTCTCTGGATCCTCAAAGCA
right   ----------CTACAGGCGTGCGCCACCACTCCTGGCTAATTTTTGTATTTTTAGTAGAGACG----GGG
                  2**111111*1111*****1*11**11***111**111****111*1**11*11111*11

left    TGTTACTTTTTCTTTTTATCAATAGCCATTCTAACAGGGGTAAAATGGTATCTCATTGTGGTTTTAATTT
chrom   TGTTACTTTTTCTTTTTATCAATAGCCATTCTAACAGGGGTAAAATGGTATCTCATTGTGGTTTTAATTT
right   TTTCACCGTGTC-----------AGCCA---------GGATGGTCTCG-ATCTCCT-GACCTCGTGATCC
        *1*1**11*1**11111111111*****111111111**1*1111*1*1*****1*1*111*11*1**11

left    GAATGTCCTTGATAATTAGTGATGTTCAGTATTTTTTT---------------TTTCTTTTTTGT---TT
chrom   GAATGTCCTTGATAATTAGTGATGTTCAGTATTTTTTACATACCTGTTGGCCATTTCTATTTCTTATTTT
right   GCCCGTCTCGGCC---TCCCGATGTTCAGTATTTTTTACATACCTGTTGGCCATTTCTATTTCTTATTTT
        *111***111*11111*111****************2222222222222222*****2***22*222**

left    GAGACGGAGTTTCGC------TCTGTCGCCC--AGGCTGGAGCGCAGTGGCGCGATC-TCGACTCACTGC
chrom   AAGAAATATTTTTTCATGTTATCTGTCCACTTTATAATGG-GATTATTAGTGGGTTGGTTTTCTTGTTGA
right   AAGAAATATTTTTTCATGTTATCTGTCCACTTTATAATGG-GATTATTAGTGGGTTGGTTTTCTTGTTGA
        2***222*2***22*222222******22*222*222***2*222*2*2*2*2*2*22*222**222**2

left    AAGCTCCGCCTCCCGGGTTCAC----GCCATTCT---CCTGCCTCA------------
chrom   TTTGTTTGAGTTCCTTGTATATTTTGGATATTAGTTCCCTGTCTGATGAATTGGGTAC
right   TTTGTTTGAGTTCCTTGTATATTTTGGATATTAGTTCCCTG-----------------
        2222*22*22*2**22**22*22222*22***22222****N11N1
```

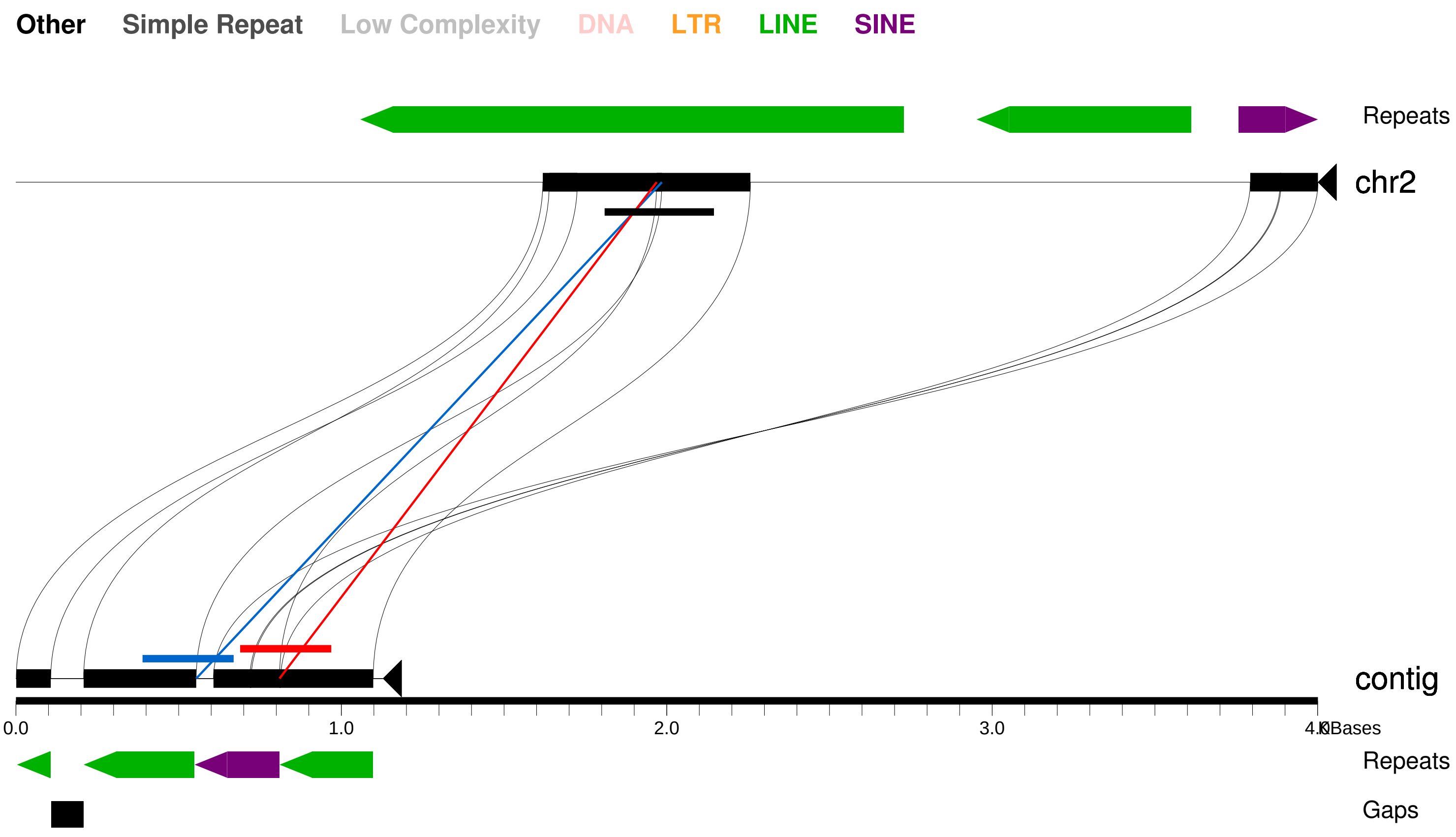

siteID: chr2_112447442 Scafftig8 - Breakpoint: chr2:112447423
chr2:112447263-112447596
Overlap Length: 14 INS size: 311 Genotyped: Yes

```
left     ---------GCAGGGGTGGGAGGAGGCCCCCTCAGAGAAACTTCGAAAATCAGTAGGGCCTTTTCTGTCA
chrom    TTTCTCAGGGCAGGGGTGGGAGGAGGCCCCCTCGGAGAAACTTCGAAAATCAGTAGGGCCTTTTCTGTCA
right    TTTAGTAGAGACGGGGTTTCACCGTGTTAGCCAGGATGGTCT-CGA--------------TCTCCTGACC
         222NNN22N*11*****111*1111*1111*112**1111**1***11111111111111*1*1***1*1

left     TCACCATGACAGGGAAGGGACACTGACAGAATTTGGCAGATGCAGTAAGAAATCCAGAACAATCCCTGAC
chrom    TCACCATGACAGGGAAGGGACACTGACAGAATTTGGCAGATGCAGTAAGAAATCCAGAACAATCCCTGAC
right    TCATGATCCGCCCGCCTCGGC-CTCCCAAAGT---GCTG--GGATTAC--AAGCGTGAGCCACCGCG---
         ***11**111111*1111*1*1**11**1*1*111**1*11*1*1**111**1*11**1*1*1*1*1111

left     AACAAAGAATCATCCCACATCCCAAATGATTTTCTTTTTTTTTTTTT--------------------TTT
chrom    AACAAAGAATCATCCCACATCCCAAATGATTTTCAAACATCCTTGTTGACAGCCTCATGGGTAAAAATCT
right    -------------CCCGGCCCCCAAATGATTTTCAAACATCCTTGTTGACAGCCTCATGGGTAAAAATCT
         1111111111111***1111**************22222*22**2**2222222222222222222222*2*

left     TTTTTTTTTTTGAGAC-GGAGTCTCGCTC---------TGT-------GGCCCAGGTGGGAGTGCAGTGG
chrom    ATTAATTACCTGAGCCTGGAACCTAATTCCATTTTATATGTAAACATTGTACGGGGTGTTTTTTACATGG
right    ATTAATTACCTGAGCCTGGAACCTAATTCCATTTTATATGTAAACATTGTACGGGGTGTTTTTTACATGG
         2**22**222****2*2***22**222**222222222***2222222*22*22****2222*2222***

left     CGCAATCTCGGCTCACTG--CAAGCTCCGCCTCCAGGGT-----TCACGCCATT
chrom    CTTAAAATACACTGAATTTCCCAGGAATGCAACCATTGTACCCATCAAGAGAAG
right    CTTAAAATACACTGAATTTCCCAGGAATGCAACCATTGTA--------------
         *22**22*222**2*2*222*2**2222**22***22**2    111N1NN1NN
```

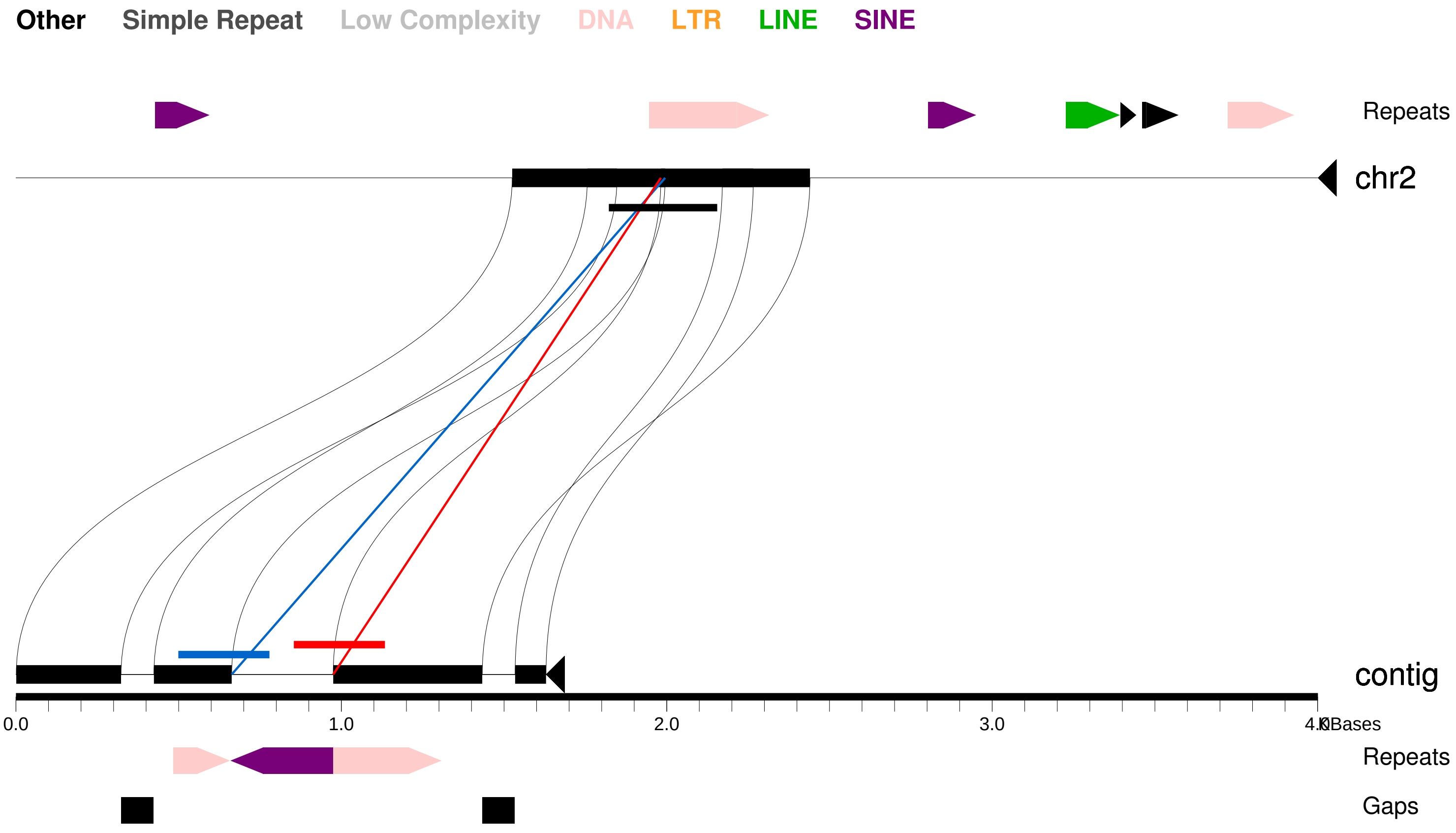

siteID: chr2_11353695 Scafftig2 + Breakpoint: chr2:11353692
chr2:11352927-11353852
Overlap Length: 16 INS size: 296 Genotyped: Yes

```
left   ------------------------------------------------------------------------
chrom  TGGGCTATTCCACTCACATGATTTCAGCTACTTACATACCAATGACTCAAATATTTCTAACTTCTACTAC
right  ------------------------------------------------------------------------

left   ------------------------------------------------------------------------
chrom  TATAGCCTACTGCAGTGCAGACATGCTAATTTGGCTGTCCCATAGGTACCCTCAAACTTAAGAGGACTAA
right  ----------------------------------GCTG------AGG-------------------------
                                         2222      222

left   ------------------------------------------------------------------------
chrom  AGTGGGTTTATCACTGTCTCATGAGCTATTTTTCCTTTATTCTTTATTAACAGCATCACTATATATCTTG
right  -------------------CAGGAG-----------------------------------------------
                          22N222

left   ------------------------------------------------------------------------
chrom  TCACAACCTACAAATCTGGGACTCATCTTTGACTGCTACTCATATGCCATATCTAATCAACTATTAAGTG
right  ------------------------------------------------------------------------

left   ------------------------------------------------------------------------
chrom  CTGCCAAATATTCCTCTTCCAAATATTTCTGAGTCTTCCAATTTTTCCAACCTTTTAATGGCTGTACTAA
right  ----------------------------------------------------------AATGGCG-------
                                                                 222222N

left   ------------------------------------------------------------------------
chrom  GTCTCCCATTATTCCTTATCAGCATTACTGAAAAAGTCTACTGACCAGTCTCTCTACCTCTAGTCTTGTC
right  ------------------------------TGAA--------------------------------------
                                     2222

left   ------------------------------------------------------------------------
chrom  CTCCTCAAATCTTTTCTCCACACAGTATACAGCGATCTATCTTTAATGTACCTCTAATAAGATCATCCAA
right  ------------------------------------------------------------------------

left   ------------------------------------------------------------------------
chrom  TTTAAAGCCCTTAAGTGGCTAGCCCATTACTACATTCAAAATTTCTTAATAGAGTATTCGCTATCTTGCA
right  -------CCCGGGAGGCG----------------------------------GAG----------CTTGCA
              222NNN22NN2                                 222          222222

left   ----------------------------------------------------------------CATAACAA
chrom  -TGATCTACCTCATGCCGAGCTCTGTAGCCTCATTCTGACCACTGAATTCCTCATGTCCTTACATAACAA
right  GTGA----------GCCGAGATC-----CCGC--------CACTGCACTCC-------------------
       1222          222222N22     22N2        22222N2N222                11111111

left   CCACATTGTGATCTTCAGGCCAATCTAACCTGATGCCTTTTTCTTTTTTAAGTTTCACTGGAACGTAGCC
chrom  CCACATTGTGATCTTCAGGCCAATCTAACCTGATGCCTTTTTCTTTTTTAAGTTTCACTGGAACGTAGCC
right  --------------------------AGCCTGG-------------------------------------GCG
       11111111111111111111111111*1****1111111111111111111111111111111111111**1

left   ACATCCATTCCTTCATGAATCACAGCAGGGCTGAACAGTTGCAAGACCATATAACCCACAGAAGATAAAA
chrom  ACATCCATTCCTTCATGAATCACAGCAGGGCTGAACAGTTGCAAGACCATATAACCCACAGAAGATAAAA
right  ACAG-------------------ACCCACACT---CCCTCTCAAAA--AAAAAA-------AAAAAAAAA
       ***1111111111111111111****11*1**111*1**11***1*11*1*1**1111111**1*1****

left   TATTTATTACCAGGCCGGG---CGC---------GGTGGCTCACGC-CTGTAATCCCAGCACTTTGGGAG
chrom  TATTTATTACCATGCCTTTAAACGATTAAAATTTGCTGACTCCCACTCTATACTCCAGAAAACAAACAAT
right  TATTTATTACCATGCCTTTAAACGATTAAAATTTGCTGACTCCCACTCTATACTCCAGAAAACAAACAAT
       ***********2***222222**22222222222*2**2***2*2*2**2**2***2222*2222222*2

left   GCCGAGGCGGGCGG--ATCA-CGAGGTCA--GGAGATCGAGACCATCCTGGCTAAAACGGTGAAA---CC
chrom  GCCTTTTTTTATTTTCCATCTTCTATGTCTTTGCTCATGCTGTTCTCCCTAACTTAGAATGTGCCTTTGCC
right  GCCTTTTTTTATTTTCCATCTTCTATGTCTTTGCTCATGCTGTTCTCCCTAACTTAGAATGTGCCTTTGCC
       ***22222222222222***22*2*2***222*222**222*22*22***22**2*2*22***222222**

left   C--CGTCTCTA-CTAAA---
chrom  CTACGGACCTATCCTGC---
right  CTACGGACCTATCCTGCTGT
       *22**222***2*222111
```


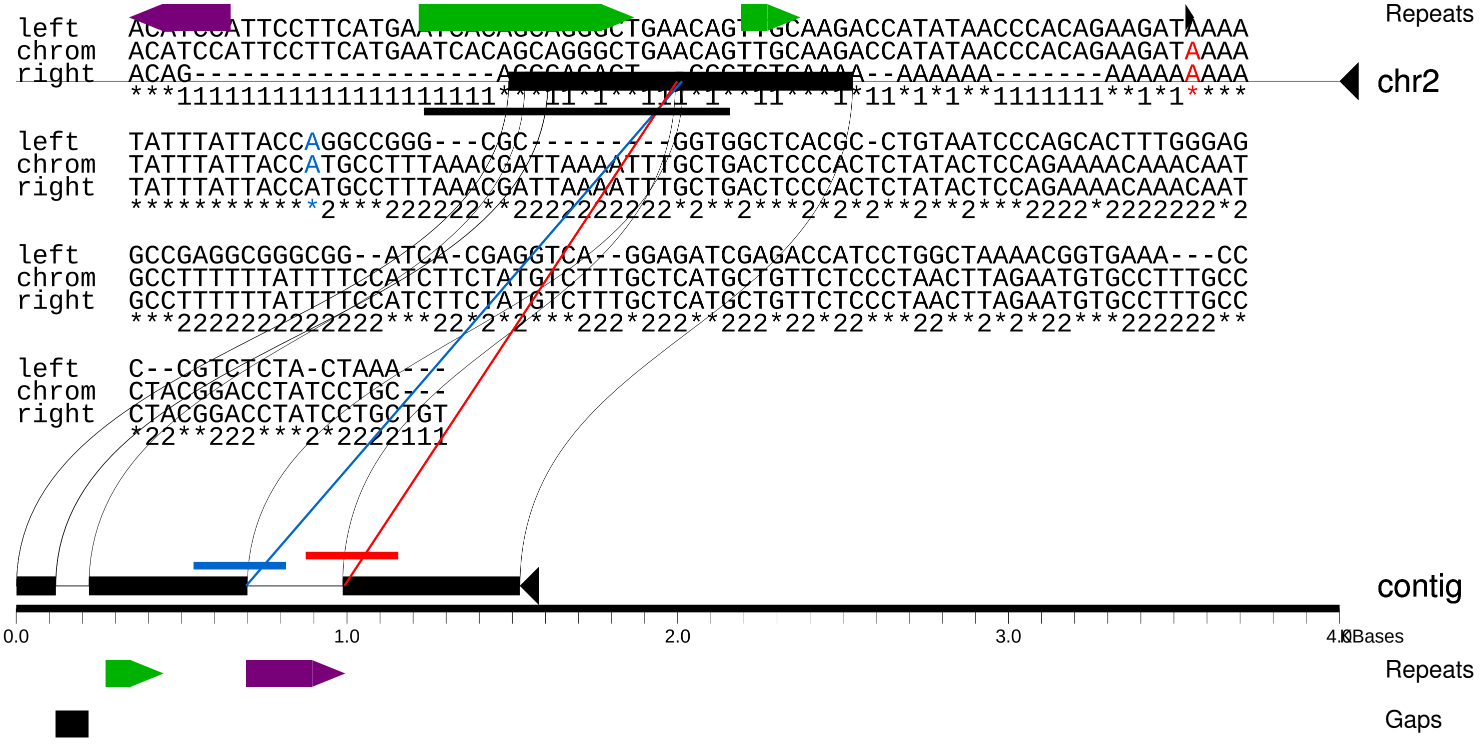

siteID: chr2_114106436 Scafftig6 - Breakpoint: chr2:114106436
chr2:114106387-114106482
Overlap Length: 11 INS size: 309 Genotyped: Yes

```
left   -------------------GGGACACAGTGTAATA----GGCTGTAGTCATAGACTCAGAAAAAGGGACA
chrom  -------------------GGGACACAGTGTAATA----GGCTGTAGTCATAGACTCAGAAAAAGGGACA
right  CGCCACTGCACTCCAGCCTGGGCGACAGAGCGAGACTCCGTCTCAAAAAAAAAAAAAAAAAAAAAAAAAA
       1111111111111111111***11****1*11*1*1111*1**11*111*1*1*111*1*****111*1*

left   AGAAATCAGAAAAGGCCGGGCGCGGTGGCTCACGCCTGTAATCCCAGCACTTTGGGAGGCCGAGGCGGGC
chrom  AGAAATCAGAAAAT-----------TGTATT---CCTTTCAT---AGCACTGGGGAAGTCACTTAC----
right  AAAAATCAGAAAAT-----------TGTATT---CCTTTCAT---AGCACTGGGGAAGTCACTTAC----
       *1***********222222222222**22*2222***2*2**222******22**2**2*22222*2222

left   GG
chrom  --
right  --
       22
```

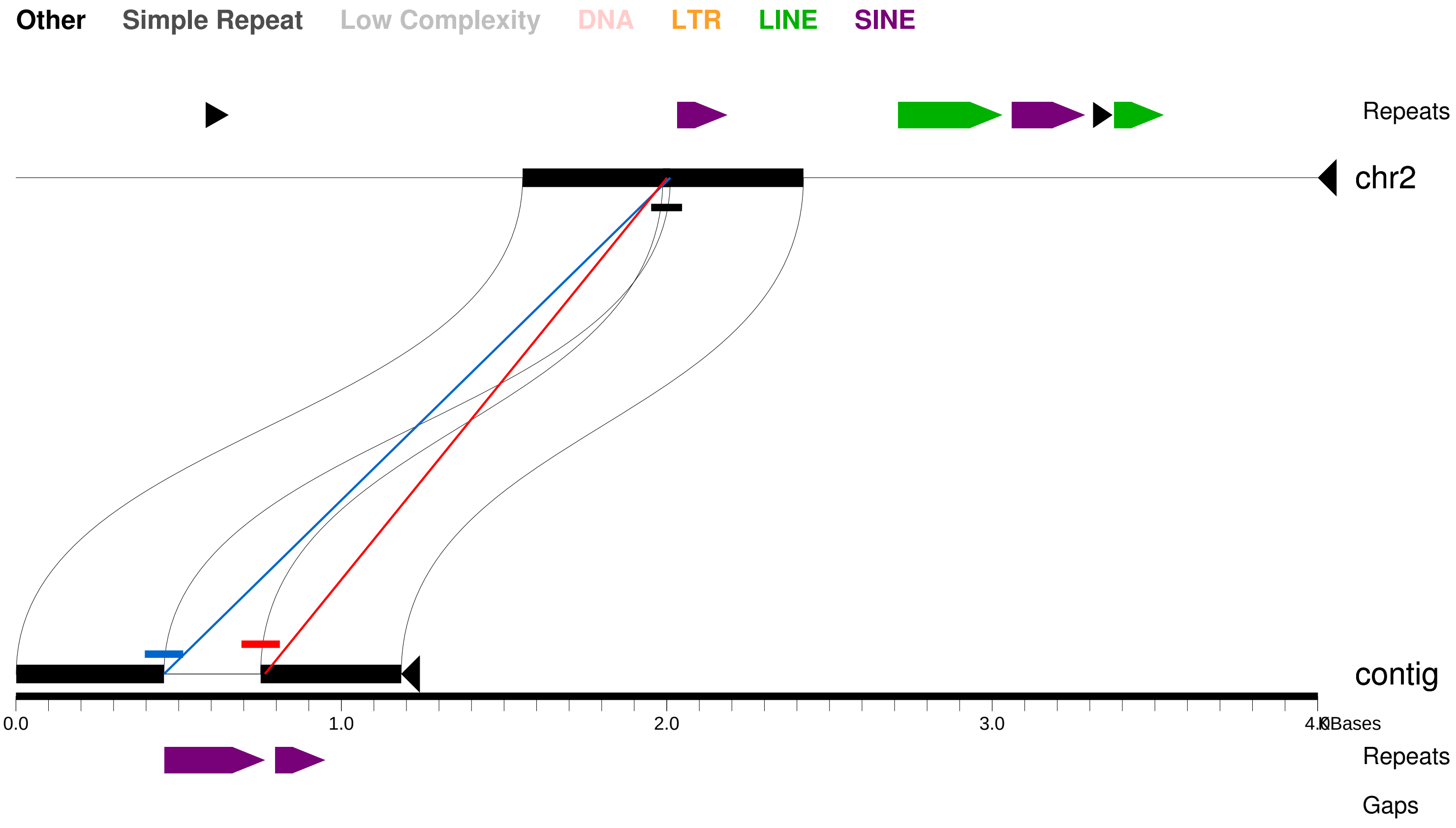

siteID: chr2_115833861 Scafftig8 + Breakpoint: chr2:115833867
chr2:115833827-115833926
Overlap Length: 14 INS size: 302 Genotyped: Yes

```
left    CAGTTTATTTT------------TTAAGGAATTAAATGCTATAAACACAATT-------GAGATCCTTTT
chrom   CAGTTTATTTT------------TTAAGGAATTAAATGCTATAAACACAATT-------GAGATCCTTTT
right   CCGCCCACCTCGGCCTCCCAAAGTGCTGGGATTACAGGCGTGAGCCACCGCGCCCGGCCGAGATCCTTTT
        *1*111*11*1111111111111*111**1****1*1**111*11***11111111111***********

left    TCTTTCTTTTTT---TTTTTTTTCCGAGACGGAGTCTCGCTCTGTCCCCCAGGCTGGAGTGCAGTGGCGC
chrom   TCTACCTTTCTCCCATATCATTTCCT----------TCCCTCC-TCCTCCA--CTATA---------CAT
right   TCTACCTTTCTCCCATATCATTTCCT----------TCCCTCC-TCCTCCA--CTATA---------CAT
        ***22****2*2222*2*22*****22222222222**2***22***2***22**22*222222222*22

left    G
chrom   A
right   A
        2
```

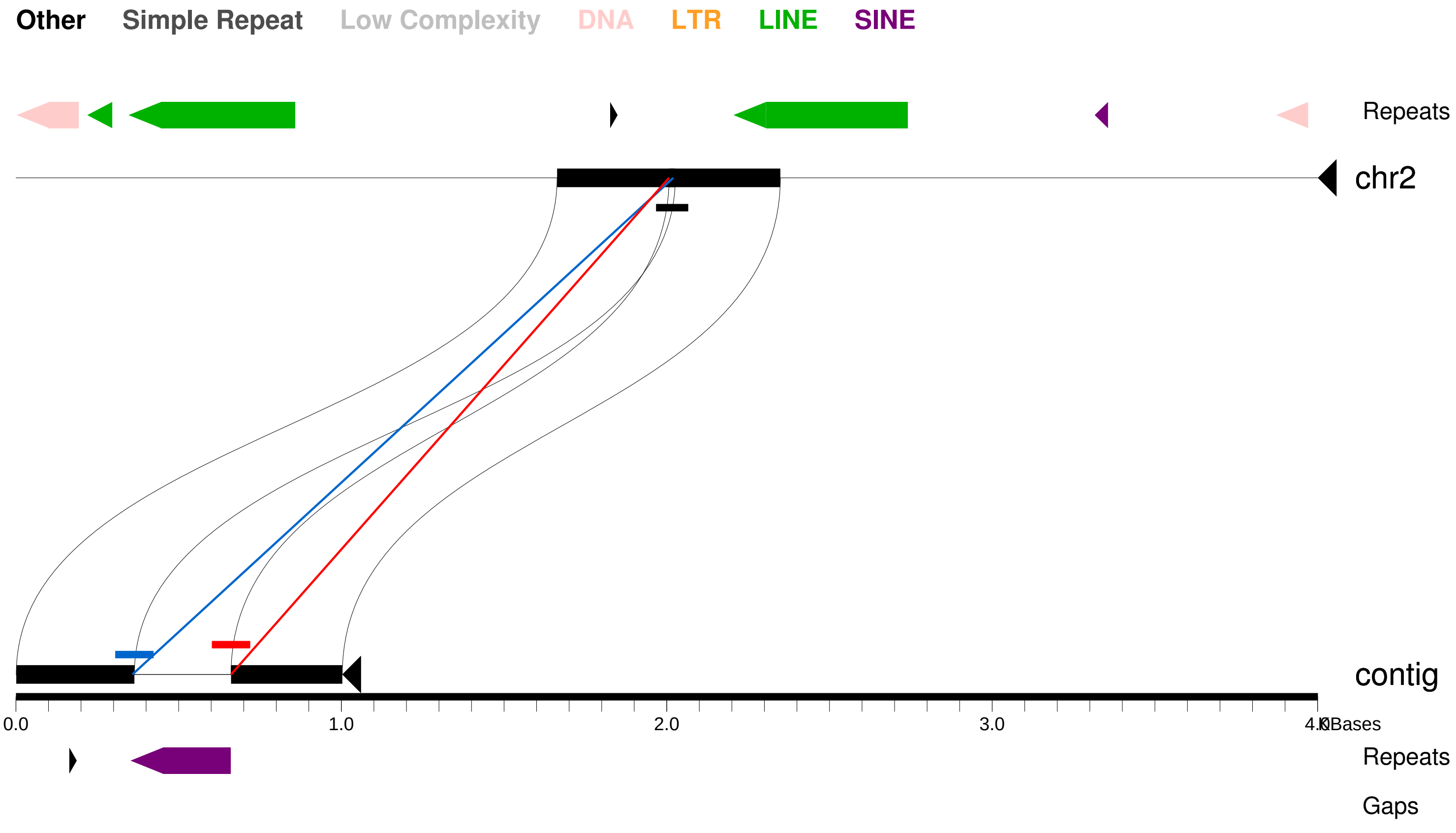

siteID: chr2_117543044 Scafftig2 - Breakpoint: chr2:117543091
chr2:117543048-117543150
Overlap Length: 17 INS size: 258 Genotyped: No

```
left     GTAATCTGAAACCAGTTTT-ATCTAATGGC-TACTGAAAAAACCT----------------GCTGAAACT
chrom    GTAATCTGAAACCAGTTTT-ATCTAATGGC-TACTGAAAAAACCT----------------GCTGAAACT
right    TTAGCCGGGATG--GTCTCGATCTCCTGACCTCGTGATCCGCCCGCCTCGGCCTCCCAAAGGCTGAAACT
         1**11*1*1*1111**1*11****11**1*1*11***11111**11111111111111111*********

left     CTTAAGACNNNNNNNNNNNNNNNNNNNNNNNNNNNNNNNNNNNNNNNNNNNNNNNNNNNNNNNNNNN
chrom    CTTAAGACTAGTTTTATTCACTGCTGTCCCTCACTAACCAGAGCTTGCCAG----------------
right    CTTAAGACTAGTTTTATTCACTGCTGTCCCTCACTAACCAGAGCTTGCCAG----------------
         *******2222222222222222222222222222222222222222222222222222222222222
```

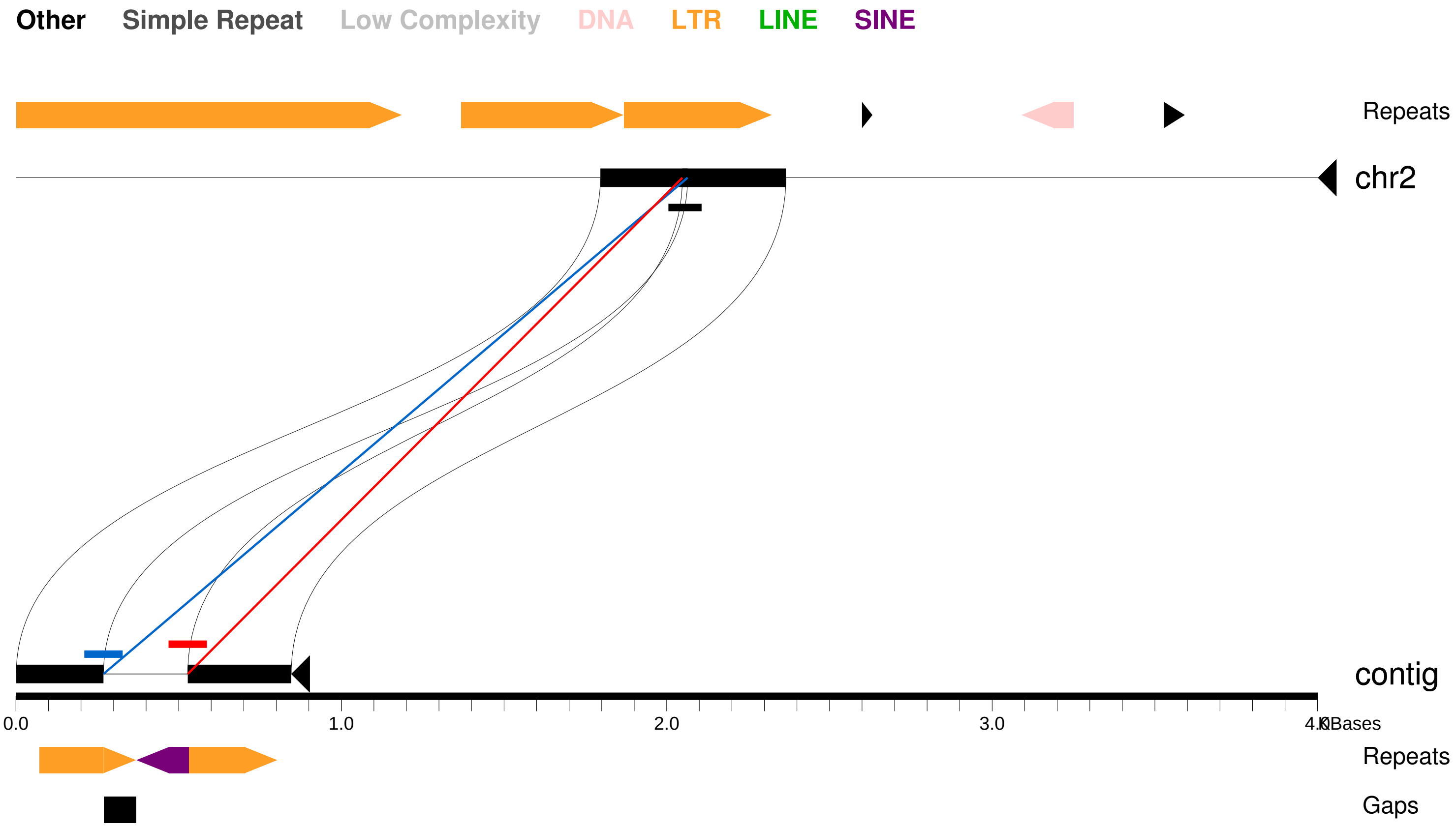

siteID: chr2_119155732 Scafftig1 - Breakpoint: chr2:119155716
chr2:119155556-119155889
Overlap Length: 14 INS size: 329 Genotyped: Yes

```
left   --------------AGAGACACGATTTAGGGTATCTGGCAGAAGAAATTTCTAAGGAGCCAGCCTTCAAG
chrom  GAACTTTGAACTTGAGAGACACGATTTAGGGTATCTGGCAGAAGAAATTTCTAAGGAGCCAGCCTTCAAG
right  CG----TGAACCCGGGAAGCG-GAGCTTG-----CAGTGAGCCGAGATTGCGCCACTGC-AGTCCGCAG-
       NN    22222NN21**11*11**11*1*11111*1*11**11**1***1*111111**1**1*11**11

left   ATGTGACTTGGGTG-CTGTTAAAGGCATTCAGTTTTATAAGGGAAGCAGAGCATCAAAGTTCAGAAAATT
chrom  ATGTGACTTGGGTG-CTGTTAAAGGCATTCAGTTTTATAAGGGAAGCAGAGCATCAAAGTTCAGAAAATT
right  -TCCGGCCTGGGCGACAGAGCGAGAC--TCCGTCTCAAAAAAAAAAAAAAAAAAAAAAAAAAAAAAAAAA
       1*11*1*1****1*1*1*1111**1*11**1**1*1*1**111**11*1*11*11***1111*1****11

left   TGCAGCCTGACAGTGTGATAGAAAAGAAACACTCAGGCCGGGCGCGGTGGCTCACGCCTGT-------AA
chrom  TGCAGCCTGACAGTGTGATAGAAAAGAAACACTCATTTTCTGAGGTGAAATTCAAGCCTGCTGTAGAAAT
right  ---------AAA-----AAAAAAAAGAAATACTCATTTTCTGAGGTGAAATTCAAGCCTGCTGTAGAAAT
       111111111*1*11111*1*1*********1*****222222*2*22*2222***2*****22222222*2

left   TCCCAGCACT--TTGGGAGGCCGAGGCGGGT--------------GGATCATGAGGTCAGGAGATC---G
chrom  TTGCATAAGTAGTGAGGAGTCCAATGTTAATCCCCAAGACAATAGGGAAAATAACTCCAGGGCATGTCAG
right  TTGCATAAGTAGTGAGGAGTCCAATGTTAATCCCCAAGACAATAGGGAAAATAACTCCAGGGCATGTCAG
       *22**22*2*22*22****2**2*2*2222*22222222222222***22**2*222****22**2222*

left   AGACCATCCTGGCTAACAAGGTGAAACCCCGTCTC------------------TACTAAA
chrom  AGGTCTTCATGGC-----AGATGGCAGCCCCTCTCATCACAGGCCCGGAGGCCTAGAAGT
right  AGGTCTTCATGGC-----AGATGGCAGCCCCTCTC-------------------------
       **22*2**2****22222**2**22*2***2****                  11NN1NN
```

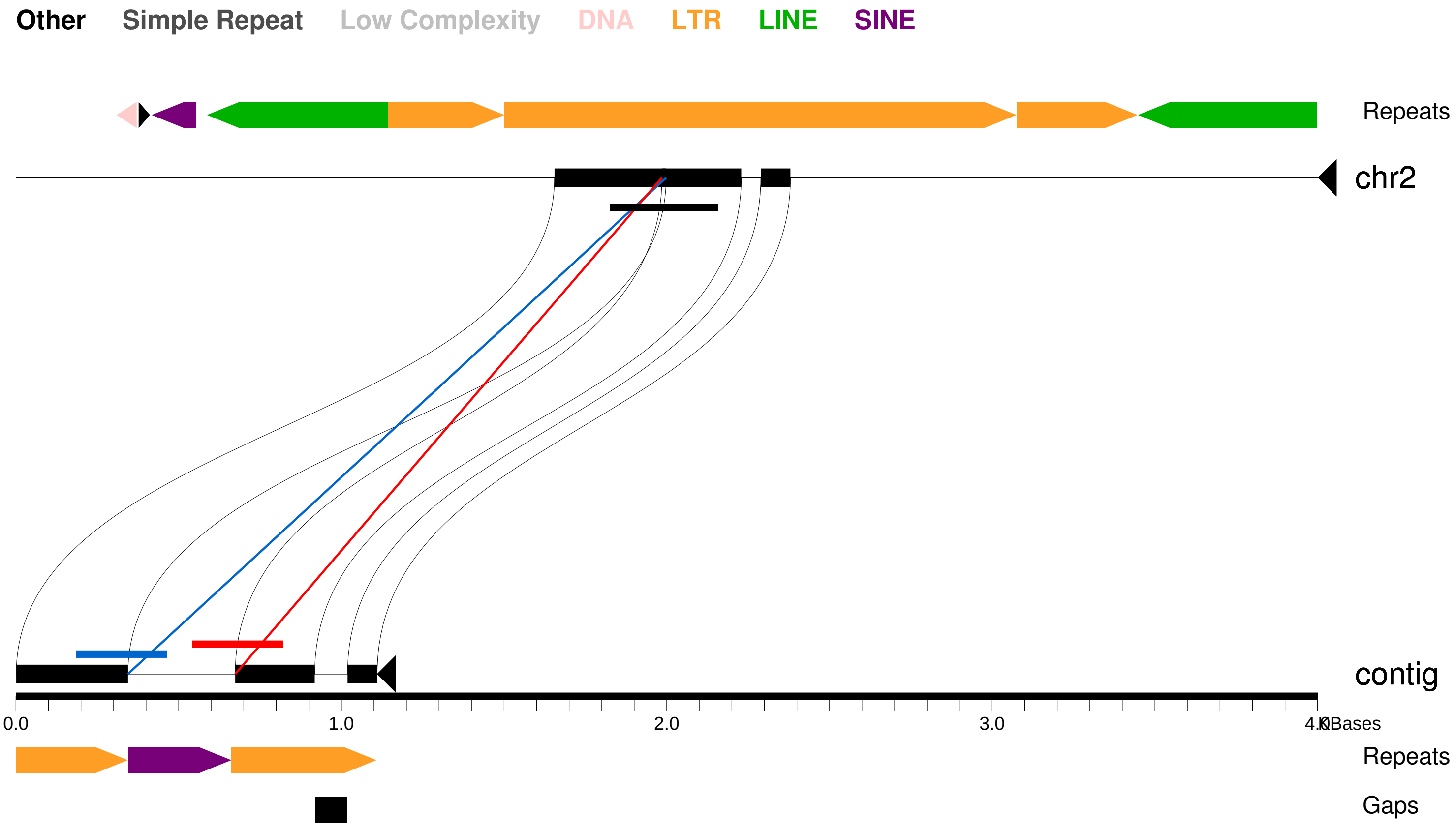

siteID: chr2_1194869 Scafftig4 - Breakpoint: chr2:1194853
chr2:1194806-1194898
Overlap Length: 13 INS size: 293 Genotyped: Yes

```
left   AAA--AACCCTCAATAAAAAAACT-------AG---------CAGACCTAA--------TCCAACAACAC
chrom  AAA--AACCCTCAATAAAAAAACT-------AG---------CAGACCTAA--------TCCAACAACAC
right  GCAGTGAGCCGAGATCGTGCCACTGCACTCCAGGCTGGGCGACAGAGCGAGACTCCGTCTCAAAAAAAAA
       11*111*1**111**111111***1111111**111111111****1*1*111111111**1**1**1*1

left   ATTAAAAAGGTAACACGGCCGGGCGCGGTGGCTCACGCCTG--TAATCCCAGCACTTTGGGAGGCCGAGG
chrom  ATTAAAAAGGTAACACCACCAT---------CATCAAGCAAGATTCATCCCAGAGAT--------------
right  AAAAAAAAGGTAACACACCAT---------CATCAAGCAAGATTCATCCCAGAGAT--------------
       *11************22*2222222222222***2**22*22*2*******222*22222222222222

left   CGGGCGG
chrom  -----GG
right  -----GG
       22222**
```

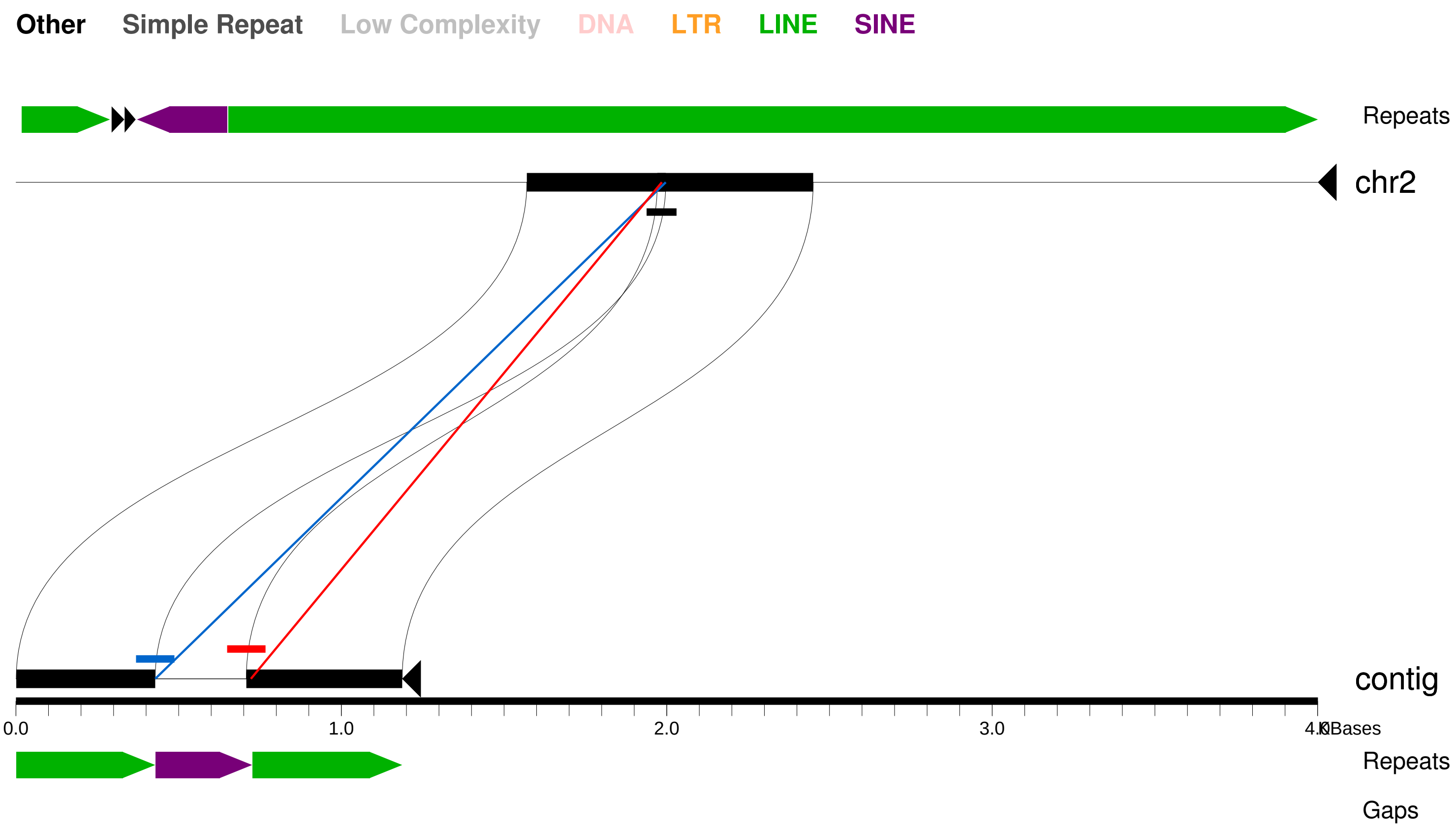

siteID: chr2_119540388 Scafftig2 + Breakpoint: chr2:119540392
chr2:119540350-119540451
Overlap Length: 18 INS size: 316 Genotyped: Yes

```
left    --------------AAAATTCTGTCC---AACAAAGAAATGTGTTAAGCAGGAATCTTTAAATAAATAAA
chrom   --------------AAAATTCTGTCC---AACAAAGAAATGTGTTAAGCAGGAATCTTTAAATAAATAAA
right   CTGGGCGACAGAGCGAGACTCCATCTCAAAAAAAAAAAAAAAAAAAAAAAAAAAAAAAAAAAAAAAAAAAA
        111111111111111*1*1**11**1111**1***1***111111**11*11**11111***1***1***

left    AATAATCGCCGGGCGCGGTGGCTCACGCCTGTAATCCCAGCACTTTGGGAGGCCGAGGCGGGCGGA
chrom   AATAATCA-----------GGCTCAG------AATAAAGGAATAATAGGTAAAGAAAATATGCAAA
right   AATAATCA-----------GGCTCAG------AATAAAGGAATAATAGGTAAAGAAAATATGCAAA
        *******222222222222******2222222***2222*2*222*2**222222*22222**22*
```

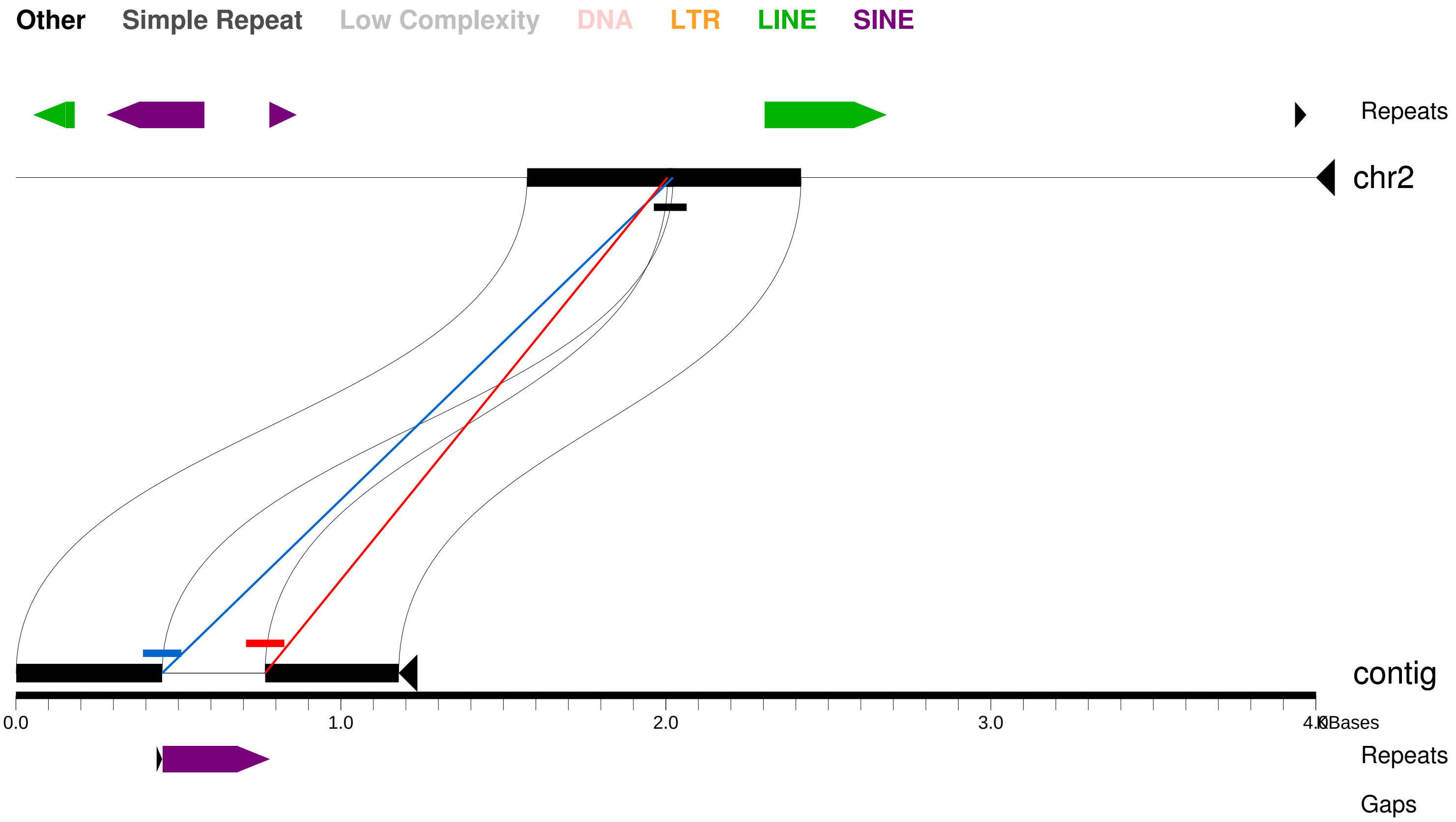

siteID: chr2_123330657 Scafftig8 - Breakpoint: chr2:123330649
chr2:123330602-123330705
Overlap Length: 13 INS size: 306 Genotyped: Yes

```
left     AGAGATAGACCCT---CTATCTGTGCAATGTTTCTTCTCTCATAATT--------------AAGAAAAAT
chrom    AGAGATAGACCCT---CTATCTGTGCAATGTTTCTTCTCTCATAATT--------------AAGAAAAAT
right    GCAC-TCCAGCCTGGGCGA-CAGAGCGAGACTCCGTCTCAAAAAAAAAAAAAAAAAAAAAAAAAAAAAAT
         11*11*11*1***111*1*1*1*1**1*111*1*1****11*1**1111111111111111**1******

left     CCACTGAGGCCGGGCGCGGTGGCTCACGCCTGTAATCCCAGCACTTTGGGAGGCCGAGGCGGGCGG
chrom    CCACTGAATTCATGCCAGG------AAGTCTGTCTGACC----CT----GATGCCTAC-CTTACTG
right    CCACTGAATTCATGCCAGG------AAGTCTGTCTGACC----CT----GATGCCTAC-CTTACTG
         *******222*22**22**222222*2*2****2222**2222**2222**2***2*22*222*2*
```

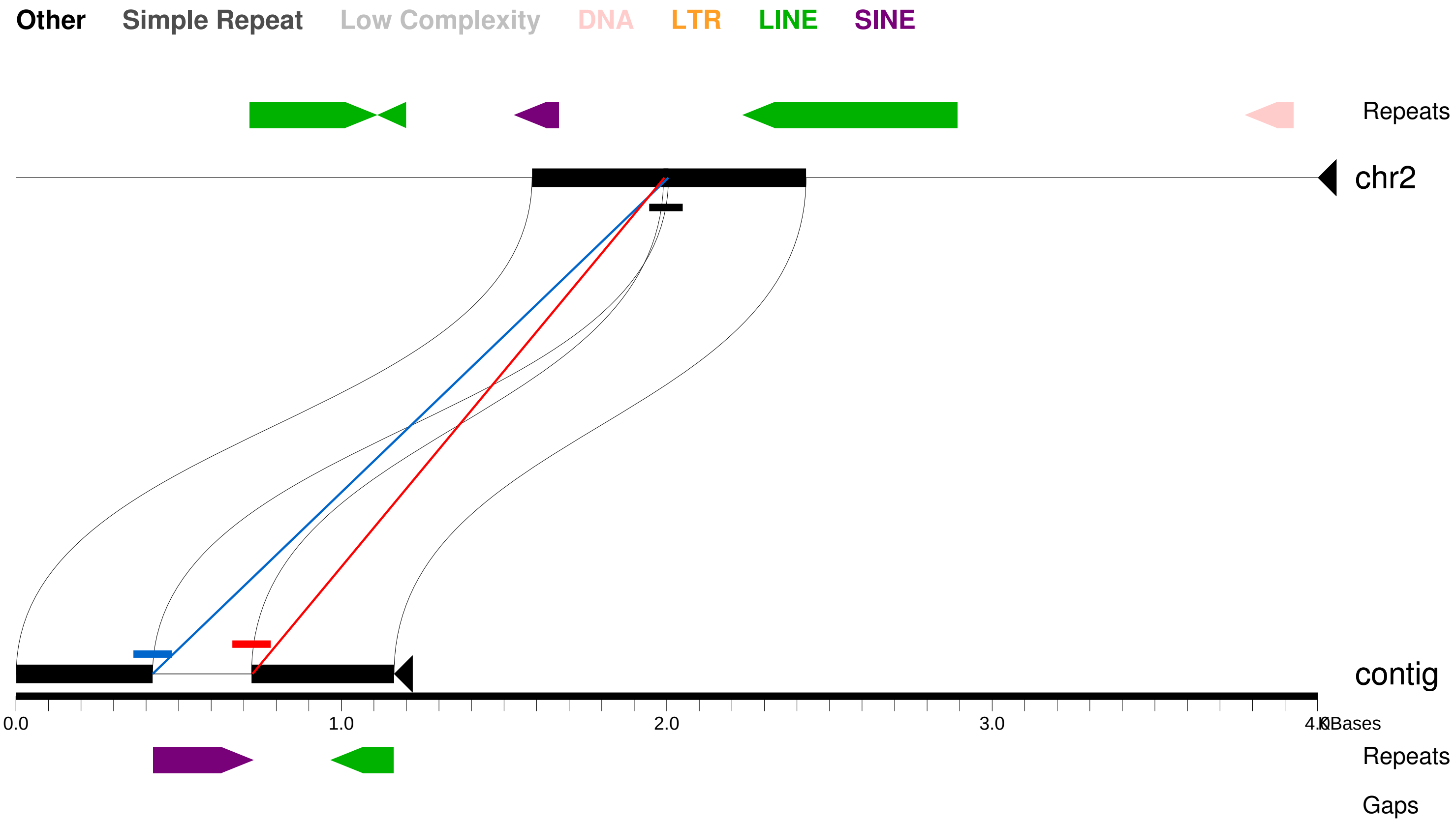

siteID: chr2_124054915 Scafftig1 - Breakpoint: chr2:124054915
chr2:124054755-124055076
Overlap Length: 0 INS size: 327 Genotyped: No

```
left   NNNNNNNNNNNNNNNNNNNNNNNNNNNNNNNNNNNNNNNNNNNNNNNNNNNNNNNNNNNNNNNNNNNNNN
chrom  ACATTAACAATTGGTTTTTTCTCAGATTCGTTTTTTCATGTATTTTTCCAGCATAGTGTTTACATTTCAA
right  -----------------TTTAGTAGAGACGGGGTTTCACCTTGTTAGCCAGGATGGTCTCGA----TCTC
       NNNNNNNNNNNNNNNNN222NNN222NN22NNN22222NN2NN22NN2222N22N22N2NN2NNNN22NN

left   NNNNNNNNNNNNNNNNNNNNNNNNNNGCTCCCCAAAAAAGGTTTAATTTAGCCAAGAAAACTTTAGTTGG
chrom  TTTATTCTATGATAAAGATTAATAT-GCTCTCCAAAAAAGGTATAATCTAGCCAAGAAAACTTCAGTAGG
right  CTGACCTCATGATCCACCCGCCTCG-GC-CTCCCAAAGTGCTGGGAT--------------TACAGGCGT
       N2N2NNNN22222NN2NNNNNN2NN2**1*2**1***11*1*N11**N1111111111111*12**1N*1

left   TTGAATTTTAGAAAGCTAGATTTTTTTTTTTTTTTTT-----------TTTTTTTTTTTTTTTTTTTTTT
chrom  TAGAATTTCAGAAAGCTAGATTGAGCTTTATTTTTAAAAAGCACTAAATTCTGTGGTATGGCCCTTAAAG
right  GAGC----CACCGCGCCCGGCCGAGCTTTATTTTTAAAAAGCACTAAATTCTGTGGTATGGCCCTTAAAG
       12*111112*1111**11*1112222***2*****2222222222222**2*2*22*2*22222**2222

left   GAGACGGAGTCTTGCTCTGTCGCCCAGGCCGGACT---GC--GGAC------TGCAGTGGC---GCAATC
chrom  GAAATAGAATAATGACAACAAACAGAGAGATCAATATAGCTAGGAGCCTTTATGTAGTAGATGGGAAATT
right  GAAATAGAATAATGACAACAAACAGAGAGATCAATATAGCTAGGAGCCTTTATGTAGTAGATGGGAAATT
       **2*22**2*22**22222222*22**22222*2*222**22***2222222**2***2*2222*2***2

left   TCG-GCTCACT-----GCA-AGCTCTCT-GCTTC---------
chrom  ACATGTTTTGTATTTAGCATATATTTATTGATTGCCAGCTAGA
right  ACATGTTTTGTATTTAGCATATATTTATTGATTGCCAGCTAG-
       2*22*2*222*22222***2*22*2*2*2*2**222222222
```

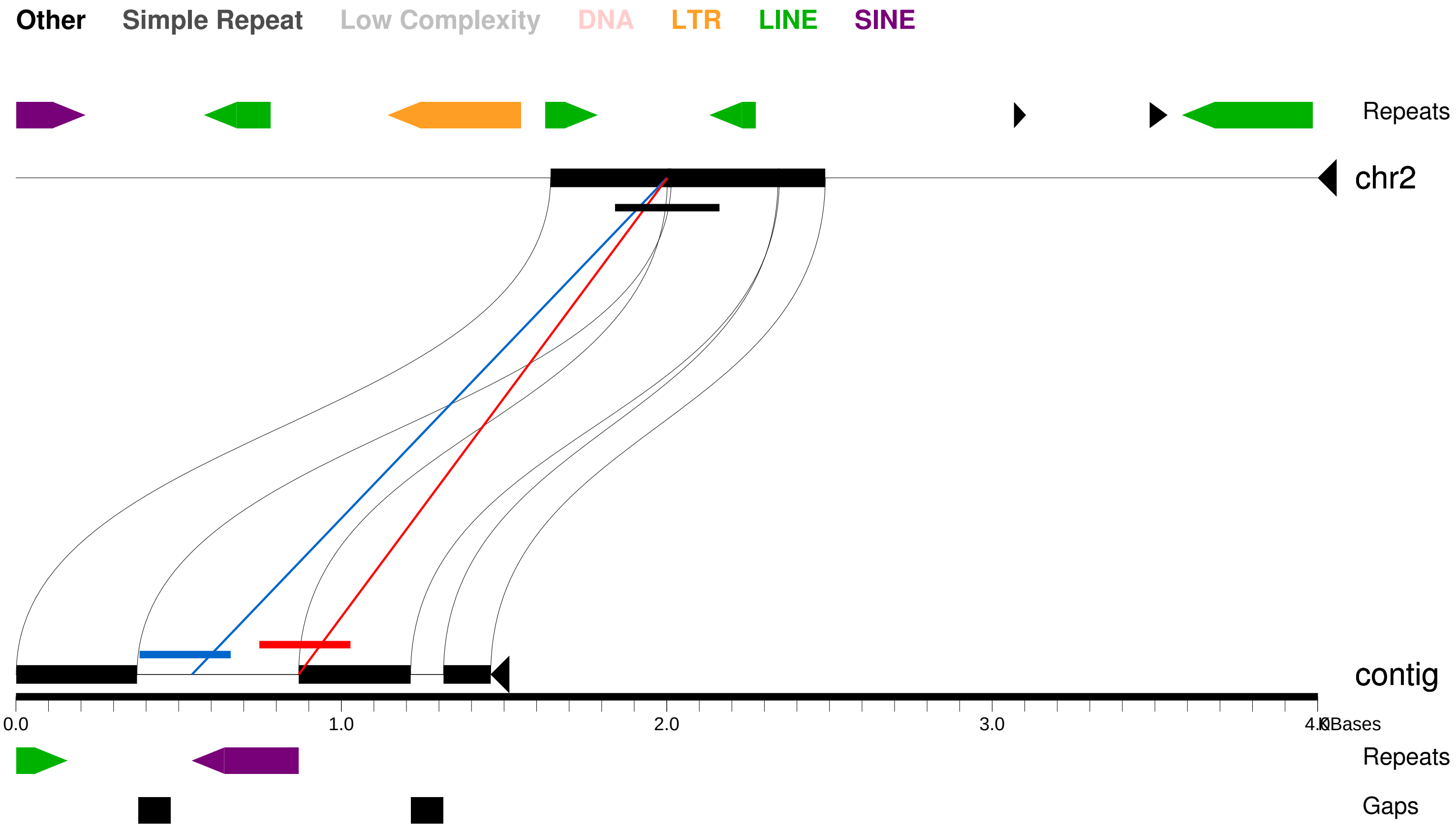

siteID: chr2_126051117 Scafftig1 - Breakpoint: chr2:126051094
chr2:126051045-126051153
Overlap Length: 11 INS size: 416 Genotyped: No

```
left     CCTCACA--TAGTCCTG---ACCTTCTTTTAATACATATATTAGCT-----GCCAGTAATAGCCAATTCT
chrom    CCTCACA--TAGTCCTG---ACCTTCTTTTAATACATATATTAGCT-----TCCAGTAATAGCCAATTCT
right    CCGCCCGCCTCGGCCTCCCAAAGTGCTGGGATTACAGGCGTGAGCCACCGCGCCCGGCCTAGCCAATTCT
         **1*1*111*1*1***1111*11*1**111*1****1111*1***111111N**1*111***********

left     NNNNNNNNNNNNNNNNNNNNNNNNNNNNNNNNNNNNNNNNNNNNNNNNNNNNNNNNNNN
chrom    TTAAAAAAATTTTTAATTAGTGAGTGTGGTGGTTAATATTGACTGTCAA----------
right    TTAAAAAAATTTTTAATTAGTGAGTGTGGTGGTTAATATTGACTGTCAA----------
         22222222222222222222222222222222222222222222222222222222222
```

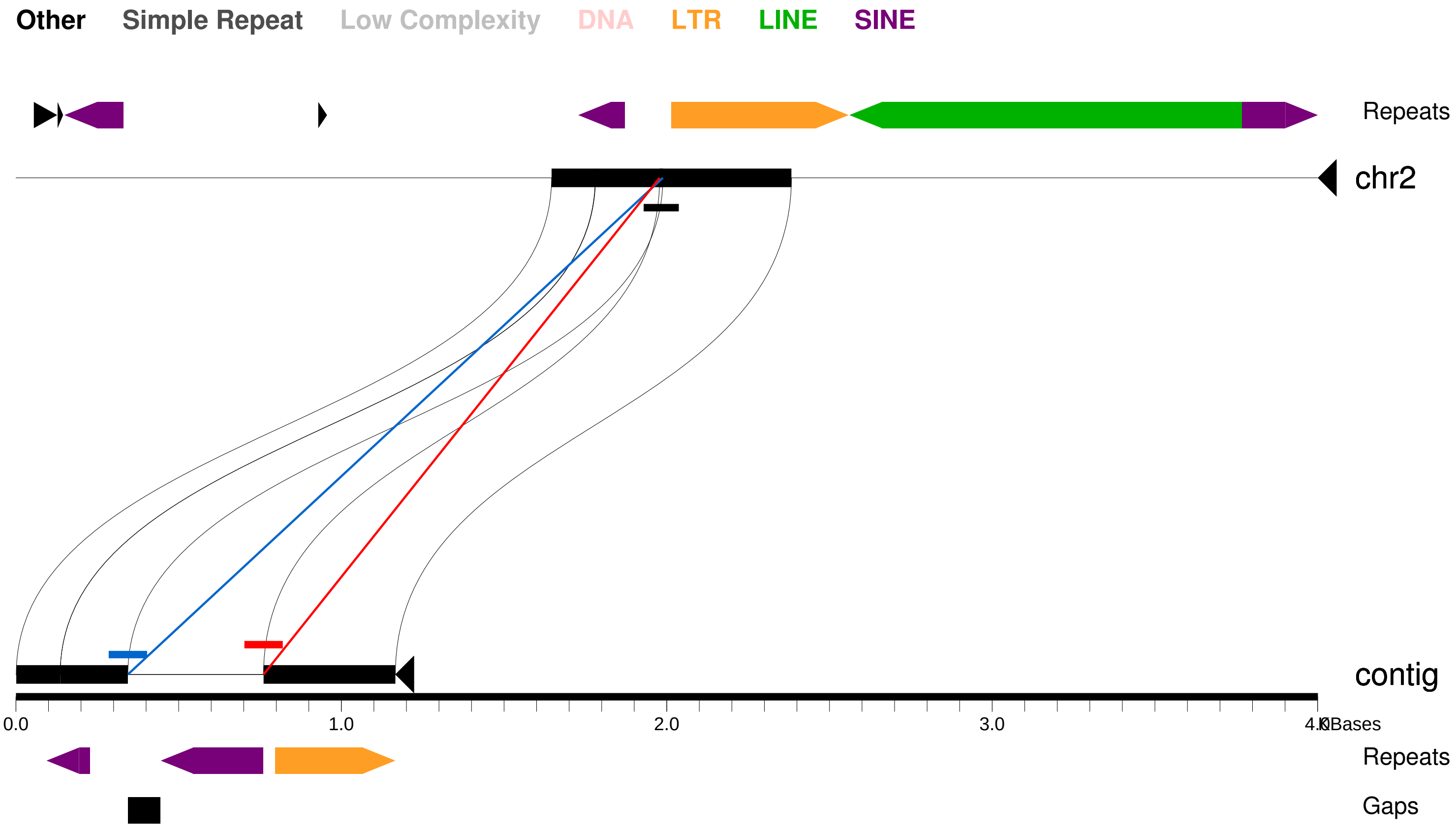

siteID: chr2_126128738 Scafftig1 + Breakpoint: chr2:126128730
chr2:126128570-126128912
Overlap Length: 23 INS size: 420 Genotyped: No

```
left   -----------------------TGAATAACCAGTAAGCTAAGTATACATAATTACTTCTACCGCTGTGG
chrom  GTGTTTCTCTTAATACTTTTCATTGAATAACCAGTAAGCTAAGTATACATAATTACTTCTACCGCTGTGG
right  NNNNNNNNNNNNAA--------------------------------------------------------
       NNNNNNNNNNNN22         111111111111111111111111111111111111111111111111

left   ACGAGATAATTTAAACATCTTTGAGTTTTAATCTCTTCAGTTGAACCTGAAAAACAGACTTAATTATAAT
chrom  ACGAGATAATTTAAACATCTTTGAGTTTTAATCTCTTCAGTTGAACCTGAAAAACAGACTTAATTATAAT
right  ----------------------------------------------------------------------
       1111111111111111111111111111111111111111111111111111111111111111111111

left   TTCATCTAATGGTTAAGAAGAAAAAAAATTAAAAGTGTGTTGTAG----------------GCCGGGCGCG
chrom  TTCATCTAATGGTTAAGAAGAAAAAAAATTAAAAGTGTGTTGTAATATACCCTATTACATAGTATAGAGCA
right  -----------------AAAAAAAAAAA--AAAAGTGTGTTGTAATATACCCTATTACATAGTATAGAGCA
       11111111111111111**1*******11**************22222222222222222*2222*2**2

left   GTGG-CTCACGCCTGTAATC-CCAGCACTTTGGGAGGCCGAGGCGGGC---------GGATCACGAGG-T
chrom  TTGTACAAACTCAGTTAATGACCTGATCATTTTCCTTCCCACATAAGCCCCATATAAGCATCATTTGGCT
right  TTGTACAAACTCAGTTAATGACCTGATCATTTTCCTTCCCACATAAGCCCCATATAAGCATCATTTGGCT
       2**22*22**2*222****22**2*22*2**222222**2*22222**222222222*2****222**2*

left   CAGGAGATC----GAGACCATCCCG--GCTAAAACGGTGAAACCCCGTCTCT--ACTGAA---
chrom  GCAGAGATTCCTTGTAACTATACAAAAGATGGAAGAGTTAAGGGCTGAATCAGAAAATAATTT
right  GCAGAGAT-------------------------------------------------------
       222*****N    1NN11N11N1NN  1N1NN11NN11N11NNN1N1NN11N  1NNN11
```

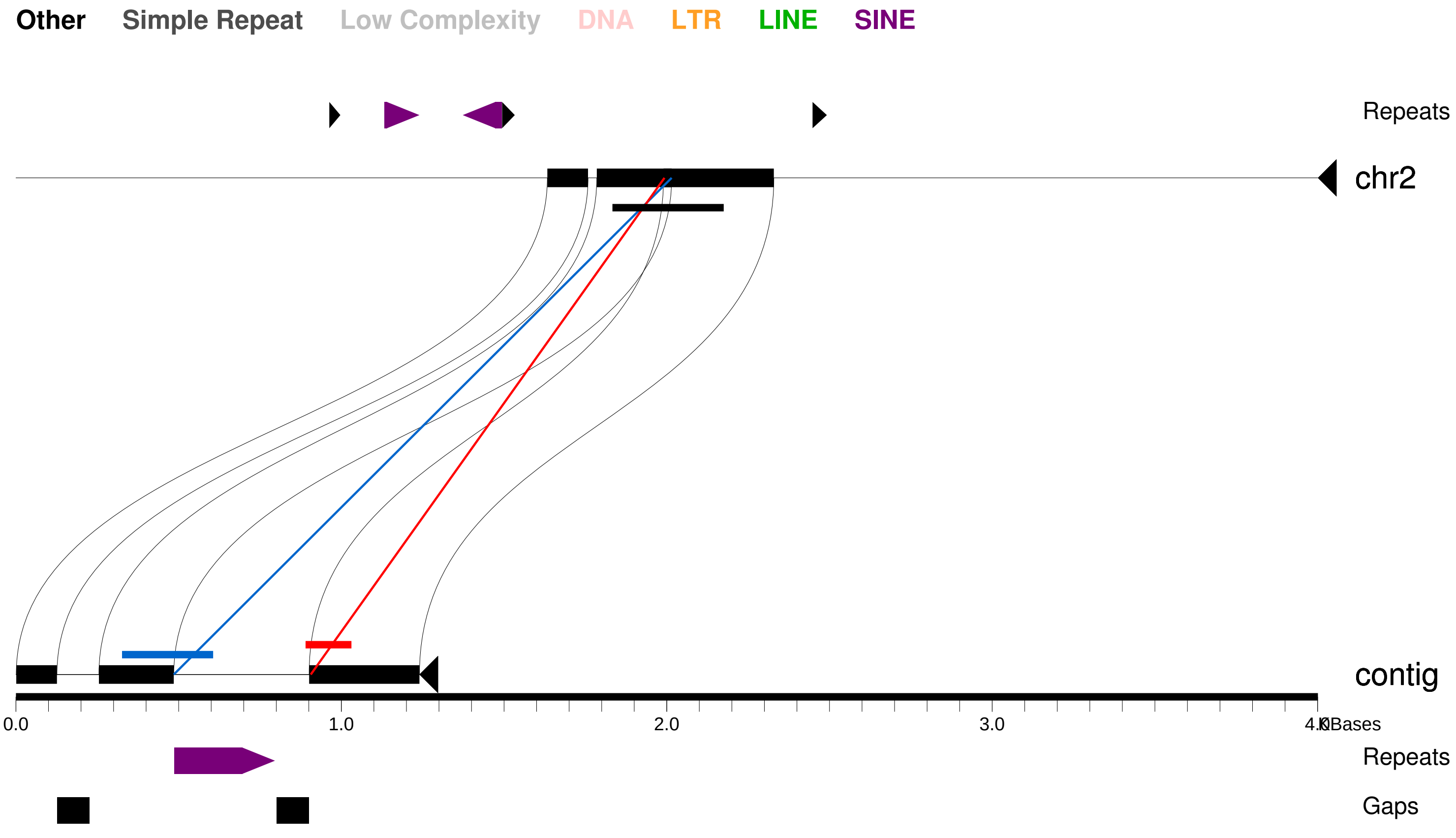

siteID: chr2_128638892 Scafftig2 + Breakpoint: chr2:128638893
chr2:128638845-128638952
Overlap Length: 12 INS size: 467 Genotyped: No

```
left     AATACAAACTATTTCTCATACACTGCTAGTATTTACA------AACCAGGTATT------GAGACTGCTA
chrom    AATACAAACTATTTCTCATACACTGCTAGTATTTACA------AACCAGGTATT------GAGACTGCTA
right    CCGCCCGCCTCGGCCTCCCAAAGTGCTGGGATT-ACAGGCGTGAGCCACCGCGCCCGGCCGAGACTGCTA
         1111*111**1111***11*1*1****1*1***1***111111*1***111111111111**********

left     TTTTTTNNNNNNNNNNNNNNNNNNNNNNNNNNNNNNNNNNNNNNNNNNNNNNNNNNNNNNNN
chrom    TT-----------GACACAAAGATGTGATGATGCCCCCACTCATGTGTCTGGCTGGGCAAA
right    TT-----------GACACAAAGATGTGATGATGCCCCCACTCATGTGTCTGGCTGGGCAAA
         **2222222222222222222222222222222222222222222222222222222222222
```

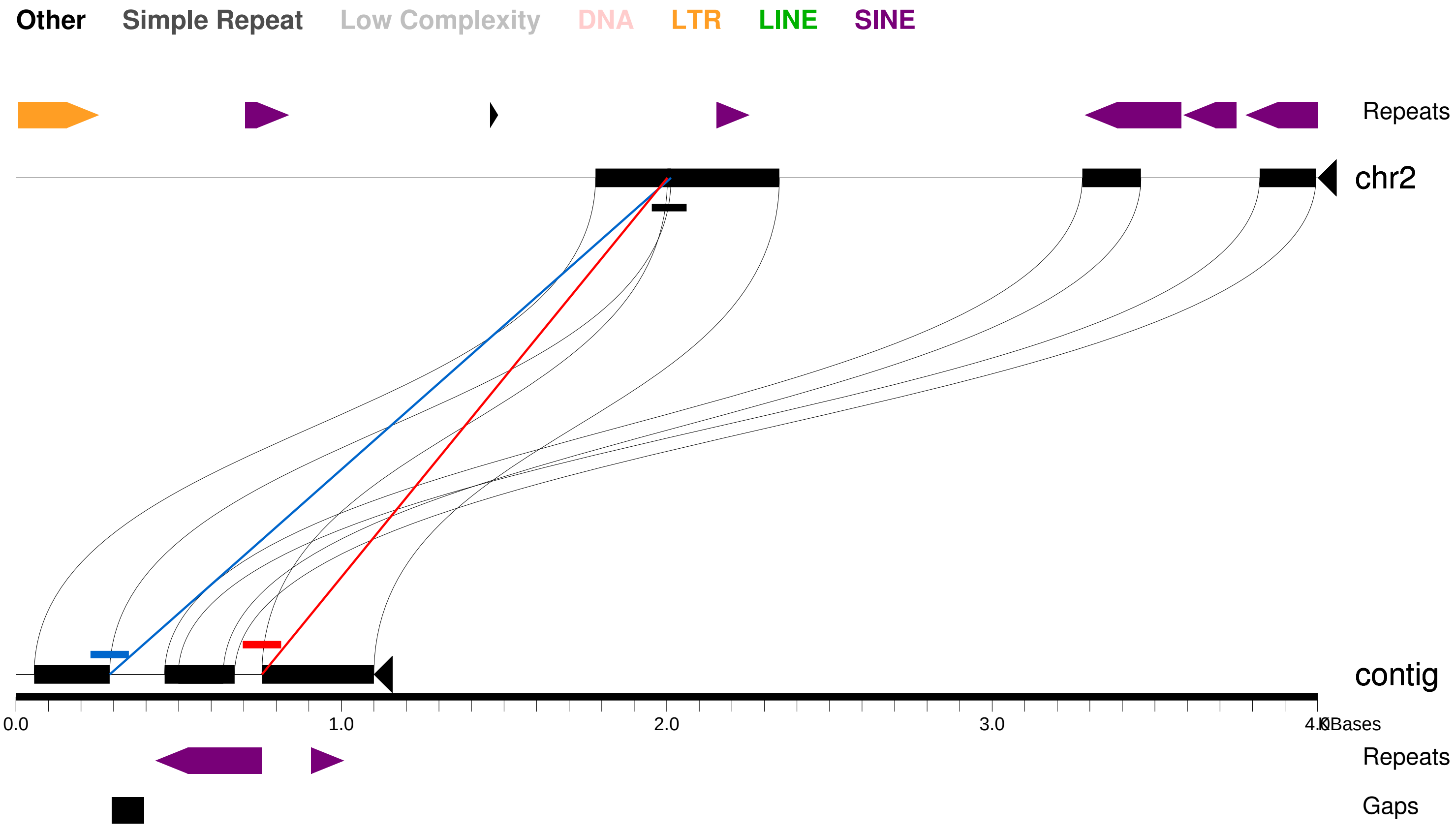

siteID: chr2_130299825 Scafftig1 + Breakpoint: chr2:130299827
chr2:130299768-130299886
Overlap Length: 1 INS size: 278 Genotyped: No

```
left   AAGAAAACAACCAGAAGCTGTTAGAACCAATATCGTAAGAGCTCTTAAAAACAAACAGGGGGCCGGGCGC
chrom  AAGAAAACAACCAGAAGCTGTTAGAACCAATATCGTAAGAGCTCTTAAAAACAATCAGGGCTTATAGCAC
right  NNNNNNNNNNNNNNNNNNNNNNNNNNNNNNNNNNNNNNNNNNNNNNNNNNNNNNNNNNNNNGCTGATAGCAC
       1111111111111111111111111111111111111111111111111111111N1111*22N222**2*

left   GGTGGCTCACGCCTGTAATCCCAGCACTTTGGGAGGCCGAGGCGGGCG---G
chrom  CCAAGCAAACACC--CAATCA-AGAAAAAAGCTATCTCCAAAATGGCAAAAG
right  CCAAGCAAACACC--CAATCA-AGAAAAAAGCTATCTCCAAAATGGCAAAAG
       2222**22**2**222****22**2*2222*22*222*2*2222***2222*
```

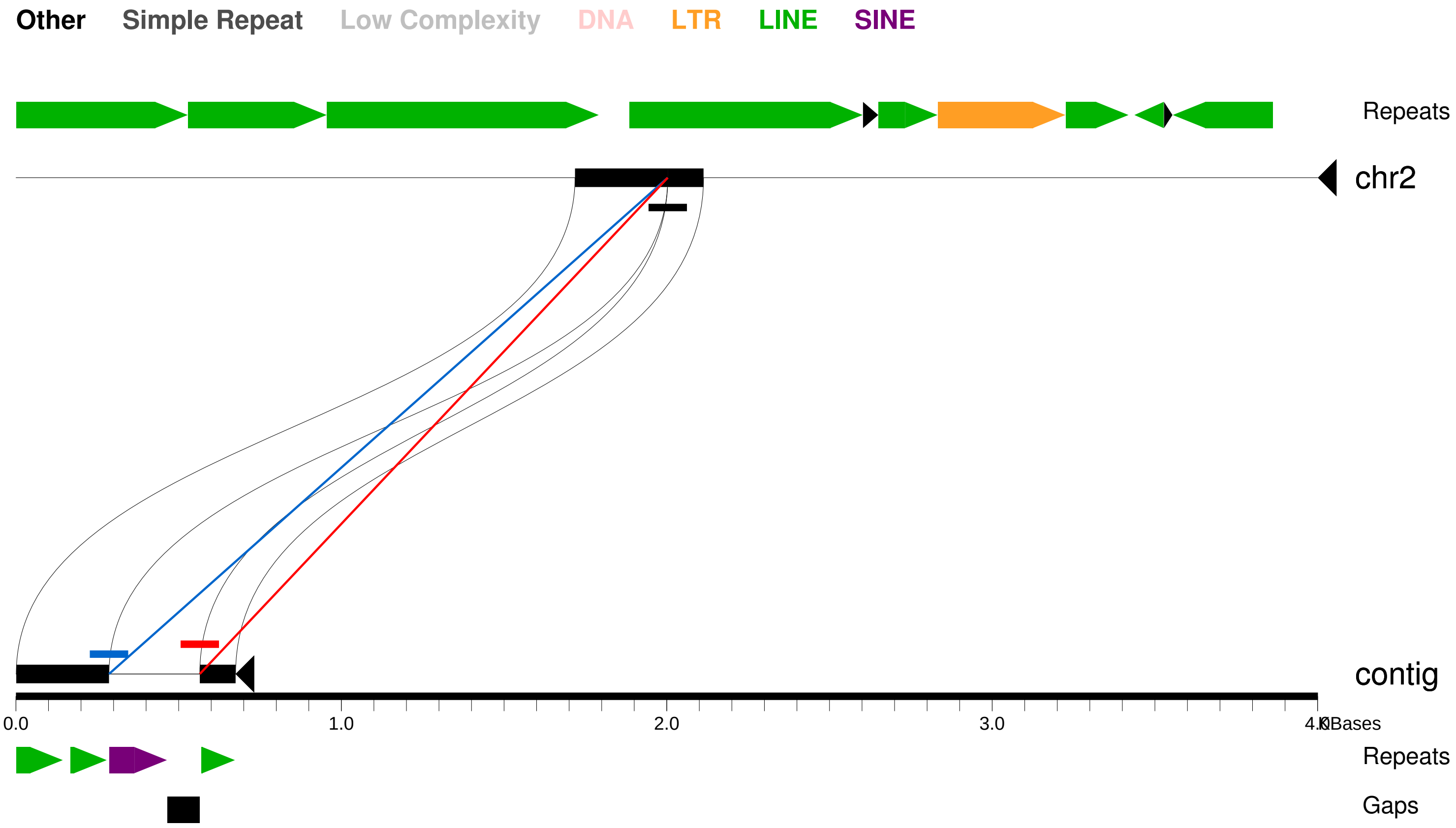

siteID: chr2_13076585 Scafftig1 + Breakpoint: chr2:13076573
chr2:13076413-13076748
Overlap Length: 16 INS size: 274 Genotyped: Yes

```
left   ------------------TACTGTCCTAGGCGTTTCAAACAGATTATCTGAAACTCATTTCATTCTCACA
chrom  G----ATTCTGTGACACATACTGTCCTAGGCGTTTCAAACAGATTATCTGAAACTCATTTCATTCTCACA
right  GGAGAATGGCGTGAACC---CGGG---AGGCGGAGCTTGCAG-TGAGCCGAGA-TCCCGCCACTG-CAC-
       2111122NNN2222NN2 11*1*1111*****111*111***1*1*1*1**1*1**1111**1*11***1

left   TCAAACTTGCATGGCACATATTATCCAAGTTTTATAGATACAGGAACTGAGGATCAAAGAGGCTAAGTAA
chrom  TCAAACTTGCATGGCACATATTATCCAAGTTTTATAGATACAGGAACTGAGGATCAAAGAGGCTAAGTAA
right  TCCAGCCTG---GGCG-----------------ACAGAGCGAG--ACTCCGTCTCAAAAAAAAAAAAAAA
       **1*1*1**111***11111111111111111*1***111**11***11*11*****1*1111**11**

left   AATGACCAAGATCATGTACCTTTTAAGAGGCTGGTGTGAGC----ACTTTGGGAGGCCGAGGC----GGG
chrom  AATGACCAAGATCATGTACCTTTTAAGAGGCTGGTGTGAGATTTGACCTCAGGCCTGCTGGGTATCAGAG
right  AAAAAAAAAAA-------------AAGAGGCTGGTGTGAGATTTGACCTCAGGCCTGCTGGGTATCAGAG
       **11*11**1*1111111111111***************22222**2*22**2222*22**22222*2*

left   CGGATCACGAGGT------------CAAGAGATCG----AGACCATCCCGGCTAAAACGGTGAAACCCCG
chrom  CCTATTTTGAGTTTTGTTCCCCCTACAACTGTTCCCATTATAATATACTG--TTAGAAGGACACACACAC
right  CCTATTTTGAGTTTTGTTCCCCCTACAACTGTTCCCATTATAATATACTG--TTAGAAGGACACACACAC
       *22**222***2*222222222222***22*2**22222*2*22**2*2*22*2*2*2**22*2**2*22

left   TCTCTACTAAAAATACAAAAAAAT---------TAGCCGGGCG------TAGTGGCGG----
chrom  ACACACACACACACACACACACACACACTGCCCTATAGTTGCACACTGCAATACTTGCCTAGCT
right  ACACACACACACACACACACAC----TGCCCTATAGTTGCACACTGCAATAC----------
       2*2*2222*2*2*2***2*2*21N  2222222***22*22*2222222**21N11NN
```

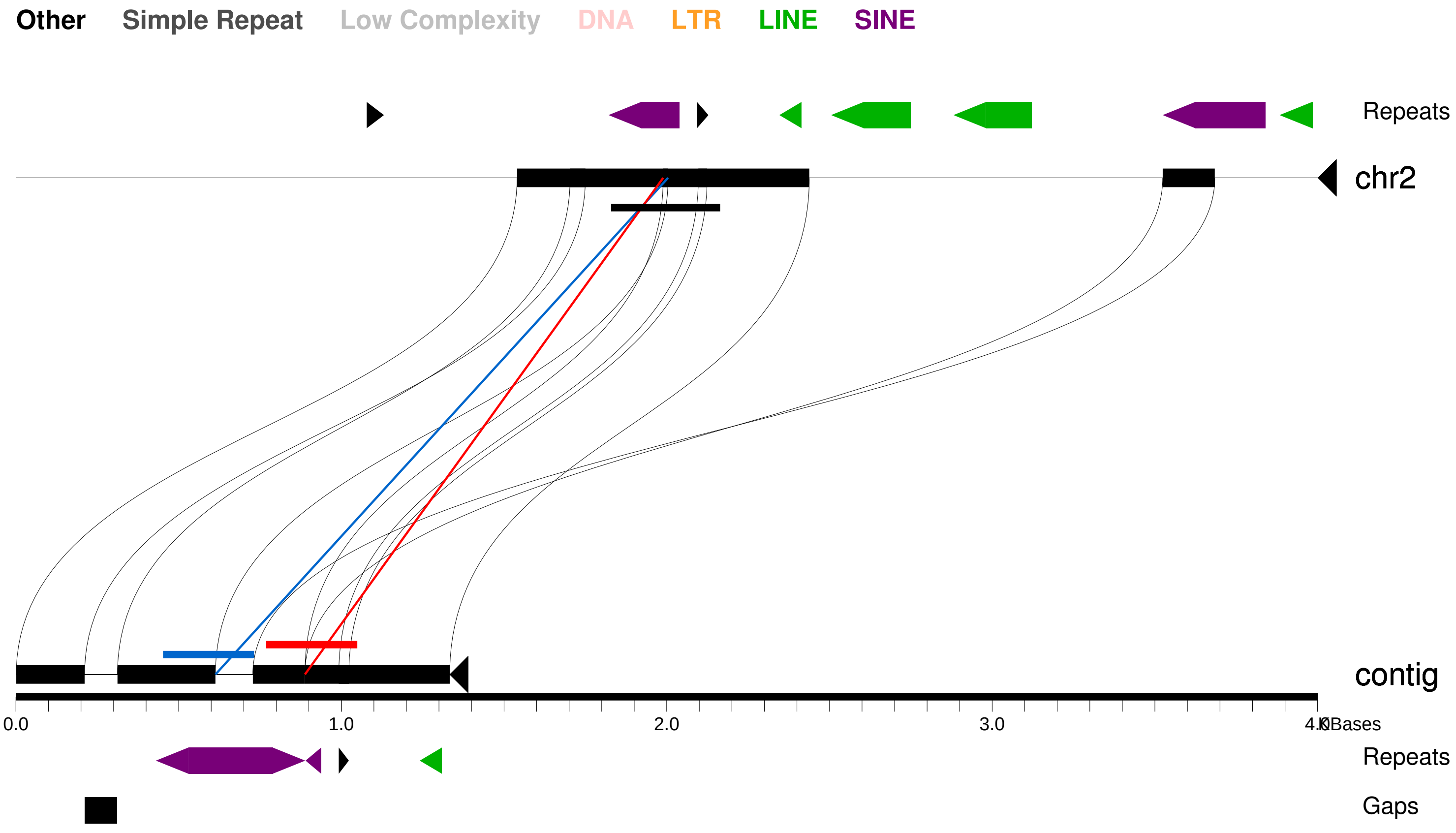

siteID: chr2_133916493 Scafftig1 - Breakpoint: chr2:133916493
chr2:133916448-133916552
Overlap Length: 15 INS size: 428 Genotyped: No

```
left     GGGAGAGGGAATAAAGAAGAAATACAATGCATGGCTTGTGCTCTT--------------AAGAACTACAG
chrom    GGGAGAGGGAATAAAGAAGAAATACAATGCATGGCTTGTGCTCTT--------------AAGGGCTACAG
right    NNNNNNNNNNNNNNNNNNNNNNNNNNNNNNNNNNNNNNNNNNNNNNNNNNNNNNNNNNNAAGAGCTACAG
         11111111111111111111111111111111111111111111111111111111111***N2******

left     TAGAGGCCGGGCGCGGTGGCTCACGCCTGTAATCCCAGCACTTTGGGAGGCCGAGGCGGGCGG
chrom    TAGATA----GAACAACAGCCTACCAAAGTTGTCCACACCCTA----ATCCCTAGAA------
right    TAGATA----GAACAACAGCCTACCAAAGTTGTCCACACCCTA----ATCCCTAGAA------
         ****222222*22*2222**22**2222**22***222*2**22222*22**2**22222222
```

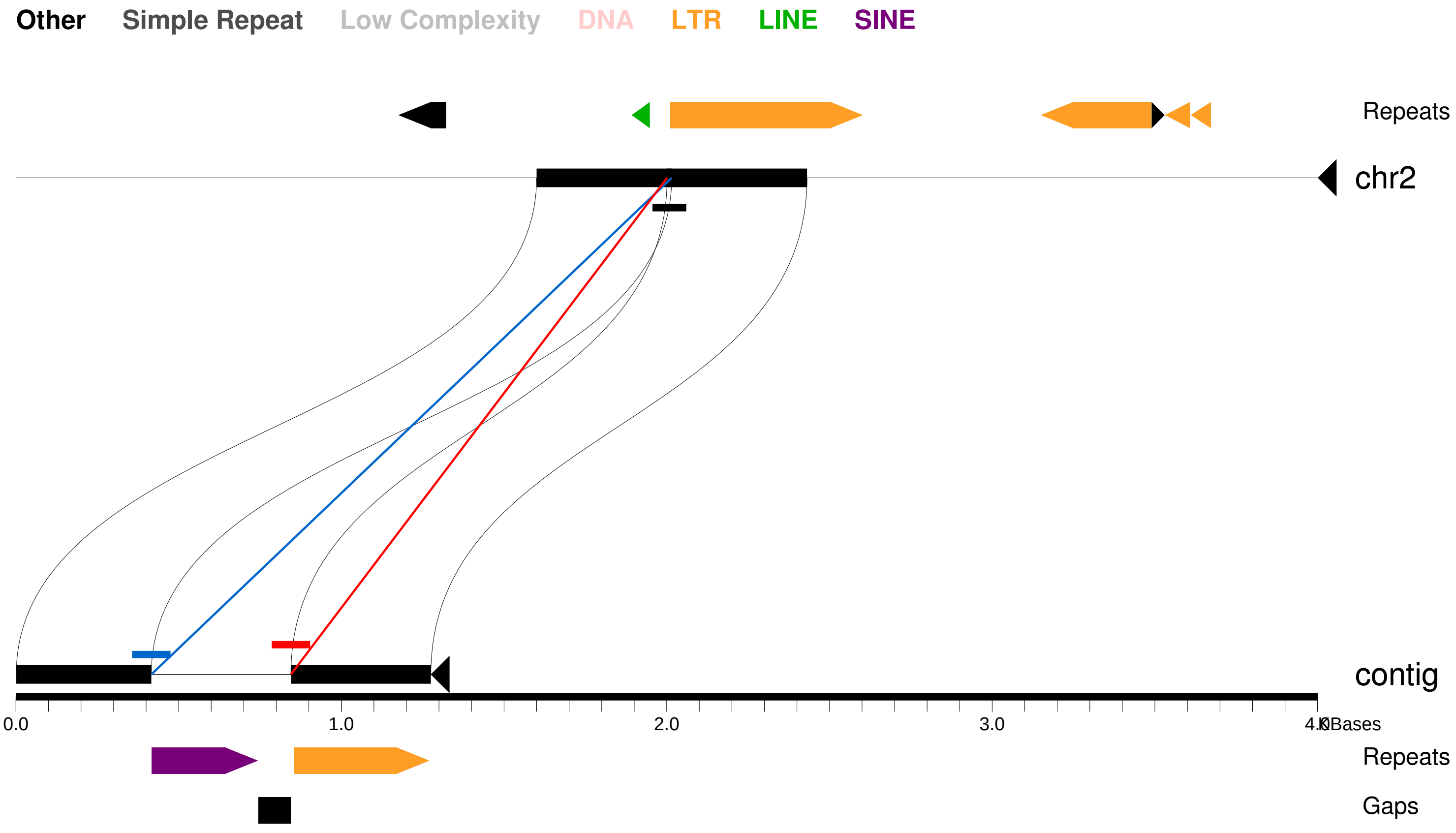

siteID: chr2_136879046 Scafftig1 - Breakpoint: chr2:136879051
chr2:136879006-136879110
Overlap Length: 15 INS size: 472 Genotyped: No

```
left     TGAGTATATGTCATACTCCTATCCCTCCAATATTTTAGGTTCCTT--------------AAAGTGAATGC
chrom    TGAGTATATGTCATACTCCTATCCCTCCAATATTTTAGGTTCCTT--------------AAAGTGAATGC
right    NNNNNNNNNNNNNNNNNNNNNNNNNNNNNNNNNNNNNNNNNNNNNNNNNNNNNNAAAAAAAAGTGAATGC
         11111111111111111111111111111111111111111111111111111111111***********

left     CCAGGCCGGGCGCGGTGGCTCACGCCTGTAATCCCAGCACTTTGGGAGGCCGAGACGGGCGGA
chrom    CCAG--------------ATATCCTCTATCCAGCTAGCCCATTGCTGAACAAATACTGGGCTC
right    CCAG--------------ATATCCTCTATCCAGCTAGCCCATTGCTGAACAAATACTGGGCTC
         ****222222222222222*22*22**2*2222*2***2*2***22222*22*2**2**2222
```

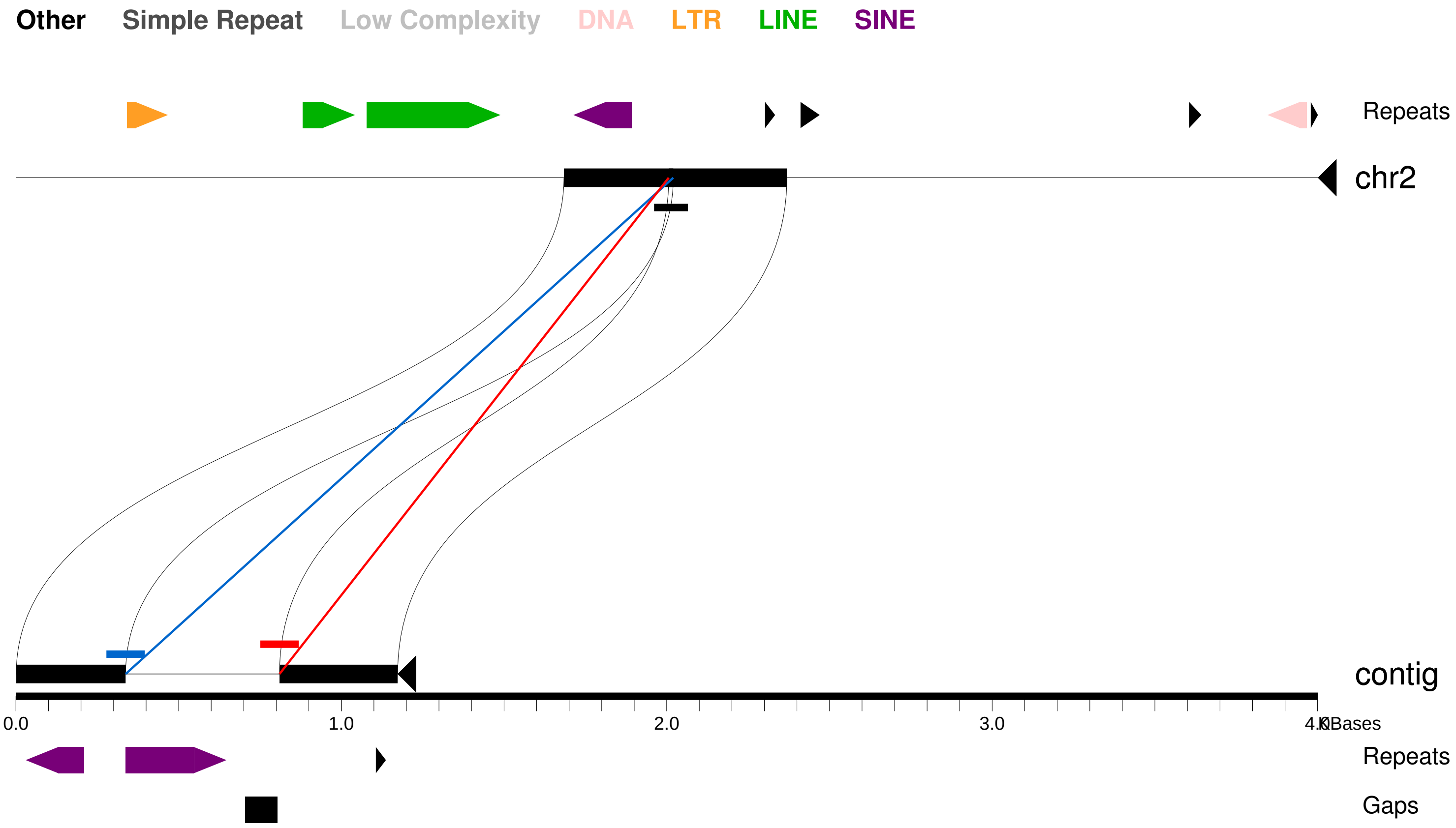

siteID: chr2_138259787 Scafftig1 + Breakpoint: chr2:138259793
chr2:138259739-138259852
Overlap Length: 3 INS size: 300 Genotyped: Yes

```
left   TTAT---TCTCCCACAATAAATATTCTTTCATTATAATAGTTTTGAACTTCAA--TGGTTATTTTTTTTT
chrom  TTAT---TCTCCCACAATAAATATTCTTTCATTATAATAGTTTTGAACTTCAA--TGGTTATCTTAGTAA
right  CCGCCCGCCTCGGCCTCCCAAAGTGCTGGGATTACAGGCGTGAGCCACCGCGCCCGGCCTATCTTAGTAA
       11111111***111*1111**11*1**111****1*111**11111**11*11111*11***2**22*22

left   TTTTTTTTTTTGAGACGGAGTCTCGCTCTGTCGCCCAGGCTGGAGTGCAGTGGC---
chrom  AT-------TAGATACTAAGTCA-GGTTAGTAGTTAACTATGCAACATAAATCCTTA
right  AT-------TAGATACTAAGTCA-GGTTAGTAGTTAACTATGCAACATAAATCCTTA
       2*2222222*2**2**22****22*2*22**2*222*222**2*2222*2222*222
```

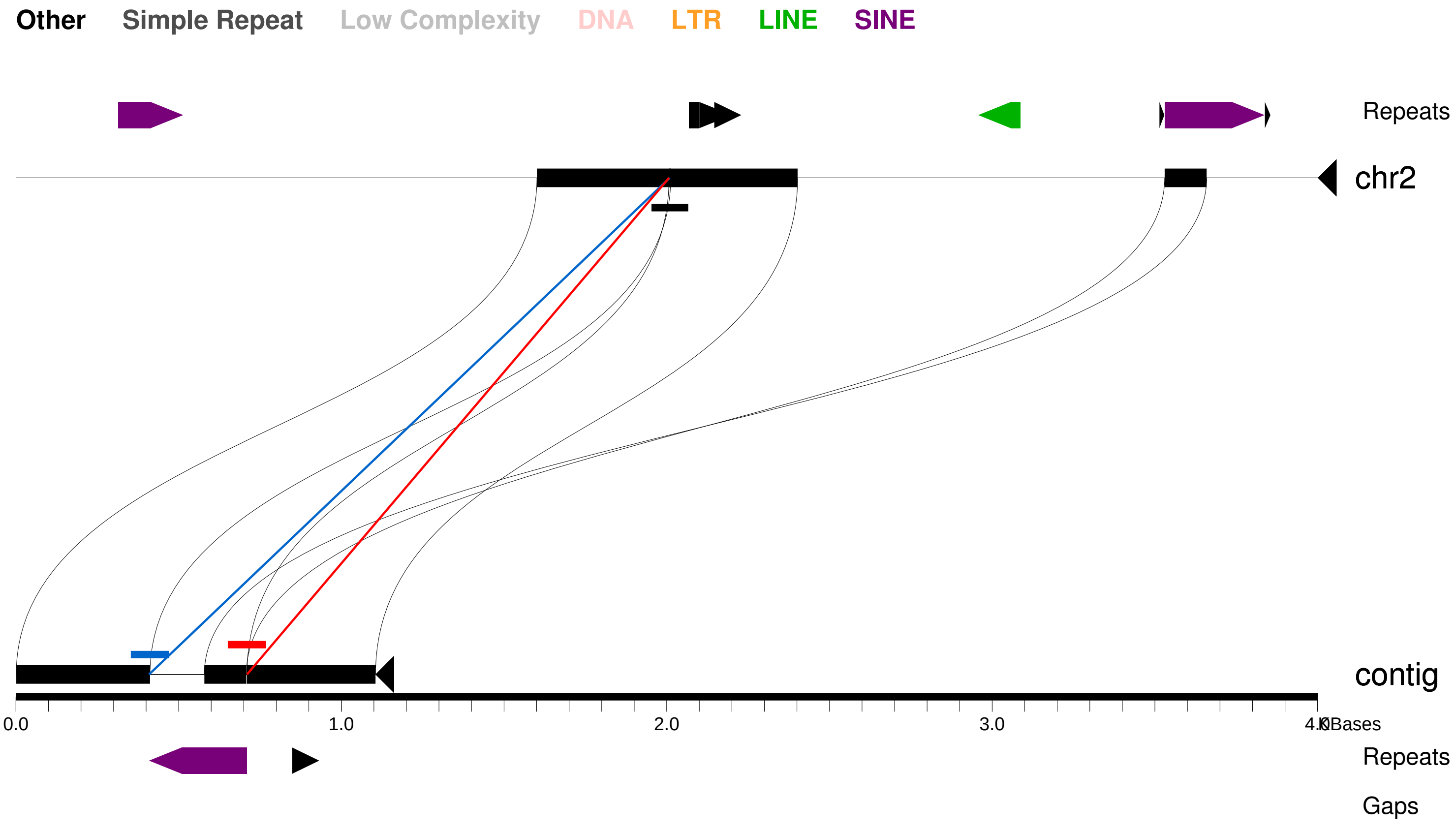

siteID: chr2_139380785 Scafftig2 - Breakpoint: chr2:139380782
chr2:139380622-139380948
Overlap Length: 7 INS size: 304 Genotyped: Yes

```
left   AA------GACATGGATTTTACAGAAAATTCTTATTTATCTGGTGCACTG--CCTGGGTCATGACTTTTG
chrom  AAAATAAAGACATGGATTTTACAGAAAATTCTTATTTATCTGGTGCACTG--CCTGGGTCATGACTTTTG
right  GCAGGAGA---ATGGCGTGAACCCGAGAGGCGGAGCT------TGCAGTGAGCCGAGATCCCGCCACT--
       112NN2N2111****11*11**111*1*11*11*11*111111****1**11**11*1**11*1*11*11

left   TGCCTTACTTCCAGGGCCTGGATATTTAGAGGGACAGAGCCAGGGCAAGCTATCCTTTTTCTATTCTGCC
chrom  TGCCTTACTTCCAGGGCCTGGATATTTAGAGGGACAGAGCCAGGGCAAGCTATCCTTTTTCTATTCTGCC
right  -GC---ACT-CCAG--CCTGG---------GCGACAGAGCGAGAC-------TCCGTCTCAAAAAAAAAA
       1**111***1****11*****111111111*1********1**111111111***1*1*111*1111111

left   TCTCTCTGTTTTCATCAACAACAGCTCTGG----CCGGGCGCGGTGGCTCACGCCTGTAATCCCAGC---
chrom  TCTCTCTGTTTTCATCAACAACAGCTCTGAATCTCTTAAGACAGTGGCTCTCAAACTAGTTCCAAGGTTA
right  AAAAA--------AAAAAAAAAAGCTCTGAATCTCTTAAGACAGTGGCTCTCAAACTAGTTCCAAGGTTA
       1111111111111*11**1**1*******22222*222222*2*******2*22222222***2**2222

left   -------ACTTTGGGAGGCCGAGGCGGGTGGATCACG---------AGG----TCAGGAGATCGAG---A
chrom  AATTCTAACAGTGGCATGCATTTACATAAAAATTAAGTTAATTTATAGAATTTTCAGAAACTAAAGTTTA
right  AATTCTAACAGTGGCATGCATTTACATAAAAATTAAGTTAATTTATAGAATTTTCAGAAACTAAAGTTTA
       2222222**22***2*2**22222*222222**2*2*222222222**22222****2*22*22**222*

left   CCATCC------CGGCTAAAACGGTGAA----ACCCCGTCTCTACTAAA
chrom  CCTCCCAAAGTGCTTTTTAAACTTTGATGTCTACCATTGCTTGCCTGTC
right  CCTCCCAAAGTGCTTTTTAAACTTTGATGTCTACCATTGCTTG------
       **22**222222*222*2****22***22222***2222**22N11NNN
```

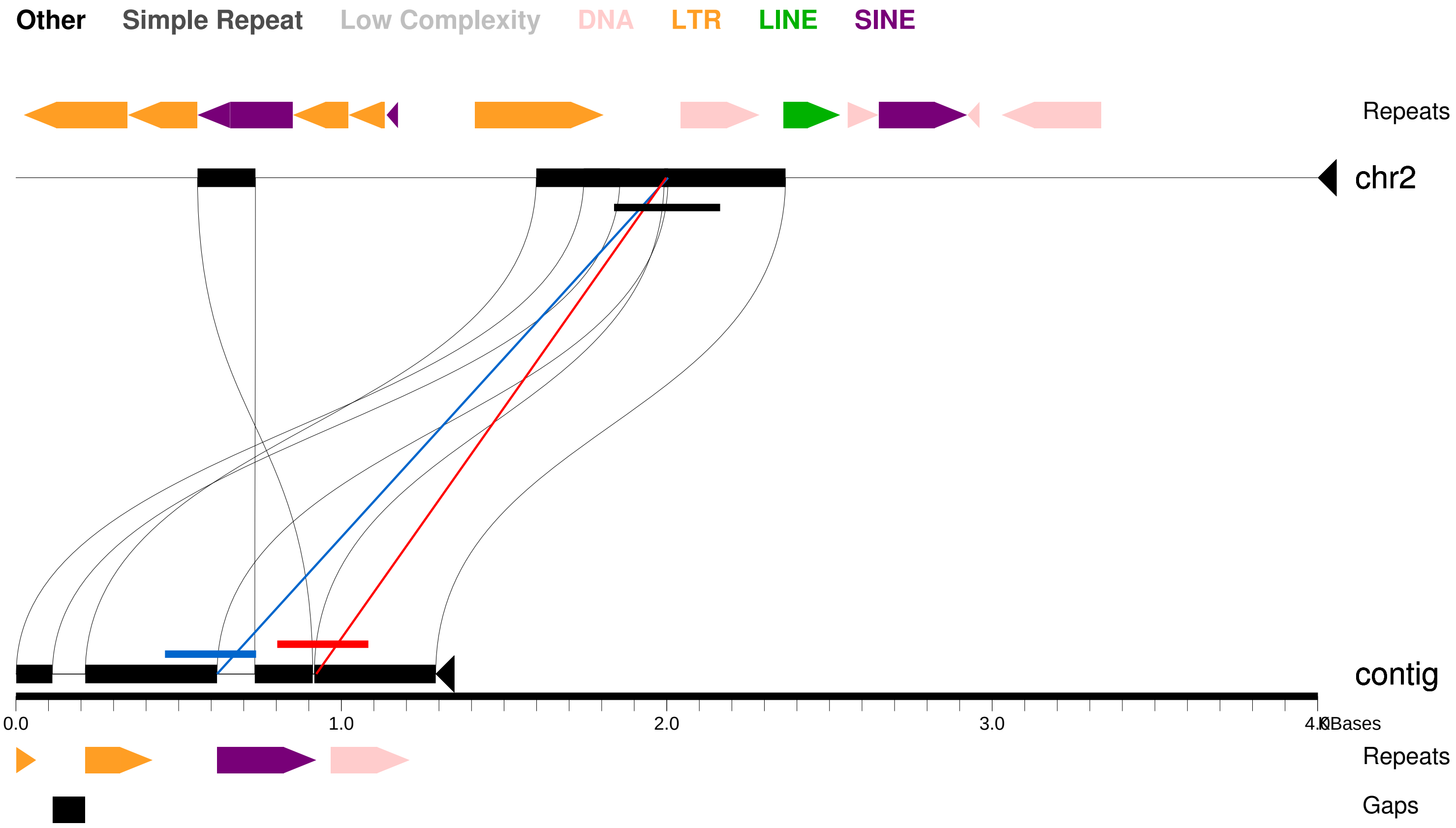

siteID: chr2_141534051 Scafftig2 + Breakpoint: chr2:141534056
chr2:141534015-141534115
Overlap Length: 12 INS size: 319 Genotyped: Yes

```
left    A-----GACTCTTCAATTTAACTT--TTCTTTTTC-----TTAGAAAAGAATA------TATAATATTCT
chrom   A-----GACTCTTCAATTTAACTT--TTCTTTTTC-----TTAGAAAAGAATA------TATAATATTCT
right   CCACCCGCCTCGGCCTCCCAAAGTGCTGGGATTACAGGCGTGAGCCACCGCGCCCGGCCTATAATATTCT
        111111*1***11*11111**11*11*1111**1*11111*1**11*111111111111***********

left    TTTTTTTTTTTTTTTTTTTTTTTTTTTTTTTTGAGACGGAGTCTCGCTCTGTCGCCCAGGCCGGACT-
chrom   TATATTTTACATAACATGTAAGTATAT------AGA-------------TGTAAATAAAAGCGTATTT
right   TATATTTTACATAACATGTAAGTATAT------AGA-------------TGTAAATAAAAGCATATTT
        *2*2****222*2222*2*222*2*2*222222***2222222222222***22222*222*12*2*2
```

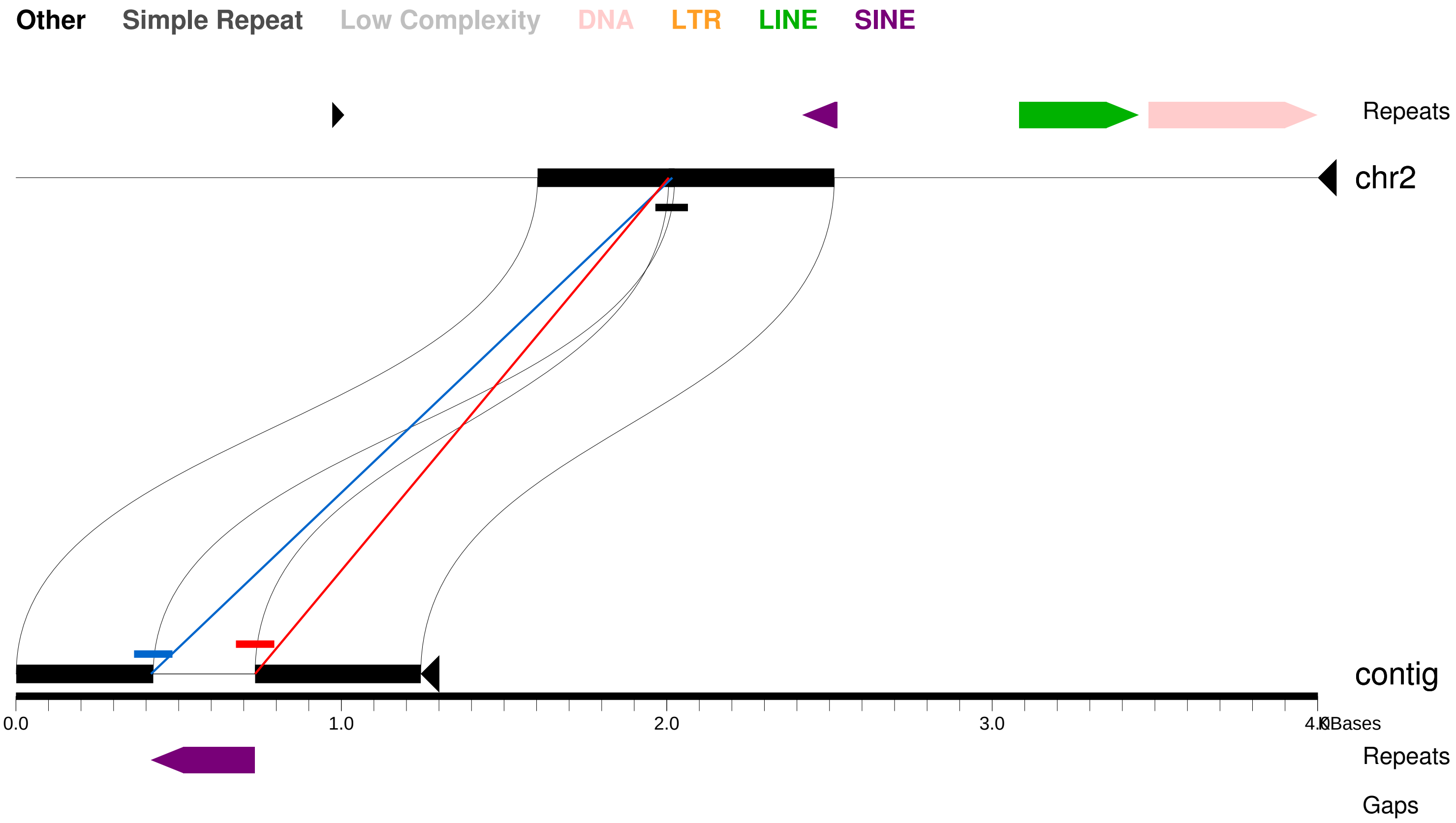

siteID: chr2_142325350 Scafftig8 - Breakpoint: chr2:142325354
chr2:142325301-142325413
Overlap Length: 7 INS size: 306 Genotyped: Yes

```
left     TGGGAAACTAA---ACAGACCTTAAATCTGG---ACTACTAAAGATTCACCTTGATTAGAATTTCTGGCC
chrom    TGGGAAACTAA---ACAGACCTTAAATCTGG---ACTACTAAAGATTCACCTTGATTAGAATTTCTTA--
right    CTCCAGCCTGGGCGACAGAGCGAGACTCCGTCTCAAAAAAAAAAAAAAAAAAAAAAAAAAATTTCTTA--
         1111*11**11111*****1*111*1**1*1111*11*11***1*111*11111*11*1*******2222

left     GGGCGCGGTGGCTCACGCCTGTAATCCCAGCACTTTGGGAGGCCGAGGCGGGCGG
chrom    GGGTTAAATGTGGAAGGGCCCCTAGGAAACCAATTTCCTAATAC-AGGCTTA---
right    GGGTTAAATGTGGAAGGGCCCCTAGGAAACCAATTTCCTAATAC-AGGCTTA---
         ***22222**2222*2*2*2222*2222*2**2***222*222*2****222222
```

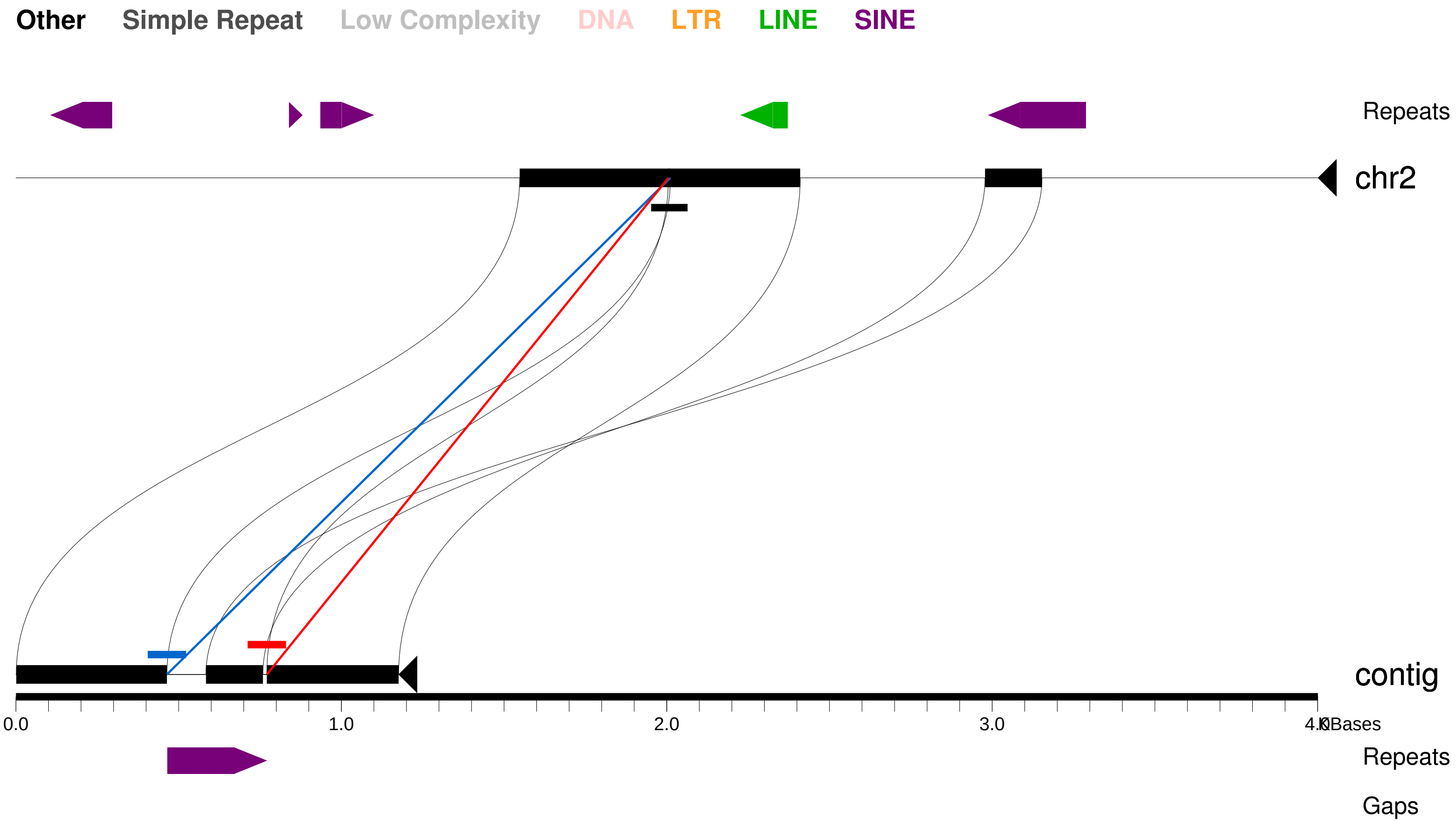

siteID: chr2_1471717 Scafftig1 + Breakpoint: chr2:1471715
chr2:1471672-1471774
Overlap Length: 17 INS size: 303 Genotyped: Yes

```
left     GATATTTTCCTGTTGGAC-----AAGGCCTTTTATCA------TTATATAATGA------CCCTCTTTGT
chrom    GATATTTTCCTGTTGGAC-----AAGGCCTTTTATCA------TTATATAATGA------CCCTCTTTGT
right    -ATCCACCCGCCTCGGCCTCCCAAAGTGCTGGGATTACAGGCGTGAGCCACCGCGCCCGGCCCTCTTTGT
         1**11111*111*1**1*11111***11**111**1*111111*1*111*11*1111111**********

left     CTTTTTTTTTTTTTTTTTTTTGAGACGGAGTCTCGCTCTGTCGCCCAGGCGGGACTGCGGACTGCAG
chrom    CTTTTTTTAACCACTGTTGCTTTAA---AGTTTATTT-TGTCT---------GATATAAGAATA---
right    CTTTTTTTAACCACTGTTGCTTTAA---AGTTTATTT-TGTCT---------GATATAAGAATA---
         *******222222*2**22*222*222***2*222*2****2222222222**22222**2*2222
```

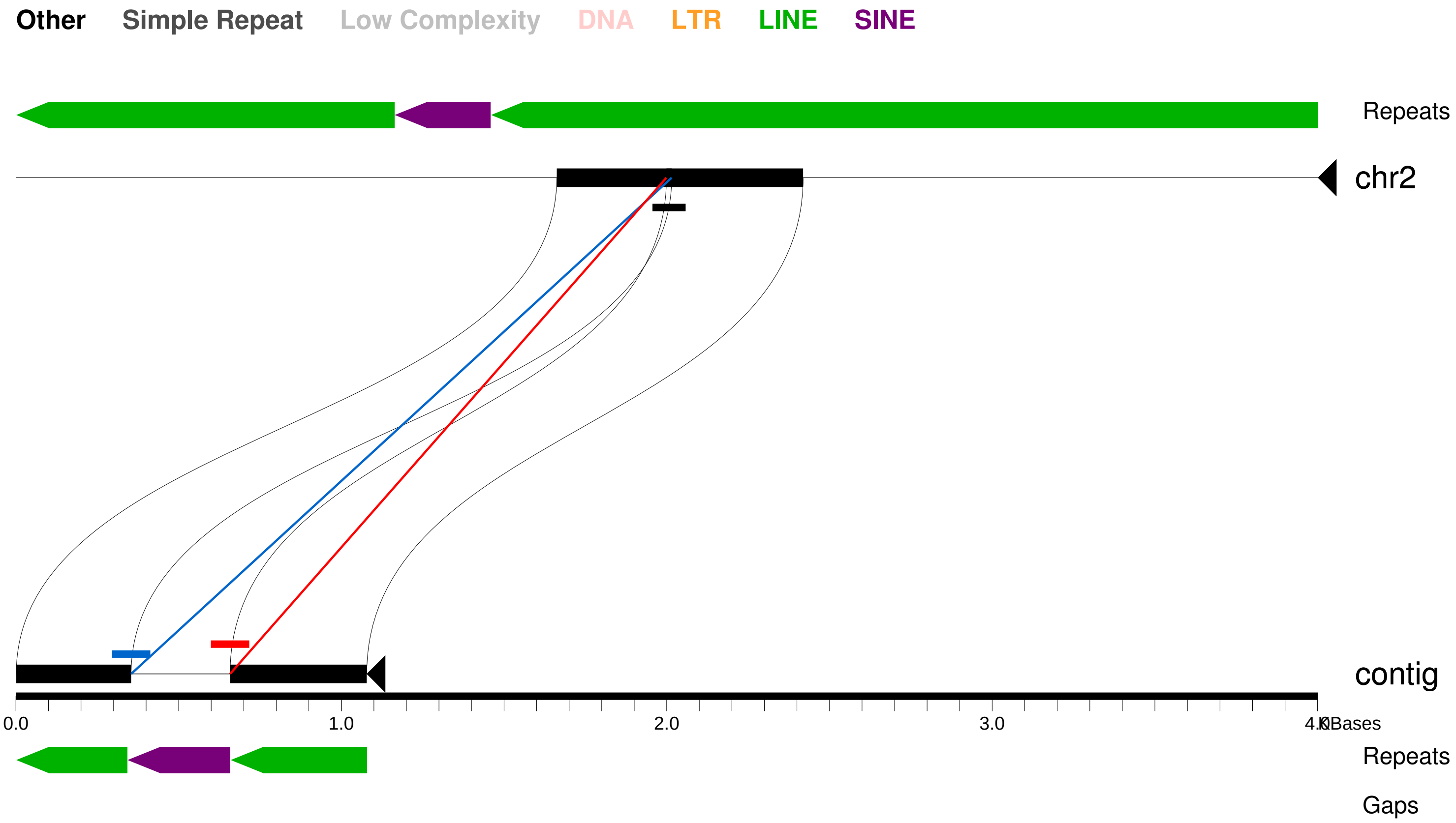

siteID: chr2_150764255 Scafftig3 + Breakpoint: chr2:150764252
chr2:150764205-150764311
Overlap Length: 13 INS size: 427 Genotyped: No

```
left   GCCAACATTTATTTAGAAATGTCTGTAGCTTAGAGTCAATTCTCT------------ATAAGAACTTACA
chrom  GCCAACATTTATTTAGAAATGTCTGTAGCTTAGAGTCAATTCTCT------------ATAAGAACTTACA
right  NNNNNNNNNNNNNNNNNNNNNNNNNNNNNNNNNNNNNNNNNNNNNNNNNNNNNNNAAAAAGAACTTACA
       111111111111111111111111111111111111111111111111111111111*1***********

left   TTGGCCGGGCGCGGTGGCTCACGCCTGTAATCCCAGCACTTTGGGAGGCCGAGGCGGGTGG
chrom  TTAAT---------TTTTAGATACAAATAATTAAAGAATTAGCTTAATTTCAATTATT---
right  TTAAT---------TTTTAGATACAAATAATTAAAGAATTAGCTTAATTTCAATTATT---
       **222222222222*22222*22*222****222**2*2*22222*22222*222222222
```

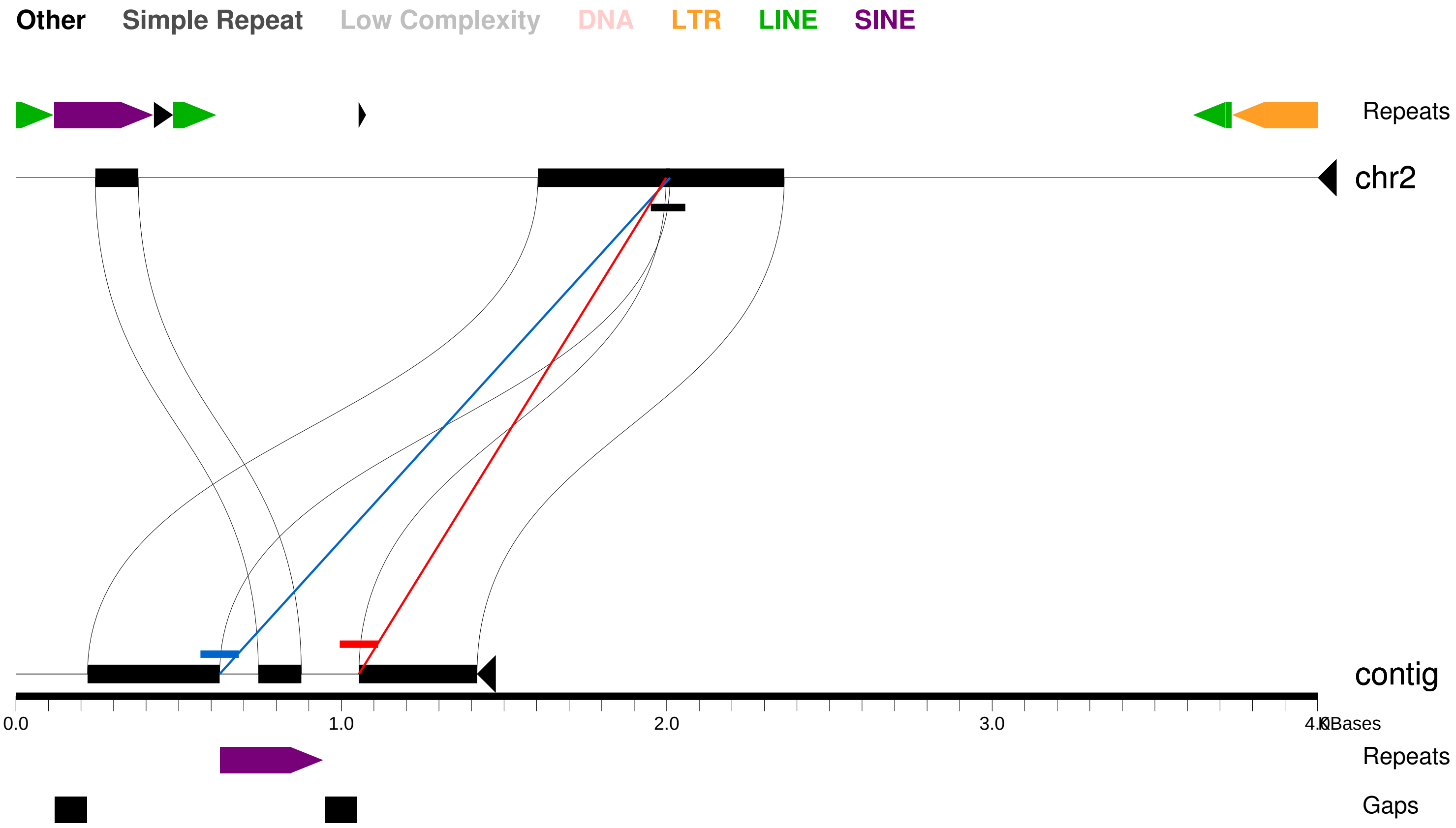

siteID: chr2_15163745 Scafftig1 + Breakpoint: chr2:15163754
chr2:15163606-15164170
Overlap Length: 13 INS size: 477 Genotyped: No

```
left   T-ATTTTAATAGATGTTGCCCATTGCTTTATAAAAAGATTATAACTTTTGACATTTCTCCAAGCTAAAAC
chrom  TTATTTTAATAGATGTTGCCCATTGCTTTATAAAAAGGTTATAACTTTTGACATTTCTCCAAGCTAAAAC
right  NNNNNNNNNNN--------------------AAAAA-----------------------------------
       1N1111111111111111111111111111111*****1N11111111111111111111111111111111

left   TAAAAGTACTGTTCTCCTCTCATCCTTGCCAGCTATCAGTTTTATGTTTCTCTTTAAATCTTGCCAATCA
chrom  TAAAAGTACTGTTCTCCTCTCATCCTTGCCAGCTATCAGTTTTATGTTTCTCTTTAAATCTTGCCAATCA
right  ----------------------------------------------------------------------
       1111111111111111111111111111111111111111111111111111111111111111111111

left   ATGGGTATAAGATTATATCTTGG-----------------------------------------------
chrom  ATGGGTATAAGATTATATCTTATAGTGATTTTCATCTATTTCCTTGAGAGTTAATTTAAACATCATTTAG
right  --------AAGATTATATCTTATAGTGATTTTCATCTATTTCCTTGAGAGTTAATTTAAACATCATTTAG
       11111111*************222222222222222222222222222222222222222222222222

left   --------CCGGGCGCGG------------------------------------TGGCTCAC--------
chrom  TAAGTTTACTGGGCACTGGTATTTGCTTTTCTCTAAGTTGAAAATGTACAACTTTTGCTCATTTTCTTAA
right  TAAGTTTACTGGGCA-------------------------------------------------------
       22222222*2****21N1                                    1N11111N

left   ---------GCCT------------GTAA-----------------------------------------
chrom  TGGTTGTTTGCCTTCTTGTCAACTTGTAAGAGATCTTTGAATATTAAAAATATTCACACTTTGTTCATTT
right  ----------------------------------------------------------------------
                1111            1111

left   ---------------TCCCAGCA----CTTTGGGA---GGCCGAGGCGGGTGGATCATGAGGT-CAGGAG
chrom  TTATCACTAATATTTTCAAAGCAATATCTTTGGTATTTGACTTATTTGTG-GGATTTTTATATACACAAA
right  ----------------------------------------------------------------------
                      11NN1111    111111N1   1N1NN1NNN1N121111NN1N1NN1 11NN1N

left   AT-----------------CGAGACCATCCTGGCT-----------AACA--------------------
chrom  ATTTTTATTTTTACATATTCAAATATATCCTCACTTTATTTTCTGGAAAATTATTTTATTTTATTTTAAT
right  ----------------------------------------------------------------------
       11                  1N1NNNN11111NN11           11N1

left   -----------------AGGT-----GAAACCCC-----GTCTCTACTAAA-------------------
chrom  ATTTTAACTTTTATTTTAGGTTCAGGGATACACGTGCAGGTTTGTTATATAGGTAAACTTGTATCTCAGG
right  ----------------------------------------------------------------------
                        1111     11N11N1N     11N1N1NN11N1

left   ------
chrom  GGTTTG
right  ------
```

Other Simple Repeat Low Complexity DNA LTR LINE SINE

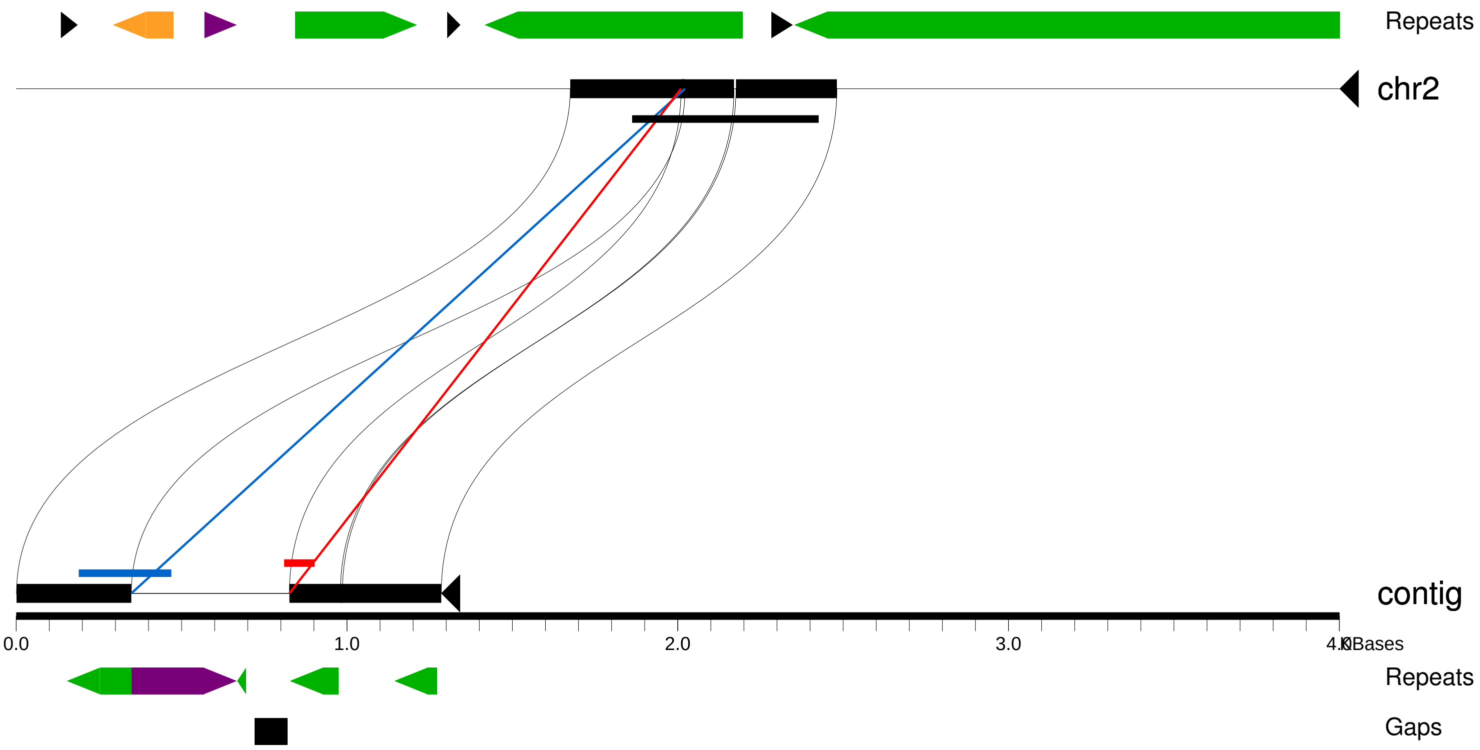

siteID: chr2_153248290 Scafftig5 - Breakpoint: chr2:153248266
chr2:153248221-153248325
Overlap Length: 15 INS size: 318 Genotyped: Yes

```
left   TATTG---GAACTATGGTATCAGATTTCCTGAAGGTCACATTAT---------TCAT--TAATAATAATG
chrom  TATTG---GAACTATGGTATCAGATTTCCTGAAGGTCACATTAT---------TCAT--TAATAATAATG
right  GACAGAGCGAAACTCCGTCTCAAAAAAAAAAAAAAAAAAAAAAAAAAAAAAAAATAATAATAATAATAATG
       1*11*111***11111**1***1*1111111**1111*1*11*1111111111*1**11***********

left   TTGCGGCCGGGCGCGGTGGCTCACGCCTGTAATCCCAGCACTTTGGGAGGCCGAGATGGGCGG
chrom  TTGCT--------CTTTGA-----GCCCTTAAAATTGGCCTTTTTCTAGACTCCTAAACTCT-
right  TTGCT--------CTTTGA-----GCCCTTAAAATTGGCCTTTTTCTAGACTCCTAAACTCT-
       *****222222222*22**222222***22***22222**22***222**2*2222*2222*22
```

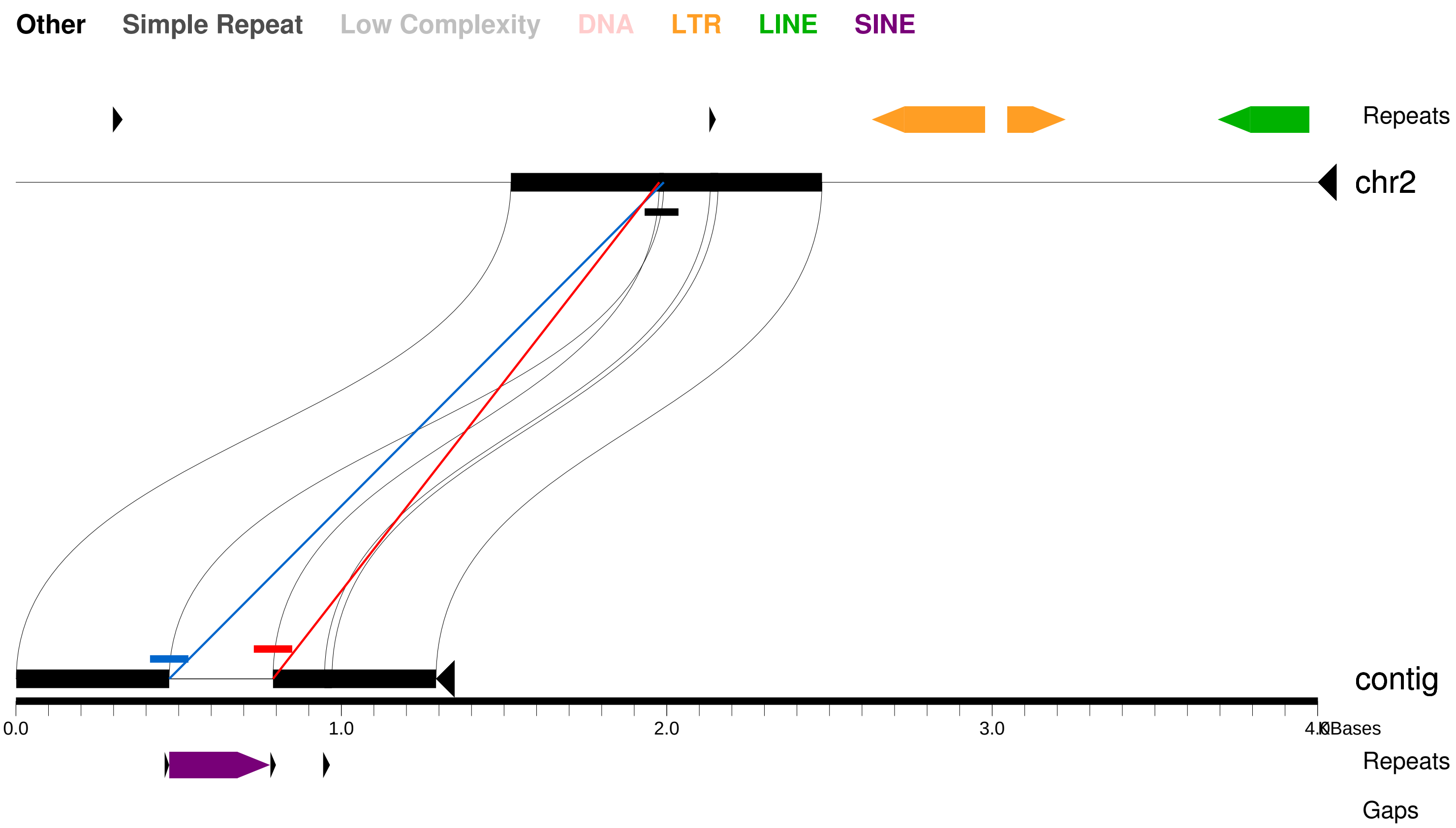

siteID: chr2_154752579 Scafftig2 + Breakpoint: chr2:154752582
chr2:154752422-154752755
Overlap Length: 14 INS size: 311 Genotyped: Yes

```
left   --------------------------GATAAAAAAAGATATTGTCACACAAGAAATTCACAATTAAGTAAA
chrom  TTAATCAGTGATCTTGCAGTGTAATGATAAAAAAAGATATTGTCACACAAGAAATTCACAATTAAGTAAA
right  TTTAGTAGAGAC---GGGGTTTCACCGTTTTAGCCGGGATGGTCTC----GATCTCCTGACCTC-GTGAT
       22N2NN22N22N   2NN22N2N2N11*111*111*11**1***1*1111**11*1*11*11*11**1*1

left   AAGAGAATATAATCATAAAATAGTTTGTATGATATAATTATTTAAATAATAGTCTGCGAGTAAGAACAGG
chrom  AAGAGAATATAATCATAAAATAGTTTGTATGATATAATTATTTAAATAATAGTCTGCGAGTAAGAACAGG
right  CCGCCCGCCTCGGCCTCCCAAAGT------GCTGGGATTAC---------AGGC-GTGAGCCACCGCGC-
       11*111111*111*1*111*1***111111*1*111****1111111111**1*1*1***11*111*111

left   ATAGTTGTTTCTGGAATTAAGGATGATTTTATATATATATATATATTTTTTTTTT---------TT----
chrom  ATAGTTGTTTCTGGAATTAAGGATGATTTTATATTTTTATGTTTTTTCCTAAATTAAAAAAAACTTGCCA
right  ----------CCGGCC----GGATGATTTTATATTTTTATGTTTTTTCCTAAATTAAAAAAAACTTGCCA
       1111111111*1**111111*************2*2***2*2*2**22*222**222222222**2222

left   -TTT---TTTTGAGACGGA---GTCT----------CGCTC-TGTCGCCCAGGCTGGAGTGCAG-TGGCG
chrom  CTTTCGATTTAGAAAACTAATTGTATAAGGAAAAACCACTTATATTGCTAATCTTTAAAGACTGATAACC
right  CTTTCGATTTAGAAAACTAATTGTATAAGGAAAAACCACTTATATTGCTAATCTTTAAAGACTGATAACC
       2***222***2**2*222*222**2*2222222222*2**22*2*2**22*222*22*222*2*2*22*2

left   GGATCTCGGCTCACTGCAAGCTCCGCCTCCCGGGTTAATGCCATTCTCCTGCCTCAGC
chrom  TTTTGTCTTTTTTCTTTAAGTCCTGACTACT--TTCACTTATTTTTTGATTAC-CAT-
right  TTTTGTCTTTTTTCTTTAAGTCCTGACTACT--TTCACTTAT----------------
       222*2**222*22**22***22*2*2**2*2222*2*2*222N11N1NN1NN1211N2
```

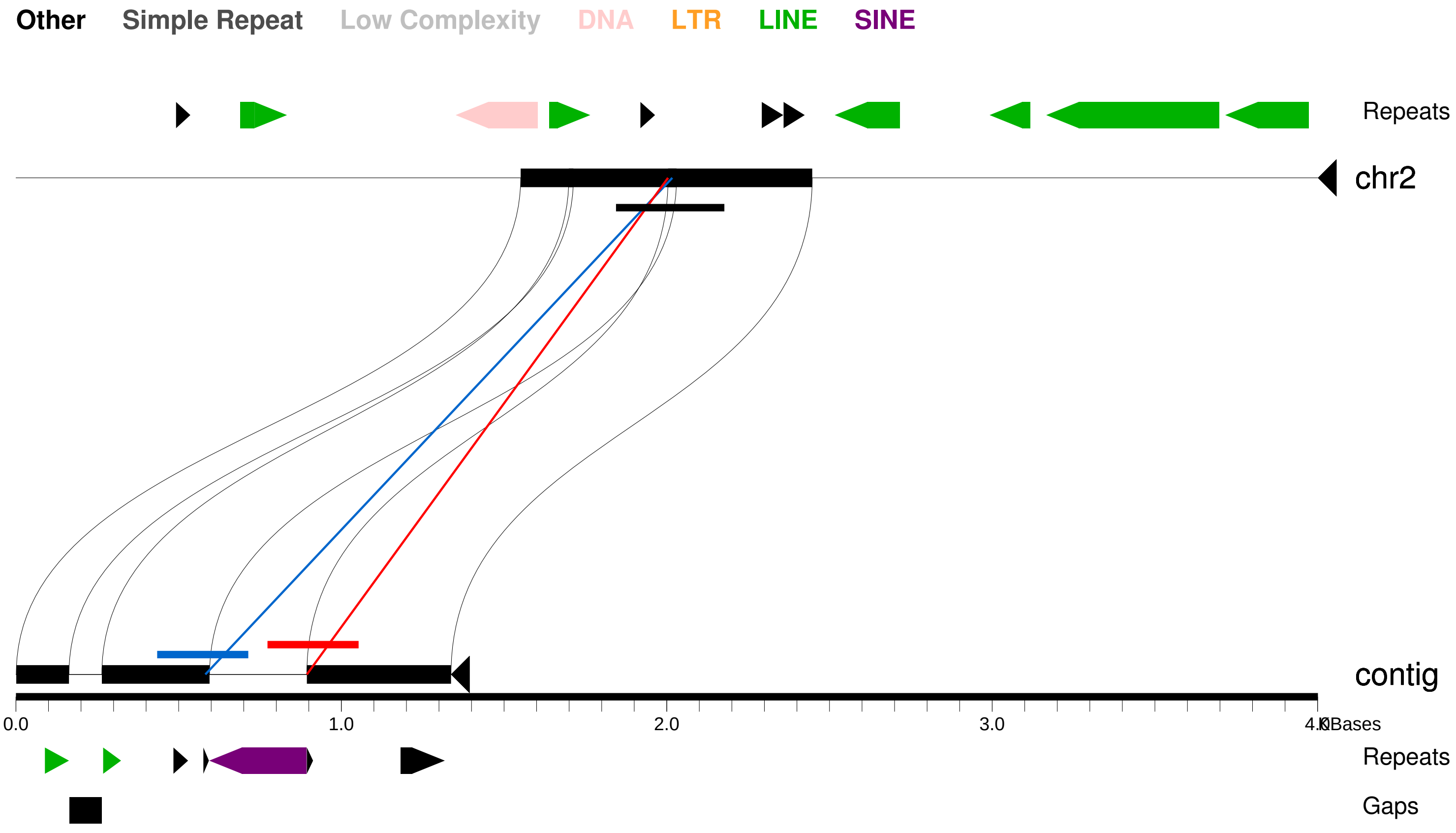

siteID: chr2_15519529 Scafftig5 - Breakpoint: chr2:15519540
chr2:15519494-15519599
Overlap Length: 14 INS size: 298 Genotyped: Yes

```
left   AATCAGCAAAAATGACCATGGGAAAGCGCG---------CTGGAGAAAAATGTTT----AAGAAGCTGAT
chrom  AATCAGCAAAAATGACCATGGGAAAGCGCG---------CTGGAGAAAAATGTTT----AAGAAGCTGAT
right  CCACTGCACTCCAGCCTGGGCGACAGAGCGAGACTCCGTCTCAAAAAAAAAAAAAAAAAGAAGAAGCTGAT
       111*1***11111*1*111*1**1**1***111111111**11*1*****111111111***********

left   GGAGGCCGGGCGCGGTGGCTCACGCCTGTAATCCCAGCACTTTGGGAGGCCGAGGCGGGCGG
chrom  GGACATTCATAACATAGTTTTTCTTTTTATATTCAAGCAAAAAATAACT-------------
right  GGACATTCATAACATAGTTTTTCTTTTTATATTCAAGCAAAAAATAACT-------------
       ***222222222*222*22*22*222*222**2*2****2222222*222222222222222
```

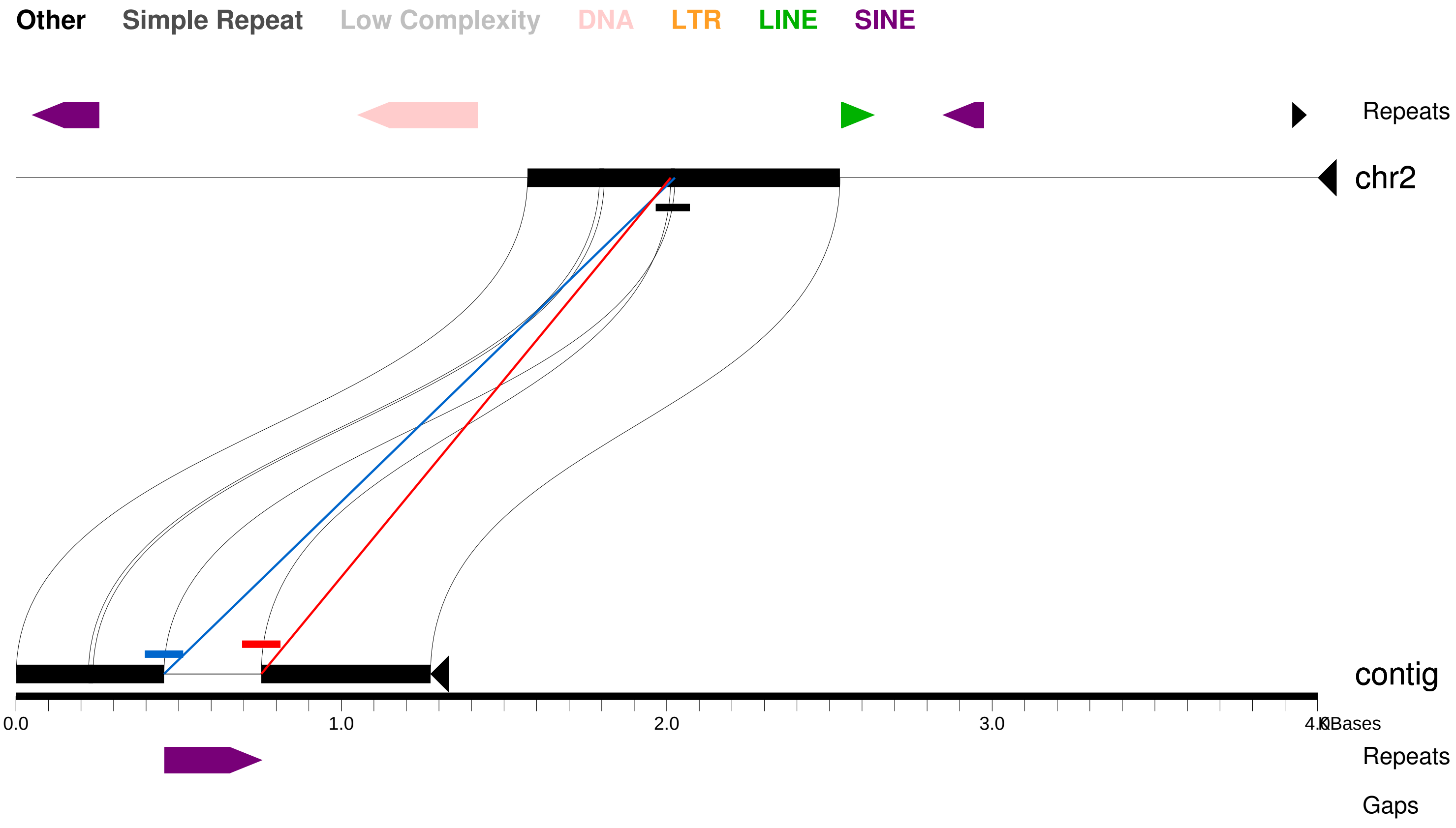

siteID: chr2_157661280 Scafftig1 - Breakpoint: chr2:157661244
chr2:157661200-157661303
Overlap Length: 16 INS size: 414 Genotyped: No

```
left    ---GTGCAGGTTAAAATATATATGTAG-GTAATTTGAAAAAT-AGCCAT----------CAATACTATTA
chrom   ---GTGCAGGTTAAAATATATATGTAG-GTAATTTGAAAAAT-AGCCAT----------CAATACTATTA
right   TCCGCCCGCCTCGGCCTCCCAAAGTGCTGGGATTACAGGCGTGAGCCACCGCGCCCGGCCAATACTATTA
        111*11*111*11111*1111*1**111*11***11*1111*1*****11111111111***********

left    CTTTTTTNNNNNNNNNNNNNNNNNNNNNNNNNNNNNNNNNNNNNNNNNNNNNNNNNNNNNNNN
chrom   CTTTTT---------------GAAATTATTTTTTTCATTTCAAATAAATAAAATTAGGAAAATGC
right   CTTTTT---------------GAAATTATTTTTTTCATTTCAAATAAATAAAATTAGGAAAATGC
        *****`*`222222222222222222222222222222222222222222222222222222222222
```

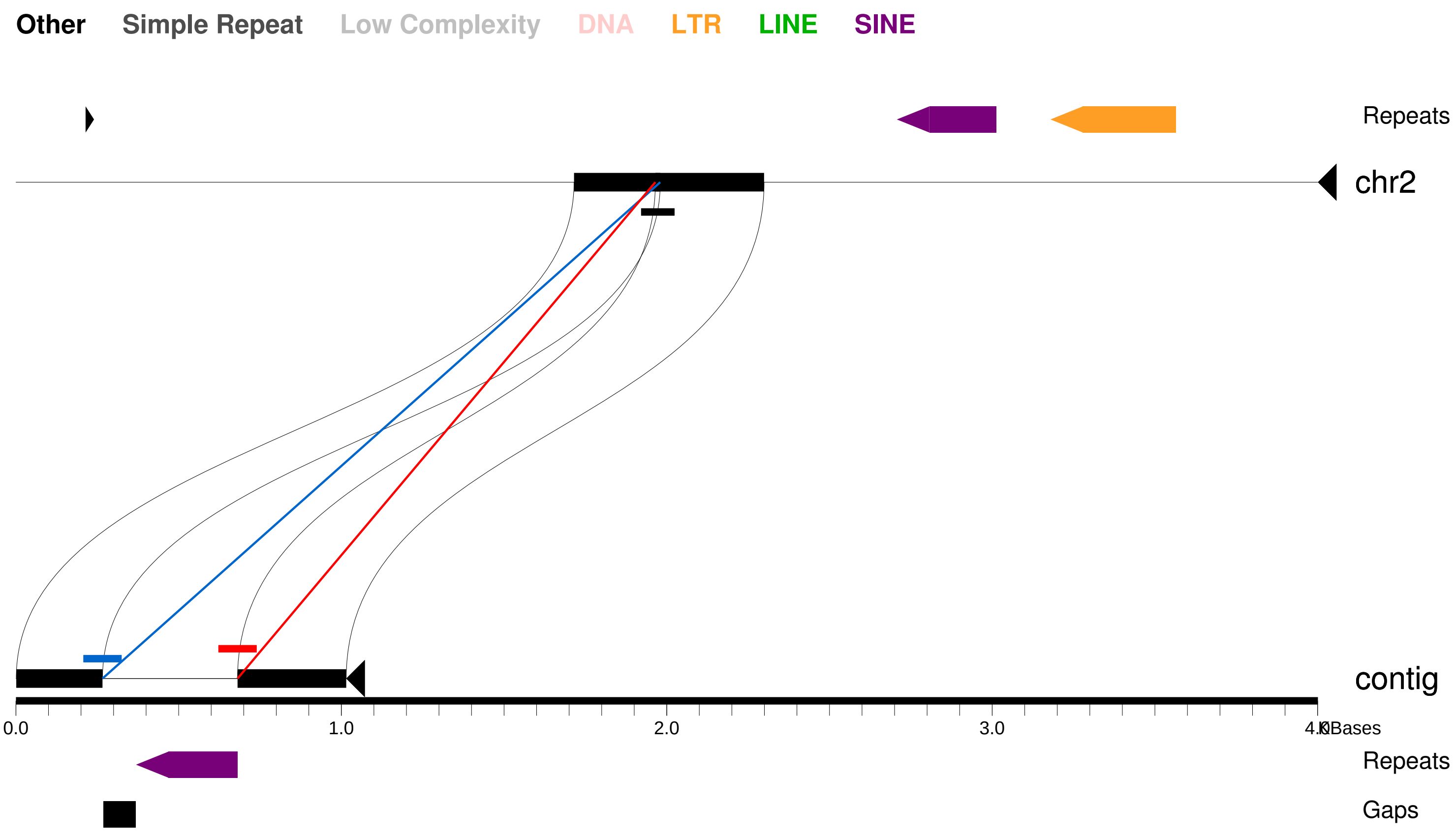

siteID: chr2_158028066 Scafftig1 + Breakpoint: chr2:158028141
chr2:158028113-158028200
Overlap Length: 32 INS size: 234 Genotyped: No

```
left    TATA----AATG-CTGCAT----GATAA----AAGTACCGC------------------AAAATTTAATG
chrom   TATA----AATG-CTGCAT----GATAA----AAGTACCGC------------------AAAATTTAATG
right   TGGAGTGCAGTGGCGGGATCTCGGCTCACTGCAAGCTCCGCCTCCCGGGTTCACGCCATAAAATTTAATG
        *11*1111*1**1*1*1**1111*1*1*1111***11****111111111111111111***********

left    TCTTAAATAGCCTTTACTAAGNNNNNNNNNNNNNNNNNNNNNNNNNNNNNNNNNNNNNNNNNNNNNNNNN
chrom   TCTTAAATAGCCTTTACTAAGCTTACAAGCCTTCAAGTCAGCAAATCAA---------------------
right   TCTTAAATAGCCTTTACTAAGCTTACAAGCCTTCAAGTCAGCAAATCAA---------------------
        *********************2222222222222222222222222222222222222222222222222

left    NNNNNNNNNN
chrom   ----------
right   ----------
        2222222222
```

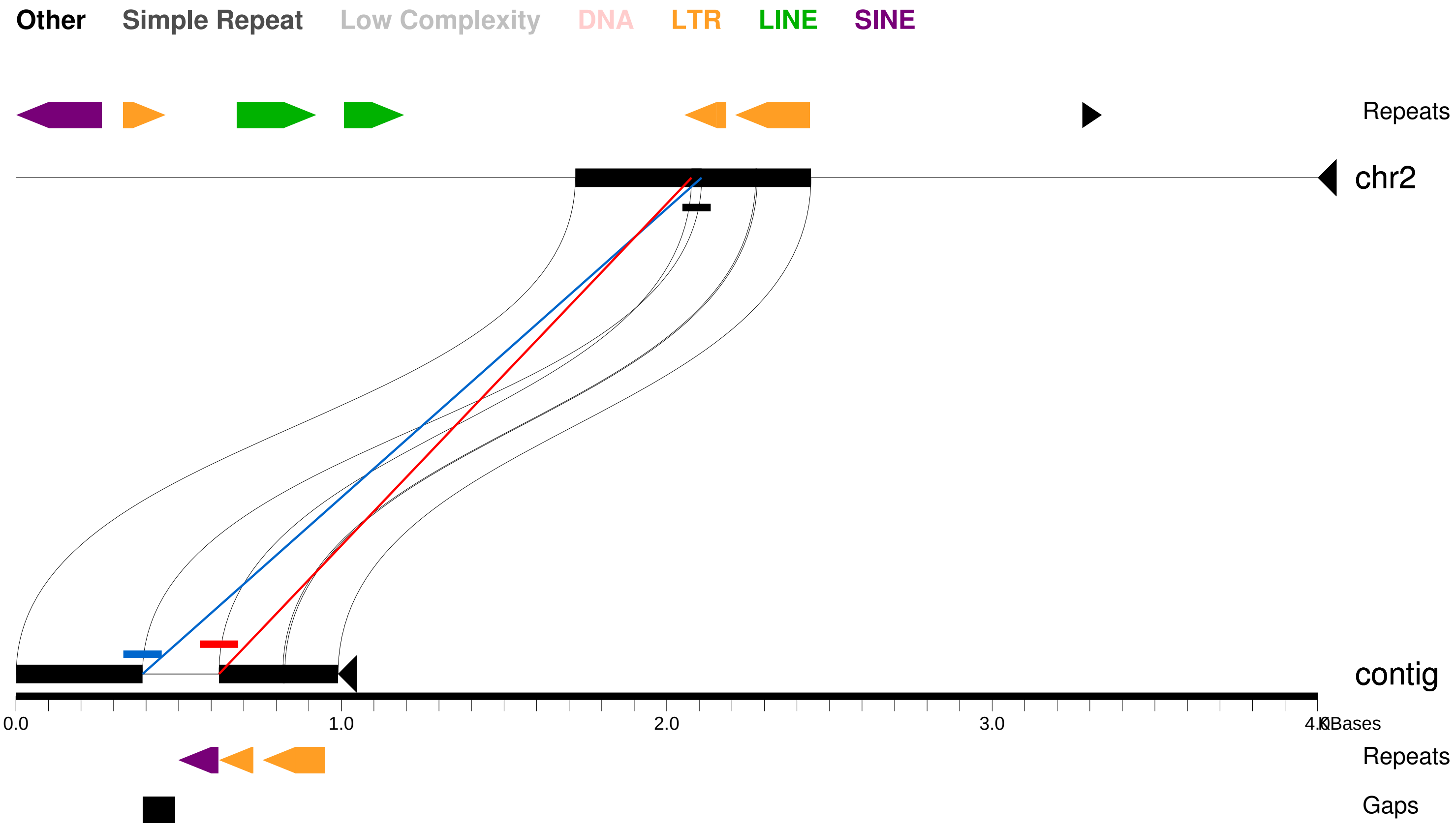

siteID: chr2_158279978 Scafftig2 - Breakpoint: chr2:158279981
chr2:158279927-158280040
Overlap Length: 11 INS size: 443 Genotyped: No

```
left   AATAAATTTAAAAACTTTGTTAACAAAAACATAAGTCGCTAAAACTGTCTTTAA-----AAAAAAAACCAG
chrom  AATAAATTTAAAAACTTTGTTAACAAAAACATAAGTCGCTAAAACTGTCTTTAA-----AAAAAA-CCAG
right  NNNNNNNNNNNNNNNNNNNNNNNNNNNNNNNNNNNNNNNNNNNNNNNNNNNNNNNNNNNNAAAAAA-CCAG
       111111111111111111111111111111111111111111111111111111111111******2****

left   AGGCCGGGCGCGGTGGCTCACGCCTGTAATCCCAGCACTTTGGGAGGCCGAGGC
chrom  AAATCCTGAGTAGT--CTCACAACT--AATAAAGTAATTGAAACAGTACATTAA
right  AAATCCTGAGTAGT--CTCACAACT--AATAAAGTAATTGAAACAGTACATTAA
       *222*22*2*22**22*****22**22***222222*2*22222**22*22222
```

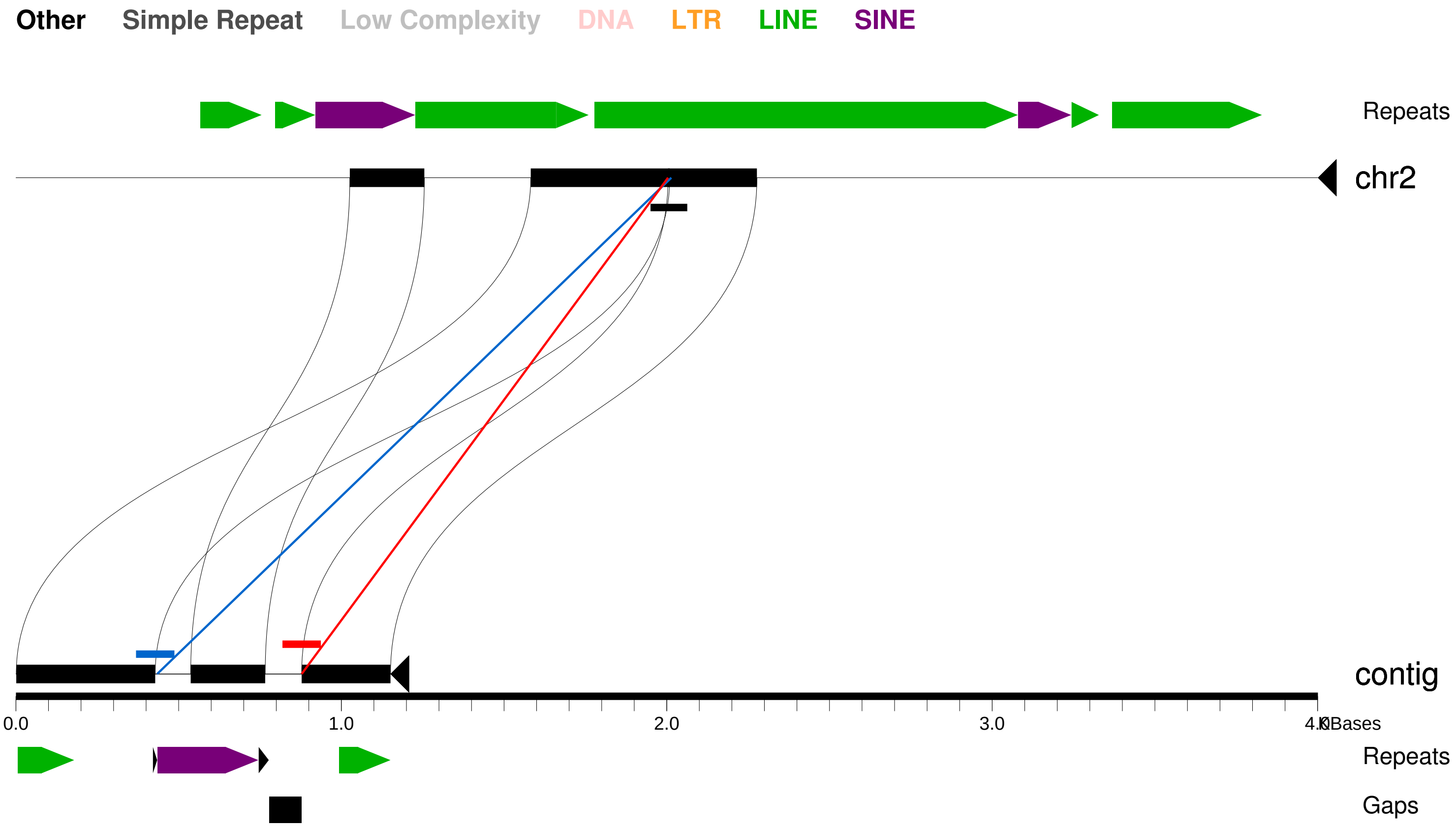

siteID: chr2_158510345 Scafftig8 - Breakpoint: chr2:158510356
chr2:158510313-158510410
Overlap Length: 16 INS size: 309 Genotyped: Yes

```
left    -----------TCTGGCAAGGGC------------CTTCTTGCTGCATTATAACACAGCAGAAGATAAGA
chrom   -----------TCTGGCAAGGGC------------CTTCTTGCTGCATTATAACACAGCAGAAGATAAGA
right   GCAGTCTGCAGTCTGGCCTGGGCGACAGAGCGAGACTCCGT-CT-CAAAAAAAAAAAAAAAAAAAAAAGA
        11111111111******11****111111111111**1*1*1**1**11*1**1*1*11*1**1*1****

left    AAGTAAGAGAGAGGGCCGGGCGCGGTGGCTCACGCCTGTAATCCCAGCACTTTGGGAGGCCGAGGCGGGT
chrom   AAGTAAGAGAGA-------GCAAGAGGGTCCA-------AATTCATTCT-TTTATAAGG-------GTAT
right   AAGTAAGAGAGA-------GCAAGAGGGTCCA-------AATTCATTCT-TTTATAAGG-------GTAT
        ************2222222**22*22**22**2222222***2*222*22***222***2222222*22*

left    GG-
chrom   GAA
right   GAA
        *22
```

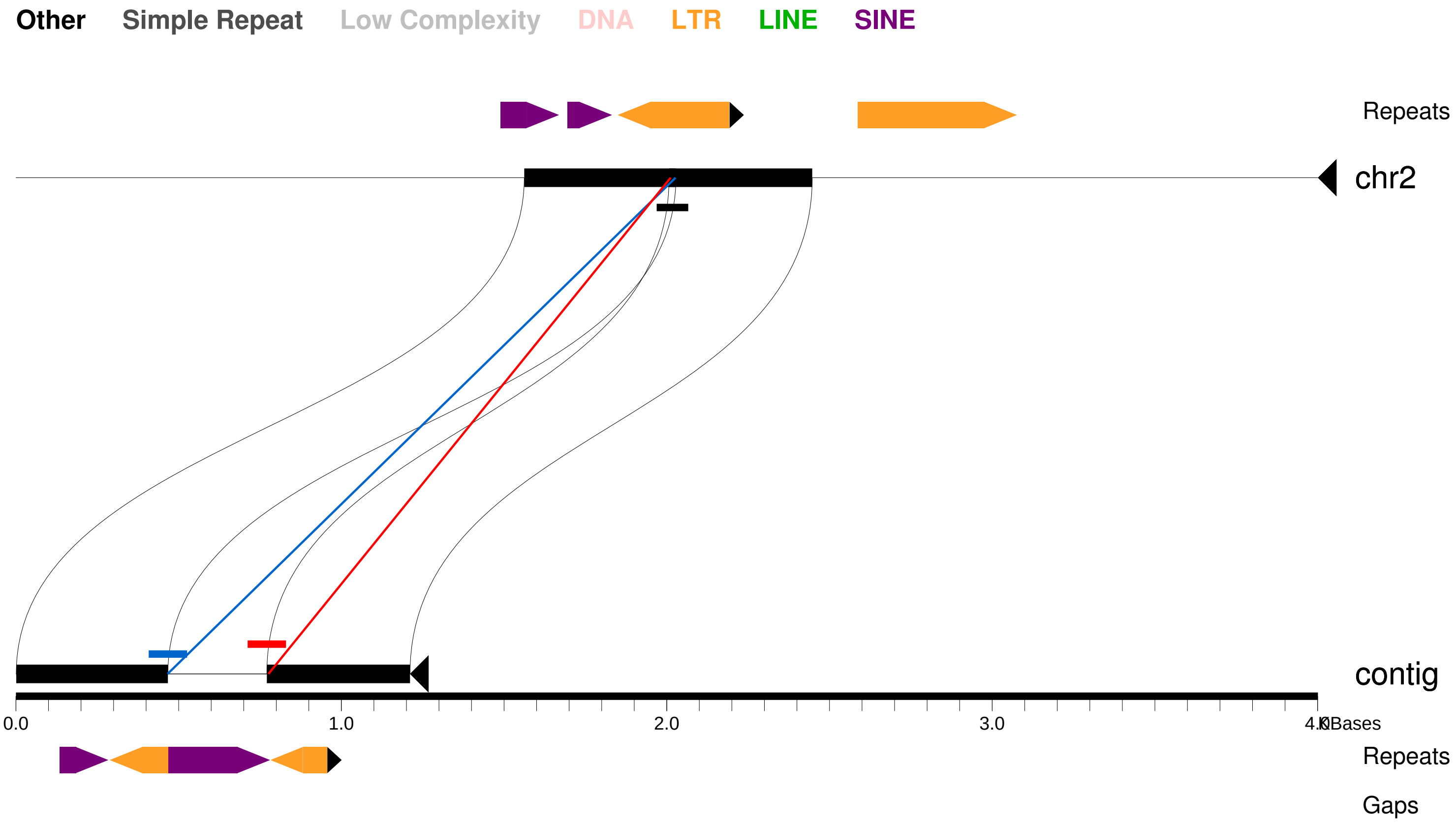

siteID: chr2_159282655 Scafftig2 + Breakpoint: chr2:159282630
chr2:159282576-159282689
Overlap Length: 6 INS size: 422 Genotyped: No

```
left    TAATTCTCTTAGTAAAAATCCAAATAAATATAGAAAAGAACACTGTAAAACTGT-----GGTAGGGGCCG
chrom   TAATTCTCTTAGTAAAAATCCAAATAAATATAGAAAAGAACACTGTAAAACTGT-----GGTAGGAACTA
right   NNNNNNNNNNNNNNNNNNNNNNNNNNNNNNNNNNNNNNNNNNNNNNNNNNNNNNNNNNNNGGTAGGAACTA
        111111111111111111111111111111111111111111111111111111111111******22*22

left    GGCGCGGTGGCTCACGCCTGTAATCCCAGCACTTTGGGAGGCCGAGGCGGGCGG
chrom   TA-GTTTAAAGTAGTGAAAGTAATTATAACACA--AGGAATTTCA--CAGATAG
right   TA-GTTTAAAGTAGTGAAAGTAATTATAACACA--AGGAATTTCA--CAGATAG
        222*2222222*222*222*****222*2***2222***22222*22*2*222*
```

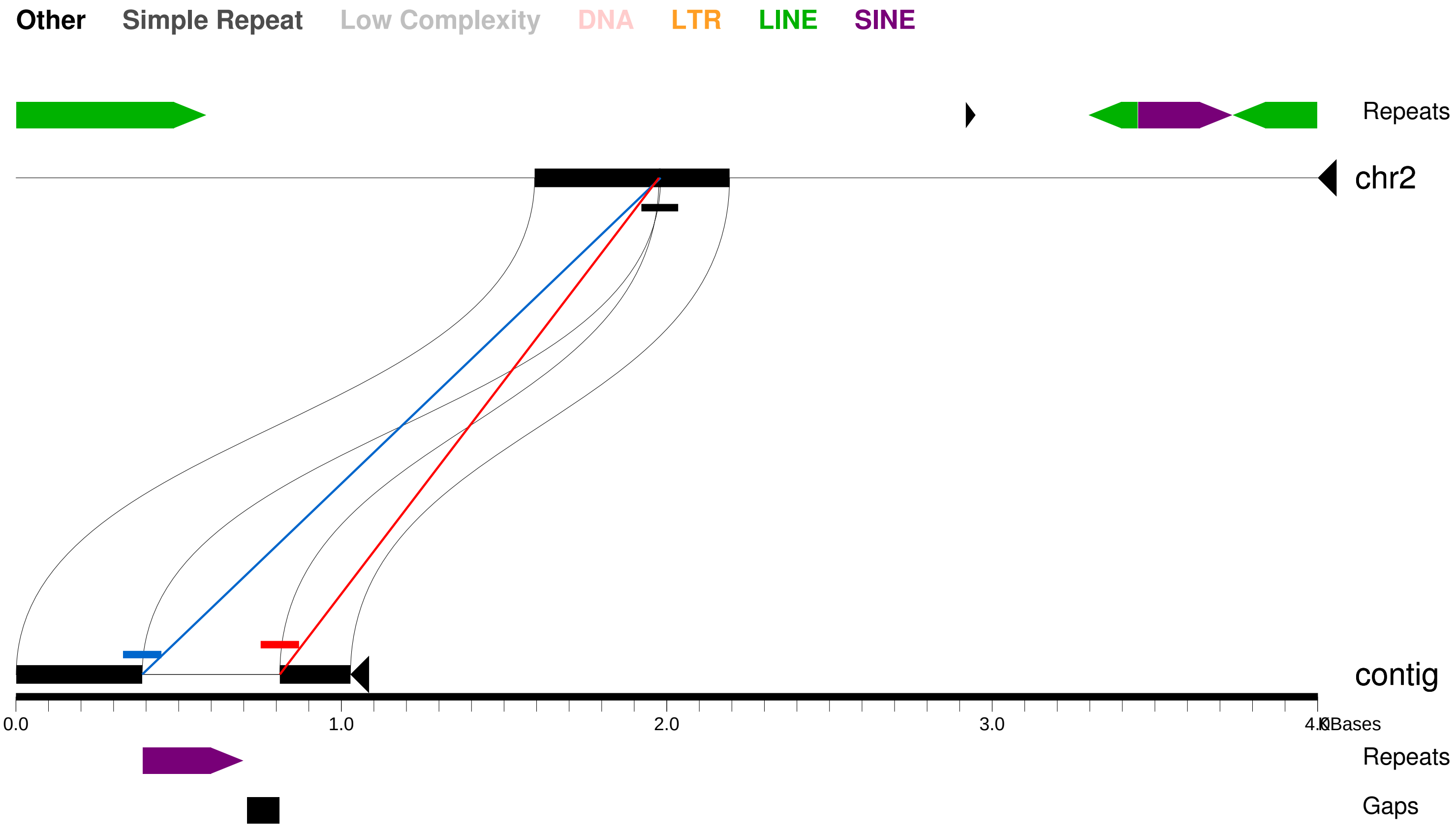

siteID: chr2_159285148 Scafftig1 - Breakpoint: chr2:159285132
chr2:159285088-159285191
Overlap Length: 16 INS size: 286 Genotyped: Yes

```
left    CCA---------CAATGAGATACCATCTCA-CACCAGTTAGAATGGTGATC-----ATTAAAAAGTCAGG
chrom   CCA---------CAATGAGATACCATCTCA-CACCAGTTAGAATGGTGATC-----ATTAAAAAGTCAGG
right   TCGAGATCGCGCCACTGCGCTCCAGCCTGGGCGACAGAGCGAAACTCCATCTCAAAAAAAAAAAGTCAGG
        1*1111111111**1**1*1*1*111**111*11***111***11111***11111*11***********

left    AAACAGGCCGGGCGCGGTGGCTCACGCCT-GTAATCCCAGCACTTTGGGAGGCCGAGACGGGCGG
chrom   AAACA--ACAGGTGCTGGAGAGGATGTAGAGAAATAGGAACACTTTT--------ACAC------
right   AAACA--ACAGGTGCTGGAGAGGATGTAGAGAAATAGGAACACTTTT--------ACAC------
        *****222*2**2**2*22*222*2*2222*2***222*2******222222222*2**222222
```

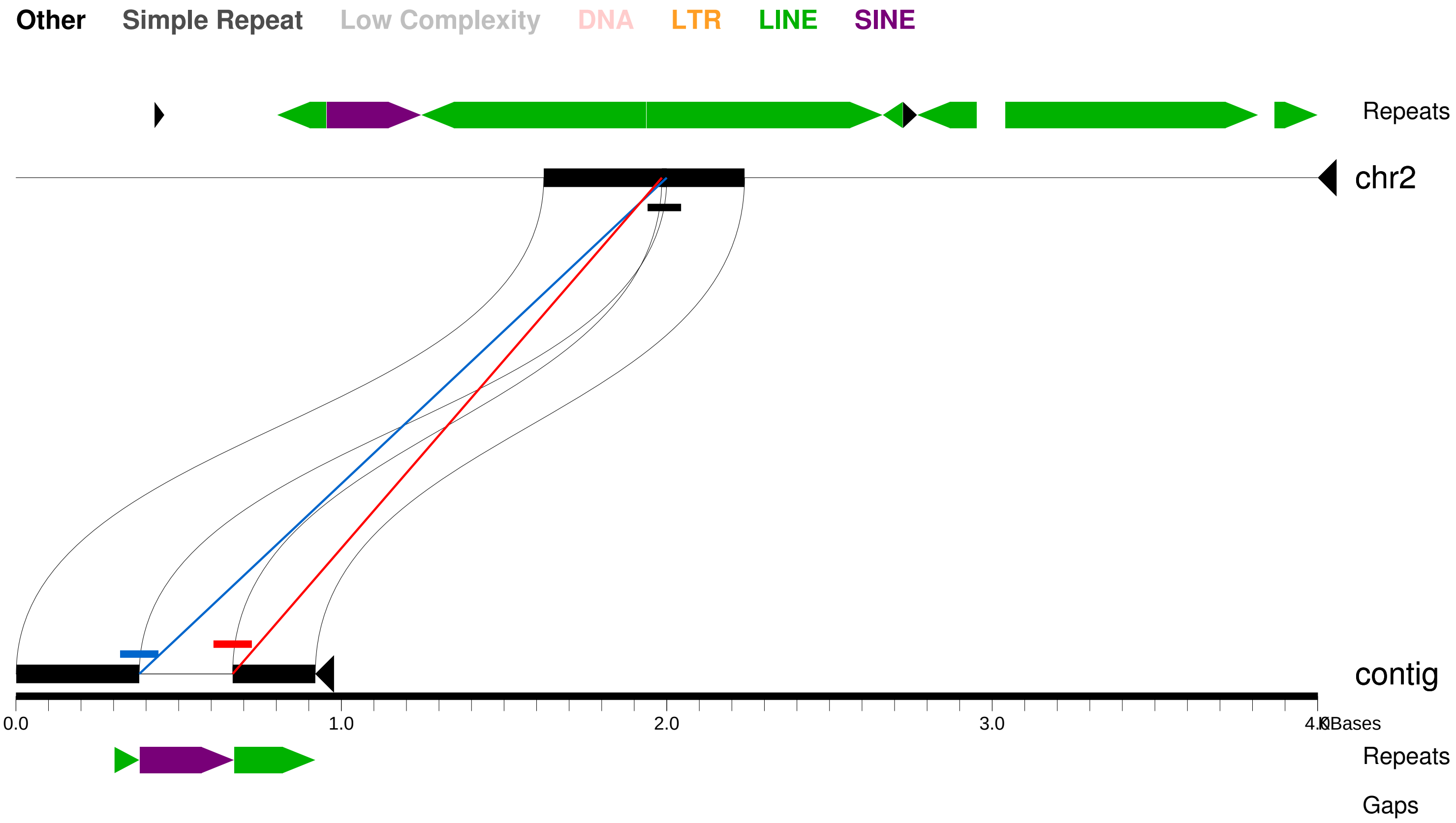

siteID: chr2_161952345 Scafftig18 - Breakpoint: chr2:161952317
chr2:161952278-161952376
Overlap Length: 21 INS size: 279 Genotyped: Yes

```
left    T--GTTGAGAA-GCACAGAGAGAAATCAATTAAAGCAA------------GTTT-----AAAAAAAAAAA
chrom   T--GTTGAGAA-GCACAGAGAGAAATCAATTAAAGCAA------------GTTT-----AAAAAAAAAAA
right   CAAGCCGAGATTGCACCACTGGATACCAGTCCAGGCAACAATGTGAGACTGTCTCAAAAAAAAAAAAAAA
        111*11****11****11111**1*1**1*11*1****111111111111**1*11111***********

left    AAAAAAAAGGCCTGCGTGGTGGCTCACGCCTGTAATCC--CAGCACTTTGGGAGGCTGAGGCGGGCAAATC
chrom   AAAAATAGGCACCAGT--------ACTCTGGAAATGCTACAAAATGTTTAAATGCTT-------------
right   AAAAATAGGCACCAGT--------ACTCTGGAAATGCTACAAAATGTTTAAATGCTT-------------
        *****2****2222**22222222**2*22*2***2*22**22*22**222*2***22222222222222

left    A
chrom   -
right   -
        2
```

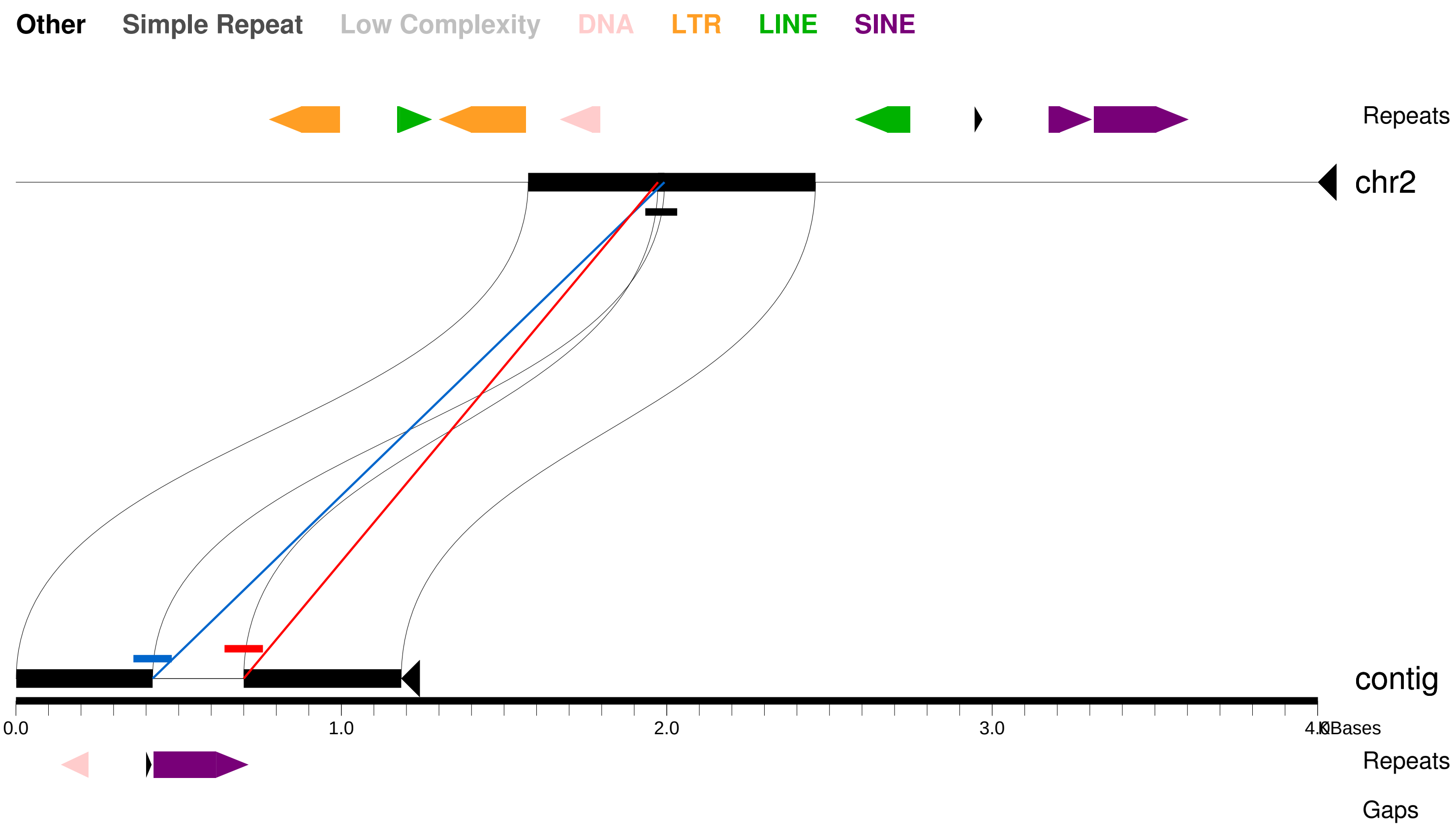

siteID: chr2_162004249 Scafftig1 + Breakpoint: chr2:162004213
chr2:162004170-162004272
Overlap Length: 17 INS size: 473 Genotyped: No

```
left    GACAGAACCTGGATACCTCCGTTGCCAGTGAAGGATTTGCGTG----------------CTGTTATAGTC
chrom   GACAGAACCTGGATACCTCCGTTGCCAGTGAAGGATTTGCGTG----------------CTGTTATAGTC
right   NNNNNNNNNNNNNNNNNNNNNNNNNNNNNNNNNNNNNNNNNNNNNNNNNNNNNNNNNNCCTGTTATAGTC
        11111111111111111111111111111111111111111111111111111111111***********

left    CTTTTTTTTTTNNNNNNNNNNNNNNNNNNNNNNNNNNNNNNNNNNNNNNNNNNNNNNNNNNNNNN
chrom   CTTTTTGAT----------------GGGAGCCTCCAATCGCTGCTCCTTCTAGTCGGCCATCATG
right   CTTTTGGGT----------------GGGAGCCTCCAATCGCTGCTCCTTCTAGTCGGCCATCATG
        *****12N*22222222222222222222222222222222222222222222222222222222
```

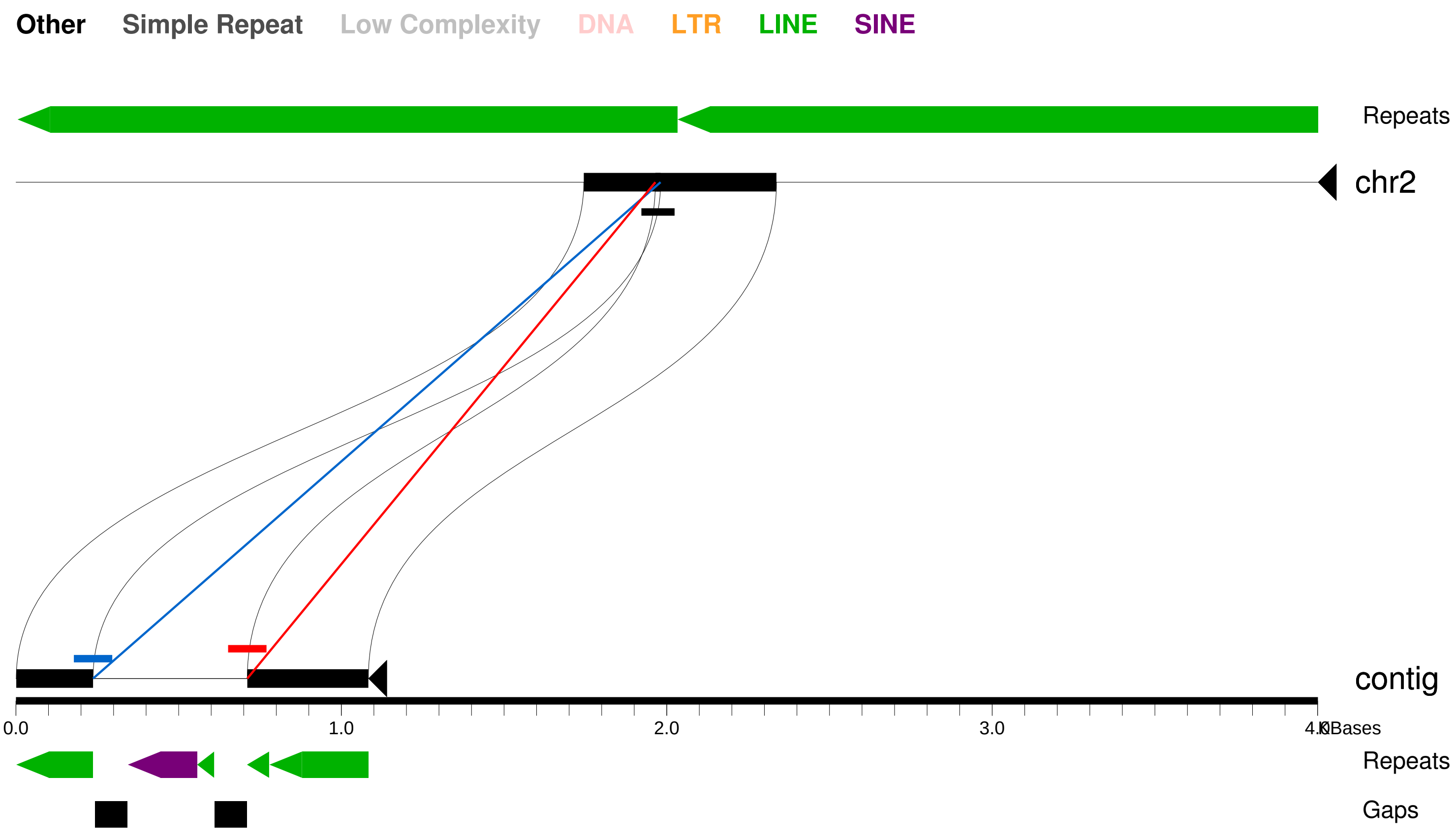

siteID: chr2_162765260 Scafftig2 - Breakpoint: chr2:162765246
chr2:162765192-162765305
Overlap Length: 5 INS size: 300 Genotyped: No

```
left   GTAAAGAAATGGAGAAAG--CTGCATAGGCC-----AGTTGGTGTCAAAGAAAGTGGGAAACAGAANNNN
chrom  GTAAAGAAATGGAGAAAG--CTGCATAGGCC-----AGTTGGTGTCAAAGAAAGTGGGAAACAGAATGCC
right  -TCCTGACCTCGTGATCCGCCCGCCTCGGCCTCCCAAAGTGCTGGGATTACAGGCGTGAG-CAGAATGCC
       1*111**11*1*1**11111*1**1*1****11111*11**1**11*1111*1*1*1**11*****2222

left   NNNNNNNNNNNNNNNNNNNNNNNNNNNNNNNNNNNNNNNNNNNNNNNNNNNNNNNN
chrom  TTTTGAGGAGGGCAATGAAATCGTAAGATTGATAATTTGTGACAGAGAAGG-----
right  TTTTGAGGAGGGCAATGAAATCGTAAGATTGATAATTTGTGACAGAGAAGG-----
       22222222222222222222222222222222222222222222222222222222
```

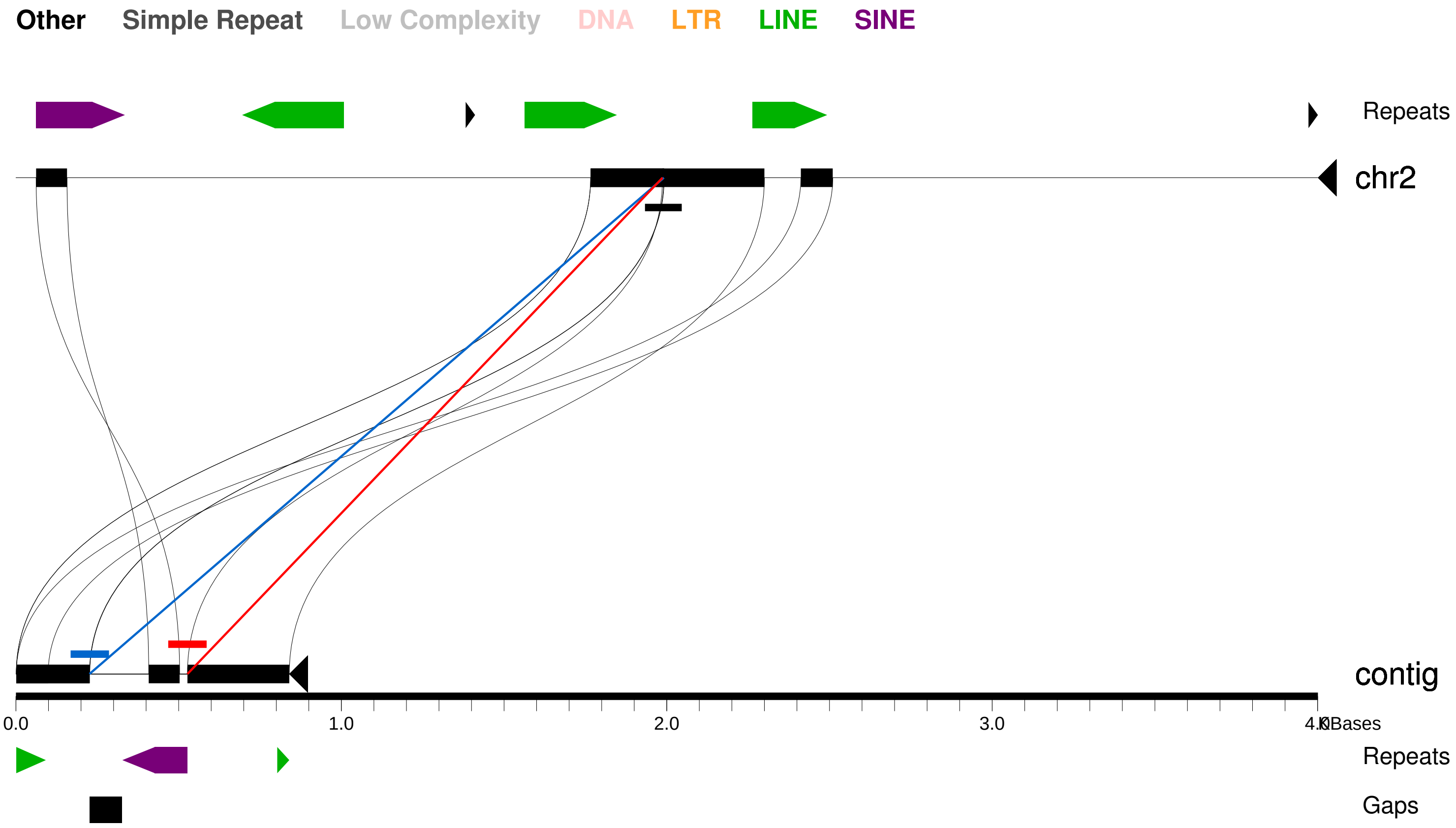

siteID: chr2_163678626 Scafftig1 + Breakpoint: chr2:163678618
chr2:163678579-163678677
Overlap Length: 14 INS size: 297 Genotyped: Yes

```
left    AATTTGATTGAGGC---CATAATCTCCACAATAAAG---------------CCC--CTAAAATTTTACTT
chrom   AATTTGATTGAGGC---CATAATCTCCACAATAAAG---------------CCC--CTAAAATTTTACAT
right   CGCCCGCCTCGGCCTCCCAAAGTGCTGGGATTACAGGCGTGAGCCACCGCGCCCGGCCCAAATTTTACAT
        11111*11*11*1*111**1*1*111111*1**1**111111111111111***11*11*********2*

left    TTTTTTTTTTTTTTTTTTGAGACGGAGTCTCGCTCTGTCGCCCAGGCTGGAGTGCAGTGGCGGGATCTC
chrom   TTTAAATTTTCAAAAACCGCTGCT-AATTTCT-TCTTTGACCTATGATGTA------------------
right   TTTAAATTTTCAAAAACCGCTGCT-AATTTCT-TCTTTGACCTATGATGTA------------------
        ***222****22222222*222*22*2*2**22***2*22**2*2*2**2*222222222222222222
```

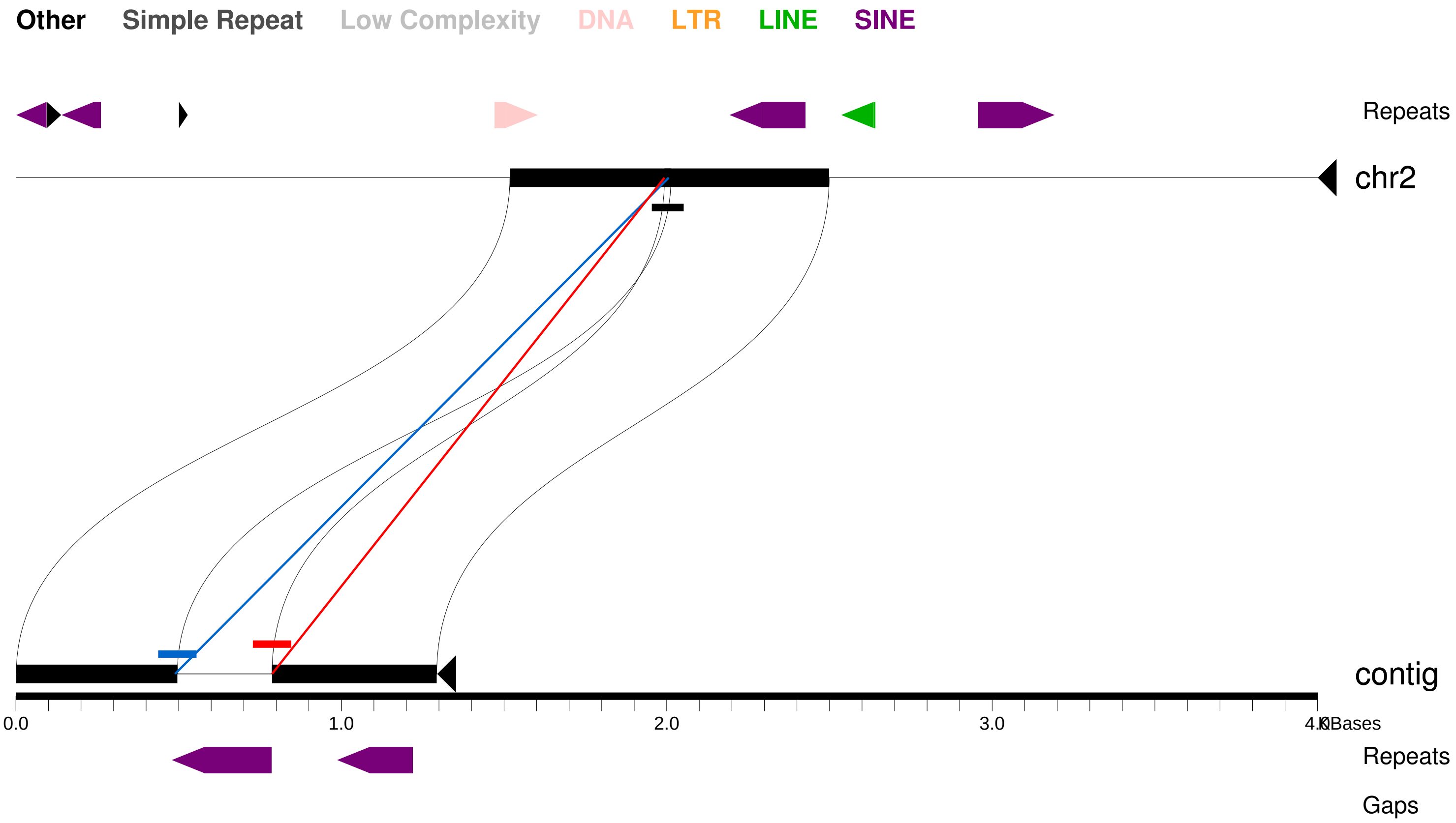

siteID: chr2_165492048 Scafftig3 + Breakpoint: chr2:165492057
chr2:165491898-165492235
Overlap Length: 19 INS size: 339 Genotyped: No

```
left   NNNNNNNNNNNNNNNNNNNNNNNNAT------------------GCCAACCTGGTACTATTTAAGTCTCAT
chrom  GATAGGAGGAAGTTAGAGATAAAATTTCAACCTCCCTTCTGATGCCAACCTGGTACTATTTAAGTCTCAT
right  NNNNNNNNNNN-------AAAAAA----------------------------------------------
       NNNNNNNNNNNNNNNNNN2N222*1                  111111111111111111111111111

left   GCCATCTAAGGCCTGCATTTCACATGTCCCCTTCCAACCCCCAAATATCTCTCTCATAAGGGGGCTTACT
chrom  GCCATCTAAGGCCTGCATTTCACATGTCCCCTTCCAACCCCCAAATATCTCTCTCATAAGGGGGCTTACT
right  ----------------------------------------------------------------------
       1111111111111111111111111111111111111111111111111111111111111111111111

left   CAATGTACAAAGGATGCCTAGATAATTTGTATGCTCTTGCCGGGCGCGGTGGCTCACGCCTGTAATCC--
chrom  CAATGTACAAAGGATGCCTAGATAATTTGTATGCTCTTTAAGGATTAAATGGATCATTCATTCATTTCTT
right  -------------------AGATAATTTGTATGCTCTTTAAGGATTAAATGGATCATTCATTCATTTCTT
       1111111111111111111*******************222**222222***2***22*2*22*2*2*22

left   ---CAGCACTTTGGGAGGCCGAGGCG---GGCGGAT--CACGAGGTCAGGAGATCGAGAC-CATCCT---
chrom  ATGCATCAATTCAG-AGTCTGTTGTCTATGGGGTATGGCATTCTATTAGAAAATCTTGTCTCAGCCTTTA
right  ATGCATCAATTCAG-AGTCTGTTGTCTATGGGGTATGGCATTCTATTAGAAAATCTTGTCTCAGCCTTTA
       222**2**2**22*2**2*2*22*22222**2*2**22**22222*2**2*2***22*2*2**2***222

left   ----------GGC------TAACACGGTGAAACCCCGTCTCTACTAAAA----------
chrom  AGAAACTTAAGTCATATATTAACAAACACATAACAATCCGCAACTTAAAAATCAGTGTG
right  AGAAACTTA--------------------------------------------------
       222222222 1N1      11111NNNNN1N1N1NNNN1N1N111N111
```

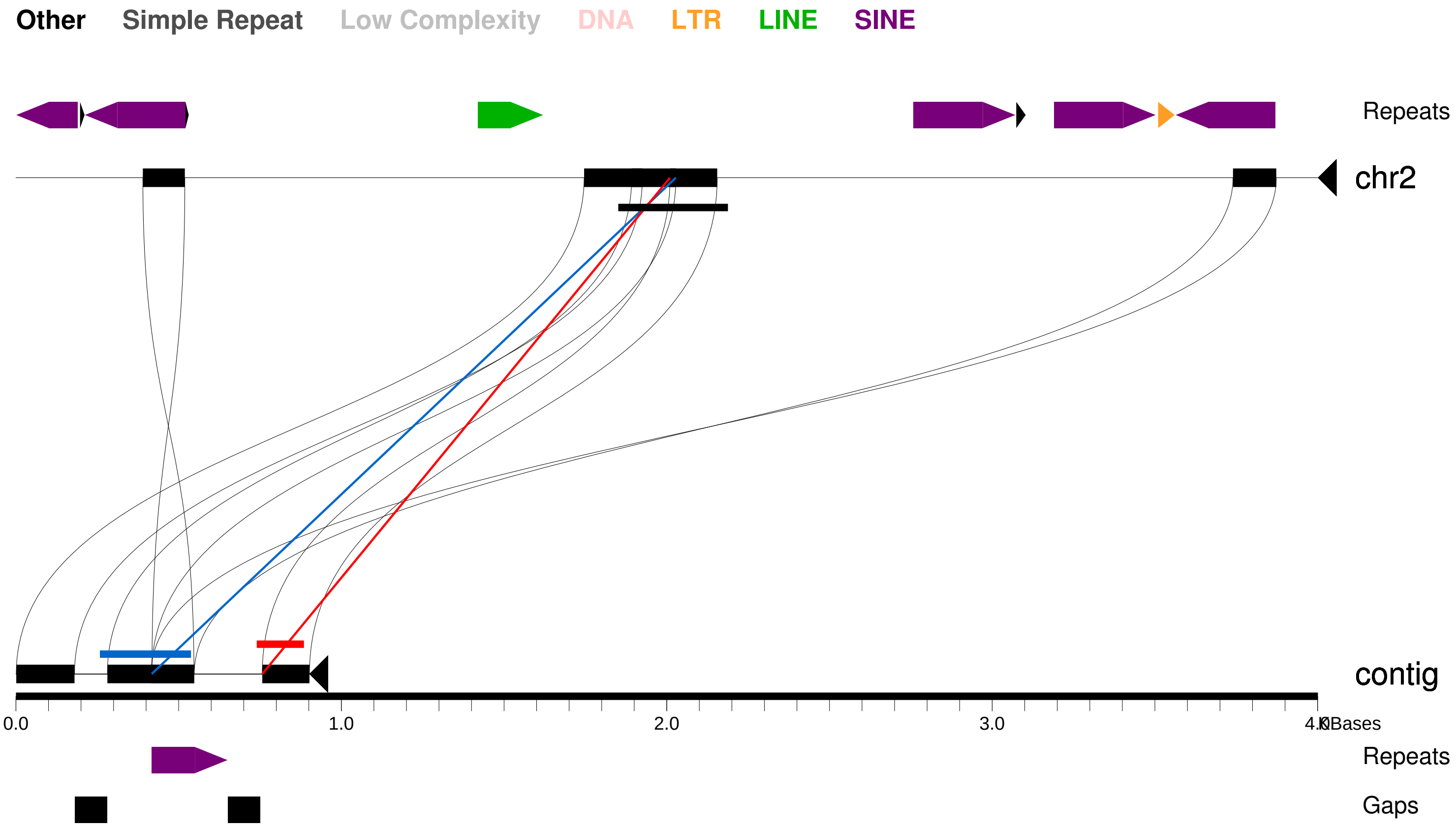

siteID: chr2_166401044 Scafftig1 - Breakpoint: chr2:166401033
chr2:166400977-166401092
Overlap Length: 4 INS size: 422 Genotyped: No

```
left    ATGATCTGACTTAGATATTTAAAAGGATCTACCTGGCAACTGTGTTAAAAGTTGGC---TGTAGGCCGGG
chrom   ATGATCTGACTTAGATATTTAAAAGGATCTACCTGGCAACTGTGTTAAAAGTTGGC---TGTAAAAGAGA
right   NNNNNNNNNNNNNNNNNNNNNNNNNNNNNNNNNNNNNNNNNNNNNNNNNNNNNNNNNNNNTGTAAAAGAGA
        11111111111111111111111111111111111111111111111111111111111****22222*2

left    CGCGGTGGCTCACGCCTGTAATCCCAGCACTTTGGGAGGCCGAG-GCGGGCGG-
chrom   AAAAATGTAGGAGATCT----TTTCAGGAGGCTTGGAC-CAGAGTGAGAGAGTA
right   AAAAATGTAGGAGATCT----TTTCAGGAGGCTTGGAC-CAGAGTGAGAGAGTA
        22222**2222*222**2222*22***2*222*2***22*2***2*2*2*2*22
```

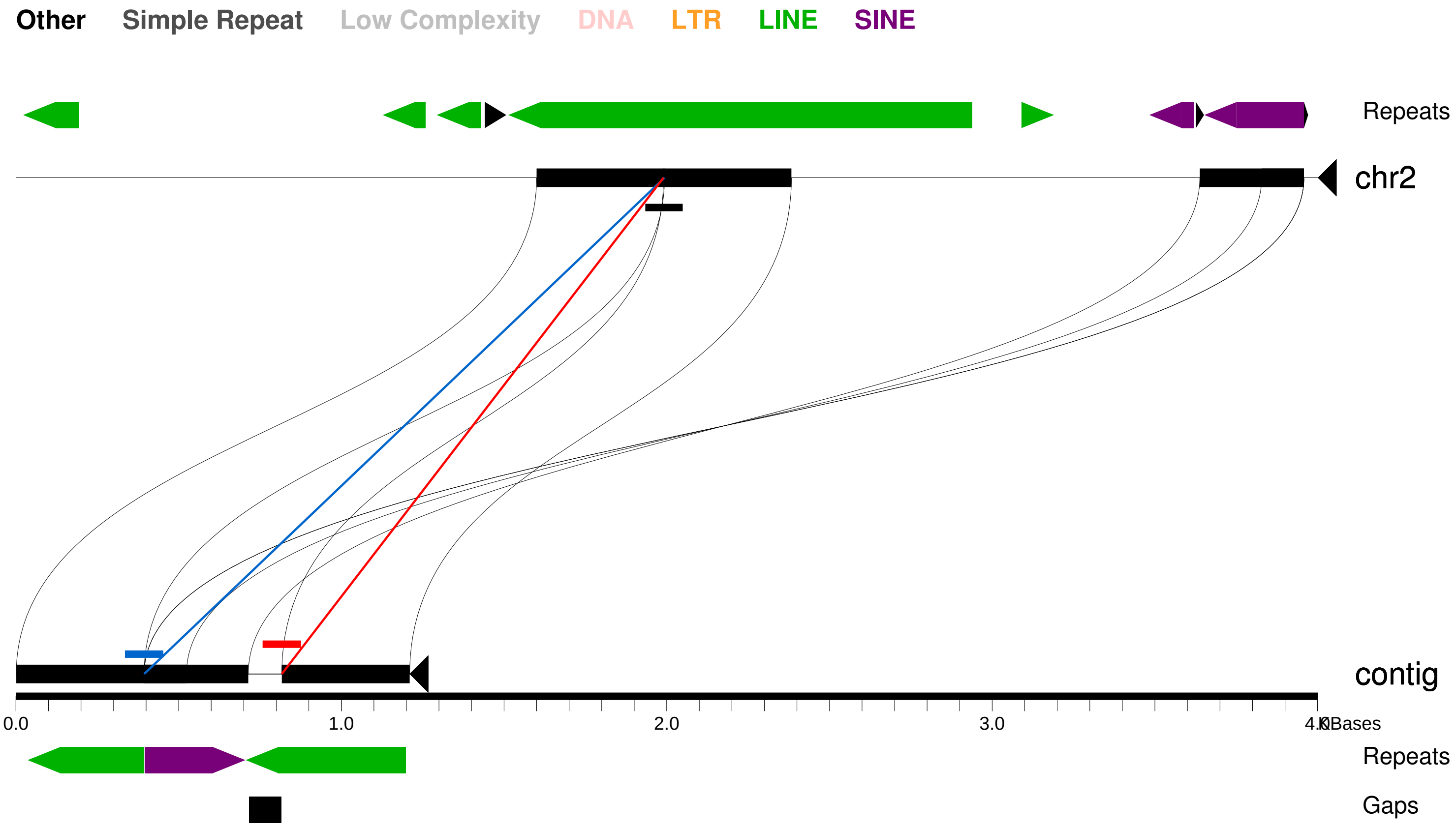

siteID: chr2_169472059 Scafftig2 + Breakpoint: chr2:169472022
chr2:169471862-169472226
Overlap Length: 45 INS size: 413 Genotyped: No

```
left   ------------------------------------------------GTCAGTAATTAAGCTAATCAAG
chrom  TCCAAACCAAGAAGAAATCCCTGATTGACCTGAAAAAGAATCCAGAAAGTCAGTAATTAAGCTAATCAAG
right  NNNNNNNNNNN-----------------------------------------------------------
       NNNNNNNNNNN                                     1111111111111111111111

left   GAGGCACCAGAGAAAGGTGAAGTCCAATTTAACGAAATAAAAAAAAAAAATGATAGAAGAAATGAGGGAA
chrom  GAGGCACCAGAGAAAGGTGAAGTCCAATTTAACGAAATAAAAAAAAAA--TGATAGAAGAAATGAGGGAA
right  ----------------------------------------------------------------------
       1111111111111111111111111111111111111111111111112211111111111111111111

left   GAAATCTTTAGTGAAATAGACAGCATAAATAAAATACAATAAAAACTTTAGGAAATACTGGATGCAC---
chrom  GAAATCTTTAGTGAAATAGACAGCATAAATAAAATACAATAAAAACTTTAGGAAATACTGGATGCACGAA
right  ----------------------GCATAAATAAAATACAATAAAAACTTTAGGAAATACTGGATGCACGAA
       1111111111111111111111*********************************************222

left   ----AGGCCGGGCGCGGTGG-----CTCACGCCTGTAATC---CCAGCAC---------TTTGGGAGGCC
chrom  AGAAATGCAAAATGCTCTGGAAAGTCTCAGCAATAGAGTCGAACAAGCAGAAAAAAGAGTGTCAGAGCTT
right  AGAAATGCAAAATGCTCTGGAAAGTCTCAGCAATAGAGTCGAACAAGCAGAAAAAAGAGTGTCAGAGCTT
       2222*2**22222**22***22222****2222*22*2**222*2****2222222222*2*22***222

left   GAGG-CGGGC----GGATCACGAG-GTC-----AGGAGATCGAGACCATCCTGGCTAACACGGTGAAACC
chrom  GAAGACAGGTTTTTGAATTAACACAGTCCAACAAAGACAAAGAAAAAAGACTAAGAAAAAATGAGCAAAG
right  GAAGACAGGTTTTTGA------------------------------------------------------
       **2*2*2**22222*211N1NN1N 111     1N11N1NN11N1NN1NN11NNNN11N1NN1N1N11NN

left   CCGTCTCTA---CTAAA
chrom  CCTCCCAGAAATCTGGG
right  -----------------
       11NN1NNN1   11NNN
```

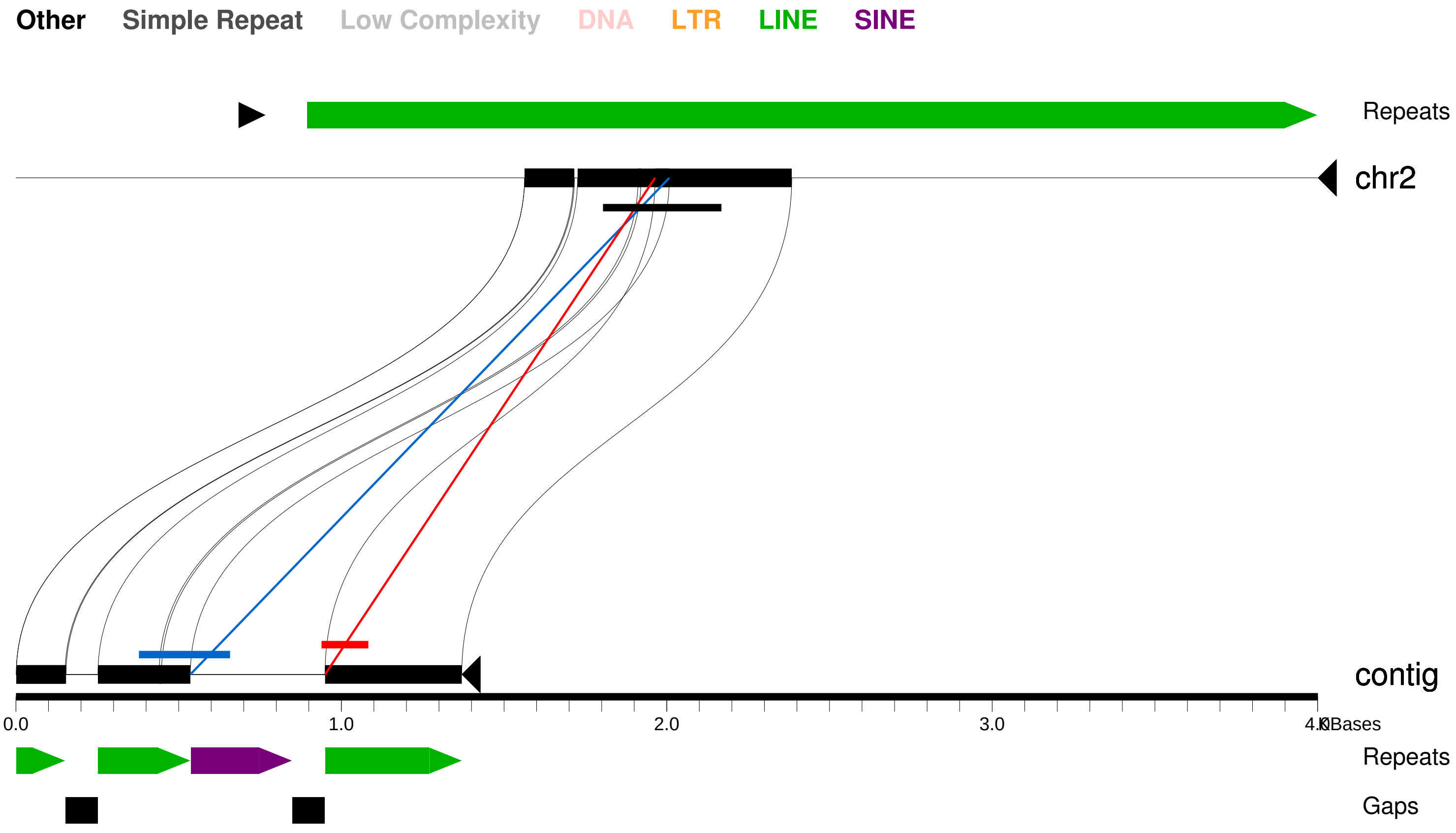

siteID: chr2_173316733 Scafftig1 - Breakpoint: chr2:173316723
chr2:173316671-173316782
Overlap Length: 8 INS size: 407 Genotyped: No

```
left    TGAAATCACATGGTACT--TAAAGATATTTATATTATCTTCTCTAT------CGTCTTTTCCTCCCTTNN
chrom   TGAAATCACATGGTACT--TAAAGATATTTATATTATCTTCTCTAT------CGTCTTTTCCTCCCTTCA
right   TCCGCCCGCCTCGGCCTCCCAAAG-TGCTGGGATTACAGGCGTGAGCCACCGCGCCCGGCCCTCCCTTCA
        *11111*1*1*1*11**111****1*11*111****1111*111*1111111**1*1111********22

left    NNNNNNNNNNNNNNNNNNNNNNNNNNNNNNNNNNNNNNNNNNNNNNNNNNNNNNNNN
chrom   TTTTTACTTTGGTAATTTTACCTATTAAGATTGTGAAGGAACTTGACATT-------
right   TTTTTACTTTGGTAATTTTACCTATTAAGATTGTGAAGGAACTTGACATT-------
        222222222222222222222222222222222222222222222222222222222
```

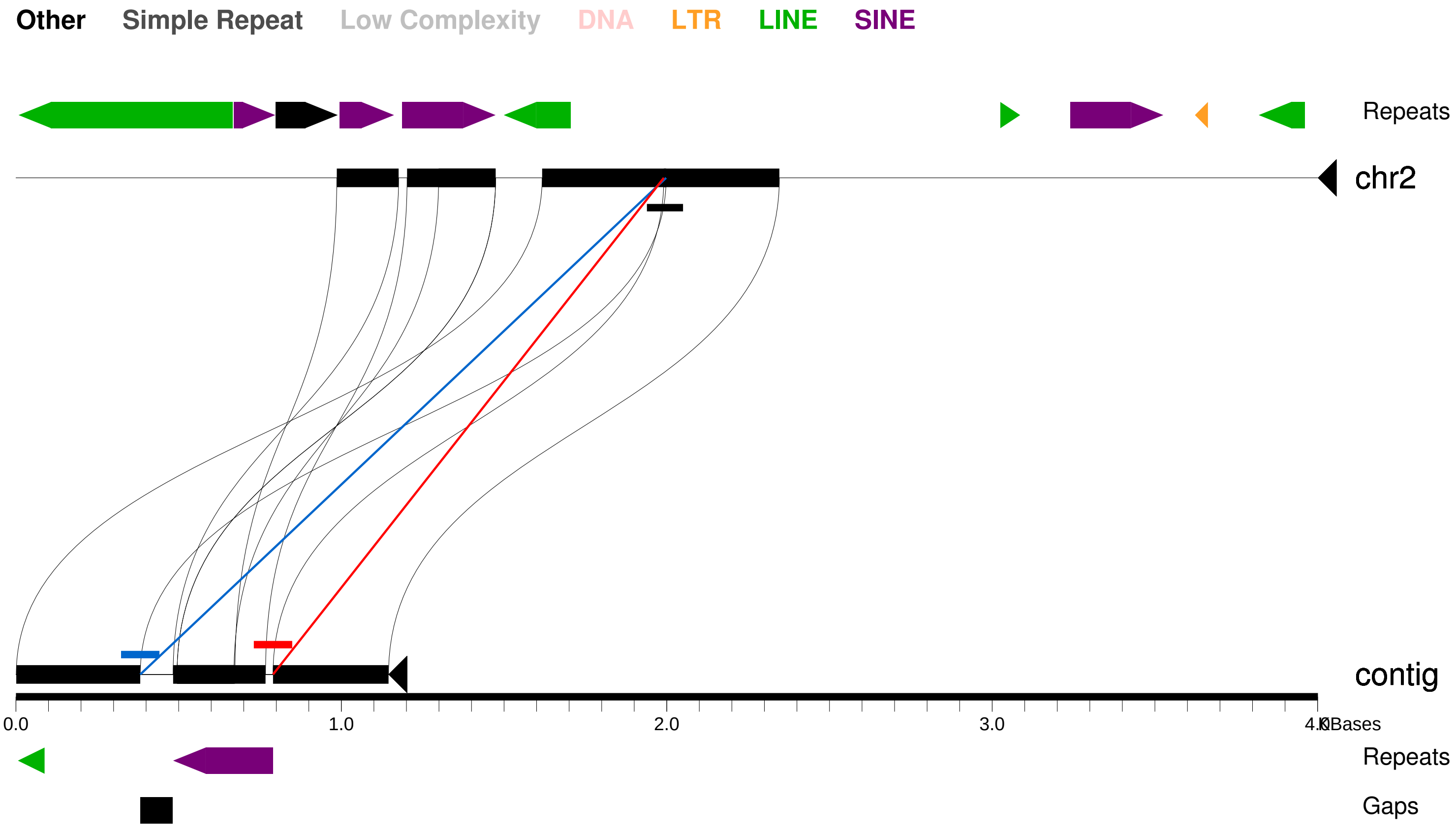

siteID: chr2_173663245 Scafftig2 + Breakpoint: chr2:173663364
chr2:173663317-173663423
Overlap Length: 13 INS size: 70 Genotyped: Yes

```
left   AAGG----------GACAAATAACCTTCTGCCAAGTGTCATATTTAAG-GCAATGTTT-TGGAGGCTTTT
chrom  AAGG----------GACAAATAACCTTCTGCCAAGTGTCATATTTAAG-GCAATGTTT-TGGAGGCTTTT
right  TTTTTTTTTTTTTTGAGACGGAGTCTTGCTCTGTCGCCCAGGCTGGAGTGCAGTGGCGCTGGAGGCTTTT
       11111111111111**1*111*11***111*1111111**111*11**1***1**1111***********

left   TCTTTTTTTTTTTTTTTTTTTTTTTTTGAGACGGAGTCTTGCTCTGTCGCCCAGGCTGGAG
chrom  TCATAGGTAGCACACTTCAAATGT---GAGAAACATTTATGA---------CACACCAAAT
right  TCATAGGTAGCACACTTCAAATGT---GAGAAACATTTATGA---------CACACCAAAT
       **2*222*2222222**2222*2*222****222*2*22**2222222222**22*222*2
```

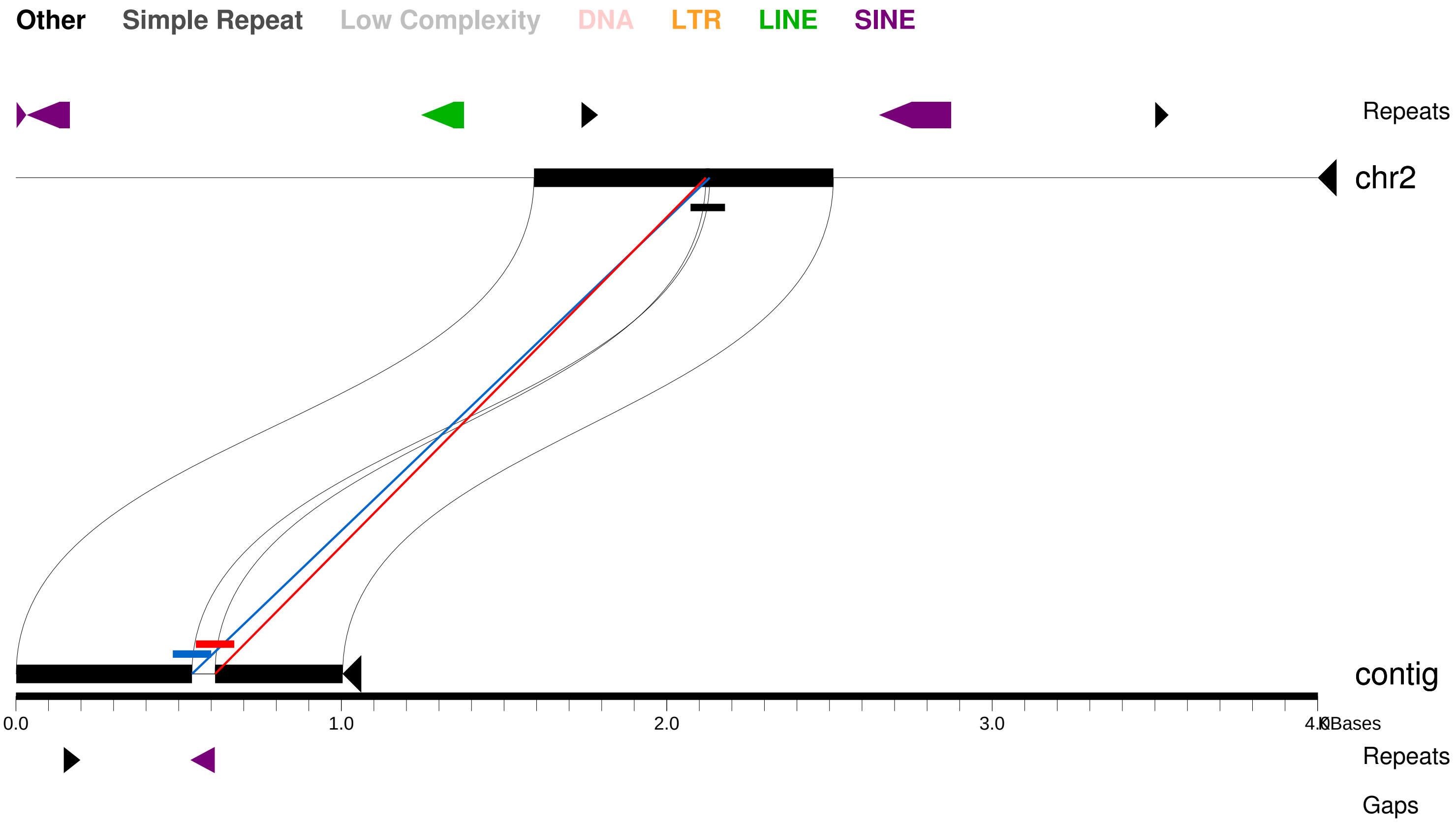

siteID: chr2_176463782 Scafftig1 + Breakpoint: chr2:176463793
chr2:176463765-176463852
Overlap Length: 32 INS size: 406 Genotyped: No

```
left    AG------------ATGCCAA-------GAATTGCAT-----ATGCATCATT-------ACAAACCGTTT
chrom   AG------------ATGCCAA-------GAATTGCAT-----ATGCATCATT-------ACAAACCGTTT
right   CTGCCCGCCTCGGCCTCCCAAAGTGCTGGGATTACAGGCGTGAGCCACCGCGCCCGGCCACAAACCGTTT
        111111111111111*1****1111111*1***1**111111*11**1*1111111111***********

left    TTAATTGTTACTTTGTTGAAANNNNNNNNNNNNNNNNNNNNNNNNNNNNNNNNNNNNNNNNNNNNNNNNNN
chrom   TTAATTGTTACTTTGTTGAAATCGAGCCCTCCTCATCTACTATCATGAA---------------------
right   TTAATTGTTACTTTGTTGAAATCGAGCCCTCCTCATCTACTATCATGAA---------------------
        *********************2222222222222222222222222222222222222222222222222

left    NNNNNNNNNN
chrom   ----------
right   ----------
        2222222222
```

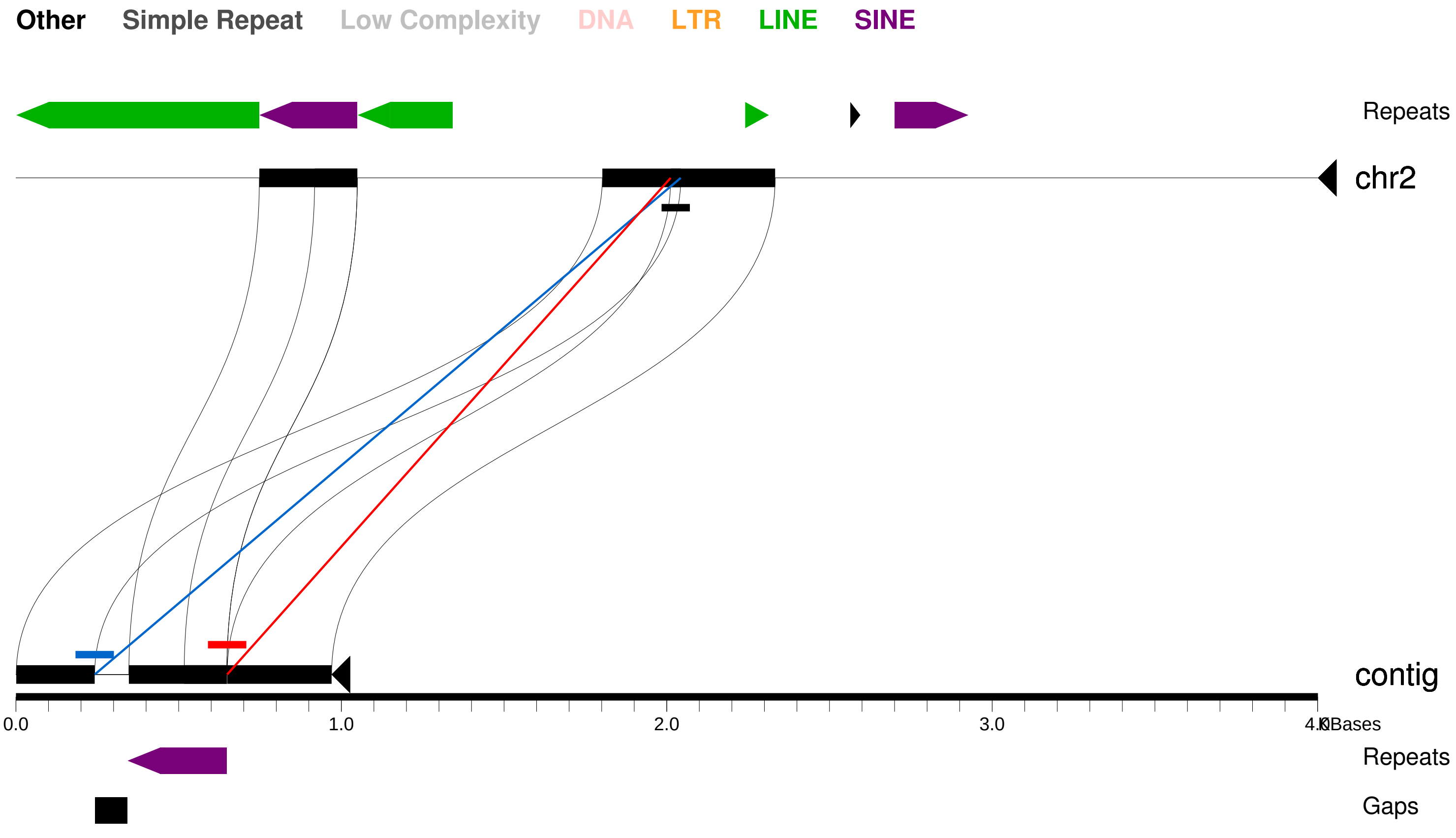

siteID: chr2_178351181 Scafftig2 - Breakpoint: chr2:178351455
chr2:178351125-178351615
Overlap Length: 12 INS size: 462 Genotyped: No

```
left    ----------------------------------------------------------------------
chrom   TGCTGCCTTCCTTTGTAATAGTGTTTTCAGGCAGAAAAATTGAAAAAATCAGGTTTTGGAACTAGTCATT
right   T----------TTAGTA-----------------------------------------------------
        2          22N222

left    ----------------------------------------------------------------------
chrom   GTTCAATTTGGGATTTGGAATTTCAG-TTGATAGCAGGGGAATTAAGATCCATTAAAGTAGATGGTACAT
right   -----------GAGACGGGGTTTCACCTTGTTAGCCAGG----------------------ATGGT----
                   22NNN22NN22222N1222N2222NN22                      22222

left    ----------------------------------------------------------------------
chrom   GGCTTATGATTGTGCCAAATTCTTGATTGAATAATGAATTGGTTGCTTGTAATTCTTCACTGTCATTTTA
right   --CT-----------CGATCTCCTGACC---TCATGA------------------TCCACCCGCCTC---
          22           2N2NN22N222NN   2N2222                  2N222NNN2N2N

left    -----------------------------------GTTGCTTTTGAAATGGAAACTGTAATTTTGGATA
chrom   TAGCACTTCCATCTTTGCAATATTTTCAGATTCAAGTTGCTTTTGAAATGGAAACTGTAATTTTGGATA
right   --GGCCTCCCA----------------------AAGT-GCT--------GGGA--------TT-------
          2NN22N222                      22**1***11111111****11111111**1111111

left    AATATCTTTTGGGAAAGAACATACATGACTGTTCACAAATAGCAAAGTGGGCTATATGTATATATATAT-
chrom   AATATCTTTTGGGAAAGAACATACATGACTGTTCACAAATAGCAAAGTGGGCTATATGTATATTTTTATT
right   ------------------ACAGGCGTGAGC---CACC----GCGCCCGGCCCTATATGTATATTTTTATT
        111111111111111111***11*1***11111***11111**11111*11************2*2***2

left    ---ATTTTT-------------------------------------------------------------
chrom   ACTATTCTTACTGAAGGAAATAAATTAAGCAACCTTATACTAGCATTAGAAGGATTCTACTAAAGTCTCT
right   ACTATTCTTACTGAAGGAAATAAATTAAGCAACCTTATACTAGCATTAGAAGGATTCTACTAAAGTCTCT
        222***2**2222222222222222222222222222222222222222222222222222222222222

left    -------TNTTT----------------------------------------TTNTNNNNNNNNNNN---
chrom   CATCCGATCTTTGTCCATGTGAGCAAGTATCCTCTTTCAATACTTCACTGCCTTGTGAGAAGCTAGAAAG
right   CATCCGATCTTTGTCCATGTGAGGAAGTATCCTCTTTCAATACTTCACTGCCTTGTGAGAAACTAGAAAG
        2222222*2***22222222222N2222222222222222222222222222**2*22222N22222222

left    --
chrom   GA
right   G-
        2
```

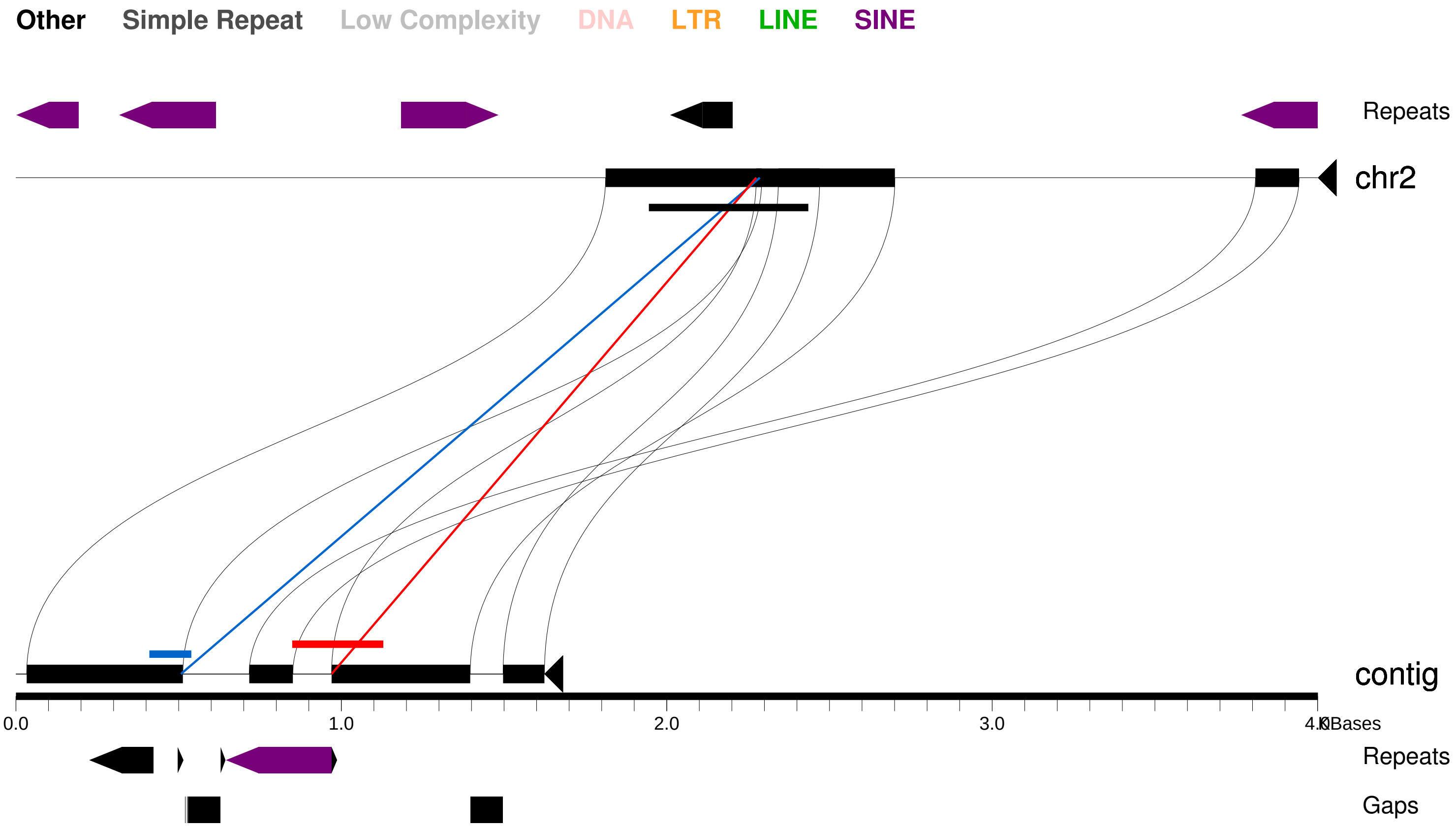

siteID: chr2_178648963 Scafftig4 - Breakpoint: chr2:178648942
chr2:178648897-178649001
Overlap Length: 15 INS size: 297 Genotyped: Yes

```
left    AGCA--GGACT------TGTTTCACA-----AGATACAGAC-CACAAAGACTGCTGATAAAACAGGATGT
chrom   AGCA--GGACT------TGTTTCACA-----AGATACAGAC-CACAAAGACTGCTGATAAAACAGGATGT
right   GCCACTGCACTCCAGCCTGGGCGACAGAGCGAGACTCCGTCTCAAAAAAAAAAAAAACGAAACAGGATGT
        11**11*1***111111**1111***11111***11*1*1*1**1***1*111111*11***********

left    GGTAGGCCGGGCGCGGTGGCTCACGCCTGTAATCCCAGCACTTTGGGAGGCCGAGGCGGGCGG
chrom   GGTAAAGAAGTC-----TGCCAAAACCTG-----CCAAAACCAA--GATGGCTATGAAAAC--
right   GGTAAAGAAGTC-----TGCCAAAACCTG-----CCAAAACCAA--GATGGCTATGAAAAC--
        ****22222*2*222222**22*22****22222***22**22222**2*2*2*2*2222*22
```

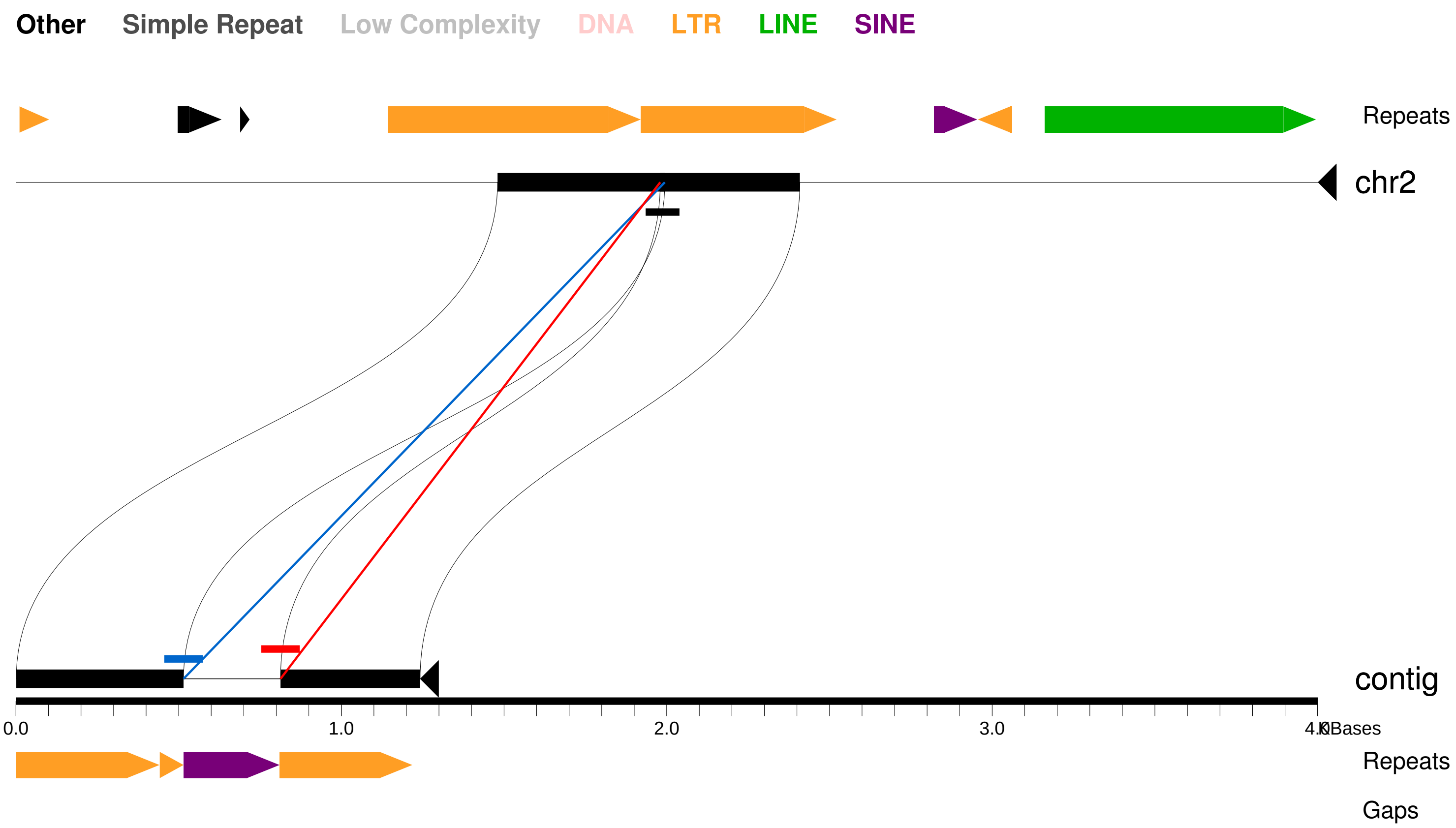

siteID: chr2_181372095 Scafftig1 - Breakpoint: chr2:181372059
chr2:181372013-181372118
Overlap Length: 13 INS size: 161 Genotyped: Yes

```
left   ---AC----TGTGTACTTAGGA---TACACTACATGTATT---TACAAATTCTTTCTTCAATAATAAACT
chrom  ---AC----TGTGTACTTAGGA---TACACTACATGTATT---TACAAATTCTTTCTTCAATAATAAACT
right  GAAACCCCGTCTCTACTAAAAAAAATACAAAAAAAAAAAAAAAATAATAATAATAATAATAATAATAAACT
       111**1111*1*1****1*11*111****11*1*111*11111**11***11*111111***********

left   TGGGGCCGGGCGCGGTGGCTCACGCTTGTAATCCCAGCACTTTGGGAGGCCGAGGCGGGCGG
chrom  TG-------GCTTACTG--TAACTTTTTTACTTAATAAACTTTTAAATTTTTTAAACTT----
right  TG-------GCTTACTG--TAACTTTTTTACTTAATAAACTTTTAAATTTTTTAAACTT----
       **2222222**2222**22*2**22**2**2*222222*****222*22222*22*222222
```

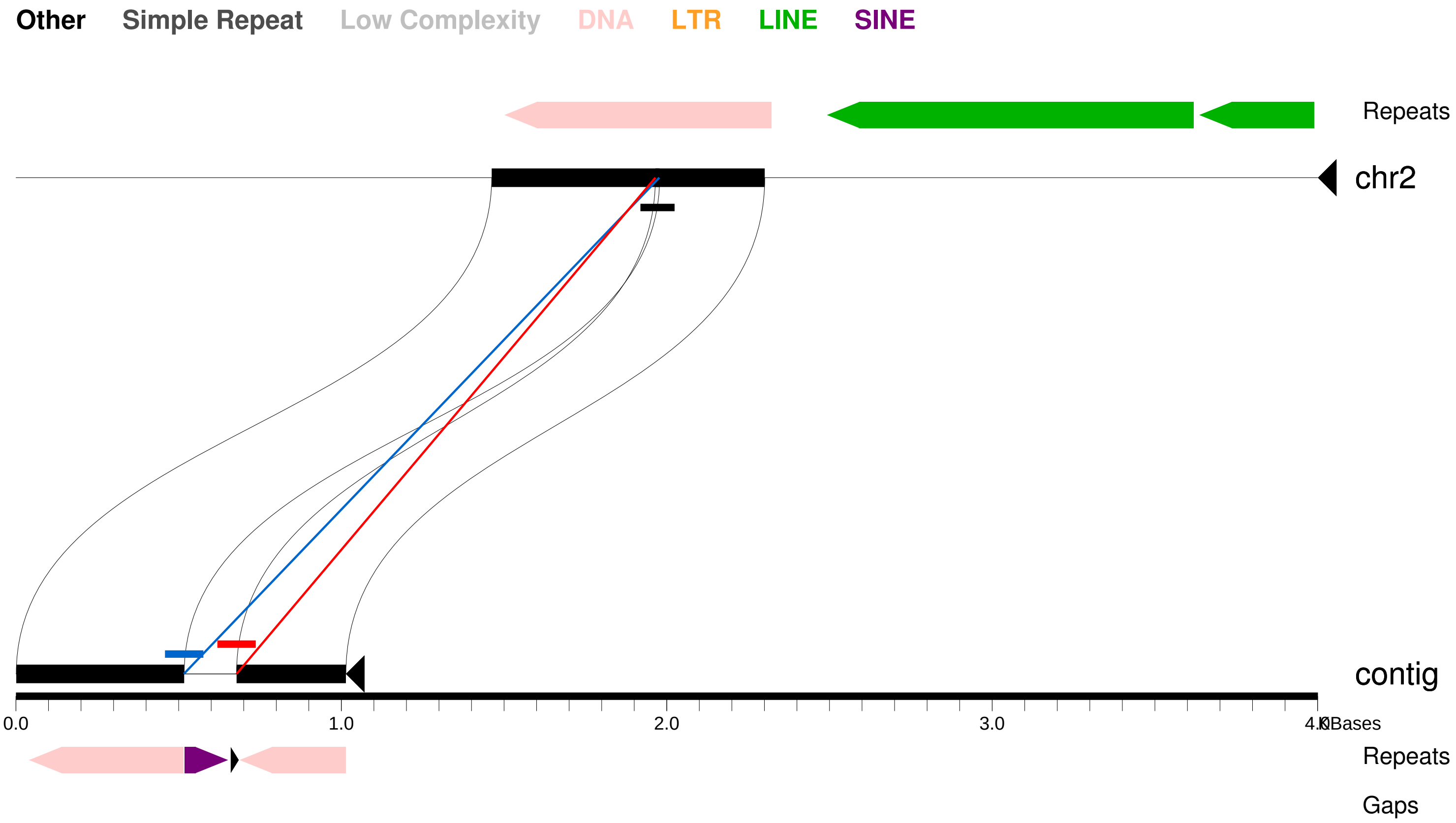

siteID: chr2_181880734 Scafftig3 - Breakpoint: chr2:181880731
chr2:181880586-181881007
Overlap Length: 16 INS size: 294 Genotyped: Yes

```
left    -AGCAATTTATAGCTAACTATGCTGCAAGTGCCCAAACTAGAAACTGGGTACTCTTCTCACCAGCAAATG
chrom   CAGCAATTTATAGCTAACTATGCTGCAAGTGCCCAAACTAGAAACTGGGTACTCTTCTCACCAGCAAATG
right   CAG-----------------TGAGCCGAGATCCC------GCCACTG---------CACTCCAGC-----
        2**11111111111111111**111*1**11***111111*11****111111111*1*1*****11111

left    TTGTTTCTGTTATATAGTACCATGGAGCATGTTAGAGCAATGAGAATGAGTC-CAGAGTAATTAGCCATG
chrom   TTGTTTCTGTTATATAGTACCATGGAGCATGTTAGAGCAATGAGAATGAGTC-CAGAGTAATTAGCCATG
right   ------CTGG----------------GC--GACAGAGC---GAGACTCCGTCTCAAAAAAA---------
        111111***11111111111111111**11*11*****111****1*11***1**1*11**111111111

left    AAGATTAAAATTTGTTCAATTC---------------------GG----------------------CCG
chrom   AAGATTAAAATTTGTTCAATTCAACTAGCTTTATAAAGTTATAGGAAATTTTAGAAAGATAAAATAACCA
right   AAAAAAAAAATTTGTTCAATTCAACTAGCTTTATAAAGTTATAGGAAATTTTAGAAAGATAAAATAACCA
        **1*11****************222222222222222222222**2222222222222222222222**2

left    GGCGC-------GG--------TGGCTCACGCCTGTAATC----CCAG----CACTTTGGGAGGCC----
chrom   CATGCTACTTCTGGCATTCATATGGCAGAC-CTTGAAATAAATACTAGTGAACAATGTAGTGGGCCCGGT
right   CATGCTACTTCTGG--------------------------------------------------------
        222**2222222**        1111NN1121N11N111N    1N11    11N1N1N1NN1111

left    ------------------------GAGGCGGGCG--GAT--CACGAGGTCAG---GAGATCGAGACCATC
chrom   TTCAAATAACTGAATTTTAACCCTGAATCTGCCACTGATTTCACTTTGTGAGCCTGGGCAAGACACTAAC
right   ----------------------------------------------------------------------
                                11NN1N1N1N  111  111NNN11N11   1N1NNN11N11N1N1

left    C-----CGGCTAAA-AC--------GGTGAAACCCCGTCTCT-----------ACTAAA-----------
chrom   CTTTACCTTCTTTATACCTTTTTGGGGTTTAAATTTTTTTTTTTAAATTTTTTAGTACAGACAGGATCTC
right   ----------------------------------------------------------------------
        1     1NN11NN1 11        111NN11NNNNN1N1N1           1N11N1

left    ----
chrom   ACTG
right   ----
```

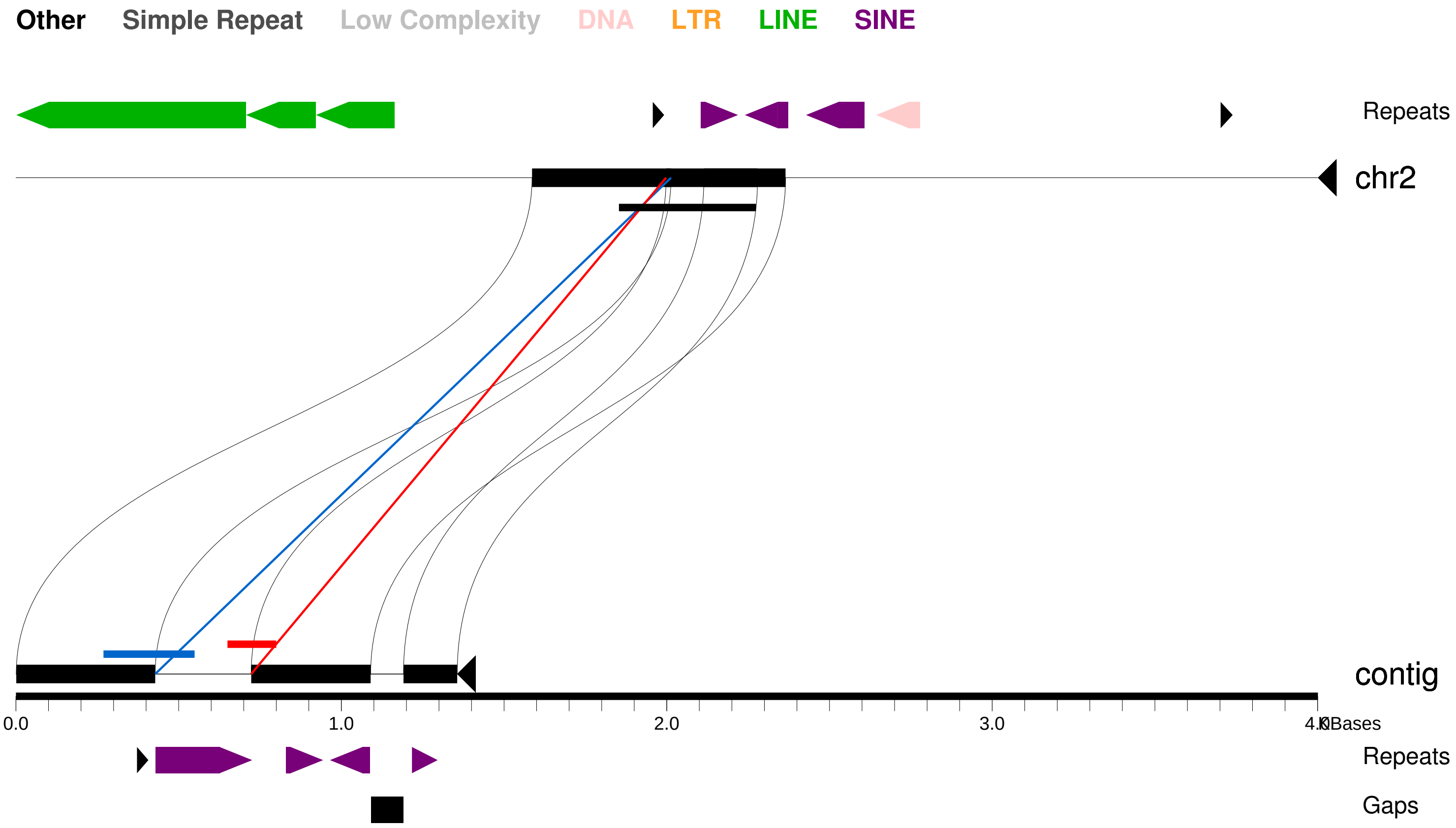

siteID: chr2_182262190 Scafftig1 - Breakpoint: chr2:182262185
chr2:182262025-182262360
Overlap Length: 16 INS size: 294 Genotyped: Yes

```
left    ---------------ACAAGTCTACTCTCTGAGGAAGCTAGAAGAGCATAATTCTCATCAAGAGGTAGCA
chrom   CCTGGAAGATCTTTTACAAGTCTACTCTCTGAGGAAGCTAGAAGAGCATAATTCTCATCAAGAGGTAGCA
right   A---GAATGGCGTGAACCCG------------GGAGGCG----GAGCTT---------------GCAGTG
        N   222NNN2N2NN**11*111111111111***1**11111****1*111111111111111*1**11

left    AATCCCTATCATGCCTCCTTTTGCCTGCTGGAACTTTAGAGGGAGAAAGAGGAACAGGCATGACTGAAGA
chrom   AATCCCTATCATGCCTCCTTTTGCCTGCTGGAACTTTAGAGGGAGAAAGAGGAACAGGCATGACTGAAGA
right   AGCCGAGATCGCGCCAC----TGCA--CTCCAGCCT----GGGCGACAGAGCGAGACTCCGTCTCAAAAA
        *11*111***11***1*1111***111**11*1*1*1111***1**1****11*1*11*1111111**1*

left    CAATAAGTAAAAGAGCCAACAAGAAGGAATACAGAG-------TGGCTC-ACG--CCTGTAATCCCAGCA
chrom   CAATAAGTAAAAGAGCCAACAAGAAGGAATACAGAGCCAAACGTGGCCAGATGAACATGTGCCCACGGGA
right   AAAAAAAAAAAAAAAAAAAAAAGAAGGAATACAGAGCCAAACGTGGCCAGATGAACATGTGCCCACGGGA
        1**1**11****1*111**1***************2222222****222*2*22*2***222*2*2*2*

left    CTTTGGGAGGCCGAGGCGGGCGGATCACGAGGTCAGGAGATCGAGACCATCCTGGCTAA---CACGG---
chrom   ATATGTGGGCATACTGAGTGGCCAGCGCCACCACAGAGGCTCTAAGCCTATCAAGCTAAAGTCACGTACC
right   ATATGTGGGCATACTGAGTGGCCAGCGCCACCACAGAGGCTCTAAGCCTATCAAGCTAAAGTCACGTACC
        2*2**2*2*222222*2*2*222*2*2*2*222***22*2**2*22**222*22****222****2222

left    TGAAACCC-CGTCTCTAC----------TAAA-AAT-ACAAAAA-----------A
chrom   TCAAGCCCTCTTCTTTACAGATGGCATCTAAAGAATGACAAGACTCACCCAATTCA
right   TCAAGCCCTCTTCTTTACAGATGGCATCTAAAGAATGACAAGACT-----------
        *2**2***2*2***2***2222222222****2***2****2*22          1
```

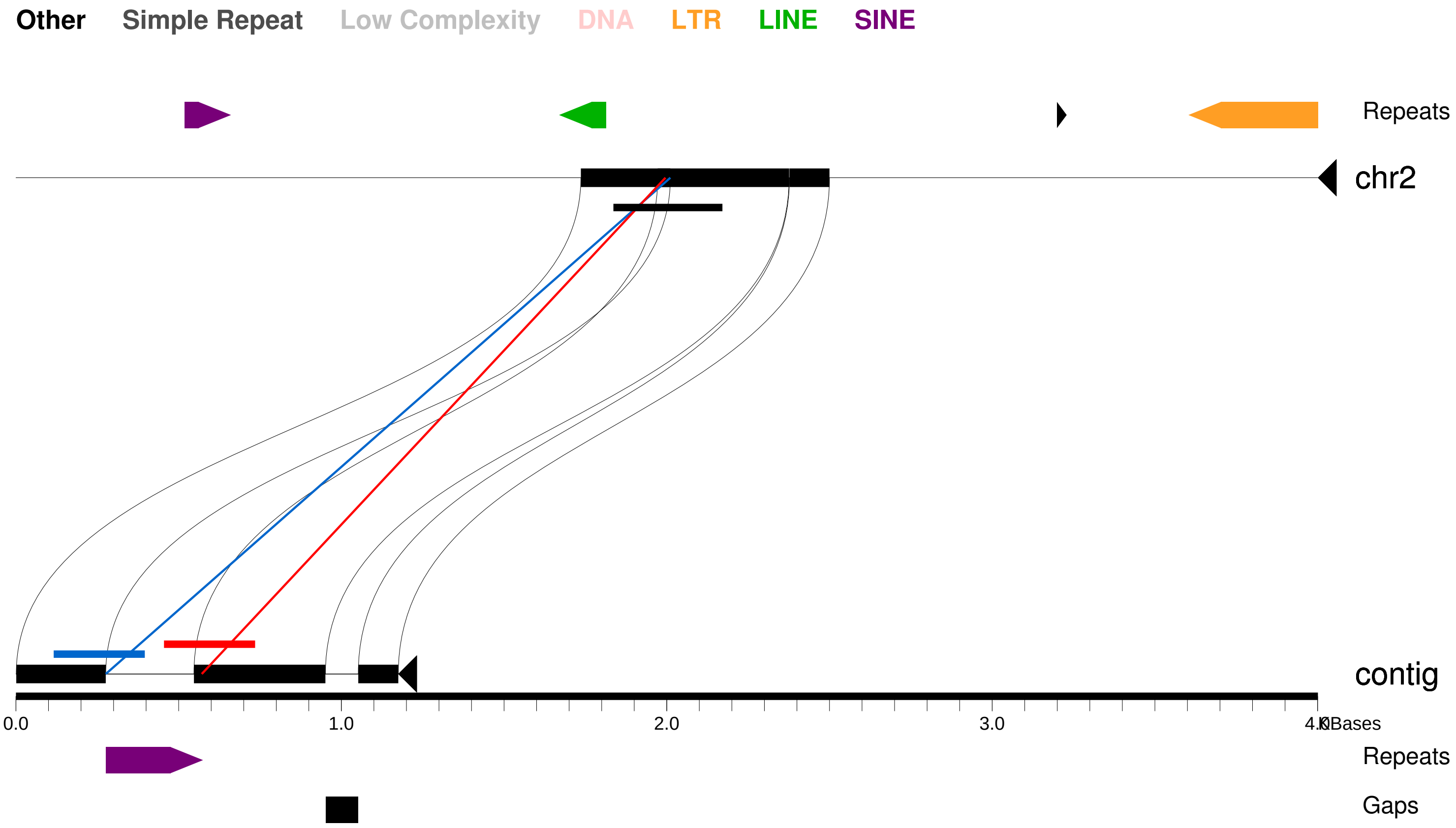

siteID: chr2_18306413 Scafftig2 + Breakpoint: chr2:18306403
chr2:18306358-18306462
Overlap Length: 15 INS size: 484 Genotyped: No

```
left    CTTCCTCCCCTCACTCACTTTTCCTCATCTTGAAAAATTAGTCTC--------------AAGAAAGGAAA
chrom   CTTCCTCCCCTCACTCACTTTTCCTCATCTTGAAAAATTAGTCTC--------------AAGAAAGGAAA
right   NNNNNNNNNNNNNNNNNNNNNNNNNNNNNNNNNNNNNNNNNNNNNNNNNNNNNNNNNNNAAGAAAGGAAA
        11111111111111111111111111111111111111111111111111111111111***********

left    AAATGGCCGGGCGCG-GTGGCTCACGCCTGTAATCCCAGCACTTTGGGAGGCCGAGATGGGCGG
chrom   AAATATATATAAACAAGTTTTTCAC------AAACTGATCAAATATG---------ACTTTTAG
right   AAATATATATAAACAAGTTTTTCAC------AAACTGATCAAATATG---------ACTTTTAG
        ****222222222*22**222****222222**2*22*2**22*22*222222222*222222*
```

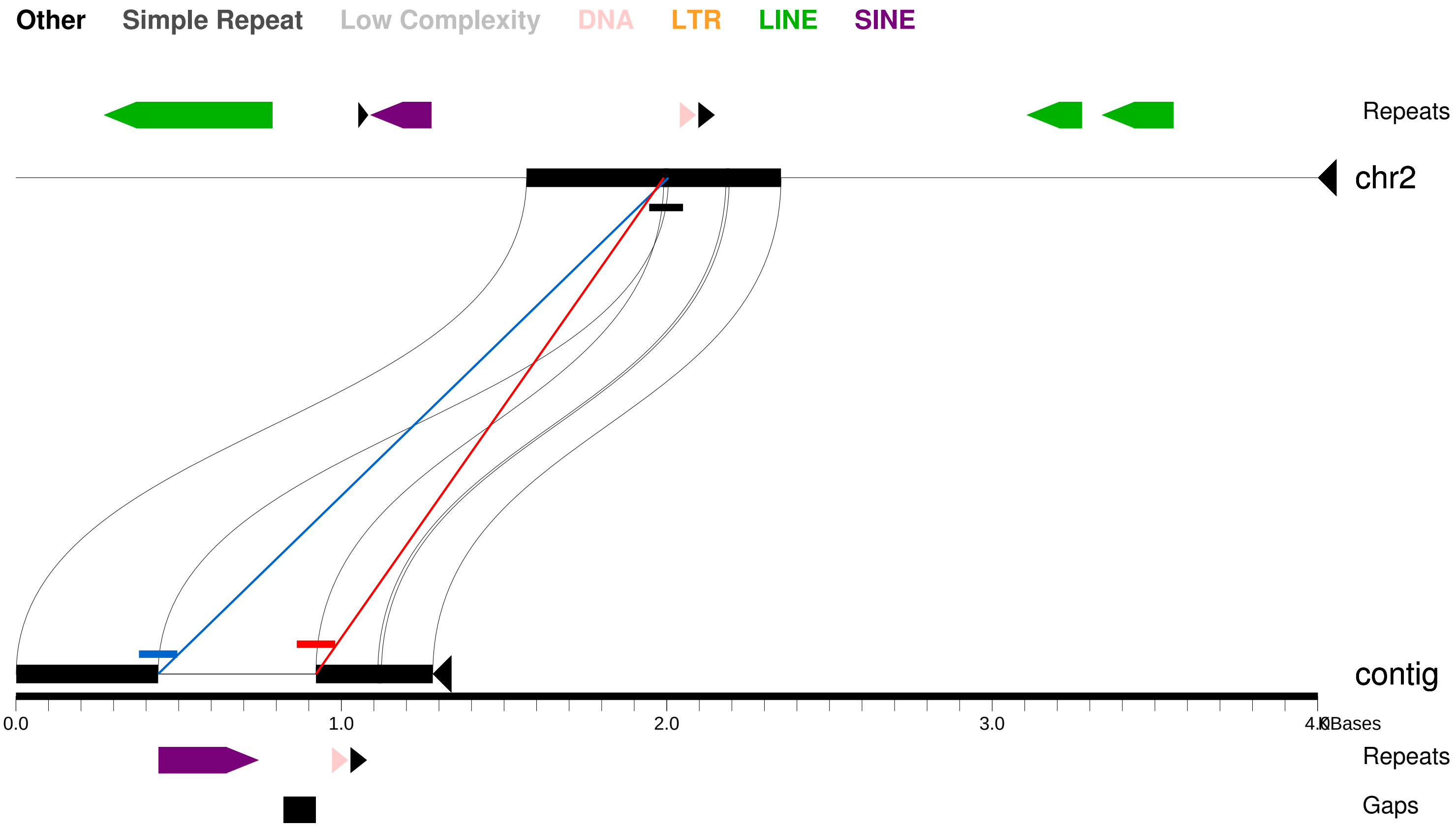

siteID: chr2_191356857 Scafftig1 + Breakpoint: chr2:191356848
chr2:191356805-191356907
Overlap Length: 17 INS size: 427 Genotyped: No

```
left     GGTTTTTGAATTAATCCAACGAAGACAAAGAAAAAAGAATTTT----------------AAAAAATGAAC
chrom    GGTTTTTGAATTAATCCAACGAAGACAAAGAAAAAAGAATTTT----------------AAAAAATGAAC
right    NNNNNNNNNNNNNNNNNNNNNNNNNNNNNNNNNNNNNNNNNNNNNNNNNNNNNNNNNAAAAAAAATGAAC
         11111111111111111111111111111111111111111111111111111111111***********

left     AAAGCCGGCTGGGCGCGGTGGCTCACGCCTGTAATCCCAGCACTTTGGGAGGCCGAGGTGGGCGG
chrom    AAAGCCTCCAAGAAGTTTAGGATTATG--TTAAATAAACAAACCTAAGAAT--------------
right    AAAGCCTCCAAGAAGTTTAGGATTATG--TTAAATAAACAAACCTAAGAAT--------------
         ******22*22*22*2222**2*2*2*22*22***222222**2*22*2*222222222222222
```

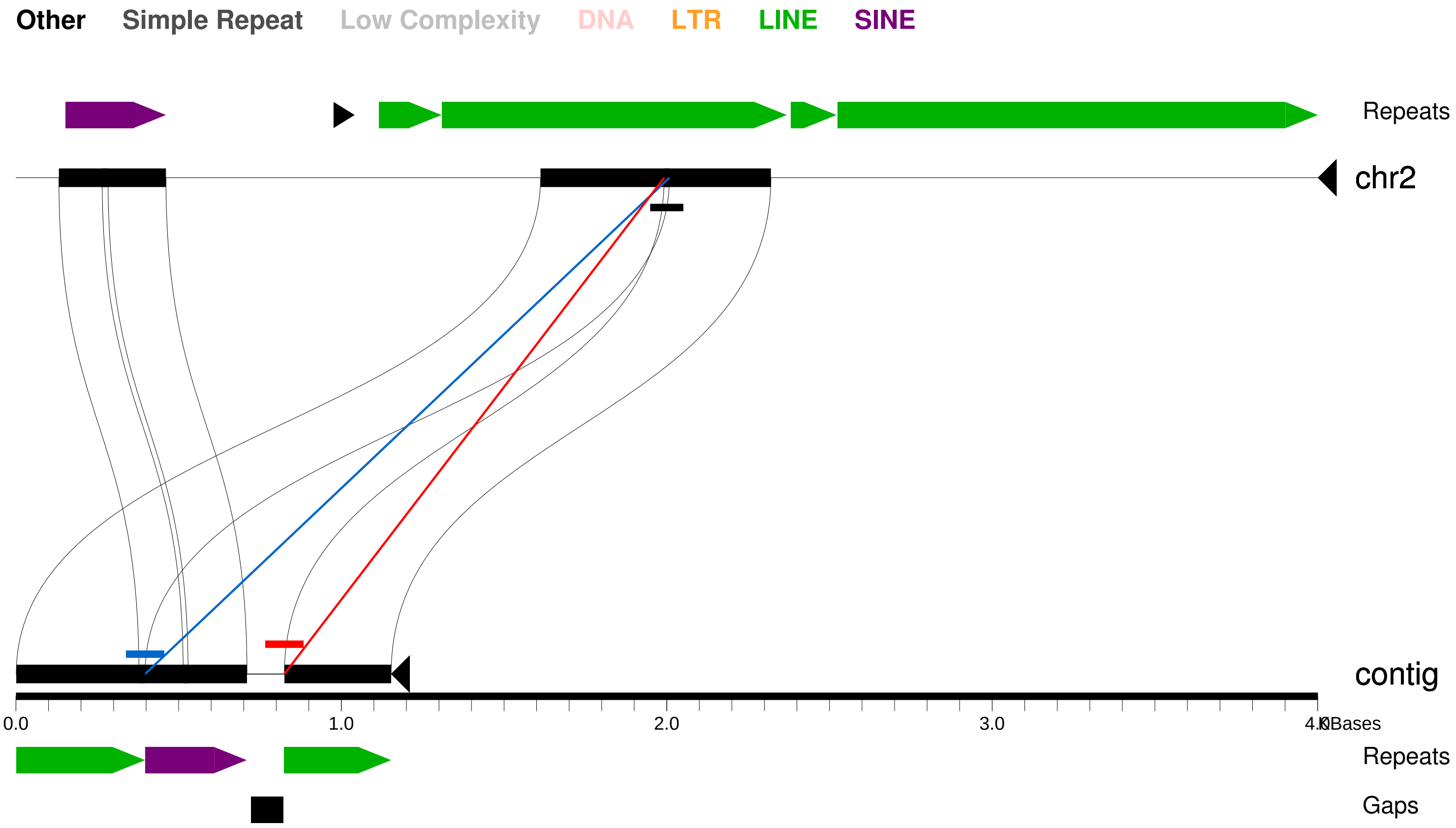

siteID: chr2_192982707 Scafftig1 - Breakpoint: chr2:192982680
chr2:192982520-192982857
Overlap Length: 18 INS size: 309 Genotyped: Yes

```
left   ----------GATCACTATTTCA----TTTACATAGGGTGTACACTAAGTAACCAATGGGAAACCTCTAG
chrom  TCTGATTGCTGATCACTATTTCA----TTTACATAGGGTGTACACTAAGTAACCAATGGGAAACCTCTAG
right  TTTAGTAGA-GA-CGGGGTTTCACCGTTTTAGCCGGGATGGTCTCGATCT--CC--TG----ACCTCGTG
       2N2NN2N2N **1*1111*****1111****1111**1**11*1*1*11*11**11**1111*****11*

left   A---GGGTGTTTAAACCCCAGAAAATTCTATAACCAGTGCTCTTGAACCACTTGCTCAAGCCCACTCCCA
chrom  A---GGGTGTTTAAACCCCAGAAAATTCTATAACCAGTGCTCTTGAACCACTTGCTCAAGCCCGCTCCCA
right  ATCTGCCCGCCTCGGCCTCCCAAAGTGCTGGGATTACAGG-CGTGAGCCAC-----CGCGCCCGGCCC--
       *111*111*11*111**1*11***1*1**111*11*11*11*1***1****11111*11****211**11

left   CTCTGTGGAGTGTACTTTTGTTTCAATAAATCGGTGCTTTTCTTTTTTTTTTTTTTTTTT----TTT---
chrom  CTCTGTGGAGTGTACTTTTGTTTCAATAAATCGGTGCTTTTCTTTCATTGCTTTGTTTGTGGGCTTTACC
right  ---------------------------AAATCGGTGCTTTTCTTTCATTGCTTTGTTTGTGGGCTTTACC
       111111111111111111111111111****************22**22***2***2*2222***222

left   ----TTTTTTTTTGAGACG----GAGTCTCGCTCTGTCGCCCAGGCTGGAGTGCAGTGGCGG--------
chrom  TAATTCTTTGTTCCAAATGCCAAGAATCTGGATACCCTCCACTGGTAACAGTTCCATTAATTAAAAAAAA
right  TAATTCTTTGTTCCAAATGCCAAGAATCTGGATACCCTCCACTGGTAACAGTTCCATTAATTTAAAAAAA
       2222*2***2**22*2*2*2222**2***2*2*222222*2*2**2222***2*22*22222N2222222

left   -----GATCTCGGCTCACTGCAAGCTCC------GCCTCCCGGGTTCACGCC-------------
chrom  AAATAGATTTGGAATAAAATTAATCTACTTGAGAGACATTTGTTTTTATTTCCTTATGATTTACT
right  AAATAGATTTGGAATAAAATTAATCTACTTGAGAGACATTTGTTTT-------------------
       22222***2*2*22*2*2222**2**2*222222*2*2222*22**N1NNN1
```

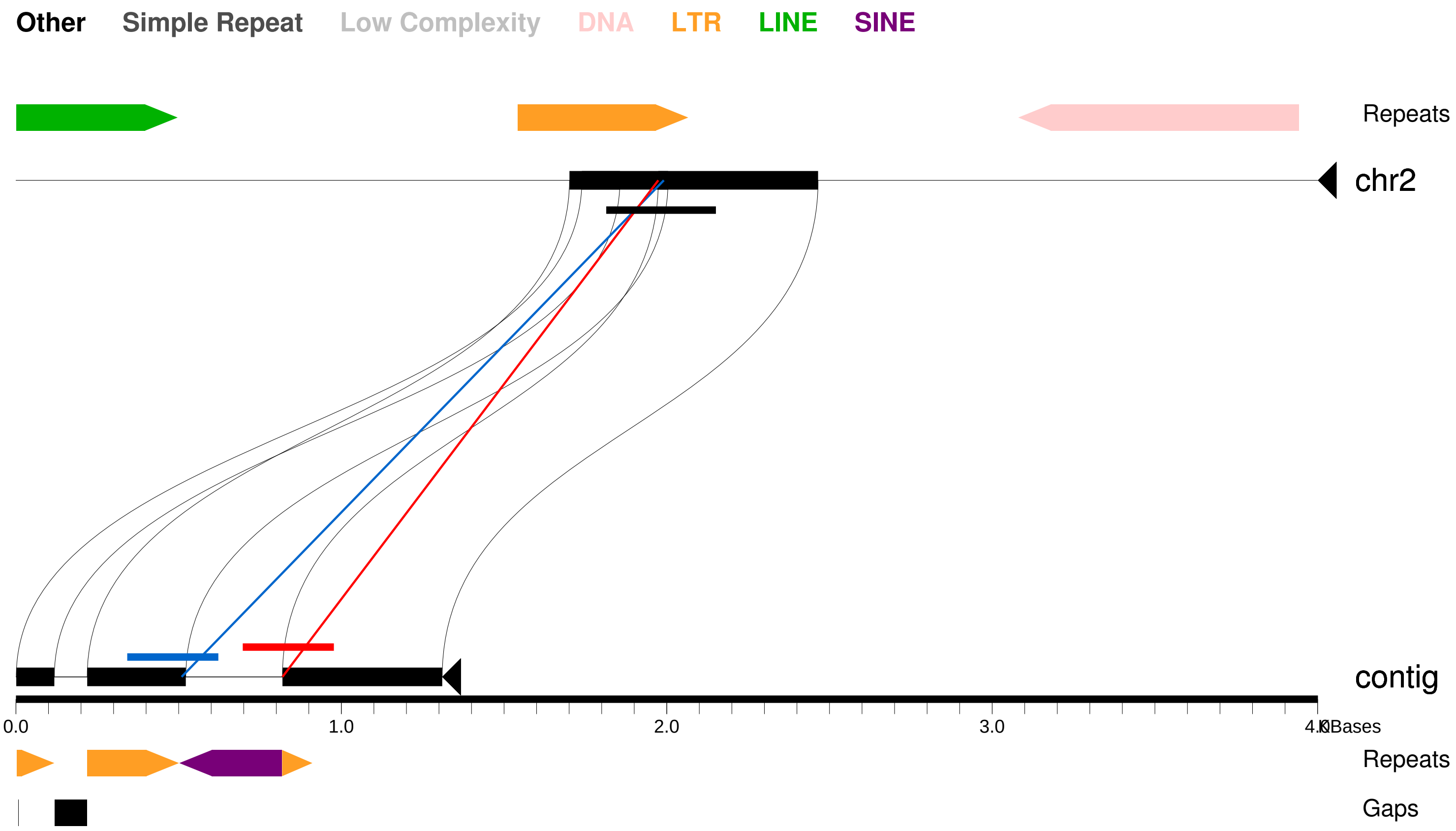

siteID: chr2_194278350 Scafftig2 - Breakpoint: chr2:194278362
chr2:194278212-194278571
Overlap Length: 60 INS size: 317 Genotyped: No

```
left   ----------------------------------------------------------------------
chrom  TTTTTTTCATGGAATTCCAAAAAACAATTTTTCCTACATTGTTACAAATAGTATAAATATTTTAAGATTT
right  TCGATCTCCTG------------------------ACCTCGT-----------------------GATCC
       2NNN2N22N22                        22N2N22                       222NN

left   ------------------------------AAATATTTTGAGCAAACTCATTTTTTAAATTTGCAGTTTA
chrom  GAGAATTTGGTAGATATTTATATTTGTAATAAATATTTTGAGCAAACTCATTTTTTAAATTTGCAGTTTA
right  GC----------------------------------------CCGCCTCGGCCTCCCAAAGTGCTGGGA-
       2N                            111111111111*111***1111*111**11***1*1111

left   TTTAAAACTTCCAGTTGGTGATCTTTTTACAAATCTGAGAAATAAATATGTATGAGCACTTTCAGTAATA
chrom  TTTAAAACTTCCAGTTGGTGATCTTTTTACAAATCTGAGAAATAAATATGTAAGAGCATTTTCAGTAATA
right  -TTACAGG--CCAGTTGGTGATCTTTTTACAAATCTGAGAAATAAATATGTAAGAGCATTTTCAGTAATA
       1***1*1111*****************************************2*****2***********

left   NNNNNNNNNNNNNNNNNNNNNNNNNNNNNNNNNNNNNNNNNNNNNNNNNNNNNNNNNNN-----------
chrom  AAAGAGATTGTAAAAAAAGTACACATTTAACTGTCTTATTCTAACTACTTTCCAATTTCCCTTGCTTAGA
right  AAAGAGATTGTAAAAAAAGTACACATTTAACTGTCTTATTCTAACTACTT--------------------
       22222222222222222222222222222222222222222222222222NNNNNNNNN

left   ----------------------------------------------------------------------
chrom  TATGCATGCAGAATTGTGAATTGTTCTTTATTAAACATTATATAGGTTTAATGTGTTATATTAAACATTG
right  ----------------------------------------------------------------------

left   ----------
chrom  TAGTGCTAAG
right  ----------
```

Other **Simple Repeat** **Low Complexity** **DNA** **LTR** **LINE** **SINE**

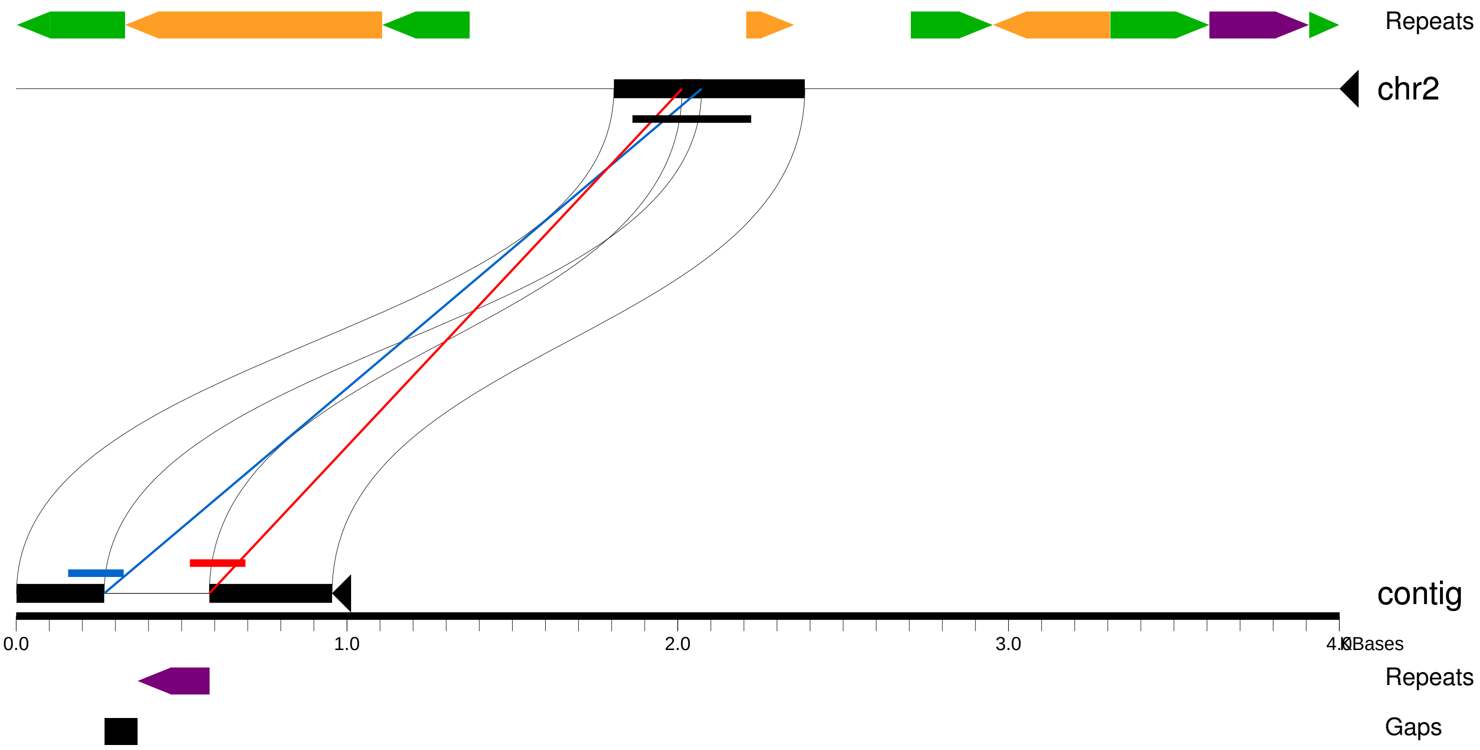

siteID: chr2_194566526 Scafftig2 - Breakpoint: chr2:194566519
chr2:194566476-194566575
Overlap Length: 17 INS size: 325 Genotyped: Yes

```
left     TAG--GTTTGGTTT--CCTCCC----TTCTGTGTTT-CAGTCCTTGGCA----------GCTGGCTGGAG
chrom    TAG--GTTTGGTTT--CCTCCC----TTCTGTGTTT-CAGTCCTTGGCA----------GCTGGCTGGAG
right    GATCCGCCCGCCTCGGCCTCCCAAAGTGCTGGGATTACAGGCGTGAGCCACCGCGCCCGGCCGGCTGGAG
         1*111*111*11*111******1111*1***1*1**1***1*1*11**11111111111**1********

left     GTTTTTTTTTTTTTTTTTTTTTTTTTTTTTTTTTTTTTTTTTTTTTTTTTTTTGAGACGGAGTCTCGC
chrom    GTTTTTTTTTCCCCGTCATCAGAATATCGAAATAGGTAATATATAATGTA-------------------
right    GTTTTTTTTTCCCCGTCATCAGAATATCGAAATAGGTAATATATAATGTA-------------------
         *********22222*22*22222*2*22222*222*22*2*2*22*2*2222222222222222222
```

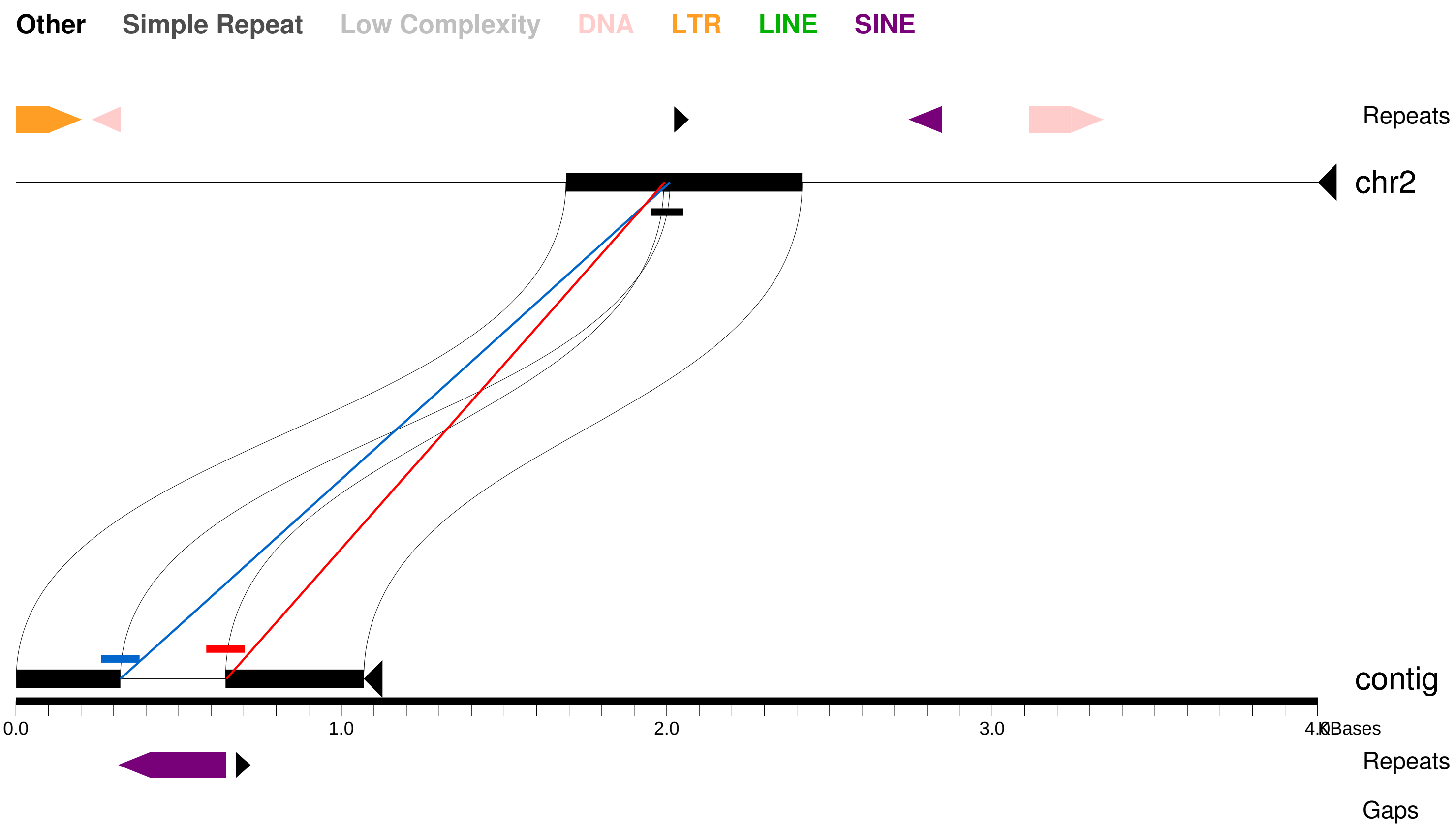

siteID: chr2_195611726 Scafftig1 - Breakpoint: chr2:195611646
chr2:195611505-195611855
Overlap Length: 69 INS size: 432 Genotyped: No

```
left   A---------------------------------------------------------------------
chrom  ATTTTGTTTTCCTTTTCTGGTTTAGCCTTATGCCAAGAAAAATAAATACATGTATAGTAAAAGAAAAAAG
right  NNNNNNNNNNNNNNNNNNNNNNNNNNNNNNNNNNNNNNNNNNNNNNNNNNNNNNNNNNNNAA--------
       1NNNNNNNNNNNNNNNNNNNNNNNNNNNNNNNNNNNNNNNNNNNNNNNNNNNNNNNNNNNN22

left   -------------------------------CCCCATCTGAGGCTTAAAATGTTATTTAAACATTTTAAA
chrom  CTTACTAGATATATGGACTTTTAAAATTCTACCCCATCTGAGGCTTAAAATGTTATTTAAACATTTTAAA
right  ----------------------------------------------------------------------
                                      111111111111111111111111111111111111111

left   TAATATGAACAAACATTTAGAATATTAGATATCATTACAGAAAAATTATTACATTAAAAACTTGTTTTCC
chrom  TAATATGAACAAACATTTAGAATATTAGATATCATTACAGAAAAATTATTACATTAAAAACTTGTTTTCC
right  ---TATGAACAAACATTTAGAATATTAGATATCATTACAGAAAAATTATTACATTAAAAACTTGTTTTCC
       111*******************************************************************

left   -----------------------------GGCCGGCG--------------CGGTGG----------CT
chrom  TAAAACATTCCCTAAAAATAGCAAATCATGGCTTTGCTATTCTTAAATGTTTCTGTTGAAATTATTTCT
right  TAAAACATTCCCTAAAAATAGCAAATCATGGCTTTGCTATT----------------------------
       22222222222222222222222222222***222**2222           1N11N1          11

left   C----ACGCCTGTAA-----TCCCAGCAC--------------TTTGGGAGGCCGAGGC-------GGGC
chrom  CTATAACAAACTTAAATAAGTCAGATAAGAAAATTATTAAATGTTTGTGAGGA-GAGGGTAGGAAAGTGA
right  ----------------------------------------------------------------------
       1    11NNNNN111     11NN1NN1N              1111N1111N21111N       1N1N

left   GG
chrom  GT
right  --
       1N
```

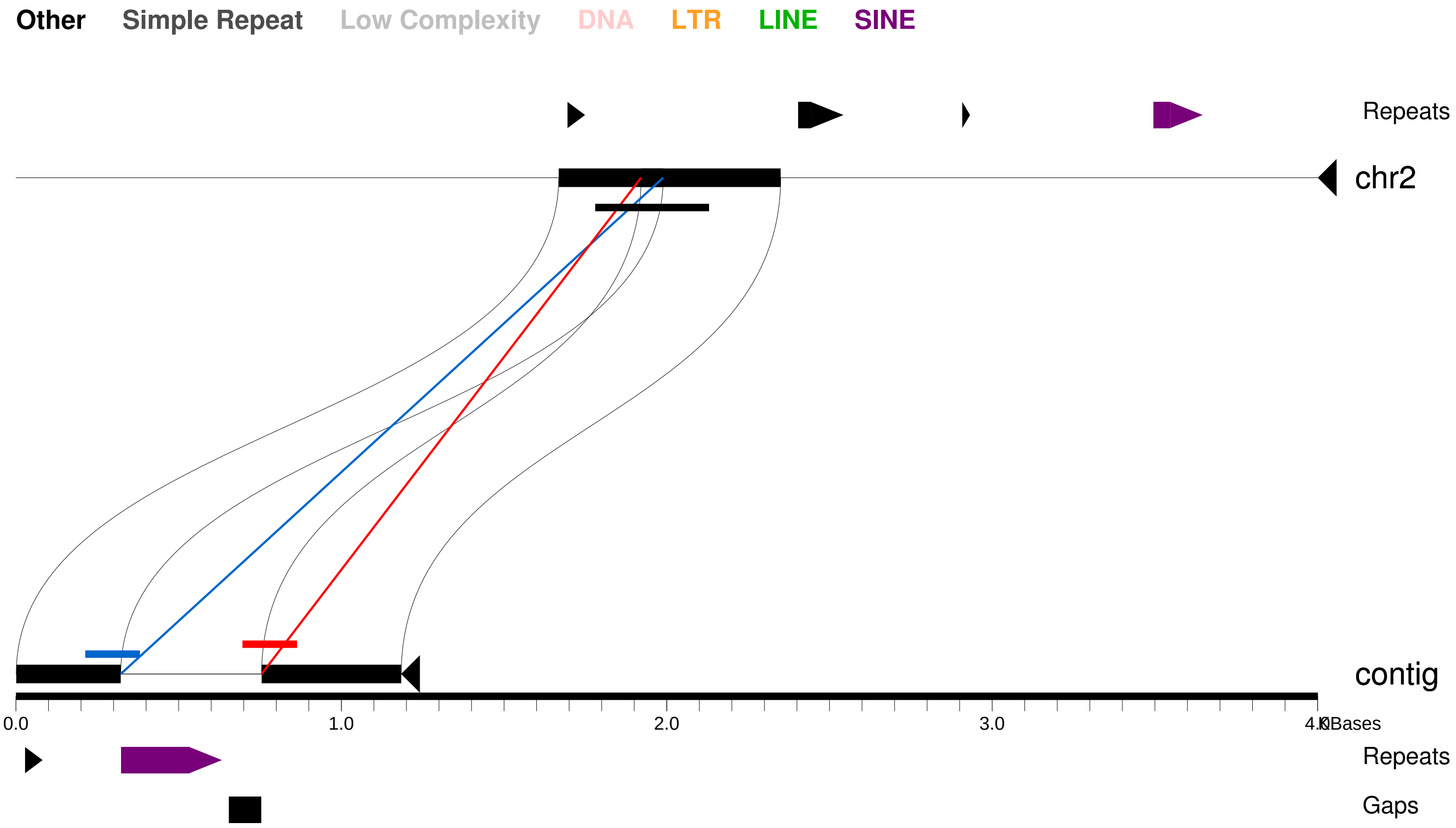

siteID: chr2_196017598 Scafftig1 - Breakpoint: chr2:196017574
chr2:196017530-196017633
Overlap Length: 16 INS size: 311 Genotyped: Yes

```
left    CGCA---ATTGTCCTTT--ATGTTCT---ATT----TATTCAACACCCAAGCGCAT---AGTCTCACAAC
chrom   CGCA---ATTGTCCTTT--ATGTTCT---ATT----TATTCAACACCCAAGCACAT---AGTCTCACAAC
right   CGCCCGCCTCGGCCTCCCAAAGTGCTGGGATTACAGGCGTGAGCCACCGCGCCCGGCCCAGTCTCACAAC
        ***11111*1*1***1111*1**1**111***1111111*1*1*11**11**N*11111***********

left    ATTTTTTTTTTTTTTTTTTTTTTTTTTTTTTTGAGACGGAGTCT---CGCTCTGTCGCCCAGGCTGGA
chrom   ATTTTAAAGCTTTC------------------AAACTGAAACTATCCCCACTAACACCTTCACCAAA
right   ATTTTAAAGCTTTC------------------AAACTGAAACTATCCCCACTAACACCTTCACCAAA
        *****22222***222222222222222222*2**2**22**222*2*2**22*2**2222*222*
```

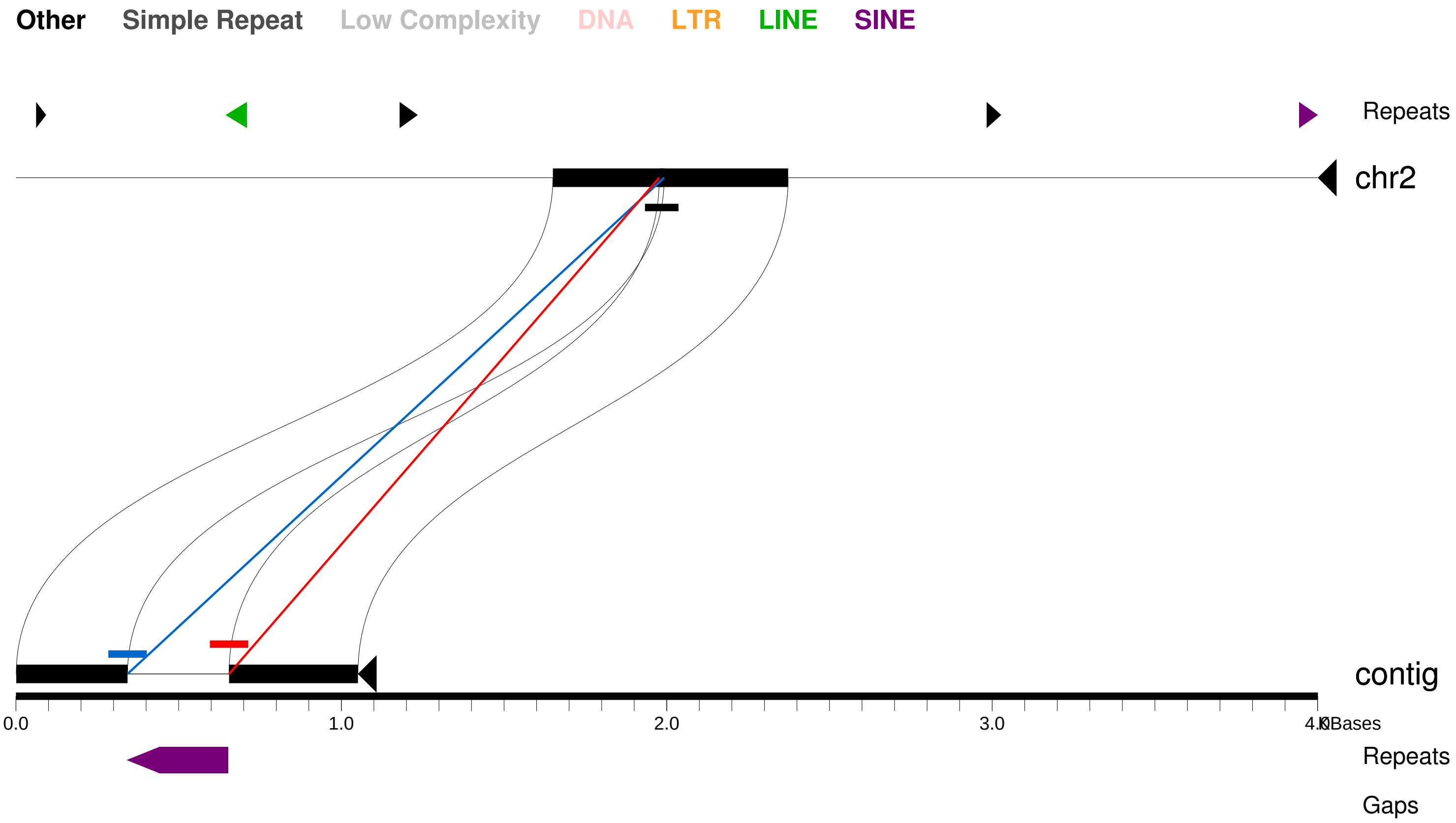

siteID: chr2_19748398 Scafftig10 - Breakpoint: chr2:19748402
chr2:19748357-19748461
Overlap Length: 15 INS size: 297 Genotyped: Yes

```
left    AGCC-CTGGGCTC-AGCCAGAG---CAGAGC-AGAGGACTGCC--------AGAGGATTAAGAGGGCAGA
chrom   AGTC-CTGGGCTC-AGCCAGAG---CAGAGC-AGAGGACTGCC--------AGAGGATTAAGAGGGCAGA
right   CGCCACTGCACTCCAGCCTGGGCGACAGAGCGAGACTCCGTCTCAAAAAAAAAAAAAAAAAGAGGGCAGA
        1*N*1***11***1****1*1*111******1***111*11*111111111*1*11*11***********

left    GAGAGGGCCGGGCGCGGTGGCTCACGCCTGTAATCCCAGCACTTTGGGAGGCCAAGGCGGGCG
chrom   GAGAAAATGGGACTA---GACAACCAGCTGCAA----AGAA------GAGTACCCTCTCTGC-
right   GAGAAAATGGGACTA---GACAACCAGCTGCAA----AGAA------GAGTACTCTCTCTGC-
        *****22222**2*22222*2*222*22***2**2222**2*222222***22*N222222**2
```

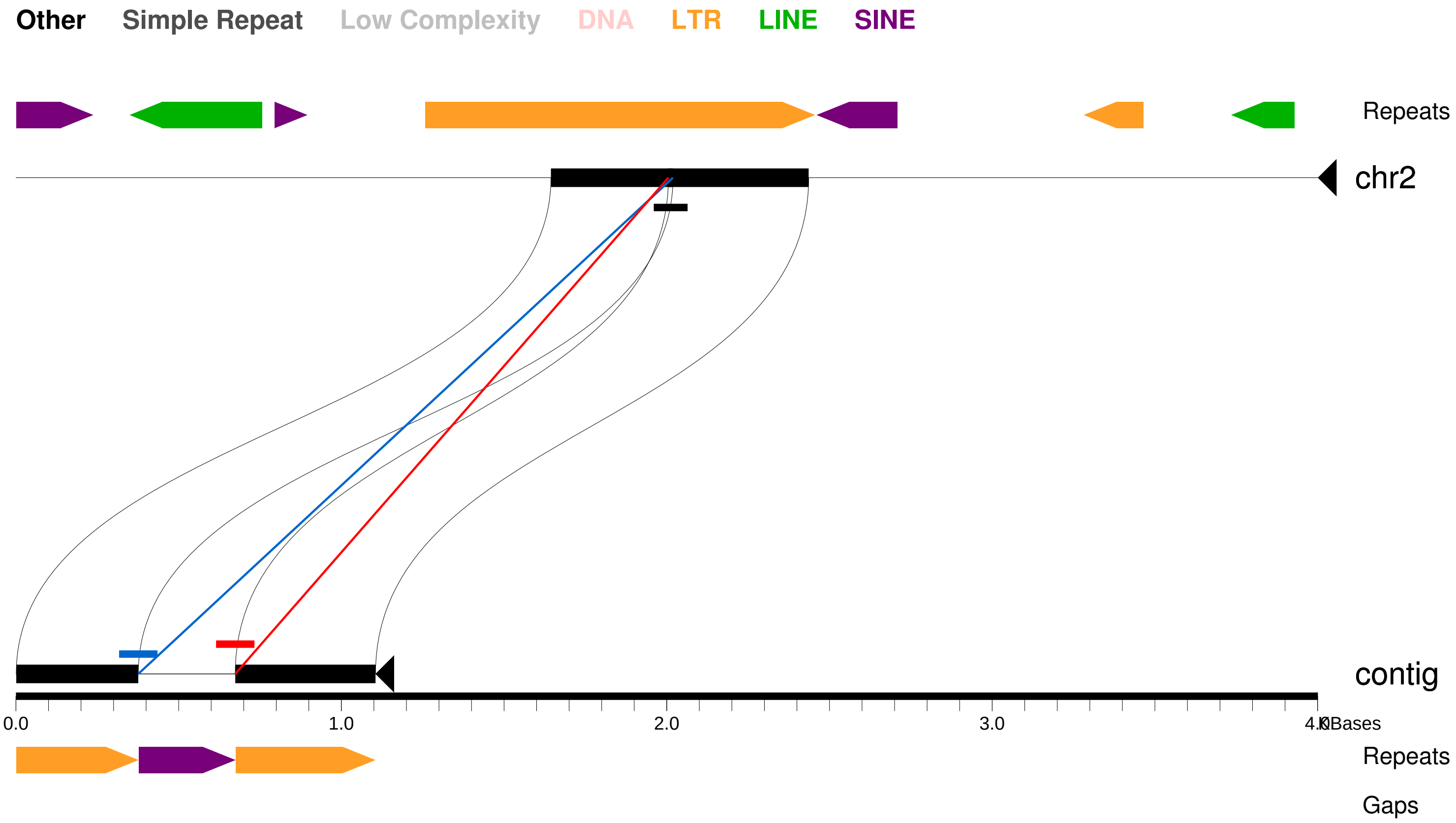

siteID: chr2_197831249 Scafftig3 - Breakpoint: chr2:197831228
chr2:197831182-197831287
Overlap Length: 14 INS size: 457 Genotyped: No

```
left    ------ACAA------CTTAATAGTATAAATTAAT-ATAAATTGGCTAATATGGTTCCC--AGGGCTAAT
chrom   ------ACAA------CTTAATAGTATAAATTAAT-ATAAATTGGCTAATATGGTTCCC--AGGGCTAAT
right   TGTTAGCCAGGATGGTCTCGATCTCCTGACCTCATGATCCACCCGCC--TCGGCCTCCCAAAGGGCTAAT
        1111111**1111111**11**1111*1*11*1**1**11*111**111*11*11****11*********

left    TCTTTTTTTTTNNNNNNNNNNNNNNNNNNNNNNNNNNNNNNNNNNNNNNNNNNNNNNNNNNNNN
chrom   TCTTT-------------ACAGATGAAATAAACTGGAAAATGAAATGAGAGAGAATGTCATTTT
right   TCTTT-------------ACAGATGAAATAAACTGGAAAATGAAATGAGAGAGAATGTCATTTT
        *****22222222222222222222222222222222222222222222222222222222222
```

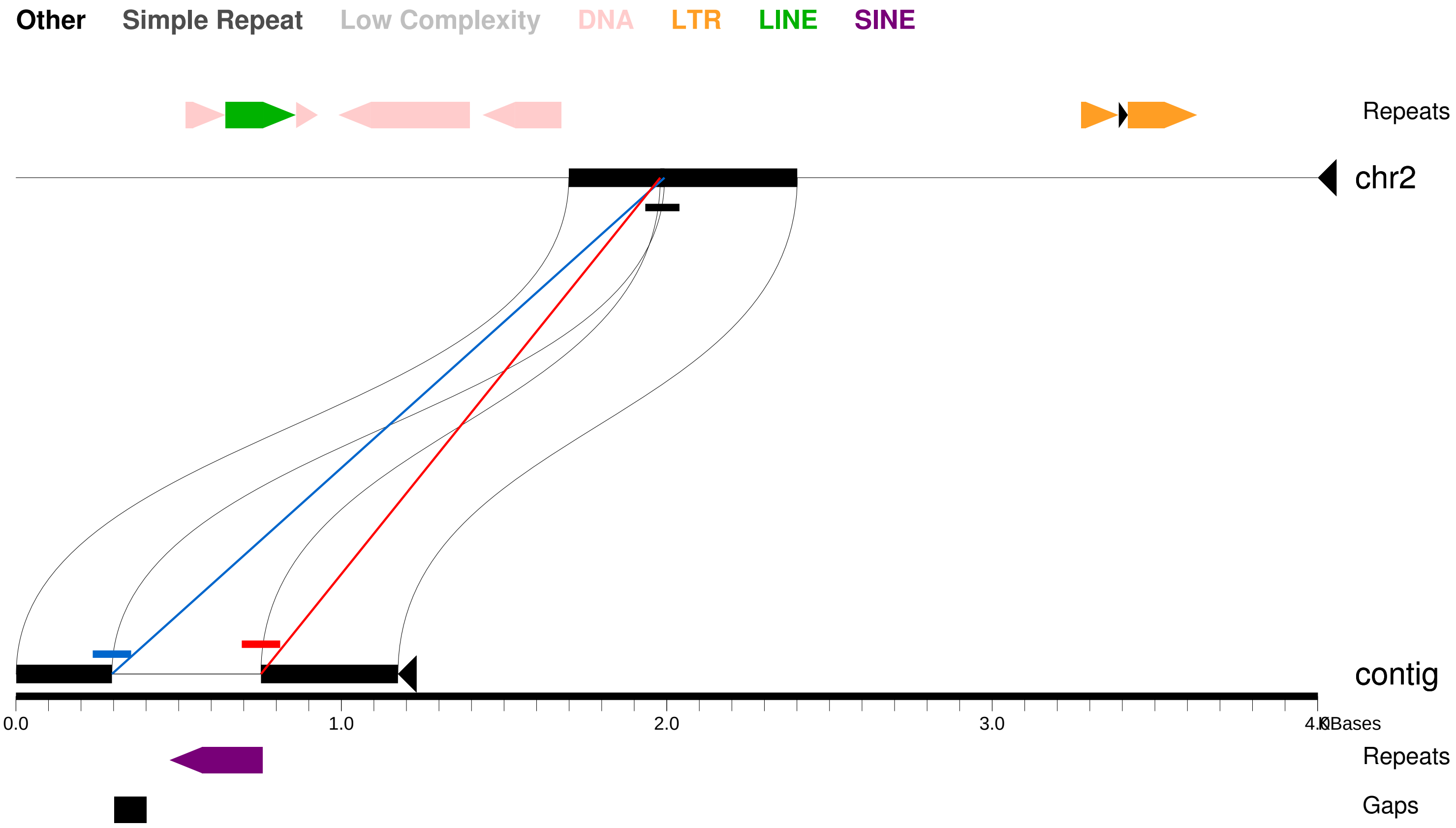

siteID: chr2_198763471 Scafftig6 + Breakpoint: chr2:198763449
chr2:198763403-198763505
Overlap Length: 14 INS size: 299 Genotyped: Yes

```
left     TTA----CAATACTTATAATTTATTAATATAAATGTGT------------GTG-TGTATGTATTTCCTAG
chrom    TTA----CAATACTTATAATTTATTAATATAAATGTGT------------GTG-TGTATGTATTTCCTAG
right    ATGGTCTCGAT-CTCCTGACCTCGTGATCCGCCCGCCTCGGCCTCCCAAAGTGCTGGGATTACTTCCTAG
         1*11111*1**1**11*1*11*11*1**111111*11*111111111111***1**1111**1*******

left     TTTCTTTCTTTTTTCTTTTTCTTTTTTCTTTTTTTTTTTTTTTTTTTGAGACGGAGTCTCGCTCTG
chrom    TTTCTTTAGTCCTTGAC--TCAAATACCCTGTGTTC-----------GAACCTGA---ACTCTGAT
right    TTTCTTTAGTCCTTGAC--TCAAATACCCTGTGTTC-----------GAACCTGA---ACTCTGAT
         *******22*22**22222**222*22*2*2*2**222222222222**22*2**2222*2**222
```

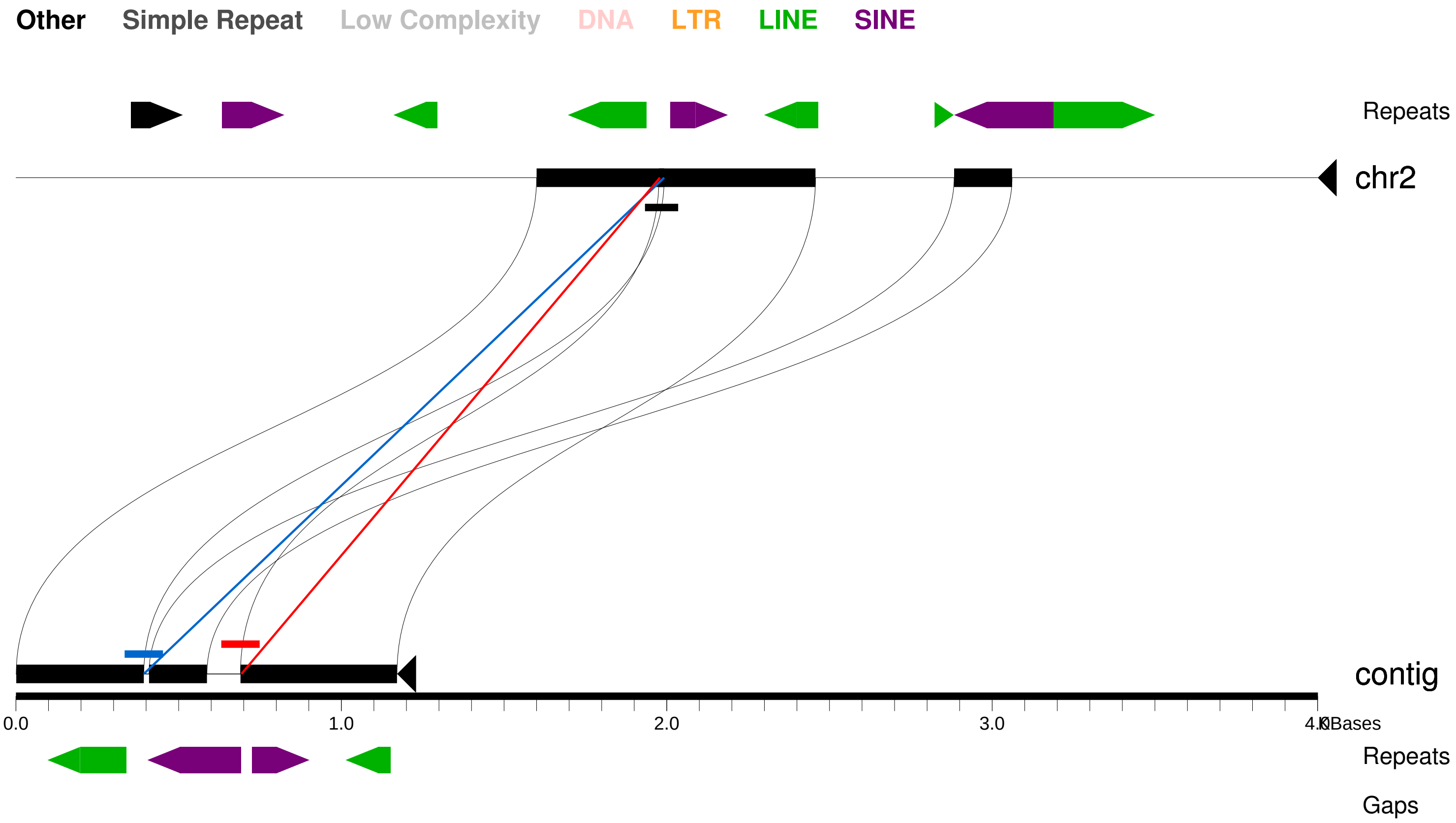

siteID: chr2_199988319 Scafftig2 - Breakpoint: chr2:199988325
chr2:199988279-199988384
Overlap Length: 14 INS size: 408 Genotyped: No

```
left     AGCTATTTAGCTTACAAAATGGACCAGCCATTTAGTCCATCCCTAA-------------AGAACTGGGGG
chrom    AGCTATTTAGCTTACAAAATGGACCAGCCATTTAGTCCATCCCTAA-------------AGAACTGGGGG
right    NNNNNNNNNNNNNNNNNNNNNNNNNNNNNNNNNNNNNNNNNNNNNNNNNNNNNNNNNNNAGAACTGGGGG
         11111111111111111111111111111111111111111111111111111111111***********

left     TCCGGCCGGGCGCGGTGGCTCACTCCTGTAATCCCAGCACTTTGGGAGGCCGAGACGGGCGG
chrom    TCCTG--------GGAATTTAACCACGTCAATGCAGTCTTTTTTATA---CTAGTA--ACTA
right    TCCTG--------GGAATTTAACCACGTCAATGCAGTCTTTTTTATA---CTAGTA--ACTA
         ***2*22222222**2222*2**22*222***2*222*22***222*222*2**22222*22
```

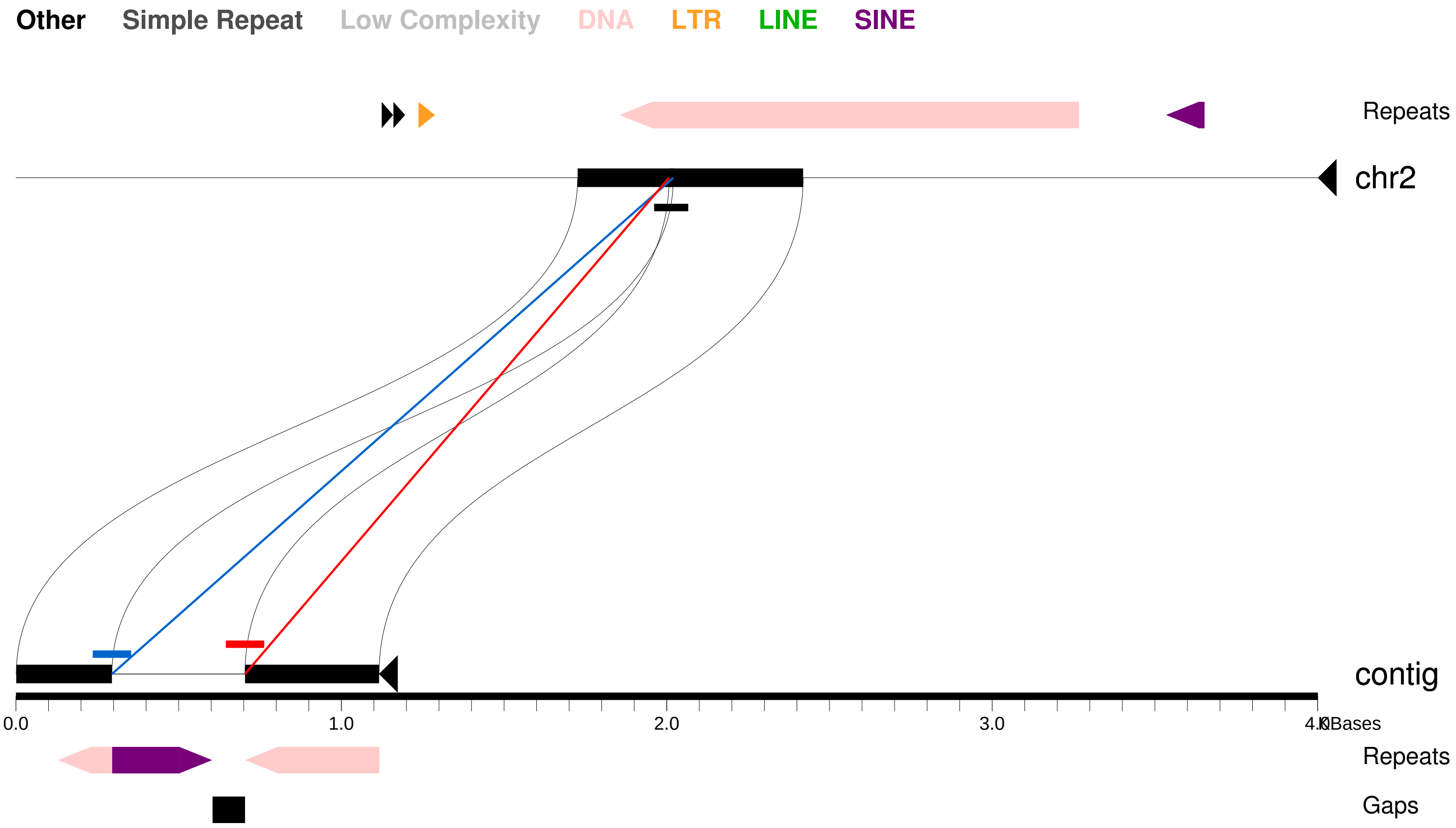

siteID: chr2_20085235 Scafftig1 - Breakpoint: chr2:20085237
chr2:20085077-20085409
Overlap Length: 13 INS size: 305 Genotyped: Yes

```
left   -------------TCTCCCCATTGAGGACCTCAGGCCCCAGTTCATTATCAGGATTTTAGAAATACCAAG
chrom  CAATATGCTGGCTTCTCCCCATTGAGGACCTCAGGCCCCAGTTCATTATCAGGATTTTAGAAATACCAAG
right  ---------GGCGTGAACCC---GGGAAGCGGAGCTTGCAGTG----AGCCGAGATTGCGCCACTGCAG-
                222N*111***111*1*1*1*11**1111****11111*1*1*111**11*11*111**11

left   AACATCAAAAGAACAGAAAAGAGGCCCTTAAGGGACTCCTAATAAGATAGAATTTGCATGTCCTCATTGG
chrom  AACATCAAAAGAACAGAAAAGAGGCCCTTAAGGGACTCCTAATAAGATAGAATTTGCATGTCCTCATTGG
right  ----TCCGCAGTCC--------GGCC--TGGGCGAC--------AGAGCGAGACTCCGTCTCAAAAAAAA
       1111**111**11*11111111****11*11*1***11111111***11**111*1*1*1**111*1111

left   ACTAGAACCTGAACTGCTTTAAAAAGCAAAAGGGGCCGGGCGC-----GGTGGCTCACGCCTGTA-ATCC
chrom  ACTAGAACCTGAACTGCTTTAAAAAGCAAAAGGCCCTTGGTACAATCTGATGGATGT-GACTGTACAGAG
right  AAAAAAA----AA-------AAAAAACAAAAGGCCCTTGGTACAATCTGATGGATGT-GACTGTACAGAG
       *11*1**1111**1111111*****1*******22*22**22*22222*2***2*222*2*****2*222

left   CAG------CACTTT----GGGAGGCCGAGGCG--GGT--GGATCATGAGGTCAGGAGAT-CGAGACCA-
chrom  AAGAGGAAACACCTCTGAGGGGAGTGAGAGGCCTTGGTAAGGAAGTTGCAATCTGGAGCCACCTGGGCAG
right  AAGAGGAAACACCTCTGAGGGGAGTGAGAGGCCTTGGTAAGGAAGTTGCAATCTGGAGCCACCTGGGCAG
       2**222222***2*22222*****222*****222***22***222**222**2****222*22*22**2

left   -TCCTGGCT----AACAAGGTGAAACCCCGTCTCTACTAAA-------------
chrom  GTCAGAGCTCTATAAGCAGGAGAAGCCAAGGGCATAACACACAGTAGTGCAGGT
right  GTCAGAGCTCTATAAGCAGGAGAAGCCAAGGGCATAACACACAGTAGTGCAG--
       2**222***2222**22***2***2**22*2222**22*2*22222222222
```

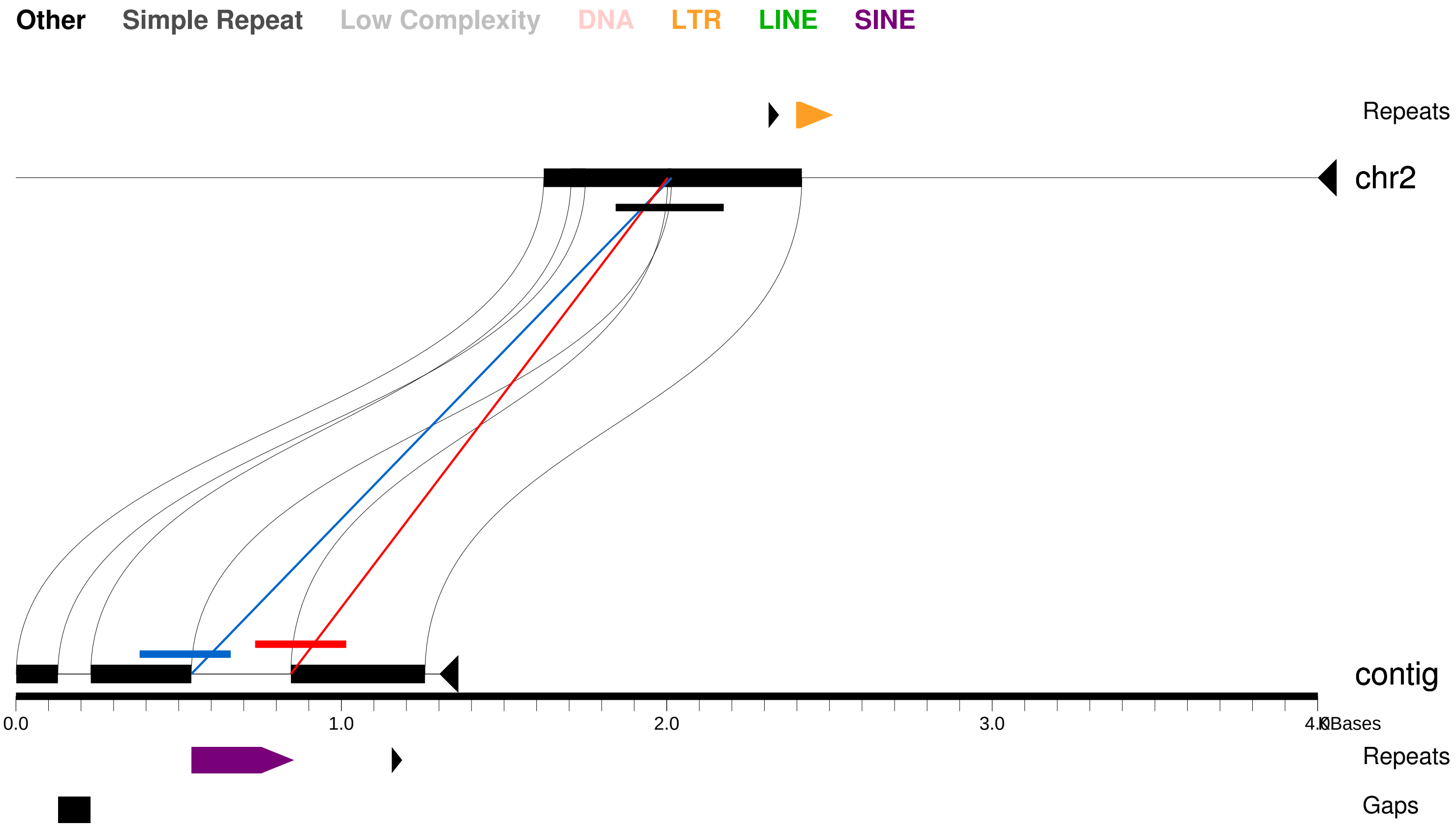

siteID: chr2_201578949 Scafftig1 - Breakpoint: chr2:201578952
chr2:201578901-201579011
Overlap Length: 9 INS size: 424 Genotyped: No

```
left    TGCATGTGTATATACATACAGACATTTTTGTGCTTTCTTTTTATAAAAGTA--------AAACATGTCGG
chrom   TGCATGTGTATATACATACAGACATTTTTGTGCTTTCTTTTTATAAAAGTA--------AAACATGTC--
right   NNNNNNNNNNNNNNNNNNNNNNNNNNNNNNNNNNNNNNNNNNNNNNNNNNNNNNNNNNNAAACATGTC--
        11111111111111111111111111111111111111111111111111111111111*********22

left    CCGGGCGCGGTGGCTCACGCCTGTAATCCCAGCACTTTGGGAGGCCGAGGCGGGCGG
chrom   -----CTTTAATGGACATTCGACAAATAC-AGAAGAATATAAAAAGAAAATCAAAAT
right   -----CTTTAATGGACATTCGACAAATAC-AGAAGAATATAAAAAGAAAATCAAAAT
        22222*222222*22**22*2222***2*2**2*222*222*22222*222222222
```

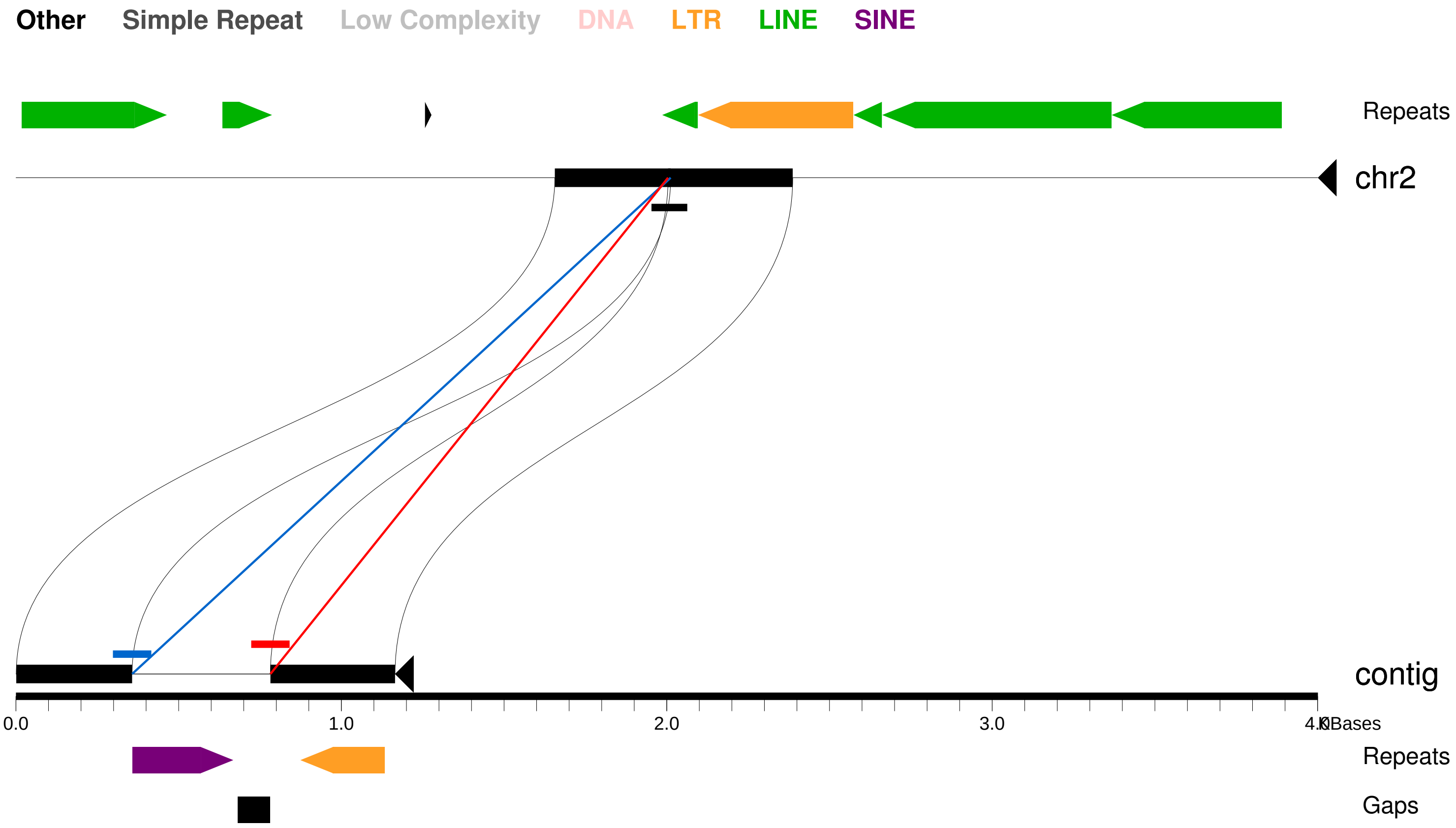

siteID: chr2_202641985 Scafftig1 + Breakpoint: chr2:202641948
chr2:202641941-202642007
Overlap Length: 53 INS size: 425 Genotyped: No

```
left   CAAGCAT----------------------------------------------------CTCTAGAATGT
chrom  CAAGCAT----------------------------------------------------CTCTAGAATGT
right  NNNNNNNNNNNNNNNNNNNNNNNNNNNNNNNNNNNNNNNNNNNNNNNNNNNNNNNNNNNCTCTAGAATGT
       11111111111111111111111111111111111111111111111111111111111***********

left   TAATTCCCCTGCAGGCTTCATGTCCTAAAGAAAGCATTTTCCGGCCGGGCGCGGTGGCTCACGCCTGTAA
chrom  TAATTCCCCTGCAGGCTTCATGTCCTAAAGAAAGCATTTTCC----------------------------
right  TAATTCCCCTGCAGGCTTCATGTCCTAAAGAAAGCATTTTCC----------------------------
       ******************************************2222222222222222222222222222

left   TCCCAGCACTTTGGGAGGCCGAGGCGGGCGG
chrom  ------------------------CAGGAGC
right  ------------------------CAGGAGC
       222222222222222222222222*2**2*2
```

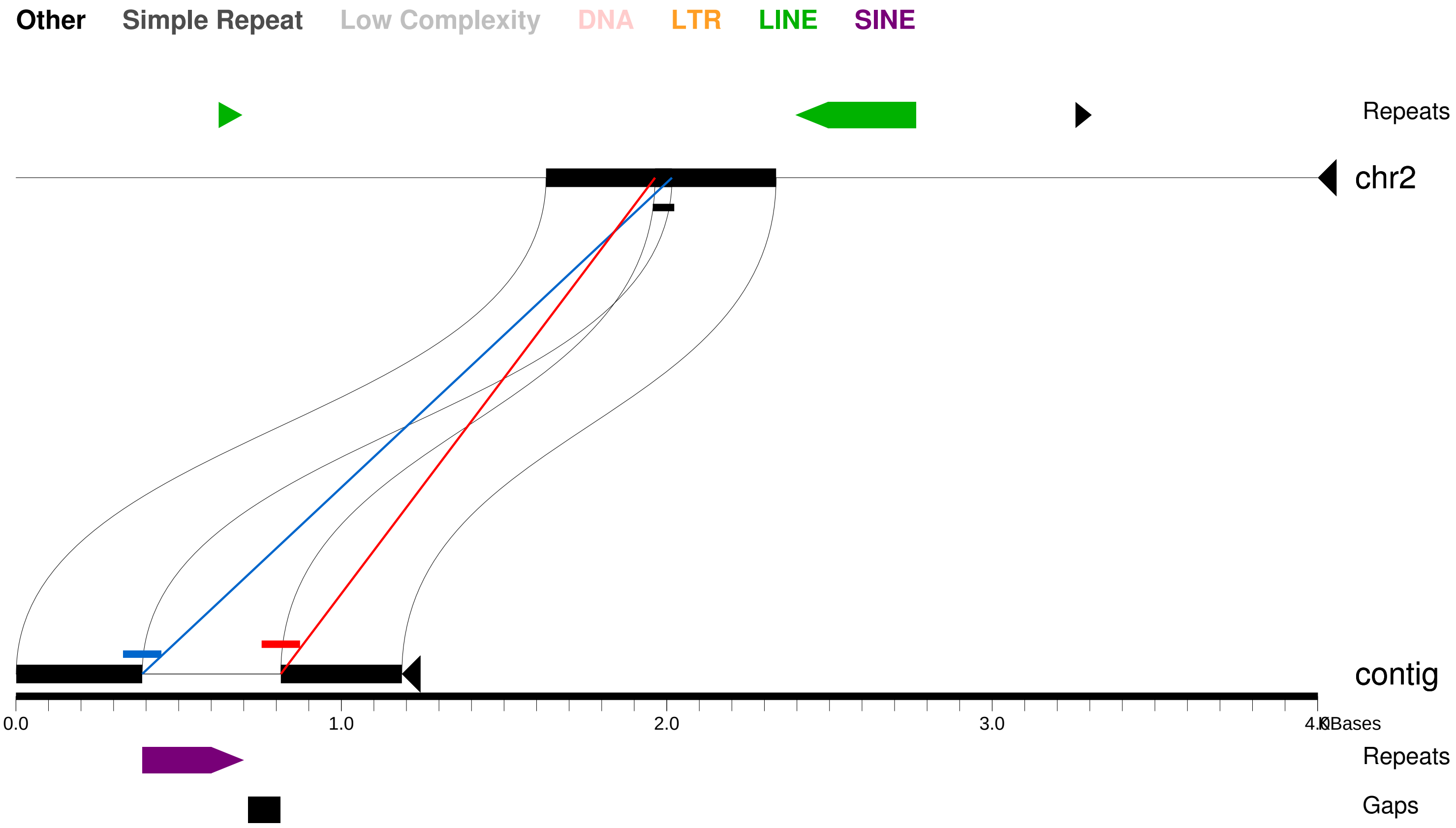

siteID: chr2_207976903 Scafftig4 - Breakpoint: chr2:207976889
chr2:207976729-207977062
Overlap Length: 14 INS size: 309 Genotyped: Yes

```
left   ----------TTGTTATTCTGACAGTCTGCTGTCAACAAAGATGAAATACTCAAACAAAACCACCTCCCT
chrom  ACTTGCAAAGTTGTTATTCTGACAGTCTGCTGTCAACAAAGATGAAATACTCAAACAAAACCACCTCCCT
right  TTTAGTAGAGACGGGGTT-TCAC---CT--TGTTAGCCAGGATGGT---CTCGATCT------CCTGAC-
       NN2N2N2N2211*111**1*1**111**11***1*1*1*1****11111***1*1*1111111***11*1

left   CTCAAAGTCAGAATATAAAACTTGTCAAGTGACAAAGGGATATTCTGACTTCTGATGGTTCTTCATAAGT
chrom  CTCAAAGTCAGAATATAAAACTTGTCAAGTGACAAAGGGATATTCTGACTTCTGATGGTTCTTCATAAGT
right  CTCATGATCC----ACCCGCCTCGGCC--TCCCAAAG---------------TGCTGGGATTACAGGCGT
       ****111**11111*11111**1*1*111*11*****111111111111111**1***111*1**111**

left   AAATGGCTAATTCATTTAAT-GGGCAAATGCTTTTTTTTTTTTT-----TTTTTTTTTTTTGAGAC---G
chrom  AAATGGCTAATTCATTTAAT-GGGCAAATGCTTTTGAGTGATTAACAACTGGTCCTTTTAACAGAGATAG
right  GA---GCCACCGCGCCCGGCCGGGCAAATGCTTTTGAGTGATTAACAACTGGTCCTTTTAACAGAGATAG
       1*111**1*111*11111111*************222*22**222222*22*22****222***2222*

left   GAGTCTCGCTCTGTTGCCCAGGCCGGAC---------TGCGGACTGCAGTGGC----GCAATCTCGGCTC
chrom  CAGAAATAATATTATACCCTCTCTGTAAGTTTTTCTTTGATAACAAGATTTTCTTTTGCAATTTGAGCAC
right  CAGAAATAATATTATACCCTCTCTGTAAGTTTTTCTTTGATAACAAGATTTTCTTTTGCAATTTGAGCAC
       2**222222*2*22*2***222*2*2*2222222222**222**222*2*22*2222*****2*22**2*

left   -----ACTGCAAGCTCCGCTTCCC--------GGGTTCAC---GCCATT-------
chrom  TACTAACGGCTAGCTG-GTTTCCTTTAGACAGGTGTTAACACTGCGATCTTCTCGC
right  TACTAACGGCTAGCTG-GTTTCCTTTAGACAGGTGTTAACAC--------------
       22222**2**2****22*2****222222222*2***2**22 11N11N
```

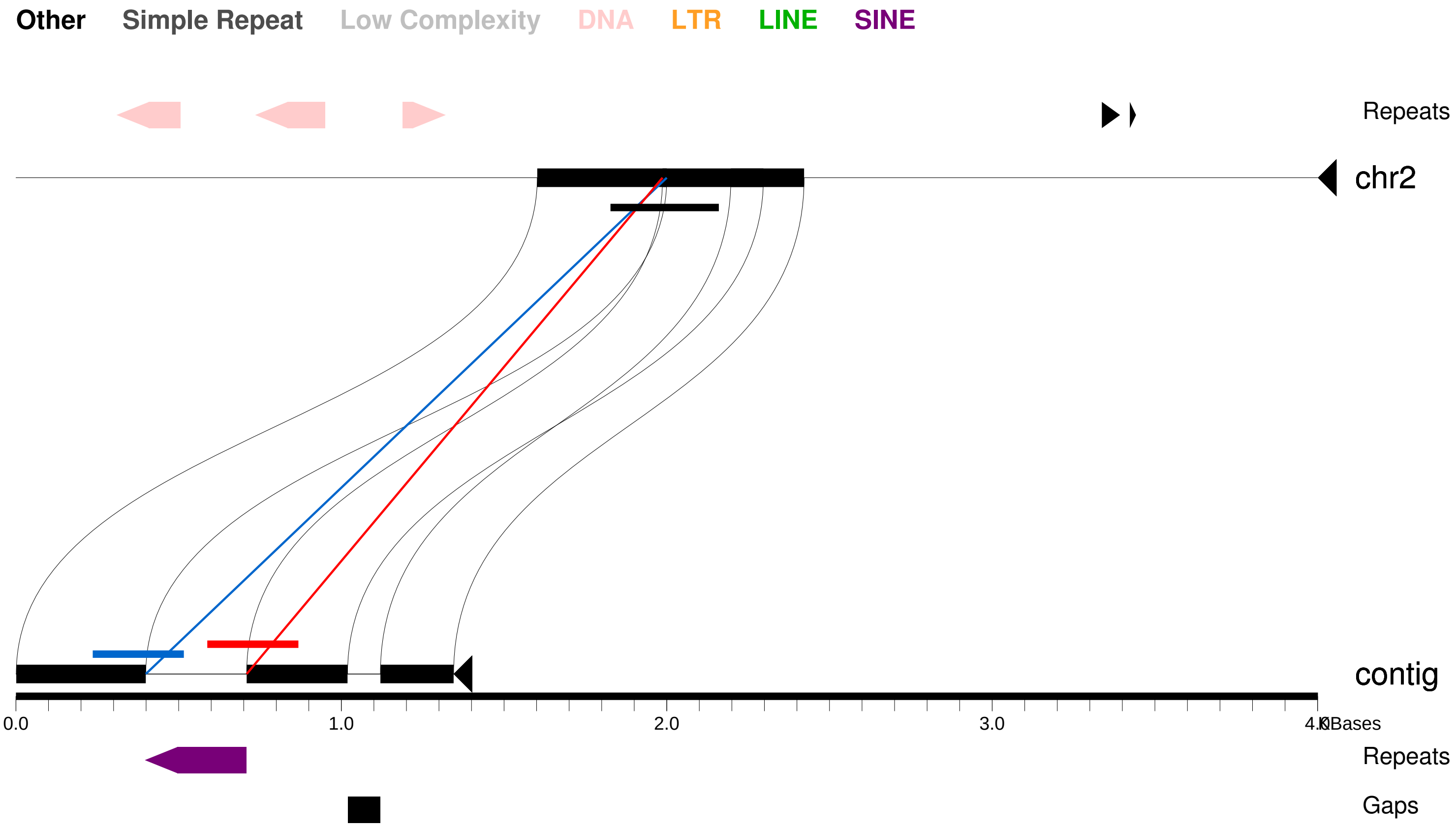

siteID: chr2_210260740 Scafftig3 - Breakpoint: chr2:210260740
chr2:210260695-210260799
Overlap Length: 15 INS size: 297 Genotyped: Yes

```
left    CCCTTTGACCATATTACCAAG----AATATCTG--CTTTG---------AGAAGGATTTTAAAACATTAT
chrom   CCCTTTGACCATATTACCAAG----AATATCTG--CTTTG---------AGAAGGATTTTAAAACATTAT
right   GCCACTG-CAATCCGGCCTAGGCTAAAGAGCGGGACTCCGTCTCAAAAAAAAAAAAAAAAAAAACATTAT
        1**11**1*1**1111**1**1111**1*1*1*11**11*111111111*1**11*1111**********

left    AACTAGGCTGGGCGCGGTGGCTCACGCCTGTAATCCCAGCACT--TTGGGAGGCCGAGGCGGGCGG
chrom   AACTAAAGAAAA-----TAGTTAGAGAAAGTACTTCATGTATAAATTTGGATTAA-----------
right   AACTAAAGAAAA-----TAGTTAGAGAAAGTACTTTATGTATAAATTTGGATTAA-----------
        *****222222222222*2*2*222*222***2*2122*2*2222**2***222222222222222
```

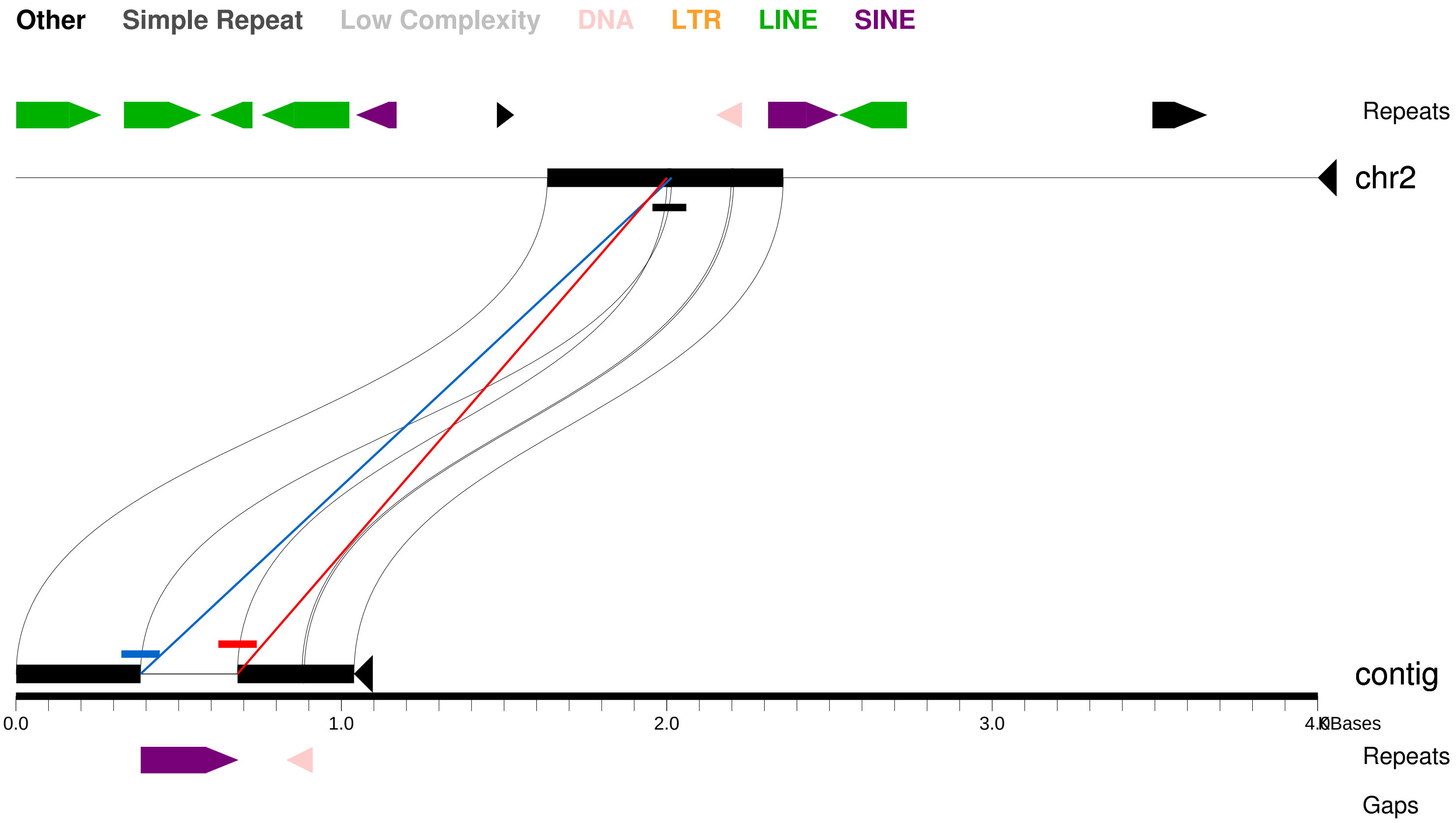

siteID: chr2_212343412 Scafftig2 + Breakpoint: chr2:212343411
chr2:212343360-212343470
Overlap Length: 9 INS size: 429 Genotyped: No

```
left    CACTTGAATCTAC-TC----ACTTCTCTCCATCCCTGCTTT---CAACATCCTAATTCAAGCTCTTCTTT
chrom   CACTTGAATCTAC-TC----ACTTCTCTCCATCCCTGCTTT---CAACATCCTAATTCAAGCTCTTCTAC
right   CACCCGCCTCGGCCTCCCAAAGTGCTGGGATTACAGGCGTGAGCCACCGCGCCCGGCCCAGCTCTTCTAC
        ***11*11**11*1**1111*1*1**11111*1*11**1*1111**1*111*11111*1*********22

left    TTTNNNNNNNNNNNNNNNNNNNNNNNNNNNNNNNNNNNNNNNNNNNNNNNNNNNNNNNN
chrom   TT--------CTTGGGTGGAATACAACATATCCCCTAATTCTTCACAGCACCATCTT
right   TT--------CTTGGGTGGAATACAACATATCCCCTAATTCTTCACAGCACCATCTT
        **22222222222222222222222222222222222222222222222222222222
```

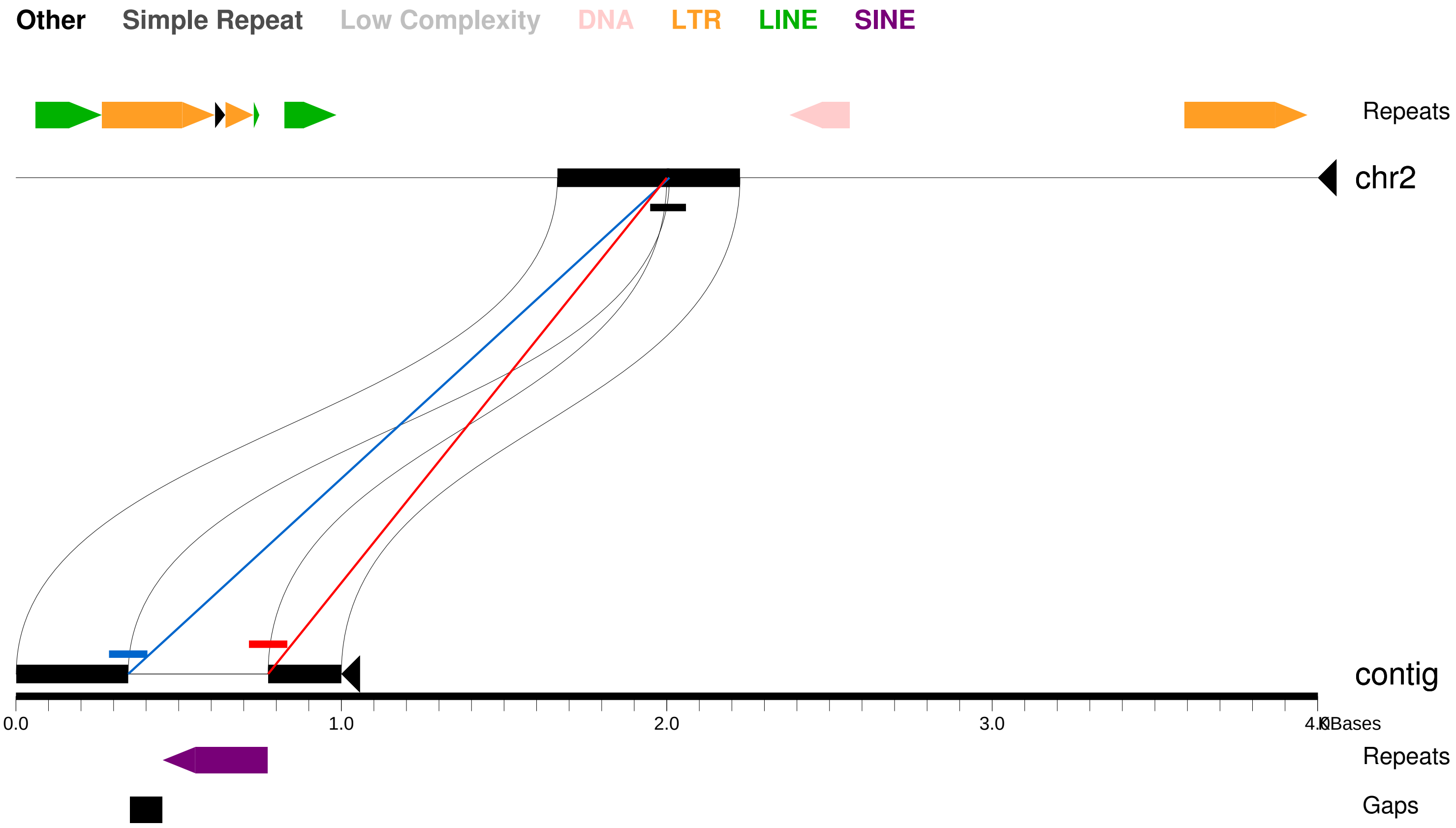

siteID: chr2_212708353 Scafftig4 - Breakpoint: chr2:212708333
chr2:212708297-212708392
Overlap Length: 24 INS size: 291 Genotyped: Yes

```
left    ----------CTAAACAAGTGATTGGATGGCAG-------------CAAAGCAGATTTTAAAAATGTTTT
chrom   ----------CTAAACAAGTGATTGGATGGCAG-------------CAAAGGAGATTTTAAAAATGTTTT
right   GATTGTGCCACTGCACTCCAGCCTGGGCGACAGAGCGAGACTCCGTCTCAAAAAAAAAAAAAAATGTTTT
        1111111111**11**1111*11***11*1***1111111111111*11*1N*1*1111***********

left    TCTAGACATCCTTGGCCGGGCGCGGTGGCTCACGCCTGTAATCCCAGCACTTTGGGAGGCCGAGGCGGGC
chrom   TCTAGACATCCTTAA----GCTAGTT---TTATGT---TTATCTTAG---TTTCGTT--CTGTG------
right   TCTAGACATCCTTAA----GCTAGTT---TTATGT---TTATCTTAG---TTTCGTT--CTGTG------
        *************222222**22*2*222*2*2*2222*2***22**222***2*2222*2*2*222222

left    GG
chrom   --
right   --
        22
```

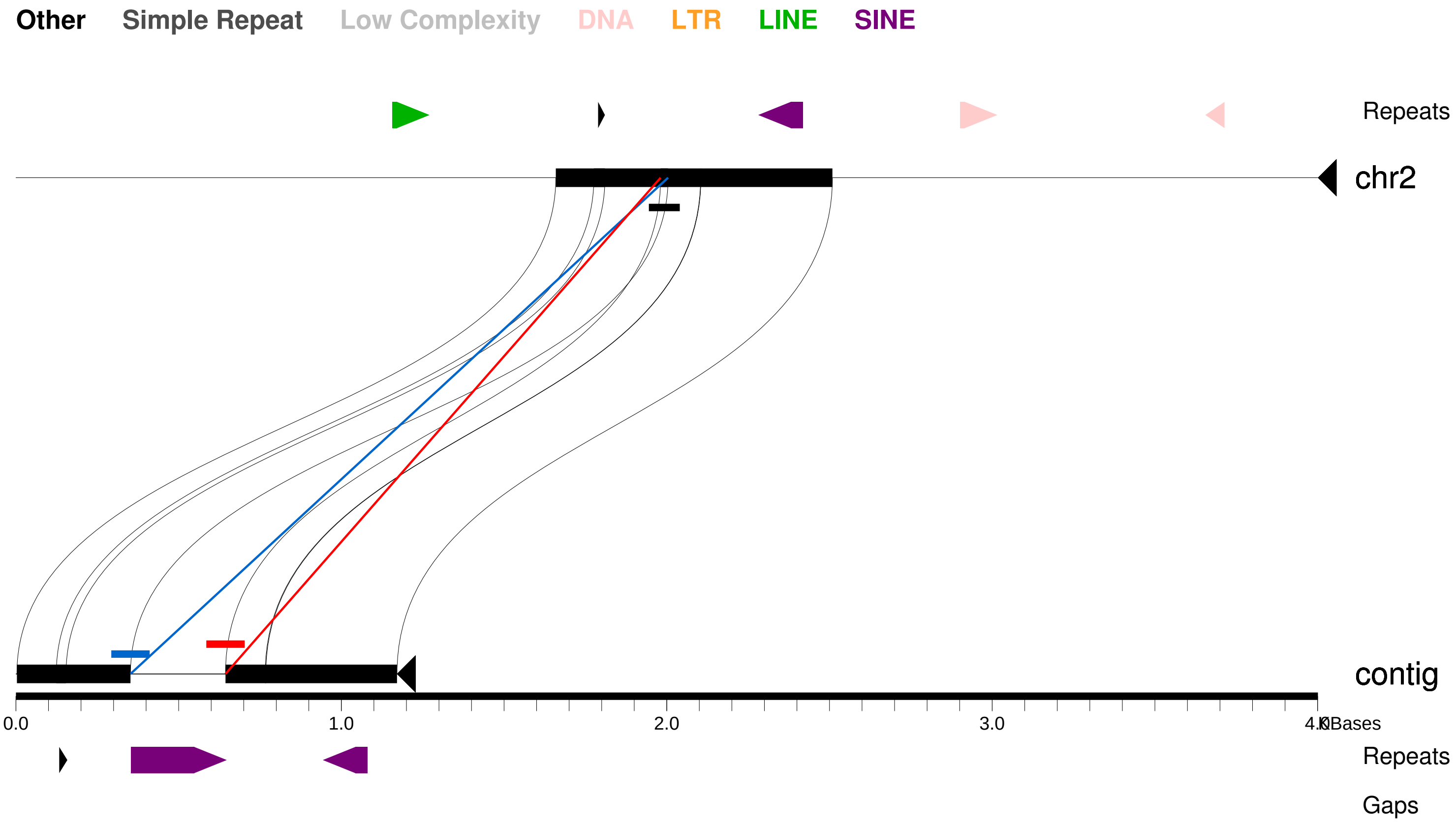

siteID: chr2_212925330 Scafftig1 - Breakpoint: chr2:212925313
chr2:212925153-212925484
Overlap Length: 12 INS size: 327 Genotyped: Yes

```
left   A-----------CCCAGCCATCCCATTACTGGTTATATACCCAAAGGATTATAAATCATGCTGCTATAAA
chrom  AATACCATTTGACCCAGCCATCCCATTACTGGTTATATACCCAAAGGATTATAAATCATGCTGCTATAAA
right  AGAATGGCGTGAACCCGGGAGGC-------GGAGCT-TGC---AGTGAGCCGAGATCCCGCCACTG----
       *NN2NNNNN2221**1*11*11*1111111**111*1*1*111*11**1111*1***11**11**11111

left   GACACATGCACACGTATGTTTATTGCGGCACTAT-TCACAATAGCAAAGACTTGGAACCCACCCCAACGT
chrom  GACACATGCACACGTATGTTTATTGCGGCACTAT-TCACAATAGCAAAGACTTGGAACCCACCCCAACGT
right  --CAC-TCCAGCCTGGGCGACAGAGCGAGACTCCGTCTCAAAAAAAAAAAAAAAAAAAAAAAAAAAA---
       11***1*1**11*11111111*11***11***111**1***1*11***1*11111**111*1111**111

left   CCATCAATGATAGACTGGATTAAGAGTATGTGGGCCGGG--CGCGGTGGCTCAC------GCCTGTAA--
chrom  CCATCAATGATAGACTGGATTAAGAGTATGTGGCACATATACACCATGCAATACTATGCAGCCATAAAAA
right  --AAAAAAAAAAAAAAAAAAAAAGAGTATGTGGCACATATACACCATGCAATACTATGCAGCCATAAAAA
       11*11**11*1*1*1111*11************22*22222*2*22**2222**222222***222**22

left   --------TCCCAGCACTTTGGGAGGCCGAGGCGGGCGGATCACGAGGTCAGGAGATCGAG---ACCATC
chrom  AGAATGAGTTCGTGTCCTTTGT-AGG--GACATGGATGAATCTGGAAACCATCATTCTCAGCAAATTATC
right  AGAATGAGTTCGTGTCCTTTGT-AGG--GACATGGATGAATCTGGAAACCATCATTCTCAGCAAATTATC
       22222222*2*22*22*****22***22**222**22*2***22**222**22*22222**222*22***

left   CC--GGCTAAAATGGTGAA-ACCCC---GTCTC--TACTA--------------AA
chrom  TCAAGGATAAAAAACCAAACACCACATGGTCTCACTCATAGATGGGAATTGAACAA
right  TCAAGGATAAAAAACCAAACACCACAT-----------------------------
       2*22**2*****22222**2***2*22 11111  1NN11              11
```

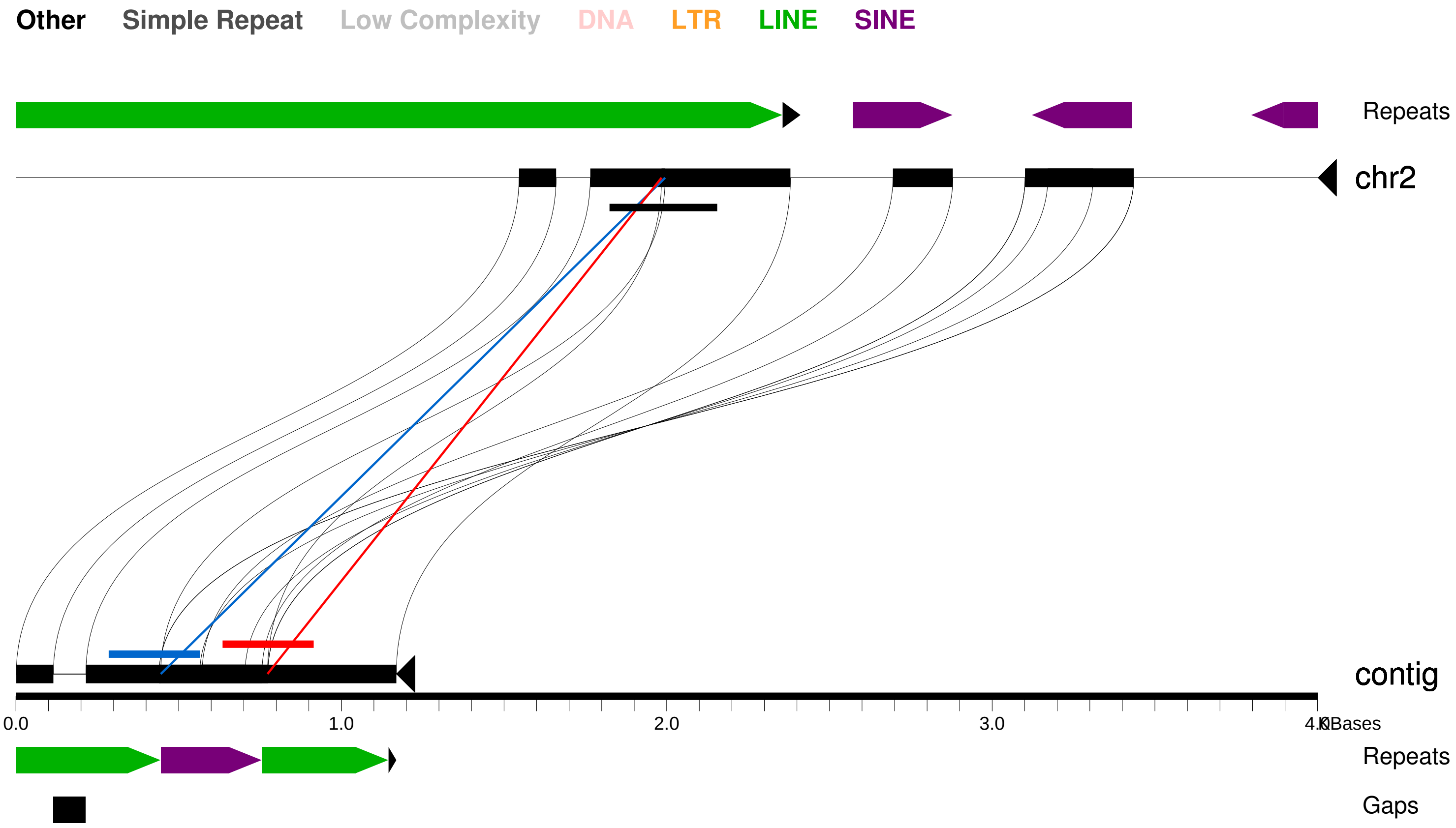

siteID: chr2_213029685 Scafftig1 + Breakpoint: chr2:213029687
chr2:213029642-213029746
Overlap Length: 15 INS size: 364 Genotyped: No

```
left     --GAACTACTTGTCCTTA--------TGG----ACAAACTCGACTTTGAAGAAATTTTTCTTTTTCGCAT
chrom    --GAACTACTTGCCCTTA--------TGG----ACAAACTTGACTTTGAAGAAATTTTTCTTTTTCGCAT
right    CCGCCCGCCTCGGCCTCCCAAAGTGCTGGGATTACAGGCGTGAGCCACCGCGCCCGGCCCTTTTTCGCAT
         11*11*11**1*N***1111111111***1111***11*12**1111111111111111***********

left     TTTTTTTTTNNNNNNNNNNNNNNNNNNNNNNNNNNNNNNNNNNNNNNNNNNNNNNNNNNNNN
chrom    TTTTAATT--------------GTCAAGTAACTTCAAAGAAAACATGAAGAAAATAAAAGACC
right    TTTTAATT--------------GTCAAGTAACTTCAAAGAAAACATGAAGAAAATAAAAGACC
         ****22**2222222222222222222222222222222222222222222222222222222
```

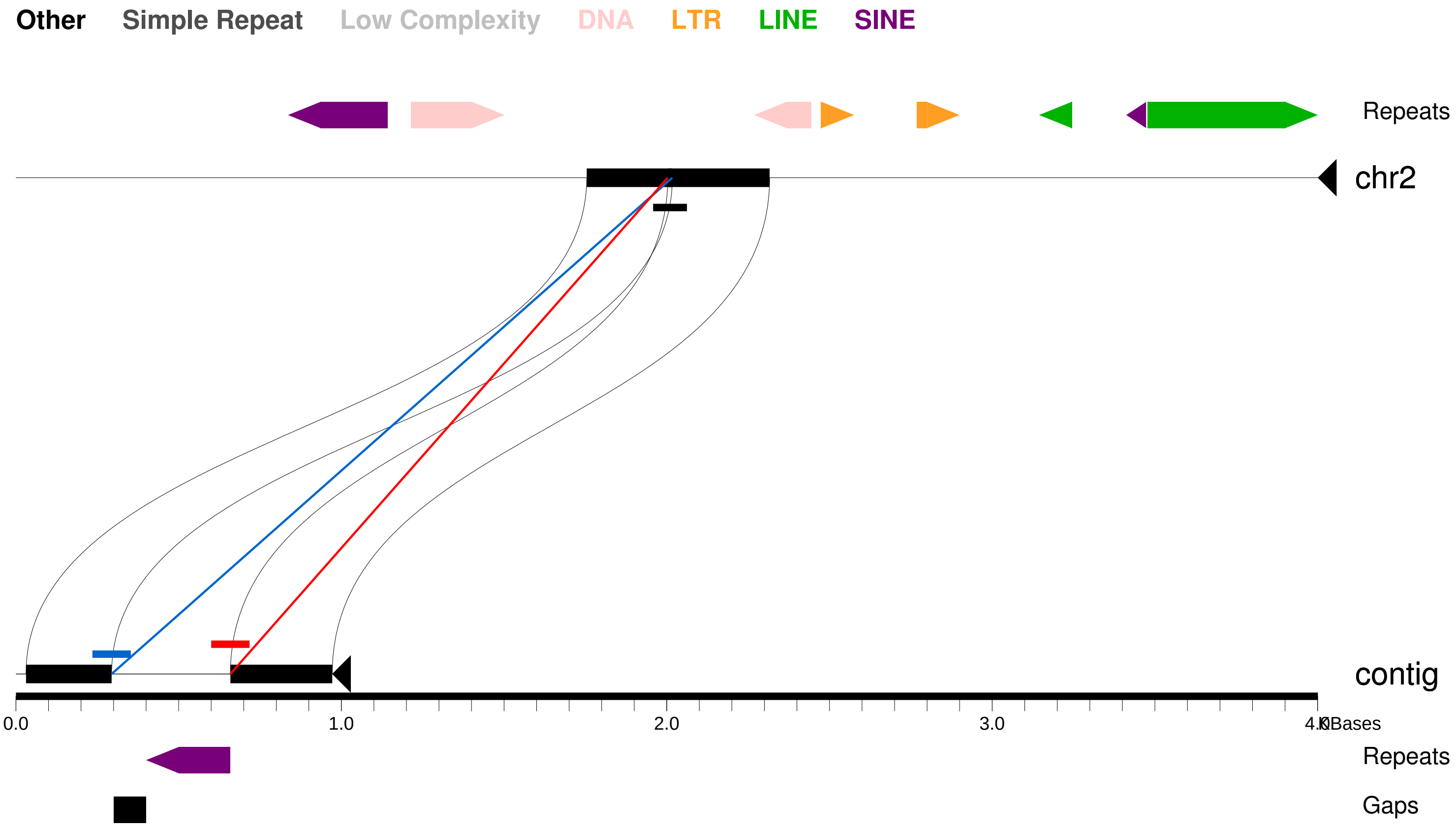

siteID: chr2_213168126 Scafftig2 + Breakpoint: chr2:213168117
chr2:213167957-213168293
Overlap Length: 17 INS size: 316 Genotyped: Yes

```
left    ----------------GATTAGGGAGCTTCAGACAGGAGGGATGTTCAGGATTTCTCCCTATTTCACTTT
chrom   CCTTCAGTGCTTAATGGATTAGGGAGCTTCAGACAGGAGGGATGTTCAGGATTTCTCCCTATTTCACTTT
right   --TTTAGTAGAGACGGGGTTTCACCGTGTTAGCCAGGATGG----TCTCGAT--CTCCTGACCTCG--TG
          22N222NNNN2NN2*1**11111*11*1**1*****1**1111**11***11****11*11**111*1

left    ATTGACAAAACACTCCAATTATGTATATTTGATTATACCCTCTGCTAGATCATGTCAGTATAGGAATGGA
chrom   ATTGACAAAACACTCCAATTATGTATATTTGATTATACCCTCTGCTAGATCATGTCAGTATAGGAATGGA
right   ATCCGC--------CCGCCTCGG---------------CCTCCCAAAGTGC-TGGGATTACAGGCGTGAG
        **111*11111111**111*11*111111111111111****1111**11*1**11*1**1***11**11

left    TCTTTATCCACAGCTATAAAGACATTTATTACTTTCTT------TTTTTTT-----------------TT
chrom   TCTTTATCCACAGCTATAAAGACATTTATTACTTTCTAACAGACTTAAGTTCACAAATTGGGAGGAACTT
right   CCACCGCGCCCGGCC-----GACATTTATTACTTTCTAACAGACTTAAGTTCACAAATTGGGAGGAACTT
        1*111111*1*1**111111****************2222222**222**22222222222222222**

left    TGTTTTTTTT---TTTGTTTTTTTT---TTTTGAGA-CGGAGTCTCGCTGTC--GCCCAGGCTGGAG--T
chrom   TGTTCTACTGATATTTGATCCTTGTAAATCCTGAGAACAGACTCTATTTATCTAGCACGTGTTTAATACT
right   TGTTCTACTGATATTTGATCCTTGTAAATCCTGAGAACAGACTCTATTTATCTAGCACGTGTTTAATACT
        ****2*22*2222****2*22**2*222*22*****2*2**2***222*2**22**2*22*2*22*222*

left    GCAGTGGCGC---AATCTCGGCTCACTGCAGGCTCCGCCCCCTGGGGTTCACGC-----
chrom   GCTTAAGCCCTGAAAGTTGTAAAAATTGGAAGTA--GTCTTATGGAAGTCTTCCTGAAT
right   GCTTAAGCCCTGAAAGTTGTAAAAATTGGAAGTA--GTCTTA-----------------
        **2222**2*222**22*222222*2**2*2*2222*2*222111NNN11NNN1
```

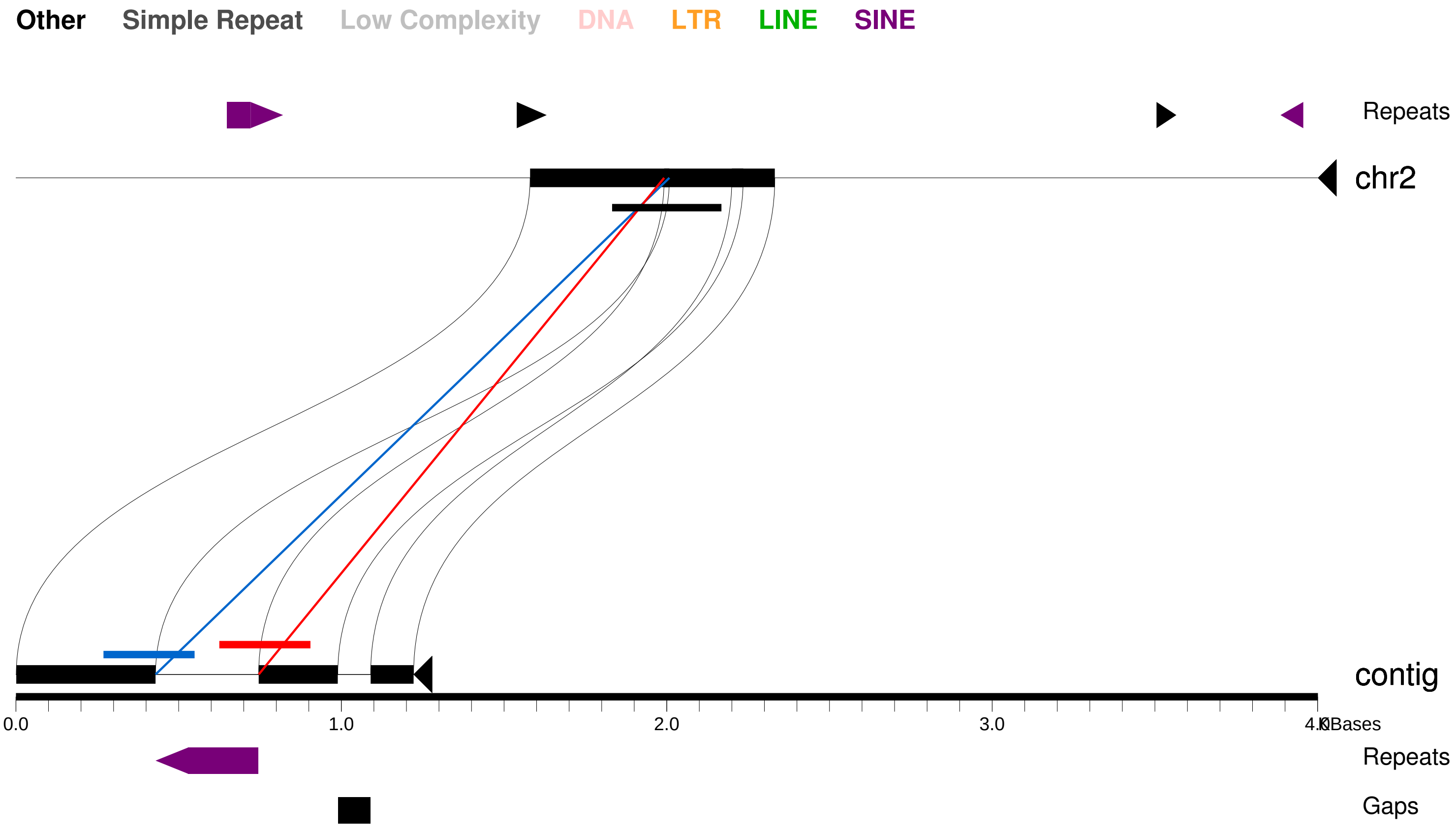

siteID: chr2_21381163 Scafftig3 - Breakpoint: chr2:21381166
chr2:21381121-21381225
Overlap Length: 15 INS size: 304 Genotyped: Yes

```
left    AAAGGGTGAATTTTAT----GGCAGGTGAATGACAGT-----------AAAGGGGTTATTAAAAATTACA
chrom   AAAGGGTGAATTTTAT----GGCAGGTGAATGACAGT-----------AAAGGGGTTATTAAAAATTACA
right   CA-GTCCGCAGTCCGGCCTGGGCAACAGAGCGAGACTCCGTCTCAAAAAAAAAAAAAAAAAAAAATTACA
        1*1*111*1*1*11111111****111**11**1*1*11111111111***111111*11**********

left    TGTATGGCCGGGCGCGGTGGCTCACGCCTGTAATCCCAGCACTTTGGGAGGCCGAGGCGGGTGG-
chrom   TGTAT------------TATACTATGAATTCA--CCTAAGACTAAAG-ATGAAAAGAAGAATGTA
right   TGTAT------------TATACTATGAATTCA--CCTAAGACTAAAG-ATGAAAAGAAGAATGTA
        *****222222222222*22222*2*22*22*22**2*22***222*2*2*222**22*22**22
```

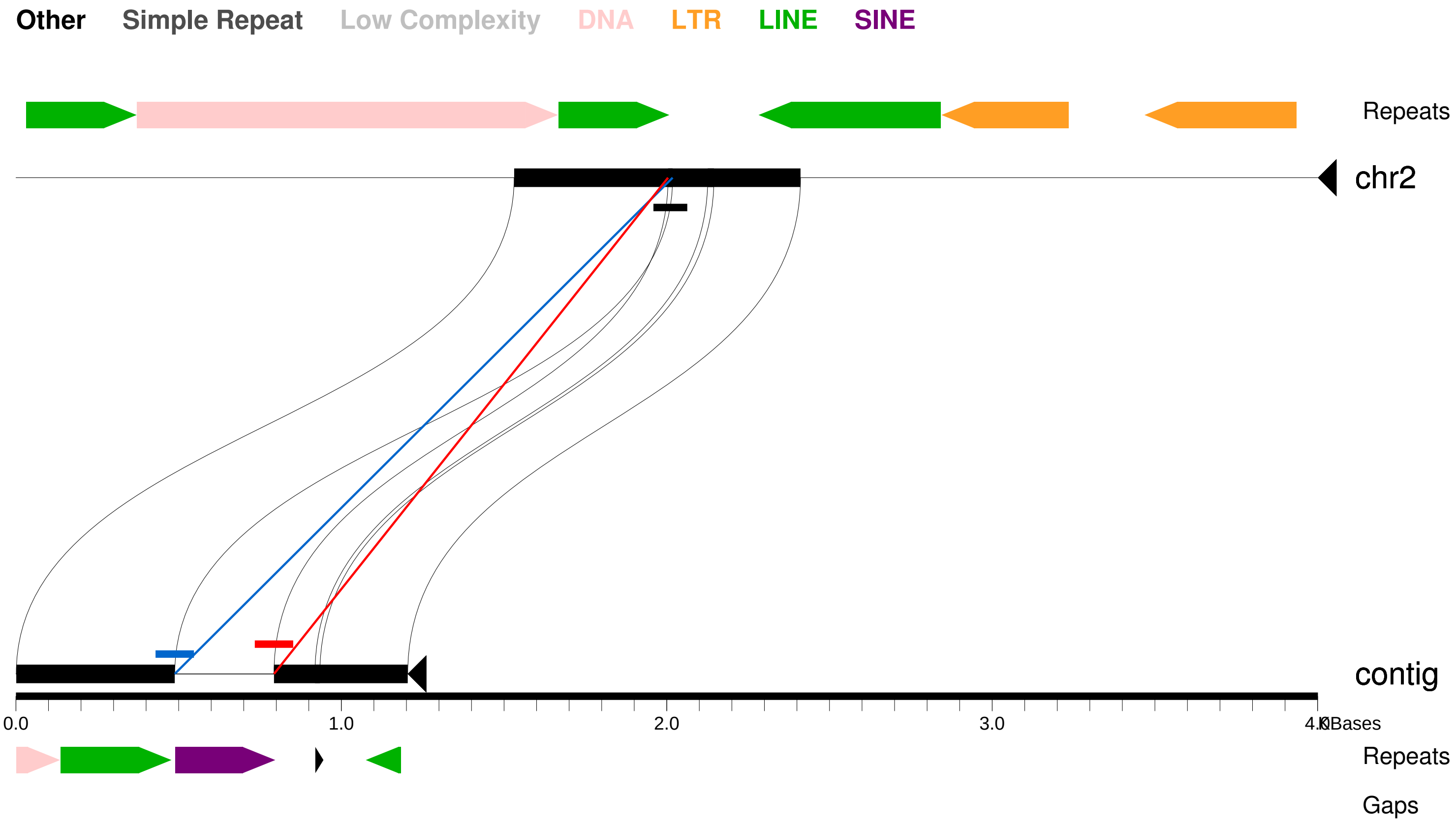

siteID: chr2_214512316 Scafftig2 + Breakpoint: chr2:214512283
chr2:214512123-214512450
Overlap Length: 8 INS size: 304 Genotyped: No

```
left   --------ACCTTTCTTTTGAGCTTCATTAGGAGCATGCTATTATTTTATTTTGCTCATTCTTTAAGACT
chrom  CTTTC---ACCTTTCTTTTGAGCTTCATTAGGAGCATGCTATTATTTTATTTTGCTCATTCTTTAAGACT
right  TTTAGTAGAGACGGGGTTTCACCGTGTTAGCCAGGATGGTCTCGATCTCCTGACCTCGT-------GA-T
       N22NN111*1111111***1*1*1*11*1111**1***1*1*111*1*11*111***1*1111111**1*

left   CAGGTCCCGCTGCTGGCATACAGTGATTCACAGAGTAGGGGATGGTGTGTTTCCTCTGCTCCATTCGGTC
chrom  CAGGTCCCGCTGCTGGCATACAGTGATTCACAGAGTAGGGGATGGTGTGTTTCCTCTGCTCCATTCGGTC
right  CCG--CCCGCCTCGGCCTCCCAAAG-TGCTGGGATTACAGGC--GTGAG---CCACCGCGCC---CGGCC
       *1*11*****11*1*1*111**11*1*1*111**1**11**111***1*111**1*1**1**111***1*

left   TTTGTTTTCACAACAGGCAGATTAGAAGTTTTTTTTTTT-----------------TTTTT--------
chrom  TTTGTTTTCACAACAGGCAGATTAGAAGTTTCTTATTGAAGCCCCAGAAGGAAACCATCTCTGCTCGCCC
right  -----------------------AGAAGTTTCTTATTGAAGCCCCAGAAGGAAACCATCTCTGCTCGCCC
       11111111111111111111111*******2**2**2222222222222222222222*2*2*22222222

left   -TTTTTTTTTTGAGACGGAGTCTCGCTC-TGTCGCCCAGGCTGGAG------TGCAGTGGCGCAATCTCG
chrom  ATCTTTTTGTAGTCTCCCAGCCTCAGTCATGCAGAGAAACTTGAAGCTTCTCTTCAAGCTCTGAAGGTCA
right  ATCTTTTTGTAGTCTCCCAGCCTCAGTCATGCAGNNNNNNNNNNNNNNNNNNNNNNNNNNNNNNNNNNNN
       2*2*****2*2*222*22**2***22**2**22*NNN1NNN11N11NNNNNN1N11NNNN1NN11NN11N

left   GCTC-ACTGCAAGCTCC-GCCTCTCGGGTTCACG---CCATTCTCCT---G
chrom  GCCAGACCAGAAACTAATGTGACATAGAGTCTCTTTTCCAATCTCTGTAGG
right  NNNNNNNNNNNNNNNNNNNNNNNNNNNNNNNNNNNNNNNNNNNNN--------
       11NNN11NNN11N11NNN1NNN1NNN1NN11N1NNNN111N1111NN   1
```

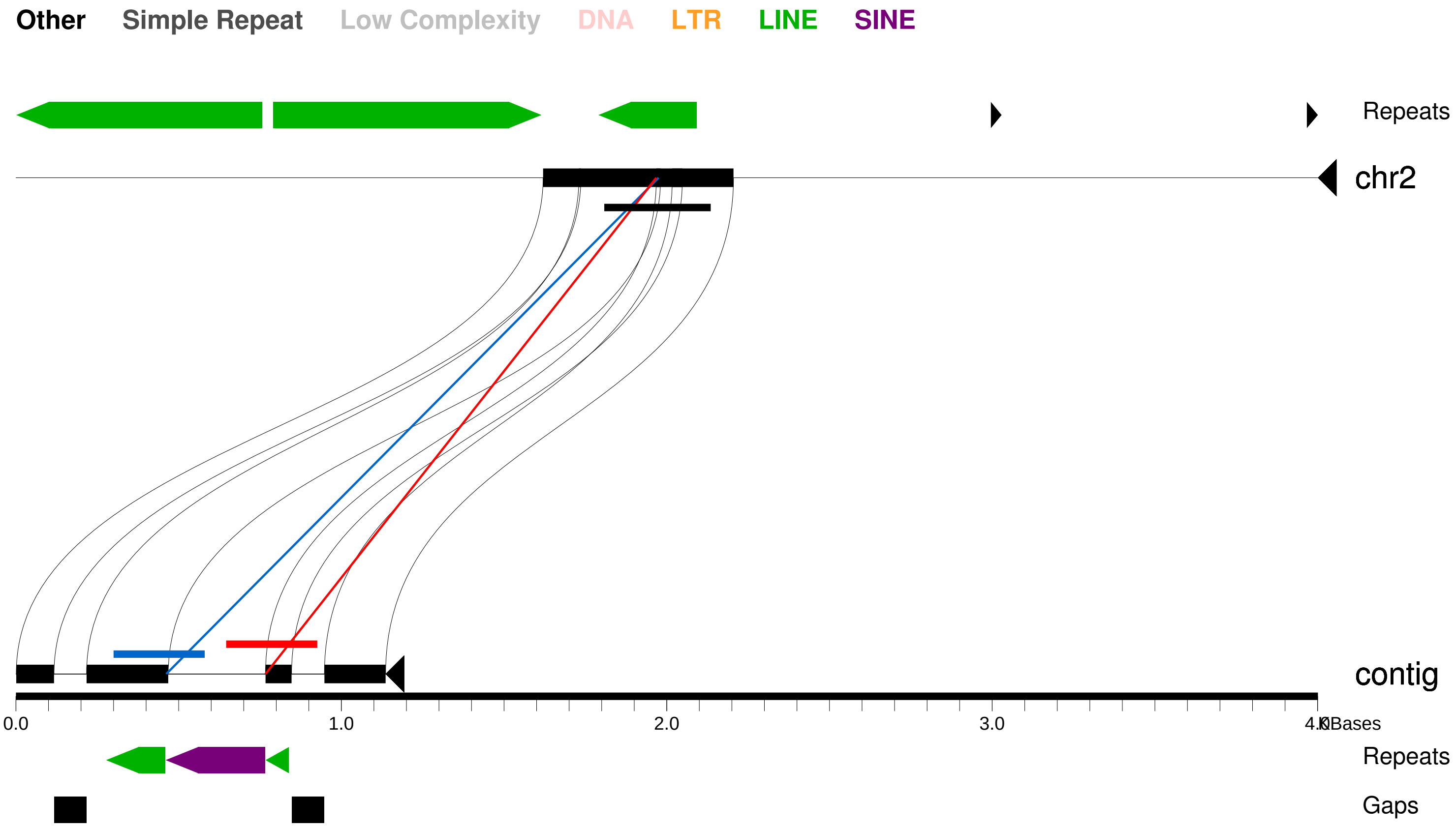

siteID: chr2_215201628 Scafftig7 + Breakpoint: chr2:215201632
chr2:215201473-215201801
Overlap Length: 10 INS size: 403 Genotyped: No

```
left    --------AAATGCATAGAAATAGAGTGTTTTTATTGTAAGCTTTTACTTAAAGAAAGTAATGTAATGAT
chrom   GGAATATCAAATGCATAGAAATAGAGTGTTTTTGTTGTAAGCTTTTACTTAAAGAAAGTAATGTAATGAT
right   NNNNNNNNNNNA----------------------------------------------------------
        NNNNNNNN111111111111111111111111N11111111111111111111111111111111111111

left    ATATCTCTACAAGTAAGATTTAAGGTAGGAGAAAGTTTTTATAAATATTCAGAAAGGATTAATGTTAGAT
chrom   ATATCTCTACAAGTAAGATTTAAGGTAGGAGAAAGTTTTTATAAATATTCAGAAAGGATTAATGTTAGAT
right   ----------------------------------------------------------------------
        1111111111111111111111111111111111111111111111111111111111111111111111

left    ATTAATTTTCTAACAGTATAAAGACTAAG-GCCGGGCGC--------GGTGGCTCACGCCTGTAATCC-C
chrom   ATTAATTTTCTAACAGTATAAAGACTAAGAGCTAGGAGTTCTCTGCTGTTGAATGAGGCTTCTAGACTAC
right   -------------------AAAGACTAAGAGCTAGGAGTTCTCTGCTGTTGAATGAGGCTTCTAGACTAC
        1111111111111111111**********2**22**2*222222222*2**22*2*2**2*2**22*22*

left    AGCACTTTGGGAGGCCGAGGCGGGCGGAT-CATG-----AG-GTCAGGAGATCGAGACCA-----TC-CT
chrom   CTCACTTCTTCCAATATAACTGATCAAAGACAAGTTTACAGAGACAGAAAATTGAAGCAAAACAGTCACT
right   CTCACTTCTTCCAATATAACTGATCAAAGACAAGTTTACAGAGACAGAAAATTGAAGCAAAACAGTCACT
        22*****2222222222*222*22*22*22**2*22222**2*2***2*2**2**22*2*22222**2**

left    GGCTAACAAGG--TGAAACCCCGTCTC----TACTAAA-----------
chrom   TGCCTGCAGGTACTTGACGCCAGTCCCCCATTTCTGAAAGAACAACCTT
right   TGCCTGCAGGTAC------------------------------------
        2**222**2*2221NN1NN11N111N1    1N11N11
```

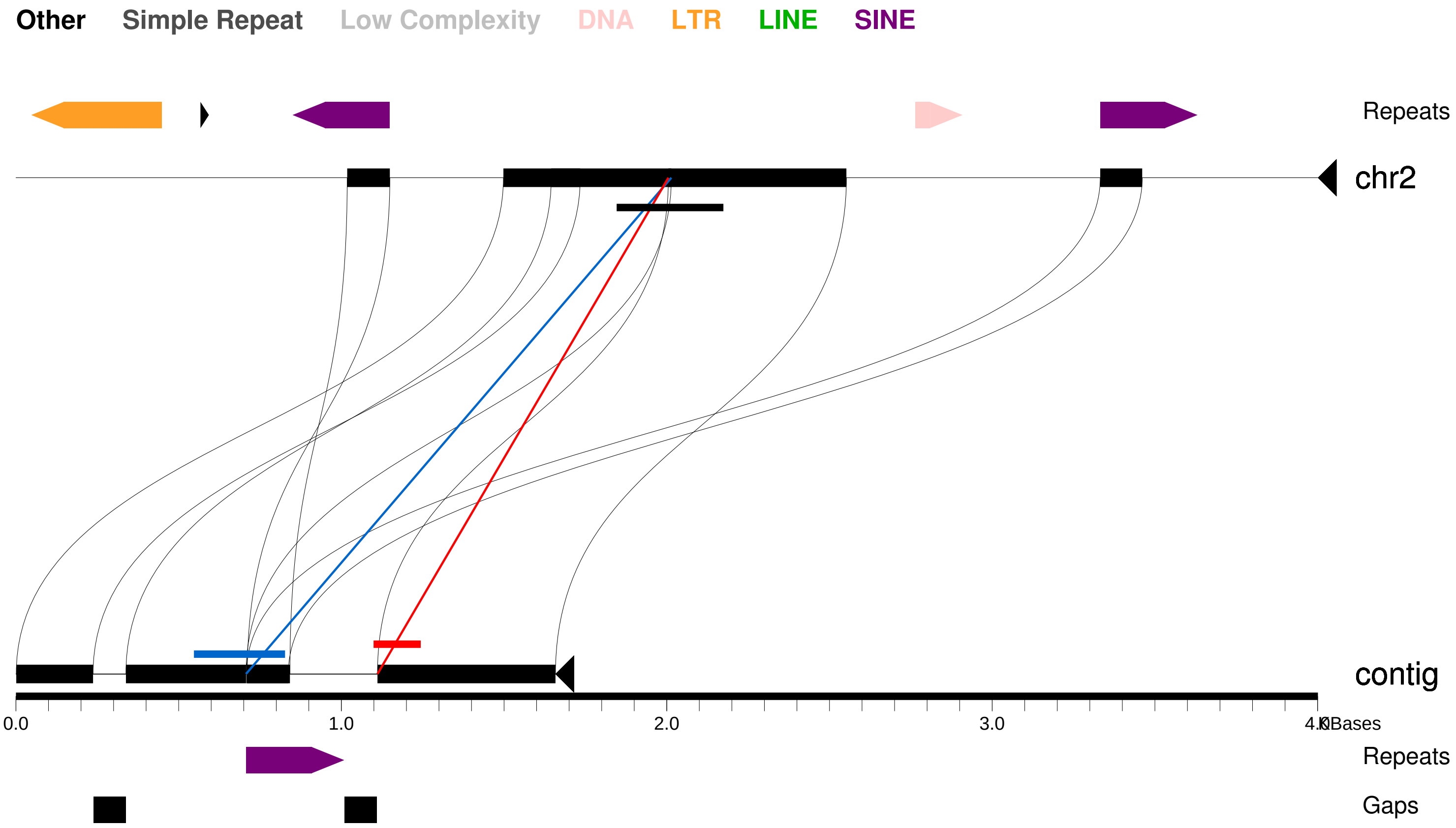

siteID: chr2_216548440 Scafftig1 + Breakpoint: chr2:216548419
chr2:216548261-216548595
Overlap Length: 17 INS size: 453 Genotyped: No

```
left   ---------------GGCAGAAATGGGAAGAAGCATTTCCAGAACACAGCTGAACAACCAAACCATCATC
chrom  CCTGTTGTCTGGACTGGCAGAAATGGGAAGAAGCATTTCCAGAACACAGCTGAACAACCAAACCATCATC
right  NNNNNNNNNNN-----------------------------------------------------------
       NNNNNNNNNNN    1111111111111111111111111111111111111111111111111111111

left   TATGTTCAAAGGGCTTCTTCTTAAATGCATTTCAAATCGATTTGCAAATTTTAAACAATGTGTCTTTAGT
chrom  TATGTTCAAAGGGCTTCTTCTTAAATGCATTTCAAATCGATTTGCAAATTTTAAACAATGTGTCTTTAGT
right  ----------------------------------------------------------------------
       1111111111111111111111111111111111111111111111111111111111111111111111

left   TTAAAAGCTCTAAATATTAAAAAATCAGTAAATTCGGCCGGGCGCGGTGGCTCACGC-CTGTAATCC---
chrom  TTAAAAGCTCTAAATATTAAAAAATCAGTAAATTCT-CCTTTAGCACTGCCCCACCCTCTGCCATTCTTC
right  --AAAA------------AAAAAATCAGTAAATTCT-CCTTTAGCACTGCCCCACCCTCTGCCATTCTTC
       11****111111111111*****************22**2222**22**2*2***2*2***22**2*222

left   -------CAGCACTTTGGGAGGCCGAGGCGGGTGGATCA------------TGAGGTC------AGGAGA
chrom  TCTTTCTCAGCACAATGGCATTACTGTTCTAACCACTCAATATTCAATATCTTATGTACTTTTAAGGAAA
right  TCTTTCTCAGCACAATGGCATTACTGTTCTAACCACTCAATATTCAATA---------------------
       2222222******22***2*222*2222*2222222***2222222222  1N1N11N      1111N1

left   TCGAGACCATCCTGGCTAACA-----------AGGTGAA--ACCCCGTCTCTACTAAA
chrom  TCTTAGGCA--CAAGAAAAAAGTTTATTTGTGAGGTGAGTTAACTCTTGTTTAAAAAA
right  ----------------------------------------------------------
       11NNNNN11221NN1NN11N1           111111N  1N1N1N1N1N11NN111
```

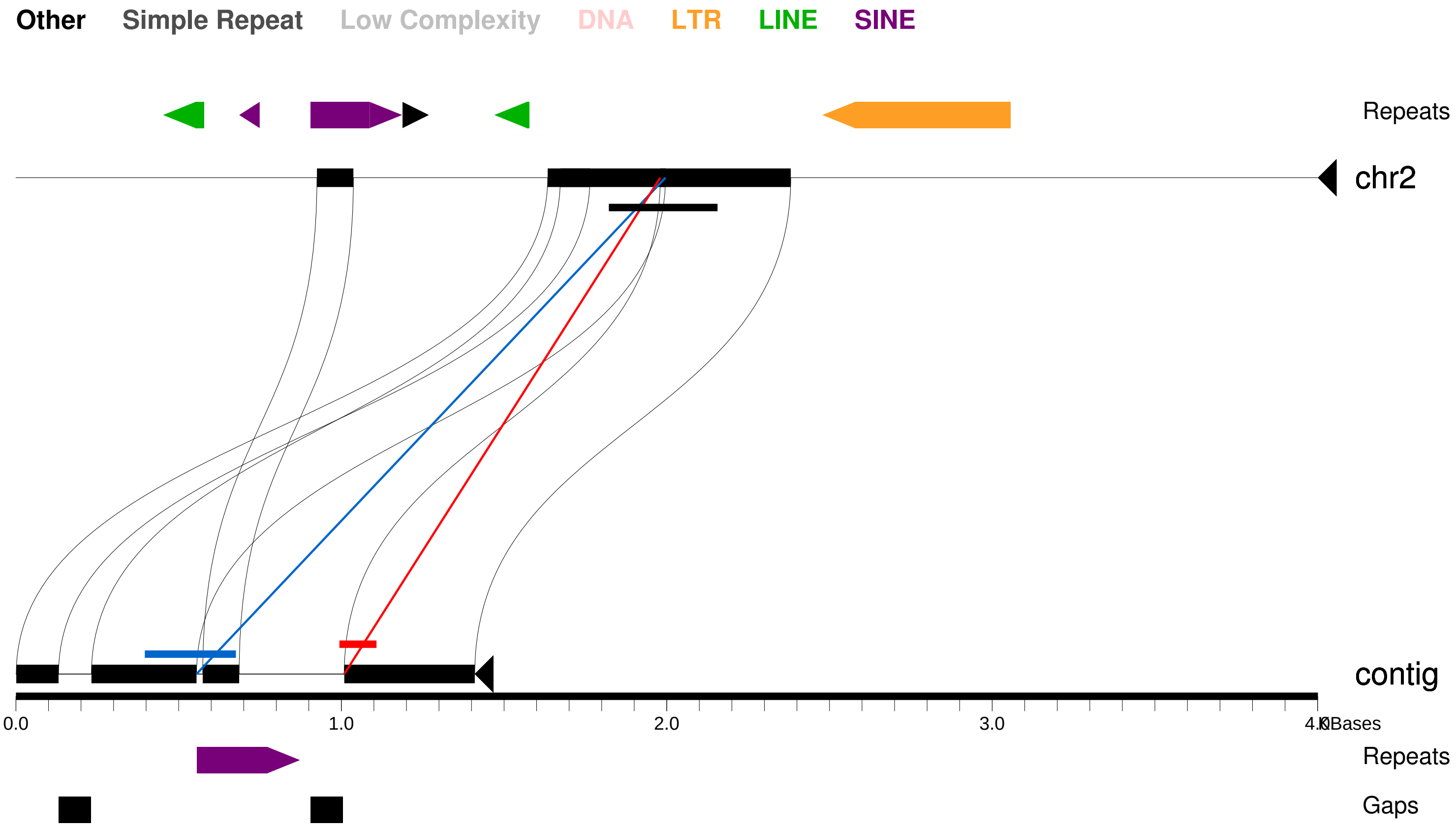

siteID: chr2_21796830 Scafftig13 - Breakpoint: chr2:21796814
chr2:21796768-21796870
Overlap Length: 14 INS size: 283 Genotyped: Yes

```
left    -----------GCAGGAAAGTCAAGA-GGCAGA--GAAGCACTTACTCACTGTAGTTAAA--AGAATAGC
chrom   -----------GCAGTAAAGTCAAGA-GGCAGA--GAAGCACTTACTCACTGTAGTTAAA--AGAATAGC
right   ATCGCGCCACAGCACTCCCGCCTGGGCGACAGAACGAGACTCCGTCTAAAAAAAAAAAAAAAAGAATAGC
        11111111111***12111*1*11*11*1****11**11*1*111****1111*111***11********

left    ATTCCTGGCCGGGCGCGGTGGCTCACGCCTGTAATCCCAGCACTTTGGGAGGCCGAGGCGGGCGG
chrom   ATTCCT------------TTCCTTAGTCCTTTCTTTCTGTC---TTTGCTTGCTGAAATATCCT-
right   ATTCCT------------TTCCTTAGTCCTTTCTTTCTGTC---TTTGCTTGCTGAAATATCCT-
        ******222222222222*22**2*22***2*22*2*222*222**2*222**2**222222*22
```

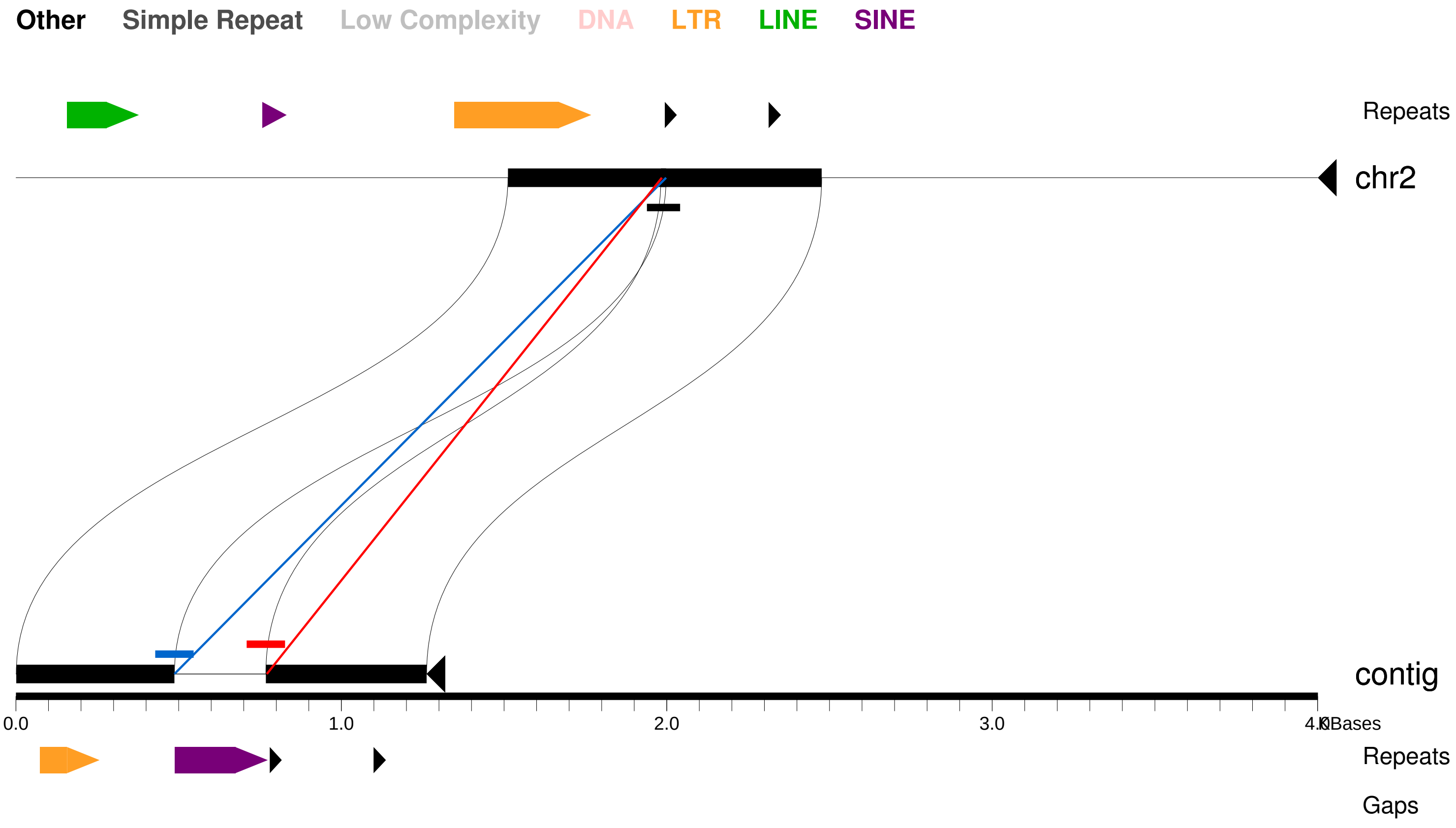

siteID: chr2_218180779 Scafftig11 + Breakpoint: chr2:218180758
chr2:218180598-218180932
Overlap Length: 15 INS size: 295 Genotyped: Yes

```
left   ---------------CAATCCTTTCATGTGACCTGATTCTTCCTGGGCACAGGACAAGAAAAGAATTCAA
chrom  GCATACACCACCCTTCAATCCTTTCATGTGACCTGATTCTTCCTGGGCACAGGACAAGAAAAGAATTCAA
right  ------------CGTGAACCC------GGGAAGCGGAGCTTGCAGTGAGCCG---------AGATTGCGC
                   2N21**1**111111*1**111*111***1*1*1*11*1*111111111***1*1*11

left   GATGCACTGGGTGCAGGAACCCAGAAAGGCTGTCACACTGACTCTTCAGTGAGTTGTTTAACACTGAAGC
chrom  GATGCACTGGGTGCAGGAACCCAGAAAGGCTGTCACACTGACTCTTCAGTGAGTTGTTTAACACTGAAGC
right  ----CACTGCAGTCCGCAGTCCGACCTGGGCGACAGAGCGAGACTCC--------GTCTCAAAAAAAA--
       1111*****1111*1*1*11**11111**11*1**1*11**11**1*11111111**1*1*1*111**11

left   CATCCAGGAATGGCAAAGCTAAAAGAAAGAGCCTT-----TCACGCCTGTAATC-------CCAGCACTT
chrom  CATCCAGGAATGGCAAAGCTAAAAGAAAGAGCCTTGTTTGTAACATATGCTTTCTGGGGCTCCAGAGGTC
right  -----AAAAAAAAAAAAAAAAAAAGAAAGAGCCTTGTTTGTAACATATGCTTTCTGGGGCTCCAGAGGTC
       11111*11**1111***111**************22222*2**222**222**2222222****222*2

left   T--GGGAGGCCGAGGCGGGTGGATCATGA-GGTCAGGAGATCG---AGACCATCCTGGCTAA--------
chrom  TCAGGCAAACCCTAGATGCTGCCACAGGCTGGTACGGGGTTTGTTTATGCCAAA-TGCCCAAAGGTACTC
right  TCAGGCAAACCCTAGATGCTGCCACAGGCTGGTACGGGGTTTGTTTATGCCAAA-TGCCCAAAGGTACTC
       *22**2*22**222*22*2**222**2*22***22**2*2*2*222*22***222**2*2**22222222

left   ---CAAGGTGAA--ACCC-----CGTCTCTACTAAAAATACAAAAAA---TTAGC-
chrom  ACCCCAGCTGCTGCACCCACTCACGTGTGTACTCCTGCTCCCACAAGGGGTTGGAG
right  ACCCCAGCTGCTGCACCCACTCACGTGTGTACTCCTGCTCCCACAAGG--------
       222*2**2**2222****22222***2*2****22222*2*2*2**22  11N1N
```

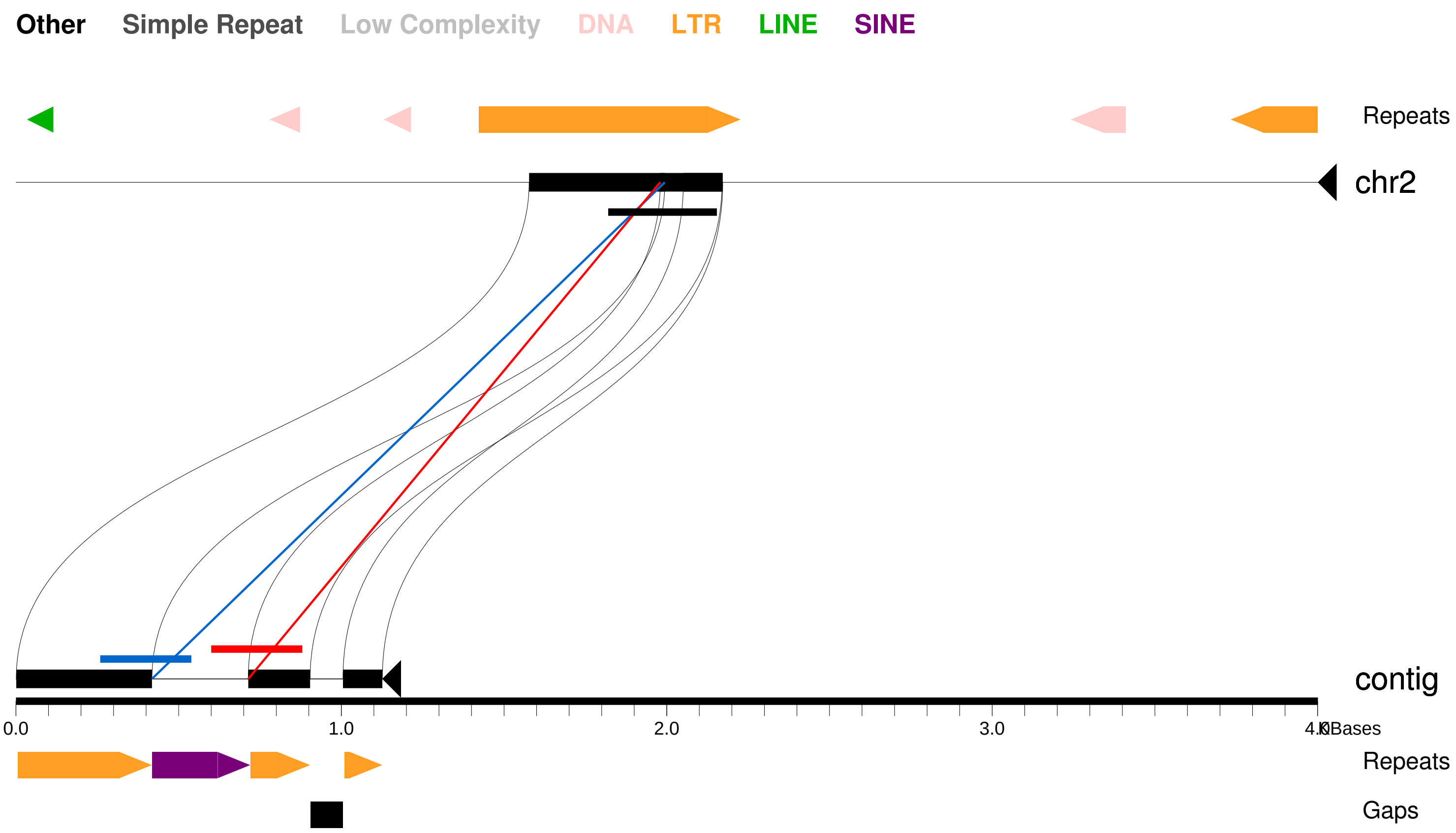

siteID: chr2_218265096 Scafftig6 - Breakpoint: chr2:218265081
chr2:218265032-218265140
Overlap Length: 11 INS size: 296 Genotyped: Yes

```
left    CCATC-CAGCA------AGATCTTGCTTTGCAAATGA---AGTTGCCTTCTTACTGTTTAAGACTGGTGG
chrom   CCATC-CAGCA------AGATCTTGCTTTGCAAATGA---AGTTGCCTTCTTACTGTTTAAGACTGGTGG
right   GCGCCACTGCAGTCCGCAGTCCAGCCTGGGCGACAGAGCGAGACTCCGTCTCAAAAAAAAAGACTGGTGG
        1*11*1*1***111111**11*111**11**1*11**111**111**1***1*111111***********

left    GGCCGGGCGCGGTGGCTCACGCCTGTAATCCCAGCACTTTGGGAGGCCGAGGCGGGTGG
chrom   AA--GTCATTGGTTCCAGAACTAAGAAAGGGAGGAAGTGTGTGGGGCCAAG--------
right   AA--GTCATTGGTTCCAGAACTAAGAAAGGGAGGAAGTGTGTGGGGCCAAG--------
        2222*22222***22*22*22222*2**22222*2*2*2**2*2****2**22222222
```

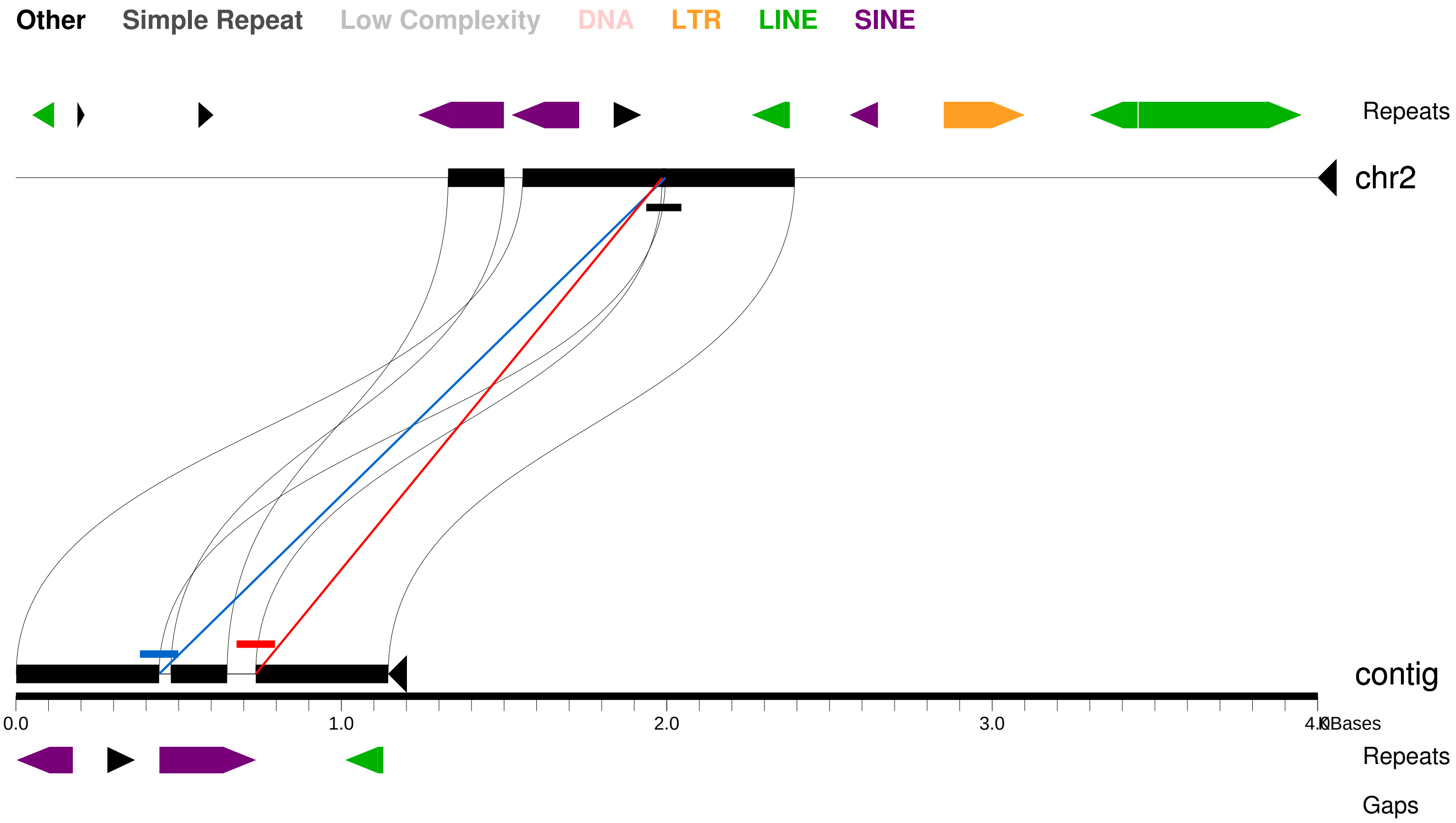

siteID: chr2_219576596 Scafftig1 - Breakpoint: chr2:219576594
chr2:219576538-219576653
Overlap Length: 4 INS size: 435 Genotyped: No

```
left   TCGGAAATATTTTAATCCTTCCTTGATGAAATCTTC-----TTTGCTTTCTCAGATACTACAAATNNNNN
chrom  TCGGAAATATTTTAATCCTTCCTTGATGAAATCTTC-----TTTGCTTTCTCAGATACTACAAATCACCT
right  -CGCCCGCCTCGGCCTCCCAAAGTGCTGGGAT-TACAGGCGTGAGCCACCGCGCCCGGCCTAAATCACCT
       1**111111*11111***11111**1**11**1*1*11111*11**111*1*111111111****22222

left   NNNNNNNNNNNNNNNNNNNNNNNNNNNNNNNNNNNNNNNNNNNNNNNNNNNNN
chrom  ATTCTTTAACTGCAGCACCTGTTGATTTAGCATTTGGGGGACCCTTTGGGA---
right  ATTCTTTAACTGCAGCACCTGTTGATTTAGCATTTGGGGGACCCTTTGGGA---
       22222222222222222222222222222222222222222222222222222
```

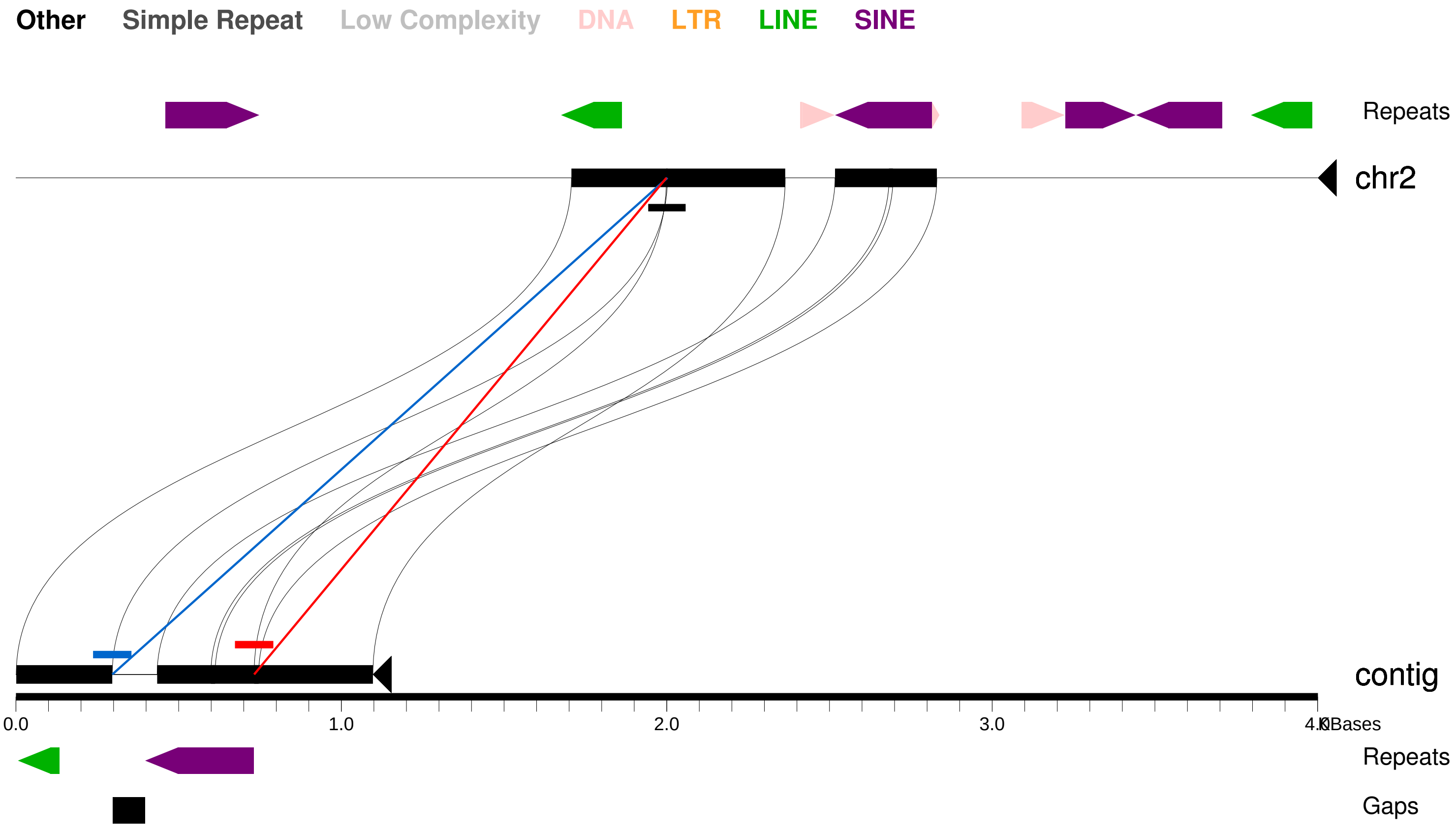

siteID: chr2_223882888 Scafftig3 - Breakpoint: chr2:223882869
chr2:223882825-223882928
Overlap Length: 16 INS size: 289 Genotyped: Yes

```
left     A--AATTCTACTTTAGTTG--GGGCATGACTTGGGC-----------ATCAGGATGTGTAAGAGTTCCTC
chrom    A--AATTCTACTTTAGTTG--GGGCATGACTTGGGC-----------ATCAGGATGTGTAAGAGTTCCTC
right    AGCACTCCCGCCTGGGCGACAGAACGAGACTCCGTCTCAAAAAAAAAAAAAAAAAAAAAAAGAGTTCCTC
         *11*1*1*11*1*11*11111*11*11****11*1*11111111111*11*11*11111***********

left     AGGTGGCCGGGCGCGGTGGCTCACGCCTGTAATCCCAGCACTTTGGGAGGCCGAGGCGGGCGGA
chrom    AGGTGACTGAT-GCATAGGCTG--GGTTGAAAACCG----CTCTGGTGAGTTGAA--------A
right    AGGTGACTGAT-GCATAGGCTG--GGTTGAAAACCG----CTCTGGTGAGTTGAA--------A
         *****2*2*222**222****222*22**2**2**22222**2***222*22**222222222*
```

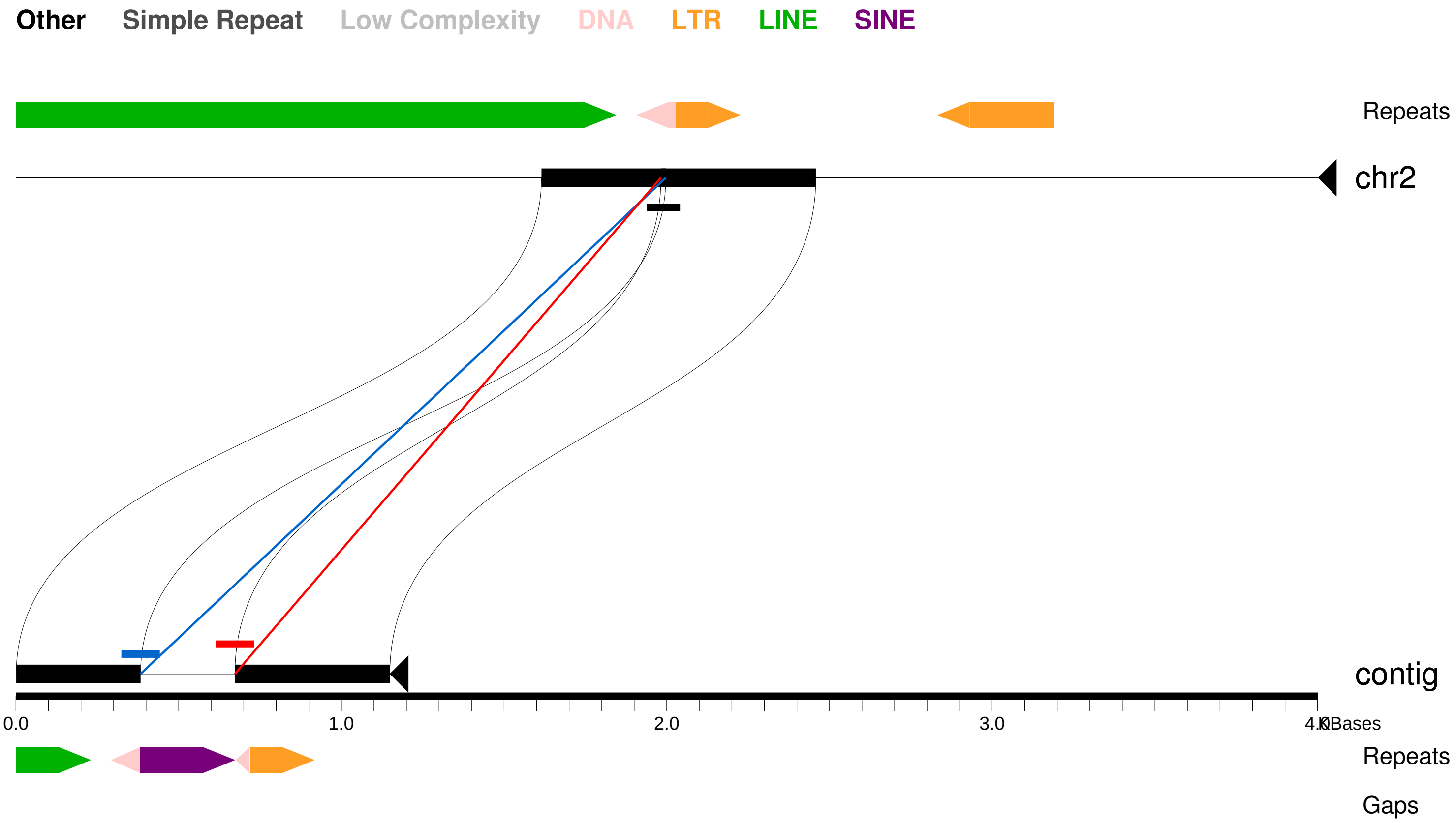

siteID: chr2_225114113 Scafftig2 - Breakpoint: chr2:225114121
chr2:225114072-225114177
Overlap Length: 11 INS size: 307 Genotyped: Yes

```
left    TG----------GATCTTTTGGAAAGATCTTGTGT---TCCAAAGTTGTTTACTGTTCTAAGAACCAAGC
chrom   TG----------GATCTTTTGGAAAGATCTTGTGT---TCCAAAGTTGTTTACTGTTCTAAGAACCAAGC
right   AGTCCGCAGTCCGGCCTGGGCGACAGAGCGAGACTCCGTCTCAAAAAAAAAAAAAAAAAAAAAACCAAGC
        1*1111111111*11**1111**1***1*11*11*111**11**1111111*1111111**1********

left    CAGGCCGGGCGCGGTGGCTCACGCCTGTAATCCCAGCACTTTGGGAGGCCGAGGCGGGTGGA
chrom   CAGT----GTACTGTTGGACACCTCATTTGAC---------TAGTATTCAGATCCCCTACAT
right   CAGT----GTACTGTTGGACACCTCATTTGAC---------TAGTATTCAGATCCCCTACAT
        ***22222*22*2**2*22***22*22*222*222222222*2*2*22*2**22*2222222
```

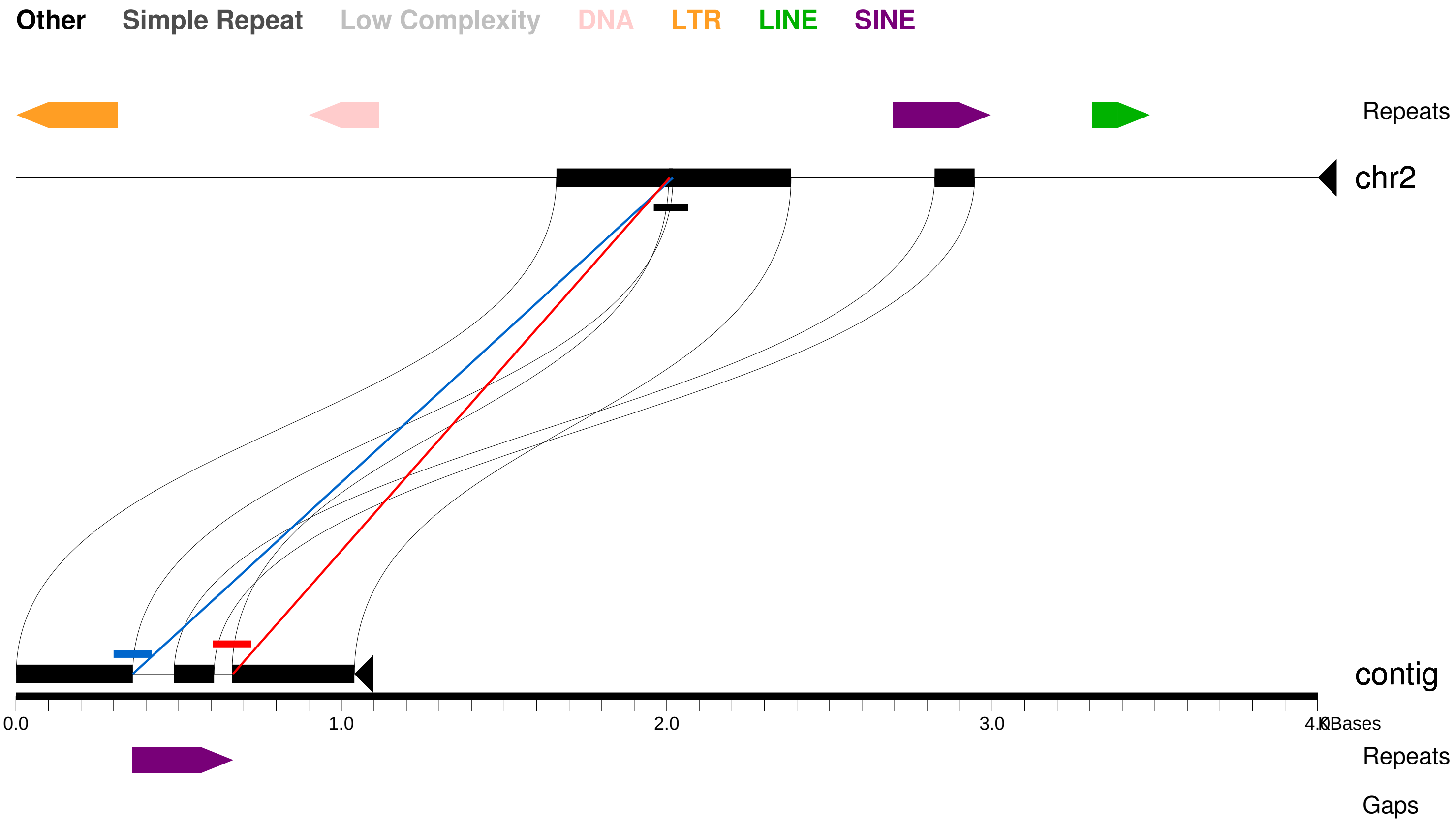

siteID: chr2_225774534 Scafftig8 - Breakpoint: chr2:225774510
chr2:225774465-225774569
Overlap Length: 15 INS size: 84 Genotyped: Yes

```
left     C-ACACAATGTATAAGCAACTGTGTACTCAACTAGAG--------CTTGATGTT-------AACATGCAG
chrom    C-ACACAATGTATAAGCAACTGTGTACTCAACTAGAG--------CTTGATGTT-------AACATGCAG
right    TGAGACAGAGTCTC-GCT-CTGTCGCCCAGGCTGGAGTGCAGTGGCGGGATCTCGGCTCACAACATGCAG
         11*1***11**1*11**11****111*1111**1***11111111*11***1*11111111*********

left     TGTTTTTTTTTTTTGTTTTGTTTTTTGTTTTTTGAGACAGAGTCTCGCTCTGTCGCCCAGGCTGGA
chrom    TGTTTTAATGGATGTGAAAT-------------AAGCCTATGCTTT-TGTGTGGGGAATGTTTAA
right    TGTTTTAATGGATGTGAAAT-------------AAGCCTATGCTTT-TGTGTGGGGAATGTTTAA
         ******22*222***2222*2222222222222*22*22*22**222*2***2*222*2*2*22*
```

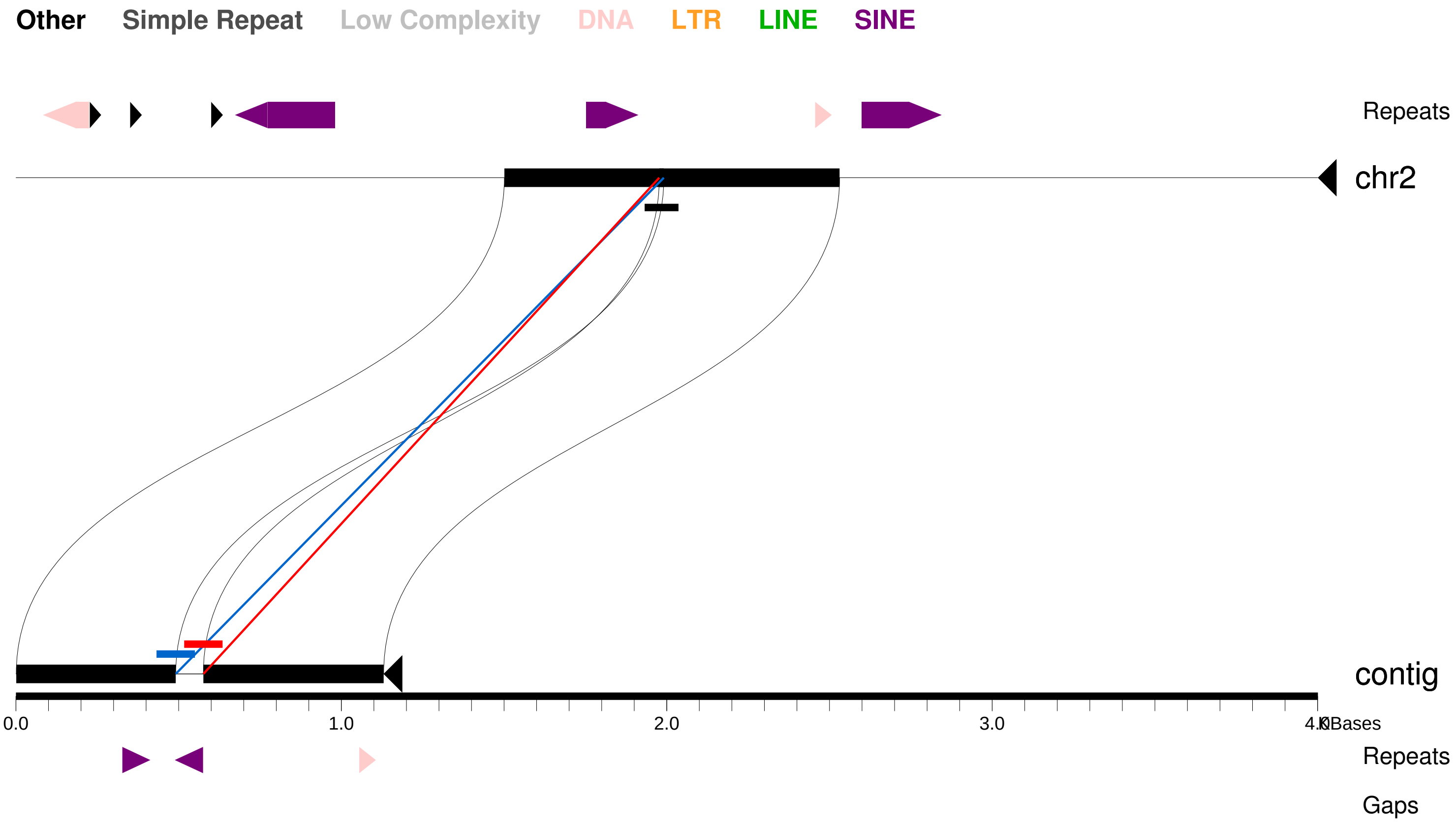

siteID: chr2_226753693 Scafftig1 + Breakpoint: chr2:226753677
chr2:226753631-226753736
Overlap Length: 14 INS size: 307 Genotyped: Yes

```
left     GCTTTC----CTTCATAGCAAT-TCCTGTAATTT--GTAATAATCCGTCCTGT------TATTGCTGCTT
chrom    GCTTTC----CTTCATAGCAAT-TCCTGTAATTT--GTAATAATCCATCCTGT------TATTGTTGCTT
right    CCACCCGCCTCGGCCTCCCAAAGTGCTGGGATTACAGGCGTGAGCCACCGCGCCCGGCCTATTGCTGCTT
         1*111*1111*11*1*11***11*1***11***111*111*1*1**21*11*1111111*****N*****

left     TTTTTTTTTTTTCTTTTTTTTTGAGACGGAGTCTCGCTCTGTCGCCCAGGTCGGACTGCGGA
chrom    TTTGAAGTCTCTCTCTCTTCATACTA--GATTATAAA-CT-----CCATGAGGG-CAG----
right    TTTGAAGTCTCTCTCTCTTCATACTA--GATTATAAA-CT-----CCATGAGGG-CAG----
         ***2222*2*2***2*2**22*222*22**2*2*2222**22222***2*22**2*2*2222
```

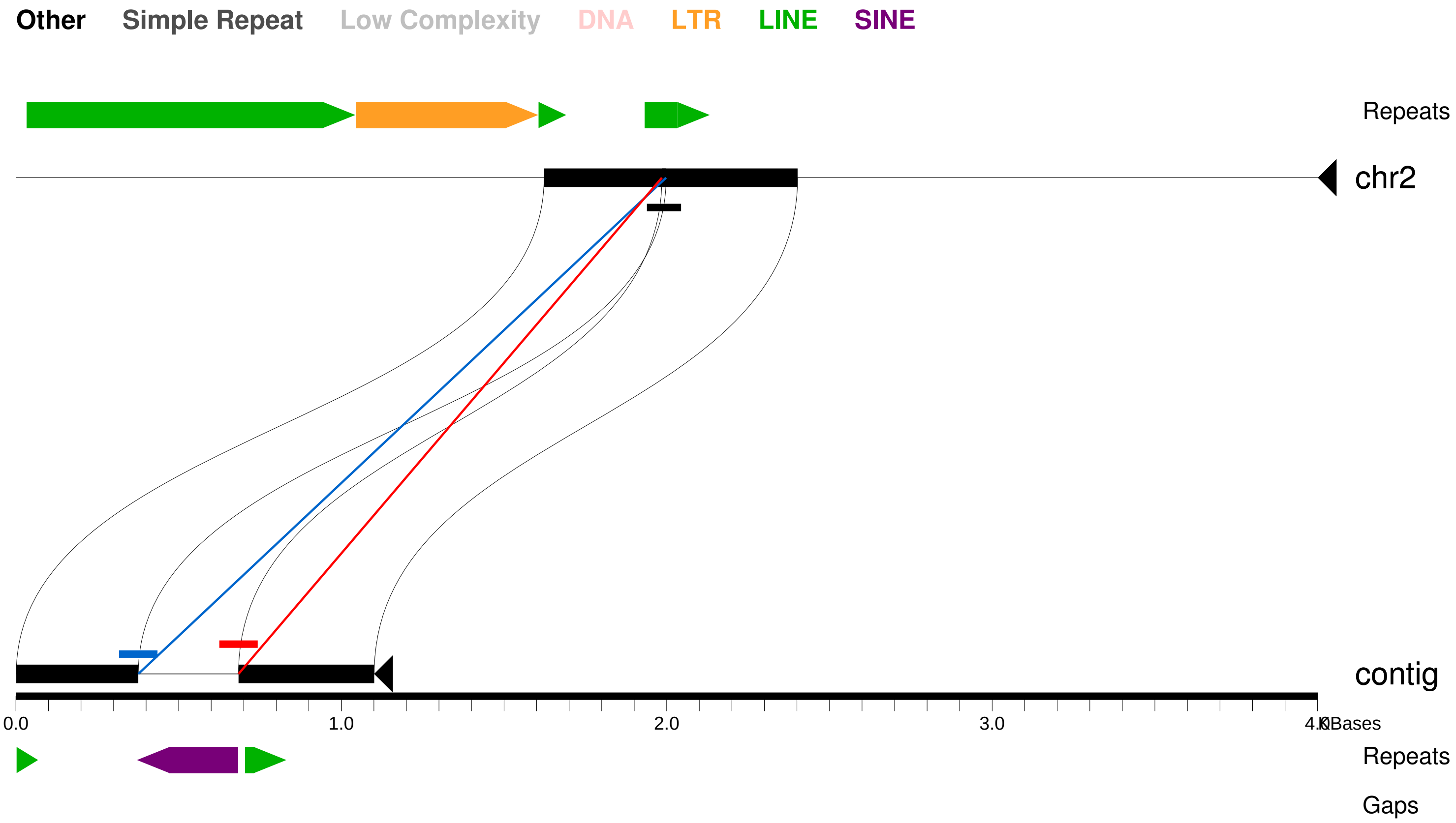

siteID: chr2_226970075 Scafftig1 - Breakpoint: chr2:226970082
chr2:226969922-226970259
Overlap Length: 18 INS size: 431 Genotyped: No

```
left   T-----------------------------------------------CCCCTAAAAATGAATGTCTGGA
chrom  TATTAATCCCCACATTCTTATAATTTCAACTCAAATTATAGATGAGTTCCCCTAAAAATGAATGTCTGGA
right  T-TTAGTAGAGACGGGGTT------TCACCG----TTTTAGCCGGGATGGTCTCGATCTC-----CTG--
       * 222N2NNNN22NNNN22      222N2N    22N222NN2N2N2111**11*11*111111***11

left   GTTTAAATAGTGAAATAAAAACATGTGAGCAGTCAACAATCAAGCAATGGGCTTTTTCAGTGTATTAAAT
chrom  GTTTAAATAGTGAAATAAAAACATGTGAGCAGTCAACAATCAAGCAATGGGCTTTTTCAGTGTATTAAAT
right  ----ACCTCGTGA------------TCCGCCCGCCTCGGCCTCCCAAAGTGCTG-------GGATTACAG
       1111*11*1****111111111111*11**111*11*111*111***1*1***11111111*1****1*1

left   AAAT-ATCTAACTCTCCTTAACCTGAAGGGGATTTTTTT------------TTTTNNNNNNNNNNN----
chrom  AAAT-ATCTAACTCTCCTTAACCTGAAGGGGATTTTTTTGACCTACTGTCATTTTTTATAATGTTTTATA
right  GCGTGAGCCACCGCGCCCGG-CCTGAAGGGGATTTTTTTGACCTACTGTCATTTTTTATAATGTTTTCTA
       111*1*1*1*1*1*1**1111******************222222222222****222222222222N22

left   ----------------------------------------------------------------------
chrom  CTTCTGTTATATTATAGTAGTACTGTTTATGTTCCTGTAGTTAAATCAGGGTCAGAGTTTCTATTAAAGT
right  CTTCTGTTATATTATAGTAGTACTGTTTATGTTCCTGTAGTTAAATCAGGGTCAGAGTTTCTATTAAAGT
       2222222222222222222222222222222222222222222222222222222222222222222222

left   -----------------------------------------------------------
chrom  GATCTCATTTGATGGAGGTAGGCAAATGACAGTCTTCTAATATACCTATGACATCCGTT
right  GATCTCATTTGATGGAGGTAGGCAAACGACAGTCTTCTAATAT----------------
       22222222222222222222222222N2222222222222222
```

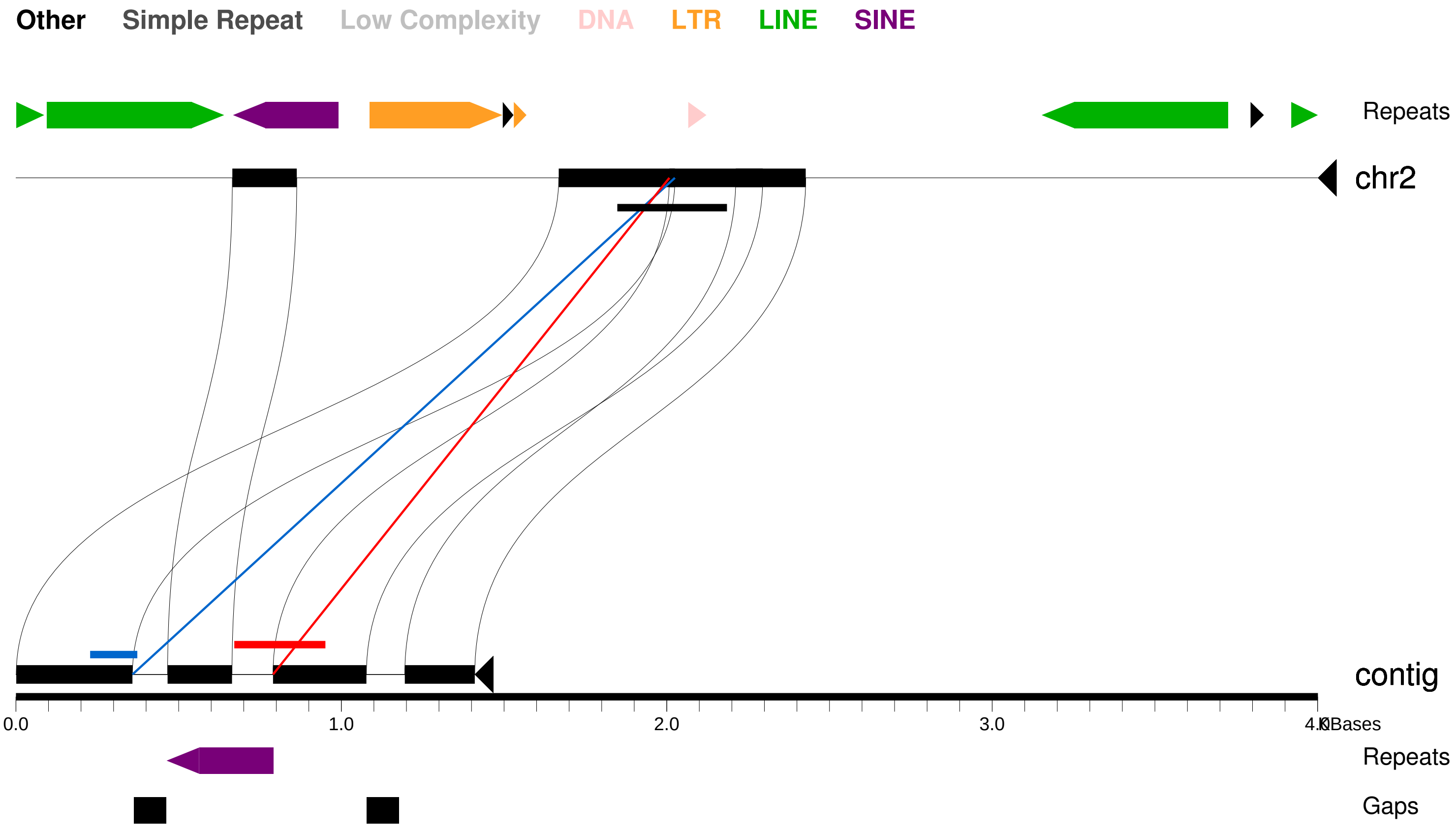

siteID: chr2_229229723 Scafftig1 - Breakpoint: chr2:229229733
chr2:229229684-229229792
Overlap Length: 11 INS size: 303 Genotyped: Yes

```
left    GAACTGGATCC----CATCAAATTTAAAACTTTGCTCTGTGAAAGACTCTGTT------AAGAGGATGAA
chrom   GAACTGGATCC----CATCAAATTTAAAACTTTGCTCTGTGAAAGACTCTGTT------AAGAGGATGAA
right   GCACTCCAGCCTGGGCGACAGAGCGAGACTCCGTCTCAAAAAAAAAAAAAAAAAAAAAAAAGAGGATGAA
        *1***11*1**1111*11**1*111*1*111111***1111***1*1111111111111***********

left    GGCCGGGCGCGGTGGCTCACGCCTGTAATCCCAGCACTTTGGGAGGCCGAGGCGGGCGG
chrom   AA---GACAAGTT---ACAAACTTGAAG----AAAATATTTGCAAATGACATCCAACAA
right   AA---GACAAGTT---ACAAACTTGAAG----AAAATATTTGCAAATGACATCCAACAA
        22222*2*22*2*2222**22*2**2*22222*22*22**2*2*22222222*222*22
```

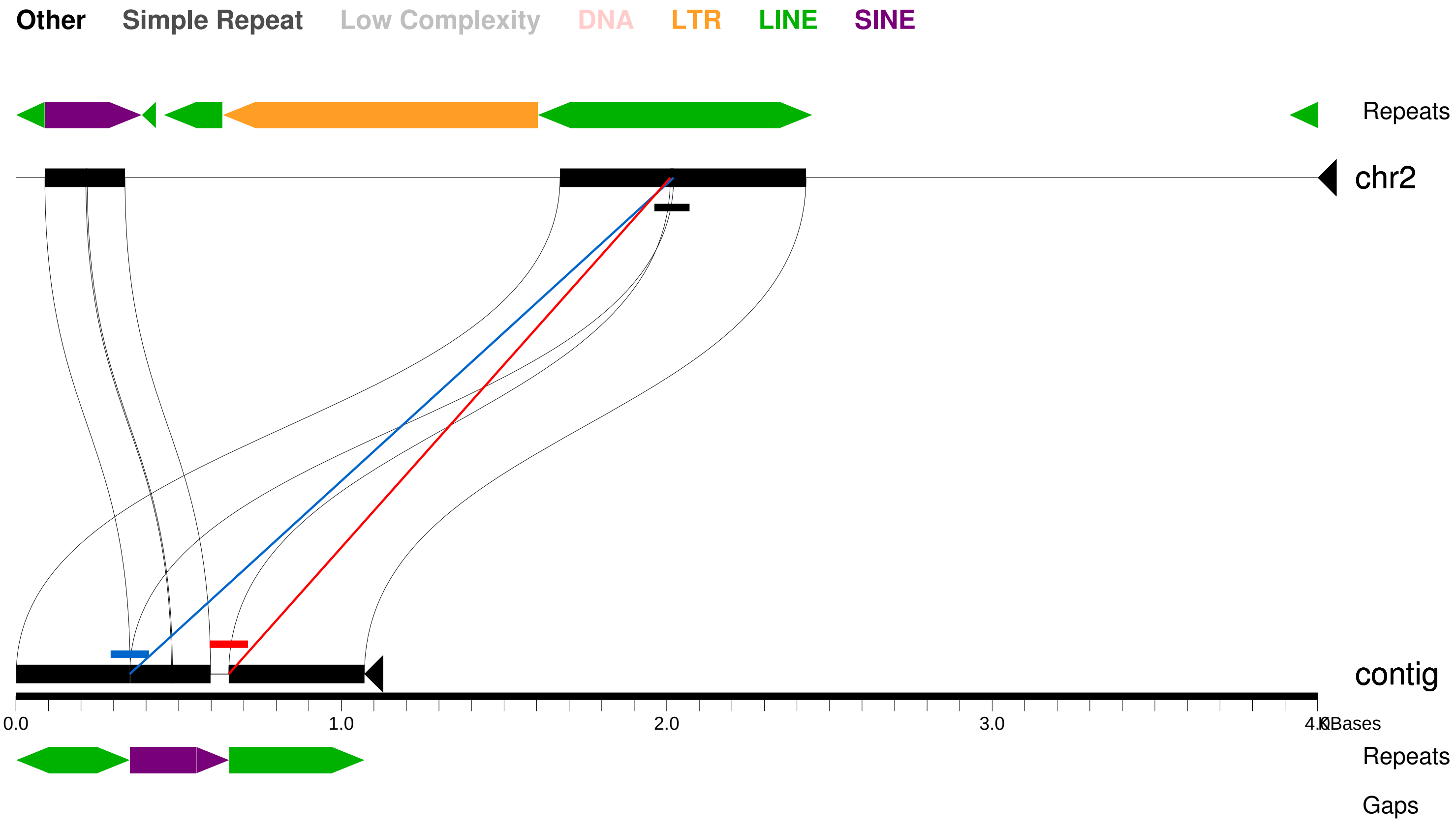

siteID: chr2_238493800 Scafftig1 + Breakpoint: chr2:238493813
chr2:238493768-238493872
Overlap Length: 15 INS size: 433 Genotyped: No

```
left    TGTTTAGCTGGTTATAGAATCCATCCCACCTAACGCAGGCCTGCA---------------AAAGGGTTCAT
chrom   TGTTTAGCTGGTTATAGAATCCATCCCACCTAACGCAGGCCTGCA---------------AAAGGGTTCAT
right   NNNNNNNNNNNNNNNNNNNNNNNNNNNNNNNNNNNNNNNNNNNNNNNNNNNNNNNNNNNNAAAGGGTTCAT
        111111111111111111111111111111111111111111111111111111111111***********

left    GCTCGGCCGGGCGCGGTGGCTCACGCCTGTAATCCCAGCACTTTGGGAGGCCGAGGCGGGTGG
chrom   GCTCAACAG-------------ATGCCTGTTAGATGAGTAAAT-GAATGAGTGAATTTGGAGA
right   GCTCAACAG-------------ATGCCTGTTAGATGAGTAAAT-GAATGAGTGAATTTGGAGA
        ****22*2*2222222222222*2******2*2222**2*22*2*222*222**2222**2*2
```

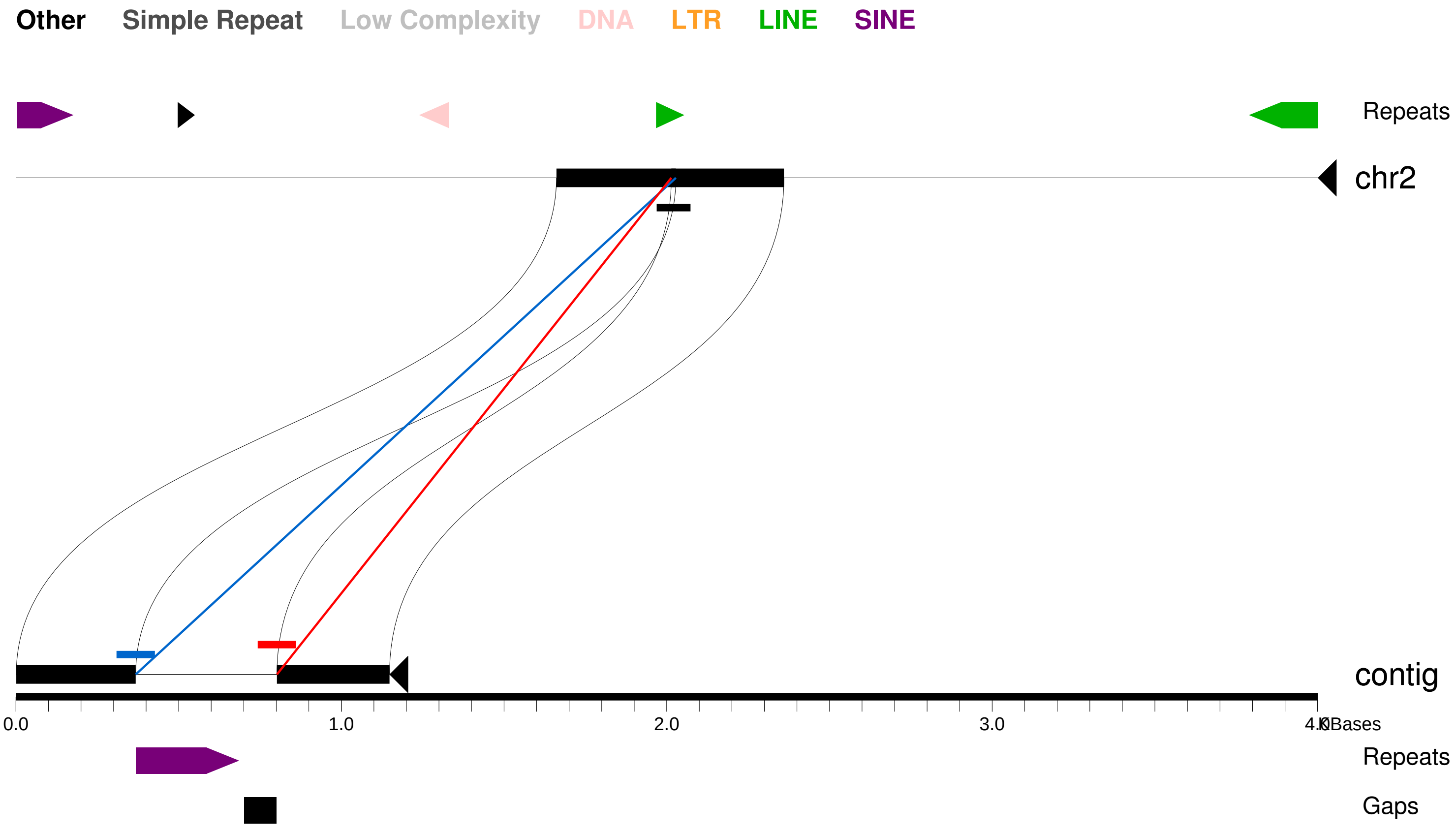

siteID: chr2_239020847 Scafftig2 + Breakpoint: chr2:239020838
chr2:239020806-239020897
Overlap Length: 16 INS size: 316 Genotyped: Yes

```
left    CTA-----------ATTC--AATTTATTTAATTATAG-----AACTACTA---------GGGCTTTCTTT
chrom   CTA-----------ATTC--AATTTATTTAATTATAG-----AACTACTA---------GGGCTTTCTAT
right   CCACCCGCCTCGGCCTCCCAAAGTGCTGGGATTACAGGCGTGAGCCACCGCGCCCGGCCGGGCTTTCTAT
        *1*111111111111*1*11**1*11*111****1**11111*1*1**11111111111*********2*

left    TTTTTTTTTTTTTTTTTTTTTTTCTTTTTTTTTGAGACGGAGTCTCGCTCTGTCGCCCAGGCCGGACTGC
chrom   TTTTTATGTGTTGGTTTCAGCAAATTATATTTTTCTA-GGATT-------TATTTCC-------------
right   TTTTTATGTGTTGGTTTCAGCAAATTATATTTTTCTA-GGATT-------TATTTCC-------------
        *****2*2*2**22***2222222**2*2****222*2***2*2222222*2*22**2222222222222

left    GGACTG
chrom   ------
right   ------
        222222
```

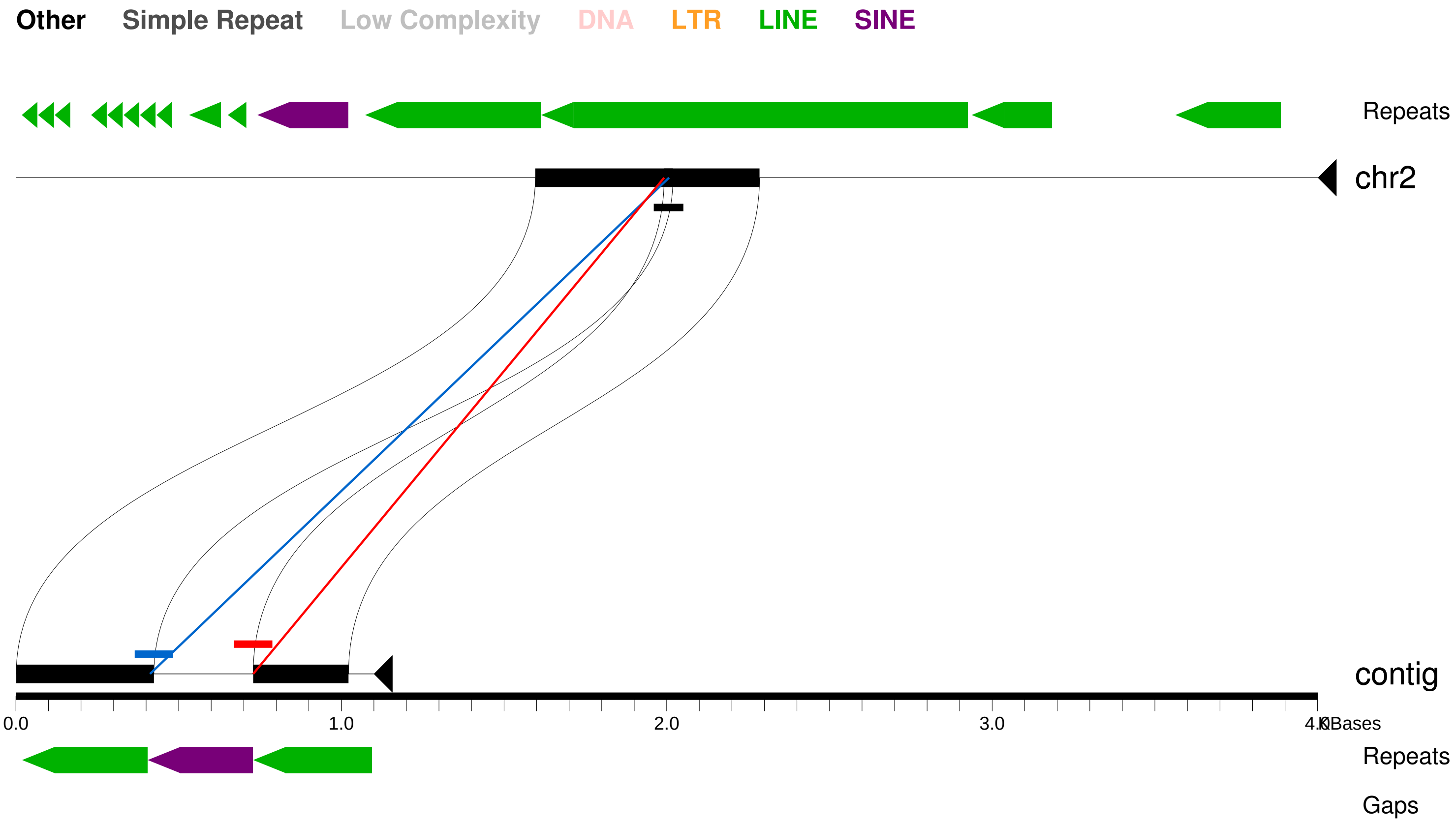

siteID: chr2_239883393 Scafftig7 - Breakpoint: chr2:239883395
chr2:239883349-239883454
Overlap Length: 14 INS size: 315 Genotyped: Yes

```
left    GACAAC----CCACAGAGTGGGA---------AAAAAATCTTCACAATCTATACATCTGAAAAAGGACTA
chrom   GACAAC----CCACAGAGTGGGA---------AAAAAATCTTCACAATCTATACATCTGAAAAAGGACTA
right   CCGGCCTGGGCGACAGAGCGAGACTCCGTCTCAAAAAAAAAAAAAAAAAAAAAAAAAAAAAAAAAGGACTA
        11111*1111*1******1*1**111111111******11111*1**111*1*1*1111***********

left    ATAGGCCGGGCGCGGTGGCTCACGCCTGTAATCCCAGCACTTTGGGAGGCCGAGGCGGGTGG
chrom   ATAT-CCAGAATCTATAAATAAC--------TCAAACAAATTAGCAAGAAAAAAAACAA----
right   ATAT-CCAGAATCTATAAATAAC--------TCAAACAAATTAGCAAGAAAAAAAACAA----
        ***22**2*222*22*222*2**22222222**22*22*2**2*22**2222*22*222222
```

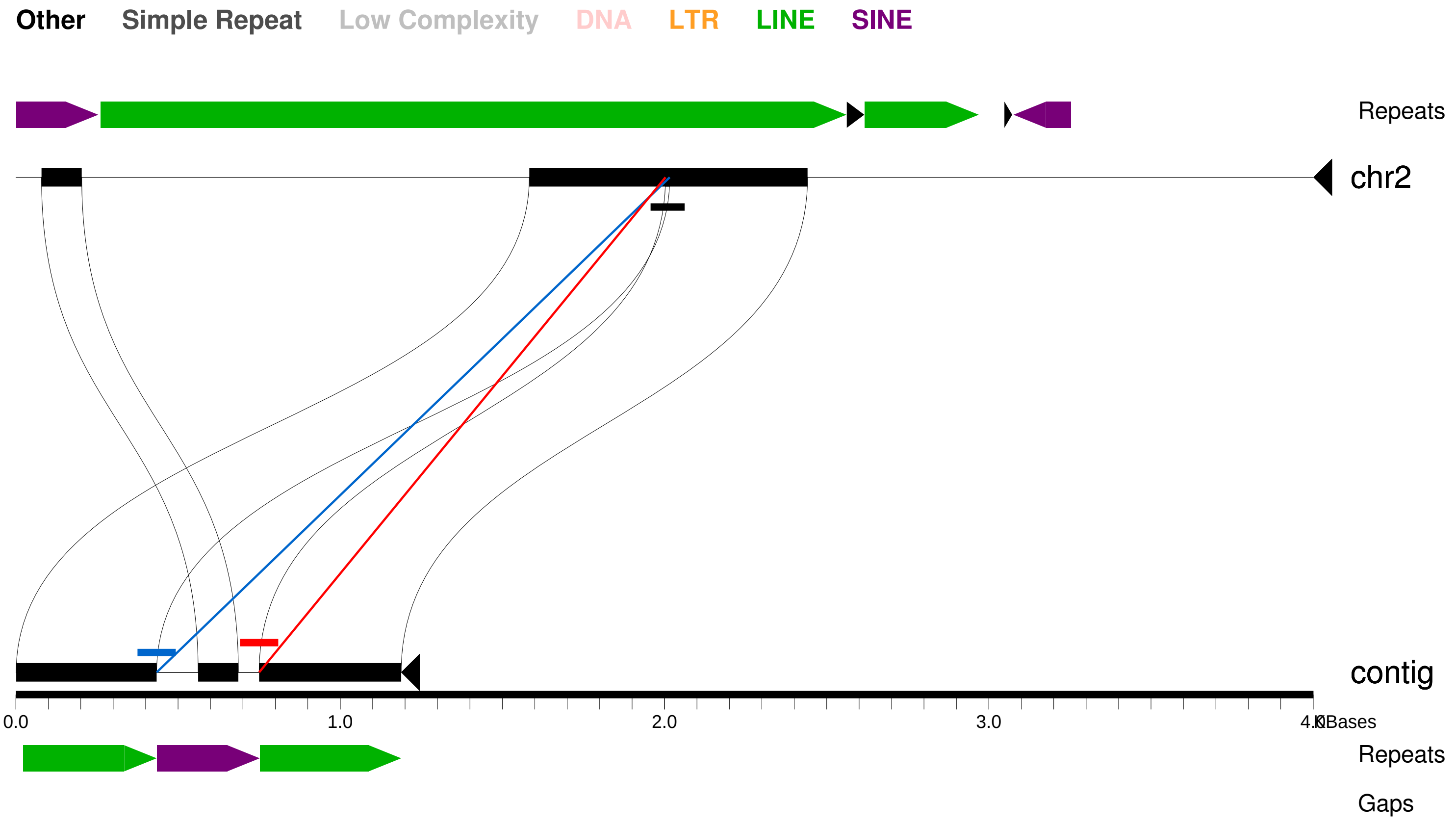

siteID: chr2_240860722 Scafftig1 - Breakpoint: chr2:240860714
chr2:240860659-240860773
Overlap Length: 5 INS size: 418 Genotyped: No

```
left   CTGGTAGGTTTGTGGTCTCA-----CTGAC-TTCAAGAACAAAGCCACGGACCTTTGCCATGAGTGNNNN
chrom  CTGGTAGGTTTGTGGTCTCA-----CTGAC-TTCAAGAACAAAGCCACGGACCTTTGCCATGAGTGTTAT
right  CCGCCCGCCTCGGCCTCCCAAAGTGCTGGGATTACAGGCGTGAGCCACCGCGCCCGGCC--GAGTGTTAT
       *1*111*11*1*111**1**11111***111**11**11111******1*11*111***11*****2222

left   NNNNNNNNNNNNNNNNNNNNNNNNNNNNNNNNNNNNNNNNNNNNNNNNNNNNNNN
chrom  AGCTCTTAAAGGTGGCACTGACCCAAAGAGTGGAGAAGGAAAGAACAAAGC----
right  AGCTCTTAAAGGTGGCACTGACCCAAAGAGTGGAGAAGGAAAGAACAAAGC----
       2222222222222222222222222222222222222222222222222222222
```

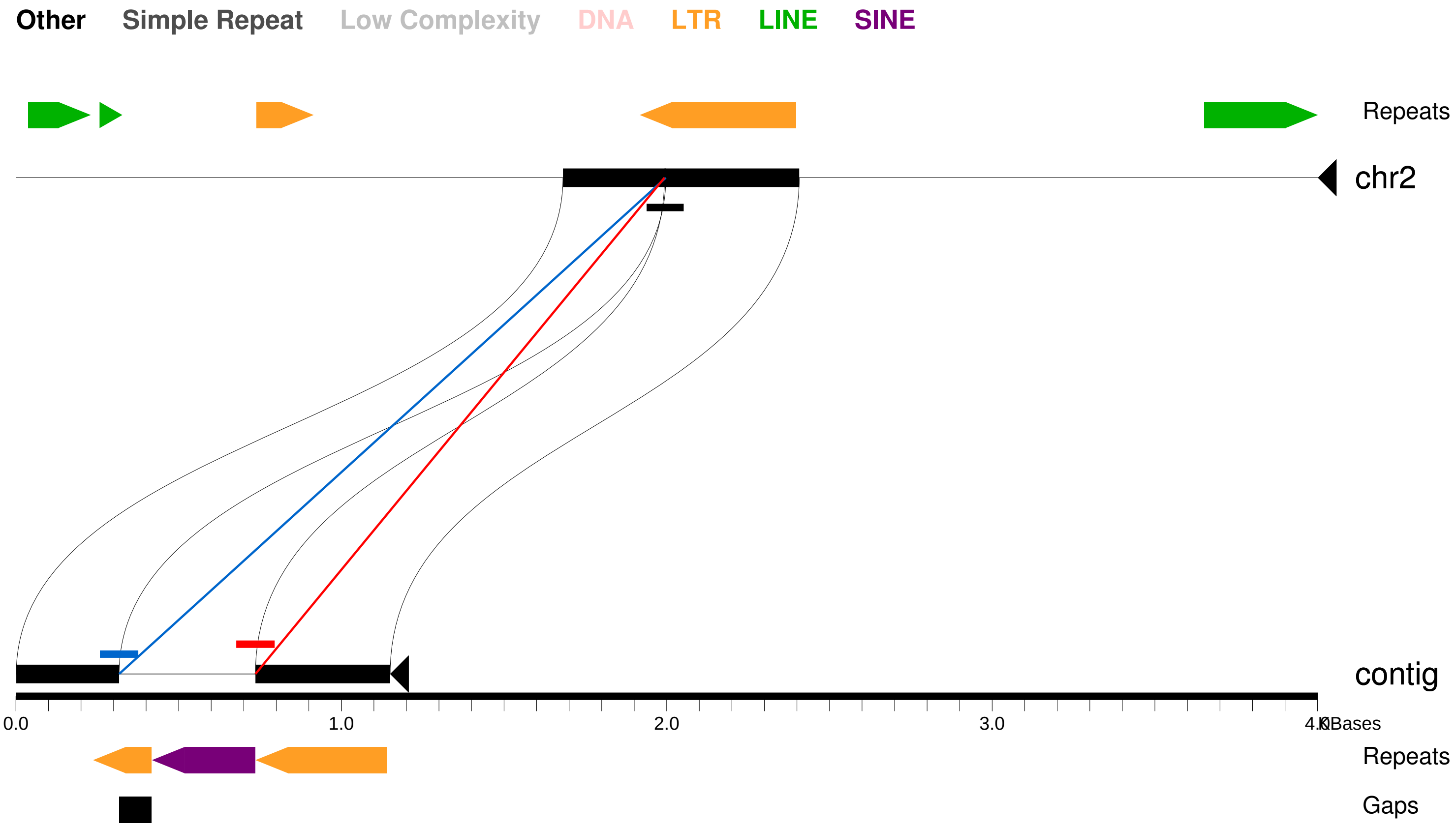

siteID: chr2_2539942 Scafftig8 - Breakpoint: chr2:2539935
chr2:2539775-2540102
Overlap Length: 8 INS size: 412 Genotyped: No

```
left   T-------------------------------------ACCCAGAATGGGGGCTAATGACCGAGGGGTGG
chrom  TTGCACCAGCTCAGGTATGTTCATCTCCTGAGTGTGGTACCCAGAATGGGGGCTAATGACCGAGGGGTGG
right  TTT-AGTAGAGACGGGGT-TTCACC--------GTGTTAGCCAGGATGGTCTCGA-TCTCC------TGA
       *2N 2NN22NNNN22NN2 2222N2        222N2*1****1****111*1*1*11**111111**1

left   CCCCATCCATTCCGAGAAGAGAAGAGCCACTGTCTCCCCTTGCTCTGGAGCTGCCACACATCACAGCACC
chrom  CCCCATCCATTCCGAGAAGAGAAGAGCCACCGTCTCCCCTTGCTCTGGAGCTGCCACACATCACAGCACC
right  CCTCGTGA--TCCGCCC--------GCCTCGGCCTCCCAAAGTGCTGGG----------ATTACAGG---
       **1*1*1111****11111111111***1*N*1*****111*11****11111111111**1****1111

left   CTGGAATGCCCGCAGCCC--TTTCAGCGGGNNNNNNNNNNN-----------------------------
chrom  CTGGAATGCCCGCAGCCC--TTTCAGCGGGTATTTTTTAATGGGATGAGGAGCGAGTTTGGGAGGGGCAC
right  CGTGAGCCACCGC-GCCCGGTCTCAGCGGGTATTTTTTAATGGGATGAGGAGCGAGTTTGGGAGGGGCAC
       *11**1111****1****11*1*******2222222222222222222222222222222222222222

left   ----------------------------------------------------------------------
chrom  GTGGGAAGACTTCATCTTTCTTTTAAATTTCAGGCTCAAGGACAAGGTGACCAAAAATCCTCTGGTCAGC
right  GTGGGAAGACTTCATCTTTCTTTTAAATTTCAGGCTCAAGGACAAGGTGACCAAAAATCCTCTGGTCAGC
       2222222222222222222222222222222222222222222222222222222222222222222222

left   --------------------------------------------------
chrom  AAGGGACAAGCCCAAAGACAGGGGCTGGGTCTGTGTGCTGTGCTAGTTCC
right  AAGGGACAAGCCCAAAGACAGGGGCTGGGTCTGTGTGCTGTG--------
       222222222222222222222222222222222222222222
```

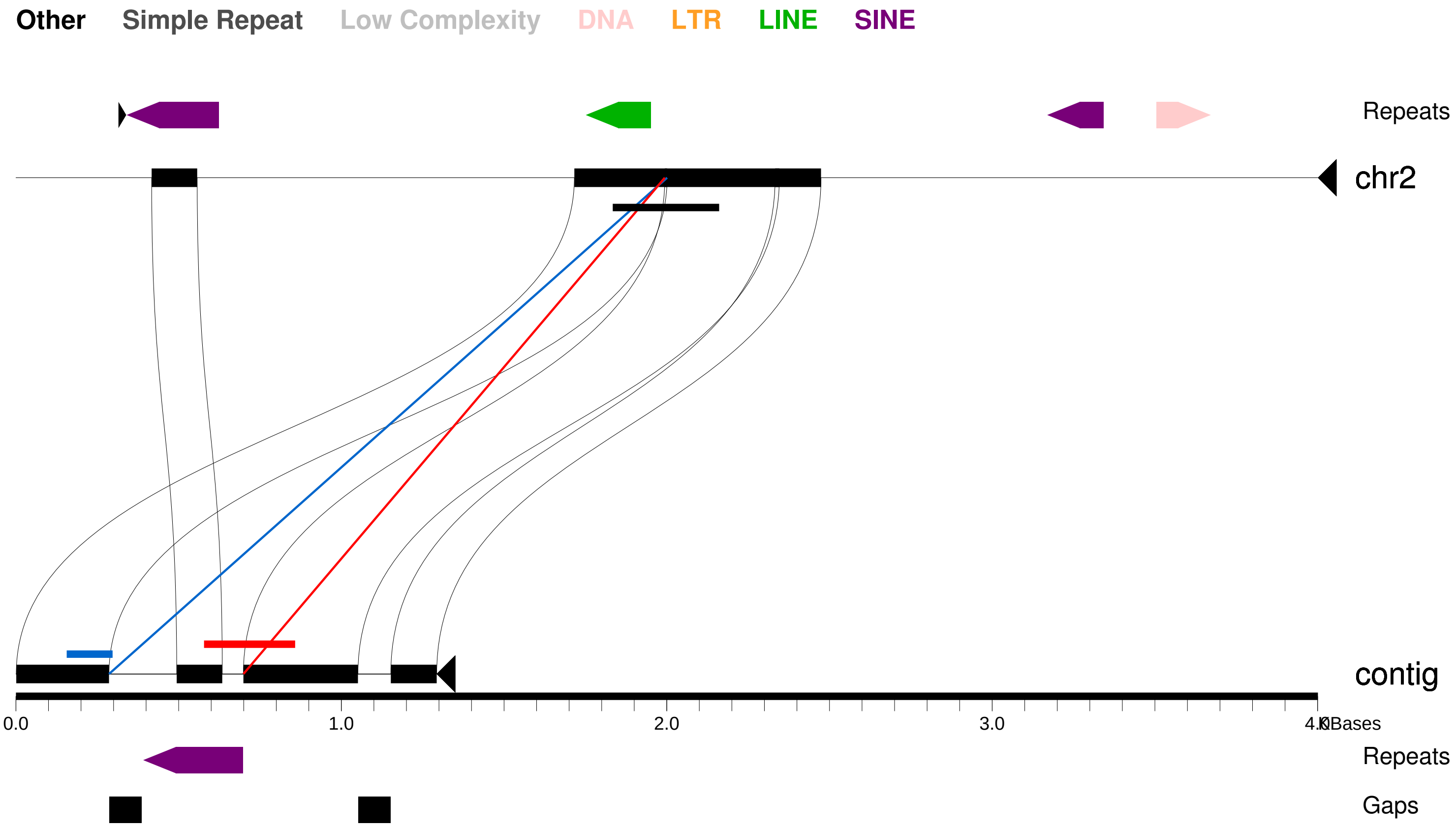

siteID: chr2_30670024 Scafftig2 - Breakpoint: chr2:30669976
chr2:30669956-30670035
Overlap Length: 18 INS size: 428 Genotyped: No

```
left    GCCTCTTTAAAGGAGCTCTC---------------------------------------AAAGAAAGGTA
chrom   GCCTCTTTAAAGGAGCTCTC---------------------------------------AAAGAAAGGTA
right   NNNNNNNNNNNNNNNNNNNNNNNNNNNNNNNNNNNNNNNNNNNNNNNNNNNNNNNNNNNAAAAGAAAGGTA
        11111111111111111111111111111111111111111111111111111111111***********

left    CCCGGGGGGCCGGGCGCGGTGGCTCACGCCTGTAATCCCAGCACTTTGGGAGGCCGAGGCGGGCGGATCA
chrom   CCCGGGGTATCCCGGGCTGTGG-----------------AGCA-------AGCGCGGGGCG---------
right   CCCGGGGTATCCCGGGCTGTGG-----------------AACA-------AGCGCGGGGCG---------
        *******222*22*2**2****22222222222222222*1**2222222**22**2****222222222

left    CGAGGTCAGGAGATCGAG
chrom   ------CCGGGGACCCGG
right   ------CCGGGGACCCGG
        222222*2**2**2*22*
```

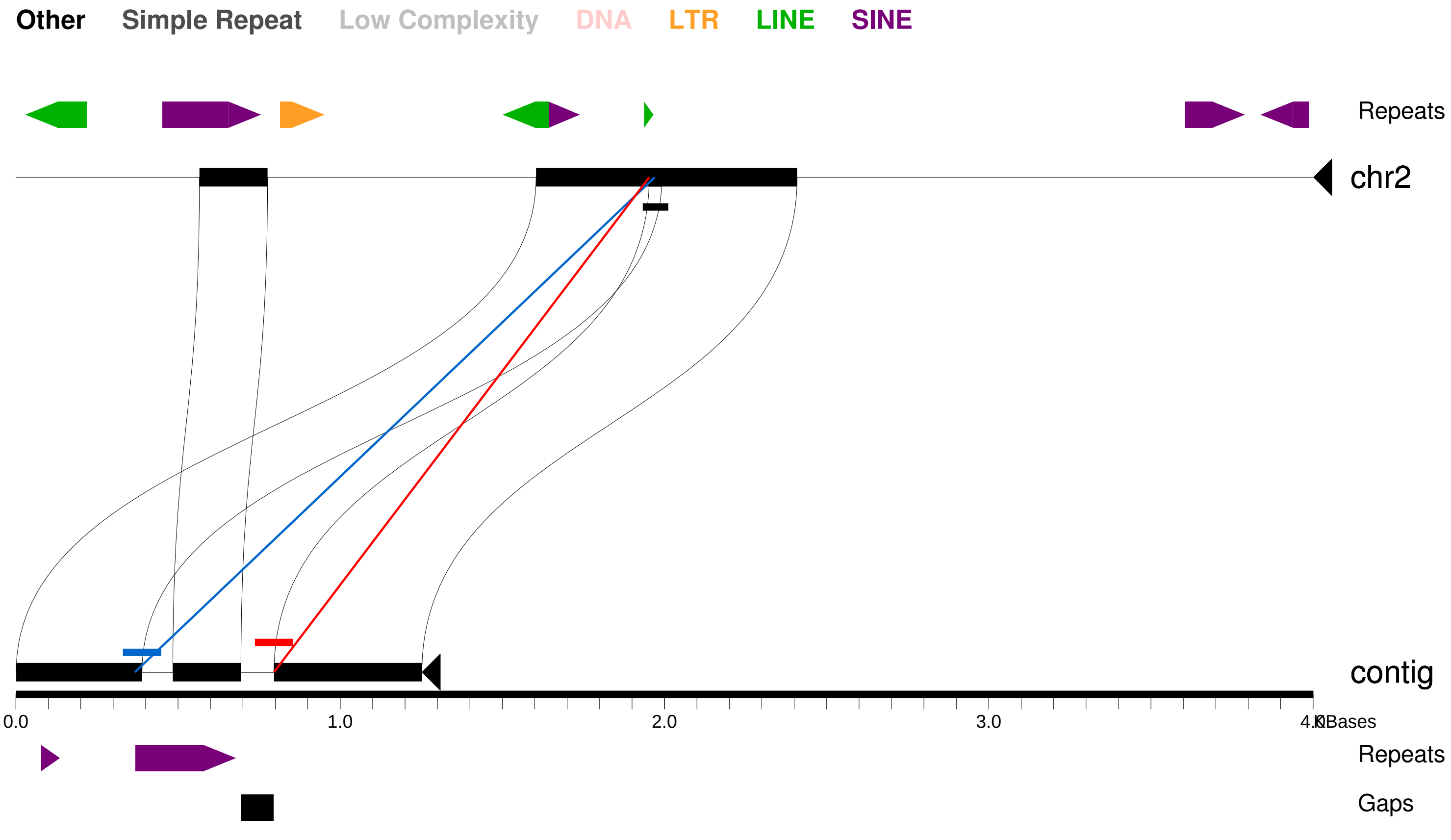

siteID: chr2_31559684 Scafftig10 + Breakpoint: chr2:31559682
chr2:31559522-31559856
Overlap Length: 15 INS size: 305 Genotyped: Yes

```
left    ---------------TG-GGGACCCATATGCCTCCTTCACCCACTGTGTTGAGGTCTGCGGATGGTGGAT
chrom   CAAGGAAGACCTGAATG-GGGACCCATATGCCTCCTTCACCCACTGTGTTGAGGTCTGCGGATGGTGGAT
right   --AGAATGGCGTGAACCCGGGAGGCGGA-GCTTGC-------AGTGAGCCGAGATC--------------
          22N2N2N2N2222111****11*11*1**1*1*1111111*1**1*11***1**11111111111111

left    TTGTCTGCCCCTGGGGTTCCTCTGTGCCCTCCCCACTTGGGACCACATGTACCCACTTTACCTATAAATC
chrom   TTGTCTGCCCCTGGGGTTCCTCTGTGCCCTCCCCACTTGGGACCACATGTACCCACTTTACCTATAAATC
right   ----CCGCCACTG-------------CACTCCAGCCTGGGCGACAGAGCGAGACTCCGTCTCAAAAAAAA
        1111*1***1***1111111111111*1****111**1**111**1*111*11*1*11*11*1*1***11

left    CAAAAAGCATATCGAATTTTAAAAAGAGGAAGCCCCGGCCGGGCGC--GGTGG--CTCACGCCTG-TAAT
chrom   CAAAAAGCATATCGAATTTTAAAAGAGAAAGCCCCACCTGAGTATCAGGAAGAACCCAGTCCTGCTAAC
right   AAAAAAAAAAAAAAAA-----AAAAAAGGAAGCCCCACCTGAGTATCAGGAAGAACCCAGTCCTGCTAAC
        1*****11*1*111**11111****1**N*******22*2*2*22222**22*22*2**22****2***2

left    CCCAG-CACTTTGG-GAGGCCGAGGC-------------GGGCGGATCACGAGGTCAGGAGATC---GAG
chrom   TCCACACAGGTTGCAGTTGCAAAGGCAATTAAAGCCACTGTACAGAGCCC-ACTTCAGCACCACTCAGAG
right   TCCACACAGGTTGCAGTTGCAAAGGCAATTAAAGCCACTGTACAGAGCCC-ACTTCAGCACCACTCAGAG
        2***22**22***22*22**22****2222222222222*22*2**2*2*2*22****2*222*222***

left    ACCATCCCGG--CTAA-------AACGGTGAAACC-CCGTCTCTACTAAA--------
chrom   GCTGGGCTGGTTCTGAGATCCCCAAAGCGGAAGCAACCGTC-CTAGCAAAGTCTTCTT
right   GCTGGGCTGGTTCTGAGATCCCCAAAGCGGAAGCAACCGTC-CTAGCAA---------
        2*2222*2**22**2*2222222**2*22***2*22*****2***22**1
```

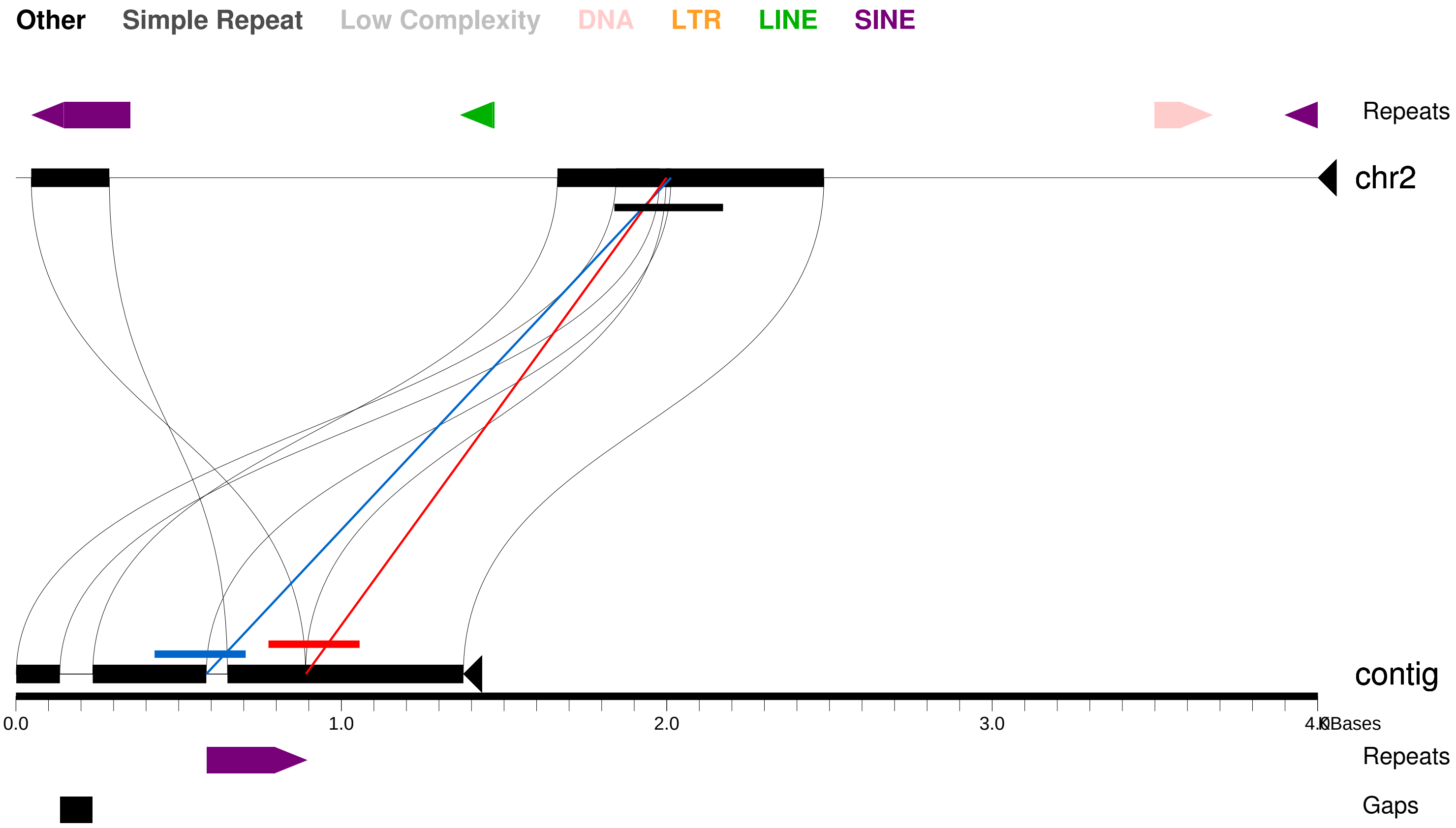

siteID: chr2_31952189 Scafftig12 + Breakpoint: chr2:31952197
chr2:31952037-31952370
Overlap Length: 14 INS size: 454 Genotyped: No

```
left   -------------TAATTAGAAAGATGGAGAAGGCACAAGAAAGAGACACTAGGGCAAAAGACTAATTTT
chrom  CACCAAATATATATAATTAGAAAGATGGAGAAGGCACAAGAAAGAGACACTAGGGCAAAAGACTAATTTT
right  NNNNNNNNNNNNAA--------------------------------------------------------
       NNNNNNNNNNNN21111111111111111111111111111111111111111111111111111111111

left   CCCTGTTTTAAAGGTGGAAGATGATACAAAAAGACTTATGGTATGATTCCACTTATATGAGGTACTTATA
chrom  CCCTGTTTTAAAGGTGGAAGATGATACAAAAAGACTTATGGTATGATTCCACTTATATGAGGTACTTATA
right  ---------------------------AAAAA--------------------------------------
       111111111111111111111111111*****11111111111111111111111111111111111111

left   GTAGTCAAAACCATAGAGACAAAAAGTAGAATGGGCCGGGCGCGGTGGCTCACGCCTGTAATCCCA---G
chrom  GTAGTCAAAACCATAGAGACAAAAAGTAGAATGGTGATTGC-CAGGGGTTAAGGGGAG-AATGGAATGGG
right  --------------------AAAAAGTAGAATGGTGATTGC-CAGGGGTTAAGGGGAG-AATGGAATGGG
       11111111111111111111**************22222**2*2*2**2*2*2*222*2***222*222*

left   CACTTTGGG----AGGCCGAGGCGGGCGGATC----------ACGAGGTCAGGAGATCGA----GACCAT
chrom  GAGTTAGTGTTTAATGGAAATGCTTTCAGTTTTGCAAGATGAAAGAGTTCTGGTGATAGATGGTGGTGAT
right  GAGTTAGTGTTTAATGGAAATGCTTTCAGTTTTGCAAGATGAAA--------------------------
       2*2**2*2*2222*2*222*2**222*2*2*22222222222*2111N11N11N111N11    1NNN11

left   CCCGGC-TAAAACGGTGAA-------ACC-CCGTC--TCTAC------TAAA----
chrom  GACTGCATAACAATATGGATGTATTTACCACCATACGTATACAAGCTTTAAAATGG
right  --------------------------------------------------------
       NN1N11 111N1NNN11N1       111 11N1N  1N111      1111
```

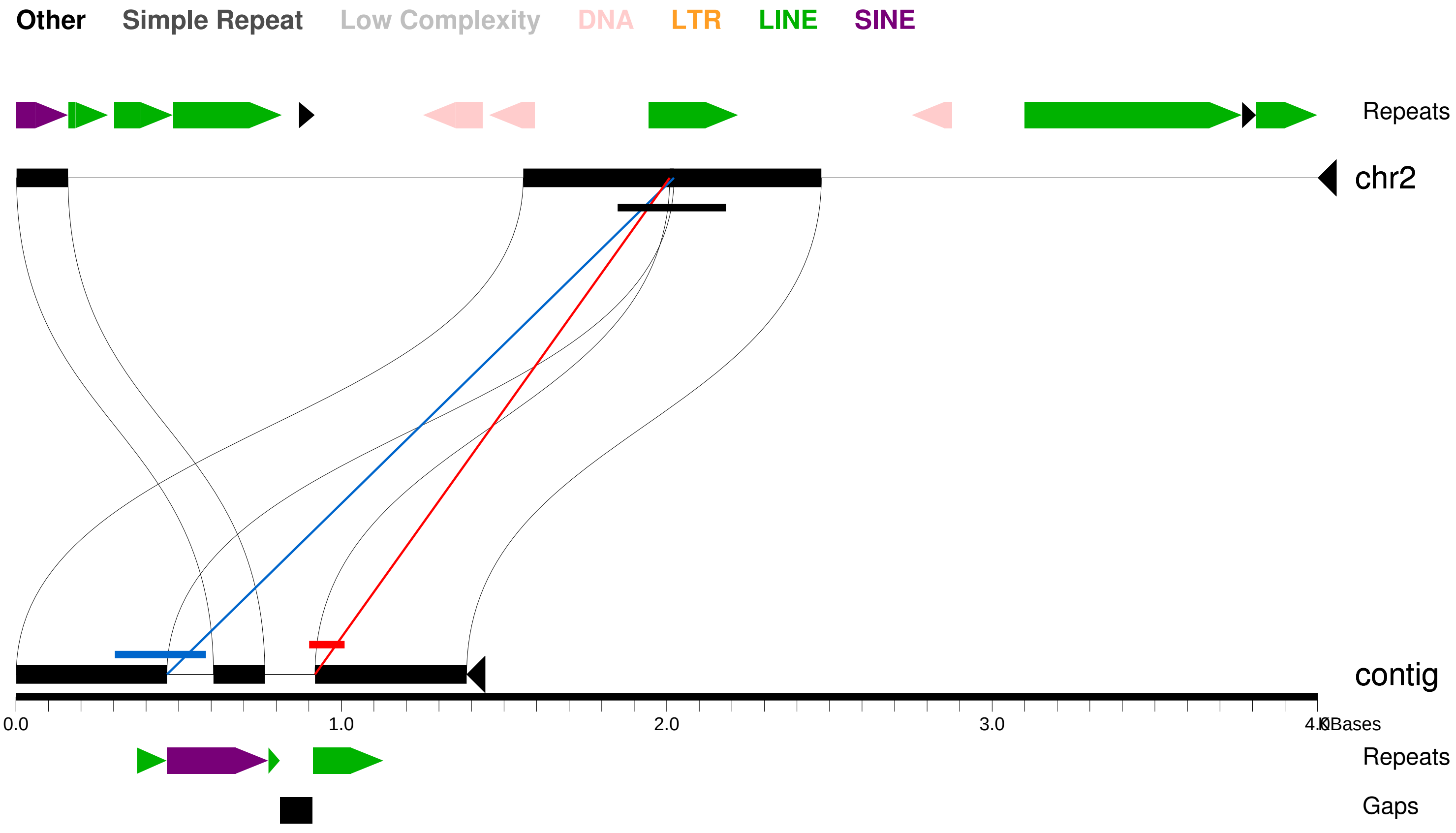

siteID: chr2_33404751 Scafftig4 - Breakpoint: chr2:33404725
chr2:33404677-33404784
Overlap Length: 12 INS size: 282 Genotyped: Yes

```
left   CCTTCATAATGATT-ATATATTTAG--TGAGTTTTAAGTTAAAACTTTTAG--------AAATTTAATTA
chrom  CCTTCATAATGATT-ATATATTTAG--TGAGTTTTAAGTTAAAACTTTTAG--------AAATTTAATTA
right  AGTCCGGCCTGGGCGACAGAGCGAGACTCCGTCTCAAAAAAAAAAAAAAAAAAAAAAAAAAAATTTAATTA
       11*1*1111**1111*1*1*111**11*11**1*1**111****11111*111111111***********

left   GCCAGCACTTTGGGAGGCCGAGGCGGGTGGATCATGAGGTCAGGAGATCGAGACCATCCT
chrom  G-----AATCTGG-----CTAATGGGTTATTTAAAAATGT-AAAATAAGGTTTAAAAACC
right  G-----AATCTGG-----CTAATGGGTTATTTAAAAATGT-AAAATAAGGTTTAAAAACC
       *22222*2*2***22222*2*222**2*222*2*22*2**2*22*2*22*22222*22*2
```

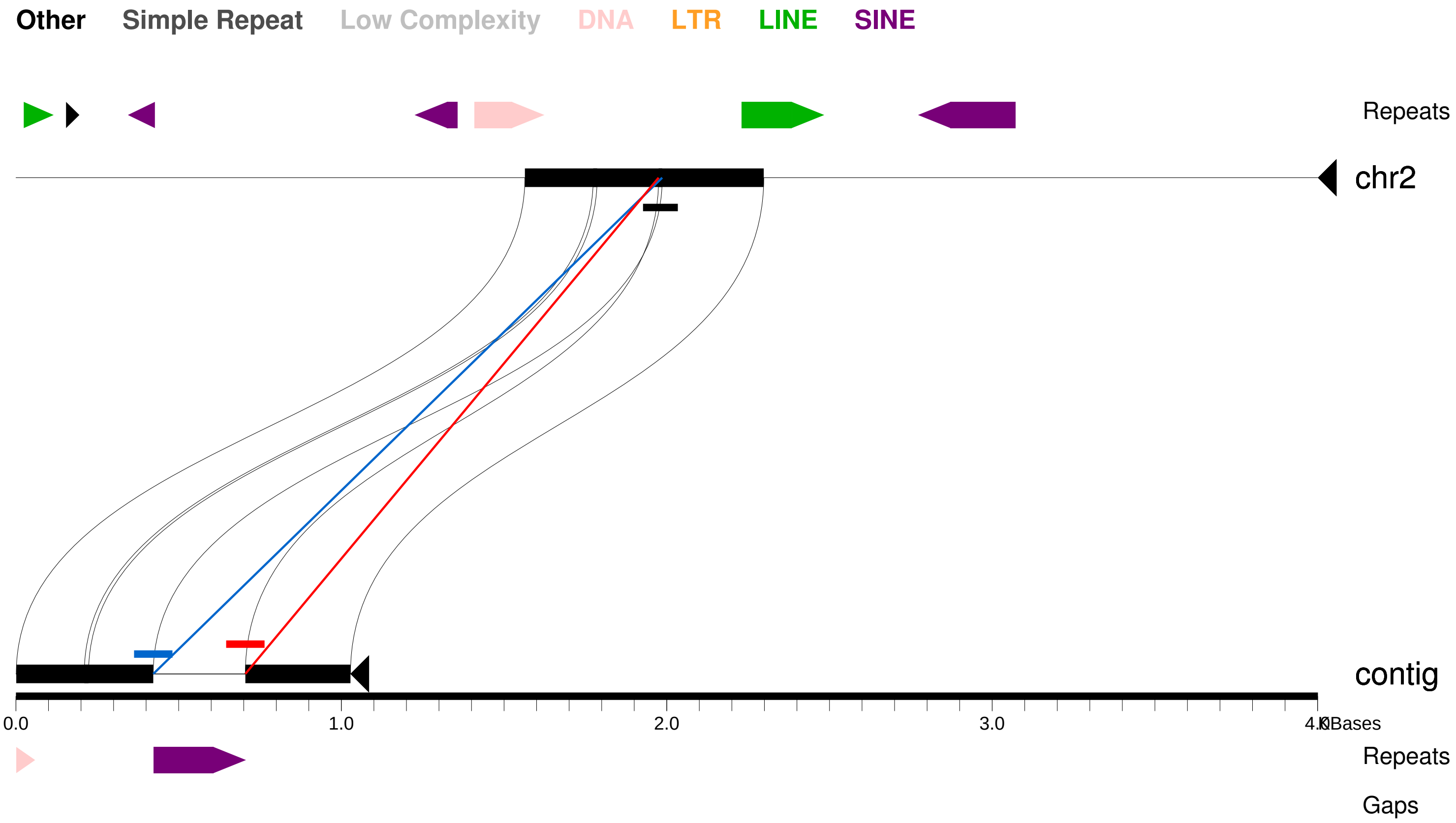

siteID: chr2_34255394 Scafftig3 + Breakpoint: chr2:34255379
chr2:34255219-34255552
Overlap Length: 14 INS size: 301 Genotyped: Yes

```
left   ----------TAAAGTCTTCAAAACACACAAAAAACCACTGCATTAGGCTACTTCATATTGCAC-CCAGT
chrom  AAAAAAGATGTAAAGTCTTCAAAACACACAAAAAACCACTGCATTAGGCTACTTCATATTGCAC-CCAGT
right  -----------------TTTAGTAGAGACGGGGTTTCACCGTTTTAGCCGGGATGGTCTCGATCTCCTGA
                 1111111**1*11*1*1**1111111***1*11****1*1111*11*1*1*11*1**1*1

left   TTTAGACTTGATTTGCAGAAGGTTGTTCCTTTAAAATCCTTTTTGTCCTTTTTGAAATTAATATACTGCA
chrom  TTTAGACTTGATTTGCAGAAGGTTGTTCCTTTAAAATCCTTTTTGTCCTTTTTGAAATTAATATACTGCA
right  CCTCG---TGATCCGCCC--GCCTCGGCCTCCCAAA--------GTGCT----GGGATTACAGGCGTGAG
       11*1*111****11**1111*11*111***111***11111111**1**1111*11****111111**11

left   ACACCTGTTAATATGGCTGGACCATTCACAGTTCTTTTTTCTTTTTTTTTTTTTTTTTGA-----GACGGA
chrom  ACACCTGTTAATATGGCTGGACCATTCACAGTTCTAATTTGAATCATTTAGCTGTTTAAAAAATGATAGG
right  CCACCGC--------GCCCGGCCATTCACAGTTCTAATTTGAATCATTTAGCTGTTTAAAAAATGATAGG
       1****1111111111**11*1*************22***222*22***222*2***2*22222**22*2

left   GTCTCGCTCTGTCGCCCAGGCTGGAGTGCAGTGGCGGGATCTCGGCTCACTGCAAGCTCCGCCTCCCG--
chrom  GAGTAGCTTTCATCCCTTTGGTGGTATGCTGAGATTAAATGCTGACTTTCTCAAGCCTTAGCAGGCAGTC
right  GAGTAGCTTTCATCCCTTTGGTGGTATGCTGAGATTAAATGCTGACTTTCTCAAGCCTTAGCAGGCAGTC
       *22*2***2*2222**222*2***22***2*2*22222**222*2**22**22*22**22**222*2*22

left   -------GG----TTCAC--GCCATTCTC---------------CCG--------
chrom  TAAGTGAGGACACTTCAGAAGAGATGCTGAACAGACCTGAGAAGCCGCAAACATC
right  TAAGTGAGGACACTTCAGAAGAGATGCTGAACAGACCTGAGAA------------
       2222222**2222****222*22**2**222222222222222 111
```

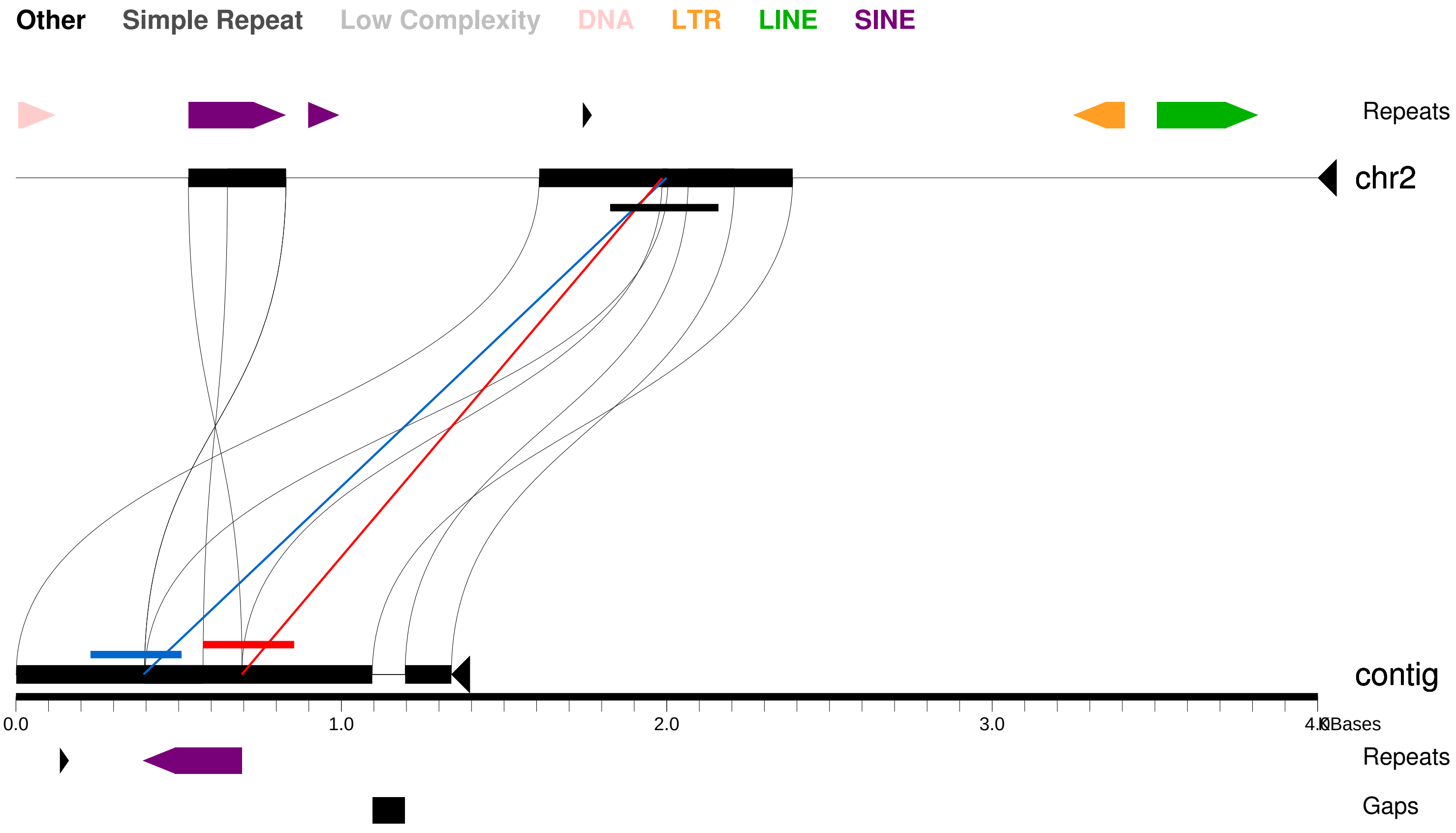

siteID: chr2_34356949 Scafftig1 + Breakpoint: chr2:34356969
chr2:34356921-34357028
Overlap Length: 12 INS size: 317 Genotyped: Yes

```
left    ATACACA---AGGTACTCCACA----TAGAATTAACTGTTATGTTTCATCAAAAT-----ACATTTTTAT
chrom   ATACACA---AGGTACTCCACA----TAGAATTAACTGTTATGTTTCATCAAAAT-----ACATTTTTAT
right   CCGCCCGCCTCGGCTTCCCAAAGTGCTGGGATTA-CAGGCGTGAGCCACCGCGCCCGGCCACATTTTTAT
        111*1*11111**1111***1*1111*1*1****1*1*111**111**1*1111111111**********

left    TTTTTTTATTTTTTTTTATTTTTTTTTTTTTTTTTGAGACGGAGTCTCGCTCTGTCGCCCA
chrom   TTATGG--AGGATTTAACAGTCAATCATAAGCATGGACA-------ACATCATGTCT--CA
right   TTATGG--AGGATTTAACAGTCAATCATAAGCATGGACA-------ACATCATGTCT--CA
        **2*2222*222***222*2*222*22*22222*2**2*22222222*2222****222**
```

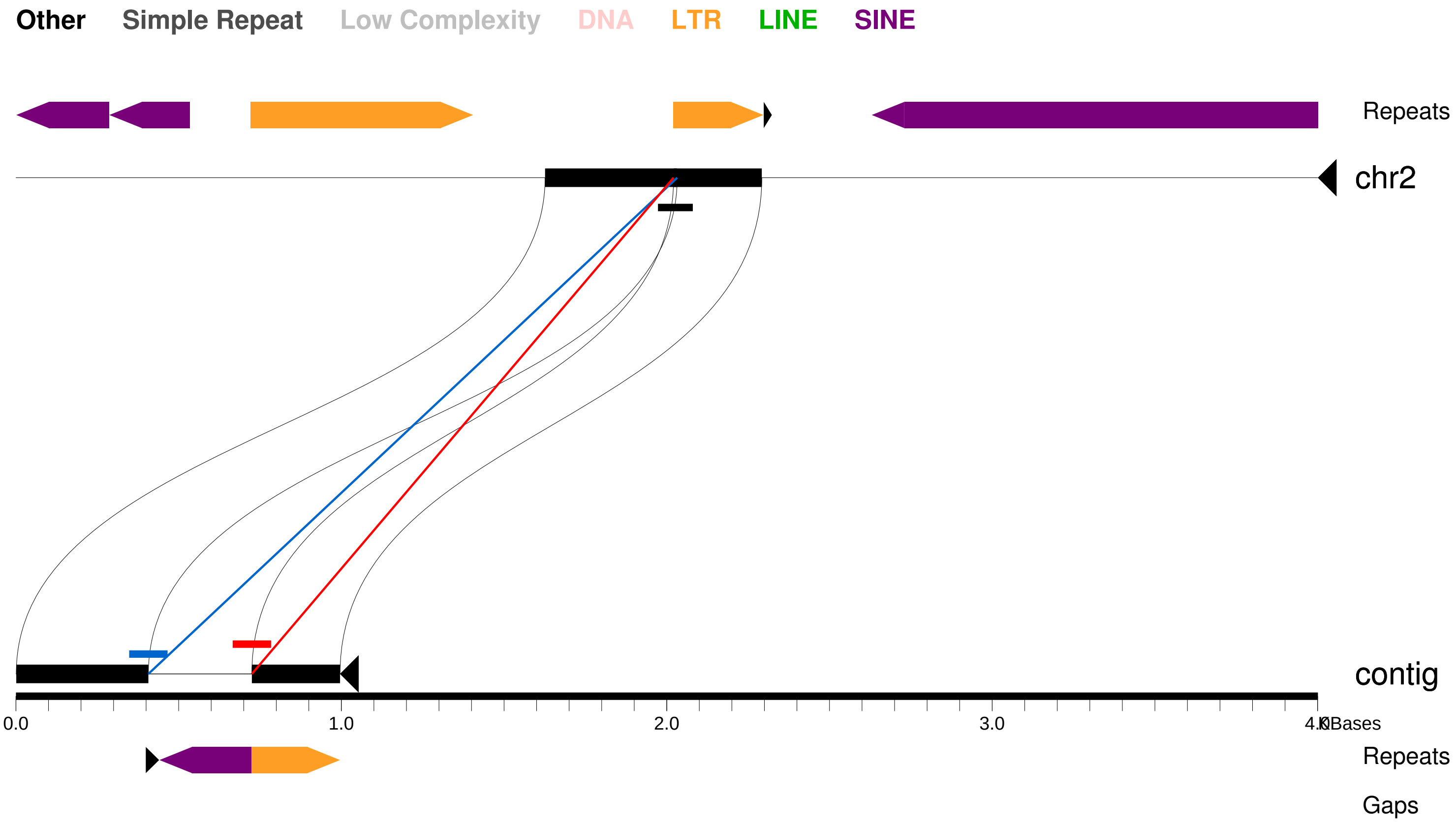

siteID: chr2_34473631 Scafftig1 + Breakpoint: chr2:34473663
chr2:34473503-34473835
Overlap Length: 13 INS size: 147 Genotyped: Yes

```
left   --------CATTGCTTATCGCATACTAAATACTCAGAAAATAGTTTTTTTCTCTTTGTAGGTGGAATTGG
chrom  GTTTGACACATTGCTTATCGCATACTAAATACTCAGAAAATAGTTTTTTTCTCTTTGTAGGTGGAATTGG
right  AGT---CTCGCTCTGTCGCCCAAGCTGGAGTG-CAGTGGCGGGATCTCGGCTCACTGCAAGC----TCCG
       NN2   2N*11*111*11*1**11**11*1111***111111*1*1*111***11**1*1*11111*11*

left   AATTGCTGTATAGCAAAACTTTCTACAATTGAAATTATTCAGACCACAGGGATACATTTTATAAGCTGAC
chrom  AATTGCTGTATAGCAAAACTTTCTACAATTGAAATTATTCAGACCACAGGGATACATTTTATAAGCTGAC
right  CCTCCCGGGTT--CACGCCATTCTCC---TG------------CCTCAGCCTCCCAAGT----AGCTGGG
       11*11*1*11*11**111*1****1*111**111111111111**1***11111**11*1111*****11

left   ACTCATCAAAGCATATACATGAGAGTAGTTCTT-------TTTTTTTTTTTT----TT------------
chrom  ACTCATCAAAGCATATACATGAGAGTAGTTCTTAAGATGCTTCTATTTCTCTAAGGTTATCTAGCTAGAA
right  ACT-----------ACAGGCGAGAGTAGTTCTTAAGATGCTTCTATTTCTCTAAGGTTATCTAGCTAGAA
       ***11111111111*1*111*************2222222**2*2***2*2*2222**222222222222

left   -TTTTTTGAGACGG--AGTCTCGCTC------TGTCGCCCAAGCTGGAGTGCAGTGGCGGGATCTCGGCT
chrom  GTTTTTAGAGATGGTCAGTTCAGCTGGTAACGTCTATCCCAAACATTTTTTTACCCCAAGA-CTTGGTT
right  GTTTTTAGAGATGGTCAGTTCAGCTGGTAACGTCTATCCCAAACATTTTTTTANNNNNNNN-NNNNNNN
       2*****2****2**22***222***2222222*2*22*****2*22222*2222NNN1NN11211N11N1

left   --CACTGCAAGCTCC-------GCCTCCCGGGTTCACGCCATTC---TCCTG-C
chrom  AACACTTCTATGTTAAAATGATGCAGAACTTGTAAACCTCATTCCATTACTGGC
right  NNNNNNNNNNNNNNNNNNNNNNNNNNNNNNNNNNNNNNNNNNN-----------
       NN1111N1N1NN1NNNNNNNNN11NNNN1NN11NN11NN11111   1N111 1
```

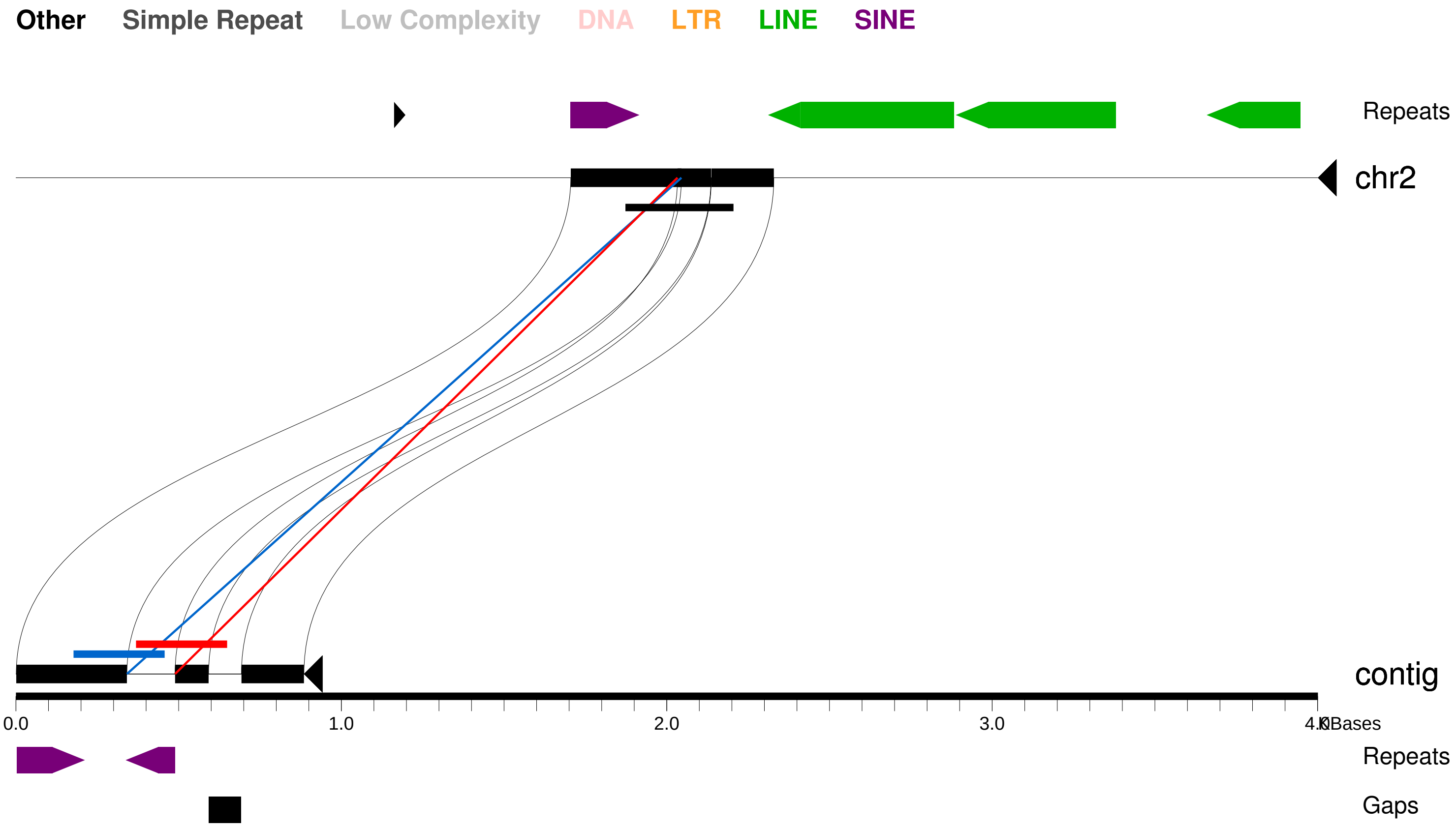

siteID: chr2_36476689 Scafftig1 - Breakpoint: chr2:36476685
chr2:36476662-36476744
Overlap Length: 37 INS size: 431 Genotyped: No

```
left    TCT-----------TTTCCATAATGGCTGTACTA-------------------------GGCTTTTATTT
chrom   TCT-----------TTTCCATAATGGCTGTACTA-------------------------GGCTTTTATTT
right   CCACCCGCCTCGGCCTCCCAAAGTGCTGGGATTACAGGCGTGAGCCACCGCGCCCGGCCGGCTTTTATTT
        1*1111111111111*1***1*1**111*1*1**1111111111111111111111111***********

left    TTTATAGCATTTTTTTCAGGCATACTNNNNNNNNNNNNNNNNNNNNNNNNNNNNNNNNNNNNNNNNNNNNN
chrom   TTTATAGCATTTTTTTCAGGCATACTGTCTCAGCAGCTCTCACTCAGTA---------------------
right   TTTATAGCATTTTTTTCAGGCATACTGTCTCAGCAGCTCTCACTCAGTA---------------------
        **************************222222222222222222222222222222222222222222222

left    NNNNNNNNNNNNNNN
chrom   ---------------
right   ---------------
        222222222222222
```

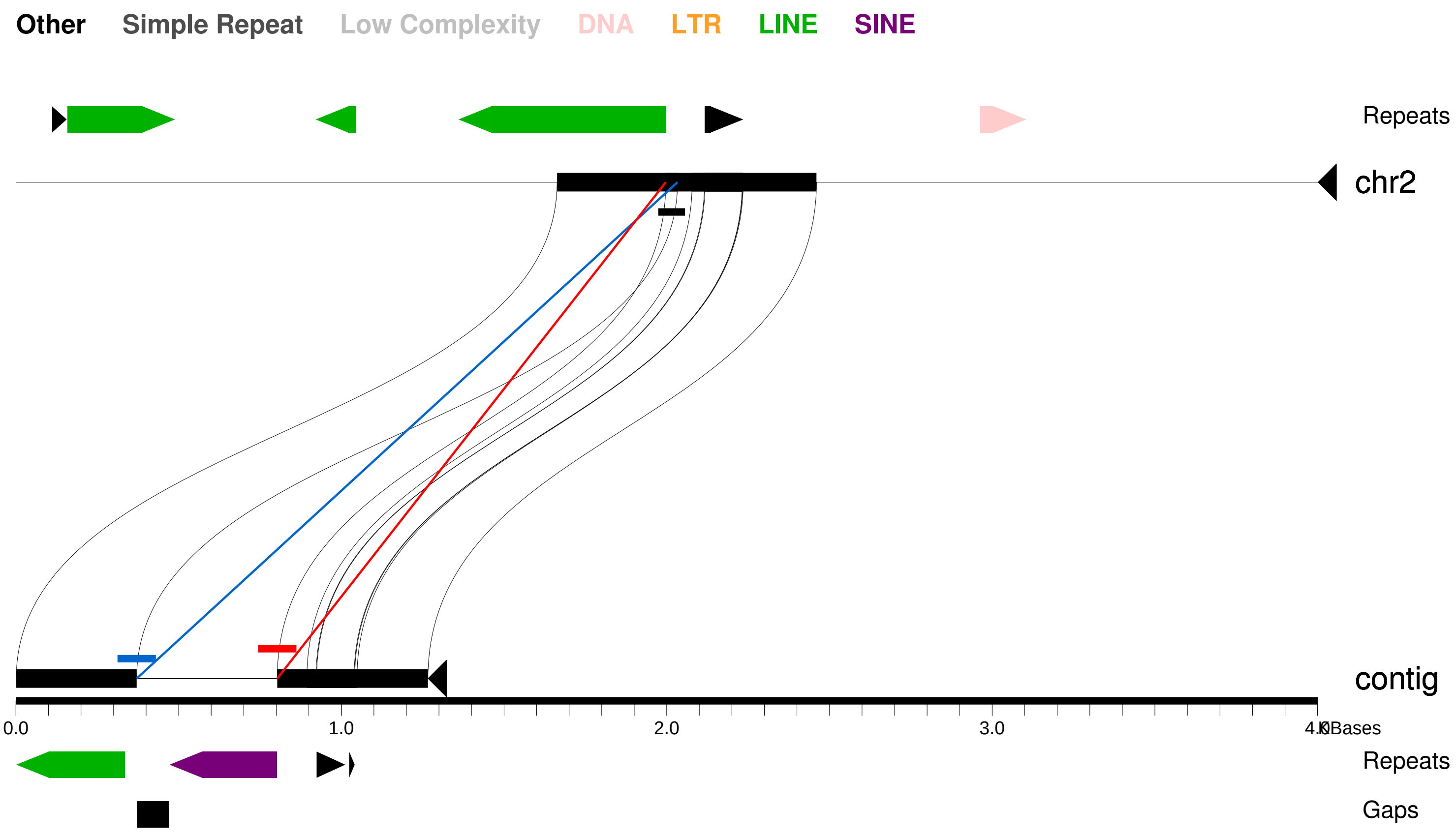

siteID: chr2_40613681 Scafftig2 + Breakpoint: chr2:40613682
chr2:40613522-40613848
Overlap Length: 7 INS size: 435 Genotyped: No

```
left   ------TGAGGCTTCCTCTCAGACCTTGGGACAAAGGTCTGAGAAATAATATGAGGCTTTTACATTATTT
chrom  CAAACCTGAGGCTTCCTCTCAGACCTTGGGACAAAGGTCTGAGAAATAATATGAGGCTTTTACATTATTT
right  NNNNNNNNNNN-----------------------------------------------------------
       NNNNNN1111111111111111111111111111111111111111111111111111111111111111

left   AATCTCACCAAAGAATGAATGCATGAAAAATAAAGCCTAAAGCTATTTTAGATCAGGATTTTTATTTAAC
chrom  AATCTCACCAAAGAATGAATGCATGAAAAATAAAGCCTAAAGCTATTTTAGATCAGGATTTTTATTTAAC
right  ----------------------------------------------------------------------
       1111111111111111111111111111111111111111111111111111111111111111111111

left   ATCAAGTAAATGAAAAAAGAGATAATGGCC---GGG----CGCGGTG----------------GCTCACG
chrom  ATCAAGTAAATGAAAAAAGAGATAATGAATAATGGGACTACCCTTTTATGATATACAAAGCAAGGTATTG
right  --------------------GATAATGAATAATGGGACTACCCTTTTATGATATACAAAGCAAGGTATTG
       11111111111111111111*******222222***2222*2*22*22222222222222222*2*222*

left   CCTGTAATCCCA----GCACTTTGGGAGGCCGAGGC---------GGGTGGATCATGAGGTCAGGAGATC
chrom  GGTCTAATTACTTGATGAAATTTAAAAATCAAAAAAAAAAATAAAGGGAAAACAATGCAGTTAAGTTTCC
right  GGTCTAATTACTTGATGAAATTTAAAAATCAAAAAAAAAAATAAAGGGAAAACAATGCAGTTAAGTTTCC
       22*2****22*22222*2*2***222*22*22*222222222222***222*22***22**2*2*2222*

left   GAGACCATCCTGGCTAACAAGGTG-----AAACCCCGTCTCTACTAAA
chrom  AAGTATATTATTAATAAATAATTGTAGGAAAAGTCAGTAAGT-CCATA
right  AAGTATATTATTAAT---------------------------------
       2**222**22*222*11NN1NN11     111NN1N11NNN121N1N1
```

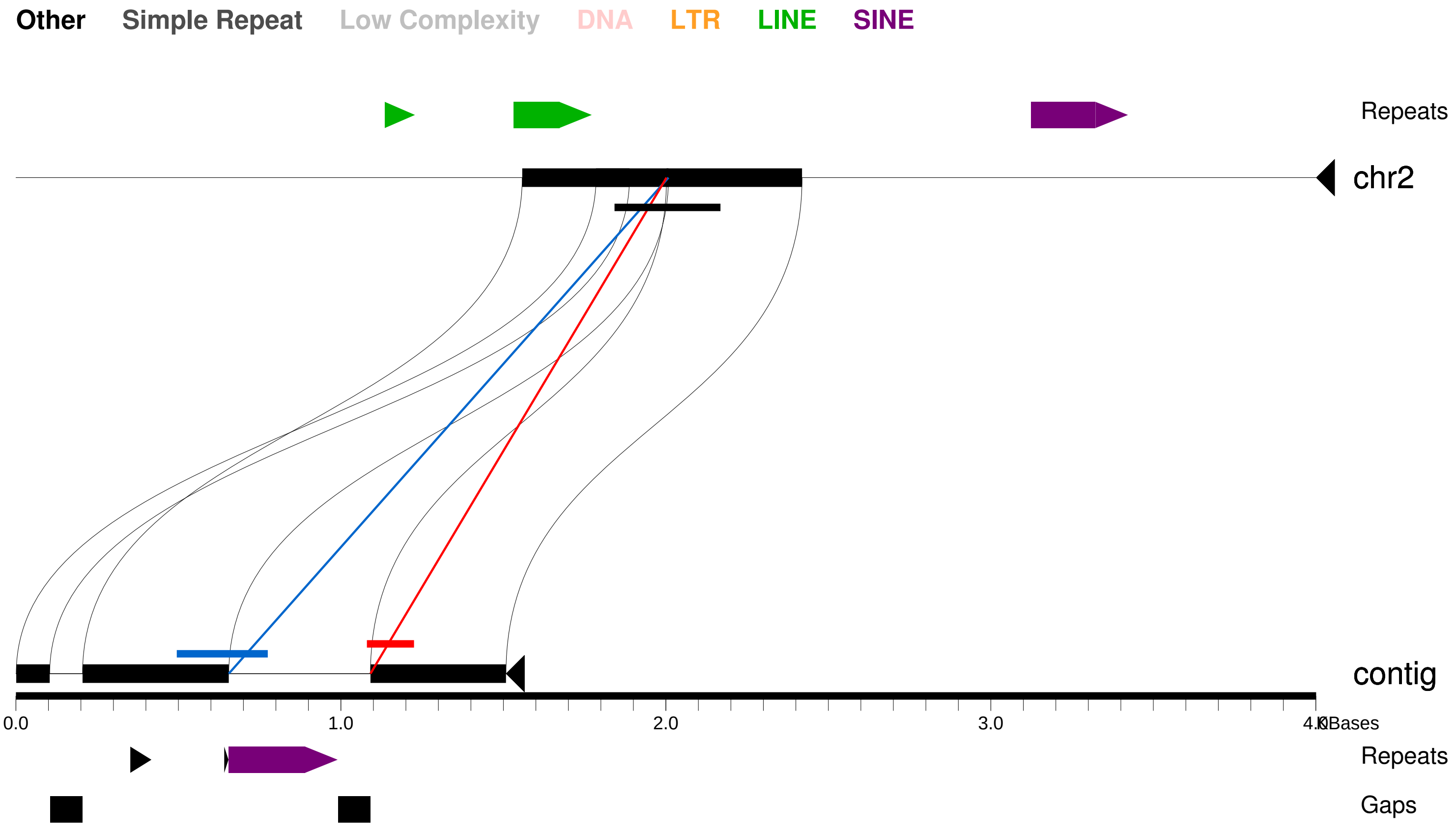

siteID: chr2_41985109 Scafftig3 - Breakpoint: chr2:41985107
chr2:41985061-41985166
Overlap Length: 13 INS size: 295 Genotyped: Yes

```
left   T------AAGAAAGTTTGAAAAT-------AACAAAACACATATGTACGGGGGTATGTCAAAGACTCAGA
chrom  T------AAGAAAGTTTGAAAAT-------AACAAAACACATATGTACGGGGGTATGTCAAAGACTCAGA
right  AGCCTGGGCGACAGAGCGAGACTCCGTCTCAAAAAAAAAAAAAAAAAAAAAAAAAAAAAAAAGACTCAGA
       111111111**1**111**1*1*1111111**1****1*1*1*111*1111111*1111***********

left   AGCGGCCGGGCGCGGTGGCTCACGCCTGTAATCCCAGCACTTTGGGAGGCCGAGGCGGGCGG
chrom  AG---CCAAATGAAAAAACTCCC------AATGGC--CAAAGCGGGAA--CAATTCAAGCAA
right  AG---CCAAATGAAAAAACTCCC------AATGGC--CAAAGCGGGAA--CAATTCAAGCAA
       **222**2222*222222***2*222222***22*22**2222****222*2*22*22**22
```

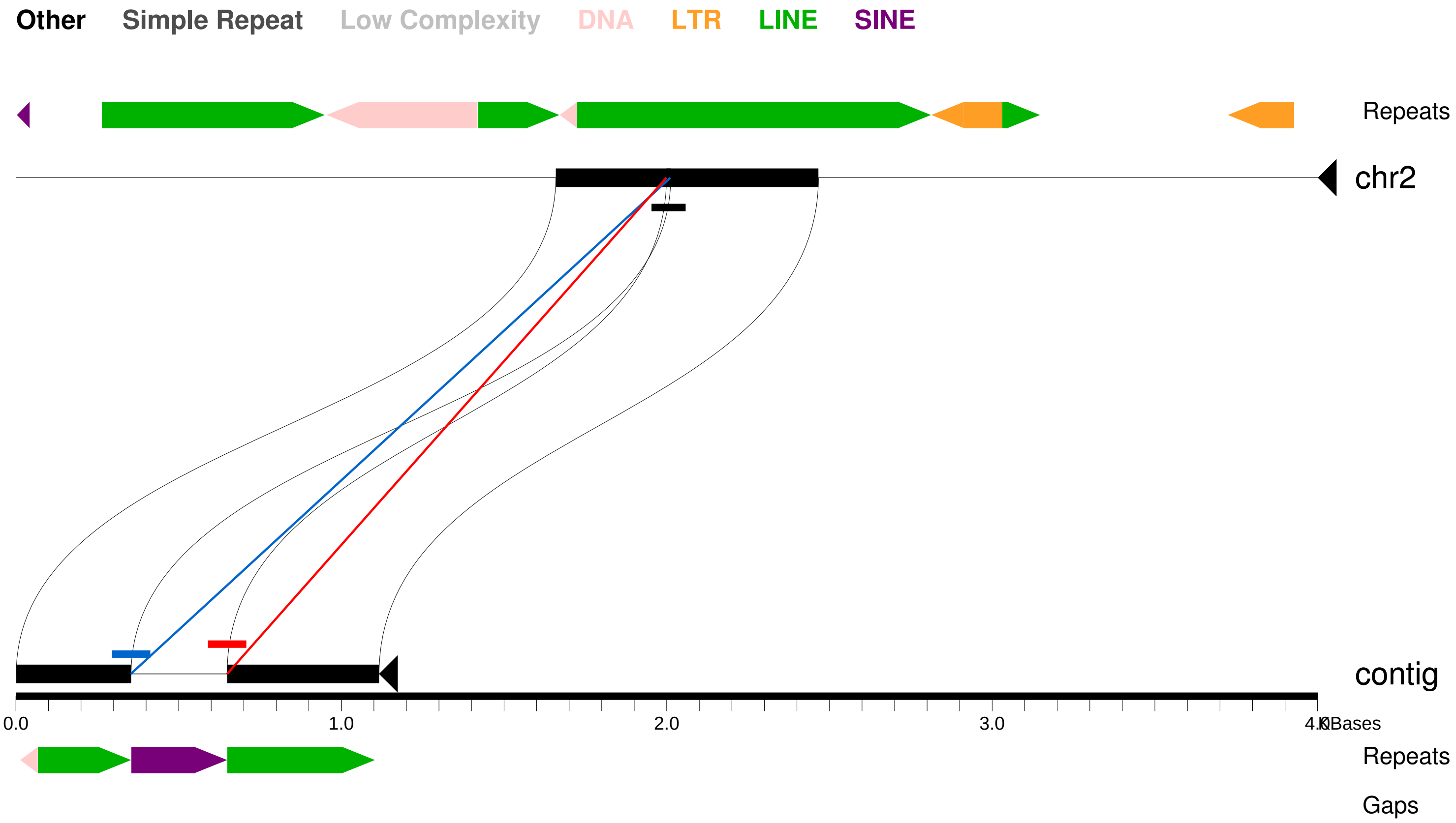

siteID: chr2_43024062 Scafftig7 + Breakpoint: chr2:43024068
chr2:43024016-43024127
Overlap Length: 8 INS size: 300 Genotyped: Yes

```
left    T-CAAAACTAGAC-----AAAACTCTCTCACTACTAAA--AACGCATATAGACAGGGAAGCATTTTTTTTT
chrom   T-CAAAACTAGAC-----AAAACTCTCTCACTACTAAA--AACGCATATAGACAGGGAAGCATTTTTTTCA
right   CGCCCGCCTCGGCCTCCCAAAGTGCTGGGATTACAGGCGTGAGCCACCGCGCCCGGCCA-CATTTTTTTCA
        11*1111**1*1*11111***111**111*1***1111111*11**1111*1*1**11*1********22

left    TTTTTTTTTTTTTTTTTTTTTTT---TTTTTTGAGACGGAGTCTCGCTCTGTCGCCCAGGC
chrom   AGTTATACTAATAAGTTGACTTGGATGCTTTAATA---------GCTTTCATGAATAAT-
right   AGTTATACTAATAAGTTGACTTGGATGCTTTAATA---------GCTTTCATGAATAAT-
        22**2*22*22*222**222**222*22***2*2*222222222***2*222*222*222
```

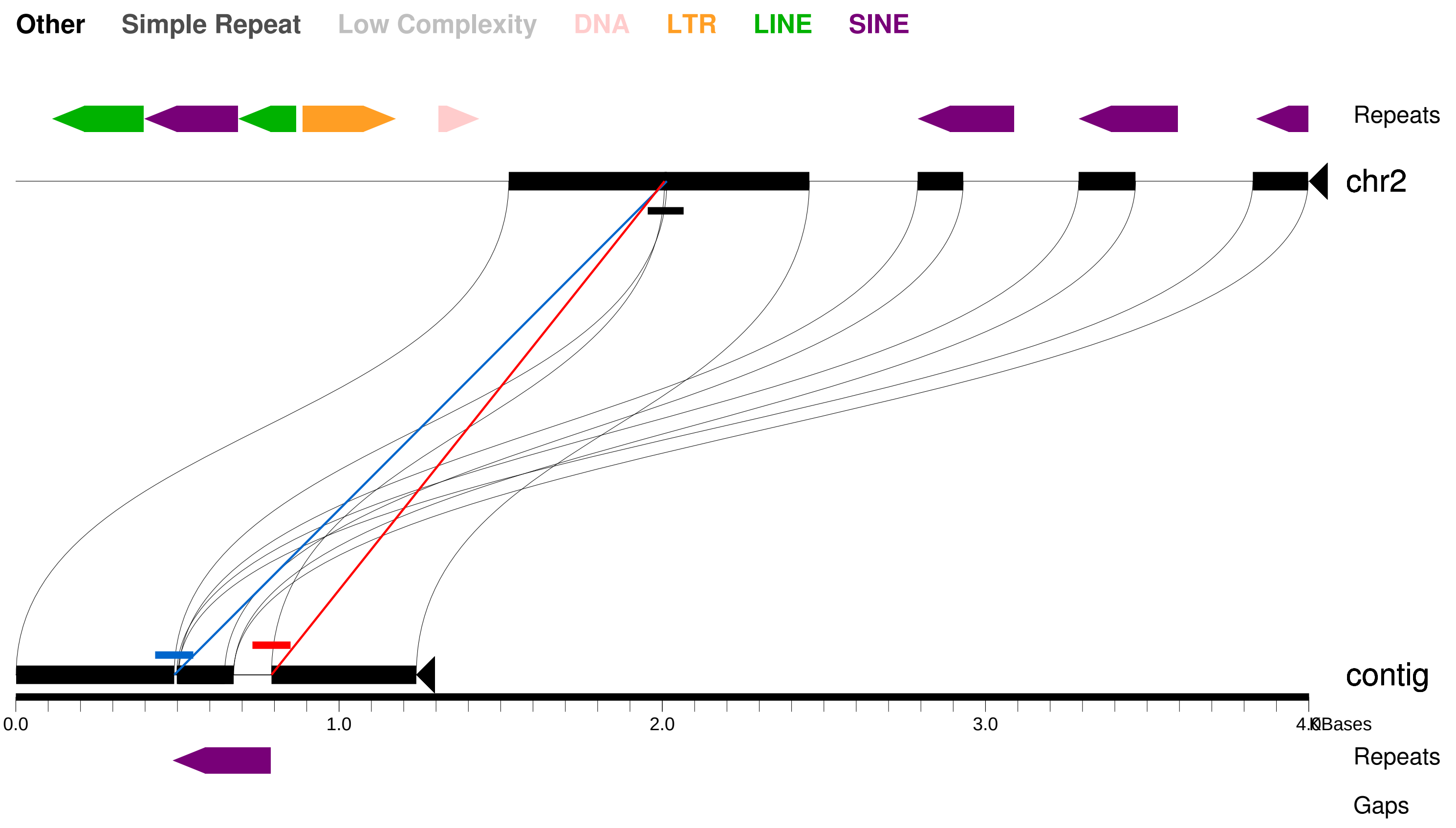

siteID: chr2_44825838 Scafftig16 - Breakpoint: chr2:44825836
chr2:44825791-44825895
Overlap Length: 15 INS size: 304 Genotyped: Yes

```
left     ATCTT-AGCT-------CAGAA-GAACCTAATTTGCAGCAAGCTTAGGACCTTT-----AAAATCAGAAG
chrom    ATCTT-AGCT-------CAGAA-GAACCTAATTTGCAGCAAGCTTAGGACCTTT-----AAAATCAGAAG
right    CACTCCAGCCTGGGCGACAGAGCGAGACTCCGTCTCAAAAAAAAAAAAAAAAAAAAAAAAAAAATCAGAAG
         11**11***11111111****11**11**111*11**11**1111*11*1111111111***********

left     TCCTGGCCGGGCGCGGTGGCTCACGCCTGTAATCCCAGCACTT-TGGGAGGCCGAGGCGGGCGG
chrom    TCCTT-----------TGGGTCTTGTCTT--AGCAACTTACTTGTTAAAGGCTCTGGGTG--GG
right    TCCTT-----------TGGGTCTTGTCTT--AGCAACTTACTTGTTAAAGGCTCTGGGTG--GG
         ****222222222222***2**22*2**222*2*22222****2*222****222**22*22**
```

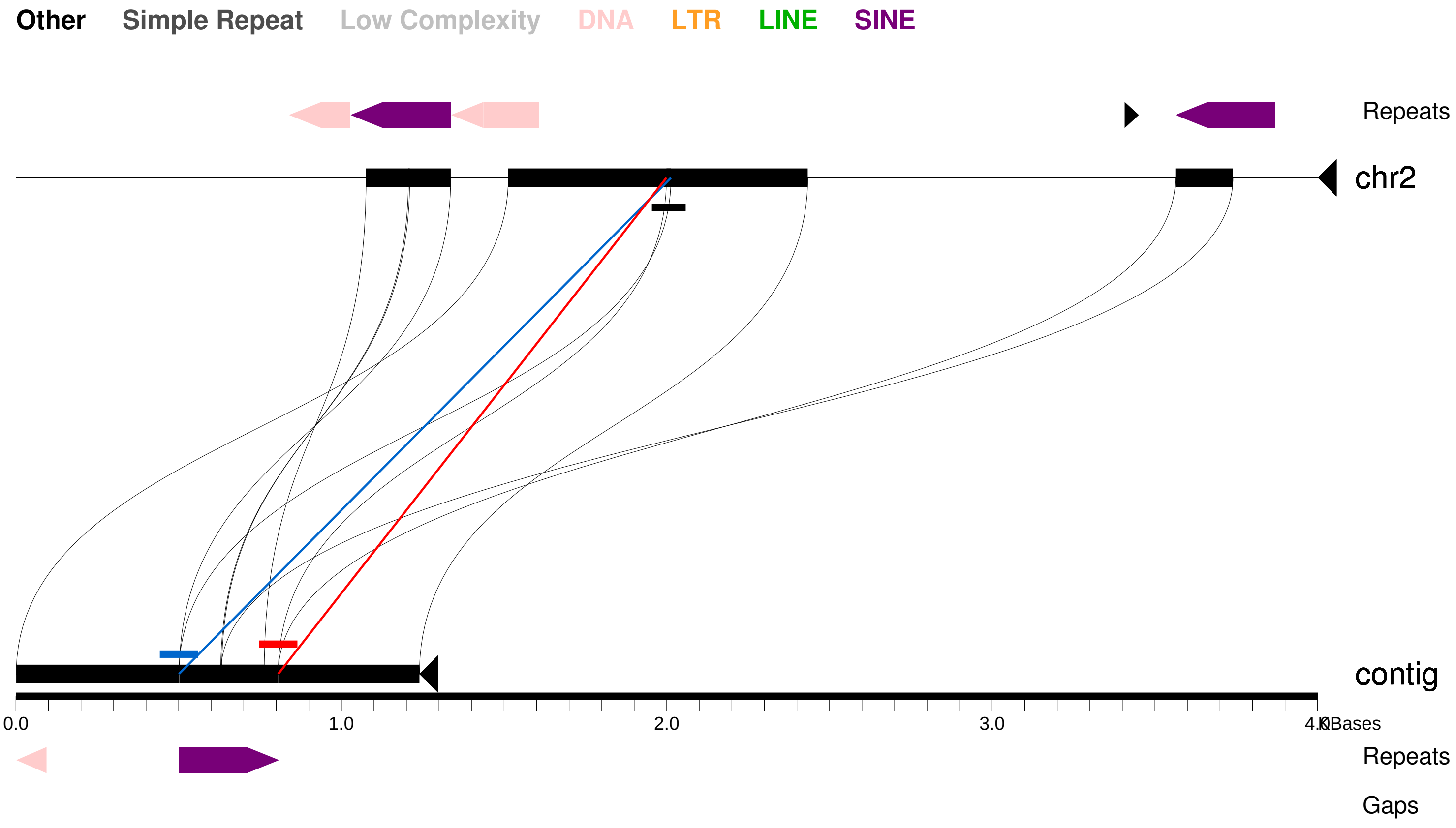

siteID: chr2_46196198 Scafftig5 - Breakpoint: chr2:46196185
chr2:46196140-46196244
Overlap Length: 15 INS size: 306 Genotyped: Yes

```
left     ACCTGCC-TACAGCCTGTGAGA------GATTATCCCGTGCATTGGGGCTGT-------AAAATATTCAT
chrom    ACCTGCC-TACAGCCTGTGAGA------GATTATCCCGTGCATTGGGGCTGT-------AAAATATTCAT
right    GTCCGCAGTCCGGCCTGGGCGACAGAGCGAGACTCCGTCTCAAAAAAAAAAAAAAAAAAAAAAAATTCAT
         11*1**11*1*1*****1*1**111111**111***1111**11111111111111111****1******

left     TGATGGCCGGGCGCGGTGGCTCACGCCTGTAATCCCAGCACTTTGGGAGGCCGAGGCGGGTGG
chrom    TGATATAAAACCT-----TCTGGAGTTTGAAGTTCATAGCCTAATTGAATCAA---------G
right    TGATATAAAACCT-----TCTGGAGTTTGAAGTTCATAGCCTAATTGAATCAA---------G
         ****2222222*2222222**222*22**2*2*2*22222**2222**22*22222222222*
```

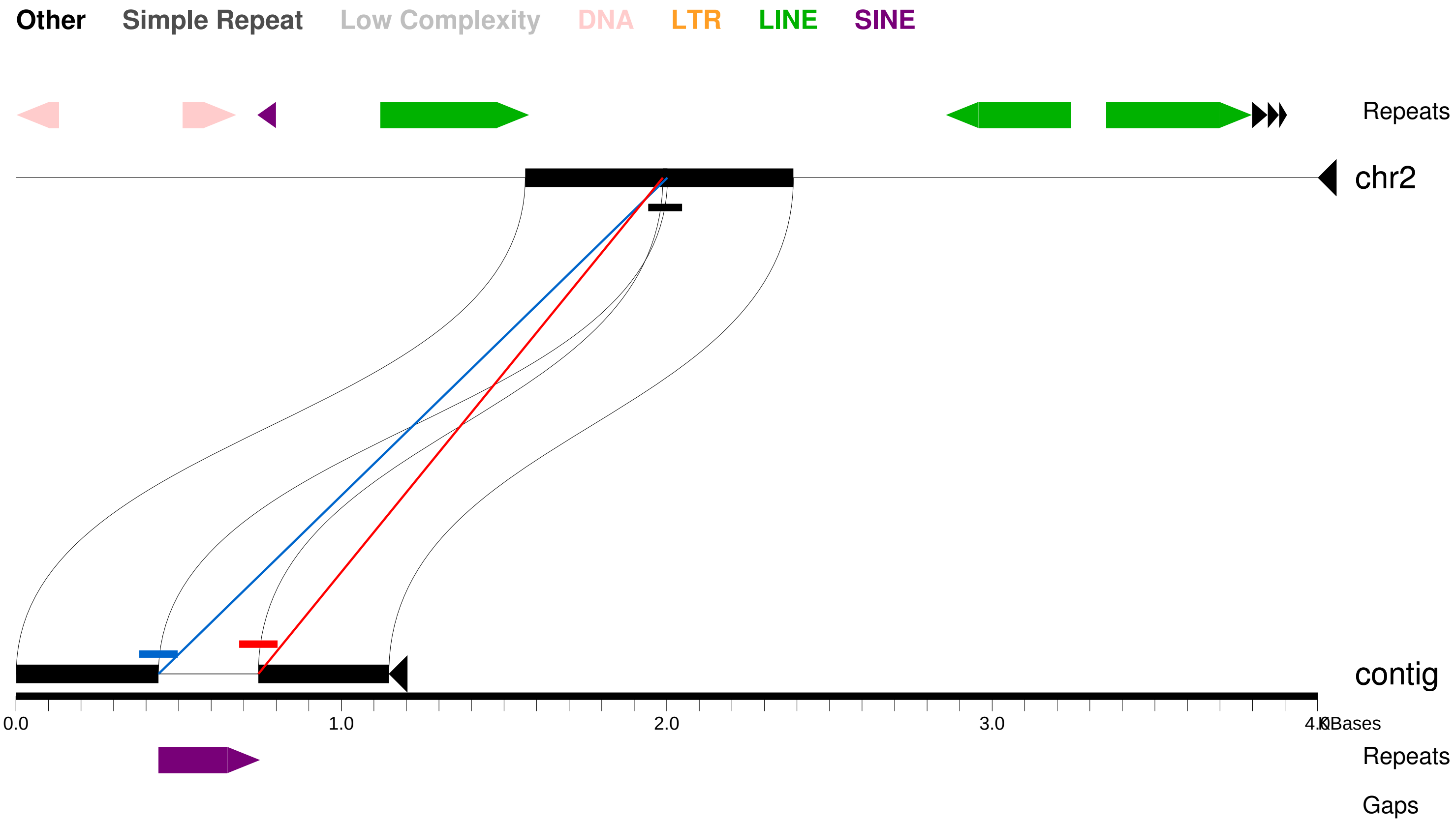

siteID: chr2_46928965 Scafftig6 - Breakpoint: chr2:46928951
chr2:46928894-46929010
Overlap Length: 3 INS size: 287 Genotyped: Yes

```
left   GTCATTTAGGTGAAAATATTAAAT--GAATGCCCTAGAG-TAGTTTAGCATTCTATAATTTTTTTTTTTTT
chrom  GTCATTTAGGTGAAAATATTAAAT--GAATGCCCTAGAG-TAGTTTAGCATTCTATAATTTTTATGCTAA
right  CTGACCTCG-TGATCCGCCCGCCTCGGCCTCCCAAAGTGCTGGGATTACAGGCGTGAGCCTTTATGCTAA
       1*1*11*1*1***1111111111*11*11*1**11**1*1*1*11*11**11*111*111***2*22*22

left   TTTTTTTTTTTTGAGACGGAGTCTCGCTCTGTCGCCCAGGCTGGAGTGCAGT--
chrom  AATTAATTGCTTAAAA--GATTTTCAAAATTCAGA--AGGCTTCTATATTTTGA
right  AATTAATTGCTTAAAA--GATTTTCAAAATTCAGA--AGGCTTCTGTATTTTGA
       22**22**22**2*2*22**2*2**2222*222*222*****222N*2222*22
```

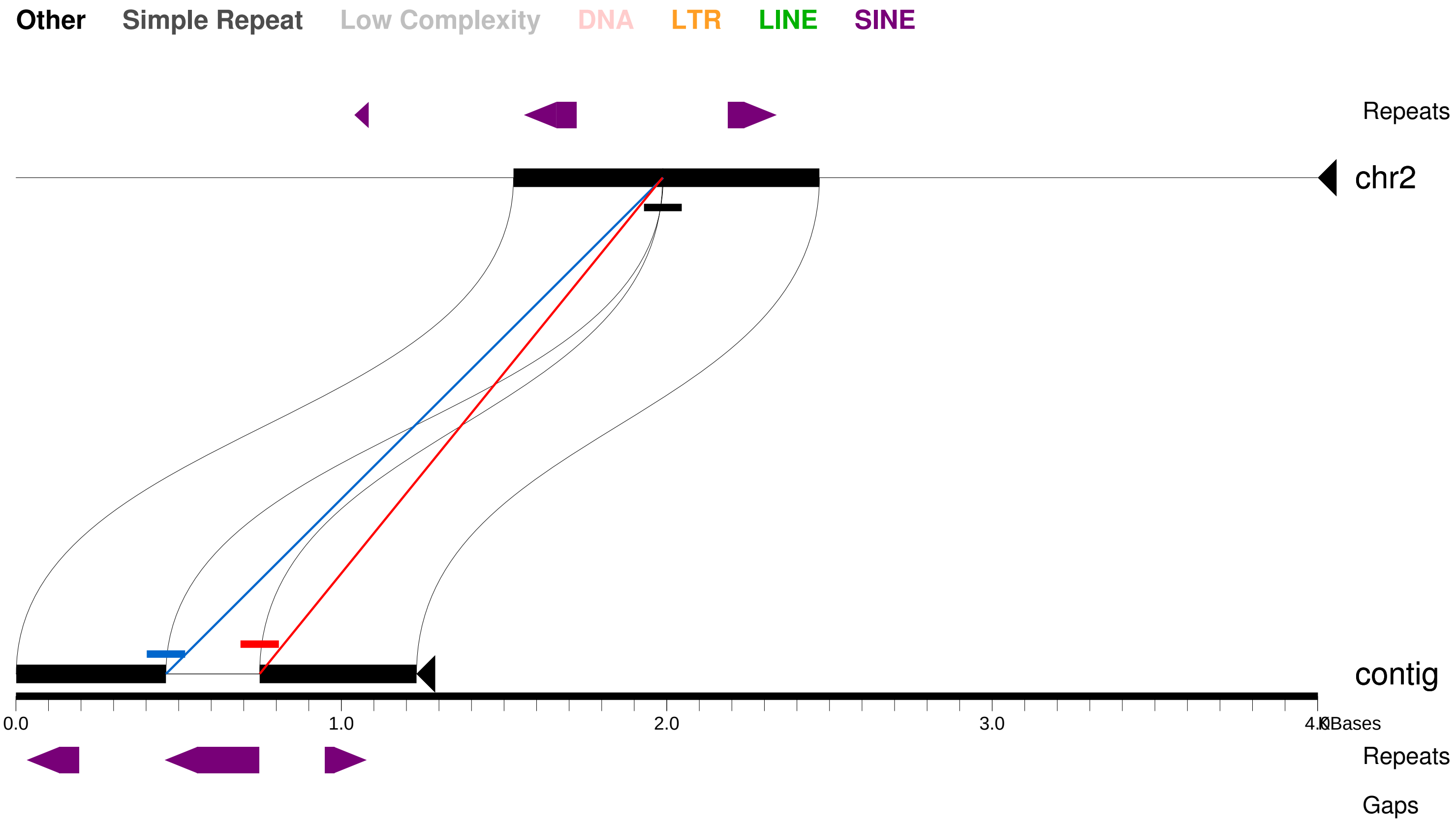

siteID: chr2_49733496 Scafftig11 + Breakpoint: chr2:49733481
chr2:49733462-49733540
Overlap Length: 14 INS size: 319 Genotyped: Yes

```
left    --------------------ATTTGTATGGAAAAAAAAT--------------------TTTAATATTTT
chrom   --------------------ATTTGTATGGAAAAAAAAT--------------------TTTAATATTCT
right   CCACCCGCCTCGGCCTCCCAAAGTGCTGGGATTACAGGCGTGAGCCACCGCGCCCGGCCTTTAATATTCT
        11111111111111111111*11**111***11*1*11111111111111111111111*********2*

left    TTTTTTTTTTTTTTTTTTTTTTTTTTTTTTTTTTGAGACGGAGTCTCGCTCTGTCGCCCAGGCCGGACTG
chrom   TTTATTCTTACCACTTTTTATTTATGTTTTGTATTTCATG---TCT------------------------
right   TTTATTCTTACCACTTTTTATTTATGTTTTGTATTTCATG---TCT------------------------
        ***2**2**22222*****2***2*2****2*2*222*2*222***222222222222222222222222

left    CGGACTGCAGTGGCGCAAT
chrom   ---AATTTA----------
right   ---AATTTA----------
        222*2*22*2222222222
```

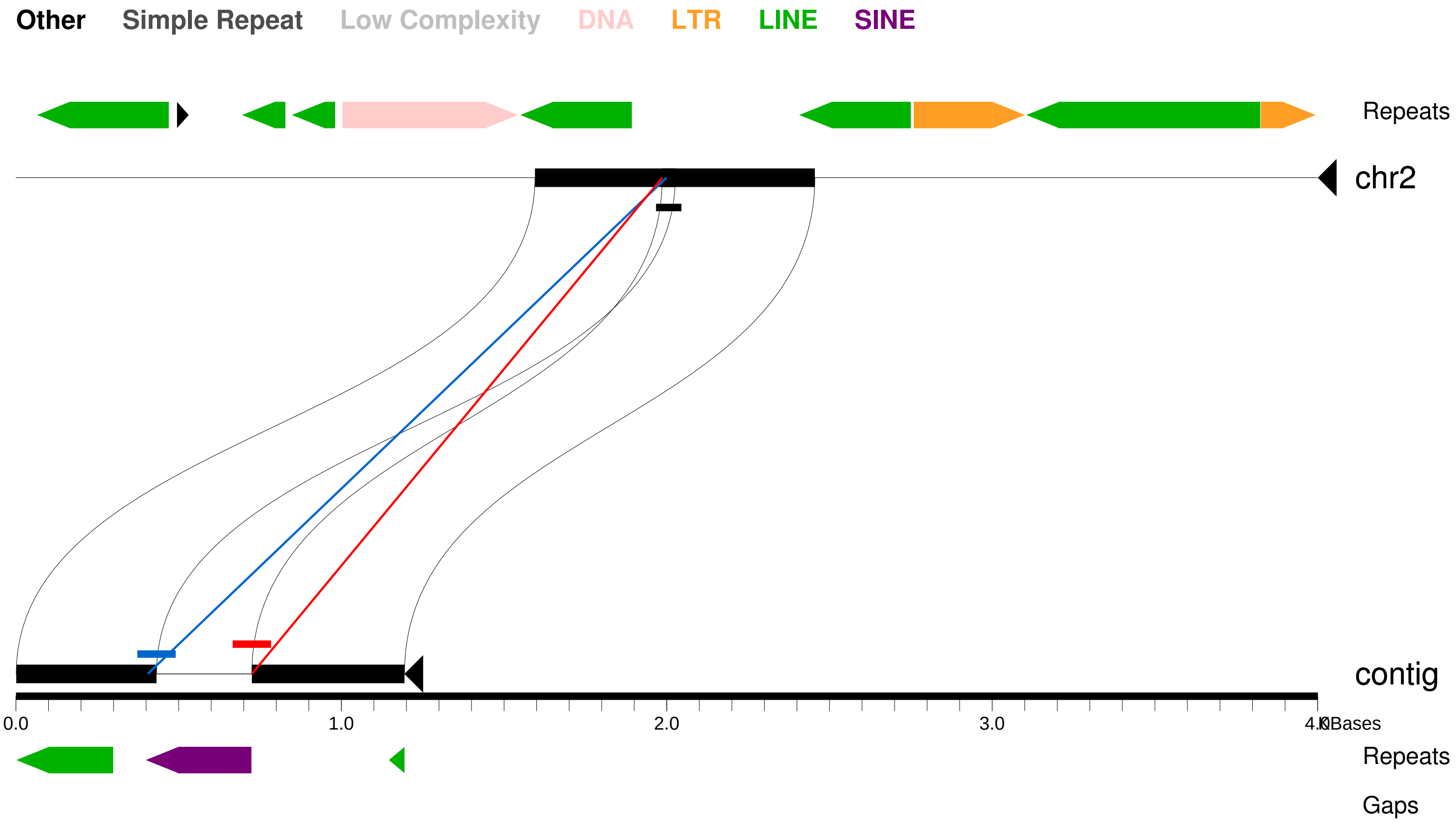

siteID: chr2_50602625 Scafftig7 + Breakpoint: chr2:50602630
chr2:50602585-50602689
Overlap Length: 15 INS size: 315 Genotyped: Yes

```
left     AC----AGCTAG-CATGCCT-----CTGAGTTAAAATTTGAAAGCTA----GTCTGTTTAATTCTGAAGC
chrom    AC----GGCTAG-CATGCCT-----CTGAGTTAAAATTTGAAAGCTA----GTCTGTTTAATTCTGAAGC
right    CCACCCGCCTCGGCCTCCCAAAGTGCTGGGATTACAGGCGTGAGCCACCGCGCCCGGCCAATTCTGAAGC
         1*111121**1*1*1*1**111111***1*1*1*1*111*11***1*1111*1*1*111***********

left     TCTTTTTTTTTTTTTTTTTTTTTTTTTTTTTTTGAGACGGAGTCTCGCTCTGTCGCCCAGGCCGG
chrom    TCTTAATCACTAAATTTTATACTACCTCTTTAGGTTGGA---TGGCA-TATCA----------
right    TCTTAATCACTAAATTTTATACTACCTCTTTAGGTTGGA---TGGCA-TATCA----------
         ****22*222*222****2*22*222*2***22*22***222*2**22*2**22222222222
```

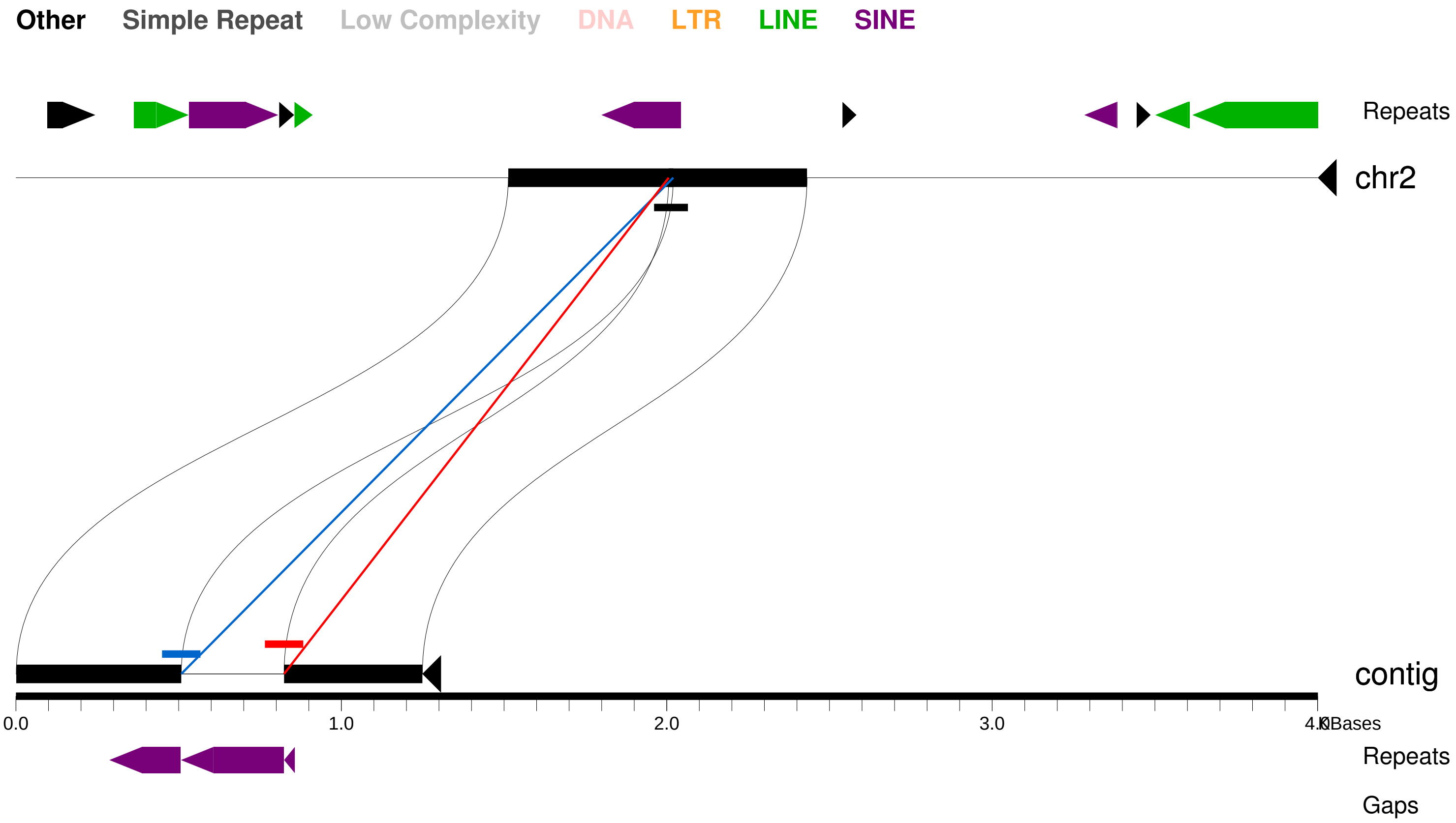

siteID: chr2_51613500 Scafftig1 - Breakpoint: chr2:51613470
chr2:51613427-51613529
Overlap Length: 17 INS size: 306 Genotyped: Yes

```
left     TTTTCTCTTG----------ATATTCCAT-----AACTTAAATACCATAATGTTAA-ATTAATAATATAT
chrom    TTTTCTCTTG----------ATATTCCAT-----AACTTAAATACCATAATGTTAA-ATTAATAATATAT
right    CTCCAGCCTGGGCGACAGCGAGACTCCGTCTCAAAAAAAAAAAAAAAAAATAATAATAATTAATAATATAT
         1*1111*1**1111111111*1*1***1*11111**111***1*11*1***11***1*1***********

left     AAACACGGCCGGGCGCGGTGGCTCAC-GCCTGTA-ATCCCAGCACTTTGGGAGGCCGAGGCGGGCGG
chrom    AAACACTA----------TGATACAAAGTCAGTATAT-------CTTATGTTACATGATAAGGGAA-
right    AAACACTA----------TGATACAAAGTCAGTATAT-------CTTATGTTACATGATAAGGGAA-
         ******222222222222**222**22*2*2***2**2222222***22*222222**222***222
```

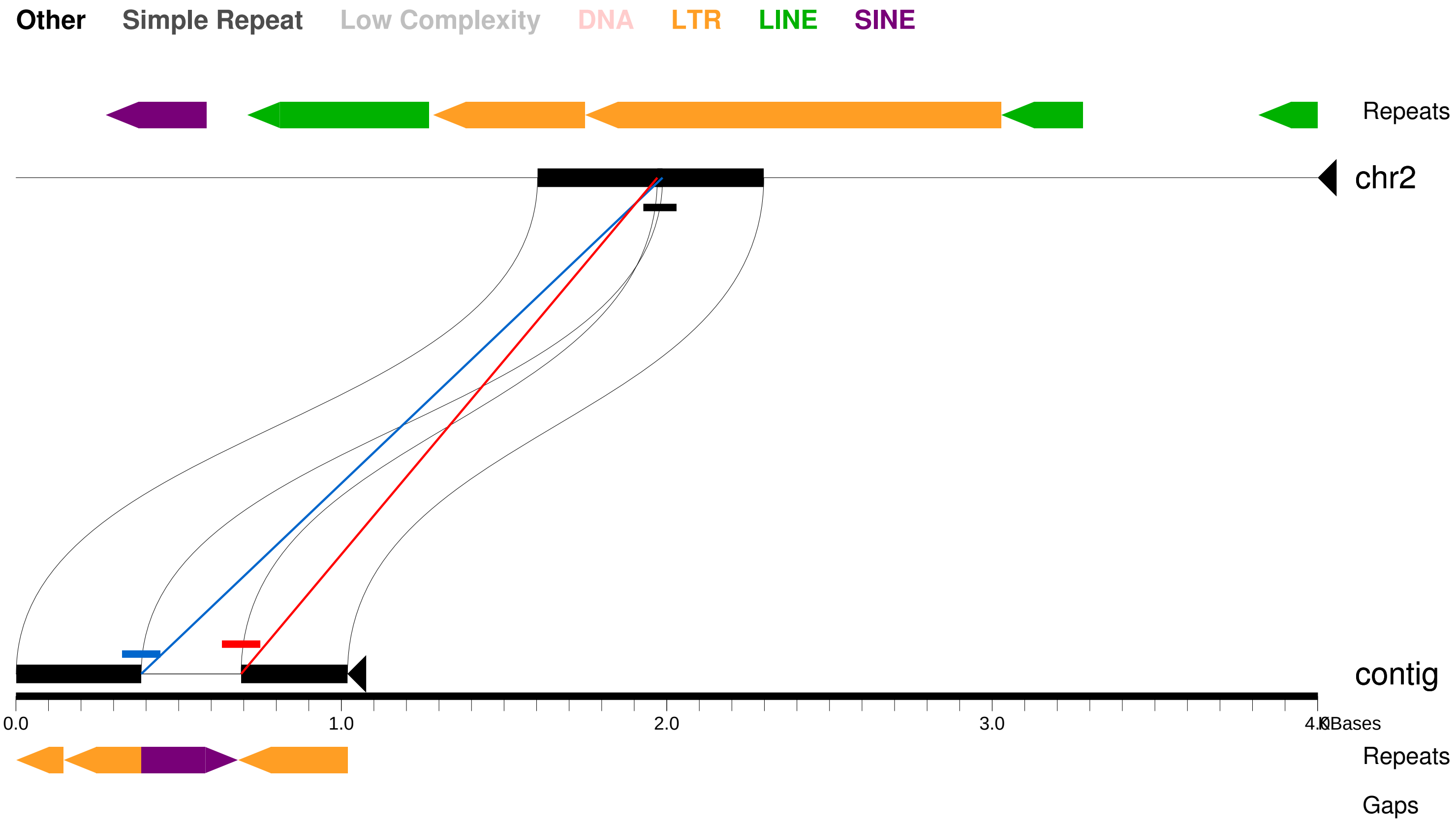

siteID: chr2_51996012 Scafftig2 + Breakpoint: chr2:51995969
chr2:51995925-51996028
Overlap Length: 10 INS size: 302 Genotyped: Yes

```
left    TA-----CTTAAATATATTTATAAGCAATAATTTTATTTTGTGAATTTT-----------GTAAAATTTT
chrom   TA-----CTTAAATATATTTATAAGCAATAATTTTATTTTGTGAATTTT-----------GTAAAATTTT
right   CCGCCCGCCTCGGCCTCCCAAAGTGCTGGGATTACAGGC-GTGAGCCACCGCGCCCGGCCGTAAAATTTT
        1111111*1*11111*1111*111**1111***11*1111****1111111111111111**********

left    TTTTTTTTTTTTTTTTTTTTTGAGAGGGAGTCTCGCTCTGTCGCCCAGGCTGGAGTGCAGTGGCG
chrom   GTATTTAAAATTATTTGACATTCCTAGTATTTGTACAATATCA-------TGGTTTT--------
right   GTATTTAAAATTATTTGACATTCCTAGTATTTGTACAATATCA-------TGGTTTT--------
        2*2***2222**2***2222*22222*2*2*2222*22*2**22222222***22*222222222
```

Other Simple Repeat Low Complexity DNA LTR LINE SINE

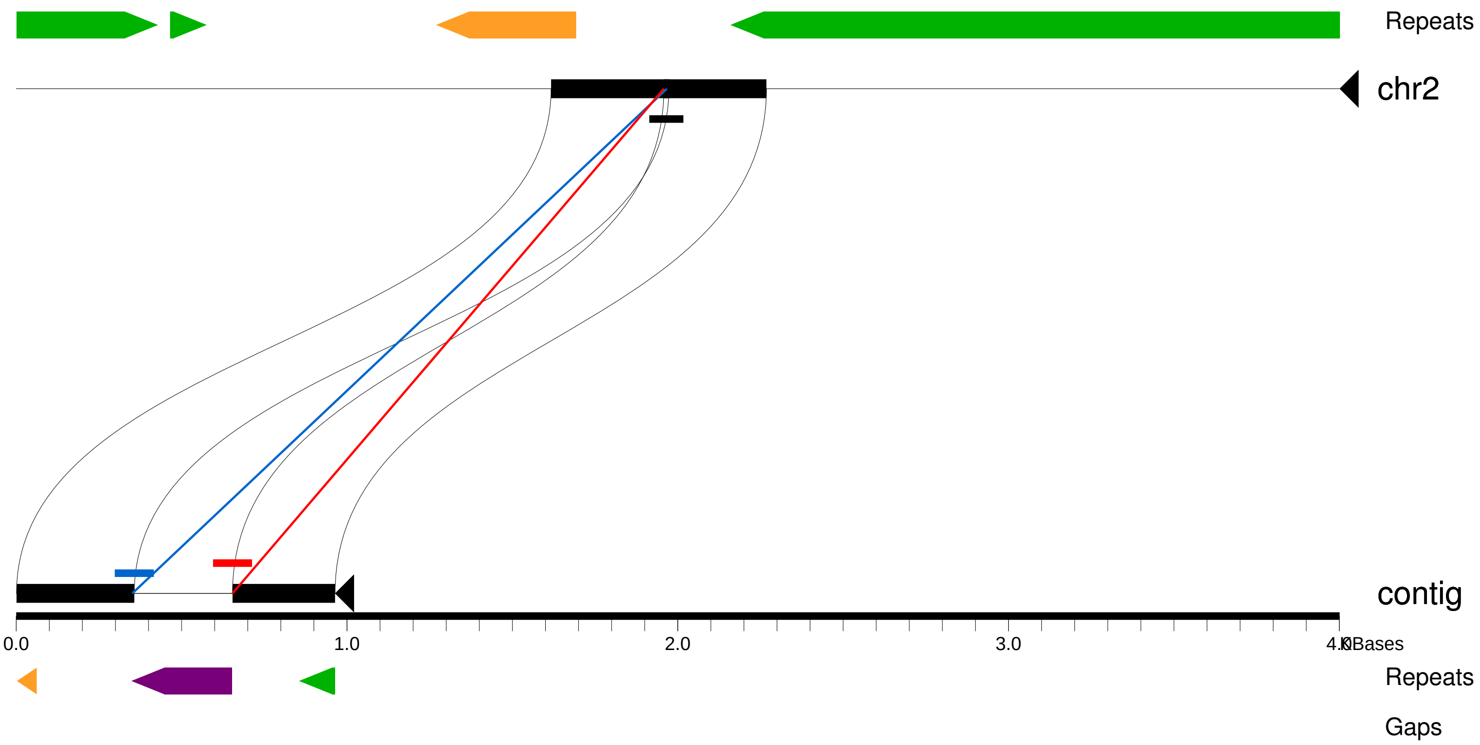

siteID: chr2_54673595 Scafftig1 - Breakpoint: chr2:54673580
chr2:54673538-54673643
Overlap Length: 18 INS size: 326 Genotyped: No

```
left    AAC-CCAGT------------AAATTCTCAAATTAACAGAG-GAACAACTGGAGAAGGCTCTGATTCCTC
chrom   AAC-CCAGT------------AAATTCTCAAATTAACAGAG-GAACAACTGGAGAAGGCTCTGATTCCTC
right   TCCGCCCGCCTCGGCCTCCCAAAGTGCTGGGATTACAGGCGTGAGCCACCGCGCCCGGC-CTGATTCCTC
        11*1**1*1111111111111**1*1**111****111*1*1**1*1**1*11111***1**********

left    TCTTTNNNNNNNNNNNNNNNNNNNNNNNNNNNNNNNNNNNNNNNNNNNNNNNNNNNNNNNNNN
chrom   TCTT-------------AAGTATGGCTAAAGATAGTAGGGCCTGCACTCCAATAGCTAAGAGA
right   TCTT-------------AAGTATGGCTAAAGATAGTAGGGCCTGCACTCCAATAGCTAAGAGA
        *****2222222222222222222222222222222222222222222222222222222222
```

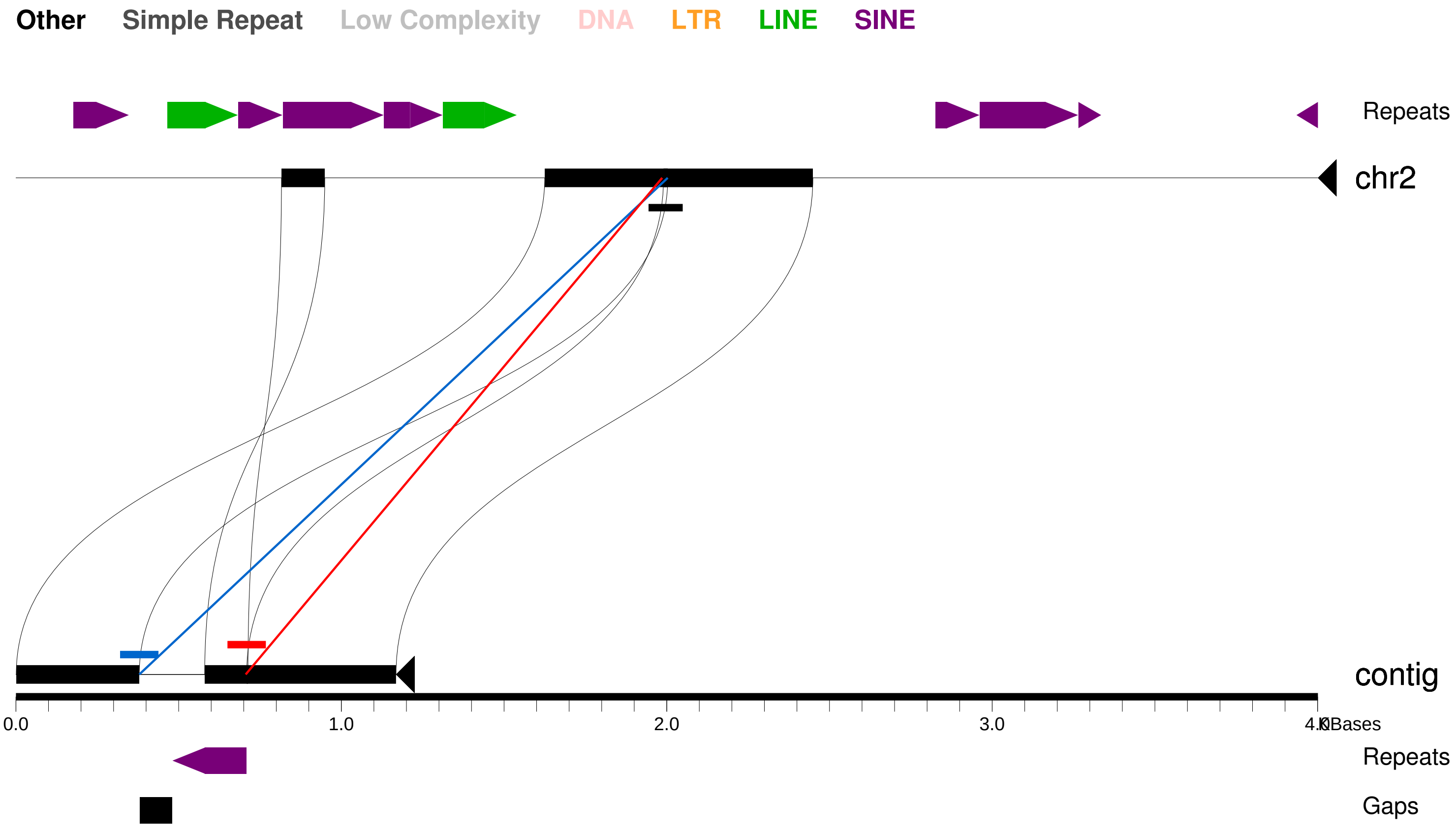

siteID: chr2_55116609 Scafftig3 - Breakpoint: chr2:55116588
chr2:55116543-55116647
Overlap Length: 15 INS size: 300 Genotyped: Yes

```
left     ATT---GAGTTTTCCTTTCCAAGAACATGGGATATAGTTCTGTTTTTT-----------AAAGTCTGCCT
chrom    ATT---GAGTTTTCCTTTCCAAGAACATGGGATATAGTTCTGTTTTTT-----------AAAGTCTGCCT
right    CCGCCCGCCTCGGCCTCCCAAAGTGCTGGGATTACAGGCGTGAGCCACCGCGCCCGGCCAAAGTCTGCCT
         111111*11*111***11*1***11*11**11**1**111**11111111111111111***********

left     TTTTTTTTTTTTTTTTT----TTTTTTTTGAGACGGAGTCTCGCTGTCGCCCAGGCTGGAGTGCAGTG
chrom    TTTTAATCTTACTTATCAGGTCTTTTTT---------TCTCTCTT-----CACTTTTATGT----TA
right    TTTTAATCTTACTTATCAGGTCTTTTTT---------TCTCTCTT-----CACTTTTATGT----TA
         ****22*2**22**2*2222*2******222222222****2**222222**222*222**2222*2
```

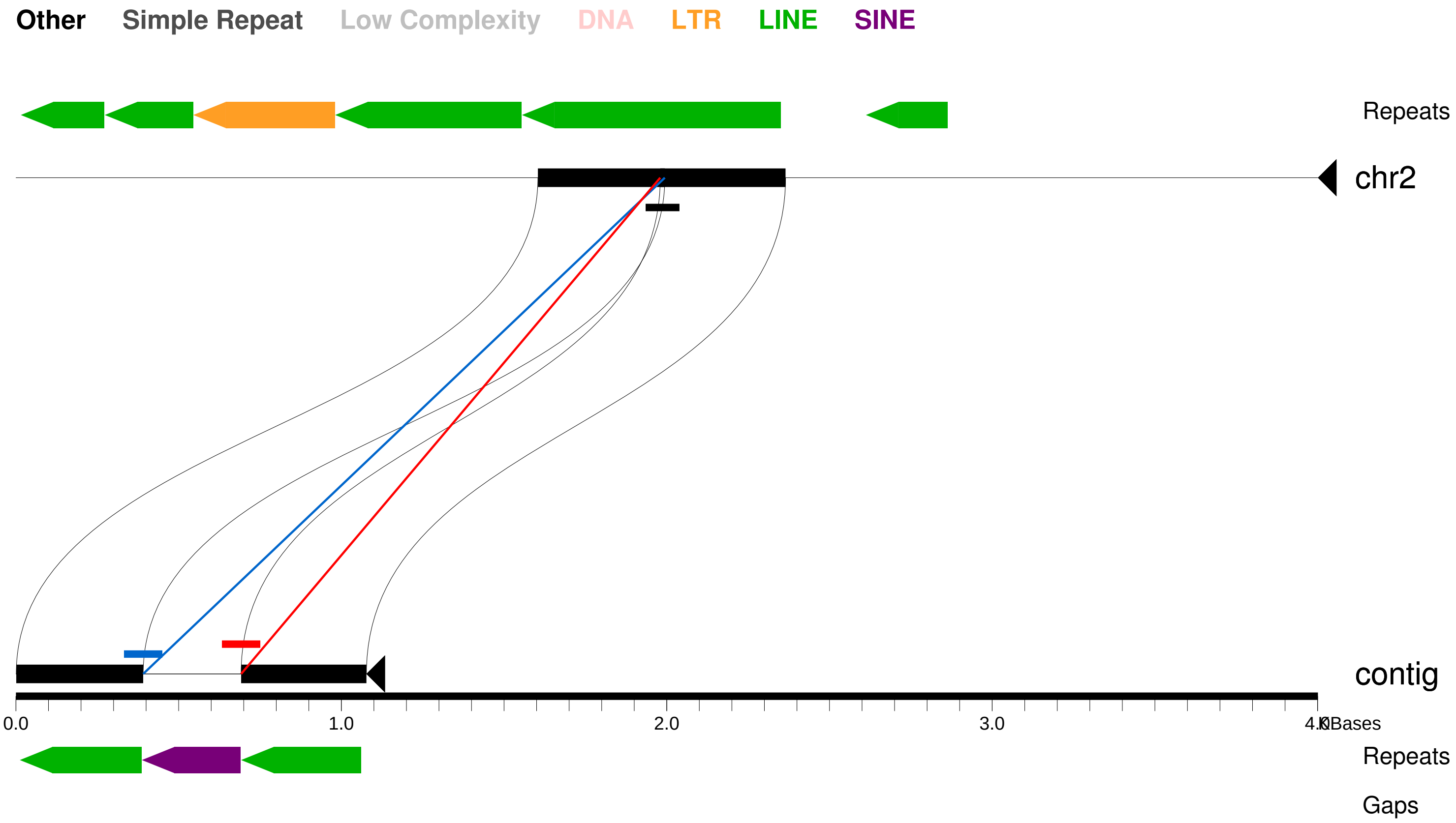

siteID: chr2_5562911 Scafftig4 - Breakpoint: chr2:5562896
chr2:5562853-5562955
Overlap Length: 17 INS size: 312 Genotyped: Yes

```
left     ATTTCTAGC----CCACTTTGGGTTAGTTCTTTTCTATTCTCCCATT------------AAAAAATTCTG
chrom    ATTTCTAGC----CCACTTTGGGTTAGTTCTTTTCTATTCTCCCATT------------AAAAAATTCTG
right    AGTCCGACCTGGGCGACAGAGCGAGACTCCGTCTCAAAAAAAAAAAAAAAAAAAAAAAAAAAAAAATTCTG
         *1*1*1*1*1111*1**111*1*11*1*1*1*1**1*1111111*11111111111111***********

left     AGTGGTGGCCGGGCGCGGTGGCTCACGCCTGTAATCCCAGCACTTTGGGAGGCCGAGGCGGGTGG
chrom    AGTGGTTAA--------GTGATTTTCTACA-TAAT------AATCTGGTTTCTATTTACTGGCA-
right    AGTGGTTAA--------GTGATTTTCTACA-TAAT------AATCTGGTTTCTATTTACTGGCA-
         ******22222222222***22*22*22*22****222222*2*2***2222222222*2**222
```

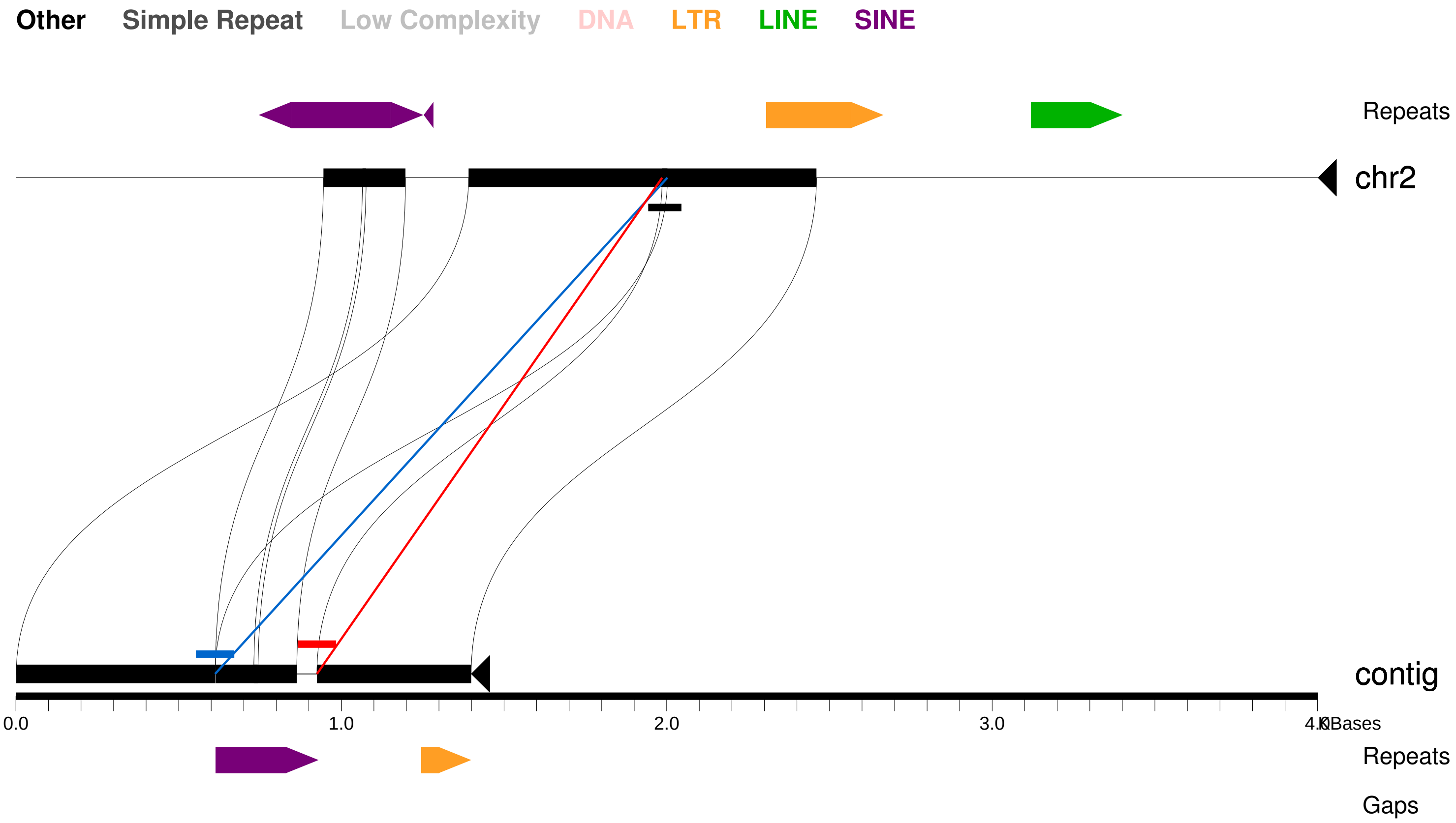

siteID: chr2_56373530 Scafftig3 + Breakpoint: chr2:56373521
chr2:56373363-56373691
Overlap Length: 11 INS size: 439 Genotyped: No

```
left   --------GAGAACAAAATGTGGGTTTGTGTGTATACTAAAGTGTCTTCATATTCCTTCCTATACTTGGG
chrom  TAAGGAGGGAGAACAAAATGTGGGTTTGTGTGTATACTAAAGTGTCTTCATATTCCTTCCTATACTTGGG
right  NNNNNNNNNNNAA---------------------------------------------------------
       NNNNNNNN111**111111111111111111111111111111111111111111111111111111111

left   TCCTAATATAACAACTGCATAAGTAAGTAACAGAAAGATGTGGATTAGCAGCAATATAAAAAAATCCTAG
chrom  TCCTAATATAACAACTGCATAAGTAAGTAACAGAAAGATGTGGATTAGCAGCAATATAAAAAAATCCTAG
right  ---------AACAA--------------------------------------------------------
       111111111*****11111111111111111111111111111111111111111111111111111111

left   GAGTTCTAACCAACAATAAACTCATTCTGGCCGGGCGCGGTGGC------TCACGCCTGTAATCCCAG-C
chrom  GAGTTCTAACCAACAATAAACTCATTCTGAGTAAGCAGTTTAGAAGCAATTAAAATCTGAAGCTACATTC
right  ------------------AACTCATTCTGAGTAAGCAGTTTAGAAGCAATTAAAATCTGAAGCTACATTC
       111111111111111111***********22222**2222*2*2222222*2*222***2*2222**22*

left   ACTTTGGG-AGGCC----GAGGCGGGCGGATCAC------GAGGTCAGGAGA-TCGAGACCATCCTGGCT
chrom  ATTATGTATAAAACAAAAGAGGGGGCAGGCACACACAACAGACGTGTTGTGGCTTCACTGCTTTTTAGCT
right  ATTATGTATAAAACAAAAGAGGGGGCAGGCACACACAACAGACGTGTTGTGGCT----------------
       *2*2**222*222*2222****2**22**22***222222**2**222*2*22*NN1NNN1N1NN1N111

left   ---------AACACGGTG------------AAACCCCGTCTCTACTAAA
chrom  TTTTTTTGGAATATGTTGCTTTCACTTTTTATAAACTGGATCTAGACCC
right  -------------------------------------------------
                11N1N1N11            1N1NN1N1NN1111NNNNN
```

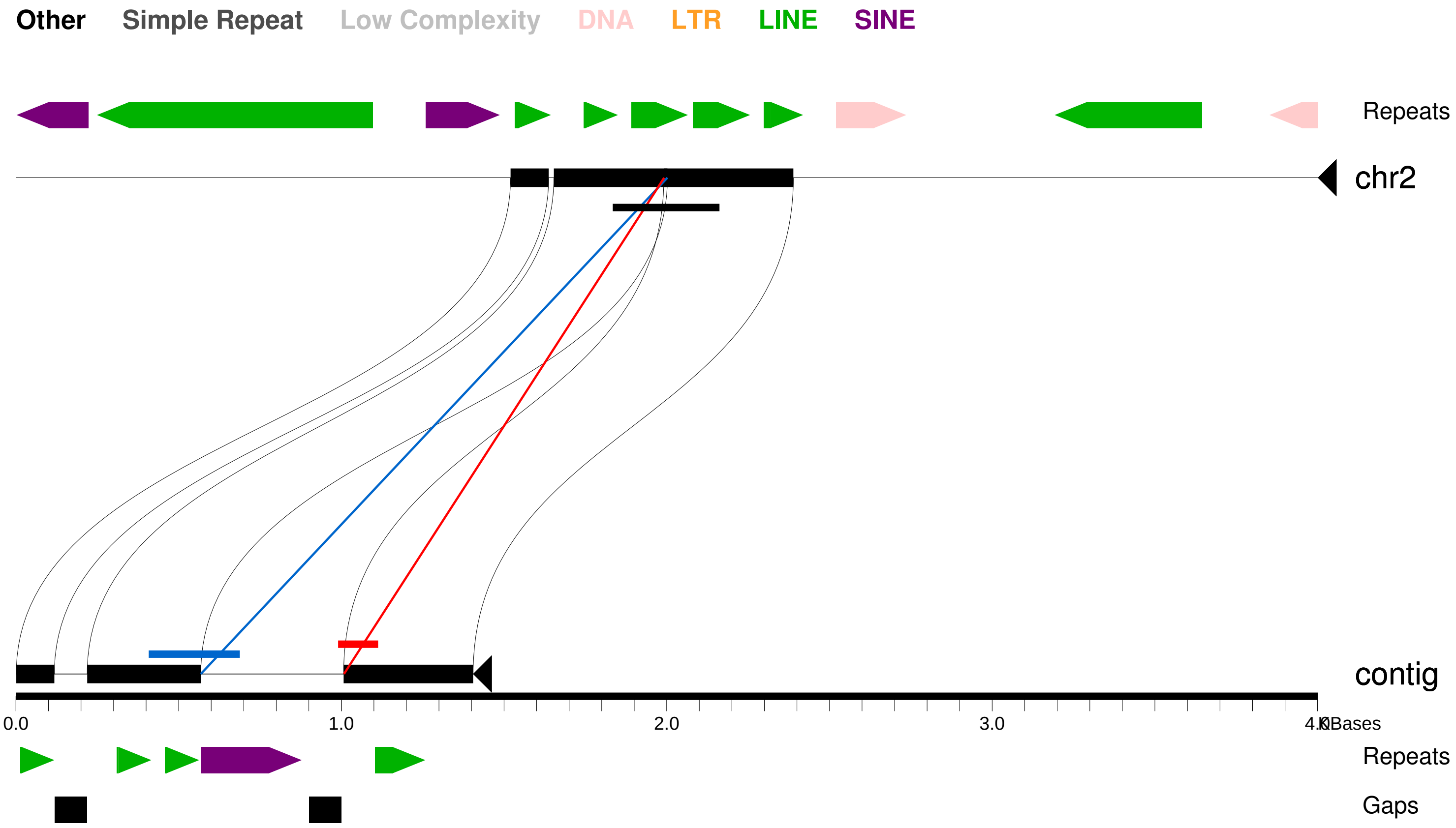

siteID: chr2_57247968 Scafftig2 + Breakpoint: chr2:57247949
chr2:57247789-57248114
Overlap Length: 6 INS size: 324 Genotyped: No

```
left   NNNNNNNNNNNNNNNNNNNNNNNNNNNNNNNNNNNNNNNNNNNNNNNNNNNNNNNNNNNNNNNNNNNNNN
chrom  TTCCCTCTTCCTTGCAGCCTCACCAACATCTGTTGTTTTTTTTACTTTTTAGTAATAGCTATTCTGACTG
right  TT---------TAGTAGAGAC-------------GGGGTTTCACCTTGTTAGCCAG-GATGGTCTCGATC
       22NNNNNNNNN2N2N22NNN2NNNNNNNNNNNNN2NNN222NNN222N2222NN2NN2N2NN222NNN2N

left   NNNNNNNNN-NNNNNNNNNNNNNNNNN-----CTTGGATTTCACTGATTGTTAGTGTAAGGAAACATTTTT
chrom  GTGTGAAAG-ATTATCTCACTGTGATTTTGACTTGGATTTCACTGATGATTAGTGACAGTGAACATTTTT
right  TCCTGACCTCATGATC-CACCC-GCCTCGGCCTCCCAAAGTGCTGG-GATTACAGGC-GTGAGCC-----
       NNN222NNN122N222N222NNN2NN2NN2N**111*11111***1122***11*N21*22*1*111111

left   CCATATGTTTTTTGGCCTCTTGTATGTTTTTTTTTT-----TTTTTTTTTTTTTTTTTTTT----TTTTGA
chrom  CCATATGTTTCTTGGCCACTTGTATGTCTTCTTTTAAGAAGTGTATATTCATGTCATTTTCCCATTTTGT
right  ----ACCGCGCCCGGCC----GTATGTCTTCTTTTAAGAAGTGTATATTCATGTCATTTTCCCATTTTGT
       1111*11111211****N111******2**2****222222*2*2*2**22*2*22****2222*****2

left   GACGGAGTCTCGCTCTGTCGCCCAGGCAGG---ACTGCG-----GA--CTGCAG-TG-GCGCAATCTCGG
chrom  AATGGGGTTGTTTTCTGTTTGTCAAATTGTTTAAGTTCCTTATAGATTCTGGATATGAGCCCATAGTAGA
right  AATGGGGTTGTTTTCTGTTTGTCAAATTGTTTAAGTTCCTTATAGATTCTGGATATGAGCCCATAGTAGA
       2*2**2**22222*****2222**2222*2222*2*2*222222**22***2*22**2**2**222*2*2

left   CTCACTG----CAAGCT------CCGCTTCCCGGGTT---------C
chrom  TGCACGGTTGTCAATATTTTGTCCCATTTATTAGGTTGTCAATTTAC
right  TGCACAGTTGTCAATATTTTGTCCCATTTATTAGGTTGTCA------
       22***N*2222***22*222222**22**2222****2222     1
```

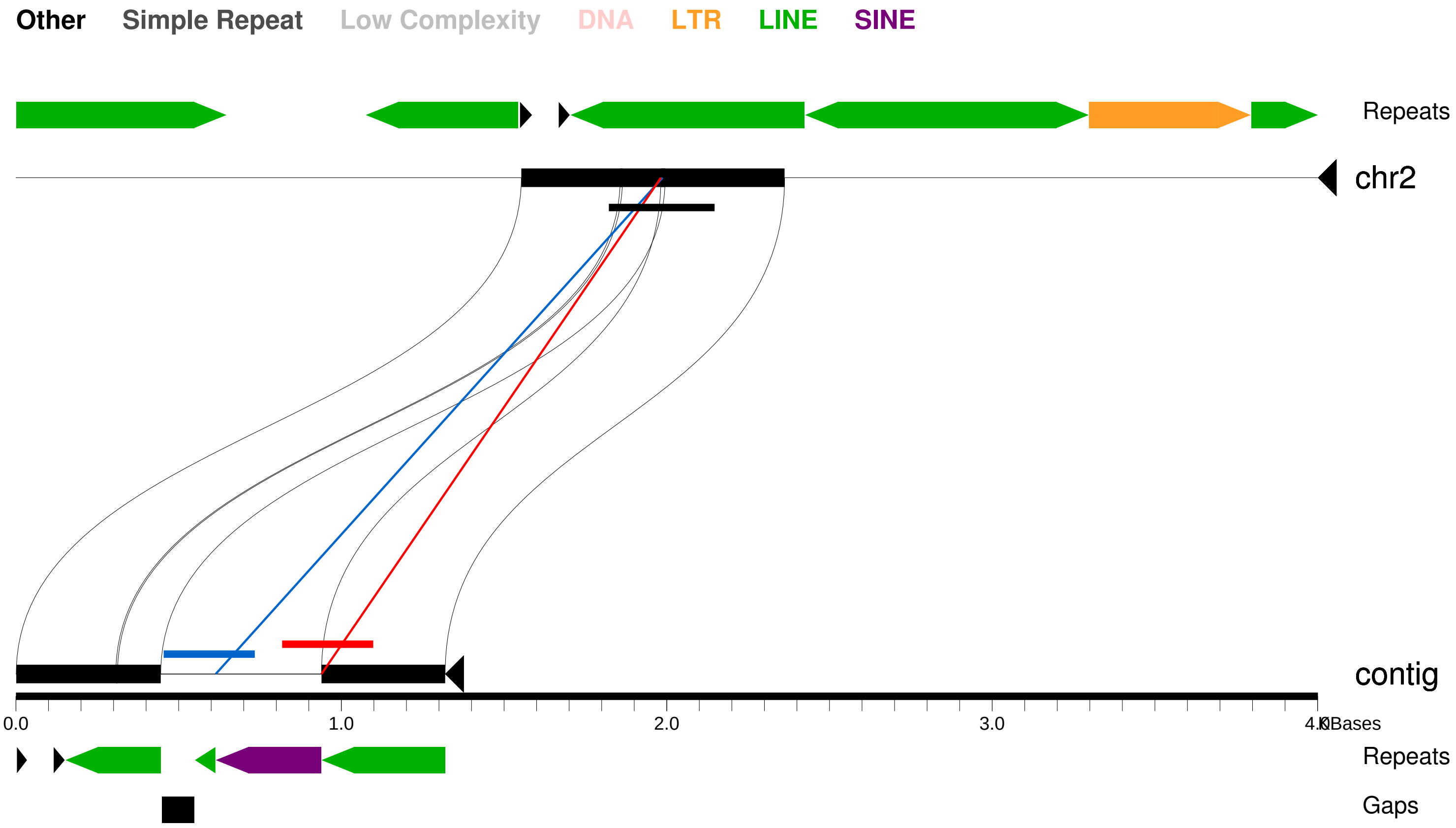

siteID: chr2_57451073 Scafftig1 + Breakpoint: chr2:57451043
chr2:57451000-57451102
Overlap Length: 17 INS size: 313 Genotyped: Yes

```
left     TTCTAATATTTATGCCTGAAGAACTCATGTTAGTATT----------------CTCTGTCTCAAACCATA
chrom    TTCTAATATTTATGCCTGAAGAACTCATGTTAGTATT----------------CTCTGTCTCAAACCATA
right    TCCACCCGCCTCGGCCTCCCAAAGTGCTGGGATTACAGGCGTGAGCCACCGCGCCCGGCCTCAAACCATA
         *1*1111111*11****1111**1*11**11*1**111111111111111111*1*1*1***********

left     TTTTTTTTTTTTTTTTTTTTTTTTTTTTTTTTGAGACGGAGTCTCGCTCTGTTGCCCAGGTCGGA
chrom    TTTTTTATCATCATCTGTTAGTACAATT-------AC--AATC---CTTTTTTA---ATTTCAG-
right    TTTTTTATCATCATCTGTTAGTACAATT-------AC--AATC---CTTTTTTA---ATTTCAG-
         ******2*22*22*2*2**22*2222**2222222**22*2**222**2*2**2222*22**2*2
```

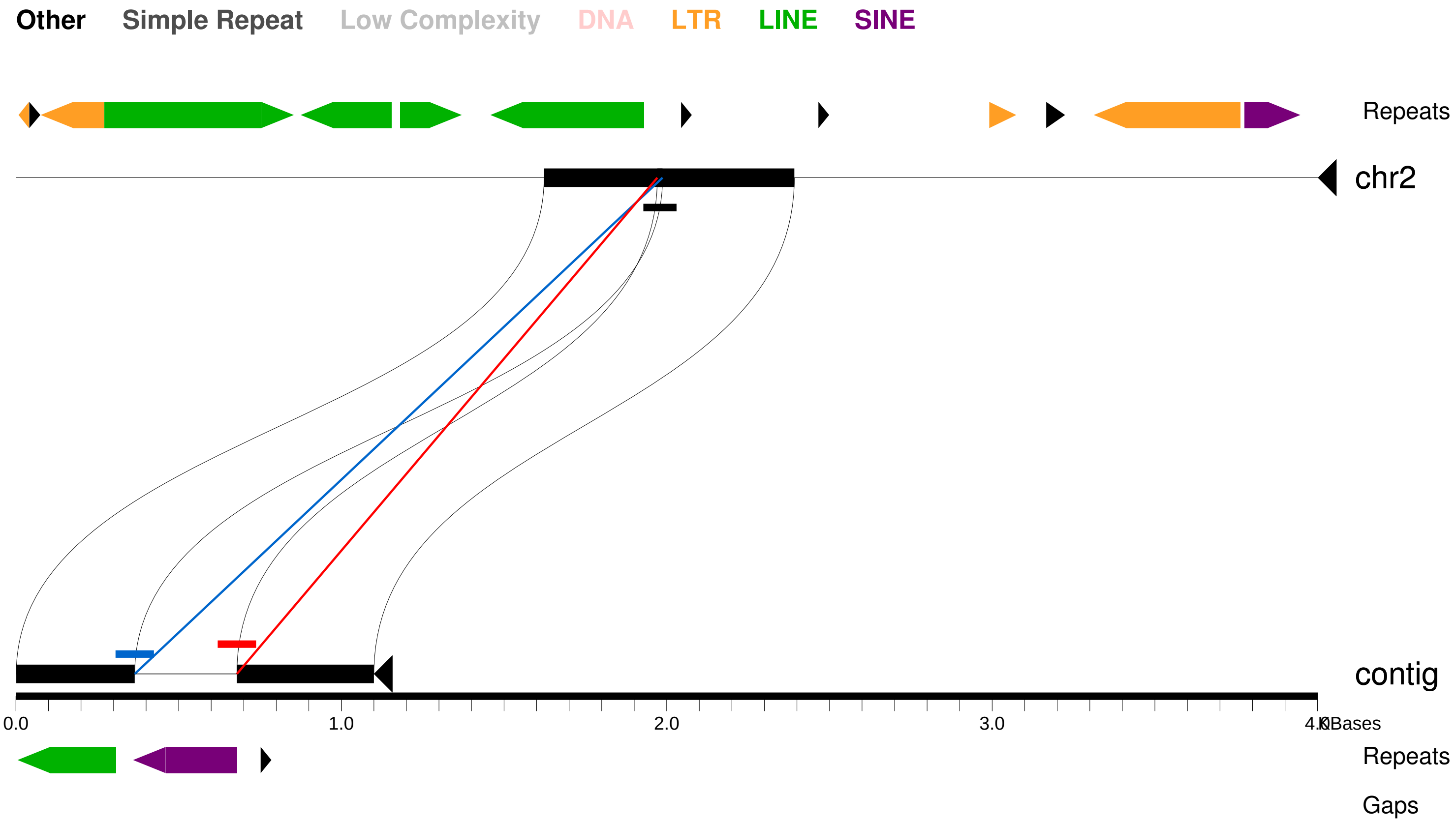

siteID: chr2_59874274 Scafftig1 - Breakpoint: chr2:59874259
chr2:59874218-59874315
Overlap Length: 19 INS size: 223 Genotyped: Yes

```
left     C--TTGAGGTGGGAAAACAGCCGGGATAA-------------------ACAAGAAGCTGAACAGAAAAAT
chrom    C--TTGAGGTGGGAAAACAGCCGGGATAA-------------------ACAAGAAGCTGAACAGAAAAAT
right    CACTGCAGTCCGCAGTCCGGCCTGGGCAACAGAGCGAGACTCCGTCTCAAAAAAAAAAAAAAAGAAAAAT
         *11*11**111*1*111*1***1**11**1111111111111111111*1**1**1111**1********

left     AACTACAAGGGAGACCATCCTGGCTAACAAGGTGAAACCCCGTCTCTACTAAAAATACAAAAAATTAGCC
chrom    AACTACAAGGGTAAAA---------AACTAGGAAAAGAGTTGT-TC--------ATAGGAGACTTTT---
right    AACTACAAGGGTAAAA---------AACTAGGAAAAGAGTTGT-TC--------ATAGGAGACTTTT---
         ***********22*22222222222***2***22**22222**2**22222222***22*2*22**2222
```

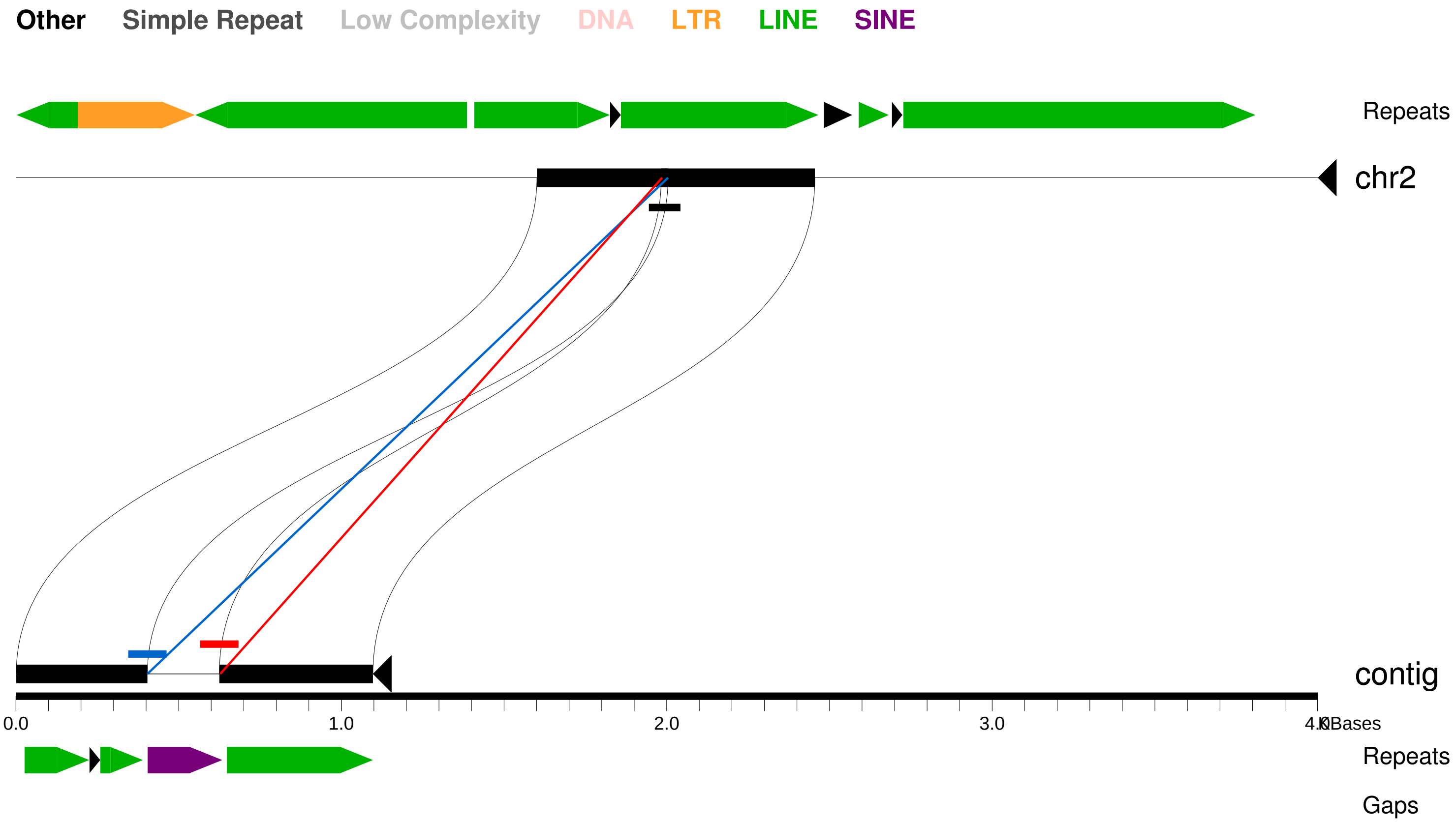

siteID: chr2_62501812 Scafftig3 + Breakpoint: chr2:62501899
chr2:62501853-62501958
Overlap Length: 14 INS size: 316 Genotyped: Yes

```
left     CAG----AAACATCCTCACAGAC----------ACACCCAGAGAGAATGTTTTACCAGTTAGCTATACAT
chrom    CAG----AAACATCCTCACAGAC----------ACACCCAGAGAGAATGTTTTACCAGTTAGCTATACAT
right    CCGCCCGCCTCGGCCTCCCAAAGTGCTGGGATTACAGGC-GTGAGCCACCGCGCCCGGCCAGCTATACAT
         *1*1111111*11****1**1*11111111111***11*1*1***111111111**1*11**********

left     TCTTTTTTTTTTTTTTTTTTTTTTTTTTTTTTTTTTTTTTTTTTTTTGAGACGGAGTCTCGCT
chrom    TCTTAAGCCCAGTCAAACTGAGACATAAAATTAACCATC-----------ACAGAATGT--AT
right    TCTTAAGCCCAGTCAAACTGAGACATAAAATTAACCATC-----------ACAGAATGT--AT
         ****22222222*22222*222222*2222**22222*222222222222**2**2*2*222*
```

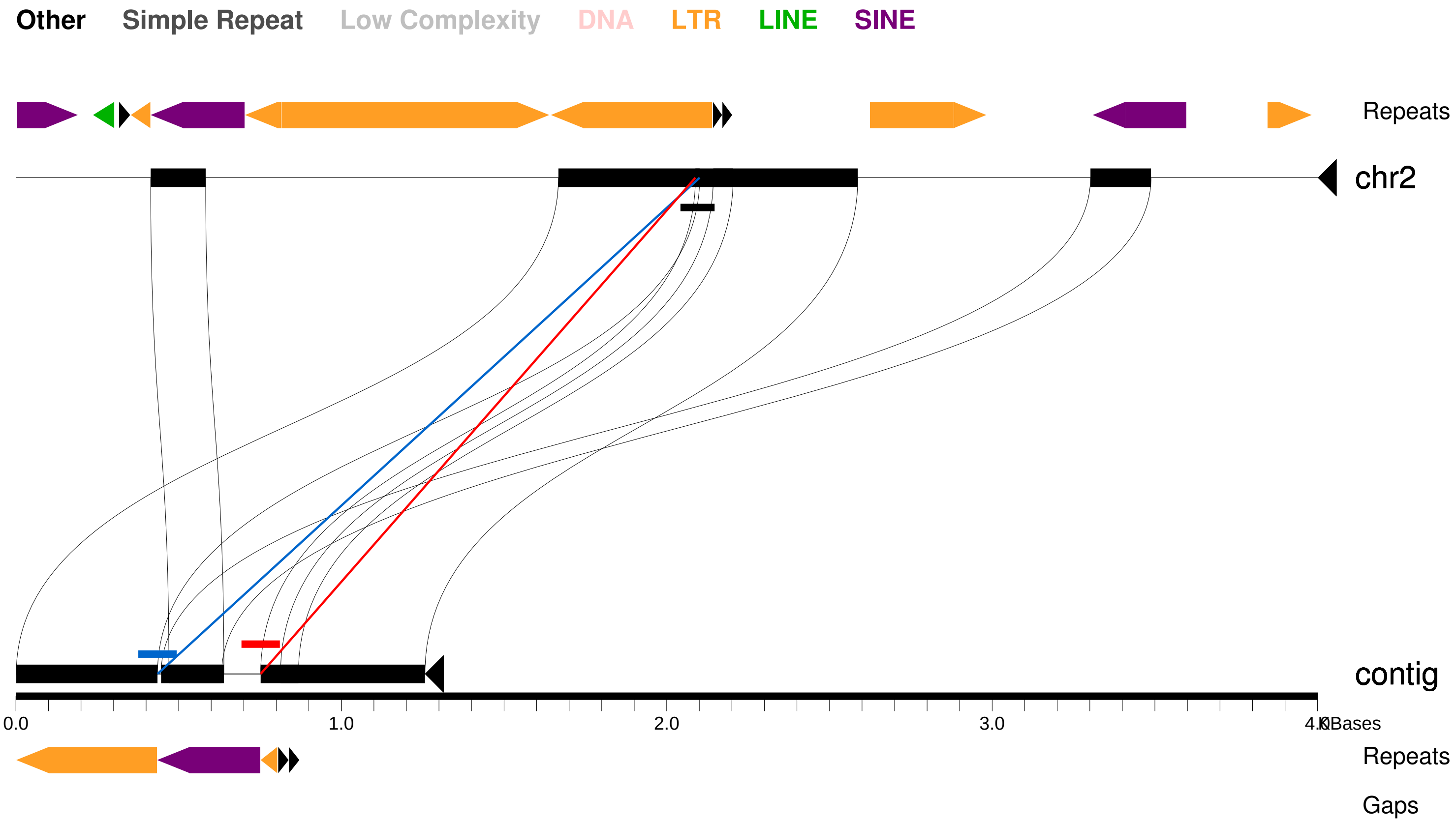

siteID: chr2_63779232 Scafftig5 + Breakpoint: chr2:63779226
chr2:63779177-63779281
Overlap Length: 11 INS size: 315 Genotyped: Yes

```
left    CA----------GCTTCCTAAGGTTCTGGATTTTCAGGTGTGAGCCACCACACTTGGCC----CTGCTTT
chrom   CA----------GCTTCCTAAGGTTCTGGATTTTCAGGTGTGAGCCACCACACTTGGCC----CTGCTTT
right   CCACCCGCCTCGGCCTCCCAAAGTGCTGGGATTACAGGCGTGAGCCACCGCGCCCGGCCGGCCCTGCTTT
        *11111111111**1***1**1**1****11**1****1*********1*1*11****1111*******

left    CTTTTTTTTTTTTTTTTTTTTTTTTTGAGACGGAGTCTCGCTCTGTCGCCCAGGTGGGACTGCG
chrom   CTTTAAAACAATATTTTATATTCT--------ATATTCTATTTAAAATCCAAGT---ATT---
right   CTTTAAAACAATATTTTATATTCT--------ATATTCTATTTAAAATCCAAGT---ATT---
        ****2222222*2****2*2**2*22222222*222**22*2*22222***2**222*2*222
```

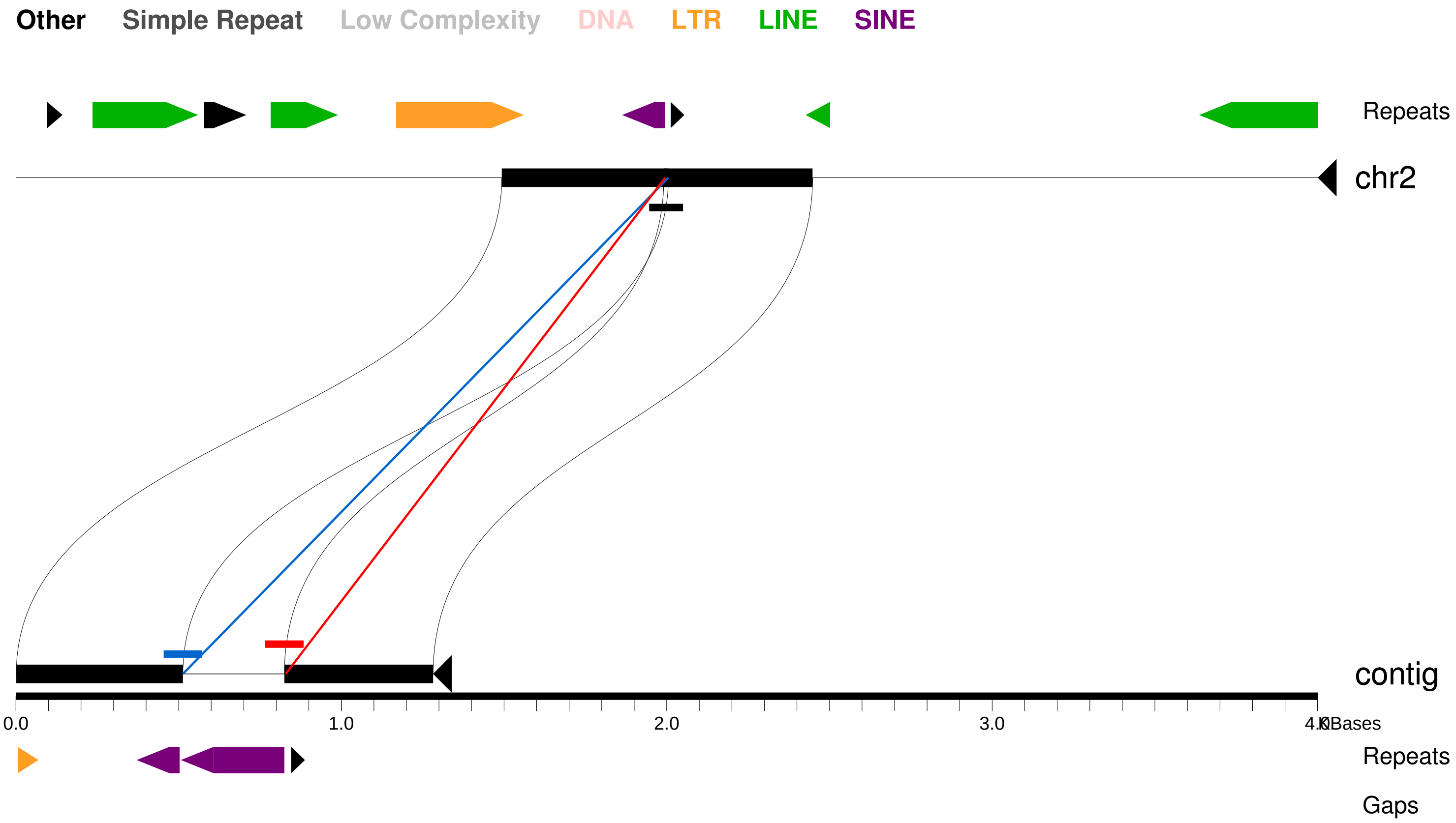

siteID: chr2_63965535 Scafftig1 - Breakpoint: chr2:63965513
chr2:63965468-63965572
Overlap Length: 15 INS size: 338 Genotyped: No

```
left    --------CTCA-----CAACAGAATGTTCAGCAAATACT-CAGGTTCTTATTTTTTTTATCAGGGCTAC
chrom   --------CTCA-----CAACAGAATGTTCAGCAAATACT-CAGGTTCTTATTTTTTTTATCAGGGCTAC
right   ACGCCATTCTCCTGCCTCAGCCTCCTGTGTAGCTGGGACTACAGGCACGCGCCACCATGATCAGGGCTAC
        11111111***111111**1*1111***11***1111***1****11*111111111*1***********

left    TATTTTTTTTNNNNNNNNNNNNNNNNNNNNNNNNNNNNNNNNNNNNNNNNNNNNNNNNNNNNNN
chrom   TATTGACATTTGCTTGTTCAGGGGACCACCCCCACTCCTTCTCCTGTGC--------------
right   TATTGACATTTGCTTGTTCAGGGGACCACCCCCACTCCTTCTCCTGTGC--------------
        *****2222**22222222222222222222222222222222222222222222222222222
```

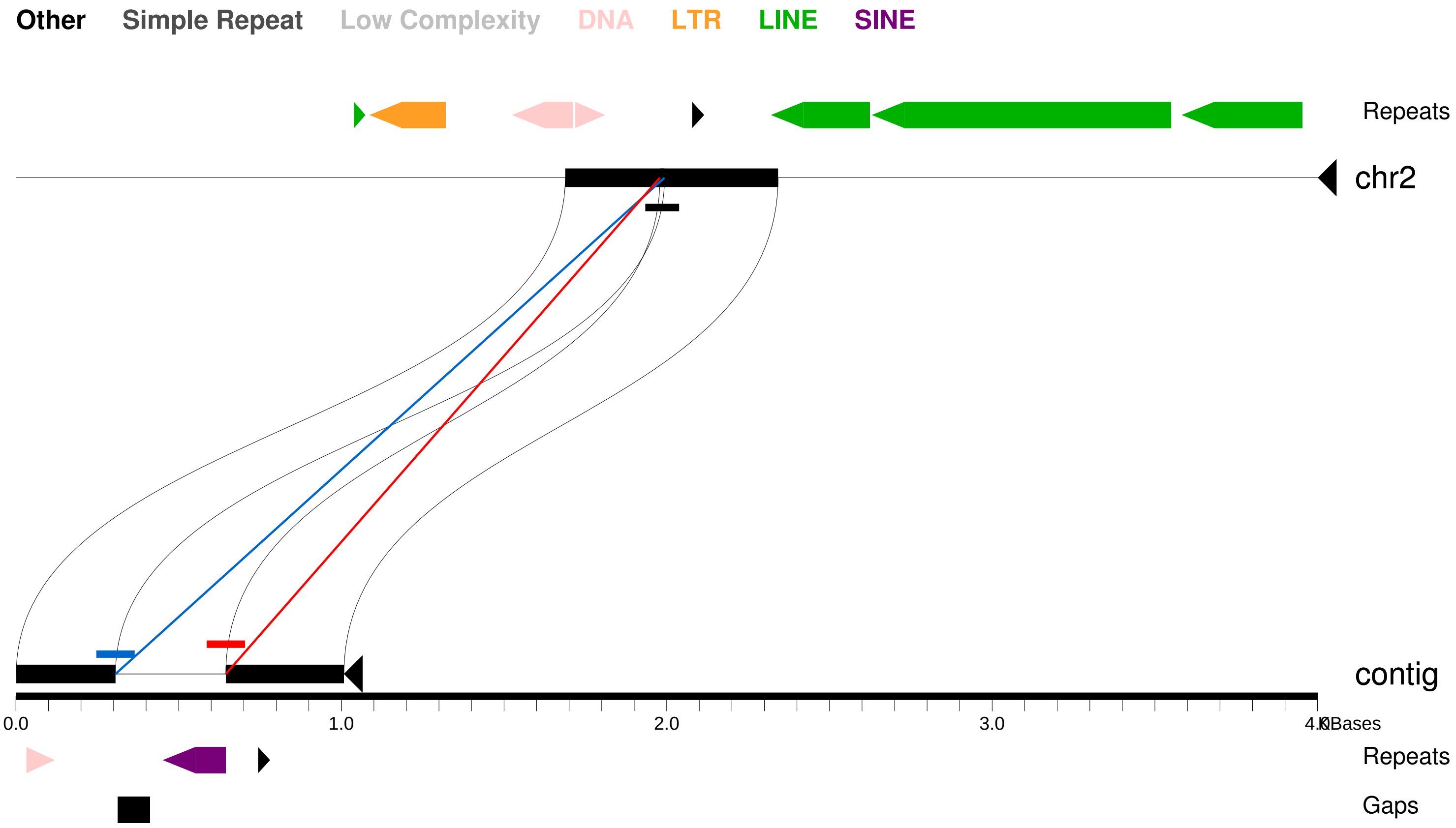

siteID: chr2_64771802 Scafftig5 - Breakpoint: chr2:64771785
chr2:64771741-64771844
Overlap Length: 16 INS size: 304 Genotyped: Yes

```
left    A-----ACAGCT----------ATTTAAAGCCCATATTTTATGTGTTAAAGTGGATTATAGAAATGAAAC
chrom   A-----ACAGCT----------ATTTAAAGCCCATATTTTATGTGTTAAAGTGGATTATAGAAATGAAAC
right   TGCACTCCAGCCTGGGCGACAGAGCGAGACTCCGTCTCAAAAAAAAAAAAAAAAAAAAAAGAAATGAAAC
        1111111****11111111111*111*1*11**1*1*111*111111***1111*11*1***********

left    TTTATGGGCCGGGCGCGGTGGCTCACGCCTGTAATCCCAGCACTTTGGGAGGCCGAGGCGGGCG
chrom   TTTATTTGTAAA------TTCATTATACAAGTAATACTT-CAATT----AACATGAAACT----
right   TTTATTTGTAAA------TTCATTATACAAGTAATACTT-CAATT----AACATGAAACT----
        *****22*2222222222*222*2*22*22*****2*222**2**2222*2222**22*22222
```

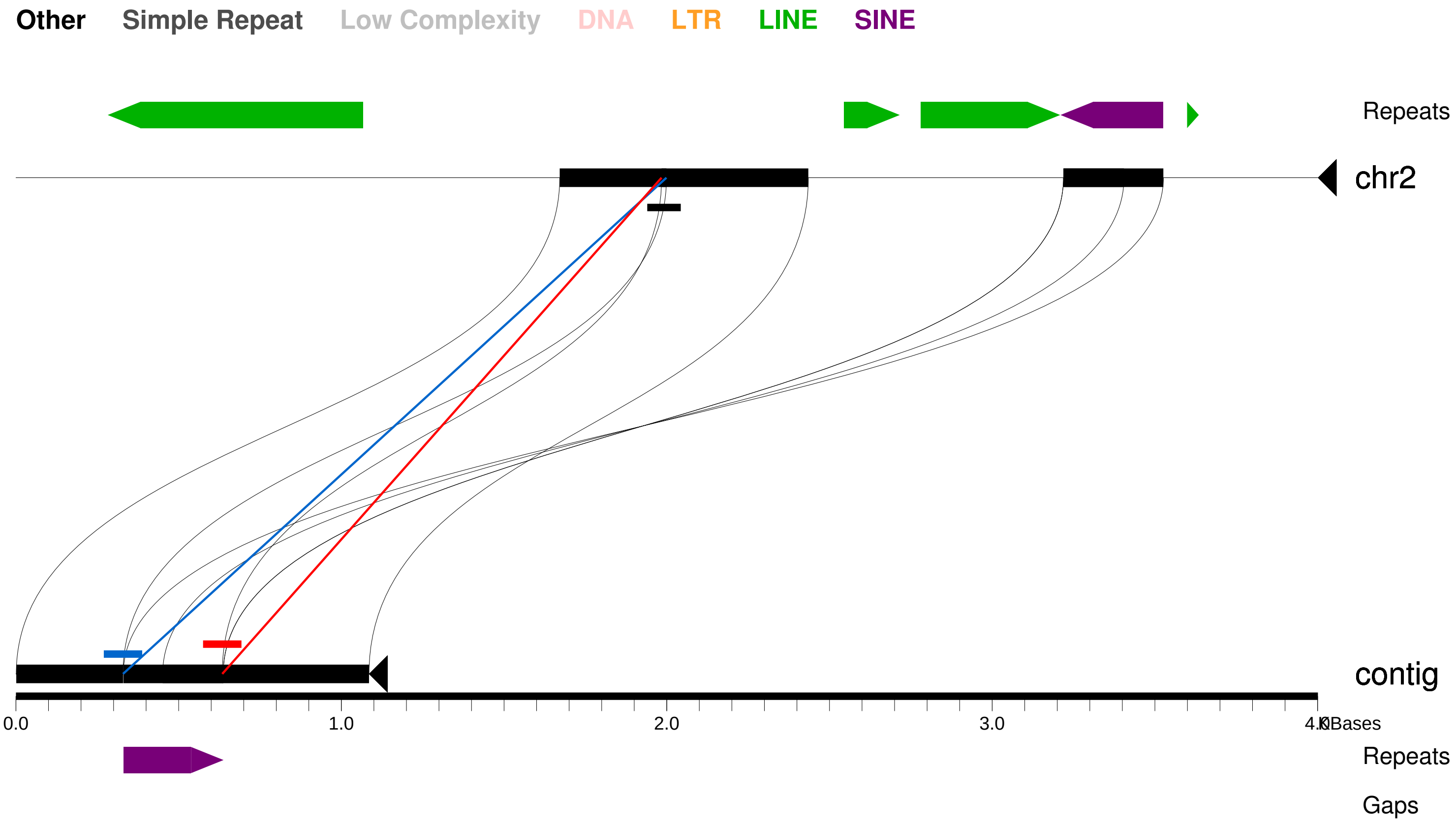

siteID: chr2_65163760 Scafftig5 - Breakpoint: chr2:65163768
chr2:65163724-65163827
Overlap Length: 16 INS size: 424 Genotyped: No

```
left    G----------GTAATGAAAATGTTCTGGAATTCATGGTGATAGTTACAGAACT-----CCATGGATATA
chrom   G----------GTAATGAAAATGTTCTGGAATTCATGGTGATAGTTACAGAACT-----CCATGGATATA
right   CCACCCGCCTCGGCCTCCCAAAGTGCTGGGATTACAGGCGTGAGCCACCGCGCCCGGCCCCATGGATATA
        11111111111*111*111**1**1****1***111**1*11**11**1*11*111111***********

left    CTTTTTTTTTNNNNNNNNNNNNNNNNNNNNNNNNNNNNNNNNNNNNNNNNNNNNNNNNNNNNNNN
chrom   CTTTTT---------------AAAAACCCCTCTGATTCGTACATTTTTAAAAGATGGTTTTATGG
right   CTTTT----------------AAAAACCCCTCTGATTCGTACATTTTTAAAAGATGGTTTTATGG
        *****2222222222222222222222222222222222222222222222222222222222222
```

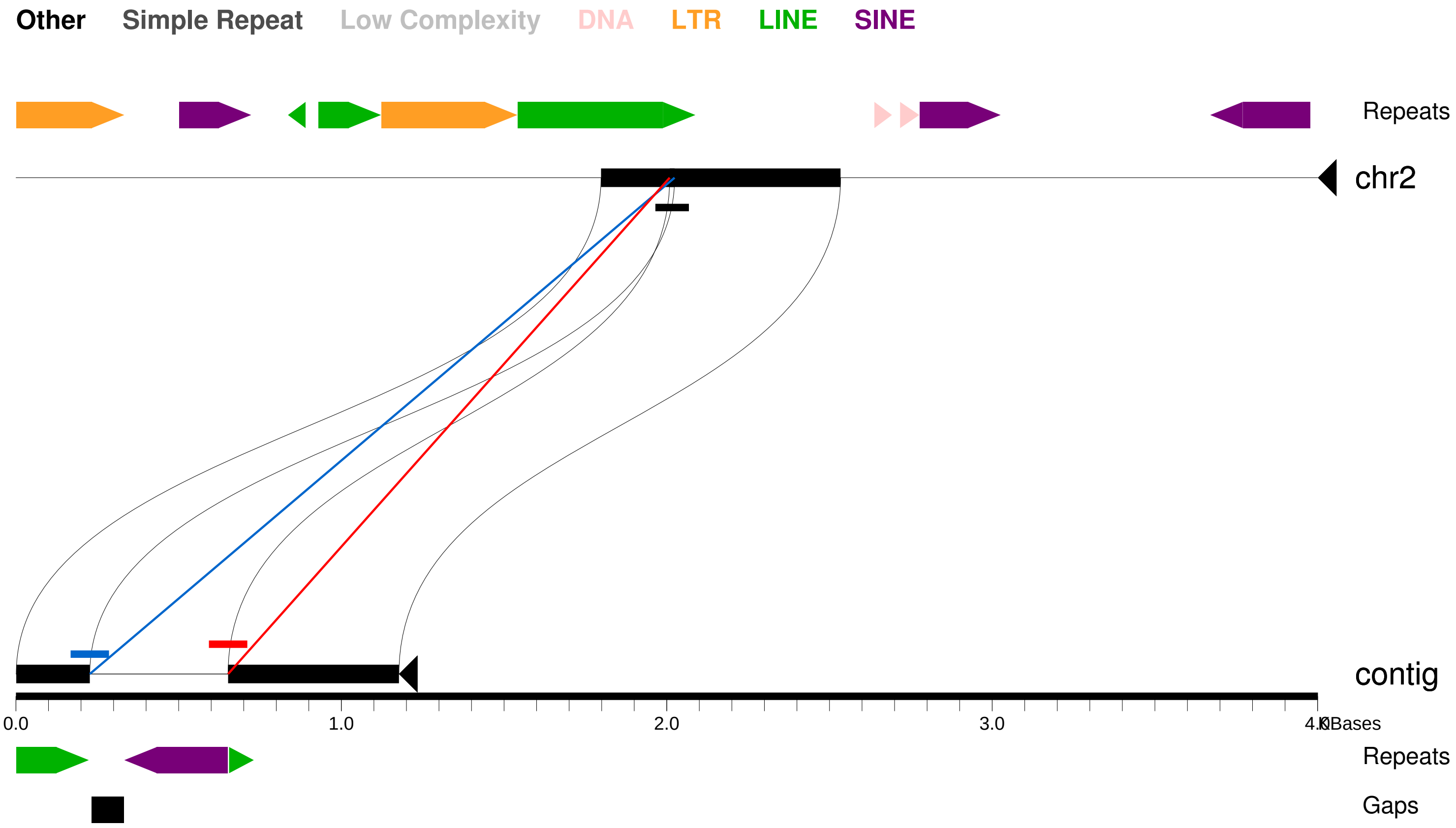

siteID: chr2_6517416 Scafftig1 - Breakpoint: chr2:6517419
chr2:6517347-6517478
Overlap Length: 15 INS size: 299 Genotyped: Yes

```
left   CTTTATGATTAAAAGCCTCAGCAAAATTGGCATACAAGGAACATACCTCAATGTAGTAAAAGCCATCCAT
chrom  CTTTATGATTAAAAGCCTCAGCAAAATTGGCATACAAGGAACATACCTCAATGTAG-AAAAGCCATCCAT
right  CACTGCACTCCA--GCCTGGGCGACAGAGCGAGACTCCGT------CTCAAAAAAA-AAAAAAAAA----
       *11*1111*11*11****11**1*1*11*11*1**111*1111111*****111*12****111*11111

left   GATAAATACACAGCCAAC--------GGCC-GGGCGCGGTG---GC--TCACGCCTGTAATCC
chrom  GATAAATACACAGCCAACACAATACTGACCAGGGAAAGTTGAAAGCATTCCCTCTGAGAACTG
right  -AAAAAAACACAGCCAACACAATACTGACCAGGGAAAGTTGAAAGCATTCCCTCTGAGAACTG
       1*1***1**********22222222*2**2***222*2**222**22**2*2*2222**222
```

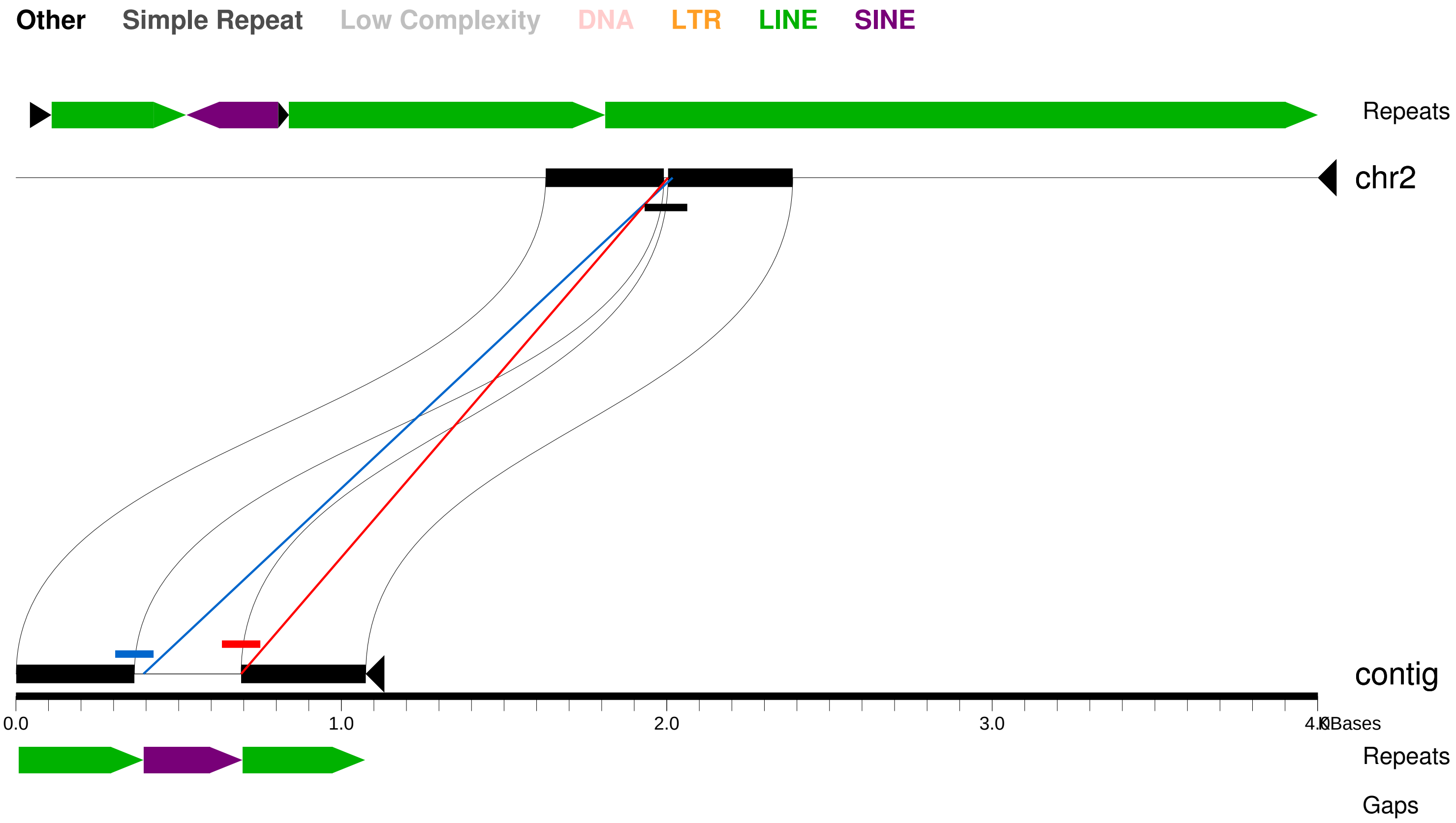

siteID: chr2_652674 Scafftig1 - Breakpoint: chr2:652594
chr2:652463-652803
Overlap Length: 79 INS size: 462 Genotyped: No

```
left   ----------------------------------------------------------------------
chrom  GGCTGGGCTTGACTCTGCAGACCAATCTGGAAACCCCTTATGTGTGACATCAGCTTTTCTTACCTGTCAA
right  NNNNNNNNNNNNNNNNNNNNNNNNNNNNNNNNNNNNNNNNNNNNNNNNNNNNNNNNNNN-----------
       NNNNNNNNNNNNNNNNNNNNNNNNNNNNNNNNNNNNNNNNNNNNNNNNNNNNNNNNNNN

left   ------------------------------ACGTCTTTTAAAACAATTTTGTAATTTTATTCATAAAAAT
chrom  TATGGCATATGTTTCCATTAAGCCATCTTTACATCTTTTAAAACAATTTTGTAATTTTATTCATAAAAAT
right  -------------------------------------------------------------CATAAAAAT
                                     11N1111111111111111111111111111*********

left   CTTGCACATATTTTAGATTAGTTTCTTGGAAACTTACAATTTTGGTTGATATTGTAAAAGACATCTTTTA
chrom  CTTGTACATATTTTAGATTAGTTTCTTGGAAACTTACAATTTTGGTTGATATTGTAAAAGACATCTTTTA
right  CTTGTACATATTTTAGATTAGTTTCTTGGAAACTTACAATTTTGGTTGATATTGTAAAAGACATCTTTTA
       ****2*****************************************************************

left   G-----GCCGGGCGCGGTGGCT---------------CA-------------------------CGCCTG
chrom  TAAAATGCATTTCTAGCTGTCTATTTTGTTATAAAAACAAATTGATGTACATTGATCTTTATATCGAGTG
right  TAAAATGCATTTCTAGCTGTCTATTTTGTTA---------------------------------------
       222222**2222*22*2**2**222222222      11                         11NN11

left   TAATCCCAGCACT---TTGGGAGGCCGAGGC--------------------GGG----CGG
chrom  ATCTCCCCCAACTCTCTTGTTAGTATTTATCATCTATCTATATCTTATTTTGGGATTTCTC
right  -------------------------------------------------------------
       NNN1111NNN111   111NN11NNNNNNN1                    111    1NN
```

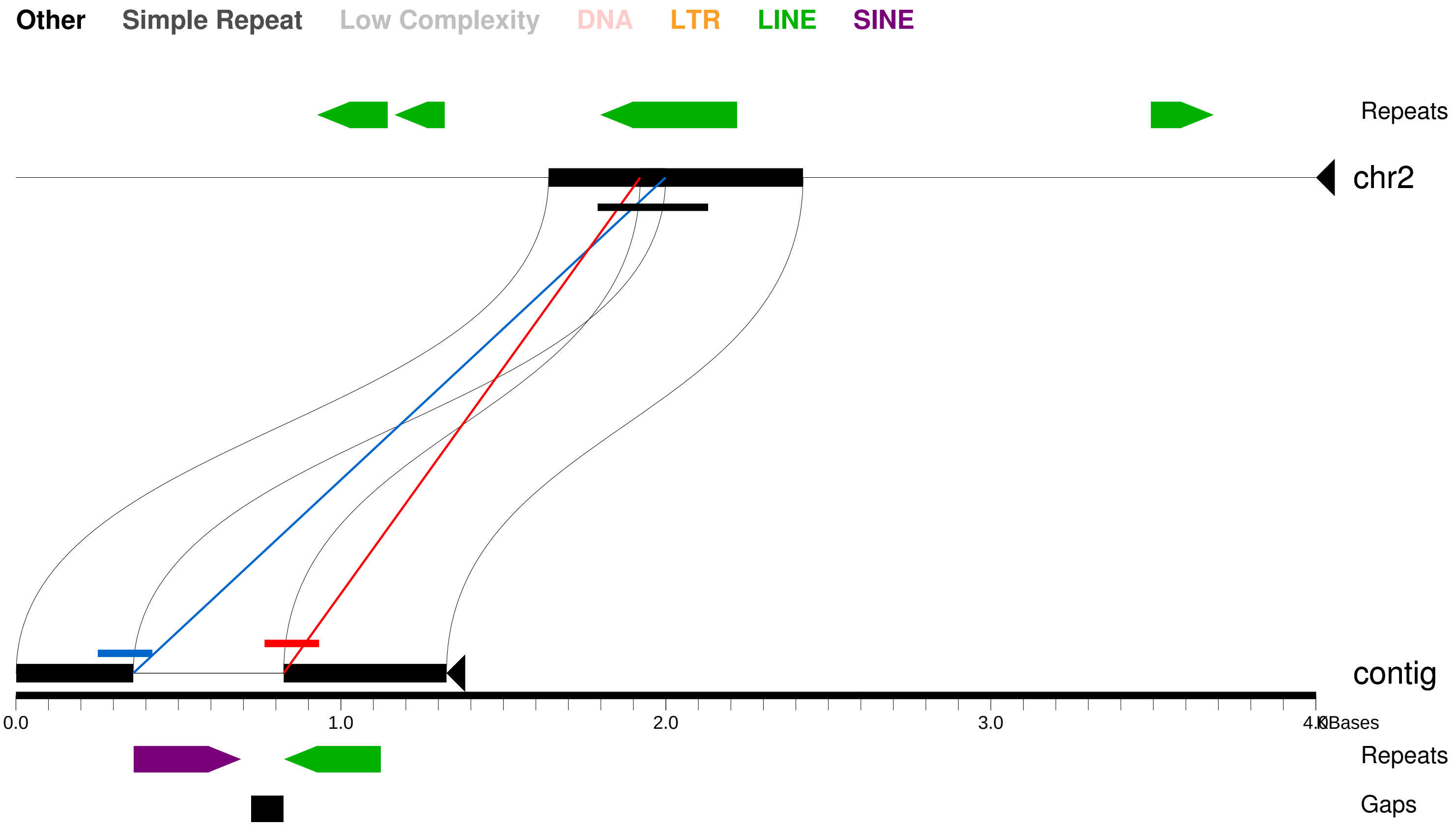

siteID: chr2_66658986 Scafftig3 - Breakpoint: chr2:66658979
chr2:66658932-66659098
Overlap Length: 13 INS size: 294 Genotyped: Yes

```
left   ------GTTTGCAAAGGGTG----ACTTACCT-----------------GAACCTTCTTTTCTCATCTGT
chrom  ------GTTTGCAAAGGGTG----ACTTACCT-----------------GAACCTTCTTTTCTCATCTGT
right  CCTGCAGTGAGCCGAGATTGCGCCACTGCACTCCAGCCTGGGCGACAGCGAGACTCCGTCTCAAAAAAAA
       111111**11**11**11**1111***111**11111111111111111**11**1*1*1**11*11111

left   GCTA--GAAAACAAAAGAAGG------CCGG---GCGCGGTGGC----TCACGCCTGT-----AAT----
chrom  GCTA--GAAAACAAAAGAAAAAGATTACCGGAAAGCAACAATACAAAATAAATCCTGTTGTCAAATATAT
right  AAAAAAGAAAACAAAAGAAAAAGATTACCGGAAAGCAACAATACAAAATAAATCCTGTTGTCAAATATAT
       111*11*************22222222****222**2222222*2222*2*22*****22222***2222

left   ---------------CCCAGC-------ACTTTGGGA----GGCCGAGGCGGGCGG
chrom  ATATATATATATATACTGACCTTTTCCAAATTTGAAAACGTGGCCGCAGAGTTGTG
right  ATATATATATATA--CTGACCTTTTCCAAATTTGAAAACGTGGCCGCAGAGTTGTG
       2222222222222  *22*2*2222222*2****22*2222*****22*2*2222*
```

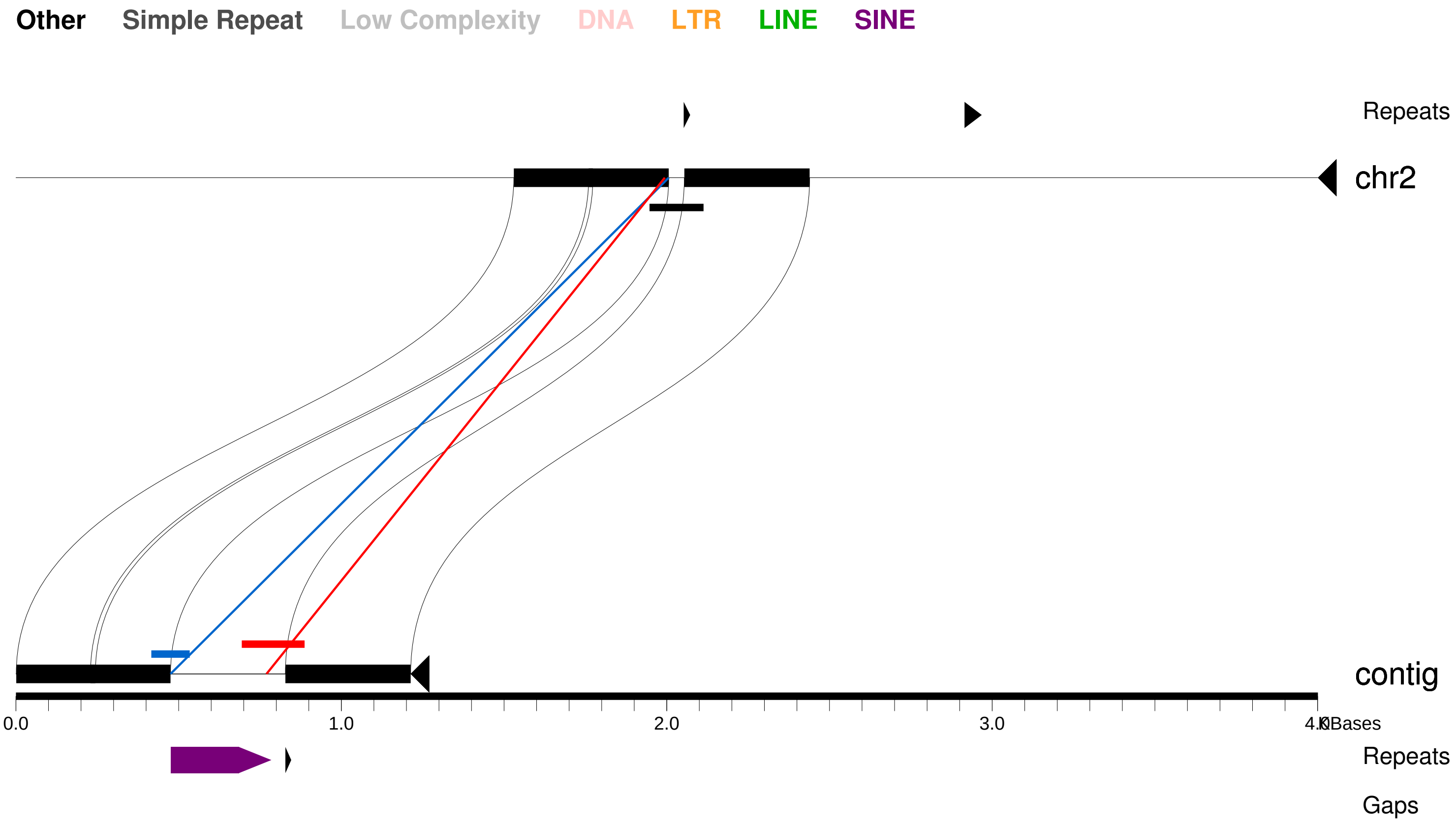

siteID: chr2_68648213 Scafftig1 - Breakpoint: chr2:68648209
chr2:68648163-68648268
Overlap Length: 14 INS size: 430 Genotyped: No

```
left     CAATAGG----GGAAT---AATGCACATTTGAAGATACTAGGGAT-------GGCAGCATCTAACCTCTA
chrom    CAATAGG----GGAAT---AATGCACATTTGAAGATACTAGGGAT-------GGCAGCATCTAACCTCTA
right    CCACCCGCCTCGGCCTCCCAAAGTGC-TGGGATTACAGGCGTGAGCCACCGCGCCCGGCCCTAACCTCTA
         *1*111*1111**11*111**1*11*1*11**11*1*111*1**11111111*1*1*111**********

left     TCTTTTTTTTTNNNNNNNNNNNNNNNNNNNNNNNNNNNNNNNNNNNNNNNNNNNNNNNNNNNNNN
chrom    TCTTGCTGCTTAAGGAATTTAGTATAAACAGTCACTTTTGCAGTCTTCTG-------------
right    TCTTGCTGCTTAAGGAATTTAGTATAAACAGTCACTTTTGCAGTCTTCTG-------------
         *****22*22**2222222222222222222222222222222222222222222222222222
```

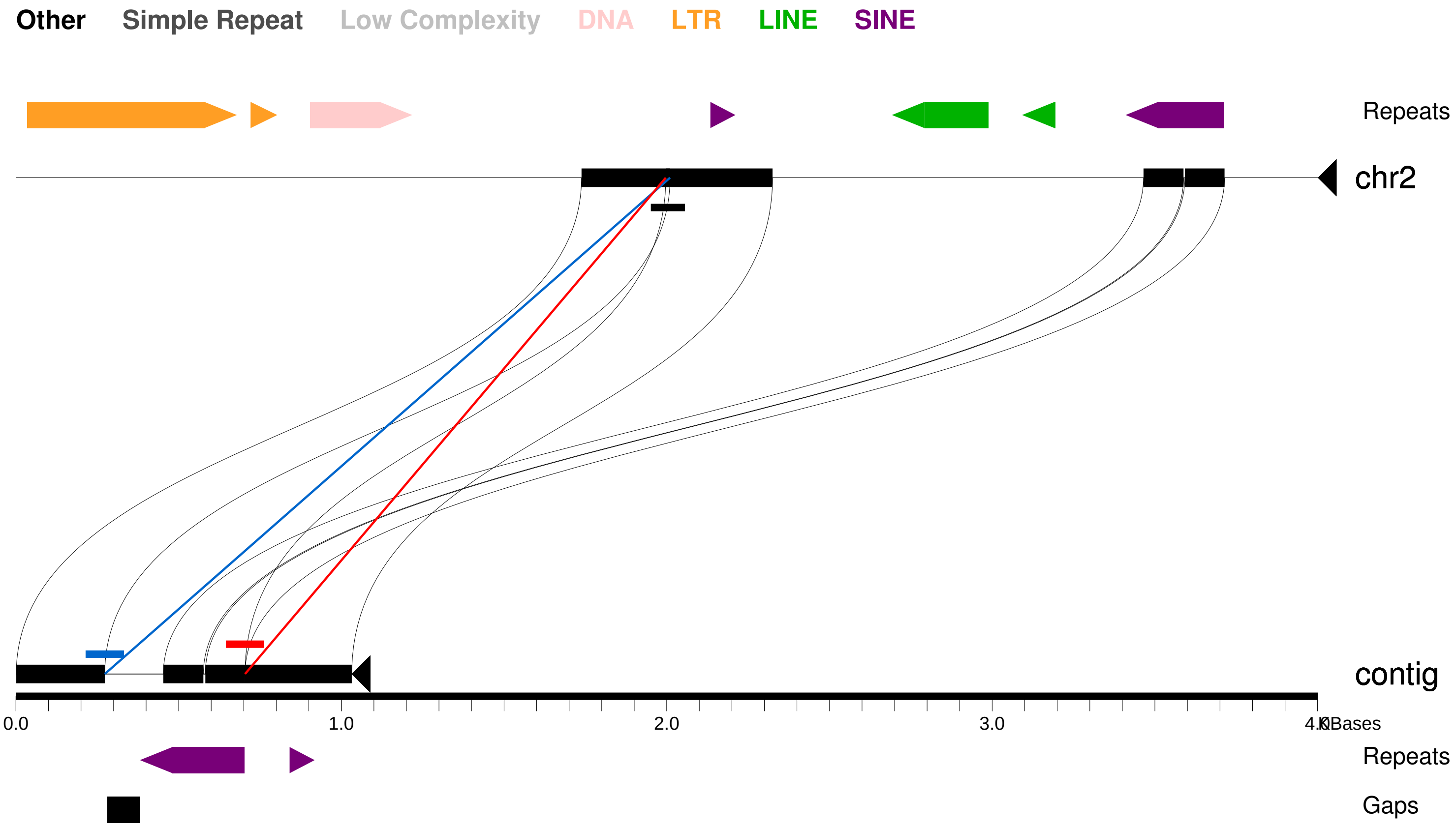

siteID: chr2_70797708 Scafftig3 + Breakpoint: chr2:70797703
chr2:70797659-70797759
Overlap Length: 11 INS size: 316 Genotyped: Yes

```
left     TGA-------GGCAACCCTACTCAC--------CAGCTATGCTCCCAATCAAAATGCCC---TCCTTCTA
chrom    TGA-------GGCAACCCTACTCAC--------CAGCTATGCTCCCAATCAAAATGCCC---TCCTTCTA
right    CGCCCGCCTCGGCCTCCCAAAGTGCTGGGATTACAGGCGTGAGCCACCGCGCCCGGCCCCCCTCCTTCTA
         1*11111111***11***1*1111*11111111***111**11**1111*11111****111********

left     ATTTTTTTTTTTTTTTTTTTTTTTTTTTTTTTTTGAGACGGAGTCTCGCTCTGTCGCCCAGGCTGGAG
chrom    ATTCTATTAGAATTATTCCCACACTTACT---------GCATTCTCATATTCTCA---------GAA
right    ATTCTATTAGAATTATTCCCACACTTACT---------GCATTCTCATATTCTCA---------GAA
         ***2*2**2222**2**2222222**22*222222222*2*2****2222*2**2222222222**2
```

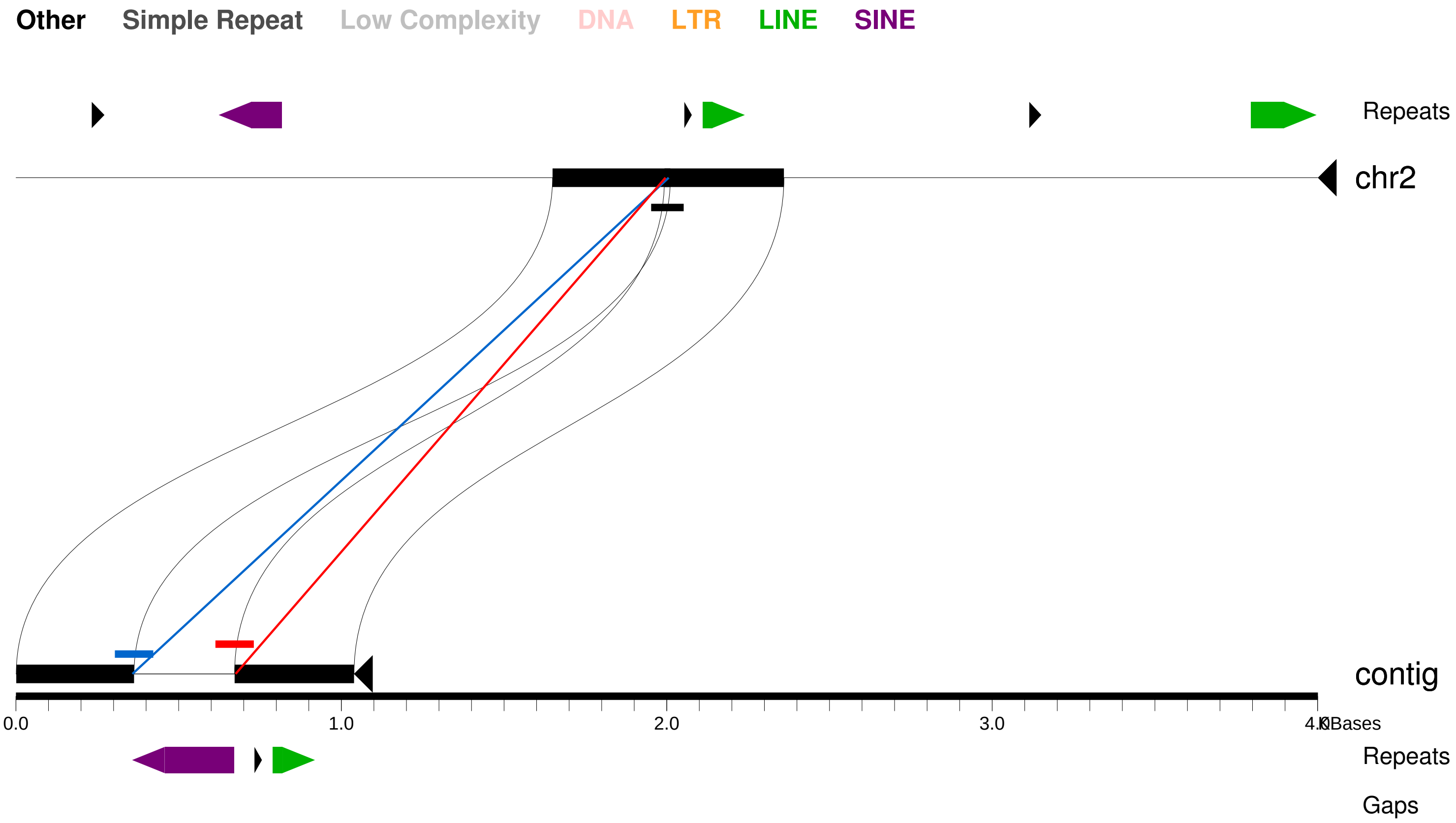

siteID: chr2_71706281 Scafftig1 + Breakpoint: chr2:71706287
chr2:71706127-71706463
Overlap Length: 17 INS size: 288 Genotyped: No

```
left   NNNNNNNNNNNNNNNNNNNNNNGG-----------------CCAGAGGCTCGAGGGACTTTCGTGGTCCAG
chrom  GCTTGGCTCTAAAGCCTACTGGGGCTCCTGGGAGATGTGGCCAGAGGCTCGAGGGACTTTCGTGGTCCAG
right  NNNNNNNNNNNNAA--------------------------------------------------------
       NNNNNNNNNNN22NNNNNNNN11                 1111111111111111111111111111111

left   GCAGGACTCTCTGAAAGCTGAGCCATCCAGGTGGAGACCAAAGGCTTTCCACTGAGTCTTGCACAGGTTG
chrom  GCAGGACTCTCTGAAAGCTGAGCCATCCAGGTGGAGACCAAAGGCTTTCCACTGAGTCTTGCACAGGTTG
right  ----------------------------------------------------------------------
       1111111111111111111111111111111111111111111111111111111111111111111111

left   GGTGGAATGAGGTGATCTTTAAGAGCCAACCCAAATACGTG----GTAGCGGGC---------------G
chrom  GGTGGAATGAGGTGATCTTTAAGAGCCGACCCAAATAAAAGTTCTGTGTCAGGCTGAACCCTTTGAATTG
right  --------------------AAGAGCCAACCCAAATAAAAGTTCTGTGTCAGGCTGAACCCTTTGAATTG
       11111111111111111111*******N*********222*2222**22*2***222222222222222*

left   CCTGTAGT----CCCAGC-----TACTCGGGAGGCTGAGGCAGGAGAATGGCGTGAACCCGGGAGG-CGG
chrom  TCTGTTGAGCGCCCCATCGTTTCTTTTCCTGATGCTTA--CTGGA-AGTGCCCAGGGCACTGCAGAACAA
right  TCTGTTGAGCGCCCCATCGTTTCTTTTCCTGATGCTTA--CTGGA-AGTGCCCAGGGCACTGCAGAACA-
       2****2*22222****2*22222*22**22**2***2*22*2***2*2**2*22*22*2*2*2**22*2N

left   AGCTTGCAGTGA--GCCGAGATCGCGCCACTGCA--CTCCAGC---------CTG-GGCGA
chrom  AGCTT-CATTAAATGCCTTCTCTTCTTCTTTGGGACCTTCAACACTCTTTTTCTGTGGATC
right  -------------------------------------------------------------
       11111211N1N1  111NNNNNNNN1NN1NN11NN  11N11N1         111 11NNN
```

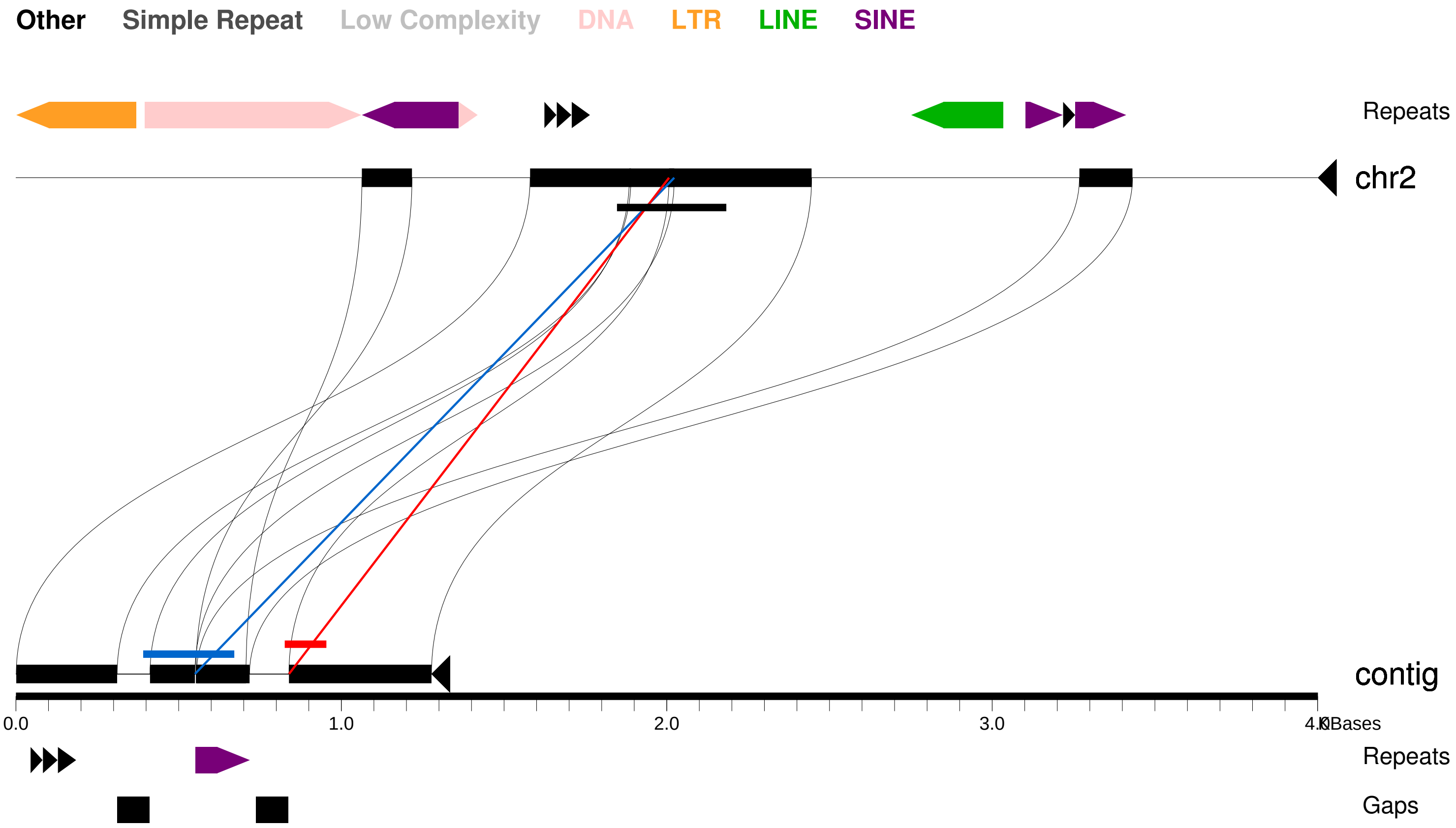

siteID: chr2_75335276 Scafftig3 + Breakpoint: chr2:75335285
chr2:75335240-75335344
Overlap Length: 15 INS size: 305 Genotyped: Yes

```
left     TTTTATGGGTAAGT-TCC---TTTGCT--AATTCCAAT-----GCTATTTTG---GAAAACAAAGACATT
chrom    TTTTATGGGTAAGT-TCC---TTTGCT--AATTCCAAT-----GCTATTTTG---GAAAACAAAGACAGT
right    CCGCCCGCCTCGGCCTCCCAAAGTGCTGGGATTACAGGCGTGAGCCACCGCGCCCGGCCACAAAGACAGT
         111111*11*11*11***11111****111***1**1111111**1*1111*111*111*********2*

left     TTTTTTTTTTTTTTTTTTTTTTTTTTTGAGACGGAGTCTCGCTCCGTCGCCCAGGCTGGAGTG
chrom    TTTTAAAAGCATCCCTAGCC-----TTGATACCAA-----AATCTGACA--AAGACAGCAC--
right    TTTTAAAAGCATCCCTAGCC-----TTGATACCAA-----AATCTGACA--AAGACAGCAC--
         *****2222222*222*222222222****2**22*2222222**2*2*2222**2*2*2*222
```

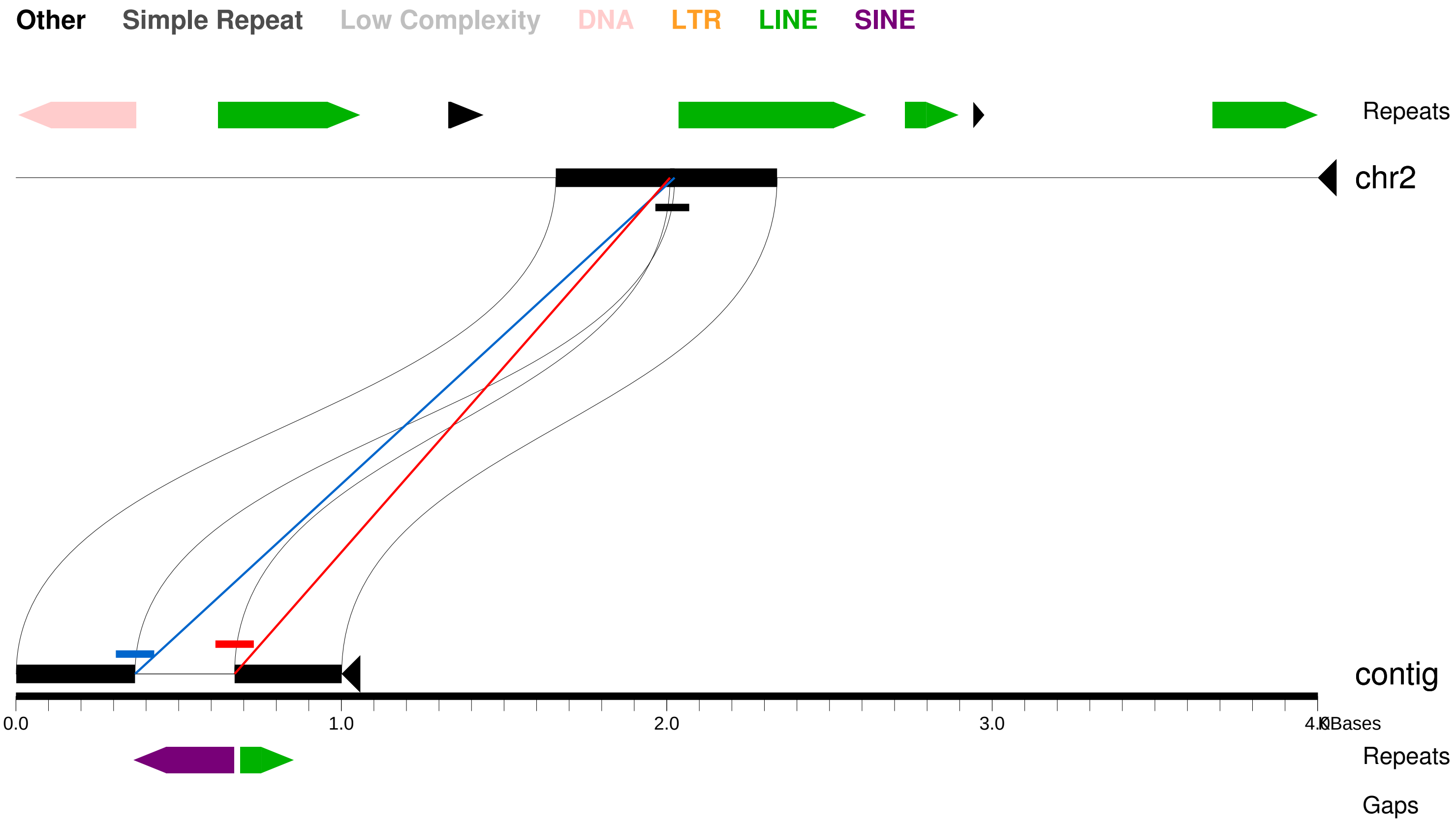

siteID: chr2_75387905 Scafftig4 - Breakpoint: chr2:75387891
chr2:75387844-75387950
Overlap Length: 13 INS size: 304 Genotyped: Yes

```
left    GATTCCTG-------GACAG---GAGA--ACTAACCAATTTGAACAGAAGTAATGATTTAAGACATAACA
chrom   GATTCCTG-------GACAG---GAGA--ACTAACCAATTTGAACAGAAGTAATGATTTAAGACATAACC
right   CACTCCAGCCTGGGCGACAGAGCGAGACTCCGTCTAAAAAAAAAAAAAAAAAAAAAAAAAAGACATAACA
        1*1***1*1111111*****111****111*1111***1111**1*1**11**11*111**********N

left    GAGGCCGGGCGCGGTGGCTCACGCCTGTAATCCCAGCACTTTGGGAGGCCGAGGCGGGCGG
chrom   GAAAAAAA----AGTTCCTTAA---TG-AATAAAAG----TTGGGATCTGCTTACACTAAG
right   GAAAAAAA----AGTTCCTTAA---TG-AATAAAAG----TTGGGATCTGCTTACACTAAG
        **2222222222**22**2*2222**2***222**2222******22222222*22222*
```

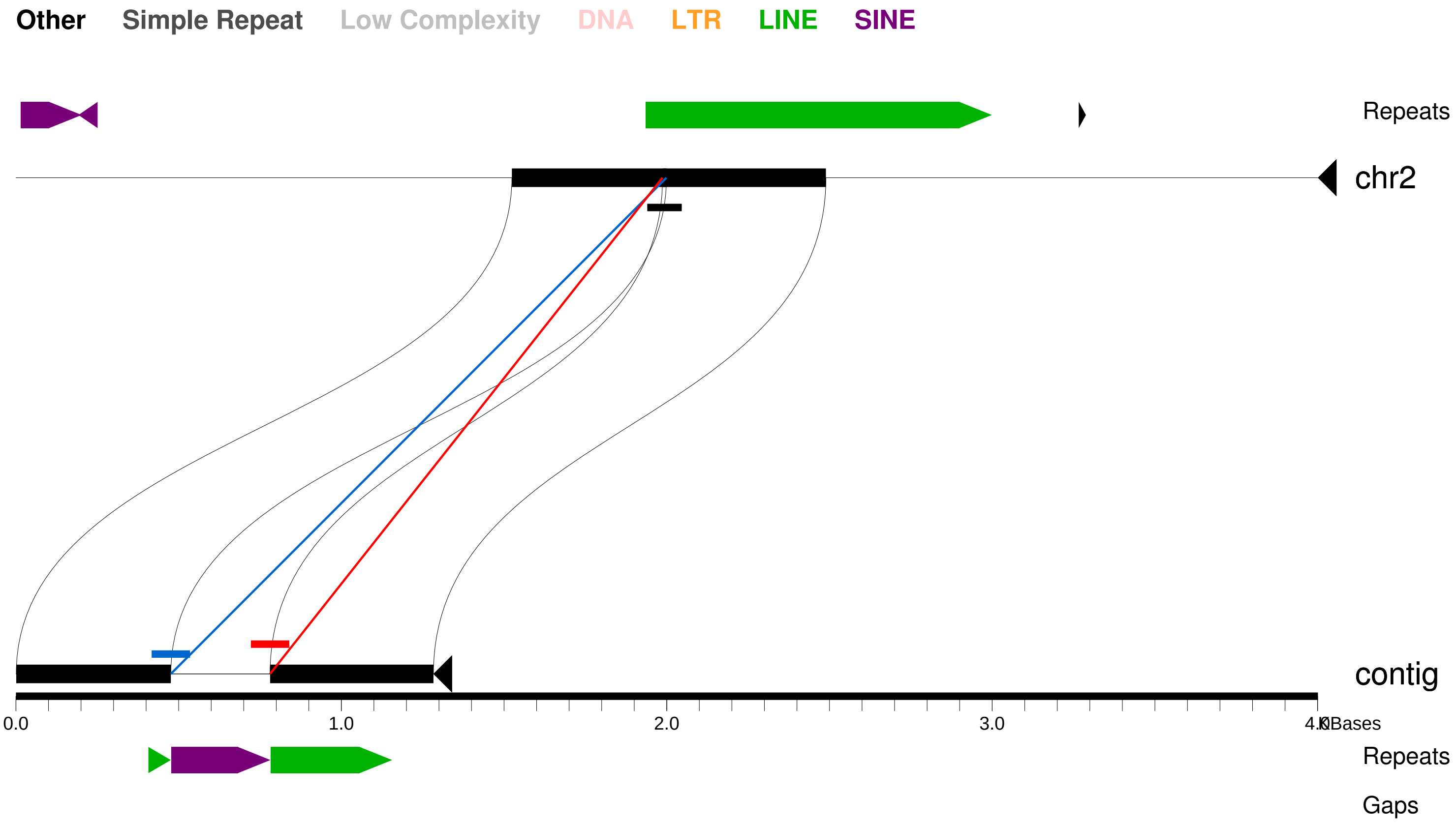

siteID: chr2_78251615 Scafftig1 + Breakpoint: chr2:78251627
chr2:78251576-78251686
Overlap Length: 9 INS size: 291 Genotyped: Yes

```
left   CTG---GAGAGGG------AGAGTGCCCA-ATGTCTCTAGTCAGCCATTTTGAACCAGAATTCCATCTTT
chrom  CTG---GAGAGGG------AGAGTGCCCA-ATGTCTCTAGTCAGCCATTTTGAACCAGAATTCCATCTTT
right  CCGCCCGCCTCGGCCTCCCAAAGTGCTGGGATTACAGGCGTGAGCCACCGTG--CCCGGCCTCCATCTTT
       *1*111*1111**111111*1*****1111**11*1111**1*****111**11**1*111*********

left   TTTTTTTTTTGAGA-CGGAGTCTCGCTCTGTCGCCCAGGCTGGAGTGCAGTGGCGGGATC
chrom  GATTCTC---GATATCTAAATATA--TTAGTAAGCCATG--GGAAAAAAAAAAACCTAA--
right  GATTCTC---GATATCTAAATATA--TTAGTAAGCCATG--GGAAAAAAAAAAACCTAA--
       22**2*2222**2*2*22*2*2*222*22**222***2*22***2222*2222*222*22
```

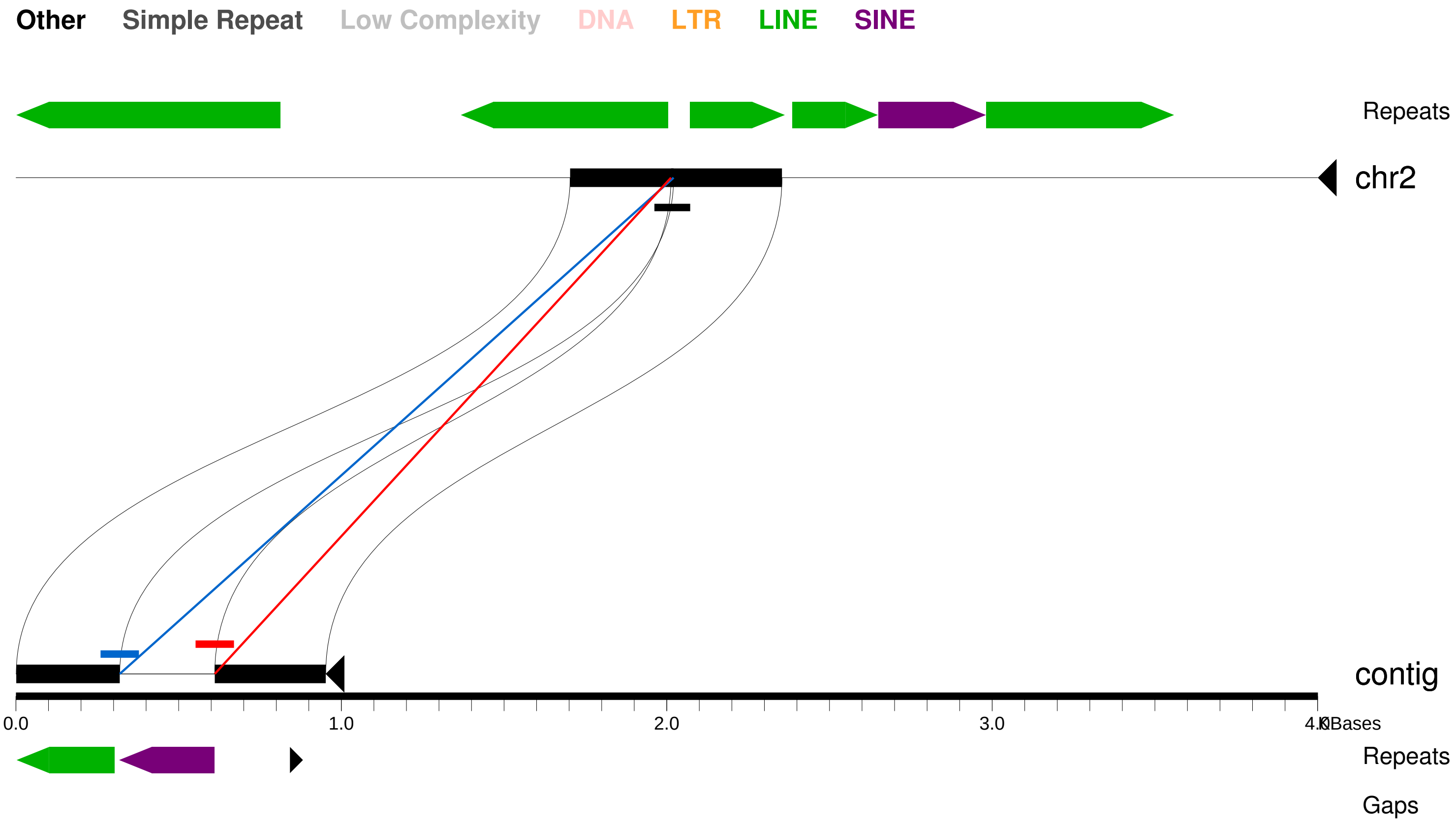

siteID: chr2_78965418 Scafftig3 - Breakpoint: chr2:78965411
chr2:78965367-78965470
Overlap Length: 16 INS size: 300 Genotyped: Yes

```
left    ---TTTACCCC---TTGTGAGGCA-----ATA----GTTTTGAAAGCCAGGCTTTGTTTAAAACAATGTT
chrom   ---TTTACCCC---TTGTGAGGCA-----ATA----GTTTTGAAAGCCAGGCTTTGTTTAAAACAATGTT
right   CACTGCACTCCAGCCTGGGCGACAGAGCGAGACTCCGTCTCAAAAAAAAAAAAAAAAAAAAAACAATGTT
        111*11**1**1111**1*1*1**11111*1*1111**1*11***111*1111111111***********

left    TTACAGGCCGGGCGCGGTGGCTCACGCCTGTAATCCCAGCACTTTGGGAGGCCGAGGCGGGCGG
chrom   TTACAAATTCTTTGAGTTACCCCTAACCT---ATCCAAGAGCAATAATTAT------------G
right   TTACAAATTCTTTGAGTTACCCCTAACCT---ATCCAAGAGCAATAATTAT------------G
        *****22222222*2*2*22*2*222***222****2**22*22*222222222222222222*
```

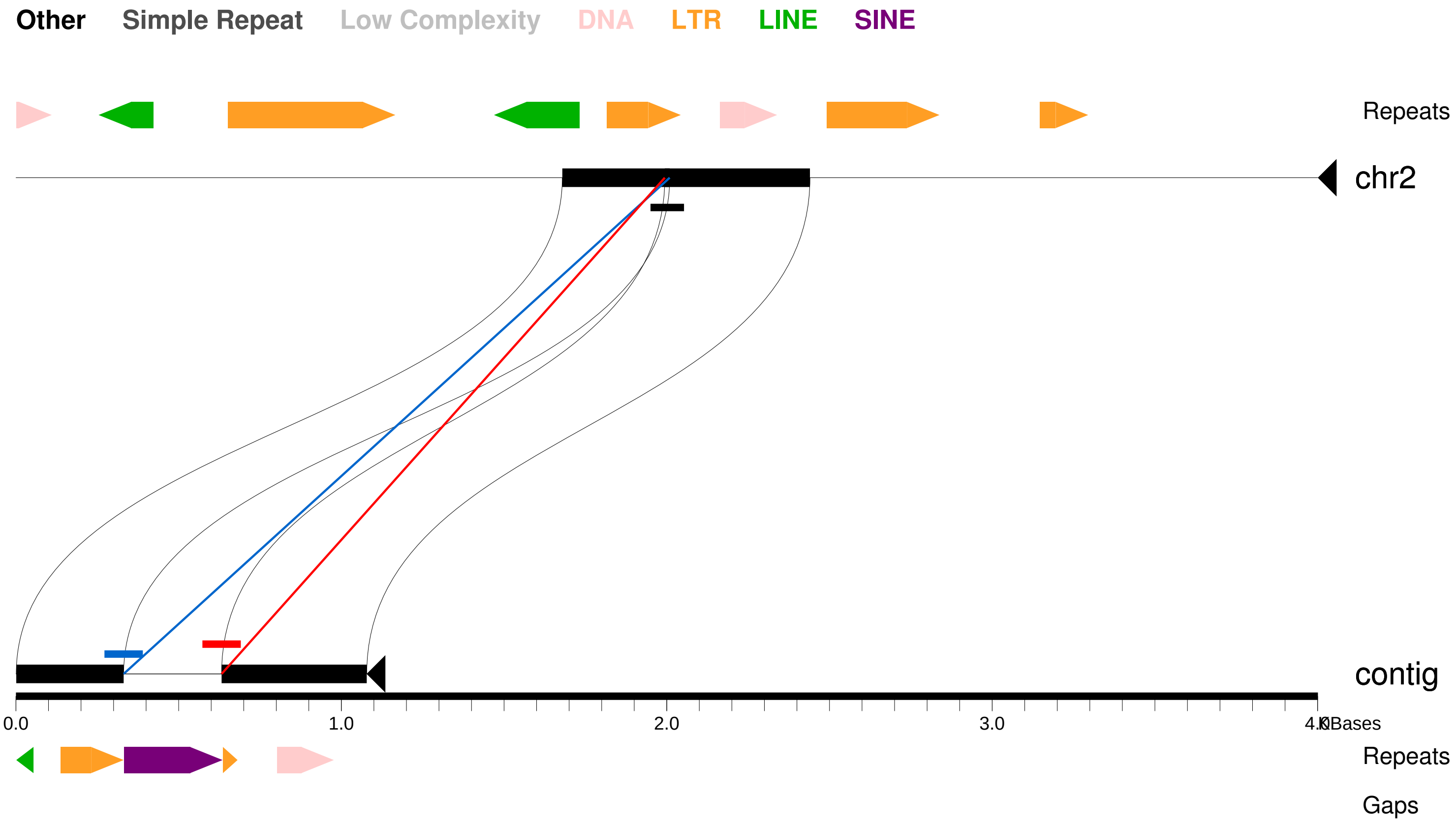

siteID: chr2_80769016 Scafftig2 - Breakpoint: chr2:80769011
chr2:80768852-80769184
Overlap Length: 14 INS size: 256 Genotyped: Yes

```
left   ACTCTTCATTTGTGCTCAGGTAGGTACCCTCTACTGTCTGCAAGTCTCAACTCCAAACCACACTCAGGTT
chrom  A-------TTTGTGCTCAGGTAGGTACCCTCTACTGTCTGCAAGTCTCAACTCCAAACCACACTCAGGTT
right  C--------------CGCCACTACGC-CCGGCTAATTTTTTGTATTTTTAG-TAGAGACGG------GGTT
       12222222111111*1*111**1*11**11***1*1*1*111*1*1*1*11*11*1**11111111****

left   TCCCTAGCTTACACTGTGCTCCCTGGAGACACGCACAGATGCAAGAAAGTGTTTTCCAGAGCTGTTGATA
chrom  TCCCTAGCTTACACTGTGCTCCCTGGAGACACGCACAGATGCAAGAAAGTGTTTTCCAGAGCTGTTGATA
right  TCACCGTTTTAG---------CCGGGATGGTCTC----------------GATCTCCTGACCTCGTGATC
       **1*1111***1111111111**1***1111*1*1111111111111111*1*1***1**1**11****1

left   TAATACTTTGTGGCTGCTACTTTT-TTTCTAAATTTTTTTTTTTTTTGAGACGG---------------A
chrom  TAATACTTTGTGGCTGCTACTTTT-TTTCTAAATGTTTTTTAATATGGAAAATGCTAAGCATATCCCAAA
right  CGCCCGCCTCGGCCTCCCAAAGTGCTGTCTAAATGTTTTTTAATATGGAAAATGCTAAGCATATCCCAAA
       11111111*11*1**1*1*111*11*1*******2******22*2*2**2*22*222222222222222*

left   GTCTCG---------------------CTCTGTCGCCCAGGCTGGAGTGC--------AGTGGCGGGATC
chrom  GTAAAGAATGTGTAATGAACCATCATACACTGTCACTCAGCTTCAACAATTATAAACTAATGGCGA-ATC
right  GTGAAGAATGTGTAATGAACCATCATACACTGTCACTCAGCTTCAACAATTATAAACTAATGGCGA-ATC
       **N22*222222222222222222222*2*****2*2***22*22*222222222222*2*****22***

left   TCGGCT------CACTGCAAGC----TCCGCCTCCTGGGTTCAC------GCCATTCTCCTG
chrom  TTGTTTTTTCTACATTTCTACCCATTTCCCCAACCCCAGATAATTGTGAAGCAAATTCCAAA
right  TTGTTTTTTCTACACTTCTACCCATTTCCCCAACCCCAGATAATTGTG--------------
       *2*22*222222**N*2*2*2*2222***2*22**222*2*2*22222  11N1N1NN1NNN
```

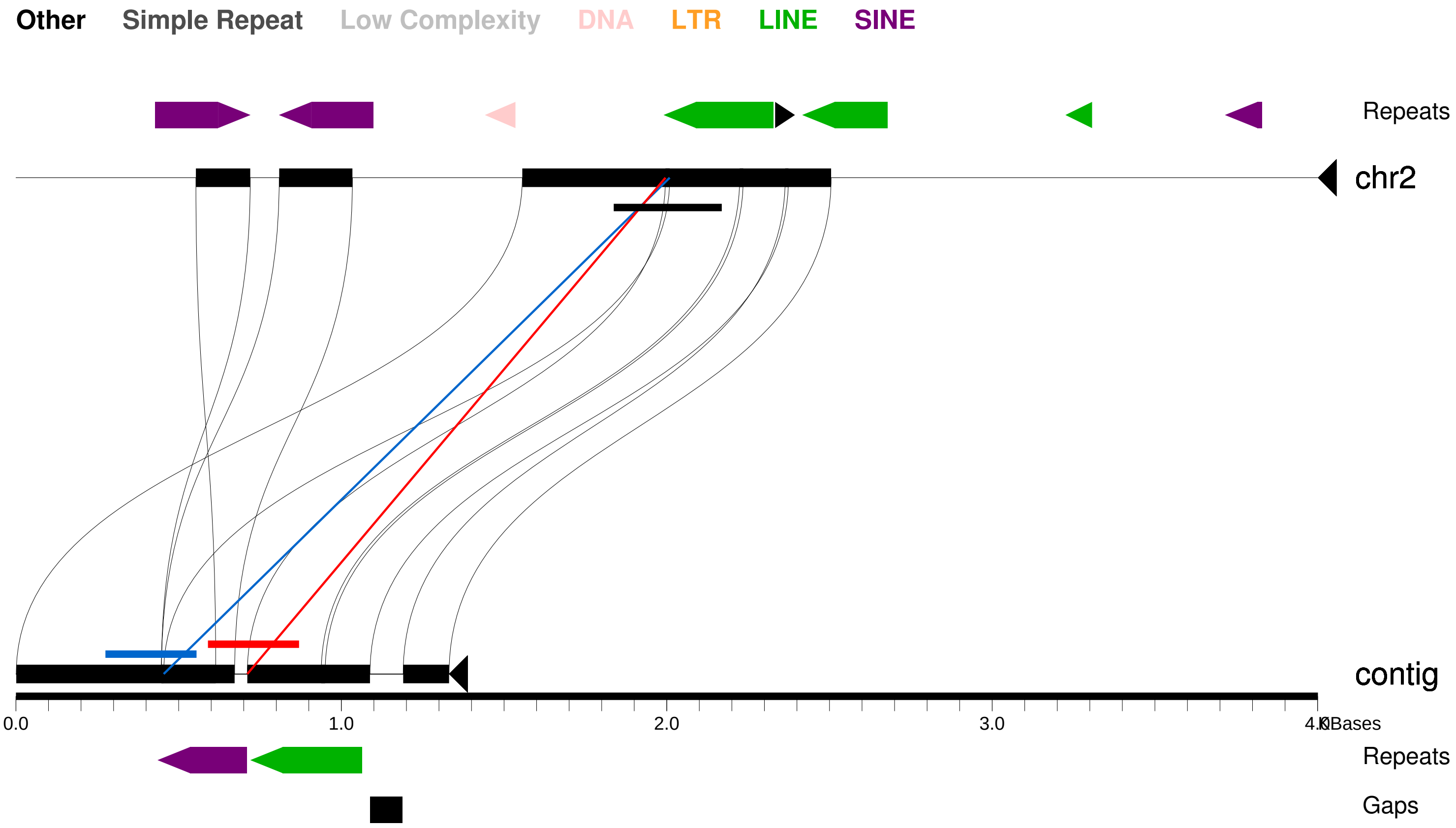

siteID: chr2_80861671 Scafftig1 + Breakpoint: chr2:80861642
chr2:80861608-80861701
Overlap Length: 26 INS size: 309 Genotyped: No

```
left    TAATGAG---AGCAAAAT-----------CAGAAGTATTGGG----CCCCAT-------GTGAAGACCAT
chrom   TAATGAG---AGCAAAAT-----------CAGAAGTATTGGG----CCCCAT-------GTGAAGACCAT
right   CACCGCGCCCGGCTAATTTTTTGTATTTTTAGTAGAGACGGGGTTTCACCTTGTTAGCCGTGAAGACCAT
        1*11*1*1111**1**1*111111111111**1**1111***1111*1**1*1111111***********

left    TTTGATAGATTTGGANNNNNNNNNNNNNNNNNNNNNNNNNNNNNNNNNNNNNNNNNNNNNNNNNNNNNNN
chrom   TTTGATAGATTTGGATTTAACTCAGTCTGTGATTGGGAAGCCACTCAAG---------------------
right   TTTGATAGATTTGGATTTAACTCAGTCTGTGATTGGGAAGCCACTCAAG---------------------
        ***************2222222222222222222222222222222222222222222222222222222

left    NNNN
chrom   ----
right   ----
        2222
```

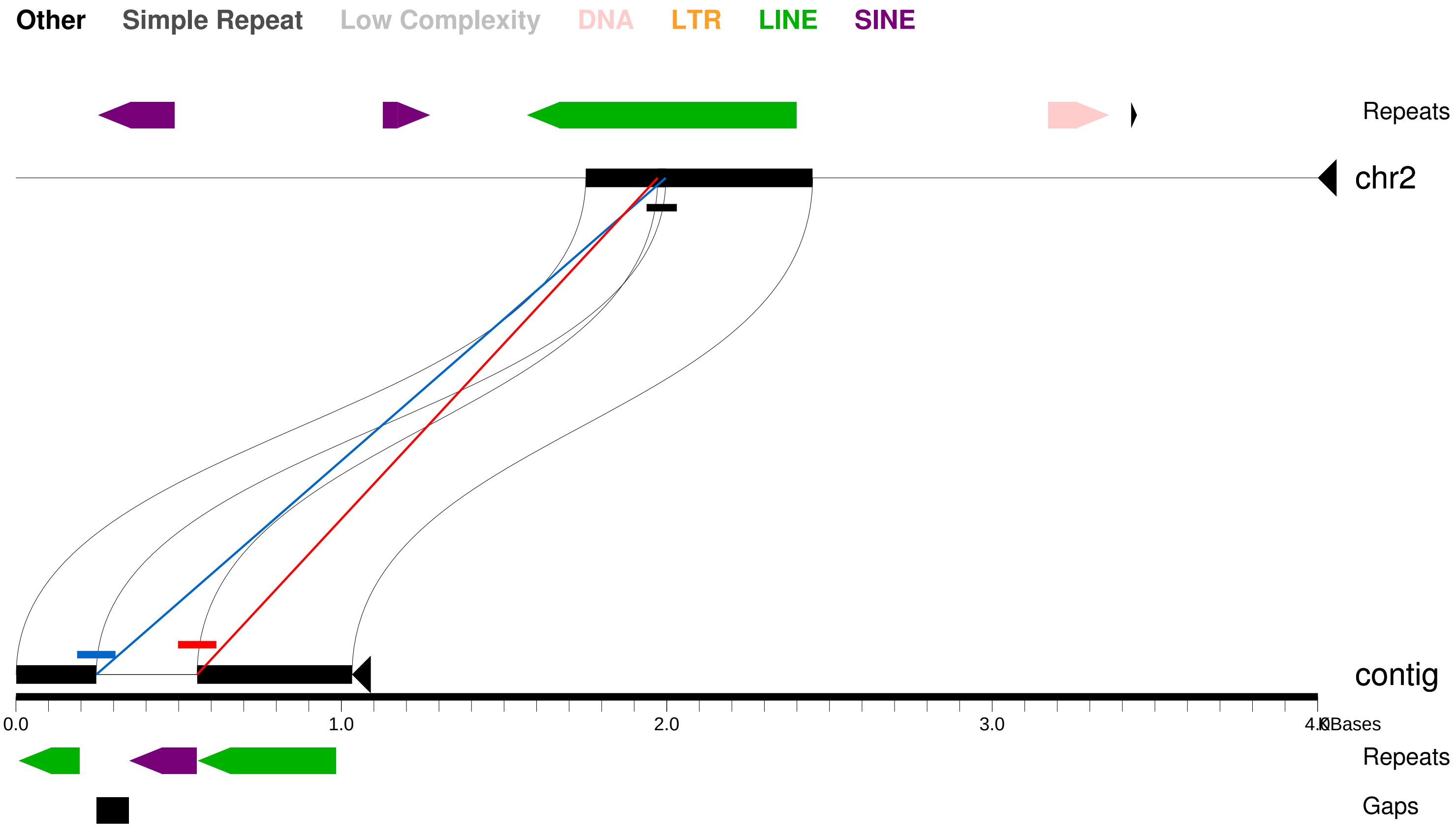

siteID: chr2_83831724 Scafftig1 - Breakpoint: chr2:83831720
chr2:83831670-83831779
Overlap Length: 10 INS size: 325 Genotyped: Yes

```
left    --------CATAGAAAAGATTGA-TGCTATCATAAAAAAACATGAAAGTATAAAACTCT--CATGACTTT
chrom   --------CATAGAAAAGATTGA-TGCTATCATAAAAAAACATGAAAGTATAAAACTCT--CATGACTTT
right   TCCGCCCGCCTCGGCCTCCCAAAGTGCTGGGATTACAGG-CGTGAGCC-ACCGCGCCCGGCCATGACTTT
        11111111*1*1*111111111*1****111**1*1*111*1***1111*11111*1*111*********

left    CTTTTTTTTTTTTTTTTTTTTTTTTTTTTTTTTTTTTTTTTTTGAGACGGAGTCTTGCTC
chrom   CAATACATTTATAAAATTGAGCCTGCAAATCAGCTATGT------AAC--AAT-TAACAT
right   CAATACATTTATAAAATTGAGCCTGCAAATCAGCTATGT------AAC--AAT-TAACAT
        *22*222***2*2222**22222*22222*2222*2*2*2222222**22*2*2*22*22
```

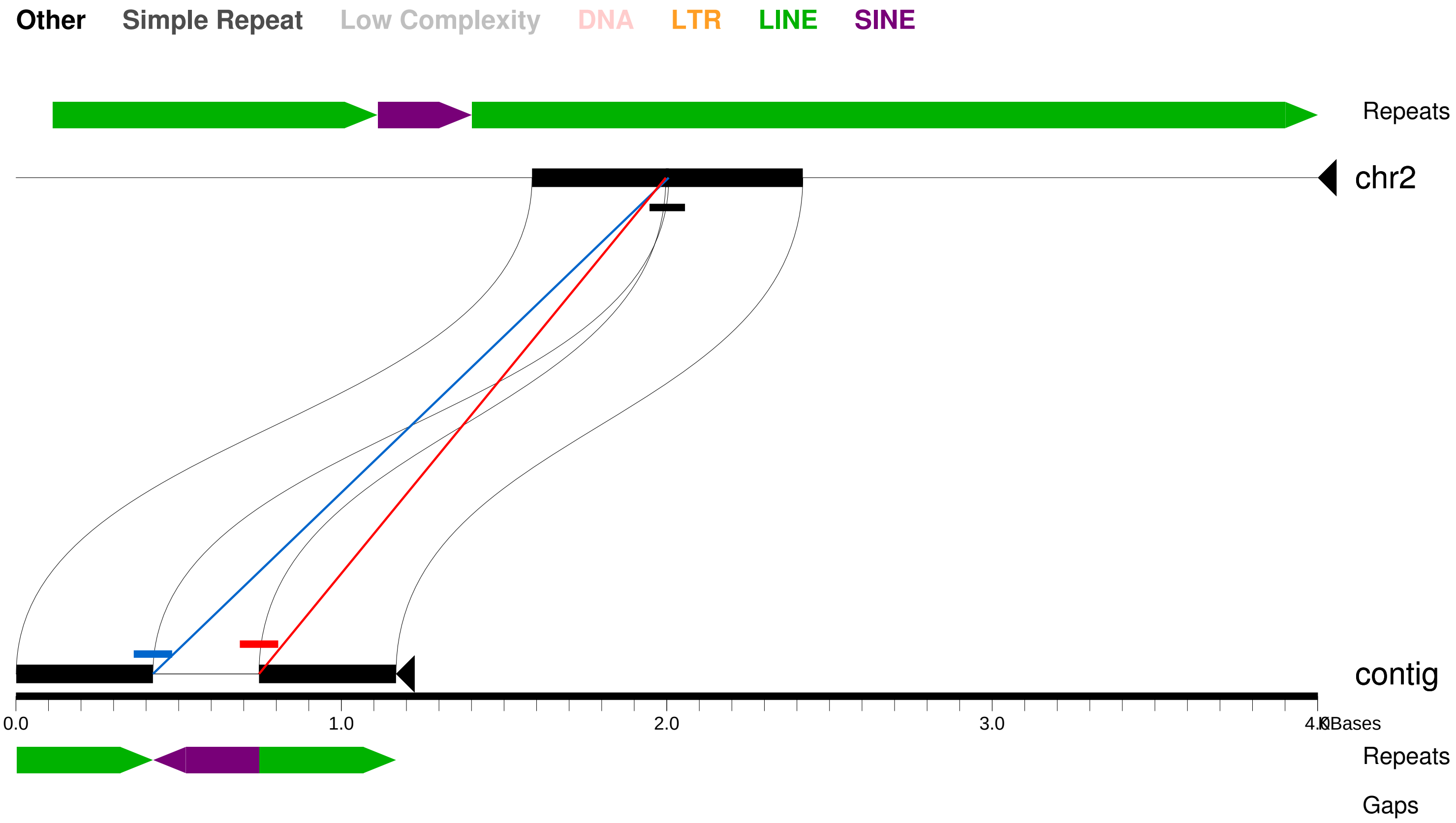

siteID: chr2_84590144 Scafftig1 - Breakpoint: chr2:84590133
chr2:84589973-84590304
Overlap Length: 12 INS size: 434 Genotyped: No

```
left   ------------------------------------------AATGACAGGGTCGAATCTACATATATAA
chrom  CAACCACACAAACAAACCTGCATAATAGCTAGCTAATAACACAATGACAGGGTCGAATCTACATATATAA
right  TTTAGT-AGAGACGGGGTTTCACCGTTT-TAGC------CGGGATG---GTCTCGA-TCTCCTGACCTCG
       NNNNNN 2N2N22NNNNN2N22NNN2NN 2222      2NN1***111*11****1***1*11*11*11

left   ATACTAATCTTCAATGTAAACAGGCTAAATGCCCCATTTGAAAGGCACAGAGTGGCAAGCTGGATAAAAT
chrom  ATACTAATCTTCAATGTAAACAGGCTAAATGCCCCATTTGAAAGGCACAGAGTGGCAAGCTGGATAAAAT
right  ----TGATCCGC--------CCGCCTCGGCCTCCCA----AAGTGCTGGGATTA-CAGGCGTGA------
       1111*1***11*11111111*1*1**111111****1111**11**111**1*11**1**11**111111

left   AGCAAGACCTAATGGTCTTCAATAGACCCATCCC------------------------------------
chrom  AGCAAGACCTAATGGTCTTCAATAGACCCATCTCACACACAATGACAGTCACAAGCTCAAAATAAAGGGA
right  -GCCACCGCGCCCGGCC---AATAGACCCATCTCACACACAATGACAGTCACAAGCTCAAAATAAAGGGA
       1**1*111*1111**1*111***********2*2222222222222222222222222222222222222

left   ------------------------------------------------------------TTTNNNNNNN
chrom  TGGAGGAAAATCTACCAAGCAAATAGAAATCAGAATAAAAGCAGGGGTTCCAATCCTAATTTCAGACAAA
right  TGGAGGAAAATCTACCAAGCAAATAGAAATCAGAATAAAAGCAGGGGTTCCAATCCTAATTTCAGACAAA
       222222222222222222222222222222222222222222222222222222222222***2222222

left   NNN-------------------------------------------------
chrom  ACAGACTTCAAACCAACAAAGATCAAAAAAGACAAAGAAGGGCATTACATAA
right  ACAGACTTCAAACCAACAAAGATCAAAAAAGACAAAGAAG------------
       2222222222222222222222222222222222222222
```

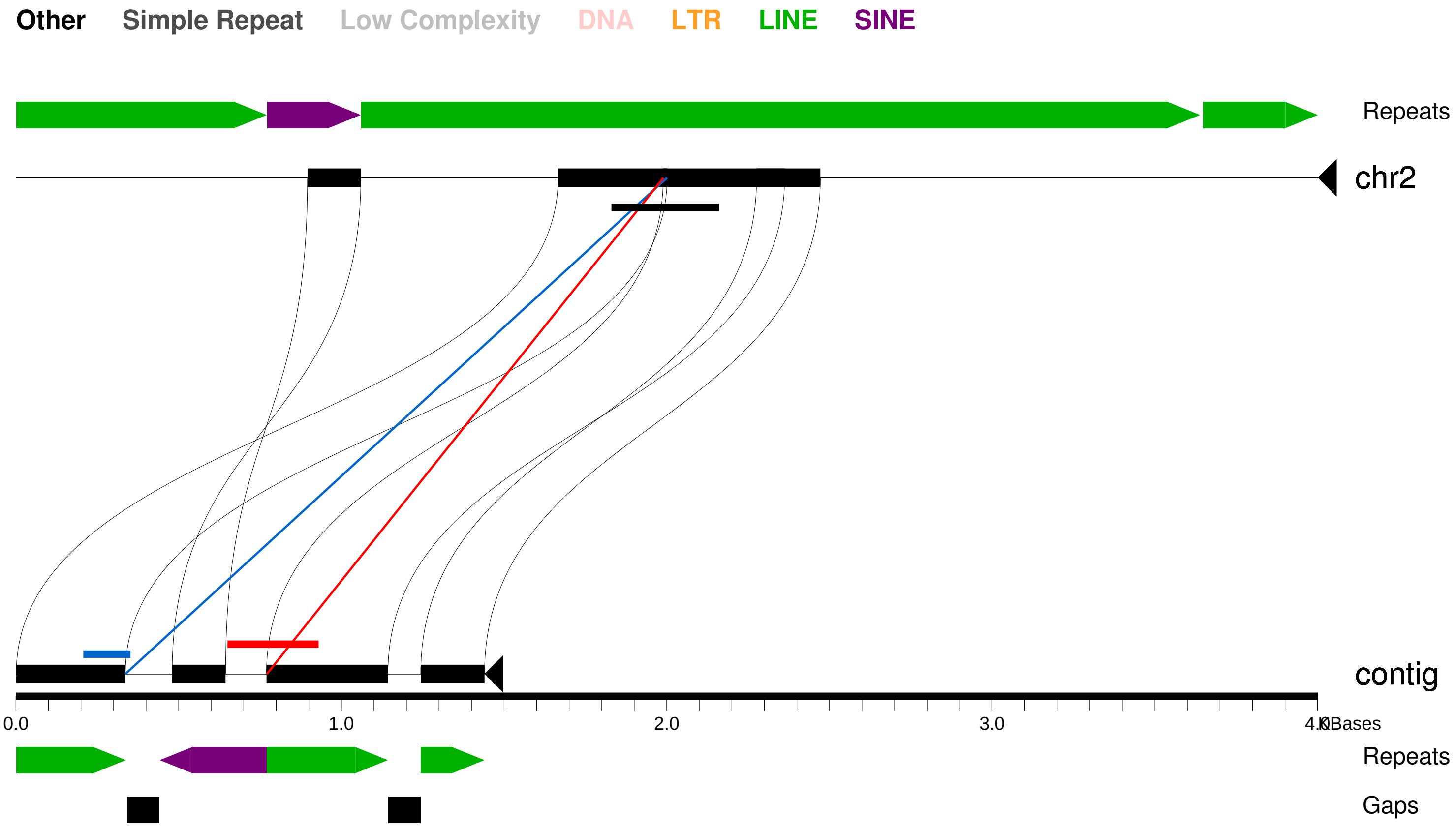

siteID: chr2_84643584 Scafftig2 + Breakpoint: chr2:84643573
chr2:84643413-84643739
Overlap Length: 7 INS size: 290 Genotyped: No

```
left   T-------GGTGAAAACTAAAGGGTCACCCAAGCCAAAATCCACTGAGGAAAGCCAACTTTCAACTACAT
chrom  TTCAGGGTGGTGAAAACTAAAGGGTCACCCAAGCCAAAATCCACTGAGGAAAGCCAACTTTCAACTACAT
right  GAGAATGGCGTGAACCCCAGGGGG----CGGAGCC----TGCAGTGAGCCGAGATTGCG--CCACTGCAC
       1NN2NN2N1*****11*1*11***1111*11****1111*1**1****111**1111*111*1***1**1

left   GGGAGAATAGGTCCTAATGTCCATACCAAGAGTCAGACCAGGAATCAAAGTAGGAGATGCAAAGCCAGAA
chrom  GGGAGAATAGGTCCTAATGTCCATACCAAGAGTCAGACCAGGAATCAAAGTAGGAGATGCAAAGCCAGAA
right  TCCAGCCTGGG---------CGACAGCGAGACTCCGTC------TCAAAAAA--------AAAAAAAAAA
       111**11*1**111111111*1*1*1*1***1**1*1*111111*****11*11111111***111*1**

left   GGCCAGAACCTAGAAAAGATAGGACCACTGTAATCCCAGCACTTTGGGAGGCCGAG-GCGGGC----GGA
chrom  GGCCAGAACCTAGAAAAGATAGGACCAAACACA-CCCAGTCAGAAACAAGCAAAAGTGCTGCCAAAAGGA
right  ----AAAAAAAAAAAAAAAAAGGCCCAAACACC-CCCAGTCAAAAACAAAAAAAAGGGCTACCAAAAGGA
       1111*1**111*1****1*1***1***2222212*****222N22222*1N222**N**212*2222***

left   TC-ACGAGGTCAGGAGATCGAGACCATCCTGGCTAACACGGTGAAAC--CCTGTCT--------CTA-CT
chrom  TTGACAATGGGAAATTATCCTCAACTTTCTAGC-AGCAGGTATAAATTGCCTGAATAATACAGACTAGCT
right  TTGAAAAGGGGAAATTATCCNNNNNNNNNNNNNN-NNNNNNNNNNNNNNNNNNNNNNNNNNNNNNNNNNN
       *22*12*N*22*2222***2NN1N1N1N11N1121N11N1NNN111NNN1111NN1NNNNNNNN111N11

left   AAAA---------ATACAAAAAAT-------TAGCCGGGC--------G
chrom  TCAAGGATGAGGGACACAATGAATCCTGTAATTCCCATGTAAGATTTAG
right  NNNNNNNNNNNNNNNNNNNNNNNNNNNNNNNNNNNNNNNNN---------
       NN11NNNNNNNNN1N1111NN111NNNNNNN1NN11NN1N        1
```

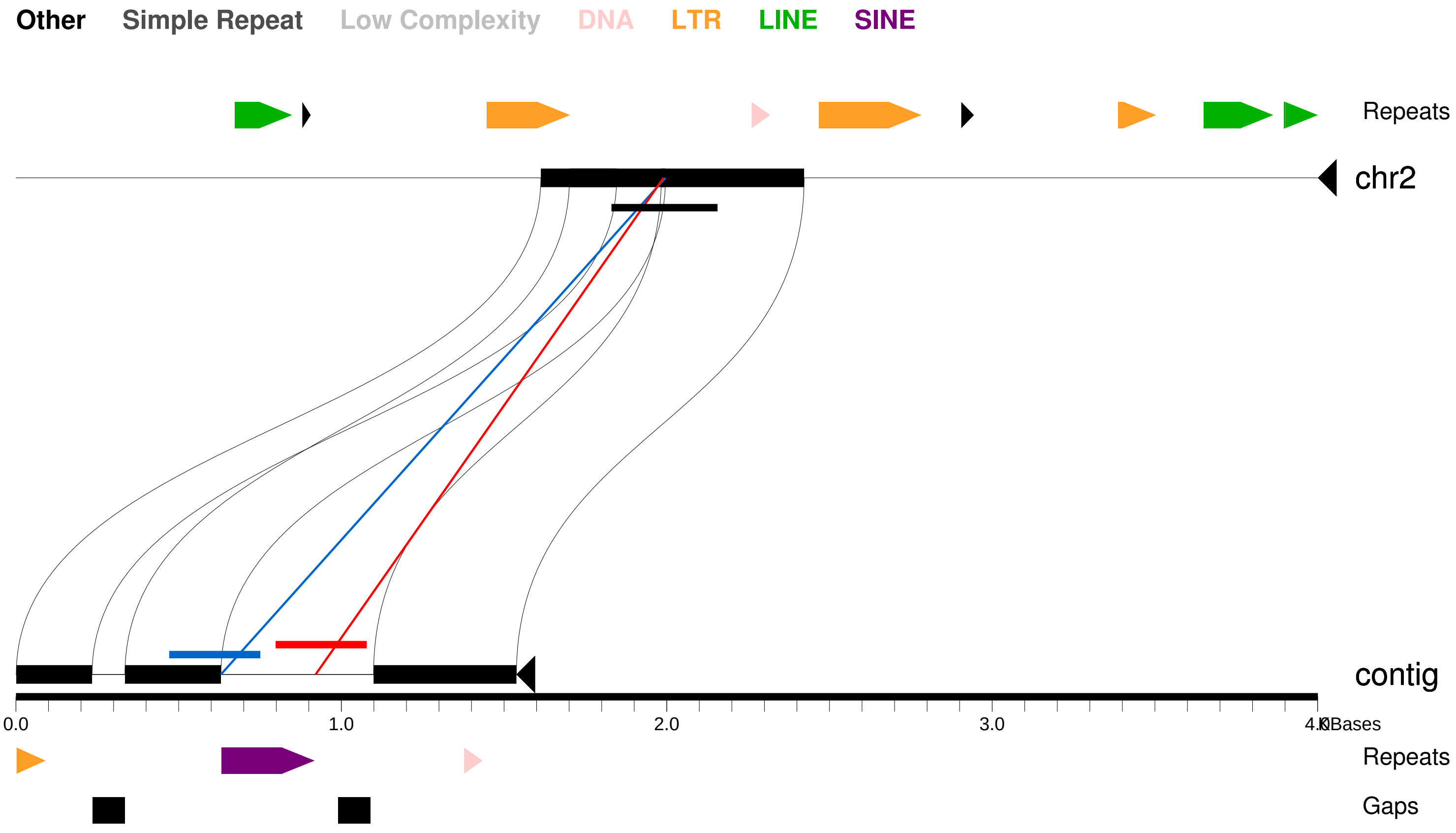

siteID: chr2_84728763 Scafftig23 - Breakpoint: chr2:84728742
chr2:84728696-84728801
Overlap Length: 14 INS size: 295 Genotyped: Yes

```
left    CTAGTTTTTGGAAT--AGTTTGAGTAGGATTGGGATTAGTTCTTCTTT--------------AAAAGTTT
chrom   CTAGTTTTTGGAAT--AGTTTGAGTAGGATTGGGATTAGTTCTTCTTT--------------AAAAGTTT
right   C--GCCACTGCACTCCAGCCTGGGCGACAGAGCGAA-ACTCCGTCTCAAAAAAAAAAAAAAAAAAAGTTT
        *11*1111**1*1*11**11**1*1111*11*1**11*1*1*1***11111111111111********

left    GGTAAAGGCCGGGCGCGGTGGCTCACGCCTGTAATCCCAGCACTTTGGGAGGCCGAGGCGGGCGG
chrom   GGTAAAATTCAG---CAGTG----AAGCCTTCAGGTCCTGGGTTTTT------CTTTACTGGGAG
right   GGTAAAGTTCAG---CAGTG----AAGCCTTCAGGTCCTGGGTTTTT------CTTTACTGGGAG
        ******N22*2*222*2***2222*2****22*222**2*222***2222222*2222*2**22*
```

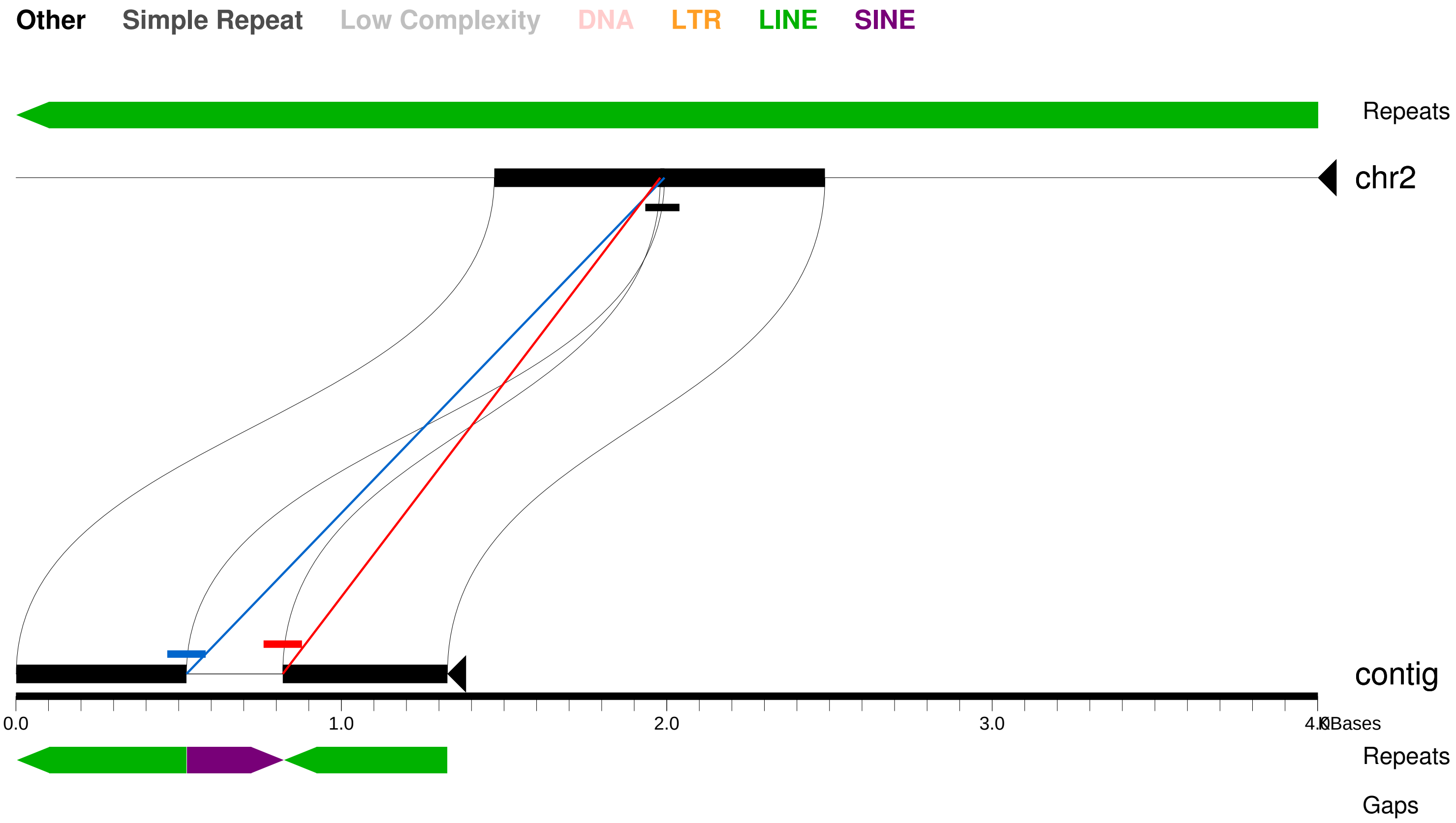

siteID: chr2_90006195 Scafftig1 + Breakpoint: chr2:90006162
chr2:90006118-90006221
Overlap Length: 16 INS size: 324 Genotyped: No

```
left    ATTAGTGAATCTACATT--AATGT-CTCTAATTACA------ATCCACATCTA------CAGTTTCTCCT
chrom   ATTAGTGAATCTACATT--AATGT-CTCTAATTACA------ATCCACATCTA------CAGTTTCTCCT
right   CCACCCGCCTCGGCCTCCCAAAGTGCTGGGATTACAGGCGTGAGCCACCGCGCCCGGCCCAGTTTCTCCT
        111111*11**11*1*111**1**1**111******111111*1****11*11111111***********

left    TTTTTNNNNNNNNNNNNNNNNNNNNNNNNNNNNNNNNNNNNNNNNNNNNNNNNNNNNNNNNNNN
chrom   TTTTT---------------ATATTTGTAGCTTTTTTTCCAAACTGAGAATCTTGGCTGTAATT
right   TTTTT---------------ATATTTGTAGCTTTTTTTCCAAACTGAGAATCTTGGCTGTAATT
        *****22222222222222222222222222222222222222222222222222222222222
```

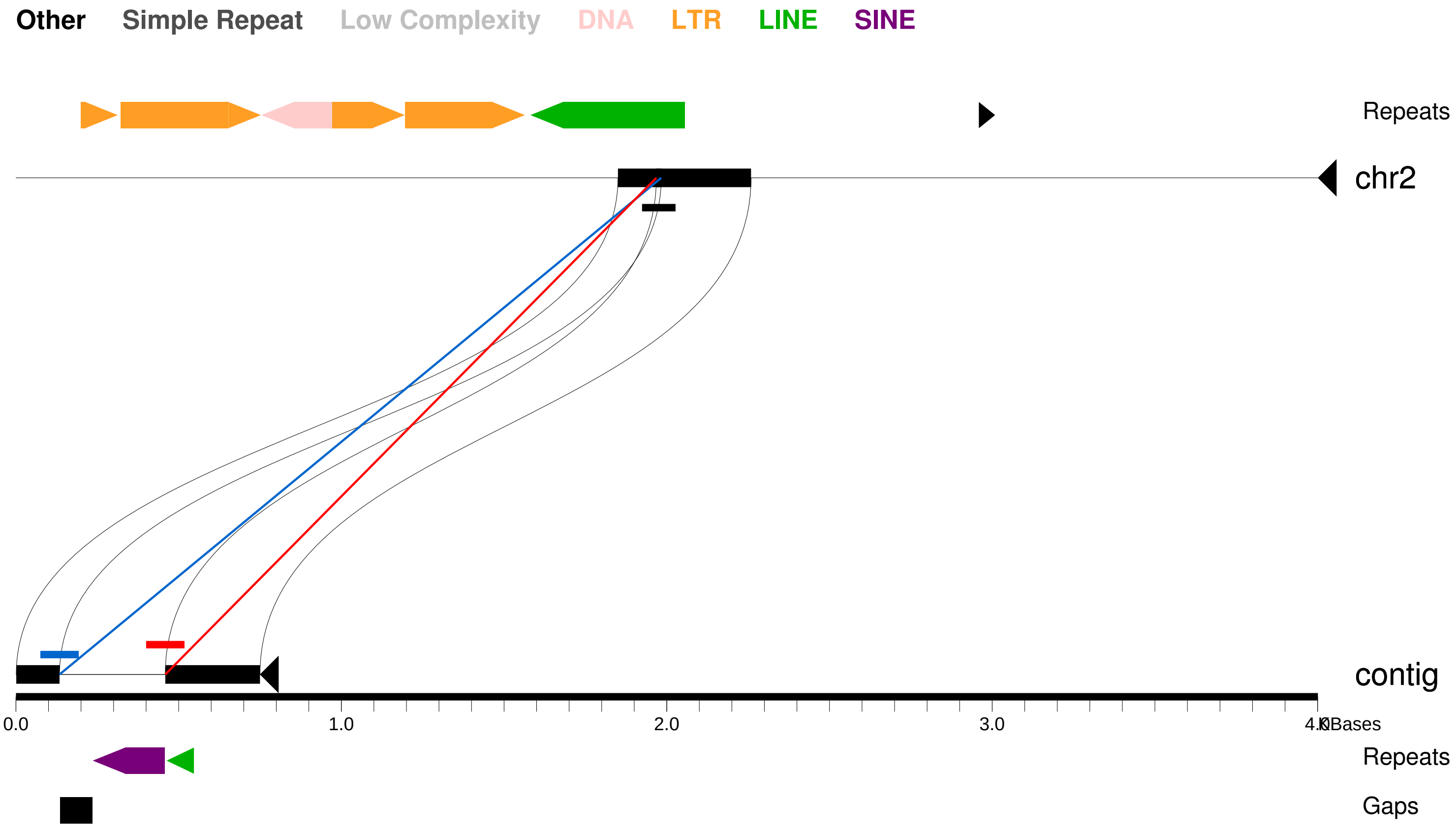

siteID: chr2_98582154 Scafftig2 - Breakpoint: chr2:98582147
chr2:98582098-98582206
Overlap Length: 11 INS size: 293 Genotyped: Yes

```
left    CATGGCTGGGCACCAATTCA-TCTAATCTCACAAACAGCAC-AGATTACTT-------------AAAAAT
chrom   CATGGCTGGGCACCAATTCA-TCTAATCTCACAAACAGCAC-AGATTACTT-------------AAAAAT
right   GATCCCG-----CCACTGCACTCCAGCCTGGGCGACAGAGCGAGACTCCGTCTCAAAAAAAAAAAAAAAT
        1**11*111111***1*1**1**1*11**11111****11*1***1*1*1*1111111111111******

left    TTCCTGGCCGGGCGCGGTGGCTCACGCCTGTAATCCCAGCACTTTGGGAGGCCGAGGCGGGCGG
chrom   TTCCTAATGAAATTAGGGA----ATG--TGTAAT----GAAAATTACTGCTCCTAAAAATCTGA
right   TTCCTAATGAAATTAGGGA----ATG--TGTAAT----GAAAATTACTGCTCCTAAAAATCTGA
        *****2222222222**222222*2*22******2222*2*22**222222**2*2222222*2
```

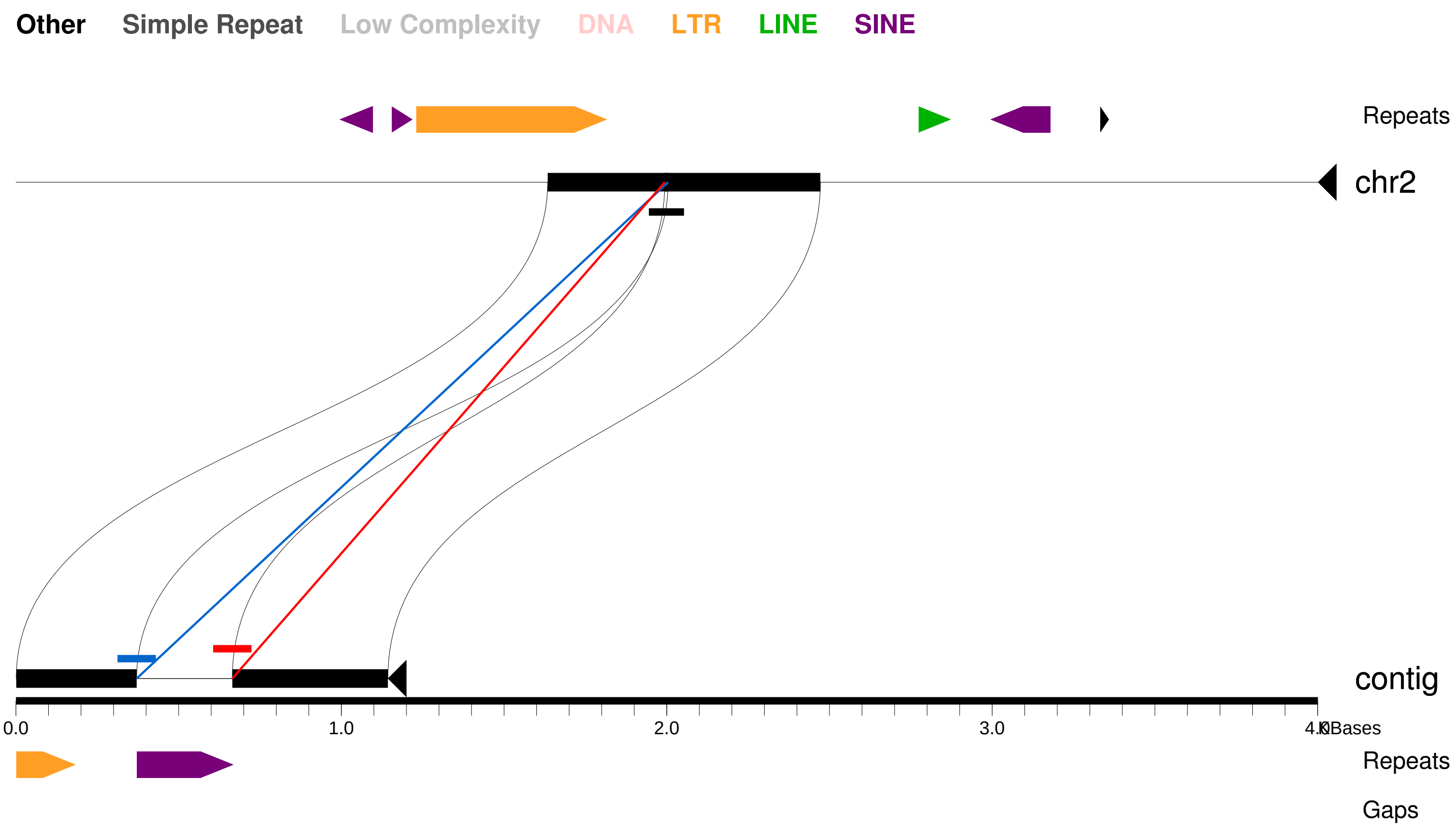

siteID: chr2_9888810 Scafftig6 + Breakpoint: chr2:9888790
chr2:9888759-9888849
Overlap Length: 12 INS size: 315 Genotyped: Yes

```
left   -----CTGCC----CCTACTAATTT------AGTAAATG-------------GCTTTGTCACGGGTGTGT
chrom  -----CTGCC----CCTACTAATTT------AGTAAATG-------------GCTTTGTCACGGGTGTGT
right  TCCACCCGCCTCGGCCTCCCAAAGTGCTGGGATTACAGGCGTGAGCCACCGCGCCCGGCCACGGGTGTGT
       11111*1***1111***1*1**11*111111*1**1*1*11111111111111**111*1***********

left   TTTTTTTTTTTTTTTTTTTTTTTTTTTTTGAGACGGAGTCTCGCTCTGTCGCCCAGGCCGGACTGCGAAC
chrom  TATTGTTTGCTTTTCTTT--------------ATGGCATCCCTCA-TGTCT--------GGGCT---AA-
right  TATTGTTTGCTTTTCTTT--------------ATGGCATCCCTCA-TGTCT--------GGGCT---AA-
       *2**2***22****2***22222222222222*2**22**2*2*22****222222222**2**222**2

left   TGCAGTG
chrom  -GCAGGT
right  -GCAGGT
       2****22
```

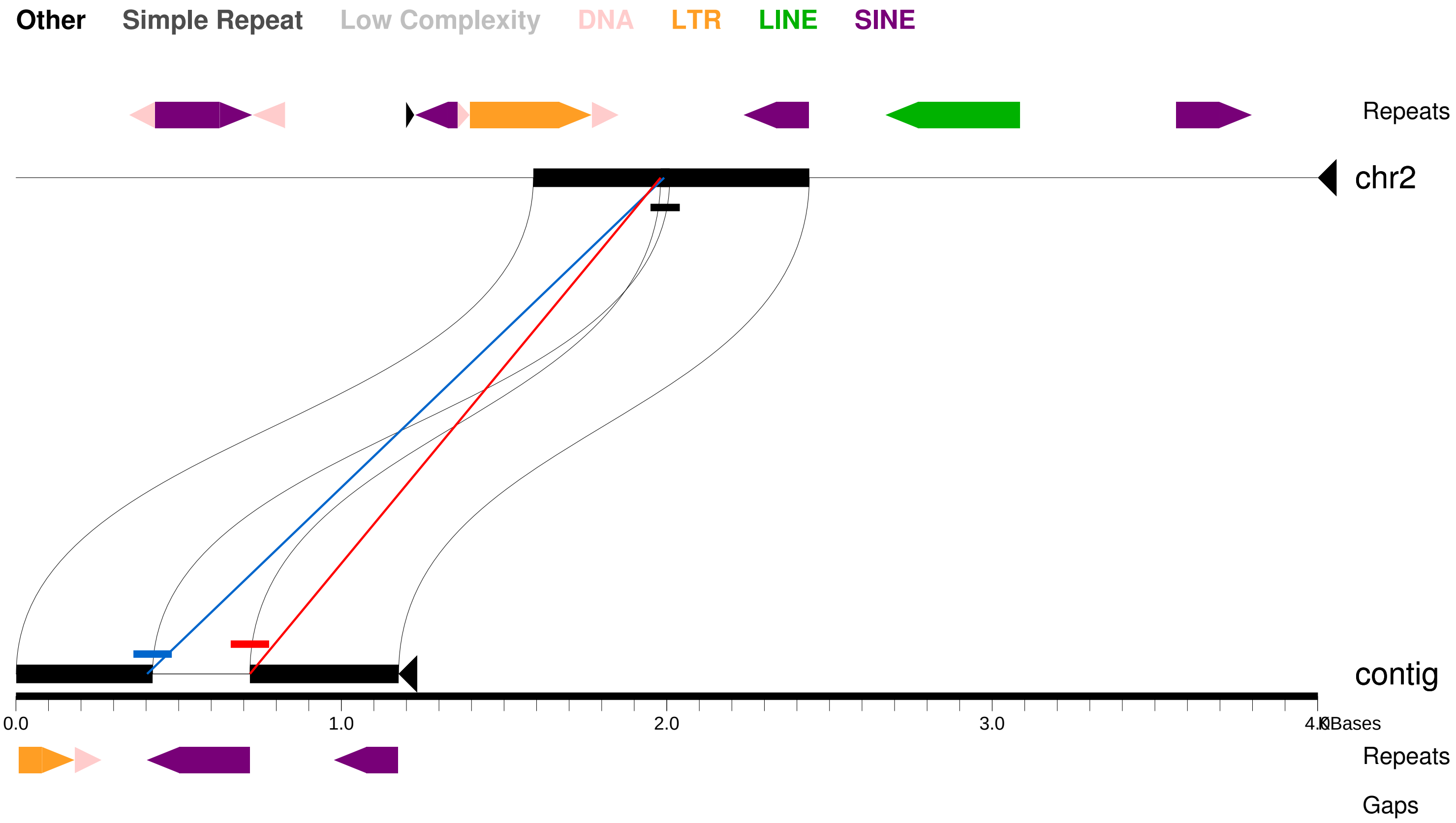

siteID: chr20_10377910 Scafftig1 - Breakpoint: chr20:10377902
chr20:10377865-10377961
Overlap Length: 20 INS size: 285 Genotyped: Yes

```
left    A-------------ACTTCAAAACAAAATA--ACAAGGAACTAATAATTGGT--------AAAATAAAAA
chrom   A-------------ACTTCAAAACAAAATA--ACAAGGAACTAATAATTGGT--------AAAATAAAAA
right   GCCGAGATCCCGCCACTGCACTCCAGCCTGGGAGACAGAGCGAG-ACTCCGTCTCAAAAAAAAAAAAAAA
        11111111111111***1**111**111*111*1*11**1*1*11*1*11**11111111****1*****

left    AGAACATAGTGCCGGGCGCGGTGGCTCACGCCTGTAATCCCAGCACTTTGGGAGGCCGAGGCGGGCGGAT
chrom   AGAACATAGTACC------TATTACTAACT--GGCAGTGGCAATAGGTAATGAGG--------------T
right   AAAACATAGTACC------TATTACTAACT--GGCAGTGGCAATAGGTAATGAGG--------------T
        *1********2**22222222*22**2**2222*2*2*22**22*22*222****22222222222222*

left    CA
chrom   TA
right   TA
        2*
```

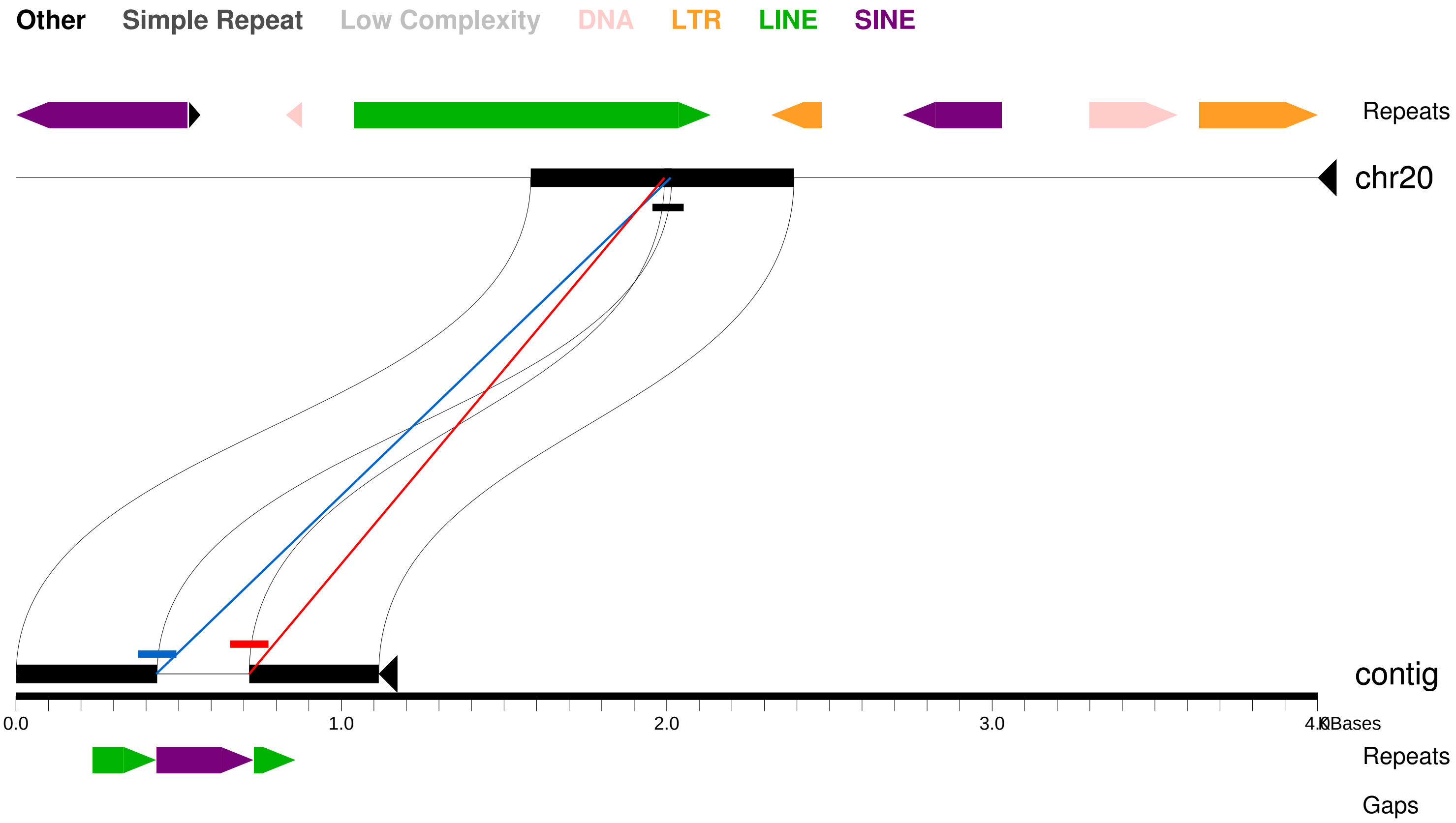

siteID: chr20_13704145 Scafftig1 + Breakpoint: chr20:13704148
chr20:13704102-13704205
Overlap Length: 14 INS size: 295 Genotyped: Yes

```
left    TT------------TTCTAATATTTCATTTCATTTTCAGC-TCATTAAT-GTGCTCATCC--TAGATGAT
chrom   TT------------TTCTAATATTTCATTTCATTTTCAGC-TCATTAAT-GTGCTCATCC--TAGATGAT
right   CCGCCCGCCTCGGCCTCCCAAAGTGC-TGGGATTACAGGCGTGAGCCACCGCGCCCGGCCCCTAGATGAT
        111111111111111**11*1*1*1*1*111***1111**1*1*111*11*1**1*11**11********

left    ATTTTTTTTTTTTTT-TTTGAGATGGAGTCTTGCTCTGTTGCCCAGGCTGGAGTGCAGTGGCGCGA
chrom   ATTTTTAAAACTCTCATTTGACACCAAGT-TTGCAATTTAG----------AAGGAAATC-----A
right   ATTTTTAAAACTCTCATTTGACACCAAGT-TTGCAATTTAG----------AAGGAAATC-----A
        ******22222*2*22*****2*222***2****22*2*2*2222222222*22*2*2*222222*
```

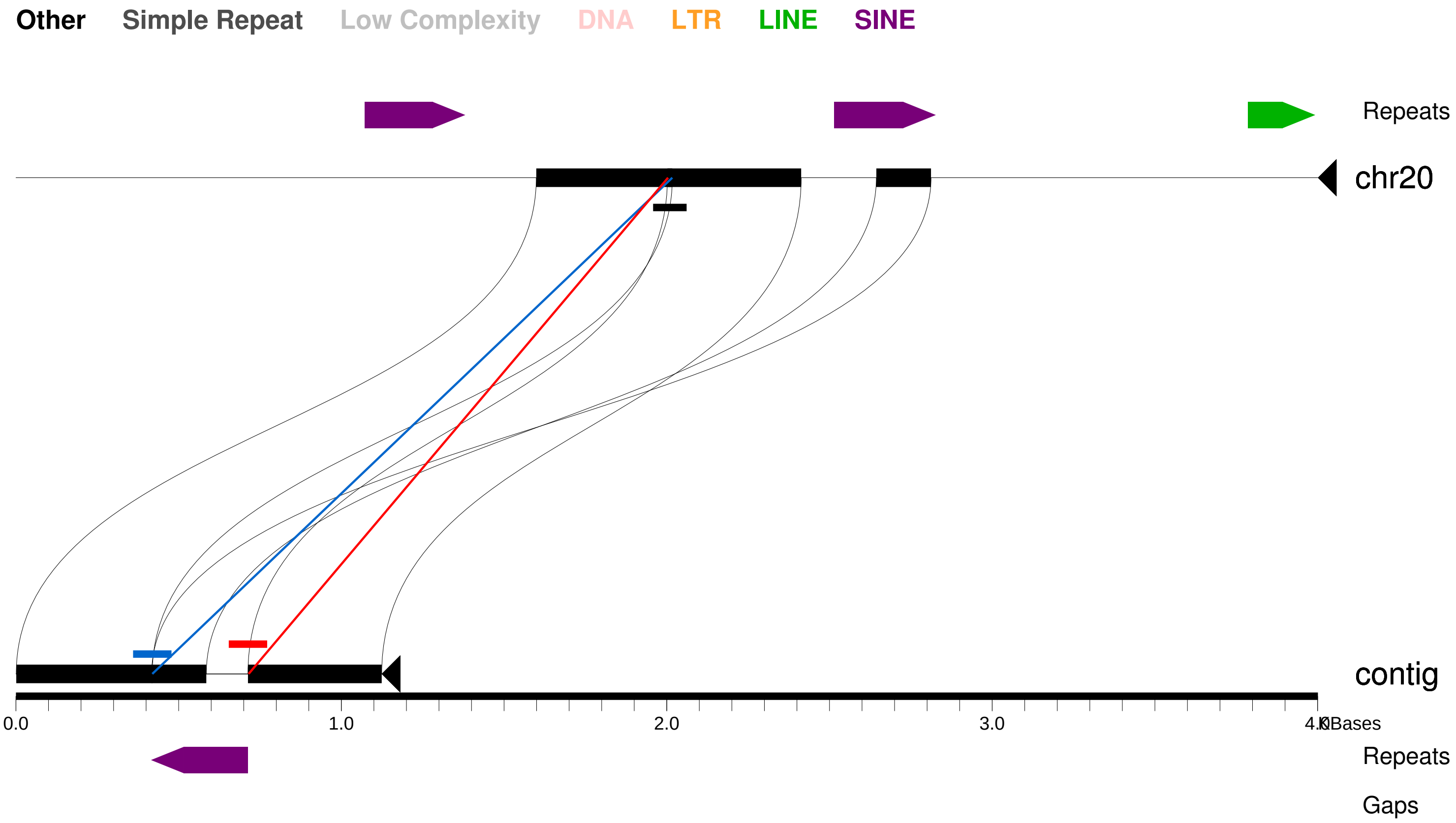

siteID: chr20_15087687 Scafftig5 + Breakpoint: chr20:15087660
chr20:15087614-15087719
Overlap Length: 14 INS size: 297 Genotyped: Yes

```
left     CCCCTAATGTATG--------ATTTATATGTATATAATA-TTTGACATCAC----AGATACACACAATCC
chrom    CCCCTAATGTATG--------ATTTATATGTATATAATA-TTTGACATCAC----AGATACACACAATCC
right    CCACCCGCCTCGGCCTCCCAAAGTGCTGGGATTACAGGCGTGAGCCACCGCGCCCGGCCACACACAATCC
         **1*11111*11*11111111*1*11*11*11**1*1111*11*1**1*1*11111*11***********

left     TTTTTTT---TTTTTG-AGACGGAGTCTCGCTCTGTCGCCCAGGTCGGACTGCGGACTGCAGTAGC
chrom    TTTATATACCTTTATGTATATAGATTGT---TCTGT------------AATTCATA--ACATTTAA
right    TTTATATACCTTTATGTATATAGATTGT---TCTGT------------AATTCATA--ACATTTAA
         ***2*2*222***2**2*2*22**2*2*222*****222222222222*2*2*22*222**2*222
```

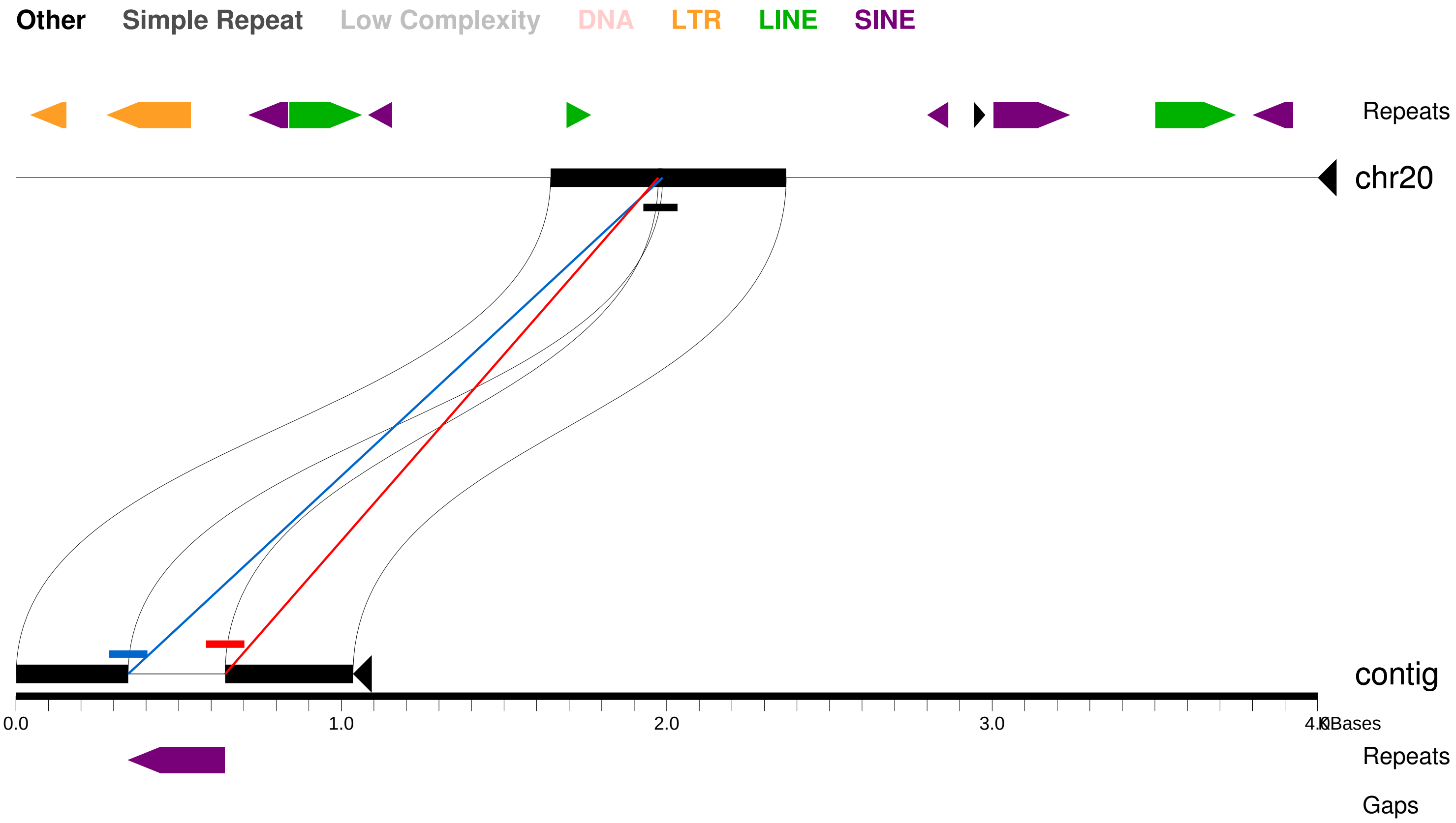

siteID: chr20_1546208 Scafftig14 - Breakpoint: chr20:1546214
chr20:1546169-1546273
Overlap Length: 15 INS size: 296 Genotyped: Yes

```
left    AGCTGT-----------GGGAAAACACATCCACATTCATTC---ATCTGCTCATCATTTAAGAAATGTTT
chrom   AGCTGT-----------TGGAAAACACATCCACATTCATTC---ATCTGCTCATCATTTAAGAAATGTTT
right   GCCACTGCACTCCAGCCTGGGCGACAGAGCGAGACTCCGTCTCAAAAAAAAAAAAAAAAAGAAATGTTT
        11*11*111111111112**111***1*1*1*1*1**11**111*1111111*11*111***********

left    CCTGGCCGGGCGCGGTGGCTCACGCCTG-TAATCCCAGCACTTTGGGAGGCCGAGGCGGGCGGA
chrom   CCTG--AGAGCCTGATATGTCCCTAGTGCTGGTCTCCTC---TTGGGATGCCTG----------
right   CCTG--AGAGCCTGATATGTCCCTAGTGCTGGTCTCCTC---TTGGGATGCCTG----------
        *****222*2**22*2*222**2*222**2*22**2*22*222******2***222222222222
```

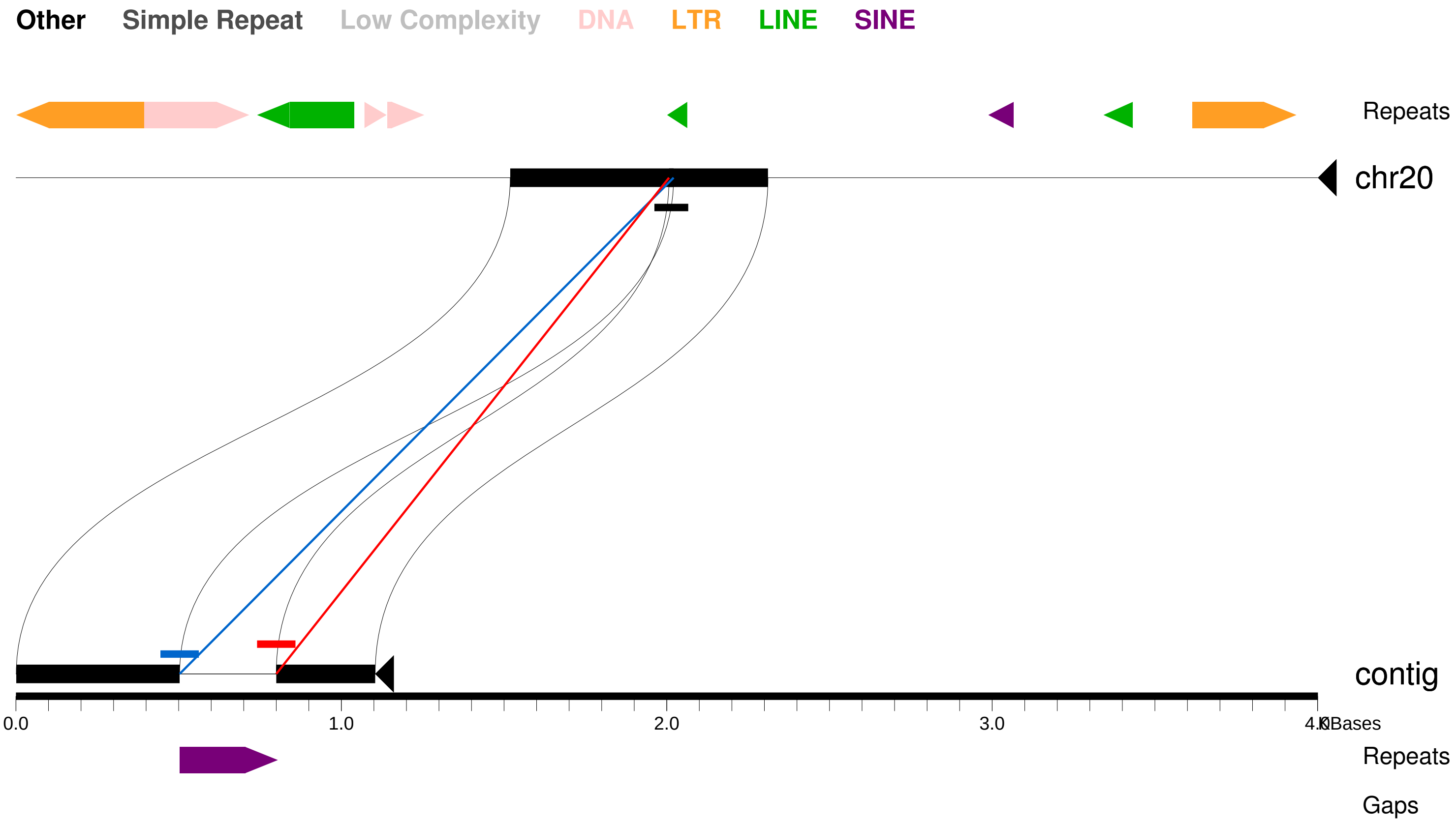

siteID: chr20_16228646 Scafftig2 - Breakpoint: chr20:16228645
chr20:16228597-16228704
Overlap Length: 12 INS size: 473 Genotyped: No

```
left   GGAGGGTGAGAATAAAGAGTCTCTGGAATTAATACTCTGTTGTTTTTT-----------AAAAGCCTGGG
chrom  GGAGGGTGAGAATAAAGAGTCTCTGGAATTAATACTCTGTTGTTTTTT-----------AAAAGCCTGGG
right  NNNNNNNNNNNNNNNNNNNNNNNNNNNNNNNNNNNNNNNNNNNNNNNNNNNNNNNNNNNAAAAGCCTGGG
       11111111111111111111111111111111111111111111111111111111111***********

left   GGCCGGGCGCGGTGGCTCACGCCTGTAA--TCCCAGCACTTTGGGAGGCCGAGGCGGGCGGA
chrom  GAACTTAAATGCTGGTTGTTGGGGTTTCGGTTCAAGAAAATT-------CTATTC------A
right  GAACTTAAATGCTGGTTGTTGGGGTTTCGGTTCAAGAAAATT-------CTATTC------A
       *22*222222*2***2*222*2222*2222*2*2**2*22**2222222*2*22*222222*
```

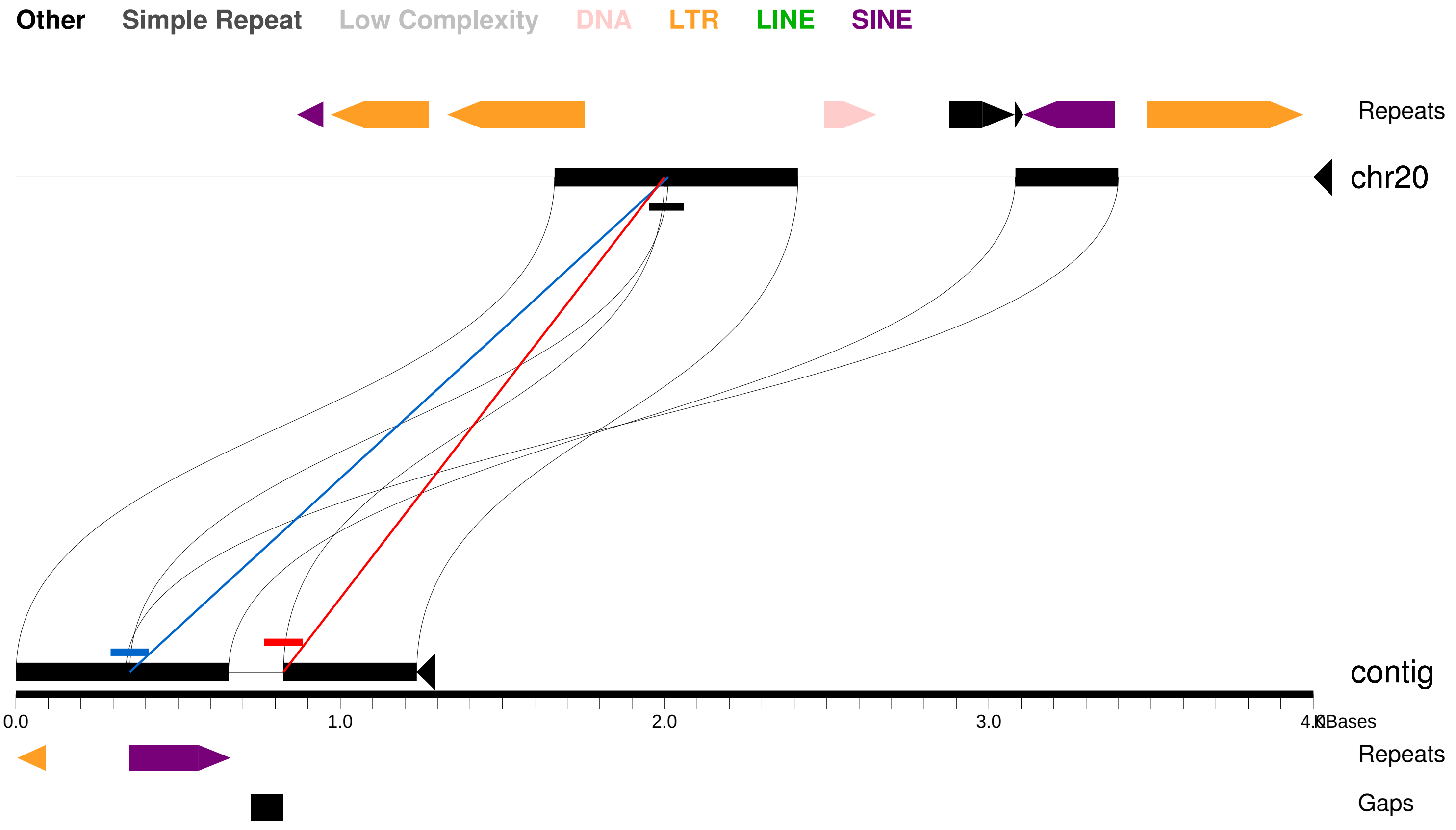

siteID: chr20_1990369 Scafftig2 - Breakpoint: chr20:1990373
chr20:1990317-1990426
Overlap Length: 4 INS size: 307 Genotyped: Yes

```
left    AAG-----------GAGAAAAATAGCATGGCTTTGTCAATGAAGTGCAGAAAGTGAGTGAGAAAGAGAGG
chrom   AAG-----------GAGAAAAATAGCATGGCTTTGTCAATGAAGTGCAGAAAGTGAGTGAGAAAGAGAGG
right   CTGCACTCCAGCCTGGGCGACAGAGCGAGACTCCGTC--TCAAAAAAAAAAAAAAAAAAAAAAAAAAAGG
        11*11111111111*1*11*1*1***11*1**11***11*1**1111*1***111*111*1***1*1***

left    CGGCCG--GGCGCGGTGGCTCACGCCTGTAATCCCAGCACTTTGGGAGGCCGAGGCGGGCGG
chrom   CACAAGATGAGGCTACAGAGCAGGCA--GAAGCCCTAGAATTAAGGA--CAAACT-------
right   CACAAGATGAGGCTACAGAGCAGGCA--GAAGCCCTAGAATTAAGGA--CAAACT-------
        *2222*22*22**2222*22**2**2222**2***222*2**22***22*22*222222222
```

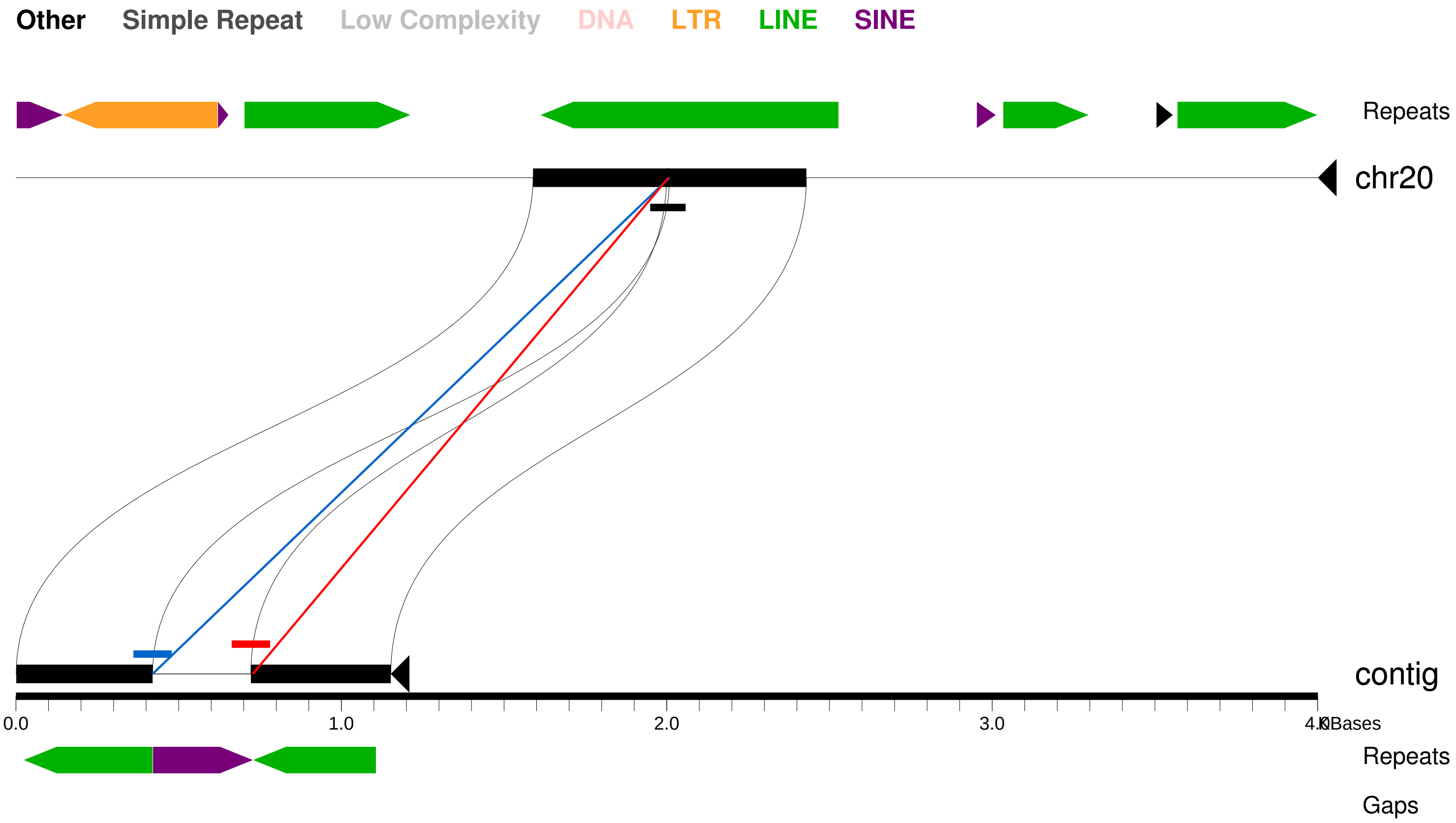

siteID: chr20_2252152 Scafftig1 + Breakpoint: chr20:2252162
chr20:2252002-2252335
Overlap Length: 14 INS size: 361 Genotyped: No

```
left   NNNNNNNN--------------GTTAGGAAACAGAATATTCATAGTCTCAAAATATGACCCCACAGTTTT
chrom  TAAAACCACTAGATGATATGCTGTTAGGAAACAGAATATTCATAGTCTCAAAATATCACCCCACAGTTTT
right  NNNNNNNNNNN-----------------------------------------------------------
       NNNNNNNNNNN           1111111111111111111111111111111111N1111111111111

left   GTTACTAATTACAAACAATAAATGTATCTTTACATTGGAGAGATCTTATAGCCCCCACCTTTACCAAATA
chrom  GTTACTAATTACAAACAATAAATGTATCTTTACATTGGAGAGATCTTATAGCCCCCACCTTTACCAAATA
right  ----------------------------------------------------------------------
       1111111111111111111111111111111111111111111111111111111111111111111111

left   ACTTGGTAACTATTGAATCTAGAAGCTAGGTGCT---GG---CCGAGGCGGGCGGATCA--CGAGGTCAG
chrom  ACTTGGTATCTATTGAATCTAGAAGCTAGGTGCTAATGGATACCACCTCTCTCTAAACAAACCAAGTATG
right  -CTAGGTA------------AGAAGCTAGGTGCTAATGGATACCACCTCTCTCTAAACAAACCAAGTATG
       1**1****N11111111111**************222**222**2222*222*22*2**22*2*2**22*

left   GA--GAT--------CGAGACC-ATCCCG-----GCTAA-----AACGGTGAAACCCCGTCT-CTACTAA
chrom  GTATGATTCCACTGACAAGAAATATCCAGAATGGGCGAATCCATAAAGATAGAAGGTAGATTACTAGTTG
right  GTATGATTCCACTGACAAGAAATATCCAGAATGGGCGAATCCATAAAGATAGAAGGTAGATTACTAGTTG
       *222***22222222*2***222****2*22222**2**22222**2*2*22**2222*22*2***2*22

left   ---AAATACAAAAAAATTAGCCGG-GCGTAGTGGCGGGCGCCTGTAG----TC-
chrom  CCTAGGTACAGGGAAATGAGTTGTTGTCTAAGAATGGGAGGGTAAAGGGTTTCT
right  CCTAGGTA----------------------------------------------
       222*22**11NNN1111N11NN1N 1NN11NNNNN111N1NN1NN11    11
```

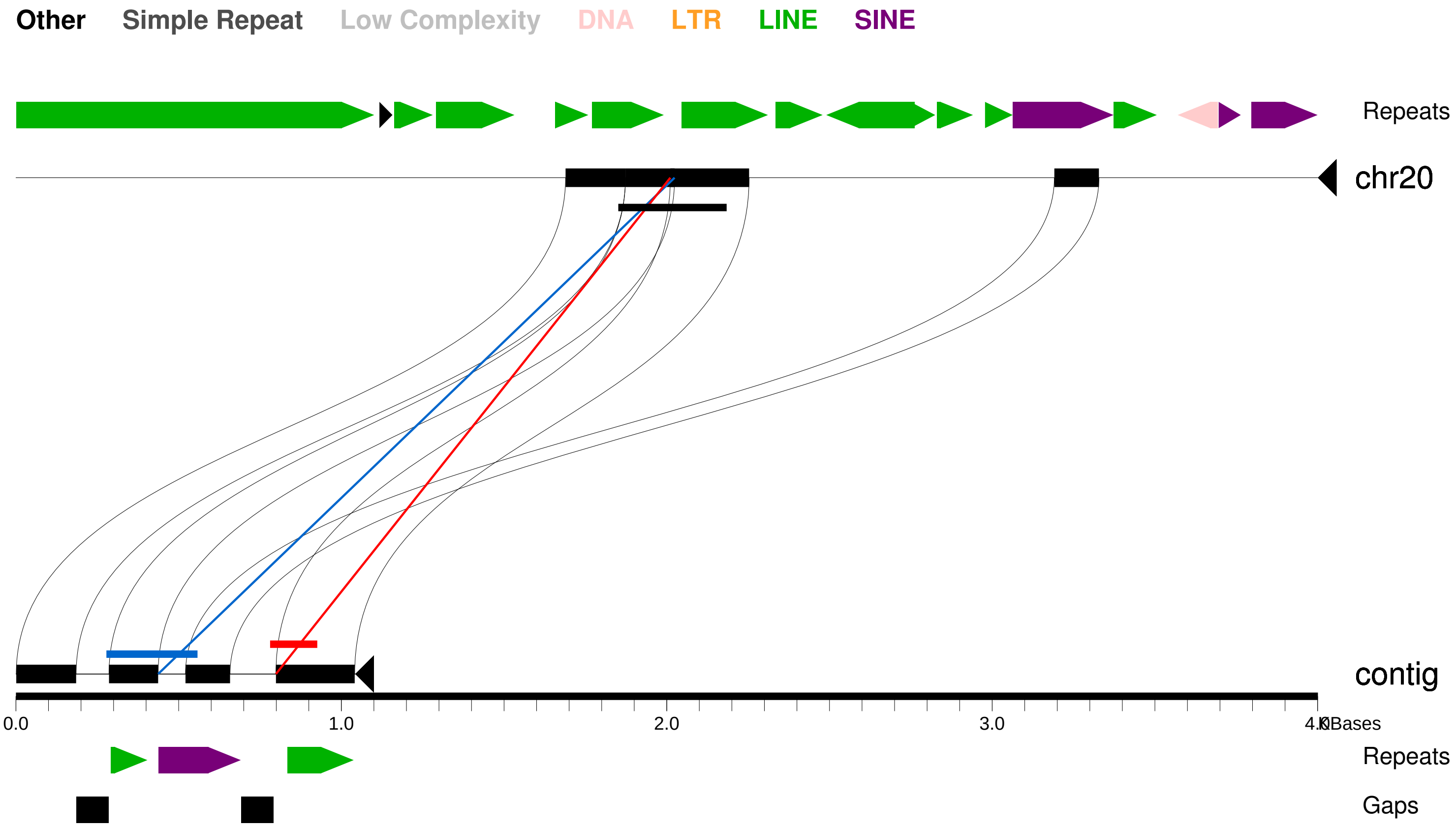

siteID: chr20_2371712 Scafftig1 + Breakpoint: chr20:2371719
chr20:2371666-2371778
Overlap Length: 7 INS size: 375 Genotyped: No

```
left   ACTTCAACTCTGCTGAGAGAGACAGGAACCAATG----GAGAGCTTGAGAA---CAACGAGTGATCTNNN
chrom  ACTTCAACTCTGCTGAGAGAGACAGGAACCAATG----GAGAGCTTGAGAA---CAACGAGTGATCTTAG
right  TTAGCCGGGATGGTCTCGATCTCCTGACCTCGTGATCCGCCCGCCTCGGCCTCCCAAAG-GTGATCTTAG
       1111*11111**1*11111111*11**1*111**1111*111**1*11*11111***1*1********222

left   NNNNNNNNNNNNNNNNNNNNNNNNNNNNNNNNNNNNNNNNNNNNNNNNNNNNNNNN
chrom  ATTTTAACAGGATCCTTCTGGCTTCCATGTTGAGAATGAGCCCTGAGGAT------
right  ATTTTAACAGGATCCTTCTGGCTTCCATGTTGAGAATGAGCCCTGAGGAT------
       22222222222222222222222222222222222222222222222222222222
```

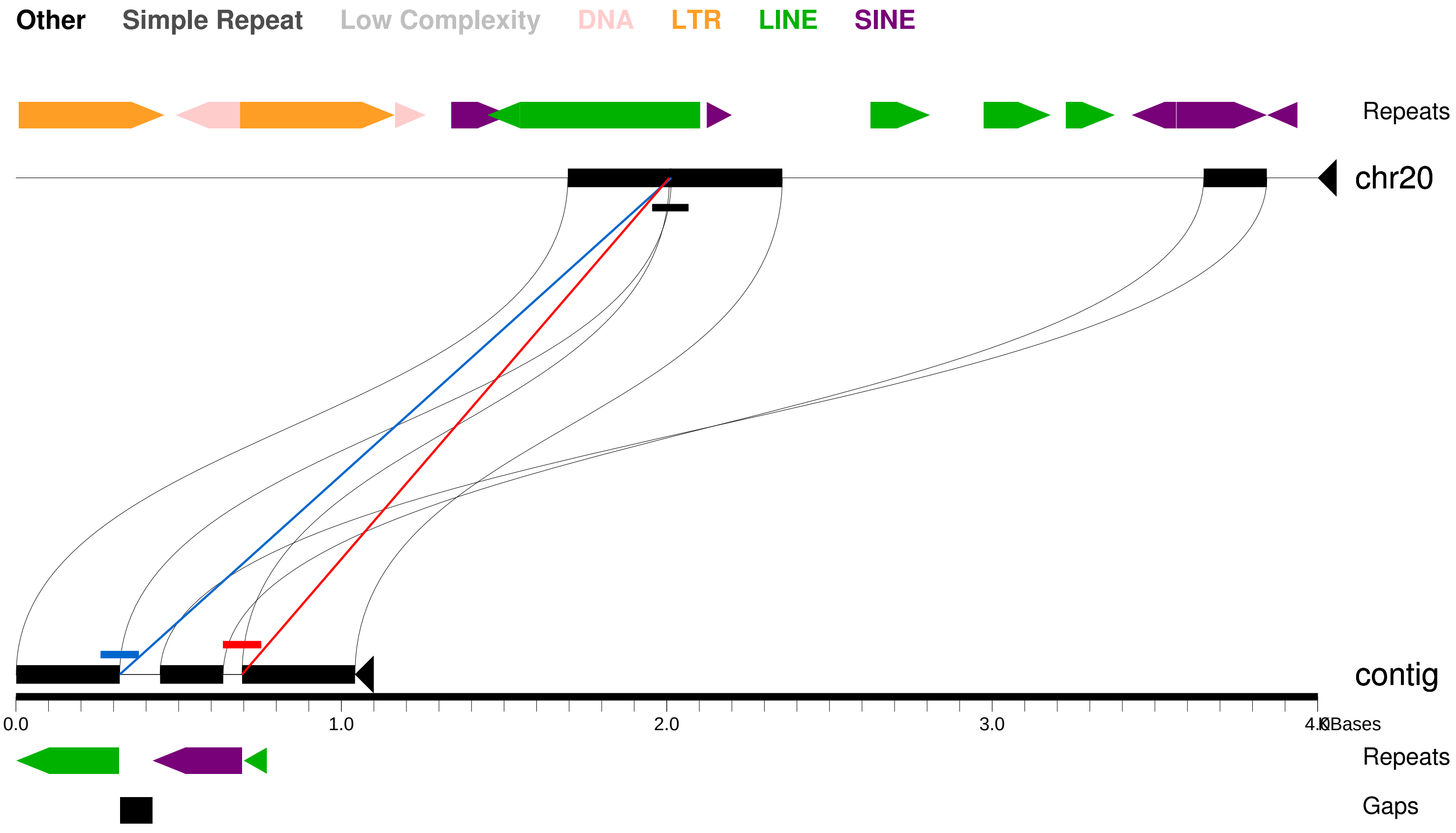

siteID: chr20_26190947 Scafftig2 + Breakpoint: chr20:26190958
chr20:26190915-26191017
Overlap Length: 17 INS size: 424 Genotyped: No

```
left    AACAAATCTGAGGGTGAACACATTTGTTAAAAAGAG----------------AGAGATTAAAAAGTTAAG
chrom   AACAAATCTGAGGGTGAACACATTTGTTAAAAAGAG----------------AGAGATTAAAAAGTTAAG
right   NNNNNNNNNNNNNNNNNNNNNNNNNNNNNNNNNNNNNNNNNNNNNNNNNNNNAAAAAAAAAAAAGTTAAG
        1111111111111111111111111111111111111111111111111111*1*1*11***********

left    ATGCTGGGCCGGGCGCGGTGGCTCACGCCTGTAATCCCAGCACTTTGGGAGGCCGAGGCGGGCGG
chrom   ATGCTGT-----------TTGTACA-GTATATAA-CTGAACAAAGTTGTAT--CTAG-CAATTTG
right   ATGCTGT-----------TTGTACA-GTATATAA-CTGAACAAAGTTGTAT--CTAG-CAATTTG
        ******222222222222*2*22**2*22*2***2*22*2**222*2*2*222*2**2*22222*
```

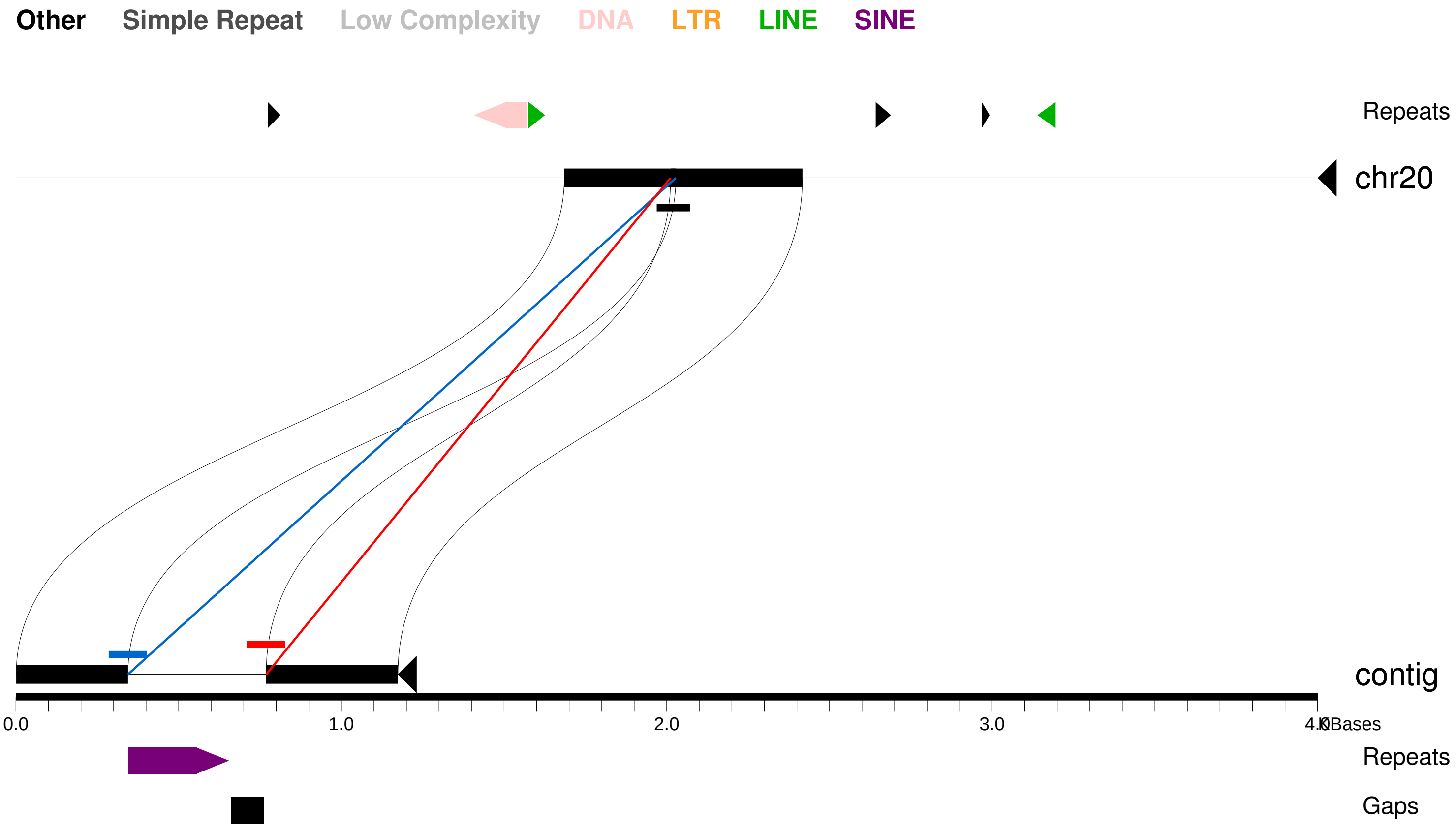

siteID: chr20_30627737 Scafftig1 + Breakpoint: chr20:30627746
chr20:30627586-30627939
Overlap Length: 34 INS size: 249 Genotyped: Yes

```
left   -----------------------------CTAATGCATGTGGGGCTTAAAACCTAGATGAC-AGGTTGAT
chrom  AAGGGGAGGGAGAGCATTAGGACAAATACCTAATGCATGTGGGGCTTAAAACCTAGATGAC-AGGTTGAT
right  C------GGGAGA--AT-------------------------GGCGTGAACCCGGGAGGCGGAGCTTGCA
       N      222222  22            1111111111111***1*1**1**11**1*111**1***11

left   GGGTGCAGCAAACCA-CCACGGCACATGTATACCTATGCAACAAACCTGCACGTTCTGCAAATGTATCTC
chrom  GGGTGCAGCAAACCA-CCACGGCACATGTATACCTATGCAACAAACCTGCACGTTCTGCAAATGTATCCC
right  GTGAGCCGAGATCCCGCCACTGCAC-TCCAG-CCTGGGCGACAGAGCGAGAC--TCCG--------TCTC
       *1*1**1*11*1**11****1****1*11*11***11**1***1*1*111**11**1*11111111**N*

left   AGAATTTAAAGTAAAAAGAAAGAAAGAAAAAAAAAAACATAGAAAAACACTTCCCCGC-ACT--TTGGGA
chrom  AGAATTTAAAGTAAAAAGAAAGAAGAAAAAAAAAAACATAGAAAAACACTTCCCCACCACCAATTCCAA
right  A---------------------AAAAAAAAAAAAAAAAAAAGAAAAACACTTCCCCACCACCAATTCCAA
       *111111111111111111111***1***********1*1***************2*2**222**222*

left   GG--CCGAGGCGGGCGGATCACAAGGTCAGG-----------------AGAT-CGAGACC---ATCCCGGC
chrom  TGTTCCTTTCCTCTTCAGTGACAACCTCTGCTTCTTGTTGGCTGTTTAGCTTCCAGACTTTTATCTACGC
right  TGTTCCTTTCCTCTTCAGTGACAACCTCTGCTTCTTGTTGGCTGTTTAGCTTCCAGACTTTTATCTACGC
       2*22**2222*2222222*2****22**2*22222222222222222**2*2*2****2222***222**

left   --TAAAAT---------GGTGAA------ACCCCGTCTCTACTAAAAATACAAAA--AATTAGCCGGGCG
chrom  ATTTACATACATGTAGAGGTAAACACAAAACCATGTTATTATTTTAAAAAGGAATGTACTCTGCGATTCT
right  ATTTACATACATGTAGAGGTAAACACAAAACCATGTTATTATTTTAAAAAGGAATGTACTCTGCGAT---
       22*2*2**222222222***2**222222***22**222**2*22***2*22**222*2*22**222N1N

left   TAGTGG
chrom  GAAAGA
right  ------
       N1NN1N
```

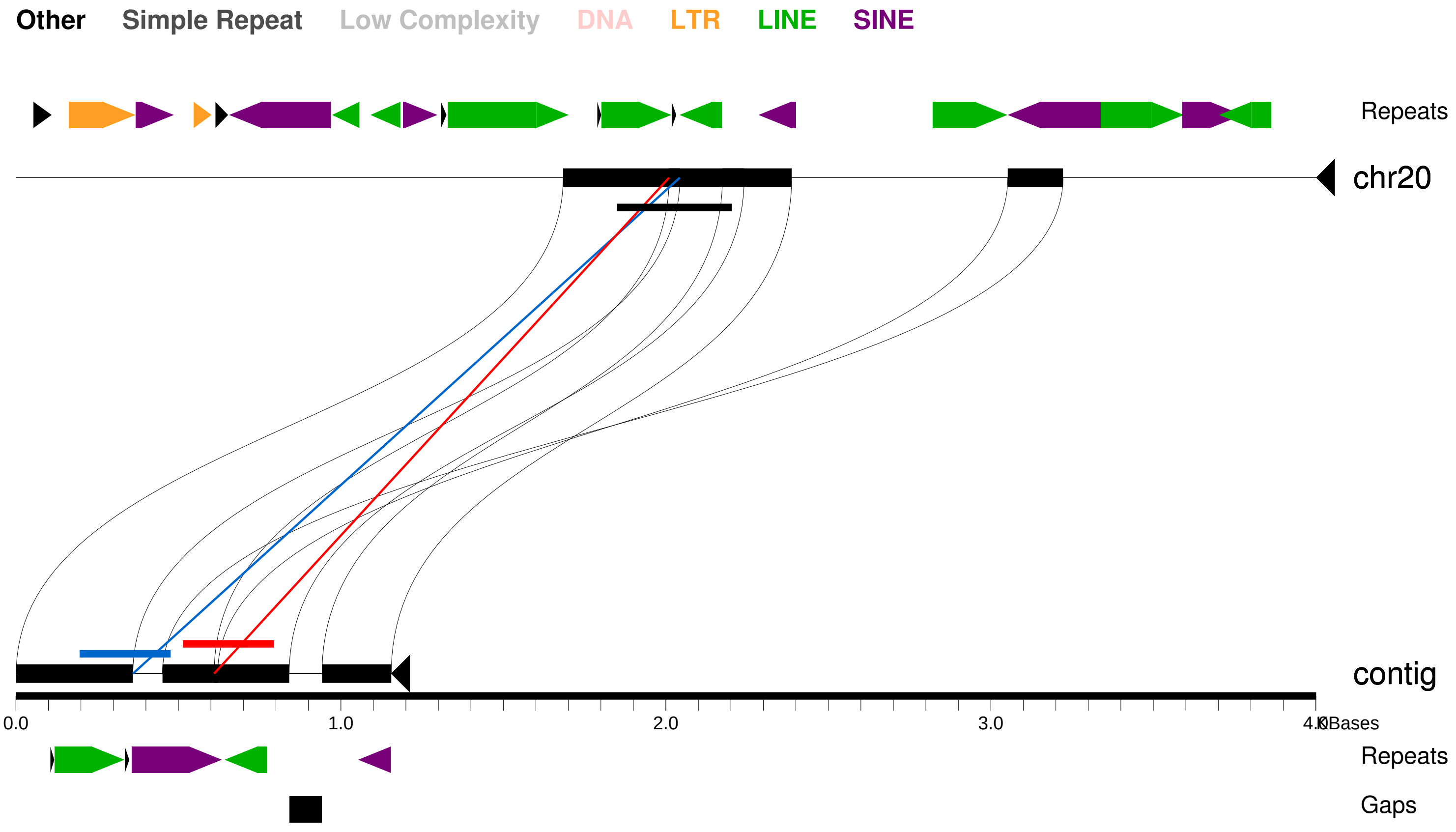

siteID: chr20_33949043 Scafftig5 - Breakpoint: chr20:33949029
chr20:33948987-33949088
Overlap Length: 18 INS size: 282 Genotyped: Yes

```
left    GGCTGAGCATAAGATTTCATTTTT-AAAAAG---GACTCTG-CTGAT------------AAAAAGTTTGA
chrom   GGCTGAGCATAAGATTTCATTTTT-AAAAAG---GACTCTG-CTGAT------------AAAAAGTTTGA
right   TGCAGTCCGCAGTCCGGCCTGGGCGACAAAGCGAGACTCCGTCTCAAAAAAAAAAAAAAAAAAAGTTTGA
        1**1*11*11*111111*1*11111*1****111*****1*1**1*1111111111111***********

left    AAACCACGCCTGTA-ATCCCAGCACTTTGGGAGGCCGAGGCGGGTGGATC-ATGAGGTCAGGAGATCG
chrom   AAACCAC-----TAGATCCCTCTACCT--GGA-----AGGCTTTTTCATCCACTCTTTCAC-------
right   AAACCAC-----TAGATCCCTCTACCT--GGA-----AGGCTTTTTCATCCACTCTTTCAC-------
        *******22222**2*****222**2*22***22222****222*22***2*22222***22222222
```

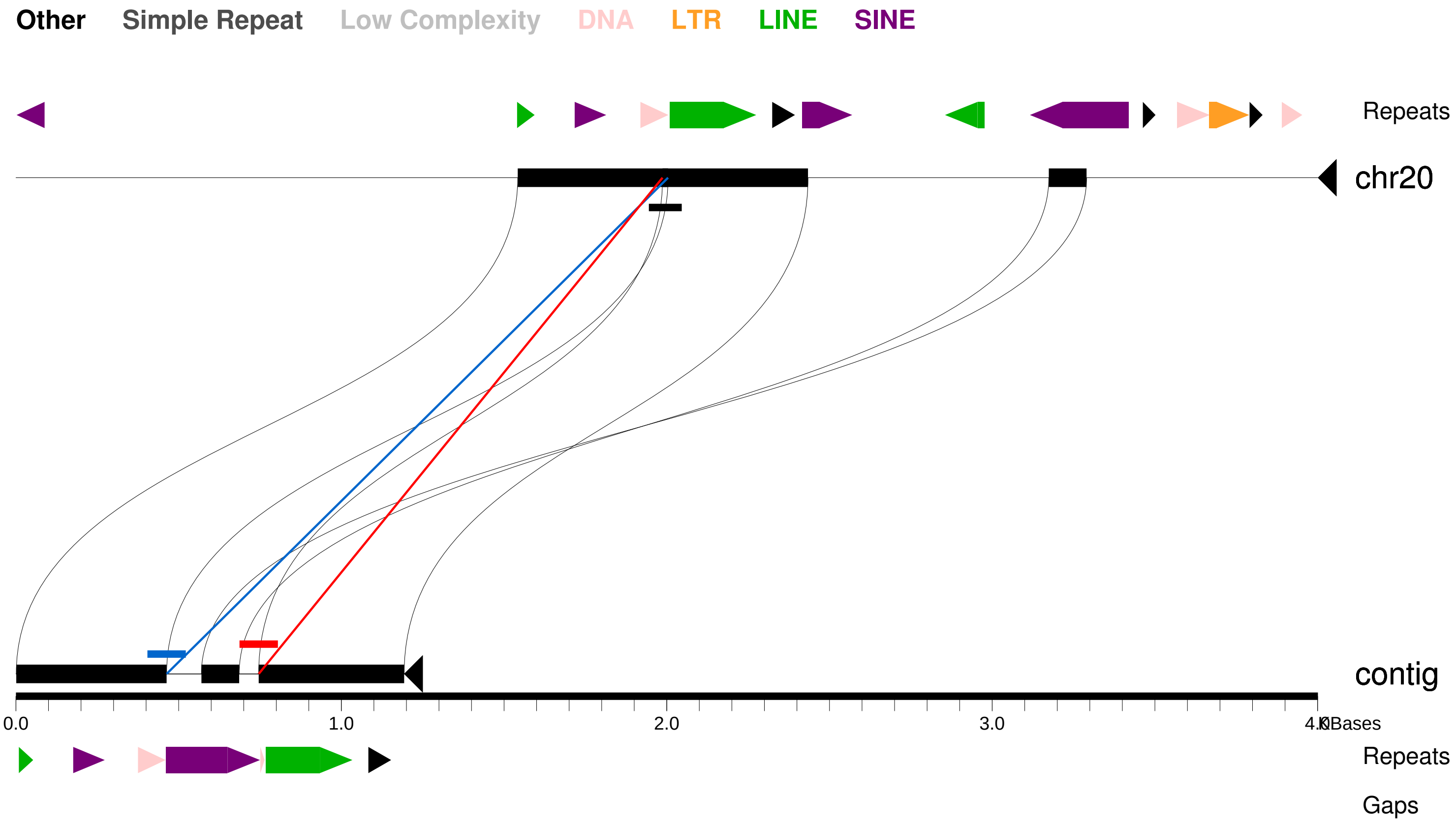

siteID: chr20_36437377 Scafftig1 + Breakpoint: chr20:36437387
chr20:36437339-36437446
Overlap Length: 12 INS size: 421 Genotyped: No

```
left   AATATTCCCCACGTGTAATTGATCATCAGCCCTATGAATTCACCTTAG-----------AAAATCTCTTG
chrom  AATATTCCCCACGTGTAATTGATCATCAGCCCTATGAATTCACCTTAG-----------AAAATCTCTTG
right  NNNNNNNNNNNNNNNNNNNNNNNNNNNNNNNNNNNNNNNNNNNNNNNNNNNNNNNNNNNAAAATCTCTTG
       11111111111111111111111111111111111111111111111111111111111***********

left   AGGCCGGGCGCGGTGGCTCACGCCTGTAATCCCAGCACTTTGGGAGGCCGAGGCGGGCGG
chrom  AA-CCAGTC-CTTTTCATTGCCACTGT---CTCTGCCCT------CATCATCTCTCCTGA
right  AA-CCAGTC-CTTTTCATTGCCACTGT---CTCTGCCCT------CATCATCTCTCCTGA
       *22**2*2*2*22*222*22*22****222*2*2**2**222222222*2222*2222*2
```

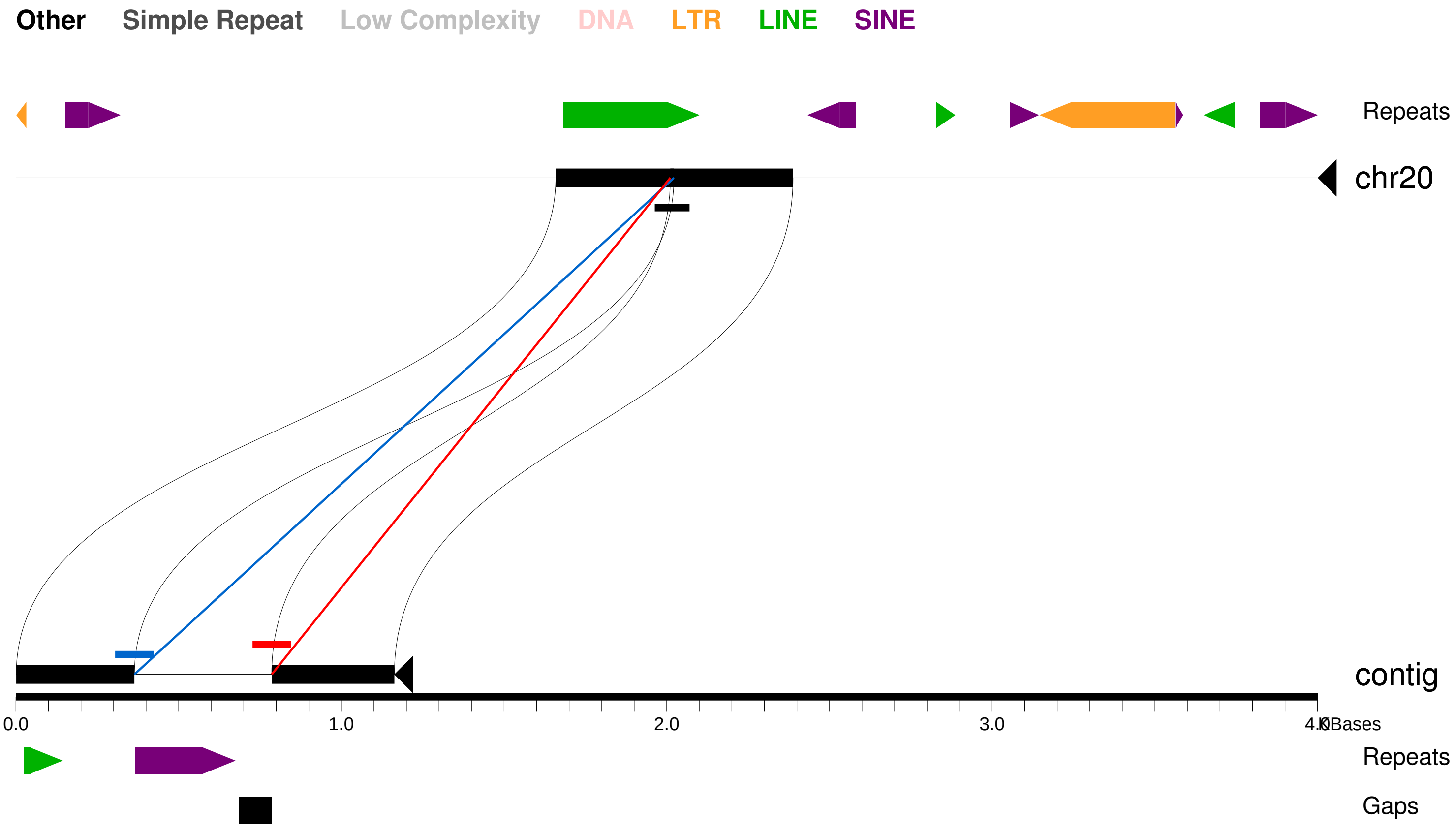

siteID: chr20_38210664 Scafftig1 - Breakpoint: chr20:38210675
chr20:38210623-38210727
Overlap Length: 8 INS size: 303 Genotyped: Yes

```
left    ----AAGGAAG-CAGAAATCAGAGATATA--ACAGA---AGAAACTTTCCTGAGTAAAAAAA----CTGA
chrom   ----AAGGAAG-CAGAAATCAGAGATATA--ACAGA---AGAAACTTTCCTGAGTAAAAAAA----CTGA
right   CGCCACTGCAGTCCGCAGTCCGACCTGGGCGACAGAGCGAGACTCCGTCTCAAAAAAAAAAAAAAACTGA
        1111*11*1**1*1*1*1**1**11*11111*****111***11*11**111*11*******1111****

left    TTCCGGCCGGGCGCGGTGGCTCACGCCTGTA--ATCCCAGCACTTTGGGAGGCCGAGGCGGGTGG
chrom   TTCCAT--------GGAAACACCACCATGTGCCATAG-AGCATTCATGAATACAAAGG-------
right   TTCCAT--------GGAAACACCACCATGTGCCATAG-AGCATTCATGAATACAAAGG-------
        ****2222222222**222*2*222*2***222**222****2*222*2*22*22***2222222
```

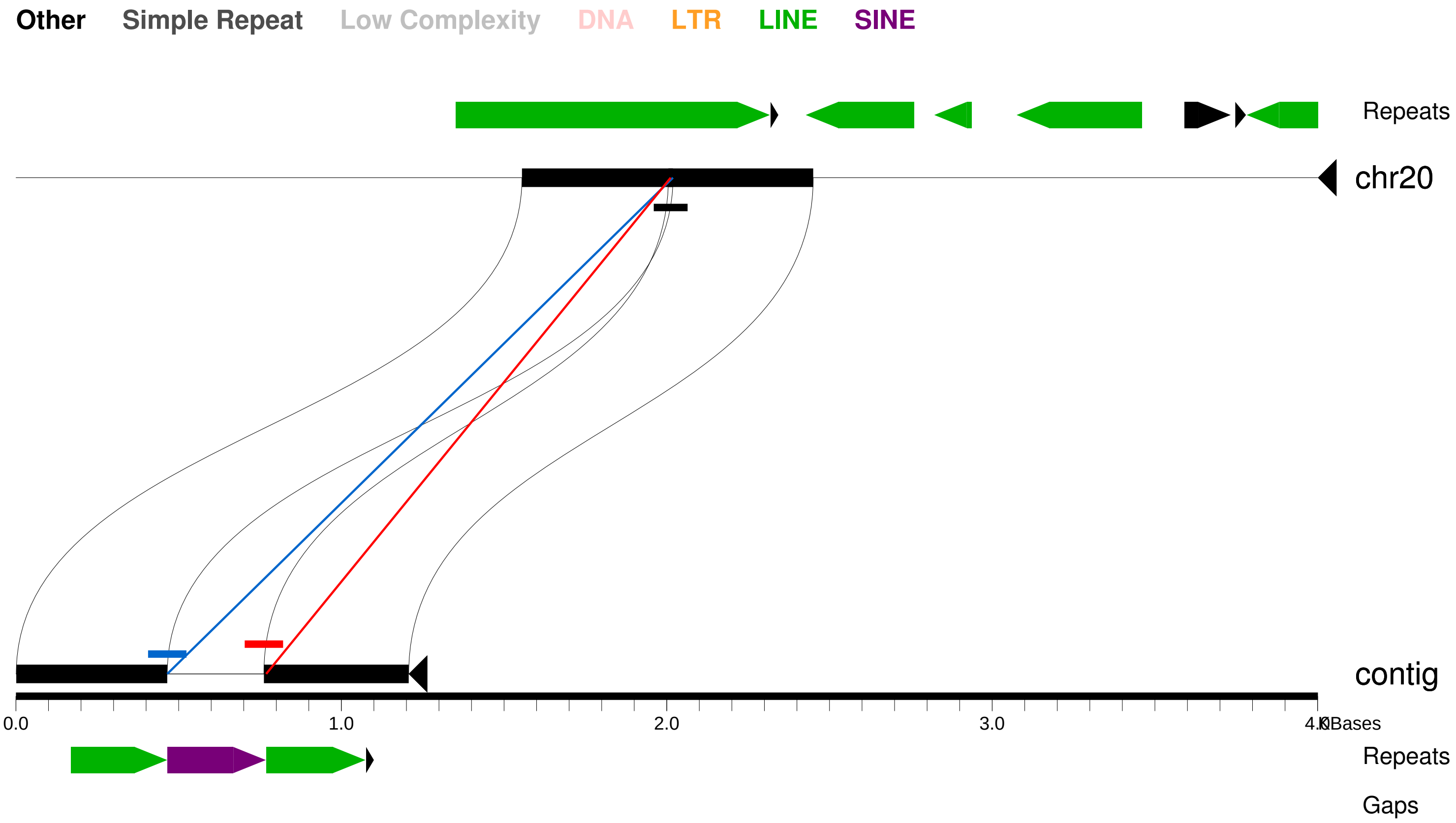

siteID: chr20_38301649 Scafftig2 - Breakpoint: chr20:38301641
chr20:38301588-38301700
Overlap Length: 7 INS size: 416 Genotyped: No

```
left    CTG---GGTAAGACCAGCAGAAGGTATTAGGTT---GGTGCGAAAGAAACTGCAGTTTTCGCAATTNNNN
chrom   CTG---GGTAAGACCAGCAGAAGGTATTAGGTT---GGTGCGAAAGAAACTGCAGTTTTCGCAATTACTT
right   CCGCCCGCCTCGGCCTCCCAAAGTGCTGGGATTACAGGCGTGAGCCACCGCGCCCGGCCCGCAATTACTT
        *1*111*1111*1**11*11***111*11*1**111**1*1**111*1111**111111*******2222

left    NNNNNNNNNNNNNNNNNNNNNNNNNNNNNNNNNNNNNNNNNNNNNNNNNNNNNNN
chrom   TCAATGGCAAAAACCACGATAACTTTTGCACGAACCTACCAACCTAATA------
right   TCAATGGCAAAAACCACGATAACTTTTGCACGAACCTACCAACCTAATA------
        2222222222222222222222222222222222222222222222222222222
```

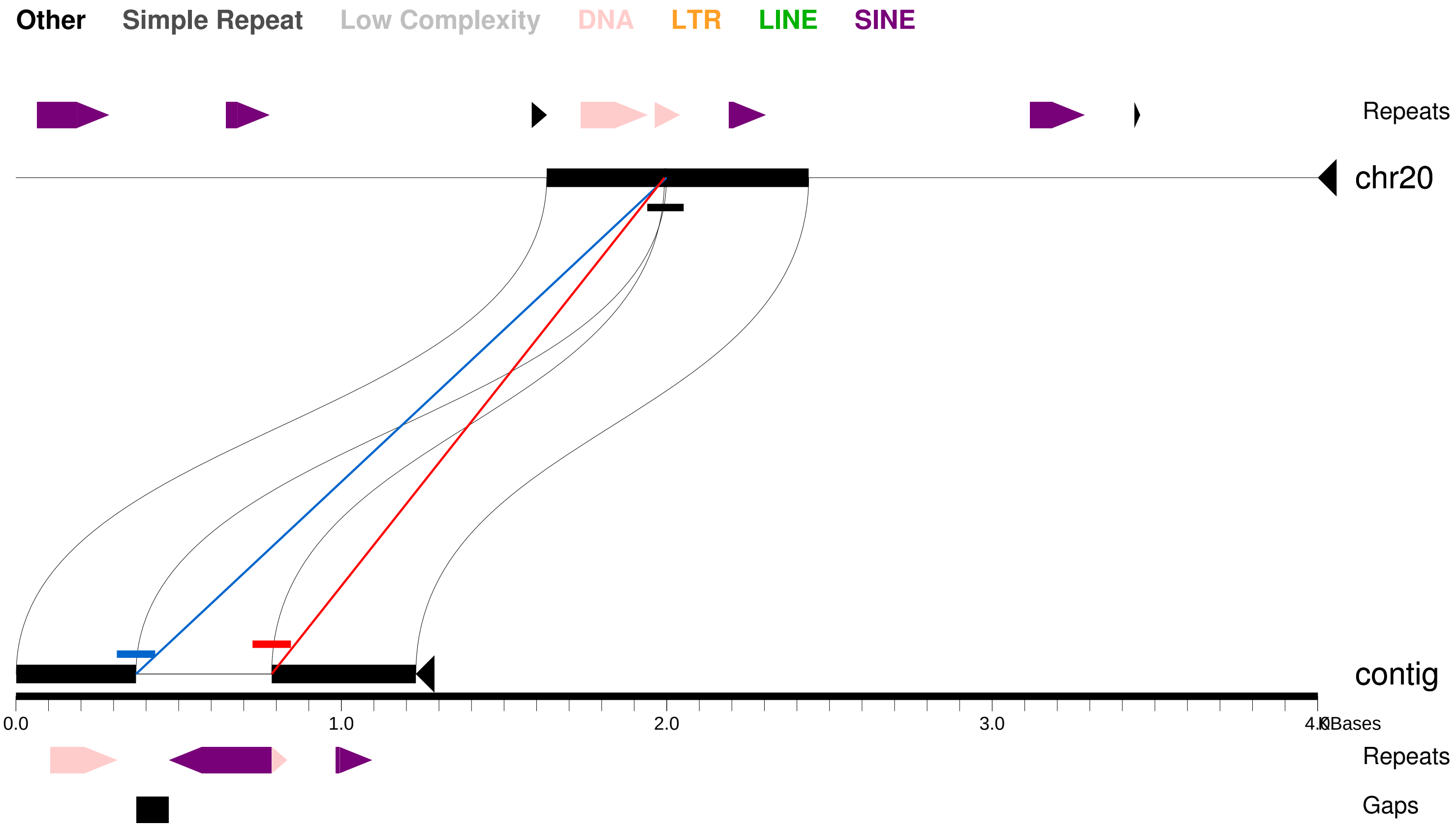

siteID: chr20_43671556 Scafftig2 - Breakpoint: chr20:43671520
chr20:43671180-43671680
Overlap Length: 19 INS size: 469 Genotyped: No

```
left    ----------------------------------------------------------------------
chrom   TCTGGATCCTCCACTACTCAAGTGAAATACAACGCAATTAGCATCTTTTGTCTTTGATAACACACCTACT
right   TTT-----------------AGT-AGAGACGGGG------------TTTCACCTTGTTAGC---------
        2N2                 222 2N2N22NNN2            222NN2N222N22N2

left    ----------------------------------------------------------------------
chrom   CCAAGCACCAGTAATGCTGGAATGGAAAGCATGTTTCATTTCCTTTCTTCATTTCTCTGTTGGAGTTTGA
right   --------CAGGA----TGGTCTCGA------------TCTCCTGACCTCAT----------------GA
                222N2    222NN2N22            2N2222NN2N2222                22

left    ----------------------------------------------------------------------
chrom   TGCTGATATTACTGAGATTTCTAATGGACAGTGTAGGGAGGAGAAGGCTGATTGATGGTAATTTATTGTA
right   TCC--ACCCGCCTCGGCCTCCCAA-----AGTGCTGGGA-----------------------TTA-----
        2N2  2NNNNN22NN2NN2N2N22     2222NN2222                       222

left    -------------------------------------------------------------------AAT
chrom   CTGCTACTGAGAAAAAAACACAACTCAGGGTATTGGTTGTTCGATACATCCAGATTTTTGACTGAAAAT
right   -------------------------CAGGC----------------------------------------
                                 2222N                                     111

left    GTTAGATTTTACTTTTTCTTTTTTAAAAATTTCCAACTTTTAAGTTCAGGGGCACATGTGCAGGATGTGC
chrom   GTTAGATTTTACTTTTTCTTTTTTAAAAATTTCCAACTTTTAAGTTCAGGGGCACATGTGCAGGATGTGC
right   GTGAG---------------------------CCACC--------------GCGCCCGGCCAGGATGTGC
        **1**111111111111111111111111111***1*1111111111111**1*11*11**********

left    AGGTTTTTT--------------------TTTTNNNNNNNNNN--------------------------
chrom   TGGTTTTTTACATAGGTAAATGTGTGCCATGTTGGTTTATGGCACAGATCATGCCATCACTTAGGTATTA
right   AGGTTTTTTACATAGGTAAATGTGTGCCATGTTGGTTTATGGCACAGATCATGCCATCACTTAGGTATTA
        N********22222222222222222222*2**2222222222222222222222222222222222222

left    ----------------------------------------------------------------------
chrom   AGCCCAGCATCCATTAGCTATTCTTCCTGATGCTCTCCCTCCTCCCAAGCCCCCACCCTCTGACAGGCCC
right   AGCCCAGCATCCATTAGCTATTCTTCCTGATGCTCTCCCTCCTCCCAAGCCCCCACCCTCTGACAGGCCC
        2222222222222222222222222222222222222222222222222222222222222222222222

left    -----------
chrom   CAGTGTGTGTT
right   CAGTGTGTGTT
        22222222222
```

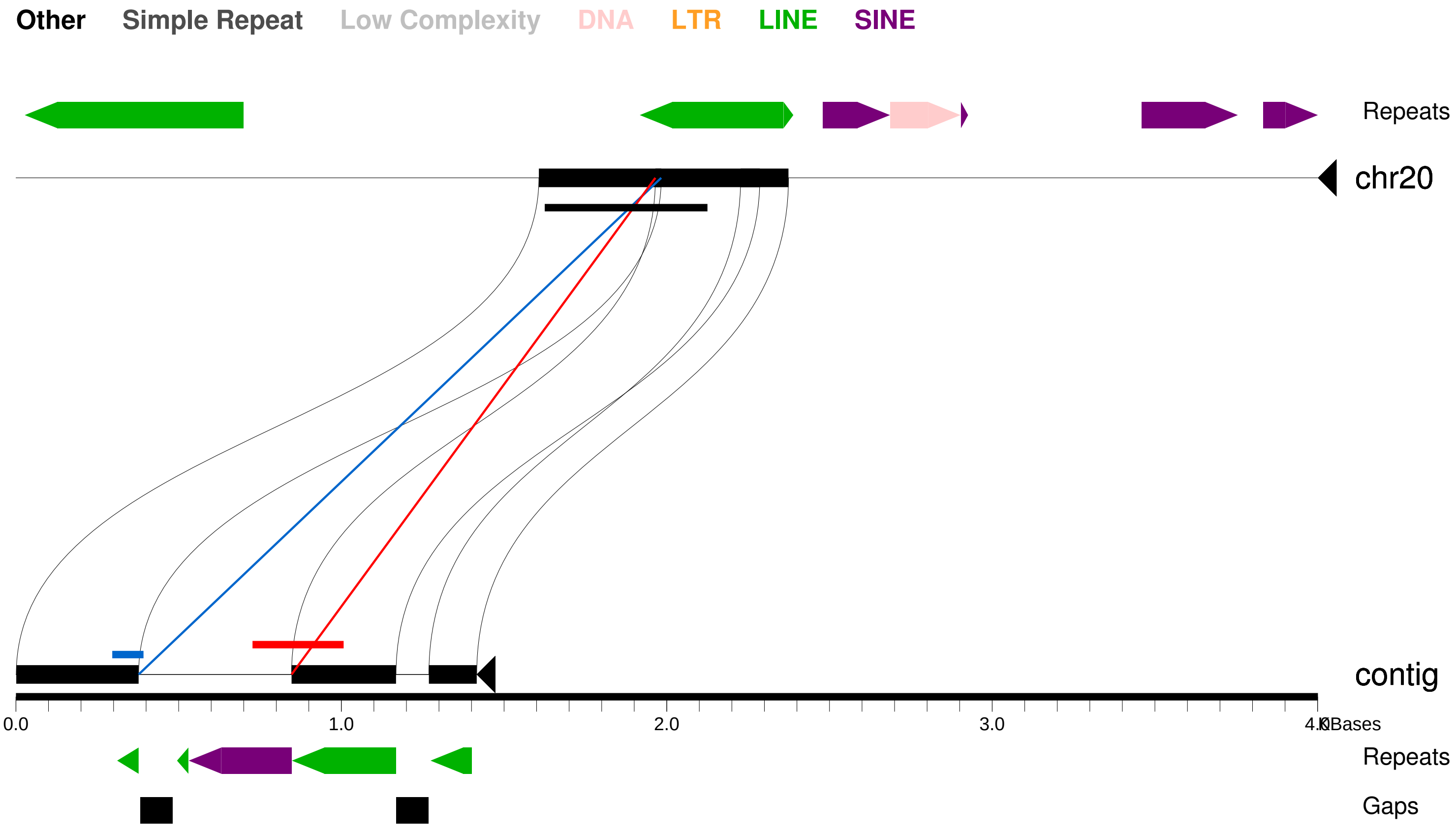

siteID: chr20_52637959 Scafftig4 + Breakpoint: chr20:52637937
chr20:52637777-52638109
Overlap Length: 13 INS size: 433 Genotyped: No

```
left   -------------TACATCGGAAATATTACACGTCCAATTCCAGGTCACTGCAATAGAGTCAATATCACA
chrom  ACATTATGACACATACATCGGAAATATTACACGTCCAATTCCAGGTCACTGCAATAGAGTCAATATCACA
right  NNNNNNNNNNNAA---------------------------------------------------------
       NNNNNNNNNNNN2111111111111111111111111111111111111111111111111111111111

left   ATAGAGTCACACACATTTTTCGGTTTCCCAGTGCACATAAAAGTTCTCCACTGTCCTGGAGTCTATTAAG
chrom  ATAGAGTCACACACATTTTTCGGTTTCCCAGTGCACATAAAAGTTCTCCACTGTCCTGGAGTCTATTAAG
right  ----------------------------------------------------------------------
       1111111111111111111111111111111111111111111111111111111111111111111111

left   TGTGCAATAGCATTACGTCTAAAAAAAAAAGTACGGCCGGGCG----------CGGTGGCTCACGCC----
chrom  TGTGCAATAGCATTACGTCTAAAAAAAAAAGTACATACTTTAATTTAAAAATACCTTGTCTCATGGTGTAT
right  --------------------AAAAAAAAAAGTACATACTTTAATTTAAAAATACCTTGTCTCATGGTGTAT
       11111111111111111111*************222*2222222222222222*22**2****2*222222

left   -TGTA----AT--------CCCAGCACTTTGG-GAGGCCGAGGCGGGCGGATCACGAGGTCAGGAGATCG
chrom  ATGTACCACATTTTCTTTACCCAGTCTATTGTTGATGGGCATTTGGGTTGATTCCATGTCTTTGCTATTG
right  ATGTACCACATTTTCTTTACCCAGTCTATTGTTGATGGGCATTTGGGTTGATTCCATGTCTTTGCTATTG
       2****2222**22222222*****2222***22**2*222*222***22***22*22*22222*22**2*

left   AGACCATCCCGGCTAAAACGGTGAAACCCTGTCTCTACTAAA---------------
chrom  TGAATAG---TGCTGCAATGGA-ATACTATGTGTCCATAAAAAGGAACAAGATCATG
right  TGAATAG---TGCTGC-----------------------------------------
       2**22*22222***2211N11N21N11NN111N11N1NN111
```

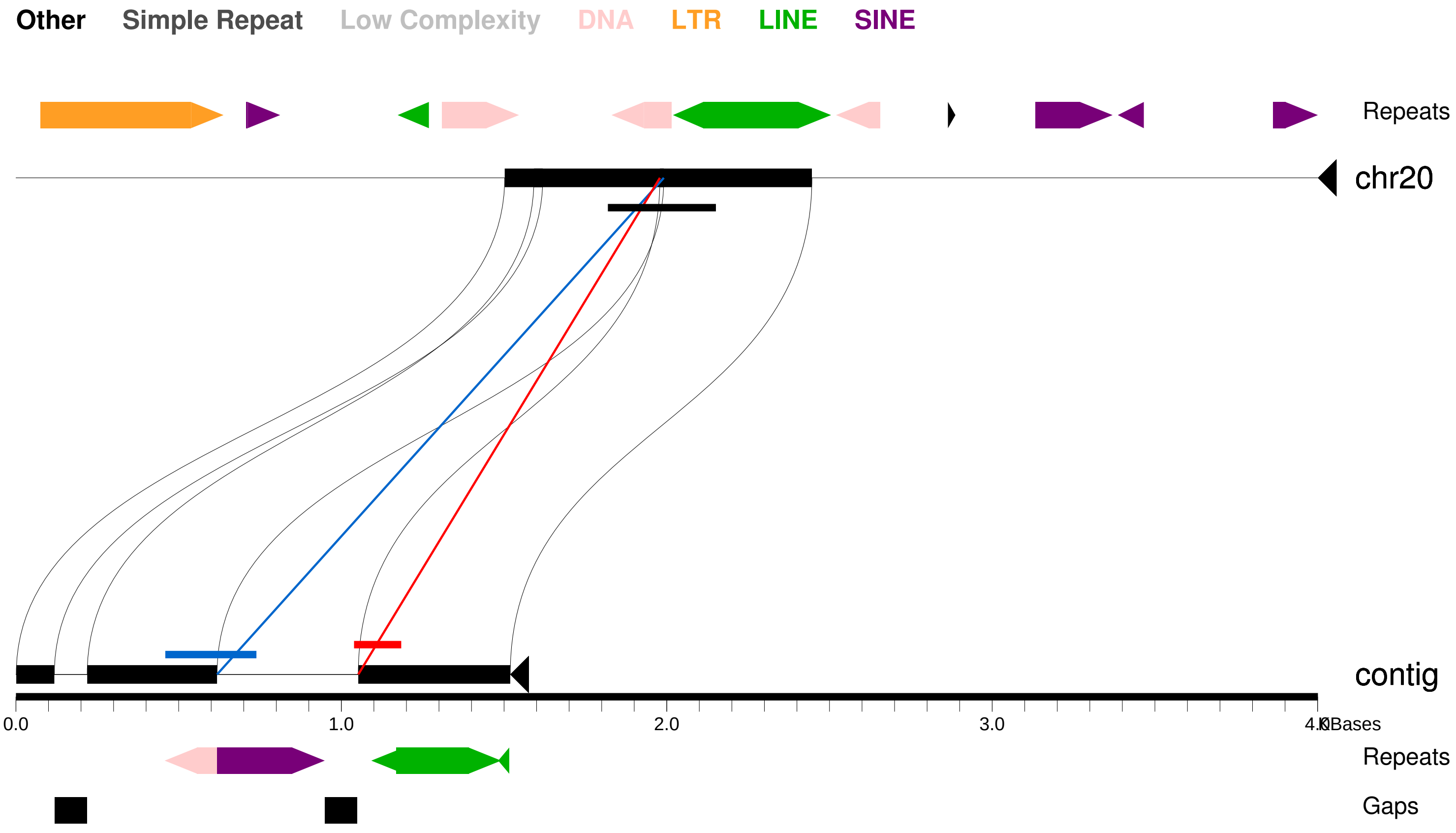

siteID: chr20_52725213 Scafftig2 - Breakpoint: chr20:52725223
chr20:52725063-52725384
Overlap Length: 2 INS size: 310 Genotyped: Yes

```
left   --CCAAACCCTGTTAACCTACATTTTATTCTTCATTTTTATGAGTTTGACTGTTTAGCTAGCTCTTATAA
chrom  TTCCAAACCCTGTTAACCTACATTTTATTCTTCATTTTTATGAGTTTGACTGTTTAGCTAGCTCTTATAA
right  TTTA-------GTAGAG--ACGGGGT-TTCACCGTTTT----AGCCGGGATGGT---CTCGATCT-----
       22111111111**11*111**1111*1***11*1****1111**111*11**1*111**1*1***11111

left   GTGGAAACATGCAGTGTTTGTCCTTCTGTGACTGGCTTGTTTCACTTGATGTAATGTTTTCAAGGTTTAT
chrom  GTGGAAACATGCAGTGTTTGTCCTTCTGTGACTGGCTTGTTTCACTTGATGTAATGTTTTCAAGGTTTAT
right  -------CCTGACCTCGTGATCCGCCCGCCTCGGCCT-----CCCAAAGTGCTGGGATTACAGGCGTGAG
       1111111*1**111*11*11***11*1*111*1*1**11111*1*1111**1111*1**1**1*11*1*1

left   TCATGTTATCACATATTTCAGGTTTTTTTTTTTTTT----------TTTTTTT---TTTTTTTTGAG---
chrom  TCATGTTATCACATATTTCAGGATTTCCTTCTTTTTAAAGGCTGAATTATATTCCGTTGTATGTGTGTGC
right  CCAC-----CGCGCCCGGCCGGATTTCCTTCTTTTTAAAGGCTGAATTATATTCCGTTGTATGTGTGTGC
       1**111111*1*111111*1**2***22**2*****2222222222**2*2**222**2*2*2**2*222

left   ------ACGGAGTCTCGCTCT----GT-CGCCCAGGC--TGGAGTGCAG-TGGCGGGATCTCGGC-----
chrom  ATGTCTATGGAATATAATTATACATGTACACACATACAATGGAATATATATATATATATATATGTATATA
right  ATGTCTATGGAATATAATTATACATGTACACACATACAATGGAATATATATATATATATAT--GTATATA
       222222*2***2*2*222*2*2222**2*2*2**22*22****2*22*22*222222**2*NN*222222

left   --TCAC---TGCAAGCTCCGCCTCCCGGGTTCACGCCATTCTC
chrom  TATCACATTTACTTTATCCATTTACCCT-TTGATGGATATTTA
right  TATCACATTTACTTTATCCATTTACCCT-TTGATGGATATTTA
       22****222*2*2222***222*2**222**2*2*2222*2*2
```

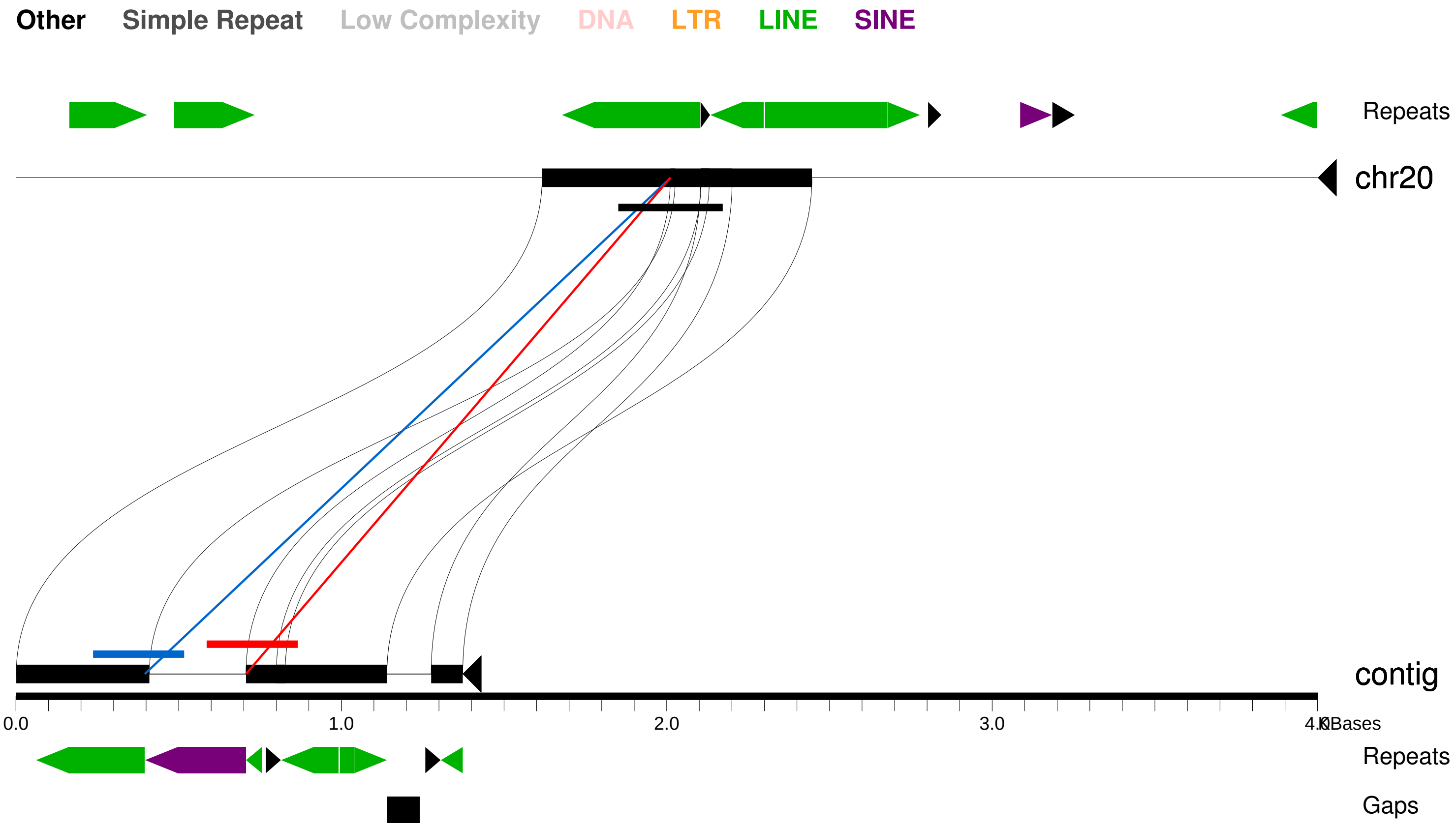

siteID: chr20_53761848 Scafftig7 - Breakpoint: chr20:53761842
chr20:53761791-53761901
Overlap Length: 9 INS size: 307 Genotyped: Yes

```
left   A--ATTC----CTACAGT---TAATGCCTTATTAAATAGCTACATATATATGGAAGTATGAAAAATTTCG
chrom  A--ATTC----CTACAGT---TAATGCCTTATTAAATAGCTACATATATATGGAAGTATGAAAAATTTC-
right  TCCAGCCTGGGCGACAGAGCGAGACTCCGTCTCAAAAAAAAAAAAAAAAAAAAAAAAAA-AAAAATTTC-
       111*11*1111*1****111111*11**1*1*1***1*111*1*1*1*1*111**11*11*********2

left   GCCGGGCGCGGTGGCTCACGCCTGTAATCCCAGCACTTTGGGAGGCCGAGGCGGGCGG
chrom  ------CAAGATTGCAAAGTAATCAAAACCCAGATTAAATCAAGGATGATTTTATAT-
right  ------CAAGATTGCAAAGTAATCAAAACCCAGATTAAATCAAGGATGATTTTATAT-
       222222*22*2*2**22*2222*22**2*****222222222***22**222222222
```

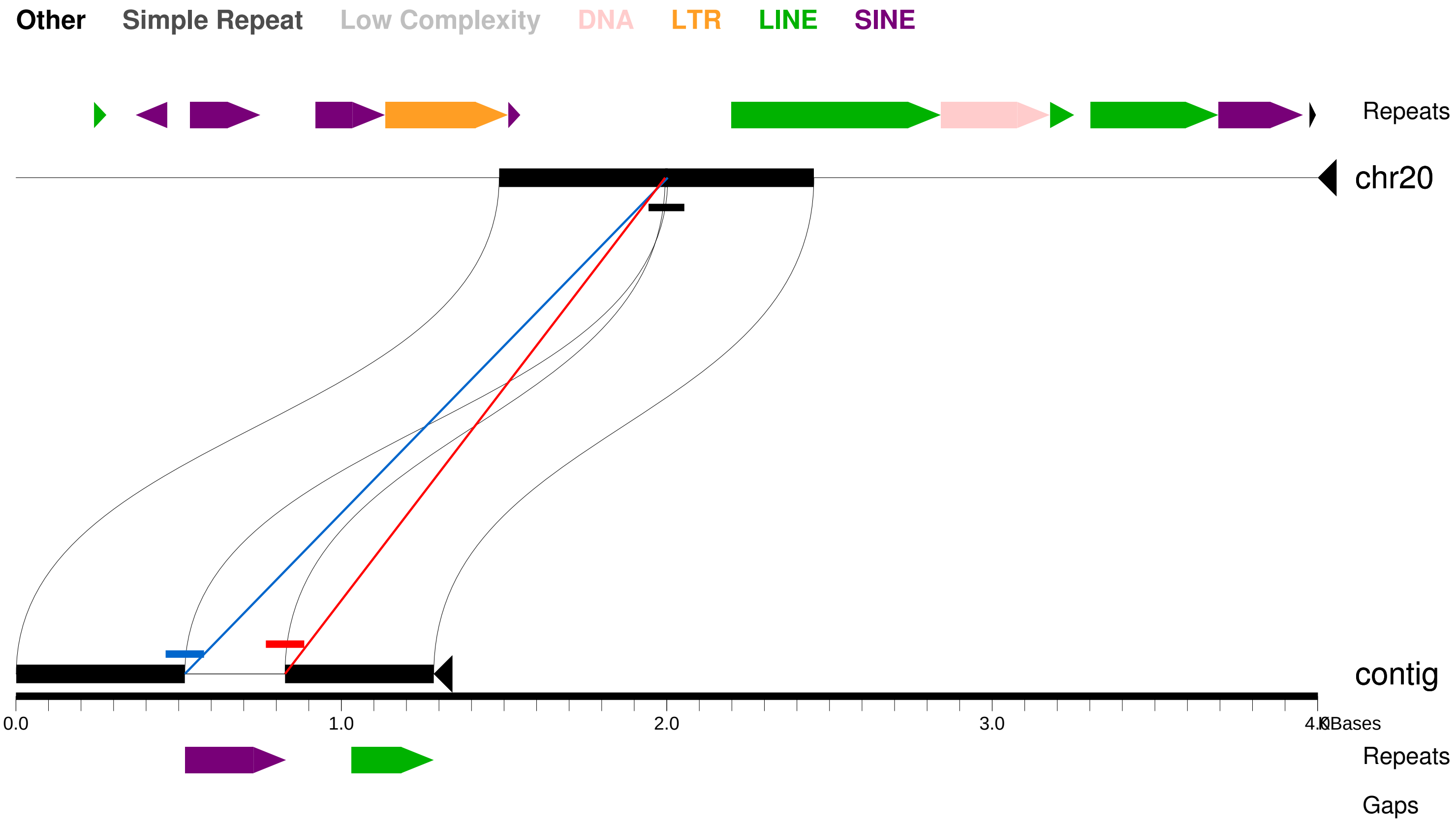

siteID: chr20_6361991 Scafftig1 + Breakpoint: chr20:6361999
chr20:6361839-6362171
Overlap Length: 13 INS size: 420 Genotyped: No

```
left   -------------ATTCACTTAGATATTTAGTTTAACTTTTGCTATTGTTATAATTTTCTGTTTTTTAGT
chrom  GCAAAATTATTTTATTCACTTAGATATTTAGTTTAACTTTTGCTATTGTTATAATTTTCTGTTTTTTAGT
right  NNNNNNNNNNN-----------------------------------------------------------
       NNNNNNNNNNN  111111111111111111111111111111111111111111111111111111111

left   TCTGGATGTACTTTCCTTATTCTTTTCTGGACCTCACTCATAATGGACTTACTACTGATGCGGGATCTGC
chrom  TCTGGATGTACTTTCCTTATTCTTTTCTGGACCTCACTCATAATGGACTTACTACTGATGCGGGATCTGC
right  ----------------------------------------------------------------------
       1111111111111111111111111111111111111111111111111111111111111111111111

left   CTTGTCTTTCTTAAAGCTAGAAAACTGTCTCGCGGCCGG---GCGC--------------------GGTG
chrom  CTTGTCTTTCTTAAAGCTAGAAAACTGTCTCGCACATGTTAAGTGCTCAAATTTTTGTTTAATGGGGCTG
right  ------------AAA---AAAAAACTGTCTCGCACATGTTAAGTGCTCAAATTTTTGTTTAATGGGGCTG
       111111111111***111*1*************2222*2222*2**22222222222222222222*2**

left   GCTCACGCCTGTAGTCCCAGC--ACTT--TGGGAGGCCGAGGCGGGTGGAT------CATGAGGTCAGGA
chrom  GCTTATAAATG-AGTCCATGATGATTTCTTAAGAATCAAATCTCTTTGGATGGAGTGCATGATATCTAAA
right  GCTTATAAATG-AGTCCATGATGATTTCTTAAGAATCAAATCTCTTTGGATGGAGTGCATGATATCTAAA
       ***2*2222**2*****22*222*2**22*22**22*22*222222*****222222*****22**222*

left   G----ATCGAGACCATCCTGGCT--AACAAGGT----GAAACCCCGTCTCTACTAAA
chrom  TTCTTATCTACAACAAAGAAGCTGAAATAAAGTTCTGGAAAGCCAGCG--GACTAG-
right  TTCTTATCTAC----------------------------------------------
       22222***2*21N11NNNNN111  11N11N11    1111N11N1NN22N1111N2
```

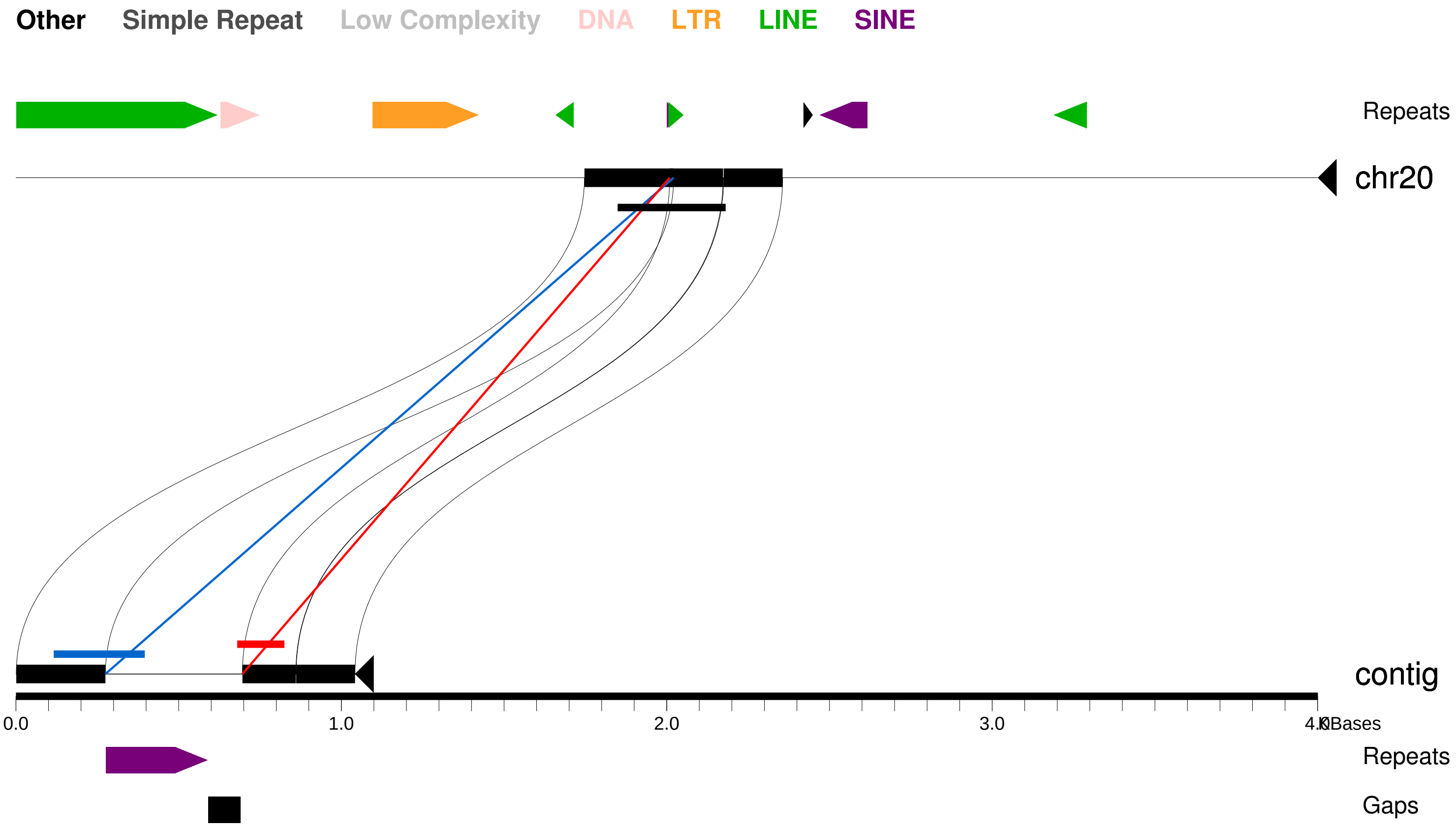

siteID: chr20_8443491 Scafftig1 + Breakpoint: chr20:8443470
chr20:8443426-8443529
Overlap Length: 16 INS size: 318 Genotyped: Yes

```
left     ATGCCTTGCCCCTTGCTTTCCTCCA-------CAATA-AGGC-TTAGACGCAA---------AACACTTC
chrom    ATGCCTTGCCCCTTGCTTTCCTCCA-------CAATA-AGGC-TTAGACGCAA---------AACACTTC
right    CCGCC---CGCCTCGGCCTCCCAAAGTGCTGGGATTACAGGCGTGAGCCACCGCGCCCGGCCAACACTTC
         11***111*1***1*111***111*11111111*1**1****1*1**1*1*11111111111********

left     TTTTTCCTTTTTTTTTTTTTTTTCTTTTCTTTTTTTTTTTTTTTTTGAGAC--GGAG-TCTCGCTCTGTCG
chrom    GTTTTCCTGATCACCACTCAGGCATGA------------------GAGACCAGGTGATCTATGGCTGGCC
right    GTTTTCCTGATCACCACTCAGGCATGA------------------GAGACCAGGTGATCTATGGCTGGCC
         2*******22*222222*2222*2*22222222222222222222*****22**2*2***2222***2*2
```

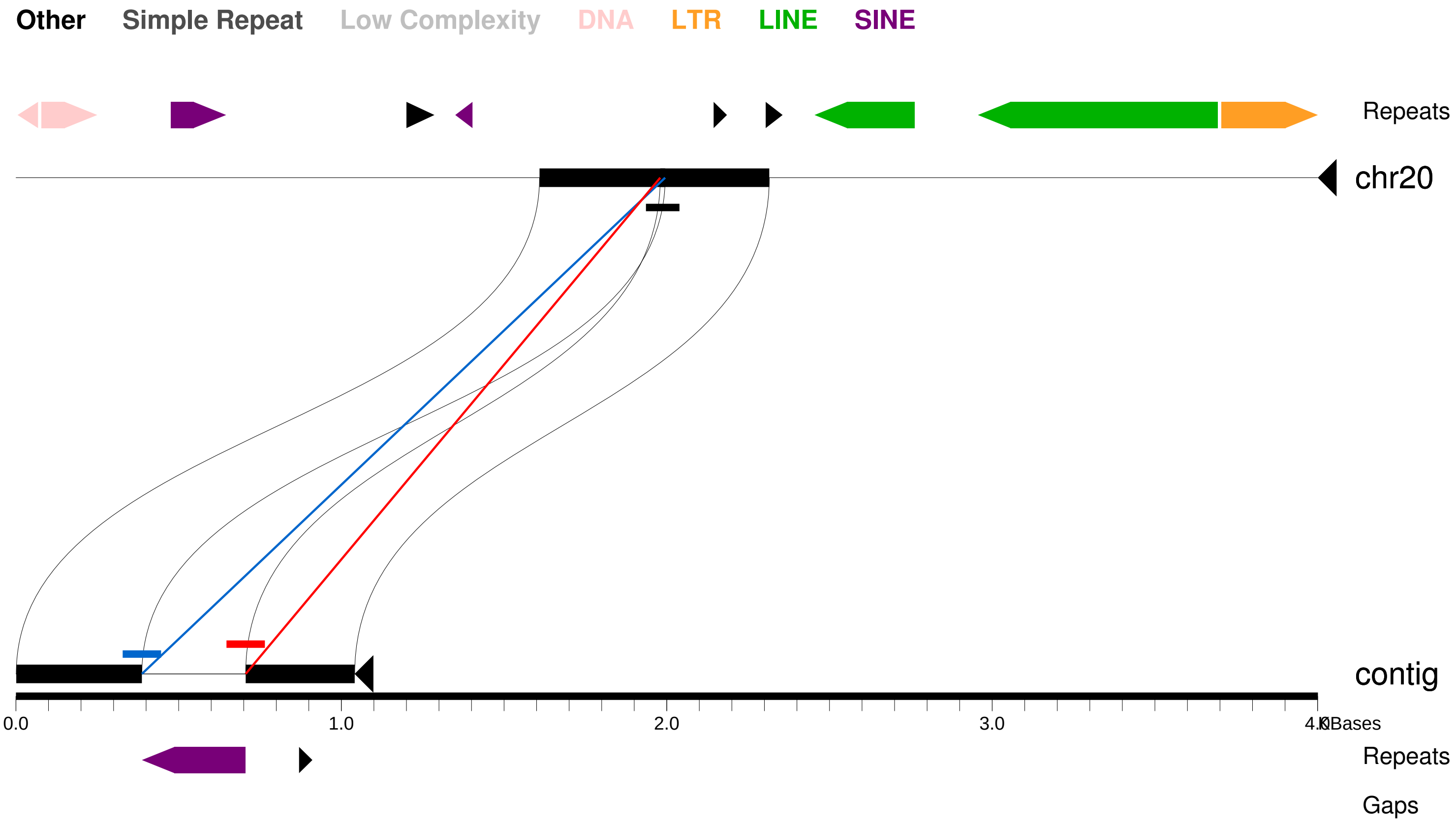

siteID: chr20_8521287 Scafftig3 - Breakpoint: chr20:8521293
chr20:8521245-8521349
Overlap Length: 12 INS size: 327 Genotyped: Yes

```
left     TCCCTCC------------CATCTTTCAAATTACTCCACATGTCTAAGTTTTCCTTTAAA--ATGTTTCC
chrom    TCCCTCC------------CATCTTTCAAATTACTCCACATGTCTAAGTTTTCCTTTAAA--ATGTTTCC
right    GGGCGACAGAGCGAGACTCCGTCTCAAAAAAAAAAAAAAAAAAAAAAAAAAAAAAAAAAAAAATGTTTCC
         111*11*111111111111*1***111***11*1111*1*11111**1111111111***11********

left     AAACGGCCGGGCGCGGTGGCTCACGCCTGTAATCCCAG---CACTTTGGGAGGCCGAGGCGGGTGG
chrom    AAACCAC---------TGGAGCACACCTCTCATCACAGACCCCCATTG--------ATGAGAGACA
right    AAACCAC---------TGGAGCACACCTCTCATCACAGACCCCCATTG--------ATGAGAGACA
         ****22*222222222***22***2***2*2***2***222*2*2***22222222*2*2*2*222
```

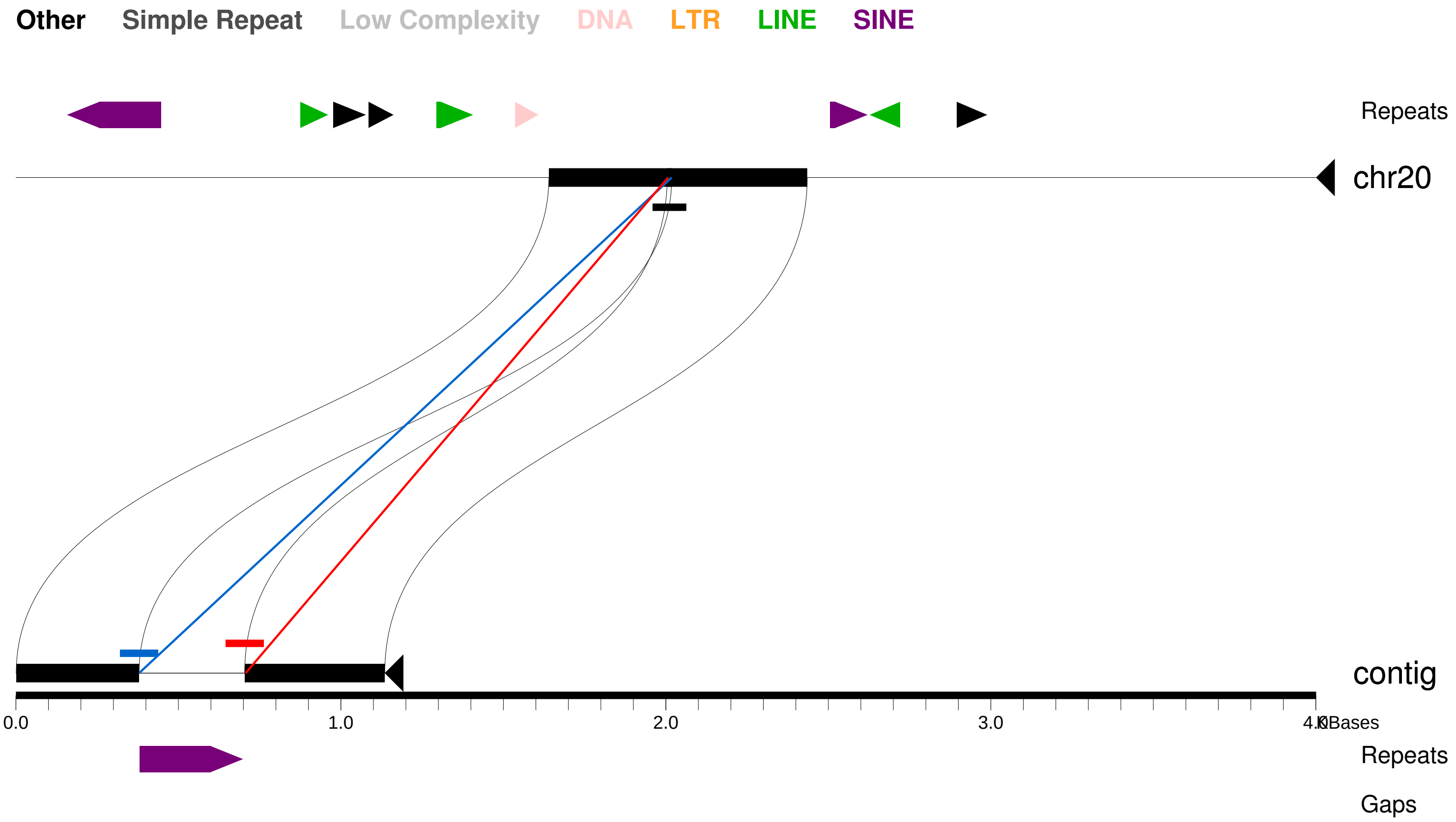

siteID: chr20_8670606 Scafftig8 + Breakpoint: chr20:8670604
chr20:8670558-8670660
Overlap Length: 14 INS size: 291 Genotyped: Yes

```
left     AAAGAAAAGGGTTTACCTT-------TTTTATGAAGAAAG---------AACTCTAATAGCTGTCTCCAA
chrom    AAAGAAAAGGGTTTACCTT-------TTTTATGAAGAAAG---------AACTCTAATAGCTGTCTCCAA
right    GATCCACCCGCCTCGGCCTCCCAAAGTGCTGGGATTACAGGCGTGAGCCACCGCGCCCGGCCGTCTCCAA
         1*111*111*11*111*1*1111111*11*11**11*1**111111111*1*1*11111**1********

left     ATTTCTTTTTTTTTTTTTTTTTTTTTTTTGAGACGGAGTCTCGTTCTGTCGCCCAGGCGGGAGTGC
chrom    ATTTCTGTACAATGGTTTTGTATCA------ACACCATCTGATTCATATACCTCG----------
right    ATTTCTGTACAATGGTTTTGTATCA------ACACCATCTGATTCATATACCTCG----------
         ******2*2222*22****2*2*22222222**2222***22***22222**22*2222222222
```

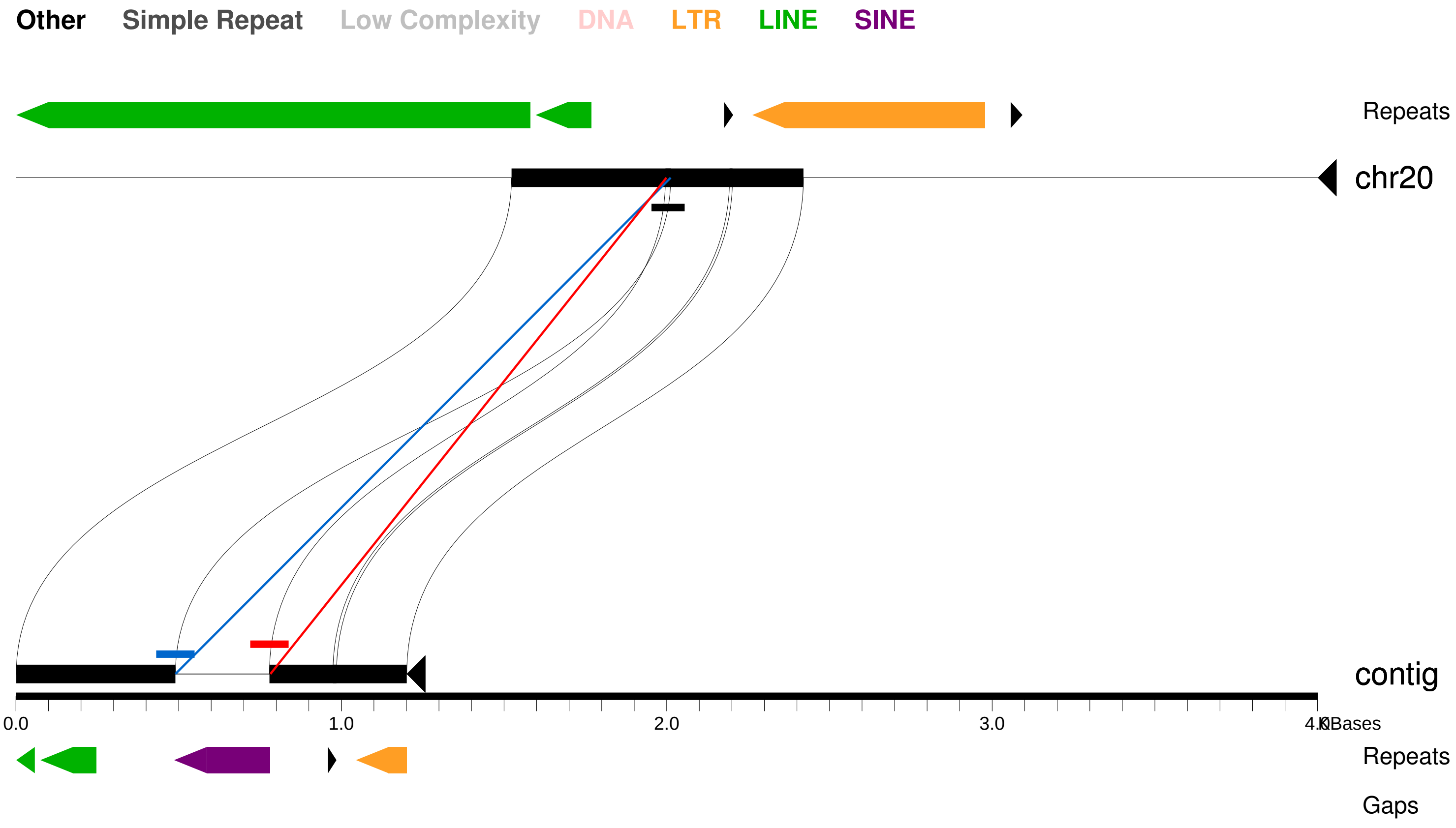

siteID: chr20_8816507 Scafftig1 + Breakpoint: chr20:8816492
chr20:8815956-8816652
Overlap Length: 13 INS size: 288 Genotyped: Yes

```
left   ----------------------------------------------------------------------
chrom  GGCTTGATAGACCATGGACATGGCTTTTTGTCTGCTTTTGGTTATTTTGTTAGACCTATTTCAGGGTCTC
right  --CTTG---------GGA---GGCTG-------------------------AGGC----------------
         2222         222    2222N                         22N2

left   ----------------------------------------------------------------------
chrom  CACCGTTGTCACATTCTTTCCTTTTTAAGAGTATTTTTTTTTTTCTGGTTATACCCATGAAACCTTATTTT
right  --------------------------AGGAGAAT-----------GG-------CGTGAACCC-------
                                 2N222N22           22       2N2222N22

left   ----------------------------------------------------------------------
chrom  AAGTCCCTAACCACAGGCTTTAGAGATCTATGCTGTATAAAAAGTAATTCATCCAATTCTATTATGACTT
right  ---------------GG-----GAG------GCGG-----------------------------------
                      22     222       22N2

left   ----------------------------------------------------------------------
chrom  ATATATAGCAATCTACTCATTTATGAAACAAAGAATTCCAGGCATATCTTGTTTTACTAGCCTGTCCTGA
right  ------AGC--------------------------TTGCAG-----------------------------
             222                          22N222

left   ----------------------------------------------------------------------
chrom  GTATTGTGGTTCGAATTCATTTGTGGACTCTTGAGTAGGGCTGTCTGTCTATAATGACAATGCATATACA
right  -------------------------------TGAGCCG--------------------------------
                                      2222NN2

left   ---------------------------------------CATAATAGATTTAAAAGTTTCTCCATACATA
chrom  TGTAGAACCAGCTTATGAGAGTTTATGTTTTATAAACTGCATAATAGATTTAAAAGTTTCTCCATACATA
right  ---AGATCCCGCC----------------------ACTGCA------------------CTCCAG-----
          222N22N22N                      2222**111111111111111111*****111111

left   TTTCATGATTCCCACCTCAGAGGGCACAGGAAATGGTCCAAAGCAGACGACTCTAACTTAAAATGACTGA
chrom  TTTCATGATTCCCACCTCAGAGGGCACAGGAAATGGTCCAAAGCAGACGACTCTAACTTAAAATGACTGA
right  --------------CCT----GGGC----GA-------CAGAGC-GA-GACTCCGTCTCAAAA-------
       11111111111111***1111****1111**1111111**1***1**1*****111**1****1111111

left   CAAGGCATCTGGGTCAGTGAGCTTTGGCTGCATAACAAACCACTTCAAAACTTAGTGACGG-----CCGG
chrom  CAAGGCATCTGGGTCAGTGAGCTTTGGCTGCATAACAAACCACTTCAAAACTTAGTGACTTGAAAACAAA
right  -----------------------------------AAA-------AAAACTTAGTGACTTGAAAACAAA
       111111111111111111111111111111111111***1111111************2222222*222

left   GCGCGGTGGC-----TCACGCCTGTAA----TCCCAGCACTTTGGGAG-GCCGAGGCGGGCGGATCACGA
chrom  TCACTGTTGAGACAATAAC-CATGTTACTGCTCACAAGTCTATGGGTCAGCTGAACCTTTCTGCTG-CTT
right  TCACTGTTGAGACAATAAC-CATGTTACTGCTCACAAGTCTATGGGTCAGCTGAACCTTTCTGCTG-CTT
       2*2*2**2*222222*2**2*2***2*2222**2**222**2****222**2**22*222*2*2*22*22

left   GGTCAGGAG---ATCGAGACCATGCCGGCTAA---[illegible]--AACGGTGAAACCCCGTC---TCTACTAAA-
chrom  TGCCTTGGGCTTCATTGATCCCAGCTGGTCTTAGGCAGGTATCTGTGTTCAGCTAGCAGATCAACTGAA-
right  TGCCTTGGGCTTCATTGATCCCAGCTGGTCTTAGGCAGGTATCTGTGTTCAGCTAGCAGATCAACTGAAG
       2*2*22*2*2222**2**22***2*22*2**2*2222222*2*2***22222*222*222**2***2**1

left   --
chrom  TC
right  11
```

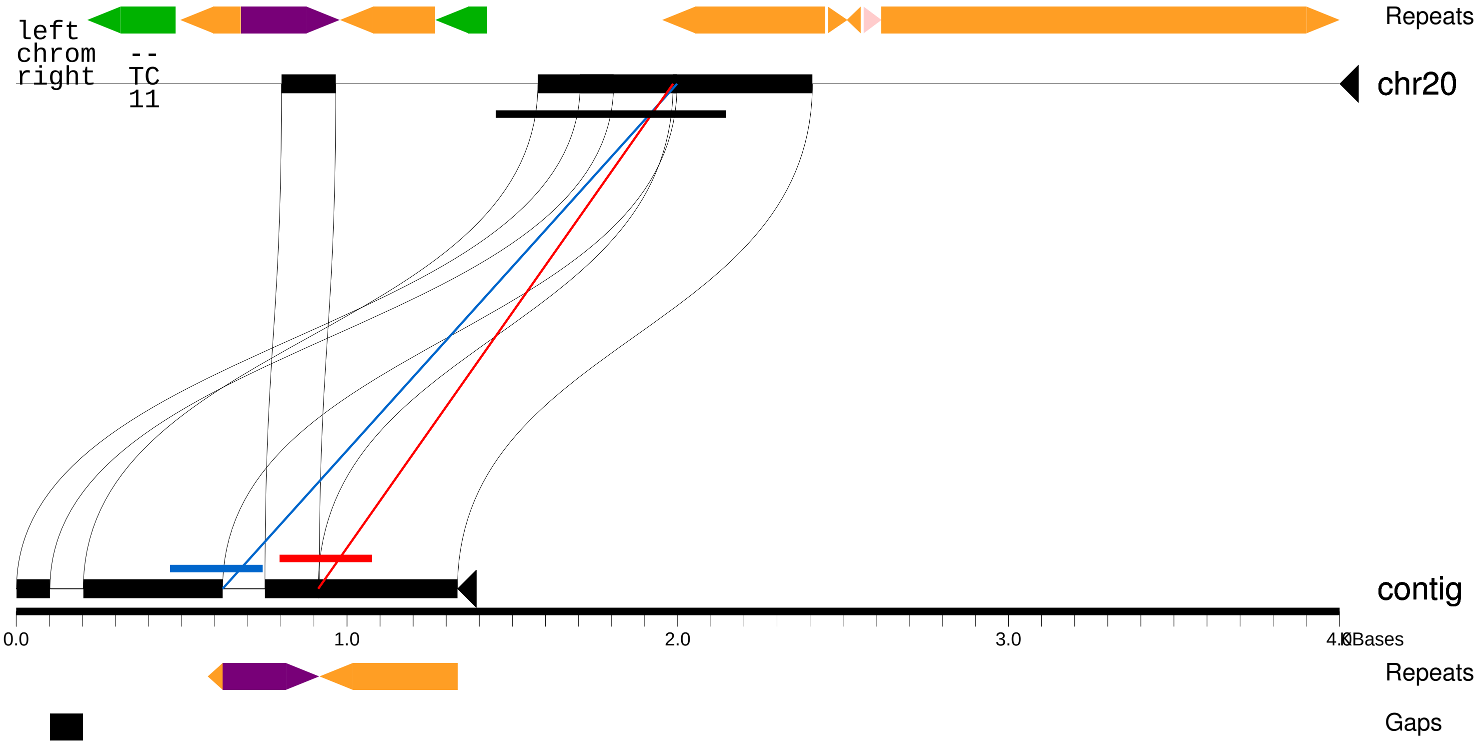

siteID: chr20_9572260 Scafftig1 - Breakpoint: chr20:9572256
chr20:9572225-9572315
Overlap Length: 29 INS size: 438 Genotyped: No

```
left    CAAAGGAAATGTTACATTGCATTTACTCAAG----------------------------ATTCTGACATT
chrom   CAAAGGAAATGTTACATTGCATTTACTCAAG----------------------------ATTCTGACATT
right   NNNNNNNNNNNNNNNNNNNNNNNNNNNNNNNNNNNNNNNNNNNNNNNNNNNNNNNNNNNNATTCTGACATT
        111111111111111111111111111111111111111111111111111111111111***********

left    TAAAAATGTAATAACTAAGGCCGGGCGCGGTGGCTCACGCCTGTAATCCCAGCACTTTGGGAGGCCGAGG
chrom   TAAAAATGTAATAACTAA----------------TAAAACATGTAGTCT----ACATATCAATTGCAAC-
right   TAAAAATGTAATAACTAA----------------TAAAACATGTAGTCT----ACATATCAATTGCAAC-
        ******************2222222222222222*2*22*2****2**22222**2*2222*222*2*22

left    CGGGCGG
chrom   -------
right   -------
        2222222
```

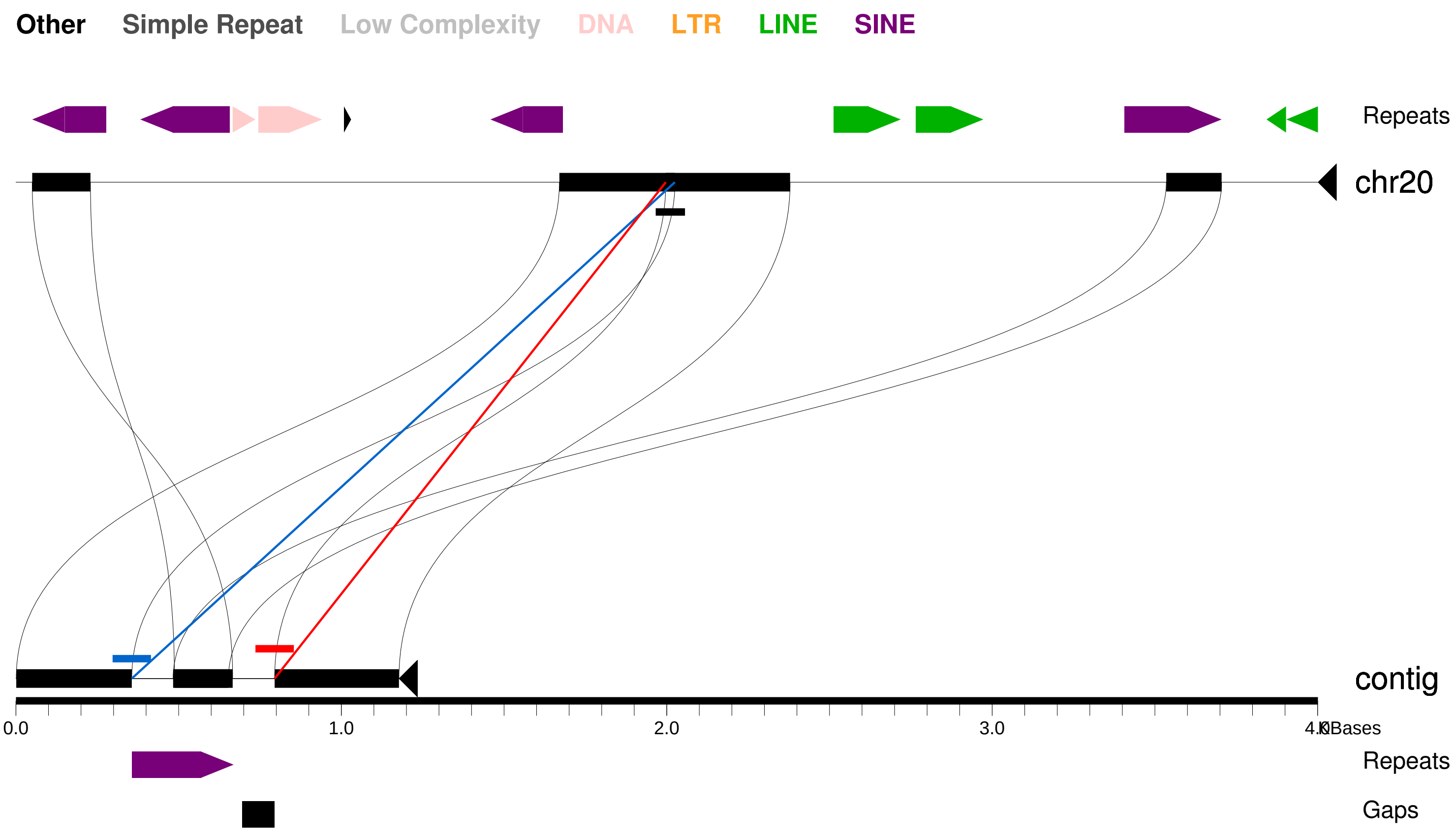

siteID: chr20_9605197 Scafftig3 - Breakpoint: chr20:9605198
chr20:9605147-9605257
Overlap Length: 9 INS size: 314 Genotyped: Yes

```
left     T--------AGAAATATTATAAAGGTAACAGAATTTCTTCCAACTTATCTGTTTTCTTGAAATTAAGGGG
chrom    T--------AGAAATATTATAAAGGTAACAGAATTTCTTCCAACTTATCTGTTTTCTTAAAATTAAGGTT
right    CTGGGCGACAGAGCGAGACTCCGTCTCAAAAAAAAAAAAAAAAAAAAAAAAAAAAAAAAGAAATTAAGGTT
         111111111***111*111*11111*1*1*1**11111111**111*11111111111N*********22

left     GCCGGGCGCGGTGGCTCACGCCTGTAATCCCAGCACTTTGGGAGGCCGAGGCGGGCG
chrom    CATGTTTTC---TCCTTACCTCTCTG-TCTTAT-AATGTGCTAGTG---TTCCCATT
right    CATGTTTTC---TCCTTACCTCTCTG-TCTTAT-AATGTGCTAGTG---TTCCCATT
         222*2222*22222**2**22**2*22**22*22*2*2**22**2222222*22222
```

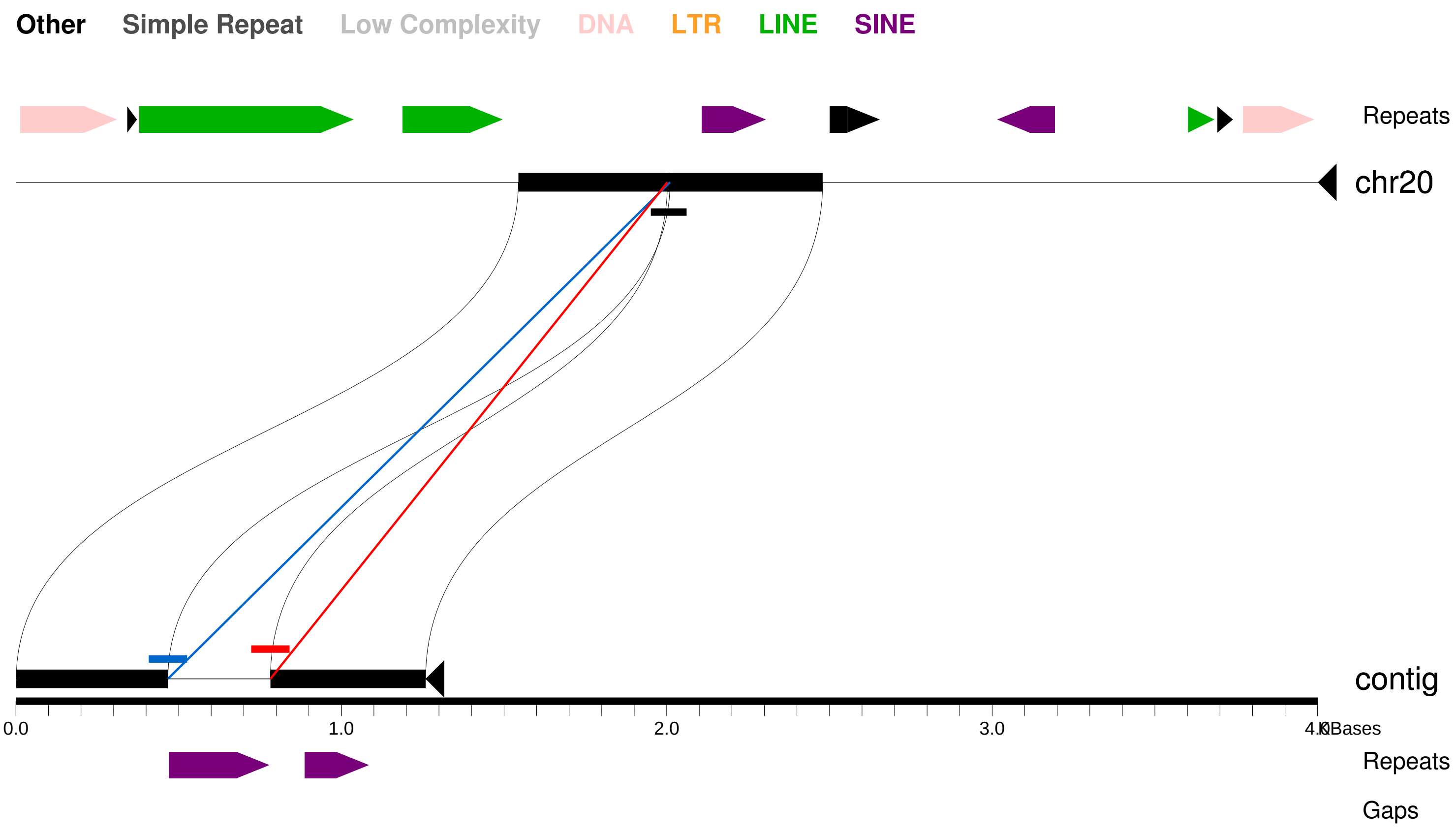

siteID: chr20_9640987 Scafftig4 - Breakpoint: chr20:9640971
chr20:9640924-9641027
Overlap Length: 9 INS size: 281 Genotyped: Yes

```
left   ---AGTGACA-AGTGATTTGCATTCC-----------AAGCAAAAATCTGTGGTAGATTAAGATTATTTT
chrom  ---AGTGACA-AGTGATTTGCATTCC-----------AAGCAAAAATCTGTGGTAGATTAAGATTATTTT
right  CTGGGCGACAGAGCGAGACTCCGTCTCAAAAAAAAAAAAAAAAAAAAAAAAAAAAAAAAAAAATTATTTT
       1111*1****1**1**1111*11**111111111111**11*****11111111*1*11**1********

left   ACTTTGGGAGGCCGAGGCGGGCGGATCACGAGGTCAAGAGATCGAGACCATCCCGGCTAAAACG
chrom  AATTT------TCTCTCCTTTC---TCTCTTCCCTTAGGGCTCTCT-CCATCTTTTCTT-----
right  AATTT------TCTCTCCTTTC---TCTCTTCCCTTAGGGCTCTCT-CCATCTTTTCTT-----
       *2***2222222*2222*222*222**2*2222222**2*2**2222*****2222**222222
```

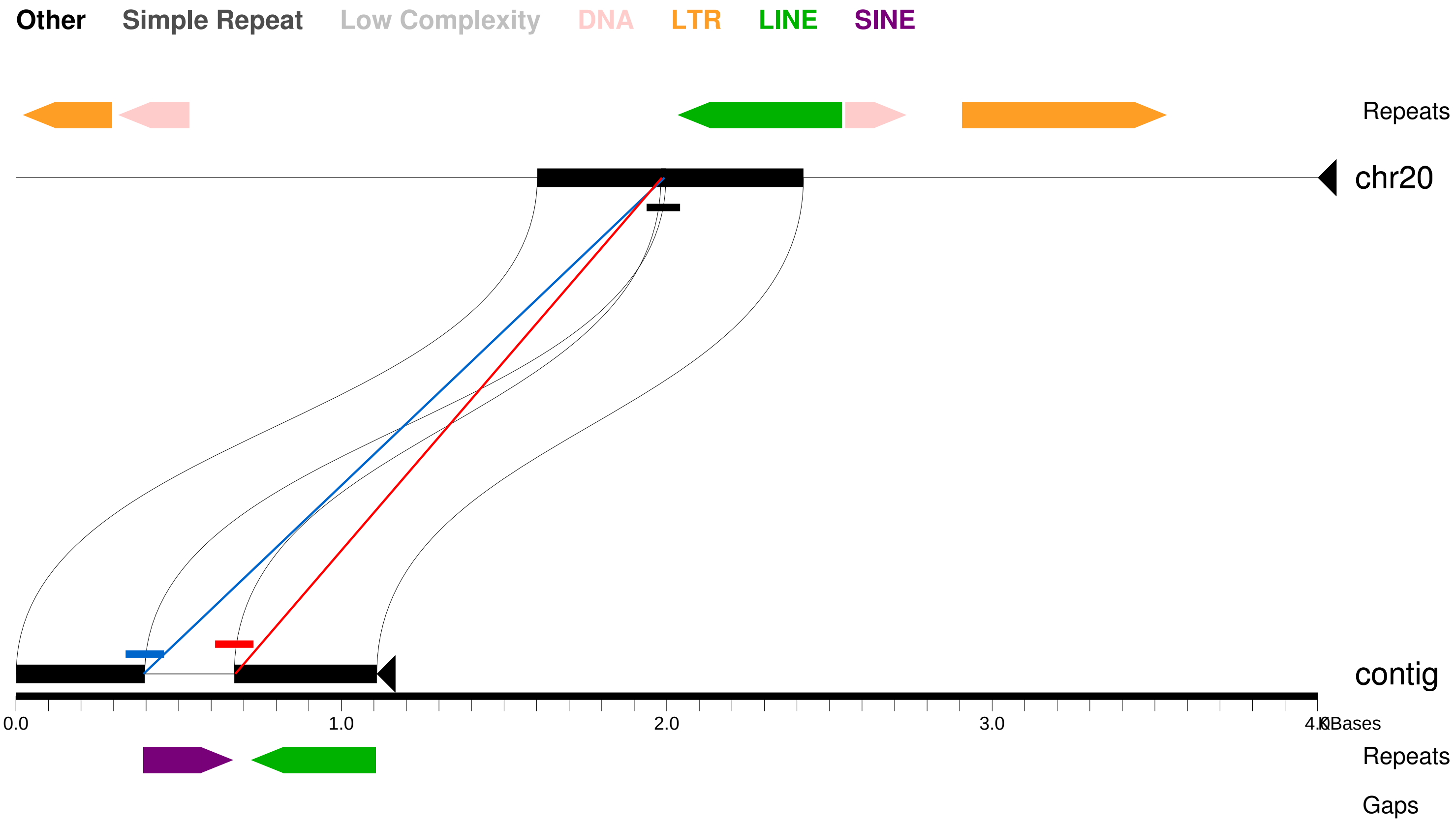

siteID: chr21_10967225 Scafftig1 + Breakpoint: chr21:10967206
chr21:10967046-10967380
Overlap Length: 15 INS size: 301 Genotyped: Yes

```
left     -----------CCTCAAAATGTGTATGTCAATGATTTGGAATCTACTATTAATATATCATTTTTTCCTAG
chrom    ACTGGCTGAATCCTCAAAATGTGTATGTCAATGATTTGGAATCTACTATTAATATATCATTTTTTCCTAG
right    TTTAGTAGA-----------------------GACGGGGTTTCACCGTTTTAGCCGGGATGGTCTC----
         NN2N2NN22  1111111111111111111111**111**11**11*11**1*111111**11*1**1111

left     AAGAAAACCTTATATTATGTAAAGGTGCTCCTGGGCCTTAACAAACAAACTAATGA-ATCACAATTTAAG
chrom    AAGAAAACCTTATATTATGTAAAGGTGCTCCTGGGCCTTAACAAACAAACTAATGA-ATCACAATTTAAG
right    --GATCTCCTGACCTCGTG-ATCCGCCCGCCTCGGCCTCC-----CAAAGTGCTGGGATTACAGGC---G
         11**111***1*11*11**1*111*11*1***1*****1111111****1*11**11**1***111111*

left     TGCAGTTCTGTATTACTTCATTTGTGAATGAATTTT-----------TTTTTTTTTTTTTTTTTTGAGAC
chrom    TGCAGTTCTGTATTACTTCATTTGTGAATGAATTTTACCAATCTTAATATATGCCTCTCTTCTTTCTCTC
right    TGAGCCACCGCGCCCGGCC--TTGTGAATGAATTTTACCAATCTTAATATATGCCTCTCTTCTTTCTCTC
         **11111*1*11111111*11**************22222222222*2*2*222*2*2**2***2222*

left     GGAGTCTCGCTCTGTCGCCCAGGCTGGAGTGCAGTGGCGGG-AT--CTCGGCTCACTGCAA----GCTCC
chrom    CGTATTTTGAAATTTCACCCATCATTGATGACTCTACCACACATTACTCTTTTTAATTCAATGAGGTAAT
right    CGTATTTTGAAATTTCACCCATCATTGATGACTCTACCACACATTACTCTTTTTAATTCAATGAGGTAAT
         2*22*2*2*222*2**2****222*2**222*22*22*2222**22***222*2*2*2***2222*2222

left     GCCTCCCGGGTTCA-----------------CGCCA----TTCTCCT-GCCTC---
chrom    AGCTAAAGGATTAAAAATGAACATAGCAAGACTCCACATGTTATCCTAGACTTAAT
right    AGCTAAAGGATTAAAAATGAACATAGCAAGACTCCACATGT---------------
         22**222**2**2*22222222222222222*2***2222*1N1111 1N11N
```

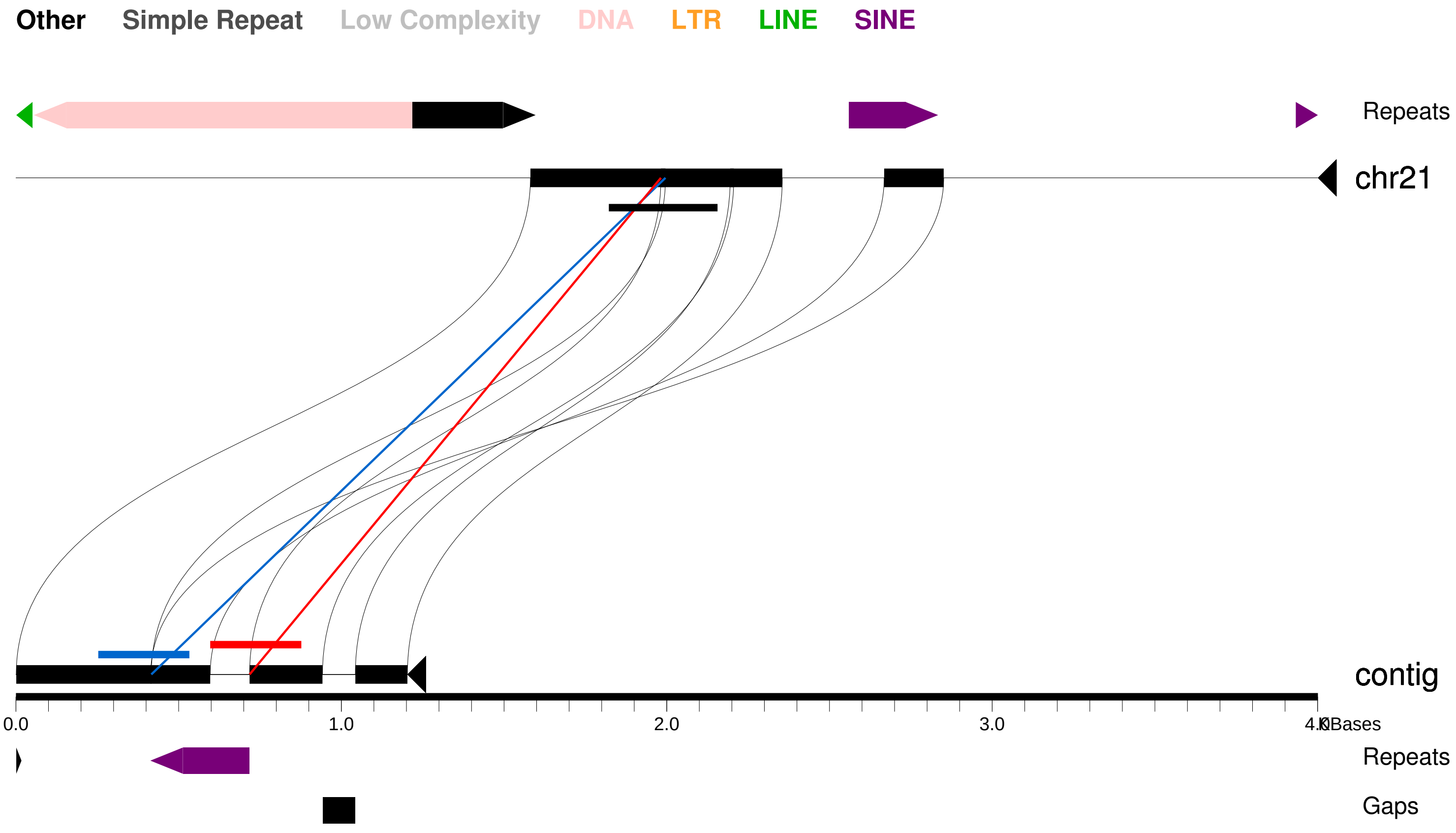

siteID: chr21_11071314 Scafftig1 - Breakpoint: chr21:11071295
chr21:11071135-11071470
Overlap Length: 16 INS size: 302 Genotyped: Yes

```
left   ----------------TTGGCCGTGATACATAAGACAAACAAAGGAAGACAACAAAATATAGAGAGAGGG
chrom  ACAGCTTTAAAAACCCTTGGCCGTGATACATAAGACAAACAAAGGAAGACAACAAAATGTAGAGAGAGGG
right  AGAA-------------TGGC-GTGA-ACCCGGGA--------GGCGGAGCTTGCAGTGAGCCGAGATCC
       2N2N            1****1****1**1111**11111111**11**111111*1*21111****111

left   CAGGCCAATTAGGGCTTCAGGTGCCAAGAAAAACAAGAGGATGAATTCCCTAGGTTTCCTTTCTGACTCA
chrom  CAGACCAATTAGGGCTTCAGGTGCCAAGAAACACAAGAGGATGAATTCCCTAGGTTTCCTTTCTGACTCA
right  CG--CCACT--GCACTCCAG---CCTGGG--CAAAAGAG----------CGAGACTCCATCTCAAAAAAA
       *11N***1*11*11**1***111**11*1112*1*****1111111111*1**11*1*1*1**11*111*

left   TATATACAAGAAAAGTTGCTAGAGAAGCAGACAACTG-GCCGGGCGCGGTGGCTCACGCCTGTAG-----
chrom  TATATACAAGAAAAGTTGCTAGAGAAGCAGACAACTCAGAAACACCAACAGGCACACACATACATAAAAC
right  AAAAAAAAAAAAAAAAG---AGAGAAGCAGACAACTCAGAAACACCAACAAGCACACACATACATAAAAC
       1*1*1*1**1****111111***************22*22222*222221**2***2*2*22*222222

left   TCCCAGCACTTTGGGAGGCC------------------GAGGCGGGCGGATCACGAGGTCAGGAGA--TC
chrom  TCCAAGAAAATTCTGCTGTCTCTTGTCAAAAAAGCAGGGAAGCGGCAGGCTACCAAGAAAAAAAAA-CTC
right  TCCAAGAAAATTCTGCTGTCTCTTGTCAAAAAAGCAGGGAAGCGGCAGGCTACCAAGAAAAAAAAAACTC
       ***2**2*22**22*22*2*222222222222222222**2****22**2*22*2**222*22*2*12**

left   G-AGAC------CATCCCGGCTAA----AACGGTGAAACCCCGTCTCTA---CTAAA
chrom  ACAGACAACAACCAACCAGTTTAAGCCAAAGGGTACAAATAAAACCCCAAGCCATAT
right  ACAGACAACAACTAACCAGTTTAAGCCAAAGGGTACAAATAAAACC-----------
       22****2222221*2**2*22***2222**2***22**222222*21N1   1NN1N
```

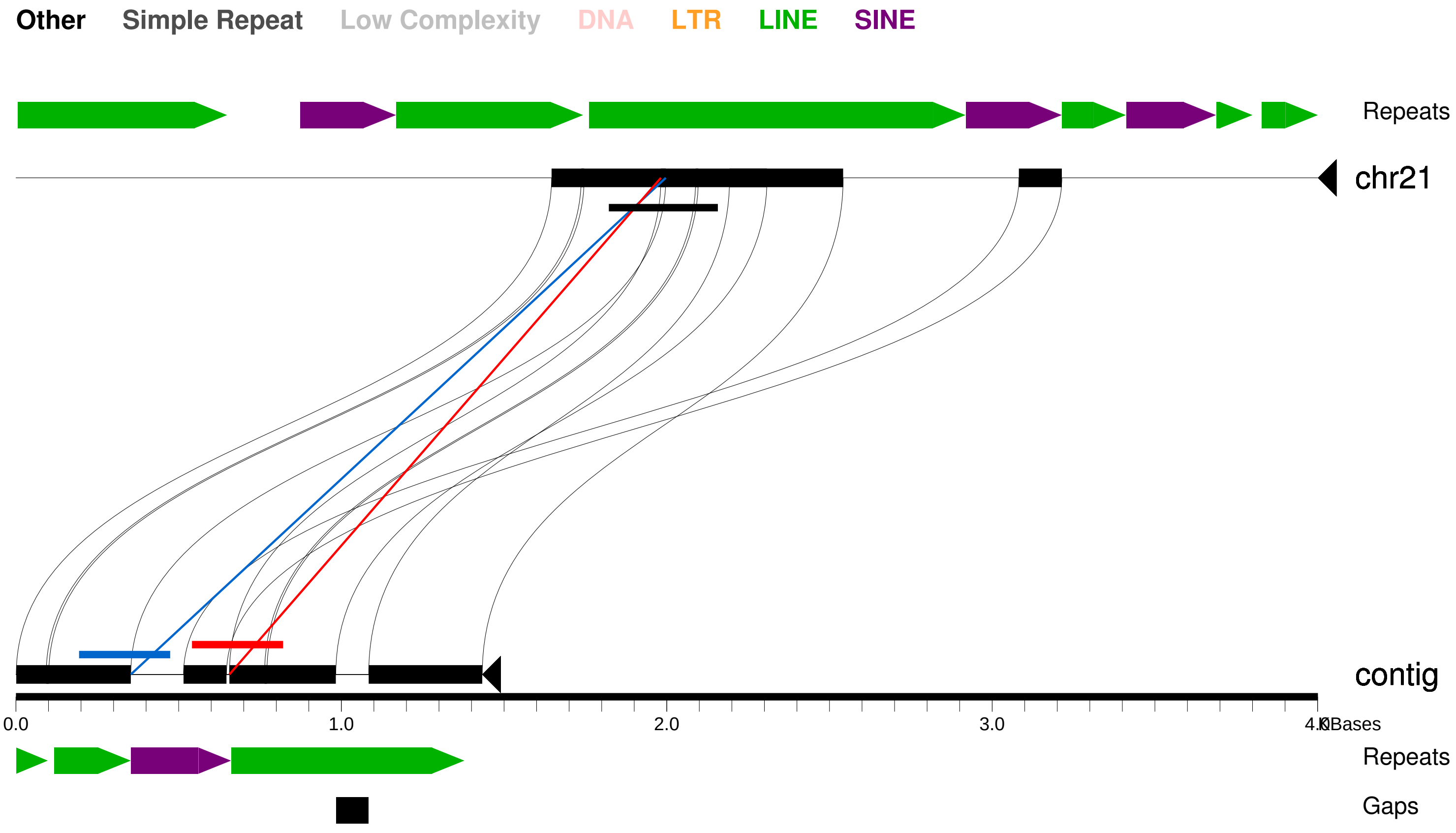

siteID: chr21_14414986 Scafftig1 + Breakpoint: chr21:14414991
chr21:14414835-14415162
Overlap Length: 12 INS size: 307 Genotyped: Yes

```
left     --------GTGCCAATGGCCATGCAGAAGTAG-TAACACTTCTGGTAGATAGAAAGTGCCAGCTTGACGT
chrom    CTGGGCCTGTGCCAATGGCCATGCAGAAGTAG-TAACACTTCTGGTAGATAGAAAGTGCCAGCTTGACGT
right    GGAGAATGGCGTGAA---CCCGGGAGGCGGAGCTTGCAGT----GAACCTAGATCGCGCCA-CTGCAC-T
         NNN2NNNN*1*11**111**11*1**11*1**1*11**1*1111*1*11****11*1****1**11**1*

left     CCTTGATGGCGAAAACAGGACAACTCTGATGAAGGTAAATGGTAGCCAGTTCTTTCAGCAGGAGATGGAT
chrom    CCTTGATGGCGAAAACAGGACAACTCTGATGAAGGTAAATGGTAGCCAGTTCTTTCAGCAGGAGATGGAT
right    CCAGCCTGGGCGACAGAGCGAGACTCCG------------------------TCTCAAAAAAAAAAAAAA
         **1111***111*1*1**1111****1*111111111111111111111111*1***11*11*1*111*1

left     TTGGTTTAAATACATAGAATAAAAATGAAGGCCGGGCGCGG------------TGGCTCACGCCTGT---
chrom    TTGGTTTAAATACATAGAATAAAAATGAATTTATCTCATTGAAATATAACTAGTTTGTGAAACCTGTGGA
right    TA-----AAAAATAAAAAATAAAA-TGAATTTATCTCATTGAAATATAACTAGTTTGTGAAACCTGTGGA
         *111111***1*1*1*1*******1****2222222*222*222222222222*222*2*22*****222

left     ------------AATCCCAGCACTTTGGGAGGCCGAGGCGGGCGGA----TCACGAGCTCAGGAGATCGA
chrom    ATATTTATTTATATTTCCTATAATTTATAATTTACTTCTTGCTTTAATACTGACAGGCTCTGCAATGCCA
right    ATATTTATTTATATTTCCTATAATTTATAATTTACTTCTTGCTTTAATACTGACAGGCTCTGCAATGCCA
         222222222222*2*2**222*2***222*2222222222*2222*2222*2**22****2*2*222*2*

left     GACCAT--CCTG-GCTAACATGGTGAAAC---CCCGT-CTCTACTAAA-
chrom    GAGGGAGGCTTGTGCAAATATTCTCATAGATTCTGGTGCTGATCCAAAT
right    GAGGGAGGCTTGTGCAAATATTCTCATAGATTCTGGTGCT---------
         **222222*2**2**2**2**22*2*2*2222*22**2**NNN1N111
```

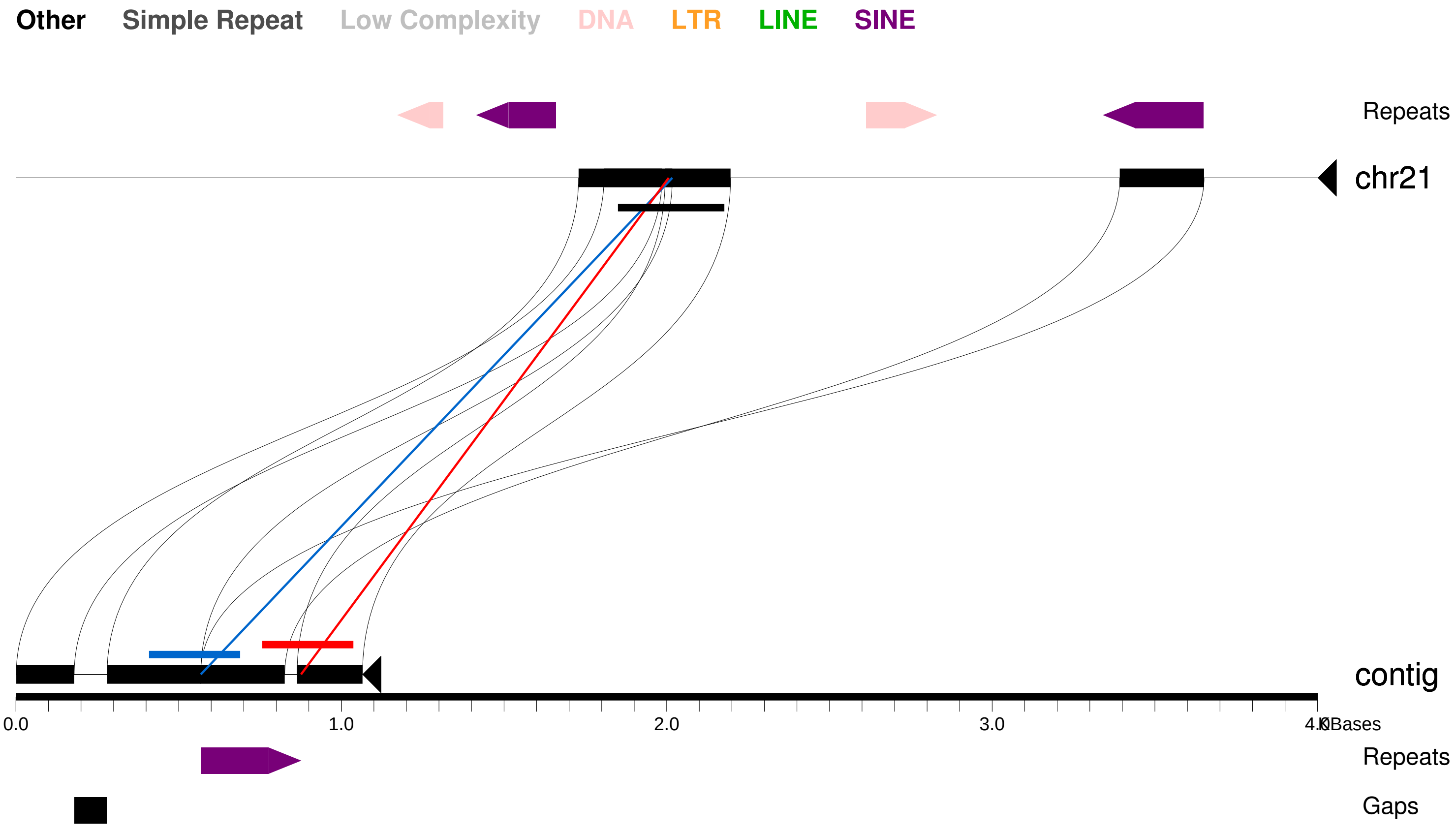

siteID: chr21_15652846 Scafftig3 + Breakpoint: chr21:15652855
chr21:15652227-15653014
Overlap Length: 13 INS size: 429 Genotyped: No

```
left   ----------------------------------------------------------------------
chrom  AACTTCTTTTTAGCATCAAGTATATGCTATCCACTTTGTGTTTATTATTTCATTTAATTCTCCTCCAAG
right  NNNNNNNNNNNNAA--------------------------------------------------------
       NNNNNNNNNNNN2

left   ----------------------------------------------------------------------
chrom  TGCTATGAGAAAGGCATTTGCATGAGAAAAATTTAGGCTCAGGAAACGAAAGCACCTGTCCATTGATACC
right  ----------------------------------------------------------------------

left   ----------------------------------------------------------------------
chrom  CATGTAGGAGTAAGTATGTGTCTTAAACCAATTTCTGATGAGTTCATGTTTCAAGCTTTATTTTTGCTAT
right  ----------------------------------------------------------------------

left   ----------------------------------------------------------------------
chrom  GTCAATCTACTTTTTATTAGAAATGCTCTCTTTTATACTAATATATGCTATTATTAAAAAACACACACAC
right  ----------------------------------------------------------------------

left   ----------------------------------------------------------------------
chrom  ACAGCCCTAAGCATTTTATATGACATATATTATGGTTTTTCTCATAGAATAATTCACCCATAACATATGT
right  ----------------------------------------------------------------------

left   ----------------------------------------------------------------------
chrom  TGAGGGCTCAGAATTTCTTCAGGAGATACCATTGTGAACTCTGATTTTTAACACAGAAATTAAATGGTAT
right  ----------------------------------------------------------------------

left   -------------------------------------------------------------TTTCTTTTT
chrom  TGAATGGGAAGAGTTGATGTTTTCTTATTTTATAAATTCAGAAGCTTTCTTTAGAAATGTATTTCTTTTT
right  ----------------------------------------------------------------------
                                                                    111111111

left   CCCCCATAACAGAAATTCCATAATAATATGAAGTTAAATTTGTGTTTCTAGCTTTATGCCCCTTGCTCTG
chrom  CCCCCATAACAGAAATTCCATAATAATATGAAGTTAAATTTGTGTTTCTAGCTTTATGCCCCTTGCTCTG
right  ----------------------------------------------------------------------
       1111111111111111111111111111111111111111111111111111111111111111111111

left   CAGTTCACAGAAACAAAACTGCTCAATTTTATCTACAGATATGTTCCCAAGAGTGGATTAATCTTTTAAA
chrom  CAGTTCACAGAAACAAAACTGCTCAATTTTATCTACAGATATGTTCCCAAGAGTGGATTAATCTTTTAAA
right  --------------------------------------------------------------------AA
       111111111111111111111111111111111111111111111111111111111111111111111**

left   AAATGATTAGGGGCCGGC-AGCGGTGGCTCCC---GCCTGTAATCC--CAGCACTTTGGGAGGCCGAGGC
chrom  AAATGATTAGGTACTAGATACCCCAAACTCAAATAGTCTTTAAAAATGCAGAGATAAATGAGTTAGTTTC
right  AAATGATTAGGTACTAGATACCCCAAACTCAAATAGTCTTTAAAAATGCAGAGATAAATGAGTTAGTTTC
       ***********22*22*22*2*22222***22222*2**2***22222***222*2222***222*222*

left   GGGC-----GGA--TCAC----GAGGTCAGGAGATCGAGACC-AT[illegible]TAAAAAAACGGTGAAA
chrom  TGTCTTCTAGGAATTCATAGTAGAATTAAAAAGATTAAAAAGTATACCACCATTACAAAAAAAGGGAAAA
right  TGTCTTCTAGGAATTCATAGTAGAATTAAAAAGATTAAAAAGTATACC----------------------
       2*2*22222***22***22222**22*2*22****22*2*222**2**NN1  11N11111NN1N1111

left   --CCCCGT---CTCTACT
chrom  GGCAACATAAGCGTAATT
right  ------------------
         1NN1N1   1NNN1N1
```

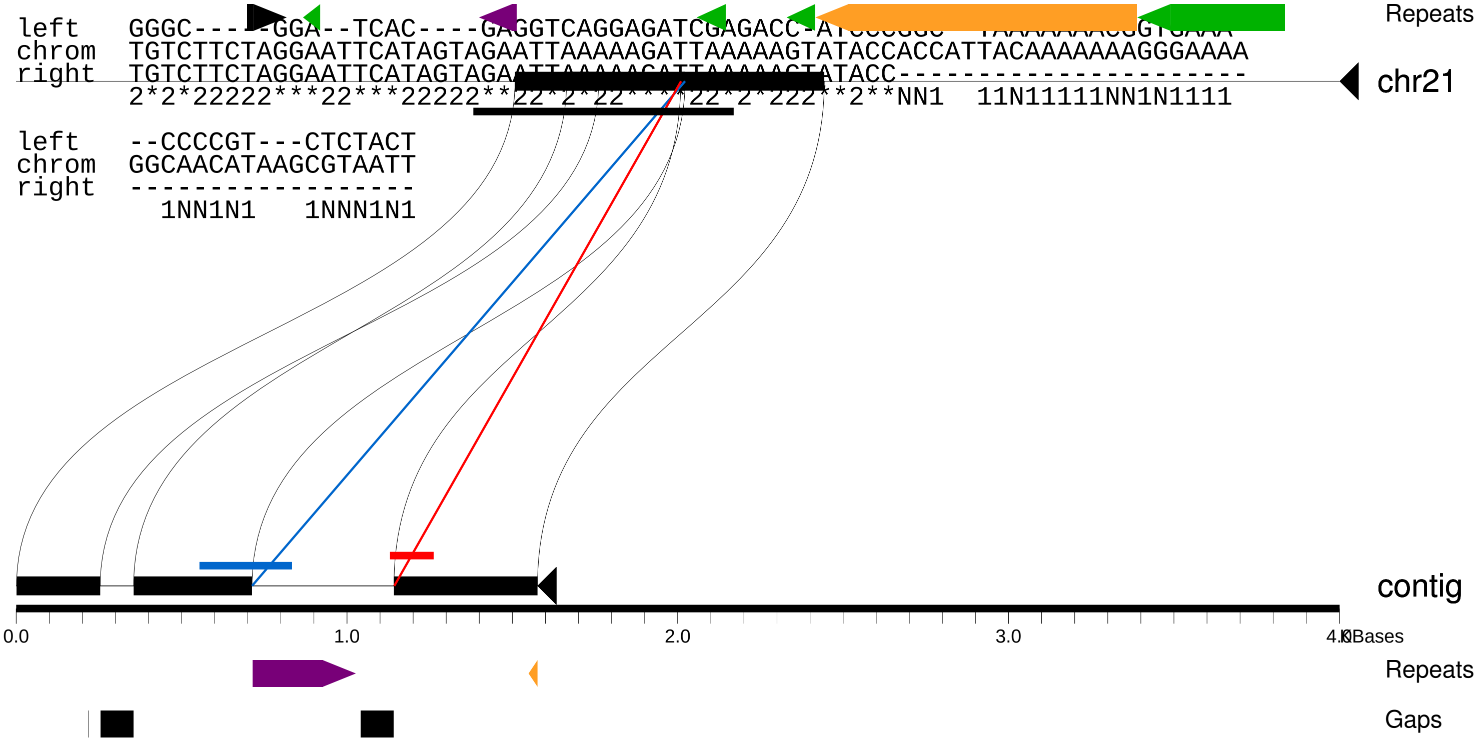

siteID: chr21_16461794 Scafftig8 - Breakpoint: chr21:16461785
chr21:16461625-16461950
Overlap Length: 6 INS size: 270 Genotyped: Yes

```
left   ------ATTCCTTTATTGCAGCATGAGAACAGACTAATACAACAACTGTATACAAATTTTGGGAAAAATT
chrom  TCAGGTATTCCTTTATTGCAGCATGAGAACAGACTAATACAACAACTGTATACAAATTTTGGGAAAAATT
right  GGAGA-ATGGCGTGAACCCGGGAGGCGGAGCTTGCAGTGAGCCGAGATTGCGCCACTGCACTCCAGCCTG
       NN22N **11*1*1*111*1*1*1*1*1*111111*1*1111*1*111*111*1*1*1111111*111*1

left   AAAAAAAAACAAAATTTACATGTAATCATAAAATAACCATTAATATTTTACTTTGTTTTTTATCCTAACC
chrom  AAAAAAAAACAAAATTTACTTGTAATCATAAAATAACCATTAATATTTTACTTTGTTTTTTATCCTAACC
right  GGCGACAGAGCGAGACTCCGTCTCAAAAAAAAAAAA-----AA---------------------------
       1111*1*1*111*111*1*N*1*1*11*1****1**11111**111111111111111111111111111

left   TTTTTCTAACACATAAACATATTCACT----TGGGAGGCCGAGG---CGG-----------GCGGATCA
chrom  TTTTTCTAACACATAAACGTATTCACACACATGTAAATACAAACAAACGTCTCCTATCTATCGCATTTCT
right  -------AAAAAAAAAAAAAATTCACACACATGTAAATACAAACAAACGTCTCCTATCTATCGCATTTCT
       1111111**1*1*1***1N1******22222**22*222*2*22222**2222222222222**222**2

left   CGAGGTCAGGAGATC-GAGACCATCCC-GGCTAAAACGGTGAAACCCCGTC---TCTACTAAAAATACAA
chrom  CTATGCATGTTTATCTGTTTGCATACATGGATATGTATGTGCATGTATATGCATTGTATTACGAATATAG
right  CTATGCATGTTTATCTGTTTGCACACATGGATATGTATGTGCATGTATATGCATTGTATTACGAATATAG
       *2*2*222*222***2*2222**12*22**2**22222***2*222222*2222*2**2**22****2*2

left   AAAATTAGCCGGGCGT--------------AGTGGCGGGCGCCTG-
chrom  TTTATTATTTTTCTTTTACTCCTTTAAAAAAATTTCAATACAGTGT
right  TTTATTATTTTTCTTTTACTCCTTTAAAAAAATTTCAATAC-----
       222****22222222*22222222222222*2*22*22222NN11
```

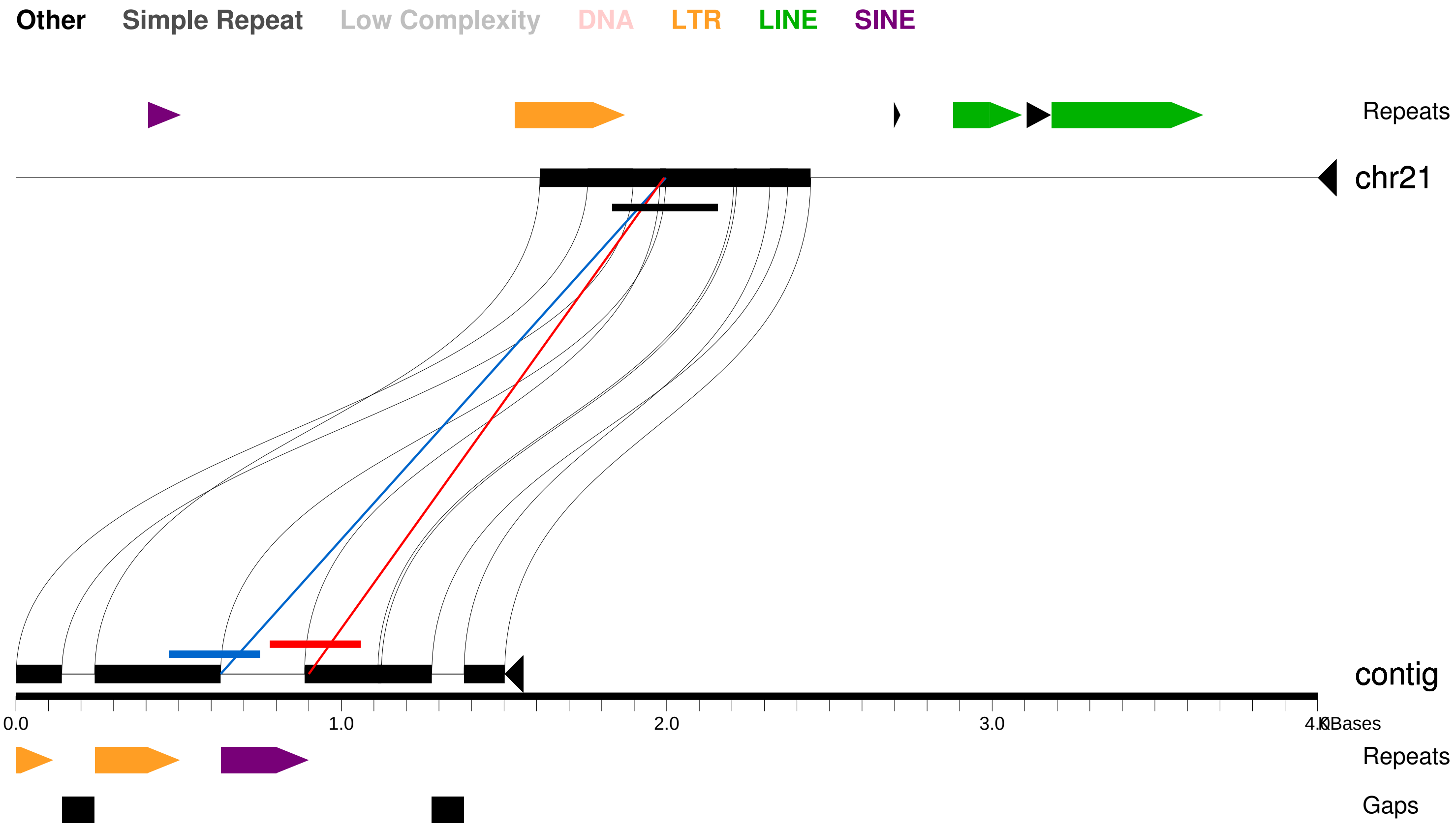

siteID: chr21_16980900 Scafftig1 - Breakpoint: chr21:16980894
chr21:16980849-16980953
Overlap Length: 15 INS size: 458 Genotyped: No

```
left    AAA--AGCATAGGA-----AAAG------GAATAAATGAAAGTAAAATTAAAATTTTT-AATTTTTTCAT
chrom   AAA--AGCATAGGA-----AAAG------GAATAAATGAAAGTAAAATTAAAATTTTT-AATTTTTTCAT
right   CCACCCGCCTCGGCCTCCCAAAGTGCTGGGATTACAGGCGTGAGCCACCGCGCCCGGCCAATTTTTTCAT
        11*111**1*1**111111****111111**1**1*1*111*1111*111111111111***********

left    TCTTTTTTTTNNNNNNNNNNNNNNNNNNNNNNNNNNNNNNNNNNNNNNNNNNNNNNNNNNNNN
chrom   TCTTAATT--------------GATCTAAAGGATAACAGTTTTTAAGATAATAACAATAATAT
right   TCTTAATT--------------GATCTAAAGGATAACAGTTTTTAAGATAATAACAATAATAT
        *****22**222222222222222222222222222222222222222222222222222222
```

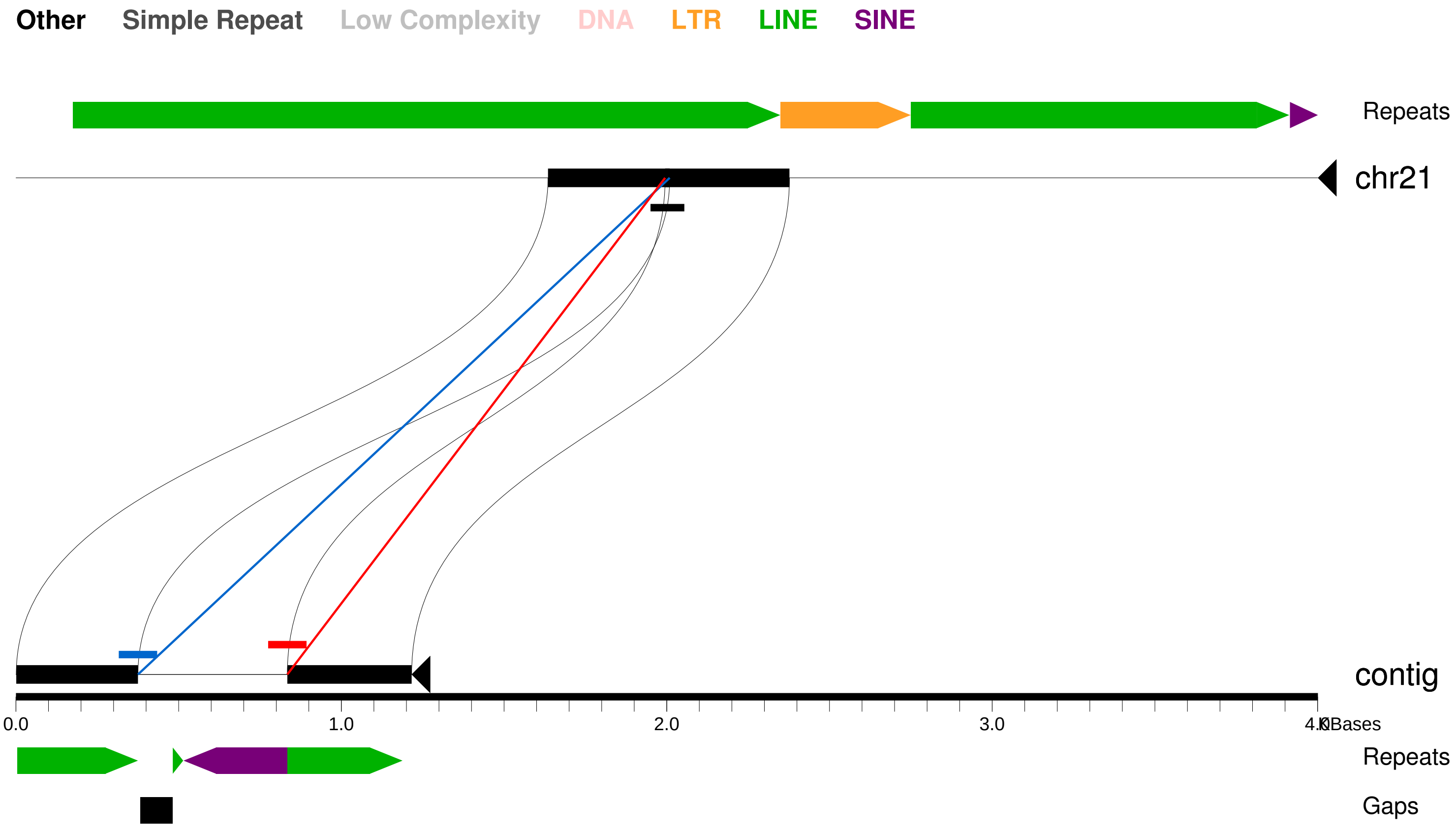

siteID: chr21_21324527 Scafftig3 - Breakpoint: chr21:21324525
chr21:21324481-21324584
Overlap Length: 16 INS size: 494 Genotyped: No

```
left     CCCAATT--CTCAG---ACCAAGATATTCTCACTA-AGGAGT-AGTTGTCA--------CCACATCCGAT
chrom    CCCAATT--CTCAG---ACCAAGATATTCTCACTA-AGGAGT-AGTTGTCA--------CCACATCCGAT
right    TCCGCCCGCCTCGGCCTCCCAAAGTGCTGGGATTACAGGCGTGAGCCACCGCGCCCGGCCCACATCCGAT
         1**111111***1*1111****11*11*111*1**1***1**1**1111*111111111***********

left     TTCTTTTTTTTTNNNNNNNNNNNNNNNNNNNNNNNNNNNNNNNNNNNNNNNNNNNNNNNNNNNN
chrom    TTCTTAAAATCTCTGGCATATCATCCCATCATTCTACTTGAGCCTGCTC---------------
right    TTCTTAAAATCTCTGGCATATCATCCCATCATTCTACTTGAGCCTGCTC---------------
         ******2222*2*2222222222222222222222222222222222222222222222222222
```

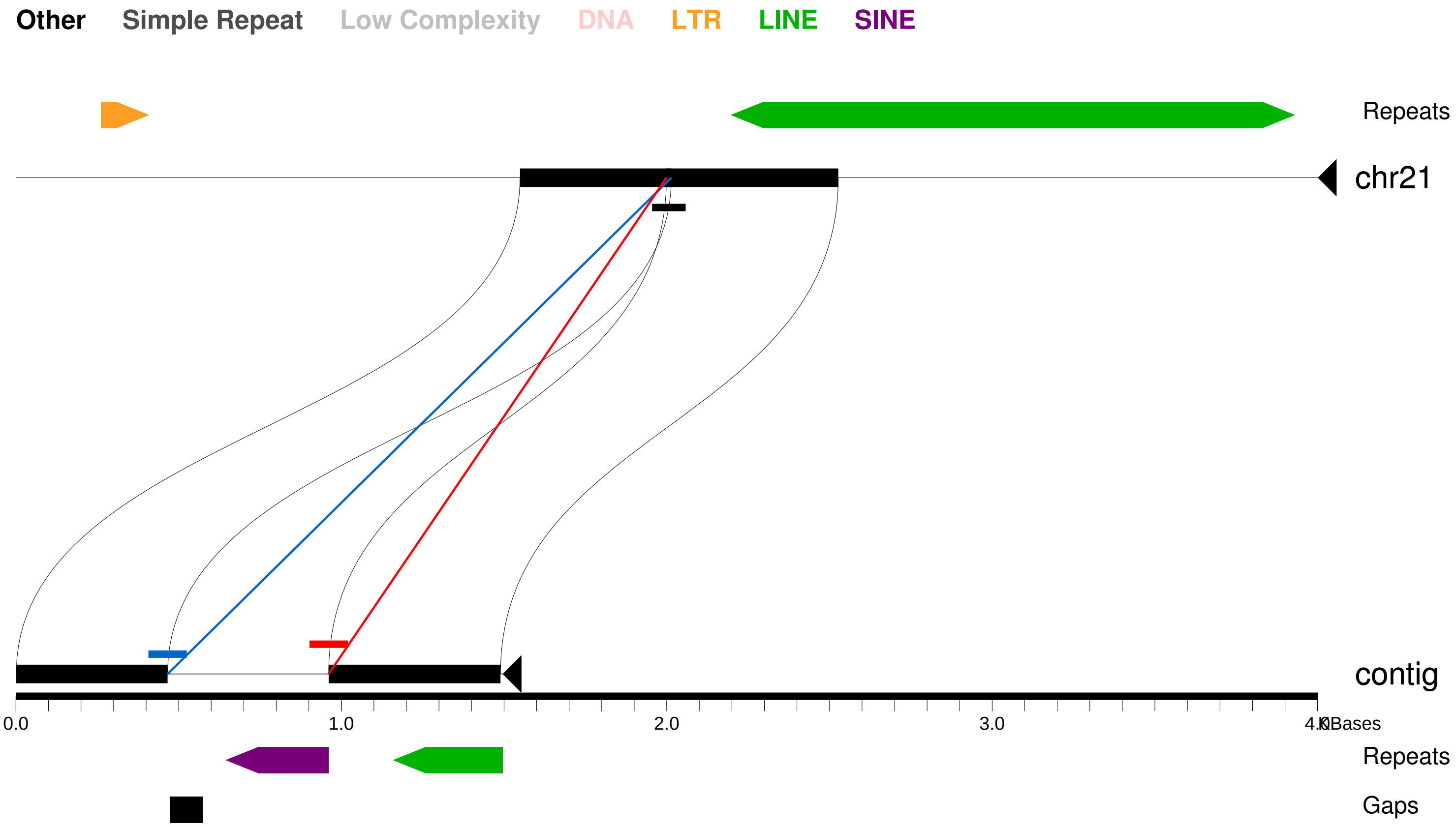

siteID: chr21_23579581 Scafftig1 - Breakpoint: chr21:23579558
chr21:23579511-23579617
Overlap Length: 13 INS size: 391 Genotyped: No

```
left    ATTTCATTAAACATTTTTAGAAGCATTTAACATGTTTTACATATCTT------------AAGATATAATT
chrom   ATTTCATTAAACATTTTTAGAAGCATTTAACATGTTTTACATATCTT------------AAGATATAATT
right   NNNNNNNNNNNNNNNNNNNNNNNNNNNNNNNNNNNNNNNNNNNNNNNNNNNNNNNNNNNAAGATATAATT
        11111111111111111111111111111111111111111111111111111111111***********

left    TTC--ACTTTGGGAGGCCGAGGCGGGCGGATCACGAGGTCAGGAGATCGAGACCATCCCGGCT
chrom   TTATTATTATATTAATTCAACTTTG------CAAGAGTGCACAAATAAATGACCG--------
right   TTATTATTATATTAATTCAACTTTG------CAAGAGTGCACAAATAAATGACCG--------
        **222*2*2*222*222*2*2222*222222**2***22**22*222222****222222222
```

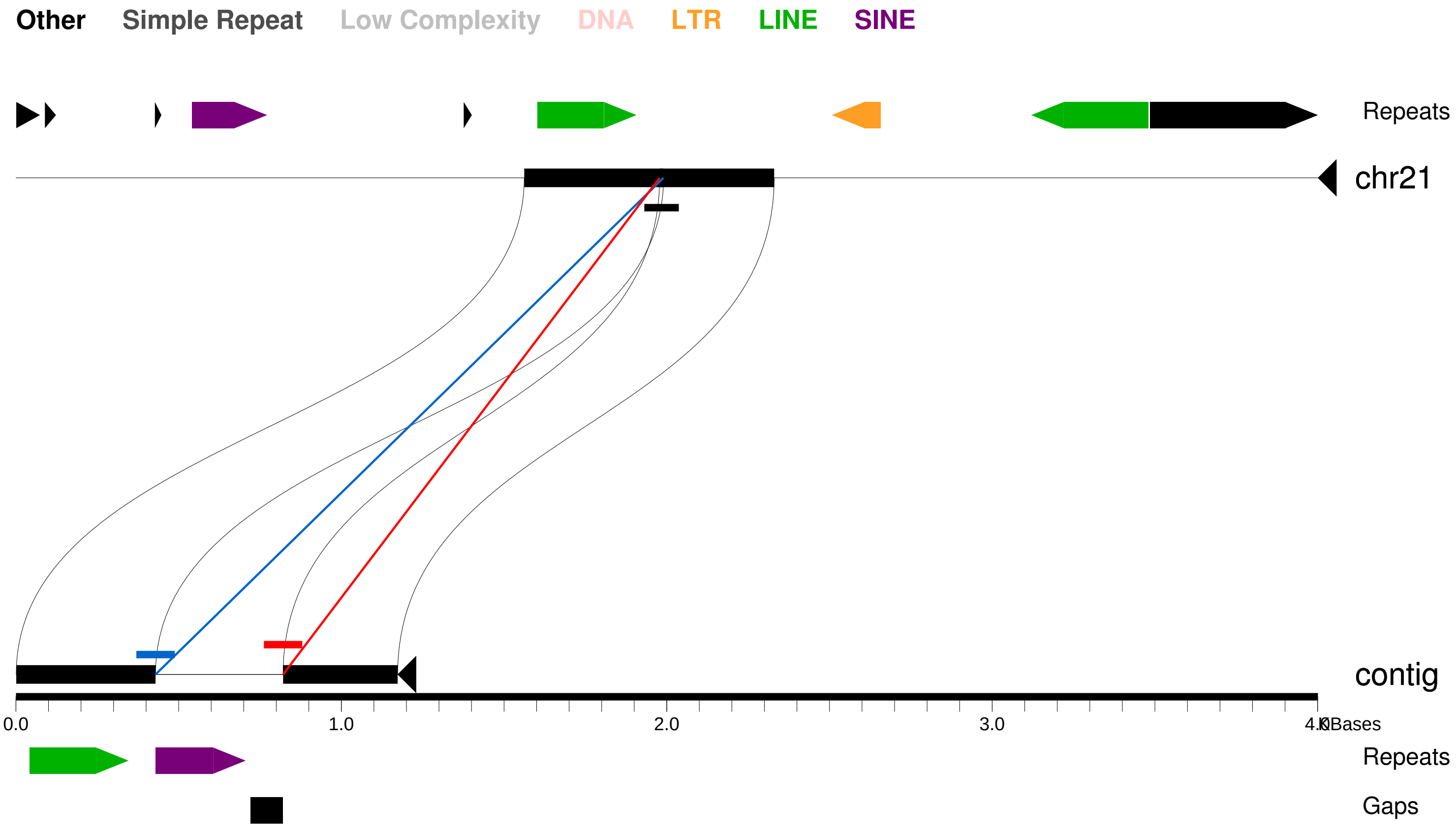

siteID: chr21_24046095 Scafftig3 + Breakpoint: chr21:24046117
chr21:24046073-24046176
Overlap Length: 16 INS size: 270 Genotyped: No

```
left     TTTAAAAAGTACTTATAAAAGGTATTGCCTTTTAATCTG---------------AAGTTAAAAGACAATA
chrom    TTTAAAAAGTACTTATAAAAGGTATTGCCTTTTAATCTG---------------AAGTTAAAAGACAATA
right    NNNNNNNNNNNNNNNNNNNNNNNNNNNNNNNNNNNNNNNNNNNNNNNNNNNNAAAAAAAAAAAGACAATA
         111111111111111111111111111111111111111111111111111111**111***********

left     CAGAGGGCCGGGCGCGGTGGCTCACGCCTGTAATCCCAGCACTTTGGGAGGCCGAGGCGGGCGG
chrom    CAGAGATACCCATAACATGATTTGTTTCAGAAAT------AATTTTTAAAATCTA---------
right    CAGAGATACCCATAACATGATTTGTTTCAGAAAT------AATTTTTAAAATCTA---------
         *****222*22222222**22*22222*2*2***222222*2***222*222*2*222222222
```

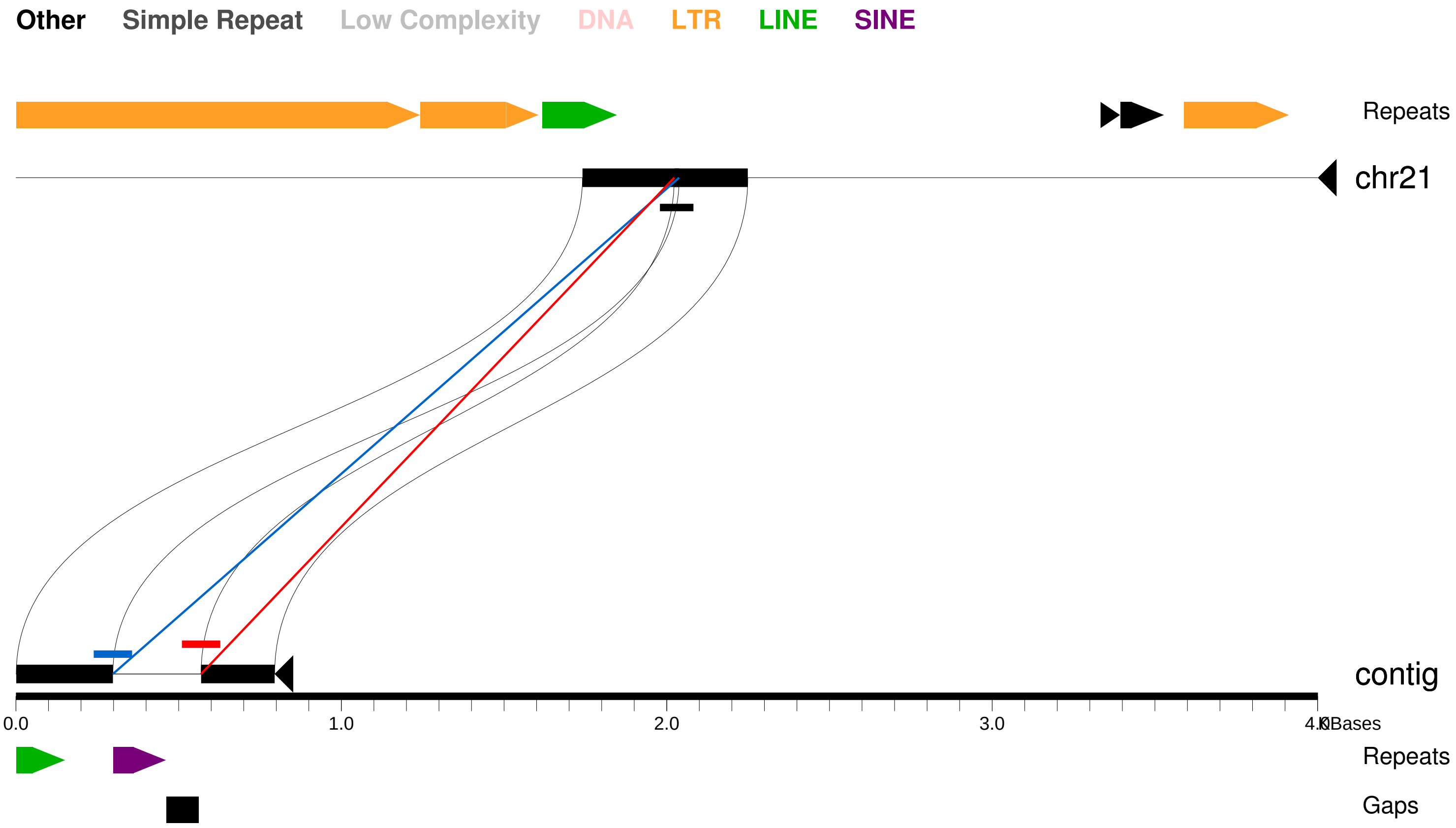

siteID: chr21_24975445 Scafftig3 + Breakpoint: chr21:24975491
chr21:24975411-24975545
Overlap Length: 12 INS size: 319 Genotyped: Yes

```
left   ----TGCACACATCTTAAGAAAAAAAAATACAGAAAATACAGCTAATACACTGATTTTTTTTTTTTTGCAAA
chrom  ----TGCACACATCTTGAGAAAAAAAAATACAGAAAATACAGCTAATACACTGATTTTTTTTTTTT-GCAAA
right  GTGATCCGCCCGCCTCGGCCT--------CCCAAAGTGCTGGGATTACAGGCGTG-------------AG
       1111*1*1*1*11**12111111111111*11***1*1*1*11*1****1111*111111111112111*1

left   GCAAATATCTTGCCTTGATTTCTCTCT--------CTTTTTTTTT---------TTTTTTTTTTTTTTTT
chrom  GCAAATATCTTGCCTTGCTTTCTCTCTTAAACACACTGAAGTTCTAGAAGAGATTTGAAAGCATTTTATT
right  CCACCGCGCCTGGCCTGATTTCTCTCTTAAACACACTGAAGTTCTAGAAGAGATTTGAAAGCATTTTATT
       1**11111*1**1*1***N*********22222222**2222**2*222222222**2222222****2**
```

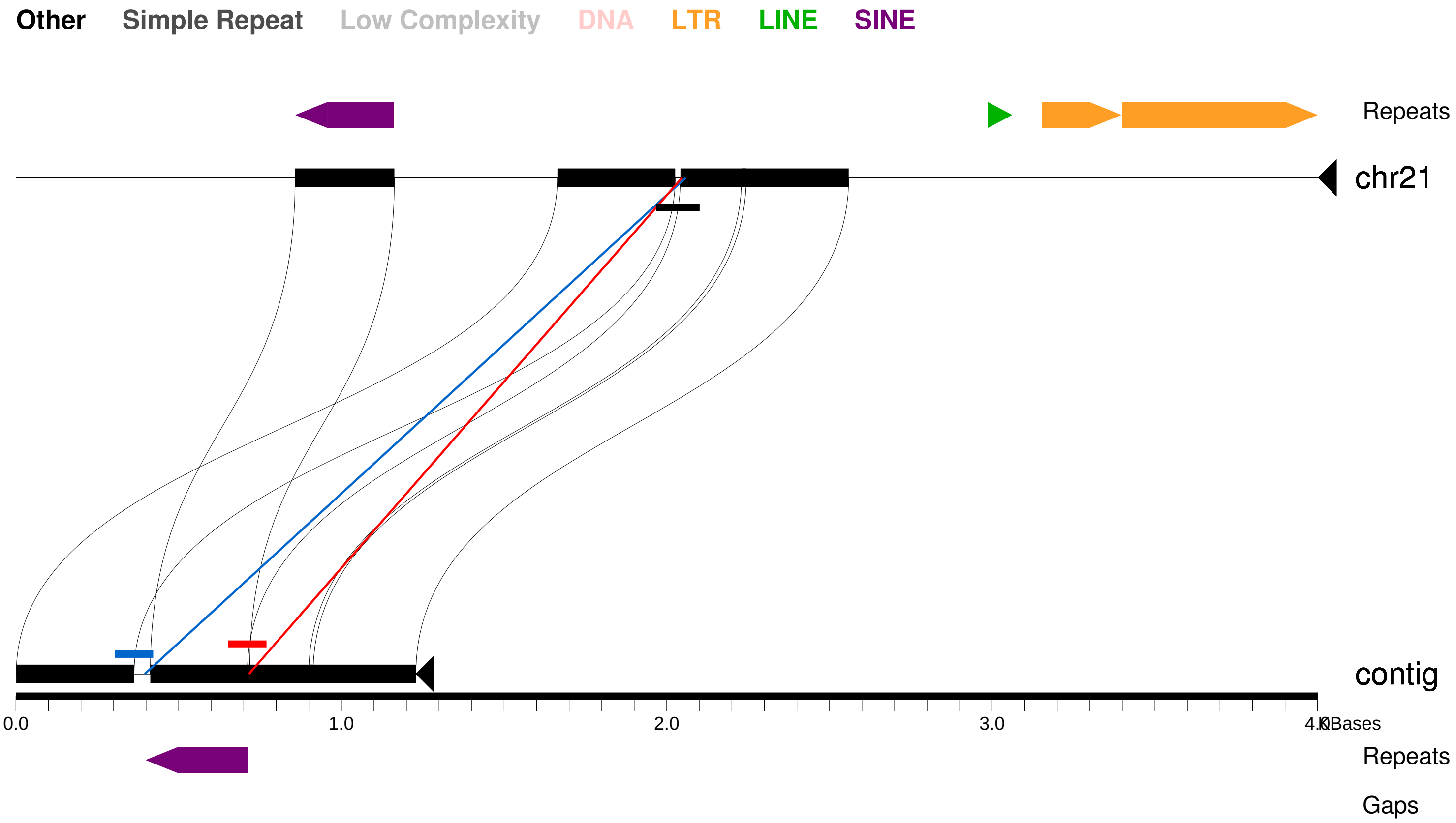

siteID: chr21_25411382 Scafftig1 - Breakpoint: chr21:25411368
chr21:25411325-25411438
Overlap Length: 17 INS size: 120 Genotyped: Yes

```
left    CAAAATTGCTCTATTGATTTATGA----TCATTGAGATAATATTATTTCAGCTATACATTTTTTTTTTTTT
chrom   CAAAATTGTTCTATTGATTTATGA----CCATTGAGATAATATTATTTCAGCTATACATTTTTTAATAAC
right   CCAGGCGGGACTGCGGACTGCAGTGGCGCAATCTCGGCTCACTGCAATCAGCTATACATTTTTTAATAAC
        *1*1111*N1**111**1*111*1111121**111*111111*1111*****************22*222

left    TTTTTTTT-TTTTTTTTTTTTTTTTTTTTTTTTTTTTTTTTTTTGAGACGGAG
chrom   CAAAAAAA-CAAAACATTTAGGGGAATCCTGAATCTAGTTT----GAGATTGT-
right   CAAAAAAAACAAAACATTTAGGGGAATCCTGAATCTAGTTT----GAGATTGT-
        222222221222222***2222222*22*222*2*22***2222****22*22
```

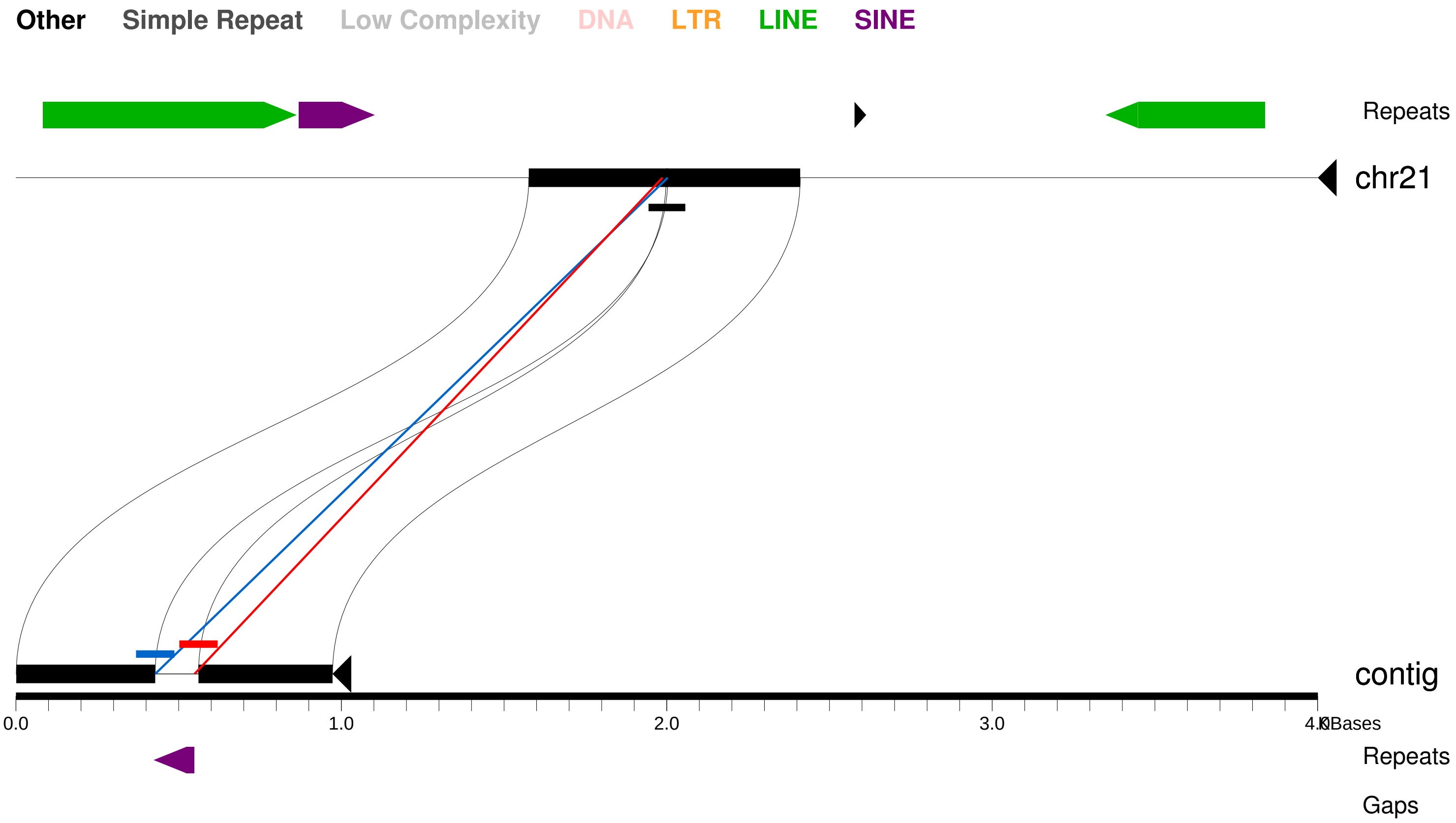

siteID: chr21_26286128 Scafftig1 - Breakpoint: chr21:26286098
chr21:26286047-26286136
Overlap Length: 9 INS size: 306 Genotyped: Yes

```
left    AAAGA-------------------ATCTTGTTCCTCTTAATGA-ATTCAG---------AAAAGAAAGAC
chrom   AAAGA-------------------ATCTTGTTCCTCTTAATGA-ATTCAG---------AAAAGAAAGAC
right   GCCGAGATCCCGCCACTGCACTCCAGCCTGGGCGACAGAGCGAGACTCCGTCTCAAAAAAAAAAAAAAAA
        111**1111111111111111111*1*1**11*11*11*11**1*1**1*111111111****1***1*1

left    CATTTAATACACATGGCAGGCCGGGCGCGGTGGCTCACGCCTGTAATCCCAGCACTTTGGGAGGCCGAGG
chrom   CATTTAATACACATGGCAG---------------------CATTATTTGTCAACATTATGTTACAAAAT-
right   AAAAAAAAAAACAAGGCCG---------------------CATTATTTGTCAACATTATGTTACAAAAT-
        1*111**1*1***1***1*22222222222222222222*2*2222*22**2**2*2**22*22222*22

left    CGGGCGGA
chrom   --------
right   --------
        22222222
```

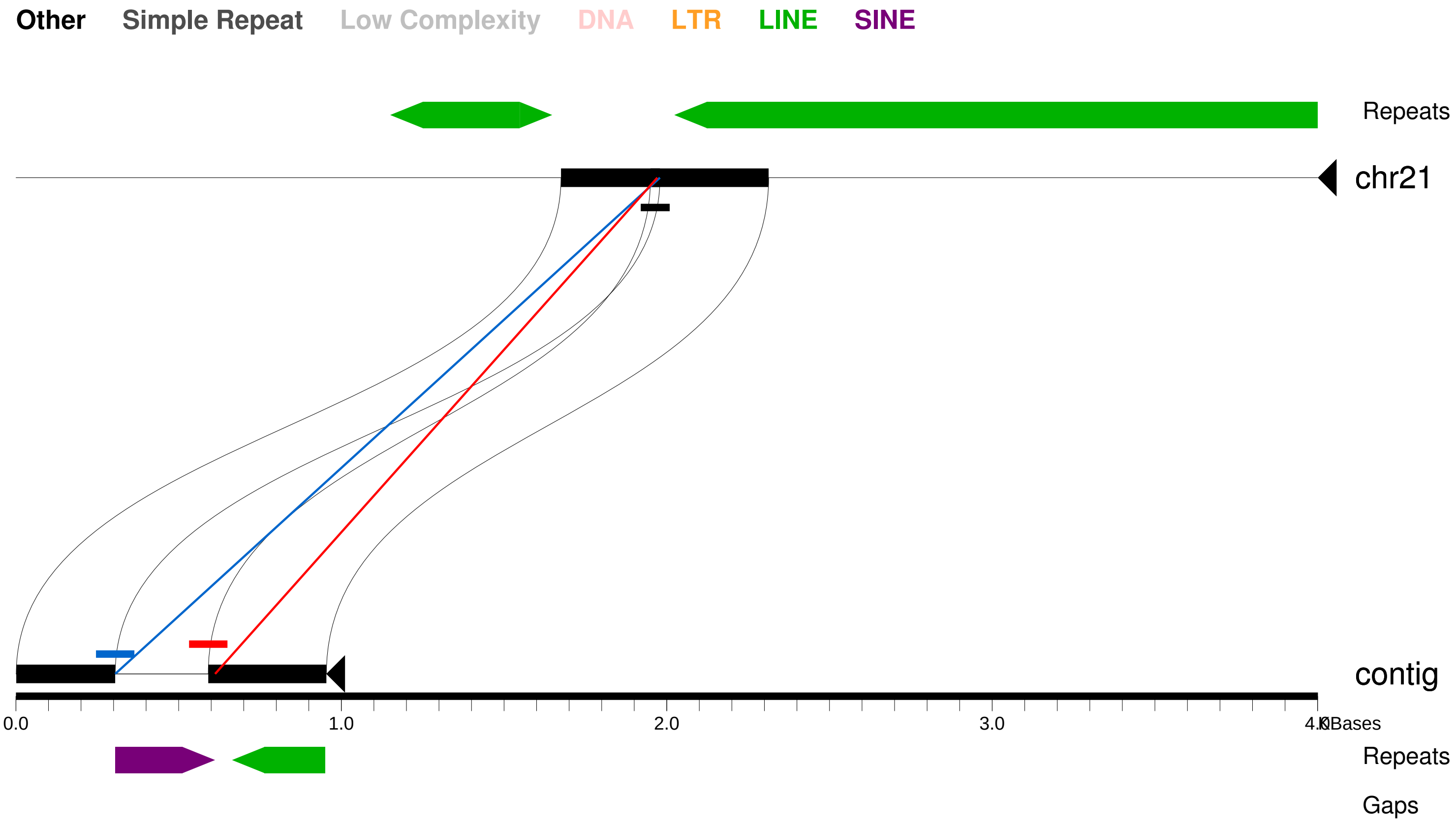

siteID: chr21_26354234 Scafftig5 + Breakpoint: chr21:26354223
chr21:26354178-26354282
Overlap Length: 15 INS size: 297 Genotyped: Yes

```
left    ---------TATGGTTGCAACAAT-CTGCACCATTATGGGTGCCTAATGGA----GGAAGCATCTGATAG
chrom   ---------TATGGTTGCAACAAT-CTGCACCATTATGGGTGCCTAATGGA----GGAAGCATCTGATAG
right   CCGCCCGCCTCGGCCTCCCAAAGTGCTGGGATTACAGGCGTGAGCCACCGCGCCCGGCCGCATCTGATAG
        111111111*11*11*1*1*1*1*1***1111111*1*1***1111*11*11111**11***********

left    TTTTTTTTTTTTTTTTTTTTTGAGACGGAGTCTCGCTCTGTCGCCCAGGCTGGAGTGCAGTGGC
chrom   TTTTAATGTAAGGTCAGTA--AAACATATTCTAGATC-------CA---TTTA-TGCTTTTG-
right   TTTTAATGTAAGGTCAGTA--AAACATATTCTAGATC-------CA---TTTA-TGCTTTTG-
        ****22*2*2222*222*222*2**22*2***2*2**2222222**222*22*2***22*2*2
```

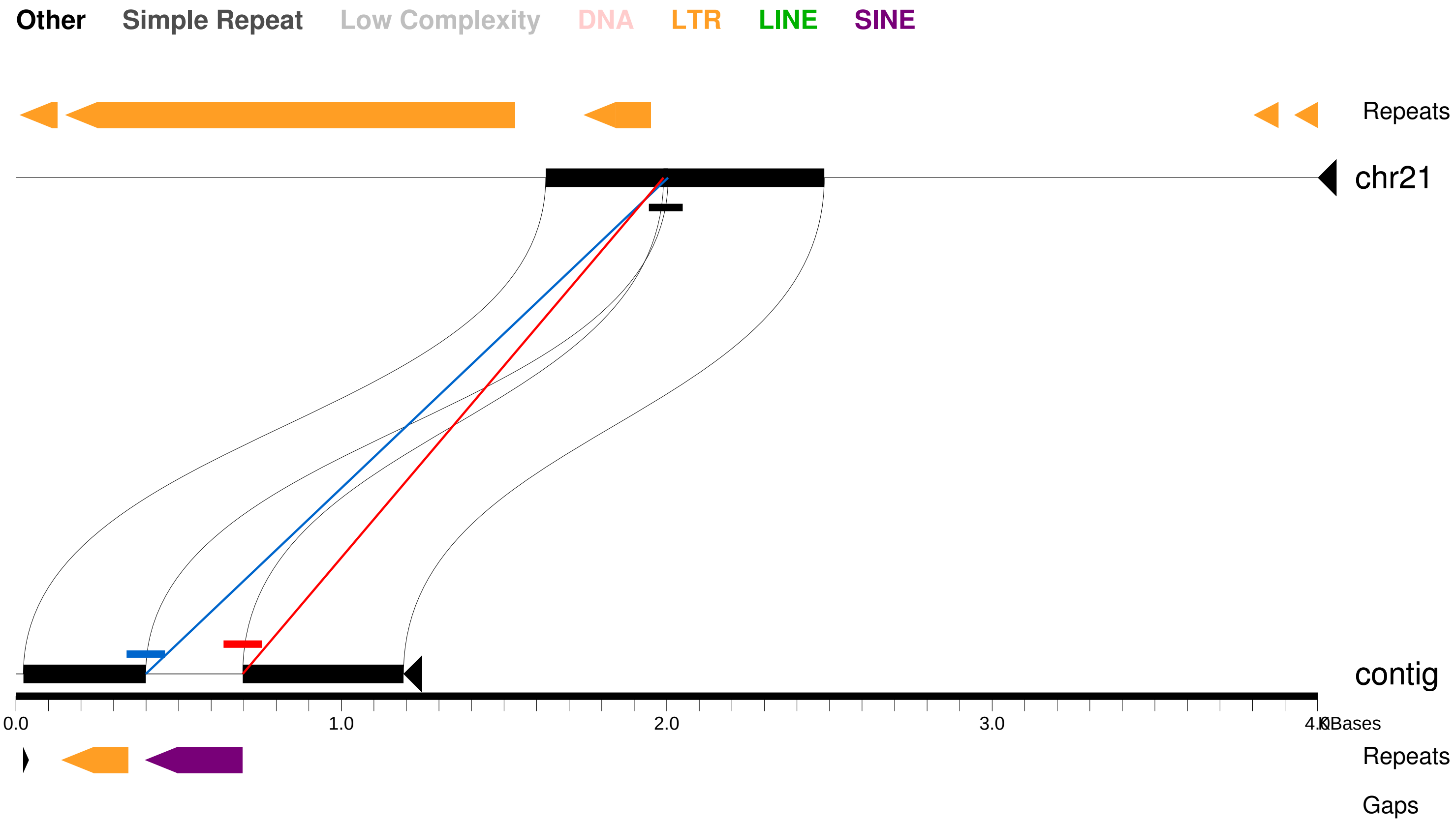

siteID: chr21_26497436 Scafftig7 - Breakpoint: chr21:26497421
chr21:26497372-26497480
Overlap Length: 11 INS size: 307 Genotyped: Yes

```
left   TT-AGCATCAACCTCTGCATGGGTTTATG---------AAGAAGATGCATTGCTTTGTTAAGGAAGGATG
chrom  TT-AGCATCAACCTCTGCATGGGTTTATG---------AAGAAGATGCATTGCTTTGTTAAGGAAGGATG
right  TCCAGCCTGGGCGACAGAGCGAGACTCCGTCTCAAAAAAAAAAAAAAAAAAAAAAAAAAAAGGAAGGATG
       *11***1*111*11*1*111*1*11*11*111111111**1**1*111*1111111111**********

left   GGCCGGGCGCGGTGGCTCACGCCTGTAATCCCAGCACTTTGGGAGGCCGAGGCGGGCGG---
chrom  ------------TATTACATAC-TGTAAGCTTAGTATTTTACCAGGAAAAAAATAGGGTAAA
right  ------------TATTACATAC-TGTAAGCTTAGTATTTTACCAGGAAAAAAATAGGGTAAA
       222222222222*2222**22*2*****2*22**2*2***222***222*22222*2*2222
```

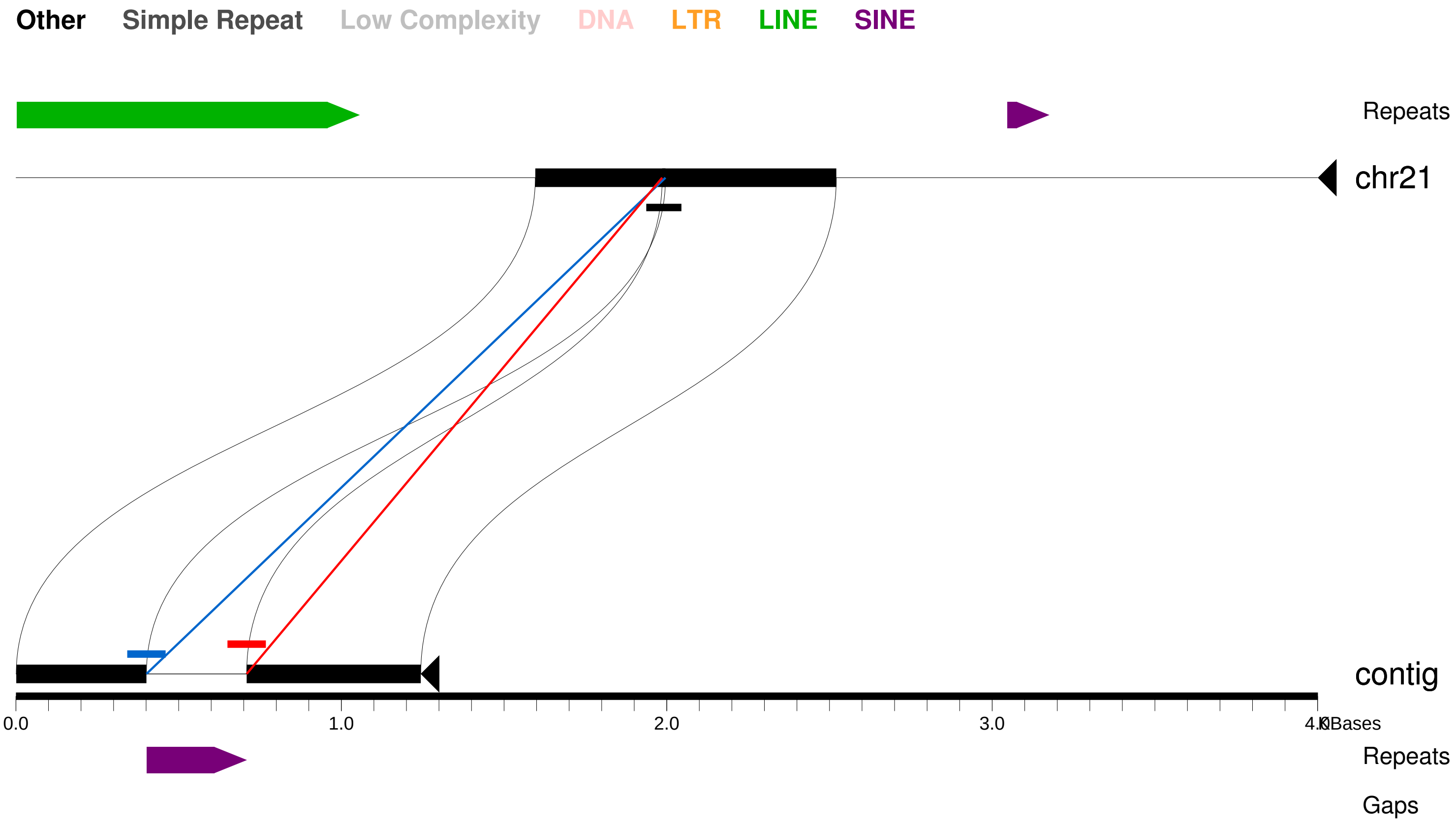

siteID: chr21_28221334 Scafftig1 - Breakpoint: chr21:28221341
chr21:28221300-28221400
Overlap Length: 16 INS size: 419 Genotyped: No

```
left     AAACATTGTCTAGAGAAACAACTAATGTGCAT--ATTTCA----TAA---------------CCGGGGAA
chrom    AAACATTGTCTAGAGAAACAACTAATGTGCAT--ATTTCA----TAA---------------CCGGGAAA
right    TCCGCCCGCCTCGGC---CTCCCAAAGTGCTGGGATTACAGGCGTGAGCCACCGCGCCCGGCCCGGGAAA
         1111111*1**1*11111*11*1**1****1111***1**1111*1*111111111111111*****2**

left     TGCTTTTTTTTTTTNNNNNNNNNNNNNNNNNNNNNNNNNNNNNNNNNNNNNNNNNNNNNNNNNNNNNNNN
chrom    TGCTTTTTATT-------------------CACATTTTAGTACCATAAGATTGACTAATTAGAAATAGGGA
right    TGCTTTTTATT-------------------CACATTTTAGTACCATAAGATTGACTAATTAGAAATAGGGA
         ********2**22222222222222222222222222222222222222222222222222222222222
```

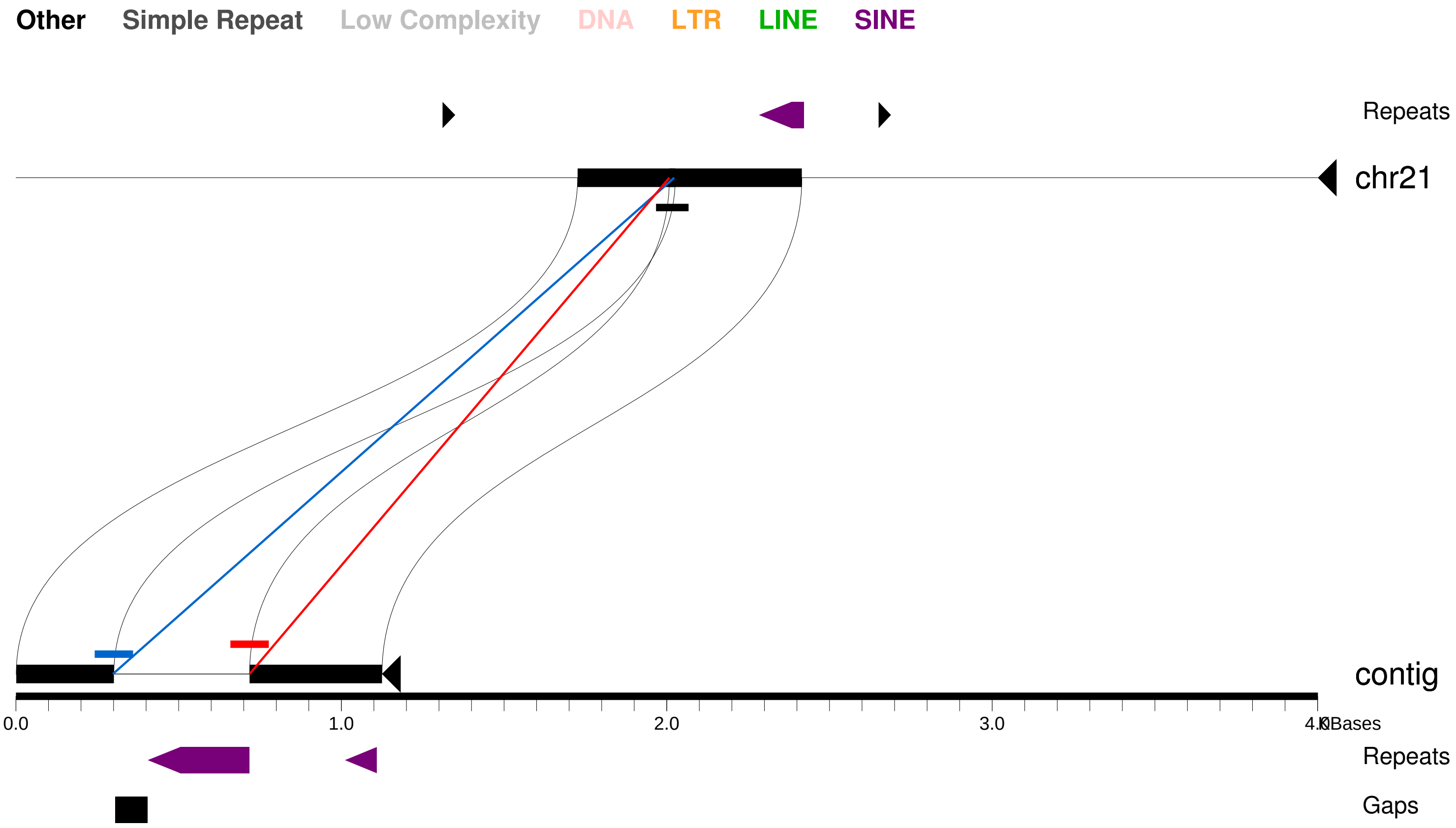

siteID: chr21_28249225 Scafftig9 - Breakpoint: chr21:28249235
chr21:28249075-28249407
Overlap Length: 13 INS size: 319 Genotyped: Yes

```
left    -------------CACTTGAAGCCCAGATTTTGATTTTCAAGTAACATT-CTCCAAAGAAAGAAACCACG
chrom   GTAGCAATAAGAACACTTGAAGCCCAGATTTTGATTTTCAAGTAACATT-CTCCAAAGAAAGAAACCACG
right   GGCGT-----GAACCCGGGAAGCGGAGCTTGCAGTGAGCCG---AGATTGCGCCACTGCAGT---CCGCA
        2NN2N     222*1*11*****11**1**1111*111*11111*1***1*1***11*1*11111**1*1

left    CTCCTTGGTAAAGAGGTTGCTTGGACAAGGAAAATATAGGACGTGCTTGAAATATCTTGTGGTACCAGAA
chrom   CTCCTTGGTAAAGAGGTTGCTTGGACAAGGAAAATATAGGACGTGCTTGAAATATCTTGTGGTACCAGAA
right   GTCCG-------------GCCTGGGCGACAGAGCGAGACTCCGT-CTCAAAAAA--------------AA
        1***11111111111111**1***1*1*111*111*1*111***1**11***1*11111111111111**

left    AGTAAAGTAGTTATAACTATAAATTACTTGTGTTGGCCG--------GGC--GCGGTGGCTCACGCCTG-
chrom   AGTAAAGTAGTTATAACTATAAATTACTTGTGTTAAACATAATTTTTGGCAAGCAAAAGTATACTGCAGA
right   AAAAAAAAAAAAAAAAAAAAACAATTACTTGTGTTAAACATAATTTTTGGCAAGCAAAAGTATACTGCAGA
        *11***11*111*1**11*11************222*222222222***22**2222*222**22*2*2

left    -TAATCCCAGCAC-------TTTGGGAGGCCGAGGCGG----------GTGGATCATGAGGTCAGGAGAT
chrom   ATACTTGCAGTATCATTCTATTTGTGTGACGTCTACAAATACCCCAAAGTAGA-CATATTGTTAAAGGAT
right   ATACTTGCAGTATCATTCTATTTGTGTGACGTCTACAAATACCCCAAAGTAGA-CATATTGTTAAAGGAT
        2**2*22***2*22222222****2*2*2*22222*222222222222**2**2***222**2*222***

left    CGAGACCATCCTGGC--TAACAGGGTGAAACCC--CGTC-TCTACTAAA------
chrom   GCAAACATACATGGTGATAATCTAAAGAAAATCGACGACATGCAATTCAGAATGC
right   GCAAACATACATGGTGATAATCTAAAGAAAATCGACGACAT--------------
        22*2**222*2***222***222222****22*22**2*2*NN1N1NN1
```

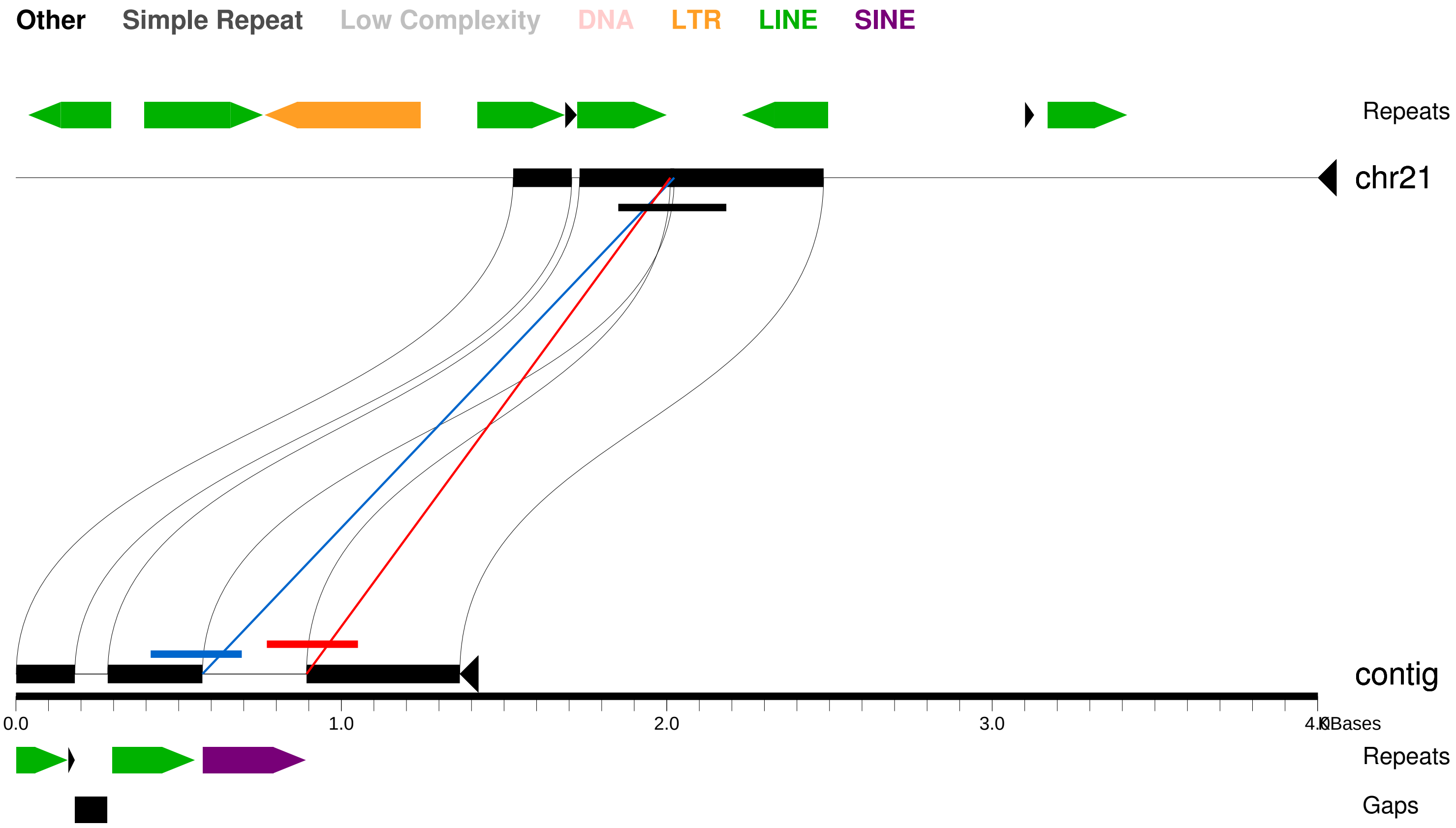

siteID: chr21_28595898 Scafftig3 - Breakpoint: chr21:28595863
chr21:28595826-28595922
Overlap Length: 23 INS size: 425 Genotyped: No

```
left   GAGTTAGATCTCCAAGAACCACAACTGAGATCCTGAA----------------------ATGTTTATTAA
chrom  GAGTTAGATCTCCAAGAACCACAACTGAGATCCTGAA----------------------ATGTTTATTAA
right  NNNNNNNNNNNNNNNNNNNNNNNNNNNNNNNNNNNNNNNNNNNNNNNNNNNNNNNNNNNATGTTTATTAA
       11111111111111111111111111111111111111111111111111111111111***********

left   AACTCTATTTTTGGCCGGGCGCGGTGGCTCACGCCTGTAATCCCAGCACTTTGGGAGGCCGAGGCGGGCG
chrom  AACTCTATTTTTA------------TGATCAGATCTGAA----CACCAATTTTAGCTTCTTAGTA-----
right  AACTCTATTTTTA------------TGATCAGATCTGAA----CACCAATTTTAGCTTCTTAGTA-----
       ************22222222222222*2***222***2*2222**2**2***22*222*22**2222222

left   G
chrom  -
right  -
       2
```

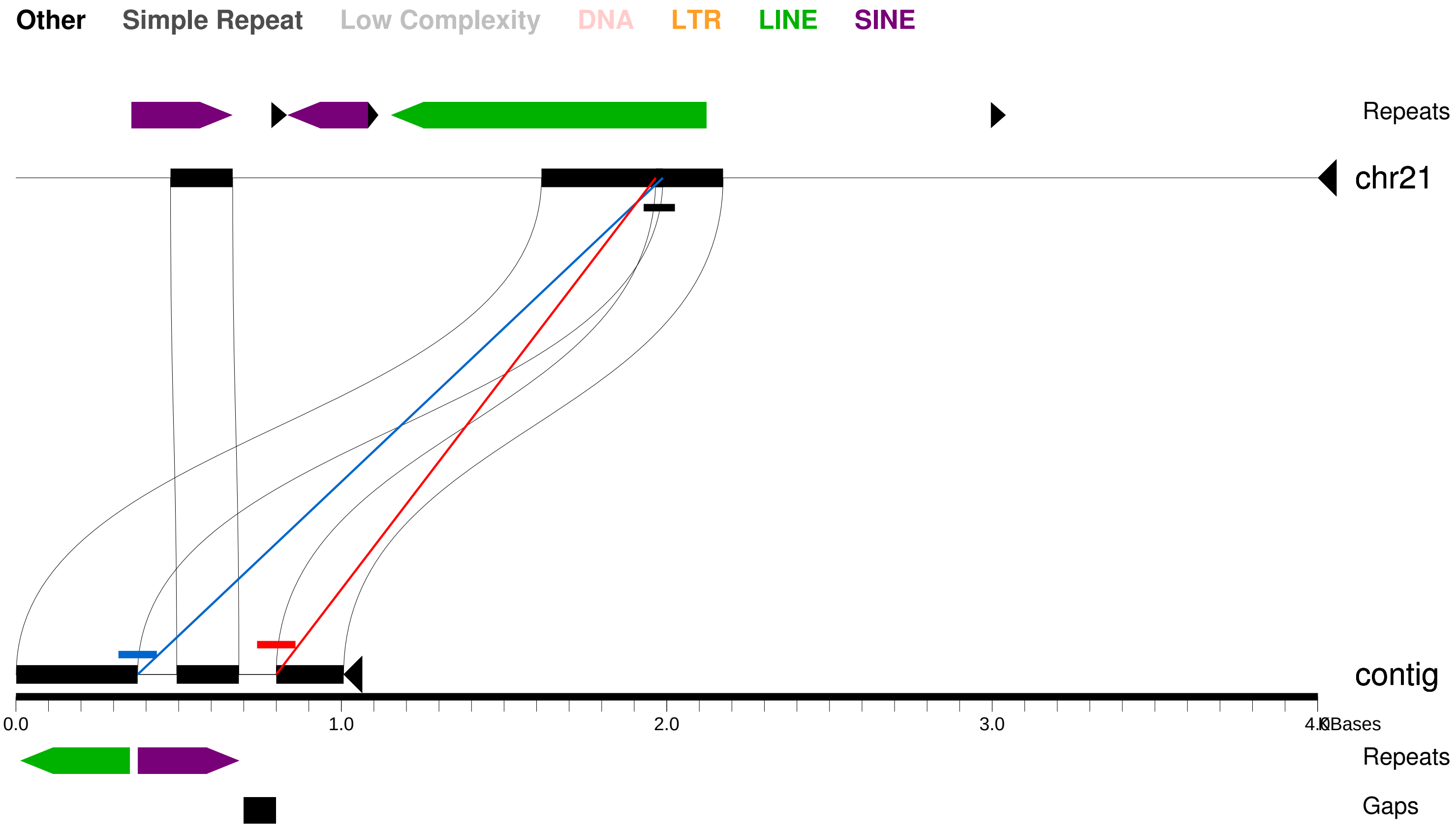

siteID: chr21_29767351 Scafftig1 - Breakpoint: chr21:29767297
chr21:29767277-29767356
Overlap Length: 40 INS size: 284 Genotyped: No

```
left    ------------GGTCTTGTTAA----TGATATTAT-----------------------CCATGGTCTTT
chrom   ------------GGTCTTGTTAA----TGATATTAT-----------------------CCATGGTCTTT
right   TCCGCCCGCCTCGGCCTCCCAAAGTGCTGGGATTACAGGCGTGAGCCACCGCGCCCGGCCCATGGTCTTT
        111111111111**1**1111**1111**11****111111111111111111111111***********

left    TTAATGCTATCAAGCTAACCTCTAATTCTNNNNNNNNNNNNNNNNNNNNNNNNNNNNNNNNNNNNNNNNN
chrom   TTAATGCTATCAAGCTAACCTCTAATTCTCTTGCAGATTTAGAATCGGC---------------------
right   TTAATGCTATCAAGCTAACCTCTAATTCTCTTGCAGATTTAGAATCGGC---------------------
        *****************************22222222222222222222222222222222222222222

left    NNNNNNNNNNNNNNNNNN
chrom   ------------------
right   ------------------
        222222222222222222
```

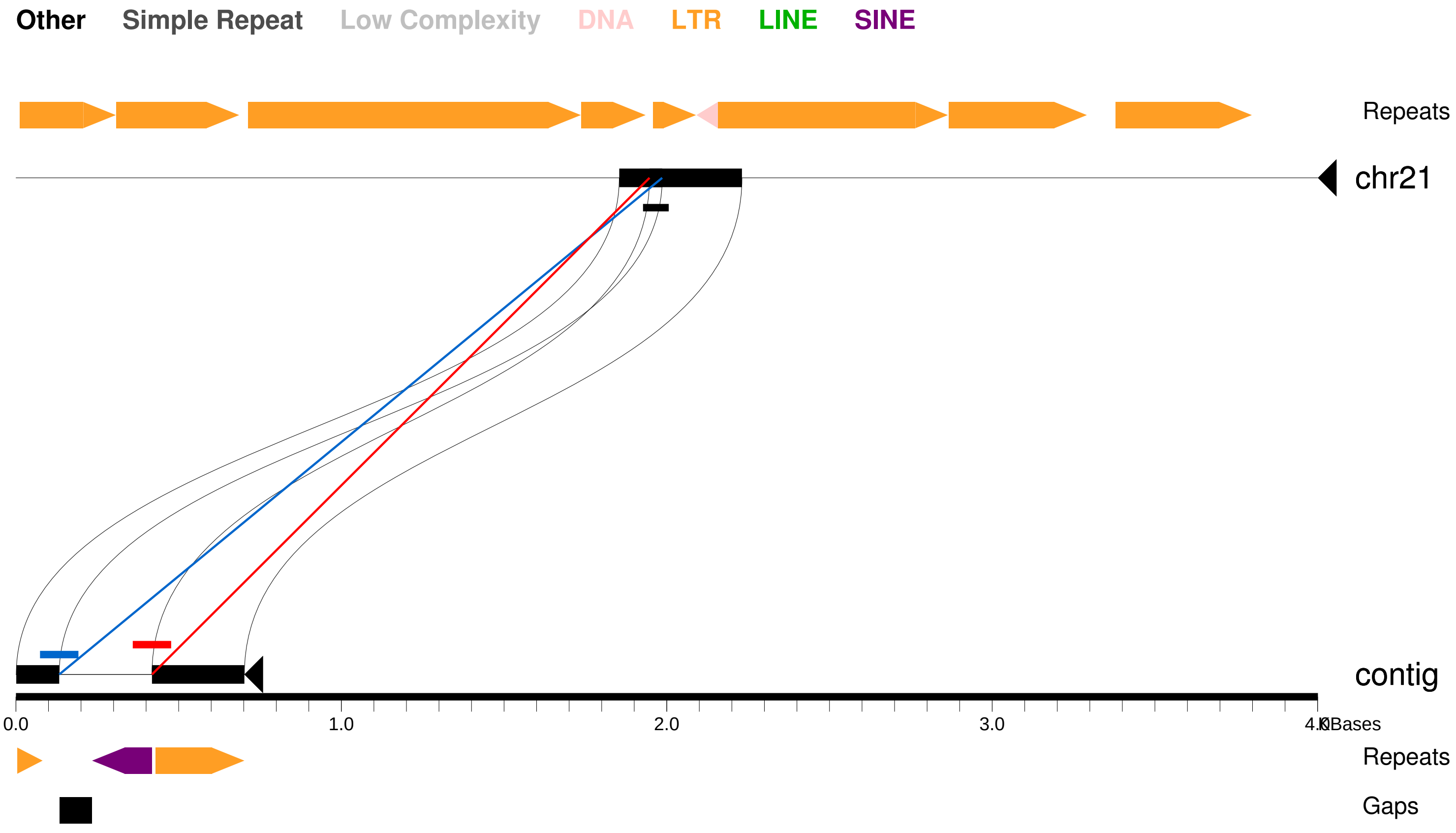

siteID: chr21_31631959 Scafftig1 - Breakpoint: chr21:31631954
chr21:31631909-31632013
Overlap Length: 15 INS size: 262 Genotyped: Yes

```
left     T----------CTC----ACA-AGCGATTTCTTGAATTCTTGGAAAAAGTTTTTCATAGTAAAAAGAAAT
chrom    T----------CTT----ACA-AGCGATTTCTTGAATTCTTGGAAAAAGTTTTTCATAGTAAAAAGAAAT
right    TGCACTCCAGCCTGGGCGACAGAGCGAAA-CTCCGTCTCAAAAAAAAAAAAAAAAAAAAAAAAAAAAAAT
         *1111111111**N1111***1*****111**11111**1111*****1111111*1*11*****1****

left     AATTCTGGGAGGCCGAGATGGGCGGATCACGAGGTCAGGAGATCGAGACCATCCTGGCTAACAC
chrom    AATTC----AAACACAGCTCTGTGTCTACCCTACTTTTCAAAAATTAAC--TCCT--------C
right    AATTC----AAACACAGCTCTGTGTCTACCCTACTTTTCAAAAATTAAC--TCCT--------C
         *****2222*22*22**2*22*2*22*22*2222*2222*2*22222**22****22222222*
```

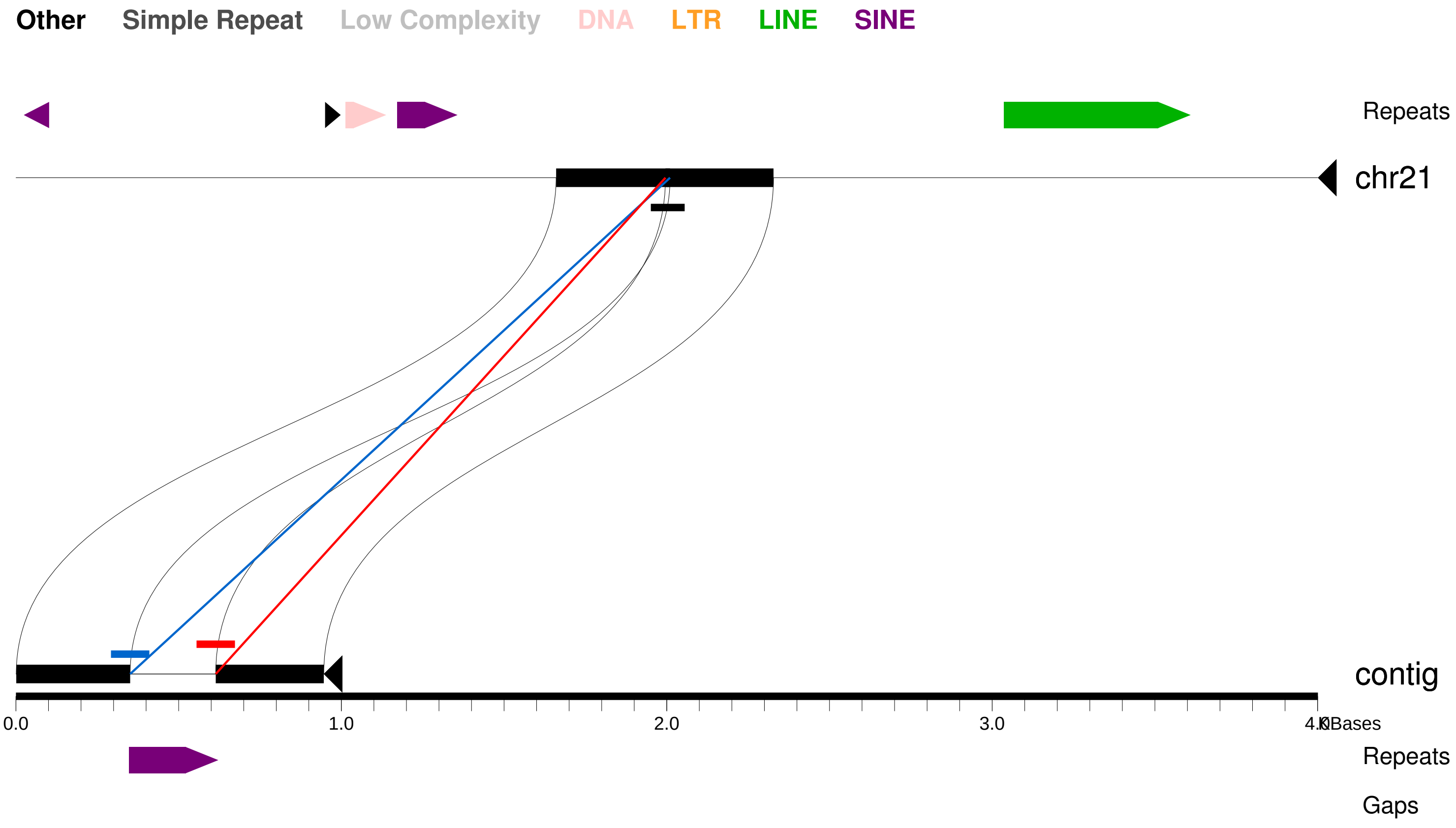

siteID: chr21_32442022 Scafftig1 - Breakpoint: chr21:32441936 chr21:32441693-32441995
Overlap Length: 15 INS size: 279 Genotyped: Yes

```
left   ----------------------------------------------------------------------
chrom  ACCACTCCAAAGTGCTTTACATTCTCAATTCATCCTCACAGGAGTCCTGCAAATGGAGATATTTTTTAGC
right  ----------------------TCTCGATC--TCCTGACC----TCGTG---------------------
                             2222N22N  2222N22N    22N22

left   ----------------------------------------------------------------------
chrom  CTTATCCTACAGATAAGAACAATGAAAATCAAAGAGGCTAAGACATGTTTCTAAAGTTGATCAGCAACTA
right  ---ATCCGCCCGC------------------------CTCGGCC----TCCCAAAGT-------------
          2222NN2N2N                        22NN2N2    2N2N22222

left   -------------CTGGAATTCCAATTTTGTTCTGGTTGACTTTGAAGCTCCTTTTCCATGTTCTGGGTA
chrom  GAAAGTGGCAAGGCTGGAATTCCAATTTTGTTCTGGTTGACTTTGAAGCTCCTTTTCCATGTTCTGGGTA
right  ------------GCTGGGATTACA----------------------------------------------
                   2****1***1**1111111111111111111111111111111111111111111111

left   CCCTGGCTTACTCTCTCCCTTGGGCTGGGGCGGGGGTGGTAGATTTTTTTTTTTTTTTTTTTTTTTTTGA
chrom  CCCTGGCTTACTCTCTCCCTTGGGCTGGGGCGGGATGGTAGATTTTTCTTACCATATTTTG--------
right  ---------------------------------GGATGGTAGATTTTTCTTACCATATTTTG--------
       111111111111111111111111111111111**2***********2**2222*2****222222222

left   GACGGAGTCTCGCTGTCGCCCAGGCTGGAGTGCAGTGGCGCAATCTCGGCTCACTGCAGGCTCCGCCCCC
chrom  --------------------CAAGATG------ACTTGCTCTTTCTCCTATATCTTC-------------
right  --------------------CAAGATG------ACTTGCTCTTTCTCCTATATCTTC-------------
       22222222222222222222**2*2**222222*2*2**2*22****222*22**2*2222222222222

left   TGGG
chrom  ----
right  ----
       2222
```

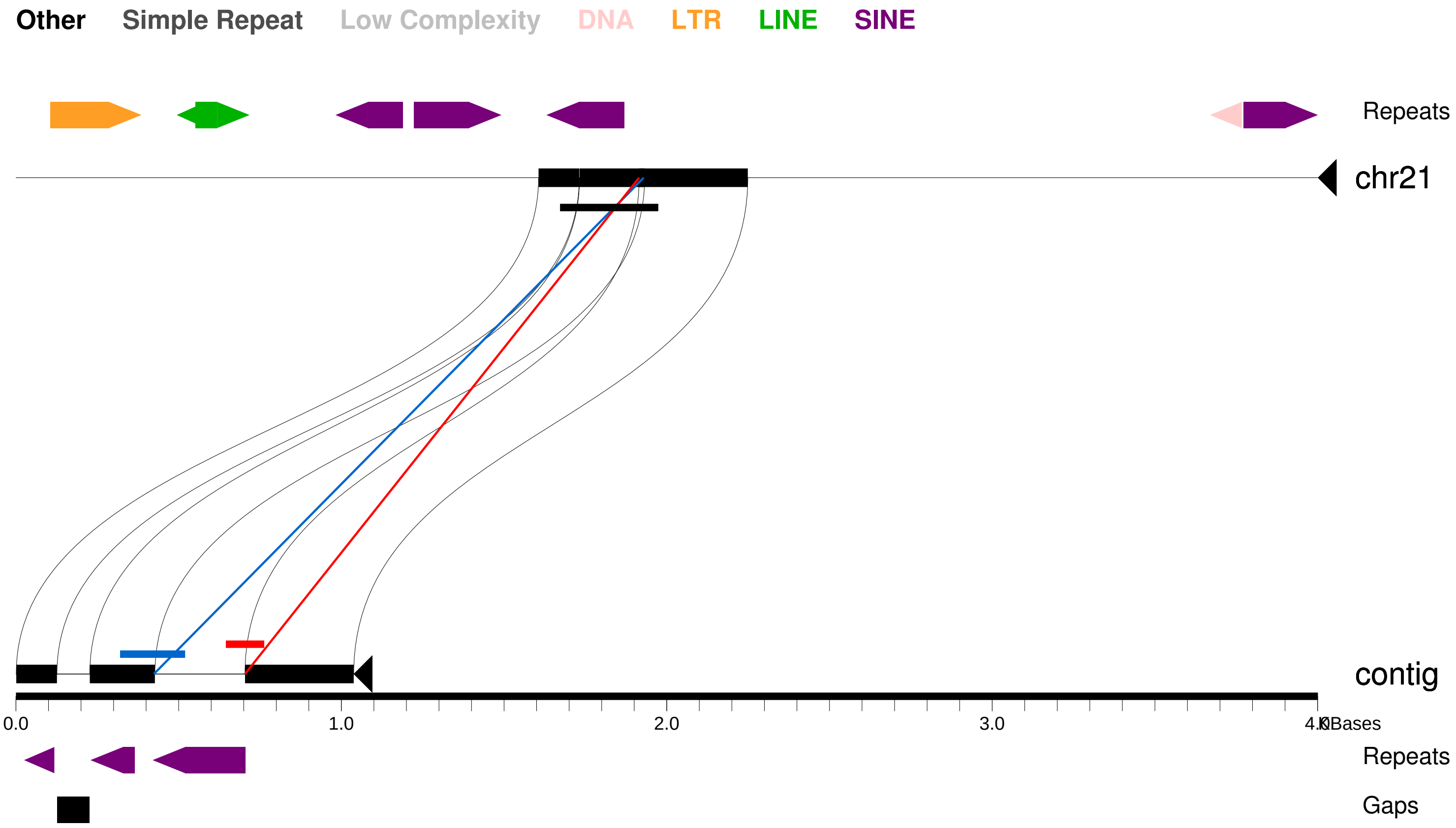

siteID: chr21_33398542 Scafftig1 + Breakpoint: chr21:33398519
chr21:33398472-33398578
Overlap Length: 13 INS size: 324 Genotyped: Yes

```
left    GTCTTTCCTTCCTTTCCATCCTT---TTCTATCTATAATTGCTCTTGCTA-----------CTCCATTCT
chrom   GTCTTTCCTTCCTTTCCATCCTT---TTCTATCTATAATTGCTCTTGCTA-----------CTCCATTCT
right   TCCG--CCCGCCTCGGCCTCCCAAAGTGCTGGGATTACAGGCGTGAGCCACCGCGCCCGGCCTCCATTCT
        11*111**11***111*1***11111*1**11111**111**1111**1*11111111111*********

left    TTTTTTTTTTTTTTTTTTTTTTTTTTTTTTTTTTTTTTTTTTTGAGACGGAGTCTCGCTCTGT
chrom   TTTTAAAAACTCACACATCTCTTACATGTCACTAACTTATTAT----------CT-GTACT-T
right   TTTTAAAAACTCACACATCTCTTACATGTCACTAACTTATTAT----------CT-GTACT-T
        ****222222*222222*2*2**222*2*222*222**2**2*2222222222**2*22**2*
```

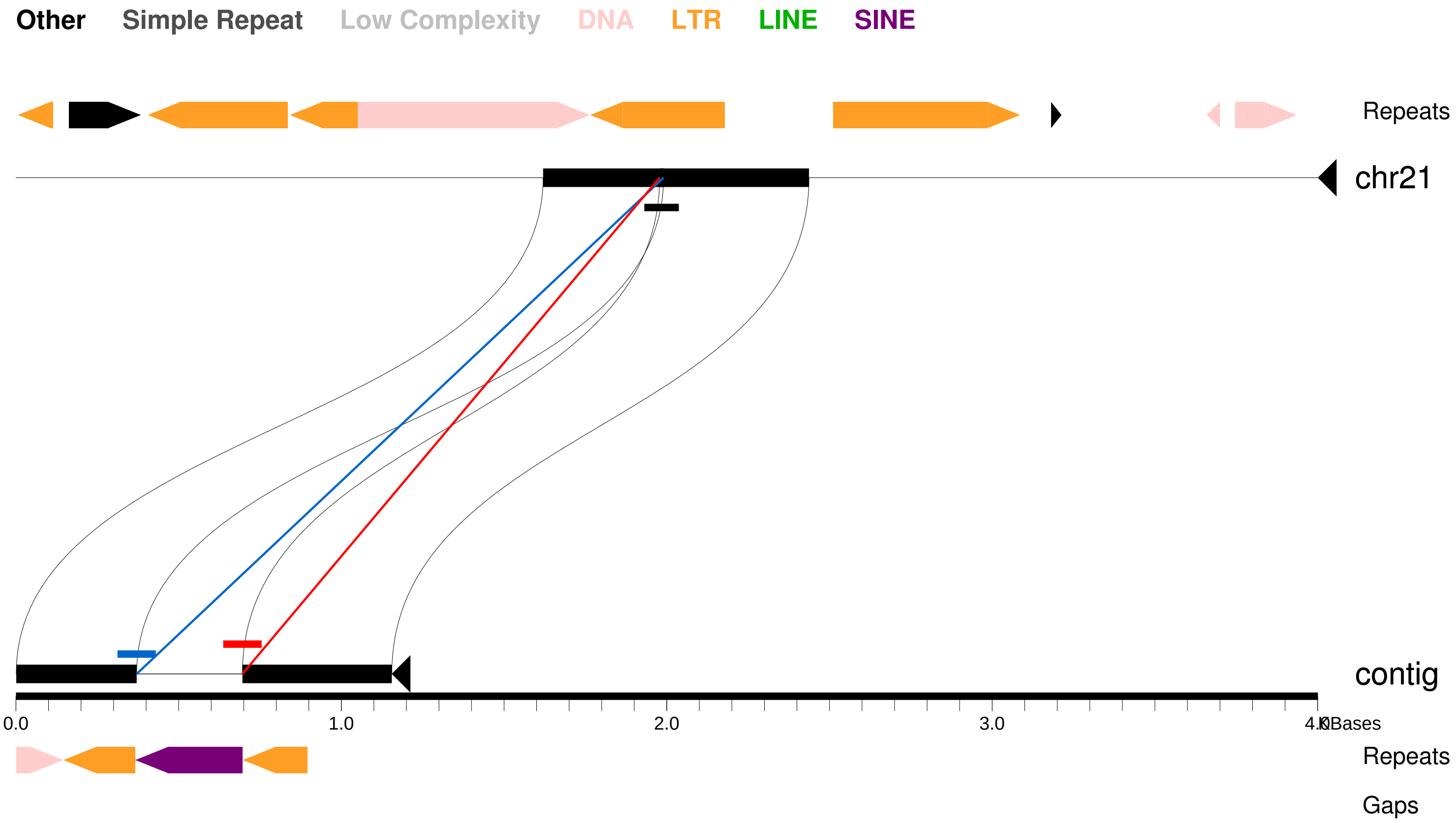

siteID: chr21_37237928 Scafftig2 + Breakpoint: chr21:37237929
chr21:37237885-37237989
Overlap Length: 16 INS size: 301 Genotyped: Yes

```
left     T-GCCCACTT-----TTTGATGGGGTTTT--TTTCAAAAATGATAAAAATGA-------AAAAAATGTTA
chrom    T-GCCCACTT-----TTTGATGGGGTTTT--TTTCAAAAATGATAAAAATGA-------AAAAAATGTTA
right    CCGCCCGCCTCGGCCTCCCAAAGTGCTGGGATTACAGGCGTGAGCCACCGCGCCCGGCCAAAAAATGTTA
         11****1*1*11111*111*11*1*1*1111**1**1111***111*111111111111***********

left     TTTTTTTTTTTTTTTTTTTTTTTTTGAGACGGAGTCTCGCTCTGTCGCCCAGGCTGGAGTGCAG
chrom    TTTTTAAAA----------GATTTTCAAAAAAAGTGAATCCATTACGATTAGG---GA-TACCA
right    TTTTTAAAA----------GATTTTCAAAAAA-GTGAATCCATTACGATTAGG---GA-TACCA
         *****2222222222222222****2*2*2221**2222*22*22**222***222**2*2*22
```

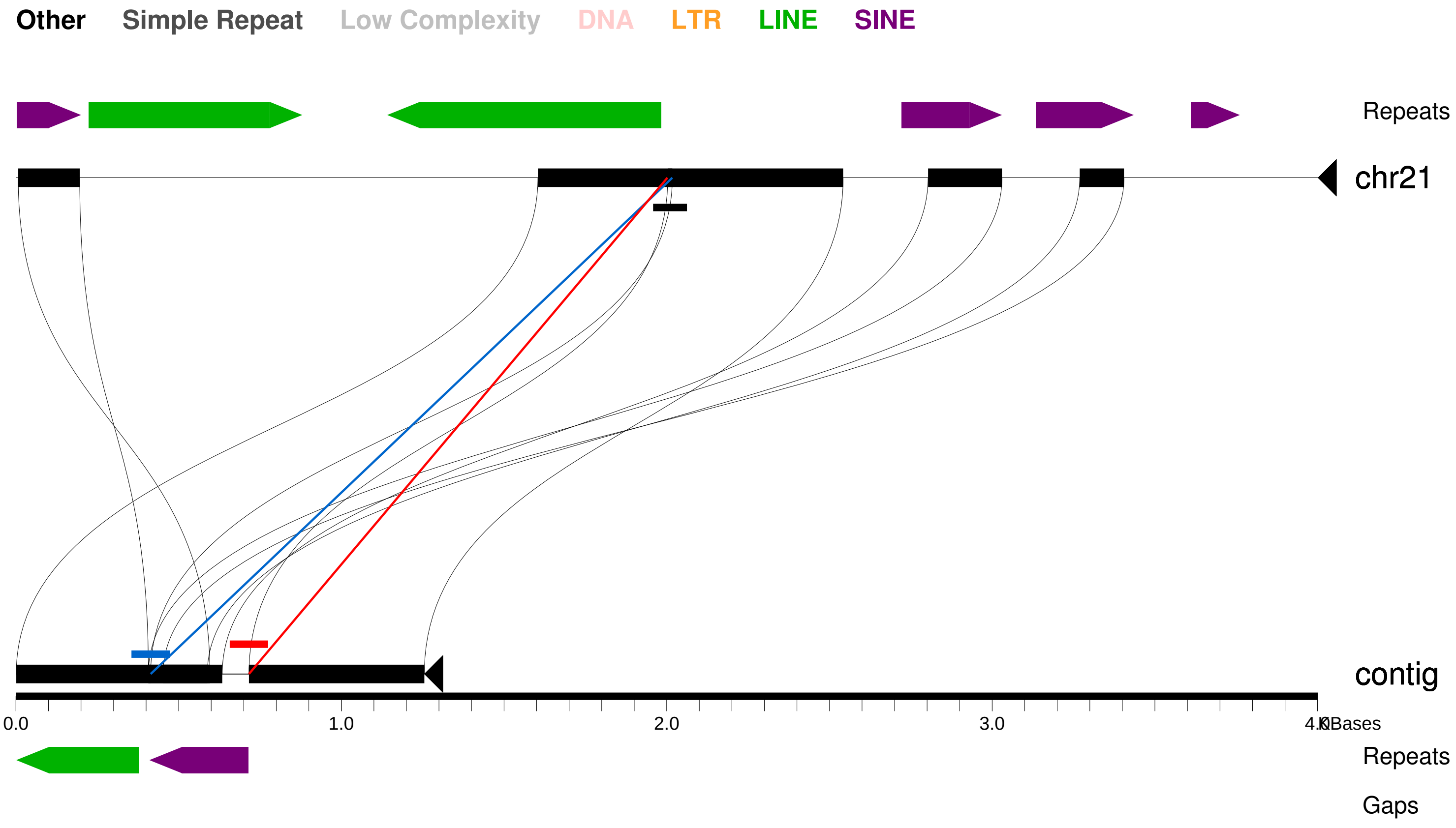

siteID: chr21_39004569 Scafftig3 - Breakpoint: chr21:39004547
chr21:39004501-39004587
Overlap Length: 14 INS size: 309 Genotyped: Yes

```
left   AAGCTT-------TGCA-TGCACAG------------GACGGA---AAAC----------AATAAGAAAA
chrom  GAGCTT-------TGCA-TGCACAG------------GACGGA---AAAC----------AATAAGAAAA
right  -AGATTGCGCCACTGCAGTCCGCAGTCCGGCCTGGGCGACAGAGCGAGACTCCGTCTCAAAAAAAAAAAA
       N**1**1111111****1*1*1***111111111111***1**111*1**1111111111**1**1****

left   GATGAACTTAAAAATTACCTCGTGGCCGGGCGCGGTGGCTCACGCCTGTAATCCCAGCACTTTGGGAGGC
chrom  GATGAACTTAAAAATTACCTCGT----------------TTTGGAATATATGACCA-CACTTCTAAA---
right  AAAAAAAAAAAAAATTACCTCGT----------------TTTGGAATATATGACCA-CACTTCTAAA---
       1*11**111**************2222222222222222*222*22*2**222***2*****2222*222

left   CGAGGCGGGTGG
chrom  ------------
right  ------------
       222222222222
```

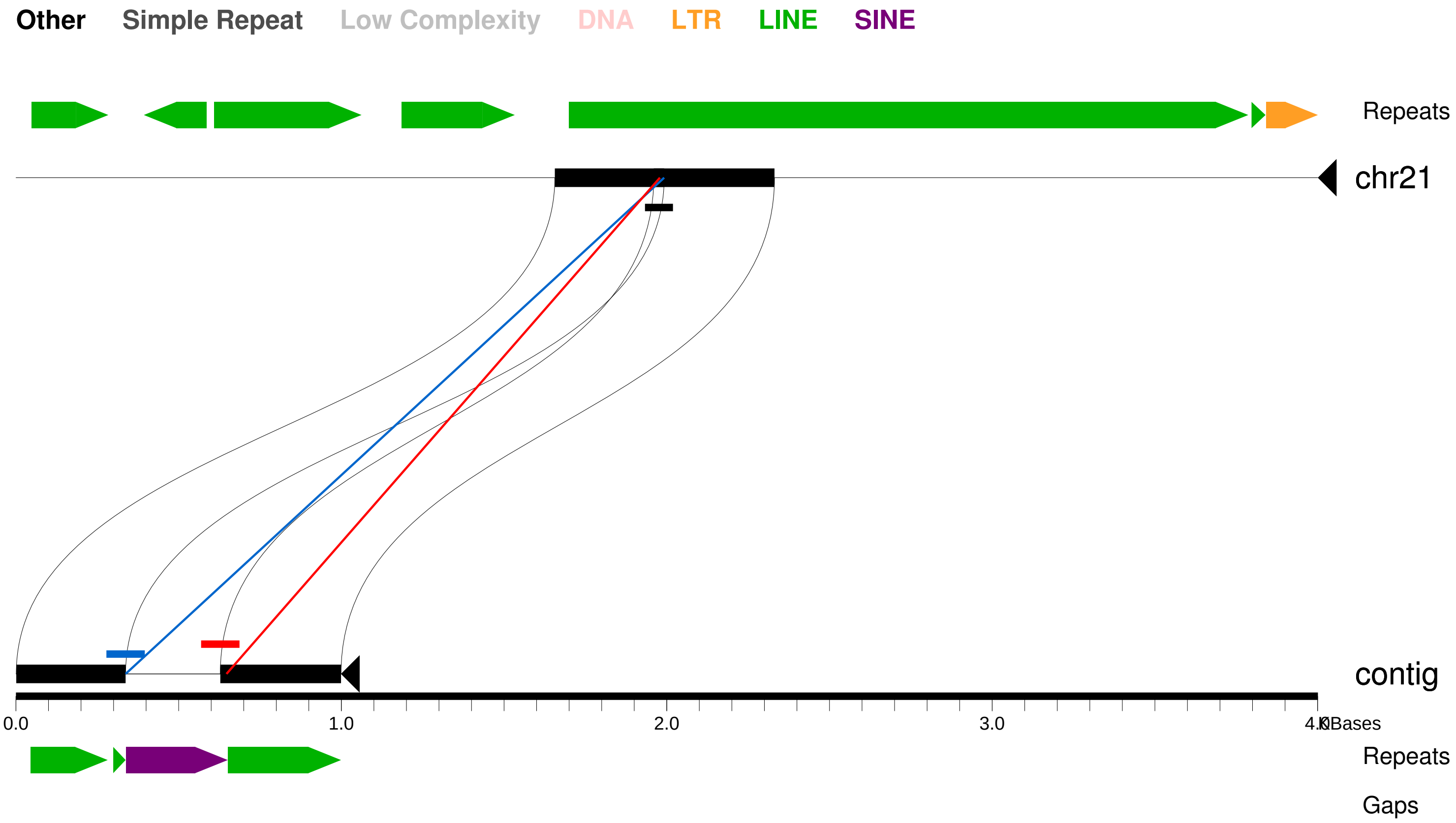

siteID: chr21_39204473 Scafftig1 + Breakpoint: chr21:39204478
chr21:39204432-39204537
Overlap Length: 14 INS size: 434 Genotyped: No

```
left     AAATATGTAATGGC------AGGAACTGAGTTGTCAAGAGTAAT-------GAGTTTAAGTCAAGTTACT
chrom    AAATATGTAATGGC------AGGAACTGAGTTGTCAAGAGTAAT-------GAGTTTAAGTCAAGTTACT
right    CCACCCGCCTCGGCCTCCCAAAGTGCTGGGATTACAGGCGTGAGCCACCGCGCCCGGCCGTCAAGTTACT
         11*111*1111***111111*1*11***1*1*11**1*1**1*11111111*1111111***********

left     TTTTTTTNNNNNNNNNNNNNNNNNNNNNNNNNNNNNNNNNNNNNNNNNNNNNNNNNNNNNNNN
chrom    TTTT-------------GAGATGATGCTATAACTACCTGTGTGAGTGTGAACCTGAGATAAGG
right    TTT-------------GAGATGATGCTATAACTACCTGTGTGAGTGTGAACCTGAGATAAGG
         ***2222222222222222222222222222222222222222222222222222222222222
```

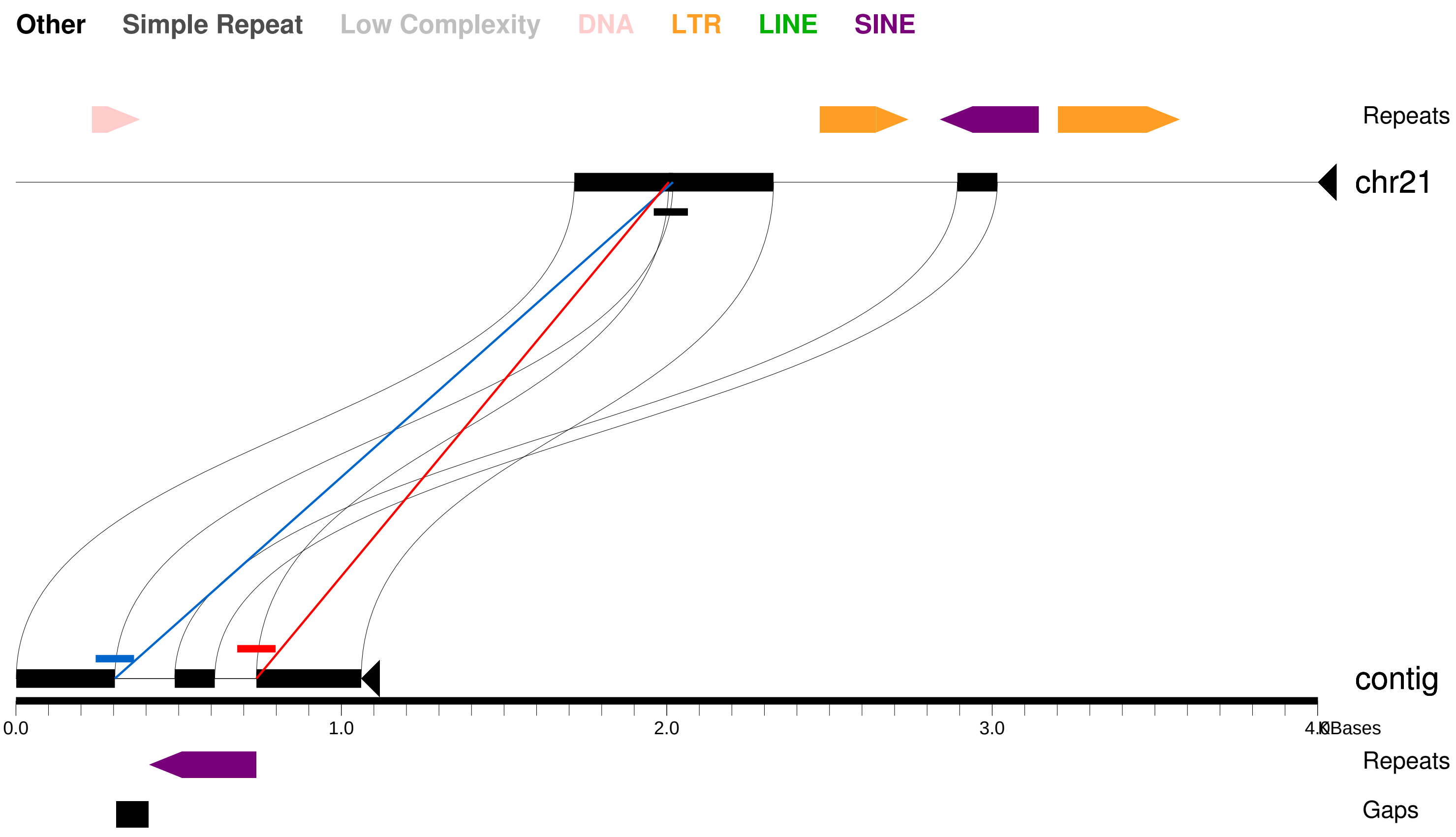

siteID: chr21_41452073 Scafftig1 - Breakpoint: chr21:41452056
chr21:41452006-41452097
Overlap Length: 10 INS size: 321 Genotyped: Yes

```
left   TTTTATGATTCCAATCTCCTCTTCTGGCCGGG--------------------------------AAAAGA
chrom  TTTTATGATTCCAATCTCCTCTTCTGGCCGGG--------------------------------AAAAGA
right  GC-----ACTCCAGCCTGGGCAACAGCGAGGCTCCGTCTCAAAAAAAAAAAAAAAAAAAAAAAAAAAAAA
       1111111*1****11**111*11*1*111**111111111111111111111111111111111****1*

left   AAGGATATTAAGAAGCGATGAGGGCCGGGCGCGGTGGCTCACGCCTGTAATCCCAGCACTTTGGGAGGCC
chrom  AAGGATATTAAGAAGCGATGAGAA-----------GGC-----------ATACCTACCTTTTCCTA----
right  AAAAAAAAAAAAAAGCGATGAGAA-----------GGC-----------ATACCTACCTTTTCCTA----
       **11*1*11**1**********222222222222***22222222222**2**22*22***222*2222

left   GAGGCGGGCGG
chrom  -AGGTCTTTGC
right  -AGGTCTTTGC
       2***22222*2
```

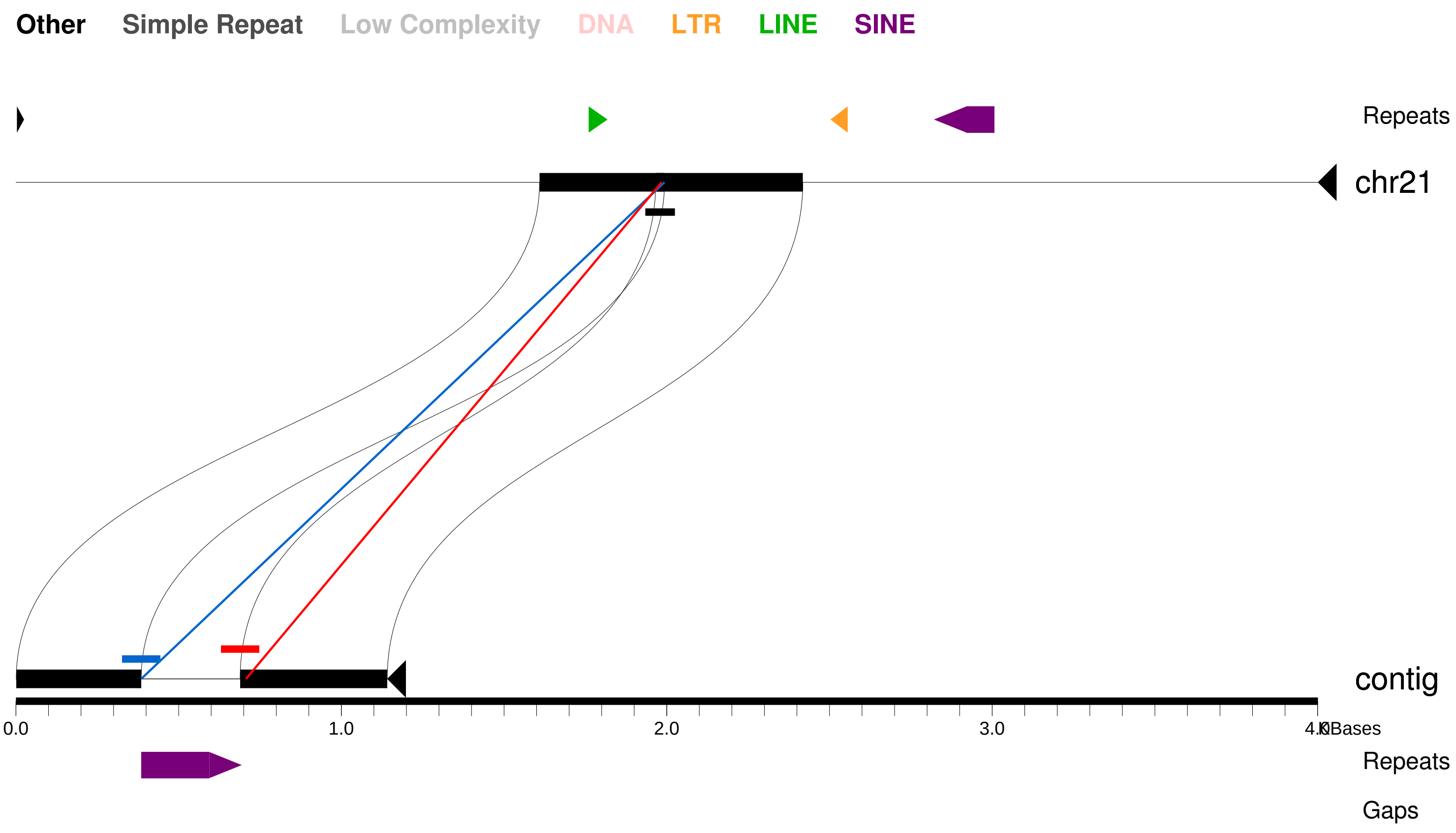

siteID: chr21_42291831 Scafftig4 + Breakpoint: chr21:42291833
chr21:42291788-42291892
Overlap Length: 15 INS size: 276 Genotyped: No

```
left     TCCAGACCCACATGTGGTCATTTCCCCCGT--------------GCTGGATGCATAATTAGAATAGATGC
chrom    TCCAGACCCACATGTGGTCATTTCCCCCAT--------------GCTGGATGCATAATTAGAATAGATGC
right    NNNNNNNNNNNNNNNNNNNNNNNNNNNNNNNNNNNNNNNNNNNNNNNNNAAAAAAAAAAAGAATAGATGC
         1111111111111111111111111111N11111111111111111111*111*1**11***********

left     ACACGGCCGGGCGCGGTGGCTCACGCCTGTAATCCCAGCACTTTGGGAGGCCGAGGCGGGTGG
chrom    ACACT-----------TAGCAGATGGCAG-AATCCCTACA--TTGGTTACCTGACCTGTGGAA
right    ACACT-----------TAGCAGATGGCAG-AATCCCTACA--TTGGTTACCTGACCTGTGGAA
         *****222222222222*2**22*2*2*2*2******22**22****2222*2**222*2*222
```

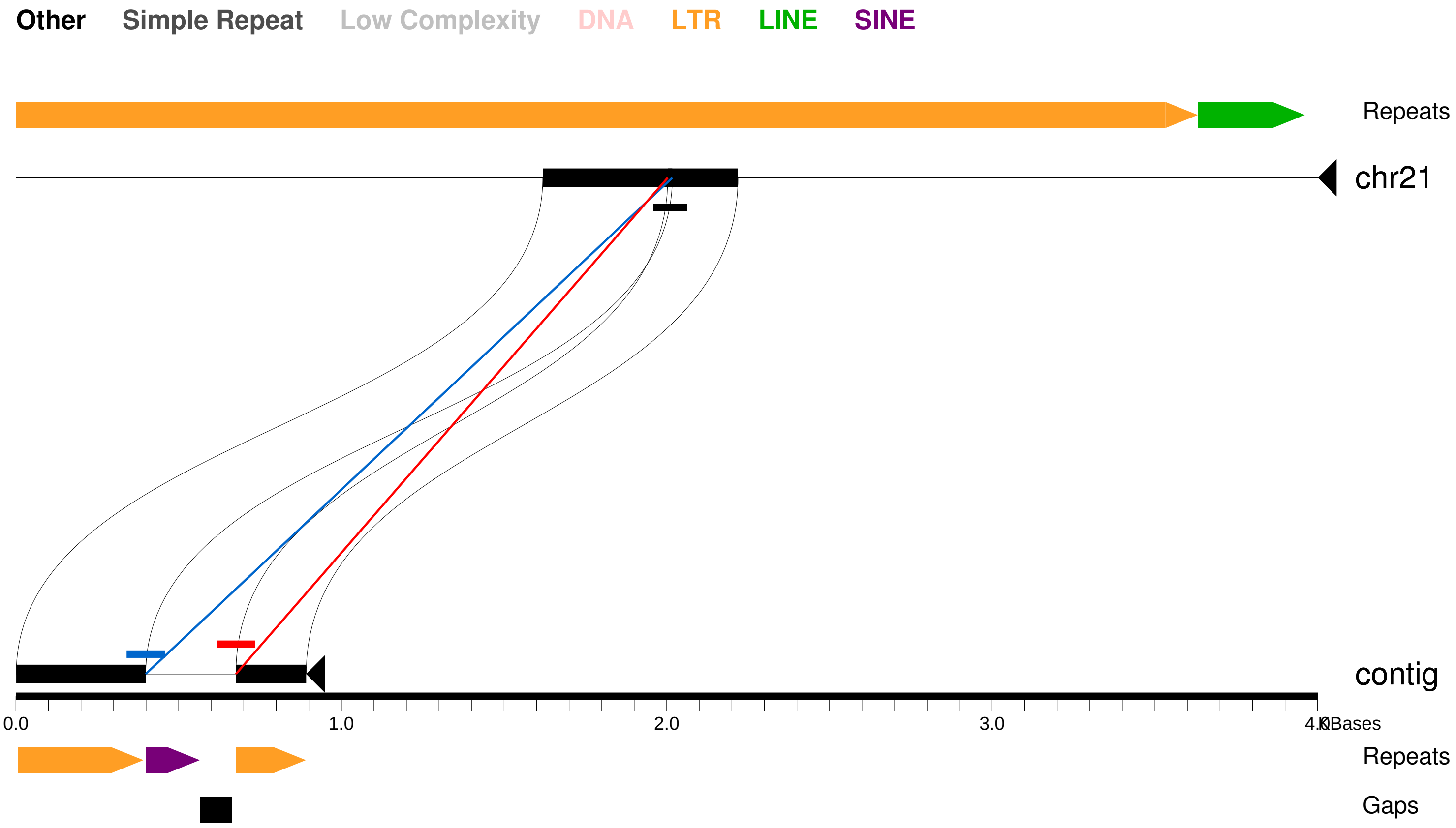

siteID: chr21_42643160 Scafftig1 - Breakpoint: chr21:42643152
chr21:42642992-42643315
Overlap Length: 4 INS size: 411 Genotyped: No

```
left    T-----------------------------CTGTCTAATAATATAGCCACTCTGGATTTCTTTTGATAAG
chrom   TTCTAGTAATTTTTTTGATCTGTATCTATTCTGTCTAATAATATAGCCACTCTGGATTTCTTTTGATAAG
right   TT-TAGTAGA------GACGGGGTTTCACCTTGT---------TAGCCAGGATGGTCTCGATCTCCTGAC
        *2 22222NN      22NNN2NN2NN2NN1***111111111******111***11*111*1*11*1*1

left    CATTAATATGATAGTTTTTTTCCATCTGTTTAGTTTTAACATTTTGAGTCTTAACATTAAAAGTGAGTTT
chrom   CATTAATATGATAGTTTTTTTCCATCTGTTTAGTTTTAACATTTTGAGTCTTAACATTAAAAGTGAGTTT
right   CTC----ATGAT---------CCACCCGCCTCGGCCTCCCAA----AGTGCTGGGATTACAGGCGTGAGC
        *111111*****111111111***1*1*11*1*111*11**11111***11*111****1*1*1*1*111

left    CCTGTAGGGAGAATATACTTGGATNNNNNNNNNNN-----------------------------------
chrom   CCTGTAGGGAGAATATACTTGGATCTTGCTTTTTATCTAATTTAGCAGTCTCTGTTTTTTAGTTGTGATG
right   C----ACCGCGCCCGGCC--GGATCTTGCTTTTTATCTAATTTAGCAGTCTCTGTTTTTTAGTTGTGATG
        *1111*11*1*111111*11****222222222222222222222222222222222222222222222222

left    ----------------------------------------------------------------------
chrom   TTTAGGCCAGAGATCCACAAACAACAGTCTATAGGCTGAATCCAGCATGTGGCTTGTTTCTGTATGGCCT
right   TTTAGGCCAGAGATCCACAAACAACAGTCTATAGGCTGAATCCAGCATGTGGCTTGTTTCTGTATGGCCT
        2222222222222222222222222222222222222222222222222222222222222222222222

left    --------------------------------------------
chrom   GTGAGGTAAGAATAGTTTTTACATTTTTAAAGGGTTGAAAAAAT
right   GTGAGGTAAGAATAGTTTTTACATTTTTAAAGGGTTGAAA----
        2222222222222222222222222222222222222222
```

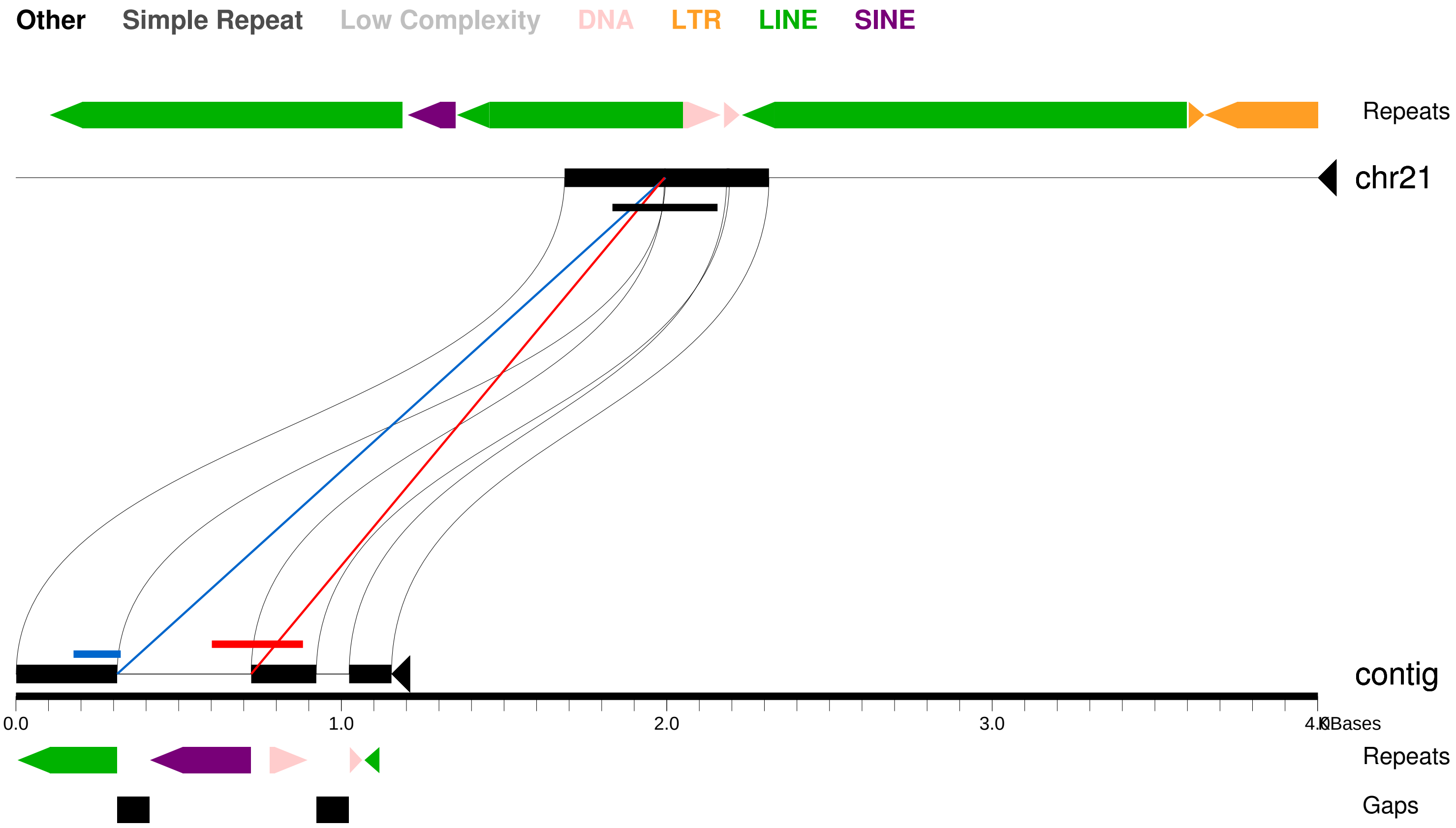

siteID: chr21_47042382 Scafftig5 - Breakpoint: chr21:47042368
chr21:47042314-47042420
Overlap Length: 6 INS size: 322 Genotyped: Yes

```
left    ATGTA--ACAAACCTGCACATTGTGC---ACATGTACTCCGGAACTTAACTTAAAAAAA-------AAAG
chrom   ATGTA--ACAAACCTGCACATTGTGC---ACATGTACTCCGGAACTTAACTTAAAAAAA-------AAAG
right   CTGGGCGACAGAGCGAGACTCCGTCTCAAAAAAAAAATAAAAAAATAAAAAAAAAAAAAAAAAAAAAAAG
        1**1111***1*1*111**111**11111*1*111*1*1111**1*1**111*******1111111****

left    CCGCGGCCGGGCGCGGTGGCTCACGCCTGTAATCCCAGCACTTTGGGAGGCCGAGGCGGGC
chrom   CCACTTATCCACCTGGAGGGGGA------TAGTTACTGAAA----GCAGAGTGAGACGT--
right   CCACTTATCCACCTGGAGGGGGA------TAGTTACTGAAA----GCAGAGTGAGACGT--
        **2*2222222*22**2**222*222222**2*22*2*2*22222*2**222***2**222
```

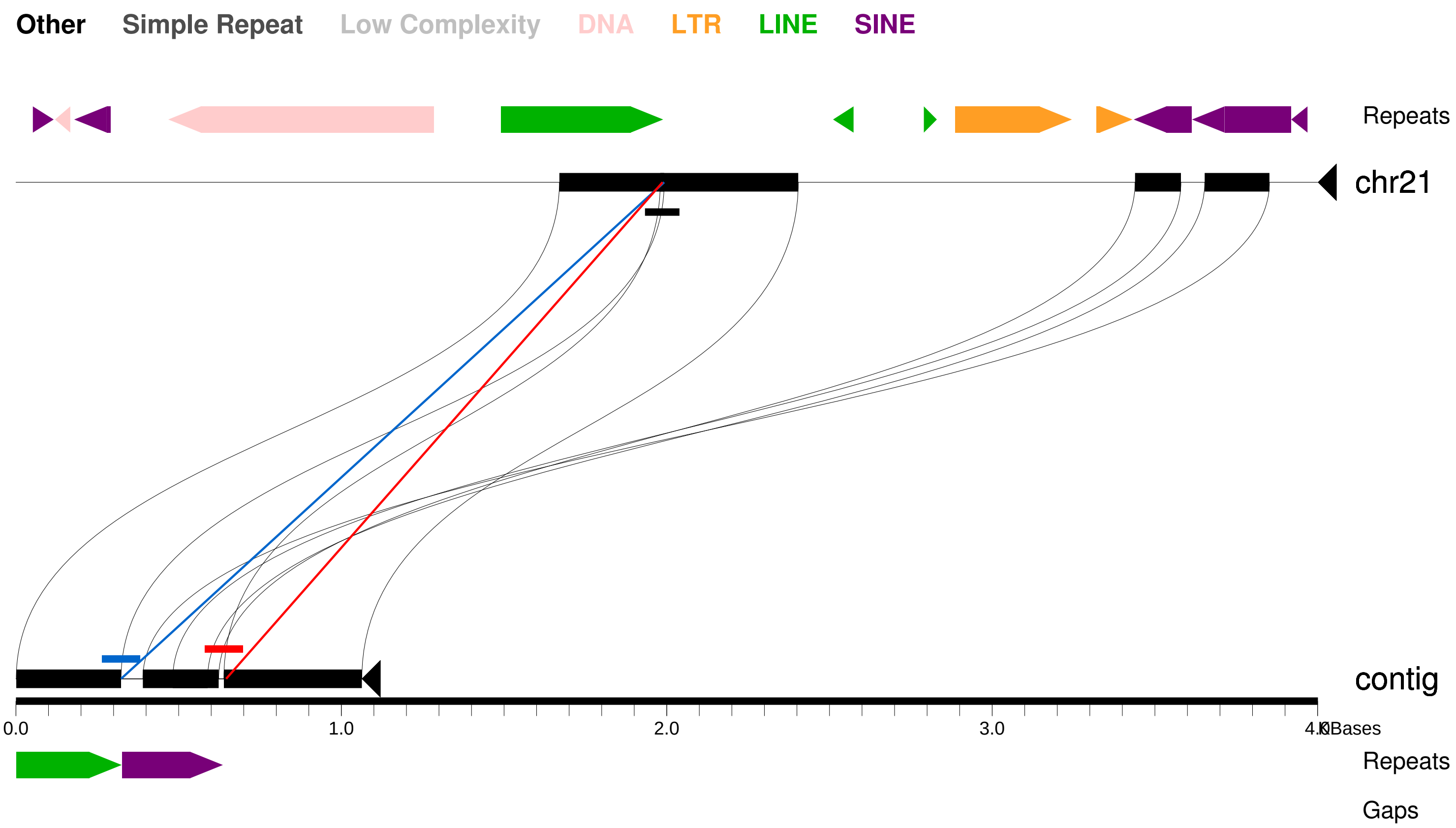

siteID: chr22_17705310 Scafftig1 - Breakpoint: chr22:17705292
chr22:17705133-17705468
Overlap Length: 17 INS size: 314 Genotyped: Yes

```
left   -------------------TGAACACATGAATTAACCTTAGTTCCTCTCCAAAACCTGCACTAAAATGAC
chrom  ------AGAGATTGCAGGCTGAACACATGAATTAACCTTAGTTCCTCTCCAAAACCTGCACTAAAATGAC
right  TTTAGTAGAGACGG--GGTTTCAC-CTTG--TTAGCCAG-GATGGTCTCGATCTCCTGACCTCATGATCC
       11111122222NN2  22N*11**1*1**11***1**111*1*11****1*111****11**1*11111*

left   AGTGGGGAAAAAAATTAAGCATATATTCACAAGGTTGAAGAAAGCAAGAAAGGAGAAAATAGCAAAAAAA
chrom  AGTGGGGAAAAAAATTAAGCATATATTCACAAGGTTGAAGAAAGCAAGAAAGGAGAAAATAGCAAAAAAA
right  ACCCGCC--------TCGGCCT-----CCCAAAGT----GCTGGGATTACAGGCGTGA------------
       *111*1111111111*11**1*11111*1***1**1111*111*1*11*1***1*11*111111111111

left   AACTTGGAAGATGGAAGGTACATAAACAAGTGCTAATAATTTTTTTTTTTTTTTTTTTTTTTTTTGAGA
chrom  AACTTGGAAGATGGAAGGTACATAAACAAGTGCTAATAATTTAGAAGACAAAAAAACAAACAAACAAACA
right  -----GCCACCGCGCCCGGCCA---ACAAGTGCTAATAATTTAGAAGACAAAAAAACAAACAAACAAACA
       11111*11*1111*111*11**111*****************222222222222222222222222*2*

left   -------CGGAGTCTCGCTCTGTCGCC--CAG-GCCGGA-----CTGCGGA---CTGC---AGTGGCGC-
chrom  AAAAAAACAAACACTGAATCTGAAACCAGCAGTGACGAAAGAAACCTCGGATACCTGTGAAAGTGAGGCT
right  AAAAAAACAAACACTGAATCTGAAACCAGCAGTGACGAAAGAAACCTCGGATACCTGTGAAAGTGAGGCT
       2222222*22*22**222****222**22***2*2**2*22222*22****222***2222****22**2

left   ------------AATCTCGGCTCACTGCAAGCTCCGCTTC----CCGGGTTCACG----CCA
chrom  GAAGGTAAGTCCAAAGCAGGAGAAATGGAGGAATGGCTGGAAAGCCTTTTTAACAAAAACCT
right  GAAGGTAAGTCCAAAGCAGGAGAAATGGAGGAATGGCTGGAAAG------------------
       222222222222**2222**222*2**2*2*2222***22222211NNN11N11N    11N
```

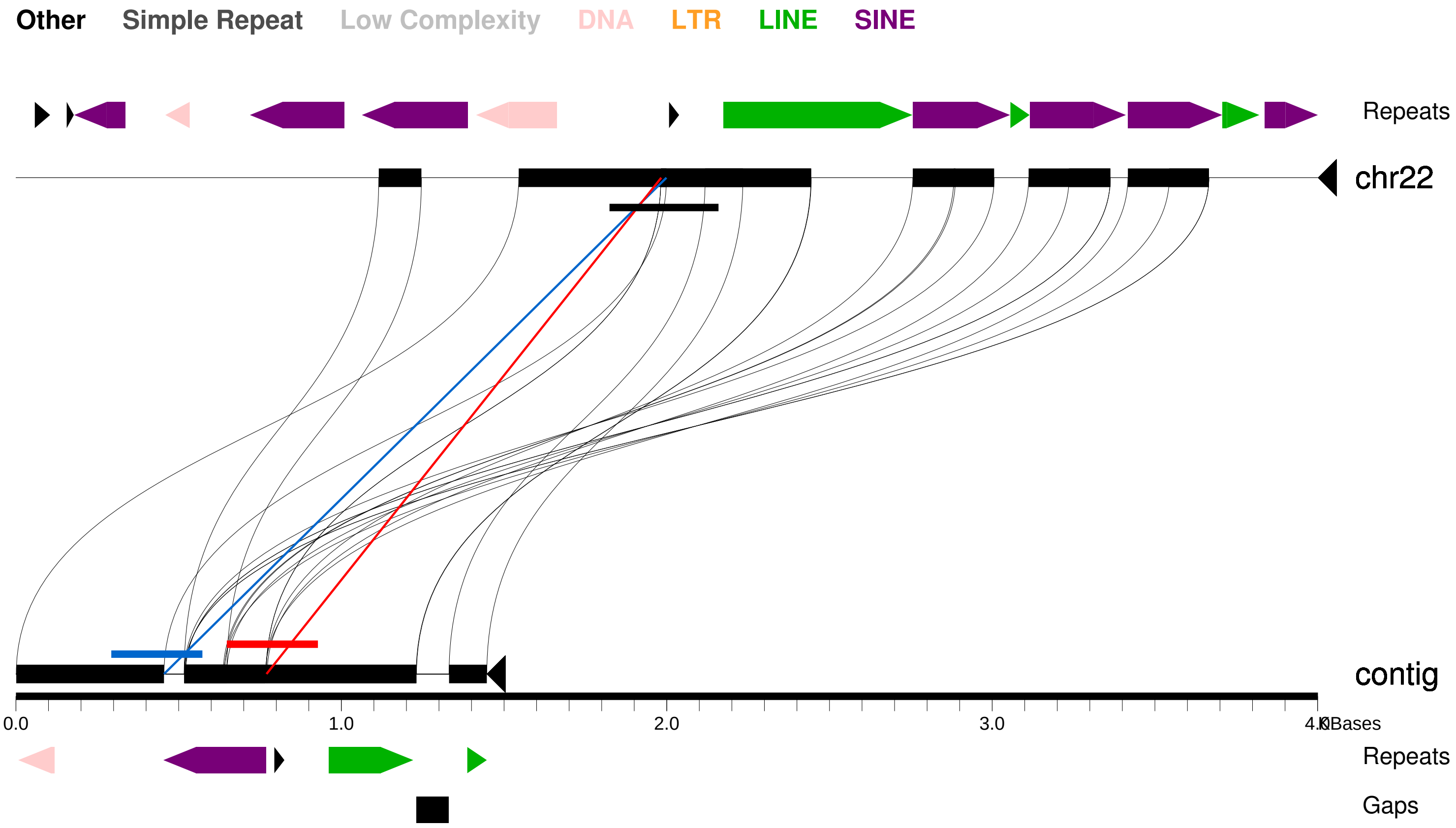

siteID: chr22_22534127 Scafftig19 - Breakpoint: chr22:22534131
chr22:22534083-22534182
Overlap Length: 6 INS size: 310 Genotyped: Yes

```
left     CATAT---------GATAACA----CTGACTCTTTCCCTGCTGAACCACATGTAATAAAA------CCAG
chrom    CATAT---------GATAACA----CTGACTCTTTCCCTGCTGAACCACATGTAATAAAA------CCAG
right    TGCACTCCAGCCTGGGCGACAGGGCGAGACTCCGTCTCAAAAGAAAAAAAAAAAAAAAAAAAAAAAACAG
         111*111111111*111***111111*****11**1*1111***11*1*111**1****1111111***

left     AGGGGCCGGGCGCGGTGGCTCACGCCTGTAATCCCAGCACTTTGGGAGGCCGAGGCGGGCGGATCACG--
chrom    AGGAGCTGG------------------TTTAAT---AAAACCTGAGGAG-CTGACTTTTCTTTTAAAAGAA
right    AGGAGCTGG------------------TTTAAT---AAAACCTGAGGAG-CTGACTTTTCTTTTAAAAGAA
         ***2**2**2222222222222222*2****222*22**2*22****2*2**22222222222*2*22
```

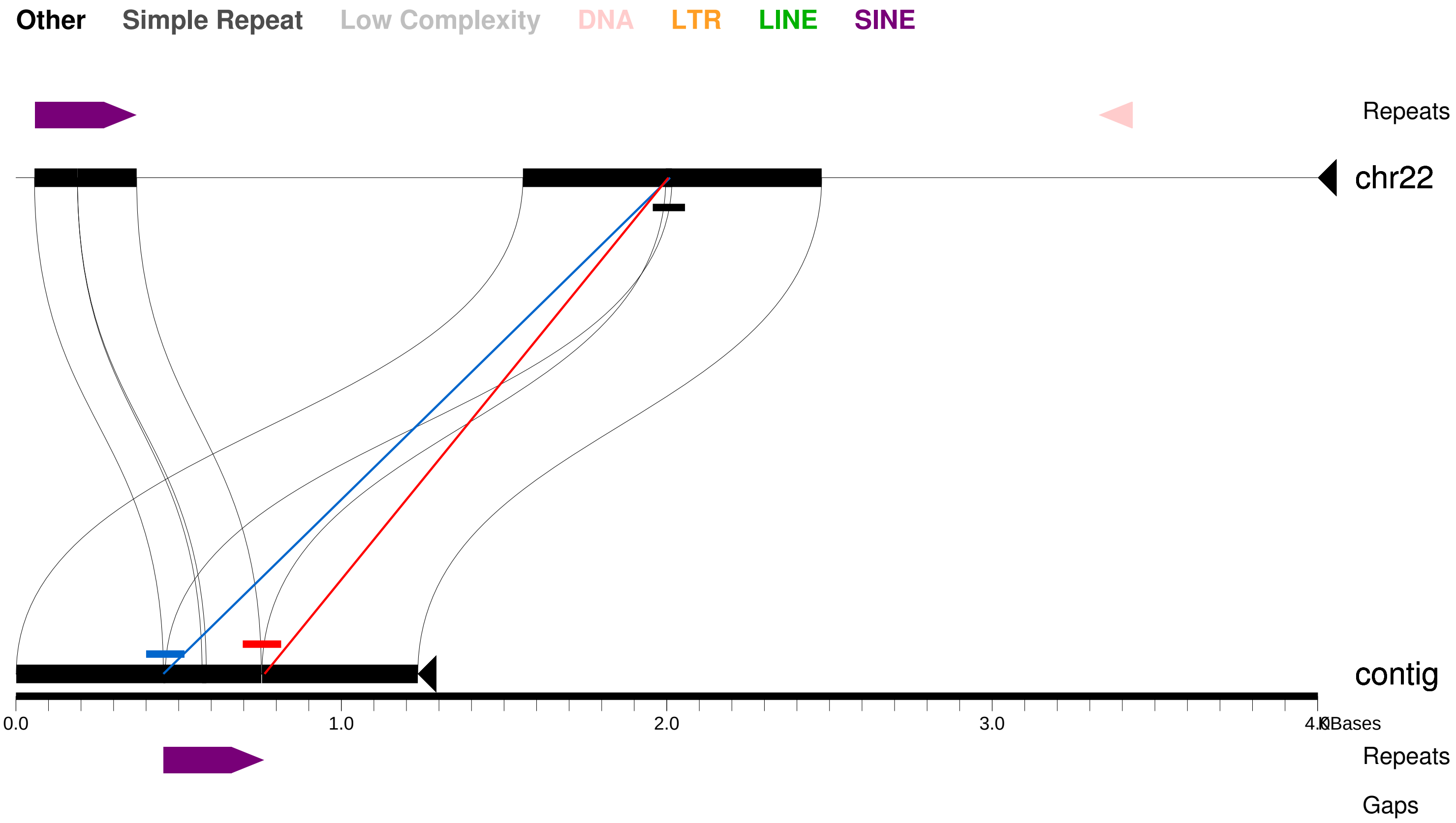

siteID: chr22_26997607 Scafftig2 - Breakpoint: chr22:26997608
chr22:26997448-26997769
Overlap Length: 2 INS size: 316 Genotyped: Yes

```
left   ------ACTCATTCTGTTCATTCTCCCAGA-AGCTCCTGATTTCCCTGAATATTCCCTGCTCCTGGATGC
chrom  CTCAGAACTCATTCTGTTCATTCTCCCAGA-AGCTTCTGATTTCCCTGAATATTCCCTGCTCCTGGATGC
right  TTTAGTAGAGACGGGGTT---TCACCGTTTTAGC--CGGGATGGTCTCGATCTCCTGACCTCGTG-ATCC
       N2N22N*111*1111***111**1**11111***1N*1*11*111**11**1*1*1111***1**1**1*

left   ATCCAGCCTCATCTATCCACATCAGCGCCGGCCCTTTTAGTTGATTACTCCTTCAACCCCAACTAGGCTT
chrom  ATCCAGCCTCATCTATCCACATCAGCGCCGGCCCTTTTAGTTGATTACTCCTTCAACCCCAACTAGGCTT
right  GCCC-GCCTCGGCCTCCCAAA---GTGCTGG-----------GATTAC-----------------AGGCGT
       11**1*****11*111***1*111*1**1**11111111111******1111111111111111****1*

left   AGCAGCTCCTATTTTCTCCATCTCACATTTTTTTTTTTTTTTTTTTTTTTTTTTTTT--TGAGA-----CG
chrom  AGCAGCTCCTATTTTCTCCATCTAACAGCTTTTTAGAGCTGATTTGTTTACACTGACCTGGAATTCCTCA
right  G--AGCCACCGCGCCCGGCCCCTAACAGCTTTTTAGAGCTGATTTGTTTACACTGACCTGGAATTCCTCA
       111***11*111111*11*11**2***22*****22222*22***2***2222*2222**22*22222*2

left   GAGTCTCGCTCTGTCGCCCAGGC---TGGAGTGCAGTGGCGGGATCTCGGCT-----CACTGCAAG-CT-
chrom  AAGTGTGACTCTATTCCCCAGAGGAATAGAGCTCTGTTGGCTGATCCCAGGAACCAGCACTGGGAGACTG
right  AAGTGTGACTCTATTCCCCAGAGGAATAGAGCTCTGTTGGCTGATCCCAGGAACCAGCACTGGGAGACTG
       2***2*22****2*22*****22222*2***22*2**2*222****2*2*2222222*****22**2**2

left   ---------------CCGCCTCCCGGGTTC--ACGCCATT-CT
chrom  TGGAAGGGGCCATGGCCTTCTCTAGGAATCTGATGTTATAACC
right  TGGAAGGGGCCATGGCCTTCTCTAGGAATCTGATGTTATA---
       222222222222222**22***22**22**22*2*22**2 1N
```

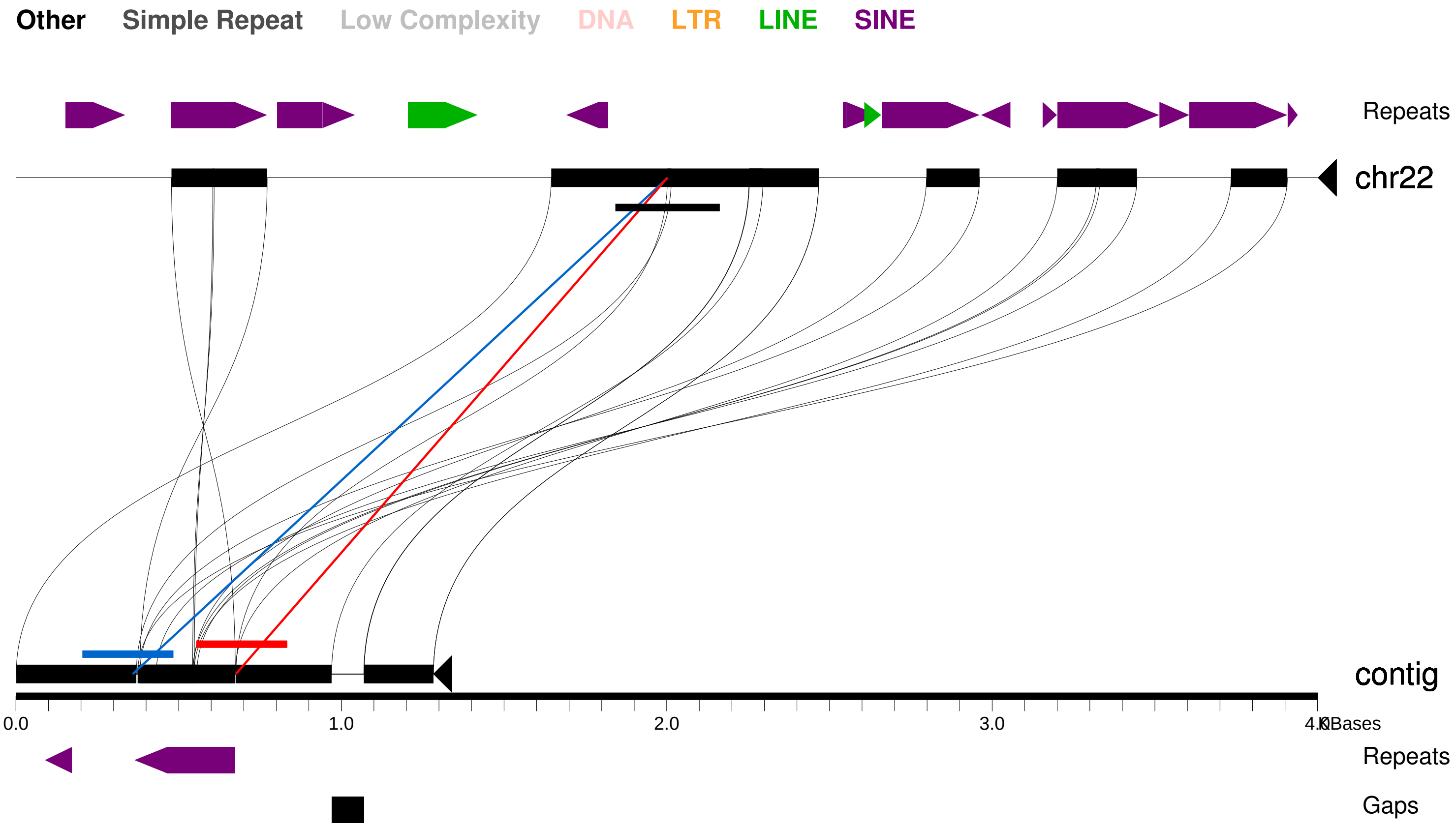

siteID: chr22_34430606 Scafftig9 + Breakpoint: chr22:34430599
chr22:34430553-34430658
Overlap Length: 14 INS size: 314 Genotyped: Yes

```
left     TGGATCTTCC----AATCACTAGTTCCTTGG-TTTCA---TTTAG-----GTCTCAGTTCAAATGGAACT
chrom    TGGATCTTCC----AATCACTAGTTCCTTGG-TTTCA---TTTAG-----GTCTCAGTTCAAATGGAACT
right    TCCACCCGCCTCGGCCTCCCAAAGTGCTGGGATTACAGGCGTGAGCCACCGCGCCCGGCCAAATGGAACT
         *11*1*11**111111**1*1*11*1**1**1**1**1111*1**11111*111*1*11***********

left     TTTTTTTTTTTTTTTTTTTTTTTTTGAGACGGAGTCTCGCTCTGTTGCCCAGGCCGGACT----GC
chrom    TTTACACAGA----------------AGCCTTCCTCAC-CACTCAAAACATGCCTGGAGTAGAGGC
right    TTTACACAGA----------------AGCCTTCCTCAC-CACTCAAAACATGCCTGGAGTAGAGGC
         ***22222222222222222222222**2*2222**2*2*2**22222*22*2*2***2*2222**
```

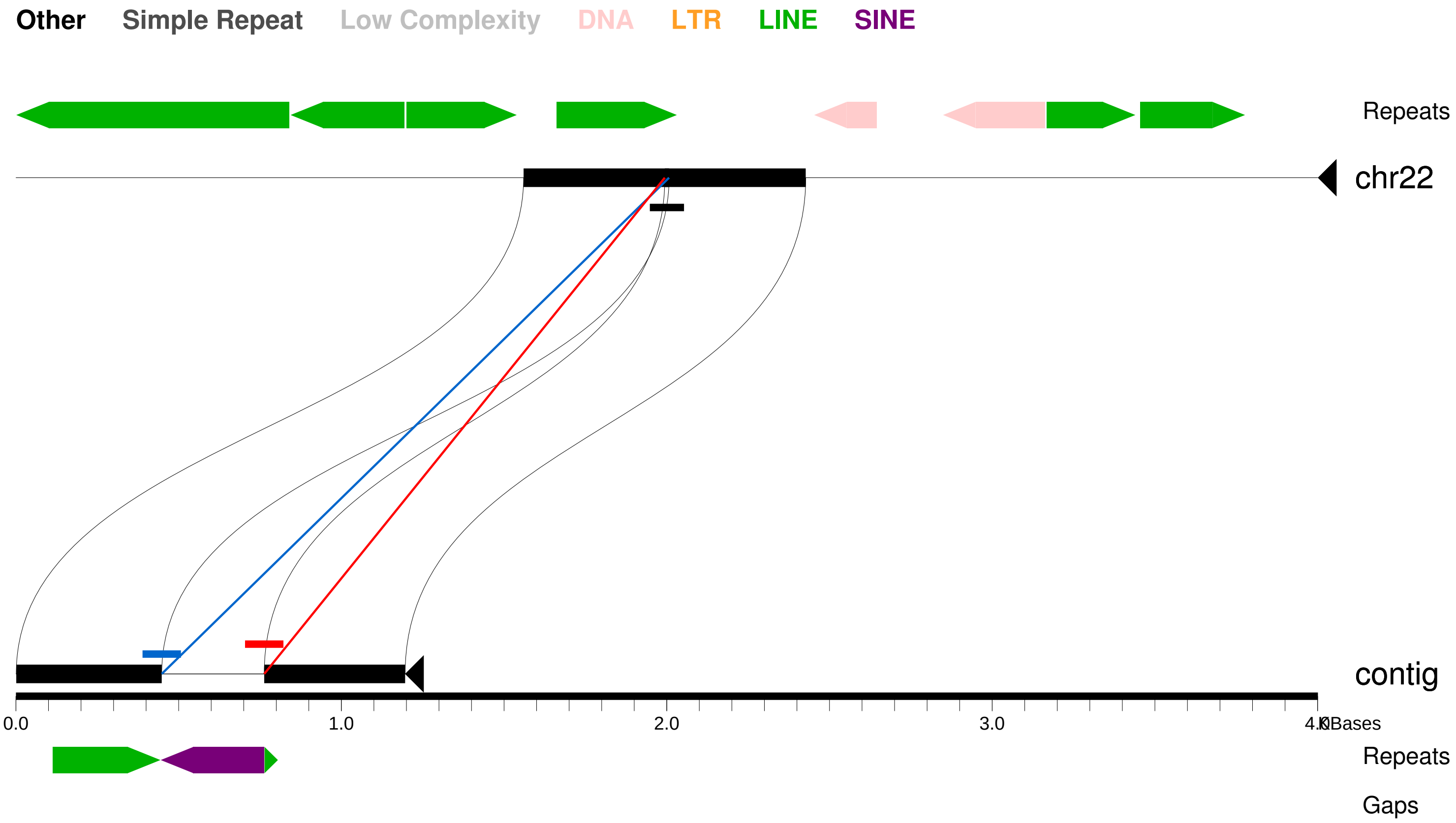

siteID: chr3_100177251 Scafftig4 - Breakpoint: chr3:100177252
chr3:100177207-100177311
Overlap Length: 15 INS size: 296 Genotyped: Yes

```
left   AGG---TCCCATACA-----------AGTCCAAAATTCAGTGGGGCAGTCATTAAATCTAAAAACCCCAA
chrom  AGG---TCCCATACA-----------AGTCCAAAATTCAGTGGGGCAGTCATTAAATCTAAAAACCCCAA
right  CGCCACTGCACTCCAGCCTGGGCGACAGAGCGAGACTCCGTCTCAAAAAAAAAAAAAAAAAAAACCCCAA
       1*1111*1*11*1**11111111111**11*1*1*1**1**11111*111*11***111***********

left   AACAGGCCGGGCGCGGTGGCTCACGCCTGTAATCCCAGCACTTTGGGAGGCCGAGGCGGGCGG
chrom  AACAATCTC----CTTTGACTC---CATGTCTT------ACATTCAG-GGCACACTGATGCAA
right  AACAATCTC----CTTTGACTC---CATGTCTT------ACATTCAG-GGCACACTGATGCAA
       ****22*222222*22**2***222*2***22*222222**2**22*2***22*22222**22
```

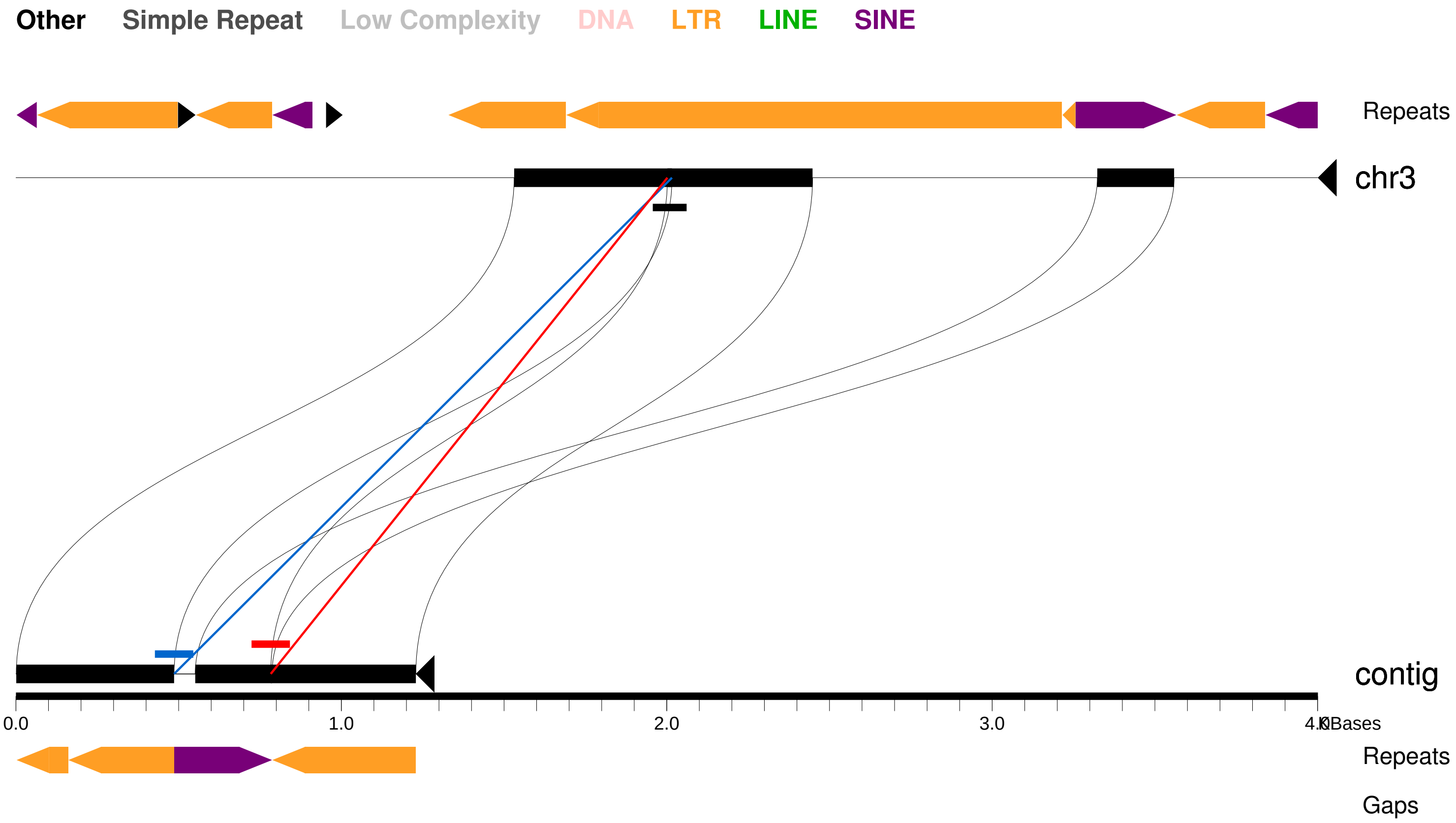

siteID: chr3_100410542 Scafftig6 - Breakpoint: chr3:100410543
chr3:100410501-100410602
Overlap Length: 18 INS size: 455 Genotyped: No

```
left     T------TATCAAAATGACAT--TGATACAATCTACATAGTTTATTTATT----------GATGGGCTTT
chrom    T------TATCAAAATGACAT--TGATACAATCTACATAGTTTATTTATT----------GATGGGCTTT
right    CCGCCCGTCTCGGCCTCCCAAAGTGCTGGGAT-TACAGGCGTGAGCCACCGCGCCCGGCCGATGGGCTTT
         1111111*1**1111*11**111**1*111**1****1111*1*111*111111111111**********

left     ATTCTTTTTTTTTTTNNNNNNNNNNNNNNNNNNNNNNNNNNNNNNNNNNNNNNNNNNNNNNNNNNNN
chrom    ATTCTTTTGTGGT-----------------GATGACTTTCATTCTAGCCTTATTAACTATCAACTAA
right    ATTCTTTTGTGGT-----------------GATGACTTTCATTCTAGCCTTATTAACTATCAACTAA
         ********2*22*222222222222222222222222222222222222222222222222222222
```

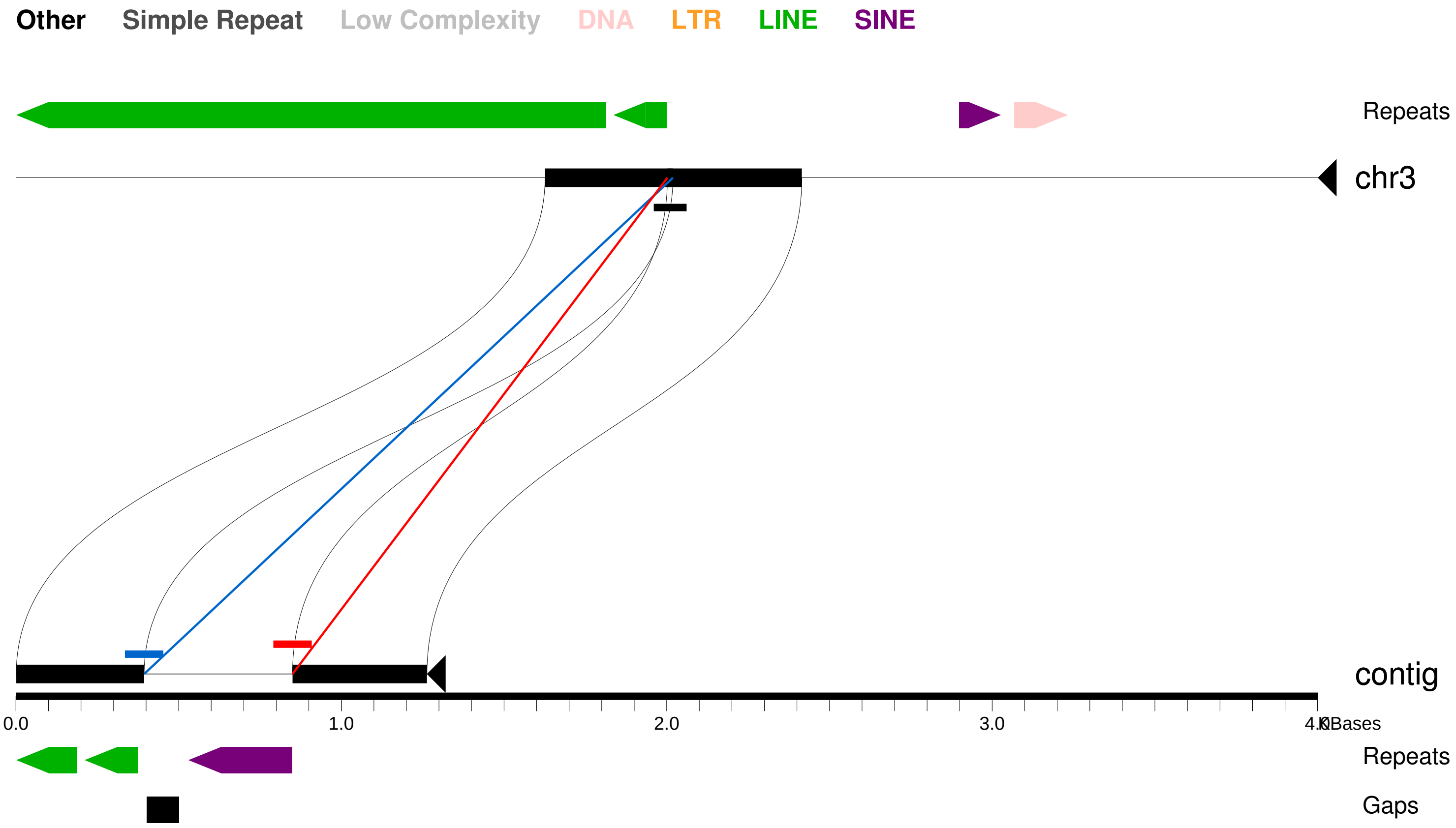

siteID: chr3_102983911 Scafftig3 - Breakpoint: chr3:102983850
chr3:102983802-102983907
Overlap Length: 9 INS size: 80 Genotyped: Yes

```
left    A-----------CTAATAAGGGAGTCTA-GTAACTTCACAGGATATGAGATCACATATAA-GAATCAATC
chrom   A-----------CTAATAAGGGAGTCTA-GTAACTTCACAGGATATGAGATCACATATAA-GAATCAATC
right   CTCCAGCCTGGGCGACAGAGCGAGACTCCGTCTAAAAAAAAAAAAAAAAAAAAAAAAAAAAGAATCAATC
        111111111111*1*111**1***1**11**11*111*1*11*1*11*1*11*1*1*1**1*********

left    CCAAGATTGCGCCACTGCACTCCAGCCTGGGCGACAGAGCGAGACTCCGTCTCAAAAAAAAA
chrom   AC---ATTT--CCA-TGTACTAAAATATGAAC-AC-GTGGAAAACAAA-----ATTAAAATC
right   AC---ATTT--CCA-TGTACTAAAATATGAAC-AC-GTGGAAAACAAA-----ATTAAAATC
        2*222***222***2**2***22*222**22*2**2*2*22*2**22222222*22****22
```

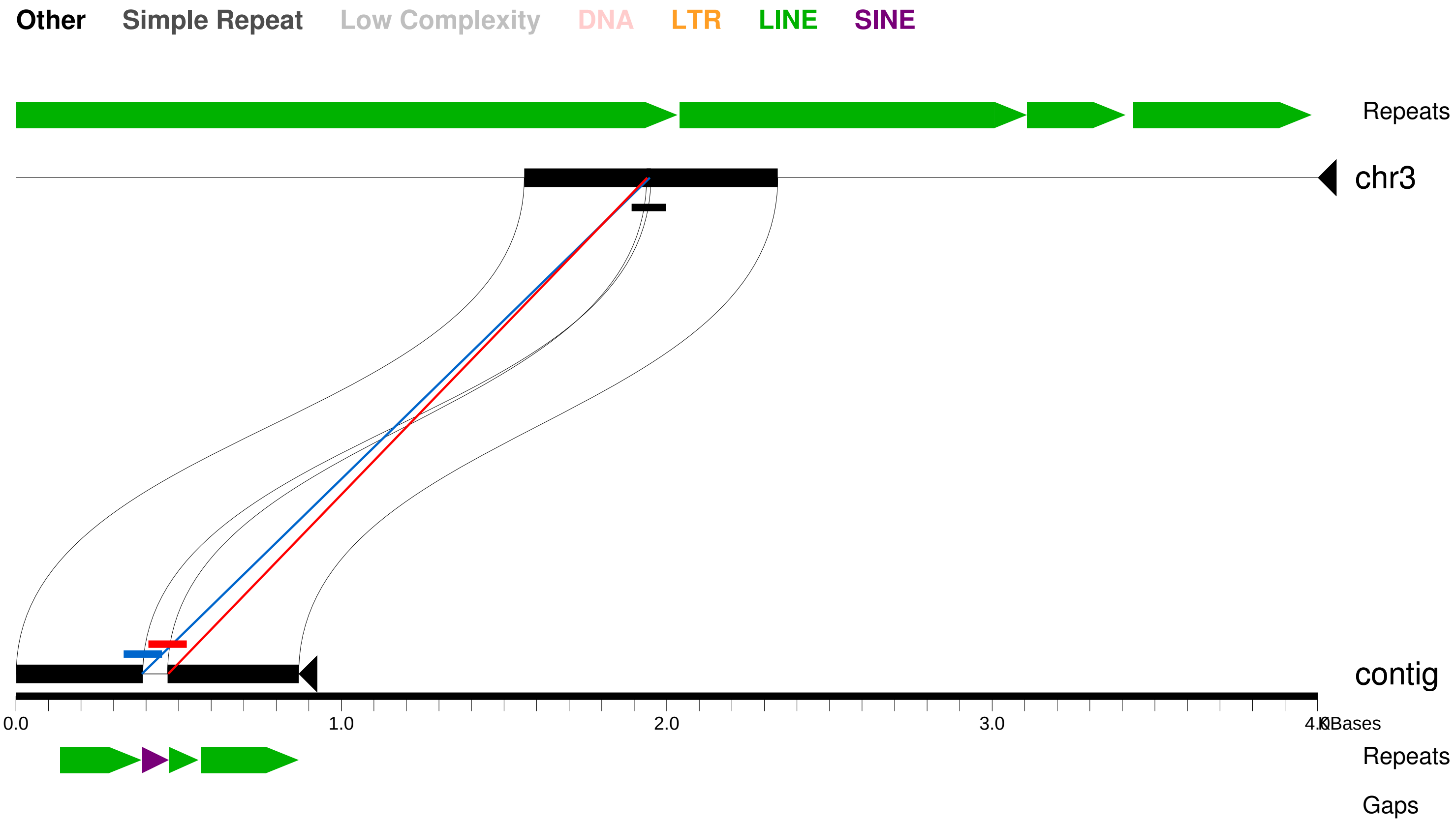

siteID: chr3_103419427 Scafftig2 + Breakpoint: chr3:103419423
chr3:103419377-103419482
Overlap Length: 14 INS size: 296 Genotyped: Yes

```
left     GAGCCTCTAATTACATTTTCACAAACTGATTGATAGATTATT----TTAT---------------GCCTG
chrom    GAGCCTCTAATTACATTTTCACAAACTGATTGATAGATTATT----TTAT---------------GCCTG
right    CCGCC-CGCCT--CGGCCTCCCAAAGTGCTGG---GATTACAGGCGTGAGCCACCGCGCCCGGCCGCCTG
         11***1*111*11*1111**1****1**1*1*111*****111111*1*1111111111111111*****

left     TTTCTCTTTTTTTTTTTTTTTTTTGAGACGGAGTCTCGCTCTGTCGCCCAGGCTGGAGTGCAGTGGCG
chrom    TTTCTCTTTAAAGACACTTTGTTGAACAATTAACATTGAACT----CACAGCCAACAGC---------
right    TTTCTCTTTAAAGACACTTTGTTGAACAATTAACATTGAACT----CACAGCCAACAGC---------
         *********22222222***2**22*2*222*222*2*22**2222*2***2*222**2222222222
```

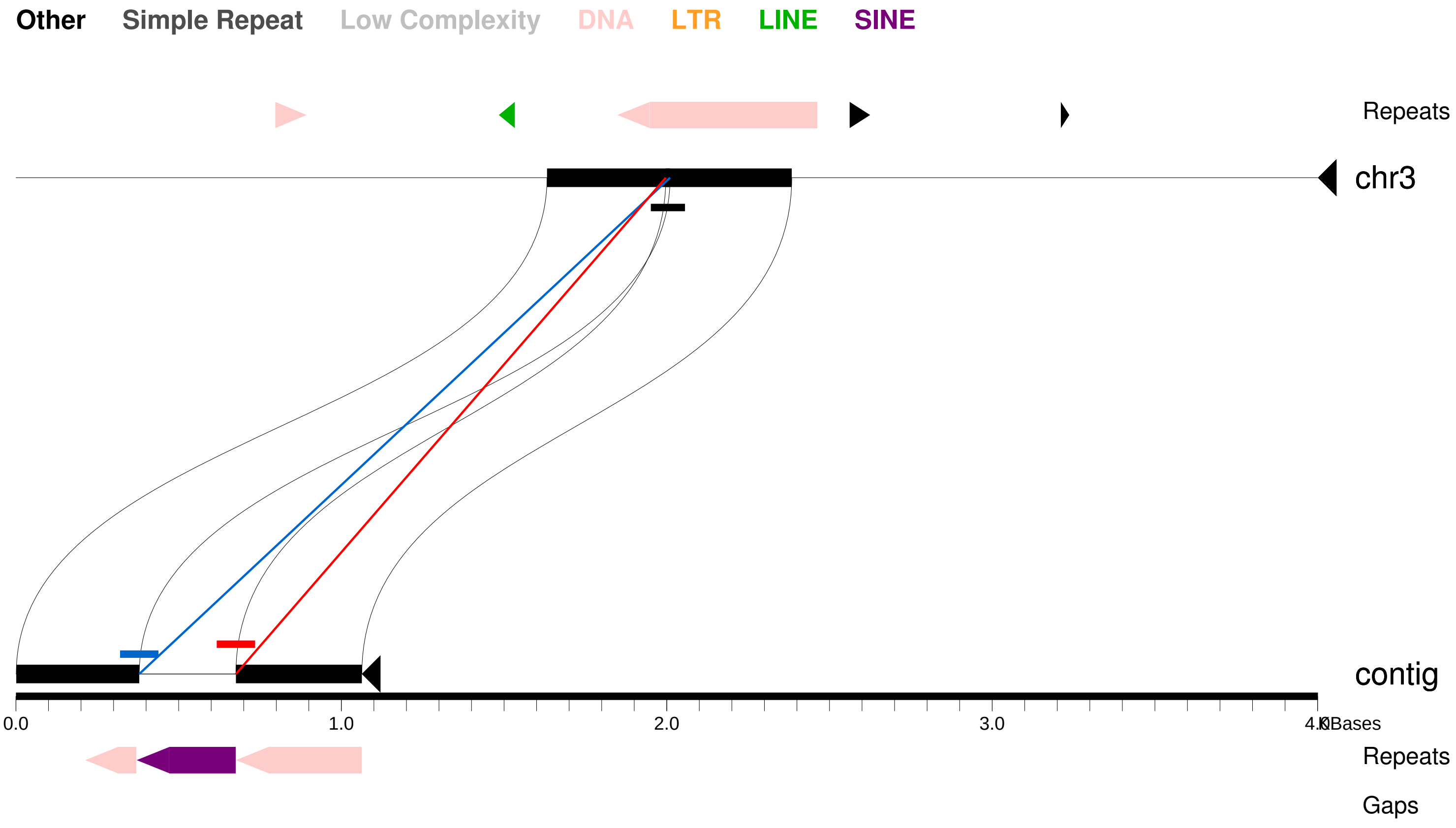

siteID: chr3_103453375 Scafftig3 + Breakpoint: chr3:103453381
chr3:103453337-103453440
Overlap Length: 16 INS size: 297 Genotyped: Yes

```
left     A-------------ATTTTATAGTCTTATTAATTTAGGAGTCAA--ATGATTGAAATTTGAGTAAAATTT
chrom    A-------------ATTTTATAGTCTTATTAATTTAGGAGTCAA--ATGATTGAAATTTGAGTAAAATTC
right    CCGCCCGCCTCGGCCTCCCAAAGTGCTGGGATTACAGGCGTGAGCCACCGCGCCCGGCCGAGTAAAATTC
         111111111111111*111*1***11*111*1*11***1**1*111*111111111111**********2

left     TTTTTTTTTTTTTTTTTTTTGAGACGGAGTCTCGCTCTGTCGCCCAGGCTGGAGTGCAGTGGC
chrom    TTTTTAAACATTTGATTAAGGGA-----AGTATAAAATT----CCCATCATTGAG------GAT
right    TTTTTAAACATTTGATTAAGGGA-----AGTATAAAATT----CCCATCATTGAG------GAT
         *****22222***22**2222**22222***2*22222*2222****222*2***222222*22
```

Other Simple Repeat Low Complexity DNA LTR LINE SINE

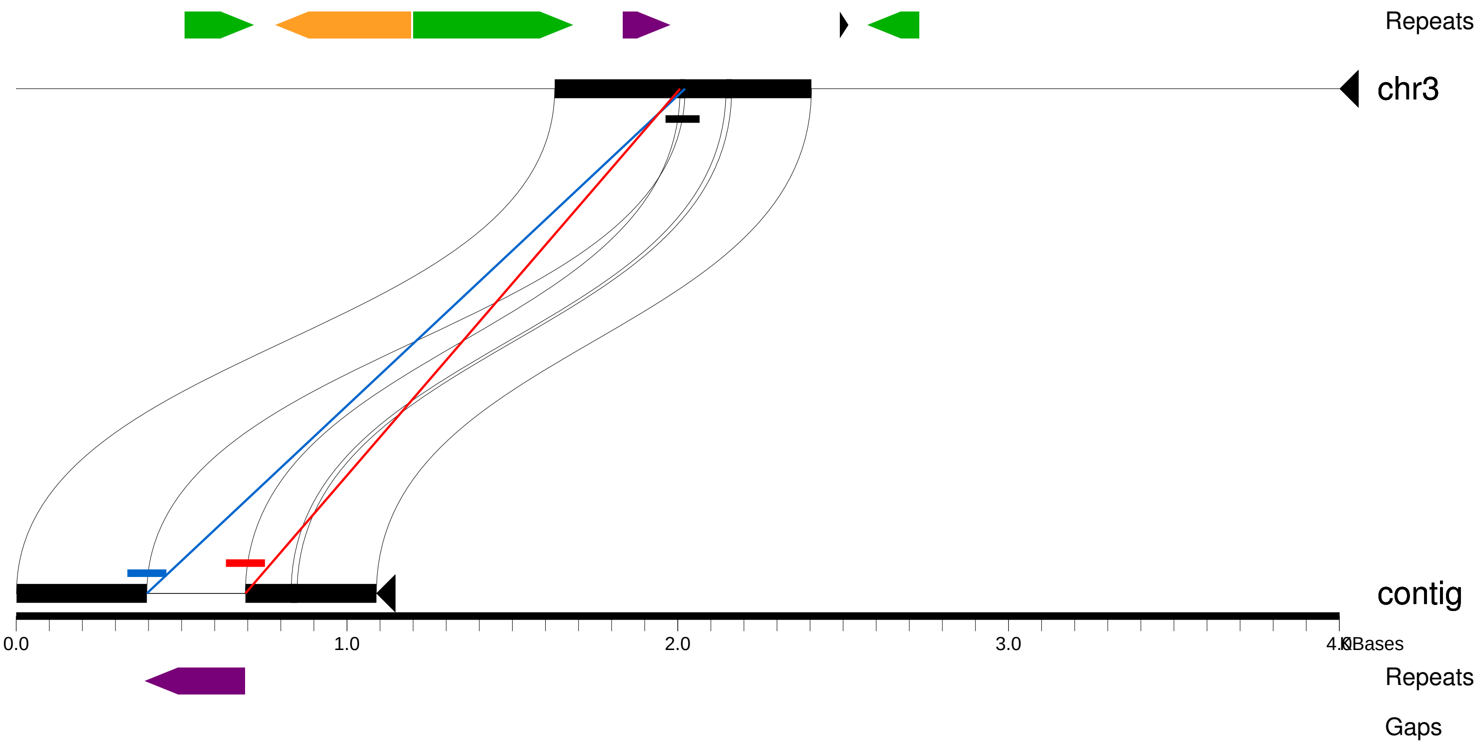

siteID: chr3_105333891 Scafftig1 - Breakpoint: chr3:105333901
chr3:105333855-105333960
Overlap Length: 14 INS size: 153 Genotyped: Yes

```
left    ----CAGCAGCAGGCATTTTTGTGAGATTTCCATGTGCTAGGCACTTTAT----------AAAGATTGGC
chrom   ----CAGCAGCAGGCATTTTTGTGAGATTTCCATGTGCTAGGCACTTTAT----------AAAGATTGGC
right   GCGACAG-AGCGAGACTCCGTCTCAAAAAAAAAAAAAAAAAAAAAAAAAAAAAAAAAAAAAAAAAAAGATTGGC
        1111***1***11*11*111*1*1*1*11111*111111*111*1111*11111111111**********

left    TCATCCAGCTACTCGGGAGGCTGAGGCAGGAGAATGGCGTGAACCCG-GAAGGCGGAGCTTGCA
chrom   TCATTTAA-TAATCAATA--------CAACAAAATGAGGTAAATATTTGTACTCTGA--TT---
right   TCATTTAA-TAATCAATA--------CAACAAAATGAGGTAAATATTTGTACTCTGA--TT---
        ****22*22**2**222*22222222**22*2****22**2**22222*2*22*2**22**222
```

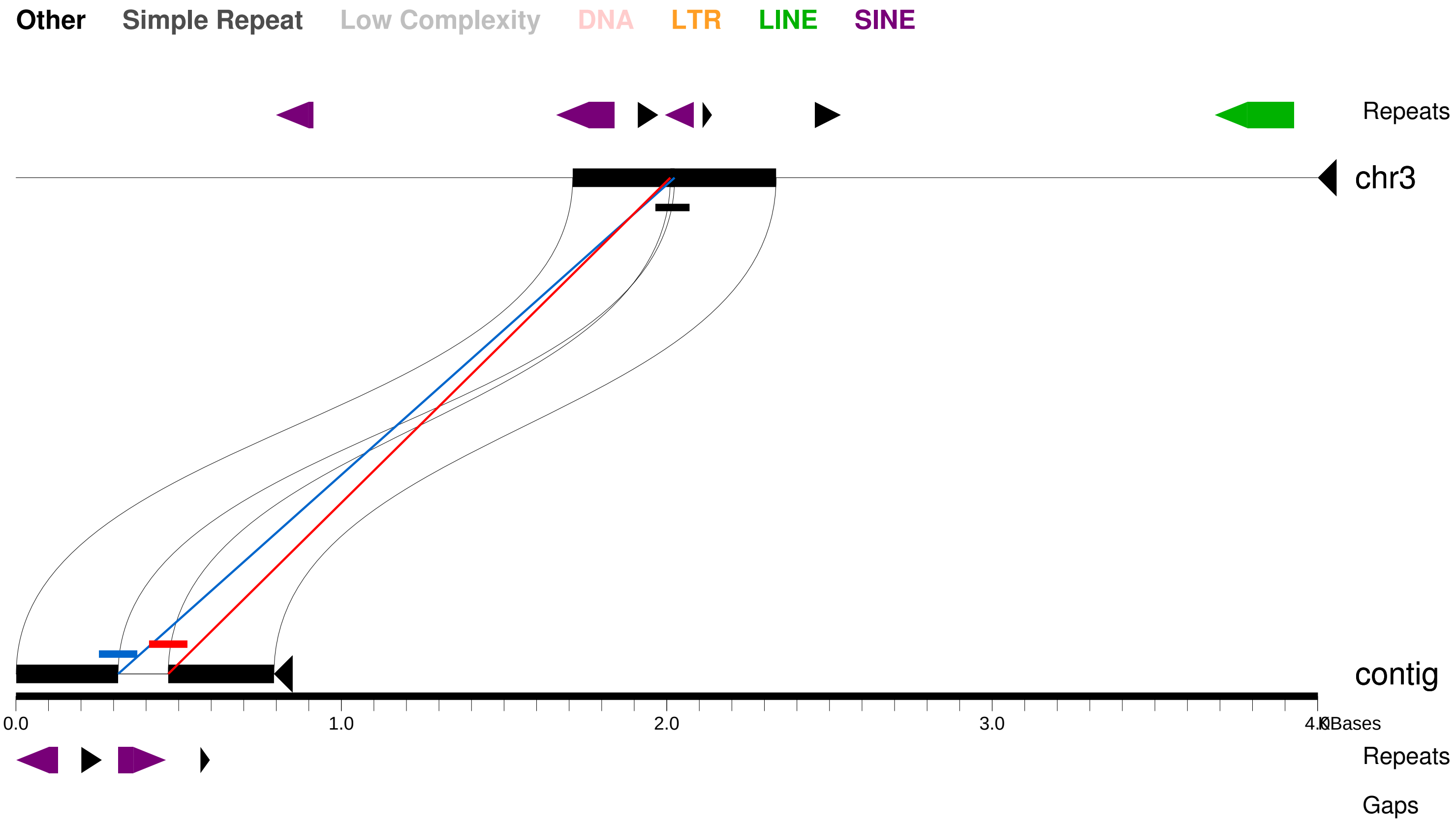

siteID: chr3_106289180 Scafftig4 + Breakpoint: chr3:106289189
chr3:106289029-106289361
Overlap Length: 13 INS size: 312 Genotyped: Yes

```
left   A--------------ACAGCTCTGTGACAGACAGTGGTGGAGATTTTTCCTTGAAGCAGAAATCCAGAGT
chrom  ATGGTAGTGATTTCAACAGCTCTGTGACAGACAGTGGTGGAGATTTTTCCTTGAAGCAGAAATCCAGAGT
right  GCG---------TGAAC----CCGGGAA--------GCGGAGCTT----------GCAGTGAGCC-GAGA
       1N2         2N2**1111*1*1**111111111*1****1**1111111111****11*1**1***1

left   TCTCTTTTGTGCA-TCTTCAGTTTGAGATGCCCATTAGAAACTCACATGCAGATCCTATATGTGGAATTT
chrom  TCTCTTTTGTGCA-TCTTCAGTTTGAGATGCCCATTAGAAACTCACATGCAGATCCTATATGTGGAATTT
right  TTGCGCCACTGCAGTCCGCAGTCCGGCCTGGGCGACAGA---------GC-GAGACTCCGTCTCAAAAAA
       *11*11111****1**11****11*111**11*111***111111111**1**11**111*1*11**111

left   GACATATGGATTTGTAGTTCTAAAAAAGGGTTCAGGGCCGGG--CGTGGTGGCTCACGCCTGTAATCCCA
chrom  GACATATGAATTTGTAGTTCTAAAAAAGGGTTCAACTGCTGAAACATATCTGATAATCATCAGAATGCAG
right  AAAAAAAAAAAAAAAAA----AAAAAAGGGTTCAACTGCTGAAACATATCTGATAATCATCAGAATGCAG
       1*1*1*112*11111*11111************2222*2*222*2*2222*2*2*2222222***2*22

left   GCACTTTGGGAGGCC----GAGGC----------------GGGTGGATCATGAGGTCAGGAGATC-----
chrom  GTAATACTTAAAGCCAGAAGAGCCTCATCTTAAAAAAAGAGGGTCTATAATAAAAATAGCAAATTTTATT
right  GTAATACTTAAAGCCAGAAGAGCCTCATCTTAAAAAAAGAGGGTCTATAATAAAAATAGCAAATTTTATT
       *2*2*22222*2***2222***2*2222222222222222****22**2**2*2222**2*2**222222

left   --GAGACCATCCT--GGCTAACA---AGGTGAAACCCCGTCTCTACTAAA-----
chrom  TTGTGGTTATGATATGCCTAACACTCACCT-AAAGGCTTTTTCCATGTTATGTTT
right  TTGTGGTTATGATATGCCTAACACTCACCT-AAAGGCTTTTTCCATGT-------
       22*2*222**22*22*2******222*22*2***22*22*2**2*222N1
```

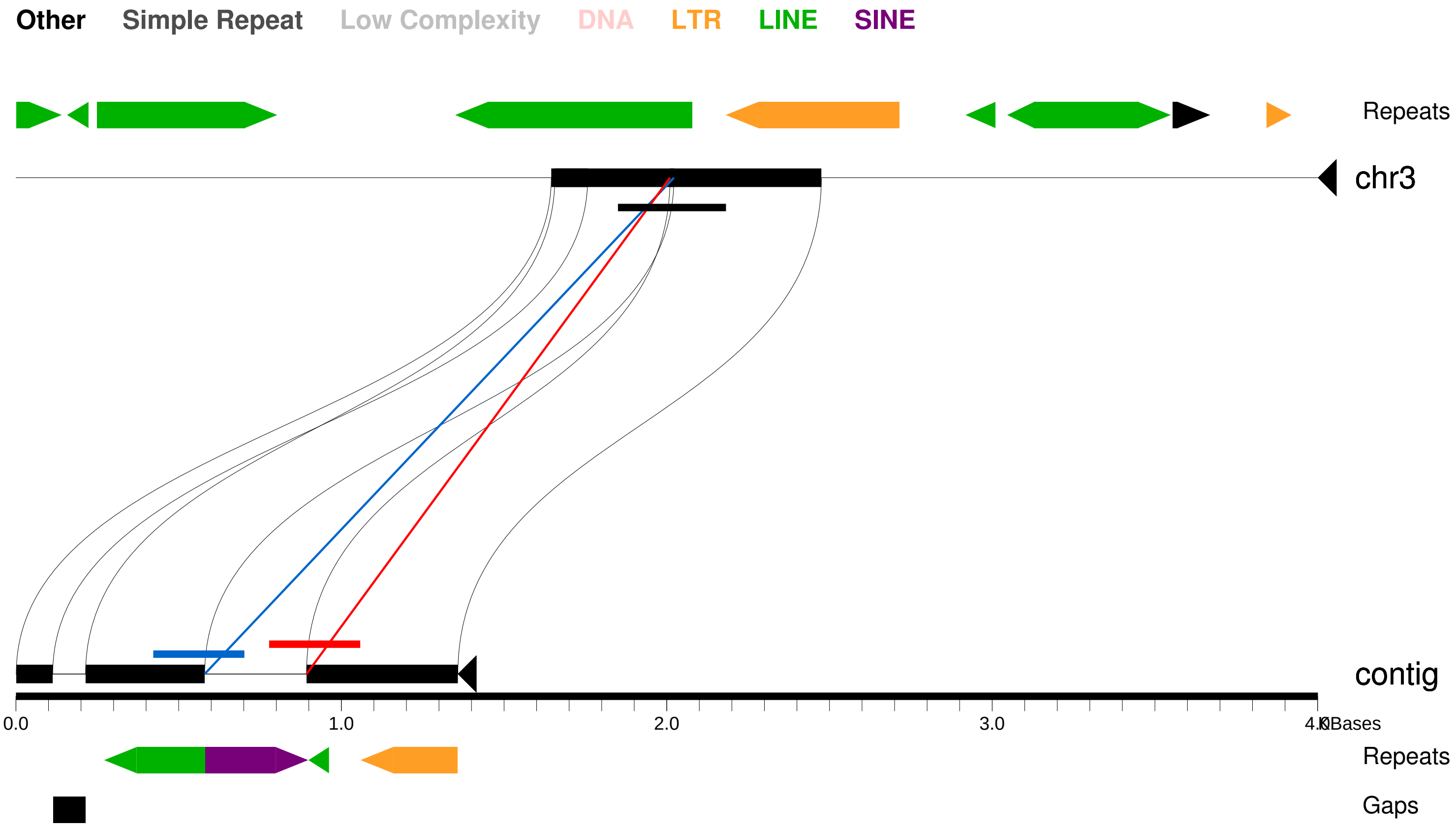

siteID: chr3_108120757 Scafftig1 - Breakpoint: chr3:108120740
chr3:108120691-108120799
Overlap Length: 11 INS size: 324 Genotyped: Yes

```
left   AC-AGAGTC---------AAGAAATCTTTAAAATGAAAACTCCCTCAAGTACATTCTTTAAGAAACATGA
chrom  AC-AGAGTC---------AAGAAATCTTTAAAATGAAAACTCCCTCAAGTACATTCTTTAAGAAACATGA
right  GCGAGACTCCGTCTCAAAAAAAAAAAAAAAAAAAAAAAAAAAAAAAAAAAAAAAAAAAAAAAAGAAACATGA
       1*1***1**111111111**1***11111****11****1111111**11*1*111111***********

left   GCCGGGCGCGGTGGCTCACGCCTGTAATCCCAGCACTTTGGGAGGCCGAGGCGGGCGGA
chrom  TGCATTCAGGA---CCAAGGGCAATATAGGAAGAAGT-------ACCTTCTTTTTCCTA
right  TGCATTCAGGA---CCAAGGGCAATATAGGAAGAAGT-------ACCTTCTTTTTCCTA
       22*222*22*2222*22*2*2*22**22222**2*2*22222222**22222222*22*
```

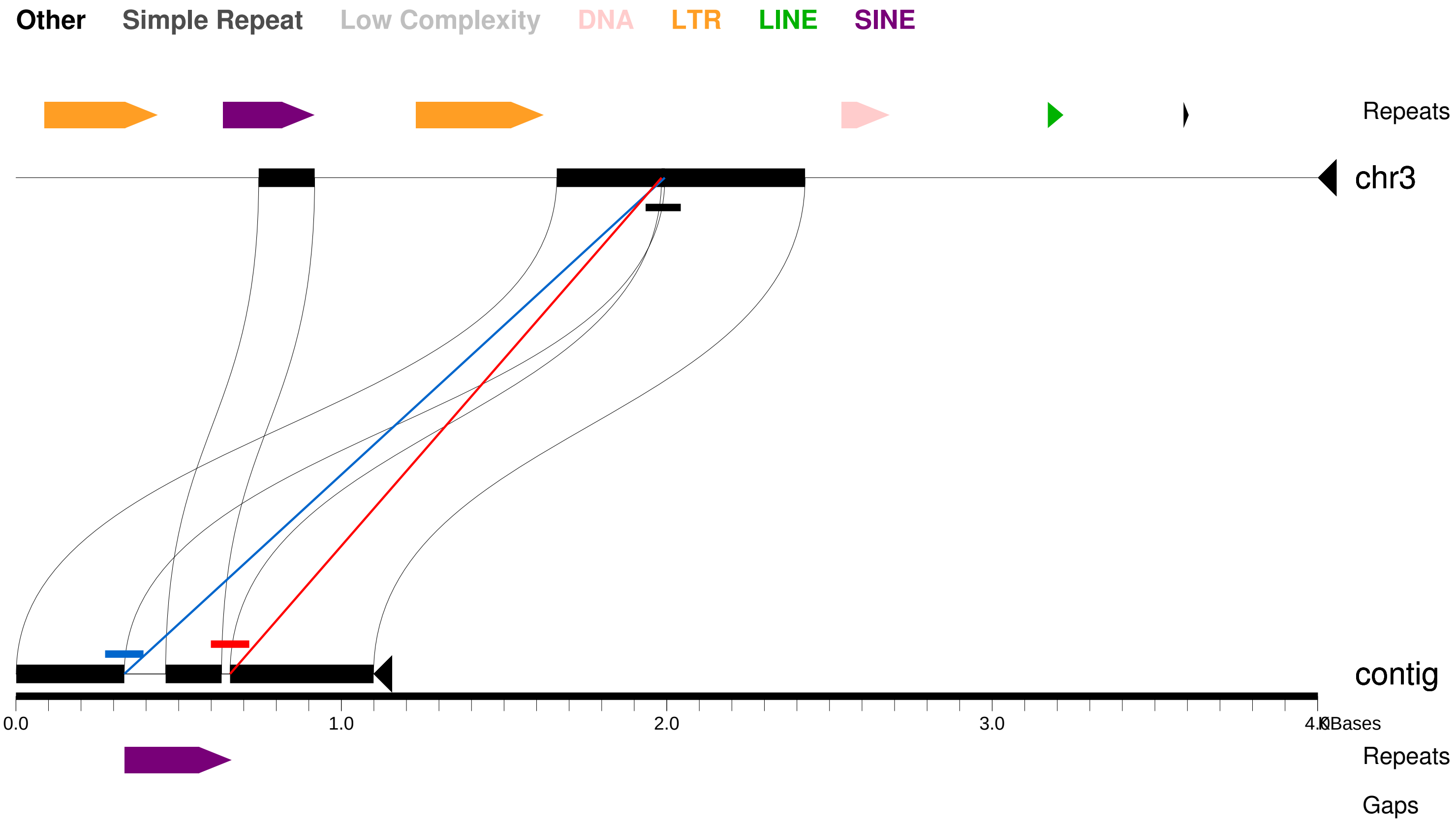

siteID: chr3_109728171 Scafftig1 - Breakpoint: chr3:109728145
chr3:109728101-109728204
Overlap Length: 16 INS size: 277 Genotyped: Yes

```
left    CTGAAGC---ATATTTACATGGAGACTCAAGAATCTGTTAGACTGTT------------AGAAAATACAC
chrom   CTGAAGC---ATATTTACATGGAGACTCAAGAATCTGTTAGACTGTT------------AGAAAATACAC
right   CCTGGGCGACAGAGCGAGACTCCGTCTCAAAAAAAAAAAAAAAAAAAAAAAAAAAAAAAAGAAAATACAC
        *1111**111*1*111*1*1111*1*****1**111111*1*11111111111111111***********

left    TGTAACTTTGGGAGGCAGAGGCGGGCGGATCATGAGGTCAGGAGATTGAGACCATCCTGGCTAA
chrom   TGTAA------------GAGGAGAAC--ACCACAAAGATACTCC-TTGAGAAGAGCAATCCCAA
right   TGTAA------------GAGGAGAAC--ACCACAAAGATACTCC-TTGAGAAGAGCAATCCCAA
        *****222222222222****2*22*22*2**22*2*22*22222******22*2*2222*2**
```

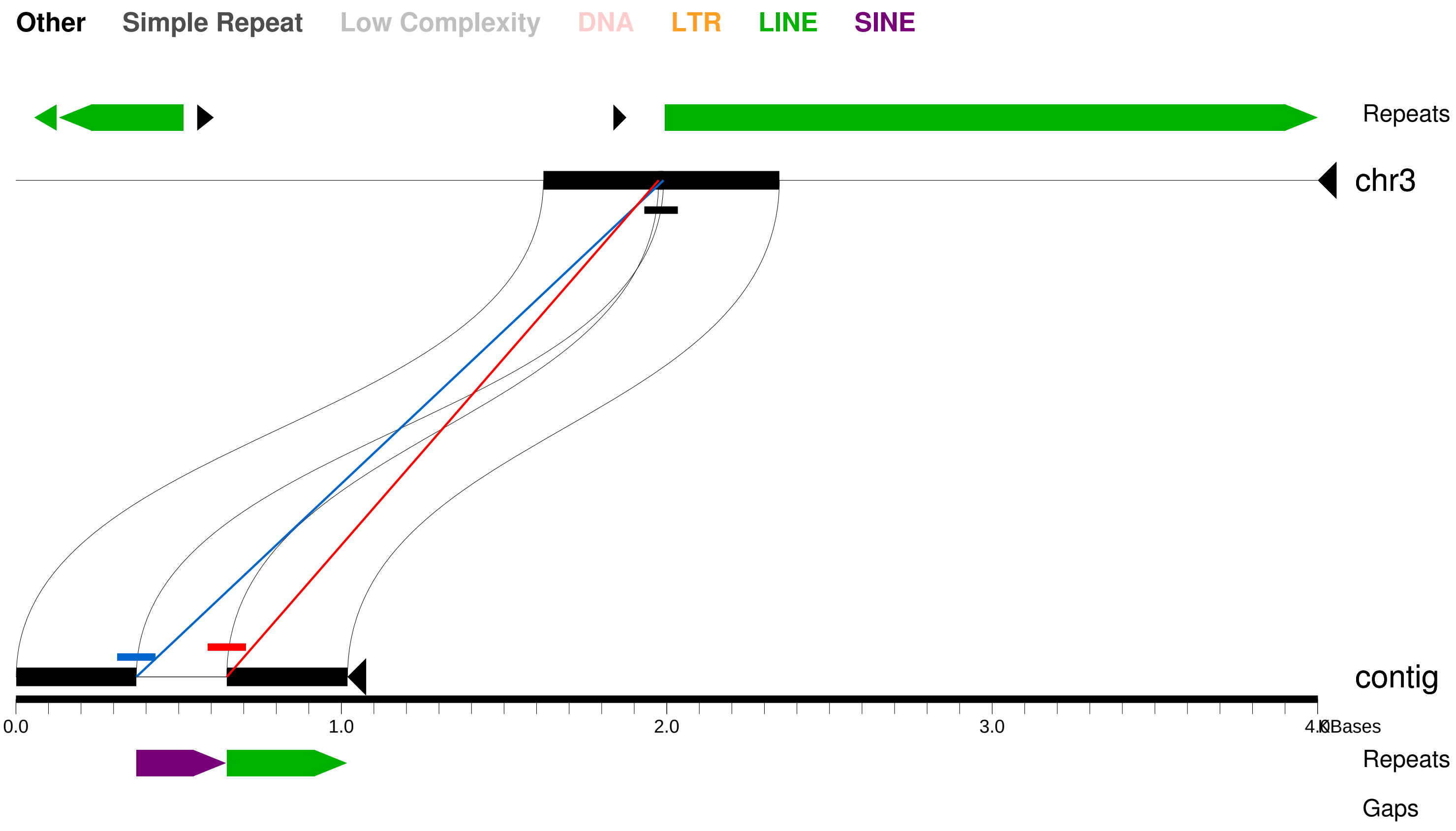

siteID: chr3_110113837 Scafftig2 - Breakpoint: chr3:110113835
chr3:110113786-110113891
Overlap Length: 11 INS size: 303 Genotyped: Yes

```
left    C----------AGTAAAGCAAGTCACACATATTTTTTTTT-GTTTCCCAATGCATATAAA--AGTTATGT
chrom   C----------AGTAAAGCAAGTCACACATATTTTTTTTT-GTTTCCCAATGCATATAAA--AGTTATGT
right   CTGCAGTCCGCAGTCCGGCCTGGGCGACAGAGCGAGACTCCGTCTCAAAAAAAAAAAAAAAAAGTTATGT
        *1111111111***111**11*1111***1*1111111*11**1**11**111*1*1***11********

left    TACGCCGGGCGCGGTGGCTCACGCCTGTAATCCCAGCACTTTGGGAGGCCGAGGCGGGTGGA
chrom   TACACTATATG-GTAGCCTATTGAGTGTAA-------AAT----GATACC-ATGTCTAAAAA
right   TACACTATATG-GTAGCCTATTGAGTGTAA-------AAT----GATACC-ATGTCTAAAAA
        ***2*22222*2*22*2**222*22*****2222222*2*2222**22**2*2*2222222*
```

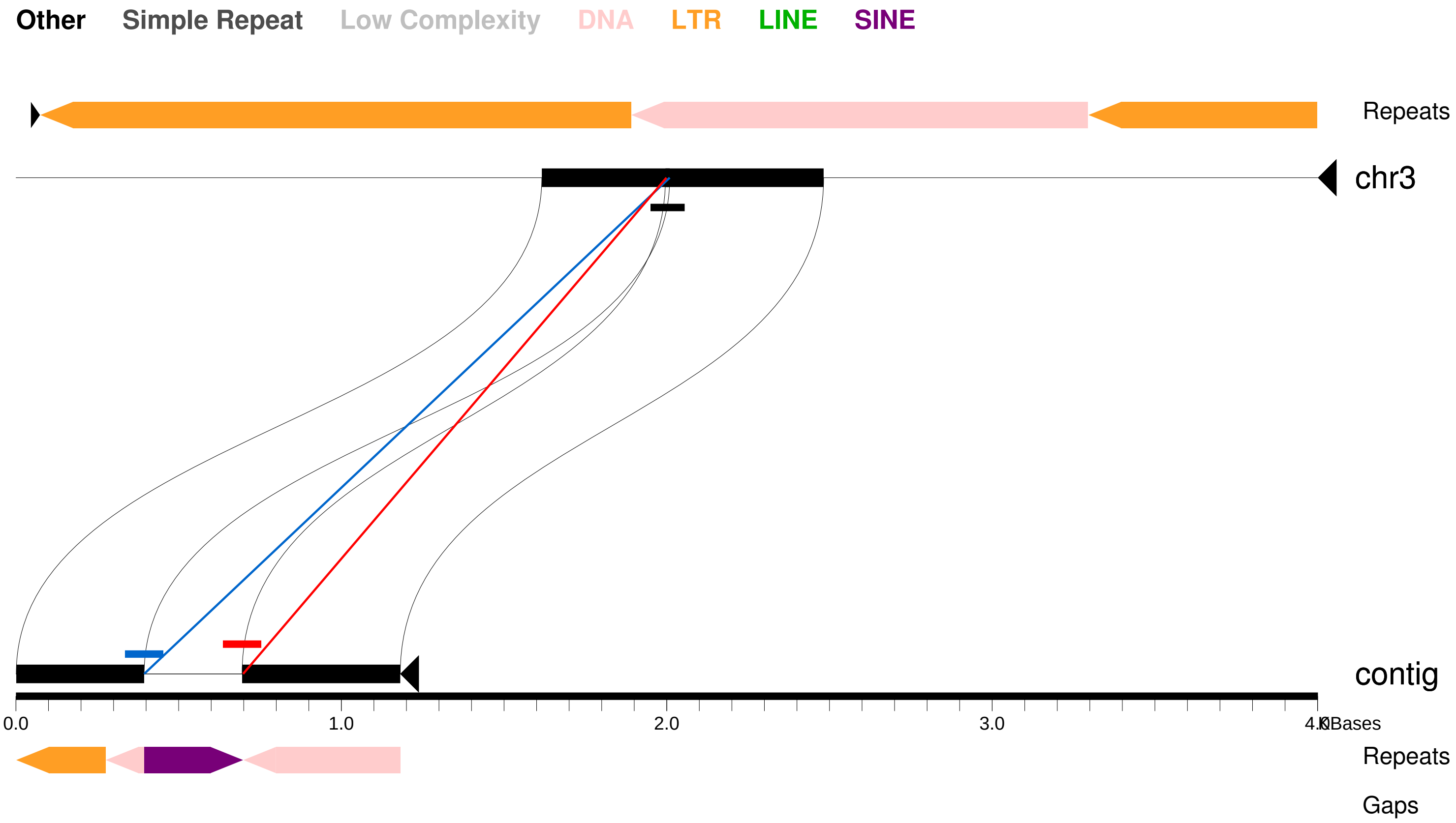

siteID: chr3_110413402 Scafftig2 + Breakpoint: chr3:110413394
chr3:110413234-110413555
Overlap Length: 0 INS size: 282 Genotyped: Yes

```
left   --GGAAGATGTAAGAAAAAATAAATAAATAAAAACATGCTCTGTAGGAAAAGAAATTAAGAGTTGTAG--
chrom  TTGGAAGATGTAAGAAAAAATAAATAAATAAAAACATGCTCTGTAGGAAAAGAAATTAAGAGTTGTAG--
right  GT---ATTTTTAGTAGAGATGGGATTTCTCCATGTTGGTCAGGCTGGTCTCGAACTCCCGACCTCCACCC
       N2111*11*1**11*1*1*1111**111*11*11111*1111*11**1111***1*111**11*11*111

left   -ATTATGAAGTGATGATTTGAATAGATTT--TGCA-GACTGAAGATTCTTGGGTATTGCTAATGTCTACA
chrom  -ATTATGAAGTGATGATTTGAATAGATTT--TGCA-GACTGAAGATTCTTGGGTATTGCTAATGTCTACA
right  CACCTTGGCCTCCCAAAGTACTGGGATTACAGGCATGAGCCACAGCGCTTGGCCCCCCCTGTTTTTTTTT
       1*111**111*1111*11*11111****1111***1**111*11111*****111111**11*1*1*111

left   TCCATTTGGTATGTTGCAAGTTTTTACTT--GCAG-----TGGCGCAATCTC-------GGCTCACTGCA
chrom  TCCATTTGGTATGTTGCAAGTTTTTACATATGTATATATATGTGTAAATATGTATATATGTGTAAATATA
right  AACAAATGAGAAATAAGAAAAGTGGA--TATGTATATATATATGTGTAAATATGTATATATGTGTAAATATA
       11**11**11*11*111**111*11*1N*22*2*222222**2222***2*22222222*22*2*2*22*

left   -----ACCTCTGCCTCC----CAGGT---TCAAGC---GATTCTCCTCCCTCAGCCTCCCAA-------G
chrom  TATGTATATATGTATAAAATACATCTATATCTAGTATAGAGTATTAGAATTAAATTTCTTAATTACTTTG
right  TATGTATATATGTATAAAATACATCTATATCTAGTATAGAGTATTAGAATTAAATTTCTTAATTACTTTG
       22222*22*2**22*222222**22*222**2**2222**2*2*222222*2*222**22**2222222*

left   TGGCTGGAATTACAGGCATGGGCCACCATACTTGGCTA--ATTTT-GT
chrom  TGAATAGACTGCTTACCCTGTGTTTCAAATGTTATACAGAATTTTAGT
right  TGAATA------------------------------------------
       **22*211N1NNNNNN1N11N1NNN1N1NNN11NNNN1  11111 11
```

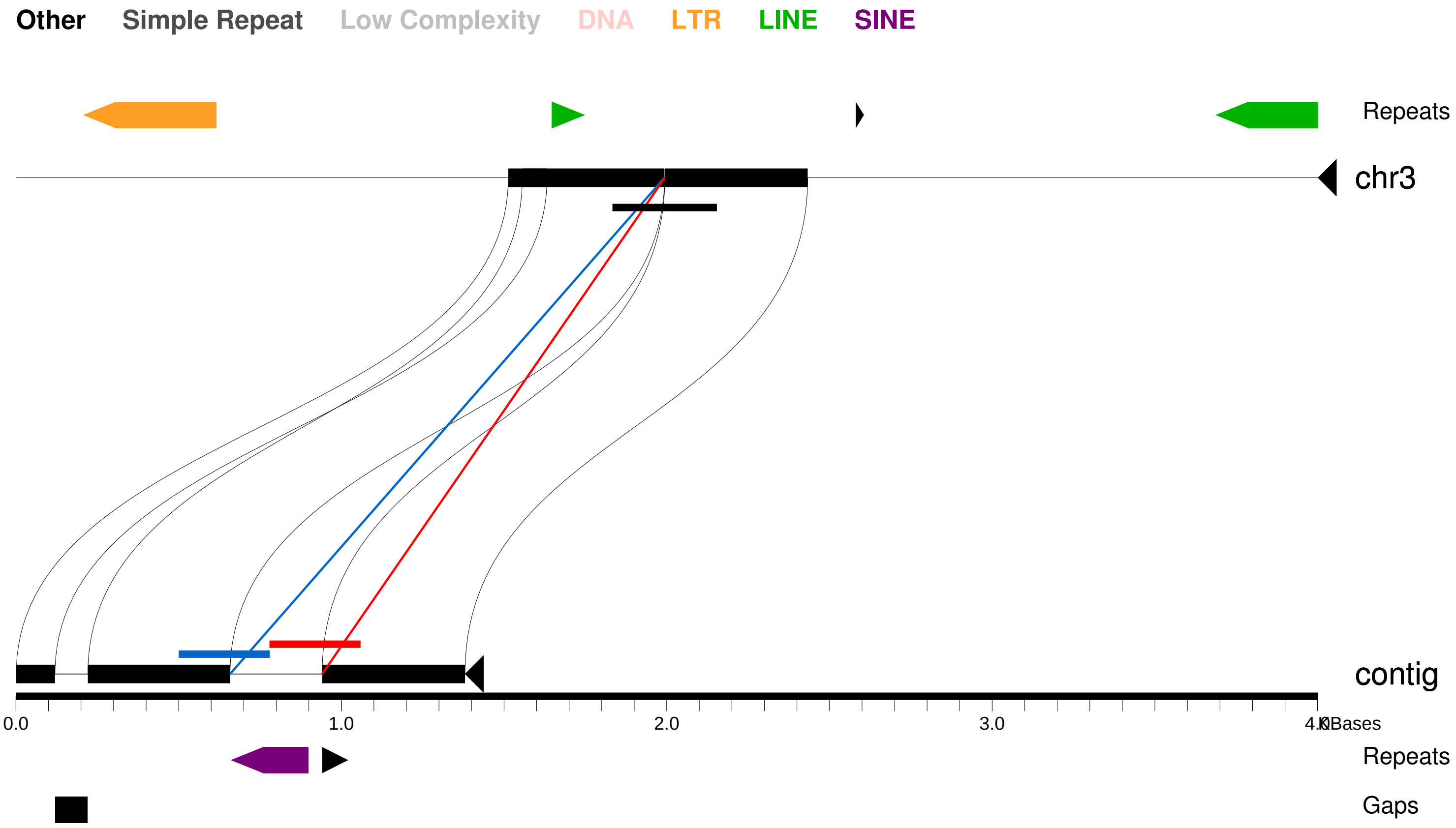

siteID: chr3_110987485 Scafftig7 - Breakpoint: chr3:110987468
chr3:110987419-110987527
Overlap Length: 11 INS size: 296 Genotyped: Yes

```
left    GCACTTATGTATTGAAGTGTGTCCTCTTCAAAGGCAG---CTGCCTCAATGT---------AAGAAATGT
chrom   GCACTTATGTATTGAAGTGTGTCCTCTTCAAAGGCAG---CTGCCTCAATGT---------AAGAAATGT
right   GCGCCACTGCACTCCAGCCTGGGCGA--CAGAGCGAGACTCCGTCTCAAAAAAAAAAAAAAAAGAAATGT
        **1*111**1*1*11**11**11*1111**1**11**111*1*1*****111111111111*********

left    GGGGGCCGGGCGCGGTGGCTCACGCCTGTAATCCCAGCACTTTGGGAGGCCGAGGCGGGCG
chrom   GGAAAAGTCATGTGGAAGAGAACCAATGTGC-CCCAG----TTGGCAGATCTAGTT-----
right   GGAAAAGTCATGTGGAAGAGAACCAATGTGC-CCCAG----TTGGCAGATCTAGTT-----
        **222222222*2**22*222**222***222*****2222****2**22*2**2222222
```

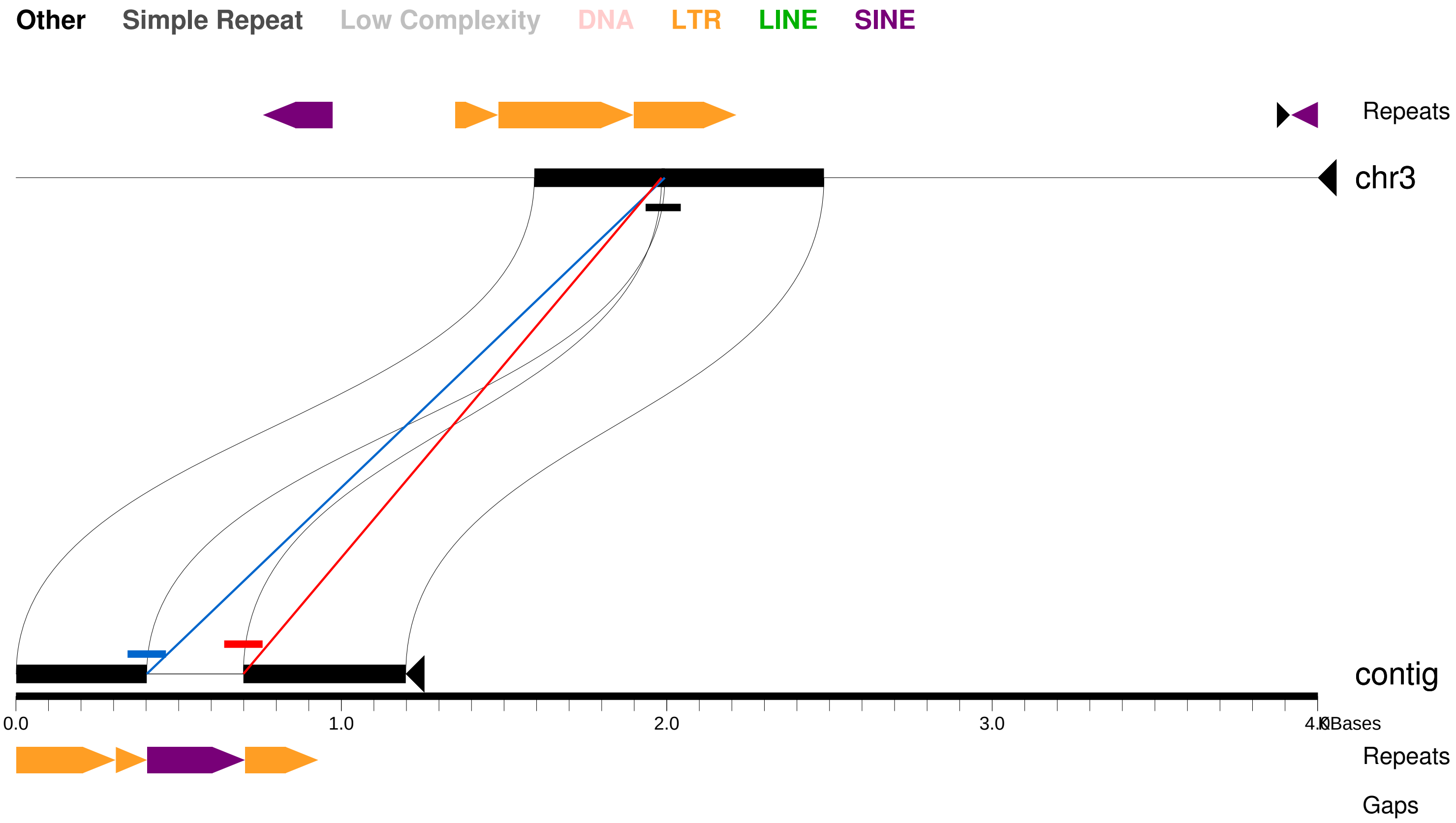

siteID: chr3_111226375 Scafftig1 + Breakpoint: chr3:111226364
chr3:111226316-111226423
Overlap Length: 12 INS size: 495 Genotyped: No

```
left    AATAGTTGTCTTCTGGAGCTAGACAGTTC---------ATTTAAAAGTTTATATGCA--AAAATAAAATC
chrom   AATAGTTGTCTTCTGGAGCTAGACAGTTC---------ATTTAAAAGTTTATATGCA--AAAATAAAATC
right   NNNNNNNNNNNNNNNNNNNNNNNNNNNNNNNNNNNNNAAAAAAAAAAATAAAATAAAATAAAATAAAATC
        11111111111111111111111111111111111111*111****11*1*1**11*11***********

left    A-GGCCGGGCGCGGTGGCTCACGCCTGTAATCCCAGCACTTTGGGAGGCCGAGGCGGGTGG
chrom   AAGGAATAACAAATAGGAAAACAC---TAAACAAAGCAGTAAGGCAGGACTA---------
right   AAGGAATAACAAATAGGAAAACAC---TAAACAAAGCAGTAAGGCAGGACTA---------
        *2**22222*22222**222**2*222***2*22****2*22**2***2*2*222222222
```

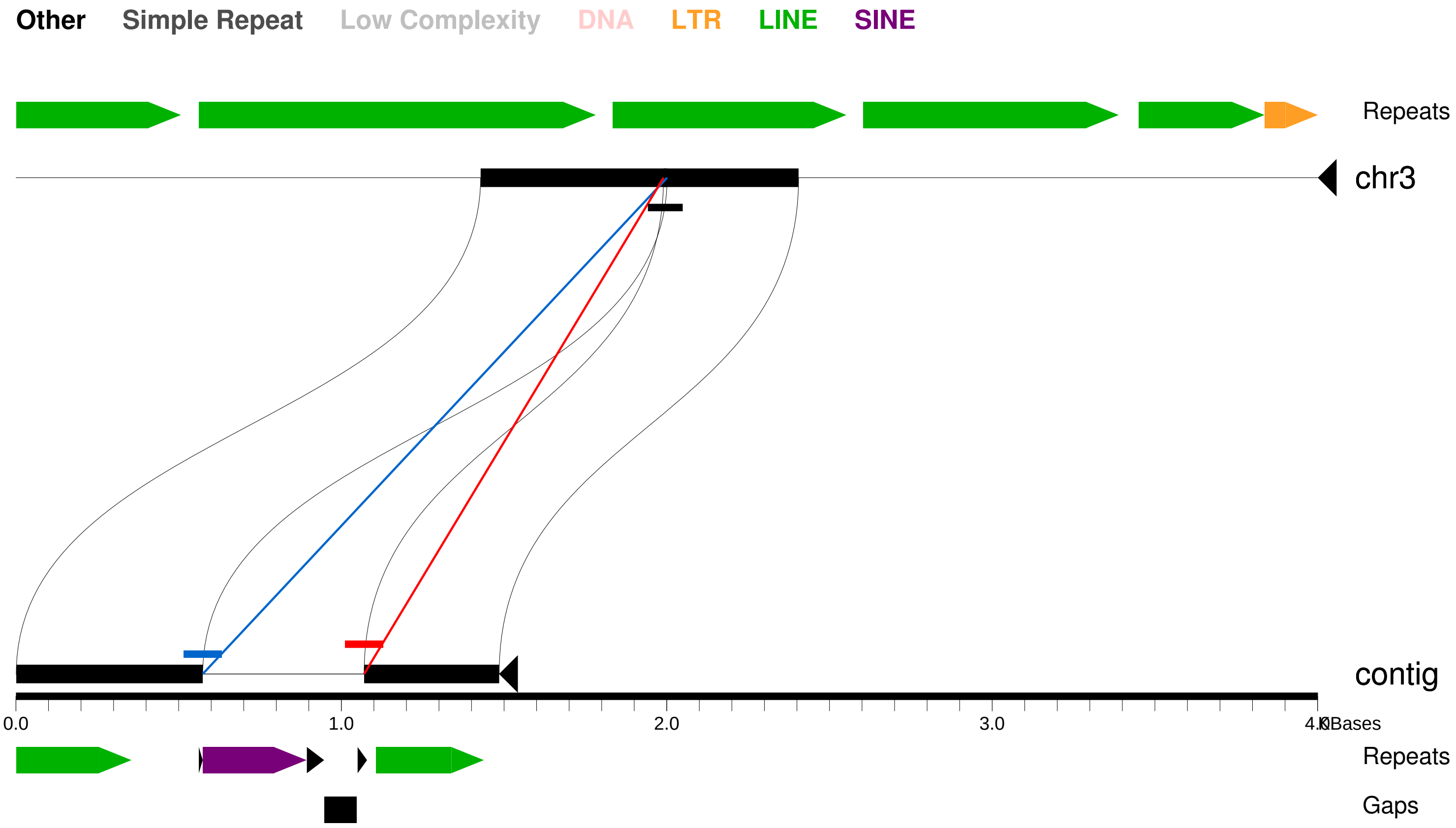

siteID: chr3_11204171 Scafftig2 - Breakpoint: chr3:11204166
chr3:11204006-11204340
Overlap Length: 15 INS size: 301 Genotyped: Yes

```
left    ----------------AGCATTTTCCTCTCCTTGGTGGGGAGTGCGGGCAAGGGGGTTGGGGCGATCCA
chrom   GATGA-ATGGCAAGAAGCATTTTCCTCTCCTTGGTGGGGAGTGCGGGCAAGGGGGTTGGGGCGATCCA
right   CAGGAGAATGGCGTGAA------------CCCGGGAGGCGGAGC-------------TTGCAGTGAGCC-
        N2N2212NN222NN22*111111111111**11**1**1****11111111111111***11*1**1**1

left    GAGGTCCCTAGCACCTGCTATTATCTGGACCAGCAGGGTATGGGAGAAATAGCCCCTGACCTTTCTGACC
chrom   GAGGTCCCTAGCACCTGCTATTATCTGGACCAGCAGGGTATGGGAGAAATAGCCCCTGACCTTTCTGACC
right   GAGATCGC--GCCACTGCACT--------CCAGCC-----TGGGCGACAGAGCGA--GAC---TCCGTCT
        ***1**1*11**11****11*11111111*****111111****1**1*1***1111***111**1*1*1

left    CTCCTGTCCTGATGACTGATGAAAAGAACTACTGTCGGCCGGG------CGCGGTGGCT-----------
chrom   CTCCTGTCCTGATGACTGATGAAAAGAAGTACTGTCTGTTGAGAAAGCACTCCAGGGCTTTCCATTTGTG
right   CAAAAAAAAAAAAAAAAAAAAAAAA-AACTACTGTCTGTTGAGAAAGCACTCCAGGGCTTTCCATTTGTG
        *1111111111*11*111*11****1**N*******2*22*2*222222*2*222****22222222222

left    ----CACGCCTGTAATCC-CAGCACTTTGGGAGGCCGAGGC--GGGCGGATCACG-AGGTCAGGAGATCG
chrom   ATGTCAC-CCAGTCCTCAACAGCTCTACGAGATGCATATTCATGTGCCCATTTTATAGATGAGACAACTG
right   ATGTCAC-CCAGTCCTCAACAGCTCTACGAGATGCATATTCATGTGCCCATTTTATAGATGAGACAACTG
        2222***2**2**22**22****2**22*2**2**22*22*22*2**22**22222**2*2**222*22*

left    AGACCAT---CCTGGC-TAAC-------ACGGTGAAACCCGGT----CTCTACTAAA
chrom   AGGCTAGAGACATGGAGTAACTTATGCTAGAGTGTAACTGGGGAGACCAGTAACAAG
right   AGGCTAGAGACATGGAGTAACTTATGCTAGAGTGTAACTGGGGAGACCA--------
        **2*2*2222*2***22****2222222*22***2***22**22222*2N11NN11N
```

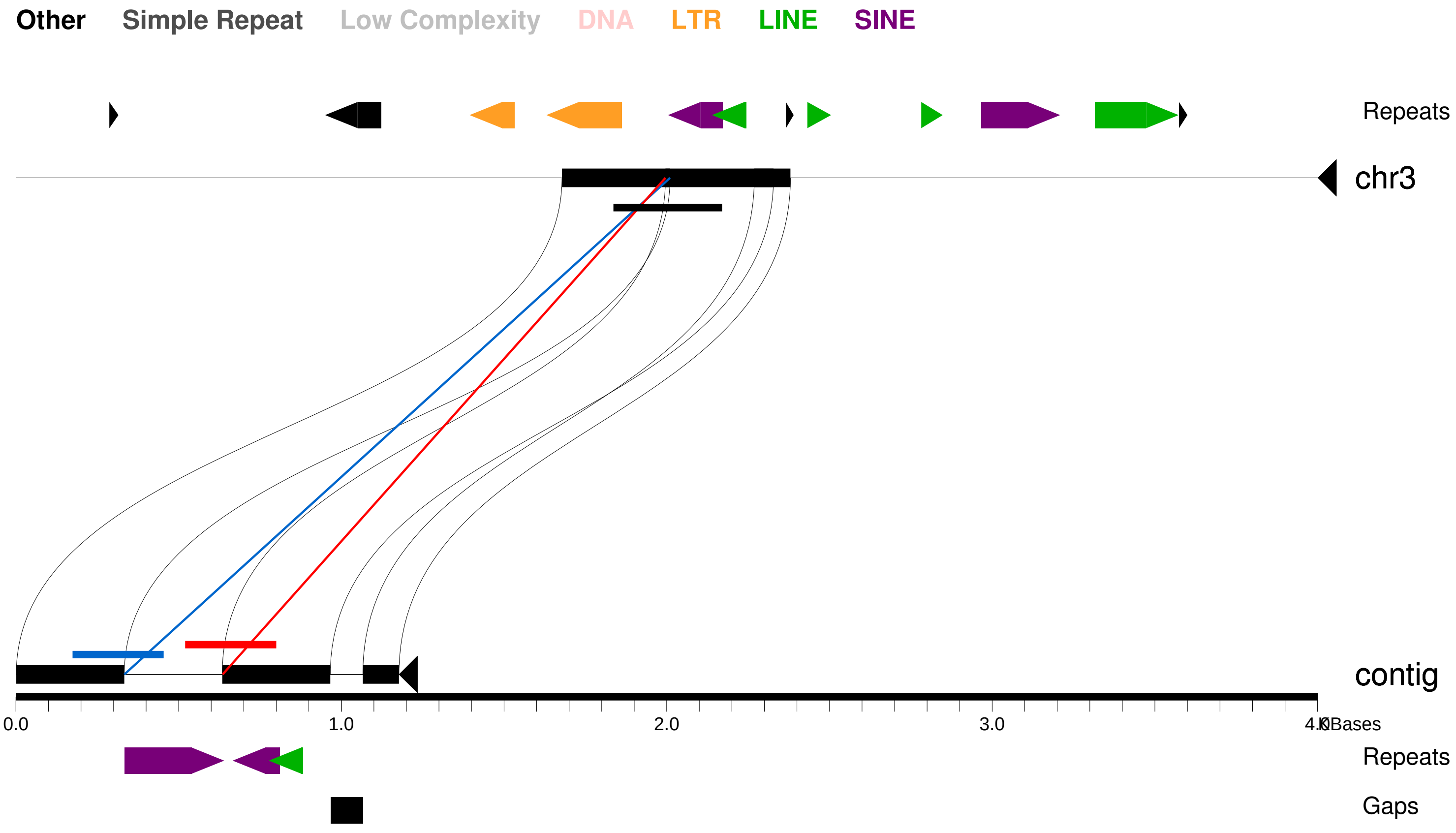

siteID: chr3_112453490 Scafftig1 - Breakpoint: chr3:112453493
chr3:112453446-112453526
Overlap Length: 13 INS size: 285 Genotyped: Yes

```
left   ACA-----CATTAATT----------AATATATC-----------------------TGTCATAAAAGAT
chrom  ACA-----CATTAATT----------AATATATC-----------------------TGTCATAAAAGAT
right  GCAGTGAGCCGAGATTGCGCCACTGCACTCCAGCCTGGGCGACAGAGCGAGACTCCGTCTCAAAAAAAAA
       1**11111*1111***1111111111*1*11*1*11111111111111111111111*1***1****1*1

left   AAAAATACTCACATTAAGATAATAAAGAGCCTGTAATCCCAGCACTTTGGGAGGCCGAGGCGGGCGGATC
chrom  AAAAATACTCACATTAAGATAATAAAGACTATGTCCTCATA-----TTGC--------------------
right  AAAAAAAAAAAAAAAAAGATAATAAAGACTATGTCCTCATA-----TTGC--------------------
       *****1*111*1*11*************222***22**22*22222***222222222222222222222

left   ACGAGGTCAGGAGATCG
chrom  ---AGGT----------
right  ---AGGT----------
       222****2222222222
```

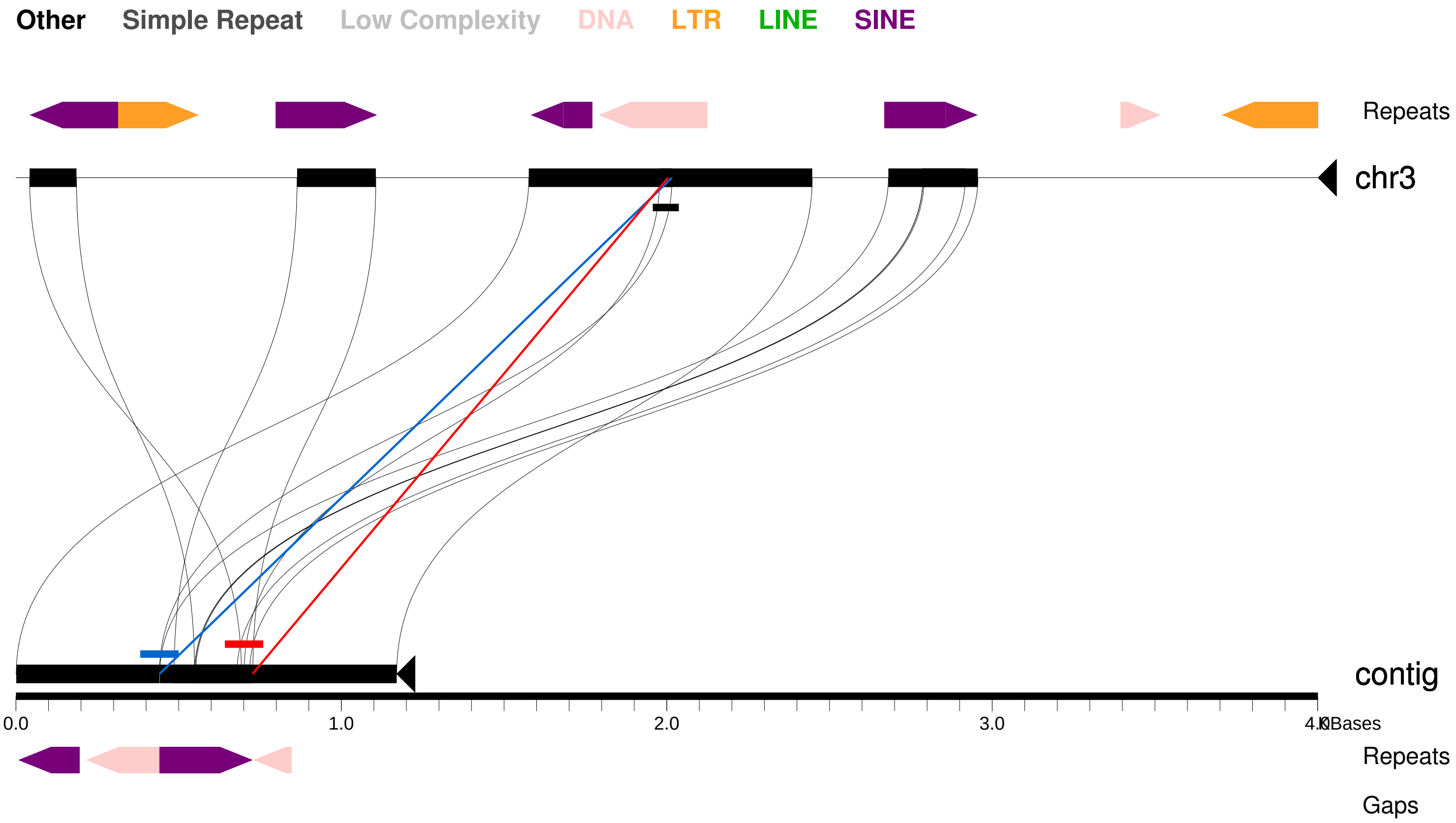

siteID: chr3_112573663 Scafftig1 - Breakpoint: chr3:112573658
chr3:112573617-112573717
Overlap Length: 14 INS size: 293 Genotyped: Yes

```
left    AAGG---TATTTCAATCAA----------CA-AGCAAAAAACAAAC----AACCTCATTAAAAAGTGGGC
chrom   AAGG---TATTTCAATCAA----------CA-AGCAAAAAACAAAC----AACCTCATTAAAAAGTGGGC
right   ATCGCGCCACTGCACTCCAGCCTGGGCGACAGAGCCAGACGCTGTCTCAAAAAAAAAAAAAAAAGTGGGC
        *11*1111*1*1**1**1*1111111111**1***1*1*11*111*1111**1111*11***********

left    AGACGGCCGGGCGTGGTGGCTCACGCCTGTAATCCCAGCACTTTGGGAGGCCGAGGCGGGTGGATCA
chrom   AGAGGACCTGAACAGAGAG---AAGGC----ATACATGCA---------GCCAA--CAAGCATTTGA
right   AGAGGACCTGAACAGAGAG---AAGGC----ATACATGCA---------GCCAA--CAAGCATTTGA
        ***2*2**2*2222*222*222*2*2*2222**2*22***222222222***2*22*22*2222*2*
```

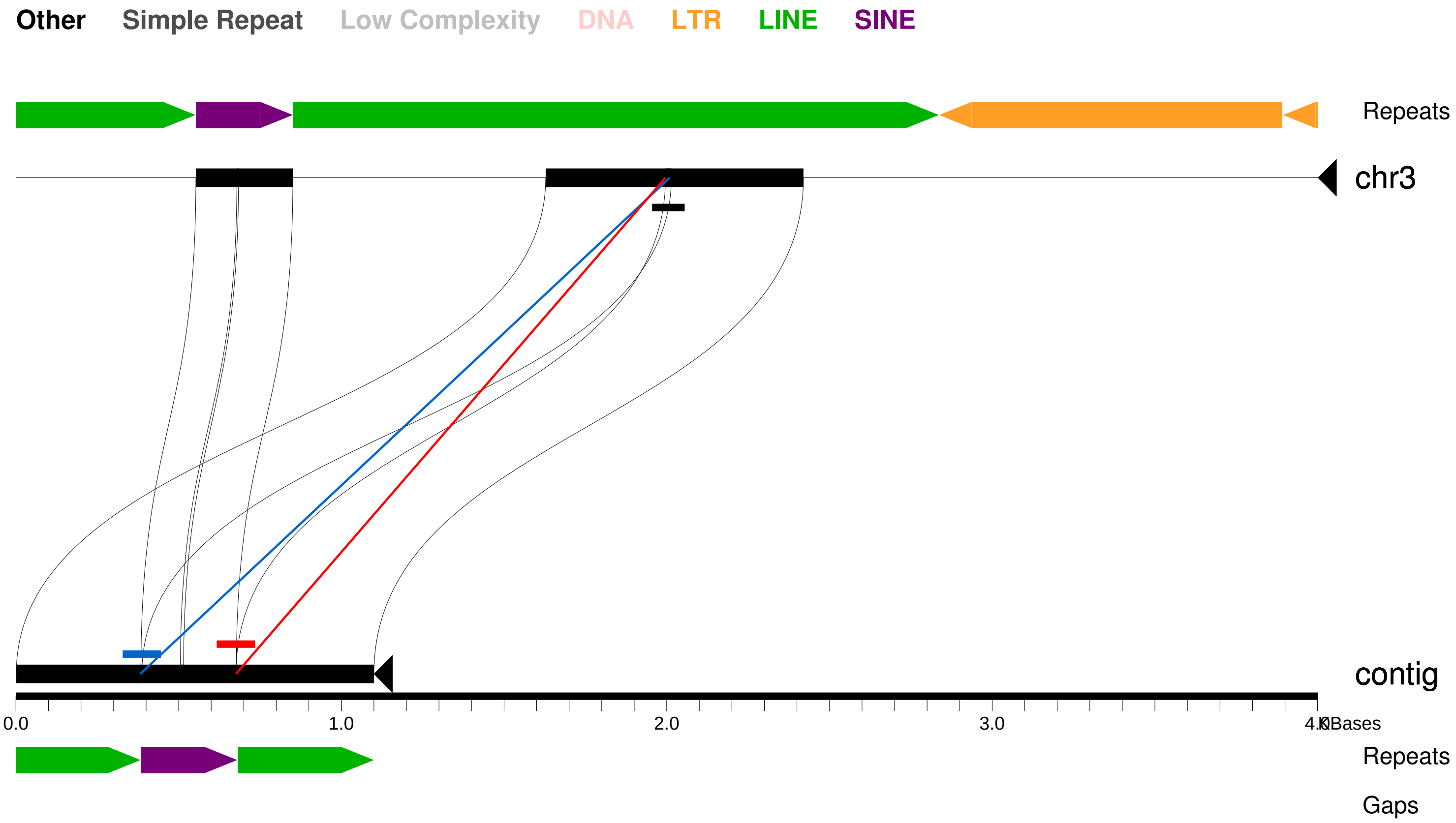

siteID: chr3_113047164 Scafftig3 - Breakpoint: chr3:113047145
chr3:113047097-113047204
Overlap Length: 12 INS size: 460 Genotyped: No

```
left     AATAT-----------TTTTAAATAGATAAAATAATCATG---GAGCCACAAAAAACCTGTTCAATCTCT
chrom    AATAT-----------TTTTAAATAGATAAAATAATCATG---GAGCCACAAAAAACCTGTTCAATCTCT
right    TCCACCCGCCTCGGCCTCCCAAAGTGCTGGGATTA-CAGGCGTGAGCTACCGCG--CCCGGCCAATCTCT
         111*111111111111*111***11*1*111**1*1**1*111****1**111111**1*11********

left     TTCTTTTTTTTNNNNNNNNNNNNNNNNNNNNNNNNNNNNNNNNNNNNNNNNNNNNNNNNNNNN
chrom    TTCTAAGATT-----------GAAGAAATTAAGGTCAAAGTTACTTTTCCTGATAAGAGCCGA
right    TTCTAAGATT-----------GAAGAAATTAAGGTCAAAGTTACTTTTCCTGATAAGAGCCGA
         ****2222**22222222222222222222222222222222222222222222222222222
```

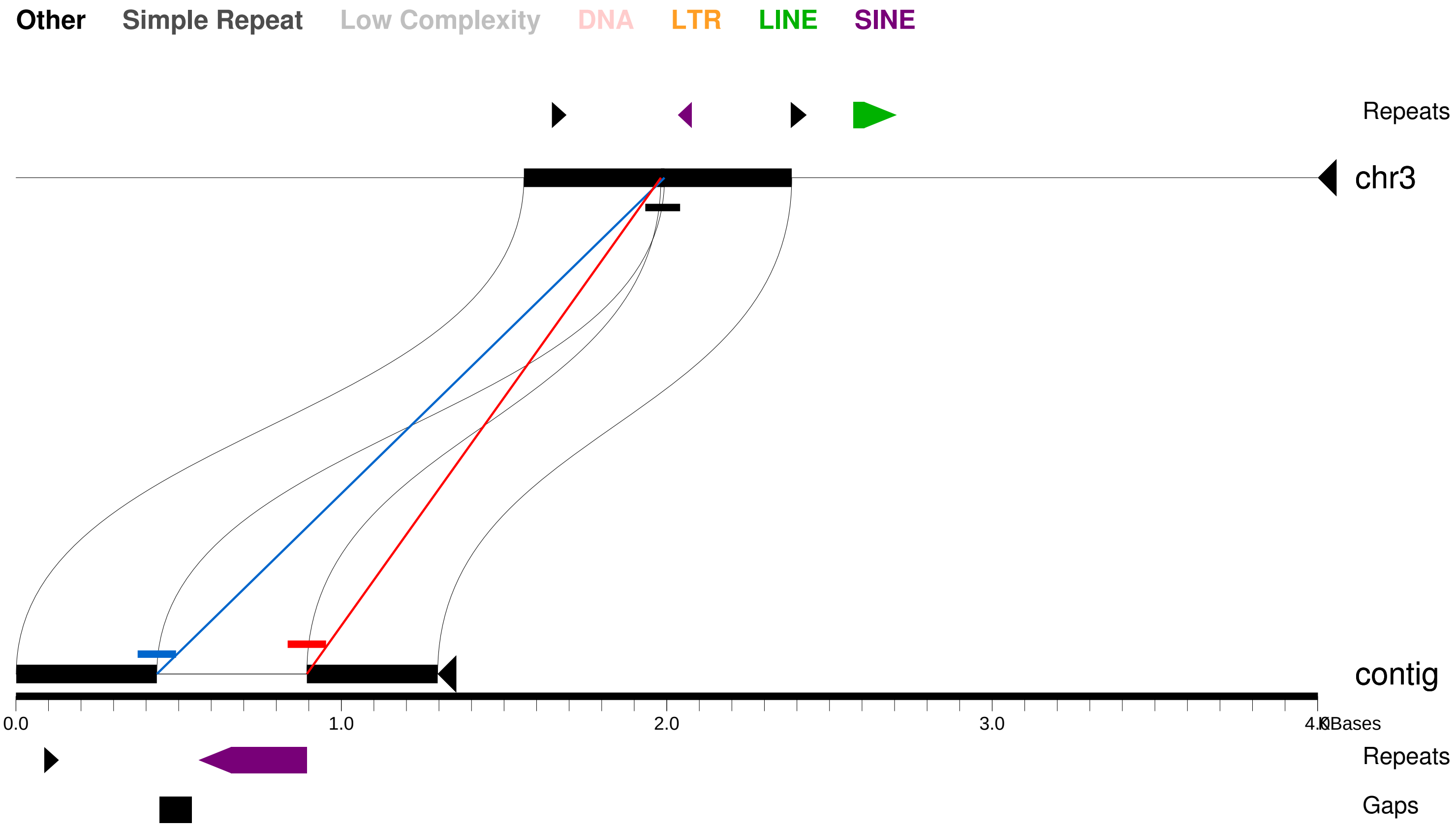

siteID: chr3_114364954 Scafftig1 - Breakpoint: chr3:114364935
chr3:114364879-114364984
Overlap Length: 4 INS size: 311 Genotyped: Yes

```
left   GAAGAGTCAACTTTTTGGCCTTAGTCATTTACTGAGTTCCTTTATT--------------AAAAATAGAG
chrom  GAAGAGTCAACTTTTTGGCCTTAGTCATTTACTGAGTTCCTTTATT--------------AAAAATAGAG
right  CTGCACTCCAGCCTG-GGCGACAGAGCGAGACTCCGTCTCAAAAAAAAAAAAAAAAAAAAAAAAAAAAAA
       1111*1**1*111*11***111**111111***11**11*111*1111111111111111*****1*1*1

left   CATAGGCCGGGCGCGGTGGCTCACGCCTGTAATCCCAGCACTTTGGGAGGCCGAGGCGGGCGG
chrom  CATAAACAT-------TTTAGAAATAATTTTTTCTCAATCTTTTTTTA---TAAAAC---CTC
right  CATAAACAT-------TTTAGAAATAATTTTTTCTCAATCTTTTTTTA---TAAAAC---CTC
       ****22*222222222*22222*2222*2*22**2**2222***222*22222*22*222*22
```

Other Simple Repeat Low Complexity DNA LTR LINE SINE

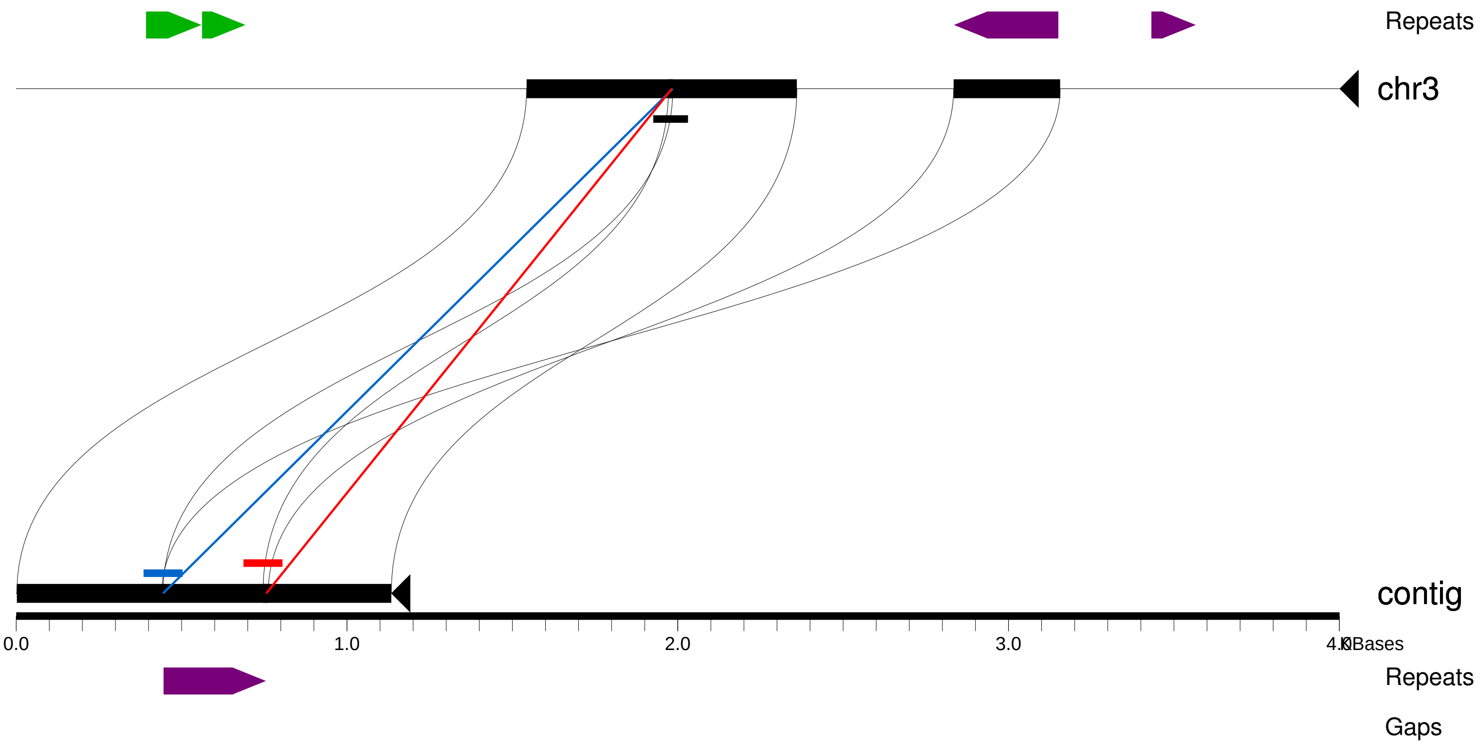

siteID: chr3_114915074 Scafftig1 - Breakpoint: chr3:114915088
chr3:114915035-114915147
Overlap Length: 7 INS size: 473 Genotyped: No

```
left    CTACGTAATTTTAAAACCCTTAGTACATGTATCTTTAAAAACCATATTTTAAG------AATTTTAGGCC
chrom   CTACGTAATTTTAAAACCCTTAGTACATGTATCTTTAAAAACCATATTTTAAG------AATTTTAATTT
right   NNNNNNNNNNNNNNNNNNNNNNNNNNNNNNNNNNNNNNNNNNNNNNNNNNNNNNNNNNNAATTTTAATTT
        11111111111111111111111111111111111111111111111111111111111*******2222

left    GGGCGCGGTGGCTCACGCCTG------TAATCCCAGCACTTTGGGAGGCCGAGGCGGGCGG
chrom   ATGAAAGGTGTCTTCTCTCTGAGTTTTTTATCCC--CATGTTGCTAAGTCT----------
right   ATGAAAGGTGTCTTCTCTCTGAGTTTTTTATCCC--CATGTTGCTAAGTCT----------
        22*222****2**22222***222222*2*****22**22***22*2*2*22222222222
```

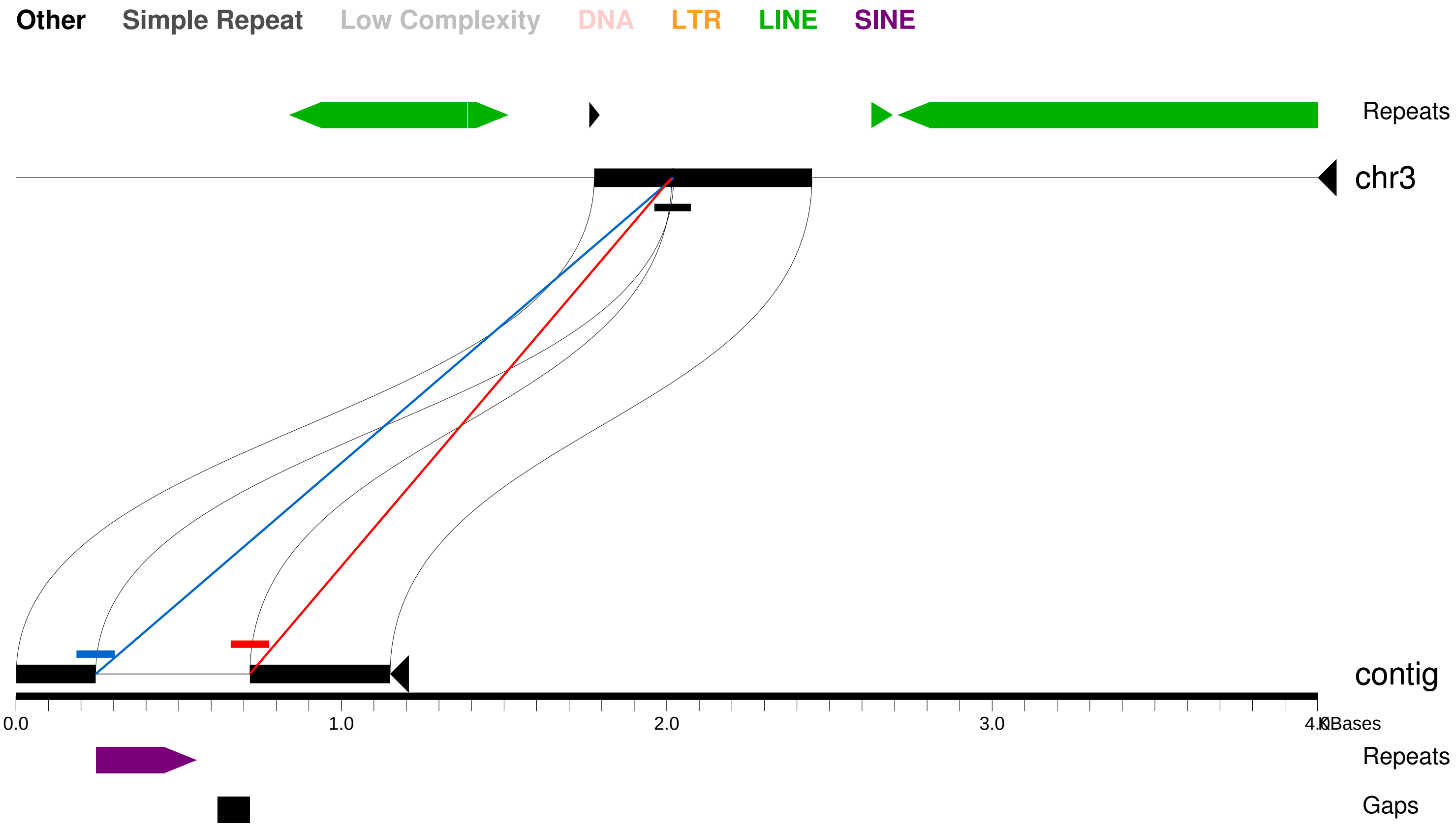

siteID: chr3_115383099 Scafftig1 + Breakpoint: chr3:115383078
chr3:115383032-115383137
Overlap Length: 14 INS size: 431 Genotyped: No

```
left    TTA---GAATTTGCTATTATTATGTTCTCCAAGCATA----TGAG-------GGTATTTTATGTCATTAC
chrom   TTA---GAATTTGCTATTATTATGTTCTCCAAGCATA----TGAG-------GGTATTTTATGTCATTAC
right   CCACCCGCCTCGGCC-TCCCAAAGTGCTGGGATTACAGGCGTGAGCCACCGCGCCCGGCCATGTCATTAC
        11*111*11*11**11*1111*1**1**111*11*1*1111****1111111*1111111**********

left    TTTTTTTTTTNNNNNNNNNNNNNNNNNNNNNNNNNNNNNNNNNNNNNNNNNNNNNNNNNNNNN
chrom   TTTT-------------AAAGGTCGCTTTTTTATATGTGTTAGTGGGAGGCATTTATTATTTT
right   TTTT-------------AAAGGTCGCTTTTTTATATGTGTTAGTGGGAGGCATTTATTATTTT
        ****22222222222222222222222222222222222222222222222222222222222
```

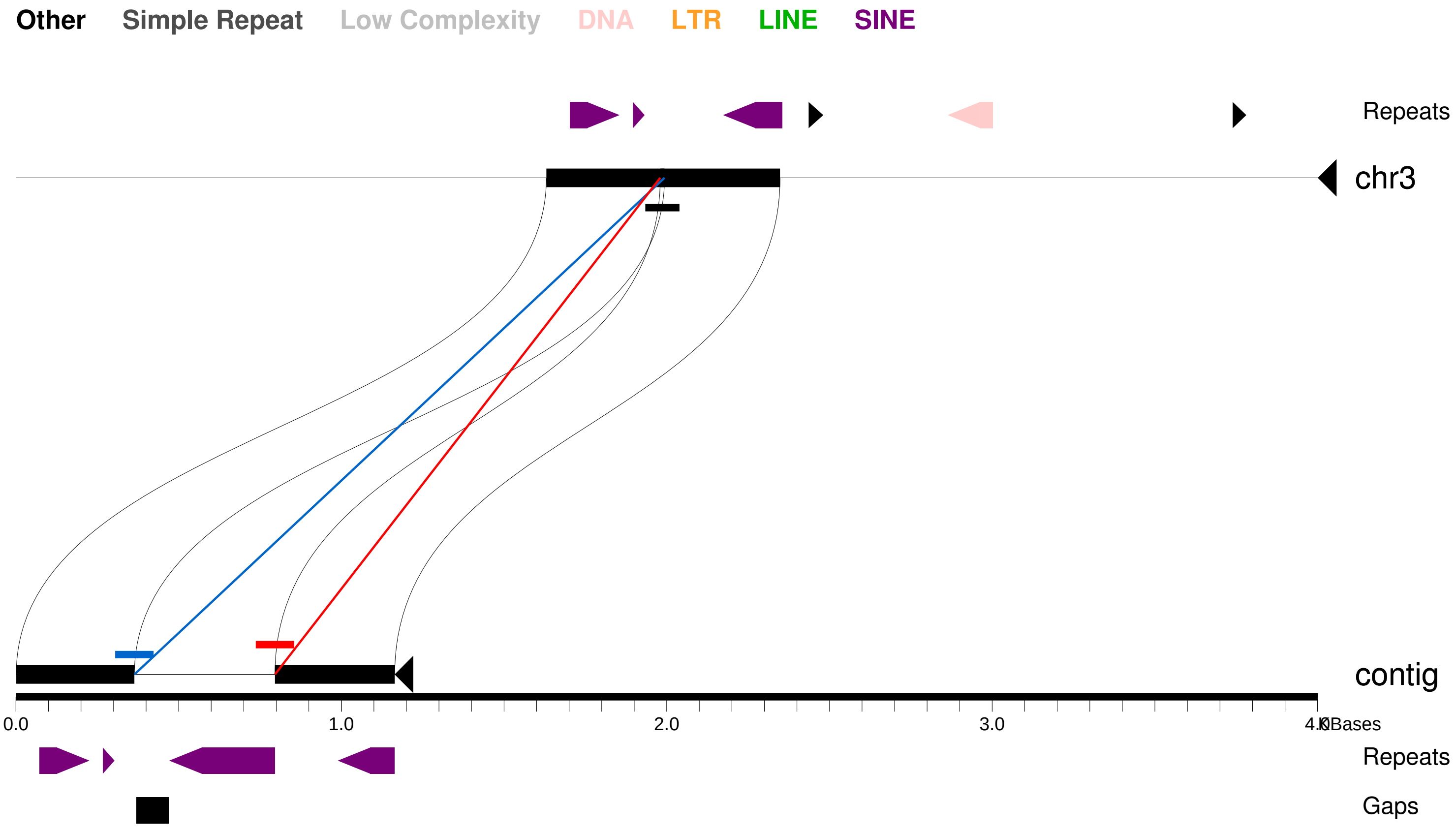

siteID: chr3_115604503 Scafftig1 + Breakpoint: chr3:115604454
chr3:115604294-115604628
Overlap Length: 15 INS size: 153 Genotyped: Yes

```
left   ---------------TAGAAGACTAGTGGGGAAGCTAGGTTATATTGACCAACAAAATATGAGGACAGAC
chrom  GTTCTGAAGAAAAACTAGAAGACTAGTGGGGAAGCTAGGTTATATTGACCAACAAAATATGAGGACAGAC
right  -------AGAATGGCGTGAACCCGGGAGGCGGAGCTTGC----AGTGAGCCG----AGATCCCGCCA--C
              2222NNN211***11*11*1**1*1****1*11111*1***1*111111*1**111*1**11*

left   TGAAAGAGGTAAGTTTAATATCAGCTAAAGTCTTGGGCAGCTGGTCAGAATCCAGACTGGATTCATGCTG
chrom  TGAAAGAGGTAAGTTTAATATCAGCTAAAGTCTTGGGCAGCTGGTCAGAATCCAGACTGGATTCATGCTG
right  TGCAC--------TCCAGCCTGGGCGACAGAGCGAGACTCCGTCTCAAAAAAAAAAAAAAA---------
       **1*11111111*11*111*11**1*1**11111*1*11*111***1**111*1*1111*111111111

left   TCATCAGCAGAGGTCAATTTGAAAGGTAAACAACTGTCCCAGCTACTTGGGAGGCTGAGGC-AGGAGAAT
chrom  TCATCAGCAGAGGTCAATGTGAAAGGTAAACAACTTTTGTGTGTGTGGGGGTGGGTGGGGGGACAAGTAT
right  --AAAAAAAAAAAAAAAAAAGAAAGGTAAACAACTTTTGTGTGTGTGGGGGTGGGTGGGGGGACAACTAT
       11*11*11*1*1111**1N1**************2*222222*2222***2**2**2**22*22*12**

left   G----GCGTGAACCCGGG-AGGCGGAGCTT------------GCAGTGAGCCGAG--------ATCCCGC
chrom  CTGCTGCATAAAGCAAGATAGATGCTTCTTATCAATCAAGAAGCATTTAGATGCTTCTTATCAATCGGAA
right  CTGCTGCATAAAGCAAGATAGATGCTTCTTATCAATC-----------------------------GGAA
       22222**2*2**2*22*22**22*222***2222222     111N1N11NN1NN        1112222

left   CACTGCACTCCAGCCTG----GGCGACAG---AGCGAGACTCCGT-CTCA----------------
chrom  TACTGGAAACAACTTAGAAATGGCAAGGCTTAAGTTAGAGTCAATACTCATGCAG-----------
right  TACTGGAAACAACTTAGAAATGGCAAGGCTTAAGTTAGAGTCAATACTCATGCAGGCTAATGCTAC
       2****2*22*2*2222*2222***2*222222**22***2**22*2****2222211111111111
```

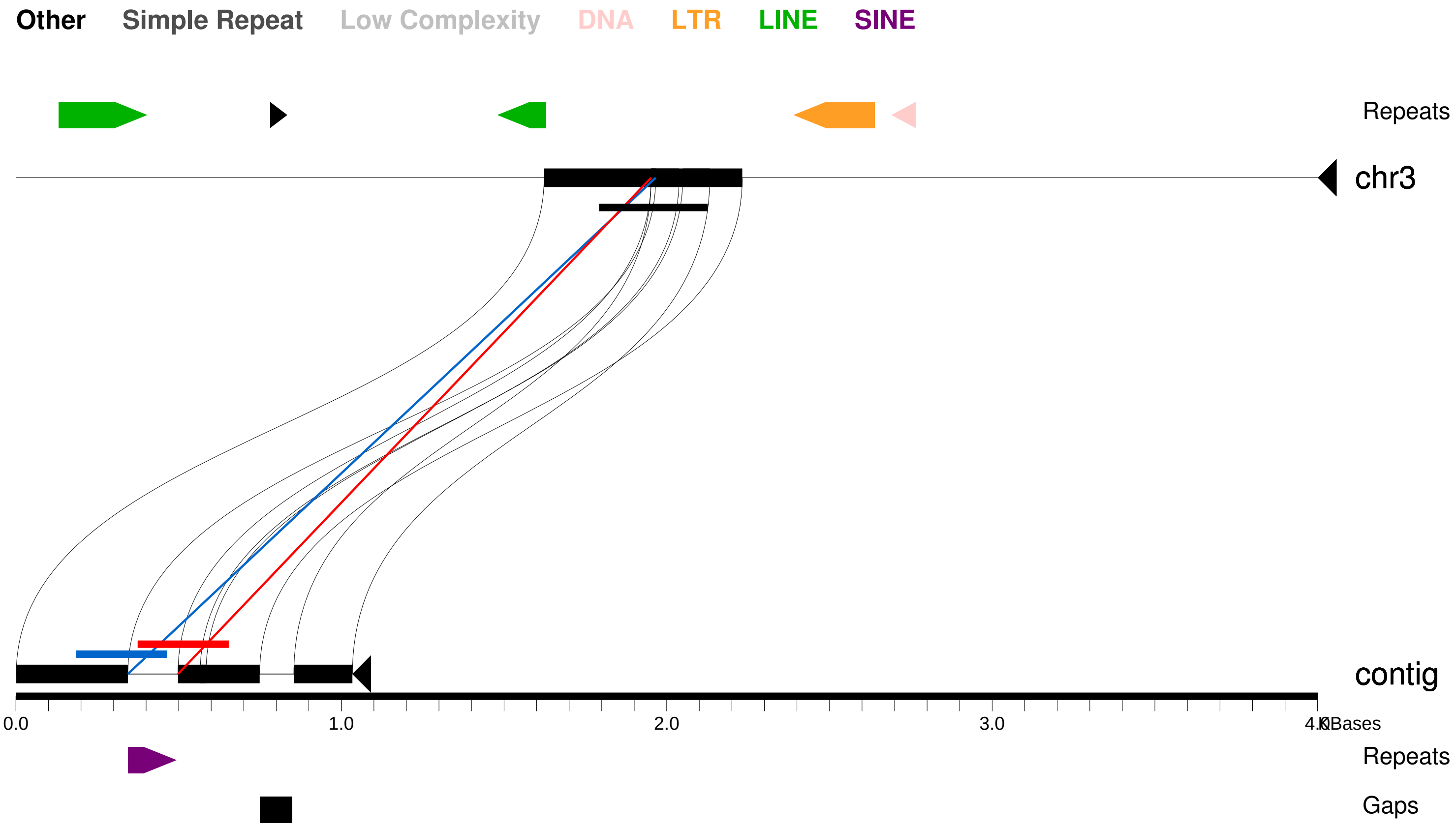

siteID: chr3_120318963 Scafftig1 + Breakpoint: chr3:120318953
chr3:120318924-120319017
Overlap Length: 31 INS size: 281 Genotyped: Yes

```
left   -----------------ATTAAAAGGAACGGGAGCATTT-CCATCAT--------AAGGTTTTTTTTTTTT
chrom  -----------------ATTAAAAGGAACGGAAGCATTT-CCATCAT--------AAGGTTTTTTTTTTTT
right  CCGCCTCGGCCTCCCAAAGTGCTGGGATTACAGGCGTGAGCCACCGCGCCCGGCCAAGGTTTTTTTTTTTT
       11111111111111111*1*1111***111121**1*111***1*1111111111***************

left   TTTTTTTTTTTTTTTTGAGACGGAGTCTCGCTCTGTCGCCCAGGCTGGAGTGCAGTGGCGGGATCTCGGC
chrom  GTTTTTTTTTTTTTTTTAA-AC---------TTCTGA-------GCTGCAGCTGATT------ATCTCAGG
right  -TTTTTTTTTTTTTTTTAA-AC---------TTCTGA-------GCTGCAGCTGATT------ATCTCAGG
       N**************2*2**2222222222****22222222****2**222*2*222222*****2*2

left   TCACT
chrom  GTA--
right  GTA--
       22*22
```

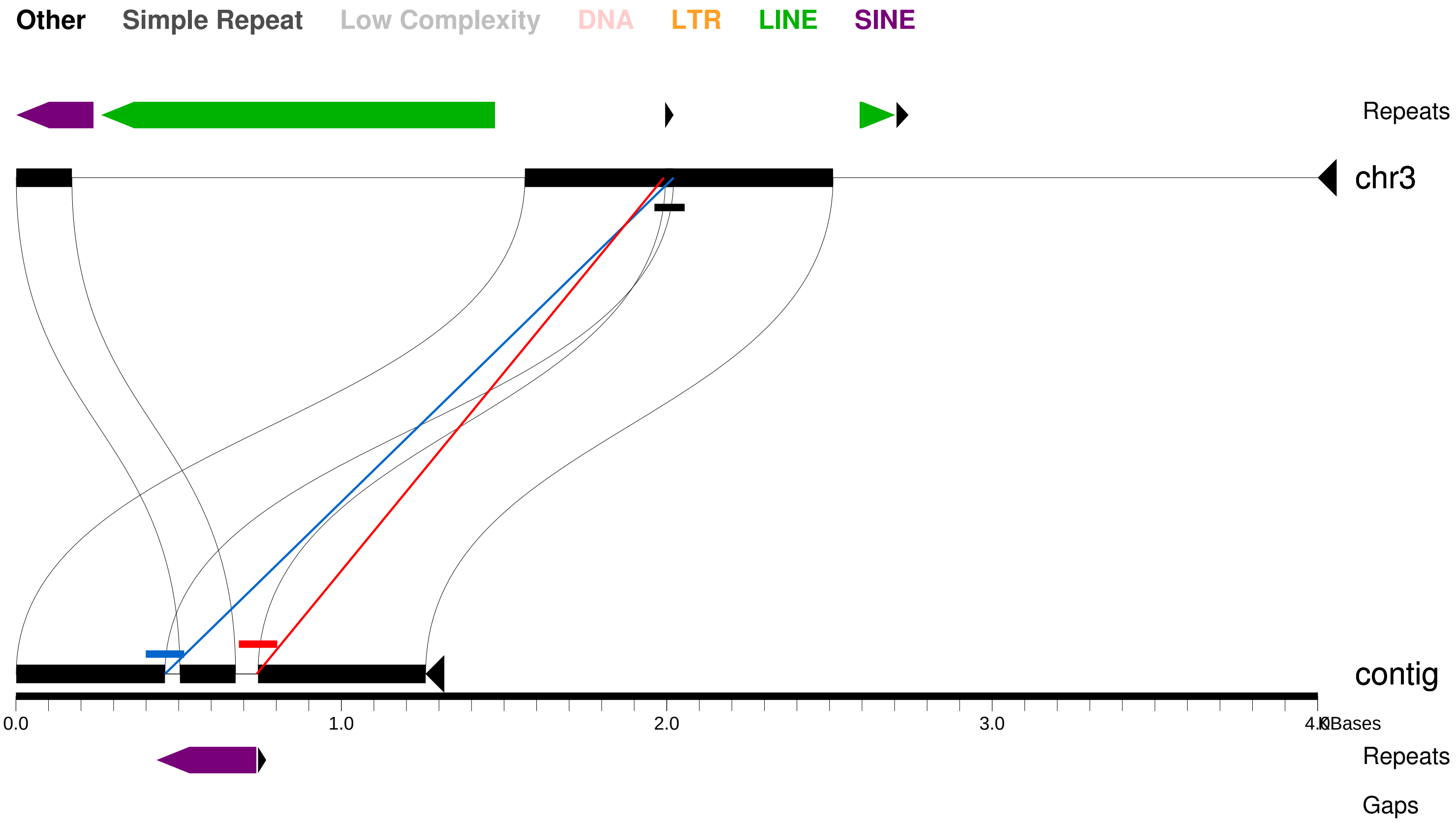

siteID: chr3_120425479 Scafftig2 + Breakpoint: chr3:120425461
chr3:120425416-120425520
Overlap Length: 15 INS size: 283 Genotyped: Yes

```
left     TAA-----TATTA----GGTAATGAAAATCTACTAAAATAGTACTGATTTGATA-------CAATTGCTA
chrom    TAA-----TATTA----GGTAATGAAAATCTACTAAAATAGTACTGATTTGATA-------CAATTGCTA
right    AGACGGGGTTTCACCTTGTTAGCCAGGATGGTCTCGATC--TCCTGACCTCATGATCCACCCAATTGCTA
         11*11111*1*1*1111*1**111*11**111**11*1111*1****11*1**11111111*********

left     TTCTTTTTTTTTTTTTTTTTTTTTTTTTTTTTTTTTTTTTTTTTTTTTTTTTTTTTTTGAGACGGAGT
chrom    TTCTTTAGCTGACAAAATAGTGTCCTGTAGATCATCTGAGACTAATCTATT--------------
right    TTCTTTAGCTGACAAAATAGTGTCCTGTAGATCATCTGAGACTAATCTATT--------------
         ******222*2222222*22*2*22*2*222*22*2*22222*22*2*2**22222222222222
```

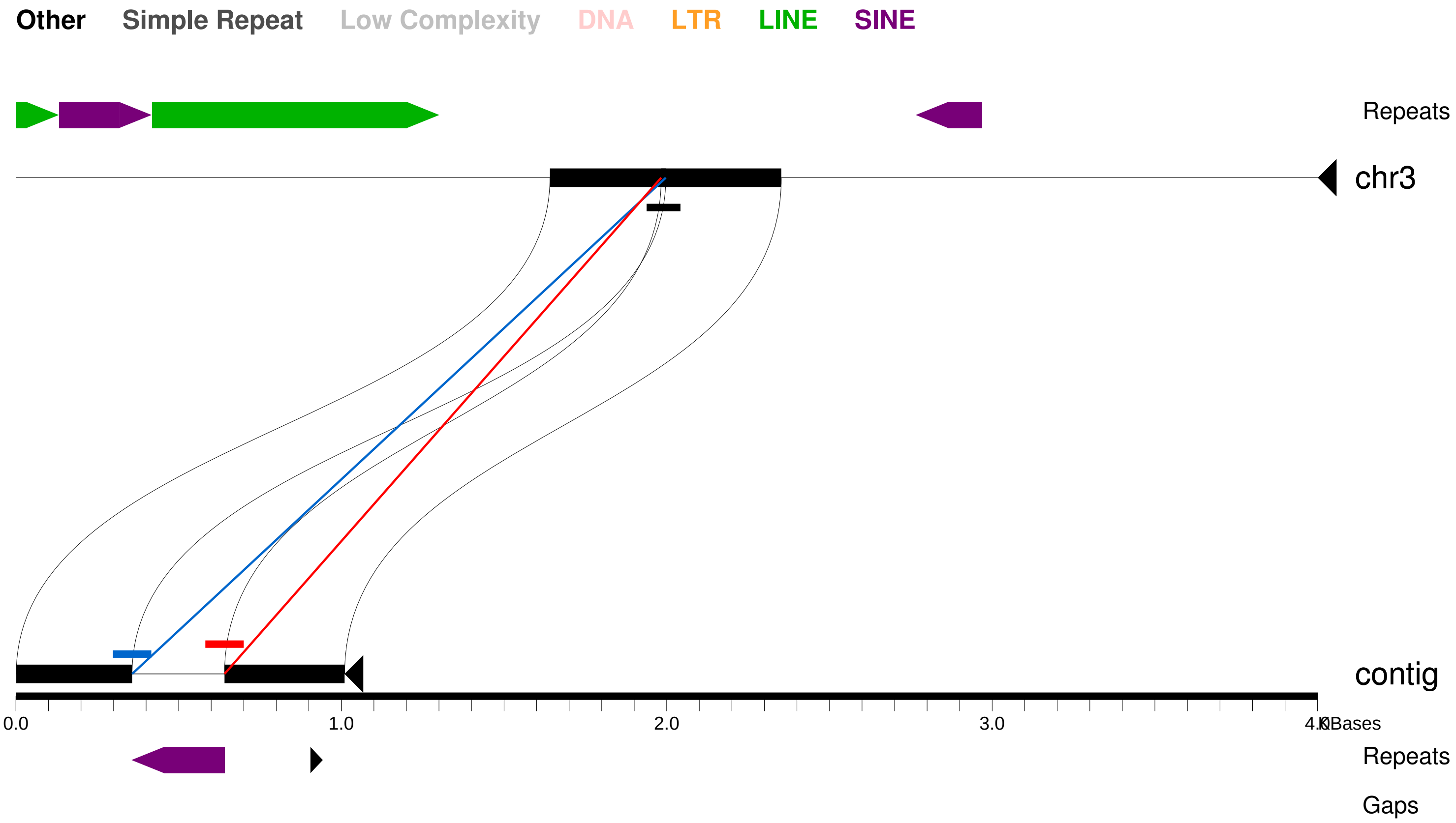

siteID: chr3_124568679 Scafftig2 + Breakpoint: chr3:124568673
chr3:124568245-124568833
Overlap Length: 12 INS size: 313 Genotyped: Yes

```
left   AA------------------------------------------------------------------
chrom  AATACCCCAATGGGCAAAAATATGCATGCTTATGCCACATCACCTGTGTTTGTTTCTATTTCTCTCGCCA
right  TTTT------------AGTAGAGACGGGGTT--------TCACCT-TGTTAG------------------
       112N            2NN2N2NN2NN2N22        222222 2222N2

left   ----------------------------------------------------------------------
chrom  TTTACCAGACTAGTATTTATTTGGGGGGCATATTTGATGTTTTGGGACATCACAGGCACAACTCATTATC
right  ----CCAG--------------------------GATGGTCTCG---ATCTC---CTAACCTCATGATC
           2222                          2222N2N2N2   222N2   2NN2N22222N222

left   ----------------------------------------------------------------------
chrom  ATTGGAAACTACATATATAAATACAGGAAGACAGATTCCAAATTTTAGCTCTATCGAAAAAAGAAAACAA
right  -------------------------------CACCCGCC--------------TCG--------------
                                      22NNNN22              222

left   -------------------------------------------------------------------AAG
chrom  GAAGAAAGAGAAAAGACAGCACTAGATTTGTCTACCAGCTACAATAAGATAGCCAACAGGCAGAGAAAAG
right  -----------------------------GCCTCCCA--------AAGT----------GCTGGGA----
                                    2N22N222        222N          22N2N22 111

left   GGGAATTAATTCAGAGGAAAAGAACAAAGAGAAAAGCTAGTGTCTGGCAGGTGTGGCCTTTTCTGTCTCA
chrom  GGGAATTAATTCAGAGGAAAAGAACAAAGAGAAAAGCTAGTGTCTGGCAGGTGTGGCCTTTTCTGTCTCA
right  -----TTA----------------------------------------CAGGCGTG---------------
       11111***1111111111111111111111111111111111111111****1***111111111111111

left   CCCTATAGAGTTCAAAGAGAGGCTCAAGTCAGGGCAATAACTGGAAGACCCAGGCCACGATAATGATCTT
chrom  CCCTATAGAGTTCAAAGAGAGGCTCAAGTCAGGGCAATAACTGGAAGACCCAGGCCACGATAATGATCTT
right  --------------------------AGCCACCGCG-------------CCCGGC---------------
       11111111111111111111111111**1**11**11111111111111**1***111111111111111

left   GAAATCATTTCATAATTTCTTTTTTTTTTTTTTTTTTTTT-----TTTGAGACGGAG-TC--------TCG
chrom  GAAATCATTTCATAATTTCTAAAAACAAATCACTTAGAACCAACTTTAAGGCTCAACTCATACCAGATCA
right  --------TTCATAATTTCTAAAAACAAATCACTTAGAACCAACTTTAAGGCTCAACTCATACCAGATCA
       11111111************222222222*222**222222222***2**2*22*22**22222222**2

left   CTCTGTC-GCCCAGGCCGGACTGC-------GGACTGC----AGTGGCGCAATCTCGGCT-CACTGCAAG
chrom  TTCTATCTGCCTTGGGCTTATTAACCTTTCAGGAGTGCCAAGAGATGCCCTATTTATCCTGCAGTAGAAC
right  TTCTATCTGCCTTGGGCTTATTAACCTTTCAGGAGTGCCAAGAGATGCCCTATTTATCCTGCAGTAGAAC
       2***2**2***22**2*22*2*222222222***2***2222**22**2*2**2*222**2**2*22**2

left   ---CTCCGCTTCCCGGG---TTCACGCCA
chrom  TGTCTACACATACTTGAAATTTGGATAGA
right  TGTCTATACATACTTGAAATTTGGATAG-
       222**212*2*2*22*2222**2222221
```

Other Simple Repeat Low Complexity DNA LTR LINE SINE

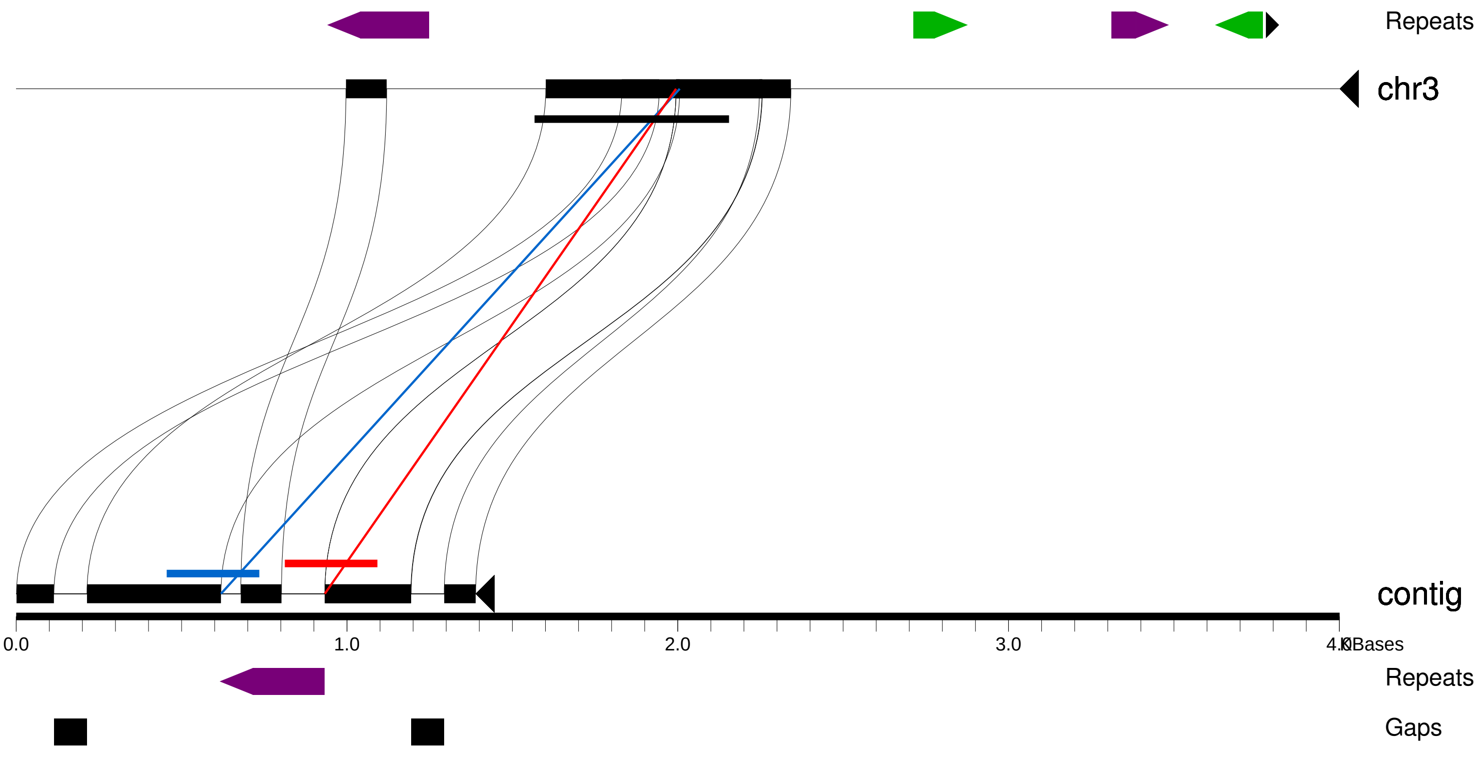

siteID: chr3_12818456 Scafftig2 - Breakpoint: chr3:12818453
chr3:12818409-12818512
Overlap Length: 16 INS size: 323 Genotyped: Yes

```
left     G--CCTGTCTCTGTTTCTCAA--TTC----ATTCCA--CATTGTGAAAGG-----GAAAGGGGCTATTGT
chrom    G--CCTGTCTCTGTTTCTCAA--TTC----ATTCCA--CATTGTGAAAGG-----GAAAGGGGCTGTTGT
right    CCACCTGCCTCGGCCTCCCAAAGTGCTGGGATTACAGGCGTGAGGCACCGCGCCCGGCTGGGGCTGTTGT
         111****1***1*11**1***11*1*1111***1**11*1*111*1*11*11111*111******2****

left     TTATTTATTTATTTATTTATTTATTTTTTTATTTTTTTTTGAGACGGAGTCTCGCTCTGTCGCC
chrom    TTATTAACACCCTAA---ATAGAAATCACCAAATAGTATGAAGAA------------TTTGACA
right    TTATTAACACCCTAA---ATAGAAATCACCAAATAGTATGAAGAA------------TTTGACA
         *****2*22222*2*222**22*22*2222*22*22*2*22***2222222222222*2*22*2
```

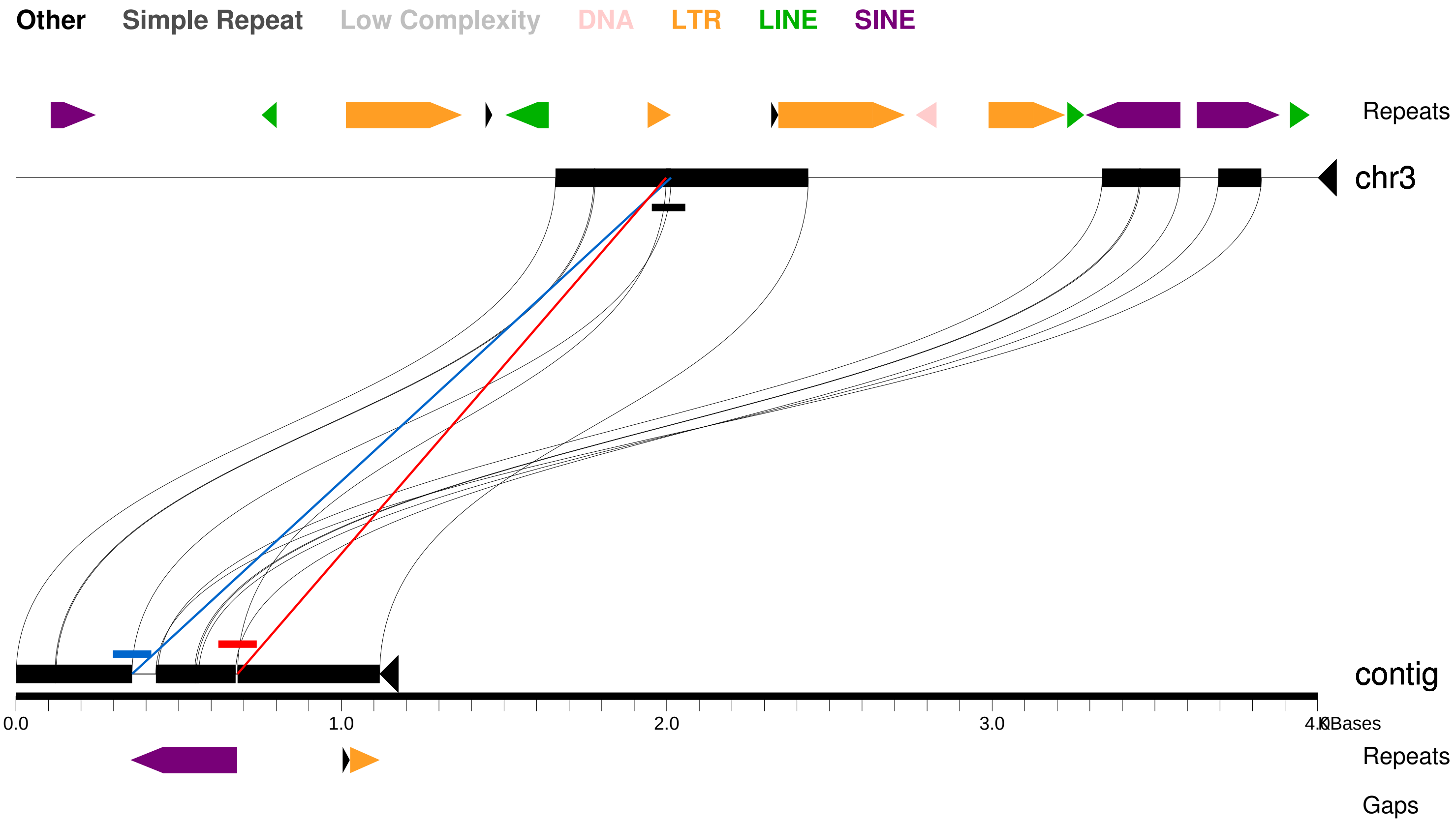

siteID: chr3_129938410 Scafftig9 - Breakpoint: chr3:129938414
chr3:129938375-129938473
Overlap Length: 18 INS size: 302 Genotyped: Yes

```
left   ATATGAGATAGGATAAGCTACAG---GAT---GT--------------AACAATGCTGCAAATAAAGAAT
chrom  ATATGAGATAGGATAAGCTACAG---GAT---GT--------------AACAATGCTGCAAATAAAGAAT
right  CTGCACTCCAGCCTGGGCGACAGCGAGACTCCGTCTCAAAAAAAAAAAAAAAAAAAAAAAAAAAAAAAAAT
       1*1111111**11*11**1****111**1111**11111111111111**1**111111***1***1***

left   CAATGGGGGCCGGGCGTGGTGGCTCACGCCTGTAATCCCAGCACTTTGGGAGGCCGAGGCGGGCGGATC
chrom  GAATGGGAGCACAGTGACTTGGAG------AGGAAAC-----------GAAAATAGATTC---CCTTTT
right  GAATGGGAGCACAGTGACTTGGAG------AGGAAAC-----------GAAAATAGATTC---CCTTTT
       2******2**222*2*222***222222222*2**2*22222222222*2*2222**22*222*222*2
```

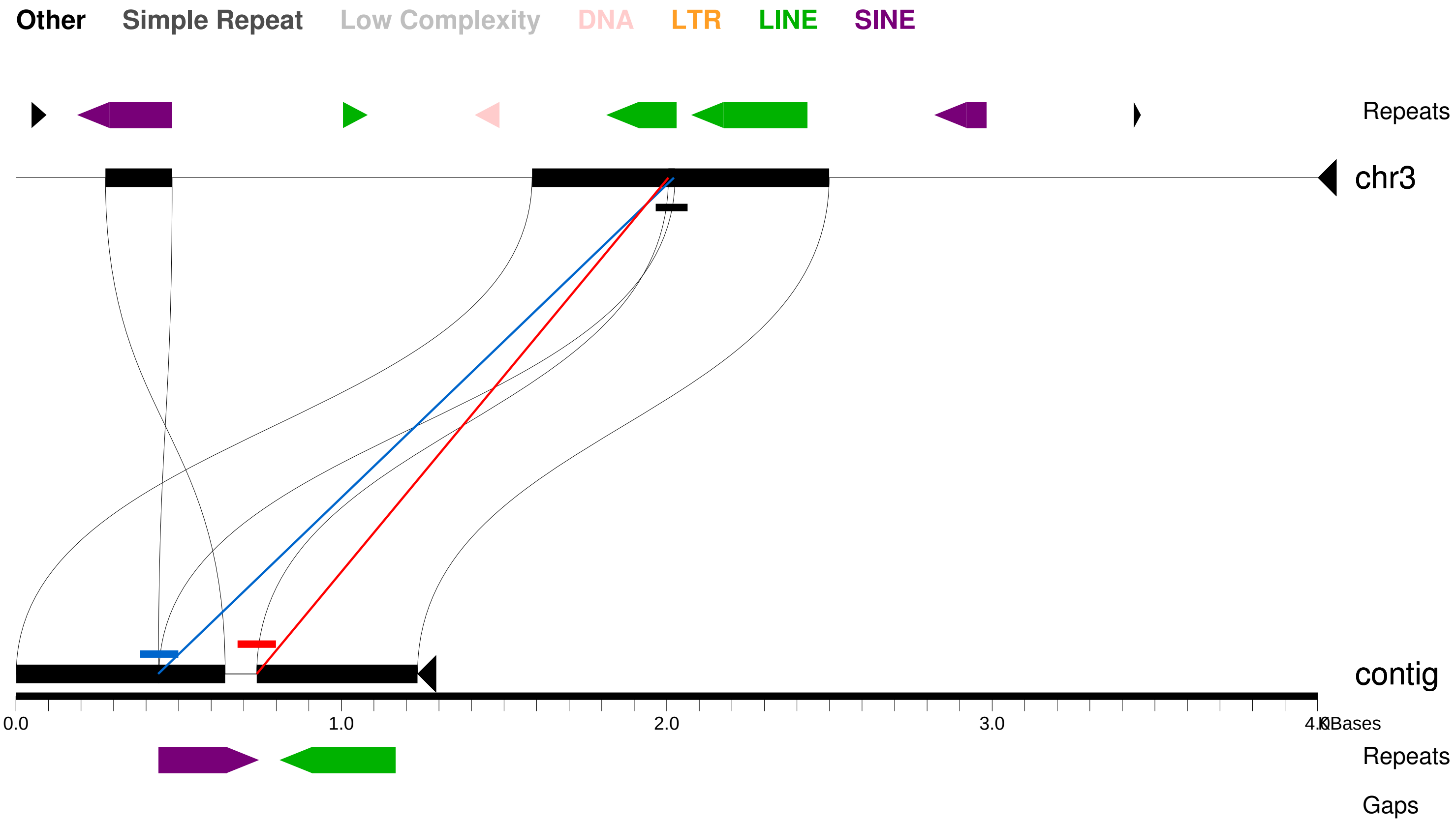

siteID: chr3_132577741 Scafftig1 - Breakpoint: chr3:132577724
chr3:132577564-132577897
Overlap Length: 14 INS size: 416 Genotyped: No

```
left    --------------------------------------------CATAAATATGTACTTACCTAAGTCCTGT
chrom   TT-ACTGTTGCTTGTGTAAG-CCAAGACAACCTTGATGTTATCATAAATATGTACTTACCTAAGTCCTGT
right   TTTAGTAGAGACGGGGTTTCACCGTGTTAGCCAGGATGGTCTCG-----ATCTCCTGACCTC------GT
        2212N2NNN2NNN2N22NNN122NN2NN2N22NN2222N2N2*111111**1*1**1****1111111**

left    CTTTTGGCAAATTATGGGCTATGAGGCACATAGCGTTCTTGCCTTTCCCTGAGGGGTTAATTTCAGTGAT
chrom   CTTTTGGCAAATTATGGGCTATGAGGCACATAGCGTTCTTGCCTTTCCCTGAGGGGTTAATTTCAGTGAT
right   GATCCGCCC-------GCCTCGGCCTCCCAAAGTGCT---------------GGGATTACAGGC-GTGAG
        11*11*1*11111111*1**11*111*1**1**1*1*111111111111111***1***1111*1****1

left    CCTACACATTACTTCTGAAGCACATATGCTCTTCTTTNNNNNNNNNNN----------------------
chrom   CCTACACATTACTTCTGAAGCACATATGCTCTTCTTATATGGCATGTAAGCCCTGGGTCTAGGGGGTAAC
right   CC--------ACCGCGCCCGGCCATATGCTCTTCTTATATGGCATGTAAGCCCTGGGTCTAGGGGGTAAC
        **11111111**11*1111*11*************2222222222222222222222222222222222

left    ----------------------------------------------------------------------
chrom   AGTGCAAAGATCTACCTGTCGTGCTGCTACATGCTTCTAAATTTGCTAATAAATCACCTTCCACTGACCA
right   AGTGCAAAGATCTACCTGTCGTGCTGCTACATGCTTCTAAATTTGCTAATAAATCACCTTCCACTGACCA
        2222222222222222222222222222222222222222222222222222222222222222222222

left    --------------------------------------------------------
chrom   AAAAAACACATTTTTCTCTCTAATCAGTATGACAATGATGGTGAGTAAAGATATAG
right   AAAAAACACATTTTTCTCTCTAATCAGTATGATAATGATGGTG-------------
        2222222222222222222222222222222N2222222222
```

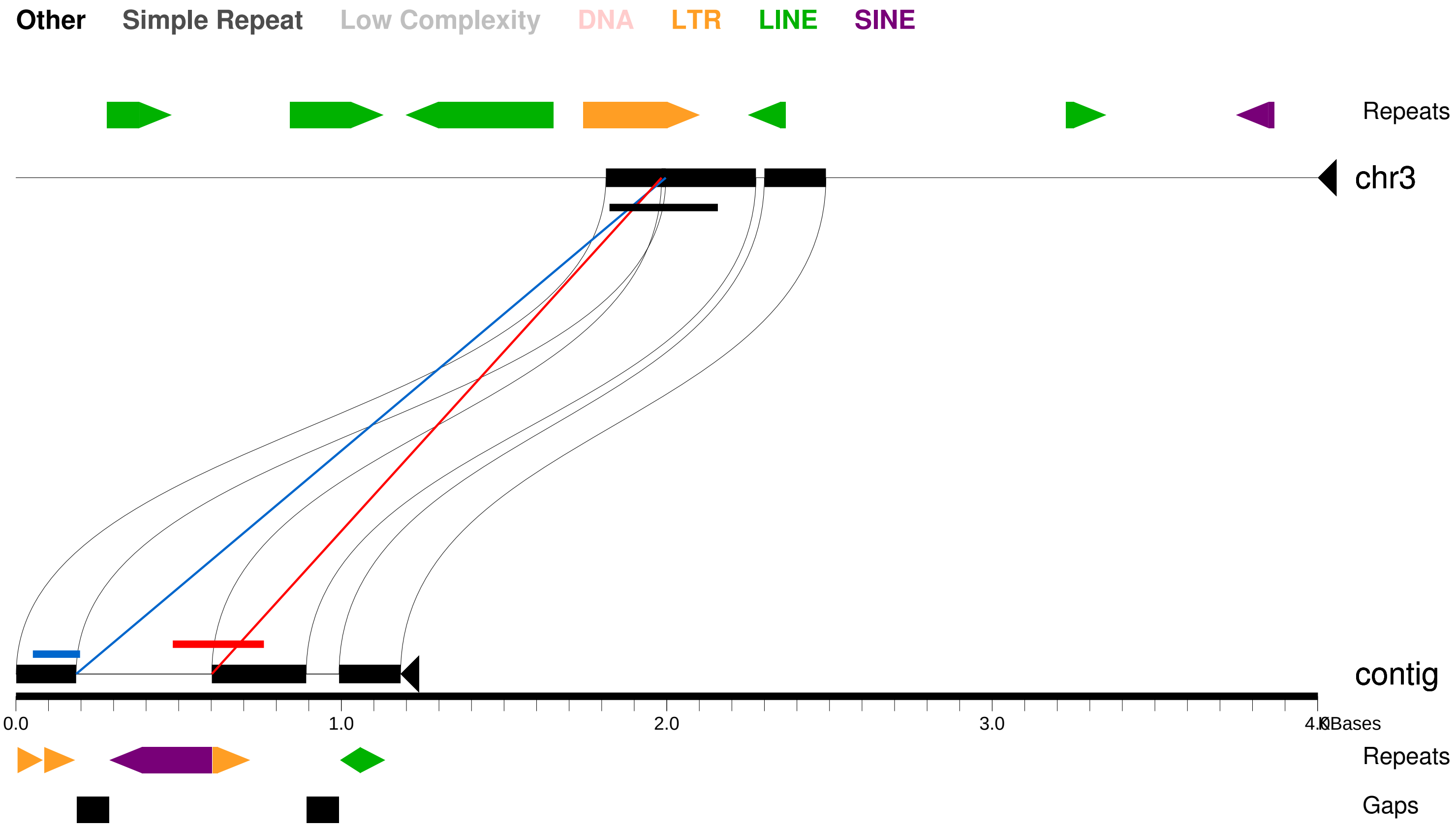

siteID: chr3_135080998 Scafftig1 - Breakpoint: chr3:135080991
chr3:135080831-135081160
Overlap Length: 10 INS size: 345 Genotyped: No

```
left   -----------------------------------TCCTTTTCAGAGATCGTGGTGTGTAAAAGTCTGCC
chrom  AGGACCAGGAACTACAATGTTTTCTACCCAATGGTTCCTTTTCAGAGATCATGGTGTGTAAAAGTCTGCC
right  TTTAGTAGAGACGG----GGTTTCA-----------CCGTGTTAGCCAGGATGGTCTCGATCTCCTGACC
       NNN2NN22NN22NN    2N2222N           1**1*1*1**11*112****1*11*11111111**

left   AATGCATCTGCACCTCTCTGGGTCATGATGCCTTTGGGGTATGAGGACTTATTATGACAATGCAACTTCC
chrom  AATGCATCTGCACCTCTCTGGGTCATGATGCCTTTGGGGTATGAGGACTTATTATGACAATGCAACTTCC
right  TCGTGATCCGCCCGCCTCGGCCTCCCAAAGTGCTGGGATTAC-AGGCGTGA----------GCCACCGCG
       11111***1**1*11***1*11**111*1*111*1**11**11***11*1*1111111111**1**11*1

left   CCATGGGACAAGAAGGATAATGAATGGTCANNNNNNNNNNN-----------------------------
chrom  CCATGGGACAAGAAGGATAATGAATGGTCATTTTTTGAGTGTTTACCCTGAAACAAATTTTGACAGGTAG
right  CCC--GGCC-----------TGAATGGTCATTTTTTGAGTGTTTACCCTGAAACAAATTTTGACAGGTAG
       **111**1*11111111111**********222222222222222222222222222222222222222222

left   ----------------------------------------------------------------------
chrom  AGTTTTGTTGACTTGAGAACAAGACCTTTCAGCCCTAGGATGGAGACCTAAAGGTATGTGGGCTCCCCAA
right  AGTTTTGTTGACTTGAGAACAAGACCTTTCAGCCCTAGGATGGAGACCTAAAGGTATGTGGGCTCCCCAA
       2222222222222222222222222222222222222222222222222222222222222222222222

left   --------------------------------------------------
chrom  AGGCACAGGAAGAGTATAGCTGAAAAGGGCACTAAGGTAGTGTAGGCTGA
right  AGGCACAGGAAGAGTATAGCTGAAAAGGGCACTAAGGTAG----------
       2222222222222222222222222222222222222222
```

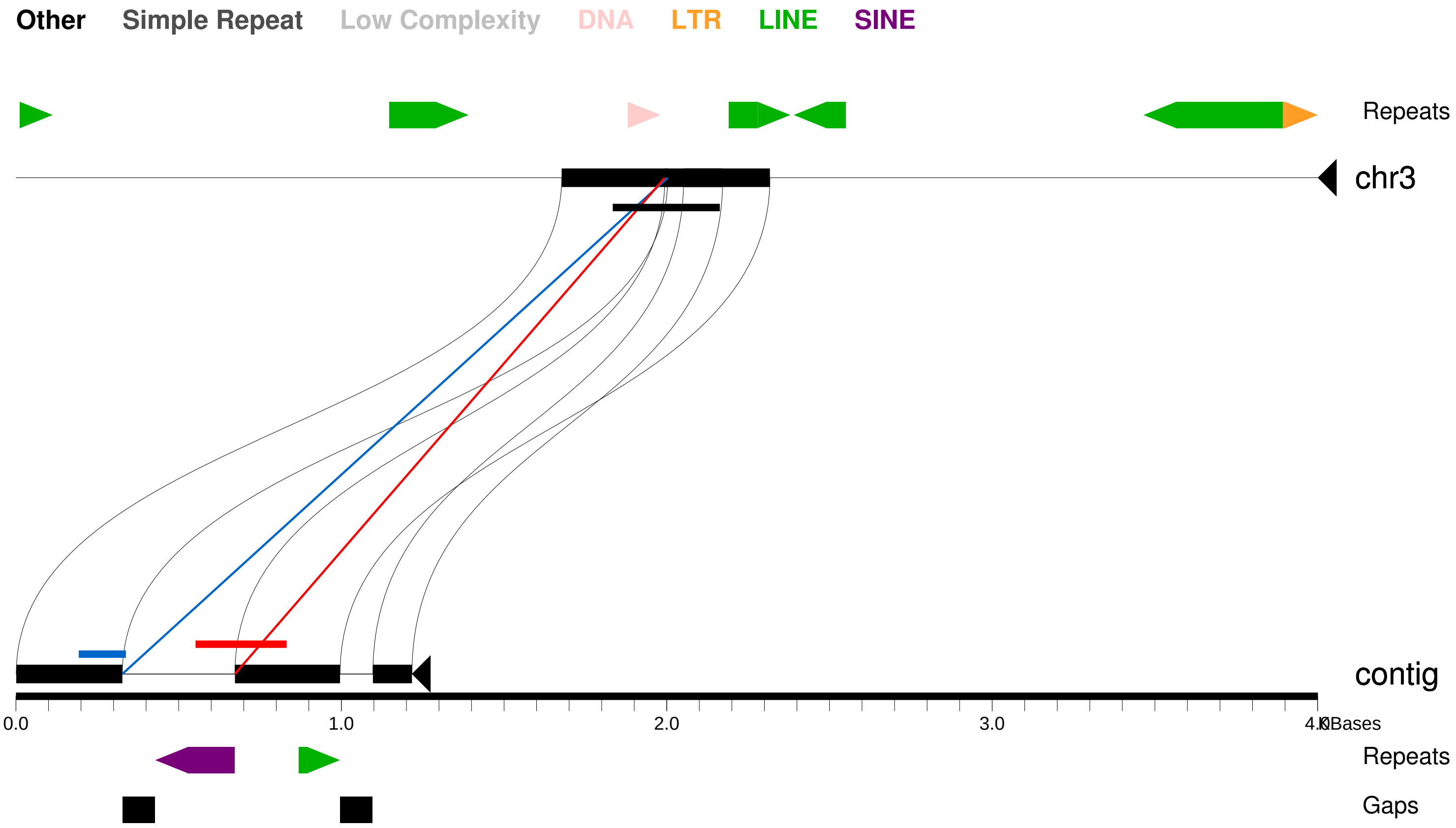

siteID: chr3_140524436 Scafftig3 - Breakpoint: chr3:140524424
chr3:140524377-140524483
Overlap Length: 13 INS size: 308 Genotyped: Yes

```
left    T------TTTAATTCTGTTTATGTGGTGT-ATTCCAT---TTACTGATTTCGTATGT--TGAACCATTTC
chrom   T------TTTAATTCTGTTTATGTGGTGT-ATTCCAT---TTACTGATTTCGTATGT--TGAACCATTTC
right   CCGCCCGCCTCGGCCTCCCAAAGTGCTGGGATTACAGGCGTGAGCCACCGCGCCCGGCCTGAACCATTTC
        111111111*1111**1111*1***1**11***1**1111*1*111*111**111*111***********

left    TTTTTTTTTTTTTTTTTTTTTTTTTTTTTTTGAGACGGAGTCTCGCTCTGTCGCCCAGGCTGG
chrom   TTAATACCTGATAGGAAACCCACTTGATCATAGTTGATTATC---CTTT---------TGA
right   TTAATACCTGATAGGAAACCCACTTGATCATAGTTGATTATC---CTTT---------TGA
        **22*222*22*22222222222**22*2222222**2*2**222**2*222222222**2
```

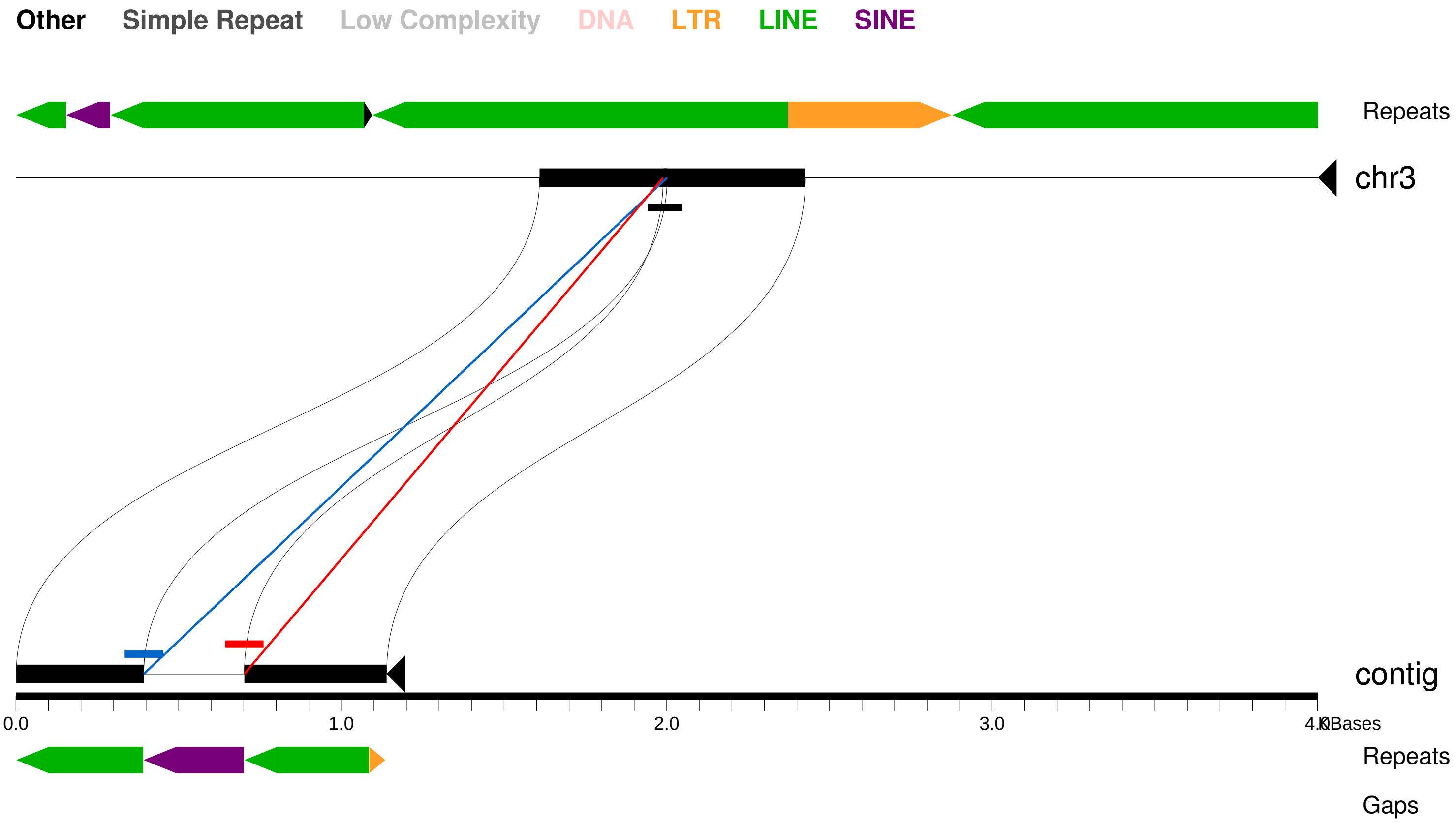

siteID: chr3_141310662 Scafftig1 + Breakpoint: chr3:141310635
chr3:141310585-141310694
Overlap Length: 10 INS size: 407 Genotyped: No

```
left    ATAGCCAGCC-----CTACCCTAGCTCA-----TATAGCCAGTGGACATTGTAAAAGAAATGTCCATGAT
chrom   ATAGCCAGCC-----CTACCCTAGCTCA-----TATAGCCAGTGGACATTGTAAAAGAAATGTCCATGAT
right   CC-GCCCGCCTCGGCCTCCCAAAGTGCTGGGATTACAGGCGTGAGCCACCGCGCCCGGCCTGTCCATGAT
        111***1***11111**1**11**11*111111**1**1*1111*1**11*11111*111**********

left    NNNNNNNNNNNNNNNNNNNNNNNNNNNNNNNNNNNNNNNNNNNNNNNNNNNNNNNNNNN
chrom   ATTTTAAACATAAATGTTCAATACACTGAAGTTGACACGGTTTTCACCTA---------
right   ATTTTAAACATAAATGTTCAATACACTGAAGTTGACACGGTTTTCACCTA---------
        22222222222222222222222222222222222222222222222222222222222
```

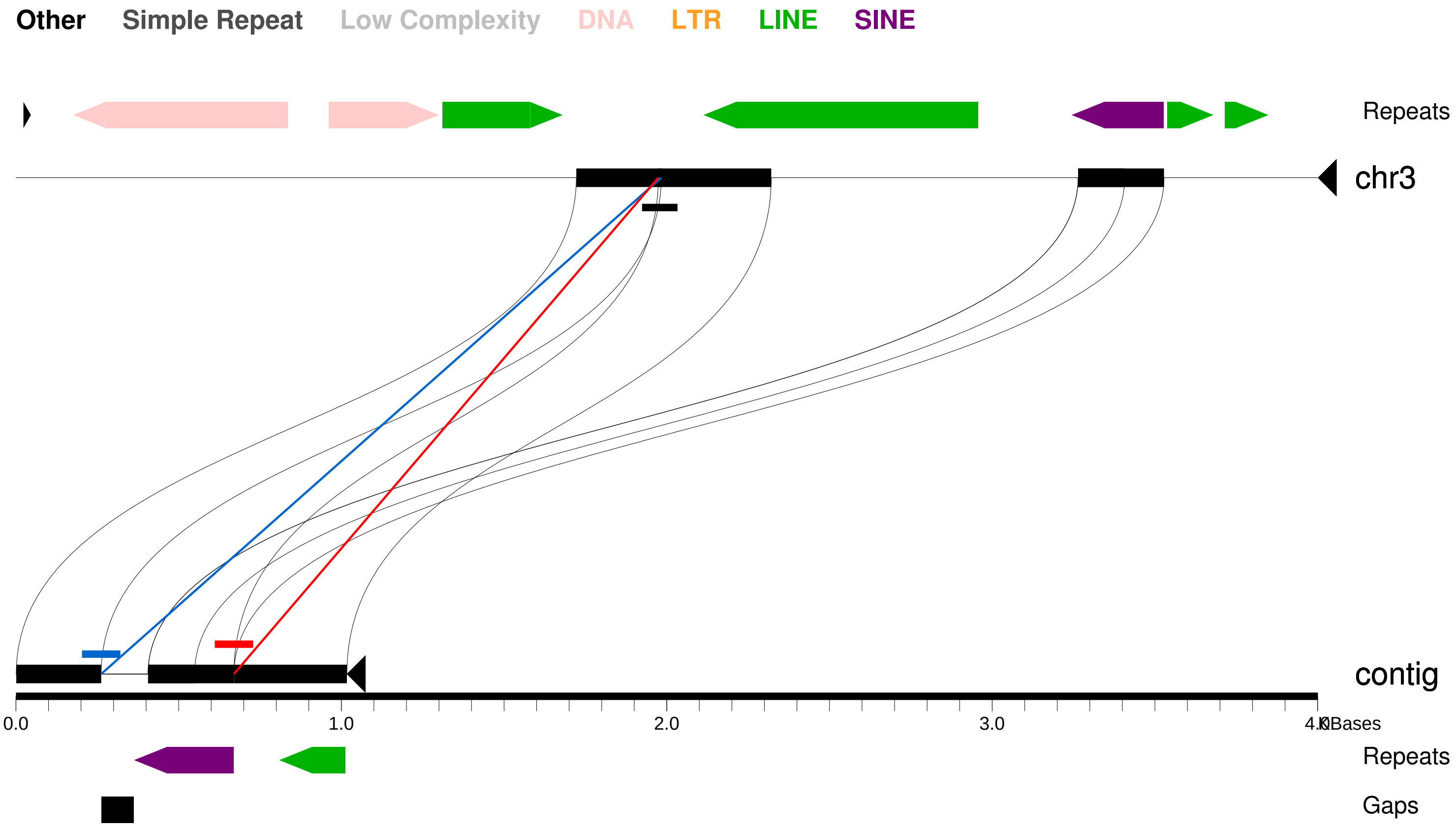

siteID: chr3_142460042 Scafftig2 + Breakpoint: chr3:142460024
chr3:142459980-142460083
Overlap Length: 16 INS size: 298 Genotyped: Yes

```
left    AATAT---------CTGAGAAATTGGAAAGCAAA-ACTAGTGAG-----GATTTTTTGTATAACTATGTA
chrom   AATAT---------CTGAGAAATTGGAAAGCAAA-ACTAGTGAG-----GATTTTTTGTATAACTATGTA
right   CCGCCCGCCTCGGCCTCCCAAAGTGCTGGGATTACAGGCGTGAGCCACCGCGCCCGGCCATAACTATGTA
        11111111111111**111***1**1111*111*1*111*****11111*111111111***********

left    TTTTTTTTTTTTTTTTTTTTTTGAGACGGAGTCTCGCTCTGTCGCCCAGGCTGGAGTGCAGTGG
chrom   TTTTTAAAATAATTT--------AAAACATTTATTTCTCT-----CATAACTGCCATTGATT--
right   TTTTTAAAATAATTT--------AAAACATTTATTTCTCT-----CATAACTGCCATTGATT--
        *****2222*22***22222222*2*22222*2*22****22222*2222***222*22*2*22
```

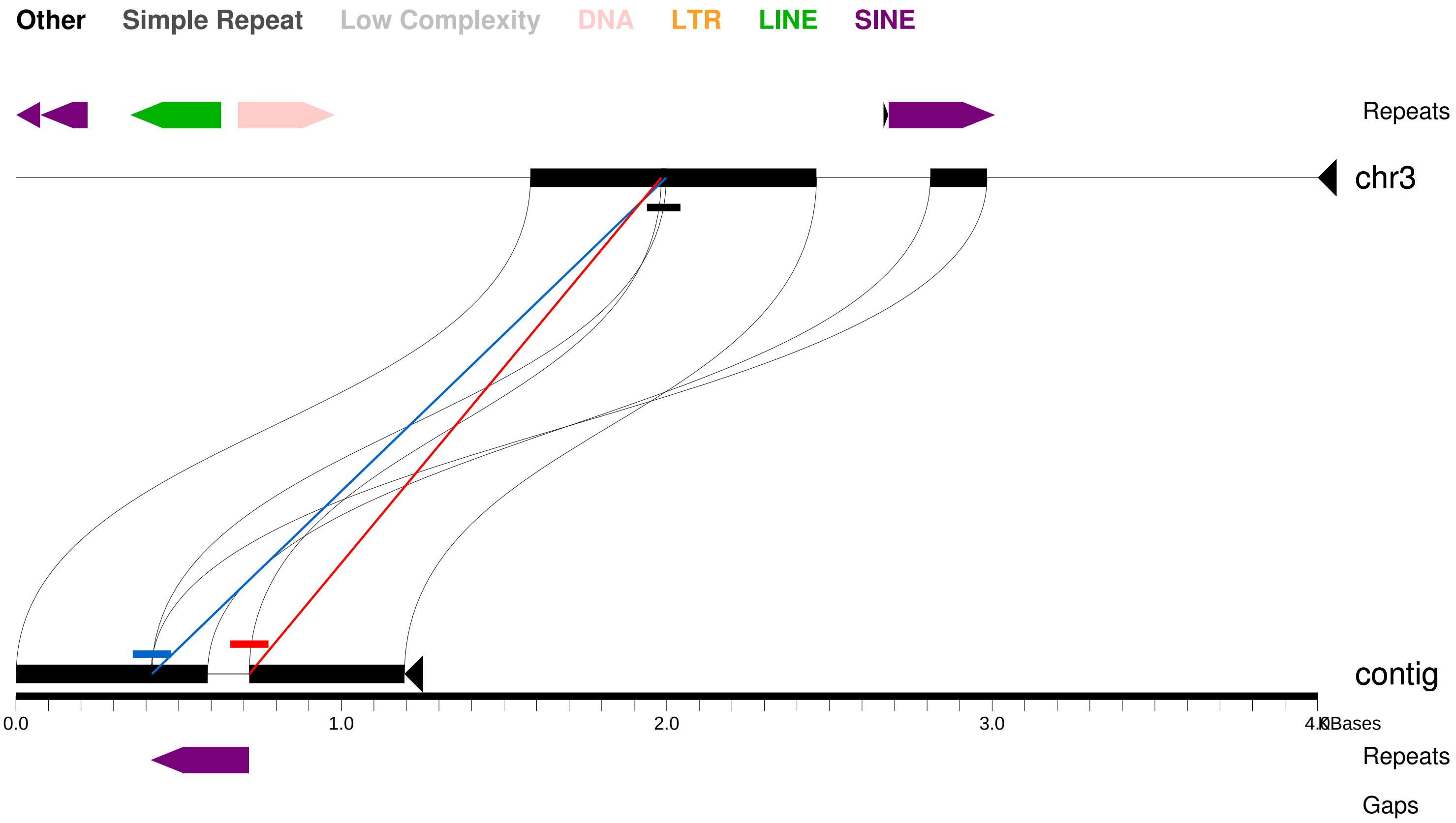

siteID: chr3_142647422 Scafftig1 + Breakpoint: chr3:142647434
chr3:142647389-142647493
Overlap Length: 15 INS size: 292 Genotyped: Yes

```
left     TTA----ATGTTTTA-TATCACTTATCTTTGA----AAG-----GCCATCTTAGGTGAGCTGCTCAGAGT
chrom    TTA----ATGTTTTA-TATCACTTATCTTTGA----AAG-----GCCATCTTAGGTGAGCTGCTCAGAGT
right    TCCGCCCATCTCGGCCTCCCAAAGTGCTGGGATTACAGGCGTGAGCCACCGCGCCCGCCCTGCTCAGAGT
         *111111**1*11111*11**11111**11**1111*1*11111****1*111111*11***********

left     CTTTTTTTTTTTTTTTTGAGACGGAGTTTCGCTCTGTCGCCCAGGCTGGAGTGCAGTGGCGCGA
chrom    CTTTAA-----------AAGCTACATTTCCCCCTCCTTTCTTACTTTAAATATAATGGT---A
right    CTTTAA-----------AAGCTACATTTCCCCCTCCTTTCTTACTTTAAATATAATGGT---A
         ****2222222222222*22*2222****2*2**22222*22222*22*2*22*2***2222*
```

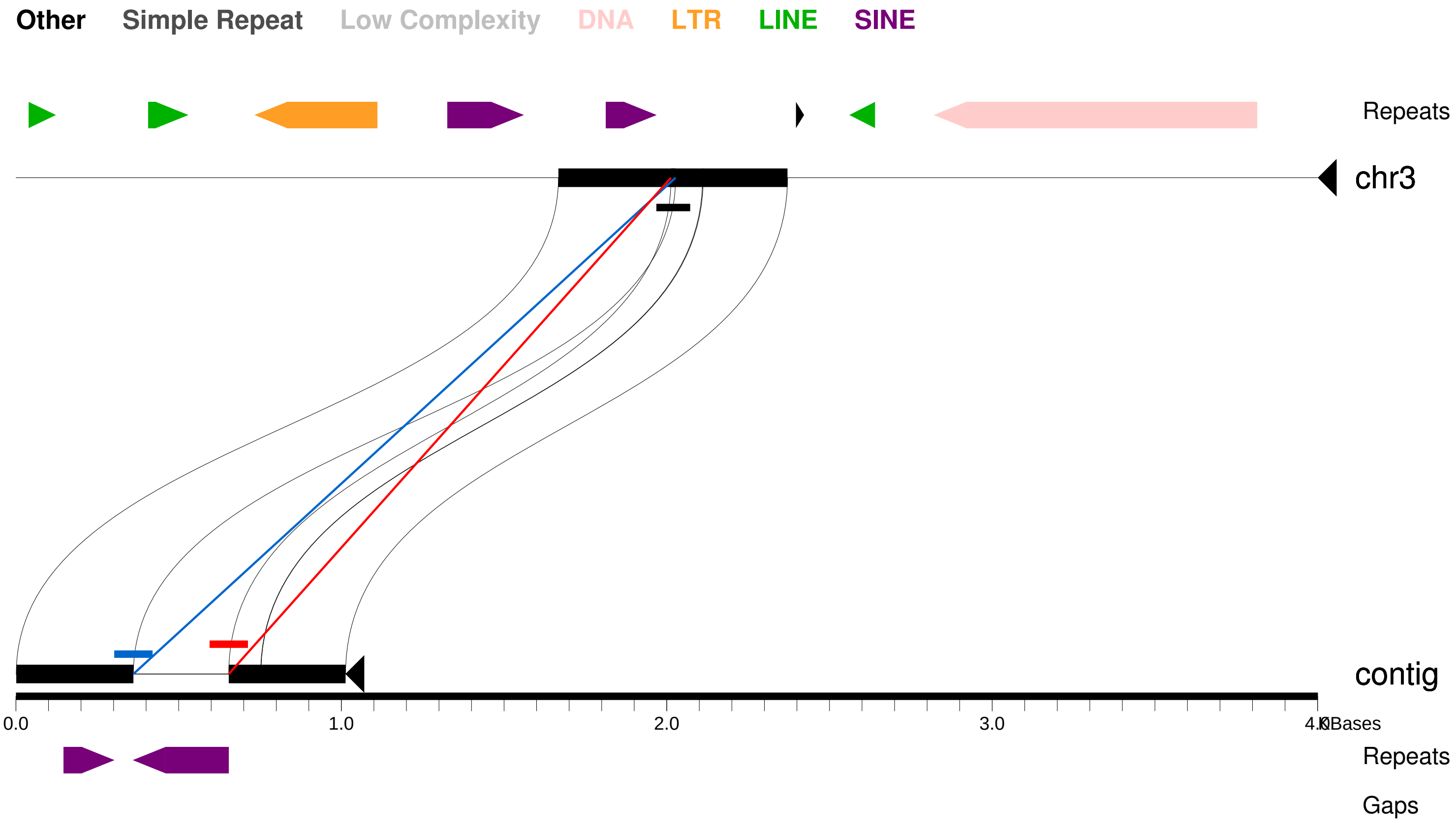

siteID: chr3_144503149 Scafftig1 + Breakpoint: chr3:144503130
chr3:144503083-144503189
Overlap Length: 13 INS size: 463 Genotyped: No

```
left   CTCTTTGTTTAGACTGCCT----TGCAGACATACCATGC-TGAATTAGTTTT-------TAGTGCAATTC
chrom  CTCTTTGTTTAGACTGCCT----TGCAGACATACCATGC-TGAATTAGTTTT-------TAGTGCAATTC
right  CCGCCCGCCTCGGCCTCCCAAAGTGCTGGGATTACAGGCGTGAGCCACCGCGCCCGGCCTAGTGCAATTC
       *11111*11*1*1*11**11111***1*11**11**1**1***111*111111111111***********

left   TTTTTTNNNNNNNNNNNNNNNNNNNNNNNNNNNNNNNNNNNNNNNNNNNNNNNNNNNNNNN
chrom  TTAAT------------ACAAATTTTGAATAGCCCCAAATAGTCAACTCAAAATTTATGGC
right  TTAAT------------ACAAATTTTGAATAGCCCCAAATAGTCAACTCAAAATTTATGGC
       **22*22222222222222222222222222222222222222222222222222222222
```

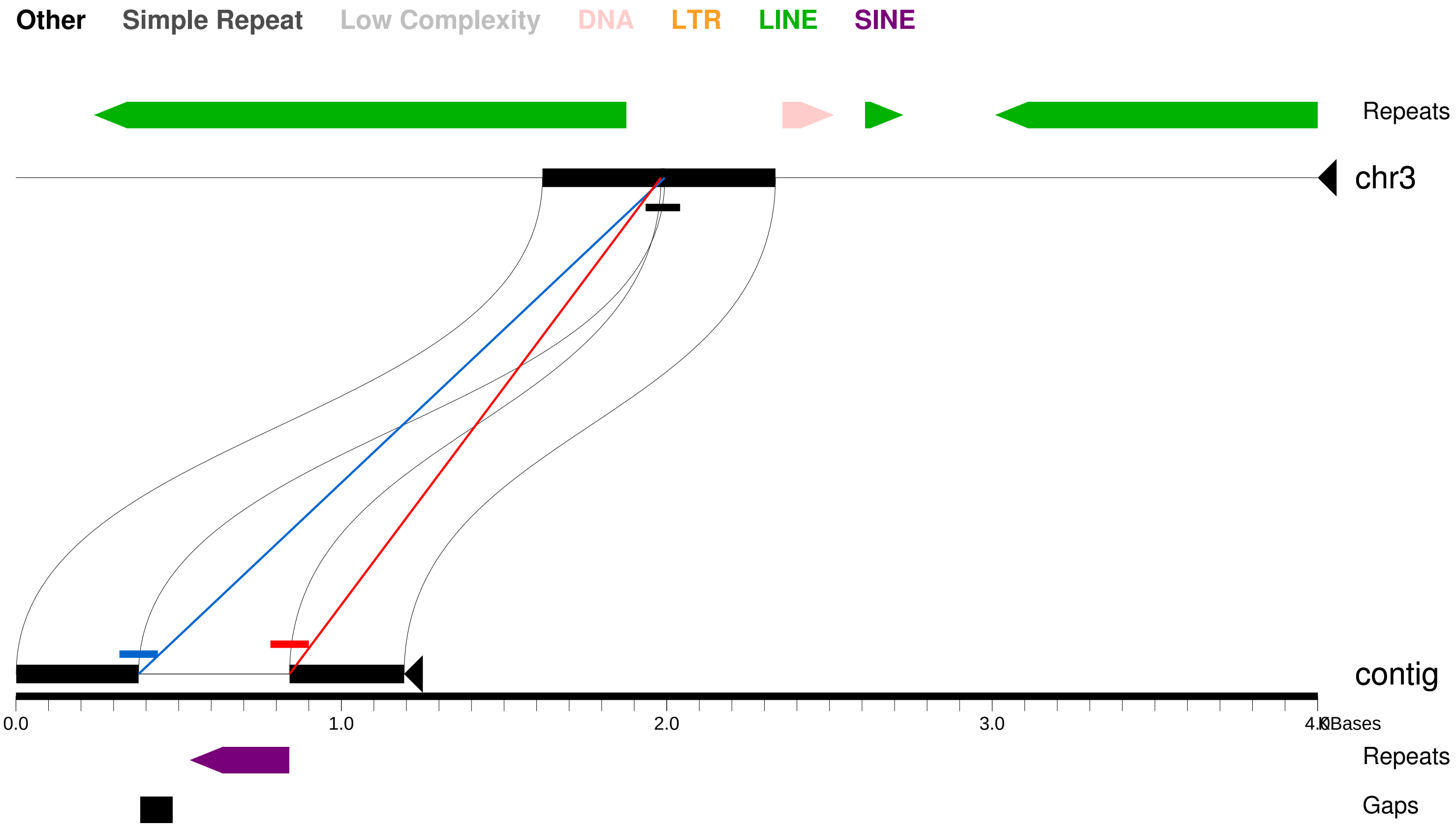

siteID: chr3_144687863 Scafftig3 - Breakpoint: chr3:144687869
chr3:144687709-144688044
Overlap Length: 16 INS size: 309 Genotyped: Yes

```
left   ----------------------------------------------------------------TGAATA
chrom  ATGGGCATATTACATGATTTCTCTGTGACTAAGACAACAATGTAAAAACCATTTTTTATTGAACTGAATA
right  TACCACGCCCGGCTAATTTTTTGTATTTTTA----------GTAGAGACGGGGTTTCACCGTGTTAGCCA
       NNNNN2NNNNNN2NNNN222N2N2N2NNN22          222N2N22NNNN222N2NN2NNN*1111*

left   TATTGGTGAGCCATCTGCCTCACACACTGGACAGCCCATTAATATGTTTCTTGAAAGTTATTTATATGGT
chrom  TATTGGTGAGCCATCTGCCTCACACACTGGACAGCCCATTAATATGTTTCTTGAAAGTTATTTATATGGT
right  GGATGGTCT-CGATCT-CCTGACCTCGTGATCCGCCCGCC--TCGGCCTCCC-AAAGTGCT------GG-
       111****111*1****1***1**1111**11*1****11111*11*11**111*****11*111111**1

left   CTTTTTTGACATTTTGCCATTGGTTTGTAATTTATT-TATTTATTTATTTAT-----TTATTTATTTATT
chrom  CTTTTTTGACATTTTGCCATTGGTTTGTAATTTATTATCAACAATTAAACATGAGGATGAGCCATAGATG
right  -------GA-----------TGGTTTGTAATTTATTATCAACAATTAAACATGAGGATGAGCCATAGATG
       1111111**11111111111***************2*2222*2***222**22222*2*222**22**2

left   TATTTTATTTTATTTTATTTTTTTTT-TTGAGACGGAGTCTCGC-TCTGTCGCCCAGGCTGGAGTGCAGT
chrom  GATC--AAGGCATTTTGTAGCCTTCTGTTGATATTCTCTCAGGCCTGAGTTATGCAGCCTTGGCTGGGTT
right  GATC--AAGGCATTTTGTAGCCTTCTGTTGATATTCTCTCAGGCCTGAGTTATGCAGCCTTGGCTGGGTT
       2**222*2222*****2*2222**2*2****2*22222**22**2*22**2222***2**2*22**222*

left   GGCGCGATCTCGGCTCACTGCAAGCTCCGCCTCCCGGGTTCACGCCATTCTCCTGCCTCAGCCTCCCGAG
chrom  TGC-CACTGGCTTCTCAGTCATAGC----CTTCAGAGGTGTAAAATAT------GCATGATGGTTTACA-
right  TGC-CACTTGCTTCTCAGTCATAGC----CTTCAGAGGTGTAAAATAT----------------------
       2**2*22*N2*22****2*222***2222*2**222***22*2222**22222211N1N1NNN1NNNN12

left   TAG
chrom  ---
right  ---
       222
```

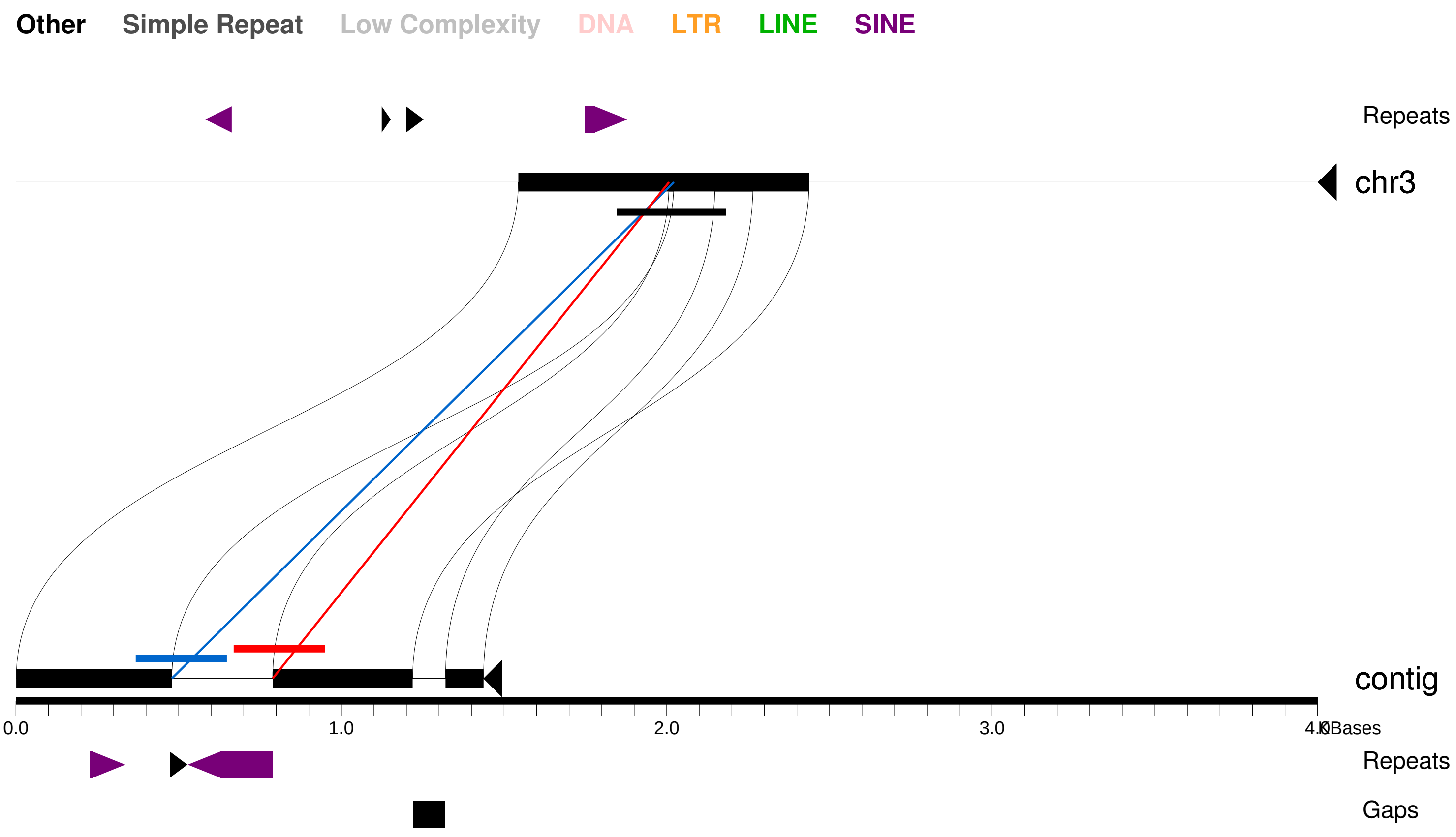

siteID: chr3_145244356 Scafftig1 + Breakpoint: chr3:145244337
chr3:145244292-145244396
Overlap Length: 15 INS size: 312 Genotyped: Yes

```
left    TCTTC---------TCTTCTAAATAGCATATGGTTAGTT-CGTG---CTTTTTTATTG--CGATATTTTA
chrom   TCTTC---------TCTTCTAAATAGCATATGGTTAGTT-CGTG---CTTTTTTATTG--CGATATTTTA
right   TCCGCCCGCCTCGGCCTCCCAAAGTGC-TGGGATTACAGGCGTGAGCCACCGCGCCTGGCCGATATTTTA
        **11*1111111111**1*1***11**1*11*1***1111****111*11111111**11**********

left    CTTTTTTTTTTTTTTTTTTTTTTTTTTTTTTTTTTT-TGAGACGGAGTCTCGCTCTGTCGCCCAG
chrom   CTTTTAATTAGAGTGTTGCATAGAACAATAACAATAATGAGATTGAAGCT---------------
right   CTTTTAATTAGAGTGTTGCATAGAACAATAACAATAATGAGATTGAAGCT---------------
        *****22**2222*2**222*2222222*22222*22*****22**22**222222222222222
```

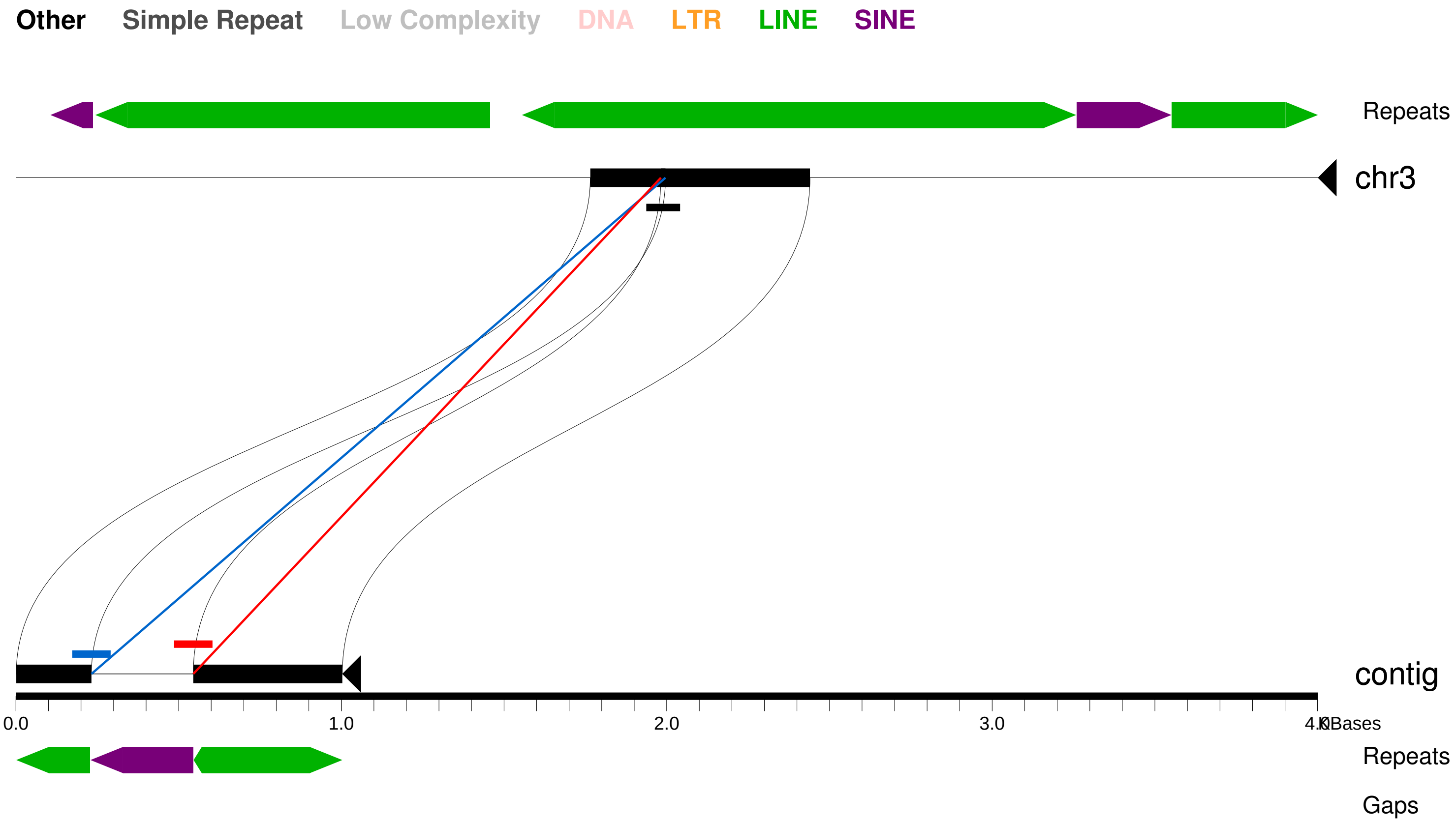

siteID: chr3_145523375 Scafftig3 + Breakpoint: chr3:145523377
chr3:145523318-145523436
Overlap Length: 1 INS size: 385 Genotyped: No

```
left    AAATACCAAAAAAGTATCATTTTTCTCTAAGAAACATTAATCTAGAAAGAACATATTCTCTGGCTCACGC
chrom   AAATACCAAAAAAGTATCATTTTTCTCTAAGAAACATTAATCTAGAAAGAACATATTCTCCACATGACAA
right   NNNNNNNNNNNNNNNNNNNNNNNNNNNNNNNNNNNNNNNNNNNNNNNNNNNNNNNNNNNCCACATGACAA
        11111111111111111111111111111111111111111111111111111111111*2222*2**22

left    T--TGTAATCCCAGCACTTTGGGAGGCCGAGGCGGGTGG-ATCACGAGGTCA
chrom   AAATGTA-TCAATGGAGAAGGAAAAACAAAGAAATGTAATAATATAAGGT--
right   AAATGTA-TCAATGGAGAAGGAAAAACATAGAAATGTAATAATATAAGGT--
        222****2**222*2*2222*22*22*2N**2222**222*22*22****22
```

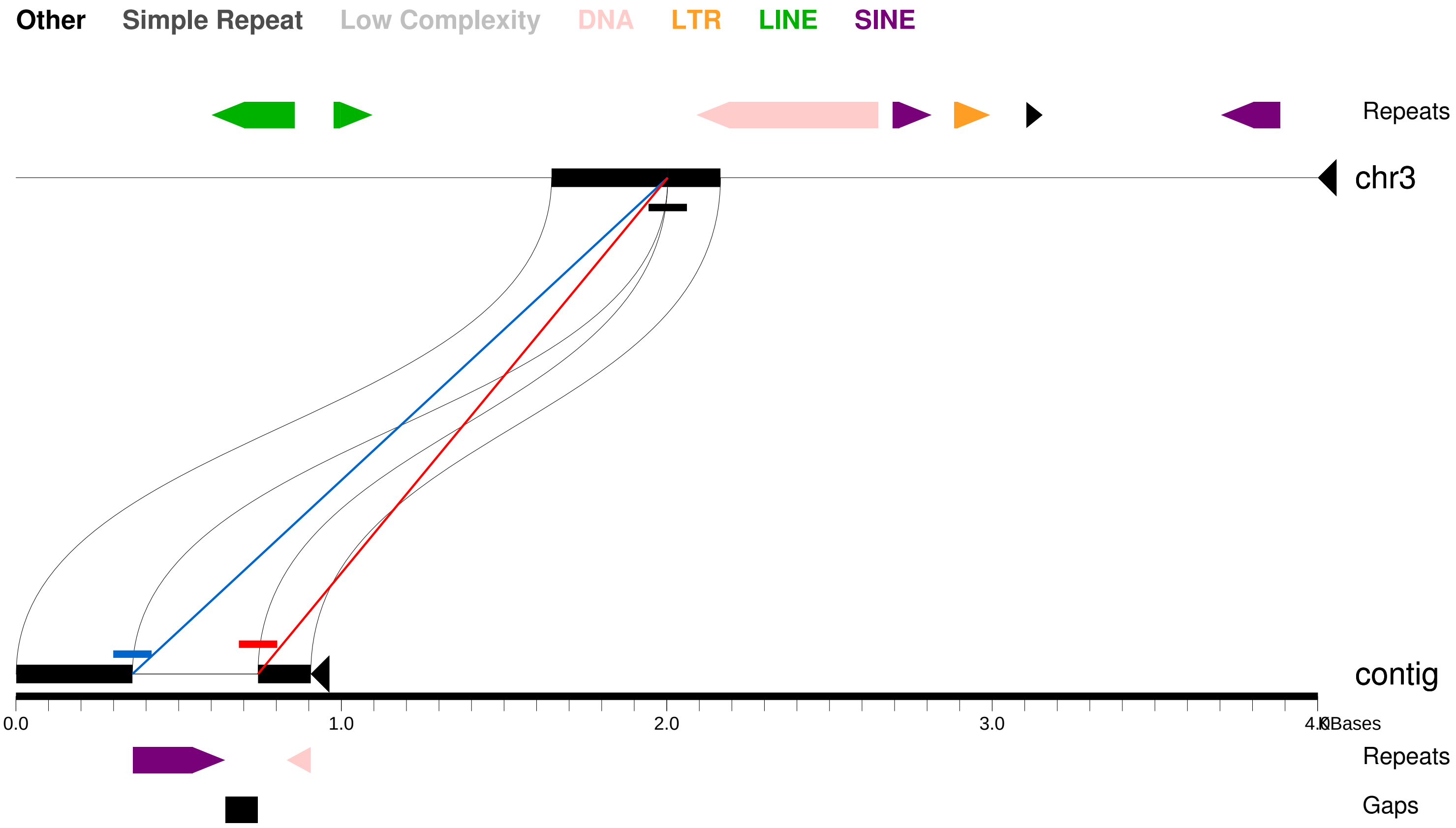

siteID: chr3_145997653 Scafftig4 + Breakpoint: chr3:145997652
chr3:145997569-145997717
Overlap Length: 8 INS size: 372 Genotyped: No

```
left   GTATAAAATTGTGCTTAAAGTTTACAATACTGTATTAAAACTTACACATTTGTTTAGAAG-TAGATCTCA
chrom  GTATAAAATTGTGCTTAAAGTTTACAATACTGTATTAAAACTTACACATTTGTTTAGAAGGTAGATCTCA
right  CCTCGGCCT----CCCAAAGTG-------CTGG------------------GATTACAGGCGTGAGCC--
       11111111*1111*11*****11111111***11111111111111111111*1***1*1*N11**1*111

left   TTAACTGTTCTTG--CCACAGTTNNNNNNN--NNNNNNNNNNNNNNNNNNNNNN----------------
chrom  TTAACTGTTCTTG--CCACAGTTTTTTTGT--AAAGCTACAATTTTGCCTCAGTTATTCCACAATGACTT
right  ---ACCGCGCCCGGCCCACAGTTTTTTTTTTTAAAGCTACAATTTTGCCTCAGTTATTCCACAATGACTT
       111**1*11*11*11********22222N2112222222222222222222222222222222222222

left   -------------
chrom  ACTAAGATTTCTC
right  ACTAAGATTTCTC
       2222222222222
```

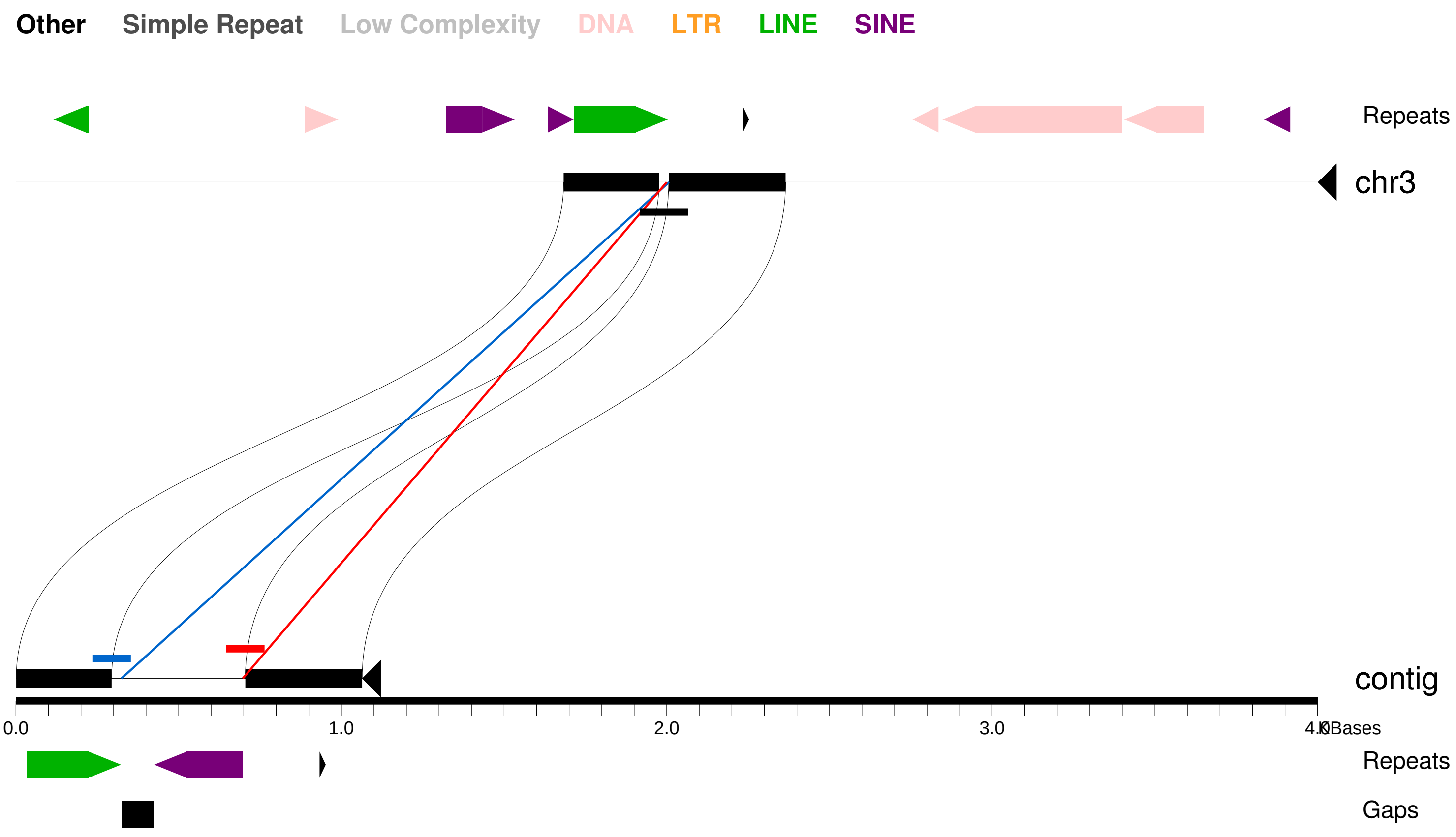

siteID: chr3_149142397 Scafftig1 - Breakpoint: chr3:149142378
chr3:149142330-149142437
Overlap Length: 12 INS size: 345 Genotyped: No

```
left   CGTATTATTTCTGCTTTAAAATGTTCATGTCCTTTGCCCTTCTATTAA-----------GATAATTTTGC
chrom  CGTATTATTTCTGCTTTAAAATGTTCATGTCCTTTGCCCTTCTATTAA-----------GATAATTTTGC
right  NNNNNNNNNNNNNNNNNNNNNNNNNNNNNNNNNNNNNNNNNNNNNNNNNNNNNNNNNNNGATAATTTTGC
       11111111111111111111111111111111111111111111111111111111111***********

left   TGGCCGGGCGTGGTGGCTCACGCCTGTAATCCCAGCA-CTTTGGGAGGCCGAGGCGGGTGG
chrom  TTTTATTAATT--TGAAATAACTTTTTAATAGCAAAAACATTAATATT----------TGC
right  TTTTATTAATT--TGAAATAACTTTTTAATAGCAAAAACATTAATATT----------TGC
       *222222222*22**2222*2222*2****22**22*2*2**222*222222222222**2
```

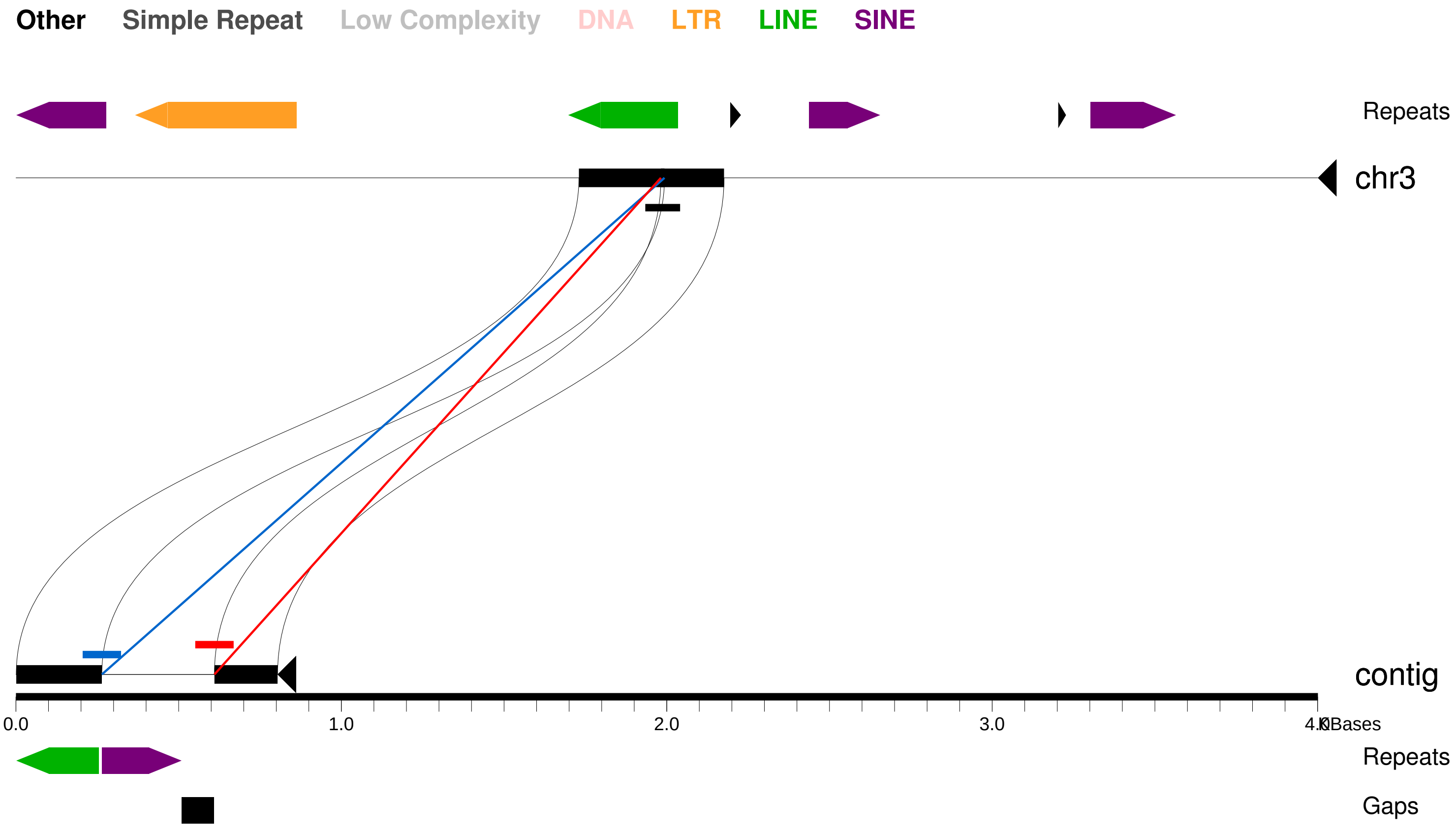

siteID: chr3_151748824 Scafftig1 - Breakpoint: chr3:151748834
chr3:151748791-151748886
Overlap Length: 17 INS size: 466 Genotyped: No

```
left    TACACAACCTCTAAAAAGAAATGATTAAATAATT-----------------------ATAAACATTAAGA
chrom   TACACAACCTCTAAAAAGAAATGATTAAATAATT-----------------------ATAAACATTAAGA
right   NNNNNNNNNNNNNNNNNNNNNNNNNNNNNNNNNNNNNNNNNNNNNNNNNNNNNNNNNAAAAAAAAAAAGA
        111111111111111111111111111111111111111111111111111111111*1***1*11****

left    ATAGCTTAACATGGCCGGGCGCGGTGGCTCACGCTTGTAATCCCAGCACTTTGGGAGGCCGAGGCGGGCG
chrom   ATAGCTTAACATGATA----------------------AATACAAATAAGCTGAT-GGCCAGTTTTTATA
right   ATAGCTTAACATGATA----------------------AATACAAATAAGCTGAT-GGCCAGTTTTTATA
        *************2222222222222222222222222***2*2*22*222**222****2222222222

left    GA
chrom   TT
right   TT
        22
```

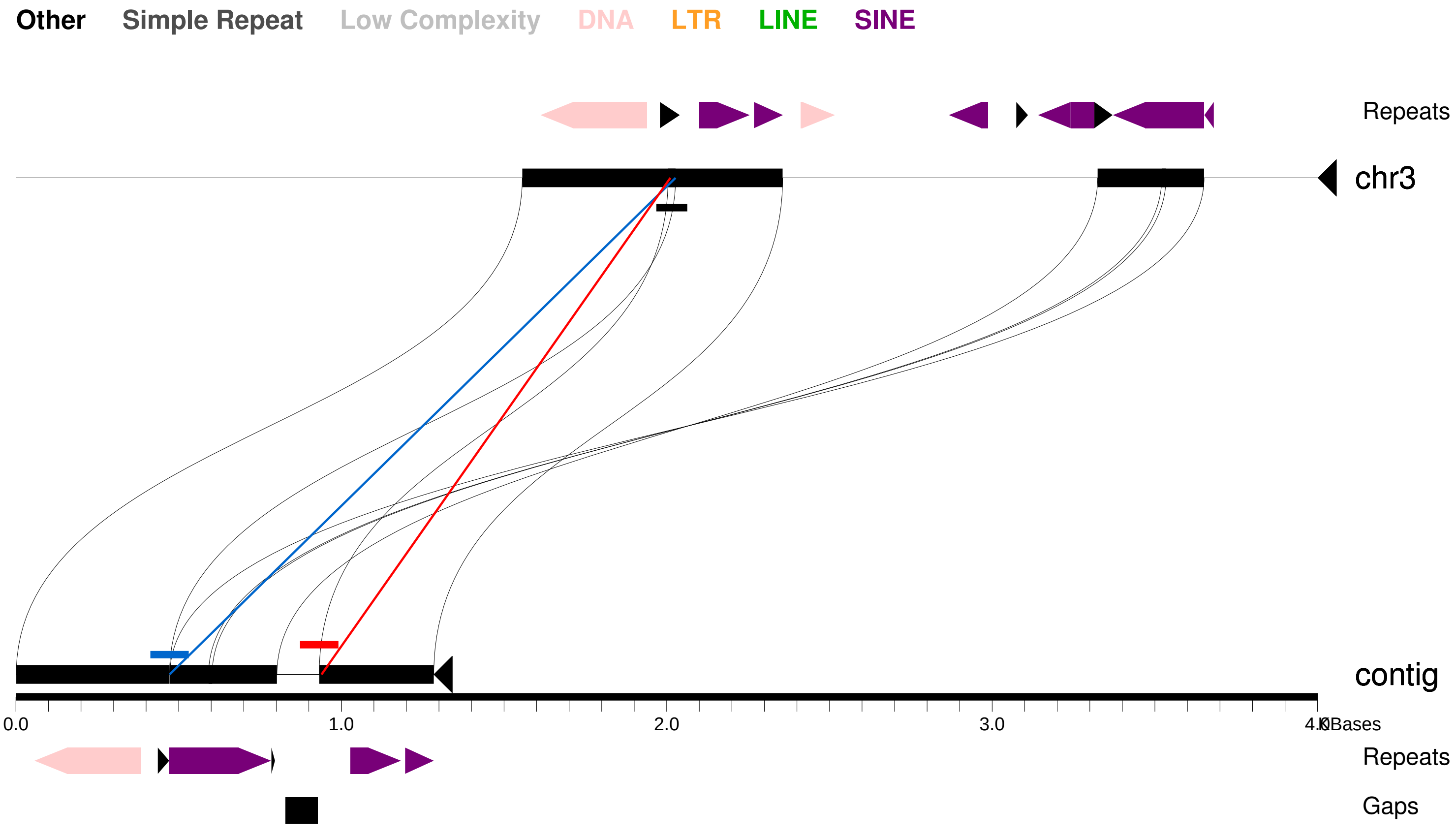

siteID: chr3_15294338 Scafftig1 + Breakpoint: chr3:15294334
chr3:15294286-15294386
Overlap Length: 9 INS size: 307 Genotyped: Yes

```
left    A---------AGAATTG-----ATACTTTAACTTGGACCTTGAAGGTAAAGCTTCAAAAGAC----AGAT
chrom   A---------AGAATTG-----ATACTTTAACTTGGACCTTGAAGGTAAAGCTTCAAAAGAC----AGGT
right   ACTGCACTCCAGCCTGGGCGACAGAGCGAGACTCCGTCTCAAAAAAAAAAAAAAAAAAAAAAAAAAAAGGT
        *111111111**11*1*11111*1*11111***11*1*1111**111***11111****1*11111**2*

left    TACTGGCCGG-GCGCGGTGGCTCACGCCTGTAATCCCAGCACTTTGGGAGGCCGAGGCGGGCGGATCA
chrom   TACTGACCATTGAGTGTTTACTA-----TGTA--CCCAA-----TGTGTATATTTTTC-------TTT
right   TACTGACCATTGAGTGTTTACTA-----TGTA--CCCAA-----TGTGTATATTTTTC-------TTT
        *****2**222*2*2*2*22**222222****22****222222**2*222222222*2222222*22
```

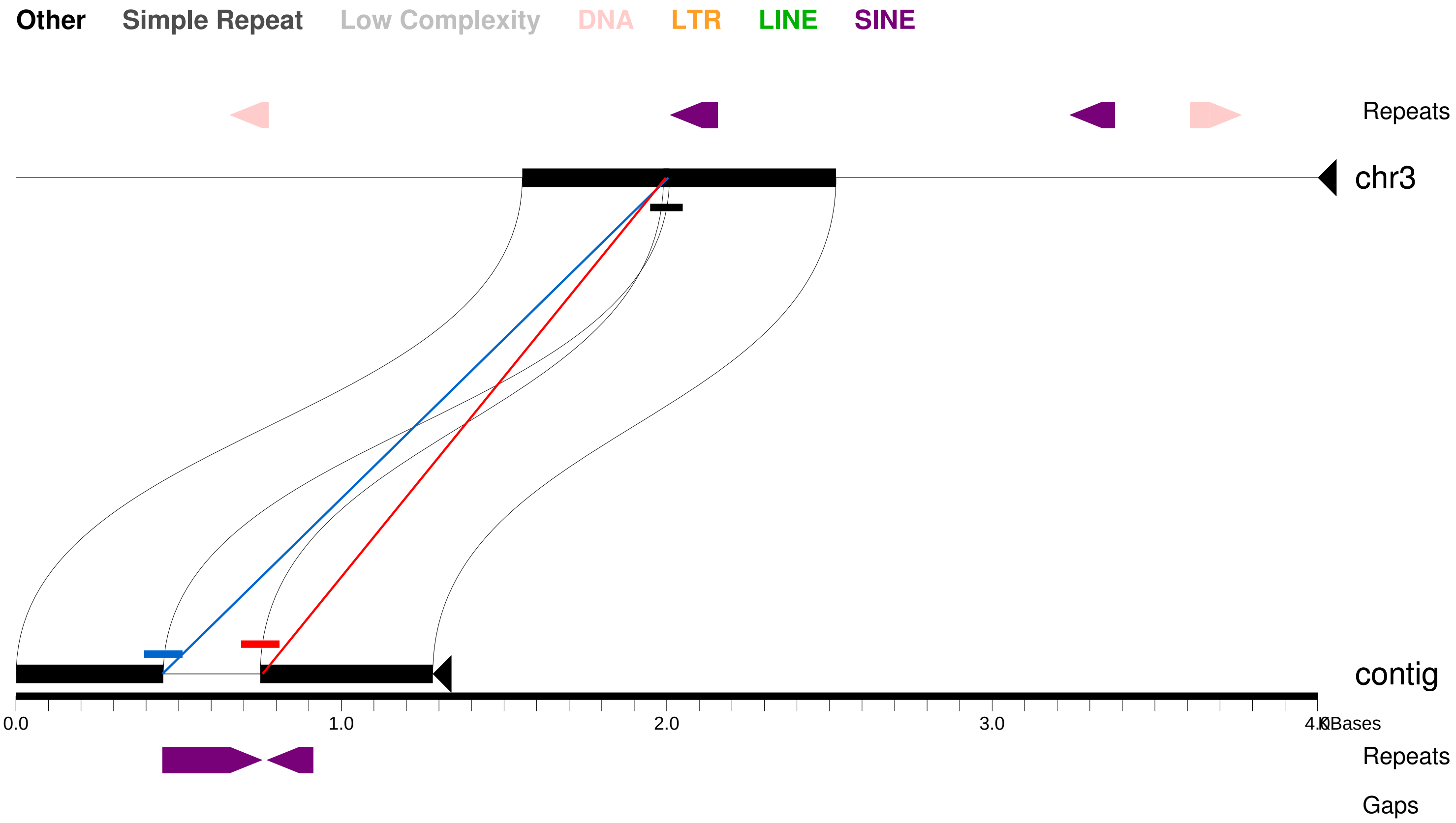

siteID: chr3_154966222 Scafftig2 + Breakpoint: chr3:154966214
chr3:154966155-154966276
Overlap Length: -2 INS size: 306 Genotyped: Yes

```
left    TCTTTCATAGTGTTGACAATTCTTCCATAGAGTAGCATGTGGGATGAACCTTAAAAGTCTCGGCCGGGCG
chrom   TCTTTCATAGTGTTGACAATTCTTCCATAGAGTAGCATGTGGGATGAACCTTAAAAGTCTTTAT--GACA
right   ACTCC---AGCCTGGGCGACAGAGCGAGACTCCGTCTCAAAAAAAAAAAAAAAAAAAAATAAAT--GACA
        1**11111**11*1*1*1*11111*1*1*111111*1111111*11**1111****111*NN2222*2*2

left    CGGTGGCTCAC-GCCTGTAATCCCAGCACTT----TGGGAGGCCGAGGCGGGCG--
chrom   CTGTGACCTAGAGACTG-ACTTAGAGATGTTGTCTTTGGAGGC-GAGTAGACCACT
right   CTGTGACCTAGAGACTG-ACTTAGAGATGTTGTCTTTGGAGGC-GAGTAGACCACT
        *2***2*22*22*2***2*2*222**222**2222*2******2***22*22*222
```

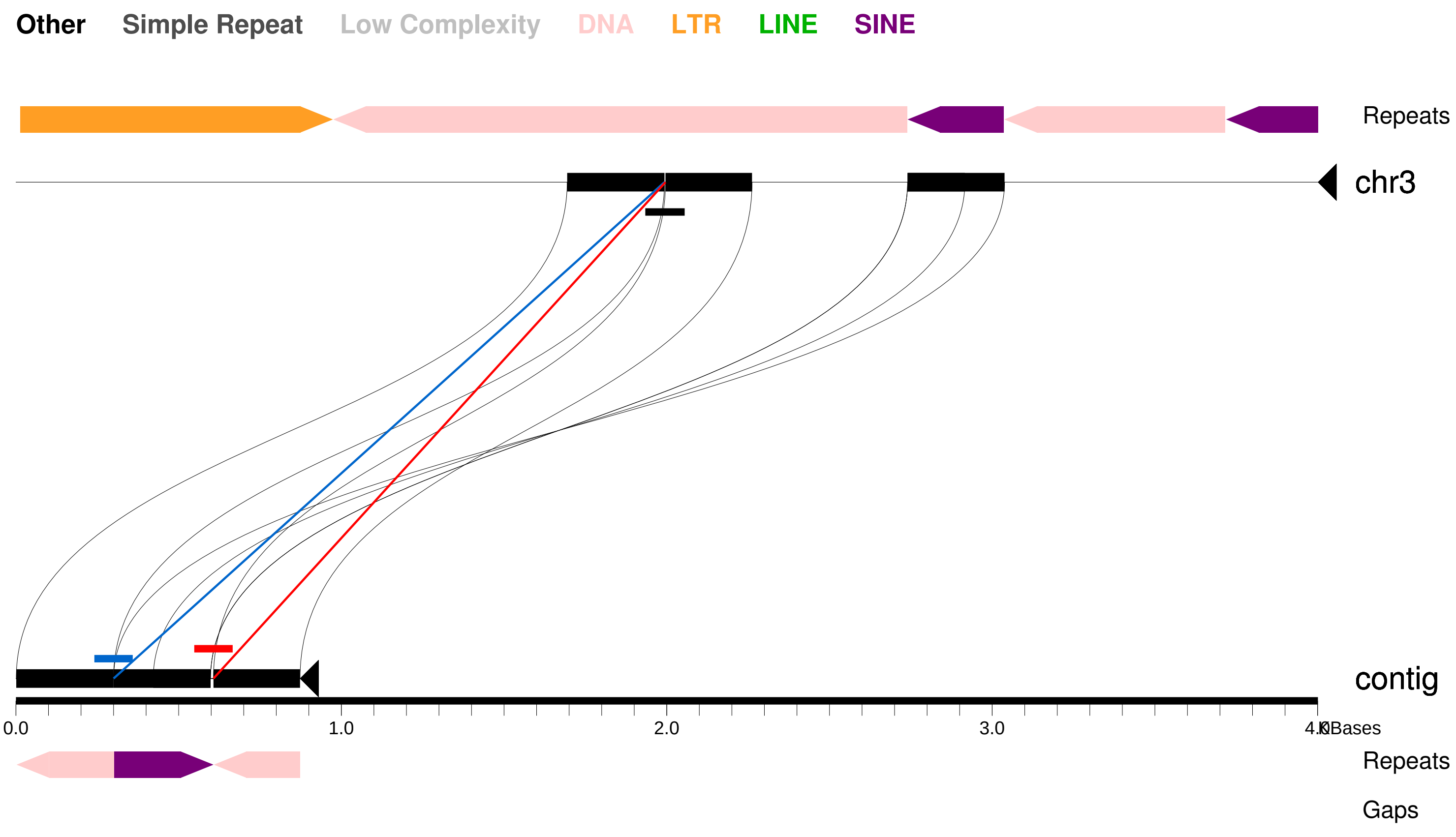

siteID: chr3_155040659 Scafftig11 + Breakpoint: chr3:155040661
chr3:155040616-155040720
Overlap Length: 15 INS size: 158 Genotyped: Yes

```
left    TTCATACATAAATATTTAATTCACAATCTCTCTA-----TAGTATTAAAT------------GAAAAGCA
chrom   TTCATACATAAATATTTAATTCACAATCTCTCTA-----TAGTATTAAAT------------GAAAAGCA
right   TTCACGCC---ATTCTCCTGCCTCAGCCTCCCGAGTAGCTGGGACTACAGGCGCCCGCCACCGAAAAGCA
        ****11*1111**11*11111*1**11***1*1*11111*1*1*1**1*1111111111111********

left    AATTCTTTTTTTTTTTTTTTTGAGACGGAGTCTCGCTCTGTCGCCCAGGCCGGACTGCGGACTGCA
chrom   AATTCTTATCACTGTAT-------ACGCA-------TAGGAAAAAGAGACAGATACCAAAATAGTA
right   AATTCTTATCACTGTAT-------ACGCA-------TAGGAAAAAGAGACAGATACCAAAATAGTA
        *******2*222*2*2*2222222***2*2222222*22*222222**2*2*22222222*22*2*
```

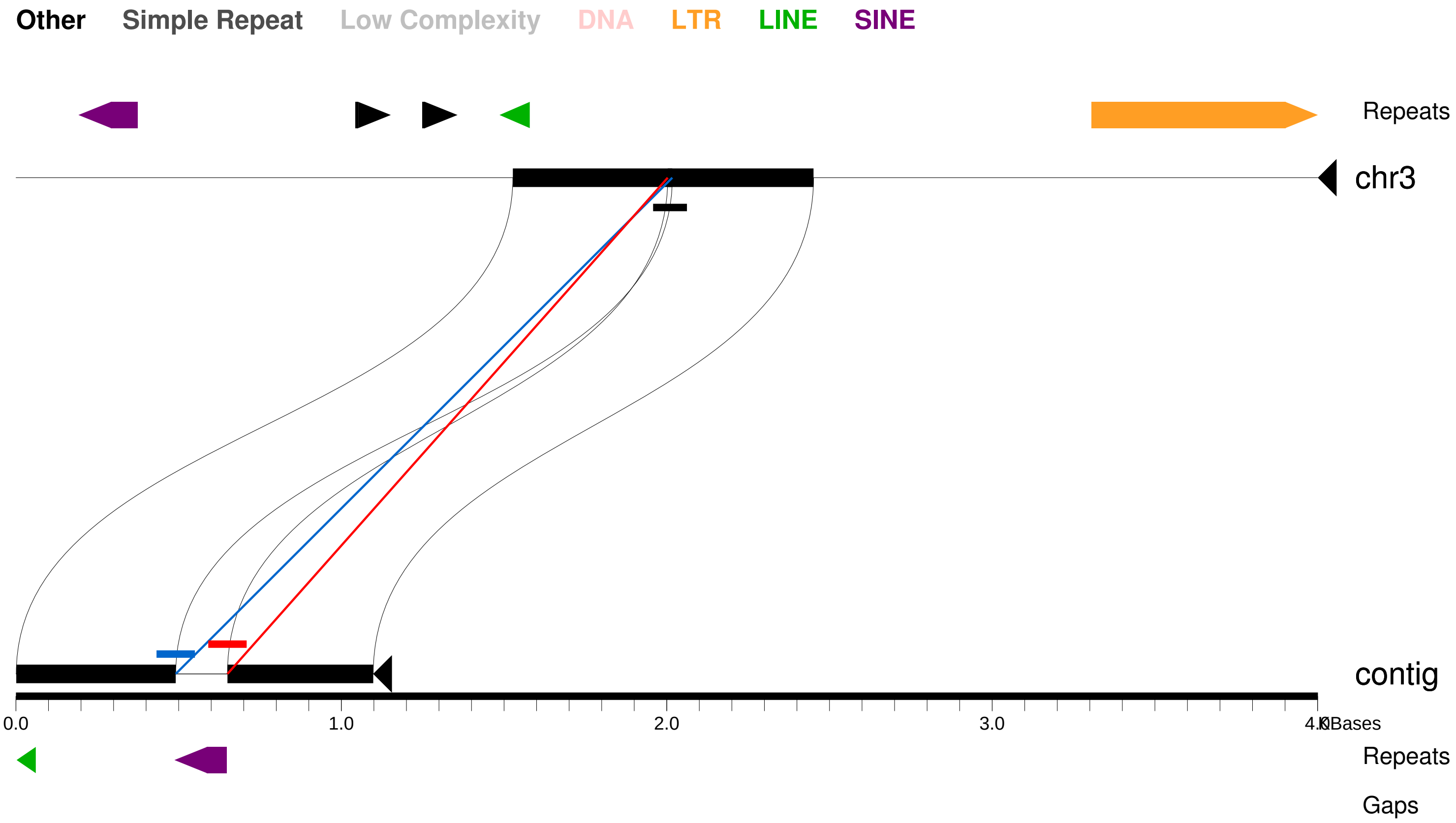

siteID: chr3_156239864 Scafftig5 + Breakpoint: chr3:156239874
chr3:156239714-156240042
Overlap Length: 9 INS size: 299 Genotyped: Yes

```
left   -----CAATAAATAATTGCTTTTTTCTTTTTAATTTTTTTTTA-ACTTTTGGAAGTCTGTCTCTGAAAATG
chrom  GGACTCAATAAATAATTGCTTTTTTCTTTTTAATTTTTTTTTTTACTTTTGGAAGTCTGTCTCTGAAAATG
right  T---TTAGTAGAGACGGGGTTTCACCGTTTTAGCCG-------------GGATG---GTCTCG----ATC
       N   21*1**1*1*111*1***111*1*****111111111N 111111***1*111*****11111**1

left   GTGTGTATGTGTGTGTCACACACATACACATATACACACACACATAAATAGAACTTGTAACCCAATCCTA
chrom  GTGTGTATGTGTGTGTCACACACATACACATATACACACACACATAAATAGAACTTGTAACCCAATCCTA
right  TCCTGACCTCGTGATCCGCCCGCCTCGGCCT-----------CCCAAAGTG--CTGGGATTACAGGCGTG
       111**11111***111*1*1*1*1*111*1*11111111111*11***11*11**1*1*111**11*1*1

left   AGAAAGAGAATGCACTGCATACAAGATTTTT------------TTTTTTTTTTTTTTTTGAGACGGAGTC
chrom  AGAAAGAGAATGCACTGCATACAAGATTTCAAAAACGGAAAAGTTACAAAAGATTGATTGATA---AACC
right  AGCCACCGC--GCCCGGCC-ACAAGATTTCAAAAACGGAAAAGTTACAAAAGATTGATTGATA---AACC
       **11*11*111**1*1**11*********22222222222222**22222222**22****2*222*22*

left   TCGCTCTGTCGCCCAGGCTGGAGTGCAGTGG----CGGGATCTCGG---CTCACTGCAAGC---------
chrom  AACCACTCTTTACATGCATGGTATATACTGAAACTCAGAAATTCTAATACTAATAAGAAGCAATTGTTCA
right  AACCACTCTTTACATGCATGGTATATACTGAAACTCAGAAATTCTAATACTAATAAGAAGCAATTGTTCA
       222*2**2*222*22*22***22*22*2**22222*2*2*22**22222**2*2222****222222222

left   --------TCCGCCTCCCGGGTTCACGCCATTCTCCTGCCT--------CA-
chrom  AAAAGCAGTTGGCTATTAGTTTTCAATCTTTGTTAGTCGGTTTGAATAACAT
right  AAAAGCAGTTGGCTATTAGTTTTCAATCTTTGTTAGTCGGTTT---------
       22222222*22**22222*22****22*22*22*22*222*22      11
```

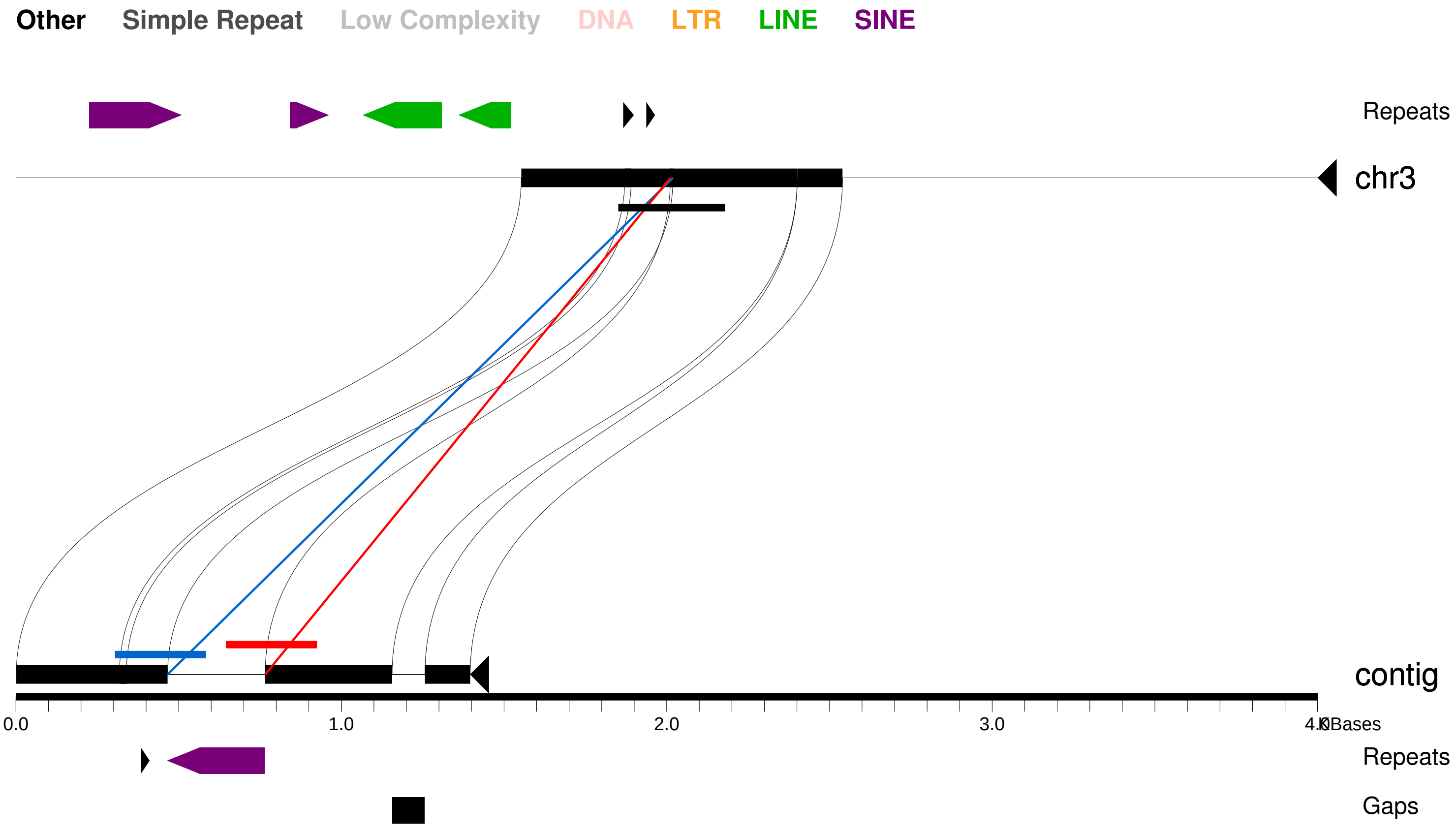

siteID: chr3_157369116 Scafftig1 - Breakpoint: chr3:157369128
chr3:157369081-157369170
Overlap Length: 13 INS size: 370 Genotyped: No

```
left    ACAGAATCTCAGGAATAAAGTGA------------------------------TGAGAGGAAAATAAAACT
chrom   ACAGAATCTCAGGAATAAAGTGA------------------------------TGAGAGGAAAATAAAACT
right   NNNNNNNNNNNNNNNNNNNNNNNNNNNNNNNNNNNNNNNNNNNNNNNNNNNNNGTCTCAAAAAAAAAAAAAA
        11111111111111111111111111111111111111111111111111111*111*11****1****11

left    GTGGAAAGAGAGGGACAGGTGAAACCCCGTCTCTACTAAAAATACAAAAAATTAGCCGGGCGCGGTGGTG
chrom   GTGGAAAGAGAGGGACAGGC------------CTACTTCAGAGA----------GGCTGGCCAG--GGAG
right   AAAAAGAGAGAGGGACAGGC------------CTACTTCAGAGA----------GGCTGGCCAG--GGAG
        1111*1*************2222222222222*****22*2*2*2222222222*2*2***22*22**2*

left    GGCGCCTG
chrom   GAC-----
right   GAC-----
        *2*22222
```

Other Simple Repeat Low Complexity DNA LTR LINE SINE

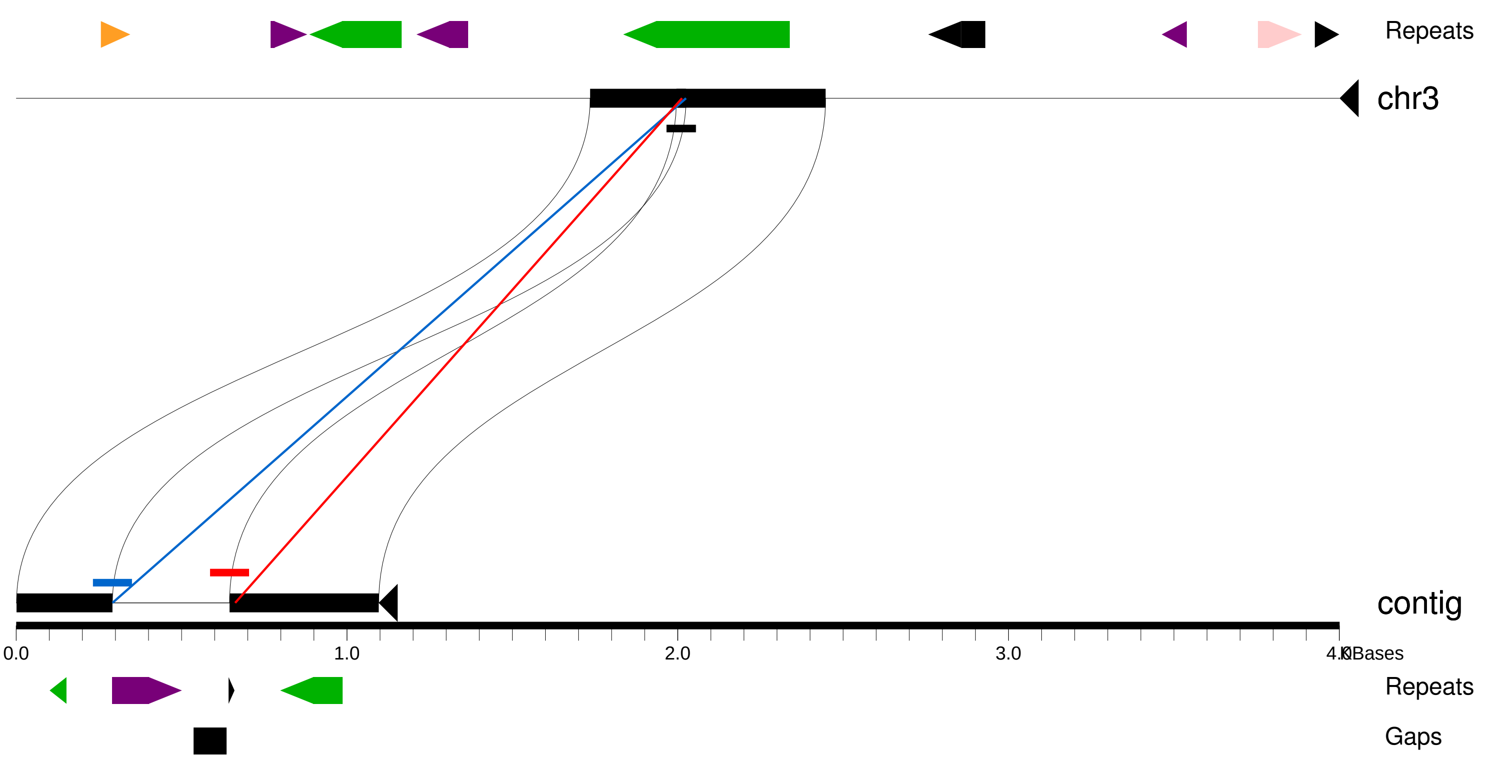

siteID: chr3_157962941 Scafftig1 + Breakpoint: chr3:157962921
chr3:157962875-157962980
Overlap Length: 14 INS size: 325 Genotyped: Yes

```
left     TTGTTTTCTCTTGG--TCCTCTAGTTCTTTTAGTTATTAATTTGAGAT-------------GTTTCTGGC
chrom    TTGTTTTCTCTTGG--TCCTCTAGTTCTTTTAGTTATTAATTTGAGAT-------------GTTTCTGGC
right    CCACCCGC-CTCGGCCTCCCAAAGTGCTGGGA-TTACAGGCGTGAGCCACCGCGCCCGGCCGTTTCTGGC
         1111111*1**1**11***111***1**111*1***111111****111111111111111*********

left     CTTTTTTTTTTTTTTTTTTTTTTTTTTGTTTTTTTTTTTTTTTTGAGACGGAGTCTCGCTCTGTCG
chrom    TTTTTGATGTGGGCATTTAGTGCTATAAATTTCCCTCTT----------AACATTGCTTTA---
right    TTTTTGATGTGGGCATTTAGTGCTATAAATTTCCCTCTT----------AACATTGCTTTA---
         2****22*2*22222***22*22*2*222***222*2**2222222222*222*2***2*2222
```

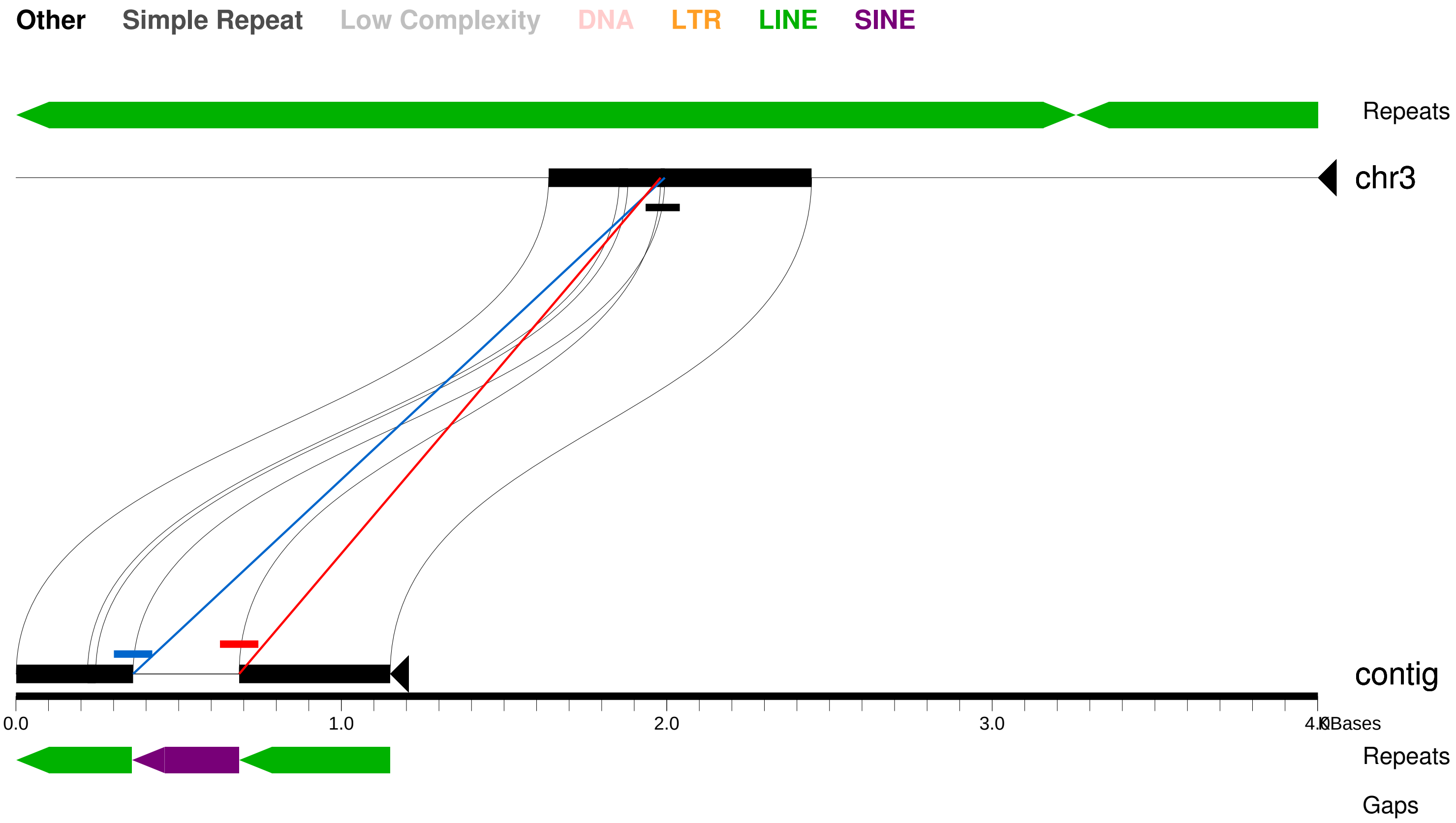

siteID: chr3_162637606 Scafftig1 - Breakpoint: chr3:162637593
chr3:162637550-162637652
Overlap Length: 17 INS size: 431 Genotyped: No

```
left     TATCACAGGACCTAGTACTATATAGGAGATGCACCACTTCCTT----------------AAAACTGTGAG
chrom    TATCACAGGACCTAGTACTATATAGGAGATGAACCACTTCCTT----------------AAAACTGTGAG
right    NNNNNNNNNNNNNNNNNNNNNNNNNNNNNNNNNNNNNNNNNNNNNNNNNNNNNNNNAAAAAAACTGTGAG
         1111111111111111111111111111111N111111111111111111111111111***********

left     TAAGGCGGCCGGGCGCGGTGGCTCACGCCTGTAATCCCAGCA-CTTTGGGAG-GCCGAGGCGGGCGG
chrom    TAAGGCAACACAGC-------CTCA----TCTA--TCCTGCAGCTTTGCTATCGCAGAGCCC-----
right    TAAGGCAATACAGC-------CTCA----TCTA--TCCTGCAGCTTTGCTATCGCAGAGCCC-----
         ******221222**2222222****2222*2**222**2***2*****22*22**2***2*222222
```

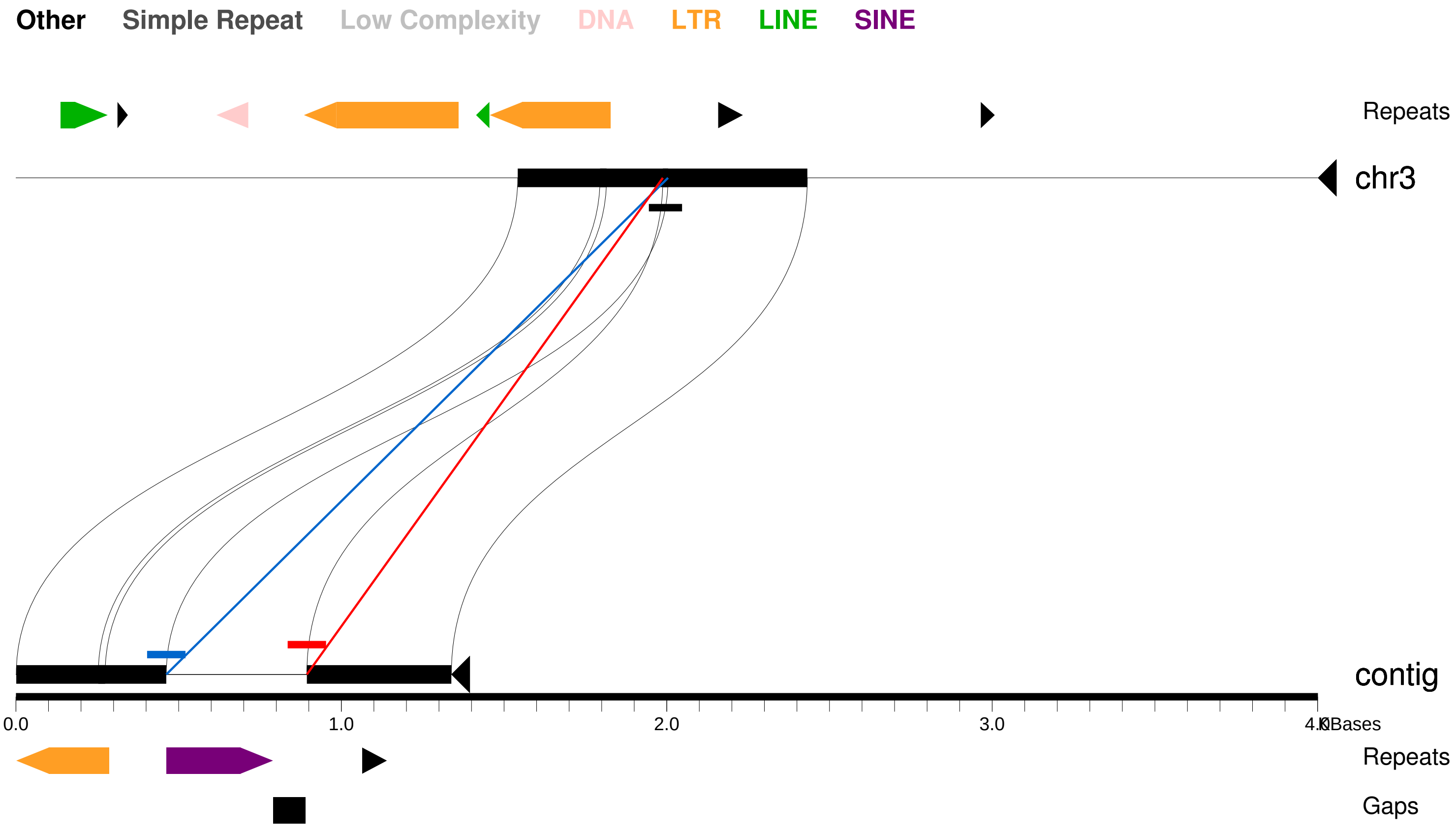

siteID: chr3_162848534 Scafftig1 - Breakpoint: chr3:162848539
chr3:162848488-162848634
Overlap Length: 9 INS size: 311 Genotyped: Yes

```
left     CACAATGCGATACCACCTTCATCCTGCAAGAATGGCCATAATTGAAAAATCAAAAAACAGGGCCGGGCGC
chrom    CACAATGCGATACCACCTTCATCCTGCAAGAATGGCCATAATTGAAAAATCAAAAAACAGTAGATGTTGG
right    AAAAA---------------------CAA--------ACAAACAAACAA--AAAAGACAGTAGATGTTGG
         1*1**111111111111111111111***11111111*1**111**1**11*****1****22222*22*2

left     AGTGGCTCACGCCTGTAATCCC---AGCACTTTGGGAG-GCCGAGGCGGGTG------------------
chrom    CATGAATGT--GCTGAAACAGG---AACACTTTTACACTGTTGATGGGAATGTAAAATAGTACAACCACT
right    CATGAATGT--GCTGAAACAGGAGGAACACTTTTACACTGTTGATGGGAATGTAAAATAGTACAACCACT
         22**22*22222***2**2222111*2******222*22*22**2*2*22**222222222222222222

left     -----------G
chrom    TGGTAAACAGTG
right    TGGTAAACAGTG
         22222222222*
```

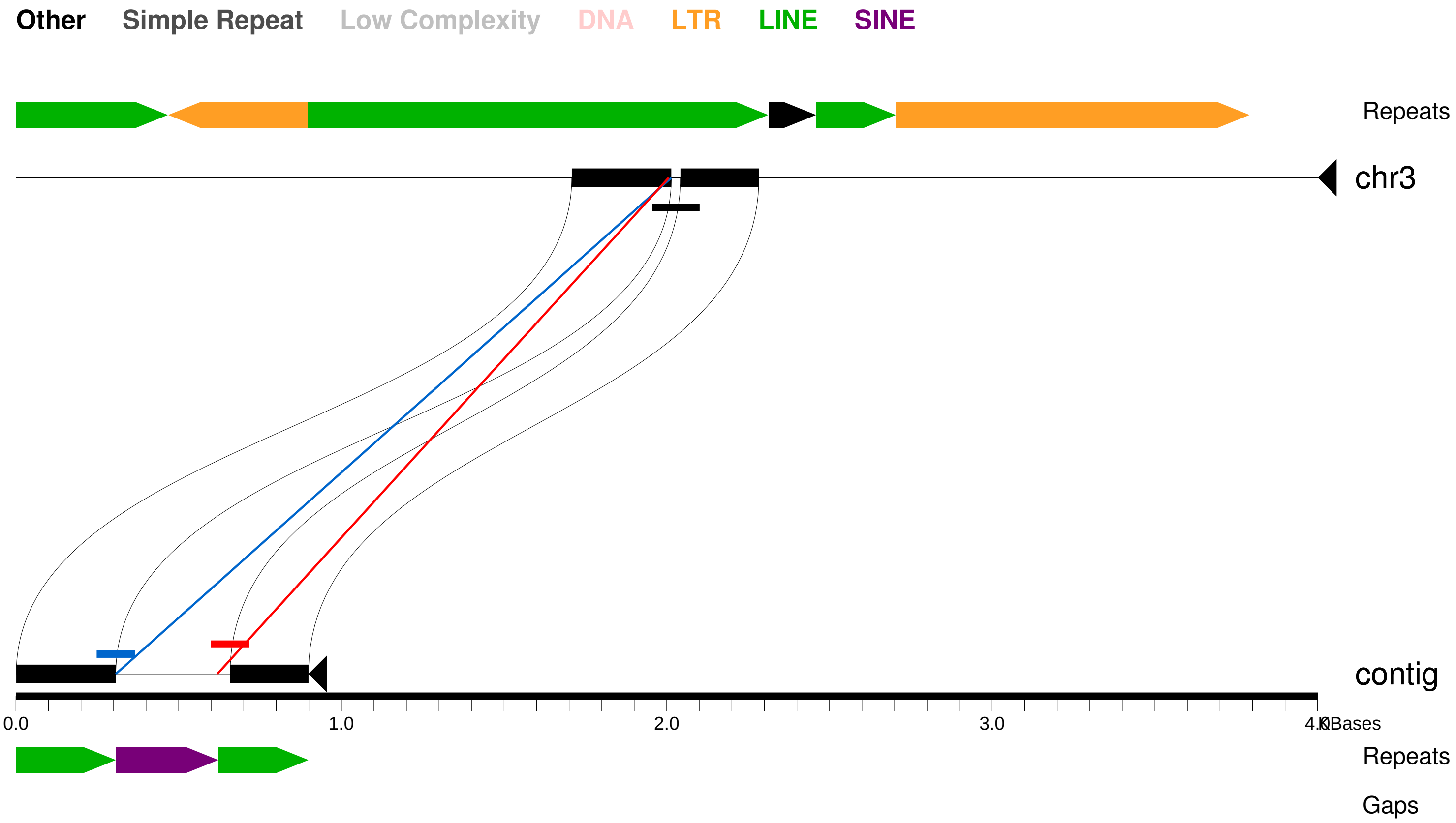

siteID: chr3_165336813 Scafftig5 + Breakpoint: chr3:165336810
chr3:165336767-165336869
Overlap Length: 17 INS size: 297 Genotyped: Yes

```
left     ATT-ACAATTCA--AGATGCG--------ATTTGTTTGGAG-ACACA---GAGACAAA-CCGTATCAGTA
chrom    ATT-ACAATTCA--AGATGCG--------ATTTGTTTGGAG-ACACA---GAGACAAA-CCGTATCAGTA
right    CCTCATGATCCACCCGCCTCGGCCTCCCAAAGTGCTGGGATTACAGGCGTGAGCCACCGCCGTATCAGTA
         11*1*11**1**111*111**11111111*11**1*1***11***11111***1**111***********

left     GTCTTTTTTTTTTTTTTTGAGACGGAGTCTCGCTCTGTCGCCCAGGCGGGACTGCGGACTGCAG-T
chrom    GTCTTTAA-----------AGAAAGACGACAGCGTGGTCATC--AGCAAGACT----TATTCAGAT
right    GTCTTTAA-----------AGAAAGACGACAGAGTGGTCATC--AGCAAGACT----TATTCAGAT
         ******2222222222222***22**22222*1222***22*222**22****222222*2***2*
```

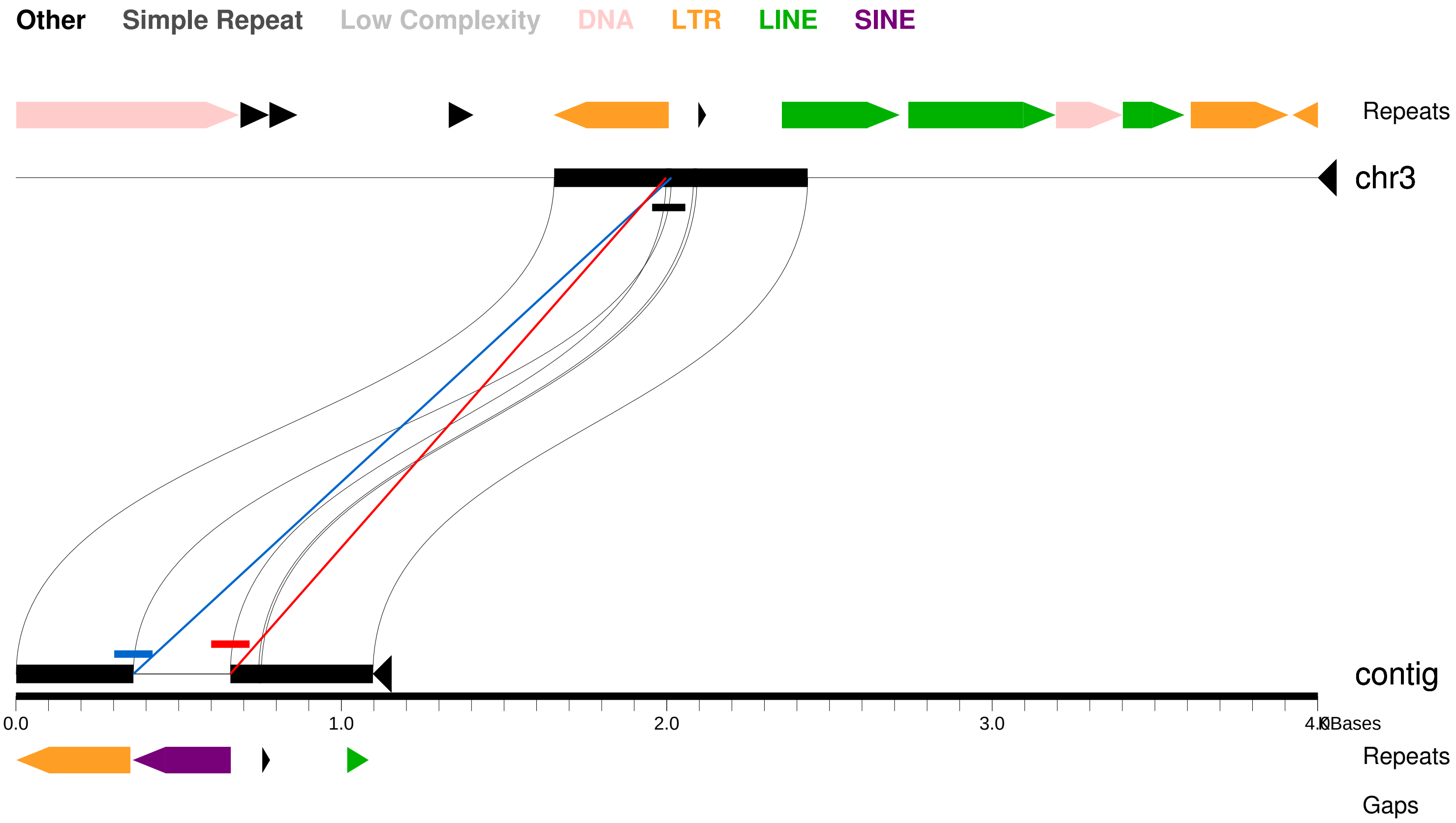

siteID: chr3_166362327 Scafftig3 + Breakpoint: chr3:166362310
chr3:166362255-166362364
Overlap Length: 5 INS size: 336 Genotyped: Yes

```
left    TGTTGAGCATGGTTTATTTGG-TTGGTATTCAGCATTTTCTACTT------TCCTTTAAAAA--GGGGGG
chrom   TGTTGAGCATGGTTTATTTGG-TTGGTATTCAGCATTTTCTACTT------TCCTTTAAAAA--GGGGGA
right   TCCGTCTCAAAAAAAAAAAAAAATTAAAATTAAAAAAAAAATAAATAAAAAATAAATAAAAAAAAGGGGGA
        *111111**111111*111111**111***1*11*11111**11*111111*111*1*****11*****2

left    CCGGGCGCGGTGGCTCACGCCTGTAATCCCAGCACTTTGGGAGGCCGAGGCGGGCGGA
chrom   GAGAG-GCACTACATTTCACATG-------AG-ACTCTACAATGACTATATCTTTCTC
right   GAGAG-GCACTACATTTCACATG-------AG-ACTCTACAATGACTATATCTTTCTC
        22*2*2**22*222*22*2*2**2222222**2***2*222*2*2*2*2222222222
```

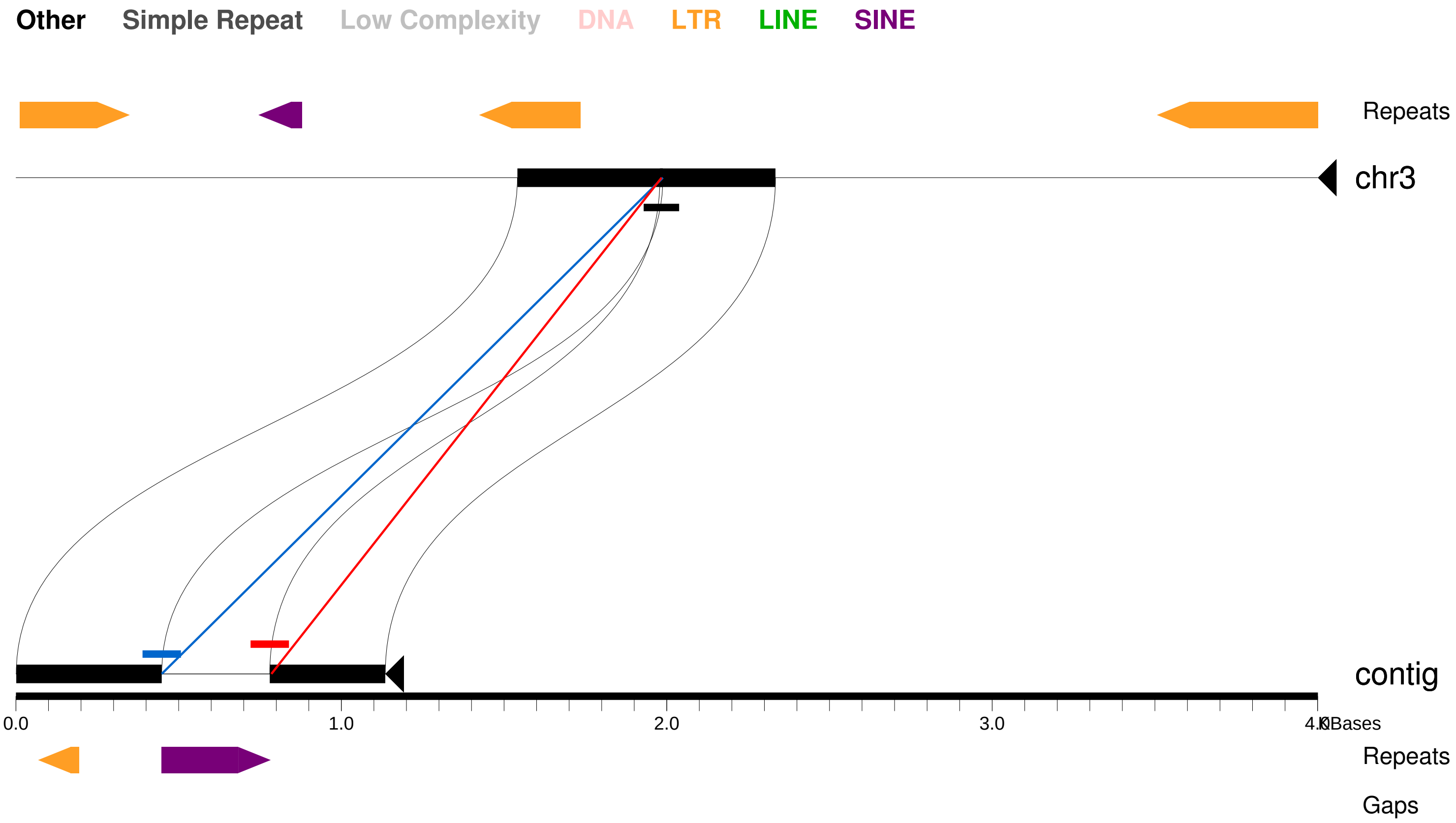

siteID: chr3_166544607 Scafftig1 - Breakpoint: chr3:166544589
chr3:166544539-166544648
Overlap Length: 10 INS size: 429 Genotyped: No

```
left    GA---------GAGTTTATAGGTTTTAGAAGCCCATGCTGTAGCAGGCGGGTGGTAACAGGCTTTAATCN
chrom   GA---------GAGTTTATAGGTTTTAGAAGCCCATGCTGTAGCAGGCGGGTGGTAACAGGCTTTAATCC
right   CACCCGCCTCGGCCTCCCAAAGTGCTGGGATTACAGGCGTGAGCCACCGCGCCCGGCCTGGCTTTAATCC
        1*111111111*11*1111*1**11*1*1*111**1**111***111**1*111111*1**********2

left    NNNNNNNNNNNNNNNNNNNNNNNNNNNNNNNNNNNNNNNNNNNNNNNNNNNNNNNNNN
chrom   TTTTAAAGCATGCTGTGGGATGGGATATTGGTGTTGAGCCGGGTAAGAG---------
right   TTTTAAAGCATGCTGTGGGATGGGATATTGGCGTTGAGCCAGGTAAGAG---------
        2222222222222222222222222222222N22222222N22222222222222222
```

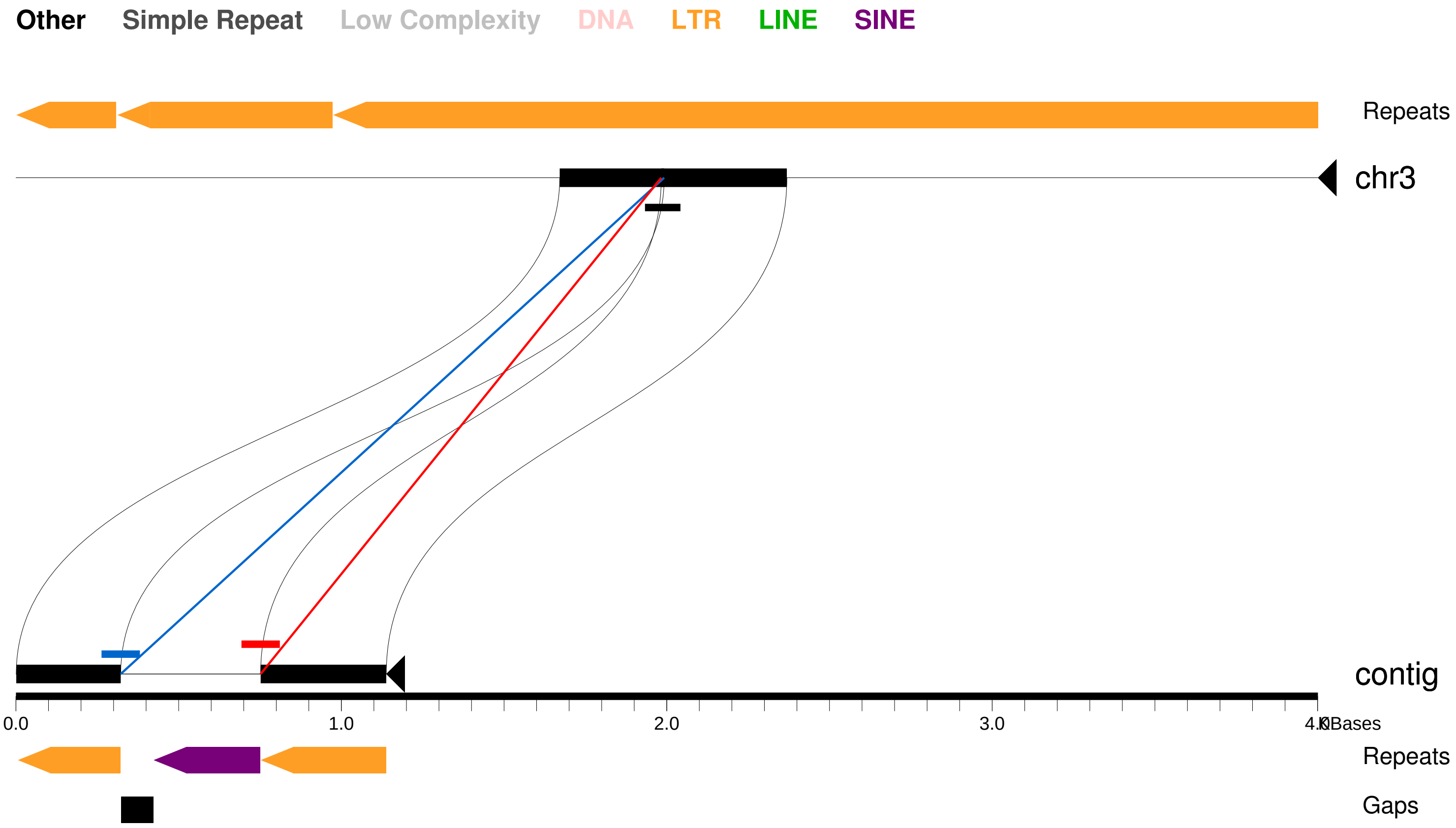

siteID: chr3_166595297 Scafftig1 + Breakpoint: chr3:166595344
chr3:166595295-166595403
Overlap Length: 11 INS size: 431 Genotyped: No

```
left    AATGTACCTCTTTAACTTAATCATTTCTAACCTTTCAAAAACTGTCTTT----------AAAAAATTATT
chrom   AATGTACCTCTTTAACTTAATCATTTCTAACCTTTCAAAAACTGTCTTT----------AAAAAATTATT
right   NNNNNNNNNNNNNNNNNNNNNNNNNNNNNNNNNNNNNNNNNNNNNNNNNNNNNNNNAAAAAAAAAATTATT
        11111111111111111111111111111111111111111111111111111111111***********

left    GG-CTGGGCGCGGTGGCTCACGCCTGTAATCCCAGCACTTTGGGAGGCCGAGGCGGGTGG
chrom   TGTCTAATAATAACTCTTCTGGTGTGTACGGCTAGCAA-----------GAGGAGAAAGA
right   TGTCTAATAATAACTCTTCTGGTGTGTACGGCTAGCAA-----------GAGGAGAAAGA
        2*2**222222222222**22*22****222*2****222222222222****2*222*2
```

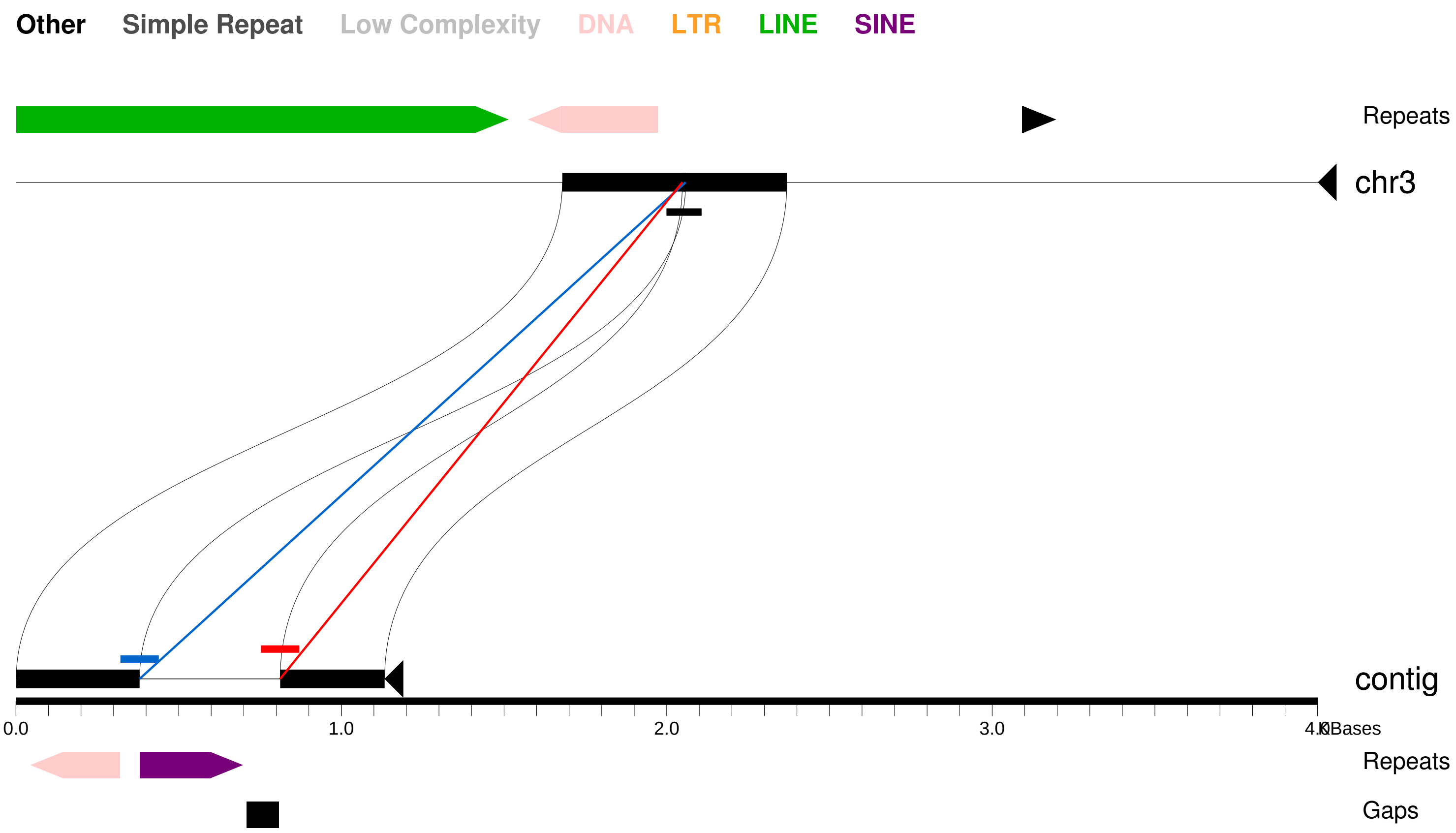

siteID: chr3_168199063 Scafftig1 + Breakpoint: chr3:168199063
chr3:168199011-168199122
Overlap Length: 6 INS size: 412 Genotyped: No

```
left     AGTGGATCTGTTTTGG---CCCATAG-GCTGC-----CTGTTTATAGCCACTGGTATACTGCATGCCNNN
chrom    AGTGGATCTGTTTTGG---CCCATAG-GCTGC-----CTGTTTATAGCCACTGGTATACTGCATGCCTGA
right    TCCGC--CCGCCTCGGCCTCCCAAAGTGCTGGGATTACAGGCGTGAGCCACCGCGCCCGGCCATGCCTGA
         111*111*1*11*1**111****1**1****111111*1*11111******1*11111111******222

left     NNNNNNNNNNNNNNNNNNNNNNNNNNNNNNNNNNNNNNNNNNNNNNNNNNNNNNNNNNN
chrom    CTCTATGCAGAGTGTTTGTGTCTTCTCACTCATGGTTTTTAAATCTAATTG-------
right    CTCTATGCAGAGTGTTTGTGTCTTCTCACTCATGGTTTTTAAATCTAATTG-------
         22222222222222222222222222222222222222222222222222222222222
```

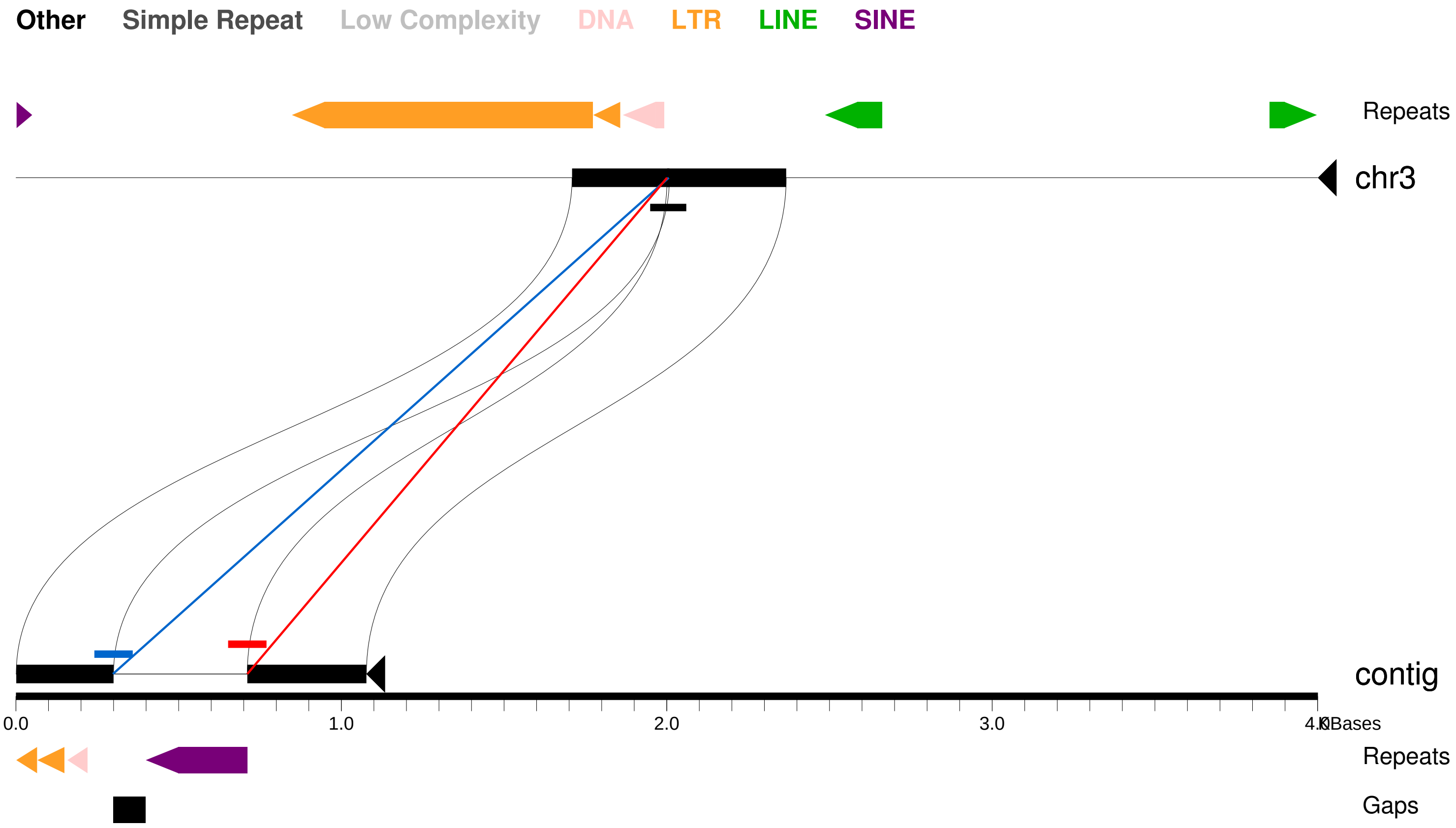

siteID: chr3_168885762 Scafftig2 - Breakpoint: chr3:168885750
chr3:168885701-168885809
Overlap Length: 11 INS size: 386 Genotyped: No

```
left   TAGTATCCACCCAGTGACTGACTCTGATTCCGTAAAAGAATATATCTTT----------AAAACATTCTT
chrom  TAGTATCCACCCAGTGACTGACTCTGATTCGGTAAAAGAATATATCTTT----------AAAACATTCTT
right  NNNNNNNNNNNNNNNNNNNNNNNNNNNNNNNNNNNNNNNNNNNNNNNNNNNNNAAAAAAAAAACATTCTT
       111111111111111111111111111111N1111111111111111111111111111***********

left   GGCCGGGCGCGGTGGCTCACGCCTGTAATCCCAGCACTTTGGGAGGCCGAGGCGGGCGG
chrom  CTACATCCATGA--GCAATGAGAGGGAAT---AGCAGCTTGTCACATGTAAACA-----
right  CTACATCCATGA--GCAATGAGAGGGAAT---AGCAGCTTGTCACATGTAAACA-----
       222*222*22*222**22222222*2***222****22***22*22222*22*222222
```

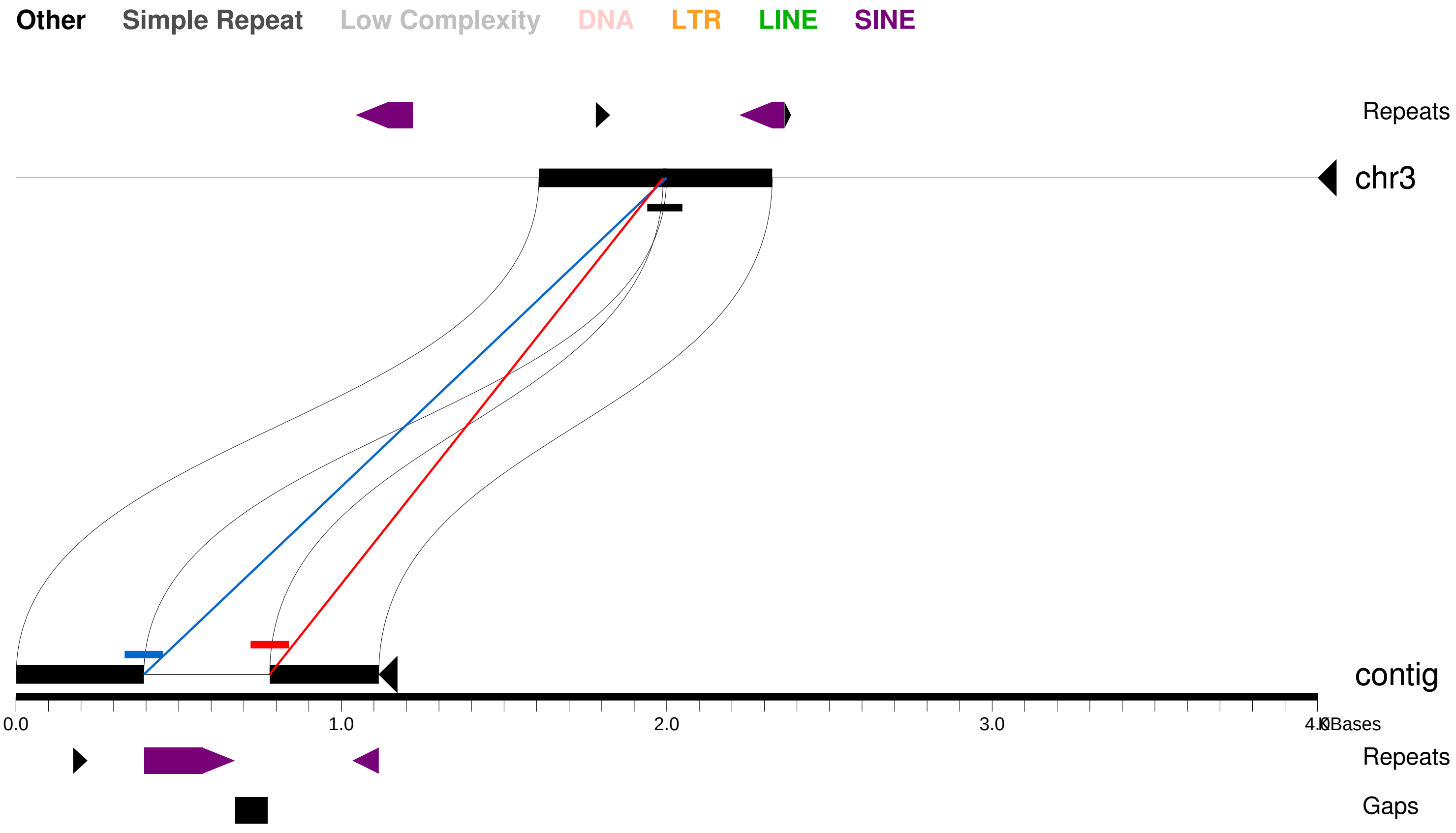

siteID: chr3_169145007 Scafftig1 - Breakpoint: chr3:169144988
chr3:169144941-169145044
Overlap Length: 13 INS size: 291 Genotyped: Yes

```
left     T-----------ACTAATATGTAAACTTTTTAGTATTTCCA-ATTCATTTTTTAGTAAA---AATTATGA
chrom    T-----------ACTAATATGTAAACTTTTTAGTATTTCCA-ATTCATTTTTTAGTAAA---AATTATGA
right    CGAGATCCCGCCACTGCACTCCAGCCTGGGCGACAGAGCGAGACTCCGACTCAAAAAAAAAAAATTATGA
         111111111111***1111*11*11**1111111*111*1*1*1**1111*11*11***111********

left     TGTTTGGCCGGGCGCGGTGGCTCACGCCTGTAATCCCAGCACTTTGGGAGGCCGAGGCGGGCGG
chrom    TGTTTAA----ATGCATATATTATCATTTAAAGTCACAGAGCTATGCTAGTCT-----------
right    TGTTTAA----ATGCATATATTATCATTTAAAGTCACAGAGCTATGCTAGTCT-----------
         *****22222222**222222*22*222*22*2**2***22**2**22**2*222222222222
```

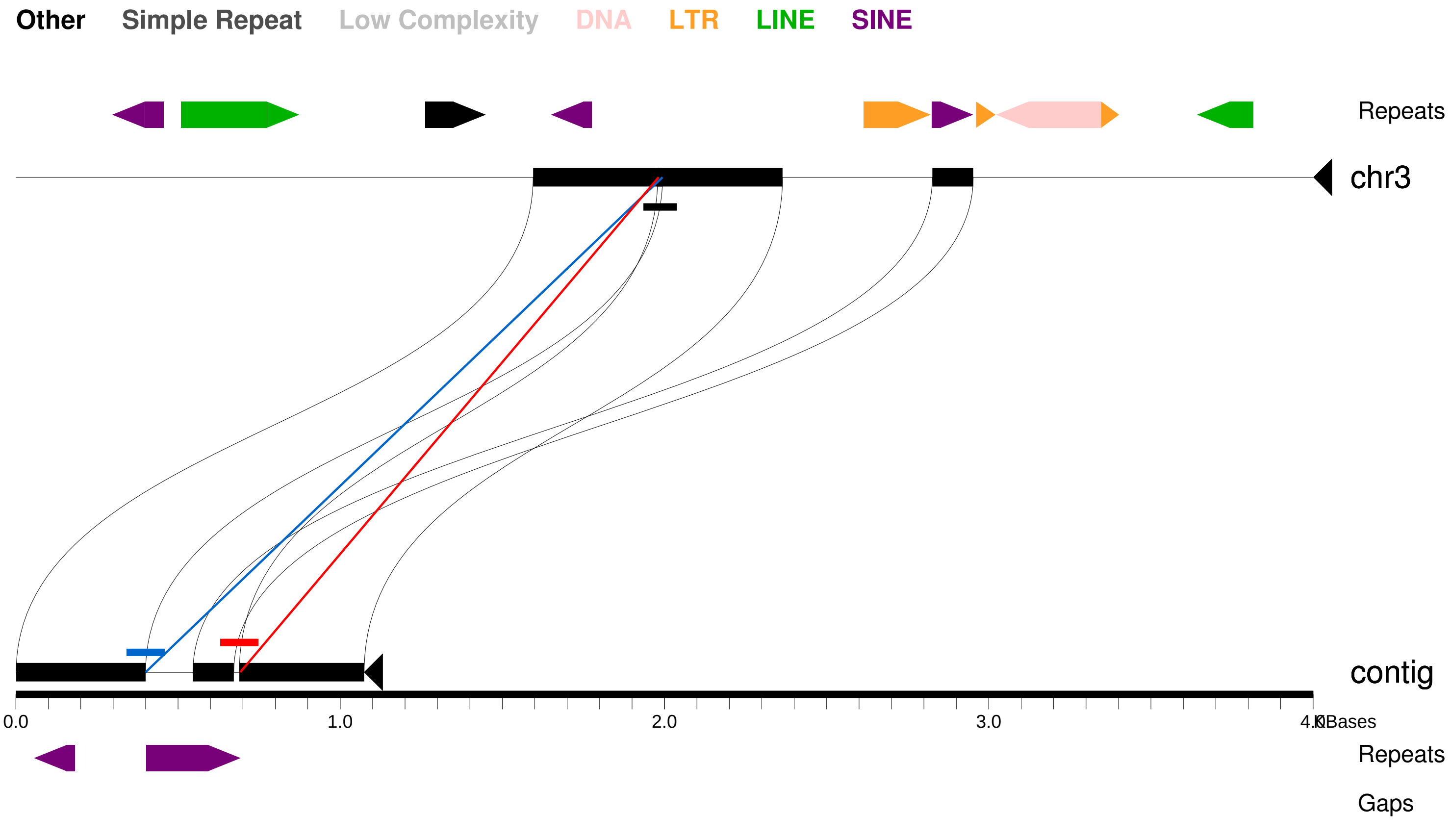

siteID: chr3_169270584 Scafftig5 - Breakpoint: chr3:169270590
chr3:169270541-169270649
Overlap Length: 11 INS size: 427 Genotyped: No

```
left     T------GTTTTGCCACTTGCAAGCTCTGTAAGACCAGTATT--TCCTTCTAGCCTG--CTCCTCCTGAT
chrom    T------GTTTTGCCACTTGCAAGCTCTGTAAGACCAGTATT--TCCTTCTAGCCTG--CTCCTCCTGAT
right    TCCACCCGCCTCGGCCTCCCAAAGTGCTGGGATTACAGGCGTGAGCCACCGCGCCCGGCCTCCTCCTGAT
         *111111*11*1*1*111111***11***11*111***111*111**11*11***1*11***********

left     NNNNNNNNNNNNNNNNNNNNNNNNNNNNNNNNNNNNNNNNNNNNNNNNNNNNNNNNNNN
chrom    TTTTAAAGTGAGGAAGTGGGGCGGTGCCATCCCTAAGTCCCCAGTCACT----------
right    TTTTAAAGTGAGGAAGTGGGGCGGTGCCATCCCTAAGTCCCCAGTCACT----------
         22222222222222222222222222222222222222222222222222222222222
```

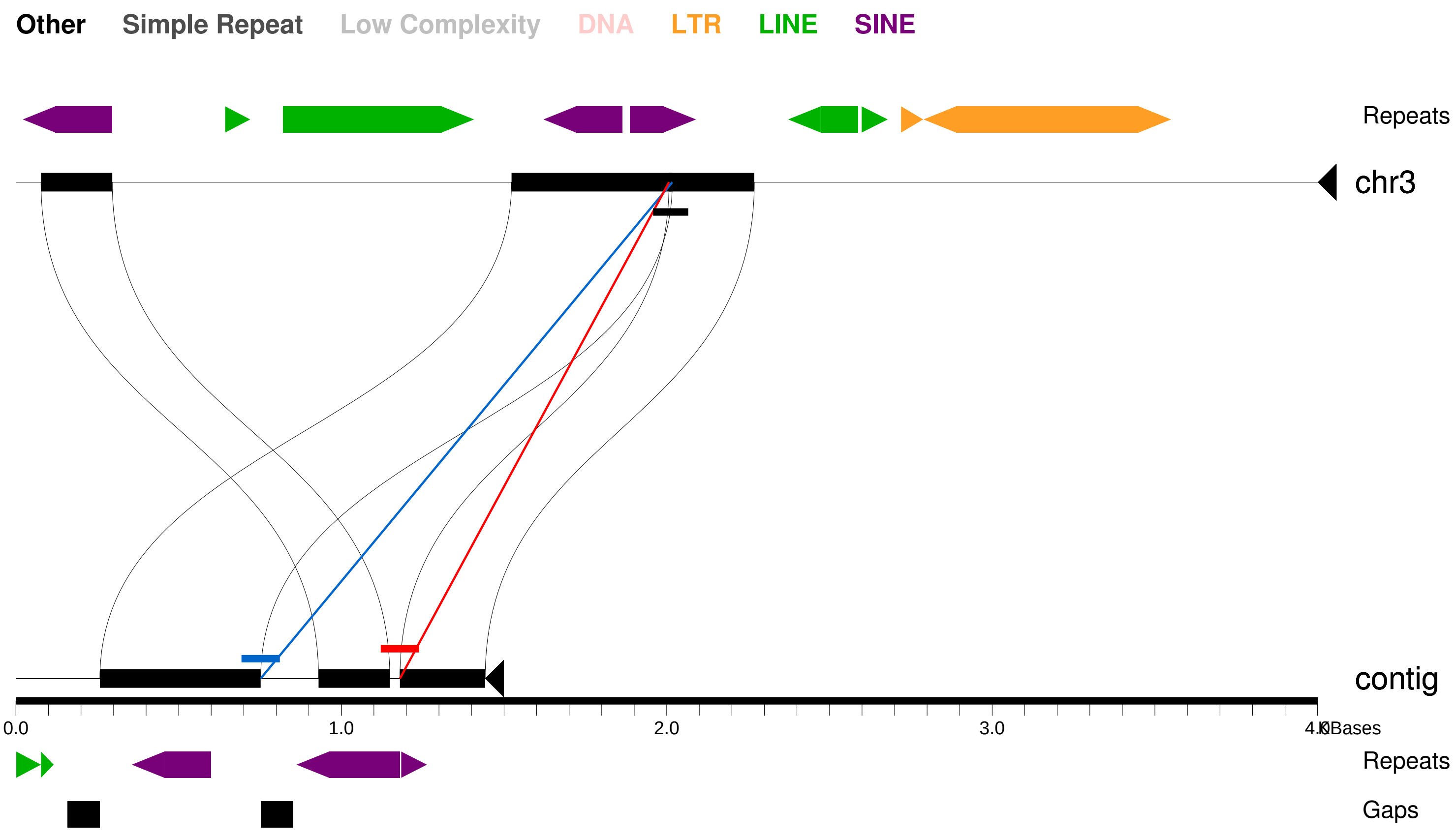

siteID: chr3_169474327 Scafftig4 - Breakpoint: chr3:169474313
chr3:169474264-169474372
Overlap Length: 11 INS size: 304 Genotyped: Yes

```
left    TGCTTCAA------AAGGGGGTCAGAGAG-GA----TTCTGAGGAGGTGATATCTAAGTTAAGACTTGAG
chrom   TGCTTCAA------AAGGGGGTCAGAGAG-GA----TTCTGAGGAGGTGATATCTAAGTTAAGACTTGAG
right   GTCCGCAGTCCGACCTGGGCGACAGAGCGAGACTCCATCTCAAAAAAAAAGAAAAAAAA-AAGACTTGAG
        11*11**111111111***1*1*****1*1**11111***1*11*1111*1*111**111*********

left    GCCGGGCGCGTTGGCTCACGCCTGTAATCCCA---GCACTTTGGGAGGCCGAGGCGGGTGGAT
chrom   G--AGGAGAGAAGGG----GCCAGGAACTACAAGTGCAATTT---AAGCAGAGAACCCA----
right   G--AGGAGAGAAGGG----GCCAGGAACTACAAGTGCAATTT---AAGCAGAGAACCCA----
        *222**2*2*22**22222***2*2**222**222***2***222*2**2***2222222222
```

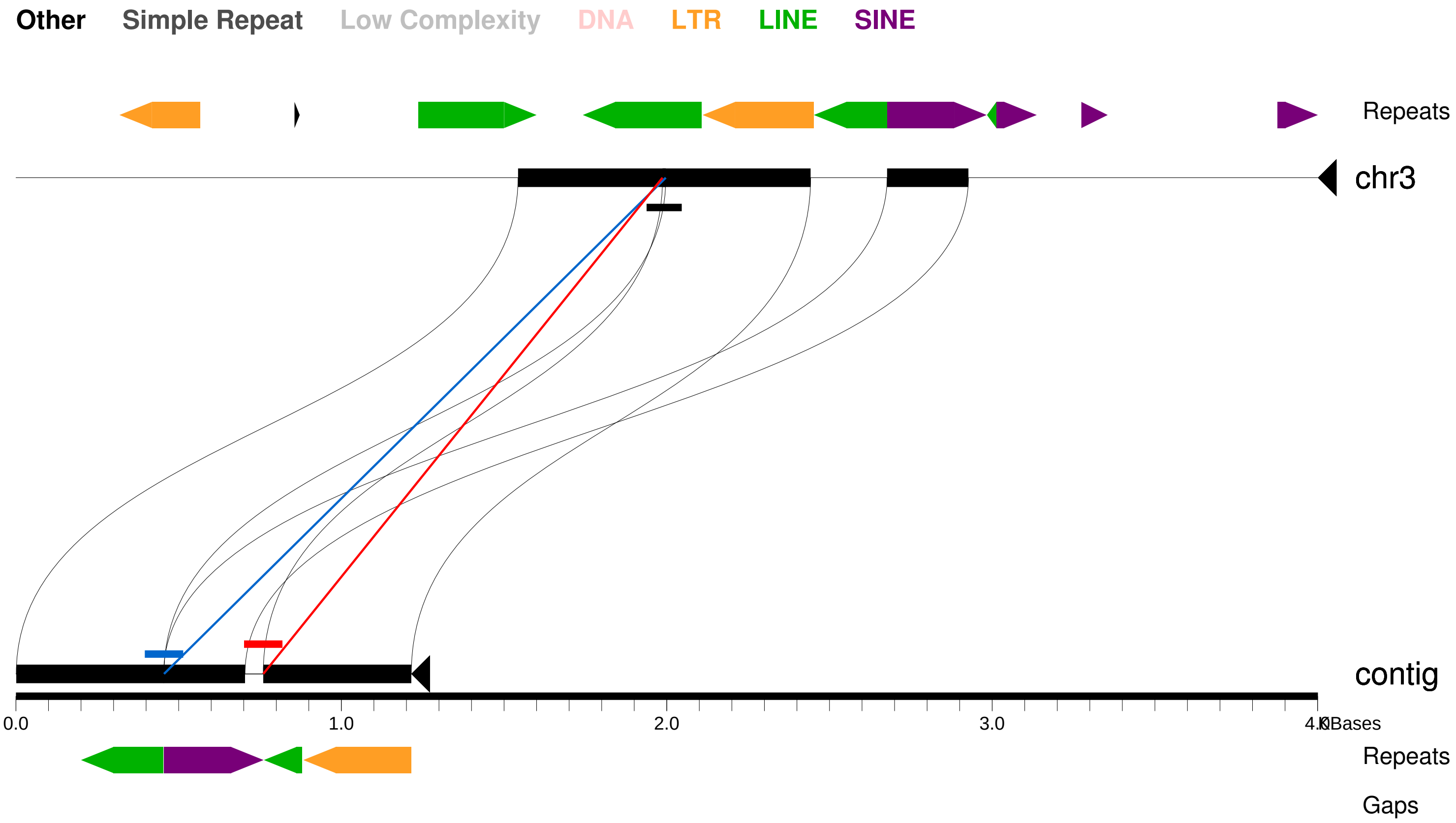

siteID: chr3_170667528 Scafftig1 - Breakpoint: chr3:170667544
chr3:170667496-170667603
Overlap Length: 12 INS size: 412 Genotyped: No

```
left     ATGAGTGGCCCAGGGAAGCCAAAA------GATTGACACCGCTGGTCTATAGTT-------TGCTTATAA
chrom    ATGAGTGGCCCAGGGAAGCCAAAA------GATTGACACCGCTGGTCTATAGTT-------TGCTTATAA
right    CCGCCCGCCTC-GGCCTCCCAAAGTGCTGGGATT-ACAGGCGTGAGCCACCGCGCCCGGCCTGCTTATAA
         11*111*1*1*1**1111*****1111111****1***1111**11*1*11*111111111*********

left     TAGNNNNNNNNNNNNNNNNNNNNNNNNNNNNNNNNNNNNNNNNNNNNNNNNNNNNNNNNNNNN
chrom    TAGCTTTATCTGGTTTGATGTCAGGGTAATGCTAGCTTCATAGAATGAATT-----------
right    TAGCTTTATCTGGTTTGATGTCAGGGTAATGCTAGCTTCATAGAATGAATT-----------
         ***222222222222222222222222222222222222222222222222222222222222
```

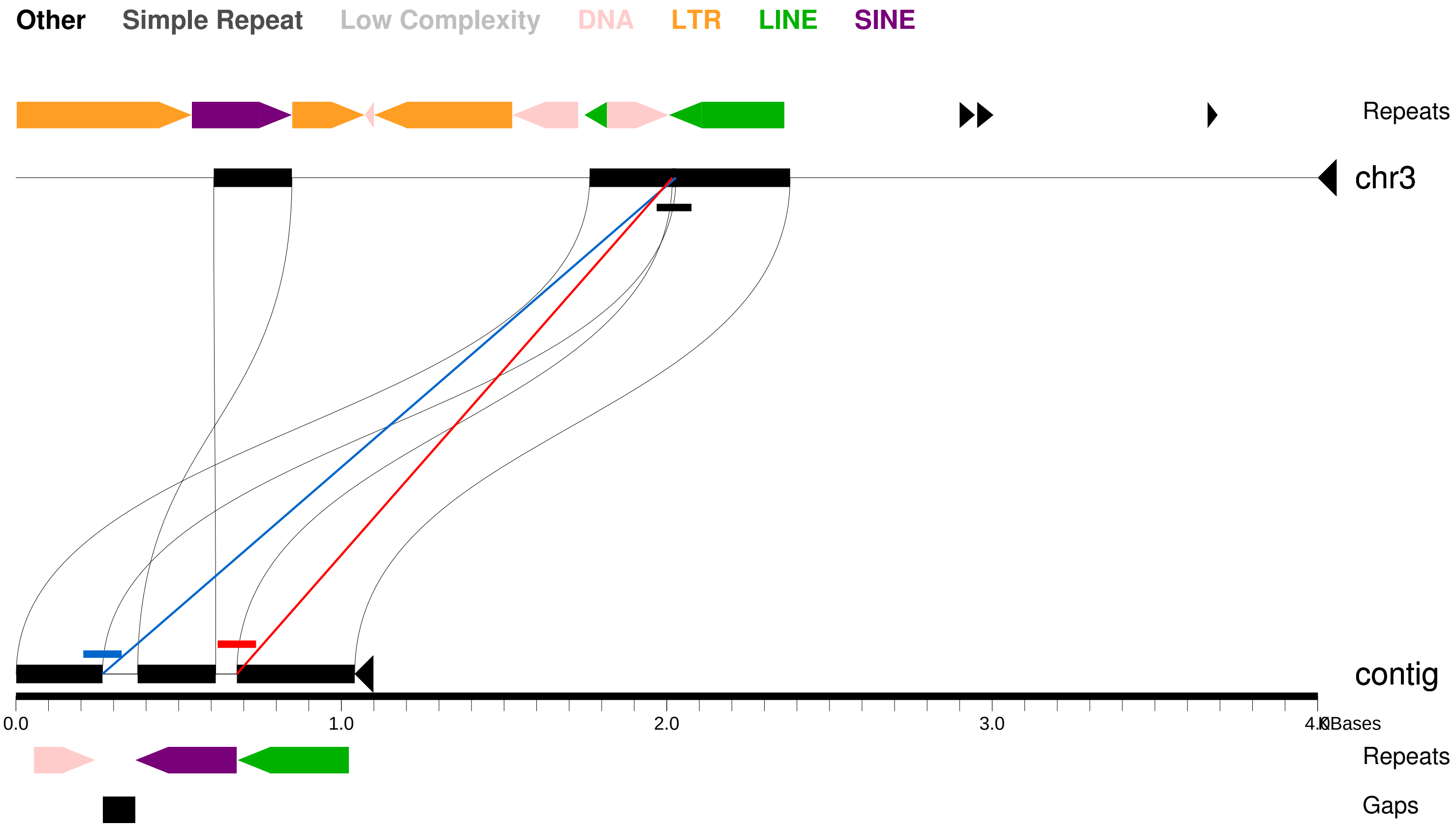

siteID: chr3_171319267 Scafftig2 - Breakpoint: chr3:171319286
chr3:171319241-171319345
Overlap Length: 15 INS size: 294 Genotyped: Yes

```
left    C-----ATTTTA--CCATCTTTAGC--CATATGAAAAATAAGTT-----ATTTACTTATAAAAACATTCA
chrom   C-----ATTTTA--CCATCTTTAGC--CATATGAAAAATAAGTT-----ATTTACTTATAAAAACATTCA
right   CCCGCCACTGCACTCCAGCCTGGGCGACAGAGCGAGACTCCGTCTCAAAAAAAAAAAAAAAAAAACATTCA
        *11111*1*11*11***1*1*11**11**1*111*1*1*11**111111*111*111*1***********

left    TTTTGGCCGGGCGCGGTGGCTCACGCCTGTAATCCCAGCAC-TTTGGGAGGCCGAGGCGGGCGG
chrom   TTTTTATTA-------TTGGTTACA----AAATCTCACCTTATTTGTGAA----ATGTTCCCTA
right   TTTTTATTA-------TTGGTTACA----AAATCTCACCTTATTTGTGAA----ATGTTCCCTA
        ****222222222222*2*2*2**222222****2**2*222****2**22222*2*2222*22
```

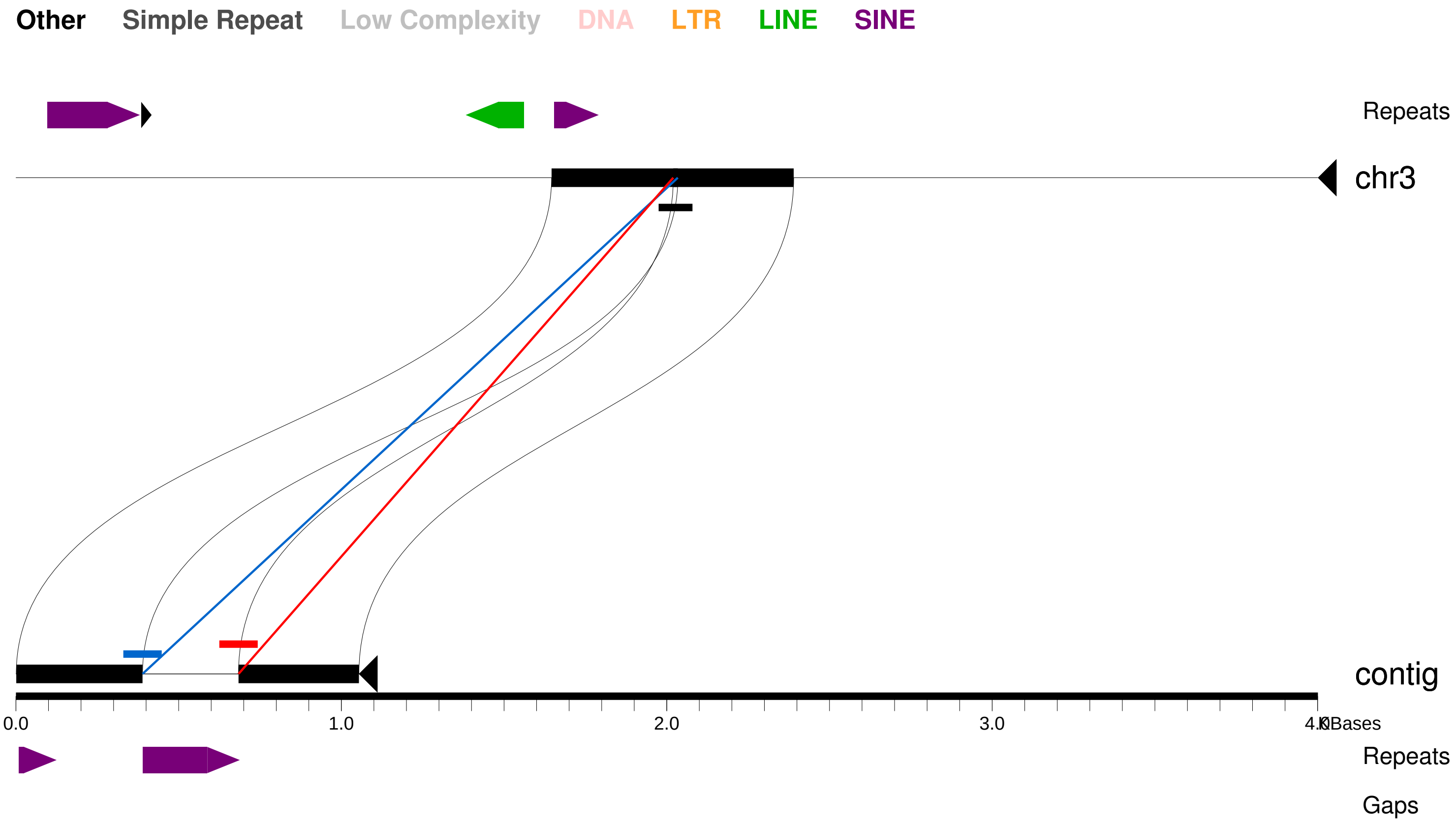

siteID: chr3_171626378 Scafftig2 + Breakpoint: chr3:171626374
chr3:171626329-171626433
Overlap Length: 8 INS size: 305 Genotyped: Yes

```
left    TT------------ATTACAGTATATTGTTATTATT--CTATTTTCATTATTAAGTTATTCCTGTTATTT
chrom   TT------------ATTACAGTATATTGTTATTATT--CTATTTTCATTATTAAGTTATTCCTGTTAATT
right   CCGCCCGCCTCGGCCTCCCAAAGTGCTGGGATTACAGGCGTGAGCCACCGCGCCCGGCCTCCTGTTAATT
        111111111111111*11**111*11**11****1111*111111**111111111111********2**

left    TTTTTTTTTTTTTTTTTTTGAGACGGAGTCTCGCTCTGTCGCCCAGGCTGGAGTGCAGTGGCG
chrom   TCTTACTATGTCTAATTTATAAA-------TTAAACTTTCTCATAGGT----GTGTA-TGT--
right   TCTTACTATGTCTAATTTATAAA-------TTAAACTTTCTCATAGGT----GTGTA-TGT--
        *2**22*2*2*2*22***22*2*2222222*2222**2**2*22***22222***2*2**222
```

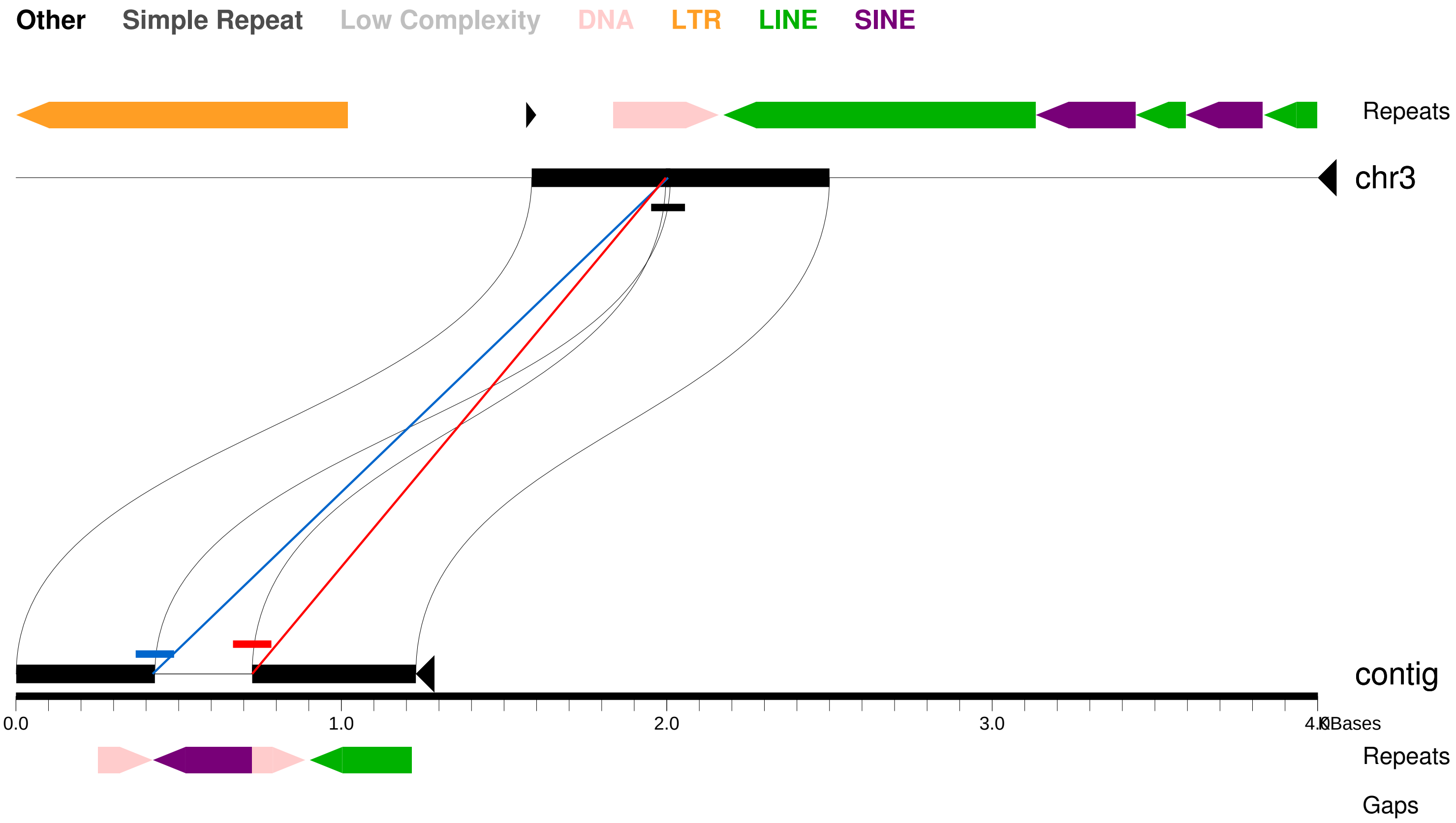

siteID: chr3_171883793 Scafftig2 + Breakpoint: chr3:171883794
chr3:171883749-171883853
Overlap Length: 15 INS size: 301 Genotyped: Yes

```
left     ATGG---TTG--CTTTTATTCTTTTGAGAATA------TTTGTTTCCTAG---GTGAATGAGAGCTAATC
chrom    ATGG---TTG--CTTTTATTCTTTTGAGAATA------TTTGTTTCCTAG---GTGAATGAGAGCTAATC
right    ACCCGCCTCGGCCTCCCAAAGTGCTGGGATTACAGGCGTGAGCCACCGCGCCCGGCCAAGAGAGCTAATC
         *111111*1*11**111*111*11**1**1**111111*11*111**11*111*111*1***********

left     TTTTTTTTCTTTTTTTTTTTTTTGAGACGGAGTCTCGCTCTGTCGCCCAGGCTGGAGTGCAG-TG
chrom    TTTTAT------------GCATGA-AGGGCCTAAAGTGCTCTGATTTATATT--AATAAATATA
right    TTTTAT------------GCATGA-AGGGCCTAAAGTGCTCTGATTTATATT--AATAAATATA
         ****2*222222222222222***2*2**22*222*22**2*22222*222*22*2*22*22*2
```

Other Simple Repeat Low Complexity DNA LTR LINE SINE

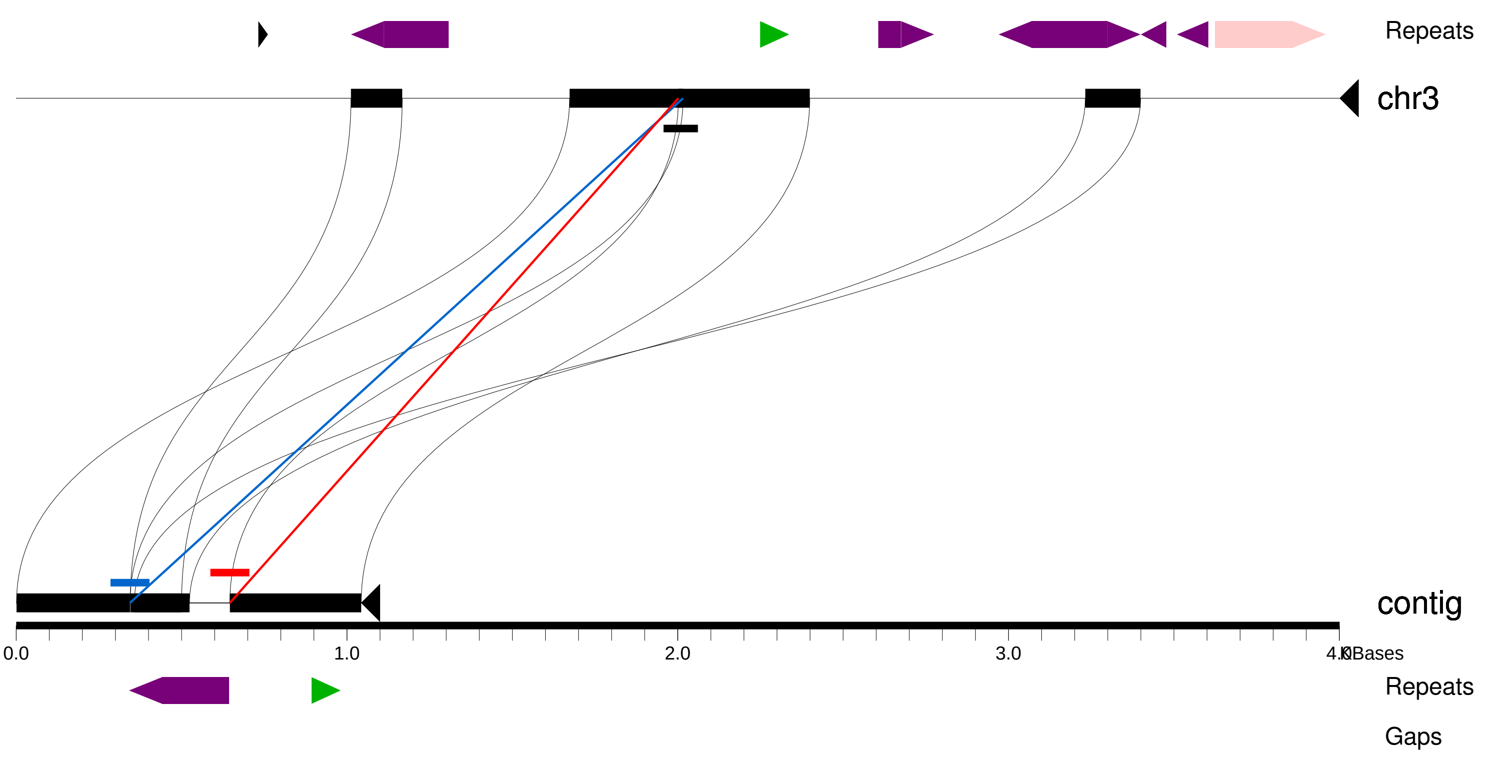

siteID: chr3_172322862 Scafftig2 - Breakpoint: chr3:172322937
chr3:172322891-172322996
Overlap Length: 14 INS size: 141 Genotyped: Yes

```
left     T--GCAGACCACA----CTGACCACAGTGTGCATAAGCAATT-----------GCTTTCCAGTTGAGTTT
chrom    T--GCAGACCACA----CTGACCACAGTGTGCACAAGCAATT-----------GCTTTCCAGTTGAGTTT
right    TCGGCTCACTGCAAGCTCCGCCTCCCAGGTTCAC--GCCATTCTCCTGCCTCAGCCTCCCA--TGAGTTT
         *11**11**11**1111*1*1*11*111**1**211**1***11111111111**1*1***11*******

left     AACTTTTTTTTTTTTTTTTTTTTTTTTTTTTTTTTTTTTTGAGACGGAGTCTCGCTCTGTCGCCCA
chrom    AACTTTTAATTCCAATATGGCCTCTCTGGTTTCTCCTCT-------GATCTGTGCTGGGT------
right    AACTTTTAATTCCAATATGGCCTCTCTGGTTTCTCCTCT-------GATCTGTGCTGGGT------
         *******22**2222*2*2222*2*2*22***2*22*2*2222222**22222***22**222222
```

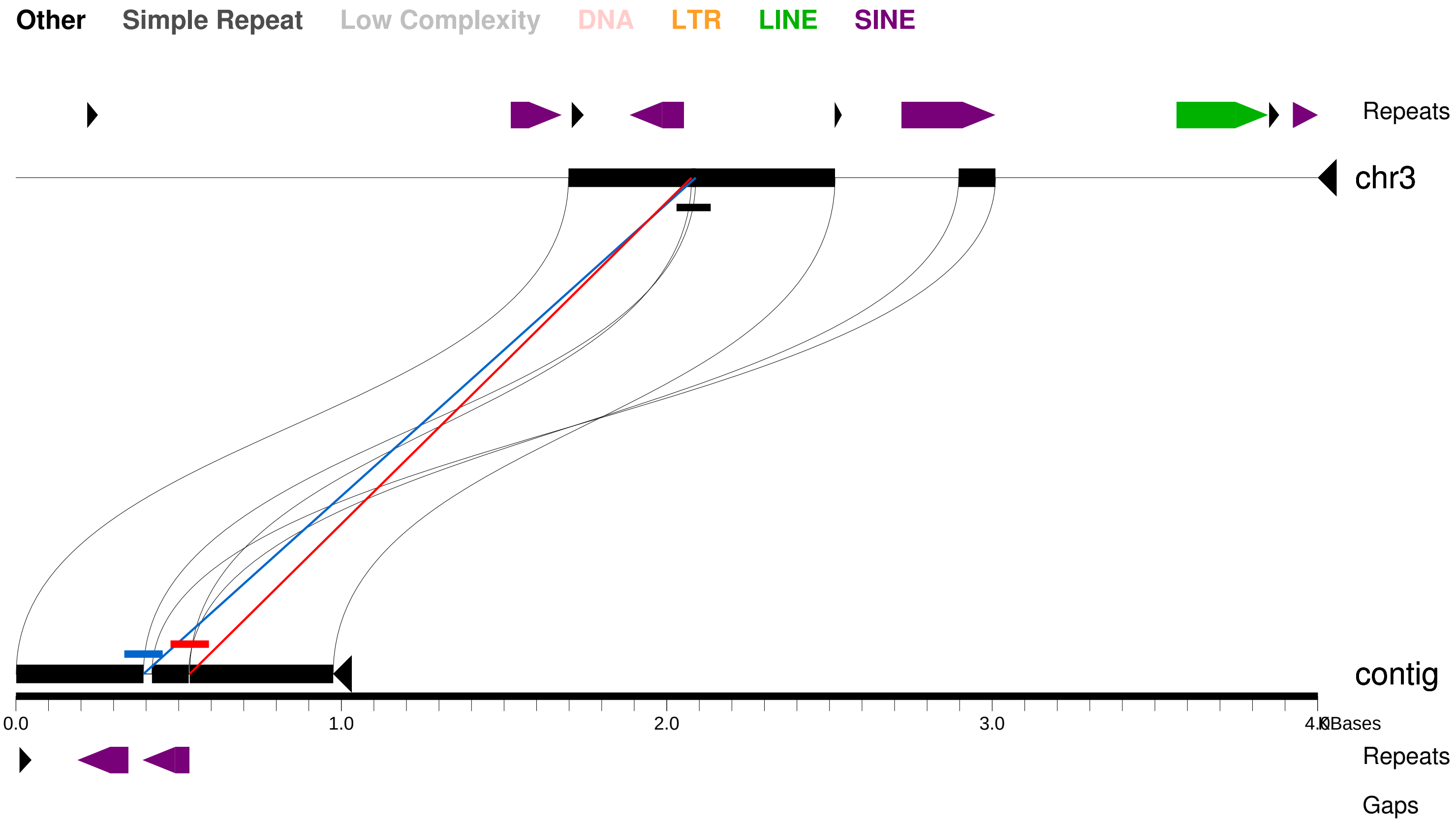

siteID: chr3_174250916 Scafftig2 - Breakpoint: chr3:174250897
chr3:174250852-174250956
Overlap Length: 15 INS size: 321 Genotyped: Yes

```
left     CCTT-ACCCTTTCCTCTCCACTTTCTTTTACGTGCCCACCTTATCT-------------AAAAAAAGCAA
chrom    CCTT-ACCCTTTCCTCTCCACTTTCTTTTACGTGCCCACCTTATCT-------------AAAAAAAGCAA
right    AGAGCAAAACTCCGTCTCAAAAAAAAAAAAAAAAAAAAAAAAAAAAAAAAAAAAAAAAAAAAAAAAAGCAA
         11111*1111*1*1****1*111111111*1111111*1111*1111111111111111***********

left     ATGTG--GCCGGGCGCGGTGGCTCACGCCTGTAAT-CCCAGCACTTTGGGAGGCCGAGACGGGCGG
chrom    ATGTTTAGCCAACCGGGATTAGTTTAGGTTGTATGACCCAAC-----------CCCAGCC------
right    ATGTTTAGCCAACCGGGATTAGTTTAGGTTGTATGACCCAAC-----------CCCAGCC------
         *****222***222**2*2*222*222*22****222****2*22222222222**2**2*222222
```

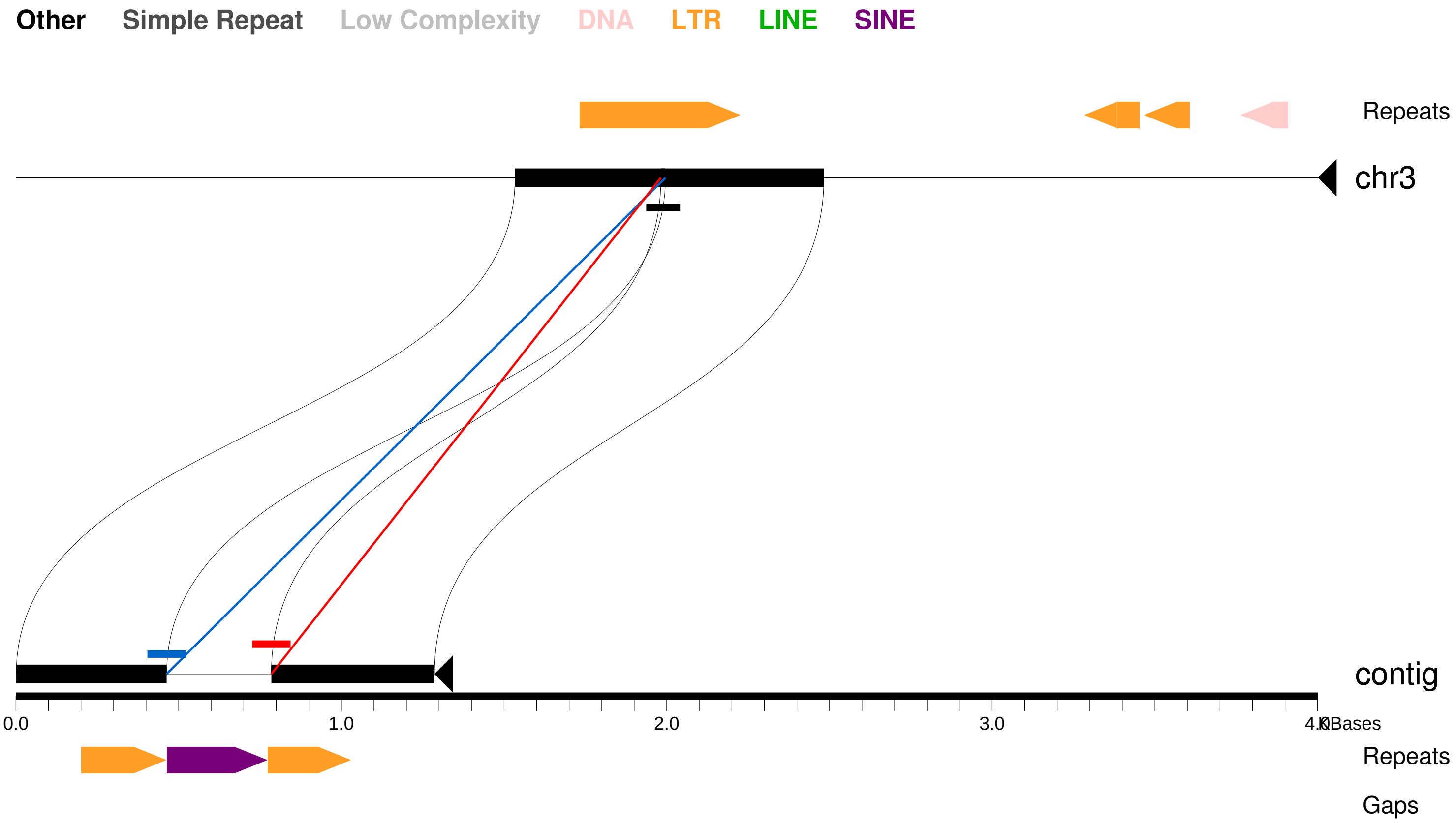

siteID: chr3_174410759 Scafftig7 + Breakpoint: chr3:174410737
chr3:174410691-174410796
Overlap Length: 14 INS size: 309 Genotyped: Yes

```
left    ATAAAATTATTGGTAT---AAAAGGTTCTATTTGTAGG---GACCCATATGA-------ACTCATACCTT
chrom   ATAAAATTATTGGTAT---AAAAGGTTCTATTTGTAGG---GACCCATATGA-------ACTCATACCTT
right   CCACCCGCCTCGGCCTCCCAAAGTGCTGGGATTACAGGCGTGAGCCACCGCGCCCGGCCACTCATACCTT
        11*111111*1**11*111***11*1*1111**11***111**1***111111111111***********

left    TCTCTTTTTTTTTTTTTTTTTTTTTGAGACGGAGTCTCGCTCTGTCGCCCAGGTCGGACTGCG
chrom   TCT-----------TGAGATTCAACATACAGAATATAAATA--CCAACCAGTAAAATAATAA
right   TCT-----------TGAGATTCAACATACAGAATATAAATA--CCAACCAGTAAAATAATAA
        ***22222222222*2222**2222*2**2**2*2*222*2222*22****22222222222
```

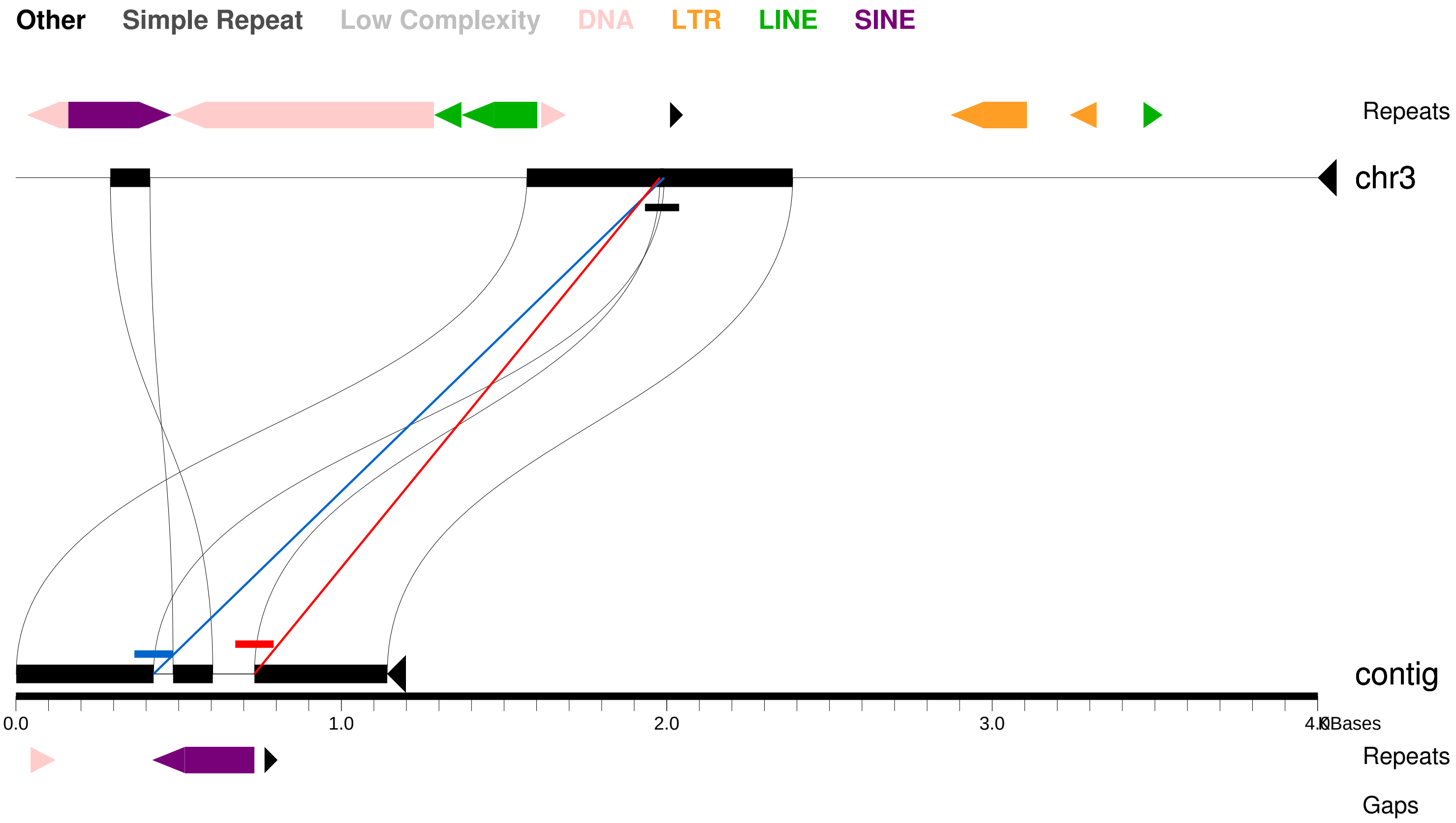

siteID: chr3_175673955 Scafftig3 + Breakpoint: chr3:175673930
chr3:175673884-175673989
Overlap Length: 14 INS size: 308 Genotyped: Yes

```
left    ------GAAT-GAAATGCAAAATATTAGAAAGTTCA------AATAAAGGGGATAAGGTCTAATTTGTGA
chrom   ------GAAT-GAAATGCAAAATATTAGAAAGTTCA------AATAAAGGGGATAAGGTCTAATTTGTGA
right   CCGCCCGCCTCGGCCTCCCAAAGTGCTGGGATTACAGGCGTGAGCCACCGCGCCCGGCCCTAATTTGTGA
        111111*11*1*111*1*1***11111*11*1*1**111111*111*11*1*1111*11***********

left    TTTTTTTTTTTTTTTTTTTTTTTTTTTTTTGAGACGGAGTCTCACTCTGTCGCCCAGGCTGG
chrom   TTTAAAAGGCATATTCTCTCTGCTGTGTAT-AGATGA------------TAGGTGTGAAAAG
right   TTTAAAAGGCATATTCTCTCTGCTGTGTAT-AGATGA------------TAGGTGTGAAAAG
        ***22222222*2**2*2*2*22*2*2*2*2***2*2222222222222*2*2222*2222*
```

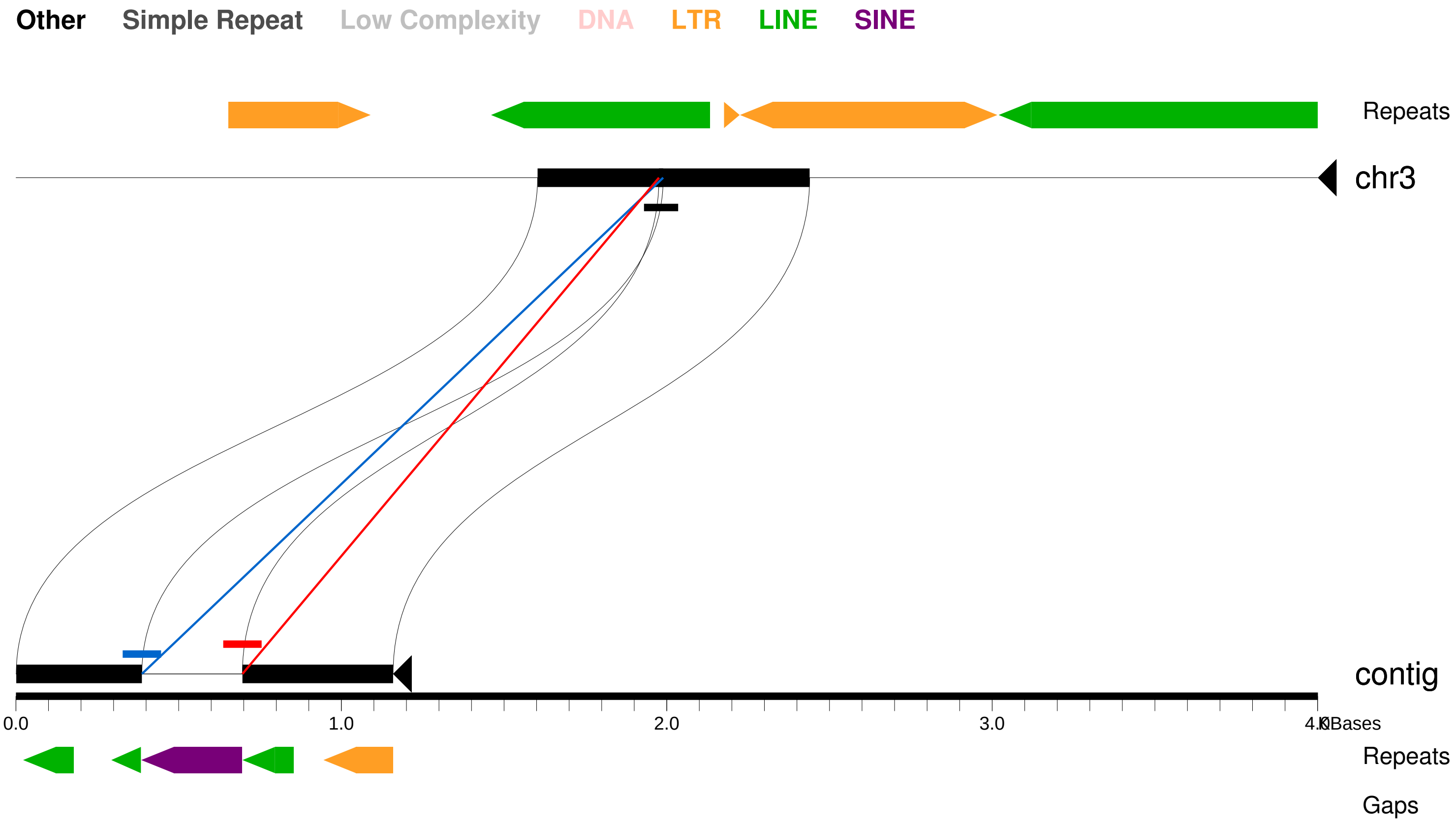

siteID: chr3_176913317 Scafftig5 - Breakpoint: chr3:176913232
chr3:176913188-176913291
Overlap Length: 16 INS size: 89 Genotyped: Yes

```
left     TCGGGGAGACAAAGGTGCCCTACTTCCCCAGACTATAAACTATT-------------------AAAATGG
chrom    TCGGGGAGACAAAGGTGCCCTACTTCCCCAGACTATAAACTATT-------------------AAAATGG
right    ----GGTGACAGAGCGAGACTCCGTCTCAAAAAAAAAAAAAAAAAAAAAAAAAAAAAAAAAAAAAAAATGG
         1111**1****1**11111**1*1**1*1*1*11*1***11*111111111111111111111*******

left     TGAGAACATGCCGAGATCCCGCCACTGCACTCCAGCCTGGGTGACAGAGCGAGACTCCGTCTCAAAAA
chrom    TGAGAACATACA---ATCCCAGGA---------AGTCGGCATTACTAAAATACA-TCAATGT--AGCA
right    TGAGAACATACA---ATCCCAGGA---------AGTCGGCATTACTAAAATACA-TCAATGT--AGCA
         *********2*2222*****222*222222222**2*2*22*2**22*222*2*2**22*2*22*22*
```

Other **Simple Repeat** **Low Complexity** **DNA** **LTR** **LINE** **SINE**

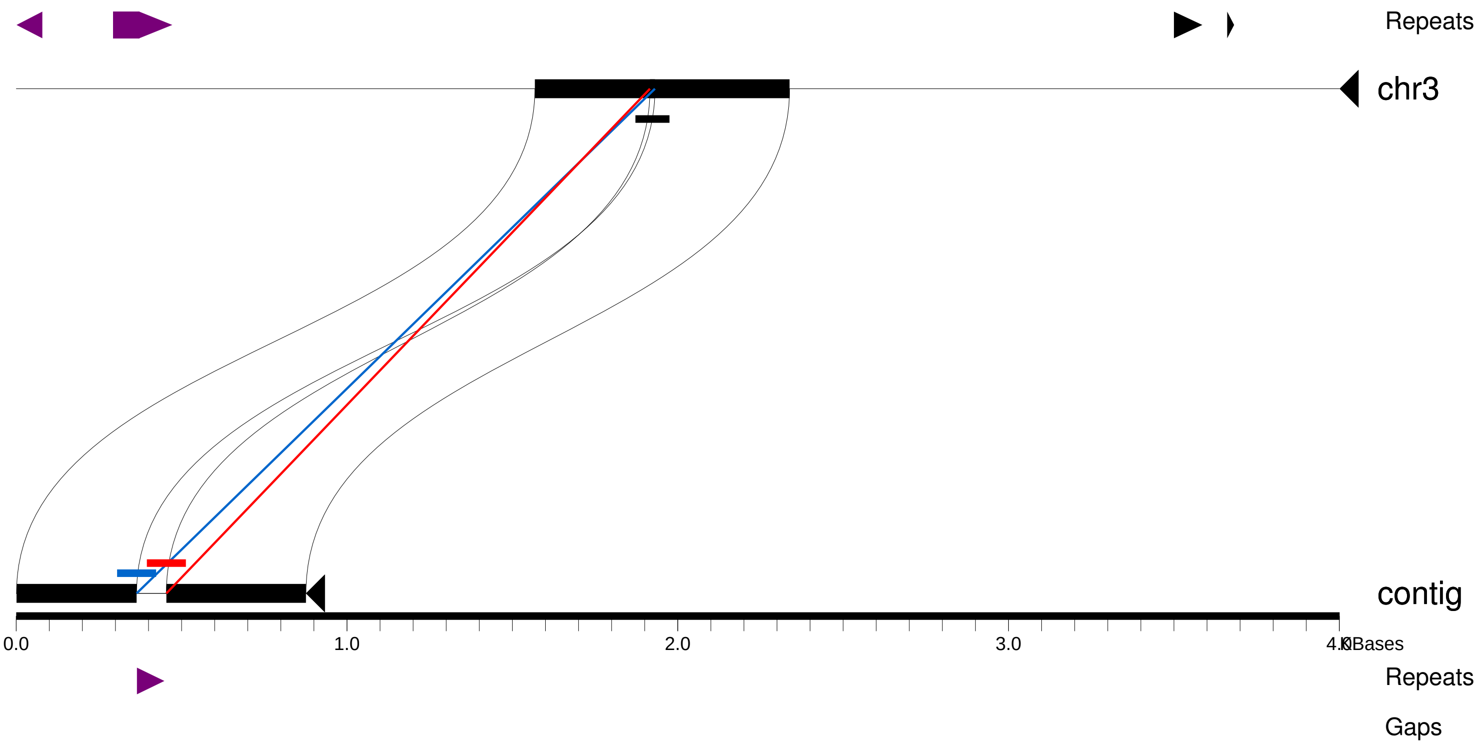

siteID: chr3_178111052 Scafftig3 - Breakpoint: chr3:178111037
chr3:178110986-178111096
Overlap Length: 9 INS size: 322 Genotyped: Yes

```
left    GCTGATA---------TCAGTGTACATCAATCCAAAATATGCTGTGGTAGATTTTTGCTTAAAAATGGGG
chrom   GCTGATA---------TCAGTGTACATCAATCCAAAATATGCTGTGGTAGATTTTTGCTTAAAAATGGGA
right   GGCGACAGAGCGAGACTCCGTCTCAAAAAAATAAAAAAATAAAAAAATAAAAAAAAAAA-AAAAATGGGA
        *11**1*111111111**1**1*11*11**111****1**1111111**1*111111111*********2

left    CCGGGCGCGGTGGCTCACGCCTGTAATCCCAGCACTTTGGGAGGCCGAGGCGGGTGGA
chrom   AGAAGTAGATTTCGGCAGGCC---AATGCT-GCCCCTGAGAATAAAAAGA----AAAA
right   AGAAGTAGATTTCGGCAGGCC---AATGCT-GCCCCTGAGAATAAAAAGA----AAAA
        2222*22222*2222**2***222***2*22**2*2*22*2*22222**22222222*
```

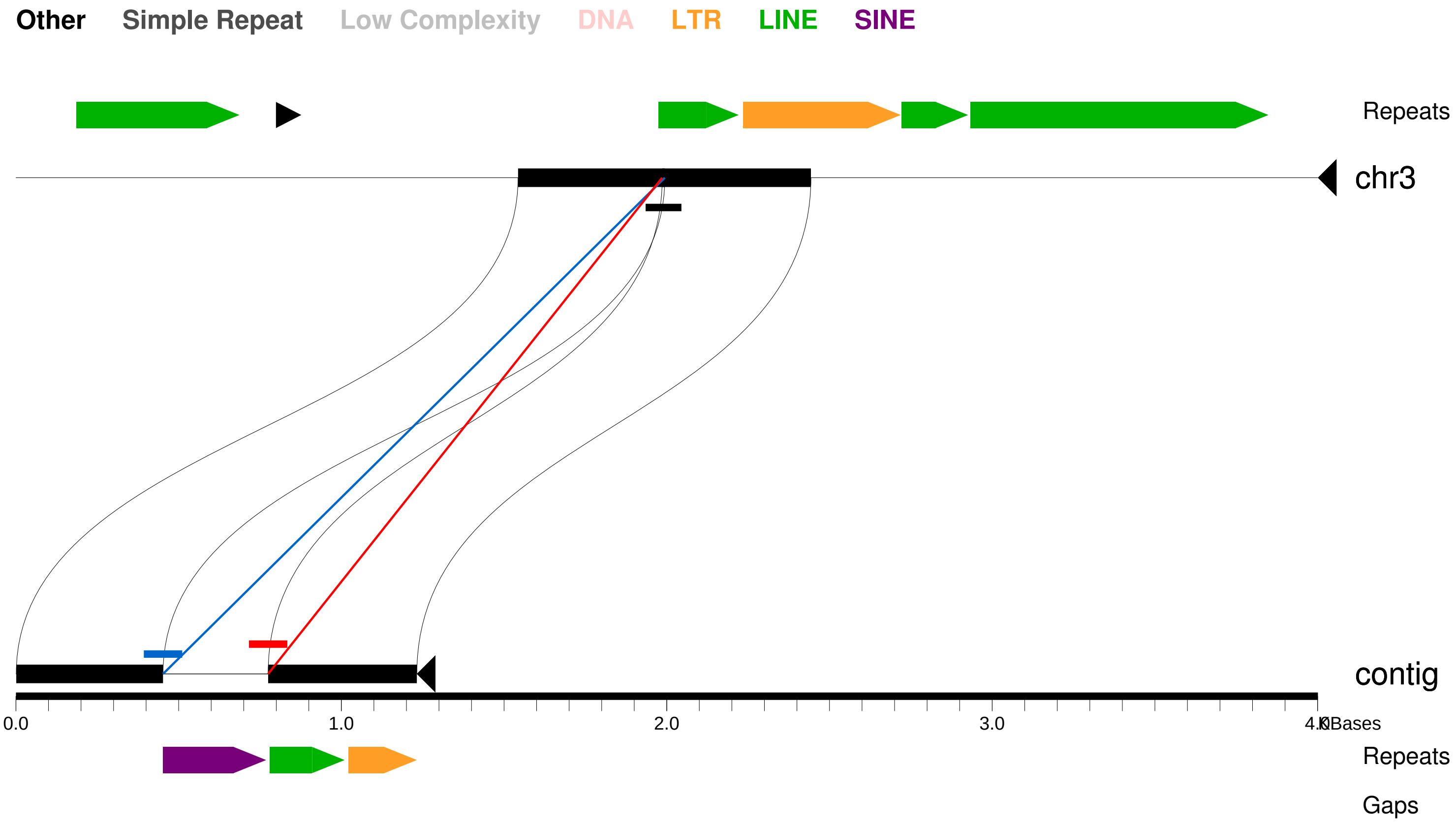

siteID: chr3_182299005 Scafftig4 - Breakpoint: chr3:182298997
chr3:182298954-182299056
Overlap Length: 17 INS size: 305 Genotyped: Yes

```
left     AA-ATTGC--CCTGT--GATTATTTAAGAT--CATCTCCTTGTTCTTTGG---------AAATATATAAC
chrom    AA-ATTGC--CCTGT--GATTATTTAAGAT--CATCTCCTTGTTCTTTGG---------AAATATATAAC
right    TGCAGTCCAGCCTGGGCGACAGAGCGAGACTCCGTCTCAAAAAAAAAAAAAAAAAAAAAAAATATATAAC
         111*1*1*11****111**1111111***111*1****111111111111111111111***********

left     TATGTAGAGGCCGGGCGCGGTGGCTCACGCCTGTAGTCCCAGCACTTTGGGAGGCCGAGGCGGGC
chrom    TATGTATCAAGCATGCTTCGGAGAAAAAGTATATAC--------CTTT---ATGTAAAT-----
right    TATGTATCAAGCATGCTTCGGAGAAAAAGTATATAC--------CTTT---ATGTAAAT-----
         ******22222*22**222*22*222*2*22*2**222222222****222*2*222*2222222
```

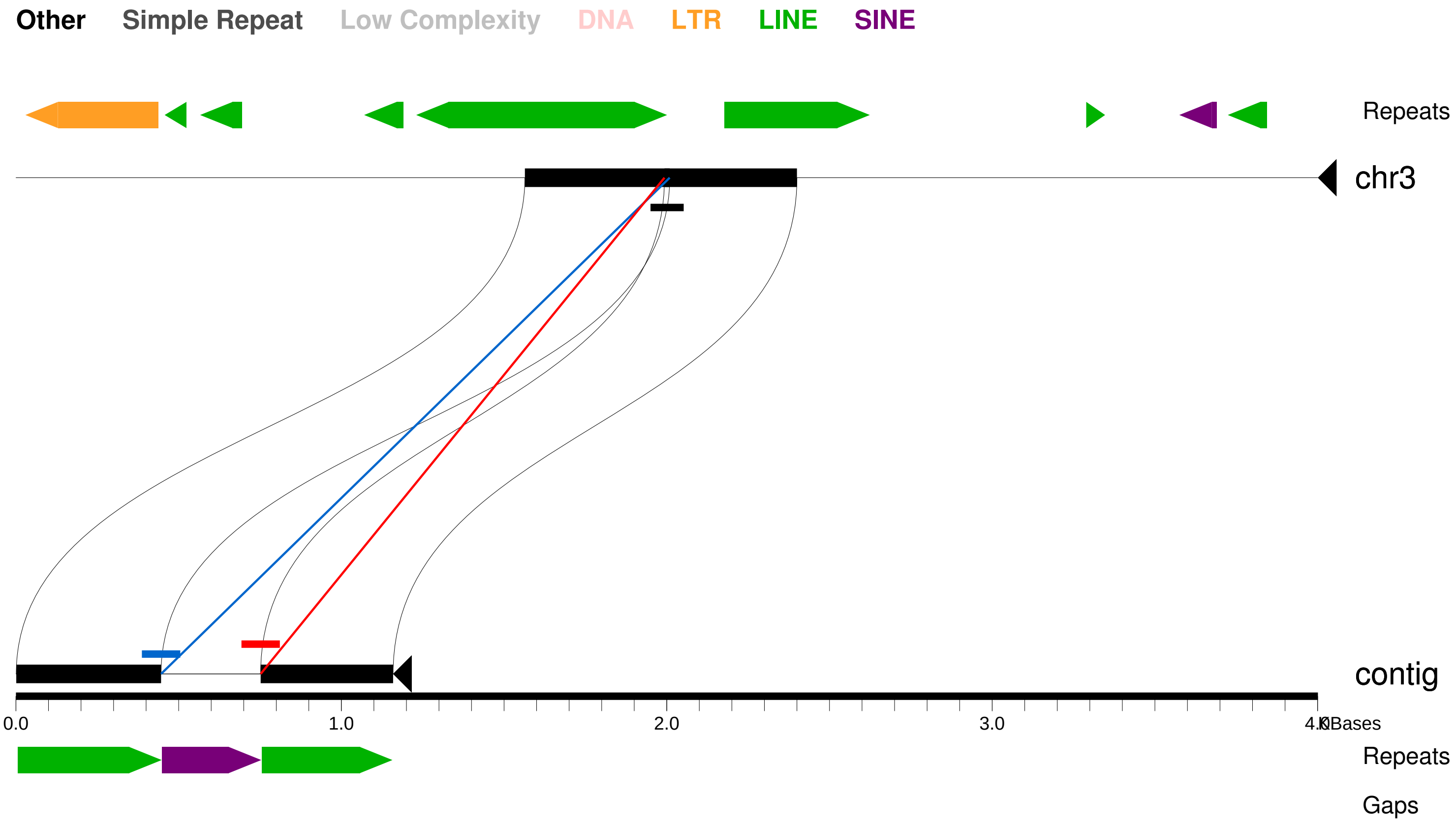

siteID: chr3_187558291 Scafftig1 - Breakpoint: chr3:187558299
chr3:187558242-187558358
Overlap Length: 3 INS size: 410 Genotyped: No

```
left   CATAATCTATTGGCATGGCTCTACACACAGGCACACACATGTACCATAAATCTATCG--AGGGCCGGGCG
chrom  CATAATCTATTGGCATGGCTCTACACACAGGCACACACATGTACCATAAATCTATCG--AGGT-------
right  NNNNNNNNNNNNNNNNNNNNNNNNNNNNNNNNNNNNNNNNNNNNNNNNNNNNNNNNNNNNAGGT-------
       111111111111111111111111111111111111111111111111111111111111***22222222

left   CGGTGGCTCACGCCTGTAATCC-CAGCACTTTGGG-----AGGCCGAGGCGGGTGGA----
chrom  -----GCACATGCCAGTCTTCTTCAGCACTAGGGTTACATATTTAGAGAAGTTTGAAGATT
right  -----GCACATGCCAGTCTTCTTCAGCACTAGGGTTACATATTTAGAGAAGTTTGAAGATT
       22222**2**2***2**22**22*******22**222222*2222***22*22**2*2222
```

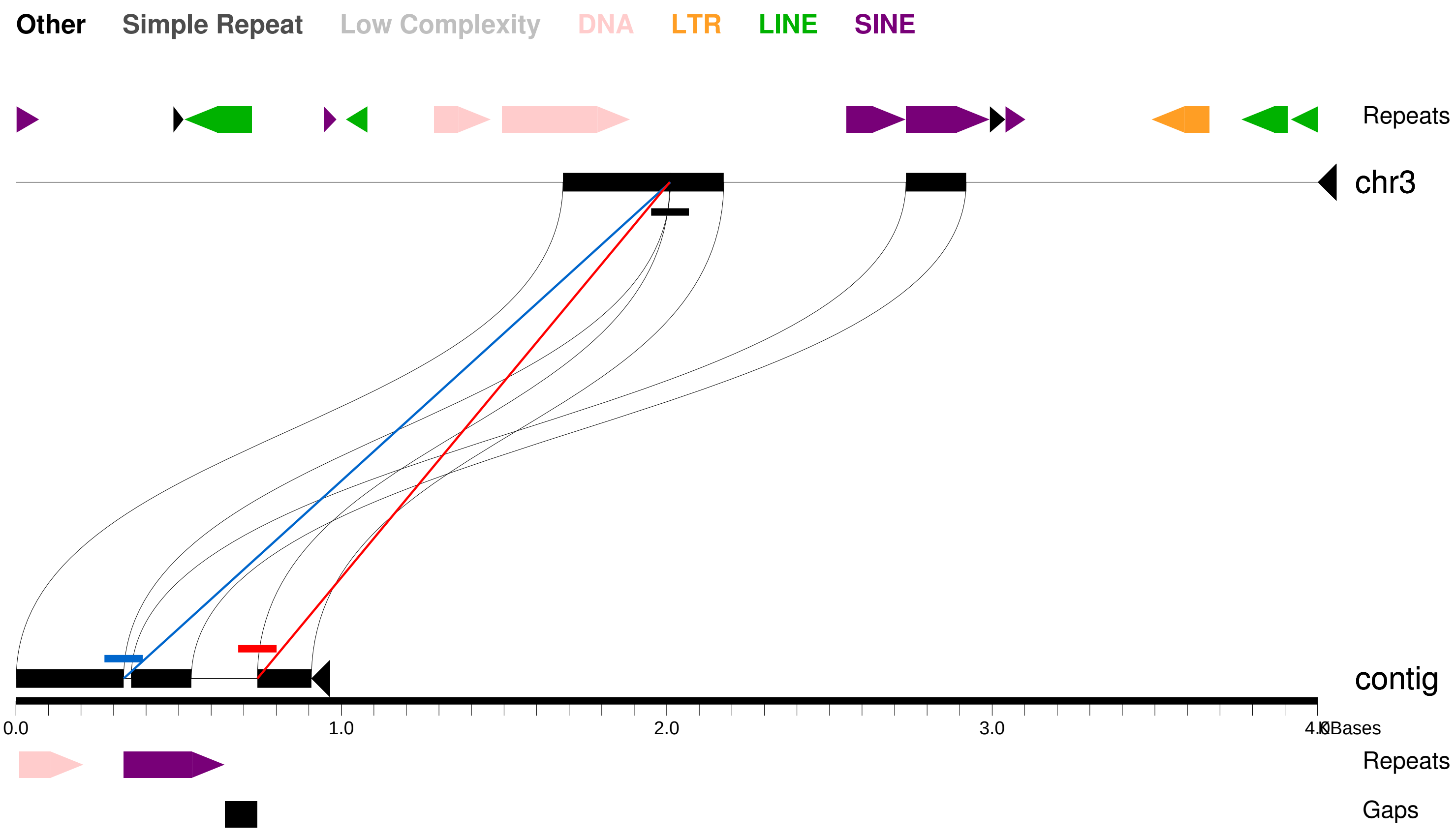

siteID: chr3_188883226 Scafftig4 + Breakpoint: chr3:188883212
chr3:188883052-188883386
Overlap Length: 15 INS size: 307 Genotyped: Yes

```
left   -----------GTGTTCCAGACACACAGGTTTTACTGCTGTTTCCTTACAGCAAGACTGATTCACCCTAC
chrom  CCTGCCGCCCAGTGTTCCAGACACACAGGTTTTACTGCTGTTTCCTTACAGCAAGACTGATTCACCCTAC
right  TTT-------AGT-----AGAGACGG-GGTTTCACCT-TGTT-------AGCCAGGATGGT--------C
       NN2       2**11111***1**111*****1**111****1111111***1**11**1*11111111*

left   AGGTTCTTGCCCCTGAGCTTTTGTACAGTCTATTCTGGGTTGGAGTTTTCTAAATGACTGTCACGATAGA
chrom  AGGTTCTTGCCCCTGAGCTTTTGTACAGTCTATTCTGGGTTGGAGTTTTCTAAATGACTGTCACGATAGA
right  TCGATCT----CCTGACCTCATGA----TCCACCC-GCCTCGGCCTC--CCAAAGTGCTGGGATTACAGG
       11*1***1111*****1**11**11111**1*11*1*11*1**11*111*1***111***11*11*1**1

left   TG-GAGAAACTGTAACACTCTGGTTCTCAAACTTTTTTTTTTTTTTT-----TTT--------TTTTTGA
chrom  TG-GAGAAACTGTAACACTCTGGTTCTCAAACTTTTATGTGCATAACAGCCATCTGGCAAGGCTTGTTGA
right  CGTGAGCCACCGCGCCCGGCCGGTTCTCAAACTTTTATGTGCATAACAGCCATCTGGCAAGGCTTGTTGA
       1*1***11**1*111*111*1***************2*2*222*22222222*2*22222222**2****

left   GACGGAG---TCTCGCTCTGTCGCCCAGG---------CCGG--ACT---GCGGACTGCAGTGGCGCAAT
chrom  CATGGAGATTTCTAGGCCTA-CCCCCAGAGATTCTAATCCAGTAAATCAAGGGGCCTGGGCATGTGCATT
right  CATGGAGATTTCTAGGCCTA-CCCCCAGAGATTCTAATCCAGTAAATCAAGGGGCCTGGGCATGTGCATT
       2*2****222***2*22**22*2*****2222222222**2*22*2*222*2**2***22222*2***2*

left   CTC-------GGCTCACTGCAAGCT---CCGCTTCCCGGG--TTCACGCCATTCT--
chrom  TTTAATATGAGGTTCTCAGGCAGGTGTCCAGGGACCTGCGCATCAAATGCTGTCTTC
right  TTTAATATGAGGTTCTCAGGCAGGTGTCCAGGGACCTGCGCA---------------
       2*22222222**2**2*2*22**2*222*2*222**2*2*221NN1NNN1NN111
```

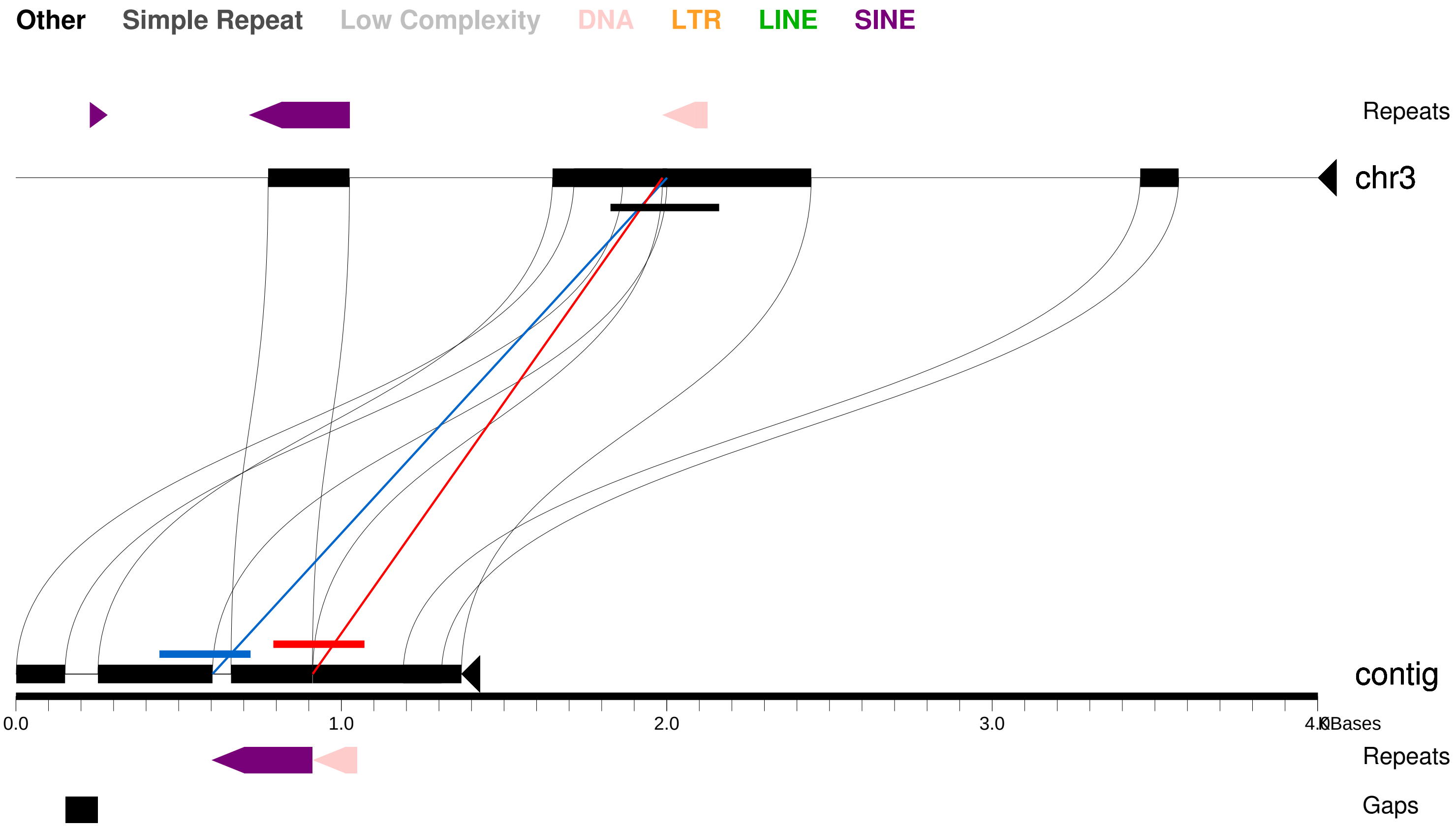

siteID: chr3_190625831 Scafftig1 + Breakpoint: chr3:190625850
chr3:190625804-190625909
Overlap Length: 14 INS size: 407 Genotyped: No

```
left    TCAATATTTATGGATCATTCATTATACTTTCATCACTGTCACTACT-------------AAAGAATAAAC
chrom   TCAATATTTATGGATCATTCATTATACTTTCATCACTGTCACTACT-------------AAAGAATAAAC
right   NNNNNNNNNNNNNNNNNNNNNNNNNNNNNNNNNNNNNNNNNNNNNNNNNNNNNNNNNNNAAAGAATAAAC
        11111111111111111111111111111111111111111111111111111111111***********

left    TAATCACGCCTGTAATCCCAGCACTTTGGGAGGCCGAGGCGGGTGGATCATGAGGTCAGGAG
chrom   TAA--AAG---GTAA----ATCATTTATTCAAGATCACACAA----AGTTTGAGGTTATTAG
right   TAA--AAG---GTAA----ATCATTTATTCAAGATCACACAA----AGTTTGAGGTTATTAG
        ***22*2*222****2222*2**2**2222*2*222*22*222222*222******2*22**
```

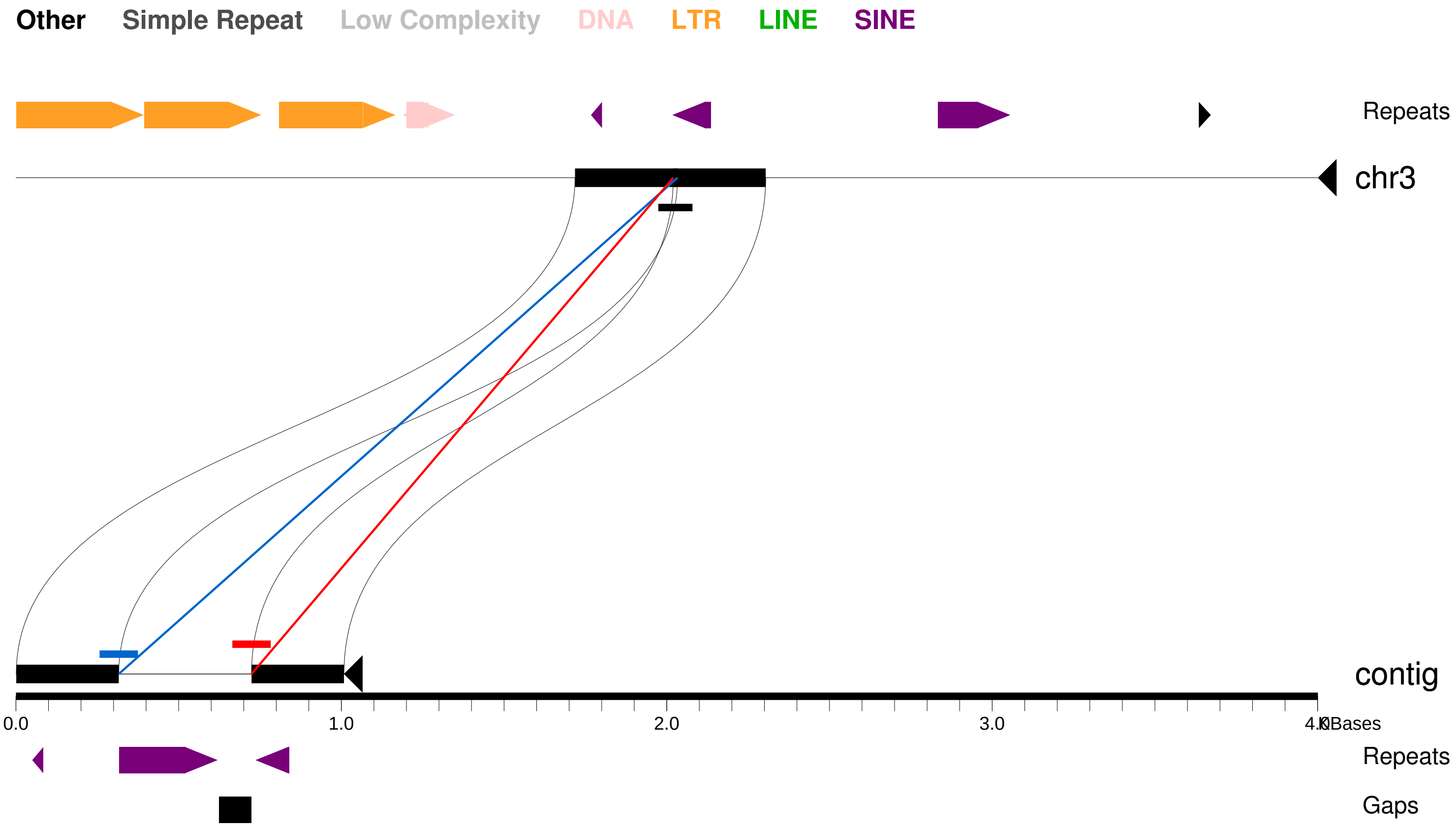

siteID: chr3_190999466 Scafftig6 + Breakpoint: chr3:190999451
chr3:190999374-190999510
Overlap Length: 14 INS size: 310 Genotyped: Yes

```
left   CTATGATTTCCCCCAATGCTTTACTTTCCTCAAATCTCTGCAAATACAAGTTTCTATCTTCTTGATGTAT
chrom  CTATGATTTCCCCCAATGCTTTACTTTCCTCAAATCTCTGCAAATACAAGTTTCTATCT---TGATGTAT
right  C--------CGCCC---GCCTCGGCCTCC-CAAAGTGCTGGGATTACAGG-------CG---TGAGCCAC
       *11111111*1***111**1*11111***1****111***11*1****1*1111111*1222***111*1

left   TTTTCATTGT-TGTACTCTATTTTCTTTT----CTTTTTTTTTTTTTTT-----------------TTTTT
chrom  TTTTCATTGT-TGTACTCTATTTTCATAAAGAACTTACTAATAGTCTTGTAAGATGAAAATCTCCTTTTC
right  CGCGCCCGGCCTGTACTCTATTTTCATAAAGAACTTACTAATAGTCTTGTAAGATGAAAATCTCCTTTTC
       1111*111*11*************2*222222***22*22*22*2**22222222222222222****2

left   T
chrom  C
right  C
       2
```

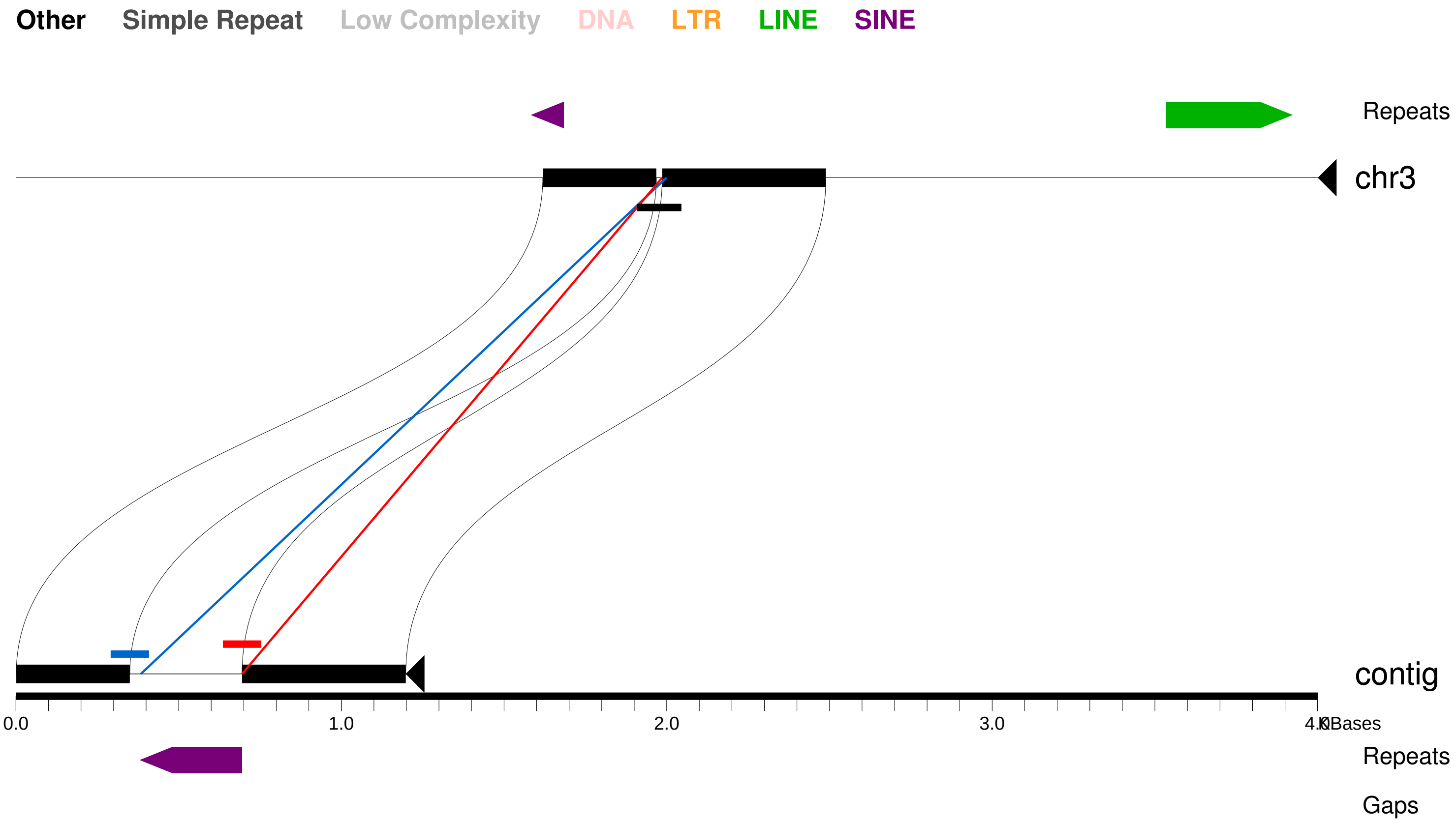

siteID: chr3_21577503 Scafftig1 - Breakpoint: chr3:21577519
chr3:21577476-21577578
Overlap Length: 17 INS size: 116 Genotyped: Yes

```
left    CCC------ACA-ATCATAAC---------AAGAAGATGGAGCTGGATCTATTATGTTTAATAAATATTC
chrom   CCC------ACA-ATCATAAC---------AAGAAGATGGAGCTGGATCTATTATGTTTAATAAATATTC
right   GCCTGGGCGACAGAGCGAGACTCCGTCTCAAAAAAAAAAAAAAAAAAAAAAAAAAAAAAATAAATATTC
        1**111111***1*1*111**111111111**1**1*111*11111*111*11*11111***********

left    CCCTATTGGCGTGAACCCGGGAGGCGGAGCTTGCAGTGAGCCGAGATCCCGCCACTGCACTCCAGC
chrom   CCCTATTAT--TTTACAAGGTTAG--------GCACTG---CCAAAGCCTTTGATTA---TCCAAA
right   CCCTATAT--TTTACAAGGTTAG--------GCACTG---CCAAAGCCTTTGATTA---TCCAAA
        ******2222*22**22**222*22222222***2**222*2*2*2**2222*2*2222****22
```

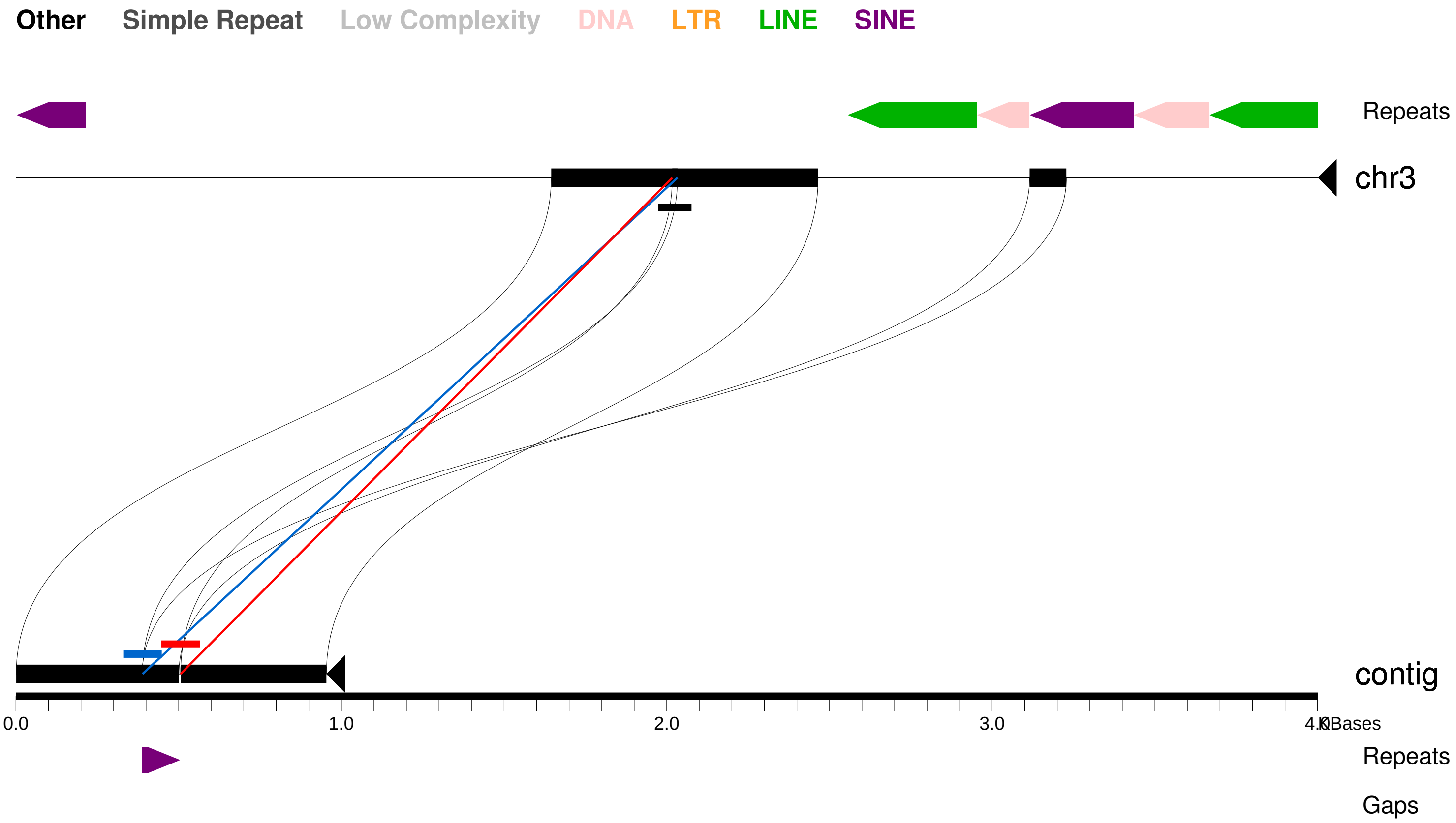

siteID: chr3_24653516 Scafftig1 - Breakpoint: chr3:24653529
chr3:24653508-24653588
Overlap Length: 37 INS size: 421 Genotyped: No

```
left    ACA------TAAGCCTAGAAAAG----------ACAA----------------------AGAAGCTTTTT
chrom   ACA------TAAGCCTAGAAAAG----------ACAA----------------------AGAAGCTTTTT
right   CCACCCGCCTCGGCCTCCCAAAGTGCTGGGATTACAGGCGTGAGCCACCGCGCCCGGCCAGAAGCTTTTT
        1**111111*11****111****1111111111***11111111111111111111111***********

left    AAATTAAAAAAAAGAAACACCAAATCNNNNNNNNNNNNNNNNNNNNNNNNNNNNNNNNNNNNNNNNNNNN
chrom   AAATTAAAAAAAAGAAACACCAAATCAGAAAAAATAACTTCACAAAAAA---------------------
right   AAATTAAAAAAAAGAAACACCAAATCAGAAAAAATAACTTCACAAAAAA---------------------
        *************************22222222222222222222222222222222222222222222

left    NNNNNNNNNNNNNNNNN
chrom   -----------------
right   -----------------
        22222222222222222
```

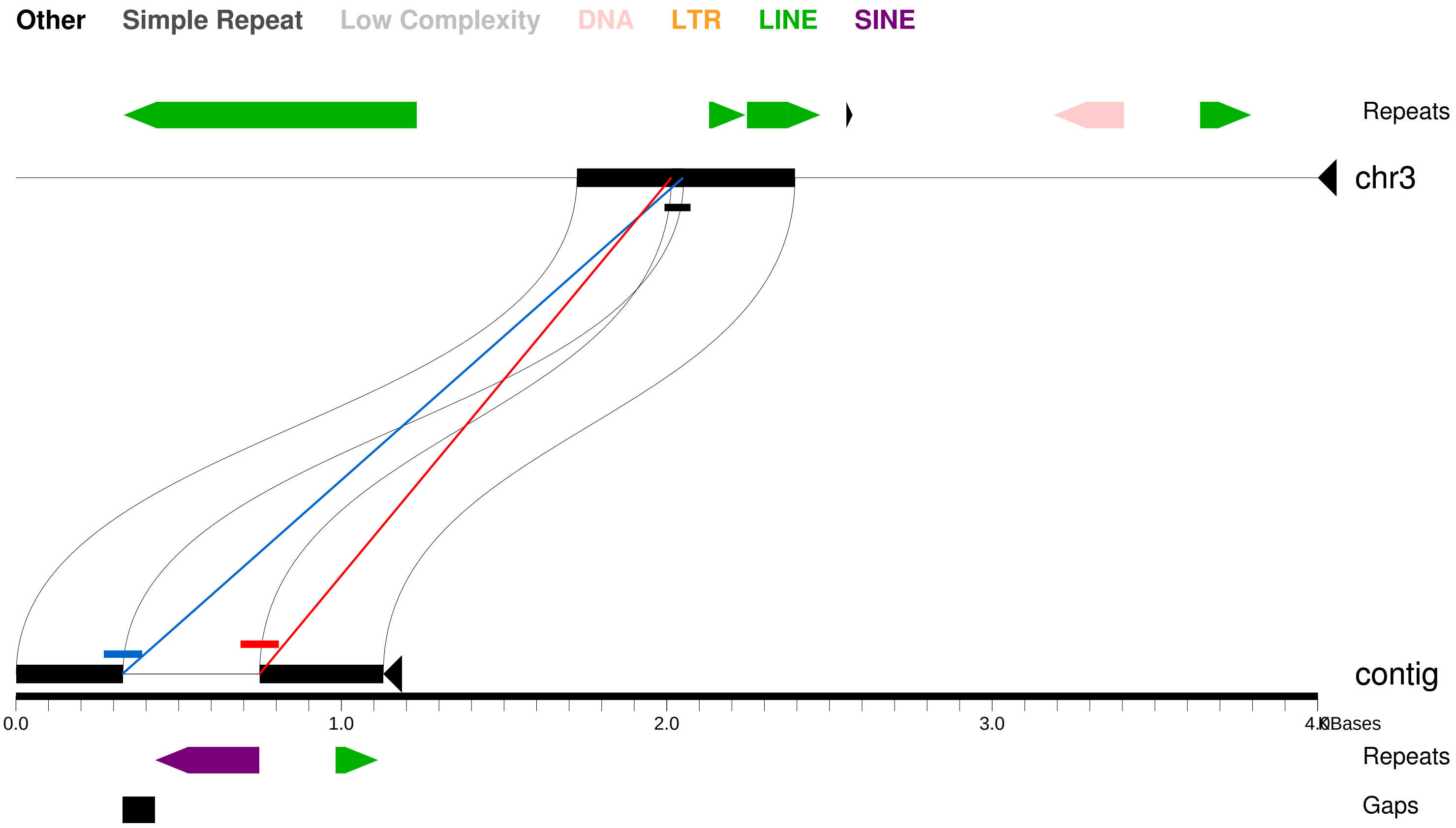

siteID: chr3_25278469 Scafftig8 + Breakpoint: chr3:25278448
chr3:25278400-25278507
Overlap Length: 12 INS size: 312 Genotyped: Yes

```
left    CCCCGGAAAT-----TCAGAGAATATCATTATTC------TGATAATTCTGTTCTCTTATCCTGTTTCTT
chrom   CCCCGGAAAT-----TCAGAGAATATCATTATTC------TGATAATTCTGTTCTCTTATCCTGTTTCTT
right   CCGCCCGACTCGGCCTCCCAAAGTGCTGGGATTACAGGCGTGAGCCACCGCGCCCGGCCTCCTGTTTCTT
        **1*111*1*11111**11*1*1*111111***1111111***11111*1111*11111***********

left    TTTTTTTTTTTTTTTTTTTTTTTTTTTTTTTTGAGA-CGGAGTCTCGCTCTGTCGCCCAGG
chrom   TACCTAACTCTAAACACAAGTTGTTGGTCTTCCAGAACTGAGTCA------GTCA------
right   TACCTAACTCTAAACACAAGTTGTTGGTCTTCCAGAACTGAGTCA------GTCA------
        *222*222*2*222222222**2**22*2**22***2*2*****2222222***2222222
```

Other **Simple Repeat** **Low Complexity** **DNA** **LTR** **LINE** **SINE**

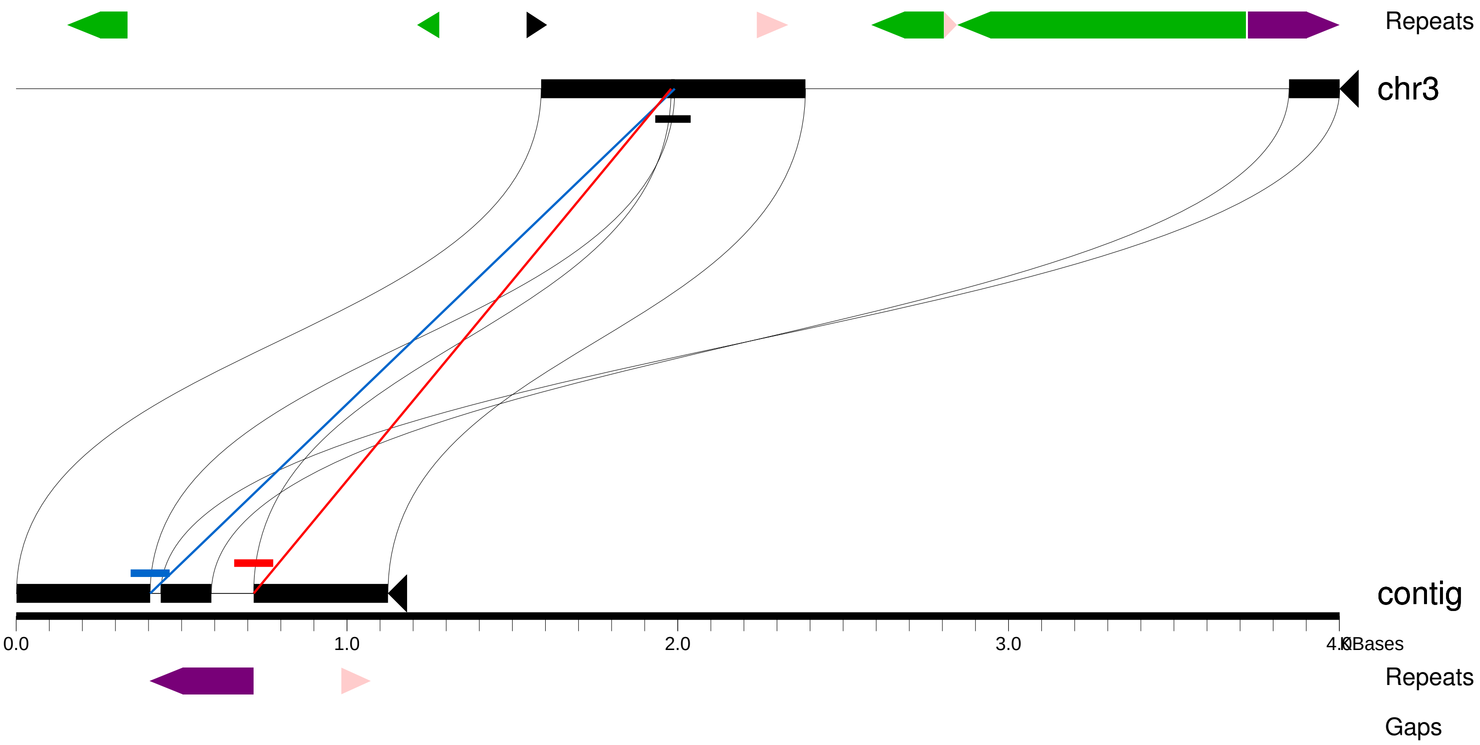

siteID: chr3_27899258 Scafftig1 + Breakpoint: chr3:27899267
chr3:27899107-27899429
Overlap Length: 3 INS size: 308 Genotyped: Yes

```
left   ---CTACAACCATTTGGTCCTTGACAAAGCTCACAAAAACAAGCAATGAGGAAAGGATTCCTTATTTAAT
chrom  GACCTACAACCATTTGATCCTTGACAAAGCTCACAAAAACAAGCAATGAGGAAAGGATTCCTTATTTAAT
right  AGG--AGAATGGCGTGAACCC---CAGGGGGCGGAGGCT---GCAGTGAGCCGAG-ATTGC---------
       NNN11*1**11111**21**1111**11*11*11*1111111***1****111**1***1*111111111

left   AAATGATGCTGGGATAACTGGCTAGCTATATGAAGAAAATTGAAACTGGACCCCTTCCTTTTACCTTATA
chrom  AAATGATGCTGGGATAACTGGCTAGCCATATGAAGAAAATTGAAACTGGACCCCTTCCTTTTACCTTATA
right  ----GCCACTGC----ACT--CCAGCC---TGGGCGACAGCGAGACT---------CCGTCTCAAAAAAA
       1111*111***11111***11*1***2111**1111*1*11**1***111111111**1*1*11111*1*

left   CAAAAATTAACTCAAGATGGATT---GGCCGGGCGCGGTGGCTCACGCCTGTAAT---CCCAGCA-----
chrom  CAAAAATTAACTCAAGATGGATTAAAGACTTAAATGTAAAACCCAAAACTATAAAAACCCTAGAAGAAAA
right  AAAAAAAAAAAAAAAAAAAAATTAAAGACTTAAATGTAAAACCCAAAACTATAAAAACCCTAGAAGAAAA
       1*****11**111**1*111***222*2*222222222222*2**222**2***2222**2**2*22222

left   -CTTTGGGAGGCC------GAGGTGGGC--GGATCACGAGGTCAGGAGATCGAGACCATCC----TGGCT
chrom  TCTAGGCAATACCATTCAGGACATAGGCATGGGCAAAAATTTCATGACAAAAATGCCAAAAGCAATTGCA
right  TCTAGGCAATACCATTCAGGACATAGGCATGGGCAAAAATTTCATGACAAAAATGCCAAAAGCAATTGCA
       2**22*22*22**222222**22*2***22**222*22*22***2**2*222*22***2222222*2**2

left   AACACGG-------TGAAACCCCGTCTCTA------CTAAA----
chrom  ACAAAAGCAAAAATTGATAAATGGGATTTAATTAAACTAAAGA--
right  ACAAAAGCAAAAATTGATAAATGGGATTTAA----ACTAAAGAGC
       *22*22*2222222***2*2222*22*2**2    2*****2211
```

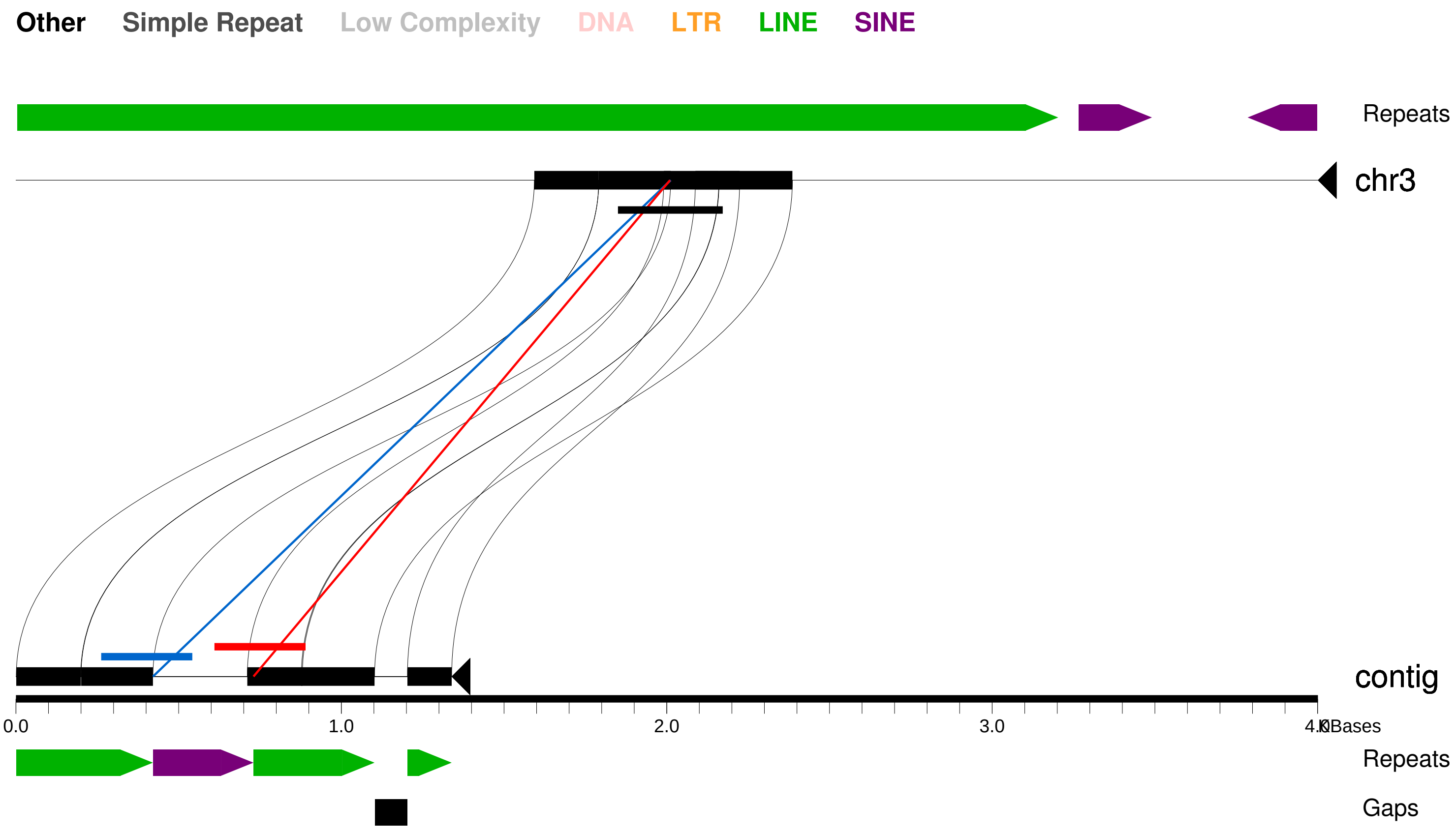

siteID: chr3_28707036 Scafftig5 - Breakpoint: chr3:28707045
chr3:28706885-28707218
Overlap Length: 14 INS size: 258 Genotyped: Yes

```
left    ------------TAAAAACTGTGAAAGATTTTACAGCTGGATATTACTCTTCCCAAGCCTTTCTGGTTAG
chrom   CATTCTTCACTTTAAAAACTGTGAAAGATTTTACAGCTGGATATTACTCTTCCCAAGCCTTTCTGGTTAG
right   AGAA--TGGCGTGAACCCGGGAGGCGGAGCTTGCAGTGAGCCGAGATCCCGCCACTGCACTCCAGCCTGG
        NNNN  2NN2N21**11111*1*111**11**1***111*11111*11*11**111**11*1*1*11*1*

left    GATTTGTCTTTCAAAGTGATCAAAGAATGTTGGAGATACAAATAATCTGAGAAGATTCATCTAGTCTGGA
chrom   GATTTGTCTTTCAAAGTGATCAAAGAATGTTGGAGATACAAATAATCTGAGAAGATTCATCTAGTCTGGA
right   GCGA-------CAGAGCGAG---ACTCCGTCTCAAAAAAAAAAAAAAAAAAAAAA--------------A
        *1111111111**1**1**1111*1111**111*1*1*1***1**1111*1**1*11111111111111*

left    ATGTCTTCCATTCCACAGATAAAAGCCCCAGAGACGGATCACGAGGTC---AGGAGATCG---AGACCAT
chrom   ATGTCTTCCATTCCACAGATAAAAGCCCCAGAGAT--ATCACGTGGCTTATACAAGCTTCCACAGAATAT
right   A-------------AAAAAAAAAACCCCCAGAGAT--ATCACGTGGCTTATACAAGCTTCCACAGAATAT
        *1111111111111*1*1*1****1********222******2**22222*22**2*22222***22**

left    CCCGGCTAAA--ACGGTGAA-----------ACCCCG---TCTCTACT--AAAAATACAAAAAAAAATTA
chrom   TAGAGATAGACTAGAGTGCCCTTTATTTCCTACACAGGTTTGTGTATTGGAAGAGTACCTAAACATACTG
right   TAGAGATAGACTAGAGTGCCCTTTATTTCCTACACAGGTTTGTGTATTGGAAGAGTACCTAAACATACTG
        2222*2**2*22*22***2222222222222**2*2*222*2*2**2*22**2*2***22***2*2*2*2

left    GC-------CGGGCGTAGTGGCGGG----CGCCTGTAGTCCCAGCTAC--------
chrom   TCACCAAAACAGGATCATTTCCCTCATCCCTCCTCAAGGACCTTATTCTGTCAGTT
right   TCACCAAAACAGGATCATTTCCCTCATCCCTCCTCAAGGACC--------------
        2*2222222*2**222*2*22*2222222*2***22**22**NNN1N1
```

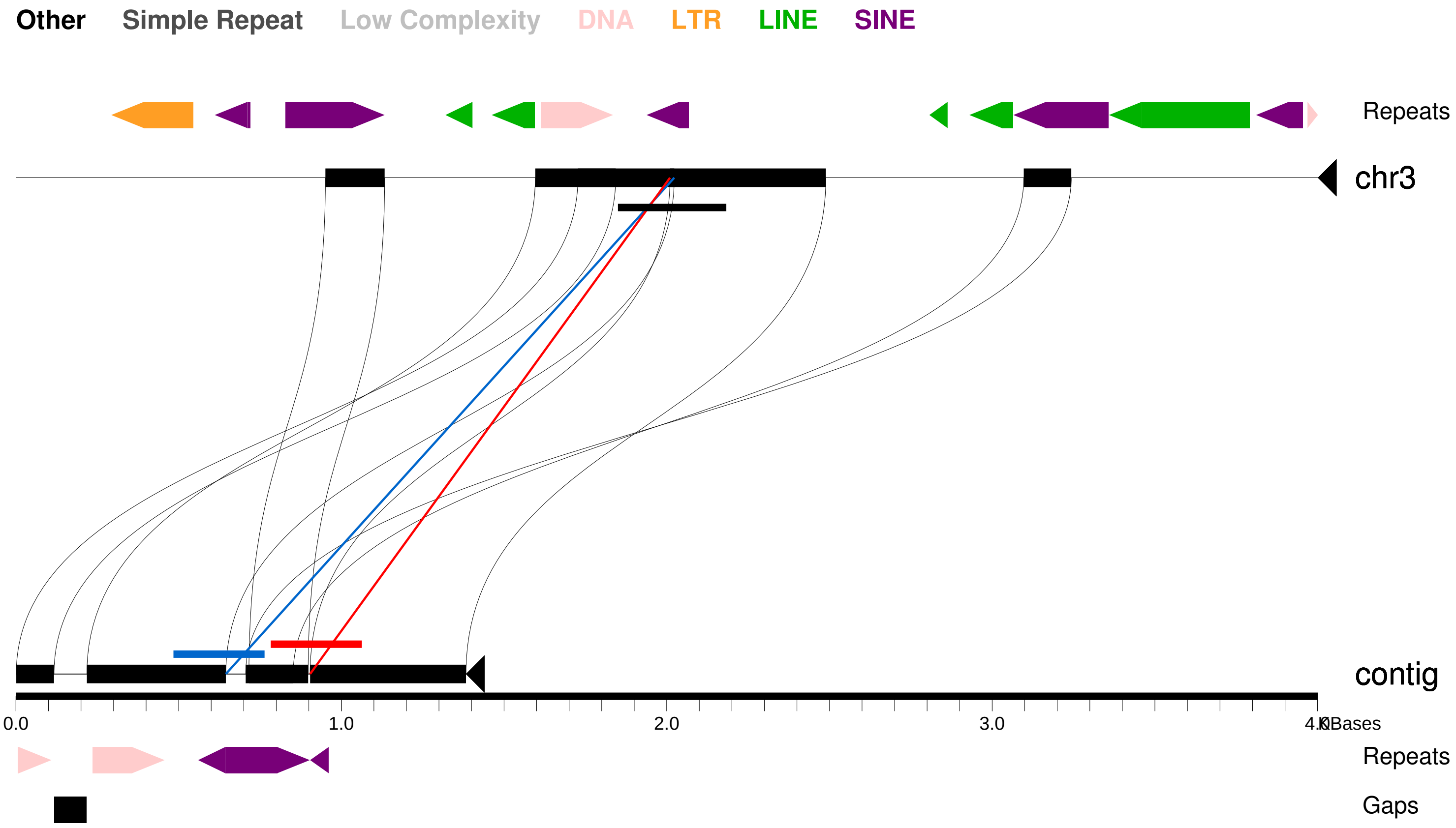

siteID: chr3_28840189 Scafftig10 - Breakpoint: chr3:28840164
chr3:28840120-28840223
Overlap Length: 16 INS size: 323 Genotyped: Yes

```
left    ---------------AATAGTAGTGTGAGTAGGTATTGATGAGGAAAATTCATCTTTATAAAAAACAGAA
chrom   ---------------AATAGTAGTGTGAGTAGGTATTGATGAGGAAAATTCATCTTTATAAAAAACAGAA
right   CTCCGTCTCAAAAAAAAAAAAAACAAAAAAAAAAAAAAAAAAAAAAAAAACAAAAAAAAAAAAAACAGAA
        111111111111111**1*11*11111*11*111*111*11*11****11**11111*1***********

left    CTTTGGCCGGGCGCGGTGGCTCACGCTTGTAATCCCAGCACTTTGGGAGGCCGAGGCGGGTGGA-
chrom   CTTTGCAT---CACTTATCCTAGAAGTTAAAATCTCAAAA-------AAACCAA------TAAAT
right   CTTTGCAT---CACTTATCCTAGAAGTTAAAATCTCAAAA-------AAACCAA------TAAAT
        *****222222*2*22222**22222**22****2**22*2222222*22**2*222222*22*2
```

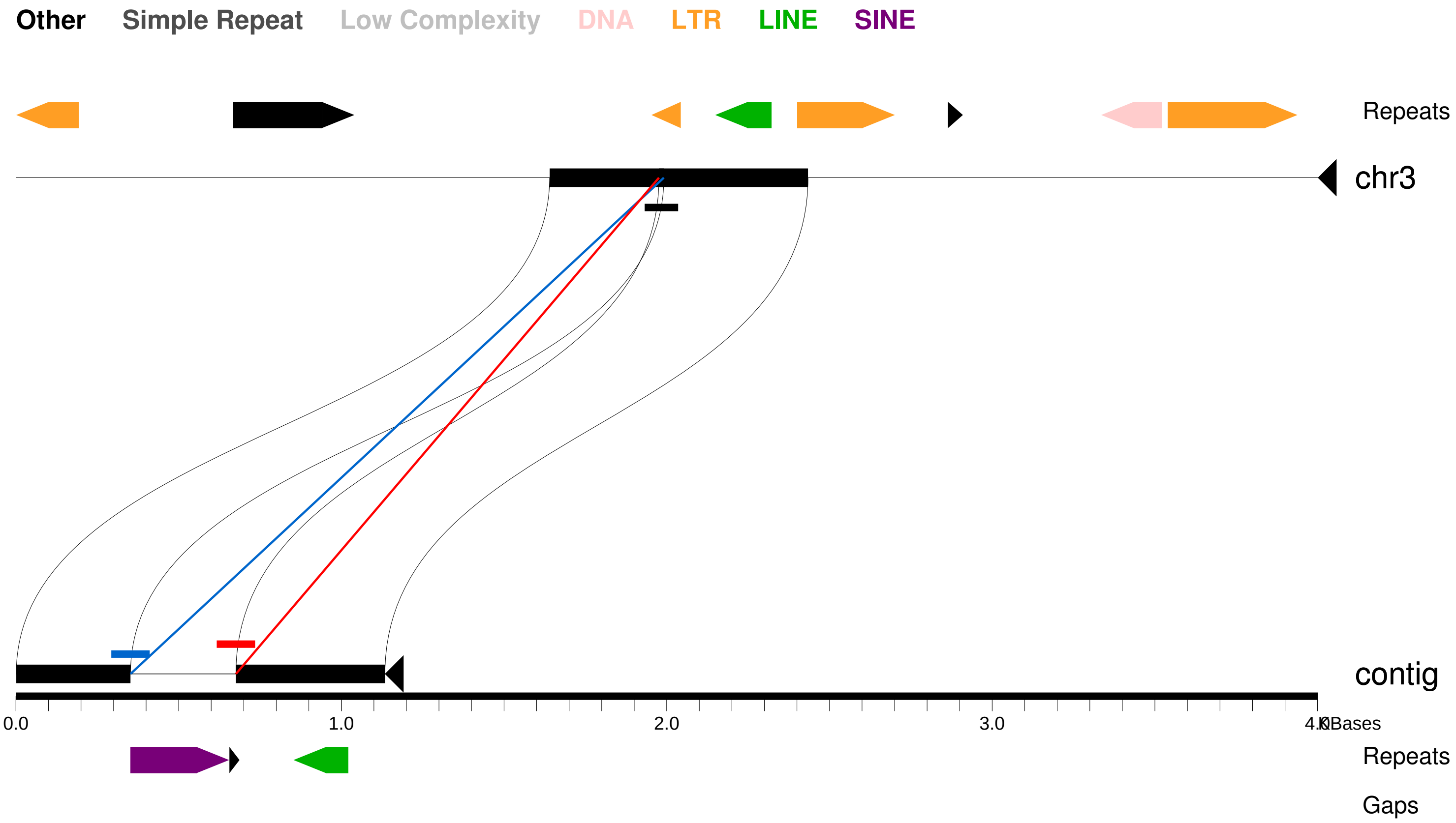

siteID: chr3_29239870 Scafftig4 + Breakpoint: chr3:29239885
chr3:29239725-29240059
Overlap Length: 15 INS size: 309 Genotyped: Yes

```
left    GT---------------TCTACCCAGTTTCGTATGTTTGATTTTTAAAAAAATCTCCTGCCGTGATAATT
chrom   GTGGTATTTTATGCTGTTCTACCCAGTTTCGTATGTTTGATTTTTAAAAAAATCTCCTGCCGTGATAATT
right   AGA-------ATGGAGTGAACCCGGGAGGCGGAGCTTGCAGTGAGCCGAGA---TCCCGCC---------
        11N       222NN221111**11*111**1*11**11*1*111111*1*111***1***111111111

left    ATTAAAAATTAACATTGAAGTTGATTAAATGCAATAAATGTCACATTAAATAGTAAATTTTAAAATTATC
chrom   ATTAAAAATTAACATTGAAGTTGATTAAATGCAATAAATGTCACATTAAATAGTAAATTTTAAAATTATC
right   -------------ACTGCACTCCAGCCTGGGCGACAGA-GCGAGACTCCGTCTCAAA----AAAAAAAAA
        1111111111111*1**1*1*11*111111**1*1*1*1*11*1*1*111*111***1111****11*11

left    AATAAAATATTAACTTATAGAAATAAGCTAGATTCGGC-----------CGGGCGCGGTGGCTCACGCCT
chrom   AATAAAATATTAACTTATAGAAATAAGCTAGATTCAAATATTTTAGTTGCTAGCATGGTGTCTTATACAG
right   AAAAAAAAAAAAA-----AAAAATAAGCTAGATTCAAATATTTTAGTTGCTAGCATGGTGTCTTATACAG
        **1****1*11**11111*1**************22222222222222*22**22****2**2*22*22

left    GTAA--TCCCAGCACTTTGGGAGGCCGAGGCGGGCGGATCACGAGGT---------CAAGAGA------T
chrom   TTTGGTTATGATCACTGGGGCAAGATTAAACTCTCTTTATAATAGGGAATCTGACACATGAGAAGAATTT
right   TTTGGTTATGATCACTGGGGCAAGATTAAACTCTCTTTATAATAGGGAATCTGACACATGAGAAGAATTT
        2*2222*222*2****22**2*2*222*22*222*22222*22***2222222222**2****222222*

left    CGAGACCATCCCGGCTAAAACGGTGAAACCCCGTCTCTACTAAA--------------
chrom   CAAGATTTTTTTTTTCCAAACATGGCA--CCATTCT-TACAAAAGGCACATGAGATGG
right   CAAGATTTTTTTTTTCCAAACATGGCA--CCATTCT-TACAAAAGG------------
        *2***222*22222222****222*2*22**22***2***2***22
```

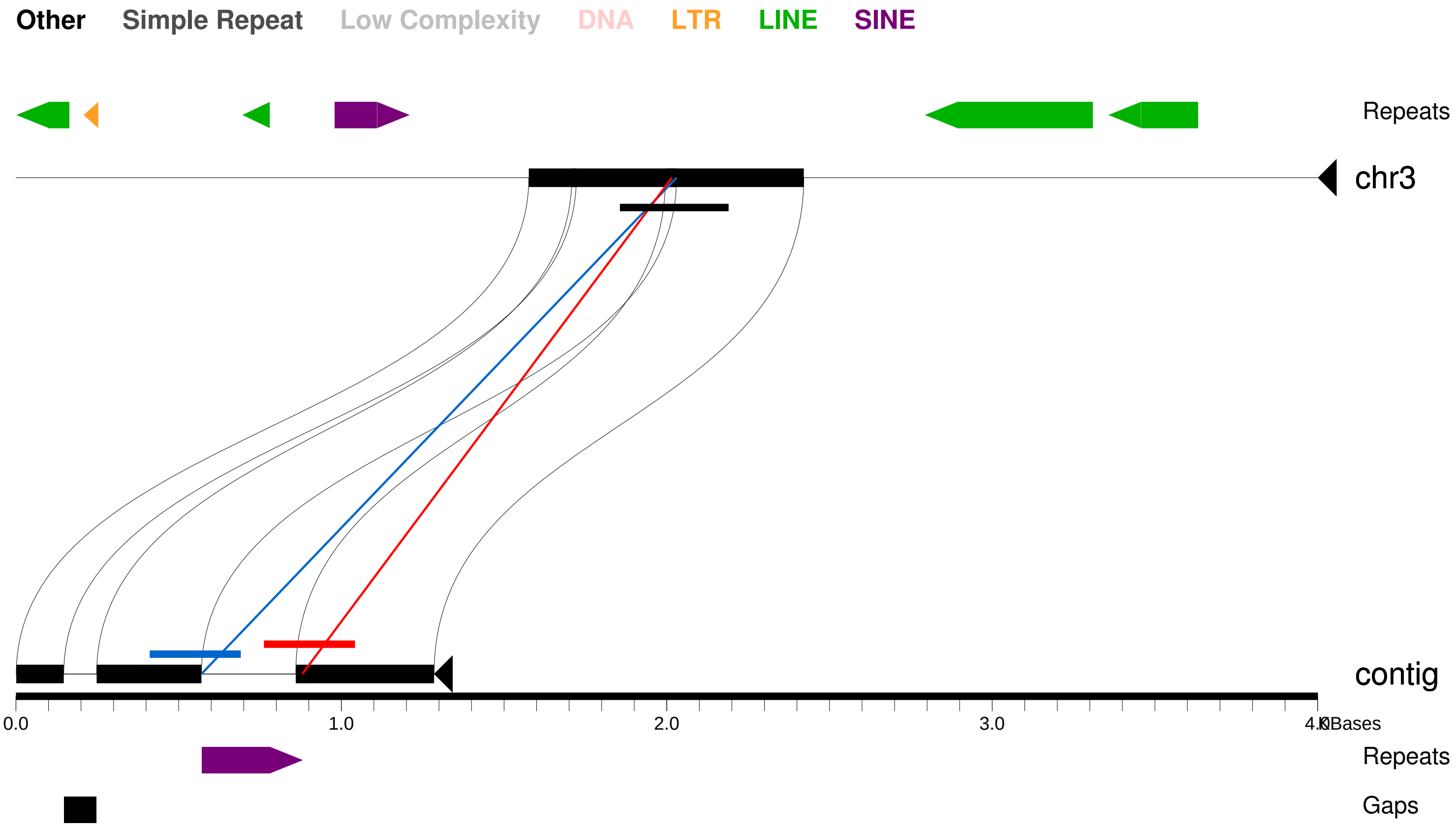

siteID: chr3_2961985 Scafftig1 + Breakpoint: chr3:2961992
chr3:2961832-2962164
Overlap Length: 13 INS size: 310 Genotyped: Yes

```
left    ---------------CCAAAAGGACACATACTGTA-TGATTACACACCACACACACACAGACTCTCTCTC
chrom   A-AGGAACAAAAACTCCAAAAGGTCACATACTGTA-TGATTACACACCACACACACACAGACTCTCTCTC
right   AGAATGGCGGGAAC-CCGGGAGG-CGGAGCTTGCAGTGAGCCGAGATCGCGC-CAC-CGCACTC-----C
        212NNNN2NNN222 **111***N*11*111**1*1***1111*1*1*1*1*1***1*11****11111*

left    ACACACATATGTATCTAAAACATTCTCAGAATGACTAAACTATAGTAATGGGAAGAGGTCAGTGGTTTCC
chrom   ACACACATATGTATCTAAAACATTCTCAGAATGACTAAACTATAGTAATGGGAAGAGGTCAGTGGTTTCC
right   AGCC---TGGGCGACAGAGCCAGACTCCGTCTCAAAAAAAAAAAAAAAAAAAAAAAA-------------
        *11*111*11*111*11*11**11***1*11*1*11***11*1*11**1111**1*1111111111111

left    AGGGGTTACAGAGGGGAGGTAGAAAGAGGCTGGATTGGCCGGGCGCGGTGGCTCACG---------CCTG
chrom   AGGAGTTACAGAGGGGAGGTAGAAGGAGGCTGGATATGACTGTGAAGGGGAAGCAAGAGGAAGATCCTTG
right   ----------------AAAAAAAAAGAGGCTGGATATGACTGTGAAGGGGAAGCAAGAGGAAGATCCTTG
        111N11111111111*111*1**N**********22*2*2*2222**2*222**2*22222222*2**

left    TAA------------TCCCAG---CACTTTG---GGAG-----GCCGAG---ACGGGCGGATCACGAGGT
chrom   TGACAATAGAATAGTTCCCTATCTCAGTTTCCATGGTGCTTTTGCCCAGTCTACATGTGTGATACATACA
right   TGACAATAGAATAGTTCCCTATCTCAGTTTCCATGGTGCTTTTGCCCAGTCTACATGTGTGATATATACA
        *2*222222222222****22222**2***2222**2*22222***2**222**22*2*2222*122222

left    CAGGAGATCGAG-ACCATCCTGGCTAACACGGTGAAACCCCGTCTCTAC-TAA-A
chrom   CATTATACCAAGTATCAGTTTCTTTGTTTTTGTATTTAACTGTATCTACATAAGA
right   CATTATACCAAGTATCAGTTTCTTTGTTTTTGTATTTAACTGT------------
        **22*2*2*2**2*2**222*222*222222**222222*2**N11111 111 1
```

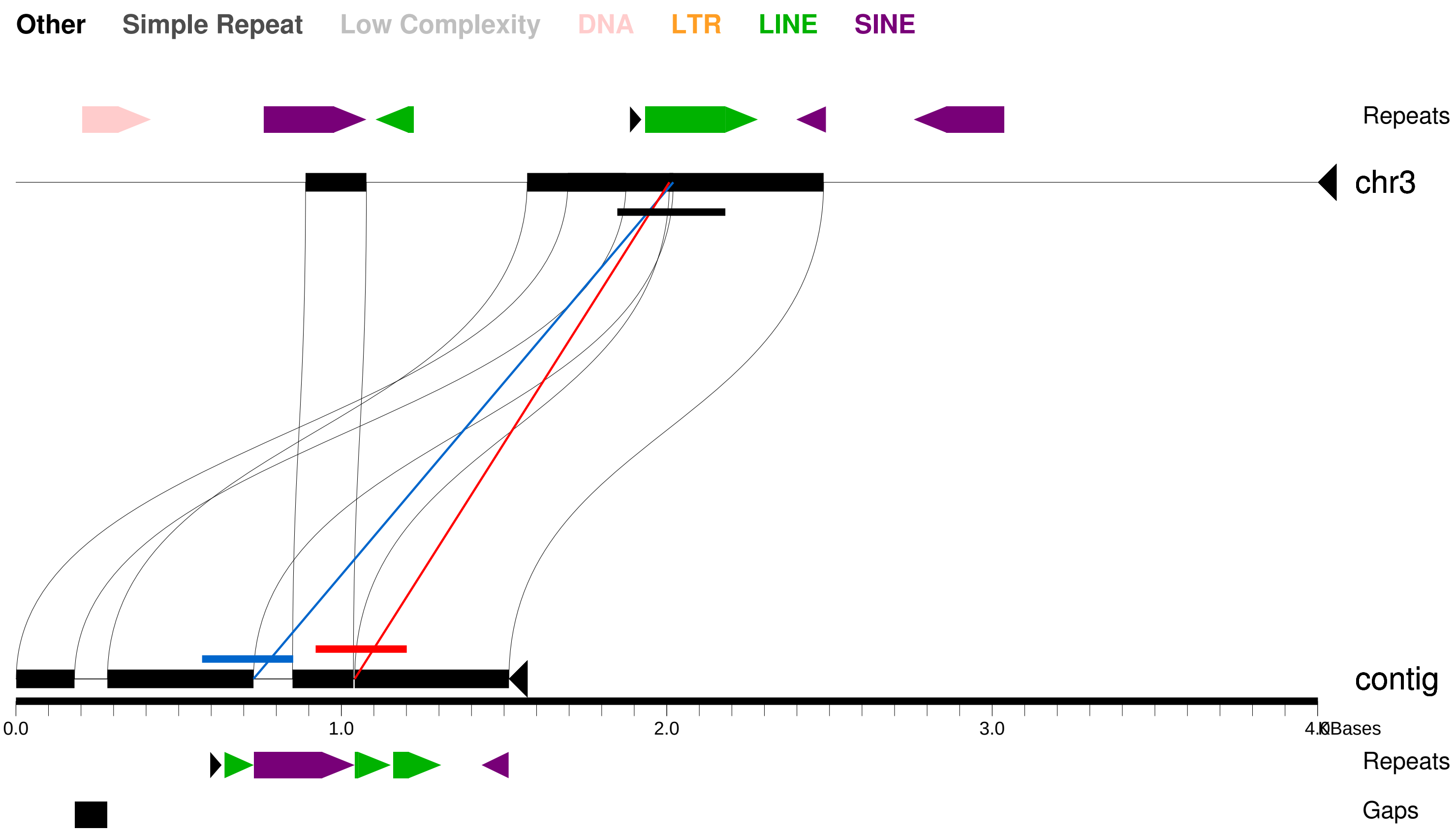

siteID: chr3_31210597 Scafftig4 - Breakpoint: chr3:31210585
chr3:31210539-31210632
Overlap Length: 13 INS size: 299 Genotyped: Yes

```
left   A-----------------------AGACTGGGGAAAACAG-GAC-CTTTCTATGGGGGTAAATAAAATCT
chrom  A-----------------------AGACTGGGGAAAACAG-GAC-CTTTCTATGGGGGTAAATAAAATCT
right  GCCGAGATCCCGCCACTGCACTCCAGCCTGGGCGACAGAGCGAGACTCCGTCTCAAAAAAAAAAAAAAAA
       111111111111111111111111**1*****11*1*1**1**11**111*1*111111***1****111

left   TAAGAGTAGGATGTGGGCCGGGCGCGGTGGCTCACGCCTGTAATCCCAGCACTTTGGGAGGCCGAGGCGG
chrom  TAAGAGTAGGATGT---------------GATCTCTCTATTCATAGGTACAATGTCGGA-----ATGTG-
right  AAAGAGTAGGATGT---------------GATCTCTCTATTCATAGGTACAATGTCGGA-----ATGTG-
       1*************222222222222222*2**2*2*222*2**22222**2*2*2***22222*2*2*2

left   GCGG
chrom  ----
right  ----
       2222
```

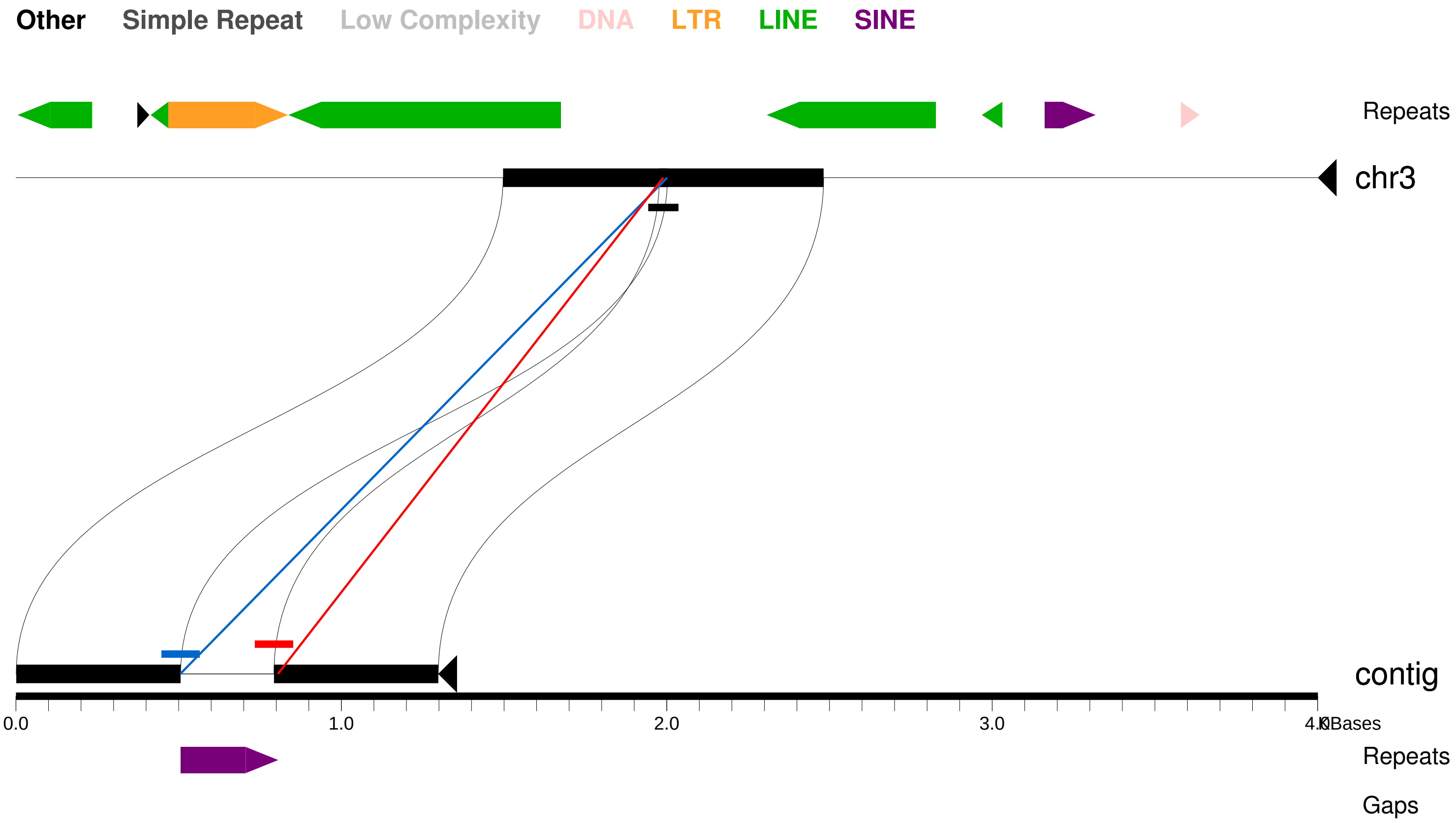

siteID: chr3_31400682 Scafftig2 + Breakpoint: chr3:31400657
chr3:31400628-31400716
Overlap Length: 15 INS size: 317 Genotyped: Yes

```
left    GCCCAAATGTAGACCT-------TG-TGGCAT-ATATT---------------------TGTAATTAACT
chrom   GCCCAAATGTAGACCT-------TG-TGGCAT-ATATT---------------------TGTAATTAACT
right   CCGCCCGCCTCGGCCTCCCAAAGTGCTGGGATTACAGGCGTGAGCCACCGCGCCCGGCCTGTAATTAACT
        1*1*11111*1*1***1111111**1***1**1*1*11111111111111111111111***********

left    TTTTTTTTTTTTTTTTTTTTTTTTTTTTTTTTTTTTTTTGAGACGGAGTCTCGCTCTGTCGCCCAGGCTG
chrom   TTTTACTTCATCTTGTTATTGCAAAAGTTGTTAG-----GAAAAAAAATGT------------------G
right   TTTTACTTCATCTTGTTATTGCAAAAGTTGTTAG-----GAAAAAAAATGT------------------G
        ****22**22*2**2**2**2222222**2**2222222**2*222*2*2*22222222222222222*

left    GAGTGCAGT
chrom   GA-------
right   GA-------
        **2222222
```

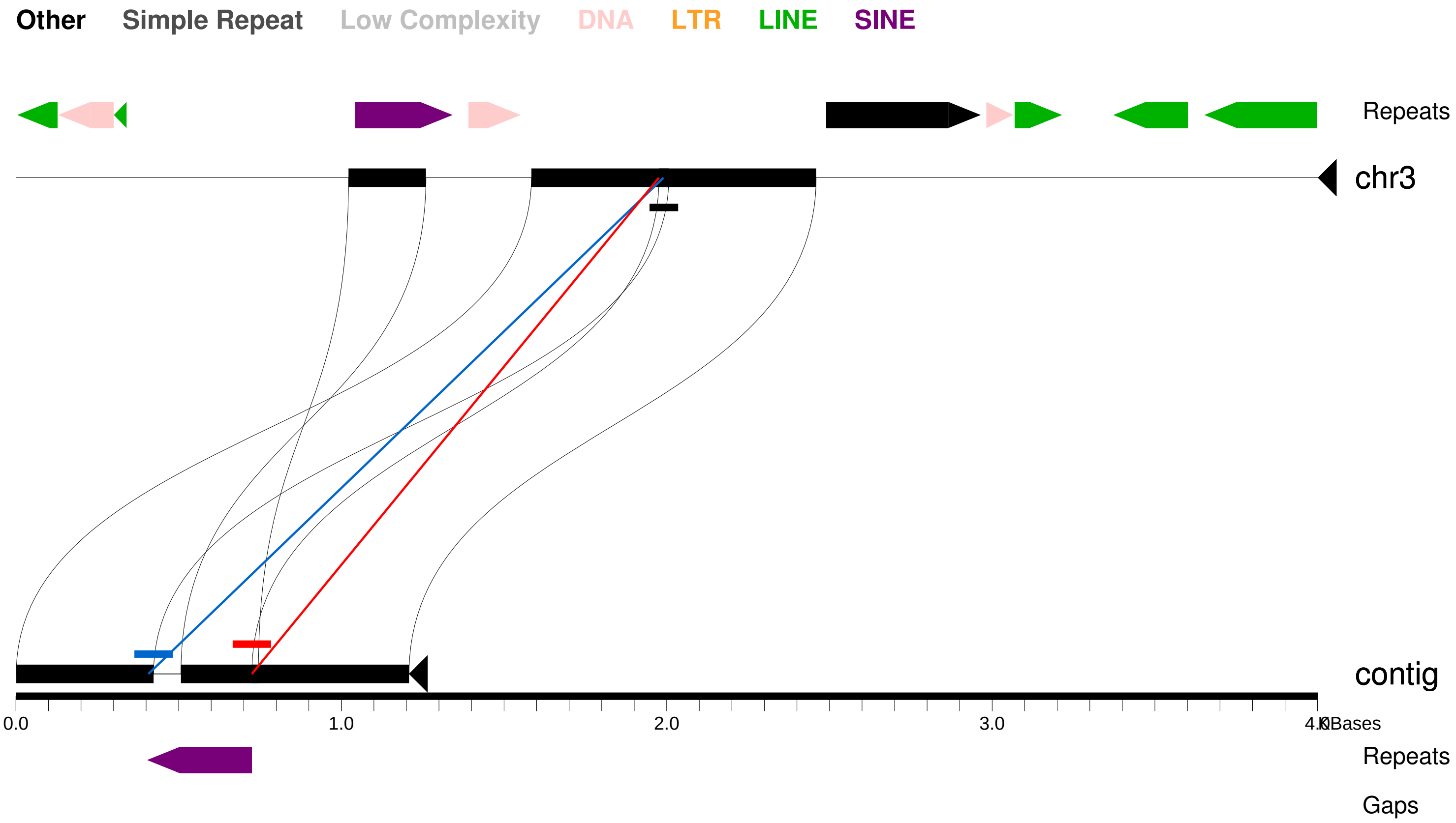

siteID: chr3_31881405 Scafftig29 - Breakpoint: chr3:31881383
chr3:31881339-31881437
Overlap Length: 16 INS size: 314 Genotyped: Yes

```
left   -----TACCACCTA------------AGGCTCATGC---AGACACAGAGAATGACAAGCAAATTAGAAGA
chrom  -----TACCACCTA------------AGGCTCATGC---AGACACAGAGAATGACAAGCAAATTAGAAGA
right  CGCAGTCCGACCTGGGCGACAGAGCGAGACTCCGTCTCAAAAAAAAAAAAAAAAAAAAAAAAAAGAAGA
       11111*1*1****1111111111111**1***111*111*1*1*1*1*1**11*1**11***11******

left   ATGGGTTTCAGGCCGGGCGCGGTGGCTCACGCCTGTAATCCCAGCACTTTGGGAGGCCGAGGCGGGTGG
chrom  ATGGGTTTCAATA----------AGCTTTCTCCT------CAGGGTCATAGTCAGGT--ATTAAATT--
right  ATGGGTTTCAATA----------AGCTTTCTCCT------CAGGGTCATAGTCAGGT--ATTAAATT--
       *********22222222222222***22*2***222222*22*22*2*2*22***222*222222*22
```

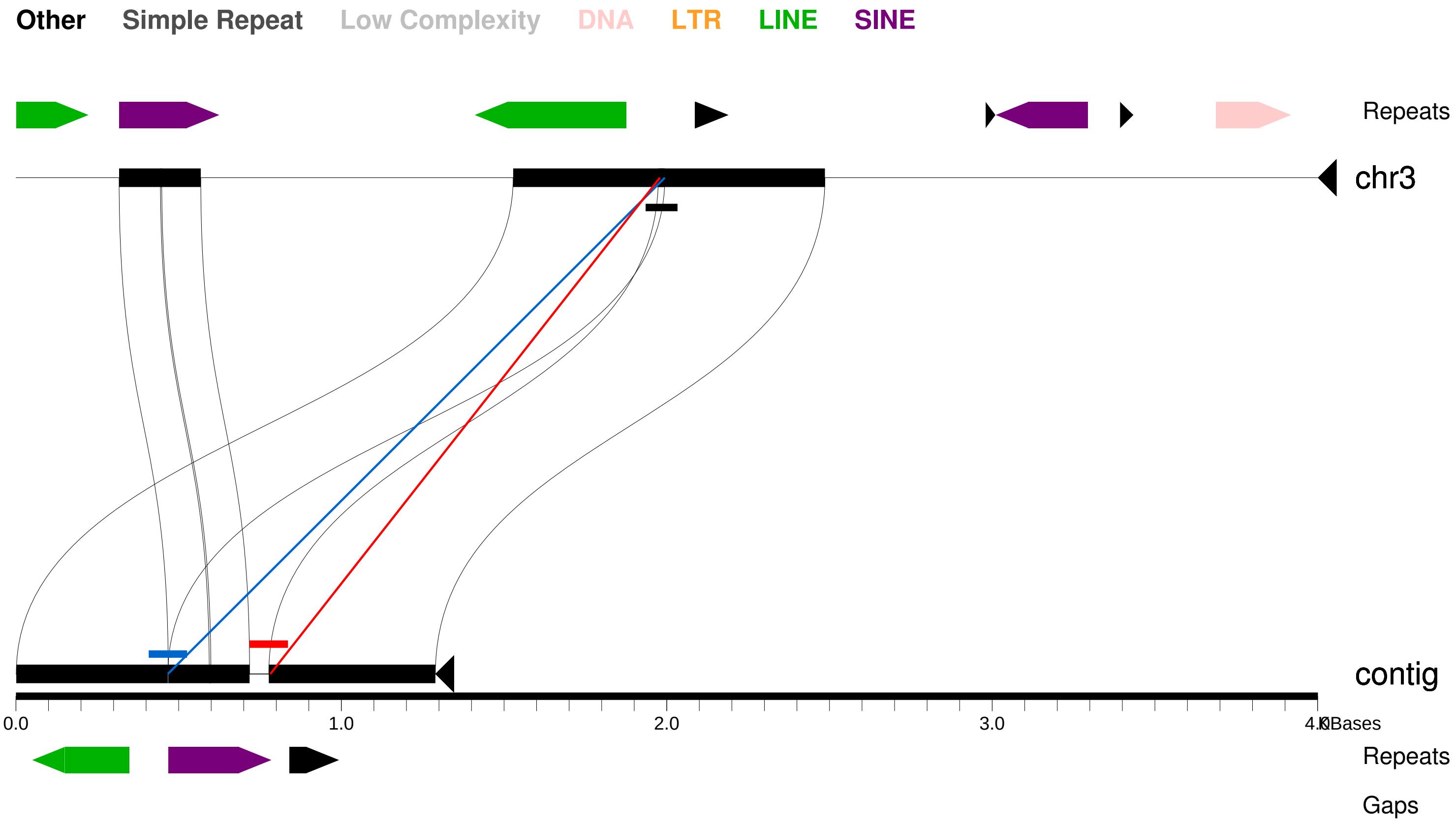

siteID: chr3_31935164 Scafftig1 - Breakpoint: chr3:31935193
chr3:31935144-31935245
Overlap Length: 11 INS size: 325 Genotyped: Yes

```
left    AAAGA--AAATGTGGCATA-TCCATCCCATGGCATACTA-----------TTCAGCAATAAAA---GTAC
chrom   AAAGA--AAATGTGGCATA-TCCATCCCATGGCATACTA-----------TTCAGCAATAAAA---GTAC
right   CCTGGGCAACAGAGCGAGACTCCGTCTCAAAAAAAAAAAAAAATAAATAAATAAAAAAATAAAAAAAGTAC
        111*111**11*1*11*1*1***1**1**1111*1*11*11111111111*11*11*******111****

left    AACATGGGCCAGGCGCGGTGGCTCACGCCTGTAATCCCAGCACTTTGGGAGGCCGAGGCGGGTGGA
chrom   AACATGG---ATGAAC-----CTCAAAAACATTATGC------TAAAGGAGCCAGATGAAAA---A
right   AACATGG---ATGAAC-----CTCAAAAACATTATGC------TAAAGGAGCCAGATGAAAA---A
        *******222*2*22*22222****222222*2**2*222222*222****2*2**2*2222222*
```

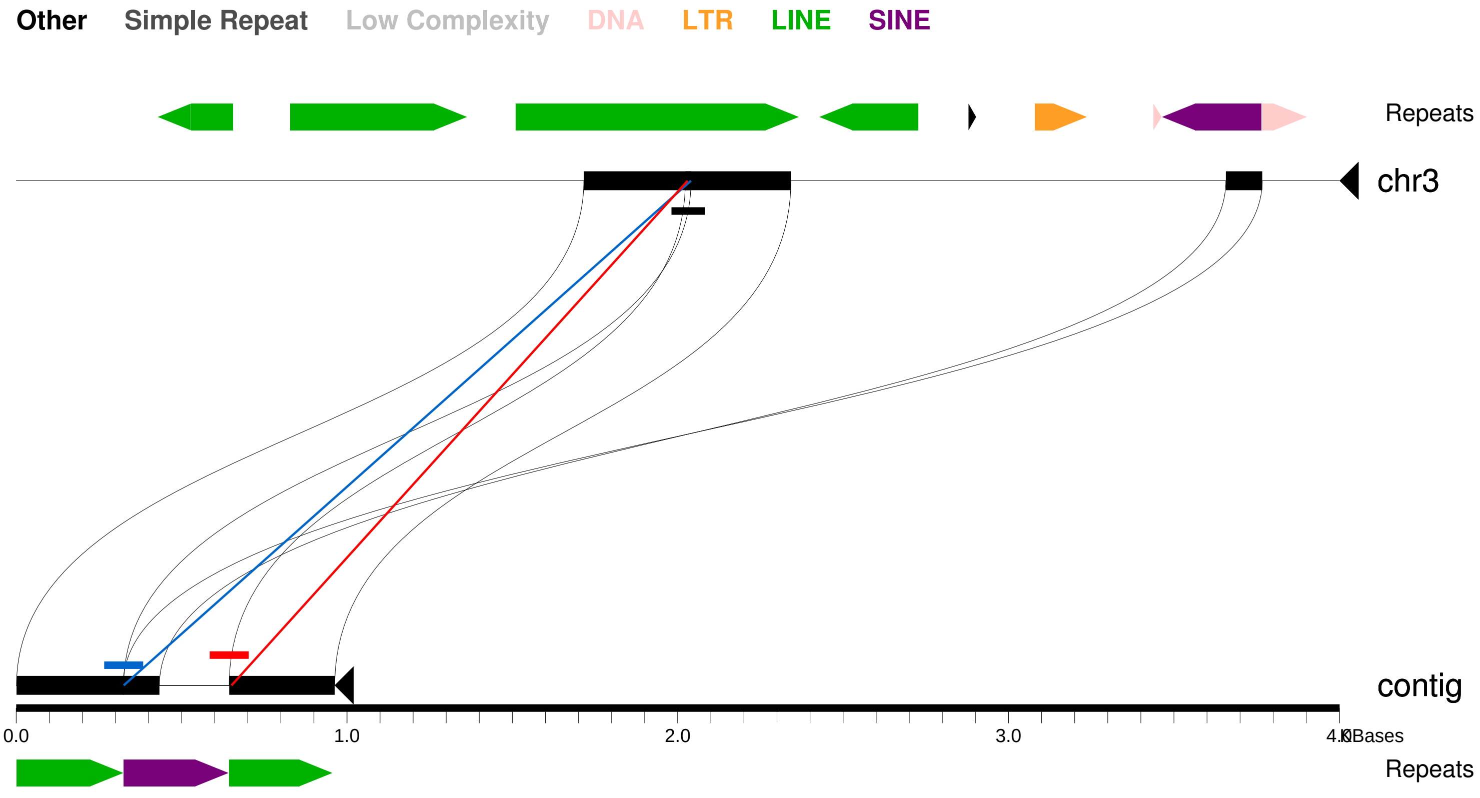

siteID: chr3_32229423 Scafftig3 - Breakpoint: chr3:32229401
chr3:32229241-32229575
Overlap Length: 15 INS size: 305 Genotyped: Yes

```
left    --------GGTTTTCTTTTCATTTTCCGGAAGATGTCCTTG--AAGCATAATAGTTTTTAATTTTGATGA
chrom   CATTCAGTGGTTTTCTTTTCATTTTCCGGAAGATGTCCTTG--AAGCATAATAGTTTTTAATTTTGATGA
right   --TTTAGTAGAGA-------------CGGAGTTTCACCGTGTTAGCCAGGATGGTCTCGA--TCTCCTGA
          22N2221*1111111111111111****111*11**1**11*11**11**1**1*11*11*1*11***

left    AGTCCCATTTATCTATTTTTTCTTTTGTTGCTTGTGCTGTTGGTGTTATAGCTAAGAAACCATTGCCTAA
chrom   AGTCCCATTTATCTATTTTTTCTTTTGTTGCTTGTGCTGTTGGTGTTATAGCTAAGAAACCATTGCCTAA
right   CCTCG---TGATCCGCCCG--CCTCGGCCTCCCAAAGTGCTGGGATTACAGGCGTGAG-CCACCGCG---
        11**1111*1***11111111*1*11*111*111111**1***11***1**1111**11***11**1111

left    TCCAAGATCATGAAGATTGAAGCCTATGCATTCTTTT-----TTTTTTTTTTT----------TTTTTTT
chrom   TCCAAGATCATGAAGATTTAGGCCTATGCATTCTTTTAAGAGTTGTATAGTTTAGCTCTCACATTTATTT
right   -CCCGGCC---GAAG-------CCTATGCATTCTTTTAAGAGTTGTATAGTTTAGCTCTCACATTTATTT
        1**11*11111****111N1N1***************22222**2*2*22***2222222222***2***

left    --------------TTGAGACGGAGTCTCGCTCTGTCGCCC-AGGCTGGAGTGCAGTGGCGCGACCTCGG
chrom   AGGCCTTTGCTCCATTTTGATGAAGTTTTG-TATATGGCATGAAGCAGGAGTTCAATTTCGTT-CTTTTG
right   AGGCCTTTGCTCCATTTTGATGAAGTTTTG-TATATGGCATGAAGCAGGAGTTCAATTTCGTT-CTTTTG
        22222222222222**22**2*2***2*2*2*2*2*2**222*2**2*****2**2*22**222*2*22*

left    CTCACTGCAAGC-------TCCGCCTCCCGGGTTCACGCCA-----TTCTCC------T
chrom   CATATGGCTAGCCAGTTGTTTCACCATCATTTTTGAAGAAACTTCCTTGTCCACTGAAT
right   CATATGGCTAGCCAGTTGTTTCACCATCATTTTTGAAGAA-------------------
        *22*22**2***2222222*2*2**22*2222**2*2*221     11N111      1
```

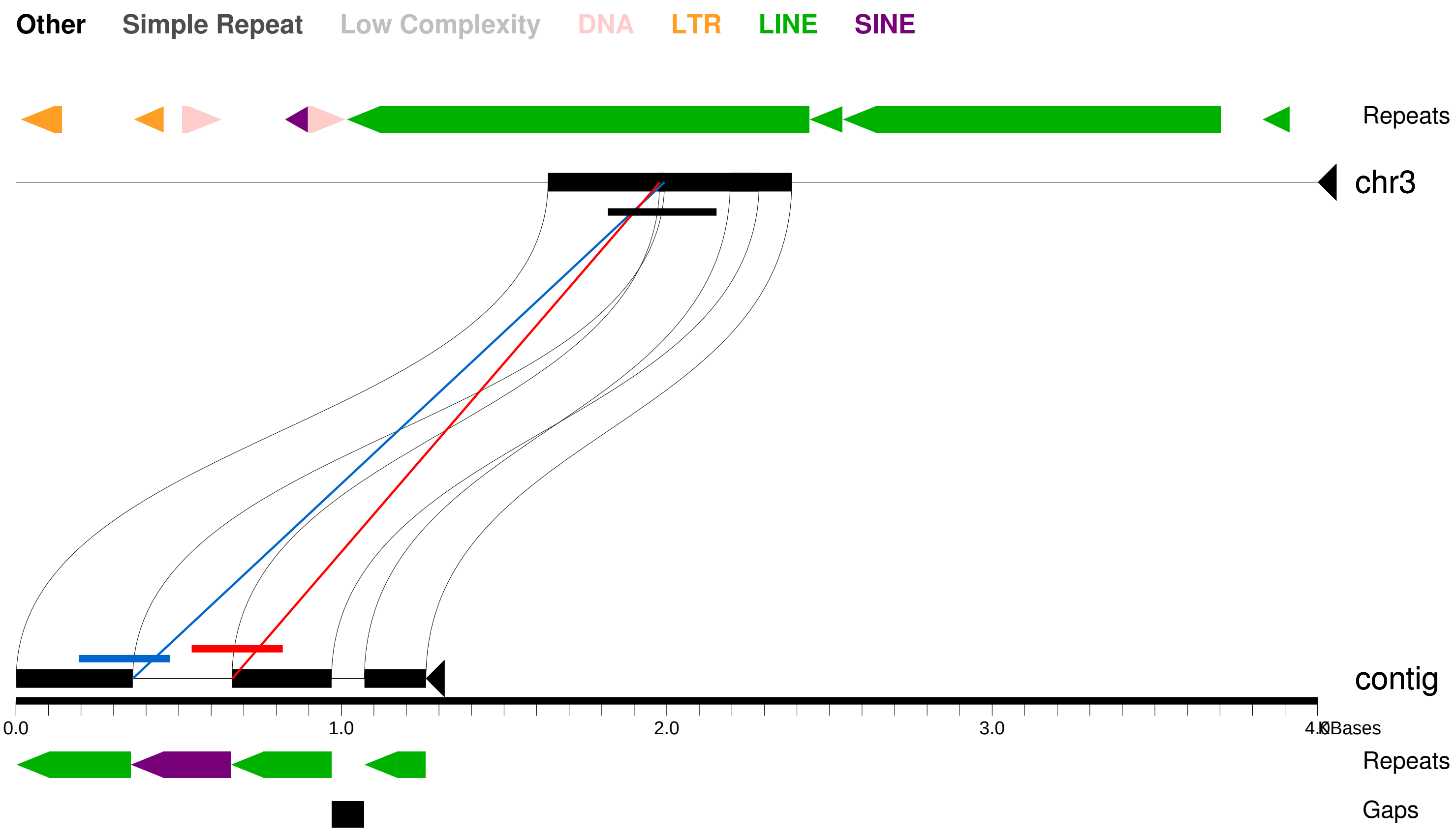

siteID: chr3_35159317 Scafftig1 - Breakpoint: chr3:35159375
chr3:35159250-35159533
Overlap Length: 9 INS size: 383 Genotyped: No

```
left   ---------------------------------------------------------------GGAGGTT
chrom  TAGAAGATAAGCTTTAGTCATTTACCATTGAATATATGATGTTCATTGGTATTTTATGTACATGGAGGTT
right  NNNNNNNNNNNC-----------------------------------------------AGAGCGAG---
       NNNNNNNNNNN2                                               2N2N1***111

left   TTCTTATGTTGAGTTAGTTCTTTCTATTCCTAGTTTGATGAGTGCTTTTTATCATAAAAAAGTTGGCCGG
chrom  TTCTTATGTTGAGTTAGTTCTTTCTACTCCTAGTTTGATGAGTGCTTTTTATCATAAAAAAGTTTTGAAT
right  -------------------------ACTCC--GTCTCAAAA------------AAAAAAAAGTTTTGAAT
       1111111111111111111111111*2***11**1*1*11*111111111111*1*********222222

left   GCGCGGTGGCTCACGCCTGTAATCCCAGCACTTTGGGAGGCCGAGGCGGGTG----------------GA
chrom  TTTTTTTTCAACTGTCTTTTTTTTCTGCATCTTTTGAGATAATTATGTGTTTTTTCCCCCTTTAACTCTGT
right  TTTTTTTTCAACTGTCTTTTTTTTCTGCATCTTTTGAGATAATTATGTGTTTTTTCCCCCTTTAACTCTGT
       222222*2222*222*2*2*22**2222222****2**22222*2*2*22*22222222222222222*2

left   TCATGAGGTC-AGGAGATCGAGACCATCCTGGCT-AACAAGG--TGAAACCCCGTCTCTACTAAAAATAC
chrom  TAATCTGGTGTATTACATATATATTTTCTGTGTTGAACAATCCTTGCATTCCAGGAATAAATCTCACTTG
right  TAATCTGGTGTATTACAT----------------------------------------------------
       *2**22***22*22*2**NN1N1NNN11NNN1N1 11111NN  11N1NN11N1NNNNN1N1NNN1N1NN

left   AAAA
chrom  CTCT
right  ----
       NNNN
```

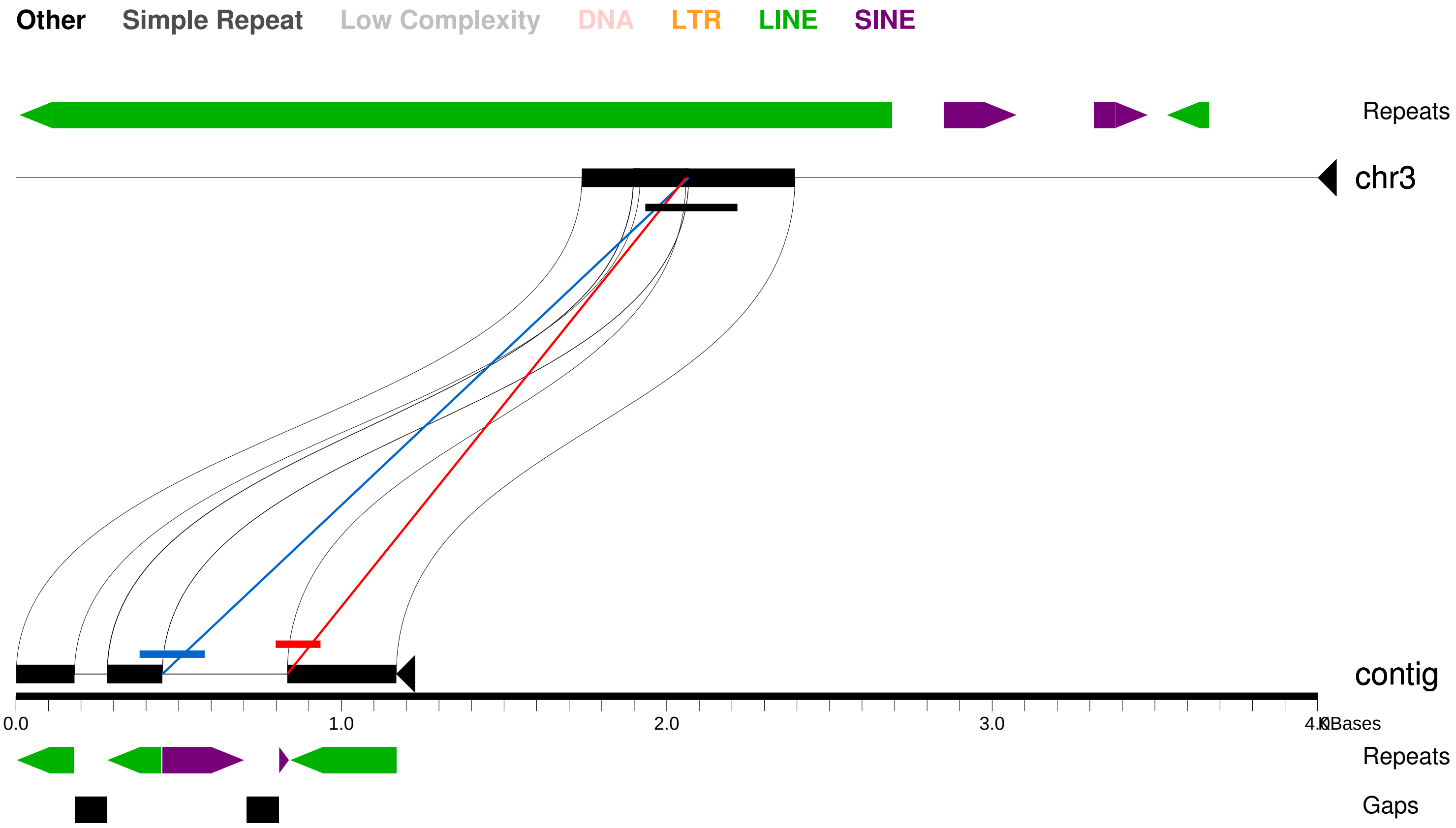

siteID: chr3_35346629 Scafftig4 - Breakpoint: chr3:35346624
chr3:35346578-35346669
Overlap Length: 14 INS size: 313 Genotyped: Yes

```
left    T--AATGGA-----------------------AAAGAAATCTTCC--CATGTATATTTCAAGGAAACTAT
chrom   T--AATGGA-----------------------AAAGAAATCTTCC--CATGTATATTTCAAGAAAACTAT
right   GCCACTGCAGTCCGCAGTCCGGCCTGGGCGACAGAGCGAGACTCCGTCTCAAAAAAAAAAAAAAAAAAAA
        111*1**1*111111111111111111111111*1**11*111***11*1111*1*1111**12***11*1

left    TAGAAAAGTAAAATAGAGGGGCCGGGCGCGGTGGCTCACGCCTGTAATCCCAGCACTTTGGGAGGCCGAG
chrom   TAGAAAAGTAAAATAGATGCTTAAAA-----TTGATCA------TTATTTCATTTCTT-------CC---
right   AAAAAAAGTAAAATAGATGCTTAAAA-----TTGATCA------TTATTTCATTTCTT-------CC---
        1*1**************2*222222222222*2*2***222222*2**22**222***2222222**222

left    GCGGGT
chrom   ------
right   ------
        222222
```

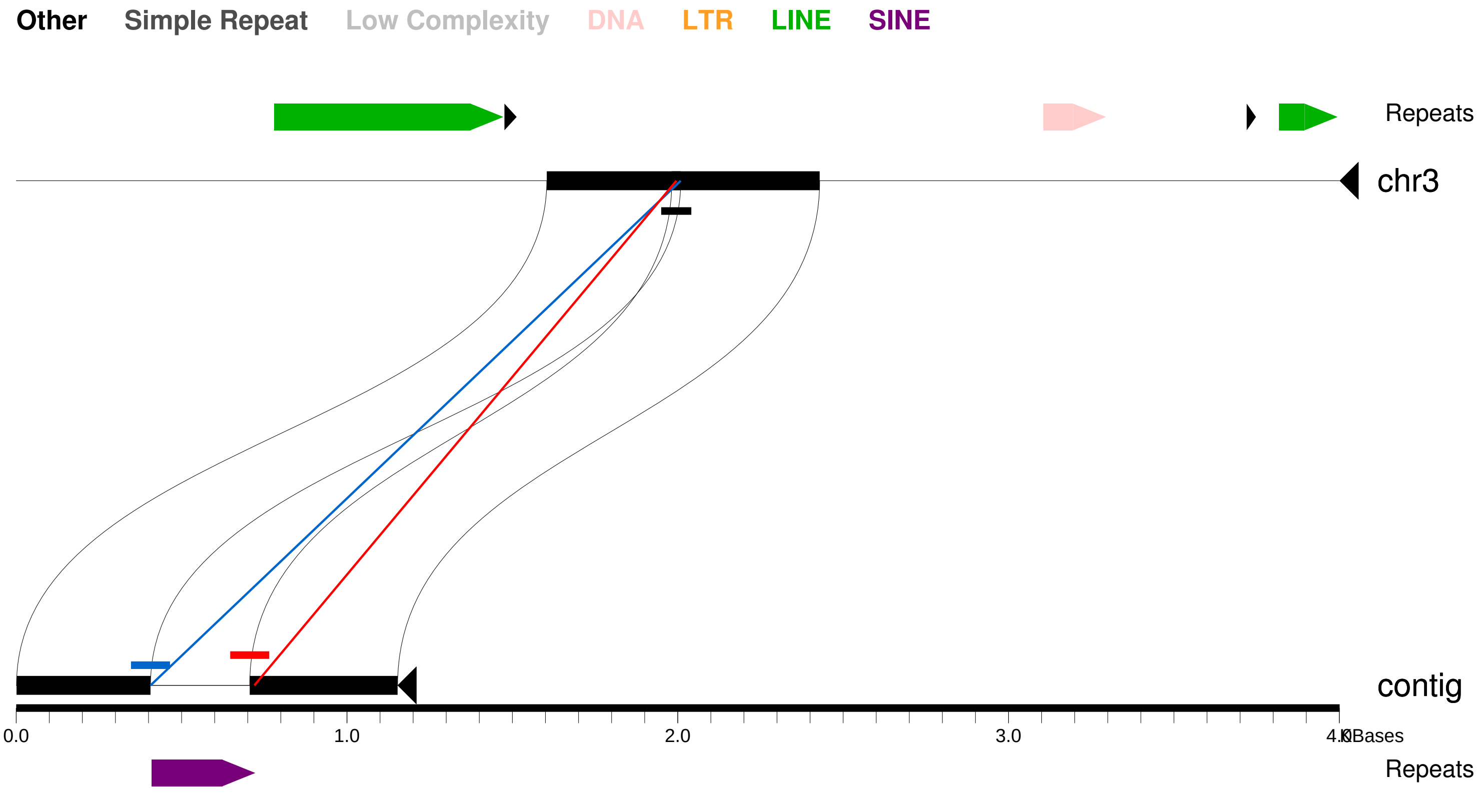

siteID: chr3_36839167 Scafftig2 - Breakpoint: chr3:36839146
chr3:36839095-36839205
Overlap Length: 9 INS size: 400 Genotyped: No

```
left    CCTTTAACTGATCATTCCATCATAGTTTTCTGCAATAAAAATATATAATAT----------GAAATCAGT
chrom   CCTTTAACTGATCATTCCATCATAGTTTTCTGCAATAAAAATATATAATAT----------GAAATCAGT
right   TCCGCC-CGCCTCGGCCTCCCAAAGTGCTGGG-ATTACAGGCGTGAGCCACCGCGCCCGGCGAAATCAGT
        1*11111*111**111*111**1***11*11*1*1**1*1111*11111*11111111111*********

left    NNNNNNNNNNNNNNNNNNNNNNNNNNNNNNNNNNNNNNNNNNNNNNNNNNNNNNNNNNNNNN
chrom   CTTTTAAATTTAATTATGTTAATGATTCATGTAATATGTAACTAGTGATCT--------
right   CTTTTAAATTTAATTATGTTAATGATTCATGTAATATGTAACTAGTGATCT--------
        222222222222222222222222222222222222222222222222222222222222
```

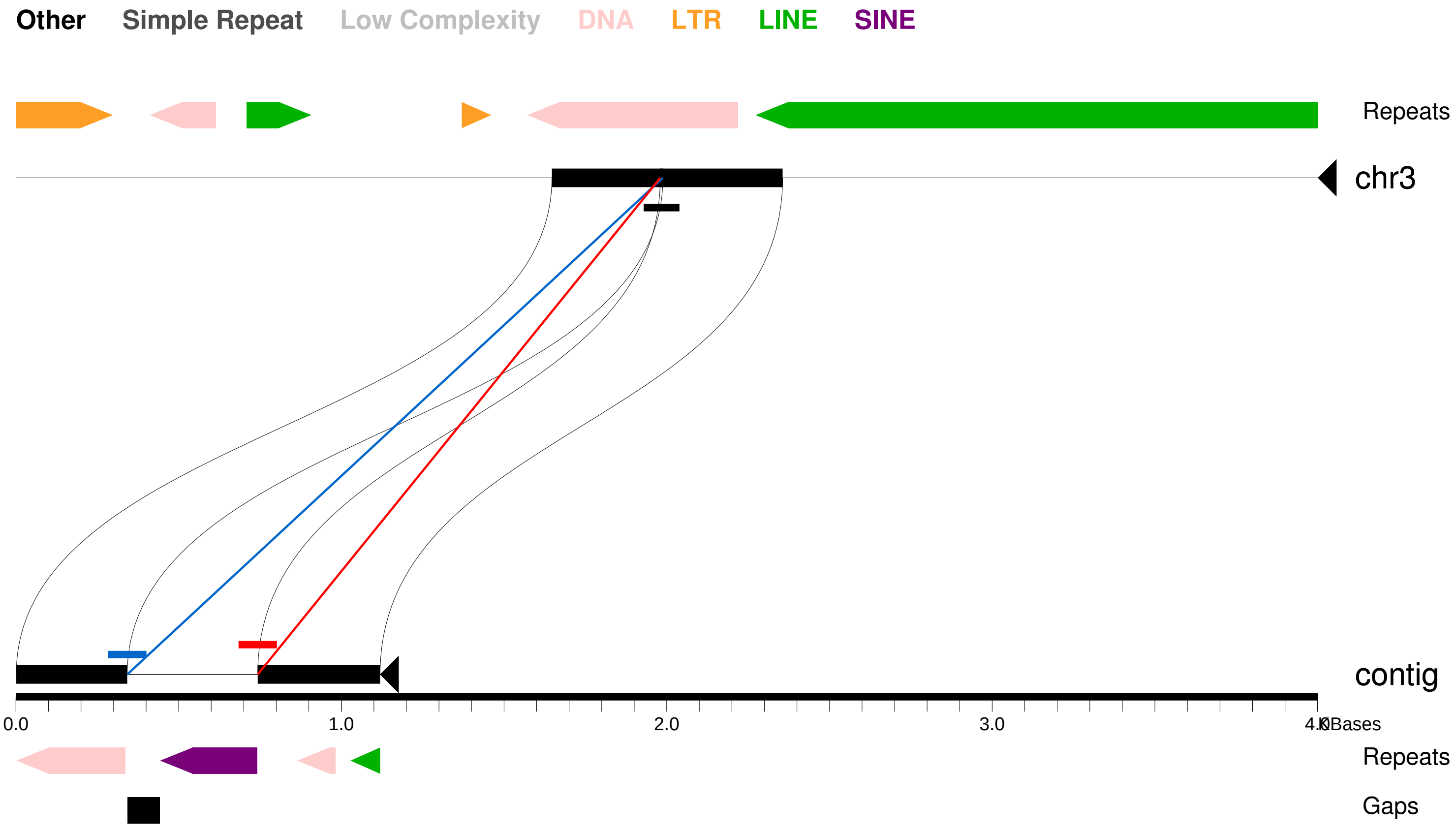

siteID: chr3_37604437 Scafftig1 - Breakpoint: chr3:37604432
chr3:37604381-37604491
Overlap Length: 9 INS size: 396 Genotyped: No

```
left   GCAAGAAATATACATGAATTCAAAGAGGTTTGGGATAATTTTATT--------AATGATAAAACCATAGG
chrom  GCAAGAAATATACATGAATTCAAAGAGGTTTGGGATAATTTTATT--------AATGATAAAACCATAAA
right  NNNNNNNNNNNNNNNNNNNNNNNNNNNNNNNNNNNNNNNNNNNNNNNNNNNNNAAAAAAAAAAAACCATAAA
       11111111111111111111111111111111111111111111111111111**11*1*********22

left   CCGGGCGCGGTGGCTCACGCCTGTAATCCCAGCACTTTGGGAGGCCGAGGCGGGCGG---
chrom  GA----------ACTCA-GAAAATATTTTAAGATGCTTGGCATCAGGAACTATTTCATTT
right  GA----------ACTCA-GAAAATATTTTAAGATGCTTGGCATCAGGAACTATTTCATTT
       2222222222222****2*2222**2*222**2222****2*2222**222222222222
```

Other **Simple Repeat** **Low Complexity** **DNA** **LTR** **LINE** **SINE**

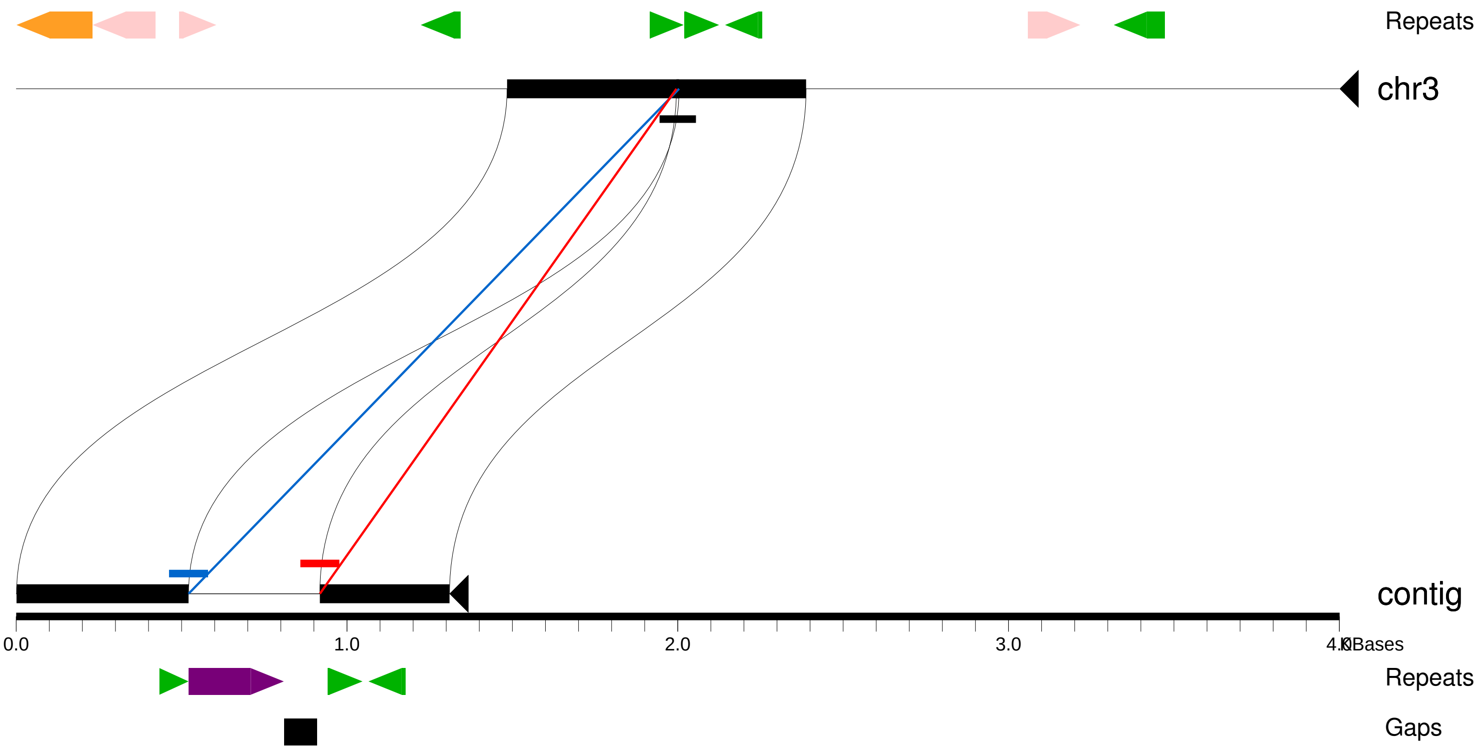

siteID: chr3_40241623 Scafftig1 + Breakpoint: chr3:40241609
chr3:40241582-40241668
Overlap Length: 33 INS size: 407 Genotyped: No

```
left     AGAC----------------ATTTGCTGAAA----AATTGTG------------CCTCTTACATTTTCTT
chrom    AGAC----------------ATTTGCTGAAA----AATGGTG------------CCTCTTACATTTTCTT
right    CCGCCCGCCTCGGCCTCCCAAAGTGCTGGGATTACAGGCGTGAGCCACCACGCCCGGCCTACATTTTCTT
         111*1111111111111111*11*****11*1111*11N***111111111111*11*1***********

left     TTTATCATAGGCCCTTTTGAGGNNNNNNNNNNNNNNNNNNNNNNNNNNNNNNNNNNNNNNNNNNNNNNNN
chrom    TTTATCATAGGCCCTTTTGAGGACCTAATGGGTACACTAGGTCAGGGTA---------------------
right    TTTATCATAGGCCCTTTTGAGGACCTAATGGGTACACTAGGTCAGGGTA---------------------
         **********************222222222222222222222222222222222222222222222222

left     NNNNNNNNNNN
chrom    -----------
right    -----------
         22222222222
```

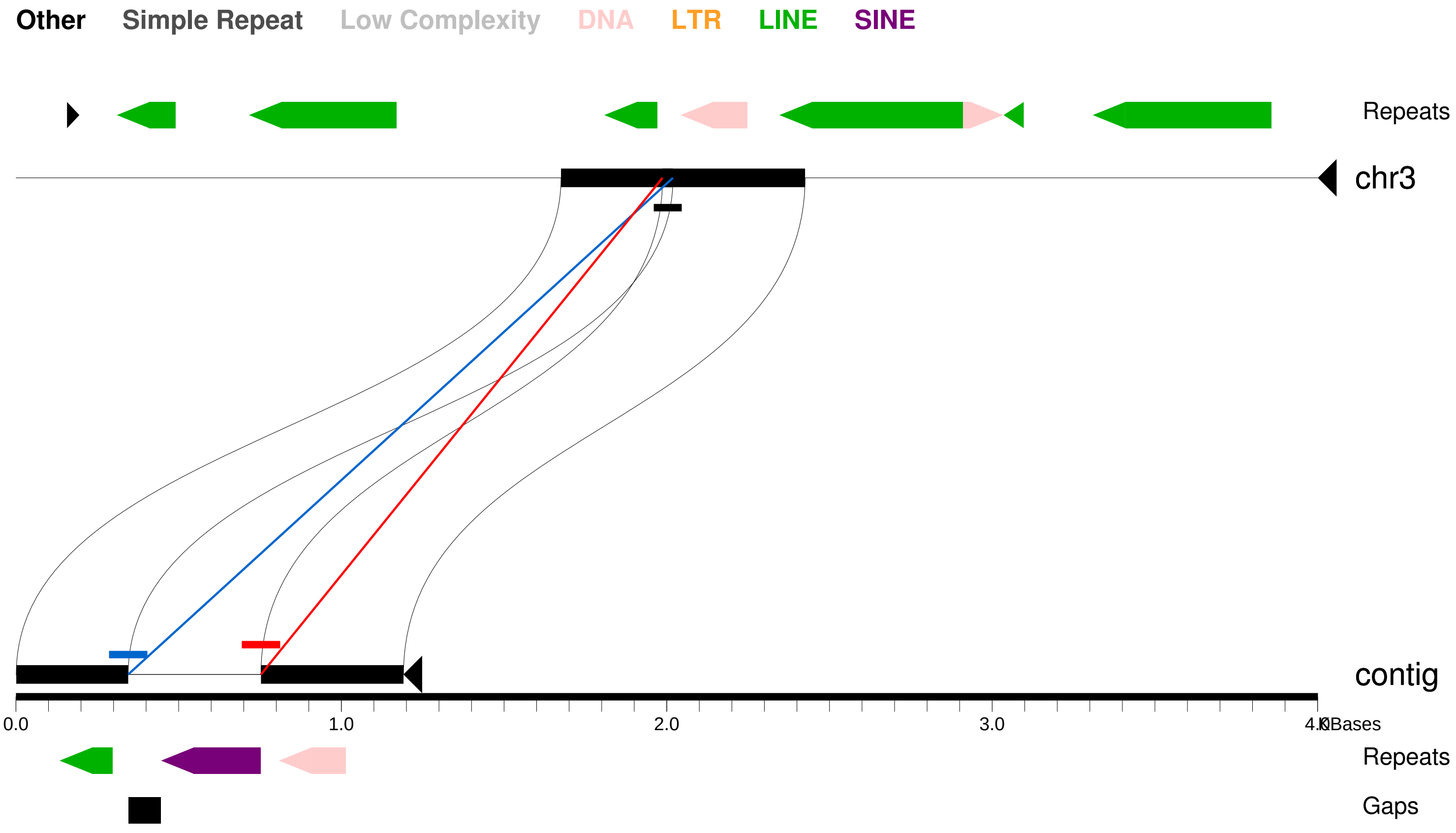

siteID: chr3_41511248 Scafftig1 + Breakpoint: chr3:41511239
chr3:41511079-41511405
Overlap Length: 7 INS size: 430 Genotyped: No

```
left   NNNNNNNNNNNNNNNNNNNNNNNNNNAAAAAAAA------CACCTTAAAAGCCTAAAAAGATGGCTATCT
chrom  AGGCCTACACCTGCCAAAAAAAAAAAAAAAAAAAAAAAAACACCTTAAAAGCCTAAAAAGATGGCTATCT
right  NNNNNNNNNNN-----------------------------------------------------------
       NNNNNNNNNNNNNNNNNNNNNNNNNN11111111      111111111111111111111111111111

left   CCTCAACTGCACAAACACCAATGCAAGAACACAAGAATTACAAAGAATCAGGAAATCATGATATATCCAA
chrom  CCTCAACTGCACAAACACCAATGCAAGAACACAAGAATTACAAAGAATCAGGAAATCATGATATATCCAA
right  ----------------------------------------------------------------------
       1111111111111111111111111111111111111111111111111111111111111111111111

left   AAGAAACTAAGAAAGCTCCAACAATAGGCCGGGCGCAGTGGCTCACGCCTGTAATCCCAGCACTTTGGGA
chrom  AAGAAACTAAGAAAGCTCCAACAATAGACCCTAAAGAAAGGGTCAAAT-TTTATAAACAGAAATACTAGA
right  --------------------ACAATAGACCCTAAAGAAAGGGTCAAAT-TTTATAAACAGAAATACTAGA
       11111111111111111111*******2**222222*22**2***2222*2**2222***2*2*2222**

left   GGCCGAGGCGGGCGGAT---------CACGAGGTCAGGAGATCGAGACCATC---CTG--------GCTA
chrom  GACAATACTGAACTGAAAAATACAAACAGGAAGCTTCAACATAGGCTCCCTCACACTGAAGAATCAGTGA
right  GACAATACTGAACTGAAAAATACAAACAGGAAGCTTCAACATAGGCTCCCTCACACTGAAG---------
       *2*222222*22*2**2222222222**2**2*22222*2**2*222**2**222***222     1NN1

left   ACACGGTGAAACCCCATCT---------------------CTACTAAA
chrom  ACTTGAAGATAGAACATCTGAAATTATCCAATCAGAGCTGCAAATAAA
right  ------------------------------------------------
       11NN1NN11N1NNN11111                     1N1N1111
```

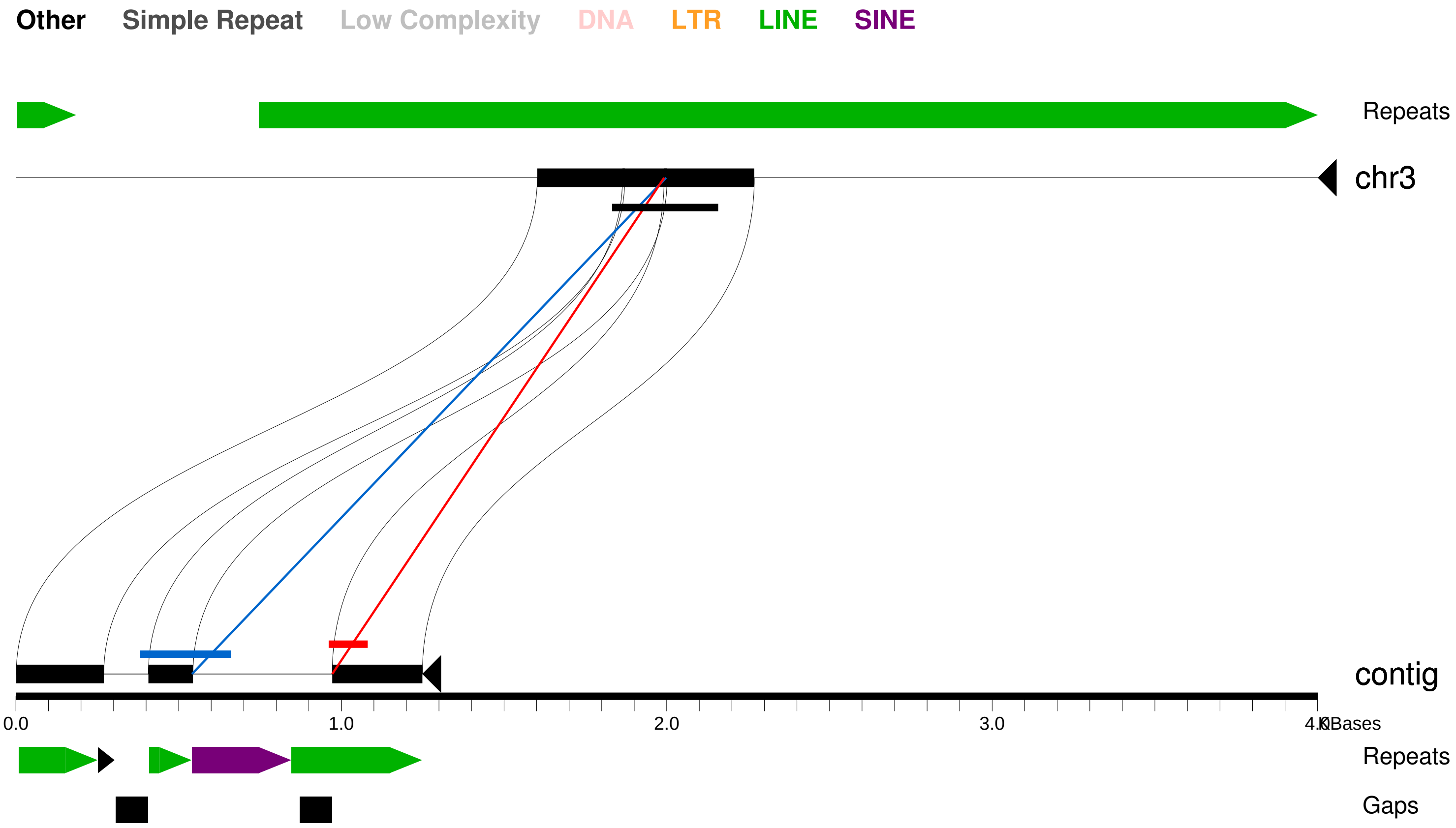

siteID: chr3_42898383 Scafftig4 - Breakpoint: chr3:42898408
chr3:42898361-42898467
Overlap Length: 10 INS size: 317 Genotyped: Yes

```
left     AA----GTCTCTCTCTCTC------CCTGTCTTTCT--CCTGGAACACACTACAGGTTTGAGCCTAATTT
chrom    AA----GTCTCTCTCTCTC------CCTGTCTTTCT--CCTGGAACACACTACAGGTTTGAGCCTAATTC
right    CCACCCGCCTCGGCCTCCCAAAGTGCTGGGATTACAGGCGTGAGCCACCGCGCCCGGCCGAGCCTAATTC
         111111*1***111***1*111111*11*11**1*111*1**111***1111*11*111*********2

left     TTTTTTTTTTTTTTTTTTTTTTTTTTTTGAGACGGAGTCTCGCTCTGTCGCCCAGGCCGGA
chrom    TTAGTTCATGACATACTTGTATC--------ACTG-GTTT--ATGTGAAGCTCTCATCA-A
right    TTAGTTCATGACATACTTGTATC--------ACTG-GTTT--ATGTGAAGCTCTCATCA-A
         **22**22*2222*22**2*2*222222222**2*2**2*222*2**22**2*2222*22*
```

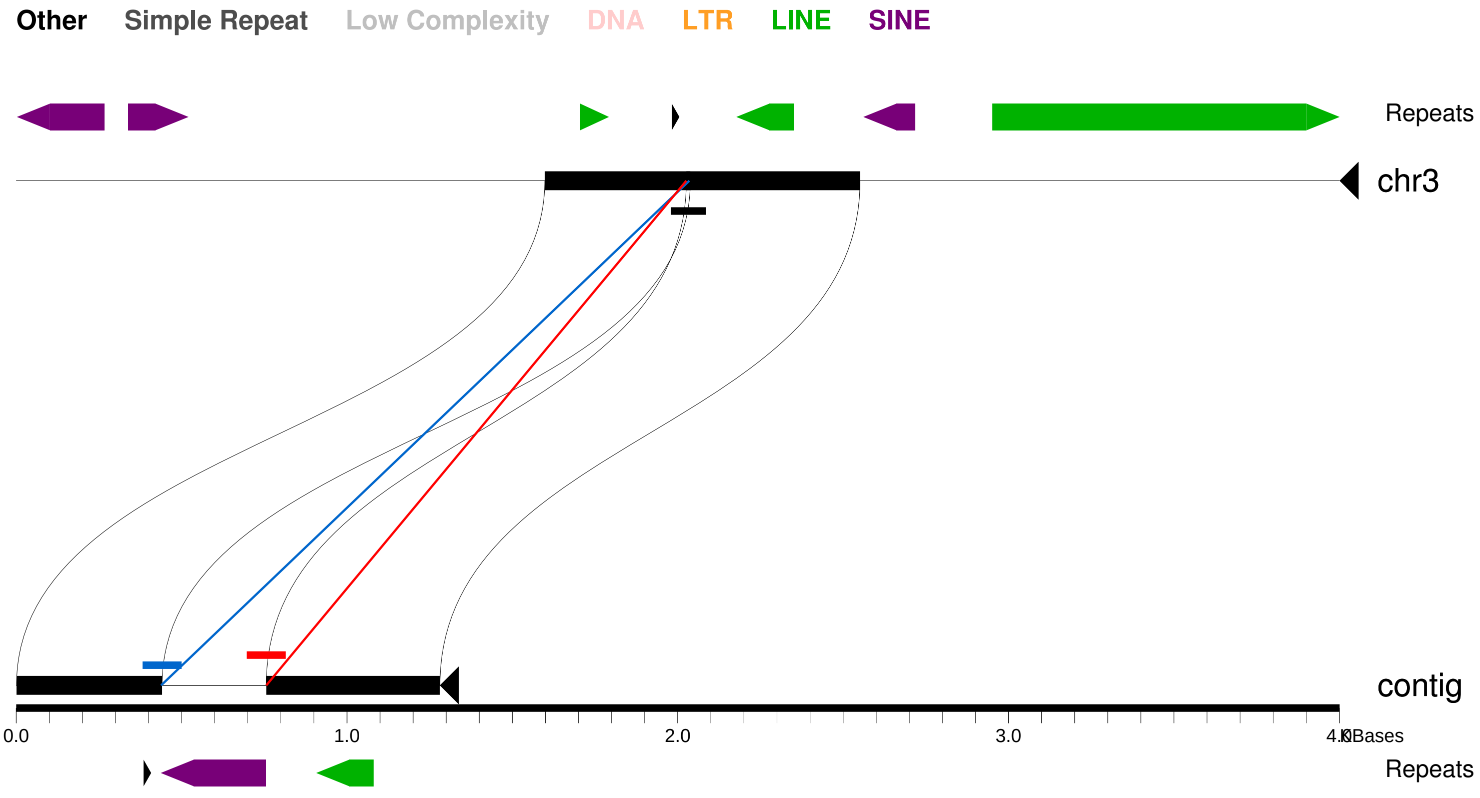

siteID: chr3_45542661 Scafftig13 + Breakpoint: chr3:45542653
chr3:45542603-45542712
Overlap Length: 10 INS size: 308 Genotyped: Yes

```
left    ---------TAAAAATTACATATTTAATGTATTTAAAGAATGGTACAACACATTAAATATGGAATTTTTT
chrom   ---------TAAAAATTACATATTTAATGTATTTAAAGAATGGTACAACACATTAAATATGGAATTTTTA
right   CCGCCCGCCTCGGCCTCCCAAAGTGCTGGGATTACAGGCGTGAGCCACCGCGCCCGGCCTGGAATTTTTA
        111111111*11111*11**1*1*1111*1***11*1*11**111**1*1*11111111**********2

left    TTTTTTTTTTTTTTTTTTGAGACGGAGTCTCGCTCTGTCGCCCAGGCTGGAGTGCAGT
chrom   TATATGAACATACAT-----GCCCCACTC---CTCAGTTTAAGAAACAG-AGTATTTC
right   TATATGAACATACAT-----GCCCCACTC---CTCAGTTTAAGAAACAG-AGTATTTC
        *2*2*22222*222*22222*2*22*2**222***2**22222*22*2*2***22222
```

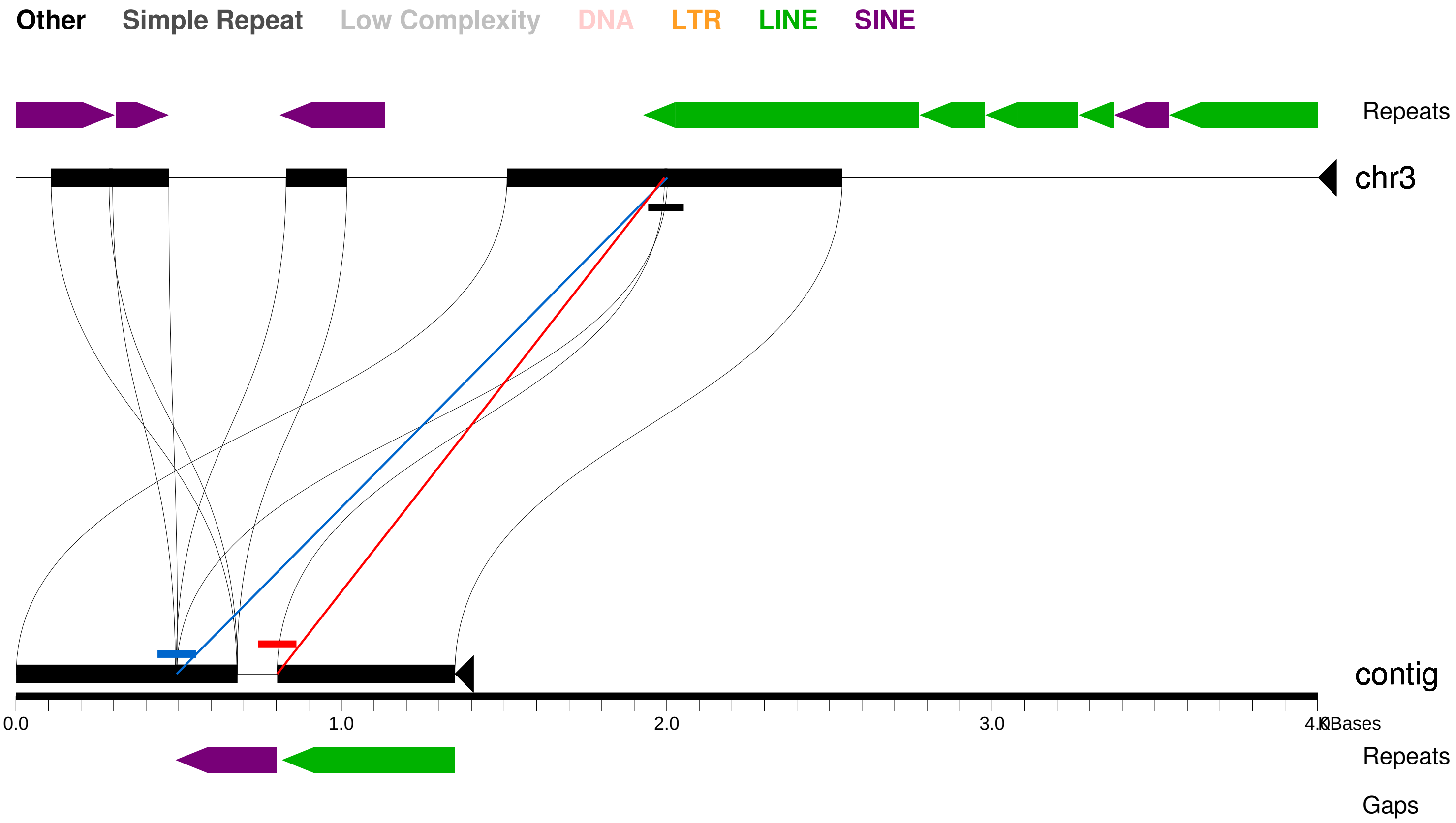

siteID: chr3_46550782 Scafftig1 + Breakpoint: chr3:46550792
chr3:46550632-46550966
Overlap Length: 15 INS size: 157 Genotyped: Yes

```
left   --------------------------------------------------------ATGTGAAGTCCTAA
chrom  TCATAAATTGAACCATTCTAAGTCAGGGGCCATCTGTATATAGGTTTATAAGGGGAATGTGAAGTCCTAA
right  TT----------CCG------GCCGGGCGCGGT---------GGTTCACGCCTGTAATCCCAGCAC----
       2N          22N      2N2N22N22NN2         2222N2NNNNN2N2**111*111*1111

left   TTAGGGAAAAGGAGTCAGGCTGGTGGGACCAGGGGAAAACAAAGAGATAAAGCAAATGAGCTA--TAGGT
chrom  TTAGGGAAAAGGAGTCAGGCTGGTGGGACCAGGGGAAAACAAAGAGATAAAGCAAATGAGCTA--TAGGT
right  TTTGGGAGGCCGAG----GCGGGTGGATCATGAGGTCAGGAGATCGAGAC--CATCCTGGCTAACAAGGT
       **1****1111***1111**1*****11*11*1**11*11*1*11**1*111**11111****111****

left   CTGCCTTTTTT-ATGGCCCAGGACATATGGCCATATGATCATATGATTTTGGCCATAT-GATGGCCAAAA
chrom  CTGCCTTTTTTTATGGCCCAGGACATATGGCCATATGTCC--------TGGGCCATAAAGATAAGTCTTT
right  GAAACCCCATCTCT--------ACATATGGCCATATGTCC--------TGGGCCATAAAGATAAGTCTTT
       1111*1111*121*11111111***************22*22222222*2*******22***22222222

left   GATCATT---TCCGGCCGGGCGCGGTGGTTCACGCCTGTAATCCCAGCACTTTGGGAGGCCGAGGCGGGT
chrom  TATCCCTAAGTCACTCCTATCTCCTTTAGTTACAAGATTTATCTGTGCAGGACATATGGCC-ATATGTCT
right  TATCCCTAAGTTACTCCTATCTCCTTTAGTTACAAGATTTATCTGTGCAGGACATATGGCC-ATATGTCT
       2***22*222*1222**222*2*22*222*2**22222*2***222***22222222****2*222*22*

left   GGATCATGAGGTCAGGAGATCGAGACCATCCTGGCTAACAAGGTGAAACCCCATCTCTACATAT------
chrom  TG---ATGACGTACATAACTCAAAAAATTCCTG-CTTACCATCAAATGCCTCAATTTATCAAACACTCCG
right  TG---ATGACGTACATAACTCAAAAAATTCCTG-CTTACCATCAAATGCCTCAATTT-------------
       2*222****2**2222*22**2*2*222*****2**2**2*2222*22**2**22*2NN11N1N
```

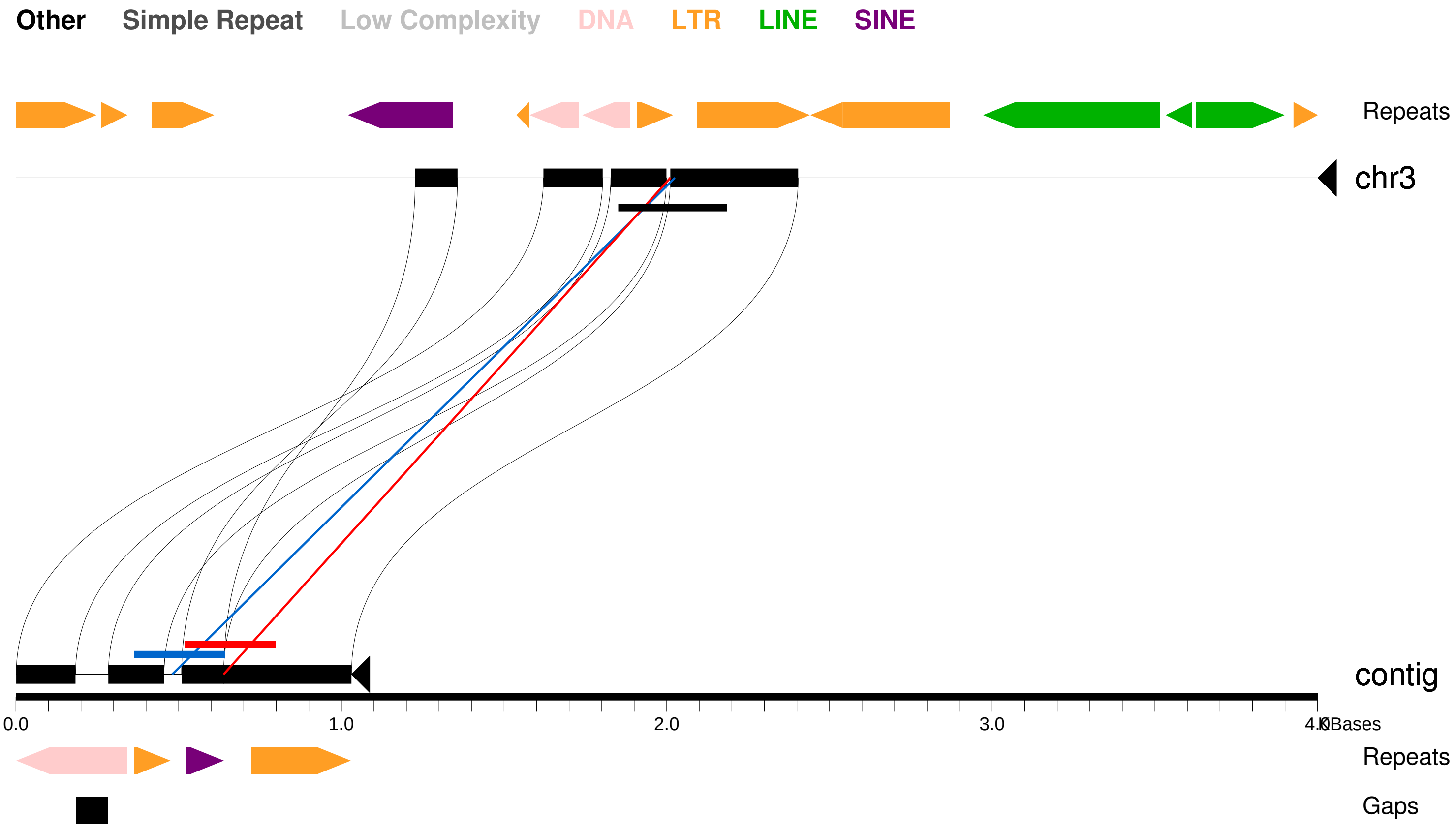

siteID: chr3_46661855 Scafftig5 - Breakpoint: chr3:46661845
chr3:46661685-46662019
Overlap Length: 15 INS size: 304 Genotyped: Yes

```
left   G----------------TCTGCCTATTTCTTCCCCACACCCTTCCTGCCCTGGGTCCTGTTTTCTAGCAG
chrom  GAGGACTGGAGGCTAAGTCTGCCTATTTCTTCCCCACACCCTTCCTGCCCTGGGTCCTGTTTTCTAGCAG
right  GAGAA-TGGCG--TGA---------------------ACCCGGGAGGCGGAGCTTGCAGTG----AGCCG
       *22N2 222N2  2N2 111111111111111111****11111**111*11*1*1**11111***1*

left   TGACTTCATCTCTCCTGGACTACAGCTCCTGCTGCAGAGCCCCTCCCCCATGACTCCAGGCTCCAAAACA
chrom  TGACTTCATCTCTCCTGGACTACAGCTCCTGCTGCAGAGCCCCTCCCCCATGACTCCAGGCTCCAAA-CA
right  AGA-TCC--CGCCACTGCACTCCAGCCTGGGCGACAGAGCGA---------GACTCC-GTCTCAAAA-AA
       1**1*1*11*1*11***1***1****1111**11******11111111111******1*1***1***21*

left   CAAGTGAAAAGGGATTGGTAGAAAGAAGCTATTGAC----GGCCGGGCGCGGTGG-------------CT
chrom  CAAGTGAAAAGGGATTGGTAGAAAGAAGCTATTGACATTTGGCAACACCTAATGAAAGTAAATTGGATCT
right  AAAAAAAAAAAAAAAA----AAAAAGAAGCTATTGACATTTGGCAACACCTAATGAAAGTAAATTGGATCT
       1**111****111*11111*1**************2222***2222*2222**22222222222222**

left   CACGC-CTGTAATCCCAGC--ACTTT-------GGGAGGCCGAGGCGGGCGGATCACGAGGTCAGGAGAT
chrom  AATGATCATTAATATCTGCTAACATCAAAAAAGGTGATCTATAACCAGACAGATTT-GATGGCCGTCCAT
right  AATGATCATTAATATCTGCTAACATCAAAAAAAGTGATCTATAACCAGACAGATTT-GATGGCCGTCCAT
       2*2*22*22****22*2**22**2*2222222N*2**22222*22*2*2*2***222**2*2*2*222**

left   CGAGACCATCCCGGCT-------------AAAACGGTGAAACCCCGTCTCTACTGAA-
chrom  T-ATATCACCCGTACTGTAGTTTTGTCAAAAAAAAAAAAAAAATCCGGATCTAATCAAA
right  T-ATATCACCCGTACTGTAGTTTTGTCAAAAAAAAAAAAA--TCCGGATCT-------
       22*2*2**2**222**2222222222222****22222**1N2***22***1N1N11
```

Other **Simple Repeat** **Low Complexity** **DNA** **LTR** **LINE** **SINE**

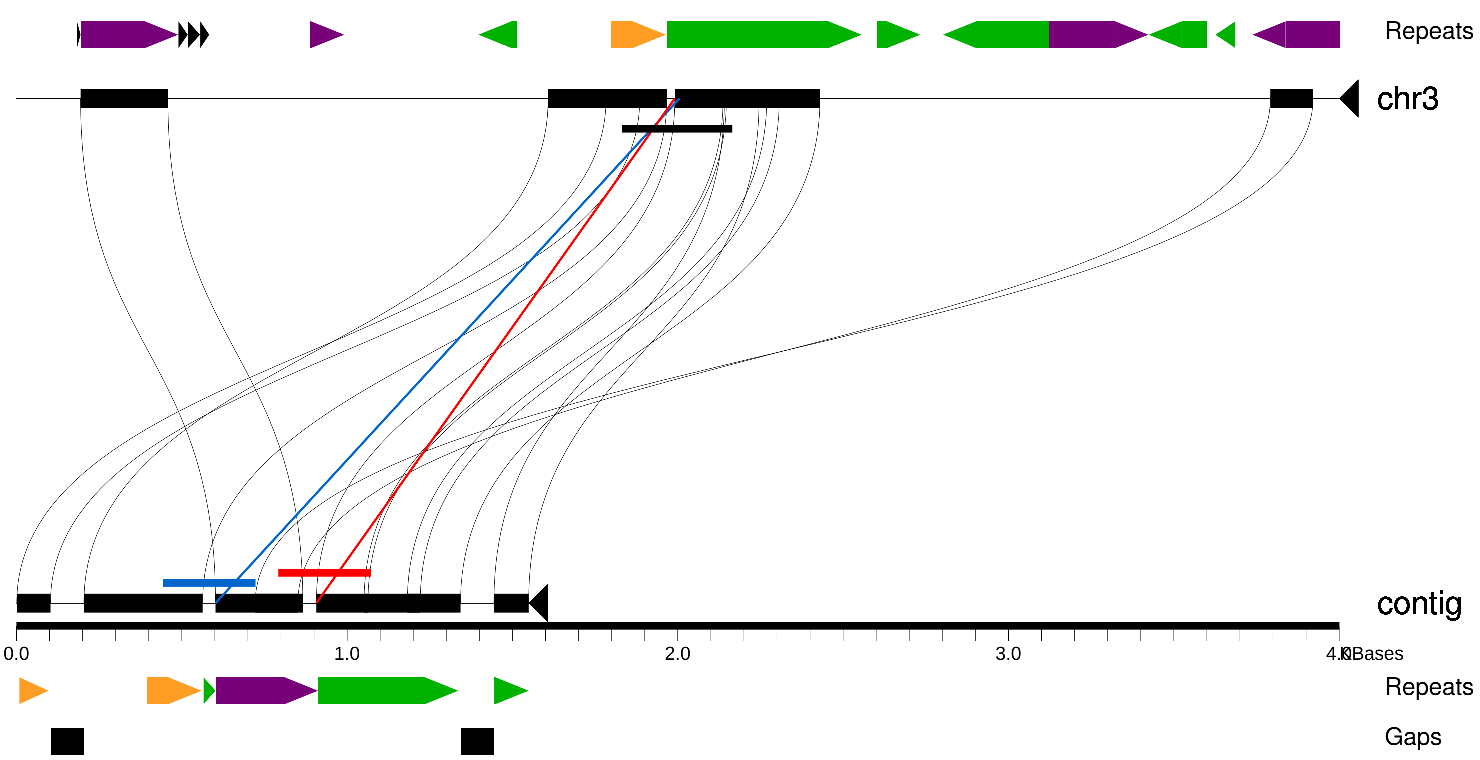

siteID: chr3_47103268 Scafftig1 + Breakpoint: chr3:47103327
chr3:47103285-47103386
Overlap Length: 18 INS size: 386 Genotyped: No

```
left     AGTATGGCACAAAACTCATAAGAAAACAAAGTTCTAGCTTTT-----------------AAAGAATAACC
chrom    AGTATGGCACAAAACTCATAAGAAAACAAAGTTCTAGCTTTT-----------------AAAGAATAACC
right    NNNNNNNNNNNNNNNNNNNNNNNNNNNNNNNNNNNNNNNNNNNNNNNNNNNNNNNAAAAAAAGAATAACC
         11111111111111111111111111111111111111111111111111111111111***********

left     ATTATCTGGCCGGGCGCGGTGGCTCACGCCTGTAATCCCAGCACTTTGGGAGGCCGAGGCGGGCGG
chrom    ATTATCTTT---GAAGACTTATCTCCCACC---AACCCC----CATTGCGC-------TCGAAAGA
right    ATTATCTTT---GAAGACTTATCTCCCACC---AACCCC----CATTGCGC-------TCGAAAGA
         *******22222*22*222*22***2*2**222**2***2222*2***2*222222222**222*2
```

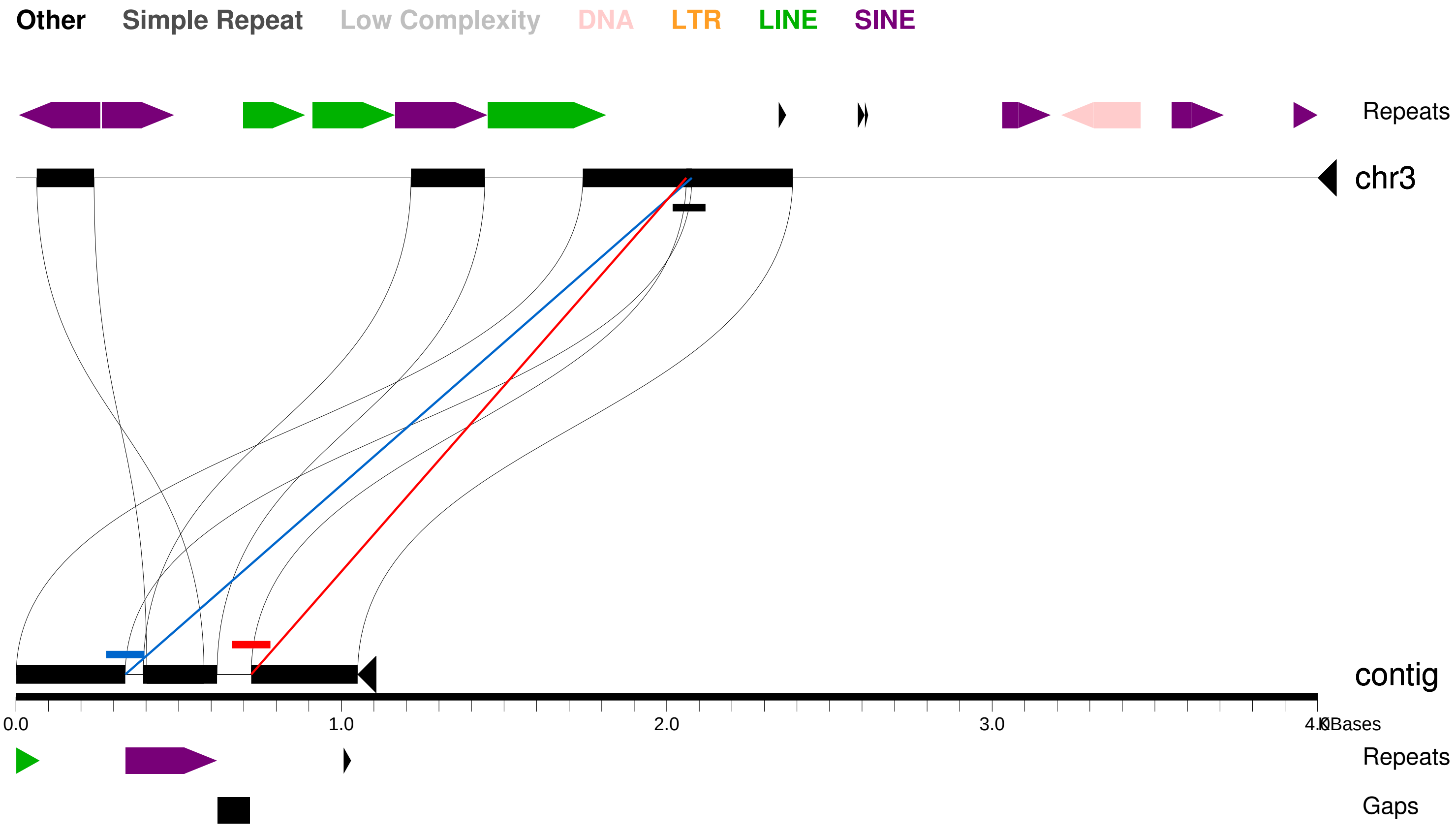

siteID: chr3_50879176 Scafftig1 + Breakpoint: chr3:50879160
chr3:50879000-50879335
Overlap Length: 16 INS size: 324 Genotyped: Yes

```
left   ----------AAGATGTGCATTTATCTATTTTTTTCAAAAAATACTATTGTGTCTCTTATTGAACATGAC
chrom  GTTTTAAGGAAAGATGTGCATTTATCTATTTTTTTCAAAAAATACTATTGTGTCTCTTATTGAACATGAC
right  TTT---AGTAGAGACGGGGTTTCACCT-TGTTAGCCAGGA--------TG-GTCTCGATCT---CCTGAC
       N22   22N21***1*1*11**1*1**1*1**111**11*11111111**1*****1111*111*1****

left   ATTGTGATTTTAATATTCTCTTATGCTTTGTTTTAGGTTGGTAC-AGAGGAGTTTCAACAAAGAAGCCAA
chrom  ATTGTGATTTTAATATTCTCTTATGCTTTGTTTTAGGTTGGTAC-AGAGGAGTTTCAACAAAGAAGCCAA
right  CTCATGATCC----ACCCGCCTCGGCCTCCCAAAGTGCTGGGATTACAGGCGTG----------AGCCAC
       1*11****111111*11*1*1*11**1*11111111*1***1*11*1***1**11111111111*****1

left   ATGTGAAGGTAATGAAAAGTTTATTGTTACCTTTTCTTTTT-------------CTTTTTTTTTTTTTTT
chrom  ATGTGAAGGTAATGAAAAGTTTATTGTTACCTTTTCTAATGTAGGAAGGAACTACCTTGTATGTTTGTTT
right  C-GCGCCCG---------GCCTATTGTTACCTTTTCTAATGTAGGAAGGAACTACCTTGTATGTTTGTTT
       11*1*111*111111111*11***************22*22222222222222*2**2*2*2***2***

left   ---------------------TTTTTTTTTTTTTTTTTGAGACGGAGTCTCGCTCTGTCG--CCCAGGCCG
chrom  GATATAACAACTCTTAGTGGTTCATCTTTTAATCTTTTACA-GTAGCCACAATGATTCTTTCCTTTACAA
right  GATATAACAACTCTTAGTGGTTCATCTTTTAATCTTTTACA-GTAGCCACAATGATTCTTTCCTTTACAA
       2222222222222222222**22*2****22*2***2*2*2*2**2*2*22*222**222**2222*22

left   GACTGCGG---ACTGCAGT--GGCGCA--ATCTC-----GGCTCA-CTGCAAGCTCCGCT
chrom  AACTGTAGCAAACTCTTGTCTGTCTCAGCATATTAAAGTGTTTCTTCTGAAAGGACAG--
right  AACTGTAGCAAACTCTTGTCTGTCTCAGCATATTAAAGTGTT------------------
       2****22*222***222**22*2*2**22**2*222222*2211N 111N111NN1N122
```

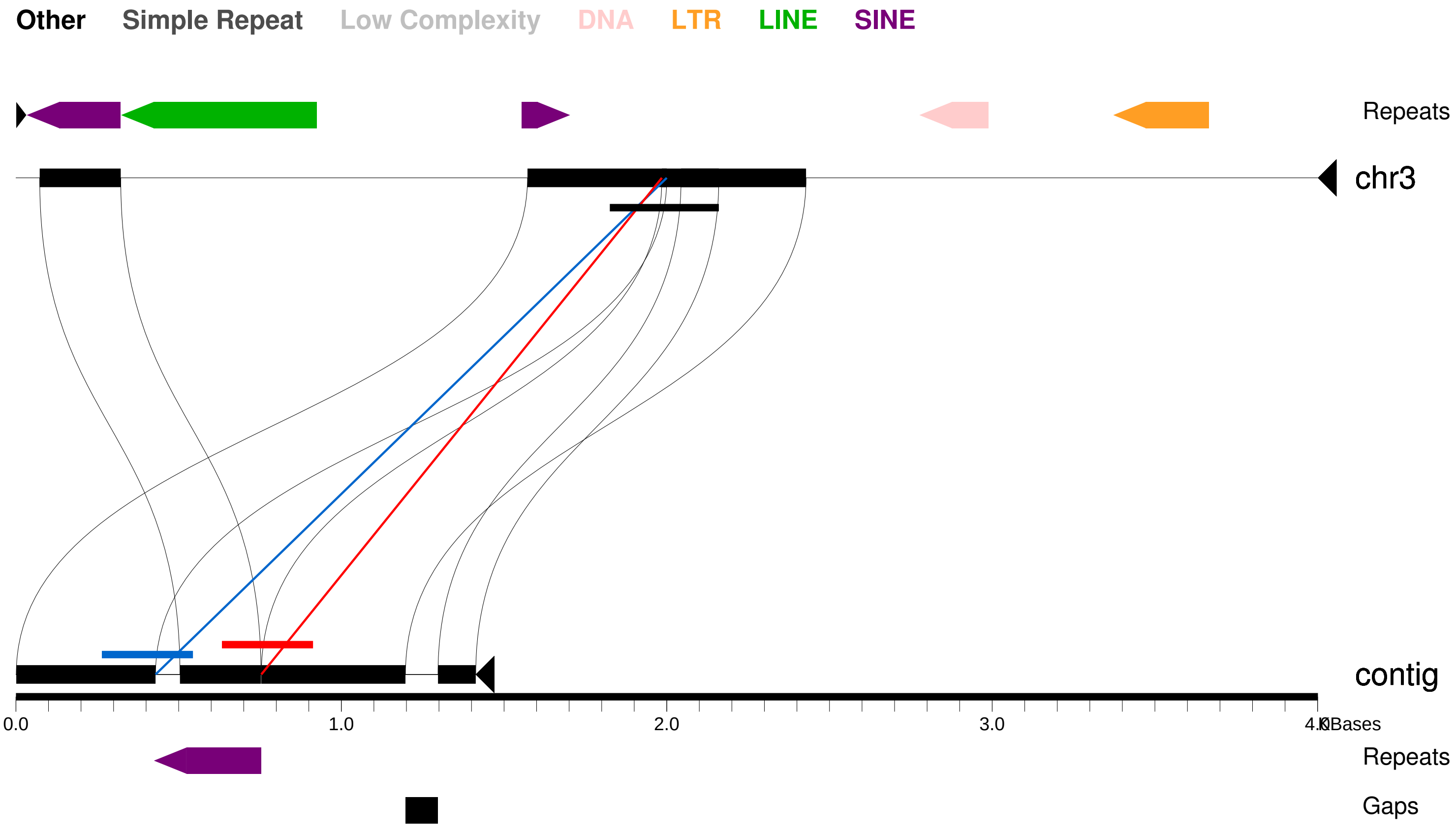

siteID: chr3_55885202 Scafftig2 + Breakpoint: chr3:55885199
chr3:55885157-55885258
Overlap Length: 18 INS size: 294 Genotyped: Yes

```
left    G------ACCTTGGGAAGAAAACTTACTTCCATCTC-----TAAGAAAAAATA------CTAGATAATGA
chrom   G------ACCTTGGGAAGAAAACTTACTTCCATCTC-----TAAGAAAAAATA------CTAGATAATGA
right   TCCGCCCGCCTCGGCCTCCCAAAGTGCTGGGATTACAGGCGTGAGCCACCGCGCCCGGCCTAGATAATGA
        11111111***1**111111**11*1**111**11*11111*1**11*11111111111***********

left    TTTTTTTTTTTTTTTTTTTTTTGAGACGGAGTCTCGCTCTGTCGCCCAGGCTG-GAGTGCAGTGGCGG
chrom   TTTTTTTAAGCCCATGCTG-------GGAG------TTGGTGG--CAGGATGAGAATGCACATG---
right   TTTTTTAAAGCCCATGCTG-------GGAG------TTGGTGG--CAGGATGAGAATGCACATG---
        ******12222222*22*22222222****222222*22**2*22****2**2**2****222*222
```

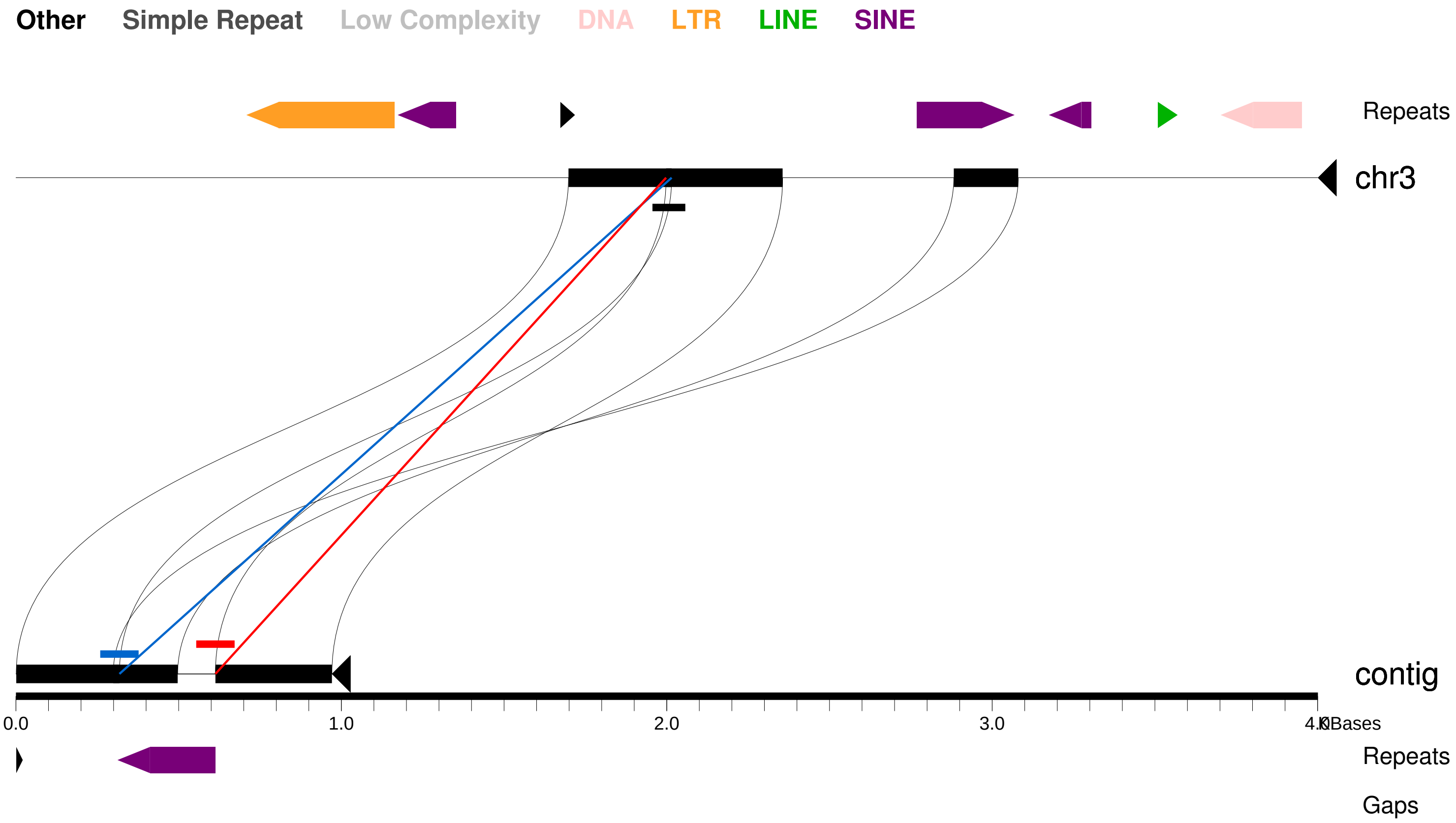

siteID: chr3_6103031 Scafftig1 + Breakpoint: chr3:6103035
chr3:6102875-6103206
Overlap Length: 12 INS size: 437 Genotyped: No

```
left    -------------------------------------CTCGGAAAGCTGTGGATATGTGCTTTACTAAAA
chrom   AAGAAAAATCTCAGACATTTTTACTTTCGGAAACTTTCTCGGAAAGCTGTGGATATGTGCTTTACTAAAA
right   TTTAGTAGAGACGGGG--TTTCACCTTG------TTAGCCAGGATGGTCTCGATCTC--CTGACCTCAT-
        NNN2NN2NNNN2N2NN  222N22N22N      22N11*1*1*1*1*1***1*111**111**1*11

left    TGGGGGAACAAATCAAGACAAAGGAAAACATCCTATTCAAGAAACAGAGCATTCTCGAGGGGGAAGTTCA
chrom   TGGGGGAACAAATCAAGACAAAGGAAAACATCCTATTCAAGAAACAGAGCATTCTCGAGGGGGAAGTTCA
right   -----GATCCACCCGCCTC---GG----CCTCC----CAAAGTGCTGGGATTAC---AGGCGTGAGCC--
        11111**1*1*11*1111*111**1111*1***1111***1111*1*1*11*1*111***1*11**1111

left    GTTGATGGTGACAGAAAGACCCAGGATGGAATNNNNNNNNNNN---------------------------
chrom   GTTGATGGTGACAGAAAGACCCAGGATGGAATCTTTATAGCAGGCCCACGGAGACACCAGTCCCATTTGG
right   ----ACCGCGCCCGG-----CCAGGATGGAATCTTTATAGCAGGCCCACGGAGACACCAGTCCCATTTGG
        1111*11*1*1*1*111111************22222222222222222222222222222222222222

left    ----------------------------------------------------------------------
chrom   AACAGAAGGACAAAAGGCTCCAGGAGGAAGTTCTCCAAGGACAACAATTGAGATGATATAAATGGCATAA
right   AACAGAAGGACAAAAGGCTCCAGGAGGAAGTTCTCCAAGGACAACAATTGAGATGATATAAATGGCATAA
        2222222222222222222222222222222222222222222222222222222222222222222222

left    ----------------------------------------------------
chrom   CGGAAAACCATATGGAGAGGCTTTTGTATGTTGCATGTATTTTGAATGTTTT
right   CGGAAAACCATATGGAGAGGCTTTTGTATGTTGCATGTATTT----------
        222222222222222222222222222222222222222222
```

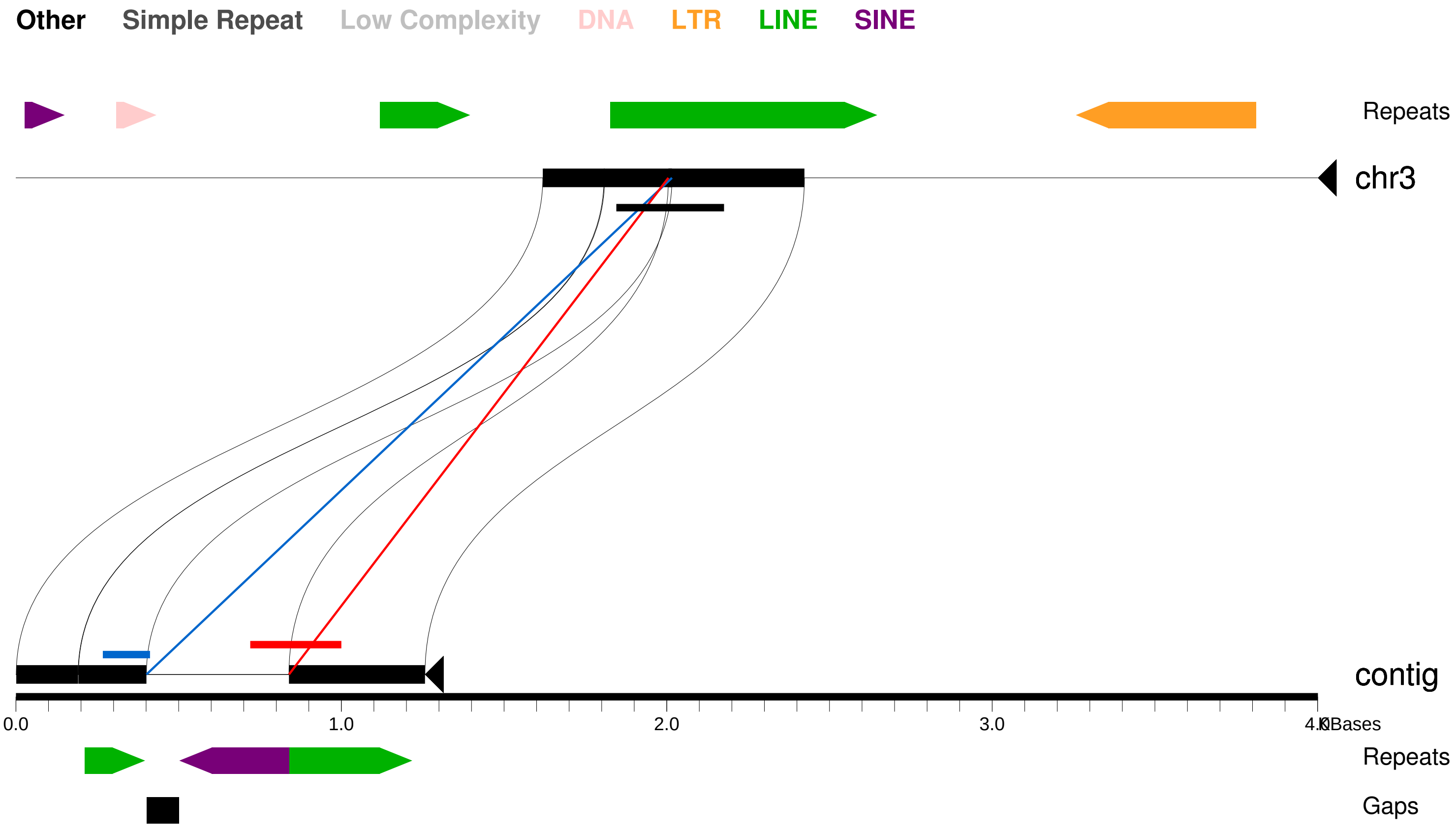

siteID: chr3_61973380 Scafftig1 - Breakpoint: chr3:61973380
chr3:61973335-61973439
Overlap Length: 15 INS size: 195 Genotyped: Yes

```
left     TTT-------------AGATTCCATAATTTGGATTTACAAACTCAATTGTGGCTAAGTAAAA-TTCCTAT
chrom    TTT-------------AGATTCCATAATTTGGATTTACAAACTCAATTGTGGCTAAGTAAAA-TTCCTAT
right    CCTGGGCGACAGAGCGAGACTCCGTCTCAAAAAAAAAAAAAAAAAAAAAAAAAAAAAAAAAAATTCCTAT
         11*1111111111111***1***1*1111111*111*1***111**11111111**11****1*******

left     TTTGTACAAAAAATTAGCCGGGCGCGGTGGCGGGCGCCTGTAGTCCCAGCTACTCGGGAGGCT
chrom    TTTGGTCAGCCATCTAATAAATCAA-----CAATCAC-TTTATTTG--GTTTTTCAA------
right    TTTGGTCAGCCATCTAATAAATCAA-----CAATCAC-TTTATTTG--GTTTTTCAA------
         ****22**222*22**222222*2222222*222*2*2*2**2*2222*2*22**22222222
```

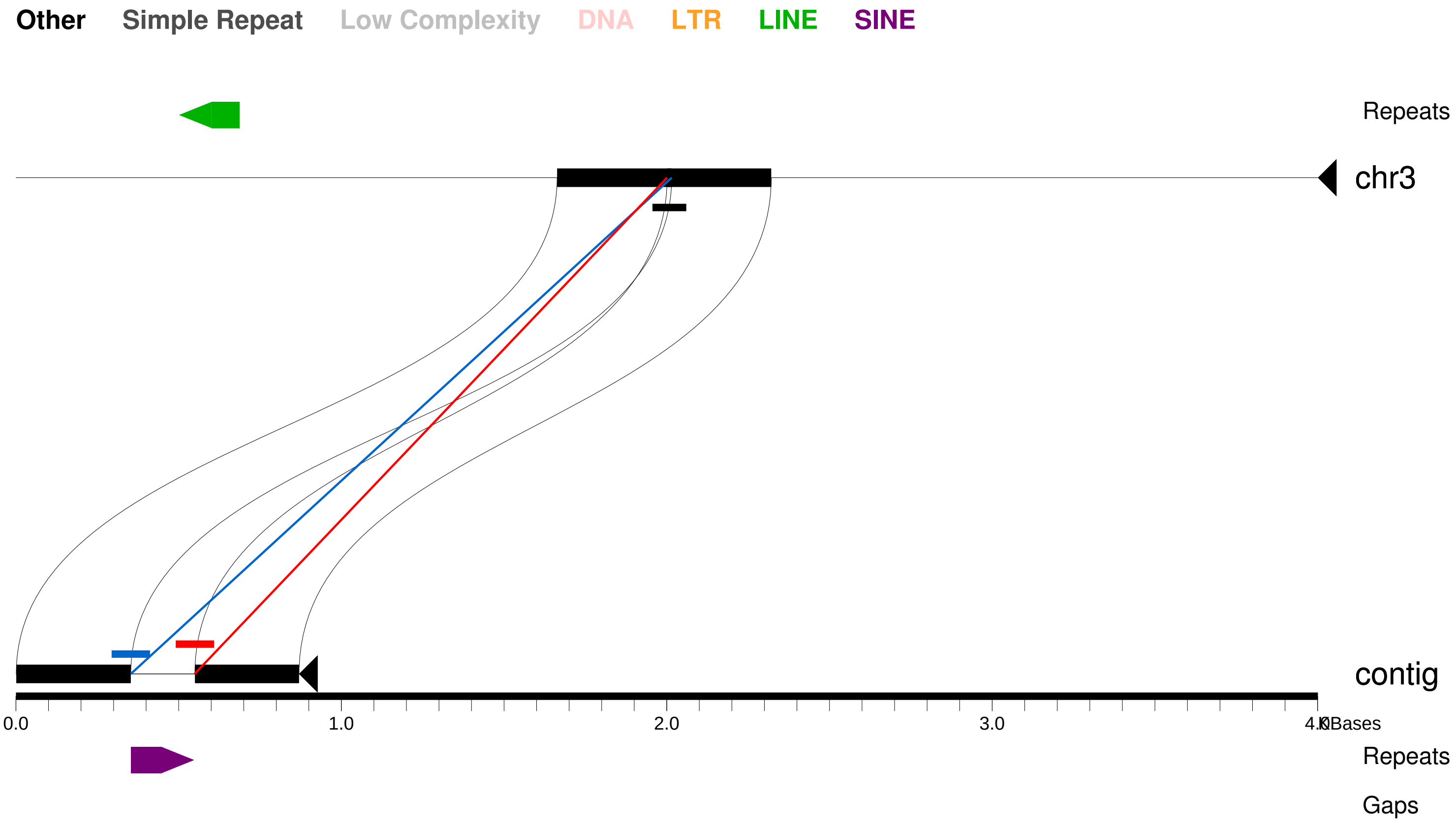

siteID: chr3_63540963 Scafftig2 + Breakpoint: chr3:63540951
chr3:63540906-63541010
Overlap Length: 15 INS size: 297 Genotyped: Yes

```
left   -----------GACATCTAACAGATCCGTTTTAAAGGGATTGAGTTTT---GTTTAGTAAGAAAAACACT
chrom  -----------GACATCTAACAGATCCGTTTTAAAGGGATTGAGTTTT---GTTTAGTAAGAAAAATACT
right  CCACCCGCCTCGGCCTCCCAAAGTGCTGGGATTACAGGCGTGAGCCACCGCGCCCGGCCAGAAAAACACT
       11111111111*1*1**11*1**11*1*111*1*11**11****1111111*1111*11*******N***

left   TCCTTTTTTTTTTGAGACGGAGTCTCGCTCTGTCGCCCAGGCCGGACTGCGGACTGCAGTGGC
chrom  TCCTAAGTATA---AGTTGGAATTTATGAGGTTCGT--------GAAT---GGCTTTATCATC
right  TCCTAAGTATA---AGTTGGAATTTATGAGGTTCGT--------GAAT---GGCTTTATCATC
       ****222*2*2222**22***2*2*2222222***222222222**2*222*2**22*2222*
```

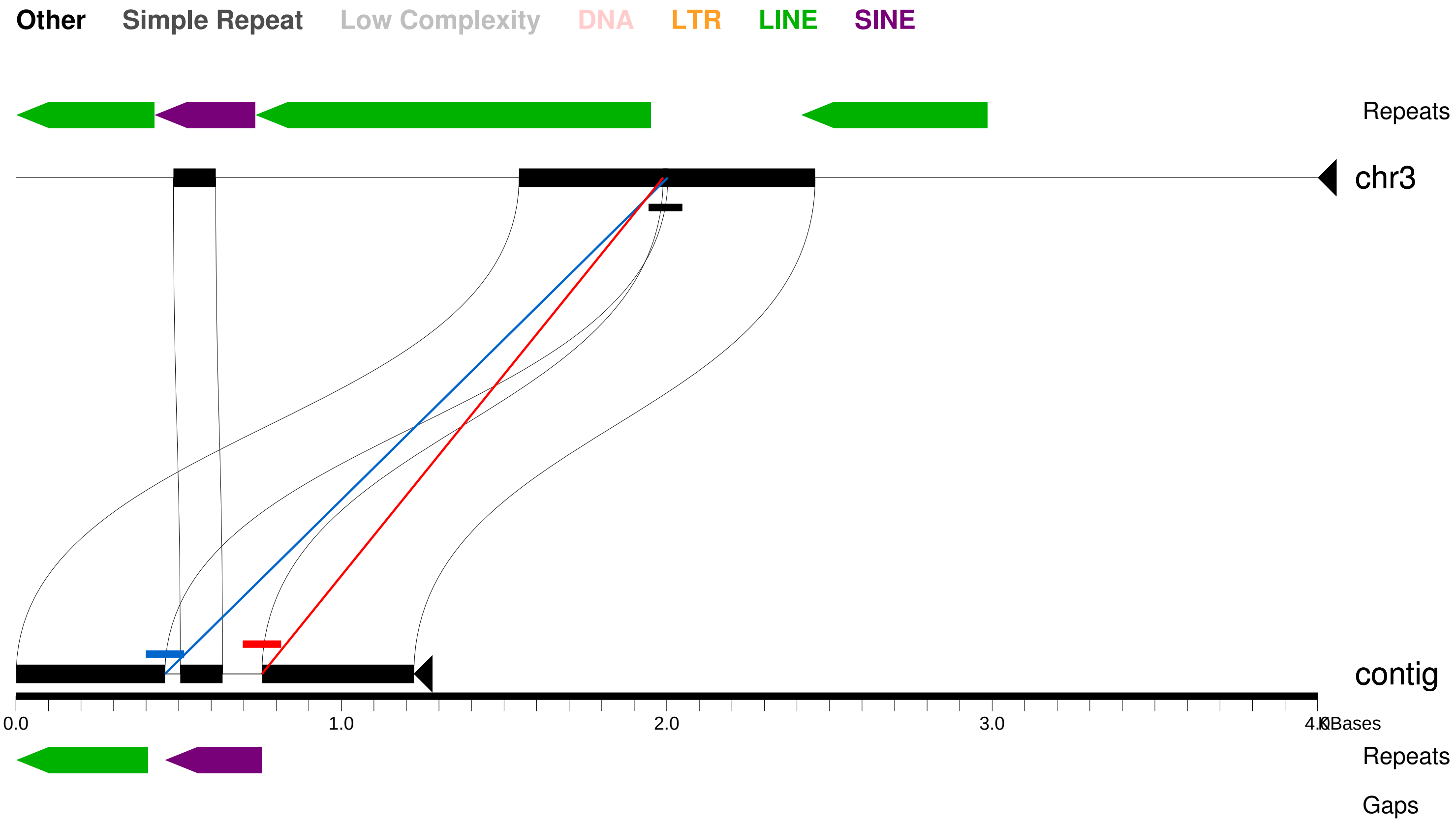

siteID: chr3_6395813 Scafftig1 - Breakpoint: chr3:6395820
chr3:6395774-6395879
Overlap Length: 14 INS size: 392 Genotyped: No

```
left     --AGC--AGAT--TCAAAAAAAGAGTAACTGAGACTCCTATCCGTCT--GACTT------GATCTCAAAC
chrom    --AGC--AGAT--TCAAAAAAAGAGTAACTGAGACTCCTATCCGTCT--GACTT------GATCTCAAAC
right    TTAGCCAGGATGGTCTCGATCTCCTGACCTCATGATCC-ACCCGCCTCGGCCTCCCAAAGGATCTCAAAC
         11***111***11**111*1111111*1**1*111***1*1***1**11*1**1111111**********

left     TCTTNNNNNNNNNNNNNNNNNNNNNNNNNNNNNNNNNNNNNNNNNNNNNNNNNNNNNNNNNNN
chrom    TCTTATCTACTATTCTATCTTCTTAAAGCTGATAATGCTCTGGCAAGTTT-------------
right    TCTTATCTACTATTCTATCTTCTTAAAGCTGATAATGCTCCGGCAAGTTT-------------
         *****2222222222222222222222222222222222N2222222222222222222222
```

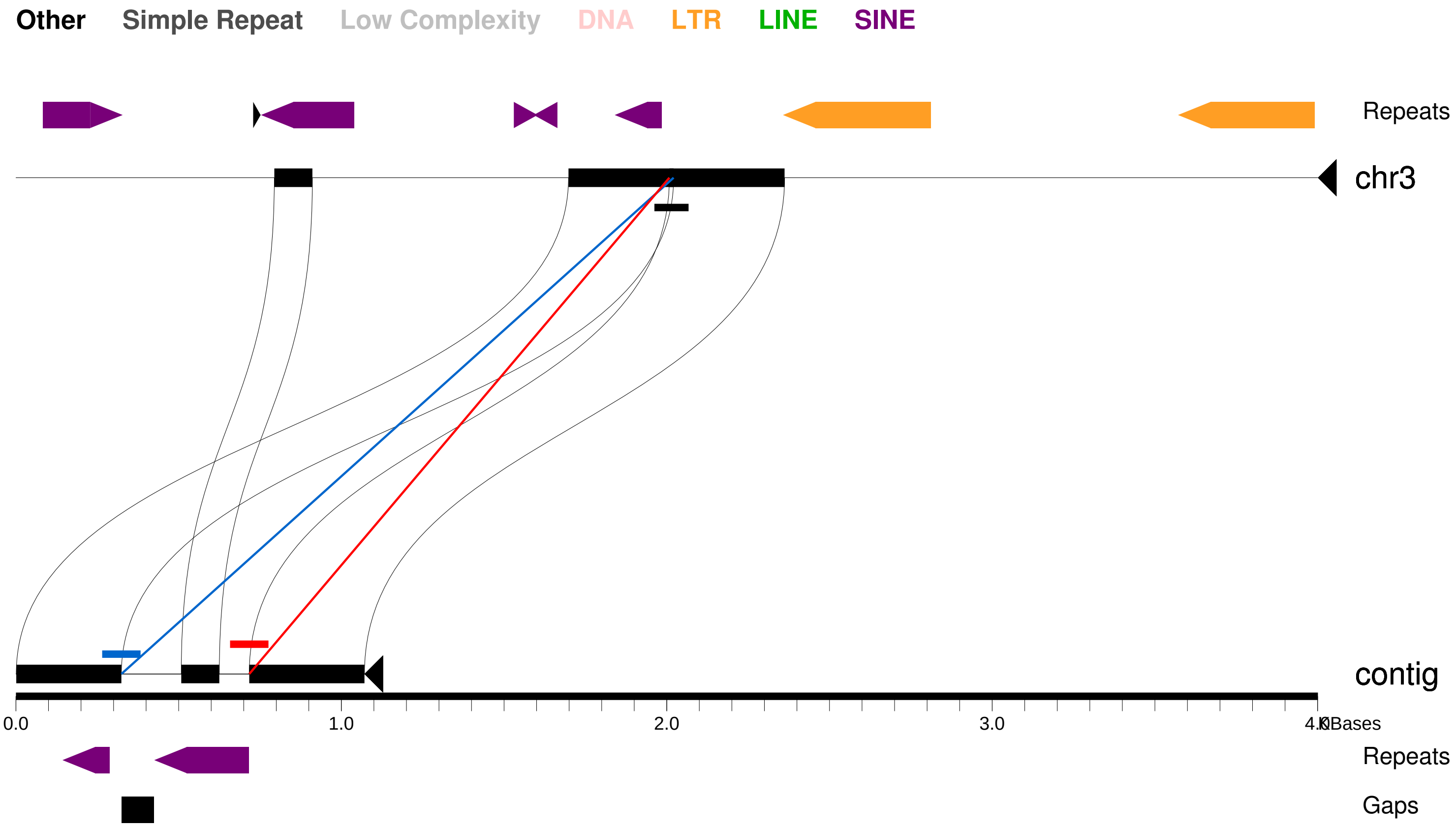

siteID: chr3_6525442 Scafftig1 - Breakpoint: chr3:6525398
chr3:6525352-6525457
Overlap Length: 14 INS size: 386 Genotyped: No

```
left   ACAGA-TATTATTCAG-----TTTCAGTCCCTTAATGT--TTATTT-----TCTTTTTTTGGCCTTCTTT
chrom  ACAGA-TATTATTCAG-----TTTCAGTCCCTTAATGT--TTATTT-----TCTTTTTTTGGCCTTCTTT
right  TCACCGTTTTAGCCGGGATGGTCTCGATCTCCTGACCTCGTGATCCGCCCGCCTCGGCCTGGCCTTCTTT
       1**111*1***11*1*11111*1**11**1*1*1*11*11*1**11111111**11111***********

left   TTTTTTTNNNNNNNNNNNNNNNNNNNNNNNNNNNNNNNNNNNNNNNNNNNNNNNNNNNNNNNN
chrom  TTTAT-------------GAAGTGTTTTTATTTTTTTCATTGTCTTCTATTATGTTTTTACT
right  TTTAT-------------GAAGTGTTTTTATTTTTTTCATTGTCTTCTATTATGTTTTTACT
       ***2*222222222222222222222222222222222222222222222222222222222
```

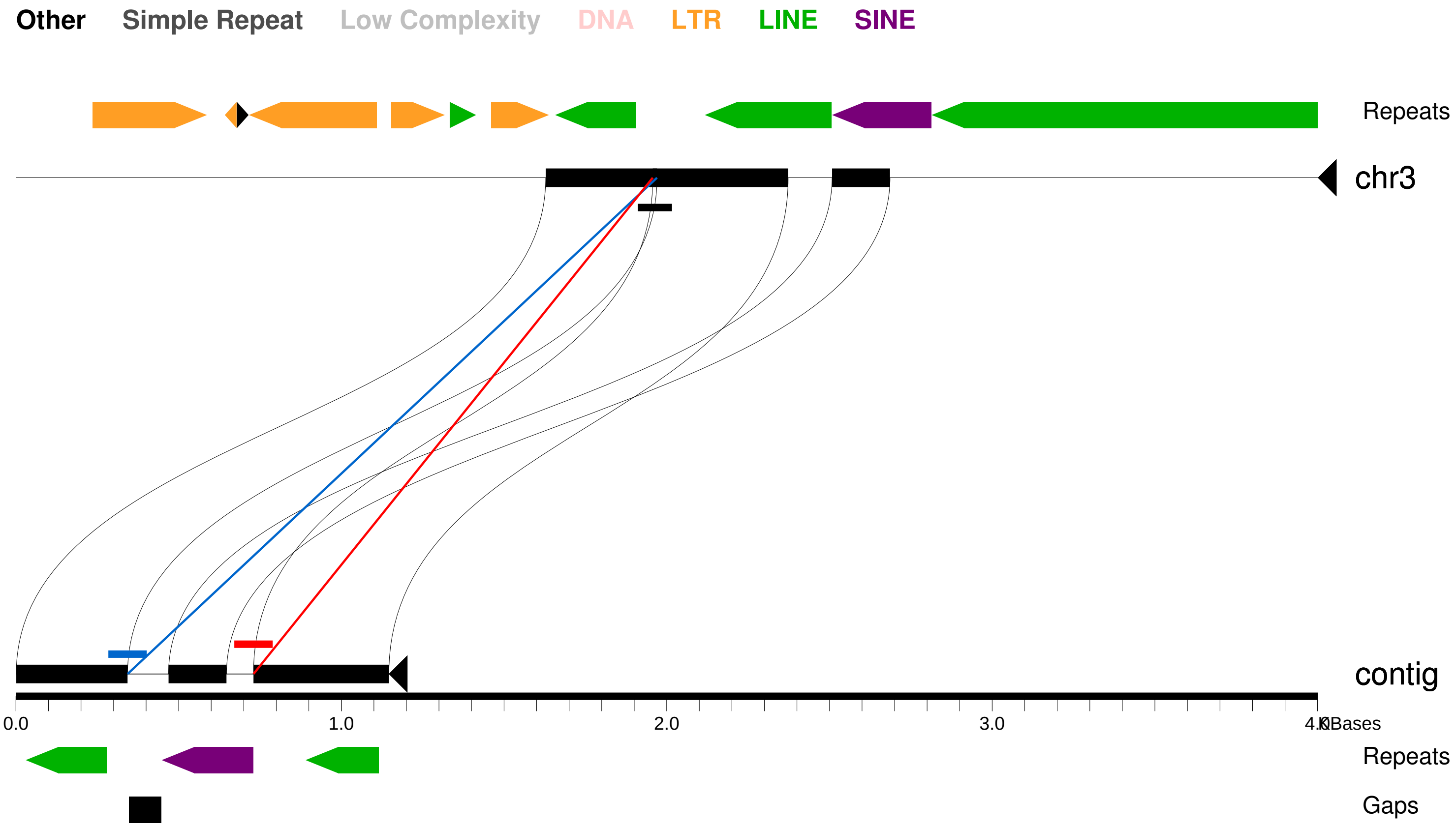

siteID: chr3_6636948 Scafftig1 - Breakpoint: chr3:6636917
chr3:6636883-6636976
Overlap Length: 15 INS size: 430 Genotyped: No

```
left   ----CAGGTGTATGATACCTGCAGCTCTGTTGTTTTTG---------------------CTCATGATTGC
chrom  ----CAGGTGTATGATACCTGCAGCTCTGTTGTTTTTG---------------------CTCATGATTGC
right  TCCGCCCGCCTCAGCCTCCCAAAGTGCTGGGATTACAGGCGTGAGCCACCGCACCCGGCCTCATGATTGC
       1111*11*11*11*111**111**11***111**111*111111111111111111111***********

left   TTTTTTTTTTTTTTTNNNNNNNNNNNNNNNNNNNNNNNNNNNNNNNNNNNNNNNNNNNNNNNNNNNNNNN
chrom  TTTTGCTTTTTTGTTTTATTTGTGGCTCCATATAGATATTAAGATTGTTT---------------------
right  TTTTGCTTTTTTGTTTTATTTGTGGCTCCATATAGATATTAAGATTGTTT---------------------
       ****22*****2***2222222222222222222222222222222222222222222222222222222

left   NNNN
chrom  ----
right  ----
       2222
```

Other Simple Repeat Low Complexity DNA LTR LINE SINE

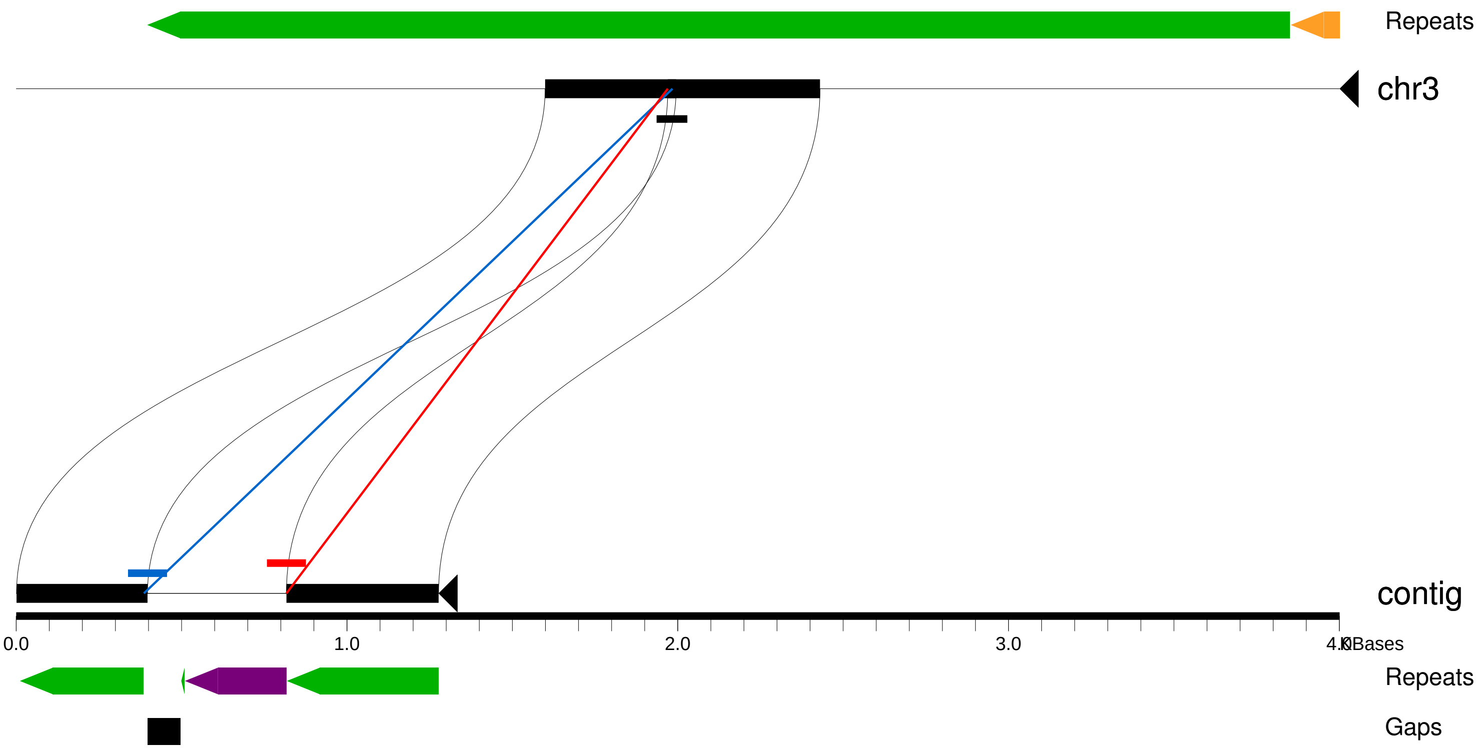

siteID: chr3_66729524 Scafftig1 + Breakpoint: chr3:66729520
chr3:66729465-66729579
Overlap Length: 5 INS size: 405 Genotyped: No

```
left     TTCTGTGGGCACTTTTGTCAGATTCCT----TTTCTGCATCAATTGATATGATCATGTAATTTTNNNNNN
chrom    TTCTGTGGGCACTTTTGTCAGATTCCT----TTTCTGCATCAATTGATATGATCATGTAATTTTTCTTCT
right    CCGCCCGCCTCGGCCTCCCAAAGTGCTGGGATTACAGGCGTGAGCCACCGCGCCCGGCCATTTTTCTTCT
         111111*11111111*11**1*1*1**1111**1*1*11111*111*111111*11*11*****222222

left     NNNNNNNNNNNNNNNNNNNNNNNNNNNNNNNNNNNNNNNNNNNNNNNNNNNNN
chrom    TTAGCCTATTAATATTTCAGATTACAATGACTGATTTTTGAATATTGAA----
right    TTAGCCTATTAATATTTCAGATTACAATGACTGATTTTTGAATATTGAA----
         22222222222222222222222222222222222222222222222222222
```

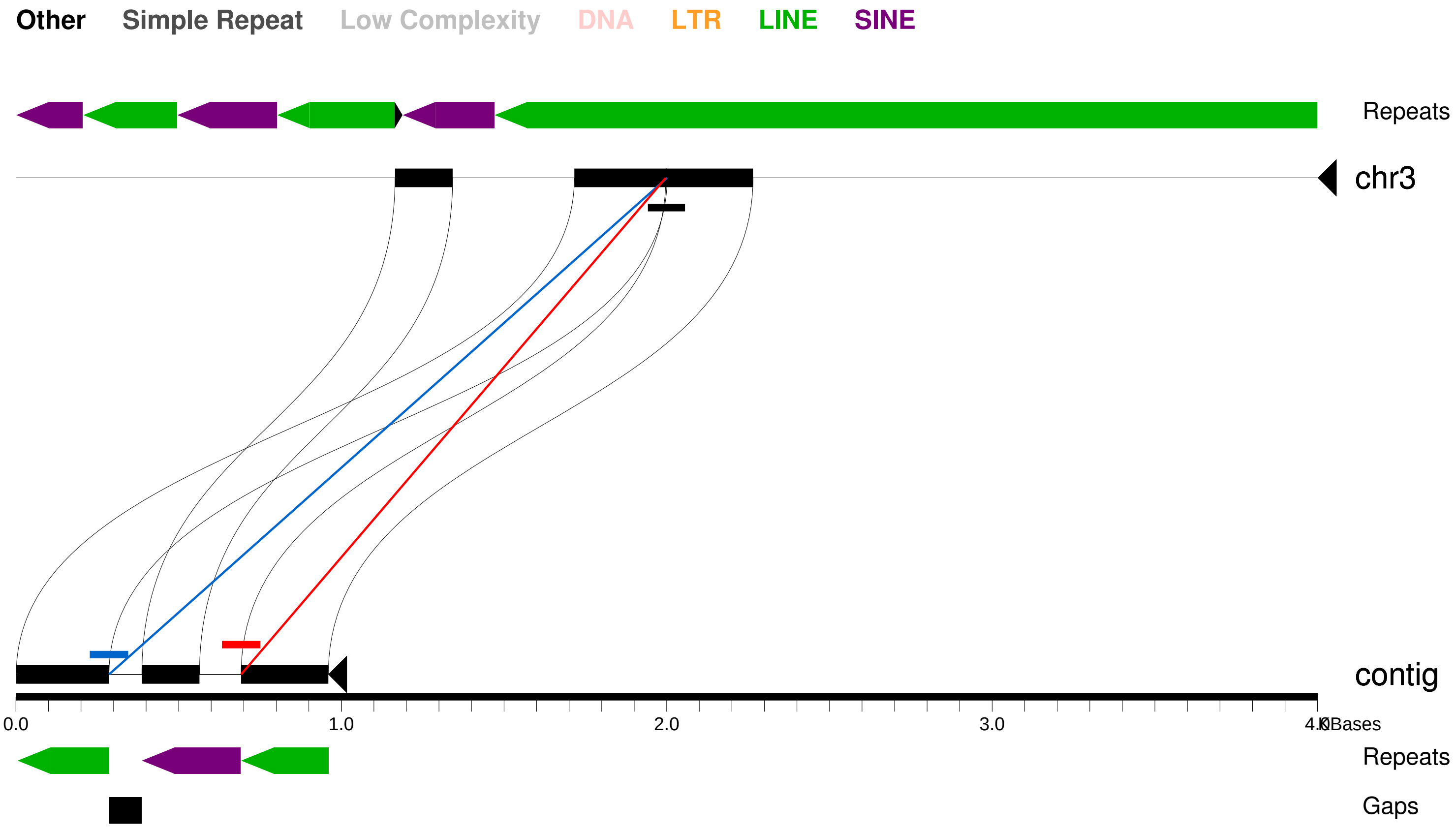

siteID: chr3_66951653 Scafftig1 + Breakpoint: chr3:66951636
chr3:66951091-66951800
Overlap Length: 15 INS size: 440 Genotyped: No

```
left   A---------------------------------------------------------------------
chrom  AGTATCACACTGTCTTGATTACTATAGCTTTGTAGTAAGTTTTGAAGTTGAAAAATGTAAGTTCTGTAAT
right  TTTA---------------------------GTAG-----------------------------------
       1N22                           2222

left   ----------------------------------------------------------------------
chrom  TTTTGTTCTTCTTTTTCAAGATTGTTTTGAAGCTCTTCTGGGTCTCCTTCATTCCTATATGAATATTTGG
right  ------------------AGA----------------CGGGGT----TTCA--CCTT-------------
                         222                2N2222    2222  222N

left   ----------------------------------------------------------------------
chrom  ATAAGTTTGCCAATTTCTGTAAAAAAAAAAAAAACCATCTGGGGTTTTGATCAAGATTTTGTTGAGTCCA
right  ----GTTAGCCAGGAT--------------------------GGTCTCGATC------------------
           222N2222NNN2                          222N2N2222

left   ----------------------------------------------------------------------
chrom  AGATCAATTTGGGAAGTATTGCCATTTTAACAATAGTAAGCCTACTGATCTAGGTACATGCATTGCTTTG
right  ------------------------------------------TCCTGACCT-----CATG----------
                                                 2N2222N22     2222

left   ----------------------------------------------------------------------
chrom  TTGAATATATTCATTGGAAATTCTTTTACAGCAATTCTGTCCCACAGCTTATTTTCTTTCATTCACTTCA
right  --------ATCCACCCG------------------------CCTCGGCCT--------------------
               22N22NNN2                        22N2N22N2

left   ----------------------------------------------------------------------
chrom  AATTATTTCATATCCATCTACTGATTATTTTAAAACTAATATTTTGAGAAAAATCATATGTATTTTCATG
right  ----------------------------------------------------------------------

left   ------CCACTTATATATTTTTCATTTTTATTGAGTGTCTACTATGTTCTCTGCATGTTGTTAAGCATTC
chrom  ACACAACCACTTATATATTTTTCATTTTTATTGAGTGTCTACTATGTTCTCTGCATGTTGTTAAGCATTC
right  -CCCAA---------------------------AGTG-------------CTGG----------------
        2N222111111111111111111111111111****1111111111111***11111111111111111

left   CTGATTTTAGTAATGAGTCTTCTCATTCTTCTGGTCAGTATAGCTAAAGGTTTGTTGATTTTGTTGATCT
chrom  CTGATTTTAGTAATGAGTCTTCTCATTCTTCTGGTCAGTATAGCTAAAGGTTTGTTGATTTTGTTGATCT
right  --GATTACAGGCGTGAGCCACCGCGCCC----GGCC-------------------TGATTTTGTTGATCT
       11****11**111****1*11*1*111*1111**1*1111111111111111111***************

left   NNNNNNNNNNN-----------------------------------------------------------
chrom  TTTAAAAGAACCAACTTTTAGTTTCATATATTTTTTTTGTATTTCCTATTCTCTAGTTCATTAATTTCTG
right  TTTAAAAGAACCAACTTTTAGTTTCATAGATTTTTTTTGTATTTCCTATTCTCTAGTTCATTAATTTCTG
       2222222222222222222222222222N22222222222222222222222222222222222222222

left   ----------------------------------------------------------------------
chrom  CTCTAATCTTTATTATTTCCTTCCTTCTGCCTACTCTAGGTTTAGTTGGCTCTTTTTTCCCCAGTGTCTT
right  CTCTAATCTTTATTATTTCCTTCCTTCTGCCTACTCTAGGTTTAGTTGGCTCTTTTTTCCCCAGTGTCTT
       2222222222222222222222222222222222222222222222222222222222222222222222

left
chrom  AAGGTGGAAA
right  AAGGT-----
       22222
```

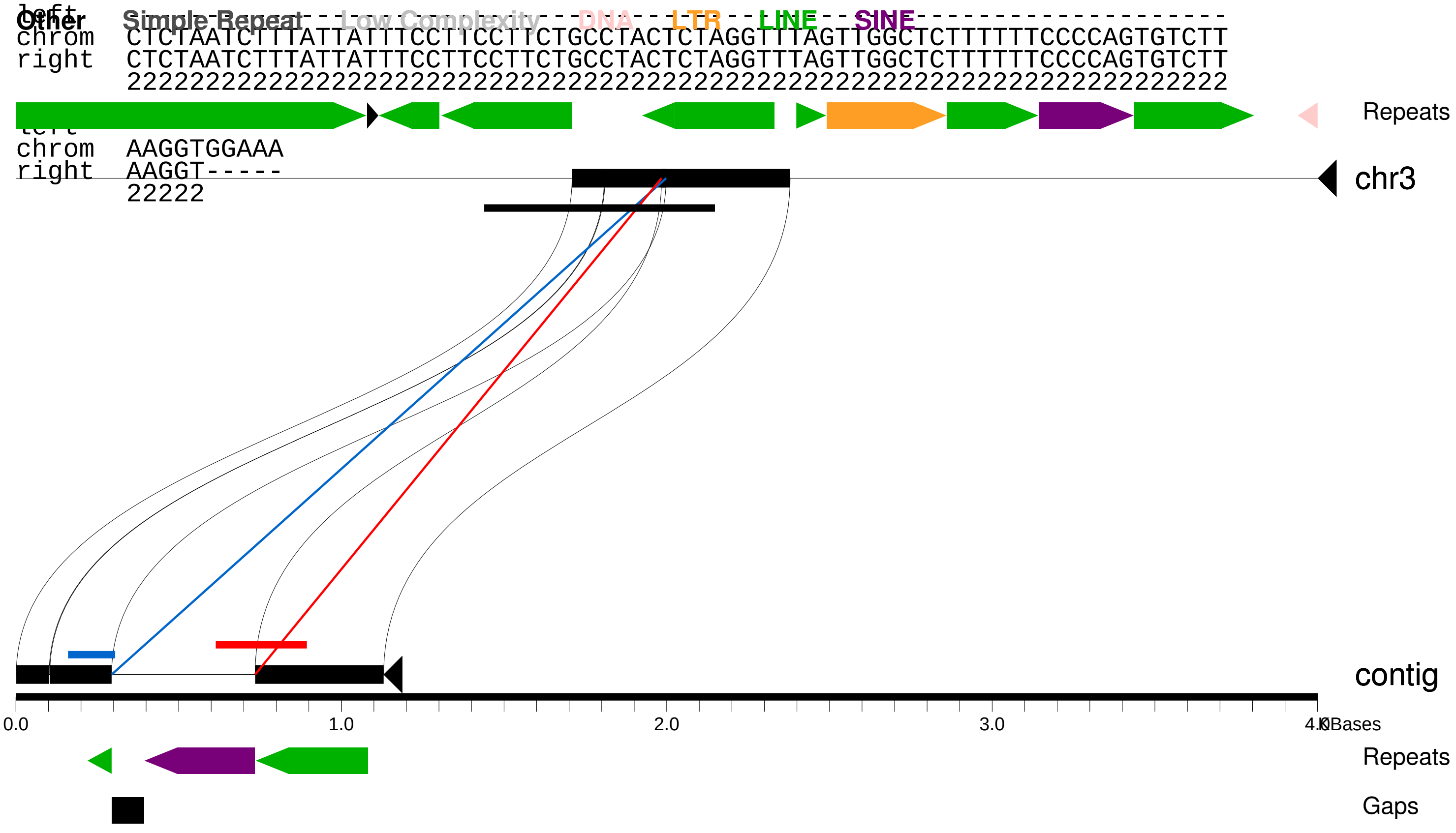

siteID: chr3_67902949 Scafftig1 - Breakpoint: chr3:67902945
chr3:67902785-67903114
Overlap Length: 10 INS size: 313 Genotyped: Yes

```
left   TT----------CCCTATCTCTAGAATTTTCAGATATGTAAGCCAGTAAATTTCCTTGTTGTTTAAGCTG
chrom  TTAATTAGTCTTCCCTATCTCTAGAATTTTCAGATATGTAAGCCAGTAAATTTCCTTGTTGTTTAAGCTG
right  C------GTGAACCCGGGAAGTGGAGCTTGCAG-----TGAGCCG--AGATTGCGCCACTG------CAG
       11     22NNN***111111*1**11**1***11111*1****111*1***1*11111**111111*1*

left   GTTTGAGATGGGTTTTCTGTTCCTTGCCTCCAAAGCCATCAAACTCTCACATGGATATCAAATGTTATGT
chrom  GTTTGAGATGGGTTTTCTGTTCCTTGCCTCCAAAGCCATCAAACTCTCACATGGATATCAAATGTTATGT
right  TCCGCAGTCCGG---------CCTGGGTGACAGAGCGAG---ACTCCGTCTTAAAAAAAAAAA-------
       11111**111**111111111***1*1111**1***1*1111****111*1*11*1*11***11111111

left   TATATGTTAGAATTAGATATAAGAAGATACGGCCGGGCGCGGTGGCTCACGCCT---GTAATCCCAGCAC
chrom  TATATGTTAGAATTAGATATAAGAAGATACACCAGCAATTGAAATATGACAATTACAGAAATGCTATCAT
right  ----------AAAAAAAAAAAAGAAGAAACACCAGCAATTGAAATATGACAATTACAGAAATGCTATCAT
       1111111111**11*1*1*1*******1**22*2*22222*22222*2**222*222*2***2*2*2**2

left   ------TTTGGG-AG-GCCG-AGGCGGGTGG----ATCATGAGGTC--AGGAGATCGAGACCATCC----
chrom  ATGACATTTGCGTAGTGCTGTACAAGGGTTGCCAAAGCATTATGTGTAACAAGCTCCTGATATTTCAGTT
right  ATGACATTTGTGTAGTGCTGTACAAGGGTTGCCAAAGCATTATGTGTAACAAGCTCCTGATATTTCAGTT
       222222****N*2**2**2*2*222****2*2222*2***2*2**222*22**2**22**222*2*2222

left   --------TGGCTA---ACAAGGTGAAACC----CCGTCTCTACT---AAA
chrom  GTAAACTATGGCTATTGAAAAAGAGAAAACATTGCCGAC-CAACATGGACC
right  GTAAACTATGGCTATTGAAAAAGAGAAAACATTGCCGAC-CAACATGGAC-
       22222222*****222*2**2*2****2*2222***2*2*2**2222*2N
```

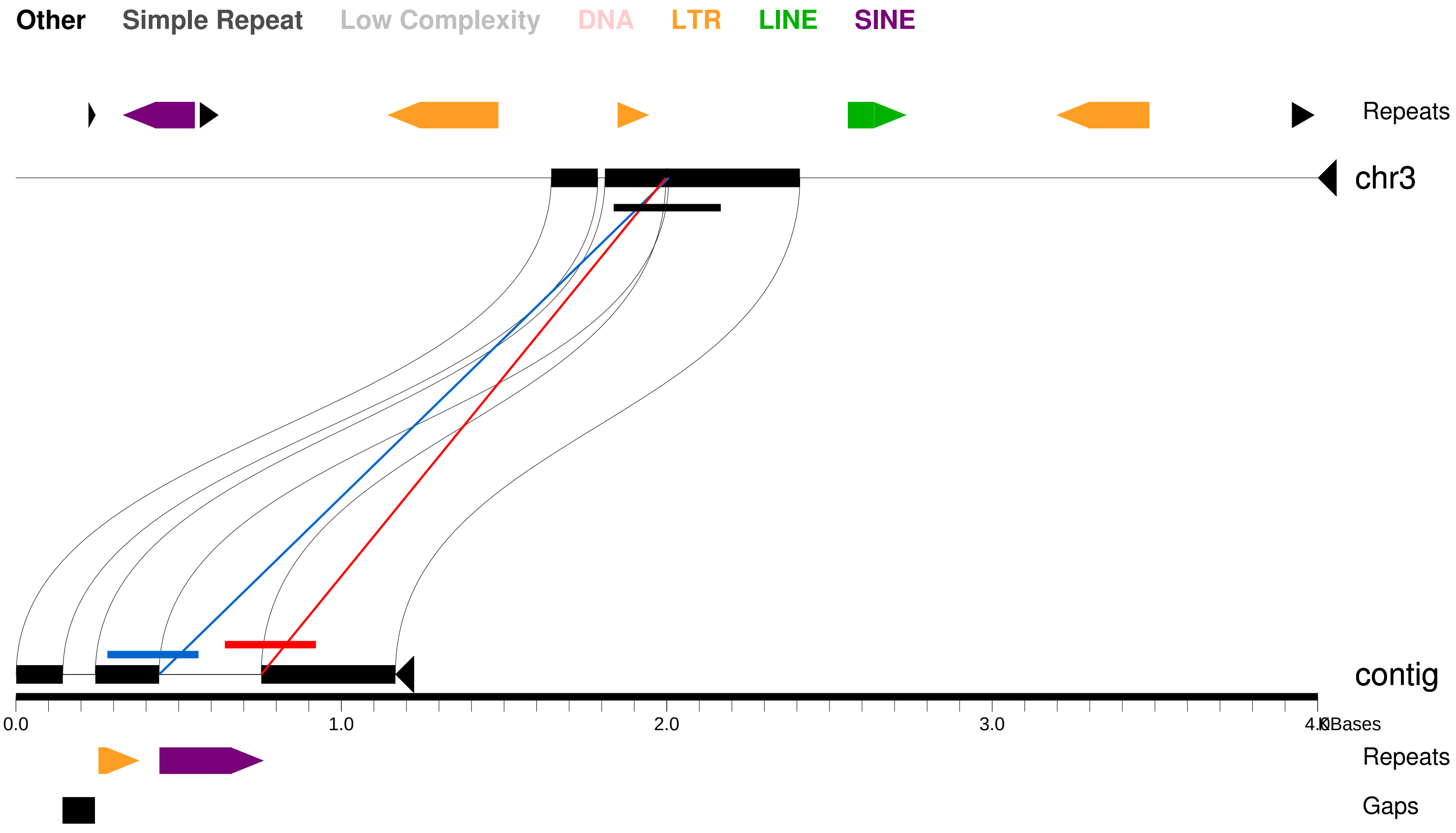

siteID: chr3_68830364 Scafftig1 + Breakpoint: chr3:68830336
chr3:68830297-68830395
Overlap Length: 21 INS size: 435 Genotyped: No

```
left    GACCAAGGGAAATTTACAAAAATAGATCCTTATATTCAA--------------------AAACAAGAAAG
chrom   GACCAAGGGAAATTTACAAAAATAGATCCTTATATTCAA--------------------AAACAAGAAAG
right   NNNNNNNNNNNNNNNNNNNNNNNNNNNNNNNNNNNNNNNNNNNNNNNNNNNNNNNNNNNAAAAAAGAAAG
        11111111111111111111111111111111111111111111111111111111111***1*******

left    GTCCTAAGTCGGCCGGGCGCGGTGGCTCACGCCTGTAATCCCAGCACTTTGGGAGGCCGAGGCGGGCGG
chrom   GTCCTAAGTCAAAAACTTAAGAT---TC---CATATAA-CTTAAAACTATATAAAG-------------
right   GTCCTAAGTCAAAAACTTAAGAT---TC---CATATAA-CTTAAAACTATATAAAG-------------
        **********2222222222*2*222**222*2*2***2*22*22***2*222*2*2222222222222
```

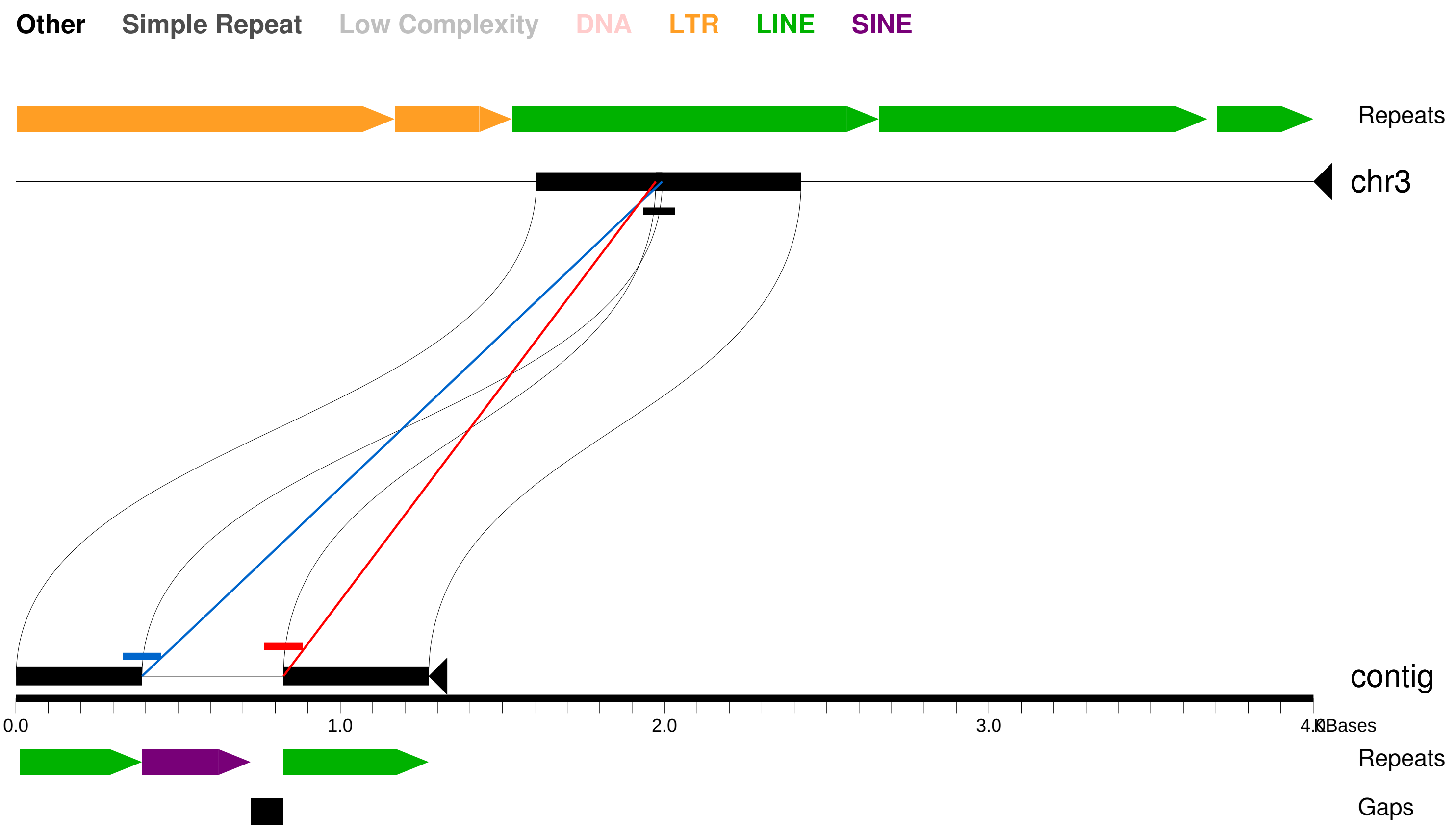

siteID: chr3_7130368 Scafftig13 + Breakpoint: chr3:7130371
chr3:7130326-7130430
Overlap Length: 9 INS size: 307 Genotyped: Yes

```
left     TT--------TCTACCTCTAAAATT------ATCTGAAGCCACAAGGACATTGGAGGTTCACTGATTTTT
chrom    TT--------TCTACCTCTAAAATT------ATCTGAAGCCACAAGGACATTGGAGGTTCACTGATTTCT
right    TCCACCCGCCTCGGCCTCCCAAAGTGCTGGGATTACAGGCGTGAGCCACCGCGCCCGGCCACTGATTTCT
         *111111111**11****11***1*111111**111*1**111*111**111*111*11*********2*

left     TTTTTTTTTTTTTTTTTTGAGACGGAGTCTCGCTCTGTCGCCCAGGCCGGACTGCGGACTGCA
chrom    TATTCATCG---------AAGCACCAGTGGTGCGA-GTTG----GAACGGCAGGGGTAAGAGG
right    TATTCATCG---------AAGCACCAGTGGCGCGA-GTTG----GAACGGCAGGGGTAAGAGG
         *2**22*222222222222**2222***22N**222**2*2222*22***222*2*2*22222
```

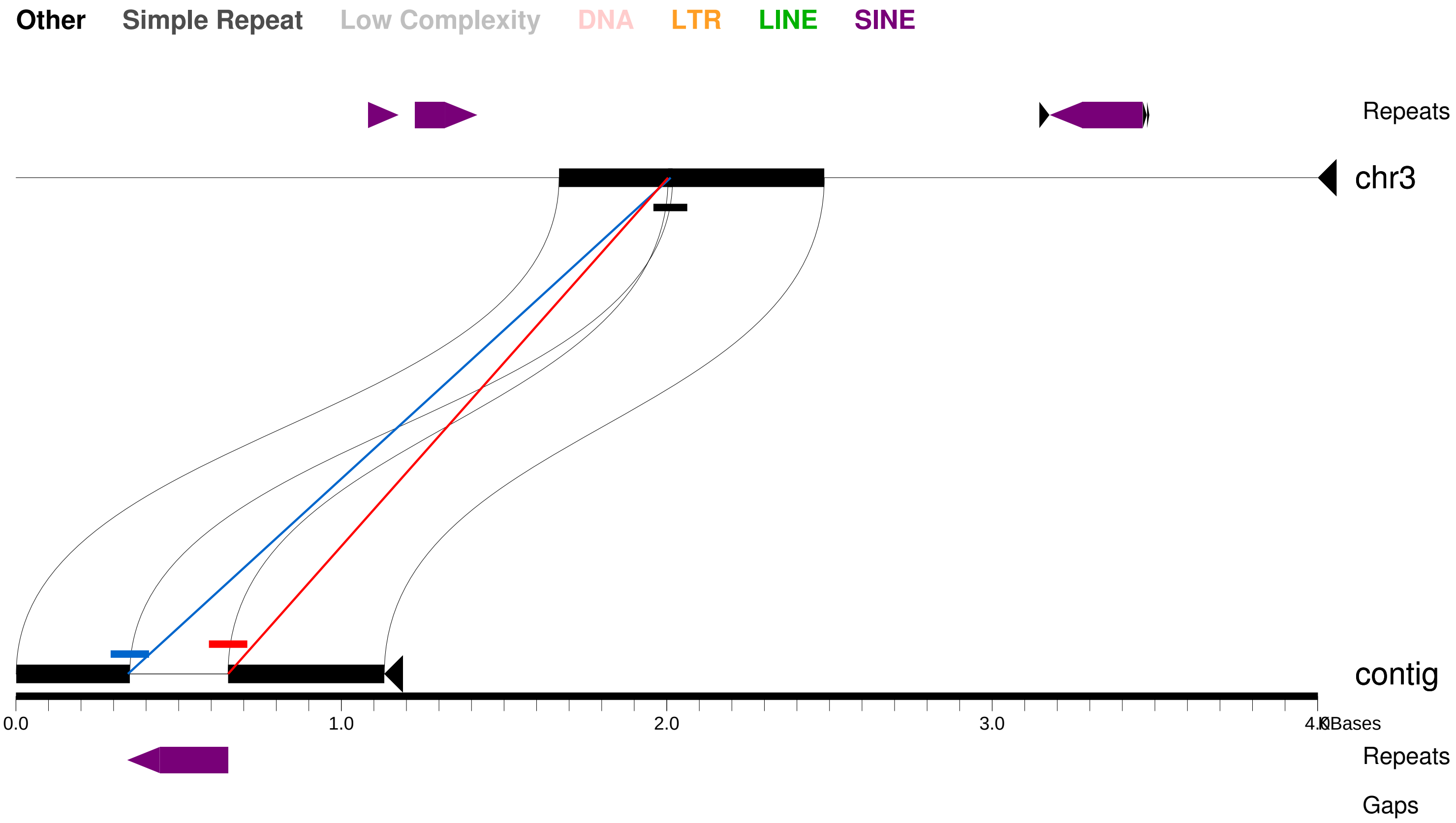

siteID: chr3_76222295 Scafftig4 - Breakpoint: chr3:76222298
chr3:76222250-76222350
Overlap Length: 12 INS size: 295 Genotyped: Yes

```
left    --------------TGCATTTCATTTTGAGC---ATAACTAG-CTTTATGACTTTGGACAAGATACAAAA
chrom   --------------TGCATTTCATTTTGAGC---ATAACTAG-CTTTATGACTTTGGACAAGATACAAAA
right   CGAGATCGCGCCACTGCACTCCAGCCTGGGCGACAGAGCGAGACTCCGTCTCAAAAAAAAAAAAAAAAAA
        11111111111111****1*1**111**1**111*1*1*1**1**111*11*11111*1**1*1*1****

left    TTCTTTGAGGCCGGGCGCGGTGGCTCACGCCTGTAATCCCAGCACTTTGGGAGGCCGAGGCGGGCGG
chrom   TTCTTTGAATCCA----CTTTTATTCATCTTCATAAG-------CTGTTGTAAG-------GATTTA
right   TTCTTTGAATCCA----CTTTTATTCATCTTCATAAG-------CTGTTGTAAG-------GATTTA
        ********22**22222*22*222***222222***22222222**2*2*2*2*2222222*22222
```

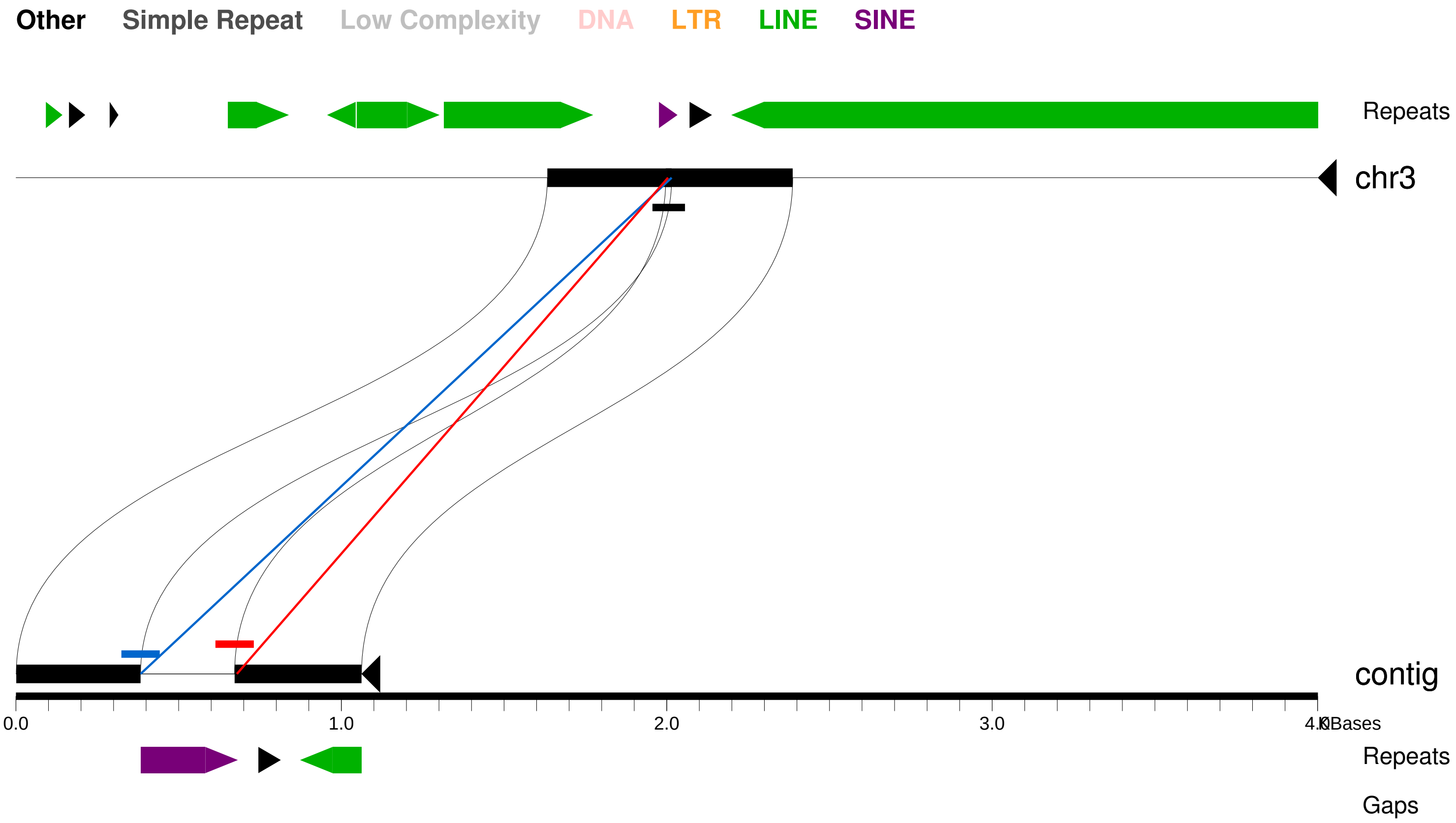

siteID: chr3_76985877 Scafftig5 + Breakpoint: chr3:76985871
chr3:76985711-76986043
Overlap Length: 13 INS size: 307 Genotyped: Yes

```
left   -------------TATTTGTGGGAGCCAACACATTACAATTACCACTAACATGTTAACCTTAGTAGGTTT
chrom  CTGATAAATTAATTATTTGTGGGAGCCAACACATTACAATTACCACTAACATGTTAACCTTAGTAGGTTT
right  --GGAGAATGGCGTGAACCCGGGAGGCGGAGC-TTGCA--------------GTGAGCCG-AG-------
         2NNN222NNNN*111111*****1*1111*1**1**11111111111111**1*1**11**1111111

left   TCTTTATAAATACACATGTCTACTATATATAAATGTTTCCAGATATGTAATTGGGATCATTCTTATAACA
chrom  TCTTTATAAATACACATGTTTACTATATATAAATGTTTCCAGATATGCAATTGGGATCATTCTTATAACA
right  -----ATCCCGCCAC-----TGCACTCCAGCCTGGGCGACAGA---GCGA---GACTCCGTCTCAAAAAA
       11111**11111***1111N*1*11*11*11111*1111****111*21*111*11**11***1*1**1*

left   CTGATTGAGAAACTGGTTCTAAGAATTCATTTTGGCCGGGC--GCGGTGGCTC-------ACGCCTGTAA
chrom  CTGATTGAGAAACTGGTTCTAAGAATTCATTTTATTCACAACAGCAGTGGATATTTTTAGACTACAG-AG
right  AAAAAAGAAAAGAAAAAAAAAAGAATTCATTTTATTCACAACAGCAGTGGATATTTTTAGACTACAG-AG
       111*11**1**111111111************222*222222**2****2*22222222**22*2*2*2

left   TCCCAGCACTTTGGGAGGCCGAGGCGG-------------------GCGGATCACG--AGGTCA----GG
chrom  TAATTGCATTTTGAGTCTTTAACTCAATTTTACTTTATTACATTCTGCCTTTAACCTTAGTTCCTTTTGT
right  TAATTGCATTTTGAGTCTTTAACTCAATTTTACTTTATTACATTCTGCCTTTAACCTTAGTTCCTTTTTT
       *2222***2****2*222222*22*2222222222222222222222**222*2**222**2**2222212

left   AGATCGAGACCATCCTGGCTAA-------CACGGTGA--AACCCCGTCTCTACTAAA
chrom  TAATTGAAAC--ACCTAGTTAAATATATACATTCTTATGAAGACATTTGTTTCTGA-
right  TAATTGAAAC--ACCTAGTTAAATATATACATTCTTATGAAGACA------------
       22**2**2**222***2*2***2222222**222*2*22**22*2N1NNN1N11N12
```

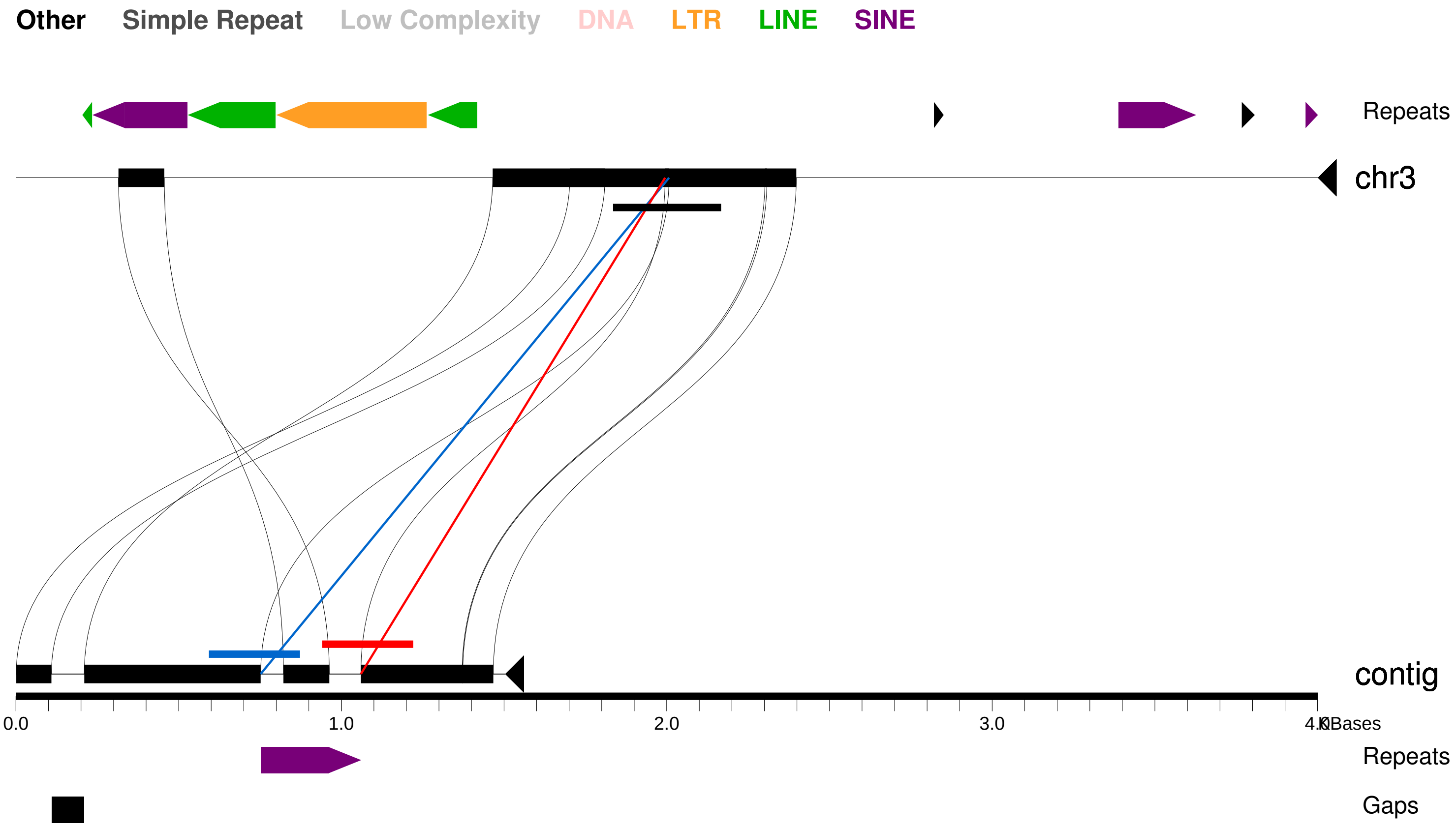

siteID: chr3_80964885 Scafftig4 - Breakpoint: chr3:80964876
chr3:80964823-80964935
Overlap Length: 7 INS size: 302 Genotyped: Yes

```
left   ---ATACCCAAATAAGGCT-----GAGAATTGTAGTTTACATATAAAAATGTAATGGGTTTAAAATTTGG
chrom  ---ATACCCAAATAAGGCT-----GAGAATTGTAGTTTACATATAAAAATGTAATGGGTTTAAAATTTAA
right  TGCAGTCCGCAGTCCGGCCTGGGCGACAGAGCGAGACTCCGTCTCAAAAAAAAAAAAAA--AAAATTTAA
       111*11**11*1*11***111111**1*11111**11*1*1*1*1****111**1111111*******22

left   --CCGGGCGCGGTGGCTCACGCCTGTAATCCCAGCACTTTGGGAGGCCGAGGCGGGTGG
chrom  TACCATCCACA-TAATTAATTCA--AAAACCAAACA---TAGCTGTAAAAATTAAAT--
right  TACCATCCACA-TAATTAATTCA--AAAACCAAACA---TAGCTGTAAAAATTAAAT--
       22**222*2*22*222*2*22*2222**2**2*2**222*2*22*2222*222222*22
```

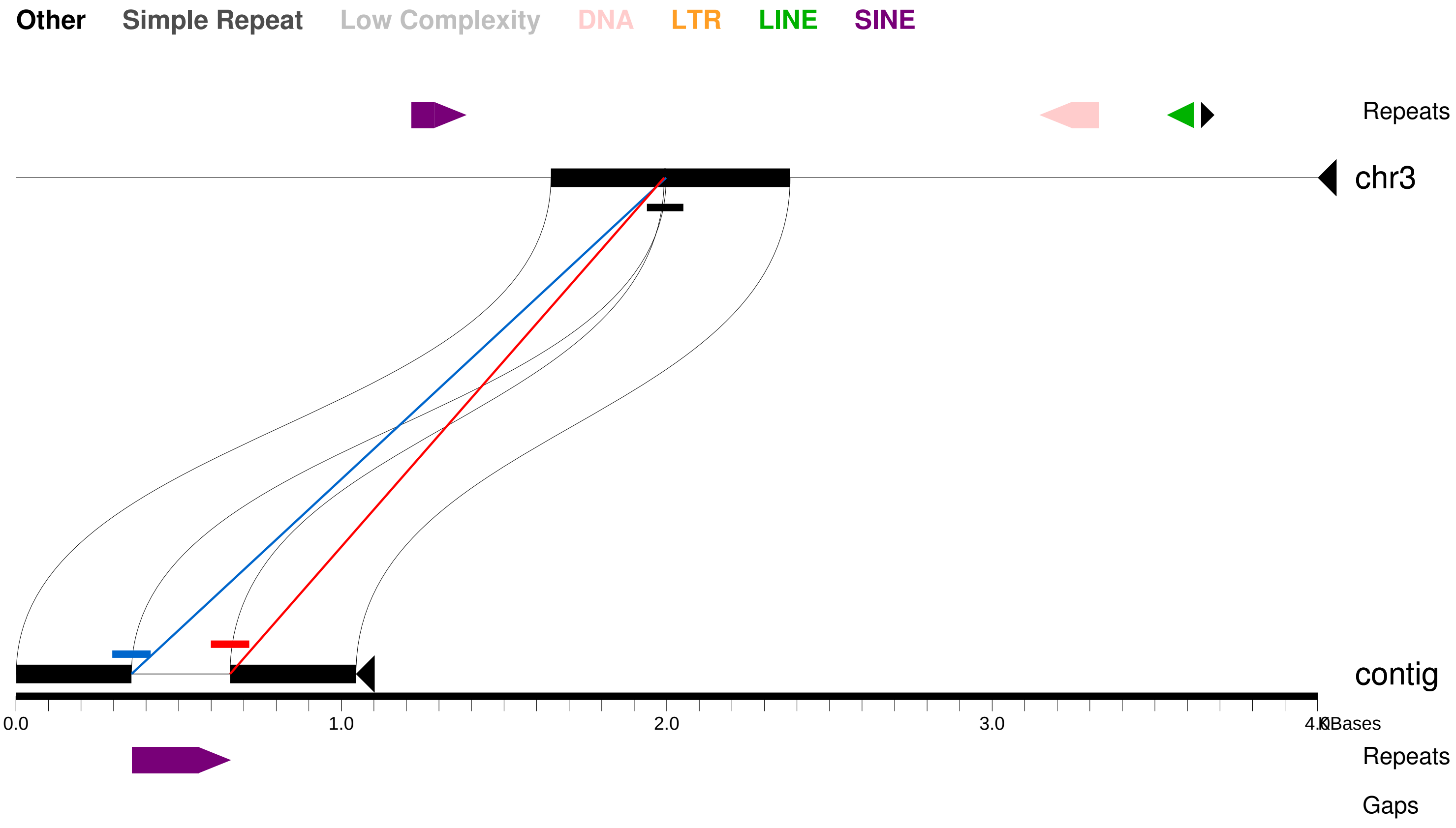

siteID: chr3_82056153 Scafftig1 + Breakpoint: chr3:82056135
chr3:82056086-82056194
Overlap Length: 11 INS size: 423 Genotyped: No

```
left   AGGGCACA-----GAAATATAAATTATAG--ACTAAATCCGCTTTACATCCTG---GATATGAATATCTT
chrom  AGGGCACA-----GAAATATAAATTATAG--ACTAAATCCGCTTTACATCCTG---GATATGAATATCTT
right  TCCGCCCGCCTCGGCCTCCCAAAGTGCTGGGATTACAGGCGTGAGCCACCGCGCCCGGCATGAATATCTT
       111**1*111111*111111***1*111*11*1**1*11**11111**1*11*111*11***********

left   TTTTTTNNNNNNNNNNNNNNNNNNNNNNNNNNNNNNNNNNNNNNNNNNNNNNNNNNNN
chrom  AAT----------AAAATATCAAGAAAACAAATTAAAGGTTAGATTTTAAAATCGTTTG
right  AAT----------AAAATATCAAGAAAACAAATTAAAGGTTAGATTTTAAAATCGTTTG
       22*222222222222222222222222222222222222222222222222222222222
```

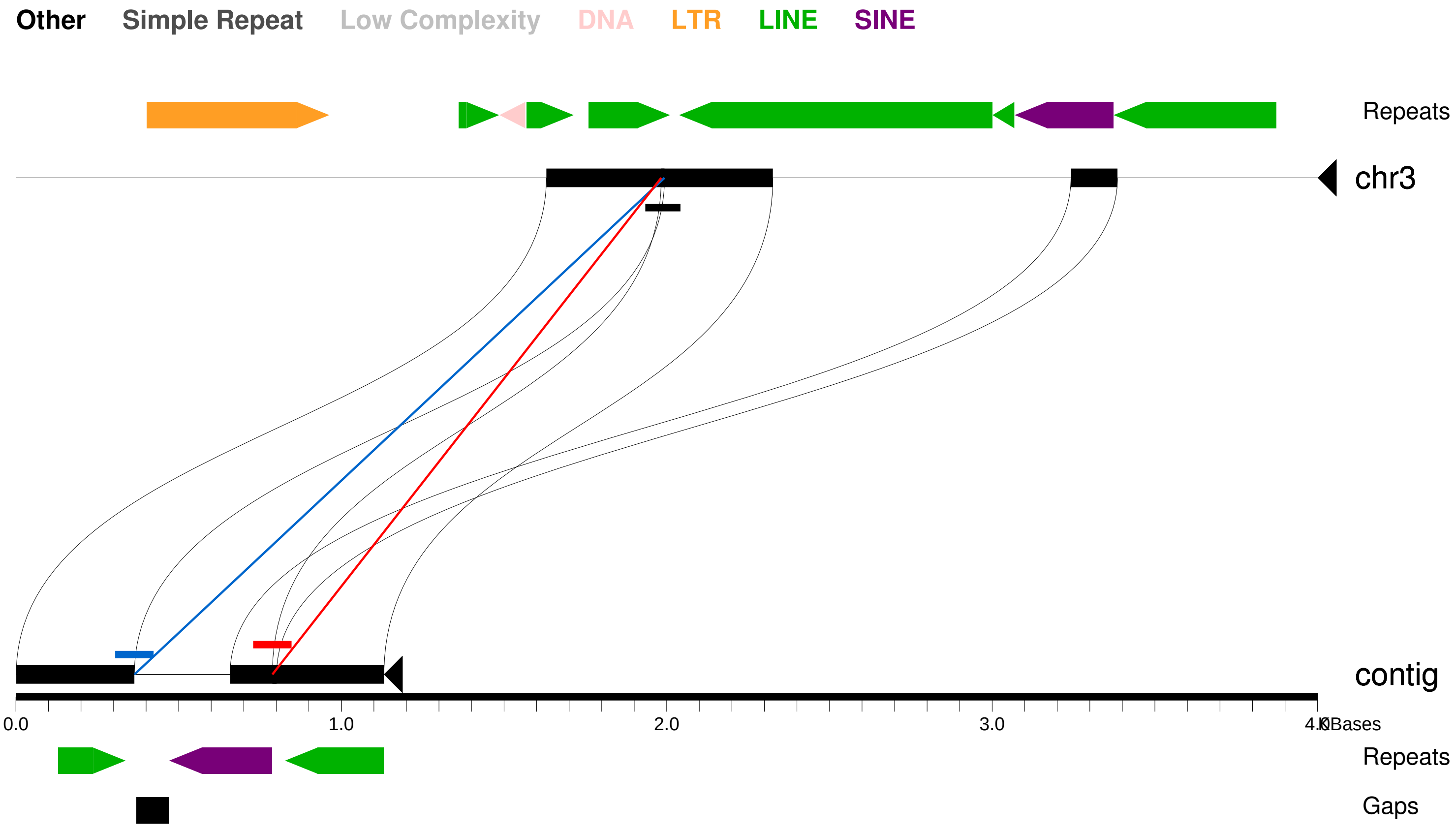

siteID: chr3_82794977 Scafftig1 + Breakpoint: chr3:82794981
chr3:82794937-82795040
Overlap Length: 16 INS size: 432 Genotyped: No

```
left    CCACAGTGCAATACAAATAGAAATCAAAGCTACAAATGTCTCTT---------------AAAACCACACA
chrom   CCACAGTGCAATACAAATAGAAATCAAAGCTACAAATGTCTCTT---------------AAAACCACACA
right   NNNNNNNNNNNNNNNNNNNNNNNNNNNNNNNNNNNNNNNNNNNNNNNNNNNNNNNNNNNAAAAAAACCACACA
        11111111111111111111111111111111111111111111111111111111111***********

left    ATTACGGCCGGGCGCGGTGGCTCA--CGCCTGTAATCCCAGCACTTTGGGAGGCCGAGGCGGGCGG
chrom   ATTACAA--------GGAAACTAAACCACCTGTTTCTGAATGACTTC--TAGGTAAATA-------
right   ATTACAA--------GGAAACTAAACCACCTGTTTCTGAATGACTTC--TAGGTAAATA-------
        *****2222222222**222**2*22*2*****222222*22****2222***222*222222222
```

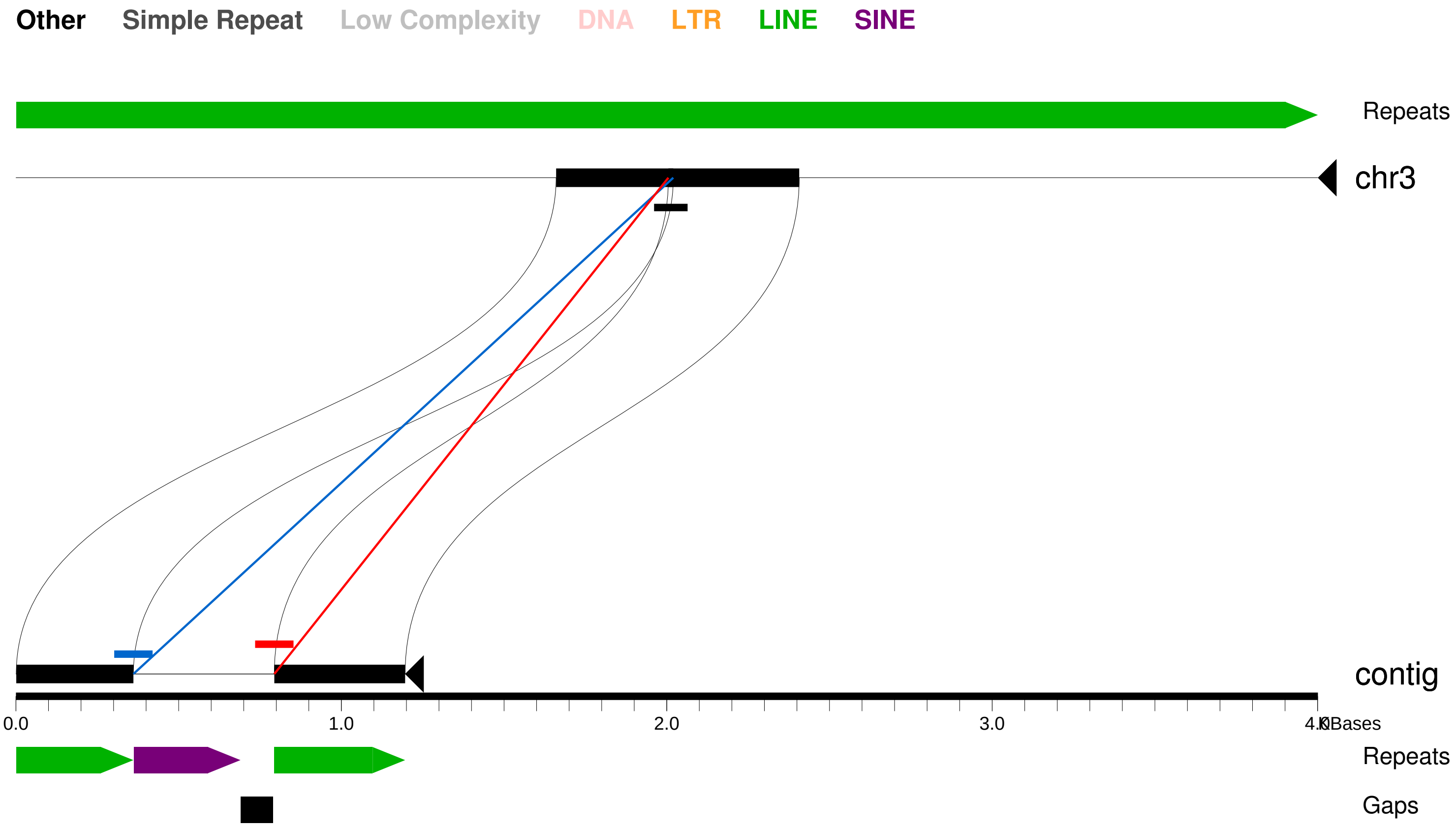

siteID: chr3_83202799 Scafftig1 + Breakpoint: chr3:83202789
chr3:83202631-83202964
Overlap Length: 16 INS size: 264 Genotyped: No

```
left   NNNNNNNNNNNNNNNNNNNNNNNNNNNNNNNNNNNNNNNN-------------AAATAGTGATATATCTA
chrom  TGTGTTACATAAATGAAGCATAACAAAAAATATGATTGGATAAAATAGTTATTAAATAGTGATATATCTA
right  NNNNNNNNNNNAA-----------AAAAAA----------------------------------------
       NNNNNNNNNNN22NNNNNNNNNNN222222NNNNNNNNNN             11111111111111111

left   AATTTAAACATTAGTCATGATGCAAAATAAAAGAAAGCCTAAAGTACAATATCTGAACATTGAAGCTTGC
chrom  AATTTAAACATTAGTCATGATGCAAAATAAAAGAAAGCCTAAAGTACAATATCTGAACATTGAAGCTTGC
right  ----------------------------------------------------------------------
       1111111111111111111111111111111111111111111111111111111111111111111111

left   AGGTGTTTCCCTAAAGACAAAAATGACAGAAGAGGCCGGGCGCGGTGGCT--CACGCC-TGTA-ATCCCA
chrom  AGGTGTTTCCCTAAAGACAAAAATGACAGAAGAGTCAGTGACCTGATAATTGCACCCCATGAAGATCAAA
right  ------------------AAAAATGACAGAAGAGTCAGTGACCTGATAATTGCACCCCATGAAGATCAAA
       111111111111111111***************2*2*2*22*2*2222*22***2**2**2*2***22*

left   GCACTTTGGGAGGCCGAGGCGGG-CGGATCAC-GAGG-------------TCAGGA----GATCGAGACC
chrom  GACCT--GAAAGATAAAAACGGATCCTCTCACTGAGCCTAAACTGGTCCTTCAGGACAGTGATTGACAAA
right  GACCT--GAAAGATAAAAACGGATCCTCTCACTGAGCCTAAACTGGTCCTTCAGGACAGTGATTGACAAA
       *22**22*22**2222*22***22*222****2***22222222222222*****2222***2**2*22

left   ATCCCGGCTAAAACGGTGAA----ACCCCGTCTCTA-------CTAAA--------
chrom  ATTCAGACACACTTTGGGAAGATAATGAGGTCACCAGAGAGGACTGAGCATGCTTG
right  ATTCAGA-------------------------------------------------
       **2*2*21NN1NNNN1N111    1NNNN111N1N1       11N1N
```

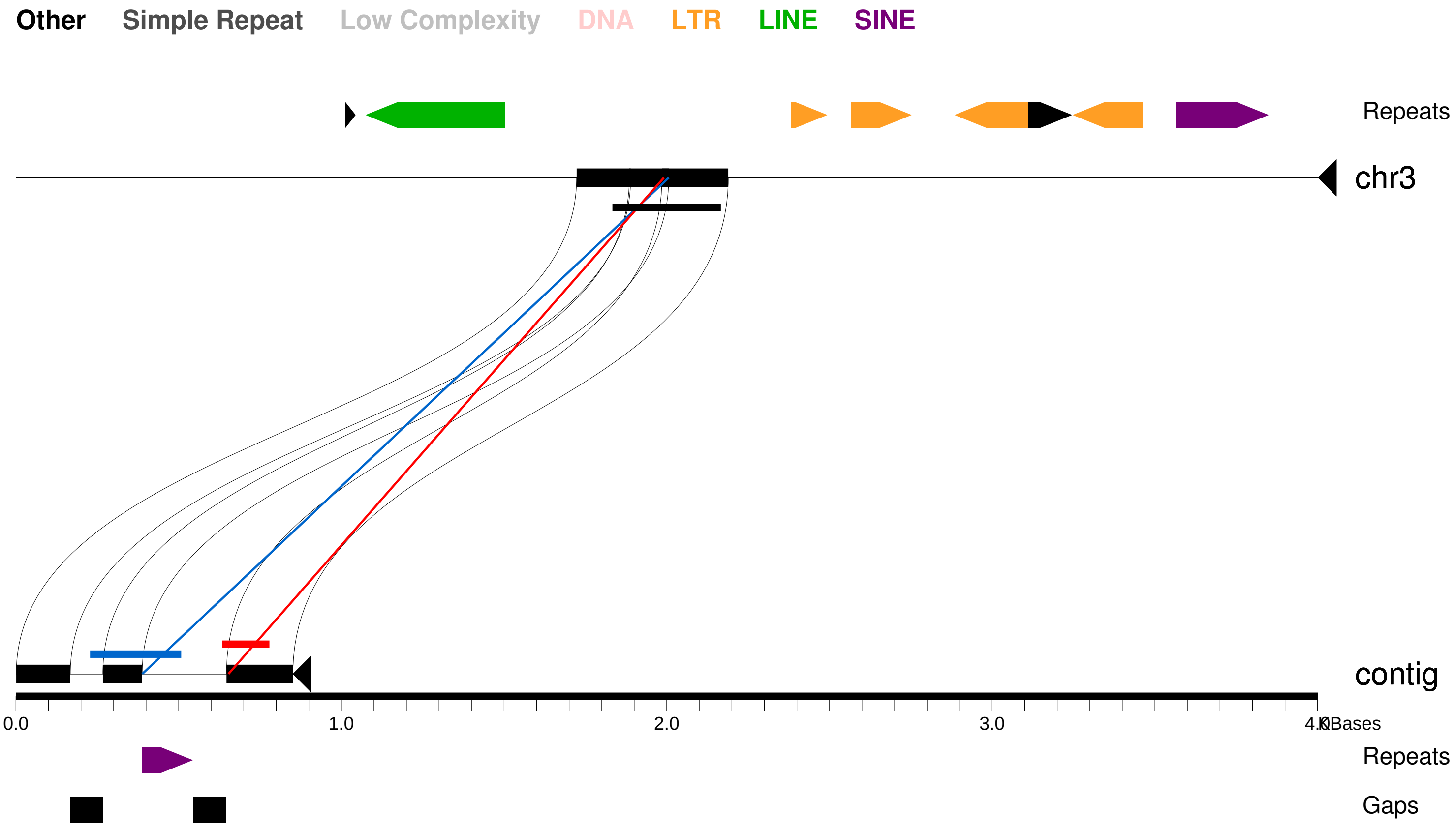

siteID: chr3_84845477 Scafftig1 - Breakpoint: chr3:84845506
chr3:84845346-84845674
Overlap Length: 9 INS size: 316 Genotyped: No

```
left    ----------------------------------------------------------CTTTAAATTATG
chrom   GAATAGCCAAAGCTACCCTGAACAAAAAGAGCAAAACTAGAGGAATCACATTACCTGACTTTAAATTATG
right   TT-TAGTAGAGACGGGGTTTCACCGTGTTAGCCAGGAT---GGTCTCG-ATCTCCTGACCTCG--TGATC
        NN 222NNN2NN2NNNNN2NN22NNNNNN222N2NNN2   22NN22N 22NN22222*1*1111*1**1

left    CTATAGAGCTATAGTAACCAAACCAGTGTGGTACTGGCATAAAAACGGACAAGTAGACCAATGGAGCAGG
chrom   CTATAGAGCTATAGTAACCAAACCAGTGTGGTACTGGCATAAAAACCGACAAGTAGACCAATGGAGCAGG
right   CGCCCGC-CTCGGCCTCCCAAA--------GTGCTGGGATTACAGGCGT------GAGCCACCGCGCCCG
        *1111*11**1111111*****11111111**1****1**1*1*112*1111111**1*1*11*1**11*

left    TCCATACACCTACAGTGAGCTCATTTTTG-----------------------------------------
chrom   TCCATACACCTACAGTGAGCTCATTTTTGACAATGGTGCCAAGAACATACATCAGGGAAAGGACAGTCTC
right   GCC-----------------TCATTTTTGACAATGGTGCCAAGAACATACATCAGGGAAAGGACAGTCTC
        1**11111111111111111********22222222222222222222222222222222222222222

left    ------------TTTT------------------------------------TTTTTTTTT---------
chrom   CTCAATAAGTGGTGTTGGTAAAAATAGATATGCAGAAGAATGAAAATAGACCTCTATCTCTCACCATATA
right   CTCAATAAGTGGTGTTGGTAAAAATAGATATGCAGAAGAATGAAAATAGACCTCTATCTCTCACCATATA
        222222222222*2**222222222222222222222222222222222222*2*2*2*2*222222222

left    ---------------------TT------TTTTTTTTTNNNNNNNNNNN
chrom   CAATAATCAAATCAAAATGGATTACATACTTAAATCTAAGACCTCAAAC
right   CAATAATCAAATCAAAATGGATTACATACTTAAANNNNNN---------
        222222222222222222222**222222**2221N1NNNNNNNNNNNN
```

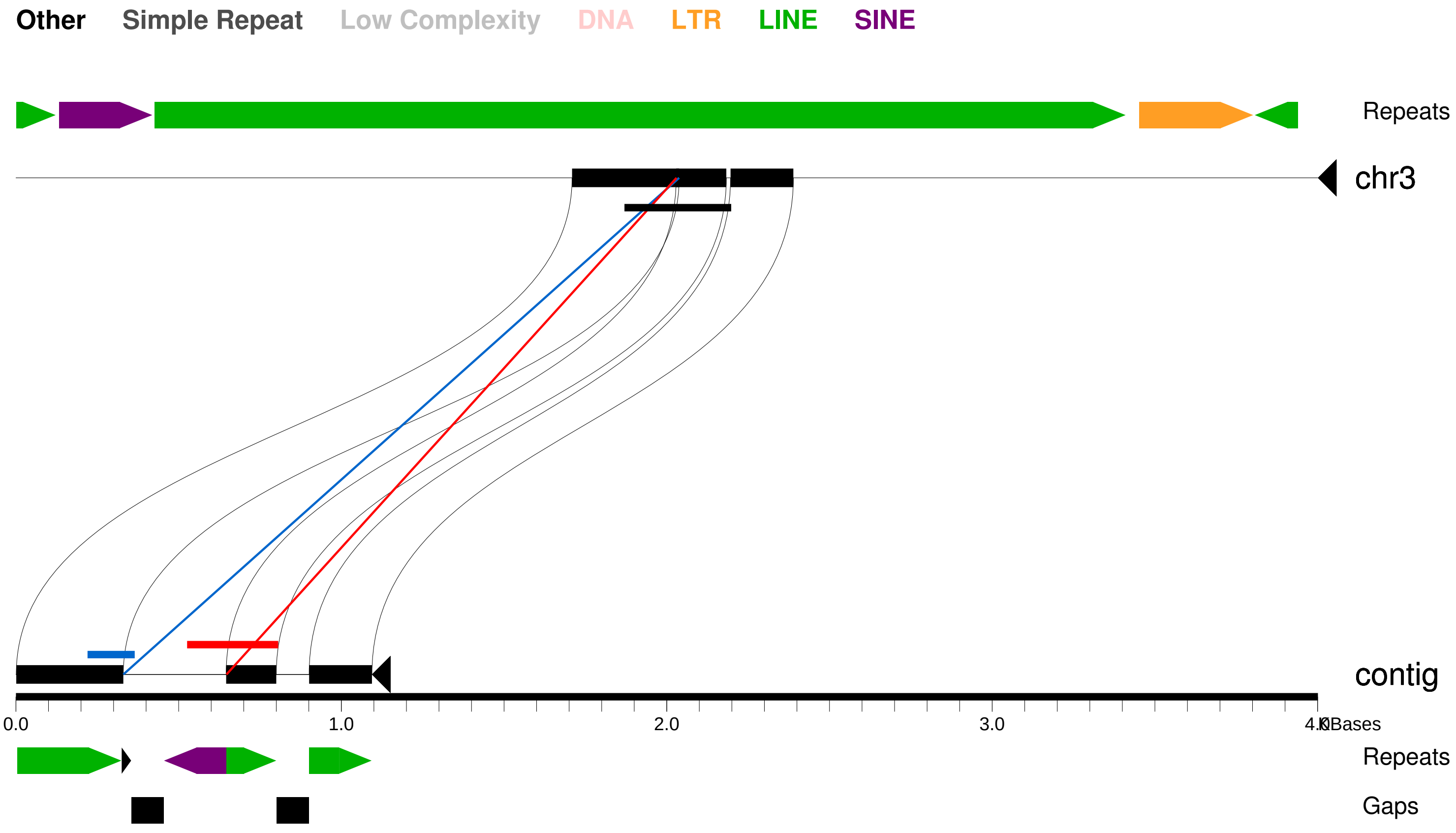

siteID: chr3_87520481 Scafftig13 - Breakpoint: chr3:87520469
chr3:87520424-87520574
Overlap Length: 15 INS size: 265 Genotyped: Yes

```
left    TTATGTCATTAGCTACCCCTGAAGTTGCTGAAAAAATGAAGTGTTAAGAGATAAAGTTGG--TTGGGAAG
chrom   TTATGTCATTAGCTACCCCTGAAGTTGCTGAAAAAATGAAGTGTTAAGAGATAAAGTTGGAATTTGGTAG
right   ---------------------AA-------AAAAAAAAAA-----AAGAGATAAAGTTGGAATTTGGTAG
        111111111111111111111**1111111******11**11111**************22**2**2**

left    CCGAGGCGGGCGGATCA-----------------CGAGGTCAGGAGATCGAGACCATCCCG-----GC--
chrom   AAGACTCCAG-GAATAAAGGTATTTATTT-GTGGCGGGGAGGGGATATCTGTCAGATCTCTATAGTGCAG
right   AAGACTCCAG-GAATAAAGGTATTTTTTTTGTGGCGGGGAGGGGATATCTGTCAGATCTCTATAGTGCAG
        22**22*22*2*2**2*22222222N22212222**2**222***2***222222***2*222222**22

left    -------TAAA-A
chrom   AGAATTTTACAGA
right   AGAATTTTACAGA
        2222222**2*2*
```

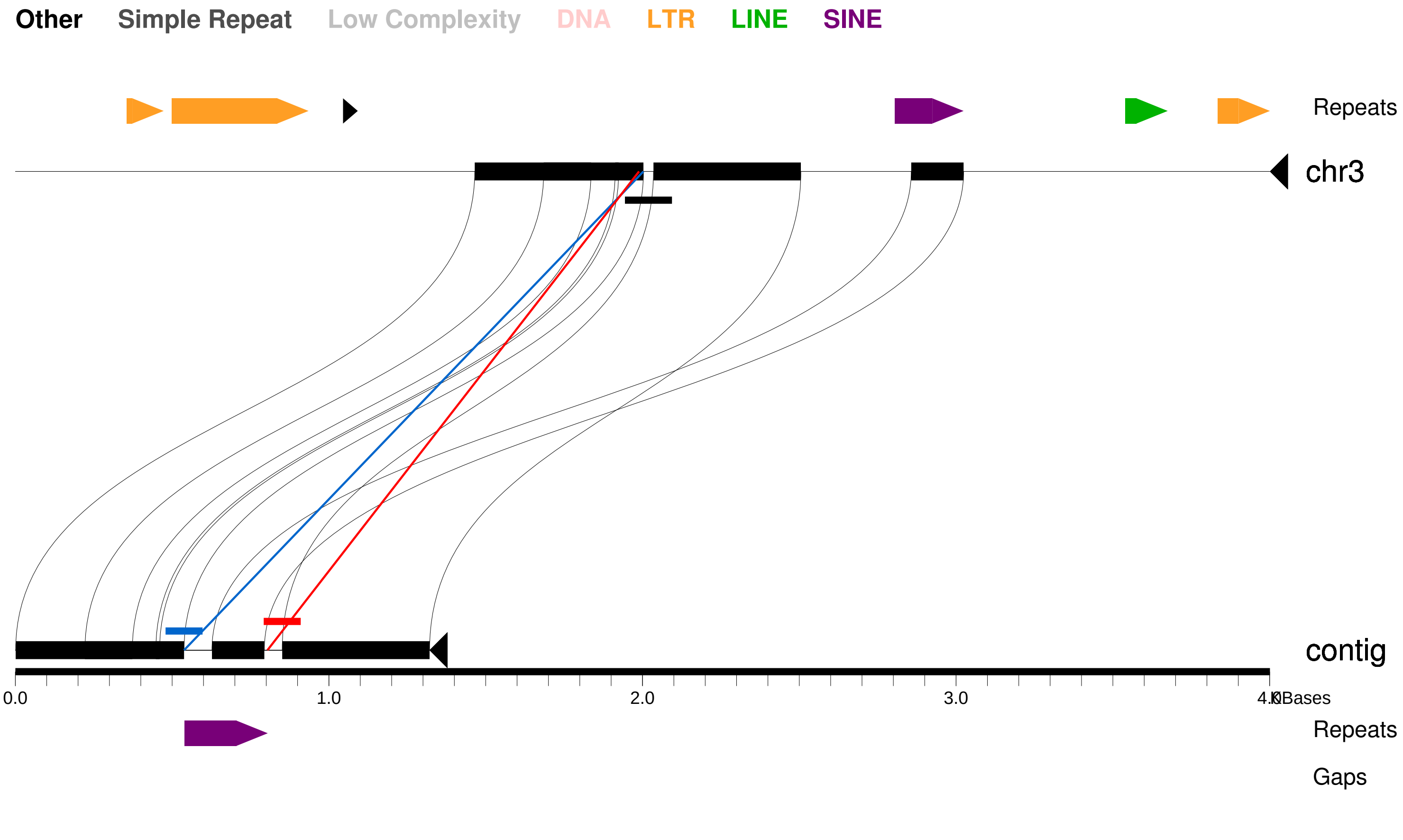

siteID: chr3_88478162 Scafftig7 - Breakpoint: chr3:88478143
chr3:88478103-88478202
Overlap Length: 15 INS size: 277 Genotyped: Yes

```
left     TGTGGTCC-AGCTTATTTCCCCTTGGGTCAGAACATCTGTT------------------AAAATTTTAGC
chrom    TGTGGTCC-AGCTTATTTCCCCTTGGGTCAGAACATCTGTT------------------AAAATTTTAGC
right    GGGCGACAGAGCGAGACTCCGTCTCAAAAAAAAAAAAAAAAAAAAAAAAAAAAAAAAAAAAAATTTTAGC
         1*11*1*11***11111***111*11111*1**1*111111111111111111111111***********

left     AAATTTGGGAGGCCGAGGCGGGCGGATCACGAGGTCAGGAGATCGAGACCATCCCGGCTAAAACGGTG
chrom    AAATATTG---GCC-------------CTTGATTTTAG-ATATGGGGTT--TCATTGGTAACACAATT
right    AAATATTG---GCC-------------CTTGATTTTAG-ATATGGGGTT--TCATTGGTAACACAATT
         ****2*2*222***2222222222222*22**22*2**2*2**2*2*2222**222*2***2**22*2
```

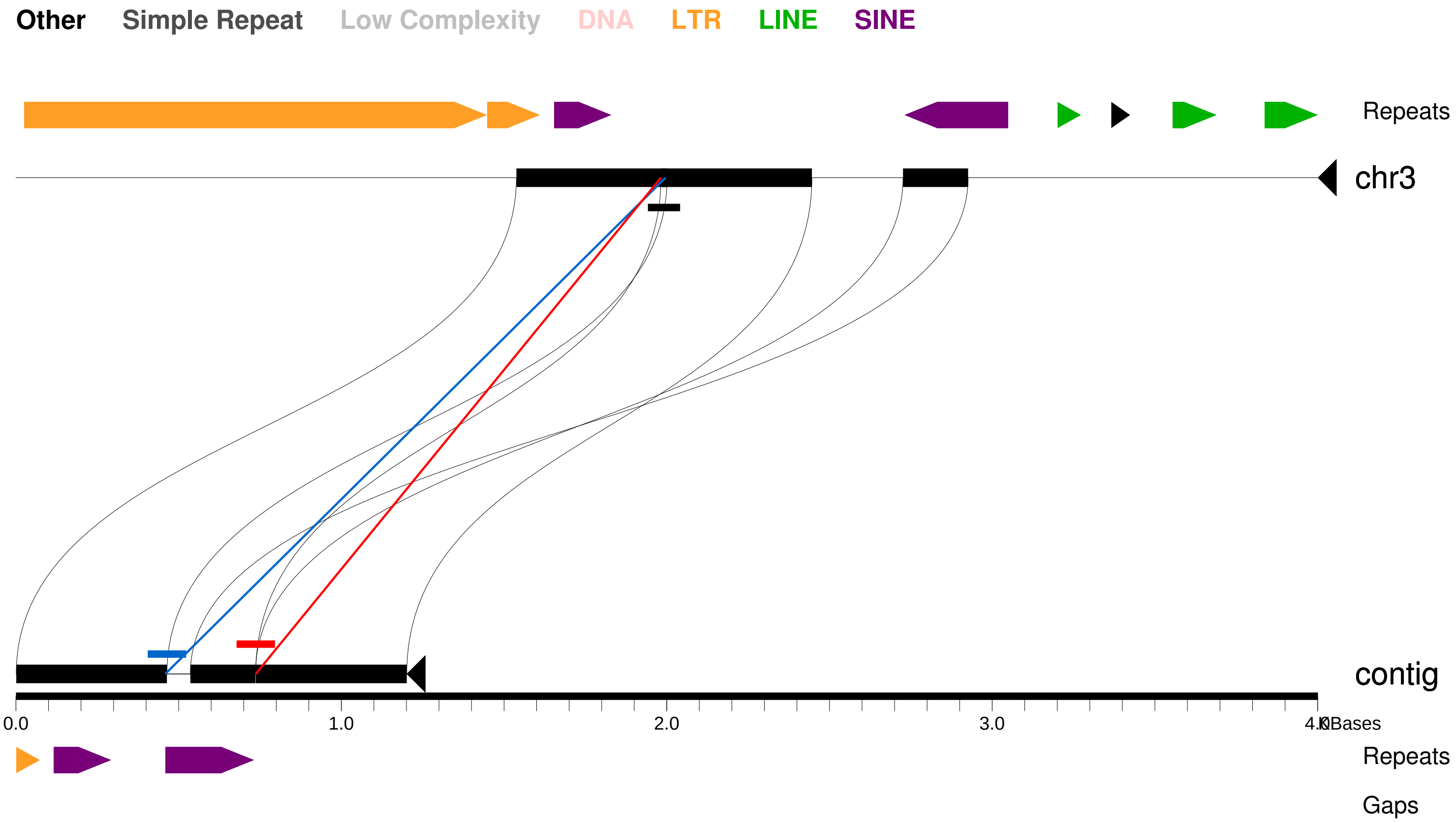

siteID: chr3_89306821 Scafftig2 + Breakpoint: chr3:89306824
chr3:89306780-89306883
Overlap Length: 16 INS size: 422 Genotyped: No

```
left    CTTGACAATTAAAAGAAAAAAAATACAACATGTTAGGAAATAAT---------------AAAAATAACAA
chrom   CTTGACAATTAAAAGAAAAAAAATACAACATGTTAGGAAATAAT---------------AAAAATAACAA
right   NNNNNNNNNNNNNNNNNNNNNNNNNNNNNNNNNNNNNNNNNNNNNNNNNNNNNNNNNNNAAAAAAGAACAA
        11111111111111111111111111111111111111111111111111111111111*****1*****

left    CTGGAGGCCGGGCGCGGTGGCTCACGCCTGTAATCCCAGCACTTTGGGAGGCCGAGGCGGGCGG
chrom   CTGGAAT--GACAGTAGTAATACA-------AATATCAACATTAT------CAAAAACTCTTAA
right   CTGGAAT--GACAGTAGTAATACA-------AATATCAACATTAT------CAAAAACTCTTAA
        *****2222*222*22**2222**2222222***22**2**2*2*222222*22*22*222222
```

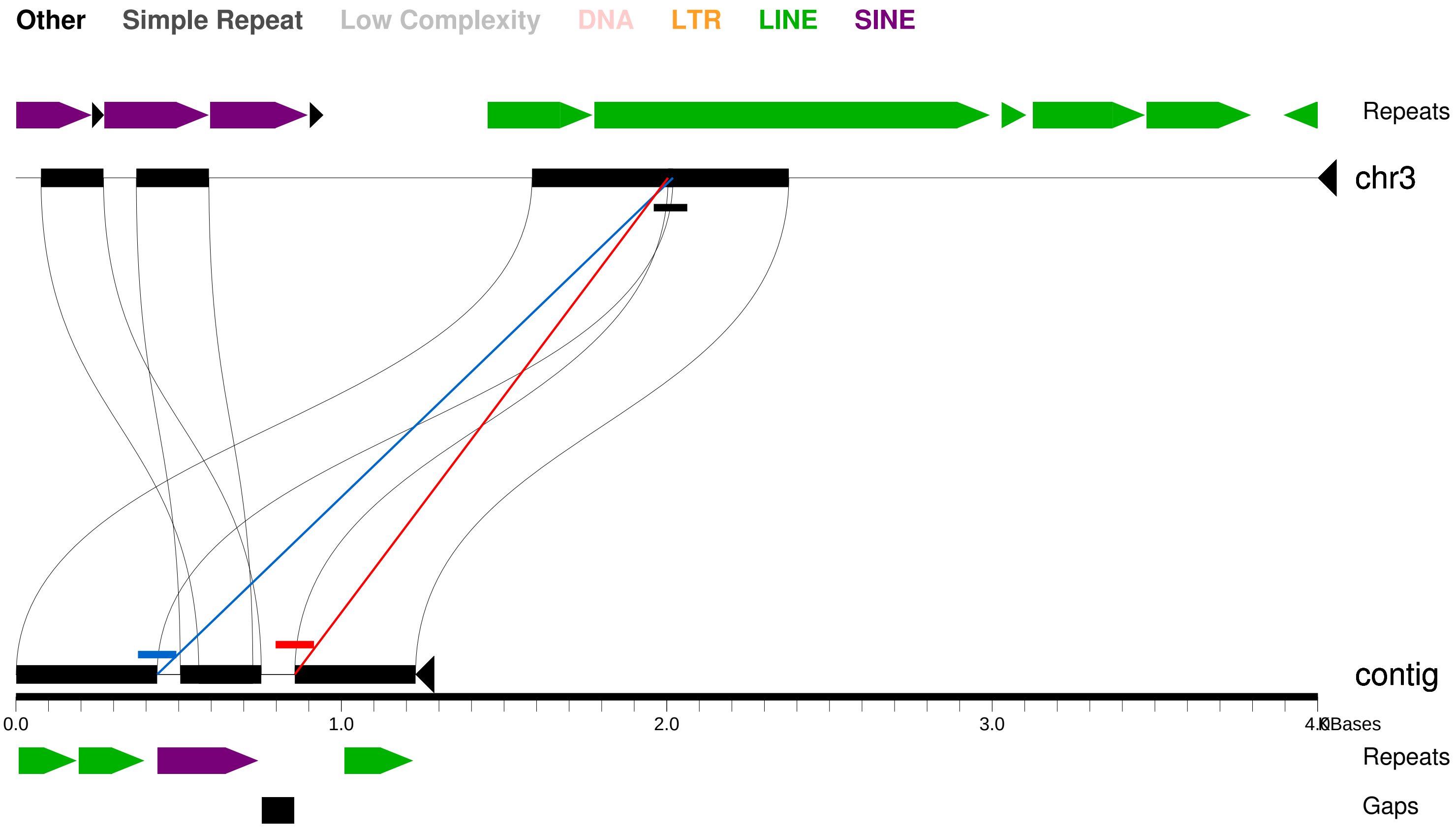

siteID: chr3_96596485 Scafftig4 + Breakpoint: chr3:96596488
chr3:96596435-96596547
Overlap Length: 7 INS size: 313 Genotyped: Yes

```
left   TTTAGAGAATTA--ATACTAAC---CAGTGAA-ACTTTCAAAAAACACAGGTTGGCGTTAAATGTTTTAT
chrom  TTTAGAGAATTA--ATACTAAC---CAGTGAA-ACTTTCAAAAAACACAGGTTGGCGTTAAATGTTAAA-
right  CCACCCGCCTCGGCCTCCCAAAGTGCTGGGATTACAGGCGTGAGCCACCGCGCCCGGCCAAATGTTAAA-
       111111*11*11111*1*1**1111*1*1**11**111*111*11***1*111111*11*******22*2

left   TTTATTTTATTTTATTTTTTTGAGACGGAGTCTCGCTCTGTCGCCCAGGCCGGAC
chrom  --TAGGTTTTTGTAATCTGTATCCATGCCAT-TAGTATTTTGAATTAAT--GTAC
right  --TAGGTTTTTGTAATCTGTATCCATGCCAT-TAGTATTTTGAATTAAT--GTAC
       22**22**2**2**2*2*2*2222*2*222*2*2*222*2*22222*2222*2**
```

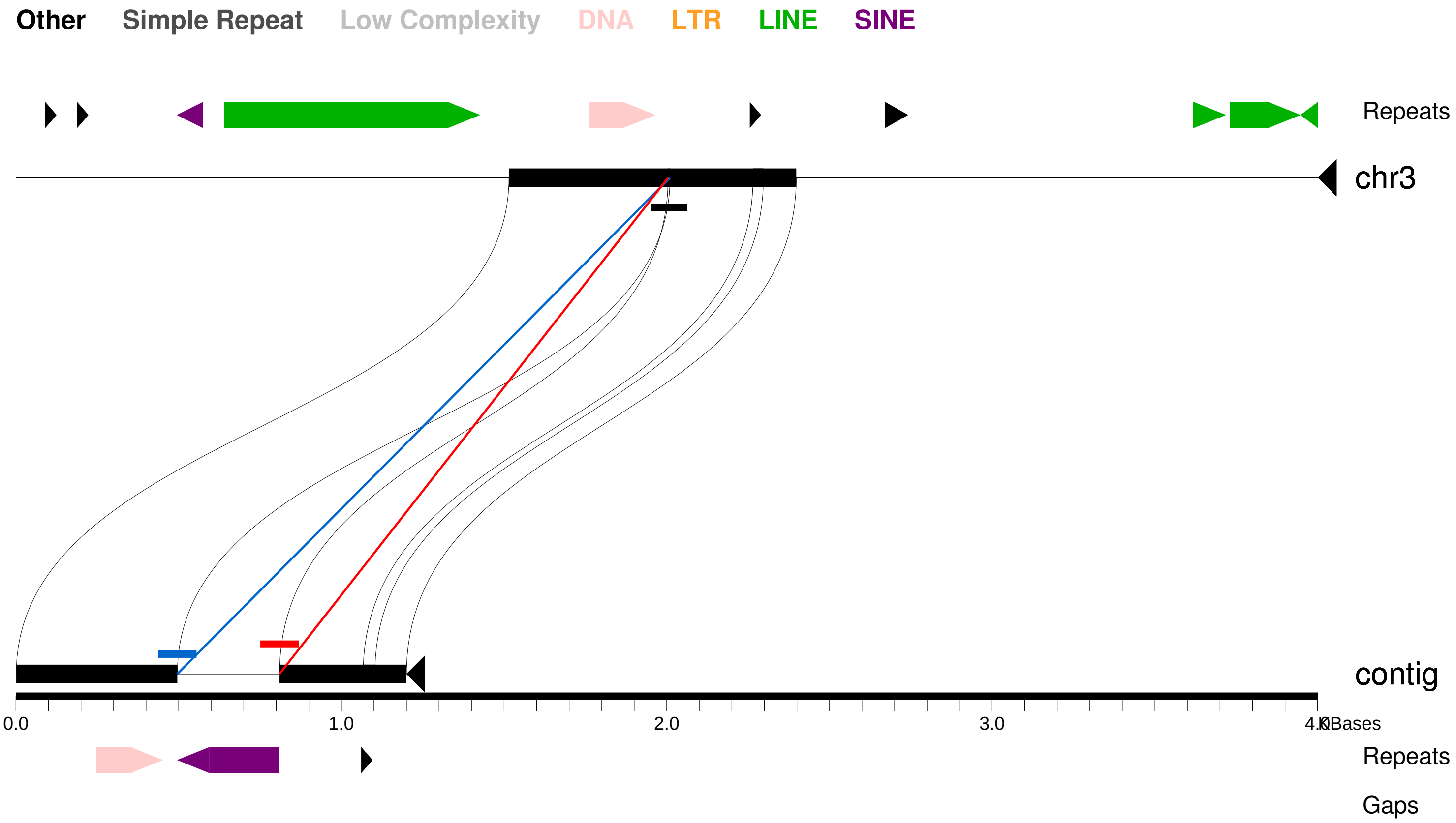

siteID: chr3_98726756 Scafftig13 - Breakpoint: chr3:98726761
chr3:98726601-98726936
Overlap Length: 16 INS size: 296 Genotyped: Yes

```
left   ----------------GTGTAGACAGGAGAGAACCCACCCTACACCAGGATCTTTTTATTAGCCAGATGC
chrom  CTAGTGATCTGCCTGGGTGTAGACAGGAGAGAACCCACCCTACACCAGGATCTTTTTATTAGCCAGATGC
right  CAGGAGAATGGC----GTGAACCCGGGAGGC----------------GGAGCTTGCAGTGAGCC-GAGAC
       2NN2N22NNN22    ***1*11*1****111111111111111111***1***1111*1****1**11*

left   TGTCACAGGGGCTGTTTGCCCATGTTCTCATGCCCAGGATTTTGGATGTCCTTCACAATTCTGGTGGATT
chrom  TGTCACAGGGGCTGTTTACCCATGTTCTCATGCCCAGGATTTTGGATGTCCTTCACAATTCTGGTGGATT
right  TGCGCCACTG-CACTCCAGCCTGGGTGACAGAGCGAGACT---------CCGTCTCAAAACAA----AAA
       **111**11*1*11*1121**11*1*11**111*1**11*111111111**1**1***11*111111*11

left   CCAATTTTCCTTCTTGAATTAAAACTCACAGAGATGGGC--------CGGGCGCG---------GTGGC
chrom  CCAATTTTCCTTCTTGAATTAAAACTCACAGAGATGATCTTTATGTACTGTCTTGCTATTTACAAGTGTC
right  ACAAAAAAC-------AAAAAAACCTCACAGAGATGATCTTTATGTACTGTCTTGCTATTTACAAGTGTC
       1***1111*1111111**11****1***********22*22222222*2*2*22*2222222222***2*

left   TCACGCTTG-TAA----TCCCAG-----CACTTTGGGAGGCCGAGGCGGGTGGATCACGAGGTCAGGAGA
chrom  TGAGGCATGCTAAAAGCTTCTAATCTGTCATCTTTGAAAAAAACTTTATTTACAAAACTTGGTGCCAACC
right  TGAGGCATGCTAAAAGCTTCTAATCTGTCATCTTTGAAAAAAACTTTATTTACAAAACTTGGTGCCAACC
       *2*2**2**2***2222*2*2*222222**22**2*2*222222222222*22*22**22***2222*22

left   TCGAGACCACGGTGA---AACCCCGTCTCTA--CTAAAAATA------AAAAAAAA
chrom  TGCAGGCTGCAGTTTGTCAACTCCTGATATATGCTATAATGTTCTGGCAGTAATCC
right  TGCAGGCTGCAGTTTGTCAACTCCTGATATATGCTATAATGTT-------------
       *22**2*22*2**22222***2**222*2**22***2**2222     1NN11NNN
```

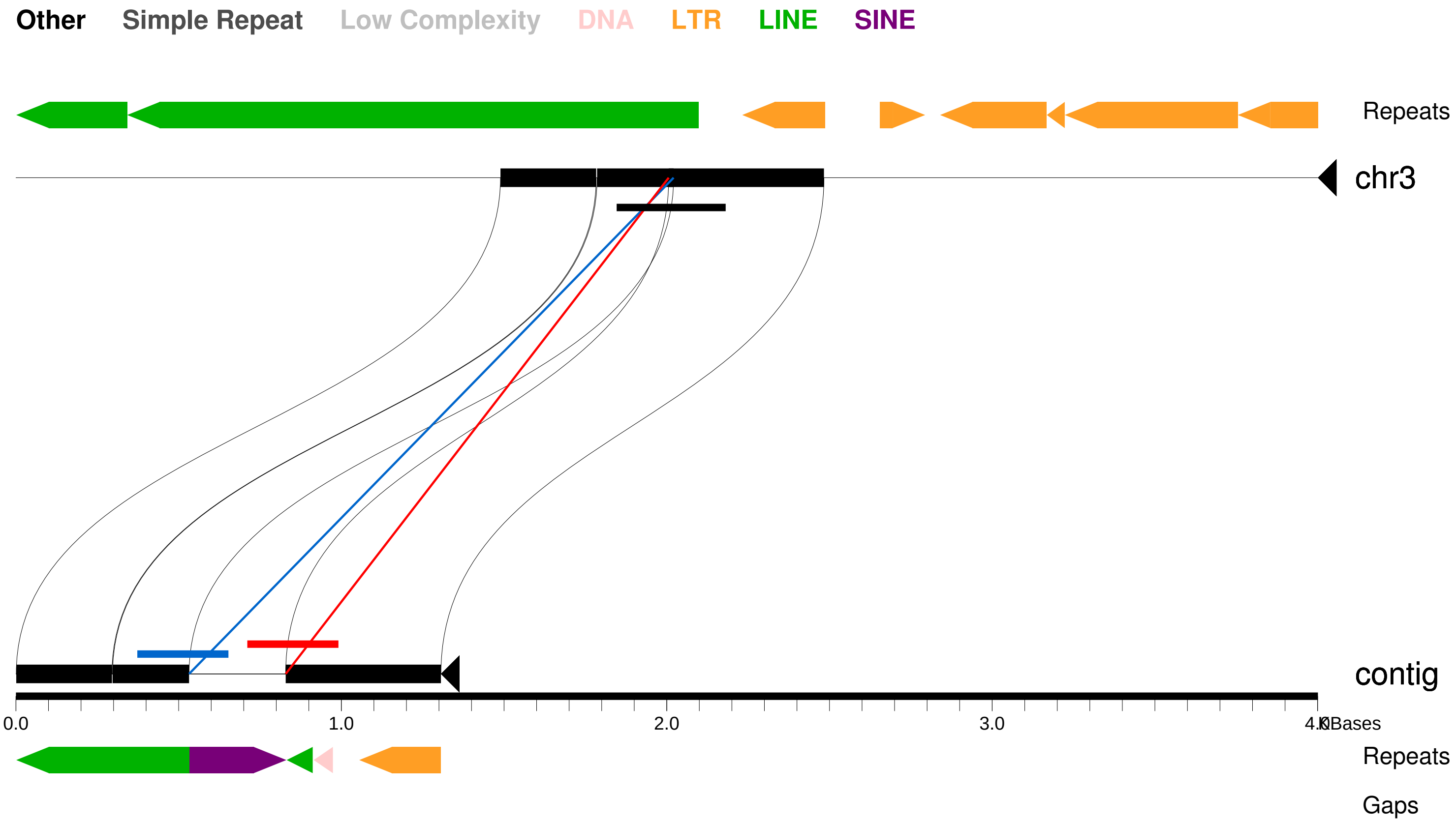

siteID: chr4_100376741 Scafftig1 + Breakpoint: chr4:100376795
chr4:100376748-100376854
Overlap Length: 13 INS size: 295 Genotyped: Yes

```
left   -----------GTTATGATGTAGTT-TCTGTTCCAAGAAGTTTACAATATAATACATGTTTAATCAACTT
chrom  -----------GTTATGATGTAGTT-TCTGTTCCAAGAAGTTTACAATATAATACATGTTTAATCAACTT
right  CCGCCCGCCTCGGCCTCCCAAAGTGCTGGGATTACAGGCGTGAGCCACCGCGCCCGGCCTTAATCAACTT
       11111111111*111*11111***11*11*1*111**11**111*1*1111111*1111***********

left   TTTTTTTTTTTTTTTTGAGACGGAGTCTCGCTCTGTCGCCCAGGCTGGAGTGCAGTGGCGG
chrom  TTATATCAATAATCTTTTGTCCCCAAATAACTCTACTTTCCAAAAAGAA------------
right  TTATATCAATAATCTTTTGTCCCCAAATAACTCTACTTTCCAAAAAGAA------------
       **2*2*222*22*2**22*2*222222*22****22222***2222*2*222222222222
```

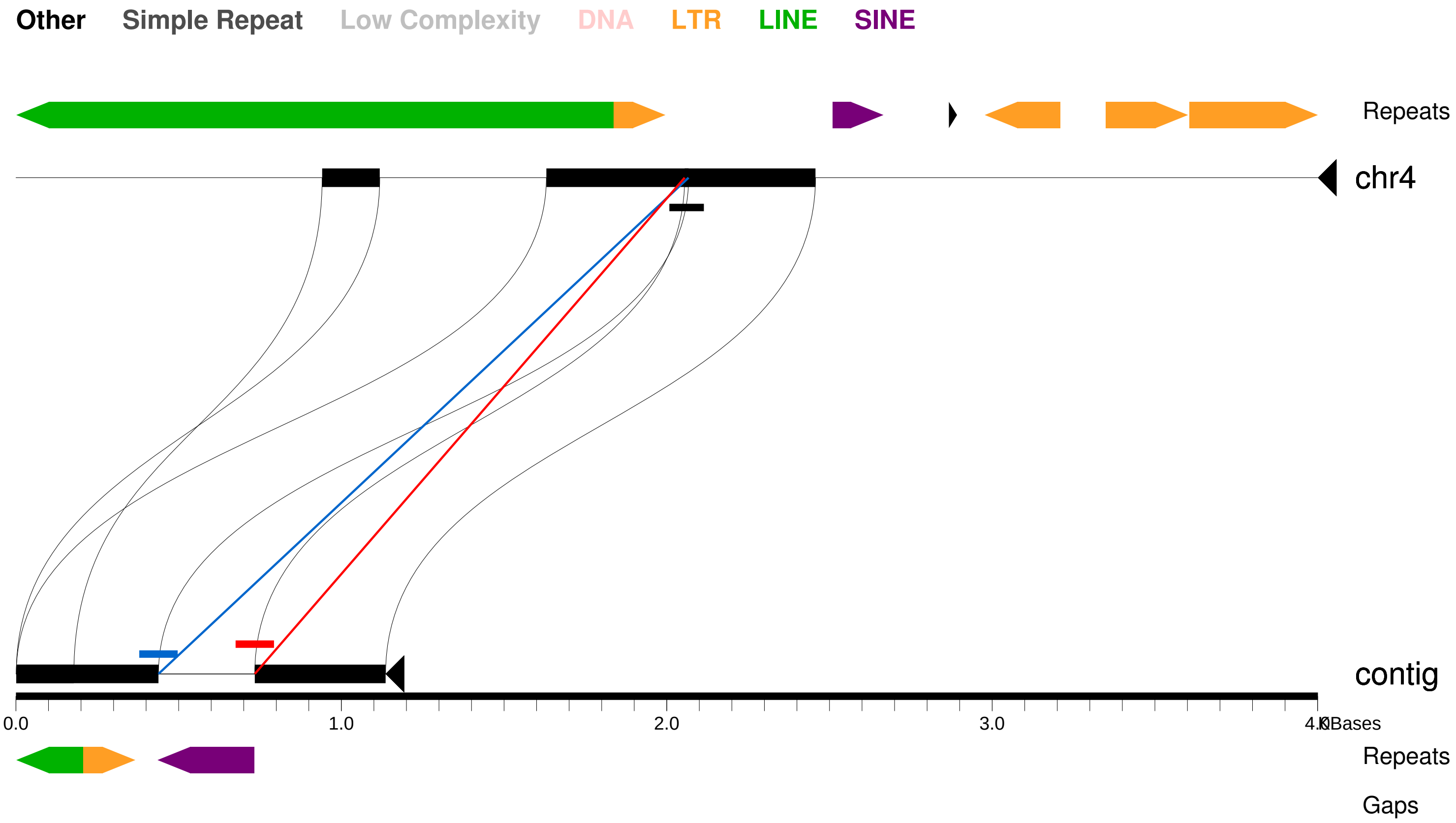

siteID: chr4_101891526 Scafftig2 + Breakpoint: chr4:101891537
chr4:101891484-101891596
Overlap Length: 7 INS size: 465 Genotyped: No

```
left   GAGATGAGGAAGACACTACAGATAATTCCAAATGATTTGATGATTCAAAAAAT------TTCTTTAGGCC
chrom  GAGATAAGGAAGACACTACAGATAATTCCAAATGATTTGATGATTCAAAAAAT------TTCTTTATTTT
right  NNNNNNNNNNNNNNNNNNNNNNNNNNNNNNNNNNNNNNNNNNNNNNNNNNNNNNNNNNNNTTCTTTATTTT
       11111N111111111111111111111111111111111111111111111111111*******2222

left   GGGCGCGGTGGCTCACGCCTGTAATCCCAGCACTTTGGGAGGCCGAGGCGGGTGG
chrom  CAGC-CTTAAATATATTCCT---ATGCTAATATTTAGGTTTGAATAT--AGGTGT
right  CAGC-CTTAAATATATTCCT---ATGCTAATATTTAGGTTTGAATAT--AGGTGT
       22**2*22222222*22***222**2*2*22*2**2**222*222*2222****2
```

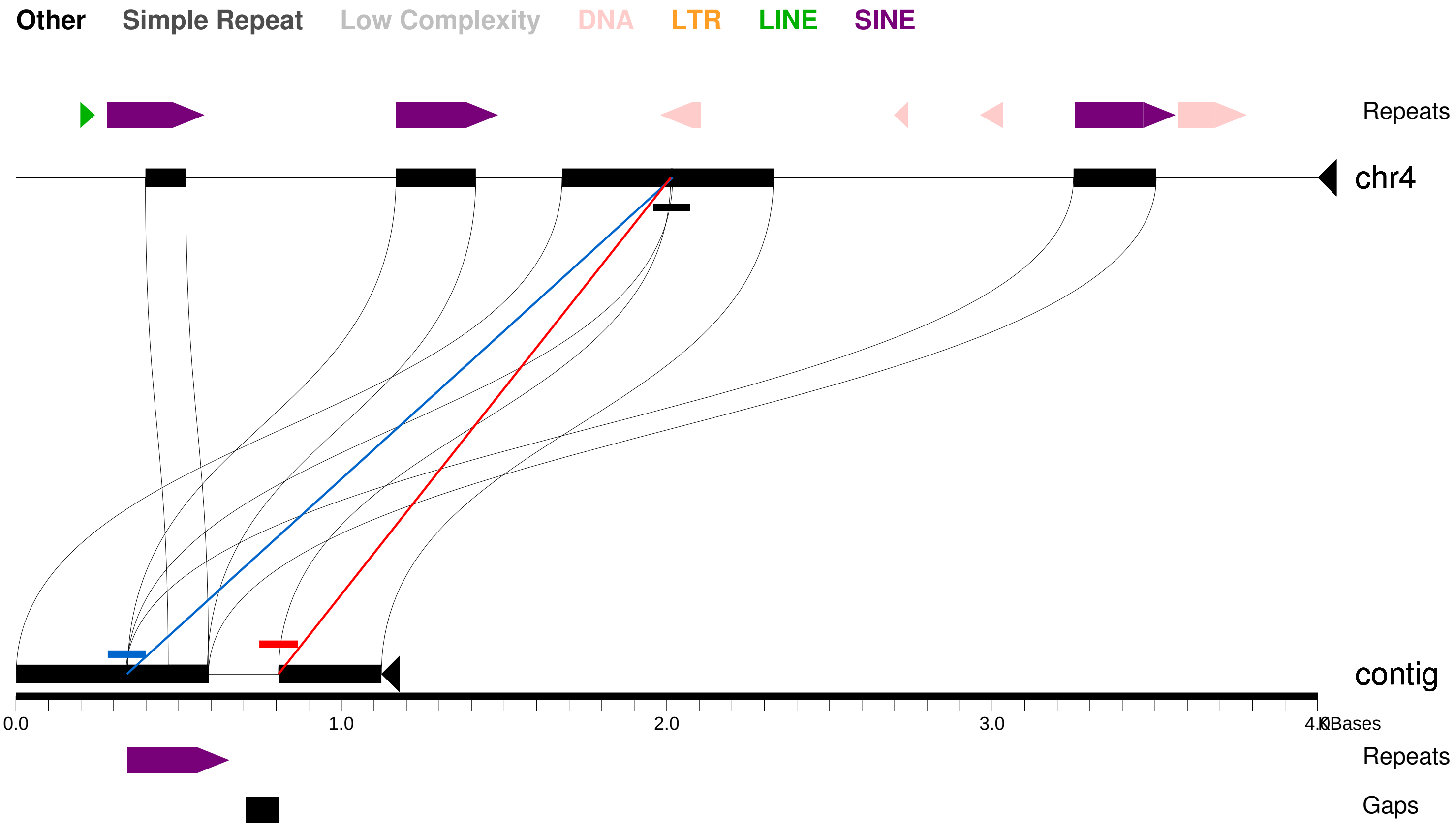

siteID: chr4_102041751 Scafftig1 - Breakpoint: chr4:102041736
chr4:102041576-102041897
Overlap Length: 2 INS size: 320 Genotyped: Yes

```
left   CCTCTCTGTACTGGAAACTCCTACGGGTTTAGGCCTTAGGTCTCCCATTGATG-TCT-GATATGGGCAAA
chrom  C-TCTCTGTACTGGAAACTCCTACGGGTTTAGGCCTTAGGTCTCCCGTTGATG-TCT-GATATGGGCAAA
right  T-T-----TAGTAGAGAC------GGGGTTTCACCTT-GTTAGCCAG--GATGGTCTCGATCTCCTGACT
       12*11111**1*1**1**111111***1**111****1*1*11**1211****1***1***1*1111*11

left   TCATTTTATTGCCCATTTCCATCCATCACATTACTCAACAGAGGCAGTAGCATATATAATTTTTTTAAAA
chrom  TCATTTTATTGCCCATTTCCATCCATCACATTACTCAACAGAGGCAGTAGCATATATAATTTTTCTAAAA
right  TCATGAT-----------CCACCCGCCTCAGCCTCCCAAAGTGCTGGGAT--TACAGGCGTGAGCCACCG
       ****11*11111111111***1**11*1**11111*1*1**1*111*1*111**1*1111*11121*111

left   AAAATTTTTTAAATTTAAAAACTTTTTATTTTTTTTTTTTTTTTTTTTTTT---TTT-------------
chrom  ATAATTGTTAAATTATAAATACTTTACATTTACTTTTATAATGATGTTTTCACTTTGAAATTTCAAATTA
right  CGCCCGG--------------CCTTACATTTACTTTTATAATGATGTTTTCACTTTGAAATTTCAAATTA
       1N1111211N11N1N1111N1*1**22****22****2*22*22*2****222***22222222222222

left   --TTTTGAGACGGAGTC-------TCGCTCTGTC--------GCCCAGGCCGGA----CTGCGGACTGCA
chrom  CATTAAGAAAAGGCATGAGATTGTTTTCTTTGTCCAAAAGAAGTACAAATCTAAAAATCTGAGAACTTAG
right  CATTAAGAAAAGGCATGAGATTGTTTTCTTTGTCCAAAAGAAGTACAAATCTAAAAATCTGAGAACTTAG
       22**22**2*2**22*22222222*22**2****22222222*22**222*22*2222***2*2***222

left   GTGGCGCAATCTCGGCT---CACTGCAAG-CTCCGCTTCCTGGGT
chrom  ATTAGATTATCTTAAATTTACATGTCAGGACTAATGTTCAATTGA
right  ATTAGATTATCTTAAATTTACATGTCAGGACTAATGTTCAATT--
       2*222222****2222*222**222**2*2**2222***22221N
```

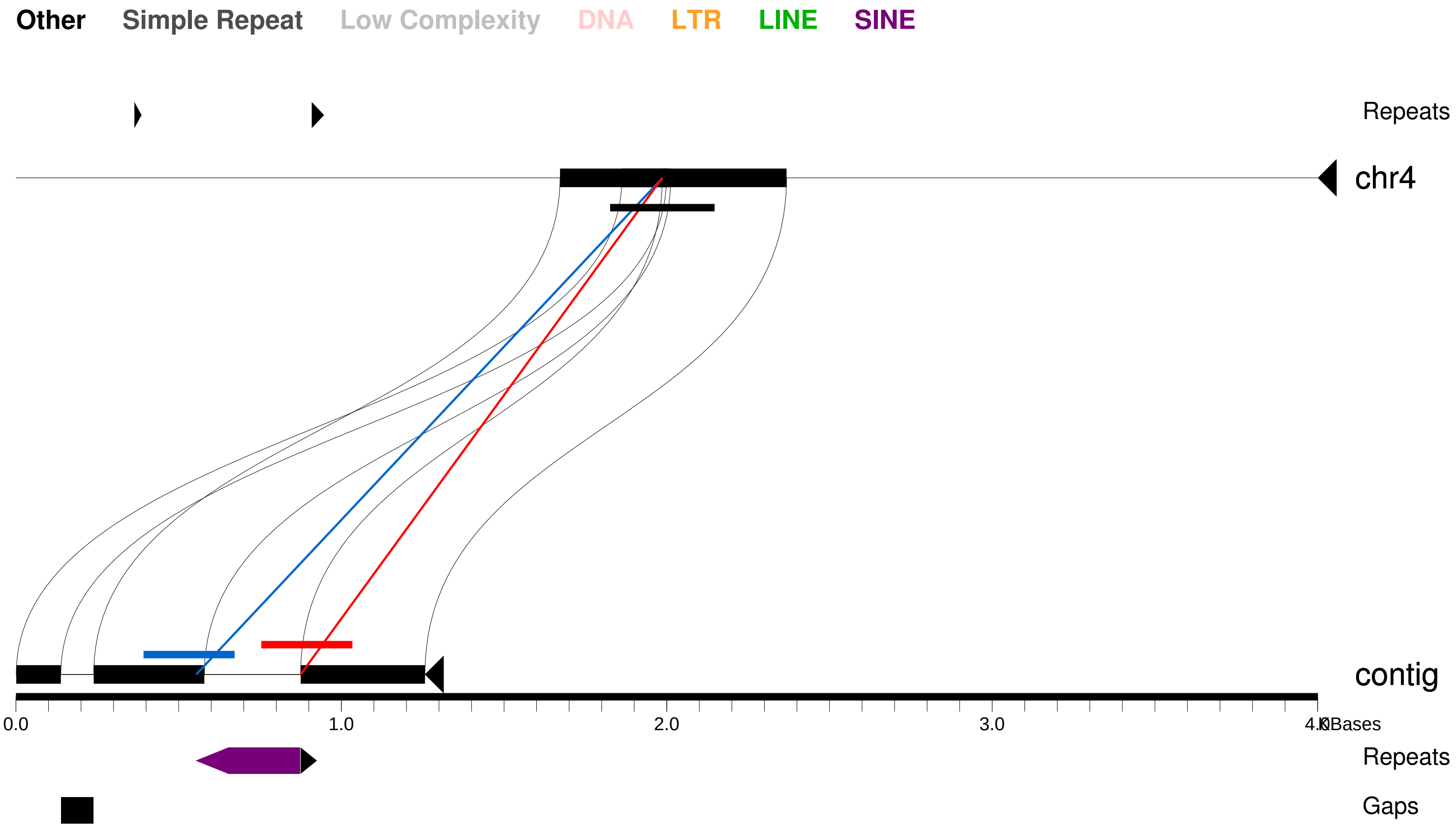

siteID: chr4_105597114 Scafftig1 + Breakpoint: chr4:105597120
chr4:105596960-105597294
Overlap Length: 15 INS size: 297 Genotyped: Yes

```
left     -------------CAATAGGAATTTATTAGCTAGACACTCAGAAGGTTTA-TGAACCCTCTTTTTGCCTA
chrom    AGTAATGAACATACAATAGGAATTTATTAGCTAGACACTCAGAAGGTTTA-TGAACCCTCTTTTTGCCTA
right    GCTGAGG------CAGGAGAATGGCGTGAACCCGGGAGGCGGAGCTTGCAGTGAGCCGAGATCCCGCCAC
         NN2N2N2      **11**1*11111*1*1*11*11*11*1**111*11*1***1**1111*111***11

left     TTTA-TATTTTCTAAAAGATATTGATCTTTTATTTATTTCAGTAAAATATTACATGCATGTATAGTATGA
chrom    TTTA-TATTTTCTAAAAGATATTGATCTTTTATTTATTTCAGTAAAATATTACATGCATGTATAGTATGA
right    TGCACTCCAGCCTGGGCGACA--GAGCGAGACTCCGTCTCAAAAAAAAAAAAAA---------------A
         *11*1*11111**1111**1*11**1*11111*111*1***11****1*11*1*111111111111111*

left     AATAGGTTTATAGCCTCTATTTAAGAGATCTTTCTGGCCGG-----------------GCGCGGTGGCT
chrom    AATAGGTTTATAGCCTCTATTTAAGAGATCTTTCTGGGACGCTCAAAACAATCTAATAAGCACTTAGAAT
right    AA--------------------AAGAGATCTTTCTGGGACGCTCAAAACAATCTAATAAGCACTTAGAAT
         **1111111111111111111*************222*2222222222222222**2*222*22*

left     CACGCCTGTAATCC----------CAGCACTTTGGGAGGCCGAGGCGGGCGG----ATCACGAGGTCAGG
chrom    GATACTTTTATTTCTTCTCACTTTCTCTTCTTTTCTATACAGAGAACCTTGGCAATATCCTGAATTTAGT
right    GATACTTTTATTTCTTCTCACTTTCTCTTCTTTTCTATACAGAGAACCTTGGCAATATCCTGAATTTAGT
         2*22*2*2**2*2*2222222222*2222****222*22*2***222222**2222***22**22*2**2

left     AGATCGAGACCATCCCGGCTAAA-ACGGTGAAACCCCGTCTCTACTAAA------------
chrom    ACTAAAAGCAAA----GGATAAGGAAGGTGTTATGTGGCCTCTGTATAACAGAGAATGTTC
right    ACTAAAAGCAAA----GGATAAGGAAGGTGTTATGTGGCCTCTGTATA-------------
         *22222**222*2222**2***22*2****22*2222*2****2222*1
```

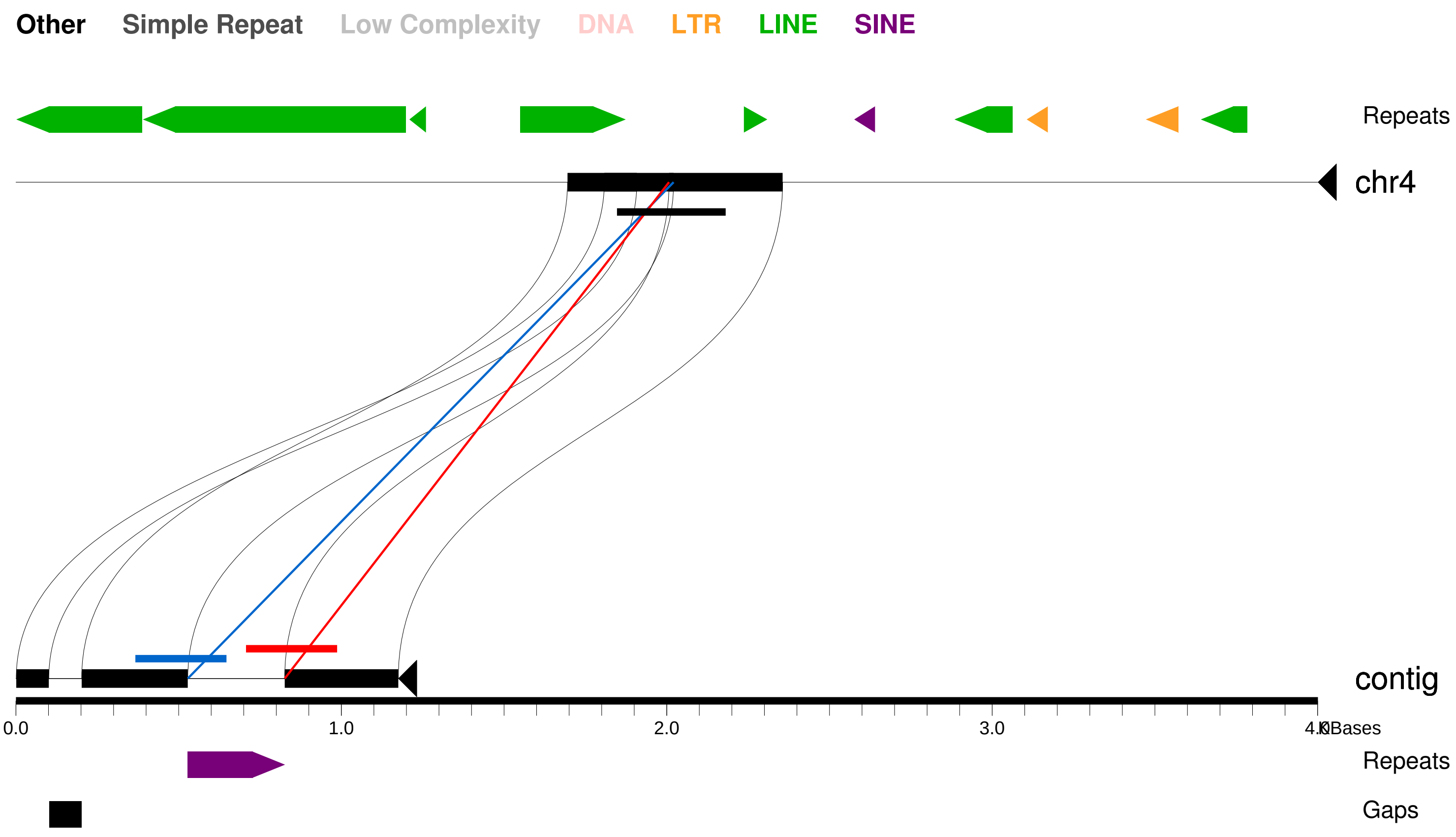

siteID: chr4_106550729 Scafftig1 - Breakpoint: chr4:106550747
chr4:106550701-106550806
Overlap Length: 14 INS size: 461 Genotyped: No

```
left     TAT-------TCAGCCATCCCAATT------AATTCAGCCACCAAATCATTATTAGTCTCAAATCTGCAA
chrom    TAT-------TCAGCCATCCCAATT------AATTCAGCCACCAAATCATTATTAGTCTCAAATCTGCAA
right    TCCGCCCGCCTCGGCCTCCCAAAGTGCTGGGATTACAGGCGTGAGCCACCGCGCCCGGCCAAATCTGCAA
         *111111111**1***11**1**1*111111*1*1***1*111*1111111111111111**********

left     TGTNNNNNNNNNNNNNNNNNNNNNNNNNNNNNNNNNNNNNNNNNNNNNNNNNNNNNNNNNNNNN
chrom    TGTTTTGAGAGCTGACCCTGTCACATATTGCTTGGATATCTGCCAAATA-------------
right    TGTTTTGAGAGCTGACCCTGTCACATATTGCTTGGATATCTGCCAAATA-------------
         ***2222222222222222222222222222222222222222222222222222222222222
```

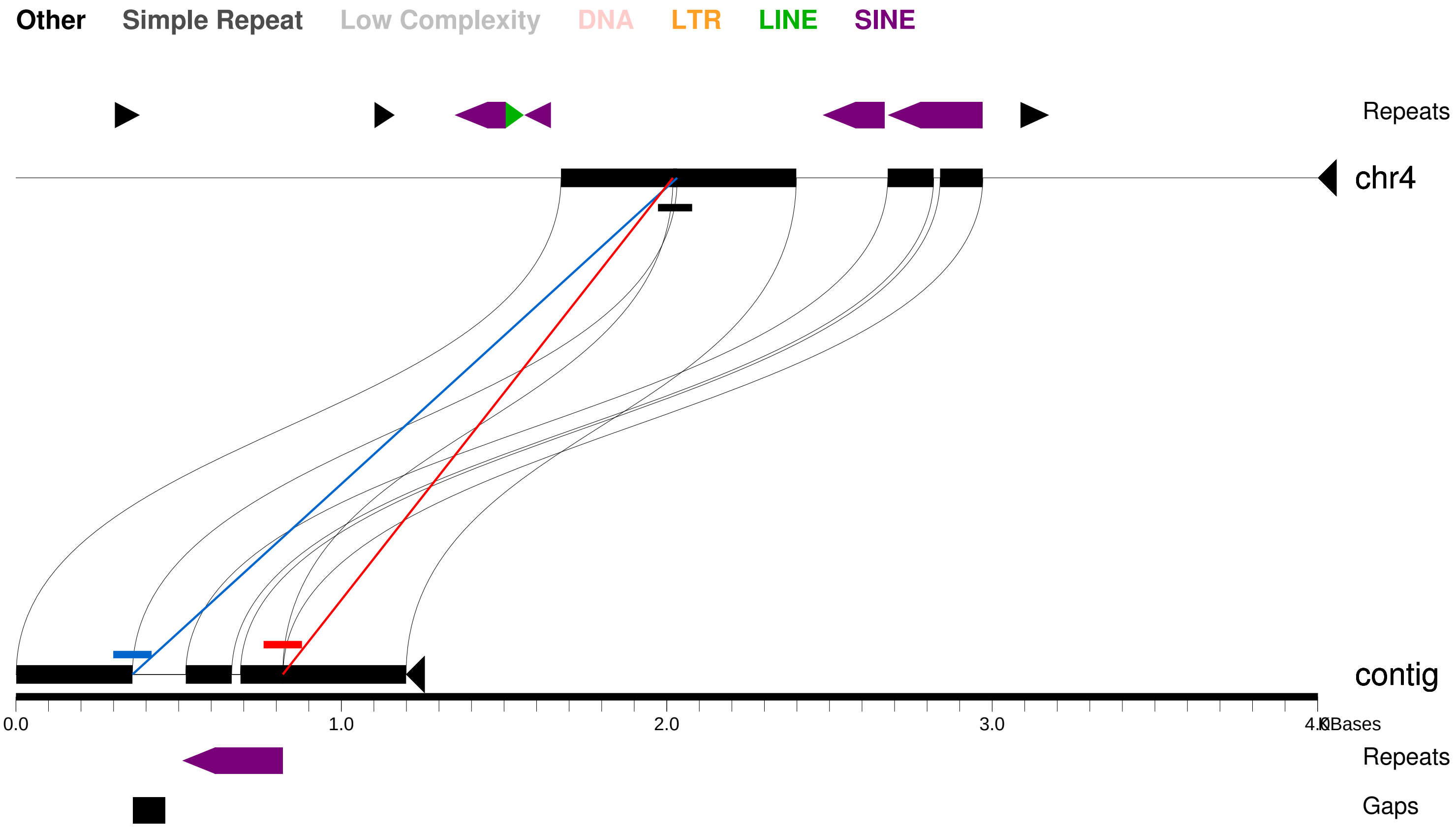

siteID: chr4_106636849 Scafftig2 + Breakpoint: chr4:106636850
chr4:106636803-106636909
Overlap Length: 13 INS size: 464 Genotyped: No

```
left    CTTAAAGTGTTTAGGTCTTGTTTCAGATTTTTTTAAAAAAGGGATTT------------AAAATATTAAA
chrom   CTTAAAGTGTTTAGGTCTTGTTTCAGATTTTTTTAAAAAAGGGATTT------------AAAATATTAAA
right   NNNNNNNNNNNNNNNNNNNNNNNNNNNNNNNNNNNNNNNNNNNNNNNNNNNNNNNNNNNNAAAAATATTAAA
        111111111111111111111111111111111111111111111111111111111111***********

left    GAGGCCGGGCGCGGTGGCTCACGCCTGTAATCCCAGCACTTTGGGAGGCCGAGGCGGGCGG
chrom   GAAAATTTA------GTATGATGAAGGTTTTCTAA--AATT----AAGTGTACACTAACAT
right   GAAAATTTA------GTATGATGAAGGTTTTCTAA--AATT----AAGTGTACACTAACAT
        **2222222222222*22*2*2*222**22**22*22*2**2222*2*222*22*222*22
```

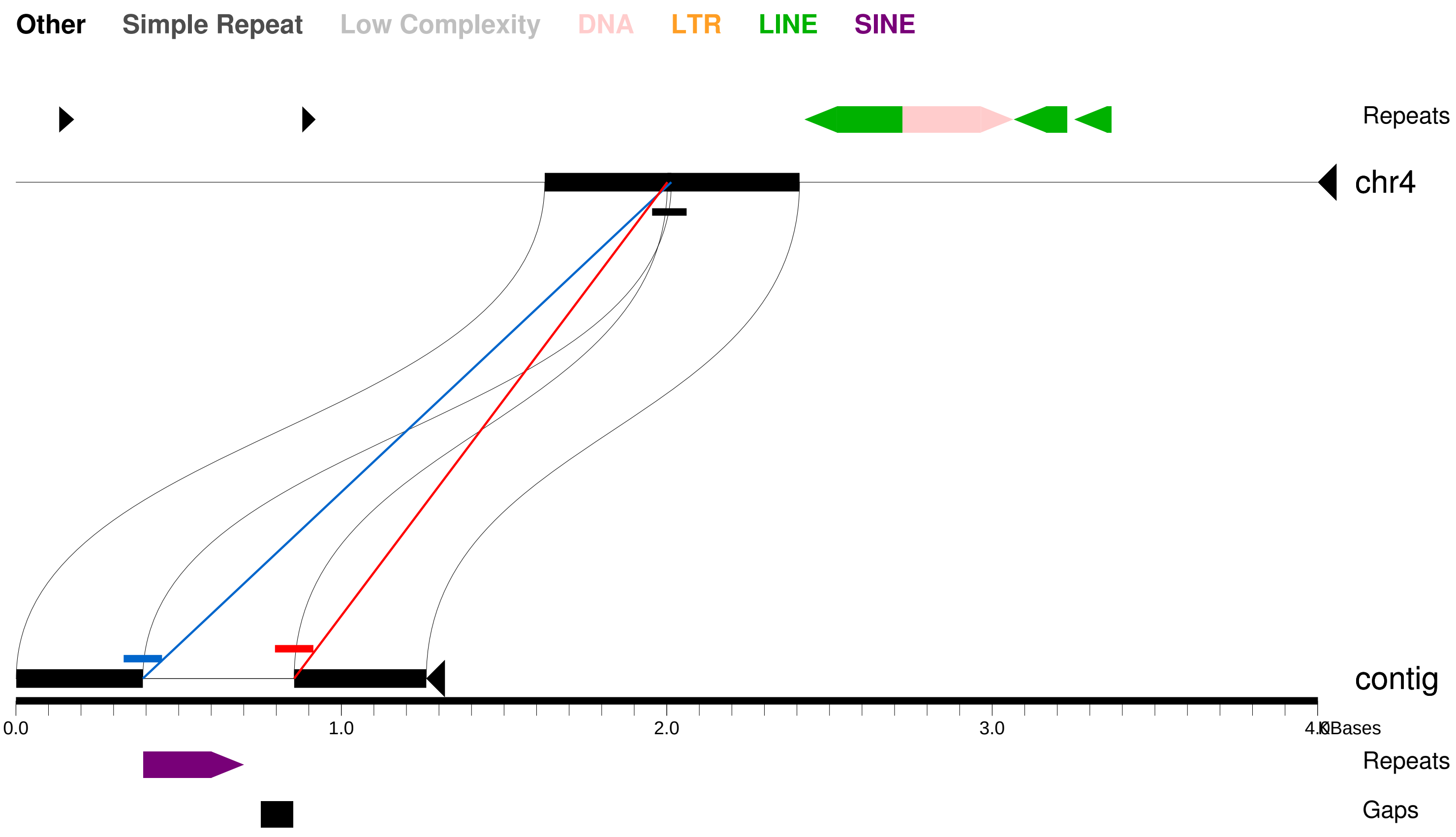

siteID: chr4_106897036 Scafftig1 - Breakpoint: chr4:106897016
chr4:106896971-106897075
Overlap Length: 15 INS size: 414 Genotyped: No

```
left    AGAGAGCAGAAAG-CTTAACATATT--TAGCAATAAA---TTTAAAAATAT--------CCATTTTTCTT
chrom   AGAGAGCAGAAAG-CTTAACATATT--TAGCAATAAA---TTTAAAAATAT--------CCATTTTTCTT
right   TCCGCCCGCCTCGGCCTCCCAAAGTGCTGGGATTACAGGCGTGAGCCACCGCGCCCGGCCCATTTTTCTT
        111*11*11111*1*1*11**1*1*11*1*1*1**1*1111*1*111*11111111111***********

left    TTTTTTTTNNNNNNNNNNNNNNNNNNNNNNNNNNNNNNNNNNNNNNNNNNNNNNNNNNNNNNNNN
chrom   TTTT--------------AAAGAAAGCAAATGCACAGCCATGAGCTGCCCTTTCCAGCCTTAT
right   TTTT--------------AAAGAAAGCAAATGCACAGCCATGAGCTGCCCTTTCCAGCCTTAT
        ****2222222222222222222222222222222222222222222222222222222222222
```

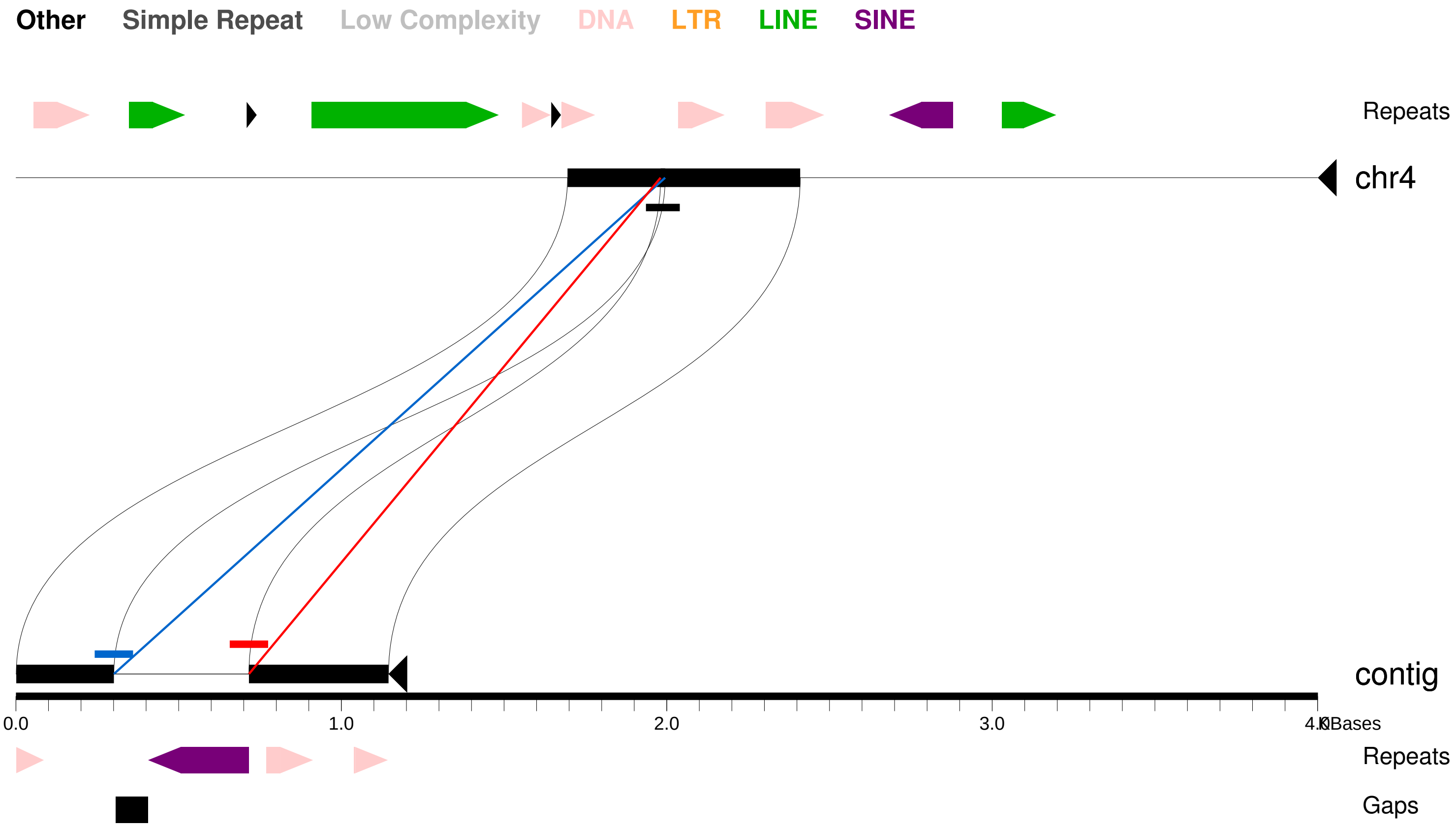

siteID: chr4_10722292 Scafftig2 - Breakpoint: chr4:10722274
chr4:10722114-10722453
Overlap Length: 20 INS size: 206 Genotyped: Yes

```
left   -----------------GATAATGAAAATTAAAAAACTGCAATGAGTTATCCCTACATACCTATTAGAAT
chrom  ACATCATTAGGTATTAAGATAATGAAAATTAAAAAACTGCAATGAGTTATCCCTACATACCTATTAGAAT
right  AGA--AT--GGCGTGAACCCGGG----AGGCGGAGCTTGCAGTGAG-----CCGAGATCGCGCC---ACT
       2N2  22  22NN2N221111111111*11111*111****1****11111**1*1**11*111111*1*

left   G-ACTTTAAAAACCTCCGCAAAACGTGTACCATTTACTTTCTACTGTACTATGAAAACGTAATGAGGTAC
chrom  G-ACTTTAAAAACCTCCGCAAAACGTGTACCATTTACTTTCTACTGTACTATGAAAACGTAATGAGGTAC
right  GCACTCCAG---CCTGGGCGACA---GAGCGAGACTCCGTCT------CAAAAAAAA-----------AA
       *1***11*1111***11**1*1*111*11*1*1111*11***111111*1*11****11111111111*1

left   AGAAAAACCACAAGAGAGATGAAAATAAAGAACTGAACAAACCCCGTCTCTACTAAAAATA---------
chrom  AGAAAAACCACAAGAGAGATGAAAATAAAGAACTGAACAAAAATGCATACAAAAAAAAATAGTGACACCA
right  AAAAAAAAAAAAAAAAAAAAAAAAAAAAAAGAACTGAACAAAAAATGCATACAAAAAAAAATAGTGACACCA
       *1*****11*1**1*1*1*11*****1**************22222222*2*22*******222222222

left   -CAAAAATTAGCCGGGC---GTGG----TGGCGGGCGCCTGTAGTCCCA--------GCTACTTGGGAGG
chrom  CCAAATGCTGACAAGGATATGTTGAAAATGGATCACACATACATTGCTGATGGGAATGTAAAATTGTACG
right  CCAAATGCTGACAAGGATATGTTGAAAATGGATCACACATACATTGCTGATGGGAATGTAAAATTGTACG
       2****222*22*22**2222**2*2222***2222*2*2*22*2*2*2222222222*22*22*2*2*2*

left   CTGAGGCAGGAGAATGGCGTGAAC----CCGGGAGGCGGAGC---------TTGCAG----
chrom  GGGACGCTGGAAAACAGCTTGACAGTTTCTTAAAAAAATAACAACTAACAATTGCACTCCT
right  GGGACGCTGGAAAACAGCTTGACAGTTTCTTAAAAAAATA---------------------
       22**2**2***2**22**2***222222*2222*22222*N1         11111N
```

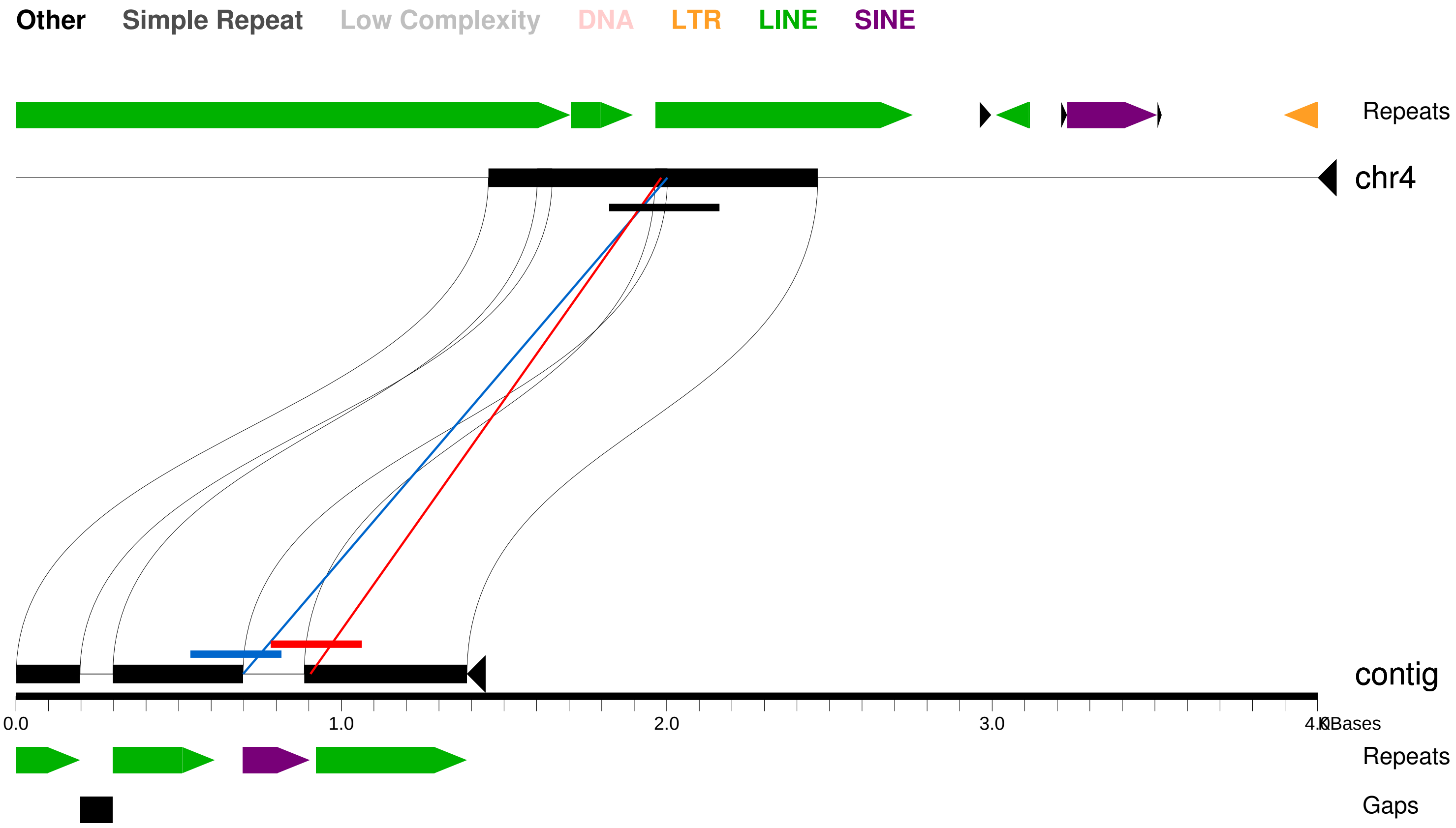

siteID: chr4_110000962 Scafftig1 + Breakpoint: chr4:110000952
chr4:110000905-110001011
Overlap Length: 13 INS size: 321 Genotyped: Yes

```
left    TCACAG---GCTTCCAGTCTTCCTGAAAAGTCTTATTTATTTACATTTCT----------AAGAGTTCAA
chrom   TCACAG---GCTTCCAGTCTTCCTGAAAAGTCTTATTTATTTACATTTCT----------AAGAGTTCAA
right   ACAGAGCGAGACTCC-GTCTCAAAAAAAAAAAAAAAAAAAAAAAAAAAAAAAAAAAAAAAAAGAGTTCAA
        1**1**111*11***1****11111****11111*111*111*1*1111111111111111*********

left    GCGGCCGGGCGC-GGTGGCTCACGCCTGTAATCCCAGCACTTTGGGAGGCCGAGGCGGGCGGA
chrom   GCGAGTTAAAACTGGTG--------CTGTTTTG--AGAAATTCATCATATCTACT---GAAAT
right   GCGAGTTAAAACTGGTG--------CTGTTTTG--AGAAATTCATCATATCTACT---GAAAT
        ***22222222*2****22222222****22*222**2*2**2222*222*2*22222*2222
```

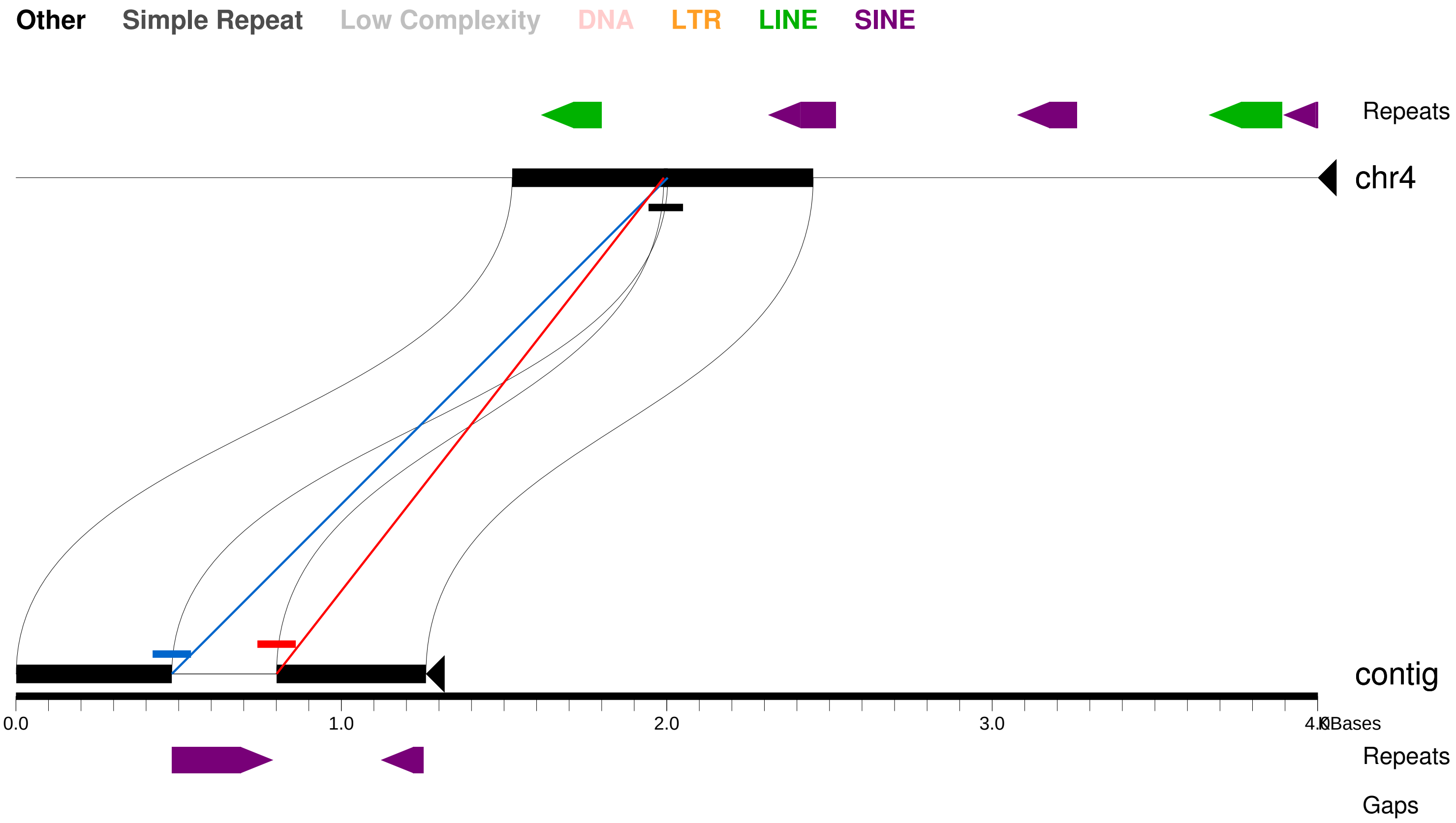

siteID: chr4_110934950 Scafftig1 - Breakpoint: chr4:110934960
chr4:110934926-110935019
Overlap Length: 4 INS size: 309 Genotyped: Yes

```
left    T---------TTTGAAACC--ATATGATTCCAGTCCAACTATTAG--------------CCTTTTTTTTTT
chrom   T---------TTTGAAACC--ATATGATTCCAGTCCAACTATTAG--------------CCTTGATTTCT
right   TCCGCCCGCCTCGGCCTCCCAAAGTGCTGGGATTACAGGCGTGAGCCACCGCGCCCGGCCCTTGATTTCT
        *111111111*11*111**11*11**1*111*1*1**1111*1**11111111111111****22***2*

left    TTTTTTTTTTTTTTTTTTTTTTGAGACGGAGTCTCGCTCTGTCGCCCAGGCTGGAGTGCAGTGGCGGGAT
chrom   AACTGATGTTCTTTTCA----TGAGAATAA--------CTG------ATTATTGTGTGATCT-------T
right   AACTGATGTTCTTTTCA----TGAGAATAA--------CTG------ATTATTGTGTGATCT-------T
        222*22*2**2****222222*****222*22222222***222222*222*2*2***222*2222222*

left    CTCG
chrom   TGAA
right   TGAA
        2222
```

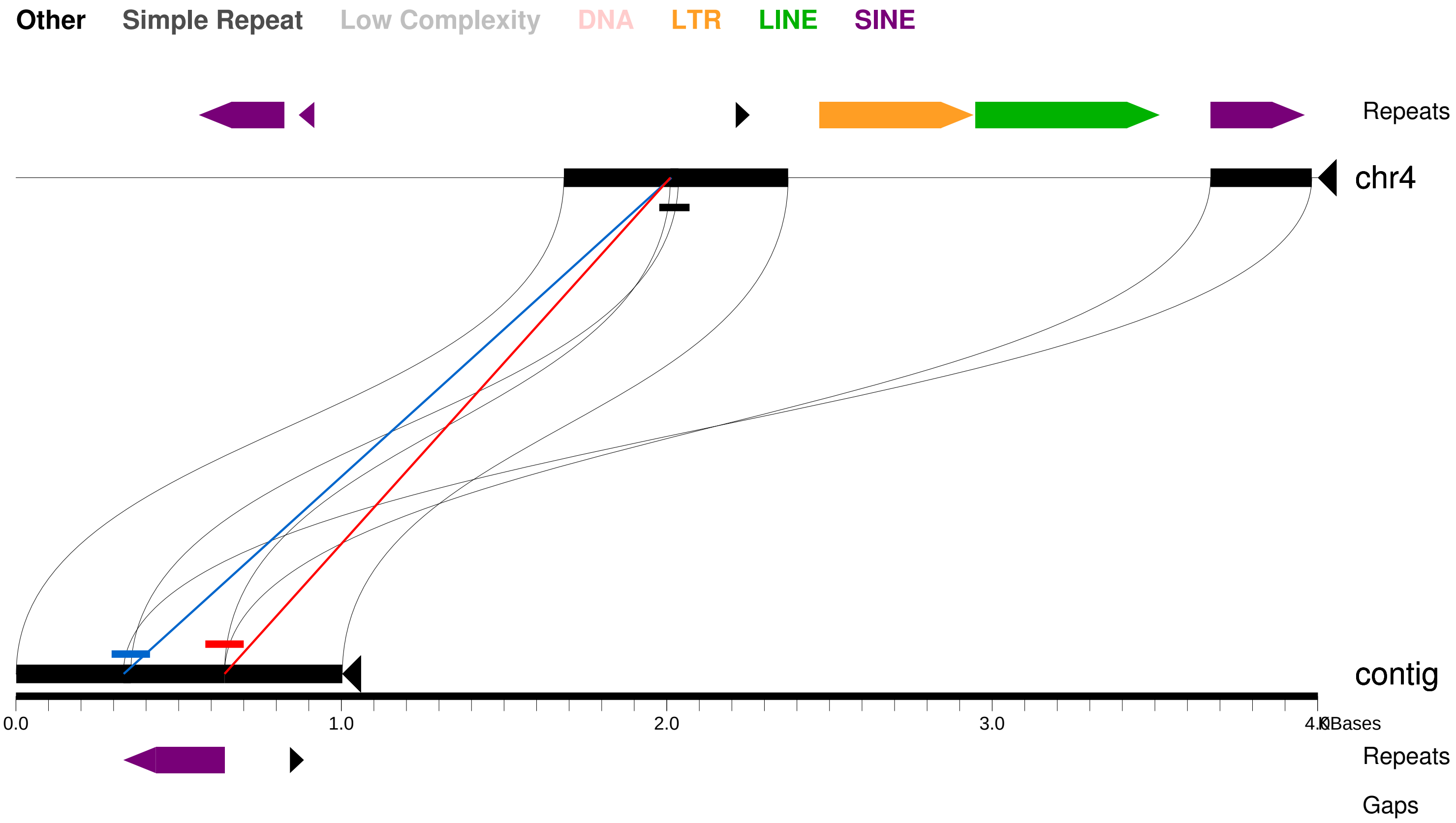

siteID: chr4_112698436 Scafftig1 - Breakpoint: chr4:112698425
chr4:112698265-112698593
Overlap Length: 9 INS size: 300 Genotyped: Yes

```
left    --------GGACCATTCTGACTACGCCCCAGCATCTAGGAAGGTAGTGATGGGATAAACAGGAGGCTGGA
chrom   ACTTTCTAGGACCATTCTGACTACGCCCCAGCATCTAGGAAGGTAGTGATGGGATAAACAGGAGGCTGGA
right   ----TTTAGTAGAGACGGGGTTTCACCGTTTTAGCCGGGATGGTCTCGATCTCCTGACCTCGTGATCCGC
            2N22*1*1111111*11*1*1**11111*1*11***1***111***1111*1*1*11*1*1111*1

left    CATTCTTTGCGTTCTCCCTTGATTGATGGGGGTTATTGGATTGCTCGCATGCTCTTTACTTATTTAACAT
chrom   CATTCTTTGCGTTCTCCCTTGATTGATGGGGGTTATTGGATTGCTCGCATGCTCTTTACTTATTTAACAT
right   CCGCCTCGGC---CTCCCAA-AGTGCTGGG---------ATTACAGGCGTG-----------------AG
        *111**11**111*****111*1**1****111111111***1*11**1**111111111111111111*1

left    CCAAAAAGTCAACCACTATTAAAAGATATTTTTTTTTTTTTTTTTTTTGAGACGGAGTCTCGCTCTGTCGC
chrom   CCAAAAAGTCAACCACTATTAAAAGATATGTTTAATTAGATGTCAGTGA--AGTAAGCTGAAT---TCAT
right   CCACCGCGCCCGGCC-----AAAAGATATGTTTAATTAGATGTCAGTGA--AGTAAGCTGAAT---TCAT
        ***1111*1*111*111111*********2***22**222*2*222***222*2*22**222*222**22

left    CCAGGCTGGAGTGCAG--TGGCGGG----ATCTCGGCTCACTGCAA-GCTC----CGCCTCCCGGGTTCA
chrom   GCAGCCATAAATTTAAATTGGCAACTCTAACCTTAGGTTTCTTAAATGGTTTAGACAACTATTTTGTTCA
right   GCAGCCATAAATTTAAATTGGCAACTCTAACCTTAGGTTTCTTAAATGGTTTAGACAACTATTTTGTTCA
        2***2*222*2*22*222****2222222*2**22*2*22**22**2*2*22222*22**22222*****

left    CGC-------------CATTCTC------------CTGCCTCAGC---------
chrom   GGCGGTAATGTTAGTACATGTTCTGTCTTACATTACTACTTCAAGACGAAAATG
right   GGCGGTAATGTTAGTACATGTTCTGTCTTACATTACTACTTCAAG---------
        2**2222222222222***22**222222222222**2*2***22
```

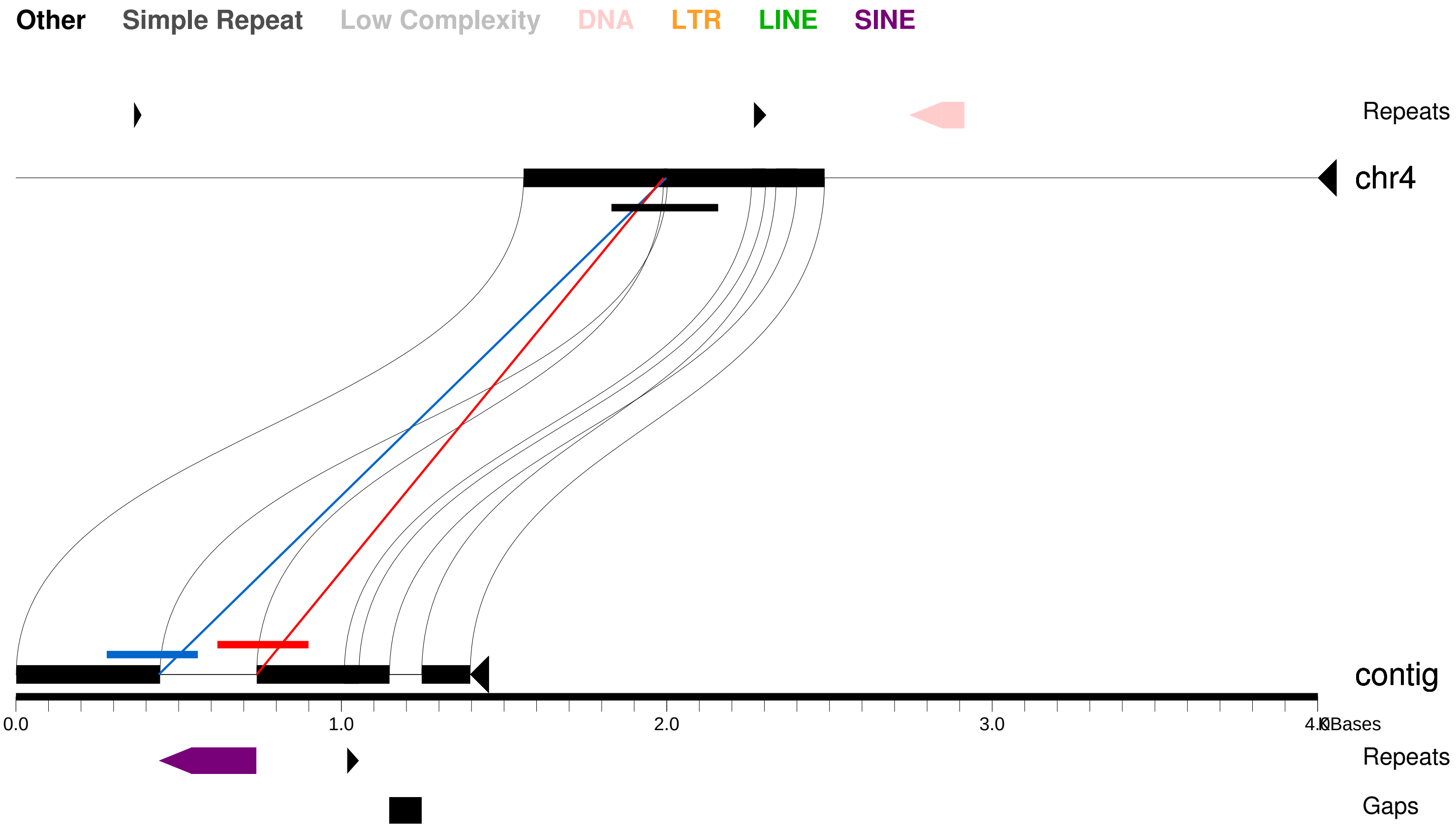

siteID: chr4_114018266 Scafftig5 + Breakpoint: chr4:114018250
chr4:114018223-114018309
Overlap Length: 15 INS size: 299 Genotyped: Yes

```
left    ---------TCTGTC--------------ATTTACAGAGTTGT---------CCAGTGTCACTTTTCCTT
chrom   ---------TCTGTC--------------ATTTACAGAGTTGT---------CCAGTGTCACTTTTCCTT
right   CCACCCGCCTCGGCCTCCCAAAGTGCTGGGATTACAGGCGTGAGCCACCGCGCCCGGCCCACTTTTCCTT
        111111111**1*1*1111111111111111******111**1111111111**1*111***********

left    TTTTTTTTTTTTTTTTTTTTTTTTTGAGACGGAGTCTCGCTGTCGCCCAGGCTGGAGTGCAGTGGCGCAATC
chrom   TTTTATATCATTAGATTTTCTT-----------------------------AAAGTG-AGTCACTCATAC
right   TTTTATATCATTAGATTTTCTT-----------------------------AAAGTG-AGTCACTCATAC
        *****2*2*22**222****2**2222222222222222222222222222222****2***22*2**22*

left    TCGGCTCACTG
chrom   TCT-CTCTCT-
right   TCT-CTCTCT-
        **22***2**2
```

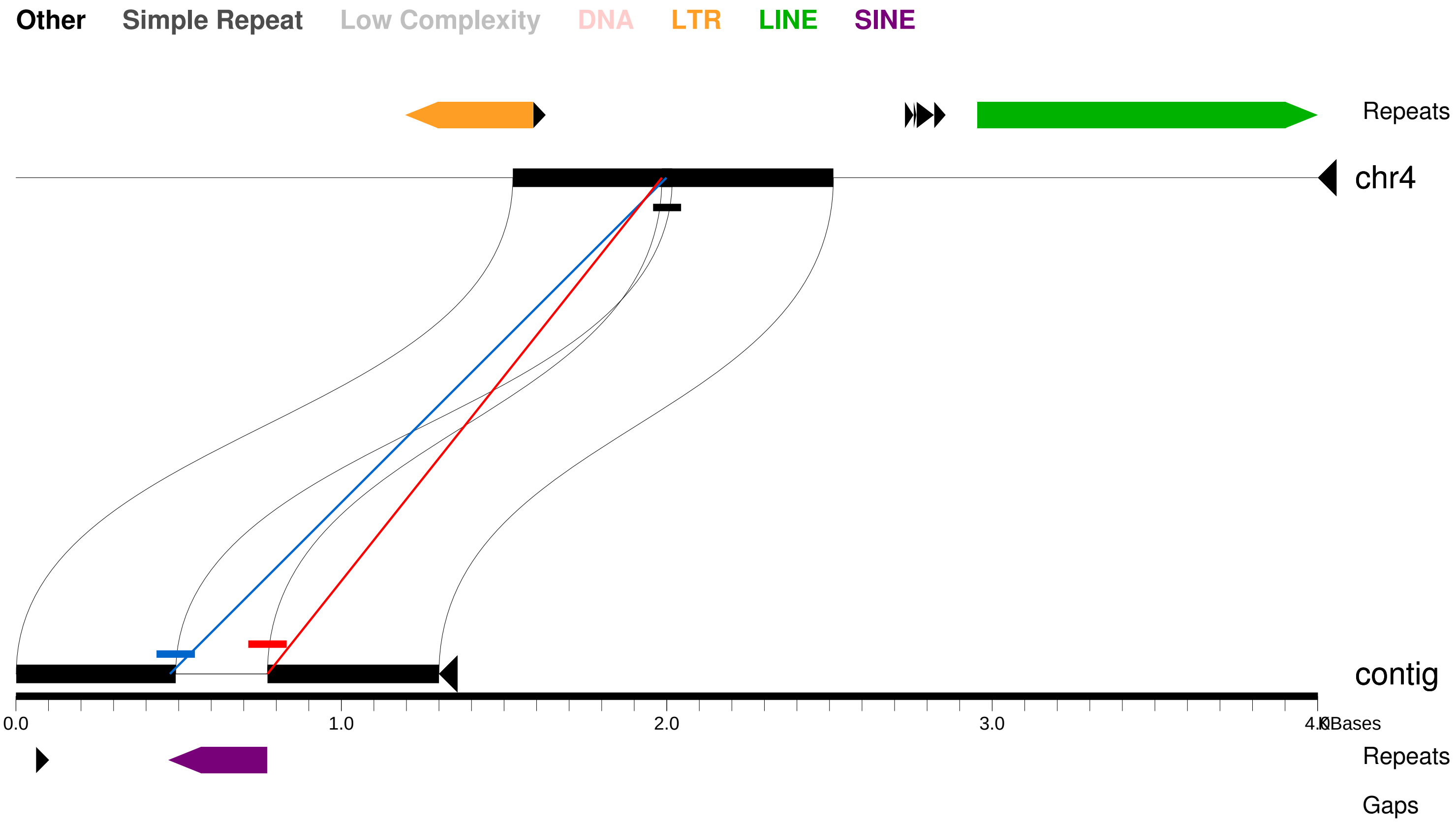

siteID: chr4_114422426 Scafftig1 - Breakpoint: chr4:114422450
chr4:114422290-114422619
Overlap Length: 10 INS size: 417 Genotyped: No

```
left     -------------------------------------------GCTGTTATTGATAAGAAACCT-ACTCC
chrom    ATT-----AGACTTT-TTTCATTCTTTTCAAAAATAGCAACTGGCTGTTATTGATAAGAAACCT-ACTCC
right    TTTAGTAGAGACGGGGTTTCACCGTTTT-------AGC--CGGGATGGTCTCGATCT----CCTGACCTC
         N22111112222NNN122222NNN2222       222  2N2*1**1*1*1***111111***1**11*

left     ATAATAAAAAGACAAAGCTCTTTCTTTGTAAAACTACAAATTAAGTGGAAGATTGTTAAGCACACTTAAA
chrom    ATAATAAAAAGACAAAGCTCTTTCTTTGTAAAACTACAAATTAAGTGGAAGATTGTTAAGCACACTTAAA
right    GTGATCCGCCCGCCTCGGCCTCCC------AAAGTGC--------TGG--GATTA-----CAGGCGTGAG
         1*1**1111111*111*11**11*111111***1*1*11111111***11****111111**11*1*1*1

left     AGTACACTGTAACTGCCAGAATTAAGATACATTTCTTT-------------------------------
chrom    AGTACACTGTAACTGCCAGAATTAAGATACATTTCTTAAAATGGTGTGATAAATGTGCTCATTAATAATG
right    C---CACCGCGCCCGGCCGA-------TACATTTCTTAAAATGGTGTGATAAATGTGCTCATTAATAATG
         1111***1*111*1*1*1**1111111**********22222222222222222222222222222222

left     ----------------------------------------------------------------------
chrom    TATGAGTAAAATATTTTCTAGCAAATAAATTTTGCAATATTCATAAGAAAGACATAGTTTTCTACTTGGA
right    TATGAGTAAAATATTTTCTAGCAAATAAATTTTGCAATATTCATAAGAAAGACATAGTTTTCTACTTGGA
         2222222222222222222222222222222222222222222222222222222222222222222222

left     ---------------------------------------------TNNNNNNNNNNN
chrom    ATGATTAAATGCTAGAGCCAAAAATAACTGGAAACAAACTTCCAATCAATAGCTCAG
right    ATGATTAAATGCTAGAGCCAAAAATAACTGGAAACAAACTTCCAA------------
         2222222222222222222222222222222222222222222221NNNNNNNNNNN
```

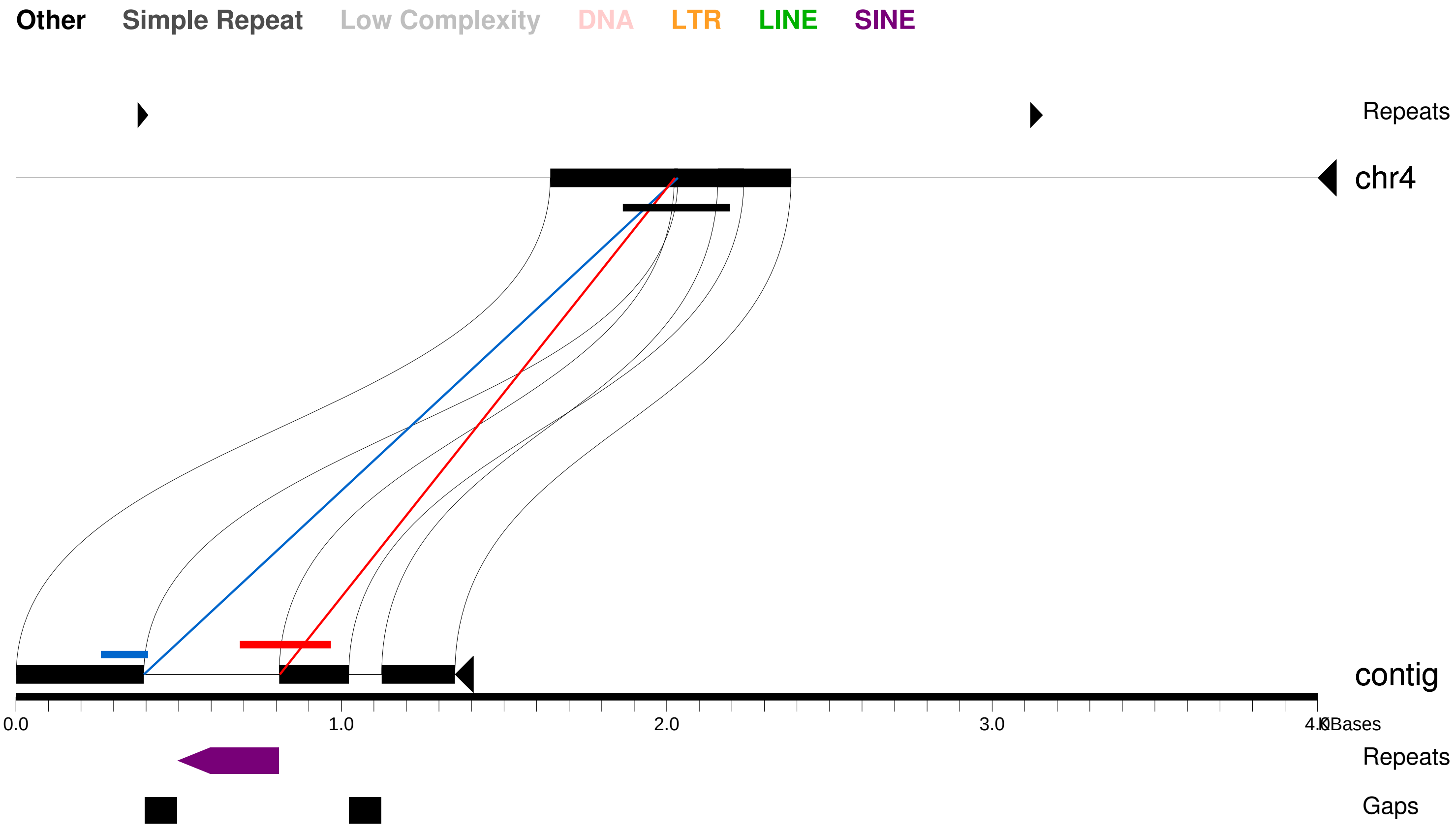

siteID: chr4_119774709 Scafftig1 + Breakpoint: chr4:119774719
chr4:119774559-119774896
Overlap Length: 18 INS size: 299 Genotyped: Yes

```
left     ---------------ATTGCAACAAAAT-GTCGGCATGAATTTGATGGACCTAAACTTGGCAATTGCAAT
chrom    GT-AGTATTAA--GCATTGCAACAAAAT-GTCGGCATGAATTTGATGGACCTAAACTTGGCAATTGCAAT
right    TTTAGTAGAGACGGGGTTTCACCGTTTTAGCCAGGATGGTCTCGATCT-CCTGACCTCGTGATCCGCCCG
         N212222NNN2112N1**1**1*1111*1*1*1*1***111*1***111***1*1**1*11*111**111

left     TTCTATTCTAGGTTCACCCAGAAGTGTGAGACTATGGTTCAGAAAACAGAACTCTCATCTTAACCCATAT
chrom    TTCTATTCTAGGTTCACCCAGAAGTGTGAGACTATGGTTCAGAAAACAGAACTCTCATCTTAACCCATAT
right    C------CTCGGC-CTCCCA-AAGTG-----CTGGGATT------ACAGG--------CGTGAGCCA---
         1111111**1**11*1****1*****11111**11*1**111111****111111111*1*1*1***111

left     AAAGACAGAGATAAGCCTATCTCTCTAGTTTAGAACTTTTCTTT-----------TTTTTTTTTTTTTTT
chrom    AAAGACAGAGATAAGCCTATCTCTCTAGTTTAGAACTTTTCTAACTGCAGAATAATTAATGCCTCCTGTT
right    -----CCGCGCCCGGCCT------CTAGTTTAGAACTTTTCTAACTGCAGAATAATTAATGCCTCCTGTT
         11111*1*1*1111****111111****************2222222222222**22*222*22*2**

left     GAGA--------CGGAGTC-TCGCTCTGTCGCCCAGGC-----TGGAGTGCAGTGGC--GGGA-------
chrom    CAGGACAGTATGCAGAGTCATAACTGGTAAAACAATGTACATTTGGACTCAAGTGGCTAGGGTGAGTTAA
right    CAGGACAGTATGCAGAGTCATAACTGGTAAAACAATGTACATTTGGACTCAAGTGGCTAGGGTGAGTTAA
         2**222222222*2*****2*22**2222222*2*2*222222****2*22******22***22222222

left     -----TCTCGGCTCACTGCAAGCTCCGCCTCCCGGGTTCA-CGCCATT--CTCCTGC---CT---
chrom    ATCCTTCTGGTCTTAGGGGTAGC---GGGAGAAGTATTCAACATCATTGCCTCCTACAGTCTTGG
right    ATCCTTCTGGTCTTAGGGGTAGC---GGGAGAAGTATTCAACATCA-------------------
         22222***2*2**2*22*22***222*222222*22****2*22**11  11111N1   11
```

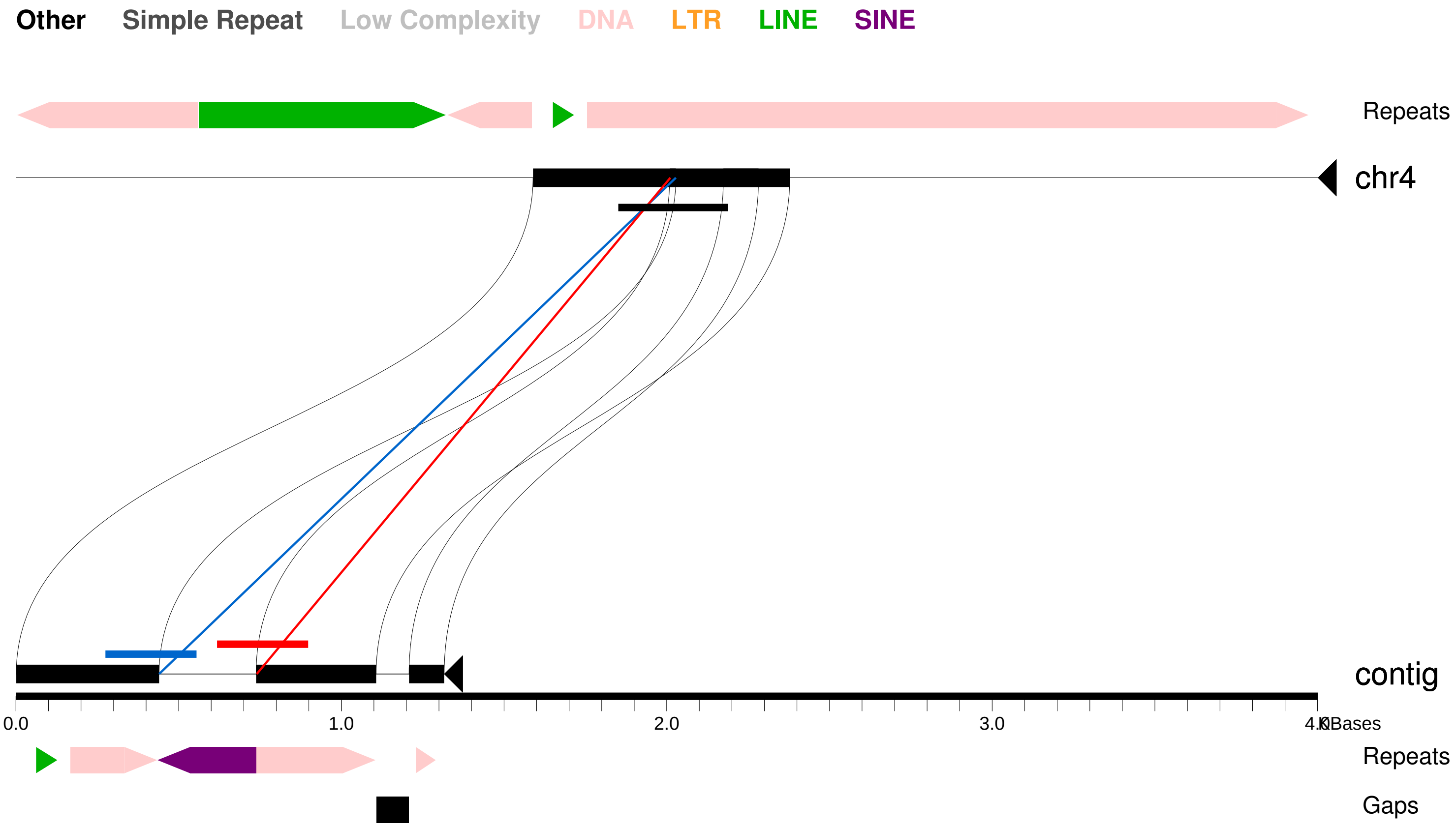

siteID: chr4_120639911 Scafftig1 - Breakpoint: chr4:120639894
chr4:120639845-120639953
Overlap Length: 11 INS size: 308 Genotyped: Yes

```
left   --AACCTTTGGGATACAGCAAGAGTG-GTGCCAATATTAAAGTTCATACATT-------AAACACCTTCA
chrom  --AACCTTTGGGATACAGCAAGAGTG-GTGCCAATATTAAAGTTCATACATT-------AAACACCTTCA
right  CCAGCCTGGGCGACAGAGCAAGACTCCGTCTCAAAAAAAAAAAAAAAAAAAAAAAAAAAAAAACACCTTCA
       11*1***11*1**1*1*******1*11**11***1*11***1111*1*1*111111111***********

left   GGCCGGGCGCGGTG-GCTCACGCCTGTAATCCCAGCACTTTGGGAGGCCGAGGCGGGCGG
chrom  TCAAAAAGTCTGAAAGAGCACAAATCTAAGGTCA-CACTC----------AAACAAACTA
right  TCAAAAAGTCTGAAAGAGCACAAATCTAAGGTCA-CACTC----------AAACAAACTA
       222222222*2*222*22***222*2***222**2****22222222222*22*222*22
```

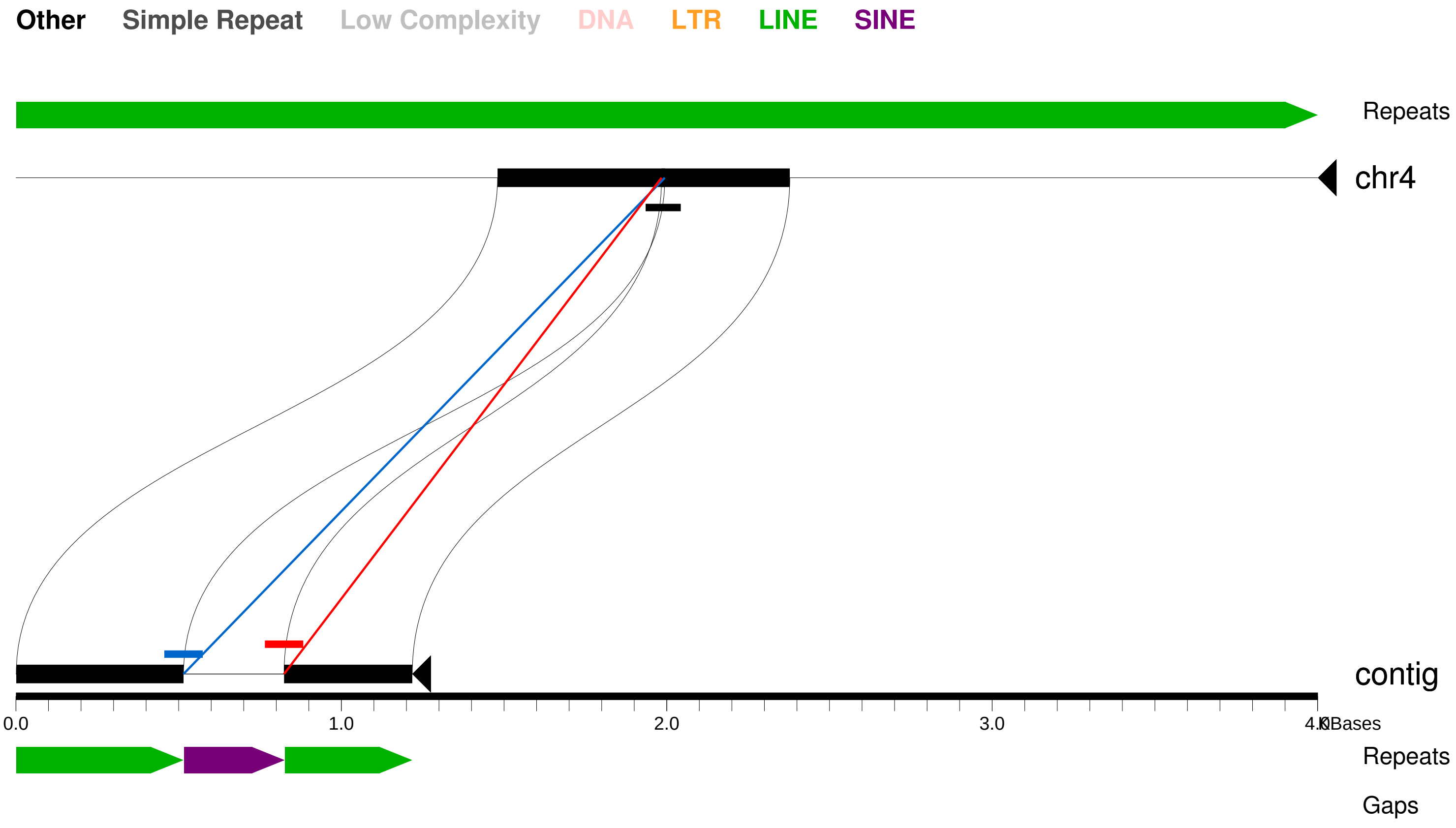

siteID: chr4_122413795 Scafftig6 + Breakpoint: chr4:122413803
chr4:122413758-122413861
Overlap Length: 15 INS size: 297 Genotyped: Yes

```
left    CTTCCATCAAGCTTTGTATC-----------ATATTATA--CATCAGATA---AGCTCATC-AACTGTGA
chrom   CTTCCATCAAGCTTTGTATC-----------ATATTATA--CATCAGATA---AGCTCATC-AACTGTGA
right   --TCCACCCGCCTCGGCCTCCCAGAGTGCTGGGATTACAAGCGTGAGCCACCGCGCCCGGCCAACTGTGA
        11****1*111**11*11**1111111111111****1*11*1*1**11*1111**1*11*1********

left    AAATTTTTTTTTTTTTTTTTTTTTTTTTTTTTTTTTGAGACGGAGTCTTGCTCTGTCCCCCAGGCTG
chrom   AAATTTTAAGCTTTATGCCTCCCTACTT---------GAAGGC-TCTT---TTGAACAACAT-CT-
right   AAATTTTAAGCTTTATGCCTCCCTACTT---------GAAGGC-TCTT---TTGAACAACAT-CT-
        *******2222***2*222*222*22**222222222**2**22****2222**22*22**22**2
```

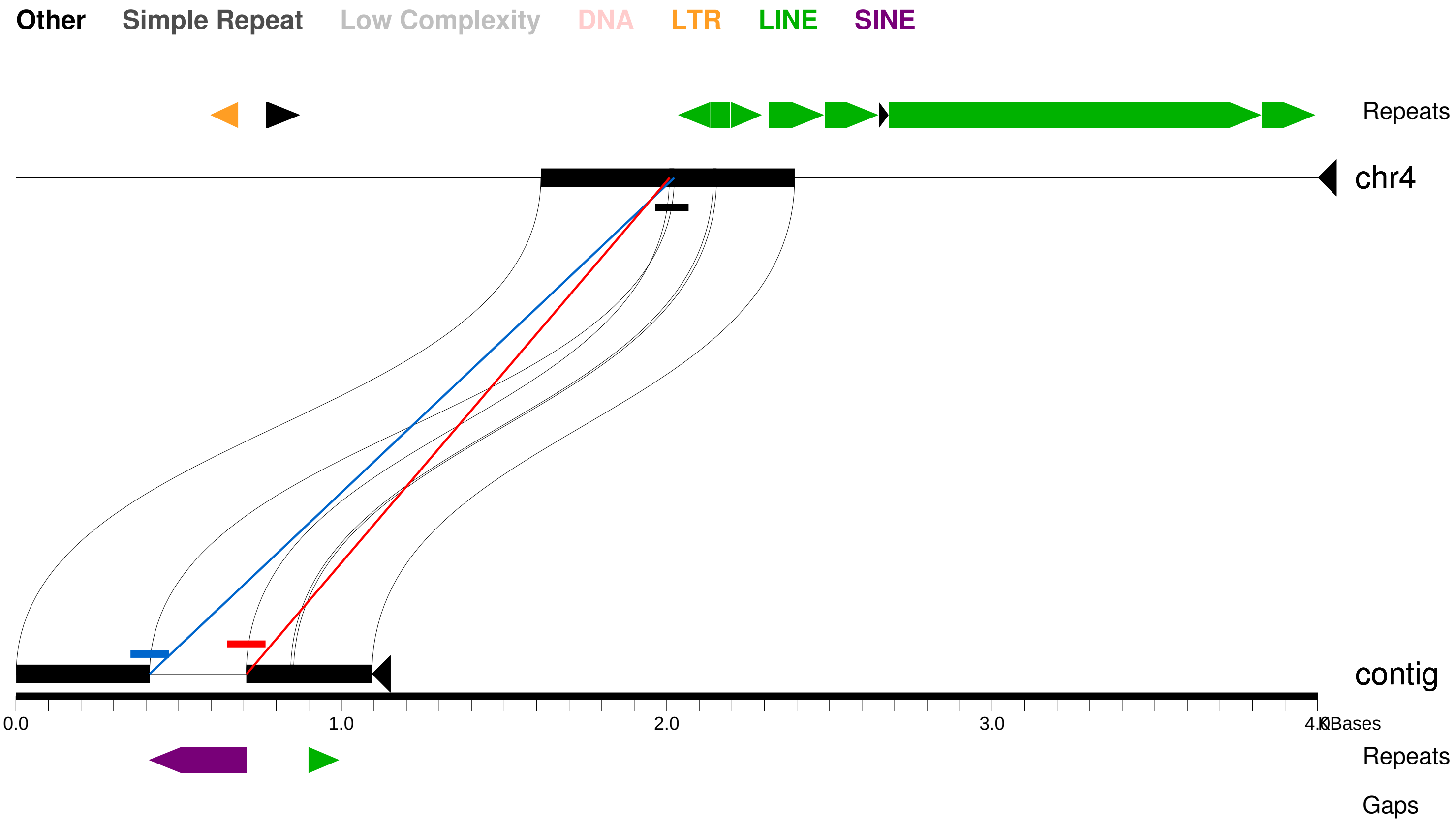

siteID: chr4_124947244 Scafftig1 + Breakpoint: chr4:124947215
chr4:124947054-124947392
Overlap Length: 18 INS size: 276 Genotyped: Yes

```
left   T-------------------AAAATCCATACAATTTCATCTCTGAAAAATGTAATAATAAATTGCTTTTA
chrom  TGTGCATAAAAATTAATTTTAAAATCCATACAATTTCATCTCTGAAAAATGTAATAATAAATTGCTTTTA
right  AGG--------------------------AGAATGGCGT----GAACCCGGGAAGCGGAGCTTGCAGTGA
       12N                 111111111*1***11*1*1111***1111*1**1111*11****11*1*

left   ATATTTTAGCAAAGATTAATTAAGTT-AGTT-GCTTGCTGGTAATTTTCCTTTCTTATTTTTTGGCAGGA
chrom  ATATTTTAGCAAAGATTAATTAAGTT-AGTT-GCTTGCTGGTAATTTTCCTTTCTTATTTTTTGGCAGGA
right  --------GCCGAGATTGCGCCACTGCAGTCCGCAGTCCGG-------CCTGGGCGACAGAGCGAGACTC
       11111111**11*****11111*1*11***11**111*1**1111111***11111*111111*11*111

left   TTTGTAAAAAACACATTCTCATAAAAAACAAGAATATTGTTCCTGT------AA--TCCCAGCACTTTG
chrom  TTTGTAAAAAACACATTCTCATAAAAAACAAGAATATTGTTTATGTGTTTGCTAAGTTACCATTACTATA
right  CGTCTCAAAAAAAAA--------AAAAAAAAAAATATTGTTTATGTGTTTGCTAAGTTACCATTACTATA
       11*1*1*****1*1*11111111*****1**1*********22***2222222**22*2***22***2*2

left   GGAGGCCGAGGCGGGTGGATCATGAGGTCAGGAGATCG----AGACC-AACCTGGCTAACA--AGGTG--
chrom  AGA--CATAACAACATAAAATACACATTCAGAAAATATTTTAACACATAAACAGTTTTAAATGAGGTCTC
right  AGA--TATAACAACATAAAATACACATTCAGAAAATATTTTAACACATAAACAGTTTTAAATGAGGTCTC
       2**22122*222222*22*22*22222****2*2**222222*2**22**2*2*22*2*2*22****222

left   AAACCCCGTCTC-TACTAAAAATAC-----AAAAAATTA---GCCGGGC--------------
chrom  AAGACTTGCTTCATAATAGGCTTATTTTTAACACAATTATATGCTCAGCTTCATTTTTCTAAT
right  AAGACTTGCTTCATAATAGGCTTATTTTTAACACAATTATATGCTCAGCTTCATTT-------
       **22*22*22**2**2**2222**222222*2*2*****222**222**2222222
```

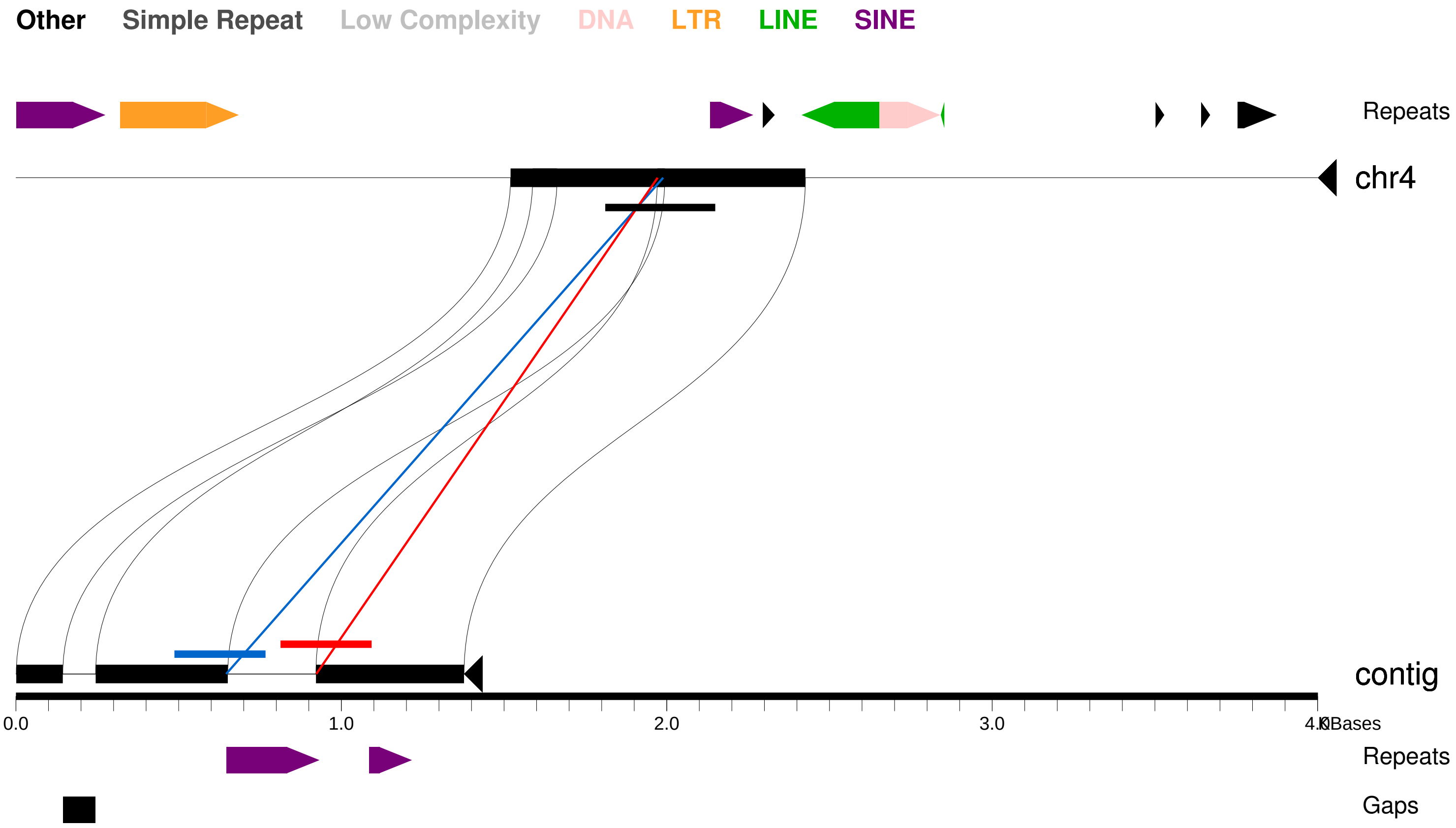

siteID: chr4_125659017 Scafftig7 + Breakpoint: chr4:125659009
chr4:125658963-125659068
Overlap Length: 14 INS size: 432 Genotyped: No

```
left    A--------TCAGAGTTTCAGGATCTTGAGGCTAACCATTGTCAAC------ATAATGAAAAGATTTTGT
chrom   A--------TCAGAGTTTCAGGATCTTGAGGCTAACCATTGTCAAC------ATAATGAAAAGATTTTGT
right   CCGCCCGCCTCGGCCTCCCAAAGTGCTG-GGATTACAGGCGTGAGCCACCGCGCCCGGCCAAGATTTTGT
        111111111**1*11*11**111*11**1**1*1**1111**1*1*11111111111*11**********

left    AAATNNNNNNNNNNNNNNNNNNNNNNNNNNNNNNNNNNNNNNNNNNNNNNNNNNNNNNNNNNN
chrom   AAATTCTAATAGTAATACCAAACAATAATATGGTGCTAGGCACTACTCTA-------------
right   AAATTCTAATAGTAATACCAAACAATAATATGGTGCTAGGCACTACTCTA-------------
        ****22222222222222222222222222222222222222222222222222222222222
```

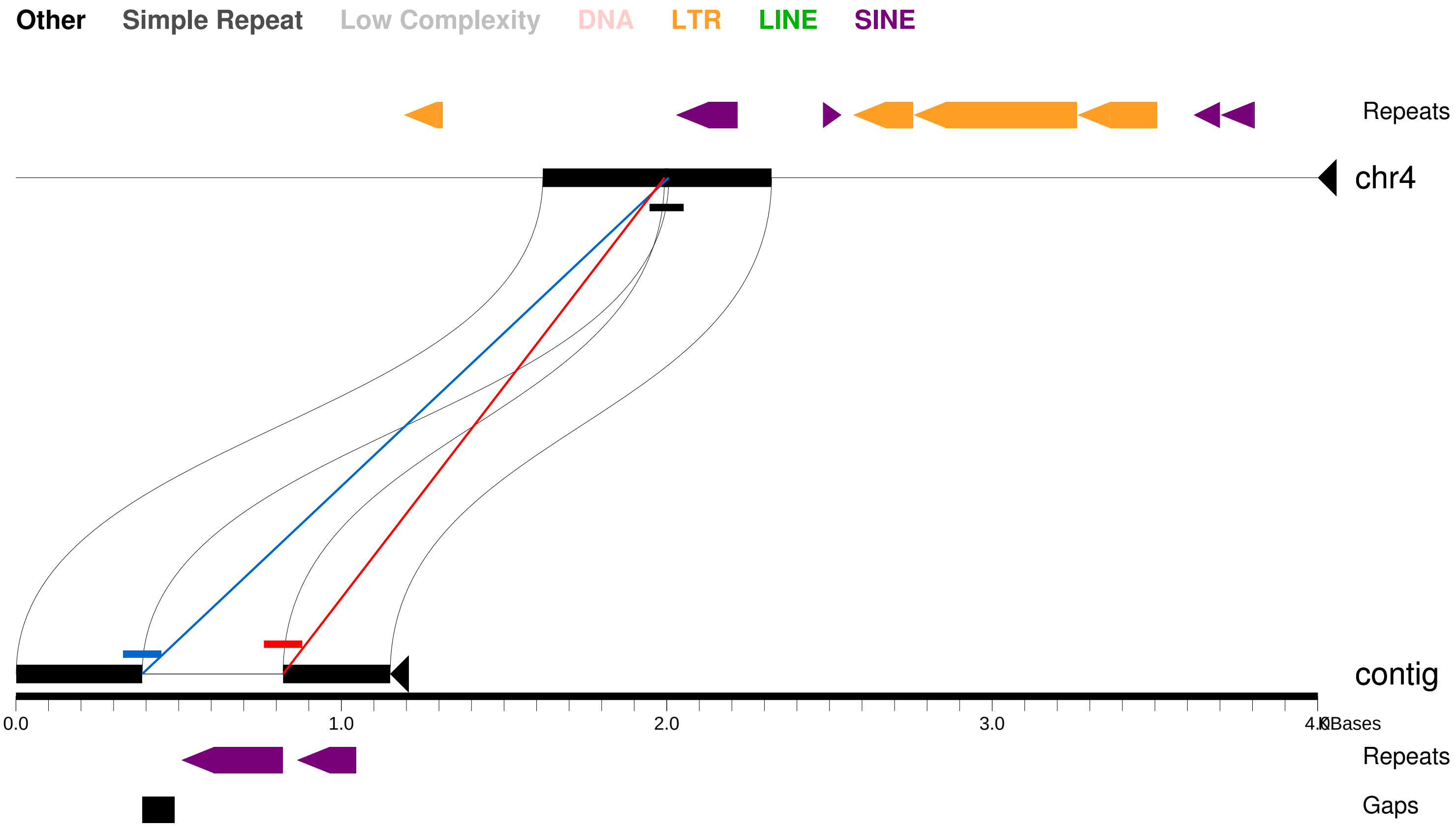

siteID: chr4_125977743 Scafftig2 + Breakpoint: chr4:125977703
chr4:125977555-125977912
Overlap Length: 62 INS size: 442 Genotyped: No

```
left   ---------------------------------------------------------------------
chrom  GAAGCTCAGCAATTGTATTACACTAAACACTATTAATGACTAAGTCTGTACATAGTGAAAGCAATTCAAT
right  NNNNNNNNNNNNNNNNNNNNNNNNNNNNNNNNNNNNNNNNNNNNNNNNNNNNNNNNNNNNNNA----------
       NNNNNNNNNNNNNNNNNNNNNNNNNNNNNNNNNNNNNNNNNNNNNNNNNNNNNNNNNNNN2

left   ------------------------------TCCTCTATTGTACCTCCATGTTACTTATCAGCATAATAAA
chrom  TCTATTTTCTTTTTCTGAAACAGTTATAATTCCTCTATTGTACCTCCATGTTACTTATCAGCATAATAAA
right  ----------------------------------------------------------------------
                                     1111111111111111111111111111111111111111

left   CTTTCTCAAAGTAAAACATTTTCAGAAAAAATATGAGTAATATATATGAGCTTTAAAAATGTCAAATGAG
chrom  CTTTCTCAAAGTAAAACATTTTCAGAAAAAATATGAGTAATATATATGAGCTTTAAAAATGTCAAATGAG
right  ---------AGTAAAACATTTTCAGAAAAAATATGAGTAATATATATGAGCTTTAAAAATGTCAAATGAG
       111111111*************************************************************

left   GGGGCCGGGCGCGGTGGCTCA---------------------CGCCTGTAA-------TCCCA---GCAC
chrom  CCTTTCTGTTATGGCCAATTAACTTGTATATTCCTTTTTTTCCCCCTCAAAAAAGAGATTCCACTTGAAC
right  CCTTTCTGTTATGGCCAATTAACTTGTATATTCCTTTTTTTCCCCCTC----------------------
       22222*2*2222**2222*2*2222222222222222222222*2***2N11       1N111   1N11

left   TTTGG-----------------GAGGCC-----------------------------------GAGGCGGG
chrom  TAAGGAAAAATTTTCAGGATATGAGACACAACTACACATTTGAATATTTTTTCTTGTATAAGAAATTGA
right  ----------------------------------------------------------------------
       1NN11                 111N1N                                  11NNNN1N

left   C-------
chrom  CTTTACAA
right  --------
       1
```

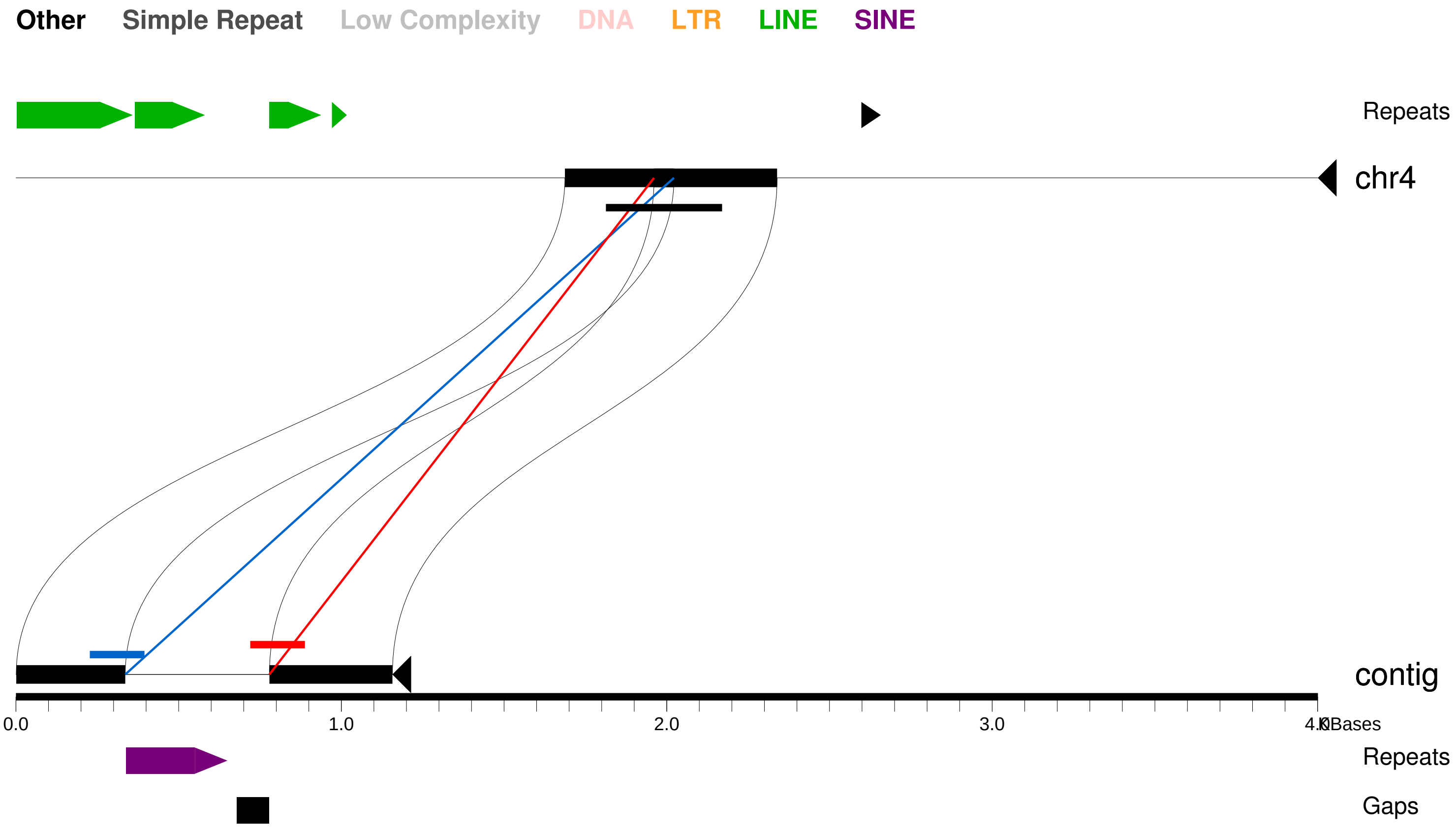

siteID: chr4_127514848 Scafftig3 - Breakpoint: chr4:127514852
chr4:127514807-127514911
Overlap Length: 15 INS size: 109 Genotyped: Yes

```
left     CTCCCAGACATTTA--CTCGAAGGACTAGAAAACATAGGTCC------------ACATTAAAACTTCACA
chrom    CTCCCAGACATTTA--CTCGAAGGACTAGAAAACATAGGTCC------------ACATTAAAACTTCACA
right    GCCACTGCACTCCAGCCTGGGCGACAGAGCGAAACTCCGTCTCAAAAAAAAAAAAAAAAAAAAACTTCACA
         11*1*1*111*11*11**1*11*1111**11**11*11***1111111111111*1*11***********

left     CAGAGGAGAATGGCGTGAACCCGGGAGGCGGAGCTTGCAGTGAGTCGAGATCGCGCCACTGCA
chrom    CAGATGTTTATAGCAG-----CTTTATTCATAATTGCCAA--AACCTAGA-------AGTAAC
right    CAGATGTTTATAGCAG-----CTTTATTCATAATTGCCAA--AACCTAGA-------AGTAAC
         ****2*222**2**2222222*222*22*22*22*22**222*22*2***2222222*2*222
```

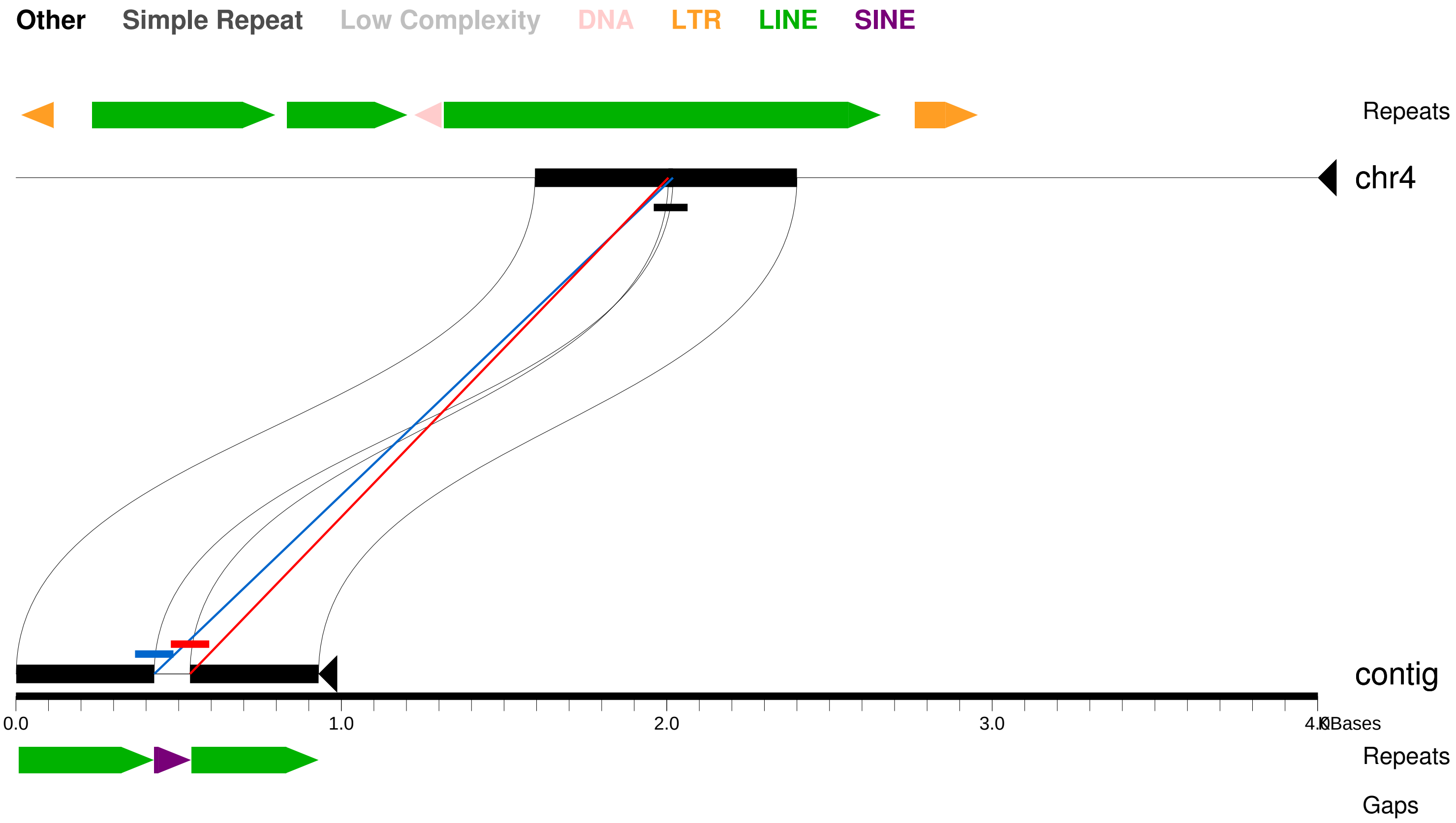

siteID: chr4_129207234 Scafftig2 - Breakpoint: chr4:129207236
chr4:129207193-129207295
Overlap Length: 17 INS size: 307 Genotyped: Yes

```
left    CAAAGC--------ACTTCTAAAAT----ATCTTCCAGCTATCAAGCACTGAAAT----CAGAAGTATAT
chrom   CAAAGC--------ACTTCTAAAAT----ATCTTCCAGCTATCAAGCACTGAAAT----CAGAAGTATAT
right   TCCACCCGCCTCGGCCTCCCAAAGTGCTGGGATTACAGGCGTGAGCCACCGCGCCCGGCCAGAAGTATAT
        111*1*111111111**1*1***1*1111111**1***111*1*11***1*11111111***********

left    ATATTTTTTTTTTTTTTTTTTTTTTTTGAGACGGAGTCTCGCTCTGTCGCCCAGGCCGGACTGCG
chrom   AT-TTTTAAGTGTCAATAAATCTTATACACCTACAGATTAAATT---------------AAGGAG
right   AT-TTTTAAGTGTCAATAAATCTTATACACCTACAGATTAAATT---------------AAGGAG
        **2*****222*2*222*222*2**2*22*22222**22*222*2222222222222222*22*2*
```

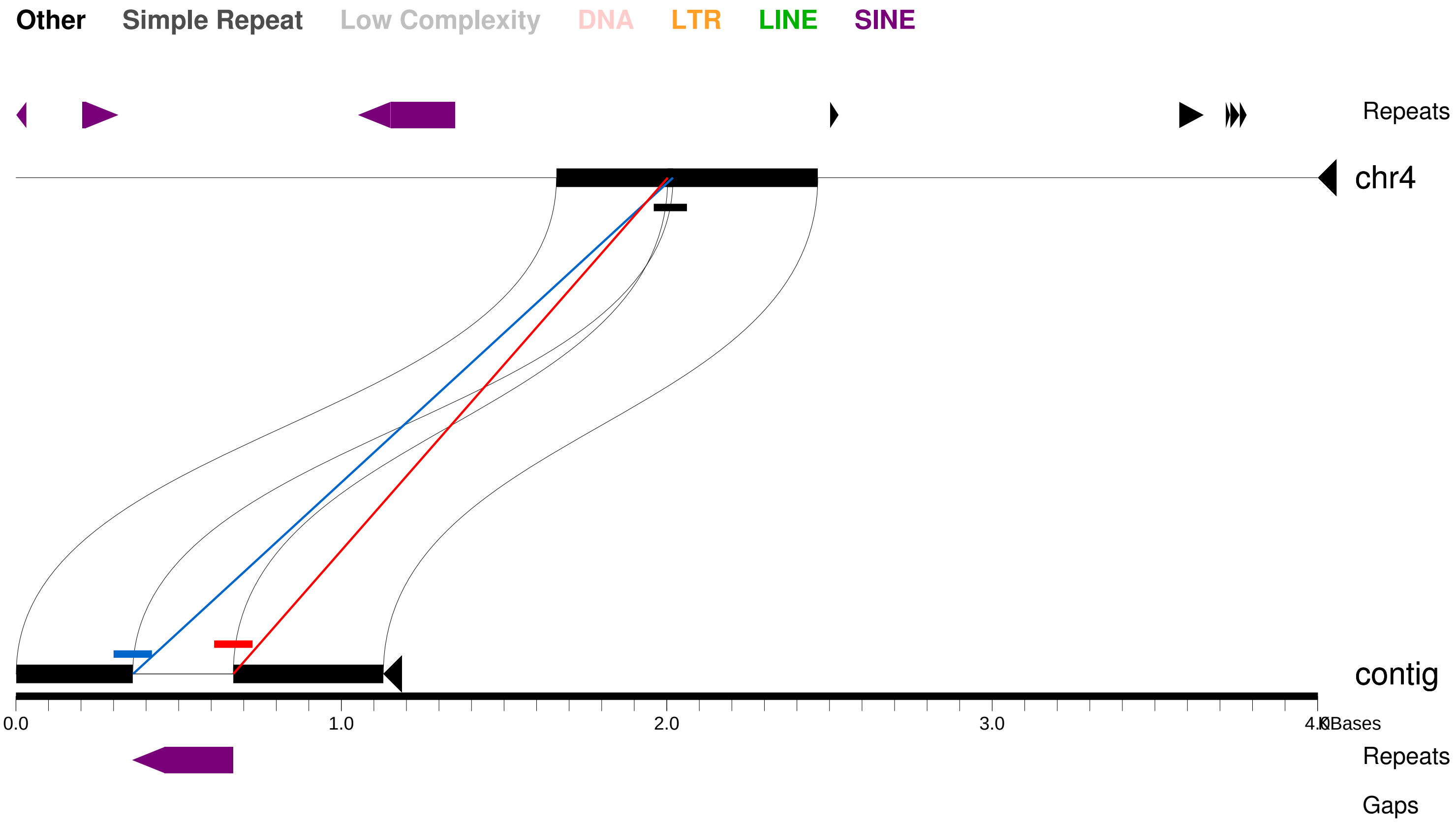

siteID: chr4_1301840 Scafftig4 - Breakpoint: chr4:1301817
chr4:1301774-1301876
Overlap Length: 17 INS size: 299 Genotyped: Yes

```
left   ---GAACCATTACCTGTGC---------AGATACGGTC----ATGTGTCACTTACATGGAAAAACTCTCA
chrom  ---GAACCATTACCTGTGC---------AGATACGGTC----ATGTGTCACTTACATGGAAAAACTCTCA
right  ACTGCACTCCAGCCTGGGCGACAGAGCGAGACTCCGTCTCAAAAAAAAAAAAAAAAAAAAAAAAACTCTCA
       111*1**11111****1**111111111***11*1***1111*111111*111*1*111***********

left   GAAATGGCCGGGCGCGGTGGCTCACGCCTGTAATCCCAGCACTTTGGGAGGCCGAGGCGGGCGGA
chrom  GAAATG-CATCGTTAGGCGACTTTGCGGTGTGA-------ACAT--------CGTGGAATGAACT
right  GAAATG-CATCGTTAGGCGACTTTGCGGTGTGA-------ACAT--------CGTGGAATGAACT
       ******2*222*222**2*2**222222***2*2222222**2*22222222**2**222*2222
```

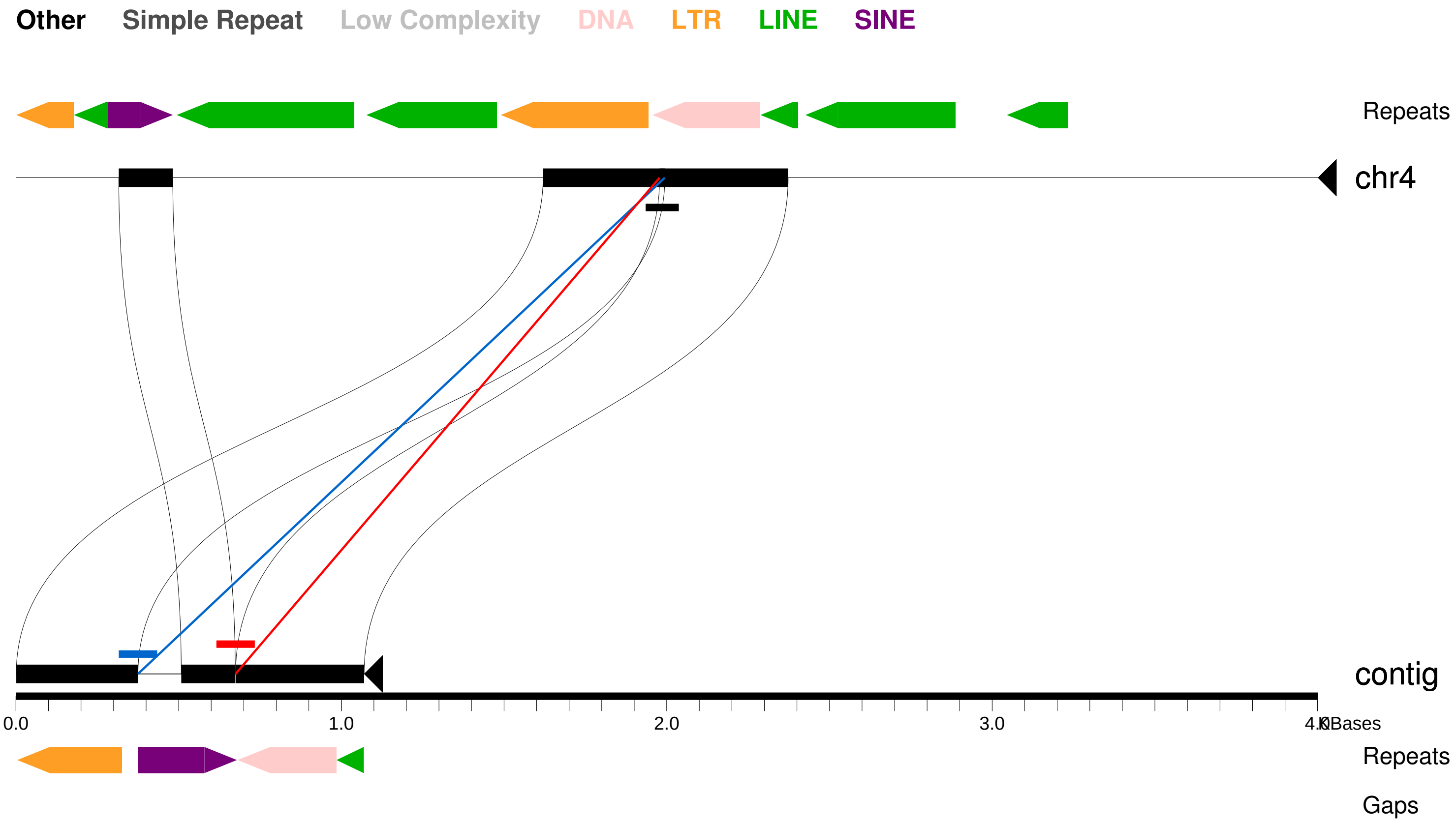

siteID: chr4_130812800 Scafftig2 + Breakpoint: chr4:130812814
chr4:130812766-130812873
Overlap Length: 12 INS size: 299 Genotyped: Yes

```
left    ----TGCTCTC-ATATTAAGACATGAAGCTT-ACT-----TTAGCATTTAGTTTTCTTTAAAAATGCAGT
chrom   ----TGCTCTC-ATATTAAGACATGAAGCTT-ACT-----TTAGCATTTAGTTTTCTTTAAAAATGCAGT
right   CCACTGCACTCCAGCCTGGGCGACAGAGCGAGACTCCGTCTCAAAAAAAAAAAAAAAAAAAAAAATGCAGC
        1111***1***1*111*11*11*111***111***11111*1*11*111*111111111**********1

left    CGGCCGGGCGCGGTGGCTCACGCCTGTAATCCCAGCACTT-----TGGGAGGCCGAGGCGGGCGG
chrom   CA-----------TATTTCACTAAT---ATGACAGAATTTATAAATTGAAGTT--AGCCATTGGT
right   CA-----------TATTTCACTAAT---ATGACAGAATTTATAAATTGAAGTT--AGCCATTGGT
        *222222222222*222****222*222**22***2*2**22222*2*2**2222**2*2222*2
```

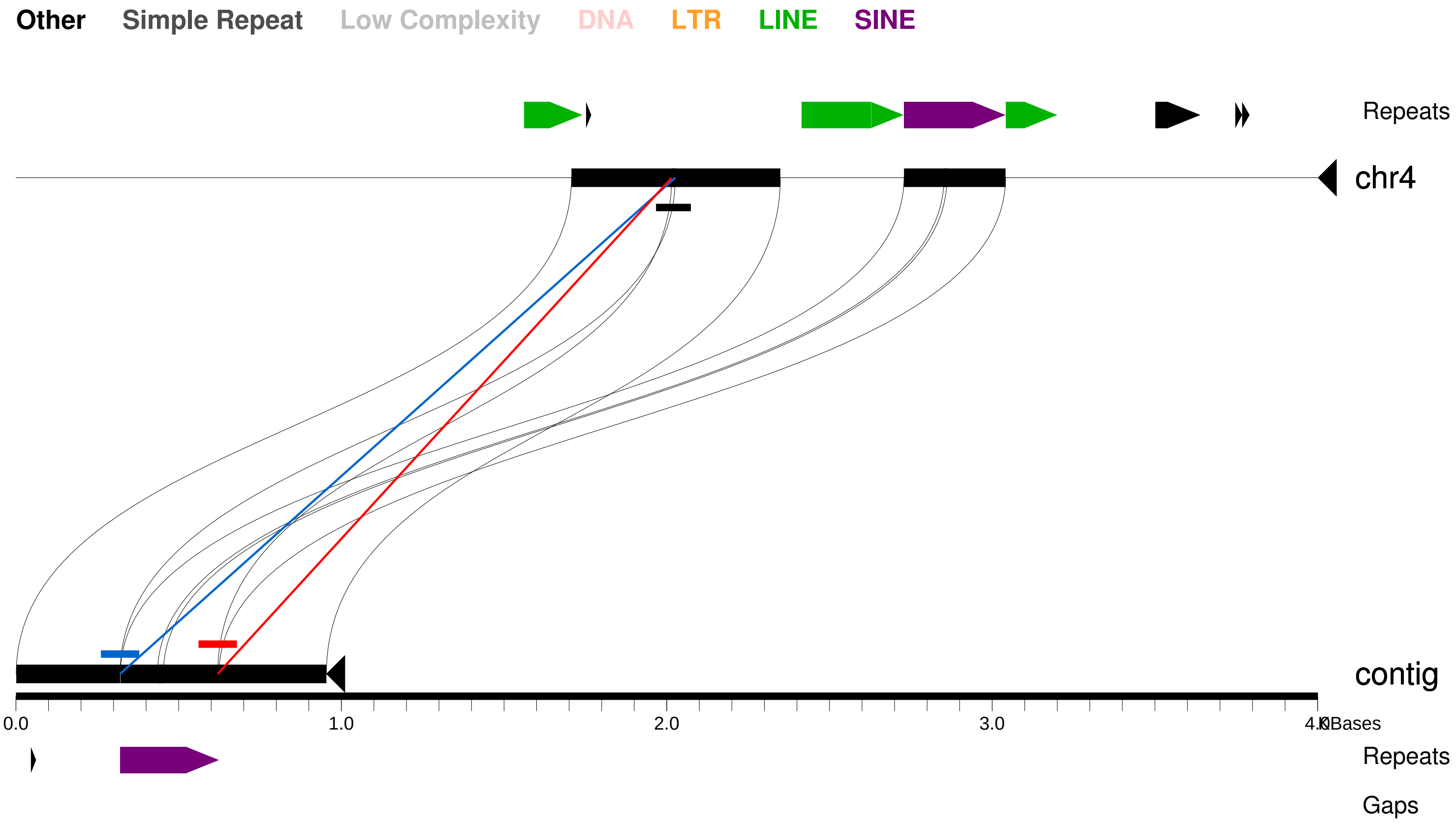

siteID: chr4_131421305 Scafftig1 - Breakpoint: chr4:131421300
chr4:131421258-131421359
Overlap Length: 14 INS size: 422 Genotyped: No

```
left   AACAGATTTCAAGACTGACAAAGAAGAACTTTTGTAGCAATA-----------------AGATATCAGAT
chrom  AACAGATTTCAAGACTGACAAAGAAGAACTTTTGTAGCAATA-----------------AGATATCAGAT
right  NNNNNNNNNNNNNNNNNNNNNNNNNNNNNNNNNNNNNNNNNNNNNNNNNNNNNNNNNNNAGATATCAGAT
       11111111111111111111111111111111111111111111111111111111111***********

left   TCCGGCCGGGCGCGGTGGCTCACGCCTGTAATCCCAGCACTT-TGGGAGGCCGAGGCGGGCGGATCA
chrom  TCCAGCC--------TGACTCTAGAATAGCATCACATGACAGATAGCAGACC--------CGGA--A
right  CCCAGCC--------TGACTCTAGAATAGCATCACATGACAGATAGCAGACC--------CGGA--A
       1**2***22222222**2***22*22*222***2**22**222*2*2**2**22222222****22*
```

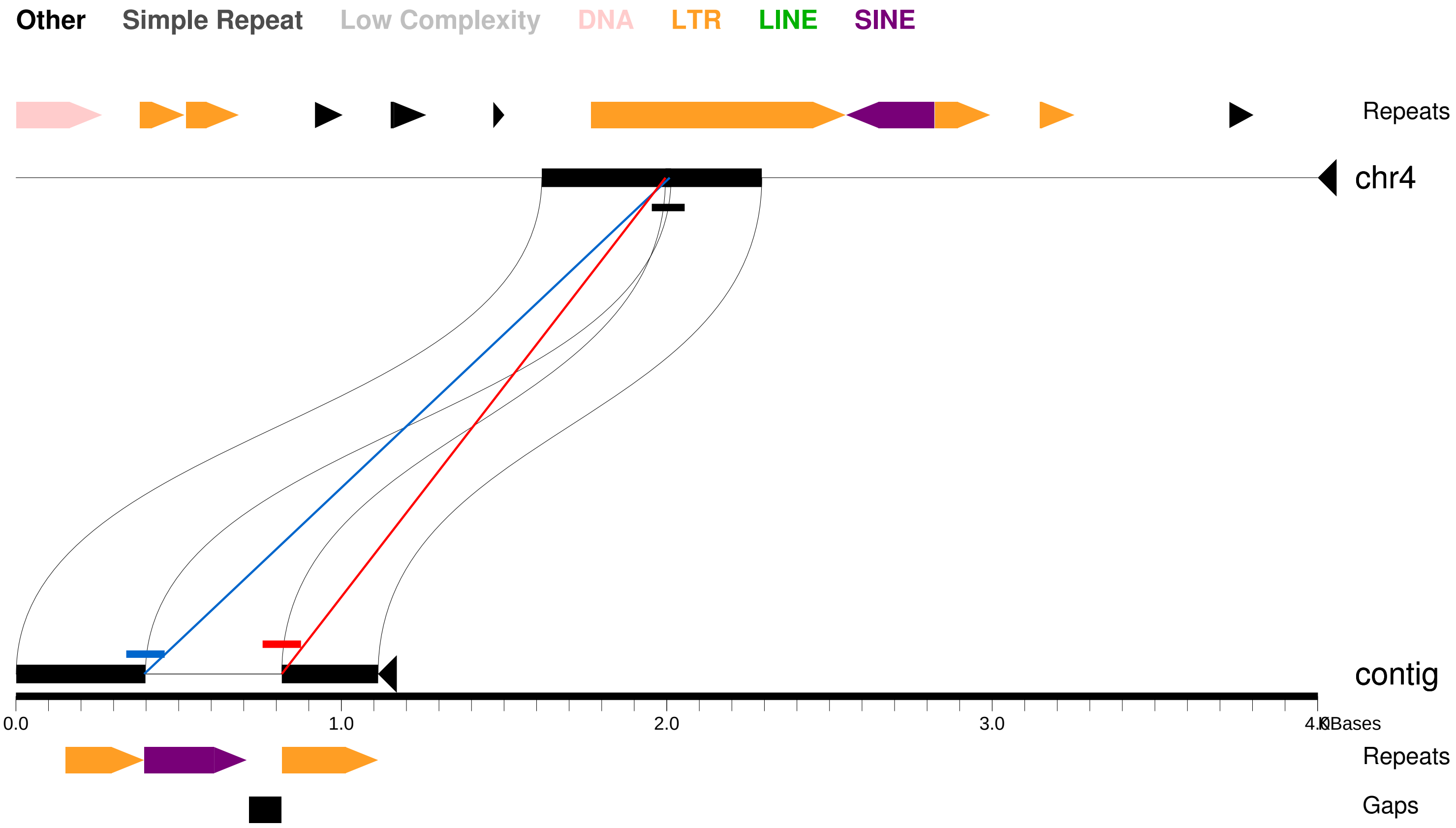

siteID: chr4_132287439 Scafftig2 - Breakpoint: chr4:132287464
chr4:132287304-132287632
Overlap Length: 9 INS size: 433 Genotyped: No

```
left    A-----------------------------------ATAGAGGCACAATCCAAATCGAGTAGAAAATAAAA
chrom   ATGTAATCCCTGATGGATGCAAAATCAAGATTGAAATAGAGGCACAATCCAAATCGAGTAGAAAATAAAA
right   TT-TAGT-----AGAGACGGGGTTTCACC-TTGTTAGCCAGG-ATGGTCTCGATCTCCTG--ACCTCA--
        12 22N2     2NN22N2NNNNN222NN 222NN*111***1*111**111***111*111*11*1*11

left    TGTGTTCAACCATAAAGATTAAAGTCAATACTTTCATTTTATGTTGACATTAATTTACGTACCATTCAAC
chrom   TGTGTTCAACCATAAAGATTAAAGTCAATACTTTCATTTTATGTTGACATTAATTTACGTACCATTCAAC
right   --TGATCCACCC----GCCTC--GGC----CTCCCAAAGT--GCTGGGATTAC-------AGGCGTGAGC
        11**1**1***11111*11*111*1*1111**11**111*11*1**11****11111111*1111*1*1*

left    CTCTGTAACTTTTGCATTTACAAAATGACNNNNNNNNNNN------------------------------
chrom   CTCTGTAACTTTTGCATTTACAAAATGACTTTTTCTATTGCTCTTAGAAATTAAGAGCAATAGAAACTAC
right   CACCGCGCCCG--GCC----CAAAATGACTTTTTCTATTGCTCTTAGAAATTAAGAGCAATAGAAACTAC
        *1*1*111*1111**11111*********222222222222222222222222222222222222222222

left    ----------------------------------------------------------------------
chrom   AGTCCGAGAAAAATTAGCTAACAATAAAGTTTAATAAAACATCCATTTCTACTCCGCAGTTTACACATAC
right   AGTCTGAGAAAAATTAGCTAACAATAAAGTTTAATAAAACATCCATTTCTACTCCGCAGTTTACACATAC
        2222N22222222222222222222222222222222222222222222222222222222222222222

left    -----------------------------------------------------
chrom   ACATAAGCACATTCACAAATATGTAAAGATTTAATAACAATACTAATAA
right   ACATAAGCACATTCACAAATATGTAAAGATTTAATAACAA---------
        2222222222222222222222222222222222222222
```

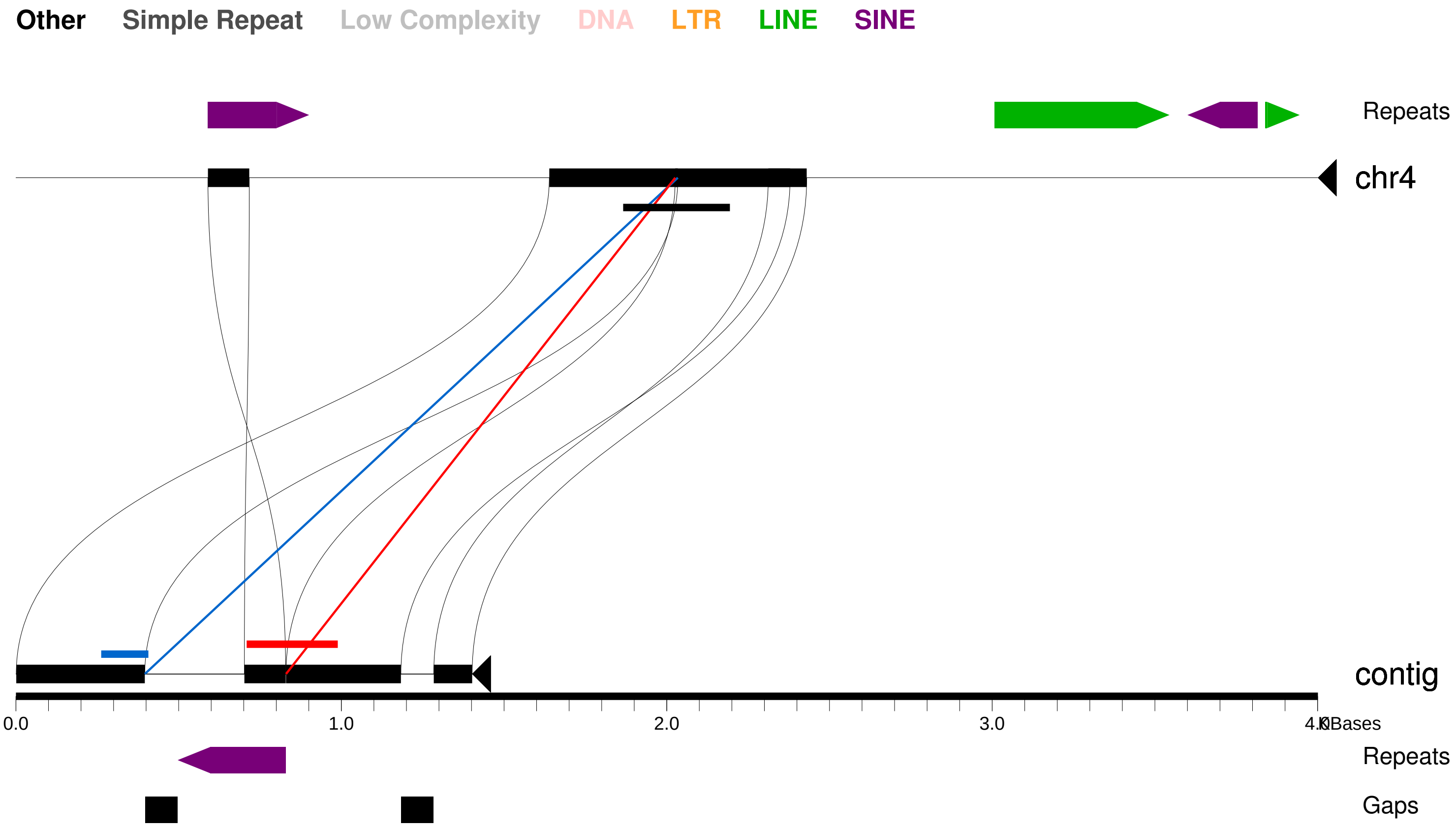

siteID: chr4_133117823 Scafftig1 - Breakpoint: chr4:133117830
chr4:133117783-133117889
Overlap Length: 13 INS size: 414 Genotyped: No

```
left    TACATAATAAAATTCTTATTAATTAAAAGTCAATTCCAAAGGCATGT------------AAAACATAAGG
chrom   TACATAATAAAATTCTTATTAATTAAAAGTCAATTCCAAAGGCATGT------------AAAACATAAGG
right   NNNNNNNNNNNNNNNNNNNNNNNNNNNNNNNNNNNNNNNNNNNNNNNNNNNNNNNNNNNNAAAAACATAAGG
        111111111111111111111111111111111111111111111111111111111111***********

left    AAGGCCGGGCGCGGTGGCTCACGCCTGTAATCCCAGCACTTTGGGAGGCCGAGACGGGCGG
chrom   AAA-----GTGCATTAAAA-ACAACTGGCTTCTATTCAGGTAAAGTATTTATTAT------
right   AAA-----GTGCATTAAAA-CCAACTGGCTTCTATTCAGGTAAAGTATTTATTAT------
        **222222*2**22*222221*22***222**2222**22*222*22222222*2222222
```

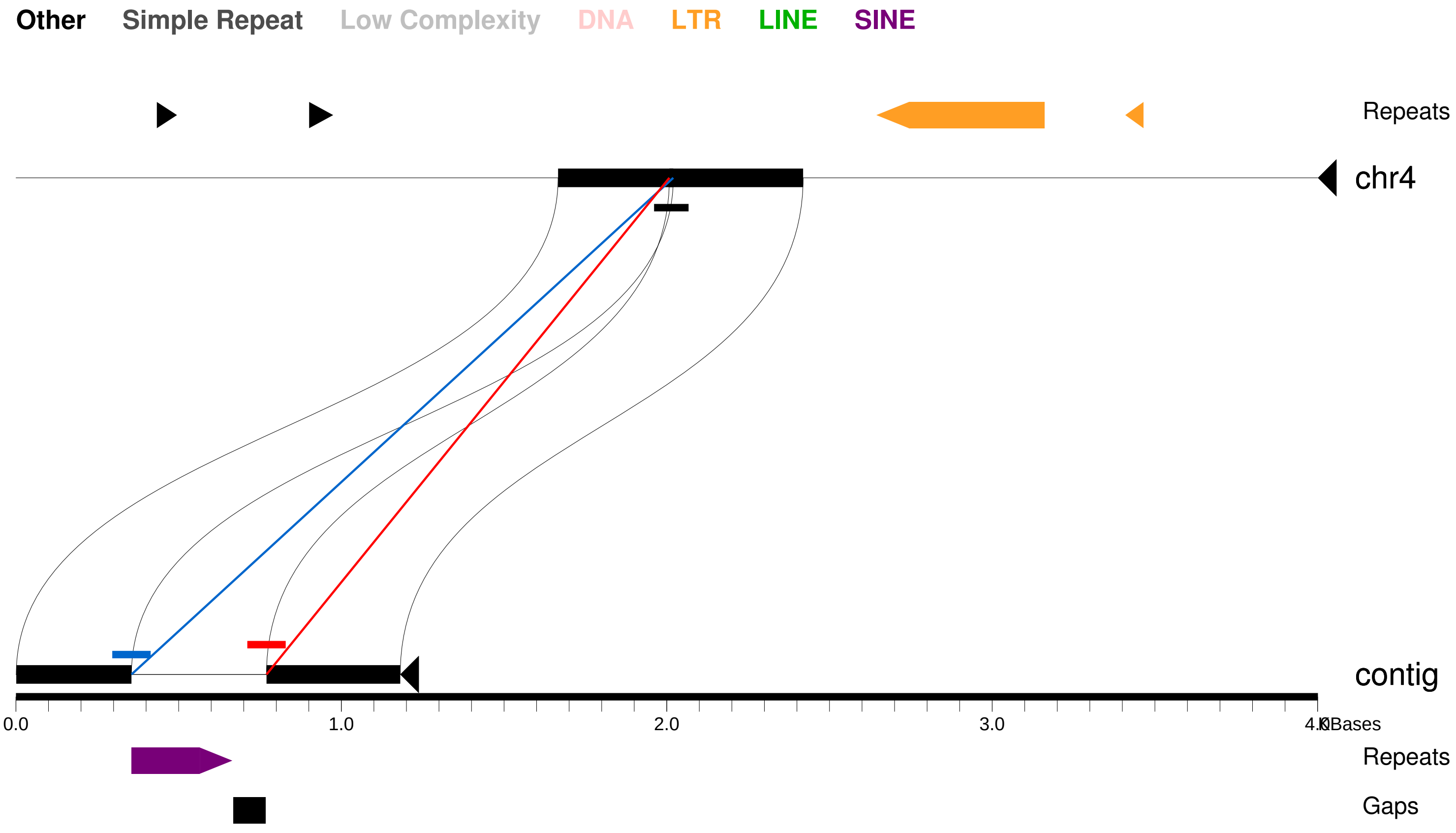

siteID: chr4_134488146 Scafftig2 - Breakpoint: chr4:134488132
chr4:134487972-134488299
Overlap Length: 8 INS size: 302 Genotyped: Yes

```
left   TA--------GTCTTTCCCCTAAAACCACATAACCCCAGTTTAATCTTATGAGGAACATTGGACAAAAAA
chrom  TACCTCTGTAGTCTTTCCCCTAAAACCACATAACCCCAGTTTAATCTTATGAGGAACATTGGACAAAAAA
right  T---TTAGTAG-----------AGACGGGGTTTCACCGTTTTAGCCG-----GGA----TGGTCTCGA--
       *1  2NN222*11111111111*1**1111*11*1**11****11*111111***1111***1*111*11

left   CAATTAAGGGAAATTCTACAAAACAAATGACAAATACTCTGCAAAACTGTCAAGGCCATCAGAAACAAGG
chrom  CAATTAAGGGAAATTCTACAAAACAAATGACAAATACTCTGCAAAACTGTCAAGGCCATCAGAAACAAGG
right  --------------TCTCCTGACCTCGTGATCCGCCCGCCTCGGC-CTCCCAAAGTGCTGGGATTACAGG
       11111111111111***1*11*1*111***111111*1*11*1111**11***1*111*11**1111***

left   CCAGACTTA--GAAACTATCATGGCAAAGATTTTTTTTTTTTTTTTTT----TTTGAGACGGAGTCTCGC
chrom  CCAGACTTA--GAAACTATCATGGCAAAGAATATCTTAAGGAGACATGACAATTGAATATAATGTAGTGA
right  CGTGAGCCACCGCGCCCGGCC-GGCAAAGAATATCTTAAGGAGACATGACAATTGAATATAACGTAGTGA
       *11**111*11*111*111*11********2*2*2**222222222*22222**22*2*222N**222*2

left   TCTGTCGCCCAGGCTG-GAGTGCAG---------TGGCGGGACCTCGGC---TCACTGCAAGCTCCGCCT
chrom  TCTGAATGGGATGCTGAGATAGAAAAATAACTTTTGTTAAAAACTGGGAAAATCTAAATAAATTAAGACT
right  TCTGAATGGGATGCTGAGATAGAAAAATAACTTTTGTTAAAAACTGGGAAAATCTAAATAAATTAAGACT
       ****222222*2****2**22*2*2222222222**22222*2**2**2222**22222**22*22*2**

left   CCCGGGTTCACGCCATTC------TCCTGCCTCA------------------
chrom  T--GGATTAATAACAATGAGTTAATATTGGTTCATTAATTCTAACAAATGTC
right  T--GGATTAATAACAATGAGTTAATATTGGTTCATTAATTCTAA--------
       222**2**2*222**2*2222222*22**22***2222222222
```

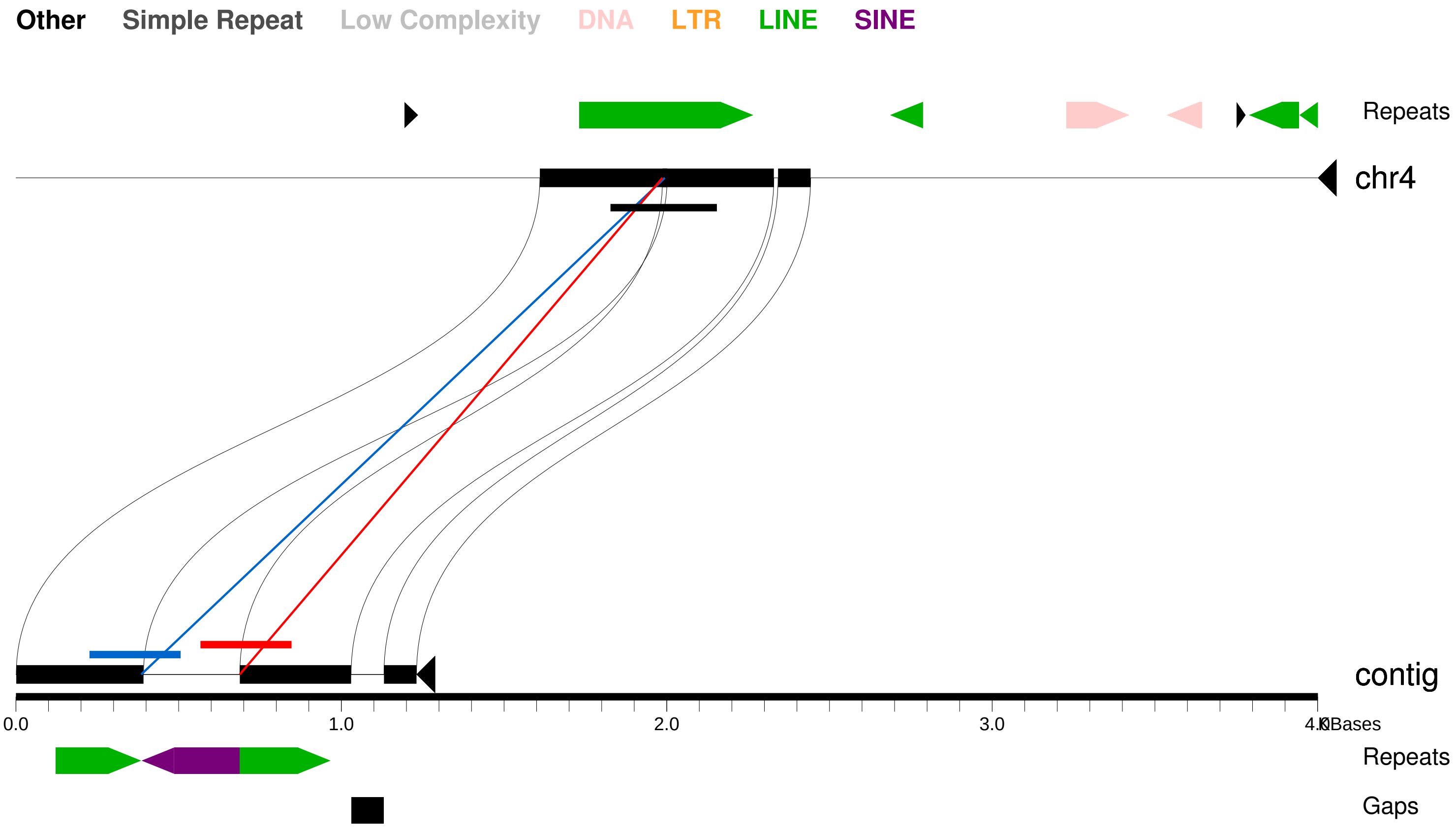

siteID: chr4_134922337 Scafftig2 - Breakpoint: chr4:134922347
chr4:134922300-134922406
Overlap Length: 13 INS size: 294 Genotyped: Yes

```
left     ACCATCTGAGTAATTGACCTCTA-TGTTATGCTAACAGGTTTAAGCAT------------GACAAACATT
chrom    ACCATCTGAGTAATTGACCTCTA-TGTTATGCTAACAGGTTTAAGCAT------------GACAAACATT
right    -CCACCCGCCTCGGCCTCCCAAAGTGCTGGGATTACAGGCGTGAGCCACCGCGCCCGGCCGACAAACATT
         1***1*1*11*111111**111*1**1*11*1*1*****11*1***111111111111111**********

left     TTTTTTTTTTTGAC----GGAGTCTCGCTCTGTCGCCCAGGCCGGACTGCGGACTGCAGTGGCGCA
chrom    TTTACAAGCTGAAAAATGGGAGTAA-GCACTC-CACCCAGA--AGA-----AACAGCAT-------
right    TTTACAAGTTGAAAAATGGGAGTAA-GCACTC-CACCCAGA--AGA-----AACAGCAT-------
         ***22222N*22*22222*****222**2**22*2*****2222**222222**2***22222222
```

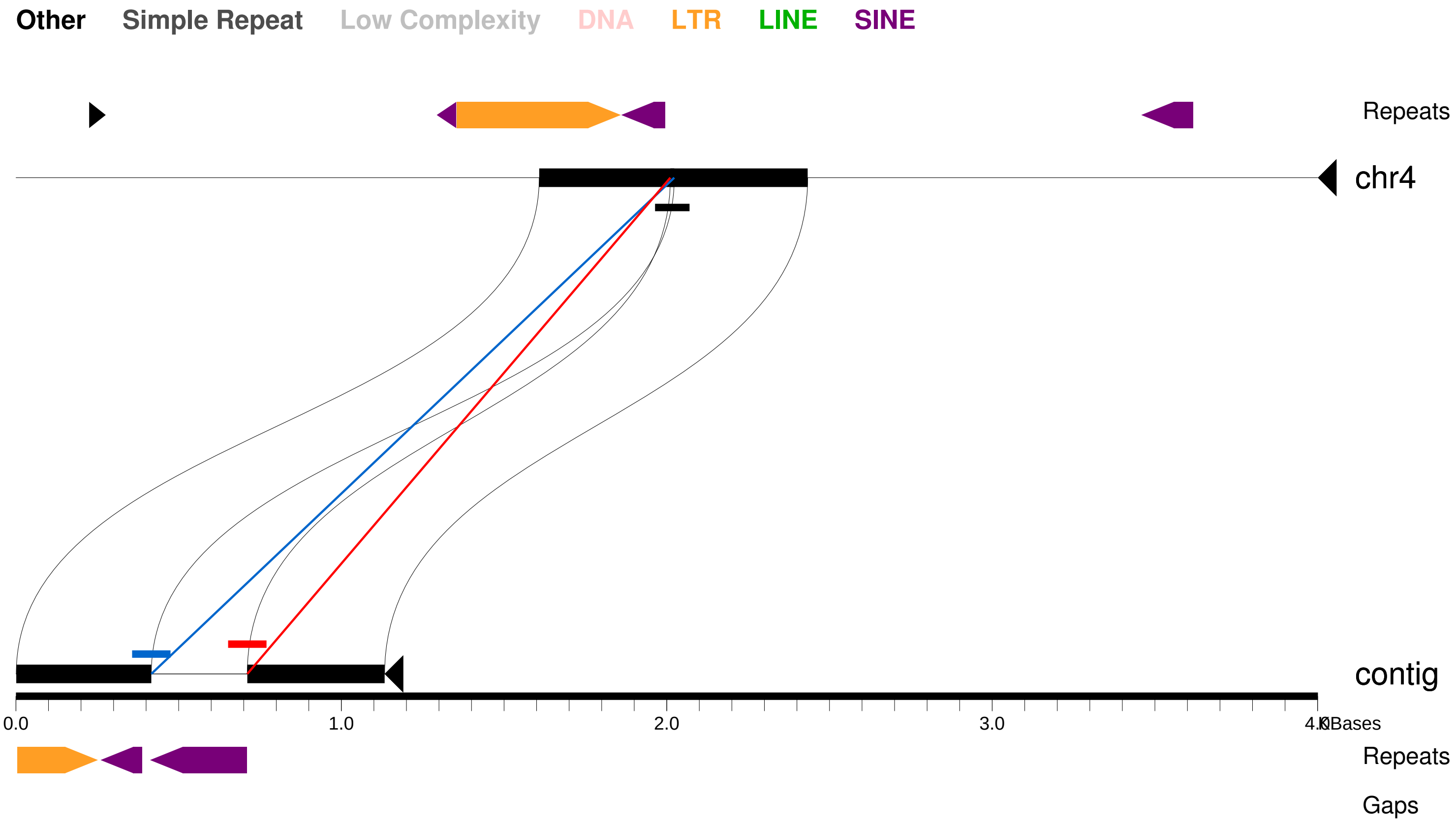

siteID: chr4_136727591 Scafftig2 - Breakpoint: chr4:136727573
chr4:136727529-136727632
Overlap Length: 16 INS size: 428 Genotyped: No

```
left     TGTATTTTCAAAGTAACCACATGTGGTGTATATATAATAATCAT---------------AGACAAGGACA
chrom    TGTATTTTCAAAGTAACCACATGTGGTGTATATATAATAATCAT---------------AGACAAGGACA
right    CCACCCGCCTCGGCCTCCCAAAGTGCTGGGATTACAGGCGTGAGCCACCGCGCCCGGCCAGACAAGGACA
         11111111*111*111**11*1***1**1111**1*1111*1*1111111111111111***********

left     TTTCTTTTTTTNNNNNNNNNNNNNNNNNNNNNNNNNNNNNNNNNNNNNNNNNNNNNNNN
chrom    TTTCTATAATTAGTTCAATTTGTCTGTTAAGAGGAAAAGACCTATGGAT---------------
right    TTTCTATAATTAGTTCAATTTGTCTGTTAAGAGGAAAAGACCTATGGAT---------------
         *****2*22**22222222222222222222222222222222222222222222222222
```

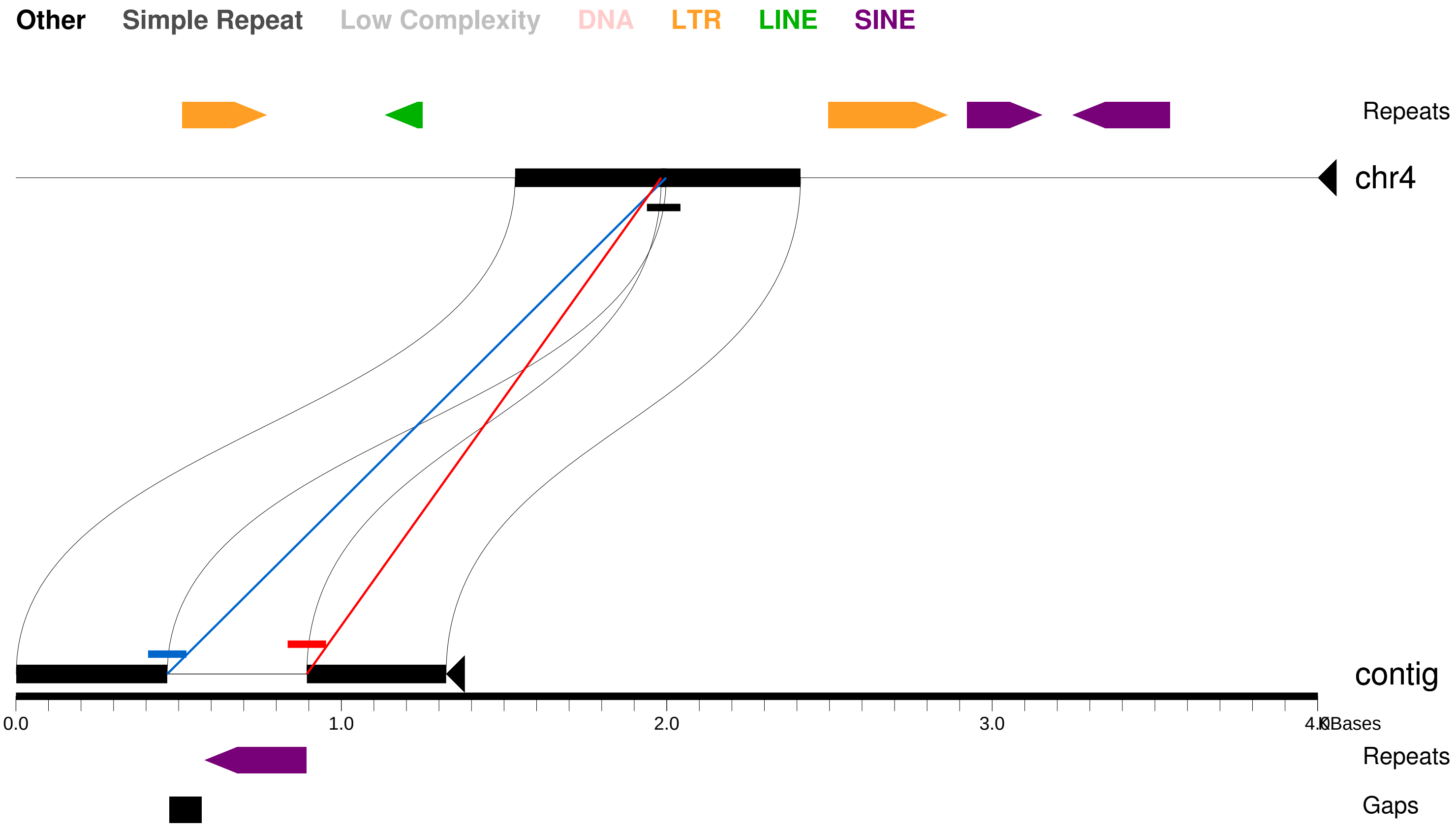

siteID: chr4_137019282 Scafftig2 - Breakpoint: chr4:137019269
chr4:137019109-137019437
Overlap Length: 9 INS size: 427 Genotyped: No

```
left   A----------------------------------GTAACCGAATGCAGTTATGACTGCACATTCATTTA
chrom  AAATAAATGCAATTTATGAATTATTAGTTCCTTTAGTAACCGAATGCAGTTATGACTGCACATTCATTTA
right  T--TTAGTAGAG---ACGGGGTTTCA---CCTT--GTTAGC-----CAGGATGGTCTCGAGCTCC--TGA
       1  2N2N2NN2N   2N2NNN2N2N2   2222  **1*1*11111***1111*1**11*11*1*11*1*

left   AGACATGTTATACAAAACTTTATGGAAATAAAAGTTTTCATTTTAGATGGGCAGACATAAGAAATGTATT
chrom  AGACATGTTATACAAAACTTTATGGAAATAAAAGTTTTCATTTTAGATGGGCAGACATAAGAAATGTATT
right  CCTCATGATCCACCCGCCTCG--GCCTCCCAAAGTGCTGGGATTA------CAGGCGTGAGCCACC----
       111****1*11**1111**1111*111111*****11*1111***111111***1*1*1**11*111111

left   AAAATACTTTGATAACAAATAGCCATTATNNNNNNNNNNN------------------------------
chrom  AAAATACTTTGATAACAAATAGCCATTATTCTTTTAAATTAAATGTGGGACAGAGAACTATAACCAAAAT
right  ----------GCGCCCGGCCAGCCATTATTCTTTTAAATTAAATGTGGGACAGAGAACTATAACCAAAAT
       1111111111*1111*1111*********2222222222222222222222222222222222222222

left   ----------------------------------------------------------------------
chrom  TTCCTTTCTATTTTTGATAGCTAAATATGGTTACAGTTAAGGTTCTTTGTGATCATCTGTTCAACTTGAG
right  TTCCTTTCTATTTTTGATAGCTAAATATGGTTACAGTTAAGGTTCTTTGTGATCATCTGTTCAACTTGAG
       2222222222222222222222222222222222222222222222222222222222222222222222

left   -------------------------------------------------
chrom  TAGCTATATAATCCAGGTTCTTTTATTAGAAACTATTTTTGAAATAAGG
right  TAGCTATATAATCCAGGTTCTTTTATTAGAAACTATTTTT---------
       2222222222222222222222222222222222222222
```

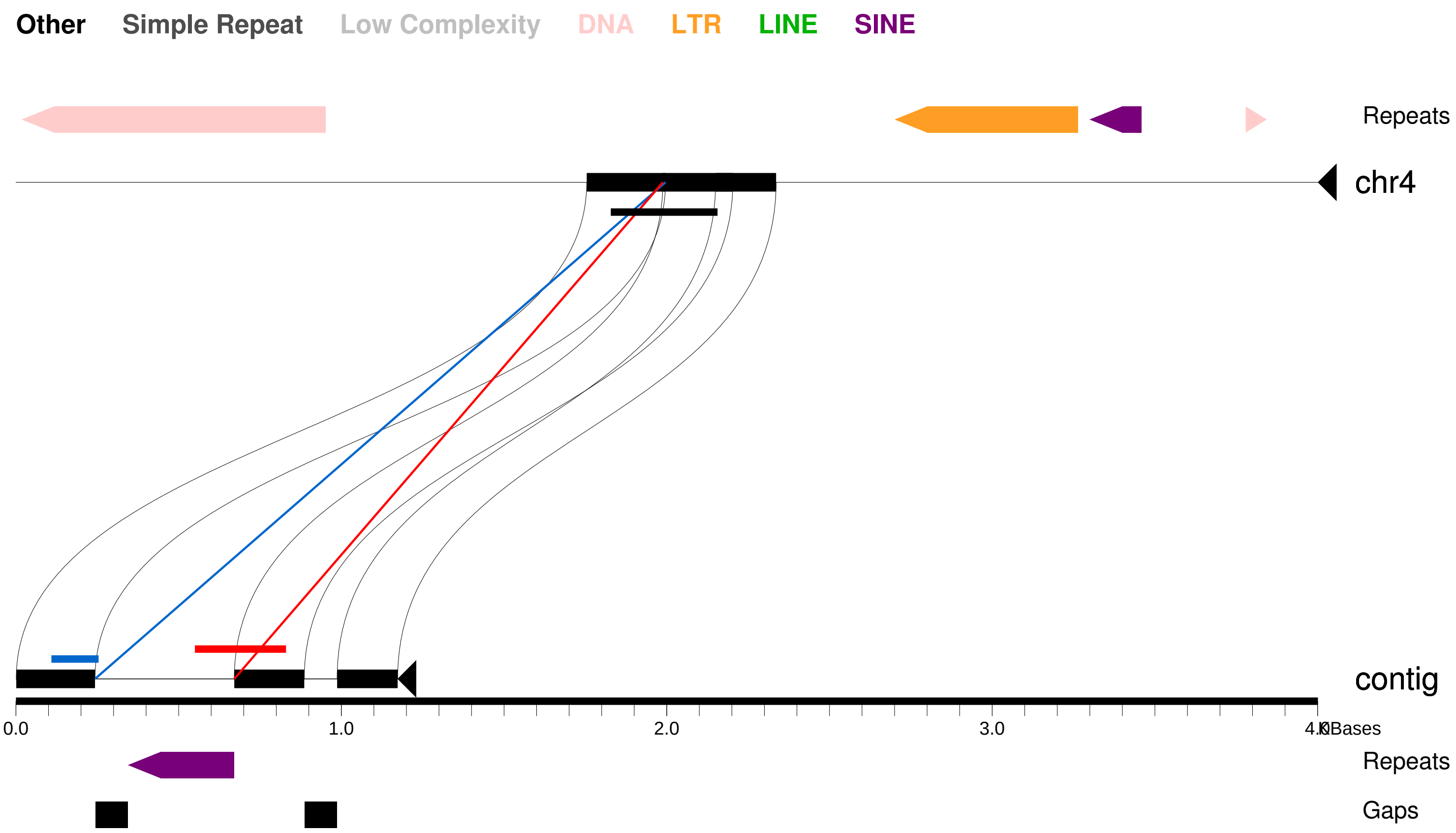

siteID: chr4_137806434 Scafftig1 + Breakpoint: chr4:137806516
chr4:137806465-137806548
Overlap Length: 9 INS size: 139 Genotyped: Yes

```
left    T-----------TTCAC------------------------CTTTTACTTACTAGAATTAAAAAAATATA
chrom   T-----------TTCAC------------------------CTTTTACTTACTAGAATTAAAAAAATATA
right   AGATCCCGCCACTGCACTCCAGCCTGGGCGACAGAGCGAGACTCCGTCTCAAAAAAAAAAAAAAAAAAAA
        111111111111*1***111111111111111111111111**1111**1*11*1**11*******1*1*

left    TATTTACATTATCTATAAAAATGATGGCTGAGGCAGGAGAATGGCGTGAACCCGGGAGGCGGAGTTTGCA
chrom   TATTTACATTATCTATAAAAATGAT--------------AAAGTAGT--ACCTAAAAGT-----TTTACA
right   AAAAAAAAAAAAAAAAAAAAATGAT--------------AAAGTAGT--ACCTAAAAGT-----TTTACA
        1*111*1*11*111*1*********22222222222222**2*22**22***2222**222222***2**

left    GTGAGCCGAGATCC
chrom   --------------
right   --------------
        22222222222222
```

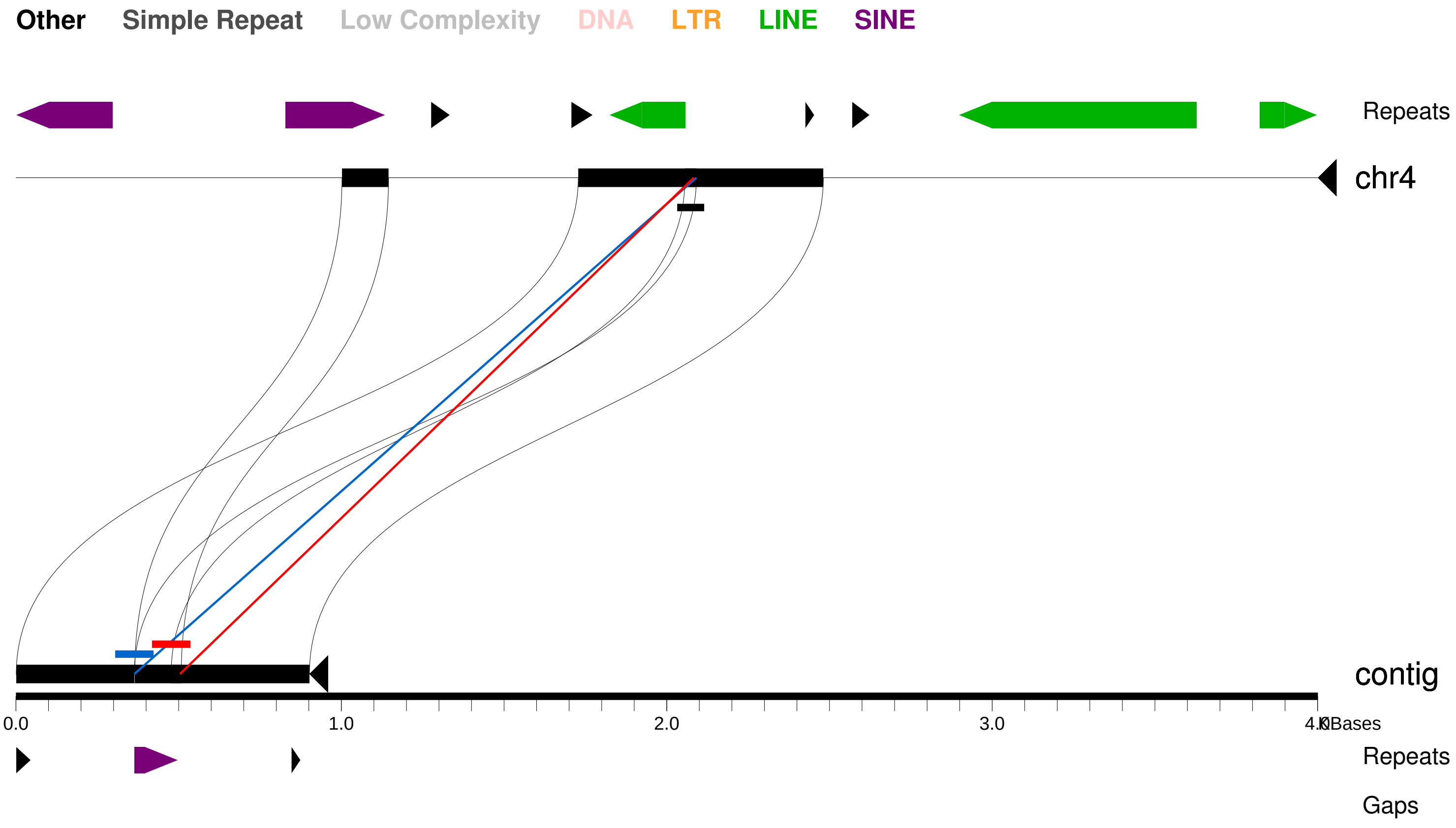

siteID: chr4_138298990 Scafftig7 - Breakpoint: chr4:138298990
chr4:138298946-138299049
Overlap Length: 16 INS size: 297 Genotyped: Yes

```
left    CCACACTTC-CCACAGGCTGCTCC-CAACTGGTGACTGGGCATGTC---------------AAGAATAAT
chrom   CCACACTTC-CCACAGGCTGCTCC-CAACTGGTGACTGGGCATGTC---------------AAGAATAAT
right   CGCCACTGCACTCCAGCCTGGGCGACAGC--GAGACTCCGTCTCAAAAAAAAAAAAAAAAAAAGAATAAT
        *11****1*1*11***1***11*11**1*11*1****11*11*111111111111111111*********

left    GTTACCAG---GCCGGGCGCAGTGGCTCACGCCTGTAATCCCAGCACTTTGGGAGGCCGAGGCGGGCGG
chrom   GTTACCAAAATGCCAAGGGTTTGGTCTAGGTCCTGTGG-CTCATCACA--------CATA---------
right   GTTACCAAAATGCCAAGGGTTTGGTCTAGGTCCTGTGG-CTCATCACA--------CATA---------
        *******2222***22*2*2222*2**2222*****222*2**2***222222222*22*222222222
```

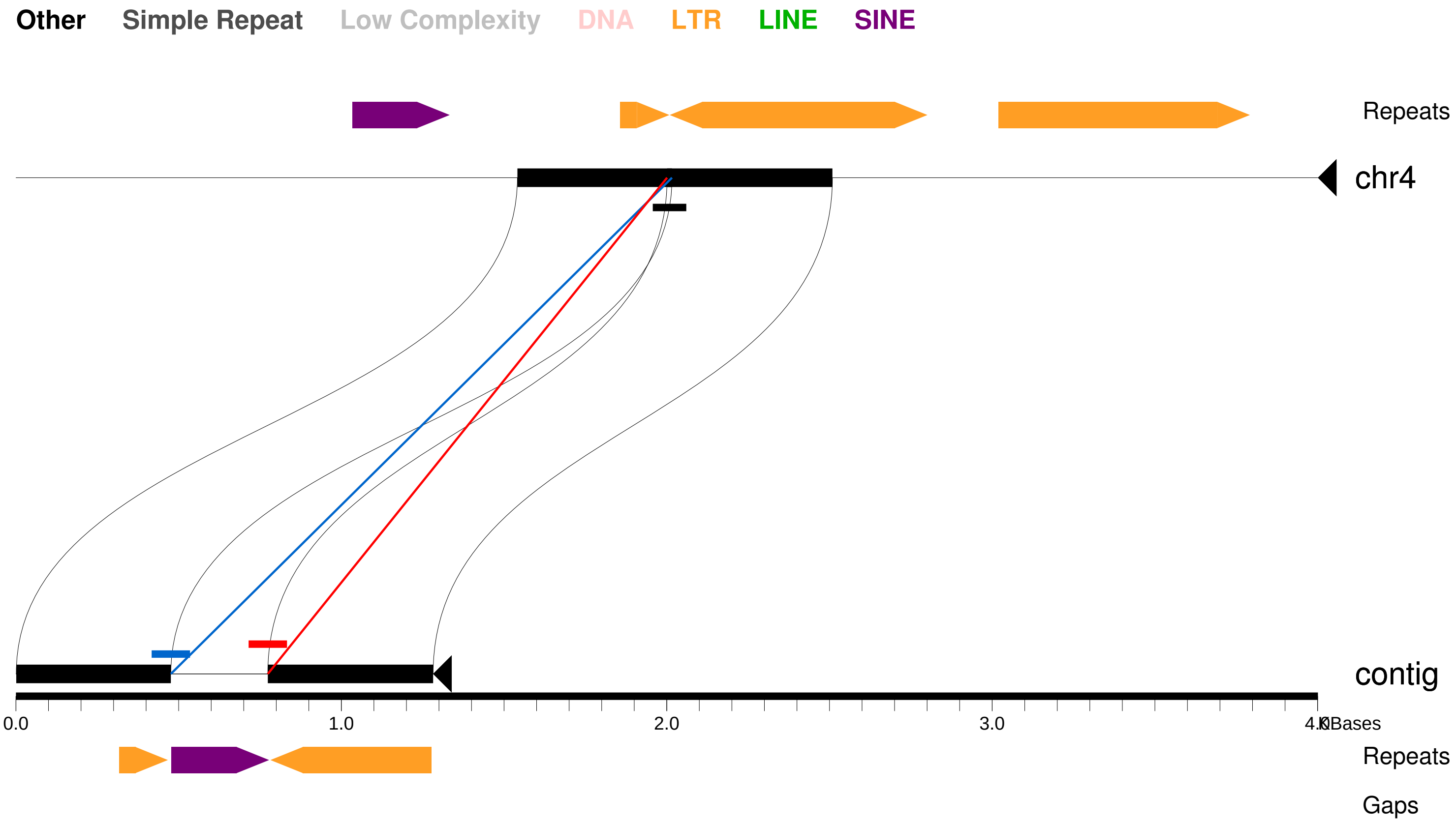

siteID: chr4_139225219 Scafftig6 - Breakpoint: chr4:139225200
chr4:139225040-139225377
Overlap Length: 18 INS size: 428 Genotyped: No

```
left   --------------------------------------------TCTGCATACTGTATACTACACCTAAC
chrom  GGTGATCATTGGAGGCCATCTTAAAAGGCTACCTATGGCTGACTTCTGCATACTGTATACTACACCTAAC
right  TTTAGTAGA--GACGGGGTTTCACCGTGTTAGCCA-GGATGG--TCTCGAT-----------CTCCTGAC
       NN2NN2NNN  22N2NNN2N2N2NNNN2N22N2N2 22N22N  ***11**11111111111*1***1**

left   TTCCTTTCCAACATTCCTGAGAGTCA-AGAGTGTTTTTTAGTATTAGCATATGCTTTATTTCCCAGTAAA
chrom  TTCCTTTCCAACATTCCTGAGAGTCA-AGAGTGTTTTTTAGTATTAGCATATGCTTTATTTCCCAGTAAA
right  CTCGTGATCCGCCCGCCTCGGCCTCCCAAAGTGCTG----GGATTACAA---GCGTGAG---CCAC----
       1**1*111*11*111***11*11**11*1****1*11111*1****11*111**1*1*1111***11111

left   TACTTGTATGACATTGCTATAAGATGTTTAAAAACTCTT-------------------------------
chrom  TACTTGTATGACATTGCTATAAGATGTTTAAAAACTCTTAAAGATAACAGTATATGTTAAAAAAAAAAAA
right  --CGCGCCCGGCCT-------AGATGTTTAAAAACTCTTAAAGATAACAGTATATGTTAAAAAAAAAAAA
       11*11*111*1*1*1111111*****************2222222222222222222222222222222

left   ----------------------------------------------------------------------
chrom  AAAGGACAAGAGTTAAGCAAATCATTCGGAGATGTGTCACTAACGGTCTGGGGGTCTGTGTAAAGGACAA
right  AA-GGACAAGAGTTAAGCAAATCATTCGGAGATGTGTCACTAACGGTCTGGGGGTCTGTGTAAAGGACAA
       22 2222222222222222222222222222222222222222222222222222222222222222222

left   -----------------------------------------------TNNNNNNNNNNN
chrom  GGCAGCATTATCCTGTGACAAGAGCAACGACTTGTTTTATAATTCCTTCAGACTGATTC
right  GGCAGCATTATCCTGTGACAAGAGCAACGACTTGTTTTATA------------------
       22222222222222222222222222222222222222222      1NNNNNNNNNNN
```

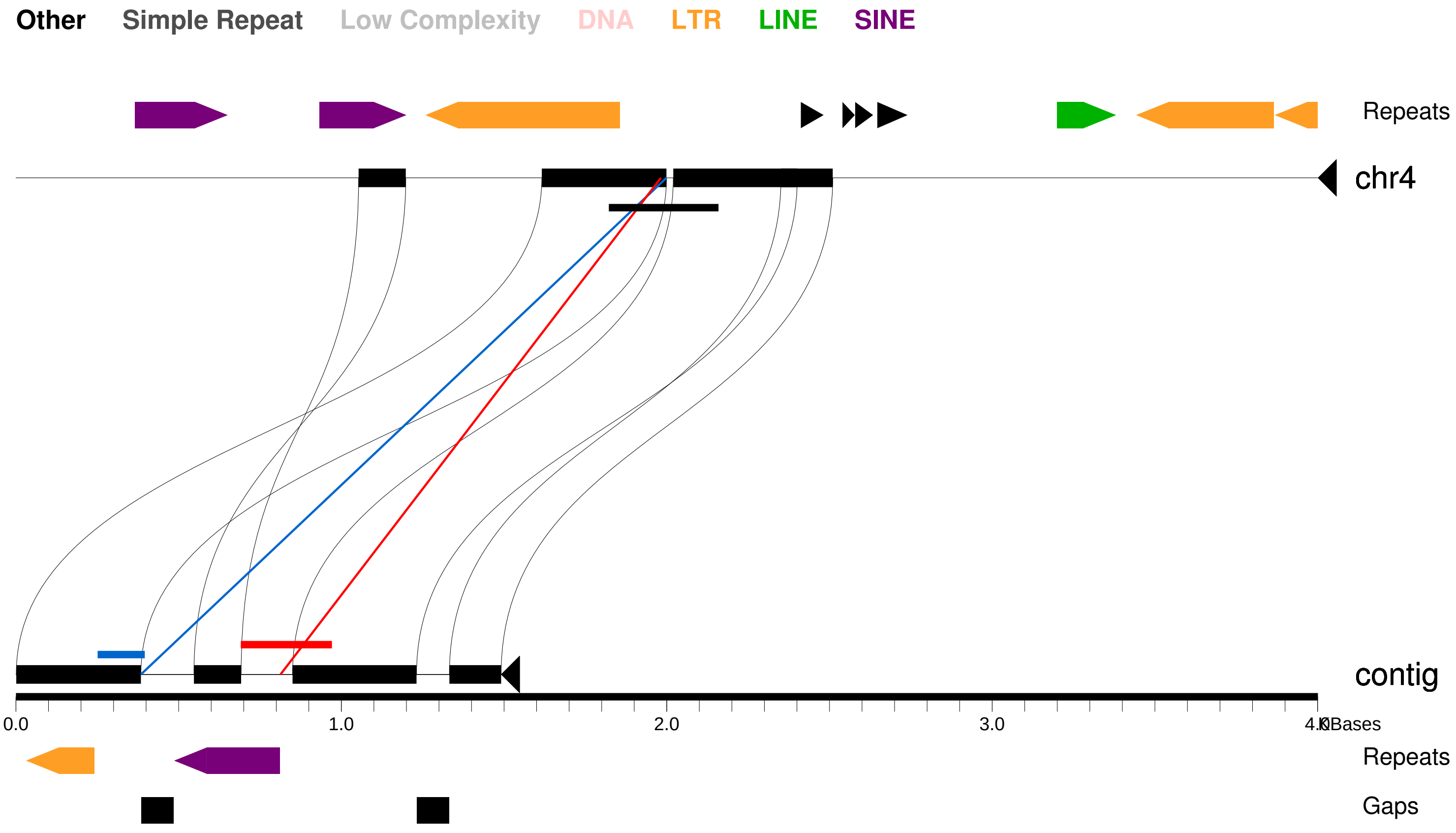

siteID: chr4_140938924 Scafftig16 - Breakpoint: chr4:140938921
chr4:140938876-140938980
Overlap Length: 15 INS size: 136 Genotyped: Yes

```
left     A--CATTCTTGAAATGACAAAAT------------TATAAAAATGAGTATCTAGTGGTTAAGAAAGGGGG
chrom    A--CATTCTTGAAATGACAAAAT------------TATAAAAATGAGTATCTAGTGGTTAAGAAAGGGGG
right    CTGCACTCCAGCCTGGGCGACAGAGCGAGACTCCGTCTCAAAAAAAAAAAAAAAAAAAGAAAGAAAGGGGG
         111**1**11*1111*1*1*1*1111111111111*1*1****11*11*111*111111***********

left     ATAGCAGCTACTTGGGAG--GCTGAGGCAGGAGAATGGCGTGAACCCGGGAGGCGGAGCTTGCAG
chrom    ATAGAGGAAAAATAGGTGTAGCTATAACAGGGCAATA---TGA------------GAGATT-CTA
right    ATAGAGGAAAAATAGGTGTAGCTATAACAGGGCAATA---TGA------------GAGATT-CTA
         ****22*22*22*2**2*22***2222****22***2222***222222222222***2**2*22
```

Other Simple Repeat Low Complexity DNA LTR LINE SINE

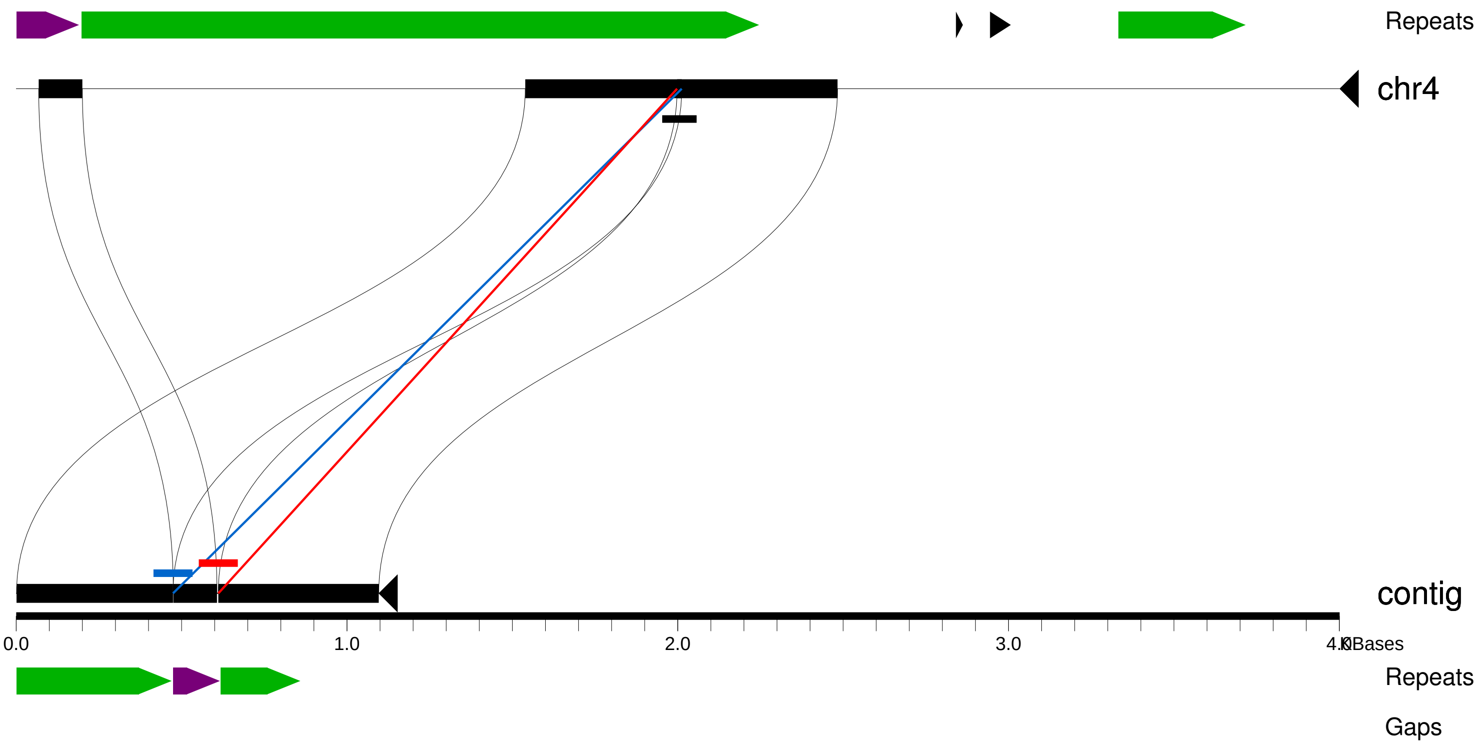

siteID: chr4_143258314 Scafftig1 + Breakpoint: chr4:143258301
chr4:143258256-143258360
Overlap Length: 15 INS size: 298 Genotyped: Yes

```
left     TAACTCAATTT---CTTTTAGATT-----TATCCATGTTGTTGCATACAGCTA------AAGTACATTTA
chrom    TAACTCAATTT---CTTTTAGATT-----TATCCATGTTGTTGCATACAGCTA------AAGTACATTTA
right    CCGCCCGCCTCGGCCTCCCAAAGTGCTGGGATTACAGGCGTGAGCCACCGCGCCCGGCCAAGTACATTTA
         111*1*111*1111**111*1*1*111111**1111*11**11111**1**11111111***********

left     TTTTTTTTTTTTTTTTTTTTTGAGATGGAGTCTCGCTCTGTCGCCCAGGCTGGAATGCAGTGG---
chrom    TTTTCAT-----------TGTCAGAT--AGTAATTCATTG----CCTGACTGTACTACAATTTATC
right    TTTTCAT-----------TGTCAGAT--AGTAATTCATTG----CCTGACTGTACTACAATTTATC
         *****22*22222222222*2*2****22***2222*22**2222**2*2***2*2*2**2*22222
```

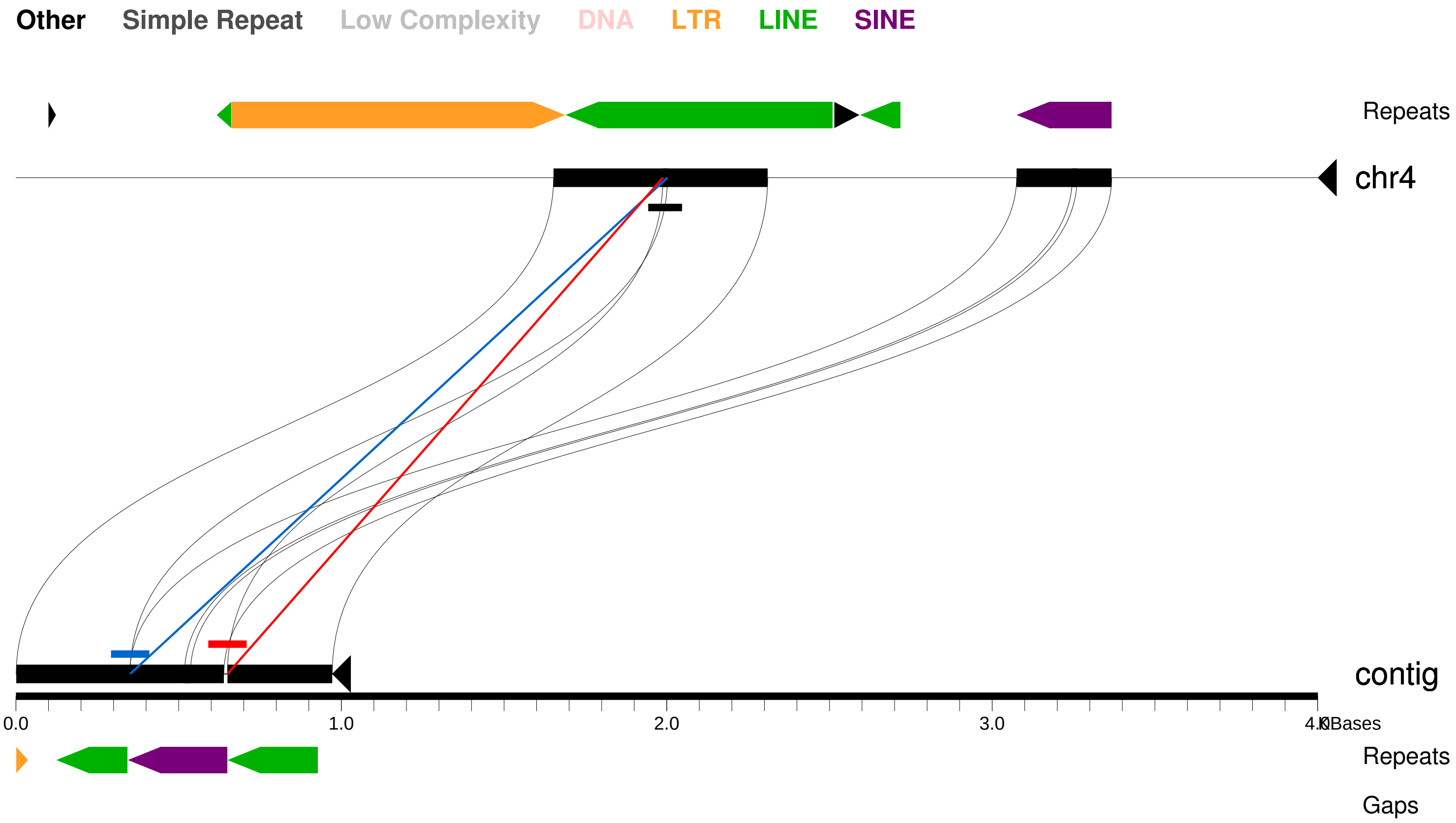

siteID: chr4_145395313 Scafftig6 - Breakpoint: chr4:145395297
chr4:145395249-145395351
Overlap Length: 12 INS size: 322 Genotyped: Yes

```
left    C------------ATTTTGCCCCAGCAGTATGTTAGCTATTTATCTGAGGAGGATAAAAA----CACATT
chrom   C------------ATTTTGCCCCAGCAGTATGTTAGCTATTTATCTGAGGAGGATAAAAA----CACATT
right   GCGACAGAGCGAGACTCCGTCTCAAAAAAAAAAAAAAAAAAAAAAAAAAAAAAAAAAAAAAAAAACACATT
        1111111111111*1*11*1*1**11*11*1111*111*111*1111*11*11*1*****1111******

left    ACCATAGGCCGGGCGCGGTGGCTCACGCCTGTAATCCCAGCACTTTGGGAGGCCGAGGCGGGCGG
chrom   ACCATAA-----AAACCATATGTAAATGTTTATAGCAATGCACAATTGGAAGCT-----------
right   ACCATAA-----AAACCATATGTAAATGTTTATAGCAATGCACAATTGGAAGCT-----------
        ******222222222*22*222*2*2222*222*2*222****22*2***2**222222222222
```

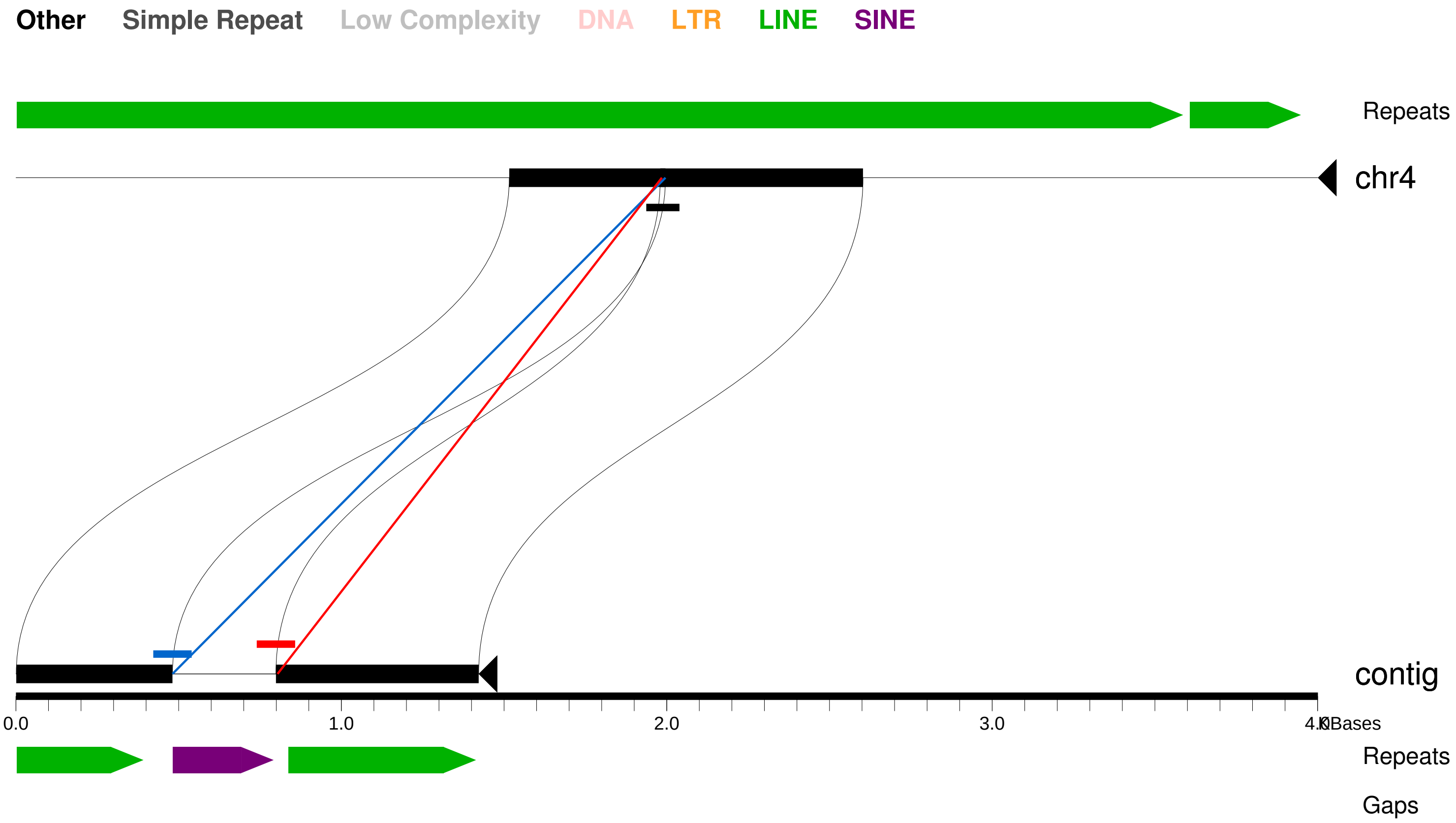

siteID: chr4_147351169 Scafftig1 - Breakpoint: chr4:147351174
chr4:147351129-147351195
Overlap Length: 15 INS size: 321 Genotyped: Yes

```
left    ------------------ACTATAC----------------------------------AAAAAAATTCA
chrom   ------------------ACTATAC----------------------------------AAAAAAAATCA
right   GTGAGCCGAGATCCCGCCACTGCACTCCAGCCTGGGCGACAGAGCGAGACTCCGTCTCAAAAAAAAAAAA
        111111111111111111***11**1111111111111111111111111111111111*******211*

left    TTCAAAACGGATCACAGGACTAAATGTAAAAACAAAAATTAAGGGCCGGGCGCGGTGGCTCACGCCTGTA
chrom   TTCAAAACGGATCACAGGACTAAATGTAAAAACAAAAATTAAA---------------------------
right   AAAAAAAAAAAAAAAAAAAAAAAAAAAAAAAAAAAAAATTAAA---------------------------
        111****111*11*1*11*11***111*****1*********2222222222222222222222222222

left    ATCCCAGCACTTTGGGAGGCCGAGGCGGGCG
chrom   --------ACTTAT-----------------
right   --------ACTTAT-----------------
        22222222****2222222222222222222
```

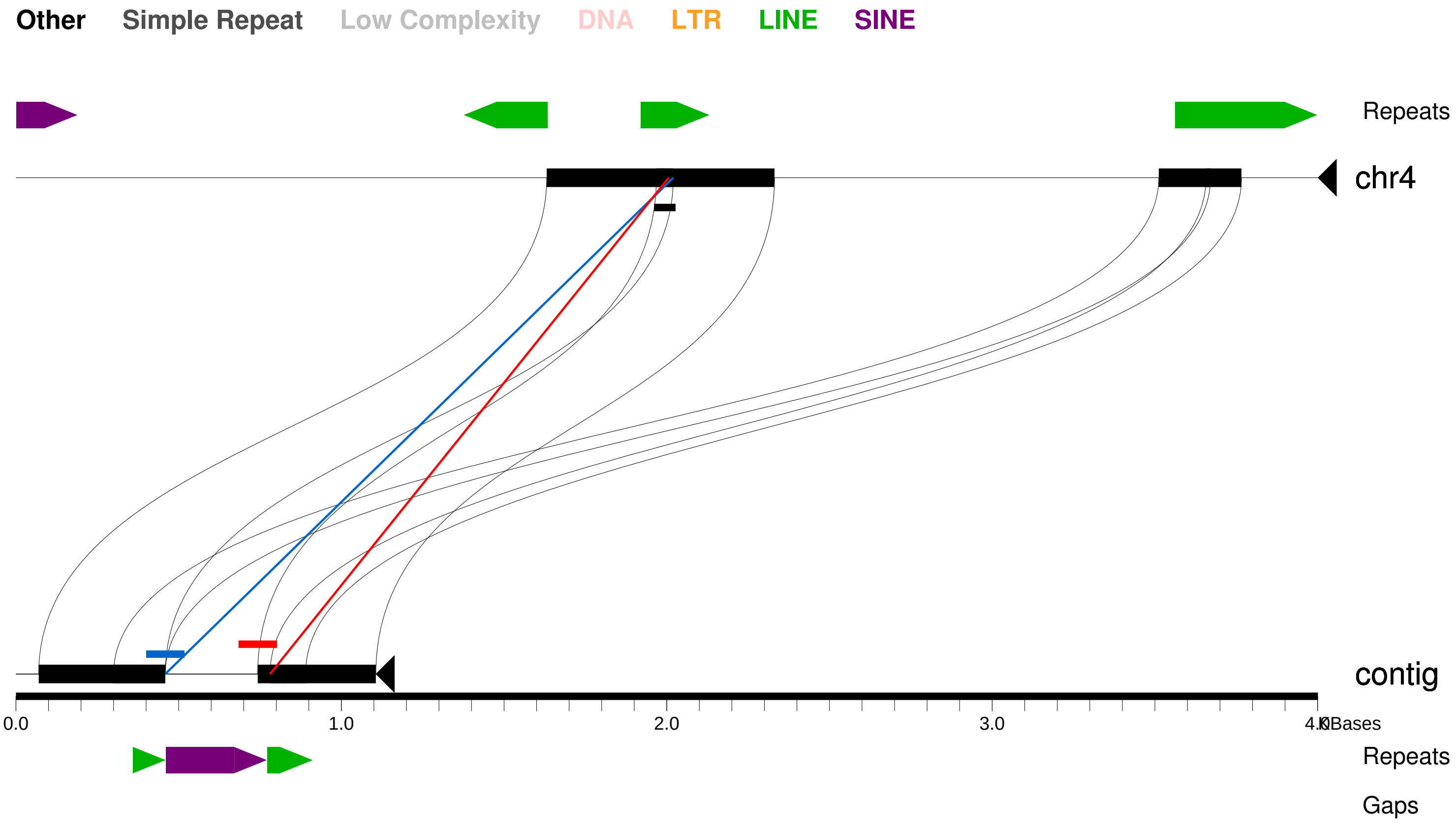

siteID: chr4_150245284 Scafftig1 + Breakpoint: chr4:150245258
chr4:150245122-150245466
Overlap Length: 74 INS size: 484 Genotyped: No

```
left   A---------------------------------------------------------------------
chrom  AAGTAGATGCAATTTGACAATTTTGGCTTTTATTGCAATTGCTTTTGGTGTTTTAGTCATGAAGTCTTTG
right  CC------------TCCCGCCTCTGCCTCCCA----AAGTGCT---GGGATTACAGGCGTGA-------G
       1N            2NN2NNN2N22N22NNN2    22N2222   22NN22NN22N2N222       2

left   -------------------------------GGTTTTCATCTAGGGTTTTTATGATTTTGGGTTTTACAT
chrom  CCCACGCCTATGTCCTGAATGCTATTGCGTAGGTTTTCATCTAGGGTTTTTATGATTTTGGGTTTTACAT
right  CCACCGCGCCCGGCCT--------------------------------------------------ACAT
       22NN222NNNN2N222               11111111111111111111111111111111111****

left   TTAAGTCTTTAATCCATCTCGAGTTAATTTTTGTACAAAGTGTAAGGAAGTGGTCCAGTTTCAGTTTTCT
chrom  TTAAGTCTTTAATCCATCTCGAGTTAATTTTTGTACAAAGTGTAAGGAAGTGGTCCAGTTTCAGTTTTCT
right  TTAAGTCTTTAATCCATCTCGAGTTAATTTTTGTACAAAGTGTAAGGAAGTGGTCCAGTTTCAGTTTTCT
       **********************************************************************

left   NNNNNNNNNNNNNNNNNNNNNNNNNNNNNNNNNNNNNNNNNNNNNNNNNNNNNNNNNNN-----------
chrom  GCATATGGCTAGCCAGTTTTCCCAGCACAATTTACTGAATAGAAGAACCTTTACCCATTGCTCGTTCTTG
right  GCATATGGCTAGCCAGTTTTCCCAGCACAATTTAC-----------------------------------
       22222222222222222222222222222222222NNNNNNNNNNNNNNNNNNNNNNNN

left   -----------------------------------------------------------------
chrom  TCAGGTTTGTTGAAGATCAAATTGTTGTAGATCTGTGGTGTTATTTCTGAGGTCTCTGTTCTGCT
right  -----------------------------------------------------------------
```

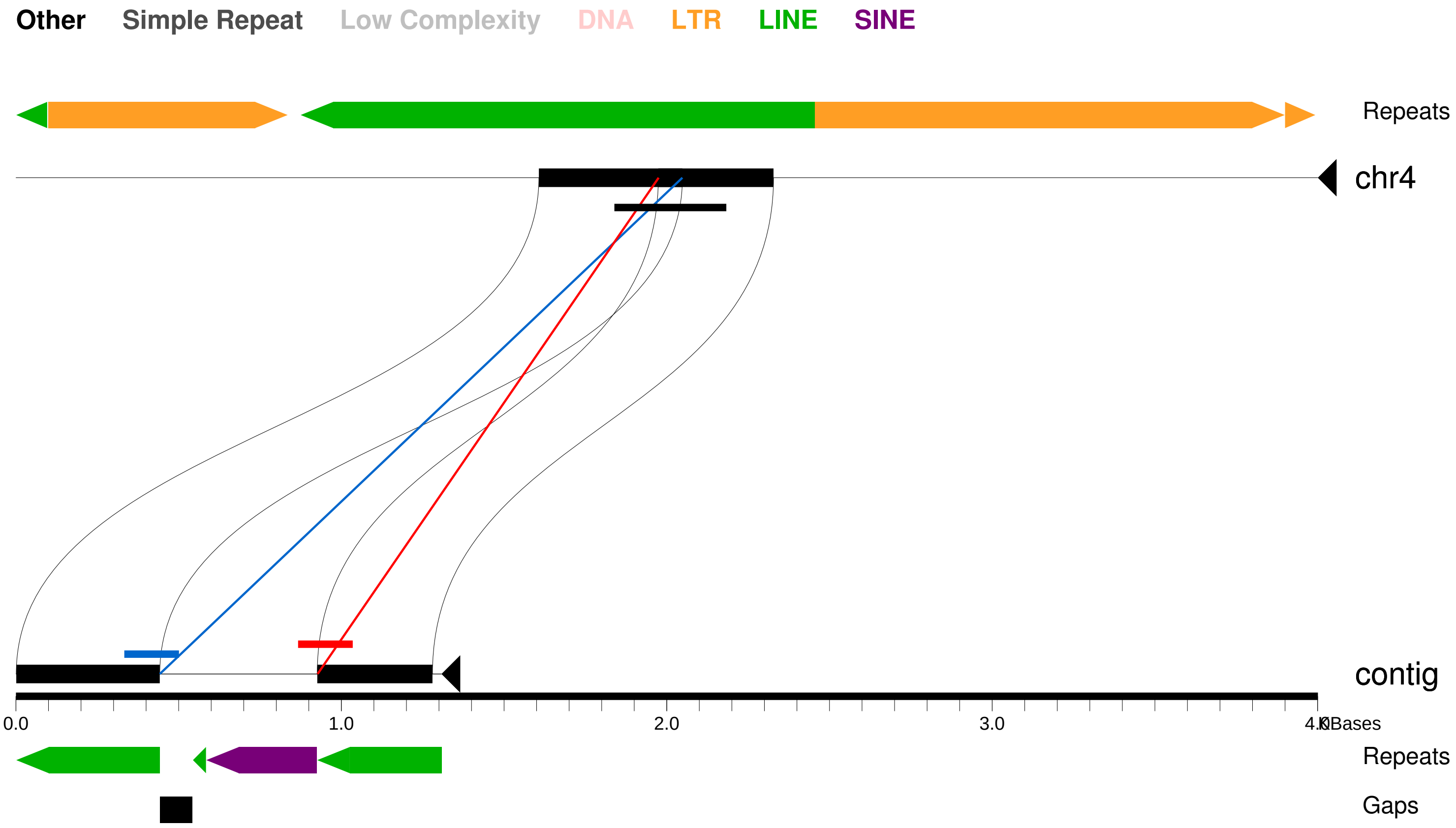

siteID: chr4_154445644 Scafftig3 + Breakpoint: chr4:154445641
chr4:154445481-154445802
Overlap Length: 2 INS size: 226 Genotyped: No

```
left    ---------------------------------------------------------CTTTGTAAATTTT
chrom   TTGAGGCCATAAGCGAGGGCTACTCCCTGCTGGCAGTCCAGGTCTTCTCCCACAACACTTTGTAAATTTT
right   TACAGGCGCCC-GCCA---CTACGCCCGGCT----------------------AATTTTTTGTATTTTTA
        2NN2222NNNN 22N2   2222N222N222                      22NN1******11***1

left    AAAAAGGCCAAAAAGAAAAATAGTTGCTAATATCAAGGACACAGATATATGTTTTAATGACTGACATTGT
chrom   AAAAAGGCCAAAAAGAAAAATAGTTGCTAATATCAAGGACACAGATATATGTTTTAATGACTGACATTGT
right   GTAGAGACGGGGTTTCACCATTTTAGCCGGGAT---GGTCTC-GATCTC-----------CTGACCTCGT
        11*1**1*11111111*11**11*1**1111**111**1*1*1***1*111111111111*****1*1**

left    TTGCAGGTAAATATGTCAAA--GGNNNNNNNNNNN-----------------------------------
chrom   TTGCAGGTAAATATGTCAAA--GGGAGAGTTATATTTTTATGAGCTTTATGTGTGTCTTATTTTGAAGAC
right   GATCCGCCCGCCTCGGCCTCCCGGGAGAGTTATATTTTTATGAGCTTTATGTGTGTCTTATTTTGAAGAC
        111*1*11111111*1*11111**2222222222222222222222222222222222222222222222

left    ----------------------------------------------------------------------
chrom   AGTTTATACAATAATGATGCATGCTTTTTATTTTAAGGGACTTTGTAAAGAAGATAAGCTTGTCTAATAG
right   AGTTTATACAATAATGATGCATGCTTTTTATTTTAAGGGACTTTGTAAAGAAGATAANNNNNNNNNNNNN
        222222222222222222222222222222222222222222222222222222222NNNNNNNNNNNNN

left    --------------------------------------------
chrom   TGCATGGGATTGCTTGGTAGGAGGCCTAGGTTTGTTTCTTAGCT
right   NNNNNNNNNNNNNNNNNNNNNNNNNNNNNNNNNNNNNNNNNN--
        NNNNNNNNNNNNNNNNNNNNNNNNNNNNNNNNNNNNNNNNNN
```

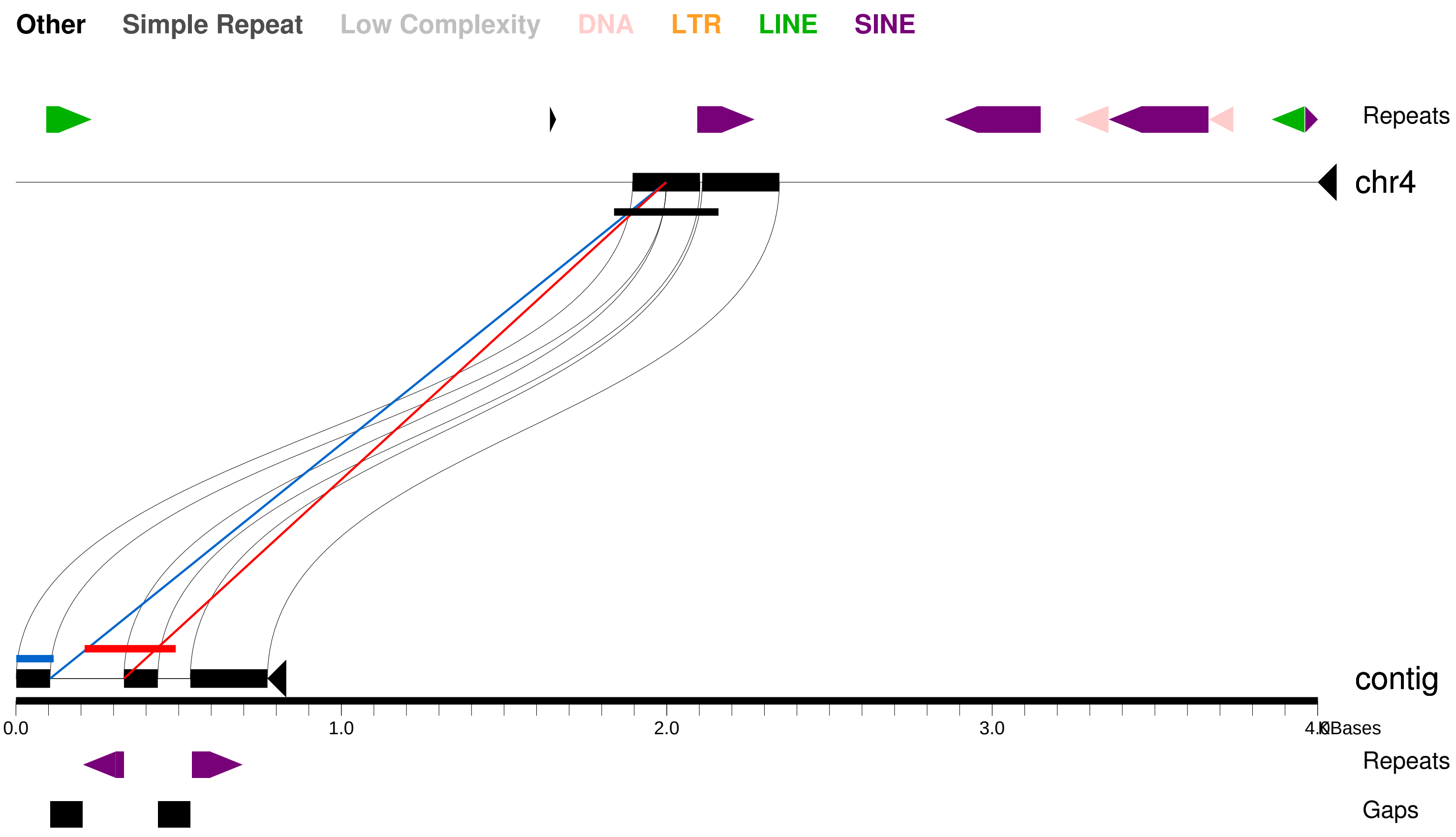

siteID: chr4_156534792 Scafftig1 - Breakpoint: chr4:156534800
chr4:156534756-156534859
Overlap Length: 16 INS size: 313 Genotyped: Yes

```
left    C----CAATTAATATGTAC-CTGGCACACACACAAGTGAGCAGATTTTT----------AAAAAATCTGA
chrom   C----CAATTAATATGTAC-CTGGCACACACACAAGTGAGCAGATTTTT----------AAAAAATCTGA
right   CTGGCGACAGAGCGAGACTCCGTCTCAAAAAAAAAAAAAAAAAAAAAAAAAAAAAAAAAAAAAAATTTGA
        *1111*1*111*11111**1*1*1*1**1*1*1**111*11*1*111111111111111*******1***

left    GTAACGGCCGGGCGCGGTGGCTCACGCCTGTAATCCCAGCACTTTGGGAGGCCGAGGCGGGCGG
chrom   GTAACACTTTCGACAGGA-----AAGAAGATAAACCTAACATCTTAT--GGCATAA--------
right   GAAACACTTTCGACAGGA-----AAGAAGATAAACCTAACATCTTAT--GGCATAA--------
        *1***222222*222**222222*2*2222***2**2*2**22**2222***22*222222222
```

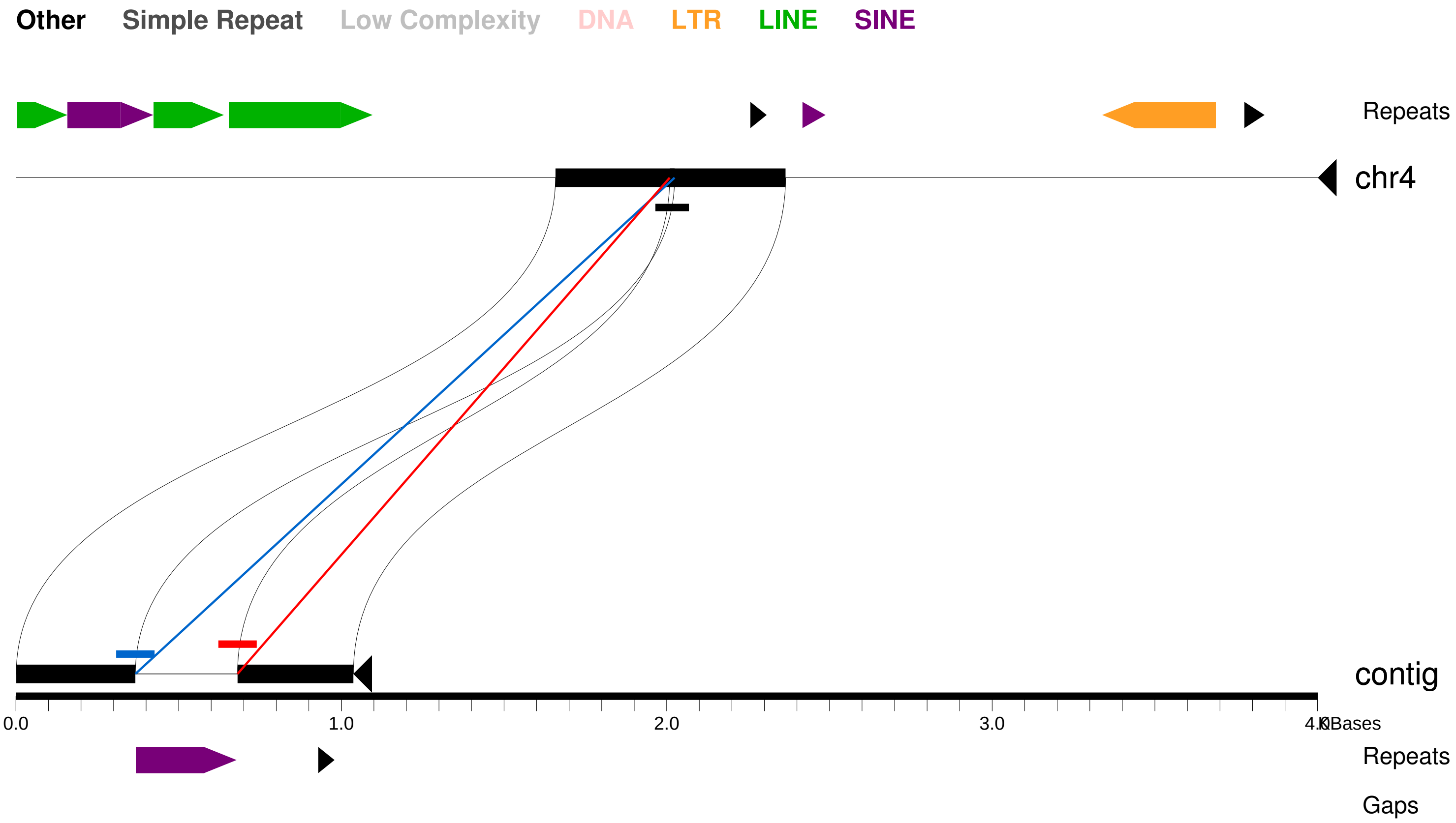

siteID: chr4_160626066 Scafftig1 + Breakpoint: chr4:160626037
chr4:160625989-160626096
Overlap Length: 12 INS size: 443 Genotyped: No

```
left    AG--------TGAGGTGCGGTATGTGCTCCATGTATCCAGGACAGAAACACT-----CTTTCTCTCATTT
chrom   AG--------TGAGGTGCGGTATGTGCTCCATGTATCCAGGACAGAAACACT-----CTTTCTCTCATTT
right   TCCACCCGCCTCGGCCTCCCAAAGTGCTGGGATTA--CAGGCGTGAGCCACCGCGCCCGGCCTCTCATTT
        1111111111*11*111*111*1*****11111**11****111**11***111111*111*********

left    CTTTTTNNNNNNNNNNNNNNNNNNNNNNNNNNNNNNNNNNNNNNNNNNNNNNNNNNNNNNN
chrom   CTTAT-----------AATCACAATTTTTGCCATAGTAATTGATACTGAATATCCTAAAGGA
right   CTTAT-----------AATCACAATTTTTGCCATAGTAATTGATACTGAATATCCTAAAGGA
        ***2*222222222222222222222222222222222222222222222222222222222
```

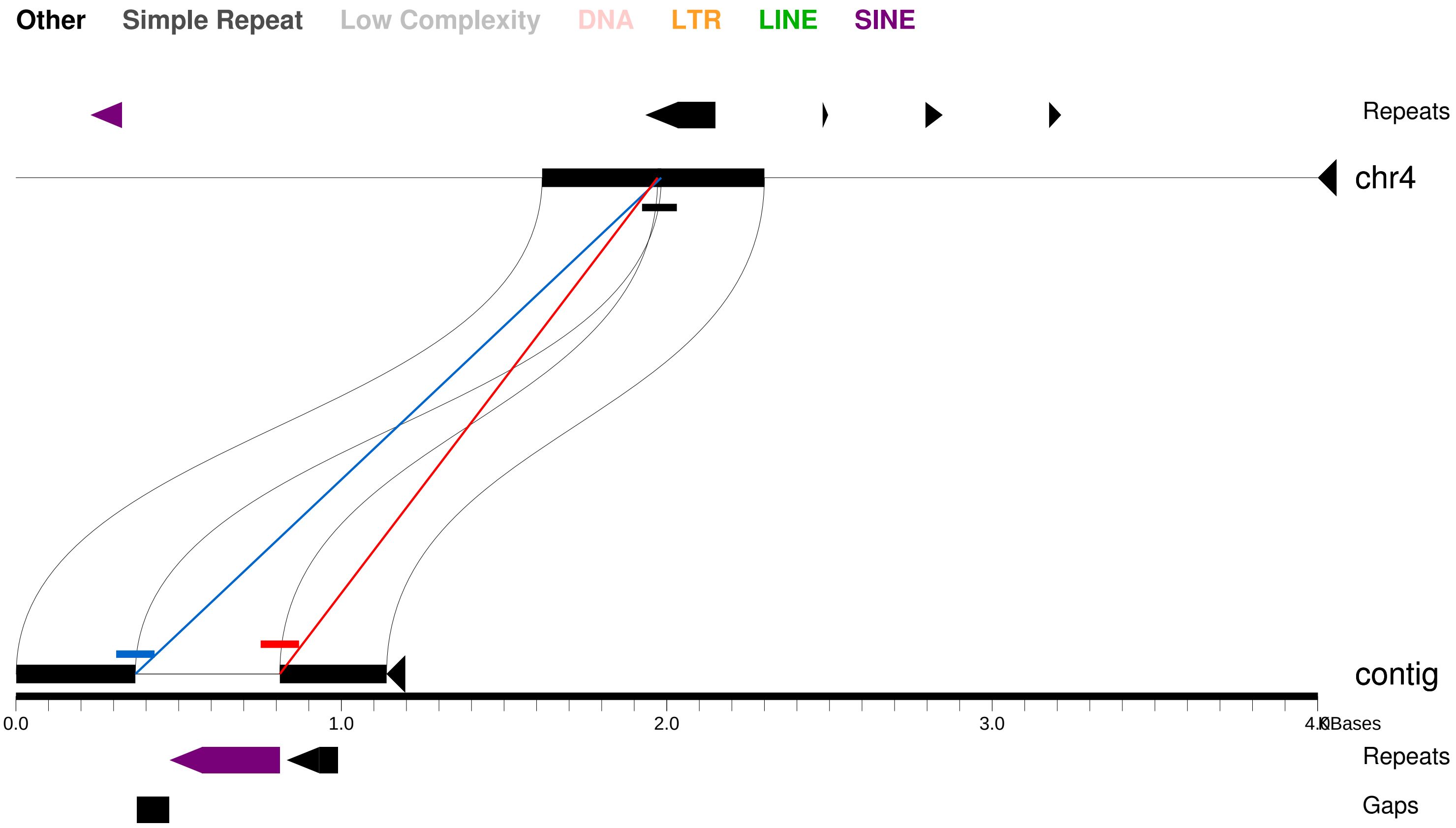

siteID: chr4_162052450 Scafftig1 - Breakpoint: chr4:162052428
chr4:162052376-162052487
Overlap Length: 8 INS size: 330 Genotyped: No

```
left    ----GAAACATCATGTGCAAAATCA--AGAAGCTGCTATT-ATAGCTGCAGAAGTCTATACCATGCCNNN
chrom   ----GAAACATCATGTGCAAAATCA--AGAAGCTGCTATT-ATAGCTGCAGAAGTCTATACCATGCCACA
right   CCCGGCTAATTTTTTTGTATTTTTAGTAGAGACGGGGTTTCACCGTTTTAGCTGGGATGACCATGCCACA
        1111*11*11*11*1**1*111*1*11***11*1*111**1*11*1*11**11*11111********222

left    NNNNNNNNNNNNNNNNNNNNNNNNNNNNNNNNNNNNNNNNNNNNNNNNNNNNNNN
chrom   TTTTCTACTATCAAGAAGCCATCTCTGCCAAATCAGCTAGCCTCAGAGA-------
right   TTTTCTACTATCAAGAAGCCATCTCTGCCAAATCAGCTAGCCTCAGAGA-------
        22222222222222222222222222222222222222222222222222222222
```

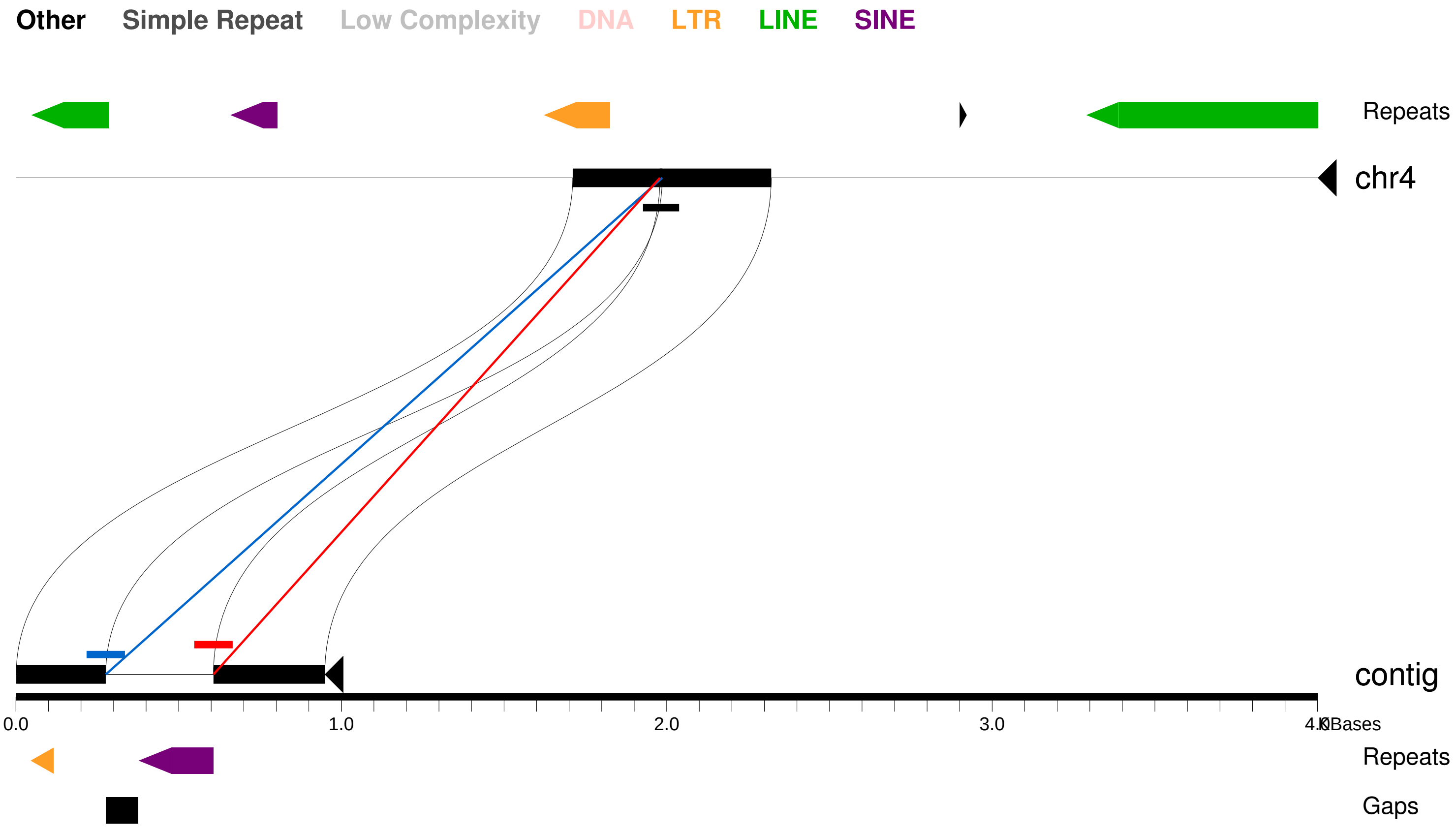

siteID: chr4_162593899 Scafftig1 + Breakpoint: chr4:162593911
chr4:162593864-162593970
Overlap Length: 13 INS size: 429 Genotyped: No

```
left    AAAAA---------AACCCAAAAATCTAAAAAAAAAAA-GAAAGGTGTTTATT--GATTTTAAGTAATTT
chrom   AAAAA---------AACCCAAAAATCTAAAAAAAAAAA-GTAAGGTGTTTATT--GATTTTAAGTAATTT
right   CCGCCCGCCTCGGCCTCCCAAAGTGCTGGGATTACAGGCGTGAGCCACCGCGCCCGGCCTTAAGTAATTT
        1111111111111111******111**111*11*1*111*21**11111111111*111***********

left    TTNNNNNNNNNNNNNNNNNNNNNNNNNNNNNNNNNNNNNNNNNNNNNNNNNNNNNNNNNNN
chrom   TTTAAATTAGAAACTGAACATTTCTTAAGCCTACAATTGTTGCCATACA------------
right   TTTAAATTAGAAACTGAACATTTCTTAAGCCTACAATTGTTGCCATACA------------
        **22222222222222222222222222222222222222222222222222222222222
```

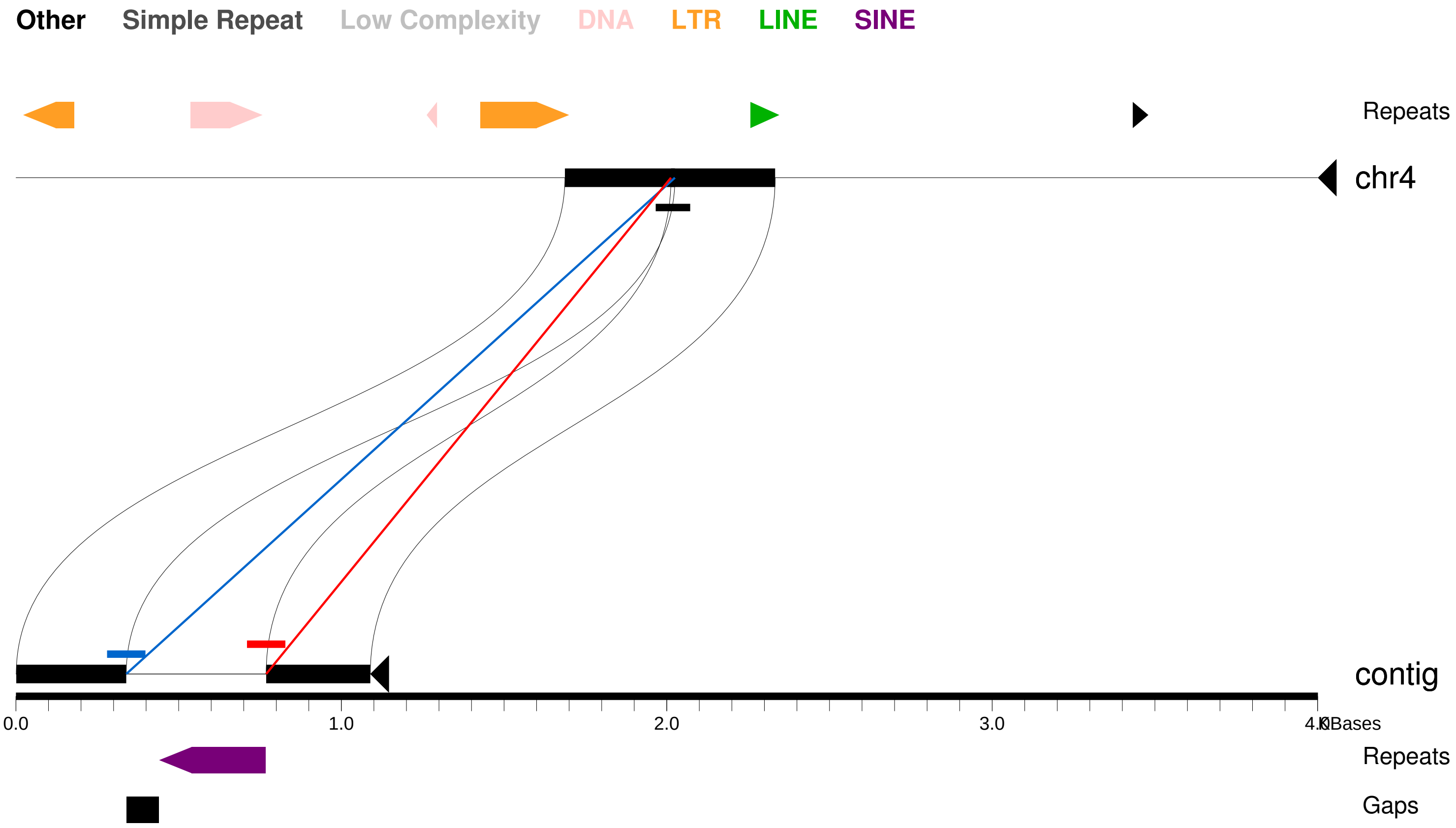

siteID: chr4_164084637 Scafftig1 - Breakpoint: chr4:164084784
chr4:164084624-164084952
Overlap Length: 9 INS size: 352 Genotyped: No

```
left    A---------CATGCAGGGGTAGAAAAAAAACAATGTGATAATATGGCCTACATTCTGAAATTCTGAGAA
chrom   ATTAAAACTACATGCAGGGGTAGAAAAAAAACAATGTGATAATATGGCCTACATTCTGAAATTCTGAGAA
right   NNNNNNNNNNN-----------------------------------------------------------
        1NNNNNNNNN111111111111111111111111111111111111111111111111111111111111

left    GGCTTTCCTCTTGGGTCATAGGTTTCAATCAGACTAACATCATCCTCCTAAGGATGTTTACAGCATAAGA
chrom   GGCTTTCCTCTTGGGTCATAGGTTTCAATCAGACTAACATCATCCTCCTAAGGATGTTTACAGCATAAGA
right   ----------------------------------------------------------------------
        1111111111111111111111111111111111111111111111111111111111111111111111

left    GGAAATAACCTTGCAAGAGTTAACTTTGCGGCCGGGCGCGGTGGCTCACGCCTGTAA-TCCCAG----CA
chrom   GGAAATAACCTTGCAAGAGTTAACTTTGCAAACCAAACCTATCGAG--TGCCAATAAATACCATTCTACA
right   --------------------TAACTTTGCAAACCAAACCTATCGAG--TGCCAATAAATACCATTCTACA
        11111111111111111111*********222*22222*22*2*22222***22***2*2***22222**

left    CTTTGGGAGGCCGAGGCGGGTGGA-TCATGAGGTCAGGAGATCGAGACCATCCTGG---CTAACAAGGTG
chrom   ATTAACAAGTTTTCTCCAACTGTAATAATAAAAACACTCCAAAAACTTCAATAGGAATCCTAACAAGTTC
right   ATTAACAAGTTTTCTCCAACTGTAATAATAAAAACACTCCAAAAACTTCAATAGGAATCCTAACAAGTTC
        2**2222**2222222*222**2*2*2**2*222**2222*222*222**2222*2222*******2*2

left    AAACCCCG-----------------TCTCTAC-----------TAAA----
chrom   AACCCTCTGAATTTCATCTTCTACTTCTCTAAATGCAGTCAATTAAAAAGT
right   AACCCTCTGAATTTCA-----------------------------------
        **2**2*222222222         111111N           1111
```

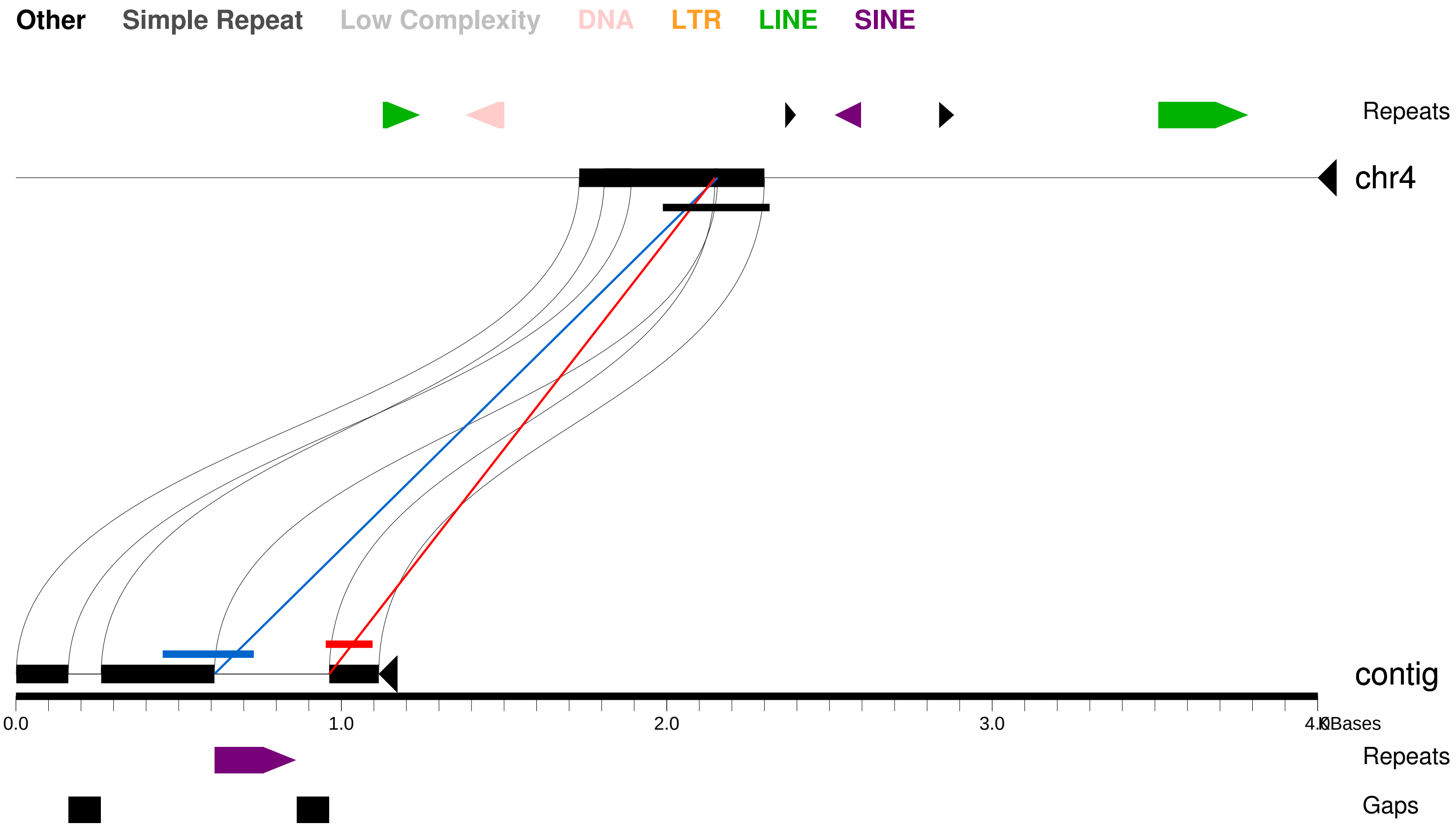

siteID: chr4_165034138 Scafftig5 - Breakpoint: chr4:165034142
chr4:165034089-165034201
Overlap Length: 7 INS size: 272 Genotyped: Yes

```
left   ATGTATTTTCAAATTCTG--AAAATAAGATATTACCAATCAAATACCCATTAAAC-------AATTGCAC
chrom  ATGTATTTTCAAATTCTG--AAAATAAGATATTACCAATCAAATACCCATTAAAA-------AATTGCAA
right  --GCACTC-CAGCCTGGGCGACAGAGCGAGACTCCGTCTCAAAAAAAAAAAAAAAAAAAAAGCAATTGCAA
       11*1*1*11**111*11*11*1*1111**1*1*1*111*****1*111*11***21111111*******2

left   -----TTTGGGAGGCCGAGGCGGGCGGA---TCACGAGGTCAGGAGATCGAGACCATCCCGGCTAA
chrom  ATAAATTTGACAAATTAAAGAATGATATCTTTCCTTGGCTCAGGATAGTGAG--------------
right  ATAAATTTGACAAATTAAAGAATGATATCTTTCCTTGGCTCAGGATAGTGAG--------------
       22222****22*22222*2*222*2222222**2222*2******2*22***22222222222222
```

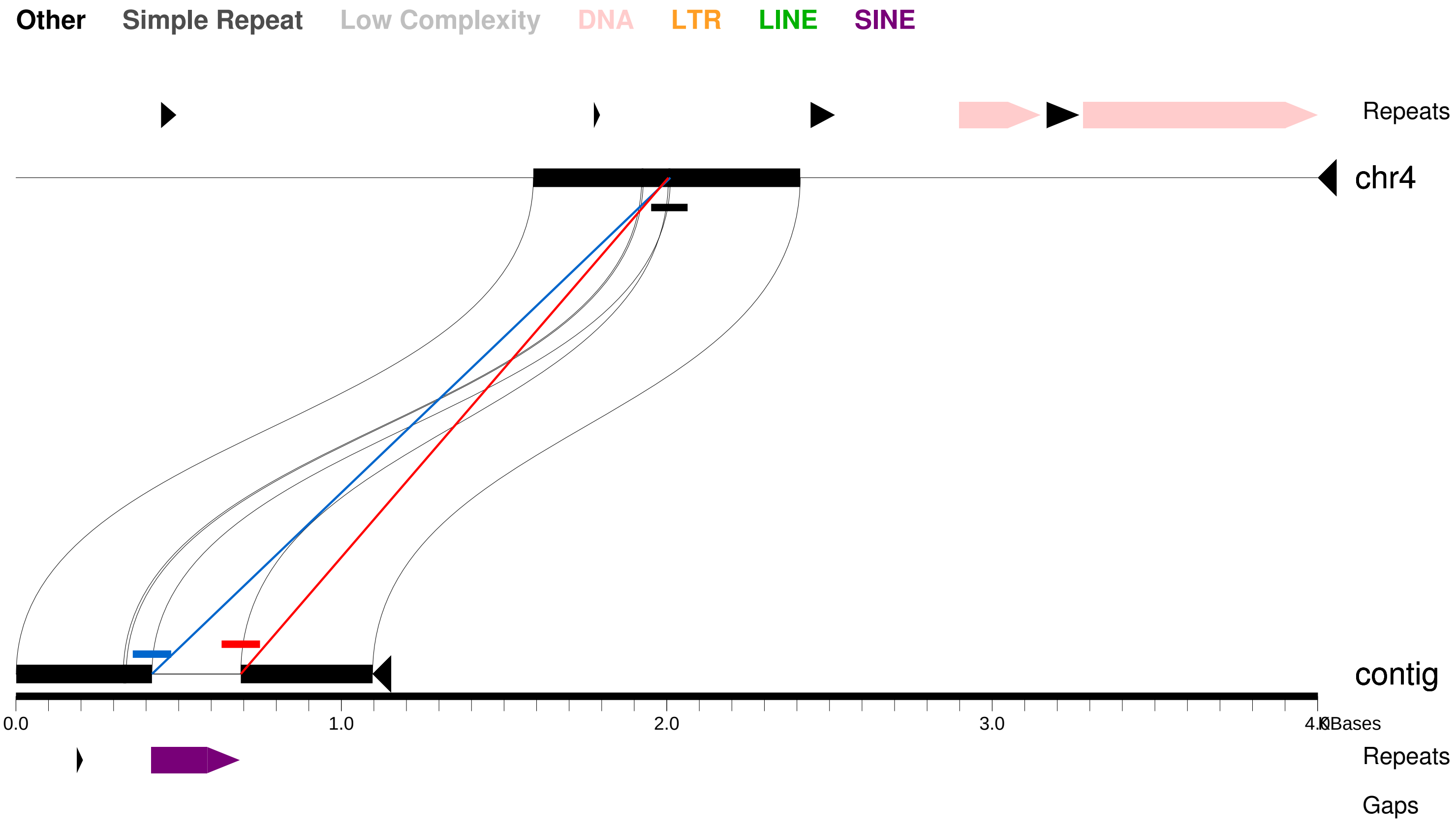

siteID: chr4_16728121 Scafftig1 - Breakpoint: chr4:16728066
chr4:16728019-16728125
Overlap Length: 13 INS size: 372 Genotyped: No

```
left   ATTTATGCATG-----CACCAGATCCAGGCAG-GTGCTCTATCCTCAATAA------ATACATTTATATT
chrom  ATTTATGCATG-----CACCAGATCCAGGCAG-GTGCTCTATCCTCAATAA------ATACATTTATATT
right  TGTTAGCCAGGATGGTCTCGATCTCCTGACCTCGTGATCCGCCCGCCTCGGCCTCCCAAACATTTATATT
       11***11**1*11111*1*1*11***1*1*111***1**111**1*11111111111*1***********

left   TTTTNNNNNNNNNNNNNNNNNNNNNNNNNNNNNNNNNNNNNNNNNNNNNNNNNNNNNNNNN
chrom  TTAGTACATGCAAAATAACAGGAGGCAAGGAATATGAGAAGAAAACTGC------------
right  TTAGTACATGCAAAATAACAGGAGGCAAGGAATATGAGAAGAAAACTGC------------
       **22*22222222222222222222222222222222222222222222222222222222
```

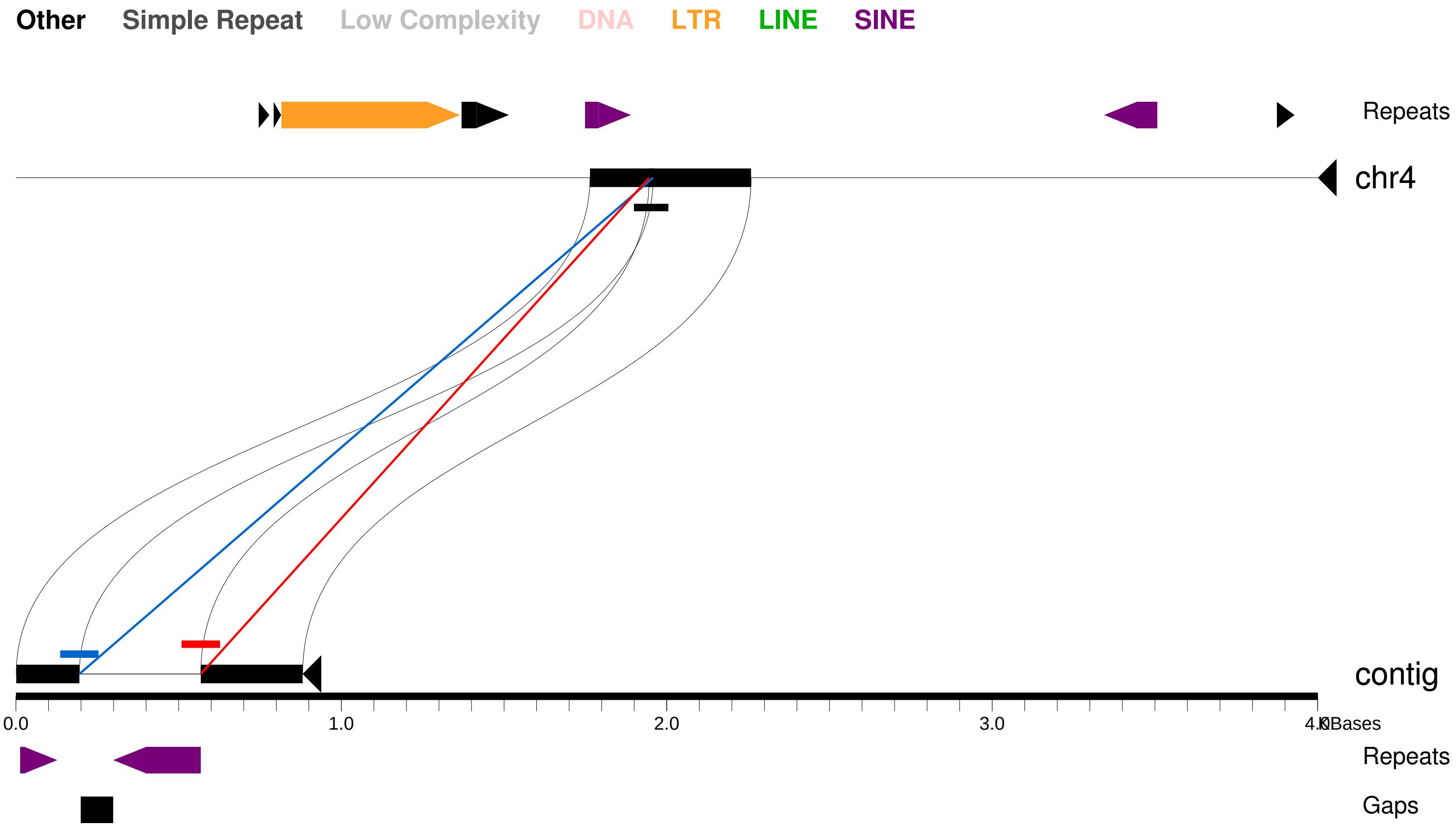

siteID: chr4_167338790 Scafftig2 - Breakpoint: chr4:167338784
chr4:167338735-167338843
Overlap Length: 11 INS size: 334 Genotyped: Yes

```
left   CTT----TTTCAGTTCTTTAAAAG-----GTATTATTAGGTTGTTT-ACCT-GACATTTTTCTACTTTTT
chrom  CTT----TTTCAGTTCTTTAAAAG-----GTATTATTAGGTTGTTT-ACCT-GACATTTTTCTACTTTTT
right  CCGCCCGCCTCGGC-CTCCCAAAGTGCTGGGATTACAGGCGTGAGCCACCGCGCCCGGCCTCTACTTTTT
       *11111111**1*11**111****11111*1****111*11**1111***11*1*11111**********

left   CTTTTTTTTTTTTTTTTTTTTTTTTTTTTTTTTTTTTTTTTTTTTTTTTTTTTTGAGACG
chrom  CAATGCAGGAGTTTATAGCTGTAAACTCTCCTCTTAGTACTGCTTTC----------ACT
right  CAATGCAGGAGTTTATAGCTGTAAACTCTCCTCTTAGTACTGCTTTC----------ACT
       *22*2222222***2*222*2*2222*2*22*2**22*22*22***22222222222**2
```

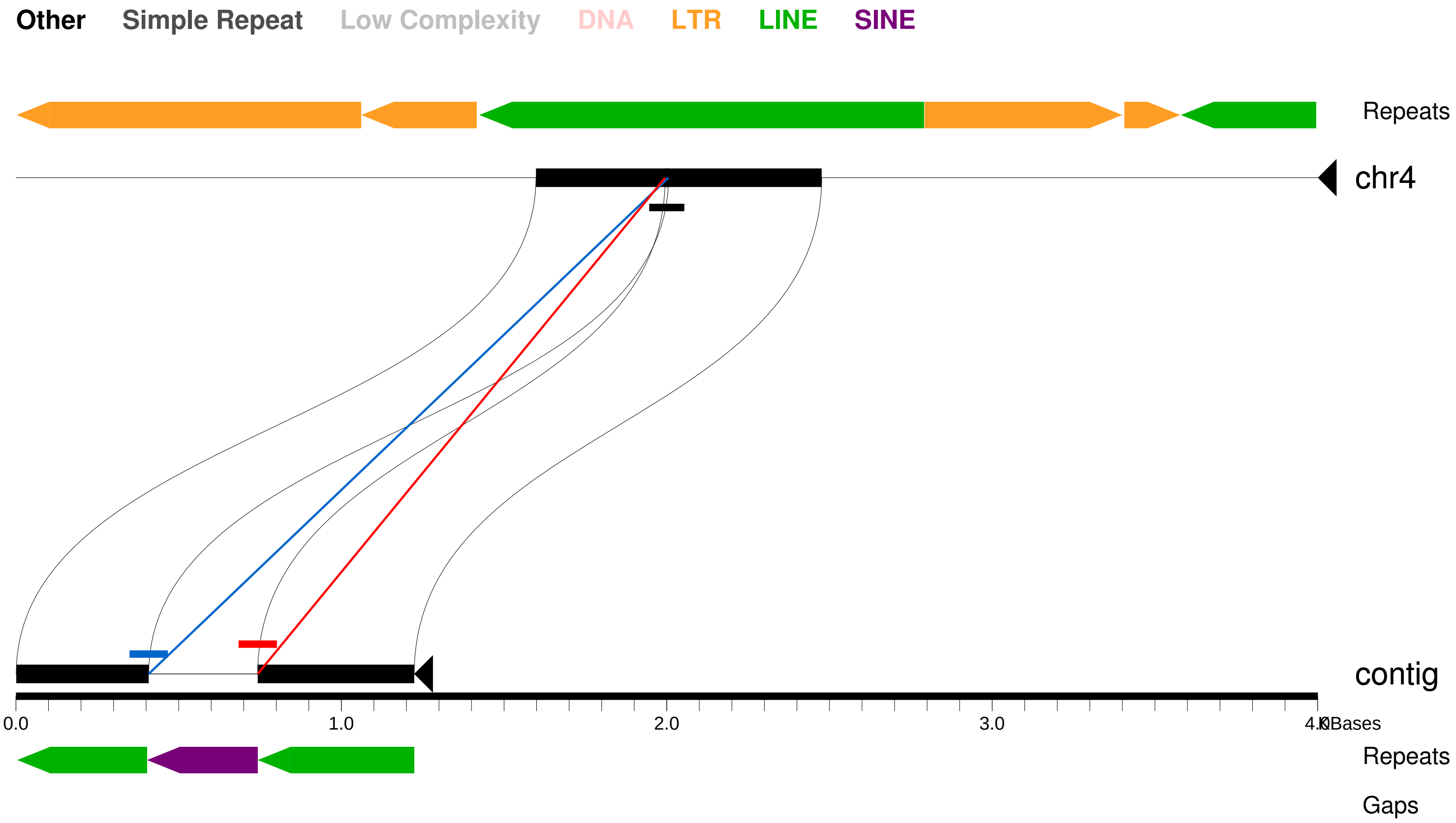

siteID: chr4_168889157 Scafftig3 - Breakpoint: chr4:168889166
chr4:168889123-168889225
Overlap Length: 17 INS size: 342 Genotyped: No

```
left     CAGGTGAGGCTTCATAGTACCTTGTTTCATCATCACATC----------------AATGAAAAAGACACC
chrom    CAGGTGAGGCTTCATAGTACCTTGTTTCATCATCACATC----------------AATGAAAAAGACACC
right    NNNNNNNNNNNNNNNNNNNNNNNNNNNNNNNNNNNNNNNNNNNNNNNNNNNNNNNAAAAAAAAAGACACC
         1111111111111111111111111111111111111111111111111111111**11***********

left     GAAGCTGGCCGGGC-GCGGTGGCTCACGCCTGTAATCCCAGCACTTTGGGAGGCCGAGGCGGGCGG
chrom    GAAGCTAAAAAAACAGTCTTGAATCACCGATGCTACCCCTCCACCATTG-----------------
right    GAAGCTAAAAAAACAGTCTTGAATCACCGATGCTACCCCTCCACCATTG-----------------
         ******2222222*2*222**22****222**22*2***22***22*2*22222222222222222
```

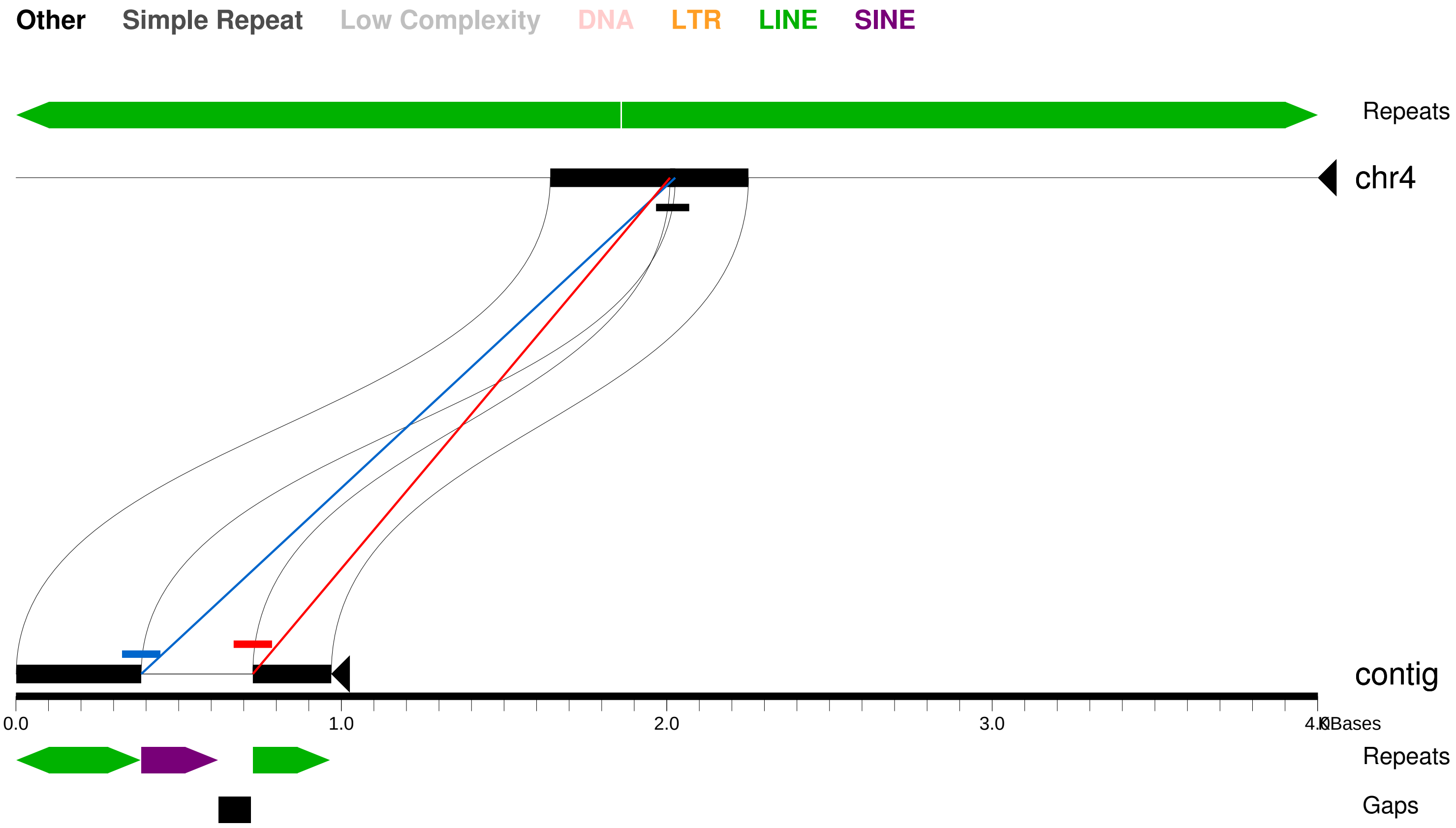

siteID: chr4_170834594 Scafftig3 - Breakpoint: chr4:170834592
chr4:170834538-170834646
Overlap Length: 6 INS size: 294 Genotyped: Yes

```
left    ATGTTTTGGGAATA------ATGCTCAGAAATTTTTTAATAATGCACTTTATGATAAAAA----TTTTGG
chrom   ATGTTTTGGGAATA------ATGCTCAGAAATTTTTTAATAATGCACTTTATGATAAAAA----TTTTGG
right   AGATCCCGCCACTGCACTCCAGCCTGGGCGACAGAGCGAGACTCCGTCTCAAAAAAAAAAAAAATTTTTGG
        *11*111*11*1*1111111*11**11*11*1111111*1*1*1*111*1*11*1*****1111******

left    GCCGGGCGCGGTGGCTCACGCCTGTAATCCCAGCACTTTGGGAGGCCGAG-GCGGGCGGA
chrom   --AGGCAACTGGGGTTAA-------AATACAA--AATTTATGAGGGCAAGCGCCATATTC
right   --AGGCAACTGGGGTTAA-------AATACAA--AATTTATGAGGGCAAGCGCCATATTC
        222**222*2*2**2*2*2222222***2*2*22*2***22****2*2**2**2222222
```

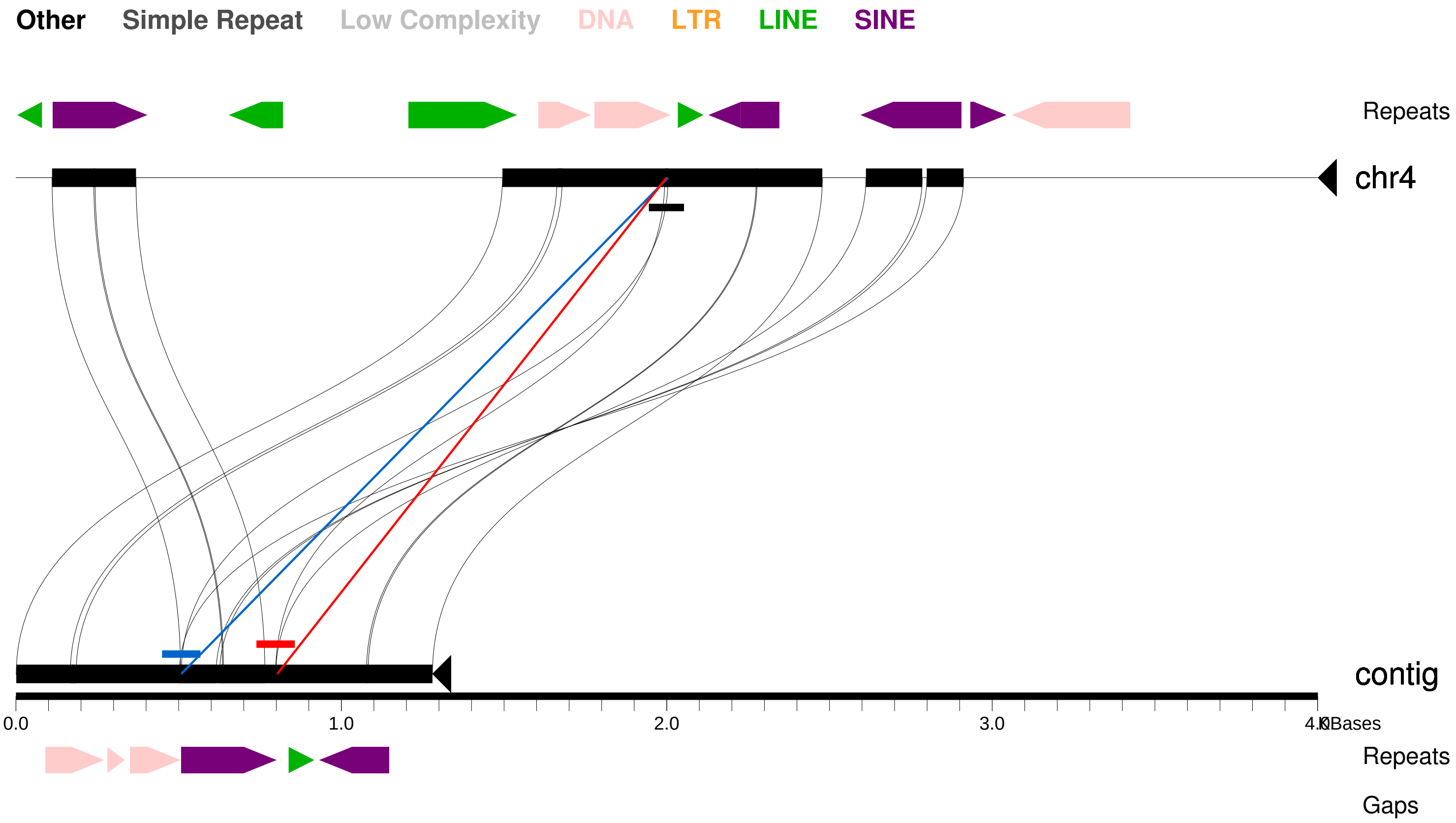

siteID: chr4_171428775 Scafftig7 - Breakpoint: chr4:171428785
chr4:171428733-171428844
Overlap Length: 8 INS size: 309 Genotyped: Yes

```
left    AA-----TGAATTAGG-----GAGTATTCCTTCTTTTTCTATTGATTGGAATAGTTTCAGAAGGAATGGT
chrom   AA-----TGAATTAGG-----GAGTATTCCTTCTTTTTCTATTGATTGGAATAGTTTCAGAAGGAATGGT
right   CGCAGTCTGGCCTGGGCGACAGAGCAAGACTCCGTCTCAAAAAAAAAAAAAAAAA---AAACGGAATGGT
        1111111**111*1**11111***1*111**1*1*1*111*111*1111**1*11111*1*1********

left    GGCCGGGCGCGGTGGCTCACGCCTGTA-ATCCCAGCACTTTGGGAGGCCAAGGCGGGTGG
chrom   ACCAGCTCCTCCTTGTACAT-CTTGTAGAATTCGGCTAT----GAATCCA--TCTGGTC-
right   ACCAGCTCCTCCTTGTACAT-CTTGTAGAATTCGGCTAT----GAATCCA--TCTGGTC-
        22*2*22*2222*2*22**22*2****2*222*2**22*2222**22***222*2***22
```

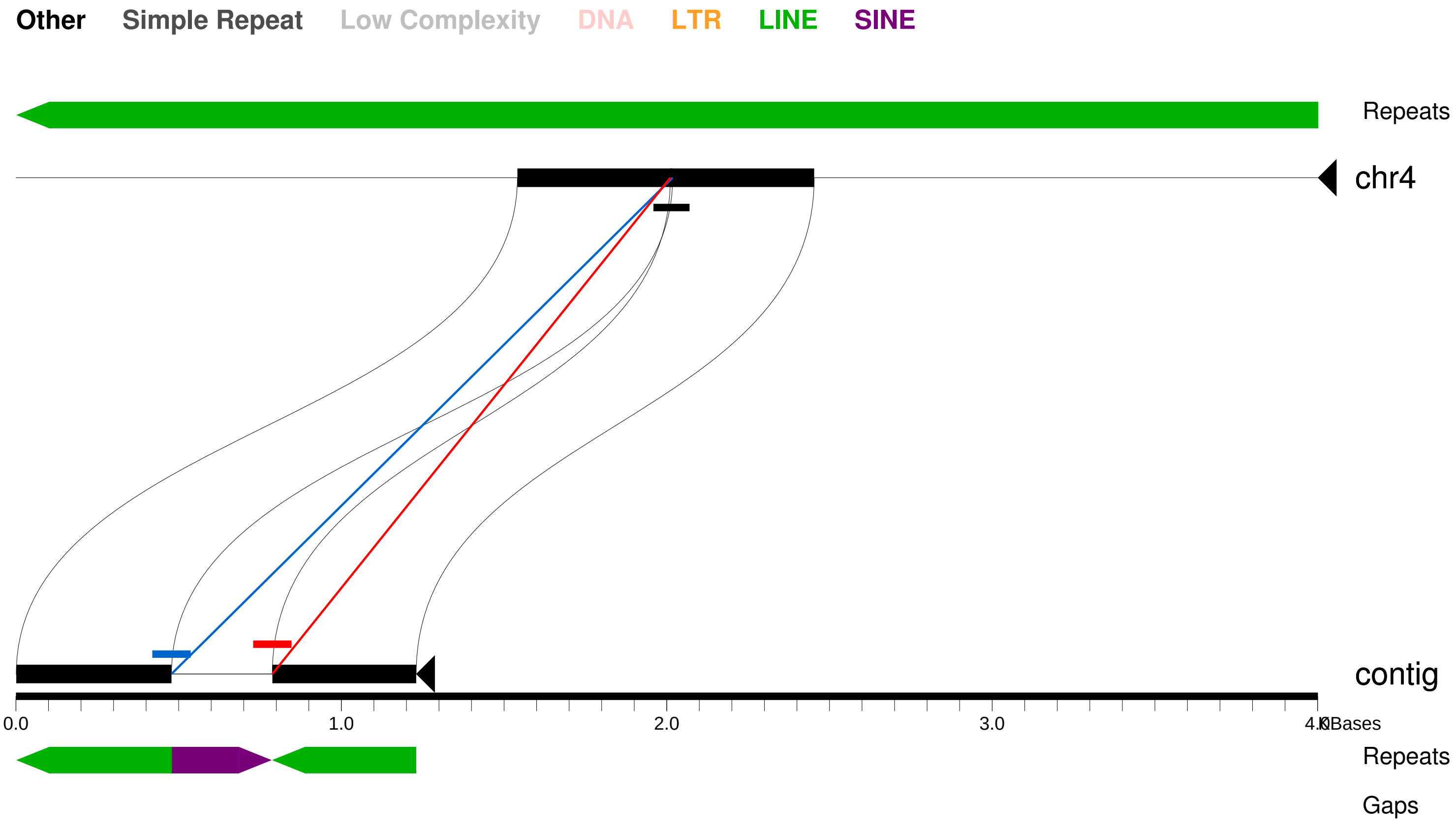

siteID: chr4_174149435 Scafftig2 + Breakpoint: chr4:174149441
chr4:174149408-174149500
Overlap Length: 27 INS size: 423 Genotyped: No

```
left    -------CATCACCAGAGAAAACTGCTT---TTACT----------------CTCTCTTCTTCTAGTCAT
chrom   -------CATCACCAGAGAAAACTGCTT---TTACT----------------CTCTCTTCTTCTAGTCAT
right   CCGCCCGCCTCGGCCTCCCAAAGTGCTGGGATTACAGGCGTGAGCCACCGCGCCCGGCCCTTCTAGTCAT
        1111111*1**11*11111***1****1111****11111111111111111*1*1111***********

left    TTCTATGCCCCATTATNNNNNNNNNNNNNNNNNNNNNNNNNNNNNNNNNNNNNNNNNNNNNNNNNNNNNN
chrom   TTCTATGCCCCATTATTATTTTTGCATATTTGAGGTGATGTGGTGTAAA---------------------
right   TTCTATGCCCCATTATTATTTTTGCATATTTGAGGTGATGTGGTGTAAA---------------------
        ****************2222222222222222222222222222222222222222222222222222222

left    NNNNN
chrom   -----
right   -----
        22222
```

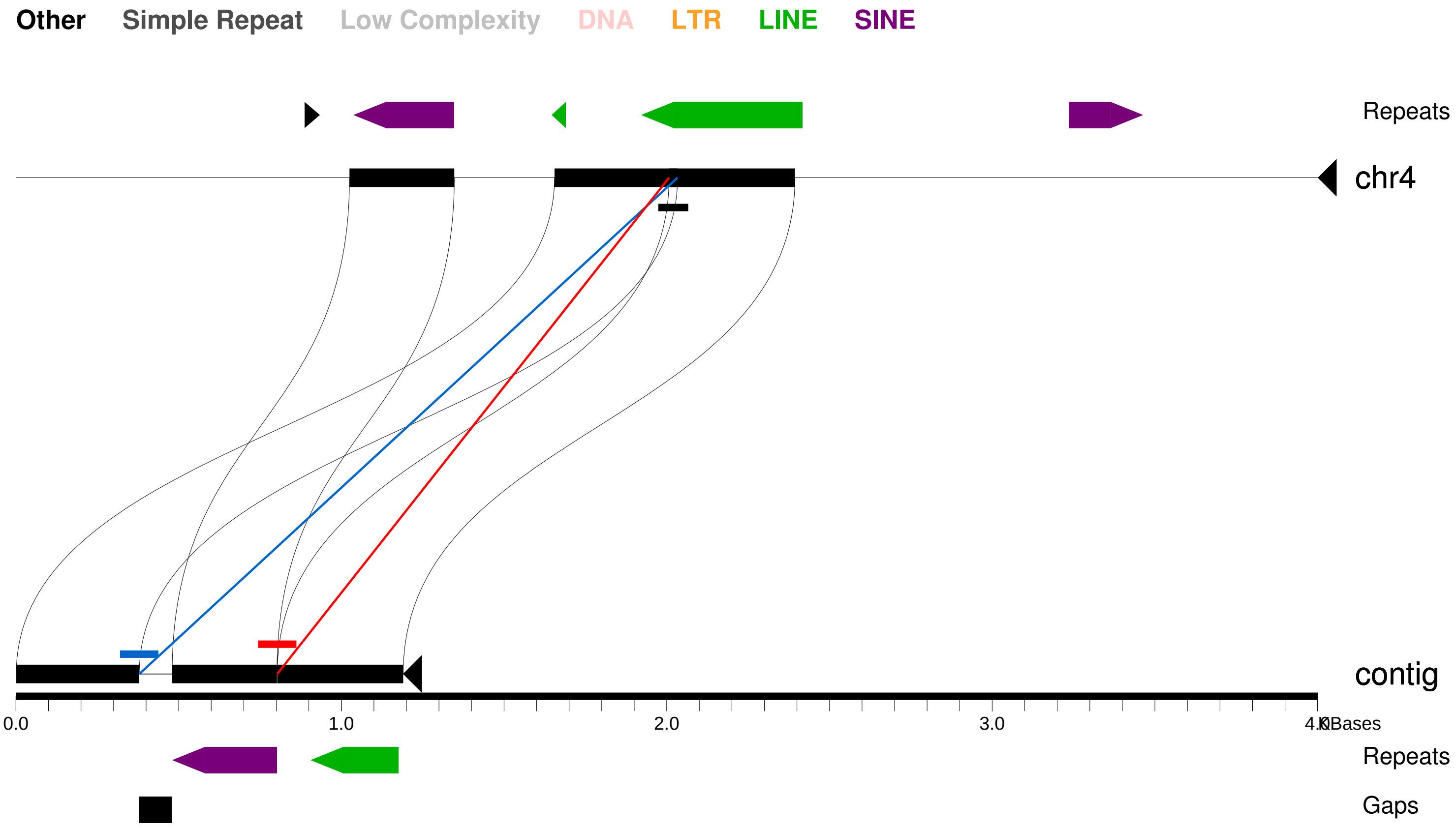

siteID: chr4_174786703 Scafftig2 + Breakpoint: chr4:174786705
chr4:174786649-174786764
Overlap Length: 4 INS size: 412 Genotyped: No

```
left    GTGTACCTTAAATCCATTGGAAAGCTAGAATAAGCTTTCATCTTAAGAAAGTTCTT---TCTGGGCCGGG
chrom   GTGTACCTTAAATCCATTGGAAAGCTAGAATAAGCTTTCATCTTAAGAAAGTTCTT---TCTGAAGATAA
right   NNNNNNNNNNNNNNNNNNNNNNNNNNNNNNNNNNNNNNNNNNNNNNNNNNNNNNNNNNNTCTGAAGATAA
        11111111111111111111111111111111111111111111111111111111111****2222222

left    CGCGGTGGCTCACGCCTGTAATCCCAGCACTTTGGGAGGCCGAGGCGGGCGG
chrom   TG-GGAGGCTTCTGTTTATGGACC-AACAATTTGAG-GAATGTAGTGATCAC
right   TG-GGAGGCTTCTGTTTATGGACC-AACAATTTGAG-GAATGTAGTGATCAC
        2*2**2****222*22*2*222**2*2**2****2*2*222*22*2*22*22
```

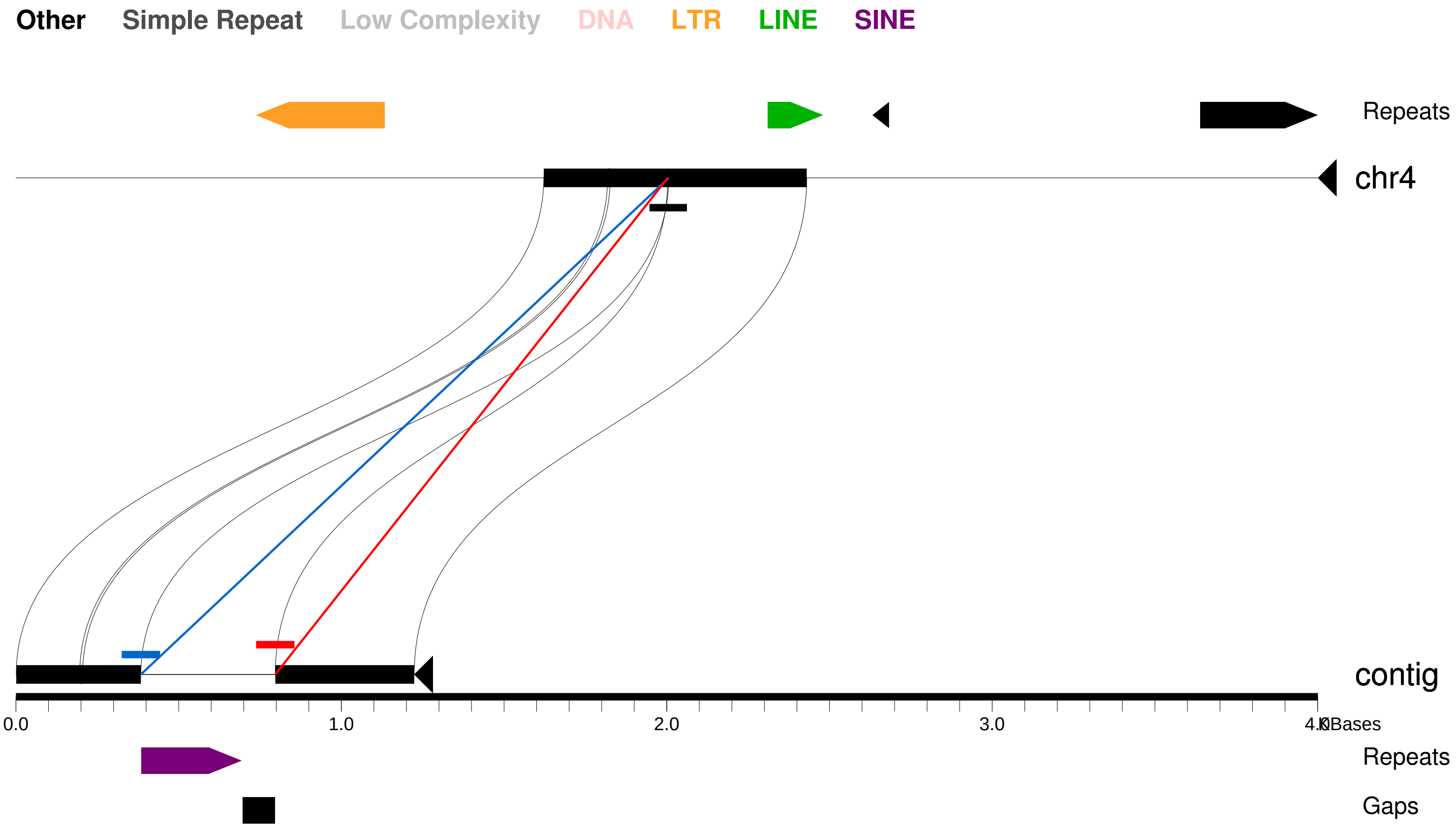

siteID: chr4_175534313 Scafftig21 + Breakpoint: chr4:175534292
chr4:175534246-175534351
Overlap Length: 14 INS size: 313 Genotyped: Yes

```
left    ATCTCACATGG---TGGCAGACAGCAG--AAGAGAAT-GAGAGCCAA-------GAGAAAGGAGTTTCCC
chrom   ATCTCACATGG---TGGCAGACAGCAG--AAGAGAAT-GAGAGCCAA-------GAGAAAGGAGTTTCCC
right   CGCCCGCCTCGGCCTCCCAAAGTGCTGGGATTACAGGCGTGAGCCACCGCGCCCGGCCTAGGAGTTTCCC
        11*1*1*1*1*111*11**1*11**1*11*11*1*111*1******11111111*1111***********

left    CTTTTTTTTTTTTTTTTTTTTTTTTTTTTGGAGACGGAGTCTCGCTCTGTCGCCCAGGCTGGA
chrom   CTTATAAAACCATCAGATCTTGT------GAGACTTATTC-------AATACCATGAGAACA
right   CTTATAAAACCATCAGATCTTGT------GAGACTTATTC-------AATACCATGAGAACA
        ***2*2222222*2222*2**2*222222*****22*2**22222222222**22*22222*
```

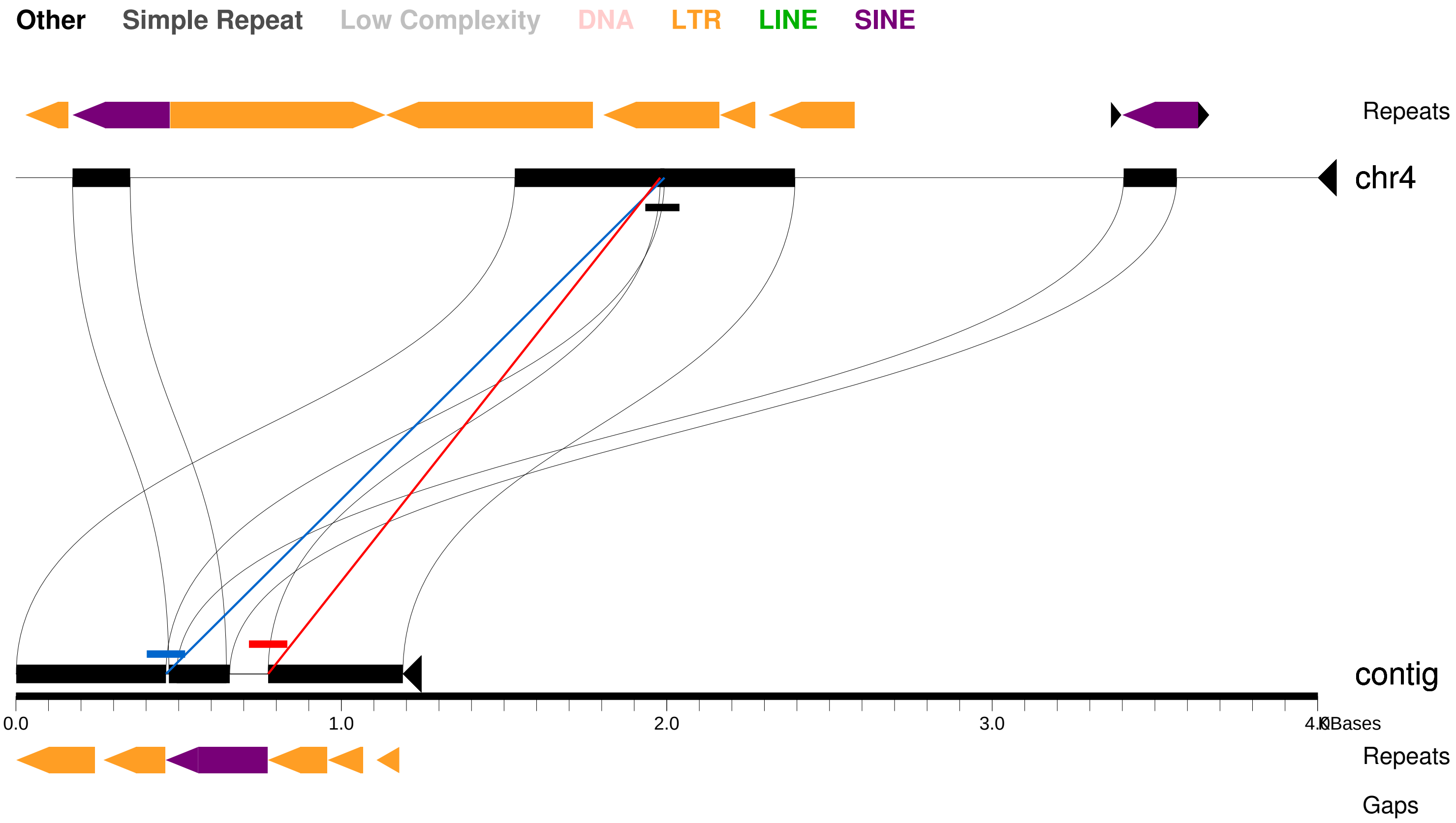

siteID: chr4_176281833 Scafftig4 - Breakpoint: chr4:176281823
chr4:176281777-176281882
Overlap Length: 14 INS size: 231 Genotyped: Yes

```
left    TACAATAAGAATTTGGGACTTTTTCTTTACAATCATAGGAAGGCAT-------------AAAAGGCTTTA
chrom   TACAATAAGAATTTGGGACTTTTTCTTTACAATCATAGGAAGGCAT-------------AAAAGGCTTTA
right   CTGGGCGACAGAGCGAGACTCCGTCTCAAAAAAAAAAAAAAAAAAAAAAAAAAAAAAAAAAAAGGCTTTA
        1111111*1*1111*1****111***11*1**11*1*11**111*11111111111111***********

left    GGCCCATCCTGGCTAACACG-GTGAAACCCCGTCTCTACTAAAAATACAAAAAATTAGCCGGG
chrom   GGC--ATAATTGATATGACTAGTTATAT---GT-TGTAGAACAATAACAAAAAAA--------
right   GGC--ATAATTGATATGACTAGTTATAT---GT-TGTAGAACAATAACAAAAAAA--------
        ***22**22*2*2**22**22**2*2*2222**2*2**22*2**22*******222222222
```

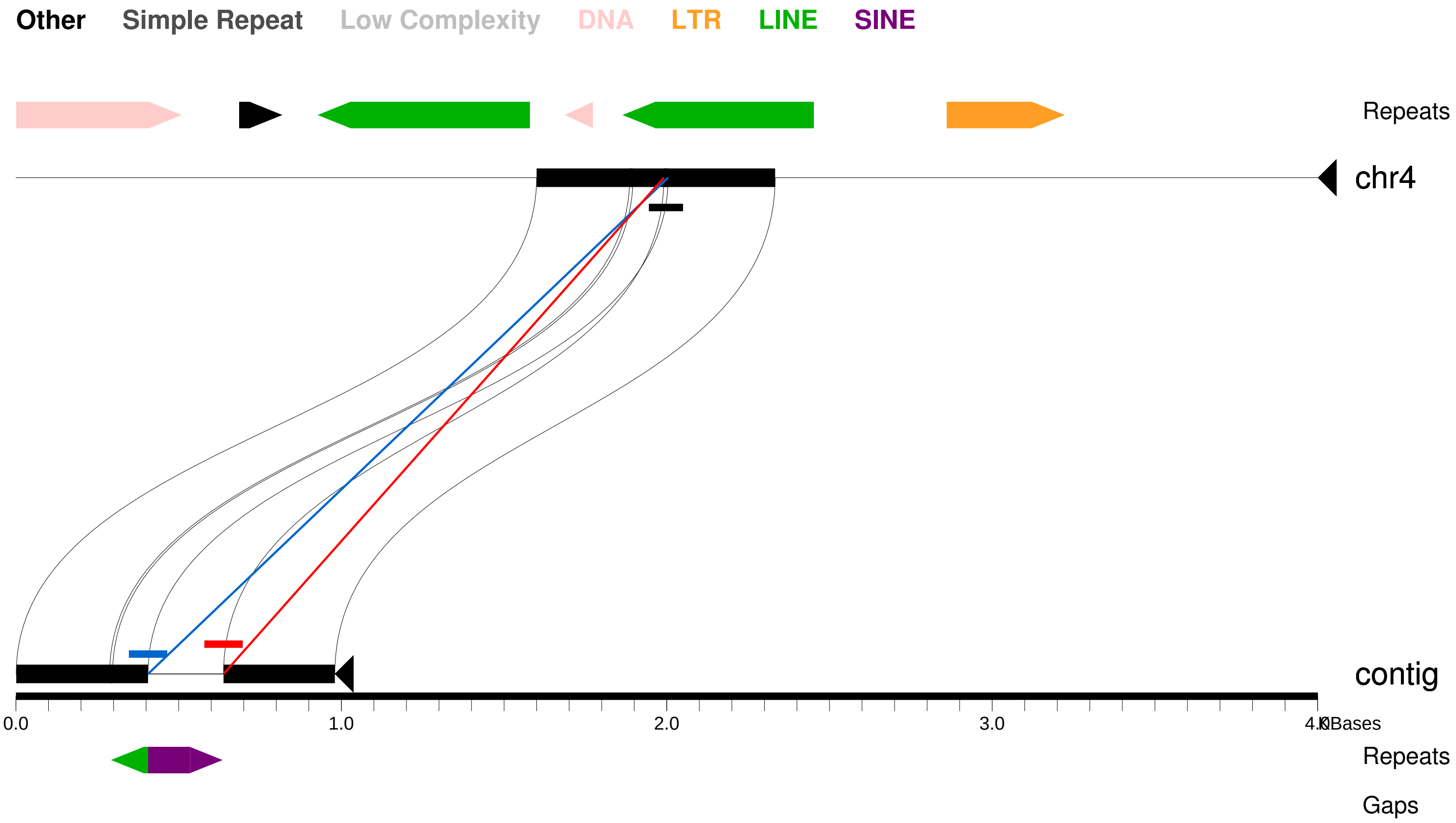

siteID: chr4_178789725 Scafftig2 - Breakpoint: chr4:178789746
chr4:178789693-178789793
Overlap Length: 7 INS size: 297 Genotyped: Yes

```
left    -------CTAAGTCAAAGCTCTGAGA---AGGGAAAACACCTATGACTACAAAAAAGTTTAAA-------
chrom   -------CTAAGTCAAAGCTCTGAGA---AGGGAGAATACCTATGACTACGAAAAAGTTTAAA-------
right   TGCAGTCCGCAGTCCGGCCTGGGCGACAGAGCGAGACTCCGTCTCAAAAAAAAAAAAAAAAAAAAAAAAA
        1111111*11****1111**11*1**111**1**2*121*1*1*1*11*1N*****1111***1111111

left    -GTTTAGTCACGCCTGTAATCCCAGCACTTTGGGAGGCCGAGGCGGGTGGATCATGAGGTCAGGAGA
chrom   -GTTTAGTTAT---TGTTATG--------------GGCTGAATTGTGTCCCCCCCAAAATTCACAT-
right   AGTTTAGTTAT---TGTTATG--------------GGCTGAATTGTGTCCCCCCCAAAATTCACAT-
        1*******2*2222***2**222222222222222***2**222*2**2222*222*22*2222*22
```

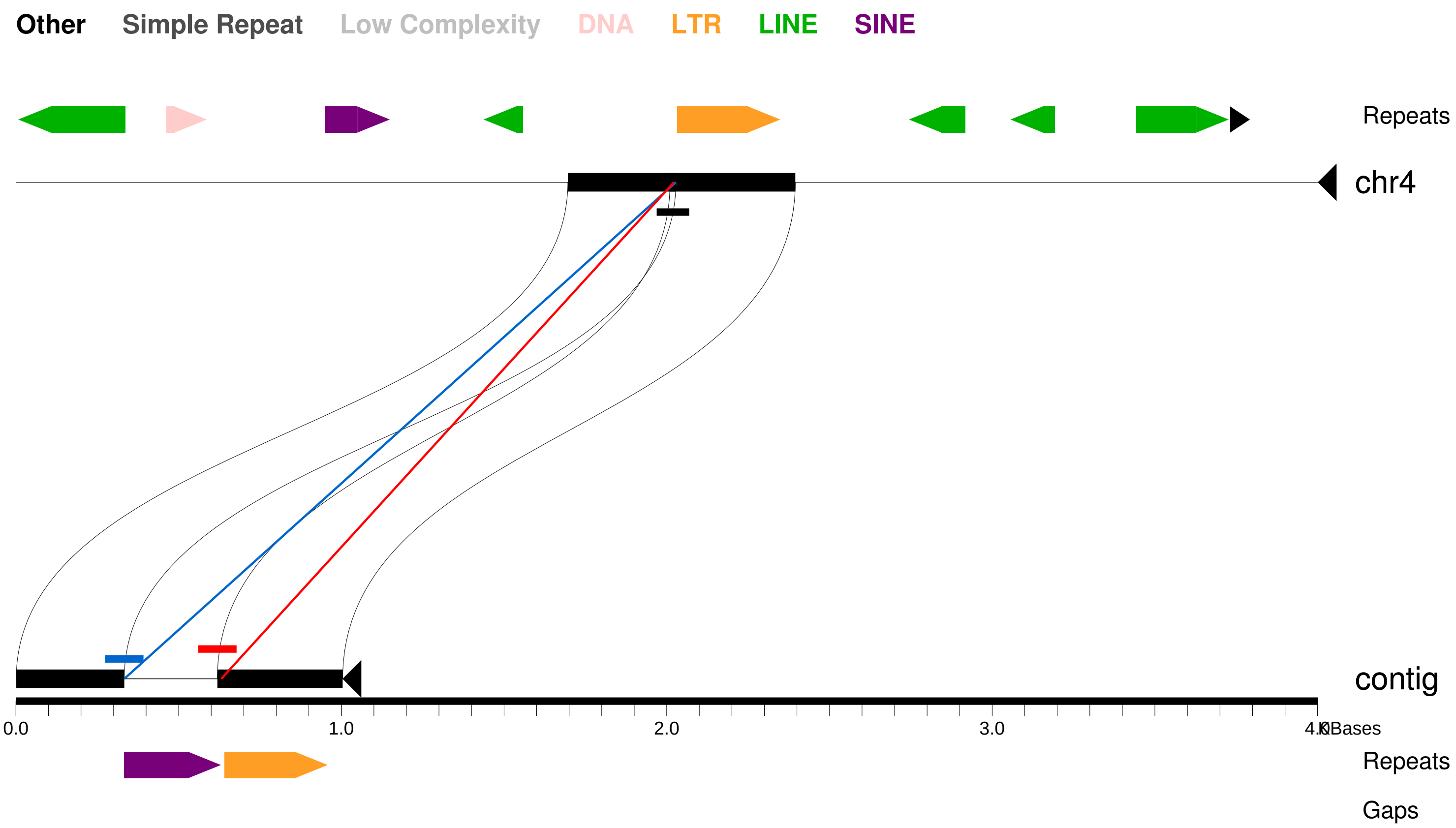

siteID: chr4_179198329 Scafftig1 + Breakpoint: chr4:179198313
chr4:179198153-179198489
Overlap Length: 17 INS size: 304 Genotyped: Yes

```
left   --------------CCCAGAATGTTTAATTTAAAATGACTTCGCATTTTTCCTCATTTAAAGACACACAT
chrom  TGTTTTTTTTTTCTCCCAGAATGTTTAATTTAAAATGACTTCGCATTTTTCCTCATTTAAAGACACACAT
right  T-----------------------TTAGTAGAGACGGAGTT--------TCACCATTTTAG--CCGGGAT
       2             1111111111***1*11*1*11**1**11111111**11*****1*111*1111**

left   GGACACTTATTTTTTGTAAAACTCACCAACACCCTTAAGAAATTTCAAAACCCTAC-TTTCTACTTCTGC
chrom  GGACACTTATTTTTTGTAAAACTCACCAACACCCTTAAGAAATTTCAAAACCCTAC-ATTCTACTTCTGC
right  GGTCTCA-ATCTCCTGACCTCGTGATCCGCCCGCCTCGGCC-TCCCAAAGTGCTGGGATTACAGGCGTGA
       **1*1*11**1*11**111111*1*1*11*1*1*1*11*111*11****111**1112**11*1111**1

left   GCTATCTGAATGCGTGTCGTC-ATGGTCATCCCTTCTTTTTTTTTTTTTTTTTTTTTTTTGAGACGGAG
chrom  GCTATCTGAATGCGTATCGTC-ATGGTCATCCCTTCTTTAAAAATGATTTTTTACATGCCTCTCACCTCT
right  GCCACC-----GCGCCCGGCCCATGGTCATCCCTTCTTTAAAAATGATTTTTTACATGCCTCTCACCTCT
       **1*1*11111***1N11*1*1****************22222*22******222*222*222**2222

left   TCTCGCTCT--GTC-------GCCCAGGCTGGAGTGCAGTGGCGG-----------GATCTCGGCTCACT
chrom  TTGTGCTATCAGCCCTTCAGGGCACAAGTTGTTTTTCATTATCCTTTACCTGCACAGAGCCTAGAATACA
right  TTGTGCTATCAGCCCTTCAGGGCACAAGTTGTTTTTCATTATCCTTTACCTGCACAGAGCCTAGAATACA
       *222***2*22*2*2222222**2**2*2**222*2**2*22*2222222222222**2*222*222**2

left   GCAAGCTCCGCCTCCCGG---GTTCACGCCAT---TCTCC----------------TGC
chrom  GAAAGTGCTTAATTTTTATTTGTTGATGTAATGAGTGTGTGTGTGGTGTAGGGGTGTGT
right  GAAAGTGCTTAATTTTTATTTGTTGATGTAATGAGTGTGTG------------------
       *2***22*2222*22222222***2*2*22**222*2*222               11N
```

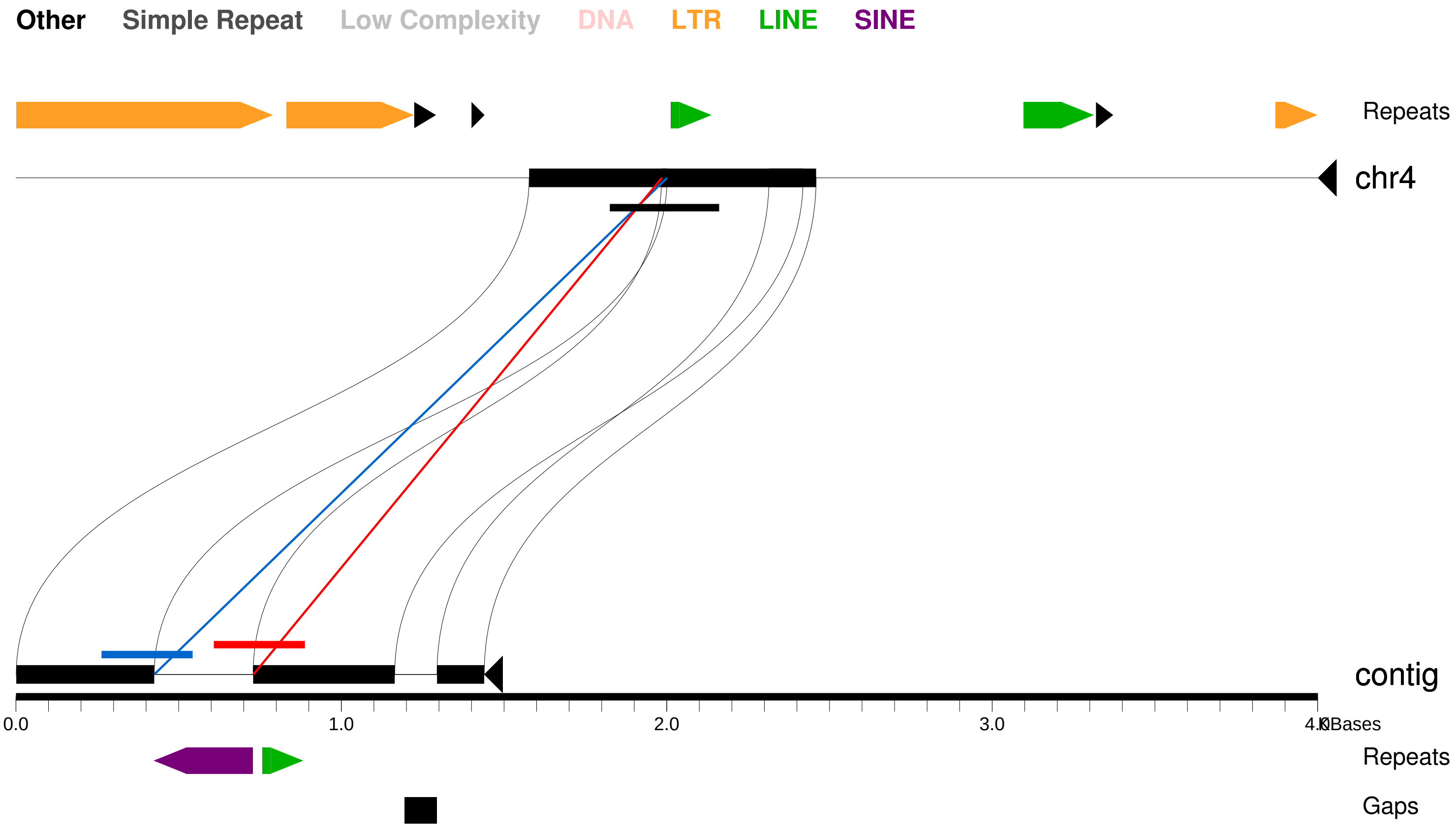

siteID: chr4_179444737 Scafftig2 + Breakpoint: chr4:179444720
chr4:179444679-179444779
Overlap Length: 16 INS size: 300 Genotyped: Yes

```
left   -------GAAAAACCATT---TATTGCT-----TA-AAACAC-ACCCATC-CAAAATATCTGATTCTCAA
chrom  -------GAAAAACCATT---TATTGCT-----TA-AAGCAC-ACCCATC-CAAAATATCTGATTCTCAA
right  TCCGCCCGCCTCAGCCTCCCAAAGTGCTGGGATTACAAGCGTGAGCCACCGCGCCCGGCCTGATTCTCAA
       1111111*1111*1*1*11111*1****11111**1**2*111*1***1*1*1111111***********

left   TTTTTTTTTTTTTTTTTTTTTTTTTGAGACGGAGTCTCGCTCTGTGGCCCAGTGGGAGTGCAGTGGC
chrom  TTTTTATTGGCTTTGAGCTATTTTA---AC--AGT-----TCTGTA----AATTGTAACTAAG----
right  TTTTTATTGGCTTTGAGCTATTTTA---AC--AGT-----TCTGTA----AAATGTAACTAAG----
       ******2**222***2222*2****2222**22***22222*****22222*212*2*2222**2222
```

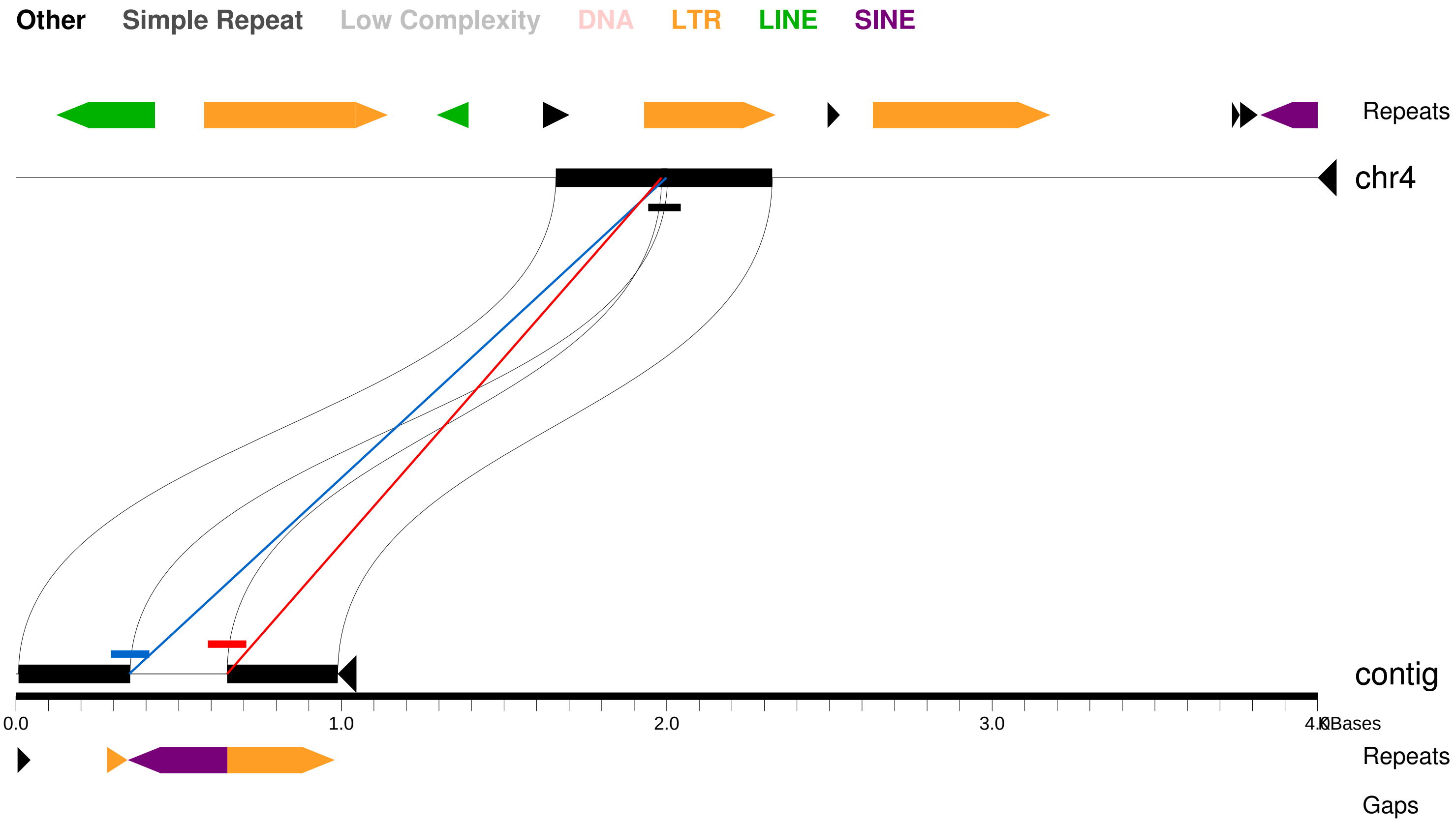

siteID: chr4_180256973 Scafftig3 + Breakpoint: chr4:180256956
chr4:180256796-180257129
Overlap Length: 14 INS size: 302 Genotyped: Yes

```
left   T--------AG-CATGCTGACATCTGGTACATACAGCTCATGTGTCTA-ATCCTCTTTCTATAAATTATT
chrom  TTTGAGAATAG-CATGCTGACATCTGGTACATACAGCTCATGTGTCTA-ATCCTCTTTCTATAAATTATT
right  TTT-AGTAGAGACGGGGTTTCACCGTGTT-AGCCAGG--ATG-GTCTCGATCTCCTGAC-----------
       *22 22N2N**1*11*1*11**1*11**11*11***111***1****11***11**11*11111111111

left   ACTTGTATTTGTTTCTTGATAA-CAGTTTTAACAAGT-CTGTGATTAATATTGGTCAGCTAGTTCCATTG
chrom  ACTTGTATTTGTTTCTTGAGAA-CAGTTTTAACAAGT-CTGTGATTAATATTGGTCAGCTAGTTCCATTG
right  -CTCGT----GATCCGCCCGCCTCGGCCTCCCAAAGTGCTGGGATTACAA-------GCGTGAGCCACCG
       1**1**1111*1*1*11112111*1*11*1111****1***1*****11*1111111**11*11***11*

left   ACCTCTTCTGCCTTTTACTCCATTAAATCAAATTTCTTTTTTTTTTTTTTTTTTTTTGAGACGGAGTCTC
chrom  ACCTCTTCTGCCTTTTACTCCATTAAATCAAATTTCTTATTGCCTAAATGTCTCC---AGAATGTGTCCA
right  CGCCCA---GCCT-----------AAATCAAATTTCTTATTGCCTAAATGTCTCC---AGAATGTGTCCA
       11*1*1111****11111111111*************2**222*222*2*2*22222***22*2***22

left   GCTCTGTGGCC--CAGGTGGGAGTGCAGTGGC-GCAATCTA----GGCT---------CACTGC----AA
chrom  GGTATATGCGTTGCATGTTAGAGTCTATTTATTGCATTCAAAAGTGGCCTGGTGTTTCCAATGCTGTCAA
right  GGTATATGCGTTGCATGTTAGAGTCTATTTATTGCATTCAAAAGTGGCCTGGTGTTTCCAATGCTGTCAA
       *2*2*2**22222**2**22****22*2*2222***2**2*2222***2222222222**2***2222**

left   GCTCCGCC-----------TCCCGGGTTCACGCCAT--------TCTCC---------TGC
chrom  GTAGTGCCAGAAAATATTTTTGAGAGTTTACCCTGTAGTGGGAATCTTTATGTGGATTTTG
right  GTAGTGCCAGAAAATATTTTTGAGAGTTTACCCTGTAGTGGGAATC---------------
       *2222***22222222222*222*2***2**2*22*22222222**1NN         1NN
```

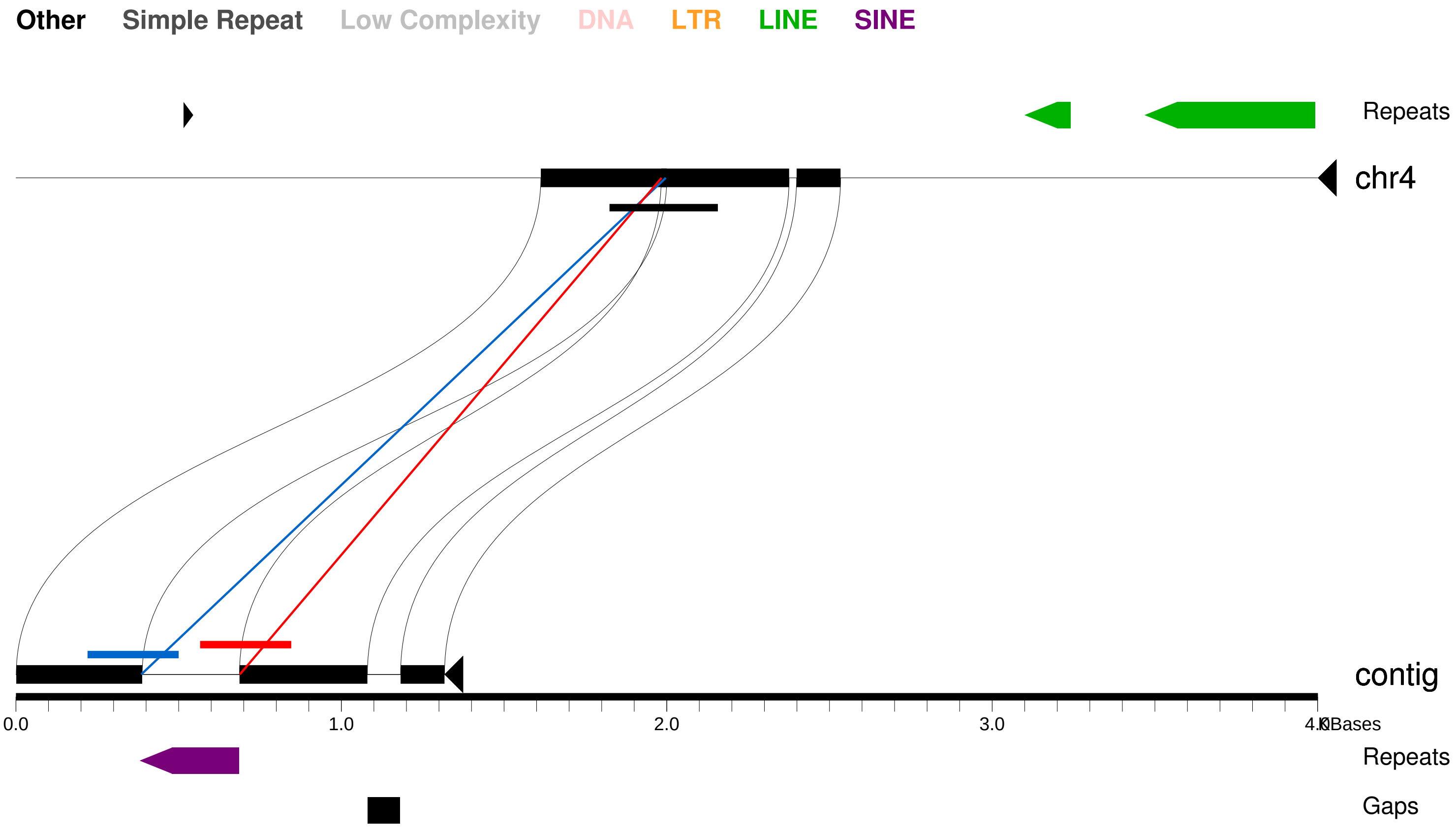

siteID: chr4_181503031 Scafftig1 - Breakpoint: chr4:181503027
chr4:181502971-181503086
Overlap Length: 4 INS size: 440 Genotyped: No

```
left    ATTGCTTTATTGTATTATTTATATTACAAATATATTGGTTTACTGTTTATTAGCAA-------------AA
chrom   ATTGCTTTATTGTATTATTTATATTACAAATATATTGGTTTACTGTTTATTAGCAA-------------AA
right   CCGCCCGCCTCGGCCTCC---------CAAAGTGCTGGGATTACAGGCG-TGAGCCACCGCGCCCGGCCAA
        1111*1111*1*111*1111111111****1111*1**1****1*1111*1***1*111111111111**

left    TGNNNNNNNNNNNNNNNNNNNNNNNNNNNNNNNNNNNNNNNNNNNNNNNNNNNNNNNNNNN
chrom   TGCTTATTTTTACTACTCTAATGAGAATAATTATTTTTGTTCTAAAGACAATAATATG---
right   TGCTTATTTTTACTACTCTAATGAGAATAATTATTTTTGTTCTAAAGACAATAATATG---
        **22222222222222222222222222222222222222222222222222222222222
```

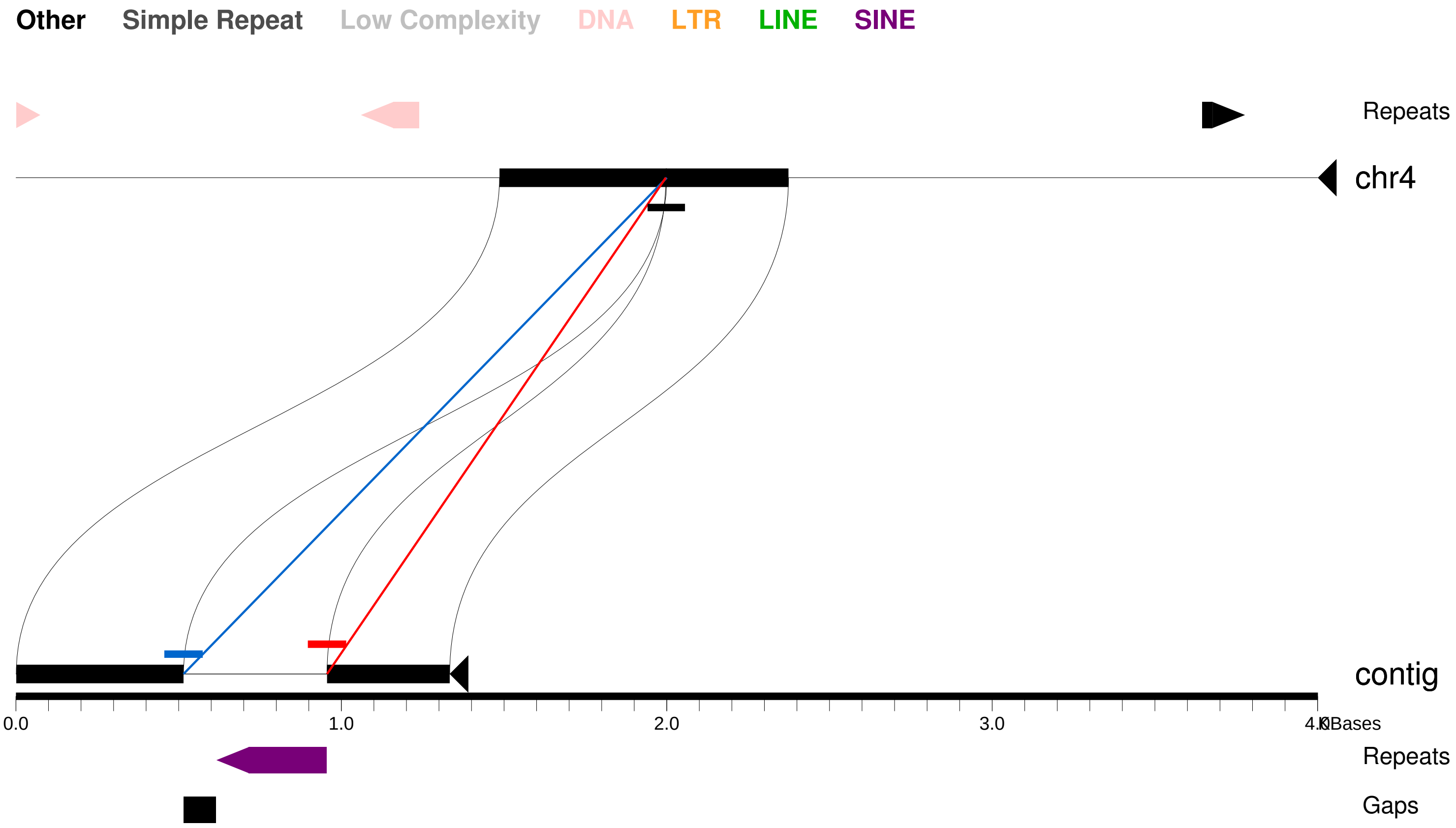

siteID: chr4_18472666 Scafftig1 + Breakpoint: chr4:18472631
chr4:18472589-18472690
Overlap Length: 18 INS size: 399 Genotyped: No

```
left    GACA----------------AAATTTAGTGGAG-ATAGGTGACTAGCAGAAGATATTTGCAACATCTATA
chrom   GACA----------------AAATTTAGTGGAG-ATAGGTGACTAGCAGAAGATATTTGCAATGTCTATA
right   TCCACCCGCCTCGGCCTCCCAAAGTGCTGGGATTACAGGCGTGAGCCACCGCGCCCGGCCAACATCTATA
        11**1111111111111111***1*1111***11*1***1*11111**11111111111***NN******

left    AATTTTTTNNNNNNNNNNNNNNNNNNNNNNNNNNNNNNNNNNNNNNNNNNNNNNNNNNNNNNNNNN
chrom   AAATTTT-----------------AAAGGCATCAATATTTAGTGTATACATGAATTGCCTGGAAAT
right   AAATTTT-----------------AAAGGCATCAATATTTAGTGTATACATGAATTGCCTGGAAAT
        **2*****22222222222222222222222222222222222222222222222222222222222
```

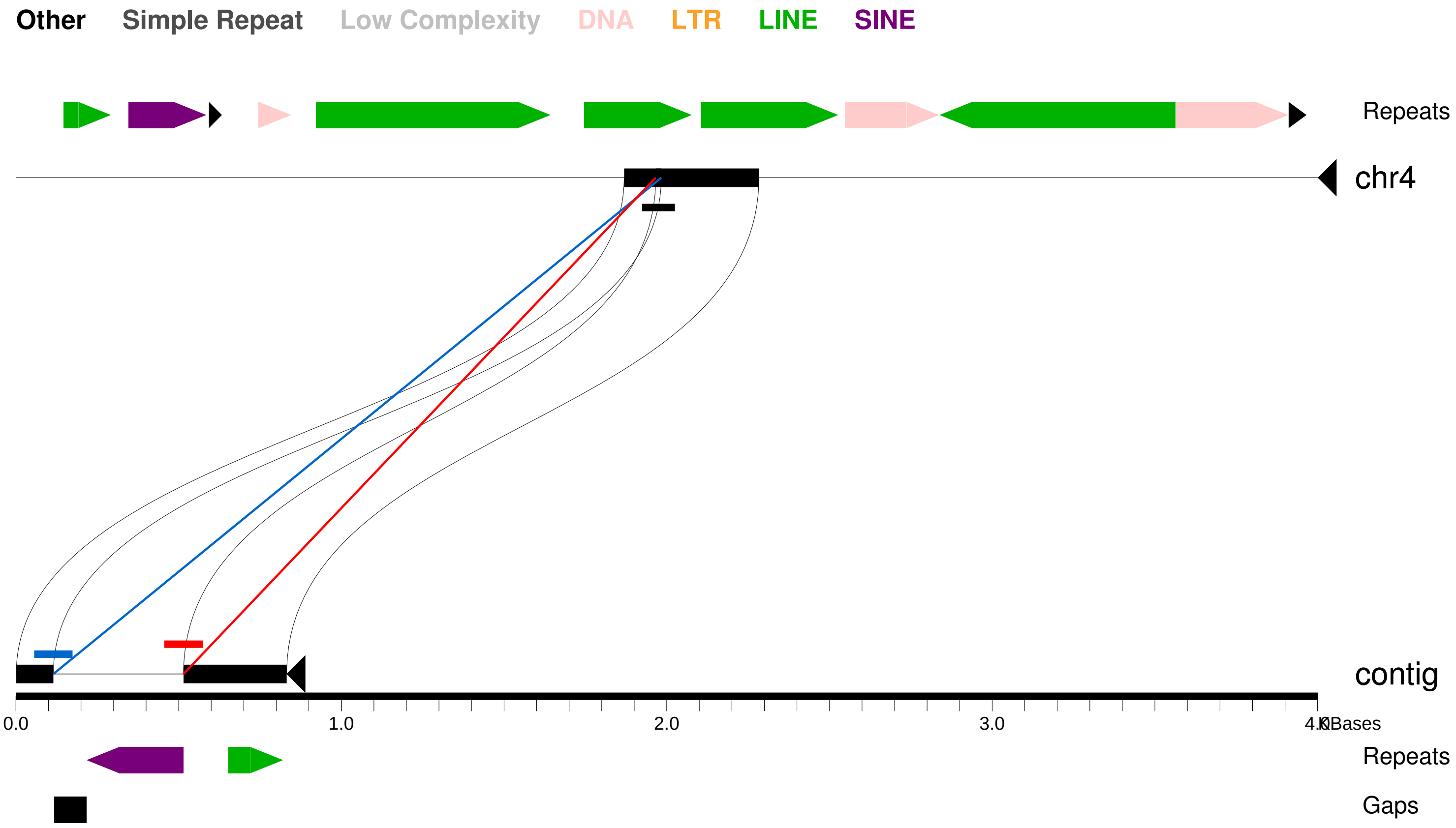

siteID: chr4_185651681 Scafftig1 + Breakpoint: chr4:185651662
chr4:185651618-185651721
Overlap Length: 16 INS size: 443 Genotyped: No

```
left   CAAGCCACTGGAAAAAAAAATTGGAATTAGCTATCAAGAACGTT----------------AAAGAAATGCA
chrom  CAAGCCACTGGAAAAAAAAACTGGAATTAGCTATCAAGAACGTT----------------AAAGAAATGCA
right  NNNNNNNNNNNNNNNNNNNNNNNNNNNNNNNNNNNNNNNNNNNNNNNNNNNNNNNNNNNAAAAGAAATGCG
       11111111111111111111N111111111111111111111111111111111111111**********1

left   CATGCGGCCGGGCGCGGTGGCTCACGCCTGTAATCCCAGCACTTTGGGAGGCCGAGGCGGTGG
chrom  CATGCC-----------TTTAACATACATTTATTCTAAG-ACTCT---AAAGAAAAGTAAACCT
right  CATGCC-----------TTTAACATACATTTATTCTAAG-ACTCT---AAAGAAAAGTAAACCT
       *****222222222222*2222**22*2*2**2**22**2***2*222*22222*2*2222222
```

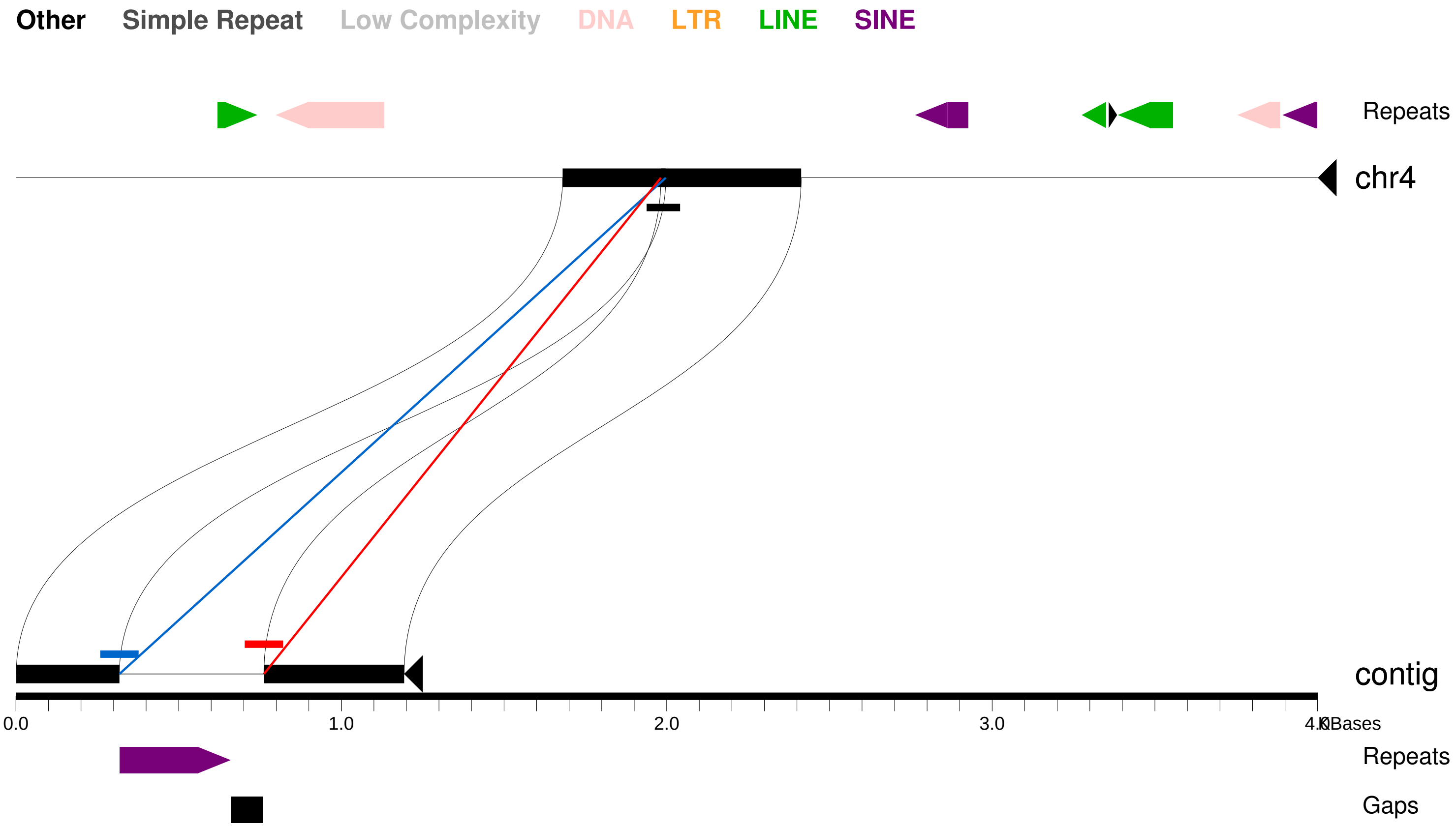

siteID: chr4_185977806 Scafftig10 + Breakpoint: chr4:185977802
chr4:185977750-185977860
Overlap Length: 8 INS size: 300 Genotyped: Yes

```
left    GTTTCTGAGCTTGAAATGTCAAAAT-----AATCAAAA---ATAAACATAGCTCACGTAC-TGAATTTTTT
chrom   GTTTCTGAGCTTGAAATGTCAAAAT-----AATCAAAA---ATAAACATAGCTCACGTAC-TGAATTTTA
right   CCGCCCGC-CTCGGCCTCCCAAAGTGCTGGGATTACAGGCGTGAGCCACCGCGCCCGGCCCTGAATTTTA
        1111*1*11**1*111*11****1*111111**1*1*111111*11**11**1*1**11*1********2

left    TTTTTTTTTT--TTTTTTTGAGACGGAGTCTCGCTCTGTCGCCCAGGCTGGAGTGCAGTG
chrom   TCATTAAAATACTTTGTTTTCCATTGAGTGTTG-------GGACATCTTTTTGTTGA---
right   TCATTAAAATACTTTGTTTTCCATTGAGTGTTG-------GGACATCTTTTTGTTGA---
        *22**2222*22***2***222*22****2*2*2222222*22**222*222**22*222
```

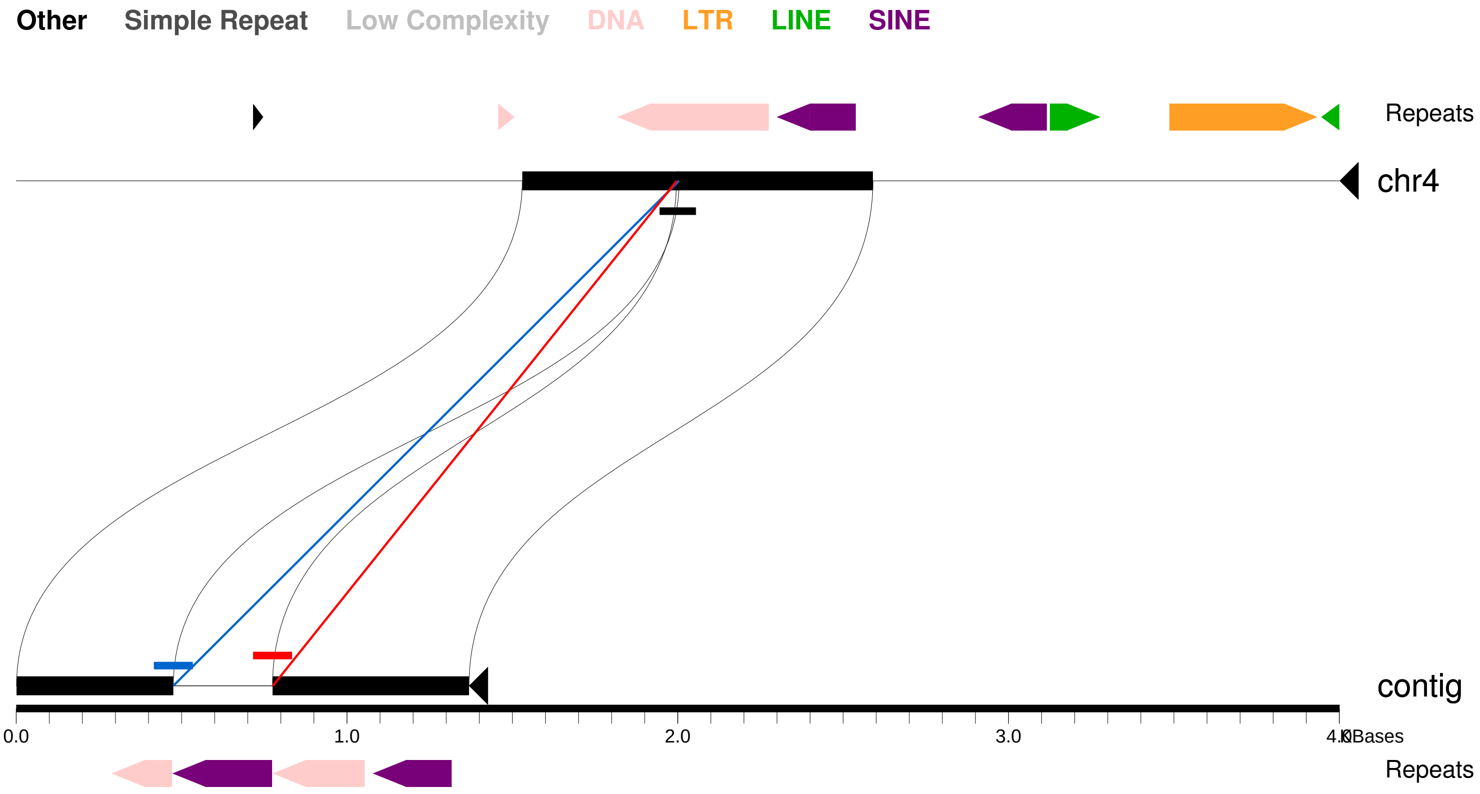

siteID: chr4_186382148 Scafftig8 + Breakpoint: chr4:186382147
chr4:186382101-186382206
Overlap Length: 14 INS size: 273 Genotyped: Yes

```
left    ATT----TTTCCTGTAACAAAGAG-----------GACCATGTAAAGTGAAATGAACTGATGGTAGTCAC
chrom   ATT----TTTCCTGTAACAAAGAG-----------GACCATGTAAAGTGAAATGAACTGATGGTAGTCAC
right   GTCTCGATCTCCTGACCTCGTGATCCGCCCGCCTCGGCCTCCCAAAGTGCTGGGA--TTACGGTAGTCAC
        1*11111*1*****1111111**111111111111*1**1111******1111**11*1*1*********

left    GTTTTTTTTTTTTTTTTTTTTGAGACGGAGTCTCACTCTTTCGCCCAAGCTGGACTGCAGTGGC
chrom   GTTTTGAAGTACCTACATATAGAAATG-ATT-----TCTGTAGAATAACAT--ACCAAA-----
right   GTTTTGAAGTACCTACATATAGAAATG-ATT-----TCTGTAGAATAACAT--ACCAAG-----
        *****2222*222*222*2*2**2*2*2*2*22222***2*2*222**22*22**222122222
```

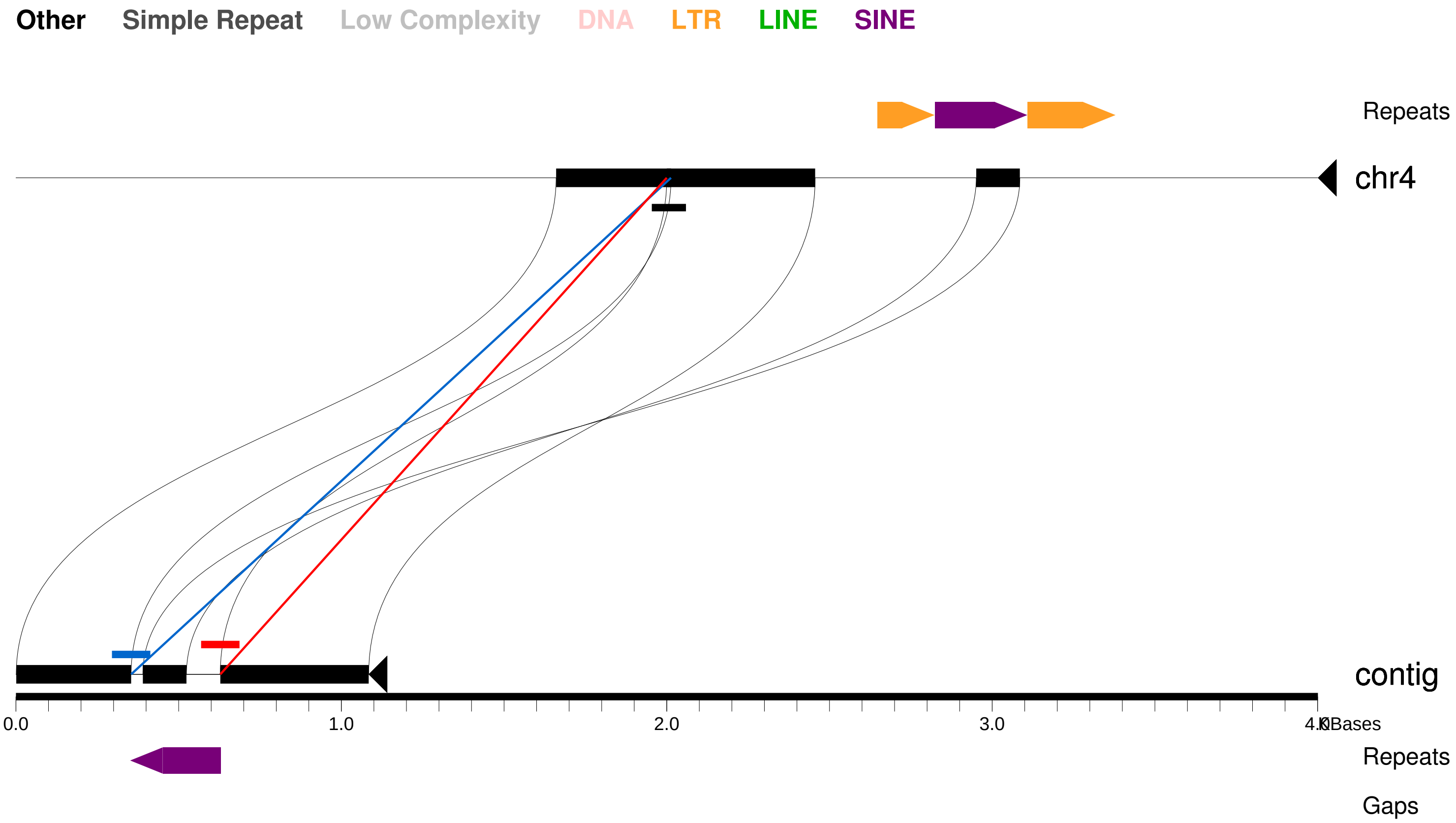

siteID: chr4_188686137 Scafftig1 - Breakpoint: chr4:188686147
chr4:188685972-188686307
Overlap Length: 11 INS size: 298 Genotyped: Yes

```
left   -------------------------------TTTTCAGGTCATCTATTGAACCCTTGAGCTGGGAGCTTA
chrom  GATGAGGCCTCCTAGAGGGCACACATTGAAGTTTTCAGGTCATCTATTGAACCCTTGAGCTGGGAGCTTA
right  TTT-AG------TAGAG---AC-----GGGGTTTCACCGT--------------TTTAGCCGGGA--TGG
       NN2 22      22222   22     2NN2***1111**11111111111111**1***1****11*11

left   TTTCTTTCTTTTCCCACATTGTCCAGTGACATTAGGAGCAAACAGCCAATATCATTATACTCAT-TATTT
chrom  TTTCTTTCTTTTCCCACATTGTCCAGTGACATTAGGAGCAAACAGCCAATATCATTATACTCAT-TATTT
right  TCTCGATCT---CC------------TGACCTCGTGATCCGCCCGCC----TCGGCCTCCCAAAGTGCTG
       *1**11***111**111111111111****1*111**1*111*1***1111**1111*1*11*11*11*1

left   TGACAAAAATATTGAAGAATATTGTATAATATTGTATACACACTCACCCTGCTTCTTTTTTTTTTTTTTT
chrom  TGACAAAAATATTGAAGAATAT----------TGTATACACACTCACCCTGCTTCTTATAAGCGGTTGAT
right  GGATTACAGGCGTGAGCCACCG----------CG------CCCGGCCCCTGCTTCTTATAAGCGGTTGAT
       1**11*1*1111***111*1112222222221*111111*1*111***********2*222222**22*

left   TTTTTGAGACGGAG-------TCTCGC-----TCTGTCGCC----CAGGCT--GGAGTG-CAGTGG----
chrom  TAGGGGAATCTAATGGTAATATTTTGCAAATTTCTACTGCCAGATCATGCTATGAACTGTCAATGCTCTC
right  TAGGGGAATCTAATGGTAATATTTTGCAAATTTCTACTGCCAGATCATGCTATGAACTGTCAATGCTCTC
       *2222**22*22*22222222*2*2**22222***222***2222**2***22*2*2**2**2**22222

left   -------CGGGATCTCGGCTCACTGCAAGCTCCGCCTCCC------GGGTTCACGCCATTCTCCTGCCT
chrom  CATACTACTGGAAATAG--TCATTGGTACCTCTAAATGTAAGTGAGGGGAAAATAGGATCTGGAGGTGA
right  CATACTACTGGAAATAG--TCATTGGTACCTCTAAATGTAAGTGAGGGGAAAATAGGATCTGGAGGTGA
       2222222*2***22*2*22***2**22*2***2222*222222222***222*2222**222222*222
```

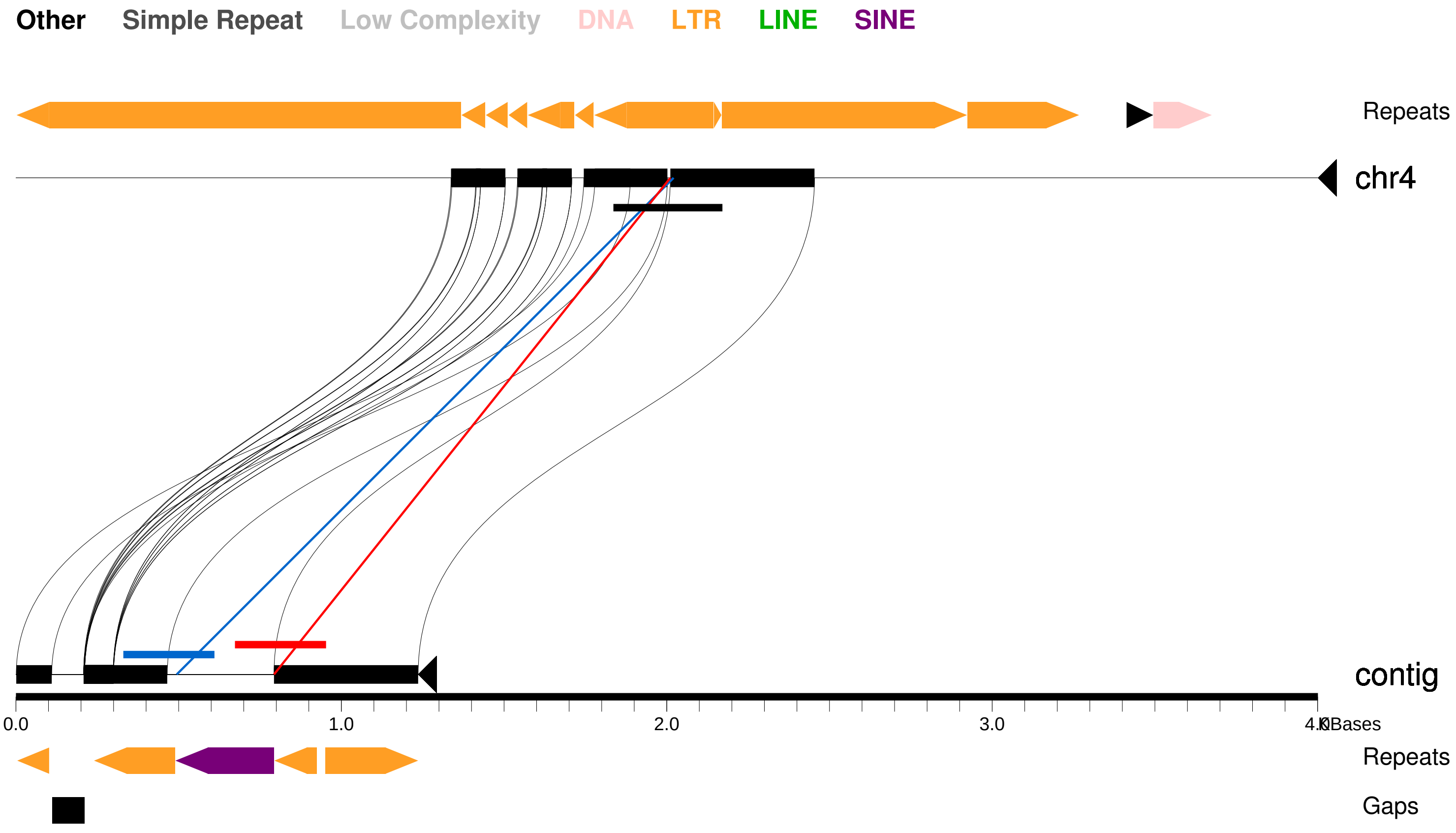

siteID: chr4_188734585 Scafftig1 - Breakpoint: chr4:188734564
chr4:188734515-188734623
Overlap Length: 11 INS size: 393 Genotyped: No

```
left   -----------GGTAATTTTACCTGATGT-ATTACCCAAGAG-GCCTCTTAAGGACAAGGGTAGCCCTCT
chrom  -----------GGTAATTTTACCTGATGT-ATTACCCAAGAG-GCCTCTTAAGGACAAGGGTAGCCCTCT
right  CCGCCCGCCTCGGCCTCCCAAAGTGCTGGGATTACAGGCGTGAGCCACC---GCGCCCGGCCAGCCCTCT
       11111111111**1111111*11**1**11*****1111*1*1***1*1111*11*11**11********

left   TTTNNNNNNNNNNNNNNNNNNNNNNNNNNNNNNNNNNNNNNNNNNNNNNNNNNNNNNNNNNN
chrom  TTTATGAAGGCAATTTTTGAGAAAAAAAGCCATTGCTTTATATATGGTACAT----------
right  TTTATGAAGGCAATTTTTGAGAAAAAAAGCCATTGCTTTATATATGGTACAT----------
       ***22222222222222222222222222222222222222222222222222222222222
```

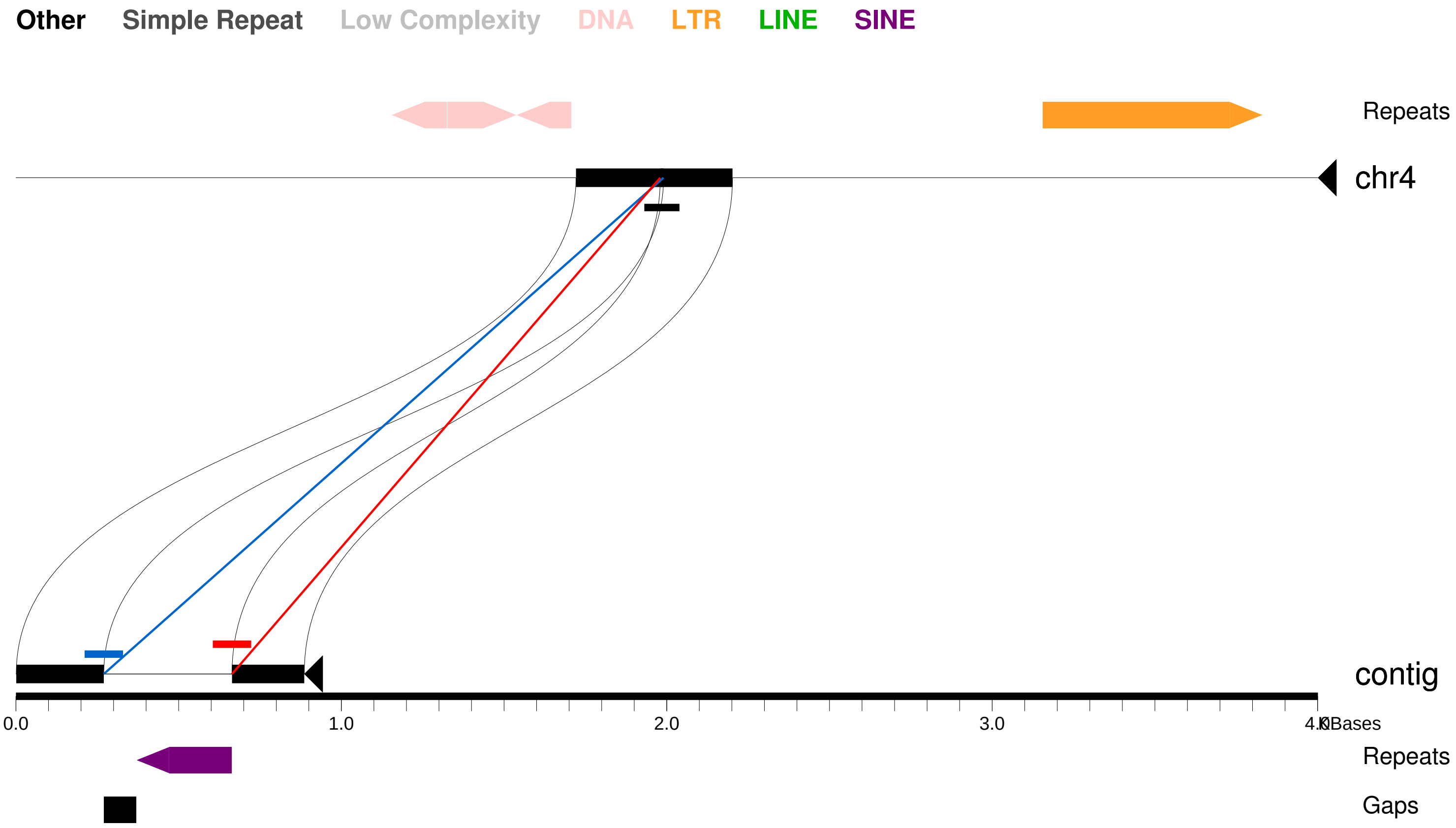

siteID: chr4_19222207 Scafftig1 + Breakpoint: chr4:19222103
chr4:19221943-19222280
Overlap Length: 18 INS size: 356 Genotyped: No

```
left   --------------------------------------------TATGGTCAAAGCGAGTTACAATTTAC
chrom  AATTAGGTCCTTAGTTTTTAATAAACATT-TTTCAAGGATATATTATGGTCAAAGCGAGTTACAATTTAC
right  T---------TTAGT-----AGAGACGGGGTTTCACCG---TGTTA--GCCAGAATG-GTCTCGATCTCC
       N         22222     2N2N22NNN122222NN2   2N2**11*1**1*11*1**11*1**1*1*

left   TAAGGAAAATATACAGATAAACAACAACAGACTAATAAATTGCCTTAATGGATCTAGAGATAAGTGCAGG
chrom  TAAGGAAAATATACAGATAAACAACAACAGACTAATAAATTGCCTTAATGGATCTAGAGATAAGTGCAGG
right  TGACCTCGTGATCCGC-----CCGCCTCGGCCTCCCAAAGTGC-----TGGGATTACAG------GCGTG
       *1*1111111**1*1111111*11*11*1*1**111***1***11111***111**1**111111**11*

left   AACAAAAACAAAAAGAGCACTGACATCTTTTTTTTTAAGTNNNNNNNNNNN-------------------
chrom  AACAAAAACAAAAAGAGCACTGACATCTTTTTTTTTAAGTTTTAAATTATGTTTTAAAATTTTTGTTGGTA
right  AGCCACCGCGCCCGG----CCGACATCTTTTTTTTTAAGTTTTAAATTATGTTTTAAAATTTTTGTTGGTA
       *1*1*111*11111*1111*1*******************222222222222222222222222222222

left   ----------------------------------------------------------------------
chrom  CATAGTAGGTGTATATATTTACCGAGCACATGAGATGTTTTGATACAGGCATGCAATGTAAAATAAGCAC
right  CATAGTAGGTGTATATATTTACCGAGCACATGAGATGTTTTGATACAGGCATGCAATGTAAAATAAGCAC
       2222222222222222222222222222222222222222222222222222222222222222222222

left   ------------------------------------------------------------
chrom  ATCAAAGAGAATGAGGTATCCATCCTCTCAAGCATTGATGGAGGGAATGGATACCCCAT
right  ATCANNNNNNNNNNNNNNNNNNNNNNNNNNNNNNNNNNNNNN-------------------
       2222NNNNNNNNNNNNNNNNNNNNNNNNNNNNNNNNNNNNNN
```

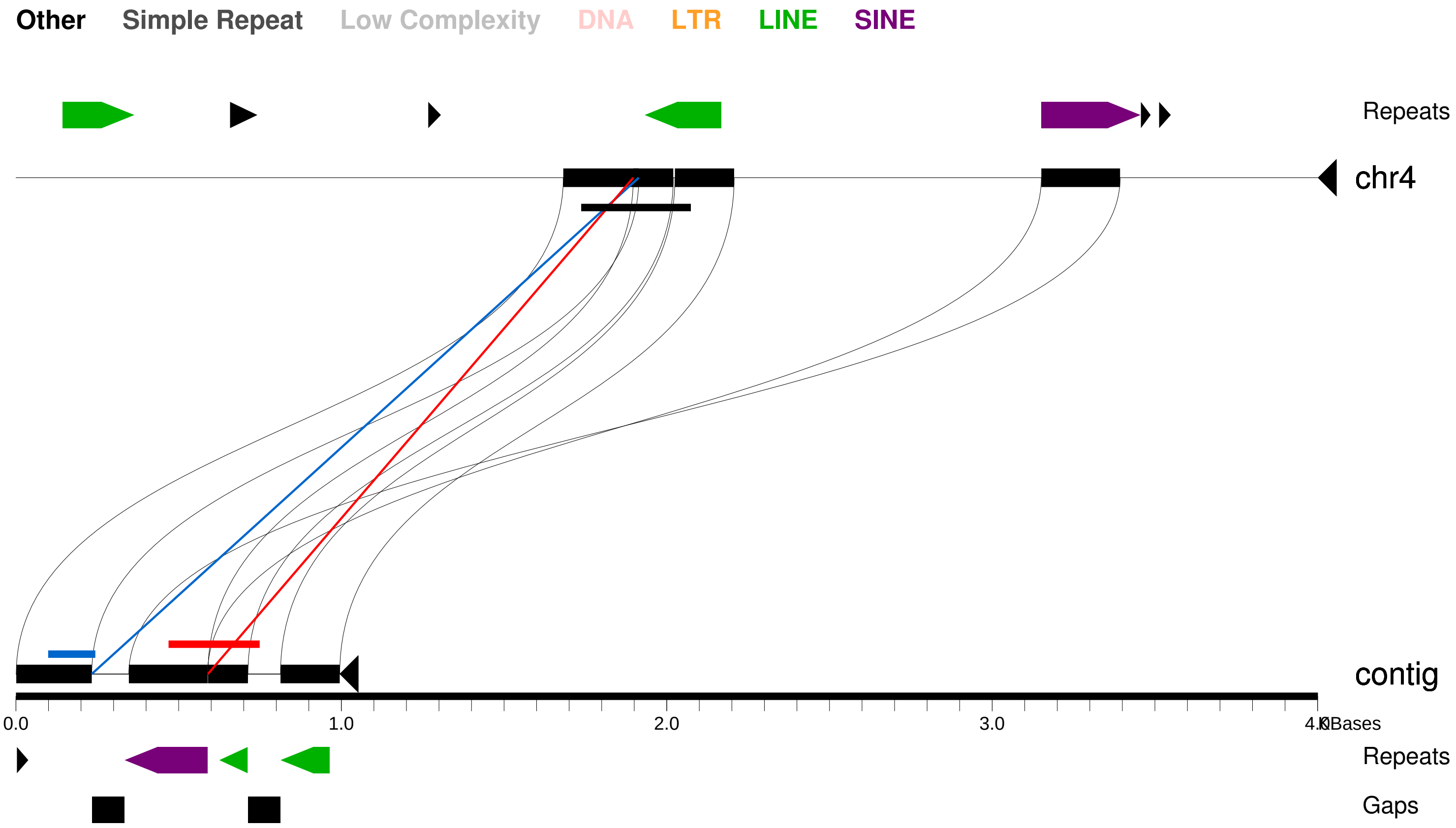

siteID: chr4_21534558 Scafftig2 - Breakpoint: chr4:21534573
chr4:21534413-21534749
Overlap Length: 17 INS size: 283 Genotyped: Yes

```
left    ----------------GGATCTCTGGAGGGGATGCATTCTACTTAACCTTGGCGTTTGTTTTAAATTACA
chrom   ATATTCTAAATTATATGGATCTCTGGAGGGGATGCATTCTACTTAACCTTGGCGTTTGTTTTAAATTACA
right   -----------TGGCGTGAACCCGGGAAGCGGAGCTTGC-AGTGAGC---GGAGATTGCGCCA-----CT
                   2NNNN1**1*1*1***1*1*11**1*1*1*1*1*1*111**1*1***1111*11111*1

left    GGAATCTACCATATTATATTATCCAAGATTTTTAAAAATAGCTTTTATTCCTTTTCTAAATTGCATCTCA
chrom   GGAATCTACCATATTATATTATCCAAGATTTTTAAAAATAGCTTTTATTCCTTTTCTAAATTGCATCTCA
right   GCAGTCCGCAGT-----------CCGGCCTGGGCGACAGAGCGAG-ACTCCGTCTCAAAAAAAAAAAAAA
        *1*1**11*11*1111111111*11*11*11111*1*1***1111*1***1*1**1***1111*1111*

left    AGGTTGTTAGATTTCCTATTAAAAGCTAATTAATTAAGCAC--------------TTTGGGAGGCCG---
chrom   AGGTTGTTAGATTTCCTATTAAAAGCTAATTAATTAAAGAGAAAATACATACATATGTGTGAGTGTGTAT
right   AAA-----AAA------AAAAAAAGCTAATTAATTAAAGAGAAAATACATACATATGTGTGAGTGTGTAT
        *1111111*1*111111*11****************22*222222222222222*2**2***222*222

left    --AGGCGGGTGGATCATGAGGTCAAGAGAT------CGAGACCATCCTGGCT-AACAAGGTGAAACCCCG
chrom   ATATATGTATATATACATATGTATATATATACTCTTCTACACCTGCCAGCCAGAACCAACAATAATATTG
right   ATATATGTATATATACATATGTATATATATACTCTTCTACACCTGCCAGCCAGAACCAACAATAATATTG
        22*222*22*22**2222*2**22*2*2**222222*2*2***22**2*2*22***2*22222**2222*

left    TCTCTA-CTAAAAA-------------AAAAATACAAAAAATTAGCTGGGCGCGGTG
chrom   TCCCAAGCTATATAGTACCTGCTTCCAAAGAAGTTTAAAATCCATTTTAGAGAGCCA
right   TCCCAAGCTATATAGTACCTGCTTCCAAAGAAGTTTAAAATCCA-------------
        **2*2*2***2*2*2222222222222**2**2222****222*NN1NN1N1N1NNN
```

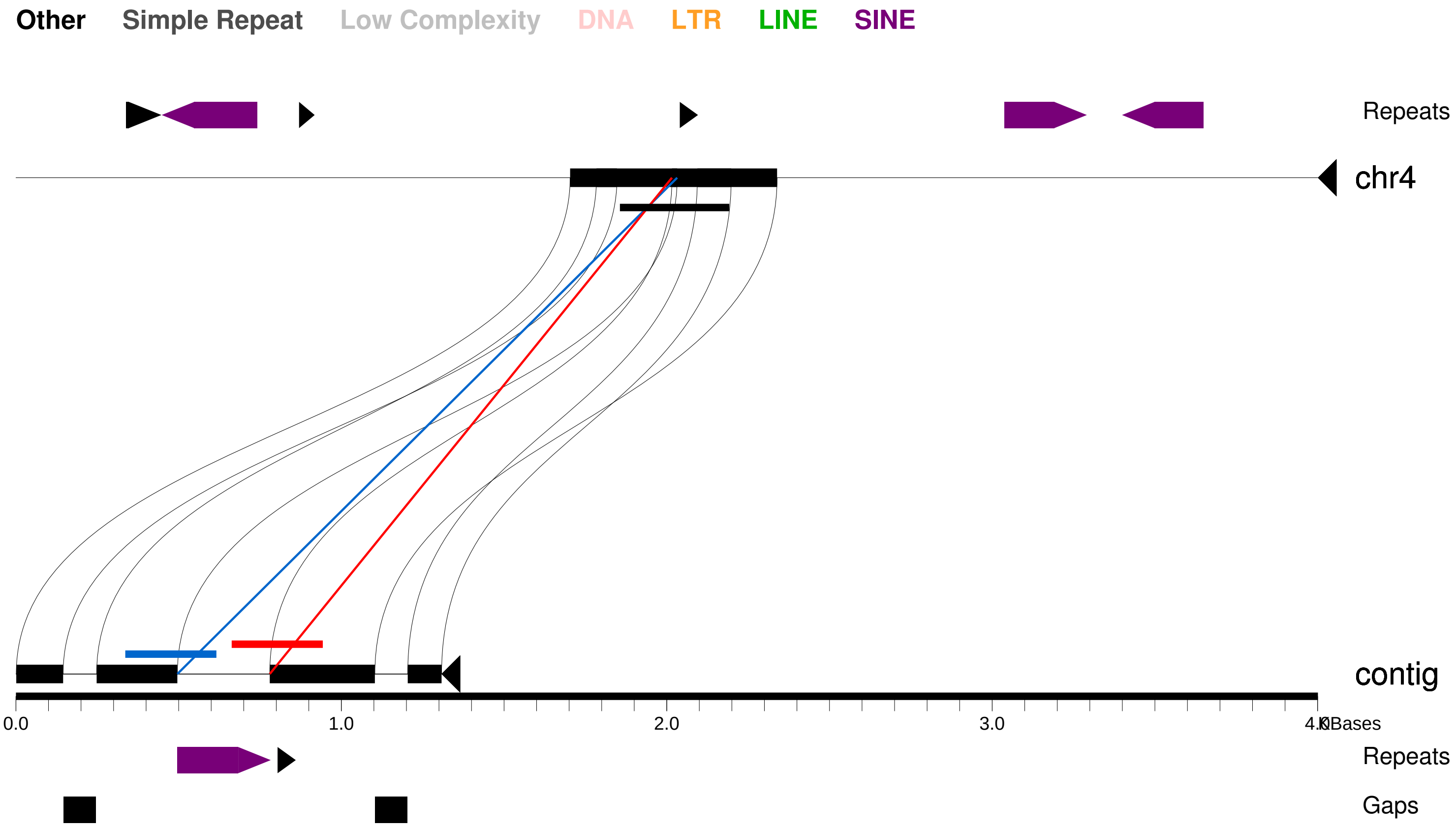

siteID: chr4_22043193 Scafftig1 - Breakpoint: chr4:22043201
chr4:22043145-22043260
Overlap Length: 1 INS size: 416 Genotyped: No

```
left    GAAACATTAGTGATCTTGATCACAGAGTTG---CTTTAGTCTTCCATCAACTTTGGCCT--CNNNNNNNN
chrom   GAAACATTAGTGATCTTGATCACAGAGTTG---CTTTAGTCTTCCATCAACTTTGGCCT--CTAGACATT
right   TCCACCCGCCTCGGCCTCC-CAAAGTGCTGGGATTACAGGCGTG-AGCCACCGCGCCCGGCCTAGACATT
        111**11111*111*1*111**1**1*1**1111*11**1*1*11*1*1**111*1**111*22222222

left    NNNNNNNNNNNNNNNNNNNNNNNNNNNNNNNNNNNNNNNNNNNNNNNNNNNNNN
chrom   TTTGAAGTACTAAGAGGGGTCTCATAAAATTTCCTTCCAATAAATTCCTAT---
right   TTTGAAGTACTAAGAGGGGTCTCATAAAATTTCCTTCCAATAAATTCCTAT---
        222222222222222222222222222222222222222222222222222222
```

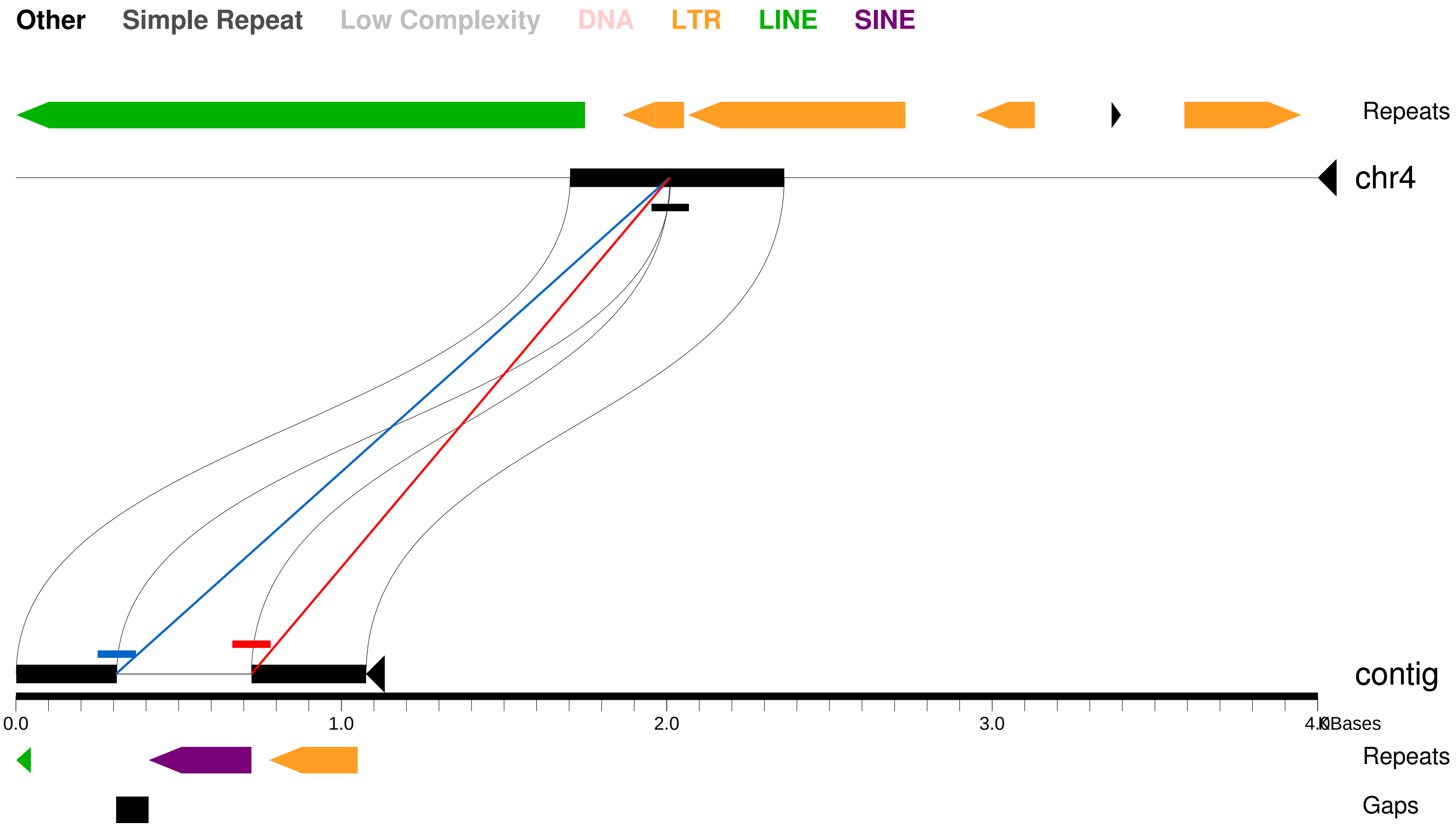

siteID: chr4_23511006 Scafftig2 - Breakpoint: chr4:23511009
chr4:23510965-23511068
Overlap Length: 16 INS size: 312 Genotyped: Yes

```
left    ---TCAATTTAGTCCAAAGGT---GACTC---------AAGCCAATAAGCCTTTCTCATAGAAAAGCCTG
chrom   ---TCAATTTAGTCCAAAGGT---GACTC---------AAGCCAATAAGCCTTTCTCATAGAAAAGCCTG
right   AGTCCGGCCTGGGCGACAGAGCCAGACTCCGTCTCAAAAAAAAAAAAAAAAAAAAAAAAAGAAAAGCCTG
        1111*1111*1*1*1*1**11111*****111111111**111**1**111111111*1***********

left    AACAAGGCCGGGCGCGGTGGCTCACGCCTGTAATCCCAGCACTTTGGGAGGCCGAGGCGGGTGG
chrom   AACAAATTACTGATA--TTGGTAATTTGTATATTTTCA-CTTTTTGTTC------------TCA
right   AACAAATTACTGATA--TTGGTAATTTGTATATTTTCA-CTTTTTGTTC------------TCA
        *****222222*22222*2*2*2*2222*2**2*22**2*22****2222222222222222*22
```

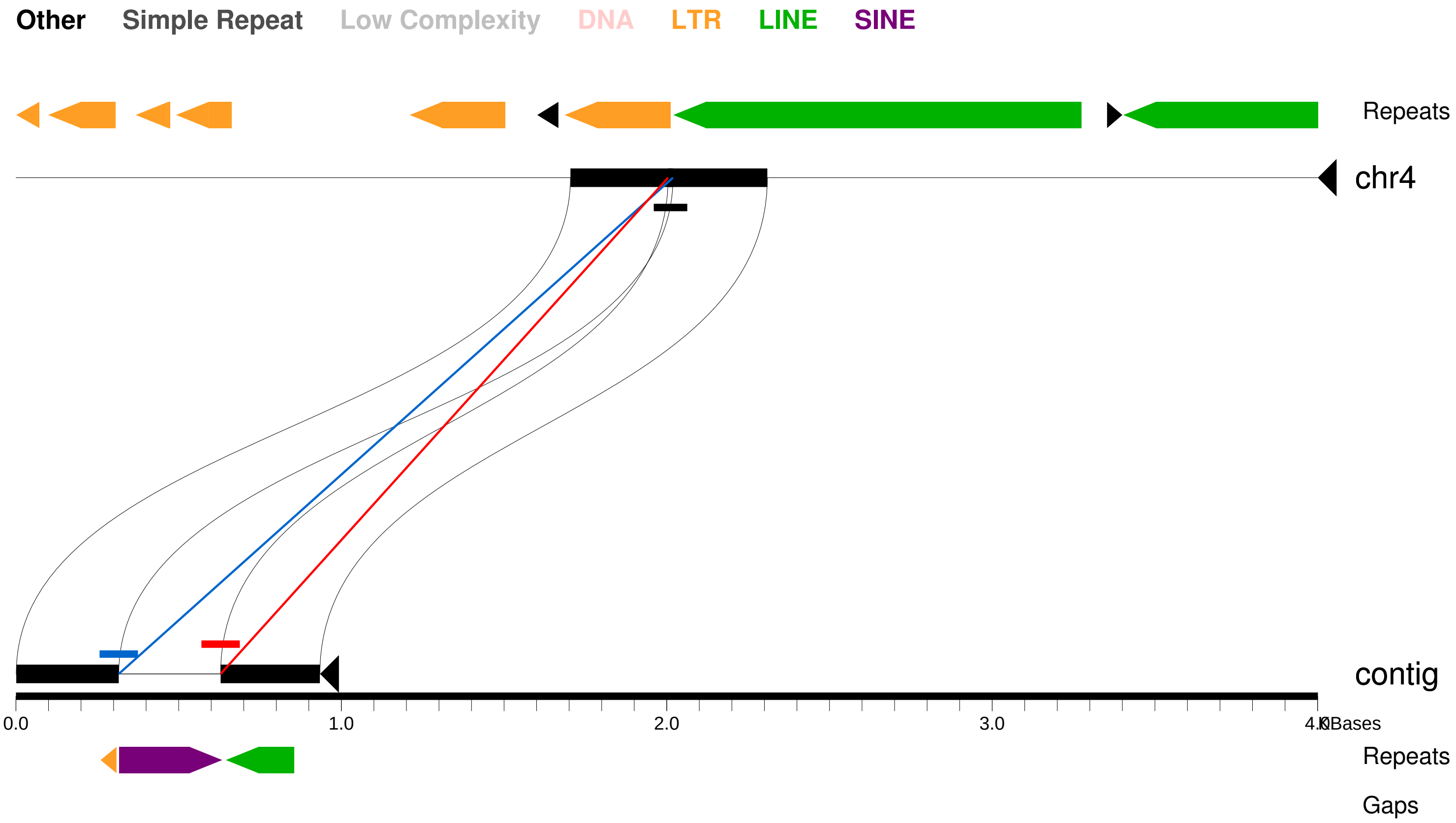

siteID: chr4_26438636 Scafftig3 + Breakpoint: chr4:26438618
chr4:26438571-26438677
Overlap Length: 13 INS size: 309 Genotyped: Yes

```
left   TATTTAGATGATACAAGGAAAAGT-CTGGG-----------GAAAAAATATGACCTCTTTGCTAACATTC
chrom  TATTTAGATGATACAAGGAAAAGT-CTGGG-----------GAAAAAATATGACCTCTTTGCTAACATTC
right  CCGCCCGCCTCGGCCTCCCAAAGTGCTGGGATTACAGGCGTGAGCCACCGCGCCCGGCCTGCTAACATTC
       111111*111111*11111*****1*****11111111111**111*1111*1**1111***********

left   TTTTTTTTTTTTTTTTTTTTTTTTTTTTTGAGACAGAGTCT-CGCTCTGTTGCCCAGGCTGG
chrom  TTAGTTCCCCTGGCCAAGTCTTACACTT-------GAATATGCGCTAGATTACACA------
right  TTAGTTCCCCTGGCCAAGTCTTACACTT-------GAATATGCGCTAGATTACACA------
       **22**2222*2222222*2**2222**2222222**2*2*2****222**2*2**222222
```

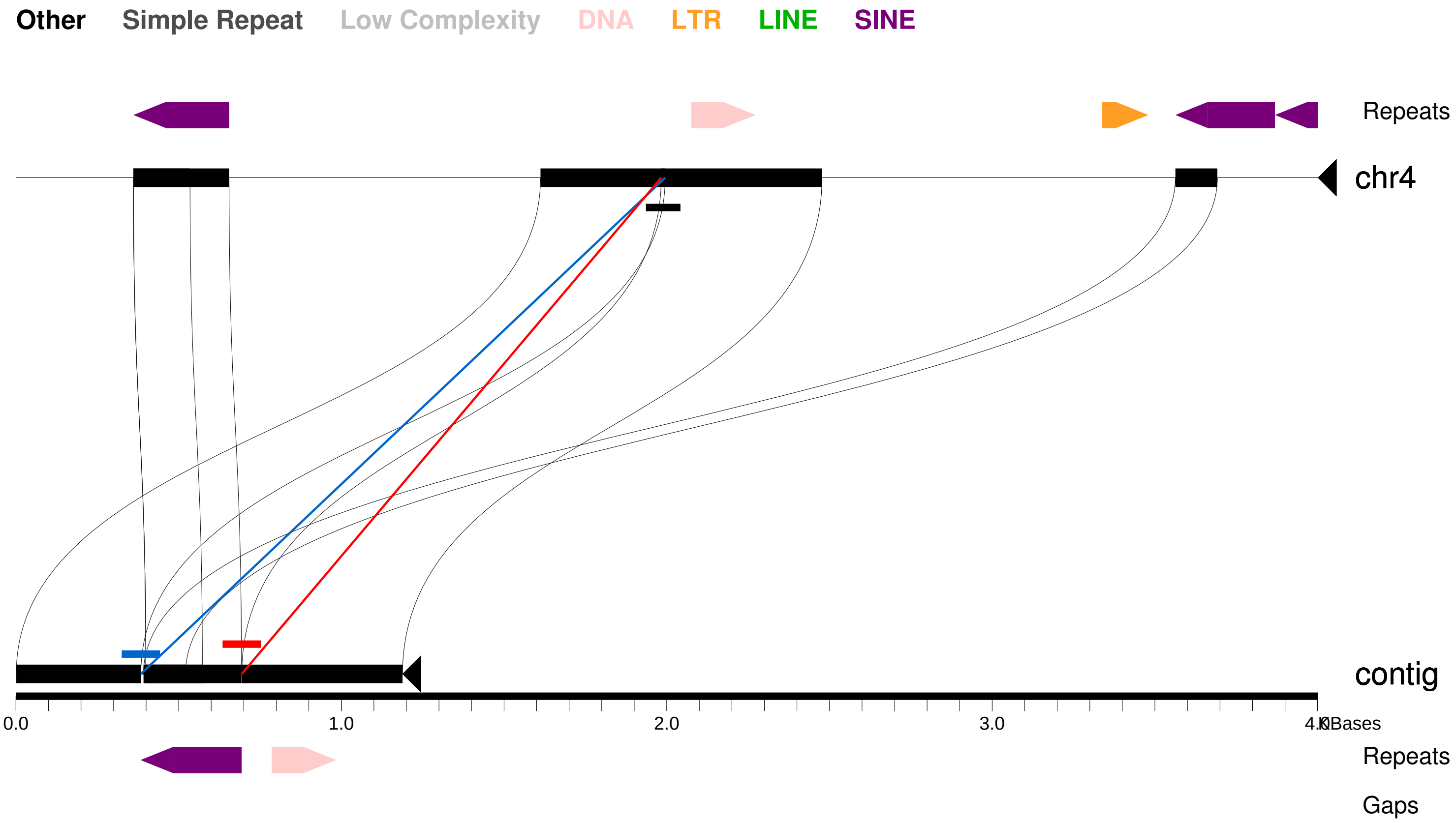

siteID: chr4_28320751 Scafftig1 - Breakpoint: chr4:28320755
chr4:28320711-28320811
Overlap Length: 16 INS size: 297 Genotyped: Yes

```
left   TTTCATATA-------------TGTTTAATTGAA---------GTTTCTAAATGGGAAATGGGAACAAAA
chrom  TTTCATATA-------------TGTTTAATTGAA---------GTTTCTAAATGGGAAATGGGAACAAAA
right  ATCCCGCCACTGCACTCCAGCCTGGGCGACAGAGCGAGACTCCGTCTCAAAAAAAAAAA----AAAAAAA
       1*1*1111*1111111111111**1111*11**1111111111**1**1***1111***1111**1****

left   AAAAGTAGACAAGGCCGGGCGCGGTGGCTCACGCCTGTAATCCCAGCACTTTGGGAGGCCGAGGCGGGCG
chrom  AAAAGTAGACAAAT------------TATAACATATATGATTC-AACA---TAGAAAAGATAGTAATTC-
right  AAAAGTAGACAAAT------------TATAACATATATGATTC-AACA---TAGAAAAGATAGTAATTC-
       ************2222222222222222*2**222*2*2**2*2*2**222*2*2*22222**22222*2

left   G
chrom  -
right  -
       2
```

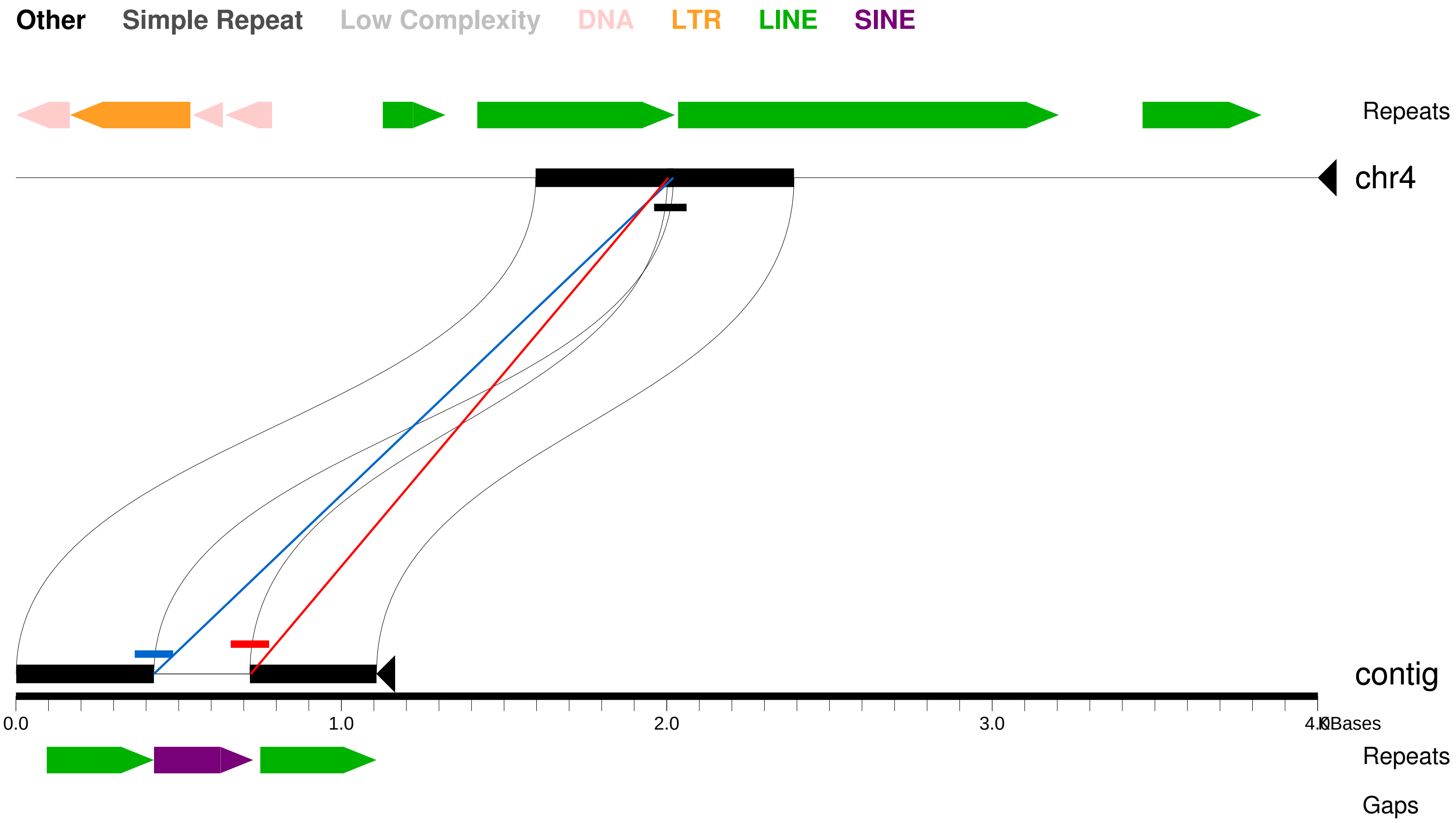

siteID: chr4_28925344 Scafftig5 - Breakpoint: chr4:28925341
chr4:28925294-28925397
Overlap Length: 13 INS size: 479 Genotyped: No

```
left    ACACAAATAACTATTCTGTGTAATAATAC---------------ATATGTATTAAGGGTAAAAGAATGAG
chrom   ACACAAATAACTATTCTGTGTAATAATAC---------------ATATGTATTAAGGATAAAAGAATGAG
right   NNNNNNNNNNNNNNNNNNNNNNNNNNNNNNNNNNNNNNNNNNNNNNNNNNNNNAAAAAAAAGAGAATGAG
        11111111111111111111111111111111111111111111111111111**1121**1********

left    CAGGGGCCGGGCACGGTGGCTCACGCCTGTAATCCCAGCACTTTGGGAGGCCGAGGCGGGTGGA
chrom   CAGGGTTTCCT-ATTTTGCCTAACATTTTTGTTTCTCACAAATATAAATG--------------
right   CAGGGTTTCCT-ATTTTGCCTAACATTTTTGTTTCTCACAAATATAAATG--------------
        *****2222222*222**2**2**222*2*22*2*222**22*2222*2*22222222222222
```

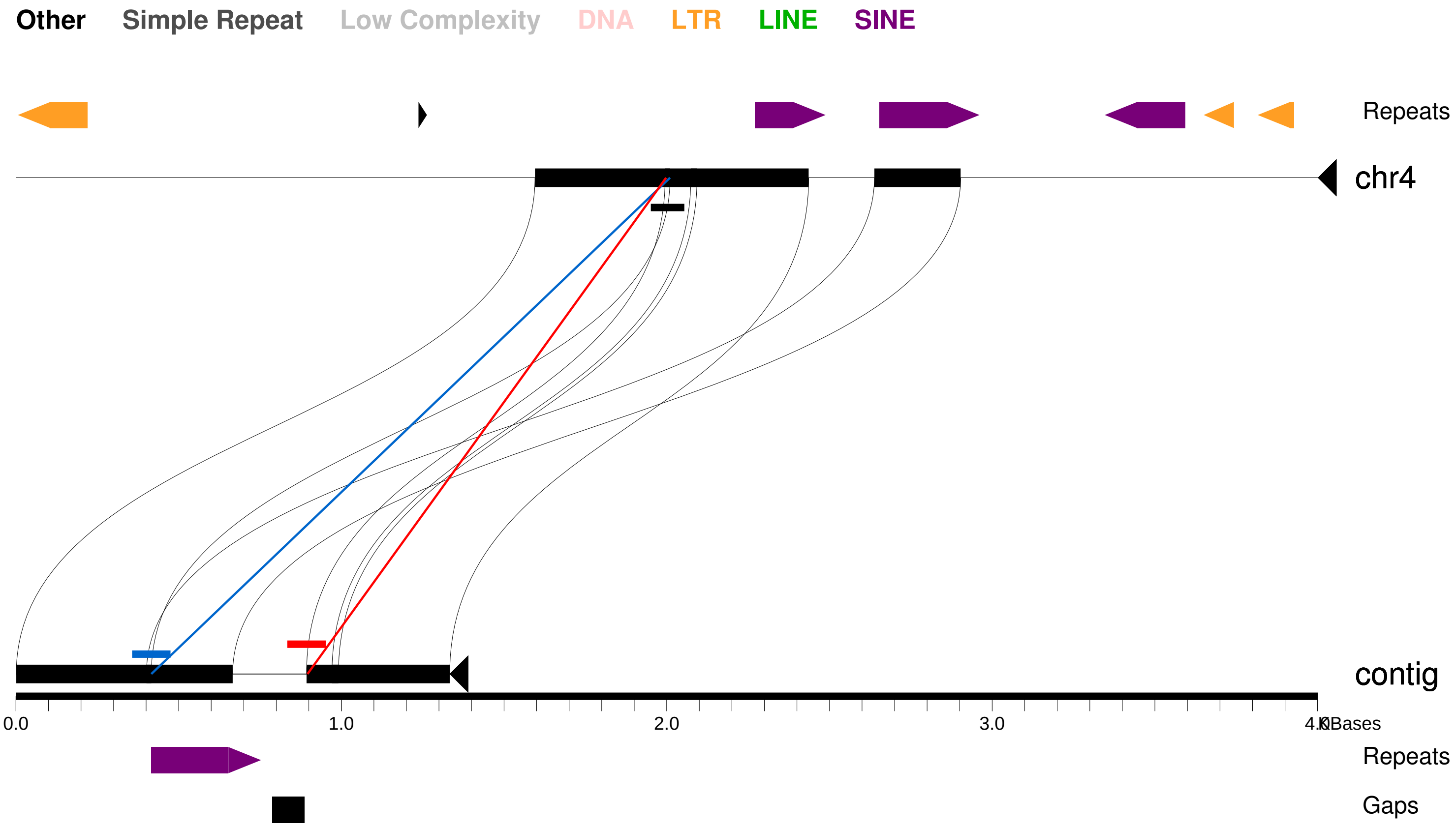

siteID: chr4_29270512 Scafftig3 + Breakpoint: chr4:29270516
chr4:29270356-29270685
Overlap Length: 10 INS size: 306 Genotyped: Yes

```
left    ----------ACTTAATTTTGAAGCTGAAATTTGATTTTGGGAAGCCTGTCAAATATGTTAAAGGTTTAG
chrom   TCTATCATTGACTTAATTTTGAAGCTGAAATTTGATTTTGGGAAGCCTGTCAAATATGTTAAAGGTTTAG
right   GG-AGAATGGC-------TTGAACCCGGGA---------GGCAGAGCTTGCA-----GTGAGCCG---AG
        NN 2NN22N211111111*****1*1*11*111111111**1*111**11**11111**1*111*111**

left    AACACTACAGAGAGCCCCTGAAGCAACTGAAAGAGATAGAGTTAAACAGGATTATTTGACATGTTAAATG
chrom   AACACTACAGAGAGCCCCTGAAGCAACTGAAAGAGATAGAGTTAAACAGGATTATTTGACATGTTAAATG
right   ATCCCGCCACTGCACTCC---AGC--CTGG--GCGACAGAGCGAGACTC----------CGTCTCAAAAA
        *1*1*11**11*11*1**111***11***111*1**1****11*1**111111111111*1*1*1***11

left    ACATGGGAAACATTATTAAGAAATAATGTTGGCCGGGCGC--GGTGGC----TCAAGCCTGTAA------
chrom   ACATGGGAAACATTATTAAGAAATAATGTTTAACCTTCTTCAGGTTATATTTTAATGAATGTTACTAATA
right   AAAAAAAAAAAAAAAAAAAAAAATAATGTTTAACCTTCTTCAGGTTATATTTTAATGAATGTTACTAATA
        *1*1111***1*11*11**1**********222*222*2222***2222222*2*2*22***2*222222

left    ----TCCCAGCACTTT--GGGAGGCCGAGGCGGGCGGATCAC-GAGGTCAGGAGATCGAG-ACCATCCCG
chrom   AGTGTTCCAAAATTGTATGGGATTTCTAAAATTCTGCATGTGTGAGTATATGATATCAAACATAATCATG
right   AGTGTTCCAAAATTGTATGGGATTTCTAAAATTCTGCATGTGTGAGTATATGATATCAAACATAATCATG
        2222*2***22*2*2*22****222*2*2222222*2**2222***222*2**2***2*22*22***22*

left    GCTAAAACGGTGAA---------ACCCCGTCTCTACTAAA----------
chrom   GTTATTATATTACATTATTATACACCACAGAAATAATCAAATTCTTTGTC
right   GTTATTATATTACATTATTATACACCACAGAAATAATCAAATT-------
        *2**22*222*22*222222222***2*22222**2*2**222
```

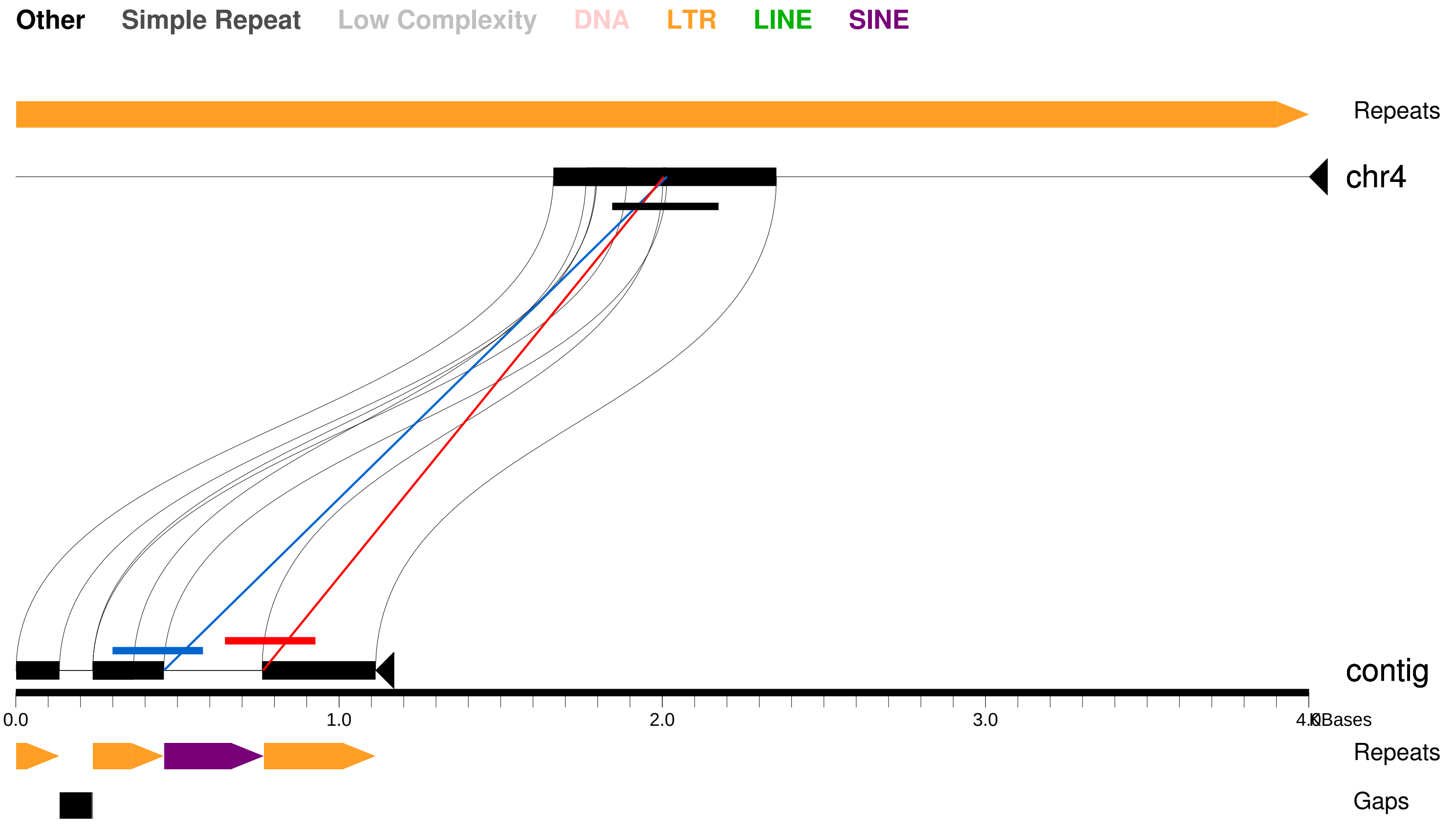

siteID: chr4_29907835 Scafftig1 + Breakpoint: chr4:29907824
chr4:29907775-29907883
Overlap Length: 11 INS size: 487 Genotyped: No

```
left    --GCAATGATGTGTGTAC---AGCTCTCTGGTCACTAT-GTCAGTAACTTG----AGATGCCATGTAATA
chrom   --GCAATGATGTGTGTAC---AGCTCTCTGGTCACTAT-GTCAGTAACTTG----AGATGACATGTAATA
right   CCGCCCGCCTCAGCCTCCCAAAGTGCTGGGATCACAGGCGTGAGCCACCGCGCCCGGCCGACATGTAATA
        11**11111*11*11*1*111**11**11*1****1111**1**11**11111111*11*2*********

left    NNNNNNNNNNNNNNNNNNNNNNNNNNNNNNNNNNNNNNNNNNNNNNNNNNNNNNNNNNN
chrom   TTTTTATCCTAATTATTGTAAATCCCAAATCACTCATCACAATGTCTGG----------
right   TTTTTATCCTAATTATTGTAAATCCCAAATCACTCATCACAATGTCTGG----------
        22222222222222222222222222222222222222222222222222222222222
```

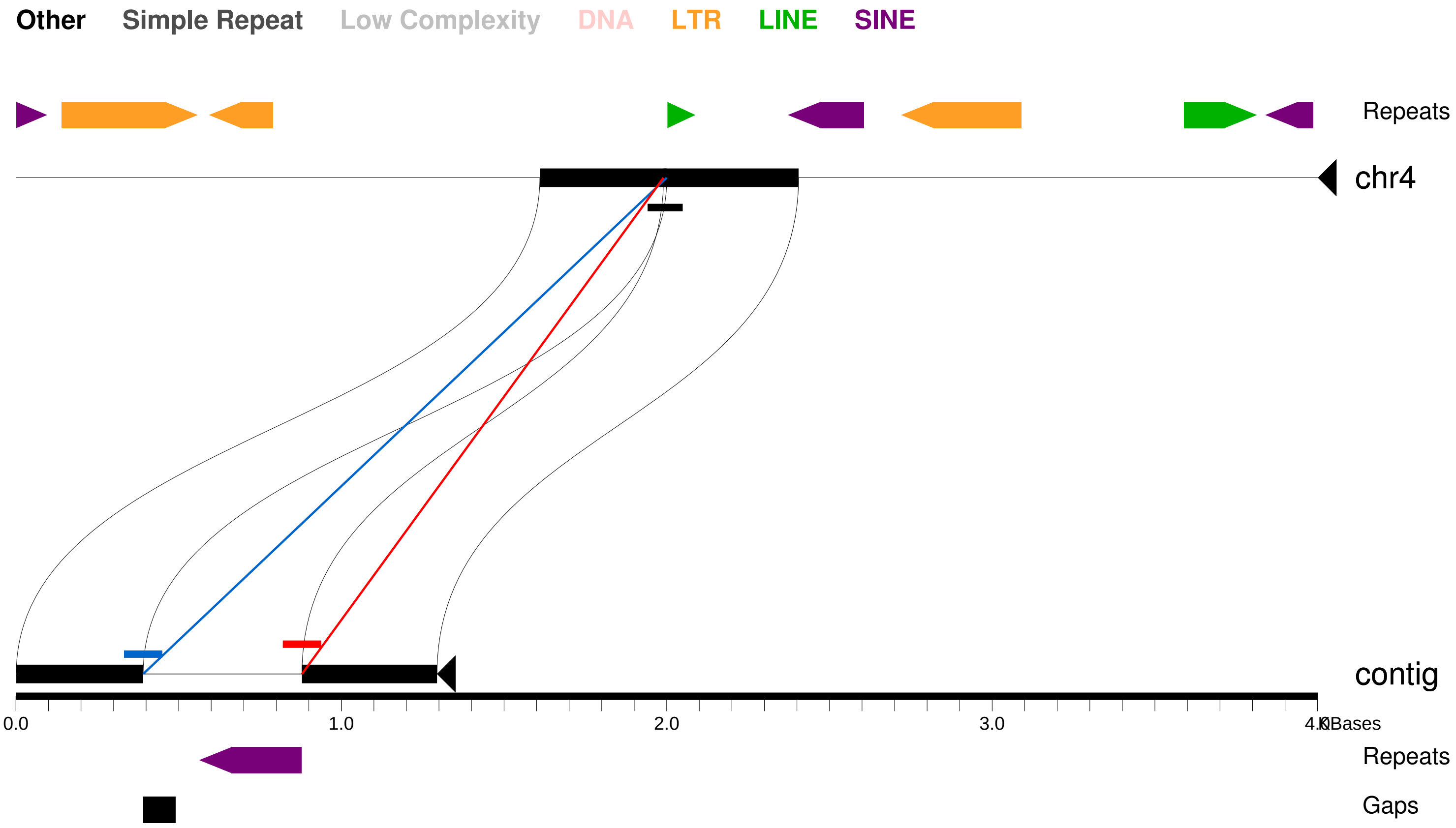

siteID: chr4_31841429 Scafftig8 + Breakpoint: chr4:31841416
chr4:31841367-31841475
Overlap Length: 8 INS size: 300 Genotyped: Yes

```
left   AAG---GTAACA--CTTTTTA--TTCAG---TTAATTTTGTGTCTAACAGGTAATCTTACATGCATTTTT
chrom  AAG---GTAACA--CTTTTTA--TTCAG---TTAATTTTGTGTCTAACAGGTAGTCTTACATGCATTCTT
right  CCGCCCGTCTCGGCCTCCCAAAGTGCTGGGATTACAGGCGTGAGCCACCGCGCCCGGCCCATGCATTCTT
       11*111**11*111**1111*11*1*1*111***11111***1111**1*111N11111********2**

left   TTTTTTTTTTTTTTTTTGAGACGGAGTTTCGCTCTGTCGCCCAGGCTGGAGTGCAGTGG
chrom  AAATCAGTTTAAATTGTTCCCTAGAC-----CACTTTCGTT---GCTAATTTTCATC--
right  AAATCAGTTTAAATTGTTCCCTAGAC-----CACTTTCGTT---GCTAATTTTCATC--
       222*222***222**2*222222**222222*2**2***22222***2222*2**2222
```

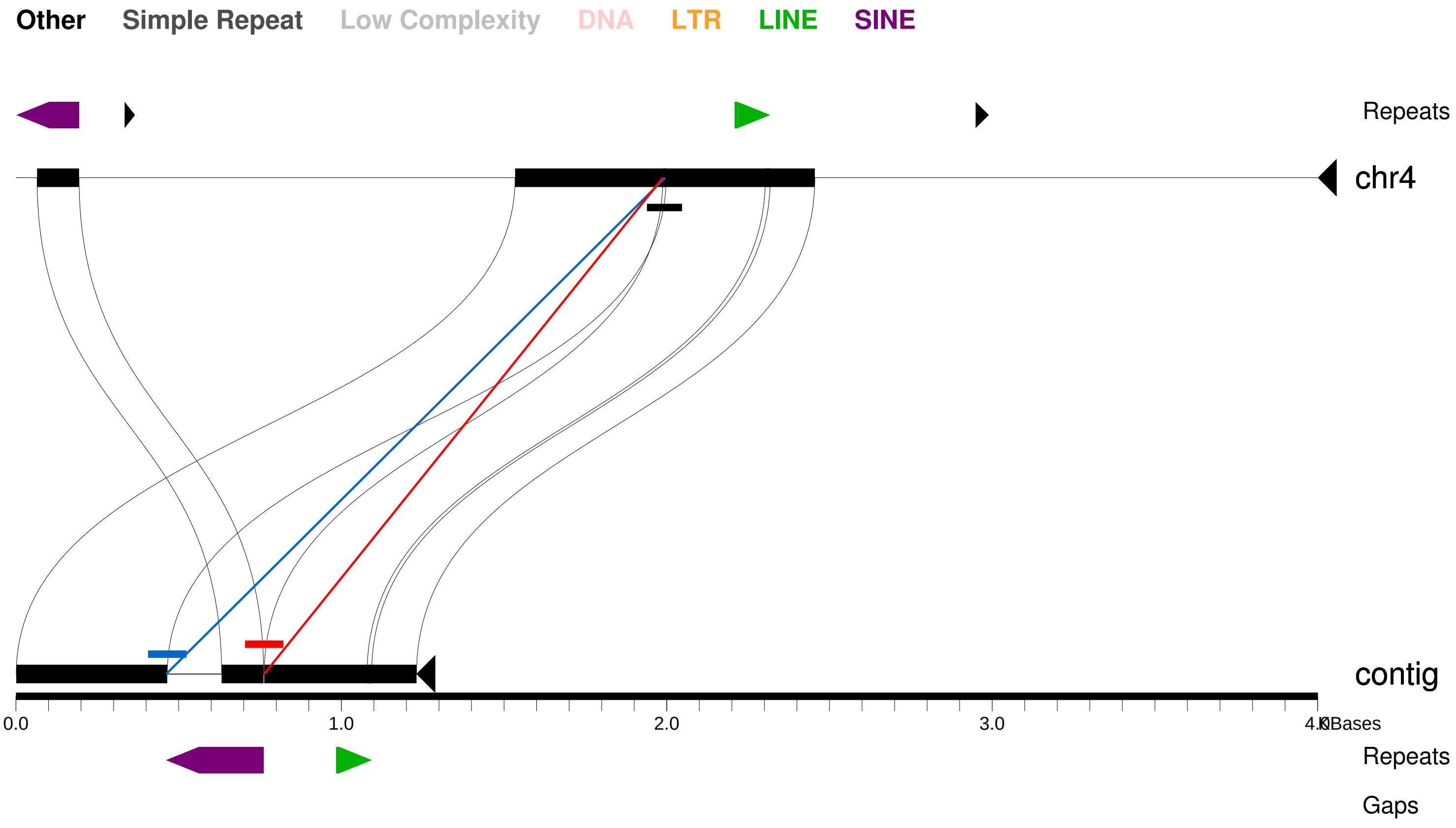

siteID: chr4_33247200 Scafftig1 - Breakpoint: chr4:33247186
chr4:33247138-33247240
Overlap Length: 12 INS size: 354 Genotyped: No

```
left   CA-----AATGAGAGAA----ATTGGCC----AAAATAAAGGGGCTGCAGGCCAAA---ACCCAGCCTGG
chrom  CA-----AATGAGAGAA----ATTGGCC----AAAATAAAGGGGCTGCAGGCCAAA---ACCCAGCCTGG
right  CTGACCTCATGATCCACCCGCCTCGGCCTCCCAAAGTGCTGGGATTACAGGCGTGAGCCACCGCGCCCGG
       *111111****111*111111*1****1111***1*111***11*1*****111*111***11****1**

left   CAGTCANNNNNNNNNNNNNNNNNNNNNNNNNNNNNNNNNNNNNNNNNNNNNNNNNNNNNNNNNNN
chrom  CAGTCATTAAATCTTAAAGCCTCAAAATGATCTCCTTTGACTCCATGTC----------------
right  CCGTCATTAAATCTTAAAGCCTCAAAATGATCTCCTTTGACTCCATGTC----------------
       *1****22222222222222222222222222222222222222222222222222222222222
```

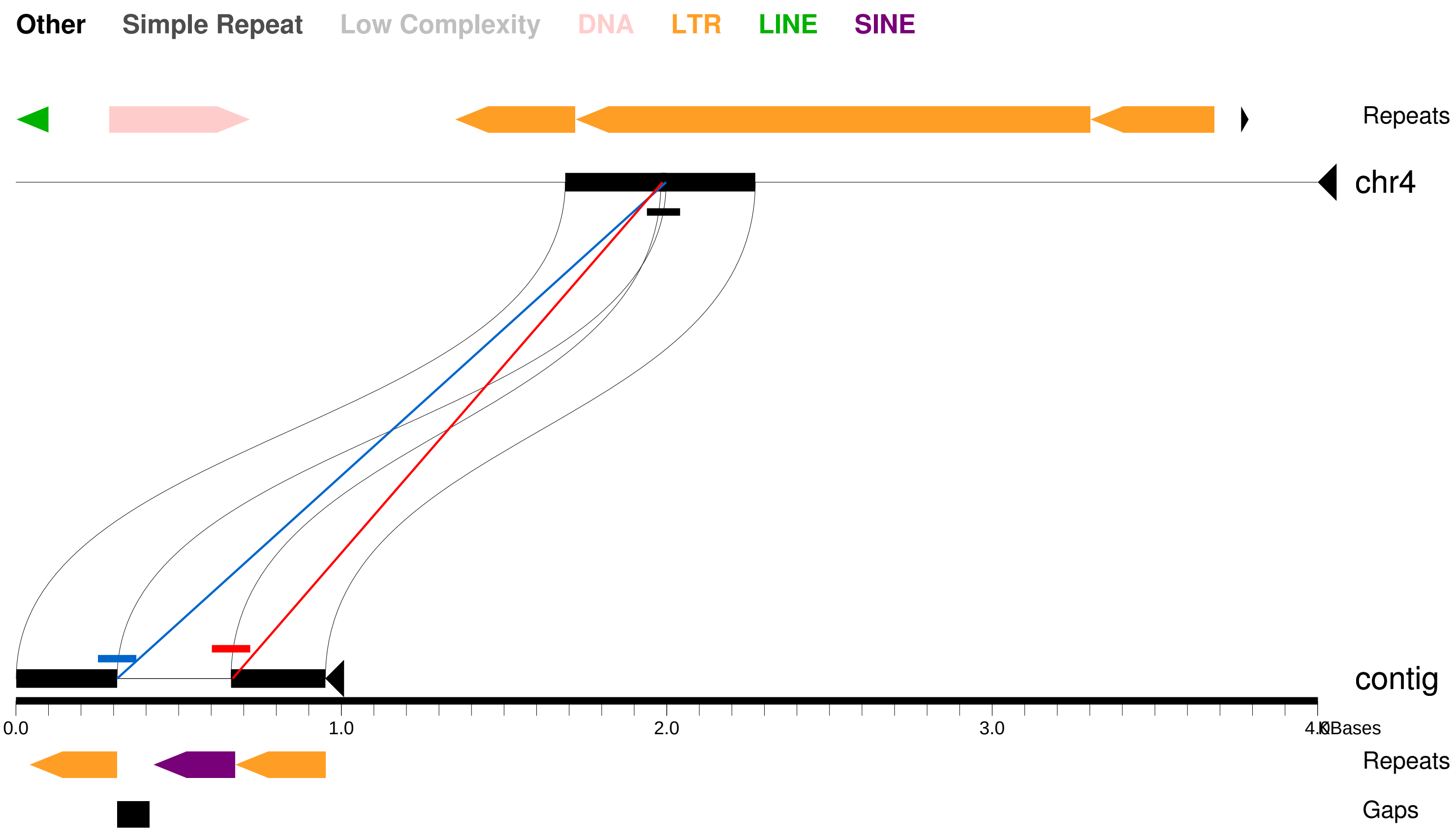

siteID: chr4_35317576 Scafftig1 - Breakpoint: chr4:35317532
chr4:35317372-35317704
Overlap Length: 13 INS size: 433 Genotyped: No

```
left    A-------------------------------------------ATAATGCTCTGTAAACCATTAATATG
chrom   ATTATCACATATTCTAAAAAAAAAAGTGTATCTTATAGTGAATAATAATGCTCTGTAAACCATTAATATG
right   TTTAGTAGAGACG------------GGGTTTCACCTTGTTAGCCAGGATGGTCTC--------------G
        1222NN2N2N2NN            2N22N22NNN2N22N2NNN*11***1***111111111111111*

left    ATTTAGTCATGGCAGTTGACAGTTGTTTCATATTTCTTAGGACCTACTAATTTTTGAATCTACTTTTCTA
chrom   ATTTAGTCATGGCAGTTGACAGTTGGTTCATATCTCTTAGGACCTACTAATTTTTGAATCTACTTTTCTA
right   ATCTCCTGACCTCA--TGA--------TCCACCCGCCTCGGCCTCCCAAAGTGCTGGGATTACAGGCGTG
        **1*11*1*111**11***111111N1**111121*1*1**1*111*1**1*11**1111***11111*1

left    ATCAATTGTTCTTAATAGTTTACCATATTTTCT---------------TTTTTNNNNNNNNNNN------
chrom   ATCAATTGTTCTTAATAGTTTACCATATTTTCTAATTATAAGTTGTAGTTATTCCATGAATATTAAGCAT
right   AGCCACCGCGCCTG---GCCTACCATATTTTCTAATTATAAGTTGTAGTTATTCAATGAATATTAAGCAT
        *1*1*11*11*1*1111*11*************22222222222222**2**2N222222222222222

left    ----------------------------------------------------------------------
chrom   TATGCATTTGTGTATGGTATTTGTTCCTGATTGATTATAAATTTATTATCAAAAGTTGGGCATCCACATC
right   TATGCATTTGTGTATGGTATTTGTTCCTGATTGATTATAAATTTATTATCAAAAGTTGGGCATCCACATC
        2222222222222222222222222222222222222222222222222222222222222222222222

left    -----------------------------------------------------
chrom   AGCACTTCATTGTGAAAGGGAAGTGGTATATATGAGGTGGGCACAAGTAGACC
right   AGCACTTCATTGTGAAAGGGAAGTGGTATATATGAGGTGG-------------
        2222222222222222222222222222222222222222
```

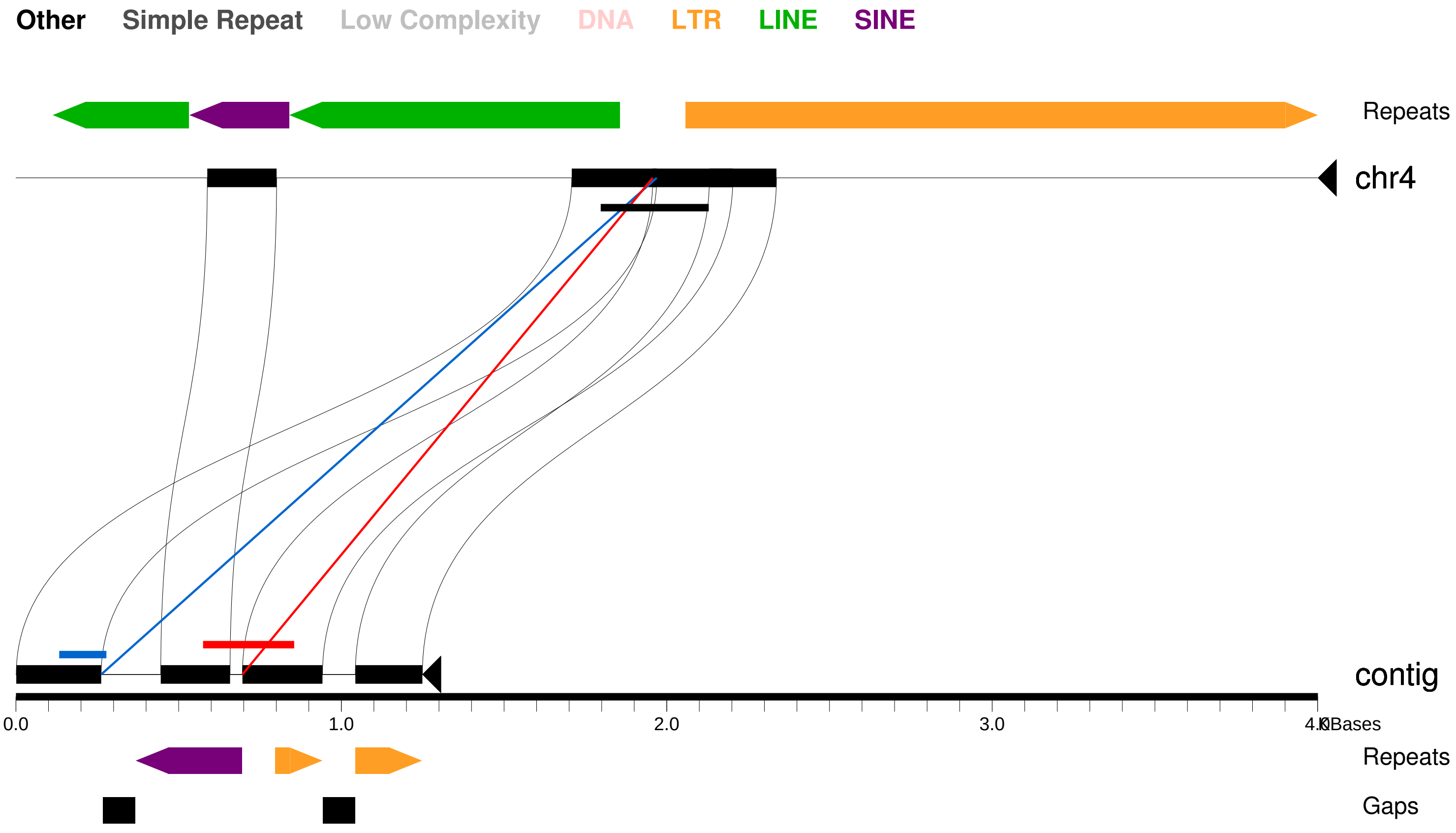

siteID: chr4_39073829 Scafftig2 - Breakpoint: chr4:39073817
chr4:39073761-39073845
Overlap Length: 4 INS size: 313 Genotyped: Yes

```
left    TC----------ATTTT-----TTAAA-------------AAAGAC-----AACCATC-CAAACAACATT
chrom   TC----------ATTTT-----TTAAA-------------AAAGAC-----AACCATC-CAAACAACATT
right   CAGTGAGCCAAGATTGTGCCACTGCAATCCGGCCTGGGCTAAAGAGCGGGACTCCGTCTCAAAAAAAAAA
        111111111111***1*11111*11**1111111111111*****11111111**1**1****1**1*11

left    ATATAAAGACAAAAGACTGTAATTGGCTGGGCGCGGTGGCTCACGCCTGTAATCCCAGCACTTTGGGAGG
chrom   ATATAAAGACAAAAGACTGTAATTA----------------CAGGCCT----TCCCA------TTGGCCA
right   AAAAAAAAAAAAAAAAAATGAAATTA----------------CAGGCCT----TCCCA------TTGGCCA
        *1*1***1*1****1*1**1****2222222222222222**2****2222*****222222*2**222

left    CCGAGGCGGGCGG
chrom   CTGAA--------
right   CTGAA--------
        *2**222222222
```

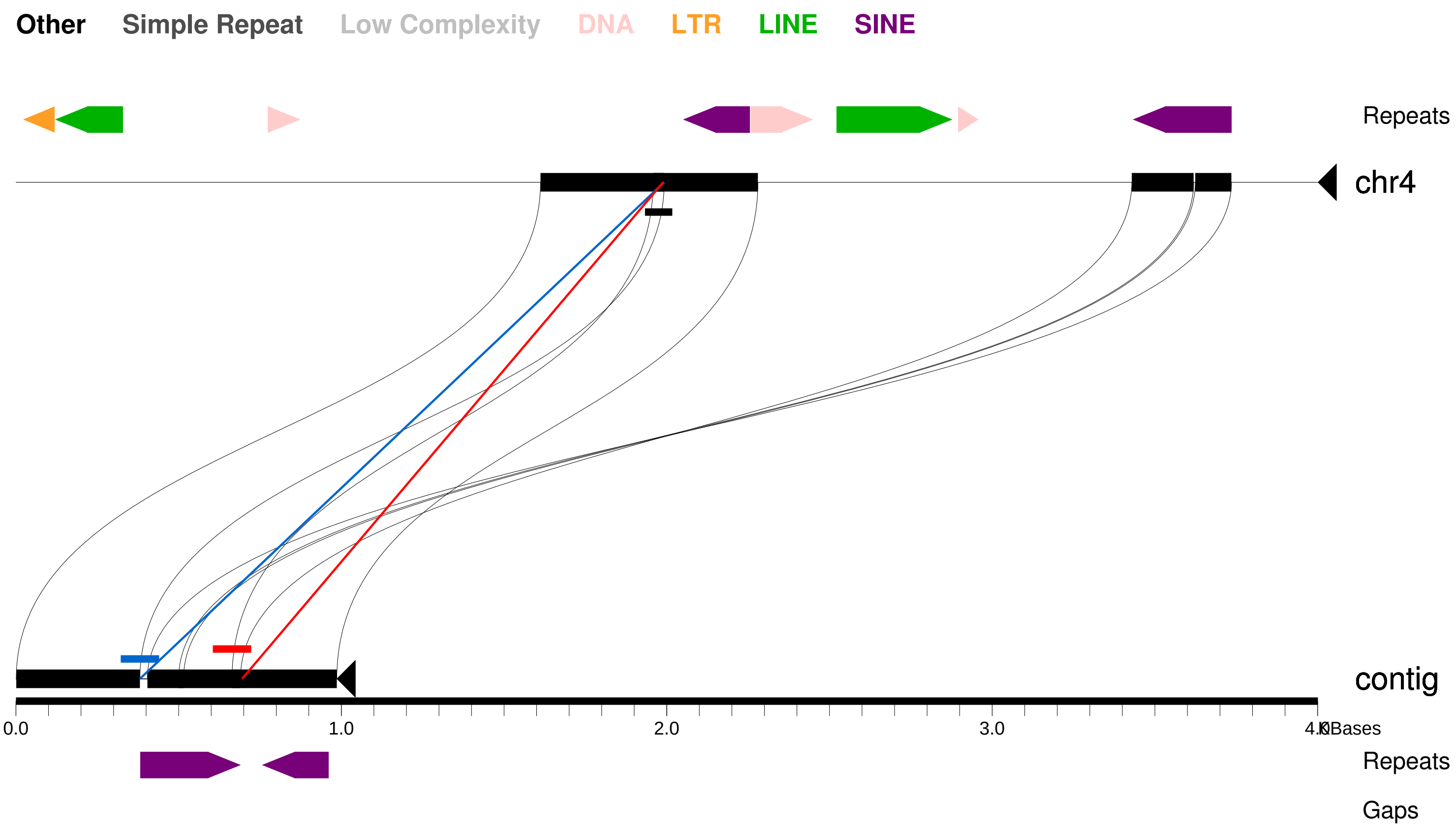

siteID: chr4_42088028 Scafftig11 - Breakpoint: chr4:42088041
chr4:42087997-42088091
Overlap Length: 16 INS size: 300 Genotyped: Yes

```
left    ATAGTTTGCAAATGTTTT----CATTGGC-----------------TCT----AATGACCAAAAGGAATA
chrom   ATAGTTTGCAAATGTTTT----CATTGGC-----------------TCT----AATGACCAAAAGGAATA
right   G-ATTGCGCCACTGCACTCCAGCCTGGGCGACAGAGCGAGACTCCGTCTCAAAAAAAAAAAAAAAAAAAA
        11*1*11**1*1**111*1111*1*1***11111111111111111***1111**11*11****11**1*

left    AAAAGAAACAAGATTGGCCGGGCGCGGTGGCTCACGCCTGTAATCCCAGCACTTTGGGAGGCCGAGGCGG
chrom   AAAAGAAACAAGATT----------------TTA---TTTTGCTACAAATAATGTGGTGGT-----GATT
right   AAAAGAAACAAGATT----------------TTA---TTTTGCTACAAATAATGTGGTGGT-----GATT
        **************2222222222222222*2*2222*2*22*2*2*22*2*2***22*222222*222

left    GCGG
chrom   TTTC
right   TTTC
        2222
```

Other Simple Repeat Low Complexity DNA LTR LINE SINE

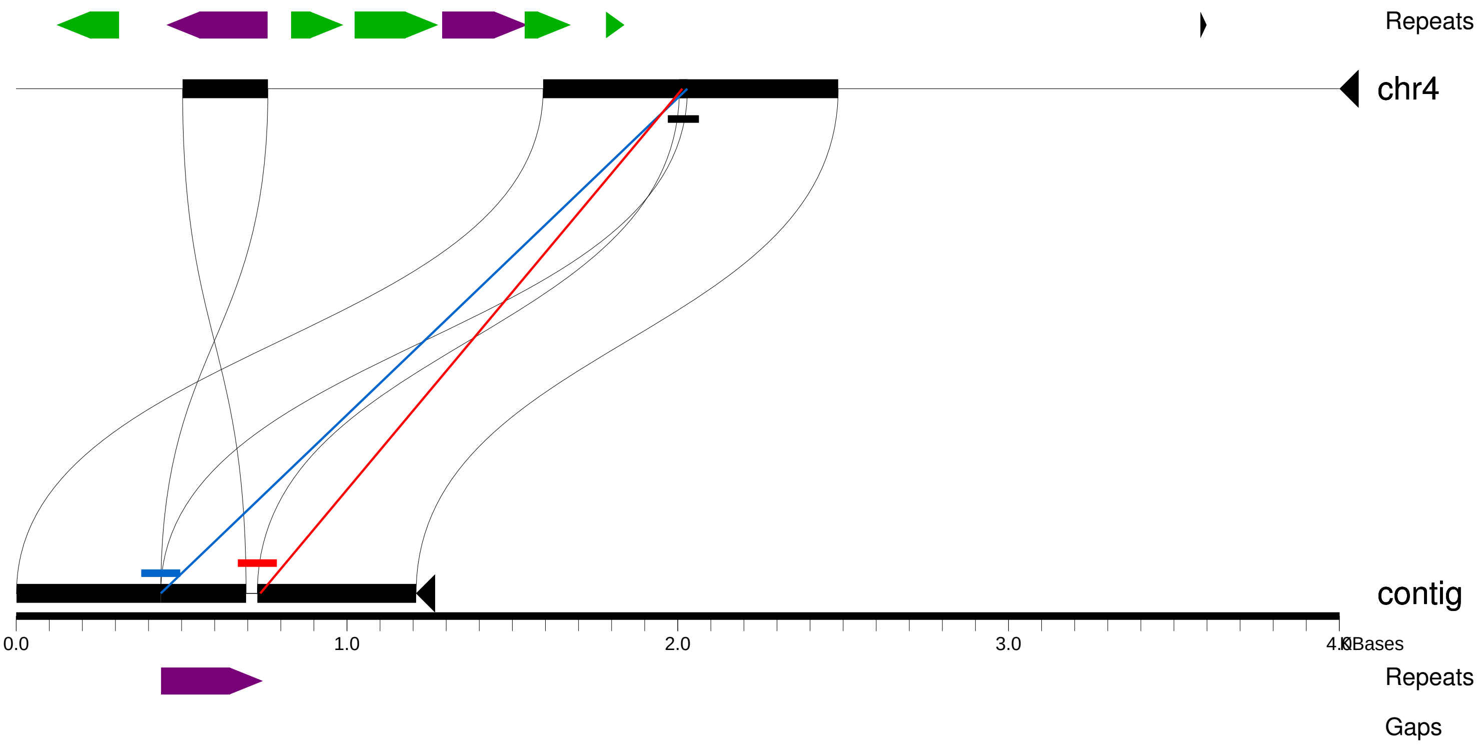

siteID: chr4_42778314 Scafftig1 - Breakpoint: chr4:42778307
chr4:42778147-42778484
Overlap Length: 18 INS size: 292 Genotyped: Yes

```
left   NNNNNNNNNNNNNNNNNNNNNNNNNNNNNNNNNNNNNNNNNNNNNNNNNNNNNNNNN----------TTG
chrom  AGGAGATTCTATGCCAAGATTTTTATTCTTCTGATTTTTTTATCATGTATTTATTCAAAACTTCTGTTTG
right  TTTAG--TAGA-GACGGGGTTTC----------ACCATTTTAGCCGGGATGGTCTCGAT-CTCCTGACCT
       NNN22NN2NN2N2N2NN2N222NNNNNNNNNNN2NNN22222N2NN2N22NNNN22N2N 22N222N111

left   GATGGAATTCATTGTATAAGACCAAGAAATTTATAATACTTTATGATTCCAAACAGTCACAATTTAATAA
chrom  GATGGAATTCATTGTATAAGACCAAGAAATTTATAATACTTTATGATTCCAAACAGTCACAATTTAATAA
right  CGTG--ATCCGCCCGCCTCGGCCTCCCAA-----AGTGCTGG--GATT----ACAGGCGTGAGCCACCGC
       11**11**1*111111111*1**1111**11111*1*1**1111****1111****1*111*111*1111

left   AAATAGACATAAAATTATTTGACAAAATAATTTTTTTTTTTTTTTTTT----TTGAG---ACGGAGTCTCG
chrom  AAATAGACATAAAATTATTTGACAAAATTATTTTTTTTACTTTGCTAGAACTTAAATTTATAAAGCCTAC
right  GCCCGGCC------------GACAAAATTATTTTTTTTTACTTTGCTAGAACTTAAATTTATAAAGCCTAC
       11111*1*111111111111********2*********22***22*22222**2*2222*222**2**22

left   CTCTGTCGCCCAG---------GCTGGAGTGCAGTGGCG---GGATCTCGGCTCAC--TG-----CAAGC
chrom  CTTCTTAGAGCATCTTATATATGATGAAATTCAGTGTTCTATGGTTTTCACTTGAGCATGTTTAACAGGA
right  CTTCTTAGAGCATCTTATATATGATGAAATTCAGTGTTCTATGGTTTTCACTTGAGCATGTTTAACAGGA
       **222*2*22**2222222222*2**2*2*2*****222222**2*2**222*2*222**22222**2*2

left   TCCGCCTCCCGGGTTCAC------GCCATTC---------------TCCCGCCTCAGC
chrom  TTGGGTTACCAGCATTACTAAAAGGACCTTAAAATATTTTATAAAATGGTCCTTCATC
right  TTGGGTTACCAGCATTACTAAAAGGACCCTAAAATATTTT------------------
       *22*22*2**2*22*2**222222*2*21*2222222222      1NNNN1N111N1
```

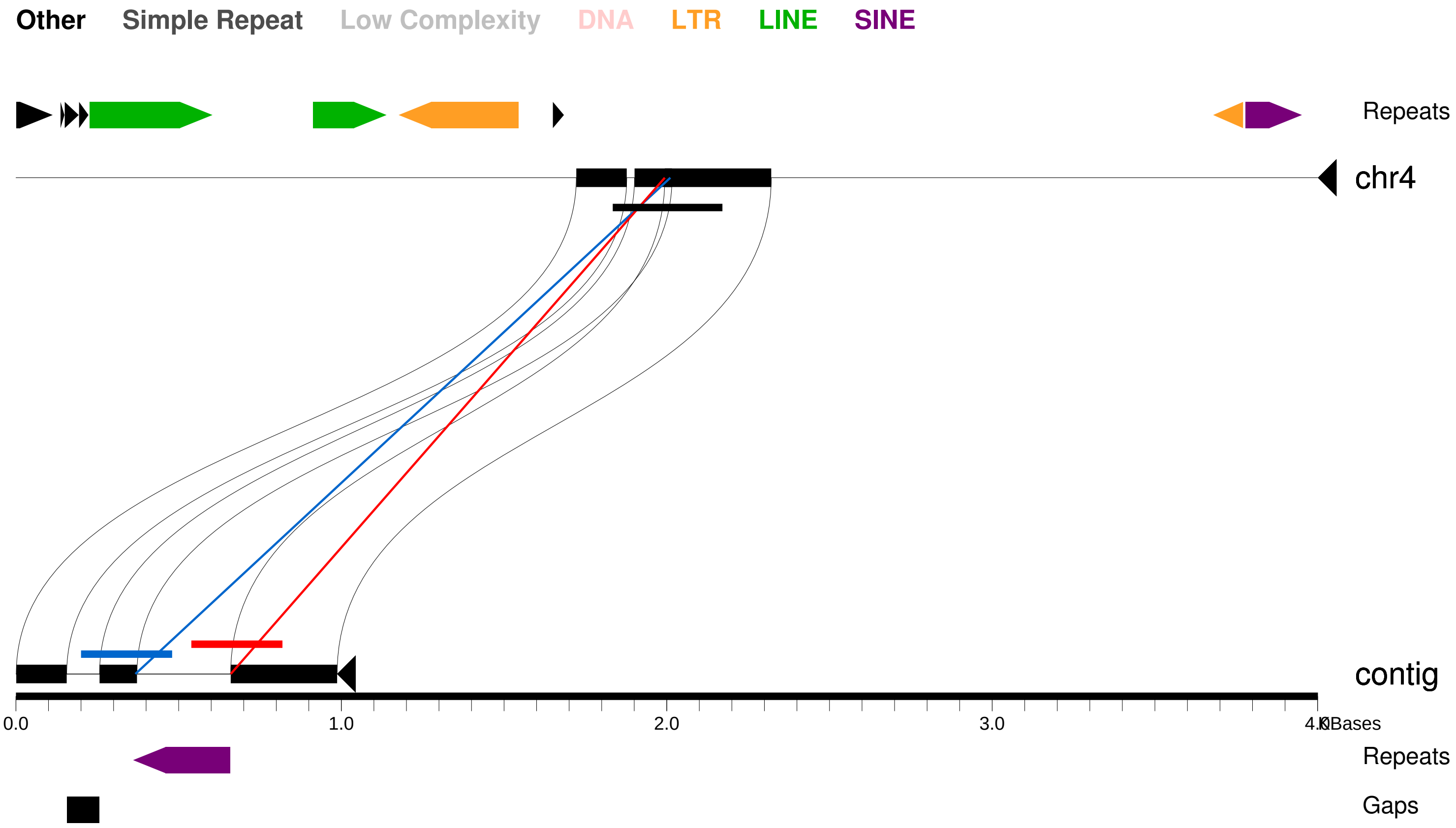

siteID: chr4_43399970 Scafftig5 - Breakpoint: chr4:43399975
chr4:43399927-43400034
Overlap Length: 12 INS size: 313 Genotyped: Yes

```
left   AGTTATCGTATGTGTG-----------CTCCTTTTTGGCTGATGCCTTTTCCTTAGTTGAAATTTAAGCT
chrom  AGTTATCGTATGTGTG-----------CTCCTTTTTGGCTGATGCCTTTTCCTTAGTTGAAATTTAAGCT
right  CAGTCCGGCCTGGGCGACAGAGCGAGACTCCGTCTCAAAAAAAAAAAAAAAAAAAAAAAAAATTTAAGCT
       111*111*11**1*1*11111111111****1*1*111111*111111111111*1111***********

left   ACAGGCCGGGCGCGGTGGCTCACGCCTGTAATCCCAGCACTTTGGGAGGCCGAGGCGGGT
chrom  AA----CTGAAAATTAGG-----GCATATCTTTCTTTTTCTTTAACCTTCCATTTTAC--
right  AC----CTGAAAATTAGG-----GCATATCTTTCTTTTTCTTTAACCTTCCATTTTAC--
       *N2222*2*2222222**22222**2*2*22*2*22222****222222**222222222
```

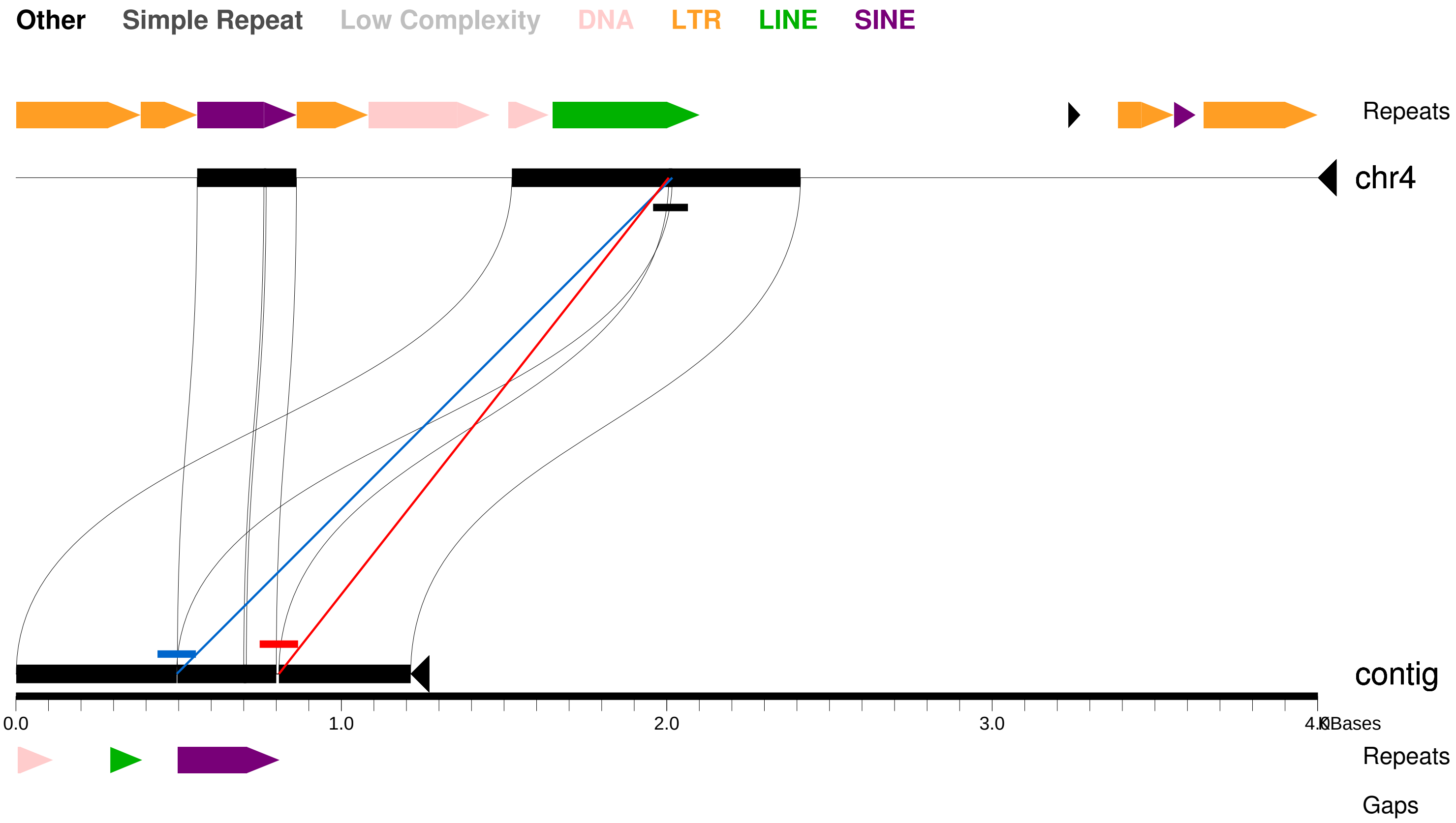

siteID: chr4_44842111 Scafftig1 - Breakpoint: chr4:44842129
chr4:44842099-44842188
Overlap Length: 16 INS size: 138 Genotyped: Yes

```
left    ATGTCAAAAAGCTTT--------------TTACT----------TTTC-----TTTTTTTATTATGGCCT
chrom   ATGTCAAAAAGCTTT--------------TTACT----------TTTC-----TTTTTTTATTATGGCCT
right   ACTGCGGACTGCGGTGGCGCAATCTCGGCTCACTGCAAGCTCCGCTTCCCGGGTTCACGTATTATGGCCT
        *111*11*11**11*11111111111111*1***11111111111***11111**1111***********

left    CTTCTTTTTTTTTTTTTTTTTTTTTTTTTTTTTTTTTTTTTTTTTTTTTTTTGAGACGGAGTCTCGCTCT
chrom   CTTCTAGAATTTTGCTTTTAATGTCTACTTGCGT-------------------AGGCTGAGA-TTGCT--
right   CTTCTAGAATTTTGCTTTTAATGTCTACTTGCGT-------------------AGGCTGAGA-TTGCT--
        *****2222****22****22*2*2*22**222*2222222222222222222**2*2***22*2***22

left    GTCGCCCA
chrom   -------A
right   -------A
        2222222*
```

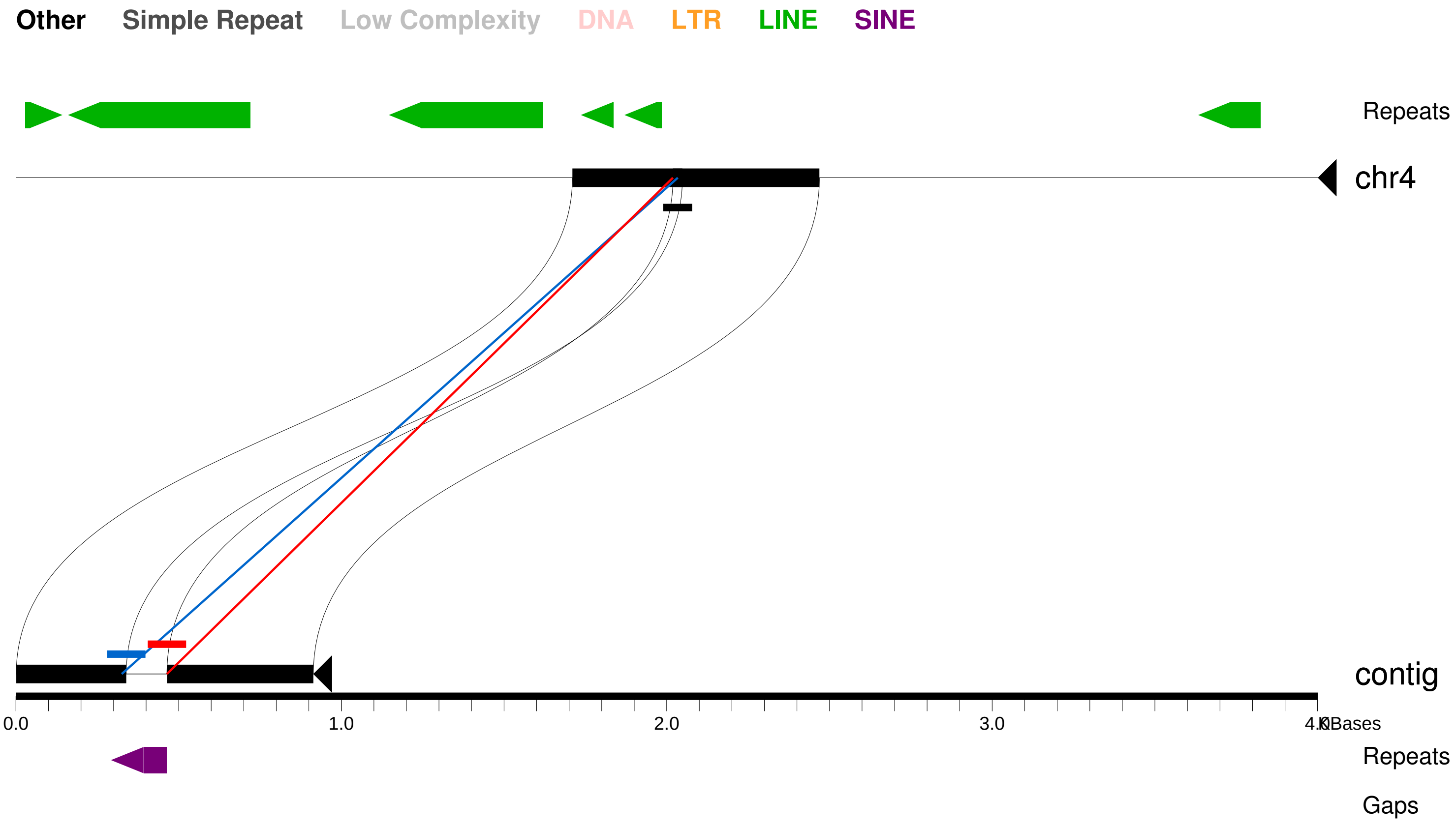

siteID: chr4_46784902 Scafftig1 - Breakpoint: chr4:46784922
chr4:46784875-46784981
Overlap Length: 13 INS size: 371 Genotyped: No

```
left    ACATCTGAACATCTGAGGGAACCAACTCTGGACACACCATCTTTAAG------------AACTGTAACAC
chrom   ACATCTGAACATCTGAGGGAACCAACTCTGGACACACCATCTTTAAG------------AACTGTAACAC
right   NNNNNNNNNNNNNNNNNNNNNNNNNNNNNNNNNNNNNNNNNNNNNNNNNNNNNNNNNNNAACTGTAACAC
        11111111111111111111111111111111111111111111111111111111111***********

left    TCGGCCGGGCGCGGTGGCTCACGCCTGTAATCCCAGCACTTTGGGAGGCCGAGGCGGGCGG
chrom   TCA-CCGTGAG----GGTCCGCGGCT-TCATTCTTGAAGTCAGCAAAACCAAGAA------
right   TCA-CCGTGAG----GGTCCGCGGCT-TCATTCTTGAAGTCAGCAAAACCAAGAA------
        **22***2*2*2222**22*2**2**2*2**2*22*2*2*22*22*22**2**22222222
```

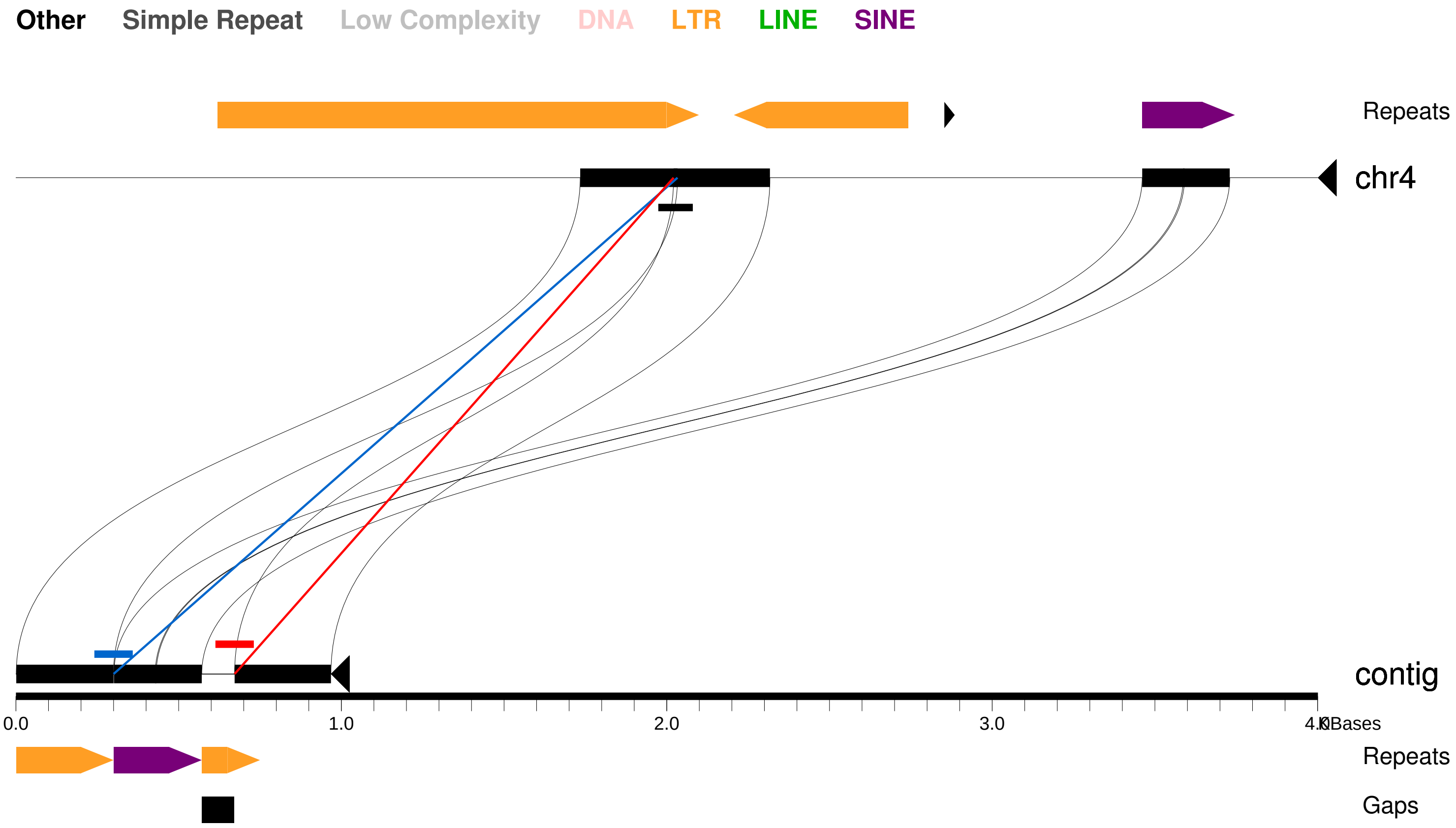

siteID: chr4_47496535 Scafftig2 - Breakpoint: chr4:47496518
chr4:47496470-47496574
Overlap Length: 12 INS size: 308 Genotyped: Yes

```
left     TCTGTAAACAACATAGGCTGTATTCTTTT------------------AATATACTTTGGATATAATAAAG
chrom    TCTGTAAACAACATAGGCTGTATTCTTTT------------------AATATACTTTGGATATAATAAAG
right    TGGG----CGACAGAGCGAGACTCCGTCTCAAAAAAAAAAAAAAAAAAAAAAAAAAAAAAAAAAAAAAAAG
         *11*1111*1***1**111*11*1*1*1*111111111111111111**1*1*111111*1*1**1****

left     TTAGATTATCACGCCTGTAGTCCCAGCACTTTGGGAGGCCGAGGCGGGTGGATCATGAGGTCAGGAG-
chrom    TTAGATTA----------AGTACCT-CATTTATTGCTGGAAATTC---TATATCCC-AGCTCACTTTA
right    TTAGATTA----------AGTACCT-CATTTATTGCTGGAAATTC---TATATCCC-AGCTCACTTTA
         *******2222222222***2**22**2**222*22*222*22*222*22***222**2***22222
```

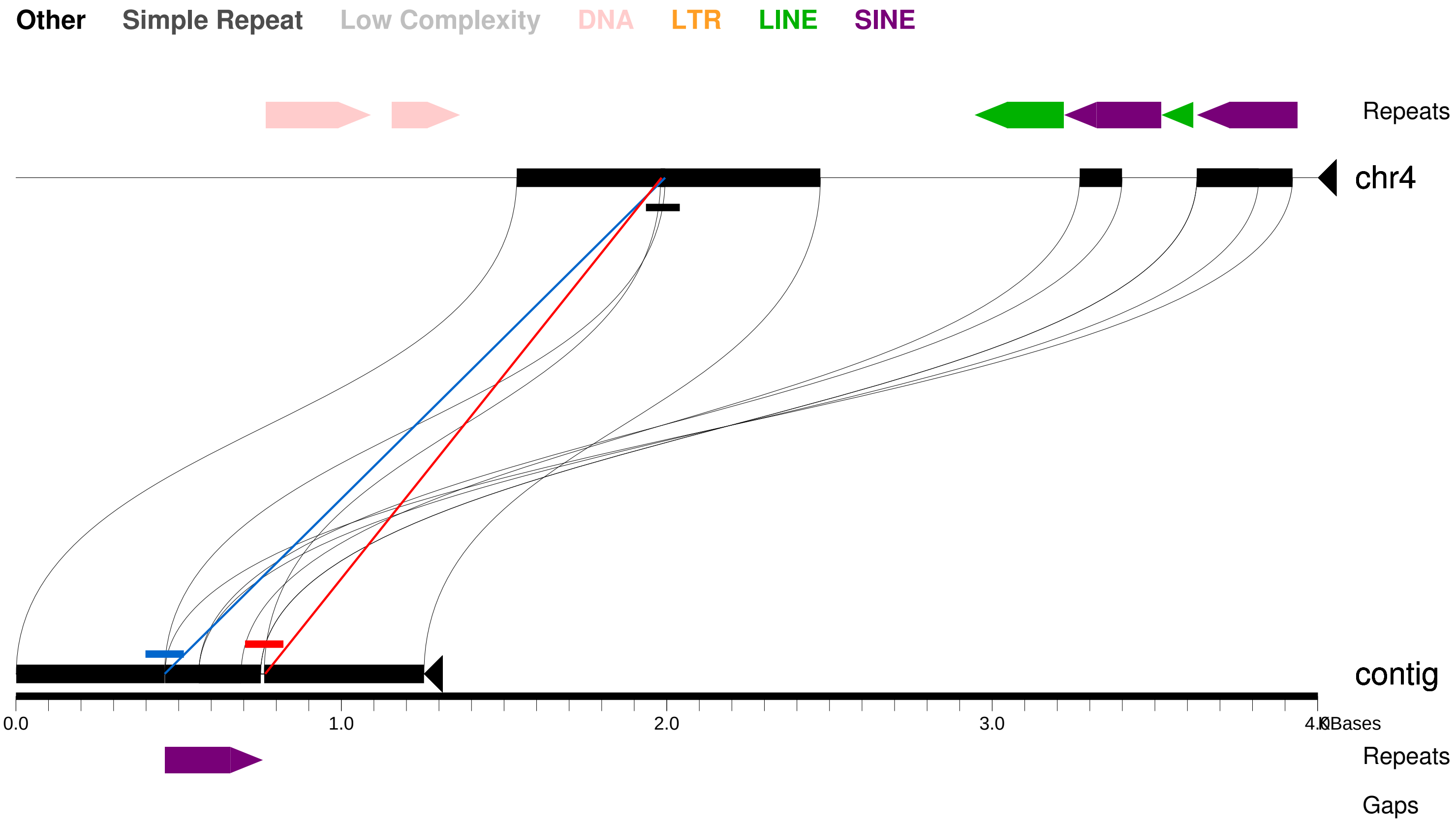

siteID: chr4_52763737 Scafftig1 + Breakpoint: chr4:52763743
chr4:52763695-52763802
Overlap Length: 12 INS size: 433 Genotyped: No

```
left    TTGATTCTCATTTTTAAGGGCTAGATATTGCAAACAAAATTAGTTTGT-----------AAAAATAACCA
chrom   TTGATTCTCATTTTTAAGGGCTAGATATTGCAAACAAAATTAGTTTGT-----------AAAAATAACCA
right   NNNNNNNNNNNNNNNNNNNNNNNNNNNNNNNNNNNNNNNNNNNNNNNNNNNNNNAAAAAAAAAATAACCA
        11111111111111111111111111111111111111111111111111111111111***********

left    AGGCCGGGCGCGGTGGCTCACGCCTGTAATCCCAGCACTTTGGGAGGCCGAGGCGGGCGG
chrom   ATAA---GTATGCTTGCTTA----TGAAAAGTAAACACTGTATCA--CTTA--CTGGTAA
right   ATAA---GTATGCTTGCTTA----TGAAAAGTAAACACTGTATCA--CTTA--CTGGTAA
        *222222*222*2*2***2*2222**2**2222*2****2*222*22*22*22*2**222
```

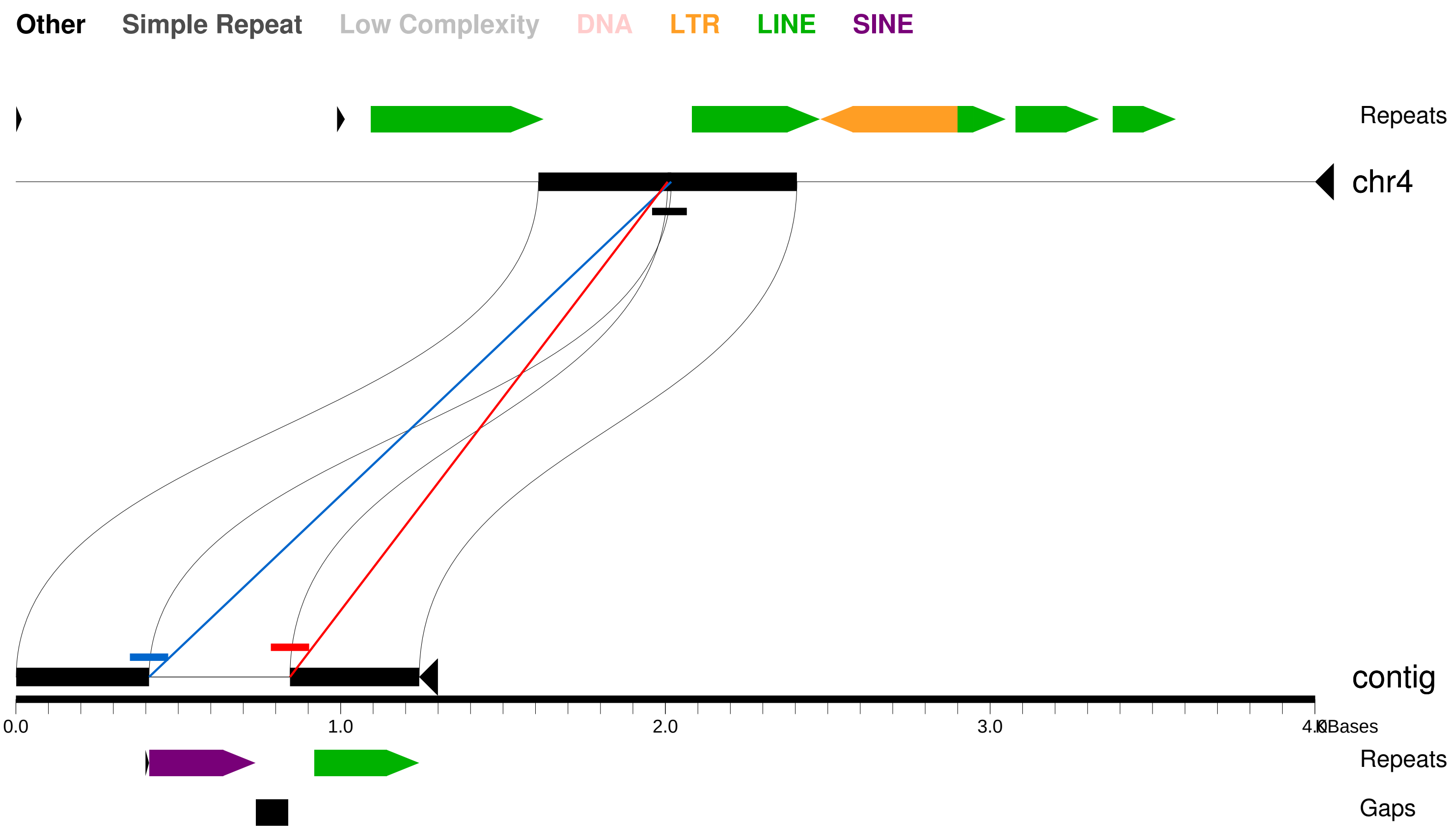

siteID: chr4_53222451 Scafftig4 - Breakpoint: chr4:53222441
chr4:53222395-53222500
Overlap Length: 14 INS size: 299 Genotyped: Yes

```
left     GAAAGAT------GAATGTTTGTCATGTGG-------GTCTTTATTCCCTTCTATGTTTAAAATGTATCT
chrom    GAAAGAT------GAATGTTTGTCATGTGG-------GTCTTTATTCCCTTCTATGTTTAAAATGTATCT
right    CCACTGCACTCCAGCCTGGGCGACAGAGCGAGACTCCGTCTCAAAAAAAAAAAAAAAAAAAAAATGTATCT
         11*1111111111*11**111*1**1111*1111111****11*111111111*11111***********

left     TTAGGCCGGGCGCGGTGGCTCACGCCTGTAATCCCAGCACTTTGGGAGGC-CGAGGCGGGCGG
chrom    TTAAAAAAA----GGTT-CTAAAG---GAAAAAGAAGAAC---AGAAGATTCAAAACATT---
right    TTAAAAAAA----GGTT-CTAAAG---GAAAAAGAAGAAC---AGAAGATTCAAAACATT---
         ***2222222222***22**2*2*222*2**2222**2**2222*2**222*2*22*222222
```

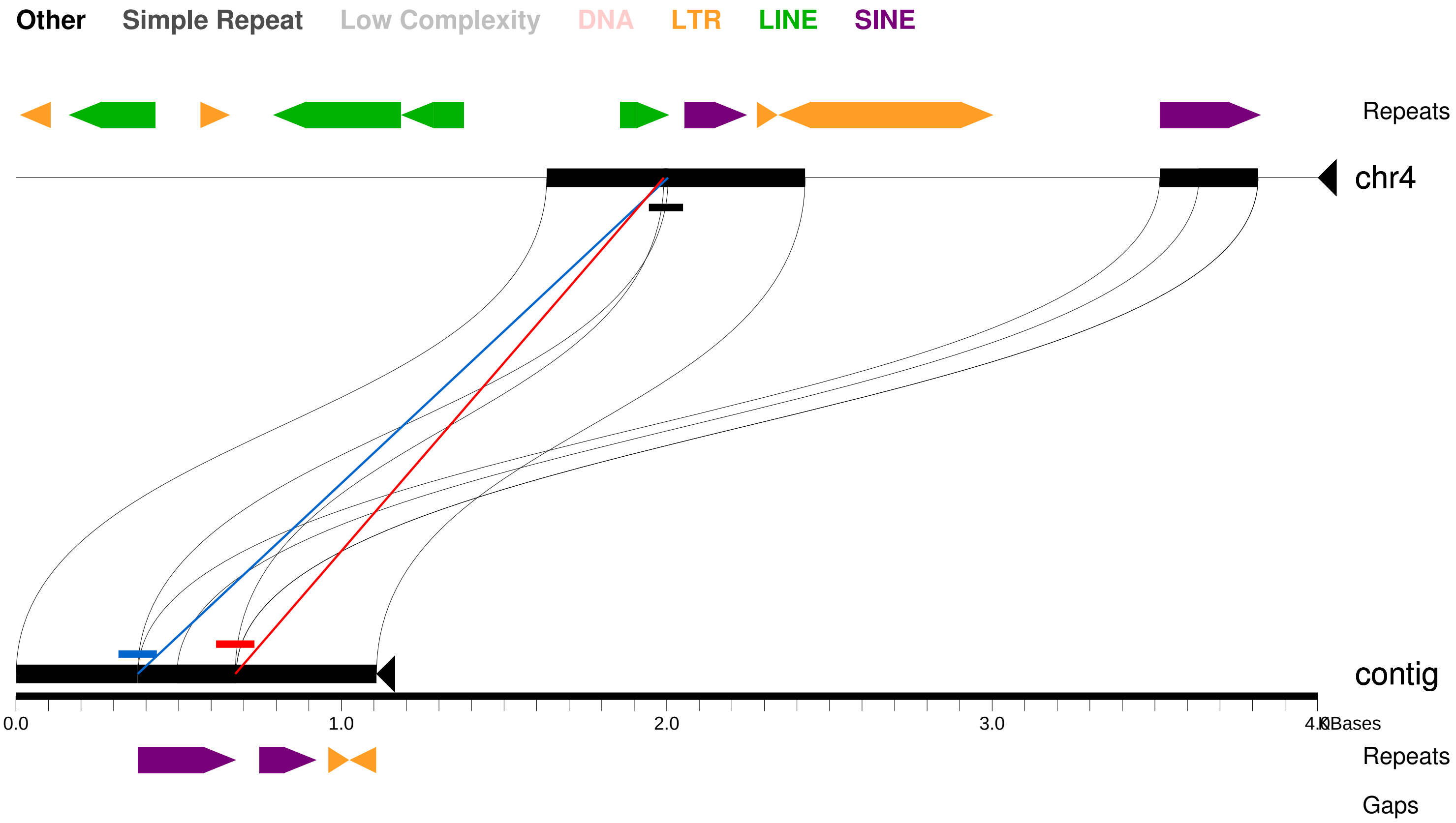

siteID: chr4_53851852 Scafftig2 - Breakpoint: chr4:53851836
chr4:53851676-53852012
Overlap Length: 17 INS size: 295 Genotyped: Yes

```
left   -----------------TGTTTTGGAGAGGTTTCTAGCACCATATAGCAGCATATTATAAAACCATGGCA
chrom  GTTGATAAATTTGAGGGTGTTTTGGAGAGGTTTCTAGCACCATATAGCAGCATATTATAAAACCATGGCA
right  CT----------GAGGCAG-----GAGAA-TGGCGTGAACCCGGGAGGCGGAGCTT-----------GCA
       N2          2222N1*11111****11*11*11*1***1111**11*1*11**11111111111***

left   GTTTTATTCTATTAAGAAATCACACCCCCAGACGTATCTGAAAAACTTCCTTTGGAAGATTGCAAAAGGC
chrom  GTTTTATTCTATTAAGAAATCACACCCCCAGACGTATCTGAAAAACTTCCTTTGGAAGATTGCAAAAGGC
right  GTG--AGCC------GAGATC---CCGCCA-------CTGCATTCCAGCCTGGGCGACAGAGCGAGACTC
       **111*11*111111**1***111**1***1111111***1*111*11***11*11*1*11**1*1*11*

left   CAGATGCAAATTTTTTGCTTAAAAAATTAGTCCAGGAGGCCGGGCG-CGGTGGCTCACGCCTGTAA----
chrom  CAGATGCAAATTTTTTGCTTAAAAAATTAGTCCAGGAATATAAATATCAAATGTTTATGATTTTAGAAGG
right  TGTCTCAAAAAAAAAAAAAAAAAAAATTAGTCCAGGAATATAAATATCAAATGTTTATGATTTTAGAAGG
       1111*11***1111111111****************2222222222*2222*2*2*2*22*2**22222

left   TCCCAGCACTT---TGGGAGGCCGAGGCGGGCGGATCACG---AGGTCAGGAGATCGAGACCATCCCGGC
chrom  TCCAAATATCTGTCTGGAATTCTGGGTTGAAGTTAGCAGGTTAAGGTCAAGTTTTTTTGAAAGTCTCTCC
right  TCCAAATATCTGTCTGGAATTCTGGGTTGAAGTTAGCAGGTTAAGGTCAAGTTTTTTTGAAAGTCTCTCC
       ***2*22*22*222***2*22*2*2*22*22222*2**2*222******2*222*222**222**2*22*

left   TAA-----AACGG-------T--GAAACCCCGTCT--------CTACT--------AAA
chrom  AAATATGCAACAGATGACACT--GCAATTCCAGCTGTGTTCACCTATTCTTAGAACAAA
right  AAATATGCAACAGATGACACTTTGCAATTCCAGCTGTGTTCACCTA-------------
       2**22222***2*2222222*11*2**22**22**22222222***N1        111
```

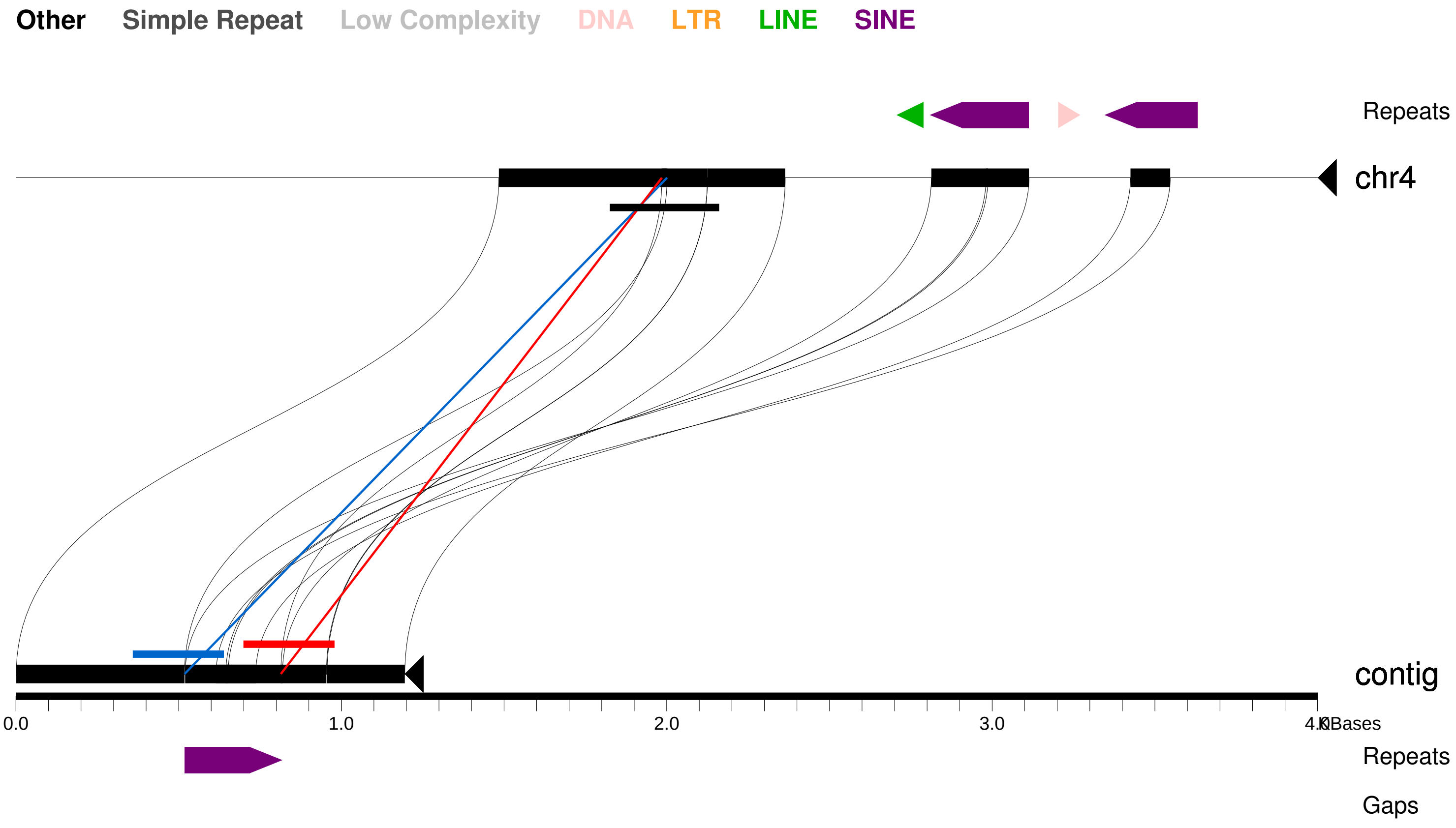

siteID: chr4_54415629 Scafftig3 + Breakpoint: chr4:54415644
chr4:54415259-54415804
Overlap Length: 11 INS size: 311 Genotyped: Yes

```
left   AA----------------------------------------------------------------------
chrom  AATAATATGTGCAAACCCCCATGACATGTATTTACCTATGTAACAAACCTTGACATGTACCCCCCAAATC
right  A-------------------------------------------------TGGCGTGAACCC--------
       *1                                                22N2N22N2222

left   ----------------------------------------------------------------------
chrom  TAAAATAAAAGTAAAAAAAATAAGTTCTCCCCTTAAAGCTTCTGGGCAACAAGGTCATAAAAGACACTCA
right  -------------------------------------------GGGAAGC--GG---------------A
                                                  222N2N2  22               2

left   ----------------------------------------------------------------------
chrom  TCTTGGGTAAAATTGTAGTTACATTCATAGACTTCAATGCTCAATGTTTTGGACCTTTGCTTTCTTACAT
right  GCTTGC-----AGTGAGCCGAGATTGCGCCACTGCAGTCCGCAGTCC----GACCTGGGC----------
       N2222N     2N22NNNNN2N222NNNNN222N22N2N2N22N2NN    22222NN22

left   ---------------------------ACCATTGTTCTATTTTTCTCTTTTTCAGTTACTAGCCCAGTAT
chrom  TTCTTTCAACTTGGACAGTAAATTAAAACCATTGTTCTATTTTTCTCTTTTTCAGTTACTAGCCCAGTAT
right  -------------GACAG----------------------------------------------------
                    22222         1111111111111111111111111111111111111111111

left   TTGGAATTTGCCAAAGCTATATTCCTCAGAATTTACTTCAAGAGCTCCTTTGTATCGGCGGAGAATGAAG
chrom  TTGGAATTTGCCAAAGCTATATTCCTCAGAATTTACTTCAAGAGCTCCTTTGTATCGGCGGAGAATGAAG
right  --------------AGCGAGACTCC--------------------------GTCTC-----AAAAAAAAA
       11111111111111***1*1*1***11111111111111111111111111**1**11111*1**11**1

left   AACGGAGAAGAGGAGGAGGAGGTTGAAAGAAACTTAAGAGATGTGG---------GCCGGGCGCGG----
chrom  AACGGAGAAGAGGAGGAGGAGGTTGAAAGAAACTTAAGAGATGTGGCAACTAATTGCAACGTGTAGATTG
right  AA---AAAAAA--------------AAAAAAA---AAGAGATGTGGCAACTAATTGCAACGTGTAGATTG
       **111*1**1*11111111111111***1***111***********222222222**222*2*22*2222

left   ----TGGCTCACGCCTGTAATC--CCAGCACTTTGGGAGGCCG--AGGCGGGTGGATCATGAGGTCAGG-
chrom  TATTTAGCTCATGATTCACACAAACTAGATATTTGTTATTAAAGAATTATGGTTGTTTTTGGTGTGATAT
right  TATTTAGCTCATGATTCACACAAACTAGATATTTGTTATTAAAGAATTATGGTTGTTTTTGGTGTGATAT
       2222*2*****2*22*222*2222*2**222****22*2222222*2222***2*2*22**22**2*222

left   -AG-ATCGAGACCATCCTGGCTAACAAGGT-------GAAACCCCGTCTCTACTAAA
chrom  TAGTATTGTGCTCACATTTCTTAAGAATCTATCTTTAGAGATAGGTGCTGAAATTC-
right  TAGTATTGTGCTCACATTTCTTAAGAATCTATCTTTAGAGATAGGTGCTGAAATTCT
       2**2**2*2*22**222*222***2**22*2222222**2*222222**22*2*22N
```

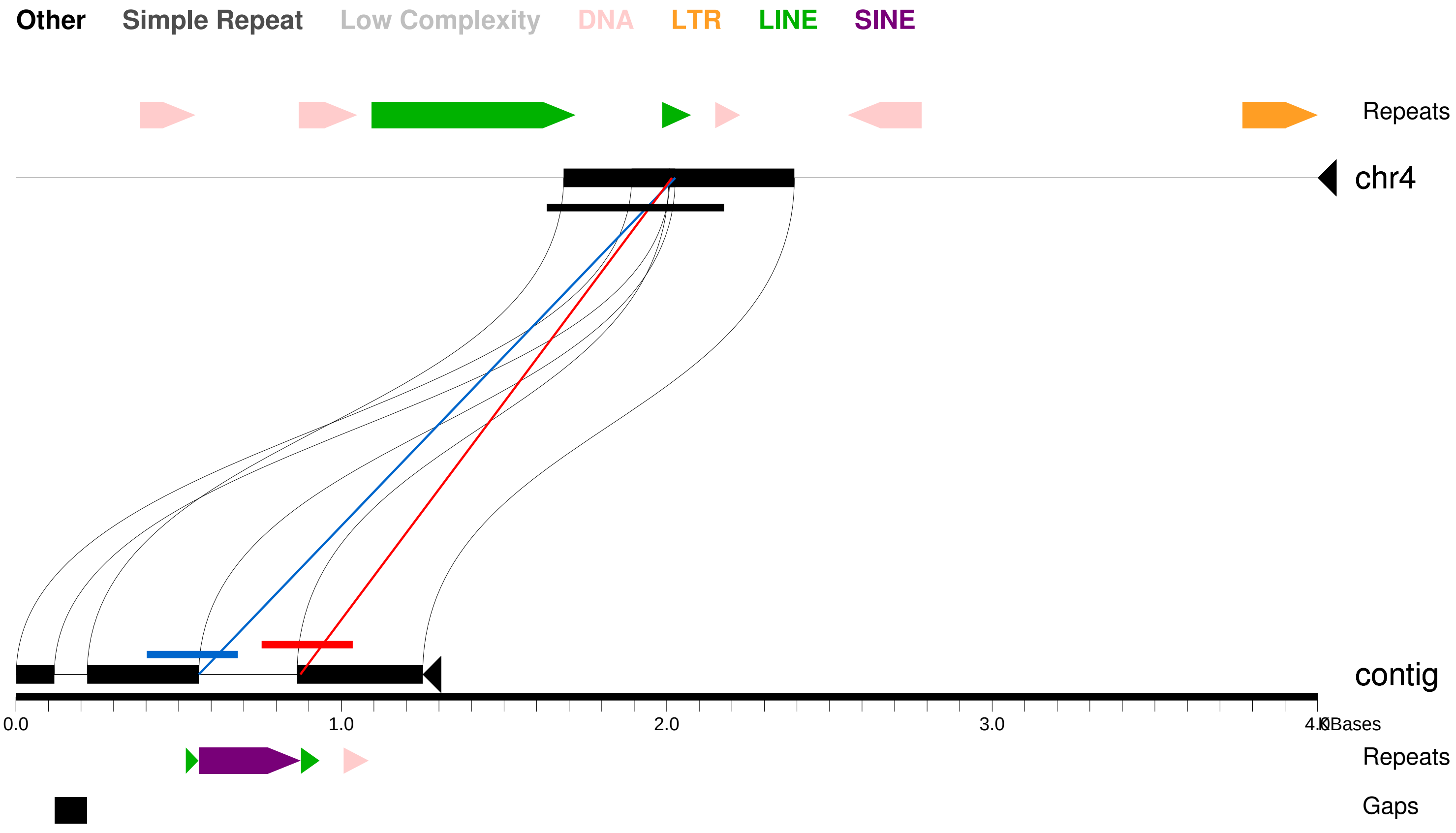

siteID: chr4_55993087 Scafftig2 - Breakpoint: chr4:55993107
chr4:55992947-55993247
Overlap Length: 14 INS size: 419 Genotyped: No

```
left   ----------------------------------------------------------------------
chrom  TGTATTCACGCTTTTACTTTTCCAAGTTGACAGGTGAAATATTTTAA-TGCTGAAATATAATGAATGTAT
right  AATTTT-----TTGTATTTTT---AGTAGAGACGGGG----TTTCACCTTGTTAGCCAGGATGG-TCTCG
       NN2N22     22N22N2222   222N22N2N2N2N    222N2N12NN2N2NNN2NN222N 2N2NN

left   ------------------------------------------------GACTTTTCTTTTGGCAGAGGCA
chrom  ATGGCAAAAAAAAATTTTGGAAAAACACAAAAAGGCTTAGAGTGAAAAGACTTTTCTTTTGGCAGAGGCA
right  ATCTCCTGACCTCAT----GATCCACCCGCCTCGGCCTCCC---AAAGTGCTGGGATT-----ACAGGC-
       22NN2NNN2NNNN22    22NNN22N2NNNNN222N2NNN   222N11**1111**11111*1****1

left   TTTTTATGTTTCCTTTTTTTTCCAAGCAGATTCTTTNNNNNNNNNNNNNNNNNNNNNNNNNNNNNNNNNN
chrom  TTTTTATGTTTCCTTTTTTTTCCAAGCAGATTCTT--------AAACACAAGTATGTAGACATGTCACCC
right  -------GTGAGC--------CCAAGCAGATTCTT--------AAACACAAGTATGTAGACATGTCACCC
       1111111**111*11111111**************22222222222222222222222222222222222

left   NNNNNNNNNNNNNNNNNNNNNNNNNNNNNNNNNNNNNNNNNNNNNNNNNNNNNNNNNNNNNNNNNNNTTT
chrom  TTCTCTTCCCACAATTGCTTGGTATAAATGCTATTTGAGCCTTGGCTTTTTTCCCTTTAAATTTGATTTA
right  TTCTCTTCCCACAATTGCTTGGTATAAATGCTATTTGAGCCTTGGCTTTTTTCCCTTTAAATTTGATTTA
       2222222222222222222222222222222222222222222222222222222222222222222**2

left   TTTTTTCCAAGCAAATTTTTTTTTTTTTTTTTTTTTTTT-----------
chrom  TTTATTTGAGACAAGGTCTCACTATGTTGC--------------------
right  TTTATTTGAGACAAGGTCTCACTATGTTGCCCAGGCTGGTCTCCAATGCC
       ***2**22*22***22*2*222*2*2**22NNNNNNNNN11111111111
```

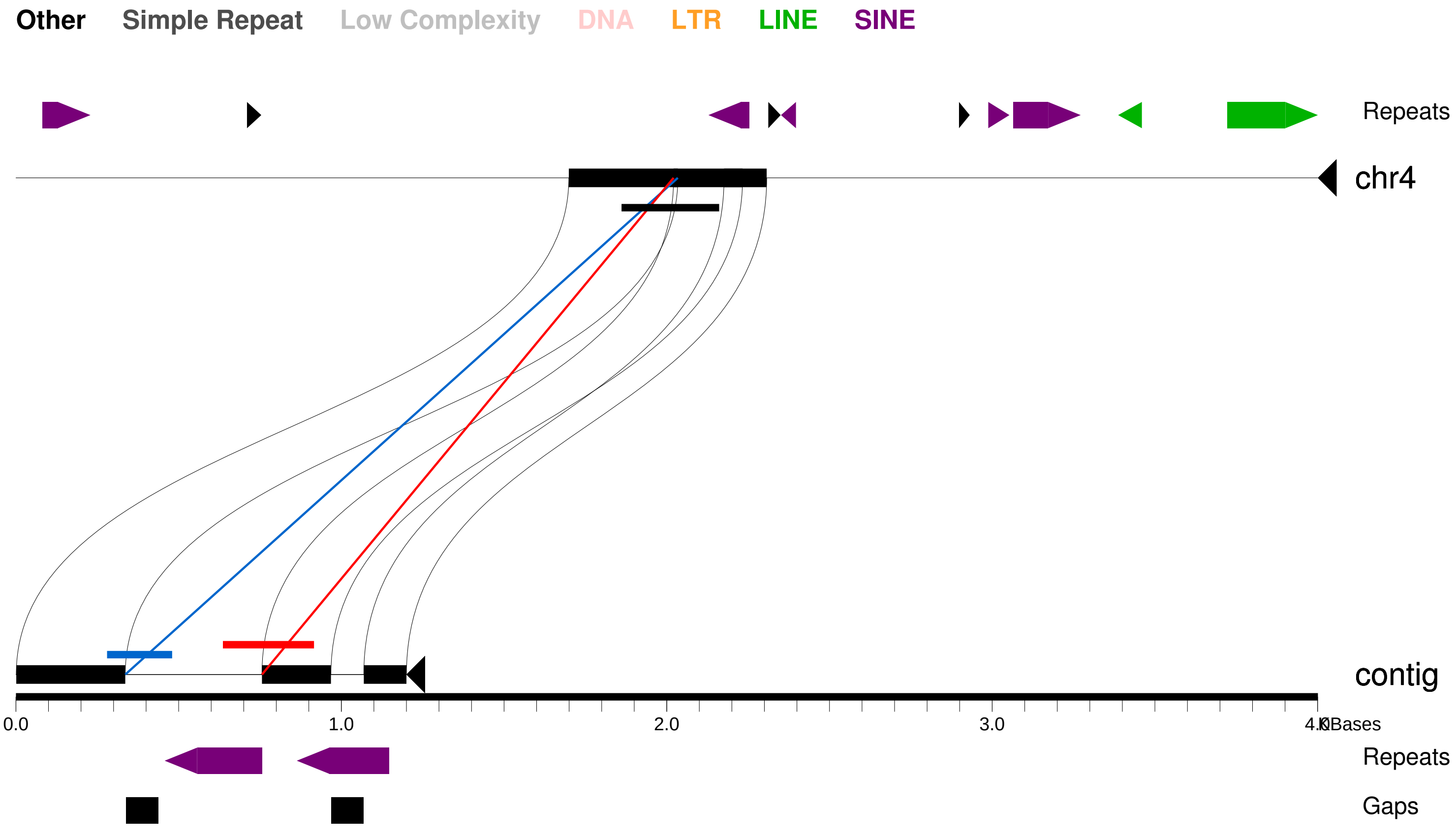

siteID: chr4_56453145 Scafftig1 + Breakpoint: chr4:56453095
chr4:56453090-56453130
Overlap Length: 36 INS size: 471 Genotyped: No

```
left   ----------------------------------------------------------------------
chrom  ----------------------------------------------------------------------
right  NNNNNNNNNNNNNNNNNNNNNNNNNNNNNNNNNNNNNNNNNNNNNNNNNNNNNNNNNNNCTTCATTATTA
       1111111111111111111111111111111111111111111111111111111111111111111111

left   --------AAGCATCTTTTCATGTGTTTATTGGCTATTTGGAGATCTTCTTTTAAGAAACGTCTATTCGG
chrom  --------AAGCATCTTTTCATGTGTTTATTGGCTATTTGGAGATCTTC---------------------
right  GTGATGTTAAGTCTCTTTTCATGTGTTTATTGGCTATGTGGAGATCTTC---------------------
       11111111***11************************1***********222222222222222222222

left   CCGGGCGCGGTGGCTCACGCCTGTAATCCCAGCACTTTGGGAGGCCGAGGCGGGCGG
chrom  ---------------------------------------------------------
right  ---------------------------------------------------------
       222222222222222222222222222222222222222222222222222222222
```

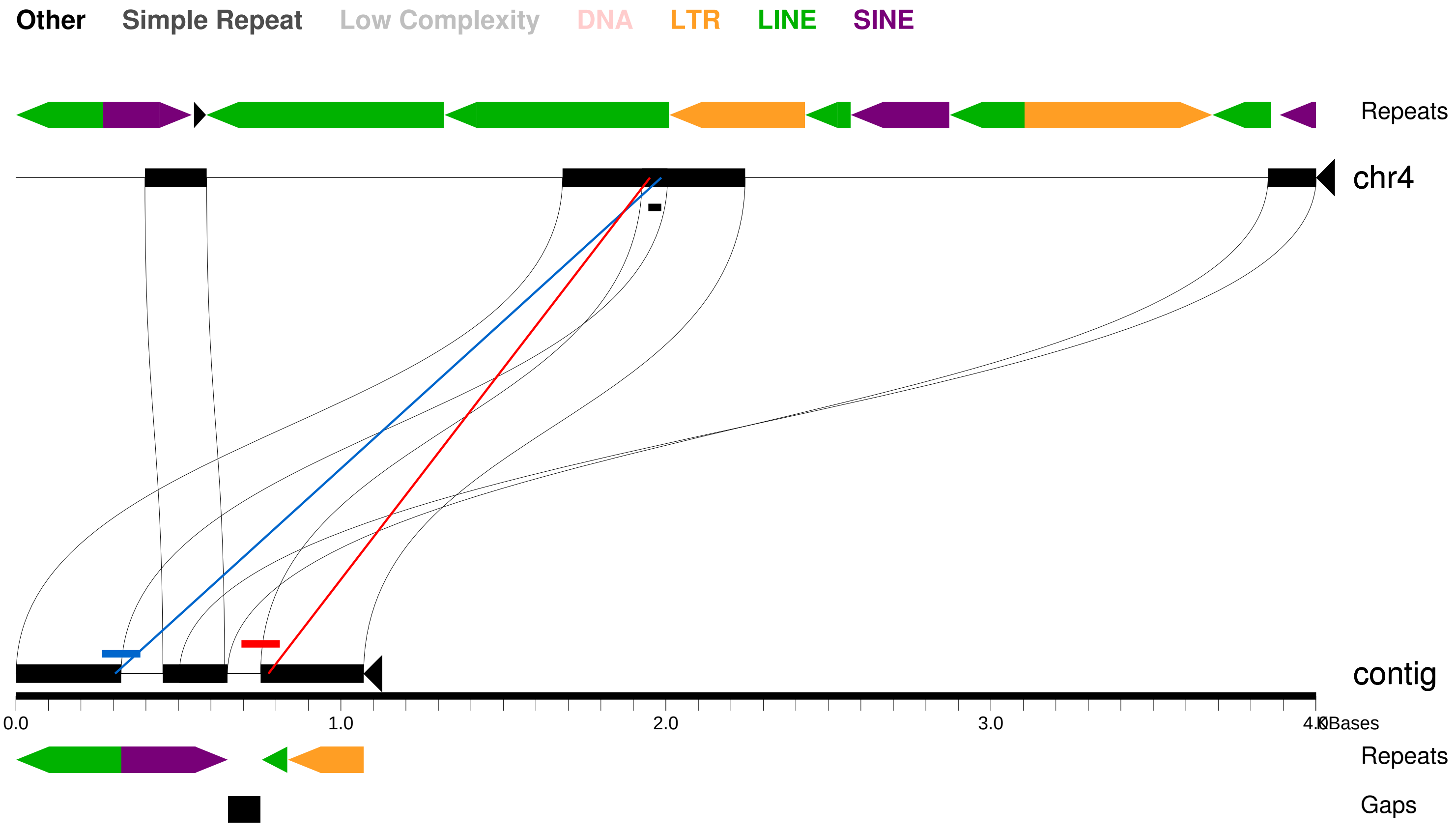

siteID: chr4_57258152 Scafftig2 + Breakpoint: chr4:57258156
chr4:57258137-57258230
Overlap Length: 41 INS size: 410 Genotyped: No

```
left   TGCAAATT-------TGACA------AGAAAC-----------AGGTATAAAGTTCTTAAAAAAAAAAAA
chrom  TGCAAATT-------TGACA------AGAAAC-----------AGGTATAAAGTTCTTAAAAAAAAAAAA
right  C-CAAAGTGCTGGGATTACAGGCGTGAGCCACCGCGCCCGGCCAGGTATAAAGTTCTTAAAAAAAAAAAA
       11****1*1111111*1***111111**11**11111111111*************************

left   A--AGAAAATACAAAGNNNNNNNNNNNNNNNNNNNNNNNNNNNNNNNNNNNNNNNNNNNNNNNNNNNNN
chrom  A--AGAAAATACAAAGTAACAATCTCAGCATAATAGTTTTCAGCCGTGAA--------------------
right  AAAAGAAAATACAAAGTAACAATCTCAGCATAATAGTTTTCAGCCGTGAA--------------------
       *11*************2222222222222222222222222222222222222222222222222222222

left   NNNNN
chrom  -----
right  -----
       22222
```

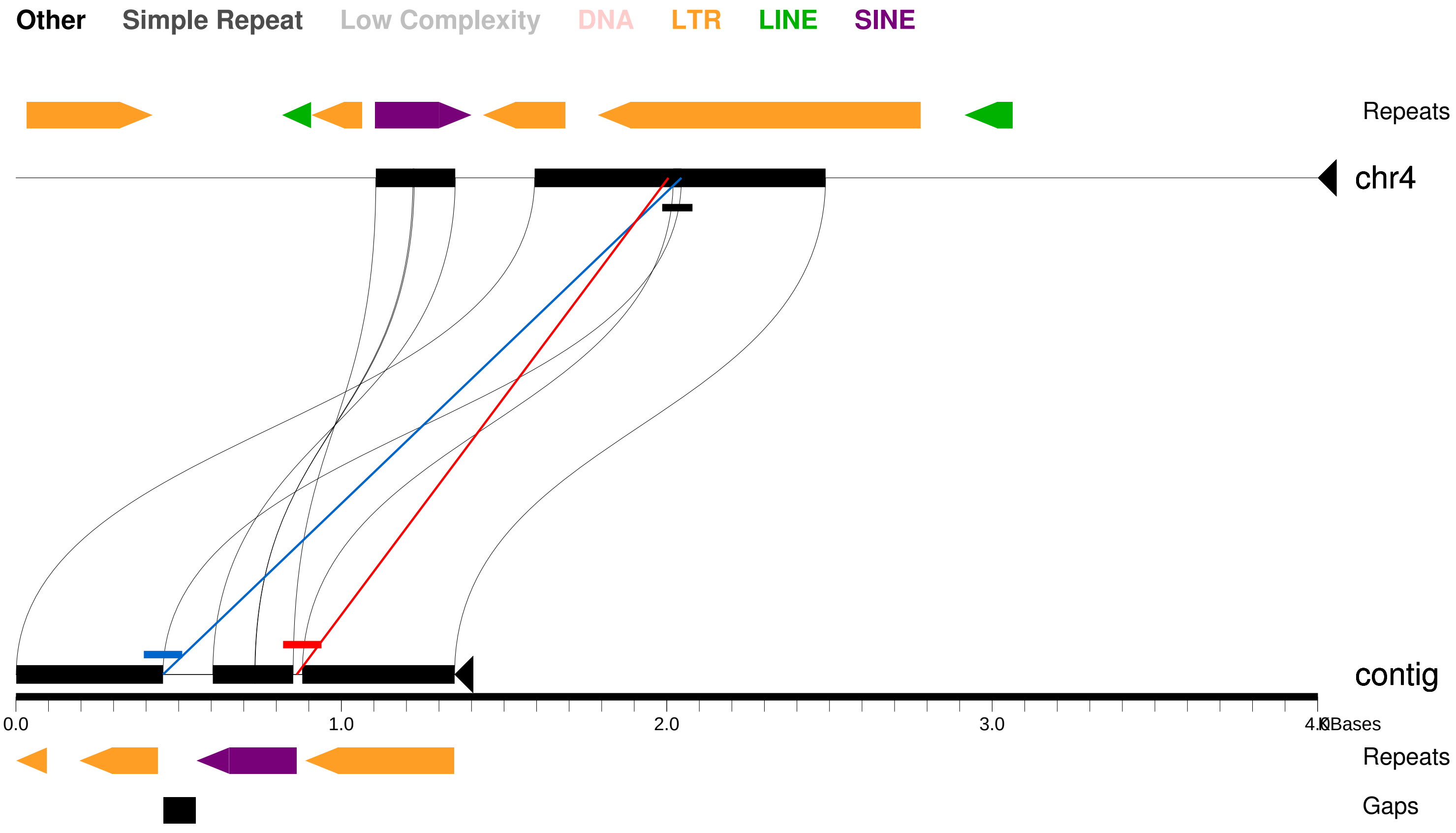

siteID: chr4_58976421 Scafftig5 - Breakpoint: chr4:58976320
chr4:58976160-58976490
Overlap Length: 11 INS size: 99 Genotyped: Yes

```
left   ---------TCAGATATATCCACACAAATATAATTTTAGTTTATATTTCTGTTACTGCAGTTCTCATCTT
chrom  CAATGGCAATCAGATATATCCACACAAATATAATTTTAGTTTATATTTTTGTTACTGCAGTTCTCATCTT
right  CAAATACT-TCATATGTATC--------TTTTTTTTTTTTTTTTTTTTTTTTT--------TTTTTTTTT
       222NNN2N ***1**1****11111111*1*11****11***1*1***2*1**11111111*1*11*1**

left   ATTCTGAGGATACGTGACAAAATAATTCTTTCAAAAATATTG--ATGCTGTGCTAGATTACTATTTTGAA
chrom  ATTCTGAGGATACGTGACAAAATAATTCTTTCAAAAATATTG--ATGCTGTGCCAGATTACTATTTTGAA
right  TTTTTGAG---ACGG---AGTCTCGCTCTGTCGCCCAGGCTGGAGTGCAGTGGCGGGAT-CTCGGCTCAC
       1**1****111***1111*111*111***1**1111*111**111***1***121*11*1**1111*1*1

left   TGAATTACTAGACAAATACTTCATATGTATCTTTTTTTTTTTTTT-----TTTTTTTTTTTTTTTTTTT
chrom  TGAATTATTAGACAAATACTTCATATGTATCTTATTATGTGGGTTTACACATTATTTATCTTATTGATTT
right  TGCA------------------ATATGTATCTTATTATGTGGGTTTACACATTATTTATCTTATTGATTT
       **1*111N11111111111111***********2**2*2*222***22222**2***2*2**2**22***

left   TTTTTGAGACGGA-------GTCTCGCTCTGTCGCCCAGGCTGG---AGTG-------------------
chrom  AACTTCAAAACTAAACTTTAGTTTAGCTCTTGGGCCCTATCTGGGAAAGGGTCATCTTTTAATCACCATT
right  AACTTCAAAACTAAACTTTAGTTTAGCTCTTGGGCCCTATCTGG-AAAGGGTCATCTTTTAATCACCATT
       222**2*2*222*2222222**2*2*****222****222**** 22**2*2222222222222222222

left   ----CAGTGGCGGGATC--TCGGCTCACTGCAATATGTATCTTATTATG-TGG
chrom  AAATCACTGAAGTCATCAGTTTATTCAAAGTACTCTGCACAAAATTAGCATTC
right  AAATCACTGAAGTCATCAGTTTATTCAAAGTACTCTGCACAAAT---------
       2222**2**22*22***22*2222***22*2*2*2**2*22221111NN 1NN
```

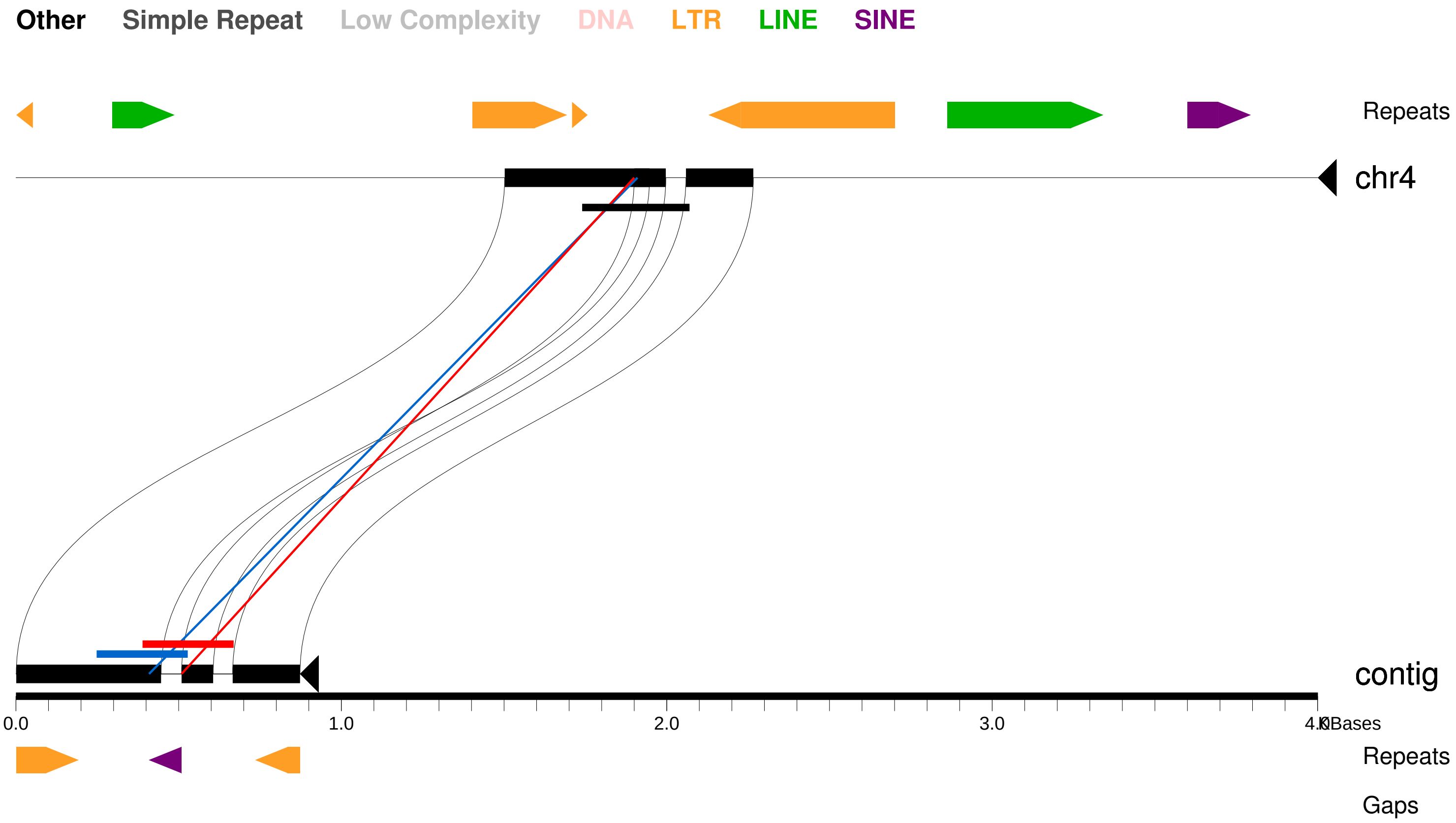

siteID: chr4_61056013 Scafftig7 + Breakpoint: chr4:61055987
chr4:61055940-61056046
Overlap Length: 13 INS size: 295 Genotyped: Yes

```
left    TGTCAAAAAACTGATGTGACAGACTCCT----TTCCAGTCATT-----CTGTGA---GGCCAGTATCATC
chrom   TGTCAAAAAACTGATGTGACAGACTCCT----TTCCAGTCATT-----CTGTGA---GGCCAGTATCATC
right   TCCGCCCGCCTCGGCCTCCCAAAGTGCTGGGATTACAGGCGTGAGCCACCGCGCCCGGCCCAGTATCATC
        *11111111111*111*11**1*1*1**1111**1***1*1*111111*1*1*1111*1***********

left    TTTTTTTTTTTTTTTTGAGACGGAGTCTCGCTCTGTCGCCCAGGCTGGAGTGCAGTGGCGG
chrom   TTGATACCAAAACTTGGAA---GAGATAGAAAGAAAACTCCACGCCAA----CATT-----
right   TTGATACCAAAACTTGGAA---GAGATAGAAAGAAAACTCCACGCCAA----CATT-----
        **22*22222222**2**2222***22222222222222***2**2222222**2*22222
```

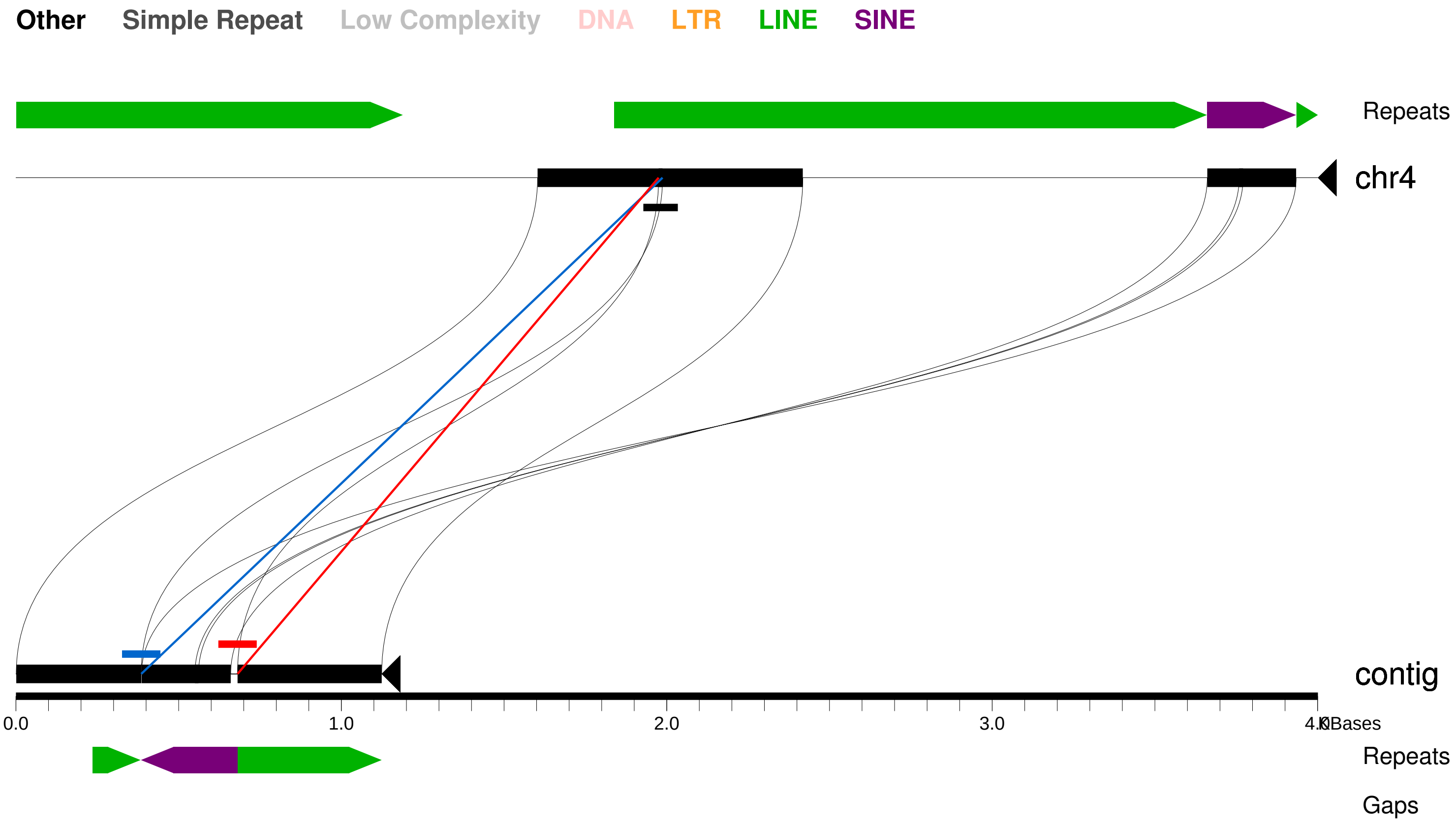

siteID: chr4_61825116 Scafftig10 - Breakpoint: chr4:61825096
chr4:61825050-61825142
Overlap Length: 14 INS size: 314 Genotyped: Yes

```
left    CAGTGGT--GCAATG----------GACAGCACG----TCTGACT----------ATGGAACAGAAGATT
chrom   CAGTGGT--GCAATG----------GACAGCACG----TCTGACT----------ATGGAACAGAAGATT
right   CTGCAGTCCGCAGTCCGGCCTGGGCGACAGAGCGAGACTCCGTCTCAAAAAAAAAAAAAAAAAAAAAAAA
        *1*11**11***1*11111111111*****11**1111**1*1**1111111111*111**1*1**1*11

left    ATAAAATTTATATATCGGCCGGGCGCGGTGGCTCACGCCTGTAATCCCAGCACT-TTGGGAGGCCGAGGC
chrom   ATAAAATTTATATATCTACCCA--------TATCA------TATTATTAGTAATATTGTTATA-------
right   AAAAAATTTATATATCTACCCA--------TATCA------TATTATTAGTAATATTGTTATA-------
        *1*************22**222222222222***222222**2*222**2*2*2***22*222222222

left    GGGTGG
chrom   ------
right   ------
        222222
```

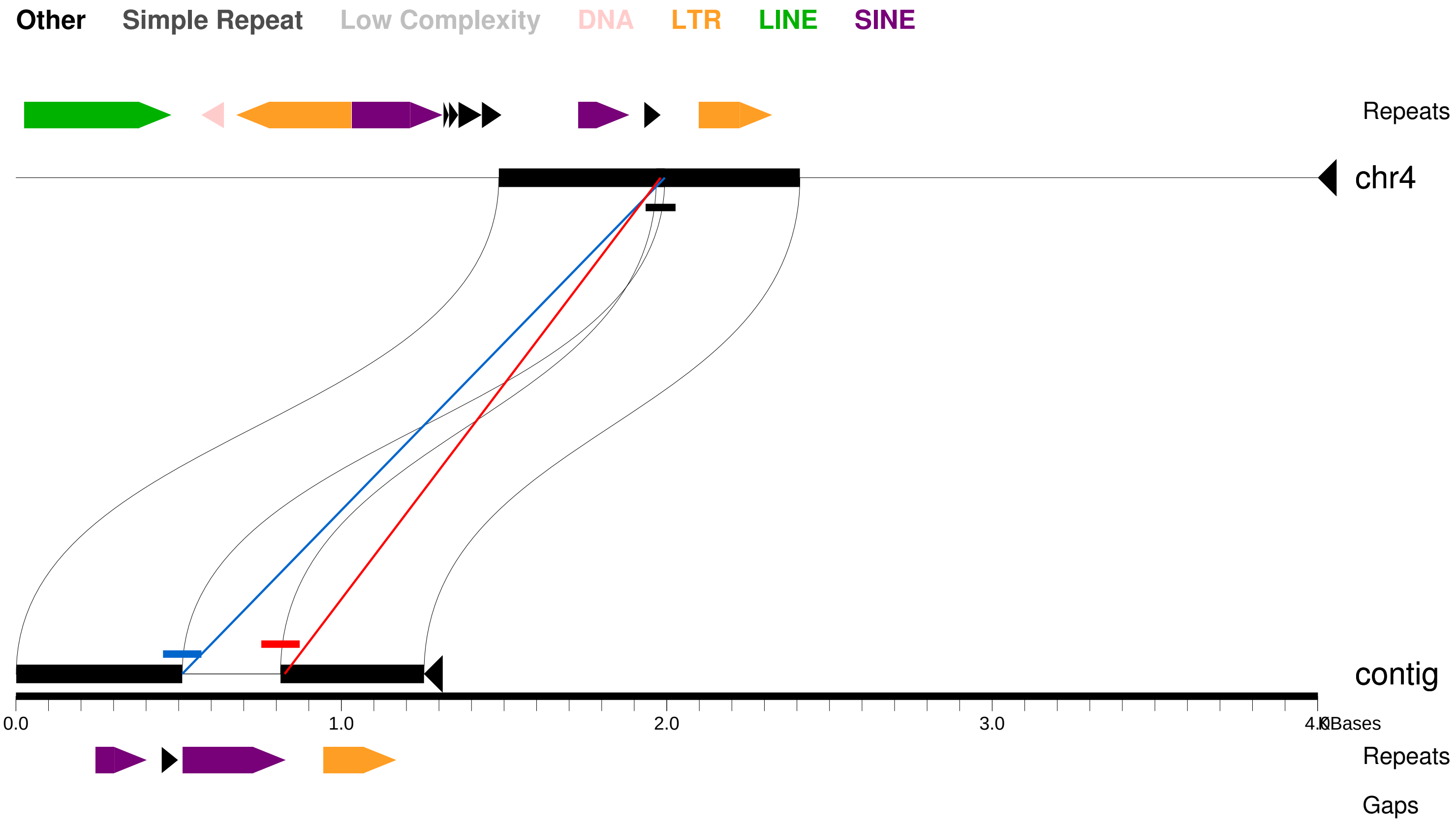

siteID: chr4_64563183 Scafftig1 - Breakpoint: chr4:64563171
chr4:64563127-64563214
Overlap Length: 16 INS size: 292 Genotyped: Yes

```
left    -------TGA----AGA-----------CAATTCA---------ACAATGTAGGAGGTTGTCACAGTAAG
chrom   -------TGA----AGA-----------CAATTCA---------ACAATGTAGGAGGTTGTCACAGTAAG
right   CTTGCAGTGAGCCGAGATCGCGCCACTGCACTCCAGCCTGGGCGACAGAGCGAGACTCCGTCTCAAAAAA
        1111111***1111***11111111111**1*1**111111111***11*111**1111***1**11**1

left    AGATTAAGAAAAAACTGTGTGGGCCGGGCGCGGTGGCTCACGCCTGTAATCCCAGCACTTTGGGAGGCCG
chrom   AGATTAAGAAAAAACTGTGTGAG------------------------------AGAACTT-GACATTTAG
right   AAAAAAAGAAAAAACTGTGTGAG------------------------------AGAACTT-GACATTTAG
        *1*11***************2*222222222222222222222222222222**2****2*22*2222*

left    AGGCGGGCGG
chrom   ATACTGTTTT
right   ATACTGTTTT
        *22*2*2222
```

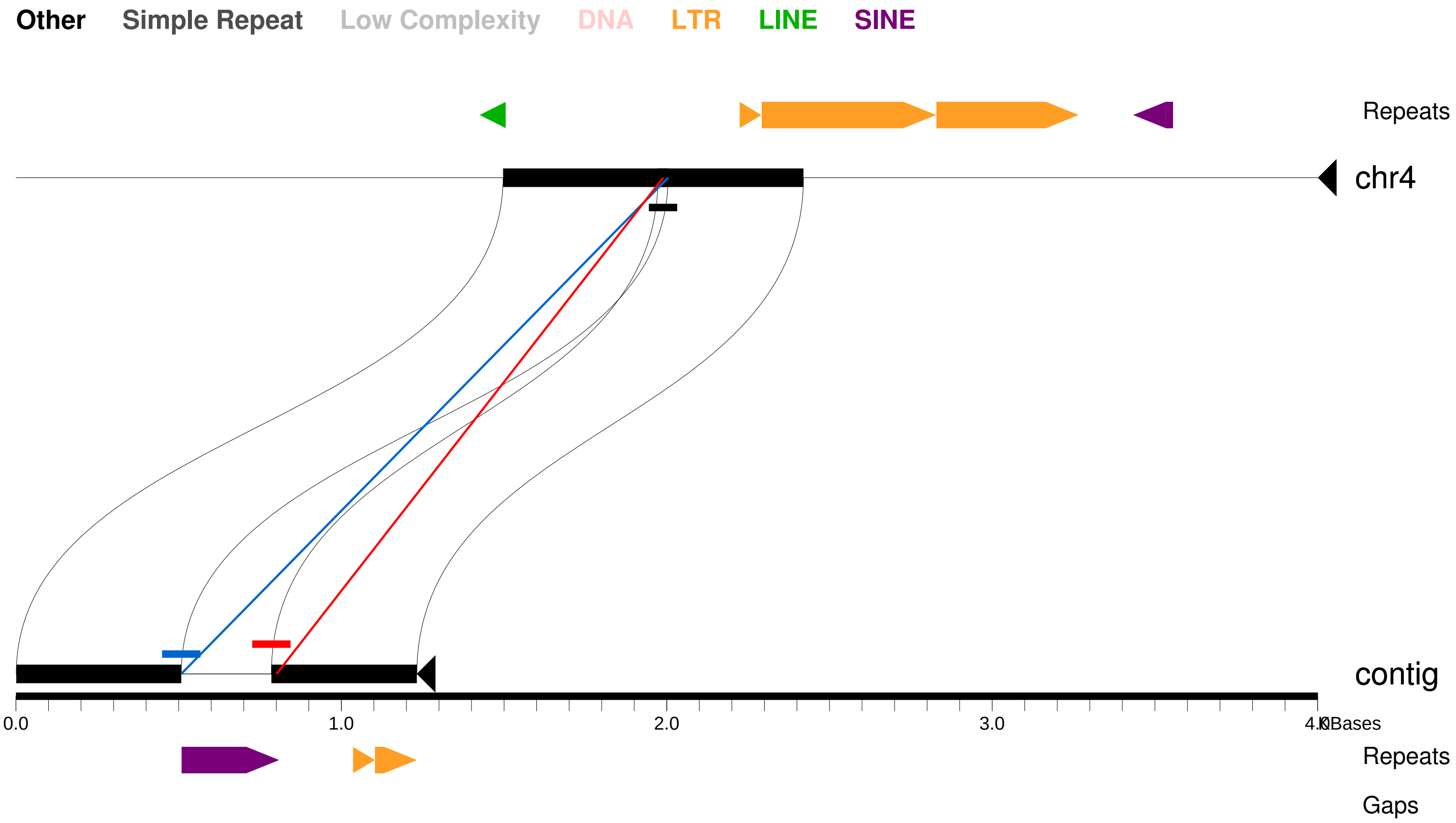

siteID: chr4_65518356 Scafftig2 - Breakpoint: chr4:65518361
chr4:65518319-65518420
Overlap Length: 5 INS size: 301 Genotyped: Yes

```
left    CTG-----------TTTGAAAACT--TTCTTTTTCAAGTCT---CCACTCCTTCCTTT-TTGTTTTTTTT
chrom   CTG-----------TTTGAAAACT--TTCTTTTTCAAGTCT---CCACTCCTTCCTTT-TTGTTGTTGTT
right   CCGCCCGCCTCGGCCTCCCAAAGTGCTGGGATTACAGGCGTGAGCCACCGCGCCCGGCCTTGTTGTTGTT
        *1*111111111111*111***1*11*1111**1**1*11*111****11*11**1111*****2**2**

left    TTTTTTTTTTTTTTGAGACGGAGTCTCGCTCTGTCGCCCAGGCTGGAGTGCAGTGGCGGATCTCG
chrom   AGTTTTTA-----AAATATTGTATC-CACAATCAAATCCA------ACTCCTGTA-----AAAATT
right   AGTTTTTA-----AAATATTGTATC-CACAATCAAATCCA------ACTCCTGTA-----AAAATT
        22*****22222222*2*22*22**2*2*22*22222***222222*2*2*2**222222*22222
```

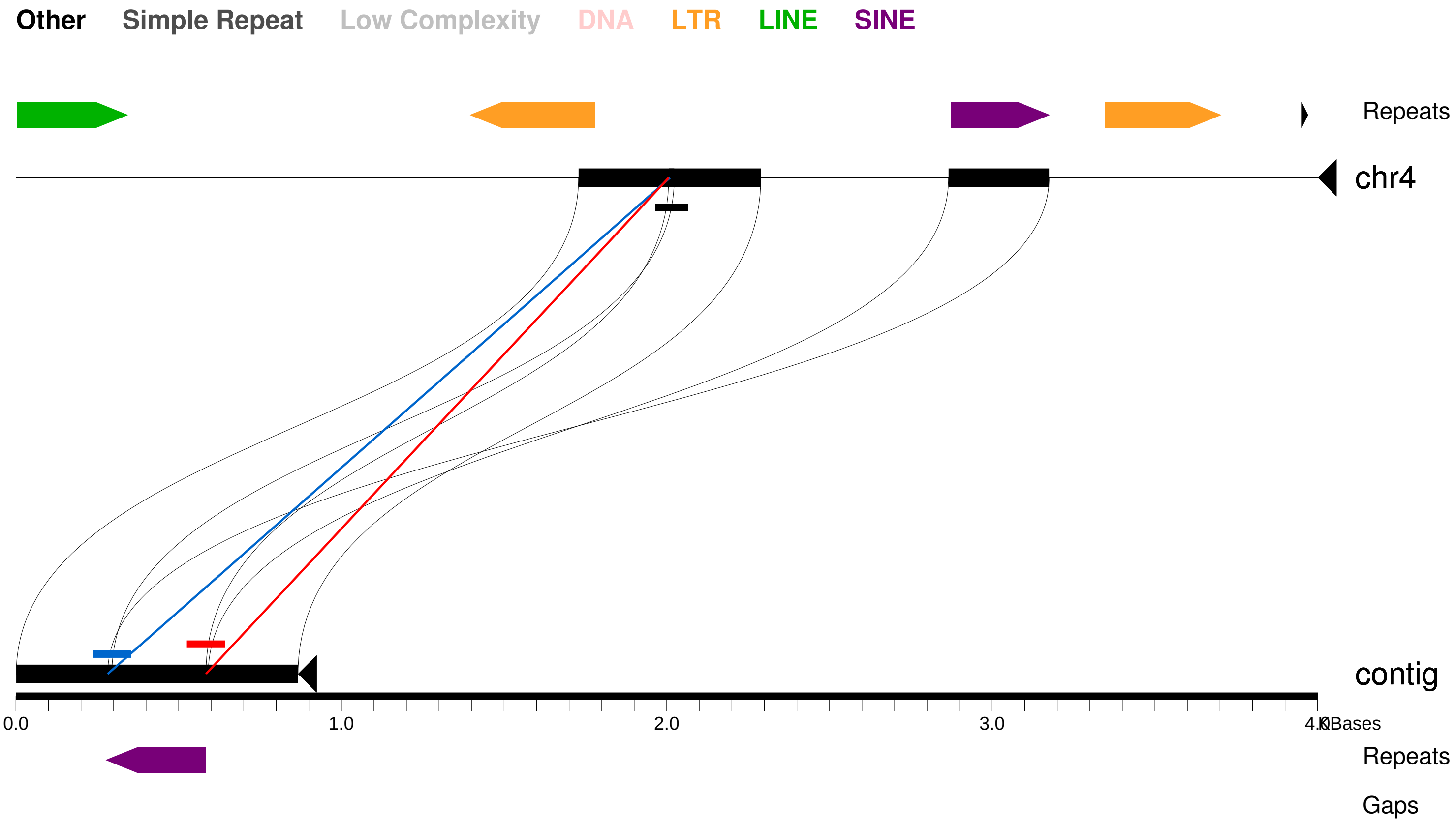

siteID: chr4_67106625 Scafftig14 - Breakpoint: chr4:67106599
chr4:67106557-67106701
Overlap Length: 18 INS size: 300 Genotyped: Yes

```
left    GATACGTCTTTGTAGGAAAAGACACTTGCAGAATTTCAAGTTAGAAAGATTAAGATTTGCGGCCGGGCGC
chrom   GATACGTCTTTGTAGGAAAAGACACTTGCAGAATTTCAAGTTAGAAAGATTAAGATTTGCTCAGGTGTCC
right   AAAA---------AAAAAAAAAAA------------------AGAAAGATTAAGATTTGCTCAGGTGTCC
        1*1*111111111*11****1*1*111111111111111111******************2222*2*22*

left    GGTGGCTCACGCCTGTAA----------------TCCC---------AGCACTTTGGGAGGCCGAGG--C
chrom   TTTATGTGTGGGCTGAAAAAAAAAAA-GTTATTATCCTGAAAAGAATAACACGGTATGAGGGAGTTCACC
right   TTTATGTGTGGGCTGAAAAAAAAAAAAGTTATTATCCTGAAAAGAATAACACGGTATGAGGGAGTTCACC
        22*222*222*2***2**2222222212222222***2222222222*2***22*22****22*22222*

left    GGGCGG
chrom   TTACAT
right   TTACAT
        222*22
```

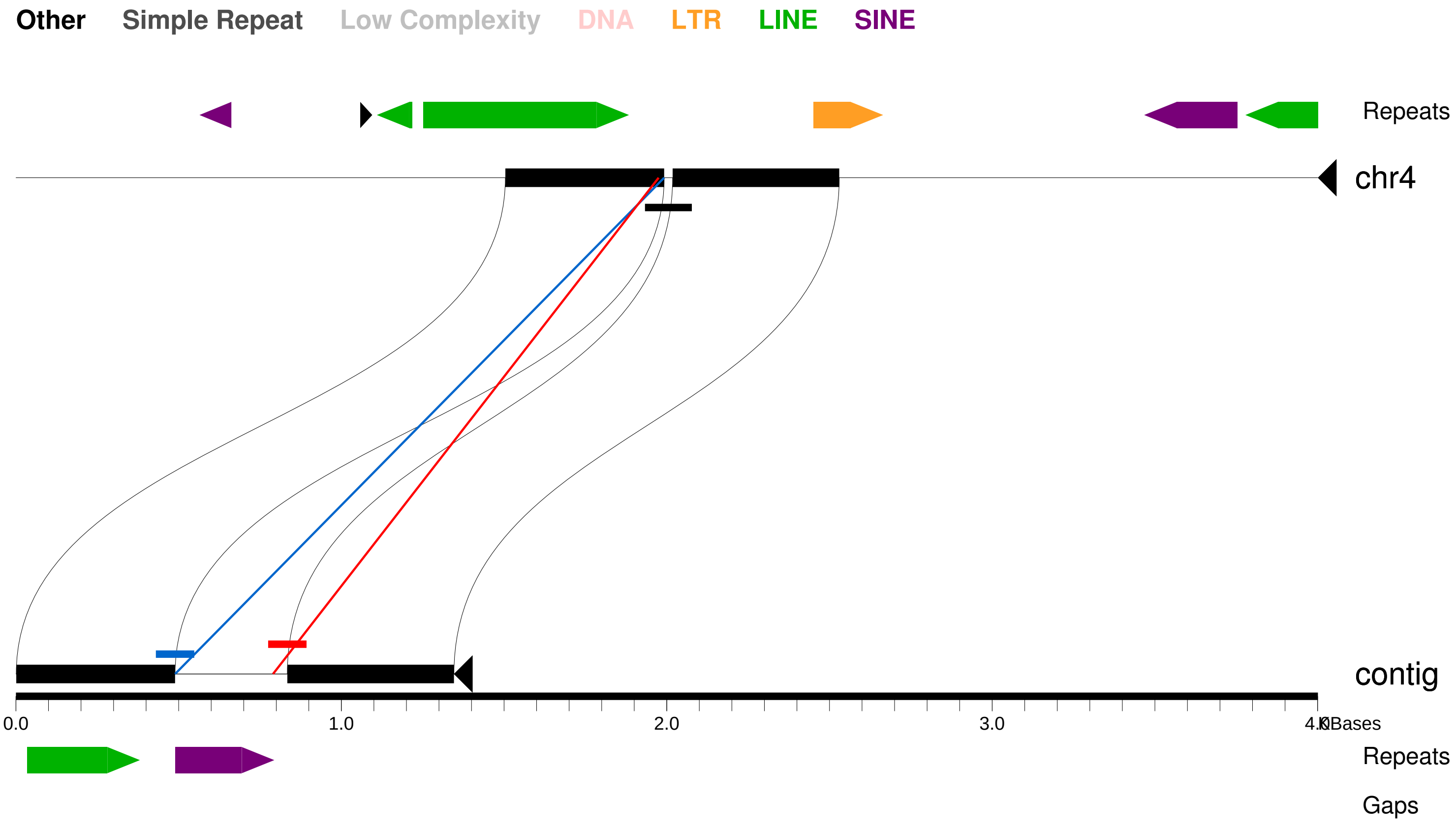

siteID: chr4_67754004 Scafftig2 - Breakpoint: chr4:67753999
chr4:67753839-67754170
Overlap Length: 12 INS size: 216 Genotyped: No

```
left   ---------ATGCTGTTTCCTACTTCATTTGATGATGCTTAGCATTTGGTTTTCAGTTGAGATTCCTTCT
chrom  GGCCCCAAAATGCTGTTTCCTACTTCATTTGATGATGCTTAGCATTTGGTTTTCAGTTGAGATTCCTTCT
right  GCTCACTGCAAGCT-----CCGCTTCCCGGGTTCACGC----CATTCTCCTGCC---TCAGCCTCCCGAG
       2NN2N2NNN*1***11111*11****1111*1*1*1**1111****1111*11*111*1**11***1111

left   AAGTTCATACCTTCAGCATCAAAACTAATACTTTTATTCCAGCAAGTAGACAGATAAATATGTTCACCTA
chrom  AAGTTCATACCTTCAGCATCAAAACTAATACTTTTATTCCAGCAAGTAGACAGATAAATATGTTCACCTA
right  TAGCTGGGAC-TACAGGCGC------------------CCGCCACCGCGCCCGGCTAATTTTTTG---TA
       1**1*111**1*1***111*111111111111111111**11**1111*1*1*111***1*1**1111**

left   AGTATAGCTGGCTCTATATAAATAGACTCTTTTTTTTTTT-------------------------TTTTT
chrom  AGTATAGCTGGCTCTATATAAATAGACTCTTTATATATATATGACCCTATAACCTAAACACCCTCTTAGT
right  TTTTTAGTAGAGACGA----AATAGACTCTTTATATATATATGACCCTATAACCTAAACACCCTCTTAGT
       11*1***11*111*1*1111************2*2*2*2*2222222222222222222222222**22*

left   TTTTTTTTTTTTTTTTTTTTTTGA------------GACGGAGTCTCGCTCTGTCGCCCAGGTCGGACTG
chrom  ATTAATAGTTATCTTTCTTTGTGATTTCTCCTCTTTGAGGATTTCTTGCTTTG-CTTTTTGGTGCCCCAG
right  ATTAATAGTTATCTTTCTTTGTGATTTCTCNNNNNNNNNNNNNNNNNNNNNNN-NNNNNNNNNNNNNNNN
       2**22*22**2*2***2***2***222222NNNNNN11N1NNN111N111N1121NNNNN111NNNN1N1

left   CGGACTGCAGTGGCGCAATCTCGGC--------TCACTGCAAGCTCCGCTTCCCG
chrom  AAGACTCCCTTGGGATTTTCTCTCAGTAAATAATTATGTCAAAATTCTCTGAC--
right  NNNNNNNNNNNNNNNNNNNNNNNNNNNNNNNNNNNNNNNN---------------
       NN1111N1NN111NNNNN1111NNNNNNNNNNN1N1NNN111NN1N1N11NN122
```

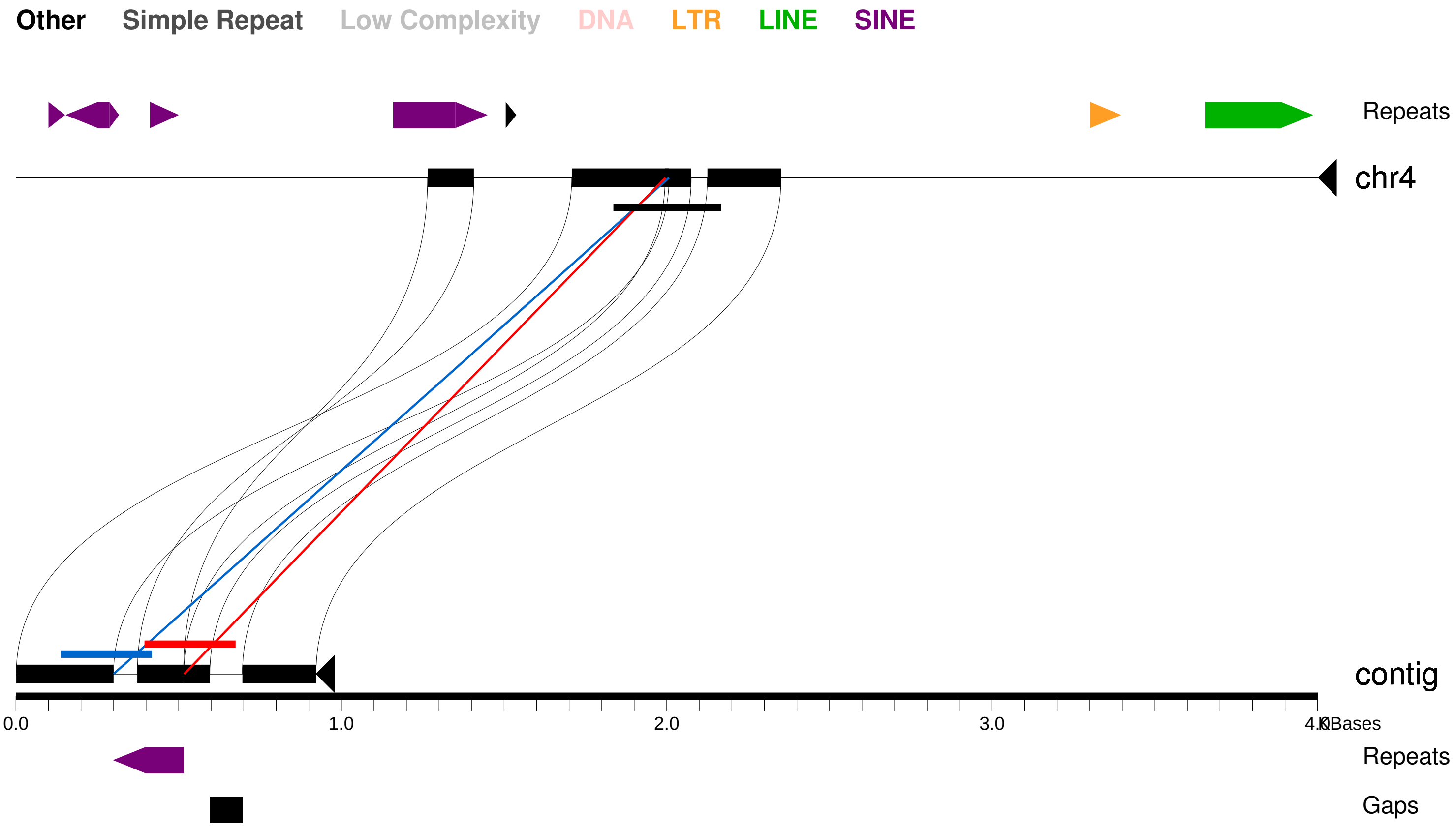

siteID: chr4_67963825 Scafftig4 - Breakpoint: chr4:67963880
chr4:67963720-67964052
Overlap Length: 13 INS size: 313 Genotyped: Yes

```
left    ---------CAGAGCCCTCAGAAATAATGCCGCATATCTACAACTATCTGATCTTCGACAAACCTGACAA
chrom   GGAACAGAACAGAGCCCTCAGAAATAATGCCGCATATCTACAACTATCTGATCTTCGACAAACCTGAGAA
right   CG-----------GCT-----AATTTTTT--GTATTTTTAGTAGAGACGGGGTTTC-----ACC-GTGTT
        N2       1111**111111**1*11*111*1**1*1**11*1111*1*111***11111***1*1211

left    AAACAAGCAATGGGGAAAGGATTCCCTATTTCAT-AAATGGTGCTGGGAAAACTGGCTAGCCATATGTAG
chrom   AAACAAGCAATGGGGAAAGGATTCCCTATTTCAT-AAATGGTGCTGGGAAAACTGGCTAGCCATATGTAG
right   AGCCAGG--ATGG---------TCTCGATCTCCTGACCTCGTGATCCGC---CCGCCTCGGCCTCCCAA-
        *11**1*11****111111111**1*1**1**1*1*11*1***1*11*1111*1*1**1*1*1*1111*1

left    AAAGCTGAAACTGGATCCCTTCCTTACAACTTTTTTTTTTTTTTTTTTTTTTTTTTTTTTTTTTTTTTTT
chrom   AAAGCTGAAACTGGATCCCTTCCTTACAACTTTTACAAAAATTAATTCGAGATGGATTAAAGACTTACAT
right   AGTGCTGGGATTACAGGCGT-CCTTACAACTTTTACAAAAATTAATTCGAGATGGATTAAAGACTTACAT
        *11****11*1*11*11*1*1*************2222222**22**22222*222**222222**222*

left    TTTTTTTTTTTTG--AGACGGAGTCTCGCTGTCGCCC-AGGC-------TGGAGTGCAGTGGC----GCA
chrom   GTTAGACCTAAAACCATAAAAACCCTAGAAGAAAACCTAGGCAATACCATTCAGGTCATAGGCATGGGCA
right   GTTAGACCTAAAACCATAAAAACCCTAGAAGAAAACCTAGGCAATACCATTCAGGTCATAGGCATGGGCA
        2**22222*222222*2*222*22**2*22*2222**2****2222222*22**22**22***2222***

left    A-----TCTCGGCTCA-----CTGCTGCA--GGCTCC-----------------
chrom   AGGACTTCATGTCTAAAACACCAAAAGCAATGGCAACAAAAGACAAAATTGACA
right   AGGACTTCATGTCTAAAACACCAAAAGCAATGGCAACAAAA-------------
        *22222**22*2**2*22222*2222***22***22*2222
```

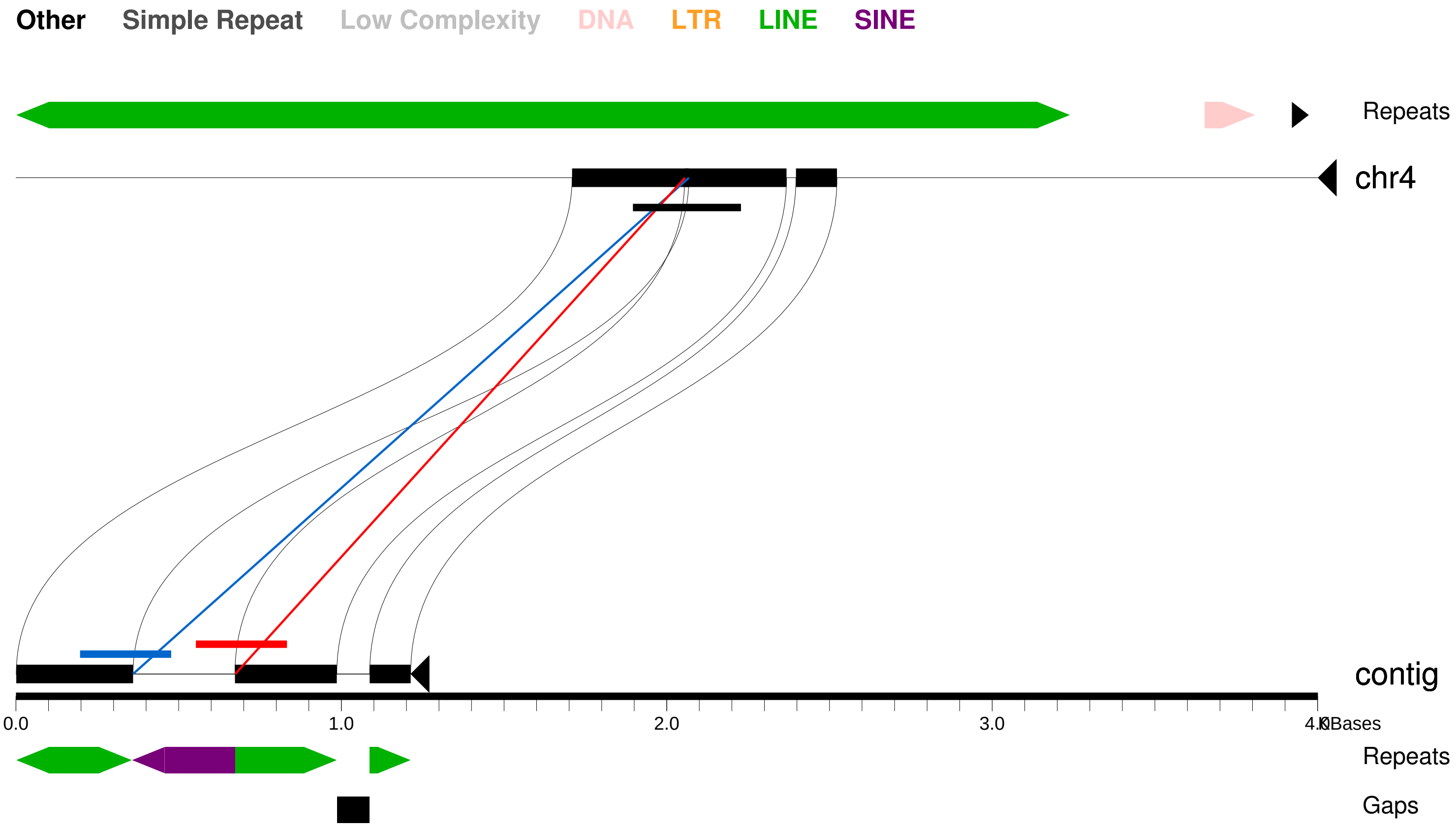

siteID: chr4_68944782 Scafftig2 + Breakpoint: chr4:68944770
chr4:68944720-68944829
Overlap Length: 10 INS size: 501 Genotyped: No

```
left   AAAATGTGATATTAACAAACCATAGTCCCCCAATGCATCAACCTCTATTT---------AAAAAATGGCG
chrom  AAAATGTGATATTAACAAACCATAGTCCCCCAATGCATCAACCTCTATTT---------AAAAAATGGCA
right  NNNNNNNNNNNNNNNNNNNNNNNNNNNNNNNNNNNNNNNNNNNNNNNNNNNNNNNNNAAAAAAAATGGCA
       11111111111111111111111111111111111111111111111111111111111**********2

left   GGC-CGGGCGCGGTGGCTCACGCCTGTAATCCCAGCACTTTGGGAGGCCGAGGCGGGTG
chrom  TATTCTGGTATAGTACAATACTGGTGTGA--CCTTTACTAAAAGCTTACAA--------
right  TATTCTGGTATAGTGCAATACTGGTGTGA--CCTTTACTAAAAGCTTACAA--------
       2222*2**2222**N2222**222***2*22**222***2222*2222*2*22222222
```

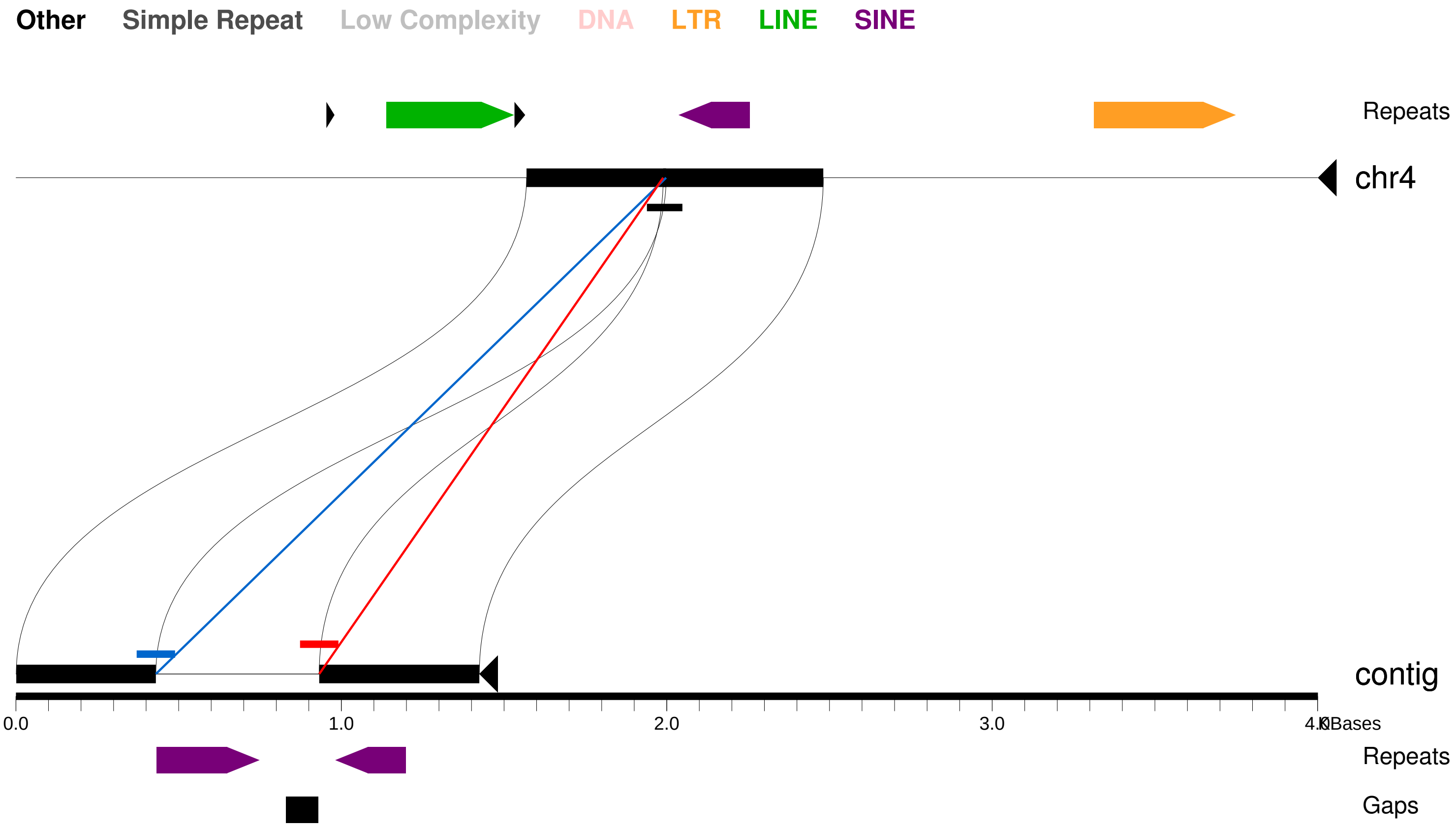

siteID: chr4_70096920 Scafftig1 + Breakpoint: chr4:70096928
chr4:70096768-70097101
Overlap Length: 14 INS size: 305 Genotyped: Yes

```
left   --------------TTTCTTAGTGATGGTACTGAATTCA-ATAGAATTTTAAATGTGCATCAATTCTG--
chrom  AGATATCATGACTATTTCTTAGTGATGGTACTGAATTCA-ATAGAATTTTAAATGTGCATCAATTCTG--
right  CGTGAACCCGGGAAGCG----GAGCTTGCAGTGAGCCGAGATTGCGCCACTGCAGTCCG-CAGTTCAGCC
       N2NN2N2NN2NNN21111111*1*1*1*1*1***1111*1**1*1111111111**1*11**1***1*11

left   TGAGCTGACATAGAG-GAATCATGATTAAGTAAATCAAAATAGTATTAATGCTCAATGATATGTGGTAGA
chrom  TGAGCTGACATAGAG-GAATCATGATTAAGTAAATCAAAATAGTATTAATGCTCAATGATATGTGGTAGA
right  TGGG-TGACAGGGCGAGACTCCGTCTCAAAAAAAAAAAAAAAAAAAAAA-----AA-----------AAA
       **1*1*****11*1*1**1**1111*1**11***11****1*11*11**11111**11111111111*1*

left   GAGAAACATTTAGTTTTAAGTTAGAAATCAGGCATTCA----CTGTAATCCCAGCACTTTGGGAGGCCGA
chrom  GAGAAACATTTAGTTTTAAGTTAGAAATCAGGCATTCAAAAACAGGAATCACAGCAATAACAAACGAACA
right  AAAAAAAA---------AA---AAAAATCAGGCATTCAAAAACAGGAATCACAGCAATAACAAACGAACA
       1*1***1*111111111**111*1*************2222*2*2****2*****2*22222*2*222*

left   GGCGGGTGGATCATG------AGGT---CAGGAGATCGA----------GACCATCCTGGCTAACAAGGT
chrom  TAACTCTGAACTATGCACACCAGATAAACATGAAATTTATTTTGCAAATGTTTTTGCAGTTTAGTCCTTT
right  TAACTCTGAACTATGCACACCAGATAAACATGAAATTTATTTTGCAAATGTTTTTGCAGTTTAGTCCTTT
       222222**2*22***222222**2*222**2**2**22*2222222222*2222*2*2*22**222222*

left   GAAACCCCGTCTCTACTA--AAAATA------CAAAA--------AATTAGCCGGGCG
chrom  GCATCAGCTTATATTGTGGGAAAGTAAGGGATCAAGAGCAGCTGAAATGATATAGCAT
right  GCATCAGCTTATATTGTGGGAAAGTAAGGGATCAA-----------------------
       *2*2*22*2*2*2*22*222***2**222222***N1        111N1NNNN1NNN
```

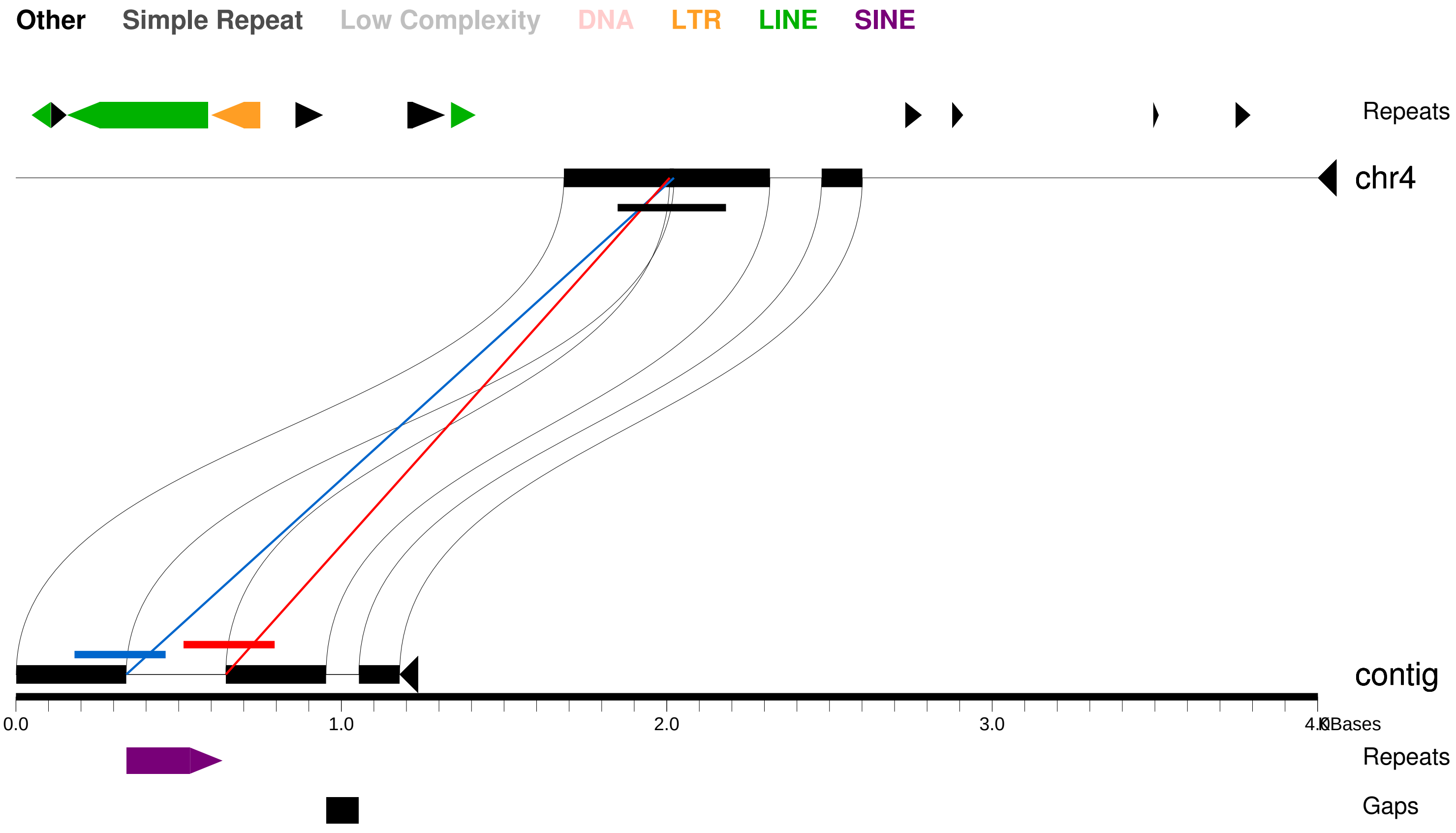

siteID: chr4_70413674 Scafftig5 + Breakpoint: chr4:70413663
chr4:70413614-70413722
Overlap Length: 11 INS size: 445 Genotyped: No

```
left    GAAATTATTTAAGGCAATCTCAATGAGCTAAGAA----------TAGTAAAAATTAATTAAAACAATTAG
chrom   GAAATTATTTAAGGCAATCTCAATGAGCTAAGAA----------TAGTAAAAATTAATTAAAACAATTAG
right   NNNNNNNNNNNNNNNNNNNNNNNNNNNNNNNNNNNNNNNNNNNNNNNNNNNNAAAAAAAAAAAAAATTAG
        1111111111111111111111111111111111111111111111111111*11**11****1******

left    GGCCGGGCGCGGTGGCTCACGCCTGTAATCCCAGCACTTTGGGAGGCCGAGGCGGGTGG
chrom   A-CAAAAAATAATACAGAATGAGGATAGTCAAAATATTTTAACAT----AGG-----GA
right   A-CAAAAAATAATACAGAATGAGGATAGTCAAAATATTTTAACAT----AGG-----GA
        22*222222222*22222*2*2222**2**22*22*2***222*22222***22222*2
```

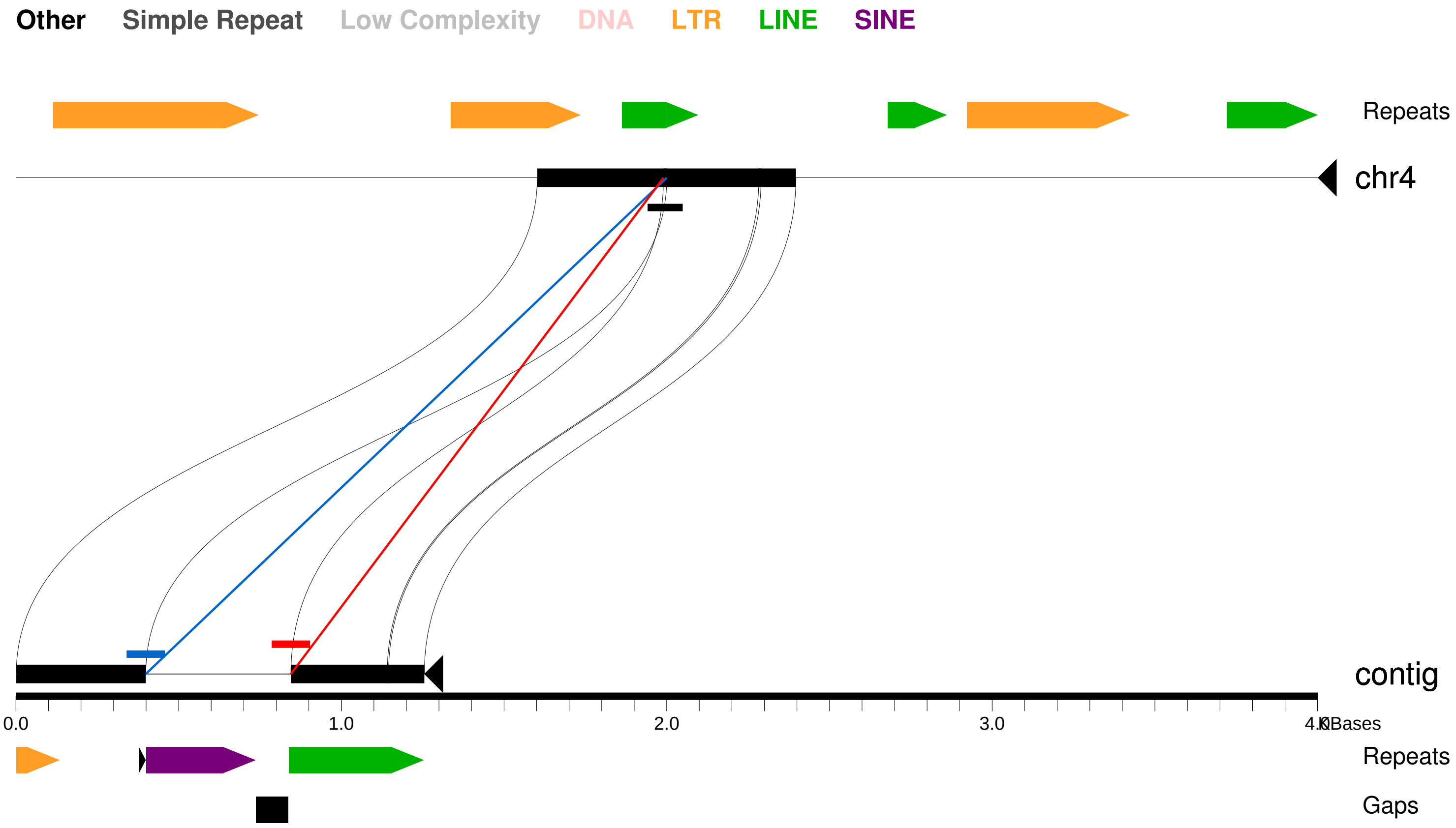

siteID: chr4_72714689 Scafftig2 + Breakpoint: chr4:72714689
chr4:72714643-72714748
Overlap Length: 14 INS size: 330 Genotyped: Yes

```
left    -----CTTTAAAATCTACAACTGACCCTTGAATAATATGGGTGTGA-ACTGT----------GCAGGTCT
chrom   -----CTTTAAAATCTACAACTGACCCTTGAATAATATGGGTGTGA-ACTGT----------GCAGGTCT
right   GCCCGCCTCGGCCTCCCAAAGTG---CTGGGATTACAGGCGTGAGCCACCGCGCCCGGCCACGCAGGTCT
        11111*1*11111**111**1**111**1*1**1*1*1*1***1*11**1*11111111111********

left    ACTTCTTTTTTTTTTTTTTTTTTTTTTTTTTTTTTTTTTTTTTTTTTTTTTTTGAGACGGAGTCT
chrom   ACTTCTAAGCAGACATTTTCCAATAAATACAATTGGCCCTCAGT------GTCCACAC-------
right   ACTTCTAAGCAGACATTTTCCAATAAATACAATTGGCCCTCAGT------GTCCACAC-------
        ******222222222****2222*222*2222**22222*222*2222222*22*2**2222222
```

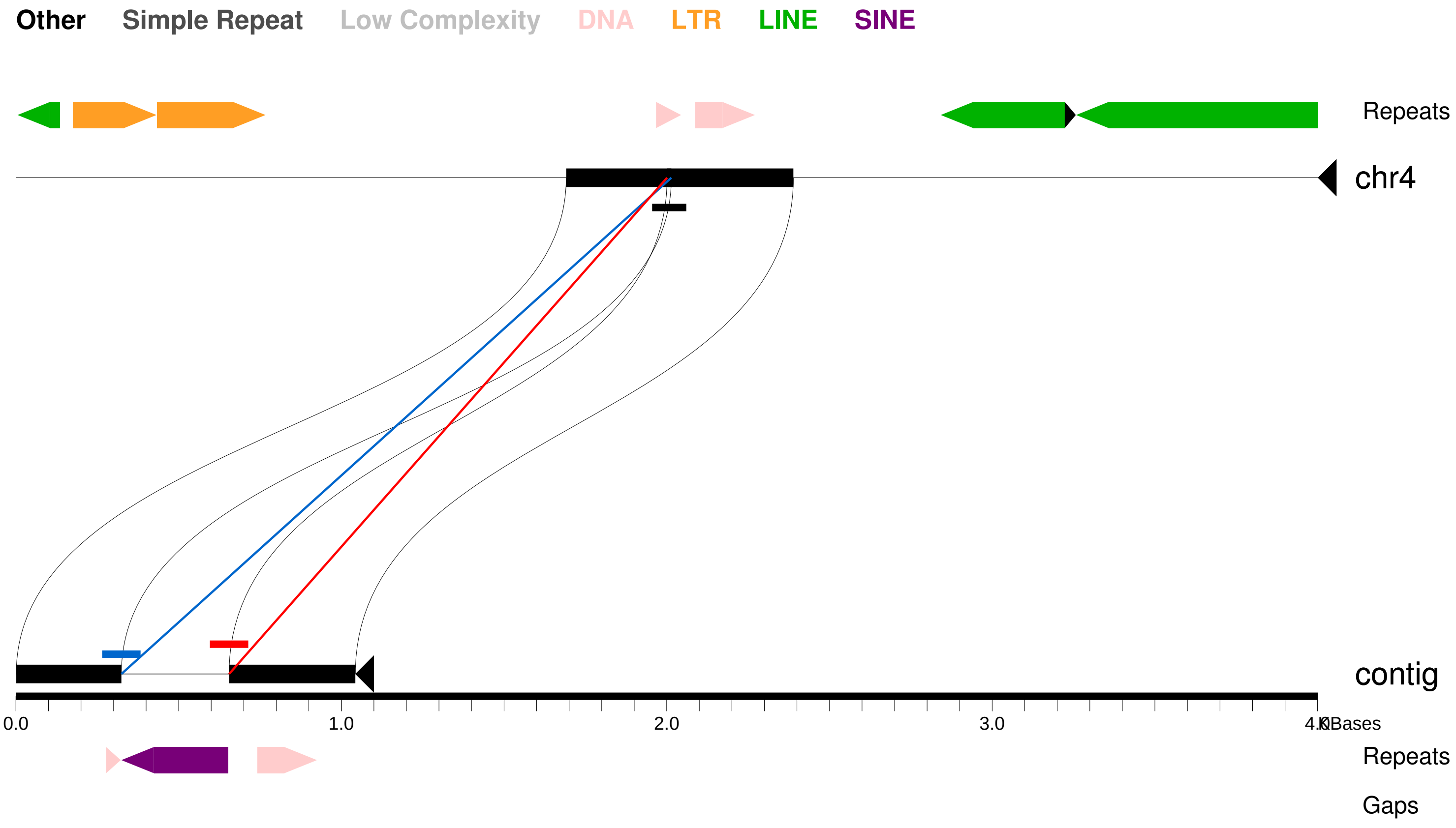

siteID: chr4_72719821 Scafftig2 - Breakpoint: chr4:72719805
chr4:72719757-72719864
Overlap Length: 12 INS size: 298 Genotyped: Yes

```
left   CT-CTGTAGCACATTCTCTG-GTAAGTCGGTGA---TATTTCAGAGCTGCTTA------AATAATACTTT
chrom  CT-CTGTAGCACATTCTCTG-GTAAGTCGGTGA---TATTTCAGAGCTGCTTA------AATAATACTTT
right  CCACTGCACTCCAGCCTGGGCGACAGAGCGAGACTCCGTCTCAAAAAAAAAAAAAAAAATAATAATACTTT
       *11***1*111**11**11*1*11**111*1**11111*1***1*1111111*111111***********

left   CGGCCGGGCGCGGTGGCTCACGCCTGTAATCCCAGCACTTTGGGAGGCCGAGGCGGGCGG
chrom  CTTACTGTT--GGTGGTGGATTTCT-TGCTTCCA------TGTGA--CTGGGGAGGGTAA
right  CTTACTGTT--GGTGGTGGATTTCT-TGCTTCCA------TGTGA--CTGGGGAGGGTAA
       *222*2*2222*****222*222**2*22*2***222222**2**22*2*2**2***222
```

Other Simple Repeat Low Complexity DNA LTR LINE SINE

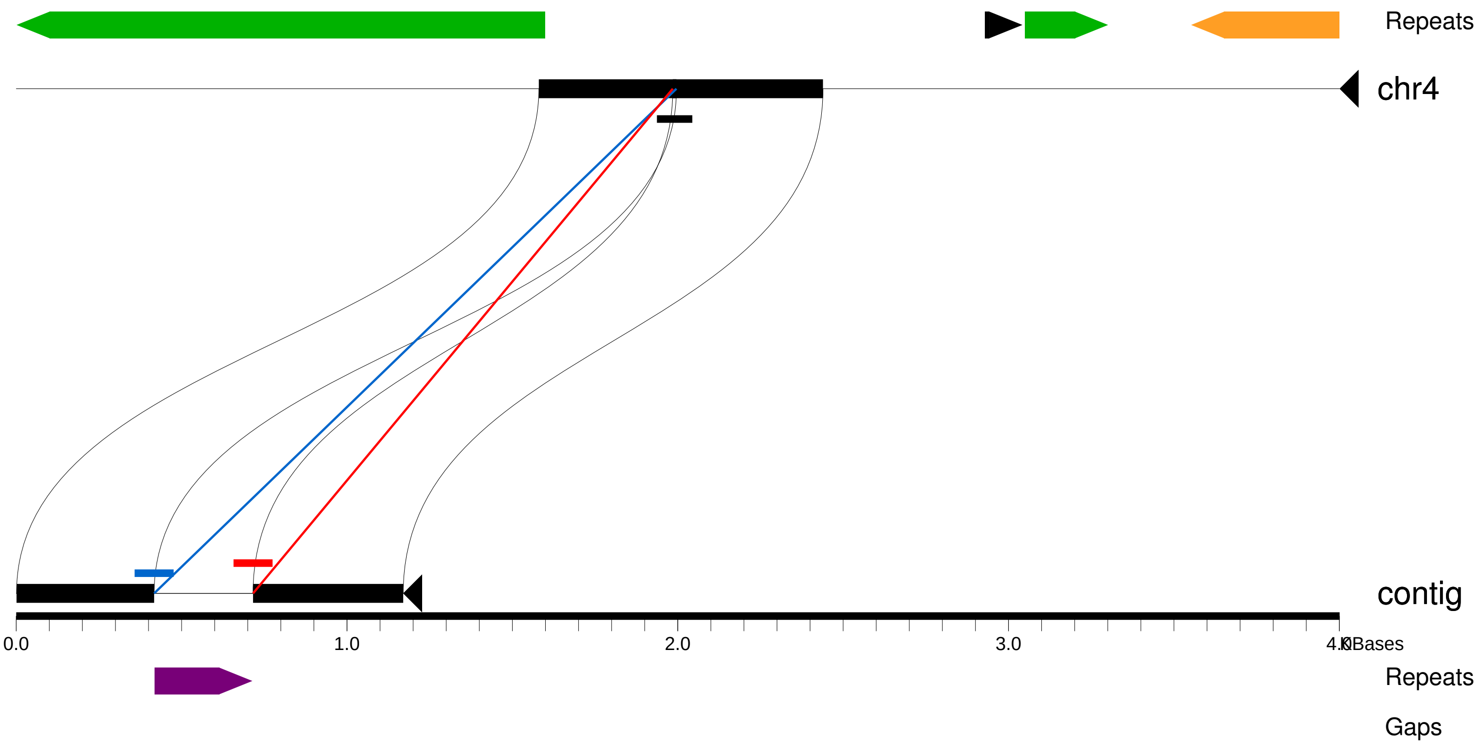

siteID: chr4_74430380 Scafftig1 - Breakpoint: chr4:74430417
chr4:74430396-74430476
Overlap Length: 39 INS size: 431 Genotyped: No

```
left   ---------TTGGG-TCCCA-------GGCTTTTCTTT---------------------GCTGCAAGACT
chrom  ---------TTGGG-TCCCA-------GGCTTTTCTTT---------------------GCTGCAAGACT
right  CCACCCGCCTCGGCCTCCCAAAGTGCTGGGATTACAGGCGTGAGCCACCGCGCCCGGCCGCTGCAAGACT
       111111111*1**11*****1111111**11**1*111111111111111111111111***********

left   TTTATTACAGCTTTGGTCTTACTACTTGNNNNNNNNNNNNNNNNNNNNNNNNNNNNNNNNNNNNNNNNNN
chrom  TTTATTACAGCTTTGGTCTTACTACTTGCTATTGGTTTGCTCAGGTTTT---------------------
right  TTCATTACAGCTTTGGTCTTACTACTTGCTATTGGTTTGCTCAGGTTTT---------------------
       **1*************************222222222222222222222222222222222222222222

left   NNNNNNNNNNNNNNNNN
chrom  -----------------
right  -----------------
       22222222222222222
```

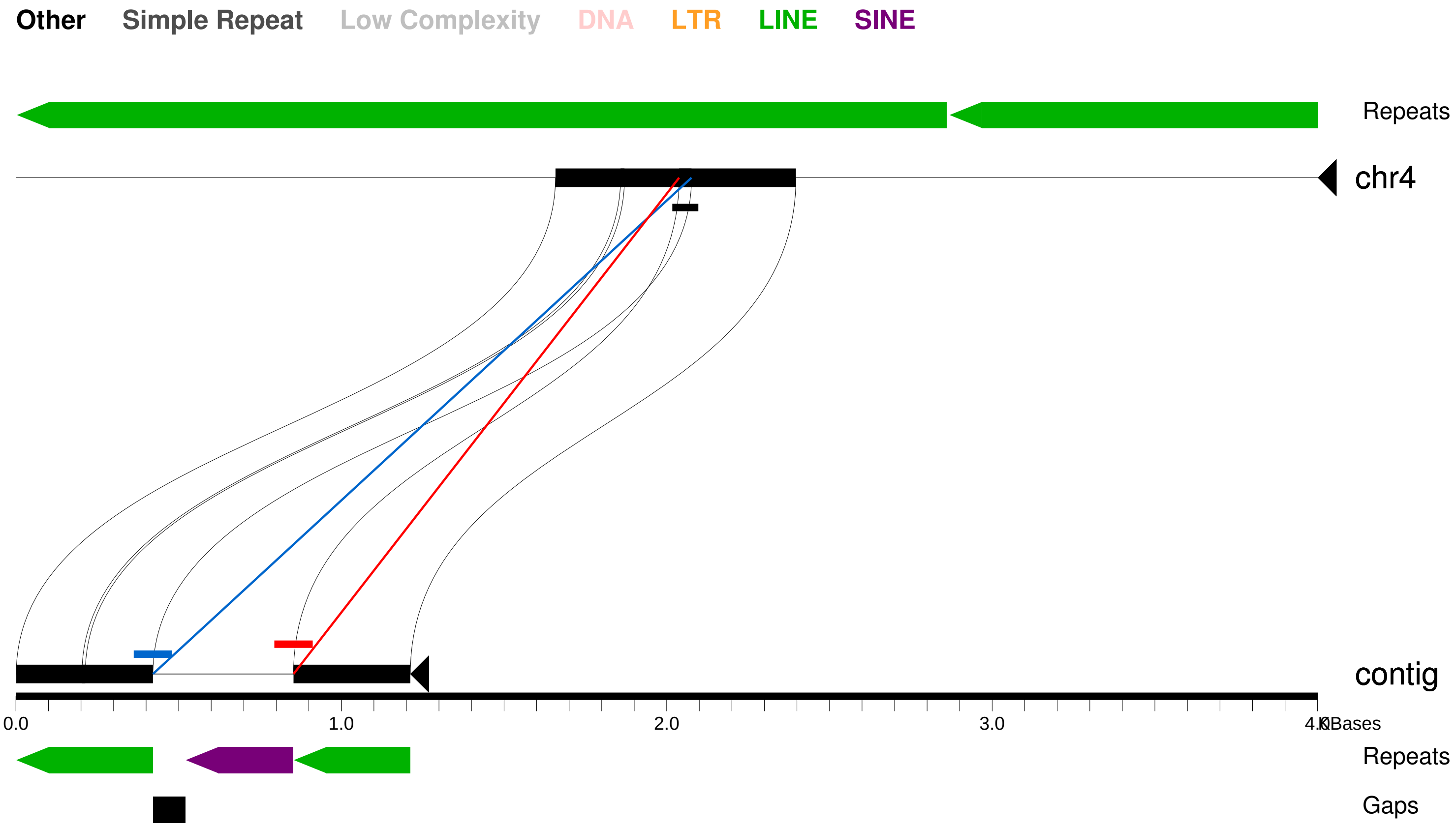

siteID: chr4_75736480 Scafftig2 + Breakpoint: chr4:75736460
chr4:75736300-75736631
Overlap Length: 12 INS size: 401 Genotyped: No

```
left   TA-------------------------------------ACATCTAAAATACTTTCCCTCATGAATCAAA
chrom  TATTTACACTGCAGTTATTTTCAACTATCTCCTTTATTAACATCTAAAATACTTTCCCTCATGAATCAAA
right  CCACTACGCC-CGGCTAATTTTTTGTATTT---TTAGTAG-----AGACGGGGTT----------TCACC
       11NN222N2N 2N2N22N222NNNN222N2   222N22111111*1*11111**1111111111***11

left   ATGATAGCTCTGCCAACAATAAATGTCTCTCATGGCTTTTCAGAGAAAATGTTCCCAAAAGCCCTTCTGT
chrom  ATGATAGCTCTGCCAACAATAAATGTCTCTCATGGCTTTTCAGAGAAAATGTTCCCAAAAGCCCTTCTGT
right  GTTTTAGCCGGG---------ATGGTCTC-----GATCTCCTGACCTCGTGATCC-----GCCCGCCTCG
       1*11****111*111111111*11*****11111*1*1*1*1**11111**1***11111****11**11

left   CCAGGGAAGAGATTCAAGCTGTCTTCACCCTCNNNNNNNNNNN---------------------------
chrom  CCAGGGAAGAGATTCAAGCTGTCTTCACCCTCTTAATAGGGACTTATGAAATGTCCTGCTCAGAAAAAAG
right  GCCTCCCAAAG-TGCTGGGAGTCTTCACCCTCTTAATAGGGACTTATGAAATGTCCTGCTCAGAAAAAAG
       1*11111*1**1*1*11*11************22222222222222222222222222222222222222

left   ----------------------------------------------------------------------
chrom  GAAATAACTAAAGATGGAAAAGATAATCTCCACCCCCCACCCCTTCACAGCCTGACTTCCCACTTCATTT
right  GAAATAACTAAGGATGGAAAAGATAATCTCCGCCCCCCACCCCTTCACAGCCTGACTTCCCACTTCATTT
       22222222222N2222222222222222222N22222222222222222222222222222222222222

left   ----------------------------------------------------
chrom  CCAATGCTATTTAACTCAGACCCTTATCATCTCATGCATGGAATAGTCTGCT
right  CCAATGCTATNNNNNNNNNNNNNNNNNNNNNNNNNNNNNN------------
       2222222222NNNNNNNNNNNNNNNNNNNNNNNNNNNNNN
```

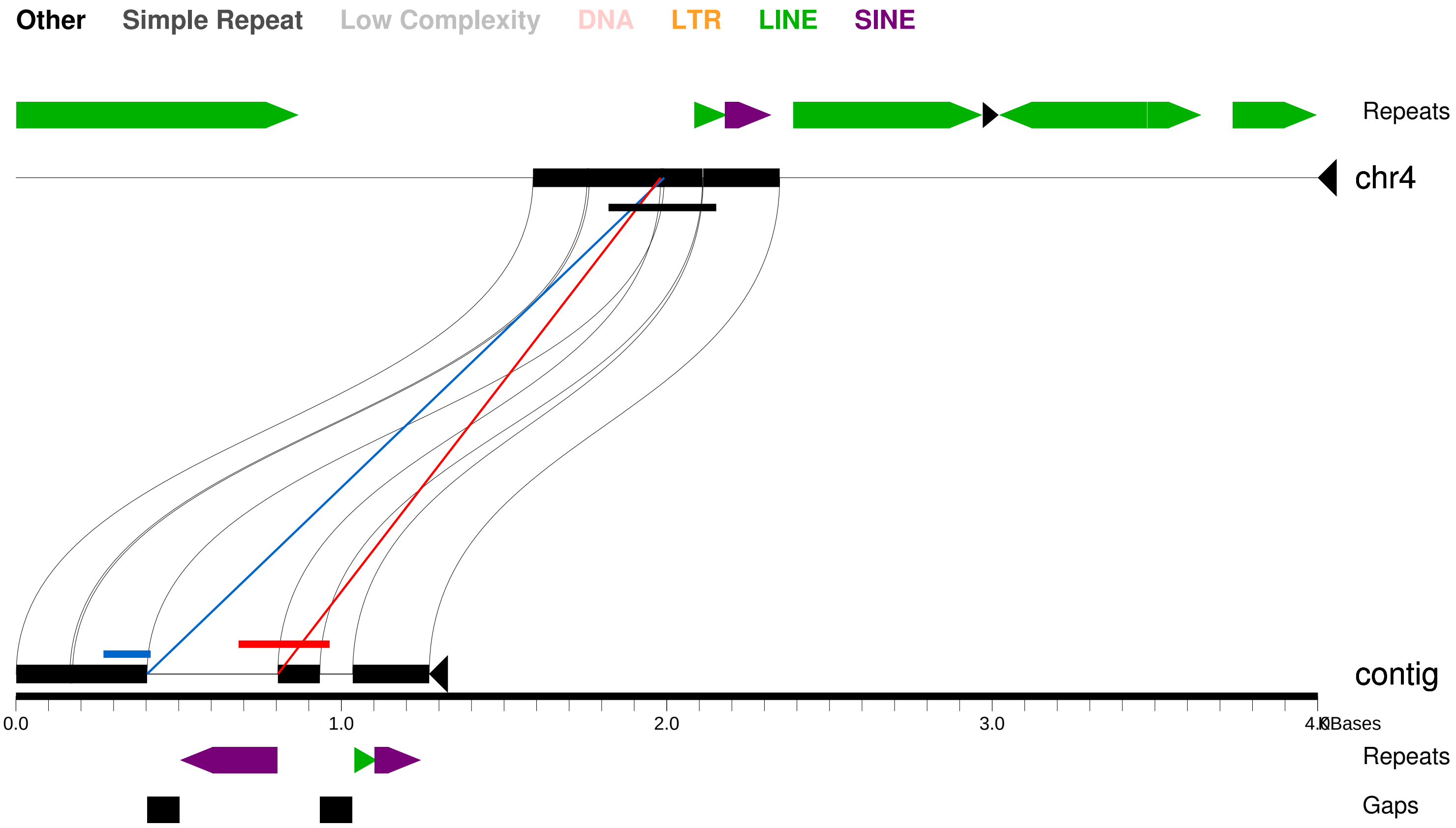

siteID: chr4_77023820 Scafftig2 + Breakpoint: chr4:77023809
chr4:77023762-77023868
Overlap Length: 7 INS size: 310 Genotyped: Yes

```
left    CTTAATACAATGGT------AAATAATTGGAT-ATAAAACTG---CATATAGA--GTATAATATTATTTT
chrom   CTTAATACAATGGT------AAATAATTGGAT-ATAGAACTG---CATATAGA--GTATAATATTAACTT
right   CCGCCCGTCTCGGCCTCCCAAAGTGCTGGGATTACAGGCATGAGCCACCGCGCCCGGCCAATATTAACTT
        *1111111111**1111111**1*11*1****1*1*2111**111**1111*111*111*******22**

left    TTTTTTTTTTTTTTTTTTTTTTTTTTGAGACGGAGTTTCGCTCTGTCGCCCAGGCTGGAGT
chrom   TTAAAAATGTATATGTATATATATGT---ATGCA---TAGCAAAAT------GATTGAAAT
right   TTAAAAATGTATATGTATATATATGT---ATGCA---TAGCAAAAT------GATTGAAAT
        **22222*2*2*2*2*2*2*2*2*2*222*2*2*222*2**2222*222222*22**2*2*
```

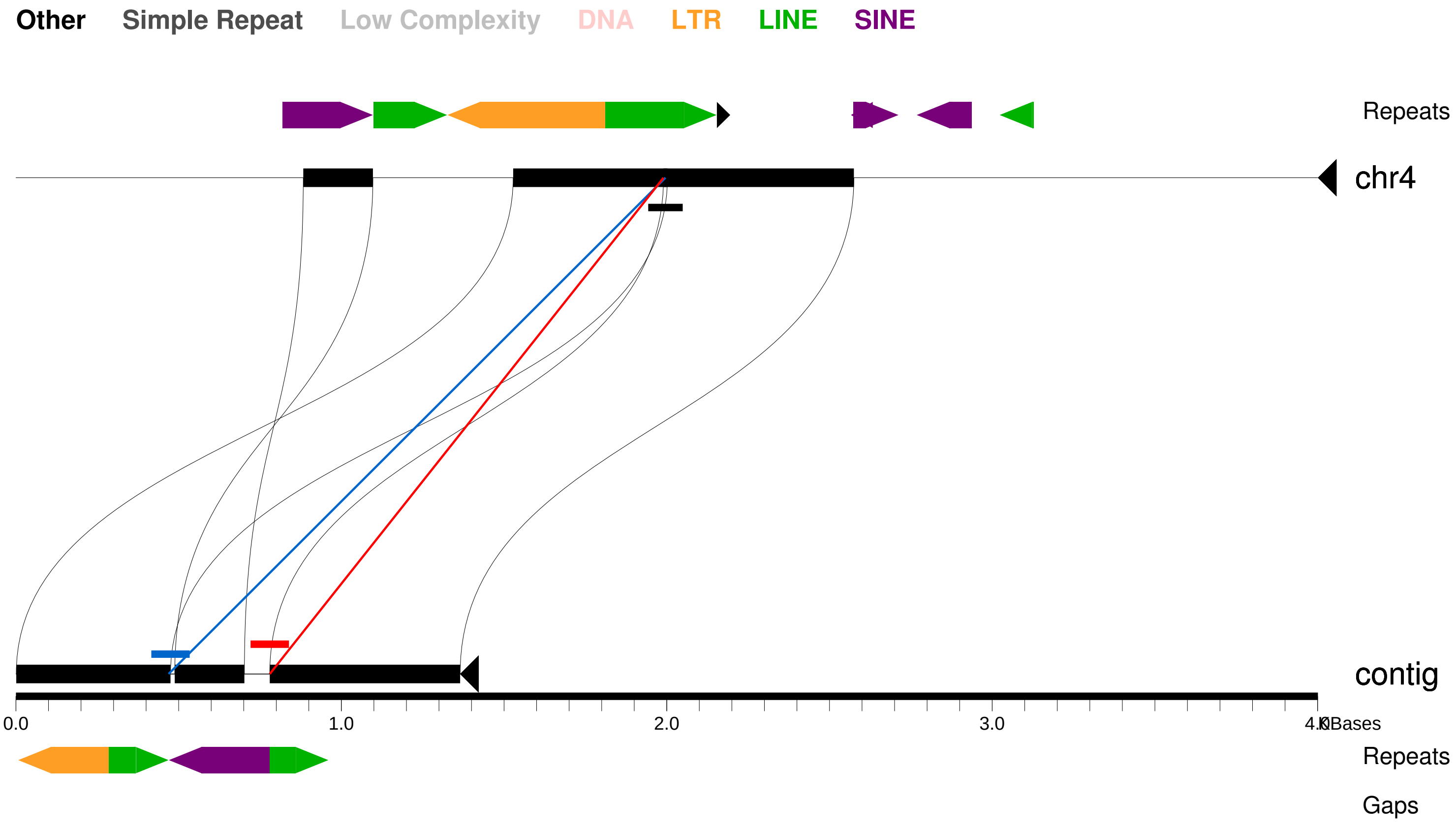

siteID: chr4_79159493 Scafftig8 - Breakpoint: chr4:79159506
chr4:79159456-79159565
Overlap Length: 10 INS size: 314 Genotyped: Yes

```
left    ATACTTTTTCTAGTTGCTGACTCTTT-TCTTTTTAAAGTGAATCTATTTTT---------TAAAAAAGCT
chrom   ATACTTTTTCTAGTTGCTGACTCTTT-TCTTTTTAAAGTGAATCTATTTTT---------TAAAAAAGCT
right   CCTGGGCGAC-AGAGCGAGACTCCGTCTCAAAAAAAAAAAAAAATAAATAAAAAAAAAAAATAAAAAAAGCT
        111111111*1**11111*****11*1**11111***111**11**11*11111111111**********

left    AGGCCAGGCGCGGTGGCTCACGCCTGTAA-TCCCAGCACTTTGGGAGGCCGAGGCGGGCG
chrom   TAAGCTTTAAATTTGA---ACTTCTGAAACTCCCAGGACA-----AGACCAATTATTG--
right   TAAGCTTTAAATTTGA---ACTTCTGAAACTCCCAGGACA-----AGACCAATTATTG--
        2222*22222222**2222**22***2**2******2**222222**2**2*22222*22
```

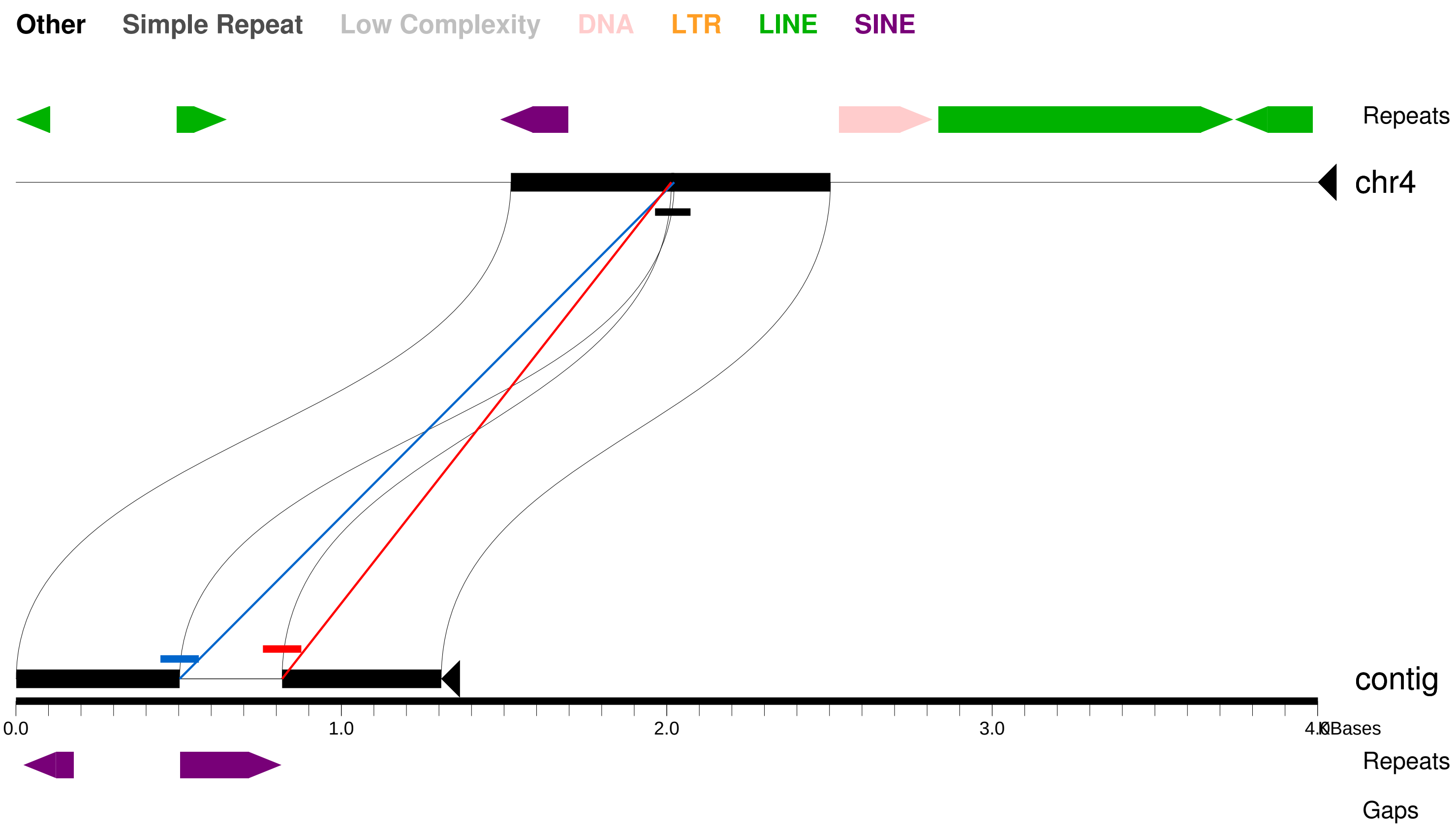

siteID: chr4_79289439 Scafftig4 + Breakpoint: chr4:79289435
chr4:79289275-79289609
Overlap Length: 15 INS size: 297 Genotyped: Yes

```
left   ------------AAAAATTCTAGCAAAAAAAAAAGGGATAAAAATTTGGTCTC-ATCTAAGCTTACAACA
chrom  TACACCATGCATAAAAATTCTAGCAAAAAAAAA-GGGATAAAAATTTGGTCTC-ATCTAAGCTTACAACA
right  T------TTTTTTTTATTTTTAGTAGAGAC----GGGGTTTCACCGTGGTCTCGATCTC--CTGACCTC-
       2      2NNN2111*1**1***1*1*1*11112***1*111*111*******1****111**1**11*1

left   GTTAAAATATTCTTTTCTAATTATTGAAAATAATTAGAACTGAAAATCCAGTCTAGAATTTTCCCAGGAC
chrom  GTTAAAATATTCTTTTCTAATTATTGAAAATAATTAGAACTGAAAATCCAGTCTAGAATTTTCCCAGGAC
right  GTGA---TCCACCCGCCTCGGCCTCCCAAA------GTGCTGGGATTACAAGCGTGAGC---CACCGCGC
       **1*111*111*1111**11111*111***111111*11***11*1*1**11*11**11111*1*1*11*

left   CTTAAATTATCAATATGCATATTCTAATGAATGTTTTTT-----TTTTTTTTTTTTT-------------
chrom  CTTAAATTATCAATATGCATATTCTAATGAATGTTTTATAACAATGTCTTTCTTTGTAGGTGAAACAATA
right  C----------------CGGCCTCTAATGAATGTTTTATAACAATGTCTTTCTTTGTAGGTGAAACAATA
       *1111111111111111*1111**************2*22222*2*2***2***2*2222222222222

left   ------TTTTTTTTTGAGACGGAG-------TCTCGCTCTGTCACCCAGGCTG-GAG----TACAGTGGC
chrom  GCATTCTTTTTTTTTTTCAGGGAGGTAATTTTCCTGCACTGTGCCATATACTTTGATATCATATAGATCT
right  GCATTCTTTTTTTTTTTCAGGGAGGTAATTTTCCTGCACTGTGCCATATACTTTGATATCATATAGATCT
       222222*********222*2****2222222**22**2****22*22*22**22**22222**2**2222

left   GCAGC---CTCGG---CTCACTGCAAGCTCCGCCTCCCGGGTTCAC-GCCATTCTCC
chrom  AAAACTCACTGGGTTTCTCTCTGTACTTTTCCTCTGACACGTACTTAGAGAAGATTT
right  AAAACTCACTGGGTTTCTCTCTGTACTTTTCCTCTGACACGT---------------
       22*2*222**2**222***2***2*222*2*22**22*22**N1NN 1NN1NNN1NN
```

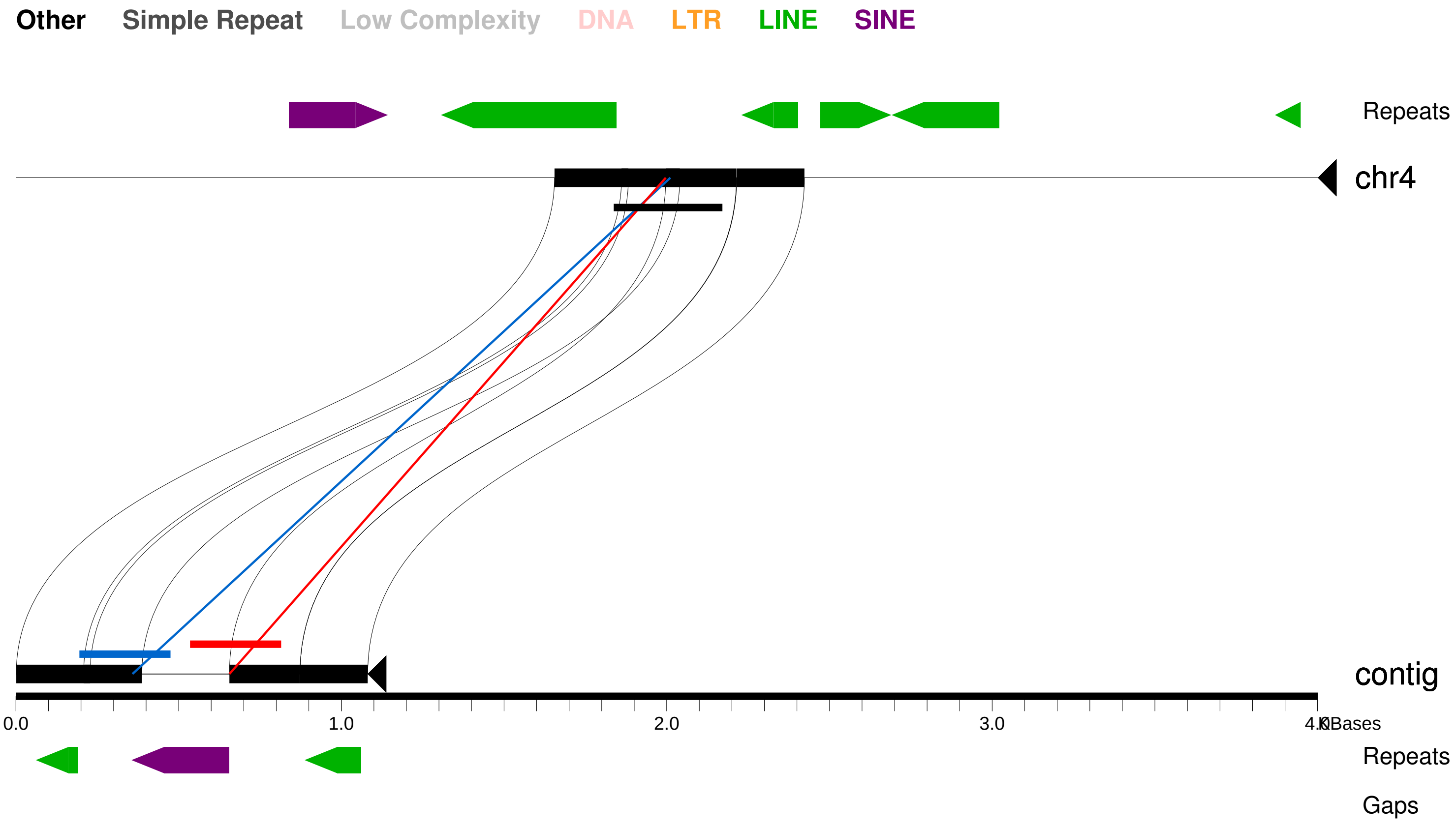

siteID: chr4_79322989 Scafftig4 - Breakpoint: chr4:79322970
chr4:79322923-79323026
Overlap Length: 13 INS size: 313 Genotyped: Yes

```
left     TTT-TCCGGTA--------------AATCTCAGGTTAAATTAGACCATGTGTCTTGTCTAAGAAACCTGA
chrom    TTT-TCCGGTA--------------AATCTCAGGTTAAATTAGACCATGTGTCTTGTCTAAGAAACCTGA
right    GCAGTCCGGCCTGGGCGACAGAGCGAGACTCCGTCTCAAAAAAAAAAAAAAAAAAAAAAAAAAAACCTGA
         1111*****1111111111111111*11***1*11*1**11*1*11*111111111111**1********

left     AGCTGGCCGGGCGCGGTGGCTCACGCCTGT-AATCCCAGCACTTTGGGAGGCCGAGGCGGGTGGA
chrom    AGCTGA--------GGTATTTTATGAAAATTAATCTCAGACCAATGATATGTC--------TTGA
right    AGCTGA--------GGTATTTTATGAAAATTAATCTCAGACCAATGATATGTC--------TTGG
         *****222222222***222*2*2*2222*2****2***22*22**22*2*2*22222222*2*1
```

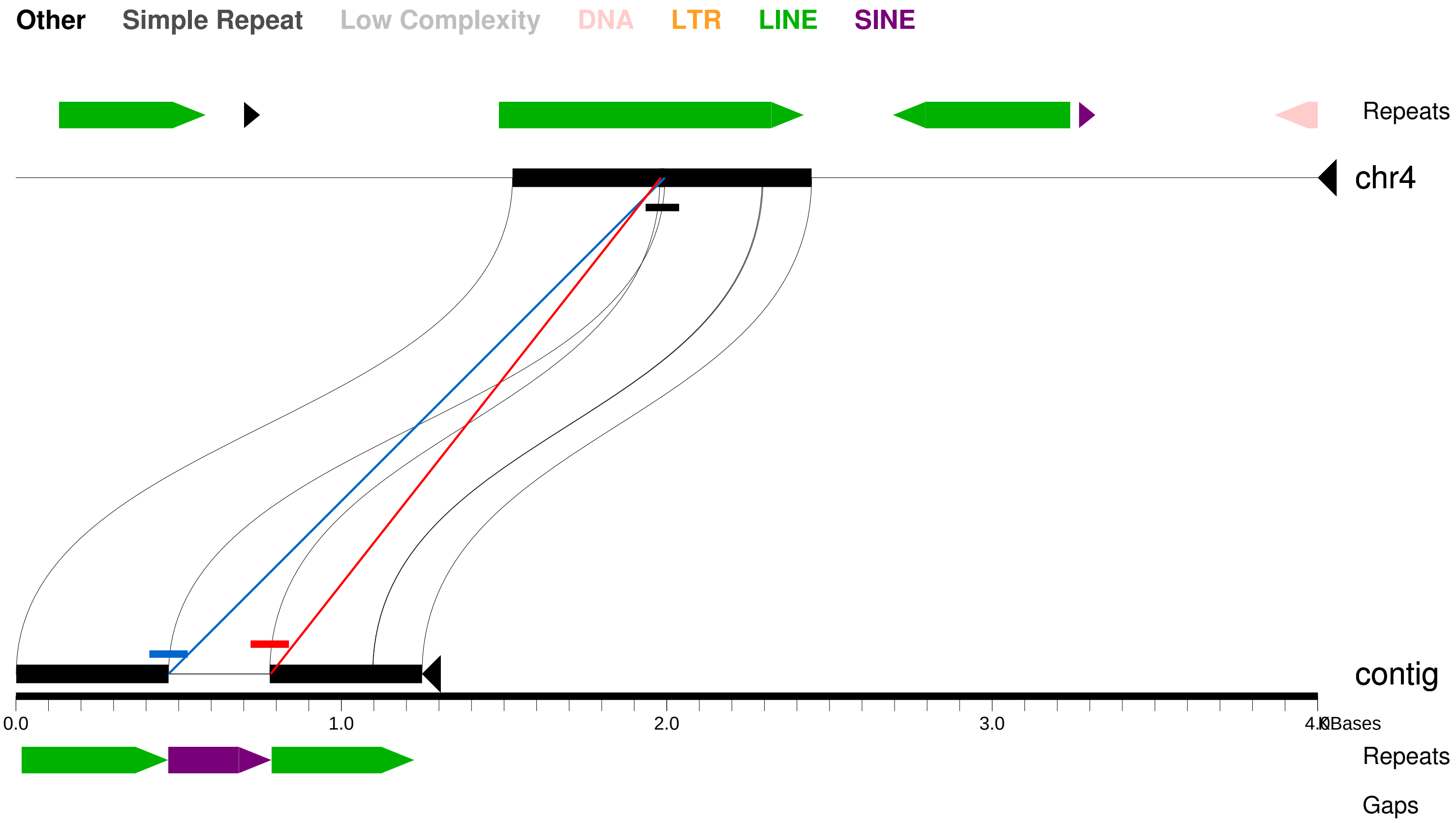

siteID: chr4_79359173 Scafftig5 + Breakpoint: chr4:79359160
chr4:79359121-79359219
Overlap Length: 18 INS size: 313 Genotyped: Yes

```
left    GAACATTCATAGATTT-----GGTGCT--GATAAAATGAA--ATCCAT-----------GACTTCATACA
chrom   GAACATTCATAGATTT-----GGTGCT--GATAAAATGAA--ATCCAT-----------GACTTCATACA
right   CCACCCGCCTCGGCCTCCCAAAGTGCTGGGATTACAGGCGTGAGCCACCGCGCCCGGCCGACTTCATACA
        11**111*1*1*111*111111*****11***1*1*1*1111*1***111111111111***********

left    TTCTTTTTTTTTTTTTTTTTTTTTTTTTTTTTGAGACGGAGTCTCGCTCTGTCGCCCAGGCCGGACTGCG
chrom   TTCTTTTGTTAATCTAAAATTTTATCACGTTGA-ACA-------------TTGCTTAA-----AGTGA-
right   TTCTTTTGTTAATCTAAAATTTTATCACGTTGA-ACA-------------TTGCTTAA-----AGTGA-
        *******2**22*2*2222****2*2222****2**22222222222222*2**22*222222*2**22
```

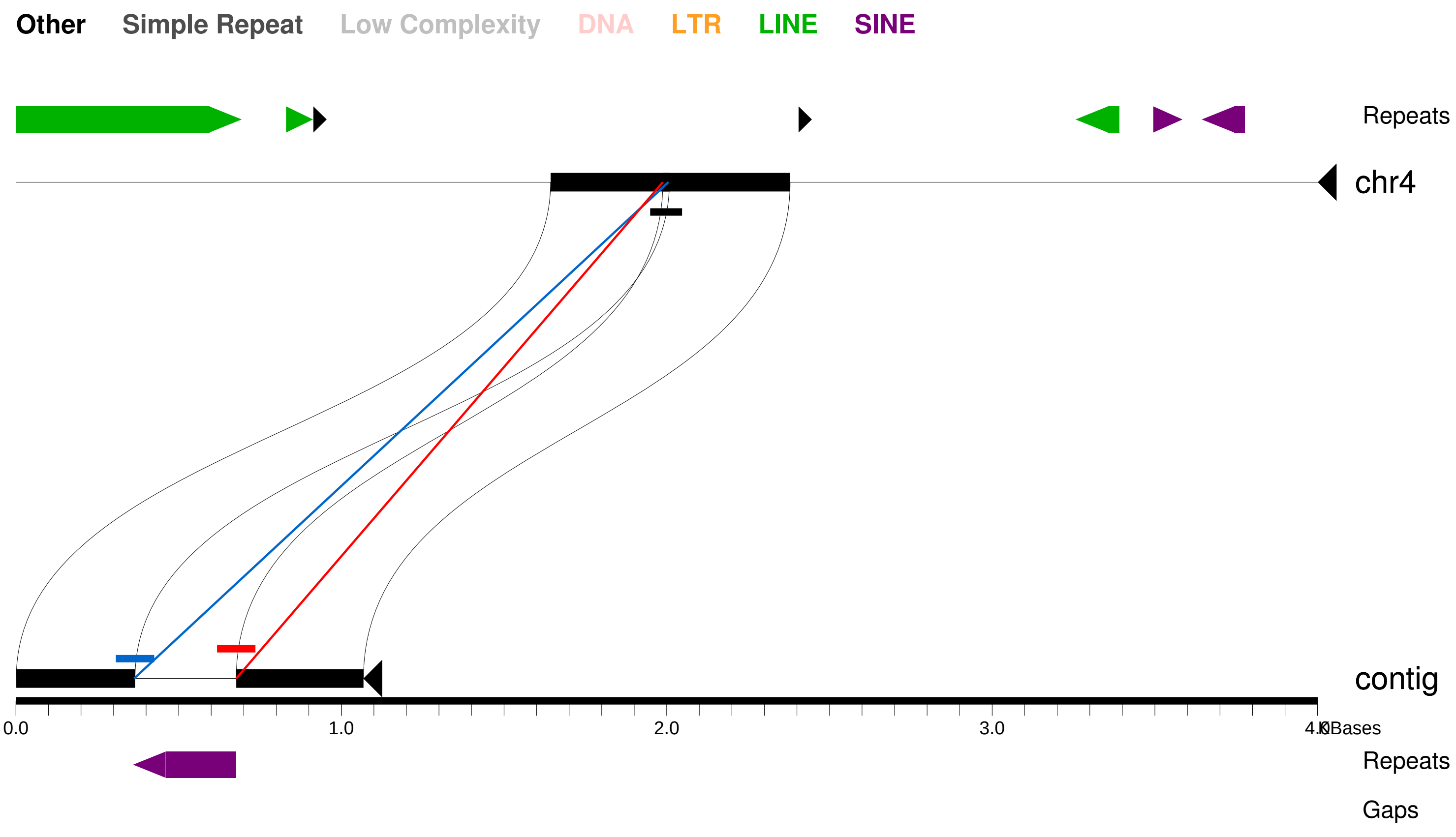

siteID: chr4_79372145 Scafftig1 - Breakpoint: chr4:79372137
chr4:79372097-79372196
Overlap Length: 17 INS size: 302 Genotyped: Yes

```
left     GT-----GCAATTCTGTTTAGAAGTG-GAAATATACCCCTGCGGAT-------------AAAAAACAAGT
chrom    GT-----GCAATTCTGTTTAGAAGTG-GAAATATACCCCTGCGGAT-------------AAAAAACAAGT
right    GCAGTCCGCAGTCCCGCCTGGGCGACAGAGCGAGACTCCGTCTCAAAAAAAAAAAAAAAAAAAAAAAAAT
         *111111***1*1*1*11*1*11*111**111*1**1**11*11*11111111111111******1**1*

left     GTCCCGGCCGGGCGCGGTGGCTCACGCCTGTAATCCCAGCACTTTGGGAGGCCAAGGCGGGTGGATCA-
chrom    GTCCCGCCCACACAC------TAACACCTAAAATT--------------GACAACAGCTGAATTCTCAT
right    GTCCCGCCCACACAC------TAACACCTAAAATT--------------GACAACAGCTGAATTCTCAT
         ******2**222*2*222222*2**2***22***22222222222222*2*2*22**2*22222***2
```

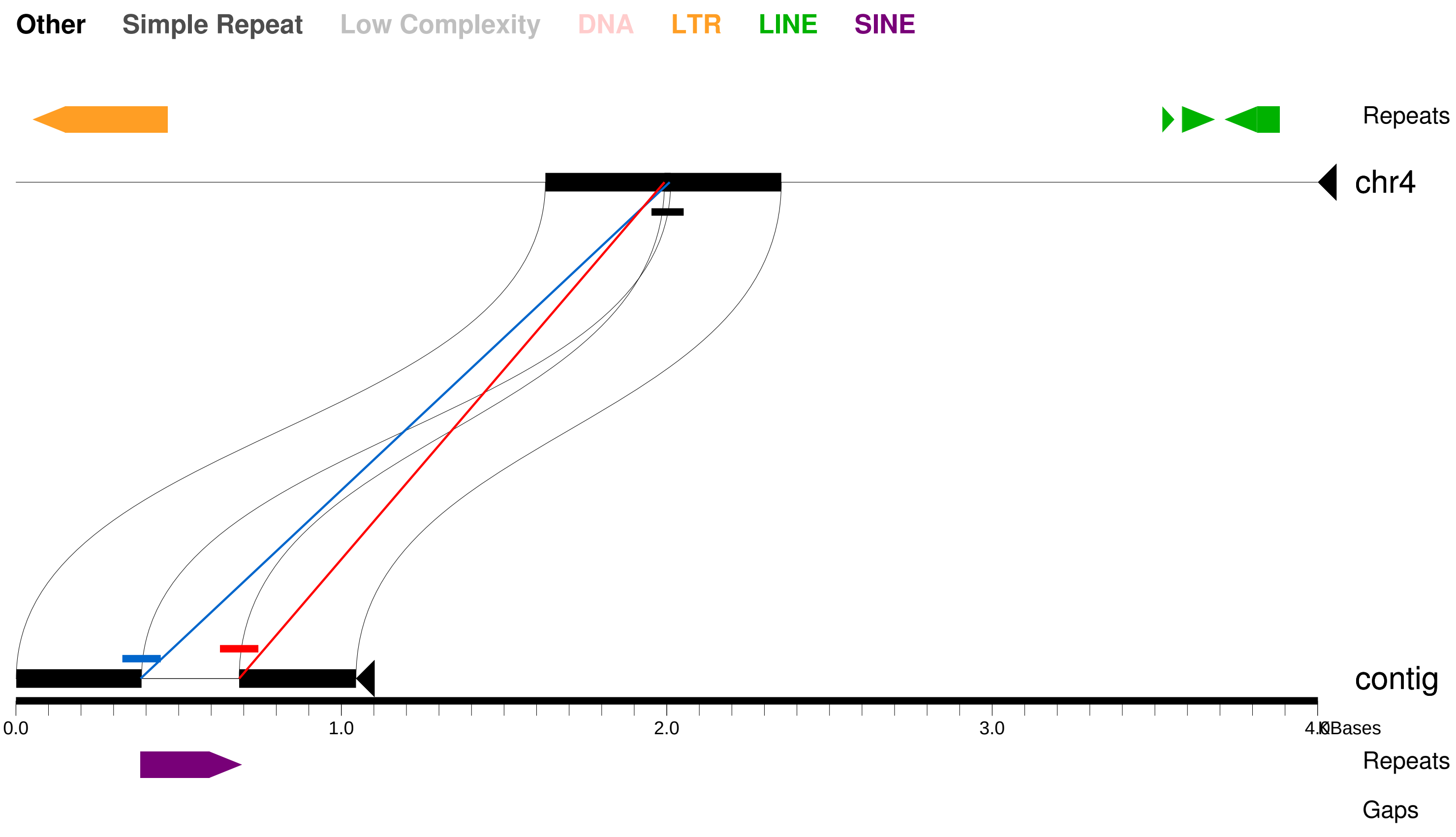

siteID: chr4_79823616 Scafftig1 - Breakpoint: chr4:79823605
chr4:79823556-79823664
Overlap Length: 11 INS size: 441 Genotyped: No

```
left    AAACCCTGTCACTTGGCCTGCCTTCTGAGAAGCAGTACAGATTTGATAA----------AACCTTCTCCT
chrom   AAACCCTGTCACTTGGCCTGCCTTCTGAGAAGCAGTACAGATTTGATAA----------AACCTTCTCCT
right   NNNNNNNNNNNNNNNNNNNNNNNNNNNNNNNNNNNNNNNNNNNNNNNNNNNNNNNNNNNAACCTTCTCCT
        11111111111111111111111111111111111111111111111111111111111***********

left    GGCCGGGCGCGGTGGCTCACGCCTGTAATCCCAGCACTTTGGGAGGCCGAG-GCGGGTGG
chrom   AATC---------CACTAATCCTTGTGA--CTAACATGGTAAATTGCTGGGAGATGATGG
right   AATC---------CACTAATCCTTGTGA--CTAACATGGTAAATTGCTGGGAGATGATGG
        222*22222222222**2*22*2***2*22*2*2**222*22222**2*2*2*22*2***
```

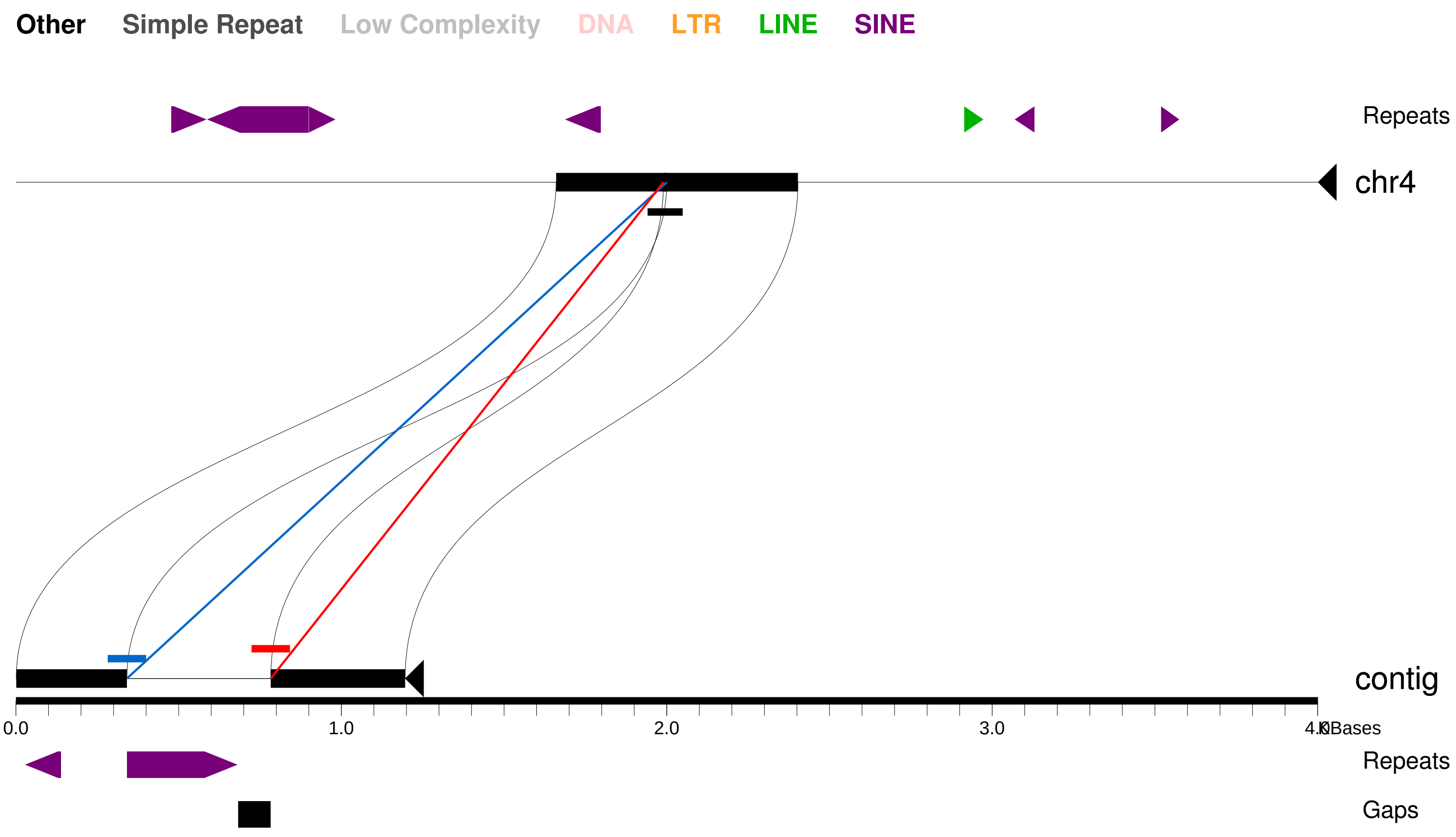

siteID: chr4_82630143 Scafftig4 - Breakpoint: chr4:82630133
chr4:82630089-82630192
Overlap Length: 16 INS size: 313 Genotyped: Yes

```
left     T------ACATATTGGGT---------ACAATGTATACTACTTGTCTGACCAGTACACTAAAATATCAGA
chrom    T------ACATATTGGGT---------ACAATGTATACTACTTGTCTGACTAGTACACTAAAATATCAGA
right    CTGGGCGACAGAGCGAGACTCCGTCTCAAAAAAAAAAAAAAAAAAAAAAAAAAAAAAAAAAAAAAATCAGA
         1111111***1*11*1*1111111111*1**111*1*11*11111111*1N*11*1*11****1******

left     CTTCAGGCCGGGCGCGGTGGCTCACGCCTGTAATCCCAG--CACTTTGGGAGGCCGAGGCGGGCGG
chrom    CTTCACTACTATA-CAAT---TCATCCATGTAACCAAAAACCACTT----ATCCCCA---------
right    CTTCACTACTATA-CAAT---TCATCCATGTAACCAAAAACCACTT----ATCCCCA---------
         *****222*22222*22*222***22*2*****2*22*222*****2222*22**2*222222222
```

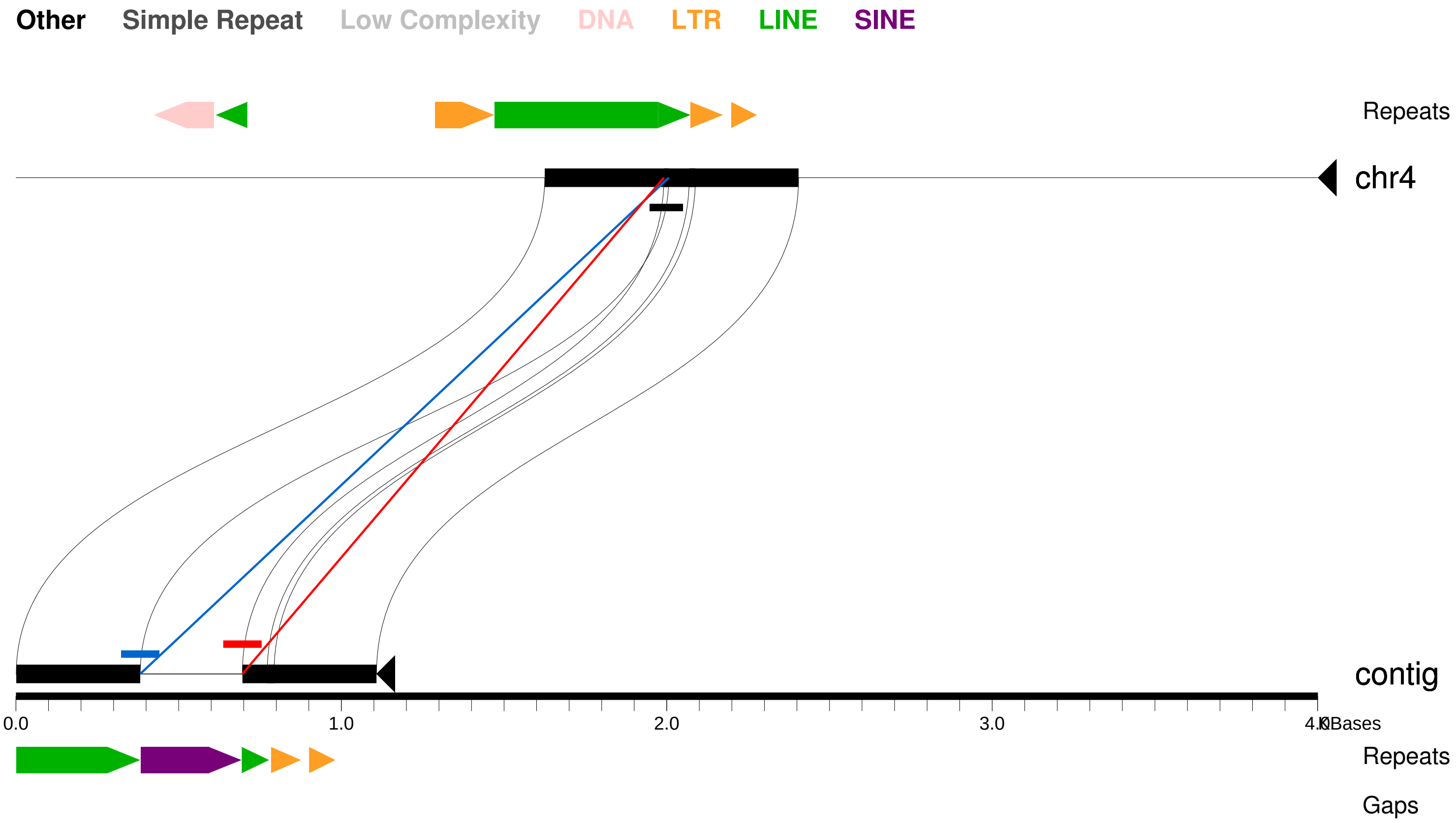

siteID: chr4_83322517 Scafftig4 + Breakpoint: chr4:83322561
chr4:83322514-83322620
Overlap Length: 13 INS size: 199 Genotyped: Yes

```
left    ATAGATCACATCTGTATTTAGTGACCAGAGGTT--TTTTTTGTTTGTTT-------------TGTTTTTG
chrom   ATAGATCACATCTGTATTTAGTGACCAGAGGTT--TTTTTTGTTTGTTT-------------TGTTTTTG
right   ---GACTACAGGCGCCCGCTACCACGCCCGGCTAATTTTTTGTATTTTTAGTAGAGACGGGGTGTTTTTG
        111**11***111*111111111**1111**1*11********1*1***1111111111111********

left    TTTTTTTTTTTTTTTTTTTTTTTTTTTTTG--AGACGGAGTCTCGC-TCTGTCGCCCAGGCTGGAGTG
chrom   TTTTTAATCACTTGACACTTTATTGAATGCAAAACAAGATTTAGCATCTTT---------------G
right   TTTTTAATCACTTGACACTTTATTGAATGCAAAACAAGATTTAGCATCTTT---------------G
        *****22*222**22222***2**222**22*2**2222*2*2**2***2*222222222222222*
```

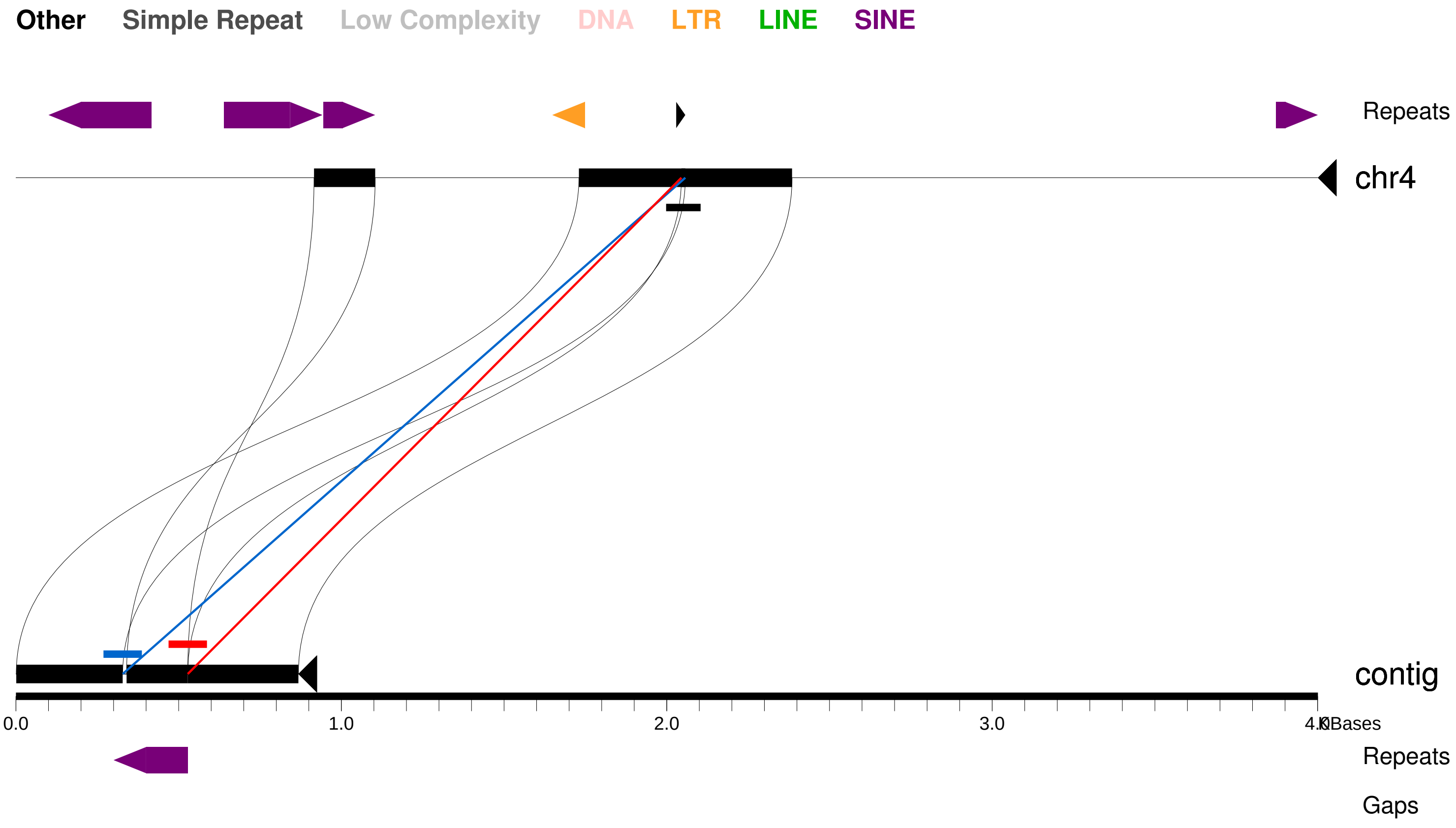

siteID: chr4_86099297 Scafftig5 + Breakpoint: chr4:86099305
chr4:86099258-86099364
Overlap Length: 13 INS size: 256 Genotyped: Yes

```
left    A-TAACA---TTACCCTTCATATTTTGTCA--GTAATTCATTATC-TCATTCTT-----TCTTTGTAAGT
chrom   A-TAACA---TTACCCTTCATATTTTGTCA--GTAATTCATTATC-TCATTCTT-----TCTTTGTAAGT
right   GGTAGCCAGGATGGTCTCGATCTCCTGACCTCGTGATCCGCCCGCCTCGGCCTCCCAAATCTTTGTAAGT
        11**1*11111*111**11**1*11**1*111**1**1*11111*1**111**111111***********

left    TT-----TTTTTTTTTTTTGAGACGGAGTCTCGCTCTGTCGCCCAGGCTGGAGTGCAGTGGCGCGA
chrom   TTAGAAGTCTTAACTTCTTG-------GTCTTGTTC---ATCTCAGATATTAGTGTTCT-------
right   TTAGAAGTCTTAACTTCTTG-------GTCTTGTTC---ATCTCAGATATTAGTGTTCT-------
        **22222*2**222**2***2222222****2*2**22222*2***22222****222*2222222
```

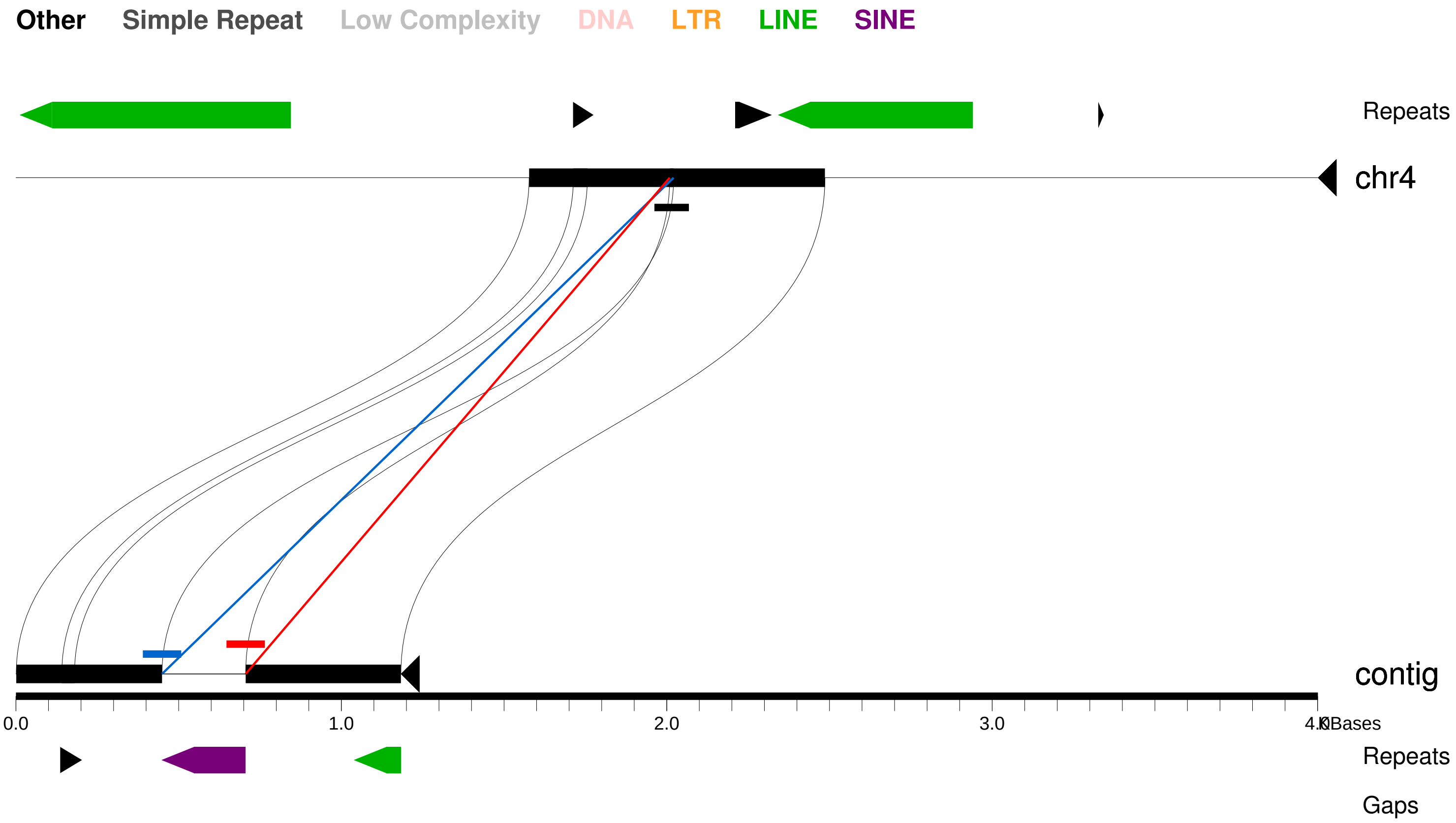

siteID: chr4_86470376 Scafftig3 - Breakpoint: chr4:86470374
chr4:86470323-86470430
Overlap Length: 9 INS size: 307 Genotyped: Yes

```
left    T--CTGAATTGGAATCA--------CTAAGTCATAGATACAGGTATATTTAATTTTATAAGAAA-TTGCC
chrom   T--CTGAATTGGAATCA--------CTAAGTCATAGATACAGGTATATTTAATTTTATAAGAAA-TTGCC
right   CTCCAGCCTGGGCGACAGAGCGAGACTCCGTCTCAAAAAAAAAAAAAAAAAAAAAAAAAAAAAATTGCC
        111*1*11*1**111**11111111**11***11*1*1*1*111*1*111**1111*1**1***1*****

left    AGCCG----GGCGCGGTGGCTCACGCCTGTAATCCCAGCACTTTGGGAGGCCGAGGCGGGCGGA
chrom   AAACATTTTGTTAAAGTGGTTGCACCATTTA-------CACTTTCACCAGCAATAT--------
right   AAACATTTTGTTAAAGTGGTTGCACCATTTA-------CACTTTCACCAGCAATAT--------
        *22*22222*22222****2*2222*2*2**2222222******22222**2222222222222
```

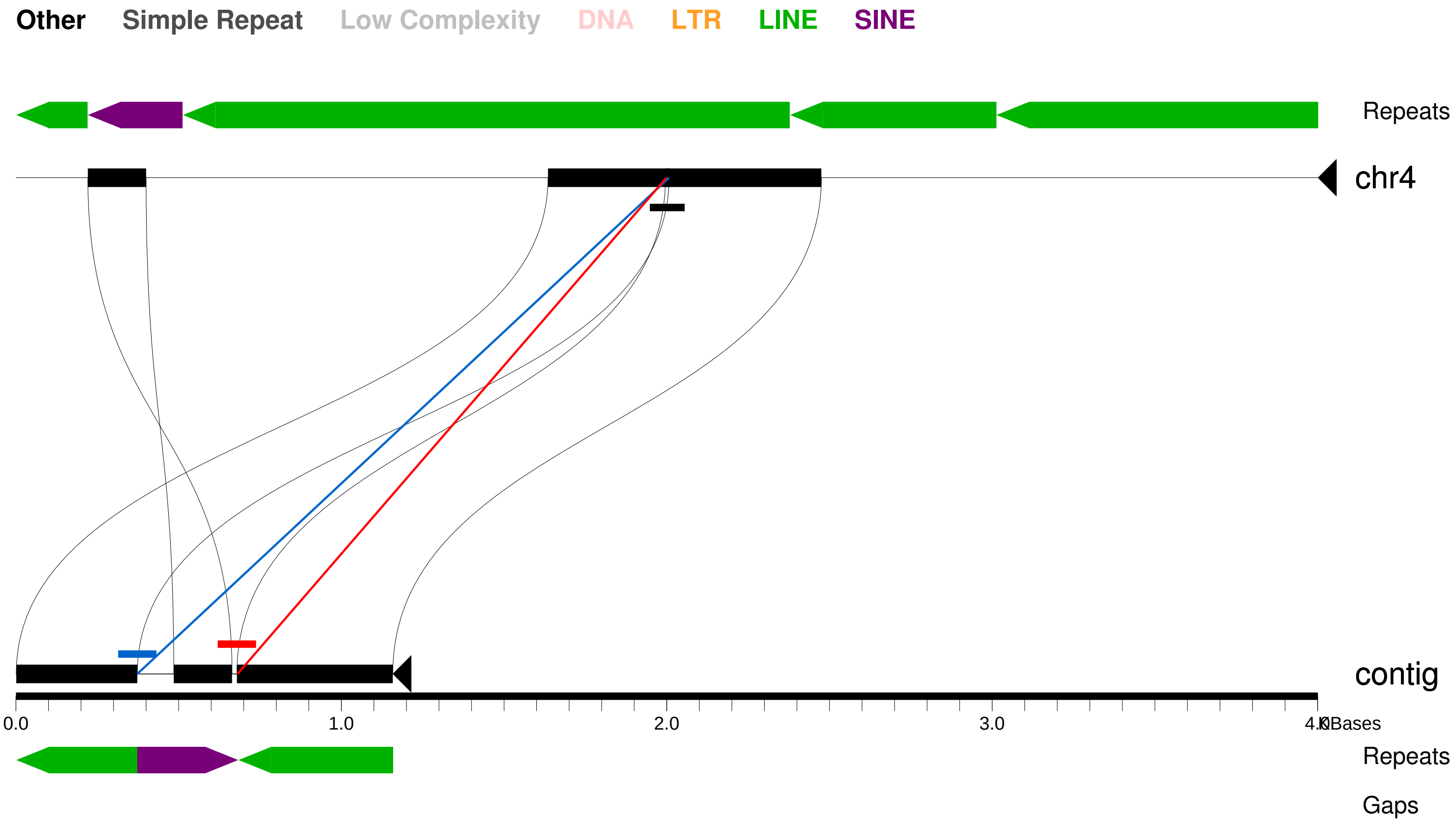

siteID: chr4_86584488 Scafftig3 - Breakpoint: chr4:86584490
chr4:86584443-86584549
Overlap Length: 13 INS size: 411 Genotyped: No

```
left    GGAGTTCACTCATGATTTGGCTCTCTGTTTGTTTGTTATTGGTGTAT------------AAGAATGCTTG
chrom   GGAGTTCACTCATGATTTGGCTCTCTGTTTGTTTGTTATTGGTGTAT------------AAGAATGCTTG
right   NNNNNNNNNNNNNNNNNNNNNNNNNNNNNNNNNNNNNNNNNNNNNNNNNNNNNNNNNNAAAGAATGCTTG
        11111111111111111111111111111111111111111111111111111111111***********

left    TGGGCCGGGCGCGGTGGCTCACGCCTGTAATCCCAGCACTTTGGGAGGCAGAGGCGGGCGG
chrom   TGATTTTTGTACATTGATTT-----TGTA-TCCTGAGACTTTG-----CTGAAGTTG-CTT
right   TGATTTTTGTACATTGATTT-----TGTA-TCCTGAGACTTTG-----CTGAAGTTG-CTT
        **222222*22*22**22*222222****2***2222******22222*2**2*22*2*22
```

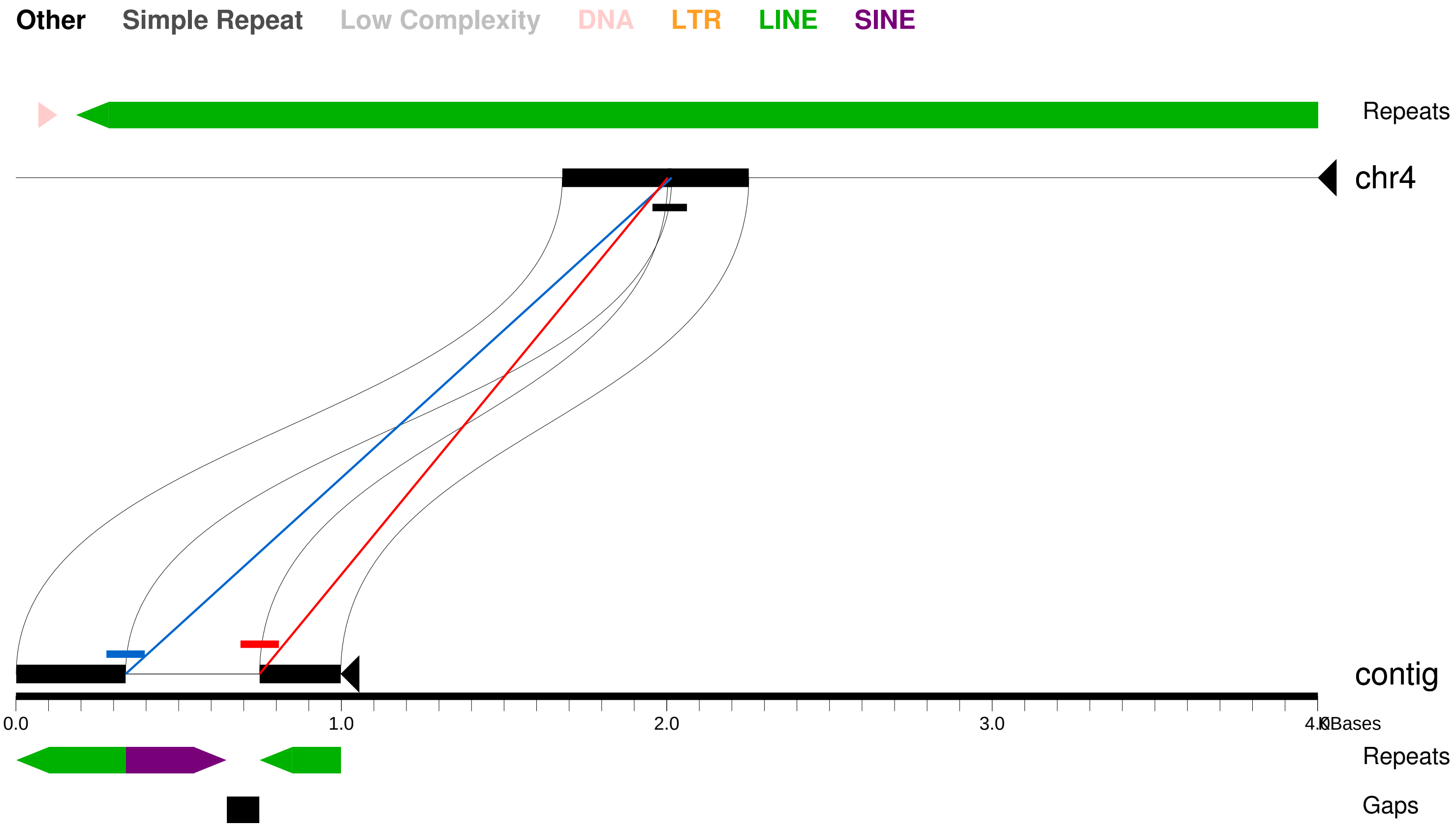

siteID: chr4_88254319 Scafftig1 - Breakpoint: chr4:88254300
chr4:88254140-88254470
Overlap Length: 11 INS size: 298 Genotyped: Yes

```
left    NNNNNNNNNNNNNNNNNNNNNNNNNNNNNNNNNNNNNNNNNNNNNN---------TTTGTTTGTTTTCCA
chrom   TTTTGGTCTTTGTTTGTTTAAAGTGTGTCTGAAATTAGAAGAGCAACCCGTGATTTTTGTTTGCTTACCA
right   TTT---------------------------------AGTAGAGACAG----GGTTTCACTGTGTTAGCCA
        222NNNNNNNNNNNNNNNNNNNNNNNNNNNNNNNNN22N2222NN2N    2N22*111*1**N*1N***

left    TTTGCTTGGTAGATTTTTCTCCATCCCTT-TACTTTGAGCCTATGGGTGTCACTGCATG-TGGGAAAGGT
chrom   TTTGCTTGGTAGATTTTTCTCCATCCCTT-TACTTTGAGCCTATGGGTGTCACTGCATG-TGGGAAAGGT
right   ---GGATGGTC--TCGATCTCCTGACCTCGTGATCCGCCCGTCTCGGCCTCCCAAAGTGCTGGGATTACA
        111*11****111*111*****111***11*11*11*11*1*1*1**11**1*1111**1*****11111

left    CTCTTGAAGACACCGTGTCATT--GAGTCTTGCTTTTTTTTTTTTTT-------------TTTTTGAGA
chrom   CTCTTGAAGACACCGTGTCATT--GAGTCTTGCTTCTTTATCCAACTTCCCACTCCAGGCCTTTCAATTA
right   GGCGTGA-GCCACCGCGCCCGGCCGAGTCTTGCTTCTTTATTCAACTTCCCACTCCAGGCCTTTCAATTA
        11*1***1*1*****1*1*11111***********2***2*N2222**2222222222222***22222*

left    CGGAGTTTCGCTCTGTCGCC--CAGGCTGGA-----------GTGCAGTGGCGCA----ATCTCGAC--T
chrom   GGGCATTTAGCCCATTTACATTCAAGTTTAATATTGATATGTGTGGATTTGATCCTGTCATTTTGTTGTT
right   GGGCATTTAGCCCATTTACATTCAAGTTTAATATTGATATGTGTGGATTTGATCCTGTCATTTTGTTGTT
        2**22***2**2*22*22*222**2*2*22*22222222222***2*2*2*22*22222**2*2*2222*

left    CACTGCAAGCTCCGC---CTCCCGGGTTCA-TGCCATTCTCCTGCC-----TCAG
chrom   AACTGGTTATTTTGCAGACTTGCTTGTGCAGTTGCTTTGTAGTGTCAATGGTCTA
right   AACTGGTTATTTTGCAGACTTGCTTGTGCAGTTGCTTTGTAGTG-----------
        2****22222*22**222**22*22**2**2*22*2**2*22**N1     11NN
```

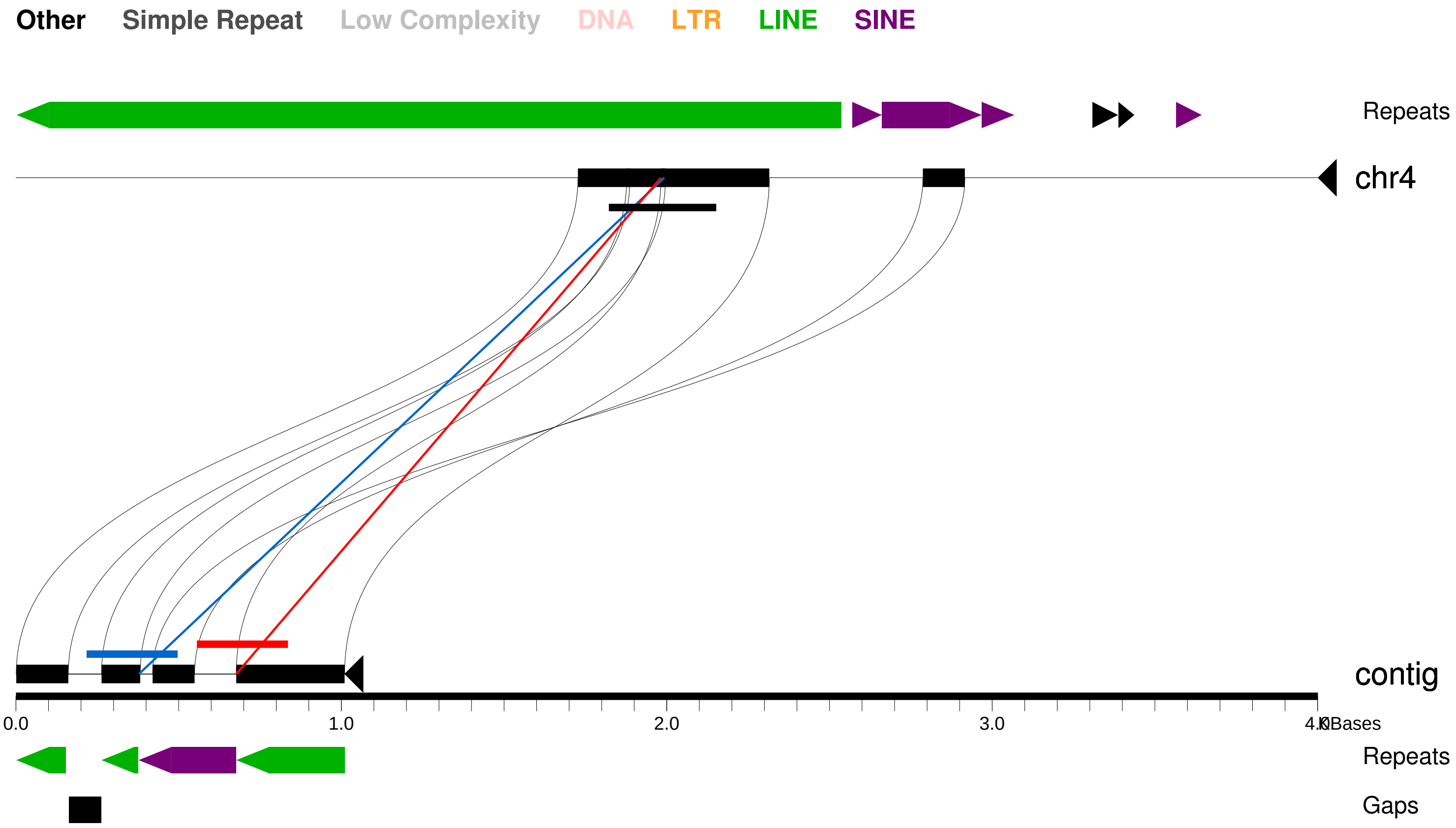

siteID: chr4_92270957 Scafftig1 - Breakpoint: chr4:92270943
chr4:92270783-92271117
Overlap Length: 15 INS size: 308 Genotyped: Yes

```
left   ----------------GTCAGGCCTTTCAAAATGGTTACTTTTTCTCTTCCCCTGCTGGAAGCAAGAGAG
chrom  AATAGTTTGTCTTGAGGTCAGGCCTTTCAAAATGGTTACTTTTTCTCTTCCCCTGCTGGAAGCAAGAGAG
right  GAGAAT--GGCGTGAACCCGGG-------AGGCGGAG--------------CTTGCAGTGAGCC-GAGA-
       N2N2N2  2N2N222N11*1**1111111*111**1111111111111111*1***1*11***11****1

left   TTTTTCTTTTTTTTTTTTCCTTTCAGGTTTTCACTGAGAGAACCCAACTGGGCTGTAGGAGGTACATTTC
chrom  TTTTTCTTTTTTTTTTT-CCTCTCAGGTTTTCACTGAGAGAACCCAACTGGGCTGTAGGAGGTACATTTC
right  --TCCCGCCACTGCACT-CCAGCCTGGGCGACAGAGCGAGACTCCGTCTCAAAAAAAAAAAAAAAAAAAA
       11*11*11111*1111*2**1N1*1**1111**11*1****11**11**1111111*11*111*1*1111

left   ATGAAAGTTTGGGGGCCTCGTAAGATTGTGTTCCCCGGCCGGG-------CGCGGTGGCTCACGCC---T
chrom  ATGAAAGTTTGGGGGCCTCGTAAGATTGAGTTCCCCTAGAATTTTTTCCACACTGTGCCTTCAGCAATTT
right  AAAAAAA--------------AAGATTGAGTTCCCCTAGAATTTTTTCCACACTGTGCCTTCAGCAATTT
       *11***111111111111111*******2*******22222222222222*2*2***2**222**2222*

left   GT-AATCCCAGCACTTTGGGAGGCCGAGGCG-------------GGCGGATCACG-----AGG-------
chrom  GTCAATTACAG--TTTAGGGTTTCCTAACAGCATTGTTTCCCATGGAGGTTTCTGCTTCTAGGCTTCTGC
right  GTCAATTACAG--TTTAGGGTTTCCTAACAGCATTGTTTCCCATGGAGGTTTCTGCTTCTAGGCTTCTGC
       **2***22***222**2***222**2*222*2222222222222**2**2*222*22222***2222222

left   -TCAGGAGATCGAGACCATCCCGG-CTAAAACG-GTGAAACCCCGTCTCTACTA--AA
chrom  TTCAGTAAATCGTGATTCTCTGTATCTTACCTGTGTGTCTCTCCATTTTAGGGAGCAA
right  TTCAGTAAATCGTGATTCTCTGTATCTTACCTGTGTGTCTCTCCA-------------
       2****2*2****2**222**22222**2*222*2***222*2**21N1NNNNN1  11
```

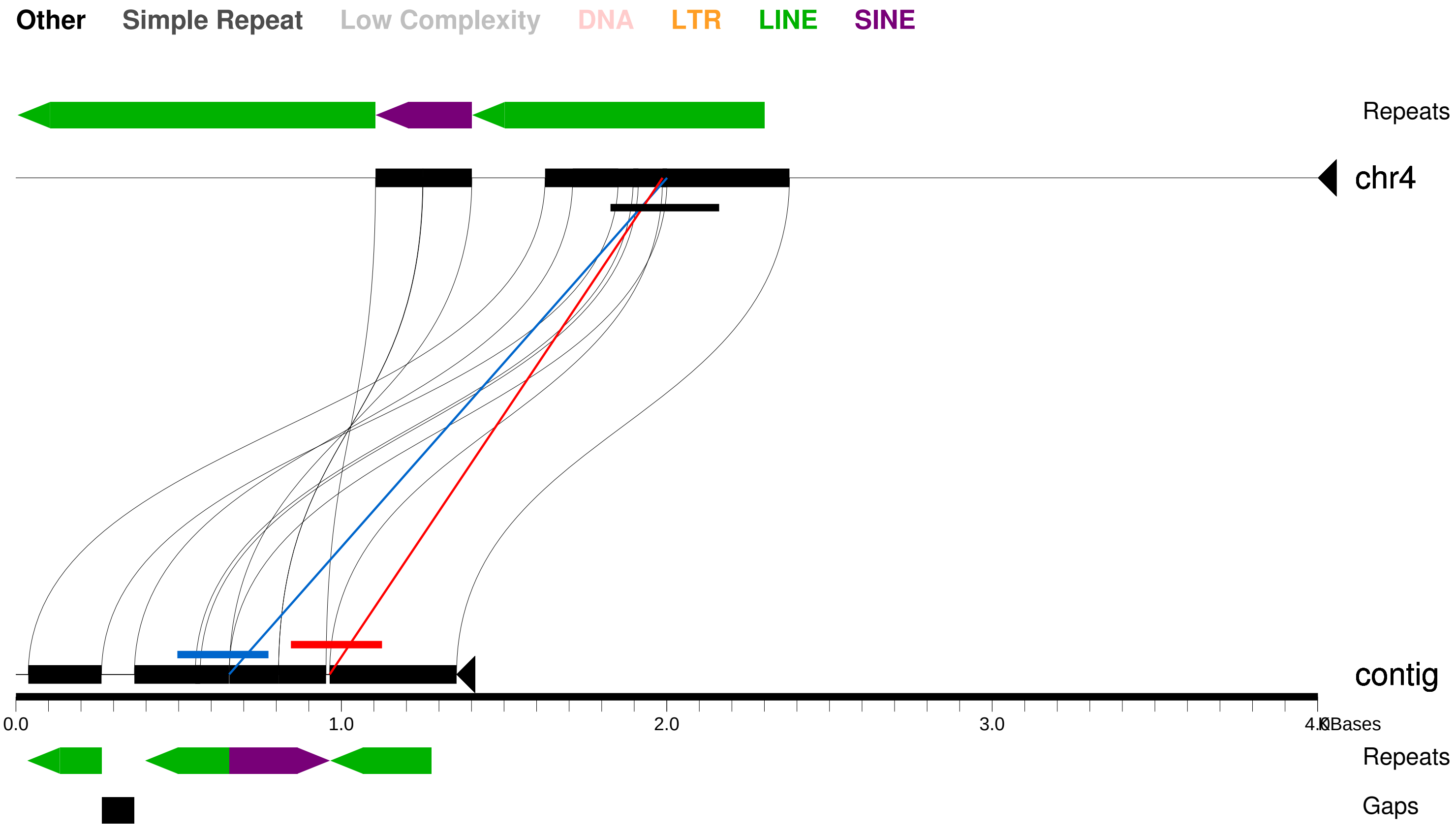

siteID: chr4_92704639 Scafftig3 - Breakpoint: chr4:92704646
chr4:92704596-92704705
Overlap Length: 10 INS size: 277 Genotyped: Yes

```
left    TTG---TGG----CATATTGATATAAGGGA--ATAATATTCAGATATTTTAAGTAAGTTAAAACATACAT
chrom   TTG---TGG----CATATTGATATAAGGGA--ATAATATTCAGATATTTTAAGTAAGTTAAAACATACA-
right   CCGACCTGGGCGACAGAGCGAGACTCCGTCTCAAAAAAAAAAAAAAAAAAAAAAAAAAAAAAAACATACA-
        11*111***1111**1*11**1*1111*1111*1**1*111*1*1*1111**11**111**********2

left    TGGGAGGCCGAGGCGGGTGGATCATGAGGTCAGGAGATCGAGACCATCCTGGCTAACA
chrom   ----ACATATAAATATTAAAATAATTA--TCCTGAGT--GATAAAATGCAAACTAAAT
right   ----ACATATAAATATTAAAATAATTA--TCCTGAGT--GATAAAATGCAAACTAAAT
        2222*22222*222222222**2**2*22**22***222**2*22**2*222****22
```

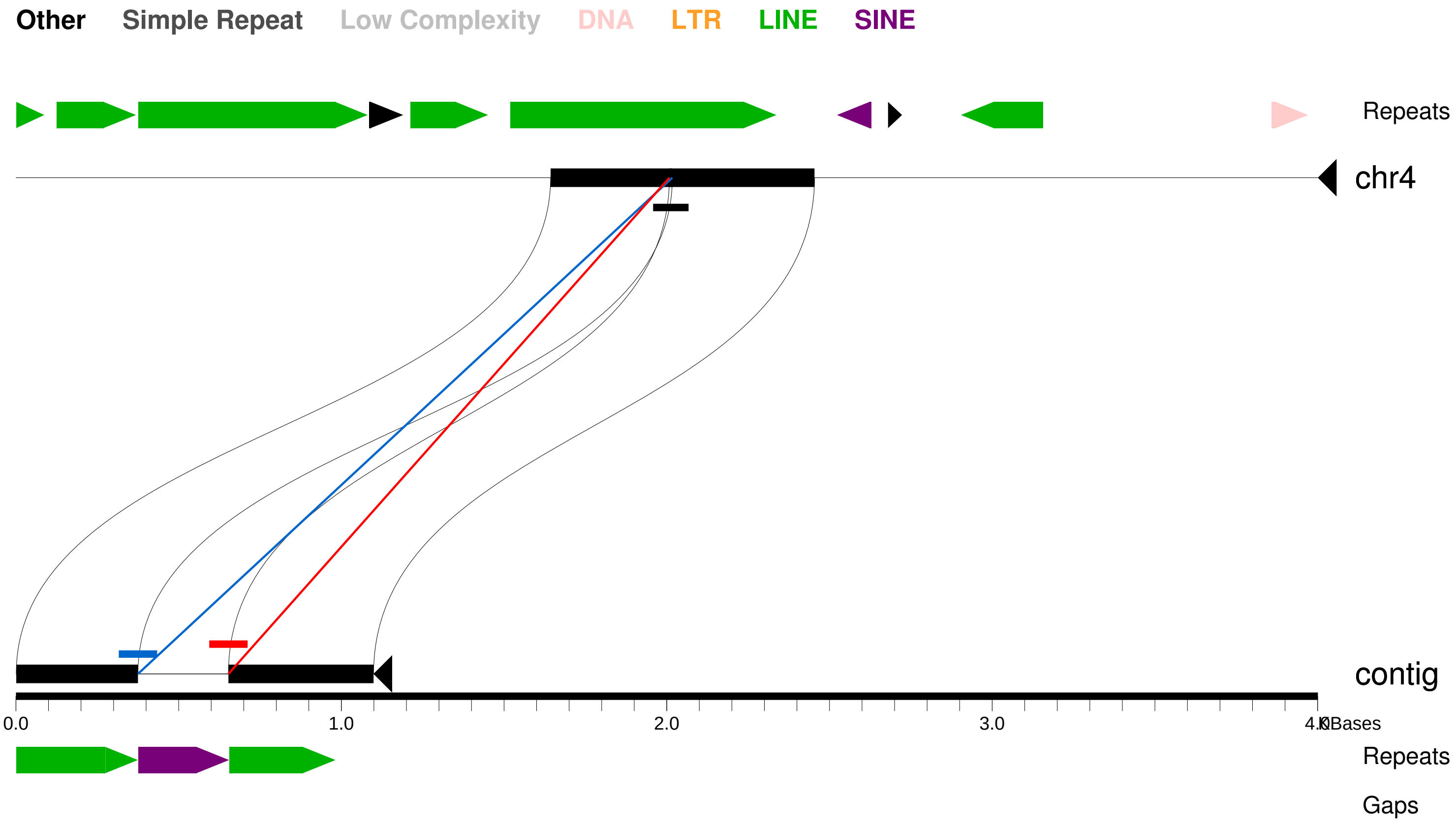

siteID: chr4_93363271 Scafftig1 + Breakpoint: chr4:93363281
chr4:93363235-93363340
Overlap Length: 14 INS size: 546 Genotyped: No

```
left    GCCCTTGAAAAGAAGGTAATGTGGAGTGGGT-------------GCCTCCATATTGATTAAGAAGGGGAC
chrom   GCCCTTGAAAAGAAGGTAATGTGGAGTGGGT-------------GCCTCCATATTGATTAAGAAGGGGAC
right   NNNNNNNNNNNNNNNNNNNNNNNNNNNNNNNNNNNNNNNNNNNNNNNNNNNNAAAAAAAAAGAAGGGGAC
        1111111111111111111111111111111111111111111111111111*111*11***********

left    GGGNNNNNNNNNNNNNNNNNNNNNNNNNNNNNNNNNNNNNNNNNNNNNNNNNNNNNNNNNNN
chrom   GGGCTTACCTTCCACTGTGAGAGTTACCTGAAGCTTGGCGTCCGTGATG-------------
right   GGGCTTACCTTCCACTGTGAGAGTTACCTGAAGCTTGGCGTCCGTGATG-------------
        ***22222222222222222222222222222222222222222222222222222222222
```

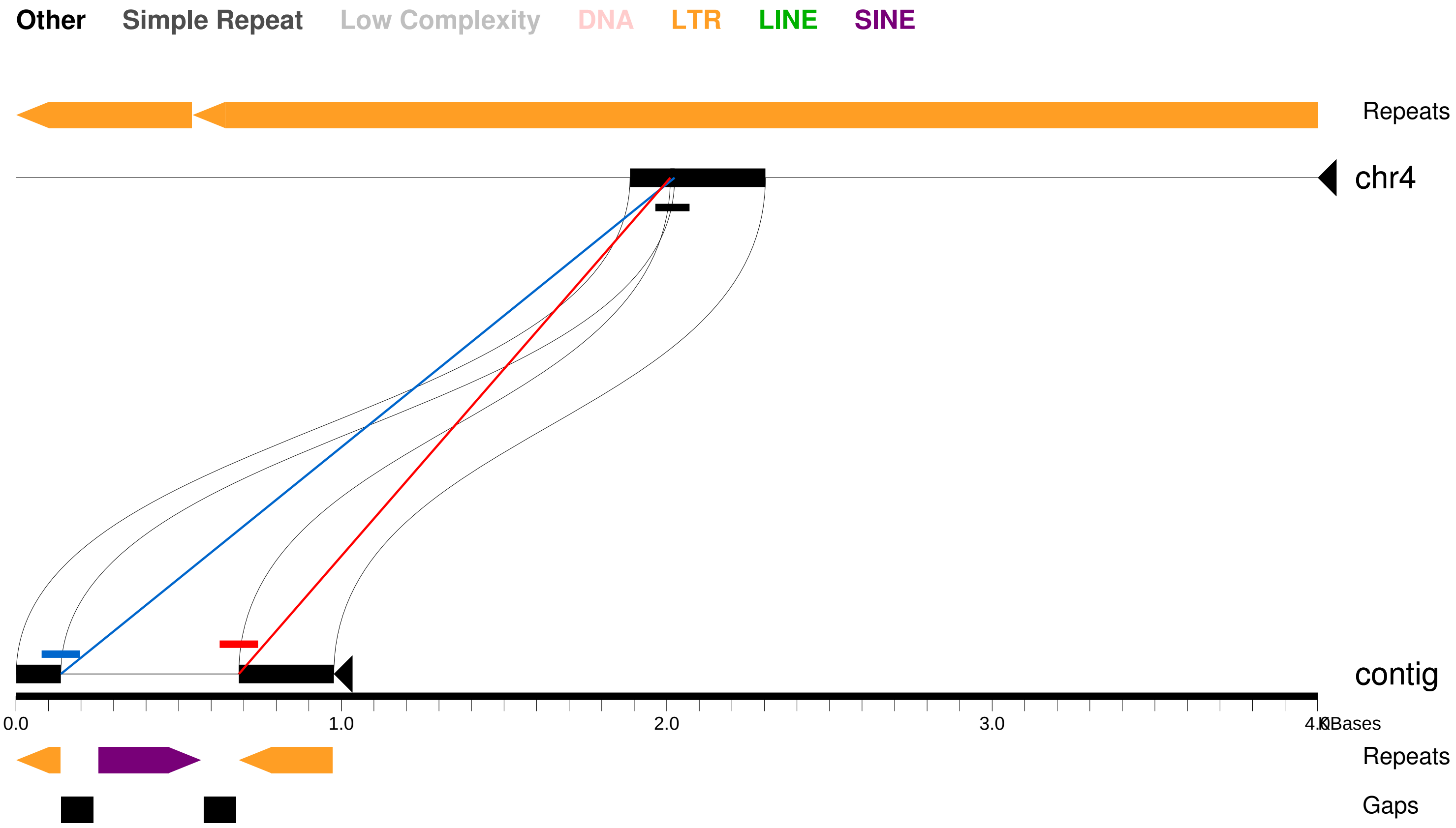

siteID: chr4_95413283 Scafftig1 + Breakpoint: chr4:95413267
chr4:95413221-95413326
Overlap Length: 14 INS size: 496 Genotyped: No

```
left    AAGTAATTTGTATAATAGTCAATGCTGATACACTTTTTTCAGCTTT-------------AAGAATTGTTT
chrom   AAGTAATTTGTATAATAGTCAATGCTGATACACTTTTTTCAGCTTT-------------AAGAATTGTTT
right   NNNNNNNNNNNNNNNNNNNNNNNNNNNNNNNNNNNNNNNNNNNNNNNNNNNNNAAAAAAAAGAATTGTTT
        11111111111111111111111111111111111111111111111111111111111***********

left    TTCGGCCGGGCGCGGTGGCTCACGCCTGTAATCCCAGCACTTTGGGAGGCCGAGGCGGGTGG
chrom   TTCTT----TTCCCATTTCCCATGAGT-TAAT---GGATAAGAGAGAGGACCAA-----TTT
right   TTCTT----TTCCCATTTCCCATGAGT-TAAT---GGATAAGAGAGAGGACCAA-----TTT
        ***222222222*22*22*2**2*22*2****2222*222222*2****2*2*222222*22
```

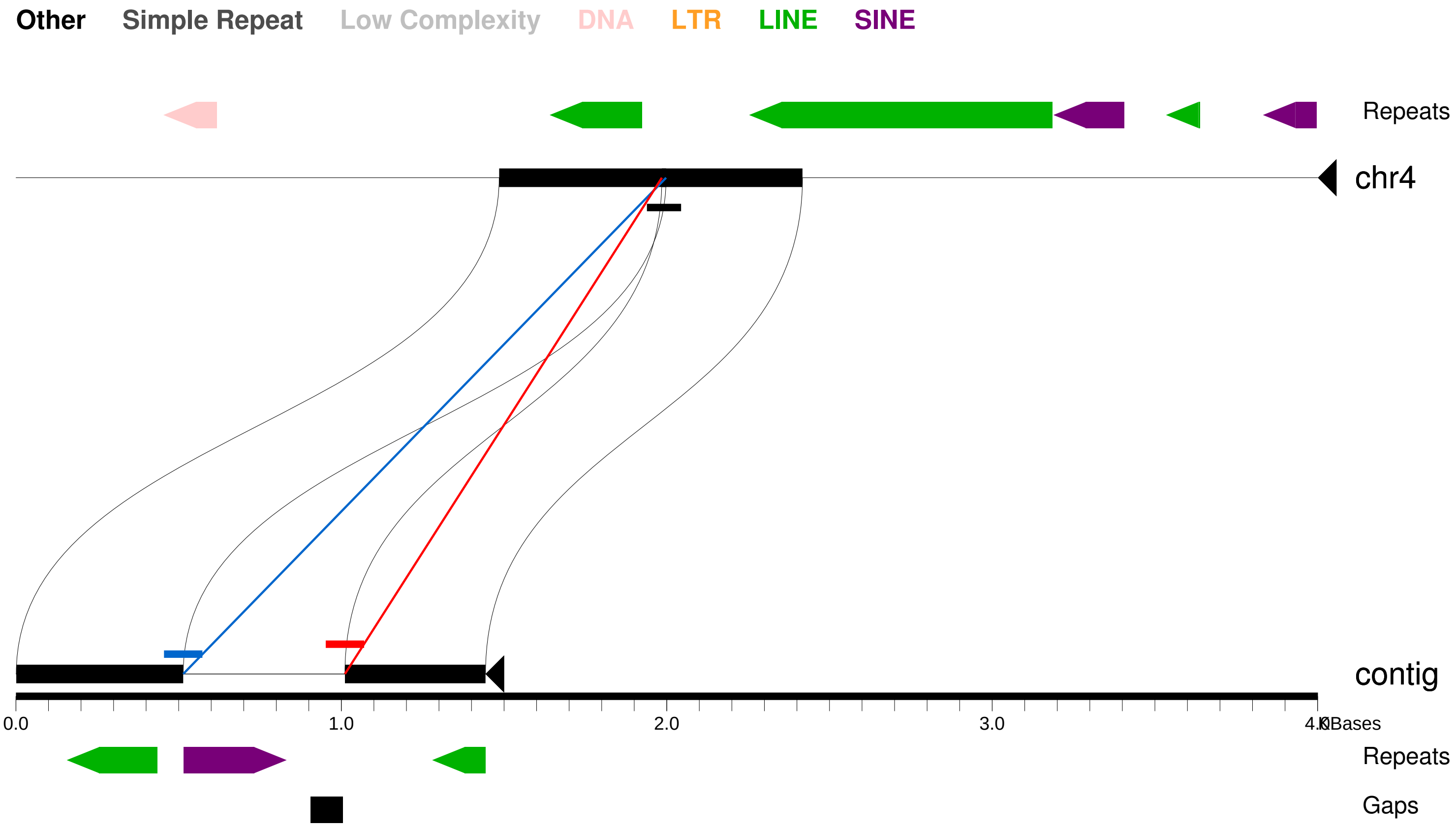

siteID: chr5_100060196 Scafftig4 + Breakpoint: chr5:100060168
chr5:100060123-100060227
Overlap Length: 15 INS size: 263 Genotyped: Yes

```
left     TGAATGATATCTTATTTAGGAAAAAGGGGTATT---TGCATGTGAAGT-----------TAATTTAAGGA
chrom    TGAATGATATCTTATTTAGGAAAAAGGGGTATT---TGCATGTGAAGT-----------TAATTTAAGGA
right    CCGCCCGCCTCGGCCTCCCAAAGTGCTGGGATTACAGGCGTGAGCCACCGCGCCCGGCCTAATTTAAGGA
         111111111**1111*1111**11111**1***1111**1**1*111111111111111***********

left     TTTTTTTTTTTTTTTTTTTTTTGAGACGGAGTCTCGCTCTGTCGCCCAGGCTGGAGTGCAGTG
chrom    TTTTAAGATAAGAAAATTATCCTGGAT------TAACTGGGTGGCCT--------TAAATCT
right    TTTTAAGATAAGAAAATTATCCTGGAT------TAACTGGGTGGCCT--------TAAATCT
         ****2222*2222222**2*2222**2222222*22**22**2*2**222222222*22*222
```

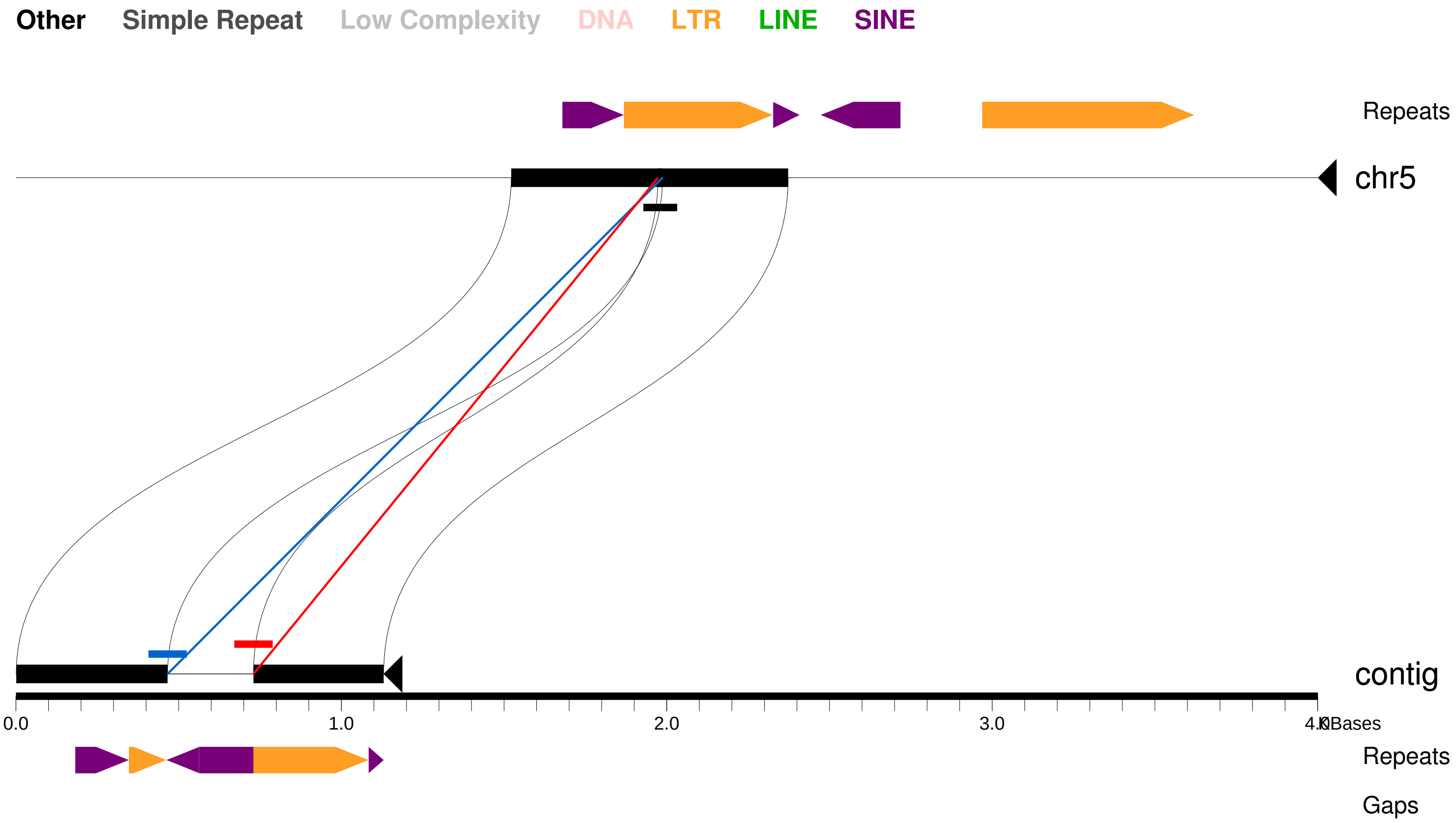

siteID: chr5_102343442 Scafftig2 - Breakpoint: chr5:102343462
chr5:102343417-102343521
Overlap Length: 15 INS size: 377 Genotyped: No

```
left    AATTGTGTT-----ATCTTA------ATGTGCTCT-TTTTATAAATT--TTCTTTGCTATATATTTCACA
chrom   AATTGTGTT-----ATCTTA------ATGTGCTCT-TTTTATAAATT--TTCTTTGCTATATATTTCACA
right   GACGGGGTTTCACCGTGTTAGCCAGGATGGTCTCGATCTCCTGACCTCGTGATCCGCCCTATATTTCACA
        1*11*1***111111*1***111111***11***11*1*11*1*11*11*11*11**11***********

left    TCTTTNNNNNNNNNNNNNNNNNNNNNNNNNNNNNNNNNNNNNNNNNNNNNNNNNNNNNNNNNN
chrom   TCTT--------------AAAAGTATCTTACAACTGTTAGTGTGTTCTAACTCAGTTTTGATT
right   TCTT--------------AAAAGTATCTTACAACTGTTAGTATGTTCTAACTCAGTTTTGATT
        ****2222222222222222222222222222222222222N222222222222222222222
```

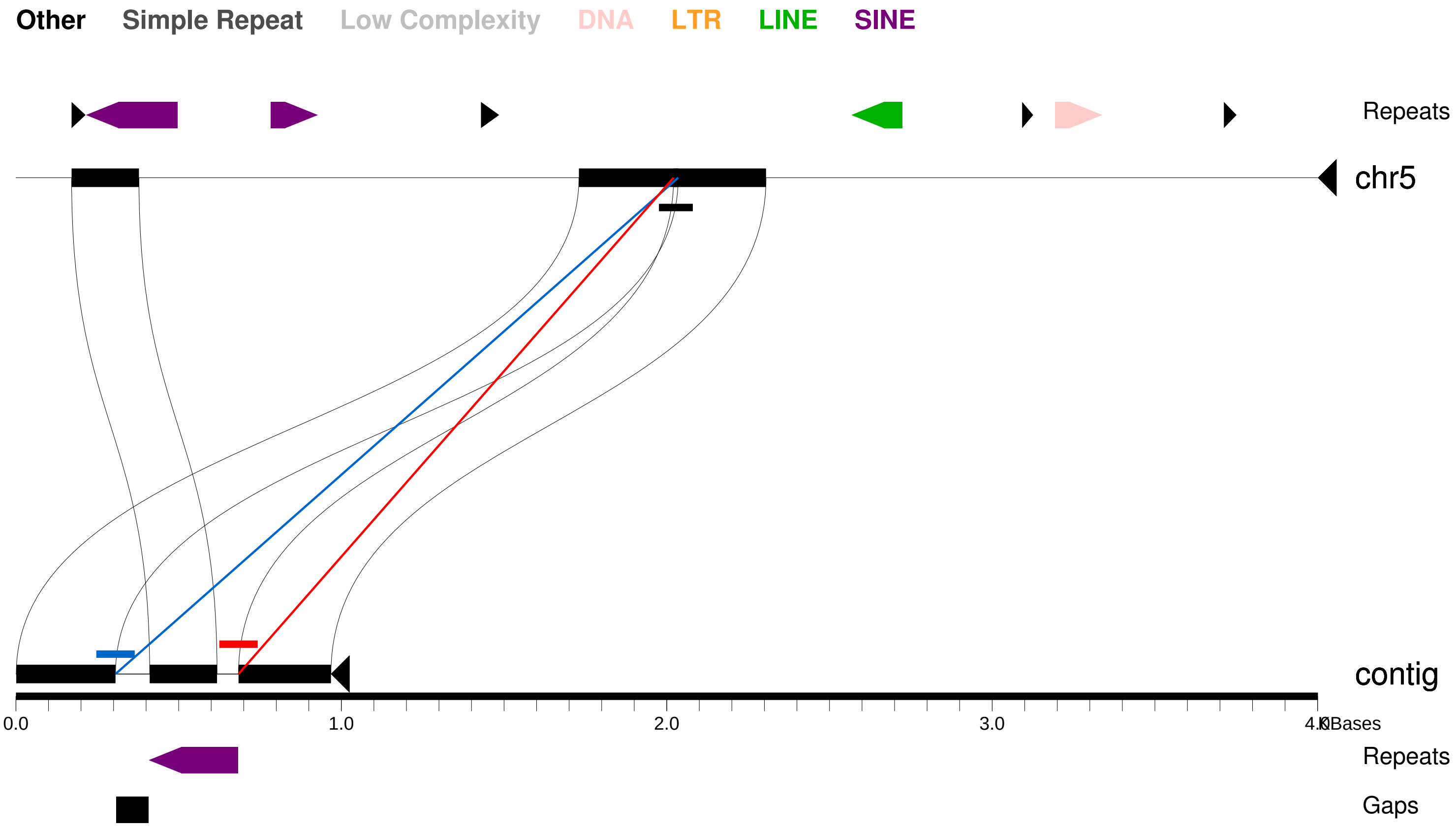

siteID: chr5_103464399 Scafftig1 + Breakpoint: chr5:103464408
chr5:103464248-103464586
Overlap Length: 19 INS size: 307 Genotyped: Yes

```
left   ---------------------TTTCAATCATATAGAGTTCTGGAAATTCAATATTATCTATCTTATTATA
chrom  AACCCTCTTACGCAATACCACTTTCAATCATATAGAGTTCTGGAAATTCAATATTATCTATCTTATTATA
right  AG-----------AATGGCG---TGAACCC----GGGAAGCGGAGCTTGCAG-TGAGCCG-------AGA
       2N           222NN2N 11*1**1*11111*1*1111***11**11*11*1*1*111111111*1*

left   TTTACAGTGGTATTTTGACTTCCCCTATAGACTGGAAATTGGCTTCCTGGCAGCTCACGAGCACCCTTGA
chrom  TTTACAGTGGTATTTTGACTTCCCCTATAGACTGGAAATTGGCTTCCTGGCAGCTCGCGAGCACCCTTGA
right  TT----GCGCCACT--GCAGTCCGC---AGTCTGGCC-TGGGC-----GACAGAGCGAGA-CTCCGTCTA
       **1111*1*11*1*11*111***1*111**1****111*1***11111*1***11*21**1*1**1*11*

left   CATTTAAAAGAGATTTAGTTAAGAAATAATATATTTTGCGGGGCC-------------GGGCGCGGTGGC
chrom  CATTTAAAAGAGATTTAGTTAAGAAATAATATATTTTGCTTGGCTTATTTGCATTTAAGGGCTTGGTGG-
right  AAAAAAAAAAAAAAAAA---AAGAAATAATATATTTTGCTTGGCTTATTTGCATTTAAGGGCTTGGTGG-
       1*111****1*1*111*111******************22***22222222222222****22*****2

left   TCACGCC--TGTAATCCCAGCACTTTGGGAGGCCGAGGCGGGTGGATC-ATGAGGTCAGGAG--ATCGAG
chrom  TGGGGACAATGCAAGTCTGGAATTTAAAAGAGCCTA--CAGCAGGAAATATCACTTTAAAAGCTATCGTA
right  TGGGGACAATGCAAGTCTGGAATTTAAAAGAGCCTA--CAGCAGGAAATATCACTTTAAAAGCTATCGTA
       *222*2*22**2**22*22*2*2**222222***2*22*2*22***222**2*22*2*22**22****22

left   ACCATCCTGGCTAACA--AGGTGAAACCCCGTCTC--TACTAA------------------A
chrom  ATATTGATAGTTAAAACCATGTTAATTGATGTCTTCTTGGTAAGGAAAGAAAAATATAGAGA
right  ATATTGATAGTTAAAACCATGTTAATTGATGTCTTCTTGGTAAGGAAAG-------------
       *222*22*2*2***2*22*2**2**22222****222*22***222222            1
```

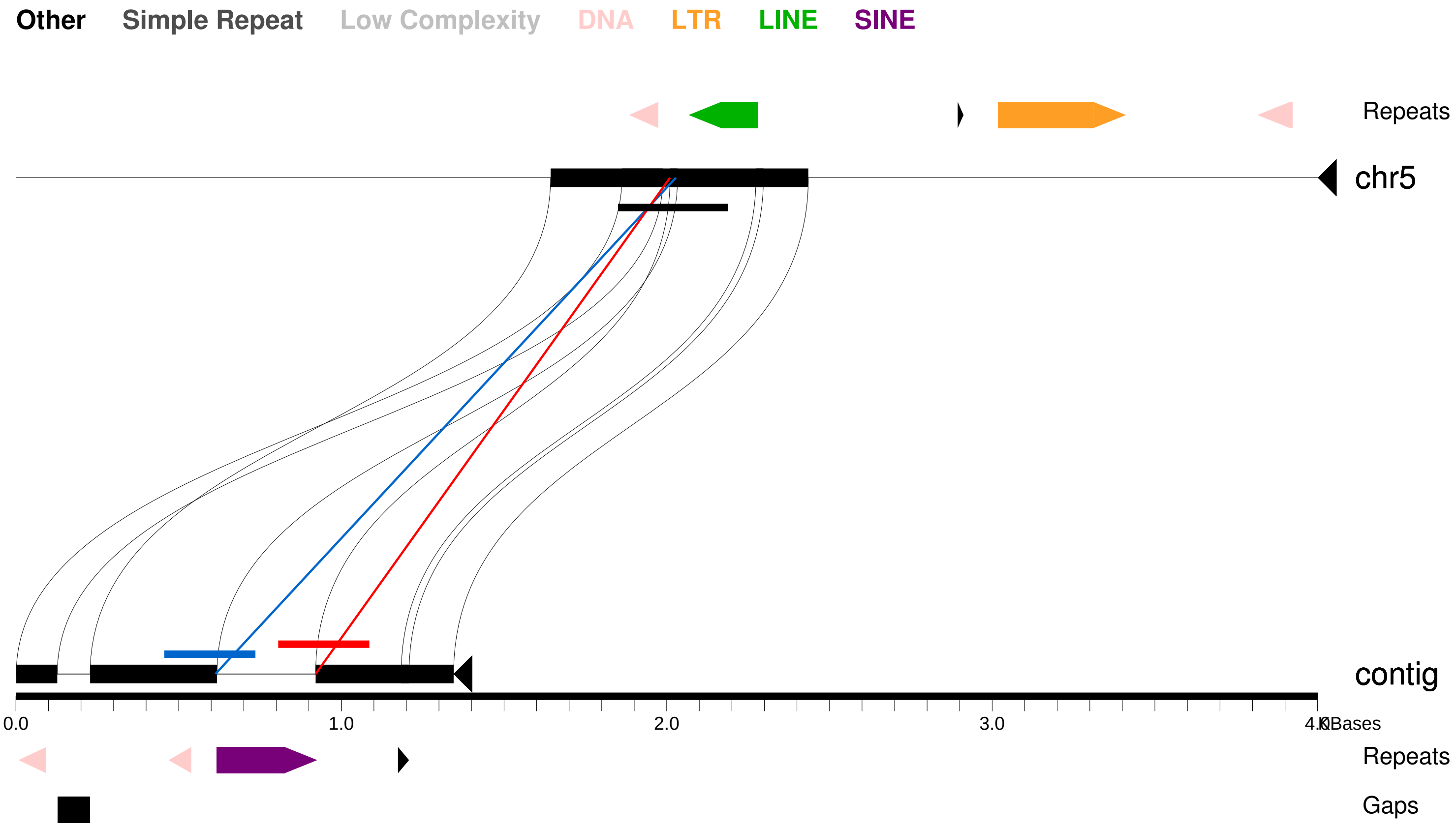

siteID: chr5_103738429 Scafftig1 + Breakpoint: chr5:103738419
chr5:103738377-103738483
Overlap Length: 18 INS size: 276 Genotyped: Yes

```
left     ACATCCTGCAGCCCCTAATGAAATT-TGATTTGA-GATTCACTA-------------AAAGTTAAAGTTA
chrom    ACATCCTGCAGCCCCTAATGAAATT-TGATTTGA-GATTCACTA-------------AAAGTTAAAGTTA
right    TTAGCCAGGATGGTCTC--GATCTCCTGACCTCATGATCCACCCGCCTCGGCCTCCCAAAGT-AAAGTTA
         11*1**1*1*1111**111**11*11***11*1*1***1***111111111111111*****1*******

left     ATTTTTTTTTTTTTTTTTTTTTTTTTTTTTTTTTG-AGACGGAGTCTCGCTCTGTCGCCCAGGCTGG
chrom    ATTTTGAAAATAACGTGAAAATTTCAAGTACAATAAATAAAATATTAAT-------------TAA
right    ATTTTGAAAATAACGTGAAAATTTCAAGTACAATAAATAAAATATTAAT-------------TAA
         *****22222*2222*22222***2222*22222*2*222*2*2*222*2222222222222*22
```

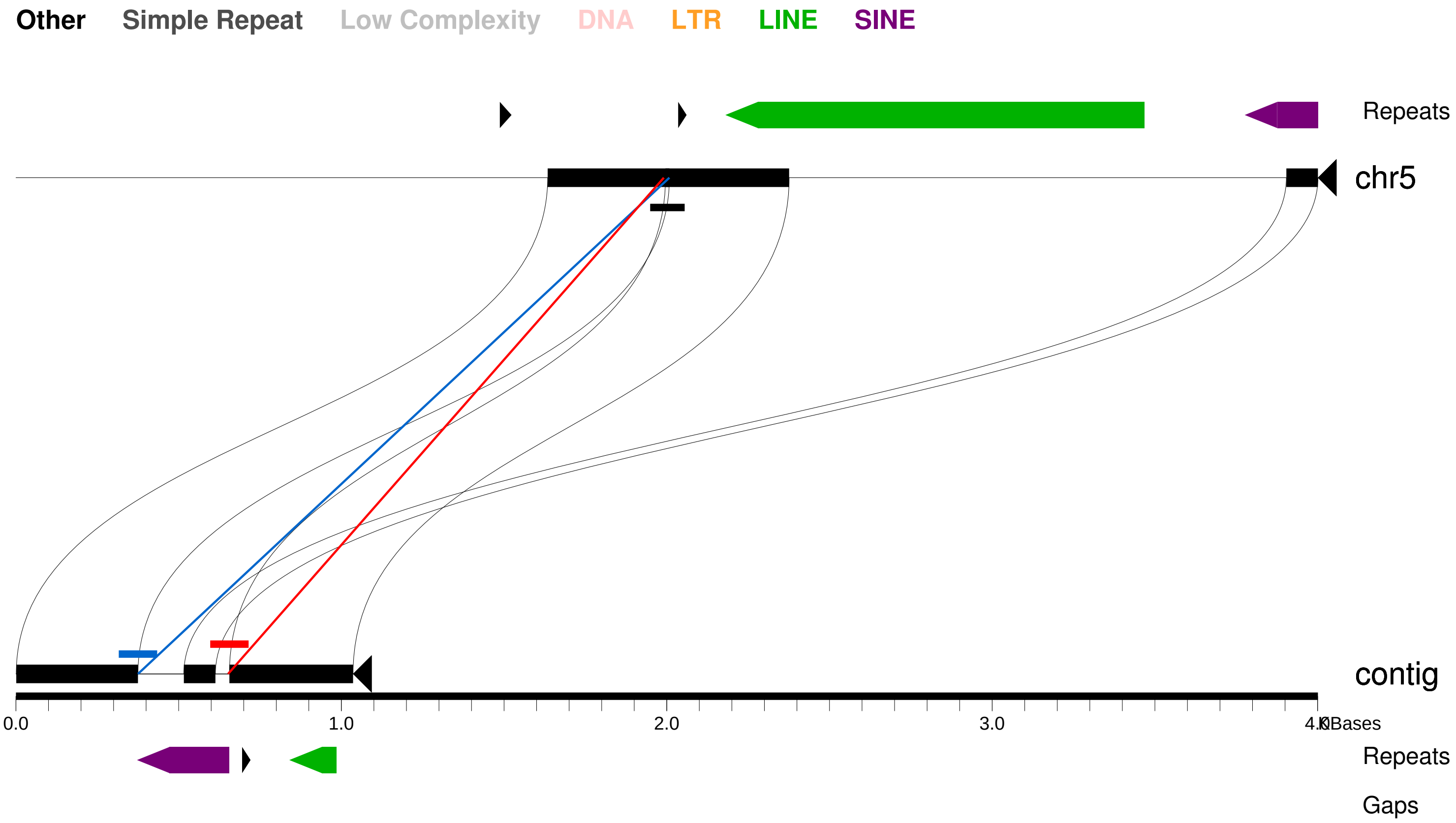

siteID: chr5_10422812 Scafftig4 - Breakpoint: chr5:10422828
chr5:10422786-10422887
Overlap Length: 18 INS size: 299 Genotyped: Yes

```
left     CC--------CCAT--TTGAAGA-AGATC----CTTAGAAT--AACCAATCAAACCAGTAAAGAAATGGG
chrom    CC--------CCAT--TTGAAGA-AGATC----CTTAGAAT--AACCAATCAAACCAGTAAAGAAATGGG
right    CCACTGCACTCCAGCCTGGGCGACAGAGCGAGACTCCGTCTCAAAAAAAAAAAAAAAAAAAAGAAATGGG
         **11111111***111*1*11**1***1*1111**11*11*11**11**11***11*11*********

left     AACATTGAGGCCGGGCGCGGTGGCTCACGCCTGTAATCCCAGCACTTTGGGAGGCCGAGGCGGGCG
chrom    AACATTG--------------GCCTCAAGATGGTAGACAGTGAA---GAGGAGAATTAACAATCTG
right    AACATTG--------------GCCTCAAGATGGTAGACAGTGAA---GAGGAGAATTAACAATCTG
         *******22222222222222*2****2*222***22*222*2*22222****2222*2222222*
```

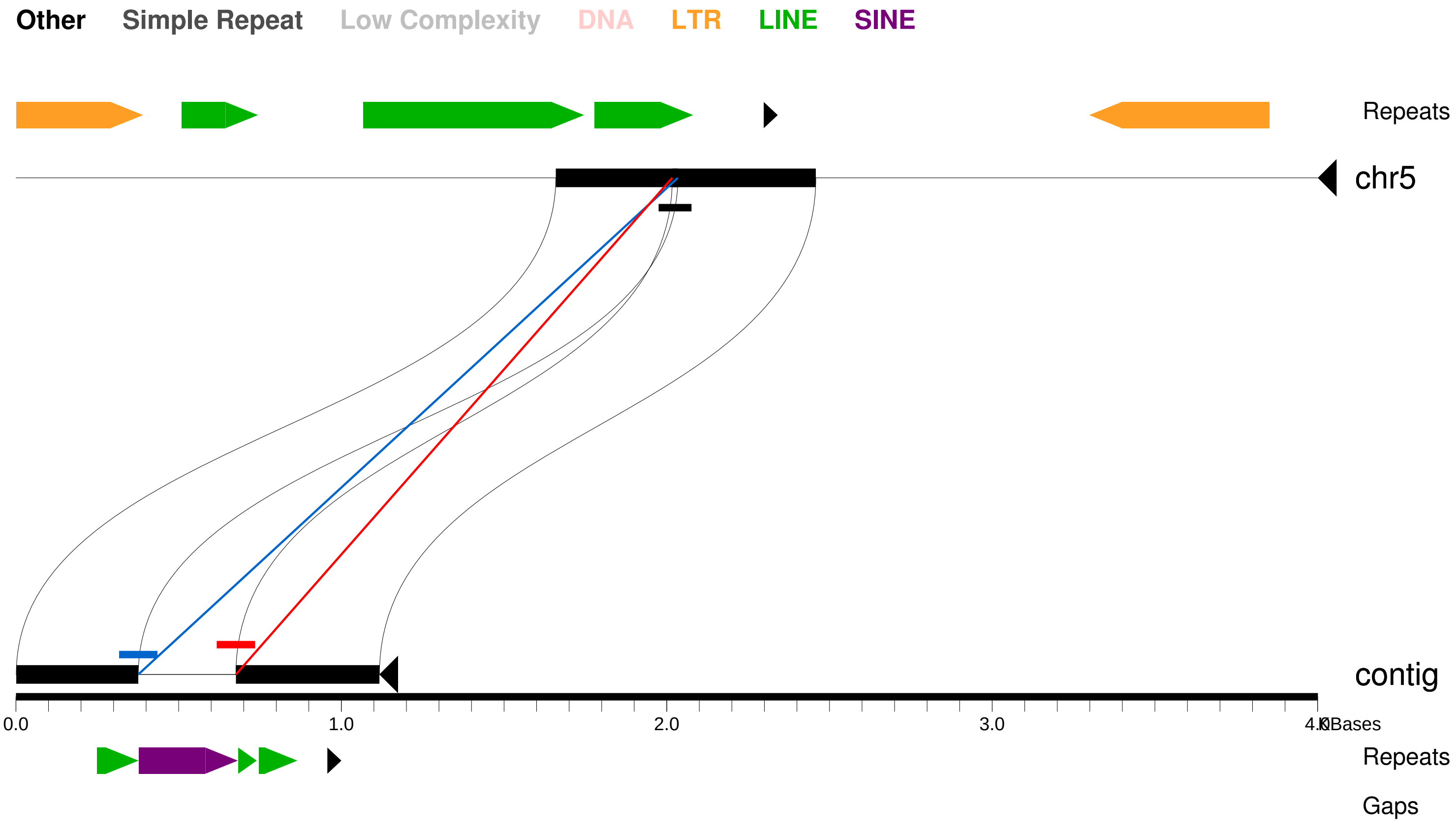

siteID: chr5_104650891 Scafftig3 + Breakpoint: chr5:104650862
chr5:104650702-104651035
Overlap Length: 14 INS size: 301 Genotyped: Yes

```
left    T--------AGTTGTTACTGAAAGTGACATCCTTTTTAATTCTATTTTTAGTAATTTAAATTGTTTTTTT
chrom   TTGGAATATAGTTGTTACTGAAAGTGACATCCTTTTTAATTCTATTTTTAGTAATTTAAATTGTTTTTTT
right   TT------TAGTAGAGACGGG--GTTTCACC-------------TTGTTAGCCAGG---ATGGTCTCGAT
        *2      2***1*11**1*111**11**1*1111111111111**1****11*11111**1**1*111*

left    TTTGTTAGTATAGCTAAATATTTGTCTATTTTATTATATTTTGAAGGAATAAACTCTTGGTTTTACTGAT
chrom   TT-GTTAGTATAGCTAAATATTTGTCTATTTTATTATATTTTGAAGGAATAAACTCTTGGTTTTACTGAT
right   CT-CCTG----ACCTCATGATCCACCCGCCTCGGCCTCCC----------AAAGTGCTGGGATTACAGGC
        1*211*11111*1**1*11**1111*1111*11111*1111111111111***1*11***11****1*11

left    ACTCTTCTACTGGTTTTATGTTATTTCATTTATTT----TTTTTTTTTTTTTGA------------GAC
chrom   ACTCTTCTACTGGTTTTATGTTATTTCATTTATTTCTACTTTGTTCTTTATTGTTTTCCTTTCTTATGAC
right   G-TGAGCCACCGCGCCCGGCCTATTTCATTTATTTCTACTTTGTTCTTTATTGTTTTCCTTTCTTATGAC
        11*111*1**1*111111111**************2222***2**2***2***22222222222222***

left    G----GAG-TC---TCGCTCTG-----TC--GCC--CAGGTCGGACTGCGGACTGCAG-TGGCGC--AAT
chrom   TTTTAGAGATCAGTTTGCTCTTCTTTTTCCAGCCATCTGCTTAGATTATTAATTTGAGATGGTTCTGAAT
right   TTTTAGAGATCAGTTTGCTCTTCTTTTTCCAGCCATCTGCTTAGATTATTAATTTGAGATGGTTCTGAAT
        22222***2**222*2*****222222**22***22*2*2*22**2*2222*2*22**2***22*22***

left    --------CTCGGCTC-ACTGCAAGCTCCGCTTCCCGGGTTCACGCCATTCTCCT-
chrom   TATTATTACTTTTTTCTAGTTTAAACTTTTATTTTAGA-TTCAAGAGATACATTTG
right   TATTATTACTTTTTTCTAGTTTAAACTTTTATTTTAGA-TTC--------------
        22222222**2222**2*2*22**2**2222**222*22***1N1NN11N1NNN1
```

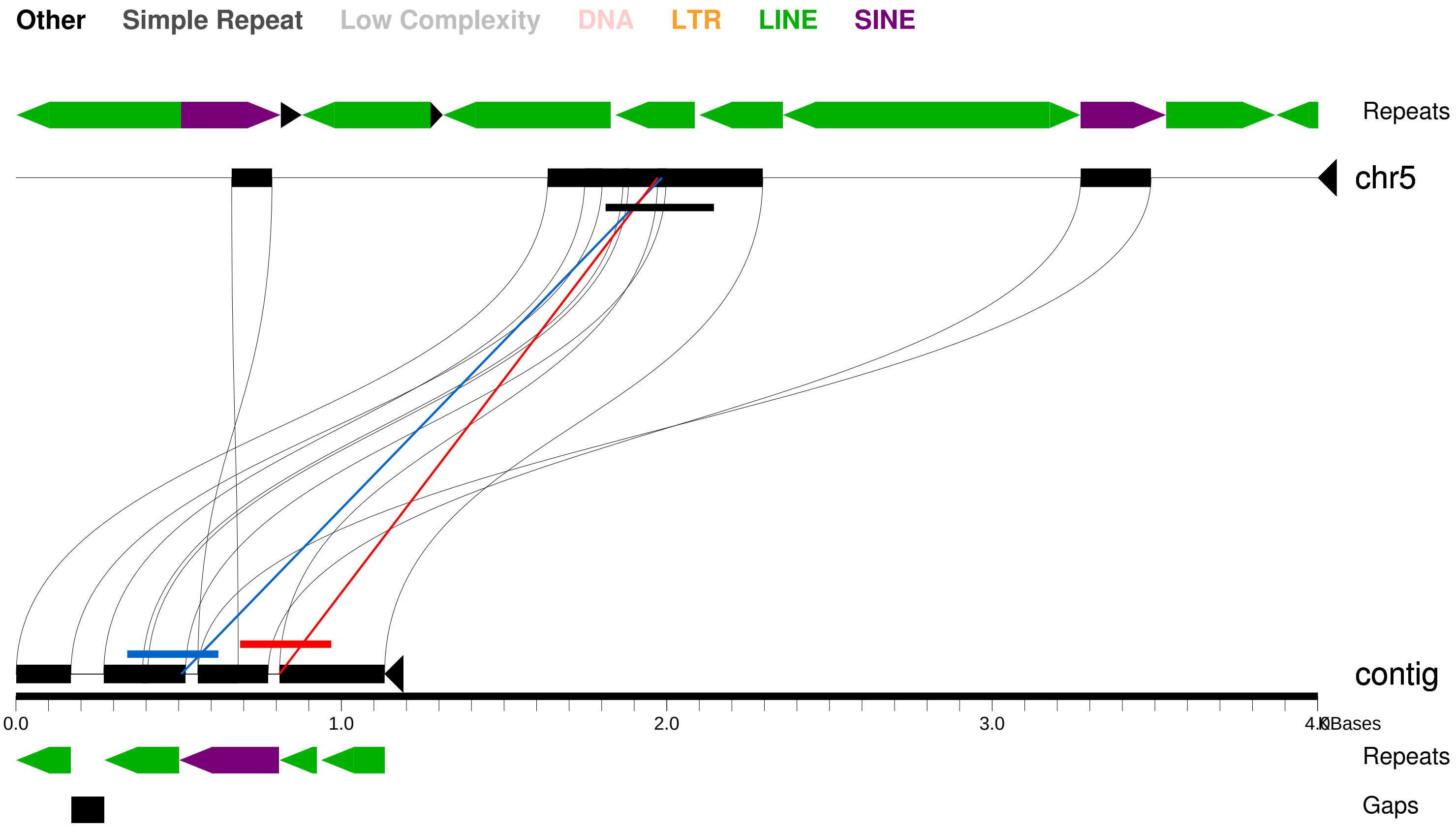

siteID: chr5_106180913 Scafftig3 + Breakpoint: chr5:106180894
chr5:106180874-106180995
Overlap Length: 40 INS size: 435 Genotyped: No

```
left     AAATGCAGACAATGAATGAATACAAACTGGATCTTACTGAACAGCAAGAATCCAAGAGATNNNNNNNNNN
chrom    AAATGCAGACAATGAATGAATACAAACTGGATCTTACTGAACAGCAAGAATCCAAGAGATGAAAAACAAA
right    GA--GCCACCGCGCCCGGCCTACAAACTGGATCTTACTGAACAGCAAGAATCCAAGAGATGAAAAACAAA
         1*11**111*1111111*11***************************************2222222222

left     NNNNNNNNNNNNNNNNNNNNNNNNNNNNNNNNNNNNNNNNNNNNNNNNN---
chrom    CCAAAAAAAAAGGCCAAAAAAAAAAAAAACACTAATGATACTACATTTGCTATTA
right    CCAAAAAAAAAGGCCAAAAAAAAAAAA-CACTAATGATACTACATTTGCTATTA
         2222222222222222222222222N22222222222222222222222222222
```

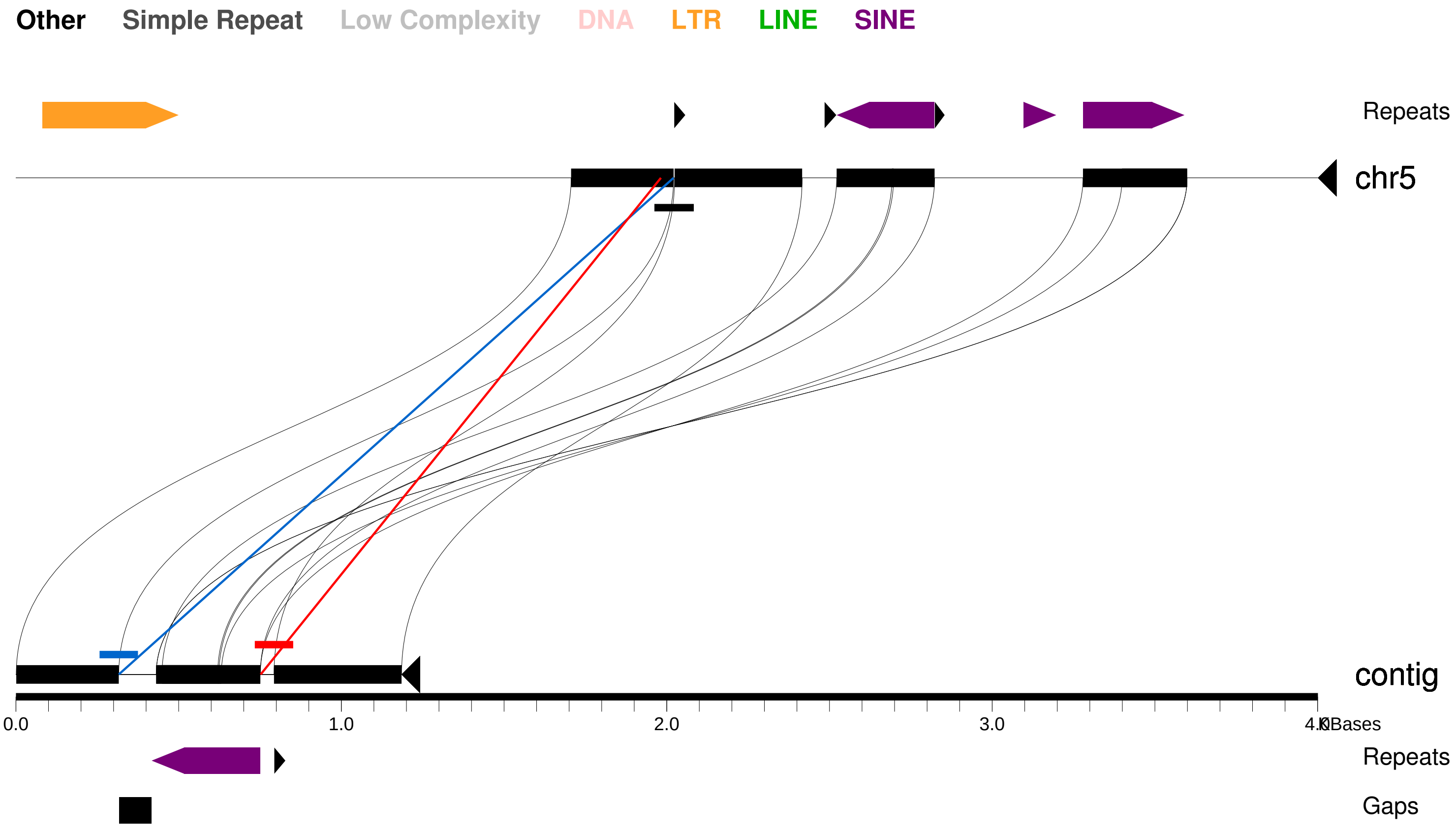

siteID: chr5_106735995 Scafftig2 + Breakpoint: chr5:106736005
chr5:106735955-106736064
Overlap Length: 10 INS size: 424 Genotyped: No

```
left     TAA-----------ATTTGAAATTTTTAGAAAGATTTAAAAAAATGTATAACTTCAATAAG--TCAAATA
chrom    TAA-----------ATTTGAAATTTTTAGAAAGATTTAAAAAAATGTATAACTTCAATAAG--TCAAATA
right    CCGCCCGCCTCGGCCTCCCAAAGTGCTGGGA----TTACAGGCGTGAGCCACCGCGCCCGGCCTCAAATA
         111111111111111*111***1*11*1*1*1111***1*1111**1111**11*11111*11*******

left     TCANNNNNNNNNNNNNNNNNNNNNNNNNNNNNNNNNNNNNNNNNNNNNNNNNNNNNNNNNNN
chrom    TCAAATTTTAAGCGTAAAATTTAAATAAACATTTTACAGATAAAACCCACCCT---------
right    TCAAATTTTAAGCGTAAAATTTAAATAAACATTTTACAGATAAAACCCACCCT---------
         ***22222222222222222222222222222222222222222222222222222222222
```

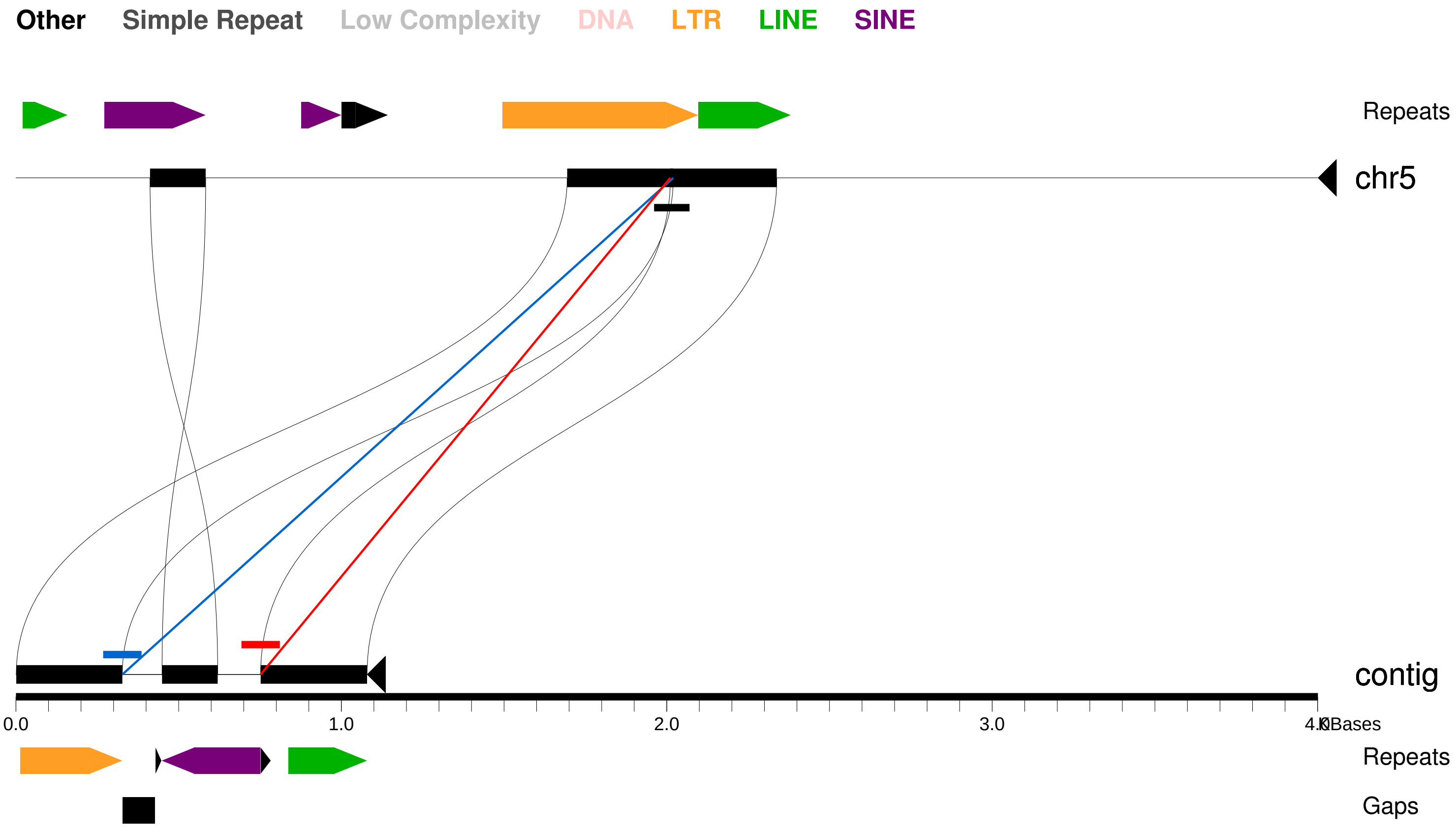

siteID: chr5_108026592 Scafftig2 + Breakpoint: chr5:108026582
chr5:108026422-108026755
Overlap Length: 14 INS size: 296 Genotyped: Yes

```
left   A--------------CAGGCATGAGCCACTGTGCCTGGCCAATGTTCTTTATATTTATTGCCAATTTATT
chrom  AAGTGCTGG-GATTACAGGCATGAGCCACTGTGCCTGGCCAATGTTCTTTATATTTATTGCCAATTTATT
right  TGAGGCAGGAGAAT---GGCGTGAACCCGGGAGGCGGAGC--------TTGCAGTGA--GCCGAG--ATC
       1NNN22N22122N2 11***1***1**111*1*1*1*11*11111111**11*1*1*11***1*111**1

left   CTGAGAAAGTCTGCATCAATTTACATTCCCATGAGCAGTATAT-GAGGGACCATTTCAATGTATCTTTAC
chrom  CTGAGAAAGTCTGCATCAATTTACATTCCCATGAGCAGTATAT-GAGGGACCATTTCAATGTATCTTTAC
right  CCGCCA----CTGCAC-----TCCAGCC---TGGGCGACAGAGCGAGACTCCGTCTCAA---------AA
       *1*11*1111*****111111*1**11*111**1**111*1*11***111**1*1****111111111*1

left   CAGCATCAAGTATTATCTTCTTAAAACTTTTTATTGGCCAGGCGCGGTGGCTCACG---CCTGTAGTCCC
chrom  CAGCATCAAGTATTATCTTCTTAAAACTTTTTATTGATATGATAAAGAAATAATAGTACCATGTTGTGAT
right  AAAAAAAAAAAA----------AAAAATTTTTATTGATATGATAAAGAAATAATAGTACCATGTTGTGAT
       1*11*11**11*1111111111****1*********2222*22222*22222222*222*2***2**222

left   AGCACTTTGGGAGGCCGAGGCGGGCGGATCACG-----------AG---------GTCAGG-AGATTGAG
chrom  ATAAGTTTGAATTTTTGTTGAGGGTTGACCATATTTTTATATATAGTATTTATTAGTCATTTATATTTAG
right  ATAAGTTTGAATTTTTGTTGAGGGTTCACCATATTTTTATATATAGTATTTATTAGTCATTTGTATTTAG
       *22*2****2222222*22*2***221*2**2222222222222**222222222****22212***2**

left   AC-------CATCCTGGCT--AAAAC----GGTGAAACCCCGTC-TCTACTAAA--
chrom  TCATCTATACATTTTCTATTCATATCCTTGGCTGATTTTTCTTAGTTTTGTTAAGT
right  TCATCTATACATTTTCTATTCATATCCTTGGCTGATTTTTCTTAGTT---------
       2*2222222***22*222*22*2*2*2222*2***22222*2*22*21NN1N11
```

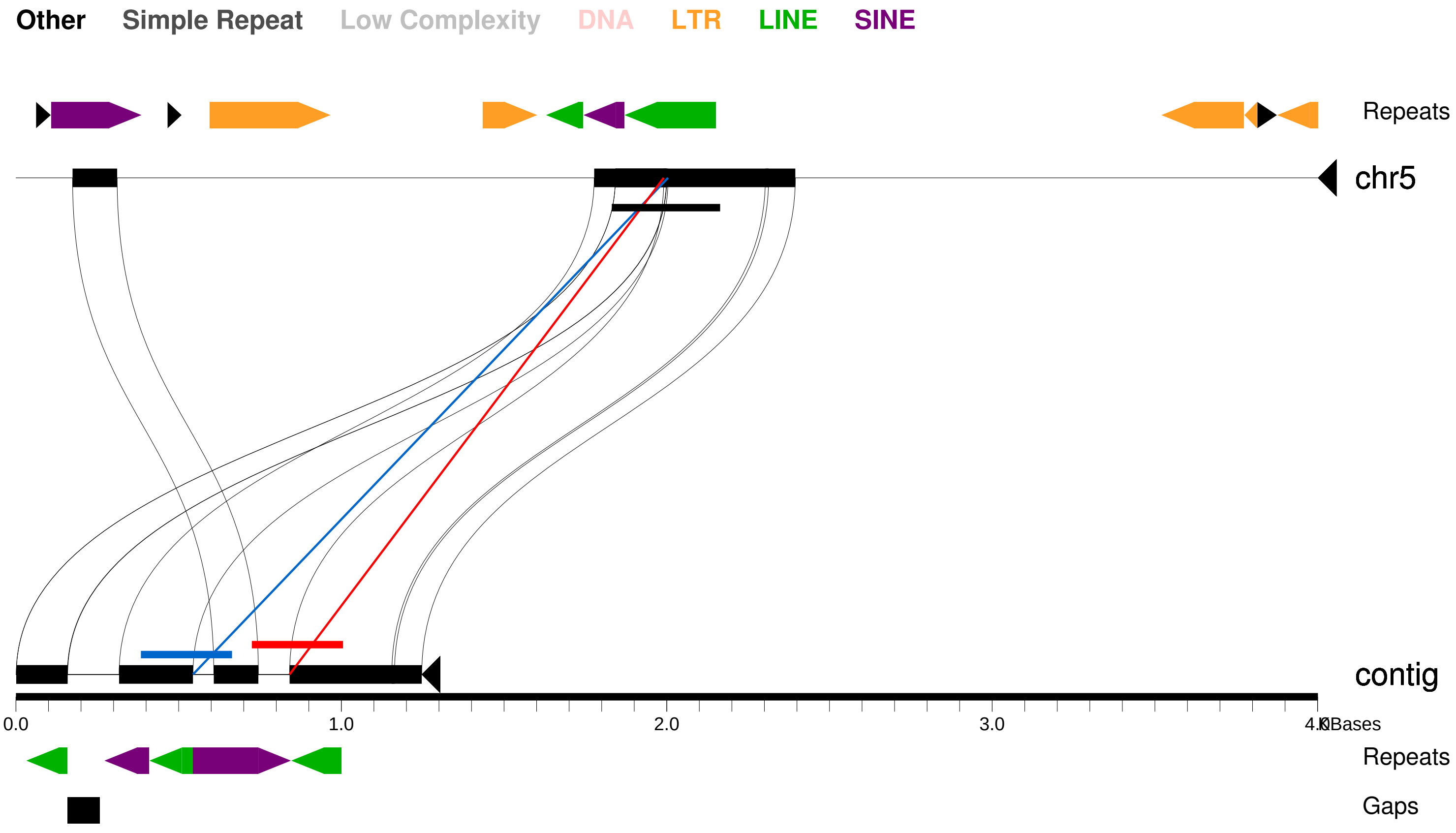

siteID: chr5_109050998 Scafftig1 + Breakpoint: chr5:109050994
chr5:109050834-109051164
Overlap Length: 11 INS size: 311 Genotyped: Yes

```
left   CT---AACACATCCTCCTGCCCAGCTTGTTTCCAAAGATTTGTCAGCTTATCCTTGAGAAGATTTTATTT
chrom  CTGCTAACACATCCTCCTGCCCAGCTTGTTTCCAAAGATTTGTCAGCTTATCCTTGAGAAGATTTTATTT
right  TT--TAGTAGAGACGGGGTTTCACCTTGTTAGCCAGGATG-GTCTCGATCTCCT------GACCTCATG-
       1*  2*11*1*11*1111111**1******11*1*1***11***1111*1****111111**11*1**11

left   TATTTATTAAACTCATATATTCCATAGGGAGATAAATATTATTGGCTTAAAATACTTTGTATTGACTTTC
chrom  TATTTATTAAACTCATATATTCCATAGGGAGATAAATATTATTGGCTTAAAATACTTTGTATTGACTTTC
right  -ATCCACCCGCCTCGGCC-TCCCAAAGTGCTGGGA---TTACAGGCGTGAGCCACCGCGCCC--------
       1**11*11111***11111*1***1**1*11111*111***11***1*1*111**111*11111111111

left   ATATAATTTGTTTGGCCTTTAAATTTTTTTTGT------TTTTTG-------TTTTT---------TTTT
chrom  ATATAATTTGTTTGGCCTTTAAATTTTTTTTATAACTTATATTTGGATGTGATTTTTGCAGTCTAGTTTT
right  -------------GGCCT--AAATTTTTTTTATAACTTATATTTGGATGTGATTTTTGCAGTCTAGTTTT
       1111111111111*****11**********2*222222*2****2222222*****222222222****

left   -------------TTTTTGAGACGGA-GTCTCGCTCTGTCGCC-CAGGCCA---------GACTGCGGAC
chrom  AACATAATTTAAATTTTACAAAAGGTTGTTTGGTTGTGTTGTCACATTTAATATTATAGAGACTATGAAG
right  AACATAATTTAAATTTTACAAAAGGTTGTTTGGTTGTGTTGTCACATTTAATATTATAGAGACTATGAAG
       2222222222222****22*2*2**22**2*2*2*2***2*2*2**2222*222222222****22*2*2

left   TGCAGTGGCGCAATCTCGGCTCACTGCA-AGCTCCGCTTCCCGGGTTCACG
chrom  AACAAAAGTAAAGTTTTAGTTTTTTTCCCACCGTTTTTTCCTTTGCATCCC
right  AACAAAAGTAAAGTTTTAGTTTTTTTCCCACCGTTTTTT------------
       22**222*222*2*2*22*2*222*2*22*2*22222**11NNN1NNNN1N
```

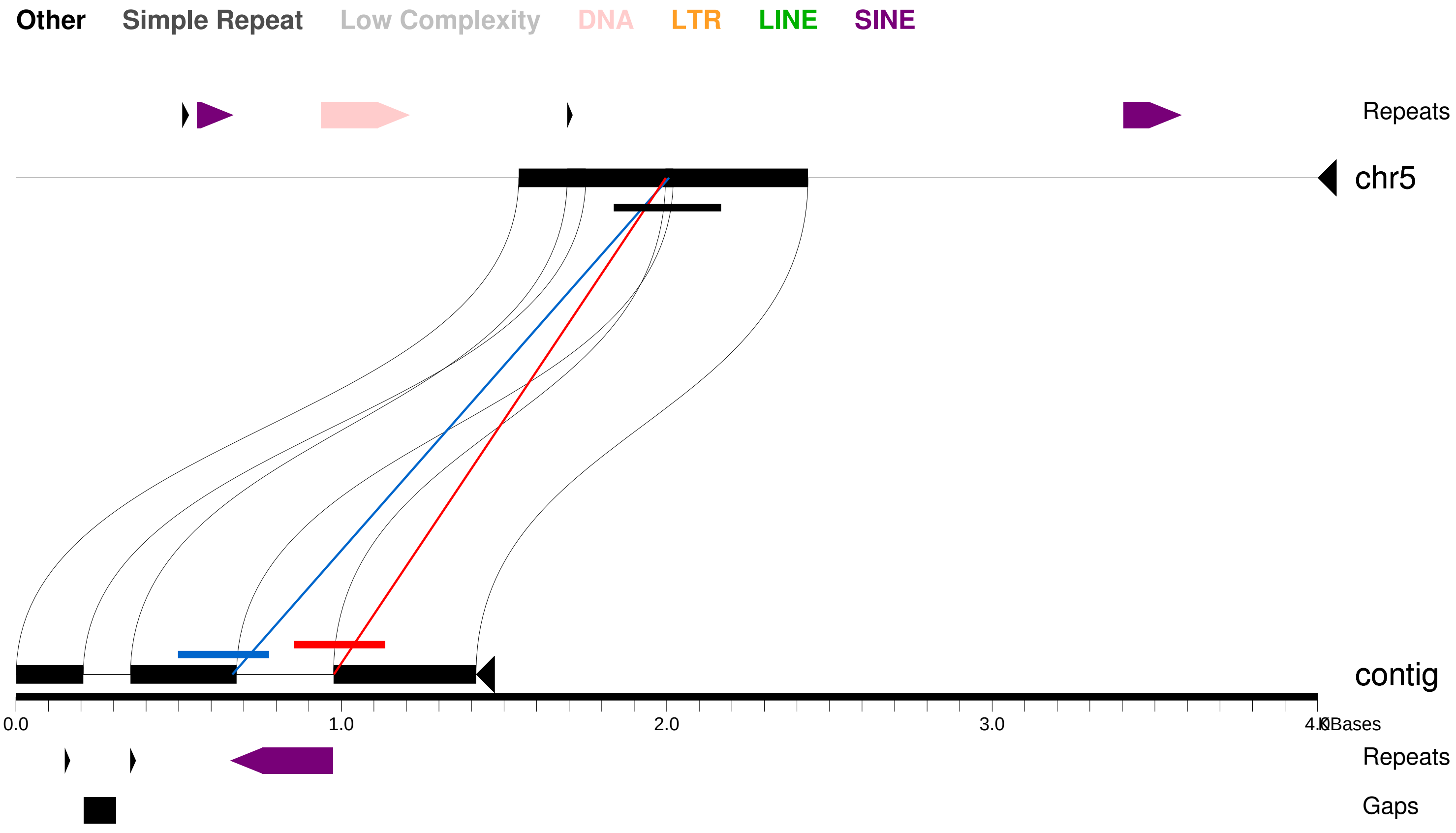

siteID: chr5_109245961 Scafftig3 + Breakpoint: chr5:109245949
chr5:109245904-109246008
Overlap Length: 15 INS size: 342 Genotyped: No

```
left    GC-----TCTTTGC------AATTCCTGT--TTCCTCTGCCTGAAGTTTCTCTTCCAG--ATTTTGAAGG
chrom   GC-----TCTTTGC------AATTCCTGT--TTCCTCTGCCTGAAGTTTCTCTTCCAG--ATTTTGAAGG
right   CCGCCCGCCTCGGCCTCCCAAAGTGCTGGGATTACAG-GCGTGAGCCACCGCGCCCGGCAATTTTGAAGG
        1*111111**11**111111**1*1***111**1*111**1***11111*1*11**1*11**********

left    ATTTTTNNNNNNNNNNNNNNNNNNNNNNNNNNNNNNNNNNNNNNNNNNNNNNNNNNNNNNNN
chrom   ATTTTT--------------ATCCAGAGTCCTTCCCTGAGCATCTGACCTAAAATGGTCCTTGAG
right   ATTTT--------------ATCCAGAGTCCTTCCCTGACCATCTGACCTAAAATGGTCCTTGAG
        *****2222222222222222222222222222222N2222222222222222222222222
```

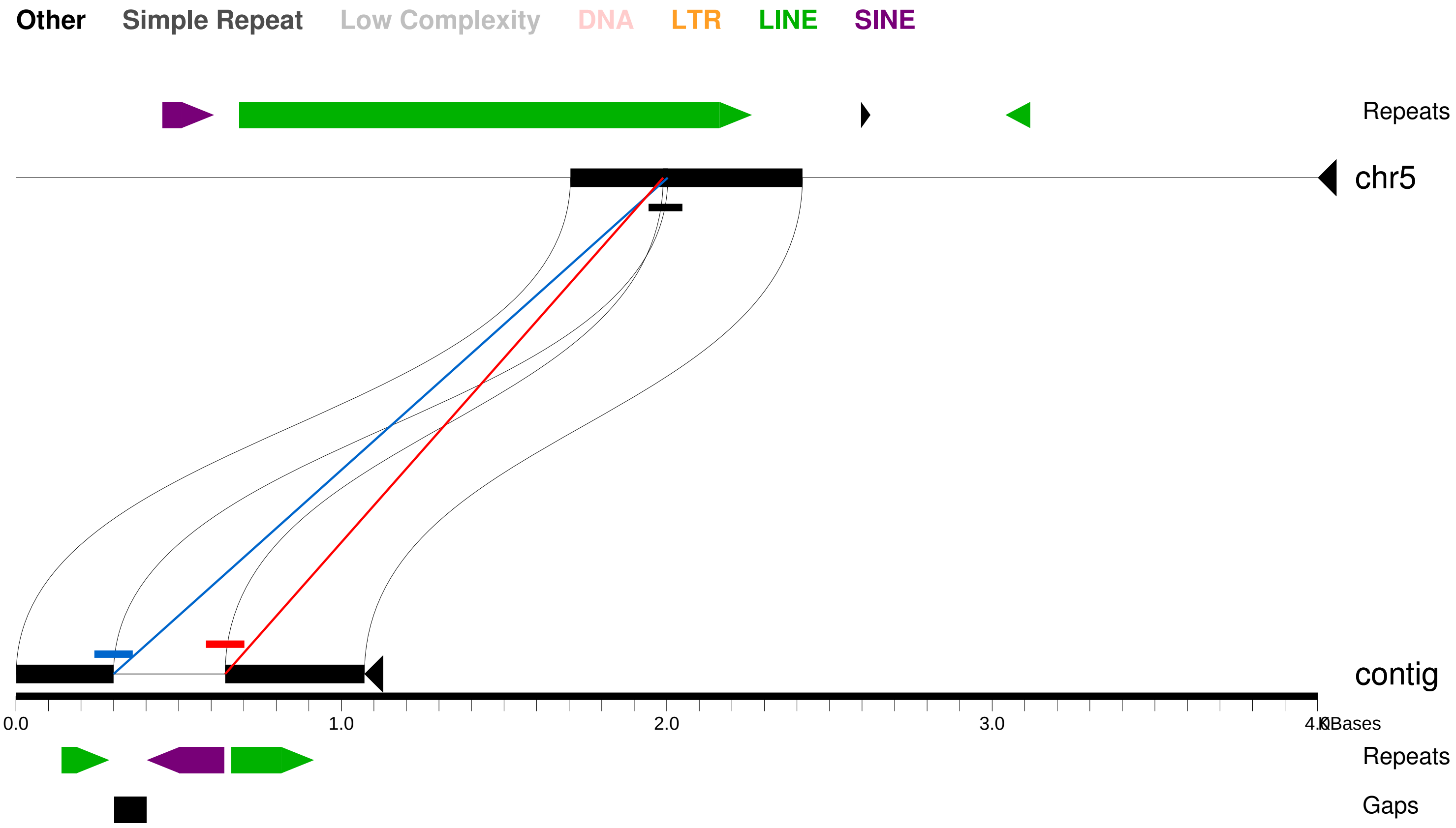

siteID: chr5_110085961 Scafftig1 - Breakpoint: chr5:110085898
chr5:110085863-110085957
Overlap Length: 25 INS size: 283 Genotyped: No

```
left    TGAAGTGCCTTAAATAAACTTTGCTAAAACGTTTG-------------------------AAACAAAACA
chrom   TGAAGTGCCTTAAATAAACTTTGCTAAAACGTTTG-------------------------AAACAAAACA
right   NNNNNNNNNNNNNNNNNNNNNNNNNNNNNNNNNNNNNNNNNNNNNNNNNNNNAAAAAAAAAAAAAAAAAA
        111111111111111111111111111111111111111111111111111111111111***1****1*

left    AAGATTAGAAAGGTCTGTAGTCCCAGCTACTCGGGAGGCTGAGGCAGGAGAATGGCGTGAACCCGGGAAG
chrom   AAGATTAGAAAGGTCAG------CAAC-ATTTGGGCAGCTCAG-----AGTTTCTTGTTTT---------
right   AAGAA-AGAAAGGTCAG------CAAC-ATTTGGGCAGCTCAG-----AGTTTCTTGTTTT---------
        ****11*********2*222222**2*2*2*2***22***2**22222**22*222**222222222222

left    CAGA
chrom   ---A
right   ---A
        222*
```

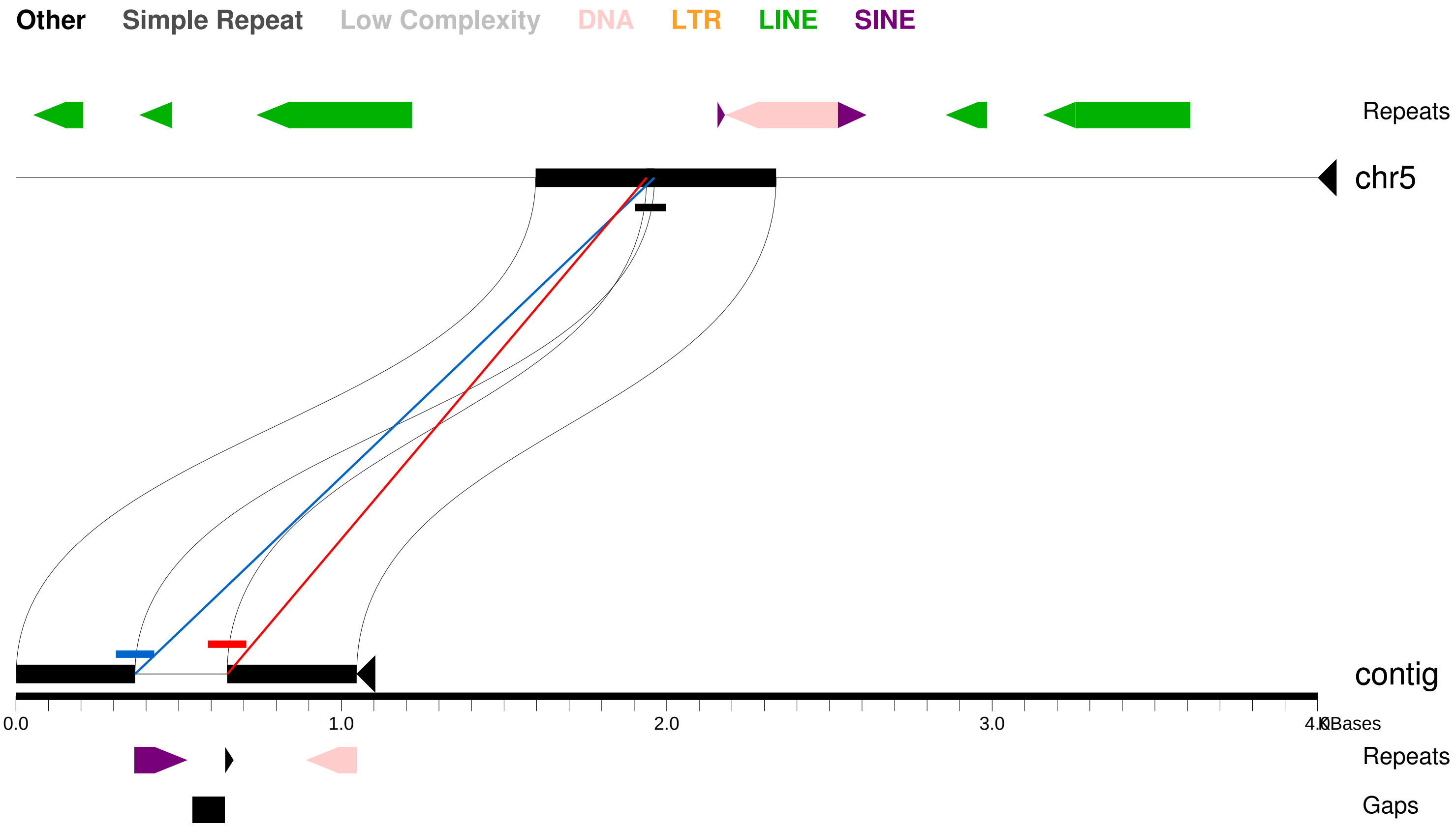

siteID: chr5_111658667 Scafftig4 - Breakpoint: chr5:111658677
chr5:111658631-111658721
Overlap Length: 14 INS size: 305 Genotyped: Yes

```
left    AGGTAAAGGGGGAAAG-------GTAATGGGGAC------------TTTCAAGAAGATACAATTA-----
chrom   AGGTAAAGGGGGAAAG-------GTAATGGGGAC------------TTTCAAGAAGATACAATTA-----
right   CGAGATTGCGCCACTGCACTCCAGCCTGGGCGACAGCGAGACTCCGTCTCAAAAAAAAAAAAAAAAAAAA
        1*11*11*1*11*11*1111111*1111**1***111111111111*1****1**1*1*1**11*11111

left    ----GAAGACAAGAAAGGTGGCCGGGCGCGGTGGCTCACGCC-TGTAATCCCAGCACTTTGGGAGGCCGA
chrom   ----GAAGACAAGAAAGGC------------TTTCTAAAGCTATTTGATTTCTGAAATTAA---------
right   AAAAGAAGACAAGAAAGGC------------TTTCTAAAGCTATTTGATTTCTGAAATTAA---------
        1111**************2222222222222*22**2*2**22*2*2**22*2*2*2**22222222222

left    GGCGGGCG
chrom   --------
right   --------
        22222222
```

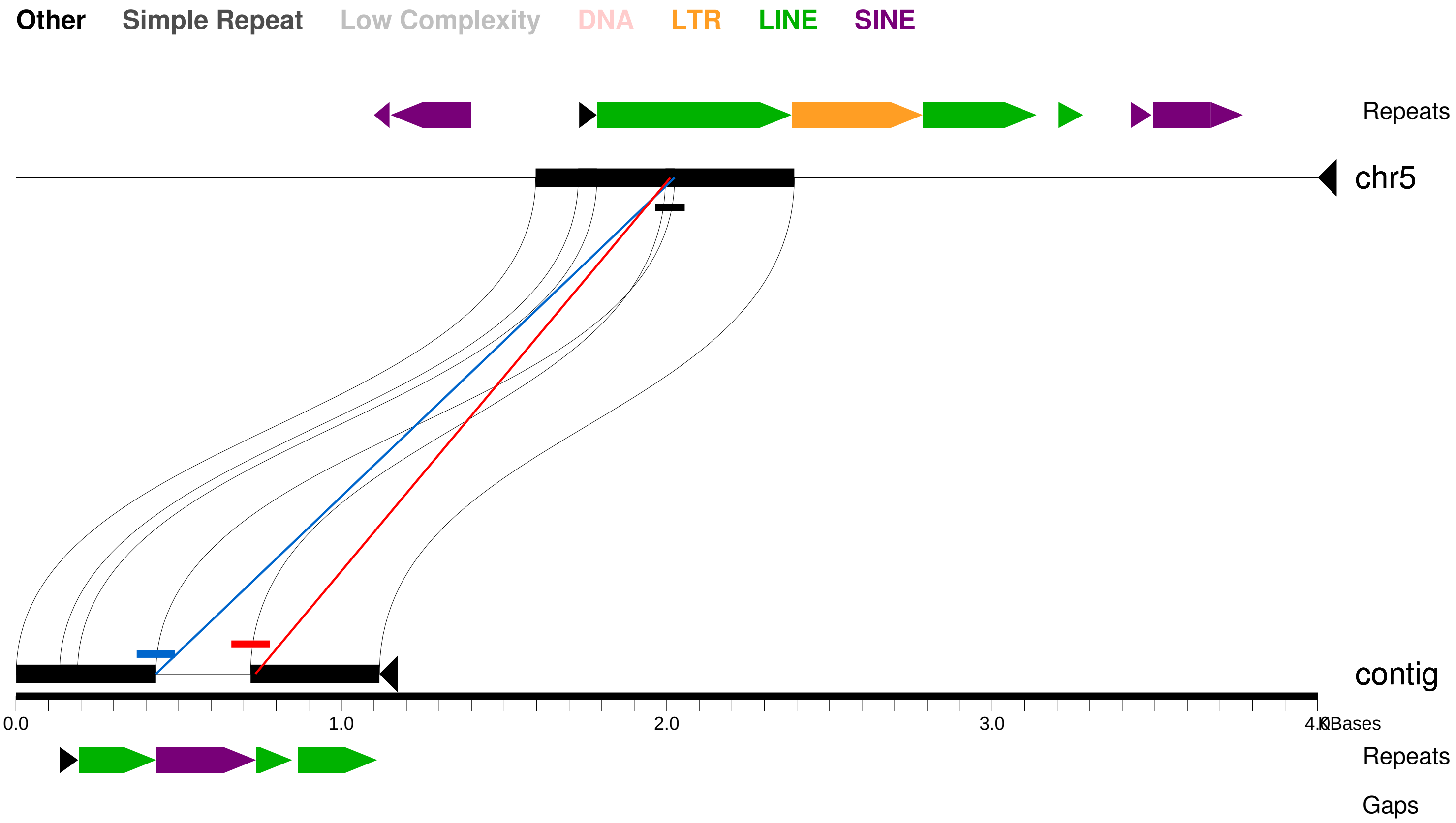

siteID: chr5_114739742 Scafftig9 + Breakpoint: chr5:114739723
chr5:114739677-114739782
Overlap Length: 14 INS size: 267 Genotyped: Yes

```
left     TTCTCT-------TTTTGATC--------TAGGTGTGC-AATATGCCTTGATGAATGACAATTAGAATGT
chrom    TTCTCT-------TTTTGATC--------TAGGTGTGC-AATATGCCTTGATGAATGACAATTAGAATGT
right    CGATCTCCTGACCTCGTGATCCGCCCGCCTCGGCCTCCCAAAGTGCTGGGATTACAGGC---TAGAATGT
         111***1111111*11*****11111111*1**11*1*1**11***111***1*11*1*111********

left     TTTTTTTTTTTTTTGAGACGGAGTCTCGCTGTCGCCCAGGCTGGAGTGCAGTGGCGCAATCTCGGC
chrom    TTTTTTA---------AAGCAGCATCAATAA----AATAATATTGTGACACTACATAAAATAAGC
right    TTTTTTA---------AAGCAGCATCAATAA----AATAATATTGTGACACTACATAAAATAAGC
         ******2222222222*2*2**22**22*2222222*222*222***222222*22**22*22**
```

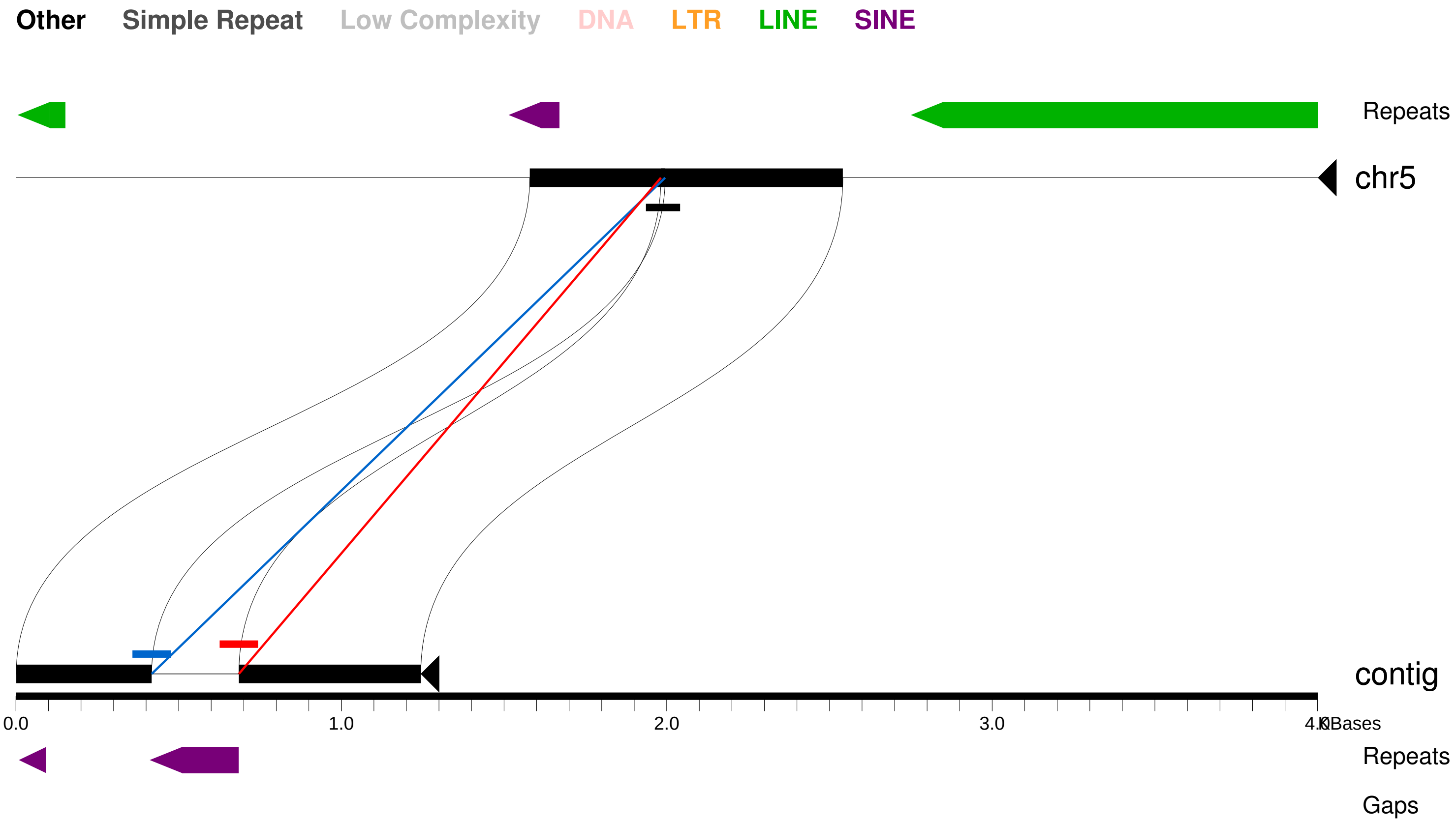

siteID: chr5_117951770 Scafftig1 + Breakpoint: chr5:117951768
chr5:117951723-117951827
Overlap Length: 15 INS size: 299 Genotyped: Yes

```
left     AAG-TCACATG--------CCAGCAT--GAATTATAAAAT------ATTTGTGGGGTAGGATCAGGCACC
chrom    AAG-TCACATC--------CCAGCAT--GAATTATAAAAT------ATTTGTGGGGTAGGATCAGGCACC
right    CTCCTGACCTCGTGATCCGCCCGCCTCGGCCTCCCAAAGTGCTGGGATTACAGGCGT--GA-CAGGCACC
         1111*1**1*211111111**1**1*11*11*111***1*111111***111**1**11**1********

left     ACTTTTTTTTTTTTTTTTTTTTTTTTTTTTTTTTTTTTTTTTTGAGACGGAGTCTCGCTCTGTCGCCC
chrom    ACTTTTTAAACAACCACTTGGCTCCCAAGTGATTCTA-----AAATATAGCCAAGAT---------
right    ACTTTTTAAACAACCACTTGGCTCCCAAGTGATTCTA-----AAATATAGCCAAGAT---------
         *******2222222222**222*222222*22**2*222222*2*222**2*22*2*222222222
```

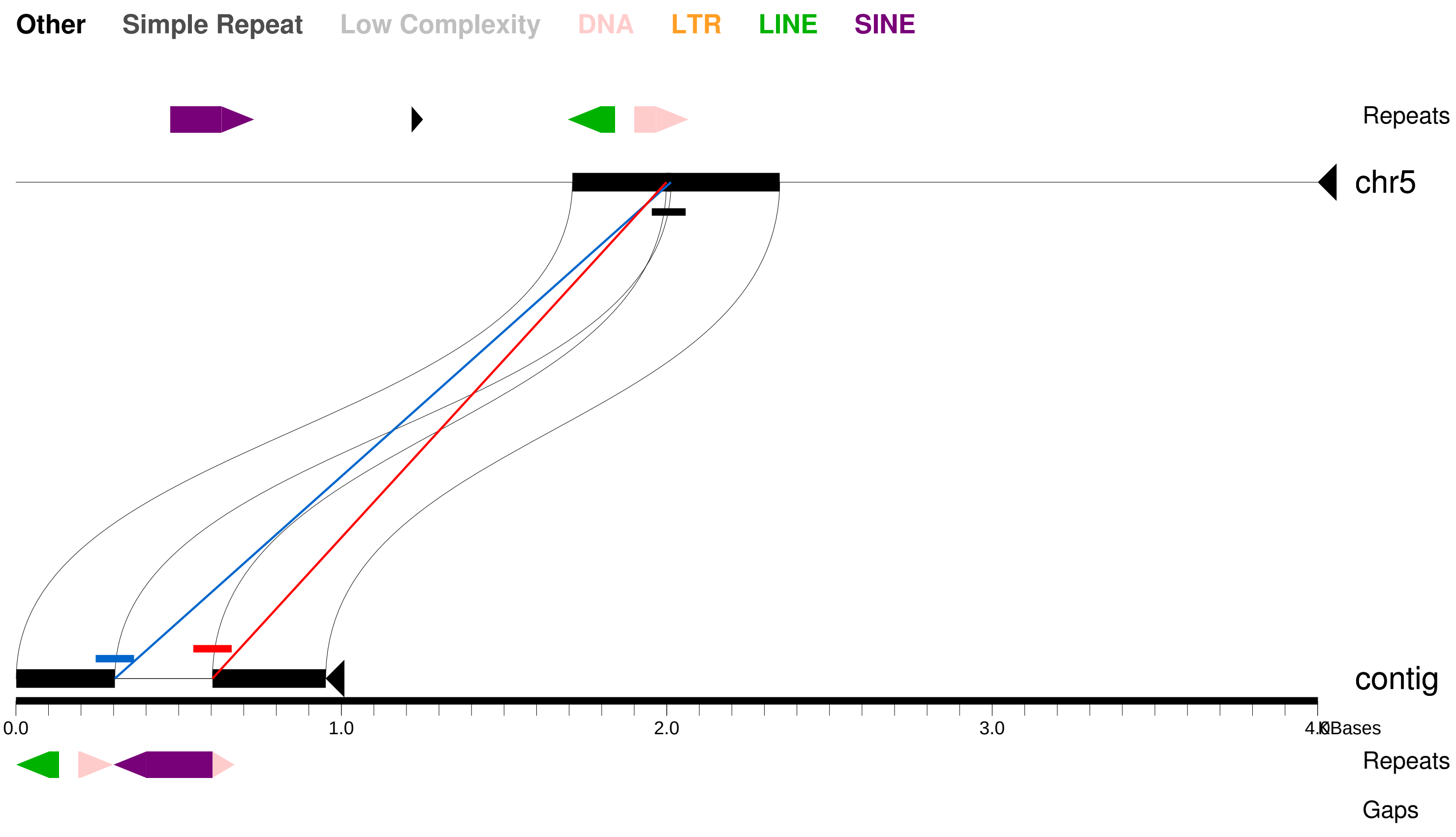

siteID: chr5_118977634 Scafftig6 - Breakpoint: chr5:118977626
chr5:118977576-118977724
Overlap Length: 10 INS size: 302 Genotyped: Yes

```
left   TATTCCATGAAGAAATTTAGTTCATTATAGTGCATCTTATATACTTTTAAAACTCATGATGGCCGGGCGC
chrom  TATTCCATGAAGAAATTTAGCTCATTATAGTGCATCTTATATACTTTTAAAACTCATGATAACTGTTTTT
right  AAAAAAAAAAAAAAA-----------------------AAAAAC------AACTCATGATAACTGTTTTT
       1*1111*11**1***11111N11111111111111111*1*1**111111*********22*2*22222

left   GGT----------GGCTCACGCCTGTAATCCCAGCACTTTGGGAG----------------GCCGAGG-C
chrom  CATACATTTTTAGGACTACCCCCCCCCACCCCCACACAGAGGTAACTAATATAAAAGATCTGCATATATC
right  CATACATTTTTAGGACTACCCCCCCC-ACCCCCACACAGAGGTAACTAATATAAAAGATCTGCATATATC
       22*2222222222*2**22*2**222N*2***22***222**2*22222222222222222**22*222*

left   GGGTG---G
chrom  CAGTCCATG
right  CAGTCCATG
       22**2222*
```

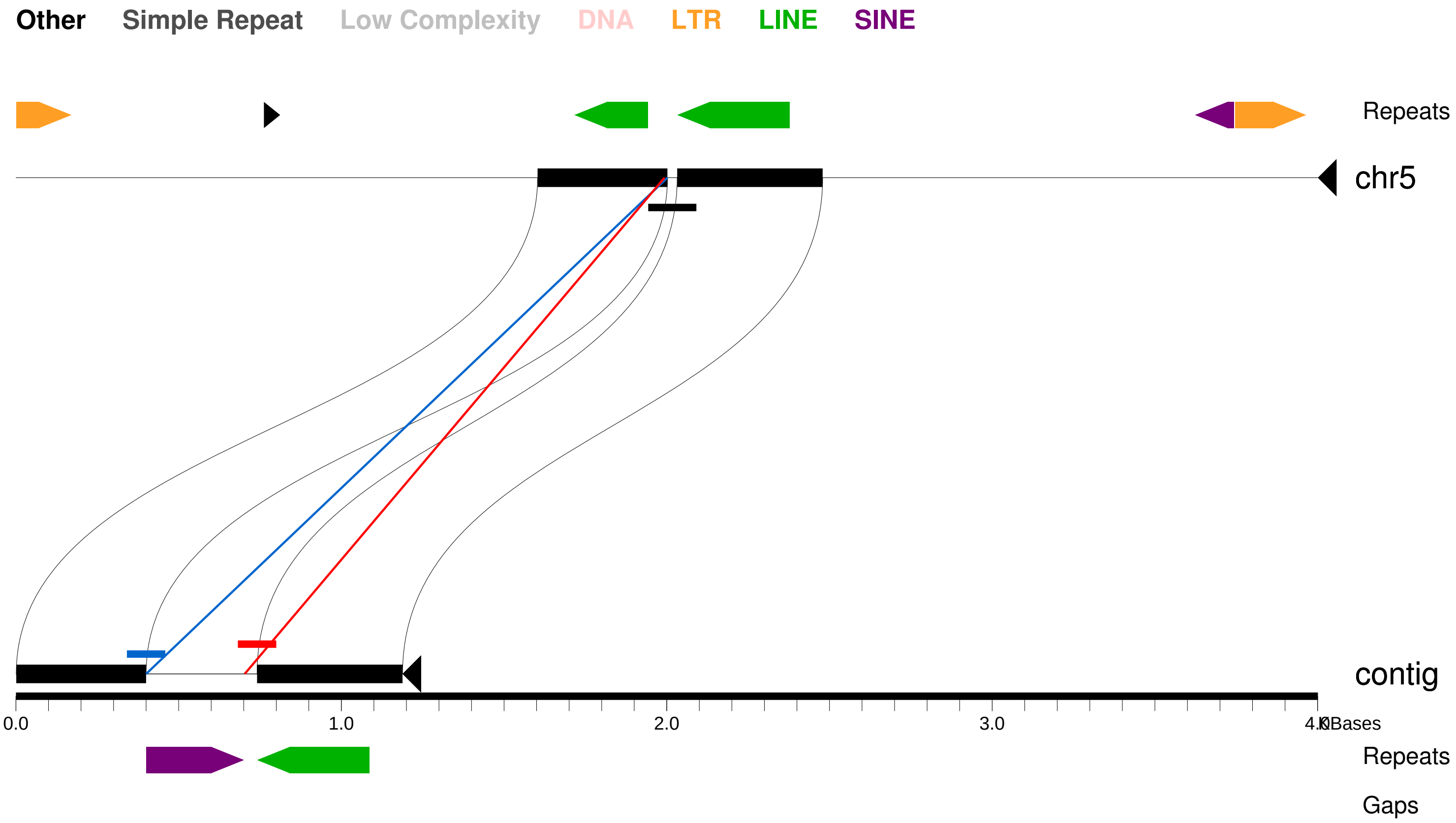

siteID: chr5_119043950 Scafftig1 - Breakpoint: chr5:119043941
chr5:119043781-119044118
Overlap Length: 18 INS size: 303 Genotyped: No

```
left     G--------------------------------------------GTAGGTGCCATCAGAGGTGGTAGTG
chrom    GAGCTGGGCAGGCCTGTGTCTAGGCCTTTTAGTTGGTTCCTGCAGGTAGGTGCCATCAGAGGTGGTAGTG
right    TTTTAGTAGAGAC---------GGGGTTTCACCTTGTT-----AGCCAGGA--------------TGGTC
         1NNNN2NNN22N2         22NN222N2NN2N222     2211***111111111111111*1**1

left     ACTATGTGTTTAGGCCCAACCTCAGGTGCTCAAGAGGAGTGCTCAGGTTTCCAAAGTGCTGGGTTGGGTT
chrom    ACTATGTGTTTAGGCCCAACCTCAGGTGCTCAAGAGGAGTGCTCAGGTTTCCAAAGTGCTGGGTTGGGTT
right    TCGATCT-------CCTGACCTCA--TGATCCACCCGC---CTCGGCCTCCCAAAGTGCTGGGATTACAG
         1*1**1*1111111**11******11**1**1*111*1111***1*11*1*************1*11111

left     GGGGAATCCCCAGCATTCCAGGCTATATGCTCTTTCTT--------------------------------
chrom    GGGGAATCCCCAGCATTCCAGGCTATATGCTCTTTCTTGGAAGGGGTAGTGAAACTAGGCTGCGTGGGTT
right    GCGTGAGCCACCGCG--CCCGGCTATATGCTCTTTCTTGGAAGGGGTAGTGAAACTAGGCTGCGTGGGTT
         *1*11*1**1*1**111**1******************22222222222222222222222222222222

left     ----------------------------------------------------------------------
chrom    TGTGTTCAGGTCCACAATGGTGAAAGCATGCACCAGCTGTTGTGGGTGGAGATGGGGCAATTCTCAGGCC
right    TGTGTTCAGGTCCACAATGGTGAAAGCATGCACCAGCTGTTGTGGGTGGAGATGGGGCAATTCTCAGGCC
         2222222222222222222222222222222222222222222222222222222222222222222222

left     ----------------------------------------------TNNNNNNNNNNN
chrom    CCAGGCAGAGTGTTCAGGTGAGGGGTGGTAGCAGTTGTGCTAAGACTCTGCCACTGGA
right    CCAGGCAGAGTGTTCAGGTGAGGGGTGGTAGCAGTTGTGCTAA---------------
         2222222222222222222222222222222222222222222   1NNNNNNNNNNN
```

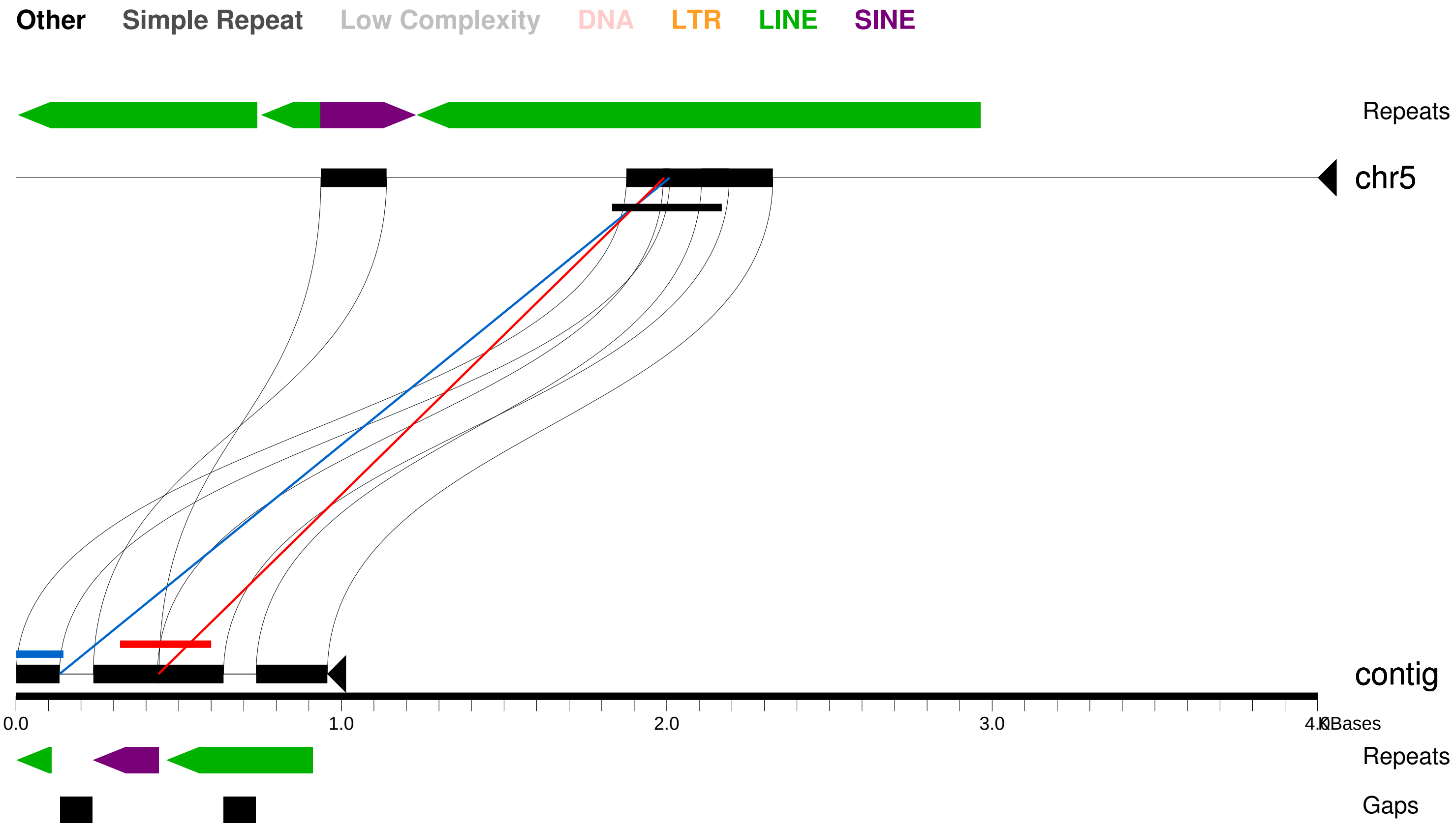

siteID: chr5_119465627 Scafftig1 + Breakpoint: chr5:119465634
chr5:119465586-119465693
Overlap Length: 12 INS size: 478 Genotyped: No

```
left   GTTATTTCTTGCCTTCTGCTAGCTTTTGAATGTGTTTGCTCTTGCTTT-----------TCTAGTTCTTT
chrom  GTTATTTCTTGCCTTCTGCTAGCTTTTGAATGTGTTTGCTCTTGCTTT-----------TCTAGTTCTTT
right  NNNNNNNNNNNNNNNNNNNNNNNNNNNNNNNNNNNNNNNNNNNNNNNNNNNNNNNNNNNTCTAGTTCTTT
       11111111111111111111111111111111111111111111111111111111111***********

left   TTTTTTTTTTTTTTTTTTTTTTTTTTTTTTT-TTTCTTTGAGACGGAGTCTCGCTCTGTCG
chrom  TAATTGTGATGTTAGGGTGTCAATTTTGTATCTTTCCT------GGTTTCTC------TTG
right  TAATTGTGATGTTAGGGTGTCAATTTTGTATCTTTCCT------GGTTTCTC------TTG
       *22**2*22*2**2222*2*222****2*2*2****2*222222**22****222222*2*
```

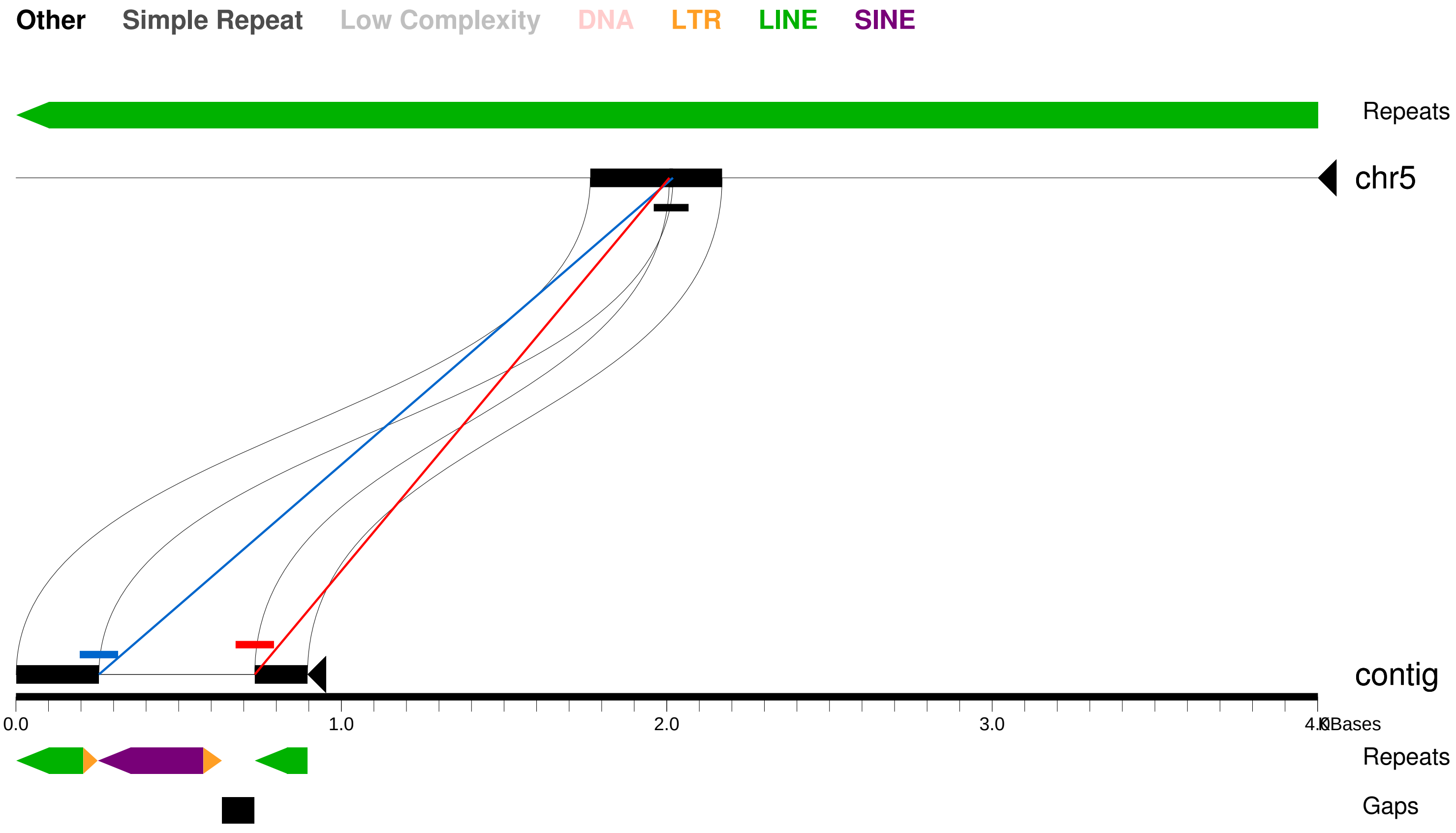

siteID: chr5_119485627 Scafftig1 - Breakpoint: chr5:119485618
chr5:119485559-119485674
Overlap Length: 1 INS size: 268 Genotyped: No

```
left   TGACACAAGTTTATCTGTATAACAGACCTG--TACA----TGTACCCCTGAACTTAAAAAAAAAAAANNN
chrom  TGACACAAGTTTATCTGTATAACAGACCTG--TACA----TGTACCCCTGAACTTAAAAAAAAAAA---C
right  CCGCCCGCCTCGGCCTCCCAAAGTGCTGGGATTACAGGCGTGAGCCACCGCGCCCGGCCAAAA------C
       111*1*111*1111**1111**11*1111*11****1111**11**1*1*11*111111****1112222

left   NNNNNNNNNNNNNNNNNNNNNNNNNNNNNNNNNNNNNNNNNNNNNNNNNNNNNNN
chrom  ATTTATTTTCTGTGCAGAGTGAGTGTTGCTATTAATTATGACTTGATGAGTTTTC
right  ATTTATTTTCTGTGCAGAGTGAGTGTTGCTATTAATTATGACTTGATGAGTTTTC
       2222222222222222222222222222222222222222222222222222222
```

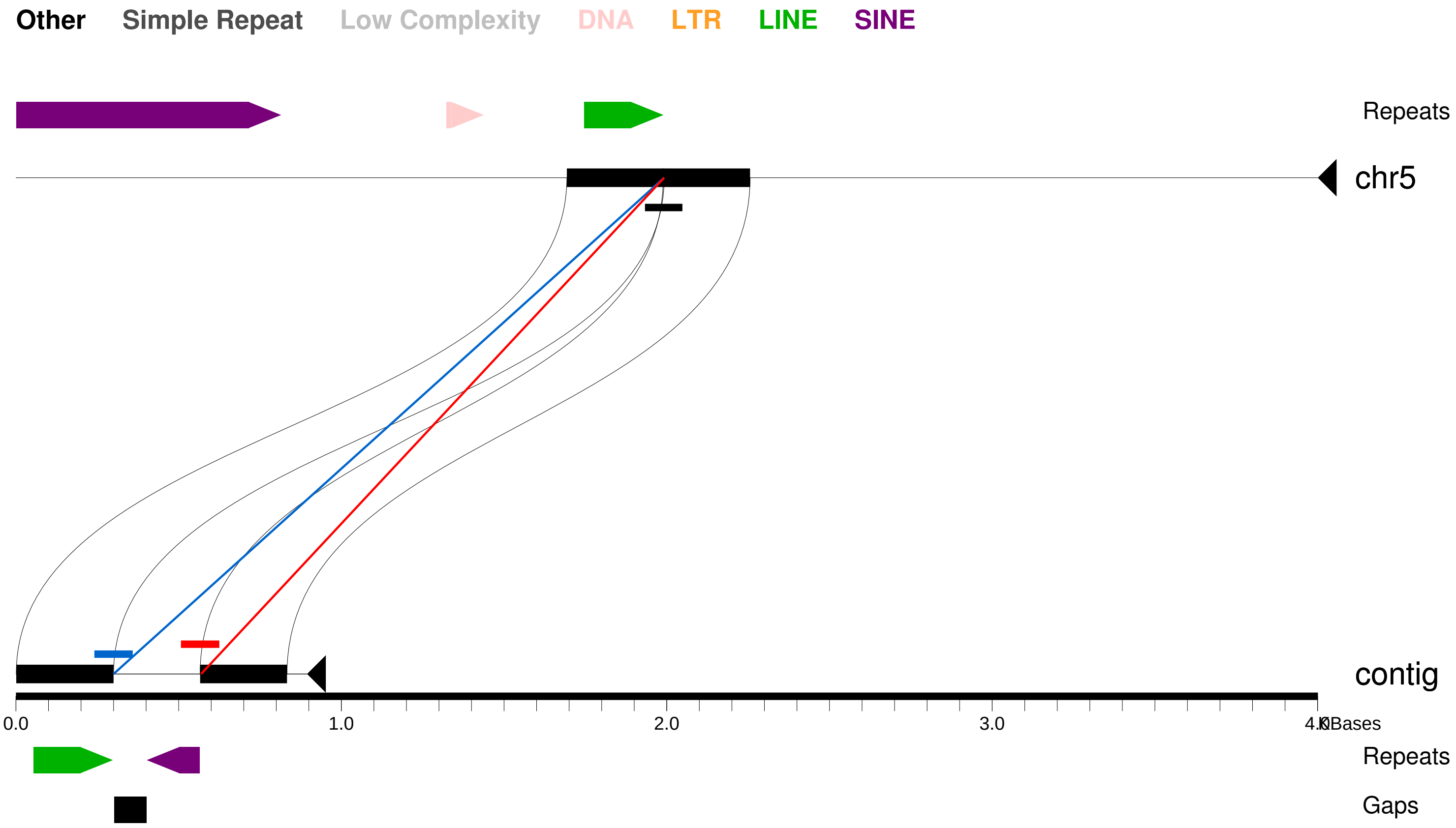

siteID: chr5_124147832 Scafftig15 - Breakpoint: chr5:124147815
chr5:124147765-124147874
Overlap Length: 10 INS size: 298 Genotyped: Yes

```
left    AAA--GC-------CTTATGACAAATTTCAAGAGAATAGCTATTTTACCTTACATTATTAATAATTTTGG
chrom   AAA--GC-------CTTATGACAAATTTCAAGAGAATAGCTATTTTACCTTACATTATTAATAATTTTGA
right   CCACTGCACTCCAGCCTGGGCGACAGAGCGAGACTCCGTCTCAAAAAAAAAAAAAAAAAAATAATTTTGA
        11*11**1111111*1*11*11*1*111*1***111111**11111*1111*1*11*11**********2

left    GCCGGGCGCGGTGGCTCAC--GCCTGTAATCCCAGC-ACTTTGG-GAGGCCGAGGCGGGCGG
chrom   T---------GTATATTACTAGAATTTTGTTGTATCTACTTTGTTGAA----AATTGTACCC
right   T---------GTATATTACTAGAATTTTGTTGTATCTACTTTGTTGAA----AATTGTACCC
        2222222222**222*2**22*22*2*22*222*2*2******22**22222*222*22*22
```

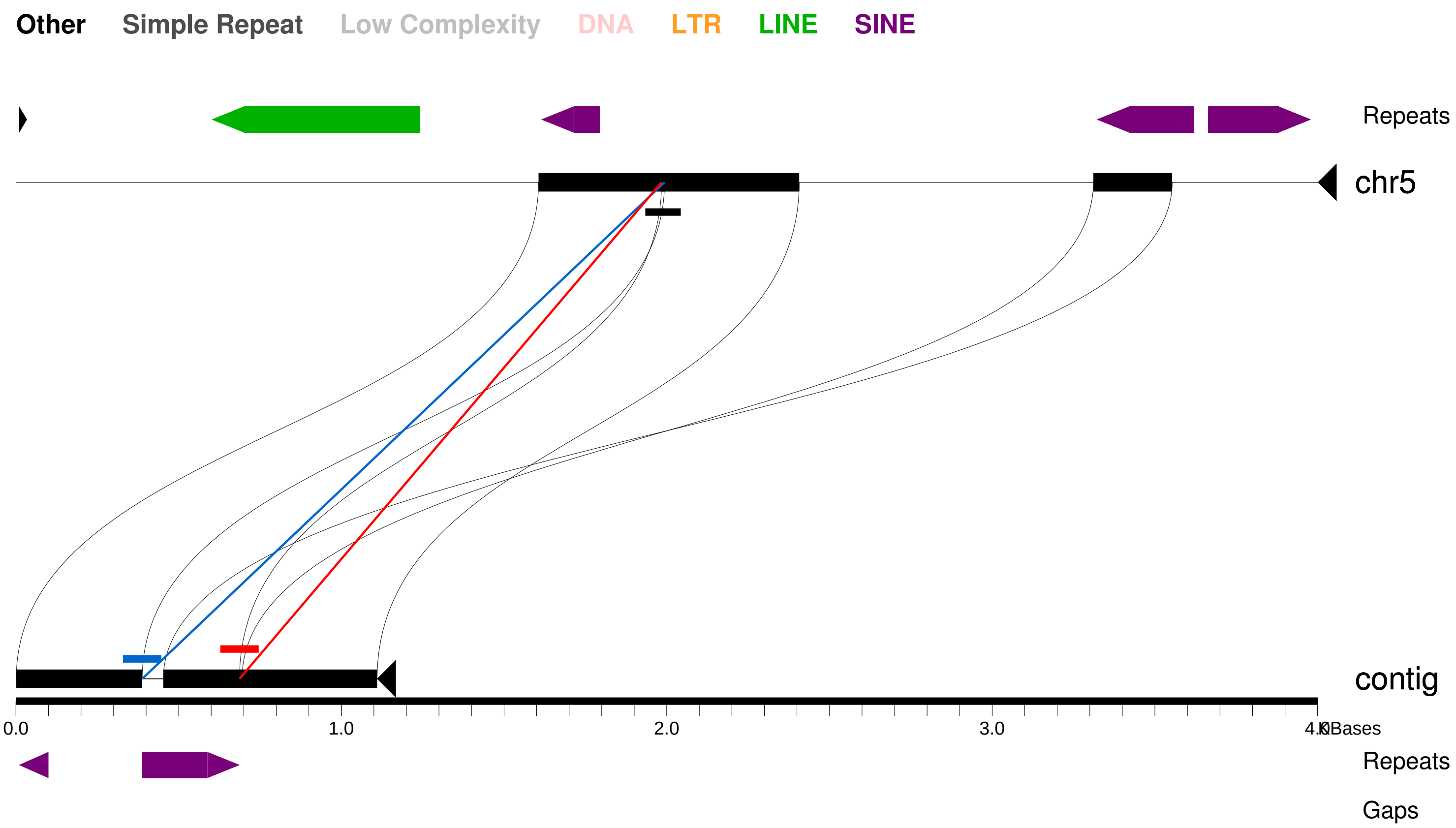

siteID: chr5_126302175 Scafftig1 - Breakpoint: chr5:126302163
chr5:126302111-126302222
Overlap Length: 8 INS size: 321 Genotyped: No

```
left   TCAGTTCCCTCCATCCCCCAAGAGTTTTCTGTATCCAATGCTTGTTTCAGAA---------AGGATGTTN
chrom  TCAGTTCCCTCCATCCCCCAAGAGTTTTCTGTATCCAATGCTTGTTTCAGAA---------AGGATGTTT
right  CCGCCCGCCTCGGCCTCCCAA-AGTGCTGGGATTACTG-GCGTGAGCCACCACGCCCGGCCAGGATGTTT
       1*11111****111*1*****1***11*11*11*1*111**1**111**11*111111111*******2

left   NNNNNNNNNNNNNNNNNNNNNNNNNNNNNNNNNNNNNNNNNNNNNNNNNNNNNNNNNN
chrom  ATATCCTGTTATTTTTCCTTAGTTGCAAGGTAGAATTTTGTTTATAAATGT-------
right  ATATCCTGTTATTTTTCCTTAGTTGCAAGGTAGAATTTTGTTTATAAATGT-------
       2222222222222222222222222222222222222222222222222222222222
```

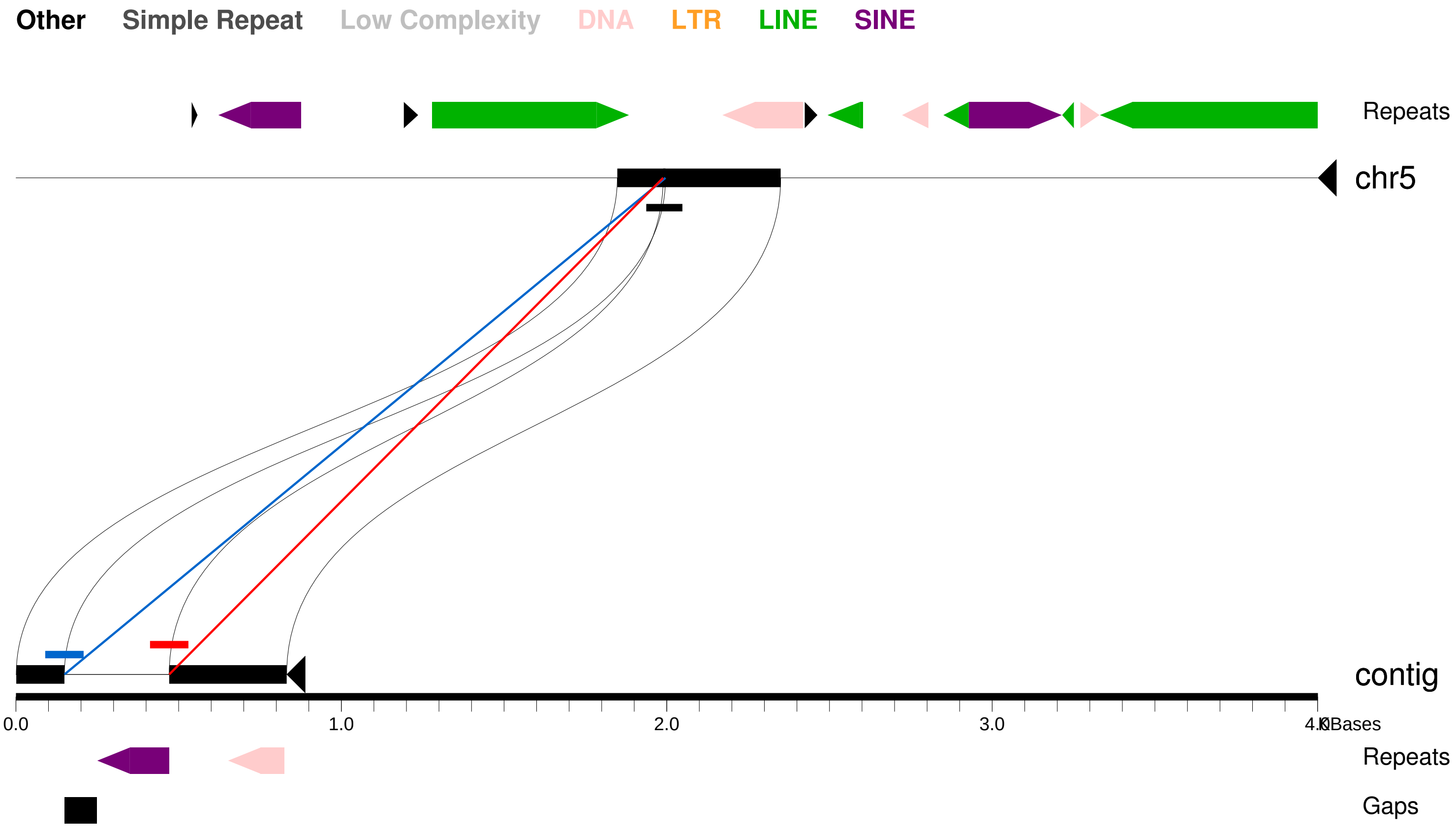

siteID: chr5_128314860 Scafftig1 - Breakpoint: chr5:128314858
chr5:128314810-128314914
Overlap Length: 12 INS size: 312 Genotyped: Yes

```
left     ATCATTATTTTTA--------------ACTGCATAATGGCAGAGACAGCCACCCATAGTAAGAAGTCACT
chrom    ATCATTATTTTTA--------------ACTGCATAATGGCAGAGACAGCCACCCATAGTAAGAAGTCACT
right    GCAGTCCGGCCTGGGCGACAGAGCGAGACTCCGTCTCAAAAAAAAAAAAAAAAAAAAAAAAAAAGTCACT
         1111*111111*111111111111111***1*1*111111*1*1*1*111*111*1*11**1********

left     TTTGGCCGGGCGCGGTGGCTCACGCCTGTA-ATCCCAGCACTTTGGGAGGCCGAGGCGGGTGGA-
chrom    TTTG--------------CTCAGGTCTCTTCATTTGAGATTTGTTGTATG--GAGGGAAACCCAC
right    TTTG--------------CTCAGGTCTCTTCATTTGAGATTTGTTGTATG--GAGGGAAACCCAC
         ****22222222222222****2*2**2*22**222**222*2*2*2*2*22****2222222*2
```

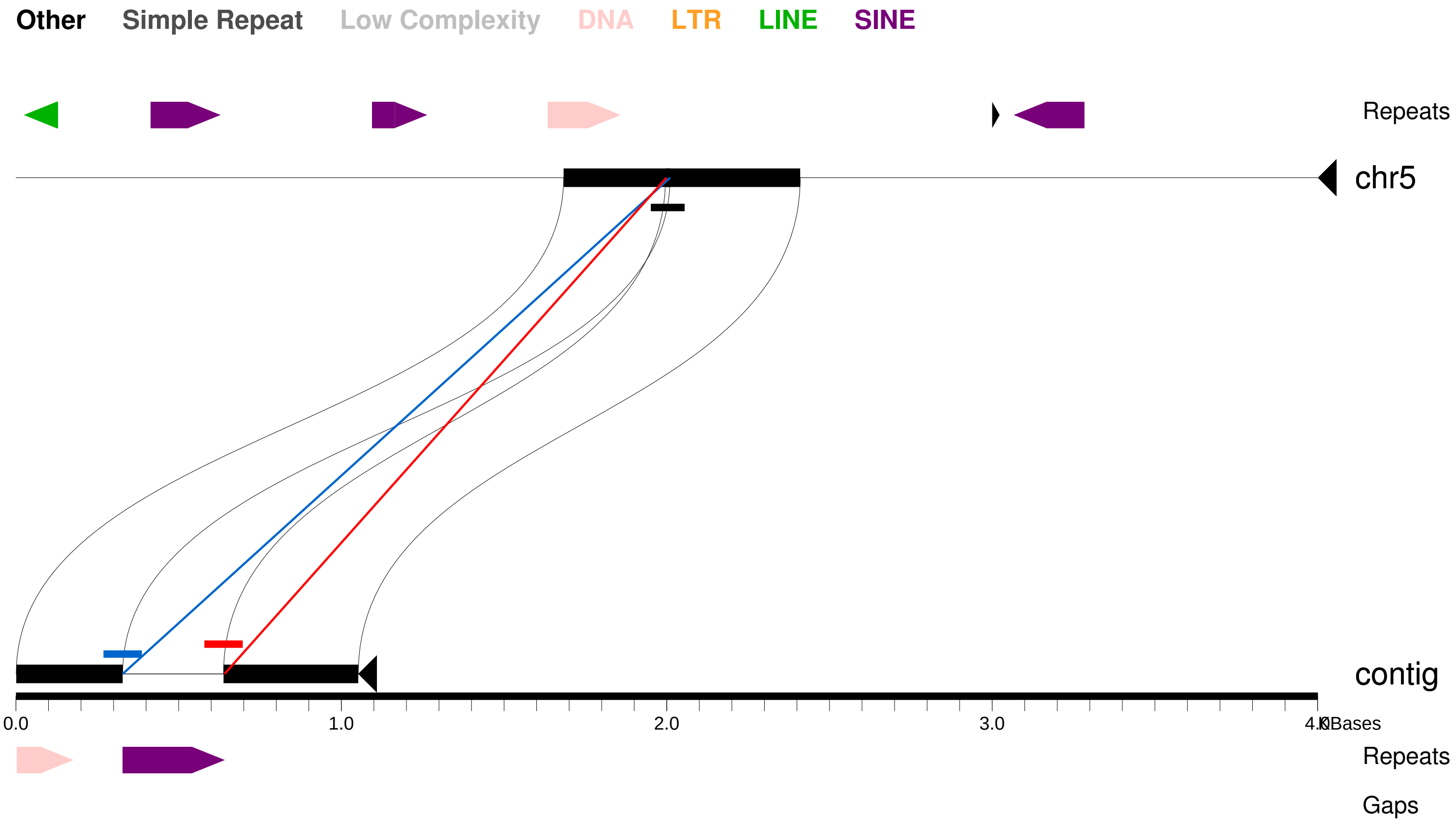

siteID: chr5_130853236 Scafftig5 - Breakpoint: chr5:130853242
chr5:130853199-130853301
Overlap Length: 17 INS size: 315 Genotyped: Yes

```
left     TCA---------------CAAGGCTTT-TCTTATGGTGTTAAAGGCTAGGTGTTCACTTAAAAAATCTCT
chrom    TCA---------------CAAGGCTTT-TCTTATGGTGTTAAAGGCTAGGTGTTCACTTAAAAAATCTCT
right    CCGGCCTGGGCGACAGAGCAAGACTCCGTCTCAAAAAAAAAAAAAAAAAAAAAAAAAAAAAAAAAATCTCT
         1*1111111111111111****1**111***1*1111111***1111*1111111*111***********

left     CAAATGGGCCGGGCGCGGTGGCTCACGCCTGTAATCCCAGCACTTTGGGAGGCCGAGGCGGGTGG
chrom    CAAATG---------CTAAAATTCAAATAACTAGACAAATCACTTCAGCTATCCCAGG-------
right    CAAATG---------CTAAAATTCAAATAACTAGACAAATCACTTCAGCTATCCCAGG-------
         ******222222222*222222***222222**22*22*2*****22*2222**2***2222222
```

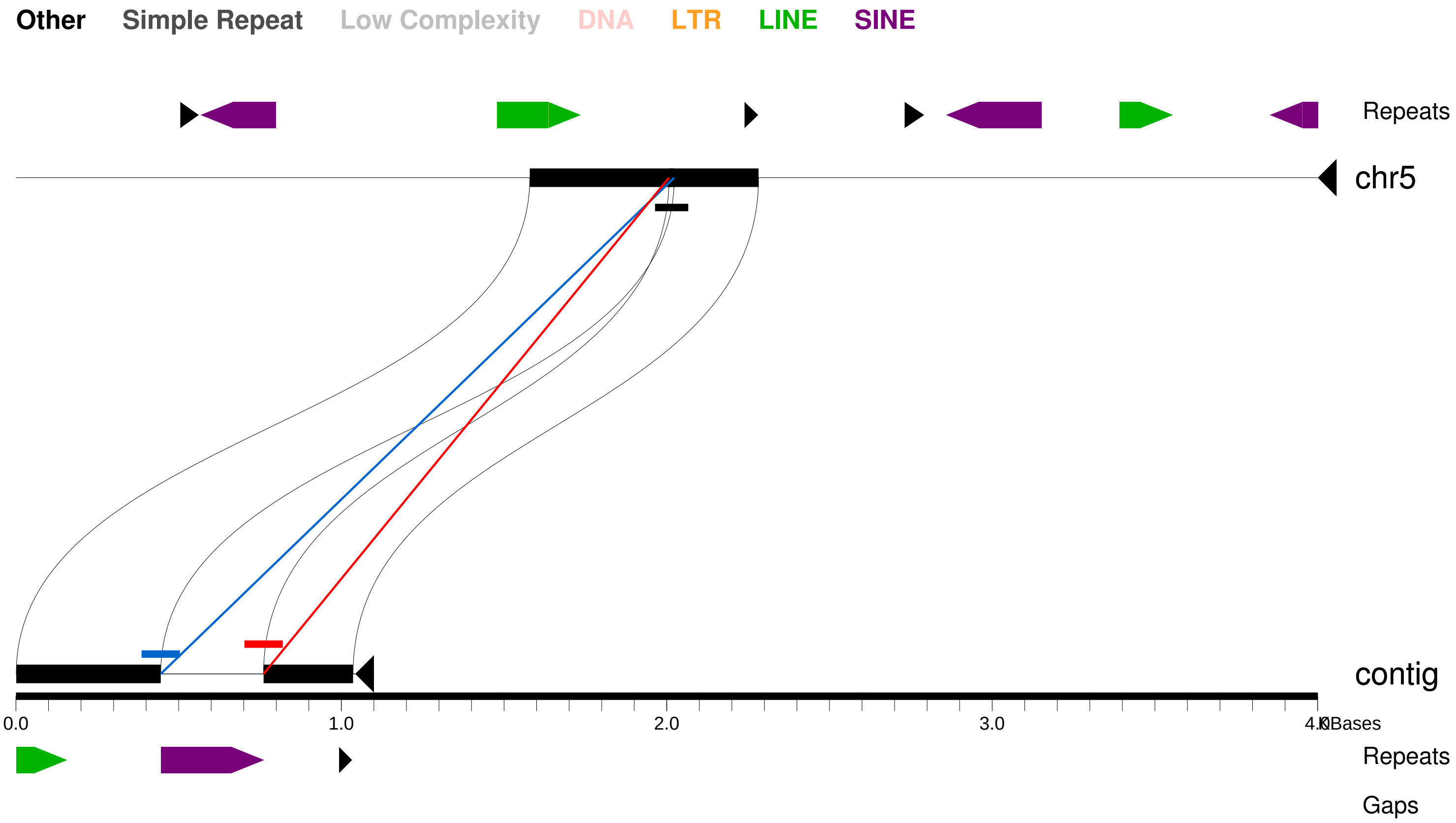

siteID: chr5_136059697 Scafftig1 + Breakpoint: chr5:136059712
chr5:136059668-136059771
Overlap Length: 12 INS size: 308 Genotyped: Yes

```
left   TTAG--------GAGAGCCTAAAG----GAGAATGATGTCATTT-CTTCCTAGAAGG--CAAGAGGACAT
chrom  TTAG--------GAGAGCCTAAAG----GAGAATGATGTCATTT-CTTCCTAGAAGG--CAAGAGGACAT
right  TCCGCCCGCCTCGGCCTCCCAAAGTGCTGGGATTACAGGCGTGAGCCACCGCGCCCGGCCAAGAGGACAT
       *11*11111111*1111**1****1111*1**1*111*1*1*111*11**11*111*11***********

left   TTTTTTTTTTTTTTTTTTTTTTTTTTTTTGAGACGGAGTCTCGCTCTGTCGCCCAGGCTGGAGTG
chrom  TGTT---------------TAGGCTCAAAAAACAATATCCCAAAATGAAGGCCACAGAAGCAGC
right  TGTT---------------TAGGCTCAAAAAACAATATCCCAAAATGAAGGCCACAGAAGCAGC
       *2**222222222222222*2222*2222*2**2222**2*2222**22*2***22222*2222
```

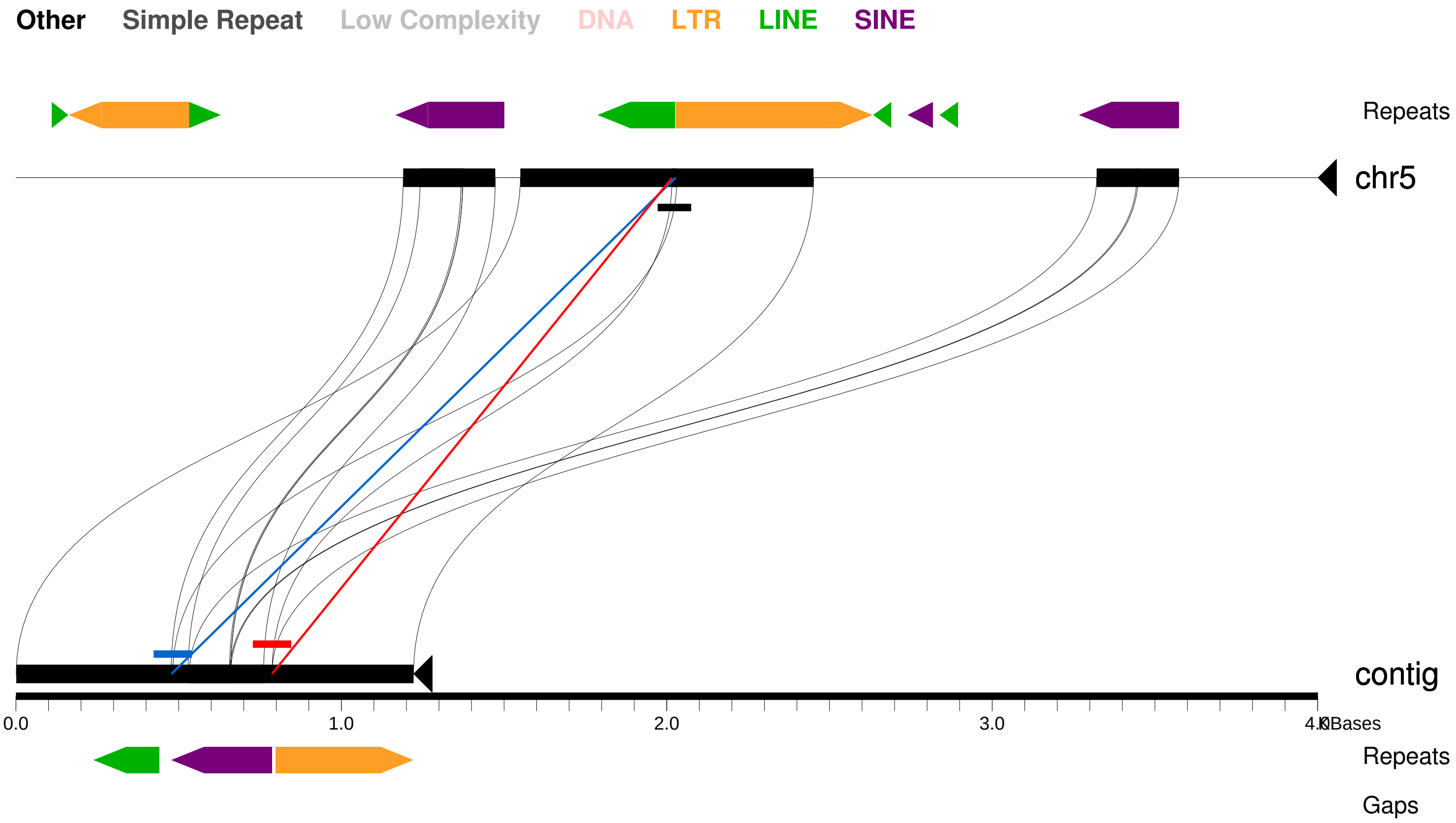

siteID: chr5_136525693 Scafftig1 - Breakpoint: chr5:136525702
chr5:136525645-136525760
Overlap Length: 3 INS size: 302 Genotyped: No

```
left    TG-TGGAGGAAATAGCAAGAGAACTAGAAGTGCA-------GCCTGAAGATGTGGCTGAATTACTATANN
chrom   TG-TGGAGGAAATAGCAAGAGAACTAGAAGTGCA-------GCCTGAAGATGTGGCTGAATTACTATAAT
right   GGATGGTCTCGATCTCCTGACCTCGTGATCCGCCCGCCTCGGCCTCCCAAAGTG-CTGGGT----ATAAT
        1*1***11111**11*11**111*11**111**11111111****1111*1***1***11*1111***22

left    NNNNNNNNNNNNNNNNNNNNNNNNNNNNNNNNNNNNNNNNNNNNNNNNNNNNNNNNN
chrom   TTCTTAATAAAACTTGAACAGATGAGGAGTTGCTTCCTATGGATGAGAAAAGAA---
right   TTCTTAATAAAACTTGAACAGATGAGGAGTTGCTTCCTATGGATGAGAAAAGAA---
        222222222222222222222222222222222222222222222222222222222
```

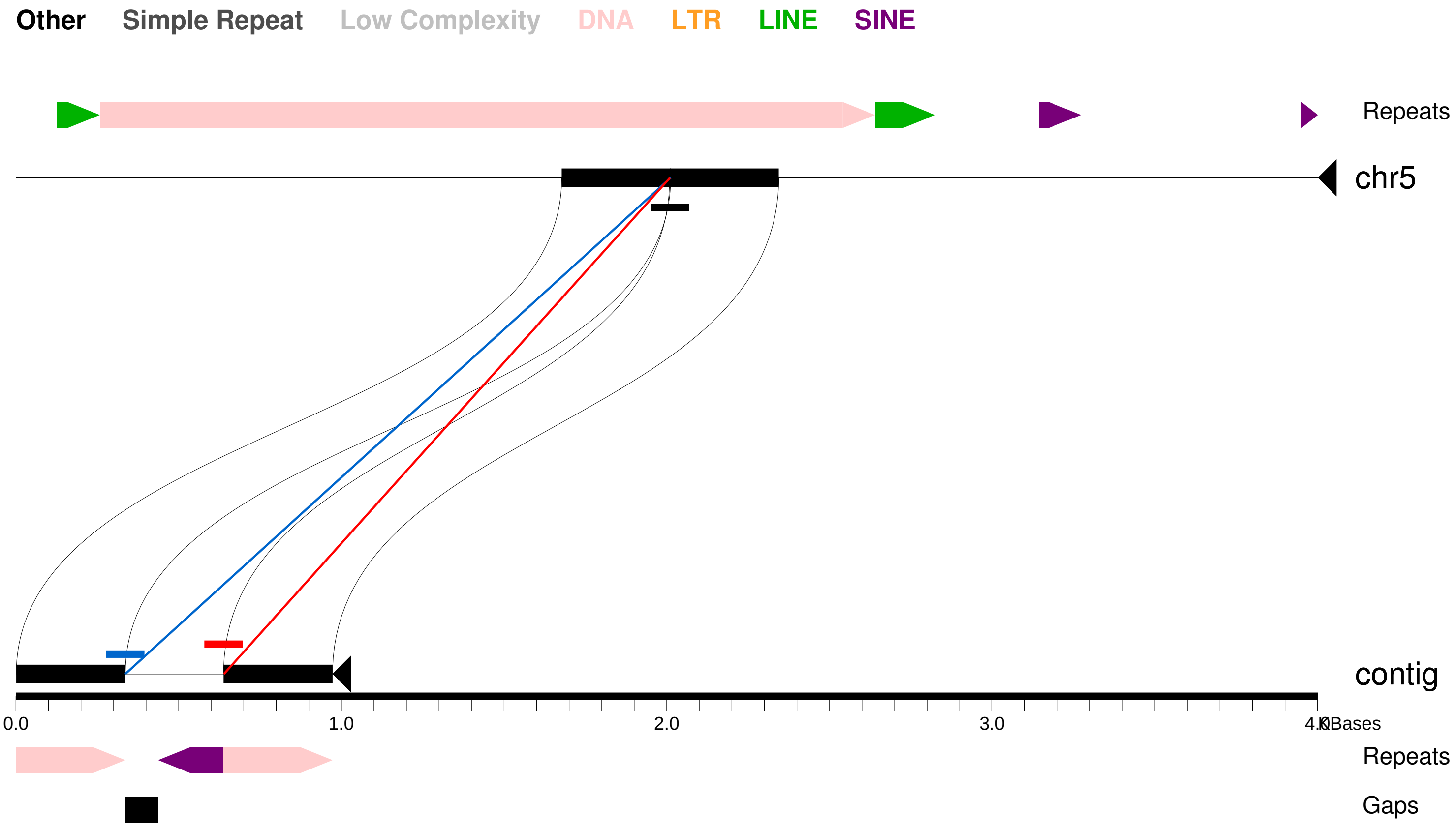

siteID: chr5_13762462 Scafftig1 - Breakpoint: chr5:13762470
chr5:13762427-13762529
Overlap Length: 14 INS size: 311 Genotyped: No

```
left    GTTCTGTCTTGACCCTACCTCACTCATAGGATTCTCTGGATAA----------------AGATCTGAAAT
chrom   GTTCTGTCTTGACCCTACCTCACTCATAGGATTCTCTGGATAA----------------AGATCTGAAAT
right   NNNNNNNNNNNNNNNNNNNNNNNNNNNNNNNNNNNNNNNNNNNNNNNNNNNNNNNNNNNNAGATCTGAAAT
        111111111111111111111111111111111111111111111111111111111111***********

left    ATCGGCCGGGCGCGGTG-GCTCACGCCTGTAATCCCAGCACTTTGGGAGGCCGAGGCGGGCGGATC
chrom   ATCAGCAGAGGATTCTGTGCTACAGTTTG------CAGCGTTTTTTGTTT-----------GTTTG
right   ATCAGCAGAGGATTCTGTGCTACAGTTTG------CAGCGTTTTTTGTTT-----------GTTTG
        ***2**2*2*22222**2***222*22**222222****22***22*22222222222222*22*2
```

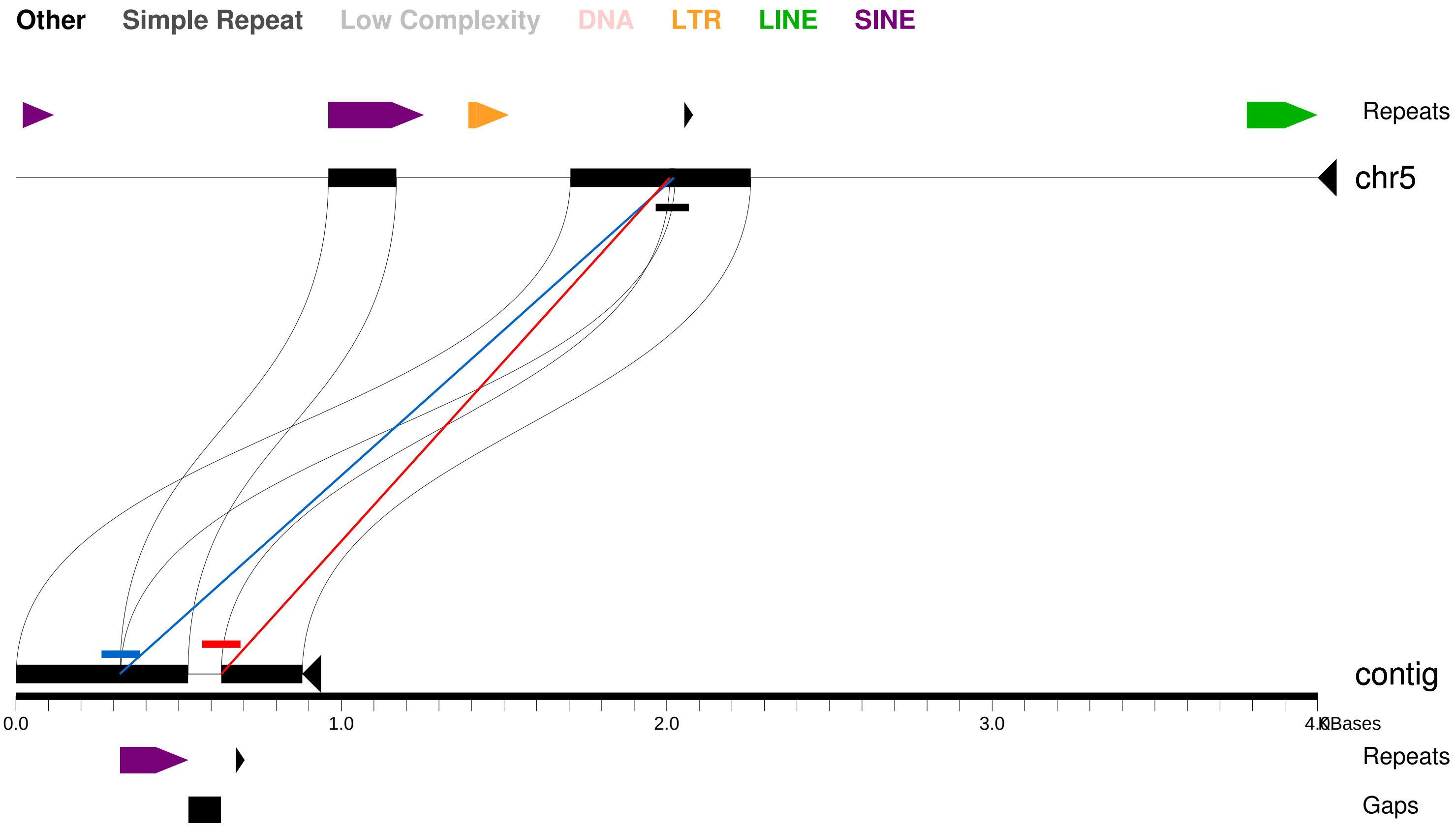

siteID: chr5_140712968 Scafftig4 - Breakpoint: chr5:140712952
chr5:140712906-140713011
Overlap Length: 14 INS size: 314 Genotyped: Yes

```
left     G----GTATGTGTGAGAT-GCCAT-CTCTGTTTGGAAAGAGCACTGCATTAG-------AAACGGCGATC
chrom    G----GTATGTGTGAGAT-GCCAT-CTCTGTTTGGAAAGAGCACTGCATTAG-------AAACGGCGATC
right    GTCTGGCCTGGGCGACAGAGCGAGACTCCGTCTCAAAAAAAAAAAAAAAAAAAAAAAAAAAACGGCGATC
         *1111*11**1*1**1*11**1*11***1**1*11***1*11*1111*11*11111111***********

left     TAGGGCCGGGCGCGGTGGCTCACGCCTGTAATCCCAGCACTTTGGGAGGCCGAGGCGGGTGG
chrom    TAGCTTCTAACATTTTCTTACTTGCCTCTGGGCTAAAAGGTTTGAT-------------TAG
right    TAGCTTCTAACATTTTCTTACTTGCCTCTGGGCTAAAAGGTTTGAT-------------TAG
         ***222*222*2222*2222*22****2*222*22*2222****222222222222222*2*
```

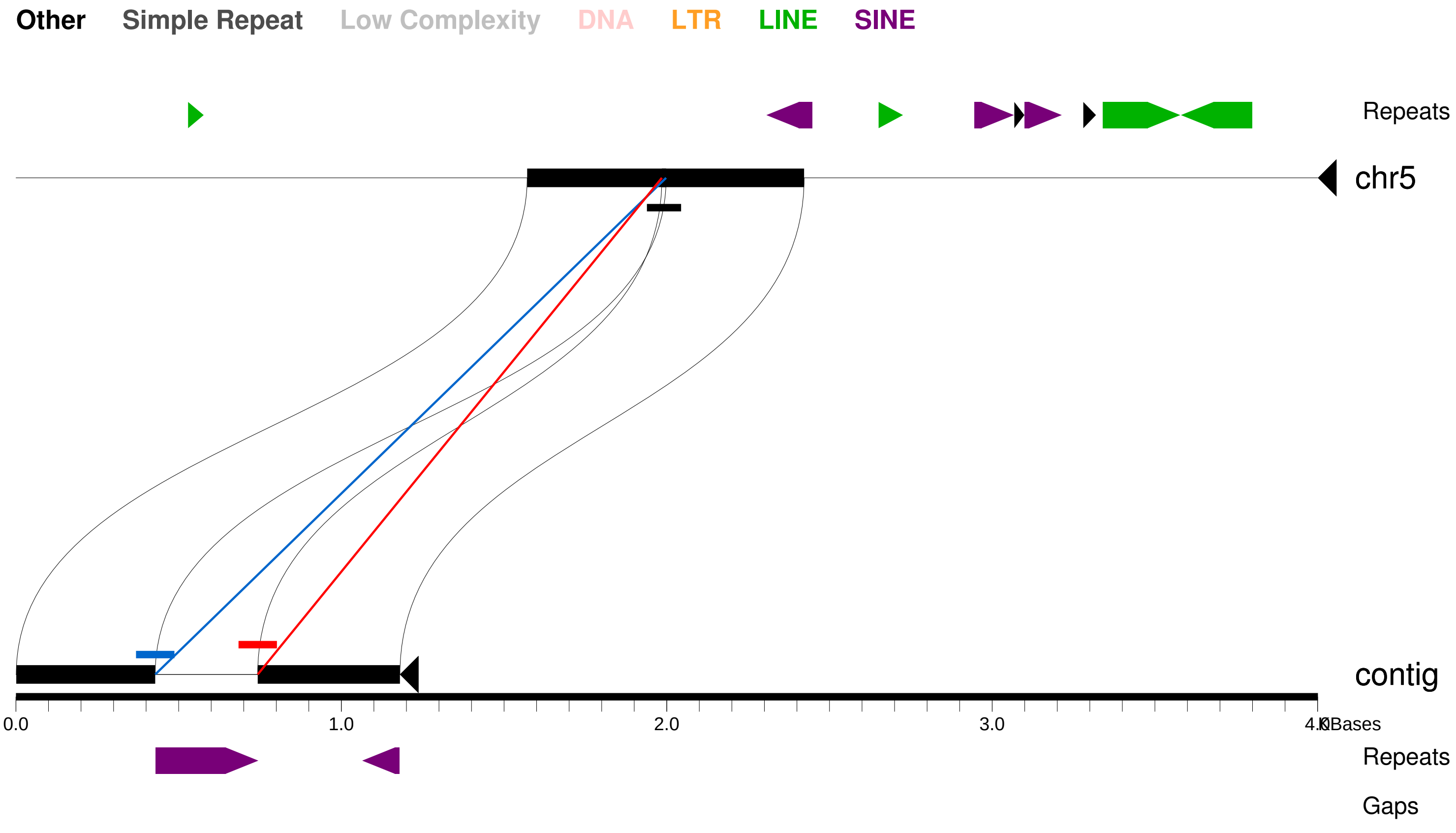

siteID: chr5_141758651 Scafftig7 + Breakpoint: chr5:141758643
chr5:141758596-141758702
Overlap Length: 13 INS size: 299 Genotyped: Yes

```
left   GGCTTAGGAG--AGGGTC------TGATGAGGACAATGAGGGTTATCAAAATTTT-----AATCCCCATT
chrom  GTCTTAGGAG--AGGGTC------TGATGAGGACAATGAGGGTTATCAAAATTTT-----AATCCCCATT
right  TCCACCCGCCTCAGCCTCCCAAAGTGCTG-GGATTACAGGCGTGAGCCACCGCGCCCGGCAATCCCCATT
       1N*1111*1111**11**111111**1**1***11*111*1**1*1*1*11111111111**********

left   TTTTTTTTTTTTT-TTGAGACGGAGTCTCGCTCTGTCGCCCAGGCCGGACTGCGGACTGCAGT
chrom  TTTAAACCCCAGTGTTTAAAC-----CTTGCTTTGT-------GAAGTGTAACCTATCACAG-
right  TTTAAACCCCAGTGTTTAAAC-----CTTGCTTTGT-------GAAGTGTAACCTATCACAG-
       ***222222222*2**2*2**22222**2***2***2222222*22*22222*22*222***2
```

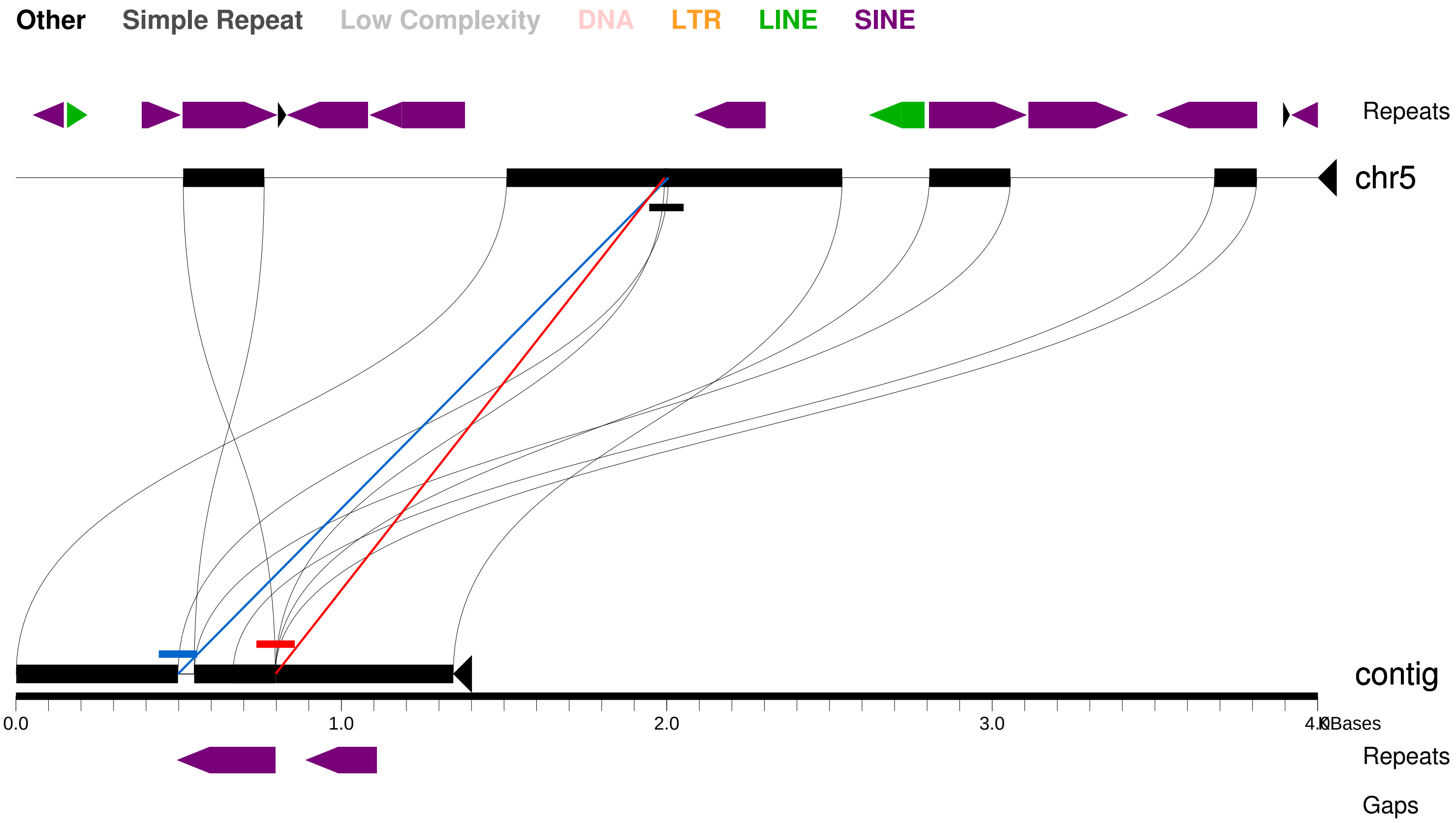

siteID: chr5_142355018 Scafftig1 + Breakpoint: chr5:142354996
chr5:142354952-142355055
Overlap Length: 16 INS size: 289 Genotyped: Yes

```
left    --------------CTTTCATCTTGCTGGTGT-ACTGGGGTTTCACATTCAATTTTTATCTGTCTGTCTA
chrom   --------------CTTTCATCTTGCTGGTGT-ACTGGGGTTTCACATTCAATTTTTATCTGTCTGTCTA
right   CCGCCCGCCTCGGCCTCCCAAAGTGCTGGGATTACAGGCGTGAGCCACCGCGCCCAGCCCTGTCTGTCTA
        11111111111111**11**111******11*1**1**1**1111**111111111111***********

left    TTTTTTTTTTTTTGAGACGGAGTCTCGCTCTGTTGCCCAGGCTGGAGTGCAGTGGCGGGATCTC
chrom   TTTTTG-----ATAAAAGATATTCT-GTACTAAT----AGTTTTAAAACCAT----CAGAT-TA
right   TTTTTG-----ATAAAAGATATTCT-GTACTAAT----AGTTTTAAAACCAT----CAGAT-TA
        *****2222222*2*2*222*2***2*22**22*2222**22*22*222**2222222***2*2
```

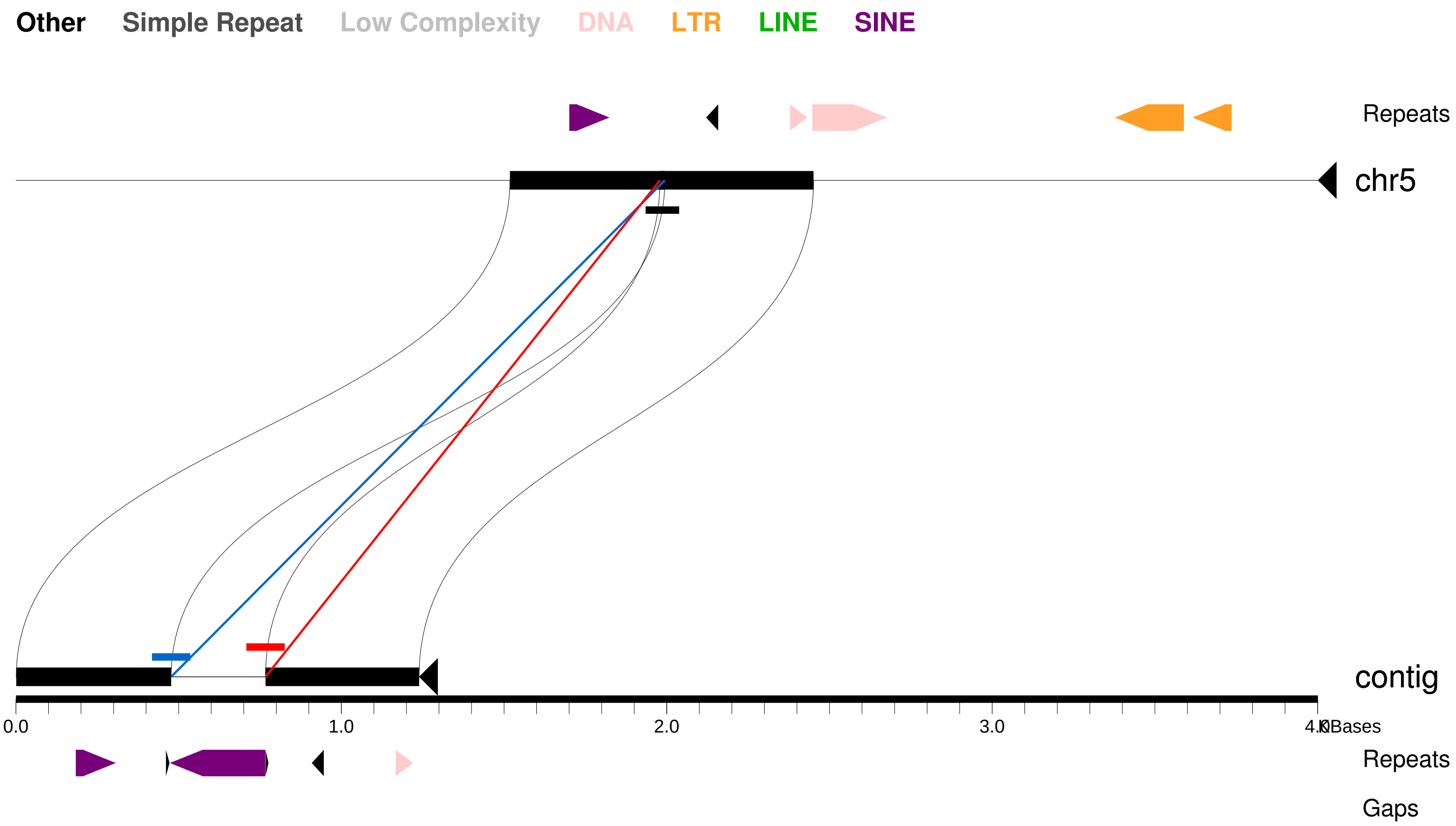

siteID: chr5_143010785 Scafftig1 + Breakpoint: chr5:143010790
chr5:143010747-143010849
Overlap Length: 17 INS size: 311 Genotyped: Yes

```
left     ATTT---CTACCCATGGAACCATCGACACAATCAAGATAATGAGTT-------------AAAAAAATCAA
chrom    ATTT---CTACCCATGGAACCATCGACACAATCAAGATAATGAGTT-------------AAAAAAATCAA
right    GCCTGGCGACAGAGCGAGACTCCGTCTAAAAAAAAAAAAAAAAAAAAAAAAAAAAAAAAAAAAAAAAAAAA
         111*111*1**11*11**11*11**1*1***11**1*1**11*1111111111111111*******11**

left     TTTCTCGGCCGGGCGCGGTGGCTCACGCCTGTAATCCCAGCACTTTGGGAGGCCGAGGCGGGCGG
chrom    TTTCTC--CCT-----------TAAATTTTGTTA---CGTTGCTTTTTAATGCATGTCTCACTTC
right    TTTCCC--CCT-----------TAAATTTTGTTA---CGTTGCTTTTTAATGCATGTCTCACTTC
         ****1*22**222222222222*2*2222***2*222*2222****222*2**222222222222
```

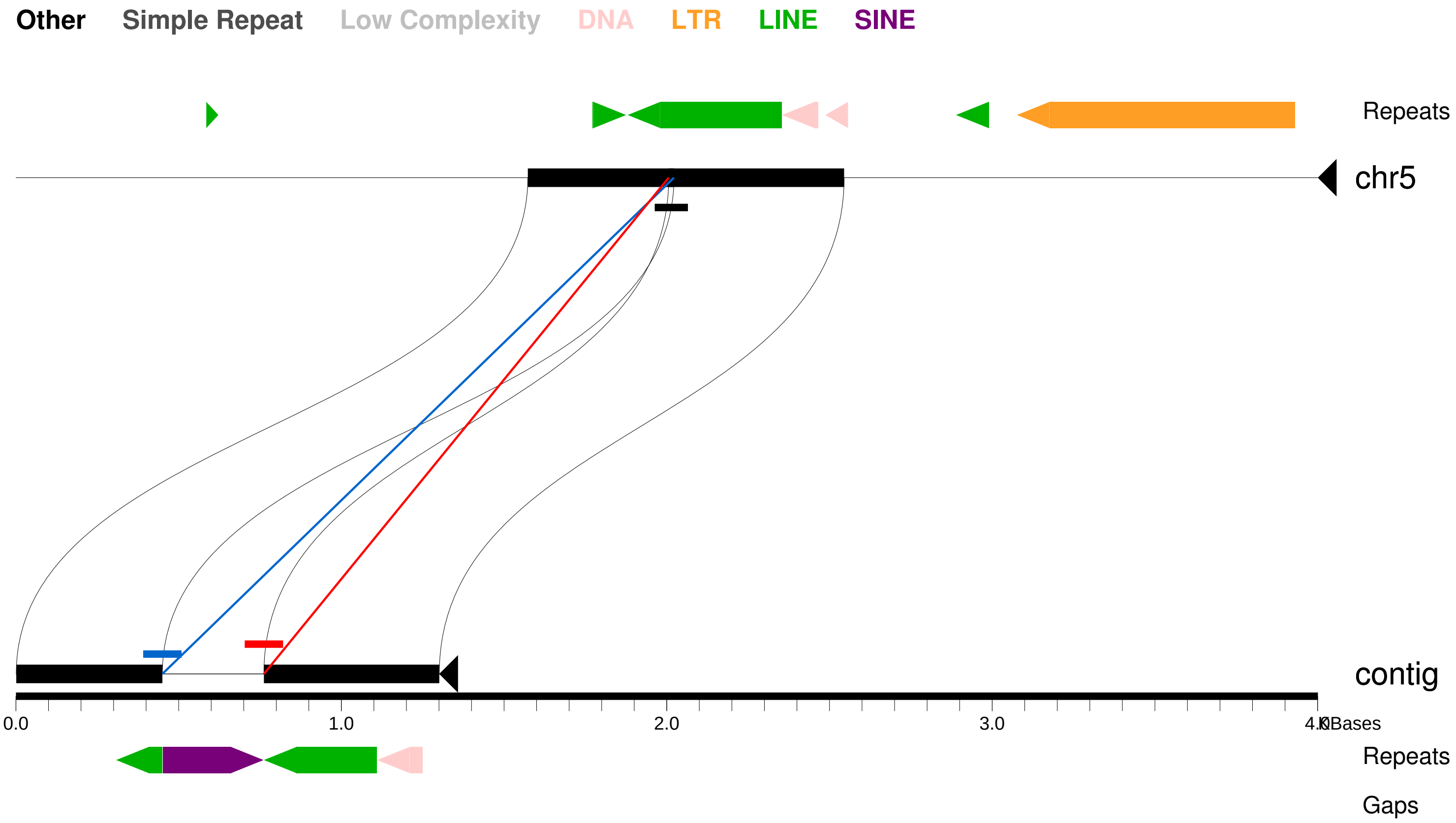

siteID: chr5_143300143 Scafftig1 + Breakpoint: chr5:143300124
chr5:143300116-143300183
Overlap Length: 52 INS size: 423 Genotyped: No

```
left    ------------------------GTTGTGAA---------------------------GGTAATATAGT
chrom   ------------------------GTTGTGAA---------------------------GGTAATATAGT
right   CCACCCGCCTCGGCCTCCCAAAGTGCTGGGATTACAGGCGTGAGCCACCGCGCCCGGCCGGTAATATAGT
        111111111111111111111111*1**1**1111111111111111111111111111***********

left    TTCTTATATGCTGAGACTATGGACCAGAGTTGGCTGTTAGTNNNNNNNNNNNNNNNNNNNNNNNNNNNNN
chrom   TTCTTATATGCTGAGACTATGGACCAGAGTTGGCTGTTAGTGCTGAGTA---------------------
right   TTCTTATATGCTGAGACTATGAACCAGAGTTGGCTGTTAGTGCTGAGTA---------------------
        *********************1*******************22222222222222222222222222222

left    NNNNNNNNNNNNNNNNNNNNNNNNNNNNNN
chrom   ------------------------------
right   ------------------------------
        222222222222222222222222222222
```

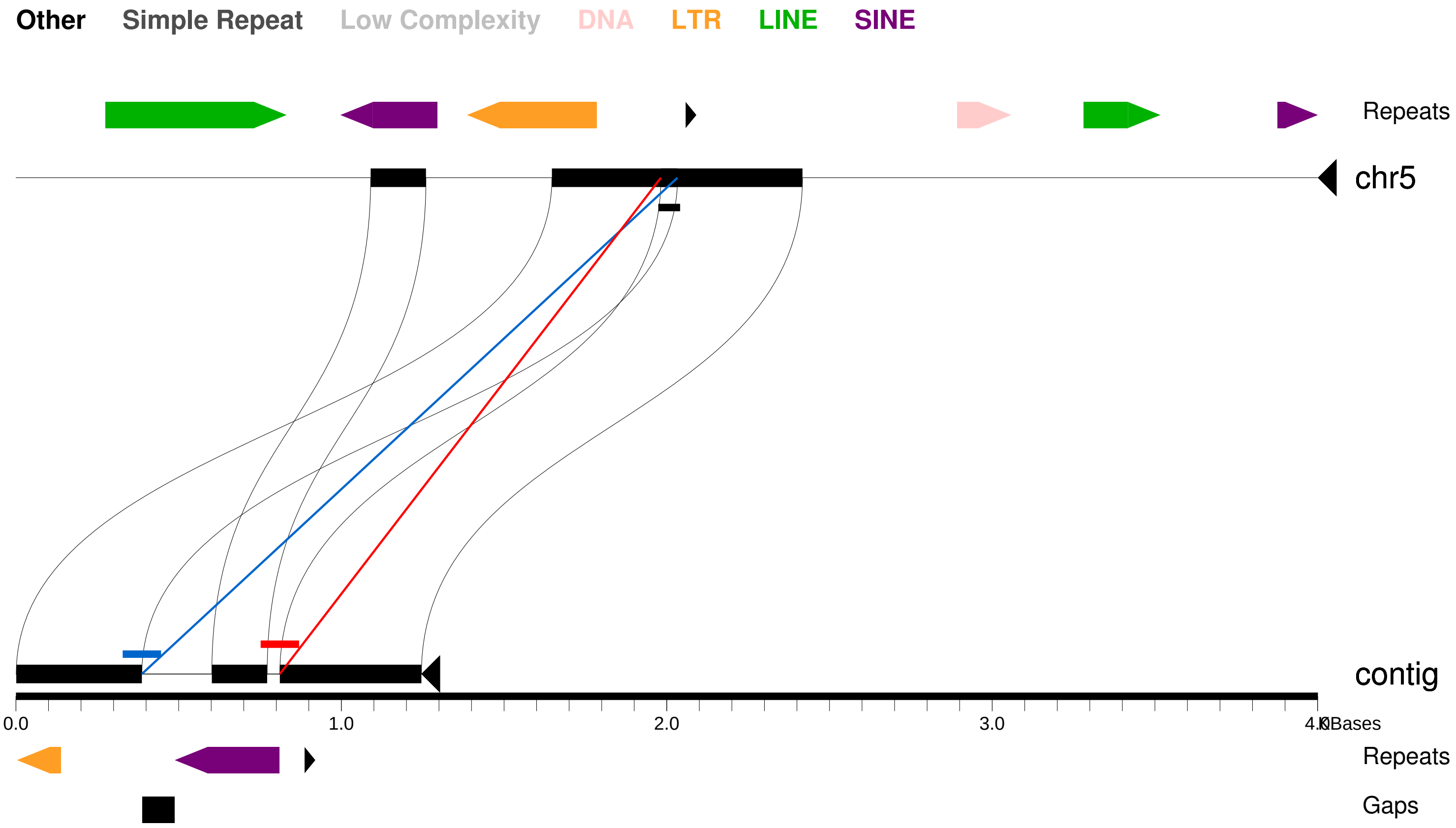

siteID: chr5_146369628 Scafftig2 - Breakpoint: chr5:146369613
chr5:146369567-146369672
Overlap Length: 14 INS size: 304 Genotyped: Yes

```
left    GA-----AATGCTAGACATAATAACCAAG-GAGG-------TGAAATATCTCTACAATTAAAACCATAAA
chrom   GA-----AATGCTAGACATAATAACCAAG-GAGG-------TGAAATATCTCTACAATTAAAACCATAAA
right   CAGTCCGCAGTCCGGCCTGGGCGACAGAGCGAGACTCCGTCTAAAAAAAAAAAAAAAAAAAAACCATAAA
        1*111111*11*11*1*111111**11**1***11111111*1***1*11111*1**11***********

left    ACAGGCCGGGCGCGGTGGCTCACGCCTGTAATCCCAGCACTTTGGGAGGCCGAGGCGGGTGG
chrom   ACATTAA--------TGAAAGAAGC-TGAAA----AAGACAAATAAATGAAAAGACATCCTG
right   ACATTAA--------TGAAAGAAGC-TGAAA----AAGACAAATAAATGAAAAGACATCCTG
        ***222222222222**2222*2**2**2**2222*22**222222*2*222**2*22222*
```

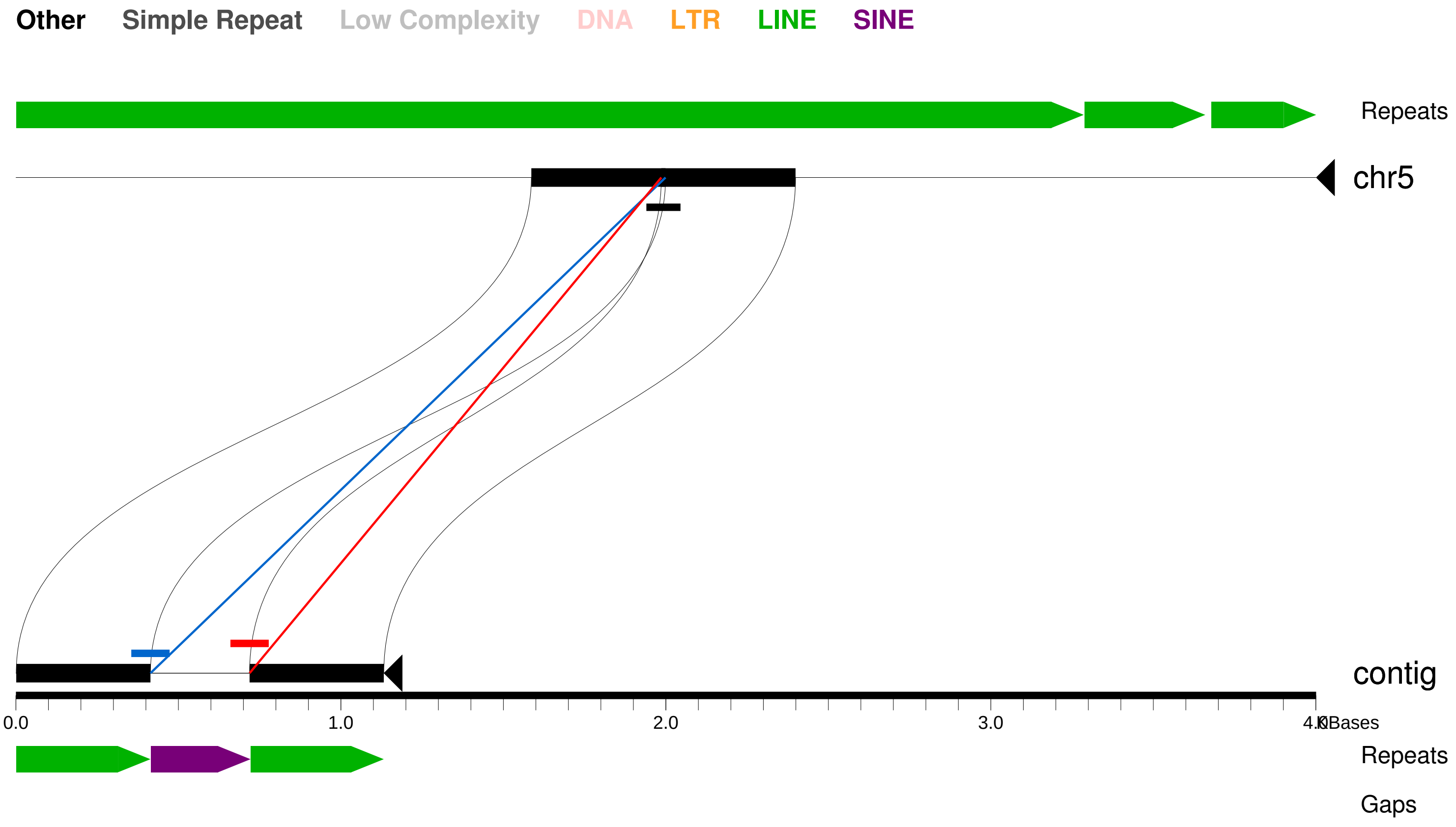

siteID: chr5_146637734 Scafftig1 + Breakpoint: chr5:146637717
chr5:146637418-146637877
Overlap Length: 82 INS size: 430 Genotyped: No

```
left   T----------------------------------------------------------------------
chrom  TTCTAAGAAATGGACCTTTTGTTTTAAATGTGGGACATCAGCAGTGGACTTTATAGTCCTTGGTGCCTT
right  CCC--------------------------------------------GGCT-------------------
       1N2                                            2N22

left   ----------------------------------------------------------------------
chrom  TTTACTGAGAAATTTCCTTTAGCACCTATTTTTATTAGATTTTAGACCAAAGAAGGCCAAACACCATTTT
right  ----------AATTT--TTTG-----TATTTTTAGTAGA------------GACGGGGTTTCACCGTTTT
                 22222  222N     22222222N2222            22N22NNNNN2222N2222

left   ----------------------------------------------------------------------
chrom  ATATTTAACAGTGCTTCCTGTATGATTCTTATACCAGATAAGCTAAGTTTCACCTTTATATTAGCAAGTT
right  AG--------------CCGGGATGGT-------CTCGAT-----------CTCCT---------------
       2N              22N2N222N2       2NN222           2N222

left   ----------------------------------------------------------------------
chrom  GTTAAACTTAATTTTAATAAAACTTTGTAGACATATTTATCCAATTTTTAATGTCTGACCATAATG-TAT
right  -----------------------------GACCTCGTGATCCGCCCGCCTCGGCCTC-CCAAAGTGCTGG
                                    222N2NN2N2222NNNNNNNNNN2N22N 222N2N2212NN

left   -------ATAGACTCTTTTTAACCTTTTATAATTTTTGTTAAAGAGCAGGTTAGTGCTTTAAGAAATACC
chrom  GATTCTTATAGACTCTTTTTAACCTTTTATAATTTTTGTTAAAGAGCAGGTTAGTGCTTTAAGAAATACC
right  GATT---ACAGGC-------AACCTTTTATAATTTTTGTTAAAGAGCAGGTTAGTGCTTTAAGAAATACC
       2222   *1**1*1111111**************************************************

left   TGTTGTGCTTTTATTTTAATGTCCAGTTCACANNNNNNNNNNN---------------------------
chrom  TGTTGTGCTTTTATTTTAATGTCCAGTTCACAGAAAAACTGTATGATACCCCTTAAACTTTAGCCAATAT
right  TGTTGTGCTTTTATTTTAATGTCCAGTTCACAGAAAAACTGTGTGATACCCCTTAAACTTTAGCCAATAT
       ********************************2222222222N222222222222222222222222222

left   -----------------------------------------
chrom  GTTTACACACAGAATTTCCTTTATAATTAACATTTCAAAAC
right  GTTTACACACAGAATTTCCTTTATAATTAACATTTCAAAA-
       2222222222222222222222222222222222222222
```

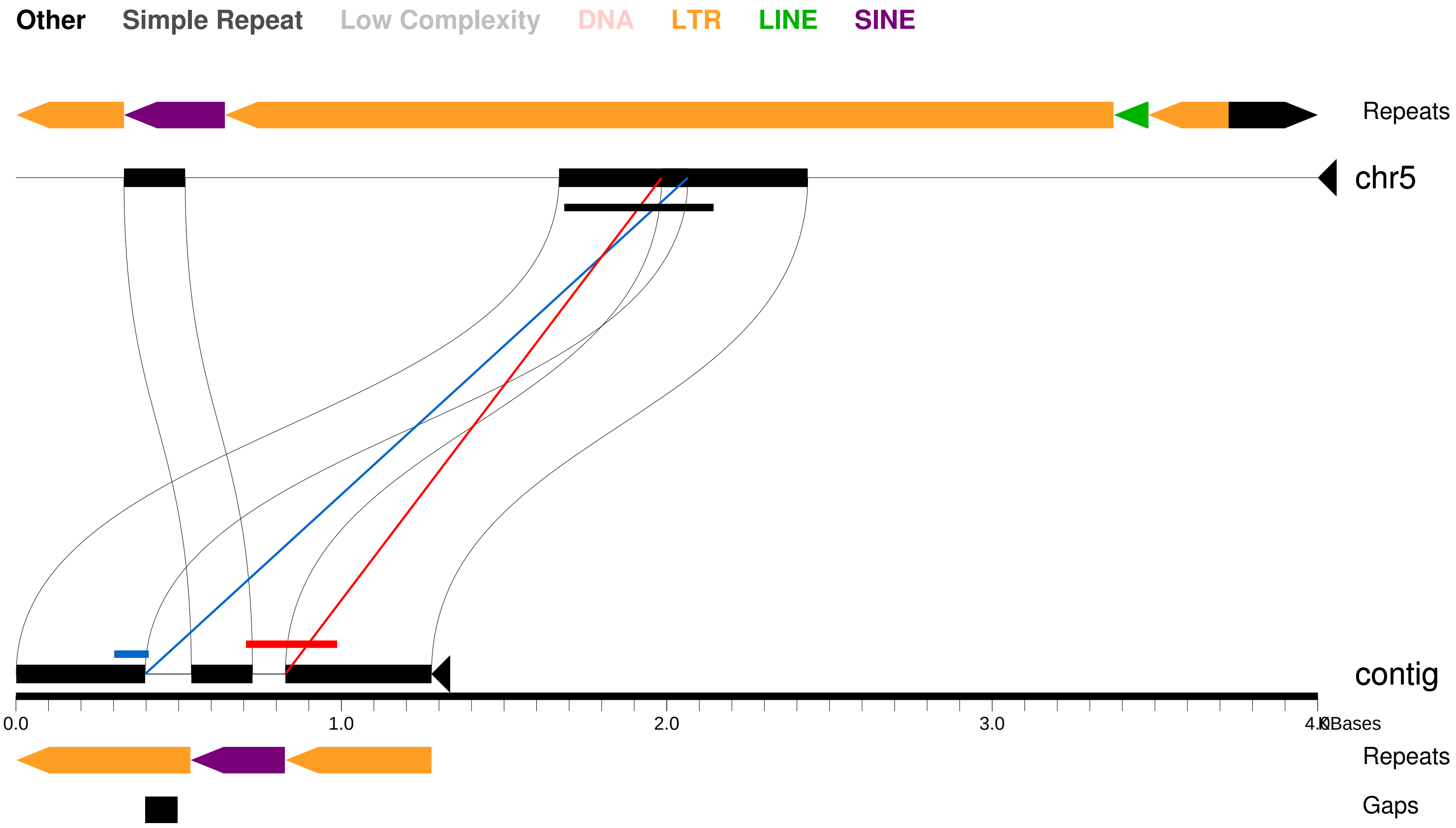

siteID: chr5_147351666 Scafftig1 - Breakpoint: chr5:147351568
chr5:147351523-147351627
Overlap Length: 15 INS size: 148 Genotyped: Yes

```
left    AAGAA--------------AAAAATAATGTGTGGTCTCTTGGACAGGTCAACTTTGTTTTAGAATGATTT
chrom   AAGAA--------------AAAAATAATGTGTGGTCTCTTGGACAGGTCAACTTTGTTTTAGAATGATTT
right   CAGAGCGAGACTCCGTCTCAAAAAAAAAAAAAAAAAAAAAAAAAAAAAAAAAAAAAAAATAGAATGATTT
        1***111111111111111*****1**111111111111111*1*1111**11111111***********

left    CTTCTGGGAGGCTGAGGCAGGAGAATGGCGTGAACCCGGGAGGCGGAGCTTGCAGTGAGCCGA
chrom   CTTC------ACAAAAATTTCAATTTTGCGTTTTCCATTAATTAAGATTTCTCAT--------
right   CTTC------ACAAAAATTTCAATTTTGCGTTTTCCATTAATTAAGATTTCTCAT--------
        ****2222222*22*222222*222*2****222**2222*2222**22*22**222222222
```

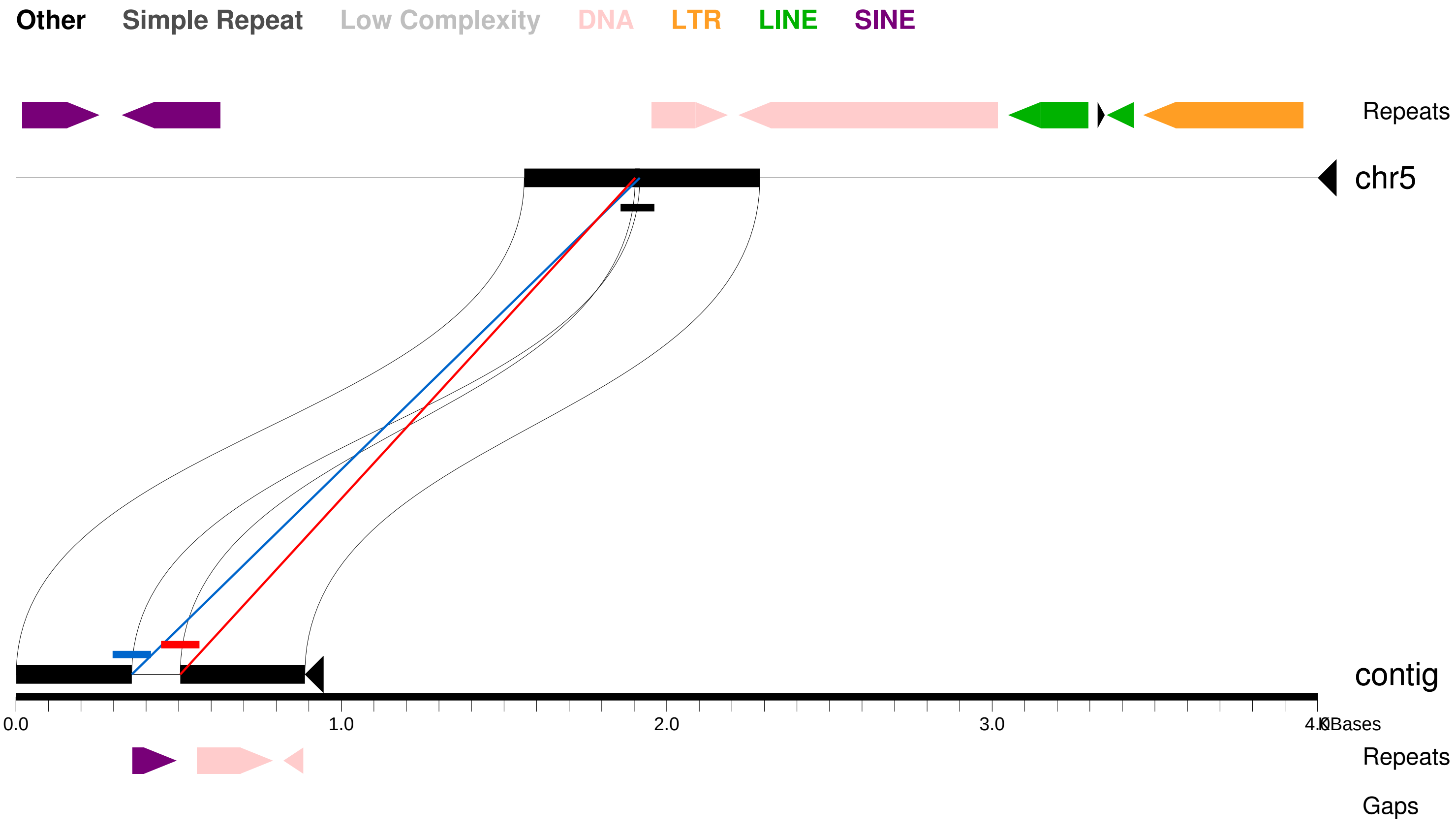

siteID: chr5_148080090 Scafftig2 + Breakpoint: chr5:148080071
chr5:148080027-148080130
Overlap Length: 16 INS size: 301 Genotyped: Yes

```
left     TAGGAAAT----GGTTAAAACAATATTTGGAAAAAAAATTTAGAGCTTT-----------ACTTGTCTTTT
chrom    TAGGAAAT----GGTTAAAACAATATTTGGAAAAAAAATTTAGAGCTTT-----------ACTTGTCTTTA
right    CCGCCCTCCTCGGCCTCCCAAAGTGCTGGGATTACAGGCGTGAGCCACCGCGCCCGGCCACTTGTCTTTA
         11*111111111*11*111*1*1*11*1***11*1*11111****11111111111111**********2

left     TTCTTTTTTTTTTTTTTTTTTTTTTTGAGACGGAGTCTCGCTCTGTCGCCCAGGCTGGAGTGCAG-
chrom    TTCTTAATAAAAT-------------AAAAGAACATTCACTATGTT-CCCA--CATCATATAAGA
right    TTCTTAATAAAAT-------------AAAAGAACATTCACTATGTT-CCCA--CATCATATAAGA
         *****22*2222*2222222222222*2*2*2*222**2**2***22****22*222*2222**2
```

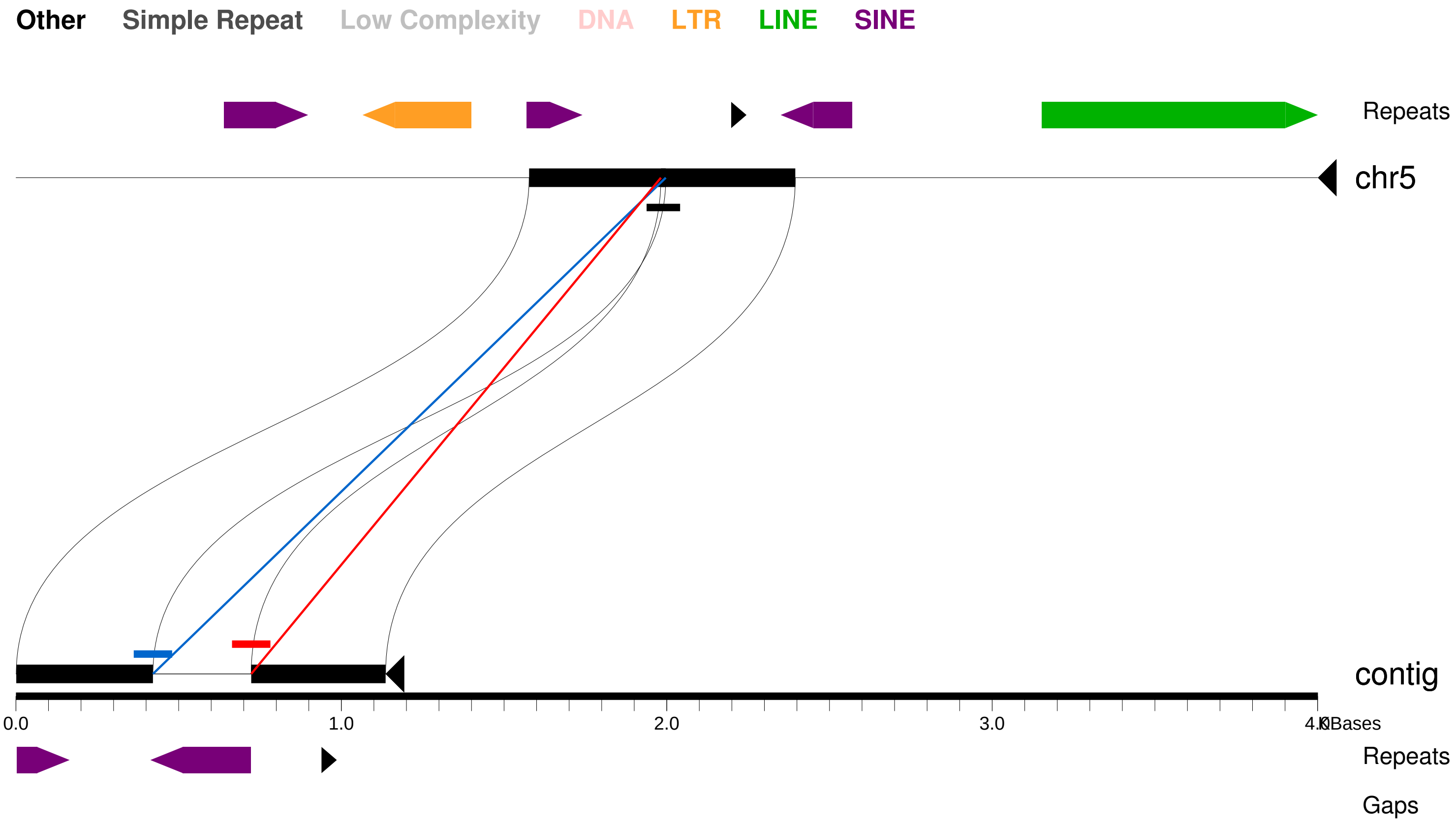

siteID: chr5_150986501 Scafftig1 + Breakpoint: chr5:150986468
chr5:150986431-150986527
Overlap Length: 15 INS size: 315 Genotyped: Yes

```
left   -----------CAGCTT-----AATGCAATAATTCTA--CAT-AGTATTTGTGACT---CCACTTTATTT
chrom  -----------CAGCTT-----AATGCAATAATTCTA--CAT-AGTATTTGTGACT---CCACTTTATTT
right  TCCGCCCGTCTCGGCCTCCCAAAGTGCTGGGATTACAGGCGTGAGCCACCGCGCCCGGCCCACTTTATTT
       1111111111*1**1*11111*1***1111***11*11*1*1**11111*1*1*1111************

left   ATTTATTTATTTATTTATTTATTTATTTATTTATTTTTTTGAGACGGAGTTTCGCTCTGTCGCCCAGGCT
chrom  ATTTTTTG--------------------------TTTTCTGAGATGGGATCTTGCTAAGTTTCCCAGGCT
right  ATTTTTTG--------------------------TTTTCTGAGATGGGATCTTGCTAAGTTTCCCAGGCT
       *****2**22222222222222222222222222****2*****2**22*2*2***22**22********

left   G----
chrom  GGTCT
right  GGTCT
       *2222
```

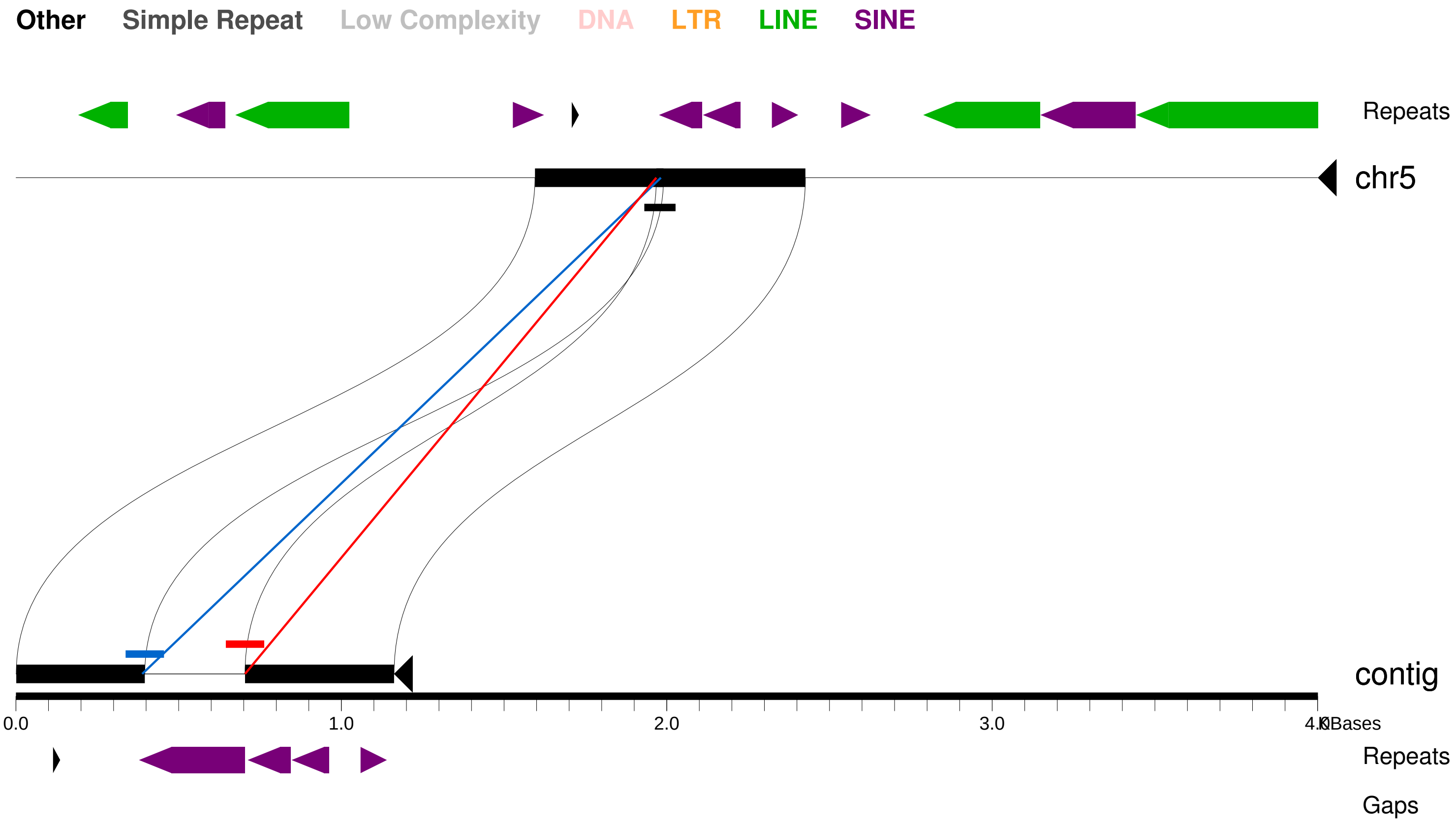

siteID: chr5_152529744 Scafftig1 - Breakpoint: chr5:152529723
chr5:152529563-152529898
Overlap Length: 16 INS size: 440 Genotyped: No

```
left   ----------------------------------------------------------------------
chrom  TGCCCATAGGCTCAAAGTAAAGAATTGAAGAAAGATTTACCAAGCAAACAGAAAATAAAAAAGAGCAGGG
right  TTT------------AGTAGAGACGGG------GTTTCACCGTGTTAGCC-AGGATGGTCTCGATCTCCT
       2NN            2222N222NNN2      2N22N222NN2NN2N2N 2NN22NNNNNN22N2NNNN

left   ------ATTCTTGTATCAGATAAAACATACTTCAAGCAAATAAAGTAAAAAAGGACCAATAAGGGCATTA
chrom  GTCCCTATTCTTGTATCAGATAAAACATACTTCAAGCAAATAAAGTAAAAAAGGACCAATAAGGGCATTA
right  GACC----TCGTG-ATCCG------CCCGCCTCGGCCTCCCAAAGTGCTG--GGAT---TACAGGCGTGA
       2N22  11**1**1***1*111111*111*1**111*1111*****111111***1111**11***1*1*

left   CCTAAAGATAAACTGTTAATTCAACACAGTATTTTTTTNNNNNNNNNNN---------------------
chrom  CCTAAAGATAAACTGTTAATTCAACACAGTATTTTTTAACTATCCTAAAAATATATTTACCTAAACTGTAG
right  GCCACCGCGCC-CGGCC---TCAACACAGTATTTTTTAACTATCCTAAAAATATATTTACCTAAACTGTAG
       1*1*11*11111*1*11111*****************222222222222222222222222222222222

left   ----------------------------------------------------------------------
chrom  CCCTCAGATTTATAAAACAAGTACTTTTAGACCTATGAAAAGACTTAGACAGTCACACAATAGTAGTGAG
right  CCCTCAGATTTATAAAACAAGTACTTTTAGACCTATGAAAAGACTTAGACAGTCACACAATAGTAGTGAG
       2222222222222222222222222222222222222222222222222222222222222222222222

left   --------------------------------------------------------
chrom  GGACTTTAACATTCCCCTGATAGCACTAGAAAAATAGCATTAGACTGATAGCATTA
right  GGACTTTAACATTCCCCTGATAGCACTAGAAAAATAGCAT----------------
       2222222222222222222222222222222222222222
```

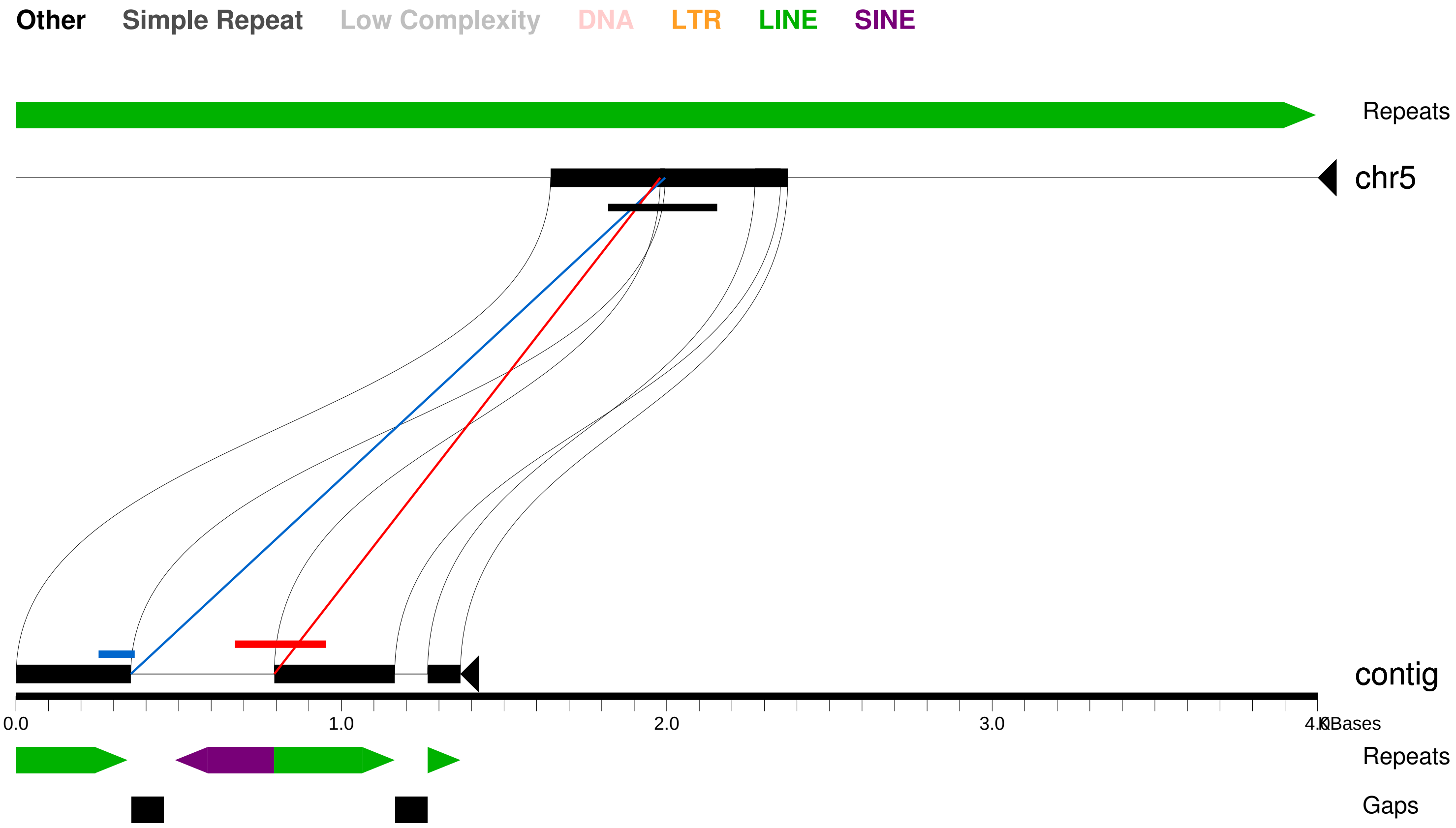

siteID: chr5_153101885 Scafftig1 - Breakpoint: chr5:153101818
chr5:153101658-153101994
Overlap Length: 17 INS size: 295 Genotyped: No

```
left   ---------------------------------------------TTGGTCACTTTTCTGTTAGGTCATCT
chrom  ATTTGAGTAGCTATCCTCTTTTATAAAGTACTTTTGTAAGTCTCTTGGTCACTTTTCTGTTAGGTCATCT
right  TTT--AGTAGAGACGGGGTTTCA-------CGGTTTTAGCCGGGATGGTCTCGATCTCCTGACCTCGT--
       N22  22222NN2NNNNN222N2       2NN22N22NNNNNN1*****1*11*1111*1*11**1*11

left   GTTCTTTTCTTATTGTTGTAC--AAGTATTCTTCATATAGCCTTGACATAAGTCCTTTTTTAGTTGTATA
chrom  GTTCTTTTCTTATTGTTGTAC--AAGTATTCTTCATATAGCCTTGACATAAGTCCTTTTTTAGTTGTATA
right  GATCCGCCCGCCTCGGCCTCCCAAAGTGCTGGGATTACAGGCGTGAGCCACCGCG---------------
       *1**1111*111*1*111*1*11****11*11111**1**1*1***111*111*1111111111111111

left   TATTGTTAATATTGTCTCTCATTCTGTGGCTTATCTTTT-------------------------------
chrom  TATTGTTAATATTGTCTCTCATTCTGTGGCTTATCTTTTCACTGTCTTGGCAGTGTGTCATGTTGAACAG
right  ---------------CCCGGCCTCTGTGGCTTATCTTTTCACTGTCTTGGCAGTGTGTCATGTTGAACAG
       111111111111111*1*1111****************22222222222222222222222222222222

left   ----------------------------------------------------------------------
chrom  AAATACATAACTTTAATGTAGTCCAGTTTCACAAGATTTTTTCTTTATGCCTAGTACTTTTTGTGTCTTG
right  AAATACATAACTTTAATGTAGTCCAGTTTCACAAGATTTTTTCTTTATGGCTAGTACTTNNNNNNNNNNN
       2222222222222222222222222222222222222222222222222N222222222NNNNNNNNNNN

left   ----------------------------------------------TTNNNNNNNNNNN
chrom  CTTAAGAAGACTTTACCTACCTCAAAGTCATAAAGATATTCTTTTATAGTTTCTTCTGA
right  NNNNNNNNNNNNNNNNNNNNNNNNNNNNNNNNNNNNNNNNNNN----------------
       NNNNNNNNNNNNNNNNNNNNNNNNNNNNNNNNNNNNNNNNNNN    1NNNNNNNNNNNN
```

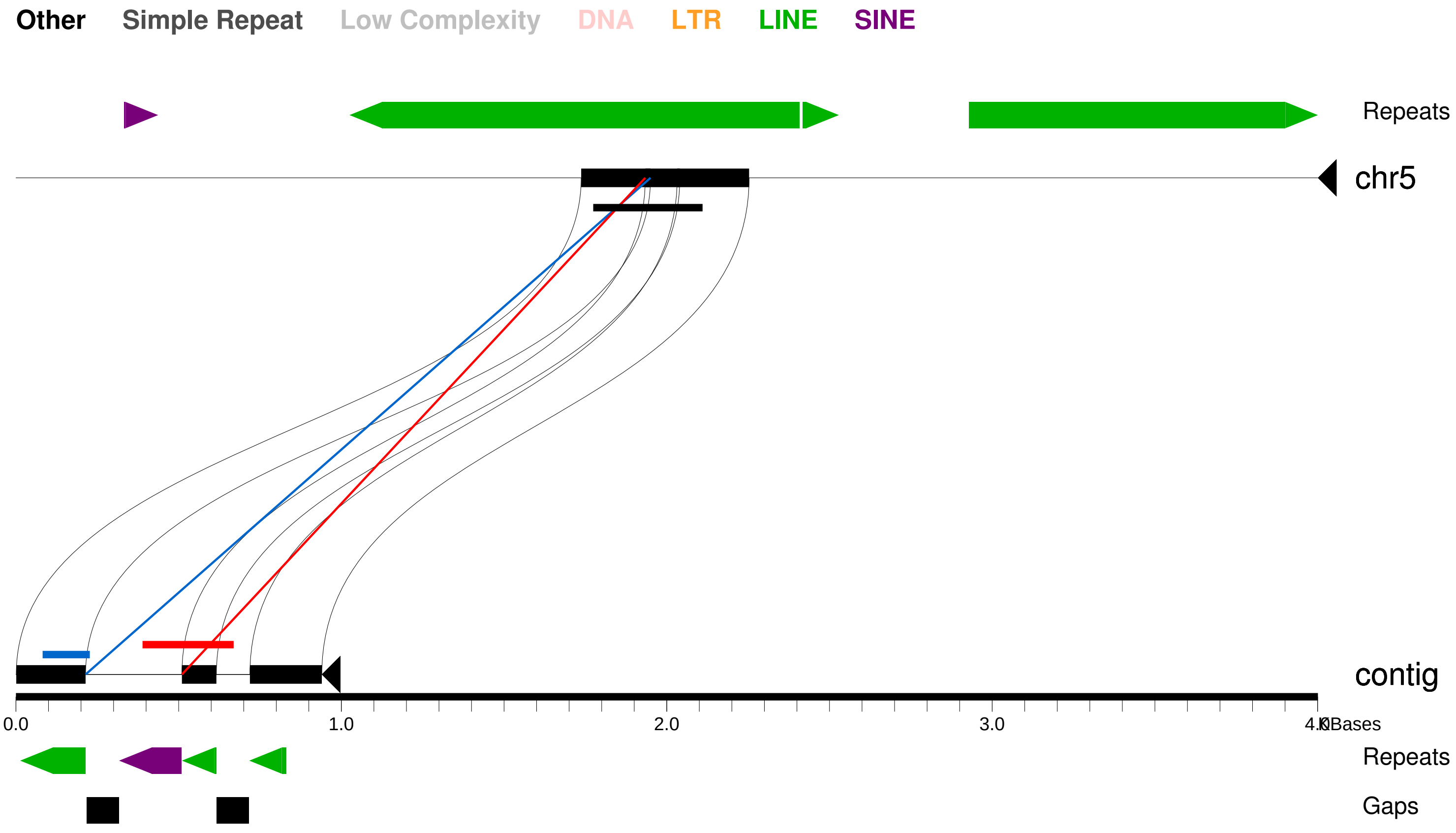

siteID: chr5_158717178 Scafftig4 + Breakpoint: chr5:158717158
chr5:158717113-158717217
Overlap Length: 15 INS size: 297 Genotyped: Yes

```
left     A----------GTGTATTTAGAGAGCTG----TACAACCATCACTGTAAGCTAATTTTAGAACCTGATTT
chrom    A----------GTGTATTTAGAGAGCTG----TACAACCATCACTGTAAGCTAATTTTAGAACCTGATTT
right    CCGCCCGCCTCGGCCTCCCAAAGTGCTGGGATTACAGGCGTGAGCCACCGCGCCCGGCCGAACCTGATTT
         11111111111*1111111*1**1****1111****11*1*1*111111**11111111***********

left     ATTTATTTTTTTTTTTTTTTGAGACGGAGTCTCGCTCTGTCGCCCAGGCTGGAGTGCAGTGGC
chrom    ATTT------------TTAAAAGGATCATTCCGGTTCT-TGTGTGAGAATGGGTTATCGAAG-
right    ATTT------------TTAAAAGGATCATTCCGGTTCT-TGTGTGAGAATGGGTTATCGAAG-
         *****222222222222**222**2222*2**22*2***2*22222**22***22*222*22*2
```

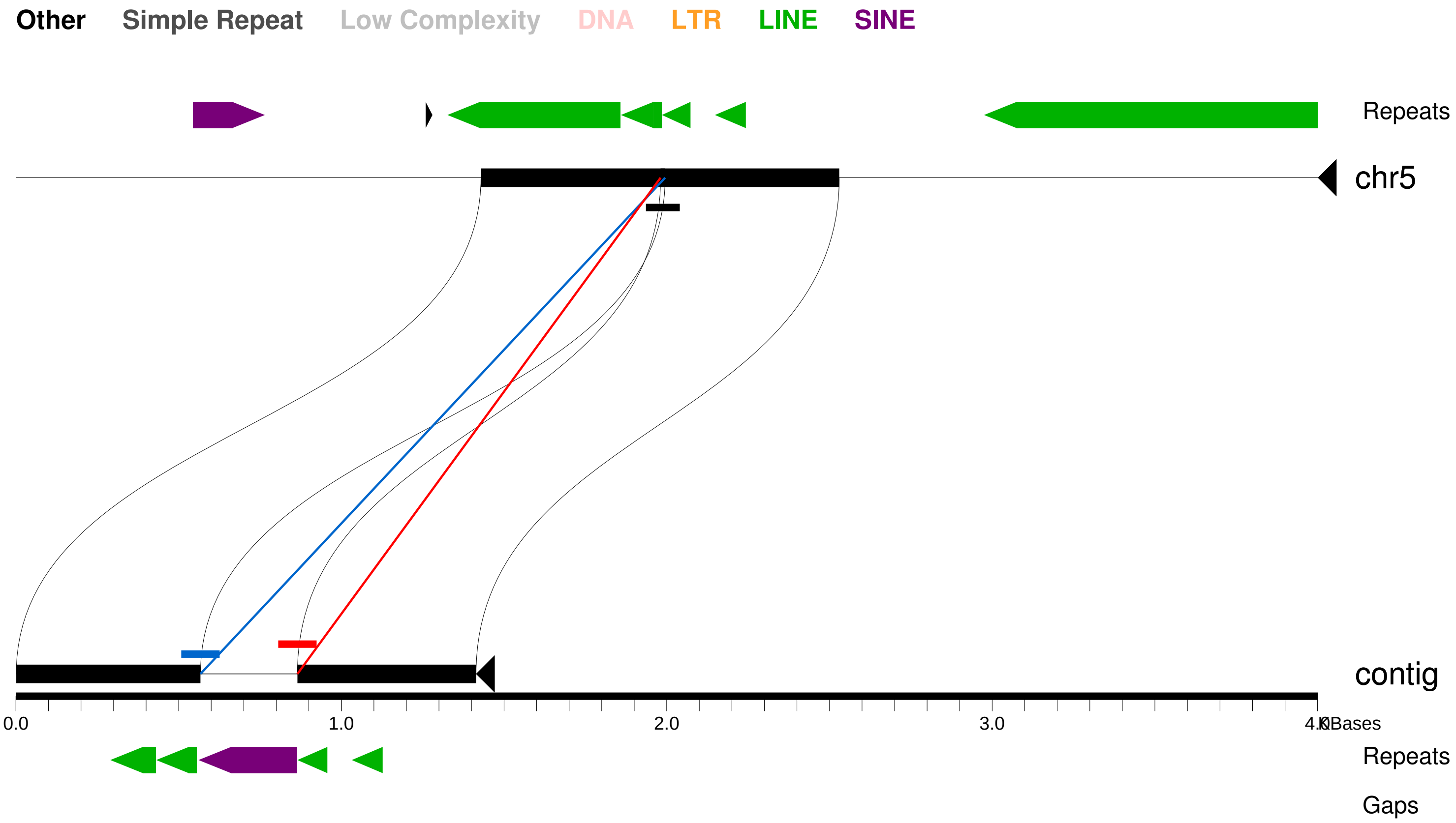

siteID: chr5_159122137 Scafftig5 - Breakpoint: chr5:159122138
chr5:159122096-159122197
Overlap Length: 18 INS size: 283 Genotyped: Yes

```
left    AT----TTGGGGGACT-----------TGGGGGAAAAGGTGGGAGGGGGTG--AGGGATAAAAGATTATA
chrom   AT----TTGGGGGACT-----------TGGGGGAAAAGGTGGGAGGGGGTG--AGGGATAAAAGATTATA
right   TCGAGATCGCGCCACTGCGCTCCAGCCTGGGCGACAGAGCGAAACTCCGTCTCAAAAAAAAAAGATTATA
        111111*1*1*11***11111111111****1**1*11*1*11*1111**111*111*1***********

left    CACTGGG--CCGGGCGCGGTGGCTCACGCCTGTAATCCCAGCACTTTGGGAGGCCGAGACGGGCGGAT
chrom   CACTGGGTACAGTGTACACTG-CTCAGG----TGAT-----------GGGTGGACCAAAATCTCA---
right   CACTGGGTACAGTGTACACTG-CTCAGG----TGAT-----------GGGTGGACCAAAATCTCA---
        *******22*2*2*22*22**2****2*2222*2**22222222222***2**2*2*2*2222*2222
```

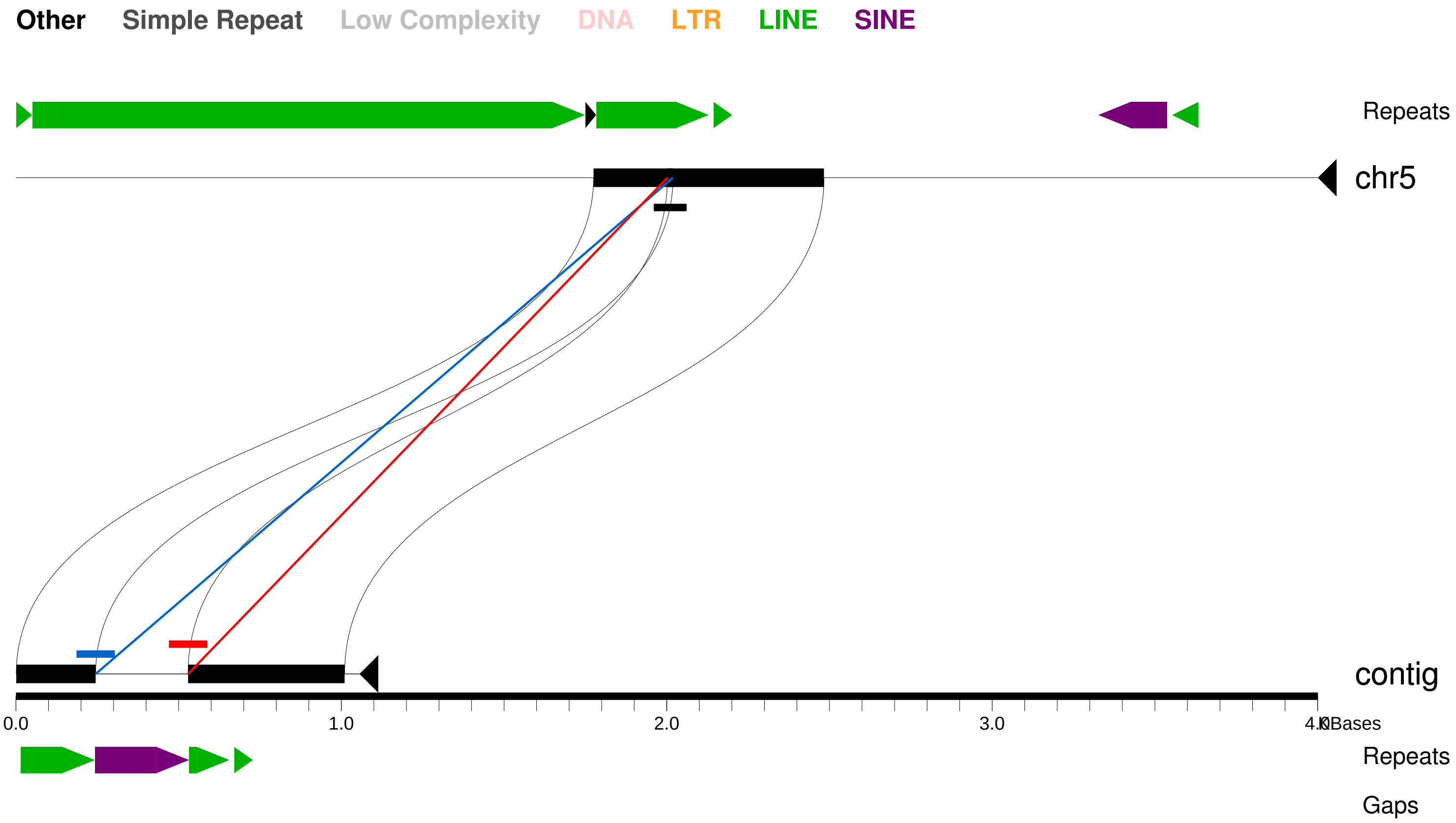

siteID: chr5_160617319 Scafftig2 + Breakpoint: chr5:160617287
chr5:160617213-160617346
Overlap Length: 12 INS size: 437 Genotyped: No

```
left    ATGAAGTAATAAATCCCTCATACACTTCTTTTTGTCTTAAGCTAGTTTGATTTTTTTTTTTTATCTGTTGC
chrom   ATGAAGTAATAAATCCCTCATACACTTCTTTTTGTCTTAAGCTAGTTTGATTTTTTTTTTT-ATCTGTTGC
right   NNNNNNNNNNNNNNNNNNNNNNNNNNNNNNNNNNNNNNNNNNNNNNNNNNNAAAA----------------
        11111111111111111111111111111111111111111111111111111111111112111111111

left    AACTGAAGAATGCCTGAG---------GCCGGGCGCGGTGGCTCACGCCT----GTAATCCC---
chrom   AACTGAAGAATGCCTGACTAATATAAAGTCTGTTGCTAATTAGCAAGCCCTCTGGCAAACATTGT
right   AAAAAAAGAATGCCTGACTAATATAAAGTCTGTTGCTAATTAGCAAGCCCTCTGGCAAACATTGT
        **111***********2222222222*2*2*22**2222222**2***22222*2**2*22222
```

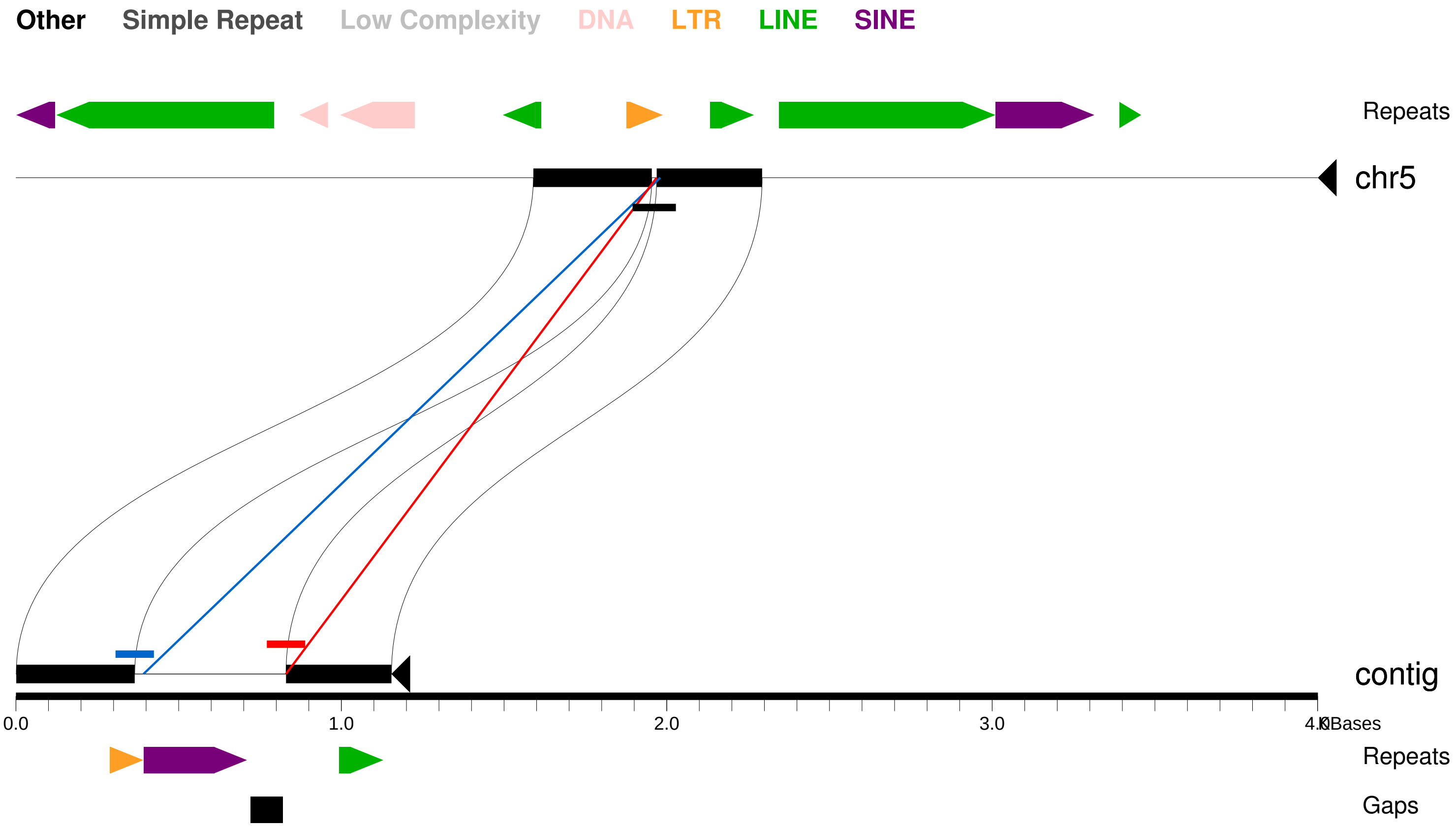

siteID: chr5_162231935 Scafftig5 + Breakpoint: chr5:162231938
chr5:162231892-162231997
Overlap Length: 14 INS size: 301 Genotyped: Yes

```
left    TC-CTCACATGTGTATTCTAACCAAAGTAGATTTTCTCAGATTTTAT---------------TAACCGTA
chrom   TC-CTCACATGTGTATTCTAACCAAAGTAGATTTTCTCAGATTTTAT---------------TAACCGTA
right   CCGCCCGCCTCGGCCTCCCAAAGTGCTGGGATTA---CAGGCGTGAGCCACCGCGCCCGGCCTAACCGTA
        1*1*1*1*1*11*11*1*1**11111111****1111***111*1*1111111111111111********

left    CATTTTTTTTTTTTTTTTTTTTTTTTGAGACGGAGTCTCGCTCTGTCGCCCAGGCTGGAGTGCAG
chrom   CATTTTAAGGAACAATCCTAAACTTTCCA--GGGGT---GGTATTT-------GATAGCAA-CAG
right   CATTTTAAGGAACAATCCTAAACTTTCCA--GGGGT---GGTATTT-------GATAGCAA-CAG
        ******222222222*22*2222***22222**2**222*2*2*2*2222222*2*2*2222***
```

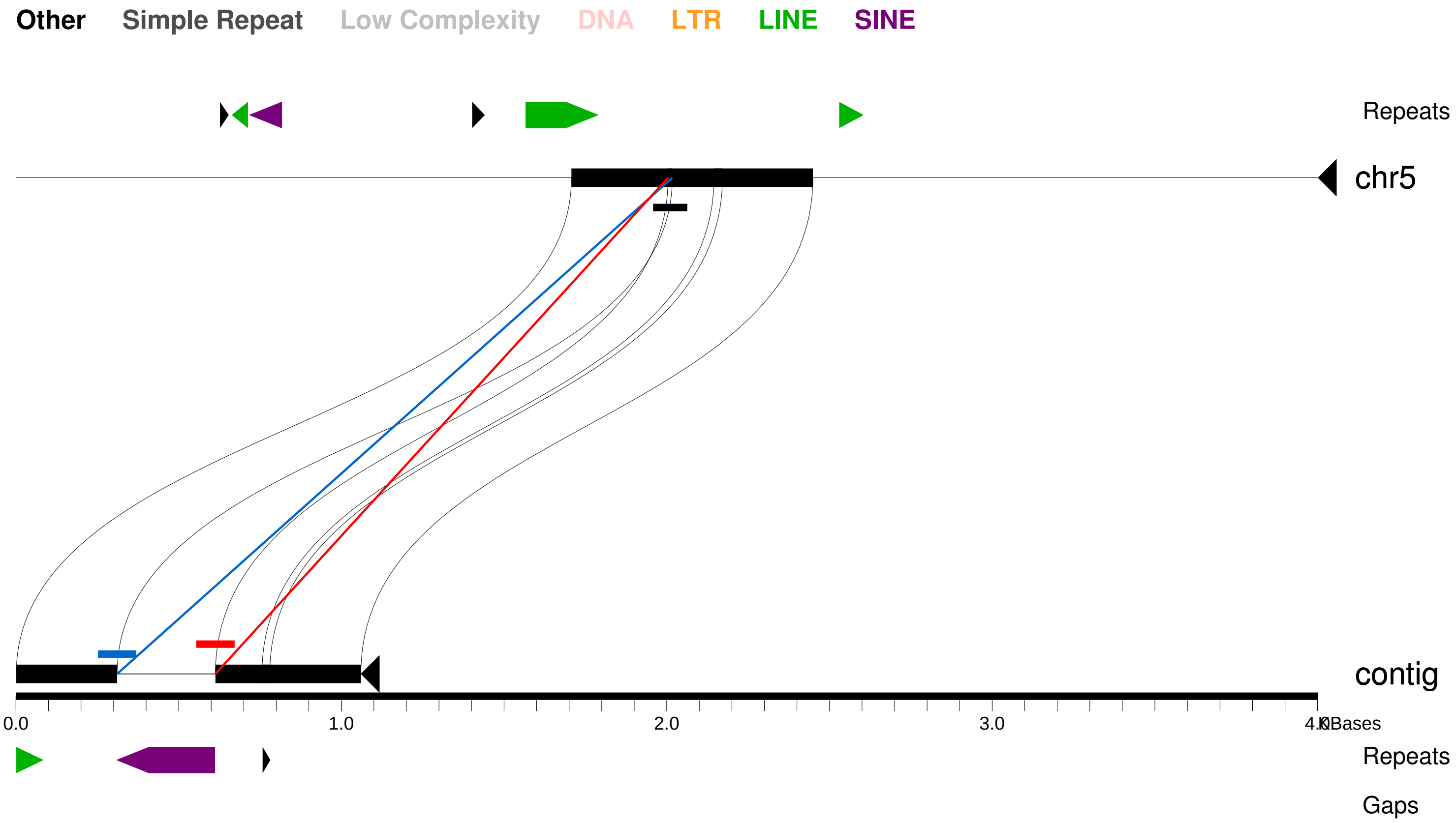

siteID: chr5_163311139 Scafftig1 + Breakpoint: chr5:163311118
chr5:163311073-163311177
Overlap Length: 15 INS size: 311 Genotyped: Yes

```
left     ACC------CTTGGT-----AATCTCCT---AAACCAGTGGTCTACAACCTTTTTGGCACCAGGGACCAG
chrom    ACC------CTTGGT-----AATCTCCT---AAACCAGTGGTCTACAACCTTTTTGGCACCAGGGACCAG
right    TCCGCCCGCCTCGGCCTCCCAAAGTGCTGGGATTACAGGCGTGAGCCACCGCGCCCGGCCCAGGGACCAG
         1**111111**1**111111**11*1**111*111***11**111*1***111111*11***********

left     TTTTTTTTTTTTTTTTTTTTTTTTTTTTTTTTTGAGACGGAGTCTCGCTCTGTCGCCCAGGCTGG
chrom    TTTTATGGAAGACAATTTTTCCATG--------GATGGGGTG------AGGTGGGAAGTGGGG
right    TTTTATGGAAGACAATTTTTCCATG--------GATGGGGTG------AGGTGGGAAGTGGGG
         ****2*222222222*****222*222222222**2**2**22222222*22*222**222**
```

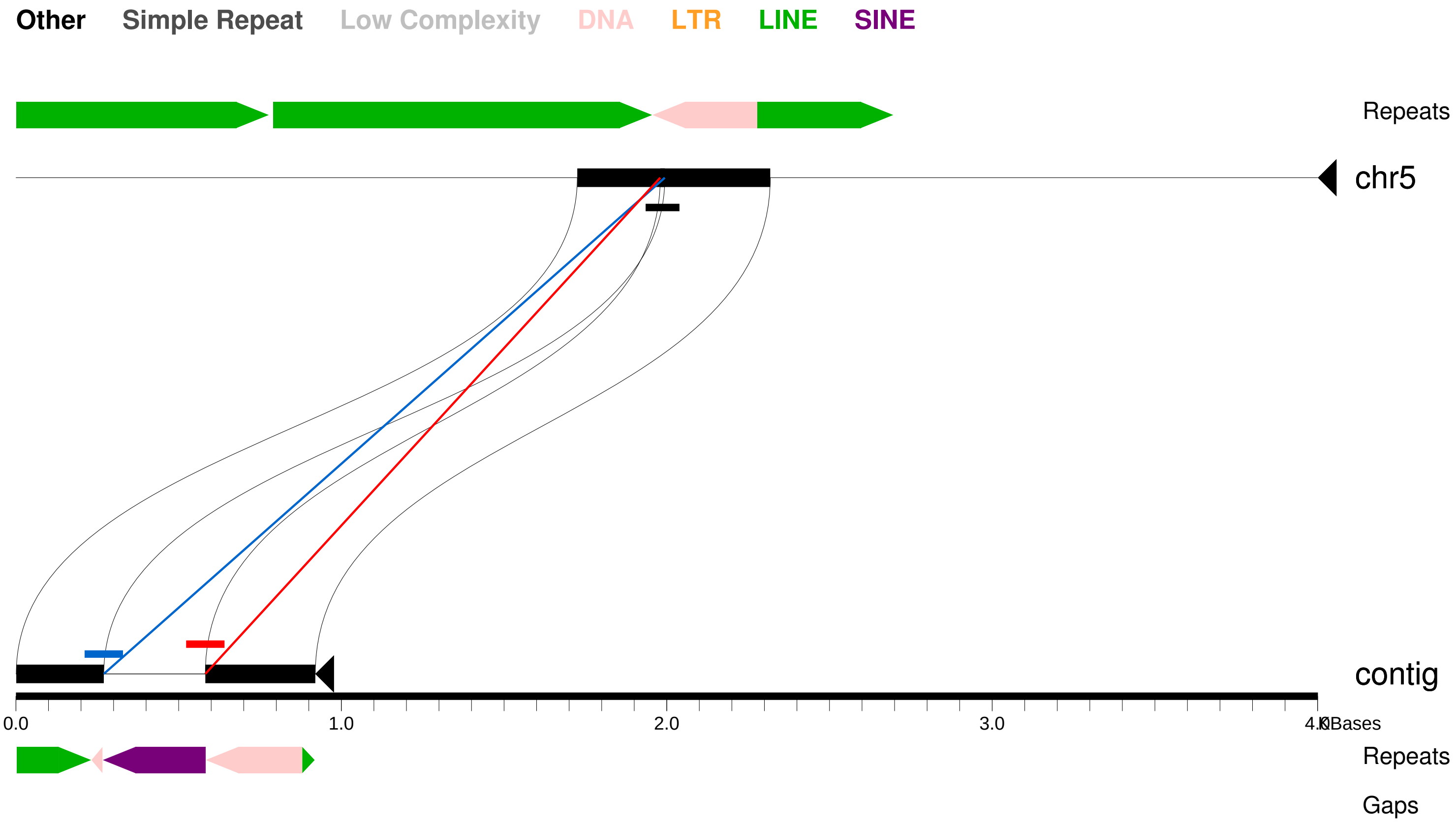

siteID: chr5_167632 Scafftig1 - Breakpoint: chr5:167518
chr5:167410-167727
Overlap Length: 102 INS size: 419 Genotyped: No

```
left   T---------------------------------------------------------------------
chrom  TATGCAGGAGTGAAGGGGACAAGTTCTGCAGCTCTGCTGTGAATGGGATGTGCGTTCAGCAGCATTTCTG
right  NNNNNNNNNNNNNNNNNNNNNNNNNNNNNNNNNNNNNNNNNNNNNNNNNNNNNNNNNNNN-----------
       1NNNNNNNNNNNNNNNNNNNNNNNNNNNNNNNNNNNNNNNNNNNNNNNNNNNNNNNNNNN

left   -------------------------------TTTTAGTTTTTCCAACTTAAAAAACAAGTAAATACAAAT
chrom  CCCACAGCTGTGCTTCCCATCATTAGCTGCTTTTTAGTTTTTCCAACTTAAAAAACAAGTAAATACAAAT
right  --------------------------------------TTTTCCAACTTAAAAAACAAGTAAATACAAAT
                                      1111111*******************************

left   AATGGTAGAAAAAAAATCTGCCAACTCCCATTGGTATCATCAGGATCGAGTTATTTAAGGAGGTTGGGGG
chrom  AATGGTAGAAAAAAAATCTGCCAACTCCCATTGGTATCATCAGGATCGAGTTATTTAAGGAGGTTGGGGG
right  AATGGTAGAAAAAAAATCTGCCAACTCCCATTGGTATCATCAGGATCGAGTTATTTAAGGAGGTTGGGGG
       **********************************************************************

left   CCGGGCGCGGT--------GG---------------CTCAC----------GCCTGTAATCCCAGCACTT
chrom  TAAGATGCCATCAAGTAGAGGAGAAAAGCAACGCACCTCACTGTTCATTAGGGCAGGGCCCCCGGCTCAC
right  TAAGATGC--------------------------------------------------------------
       222*22**NN1        11                11111          1N1N1NNNN111N11N1NN

left   --------TGGGAGGCCG--------AGGCGGGCGGAT
chrom  CATGGCCCTGGGAAATCTCCTTTGAAAGTGCAGTGGAT
right  --------------------------------------
               11111NNN1N        11NNNN1N1111
```

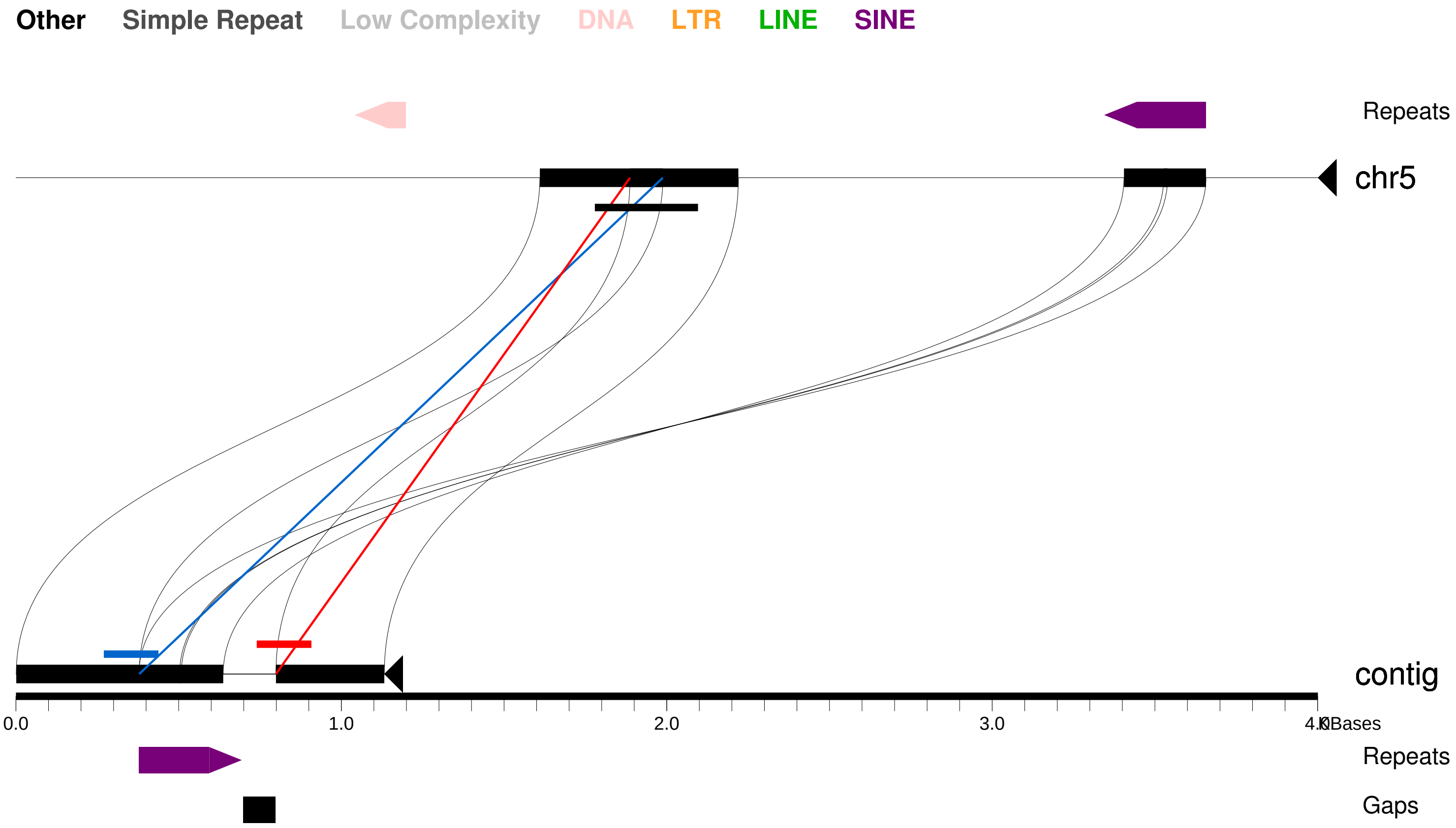

siteID: chr5_168911905 Scafftig6 - Breakpoint: chr5:168911928
chr5:168911874-168911987
Overlap Length: 6 INS size: 293 Genotyped: Yes

```
left    TTTTACCTATAGCTATGTAATAAATCCAAGAATACATGGCTATTATCATTGTTT-----AAAGAGCGCGG
chrom   TTTTACCTATAGCTATGTAATAAATCCAAGAATACATGGCTATTATCATTGTTT-----AAAGAGTACTT
right   ACTGCACTCCAGCCTGGGCGACAGAGCGAGACTCCGTCTCAAAAAAAAAAAAAAAAAAAAAAGAGTACTT
        11*111**11***111*11111*111*1***1*1*1*11*1*11*11*11111111111******22*22

left    TGGCTCACGCCTGTAATCCCAGC-ACTTTGGGAGGCCGAGGCGGGCGGATCA-CGA
chrom   TT-------TTTTTAGTGACACTTACTTAGTAAGAAAAATGGTATATAATTATCCA
right   TT-------TTTTTAGTGACATTTACTTAGTAAGAAAAATGGTATATATTTATCCA
        *2222222222*2**2*22**N22****2*22**2222*2*22222221*2*2*2*
```

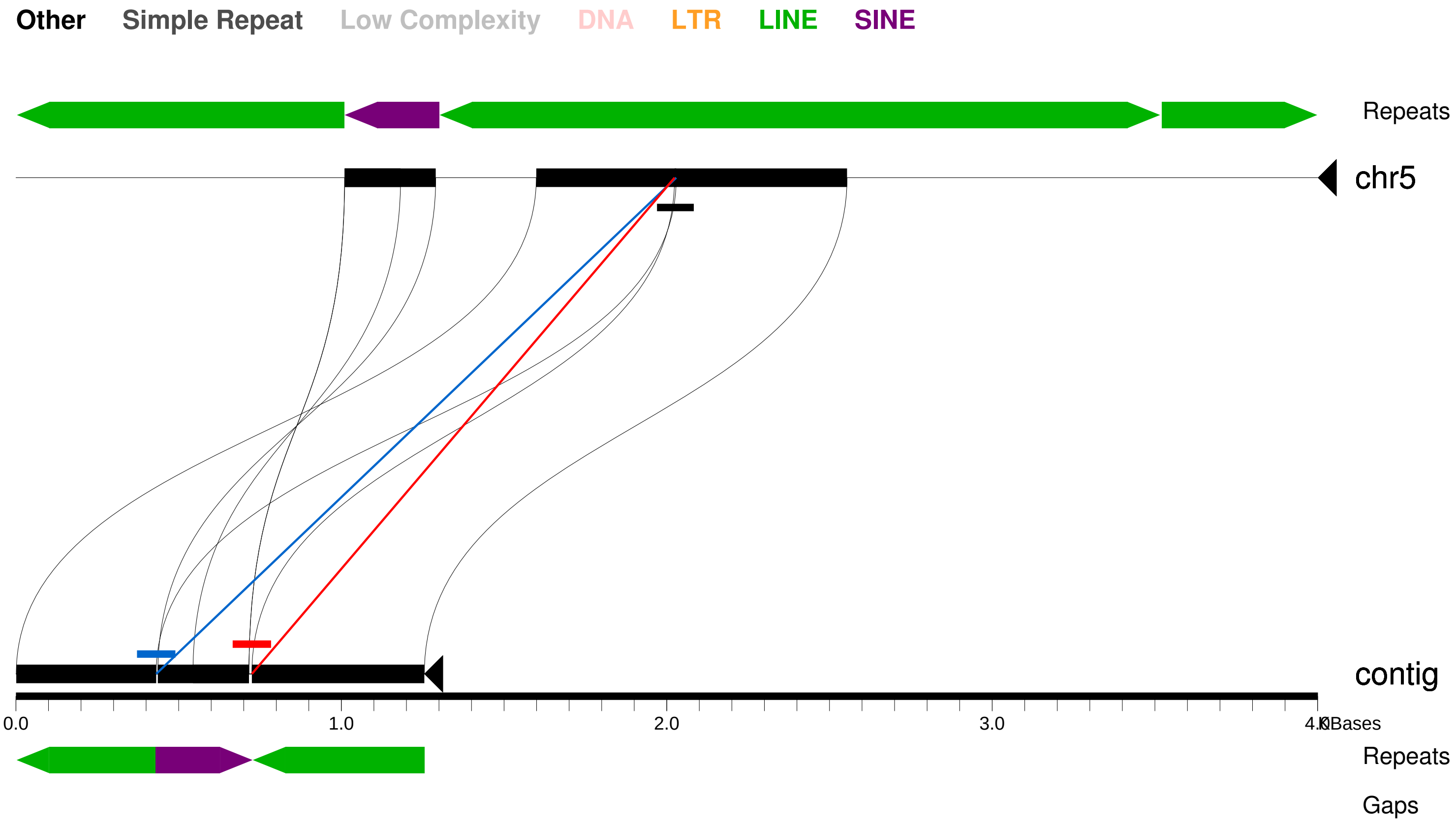

siteID: chr5_170201345 Scafftig1 + Breakpoint: chr5:170201326
chr5:170201166-170201499
Overlap Length: 14 INS size: 296 Genotyped: Yes

```
left    T--------------TTTGCAAATGAGATCCTGTCATTTCCAACATGGATGGAACTGGGGAACATGTTAG
chrom   TAGGGAAATGCTGATTTTGCAAATGAGATCCTGTCATTTCCAACATGGATGGAACTGGGGGACATGTTAG
right   ---------GCTGAG---GCAG--GAGAA--TGGCGTGAACCCGGGAGGCGGAGCTTG----CA-GTGAG
        1        22222N111***111****111**1*1*111*111111*11***1**1*11N1**1**1**

left    ATGAAATAACCCAGGCACAGAAAAGCTCACTTCCCAGGTTCTCATTCATTTGTGGGAG-CTAAAAATTAA
chrom   ATGAAATAACCCAGGCACAGAAAAGCTCACTTCCCAGGTTCTCATTCATTTGTGGGAG-CTAAAAATTAA
right   CCGAGAT---CCCGCCACTG-------CACT--CCAGCCTGGGAGACA---GAGCGAGACTCCGTCTCAA
        11**1**111**1*1***1*1111111****11****11*111*11**111*1*1***1**11111*1**

left    AACAATTTAATTCACGGGGATAGAAAAGTAGAATG------------GGCCGGGCGCGGT----GGCTCA
chrom   AACAATTTAATTCACGGGGATAGAAAAGTAGAATGATGGTTAACAGAGGCCGGGAAGGGTAGTGGGGTTG
right   AAAAAAAAAAA----------AGAAAAGTAGAATGATGGTTAACAGAGGCCGGGAAGGGTAGTGGGGTTG
        **1**111**1111111111*************222222222222*******222***2222**2*22

left    CGCC--TGTAA-------TCCCAGC-----ACTTTGGGAGGC---CGAGGC---------GGGCGGATCA
chrom   GGGGGATGTAGGGATGATTAACAGGTACAAAATATGCAATGAAAGCAATGCAATGAATATGATCTGTTAT
right   GGGGGATGTAGGGATGATTAACAGGTACAAAATATGCAATGAAAGCAATGCAATGAATATGATCTGTTAT
        2*2222****22222222*22***222222*2*2**22*2*2222*2*2**222222222*22*2*2*22

left    CGAGGTCAGGAGATCGAGACCATCC-CGGCTAAAAAACGGTGAAACCCCGTCTCTACTA
chrom   TGATAGCAGAACAAGGTGACTACAGACAGCAATAATTTGTTGTA----CATTTTCACAT
right   TGATAGCAGAACAAGGTGACTACAGACAGCAATAATTTGTTGTA----CAT--------
        2**222***2*2*22*2***2*2222*2**2*2**222*2**2*2222*2*N1NN11NN
```

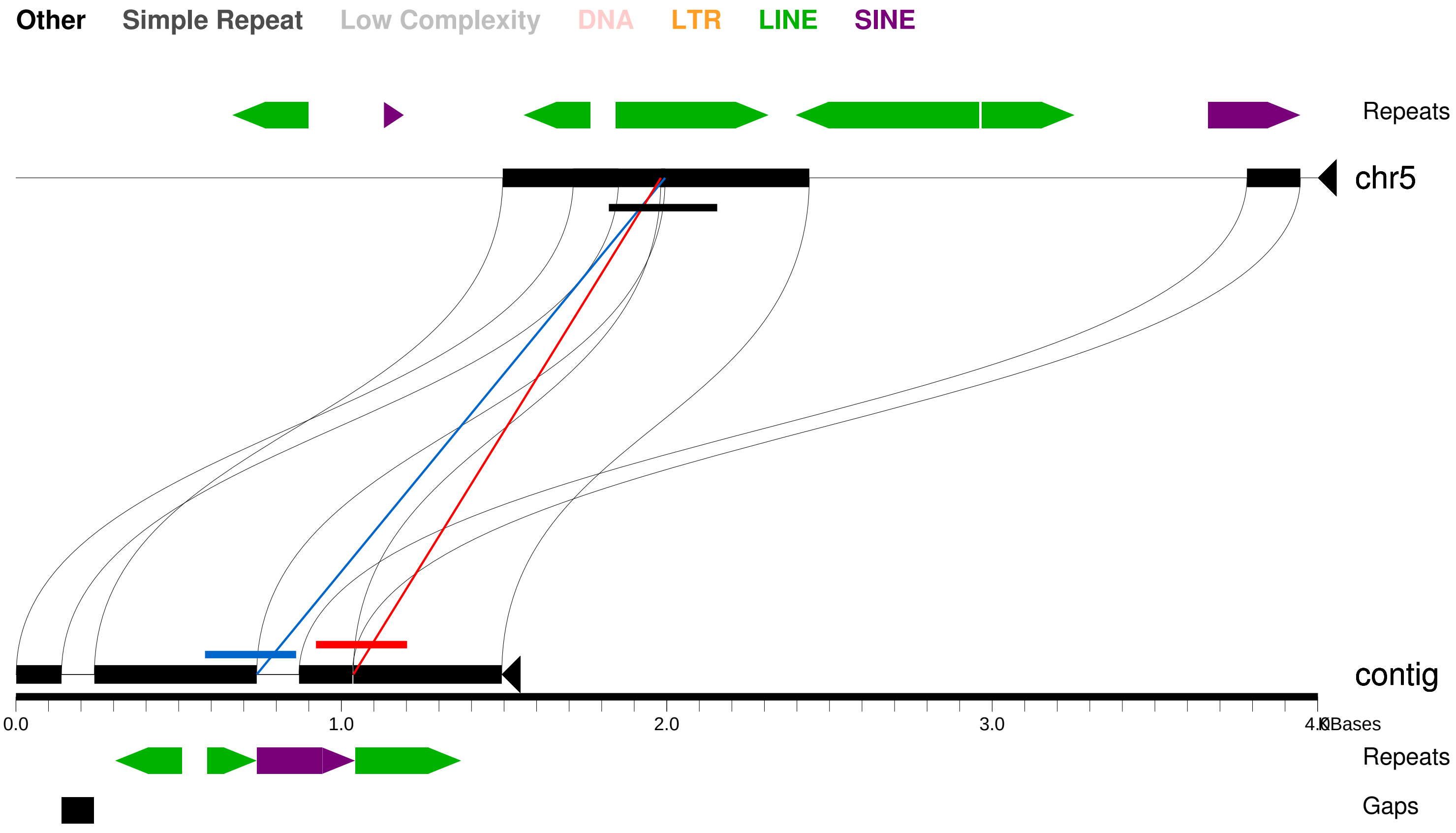

siteID: chr5_171893757 Scafftig1 - Breakpoint: chr5:171893734
chr5:171893709-171893793
Overlap Length: 34 INS size: 424 Genotyped: No

```
left    TCTA----------TTGCAAAATTTCTG------------------------CTCAGTATCCCCAGATTT
chrom   TCTA----------TTGCAAAATTTCTG------------------------CTCAGTATCCCCAGATTT
right   CCGCCCGCCTCGGCCTCCCAAAGTGCTGGGATTACAGGCGTGAGCCACCGCGCCCGGCCTCCCCAGATTT
        1*1111111111111*1*1***1*1***111111111111111111111111*1*1*11***********

left    CTAACTACTTCTTCAGTCTCATTNNNNNNNNNNNNNNNNNNNNNNNNNNNNNNNNNNNNNNNNNNNNNNN
chrom   CTAACTACTTCTTCAGTCTCATTTTCCATTAGAATCTTTCAGTTCAAGA---------------------
right   CTAACTACTTCTTCAGTCTCATTTTCCATTAGAATCTTTCAGTTCAAGA---------------------
        ***********************22222222222222222222222222222222222222222222222

left    NNNNNNNNNNNNN
chrom   -------------
right   -------------
        2222222222222
```

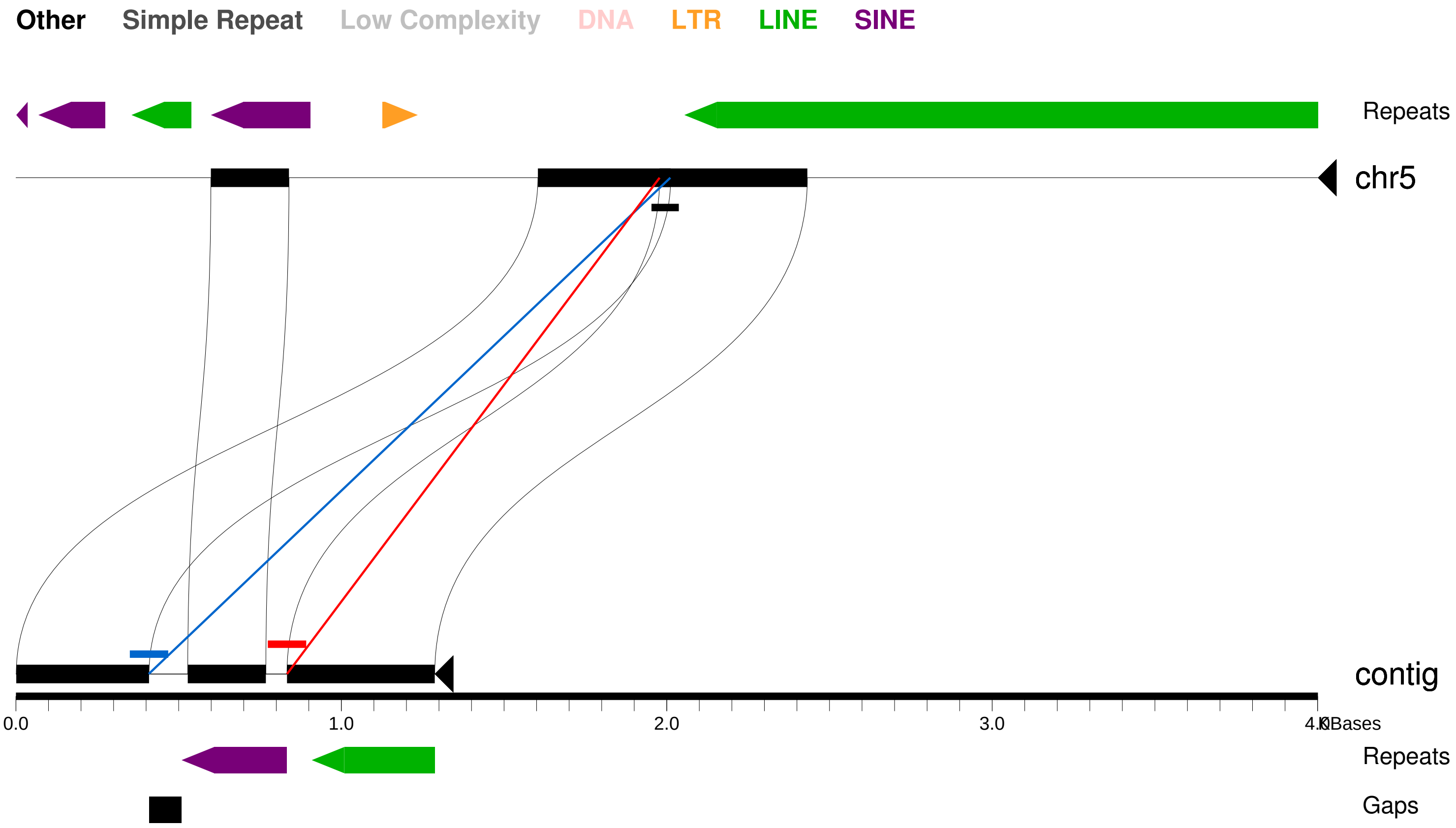

siteID: chr5_172054825 Scafftig1 - Breakpoint: chr5:172054822
chr5:172054662-172054947
Overlap Length: 1 INS size: 462 Genotyped: Yes

```
left   ----------------------------TTGCTT----TTCTTTTGA-------GTTTTATCACCTACAC
chrom  CCTGCATTTTACGAT-AGG---CATTTCTTGCTT----TTCTTTTGA-------GTTTTATCACCTACAC
right  TTT----TGTAGGACCAGGTTCCACCTGTTGCCTAGGCTGGTCTTGAACTCCTGGGCTTAAGCGATTCAC
       NN2    2N22N22N122211122NN2N****1*1111*11*1****1111111*11***11111*1***

left   ACGC--TGGGCTTTGAACATCATGTAAGTGGAGTC--------CCGTAGTATGCATTCTTTTGTGTCT--
chrom  ACGC--TGGGCTTTGAACATCATGTAAGTGGAGTC--------CCGTAGTATGCATTCTTTTGTGTCT--
right  CTGCCTTGGCCTCCTAAAATGCTGGGATTACAGGCATGAGCCACCGTGCCCGGCCCTCTTGTGTTTTAAA
       11**11***1**111**1**11**11*1*11**1*11111111****11111**11****1***1*1111

left   ---GGCTTCTTT-CACAG--AACATT-ATGTGTGTGAATCACTCGGGTCG------CTGTGATCCACATC
chrom  ---GGCTTCTTT-CACAG--AACATT-ATGTGTGTGAATCACTCGGGTCG------CTGTG-TGTA----
right  AGAGAATTCATTACATAGGCATGATTGACAACAGTGCAGAAATGTGATTGGACAAAAAGGG-TATG----
       111*11***1**1**1**11*11***1*11111***1*11*1*11*1*1*11111111*1*2*N212222

left   GGGTCCACGTGGTCACCTCTTCAGAAGCTCTCTGAACCCTGTCCTCTTGTGTGTGGTGGAGTTGTTGGCG
chrom  -GCTGTATTTTGTTTTTTCTCATTACGGCATAGTATTCCACTTTGTAAGGGTCTCCTA-ACTTATTGATC
right  -ACTGCATTTTGTTTTTTCTCATTACGGCATAGTATTCCACTTTGTAAGGGTCTCCTA-ACTTATTGATC
       212*2N*22*2**2222***2222*2*222*222*22**22*222222*2**2*22*22*2**2***222

left   GGGAACGGTTGTGGGGTTGTTTTTTGAGACAGGGTCTCACTCTGTCACCAAGGCTGGAGTGCAGT
chrom  CAGT-CTATAGTGGA-TGGACATTTGGGT--TGTTCTCAGTTTGGGGCCA------TTTTGAA--
right  CAGT-CTATAGTG----------------------------------------------------
       22*22*22*2***1N21N1NNN1111N1N22N1N11111N1N11NNN111222222NNN11N122
```

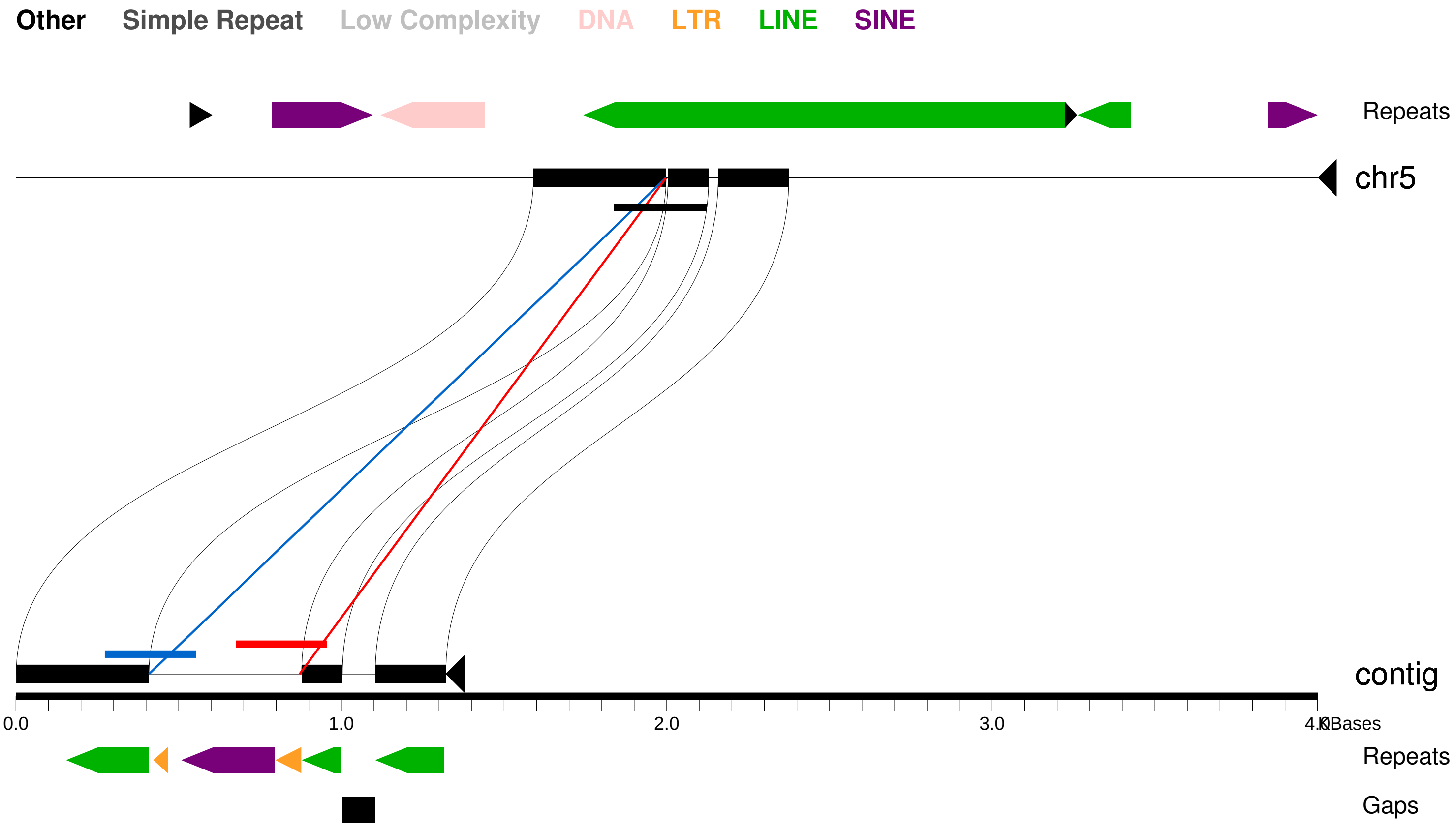

siteID: chr5_17712601 Scafftig4 - Breakpoint: chr5:17712608
chr5:17712563-17712667
Overlap Length: 15 INS size: 319 Genotyped: Yes

```
left     ATCTGAGACA-ATTTATTTTTCATTT--------------AAATTGTTTTTATCTCTTAGAAAAATATAC
chrom    ATCTGAGACA-ATTTATTTTTCATTT--------------AAATTGTTTTTATCTCTTAGAAAAATATAC
right    -TGGGCGACAGAGCGAGACTCCGTCTCAAAAAAAAAAAAAAAAAAAAAAAAAAAAAAAAAAAAAAAATAC
         1*11*1****1*111*111*1*1*1*11111111111111***11111111*111111*1*****1****

left     ACAAGGCCGGGCGCGGTGGCTCACGCCTGTAATCCCAGCACTTTGGGAGGCCGAGGCGGGCGGA
chrom    ACAAGAAATAAAGAGGTTGGATA----TGGA---GCATCATT-----AAAACTAATCTGC--AA
right    ACAAGAAATAAAGAGGTTGGATA----TGGA---GCATCATT-----AAAACTAATCTGC--AA
         *****2222222*2***2*222*2222**2*2222**2**2*22222*222*2*22*2*2222*
```

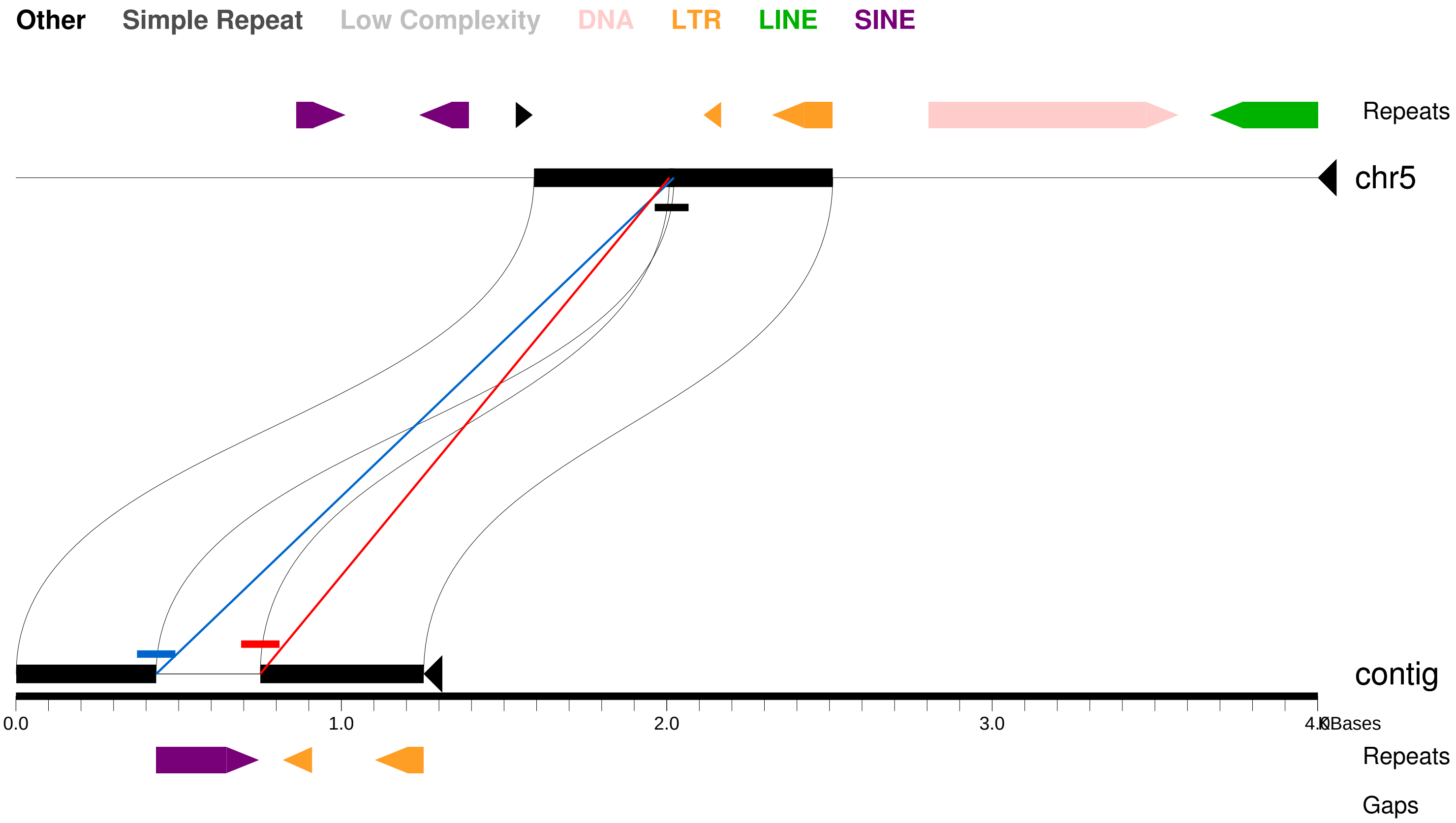

siteID: chr5_179369169 Scafftig2 + Breakpoint: chr5:179369169
chr5:179369009-179369345
Overlap Length: 17 INS size: 171 Genotyped: Yes

```
left   --------------GCACAACTCTGATACAGTTTATTTCAGTTTCTTCACATCATGCCAATAAATATTC
chrom  ATAAACTCCCATGGGCACAACTCTGATACAGTTTATTTCAGTTTCTTCACATCATGCCAATAAATATTC
right  AGAA-------TGGCGTGAACCCGGGAGGCGGAGCTTGCAGTGAGCCGAGATTGCGCCACCGCAC--TC
       2N22       2221111***1*1*11111*1111**1****111111*1**111****1111*111**

left   CATTAAATTG-AGTTG-AGTGGGATGTACACT-ATTCTAATTAGAGTCACTAATTTGTGTCCAAATAGGT
chrom  CATTAAATTG-AGTTG-AGTGGGATGTACACT-ATTCTAATTAGAGTCACTAATTTGTGTCCAAATAGGT
right  CA--ACCTGGGAGACACAGCGAGACTCCGTCTCAAAAAAAAAAAAAAAAAAAAAAAAAAAAAAAAAAAAA
       **11*11*1*1**1111**1*1**111111**1*1111**11*1*111*11**111111111***1*111

left   TTCACTTAGAACCATCTCAGTATGAAATAAAGAGGGCACTA----CTTGGGAGGCTGAGGCAG--GAGAA
chrom  TTCACTTAGAACCATCTCAGTATGAAATAAAGAGGGCACTGGCACCAAGAAACAATGAAGAAGCTGAAAA
right  AAAAAAAAAAAAAAAAAAAAAAAGAAATAAAGAGGGCACTGGCACCAAGAAACAATGAAGAAGCTGAAAA
       111*111*1**11*1111*11*1*****************22222*22*22*222***2*2**22**2**

left   T---------GGCGTGAACCCGGGAGGCGGAGCTT--GCAGTGAGCCG---------AGATTGCGCCAC
chrom  TTAACCAGCAGTTGTTATTTCTGTACCATCAGTTATGGTAGTTAGACGGCTTTAATGAAGATTACA--AA
right  TTAACCAGCAGTTGTTATTTCTGTACCATCAGTTATGGTAGTTAGACGGCTTTAATGAAGATTACA--AA
       *222222222*22**2*222*2*2*22222**2*222*2***2**2**2222222222*****2*222*2

left   CGCACTCCA-ACCTGGGAGACACAGC----GAG-ACTCCGTCTCAAAAAAA----------A
chrom  GGTTTTAAATAACTGAAACACTCATCAGTTGAGGACTGATTCAATATACAATGCATTTATCA
right  GGTTTTAAATAACTG-----------------------------------------------
       2*222*22*2*2***NN1N11N11N1    111 111NNN11NNN1N1N11          1
```

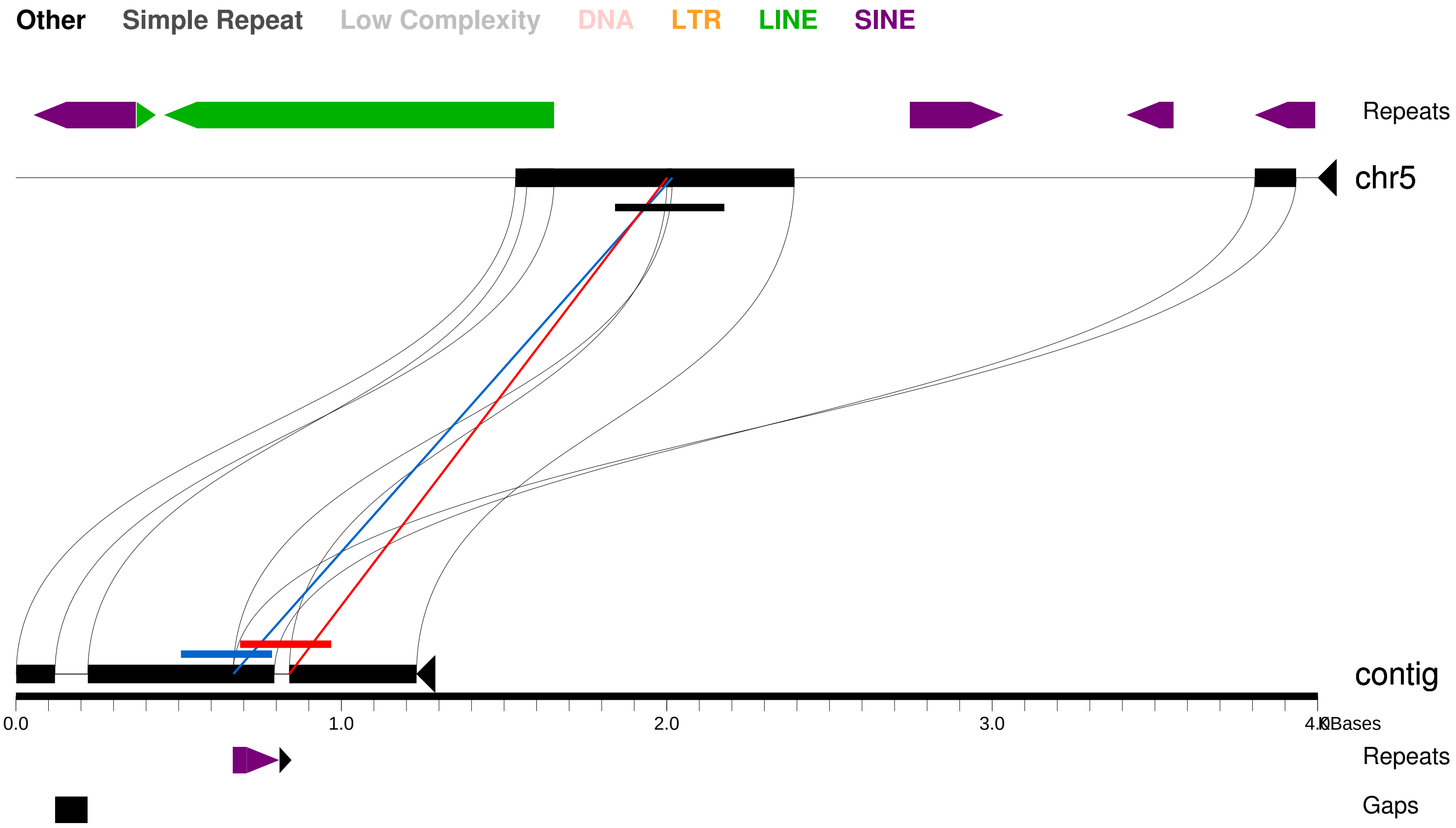

siteID: chr5_180349611 Scafftig1 - Breakpoint: chr5:180349598
chr5:180349545-180349651
Overlap Length: 7 INS size: 309 Genotyped: Yes

```
left   -----TG-------GTCTGAGAGAGCGGTTGTTATGATTTCAGTTCTTTTGCATTTGCTAAGAAGTGTTT
chrom  -----TG-------GTCTGAGAGAGCGGTTGTTATGATTTCAGTTCTTTTGCATTTGCTAAGAAGTGTTT
right  GCCACTGCACTCCAGCCTGGGCGACAGAGCGAGACTCCGTCTCAAAAAAAAAAAAAAAAAAAAAATGTTT
       11111**1111111*1***1*1**11*111*11*11111**1111111111*111111**1**1*****

left   TGGGCCGGGCGCGGTGGCTCACGCCTGTAATCCCAGCACTTTGGGAGGCCGAGGCGGGCGG
chrom  TGTGTCTGATTATGTGG-TCAG--TTTTAA----AGTA-TGTGCTATGTAGTGATAA----
right  TGTGTCTGATTATGTGG-TCAG--TTTTAA----AGTA-TGTGCTATGTAGTGATAA----
       **2*2*2*22222****2***2222*2***2222**2*2*2**22*2*22*2*22222222
```

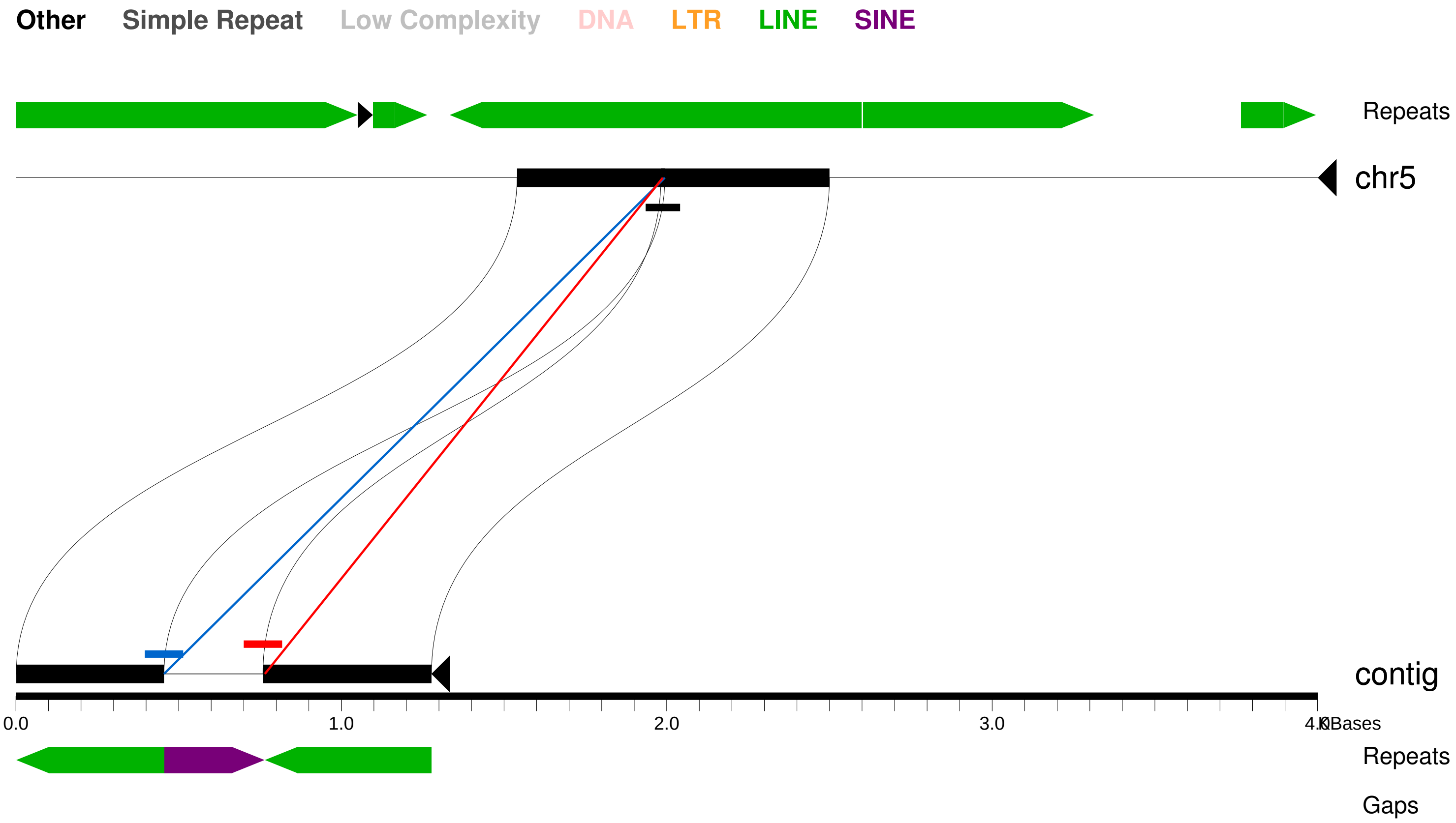

siteID: chr5_180368838 Scafftig2 - Breakpoint: chr5:180368896
chr5:180368736-180369074
Overlap Length: 19 INS size: 106 Genotyped: Yes

```
left   -----------------TGATATAAACTCTCAACAGTTTAGATGTAGAAGAAATGTACCTCA-ACACAAT
chrom  AAATTCAACATCCTGCATGATATAAACTCTCAACAGTTTAGATGTAGAAGAAATGTACCTCA-ACACAAT
right  ---TTTACTACTCTTTTTTTTTTTTTTTTTT-----TTTTTTTTGAGACGGAGTCTCGCTGTCACCCAGG
          22N2NN2NN22NNN*11*1*1111*1*111111***111*11***1*1*1*1*11**111**1**11

left   CAAGGTCACATATGACAAACTCACAGCTAACATCATACAGAATGGGGAAAAGTTGAAAGCTTTTCAATAA
chrom  CAAGGTCACATATGACAAACTCACAGCTAACATCATACAGAATGGGGAAAAGTTGAAAGCTTTTCAATAA
right  CTGGAGTGCAG-TGGCGCAATCTCGGCTCAC----------------------TGCAGGCT---------
       *11*1111**11**1*11*1**1*1***1**1111111111111111111111**1*1***111111111

left   GAACTGGAACAAGACAAGGATGCCCACTTTTACTACTCTTTTTTTTTTTTTTTTTTTTTTTTTTGAGACG
chrom  GAACTGGAACAAGACAAGGATGCCCACTTTTACTACTCTTATTCAACAAAGTACTGGAAGTCCT-AGCCG
right  ---CCGCCCCCTGGGGTTCACGCCCACTTTTACTACTCTTATTCAACAAAGTACTGGAAGTCCT-AGCCG
       111*1*111*11*111111*1*******************2**22222222*22*22222*22*2**2**

left   GAG---TCTCGC----------------TGTCACCCAGGCTGGAGTGCAGT--GGCGCAATCTCGGCTCA
chrom  GAGCAATCAGGCAAGAGCAAAAAATAAATGGCATCTAAATTGGAAAGGAGAAAGTCTAATTGTCC-CTGT
right  GAGCAATCAGGCAAGAGCAAAAAATAAATGGCATCTAAATTGGAAAGGAGAAAGTCTAATTGTCC-CTGT
       ***222**22**2222222222222222**2**2*2*222****22*2**222*2*22*2*2**22**22

left   CTGCAGGCTCCGCCCCCTGG----------------GGTTCACGCCCA-----CTTTTACT-
chrom  TTGCAGACAACATGATCTTTATATAAAAAAACTAAAGATTCCATCAAAAAAACCTCTTAGAA
right  TTGCAGACAACATGATCTTTATATAAAAAAACTAAAGATTCCATCA----------------
       2*****2*22*22222**222222222222222222*2***222*2N1     11N111NN
```

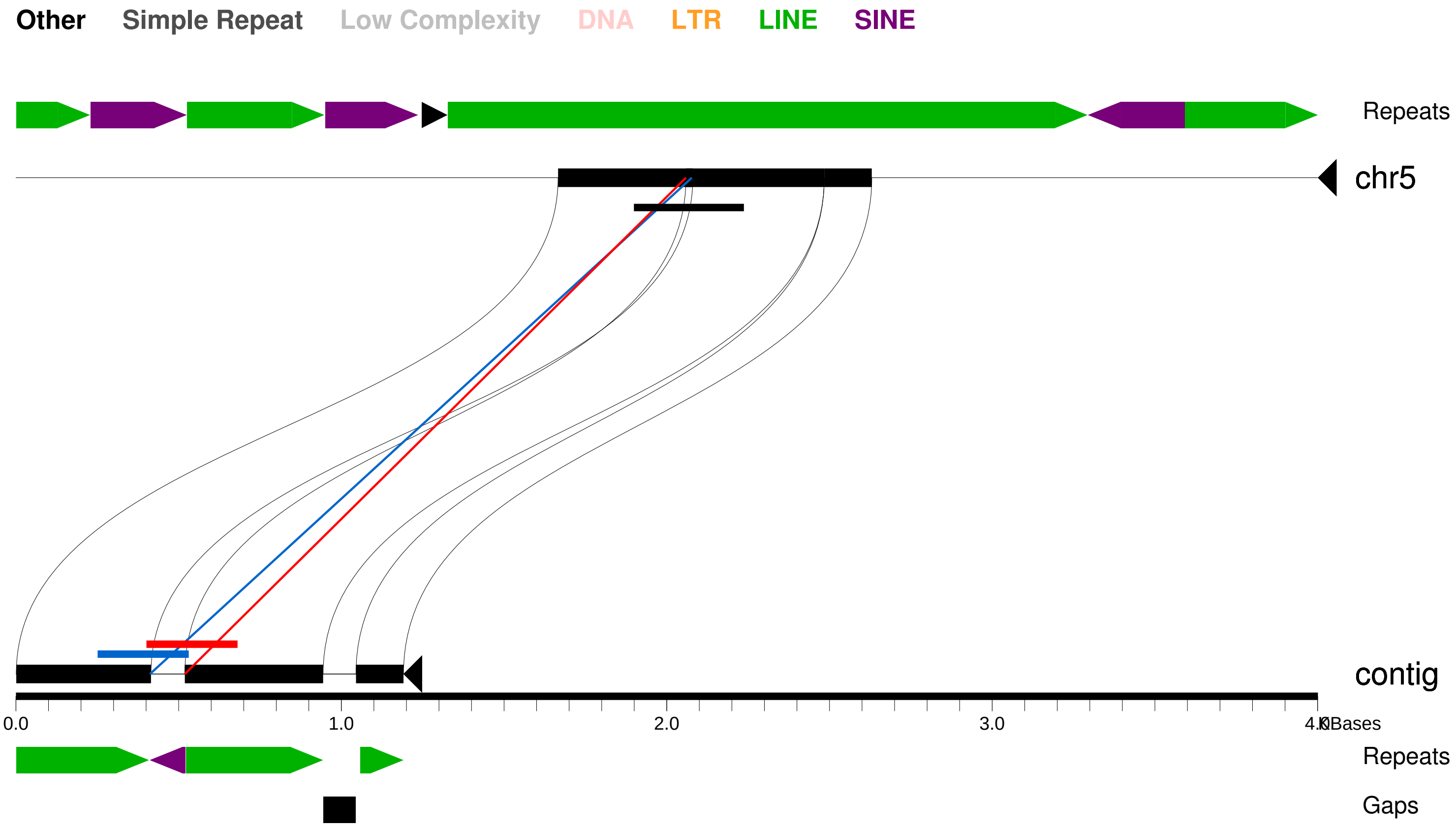

siteID: chr5_18257572 Scafftig3 - Breakpoint: chr5:18257562
chr5:18257509-18257621
Overlap Length: 7 INS size: 426 Genotyped: No

```
left   CATGAGAAGTGTTTCACATTTTCTTCTAAATGGGATAAAGAAAAGATATAA------AACATTCTGGCCG
chrom  CATGAGAAGTGTTTCACATTTTCTTCTAAATGGGATAAAGAAAAGATATAA------AACATTCTG----
right  NNNNNNNNNNNNNNNNNNNNNNNNNNNNNNNNNNNNNNNNNNNNNNNNNNNNNNNNNACCATTCTG----
       111111111111111111111111111111111111111111111111111111111*1*******2222

left   GGCGCGGTGGCTCACGCCTGTAATCCCAGCACTTTGGGAGGCCGAGGCGGGCGGA
chrom  --CAAAATAAAAACCCCATATATAAGGAGACGTGTAAATGTAATTTTAGGTTAAG
right  --CAAAATAAAAACCCCATATATAAGGAGACGTGTAAATGTAATTTTAGGTTAAG
       22*2222*222222*2*2*2**22222**222*2*2222*22222222**22222
```

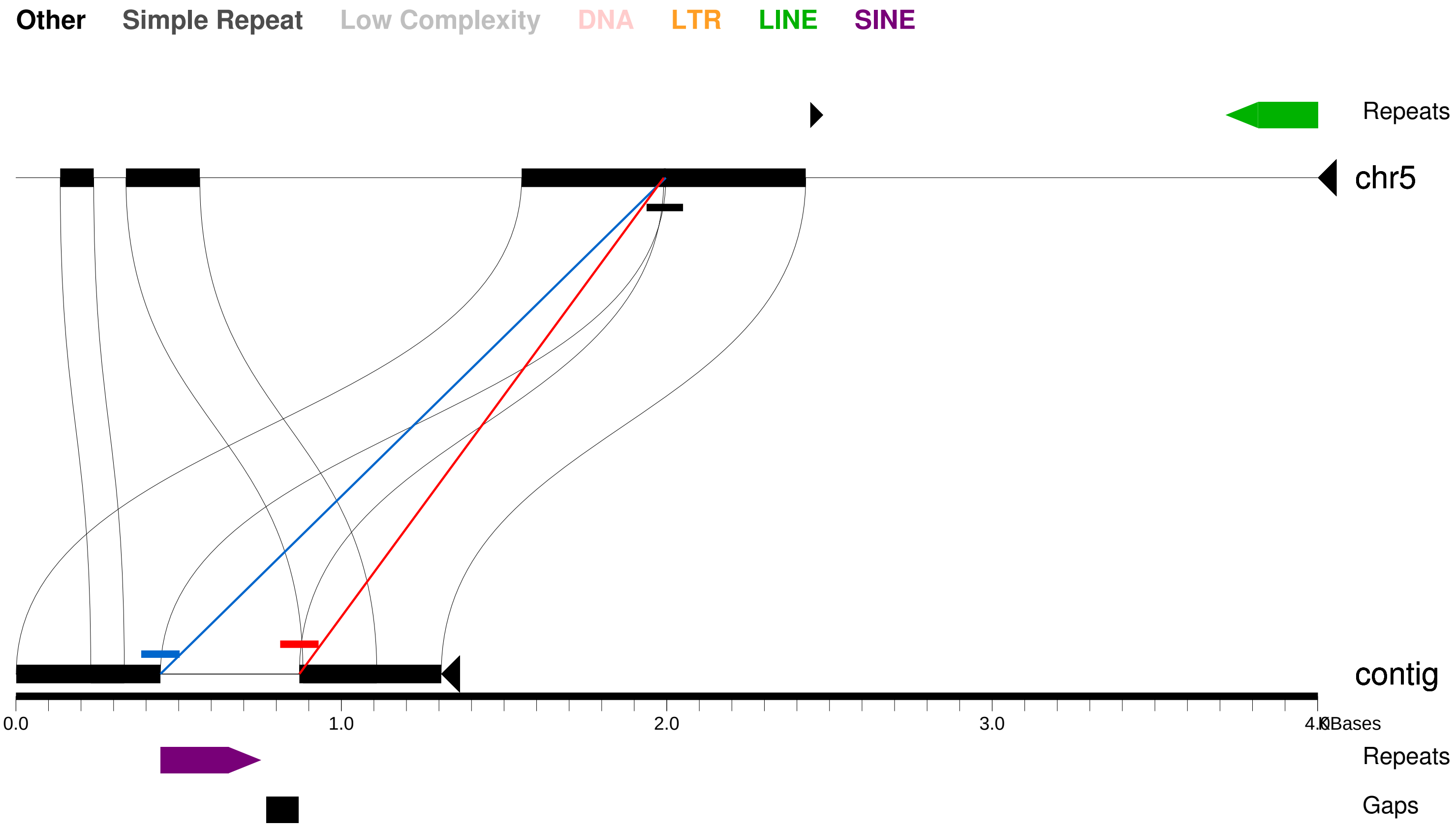

siteID: chr5_18570175 Scafftig4 + Breakpoint: chr5:18570156
chr5:18570105-18570215
Overlap Length: 9 INS size: 305 Genotyped: Yes

```
left    CAGT--------GAATGCTAATAGAATATGAATATTTTTAATTTTTTCCCCTTCCATGTGAAACTATTTTT
chrom   CAGT--------GAATGCTAATAGAATATGAATATTTTTAATTTTTTCCCCTTCCATGTGAAACTATTAA
right   CCACCCGCCTCGGCCTCCCAAAGTGCTGGGATTACAGGCGTGAGCCACCGCGCCCGGCCGAAACTATTAA
        *11111111111*11*1*1**11111*11**1**1111111111111**1*11**1111*********22

left    TTTTTTTTTTTTTTGAGACGGAGTCTCGCTCTGTCGCCCAGGCTGGACTGCGGACTG
chrom   TTGAT-------ATGAAAAAACCTCCGCAGAAAGAGAGAAAAGTGATAATGTGGAAT-
right   TTGAT-------ATGAAAAAACCTCCGCAGAAAGAGAGAAAAGTGATAATGTGGAAT-
        **22*22222222***2*222222*2*22222*222222*2*2222*2**2***2*2
```

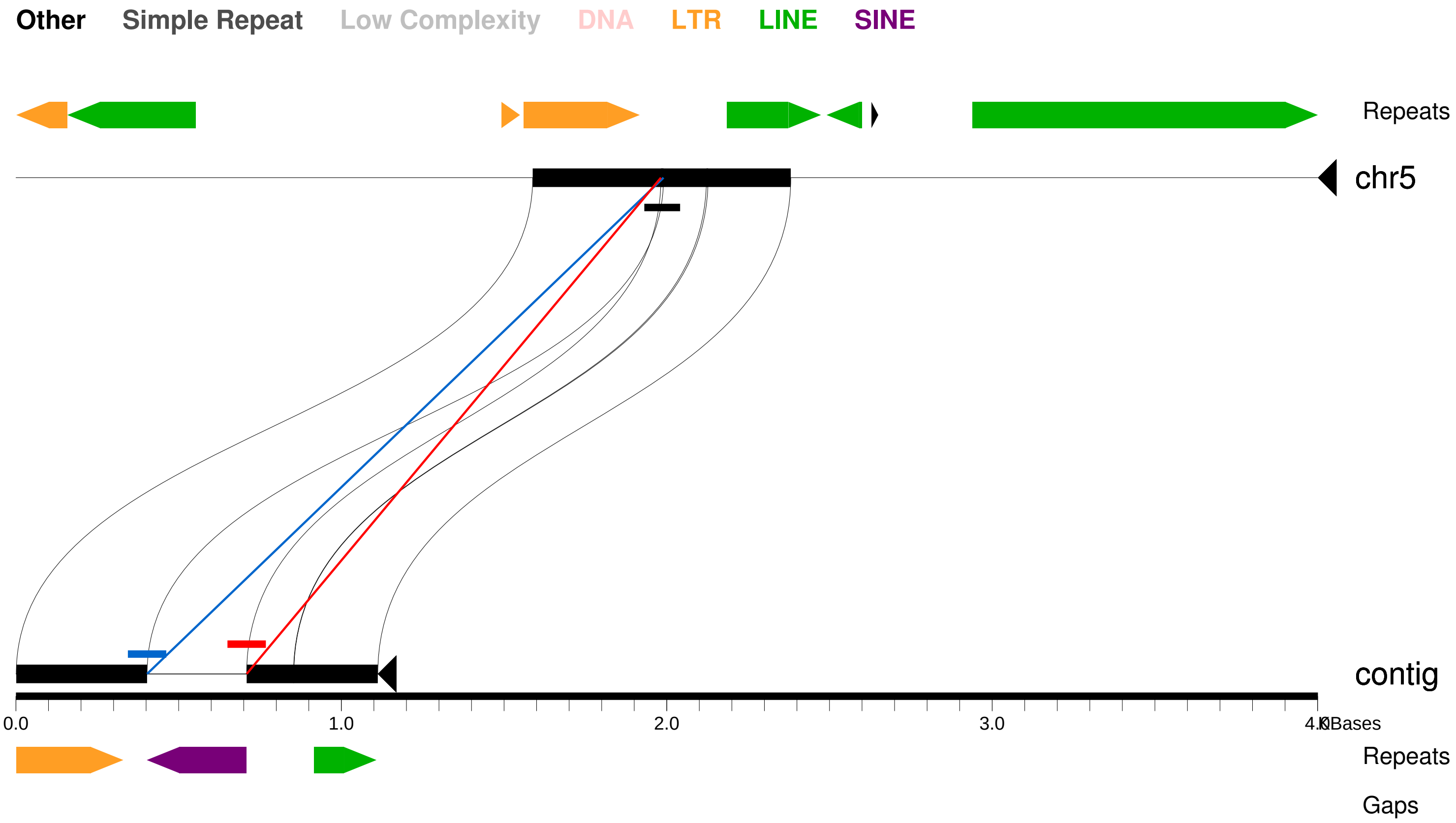

siteID: chr5_18863803 Scafftig1 + Breakpoint: chr5:18863815
chr5:18863656-18863992
Overlap Length: 18 INS size: 260 Genotyped: No

```
left    -----------------CAGTGGACATGCACAATGAAAACTGTATTTTAAATCTTTTTTGGACAATTTCA
chrom   AGATATCTTTGACAATTCAGTGGACATGCACAATGAAAACTGTATTTTAAATCTTTTTTGGACAATTTCA
right   NNNNNNNNNNNA-----------------------------------------------------------
        NNNNNNNNNNN2     11111111111111111111111111111111111111111111111111111

left    TTTCCATGAAAAATAGAATGAAGCTATATCTTTGTTCAGTGTTATTGTGAACACTAAAATCCTGGGCACG
chrom   TTTCCATGAAAAATAGAATGAAGCTATATCTTTGTTCAGTGTTATTGTGAACACTAAAATCCTGGGCACG
right   --------AAAAA---------------------------------------------------------
        11111111*****111111111111111111111111111111111111111111111111111111111

left    GAGACCGCAATAATATTACAGAAATAGACAGTGACTC--GGCCGGGCGCGGT--------GGCTCACG--
chrom   GAGACCGCAATAATATTACAGAAATAGACAGTGACTCAAGACCAGACAATTTTAACATCAGACTCCAGTG
right   -------------------AGAAATAGACAGTGACTCAAGACCAGACAATTTTAACATCAGACTCCAGTG
        1111111111111111111*****************22*2**2*2*2222*22222222*2***22*22

left    --CCTGTAATCCCA-----GCACTT----TGGGAGGCCGAGGC-----------GGGCGGATC---ACGA
chrom   AGCCTAAAAACCCATAGATGAAAATAAAATGATAAGTATATGTATTTCTTAAATGTATGTATTTTAAGTA
right   AGCCTAAAAACCCATAGATGAAAATAAAATGATAAGTATATGTATTTCTTAAATGTATGTATTTTAAGTA
        22***22**2****22222*2*22*2222**22*2*222*2*222222222222*222*2**2222*22*

left    GGTCAGGAGATCGAGACCATCCCAG---CTAAAACGGTGAAACCCCGTCTCTACTAAA
chrom   GCAAAGTCTTTCCAGAATATTAAATTGACTTGATATCTAAATATTCATTTTTAG-AAG
right   GCAAAGTC--------------------------------------------------
        *222**22NN11N111NN11NNN1N   11NN1NNNN1N11NNNN1N1N1N11N211N
```

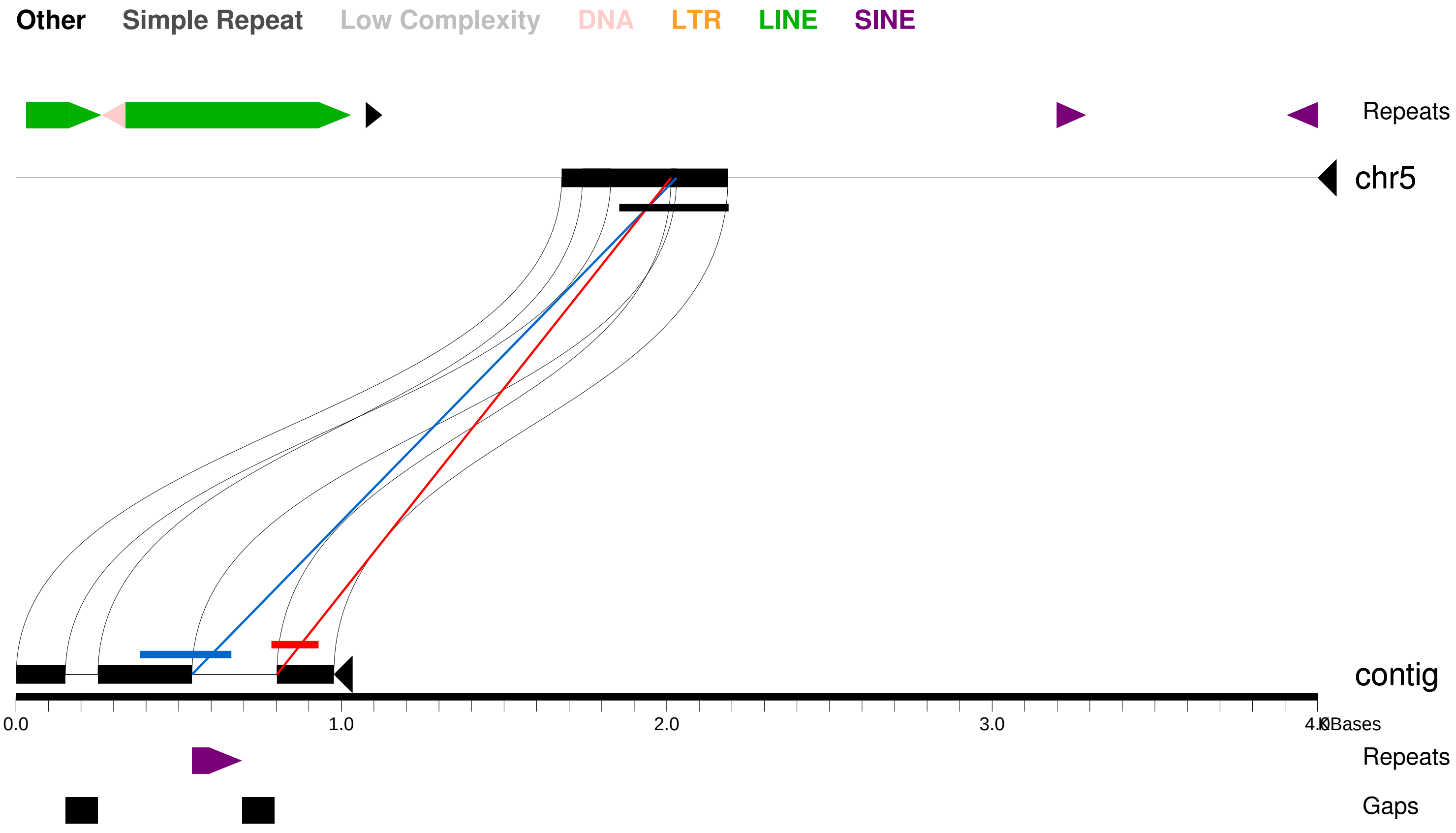

siteID: chr5_19067866 Scafftig1 + Breakpoint: chr5:19067851
chr5:19067691-19068016
Overlap Length: 6 INS size: 345 Genotyped: No

```
left   -------------------------------AAAATAAAATCCAGGTTTGTATGATCATGGAAATCAAGG
chrom  GGGTCTCAAAACAAGGGTTCAGAAATAAAATAAAATAAAATCCAGGTTTGTATGATCATGGAAATCAAGG
right  TTTAGTAGAGACGGGGTTTCACCT-----------TGTTAGCCAGG-----ATGGTC-TCGATCTCC---
       NNNNN2NN2N22NN22N2222NNN       1111*111*1*****11111***1**1*1**11**1111

left   ATTGACAGTTAACAAACTCACTGACAGTCACTGACAGTCACAAACA-CTGTCAATGCCCCACTCTTGTAA
chrom  ATTGACAGTTAACAAACTCACTGACAGTCACTGACAGTCACAAACA-CTGTCAATGCCCCACTCTTGTAA
right  --TGACC--TCATGATC-CAC---CCGCCTCGGCC--TCCCAAAGTGCTGGGATTAC---AGGCGTG-AG
       11****111*1*11*1*1***111*1*1*1*1*1*11**1****111***11*1*1*111*11*1**1*1

left   CTTCAGCACACACTTGCACATGACAAANNNNNNNNNNN--------------------------------
chrom  CTTCAGCACACACTTGCACATGACAAATTTTTTTACTTCAAACAACTATGACTCTTTTCTACGTGACTG
right  CCACCGCGCCCCG---GCC---GACAAAATTTTTTTACTTCAAACAACTATGACTCTTTTCTACGTGACTG
       *11*1**1*1*1111**1111******2222222222222222222222222222222222222222222

left   ----------------------------------------------------------------------
chrom  TGAAAACTCTTTCAGTATAGAGTAAGCCAGAGATGTGTGTTTGTGTTACAGAGAGGGAGGAGAGACAGGG
right  TGAAAACTCTTTCAGTATAGAGTAAGCCAGAGATGTGTGTTTGTGTTACAGAGAGGGAGGAGAGACAGGG
       2222222222222222222222222222222222222222222222222222222222222222222222

left   -----------------------------------------------
chrom  TGTTAGTTAAAGGCCTGGGAATAGCTCTTATTGAAGGGTGGACAGGG
right  TGTTAGTTAAAGGCCTGGGAATAGCTCTTNNNNNNNNNNNN------
       22222222222222222222222222222NNNNNNNNNNNN
```

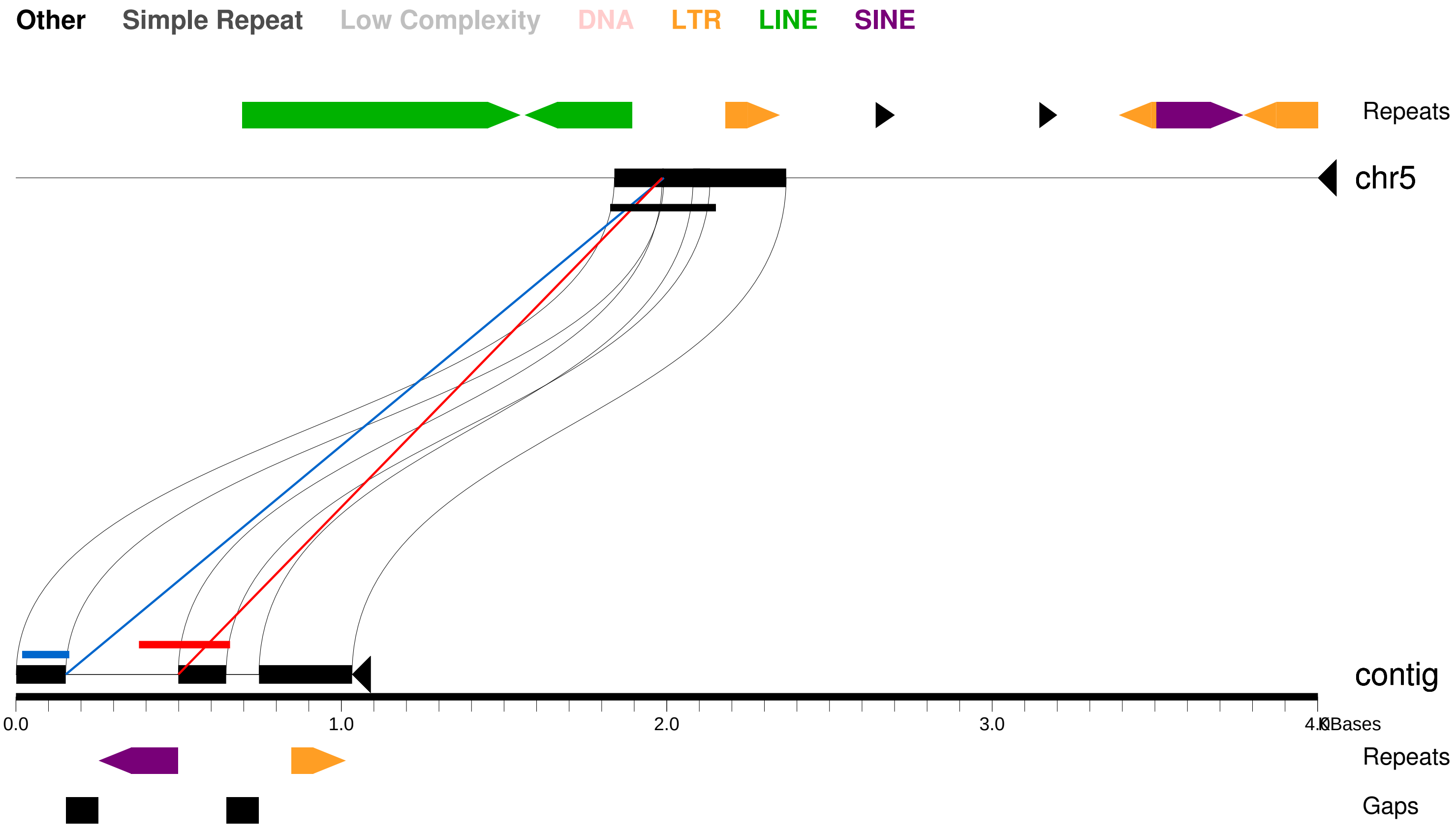

siteID: chr5_19326403 Scafftig1 + Breakpoint: chr5:19326404
chr5:19326385-19326463
Overlap Length: 41 INS size: 406 Genotyped: No

```
left    A--------------------------GAGAAAAATGGCTAATGT--------------GGAAACATTTT
chrom   A--------------------------GAGAAAAATGGCTAATGT--------------GGAAACATTTT
right   CCGCCCGCCTCGGCCTCCCAAAGTGCTGGGATTACAGGCGTGAGCCACCGCGCCCGGCCGGAAACATTTT
        111111111111111111111111111*1**11*11***1111*111111111111111***********

left    TTACAAAGGAATCAGAATACAAAGATTTTTNNNNNNNNNNNNNNNNNNNNNNNNNNNNNNNNNNNNNNNN
chrom   TTACAAAGGAATCAGAATACAAAGATTTTTATTTTTCAGAATTTTAGAA---------------------
right   TTACAAAGGAATCAGAATACAAAGATTTTTATTTTTCAGAATTTTAGAA---------------------
        ******************************2222222222222222222222222222222222222222

left    NNNNNNNNNNNNNNNNNNN
chrom   -------------------
right   -------------------
        2222222222222222222
```

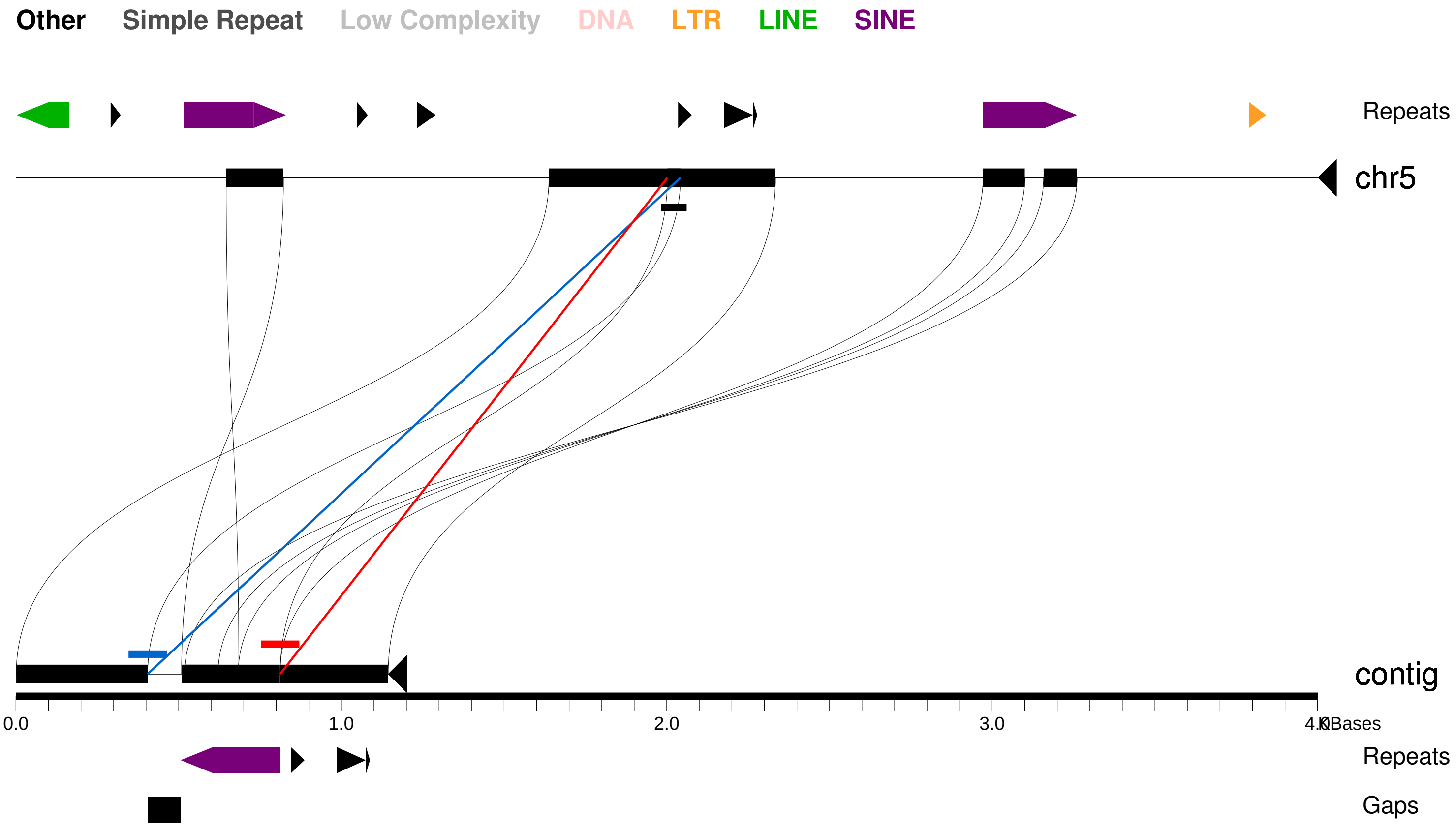

siteID: chr5_24150524 Scafftig1 - Breakpoint: chr5:24150529
chr5:24150482-24150588
Overlap Length: 13 INS size: 307 Genotyped: Yes

```
left   CCT---TTTGC-ATTGA-------TATGATTGATAGATAGATGATAGATAC-ATGCCATAAGAGTATTGC
chrom  CCT---TTTGC-ATTGA-------TATGATTGATAGATAGATGATAGATAC-ATGCCATAAGAGTATTGC
right  CCAGCCTGGGCGACAGAGCGAGACTCCGTCTCAAAAAAAAATAAAATAAAATAAAAAAAAAGAGTATTGC
       **1111*11**1*11**1111111*11*11*1*1*1*1*1**1*1*1*1*11*1111*1***********

left   AGGCCGGGCGCGGTGGCTCACGCCTGTAATCCCAGCACTTTGGGAGGCCGAGGCGGGCGGA
chrom  AGACTTCTC---CTGCTTAAAG--TG-AATGTATTCATTCT------CCCTTGAAGTTACT
right  AGACTTCTC---CTGCTTAAAG--TG-AATTTATTCATTCT------CCCTTGAAGTTACT
       **2*2222*2222**22*2*2*22**2***N2222**2*2*222222**222*22*22222
```

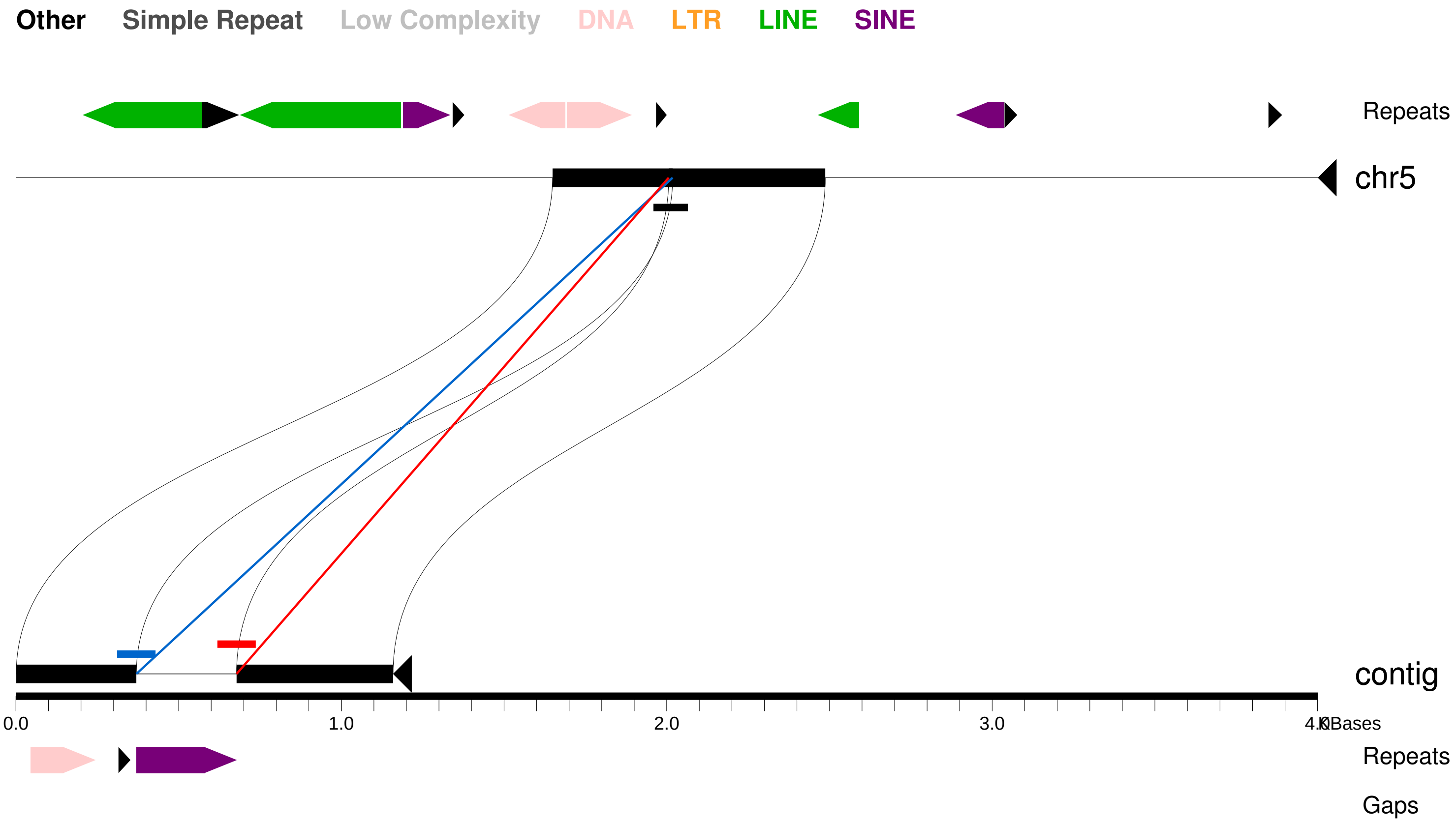

siteID: chr5_29454594 Scafftig3 - Breakpoint: chr5:29454595
chr5:29454544-29454654
Overlap Length: 9 INS size: 290 Genotyped: Yes

```
left    ATATACATTATTCTTCTGAGTTGCATTTTTGCTTAATGAAATAGAATTTTT------------AAAAAAA
chrom   ATATACATTATTCTTCTGAGTTGCATTTTTGCTTAATGAAATAGAATTTTT------------AAAAAAA
right   AGATCCCG----CCACTGCACTCCAGCCTGGGCGACAGAGCGAGACTCCGTCTCAAAAAAAAAAAAAAAAA
        *1**1*111111*11***111*1**111*1*111*11**111***1*111*111111111111*******

left    TGGGCCGGGCGCGGTGGCTCACGCCTGTAATAC-CAGCACTTTGGGAGGCCGAGGCGGGCGG
chrom   TGTTTATTTTACCTTTATTC-CTACTAGATTATACATTTCATTGGAA--------CAGCATT
right   TGTTTATTTTACCTTTATTC-CTACTAGATTATACATTTCATTGGAA--------CAGCATT
        **222222222*22*222**2*22**22*2**22**222*2****2*22222222*2*2222
```

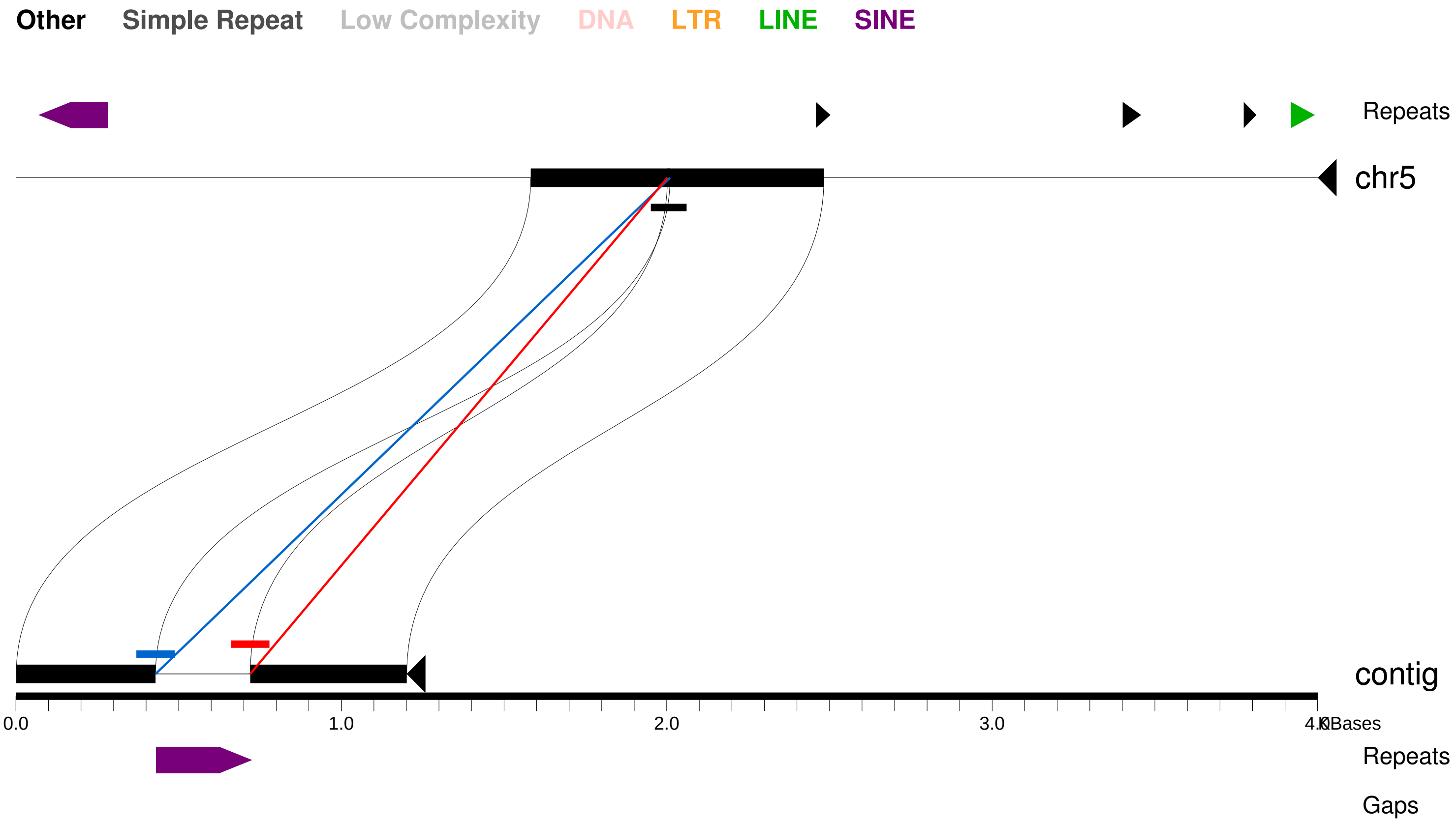

siteID: chr5_30532562 Scafftig3 + Breakpoint: chr5:30532578
chr5:30532528-30532637
Overlap Length: 10 INS size: 428 Genotyped: No

```
left   GGAAGCATTCTTCATTTTCAGATTCCTACAGGTTTGCTAACTGCATCAAA---------ACTTAGAGGAG
chrom  GGAAGCATTCTTCATTTTCAGATTCCTACAGGTTTGCTAACTGCATCAAA---------ACTTAGAGGAA
right  NNNNNNNNNNNNNNNNNNNNNNNNNNNNNNNNNNNNNNNNNNNNNNNNNNNNNNNNNNNACTTAGAGGAA
       11111111111111111111111111111111111111111111111111111111111*********2

left   GCCGGGCGCGGTGGCTCACGCCTGTAATCCCAGCACTTTGGGAGGCCGAGGCGGGTGG
chrom  TATGGTAT-----TTTCAATCCTTTATTAAAATCTAATCTTGAGAC--AGTC--TTGG
right  TATGGTAT-----TTTCAATCCTTTATTAAAATCTAATCTTGAGAC--AGTC--TTGG
       222**2222222222***22***2**2*222*2*222*222***2*22**2*222***
```

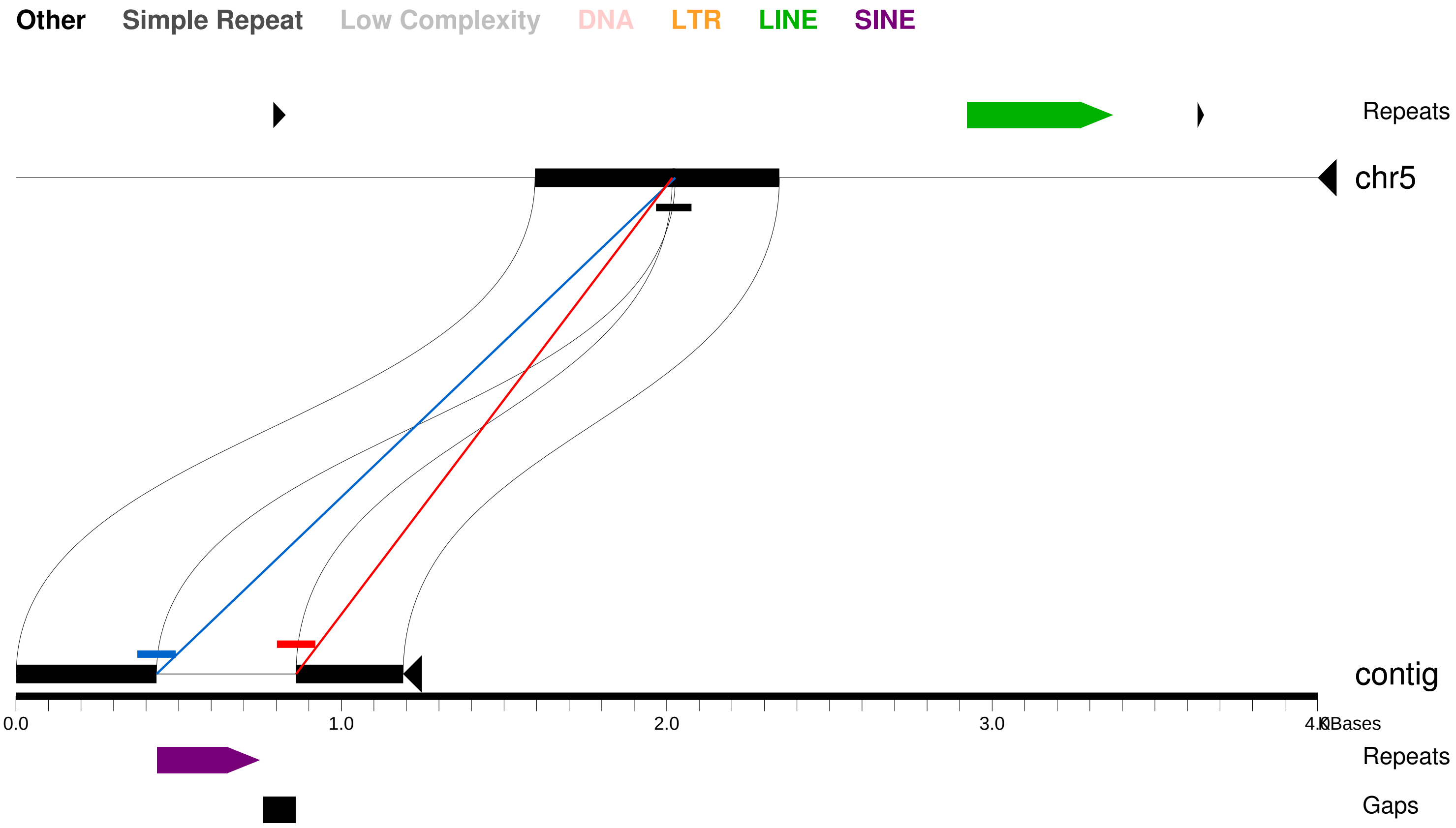

siteID: chr5_32762571 Scafftig2 + Breakpoint: chr5:32762563
chr5:32762519-32762622
Overlap Length: 16 INS size: 318 Genotyped: Yes

```
left     GT----GTAAAGGCATTCCTATTTC-------TCCA--CATCCTCTCCAGCATCTGT--TGTTTCCTGAT
chrom    GT----GTAAAGGCATTCCTATTTC-------TCCA--CATCCTCTCCAGCATCTGT--TGTTTCCTGAT
right    CCACCCGCCTCGGCCTCCCAAAGTGCTGGGATTACAGGCGTGAGCCACCGCGCCCGGCCTGTTTCCTGAT
         111111*1111***1*1**1*11*11111111*1**11*1*111*11*1**11*1*111***********

left     TTTTTTTTTTTTTTTTTTTTTTTTTTTTTTTTTTTTTTTGAGACGGAGTCTCGCTCTGTCGCCCAGGT
chrom    TTTTTAATGATCACCATTCTAACTGG--------CGTGAGGTGGTATCTCATTGTGG-------
right    TTTTTAATGATCACCATTCTAACTGG--------CGTGAGGTGGTATCTCATTGTGG-------
         *****22*22*22222**2*222*22222222222*2**2*22**2**2*22***2*2222222
```

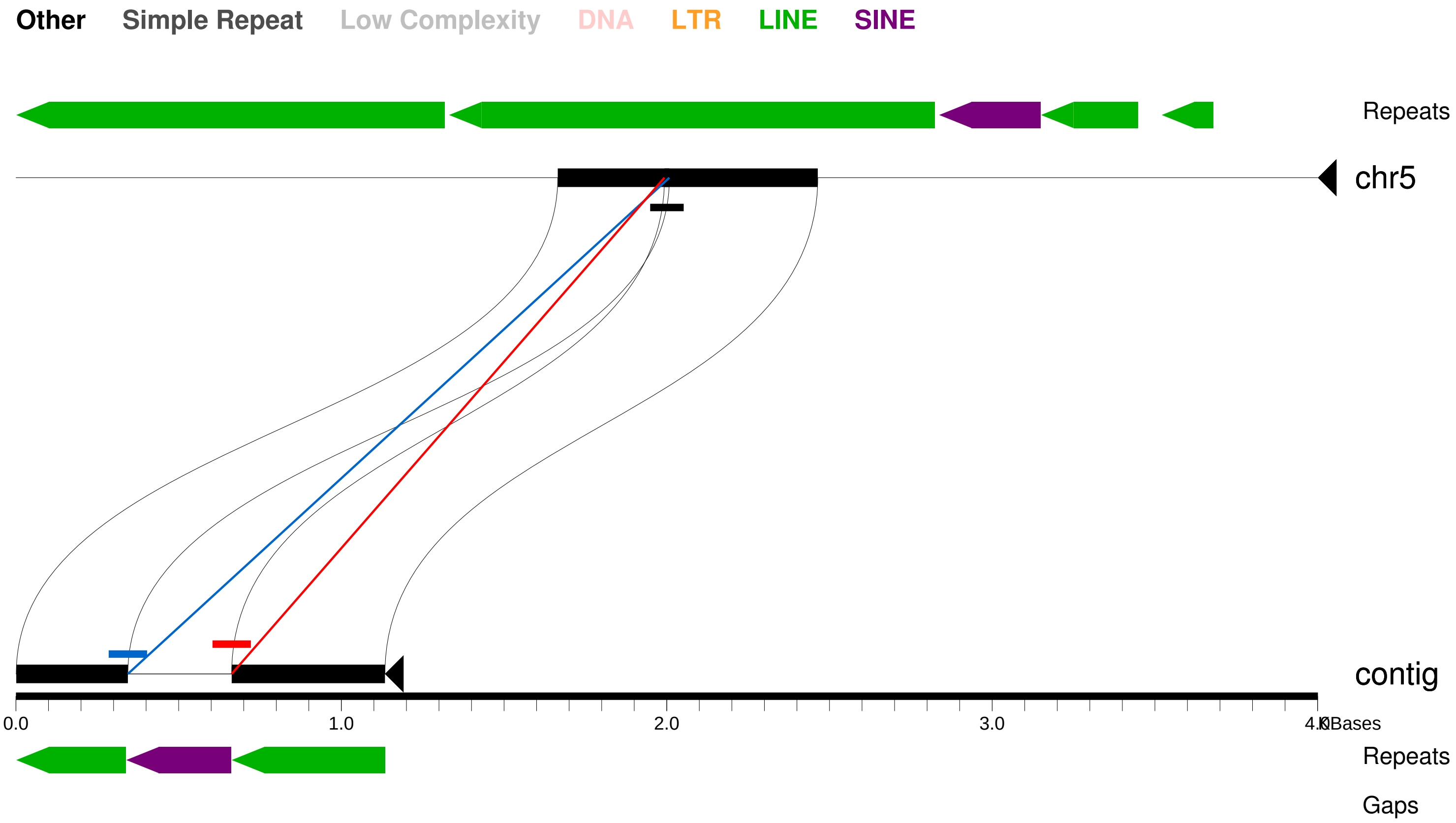

siteID: chr5_34459553 Scafftig4 - Breakpoint: chr5:34459553
chr5:34459506-34459612
Overlap Length: 13 INS size: 305 Genotyped: Yes

```
left   ATCTTCTACCAGGCCTG-ATAGTAAGGCTTT------------AATTGCTGTCAGTGTTTAAGATTTAAC
chrom  ATCTTCTACCAGGCCTG-ATAGTAAGGCTTT------------AATTGCTGTCAGTGTTTAAGATTTAAC
right  A-CTCCAGCCTGGGCGACAGAGCGAGACTCGTCTCAAAAAAAAAAAAAAAAAAAAAAAAAAAGATTTAAC
       *1**1*11**1**1*111*1**11**1**11111111111111**11111111*111111*********

left   AGGGGCCGGGCGCGGTGGCTCACGCCTGTAATCCCAGCACTTTGGGAGGCCGAGGCGGGCAG
chrom  AGGA-CTTGGTGTTGTTTTT-AGACCCAGAGTCAAAGCCCTGT---AACTCAATG-------
right  AGGA-CTTGGTGTTGTTTTT-AGACCCAGAGTCAAAGCCCTGT---AACTCAATG-------
       ***22*22**2*22**222*2*22**222*2**22***2**2*222*222*2*2*2222222
```

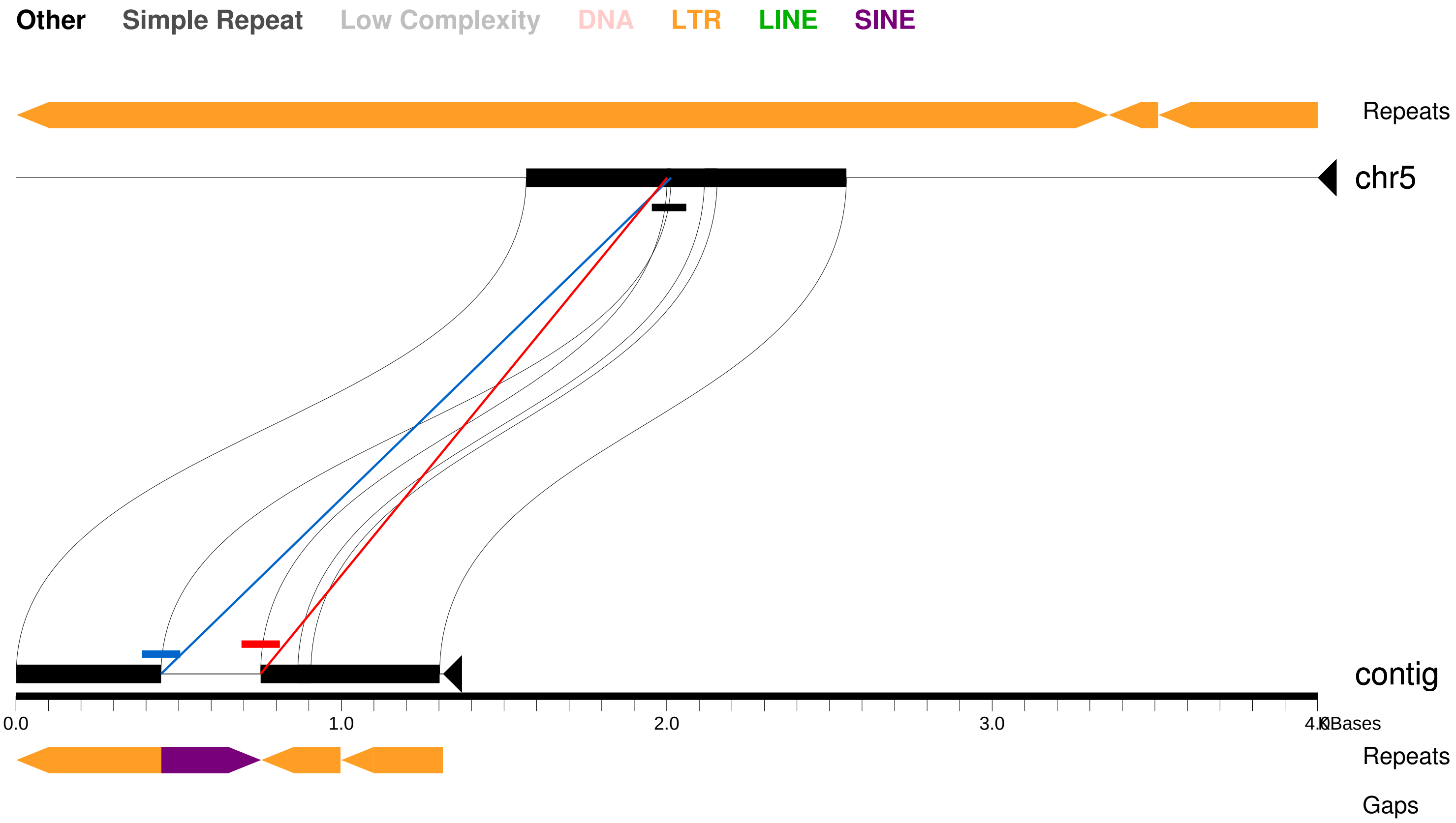

siteID: chr5_34718033 Scafftig2 - Breakpoint: chr5:34718031
chr5:34717997-34718090
Overlap Length: 14 INS size: 337 Genotyped: Yes

```
left   -------------------AAGTT----GTTCCCAAGCCTGT--CACCACCACATCCTTCCAACCCTGCT
chrom  -------------------AAGTT----GTTCCCAAGCCTGT--CACCACCACATCCTTCCAACCCTGCT
right  CGCCCGCCTCGGCCTCCCAAAGTGCTGGGATTACAGGCGTGAGCCACCGCGCCCGGCCCCCAACCCTGCT
       1111111111111111111****11111*1*11**1**1**111****1*11*111*11***********

left   CTTTTTTTTTTTTTTTTTTTTTTTTTTTTTTTTTTTTTTTTTTTTTTTTTTTTTGAGACGGAGTCTCGCT
chrom  CTTAAGCTTCTTTTTCAGAGGGAAGCAG----------------AATGTTTACTCAGTAGGACA------
right  CTTAAGCTTCTTTTTCAGAGGGAAGCAG----------------AATGTTTACTCAGTAGGACA------
       ***2222**2*****2222222222222222222222222222222*2***22*2**22***22222222

left   CTGT
chrom  ---T
right  ---T
       222*
```

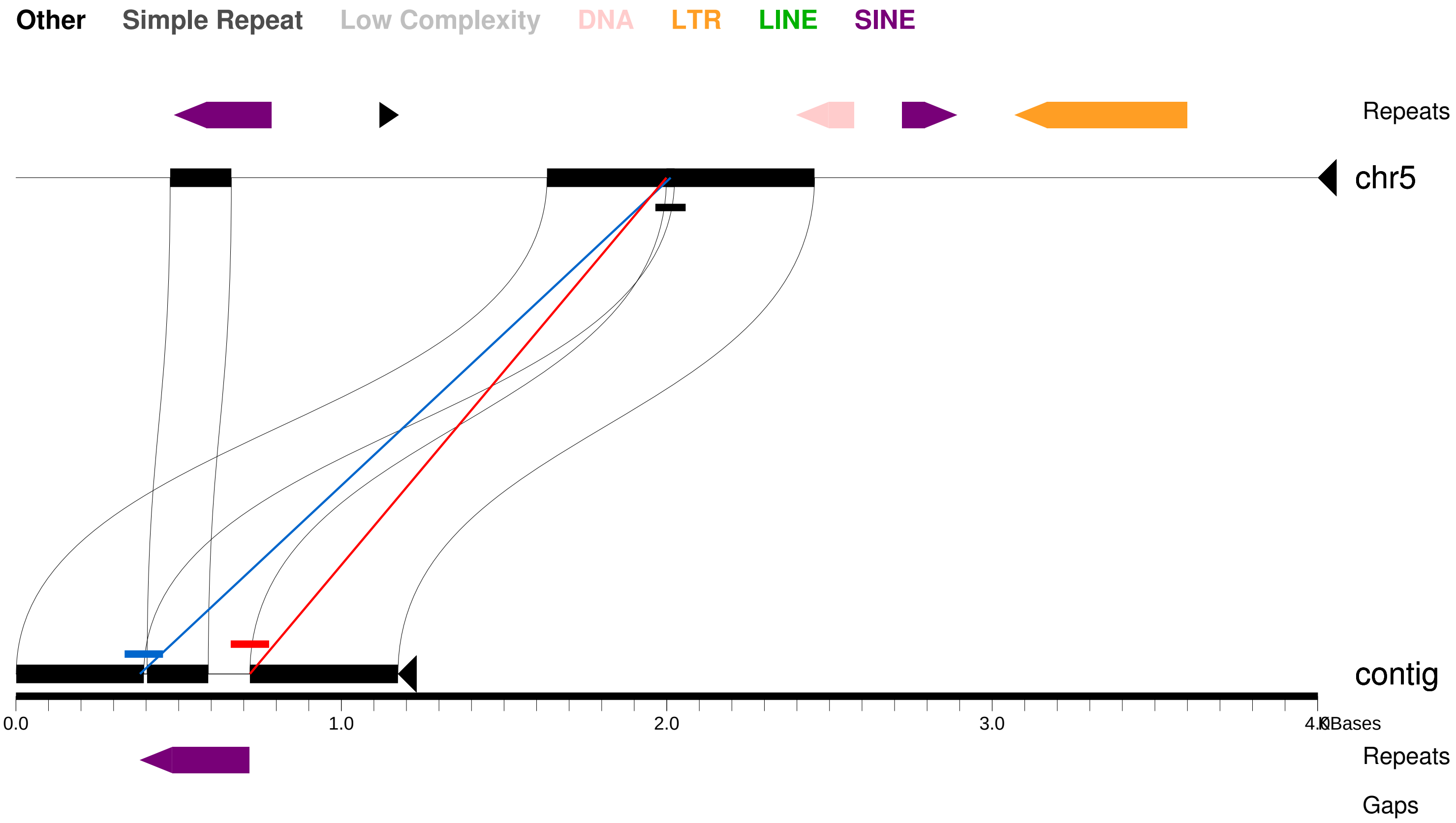

siteID: chr5_36792628 Scafftig1 + Breakpoint: chr5:36792605
chr5:36792445-36792780
Overlap Length: 16 INS size: 435 Genotyped: No

```
left   ----------------GCACAGTGGACATTGAGGAATCTTGGATATGCCACACTCCTGATTCAAGAGCAG
chrom  AGCAGGAAGCCAGCAGGCACAGTGGACATTGAGGAATCTTGGATATGCCACACTCCTGATTCAAGAGCAG
right  NNNNNNNNNNNNA---------------------------------------------------------
       NNNNNNNNNNNN2    111111111111111111111111111111111111111111111111111111

left   CAGAGAGTAAATTAAGAAGAGAGAATACAACGAGTCAGGAAAGGCTTTGCCCTGGAGAAAAAGCCAACTC
chrom  CAGAGAGTAAATTAAGAAGAGAGAATACAACGAGTCAGGAAAGGCTTTGCCCTGGAGAAAAAGCCAATTC
right  ---------------------------------------------------------AAAAA--------
       111111111111111111111111111111111111111111111111111111111*****11111N11

left   CATGGTCAGCGGGAGAAACTAGAATCAGTTCACTGGGGC------CGGGCGCG----GTGGCTCACGCCT
chrom  CATGGTCAGCAGGAGAAACTAGAATCAGTTCACTGGTCTGTTACTCATGCACGAAACGAAGTCAGGGCTT
right  --------------------AGAATCAGTTCACTGGTCTGTTATTCATGCACGAAACGAAGTCAGGGCTT
       1111111111N111111111***************2222222N2*22**2**2222*22*22222**2*

left   GTA-----ATCCCAG---CACTTTGGGAGGCTGAGGCGGGCG----GATCACG--AGGTCAGGA-GATCG
chrom  GTATCAAAACCTCATGTCCAAGTTGGTAAGTCAAGACTGACATTCAGAAGACTCAAATTGAGTATGACCC
right  GTATCAAAACCTCATGTCCAAGTTGGTAAGTCAAGACTGACATTCAGAAGACTCAAATTGACT-------
       ***22222*2*2**2222**22****2*2*222**2*2*2*22222**22**222*22*2*121 11N1N

left   AGACCATCCTG-GCTAACACGGTGAAACCCCGT-CTCT------------ACTAAA
chrom  AAACTGACTTTAGCAAAAGCAGGGAATCAAAATGCTGGGGGTTAGAGGACAGTAAT
right  --------------------------------------------------------
       1N11NNN1N1N 11N11NN1N1N111N1NNNN1 11NN            1N111N
```

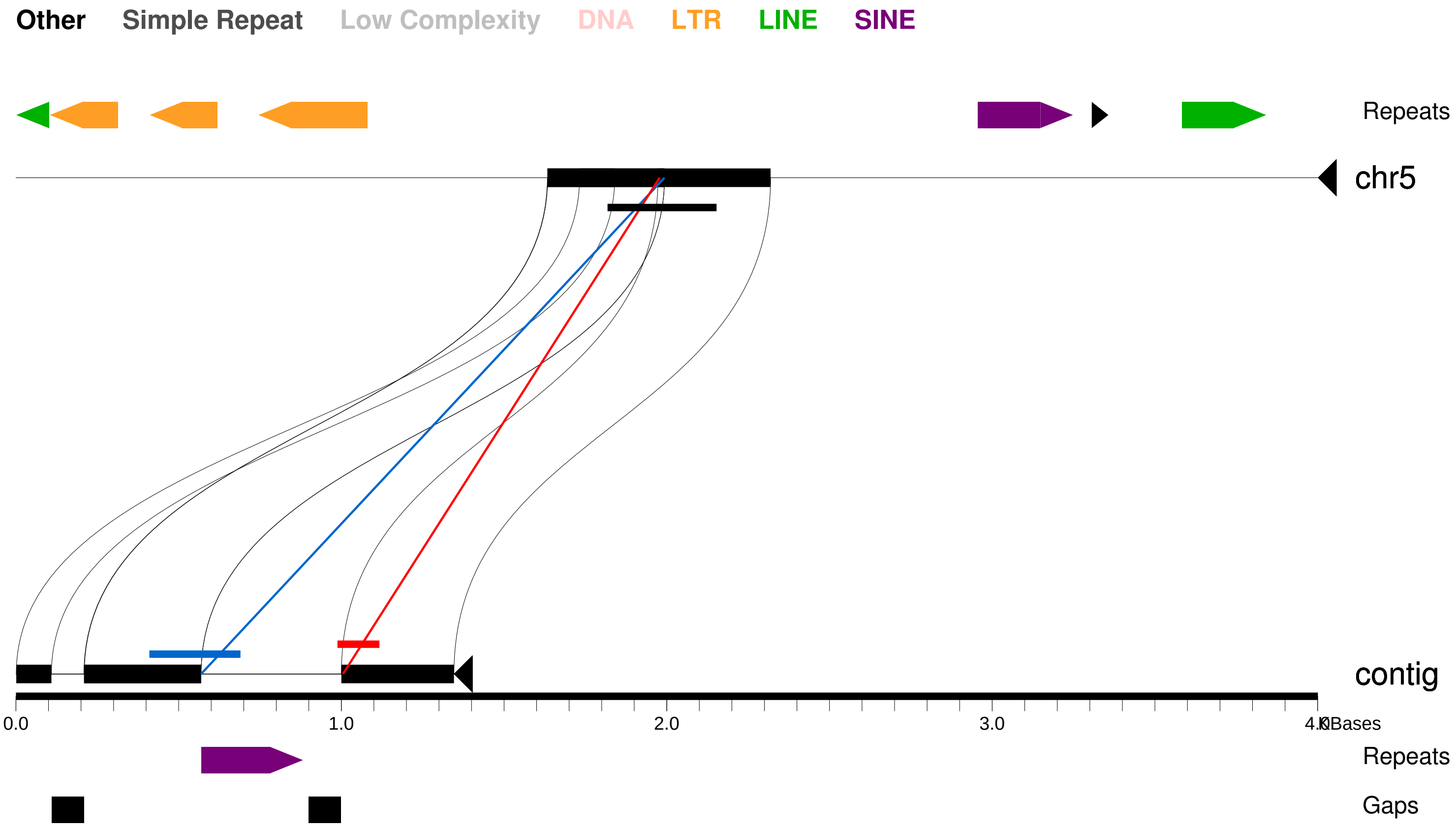

siteID: chr5_37648674 Scafftig1 - Breakpoint: chr5:37648659
chr5:37648613-37648717
Overlap Length: 14 INS size: 305 Genotyped: Yes

```
left     -AGA---------GAACTATTCTTTT-AGTGGTAATC--TCTTGGTAGTTCAGTTGTTA-GAAGTCACTG
chrom    -AGA---------GAACTATTCTTTT-AGTGGTAATC--TCTTGGTAGTTCAGTTGTTA-GAAGTCACTG
right    CAGTCCGCAGTCCGACCTGGGCGACAGAGCGAGACTCCGTCTCAAAAAAAAAAAAAAAAAGAAGTCACTG
         1**1111111111**1**111*11111**1*11*1**11***1111*1111*111111*1**********

left     TAAAGGCCGGGCGCGGTGGCTCACGCCTGTAATCCCAGCACTTTGGGAGGCCGAGGCGGGTGG-
chrom    TAAAA---------------TAACACCAACAAACATGGTTTTTACCAATCTTGTGCCATCTGGA
right    TAAAA---------------TAACACCAACAAACATGGTTTTTACCAATCTTGTGCCATCTGGA
         ****2222222222222222*2**2**222**2*222*222**2222*2222*2*2*222***2
```

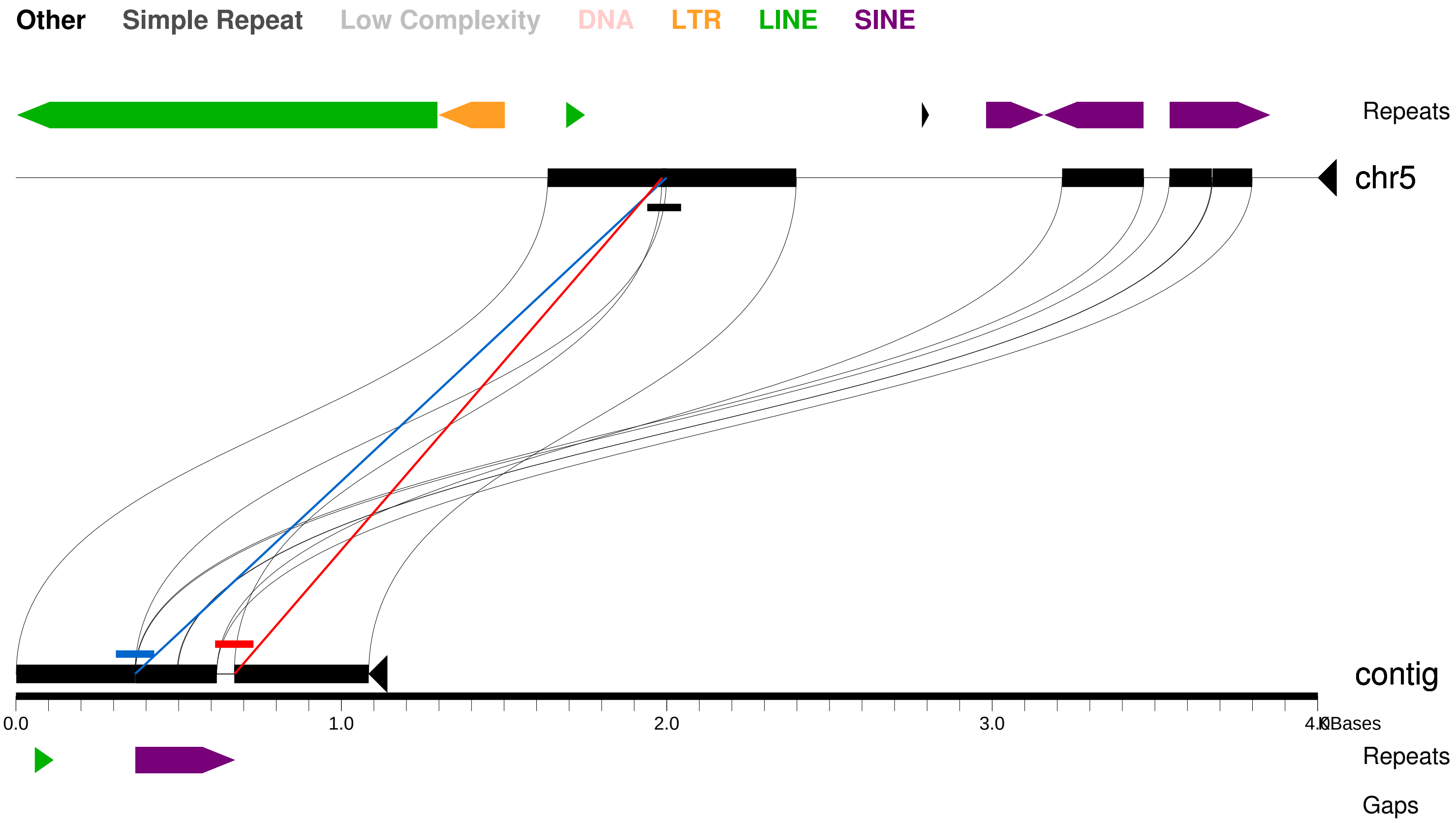

siteID: chr5_37688818 Scafftig5 - Breakpoint: chr5:37688796
chr5:37688636-37688971
Overlap Length: 16 INS size: 266 Genotyped: Yes

```
left   ----------------GTTTCATTTAGAGTTTAGTAGTTGGTAACTTGATGGGATATAAGGAAGTGGCAA
chrom  ATAAGATCTTCCAAAAGTTTCATTTAGAGTTTAGTAGTTGGTAACTTGATGGGATATAAGGAAGTGGCAA
right  GGCAGG-------AGAATGGCGT---GAACCCGGGAGGCGG-AGCTTGCAGTGAGCCGAG-ATCCCGCCA
       NNN22N       2N21*11*1*111**11111*1**11**1*1****11*1**1111**1*1111**1*

left   ATTTTCCTTATTGAGGTTGGTAG-CAGAGTTTCGTTTGTCCTAGGAACAGAATATTCCCAGTCTGTTCAC
chrom  ATTTTCCTTATTGAGGTTGGTAG-CAGAGTTTCGTTTGTCCTAGGAACAGAATATTCCCAGTCTGTTCAC
right  CTGCAC----TCCAGCCTGGGCGACAGAG-----------CGAG---------ACTCC--GTCTCAAAAA
       1*111*1111*11**11***11*1*****11111111111*1**111111111*1***11****1111*1

left   ATGTGTTCAGAATGATCATTTAAAAATAAGCAAATTTCACTTTGGGAG--GCCGAGGCGGGCGGATCACG
chrom  ATGTGTTCAGAATGATCATTTAAAAATAAGCAAATTTAGGTTTCCAAGATGGCCAAATAGGAACAGCTCT
right  A-------AAAAAAAAAAAAAAAAAATAAGCAAATTTAGGTTTCCAAGATGGCCAAATAGGAACAGCTCT
       *1111111*1**11*11*111***************222***222**22*2*2*2222**222*2*2*2

left   AGGTCAGGAGATC-----GAGACC-ACCCCG--GCTA---GAATGGTGAAACCCCGTCT-------CTAC
chrom  -GGTCTGCAGCTCCCAGTGTGATCGACGCAGAAGATAAGTGATTTCTGCATTTCCAGCTGAGGCACCTGG
right  -GGTCTGCAGCTCCCAGTGTGATCGACGCAGAAGATAAGTGATTTCTGCATTTCCAGCTGAGGCACCTGG
       2****2*2**2**22222*2**2*2**2*2*22*2**222**2*22**2*222**22**2222222**22

left   TAAA----AATACAAAAAATTAGCC---GGGCGTAG----TGGCGGGCG---------
chrom  TTCATCTCATTGGGACTGGTTGGACAGTGGGTGCAGCCCATGGAGGGGGCAAGCCAAA
right  TTCATCTCATTGGGACTGGTTGGACAGTGGGTGCAGCCCATGGAGGG-----------
       *22*2222*2*222*2222**2*2*222***2*2**2222***2***N1
```

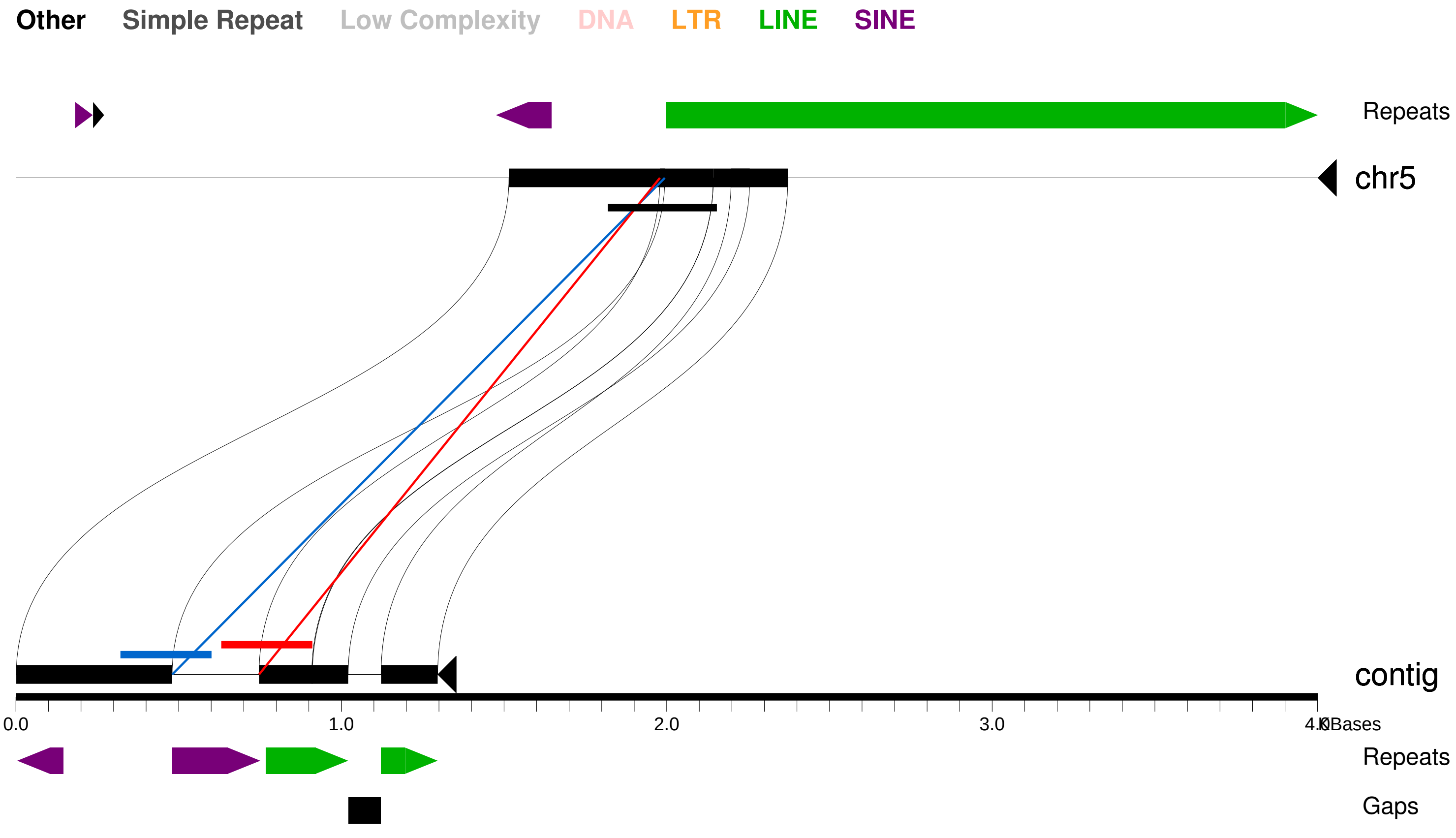

siteID: chr5_39292977 Scafftig1 - Breakpoint: chr5:39292970
chr5:39292921-39293029
Overlap Length: 11 INS size: 428 Genotyped: No

```
left   GCA---GACTATGAT----AAATTA--AGGATGTATATTGTGAATCATAT-GTTATAGAGTAAAAAATAT
chrom  GCA---GACTATGAT----AAATTA--AGGATGTATATTGTGAATCATAT-GTTATAGAGTAAAAAATAT
right  CCACCCGCCTCGGCCTCCCAAAGTGCTGGGATTACAGGCGTGAGCCACCGCGCCCGGCCGTAAAAAATAT
       1**111*1**11*111111***1*1111****1111111****11**1111*1111111***********

left   ATATNNNNNNNNNNNNNNNNNNNNNNNNNNNNNNNNNNNNNNNNNNNNNNNNNNNNNNNNNN
chrom  TTTTTAAAAGAGACATGAGTAATATATCAATACTGGAAATAAAATGGAA----------
right  TTTTTAAAAGAGACATGAGTAATATATCAATACTGGAAATAAAATGGAA----------
       2*2*222222222222222222222222222222222222222222222222222222222
```

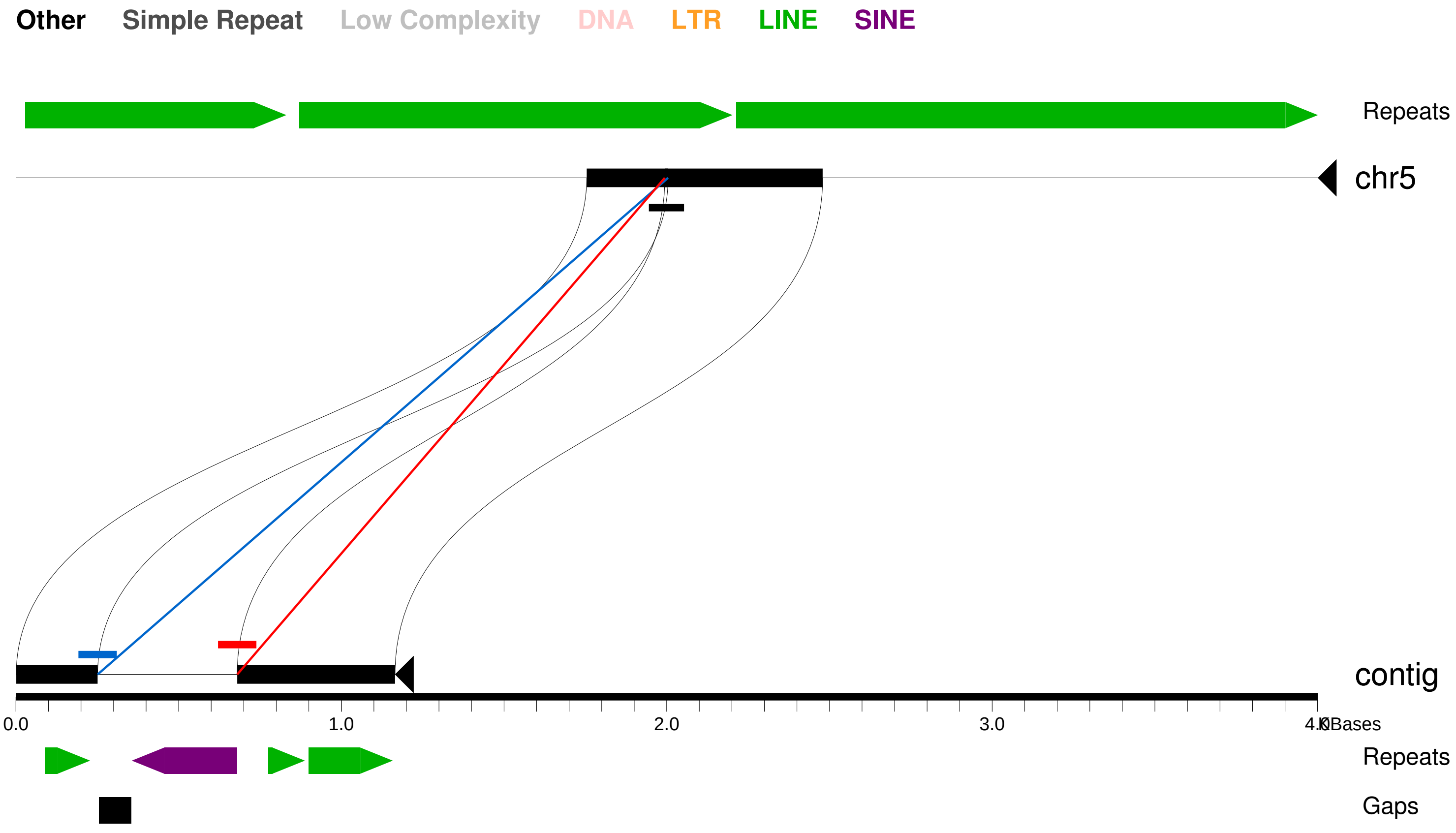

siteID: chr5_39505879 Scafftig1 - Breakpoint: chr5:39505860
chr5:39505809-39505919
Overlap Length: 9 INS size: 411 Genotyped: No

```
left    ---------TAGATGATAGAAGTTACTGAATTTGTCAAGTCCATAAGACAA---GTATGAAAAGATTAAT
chrom   ---------TAGATGATAGAAGTTACTGAATTTGTCAAGTCCATAAGACAA---GTATGAAAAGATTAAT
right   CCGCCCGCCTCGGCCTCCCAAAGTGCTGGGATTA-CAGG--CGTGAGCCACCGCGCCCGGCC-GATTAAT
        111111111*1*1111111**11*1***111**11**1*11*1*1**1**1111*111*1111*******

left    TTNNNNNNNNNNNNNNNNNNNNNNNNNNNNNNNNNNNNNNNNNNNNNNNNNNNNNNNNNNNNN
chrom   TTCTTATATCTGGGAATGGAATAAGCAACAGAGGTGGGAAGTTTGGCCTGTGA--------
right   TTCTTATATCTGGGAATGGAATGAGCAACAGAGGTGGGAAGTTTGGCCTGTGA--------
        **22222222222222222222N2222222222222222222222222222222222222222
```

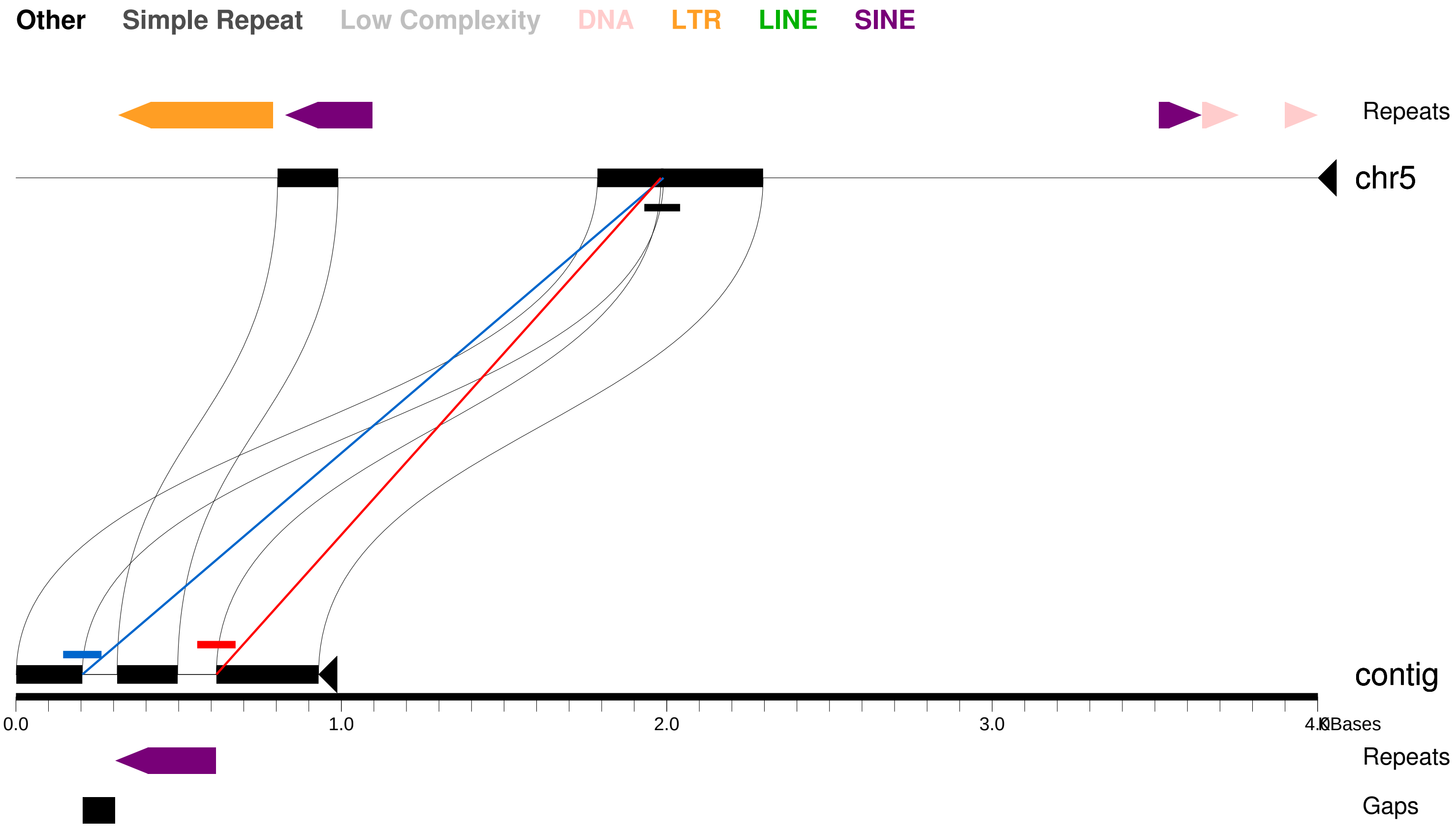

siteID: chr5_3995180 Scafftig10 - Breakpoint: chr5:3995183
chr5:3995140-3995242
Overlap Length: 17 INS size: 438 Genotyped: No

```
left     CCACTTATAAAACTCCAGCCAGGTCATGAGTGCCGCCTC----------------ACCTAGAAAACGACA
chrom    CCACTTATAAAACTCCAGCCAGGTCATGAGTGCCGCCTC----------------ACCTAGAAAACGACA
right    NNNNNNNNNNNNNNNNNNNNNNNNNNNNNNNNNNNNNNNNNNNNNNNNNNNNNAAAAAAAGAAAACGACA
         1111111111111111111111111111111111111111111111111111111*111***********

left     GAAATCGGCCGGGCGCGGTGGCTCACGCCTGTAATCCCAGCACTTTGGGAGGCCGAGGCGGGCGG
chrom    GAAATCCACTGCACACA-TGGTT----CCAGAAAAC--AACA------GAAATCCACT---GCAC
right    GAAATCCACTGCACACA-TGGTT----CCAGAAAAC--AACA------GAAATCCACT---GCAC
         ******22*2*22*2*22***2*2222**2*2**2*22*2**222222**222*2*22222**22
```

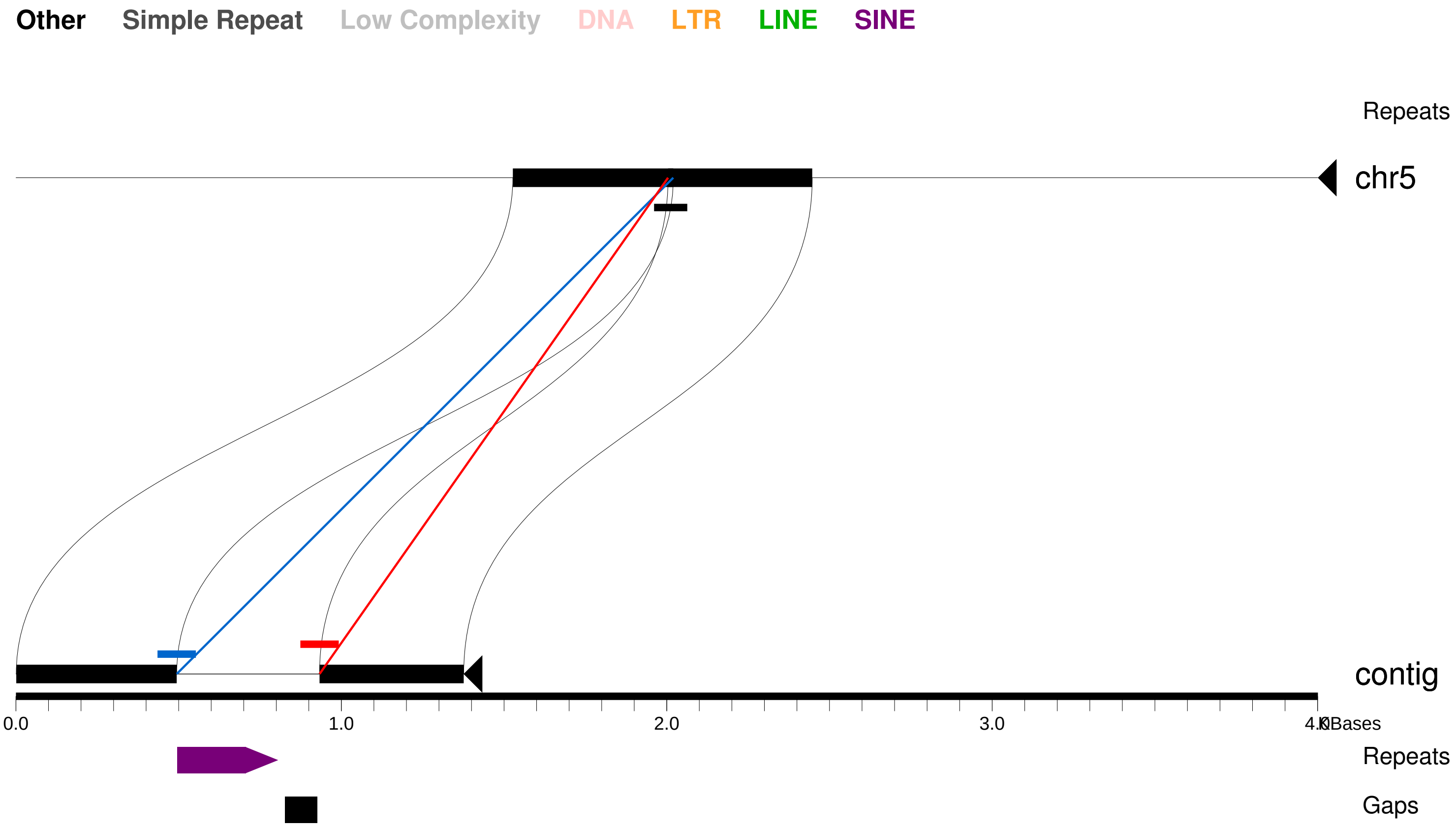

siteID: chr5_40181646 Scafftig4 + Breakpoint: chr5:40181650
chr5:40181599-40181709
Overlap Length: 9 INS size: 297 Genotyped: Yes

```
left   TATTAAAAAGTCGGGAAACAATAGATGCTGC----AGAGGA-TGTG-----GAGAAATAGGAACACTTTT
chrom  TATTAAAAAGTCGGGAAACAATAGATGCTGC----AGAGGA-TGTG-----GAGAAATAGGAACACTTTT
right  CCGCCCGCC-TCGGCCTCCCAAAG-TGCTGGGATTACAGGCGTGAGCCACCGCGCCCGGCCAACACTTTT
       1111111111****1111*1*1**1*****11111*1***11**1*11111*1*1111111*********

left   TTTTTTTTTTTTTTTTGAGACGGAGTCTCGCTCTGTCGCCCAGGCTGGAGTGCAGTGGC
chrom  ACACCATTGG----TCAAAATGTAAATTAGTTCAAT----GATTGTGGATGACAGTGTG
right  ACACCACTGG----TCAAAATGTAAATTAGTTCAAT----GATTGTGGATGACAGTGTG
       2222221*222222*22*2*2*2*222*2*2**22*22222*222****222*****22
```

Other Simple Repeat Low Complexity DNA LTR LINE SINE

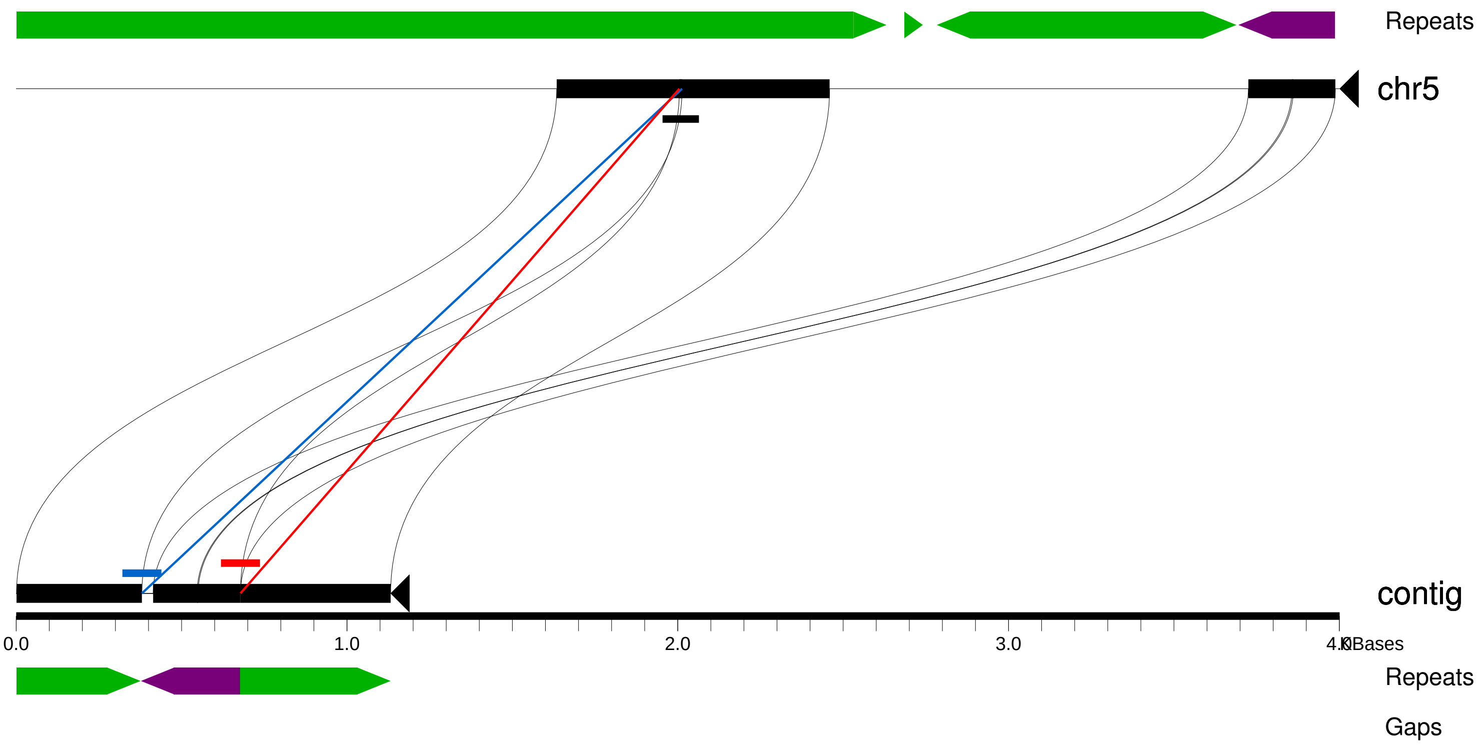

siteID: chr5_45258061 Scafftig1 - Breakpoint: chr5:45258051
chr5:45258007-45258110
Overlap Length: 16 INS size: 240 Genotyped: No

```
left    ---TCAGCAT-TCCA----TTCTATAAGGCCCATGTTTCTTTCCCATGGTAC-------AGTATGAGTAC
chrom   ---TCAGCAT-TCCA----TTCTATAAGGCCCATGTTTCTTTCCCATGGTAC-------AGTATGAGTAC
right   TGGTCTCGATCTCCTGACCTCGTGATCCGCCCGCCTCGGCCTCCCAAAGTGCTGGGATTAGTATGAGTAC
        111**111**1***11111*11*11111****111*11111*****11**1*1111111***********

left    TTTTTTNNNNNNNNNNNNNNNNNNNNNNNNNNNNNNNNNNNNNNNNNNNNNNNNNNNNNNNNNN
chrom   TTTTTT--------------AAAGTAGGTGAACATAAAATAAAAGTTTGCTAGTTGTAGTGCAT
right   TTTTT---------------AAAGTAGGTGAACATAAAATAAAAGTTTGCTAGTTGTAGTGCAT
        ******2222222222222222222222222222222222222222222222222222222222
```

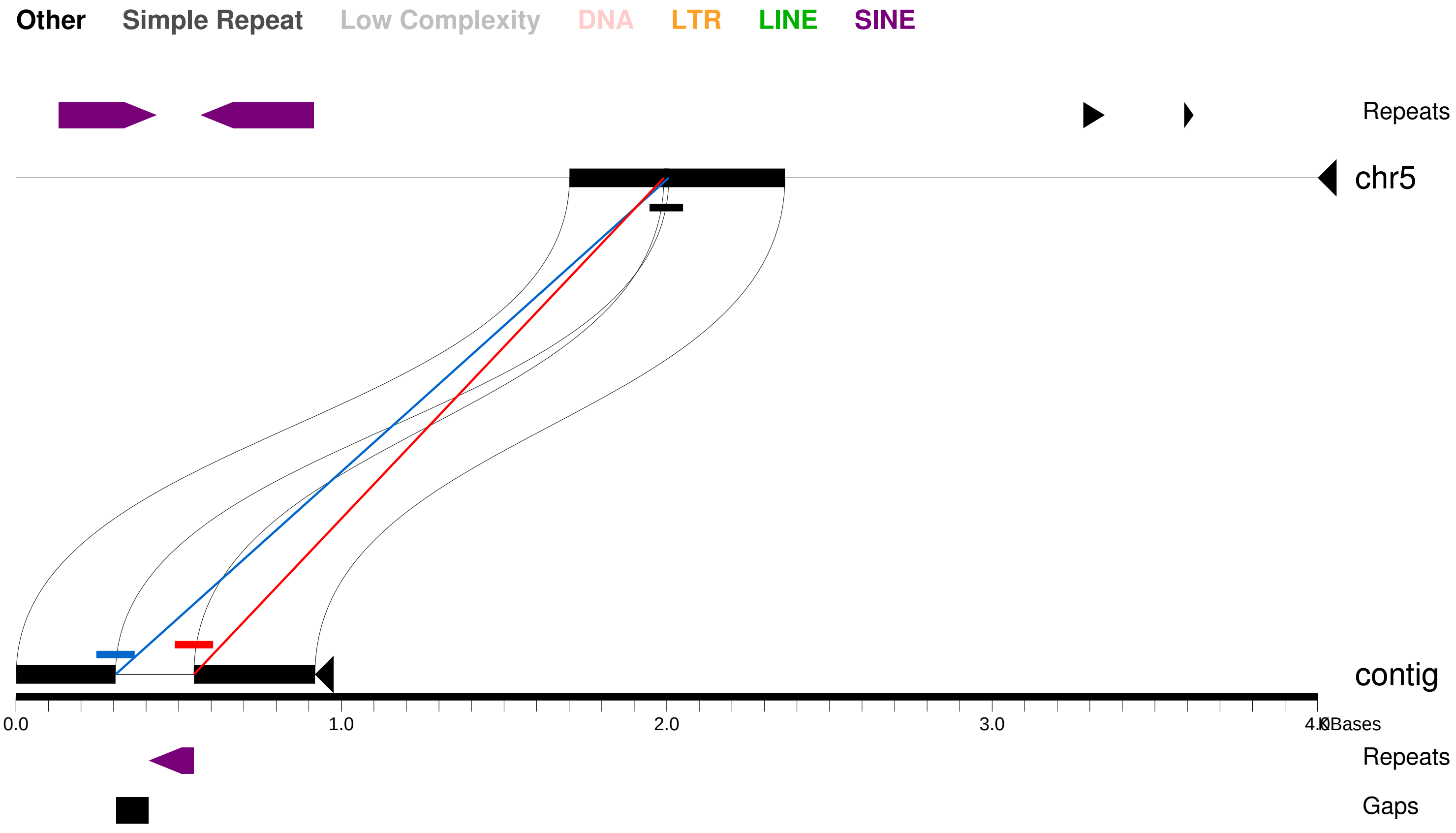

siteID: chr5_45724110 Scafftig3 + Breakpoint: chr5:45724097
chr5:45723937-45724270
Overlap Length: 14 INS size: 442 Genotyped: No

```
left   --------------GGTTTTTACTCTCAGAGGGGAAAACATTCCTTTTTTAAAAAGCAAAGGCGTGGCAT
chrom  ATAGTCAAGTTATTGGTTTTTACTCTCAGAGGGGAAAACATTCCTTTTTTAAAAAGCAAAGGCGTGGCAT
right  NNNNNNNNNNNA--------------------------------------AAAAA---------------
       NNNNNNNNNNN2  111111111111111111111111111111111111*****111111111111111

left   GAGCTGACTTATGATTTTAAAAGAATGTTTCTTGATGCTTTATTGAGACTCTGATACATGACAGAGATGG
chrom  GAGCTGACTTATGATTTTAAAAGAATGTTTCTTGATGCTTTATTGAGACTCTGATACATGACAGAGATGG
right  ----------------------------------------------------------------------
       1111111111111111111111111111111111111111111111111111111111111111111111

left   AAGCATGAATTAATTCAGTTAGAAAGCTAAAGCAGG---CCGGGCGCG--------GTGGCTCACGCC--
chrom  AAGCATGAATTAATTCAGTTAGAAAGCTAAAGCAATGCTCCAGGCATGAAACAACAGAAGTTTATACCAG
right  --------------------AGAAAGCTAAAGCAATGCTCCAGGCATGAAACAACAGAAGTTTATACCAG
       11111111111111111111**************22222**2***22*22222222*22*2*2*22**22

left   --TGTAATCC-CAGCACTTTGG--GAGGCCGAGGCGGGC-------------GGATCACGAGGT--CAGG
chrom  AATGAAATTCACAGAAGTAAAGAGGAATTATAAACTTCTAAATATTTGTTTTGAATATAGAGATAACAGG
right  AATGAAATTCACAGAAGTAAAGAGGAATTATAAACTTCTAAATATTTGTTTTGA----------------
       22**2***2*2***2*2*222*22**22222*22*222222222222222222*211NNN111N1  1111

left   AGATCGAGACC----ATCCCGGCTAAAACGGTGAAACCCCGTCTCTACTAAA--
chrom  ATTTTCAGAAGTGGAATGTAAGAGAAAGAGAGGAGGCAAGGATGATCCCAAGAT
right  ------------------------------------------------------
       1NN1NN111NN    11NNNN1NN111NN1NN11NN1NNN1NNNN1N1N11N
```

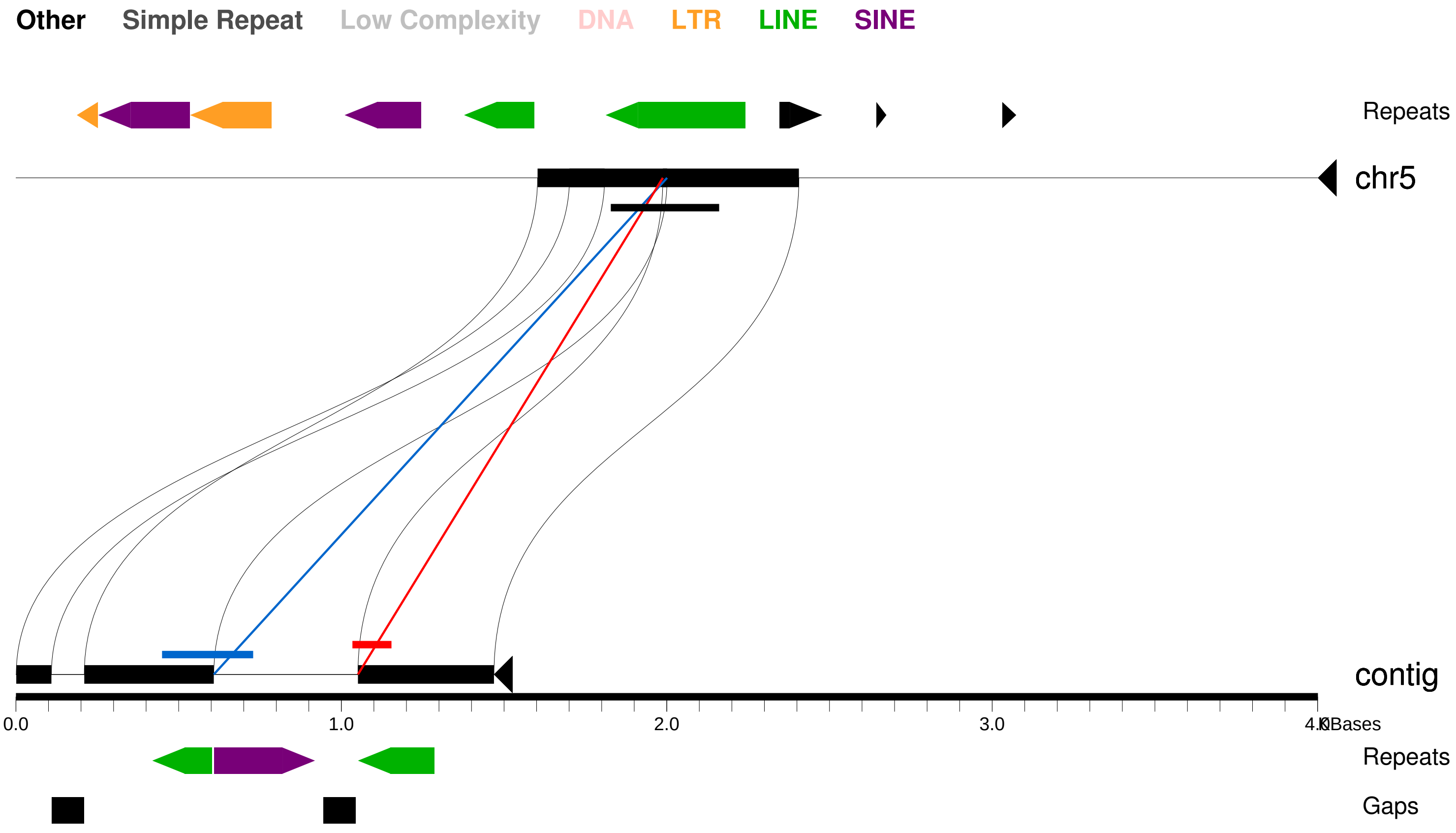

siteID: chr5_50785676 Scafftig3 + Breakpoint: chr5:50785671
chr5:50785630-50785730
Overlap Length: 16 INS size: 456 Genotyped: No

```
left   TTAAATAATCCTCCAAAATTCGGTATCTATTGTGAACTTTT------------------AATAGTTAGGA
chrom  TTAAATAATCCTCCAAAATTCGGTATCTATTGTGAACTTTT------------------AATAGTTAGGA
right  NNNNNNNNNNNNNNNNNNNNNNNNNNNNNNNNNNNNNNNNNNNNNNNNNNNNNNNNAAAAATAGTTAGGA
       11111111111111111111111111111111111111111111111111111111111***********

left   ATAGTTGGCCGGGCGCGGTGGCTCACGCCTGTAATCCCAGCACTTTGGGAG-GCCGAGGCGGGCGGAT-
chrom  ATAGTGGGT--------------------TATAAACTTTGTATTTCTGTTGTGCTGTAGCATCACTATA
right  ATAGTGGGT--------------------TATAAACTTTGTATTTCTGTTGTGCTGTAGCATCACTATA
       *****2**222222222222222222222*2***2*222*2*2**22*22*2**2*22**222222**2
```

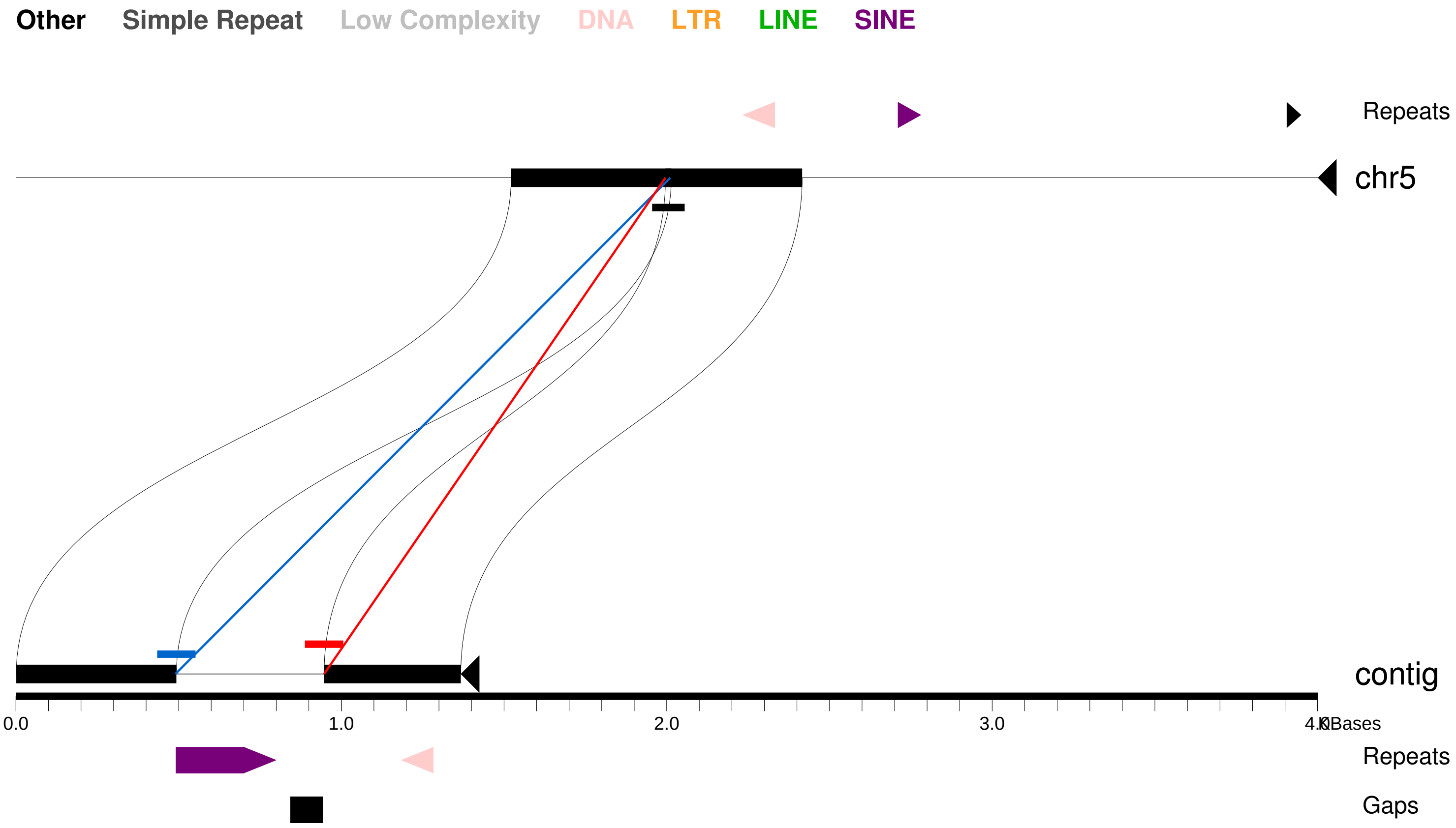

siteID: chr5_5279747 Scafftig6 + Breakpoint: chr5:5279782
chr5:5279724-5279841
Overlap Length: 2 INS size: 472 Genotyped: No

```
left    AGACAAATGTTATGATGTTCTTGTGTTTTCTTTTCAGATGTCCATCTCTTTGTCTTTT-TANNNNNNNNN
chrom   AGACAAATGTTATGATGTTCTTGTGTTTTCTTTTCAGATGTCCATCTCTTTGTCTTTT-TATCTTGGAAG
right   CCGCCCGCCTCGGCCTCCCAAAGTGCTGGGATTACAGGCGTGAGCCACCGCGCCCGGCCTATCTTGGAAG
        111*11111*11111*111111***1*1111**1***11**1111*1*111*1*11111**222222222

left    NNNNNNNNNNNNNNNNNNNNNNNNNNNNNNNNNNNNNNNNNNNNNNNNNN
chrom   TTTTTTTGAATTTTATCTTCCAGGTTTTCTGCTTTTTGTTTTGCTTTTG-
right   TTTTTTTGAATTTTATCTTCCAGGTTTTCTGCTTTTTGTTTTGCTTTTG-
        22222222222222222222222222222222222222222222222222
```

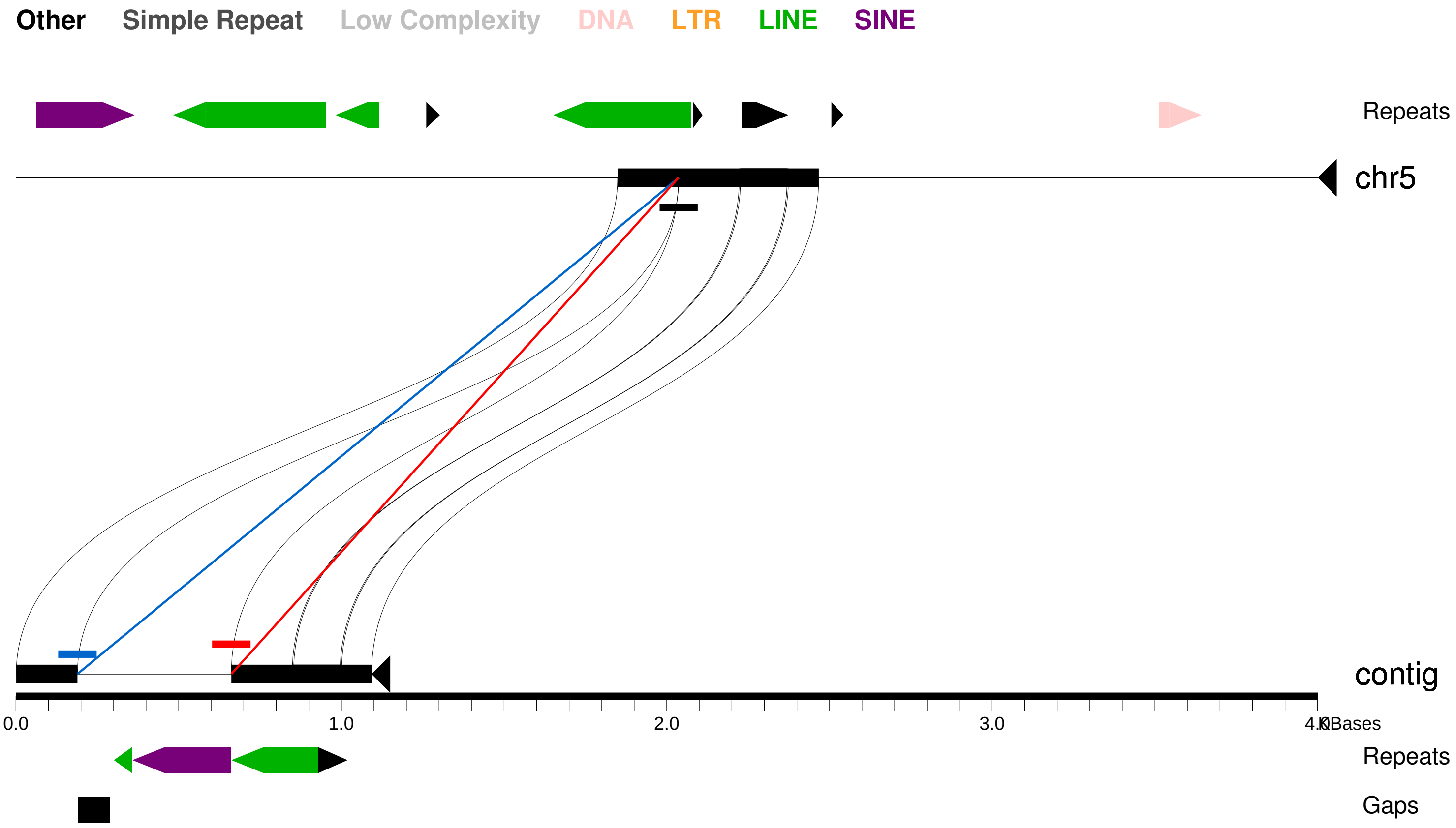

siteID: chr5_54865782 Scafftig14 - Breakpoint: chr5:54865784
chr5:54865741-54865843
Overlap Length: 17 INS size: 300 Genotyped: Yes

```
left    AC----CTGCT---CAGCAGGGAAAATAAAAAGAGGCCCTG---------AGGGATGCTAAAAAATATTA
chrom   AC----CTGCT---CAGCAGGGAAAATAAAAAGAGGCCCTG---------AGGGATGCTAAAAAATATTA
right   GCGCCACTGCATTCCAGCCTGGGCGACAGAGCGAGACTCCGTCTCAAAAAAAAAAAAAAAAAAAAAATTA
        1*1111****1111****11**111*1*1*11***1*1*1*111111111*111*1111******1****

left    ACTTGCAGGCCGGGCACGGTGGCTCACGCCTGTAATCCCAGCACTTTGGGAGGCCGAGGCGGGTG
chrom   ACTTGC----CATTAAAAGTAATTATAA---AAAACGCAATTACTTTTGCA--CCAAC-------
right   ACTTGC----CATTAAAAGTAATTATAA---AAAACGCAATTACTTTTGCA--CCAAC-------
        ******2222*2222*22**222*222222222**22*2*22*****2*2*22**2*22222222
```

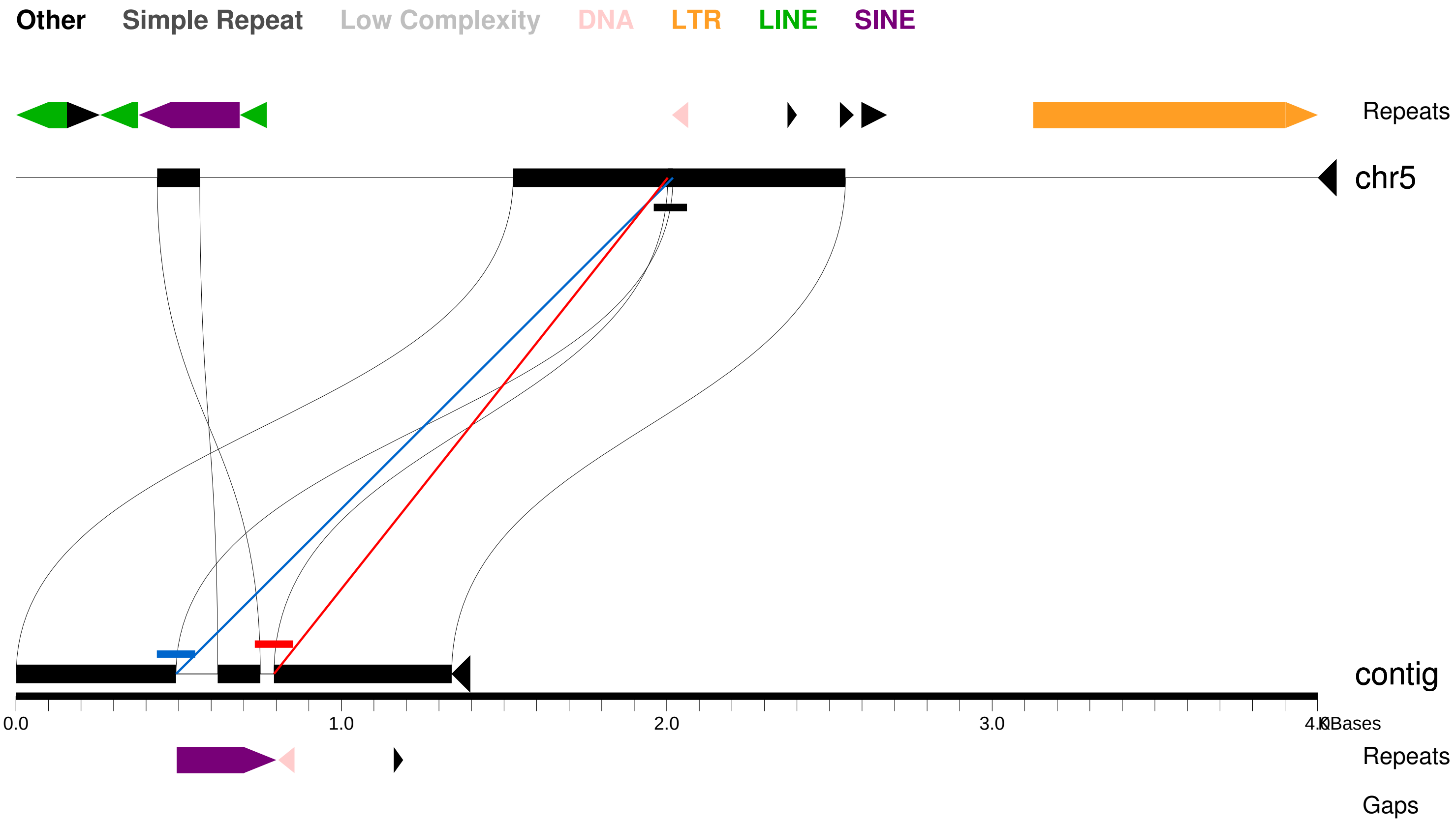

siteID: chr5_55689488 Scafftig4 - Breakpoint: chr5:55689489
chr5:55689440-55689528
Overlap Length: 8 INS size: 288 Genotyped: Yes

```
left   ATAAACAACTTTAGCA-------------AATTTTGGGATAC-----------------AAAATCAATGT
chrom  ATAAACAACTTTAGCA-------------AATTTTGGGATAC-----------------AAAATCAATGT
right  GCCACTGCAGTCCGCAGTCCGGCCTGGGCGACAGAGCGAGACTCCGTCTCAAAAAAAAAAAAAAAAAAAA
       111*111111*11***11111111111111*1111*1**1**1111111111111111****11**111

left   ACAAAAATCACTAGCATCCCAGCACTTTGGGAGGCCGAGGCGGGTGGATCATGAGGTCAAGAGATCGAGA
chrom  ACAAAAATCACTAGCATTCCTATAC---------ACCAG----------CAACA----ACCAAACTGAGA
right  AAAAAAAAAACTAGCATTCCTATAC---------ATCAG----------CAACA----ACCAAACTGAGA
       *1*****11********2**222**222222222212**2222222222**22*2222*22*2*22****

left   CCATCCTGG
chrom  GC-------
right  GC-------
       2*2222222
```

Other Simple Repeat Low Complexity DNA LTR LINE SINE

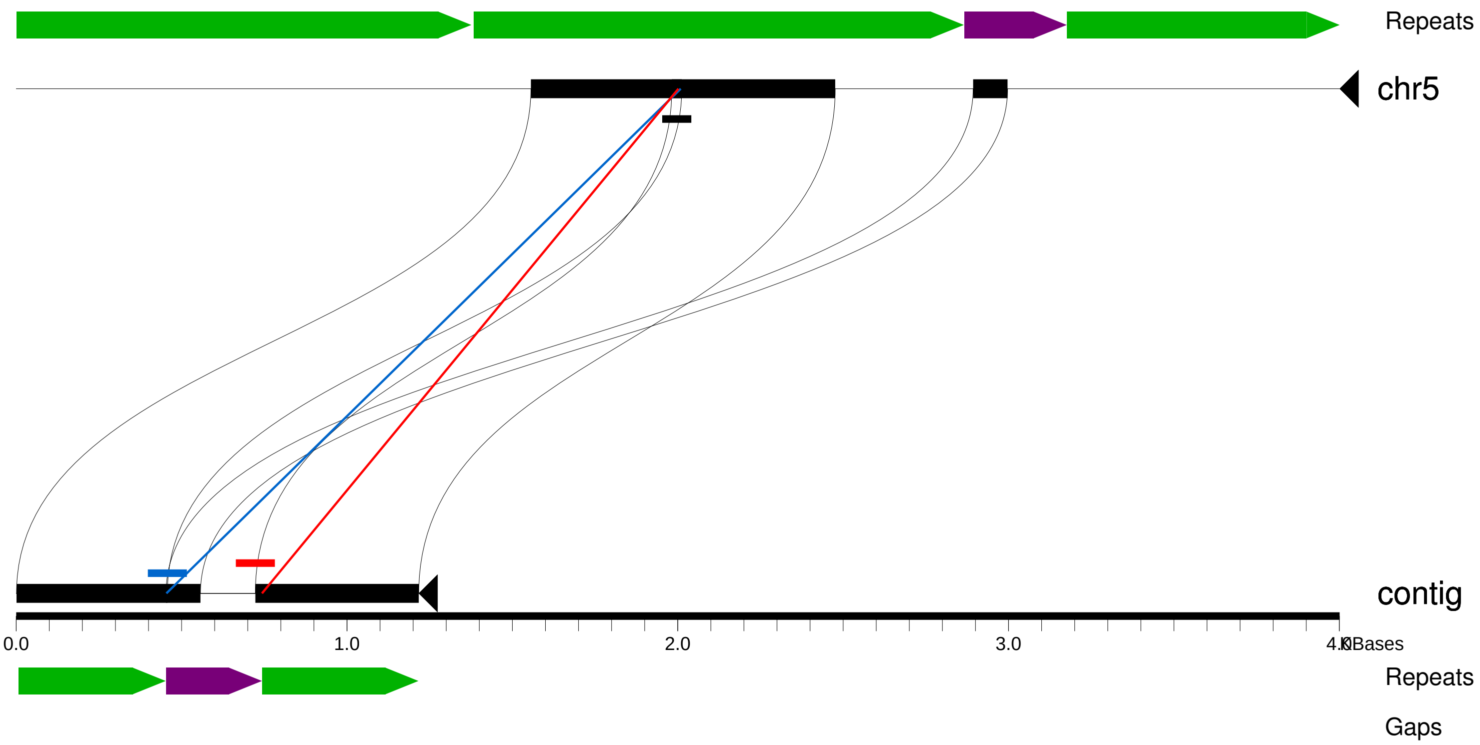

siteID: chr5_55927965 Scafftig6 - Breakpoint: chr5:55927967
chr5:55927921-55928026
Overlap Length: 14 INS size: 297 Genotyped: Yes

```
left    -------CTTATCAAAATATATTTGA---ATATATTTTCCAGGC----AGAAAGCCTTTTAAAAGATACA
chrom   -------CTTATCAAAATATATTTGA---ATATATTTTCCAGGC----AGAAAGCCTTTTAAAAGATACA
right   GCCACTGCACTCCAGAGTCTGGGCGACAGAGCGAGACTCC-GTCTCAAAAAAAAAAAAAAAAAAGATACA
        1111111*1111**1*1*1*1111**111*111*111***1*1*1111*1***1111111**********

left    GAGAGGCCGGGCGCGGTGGCTCACGCCTGTAATCCCAGCACTTTGGGAGGCCGAGGCGGGCGG
chrom   GAGACACAAAGCTT------------CTCTAAGACAAACTCTGAACCAGGGCAACA-GAGAAA
right   GAGACACAAAGCTT------------CTCTAAGACAAACTCTGAACCAGGGCAACA-GAGAAA
        ****22*222**22222222222222**2***22*2*2*2**22222***2*2*222*2*222
```

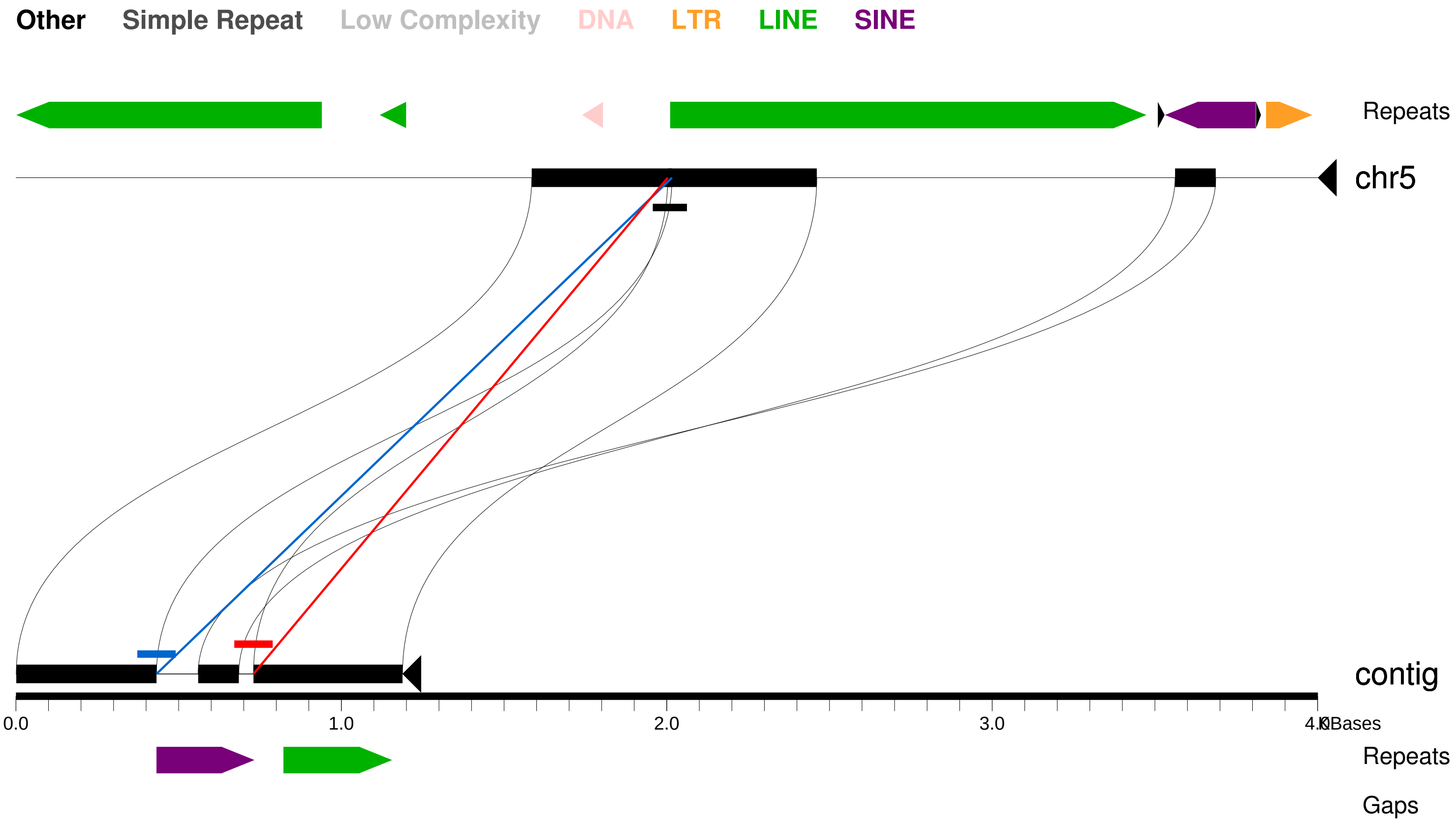

siteID: chr5_5595239 Scafftig3 + Breakpoint: chr5:5595241
chr5:5595200-5595300
Overlap Length: 8 INS size: 291 Genotyped: Yes

```
left   ------GACGTGATTATTTTTAGTCCT---ATTCTTTTCTGAATATA---------GAAGACTTTTTTTT
chrom  ------GACGTGATTATTTTTAGTCCT---ATTCTTTTCTGAATATA---------GAAGACTTTTTAAT
right  CCGCCCGCCTCGGCCTCCCAAAGTGCTGGGATTACAGGCGTGAGCCACCGCGCCCGGCCGACTTTTTAAT
       111111*1*11*111111111***1**111***11111*111*111*111111111*11*******22*

left   TTTTTTGAGACGGAGTCTCGCTCTGTCGCCCAGGCTGGAGTGCAGTGGCGCAATCTCGGCTCACTGC
chrom  ATTATAGA-----AAACACATTCT-TTGCC---TCTGTAATATAAAAACTGAAAATCA---------
right  ATTATAGA-----AAACACATTCT-TTGCC---TCTGTAATATAAAAACTGAAAATCA---------
       2**2*2**22222*22*2*22***2*2***2222***2*2*22*2222*22**22**2222222222
```

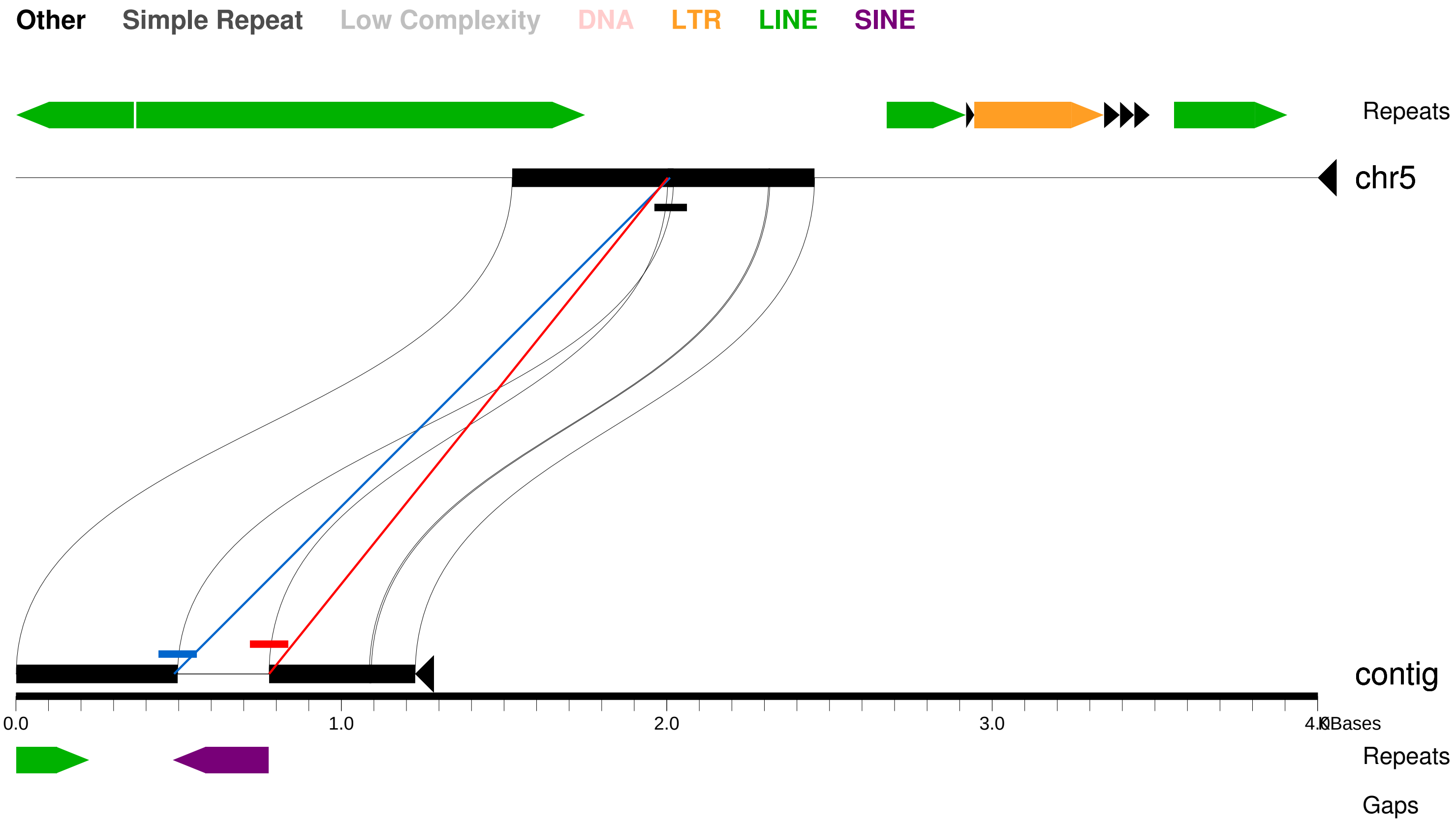

siteID: chr5_56109713 Scafftig1 + Breakpoint: chr5:56109710
chr5:56109664-56109769
Overlap Length: 14 INS size: 318 Genotyped: Yes

```
left     ATACATTTGACTGCCATTAAAATAGCATATAATTATAGCCTAAATG--------------TAGACTTAAT
chrom    ATACATTTGACTGCCATTAAAATAGCATATAATTATAGCCTAAATG--------------TAGACTTAAT
right    CCGCCCGCCTCGGCCTCCCAAAGTGC-TGGGATTACAGGCGTGAGCCACCGCGCCCGGCCTAGACTTAAT
         111*111111*1***1111***11**1*111****1**1*111*1111111111111111**********

left     TTTTTTTTTTTTTTTTTTTTTTTTTTTTTTTTTTTTTTTTGAGACGGAGTCTCGCTCTGTCGC
chrom    TTTTAACAGATTTATCTGAACTTTCAAAATTTGTACCGTTG----GGTGTAACG---------
right    TTTTAACAGATTTATCTGAACTTTCAAAATTTGTACCGTTG----GGTGTAACG---------
         ****222222***2*2*2222***22222***2*2222***2222**2**22**222222222
```

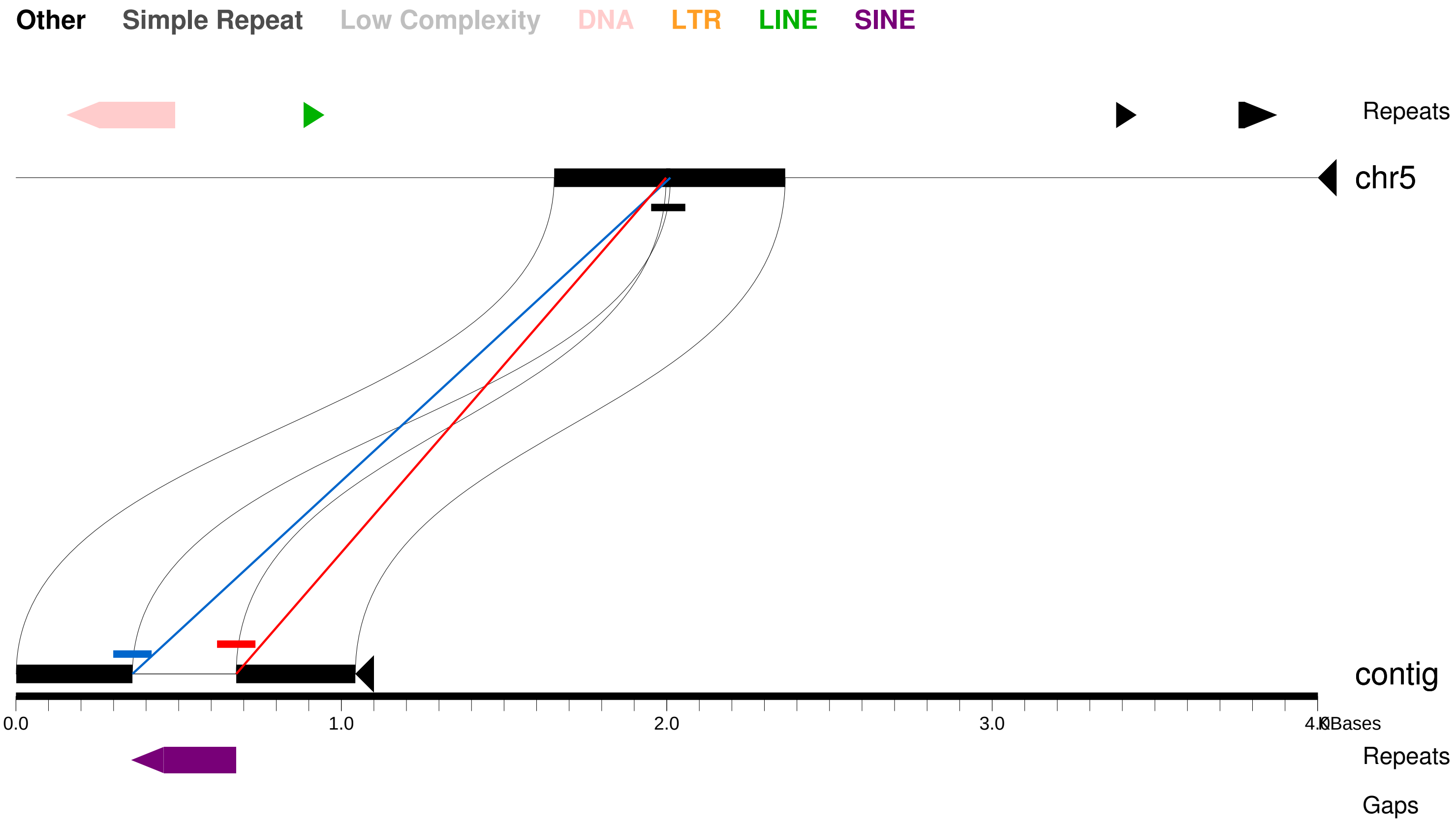

siteID: chr5_56830721 Scafftig1 + Breakpoint: chr5:56830722
chr5:56830675-56830781
Overlap Length: 13 INS size: 298 Genotyped: Yes

```
left    ATGCCCACCTTT--ATACCAA-----TACCAT-ACTATCTTGAAT-ACTGTAGCAT---TATAATCATTC
chrom   ATGCCCACCTTT--ATACCAA-----TACCAT-ACTATCTTGAAT-ACTGTAGCAT---TATAATCATTC
right   CCGCCCGCCTCGGCCTCCCAAAGTGCTGGGATTACAGGCGTGAGCCACCGCGCCCGGCCTATAATCATTC
        11****1***11111*1****11111*111**1**111*1***111**1*111*11111***********

left    TTTTTTTTTTTTTTTTTTTGAGACGGAGTCTCGCTCTGTCGCCCAGGCTGGAGTGCAGTGG
chrom   TTGAAACAAGTAGTATAT-GTCTCAAATT-TCATTCTTTATCAAA------ATT--ATT--
right   TTGAAACAAGTAGTATAT-GTCTCAAATT-TCATTCTTTATCAAA------ATT--ATT--
        **22222222*22*2*2*2*222*22*2*2**22***2*22*22*222222*2*22*2*22
```

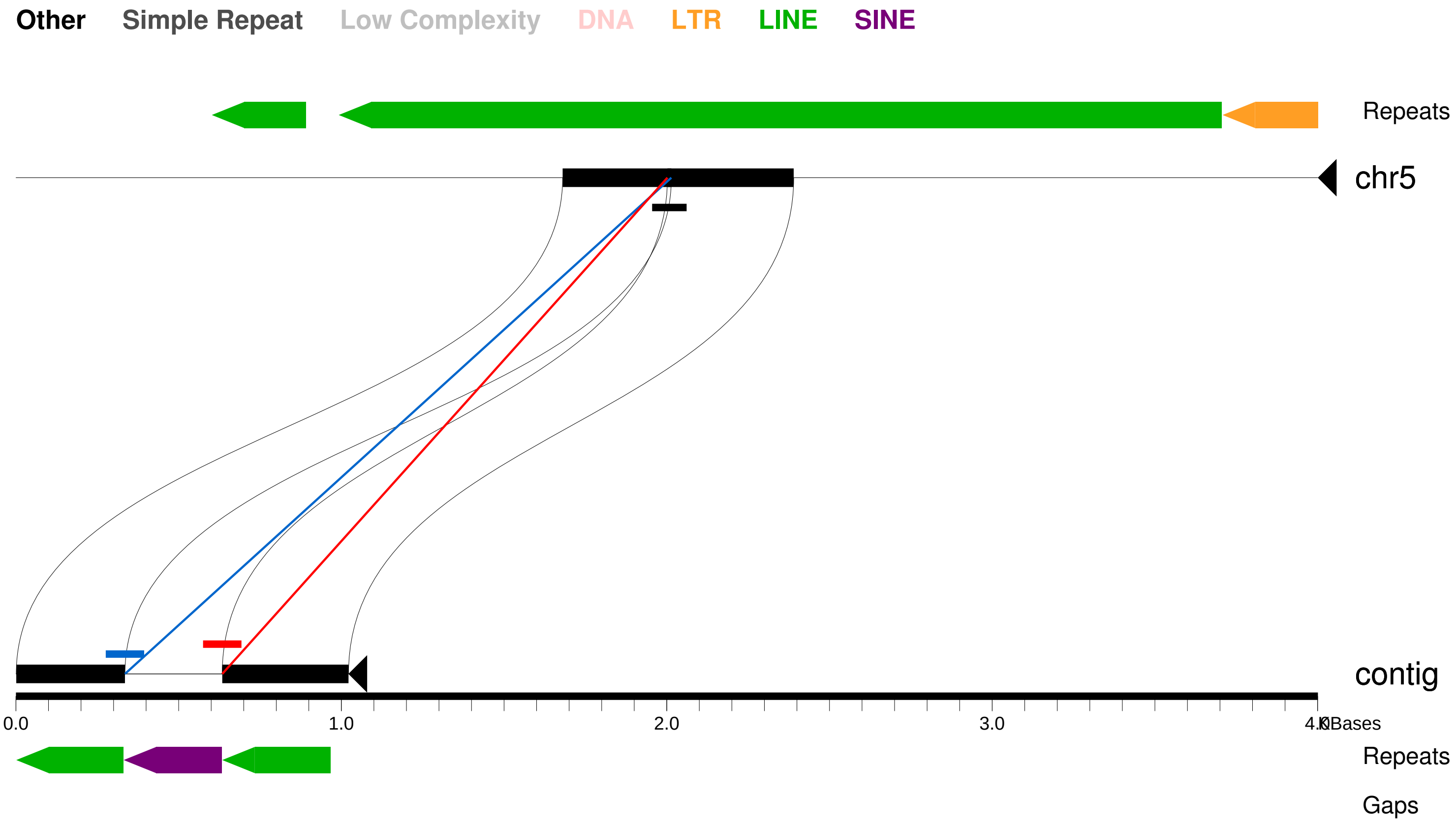

siteID: chr5_57392462 Scafftig1 - Breakpoint: chr5:57392443
chr5:57392397-57392502
Overlap Length: 14 INS size: 303 Genotyped: Yes

```
left     CAA----CCACAGGTTGCACAAGTTTT---ATTTC---CATTATTCACTGAAATTT---GGCTCTAAGTT
chrom    CAA----CCACAGGTTGCACAAGTTTT---ATTTC---CATTATTCACTGAAATTT---GGCTCTAAGTT
right    CCGCCCGCCTCGGCCTCCCAAAGTGCTGGGATTACAGGCGTGAGCCACCGCGCCCGGCCGGCTCTAAGTT
         *111111**1*1*11*1*11****11*111***1*111*1*1*11***1*111111111***********

left     TTTTTTTTTTTTTTTTTTTTTTTTTTGAGACGGAGTCTCGCTCTGTCGCCCAGGCTGGAGTGC
chrom    TTTATCATGACCCCCTTAAAT------------GACTTGCCATGAATACTTAACCATAGTT-
right    TTTATCATGACCCCCTTAAAT------------GACTTGCCATGAATACTTAACCATAGTT-
         ***2*22*2222222**222*222222222222*2**2**22**2222*2222*222***22
```

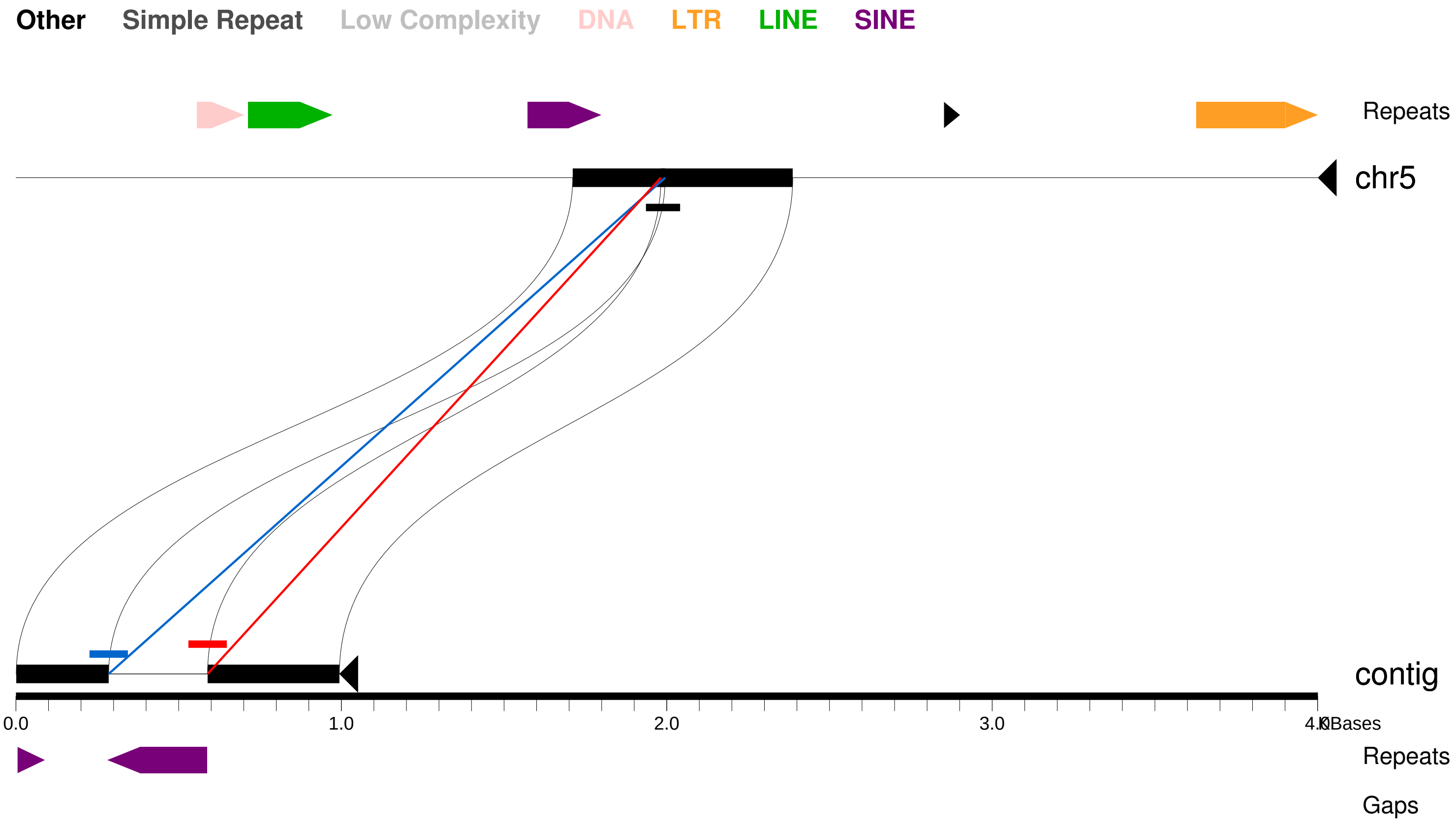

siteID: chr5_58516629 Scafftig1 + Breakpoint: chr5:58516630
chr5:58516580-58516689
Overlap Length: 10 INS size: 451 Genotyped: No

```
left    -------GTAGCCCTAACCTTAGATGCTGGTATCAAAA---TGATCAGTTGTGAATTTTC-CAAAGACAT
chrom   -------GTAGCCCTAACCTTAGATGCTGGTATCAAAA---TGATCAGTTGTGAATTTTC-CAAAGACAT
right   CGCCCGCCTCGGCCT--CCCAAAGTGCTGGGATTACAGGCGTGAGCCACCGCGCCCGGCCGCAAAGACAT
        11111111*1*1***11**11*11******1**1*1*1111***1*1111*1*111111*1*********

left    TNNNNNNNNNNNNNNNNNNNNNNNNNNNNNNNNNNNNNNNNNNNNNNNNNNNNNNNNNNN
chrom   TCTTTAGAAAACACTTGGATGTGGTGAATTCTCTTTTGACTCACAGCCAAT---------
right   TCTTTAGAAAACACTTGGATGTGGTGAATTCTCTTTTGACTCACAGCCAAT---------
        *222222222222222222222222222222222222222222222222222222222222
```

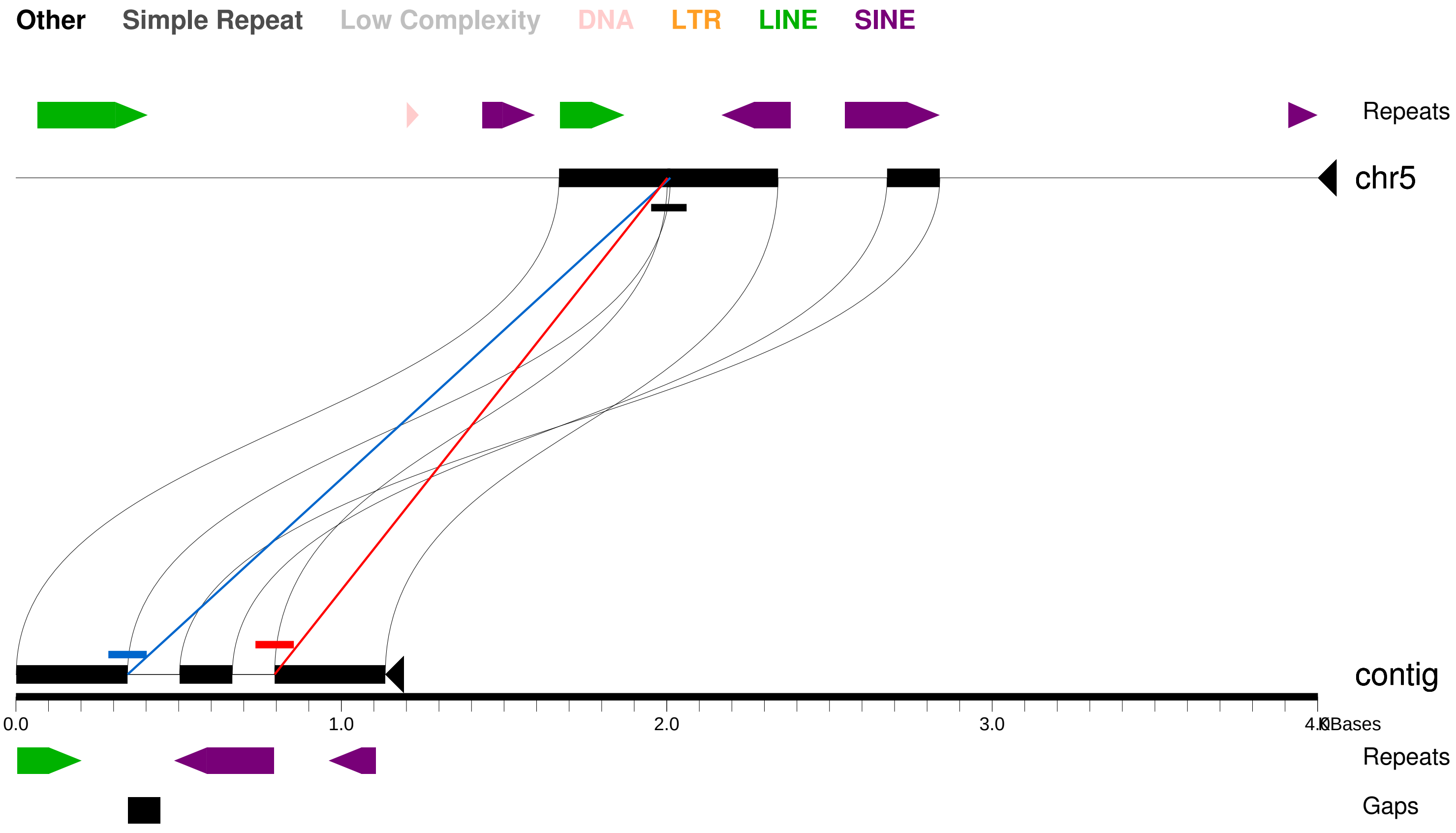

siteID: chr5_58609362 Scafftig1 + Breakpoint: chr5:58609360
chr5:58609317-58609418
Overlap Length: 17 INS size: 173 Genotyped: Yes

```
left    GAGAT------------GCCCCACACTTATTTGG----ACAAGACCCAGCAAAGAAATC-TAACCAAGCT
chrom   GAGAT------------GCCCCACACTTATTTGG----ACAAGACCCAGCAAAGAAATC-TAACCAAGCT
right   GCCATTCTCCTGCCTCAGCCTCCCAAGTAGCTGGGACTACAGGCGCCCGCCACCACGCCCTAACCAAGCT
        *11**111111111111***1*1**11**11***1111***1*11**1**1*11*111*1**********

left    ATATCTTTTTTTTTTTTTTTTTTTTTTTTTTTTTTTTTTTTGAGACGGAGTCTCGCTCTGTCGCCCAGGC
chrom   ATATCTTGAGTGGGGACAACTTCTGCTGTATGT-----AGATGG---CTTACTCAT---------C
right   ATATCTTGAGTGGGGACAACTTCTGCTGTATGT-----AGATGG---CTTACTCAT---------C
        *******222*222222222**2*22*2*2*2*22222***2**222**22***22222222222*
```

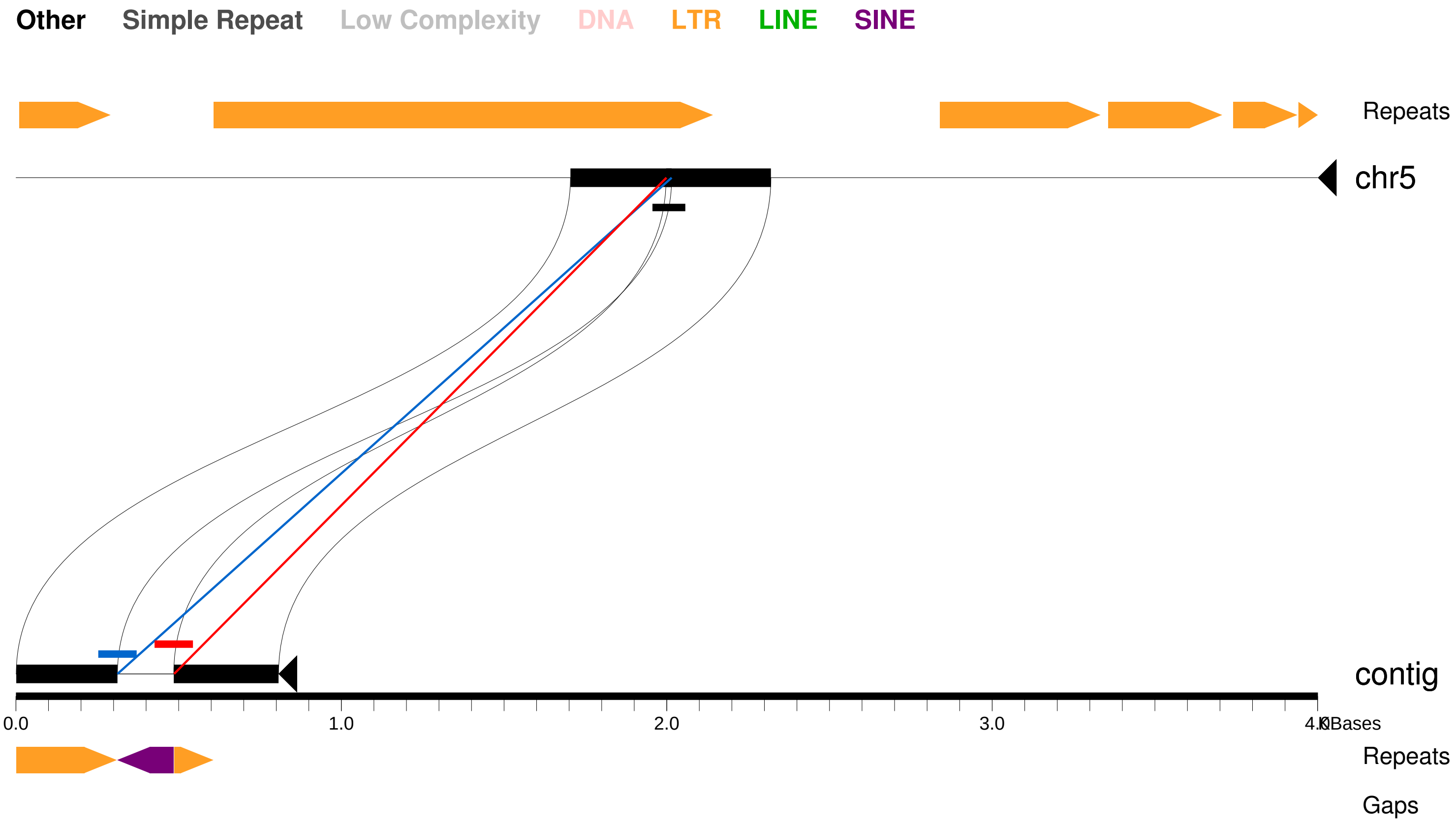

siteID: chr5_59580677 Scafftig2 - Breakpoint: chr5:59580681
chr5:59580521-59580856
Overlap Length: 16 INS size: 283 Genotyped: Yes

```
left    ----------------AATAGAGAACAGCTAGAGGCTTGTGATTTATTCTCATGTTAGAAGCCCTCTCAT
chrom   TTCATCCCCACTTTAAAATAGAGAACAGCTAGAGGCTTGTGATTTATTCTCATGTTAGAAGCCCTCTCAT
right   GCG----------TGAACCCGGGAAGCG---GAG-CTTG-----------CA-GTGAGCCGAGATTGC--
        NNN          2N2*111*1***11*111***1****11111111111**1**1**11*111*11*11

left    ATGACATATTCAGGATAGGTAGTT-GGCCAGTTTGTTAAATGAATATATTAAAGTCAAGAATCTTCAGAA
chrom   ATGACATATTCAGGATAGGTAGTT-GGCCAGTTTGTTAAATGAATATATTAAAGTCAAGAATCTTCAGAA
right   --GCCAC-TGCAG--TCCGCAGTCCGGCCTGGGCGACAGAGCGAGAC-TCCGTCTCAAAAA-----AAAA
        11*1**11*1***11*11*1***11****1*111*11*1*111*1*11*11111****1**11111*1**

left    ATAATACCTCAAGTATTCTTTATAAATGGCAACACTT----GCAC---------------TTTG--GGAG
chrom   ATAATACCTCAAGTATTCTTTATAAATGGCAACACTTAAATGCACATAGTTGGCTACTCTTTTGATGTAG
right   AAAATAAATAAATAAATAA--ATAAATGGCAACACTTAAATGCACATAGTTGGCTACTCTTTTGATGTAG
        *1****11*1**11*1*1111***************2222****222222222222222****22*2**

left    -GCCGAGGCGGGTG----GATCATGAGGTCAGGAGATCGAGACCATCC-TGGCTAACAAGGTGAA-ACCC
chrom   AGCCAAGGCCTTTGCTTTGAGTTTGAATCTCTTAGTTAGATTTCTACAATGTCTTACATCAAGAACATCC
right   AGCCAAGGCCTTTGCTTTGAGTTTGAATCTCTTAGTTAGATTTCTACAATGTCTTACATCAAGAACATCC
        2***2****222**2222**222***2222222**2*2**222*22*22**2**2***2222***2*2**

left    ------CGTC-TCTA---CTAAAAATACAAAAAATTAGCCGGGC-GTGGTGGCGGGC
chrom   ATTATGCATCATCTATGGCTTACAGTGTTTCTTTGTAATTGAGAAGTTAAGGCACTT
right   ATTATGCATCATCTATGGCTTACAGTGTTTCTTTGTAATTGA---------------
        222222*2**2****222**2*2*2*222222222**222*21N 11NNN111NNNN
```

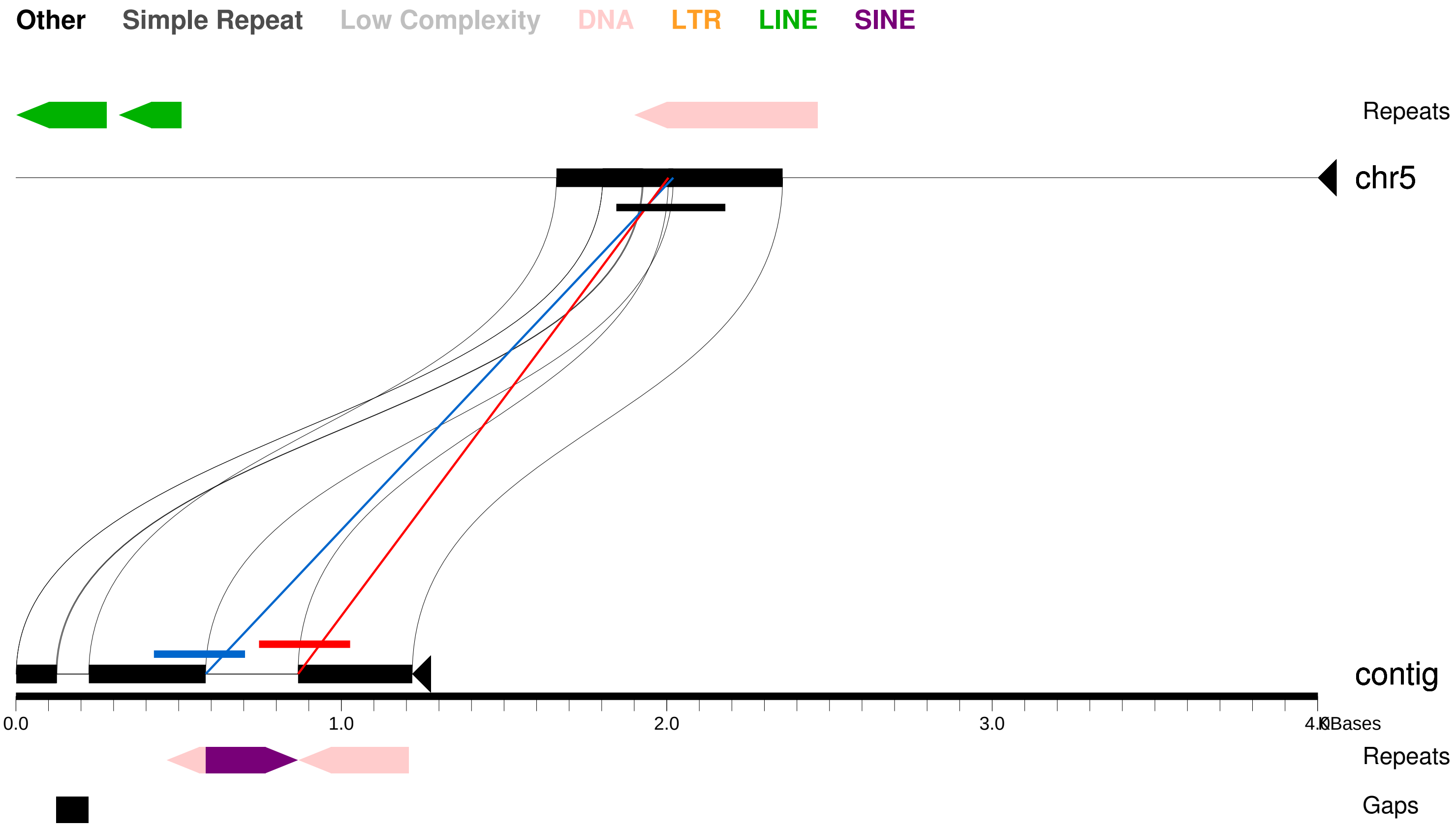

siteID: chr5_61857113 Scafftig12 - Breakpoint: chr5:61857103
chr5:61857059-61857162
Overlap Length: 16 INS size: 309 Genotyped: Yes

```
left    CTCTCTCT--TCTG-----------------CCATCTGATAATAGGTGAGGAAATGTTTTCTTAAAATTT
chrom   CTCTCTCT--TCTG-----------------CCATCTGATAATAGGTGAGGAAATGTTTTCTTAAAATTT
right   CGCAGTCCGGCCTGGGCGACAGAGCGAGACTCCGTCTCAAAAAAAAAAAAAAAAAAAAA----AAAATTT
        *1*11**1111***11111111111111111**1***1*1**1*1111*11***111111111*******

left    GTTTCTTTCGGCCGGGCGCGGTGGCTCACGCCTGTAATCCCAGCACTTTGGGAGGCCGAGGCGGGTGG
chrom   GTTTCTTTCAA-----------GCATCAAAA-TGTAAACAC--TATTTTTGGCCATC-AGTATTGCTG
right   GTTTCTTTCAA-----------GCATCAAAA-TGTAAACAC--TATTTTTGGCCATC-AGTATTGCTG
        *********2222222222222*22***2222*****2*2*222*2***2**2222*2**2222*22*
```

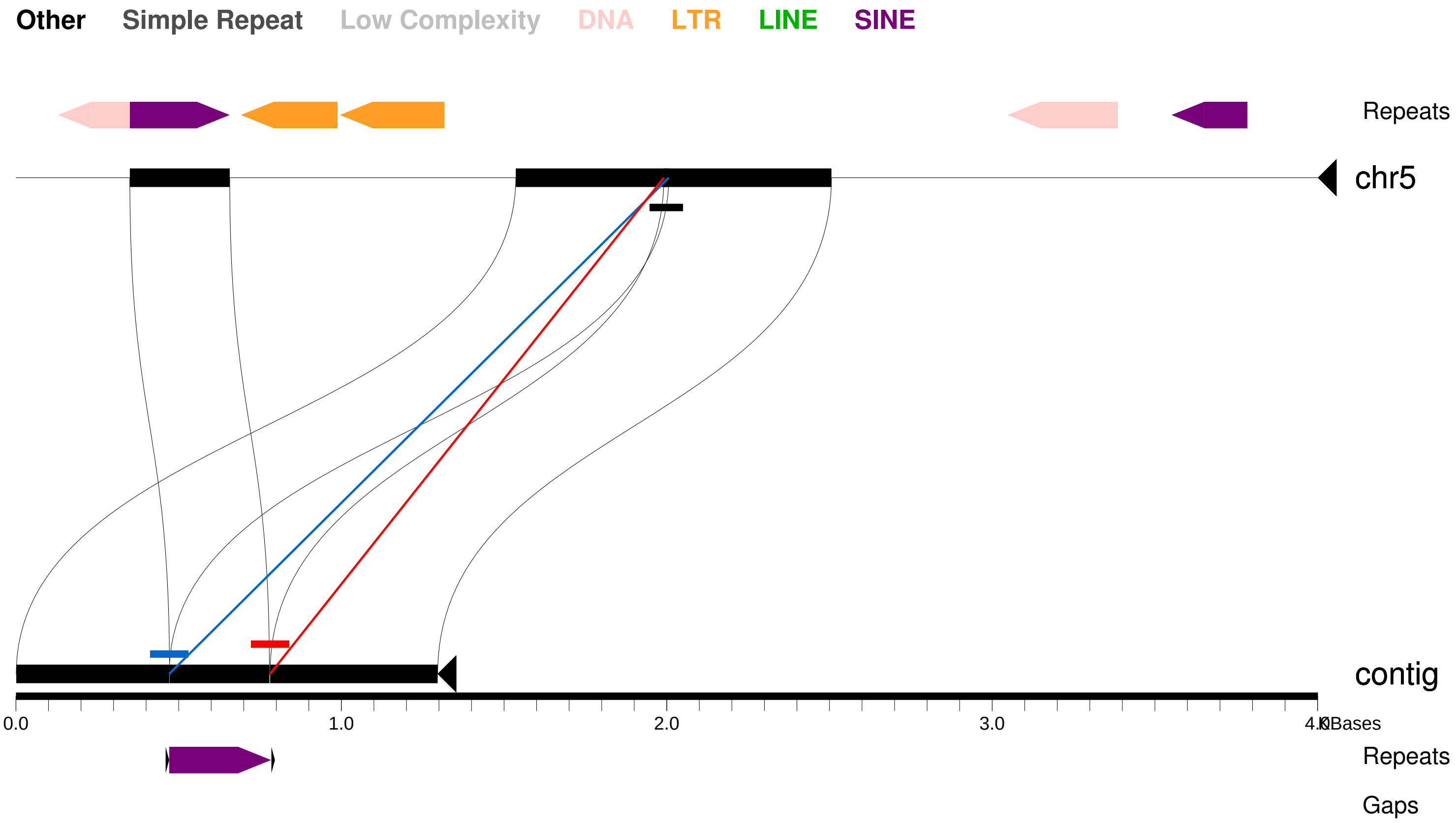

siteID: chr5_6218106 Scafftig7 + Breakpoint: chr5:6218088
chr5:6217928-6218258
Overlap Length: 11 INS size: 301 Genotyped: Yes

```
left   ------GTGGGGCTGGCCAGGTGCCATGTGATGCACACACTGAAGCAGAACAGAGGACACAATGACTCTG
chrom  ATAGCAGTGGGGCTGGCCAGGTGCCATGTGATGCACACACTGAAGCAGAACAGAGGACACAATGACTCTG
right  TTT--AGTAGAGACGG---GGT---------TTCACCTTGTTAGCCAGGAT---GGTCTCGATCTC-CTG
       N2N  2**1*1*11**111***111111111*1***1111*1*11***1*1111**1*1*1**11*1***

left   AAACAGGGAGAGATGTTCCCATACTAGGCGATGGGTCCAGGAGAGGTTGTGAGGATGCTGCCTTTTGGTA
chrom  AAACAGGGAGAGATGTTCCCATACTAGGCGATGGGTCCAGGAGAGGTTGTGAGGATGCTGCCTTTTGGTA
right  ACCTC-------ATGATCC---ACCCGCCTCGGCCTCCCAAAGTGCTGG---GATTACAGGC-----GTG
       *11111111111***1***111**11*1*111*11***111**1*1*1*111*11*1*1*1*11111**1

left   AGGACGTGTCTCAGGCCCATAGCCCATTCTTTTTTTTTTTTTTTGAGACGGA------GTCTCGCTCTGT
chrom  AGGACGTGTCTCAGGCCCATAGCCCATTCTTATGTGTCTGAGCAGGGCTGGAAAGCCTGTGTCCCTGAGG
right  AGCCACCGCGCCCGGCC---AGCCCATTCTTATGTGTCTGAGCAGGGGTGGAAAGCCTGTGTCCCTGAGG
       **11111*111*1****111**********2*2*2*2*22222*2*N2***222222**2**2**22*2

left   CGCCCAGGCC-----GGACTGCGGACTG-----CAGTGG---CGCAATCTCGGCTC----ACTGCA----
chrom  CTGCCCTTCCTAATAGGGCTGTTCACTTTGACCCAGTGCAACCCCACCCTGCCCTCTAGAACTGCACACA
right  CTGCCCTTCCTAATAGGGCTGTTCACTTTGACCCAGTGCAACCCCACCCTGCCCTCTAGAACTGCACACA
       *22**222**22222**2***222***222222*****2222*2**22**222***2222*****2222

left   --------------AG--CTCCGCTTCCTGGGTTCACGCCATTCTCCTGC-
chrom  TGGTGGCCCTATATAGGACTCTTATAGCAGAGGACGTGCCATTGGTCTGGT
right  TGGTGGCCCTATATAGGACTCTTATAGCAGAGGACGTGCC-----------
       22222222222222**22***222*22*2*2*22*22***111NNN111N
```

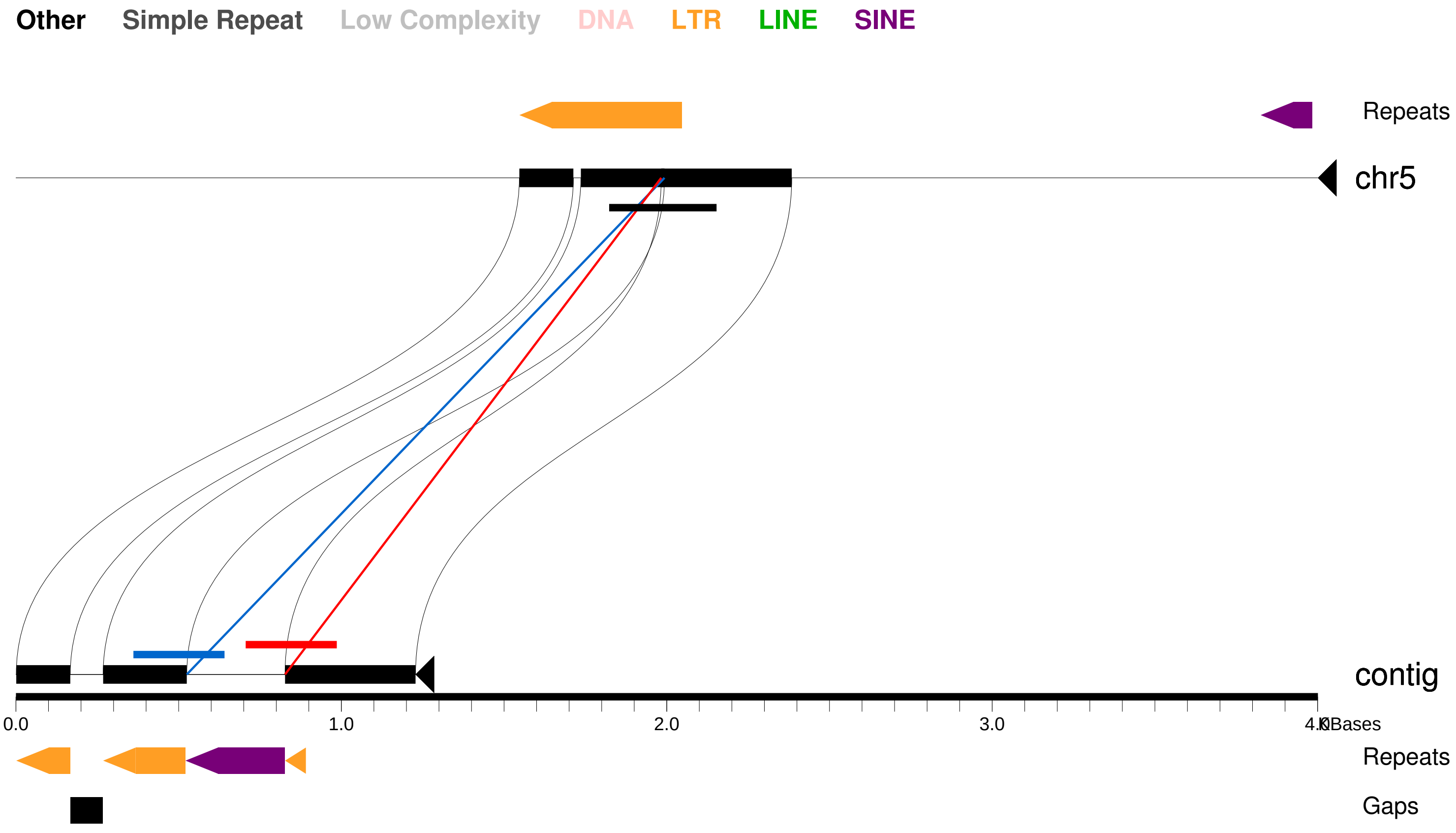

siteID: chr5_6284525 Scafftig2 + Breakpoint: chr5:6284520
chr5:6284476-6284579
Overlap Length: 16 INS size: 489 Genotyped: No

```
left     TAACTAT------------AACTTAATATTAAAACAGA----AGACAAGGTTGAAATTATGTATTCAAAC
chrom    TAACTAT------------AACTTAATATTAAAACAGA----AGACAAGGTTGAAATTATGTATTCAAAC
right    CCACCCGCCTCGGCCTCCCAAAGTGCTGGGATTACAGGCGTGAGCCACCGC-GCCCGGCCGTATTCAAAC
         11**1111111111111111**11*11*111*11****11111**1**11*11*1111111**********

left     GTTTTTTTTTTNNNNNNNNNNNNNNNNNNNNNNNNNNNNNNNNNNNNNNNNNNNNNNNNNNNNNNNNN
chrom    GTTTTTT---------------AAGGAAACAAACAGAAAACCACTTAACCTTTCTGAAGTTTGGTT
right    GTTTTTT---------------AAGGAAACAAACAGAAAACCACTTAACCTTTCTGAAGTTTGGTT
         ******2222222222222222222222222222222222222222222222222222222222222222
```

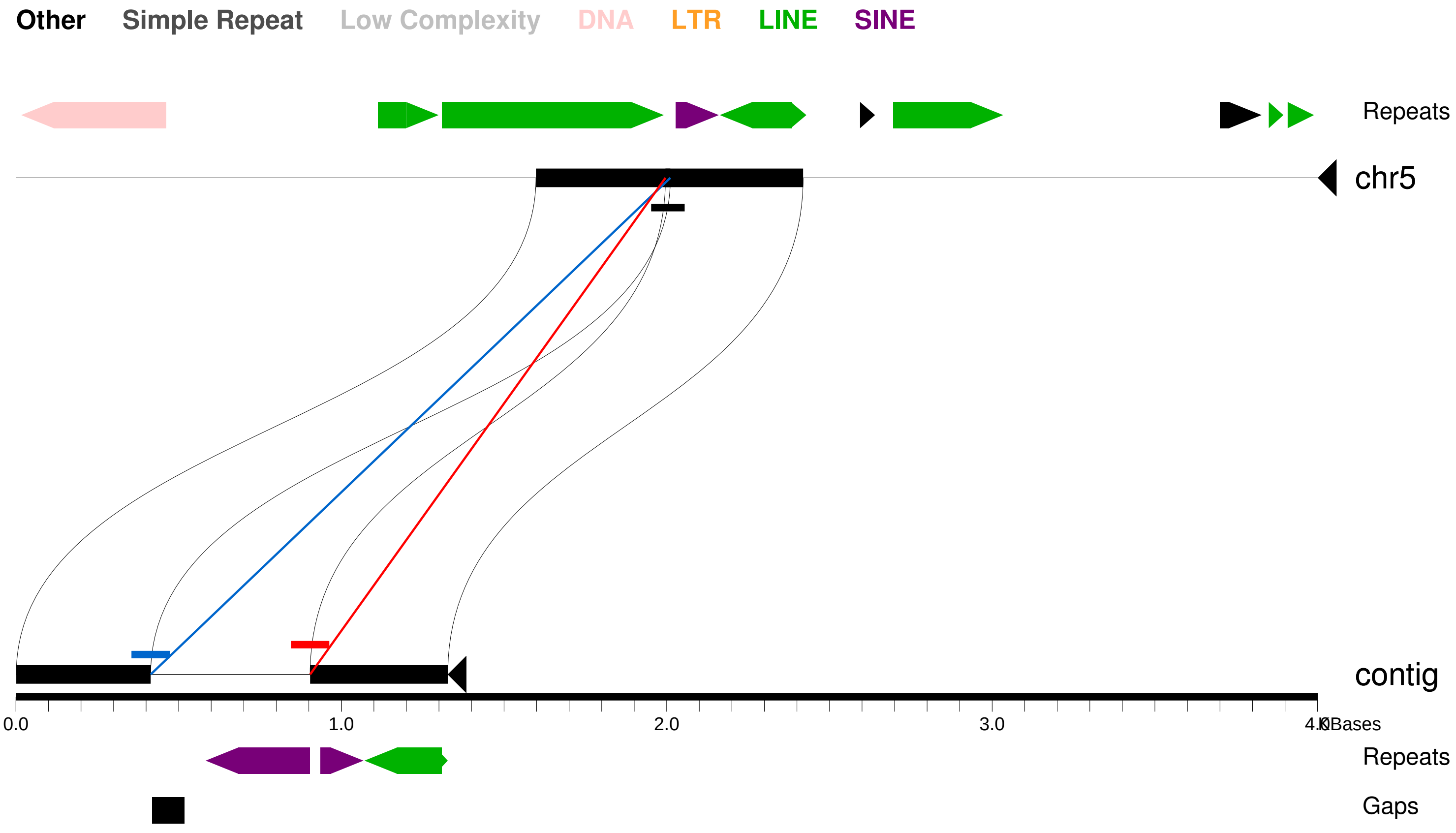

siteID: chr5_63658732 Scafftig2 + Breakpoint: chr5:63658736
chr5:63658692-63658795
Overlap Length: 16 INS size: 292 Genotyped: Yes

```
left     ---------CTTGGCCATATGTATGTCTTCT--TTTCAAGAATGT-----CTGTTCATGTCCTTTGCCCC
chrom    ---------CTTGGCCATATGTATGTCTTCT--TTTCAAGAATGT-----CTGTTCATGTCCTTTGCCCA
right    TCCGCCCGCCTCGGCC-TCCCAAAGTGCTGGGATTACAGGCGTGAGCCACCGCGCCCGGCCCTTTGCCCA
         111111111**1****1*1111*1**11*1111**1**1*11**111111*1111*11*1*********2

left     CTTTTTTTTTTTTTTTTTTTGAGACGGAGTCTCGCTCTGTCGCCCAGGCTGGAGTGCAGTGGCGCAA
chrom    CTTTTTT--------------AAAGGGGTTATTCT-TTTCTTGTAAATTTAAGTGCCTTATAGAGG
right    CTTTTTT--------------AAAGGGGTTATTCT-TTTCTTGTAAATTTAAGTGCCTTATAGAGG
         ******222222222222222*2**2**2222**2*2**2222*222*22*****22*222*222
```

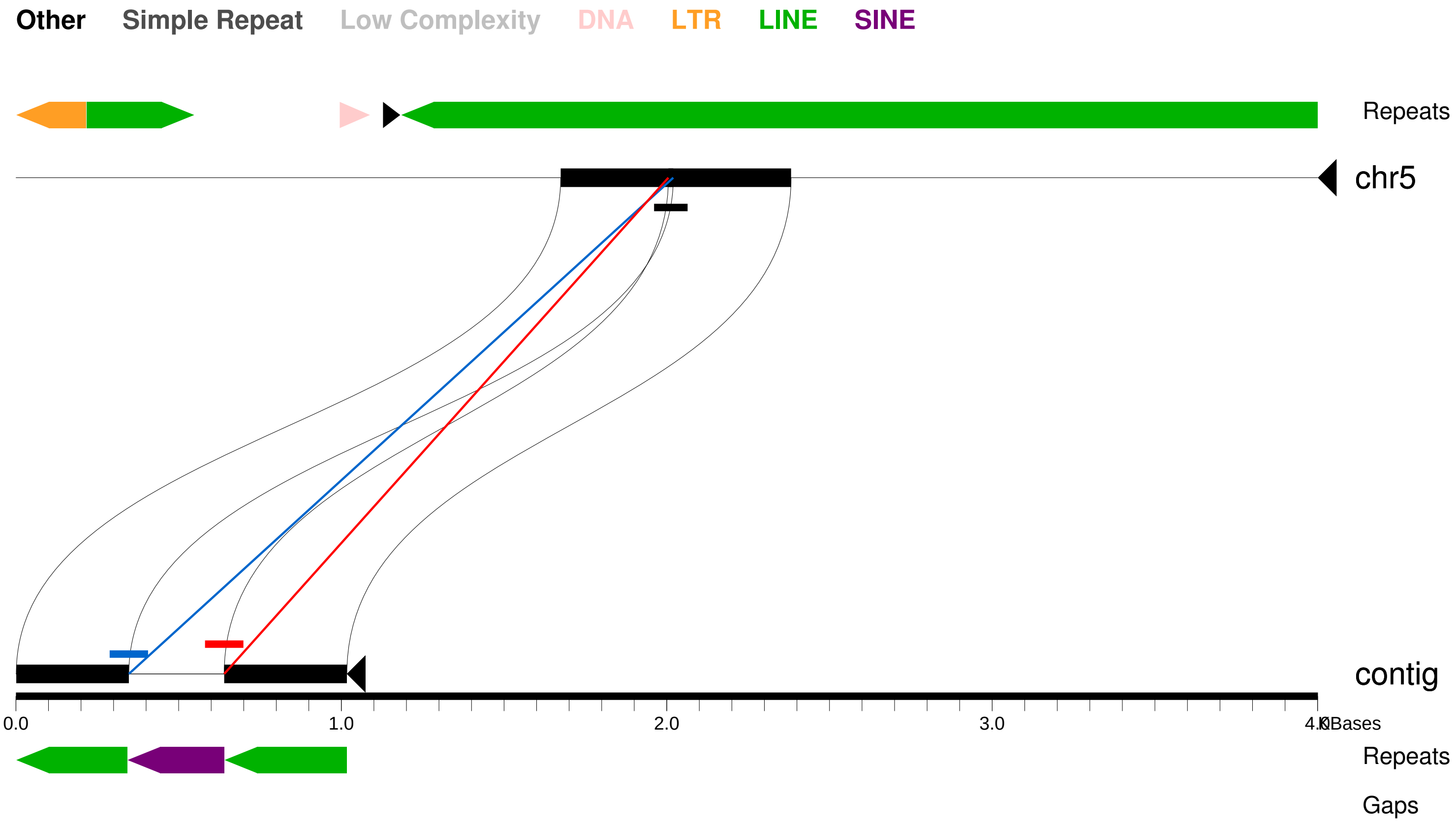

siteID: chr5_64518805 Scafftig2 + Breakpoint: chr5:64518814
chr5:64518655-64518982
Overlap Length: 9 INS size: 403 Genotyped: No

```
left    A-----AAGCACAAACCATTAAAAAATTAATAAATTGAATTTCATCAAAATTAAAAGGCACTTCAAAAAA
chrom   ACATGAAAGCACAAACCATTAAAAAATTAATAAATTGAATTTCATCAAAATTAAAAGGCACTTCAAAAAA
right   NNNNNNNNNNN---------AAAAAA--------------------------------------------
        1NNNNN11111111111111******11111111111111111111111111111111111111111111

left    TGAAGACGCAAGCAGAAACTAGGAGAAAATATCCATAAAATATATCTGACAAAGACTTATATCCAGAATA
chrom   TGAAGACGCAAGCAGAAACTAGGAGAAAATATCCATAAAATATATCTGACAAAGACTTATATCCAGAATA
right   ----------------------------------------------------------------------
        1111111111111111111111111111111111111111111111111111111111111111111111

left    TATAAAGAACTCATAACTCAAAAAAAAGGCCGAGGCGGGCGGATCACGAGGTCAGGAGATCG---AGAC-C
chrom   TATAAAGAACTCATAACTCAAAAAAAAGGAAAAAAAAAATTTTA--AATGGGTAAAATATTTGTGTAGACAC
right   -------------------AAAAAAAAGGAAAAAAAAAATTTTA--AATGGGTAAAATATTTGTGTAGACAC
        1111111111111111111*********222*222222222*22*222***2*22222*2*222****2*

left    ATCCCGGCTAAAA---CGGT---GA-AACCCCGTCTCTACTAAAAATACAAAAAATTAGCCGGGC-GTAG
chrom   TTCACTACAAAAAAATCCATATGGATAAAATTAAAAAGACTGACAATACCAAGTATTGACAAGGATGTAG
right   TTCACTACAAAAAAATCCATATGGATAAAATTAAAAAGACTGACAATA----------------------
        2**2*22*2****222*22*222**2**2222222222***2*2****1N11NN111NN1NN11N 1111

left    TG--GCGGGCGC------------CTG-TAG-----------------TC
chrom   AGCAGCTGGAACTCTTCTATATTACTGATAGAAATATAAAATAATACATC
right   --------------------------------------------------
        N1  11N11NN1            111 111                 11
```

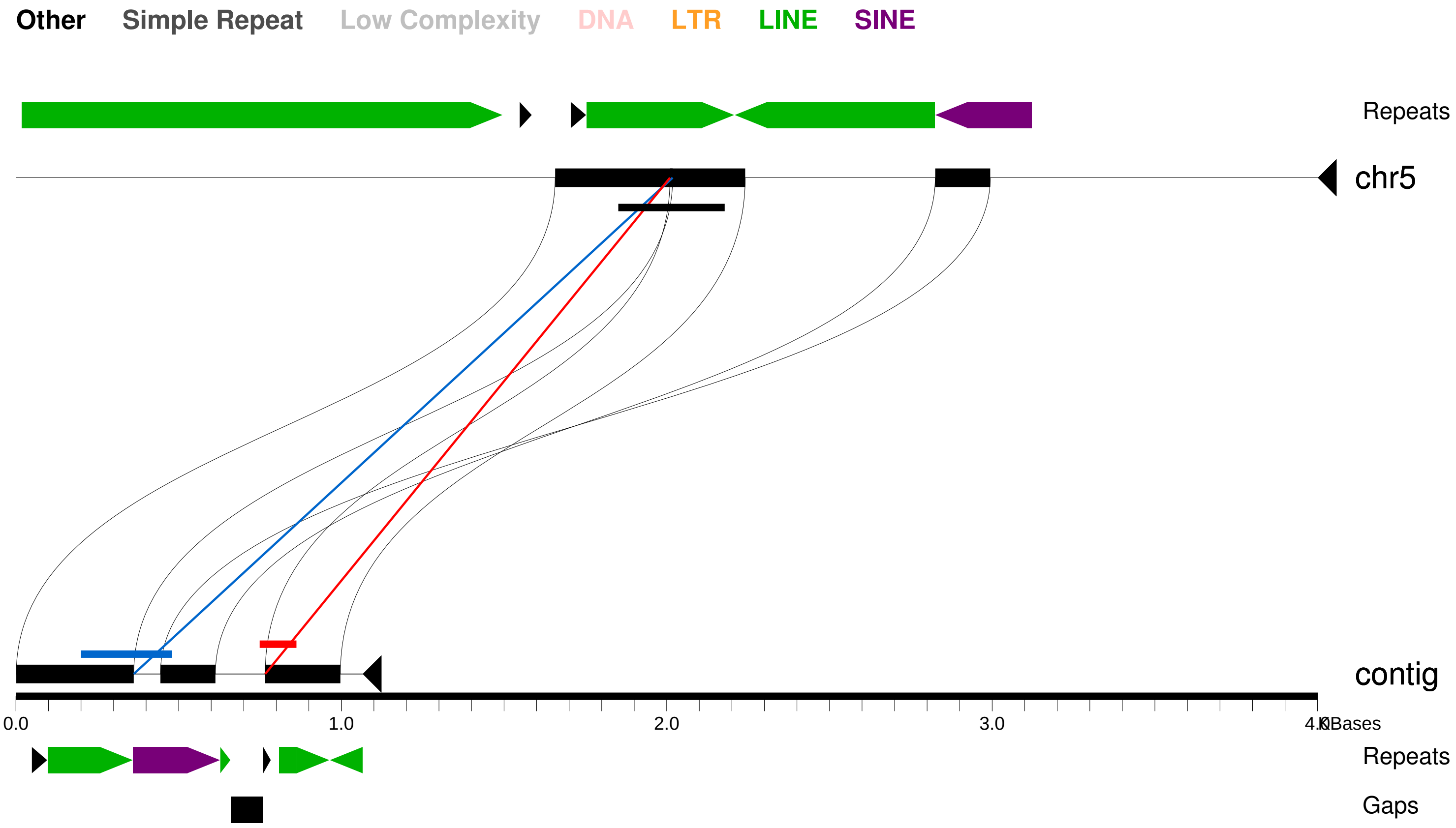

siteID: chr5_65569646 Scafftig1 - Breakpoint: chr5:65569580
chr5:65569432-65569789
Overlap Length: 58 INS size: 395 Genotyped: No

```
left     T---------------------------------------------------------------------
chrom    TGCTAATCATGTGTAAAAATTACTCCTGACCCACAATTTCATTATAGCACCTGAACCAGCCTGAAAATTG
right    NNNNNNNNNNNNNNNNNNNNNNNNNNNNNNNNNNNNNNNNNNNNNNNNNNNNNNNNNNN-----------
         1NNNNNNNNNNNNNNNNNNNNNNNNNNNNNNNNNNNNNNNNNNNNNNNNNNNNNNNNNN

left     -------------------------------AAAAAGAAGAATAAACCTTGAACATTAACAGCTACACAG
chrom    GAGAAATTAAATGGCATAAAATAGAAGCAATAAAAAGAAGAATAAACCTTGAACATTAACAGCTACACAG
right    ----------------------------------------------------------------------
                                        111111111111111111111111111111111111111

left     TGTTCTGGATGCTAAAGCACAAAGCATGTCAAACACACTTCAGTGGATTTTAAGAAATCCACCAGAGGCC
chrom    TGTTCTGGATGCTAAAGCACAAAGCATGTCAAACACACTTCAGTGGATTTTAAGAAATCCACCAGATGCC
right    --------ATGCTAAAGCACAAAGCATCTCAAACACACTTCAGTGGATTTTAAGAAATCCACCAGATGCC
         11111111*******************1**************************************2***

left     GGGCGCGGT------------GGCTCAC------GCCTGTAATCCCAGCAC-------------------
chrom    ACTTGTCCTTCAGCATCCTAGGGCTCTCTTCCTAGCCTCTTCTCTCTGTACCCACAAAAAAGGTGCCCGA
right    ACTTGTCCTTCAGCATCCTAGGGCTCTCTTCCTAGCCTCTTCTCTCTG----------------------
         2222*222*222222222222*****2*222222****2*22**2*2*N11

left     TTTGGG--AGGCCGAGG------------------------------------------CGGG-------
chrom    TTTGGATAATGACAGGGTGCTTTTTATCTTCCATAAACTACTACTTGCTTTTTCGCCTTCGGGGAGTGAC
right    ----------------------------------------------------------------------
         11111N  1N1N1NN11                                          1111

left     -CGGATCA
chrom    ACGTGGCT
right    --------
          11NNN1N
```

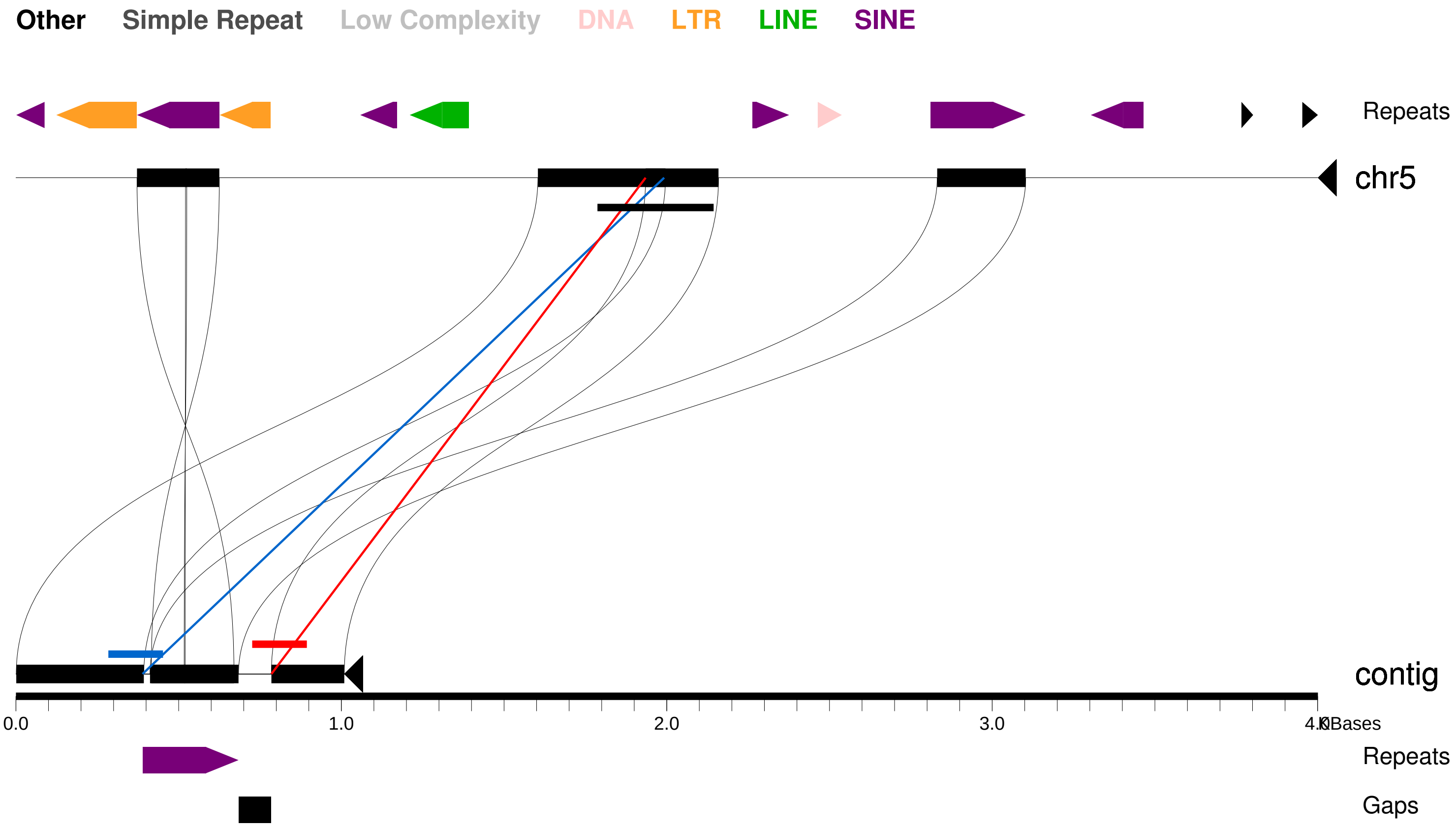

siteID: chr5_68191002 Scafftig3 - Breakpoint: chr5:68191050
chr5:68190998-68191109
Overlap Length: 8 INS size: 456 Genotyped: No

```
left    TTGACCTTTACATTTGTTCATGCAGAGTCACATCACACCCCTGTGAAATATG-------TAAGGCAGGCC
chrom   TTGACCTTTACATTTGTTCATGCAGAGTCACATCACACCCCTGTGAAATATG-------TAAGGCAGAT-
right   NNNNNNNNNNNNNNNNNNNNNNNNNNNNNNNNNNNNNNNNNNNNNNNNNNNNNNNNNNNTAAGGCAGAT-
        11111111111111111111111111111111111111111111111111111111111*********222

left    GGGCGCGGTGGCTCACGCCTGTAATCCCAGCACTTTGGGAGGCCGAGGCGGGCGGA-------
chrom   --------TTACT-AAGCCCATTTTAC----AGGTGGGGAAACTGACACCCAGAGATTTACTC
right   --------TTACT-AAGCCCATTTTAC----AGGTGGGGAAACTGACACCCAGAGATTTACTC
        22222222*22**2*2***22*22*2*2222*22*2****22*2**22*22222**2222222
```

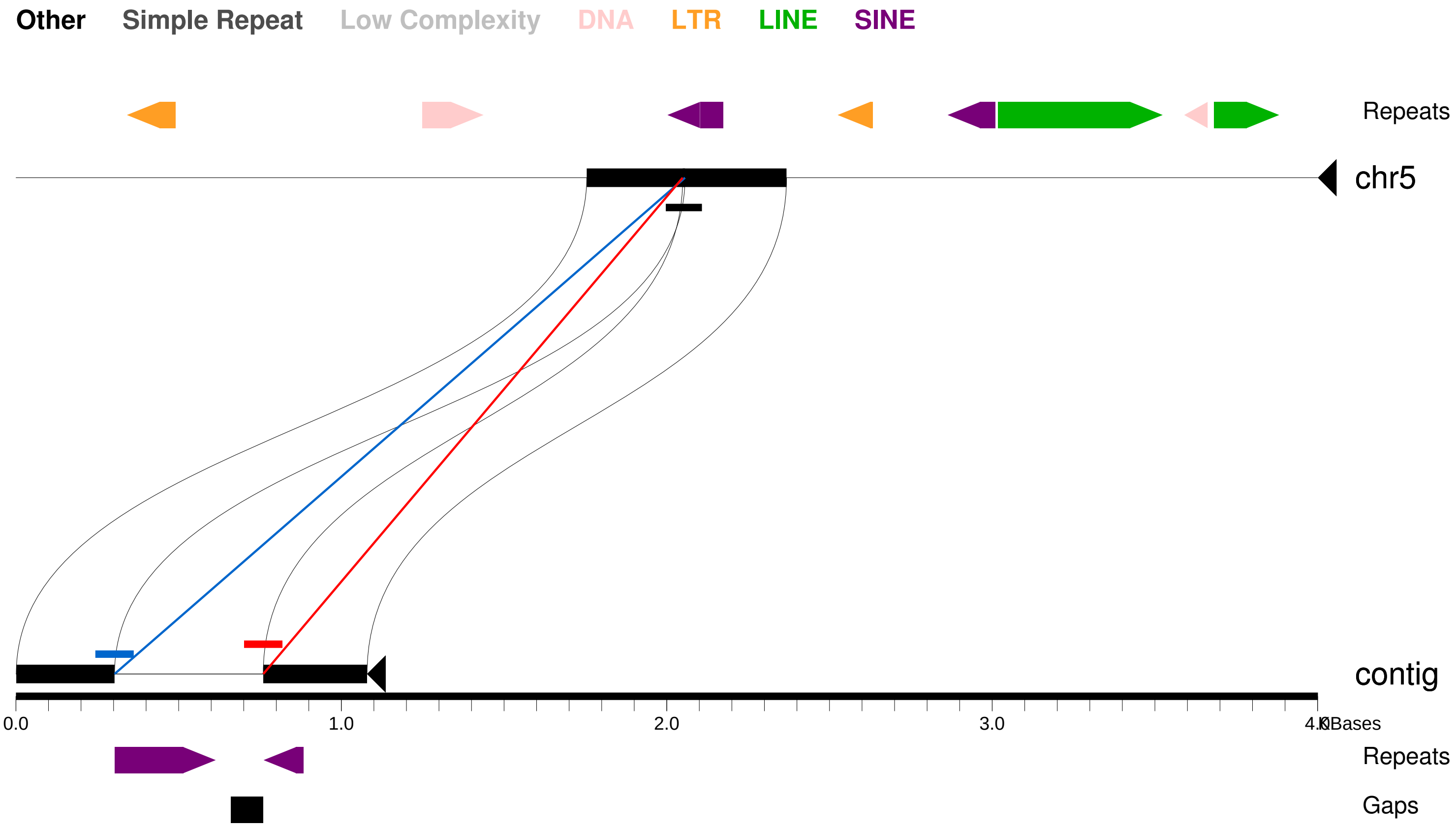

siteID: chr5_71412961 Scafftig1 - Breakpoint: chr5:71412949
chr5:71412904-71413008
Overlap Length: 15 INS size: 435 Genotyped: No

```
left     AG-CAAGAAATGGTTTATTAAAA-GTT--AACTTCCTGCAA-AGCCACAT---------GAAAAATCAGT
chrom    AG-CAAGAAATGGTTTATTAAAA-GTT--AACTTCCTGCAA-AGCCACAT---------GAAAAATCAGT
right    CCACCCGCCTCGGCCTCCCAAAGTGCTGGGATTACAGGCGTGAGCCACCGCGCCCGGCCGAAAAATCAGT
         111*11*1111**11*111***11*1*111*1*1*11**111******11111111111***********

left     TTTTTTTTTNNNNNNNNNNNNNNNNNNNNNNNNNNNNNNNNNNNNNNNNNNNNNNNNNNNNN
chrom    TTTTAAGT--------------AACTTTGCTTTGAGAAAAAAGGAATCCAGAGACTGTCAGAC
right    TTTTAAGT--------------AACTTTGCTTTGAGAAAAAAGGAATCCAGAGACTGTCAGAC
         ****222*2222222222222222222222222222222222222222222222222222222
```

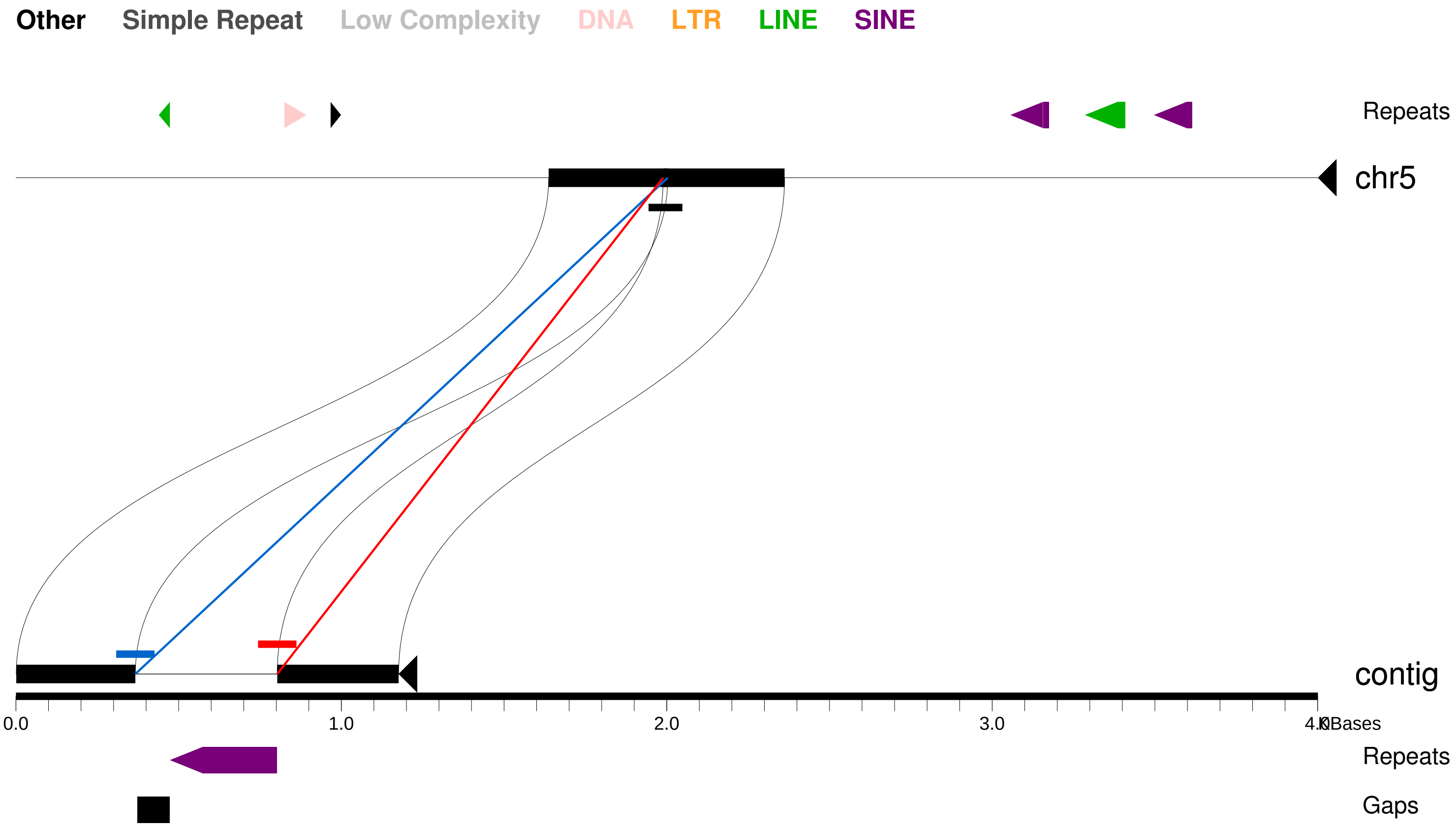

siteID: chr5_73145907 Scafftig1 + Breakpoint: chr5:73145911
chr5:73145863-73145970
Overlap Length: 12 INS size: 479 Genotyped: No

```
left    GGATTTATTTTATTCCCTCTCTTTTTTCTTATT-CTTGCTTATTAAATT----------GTTATTTTGCA
chrom   GGATTTATTTTATTCCCTCTCTTTTTTCTTATT-CTTGCTTATTAAATT----------GTTATTTTGCA
right   CCGCCCGCCTCGGCCTCCCAAAGTGCTGGGATTACAGGCGTGAGCCACCGCGCCCGGCCGTTATTTTGCA
        111111111*1111*1*1*1111*11*111***1*11**1*11111*111111111111***********

left    TNNNNNNNNNNNNNNNNNNNNNNNNNNNNNNNNNNNNNNNNNNNNNNNNNNNNNNNNNNN
chrom   TTTTTATGCCATGCAGAACAATACTGAAAACATAATCTTGGTGCTTGAA-----------
right   TTTTTATGCCATGCAGAACAATACTGAAAACATAATCTTGGTGCTTGAA-----------
        *22222222222222222222222222222222222222222222222222222222222
```

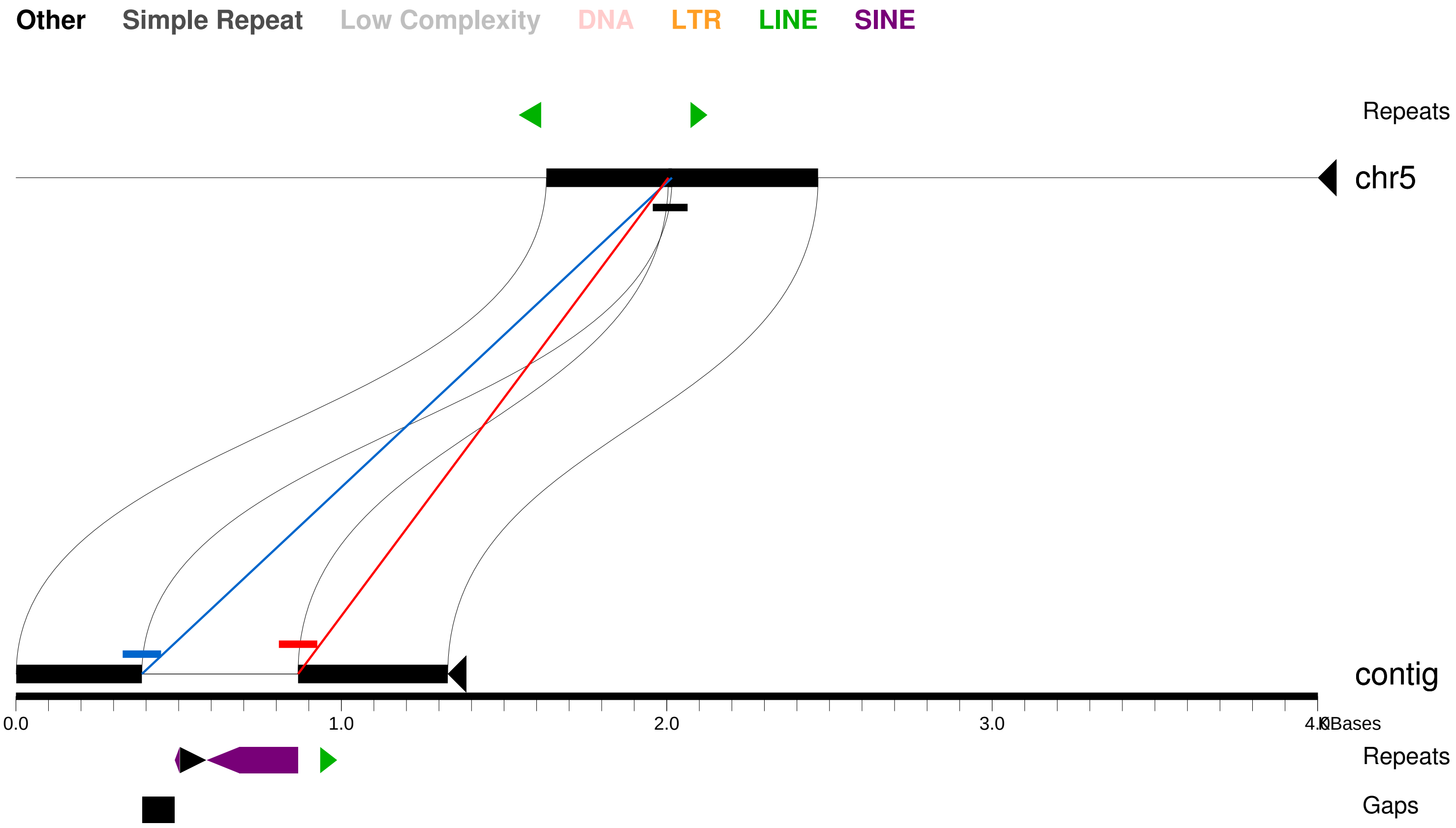

siteID: chr5_74323808 Scafftig1 + Breakpoint: chr5:74323794
chr5:74323750-74323853
Overlap Length: 16 INS size: 297 Genotyped: Yes

```
left     ATTCATGATACTGTTTTGTAACAT-----GATAAAAATGTTGTATAAAT----------CCCTTCGCTCC
chrom    ATTCATGATACTGTTTTGTAACAT-----GATAAAAATGTTGTATAAAT----------CCCTTCGCTCC
right    CCGCCCGCCTCGGCCTCCCAAAGTGCTGGGATTACAGGCGTGAGCCACCGCGCCCGGCCCCCTTCGCTCC
         111*11*111*1*11*111**11*11111***1*1*1111**1111*111111111111***********

left     TCTTTTTTTTTTTTTTTTTTTGAGACGGAGTCTCGCTCTGTCGCCCAGGCTGGAGTGCAGTGGC
chrom    TCTTTAGGTACTACCTGTCCCAACCCACATCCTCA------CCCCCTTCCTTAA---------C
right    TCTTTAGGTACTACCTGTCCCAACCCACATCCTCA------CCCCCTTCCTTAA---------C
         *****222*22*222*2*2222*22*22*22***2222222*2***222**22*222222222*
```

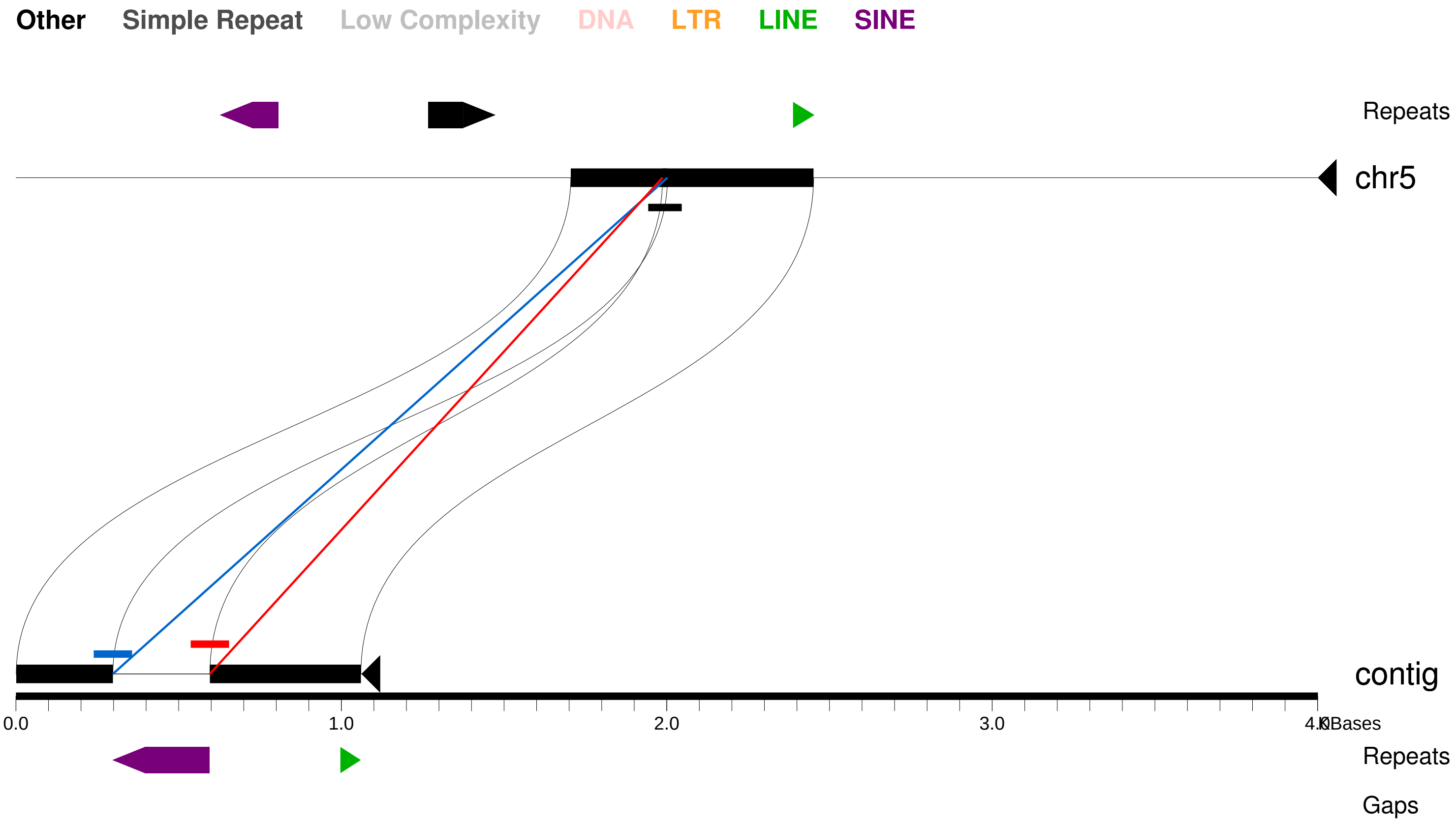

siteID: chr5_76368938 Scafftig2 + Breakpoint: chr5:76368927
chr5:76368874-76368986
Overlap Length: 7 INS size: 211 Genotyped: Yes

```
left    TGCTGAATGCT---------------GAAGGAGAGGAATTTTGTCTACAAACAAAATTTTTTTATCATAA
chrom   TGCTGAATGCT---------------GAAGGAGAGGAATGTTGTCTACAAACAAAATTTTTTTATCATAA
right   TGTGCAGTCCGCCGTCCGGCCTGGGCGACAGAGCGAGACTCCGTCTCAAAAAAAAAA---------AAAA
        **111*1*1*1111111111111111**11***1*11*1N11****11***1****1111111111*1**

left    AAGTACTAACAAGGTGAAACCCCGTCTCTACTAAAAATACAAAAAATTAGCCGGGC-----GCGGTGGC
chrom   AAGTAAT-------TCATACTTTGTGTAAA---AAATTACAACAATGCAGAAGTGTATAAAGCAGAAA-
right   AAGAAAT-------TCATACTTTGTGTAAA---AAATTACAACAATGCAGAAGTGTATAAAGCAGAAA-
        ***1*2*2222222*2*2**222**2*22*222***2*****2**222**22*2*222222**2*2222
```

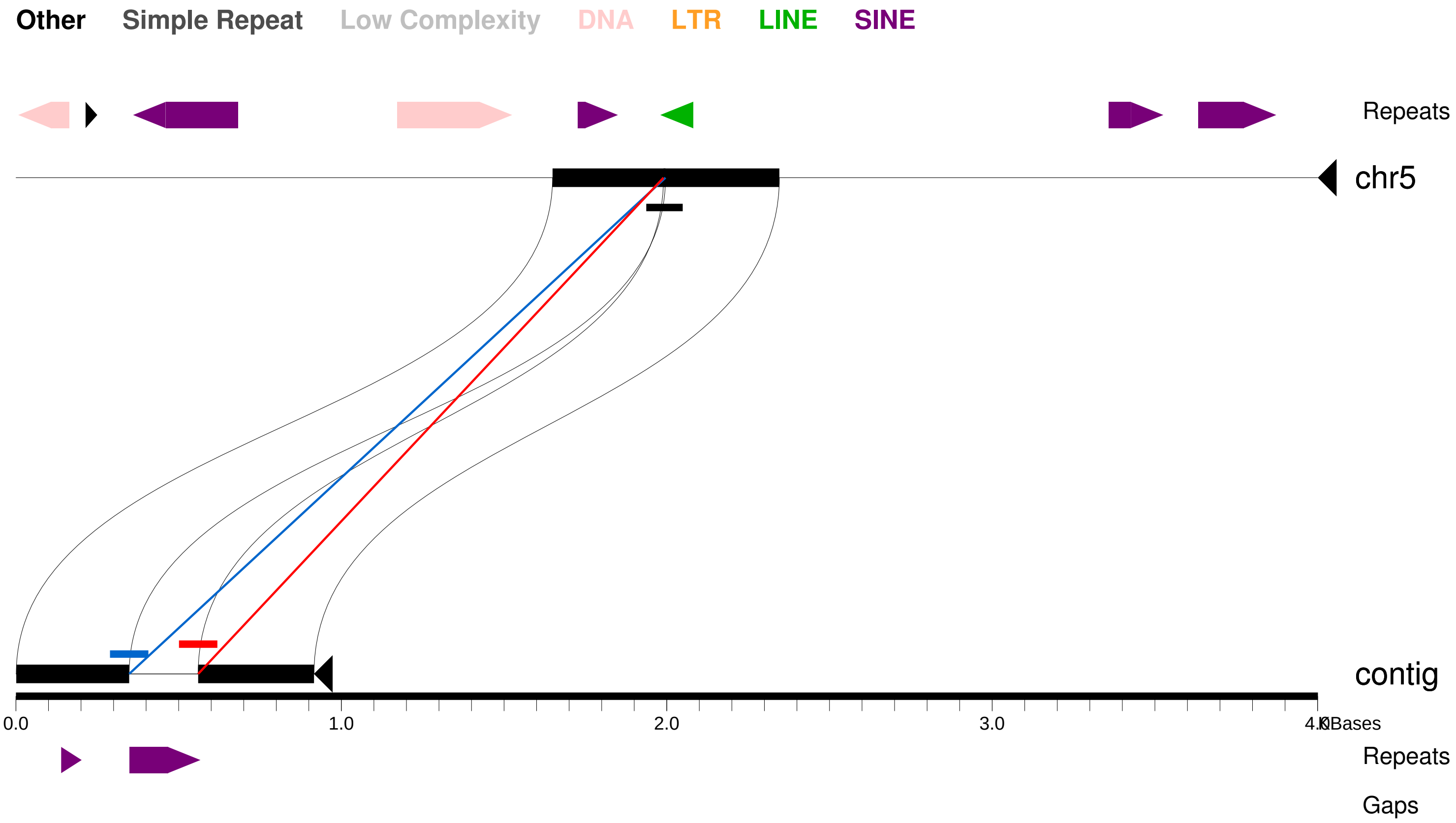

siteID: chr5_76716204 Scafftig1 - Breakpoint: chr5:76716200
chr5:76716150-76716259
Overlap Length: 10 INS size: 429 Genotyped: No

```
left   TGGGAGTGACTGGAAAGCAGAAGCATTTATTGTGTTTTCCTTTTTTAAAA---------CCTACTCATGG
chrom  TGGGAGTGACTGGAAAGCAGAAGCATTTATTGTGTTTTCCTTTTTTAAAA---------CCTACTCATGT
right  NNNNNNNNNNNNNNNNNNNNNNNNNNNNNNNNNNNNNNNNNNNNNNNNNNNNNNNNNNNNCCTACTCATGT
       111111111111111111111111111111111111111111111111111111111111**********2

left   CCGGGCGCGGTGGCTCACGCCTGTAATCCCAGCACTTTGGGAGGCCGAGGCGGGCGGA
chrom  ACCTTGGACCTGCCTTAAG---GTCCTT--ACCAATTTG----GCTTTTAGGAACATG
right  ACCTTGGACCTGCCTTAAG---GTCCTT--ACCAATTTG----GCTTTTAGGAACATG
       2*2222*222**2**2*2*222**22*222*2**2****2222**222222*22*222
```

Other **Simple Repeat** **Low Complexity** **DNA** **LTR** **LINE** **SINE**

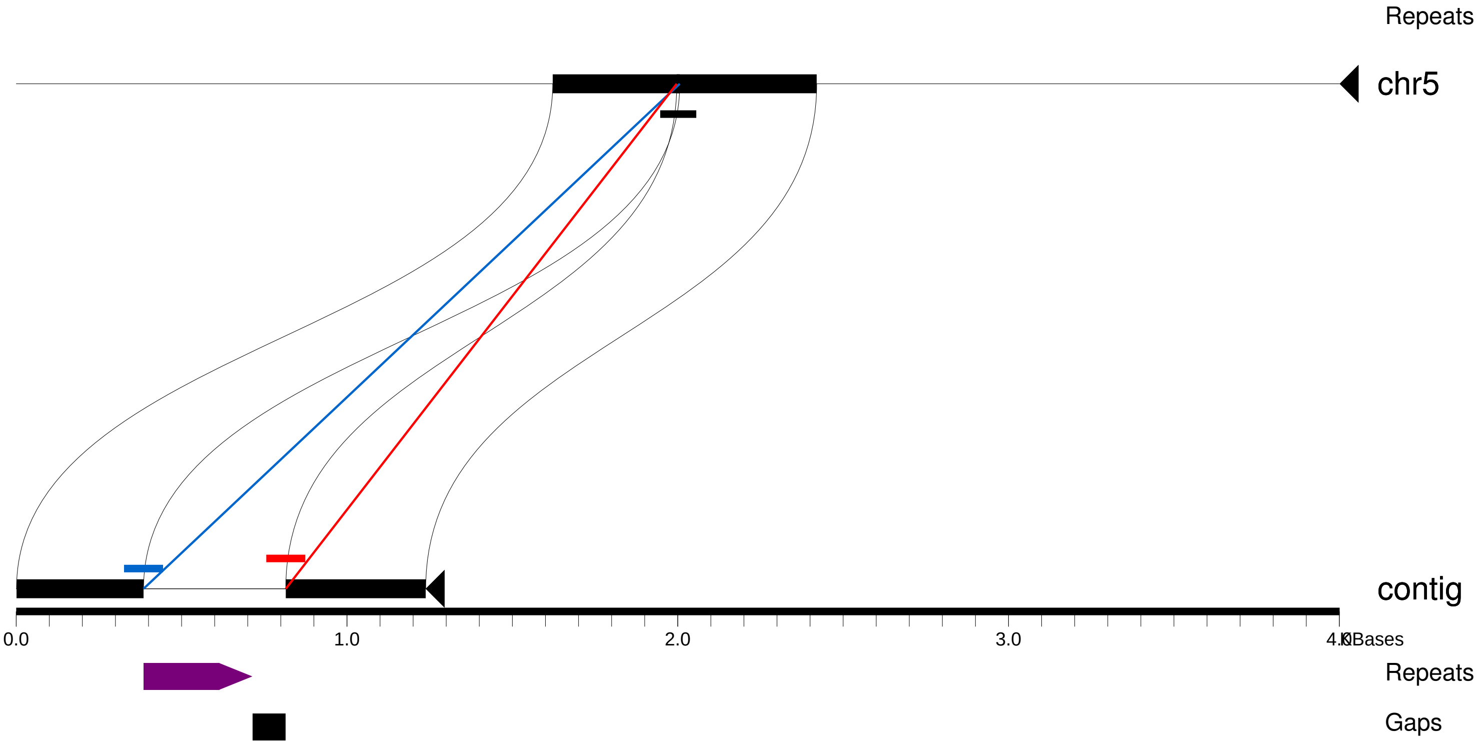

siteID: chr5_76753600 Scafftig3 - Breakpoint: chr5:76753594
chr5:76753545-76753653
Overlap Length: 11 INS size: 316 Genotyped: Yes

```
left    ---AGTATTTCTCATATTAGGGATA-------TAAATTTCTTGGATAAACTATCTATTCAAGAACAACTG
chrom   ---AGTATTTCTCATATTAGGGATA-------TAAATTTCTTGGATAAACTATCTATTCAAGAACAACTG
right   GTCCGGCCTGGGCGACAGAGCGAGACTCCGTCTCAAAAAAAAAAAAAAAAAAAAAAAAAAGAACAACTG
        1111*111*111*11111**1**1*1111111*1**11111111*1***11*111*111***********

left    GGCCGGGCGCGGTGGCTCACGCCTGTAATCCCAGCACTTTGGGAGGCCGAGGCGGGTGG
chrom   AACCA----CTGAACTAAATGAGTGCAATGCTA---TTTTTTAA---CAATTTTTATGT
right   AACCA----CTGAACTAAATGAGTGCAATGCTA---TTTTTTAA---CAATTTTTATGT
        22**22222*2*222222*2*22**2***2*2*2222***222*222*2*222222**2
```

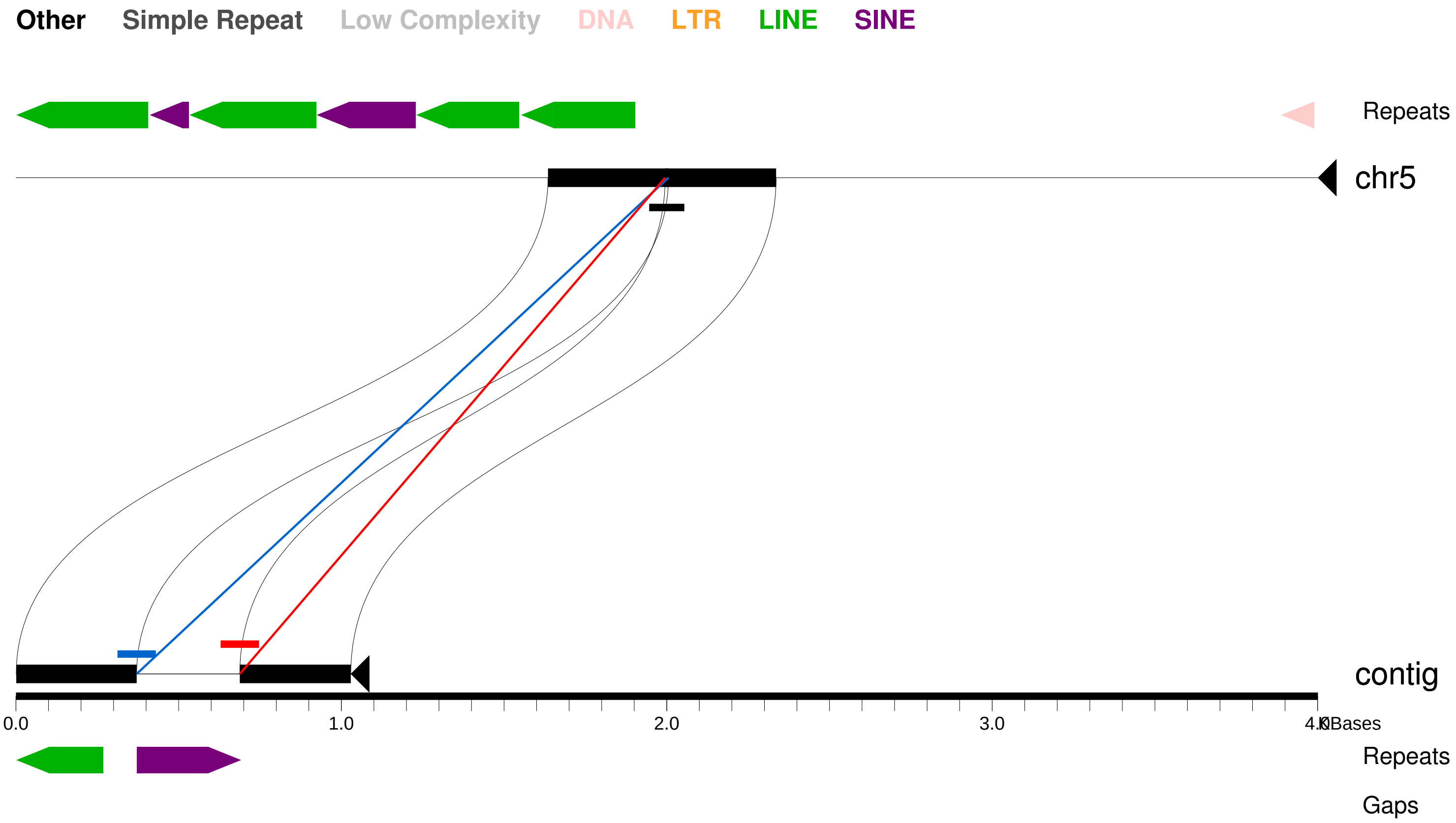

siteID: chr5_77654193 Scafftig1 + Breakpoint: chr5:77654170
chr5:77654128-77654209
Overlap Length: 18 INS size: 301 Genotyped: Yes

```
left   G------------------ACTCCATTTATACATT--------------TATGT-----AAAATAATGTA
chrom  G------------------ACTCCATTTATACATT--------------TATGT-----AAAATAATGTA
right  AGTGAGCCGAGATTGCGCCACTGCACTCCAGCCTGGGCGACAGCGAGACTCCGTCTCAAAAAAAAAAAAA
       1111111111111111111***1**1*1111*1*111111111111111*11**11111****1**111*

left   TAGAGATGTAAAAAATAAAAAATGCAGGCCGGGCGCGGTGGCTCACGCCTGTAATCCCAGCACTTTGGGA
chrom  TAGAGATGTAAAAAATAAAAAATGCAGATCCAGAGCC----------CCT---ACCTCAGAT--------
right  TAAAAAATAAAAAAATAAAAAATGCAGATCCAGAGCC----------CCT---ACCTCAGAT--------
       **1*1*111*****************22*22*2**22222222222***222*2*2***2222222222

left   GGCCGAGGCGGGCGGA
chrom  ----------------
right  ----------------
       2222222222222222
```

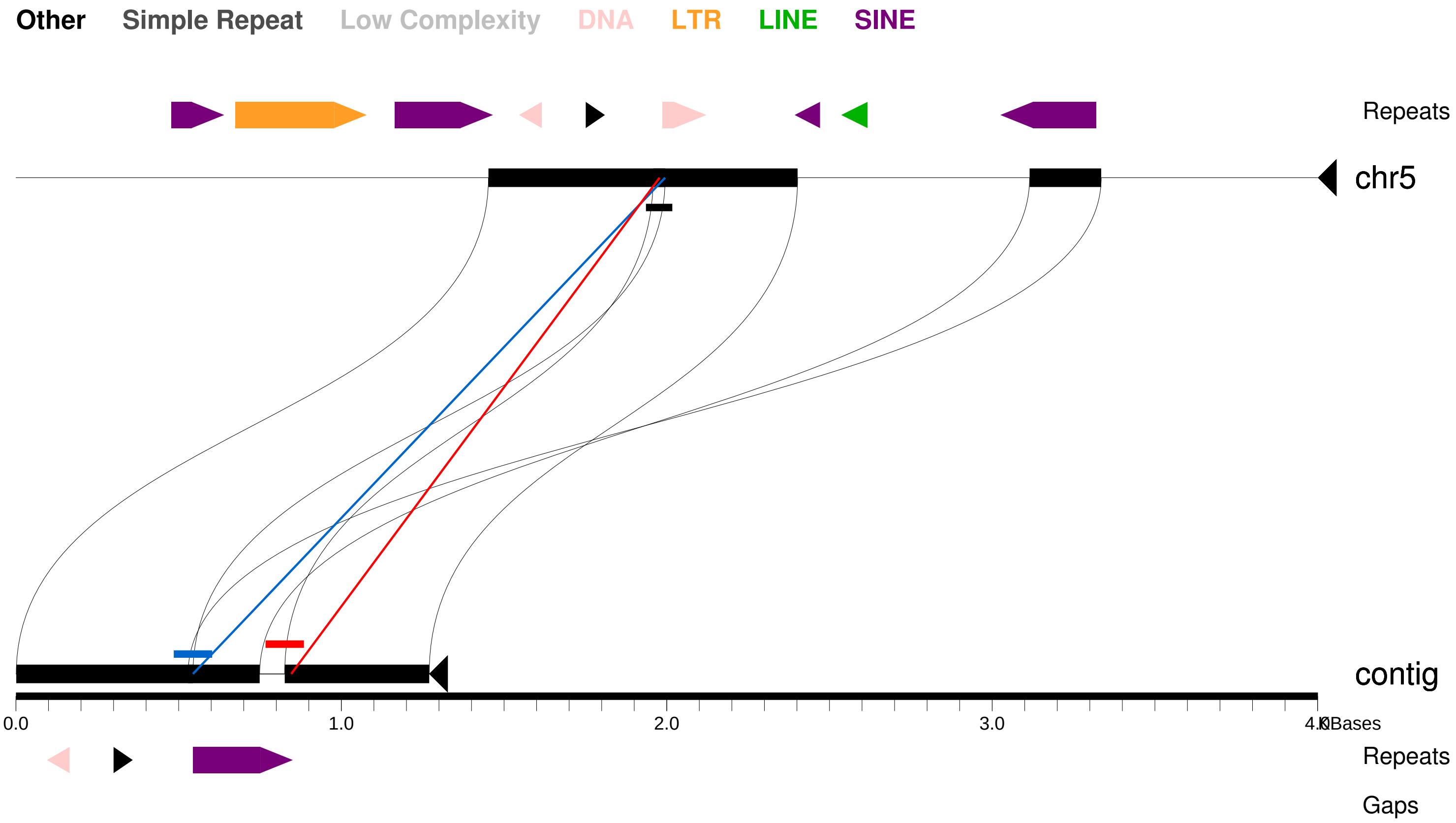

siteID: chr5_78253754 Scafftig2 - Breakpoint: chr5:78253752
chr5:78253700-78253845
Overlap Length: 8 INS size: 310 Genotyped: Yes

```
left    GAGCCAACTCCAGCCACCAGCCAAGGTCCAGATGGGGATAGGATTAAAAATCGGAAGTCTG----------
chrom   GAGCCAACTCCAGCCACCAGCCAAGGTCCAGATGGGGATAGGATTAAAAATCGGAAGTCTACAACAATTT
right   AAAAAAA--------AAAAAAAAA------------------AAAAAAAAAAGGAAGTCTACAACAATTT
        1*111**11111111*11*111**111111111111111111*11*****11********2222222222

left    GCCGGGCGCGGTGGCTC--------ACGCCTGTAATCCCAGCAC-------TTTGGGAGGCCGAGGCGGG
chrom   GCAGCCCACGTAAAGTTTTAAAAAAAATCAGGAAATATCACATCGATCTGTTTTTATATAAAAAAACTTT
right   GCAGCCCACGTAAAGTTTTAAAAAAA-TCAGGAAATATCACATCGATCTGTTTTTATATAAAAAAACTTT
        **2*22*2**22222*222222222*N2*22*2***22**222*2222222***222*22222*22*222

left    C---GG
chrom   TTTGGA
right   TTTGGA
        2222*2
```

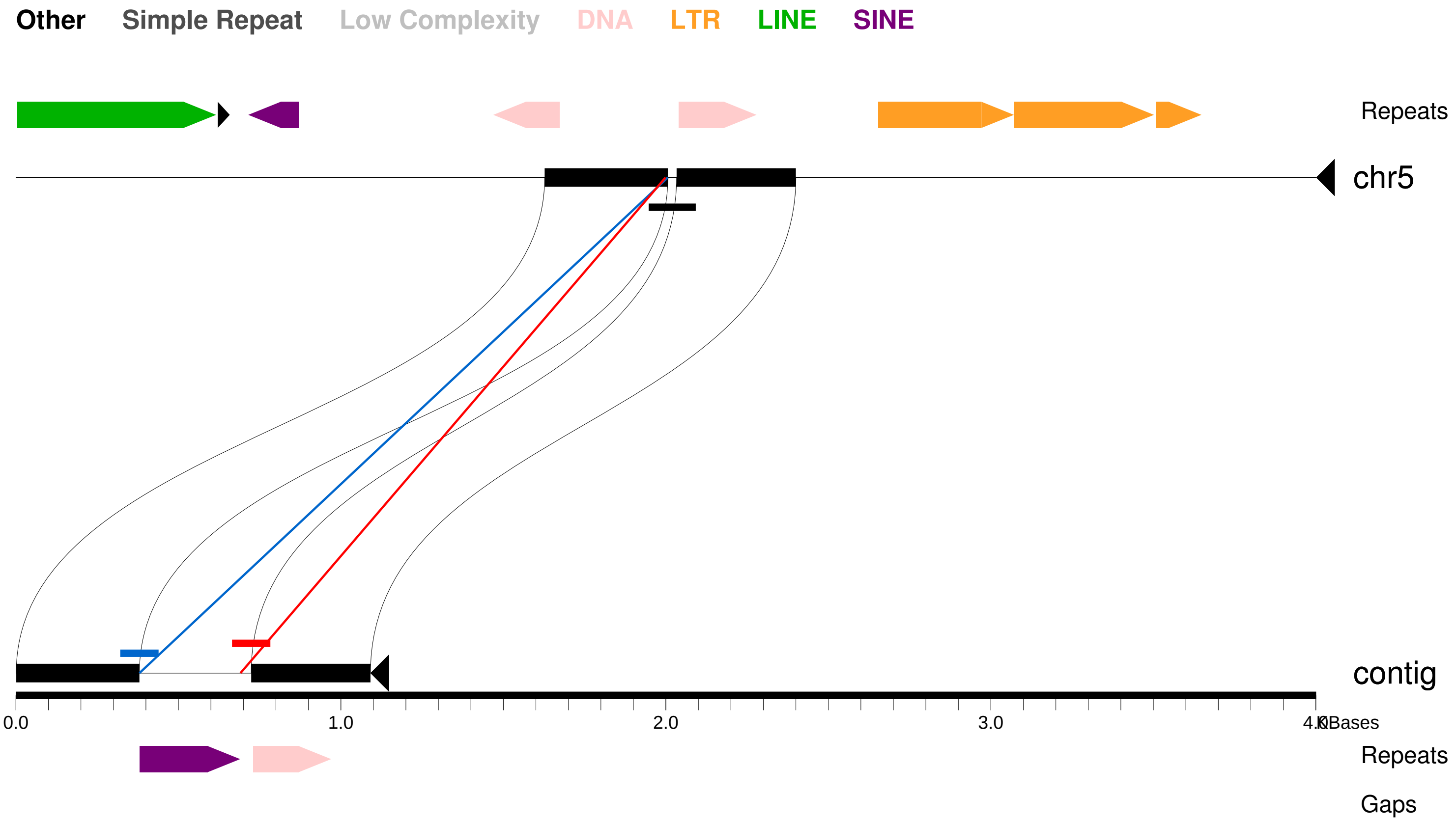

siteID: chr5_7846038 Scafftig2 + Breakpoint: chr5:7846040
chr5:7845994-7846099
Overlap Length: 14 INS size: 362 Genotyped: No

```
left     GGGCTC-CCTGAAGGGAGCTGCTTCAGAGGATTAGA------AGCCACAGATT------GACACTCACAT
chrom    GGGCTC-CCTGAAGGGAGCTGCTTCAGAGGATTAGA------AGCCACAGATT------GACACTCACAT
right    CCGCCCGCCTCGGCCTCCCAAAGTGCTGGGATTACAGGCGTGAGCCACCGCGCCCGGCCGACACTCACAT
         11**1*1***11111111*1111*1111******1*111111******1*111111111***********

left     TTTTTTTTTNNNNNNNNNNNNNNNNNNNNNNNNNNNNNNNNNNNNNNNNNNNNNNNNNNNNNN
chrom    TTTGCCTT-------------CACCTGTGTTAATTTCTGACAGCAAACGAGGGTCAGGGATA
right    TTTGCCTT-------------CACCTGTGTTAATTTCTGACAGCAAACGAGGGTCAGGGATA
         ***222**222222222222222222222222222222222222222222222222222222
```

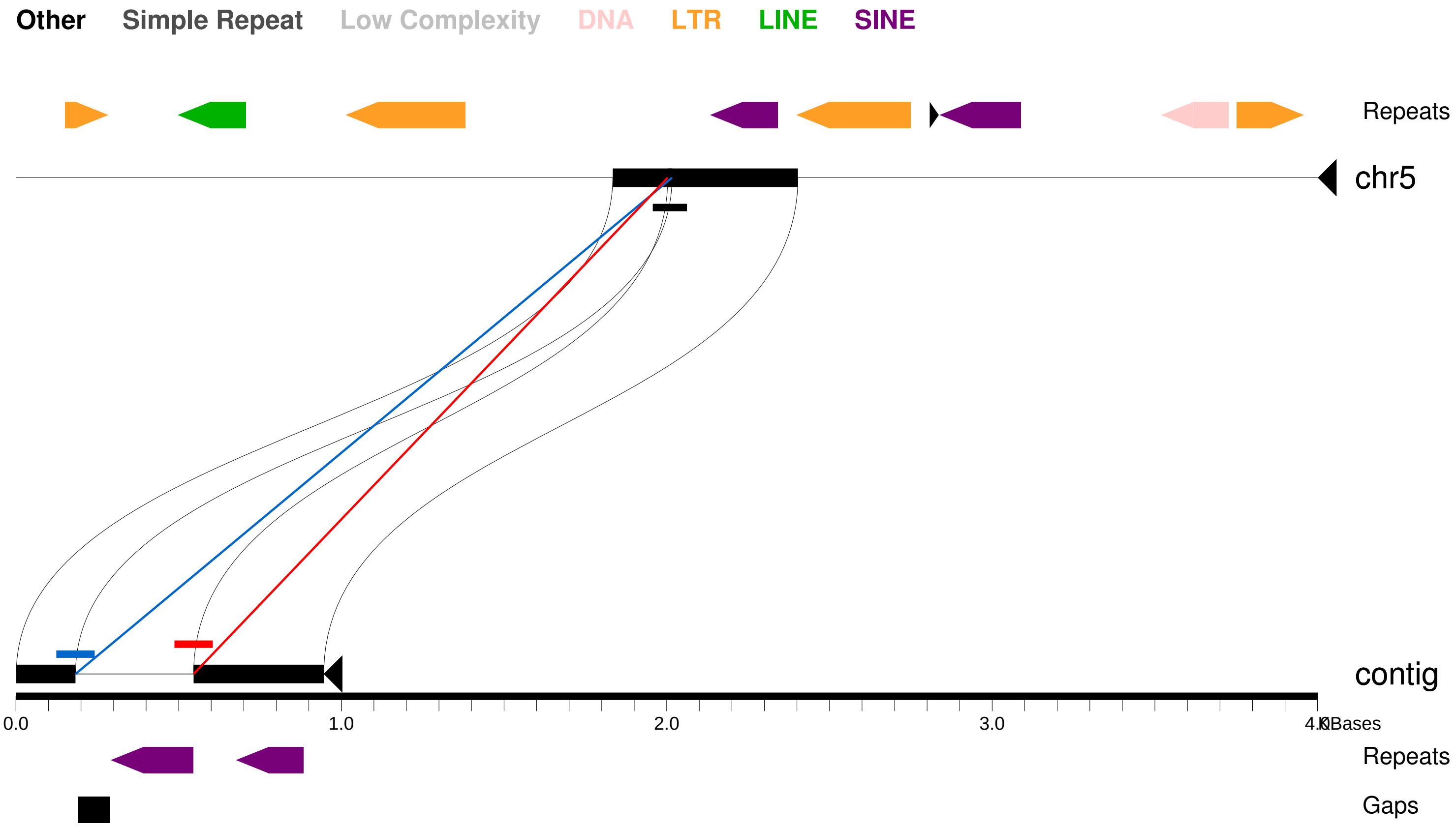

siteID: chr5_83127435 Scafftig5 - Breakpoint: chr5:83127420
chr5:83127260-83127592
Overlap Length: 13 INS size: 307 Genotyped: Yes

```
left   -------------GGACTTGCTGACTGATACACACAAATCATGTTTGTTGCTCTAACAAGTATTTTTCTT
chrom  AAAAGAGAGCAAAGGACTTGCTGACTGATACACACAAATCATGTTTGTTACTCTAACAAGTATTTTTCTT
right  G---GAGA----ATGGCGTGAACCCGGG-AGGCGCA------GCTTGC----------AGT---------
       N   2222    21*1*1**1111*1*11*11*1**111111*1***11N11111111***111111111

left   AACGAGCC-AGATTTTCCTATTGTTTAAATTGTTTCCTTAATTCTTCCAAGCGATTGGAGTGCTATGCCC
chrom  AACGAGCC-AGATTTTCCTATTGTTTAAATTGTTTCCTTAATTCTTCCAAGCGATTGGAGTGCTATGCCC
right  ---GAGCCGAGATCCCGCCACTGC----ACTCCAGCCTGGG--CGACAGAGCGAGACTCCGTCTCAAAAA
       111*****1****1111*1*1**11111*1*1111***11111*11*11*****11111111**111111

left   TGGAGTATCAAAACTGCCTTTAATGTTACCCTGAGGCCGGGCGCGGTGG-CT--CACGCCTGTAATCCCA
chrom  TGGAGTATCAAAACTGCCTTTAATGTTACCCTGATGTCCCAAATGTTATACTTCCAAAAGCGTAGTTGAG
right  AAAAAAAAAAAAAAAAAAAAAAATGTTACCCTGATGTCCCAAATGTTATACTTCCAAAAGCGTAGTTGAG
       111*11*11****11111111************2*2*222222*2*222**22**22222***2*2222

left   GCACTTTGGGAGGCCGAGGC----------GGGC--------GGATCACGAGGT----CAGGAGATCG-A
chrom  GCAGTT-GAGAAGATCTGGCTCAATTTAATGTTCAATTTTAAGTACCAGGGGCTTTTGCATGATATCTTA
right  GCAGTT-GAGAAGATCTGGCTCAATTTAATGTTCAATTTTAAGTACCAGGGGCTTTTGCATGATATCTTA
       ***2**2*2**2*2222***2222222222*22*22222222*2*2**2*2*2*2222**2**2***22*

left   GACCATCCC----GGCTAA----------AACGGTGAAACCCCGTCTCTACTAAA
chrom  TTCTCTCCCTGGAGGATTATATTAATTGGAACAGGAACACTGAAGTGGTAACACA
right  TTCTCTCCCTGGAGGATTATATTAATTGGAACAGGAACACTGAA-----------
       22*22****2222**2*2*2222222222***2*22*2**2222NNNN11NN1N1
```

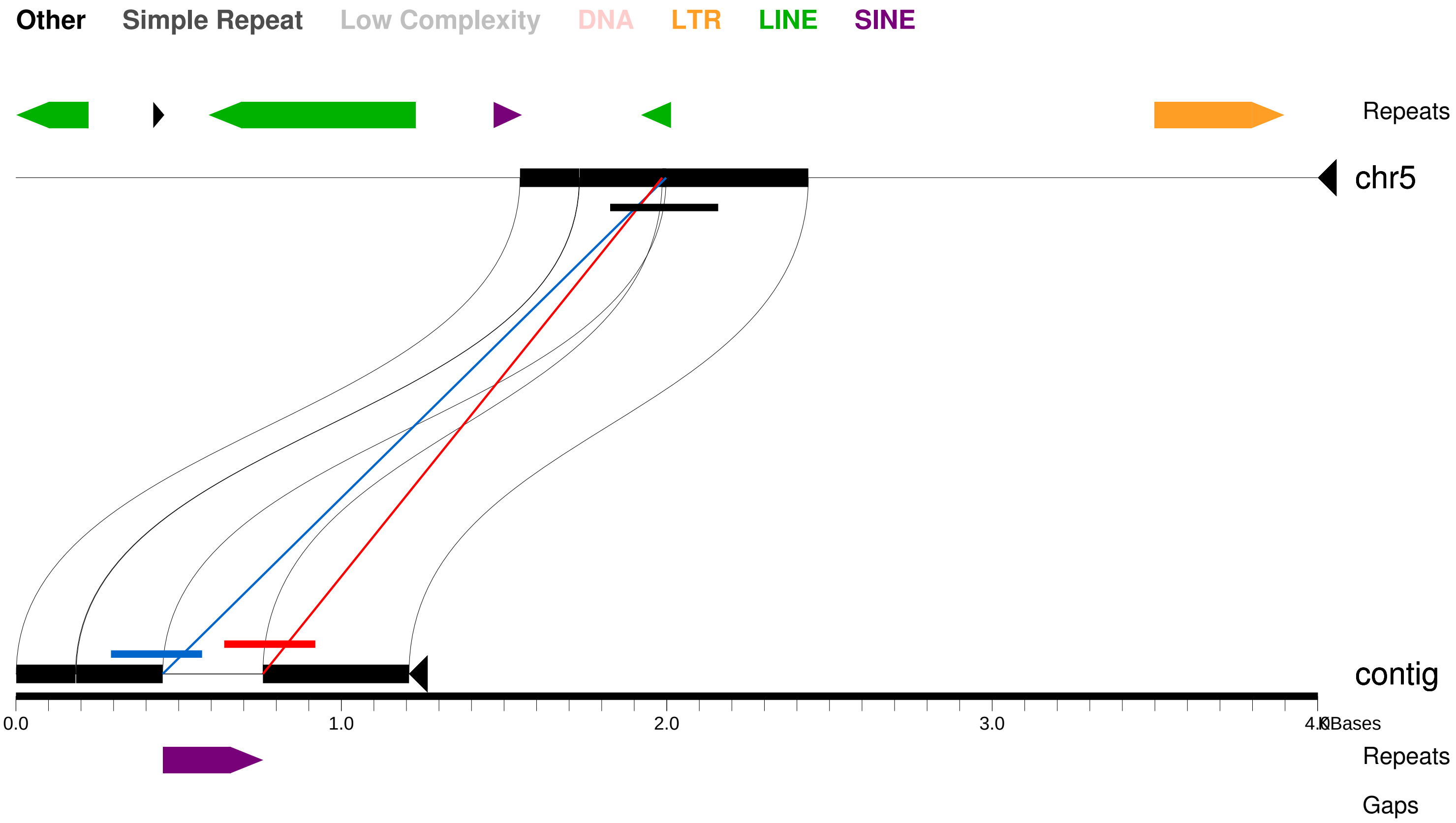

siteID: chr5_84384456 Scafftig1 - Breakpoint: chr5:84384476
chr5:84384433-84384535
Overlap Length: 17 INS size: 318 Genotyped: Yes

```
left     AGTTGAAG--AGACT--------------AACATTTGGAAATGGAAGAAAAATTTTCTTAAAAAAATTAA
chrom    AGTTGAAG--AGACT--------------AACATTTGGAAATGGAAGAAAAATTTTCTTAAAAAAATTAA
right    CGACAGAGCGAGACTCCGTCTCAAAAAAAAAAAAAAAAAAAAAAAAAAAAAAAAAAAAAAAAAATAATTAA
         1*1111**11*****11111111111111**1*11111***111**1*****1111111****1******

left     ATTGCAGGCCGGGCGCGGTGGCTCACGCCTGTAATCCCAGCACTTTGGGAGGCCGAGGCGGGCGG
chrom    ATTGCATT----------TTGTTTA----TCTAATTCCATGGATAAGTAAA--AGAGTAGTCCTT
right    ATTGCATT----------TTGTTTA----TCTAATTCCATGGATAAGTAAA--AGAGTAGTCCTT
         ******222222222222*2*2*2*2222*2****2***2222*22*22*2222***22*22*22
```

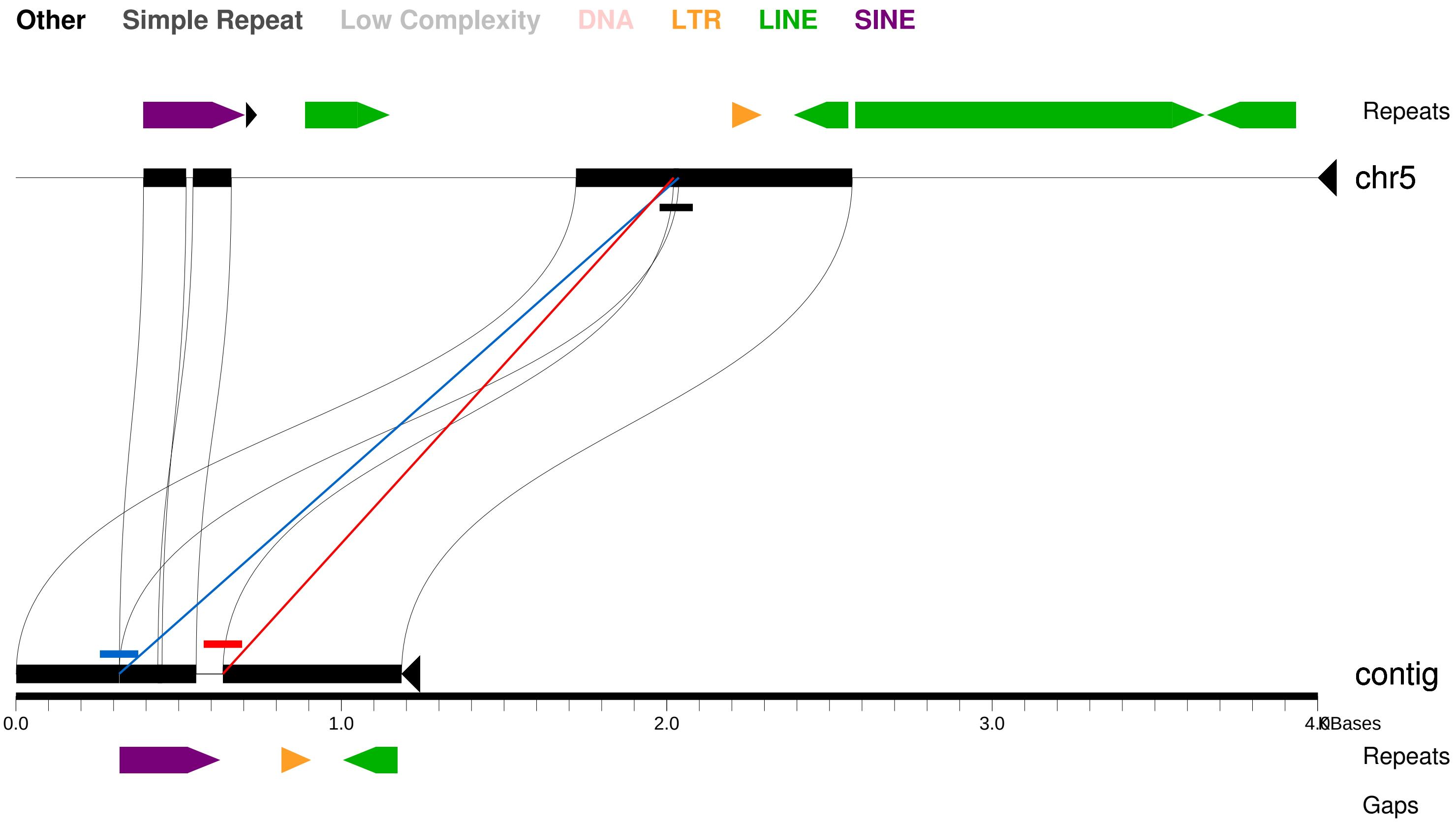

siteID: chr5_84929800 Scafftig3 - Breakpoint: chr5:84929774
chr5:84929614-84929951
Overlap Length: 18 INS size: 327 Genotyped: Yes

```
left   -----------------TC-CATTATCTATGAATAATTTGTGG-GTATGTATTTTTTAGAAACAAAGATA
chrom  AAACAGTTACACAATACTC-CATTATCTATGAATAATTTGTGG-GTATGTATTTTTTAGAAACAAAGATA
right  GG-CTGAGGCAGGAGAATGGCGTGAACCCGGGAG-----GCGGAGCTTGCAGTGAGCCGAGATTGCGCCA
       NN 2N2NNN22NN2N2N*11*1*1*1*111*1*111111*1**1*11**1*1*11111**1*1111*11*

left   TTT---TCTAACATAATCACCAGAATAAGAATATTAACATTGACACATTATTGCCATCTAATCCTCAGCC
chrom  TTT---TCTAACATAATCACCAGAATAAGAATATTAACATTGACACATTATTGCCATCTAATCCTCAGCC
right  CTGCACTCCAGCCTGGGCGACAGAGCGAGACT-----CCGTCTCAAAAAAAAAAAAAAAAAAAATAAAA-
       1*1111**1*1*1*111*11****111***1*11111*11*11**1*11*11111*111**111*1*111

left   TTCATTCAACTTTCAACAATTGTCTTAATAATTTACTTTATCGGC-------CGGGCGC-----------
chrom  TTCATTCAACTTTCAACAATTGTCTTAATAATTTACTTTATCGTAAAACTATCCAGTTCAAAATAAAACT
right  ---ATAATAATAATAATAATAATAATAATAATTTACTTTATCGTAAAACTATCCAGTTCAAAATAAAACT
       111**111*1*111**1***11*11******************222222222*22*22*22222222222

left   GGTGGCTCACGCCTGTAAT----CCCAGC------------ACTTTGGGAGGCCGAGGCGGGCGGATCAC
chrom  GTTTGCTTTTAGTTGTCATGTGTCTCATTTTCTTTGAAATTACTTTGACTTTCCTATTCTGTATAATTTC
right  GTTTGCTTTTAGTTGTCATGTGTCTCATTTTCTTTGAAATTACTTTGACTTTCCTATTCTGTATAATTTC
       *2*2***222222***2**2222*2**22222222222222******22222**2*22*2*2222**22*

left   GAGGTCAGGAGATCGAGACCATCCC--GGCTAAAAC--GGT----GAAACCCCGTCTCTACTAAA
chrom  TAAAACTATGGAACAAGTGTTTTTGTAGAATATTTCCAGGTTTGGGAAACAATTTGAGTATGC--
right  TAAAACTATGGAACAA-------------------------------------------------
       2*222*2222**2*2*1NNNN1NNN  1NN11NNN1  111    11111NNNN1NNN11NNN22
```

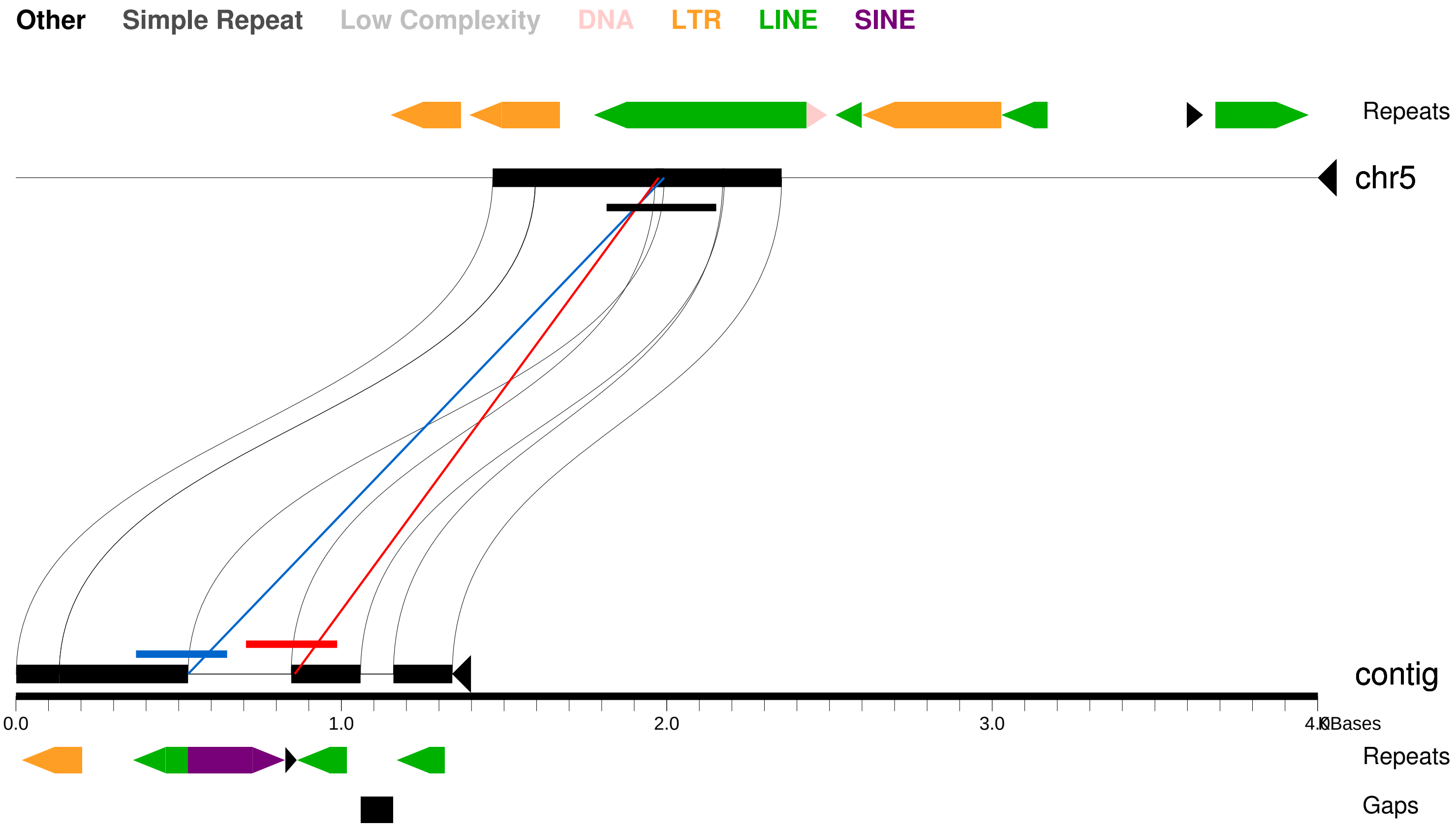

siteID: chr5_85764868 Scafftig1 - Breakpoint: chr5:85764950
chr5:85764790-85765120
Overlap Length: 11 INS size: 99 Genotyped: Yes

```
left   T-------GCCTTAATAGAATTGAAAATTAAGAAATAATTGCAGTCTAGTGTTCTTAGAGATAACAATGA
chrom  TATTTGCTGCCTTAATAGAATTGAAAATTAAGAAATAATTGCAGTCTAGTGTTCTTAGAGATAACAATGA
right  CAG-----GGCTTCATAAACTT-------------TTTTT------TTTTTTTTTTTGAGACG-------
       12N     *1***1***1*1**1111111111111*11**111111*11*1**1**1****111111111

left   TGATACACTTTTCTTGGCTTTGACCATCAATATATTACCATTAATGTTCCATTATTATATTTAAAAGCAT
chrom  TGATACACTTTTCTTGGCTTTGACCATCAATATATTACCATTAATGTTCCATTATTATATTTAAAAGCAT
right  -GAGTCTC--------GCTCTGTCGCCCAGGCTGG----AGTGCAGTGGCGCGATCTCGGCTCACTGCAA
       1**11*1*11111111***1**1*111**111*111111*1*111**11*111**111111*1*11***1

left   TATTACCTAAAGCAGGGCT----TCATAAACTTTTTTTTT--------------TTTTTTTTTGAGACGG
chrom  TATTACCTAAAGCAGGGCT----TCATAAACTTTTCTACTAAAGGGCCACATAGTAAATAATTTAGGTTG
right  GCT--CCGCCTCCCGGGTTCACGCCATAAACTTTTCTACTAAAGGGCCACATAGTAAATAATTTAGGTTG
       11*11**11111*1***1*11111**********2*22*22222222222222*222*22**2**222*

left   AG--TCTCGCTCTGTCG--------CCCAGGC--TGGAGTGCAGT---GGCGCGAT----CTCGGCTCAC
chrom  TGCATGTCAAATGGTCCTAGAACAACAAAAGCCATAGACAACACATAAGACTCAATAGGACAGTGTACCA
right  TGCATGTCAAATGGTCCTAGAACAACAAAAGCCATAGACAACACATAAGACTCAATAGGACAGTGTACCA
       2*22*2**22222***222222222*22*2**22*2**222**22222*2*2*2**2222*222*22*22

left   TGCAAGCTCCGCCTCCCGGGTTCAC--GCCATAAACTTTTCTACTAA--------
chrom  AGAAAACTTTATTTTTAAGAATGAATGGCAGTCCGGTTTTGGCCCAAAGCTCATC
right  AGAAAACTTTATTTTTAAGAATGAATGGCAGTCCGNNNNNNNNNNNNN--------
       2*2**2**22222*2222*22*2*222**22*222N1111NNN1N11
```

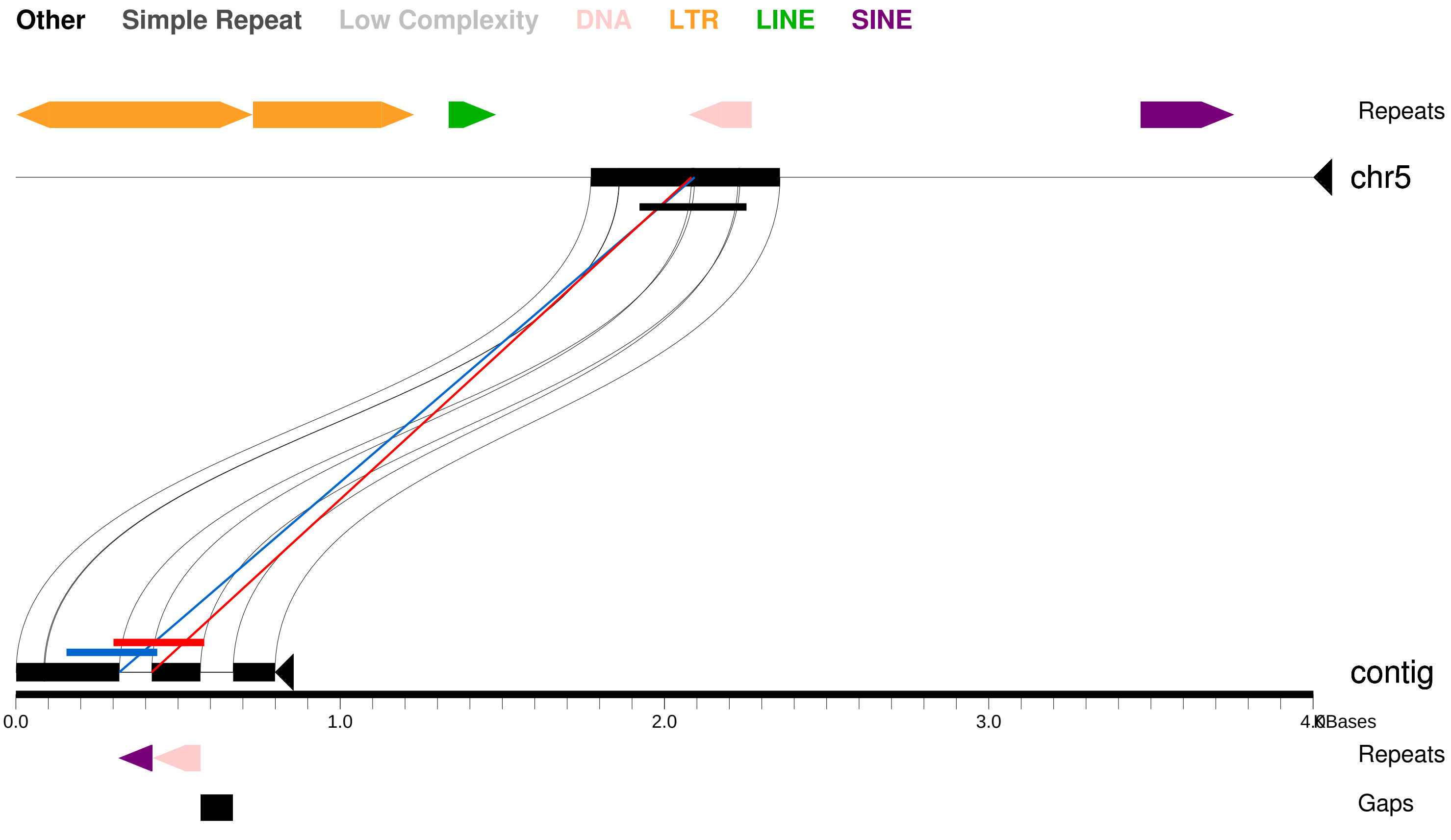

siteID: chr5_86372702 Scafftig16 - Breakpoint: chr5:86372683
chr5:86372636-86372726
Overlap Length: 13 INS size: 284 Genotyped: Yes

```
left   -AAAT--AGAAAAGGTAC----------GTGGAATAGGGAT-----GTC----------AAGATACAAAG
chrom  -AAAT--AGAAAAGGTAC----------GTGGAATAGGGAT-----GTC----------AAGATACAAAG
right  GAGATCCCGCCACTGCACTCCAGCCTGGGCGACAGAGCGAGACTCCGTCTCAGAAAAAAAAAAAAAAAAA
       1*1**111*11*11*1**1111111111*1*11*1**1**111111***1111111111**1*1*1***1

left   GATATAAAGAAAATATTTTCCTGTAATCCCAGCACTTTGGGAGGCCGAGGCGGGCGGATCACGAGGTCAG-
chrom  GATATAAAGAAAATATTTTT-TGTA----CAGC----TG--------------------CACAATGTATTT
right  AAAAAAAAGAAAATATTTTT-TCTA----CAGC----TG--------------------CACAATGTATTT
       1*1*1************22*1**2222****2222**22222222222222222222***2*2**2222

left   GAGATCGA
chrom  GTGTTTAA
right  GTGTTTAA
       *2*2*22*
```

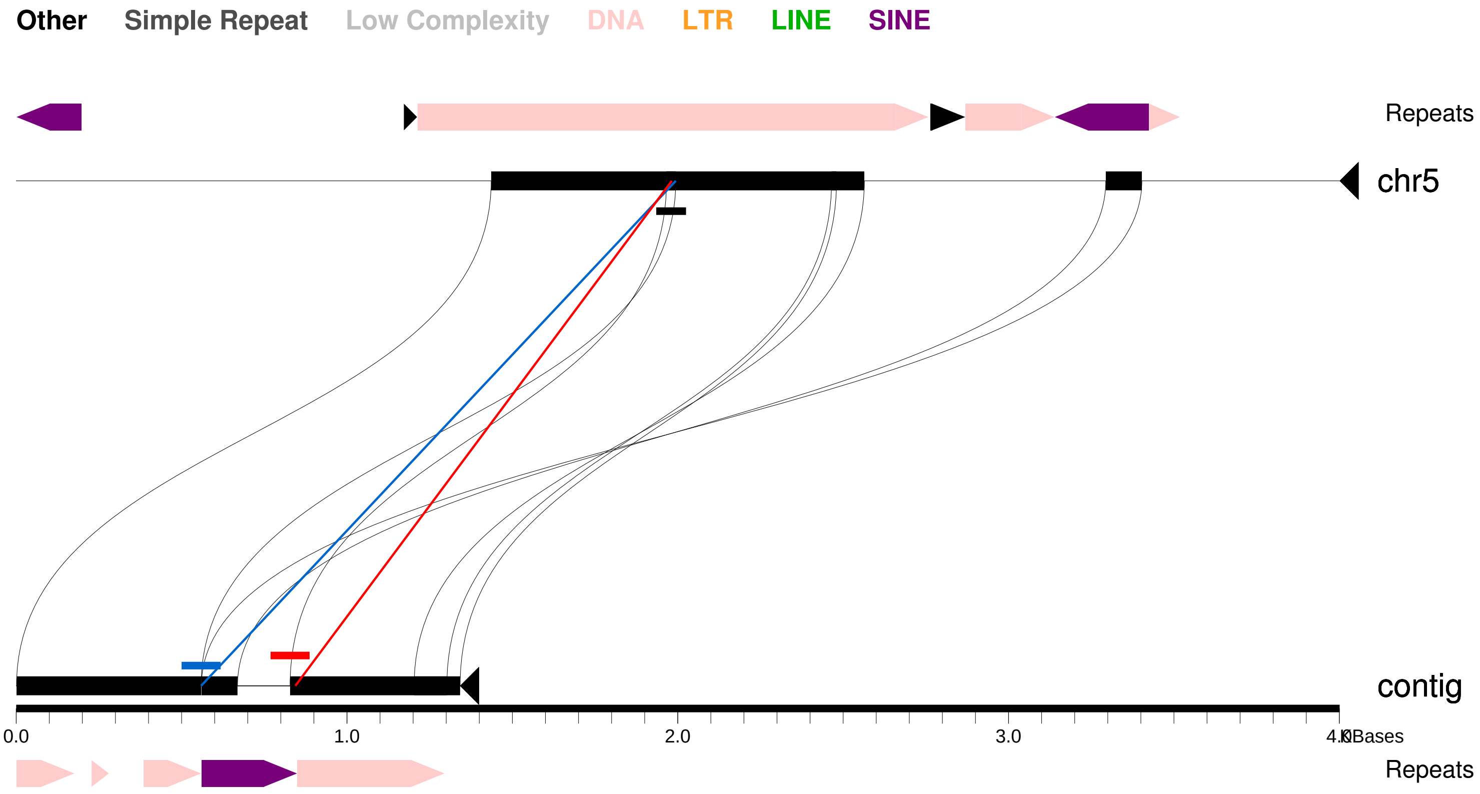

siteID: chr5_87866349 Scafftig1 - Breakpoint: chr5:87866342
chr5:87866182-87866515
Overlap Length: 14 INS size: 322 Genotyped: Yes

```
left   ---------------------GCTACCATATCCAGTGA--CTGGGAAGCATGGATATATAAAGTCCTAGC
chrom  TA-CTCCTTC-----CTGATGGCTACCATATCCAGTGA--CTGGGAAGCATGGATATATAAAGTCCTAGC
right  CAGCTACTCGGGAGGCTGA-GGCAGGAGAATGGCGTGAACCCGGGAGGC--GGAGCT-TGCAGTG--AGC
       N2122N22NN111112222 2**111111**111****11*1****1**11***11*1*11***111***

left   CTCCTTATC-CCAATGTGGAGAAACACTGAAGAGTCATCCCTGCAGGATAGTTCTCTAGGTGGCTGTGGA
chrom  CTCCTTATC-CCAATGTGGAGAAACACTGAAGAGTCATCCCTGCAGGATAGTTCTCTAGGTGGCTGTGGA
right  CGAGATCCCGCCACTGCACTCCAGC-CTGG-GCGACAGAGCG--AGACTCCGTCTCAAAAAAAATA---A
       *1111*11*1***1**111111*1*1***11*1*1**111*111**11*111****1*111111*1111*

left   TTGGCTCTGTTCTCCCCTATTTCTTGCTTAAGAATAACTATAG---GGGCCGGGCGCGGTGGCTCACGCC
chrom  TTGGCTCTGTTCTCCCCTATTTCTTGCTTAAGAATAACTATAGAATGTGCTGGGAATGCAGCATCCTGAG
right  ATAAATAAATAAATAAATAAATAAAAAAAAAGAATAACTATAGAATGTGCTGGGAATGCAGCATCCTGAG
       1*111*111*1111111**11*1111111**************222*2**2***222*22*22**22*22

left   T---GTAATCCCAGCACTTTGGGAG----GCCGAGGC---GGGCGGATC----ACGAGGTCAGGA-GA--
chrom  ATGGGTGTGGAGAGAAGCTGGCAAGAACAGCCTGGGCTCTGTCCCAATCTCCCACTAGAACAGGATGACC
right  ATGGGTGTGGAGAGAAGCTGGCAAGAACAGCCTGGGCTCTGTCCCAATCTCCCACTAGAACAGGATGACC
       2222**222222**2*22*2*22**2222***22***222*22*22***2222**2**22*****2**22

left   -TCGAGACCAT--CCCGGCTAAAACGG-----TGAAACCCCGTCT---CTACTAAA-------
chrom  TTCAACACTTTAGCCCAGTGATCATGTGGCCCTGAGACTTAGGGTAGGCTGCTTCCCAGGGTC
right  TTCAACACTTTAGC-------------------------------------------------
       2**2*2**22*22*11N1NN1NN1N1N     111N11NNN1NN1   11N11NNN
```

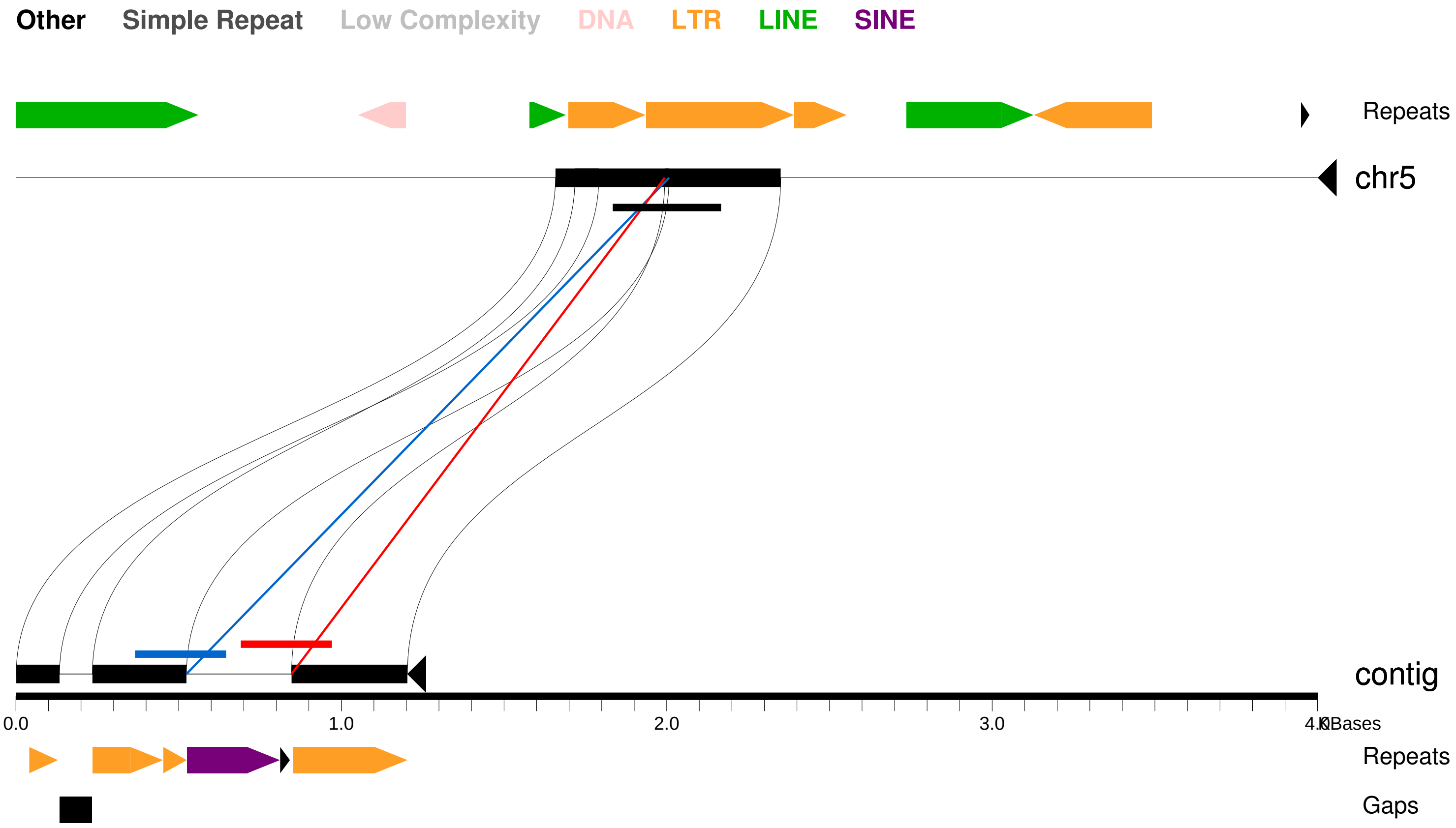

siteID: chr5_88804330 Scafftig1 - Breakpoint: chr5:88804363
chr5:88804321-88804422
Overlap Length: 18 INS size: 392 Genotyped: No

```
left     GTAAATGTGGTACATATACACCATGGAATATTATGCAGCC-----------------ACAAAAAGAACGA
chrom    GTAAATGTGGTACATATACACCATGGAATATTATGCAGCC-----------------ACAAAAAGAACGA
right    NNNNNNNNNNNNNNNNNNNNNNNNNNNNNNNNNNNNNNNNNNNNNNNNNNNNNNNNAAAAAAAAGAACGA
         111111111111111111111111111111111111111111111111111111111*1***********

left     GATTGTGGCCGGGCGCGTTGGCTCACGCCTGTAATCCCAGCACTTTGGGAGGCCGAGGCGGGTGGA
chrom    GATTGTGTCTTTT-GCG---GAACATGGATGGA-----------TCTGGAGGCTAT--CATCATCA
right    GATTGTGTCTTTT-GCG---GAACATGGATGGA-----------TCTGGAGGCTAT--CATCATCA
         *******2*22222***222*22**2*22**2*22222222222*22******22222*222222*
```

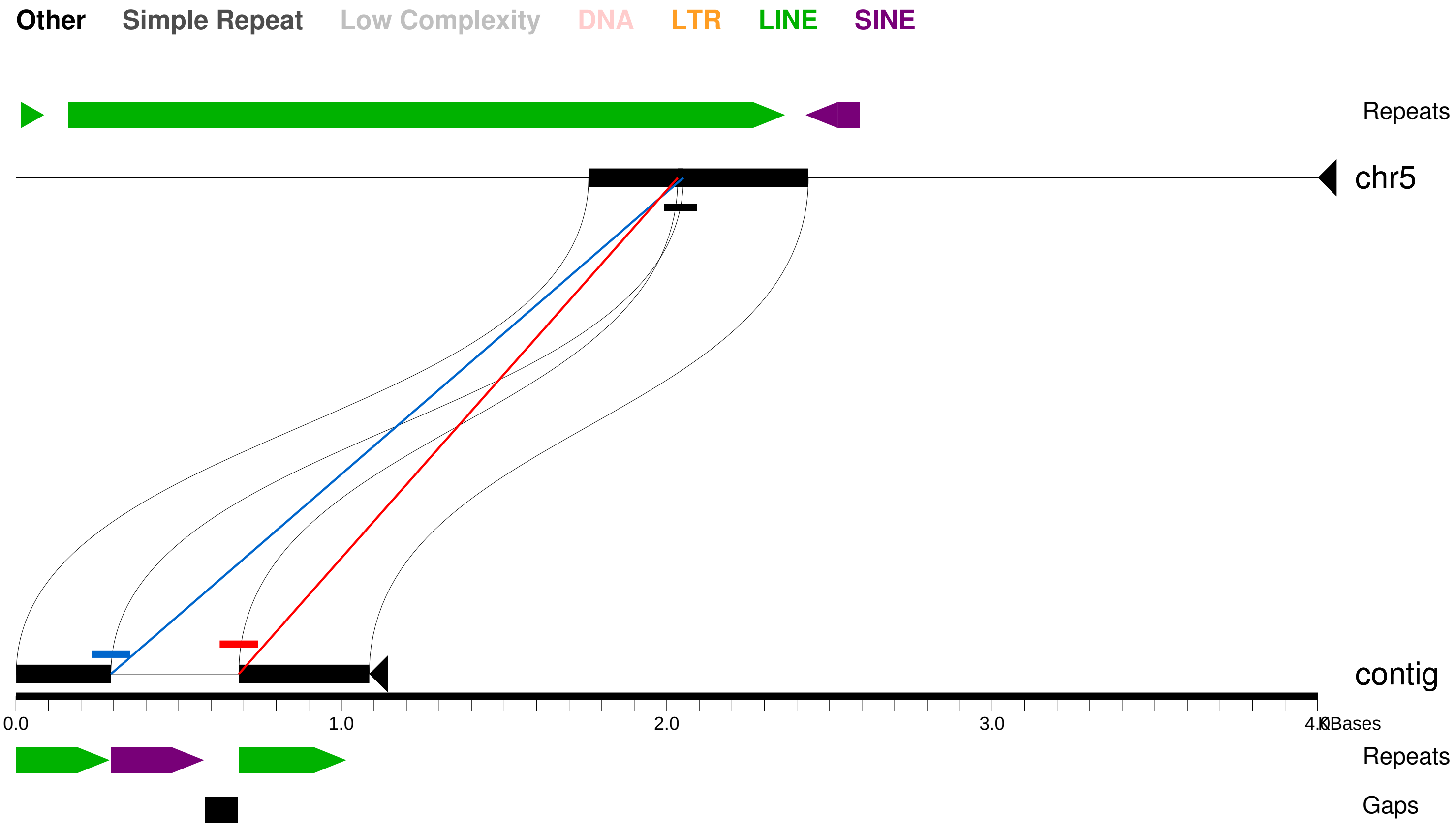

siteID: chr5_89997884 Scafftig4 - Breakpoint: chr5:89997825
chr5:89997775-89997884
Overlap Length: 10 INS size: 154 Genotyped: Yes

```
left    CCATA-ATTTTCTTTTTGAACCATTAACAAATTTTGTGATTTGAAAAGTTT--------GAAAACCATGC
chrom   CCATA-ATTTTCTTTTTGAACCATTAACAAATTTTGTGATTTGAAAAGTTT--------GAAAACCATGA
right   AGAGCGAGACTCCGTCTCAAAAAAAAAAAAAAAAAAAAAAAAAAAAAAAAAAAAAAAAAGAAAACCATGA
        11*111*111**11*1*1**11*11**1***1111111*1111****111111111111**********2

left    TACTTGGGAGGCTGAGGCAGGAGAATGGCGTGAACCCGGGAGGCGGA---GCTTGCAGTGA
chrom   CTCTTATGAA--AGAGTCACCAGAC-------AACACTGTAGGTGAAATAGCTT--AAT-A
right   CTCTTATGAA--AGAGTCACCAGAC-------AACACTGTAGGTGAAATAGCTT--AAT-A
        22***22**2222***2**22***22222222***2*2*2***2*2*222****22*2*2*
```

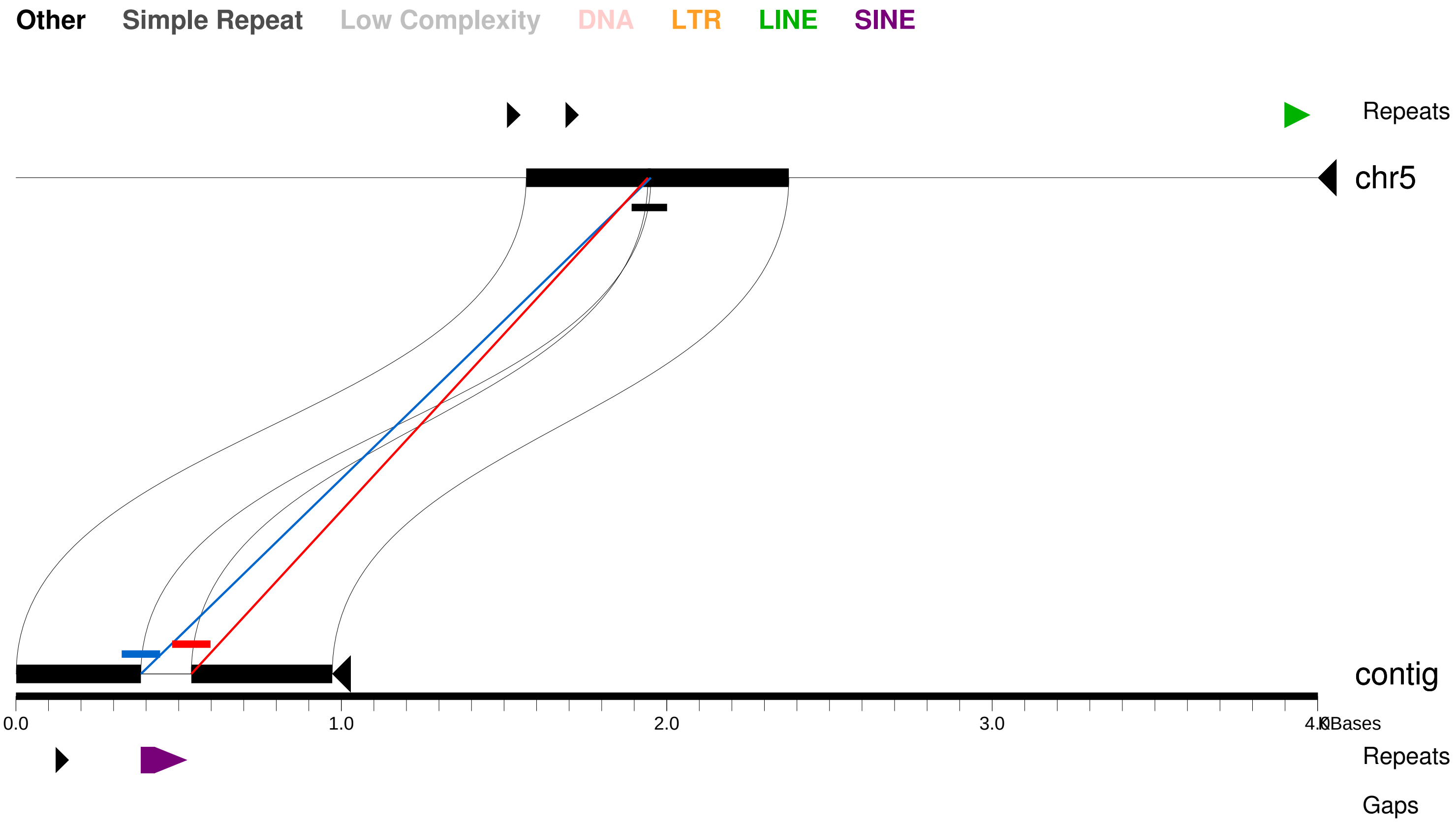

siteID: chr5_90819294 Scafftig1 - Breakpoint: chr5:90819282
chr5:90819239-90819340
Overlap Length: 10 INS size: 422 Genotyped: No

```
left    --------CGTAGG-------AATTTATGCTTTTCTTTTCTATATCT-CAACTTTTTGC-TCACATTTTT
chrom   --------CGTAGG-------AATTTATGCTTTTCTTTTCTATATCT-CAACTTTTTGC-TCACATTTTT
right   TCCACCCGCCTCGGCCTCCCAAAGTGCTGGGATTACAGGCGTGAGCCACTGCGCCCGGCCTCACATTTTT
        11111111*1*1**1111111**1*11**111**11111*111*1*11*11*11111**1**********

left    TTTTTTTTNNNNNNNNNNNNNNNNNNNNNNNNNNNNNNNNNNNNNNNNNNNNNNNNNNNNNNNNNN
chrom   AATCTTT-----------------GCTTTTTTGTTCTATATTCAAGAACTTTCTCTGAGTTTTTCT
right   AATCTTT-----------------GCTTTTTTGTTCTATATTCAAGAACTTTCTCTGAGTTTTTCT
        22*2***22222222222222222222222222222222222222222222222222222222222
```

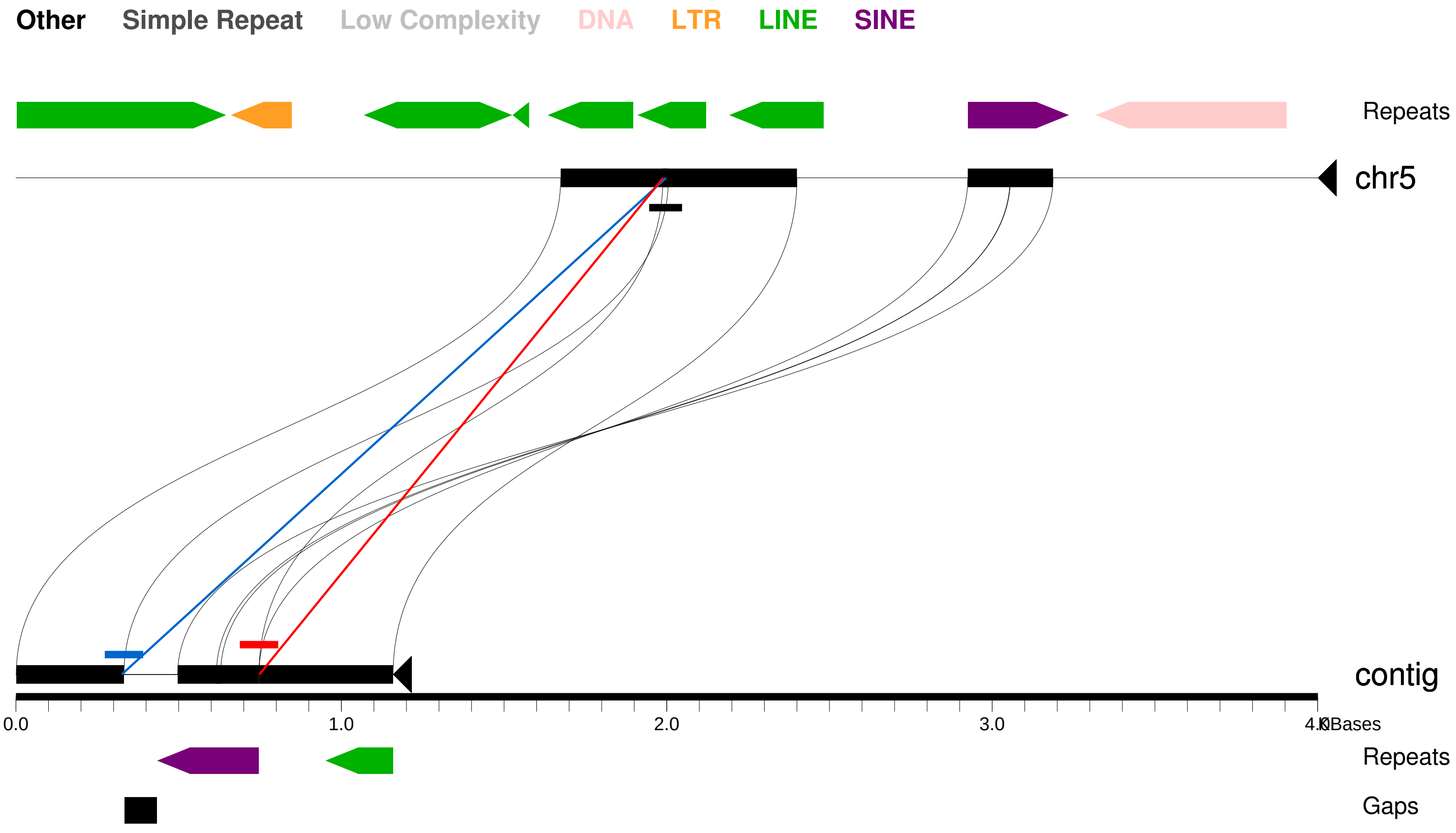

siteID: chr5_91031054 Scafftig1 - Breakpoint: chr5:91031017
chr5:91030967-91031076
Overlap Length: 10 INS size: 338 Genotyped: No

```
left   TAAGTGA--TGAATATCTTGAAGACACTGAGTTCCAGAGTTCTTATCTACCA---------GGGAACTTT
chrom  TAAGTGA--TGAATATCTTGAAGACACTGAGTTCCAGAGTTCTTATCTACCA---------GGGAACTTT
right  CCGCCCGCCTCGGCCTCCCAAAGTG-CTGGGATT-ACAGGCGTGAGCCACCGCGCCCGGCCGGGAACTTT
       111111111*11111**111***111***1*1*11*1**111*1*1*1***1111111111*********

left   TTTTT-----TCTTTTTTTTTTTTTTTTTNNNNNNNNNNNNNNNNNNNNNNNNNNNNNNNNNNNNN
chrom  TAATAGTAAATCAACACCTTGTCTTTTTGCCTAGTCCAATTGTGTTGCAGC--------------
right  TAATAGTAAATCAACACCTTGTCTTTTTGCCTAGTCCAATTGTGTTGCAGC--------------
       *22*222222**222222**2*2*****2222222222222222222222222222222222222
```

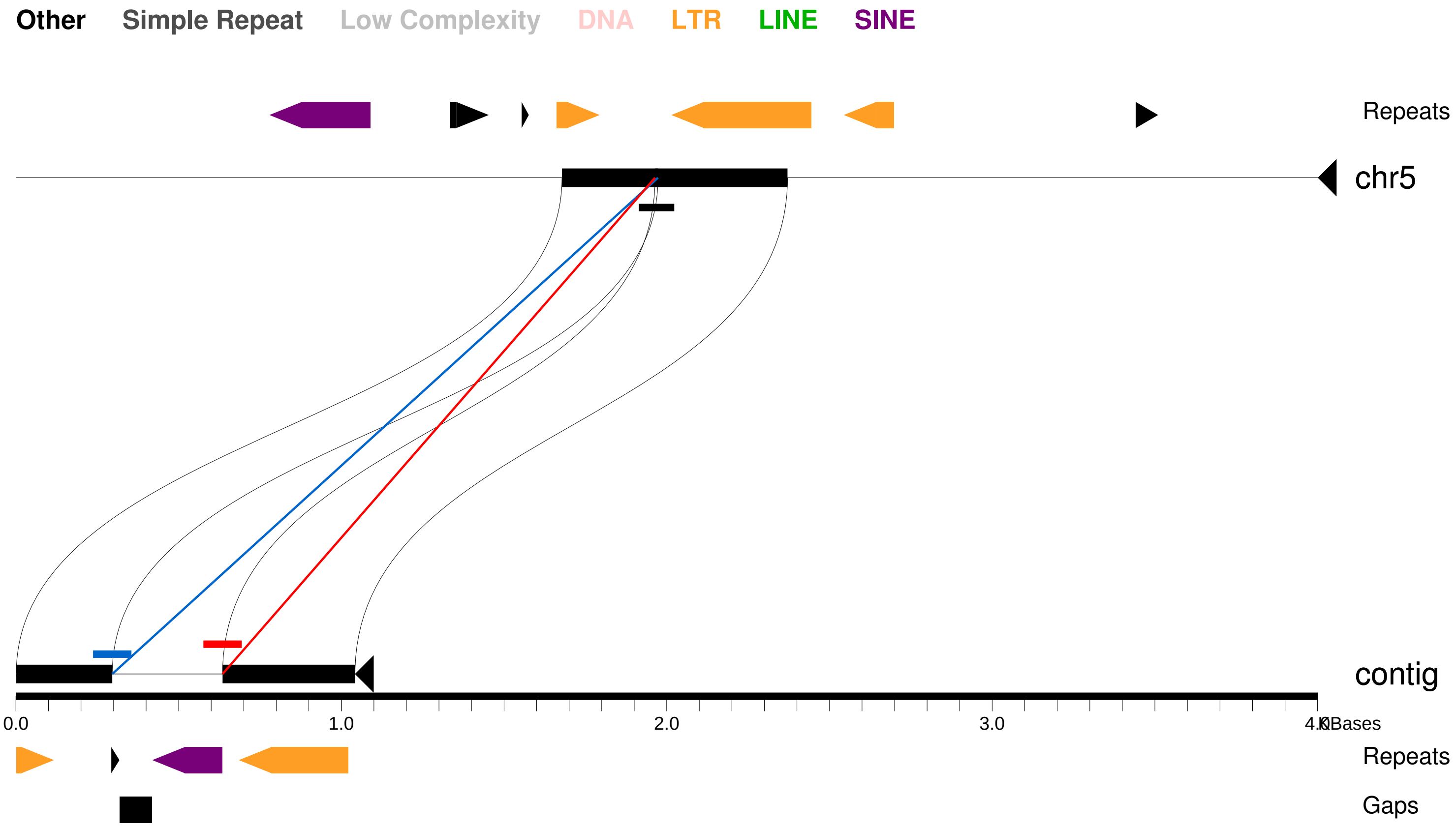

siteID: chr5_94994224 Scafftig6 - Breakpoint: chr5:94994210
chr5:94994161-94994269
Overlap Length: 11 INS size: 316 Genotyped: Yes

```
left   ACTCTATCATGTAAATACTAGAAAA-TGACATTTTAATAGT-AGCCCCCTT--------CTCTTTTTTTT
chrom  ACTCTATCATGTAAATACTAGAAAA-TGACATTTTAATAGT-AGCCCCCTT--------CTCTTTTTTTT
right  CCGCCCGCCTCGGCCTCCCAAAGTGCTGGGATTACAGGCGTGAGCCACCGCGCCCGGCCCTCTTTTTTTTT
       1*1*111*1*11111*1*1*1*1111**11***11*111**1****1**1111111111***********

left   TTTTTTTTTTTTTTTTTTTTGAGACGGAGTCTCGCTCTGTCGCCCAGGCTG-GAGTGCAGT-
chrom  AA------------GAAAGCAGAGCAATTCAGAATATGTAAACTAGACTCTGAGTTTTGTC
right  AA------------GAAAGCAGAGCAATTCAGAATATGTAAACTAGACTCTGAGTTTTGTC
       22222222222222222222***222*2**2222*2***222*2**2**22****222**2
```

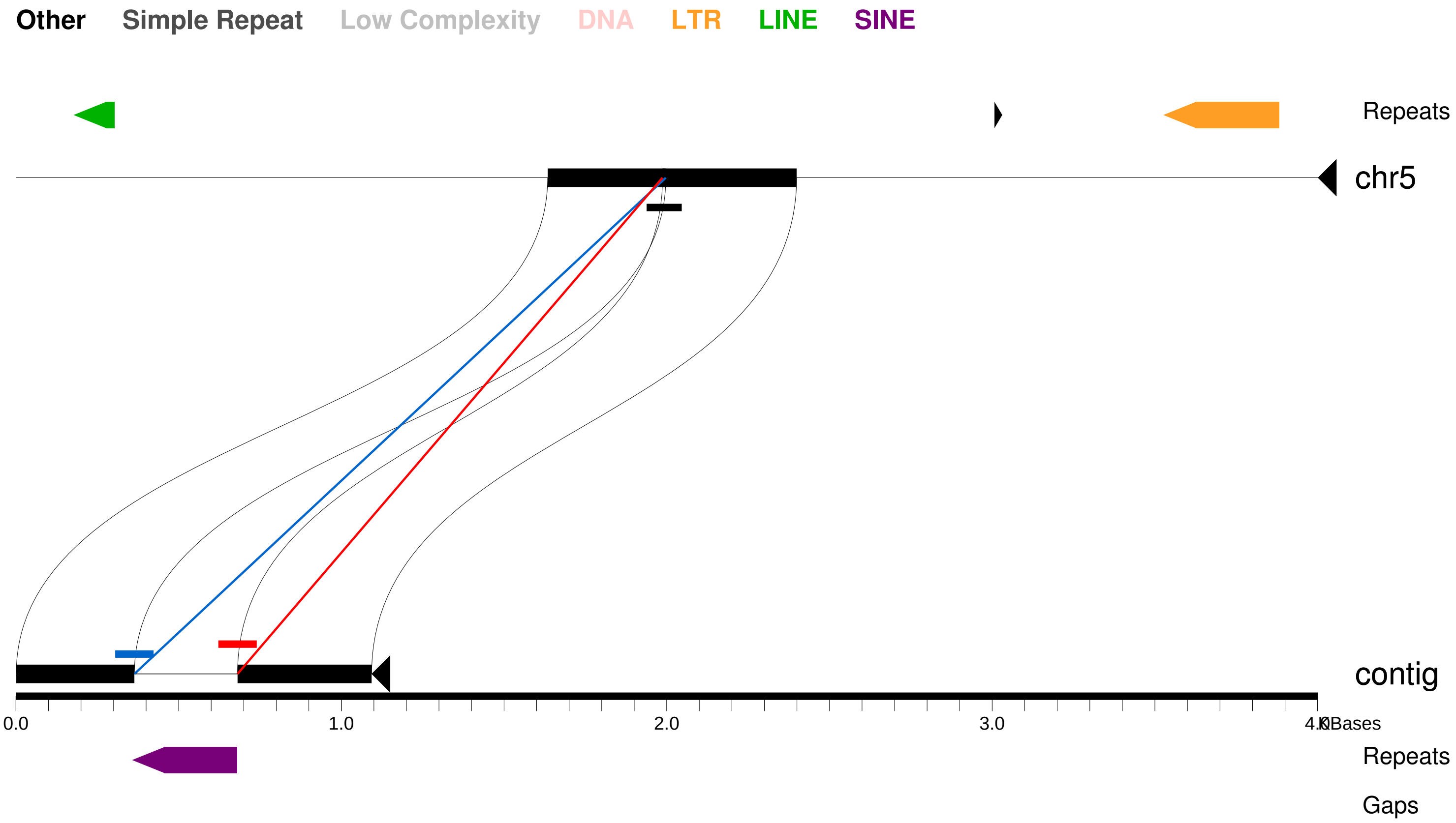

siteID: chr5_95313714 Scafftig2 + Breakpoint: chr5:95313726
chr5:95313680-95313785
Overlap Length: 14 INS size: 290 Genotyped: Yes

```
left   TTG---GTTTTTTTTT-------TTATGGC--TGCATG-GTATTCCACGGTGTGTATGTACCACTTTTTC
chrom  TTG---GTTTTTTTTT-------TTATGGC--TGCATG-GTATTCCACGGTGTGTATGTACCACATTTTC
right  CCGCCCGCCTCAGCCTCCCAAAGTGCTGGGATTACAGGCGTGAGCCACCGCGCCCGGCCACCACATTTTC
       11*111*11*11111*1111111*11***111*1**1*1**111****1*1*1111111*****2*****

left   TTTTTTTTTTTGAGACGGAGTCTCGCTCTGTCGCCCAGGCTGGAGTGCAGTGGCGGGATCTC
chrom  TTTATC-----------CAGTCTACCACTGATGGACATTTAGG-GTG-ATTCCATGTCTTTA
right  TTTATC-----------CAGTCTACCACTGATGGACATTTAGG-GTG-ATTCCATGTCTTTA
       ***2*2222222222222*****22*2***22*22**2222**2***2*2*2222*22*2*2
```

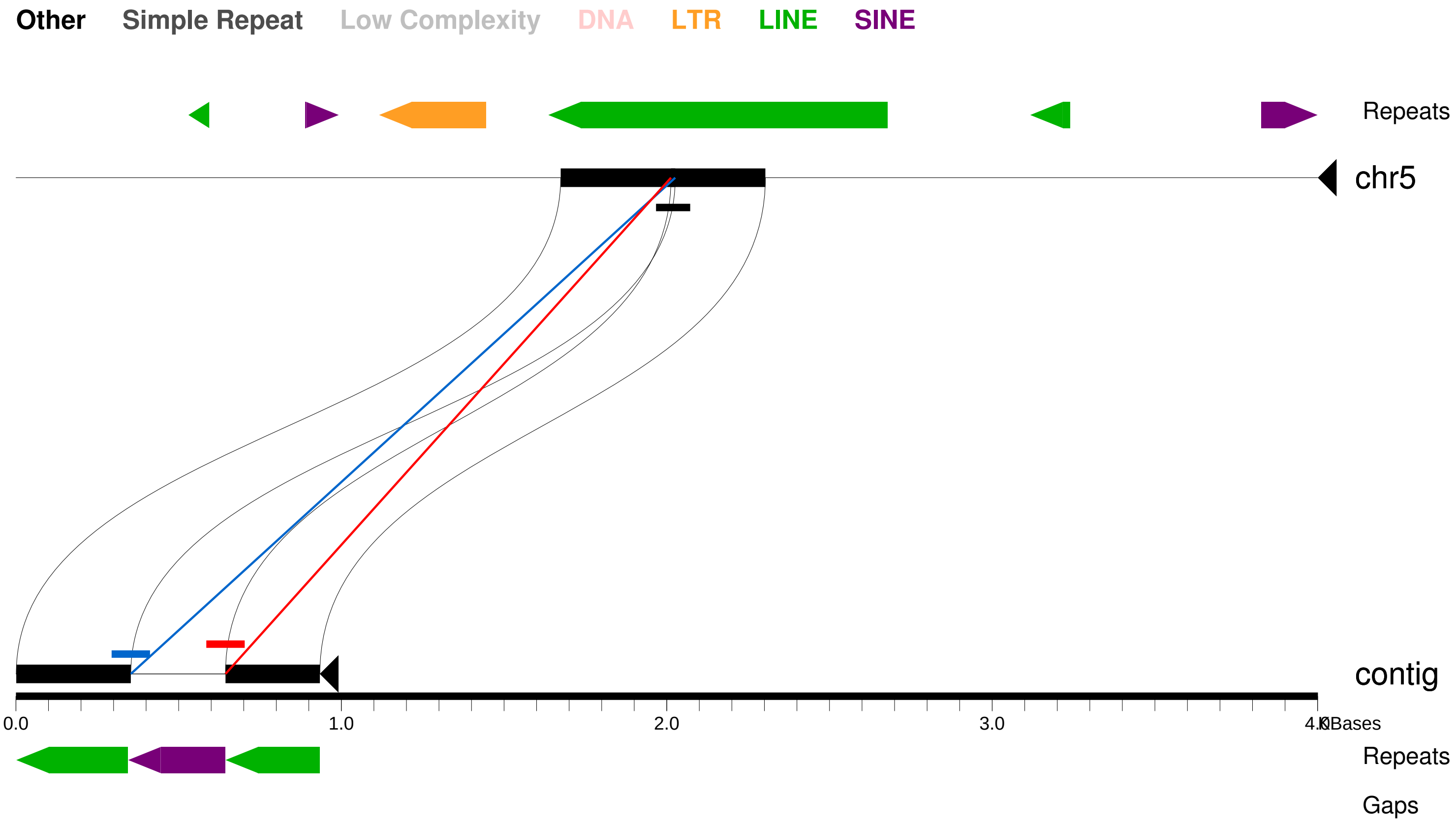

siteID: chr5_95394228 Scafftig1 - Breakpoint: chr5:95394257
chr5:95394097-95394426
Overlap Length: 10 INS size: 240 Genotyped: Yes

```
left   TTT---------CTCTGGCCACACCTACTTCCTTCATTTTCCCACAGGTGTGGAAACCAAGGGTCCTCCT
chrom  TTTAATACTTTTCTCTGGCCACACCTACTTCCTTCATTTTCCCACAGGTGTGGAAACCAAGGGTCCTCCT
right  CTGA------------GGCAGGAG--AATGGCGTGAA---CCCG-------GGAGGCGGAGCTTGC----
       1*12        1111***111*111*1*11*1*1*1111***11111111***11*11**11*1*1111

left   CAGTCAACTTCATGTTTTCAAATCTCCTTCAGAATCAGTTTTCCAGGGAAATTGACCTAAGAAATTTGGT
chrom  CAGTCAACTTCATGTTTTCAAATCTCCTTCAGAATCAGTTTTCCAGGGAAATTGACCTAAGAAATTTGGT
right  -AGTGAGC--CGAGATAGCACCACTGC----GCTCCAG----CCTGGGCGACAGAGC---GAGACTCCGT
       1***1*1*11*11*1*11**111**1*1111*111***1111**1***11*11**1*111**1*1*11**

left   ACCAGAAGGGCTCTAAGAAA---GCTGATTTTGGTCAGGAGATCGAGACCATCCTGGCTA-ACACGGTGA
chrom  ACCAGAAGGGCTCTAAGAAA---GCTGATTTTGAAGTGGGAATTTGGAGCTTGGTCACTGTAGTTGGCTA
right  CTCAAAAAAAAAAAAAAAAAAAAGCTGATTTTGAAGTGGGAATTTGGAGCTTGGTCACTGTAGTTGGCTA
       11**1**1111111**1***111**********2222**22**222**2*2*22*22**22*222**22*

left   --AACCCCG-----TCTCTACTAAAAAAAAAAATACAAAAAATTA--GCCGGGCGTGGTGGCGGGCGCCT
chrom  GTAATAGTGGATAATCTTTATTATGAAGTAGCCATCCAATGGTTAACGCTTTCACTGGTGAACTGGGATG
right  GTAATAGTGGATAATCTTTATTATGAAGTAGCCATCCAATGGTTAACGCTTTCACTGGTGAACTGGGATG
       22**2222*22222***2**2**22**22*22222*2**222***22**222222****2222*2*222

left   GTAGTCCCAGCTA--------CTCGGGAGGC----------------------
chrom  GGATACCCAGGAAGGGAATGACTTGGCTGGTGCAACTTTTCAGATTTTTGAGA
right  GGATACCCAGGAAGGGAATGACTTGGCTGGTGCAACTTTTCAG----------
       *2*22*****22*22222222**2**22**2222222222222
```

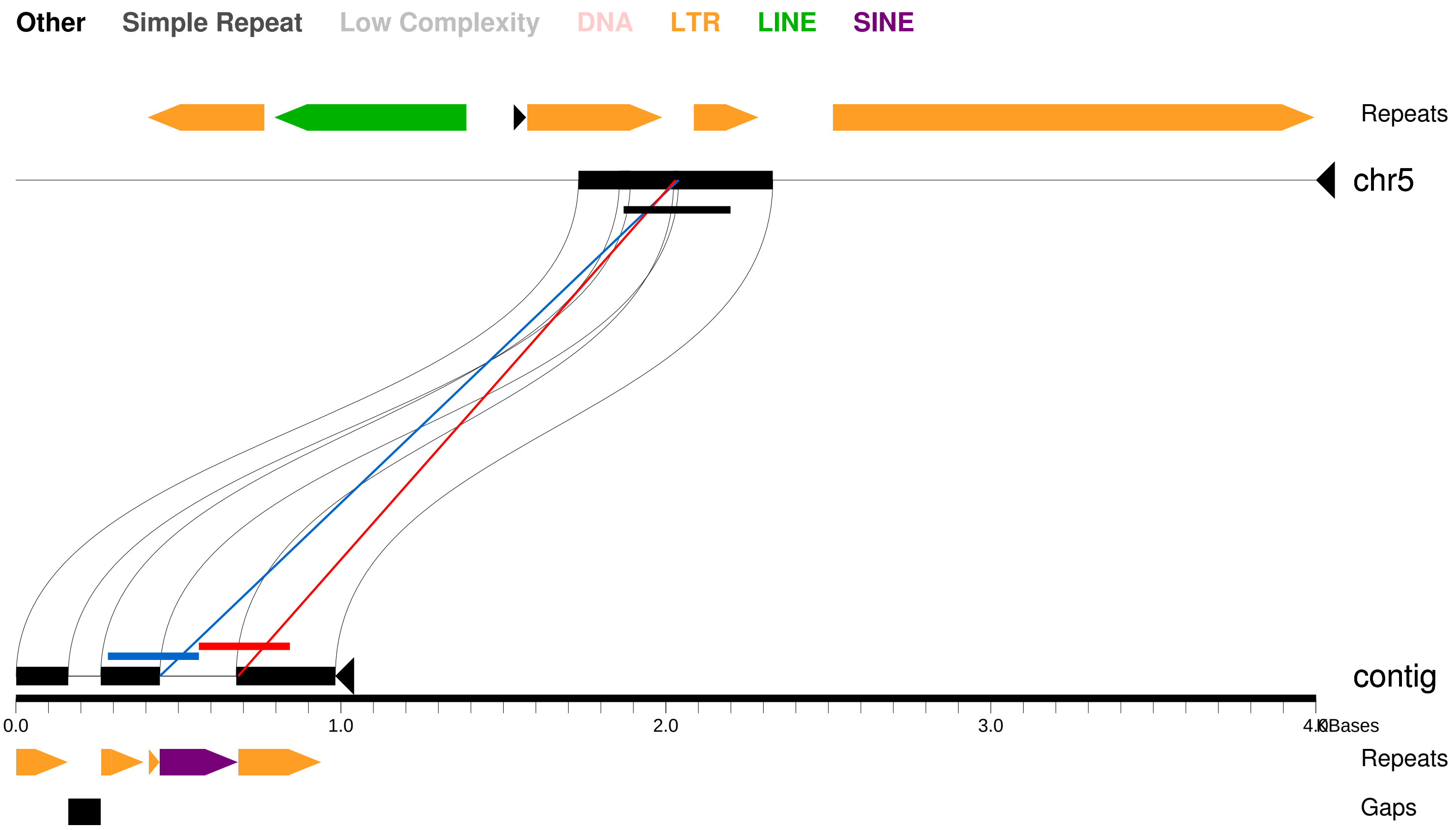

siteID: chr5_99299921 Scafftig2 - Breakpoint: chr5:99299906
chr5:99299864-99299965
Overlap Length: 14 INS size: 430 Genotyped: No

```
left     CAATCTGTGTTCAC-TCATACACAGTTTGCTTTA--GGGCTTAGT--------------GGATTTTTTTC
chrom    CAATCTGTGTTCAC-TCATACACAGTTTGCTTTA--GGGCTTAGT--------------GGATTTTTTTC
right    CCGCCCGCCTCGGCCTCCCAAAGTGCTGGGATTACAGGCGTGAGCCACCGCGCCCGGCCGGATTTTTTTC
         *111*1*11*111*1**11*1*11*1*1*11***11**11*1**111111111111111***********

left     TTTTTTTTTTTTNNNNNNNNNNNNNNNNNNNNNNNNNNNNNNNNNNNNNNNNNNNNNNNNNNNNNN
chrom    TTTCTTTGAAT-----------------ATGAGGTCATTGCTTGGAATTTCACTTAGACTTCTTTT
right    TTTCTTTGAAT-----------------ATGAGGTCATTGCTTGGAATTTCACTTAGACTTCTTTT
         ***2***222*2222222222222222222222222222222222222222222222222222222
```

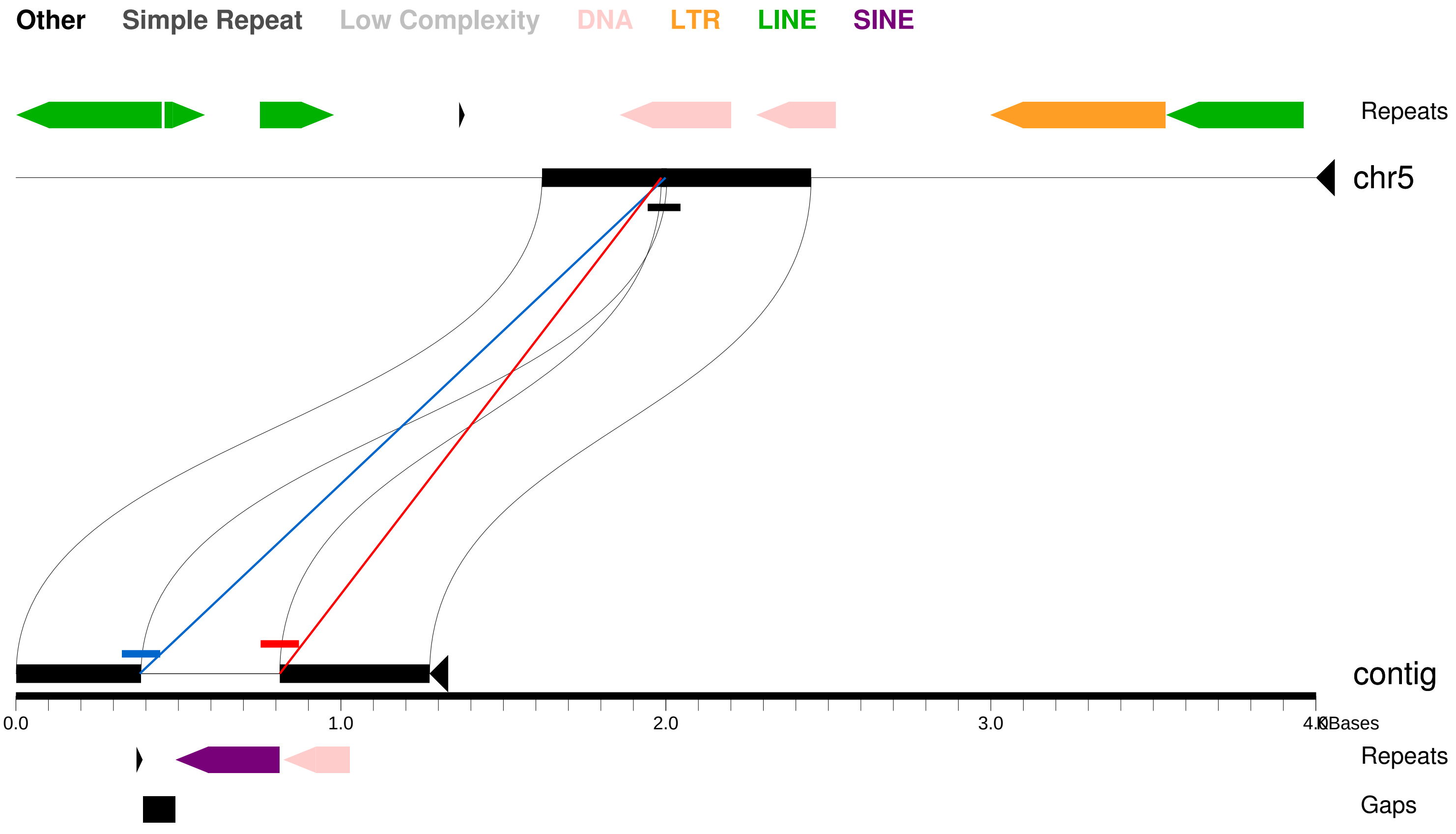

siteID: chr6_100268626 Scafftig1 + Breakpoint: chr6:100268631
chr6:100268577-100268690
Overlap Length: 6 INS size: 390 Genotyped: No

```
left   AGGGAATATATCTATATTCTATTATTACTCTATAAATAGGTGAAAGAAAACTAT-----AACATCTCACG
chrom  AGGGAATATATCTATATTCTATTATTACTCTATAAATAGGTGAAAGAAAACTAT-----AACATCATTTT
right  NNNNNNNNNNNNNNNNNNNNNNNNNNNNNNNNNNNNNNNNNNNNNNNNNNNNNNNNNNNNAACATCATTTT
       111111111111111111111111111111111111111111111111111111111111******22222

left   CCTGTAATCCCAGCACTTTGGGAGGCCGAGGCGGGCGGATCACGAGGTCAGGAG
chrom  GAGAGATTGCTATTA-TACAAAAGAATTAGGCAAGAT--TCACCAC--CACTTT
right  GAGAGATTGCTATTA-TACAAAAGAATTAGGCAAGAT--TCACCAC--CACTTT
       22222*2*2*2*22*2*22222**2222****22*2222****2*222**2222
```

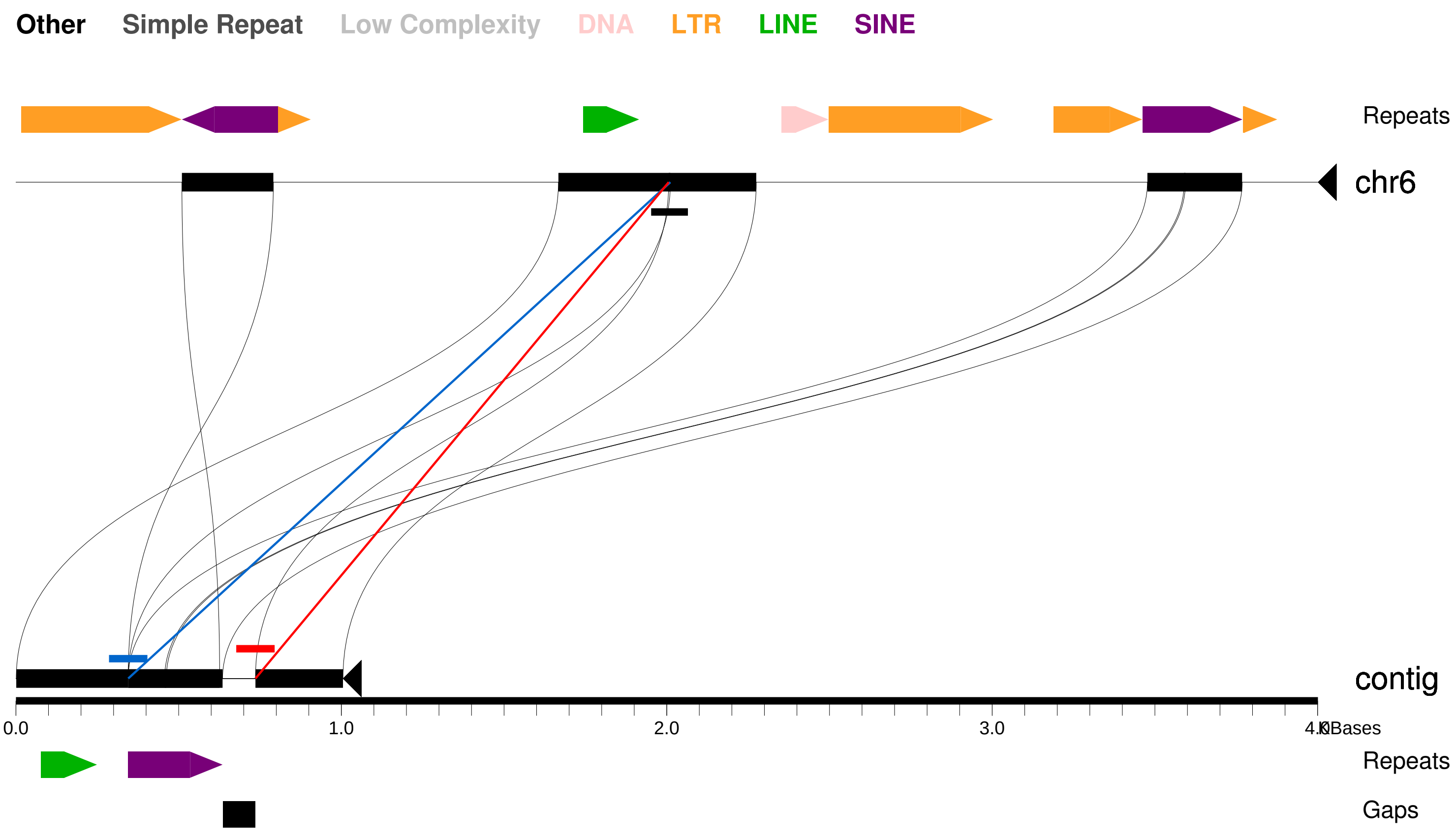

siteID: chr6_100328163 Scafftig1 + Breakpoint: chr6:100328149
chr6:100328099-100328206
Overlap Length: 10 INS size: 416 Genotyped: No

```
left    ------------GCATGTCAATGTCATAGAAGC-CAGAGTTTTACCATATGTTACTATTAGAGCAGGGTG
chrom   ------------GCATGTCAATGTCATAGAAGC-CAGAGTTTTACCATATGTTACTATTAGAGCAGGGTG
right   CCGCCCGCCTCGGCCTCCCAAAGTGCTGGGATTACAGGCGTGAGCCACCGCGCCCGGCCAG--CAGGGTG
        111111111111**1*11***1**11*1*1*111***111*111***1111111*1111**11*******

left    TATNNNNNNNNNNNNNNNNNNNNNNNNNNNNNNNNNNNNNNNNNNNNNNNNNNNNNNNNNNN
chrom   TATCTTAAAAGCAAATAGGGAGACTTGTTAGGGATATTGGTTCTTTGACAG-----------
right   TATCTTAAAAGCAAATAGGGAGACTTGTTAGGGATATTGGTTTTTTGACAG-----------
        ***222222222222222222222222222222222222222N2222222222222222222
```

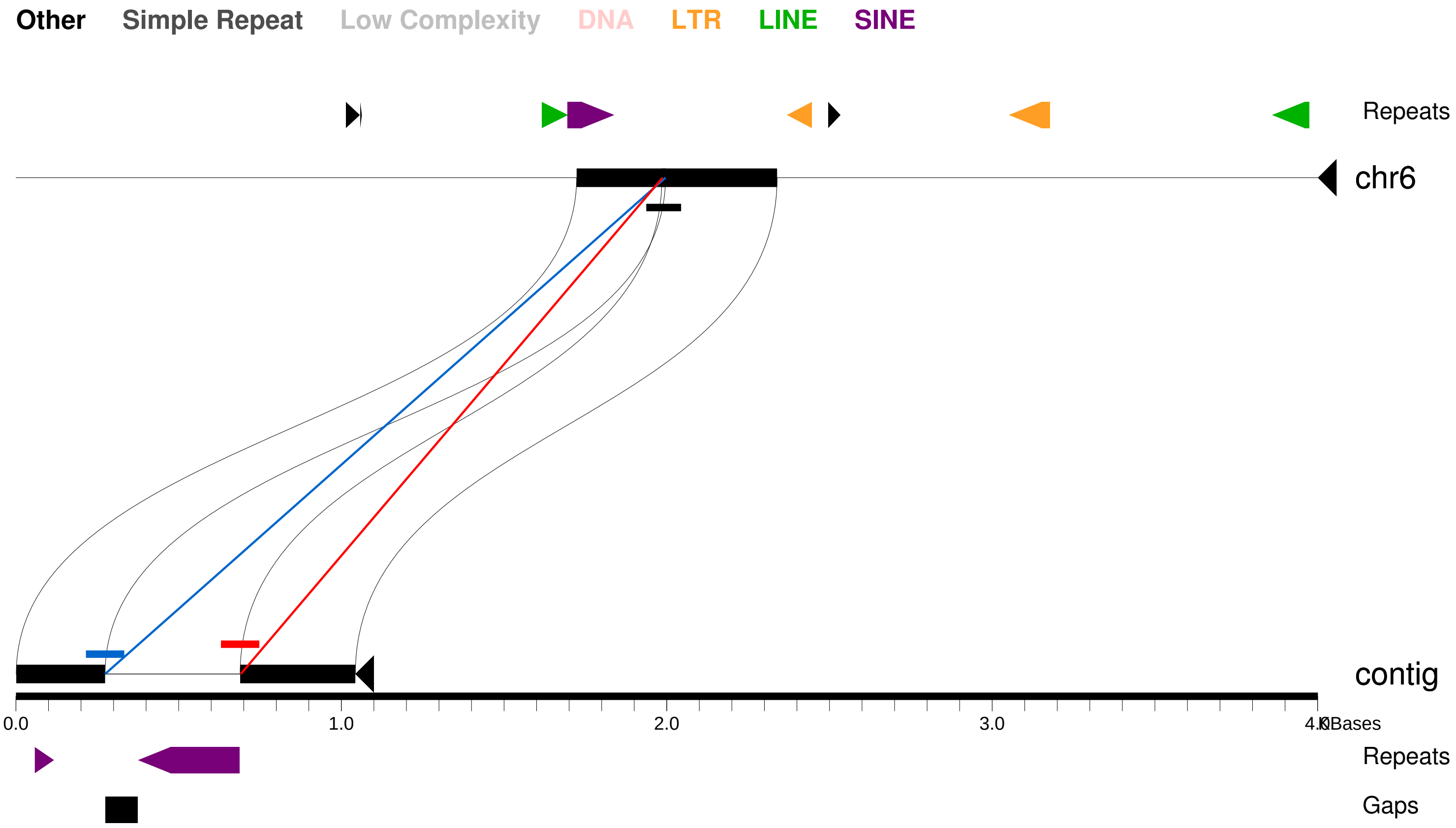

siteID: chr6_100407478 Scafftig8 + Breakpoint: chr6:100407486
chr6:100407326-100407659
Overlap Length: 14 INS size: 298 Genotyped: Yes

```
left   C----------------AAGGAAAACAAAGAAGCGGAGAAAAATTGCCCAATCAAATGAGCCAAGTAAAT
chrom  CCAA--TGCAAAGTTACAAGGAAAACAAAGAAGCAGAGAAAAATTGCCCAATCAAATGAGCCAAGTAAAT
right  TGAGGCAGGAGAATGGCGTG-AACCCCAGGGGGCGGAGCC----TGC-------AGTGAGCCGAGA----
       1N2N11N2N2N2N2NN211*1**11*1*1*11**N***111111***1111111*1******1**11111

left   TTTCAGAAATTGATACTAAAGAAACAGTTAGATACTAATTACCTGACAAATAATTCAAAATAACCACCAT
chrom  TTTCAGAAATTGATACTAAAGAAACAGTTAGATACTAATTACCTGACAAATAATTCAAAATAACCACCAT
right  -TTGCGCCACTGC-ACTCCTGCCTGGGCGACAGCGAGACTCCGTCTCAAAAAA---AAAAAAA-----AA
       1**11*11*1**11***111*11111*11*1*11111*1*1*1*11****1**111****1**11111*1

left   AAAGGTGATTGATAAACTCATTAAAACAATACAGGAGGCC---GGGCGCGGTGGCTCACGCCTGTAAT--
chrom  AAAGGTGATTGATAAACTCATTAAAACAATACAGGAACAAAATGAGAGTTTCAGCAAAGGGATAGAAAAT
right  AAA-------------------AAAACAATACAGGAACAAAATGAGAGTTTCAGCAAAGGGATAGAAAAT
       ***1111111111111111111*************2222222*2*2*22222**22*2*22*22**222

left   -----------CCCAGCACTTTGGGAGGCCGAGGCGGGCGG--------ATCACGAGGTCAGGAGA----
chrom  ATAAAAAAGAACCAAACAGATTTTGGAGCTGAGAAATGCAACAATTGAAATGAAAAATTCACTATAAAGT
right  ATAAAAAAGAACCAAACAGATTTTGGAGCTGAGAAATGCAACAATTGAAATGAAAAATTCACTATAAAGT
       22222222222**2*2**22**22*22**2***2222**2222222222**2*22*22***22*2*2222

left   -TCGAGACCATCCTGGCTAA--CACG-GTGAAACCCCG----TCTCTACTAAA---
chrom  TTCAACAGCAGACTTGATCAAGCAAGAGAGAGAATCAGAAAATTTGAAGACAACTC
right  TTCAACAGCAGACTTGATCAAGCAAGAGAGAGAATCAGAAAATTTG----------
       2**2*2*2**22**2*2*2*22**2*2*2**2*22*2*2222*2*2N1NNN11
```

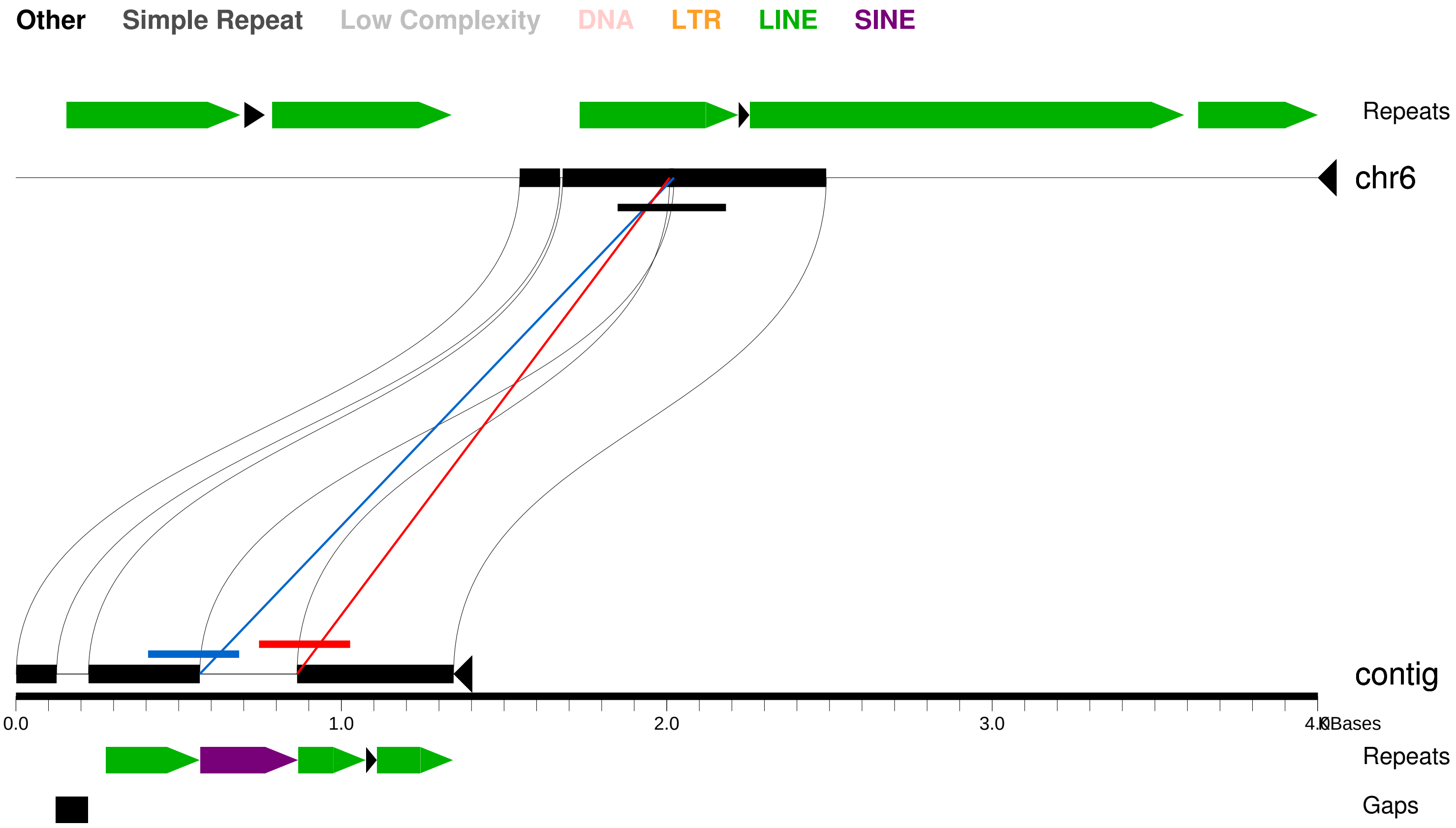

siteID: chr6_101720785 Scafftig1 + Breakpoint: chr6:101720768
chr6:101720721-101720827
Overlap Length: 13 INS size: 416 Genotyped: No

```
left     AAACTGAAAGC-TCTTTTTCTAAAAAT-CAAAGA--ACAGGACA-AGACAAA----------GACGCCTA
chrom    AAACTGAAAGC-TCTTTTTCTAAAAAT-CAAAGA--ACAGGACA-AGACAAA----------GACGCCTA
right    ---CCGCCCGCCTCGGCCTCCCAAAGTGCTGGGATTACAGGCGTGAGCCACCGCGCCCGGCCGACGCCTA
         111*1*111**1**1111**11***1*1*111**11*****1111**1**111111111111********

left     CTTTTTNNNNNNNNNNNNNNNNNNNNNNNNNNNNNNNNNNNNNNNNNNNNNNNNNNNNNNNNNN
chrom    CTTTTTATCACTGTTATACAACATAGTATTGGAAGTTCTAGCCAGAAAAAATA------------
right    CTTTTTATCACTGTTATACAACATAGTATTGGAAGTTCTAGCCAGAAAAAATA------------
         ******2222222222222222222222222222222222222222222222222222222222
```

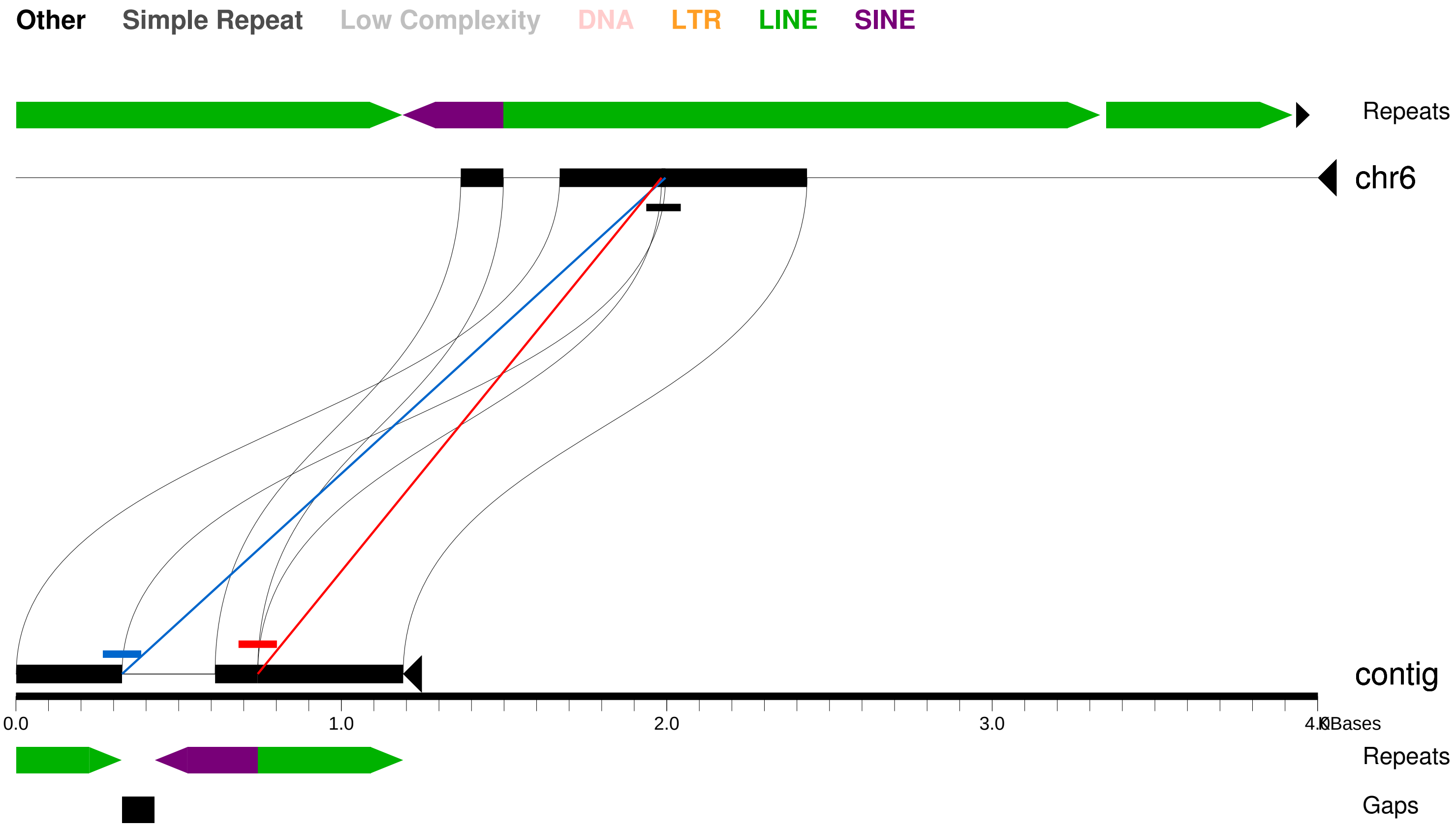

siteID: chr6_102420453 Scafftig2 + Breakpoint: chr6:102420453
chr6:102420405-102420512
Overlap Length: 11 INS size: 456 Genotyped: No

```
left    TT-----TCTTCTATTCTGTAGGTTGTCTGTTTACTCTGTCGATAGTTTCTTT--------AATTTGGTC
chrom   TT-----TCTTCTATTCTGTAGGTTGTCTGTTTACTCTGTCGATAGTTTCTTT--------AATTTGGTC
right   CCGCCCGCCTCGGCCTCCCAAAGTGCTGGGATTACA--GGCGTGAGCCACCGCGCCCGGCCAATTTGGTC
        11111111**11111**111*1**11*11*1****111*1**11**111*11111111111*********

left    CTNNNNNNNNNNNNNNNNNNNNNNNNNNNNNNNNNNNNNNNNNNNNNNNNNNNNNNNNNNNNN
chrom   CTACTTTTATTGCAATTGCTTTTGGGGACTTGGCCAAAATTATTTGCCAAG-----------
right   CTACTTTTATTGCAATTGCTTTTGGGGACTTGGCCAAAATTATTTGCCAAG-----------
        **2222222222222222222222222222222222222222222222222222222222222
```

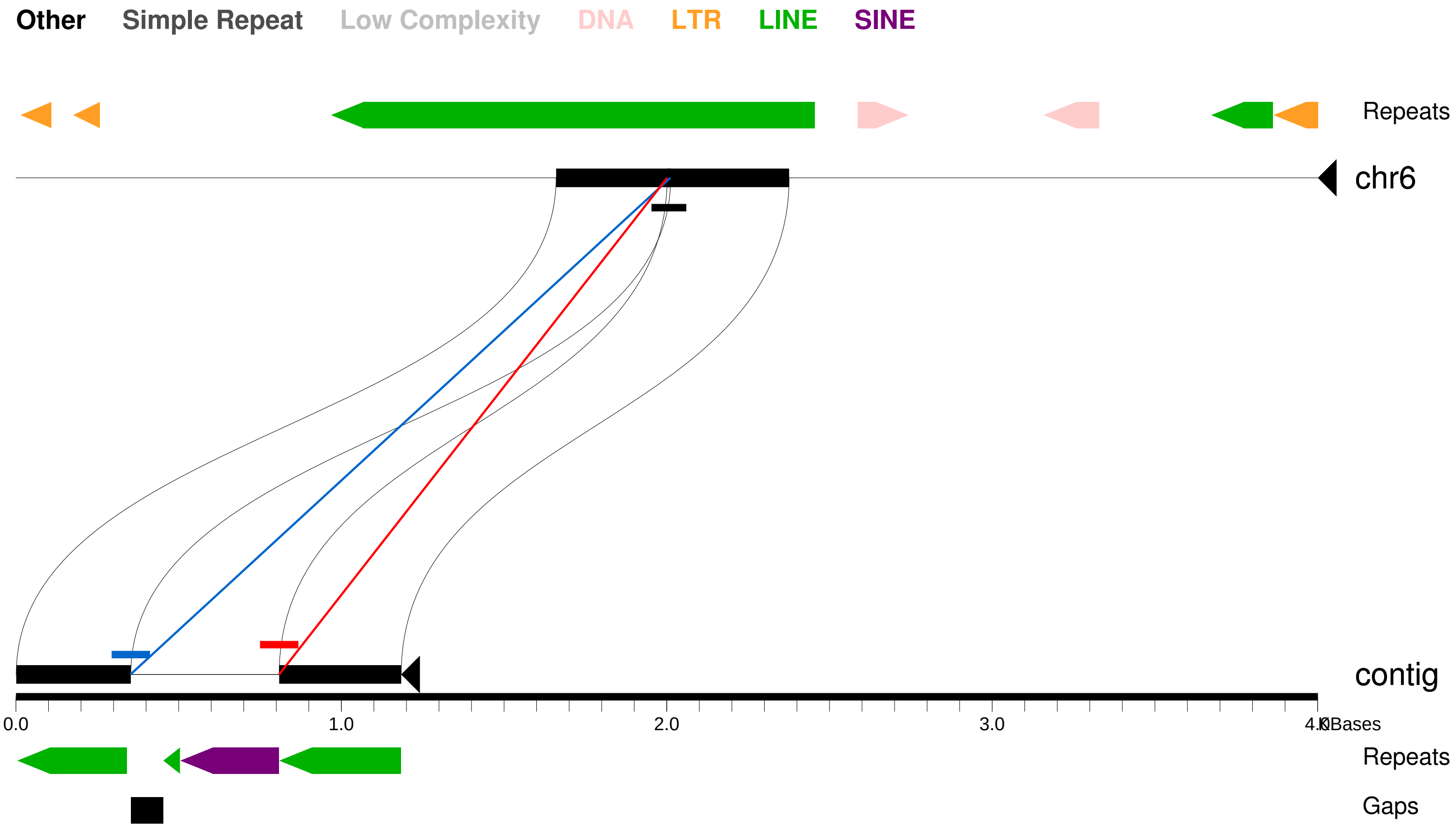

siteID: chr6_102958461 Scafftig1 + Breakpoint: chr6:102958468
chr6:102958423-102958527
Overlap Length: 15 INS size: 433 Genotyped: No

```
left    GTGTTTCTTTGTATTTGTTTGCTTTACTAAGGGAGAAAGTTTGTT--------------AAAAAGTAGAC
chrom   GTGTTTCTTTGTATTTGTTTGCTTTACTAAGGGAGAAAGTTTGTT--------------AAAAAGTAGAC
right   NNNNNNNNNNNNNNNNNNNNNNNNNNNNNNNNNNNNNNNNNNNNNNNNNNNNNNAAAAAAAAAAGTAGAC
        11111111111111111111111111111111111111111111111111111111111***********

left    TTTAGGCCGGGCGCGGTGGCTCACGCCTGTAATCCCAGCACTTTGGGAGGCCGAGGCGGGCGG
chrom   TTTA-------CACATT---TCA----TGAAAACACAGAAAACTTCGAGGTATAATTTAGTAA
right   TTTA-------CACATT---TCA----TGAAAACACAGAAAACTTCGAGGTATAATTTAGTAA
        ****2222222*2*22*222***2222**2**2*2***2*222*22****222*22222*222
```

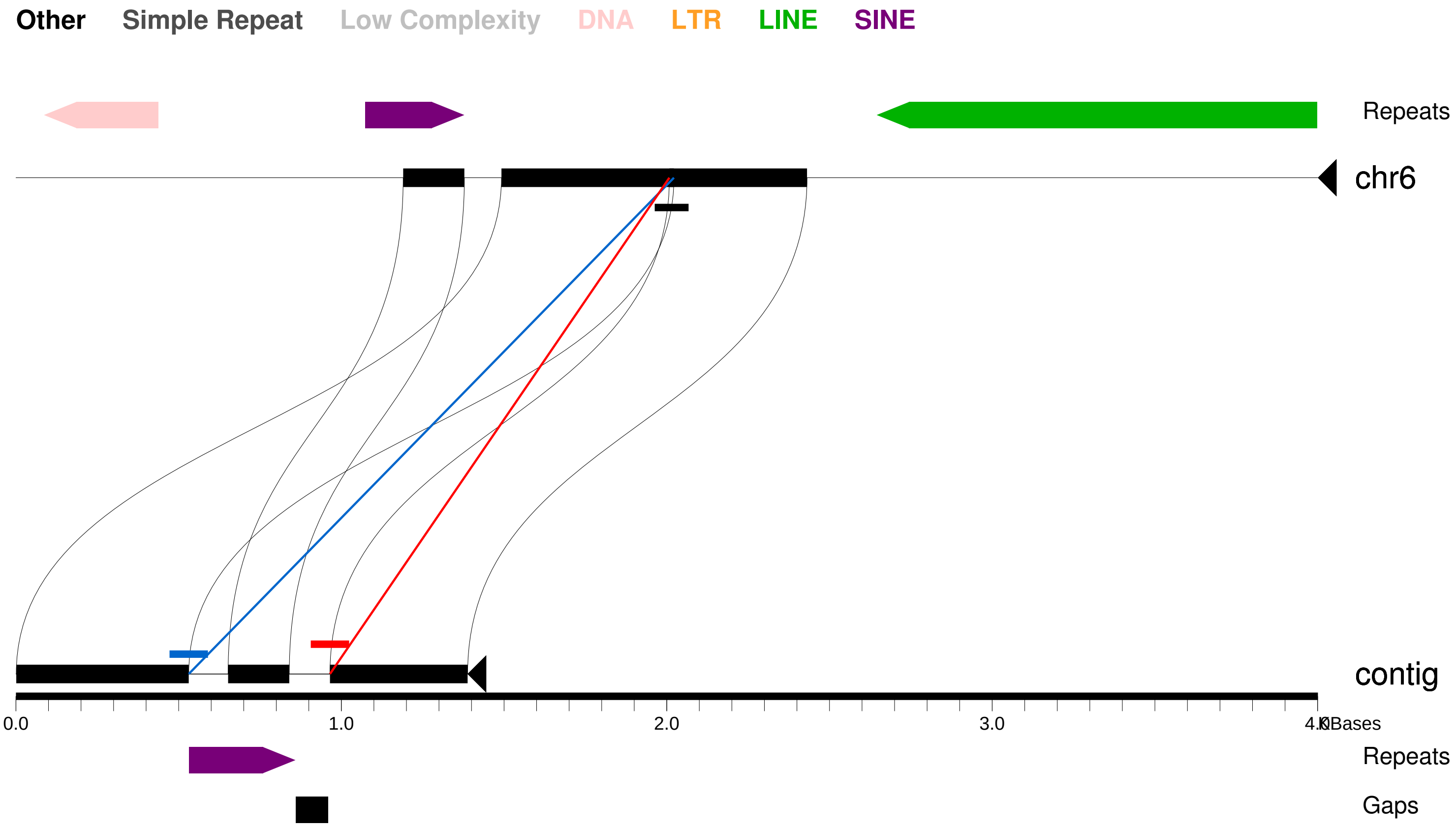

siteID: chr6_103330600 Scafftig1 - Breakpoint: chr6:103330604
chr6:103330444-103330778
Overlap Length: 15 INS size: 290 Genotyped: No

```
left    A-----GGTTTAAGCATCAAAAGAAATTATAGAATTACATATTAGAAAAGACAGATGAGTTAATTCGCTG
chrom   ATTTTAGGTTTAAGCATCAAAAGAAATTATAGAATTACATATTAGAAAAGACAGATGAGTTAATTCGCTG
right   --TTTAG--TAGAG------ACGGGGTTTCACCGTT-----TTAGCCGGGA----TG-GTC---TCGATC
        1 2222*11*11**111111*1*111**11*111**11111****1111**1111**1**1111***1*1

left    GCTTGCTTTTTTATAGTATATATACACTATAATGTTTCCATAGTAACATAGTTCTTTGTGGATTTTTCCC
chrom   GCTTGCTTTTTTATGGTATATATACACTATAATGTTTCCATAGTAACATAGTTCTTTGTGGATTTTTCCC
right   TCCTGACCTCGTG------ATCCGCCCGCCTCGGCCTCC-------CAAAGTGCTG---GGATTA--CAG
        1*1**111*11*11N1111**111*1*111111*11***1111111**1***1**1111*****111*11

left    CCTTTATGTTAAC---CGTTTTTCTACATTTTTTTTTT--------TTTTTTTTTG--------AGACGG
chrom   CCTTTATGTTAAC---CGTTTTTCTACACTTTTTTTTTAAATAGCTTGTGTGCTTGCCTTAATAAGACAG
right   GCGTGA-GCCACCGCGCCCGGCCCTACACTTTTTTTTTAAATAGCTTGTGTGCTTGCCTTAATAAGACAG
        1*1*1*1*11*1*111*111111*****2*********22222222*2*2*22***22222222****2*

left    A------------GTCTCGCT-CTGTCGC-----CCAGGCTGGAGTGCAGTGGCGGGATCTCGGCTC--A
chrom   CTACCACATTGCTGTTTCTCTTCTGTAACAATTTCCAAGCTTTC-TGAAGCCACAAGATACAGATTATAA
right   CTACCACATTGCTGTTTCTCTTCTGTAACAATTTCCAAGCTTTC-TGAAGCCANNNNNNNNNNNNNNNNN
        2222222222222**2**2**2****22*22222***2***2222**2**2221NN111NNN1NN1NNN1

left    CT-GCAA----GCTCCGCCTCCCGGGTTCACGCCATTCTCCTGCCTCAGC---------
chrom   CTTGTAATAGGGCAGATCATTCCAGAATCTTAAACTTATATTGTCAAATACGTACAATA
right   NNNNNNNNNNNNNNNNNNNNNNNNNNNNNNNNNNNNNNNNNNNNNNN--------------
        11N1N11NNNN11NNNN1N1N11N1NN11NNNNNN11N1NN11N1NN1NN
```

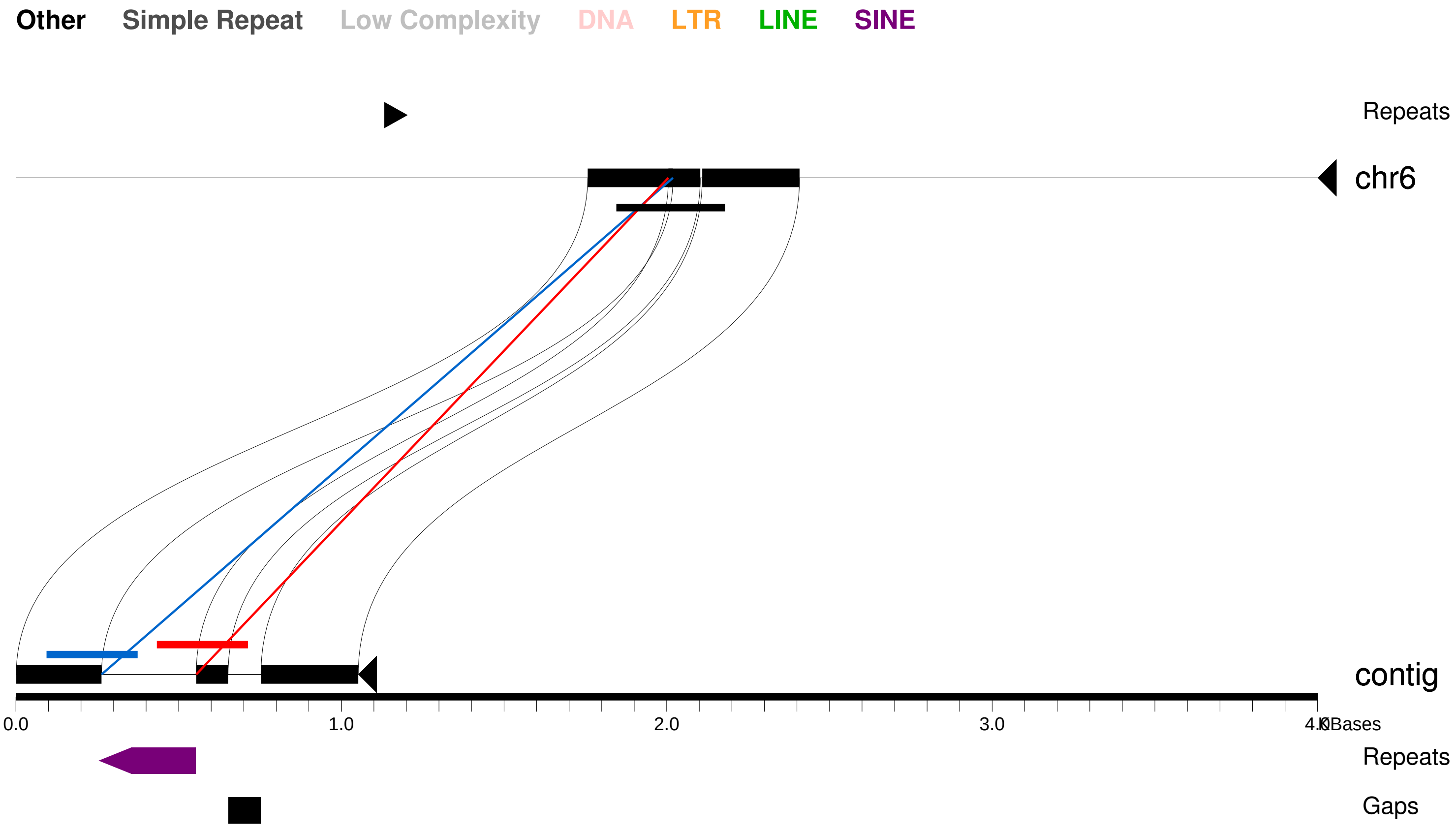

siteID: chr6_104350575 Scafftig1 - Breakpoint: chr6:104350565
chr6:104350405-104350739
Overlap Length: 15 INS size: 316 Genotyped: Yes

```
left   C----------ATTTGTTCAGTCGTGCCTGTCCTGGAGATCTTGTAGAAAGTCAGTGAAGTAGTAACATA
chrom  CCTACATGGGCATTTGTTCAGTCGTGCCTGTCCTGGAGATCTTGTAGAAAGTCAGTGAAGTAGTAACATA
right  TTTA-----------GTAGAGACGGGGTTTCACCG------TTTTAG-----CCGGGATG--GTCTC---
       1N22       1111**11**1**1*11*111*1*111111**1***11111*1*1**1*11**11*111

left   GAGTCAAAGGTCAAGTCGAGATCATTCT-CAAAGGCATGAAAAAGTCAAAGGAGACCTGGGGCTAAATGC
chrom  GAGTCAAAGGTCAAGTCGAGATCATTCT-CAAAGGCATGAAAAAGTCAAAGGAGACCTGGGGCTAAATGC
right  GA-TCTCCTGACC--TCGTGATCCGCCCGCCTCGGCCTCCCAAAGTG---------CTGGGATTACAGGC
       **1**1111*1*111***1****111*11*111***1*111*****1111111111*****11**1*1**

left   TTCTGTAGGAAATTTTGTTC--ACTCCTTCCCATTTTTTTTTTTTTTTTTTTTTTTTTTTT----------
chrom  TTCTGTAGGAAATTTTGTTC--ACTCCTTCCCATTTTTAAAGTCTTTATTCTCCTCTACCAAAGGAGAGT
right  ----GTGAGCCACCGCGCCCGGCCTCCTTCCCATTTTTAAAGTCTTTATTCTCCTCTACCAAAGGAGAGT
       1111**11*11*1111*11*111**************2222*2***2**2*22*2*2222222222222

left   --------------TTTTTTTTTTTGAGACGGAGTCTCGCTCT--GTCGCCCAGGCTG----GAGTGCAG
chrom  GACGACAAAGAAGGTCTTCAGGAAGAAGATACAGTCTGGATGTAAGTTCCACTCCCTTTTAAGAGATTAA
right  GACGACAAAGAAGGTCTTCAGGAAGAAGATACAGTCTGGATGTAAGTTCCACTCCCTTTTAAGAGATTAA
       22222222222222*2**22222222***222*****2*2*2*22**22*2*222**22222***222*2

left   TGGCGGGATCTCGGCT-------------CACTGCAAGCTCCGCCTCCCGGGTTCAC--
chrom  AGAAAATATTTCATCTAAGAATTTTTTATCATTTCT-GTTTCAACACTATAATTGACTT
right  AGAAAATATTTCATCTAAGAATTTTTTATCATTTCT-GTTTCAAC--------------
       2*22222**2**22**2222222222222**2*2*22*2*2*22*N1NNNNN11N11
```

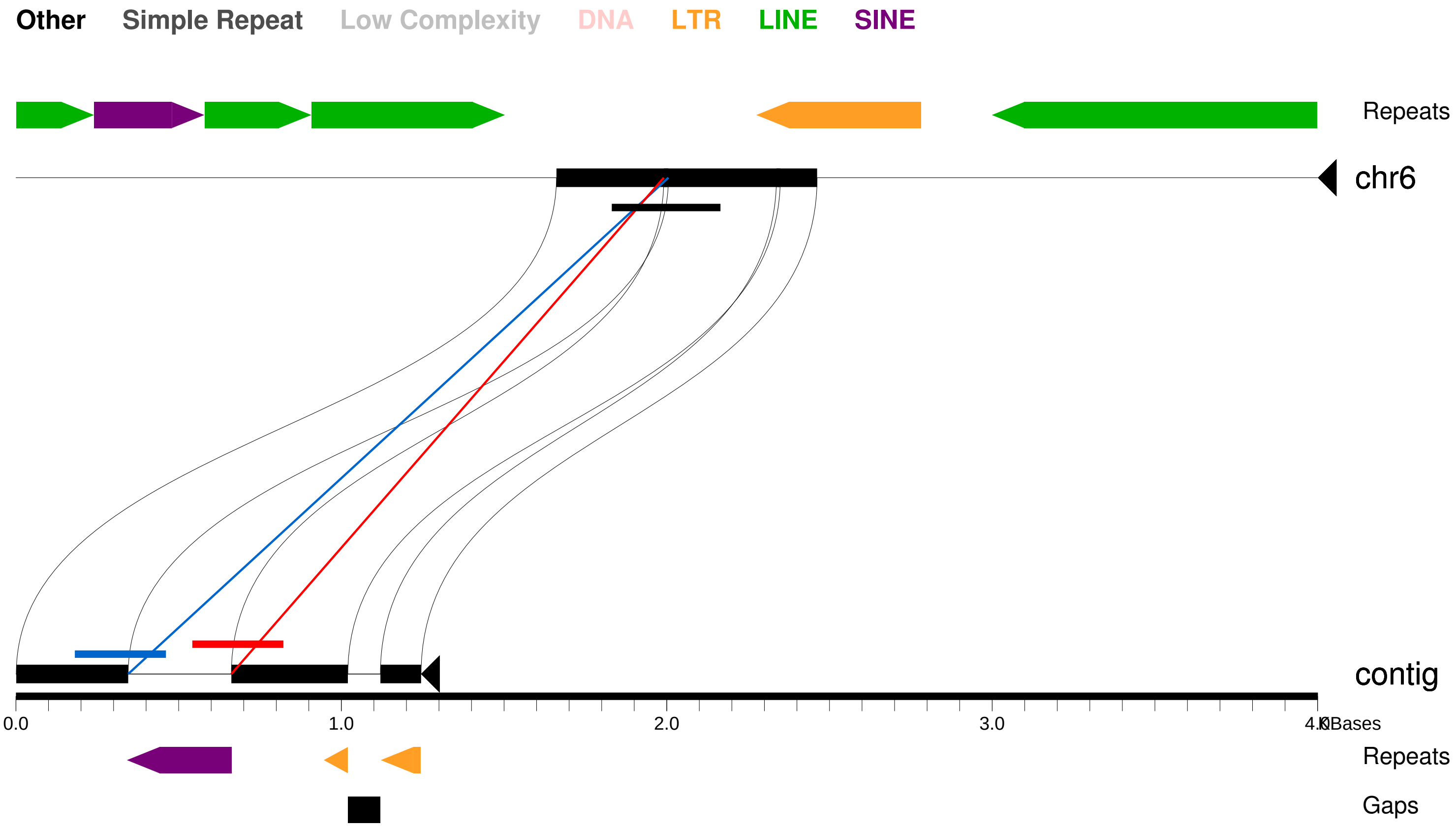

siteID: chr6_104772669 Scafftig8 + Breakpoint: chr6:104772658
chr6:104772498-104772829
Overlap Length: 12 INS size: 303 Genotyped: Yes

```
left    -----------AACACATACAGCAACCCAACTGTGCAGGCTGGAAAACAGCAGTG-TCACAGTAGACACT
chrom   CTGTCAGCTGCAACACATACAGCAACCCAACTGTGCAGGCTGGAAAACAGCAGTG-TCACAGTAGACACT
right   C----AGGAGAATGGCGTG----AACCCGGGAGGCGGAGCTTGCAGTGAGCCGAGATCCC----GCCACT
        2    22NN2N*111*1*11111*****1111*11111***1*1*111***1*1*1**1*1111*1****

left    GCAGTGAGAGGCAGCCCAGTAGAGCACACTGCACCAGACCAGCGGCTCTCAGCAGGTCTCTGAGTAGCAT
chrom   GCAGTGAGAGGCAGCCCAGTAGAGCACACTGCACCAGACCAGCGGCTCTCAGCAGGTCTCTGAGTAGCAT
right   GCACTC-----CAGCCTGGGCGA-----------CAGAGC-GAGACTCC------GTCTCAAAAAAAAAA
        ***1*111111*****11*11**11111111111****1*1*1*1***1111111*****11*11*11*1

left    GGAATATCATCCTACGCCAATGAAAGATCATGGGCCG-GGCGCGGTGGCTCACGCC------TGTAATCC
chrom   GGAATATCATCCTACGCCAATGAAAGATCATGGACATTGGCTTTGTGGTTGTAGTGAGGGGATGCCATAC
right   AAAAAAAAA---------AAAGAAAGATCATGGACATTGGCTTTGTGGTTGTAGTGAGGGGATGCCATAC
        11**1*11*111111111**1************2*222***222****2*222*22222222**22**2*

left    CAG-----------CACTTT---------GGGAGGCCGAGGCGGGCGGATCAC--------GAGGTCAGG
chrom   TAGGAGGCAGATAACAGTTTATGCGACAAGGAAAGCAGTGAGAACCGCATGATTTTCAGTGGATGCCAGC
right   TAGGAGGCAGATAACAGTTTATGCGACAAGGAAAGCAGTGAGAACCGCATGATTTTCAGTGGATGCCAGC
        2**2222222222**2***222222222**2*2**2*2*22222**2**2*222222222**2*2***2

left    A-GATCGA--GACCAT---CCCGGCTAAAACGG-TGAAACCCCGTCTCTACTAAA
chrom   ACGGAGGACTGGCCATGCTCCTGGCCCAAATATATGAATGTCCATGTCAG--AGA
right   ACGGAGGACTGGCCATGCTCCTGGCCCAAATATATGAATGTCCAT----------
        *2*222**22*2****222**2***22***2222****222**2*N11NN221N1
```

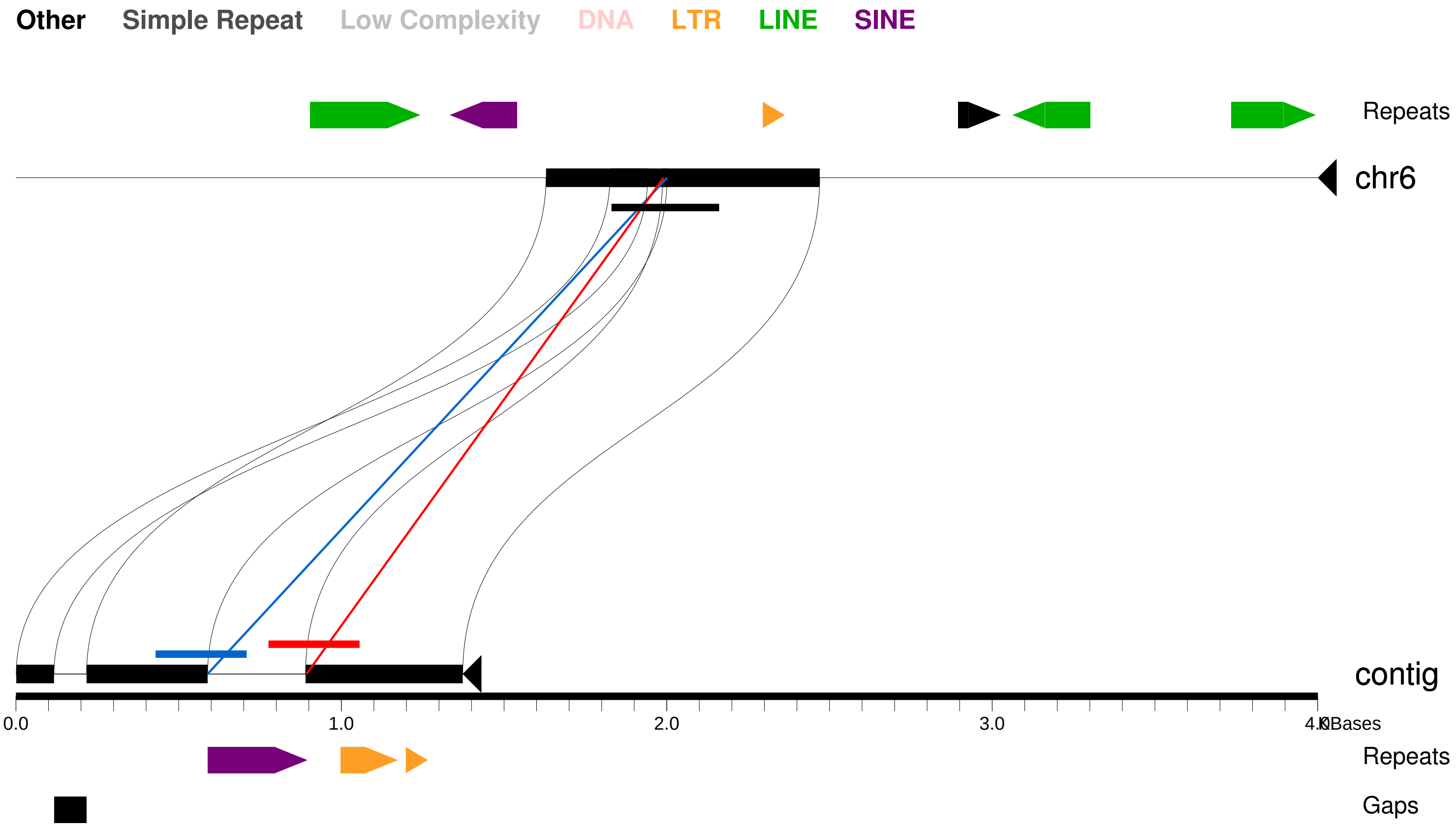

siteID: chr6_109148463 Scafftig1 + Breakpoint: chr6:109148300
chr6:109148242-109148361
Overlap Length: 2 INS size: 206 Genotyped: No

```
left   CCTAACACTCATCTCAGACTTCTGACCTCCAGAACCTTAAGTGAATATATTGGTATTGTTNNNNNNNNNN
chrom  CCTAACACTCATCTCAGACTTCTGACCTCCAGAACCTTAAGTGAATATATTGGTATTGTTTTAAGTCACA
right  ----------------GGCGTGAGCC-------ACCGTGCCCGGCCATTTTCATACT-TTTTAAGGAAC-
       1111111111111111*1*1*11*1*1111111***1*1111*111**1**11**1*1**22222NN22N

left   NNNNNNNNNNNNNNNNNNN---NNNNNNNNNNNNNNNNNNNNNNNNNNNNNNN-
chrom  AAGTTTGTAGTAATTTGTA---CTAGCAAGTTAGAATATTGATATGGCTTTGA
right  ----TTGTTGAGGTCTGCAAAGCTTTCTGCTAAATCTTTCACTGTGAACTTTC
       NNNN2222N2NNN2N22N211122NN2NNN2N2NNN2N2NNN2N22NNN22NN
```

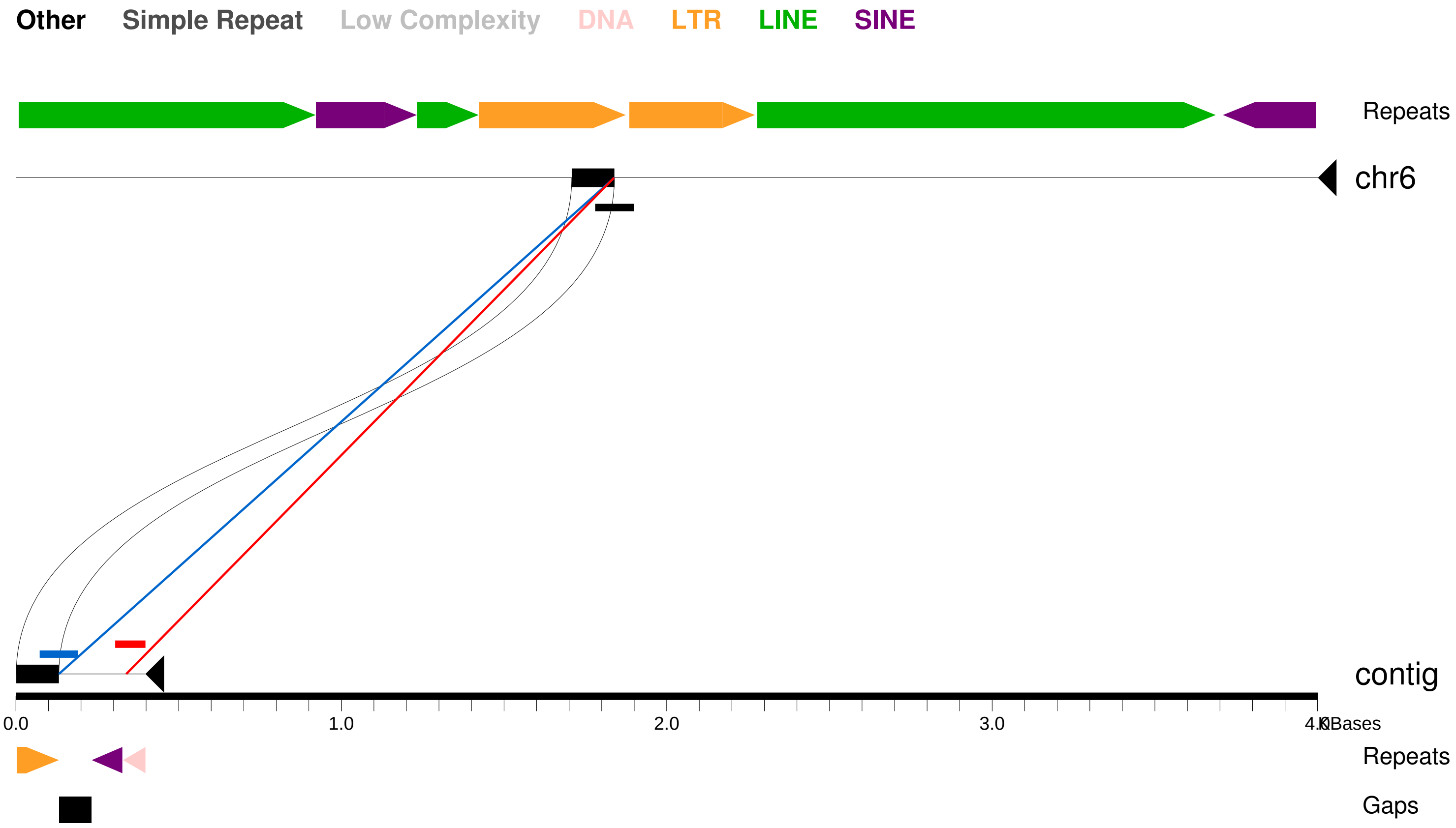

siteID: chr6_110102983 Scafftig1 - Breakpoint: chr6:110102977
chr6:110102817-110103142
Overlap Length: 6 INS size: 408 Genotyped: No

```
left    --------------------------------------TGCCGCTGCCACTCTGCCACATCTGTACCATT
chrom   CTTCCTGTCTGTTAGAACTCCTAAGTCTTTTTAACATGTGCCGCTGCCACTCTGCCACATCTGTACCATT
right   -----TGCCACCGCGCCCGGCTAATTTTTTGTA---TTTTTAGTAGAGACGGGGTTTCACCT------TG
             22N2NNNNN2NN2NN2222N2N222N22   2N*111*11*11**111*111**1**111111*1

left    CTTGTCATGTTCGTATATGCTTTGCCTACTTCCAAAAAAAGTATTGAAATGTGTACCTTTAATGAATACT
chrom   CTTGTCATGTTCGTATATGCTTTGCCTACTTCCAAAAAAAGTATTGAAATGTGTACCTTTAATGAATACT
right   TTAGCCAGGATGGTCTC-GATCTCCTGACCTCA-----------TGA----TCCACCCG---------CC
        1*1*1**1*1*1**1*11*1*1*1*11**1**111111111111***1111*11***1111111111*1

left    TCTAGCTTTATAGTTTCTTCAGATTT--------------------------------------------
chrom   TCTAGCTTTATAGTTTCTTCAGATTTAATCAGTTGACCATCTTGATCCTAATTCAAGTACTTGATGAAAA
right   TCGGCCTCCCAAAGTGCT--AGATTTAATCAGTTGACCATCTTGATCCTAATTCAAGTACTTGATGAAAA
        **111**1111*11*1**11******22222222222222222222222222222222222222222222

left    -----------------------------------TTTTTTTNNNNNNNNNNN-----------------
chrom   ATGTTGAGTACAGTAGGGTAAATGGCAGAACCCATTGATTTCTACCCACAGACAACTCCTTCCACATAAC
right   ATGTTGAGTACAGTAGGGTAAATGGCAGAACCCATTGATTTCTACCCACAGACAACTCCTTCCACATAAC
        22222222222222222222222222222222222**22***2222222222222222222222222222

left    ----------------------------------------------
chrom   ATTGTTTAGTGCCTTTGATTGTAATCATTTAAACAGCTGTAATTTC
right   ATTGTTTAGTGCCTTTGATTGTAATCATTTAAACAGCTGTAA----
        222222222222222222222222222222222222222222
```

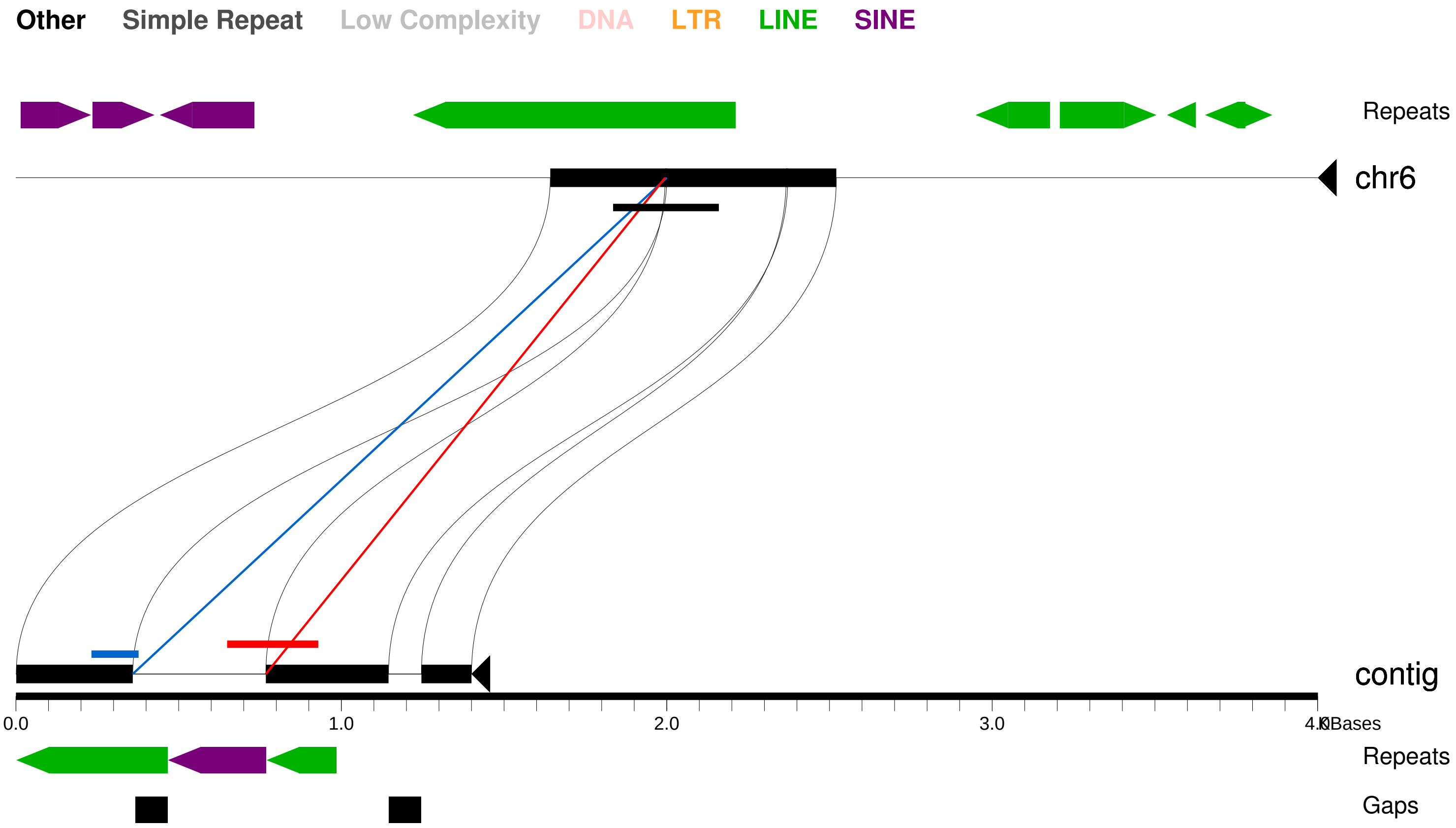

siteID: chr6_110450796 Scafftig1 - Breakpoint: chr6:110450802
chr6:110450751-110450861
Overlap Length: 9 INS size: 429 Genotyped: No

```
left    AAGAAACTCTTGGAGCTACTTCATGACAGTGGAAGCACAAAAGATAAAATG--------TTGGAAGCTGG
chrom   AAGAAACTCTTGGAGCTACTTCATGACAGTGGAAGCACAAAAGATAAAACG--------TTGGAAGCTAA
right   NNNNNNNNNNNNNNNNNNNNNNNNNNNNNNNNNNNNNNNNNNNNNNNNNNNNNNNNNNNTTGGAAGCTAA
        1111111111111111111111111111111111111111111111111N111111111**********22

left    CCGGGCGCGGTGGCTCACGCCTGTAATCCCAGCACTTTGGGAGGCCGAGGC---GGGTGG
chrom   TCCTAATTTAAAAGAAAGGAGTGTGA------CAATTTG-----CCAAGGCACAGAAAAG
right   TCCTAATTTAAAAGAAAGGAGTGTGA------CAATTTG-----CCAAGGCACAGAAAAG
        2*22222222222222*2*22***2*222222**2****22222**2****222*2222*
```

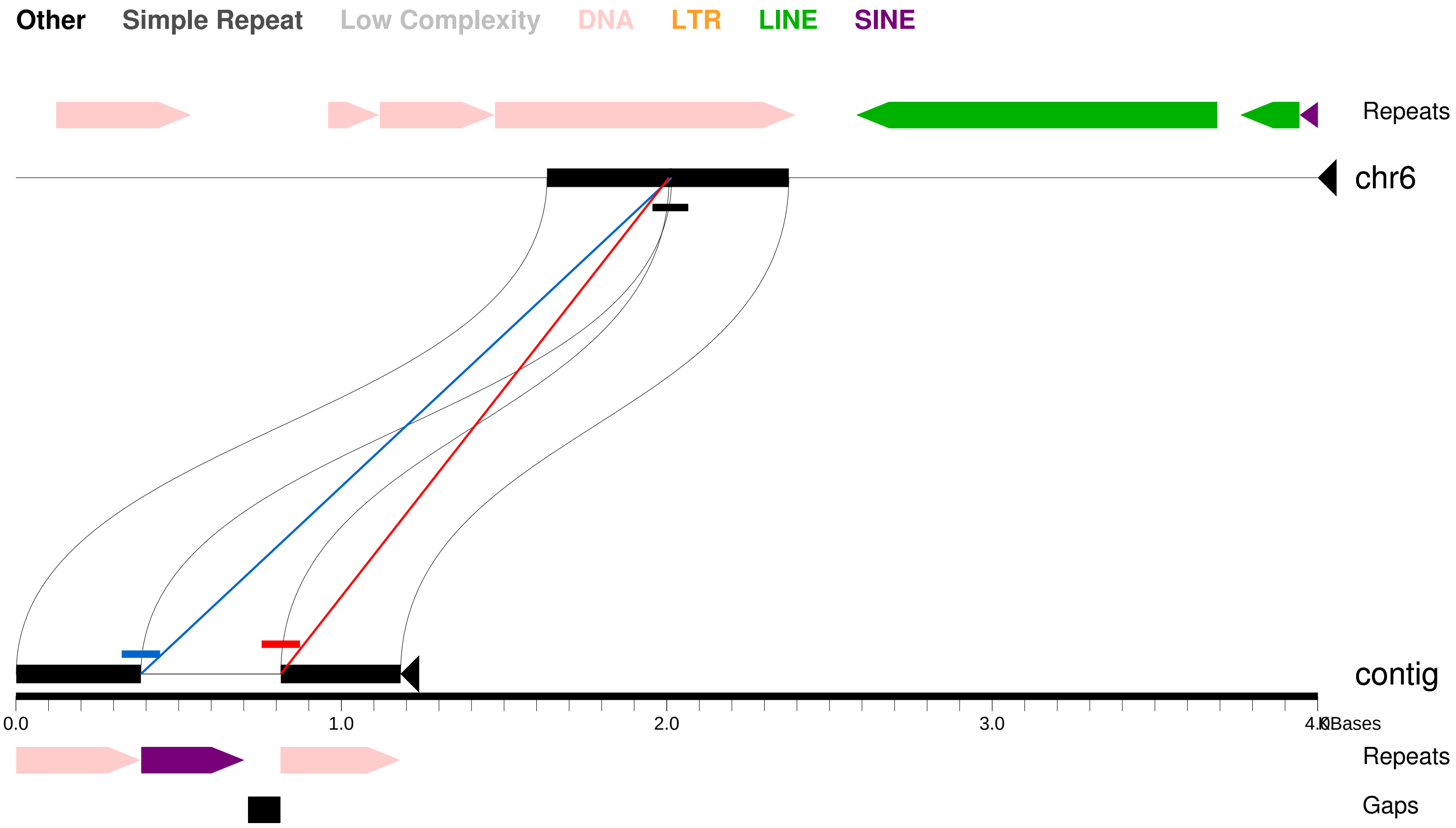

siteID: chr6_110626735 Scafftig6 - Breakpoint: chr6:110626741
chr6:110626695-110626800
Overlap Length: 14 INS size: 298 Genotyped: Yes

```
left    ACAATTCCATCAAATATTGGGCAAAGGAT--------------ATGAACAGACACTTTTCAAAAGAAGAC
chrom   ACAATTCCATCAAATATTGGGCAAAGGAT--------------ATGAACAGACGCTTTTCAAAAGAAGAC
right   CCACTGCACTCCAGCCT-GGGCAACAGAGCGAGACTCCGTCTCAAAAAAAAAAAAAAAAAAAAAGAAGAC
        1**1*1*11**1*111*1******11**111111111111111*11**1*1*1N111111*********

left    ATTTGGCCGGGCGCGGTGGCTCACGCCTGTAATCCCAGCACTTTGGGAGGCCGAGGCGGGTGG
chrom   ATTTATGTGGCCAACAAA---CATGAAAAAAAATCTCATCA---TCACTGGTCA-------TCA
right   ATTTATGTGGCCAACAAA---CATGAAAAAAAATCTCATCA---TCACTGGTCA-------TCA
        ****2222**2*222222222**2*22222****2**2**222*2222**2*22222222*22
```

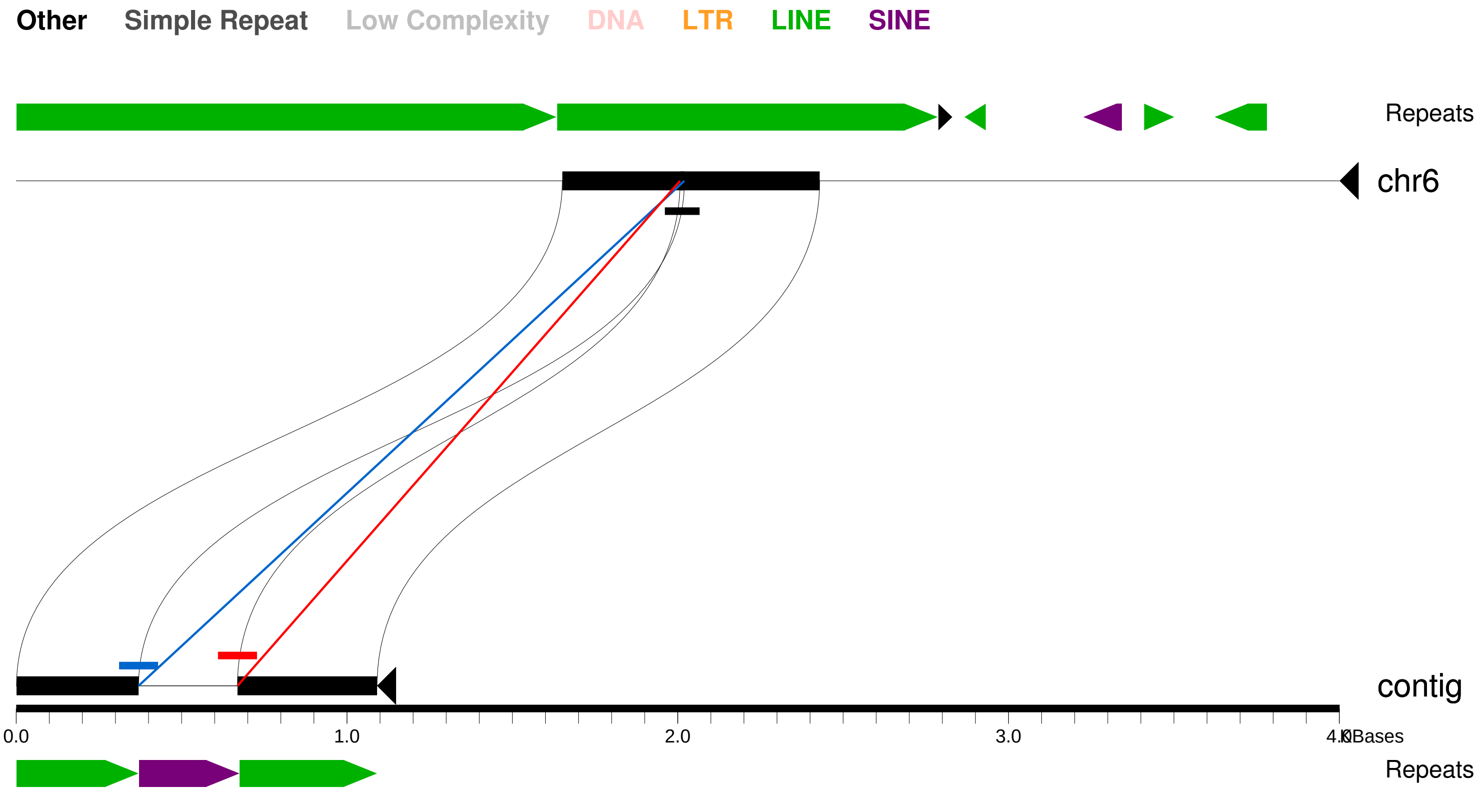

siteID: chr6_114548387 Scafftig2 - Breakpoint: chr6:114548398
chr6:114548352-114548457
Overlap Length: 14 INS size: 300 Genotyped: Yes

```
left     CTCTACATGTAAAACTA------ATAGCTAATATGTG-CT------AATTGGCACTATTAAGATATACTT
chrom    CTCTACATGTAAAACTA------ATAGCTAATATGTG-CT------AATTGGCACTATTAAGATATACTT
right    CACTGCACTCCAGCCTGGGCGACAGAGCGAGACTCCGTCTCAAAAAAAAAAAAAAAAAAAGATATACTT
         *1**1**1111*11**1111111*1***1*111*11*1**111111**11111*11*11***********

left     CTTGGCCGGGCGCGGTGGCTCACGCCTGTAATCCCAGCACTTTGGGAGGCCGAGGCGGGCGG
chrom    CTTTTTGATGAAAGGT------------TTATTGTTGCCTTTTGATACGTT-ATGGATAAAA
right    CTTTTTGATGAAAGGT------------TTATTGTTGCCTTTTGATACGTT-ATGGATAAAA
         ***222222*222***222222222222*2**2222**22****22*2*222*2*2222222
```

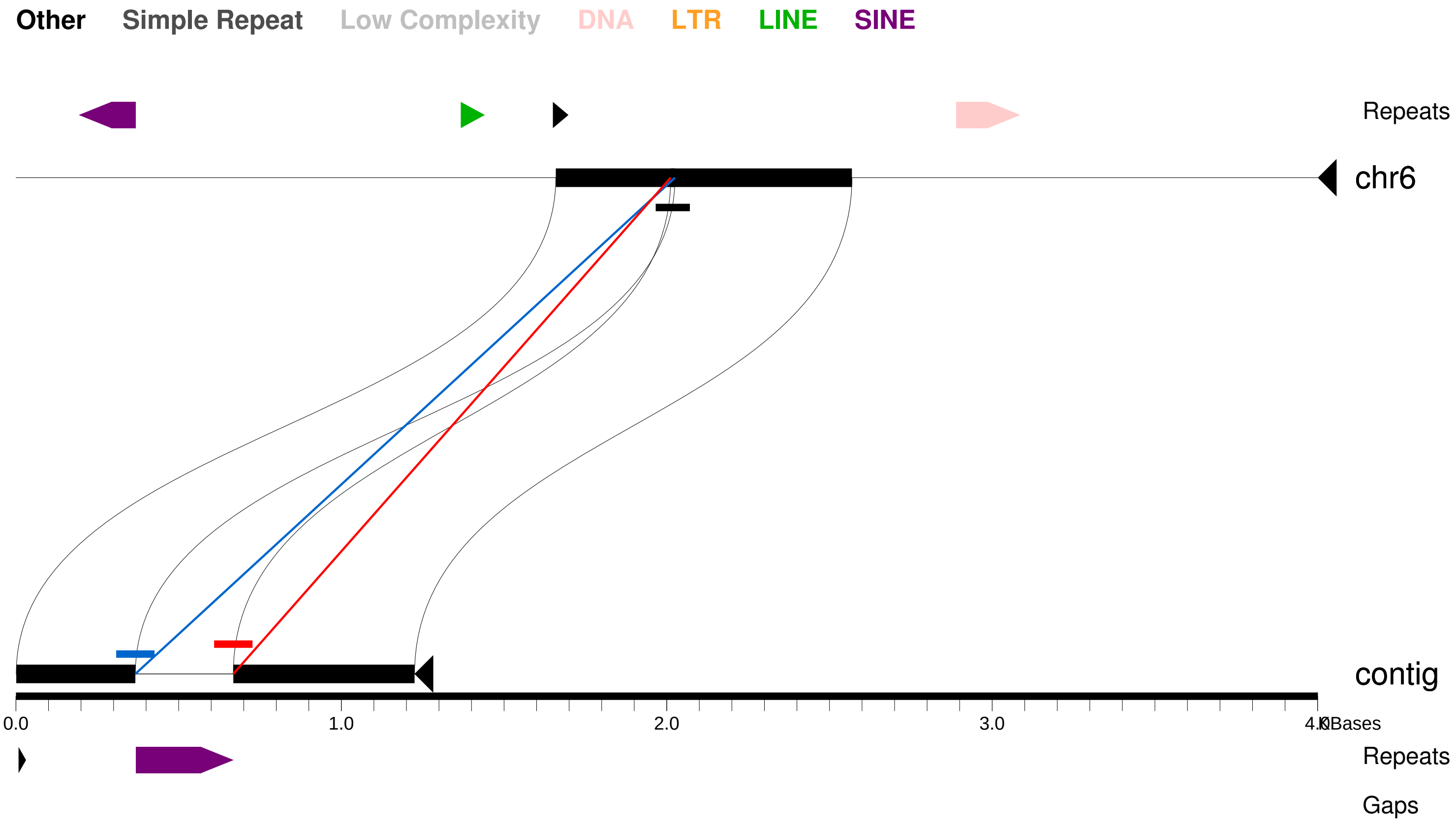

siteID: chr6_115788651 Scafftig1 - Breakpoint: chr6:115788630
chr6:115788598-115788686
Overlap Length: 17 INS size: 492 Genotyped: No

```
left    T-----------CTTGG------AAAGA-CT----TTCTAGTTATTTGAGA--------GGACTTGGGTG
chrom   T-----------CTTGG------AAAGA-CT----TTCTAGTTATTTGAGA--------GGACTTGGGTG
right   TGATCCGCCCGCCTCGGCCTCCCAAAGTGCTGGGATTACAGGCGTGAGCCACCGCGCCCGGCCTTGGGTG
        *11111111111**1**111111****11**1111**11**111*11*11*11111111**1********

left    TTGTTTTCTTTTTTTTTTTTNNNNNNNNNNNNNNNNNNNNNNNNNNNNNNNNNNNNNNNNNNNNNNNNNN
chrom   TTGTTTTCTAAGCTGTTTTTGTGTTAGGGGGCACCTCAAGCCCAGTAAT---------------------
right   TTGTTTTCTAAGCTGTTTTTGTGTTAGGGGGCACCTCAAGCCCAGTAAT---------------------
        *********2222*2*****22222222222222222222222222222222222222222222222222

left    NNNNNNNNN
chrom   ---------
right   ---------
        222222222
```

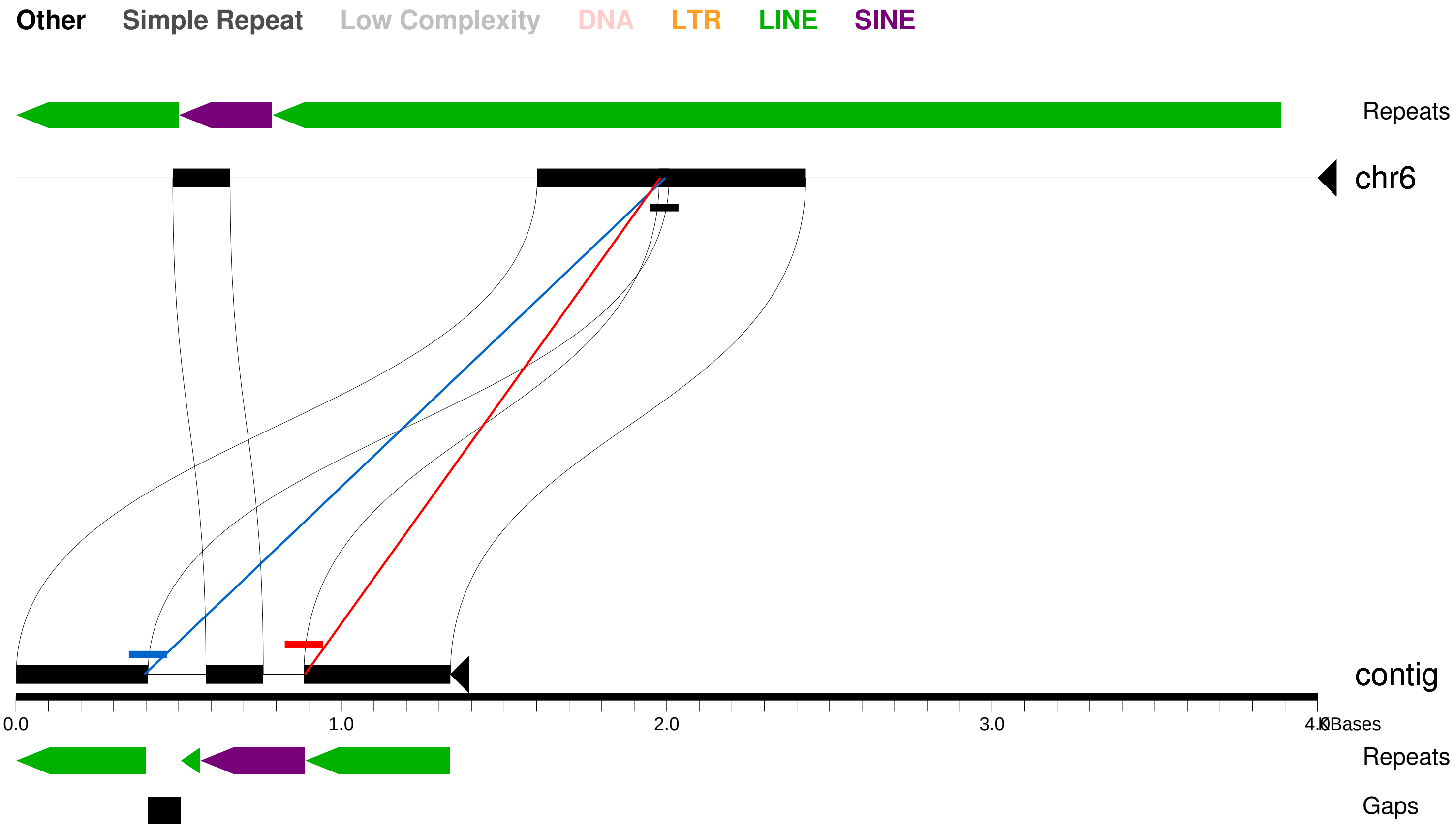

siteID: chr6_116307105 Scafftig9 - Breakpoint: chr6:116307081
chr6:116307037-116307140
Overlap Length: 16 INS size: 279 Genotyped: Yes

```
left     AGT---------ATCAATACTTTCTCCCAACAAGCAGCAAAATCATTATGATT------AGAATGCTGAG
chrom    AGT---------ATCAATACTTTCTCCCAACAAGCAGCAAAATCATTATGATT------AGAATGCTGAG
right    CCTGGGCGACAGAGTGAGACTCCGTCTCAAAAAAAAAAAAAAAAAAAAAAAAAAAAAAAAGAATGCTGAG
         11*111111111*111*1***111**1***1**11*11****11*11*11*11111111***********

left     TGCTTGCACTTTGGGAGGCCGAGGCGGGCGGATCACGAGGTCAGGAGATCGAGACCATCCCGGC
chrom    TGCTTATATTATA-----CATAT--------ATTTTATAGT--AGACAACTTTTCAATACAGTG
right    TGCTTATATTATA-----CATAT--------ATTTTATAGT--AGACAACTTTTCAATACAGTG
         *****22*2*2*222222*22*222222222**222222**222**2*2*2222*2**2*2*22
```

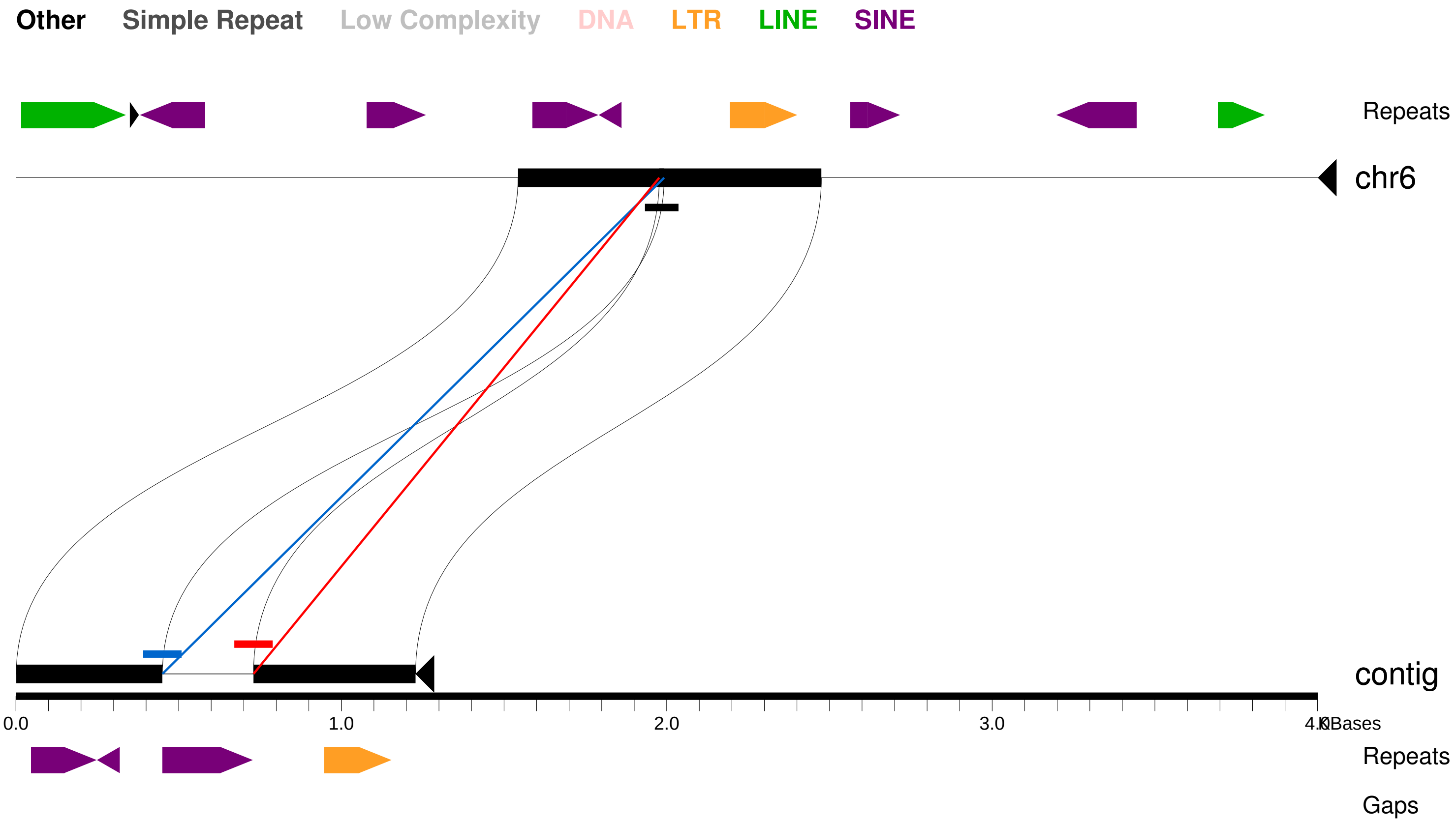

siteID: chr6_116898670 Scafftig4 + Breakpoint: chr6:116898671
chr6:116898627-116898730
Overlap Length: 16 INS size: 301 Genotyped: Yes

```
left    TTAAAGCCCAGCTCTCTTTGCCACCATTCGCCCATTTC-----TGATTG------------CATGTTCAA
chrom   TTAAAGCCCAGCTCTCTTTGCCACCATTCGCCCATTTC-----TGATTG------------CATGTTCAA
right   TCCA--CCCGCCTCGGCCTCCCAAAGTGCTGGGATTACAGGCGTGAGCCACCGCGCCCGGCCATGTTCAA
        *11*11***11***1111*1***111*1*1111***1*11111***111111111111111*********

left    CACTTCTTTTTTTTTTTTTTTGAGACGGAGTCTCGCTCTGTCGCCCAGGCCGGACTGCGGACTGCA
chrom   CACTTCTAACTGAAAGTCCTAAAG----TGTC-CTTAAAATATATCATGTCTGATT----------
right   CACTTCTAACTGAAAGTCCTAAAG----TGTC-CTTAAAATATATCATGTCTGATT----------
        *******222*22222*22*22**22222***2*222222*2222**2*2*2**2*2222222222
```

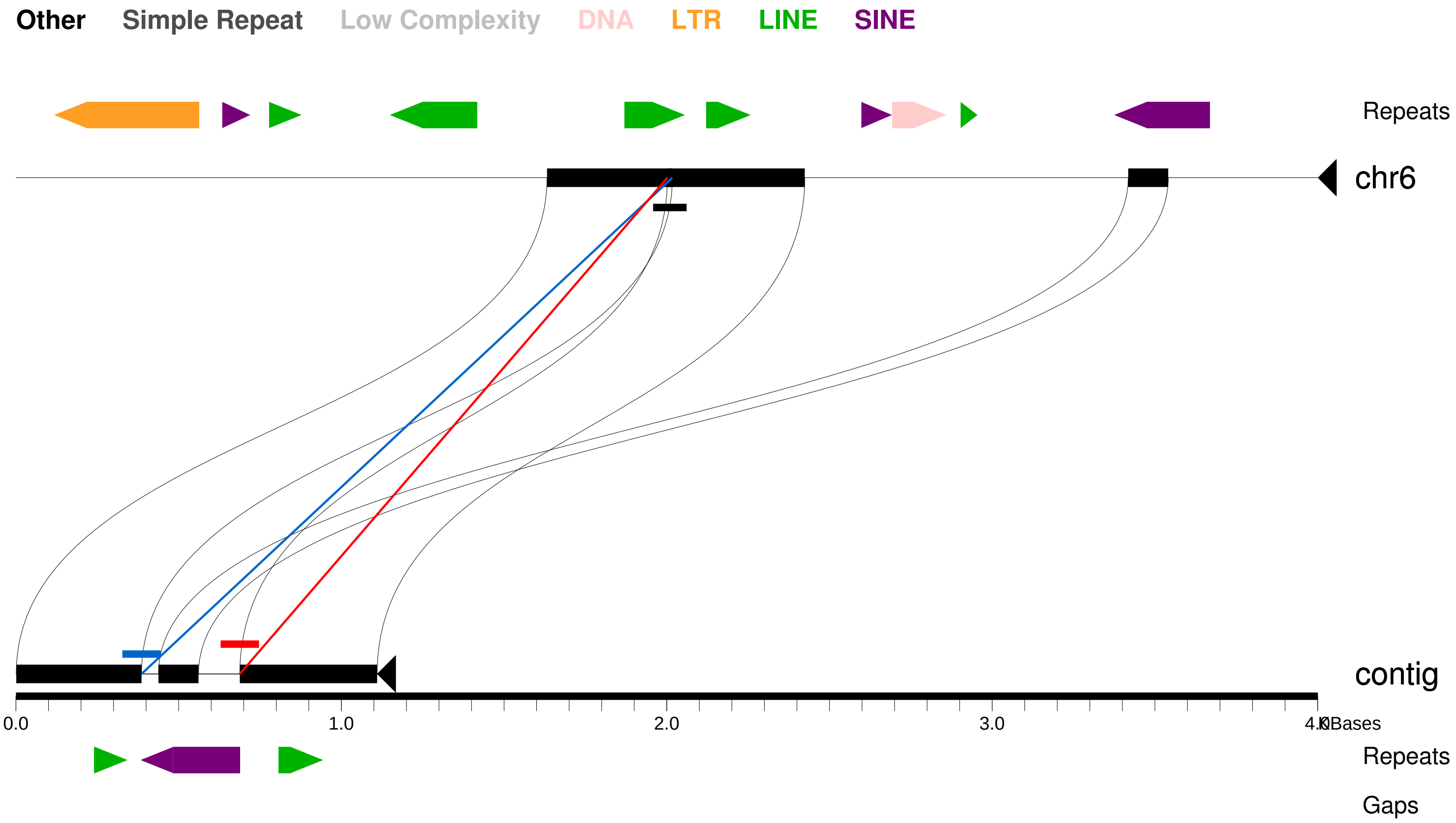

siteID: chr6_118626169 Scafftig10 + Breakpoint: chr6:118626149
chr6:118626103-118626208
Overlap Length: 14 INS size: 276 Genotyped: Yes

```
left     ACTTCTAAAAC---TTACACAGAGA-------GAAATTGTTAC---AAGTTTACATCACAGTCAAAGATC
chrom    ACTTCTAAAAC---TTACACAGAGA-------GAAATTGTTAC---AAGTTTACATCACAGTCAAAGATC
right    TGGTCTCGATCTCCTGACCTCGTGATCCGCCCGCCTCGGCCTCCCAAAGTGCTGGGATTAGTCAAAGATC
         111***11*1*111*1**111*1**1111111*11111*111*111****111111111***********

left     TTTTTTTTTTTTTTTTTT--TTCTTTGAGACGGAGTCTCGCTCTGTCGCCCAGGCTGGAGTGCA
chrom    TTTAGTGTCACAGATTGTCATTATATTAG-CAGAGAA------------ACAGAATCAACAG--
right    TTTAGTGTCACAGATTGTCATTATATTAG-CAGAGAA------------ACAGAATCAACAG--
         ***22*2*222222**2*22**2*2*2**2*2***222222222222222***22*22*22*22
```

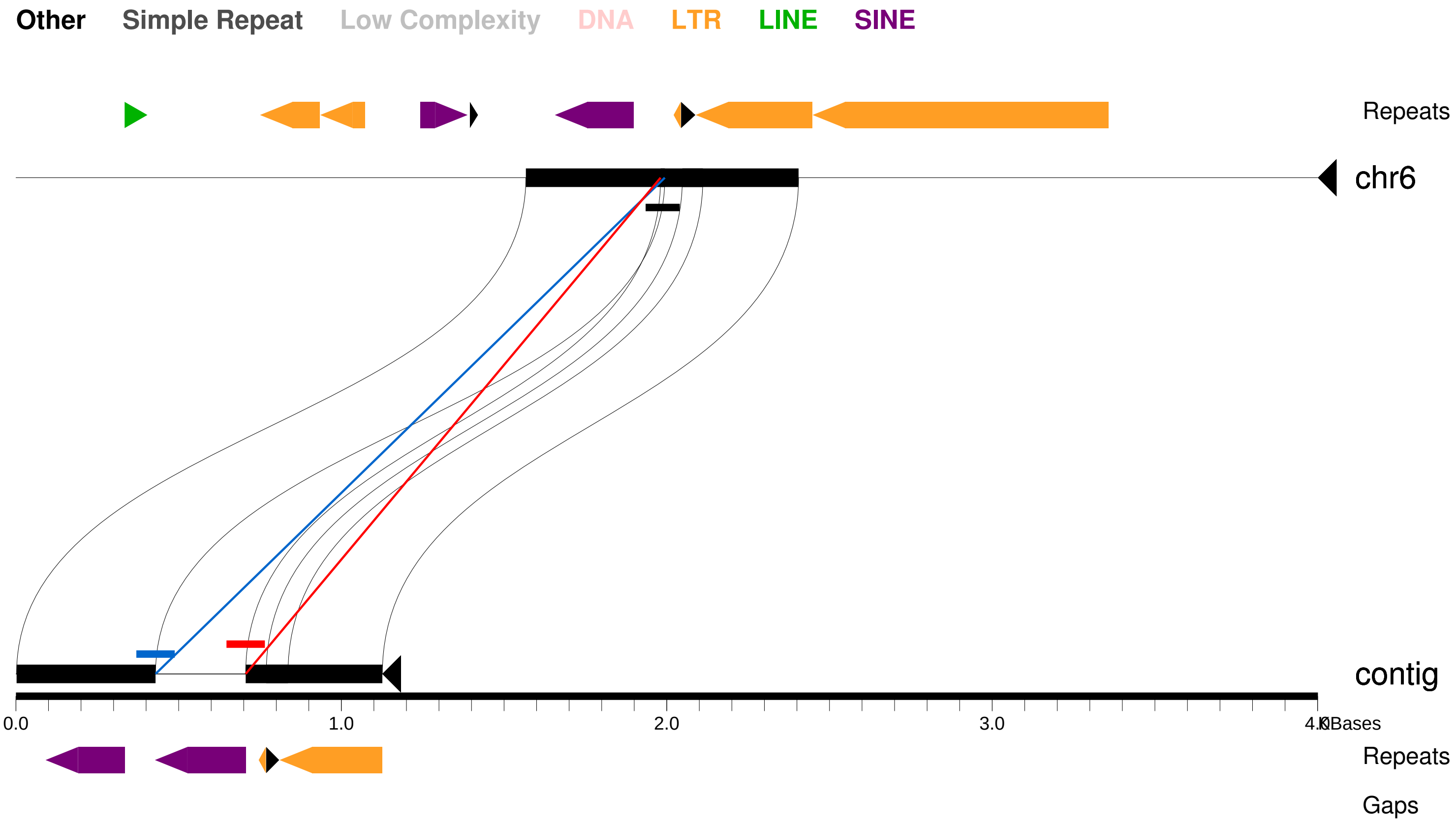

siteID: chr6_120380251 Scafftig5 + Breakpoint: chr6:120380254
chr6:120380207-120380313
Overlap Length: 7 INS size: 306 Genotyped: Yes

```
left     TAGTGATTGA-GTTCTCATGAAAT-CT--GAT----GGTGTTATAAAACAGTTTT----CCCTGCTTTTT
chrom    TAGTGAGTGA-GTTCTCATGAAAT-CT--GAT----GGTGTTATAAAACAGTTTT----CCCTGCTCTTG
right    CCGCCCGCCTCGGCCTCCCAAAGTGCTGGGATTACAGGCGTGAGCCACCGCGCCCGGCCCCCTGCTCTTG
         11*11121111*11***111**1*1**11***1111**1**1*111*1*1111111111*******2**2

left     TTTTTTTTTTTTTTTTTTTT--TGAGACGGA-GTCTCGCTCTGTCGCCCAGGCTGGAGTGCAGT
chrom    TTAGCTCTCTTTCCTGCCACCATGTGAAGAAAGTCTT----TGCTTCCC-----------CTTT
right    TTAGCTCTCTTTCCTGCCACCATGTGAAGAAAGTCTT----TGCTTCCC-----------CTTT
         **222*2*2***22*2222222**2**2*2*2****22222**222***22222222222*22*
```

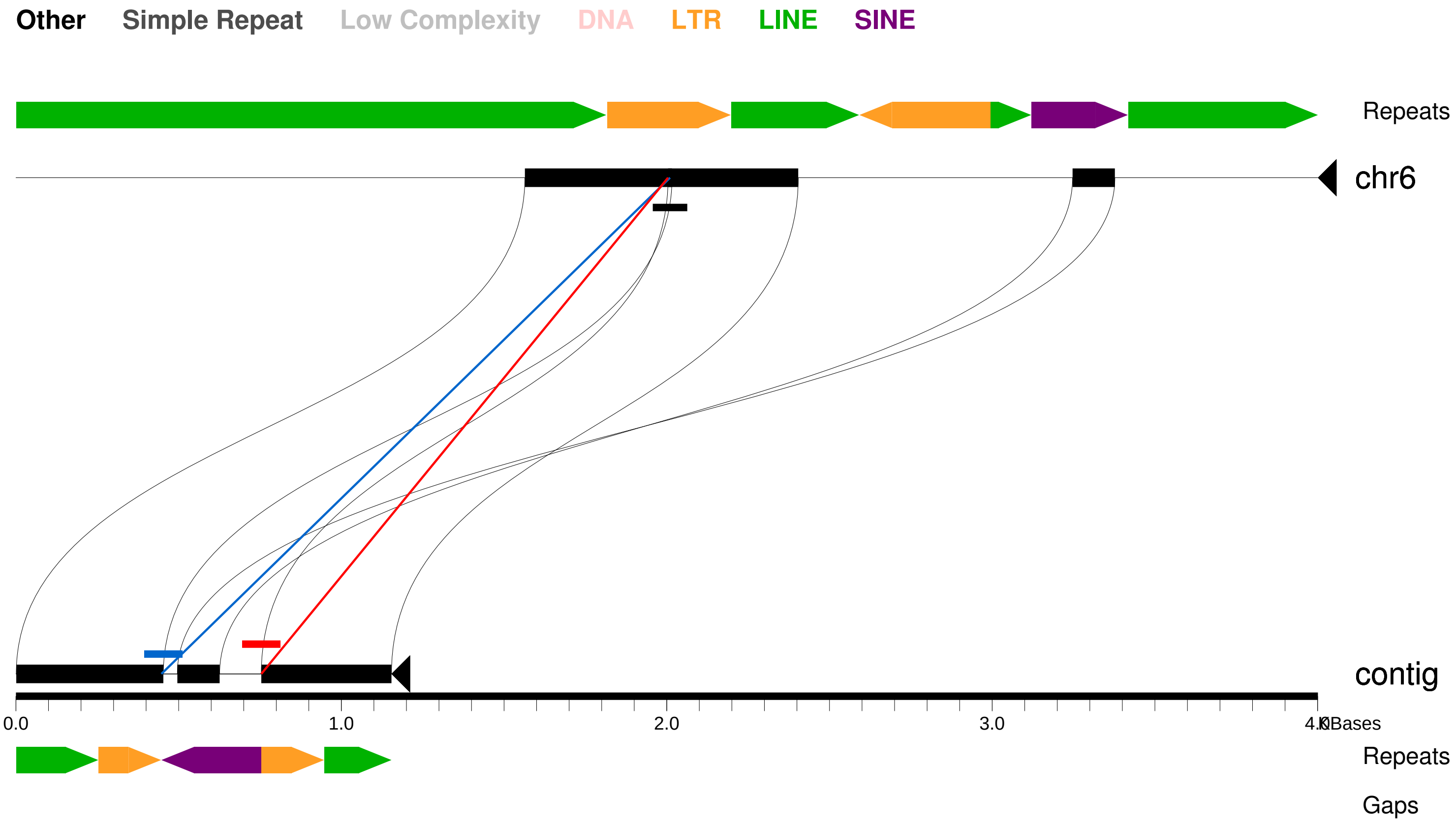

siteID: chr6_120568268 Scafftig4 + Breakpoint: chr6:120568247
chr6:120568203-120568306
Overlap Length: 16 INS size: 296 Genotyped: Yes

```
left     TAAAT---------------AAATCACATCATGTCACTCTTCAGTAAAGCCCCTGTATTGACTCTCAATT
chrom    TAAAT---------------AAATCACATCATGTCACTCTTCAGTAAAGCCCCTGTATTGACTCTCAATT
right    CCACCCGCCTCGGCCTCCCAAAGTGCTGGGATTACAGGCGTGAGCCACCGCGCCCGGCCGACTCTCAATT
         11*111111111111111111**1*111111**11**11*1*1**11*111*1*111111***********

left     TCTTTTTTTTTTTTTTTTTTGAGACGGAGTCTCGCTCTGTCGCCCAGGCTGGAGTGCAGTGGCG
chrom    TCTTTCAAAGTAAATGTCATT-------GTCTTGATAA-TGGCCTAAG-TGGC-TTCAT-----
right    TCTTTCAAAGTAAATGTCATT-------GTCTTGATAA-TGGCCTAAG-TGGC-TTCAT-----
         *****22222*222*2*22*22222222****2*2*222*2***2*2*2***22*2**222222
```

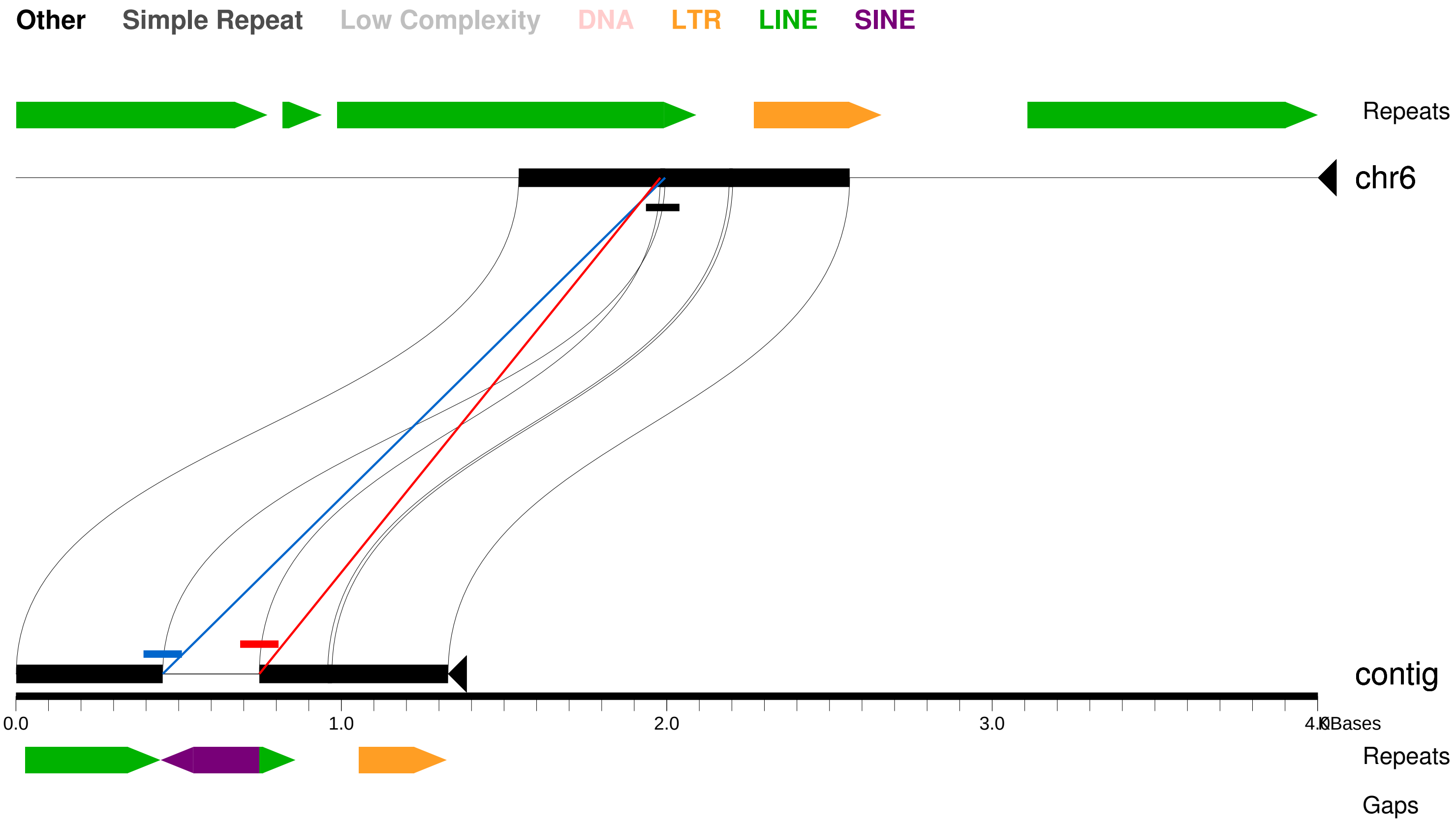

siteID: chr6_120894466 Scafftig1 + Breakpoint: chr6:120894460
chr6:120894415-120894519
Overlap Length: 15 INS size: 190 Genotyped: Yes

```
left    CAATAA-------TTTCAATCATAGAGTTGGTGGTTATAGTAGCAGAACTAC-------AAAAAGGGCTA
chrom   CAATAA-------TTTCAATCATAGAGTTGGTGGTTATAGTAGCAGAACTAC-------AAAAAGGGCTA
right   GAGCGAGACTCCGTCTCAAAAAAAAAAAAAAAAAAAAAAAAAAAAAAAAAAAAAAAAAAAAAAAAGGGCTA
        1*111*1111111*1****11*1*1*1111111111*1*11*11*1**11*11111111***********

left    AATTTAGCCGGGCGTAGTGGCGGGCGCCTGTAGTCCCAGCTACTTGGGAGGCTGAGGCAGGAG--
chrom   AATTCA--CAGCCTCAG---------CAAGTCTTCCATGCAAA-----AGTCACAGGGAATAAGT
right   AATTCA--CAGCCTCAG---------CAAGTCTTCCATGCAAA-----AGTCACAGGGAATAAGT
        *****2*22*2*2*22**222222222*22**22***22**2*222222**2*22***2*22*222
```

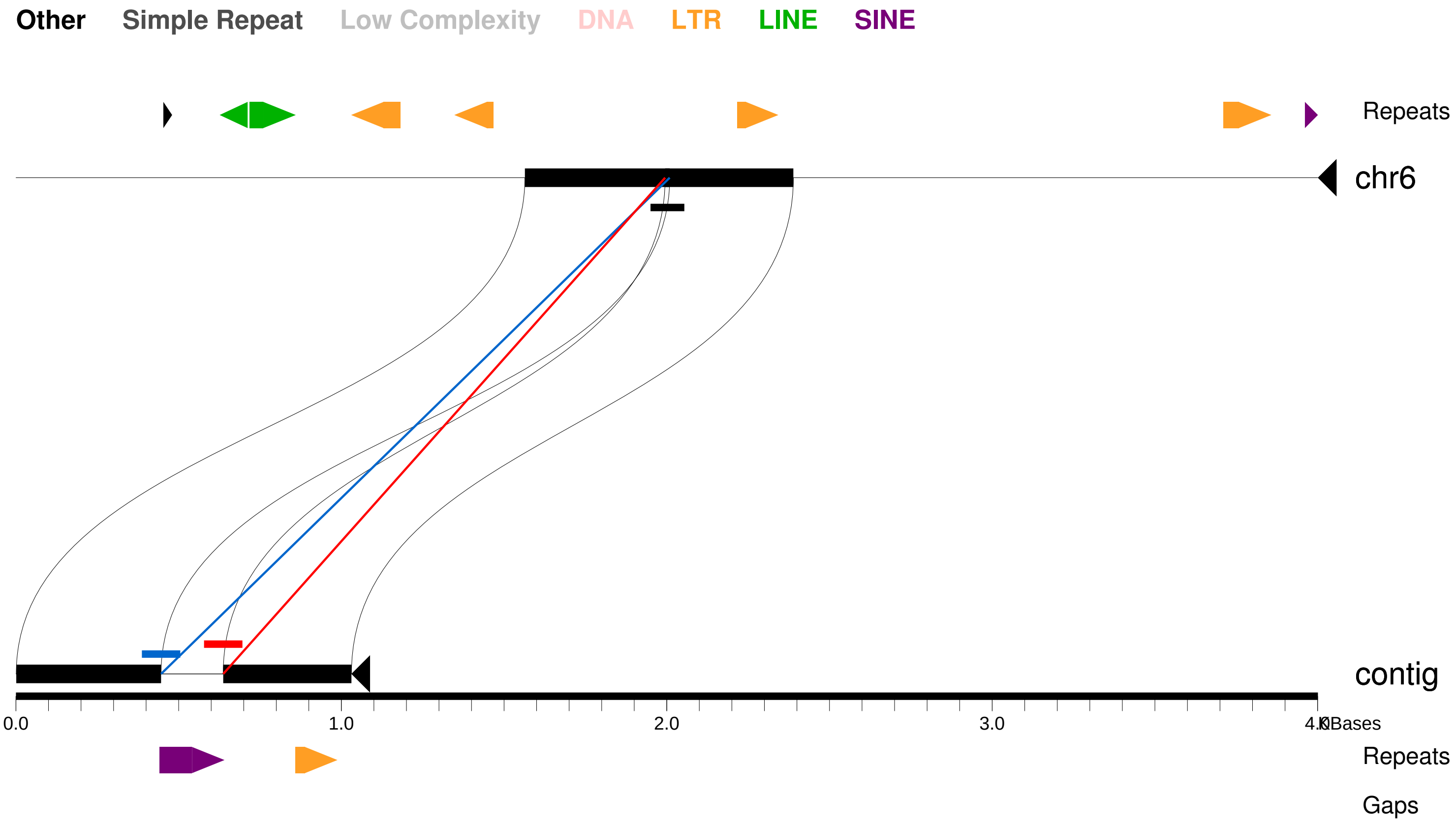

siteID: chr6_12102154 Scafftig1 - Breakpoint: chr6:12102168
chr6:12102124-12102218
Overlap Length: 16 INS size: 297 Genotyped: Yes

```
left    AAG----------TGAAA-----------GGATAGGA-AAAGATGTATCATGCAAAT--AACAAGCATAA
chrom   AAG----------TGAAA-----------GGATAGGA-AAAGATGTATCATGCAAAT--AACAAGCATAA
right   GAGATTGCGCCACTGCAGTCCGCAGTCCGGCCTGGGCGACAGAGCGAGACTCCGTCTCAAAAAAAAAAAA
        1**1111111111**1*111111111111*11*1**11*1***111*111*1*111*11**1**11*1**

left    GAAAGCTGGAGTGGGCCGGGCGCGGTGGCTCACGCCTGTAATCCCAGCACTTTGGGAGGCCGAGGCGGGT
chrom   GAAAGCTGGAGTGG--------CTATGT----------TAATATCAAAA----GGGACTTCAAAATAAA-
right   GAAAGCTGGAGTGG--------CTATGT----------TAATATCAAAA----GGGACTTCAAAATAAA-
        **************22222222*22**22222222222****22**22*2222****222*2*2222222

left    GGA
chrom   -AA
right   -AA
        22*
```

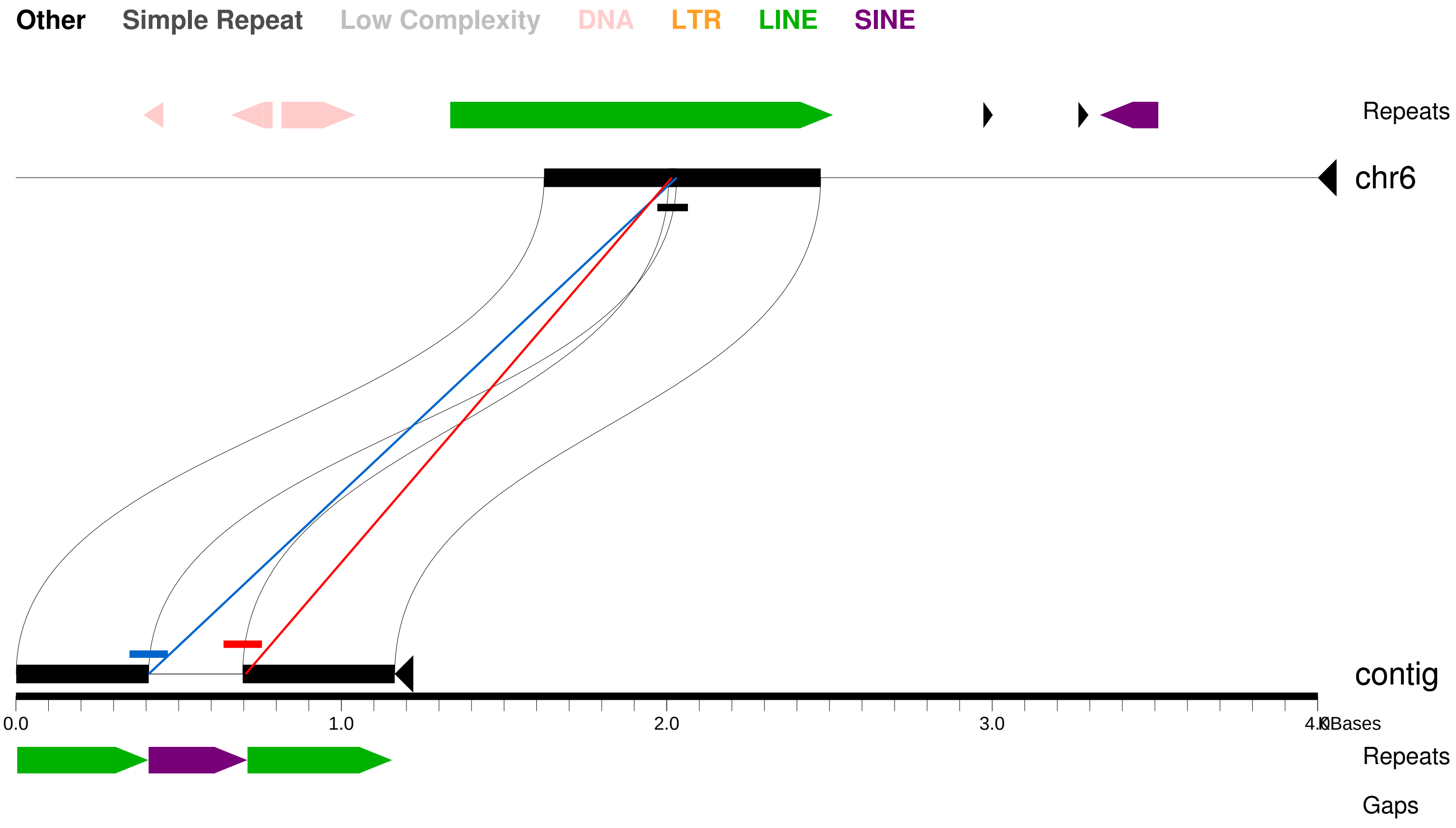

siteID: chr6_122980019 Scafftig1 + Breakpoint: chr6:122979993
chr6:122979953-122980052
Overlap Length: 17 INS size: 305 Genotyped: Yes

```
left   ------GTTTATGT---TAACAGTAGCATTTATT-CATTTGTTAAGCATT----------CATTTGACAA
chrom  ------GTTTATGT---TAACAGTAGCATTTATT-CATTTGTTAAGCATT----------CATTTGACAA
right  CCGCCCGCCTCGGCCTCCCAAAGT-GCTGGGATTACAGGCGTGAGCCACCGCGCCCGGCCCATTTGACAA
       111111*11*11*111111*1***1**1111***1**111**1*11**111111111111**********

left   ATTTTTTTTTTTTTTTTTTTTTTTTTTTTTTGAG-ACGGAGTCTCGCTCTGTCGCCCAGGC-TGGAGTGC
chrom  ATTTTTTTATT--------------------GAGTACCTACTTACCATCTGCCAGGCACTCCTCTAGATA
right  ATTTTTTTATT--------------------GAGTACCTACTTACCATCTGCCAGGCACTCCTCTAGATA
       *******2**222222222222222222222***2**22*2*22*22****2*222**22*2*22**222

left   A
chrom  C
right  C
       2
```

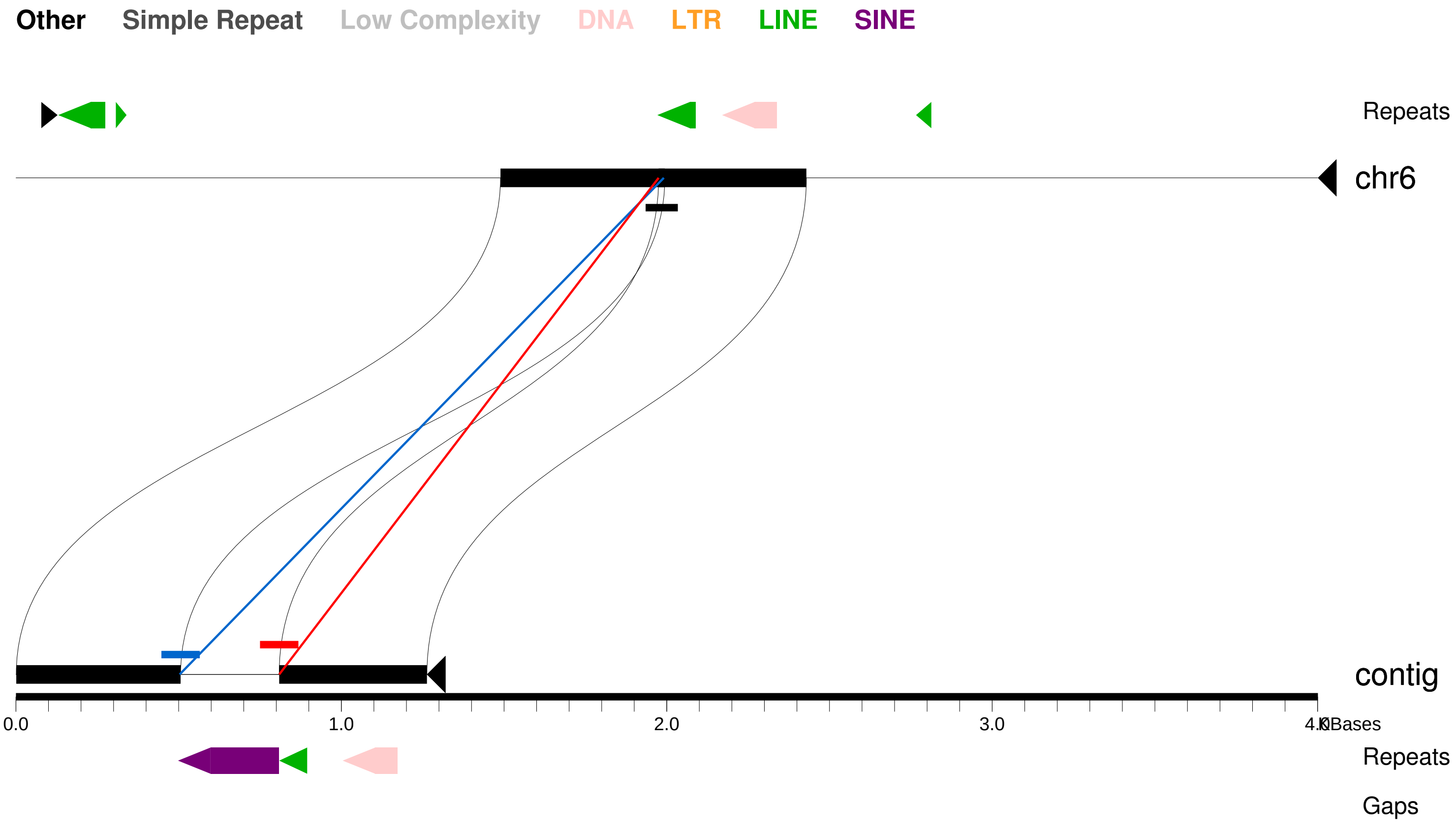

siteID: chr6_124618887 Scafftig1 - Breakpoint: chr6:124618860
chr6:124618260-124619020
Overlap Length: 25 INS size: 310 Genotyped: Yes

```
left   T---------------------------------------------------------------------
chrom  TATGAAAACTGTTATACATACTGTTGATCCTTGAACAACATAGGGGTTAGGGATGCTGACCCACACACAG
right  T----------TTA--------GTAGAGAC------------GGGGTT----------------------
       *          222        22N22NN2            222222

left   ----------------------------------------------------------------------
chrom  TCAAAAATCTGCATATAAGTTTTGACTCCCCCAAATCTTAACTACTAATAGTCTATTATTTATTGGAAGC
right  TCA------------------------------------------------------------------C
       222                                                                  2

left   ----------------------------------------------------------------------
chrom  CTTACTGATAACATAAACAATGGATTAATTAAAACATTTCATTTCTGATATGTGTTATATACTGTATTCT
right  CTT---------------------------------------------------GTTA-------------
       222                                                   2222

left   ----------------------------------------------------------------------
chrom  TACAACAAATCAAGCTAGAGAAAAGAAAATATTAAGAAAATAATAAGAACAGAAAACATATTTACTATTA
right  -------------GCCAG----------------------------------------------------
                    22N22

left   ----------------------------------------------------------------------
chrom  TGTAAAAGTGGATCATTATAAAGATCTTTATGTTCATCATCTTCATGTTGAGTAGGCTGAAGAAAAGAAG
right  ----------------------------------------------------------------------

left   ----------------------------------------------------------------------
chrom  GGTTGGTCTTGGTGTCTCAGGAGTGGCAGAGGCAGAAAAGGTGGAAACTGGCCCCCCTGCCCCGTGTGGC
right  -GATGGTCTCGATCTC-------------------------------CTGACC-----------------
        2N222222N2N2N22                                222N22

left   -----------------------------------------CCCTGGATCATACCACCCTTCTAGTCAGT
chrom  TCTCAGGTGGGCTGCAGCACCATACTGCTCTTCCTTCCTCTCCCTGGATCATACCACCCTTCTAGTCAGT
right  --TCA--------------------TGATCCACCCGCCTCGGCCTC-------CCAA----------AGT
         222                  22N22NN22NN2222N1***11111111***11111111111***

left   TCTGATGGAGAACCTGGTTACCTTGGTTGCTGGTGAAGGATTCACATGCTTATTATTGTTCTTTTCCTTG
chrom  TCTGATGGAGAACCTGGTTACCTTGGTTGCTGGTGAAGGATTCACATGCTTATTATTGTTCTTTTCCTTG
right  GCTG-------------------------------------GGATT-ACAGGCGT-------------------
       1***11111111111111111111111111111111111*****1***1**1*1111111111111111111

left   AGAGCCTCAGAACTGAGCTGTTTCCGAGTCGGCCATCTTGGCCCCGCCCCCTGTCAGTAATTTTTTTTTTT
chrom  AGAGCCTCAGAACTGAGCTGTTTCCGAGTCGGCCATCTTGGCCCCGCCCCCTGTCAGTAATTTTTTAAAGC
right  -GAGCCACCGC----------------------------GCCCGGCCCCCTGTCAGTAATTTTTTAAAGC
       1*****1*1*11111111111111111111111111111*****1*******************22222

left   [illegible]GAGA[illegible]GCAGTCTCGC[illegible]-------CCAGGT---CGGAC
chrom  CATGGCTTTCTCCGTGTTTTTATGTGGAACAAAATCGGGCATTTGGCAAGAAACCACCAGTTGTTCATAA
right  CATGGCTTTCTCCGTGTTTTTATGTGGAACAAAATCGGGCATTTGGCAAGAAACCACCAGTTGTTCATAA
       22*222***2*222*2*****2*2**22**22*2**22**2*2**2*222222222****2*222*22*2

left   TGCGGAC--TGCAGTGGCGCAAT[illegible]GCA-----AGCT[illegible]CCGCTTCCCGGGT---------
chrom  TTTAAATCATTTCTTCTCTAAAACCCTCAAAATTTCATAAGAAGTTGAGGTCCCAGAATCT-------
right  TTTAAATCATTTCTTCTCTAAAACCC[illegible]GAAGTTGAGGTCCCAGAATCTGAGGCTA
       *2222*222*2222*22*22**2*2*22222*2*2**22222**2*22*2*2**2*22*221111111
```

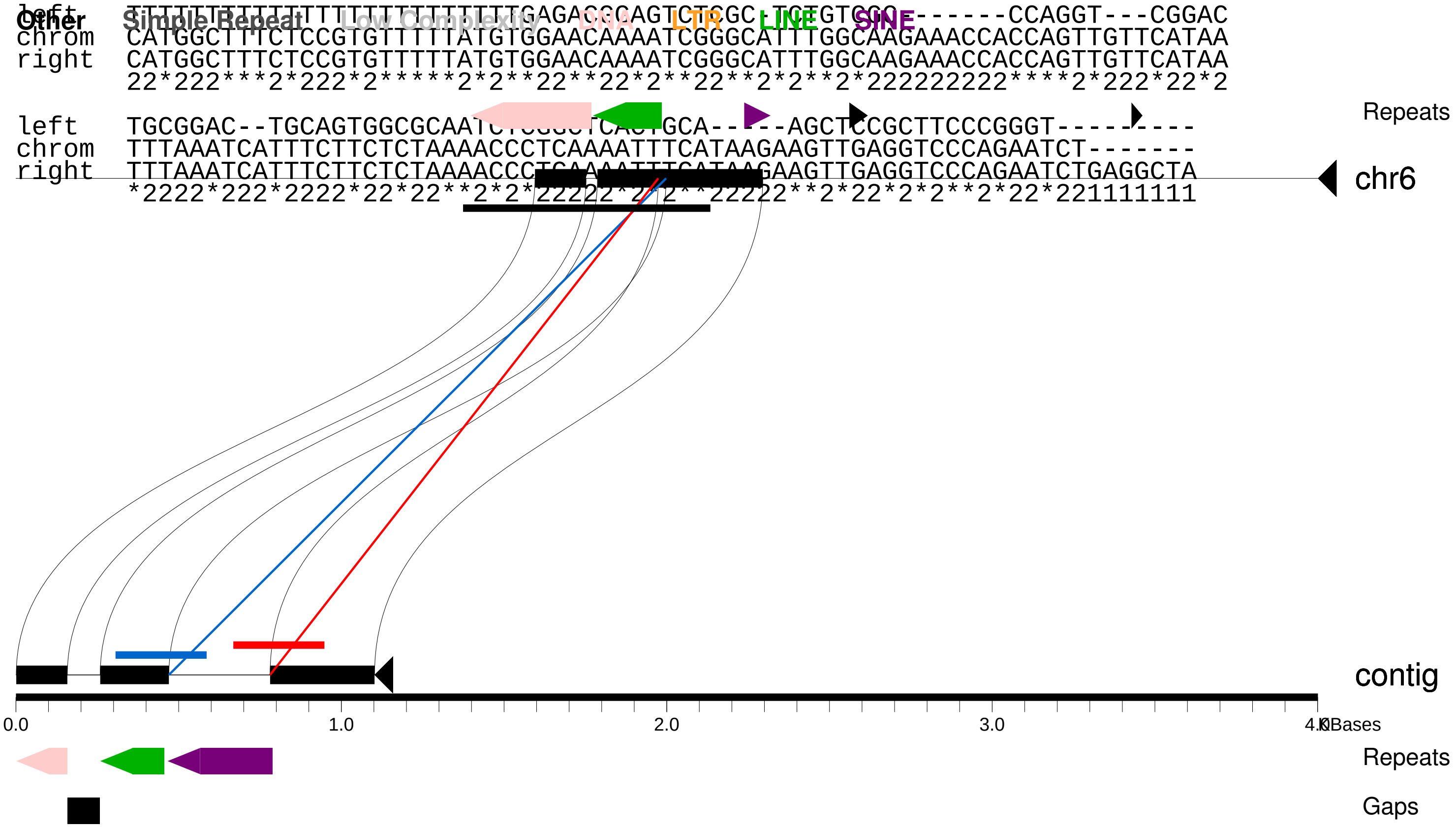

siteID: chr6_124865508 Scafftig1 + Breakpoint: chr6:124865521
chr6:124865462-124865580
Overlap Length: 1 INS size: 499 Genotyped: No

```
left    CTCCCAACTAGATCTCCAACCACAGAAGCCTCAGAACTCTTTGCAAGATGGAGGAGAAGGGCCGGGCGCG
chrom   CTCCCAACTAGATCTCCAACCACAGAAGCCTCAGAACTCTTTGCAAGATGGAGGAGAAGG------AGCT
right   NNNNNNNNNNNNNNNNNNNNNNNNNNNNNNNNNNNNNNNNNNNNNNNNNNNNNNNNNNNG------AGCT
        11111111111111111111111111111111111111111111111111111111111*2222222**2

left    GTGGCTCAC--GCCTGTAATCCCAGCACTTTGGGAGGCCGAGGCGGGCG------GA
chrom   GTGGAATACTGGCCT-TCAGCTCTCCACCTTACCAG-CATAGGTCCTCCCCTTTAGC
right   GTGGAATACTGGCCT-TCAGCTCTCCACCTTACCAG-CATAGGTCCTCCCCTTTAGC
        ****222**22****2*2*2*2*22***2**222**2*22***2222*2222222*2
```

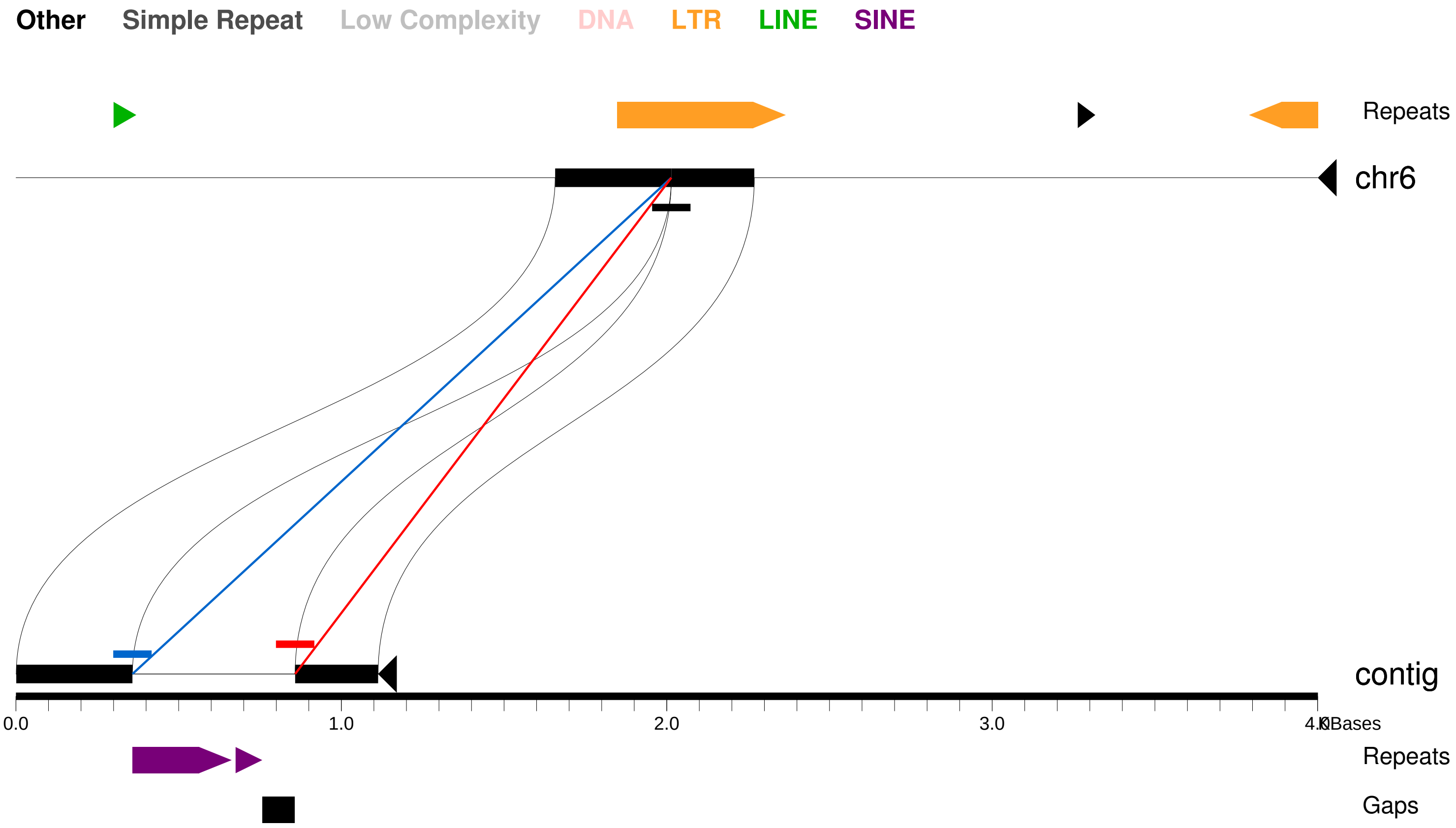

siteID: chr6_125421395 Scafftig5 - Breakpoint: chr6:125421414
chr6:125421362-125421456
Overlap Length: 8 INS size: 459 Genotyped: No

```
left    GAGAAGGAGATGAAAGGGAGAGAGAAAGGAAAG------------------------ACAAAACGAAGAG
chrom   GAGAAGGAGATGAAAGGGAGAGAGAAAGGAAAG------------------------ACAAAACGAAGAG
right   NNNNNNNNNNNNNNNNNNNNNNNNNNNNNNNNNNNNNNNNNNNNNNNNNNNNNNNNGTCTTAAAAAAAAAAAAAA
        111111111111111111111111111111111111111111111111111111111*1****11**1*1

left    AAAGTTAAAAAAAA--GGCCGGGCGCGGTGGCTCACGCCTGTAATCCCAGCACTTTGGGAGGCCGAGGCG
chrom   AAAGTTAAAAAAAATAGACTAGGAAAGAGGGCCCAC--CAGGAA--CCAGGA------------------
right   AAAAAAAAAAAAAATAGACTAGGAAAGAGGGCCCAC--CAGGAA--CCAGGA------------------
        ***111********22*2*22**222*22***2***22*2*2**22****2*222222222222222222

left    GGCGG
chrom   ----G
right   ----G
        2222*
```

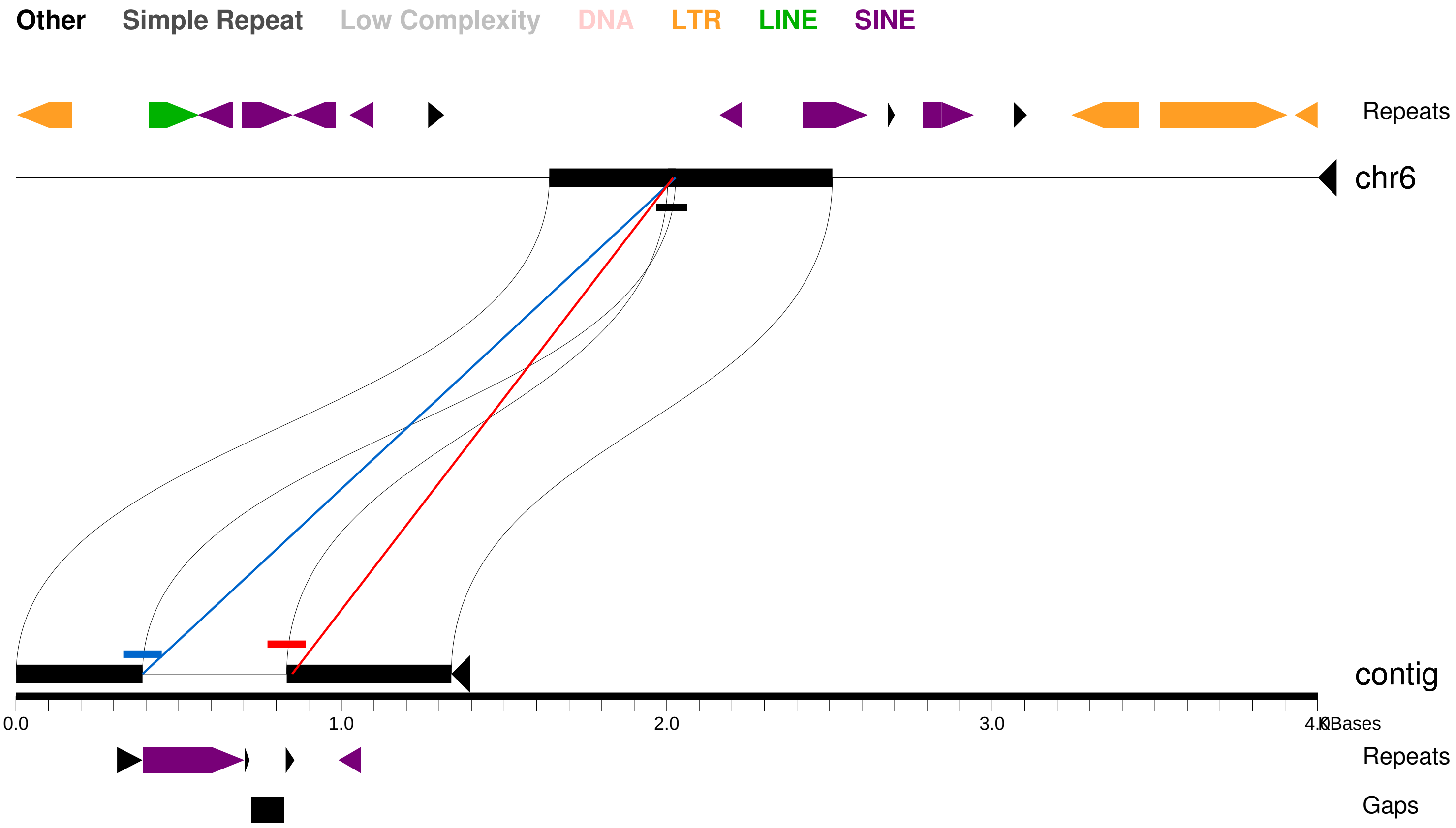

siteID: chr6_126509029 Scafftig5 - Breakpoint: chr6:126509035
chr6:126508990-126509094
Overlap Length: 15 INS size: 306 Genotyped: Yes

```
left     AGC-ACCCAG----TCAGTGGGAAATTCATT---------AGAGGAAAGGAATCTTTTTAAGAAATGGCA
chrom    AGC-ACCCAG----TCAGTGGGAAATTCATT---------AGAGGAAAGGAATCTTTTTAAGAAATGGCA
right    CTCCAGCCTGGGCGACAGAGCGAGACTCCGTCTCAAAAAAAAAAAAAAAAAAAAAAAAAAGAAATGGCA
         11*1*1**1*11111***1*1**1*1**11*111111111*1*11***11**1111111***********

left     ATGGGGCCGGGCGCGGTGGCTCACGCCTGTAATCCCAGCACTTTGGGAGGCCGAGGCGGGCGG
chrom    ATGGAACA----------ATTCACTATTCTTTT----GGAAAAGGTGACCCTTAGCTCCCTAC
right    ATGGAACA----------ATTCACTATTCTTTT----GGAAAAGGTGACCCTTAGCTCCCTAC
         ****22*2222222222222****222*2*22*2222*2*2222*2**22*22**22222222
```

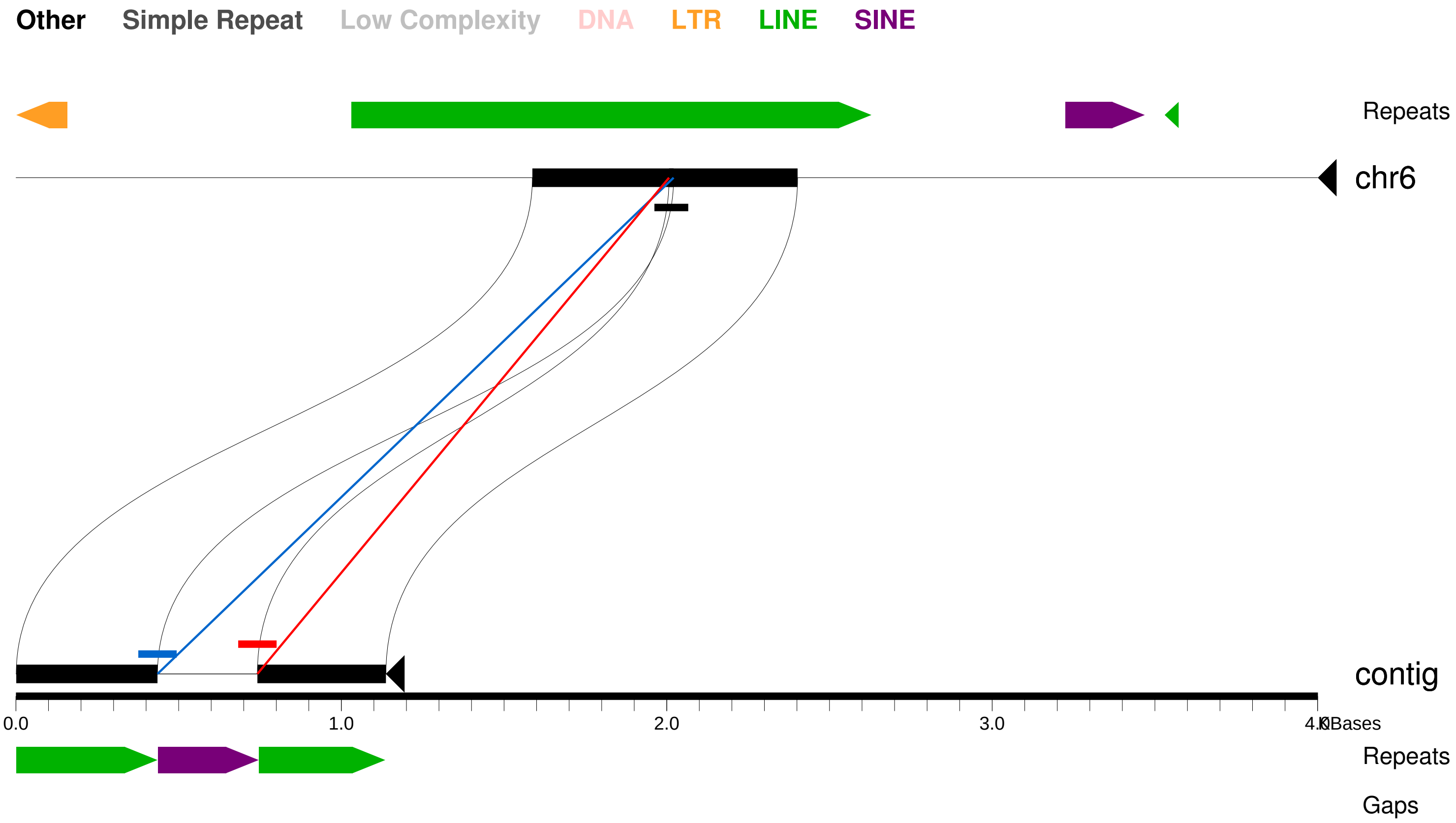

siteID: chr6_129045859 Scafftig4 - Breakpoint: chr6:129045836
chr6:129045676-129046038
Overlap Length: 43 INS size: 434 Genotyped: No

```
left   ----------------------------------------------------------------------
chrom  ACAGGATAAAGTCAGAGGAT-ATAGGAGAAAAATAAATAGAAAA-ATCTACTTGCAACCAAATTCAGGGA
right  TTTAG-TAGAGACGGGGTTTCACCGTTTTAGCCGGGATGGTCTCGATCTCCTGACCTCGTGATCCGCCCG
       NNNN2 22N22N2N2N2NN212NN2NNNN2NNNNNN22N2NNNN12222N22NN2NN2NNN22N2NNNNN

left   -----------------GACTAACACAAAATGTTTGCTGGTAGAACTGGCACTTAGAAAGCATAAGATAC
chrom  CTTTGGAGGGTTTAAATGACTAACACAAAATGTTTGCTGGTAGAACTGGCACTTAGAAAGCATAAGATAC
right  CCTCGGC------------CT--CCCAAAGTG----CTGGGATTACAGGCG-----------TGAGCCAC
       2N2N22N          11**11*1****1**1111****1*11**1***111111111111*1**11**

left   CAACTGTAAAGATCTCACCAAGCAAAAGGCTCTCTTTTCATGGAAGCAGTAGAACAGTGAGAAAANNNNN
chrom  CAACTGTAAAGATCTCACCAAGCAAAAGGCTCTCTTTTCATGGAAGCAGTAGAACAGTGAGAAAATAAAT
right  C---------GCGCCCGGC---CAAAAGGCTCTCTTTTCATGGAAGCAGTAGAACAGTGAGAAAATAAAT
       *111111111*11*1*11*111*******************************************22222

left   NNNNNN----------------------------------------------------------------
chrom  TCAAATCCTAAGAACTAACCGTGTTGTATGGATGATGGATGATGTGTCTTCAGTTATTGTTAATATAAAA
right  TCAAATCCTAAGAACTAACCGTGTTGTATGGATGATGGATGATGTGTCTTCAGTTATTGTTAATATAAAA
       2222222222222222222222222222222222222222222222222222222222222222222222

left   ----------------------------------------------------------------------
chrom  TTTCTATTGTCCTTTTAAATTCCACCTACCATATTAAATTGGCAGTTTCATCGCCATATTCCAATTCCTT
right  TTTCTATTGTCCTTTTAAATTCCACCTACCATATTCAATTGGC---------------------------
       22222222222222222222222222222222222N2222222

left   ---------------
chrom  CTCATGCTTAATTCC
right  ---------------
```

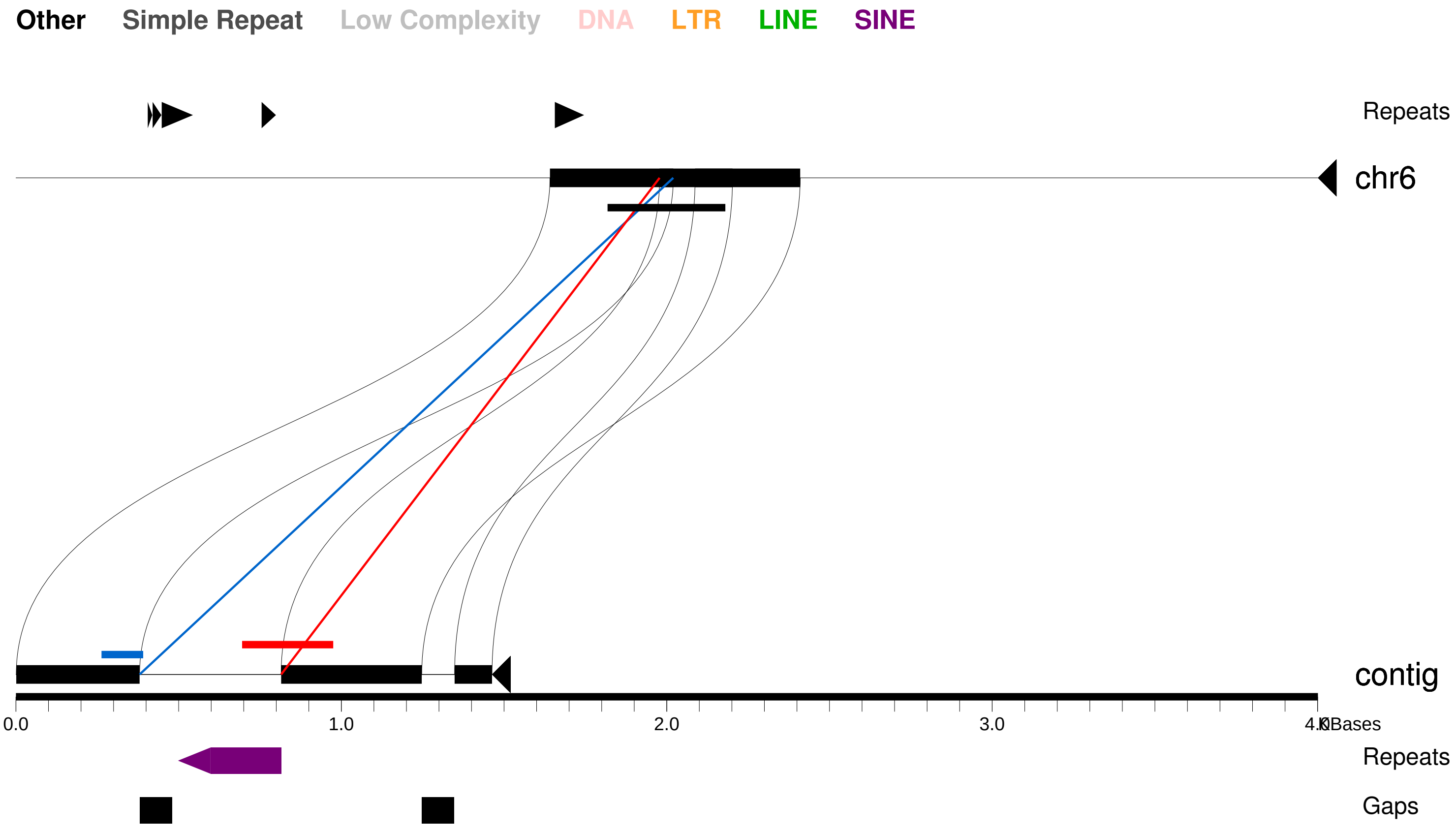

siteID: chr6_129746036 Scafftig1 - Breakpoint: chr6:129746037
chr6:129745994-129746096
Overlap Length: 17 INS size: 309 Genotyped: Yes

```
left    ---TCTGTG-----TATTTA--------CTCCAAGATAAATGATGGCATATGTTCACCCAAAAATTTGTA
chrom   ---TCTGTG-----TATTTA--------CTCCAAGATAAATGATGGCATATGTTCACCCAAAAATTTGTA
right   CAGCCTGGGCGACAGAGCGAGACTCCGTCTCAAAAAAAAAAAAAAAAAAAAAAAAAAAAAAAAAATTTGTA
        1111***1*111111*111*11111111***1**1*1***11*1111*1*11111*111***********

left    CACAGAGGCCGGGCGCGGTGGCTCACGCCTGTAATCCCAGCACTTTGGGAGGCCGAGGCGGGCGG
chrom   CACAGATGT--------GTACAT-ATCCCTATGATGAAATAATTA-------CCAATAAAAAGGA
right   CACAGATGT--------GTACAT-ATCCCTATGATGAAATAATTA-------CCAATAAAAAGGA
        ******2*222222222**222*2*22***2*2**222*22*2*22222222**2*2222222*2
```

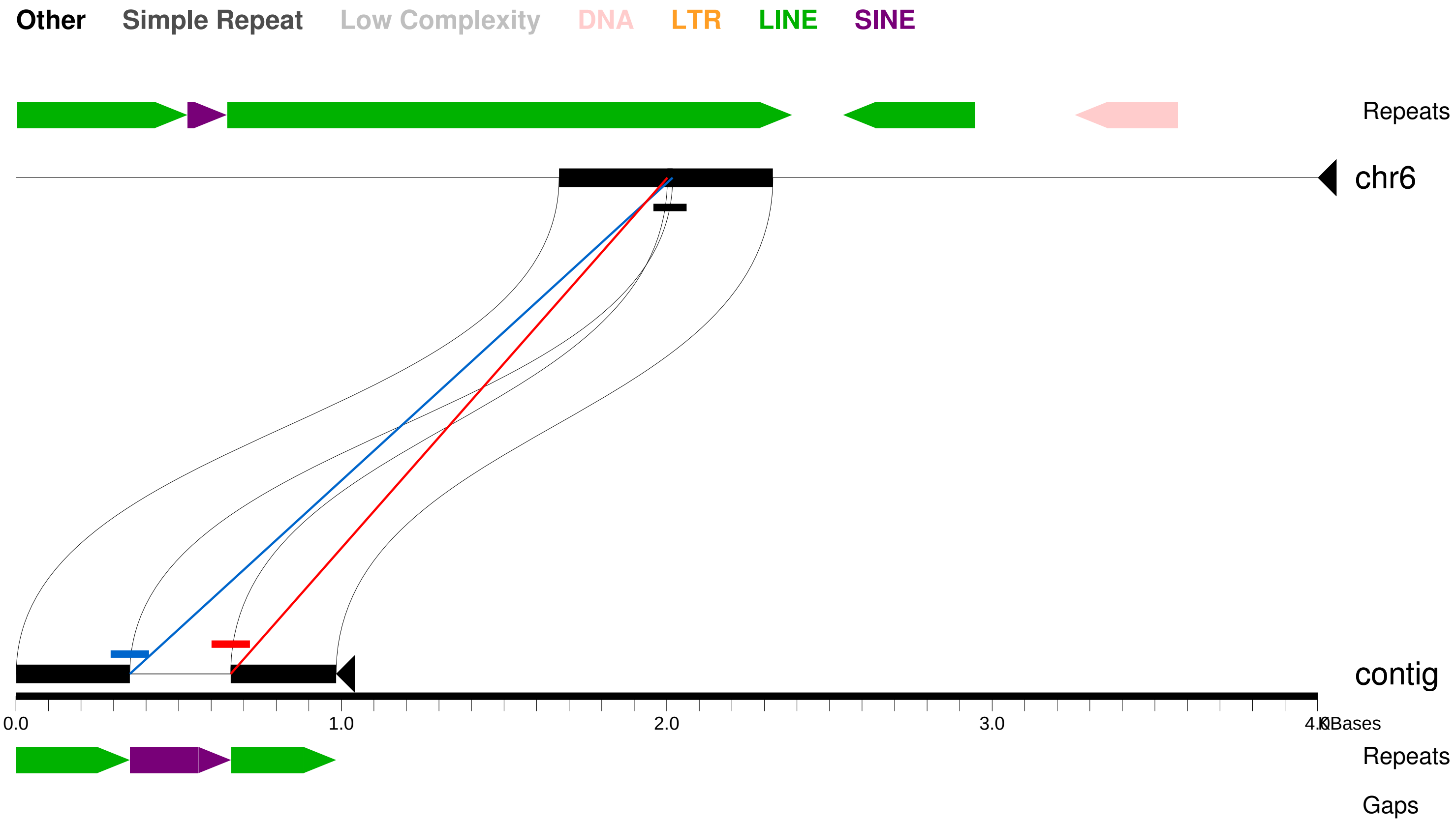

siteID: chr6_129895112 Scafftig6 - Breakpoint: chr6:129895097
chr6:129895050-129895156
Overlap Length: 13 INS size: 292 Genotyped: Yes

```
left    ----GCCATATGCAGA--AAACTG--AAACTG-----GACGCCTACCTTACACCTTATACAAAAATTAAC
chrom   ----GCCATATGCAGA--AAACTG--AAACTG-----GACGCCTACCTTACACCTTATACAAAAATTAAC
right   TCCCGCCAC-TGCACTCCAGCCTGGGCAACAGAGCGAGACTCCGTCTCAAAAAAAAAAAAAAAAAATTAAC
        1111****11****1111*11***111***1*11111***1**11*111*1*1111*1*1***********

left    TCGGCCGGGCGCGGTGGCTCAC-GCCTGTAATCCCAGCACTTTGGGAGGCCGAGGCGGGCGGA-
chrom   TCGA----------TGGATTAAAGACTTAAATATAAGATCTA----AAACCATAAAAACTGTAG
right   TCGA----------TGGATTAAAGACTTAAATATAAGATCTA----AAACCATAAAAACTGTAG
        ***22222222222***2*2*22*2**22***222**22**22222*22**222222222*2*2
```

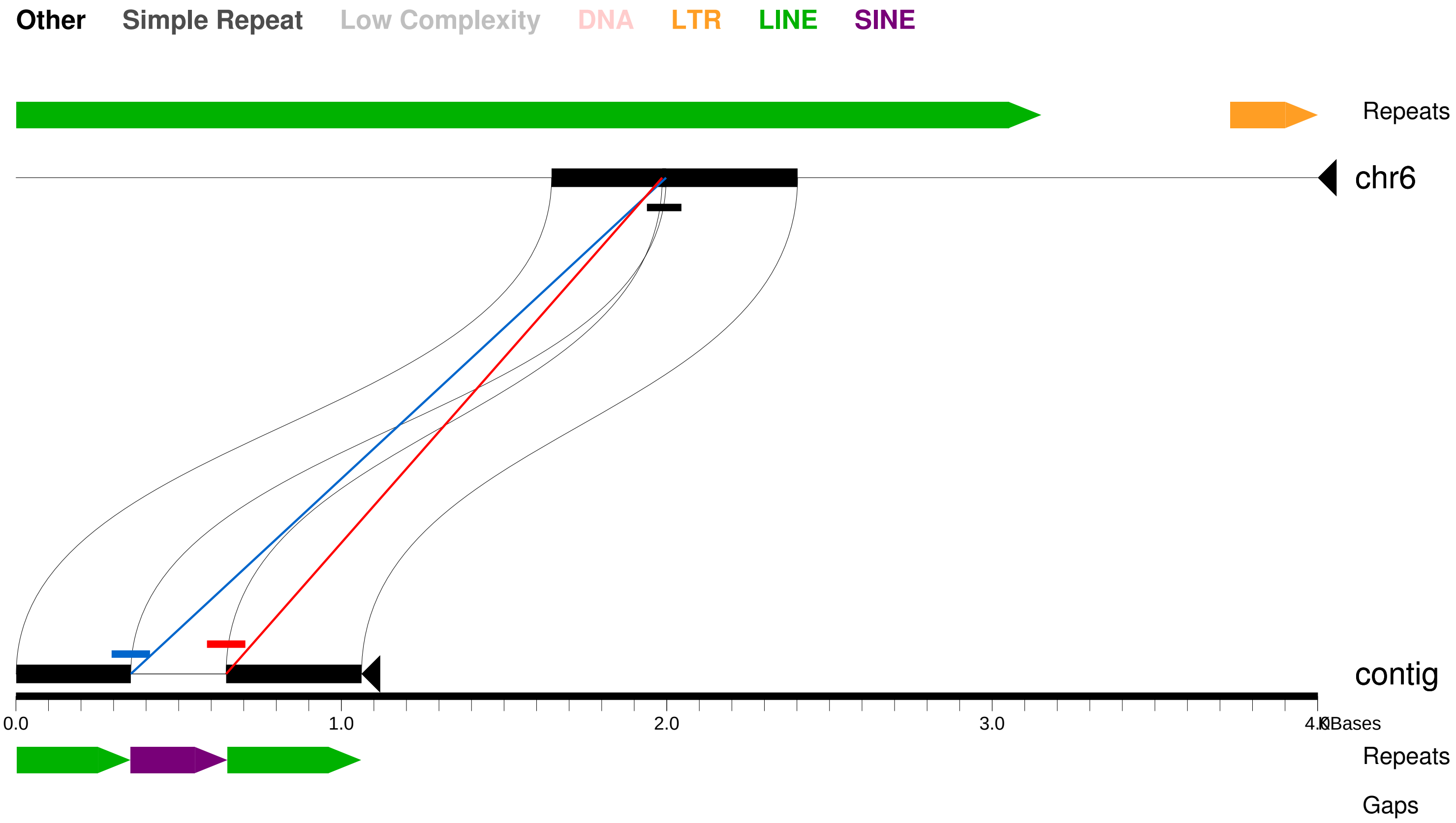

siteID: chr6_130614796 Scafftig10 - Breakpoint: chr6:130614789
chr6:130614739-130614843
Overlap Length: 10 INS size: 270 Genotyped: Yes

```
left    A--------AGAATAGCC----CCAGTGATGCTTCCACAGTTTCCATAAACATTTTTAAAAA--CCAGTC
chrom   A--------AGAATAGCC----CCAGTGATGCTTCCACAGTTTCCATAAACATTTTTAAAAA--CCAGTC
right   GCAGTCCGCAGTCCGGCCTGGGCGACAGAGCGAGACTCCGTCTCAAAAAAAAAAAAAAAAAAAACCAGTC
        111111111**1111***1111*1*11**111111*1*1**1**1*1***1*11111*****11******

left    CTGATTGGGAGGCCGAGGCGGGTGGATCATGAGGTCAGGAGATCGAGACCATCCTGGCTAACA
chrom   CTGAA----AGATGTAGGATAATATTTGATGTTGACATATGATTAATTCAATG----------
right   CTGAA----AGATGTAGGATAATATTTGATGTTGACATATGATTAATTCAATG----------
        ****22222**2222***2222*222*2***22*2**222***22*22*2**22222222222
```

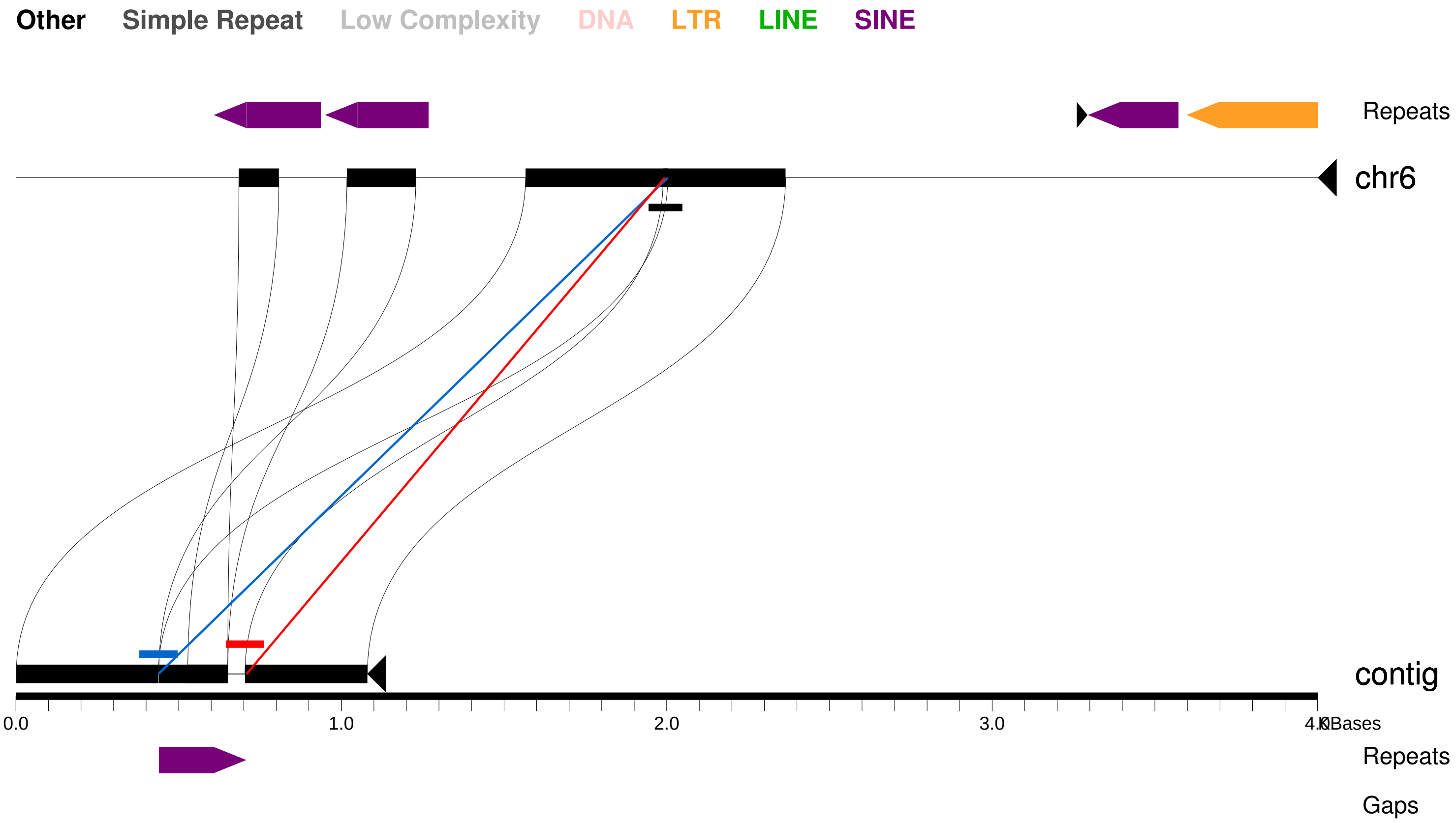

siteID: chr6_131028583 Scafftig2 - Breakpoint: chr6:131028574
chr6:131028529-131028633
Overlap Length: 15 INS size: 431 Genotyped: No

```
left    AAAGCAAATGTGGC--ACATA----TACACCATGG--------AATACTATGCAGCCATAAAAAAGGATG
chrom   AAAGCAAATGTGGC--ACATA----TACACCATGG--------AATACTATGCAGCCATAAAAAAGGATG
right   CAGTCCGGCCTGGGCGACAGAGCGAGACTCCGTCTCAAAAAAAAAAAAAAAAAAAAAAAAAAAAGGATG
        1*11*11111***111***1*11111**1**1*1111111111**1*11*111*111*1***********

left    AGTTGGCNNNNNNNNNNNNNNNNNNNNNNNNNNNNNNNNNNNNNNNNNNNNNNNNNNNNNNNN
chrom   AGTT--------------CATGTCCTTTGCAGGGACATAGATGAAGCTGGAAATCATCATTCT
right   AGTT--------------CATGTCCTTTGCAGGGACATAGATGAAGCTGGAAATCATCATTCT
        *****22222222222222222222222222222222222222222222222222222222222
```

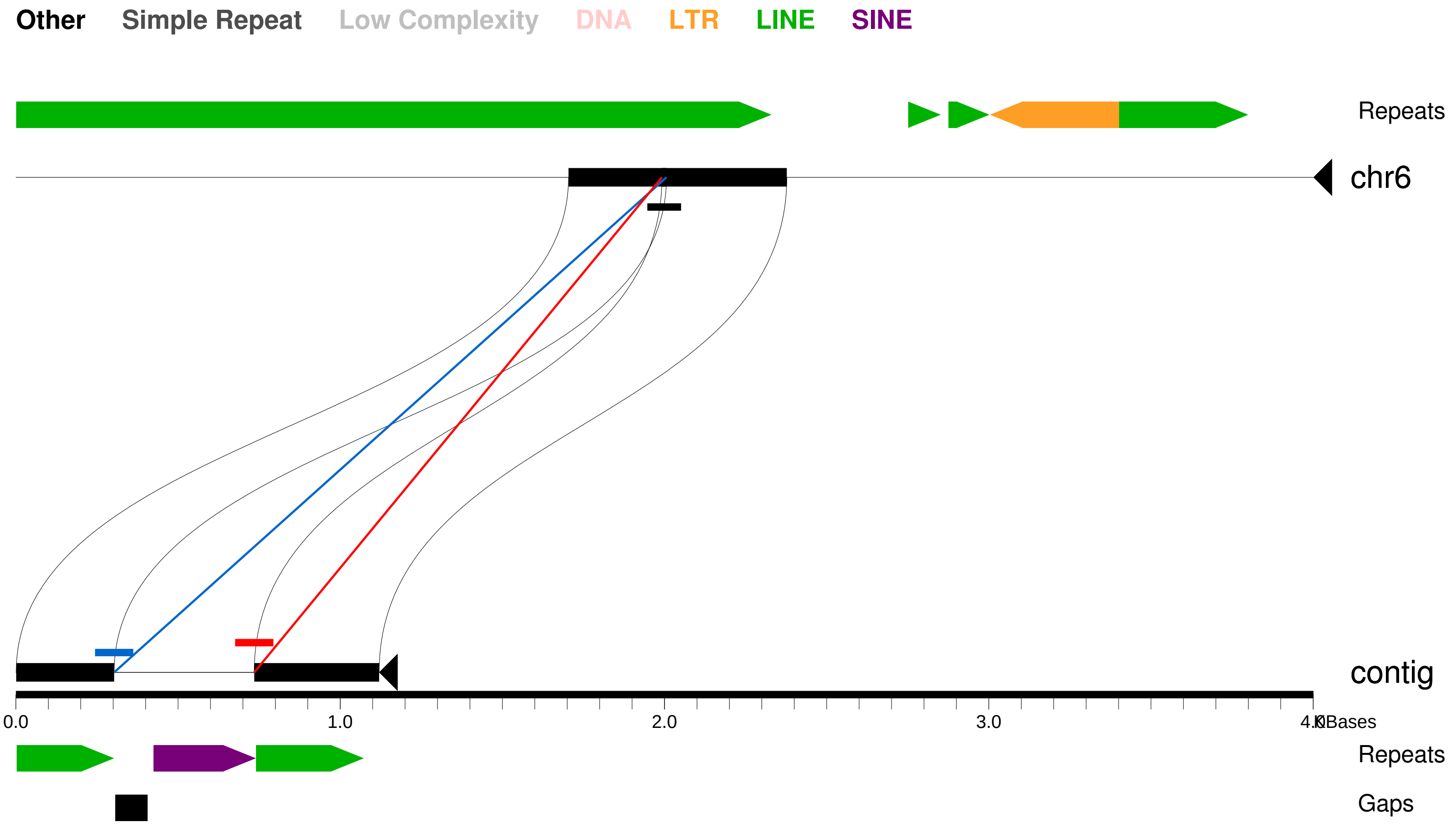

siteID: chr6_131057584 Scafftig5 - Breakpoint: chr6:131057569
chr6:131057527-131057628
Overlap Length: 18 INS size: 302 Genotyped: Yes

```
left    --TTGTTTCAAGGAC----ATACTGATTTGAGTTCAA---TTAGGGAT--------GTTGGTAGATTCTC
chrom   --TTGTTTCAAGGAC----ATACTGATTTGAGTTCAA---TTAGGGAT--------GTTGGTAGTATCTC
right   CCTCCTGCCTCTGCCTCCCAAAGTGCTGGGATTACAGGCGTGAGCCACCGCGCCCGGCCGGTAGTATCTC
        11*11*11*111*1*1111*1*1**1*11**1*1**1111*1**11*111111111*11*****22****

left    CTTTTTTTTTTTTTTTTTTTTTTTTTTTGAGACGGAGTCTCGCTCTGTCGCCCAGGCTGGAGTGCA
chrom   CTTTTTTAAGAGAACTATCTACA-----GTGCACCAGTAATGATATTTCAA--AGG----------
right   CTTTTTTAAGAGAACTATCTACA-----GTGCACCAGTAATGATATTTCAA--AGG----------
        *******22222222*2*2*22222222*2*2222***222*2*2*2**2222***2222222222
```

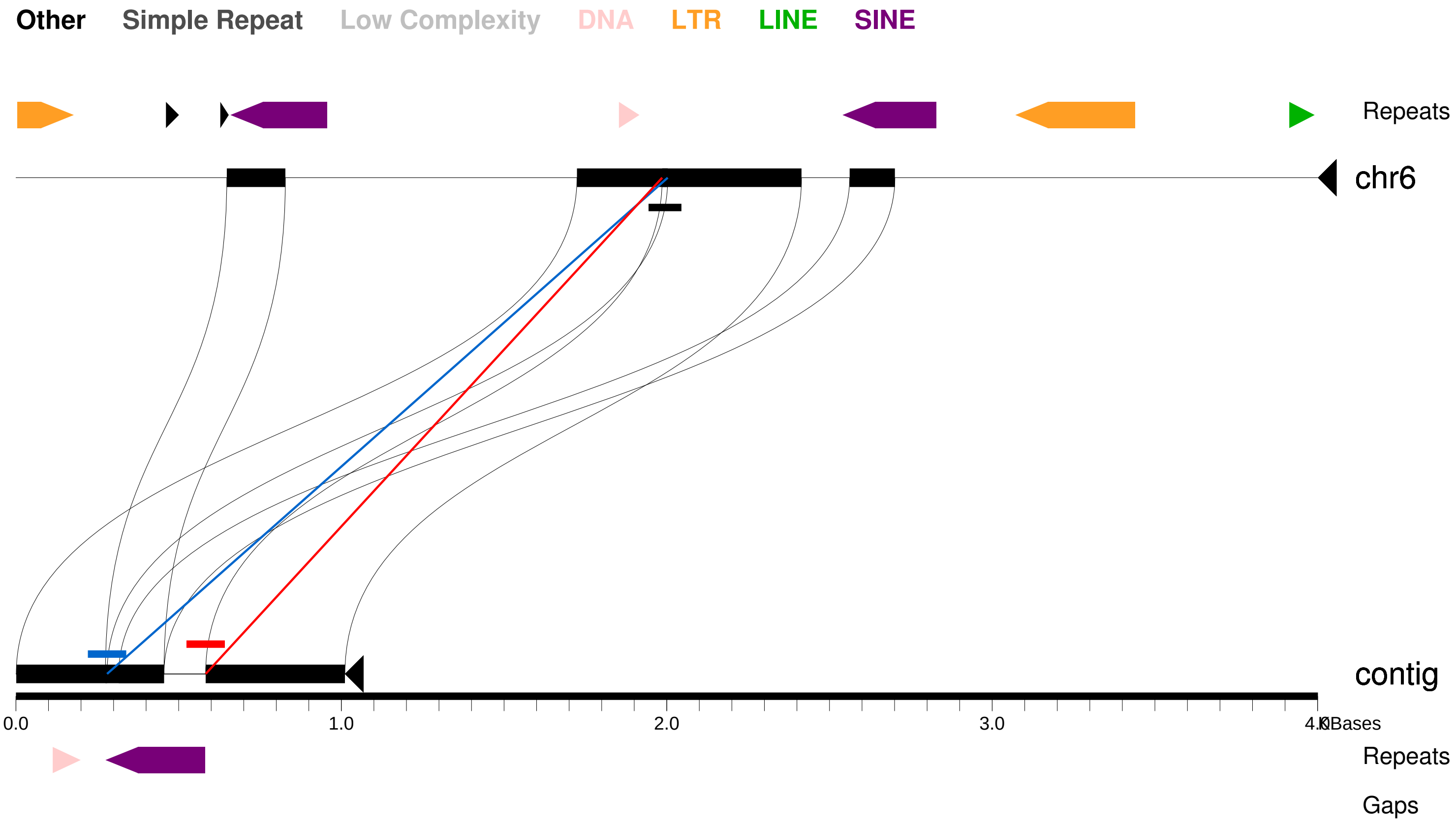

siteID: chr6_131113268 Scafftig5 - Breakpoint: chr6:131113270
chr6:131113220-131113314
Overlap Length: 10 INS size: 313 Genotyped: Yes

```
left   ACA----CCGACCG----------TGTGCTAAACAG-GATT-TGTGTATCTAAAAGAATCTCAAAA----
chrom  ACA----CCGACCG----------TGTGCTAAACAG-GATT-TGTGTATCTAAAAGAATCTCAAAA----
right  GCCACTGCAGTCCGCAGTATGGCCTGGGCGACAGAGCGAGACTCCGTCTCAAAAAAAAAAAAAAAAAAAA
       1*11111*1*1***1111111111**1**1*1*1**1**111*11**1**1****1**1111****1111

left   ----TCTCAAGGCCGGGCCGGGCGCGGTGGCTCACGCCTGTAATCCCAGCACTTTGGGAGGCCGAGGCGG
chrom  ----TCTCAAGGCCAAG----------------AATCCCGGCCTTCAAGGACTTTATGATATTGT-----
right  AAAATCTCAAGGCCAAG----------------AATCCCGGCCTTCAAGGACTTTATGATATTGT-----
       1111**********22*2222222222222222*22**2*222*2*2**2*****22**2222*222222

left   GTG
chrom  ---
right  ---
       222
```

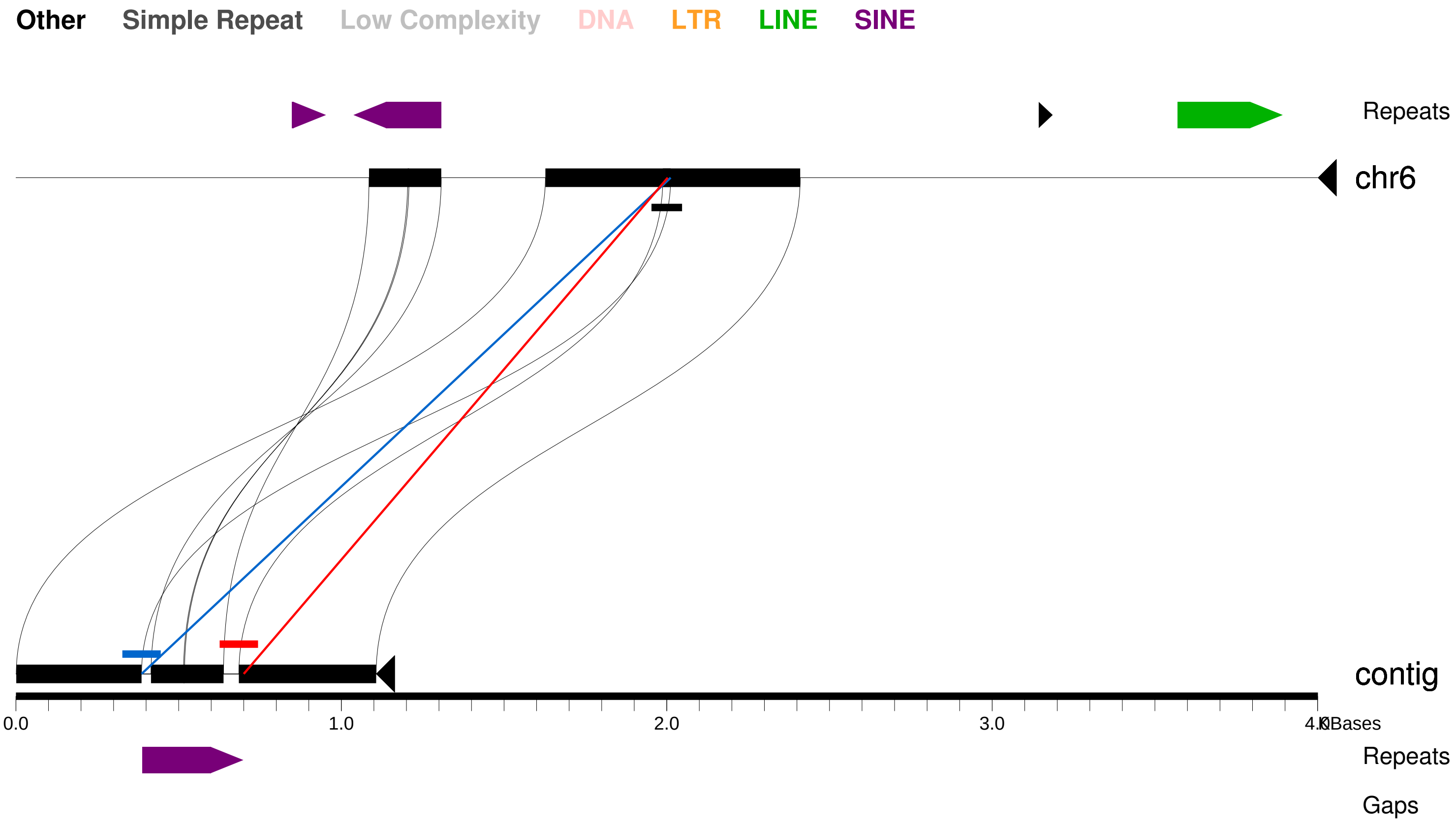

siteID: chr6_133160117 Scafftig9 + Breakpoint: chr6:133160107
chr6:133160061-133160166
Overlap Length: 14 INS size: 297 Genotyped: Yes

```
left    ---------TTAGCATTCATCTATGATGAAGAGAGCGGGCTGGTTACTCAG-----GAAATCAATACAGT
chrom   ---------TTAGCATTCATCTATGATGAAGAGAGCGGGCTGGTTACTCAG-----GAAATCAATACAGT
right   CCGCCCGCCTCGGCCTCCCAAAGTGCTGG-GATTACAGGCGTGAGCCACCGCGCCCGGCCTCAATACAGT
        111111111*11**1*1*11111**1**11**111*1***11*111*1*1*11111*111**********

left    CTTT------TTTTTTTTTTTTTTTGAGACGGAGTCTCGCTCTGTCGCCCAGGCTGGAGTGCAGTGGCG
chrom   CTTTAAACCATGGCATAGTTTTTTAAATA---AGTGT-GCATT-------AATCT----TGCAGT----
right   CTTTAAACCATGGCATAGTTTTTTAAATA---AGTGT-GCATT-------AATCT----TGCAGT----
        ****222222*2222*22******22*2*222***2*2**22*2222222*22**2222******2222
```

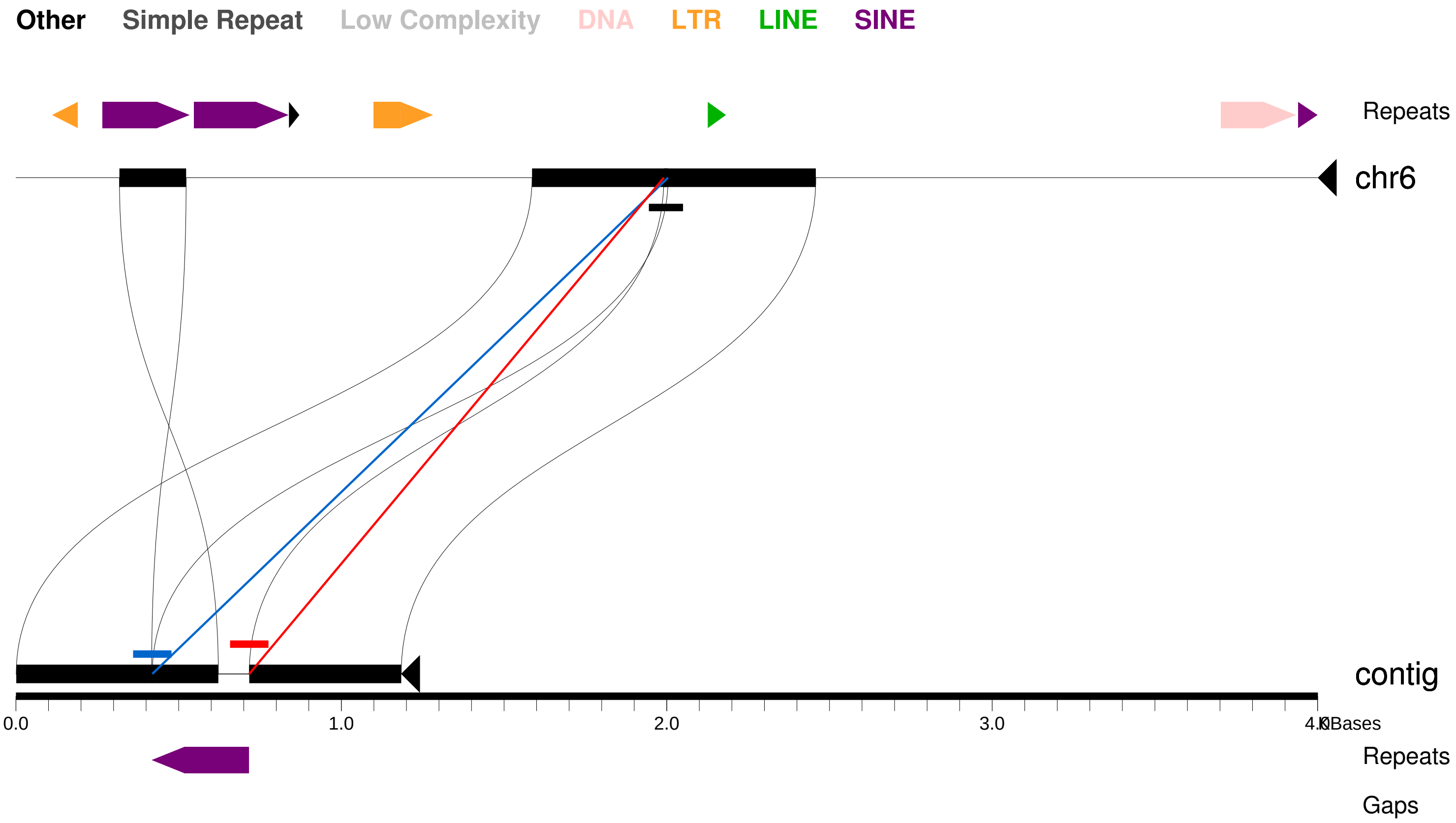

siteID: chr6_133731753 Scafftig5 - Breakpoint: chr6:133731758
chr6:133731712-133731817
Overlap Length: 14 INS size: 171 Genotyped: Yes

```
left    AGAG------------CTCCT-CCTAA----CCAAAAAAAAAAAAAACTTCTGTATTTTTCCATAGAACTA
chrom   AGAG------------CTCCT-CCTAA----CCAAAAAAAAAAAAAACTTCTGTATTTTTCCATAGAACTA
right   CCGGGTTCACGCCATTCTCCTGCCTCAGCCTCCCAAGTAGCTGGGACTACAGGCG----CCCGAGAACTA
        111*111111111111*****1***1*1111**1**11*111111***1*1*1111111**11*******

left    AGGTCTTTTTTTTTTTTTTTTTTTTTTTTTTTTTTTTTTTTTTTTTGAGACAGAGTCTCGCTCTGT
chrom   AGGTCTTAATTCCTTCTATCAATCATGTGTTTTCCCCGTTTGCATTGA-----AGGCT--------
right   AGGTCTTAATTCCTTCTATCAATCATGTGTTTTCCCCGTTTGCATTGA-----AGGCT--------
        *******22**22**2*2*222*22*2*2****22222***222****22222**2**22222222
```

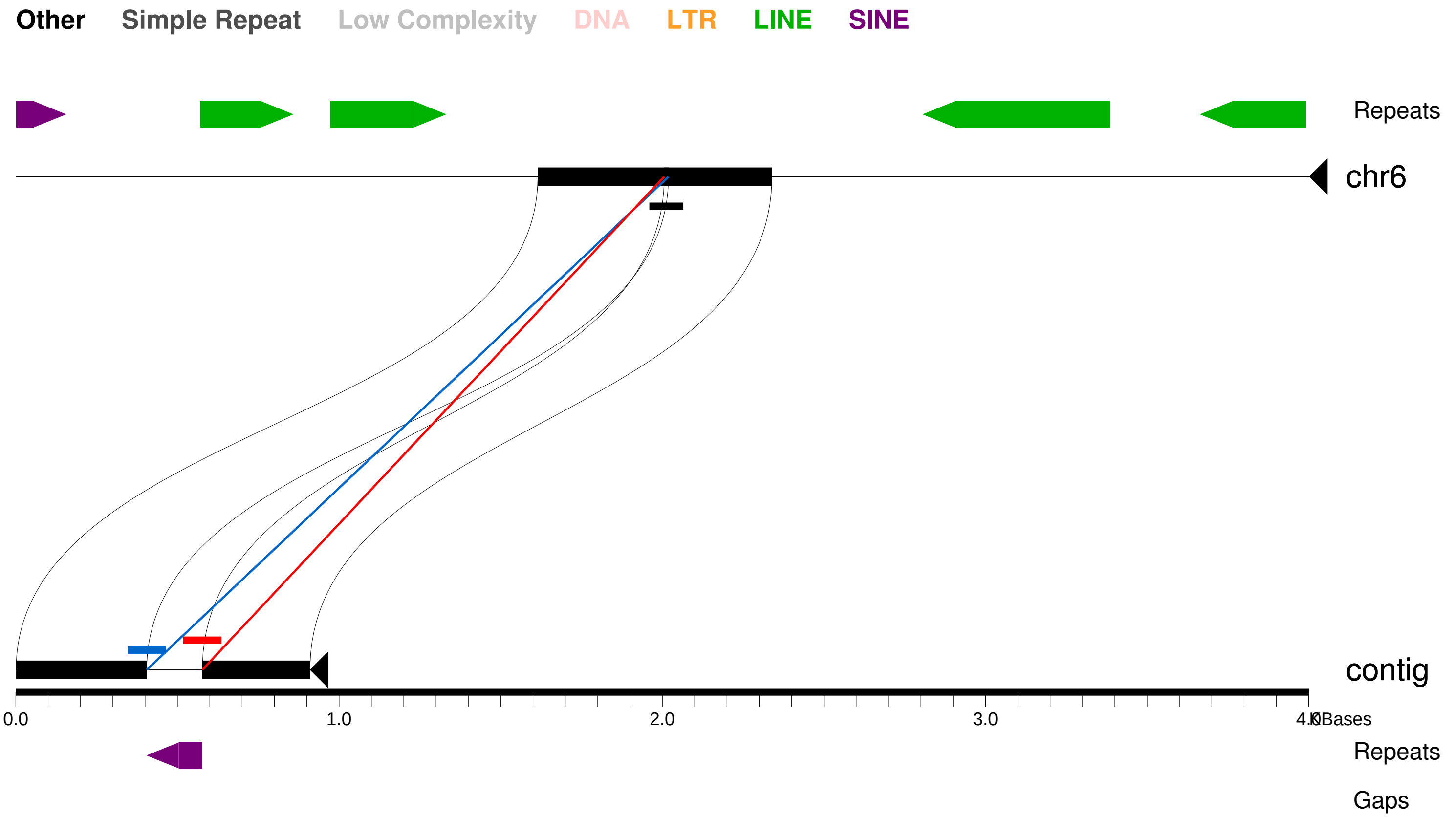

siteID: chr6_13423372 Scafftig7 - Breakpoint: chr6:13423453
chr6:13423406-13423512
Overlap Length: 12 INS size: 80 Genotyped: Yes

```
left    ---CCCAG-------------ACAGCGTTAGATTTCAAGATAAATATAGACAGTCCCCTACTTAAGAACA
chrom   ---CCCAG-------------ACAGTGTTAGATTTCAAGATAAATATAGACAGTCCCCTACTTAAGAACA
right   GTCCGCAGTCCGGCCTGGGCGACAGAGCGTGACTCCGTCTCAAAAAAAAAAAAAA----AAAAAAGAACA
        111*1***1111111111111****N*111**1*1*11111***1*1*1*1*1111111*111*******

left    GTTTGAGCCGAGATTGCGCCACTGCAGTCCGCAGTCCGGCCTGGGCGACAGAGCGTGACTCCGTC
chrom   GTTTG-------ATTT--ACAATGT--TTTGATGTTAAGA-TGGGTTTATTGGGGATATTAAATG
right   GTTTG-------ATTT--ACAATGT--TTTGATGTTAAGA-TGGGTTTATTGGGGATATTAAATG
        *****2222222***2222**2**222*22*22**222*22****2222222*2*22*2*222*2
```

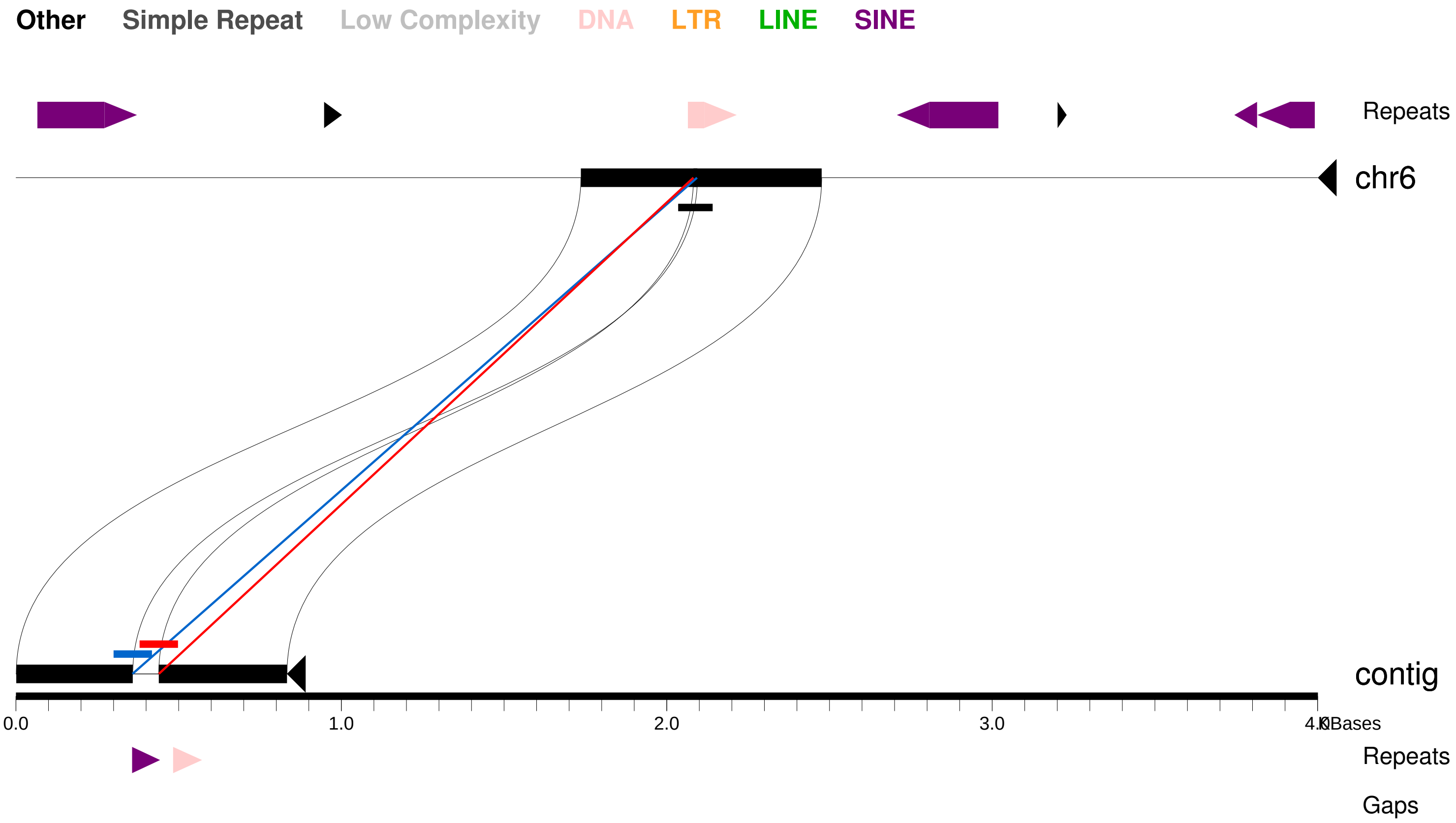

siteID: chr6_13459572 Scafftig5 - Breakpoint: chr6:13459578
chr6:13459537-13459637
Overlap Length: 19 INS size: 301 Genotyped: Yes

```
left     TGCAG--AAAACTG-------AAACTGGACCCCTTCCTTACACCTTATAC---------AAAAATTAACT
chrom    TGCAG--AAAACTG-------AAACTGGACCCCTTCCTTACACCTTATAC---------AAAAATTAACT
right    TGCACTCCAGCCTGGGCGACAGAGCGAGACTCCGTCTCAAAAAAAAAAAAAAAAAAAAAAAAAATTAACT
         ****1111*11***11111111*1*11***1**1**111*1*1111*1*1111111111***********

left     CAAGATGGGCCGGGCGCGGTGGCTCACGCCTGTAATCCCAGCACTTTGGGAGGCCGAGGCGGGCGGA
chrom    CAAGATGGATTAAA------GACTTAAATATAAAATCCAAA-ACCATAAAAACCCT-----------
right    CAAGATGGATTAAA------GACTTAAATATAAAATCCAAA-ACCATAAAAACCCT-----------
         ********222222222222*2**2*2222*22*****2*22**22*222*22**222222222222
```

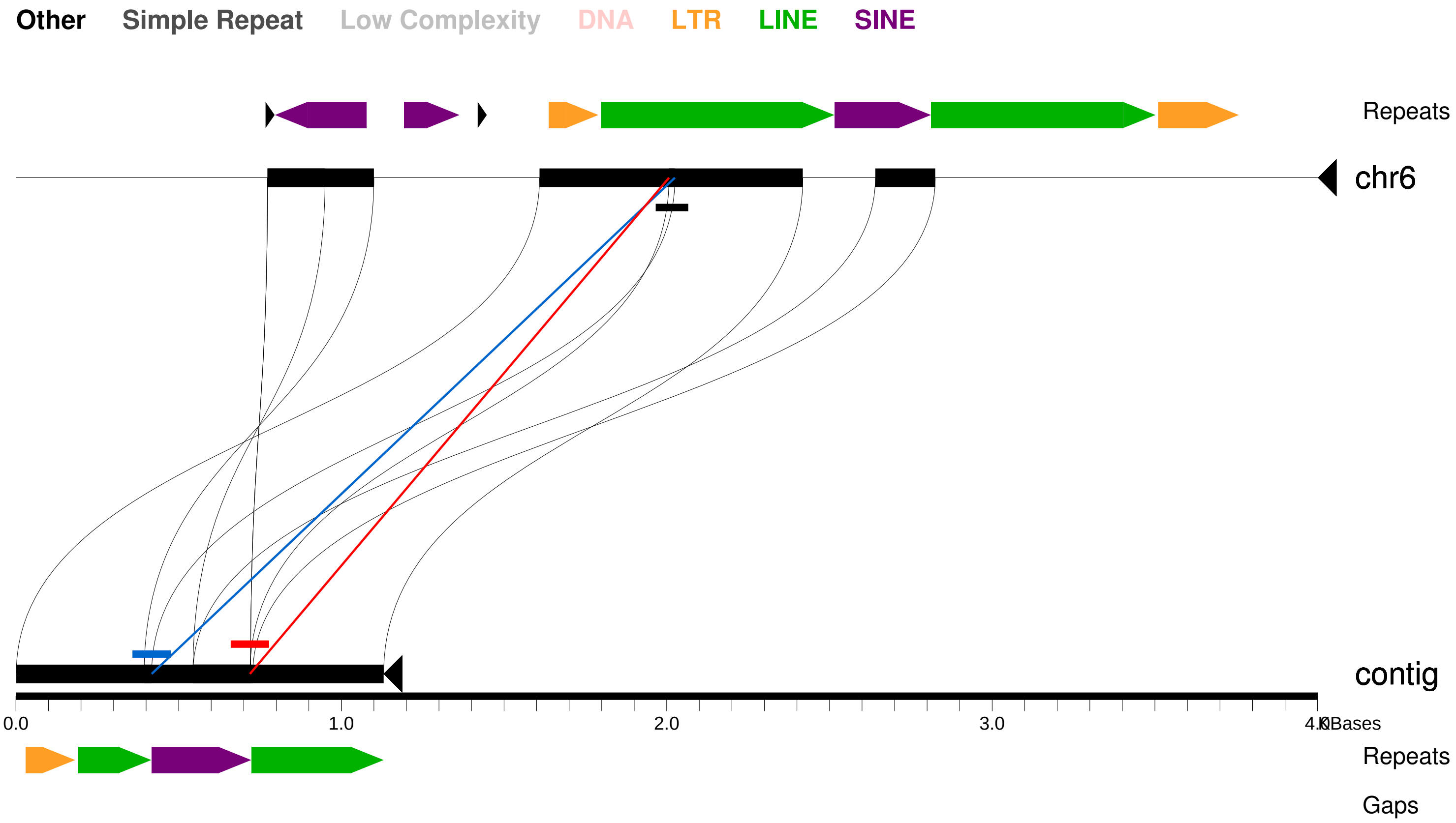

siteID: chr6_136367039 Scafftig4 - Breakpoint: chr6:136367040
chr6:136366987-136367092
Overlap Length: 7 INS size: 264 Genotyped: Yes

```
left    GA------------AAAGGAAAATTAAATA-ACTAAGGGTAATAAGACACATATCATGCAATAATTAGAA
chrom   GA------------AAAGGAAAATTAAATA-ACTAAGGGTAATAAGACACATATCATGCAATAATTAGAA
right   CACTGCACTCCAGCCTGGGCGACAGAGCGAGACTCCGTCTCAAAAAAAAAAAAAAAAAAAAAAAAAAGAA
        1*111111111111111**11*111*111*1***11*11*1*1**1*1*1*1*11*111**1**11****

left    TTTGAGGCCGAGGCGGGCGGATCACGAGGTCAGGAGATCGAGACCATCCCGGCTAAA--ACGGT
chrom   TTTTA-----ATGCT------TCTTAACCTCTGGAATTAAAGCCAAA----GCTAAAGAAAGAT
right   TTTTA-----ATGCT------TCTTAACCTCTGGAATTAAAGCCAAA----GCTAAAGAAAGAT
        ***2*22222*2**2222222**222*22**2***22*22**2*2*22222*****22*2*2*
```

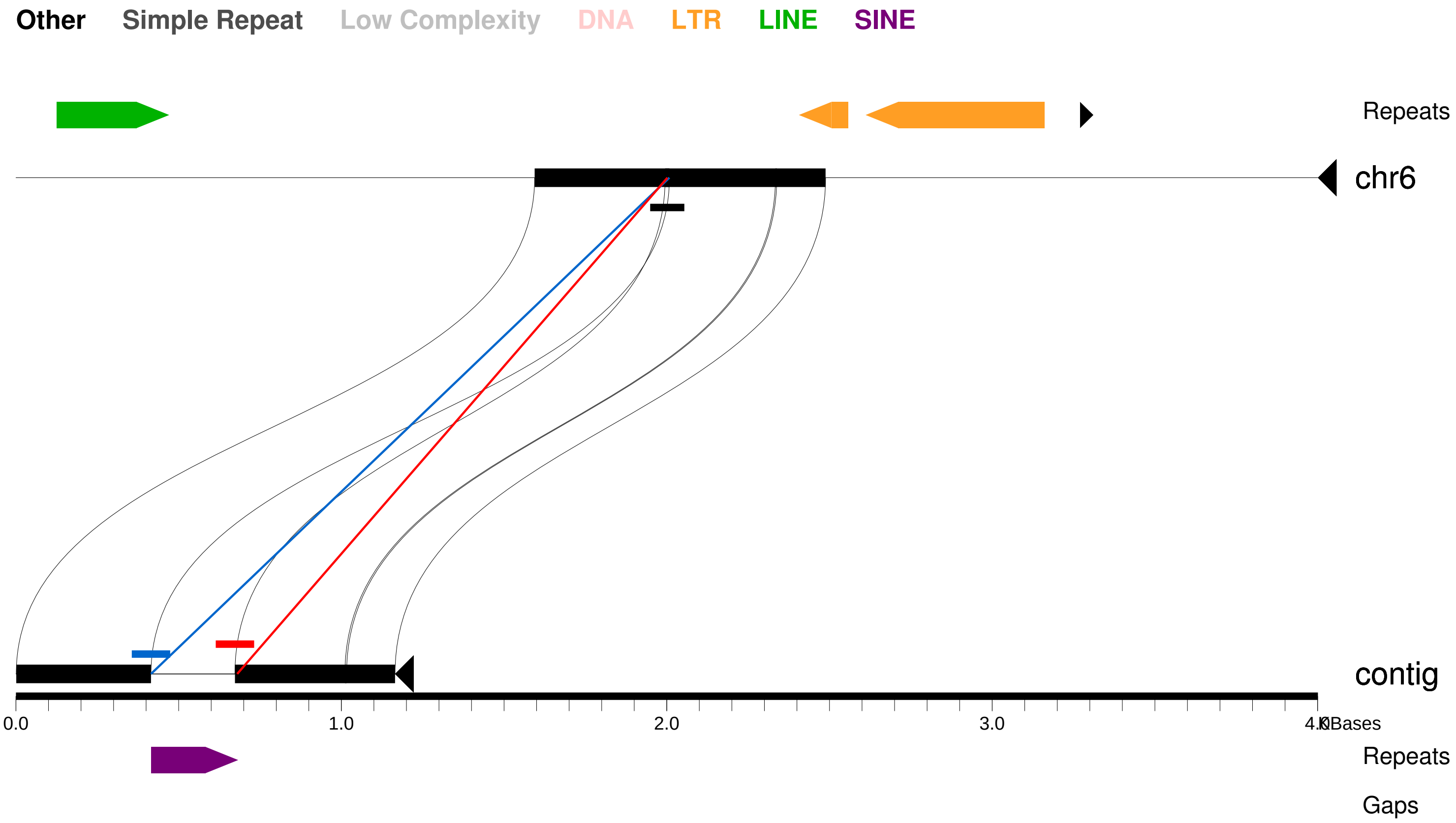

siteID: chr6_136700596 Scafftig2 - Breakpoint: chr6:136700469
chr6:136700426-136700528
Overlap Length: 17 INS size: 162 Genotyped: Yes

```
left    T----------------AATTTAAAAGAGTAAATATTTTGATCAGGCTCTGGTGAATTTAAGAATTAGTG
chrom   T----------------AATTTAAAAGAGTAAATATTTTGATCAGGCTCTGGTGAATTTAAGAATTAGTG
right   GCGAGACTCCGTCTCAAAAAAAAAAAAAAAAAAAAAAAAAAAAAAAAAAAAAAAAAAAAAAGAATTAGTG
        11111111111111111**111****1*11***1*11111*11*1111111111**111***********

left    AATTGTCCCAGCTACTTGGGAGGCTGAGGCAGGAGAATGGCGTGAACCCGGGAGGCGGAGCTTGC
chrom   AATTGT------TTGTTAG-AGTCTGTTT-ACTAGAA--GAGT-----CTTCATGGTTAAATTA-
right   AATTGT------TTGTTAG-AGTCTGTTT-ACTAGAA--GAGT-----CTTCATGGTTAAATTA-
        ******222222*22**2*2**2***2222*22****22*2**22222*222*2*222*22**22
```

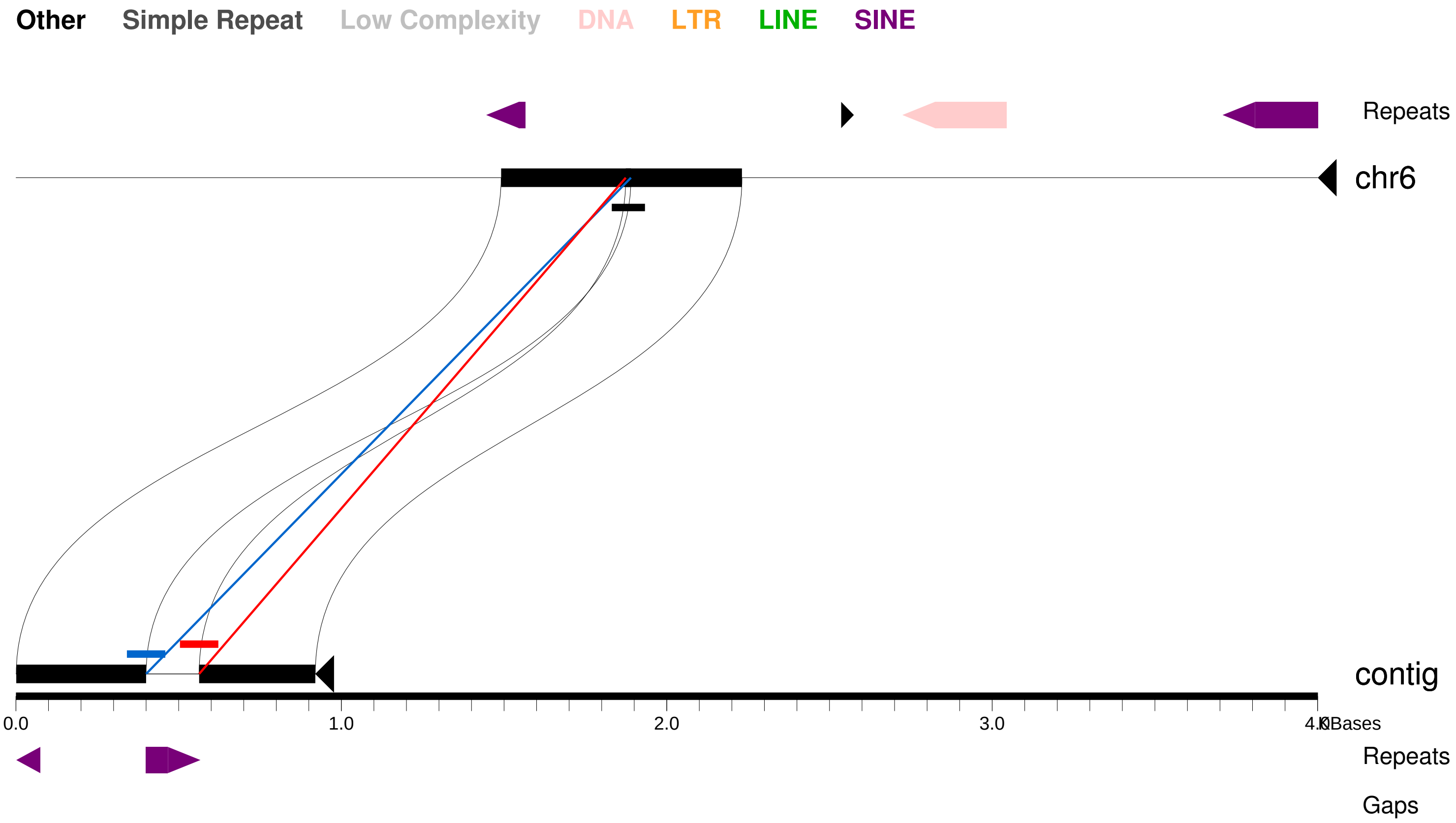

siteID: chr6_13718487 Scafftig4 - Breakpoint: chr6:13718468
chr6:13718431-13718562
Overlap Length: 23 INS size: 404 Genotyped: No

```
left    ACCTTCATGCTGTTCTCATGATAGTGAGGTCTCACCAGATCTGGTGGGTTTATAACGGTCNNNNNNNNNN
chrom   ACCTTCATGCTGTTCTCATGATAGTGAGGTCTCACCAGATCTGGTGGTTTTACAACGGTCTTTATCTGCC
right   GCGTG-AGCCACCGCGCCCGG-------------CCTGATCTGGTGGTTTTACAACGGTCTTTATCTGCC
        1*1*11*11*1111*1*11*11111111111111**1**********2****2*******2222222222

left    NNNNNNNNN-NNNNNNNNNNNNNNNNNNNNNNNNNNNNNNNNNNNNNNNNN-------------
chrom   TGTTTTTTT-CTGCACTTTTCTTTGCTGCTGTCTTGTGAGGAAGGATGTGCTTGTGTCCGTTT
right   TGTTTTTTTTCTGCACTTTTCTTTGCTGCTGTCTTGTGAGGAAGGATGTGCTTGTGTCCGTTT
        222222222122222222222222222222222222222222222222222222222222222
```

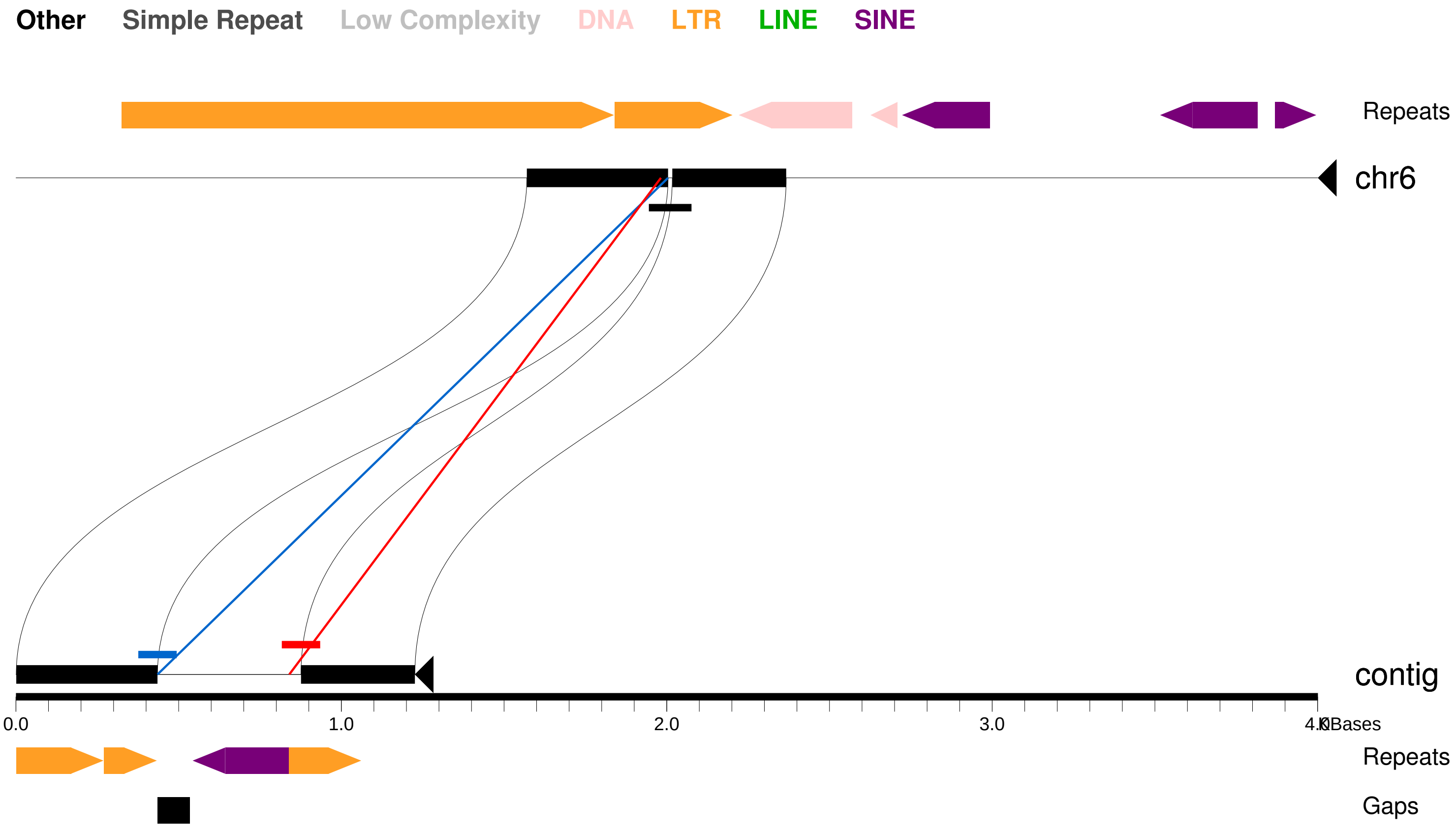

siteID: chr6_137254207 Scafftig1 + Breakpoint: chr6:137254192
chr6:137254032-137254367
Overlap Length: 16 INS size: 263 Genotyped: Yes

```
left   -------------------TCCCAGCACTTTGGGAGGCTGAGGTGGGAGGATCACTTGCGCCCAGGAGTT
chrom  GG--TGGCTACACCTGTAATCCCAGCACTTTGGGAGGCTGAGGTGGGAGGATCACTTGCGCCCAGGAGTT
right  AGGCAGGAGAATGGCGTGAACCC--------GGGAGGCGGAGTTTGCAGTGAGCCGAGATCCCGCCACTG
       N211N22NN2NNNNN22N21***11111111*******1***1*1*1**11111*11*11***111*1*1

left   TGAGACCAGGCTGGGCAACATAATGATACCTTGTCTCTATTTTTAAAAATTAAAAAAAAAAAAAAACTCA
chrom  TGAGACCAGGCTGGGCAACATAATGATACCTTGTCTCTATTTTTAAAAATTAAAAAAAAAAAAAA-CTCA
right  C-ACTCCAGCCTGGGCGACAGAGCGAGACTCCGTCTC-------AAAAA--AAAAAAAAAAAAAA-----
       11*11****1******1***1*11**1**111*****1111111*****11**************21111

left   TTGAAATATAATTTAGATACCATAAAATTTGCCTGATTT-------------------TTTGGGAGG---
chrom  TTGAAATATAATTTAGATACCATAAAATTTGCCTGATTTAAGCGTACAACTAGTGATCTTTAGTATATTT
right  -----------------------AAAATTTGCCTGATTTAAGCGTACAACTAGTGATCTTTAGTATATTT
       11111111111111111111111***************2222222222222222222***2*2*22222

left   -CCGAGGCGGGCGGATCACGAGGTCAGGAGA--TCGAGACCAT--CCTGG----CTAACAAGGTGAAACC
chrom  ACAGAGTTGTGCA-ATCATCATTACTGTCTAATTTTAGAACATTTCCTTCACCTCAAAAAATAAAAAACC
right  ACAGAGTTGTGCA-ATCATCATTACTGTCTAATTTTAGAACATTTCCTTCACCTCAAAAAATAAAAAACC
       2*2***22*2**22****22*222*2*222*22*22***2***22***222222*2**2**2222*****

left   CCGTCTCTACTAAAAAT-ACAAAAAATTAG---CCGGGCGTAGTGGCGGGCGC-----CT
chrom  CTACACCCATTGGAAGTCACTACCCATTTCTTCCCAATCCTCCAGCCCCCAGCAATTACT
right  CTACACCCATTGGAAGTCACTACCCATTTCTTCCCAATCCTCCAGCCC------------
       *22222*2*2*22**2*2**2*222***22222**222*2*222*2*2NNN11    11
```

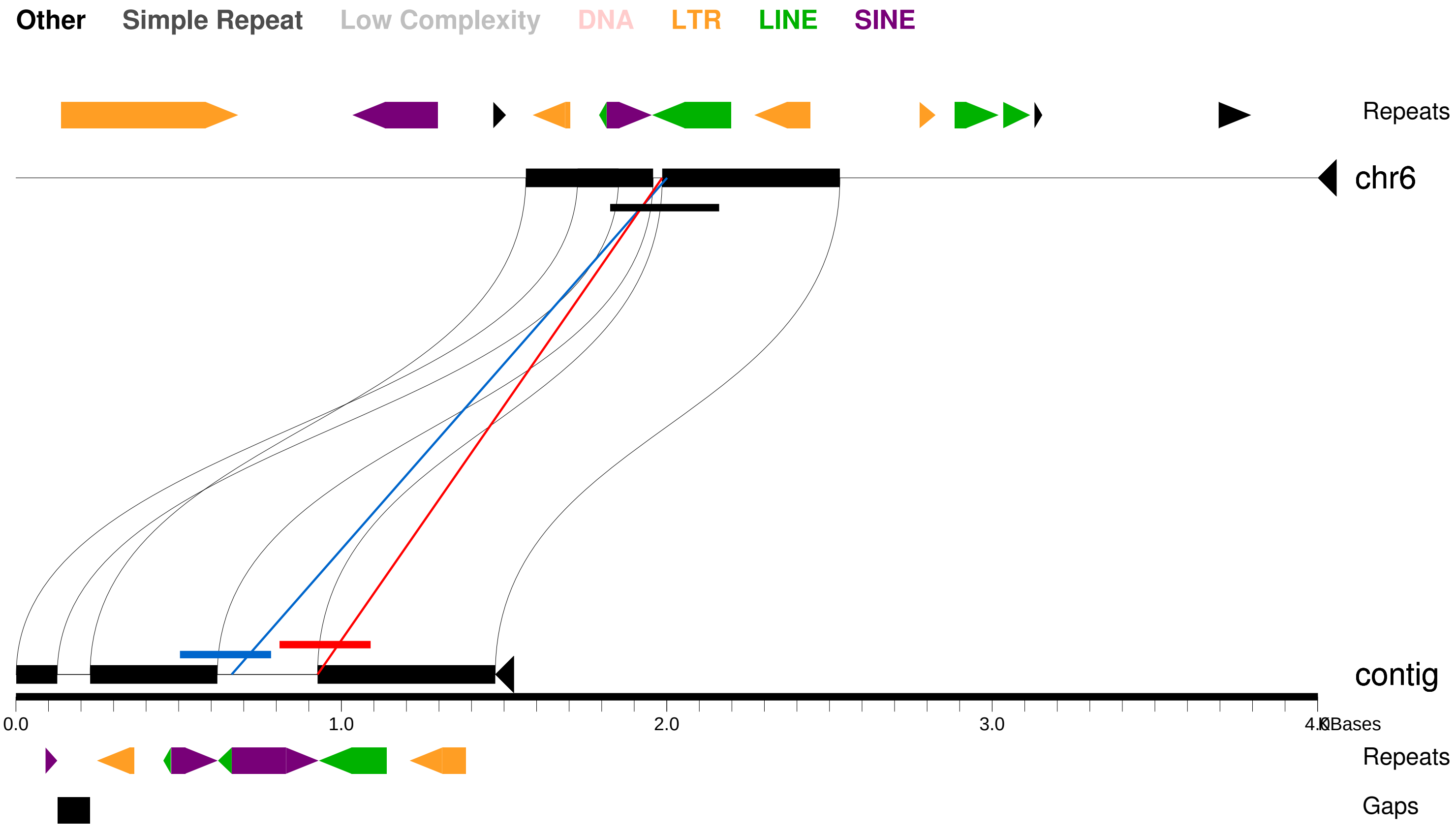

siteID: chr6_140269424 Scafftig2 + Breakpoint: chr6:140269417
chr6:140269257-140269591
Overlap Length: 15 INS size: 446 Genotyped: No

```
left   ---------------GTGGGAACCACTACCAACAGAATATACATCCTTTTCAATTGCGTAGTGAACATTC
chrom  ACCTTTTTGACATTTGTGGGAACCACTACCAACAGAATATACATCCTTTTCAATTGCGTAGTGAACATTC
right  NNNNNNNNNNNNAA--------------------------------------------------------
       NNNNNNNNNNN2N  1111111111111111111111111111111111111111111111111111111

left   ACCAAAATAGCAGACTATAGAACAAGTCTTCCAAAACTTAGAAGAATTGAAATAATATAAAATATTTTTT
chrom  ACCAAAATAGCAGACTATAGAACAAGTCTTCCAAAACTTAGAAGAATTGAAATAATATAAAATATTTTTT
right  ----------------------------------------------------------------------
       1111111111111111111111111111111111111111111111111111111111111111111111

left   GAAAGTGACAAAATATTTTCAAAAATCAACAACAAGGCCGGGCGCGGTGGCTCAC------GCCTGTAAT
chrom  GAAAGTGACAAAATATTTTCAAAAATCAACAACAAAACATTTAGAAAACTCTAAATATTTGGAATTAAAT
right  --------------------AAAAATCAACAACAAAACATTTAGAAAACTCTAAATATTTGGAATTAAAT
       11111111111111111111***************22*22222*222222**2*2222222*22*22***

left   CCCAGCACTT---------TGGGAGGCCGAGGCGGGCGGATCACGAG----GTCAGGAGATC--------
chrom  GACA-CACTTCTAAATAACTGACAGAACAAAGAAAA-GGATCACAATAAAAGTTAGAAGATATTTTTGAT
right  GACA-CACTTCTAAATAACTGACAGAACAAAGAAAA-GGATCACAATAAAAG------------------
       22**2*****222222222**22**22*2*2*22222*******2*22222*1N11N1111N

left   -----GAGACCATCCCGGCTAAAACGGTGAAACCCCGT---CTCT------ACTAAA
chrom  CAATAGTGATAATCAAAATATAATATATAAAATTTTGTGAGCTATCGATAAAGTAAG
right  ---------------------------------------------------------
            1N11NN111NNNNNNN11NNNN1N111NNNN11   11N1      1N111N
```

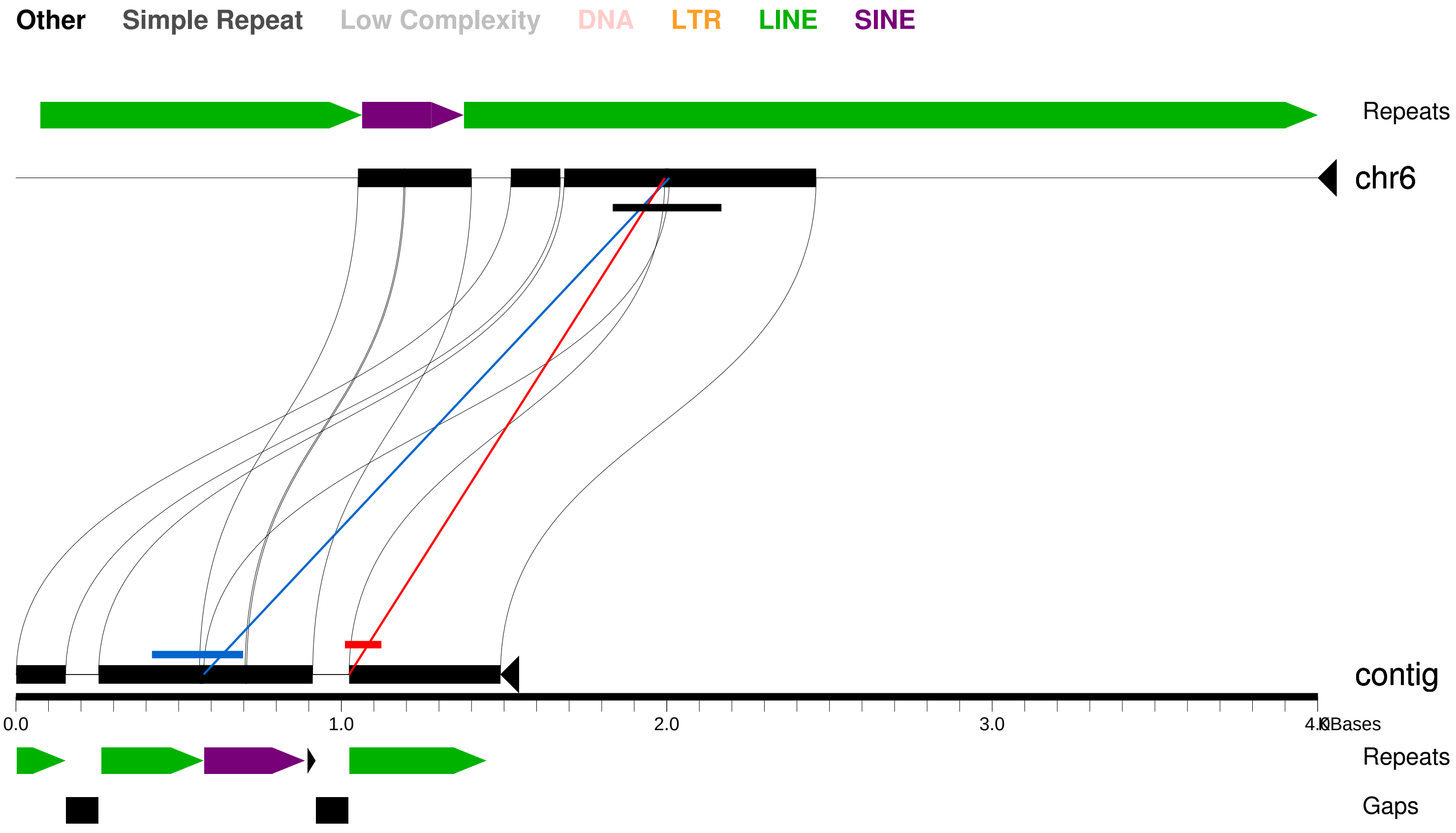

siteID: chr6_140417814 Scafftig1 - Breakpoint: chr6:140417830
chr6:140417783-140417889
Overlap Length: 13 INS size: 409 Genotyped: No

```
left    GCATTTGAAGGGAAATTGAATTAACCAAGGAGGGAAATAAAAAATTT------------AAGAATAAGTT
chrom   GCATTTGAAGGGAAATTGAATTAACCAAGGAGGGAAATAAAAAATTT------------AAGAATAAGTT
right   NNNNNNNNNNNNNNNNNNNNNNNNNNNNNNNNNNNNNNNNNNNNNNNNNNNNNNNNNNNAAGAATAAGTT
        11111111111111111111111111111111111111111111111111111111111***********

left    TAGGCCGGGCGCGGTGGCTCACGCCTGTAATCCCAGCACTTTGGGAGGCCGAGGCGGGCGG
chrom   TA--ACAAGATCAATT-----CTTTTATAATTGGAACGCTT-----GCTCTCAGCCTTGAA
right   TA--ACAAGATCAATT-----CTTTTATAATTGGAACGCTT-----GCTCTCAGCCTTGAA
        **222*22*22*22*222222*222*2****222*2*2***22222*22*222**222222
```

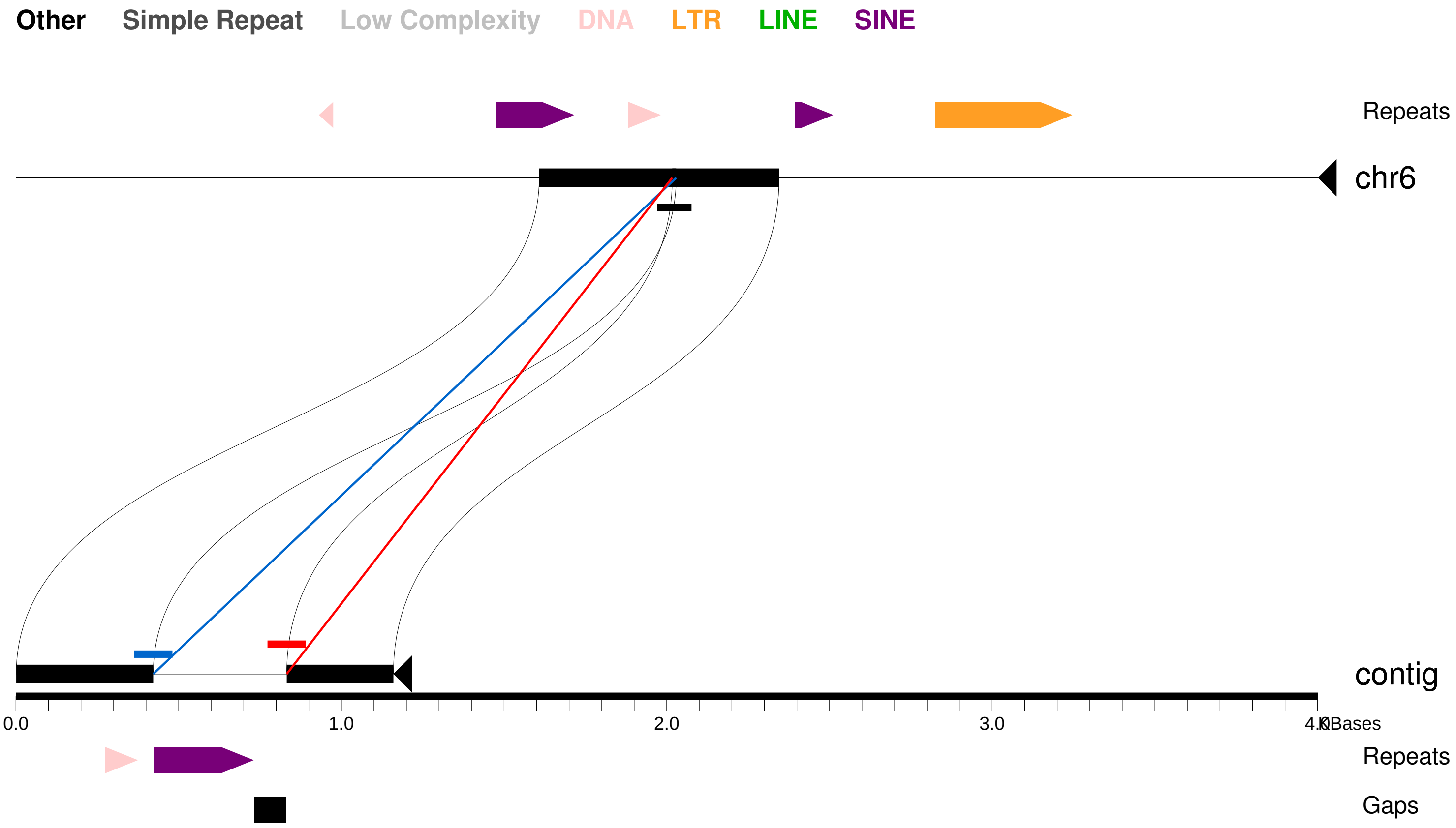

siteID: chr6_141134055 Scafftig4 + Breakpoint: chr6:141134052
chr6:141134008-141134111
Overlap Length: 16 INS size: 308 Genotyped: Yes

```
left    AAA--GGAATAGAATTT--AAACT-CTAGAAATAAACCCATGTGTCTAT----------GGTCAACTGAT
chrom   AAA--GGAATAGAATTT--AAACT-CTAGAAATAAACCCATGTGTCTAT----------GGTCAACTGAT
right   CCGCCCGCCTCGGCCTCCCAAAGTGCTGGGATTACAAGCGTGAGCCACCGCGCCCGGCCGGTCAACTGAT
        111111*11*1*111*111***1*1**1*1*1**1*11*1**1*1*1111111111111***********

left    TTTCTTTTTTTTTTTTTTTTTTTTTTTTTTTGAGACGGAGTCTCGCTCTGTGGCCCAGGTGGGA
chrom   TTTCTGAAAGAGTGCCAAATTCCATCATTG-GAGAAAAAGTCTTTCAAC--------------A
right   TTTCTGAAAGAGTGCCAAATTCCATCATTG-GAGAAAAAGTCTTTCAAC--------------A
        *****2222222*222222**222*22**22****222*****22*22222222222222222*
```

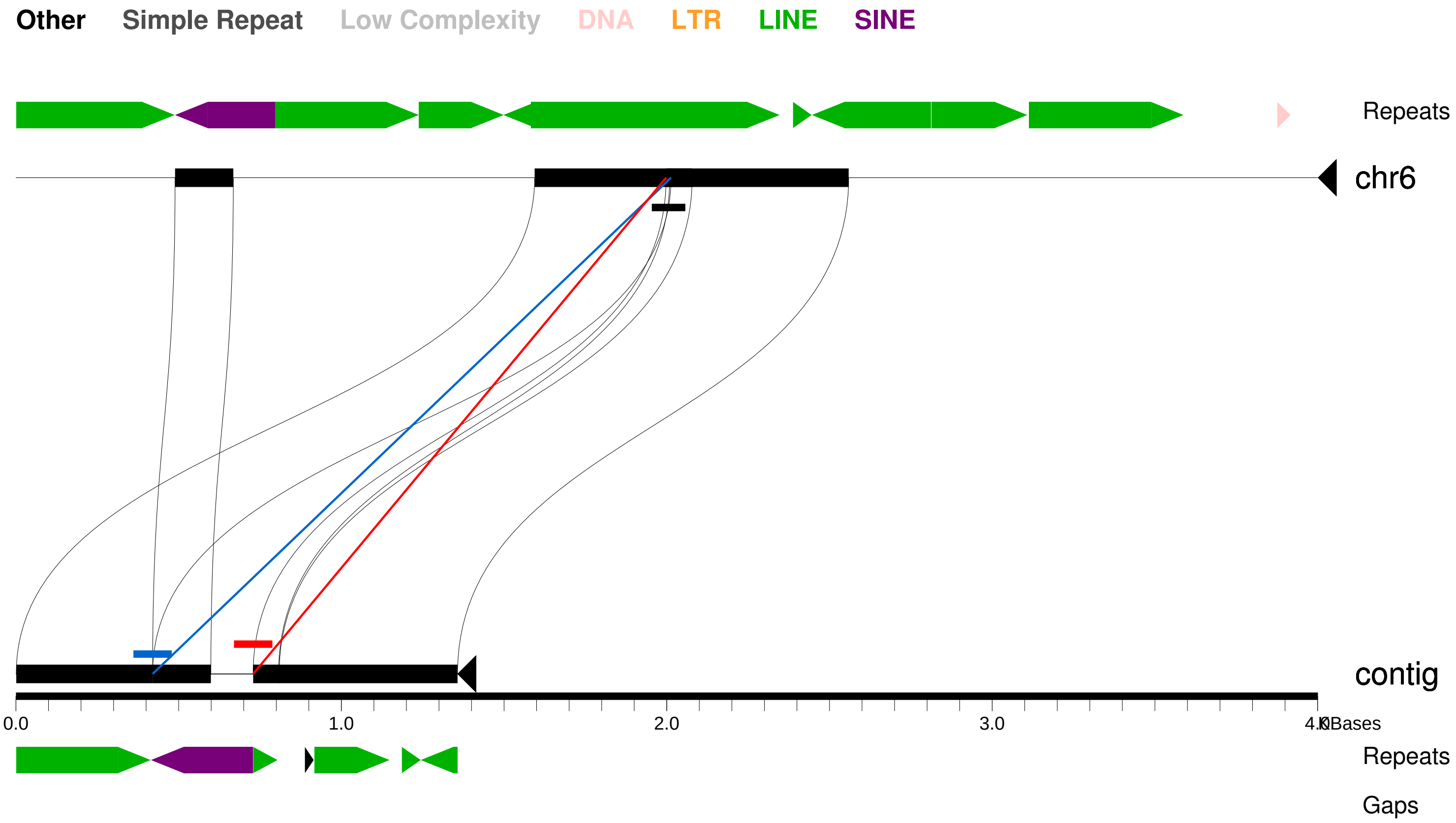

siteID: chr6_141414888 Scafftig2 + Breakpoint: chr6:141414889
chr6:141414862-141414948
Overlap Length: 16 INS size: 299 Genotyped: Yes

```
left    --------------------AAGT-CT----TTCTATCCATGAACATA-------GGATATCTCTCCATT
chrom   --------------------AAGT-CT----TTCTATCCATGAACATA-------GGATATCTCTCCATT
right   CCGCCCGCCTCGGCCTCCCAAAGTGCTGGGATTACAGGCGTGAGCCACCGCGCCCGGCCATCTCTCCATT
        11111111111111111111****1**1111**11*11*1***1*1111111111**11***********

left    TATTTTTTTTTTTTTTTTTTTTTGAGACGGAGTCTCGCTCTGTCGCCCAGGCTGGAGTGCAGTGGCGGGA
chrom   TATTTAATTCATCTTTGATTTT-------------------GTTCATCAGACTG---TGTAAT-------
right   TATTTAATTCATCTTTGATTTT-------------------GTTCATCAGACTG---TGTAAT-------
        *****22**22*2***22****2222222222222222222**2222***2***222**2*2*2222222

left    TCTCGGCTCAC
chrom   TTTC--CTCA-
right   TTTC--CTCA-
        *2**22****2
```

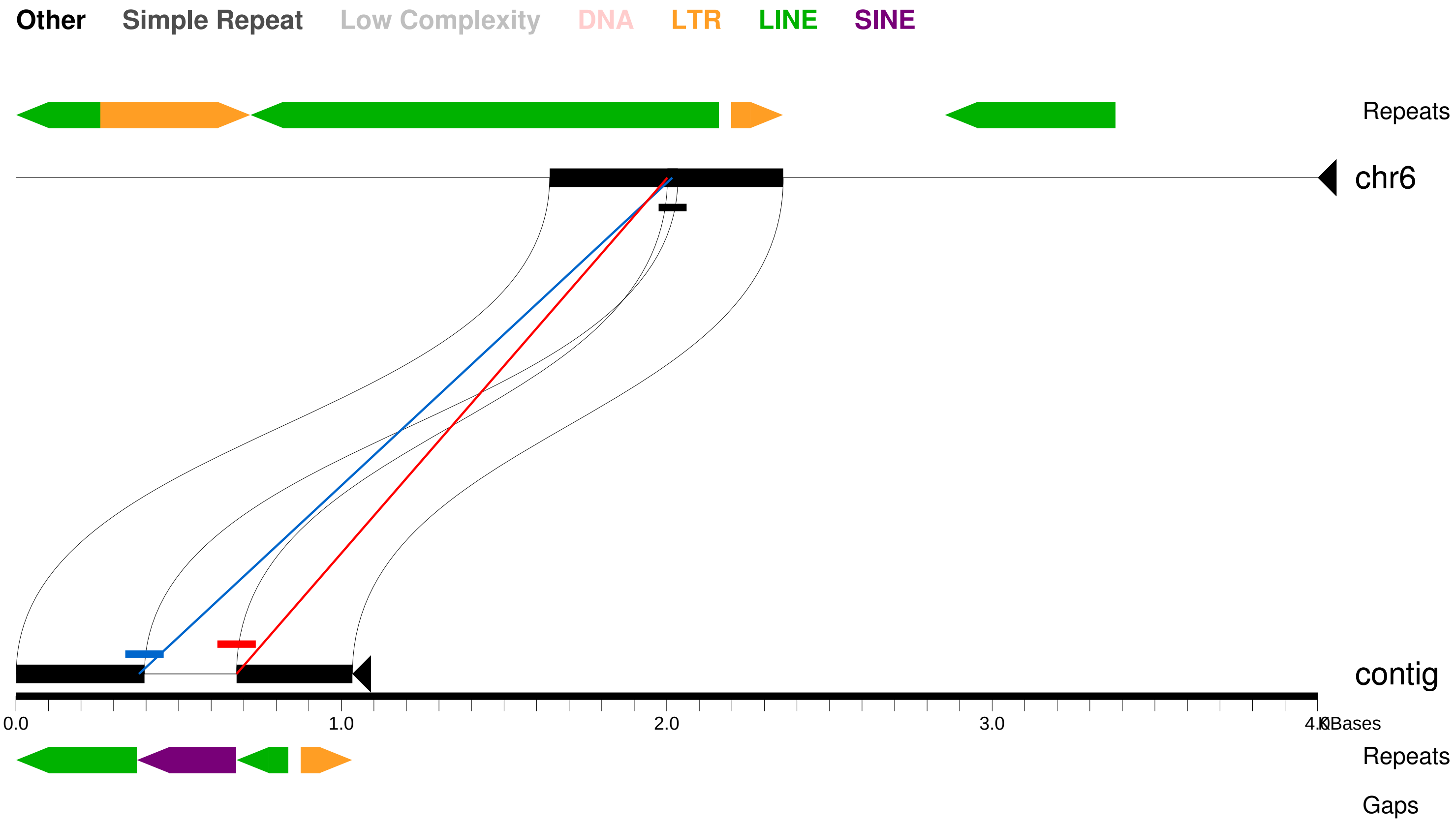

siteID: chr6_141770872 Scafftig8 + Breakpoint: chr6:141770870
chr6:141770844-141770929
Overlap Length: 13 INS size: 308 Genotyped: Yes

```
left    CCAGA---------------AACATACATATTTTATAGTTT------------------TTCTTTGCTTC
chrom   CCAGA---------------AACATACATATTTTATAGTTT------------------TTCTTTGCTTC
right   TCCGCCCGCCTCGGCCTCCCAAAGTGCTGGGATTACAGGCGTGAGCCACCGCGCCCGGCTTCTTTGCTTC
        1*1*1111111111111111**11*1*11111***1**1111111111111111111111***********

left    TTTTTTTTTTTTTTTTTTTTTTTTTTTTTTGAGACGGAGTCTCGCTCTGTCGCCCAGGCTGGAGTGCAGT
chrom   TTAAATTTTCCTTTATTTATTT-------TCAGA---AGTCTTTGTCTTTCTTT----------------
right   TTAAATTTTCCTTTATTTATTT-------TCAGA---AGTCTTTGTCTTTCTTT----------------
        **222****22***2***2***2222222*2***222*****222***2**2222222222222222222

left    GGCGGGATCTCG
chrom   -------TCACT
right   -------TCACT
        2222222**2*2
```

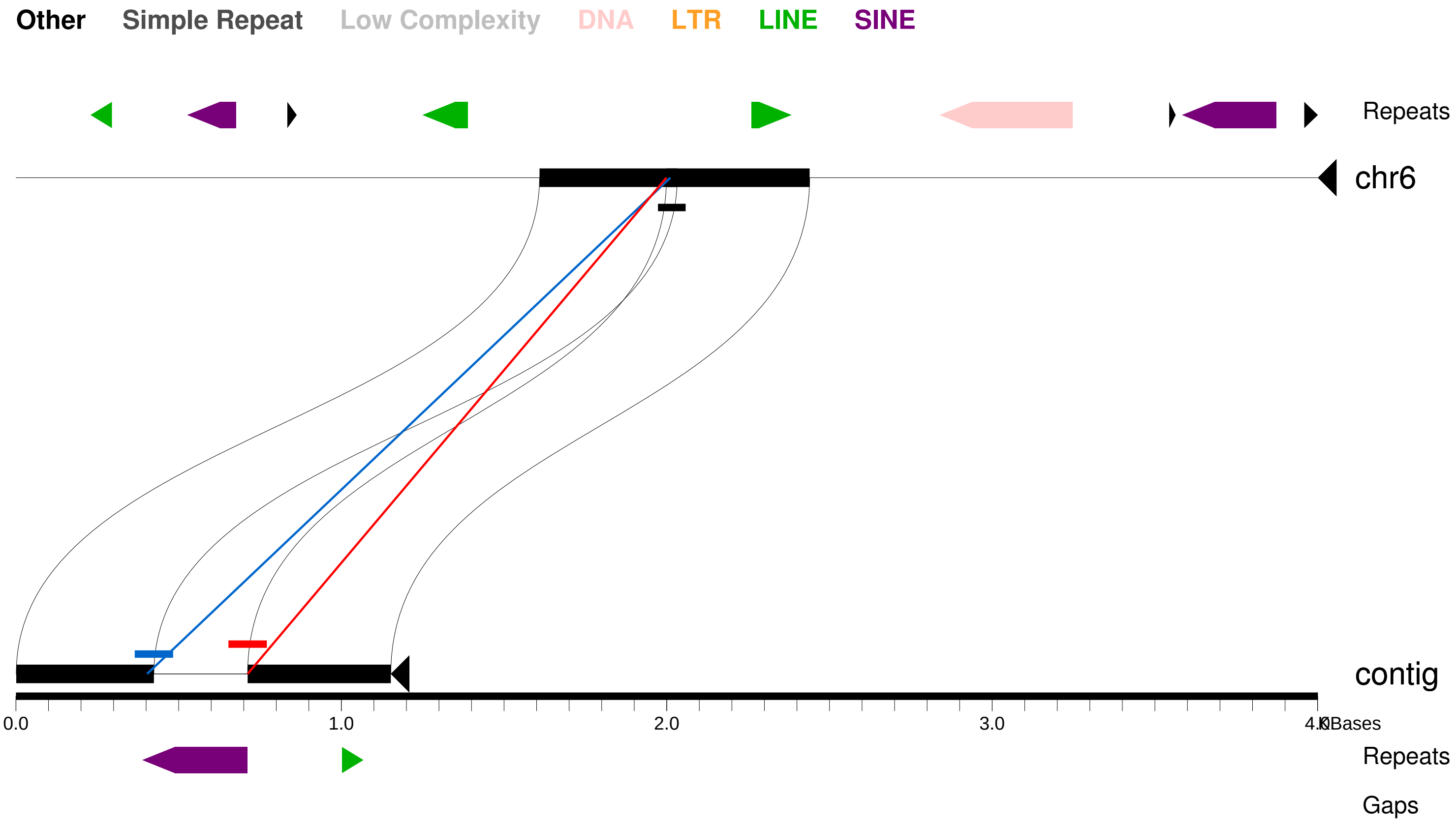

siteID: chr6_143951368 Scafftig3 - Breakpoint: chr6:143951371
chr6:143951316-143951424
Overlap Length: 5 INS size: 311 Genotyped: Yes

```
left    TTAT---TTGAATGA-AGATCAAAAAA-----TTAA-AAGACTTTATTTACTATCATTTAAAATATTTTC
chrom   TTAT---TTGAATGA-AGATCAAAAAA-----TTAA-AAGACTTTATTTACTATCATTTAAAATATTTTC
right   CTCCAGCCTGGGCGACAGAGCGAGACTCCGTCTCAAGAAAAAAAAAAAAAAAAAAAAAAAAAAATTTTTC
        1*111111**111**1***1*1*1*1111111*1**1**1*1111*111*11*11*111****11*****

left    GCCGGGCGCGGTGGCTCACGCCTGTAATCCCAGCACTTTGGGAGGCCGAGGCGGGCG-GA---
chrom   -----------TTGTTTT-GTATG-AATGCAAACA-TTGAGGAAACAAAGTATGTCTAGAAAG
right   -----------TTGTTTT-GTATG-AATGCAAACA-TTGAGGAAACAAAGTATGTCTAGAAAG
        22222222222*2*2*222*22**2***2*2*2**2**22***22*22**222*2*22**222
```

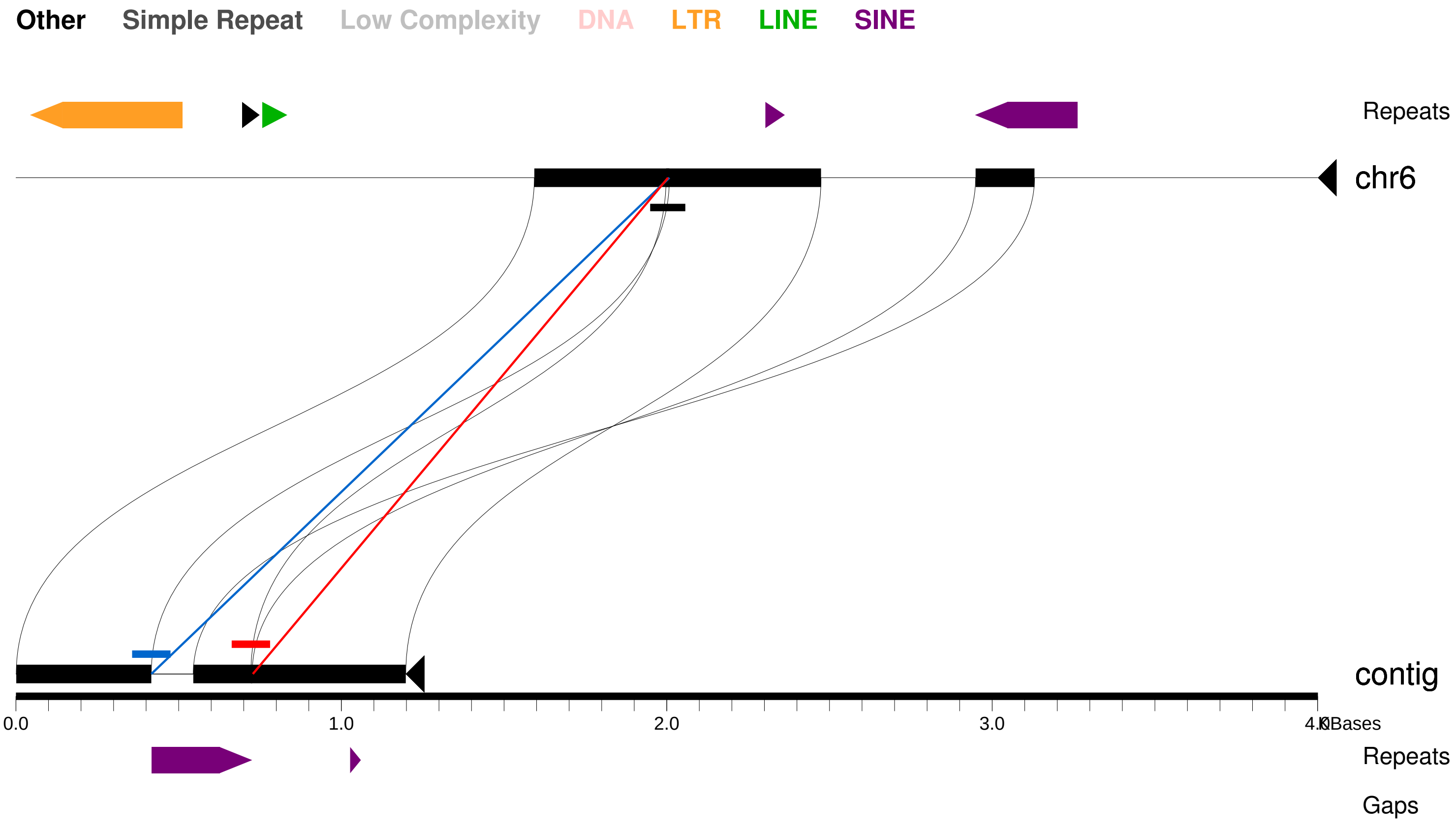

siteID: chr6_153633391 Scafftig5 + Breakpoint: chr6:153633370
chr6:153633324-153633429
Overlap Length: 14 INS size: 291 Genotyped: Yes

```
left    GTATACTTCATGCATTCTCCCACT-CTCCCAAAAGA----TATTTCACATT--------TTTTTCTCTGC
chrom   GTATACTTCATGCATTCTCCCACT-CTCCCAAAAGA----TATTTCACATT--------TTTTTCTCTGC
right   CCGCCCACCTCGGCCTCCCAAAGTGCTGGGATTACAGGCGTGAGCCACCGCGCCCAGCCTTTTTCTCTGC
        11111*11*11*111**1*11*1*1**111*11*1*1111*1111***11111111111***********

left    CTTTTTTTTTTTTTTGAGACGGAGTCTCGCTGTCGCCCAGGTTGGAGTGCAGTGGCGCGATC-
chrom   CTTCTGAACTTCT-----AACGTCTCTTCCTCTATCTCTCCTCTCAGCT---------GATCA
right   CTTCTGAACTTCT-----AACGTCTCTTCCTCTATCTCTCCTCTCAGCT---------GATCA
        ***2*2222**2*22222*22*22***22**2*22*2*222*222**22222222222****2
```

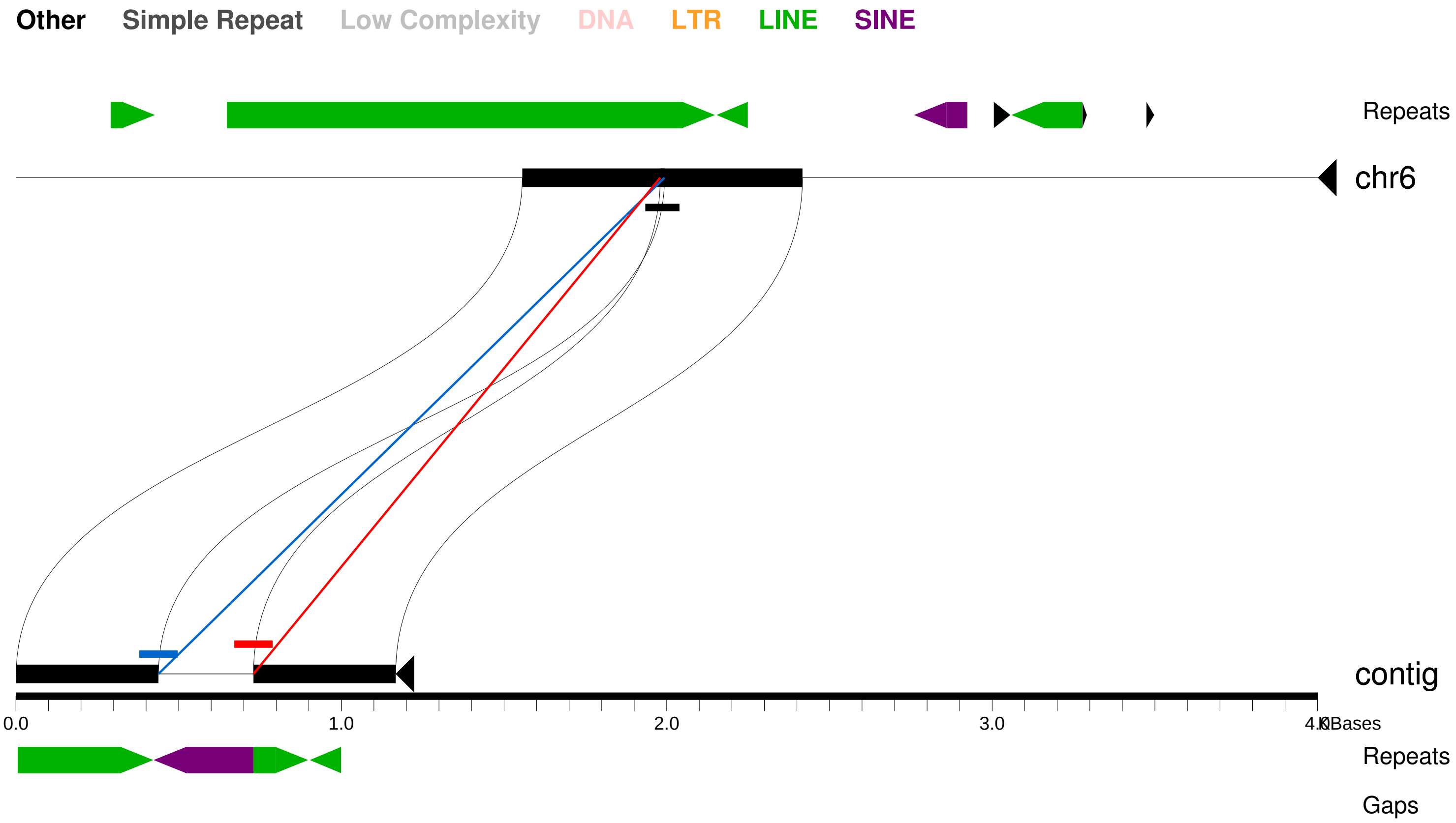

siteID: chr6_156541106 Scafftig1 - Breakpoint: chr6:156541100
chr6:156541054-156541159
Overlap Length: 14 INS size: 328 Genotyped: No

```
left   GCTGTGCTGTGTTAGGAGACATTGGAGTTCTATCCCT-------------ACGACATTCAAGAGACTACA
chrom  GCTGTGCTGTGTTAGGAGACATTGGAGTTCTATCCCT-------------ACGACATTCAAGAGACTACA
right  NNNNNNNNNNNNNNNNNNNNNNNNNNNNNNNNNNNNNNNNNNNNNNNNNNAAAAAAAAAAAAGAGACTACA
       11111111111111111111111111111111111111111111111111*11*1*111***********

left   CTAGGTGAAACCCCGTCTCTACTAAAAATACAAAAAAT-TAGCCGGGCGCGGTGGCGGGCGCC
chrom  CTA-----AGCACAGTGCACATTCAATGGATAATAATCATAGC---------TAGAGTAAGAC
right  CTA-----AGCACAGTGCACATTCAATGGATAATAATCATAGC---------TAGAGTAAGAC
       ***22222*2*2*2**2222*2*2**222*2**2**222****222222222*2*2*222*2*
```

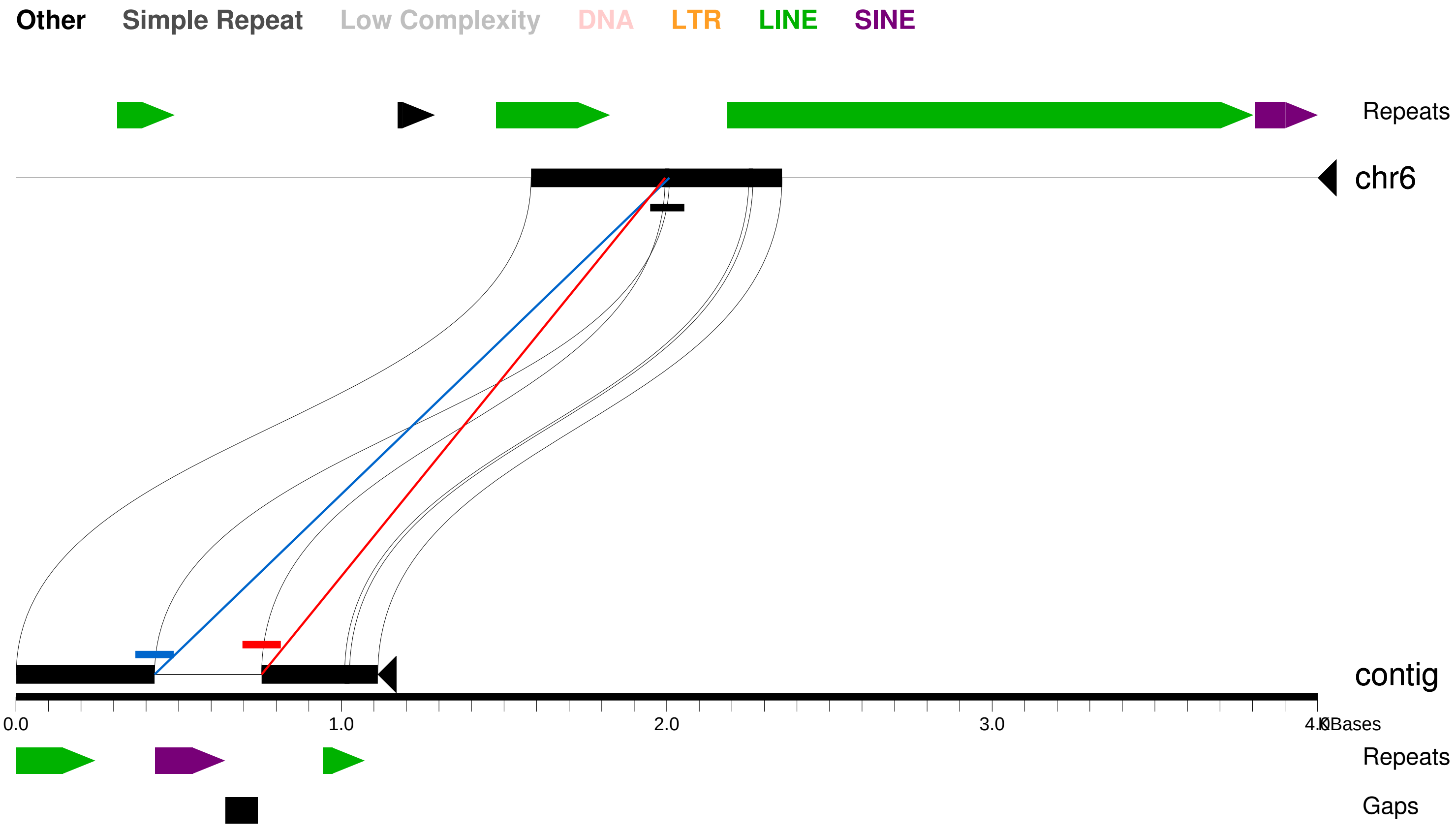

siteID: chr6_157397658 Scafftig5 + Breakpoint: chr6:157397675
chr6:157397630-157397734
Overlap Length: 12 INS size: 343 Genotyped: No

```
left    CA------TTTTACTGCTATACTACTGA------AGAAATGAATGTCTCCAGAAGGC--TCATGCCTTTT
chrom   CA------TTTTACTGCTATACTACTGA------AGAAATGAATGTCTCCAGAAGGC--TCATGCCTTTT
right   CACCCGCCTCGGCCTCCCAAAGTGCTGGGATTACAGGCGTGAGCCACCGCGCCCGGCCTTCATGCCTTTT
        **111111*1111**1*1*1*1*1***1111111**111***1111*11*1111***11***********

left    TTTTNNNNNNNNNNNNNNNNNNNNNNNNNNNNNNNNNNNNNNNNNNNNNNNNNNNNNNNNNNN
chrom   TATTGTGTTATTTTTAGAGATTCTGTGTGCTAAGCATTGTACTTCTAAG--------------
right   TATTGTGTTATTTTTAGAGATTCTGTGTGCTAAGCATTGTACTTCTAAG--------------
        *2**22222222222222222222222222222222222222222222222222222222222
```

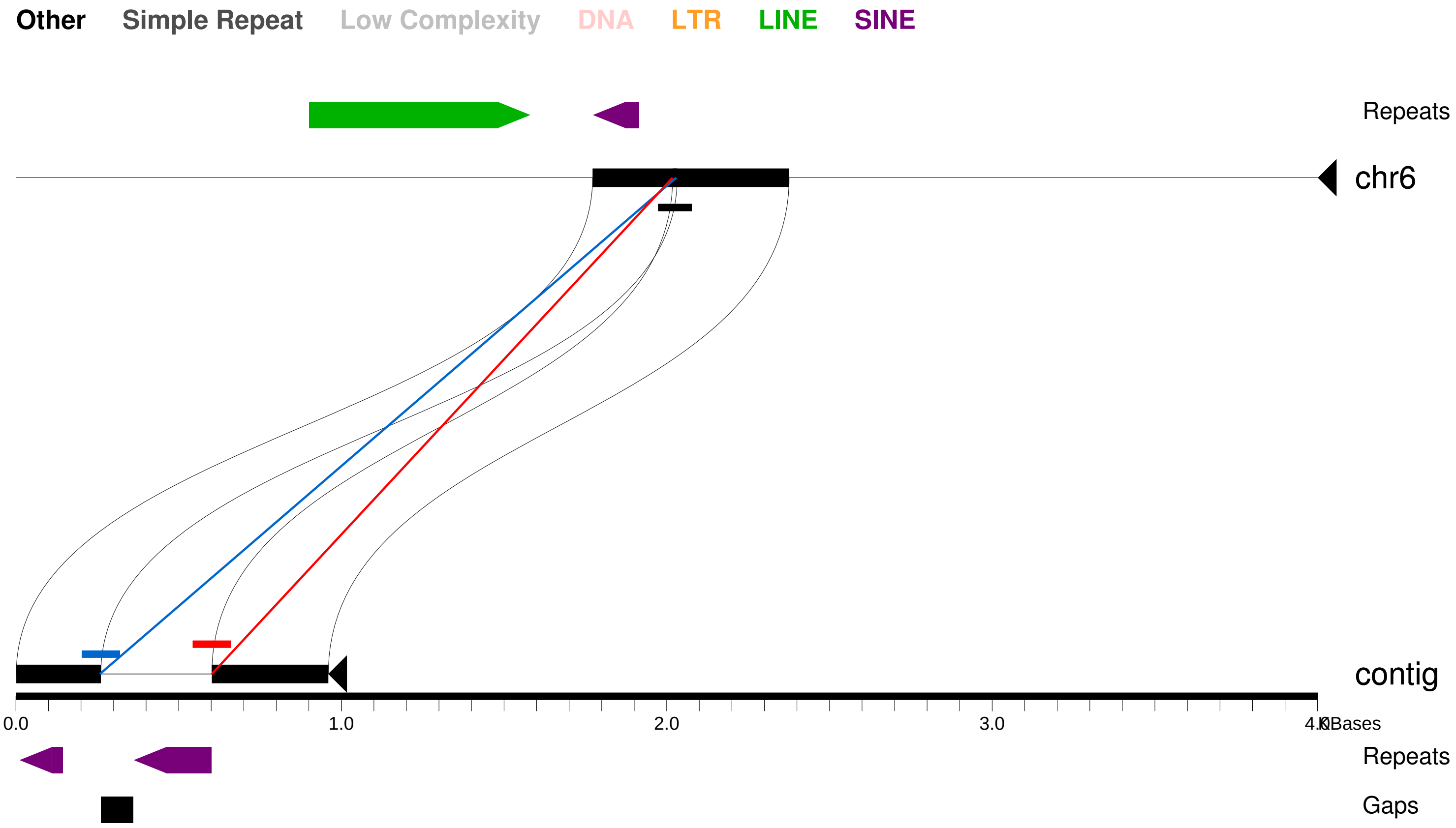

siteID: chr6_159166282 Scafftig1 - Breakpoint: chr6:159166286
chr6:159166243-159166345
Overlap Length: 17 INS size: 302 Genotyped: Yes

```
left     AT---------TTGTGTTAAAGAATGAGAT--CGTGT-----AACATTTATTGCTAGGCAAAAACTGTAT
chrom    AT---------TTGTGTTAAAGAATGAGAT--CGTGT-----AACATTTATTGCTAGGCAAAAACTGTAT
right    GCACTCCAGCCTGGGGCGACAGAGCGAGACTCCGTCTCAAAAAAAAAAAAAAAAAAAAAAAAAAAACTGTAT
         11111111111*1*1*11*1***11****111***1*11111**1*111*11111*111***********

left     CTAGGCCGGGCGCGGTGGCTCATGCCTGTAATCCCAG-CACTTTGGGAGGCCGAGGCGGGCGGATC
chrom    CTAGGCAAGA-GAGG-GCCTTCTGA--GGATTCTCTGTCTCCTTG--AGATAGAA-----------
right    CTAGGCAAGA-GAGG-GCCTTCTGA--GGATTCTCTATCTCCTTG--AGATAGAA-----------
         ******22*22*2**2*2**22**222*2*2**2*212*2*2***22**222**222222222222
```

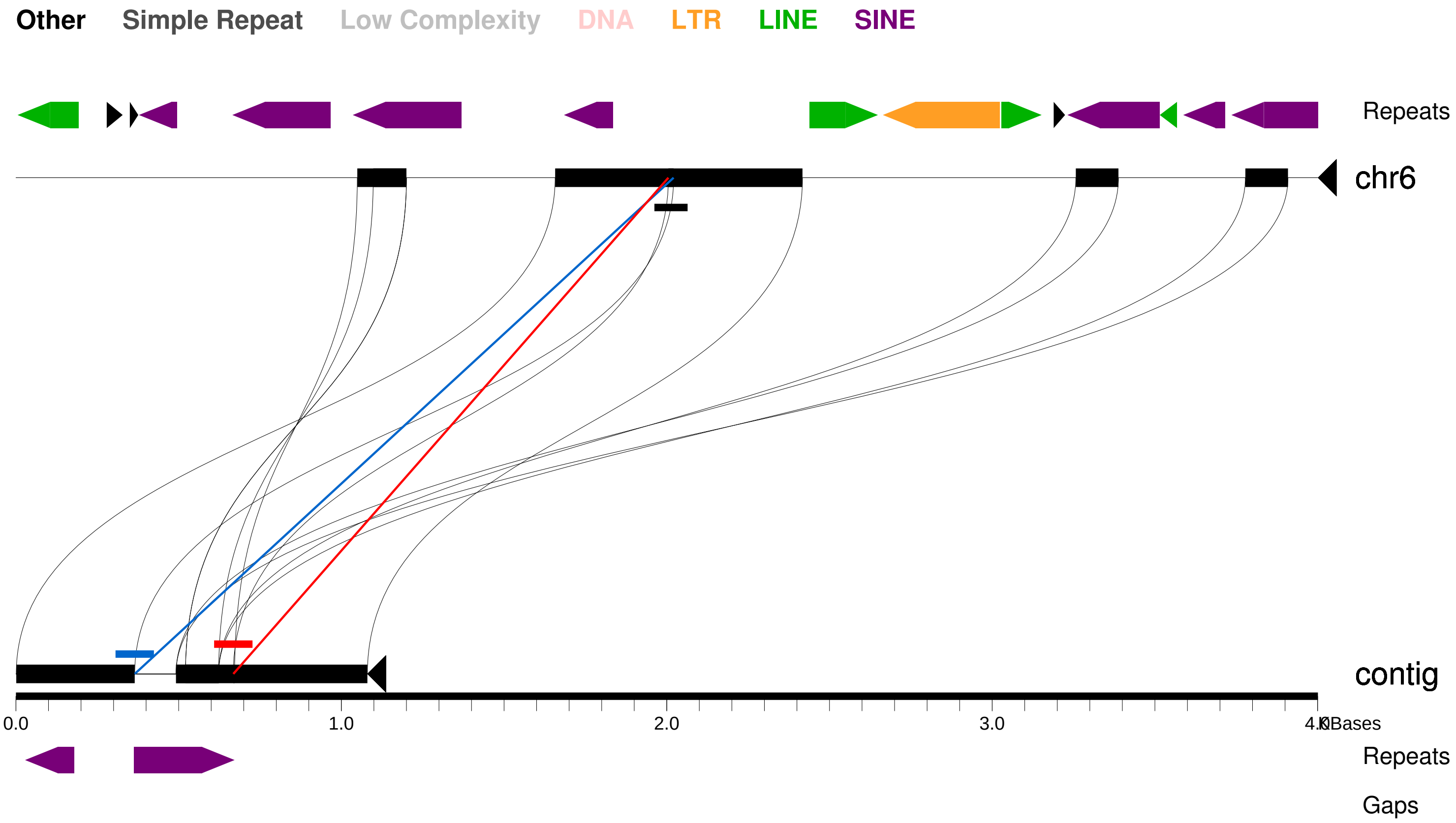

siteID: chr6_162707242 Scafftig2 - Breakpoint: chr6:162707268
chr6:162707221-162707327
Overlap Length: 10 INS size: 312 Genotyped: Yes

```
left    AT-----GTATAAAA-TGCCACATT--AGGGC--ACAGGTTTTATCA--CGTTTTTTTGTTAGCTAATTT
chrom   AT-----GTATAAAA-TGCCACATT--AGGGC--ACAGGTTTTATCA--CGTTTATTTGCTAGCTAATTA
right   TCCGCCCGCCTCGGCCTCCCAAAGTGCTGGGATTACAGGCGTGAGCCACCGCGCCCGGCCTAGCTAATTA
        1111111*11*11111*1***1*1*111***111*****11*1*1*111**111N11112*********2

left    TTTTTTTTTTTTTTTTTTTTTTTTTTTTTTTGAGACGGAGTCTCGC-TCTGTCGCCCAGGCTG
chrom   TTGAATCCAAAGCCTTTCCAAACTTGTTTTTGGATG--TTCTTGTGTTTGT-----------
right   TTGAATCCAAAGCCTTTCCAAACTTGTTTTTGGATG--TTCTTGTGTTTGT-----------
        **222*22222222***222222**2****22**2*222***2*22*2***22222222222
```

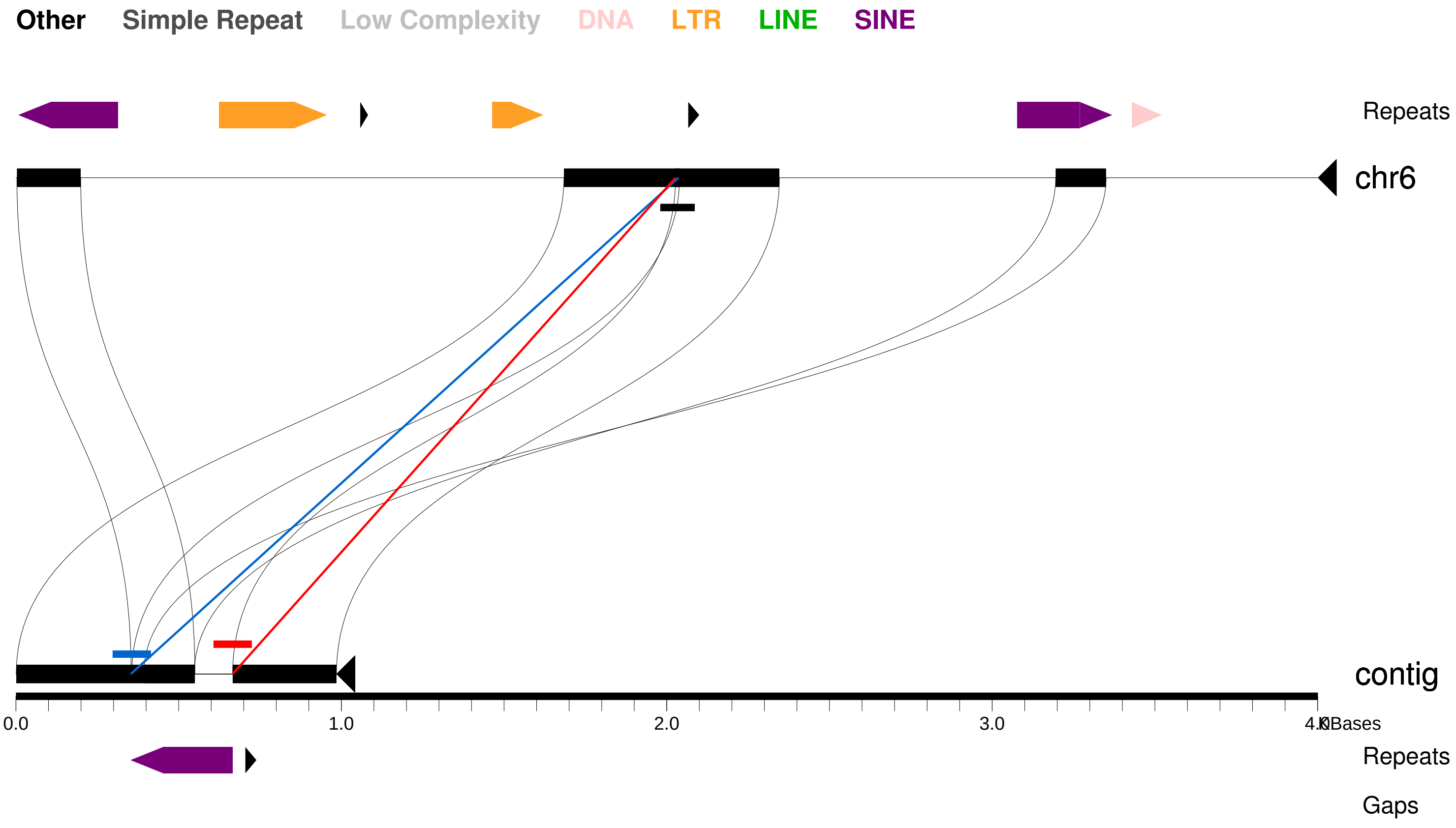

siteID: chr6_163814209 Scafftig11 - Breakpoint: chr6:163814216
chr6:163814171-163814275
Overlap Length: 15 INS size: 297 Genotyped: Yes

```
left     A--------ACTCCAT----GGTAATGACGCTGTACT--GGAAAAAAGAGAGGTTAGATAAAAGATGAGG
chrom    A--------ACTCCAT----GGTAATGACGCTGTACT--GGAAAAAAGAGAGGTTAGATAAAAGATGAGG
right    CGCCACTGCACTCCAGCCTGGGCGACAGAGCGAAACTCCGTCTCAAAAAAAAAAAAAAAAAAAGATGAGG
         111111111******11111**11*1111**111***11*1111***1*1*1111*1*1***********

left     TCTCGGCCGGGCGCGGTGGCTCACGCCTGTAATCCCAGCACTTTGGGAGGCCGAGACGGGCGG
chrom    TCTC-------CAAGTT---TTA-GAGTGAAAATGCTAAATGTTGCTT---AGATTTTGTGTG
right    TCTC-------CAAGTT---TTA-GAGTGAAAATGCTAAATGTTGCTT---AGATTTTGTGTG
         *****2222222*22*2*222*2*2*22**2**222*222*22***2222222**2222*222*
```

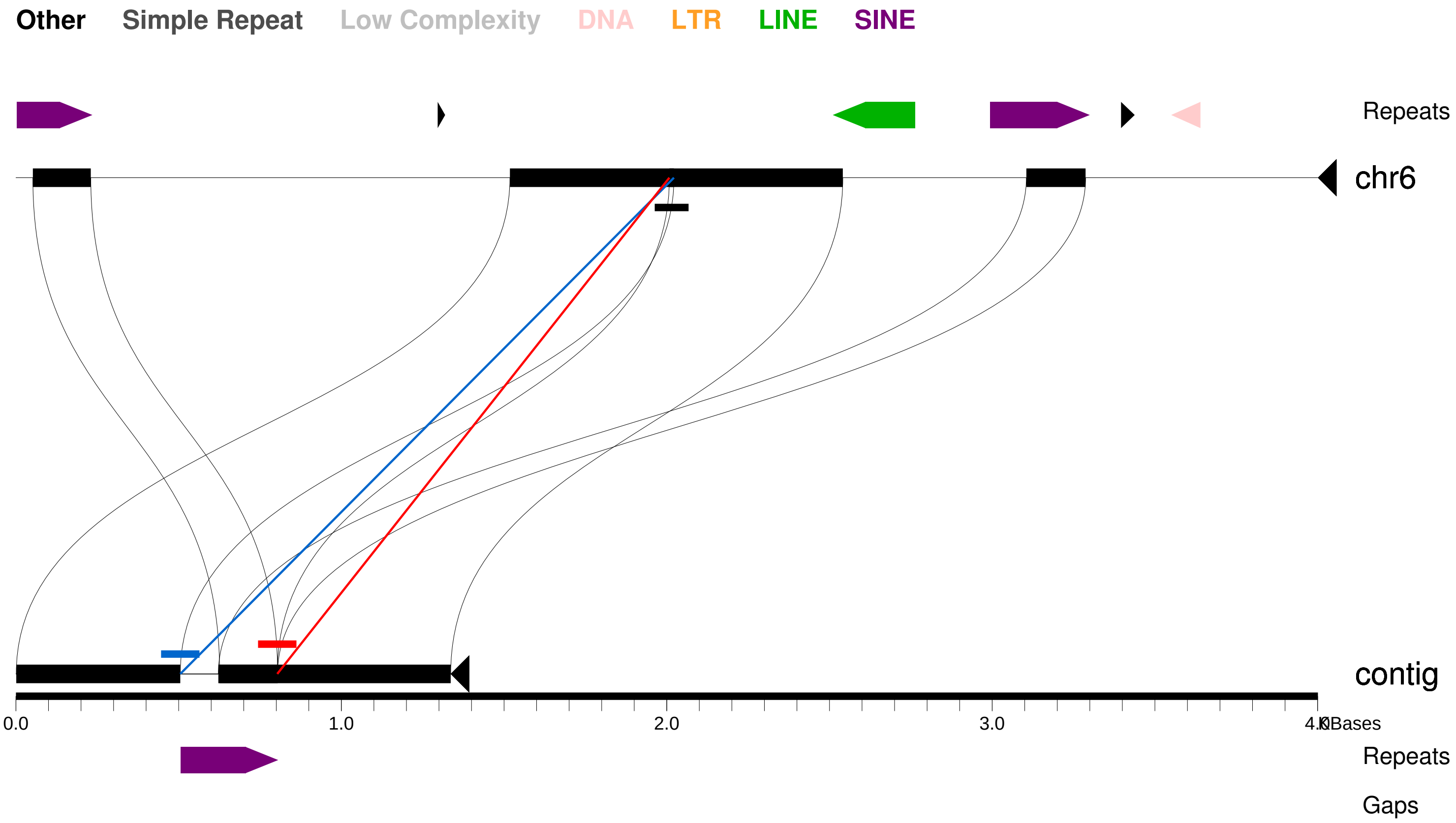

siteID: chr6_164161976 Scafftig4 - Breakpoint: chr6:164161904
chr6:164161845-164161970
Overlap Length: -6 INS size: 102 Genotyped: Yes

```
left    TTTCCACAGCCTAGGTTTCAACTCAACGCAGAGGGGAATGGGACTCATTTTCCCTTTTTTTTTTTTTTTTT
chrom   TTTCCACAGCCTAGGTTTCAACTCAACGCAGAGGGGAATGGGACTCATTTTCCCTTTTTTAATATAGCAT
right   CTGTC---GCCCAGGCTGGAGTGCAGTG--GCGGGATCTCGG-CTCACTG-CAAGCTCCGCCTCCCGCAT
        1*11*111***1***1*11*111**11*11*1***111*1**1****1*11*1111*111NN*N1N222*

left    TTTTTTTTTTTTTTTTT---GAGACGGAGTCTCGCTCTG-------TCGCCCAGGCTGG
chrom   TCTATCTCCATGATTAAAGGGATACAAAA-CACACTGTGGGAATTTTAGGAAATGCT--
right   TCTATCTCCATGATTAAAGGGATACAAAA-CACACTGTGGGAATTTTAGGAAATGCT--
        *2*2*2*222*22**22222**2**22*22*2*2**2**2222222*2*222*2***22
```

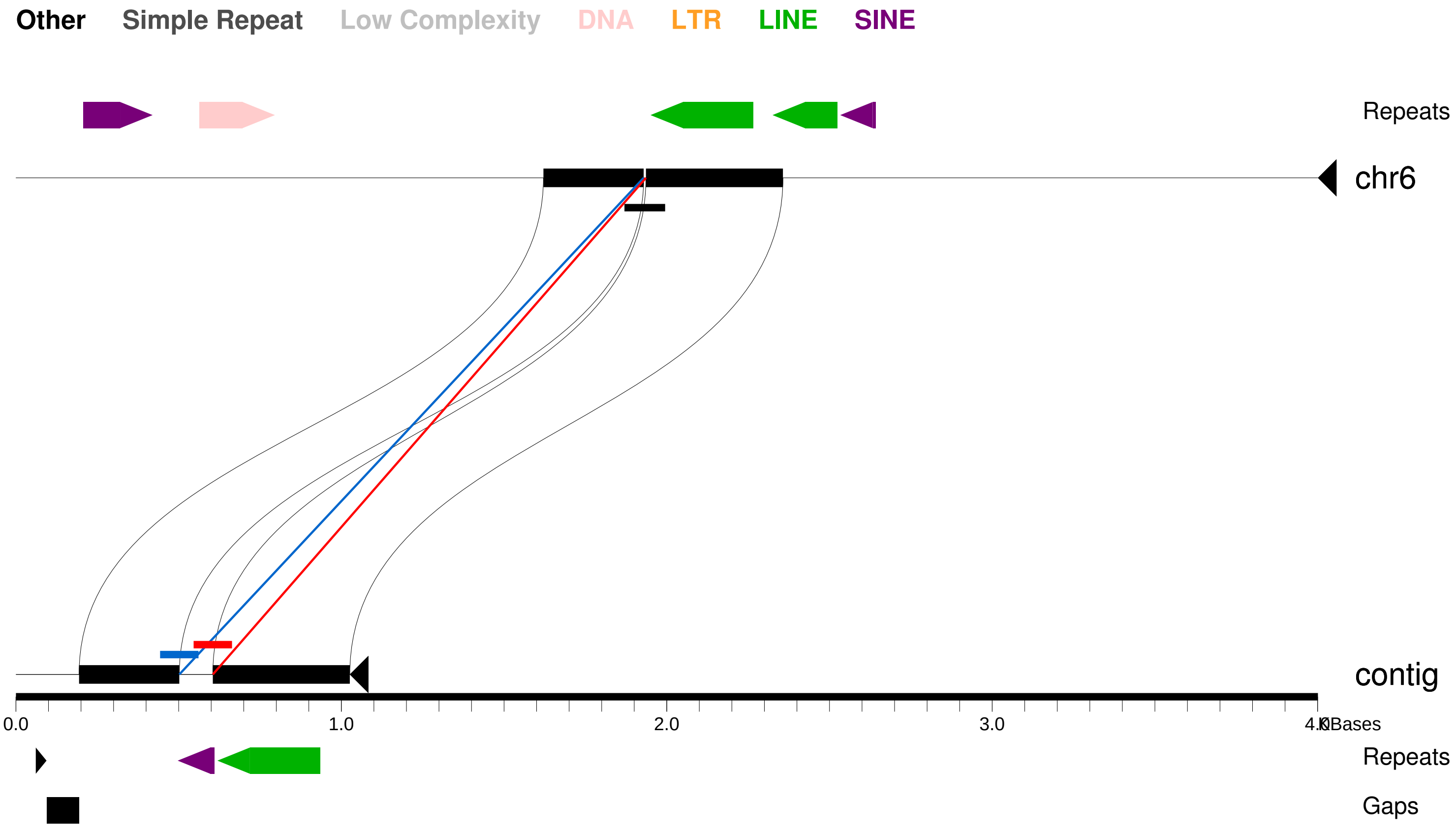

siteID: chr6_165186956 Scafftig1 + Breakpoint: chr6:165186931
chr6:165186892-165186990
Overlap Length: 15 INS size: 275 Genotyped: Yes

```
left    ---AATCCAAGAAGTAGTAAA--GATGG--TGGGG------AATTCCTG-------AATCCCAAAAGCCT
chrom   ---AACCCAAGAAGTAGTAAA--GATGG--TGGGG------AATTCCTG-------AATCCCAAAAGCCT
right   TCGATCTCCTGACCTCGTGATCCGCCCGCCTCGGCCTCCCAAAGTGCTGGGATTACAGGCCCAAAAGCCT
        111*121*11**11*1**1*111*111*11*1**1111111**1*1***1111111*11***********

left    TCTTTTTTTTTTTTTTTTTGAGACGGAGTCTCGCTCTGTCGCCCAGGCTGGAGTGCAGTGGCGCGATCT
chrom   TCTTAATTTTGACCATTG-GACATTCA-TATATGTGTGT-----------GTGTGTAGTAA-------T
right   TCTTAATTTTGACCATTG-GACATTCA-TATATGTGTGT-----------GTGTGTAGTAA-------T
        ****22****22222**22**2*222*2*2*222*2***22222222222*2***2***222222222*
```

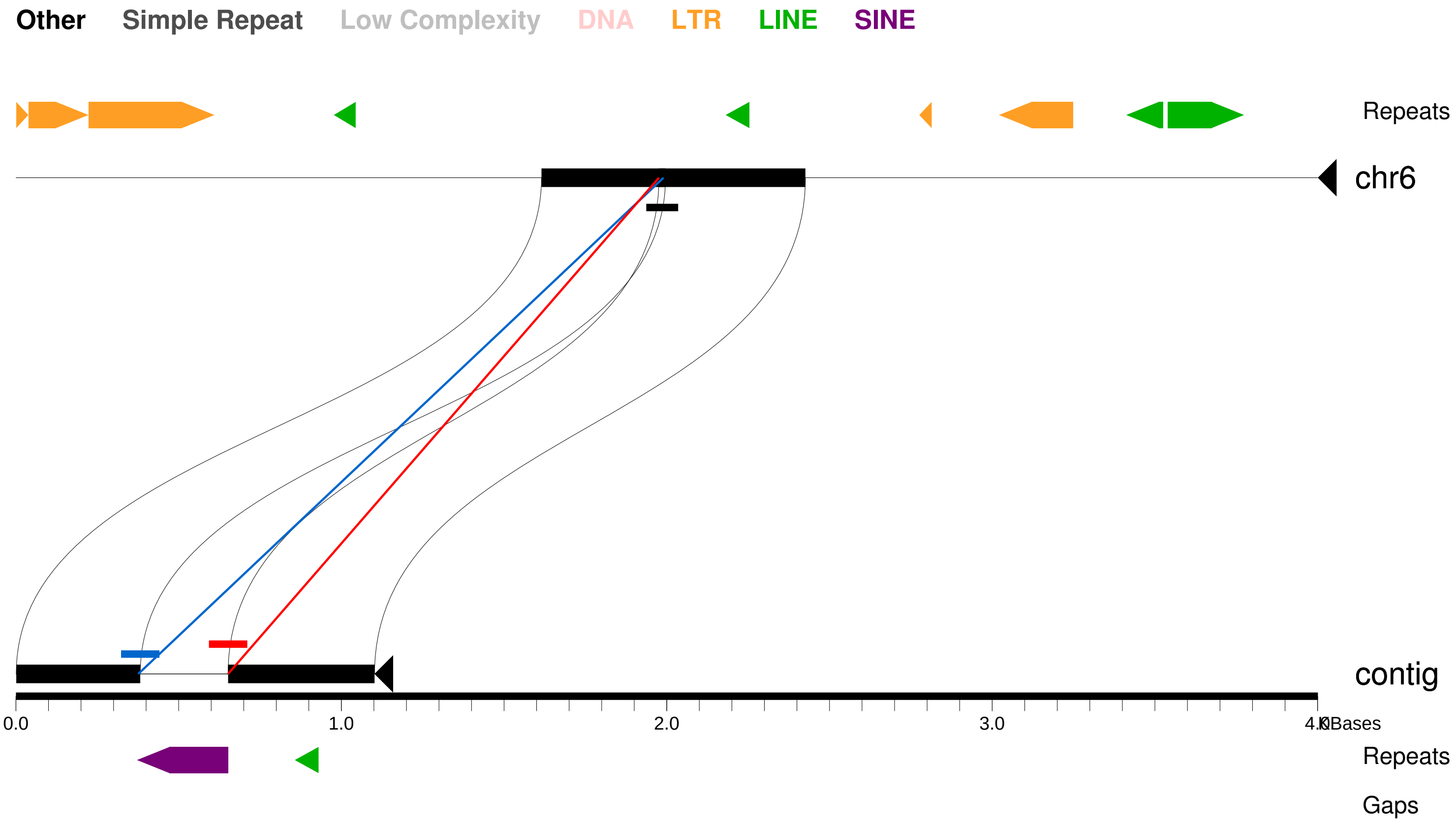

siteID: chr6_165439447 Scafftig4 + Breakpoint: chr6:165439457
chr6:165439407-165439516
Overlap Length: 10 INS size: 372 Genotyped: No

```
left   TAGACCTTATTTAGATTTAAGATATATGGAACTTC-----TTTGAAAATGTTCAT----TAAACATGTAN
chrom  TAGACCTTATTTGGATTTAAGATATATGGAACTTC-----TTTGAAAATGTTCAT----TAAACATGTAA
right  CCACCCGCCTCGGCCTCCCAAAGTGCTGGGATTACAGGCGTGAGCCACCGCGCCCGGCCTAAACATGTAA
       1111**111*11211*111*1*1111***1*1*1*11111*11*11*11*11*111111**********2

left   NNNNNNNNNNNNNNNNNNNNNNNNNNNNNNNNNNNNNNNNNNNNNNNNNNNNNNNNNNNN
chrom  TTCTAAAAATTGAGTAGTCACTGCTGGCTAGCTATGATGGAGCAATACG---------
right  TTCTAAAAATTGAGTAGTCACTGCTGGCTAGCTATGATGGAGCAATACG---------
       222222222222222222222222222222222222222222222222222222222222
```

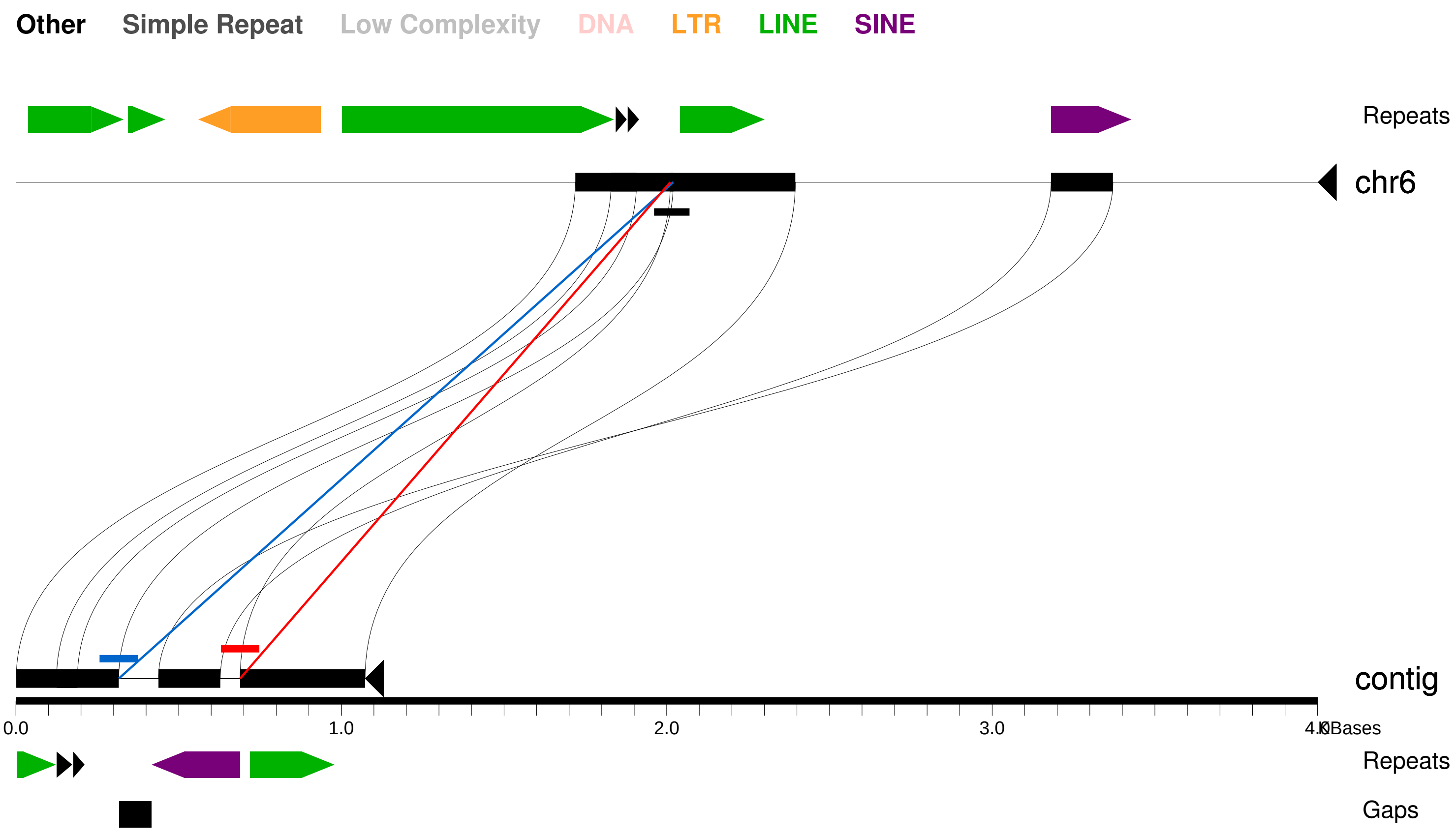

siteID: chr6_166279903 Scafftig1 + Breakpoint: chr6:166279893
chr6:166279851-166279952
Overlap Length: 15 INS size: 421 Genotyped: No

```
left     TCCTG----------------AATCACTAGGATTTTAT--TTACTGCACTTCTGGCTTTTCAGTAATGAA
chrom    TCCTG----------------AATCACTAGGATTTTAT--TTACTGCACTTCTGGCTTTTCAGTAATGAA
right    TCCGCCCACCTCGGCCTCCCAAAGTGCTGGGATTACAGGCGTGAGCCACCGC-GCCCGGCCAGTAATGAA
         ***111111111111111111**111**1*****11*1111*1111***11*1*1*1111**********

left     TTTCTTTTTNNNNNNNNNNNNNNNNNNNNNNNNNNNNNNNNNNNNNNNNNNNNNNNNNNNNNNNNNNNNNN
chrom    TTTCTGTT-----------------AATTCATTTCGTTAAGCATGATTGGGTTGACCATACACAGTC
right    TTTCTGTT-----------------AATTCATTTCGTTAAGCATGATTGGGTTGACCATACACAGTC
         *****2**222222222222222222222222222222222222222222222222222222222222222
```

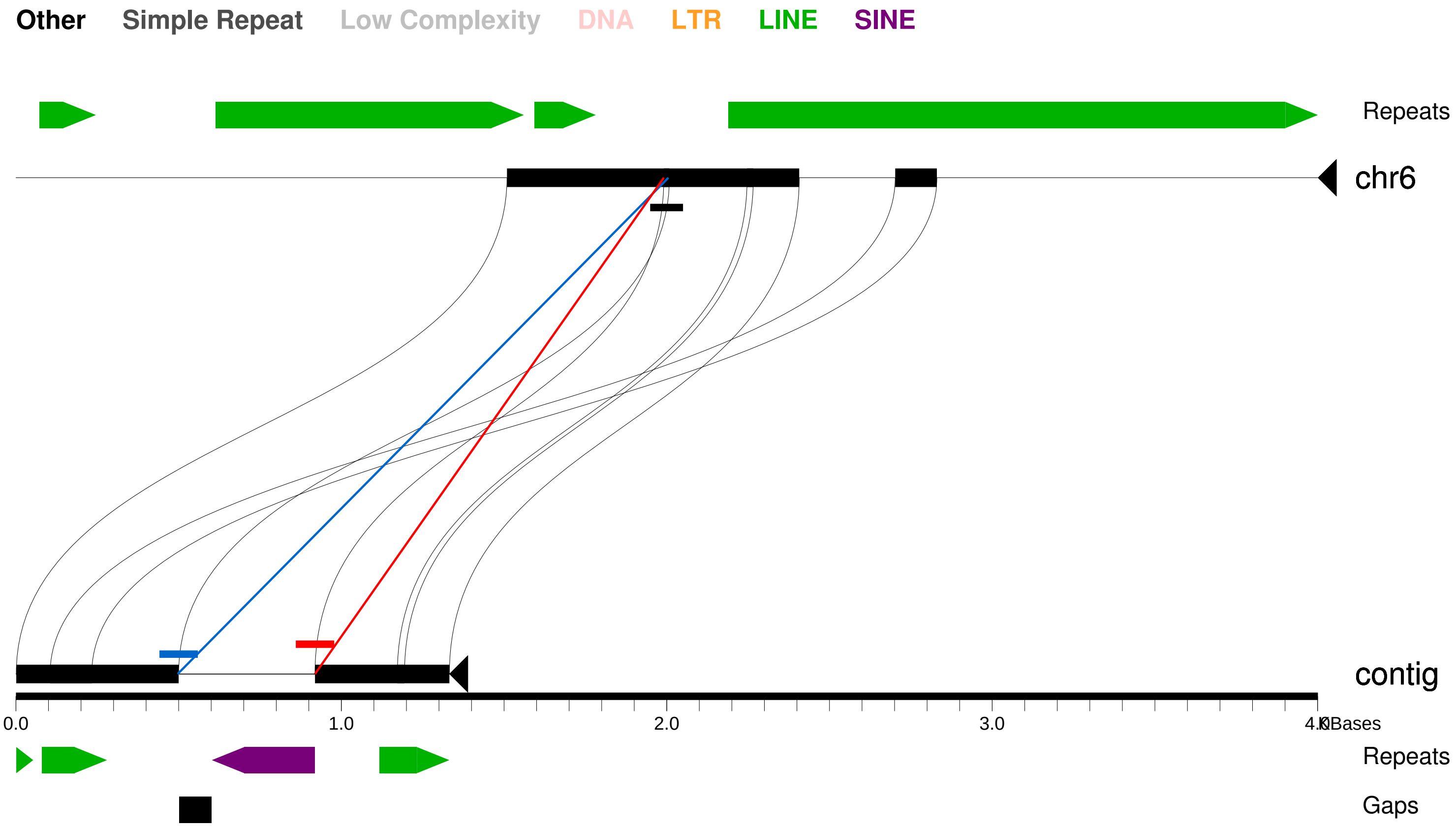

siteID: chr6_167124808 Scafftig2 + Breakpoint: chr6:167124801
chr6:167124641-167124972
Overlap Length: 12 INS size: 307 Genotyped: Yes

```
left   ------------GGAGGCCACAGAGGGAGCTGGAGCCTCCACCCCCACAAAGCAGGAATAGCCCCACAAC
chrom  CTTGGGATTAGTGGAGGCCACAGAGGGAGCTGGAGCCTCCACCCCCACAAAGCAGGAATAGCCCCACAAC
right  A---GGAGAA-TGGCGTGAACCCGGGAAGC-GGAGCTTGCAGTG------AGCCGAGATTGCGCCAC---
       N   222NN2 2**1*111**111**1***1*****1*1**111111111***1*11**1**1****111

left   TTCCTTCATTGCAGTAATGAGGTGGTGCCCCCAATTCCTAGGTTGGAGTCAC-GTGAGACCCAGTCTGTT
chrom  TTCCTTCATTGCAGTAATGAGGTGGTGCCCCCAATTCCTAGGTTGGAGTCAC-GTGAGACCCAGTCTGTT
right  ---------TGCAGT-------------CCGCAGT-CCGGCCTGGGCGACAGAGCGAGACTCCGTCTCAA
       111111111*****1111111111111**1**N*1**1111*1**1*1**11*1*****1*1****111

left   AAAACAGAAAGCTTATATAAGATCCAGAGTCTCGG--CCGGGCGCG------GT---GGCTCACGCCTGT
chrom  AAAACAGAAAGCTTATATAAGATCCAGAGTCTCATAACATAGTGCCCCAAAAGTCTAGGTTCCAATTTAA
right  AAAAAAAAAA----AAAAAAAATCCAGAGTCTCATAACATAGTCCCCCAAAAGTCTAGGTTCCAATTTAA
       ****1*1***1111*1*1**1***********2222*222*21*2222222**222**2**22222*22

left   AATCCCAGCACTT-TGGGAGGCCGAGGCGGGTGGATC---------------ATGAGGTCAGG---AGA
chrom  AAAAAAATCACTAATTACACAAAGAACCAGGAAGATCTCAAACAGAAAAAAAAAAAGATGCCAAACCAAGA
right  AAAAAAATCACTAATTACACAAAGAACCAGGAAGATCTCAAACAGAAAAAAAAAAAGATGCCAAACCAAGA
       **2222*2****22*222*2222**22*2**22****2222222222222222*2**2*2**22222***

left   TCGAGACCATCCTGGCTA------ACAAGG---TGAAACCCCGTCTCTACTAAA
chrom  T-GACAGAATGTTAGATATATCTGACAAAGATTTCAAAGCAGCCATGATAAAAA
right  T-GACAGAATGTTAGATATATCTGACAAAGATTTCAAAGCAGC-----------
       *2**2*22**22*2*2**222222****2*222*2***2*222NN1NNNNN111
```

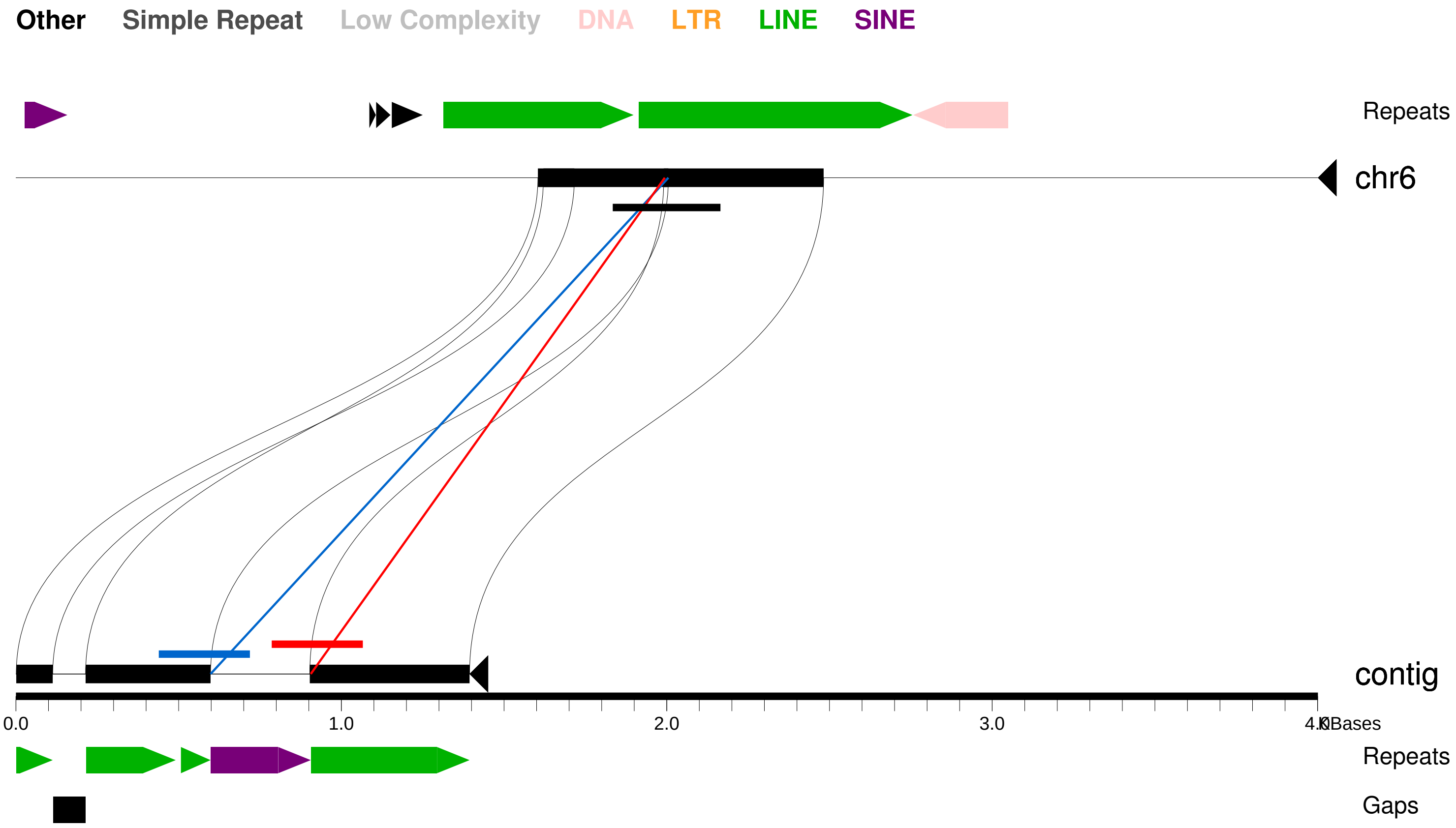

siteID: chr6_1674195 Scafftig2 + Breakpoint: chr6:1674194
chr6:1674034-1674366
Overlap Length: 13 INS size: 133 Genotyped: Yes

```
left   -------------------------------------------------------------CATGGCTTGATA
chrom  TTTTCAGACTGGCTTCTTTCATTTAGCGACATACATTTAAGGTTCCTCCATGGCCTTTCATGGCTTGATA
right  TTTTTTTTTTTTTTTTTTTTTTTTTT------TTTTTTTTAGTAGAGAC---GGGGTTTCACCG--TGTTA
       2222NNNNN2NNN22N222NN222N      2NNN222N22NNNNNN2   22NN222**11*11**1**

left   GCTCATTTCCTTTTATCACTGAAGAGCATTCCATTGGATGAACGTACCAGAAGTTGATCCATTAGAAGGA
chrom  GCTCATTTCCTTTTATCACTGAAGAGCATTCCATTGGATGAACGTACCAGAAGTTGATCCATTAGAAGGA
right  GCCAGGATGGT-------CTCGATCTCCTGACCTCG--TGATCCGCCC----GTCTCGGCCTCCCAAAG-
       **11111*11*1111111**11*111*1*11*1*1*11***1*111**1111**11111*1*111**1*1

left   TGTCTTGCTTGCTTCCAGTTTAGGTTGATTATTTTTTTTTTTTTTTTTTTTTTTTTTTTTTTTTTTTTTT
chrom  TGTCTTGCTTGCTTCCAGTTTAGGTTGATTATTAATAATGCTGCTATAAACATCTATGCGCAGGTTTTT-
right  -----TGCTGG---------TAGGTTGATTATTAATAATGCTGCTATAAACATCTATGCGCAGGTTTTT-
       11111****1*111111111************22*22*22*22*2*22222*2*2*2222222*****2

left   TTTTTTTTAGTAGAGACGGGGTTTCACCGTGTTAGCCAGGATGGTCTCGATCTCCTGACCTCGTGAT--C
chrom  -----CTGAG----GACATGTTTTCAAC---TTA------ATTGGGTAAATA-CATAGCCGCGAGGTTGC
right  -----CTGAG----GACATGTTTTCAAC---TTA------ATTGGGTAAATA-CATAGCCGCAAGGTTGC
       222222*2**2222***22*2*****2*222***222222**2*22*22**22*2*22**2*12*2*22*

left   CGCCCGTCTCGGCCTCCCAAAGTGCTGGTAGGTTGATTATTAATAATGCTGCTA------TAAACAT---
chrom  TGATTGCGTAGTGAAACCATCGTGCTATTTATGAAATTGCAAACATTCGTGCAAAGTGTCTAAACCTTTT
right  TGATTGCGTAGTGAAACCATCGTGCTATTTATGAAATTGCAAACATTCGTGCAAAGTGTC----------
       2*222*22*2*22222***22*****22*222222***222**2*2*22***2*22222211111N1

left   ---
chrom  GCA
right  ---
```

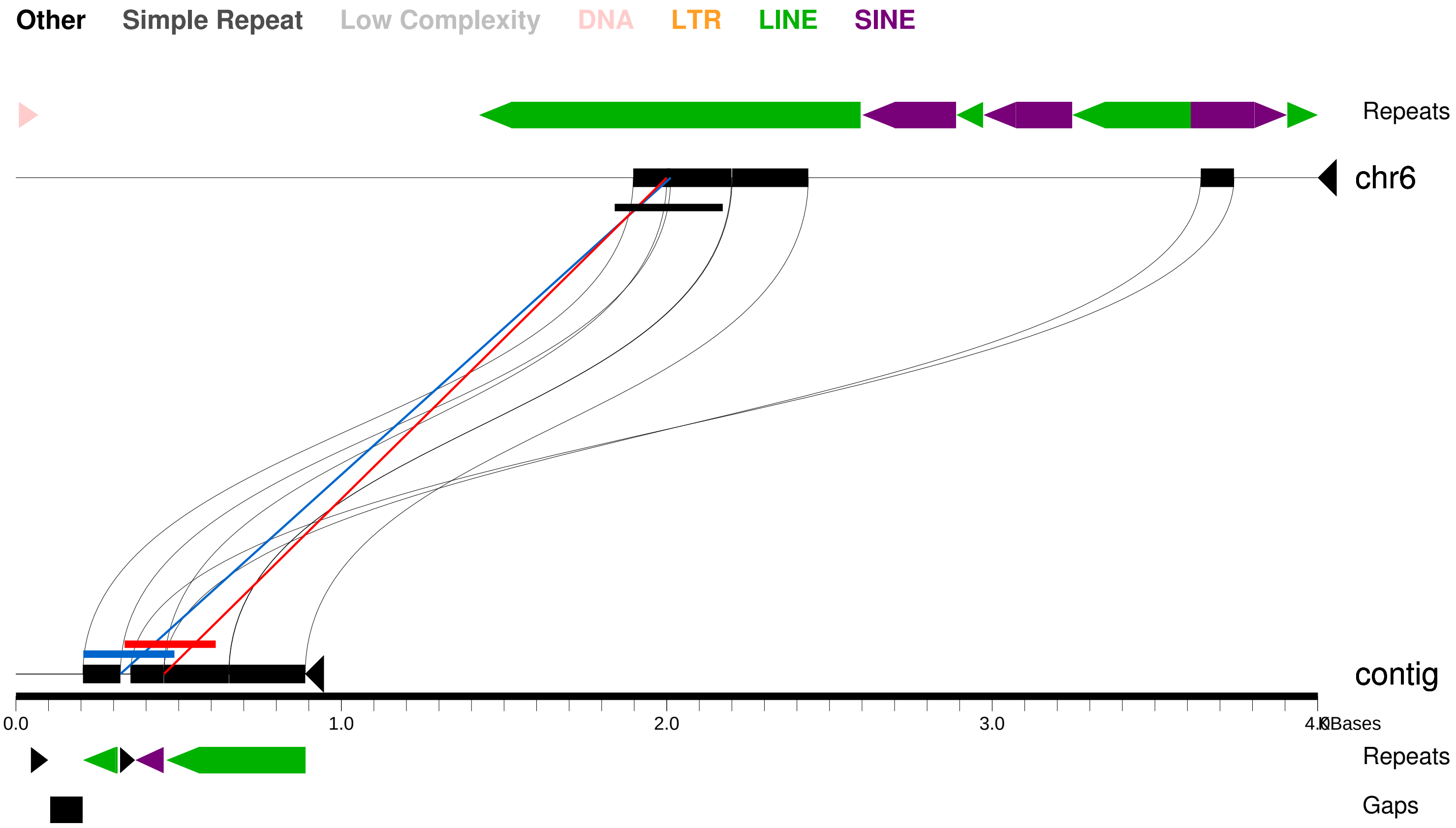

siteID: chr6_170063349 Scafftig2 - Breakpoint: chr6:170063372
chr6:170063326-170063410
Overlap Length: 14 INS size: 302 Genotyped: Yes

```
left   C-------------------------CTTCAGC--AGTAGATAT------------TATCAAATAAATAAC
chrom  C-------------------------CTTCAGC--AGTAGATAT------------TATCAAATAAATAAC
right  AGTGAGCCGAGATCCCGCCACTGCACTCCAGCCCGGGCGACAGAGCGAGACTCCATCTCAAAAAAAAAAA
       1111111111111111111111111**1****111*11**1*1111111111111*1*****1***1**1

left   AAAAACCAAGGTTTAAAACATCAGGAAAGGCCGGGCGCGGTGGCTCACGCCTGTAATCCCAGCACTTTGG
chrom  AAAAACCAAGGTTTAAAACATCAGGAAAAAATTGGTATACTGGATAAAA---GTAA--------------
right  AAAAAAAAAA----AAAACATCAGGAAAAAATTGGTATACTGGATAAAA---GTAA--------------
       *****11**11111**************22222**22222***2*2*22222****22222222222222

left   GAGGCCGAGGCGGGCGG
chrom  -----------------
right  -----------------
       22222222222222222
```

Other **Simple Repeat** **Low Complexity** **DNA** **LTR** **LINE** **SINE**

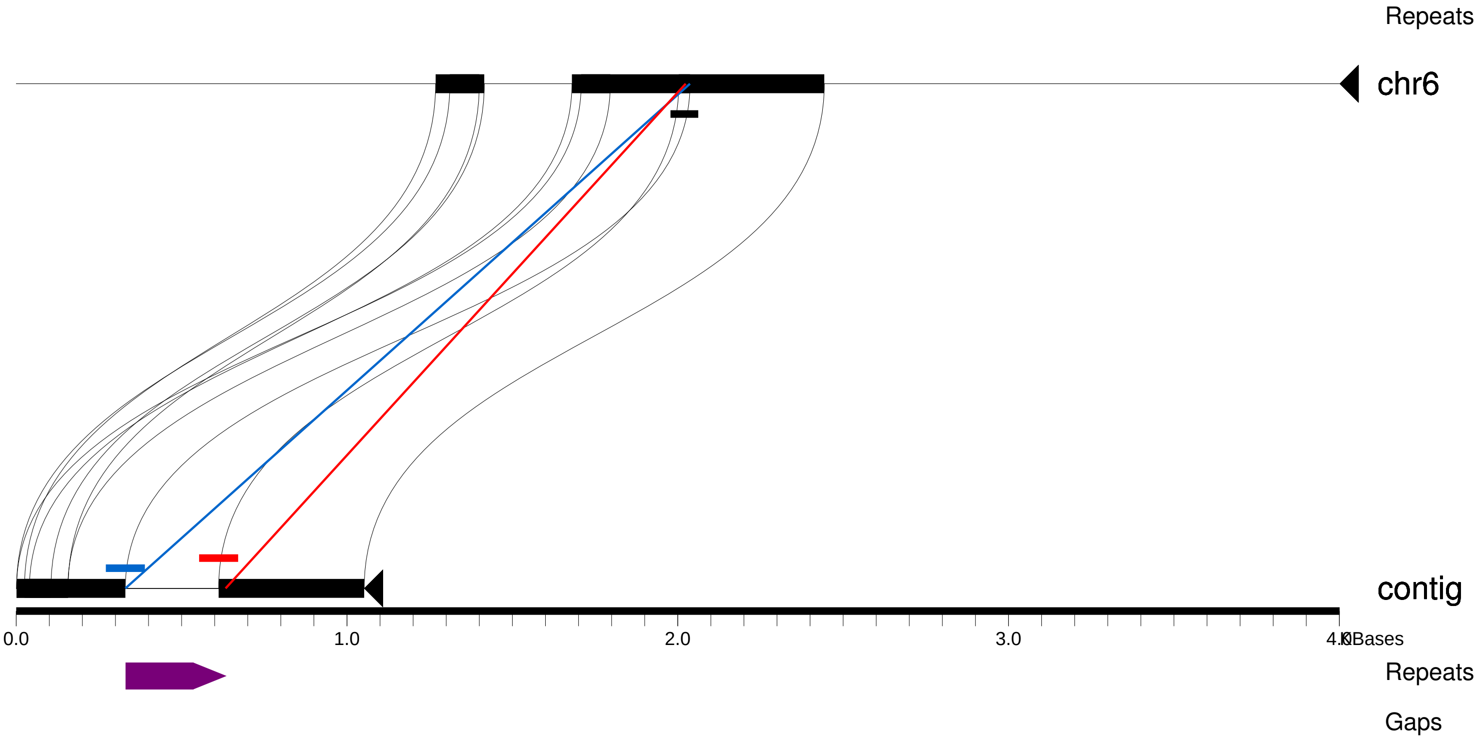

siteID: chr6_17416373 Scafftig1 + Breakpoint: chr6:17416385
chr6:17416263-17416594
Overlap Length: 88 INS size: 419 Genotyped: No

```
left   A---------------------------------------------------------------------
chrom  AGCAAAAGAGTCATTGTAAAAATGTTCAGAGGGAAAATATGTGTAAATATGTTCAGAGGGAAAAATAACG
right  C-CGCCCGCCTCGGCCTCCCAAAGTGCTG--GGATTACAGGCGT-----------GAG------------
       1 2NNNN2NN22NNNN2NNN22N22N2N2  222NN2N2N2N22           222

left   -------------------------------ACCGCACATACACGTAGACTTGAGAAAACTCTTTTAAAC
chrom  AATTACATACCACAATTTTCATTTTATGGTAACCGCACATACACGTAGACTTGAGAAAACTCTTTTAAAC
right  ---------CCAC--------------------CGCGCC----CG--GCC--GAGAAAACTCTTTTAAAC
                2222                  11***1*11111**11*1*11******************

left   ATGTTGGAGGAATAAGTACATGGGCAGAGGAAAATGAAATGTTGGGGACAAAAATAAGGCTATACTGACC
chrom  ATGTTGGAGGAATAAGTACATGGGCAGAGGAAAATGAAATGTTGGGGACAAAAATAAGGCTATACTGACC
right  ATGTTGGAGGAATAAGTACATGGGCAGAGGAAAATGAAATGTTGGGGACAAAAATAAGGCTATACTGACC
       **********************************************************************

left   NNNNNNNNNNNNNNNNNNNNNNNNNNNNNNNNNNNNNNNNNNNNNNNNNNNNNNNNNNN-----------
chrom  TGAAGTTTTCTGTTAGTCATTTCCATTGACCAGATCTTCTAGATGACTGCTTGACATTTCAAATATAACT
right  TGAAGTTTTCTGTTAGTCATTT------------------------------------------------
       2222222222222222222222NNNNNNNNNNNNNNNNNNNNNNNNNNNNNNNNNNNNN

left   ----------------------------------------------------
chrom  ATTCCCACTGACAGCAAAATGAGATGGGATGACTTAATAGCTGCAGCGAACG
right  ----------------------------------------------------
```

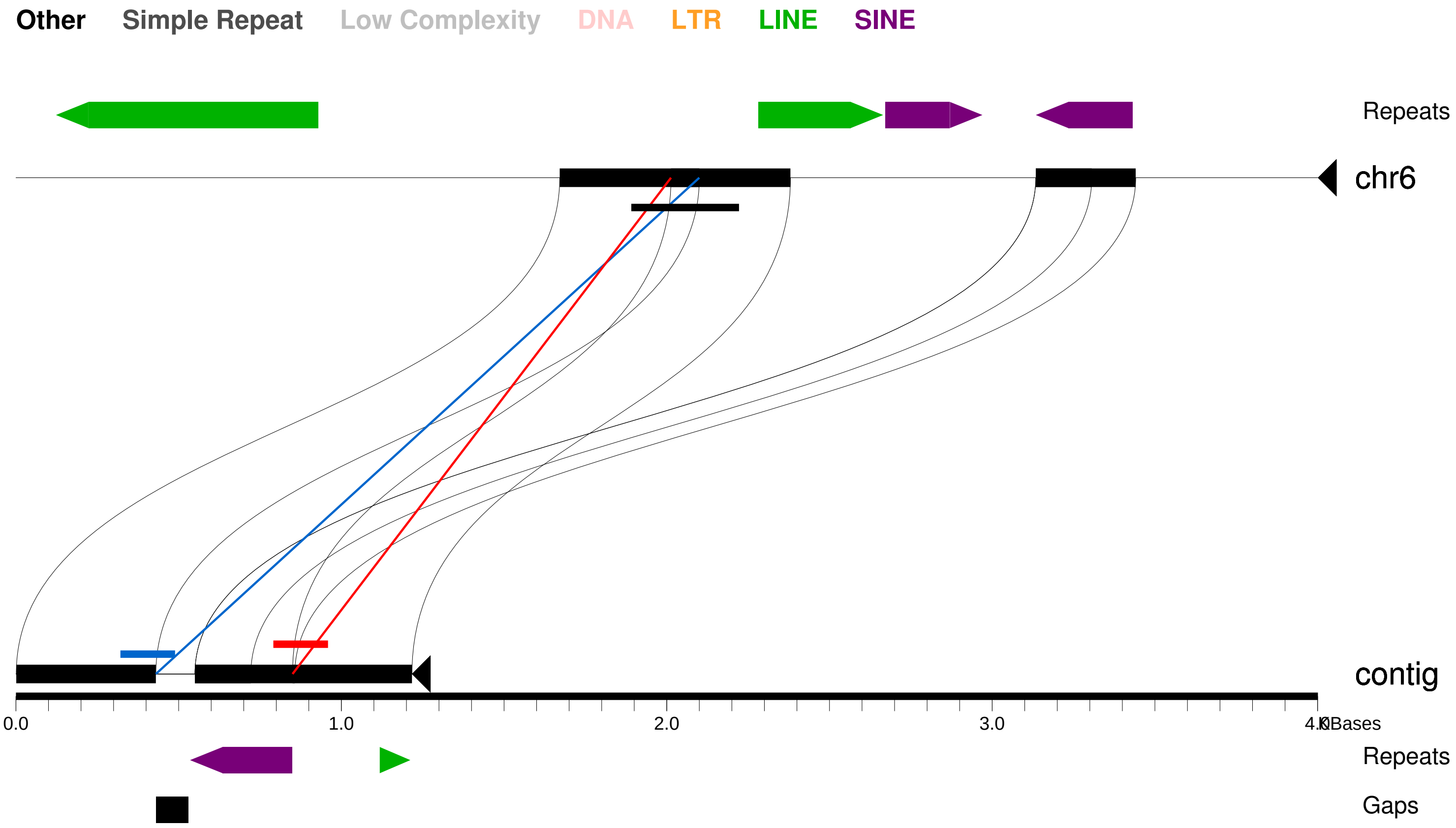

siteID: chr6_19137101 Scafftig7 + Breakpoint: chr6:19137094
chr6:19137054-19137153
Overlap Length: 16 INS size: 306 Genotyped: Yes

```
left    ACTTAATGACTG-------CAA------GGGAGTAT--GAGGGAGTTTCTGTGTG----CTGTTTCTGTT
chrom   ACTTAATGACTG-------CAA------GGGAGTAT--GAGGGAGTTTCTGTGTG----CTGTTTCTGTT
right   TCCACCCGCCTCGGCCTCCCAAAGTGCTGGGATTACAGGCGTGAGCCACCGCGCCCGGCCTGTTTCTGTT
        1*11111*1**11111111***111111****1**111*1*1***111*1*1*111111***********

left    TCTTTTTTTTTTTTTTTTTTTTTTGAGACGGAGTCTCGCTCTGTCGCCCAGGCCGGACTGCGGACTGC
chrom   TCTTTATTTGAGCGCTGGTTACATG-GA--TATACTCACTATATAAA----------------AATTT
right   TCTTTATTTGAGCGCTGGTTACATG-GA--TATACTCACTATATAAA----------------AATTT
        *****2***222222*22**222**2**222*22***2**2*2*2222222222222222222*2*22
```

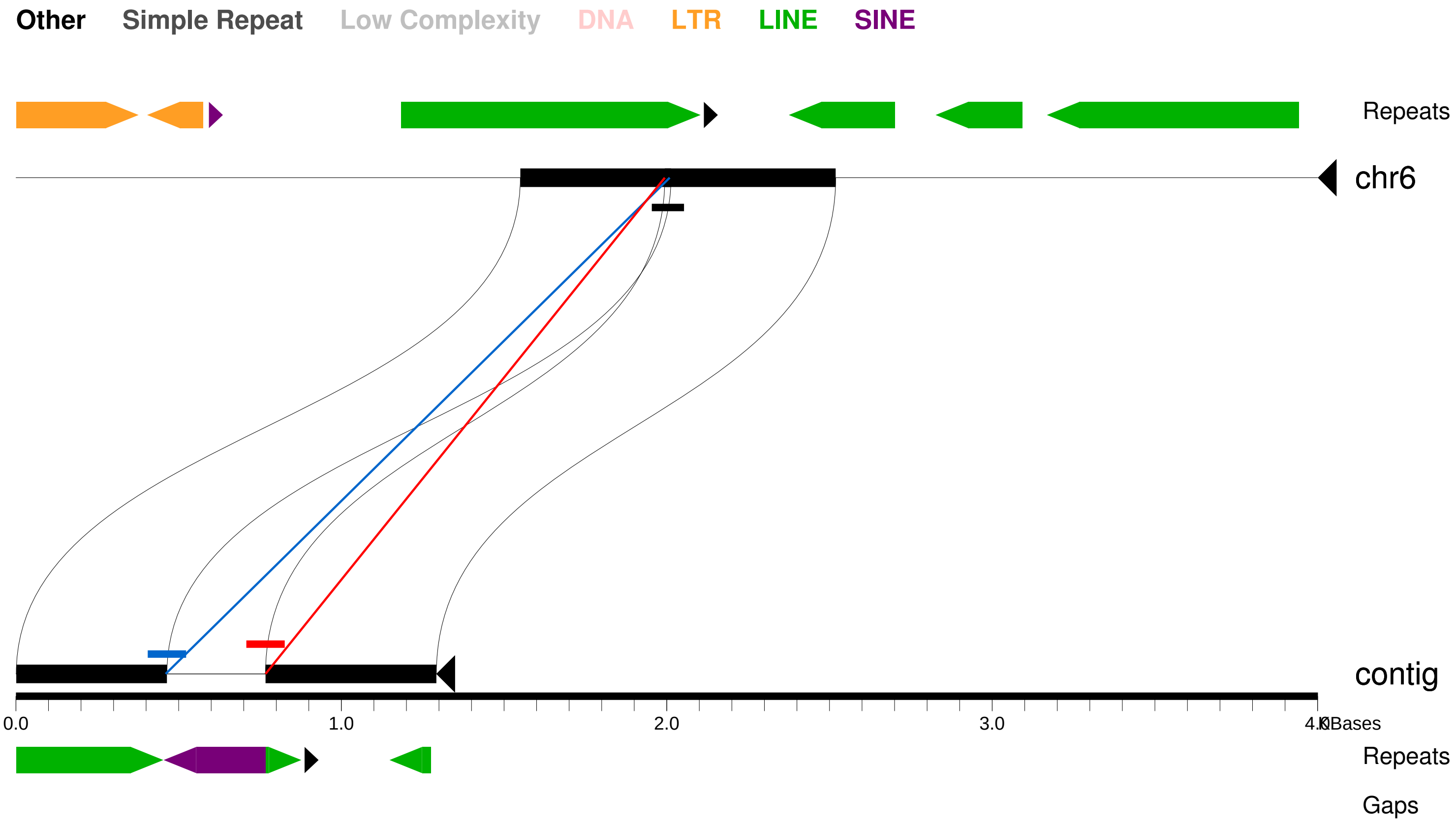

siteID: chr6_19346427 Scafftig5 - Breakpoint: chr6:19346437
chr6:19346395-19346487
Overlap Length: 18 INS size: 432 Genotyped: No

```
left    CTAACAAAATCCTAGCAAGAATTATTGCAAATT--------------------------AGTAGTCTTAA
chrom   CTAACAAAATCCTAGCAAGAATTATTGCAAATT--------------------------AGTAGTCTTAA
right   NNNNNNNNNNNNNNNNNNNNNNNNNNNNNNNNNNNNNNNNNNNNNNNNNNNNNNNNNNNAGTAGTAAAAA
        11111111111111111111111111111111111111111111111111111111111******111**

left    AATTTACATGGAAATGGGCCGGGCGCGGTGGCTCACGCCTGTAATCCCAGCACTTTGGGAGGCCGAGGCG
chrom   AATTTACATGGAAATGCAAAAGAATCAGAA-----------TAGCCAAAACAGTTTTGGA----------
right   AATTTACATGGAAATGCAAAAGAATCAGAA-----------TAGCCAAAACAGTTTTGGA----------
        ****************22222*222*2*2222222222222**22*22*2**2***2***2222222222

left    GGCGG
chrom   -----
right   -----
        22222
```

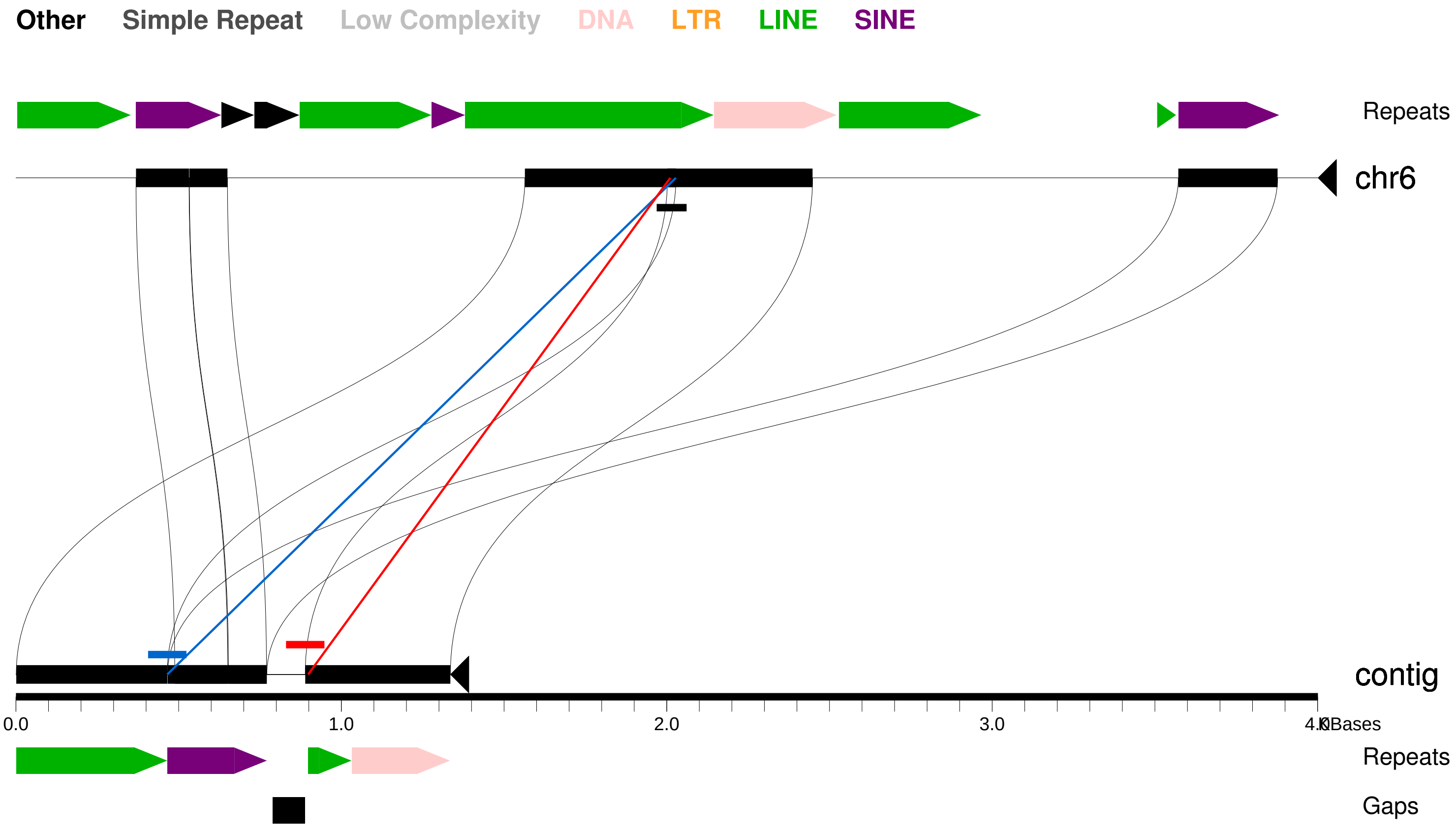

siteID: chr6_23978150 Scafftig19 - Breakpoint: chr6:23978344
chr6:23978302-23978403
Overlap Length: 14 INS size: 299 Genotyped: Yes

```
left    AAATATCGTTGTCCAA-----------GGAAACACTTCAAGATACTGTTACAG------AAAAATGACAA
chrom   AAATATCGTTGTCCAA-----------GGAAACACTTCAAGATACTGTTACAG------AAAAATGACAA
right   CGCCACTGCAGTCCGCAGTCCGGCCTGGGCGACAGAGCGAGACTCCGTCTCAAAAAAAAAAAAATGACAA
        1111*11*11****11111111111111**11***111*1***11*1**11**1111111***********

left    CCAGGCCGGGTGCGGTGGCTCAC-GCCTGTAATCCCAGCACTTTGGGAGGCCGAGGCGGGTGGATCA
chrom   CCATGCC--------TACCCCATAGGCAGTTGT--GAGAATTTAATGAGAT-------GATGGATG-
right   CCATGCC--------TACCCCATAGGCAGTTGT--GAGAATTTAATGAGAT-------GATGGATG-
        ***2***22222222*22*2**22*2*2**22*222**2*2**222***222222222*2*****22
```

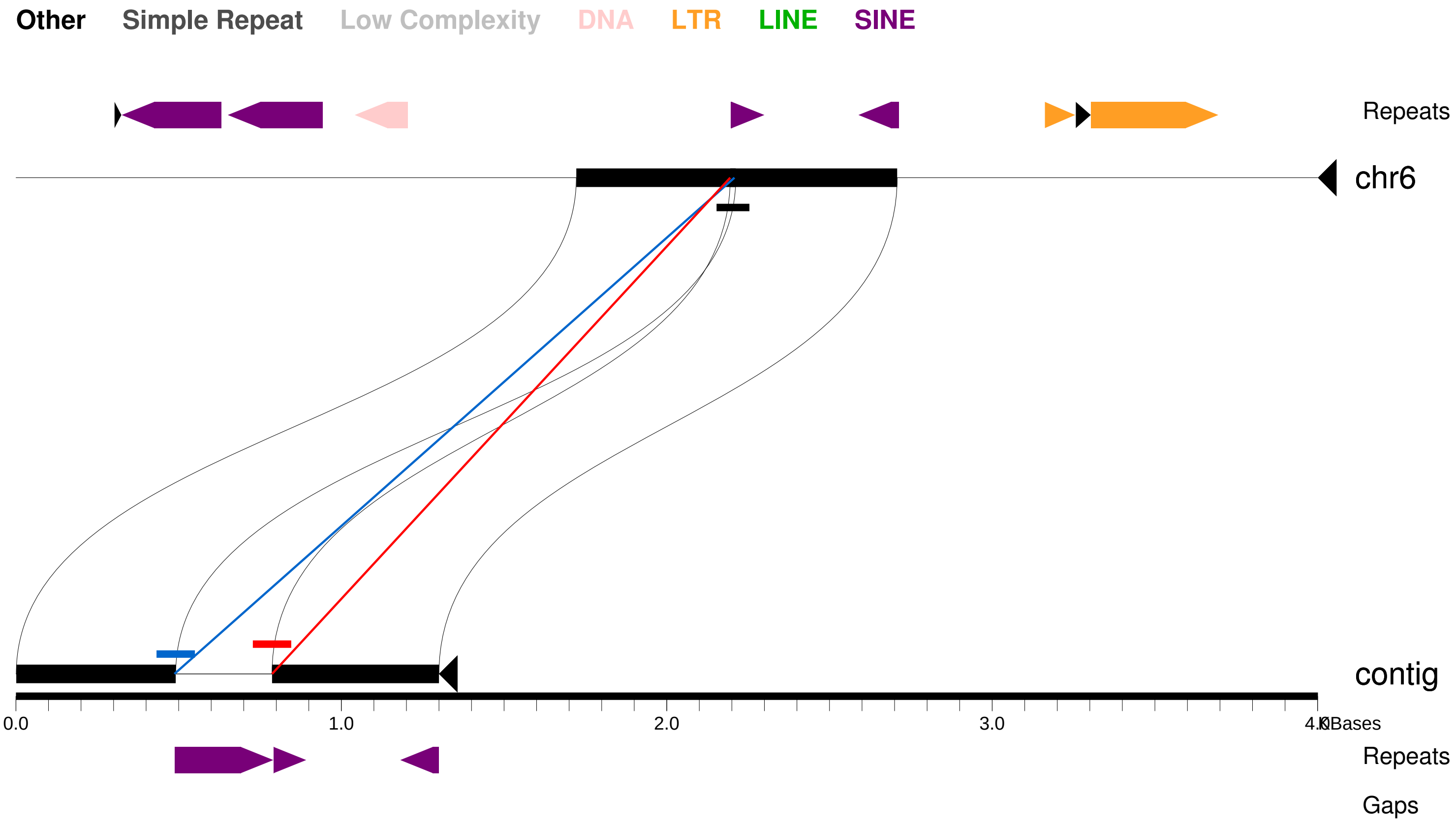

siteID: chr6_25380209 Scafftig1 + Breakpoint: chr6:25380225
chr6:25380177-25380283
Overlap Length: 12 INS size: 416 Genotyped: No

```
left    C-------------TTCTTTATGCACTTTTTTTCTTTCCTTTTCTTACCTC-CATGGAATTTATTCTGCT
chrom   C-------------TTCTTTATGCACTTTTTTTCTTTCCTTTTCTTACCTC-CATGGAATTTATTCTGCT
right   CCACCCGCCTCGGCCTCCCAAAGTGCTGGGATTACAGGCGTGAGCCACCGCGCCCGGCCT--ATTCTGCT
        *11111111111111**111*1*11**1111**11111*1*11111***1*1*11**11*11*******

left    CTTTTTTTTNNNNNNNNNNNNNNNNNNNNNNNNNNNNNNNNNNNNNNNNNNNNNNNNNNNNNNN
chrom   CTTTT------------AAGGACTCTCAAAGGCATAGTAGATCATGGCATAAATTAGGTGGCAA
right   CTTTT------------AAGGACTCTCAAAGGCATAGTAGATCATGGCATAAATTAGGTGGCAA
        ****222222222222222222222222222222222222222222222222222222222222
```

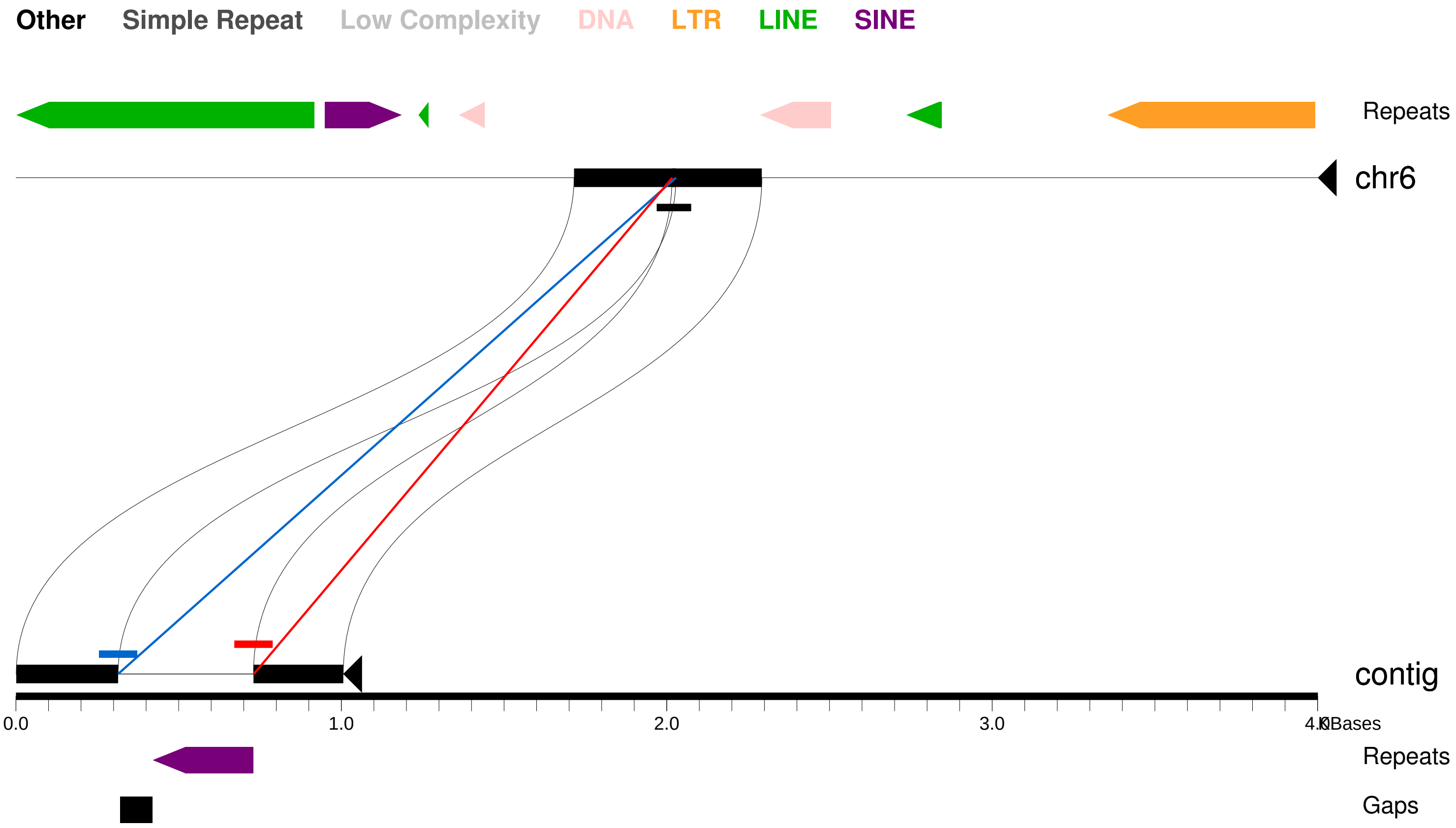

siteID: chr6_26962169 Scafftig3 + Breakpoint: chr6:26962172
chr6:26962115-26962231
Overlap Length: 3 INS size: 116 Genotyped: Yes

```
left   C------TGATTTACTTCACTAGTTGG-TCTTTTCA-ACTTAACTTGTTTCCTAATGAGATTAAATTCTT
chrom  C------TGATTTAGTTCACTAGTTGG-TCTTTTCA-ACTTAACTTGTTTCCTAATGAGATTAAATTCAT
right  TCGCCCAGGCTGGAGTGCAGTGGCGGGATCTCGGCTCACTGCAAGCTCCGCCTCCCGGG------TTCAT
       11111111*1*11*2*1**1*1*11**1***111*11***11*1111111***111*1*111111***2*

left   TTTTTTTTTTTTTTTTTTTTTTTTTTTTTTTTTTTTGAGACGGAGTCTCGCTC-------TGTC
chrom  GCATAAATAACCAAACCAAAAATTAAACC--------AAAAGAATACTCACCAGAAAGGATGT-
right  GCATAAATAACCAAACCAAAAATTAAACC--------AAAAGAATACTCACCAGAAAGGATGT-
       222*222*22222222222222**2222222222222*2*2*2*22***2*222222222***2
```

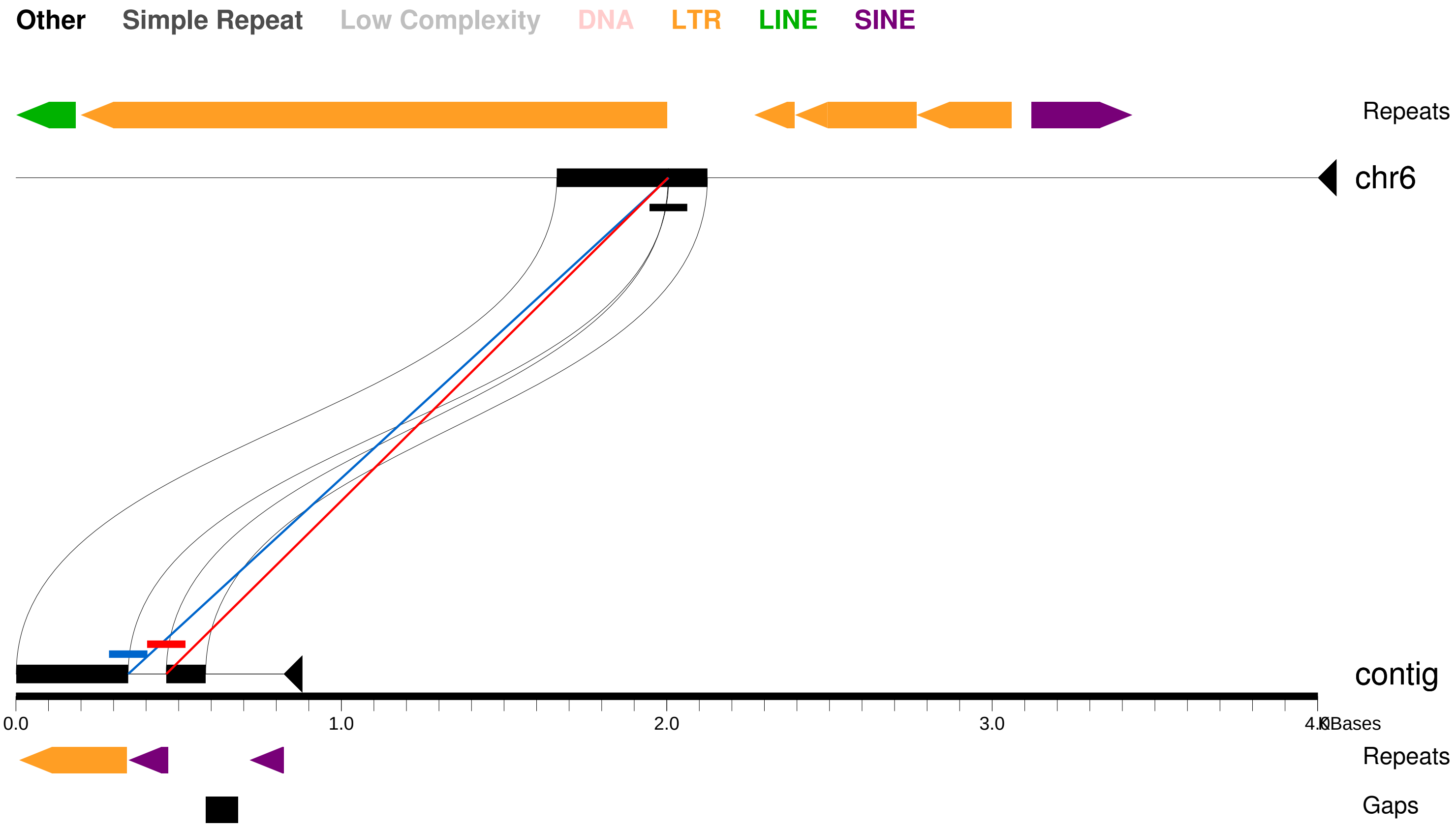

siteID: chr6_28514323 Scafftig1 - Breakpoint: chr6:28514337
chr6:28514292-28514396
Overlap Length: 15 INS size: 230 Genotyped: Yes

```
left    C--------------TTTTTTTTTTTGGCATATATGTGCACAAGTTTTACTGGGTATTAAAATAAAGAGA
chrom   C--------------TTTTTTTTTTTGGCATATATGTGCACAAGTTTTACTGGGTATTAAAATAAAGAGA
right   CAGAGCGAGACTCCGTCTCAAAAAAAAAAAAAAAAATAAAAAAAAAAAAATAAATAAATAAATAAAGAGA
        *11111111111111*1*11111111111*1*1*11*11*1**11111*1*111**111***********

left    ATAAGGTGAAACCCCGTCTCTACTAAAAATACAAAAAATTAGCCGGGCGTGGTGGCGGGCGCC
chrom   ATAAATCGATTTTTTAATGTTTGCTGACAGCTTTCAAAA----CC---CATTATT-------CT
right   ATAAATCGATTTTTTAATGTTTGCTGACAGCTTTCAAAA----CC---CATTATT-------CT
        *****222**2222222*2*2*2**2*2*222222****2222**222*2*22*22222222*2
```

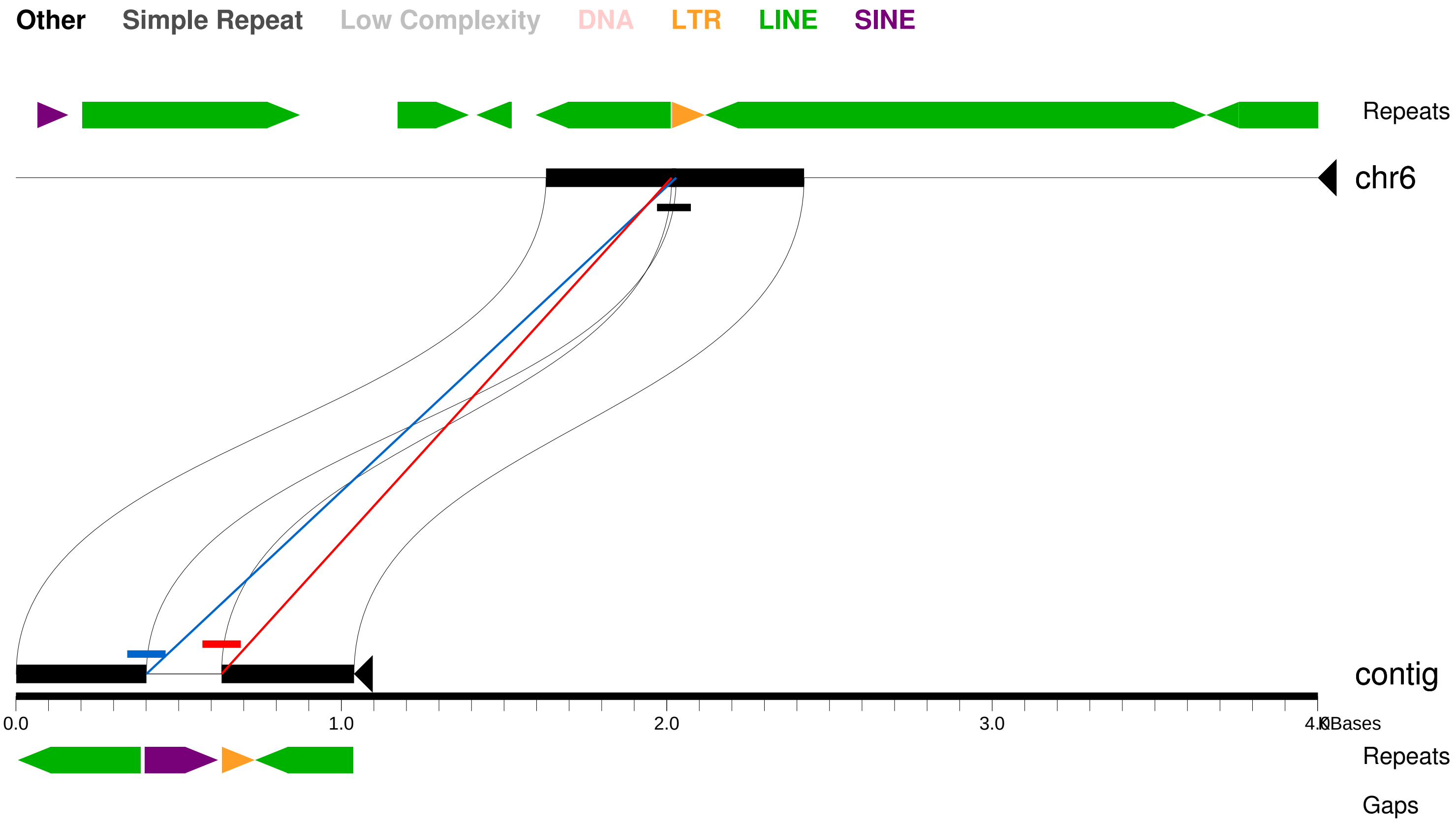

siteID: chr6_29680466 Scafftig2 - Breakpoint: chr6:29680453
chr6:29680413-29680512
Overlap Length: 16 INS size: 140 Genotyped: Yes

```
left    AGGGTTATTGTAACTGACCTGCCCTCT-------------------ATAGTATAGTCTTAAGAGATCTGA
chrom   AGGGTTATTGTAACTGACCTGCCCTCT-------------------ATAGTATAGTCTTAAGAGATCTGA
right   CCTGGACGACAGAGCGAGACTCCGTCTCAAAAAAAAAAAAAAAAAAAAAAAAAAAAAAAAAGAGATCTGA
        111*11111111*11**1111**1***1111111111111111111*1*11*1*11111***********

left    AGAACTTGGGAGGCTGAGGCAGGAGAATGGCGTGAACCCGGGAGGCGGAGCTTGCAGTGAGCCGAGAT
chrom   AGAACCTGGCATCCTAT------AGAATGG--TAAATC----------AGCTTATT-TCATCAACAAC
right   AGAACCTGGCATCCTAT------AGAATGG--TAAACC----------AGCTTATT-TCATCAACAAC
        *****2***2*22**22222222*******22*2**N*2222222222*****2222*2*2*2222*2
```

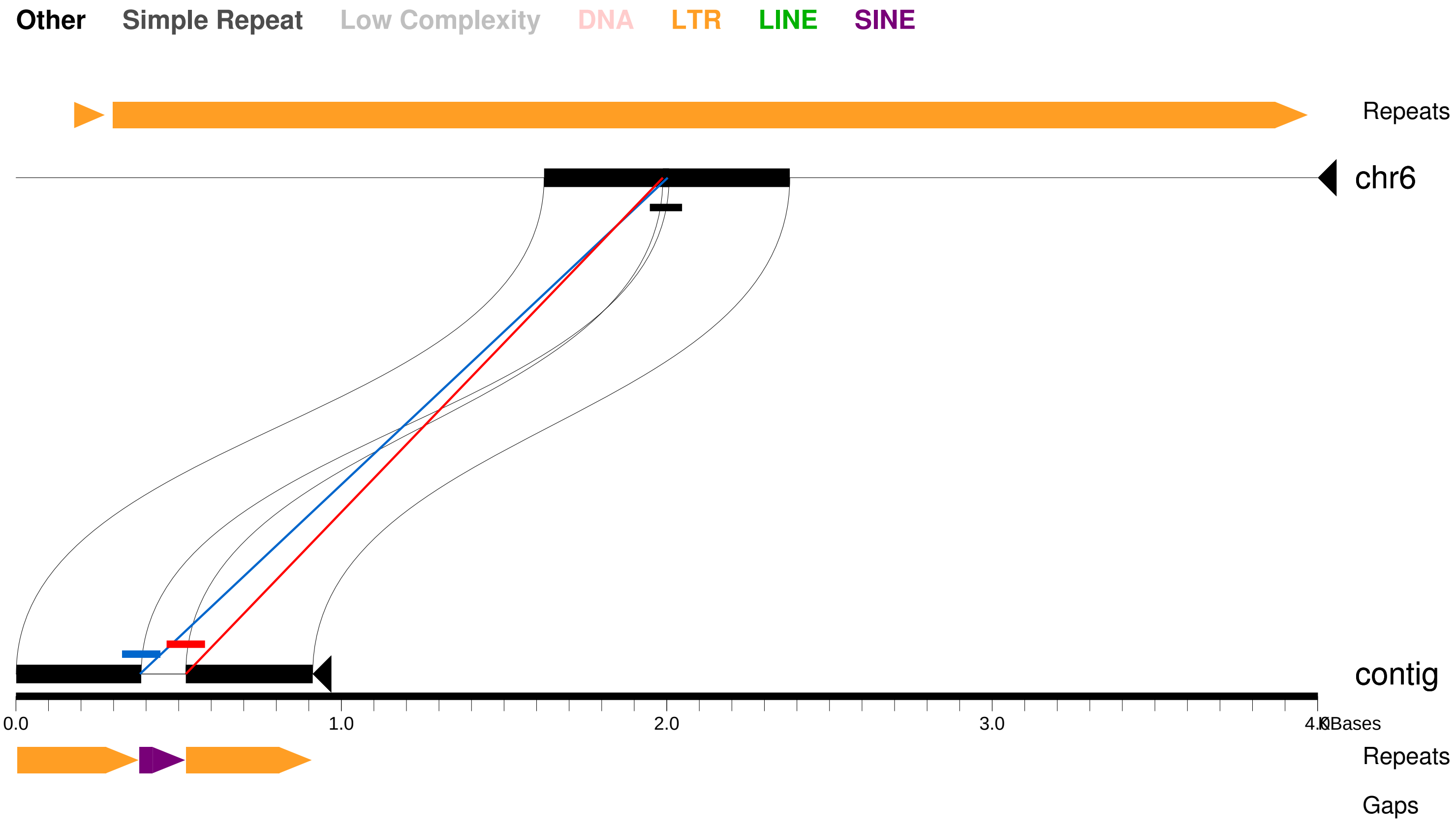

siteID: chr6_29818870 Scafftig4 - Breakpoint: chr6:29818857
chr6:29818813-29818909
Overlap Length: 16 INS size: 311 Genotyped: Yes

```
left   AAT--------GACA-------ACTTTGTTT-------AACAATGATAAAGGGCTAACCAAAATATAAAA
chrom  AAT--------GACA-------ACTTTGTTT-------AACAATGATAAAGGGCTAACCAAAATATAAAA
right  CCGACCTGGGCGACAGAGCGAGACTCCGTCTCAAAAAAAAAAAAAAAAAAAAAAAAAAAAAAAAAAAAAA
       11111111111****1111111***11**1*1111111**1**11*1***11111**11****1*1****

left   ACACTTAAACATGTGGCTCACGCCTGTAATCCCAGCACTTTGGGAGGCCGAGGCGGGTGGATCATGAGGT
chrom  ACACTTAAACAT----------------AAAACAGCA-TGTATAA-----ATGTGTATGTGACATCAACC
right  ACACTTAAACAT----------------AAAACAGCA-TGTATAA-----ATGTGTATGTGACATCAACC
       ************2222222222222222*222*****2*2*222*22222*2*2*22**222***2*222

left   C
chrom  C
right  C
       *
```

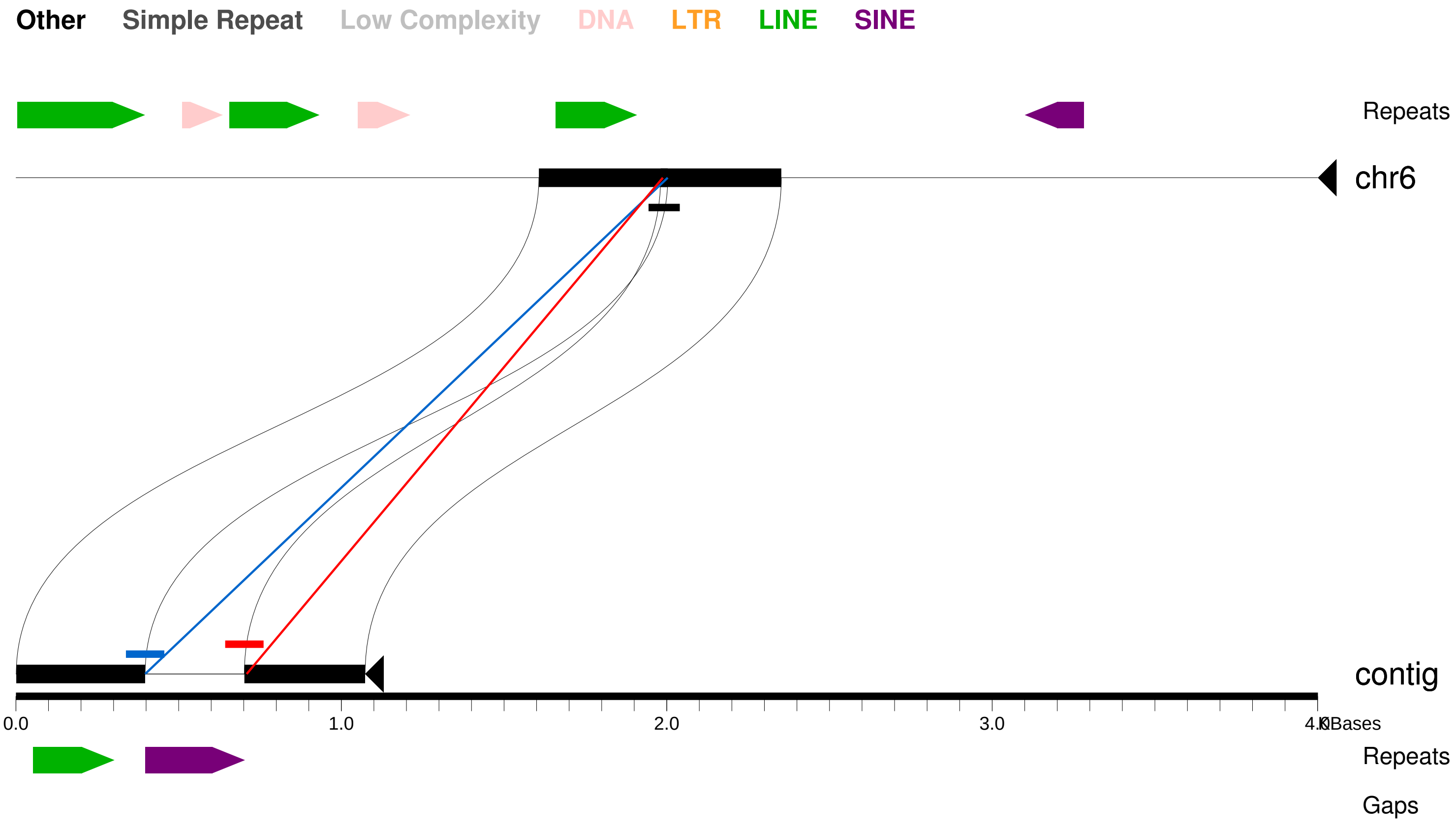

siteID: chr6_31296802 Scafftig3 + Breakpoint: chr6:31296783
chr6:31296623-31296958
Overlap Length: 16 INS size: 328 Genotyped: Yes

```
left    ---------------TAGGCCTTGGTTCAAGTACAAGAACATAGAATGTGTTTATTTCTTTACTGTATGT
chrom   AGGCAGACTACGAGTTAGGCCTTGGTTCAAGTACAAGAACATAGAATGTGTTTATTTATTTACTGTATGT
right   --------TTTTAGTAGAGGCGGGGTTTCACCATGTTAGTCAGGATGGTCTCGATCTCCTGAACTCATGA
                2NNN222111*1*11****11*11*1111*11111**11**1*11**1*N1*1*1111***1

left    AACAACTACTTGGAGCACAATAAAGAATCATTAGTAAATTAGAAGATTTGTTAGTATGAAGAGTAAGGGA
chrom   AACAACTACTTGGAGCACAATAAAGAGTCATTAGTAAATTAGAAGATTTGTTAGTATGAAGAGTGAGGGA
right   TCCACCTGCCTC-AGCCTCCCAAAGTGT--TGGG---ATTACAGG---TGTGAGC-TGCTGCGCCCGG--
        11**1**1*1*11***11111****12*11*11*111****1*1*111***1**11**11*1*1N1**11

left    AACTTAAAGAAAGATTTTAAGAGGAACTATCTTCTTT----TTTTGT---TTGTTTTTTGTTTGTTTGTT
chrom   AACTTAAAGAAAGCTTTTAAGAGGAACTATCTTCTTAACACTTATGT---TAAACCAAAAAGGAAAACTT
right   --CT----------------GAGGAACTATCTTCTTAACACTTATCATTCTTAACCAAAAAGGAAAACTT
        11**111111111N111111***************22222**2*11111*N2222222222222222**

left    TTGAGACAGAGTCTCACTCTGTAG-----CCCAGACTGGAGTG--CAGTGGCGCAATTTAGGCTCACTGC
chrom   TGGAGAGGAACTTTTATTCTTTACAGCTTCCCCCTCTTGATTTTACAGTTCTTCCTCTTCAAATCTCCTT
right   TGGAGAGGTACTTTTATTCTTTACAGTTTCCCCCTCTTGATTTTACAGTTCTTCCTCTTCAAATCTCCTT
        *2****22N*2*2*2*2***2**222N22***222**2**2*222****2222*222**2222**2*222

left    AAC-----------------CTCCGCCT---CCG------GGG----CTCAAGCGATTCT
chrom   AACATATCTTTAGTTTGTTACTCTTCTTAATCAGTTAGAAGGGACAACTTATCCGAGTA-
right   AACATATCTTGATTTTGTTNNNNNNNNNNNNNNNNNNNNN--------------------
        ***2222222N2N222222N111NN1N1NNN1N1NNNNN 111    11N1NN111N1N2
```

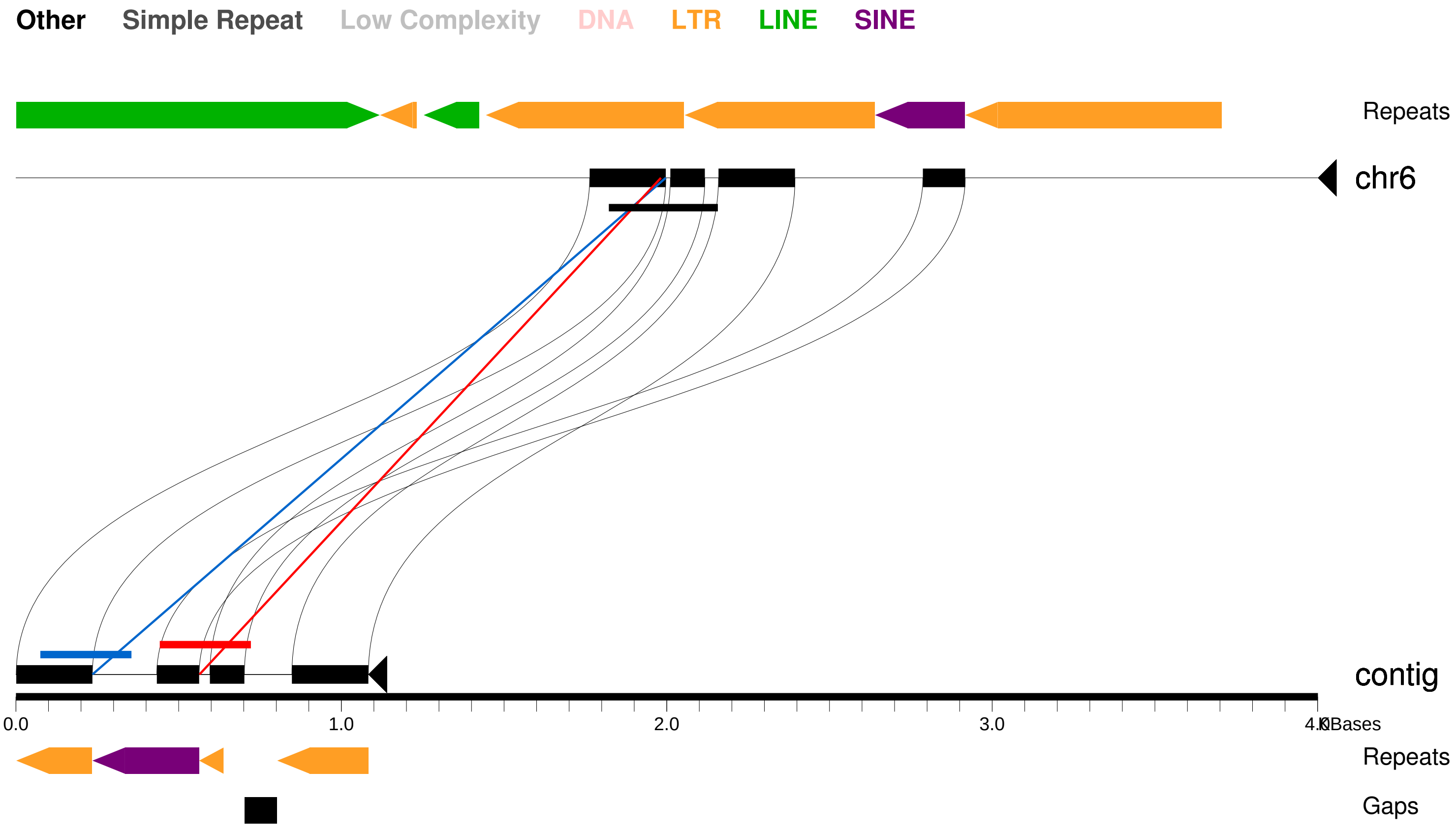

siteID: chr6_32687168 Scafftig6 + Breakpoint: chr6:32687154
chr6:32687113-32687214
Overlap Length: 14 INS size: 301 Genotyped: Yes

```
left    GTTTTGTCTTTTACACTTGA--TGCTGAAATCA-AGAAGTCTTT--------------TAAAAAATAATT
chrom   GTTTTGCCTTTTACACTTGA--TGCTGAAATCA-AGAAGTCTTT--------------TAAAAAATAATT
right   GCCC-GCCTCGGCCTCCCAAAGTGCTGGGATTACAGGCGTGAGCCACCGCGCCCGGCCTAAAAAATAATT
        *1111*2**1111*1*111*11*****11**1*1**11**111111111111111111************

left    TTTTTTTTTT-TTTTTTTTTTTTGAGACGGAGTCTCGCTCTGTCGCCCAGGCTGGAGTGCAGTGGCG
chrom   TTCCTTTAAA-CTTT--------------GAGTACAGCT-TTTCACT-AGGATTGCCAACA-TGATG
right   TTCTTTTAAAACTTT--------------GAGTACAGCT-TTTCACT-AGGATTGCCAACA-TGATG
        **2N***22212***22222222222222****222***2*2**2*22***2*2*2222**2**22*
```

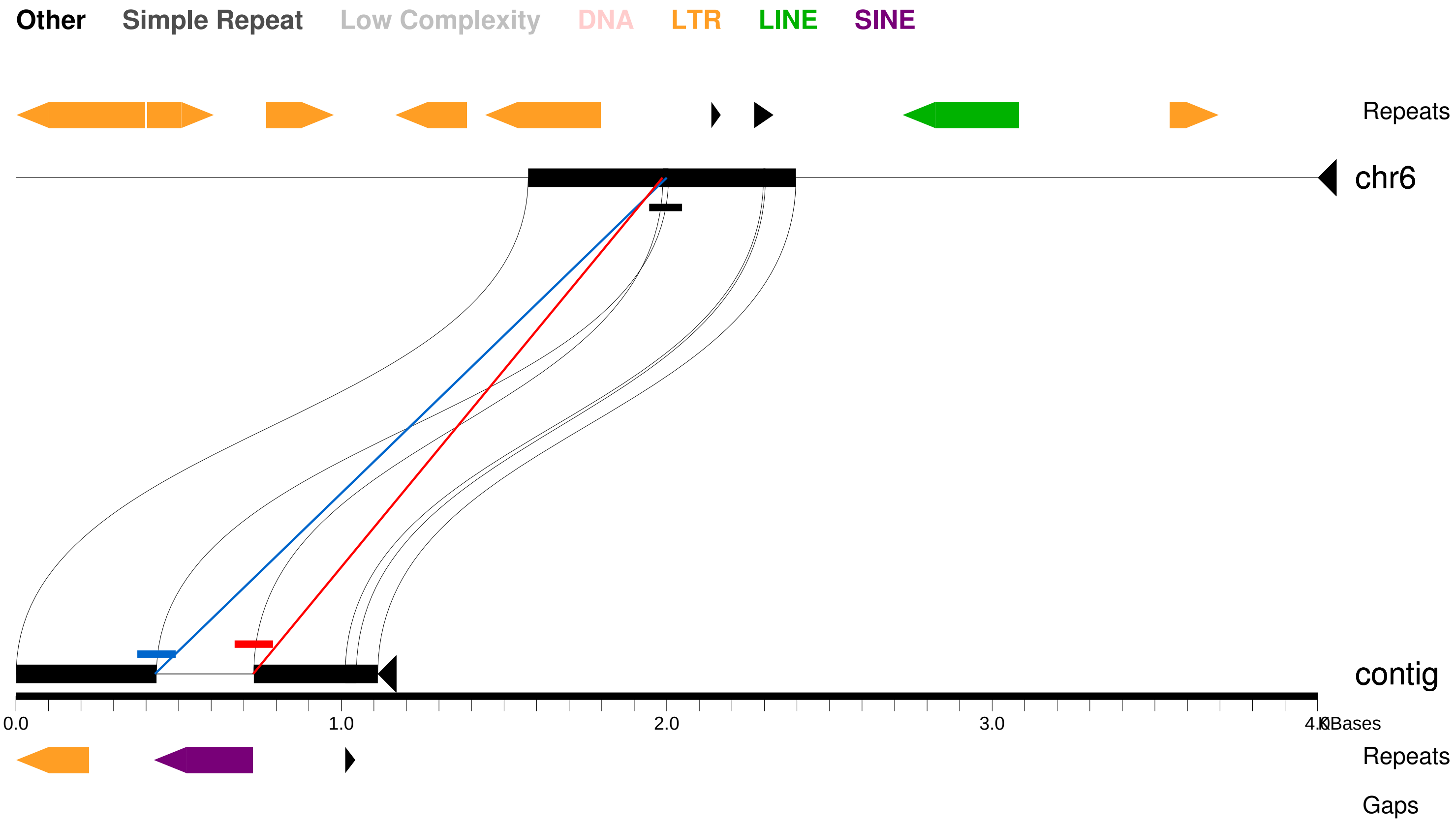

siteID: chr6_32688601 Scafftig3 - Breakpoint: chr6:32688579
chr6:32688536-32688638
Overlap Length: 17 INS size: 322 Genotyped: Yes

```
left    TTGGTGGTGGTGG----------------TATATTAAGTAGAAAAGGTCTATTGGGCCTAAAAATTATTG
chrom   TTGGTGGTGGTGG----------------TATATTAAGTAGAAAAGGTCTATTGGGCCTAAAAATTATTG
right   AGCGAGACTCCGTCTCAAAAAAAAAAAAAAAAAAAAAAAAAAAAAAAAAAAAAAAAAAAAAAAAAAATTATTG
        111*1*11111*111111111111111111*1*11**11*1****11111*11111111***********

left    ACATGTGGCCGGGCGCGGTGGCTCACGCCTGTAATC---CCAGCACTTTGGGAGGCCGAGGCGGGCGG
chrom   ACATGTA--------CATTATCTAT--TCTGTAATGATGCCATCCCT--------CCTAGTTTC-CAT
right   ACATGTA--------CATTATCTAT--TCTGTAATGAGGCCATCCCT--------CCTAGTTTC-CAT
        ******222222222*22*22**22222*******22N2***2*2**22222222**2**22222*22
```

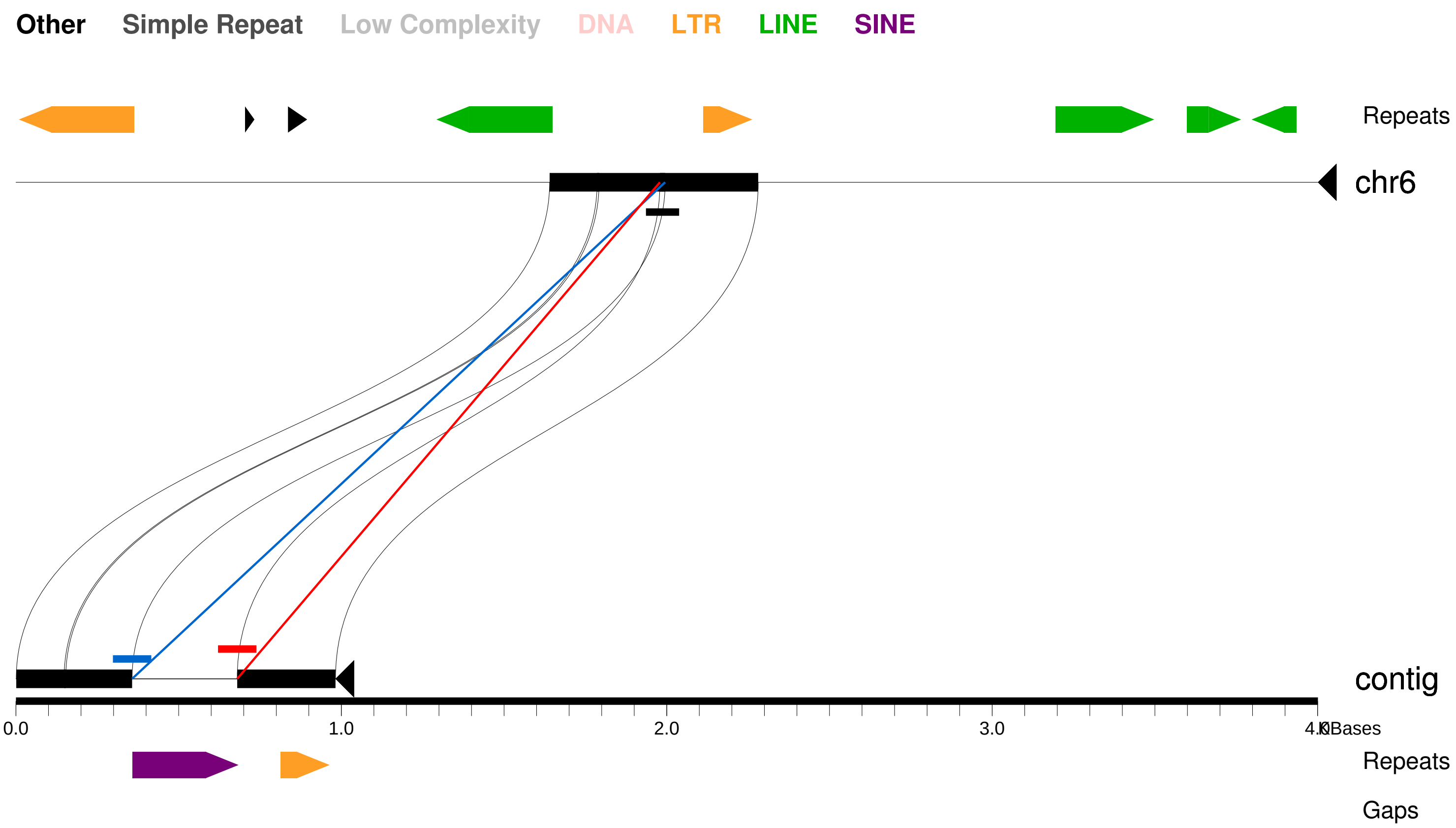

siteID: chr6_32773389 Scafftig5 + Breakpoint: chr6:32773436
chr6:32773287-32773495
Overlap Length: 9 INS size: 310 Genotyped: Yes

```
left   CATTTGCTTGGTAGATTTTTCTCCATCTGTTTATTTTGATCCTATGTGTGTCACTGCATGTGAGA--TGA
chrom  CATTTGCTTGGTAGATTTTTCTCCATCTGTTTATTTTGATCCTATGTGTGTCACTGCATGACACACATGA
right  ---------------------TCCGCCG------------CCT----------CGGCCTCCCAAA-----
       111111111111111111111***11*1*11111111111***1111111111*1**1*1N2*N*  111

left   GTCTTTTGAAGACAGCATACTAATAGGCCTTGGTTCTTTATCCAGATTACCACTCTGTGCCTTTTAATTG
chrom  GTCTTTTGAAGACAGCATACTAATAGGCCTTGGTTCTTTATCCAGATTACCACTCTGTGCCTTTTAATTG
right  GTGCTGGGATTACAGG-------------------CGTGAGCCA----------CCGTGCCC--------
       **11*11**11****11111111111111111111*1*1*1***1111111111*1*****111111111

left   GGGCACTAGCC-ATTTACTTTT----TTTTTTTT---------------------------------TT
chrom  GGGCACTAGCCCATTTACATTTAAGATTAGTATTGATATGTGTGGATTTCATCCTGTCATCATGATGTT
right  -GGC-----CCCATTTACATTTAAGATTAGTATTGATATGTGTGGATTTCATCCTGTCATCATGATGTT
       1***11111***2*****2***2222**22*2**222222222222222222222222222222222**
```

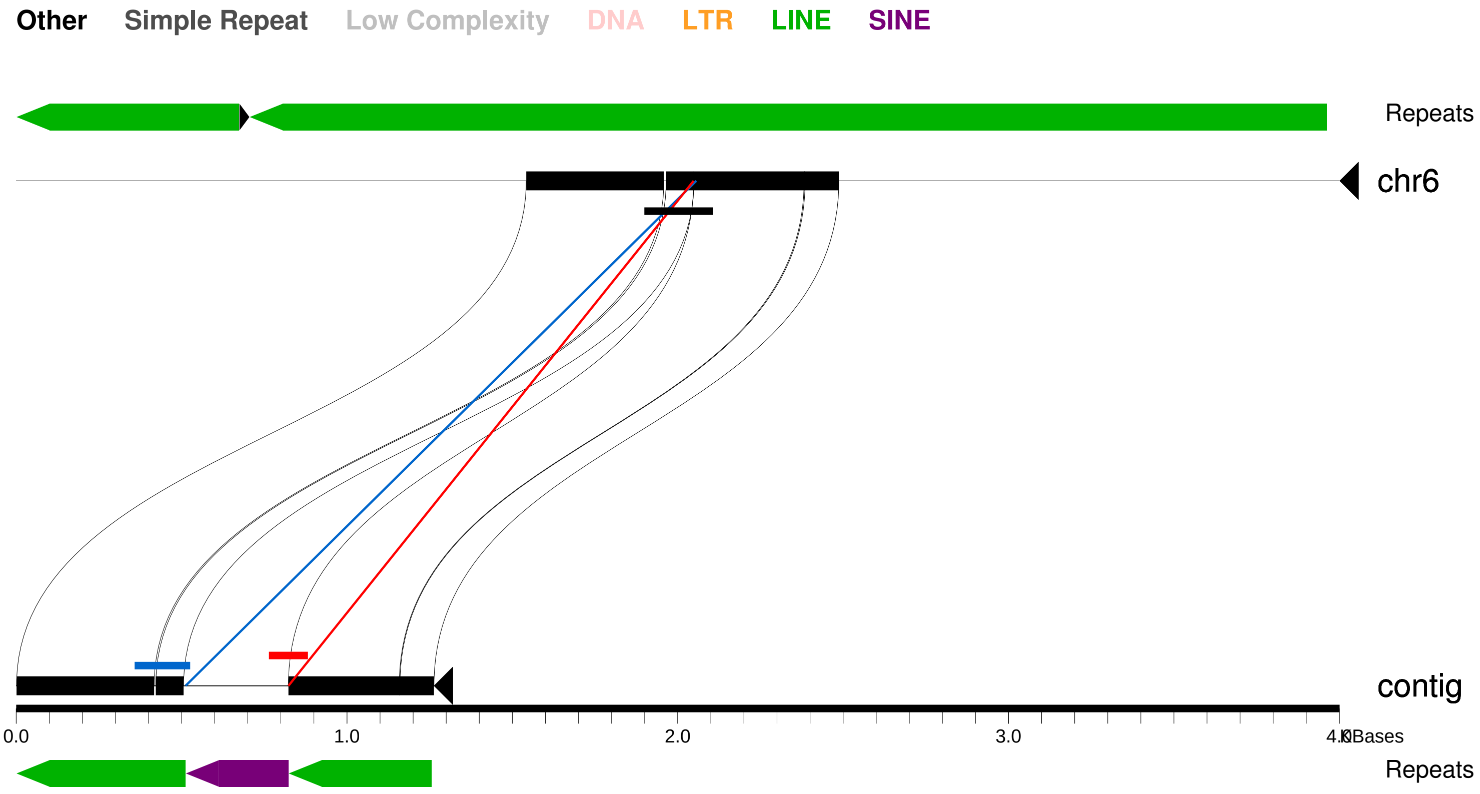

siteID: chr6_35927165 Scafftig1 + Breakpoint: chr6:35927168
chr6:35927109-35927228
Overlap Length: 11 INS size: 432 Genotyped: No

```
left    CTGAATTCTTCATGGTCCATCTAAAAGTCAAAATCCCTTAGAGGTTTTGATTTTTTTTTTTTAAGAAATG
chrom   CTGAATTCTTCATGGTCCATCTAAAAGTCAAAATCCCTTAGAGGTTTTGATTTTTTTTTT--AAGAAATG
right   NNNNNNNNNNNNNNNNNNNNNNNNNNNNNNNNNNNNNNNNNNNNNNNNNNNNNNNNNAA---AAGAAATG
        11111111111111111111111111111111111111111111111111111111111122********

left    ACGGCCGGGCGCGGTGGCTCACGCCTGTAATCCCAGCACTTTGGGA------GGC-
chrom   ACTAGTGTACTTGTTTA-TAAAATATGGAGA---AAAACATAGGAAAGAAAAGGCA
right   ACTAGTGTACTTGTTTA-TAAAATATGGAGA---AAAACATAGGAAAGAAAAGGCA
        **2222*22*22*2*222*2*2222**2*22222*22**2*2**2*222222***2
```

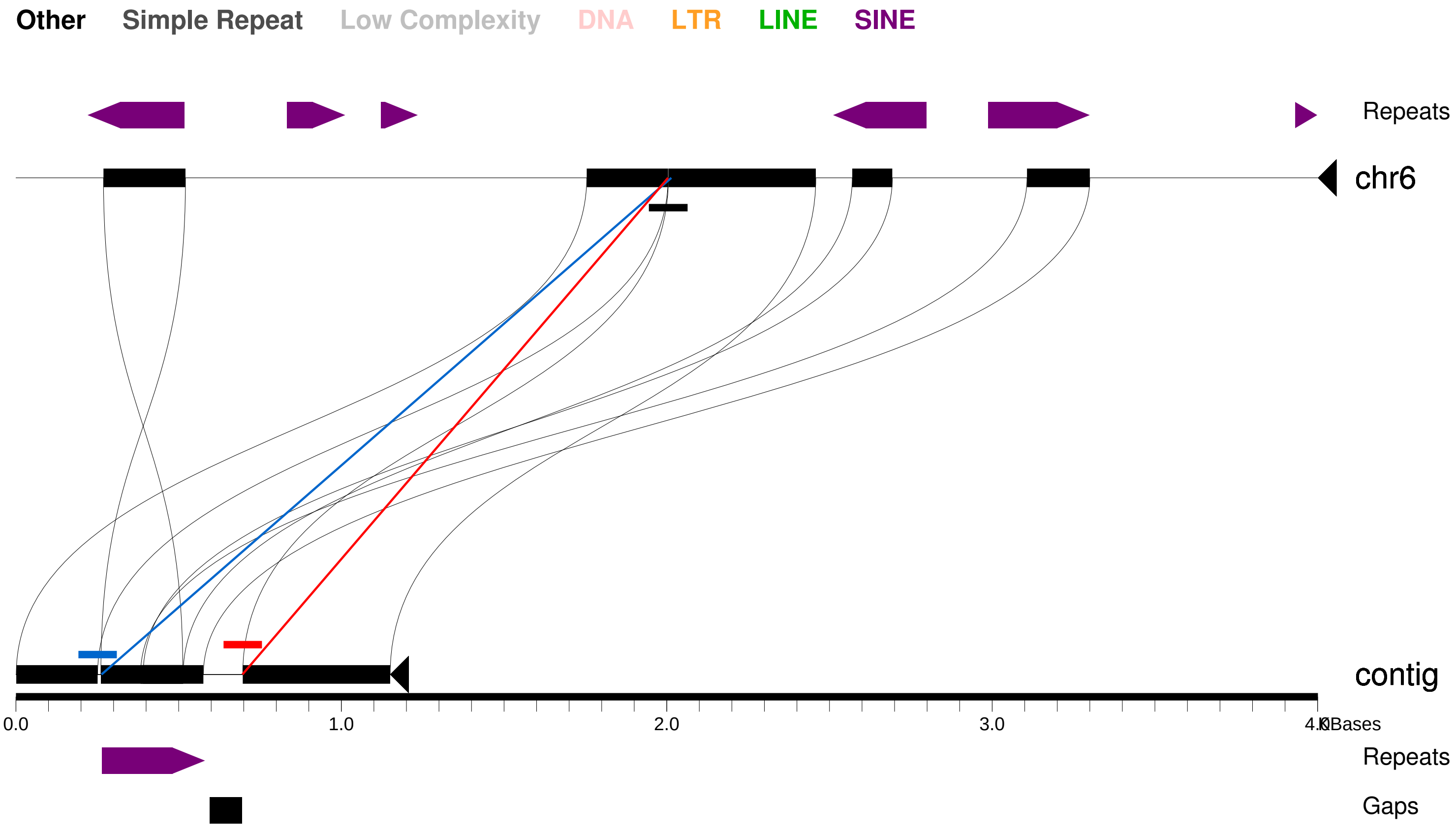

siteID: chr6_36021794 Scafftig2 - Breakpoint: chr6:36021769
chr6:36021725-36021828
Overlap Length: 16 INS size: 121 Genotyped: Yes

```
left     AGACA-ACTTCTATTCCTGAGTATAATTTTACTTAAGTAGCTATG----------------TGCAAACCA
chrom    AGACA-ACTTCTATTCCTGAGTATAATTTTACTTAAGTAGCTATG----------------TGCAAACCA
right    GGCTGGAGTGCAGTGGCGGGATCTCGGCTCACTGCA--AGCTCCGCCTCCCGGGTTCACGCTGCAAACCA
         1*1111*1*1*11*11*1*11*1*1111*1***11*11****11*1111111111111111*********

left     GAGTTTTTTTTTTTTTTTTTTTTTTTTTTTTTTTTTTTTTTTTTTGAGACGGAGTCTCGCTCTGTCGC
chrom    GAGTTTTAAGAATGTTCAGTGTGAAGCTGCAGCCTTCCTAAT---------------CAGTGACAT
right    GAGTTTTAAGAATGTTCAGTGTGAAGCTGCAGCCTTCCTAAT---------------CAGTGACAT
         *******22222*2**222*2*22222*222222**22*22*222222222222222*22**2*22
```

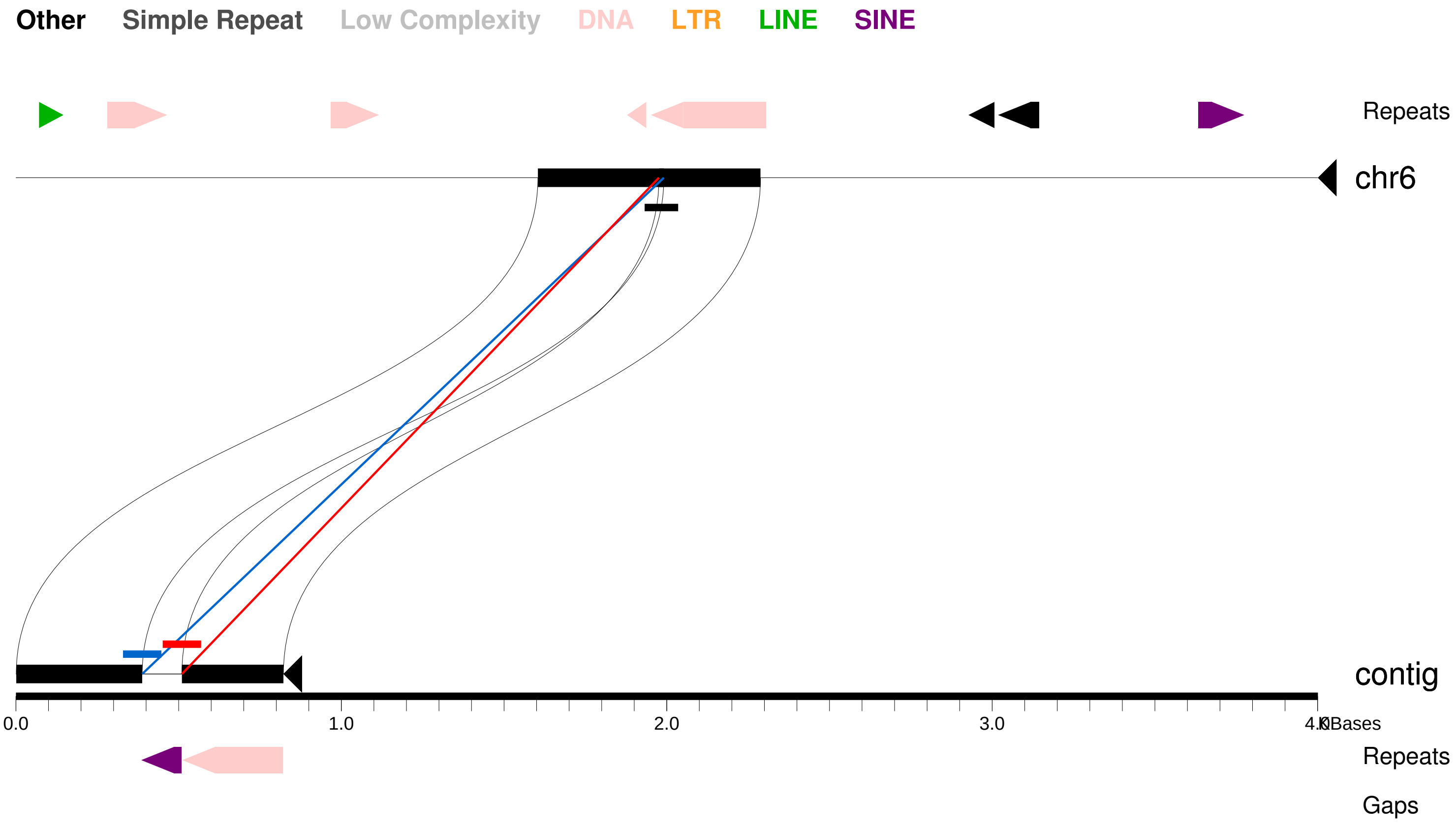

siteID: chr6_39350742 Scafftig10 + Breakpoint: chr6:39350733
chr6:39350688-39350792
Overlap Length: 15 INS size: 314 Genotyped: Yes

```
left     ATTATTACTGATGTTGAACATTGTTTTG----TATGTTTTTTGGCCACT----------TGTATGTCTTT
chrom    ATTATTACTGATGTTGAACATTGTTTTG----TATGTTTTTTGGCCACT----------TGTATGTCTTC
right    CCACCCGCCTCGGCCTCCCAAAGTGCTGGGATTACAGGCGTGAGCCACCGCGCCCGGCCTGTATGTCTTC
         1111111*1111*11111**11**11**1111**111111*11*****11111111111**********2

left     TTTTTTT---TTTTTTTTTTTTTTTTTGAGACGGAGTCTCGCTCTGTCGCCCAGGCCGGACTGCGG
chrom    TTTTGAGAAGTGTCTGTTCCTGTCTTTG-----------CCCACTTTTTAATAGG--GTTCT----
right    TTTTGAGAAGTGTCTGTTCCTGTCTTTG-----------CCCACTTTTTAATAGG--GTTCT----
         ****222222*2*2*2**22*2*2****22222222222*2*2**2*22222***22*22**2222
```

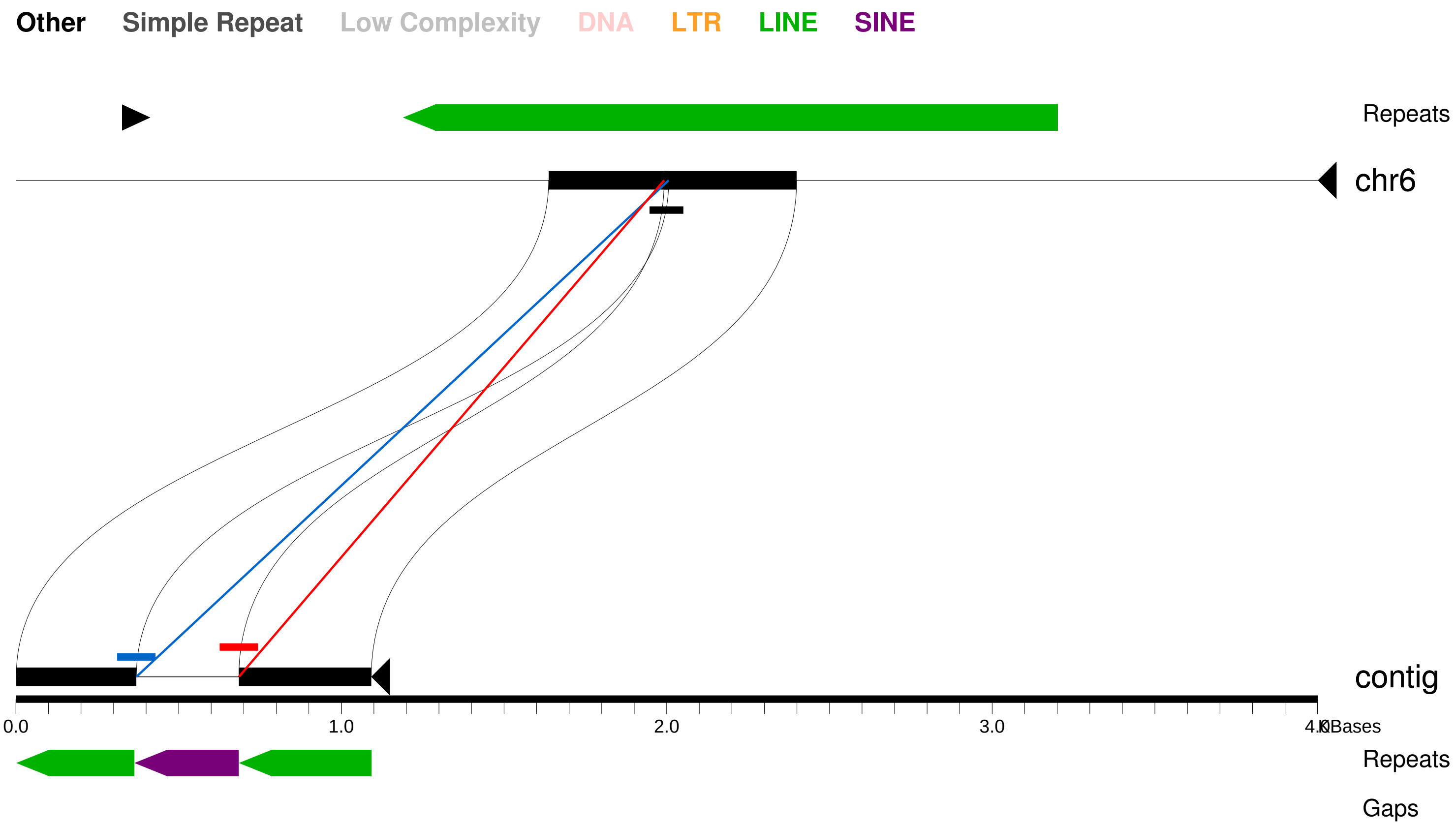

siteID: chr6_40046901 Scafftig3 - Breakpoint: chr6:40046895
chr6:40046850-40046954
Overlap Length: 15 INS size: 301 Genotyped: Yes

```
left     TTAGTGAAAGAAAATGTTTTGGGGTGAGGTATTGCTTCTGTTTAG---------------AAAGTAGTCT
chrom    TTAGTGAAAGAAAATGTTTTGGGGTGAGGTATTGCTTCTGTTTAG---------------AAAGTAGTCT
right    CT-GCACTCCAGCCTGGGCGACAGAGCGAGACTCCGTCTCAAAAAAAAAAAAAAAAAAAAAAAGTAGTCT
         1*1*111111*111**1111111*1*1*11*1*1*1***1111*1111111111111111**********

left     GTAGAGGCCGGGCGCGGTGGCTCACGCCTGTAATCCCAGCACTTTGGGAGGCCGAGGCGGGCGG
chrom    GTAGAAT---GAAGAGAACATTCGTCCCTTAAGGGCCAGGTAATTA-----------CACAATT
right    GTAGAAT---GAAGAGAACATTCGTCCCTTAAGGGCCAGGTAATTA-----------CACAATT
         *****22222*22*2*22222**222***22*222****2222**222222222222*222222
```

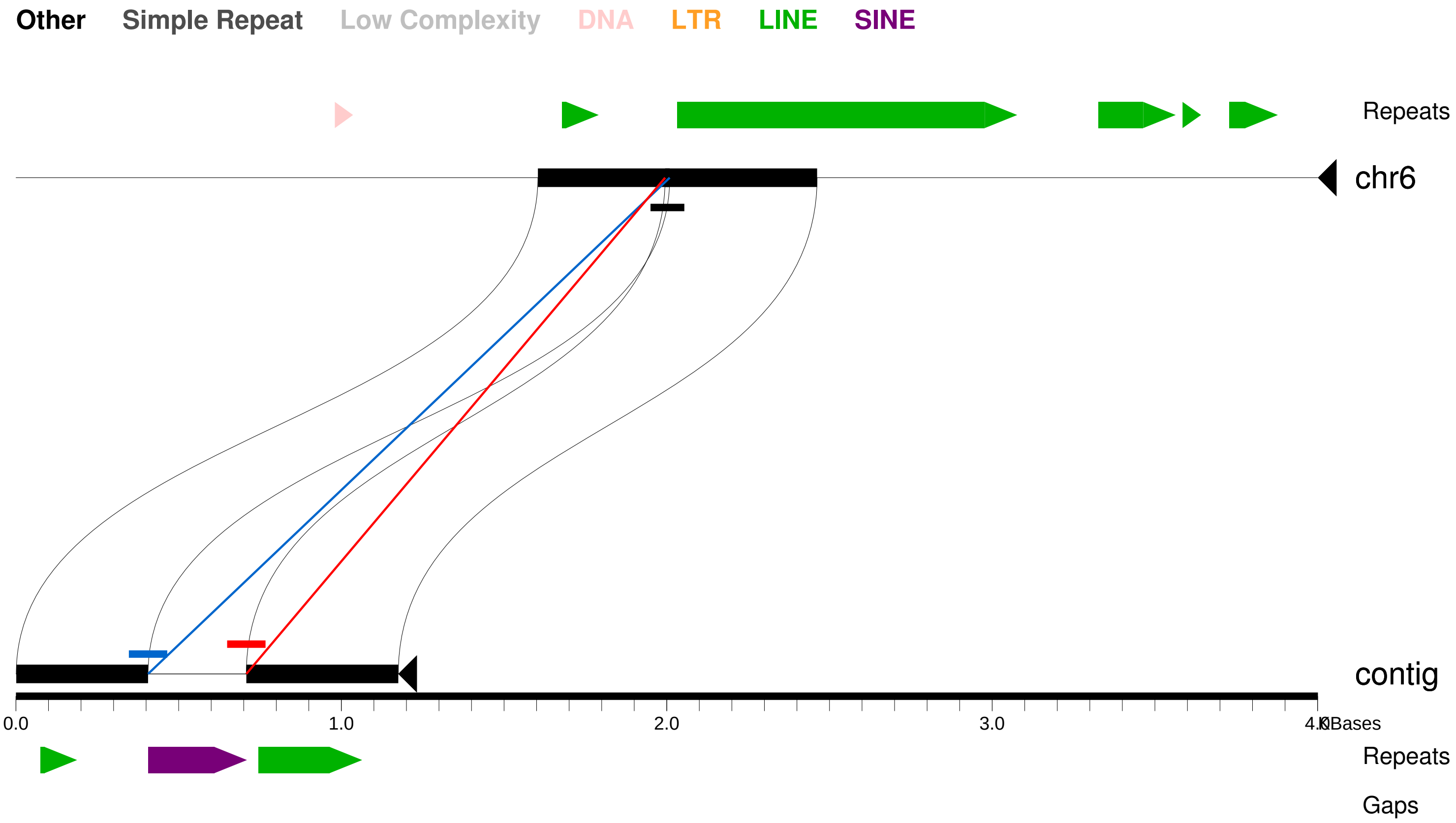

siteID: chr6_40170283 Scafftig7 + Breakpoint: chr6:40170280
chr6:40170236-40170339
Overlap Length: 16 INS size: 314 Genotyped: Yes

```
left    TGGACTGTTCAGGCTTTCTATTTT-------TTCCTGGCTCAATTTTTTGT--------TAGGCTATATT
chrom   TGGACTGTTCAGGCTTTCTATTTT-------TTCCTGGCTCAATTTTTTGT--------TAGGCTATATT
right   CCGCCCGCCTCGGCCTCCCAAAGTGCTGGGATTACAGGCGTGAGCCACCGCGCCCGGCCTAGGCTATATT
        11*1*1*1111***1*1*1*111*1111111**1*1***111*111111*111111111***********

left    TTTCTTTTTTTTTTTTTTTTTTTTTTTTTTTTTTTTTTGAGACAGAGTCTCGCTCTGTCGCCCA
chrom   TTTCTATAAAGCTATTCATTTCCTCTGGGCTATCTAATTTGCTGGAAT---------------A
right   TTTCTATAAAGCTATTCATTTCCTCTGGGCTATCTAATTTGCTGGAAT---------------A
        *****2*22222*2**22***22*2*2222*2*2*22*22*222**2*222222222222222*
```

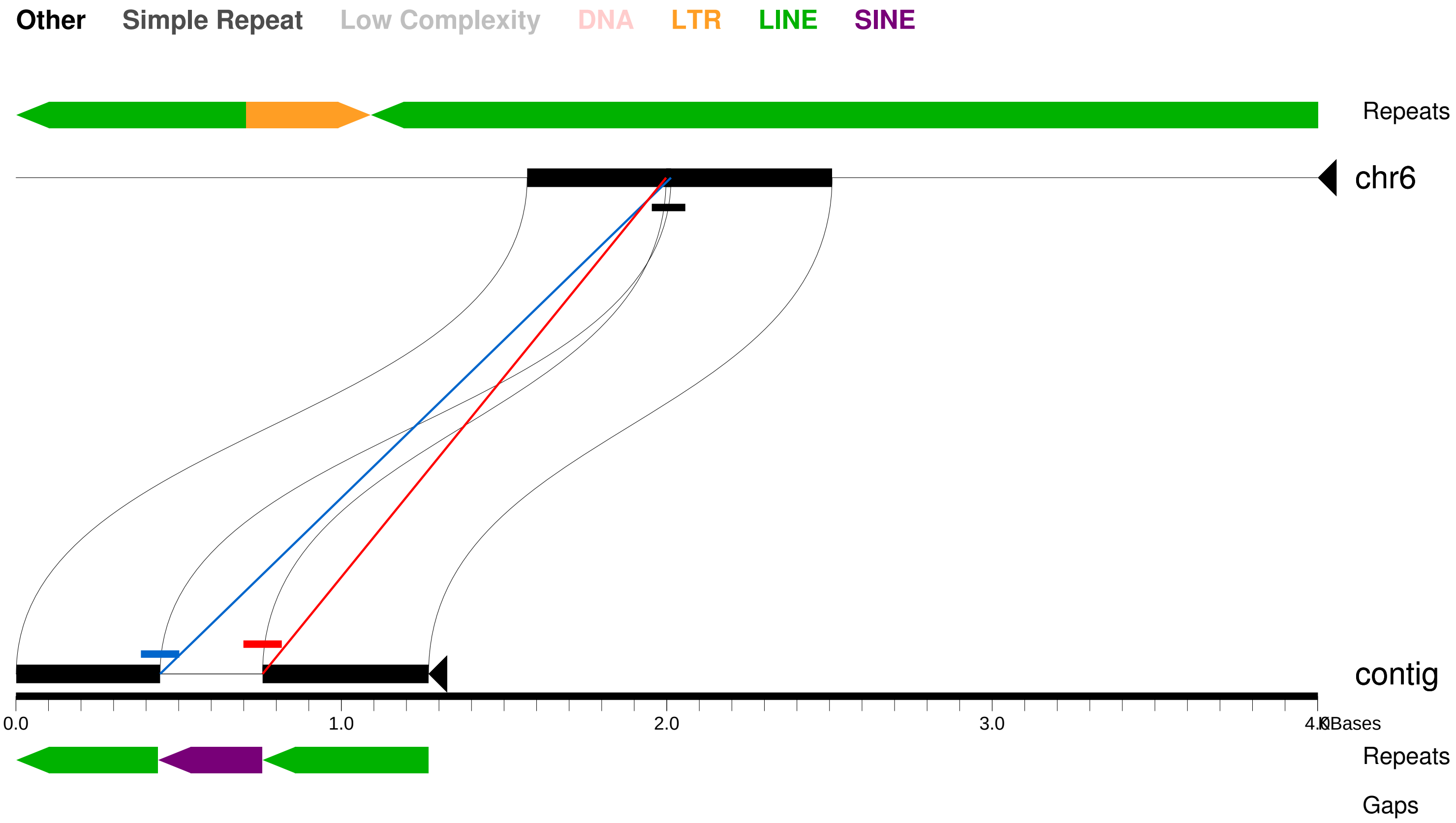

siteID: chr6_40731388 Scafftig3 - Breakpoint: chr6:40731415
chr6:40731370-40731474
Overlap Length: 15 INS size: 467 Genotyped: No

```
left    AATGTCTGCAACACACTTTGAAAACAGTAA--------------ATCAGTAGACAAGTTAAAATTGCACC
chrom   AATGTCTGCAACACACTTTGAAAACAGTAA--------------ATCAGTAGACAAGTTAAAATTGCACC
right   NNNNNNNNNNNNNNNNNNNNNNNNNNNNNNNNNNNNNNNNNNNNNNNNNNNNAAAAAAAAAAATTGCACC
        1111111111111111111111111111111111111111111111111111*1**111***********

left    TGTTGGCCGGGCGCGGTGGCTCACGCCTGTAATCCCAGCACTTTGGGAGGCCGAGGCGGGCGG
chrom   TGTTATTAAGCCAGG---ACCCAGGCAACAAAGACCCAAACC-----------AGGCAGACAA
right   TGTTATTAAGCCAGG---ACCCAGGCAACAAAGACCCAAACC-----------AGGCAGACAA
        ****22222*2*22*2222*2**2**2222**22**222**222222222222****2*2*22
```

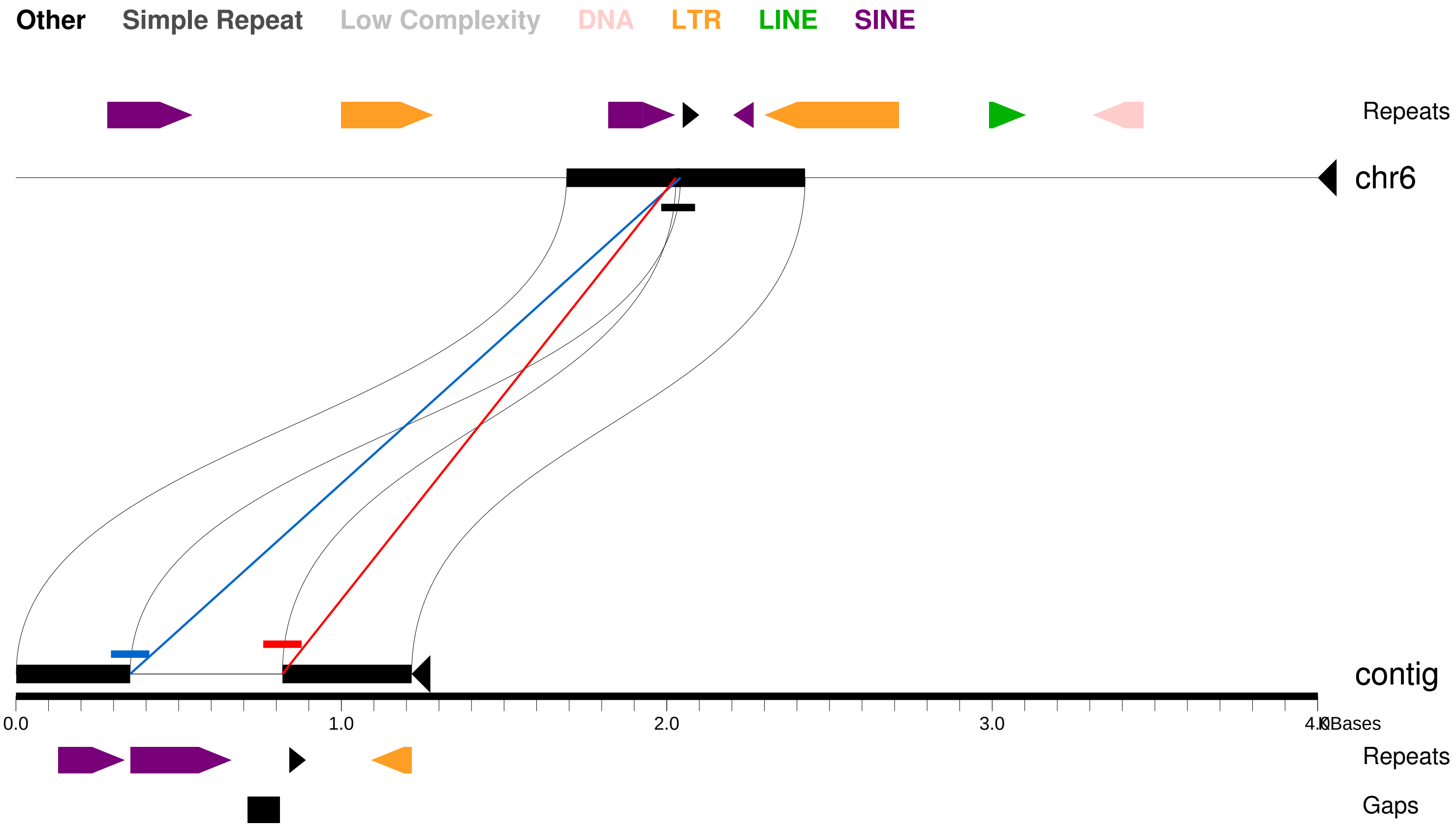

siteID: chr6_40926796 Scafftig1 + Breakpoint: chr6:40926819
chr6:40926660-40926989
Overlap Length: 11 INS size: 352 Genotyped: No

```
left    ---------ATTATATAGTCCTGGTTTGTCCAAAGCACTCTCCACAAACAGGCTGTGTGACCTTCAAATA
chrom   GGCTTTGGAATTATATAGTCCTGGTTTGTCCAAAGCACTCTCCACAAACAGGCTGTGTGACCTTCAAATA
right   NNNNNNNNNNNNAA--------------------------------------------------------
        NNNNNNNNN111*111111111111111111111111111111111111111111111111111111111

left    GCTGCTTAACCCCTCTAAGCCTGTCATCTGAAAGTAGAAATGATAATGATAACATTAGAGTAAACCACAT
chrom   GCTGCTTAACCCCTCTAAGCCTGTCATCTGAAAGTAGAAATGATAATGATAACATTAGAGTAAACCACAT
right   ----------------------------------------------------------------------
        1111111111111111111111111111111111111111111111111111111111111111111111

left    GAAAGTGTCTTTGTAGGTTAAAAATTGTTG------GCCGGGCGCGGTGGCTCACGCCTGTAATCCCAGC
chrom   GAAAGTGTCTTTGTAGGTTAAAAATTGTTGAATATTGGCAGTCTCATAGGTTC-CACCTAAAACCTTTGT
right   -------------------AAAAATTGTTGAATATTGGCAGTCTCATAGGTTC-CACCTAAAACCTTTGT
        1111111111111111111**********222222*2*2*2*2*222**2**2*2***22**2*222*2

left    ACT-TTGGGAGGCCGAGGCGGGCGGATCA--------------CGAGGTCAGGAGATCGAGACCATC---
chrom   GGGGTTCTGGGGAAGATTCGGTAAGAGAATATATGTATTACTGCTAGGGCCTCAGATCTA-ACTAGTTTA
right   GGGGTTCTGGGGAAGATTCGGTAAGAGAATATATGTATTACTGCTAGGGCCTCAGATCTA-ACTAGTTTA
        2222**22*2**22**22***222**22*22222222222222*2***2*222*****2*2**2*22222

left    ---CCGGCTAAAACGGTGAAA--CCCC-----GTCTCTACTAAA--------
chrom   GGACCACAGAAGAAGGTCACGTTCCCCTTAGTGTCTGAACCACATCTCCTGT
right   GGACCACAGAAGAA--------------------------------------
        222**2222**2*2111N1NN  1111     1111NN11N1N1
```

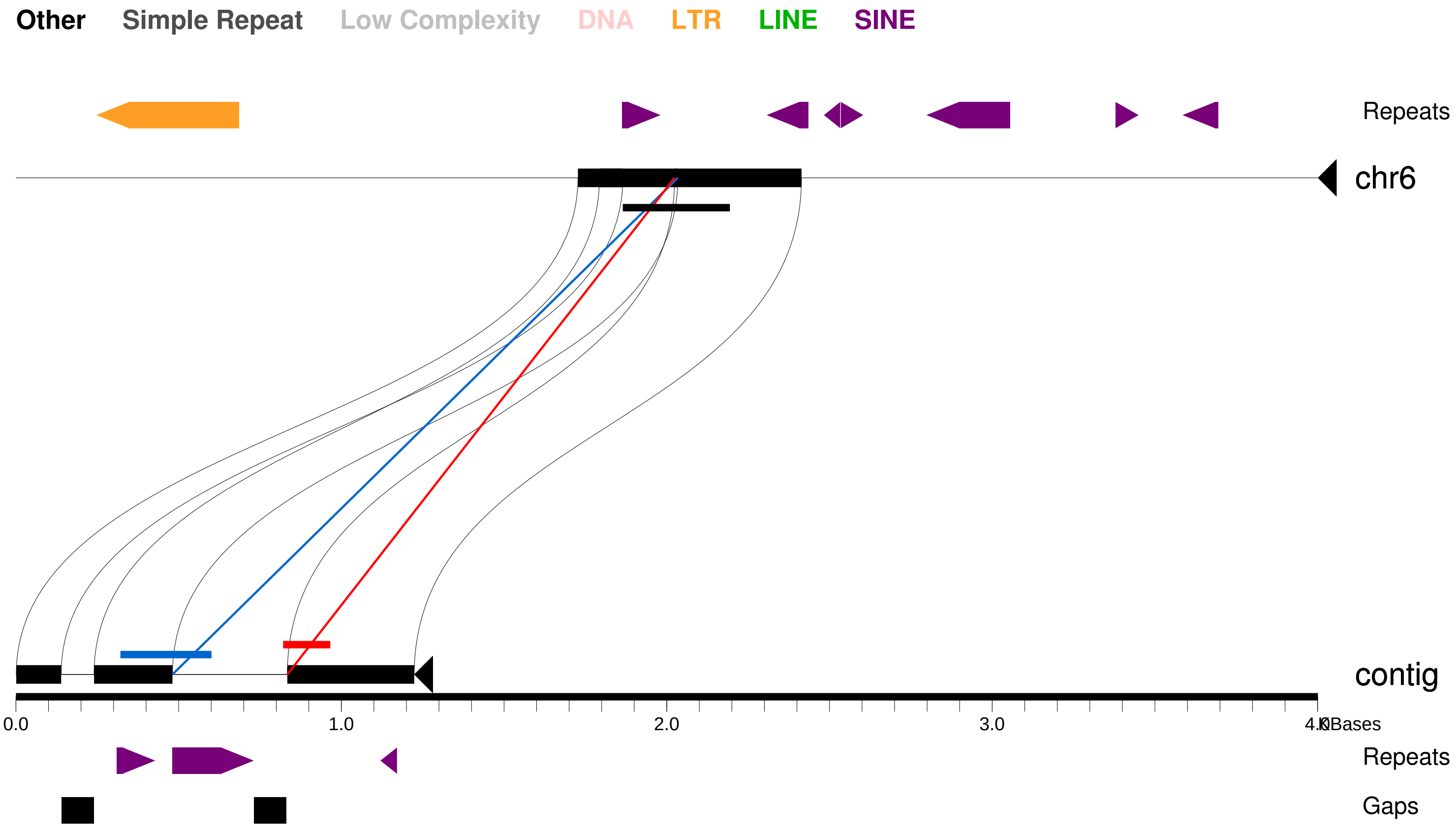

siteID: chr6_43895477 Scafftig2 - Breakpoint: chr6:43895475
chr6:43895428-43895534
Overlap Length: 13 INS size: 464 Genotyped: No

```
left    ---TCCCCCTCCGCATCCTCAGTTTCTCCATTTACAAG---GAGGAAAGTTAT------CCAGACAATCT
chrom   ---TCCCCCTCCGCATCCTCAGTTTCTCCATTTACAAG---GAGGAAAGTTAT------CCAGACAATCT
right   CCGCCCGCCTCGGCCTCCCAAAGTGCTGGGATTACAGGCGTGAGCCACCGCGCCCGGCCCCAGACAATCT
        1111**1****1**1***11*11*1**1111*****1*111***11*111111111111***********

left    TTTTTTTTNNNNNNNNNNNNNNNNNNNNNNNNNNNNNNNNNNNNNNNNNNNNNNNNNNNNNN
chrom   TTAAAGTTCAGCTGGGGCCTTCTCTTAGTCTAAGGTGAACTCTGTTTTT------------
right   TTAAAGTTCAGCTGGGGCCTTCTCTTAGTCTAAGGTGAACTCTGTTTTT------------
        **2222**22222222222222222222222222222222222222222222222222222
```

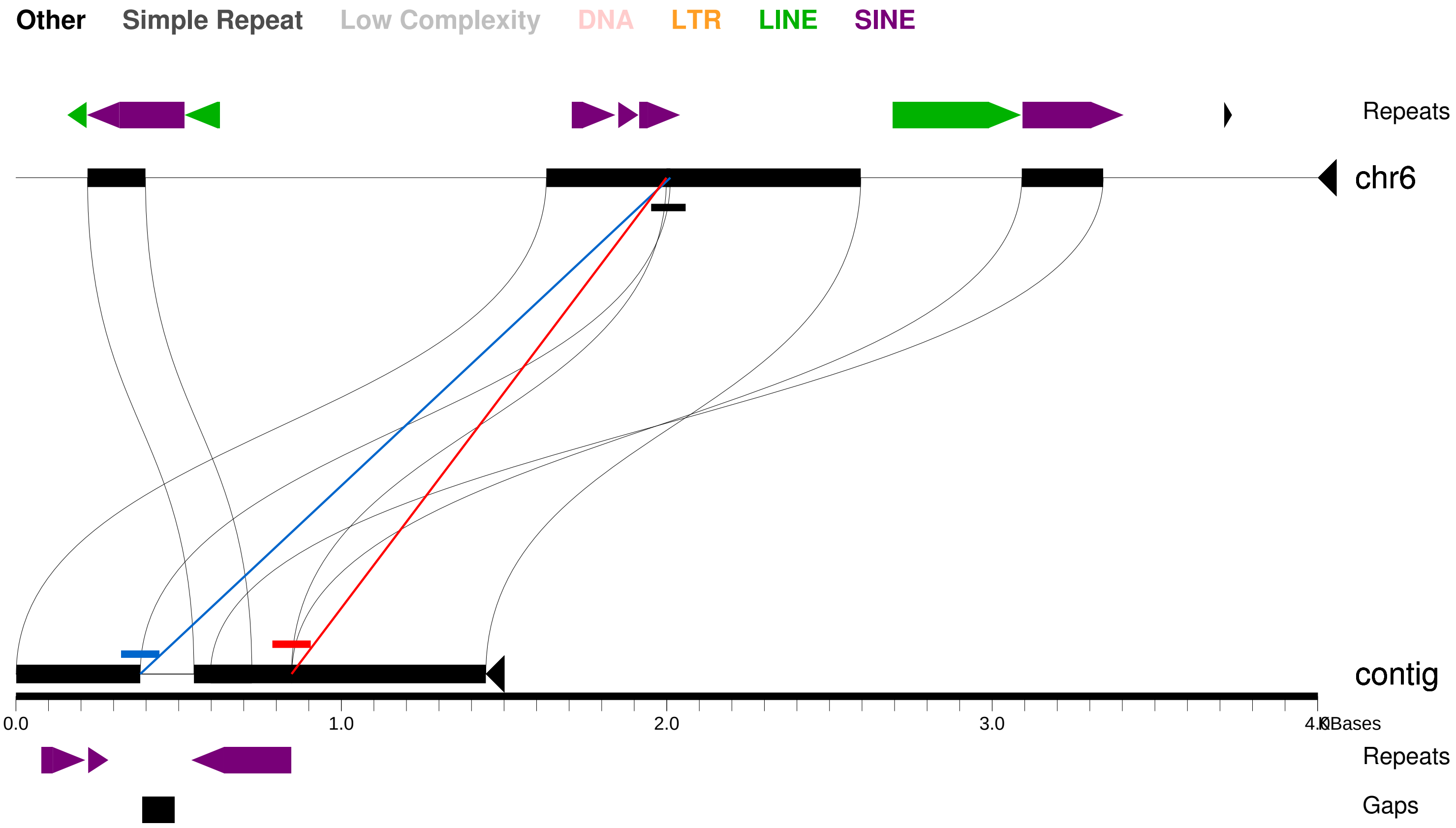

siteID: chr6_44798616 Scafftig1 + Breakpoint: chr6:44798617
chr6:44798568-44798676
Overlap Length: 11 INS size: 422 Genotyped: No

```
left    GCA---GTTCTGGAGTC------TG-TATACTTCCAGCCCAATGAATTAACCTTGAACAGCCACAGCCAT
chrom   GCA---GTTCTGGAGTC------TG-TATACTTCCAGCCCAATGAATTAACCTTGAACAGCCACAGCCAT
right   CCACCCGCCTCGGCCTCCCAAAGTGCTGGGATTACAGGCGTGAGCCACCGCGCCCGGCCGCCACAGCCAT
        1**111*1111**11**111111**1*1111**1***1*1111*111111*111111*1***********

left    NNNNNNNNNNNNNNNNNNNNNNNNNNNNNNNNNNNNNNNNNNNNNNNNNNNNNNNNNNNNN
chrom   ATTTTAAGAAATACAGAATAACCTTTTACGATCAAGATAATGCTATGGA----------
right   ATTTTAAGAAATACAGAATAACCTTTTACGATCAAGATAATGCTATGGA----------
        2222222222222222222222222222222222222222222222222222222222222
```

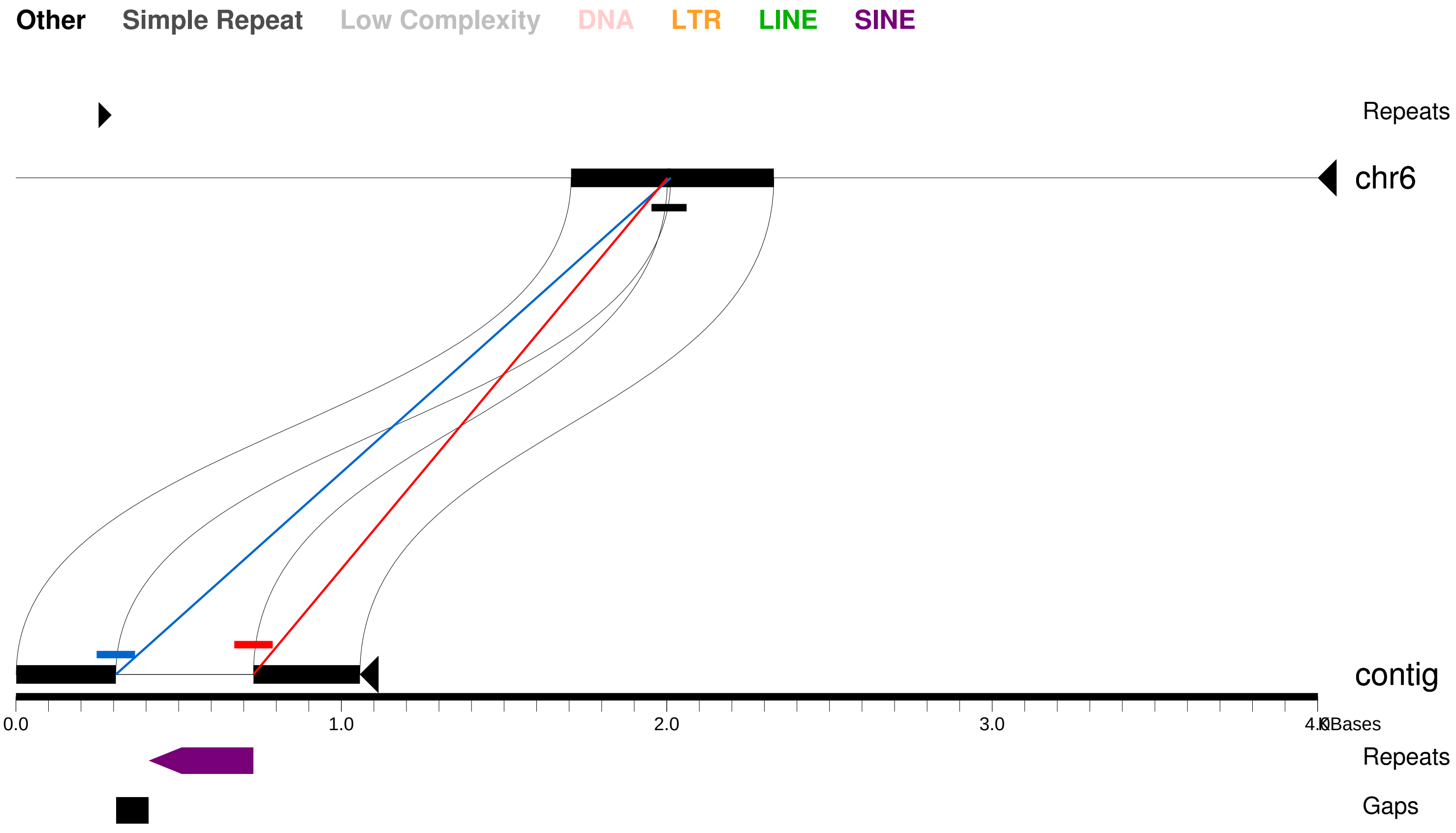

siteID: chr6_45260477 Scafftig2 - Breakpoint: chr6:45260463
chr6:45260420-45260495
Overlap Length: 17 INS size: 317 Genotyped: Yes

```
left   T-ATT------CTT-ATACCAGAC-----------------------------------AAAAGAAACTT
chrom  T-ATT------CTT-ATACCAGAC-----------------------------------AAAAGAAACTT
right  AGATTGCGCCACTGCACTCCAGCCTGGGCGACAGCGAGACTCCGTCTCAAAAAAAAAAAAAAAAAAAAAA
       11***111111**11*11****1*11111111111111111111111111111111111****1***111

left   TAAAGCAACAGCAGTTAAAAAAGACAAGGAGGGGCCGGGCGCGGTGGCTCACGCCTGTAATCCCAGCACT
chrom  TAAAGCAACAGCAGTTAAAAAAGACAAGGAGGGACA------------TTA----TATAAT---------
right  AAAAAAAAAAAAAAAAAAAAAAGACAAGGAGGGACA------------TTA----TATAAT---------
       1***11**1*11*111*****************2*2222222222222*2*2222*2****222222222

left   TTGGGAGGCCGAGGCGGCGGA
chrom  ----GAT-------------A
right  ----GAT-------------A
       2222**22222222222222*
```

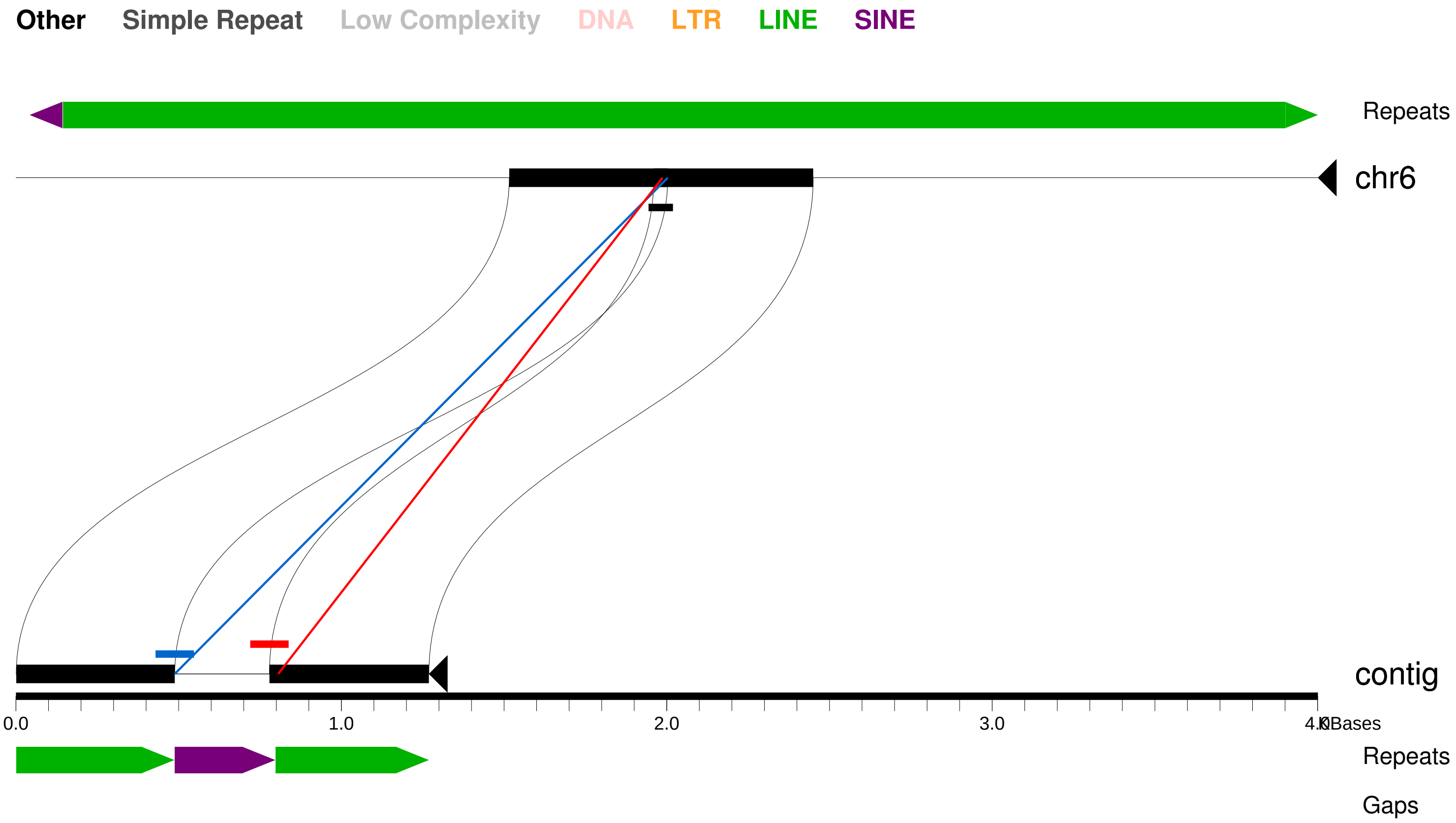

siteID: chr6_47708279 Scafftig9 - Breakpoint: chr6:47708257
chr6:47708212-47708313
Overlap Length: 15 INS size: 329 Genotyped: Yes

```
left    GTGAAT-------------AATGTGGATCCAAAGGAGAGGGTGGGGTGAGGCTCT----GACTAGAGGGG
chrom   GTGAAT-------------AATGTGGATCCAAAGGAGAGGGTGGGGTGAGGCTCT----GACTAGAGGGG
right   GAGACTCCGTCTCAAAAAAAAAAAAAAAAAAAAAAAAAAAAGAAAAAAAAGAAAAAAAAGAATAGAGGGG
        *1**1*1111111111111**11111*111****1*1*1111111111*1*11111111**1********

left    GCCAGAGGCCGGGCGCGGTGGCTCACGCCTGTAATCCCAGCACTTTGGGAGGCCGAGGCGGGCGGA
chrom   GCCAGAG--AGGGTGAGTT----------TCTAGAGCCCCCTCTTT---ACACTGATGCTGGGG--
right   GCCAGAG--AGGGTGAGTT----------TCTAGAGCCCCCTCTTT---ACACTGATGCTGGGG--
        *******222***2*2*2*2222222222*2**222**22*2****222*22*2**2**2**2*22
```

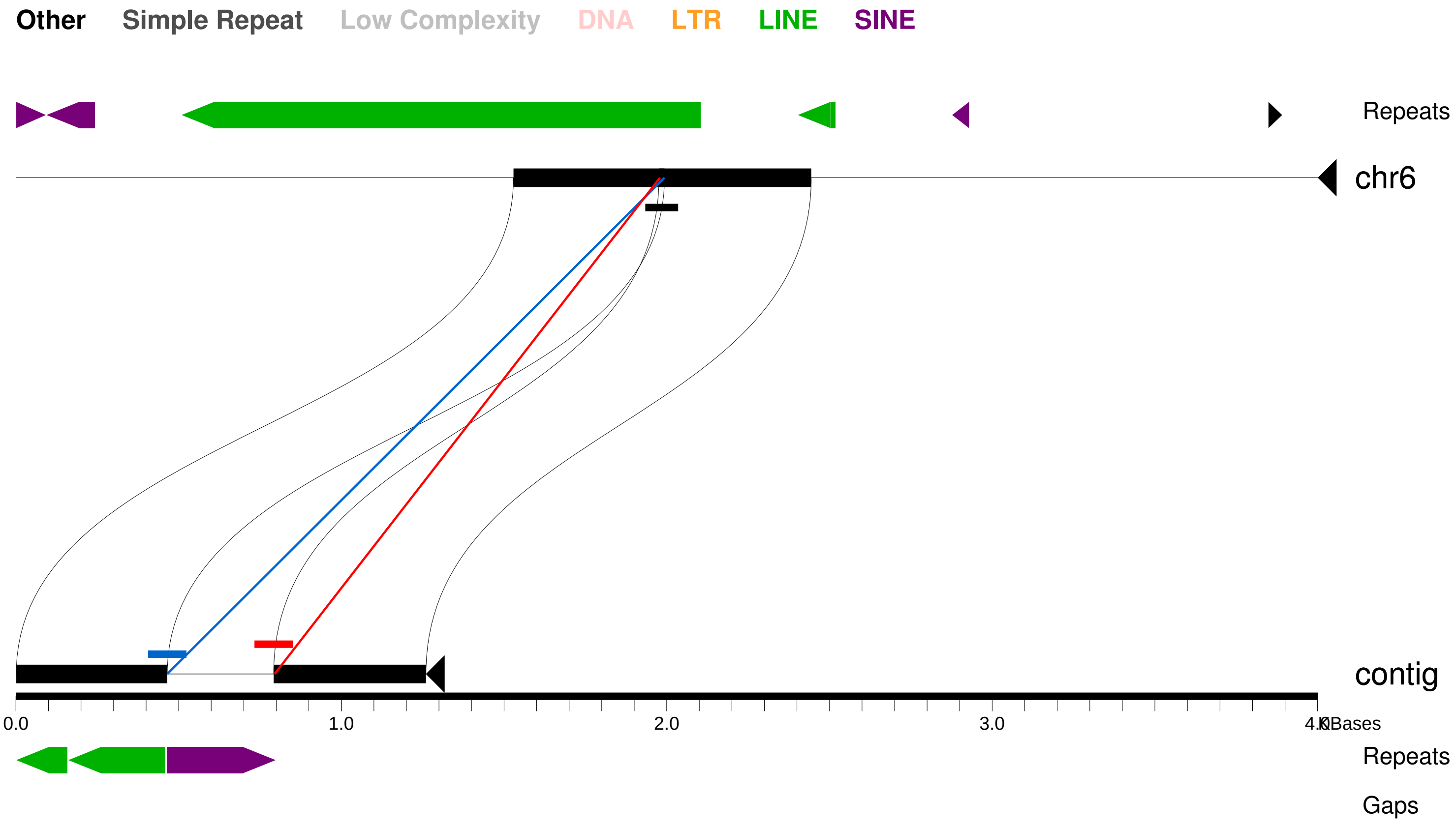

siteID: chr6_47886225 Scafftig1 + Breakpoint: chr6:47886254
chr6:47886094-47886424
Overlap Length: 11 INS size: 271 Genotyped: No

```
left    ------------AAAAAATCCAGATCAACCTGGGGAAATACAATGGTGTGGGATCCTGGACTACA-AGAC
chrom   C-TAAACTAGAAAAAAAATCCAGATCAACCTGGGGAAATACAATGGTGTGGGATCCTGGACTACA-AGAC
right   CGTGAACCCGGGAA--------GCGGAGCTTGCAGTGAGCCGAGATTGCGCCA--CTGCAGTCCGCAGTC
        212N222NN2NN**11111111*111*1*1**11*11*11*1*111**1*11*11***1*1*1*11**1*

left    AAGAACCAGGACAACAAAGTACCAAAAAAGTTGAATAAGAAATAAGTCTGAGATTGCTCTATGCCATTTT
chrom   AAGAACCAGGACAACAAAGTACCAAAAAAGTTGAATAAGAAATAAGTCTGAGATTGCTCTATGCCATTTT
right   CG--ACCTGGGCGACAGAGCGAGACTCCGTCTCAAAAAAAAAAAA-------------------------
        1111***1**1*1***1**1111*1111111*1**1**1***1**1111111111111111111111111

left    CGTTAAGGTATTCTGAGTAATTAAAAGAAAAGGTTGGG-AGGCCGAGGCGGGTGGATCA-----TGAGGT
chrom   CGTTAAGGTATTCTGAGTAATTAAAAGAAAAGGATGAGTAATGTTAGAATATTTGCACAATATTTGTAGT
right   ----AA---------AAAAAAAAAAAAAAAAAGGATGGGTAATGTTAGAAATTTTGCAAAATTTTTGTAGT
        1111**111111111*11**11****1******2**N*2*22222**22NN2*2*221*22N22**22**

left    CAGG-AGATCGAGACC-ATC---CTGGCTAACAAGGTGAAACC--CCGTCTCTACTAAAAATACAA----
chrom   TGGCTAGCTATAGGCATATTTAACTGTTCCAAATGCTGTAGCGGTCATTCATTACTGTGGATTCAATTCT
right   TGGCTAGCTATAGGCATTTTTAACTGTNNNNNNNNNNNNNNNNNNNNNNNNNNNNNNNNNNNNNNNNNNN
        22*22**2*22**2*221*2222***2NNN1N1N1N11N1N1NNN1NN11NN1111NNNN11N111NNNN

left    ---------AAAATTAGCCGGG--------CGCGGTGGCGGGCGC-----CTG
chrom   TTTCAATGGAATTTTTTCCCTTATAAAATTCTAAGTGGAAAAAAAAATCACTT
right   NNNNNNNNNNNNNNNNNNNNNNNNNNNNNNNNNNNNNNNNNNNNNNNNNNN--
        NNNNNNNNN11NN11NN11NNNNNNNNNNN1NNN1111NNNNNNNNNNNN11N
```

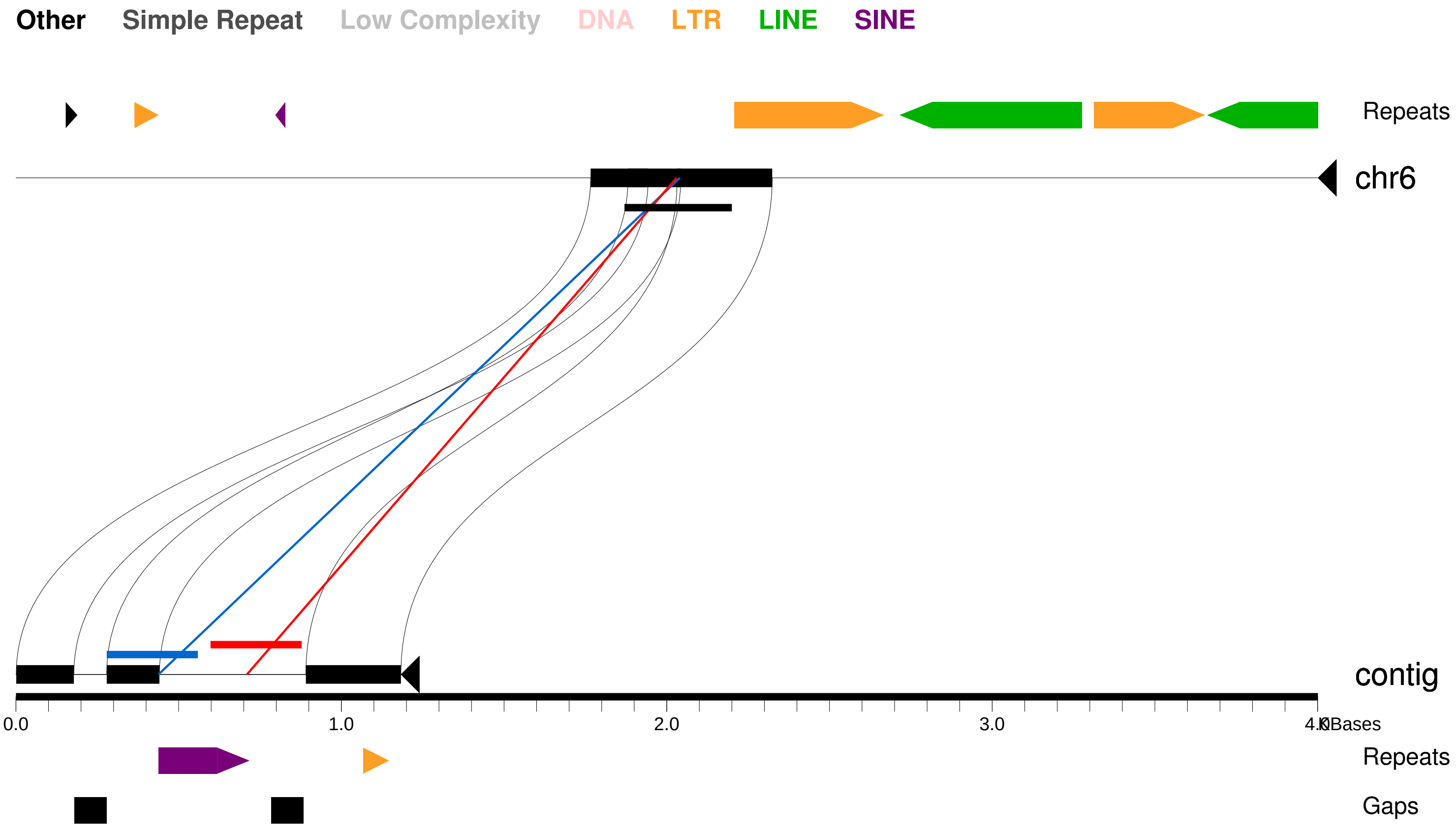

siteID: chr6_50756927 Scafftig2 + Breakpoint: chr6:50756920
chr6:50756874-50756976
Overlap Length: 14 INS size: 431 Genotyped: No

```
left    TATGCAGACCCACTACAGGATACGTCCAGAACAAACGGTTTTT----------------AATAAAAGAAA
chrom   TATGCAGACCCACTACAGGATACGTCCAGAACAAACGGTTTTT----------------AATAAAAGAAA
right   NNNNNNNNNNNNNNNNNNNNNNNNNNNNNNNNNNNNNNNNNNNNNNNNNNNNNNNNNNAAAAAAAAGAAA
        11111111111111111111111111111111111111111111111111111111111**1********

left    CAGATGGGCCGGGCGCGGTGGCTCACGCCTGTAATCCCAGCACTTTGGGAGGCCGAGGCGGGTGG
chrom   CAGATGAATGTTCAGTCTTGTTTTGGCCGCATAAT------ACTTTTGTAGC----------TTG
right   CAGATGAATGTTCAGTCTTGTTTGGGCCGCATAAT------ACTTTTGTAGC----------TTG
        ******22222222*222**22*N222*222****222222*****2*2**22222222222*2*
```

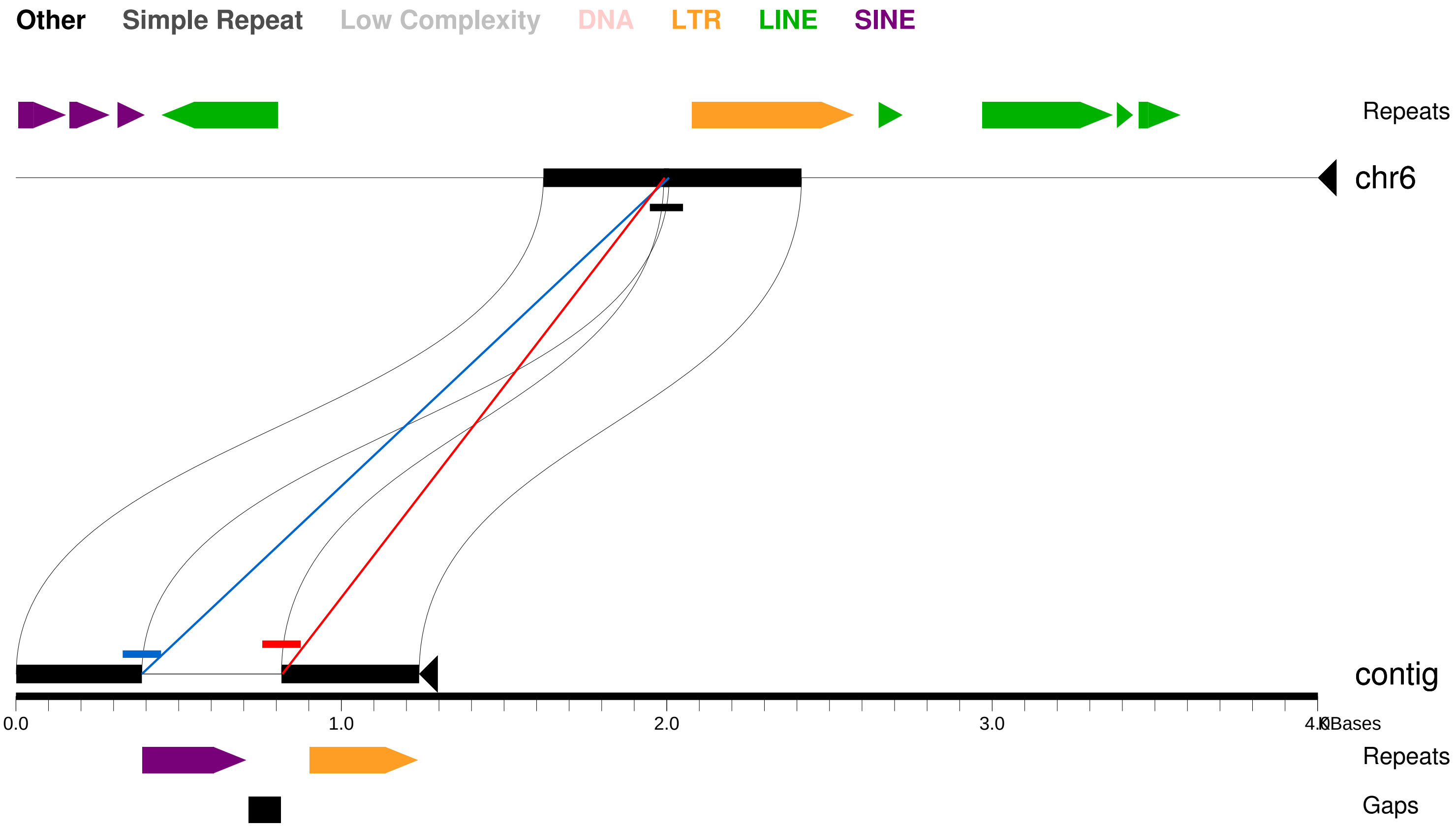

siteID: chr6_51929661 Scafftig2 - Breakpoint: chr6:51929663
chr6:51929613-51929711
Overlap Length: 10 INS size: 308 Genotyped: Yes

```
left     ----ATGGTATCATGGACCCAGGGCTC----ACTGAGTAAGCCA--GTC----------AAATAGATGAG
chrom    ----ATGGTATCATGGACCCAGGGCTC----ACTGAGTAAGCCA--GTC----------AAATAGATGAG
right    TGCCACTGCAGTCCGCAGTCCGGCCTGGGCGACAGAGCGAGACTCCGTCTCAAAAAAAAAAAAAAAAAAA
         1111*11*1*1111*1*11*1**1**11111**1***11**1*111***1111111111***1*1*11*1

left     AAAGAATTCAGGCTGGGCGCGGTGGCTCACGCCTGTAATCCCAGCACTTTGGGAGGCCGAGGCGGGTGG-
chrom    AAAGAATTCAA-----------------ACACTTTTATTTCTAC----TCGCCAGCCCATCATGATTAAA
right    AAAGAATTCAA-----------------ACACTTTTATTTCTAC----TCGCCAGCCCATCATGATTAAA
         **********222222222222222222**2*2*2**2*2*2*22222*2*22**2**22222*22*222
```

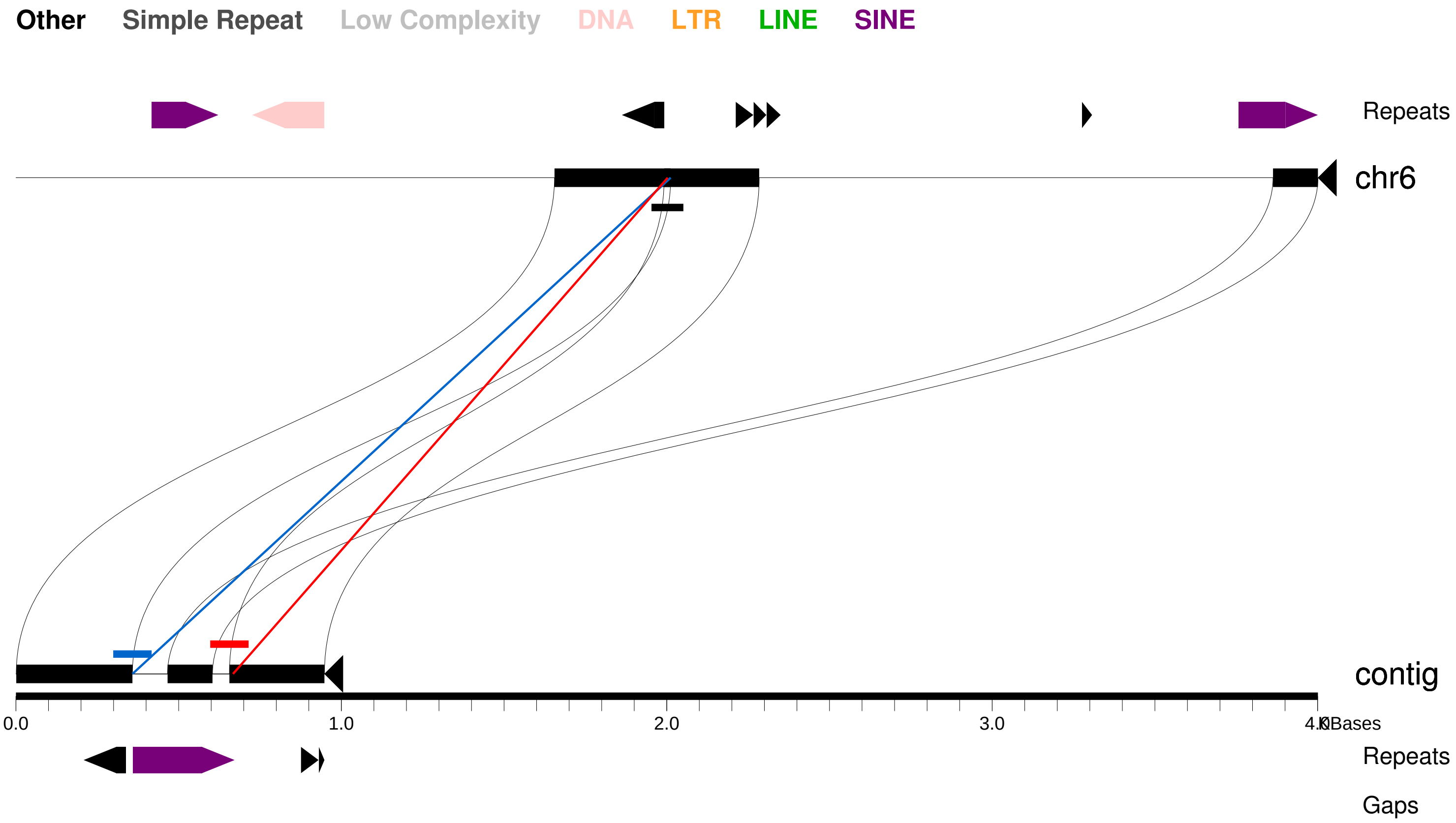

siteID: chr6_53167450 Scafftig2 - Breakpoint: chr6:53167467
chr6:53167416-53167523
Overlap Length: 9 INS size: 292 Genotyped: Yes

```
left    A--------------AGGTATCAAGATT---TTTAAAAATATACATTATAATAAAGAGTTGGTATAAGAA
chrom   A--------------AGGTATCAAGATT---TTTAAAAATATACATTATAGTAAAGAGTTGGTATAAGAA
right   ACTCCAGCCTGGGCGACAGAGCGAGACTCCGTCTCAAAAAAAAAAAAAAAAAAAAAA------AAAAAAA
        *11111111111111*111*1*1***1*111*1*1****1*1*1*11*1*N1***1*111111*1**1**

left    TTATATTTCACGCCTGT--AATCCCAGCACTTTGGGAGGCCGAGGCGGGCGGATCACGAGGTCAGGAA
chrom   TTATATTAAAGAAAAGTTTATTTCCTAAAC----CCACAACCAGGCTGTTTGCTGAC----TC-----
right   TTATATTAAAGAAAAGTTTATTTCCTAAAC----CCACAACCAGGCTGTTTGCTGAC----TC-----
        *******22*22222**22*2*2**222**222222*222*2****2*222*2*2**2222**22222
```

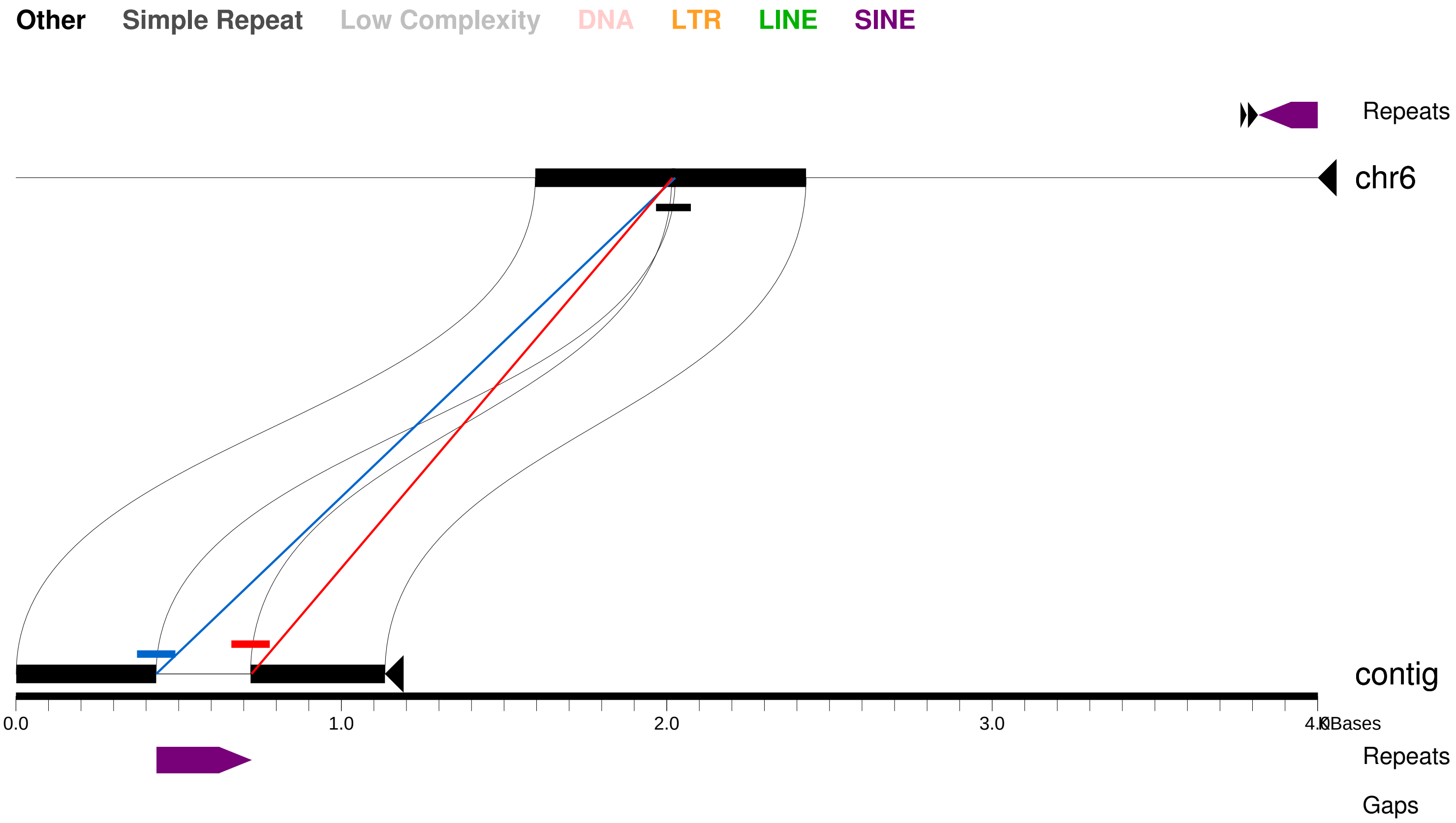

siteID: chr6_5348761 Scafftig4 + Breakpoint: chr6:5348761
chr6:5348601-5348922
Overlap Length: 2 INS size: 319 Genotyped: Yes

```
left    TGAATGGATGTATGTGTGTGCTGTGAGATAGGAGCTCTGCCTGCT-----TCTGGC--CACTCC-TTCAA
chrom   ---------------------------------------CCTGCT-----TCTGGC--CACTCC-TTCAA
right   ---------------------------------------TTTAGTAGAGATGGGGTTTCACTATGTTGGC
        222222222222222222222222222222222222222211*11*11111*11**111****111**111

left    CACAATCTCTGCTGCTGTTAC-CCTGGCTTACTGGCCATTTCCCAAATGTATATACACTCATGT-CTGTG
chrom   CACAATCTCTGCTGCTGTTAC-CCTGGCTTACTGGCCATTTCCCAAATGTATATACACTCATGT-CTGTG
right   CAGA----CTGGT-CTCAAACTCCTGACCTTGTGGTC--TGCCCACCTCAGCCTCC--TGAAGTGCTGGG
        **1*1111***1*1**111**1****1*1*11***1*11*1****11*11111*1*11*1*1**1***1*

left    --TACTTGTATGTT---GTGTTACTTCCTTACTTATTTATTTATTTATT--TTATTTATTTTTTTGAGAC
chrom   --TACTTGTATGTT---GTGTTACTTCCTTACTTATTTATTTATTTATT--TTATTTATTTTTTTGAGAC
right   ATTACAAGCGTGAGCCACTGCGCCTGGCCCACTTCTTTATTTATTGATTGCTTATTATGTGCTAGCAGCT
        11***11*11**111111**111**11*11****1**********1***11*****111*11*111**11

left    AGA----GTTTC---------GCACT----------------------GTCGCCCAGGCTGGAGT-----
chrom   AGA----GTTTCAAGTATATAGTACTAAATAAAACATAAGTAATCTGTGCCACCGTGGTTCTTGTTATGT
right   AGGATAGGTATCAAGTATATAGTACTAAATAAAACATAAGTAATCTGTGCCACCGTGGTTCTTGTTGTGT
        **11111**1**222222222*2***2222222222222222222222*2*2**22**2*222**2N222

left    -GCAATGGTG-------------------CAATCTCGGTTCACTGCA----------AGCTCTGTCT---
chrom   AGTAAGGATAGACATTAAACAAATAAACACAAATGCTCTTCTATGAATATCTGGTGAAGTTCTTTTTATC
right   AGTAAGGATAGACATTAAACAAATAAACACAAATGCTCTTCTATGAATA---------------------
        2*2**2*2*22222222222222222222***222*22***22**2*22        11N111N1N1

left    --------CCTGGGCTCATGCGATTCTC---C
chrom   CTTTAAAACTTGTCTTTACGGTATTTGGAGAC
right   --------------------------------
                1N11NNN1N1N1NN111NNN   1
```

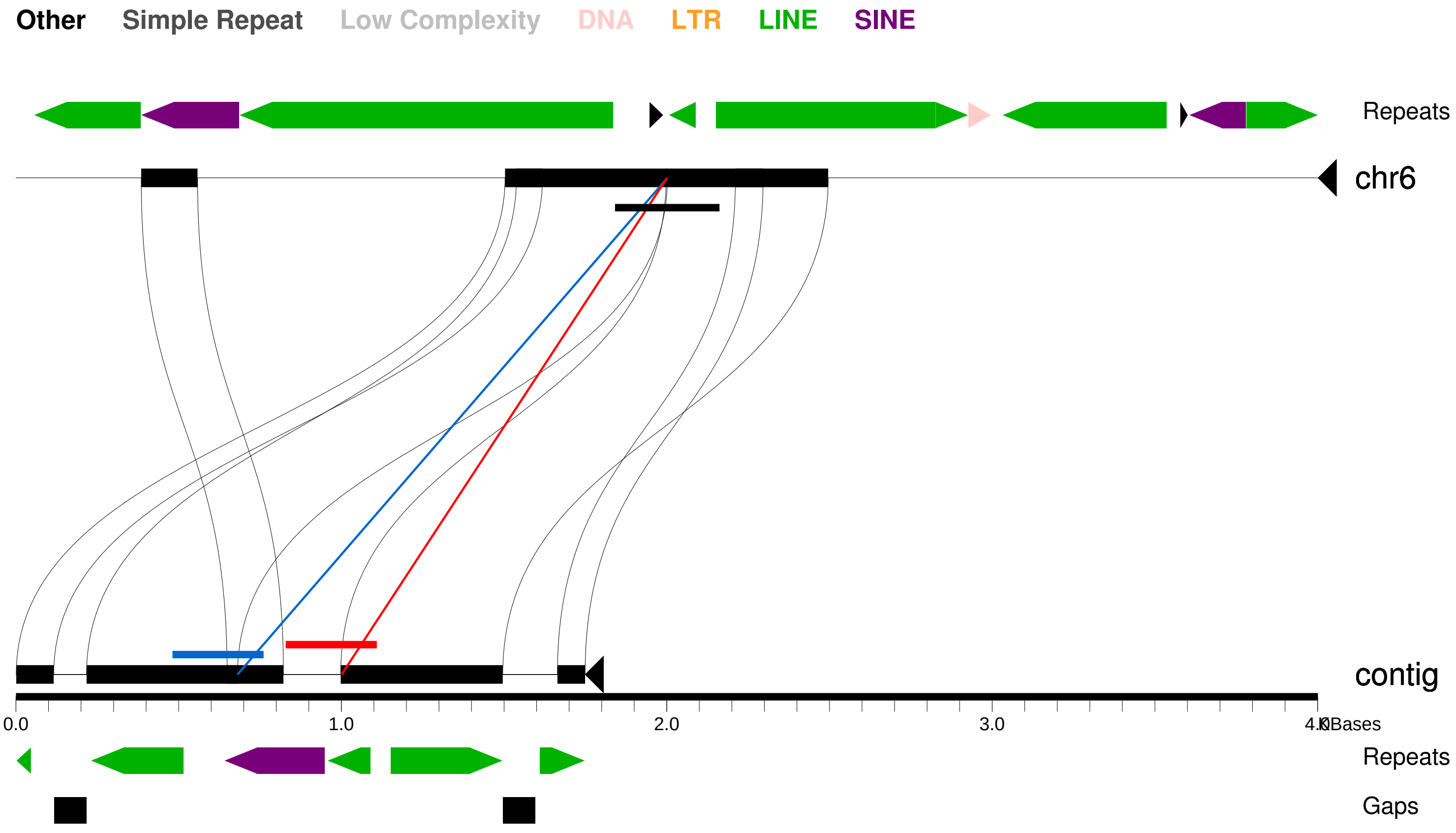

siteID: chr6_53648750 Scafftig3 - Breakpoint: chr6:53648745
chr6:53648699-53648791
Overlap Length: 14 INS size: 301 Genotyped: Yes

```
left   TAGTAT--------TAGA----AGTATTAGC--CAGA---ACAATTAGTC---------AAGAGAAGGAC
chrom  TAGTAT--------TAGA----AGTATTAGC--CAGA---ACAATTAGTC---------AAGAGAAGGAC
right  CGAGATCCCGCCACTGCACTCCAGCCTGGGCGACAGAGCGAGACTCCGTCTCAAAAAAAAAAAAAAAAAA
       1111**11111111*11*1111**11*11**11****111*1*1*11***111111111**1*1**11*1

left   ATAAAAGGCATATAAAGGCCGGGCGCGGTGGCTCACGCCTGTAATCCCAGCACTTTGGGAGGCCGAGGCG
chrom  ATAAAAGGCATATAAAT-----------TGGAATA-GCAGGAAATC-----AATTTGTT---------CT
right  AAAAAAGGCATATAAAT-----------TGGAATA-GCAGGAAATC-----AATTTGTT---------CT
       *1**************222222222222***222*2**22*2****22222*2****22222222222*2

left   GGCGG
chrom  TGTTT
right  TGTTT
       2*222
```

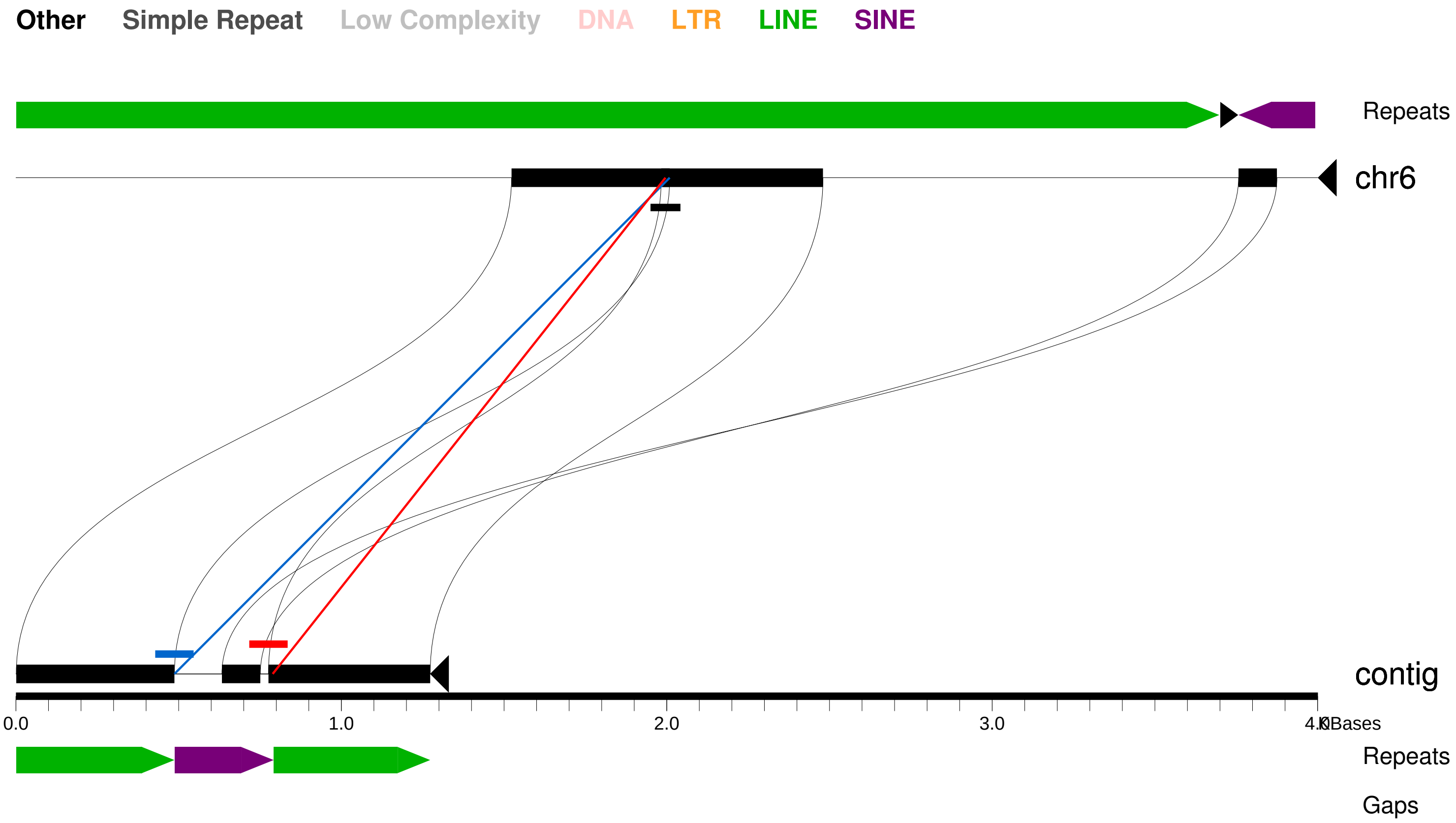

siteID: chr6_54714547 Scafftig5 - Breakpoint: chr6:54714530
chr6:54714492-54714589
Overlap Length: 22 INS size: 430 Genotyped: No

```
left    TAATTACTACCCAGTTTGTCTTTTGTTTTATTTTTACA---------------------TTGTCCTTAAG
chrom   TAATTACTACCCAGTTTGTCTTTTGTTTTATTTTTACA---------------------TTGTCCTTAAG
right   NNNNNNNNNNNNNNNNNNNNNNNNNNNNNNNNNNNNNNNNNNNNNNNNNNNNNNNNNNNNNTTGTCCTTAAG
        11111111111111111111111111111111111111111111111111111111111***********

left    AGTGGGGTGAGGCCGGGCGCGGTGGCTCACGCCTGTAATCCCAGCACTTTGGGAGGCCGAGGCGGGTGGA
chrom   AGTGGGGTGAGAAGTCCCCTAAGGGCACATGACATTTATC-------TTTT-----------CAGCT---
right   AGTGGGGTGAGAAGTCCCCTAAGGGCACATGACATTTATC-------TTTT-----------CAGCT---
        ***********222222*22222***2**2*2*22*2***2222222***222222222222*2*2*222
```

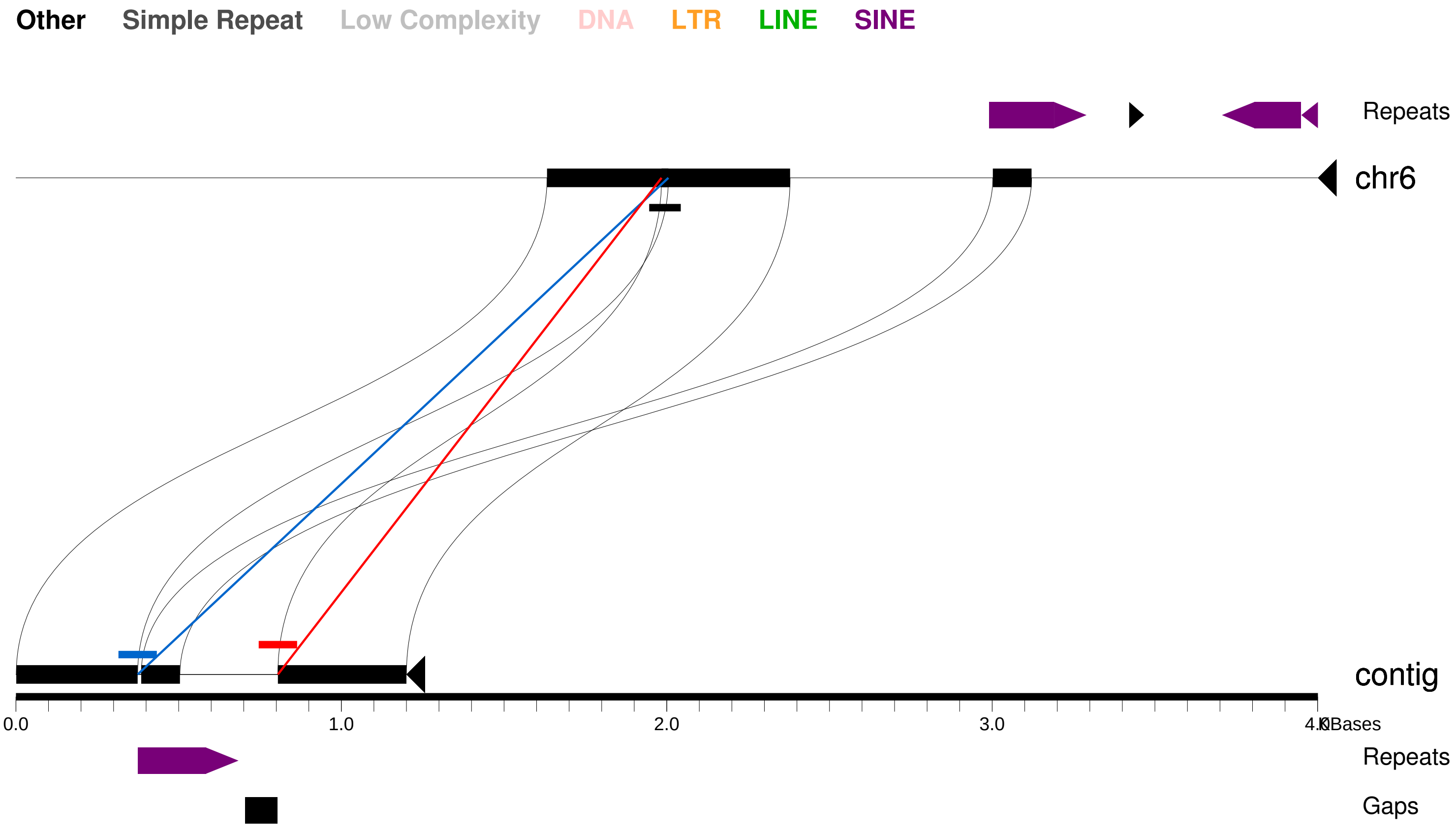

siteID: chr6_5488864 Scafftig2 - Breakpoint: chr6:5488873
chr6:5488816-5488932
Overlap Length: 3 INS size: 121 Genotyped: Yes

```
left   TTT--TTGTTTATTTGCTGTAGATCTCTTTTGTGAAAGAGAATGGGTTTAATGGAGCATAAGGGAGAATG
chrom  TTT--TTGTTTATTTGCTGTAGATCTCTTTTGTGAAAGAGAATGGGTTTAATGGAGCATAAG--AGTTTA
right  CGCAGTCCGGCCTGGGCGACAGAGCGAGACTCCGTCTCAAAAAAAAAAAAAAAAAAAAAAG--AGTTTA
       11111*111111*11**111***1*11111*11*1111*1**1111111**111*11*1***22**22*2

left   GCGTGAACCCGG-GAAGCGGAGCTTGCAGTGAGCCGAGATTGCGCCACTGCA
chrom  GTTGGGACTTGTTGAATTTGAGGTGCCTCTTAGAA-ATCTCGTGTCTTTGCA
right  GTTGGGACTTGTTGAATTTGAGGTGCCTCTTAGAA-ATCTCGTGTCTTTGCA
       *222*2**22*22***222***2*22*22*2**222*22*2*2*2*22****
```

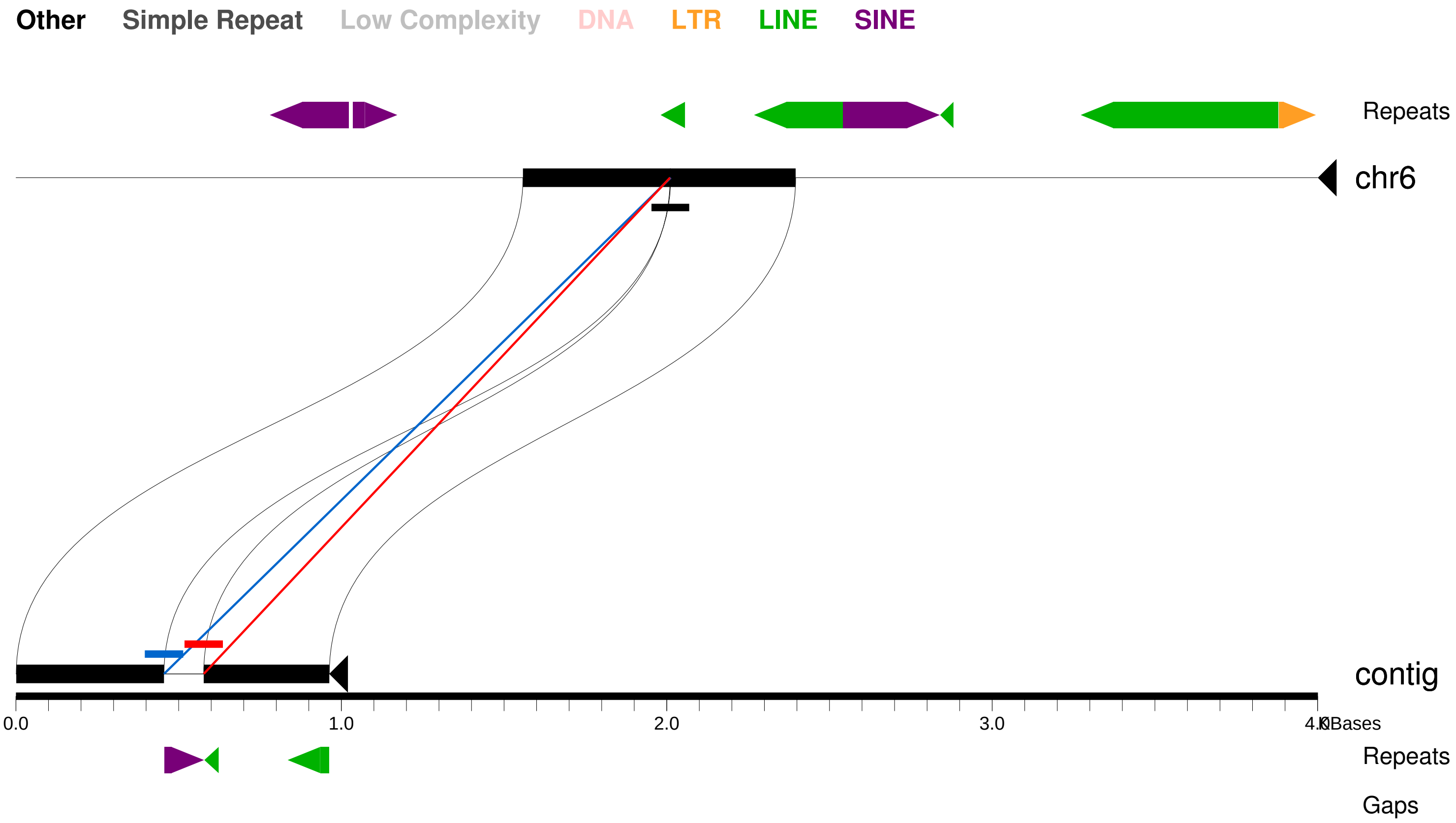

siteID: chr6_55854923 Scafftig2 - Breakpoint: chr6:55854902
chr6:55854882-55854961
Overlap Length: 40 INS size: 355 Genotyped: No

```
left    TGTC---ATG-------TCTTCT----TTTTCAT-------------------------CTCTGATTTTA
chrom   TGTC---ATG-------TCTTCT----TTTTCAT-------------------------CTCTGATTTTA
right   AGCCGGGATGGTCTCGATCTCCTGACCTCGTGATCCGCCCGCCTCGGCCTCCCAAAGTGCTCTGATTTTA
        1*1*111***1111111***1**1111*11*1**1111111111111111111111111***********

left    TTTATTTAGGTATTCTCTCTTTTTGTCTTNNNNNNNNNNNNNNNNNNNNNNNNNNNNNNNNNNNNNNNNN
chrom   TTTATTTAGGTATTCTCTCTTTTTGTCTTAACTAGTCTAGCTAAGGGTT---------------------
right   TTTATTTAGGTATTCTCTCTTTTTGTCTTAACTAGTCTAGCTAAGGGTT---------------------
        *****************************2222222222222222222222222222222222222222

left    NNNNNNNNNNNNNNNNNN
chrom   ------------------
right   ------------------
        222222222222222222
```

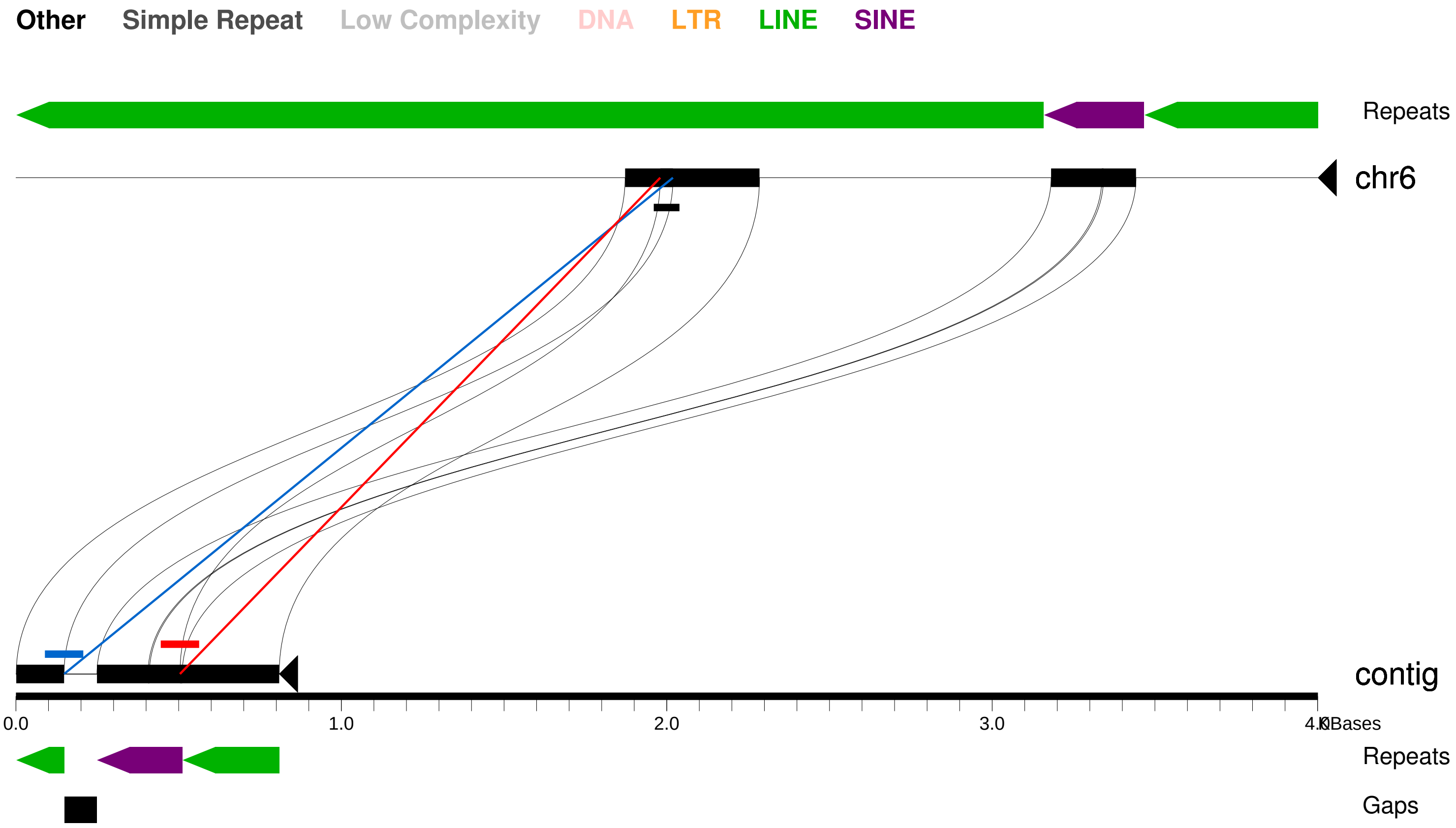

siteID: chr6_55894486 Scafftig2 - Breakpoint: chr6:55894495
chr6:55894445-55894554
Overlap Length: 10 INS size: 295 Genotyped: Yes

```
left    GAAGCATGGTGCAGACCAGGA--AGTGAAA---TGGGACAGTG------ATAACAGTTTATAAAAATTAT
chrom   GAAGCATGGTGCAGACCAGGA--GGTGAAA---TGGGACAGTG------ATAACAGTTTATAAAAATTAT
right   GCGCCAC--TGCAGTCCAGCTTGGGCGAAAGAGTGAGACTCCGTCTCAAAAAAAAAAAAAAAAAAATTAT
        *111**111*****1****11112*1****111**1***111*111111*1**1*1111*1*********

left    AGGCCGGGCGCGGTGGCTCACGCCTGTAATCCCAGCACTTTGGGAGGCCGAGGCGGGCGG
chrom   AAA----GAACTTTAAATATCTTTT-TAA----AAAACCTGAGCAATAGGAGTTTGCTAA
right   AAA----GAACTTTAAATATCTTTT-TAA----AAAACTTGAGCAATAGGAGTTTGCTAA
        *222222*22*22*222*22*222*2***2222*22**N*22*2*2222***222*2222
```

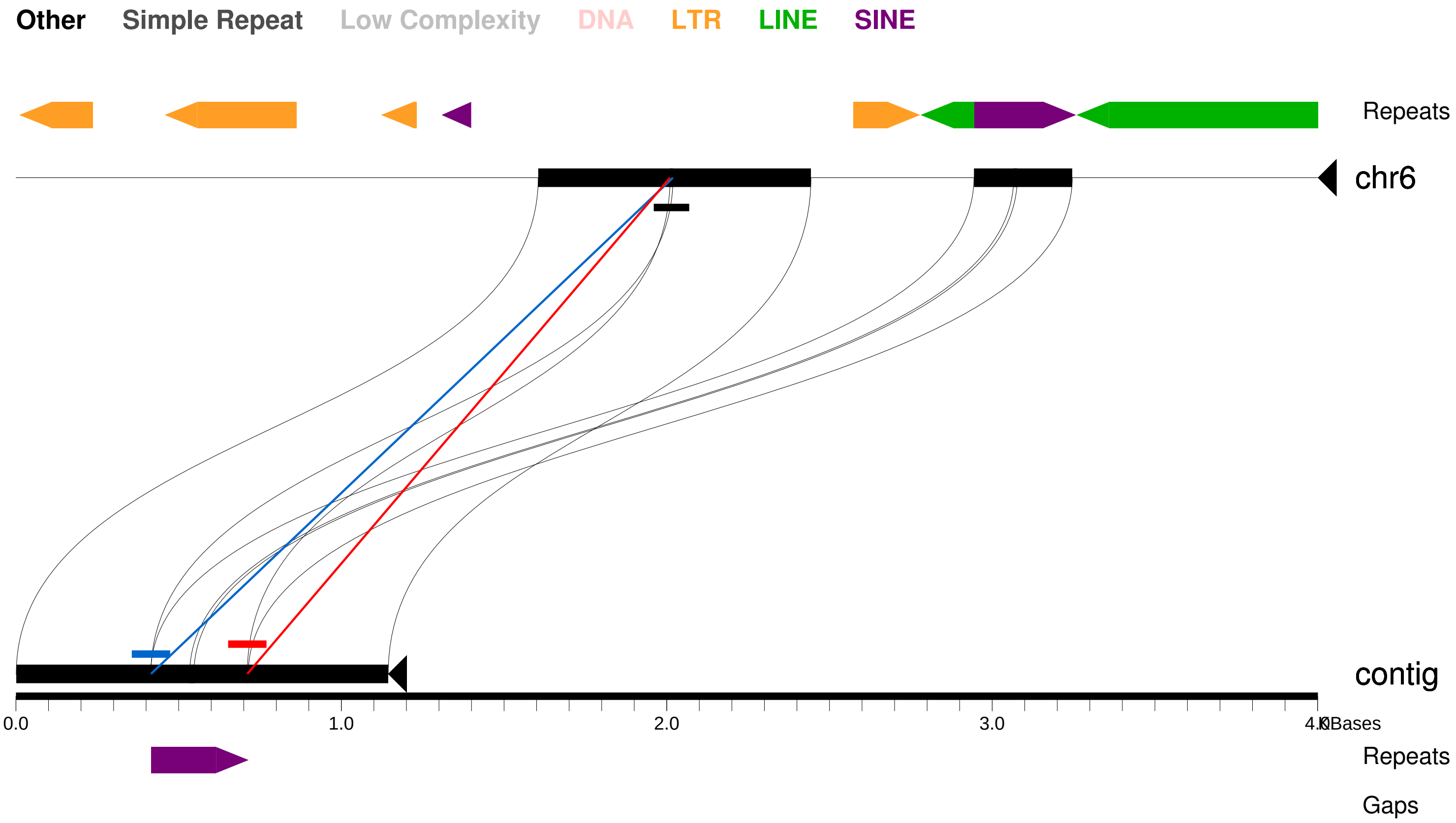

siteID: chr6_56525803 Scafftig1 + Breakpoint: chr6:56525786
chr6:56525626-56525957
Overlap Length: 12 INS size: 473 Genotyped: No

```
left   TA------------CAAAGTGACTCTCCATTAACAACAAGGTAGACAGCAAAGATGAGAAACAGTATATT
chrom  TATATTTTTTTCTACAAAGTGACTCTCCATTAACAACAAGGTAGACAGCAAAGATGAGAAACAGTATATT
right  NNNNNNNNNNNA----------------------------------------------------------
       11NNNNNNNNNN  11111111111111111111111111111111111111111111111111111111

left   TATAGCAAGCTTAGTTAGTACTTCAAGGTTAGAGTCTAACAAATTCAATAATTAAAACATATCTAATGAA
chrom  TATAGCAAGCTTAGTTAGTACTTCAAGGTTAGAGTCTAACAAATTCAATAATTAAAACATATCTAATGAA
right  ----------------------------------------------------------------------
       1111111111111111111111111111111111111111111111111111111111111111111111

left   TTATGACTTTCTCACTTCACAAAGAACAATCAGGCCG-------GGCGCGGTG-----GCTCAC---GCC
chrom  TTATGACTTTCTCACTTCACAAAGAACAATCAATCCCATTAACAGCCTAAATTTTAATGAATACAGAGAA
right  --------------------AAAGAACAATCAATCCCATTAACAGCCTAAATTTTAATGAATACAGAGAA
       11111111111111111111************22**22222222*2*2222*222222*222**222*22

left   TGTAATCCCAGCACTTTGGGAGG---CCGAGGCGG-------------GTGGATCATGAGGTCAGGAGAT
chrom  TGGTATTGATGTTGATAGAGAAGTAACTCCTCCAGTACCTATATTTTAGTGAAT-AAAAGGAAAATTTAA
right  TGGTATTGATGTTGATAGAGAAGTAACTCCTC--------------------------------------
       **22**2222*2222*2*2**2*222*222221N1             111N1121NN111NN1NNNN1N

left   CGAGACCATCCTGGCTAACAAGGTGAAACCCCGTCTCTACTAAA---------
chrom  GTAAACTTTAGTGACTAATAAGGATAAATACACCCTCCCCAAAAAGGGGGTAG
right  -----------------------------------------------------
       NN1N11NN1NN11N1111N1111NN111NN1NNN111NN1N111
```

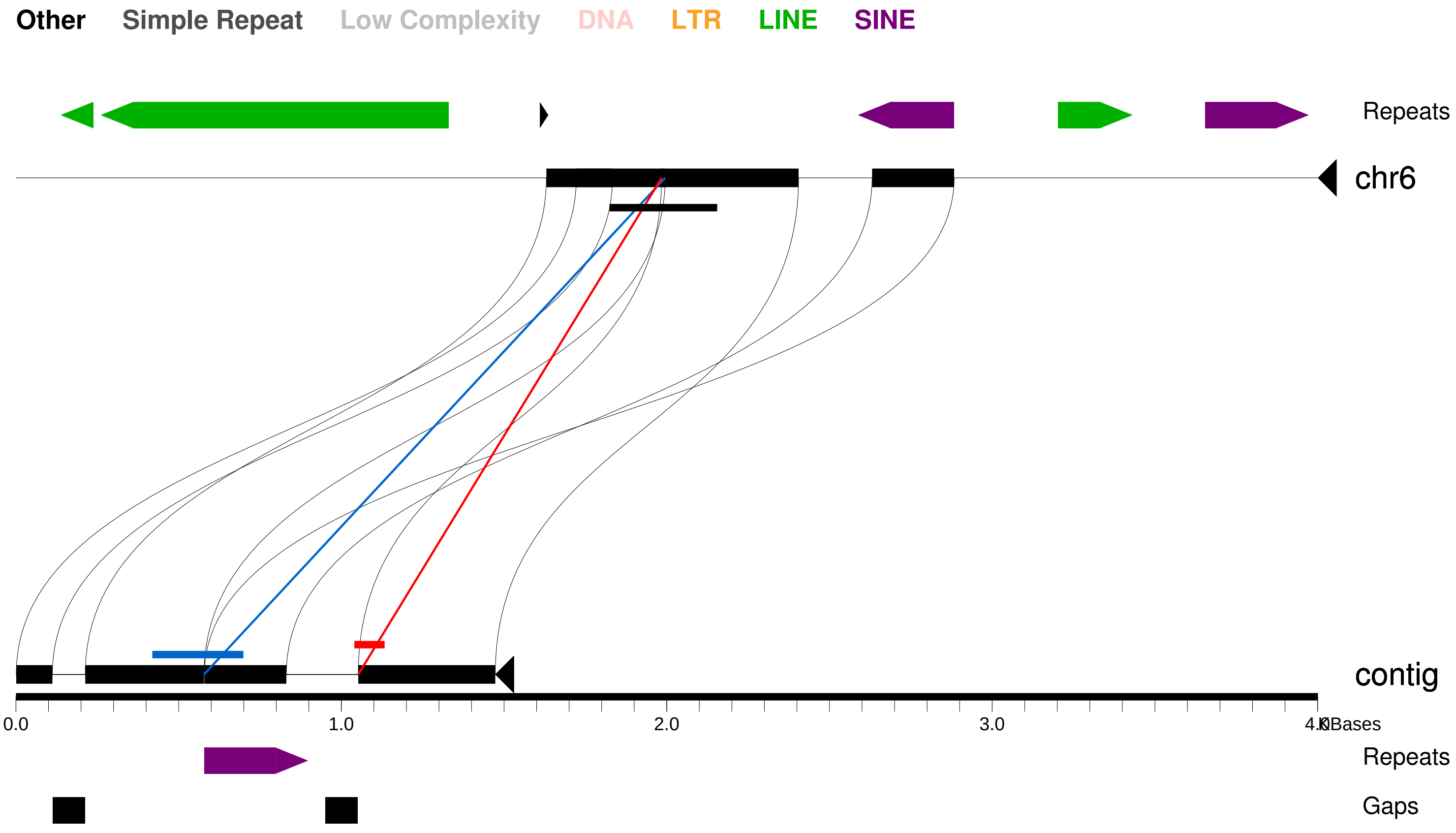

siteID: chr6_57267491 Scafftig1 - Breakpoint: chr6:57267473
chr6:57267313-57267643
Overlap Length: 11 INS size: 296 Genotyped: Yes

```
left    ----------AGAGTTGATACTGGCTTTAAATGATTTGGGTAAATGGAGATGTGCAGCTTACAGAGAGTA
chrom   GAGAAAAGATAGAGTTGATACTGGCTTTAAATGATTTGGGTAAATGGAGATGTGCAGCTTACAGAGAGTA
right   TG----AGGCAG----GAGAATGGCGTGAACCC-------------GGGAGGCGGAGCTTGCAGTGAGCC
        NN    22NN**1111**1*1****1*1**1111111111111111*1**1*1*1*****1***1***11

left    GTAGATGTAAATGGTTAGGTGCTGGAAAGCATAACTATGTTTGGGGAATGGTGTTAGTGGTATGATTGGT
chrom   GTAGATGTAAATGGTTAGGTGCTGGAAAGTATAACTATGTTTGGGGAATGGTGTTAGTGGTATGATTGGT
right   G-AGAT---------------CCCGCCACTGCACTCCAGCCTGGGCGACAGAGCGAGAC------TCCGT
        *1****111111111111111*11*11*1211*11111*11****11*11*1*11**11111111*11**

left    ATGATTTAATGTCTATGAATAAGAATAGCAGGCCGGGCGCGGTGGCTCACGCCTGTAATCCCA-GC----
chrom   ATGGTTTAATGTCTATGAATAAGAATAGCAGAA-GATCAAATTGTCAAA-GTAAGTATTACCAAGCTTAT
right   CTCAAAAAAAAAAAAAAAA-AAGAATAGCAGAA-GATCAAATTGTCAAA-GTAAGTATTACCAAGCTTAT
        1*1N111**11111*11**1**********222*22*2222**2*22*2*222***2*2***2**2222

left    -ACTTTGGG----AGGCCGAG-GCGGGCGGATC----AC----GAG---GTCAGGAGATCGAGACCAT--
chrom   GAGTTTGAATTTTAGTGTGTGTGCTGGTGGTTTCTAAACTTTTGATTGTGTCCCCTCAGTTAAAATATTA
right   GACTTTGAATTTTAGTGTGTGTGCTGGTGGTTTCCAAACTTTTGATTGTGTCCCCTCAGTTAAAATATTA
        2*N****222222**222*2*2**2**2**2*22N22**2222**2222***22222*222*2*22**22

left    -CCCGGCTAAAAC-----GG-----TGAAACCCCGTCTCTACTAA-------A
chrom   CCCCTGCCCACAGATGTAGGCAATATAAAATGGGGGTTTTAAAAAGGAGAAAA
right   CCCCTGCCCACAGATGTAGGCAATATAAAATGGGGGTTTTAAAAGG------A
        2***2**22*2*222222**22222*2***2222*22*2**22*12      *
```

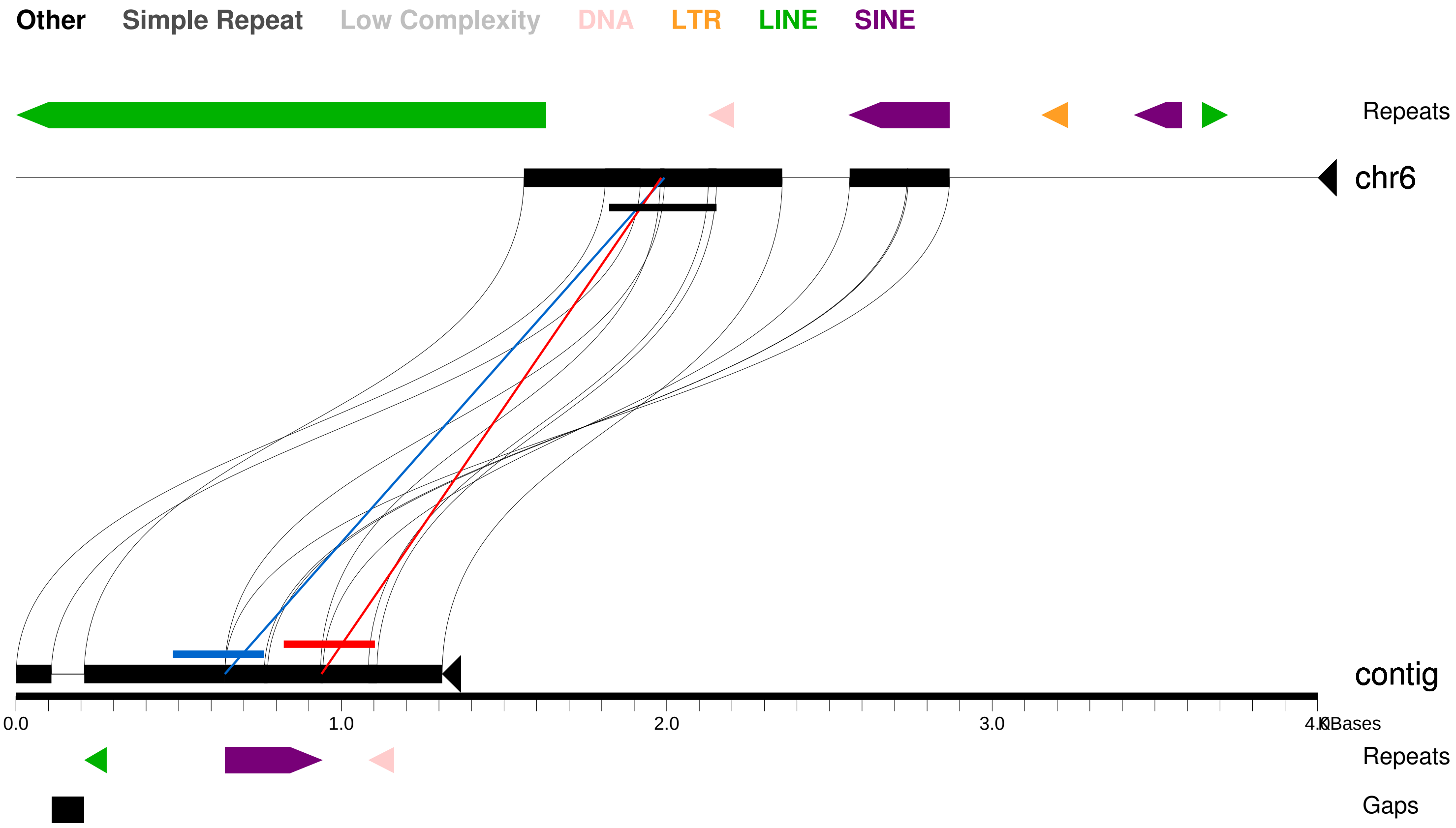

siteID: chr6_57403550 Scafftig14 + Breakpoint: chr6:57403535
chr6:57403375-57403706
Overlap Length: 12 INS size: 291 Genotyped: Yes

```
left   ----ATCGTTTACATGTTTTTTCAATGACATGTAATAAACTAAGGCATACTAAATTACAATATTCTCTAA
chrom  CTATATCATTTAAATGTTTTTTCAATGACATGTAATAAACTAAGGCATACTAAATTACAATATTCTCTAA
right  TT---TAGTAGAGACGGGGTTTCACCG---TGT--TAGCC--AGG--------------ATGGTCTC---
       N2  1*1N*11*N*1*111*****11*111***11**11*11***11111111111111**11****111

left   TTGAACCATTGTATCTATTTGTTAAGTGATACATACAATTAAAAATATTCTTAAGAGGTATAAAAATTGT
chrom  TTGAACCATTGTATCTATTTGTTAAGTGATACATACAATTAAAAATATTCTTAAGAGGTATAAAAATTGT
right  --GATCTCCTG-ACCTC--------GTGATCCGCCCGCCTCGGCCTC--CCAAAGTGCTGGGA---TTAC
       11**1*111**1*1**111111111*****1*111*111*11111*111*11***1*1*111*111**11

left   TGGCTTAAAATACTGCTTAT----AGGATTTTTTTT-------TTTT---------------TTTTTTTG
chrom  TGGCTTAAAATACTGCTTAT----AGGATATTTTTTAGGATGATTATAGAAGATGTTAAACATTTTTTAT
right  AGGCGTGAGCCACCGCGCCCGGCCAGGATATTTTTTAGGATGATTATAGAAGATGTTAAACATTTTTTAT
       1***1*1*111**1**11111111*****2******2222222**2*222222222222222******22

left   AG---------ACGGAGTCTCGCTGTCG--CCCAGGCTGGAGTGCAGTGGCGCAATCTCGGCTCACTG--
chrom  AGTGCTAATGAAAGGACT-TGGGTGTTGAACCCAG-CTCGAATGATTTGAAATAACAGCTTCTGTTTGGA
right  AGTGCTAATTAAAGGACT-TGGGTGTTGAACCCAG-CTCGAATGATTTGAAATAACAGCTTCTGTTTGGA
       **2222222N2*2***2*2*2*2***2*22*****2**2**2**222**2222**222*22**222**22

left   CAGGCTCCGCCCC--CTGGGCT-------TCACGC-----CATTCTCCTGCCTCAGCC
chrom  CAGCTTATGTCCTAACAGTGTAAGAAAAATGATGTGGTAACAGTCAATTTCAACTGGA
right  CAGCTTATGTCCTAACAGTGTAAGAAAAATGATGTGGTAACAGTCA------------
       ***22*22*2**222*2*2*222222222*2*2*222222**2**2NN1N1NN1N1NN
```

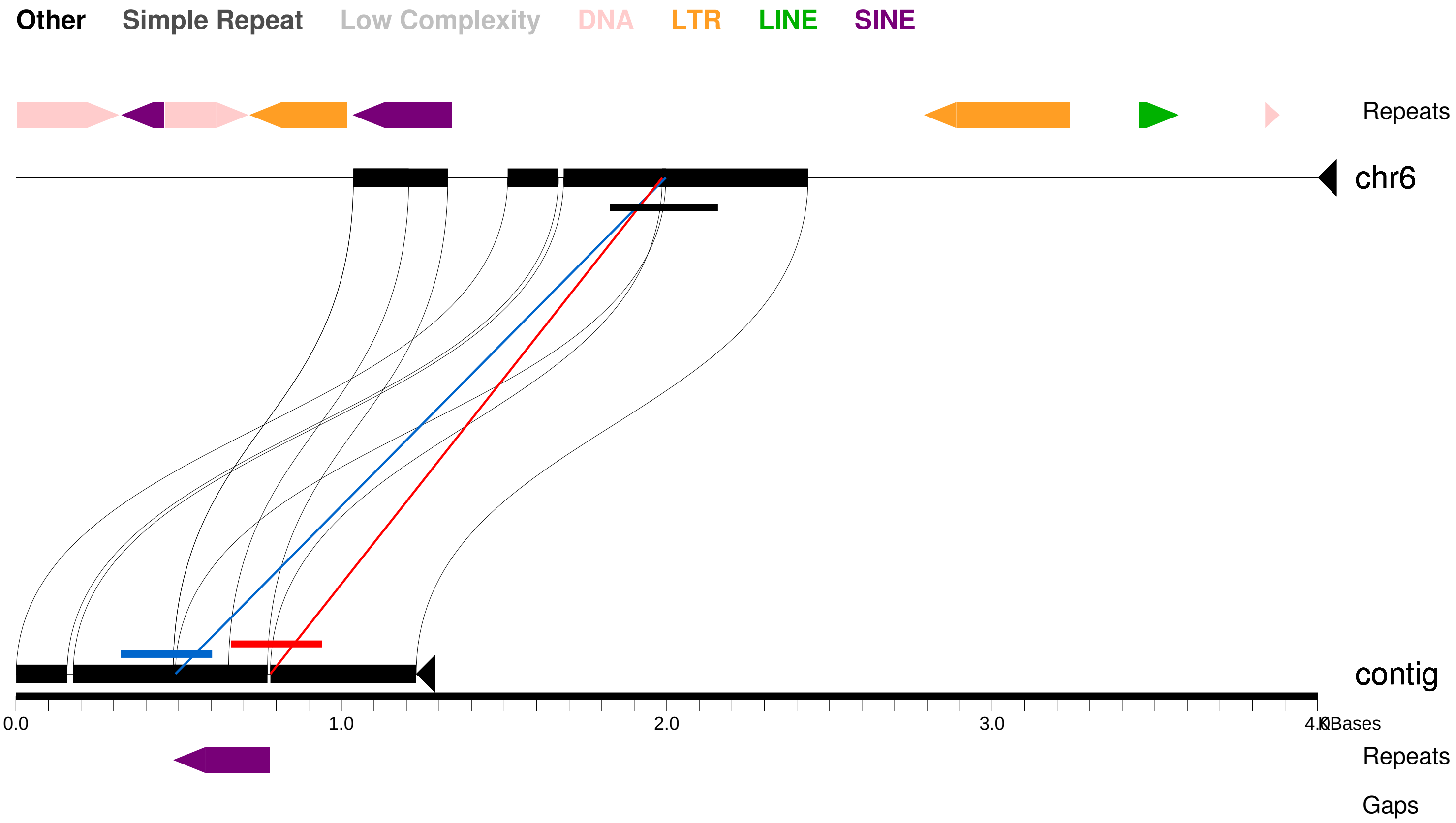

siteID: chr6_57444953 Scafftig15 + Breakpoint: chr6:57444959
chr6:57444918-57445018
Overlap Length: 19 INS size: 305 Genotyped: Yes

```
left     AC-------TGGG--------AGCTCT-----TTCAGATTGGCTCCTGTGCTTTCTTGACACACCTCCTT
chrom    AC-------TGGG--------AGCTCT-----TTCAGGTTGGTTCCTGTGCTTTTTTGACACACCTCCTT
right    CCGCCCGCCTCGGCCTCCCAAAGTGCTGGGATTACAGGCGTGAGCCACCGCGCCC--GGCCCACCTCCTT
         1*1111111*1**11111111**11**11111*1***2111*N1**111**111N11*1*1*********

left     CCTTCTTTTGTTTTTTGTTTTTTTTTTTTTTTTTTGAGACGGAGTCTCGCTCTGTCGCCCAGGCTGGAGT
chrom    CCTTCTTTTGA-----GCACATCCTC------------CCTAGCACCACAAGATGCTTCAGACTC-ATC
right    CCTTCTTTTGA-----GCACATCCTC------------CCTAGCACCACAAGATGCTTCAGACTC-ATC
         **********222222*2222*22*2222222222222*22**222*2*2222*2222***2**22*22
```

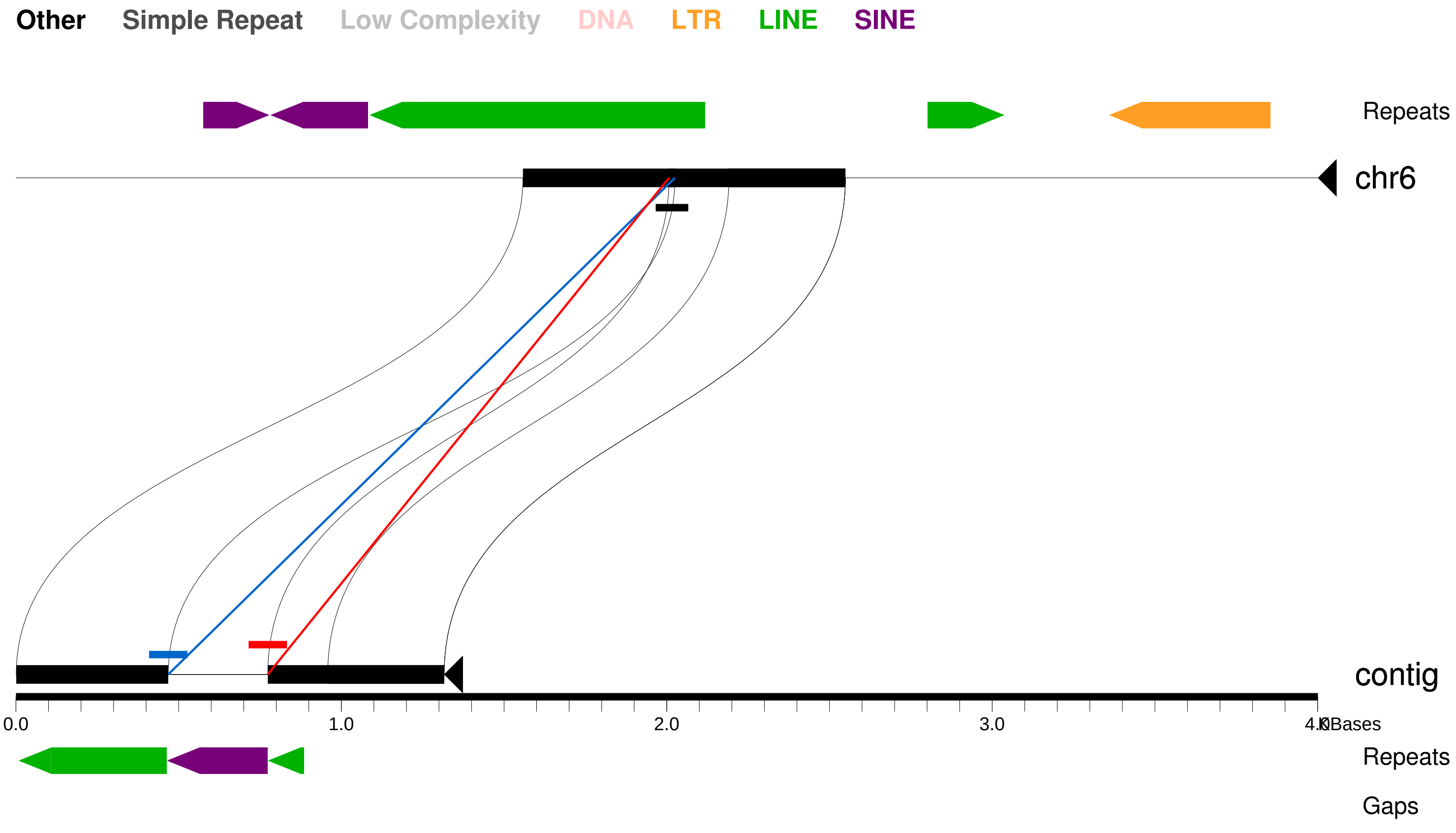

siteID: chr6_57509289 Scafftig2 - Breakpoint: chr6:57509298
chr6:57508737-57509458
Overlap Length: 17 INS size: 301 Genotyped: Yes

```
left   ------------------------------------------------------------------------
chrom  GCAATCCCATTACTGGGTTGTACCCAAAGGATTATACATCATGCTACTCTAAAGACACATGCACACGTAT
right  ------------------------------------------------------------------------

left   ------------------------------------------------------------------------
chrom  GTTTATTGTGGCATTGTTCACAATAGCAAAGACTTGGAACCAACCCAAATGCCCACCAATGATAGACTGG
right  -TTTAGT-------------------------------------------------------AGAGACGGG
        2222N2                                                      2N2222N22

left   ------------------------------------------------------------------------
chrom  GTAAAGAATATGTGGCACATATACACCATGGAATACTATGCAGCCATTAAAAAGGATGAGTTCATGTCCT
right  GT--------------------TTCACCGTG----------------TTAGCCAGGATG-------GTC--
       22                    2N2222N22                222NNN222222       222

left   ------------------------------------------------------------------------
chrom  TTGCAGGGACATGGAAGAAGCTGGAAACCATCATTCTCAGCAAACTAACACAAGAACAGAAACCAAACAC
right  ------------------------------TCAATCTC------CTGAC----------------------
                                     222N2222      22N22

left   ------------------------------------------------------------------------
chrom  TGCATGTTCTCACTCATAAGTGGGAGTTGAACAATGAGAACACATGGACACAGGGAGGGAAATATCACAC
right  --------CTC--------GTG-----------------------------------------ATCC---
               222        222                                          222N

left   -------------------------------------------------------------------AGAT
chrom  ACCAGGGCCTGTTTTGGGGGGGTGGGGTGCTAGGGGAGAGATGGCATTAGTAGAAGTCCCTAATGTAAAT
right  ------GCCCGTCTCGGC-----------CTCCCAAAGTGCTGGGATTA---------------------
             222N22N2N22N           22NNNN22N2N222N2222                   1N11

left   AACAGGTTGGTGGGCGCAGCAAACCACCATGGCATGTGTATGCCTATGTAACCTGCACGTTCTGCACATG
chrom  AACAGGTTGGTGGGCACAGCAAACCACCATGGCATGTGTATACCTATGTAACCTGCATTTTCTGCACATG
right  --CAGGC--GTGAGC---------CACCACGCC----------------------------CAGCCC---
       11****111***1**N11111111*****1*1*11111111N111111111111111NN11*1**1*111

left   GACTCCAGAACTTTAAGTATAATTTAAAAAATATTAATTAAAATGAGGATTCATTAACGATATGCACATA
chrom  GACTCCAGAACTTTAAGTATAATTTTAAAAATATTAATTAAAATGAGGATTCATTAATGATATGCACATA
right  ------------------------------------------------------------------------
       1111111111111111111111111N111111111111111111111111111111111N111111111111

left   CCTTAAATATTATATCTTTTTTTTT------TTT--------TTTTTTTTTTTGAGACGGAGTTTCGCTCT
chrom  CCTTAAATATTATATCTTAAAGTTAAGATATTTAAATGCCATTTATTAGTTAGAGCAGGGTCTTTTGAGT
right  -CTTAAATATTATATCTTAAAGTTAAGTCATTTAAATGCCATTTATTAGTTAGGGCAGGGTCTTTTGAGT
       1****************2222**222NN2***22222222***2**22**2*1*22**222**22222*

left   GTCGCCCAGGCT---GGAGTGCAGTGGCG[illegible]ATCTC[illegible]--[illegible]ACTCA[illegible]CAAGCTCCGCCTCCCGGGTT
chrom  ATCGTTTAGTGTTTGGAATTTGATTTTATCTTTCTTTTATAAACTATACCCATAATACAGCATCAGCAAT
right  ATCGTTTAGTGTTTGGAATTTGATTTTATCTTTCTTTTATAAACTATACCCATAATACAGCATCAGCAAT
       2***222**22*222*2*2*22*2*2222*22***2222222***22222**222*2*22*22*2*222*

left   [illegible]
chrom  TTCCAGTTTGGATTCCTTAAAG
right  TTCCAGTTTGGATTCCTTAA--
       22*2222**2222****222NN
```

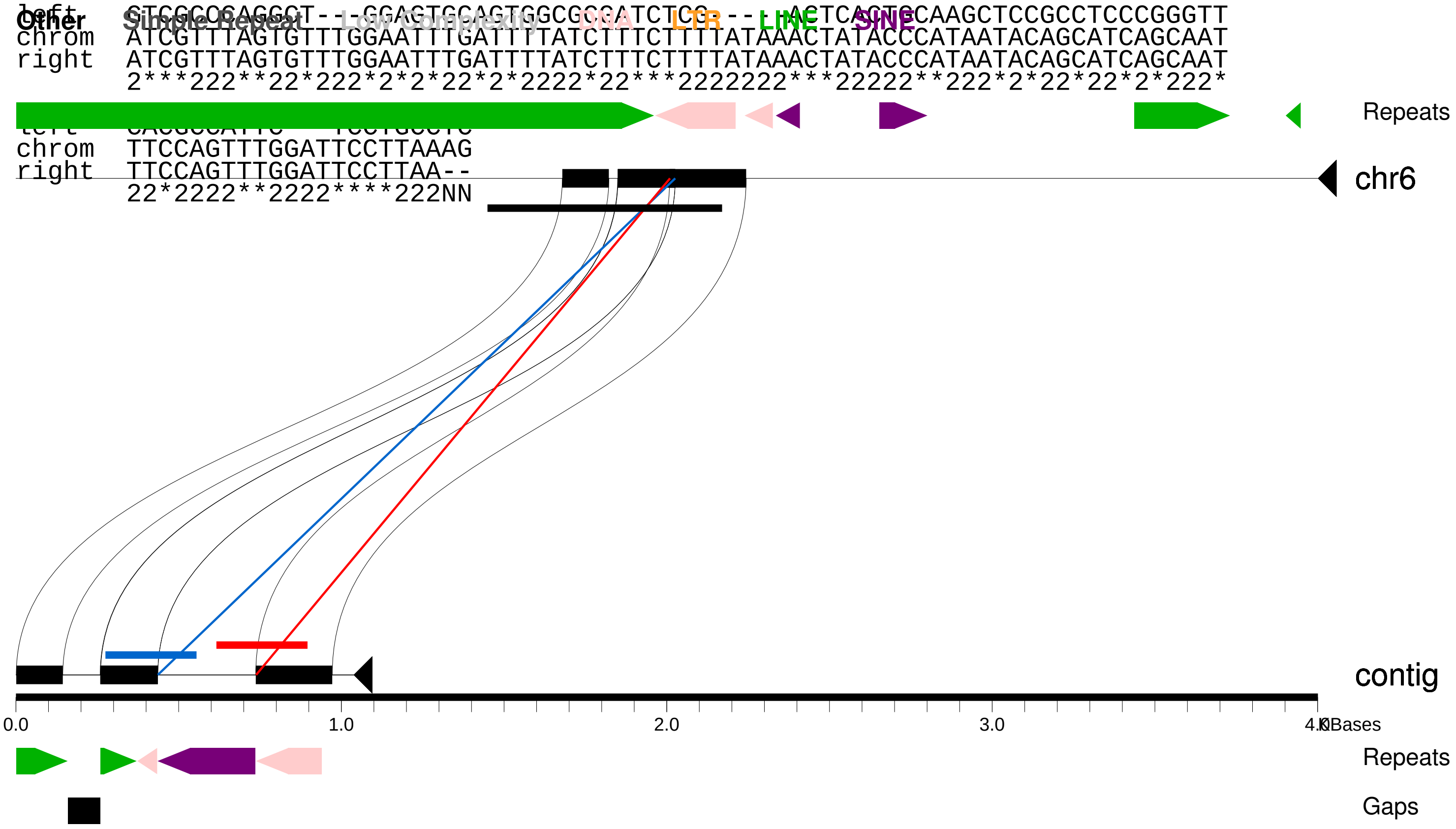

siteID: chr6_58146695 Scafftig5 + Breakpoint: chr6:58146658
chr6:58146498-58146823
Overlap Length: 1 INS size: 351 Genotyped: Yes

```
left   --------------------------------------CCCAGTACTGCCTAAAACAAGGGTGAGTCAGAG
chrom  TAAACTTGCTGAGCAAAGAGCCGTACCTCTGTGGAGTCCCAGTACTGCCTAAAACAAGGGTGAGTCAGAG
right  TTTAGT----------AGAGACGGAGTTTCGCCGTGT-----TA--GCC----AGGATGGTC--TCGGTC
       2NN2N2          2222N22N2NN2NN2NN2N2211111**11***1111*11*1***111**1*11

left   ACCAGAATTTAAAGGGTTTGGGGACTGGAGCTATTCATAGGACACTTTTAGGGGGAAAGTTAGTAAGGTC
chrom  ACCAGAATTTAAAGGGTTTGGGGACTGGAGCTATTCATAGGACACTTTTAGGGGGAAAGTTAGTAAGGTC
right  TCCTGACCTT-----GTGATCCGCCCGC--CTCGGCCTCCGAAAGTGCTGGGATTACAGAC-GTGAG---
       1**1**11**11111**11111*1*1*111**111*1*11**1*1*11*1**111*1**111**1**111

left   CTTCTCAGGAGGGACTGGACAGAATTTCTTTTTCTTTTTCTTTTCTTTTCTTTTCTTTTTCTTTTTCTTT
chrom  CTTCTGAGGAGGGACTAGACA--ATTTCTA-------AACTAGGCGAATCATAGGTTTATTCAGTGGAAC
right  CCACCGCGCACGGCC---AGA--ATTTCTA-------AACTAGGCGAATCATAGGTTTATTCAGTGGAAC
       *11*121*1*1**1*1N1*1*22******2222222222**222*222**2*222***2*2222*22222

left   TTTTTTTTTTTTTTTTTGAGA----CGGAGTCTTGCTCTGTCGTCTTGGCTGGAGTGCAGTGGCGCGATC
chrom  TTAGCTGCTGGAACATGAAGATCTGCGCAGAGTTGCTGGATCCGTTTGGCTTTGGTCTTATCTTG-GATC
right  TTAGCTGCTGGAACATGAAGATCTGCGCAGAGTTGCTGGATCCGTTTGGCTTTGGTCTTATCTTG-GATC
       **222*22*222222*22***2222**2**22*****222**222******222**2222*222*2****

left   -TCGGCTCACTGCAAGCTCCGCCTCCAGGATTCACGCCATTCT-------------
chrom  ATCGGGTCTGAGTAAGCTGGTGTTAAAACAATTAGAGCTTAGTGTAGCATGGCAGG
right  ATCGGGTCTGAGTAAGCTGGTGTTAAAACAATTAGAGCTTAGTGTAGC--------
       2****2**222*2*****22222*22*22*2*2*222*2*22*22222
```

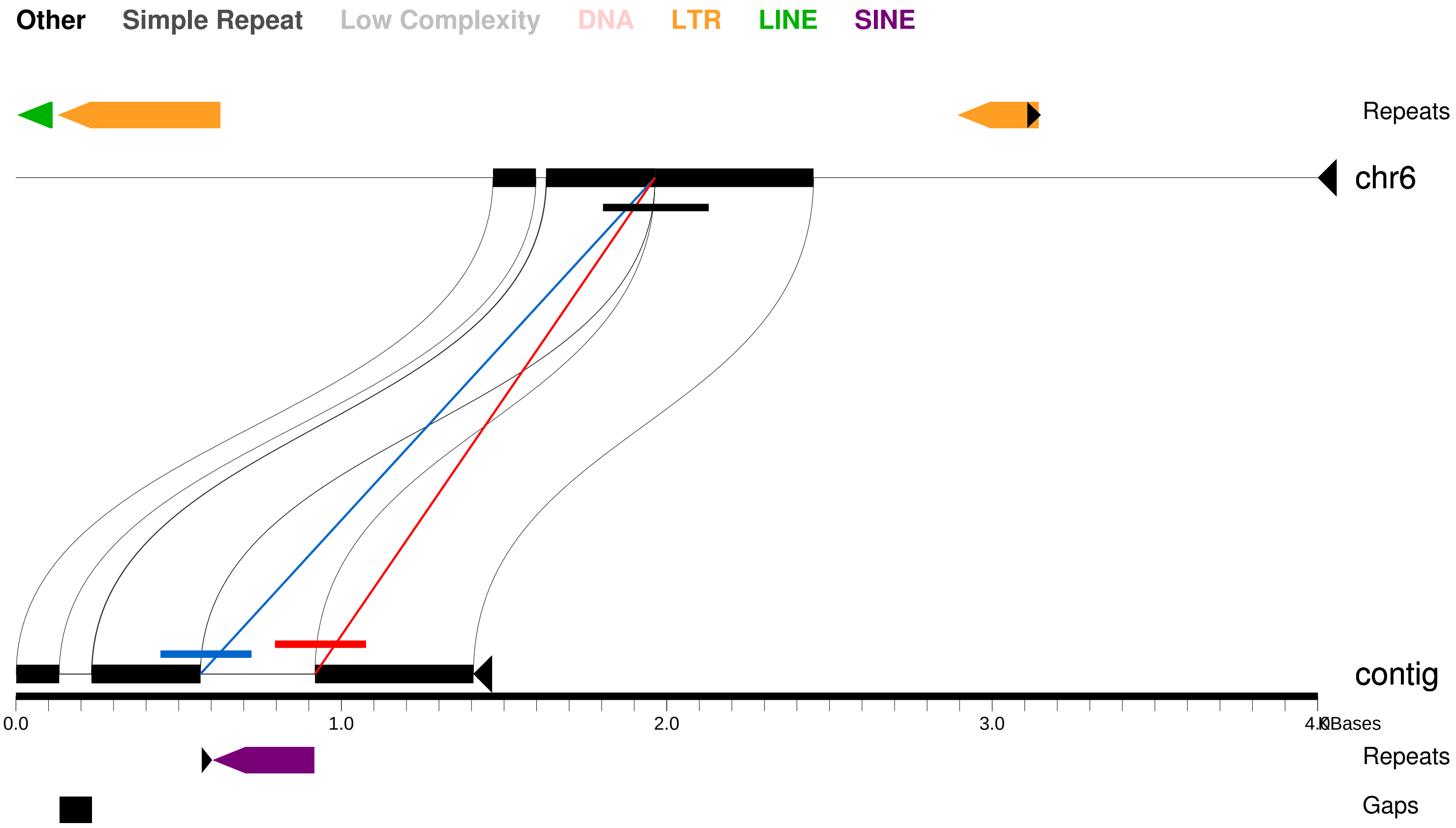

siteID: chr6_6034772 Scafftig3 - Breakpoint: chr6:6034763
chr6:6034717-6034822
Overlap Length: 14 INS size: 307 Genotyped: Yes

```
left    G-----------GATAAAGTGTG-----GTTTTTAACAGGTGATATAGGTTGTTTAATTTAGAAAAGAGA
chrom   G-----------GATAAAGTGTG-----GTTTTTAACAGGTGATATAGGTTGTTTAATTTAGAAAAGAGA
right   TCCAGCCTGGGCGACAGAGCGAGACTCCGTCTCAAAAAAAAAAAAAAAAAAAAAAAA---AAAAAAAAGA
        111111111111**1*1**1*1*11111**1*11**1*1111*1*1*11111111**111*1****1***

left    AAGCACGGCCGGGCGCGGT-GGCTCACGCCTGTAATCCCAGCACTTTGGGAGGCCGAGGCGGGCGG
chrom   AAGCACA--------CTGTAGGCCCATA---GAAGTGGAAAGATTTTTCTTCTCC-AGAAGAAA--
right   AAACACA--------CTGTAGGCCCATA---GAAGTGGAAAGATTTTTCTTCTCC-AGAAGAAA--
        **1***222222222*2**2***2**22222*2*2*222*22*2***222222**2**22*22222
```

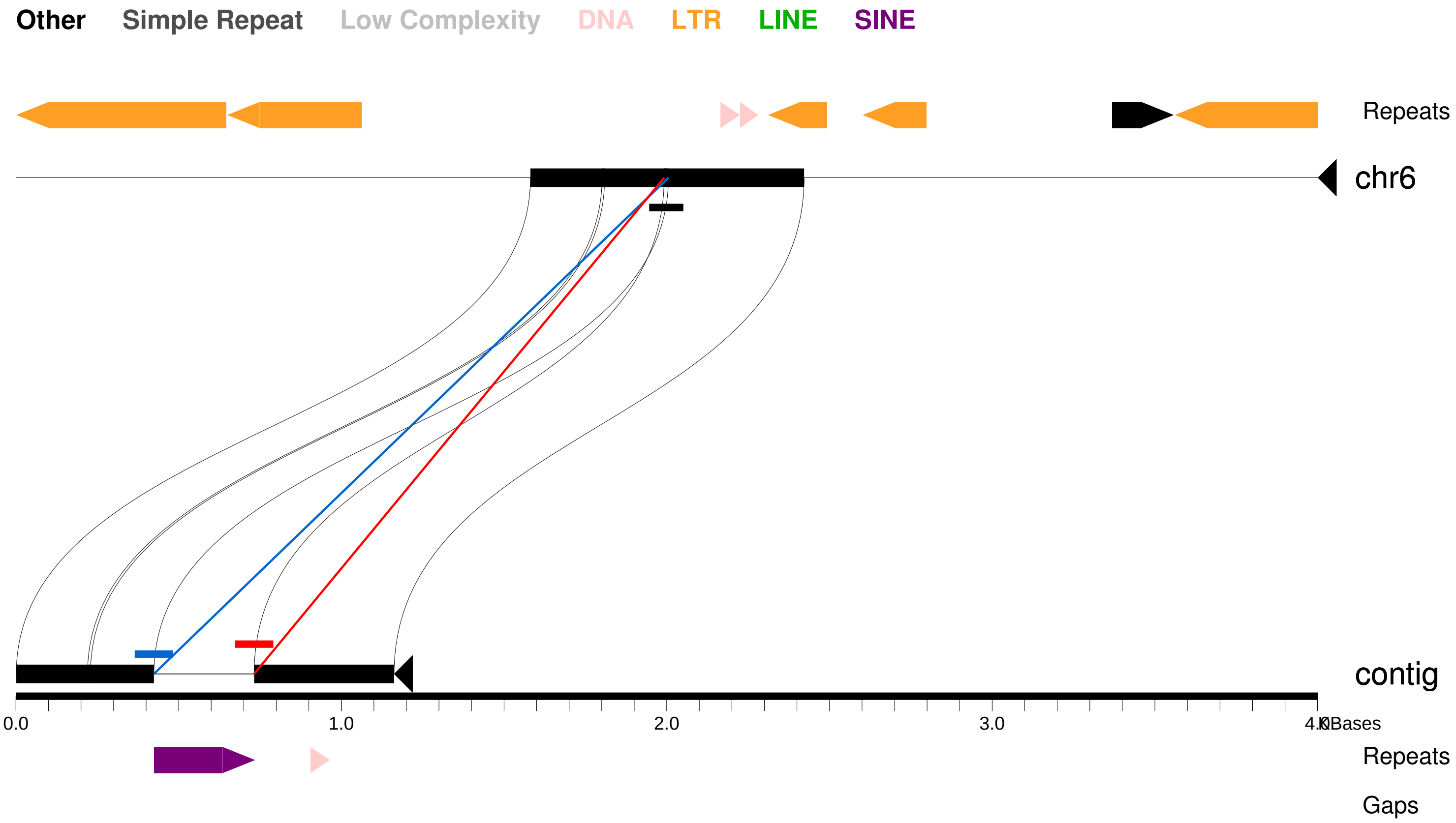

siteID: chr6_66163985 Scafftig1 + Breakpoint: chr6:66163967
chr6:66163923-66164026
Overlap Length: 16 INS size: 295 Genotyped: Yes

```
left    TTGGATACATAACCACTGGGGGATTGTTGG-ATCATATG-GTA--------------GTTCTATTTTTCT
chrom   TTGGATACATAACCACTGGGGGATTGTTGG-ATCATATG-GTA--------------GTTCTATTTTTCA
right   CCGCCCGCCTCGGC-CTCCCAAAGTGCTGGGATTACAGGCGTGAGCCACCGCGCCCGGCCCTATTTTTCA
        11*1111*1*111*1**11111*1**1***1**1*1*1*1**111111111111111*11*********2

left    TTTTTTTTTTTTTTTTTTTTGAGACGGAGTCTCGCTCTGTCGCCCAGGCTGGAGTGCAGTGGCGG
chrom   TTTTTTGAGGAACTTCTAT----AC---TTCTTTCCATAATGTCTCTACTAGATTAC--------
right   TTTTTTGAGGAACTTCTAT----AC---TTCTTTCCATAATGTCTCTACTAGATTAC--------
        ******2222222**2*2*2222**2222***22*22*222*2*2222**2**2*2*22222222
```

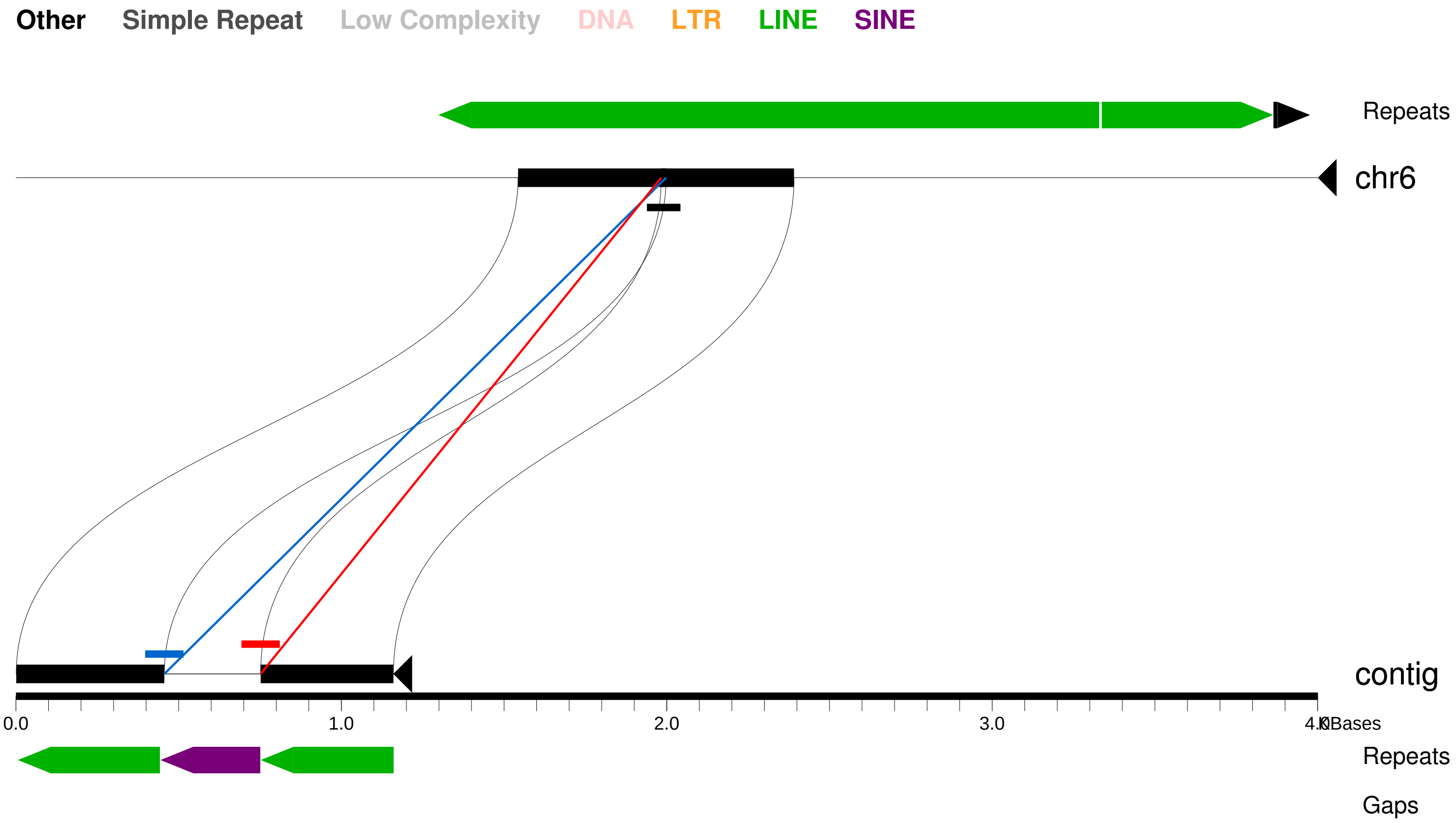

siteID: chr6_66816126 Scafftig10 + Breakpoint: chr6:66816117
chr6:66816090-66816176
Overlap Length: 8 INS size: 307 Genotyped: Yes

```
left    ----CTCA------ATTATTCT-------CCA------TGAGATAAAATC---------TTTATTTCTTT
chrom   ----CTCA------ATTATTCT-------CCA------TGAGATAAAATC---------TTTATTTCATT
right   TGACCTCATGATCCACTCGCCTCGGCCTCCCAAAGTGCTGGGATTACAGGCGTGAGCCATTTATTTCATT
        1111****111111*1*111**1111111***111111**1***1*1*11111111111********2**

left    TTTTTTTTTTTTTTTTTTTTTTTTTTTTGAGACGGAGTCTCGCTCTGTCGCCCAGGCCGGACTGCGGACT
chrom   CTTATGTGTAAAATATATTTTT---------------------------------GCTGGATATAGAACT
right   CTTATGTGTAAAATATATTTTT---------------------------------GCTGGATATAGAACT
        2**2*2*2*2222*2*2*****222222222222222222222222222222222**2***2222*2***

left    GC-AGTGGCGCA
chrom   GCTAGTTGGCAG
right   GCTAGTTGGCAG
        **2***2*2222
```

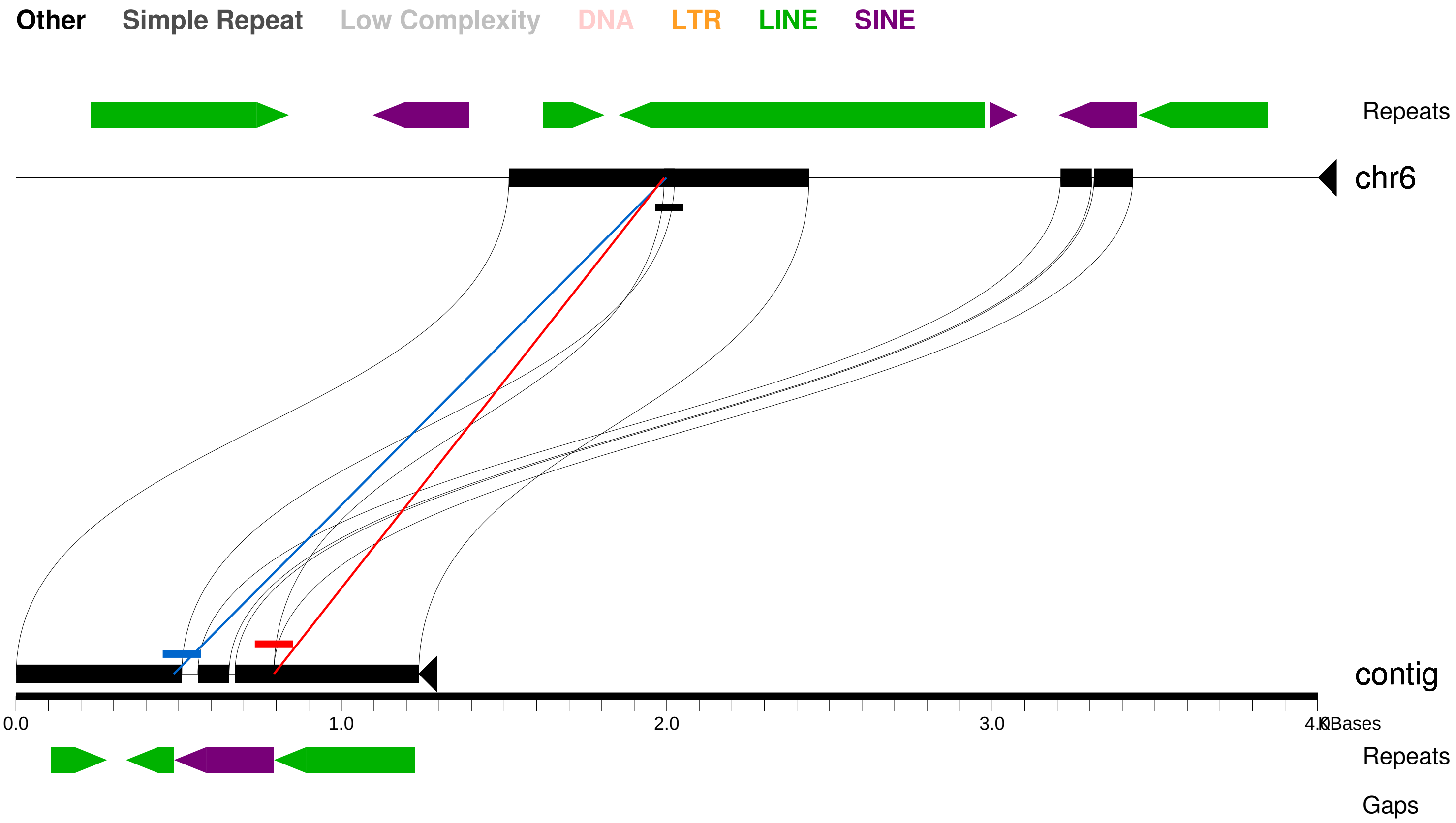

siteID: chr6_67194338 Scafftig2 - Breakpoint: chr6:67194377
chr6:67194318-67194440
Overlap Length: 1 INS size: 414 Genotyped: No

```
left    CTTTTACGTAATGAACTATCTCAAGGTATGTATAATCTAAAGGGAAGTAGGGATTGAAG--GNNNNNNNN
chrom   CTTTTACGTAATGAACTATCTCAAGGTATGTATAATCTAAAGGGAAGTAGGGATTGAAG--GGGAAACAT
right   CCGCC-CGCCTCGGCCT--CCCAAAGTGC-TGGGAT-TACAGGCGTG-AGCCACCGCGCCCGGCCAACAT
        *11111**1111*11**11*1***1**111*111**1**1***111*1**11*11*11111*2NN22222

left    NNNNNNNNNNNNNNNNNNNNNNNNNNNNNNNNNNNNNNNNNNNNNNNNNNNNNNN----
chrom   TTTGAAATAATTAATTTTAAACTTCATTCTGTTAATGATTGTTGCTAAAATAATC
right   TTTGAAATAATTAATTTTAAACTTCATTCTGTTAATGATTGTTGCTAAAATAATC
        22222222222222222222222222222222222222222222222222222222222
```

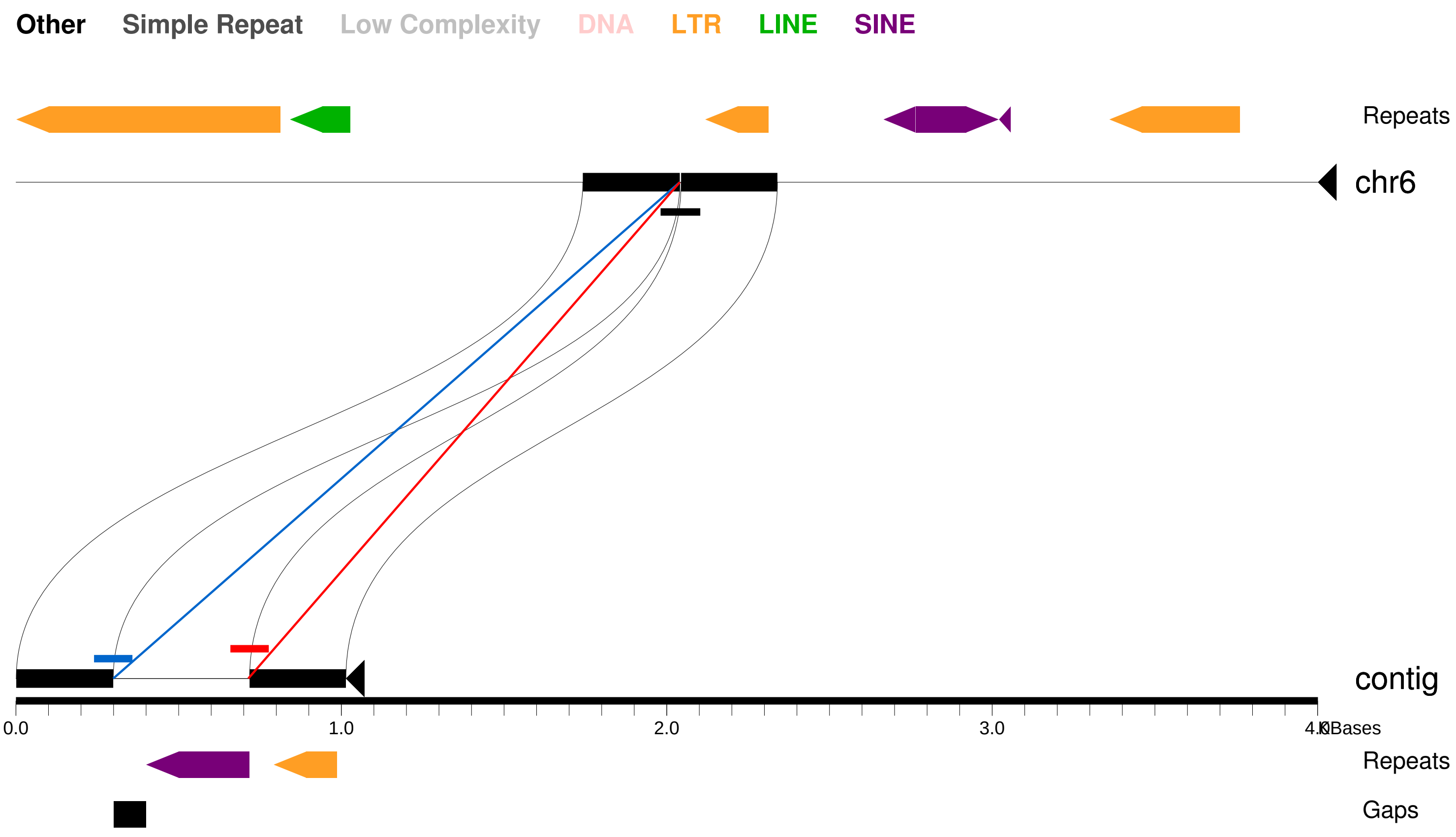

siteID: chr6_68311289 Scafftig3 + Breakpoint: chr6:68311296
chr6:68311252-68311355
Overlap Length: 16 INS size: 435 Genotyped: No

```
left    TA---------CAGGGCCTCGATATTTTCTG---TATAATTTTTTAGGAATTTTTA---TTTTTGCCAGT
chrom   TA---------CAGGGCCTCGATATTTTCTG---TATAATTTTTTAGGAATTTTTA---TTTTTGCCAGT
right   GATCCACCCGCCTCGGCCTCCCAAAGTGCTGGGATTACAGGCGTGAGCCACCGCGCCCGTTTTTGCCAGT
        1*111111111*11******111*11*1***111*111*1111*1**11*111111111***********

left    ATTTTTTTNNNNNNNNNNNNNNNNNNNNNNNNNNNNNNNNNNNNNNNNNNNNNNNNNNNNNNNN
chrom   ATTTTT--------------AAATGAAGTTTTAAAATGATCTCTAATTGTTGTTGCTGCCTTAT
right   ATTTT---------------AAATGAAGTTTTAAAATGATCTCTAATTGTTGTTGCTGCCTTAT
        ******2222222222222222222222222222222222222222222222222222222222
```

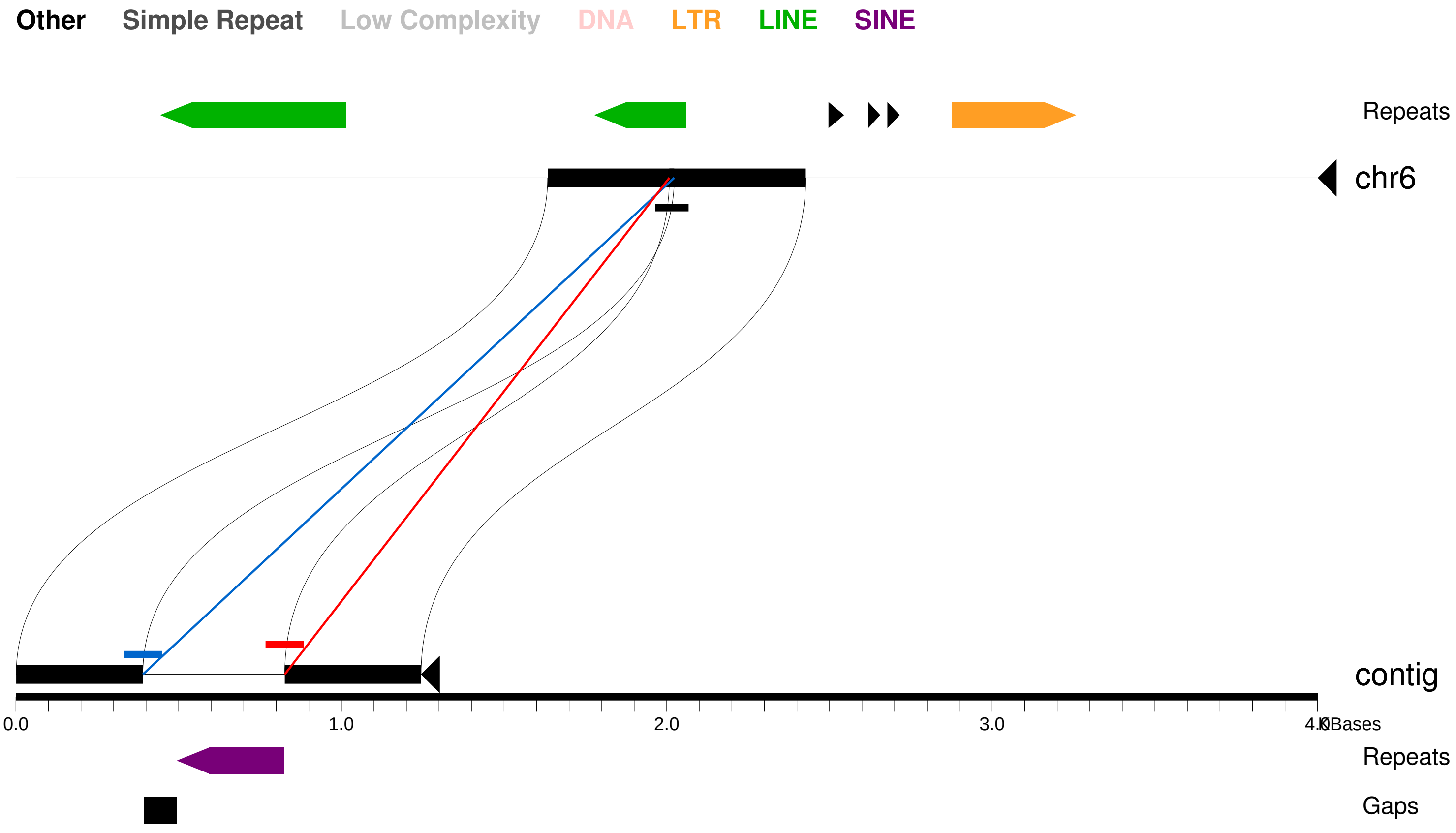

siteID: chr6_68314076 Scafftig7 + Breakpoint: chr6:68314050
chr6:68314009-68314109
Overlap Length: 19 INS size: 299 Genotyped: Yes

```
left     C-------------ATATTTATTTTGTTTTTTTATGGAACTGA--CTTCTAGAATT---TGATTTGGTAT
chrom    C-------------ATATTTATTTTGTTTTTTTATGGAACTGA--CTTCTAGAATT---TGATTTGGTAT
right    CCGCCCGCCTCGGCCTCCCAAAGTGCTGGGATTACAGGCGTGAGCCACCGCGCCCGGCCTGATTTGGTAT
         *11111111111111*1111*11*11*1111***11*111***11*11*11*1111111***********

left     ATACATTTTTTTTTTTTTTTTTTTTTGAGACGGAGTCTCGCTCTGTCGCCCAGGCTGGAGTGCAGTG
chrom    ATACATTTAAGTTTAAATACACATTTA----------TCAATCT-------AAAATAGA-TAAGAAA
right    ATACATTTAAGTTTAAATACACATTTA----------TCAATCT-------AAAATAGA-TAAGAAA
         ********222***222*22222***22222222222**22***2222222*222*2**2*222222
```

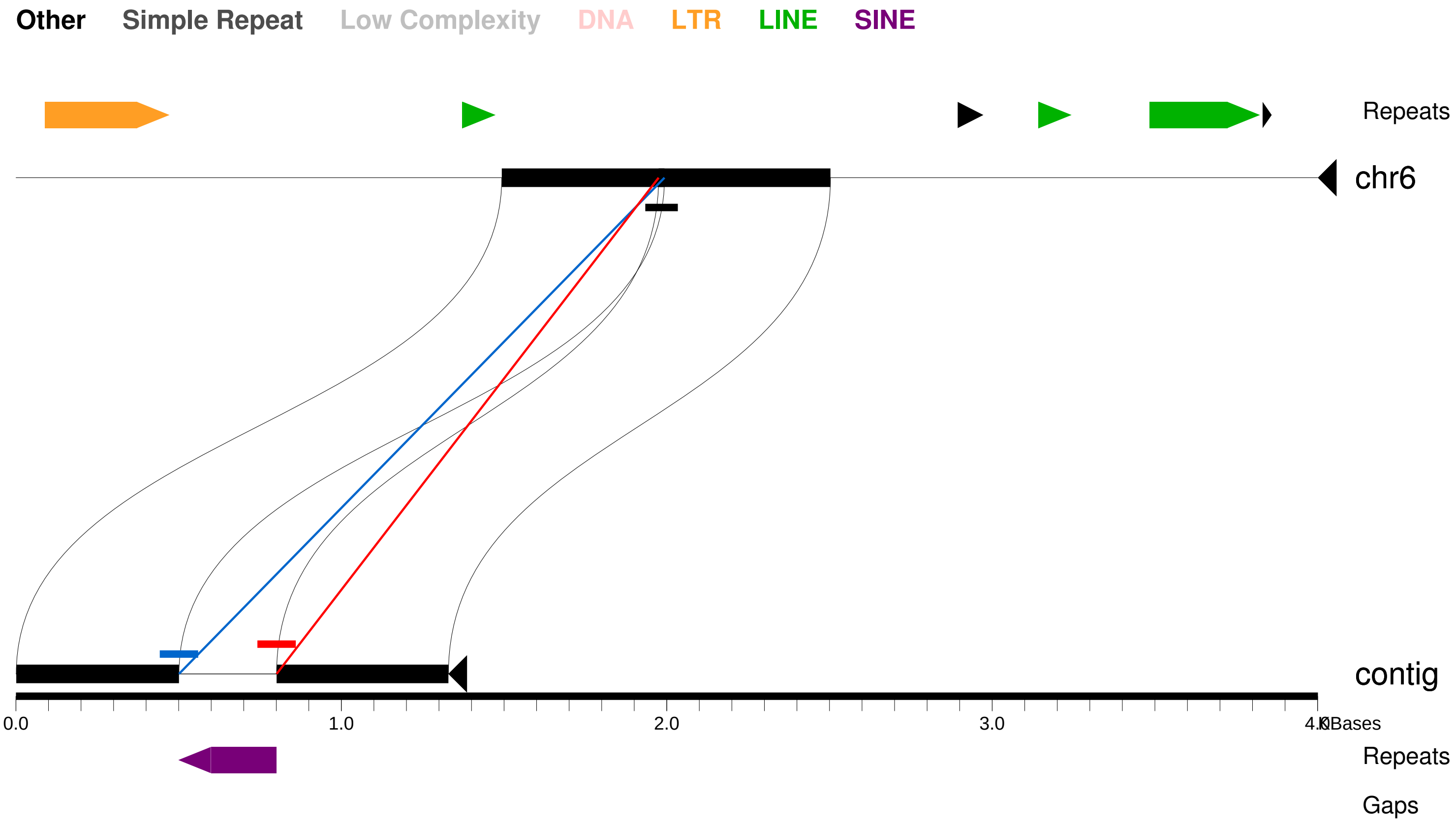

siteID: chr6_68502419 Scafftig5 + Breakpoint: chr6:68502428
chr6:68502268-68502605
Overlap Length: 18 INS size: 320 Genotyped: Yes

```
left   --------------GTGCTACAGAGATTTCCAAAAGTAGAATATCCAGGTCAAAGAGTATTAAGGATAGT
chrom  CTTC--AGACACTGGTGCTACAGAGATTTCCAAAAGTAGAATATCCAGGTCAAAGAGTATTAAGGATAGT
right  TTTAGTAGAGACGGG-GTTTCACCGTTTT-------------AGCCGGG----ATGGTCTC---GATCTC
       N22N11222N22N2*1*1*1**11*1***1111111111111*1**1**1111*11**1*1111***111

left   TAACATTTTTTTTTTTTACCTGATCACTTTCCAGAAAGTTTGTAGCAATTTACACTCAGAAATGAGGTTGC
chrom  TAACATTTTTTTTTTTTACCTGATCACTTTCCAGAAAGTTTGTAGCAATTTACACTCAGAAATGAGGTTGC
right  TTGACCTCGTGATCCGCCCGCCTCGGCCTCCC-AAAGT--GCTGGGATTA-----CAGGCGTGAG-----
       *11111*11*11*1111**111**1111***11*****11*11*11***111111***111****11111

left   TGTTTCCCTCTACGATCATCATCGAAAGTGATTATTTTTTTTTTTATTTTTTTTTTTATTTTTTATTTTTT
chrom  TGTTTCCCTCTACGATCATCATCGAAAGTGATTATTTTTTAAATTGTCATAATCTTATGGTAAAAATTAT
right  ------CCACCGCGCCCGGC--CGAAAGTGATTATTTTTTAAATTGTCATAATCTTATGGTAAAAATTAT
       111111**1*11**11*11*11******************222**2*22*22*2****22*22*22**2*

left   TTT---------TTTTTTGAGACGGAGTCTCGCTC-TGTCGCCCAGGCTG---------------GAGTG
chrom  GTTACCTAAATGTCATCTAATTTACATTTCCCCTCATCACACTTAGGTTGAACATTATTTCATAAGAATA
right  GTTACCTAAATGTCATCTAATTTACATTTCCCCTCATCACACTTAGGTTGAACATTATTTCATAAGAATA
       2**222222222*22*2*2*22222*2*22*2***2*22*2*22***2**222222222222222**2*2

left   CAGTGGC----GGGAT--CTC-----GGCTCACTG---------CAAGCTCCGCCTCCCG
chrom  GAGAGGCCACAGTTATTACTCATAACGTATTTCAGAGGGAATATCAATTGCTGTAGTTAG
right  GAGAGGCCACAGTTATTACTCATAACGTATTTCAGAGGGAATA-----------------
       2**2***2222*22**22***22222*22*22*2*22222222 111NNN1N1NNNNNN1
```

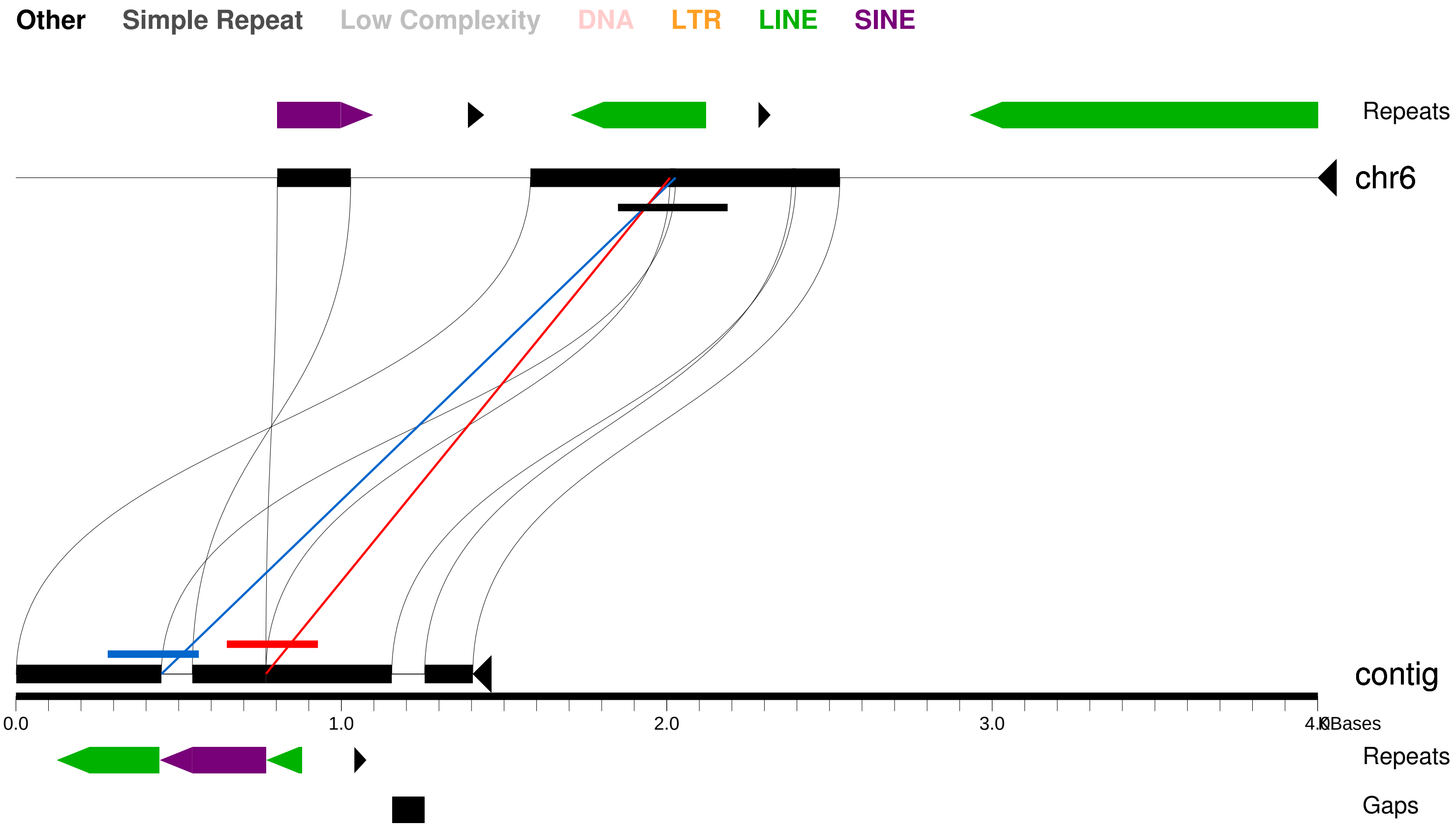

siteID: chr6_69543470 Scafftig5 - Breakpoint: chr6:69543454
chr6:69543408-69543513
Overlap Length: 9 INS size: 437 Genotyped: No

```
left   A-AGCAG--------TTAAAAAGAAAATCAATTTCCTTATTGTTTTAACTGGATA----AGTATTCTTTT
chrom  A-AGCAG--------TTAAAAAGAAAATCAATTTCCTTATTGTTTTAACTGGATA----AGTATTCTTAT
right  CCACCCGCCTCGGCCTCCCAAAGTGCTGGGATTACAGGCGTGAGCCACCGCGCCCGGCCAGTATTCTTAT
       11*1*1*11111111*111****1111111***1*11111**1111*1*11*1111111*********2*

left   TTTTTNNNNNNNNNNNNNNNNNNNNNNNNNNNNNNNNNNNNNNNNNNNNNNNNNNNNNNNNNN
chrom  ATT-------------AAAGAATGAAGTATTTGAACTTAGAATCTTCATTATCTTCTTGTTT
right  ATT-------------AAAGAATGAAGTATTTGAACTTAGAATCTTCATTATCTTCTTGTTT
       2**222222222222222222222222222222222222222222222222222222222222
```

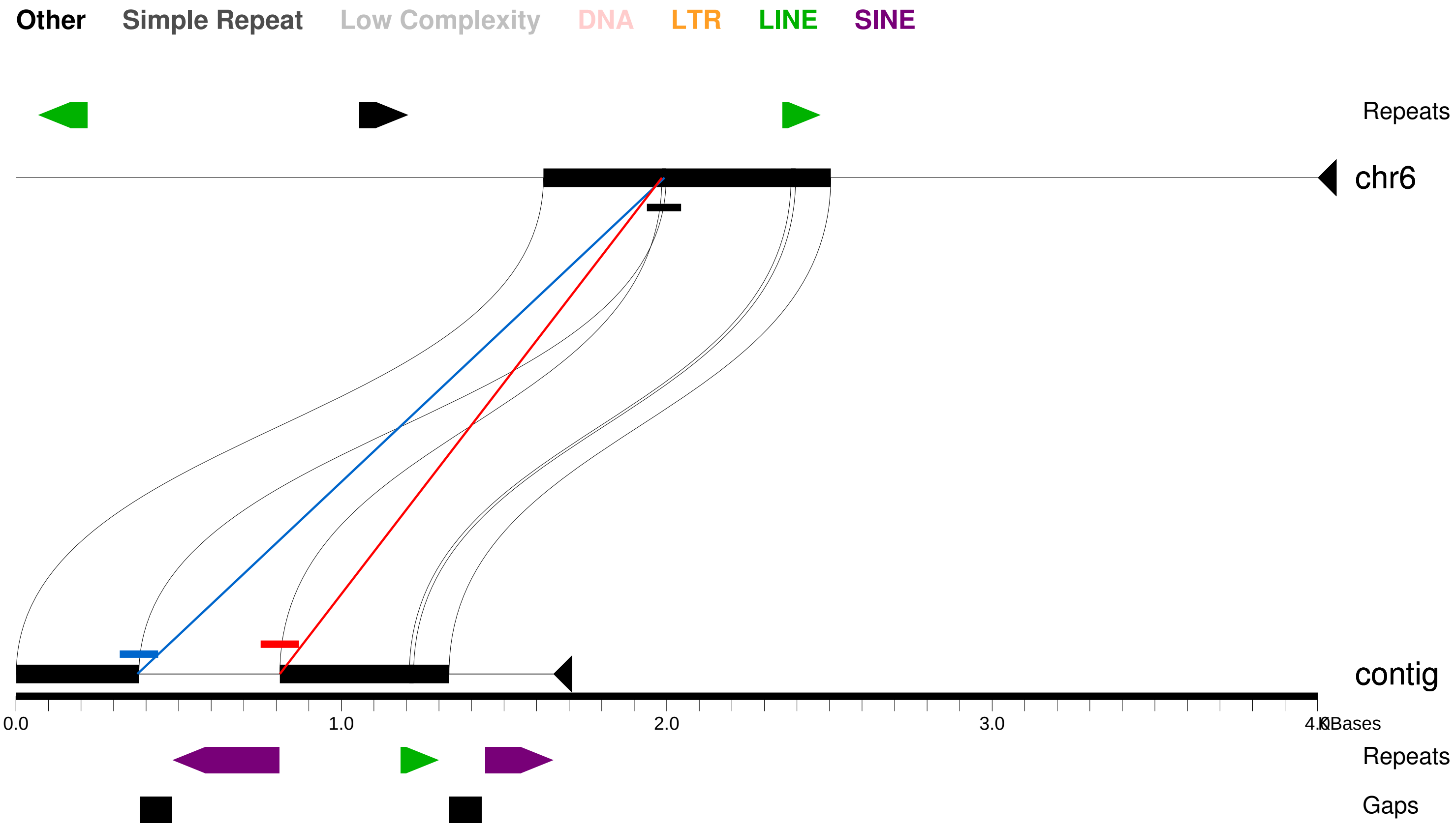

siteID: chr6_71729306 Scafftig5 + Breakpoint: chr6:71729308
chr6:71729257-71729367
Overlap Length: 9 INS size: 452 Genotyped: No

```
left    AATAAATGGAAAGATATCCCACGTACATGGACTGGAAGAATTAATATTGTT--------AAAATGGTCGG
chrom   AATAAATGGAAAGATATCCCACGTACATGGACCGGAAGAATTAATATTGTT--------AAAATGGTCAT
right   NNNNNNNNNNNNNNNNNNNNNNNNNNNNNNNNNNNNNNNNNNNNNNNNNNNNNNNNNNNNAAAAAATGGTCAT
        11111111111111111111111111111111N1111111111111111111111111*********22

left    CCGGGCGCGGTGGCTCACGCCTGTAATCCCAGCACTTTGGGAGGCCGAGGCGGGTGG
chrom   ATTACTGAAAGTGATCTACAGATTAATGCAATCACTATCAAAATACCAA--------
right   ATTACTGAAAGTGATCTACAGATTAATGCAATCACTATCAAAATACCAA--------
        222222*22222*2**2222222****2*2*2****2*222*222*2*222222222
```

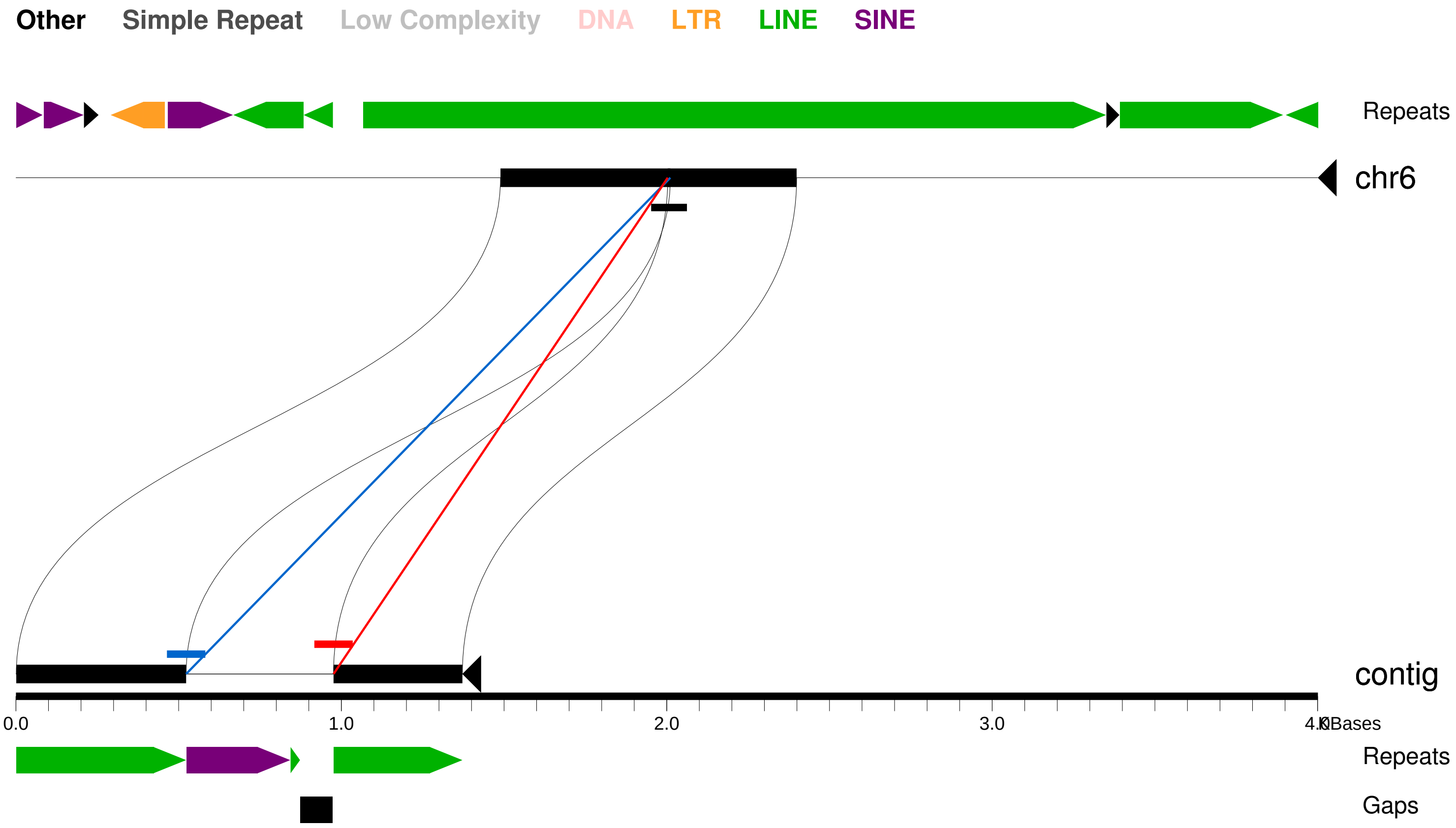

siteID: chr6_72254381 Scafftig1 + Breakpoint: chr6:72254372
chr6:72254212-72254542
Overlap Length: 11 INS size: 332 Genotyped: Yes

```
left   -------TAATCTTTTATTTTTCTCATAATAGGTATCAGTACAAAAATAATGTCTTACATTTGCACAGTG
chrom  CTTGAATTAATCTTTTATTTTTCTCATAATAGGTATCAGTACAAAAATAATGTCTTACATTTGCACAGTG
right  TTT--AGTAG---------------AGACGGGGTTTCACCT---------TGTTAGCCA---GGATGGT-
       N22  2N**1111111111111111*1*111***1***111111111111***1111**111*1*11**1

left   CTCAGAGATTTACAAAATGCCTTCATACACTTTATCTCATTTTATCCTTCTAATAAAATAATAATGGAGG
chrom  CTCAGAGATTTACAAAATGCTTTCATACACTTTATCTCATTTTATCCTTCTAATAAAATAATAATGGAGG
right  CTC---GATCTCCTGACCTCGTG-ATCCACC------CGCCTCGGCCTCCCAAAGTGCTGGGATTACAGG
       ***111***1*1*11*111*N*11**1***1111111*111*111***1*1**11111*111*1*11***

left   CA-GAGTTTGCCTGCATCTGTCTCTCCATTTTTTTTTTTTTTTTTTTTTTTTTTTTTTTTTTTTTTTTTT
chrom  CA-GAGTTTGCCTGCATCTGTCTCTCCATTTTAAAGTTGTAAAATTTGAGGTTTAATGTGTGTAAATATT
right  CGTGAGCCACCGCGCCCGGCCCTCTCCATTTTAAAGTTGTAAAATTTGAGGTTTAATGTGTGTAAATATT
       *11***1111*11**111111***********2222**2*2222***2222***22*2*2*2*222*2**

left   T-----------------TTTTTGAGGCGGA------GTCTCGCTCTGTCGCCC--AG-GCCGGACTGCG
chrom  TGGCCAAAGGTCACAAAGTGTGTAAGTGACATATTTGGTATCGATTTCTGGGCCTAAGTGCAGCACCATT
right  TGGCCAAAGGTCACAAAGTGTGTAAGTGACATATTTGGTATCGATTTCTGGGCCTAAGTGCAGCACCATT
       *222222222222222222*2*2*2**2222*222222**2***2*2*2*2*2**22**2**2*2**2222

left   GACTGCAGTGGCG-----CAA-----TCTCGGCTCACTGCAAGCT-------
chrom  TTTTGCTATAGCATATTTCAAGGGTTTTTTGTTTGTTTGTTTGTTTTTCTCG
right  TTTTGCTATAGCATATTTCAAGGGTTTTTTGTTTGTTTGTT-----------
       222***22*2**222222***22222*2*2*22*222**22N1N1
```

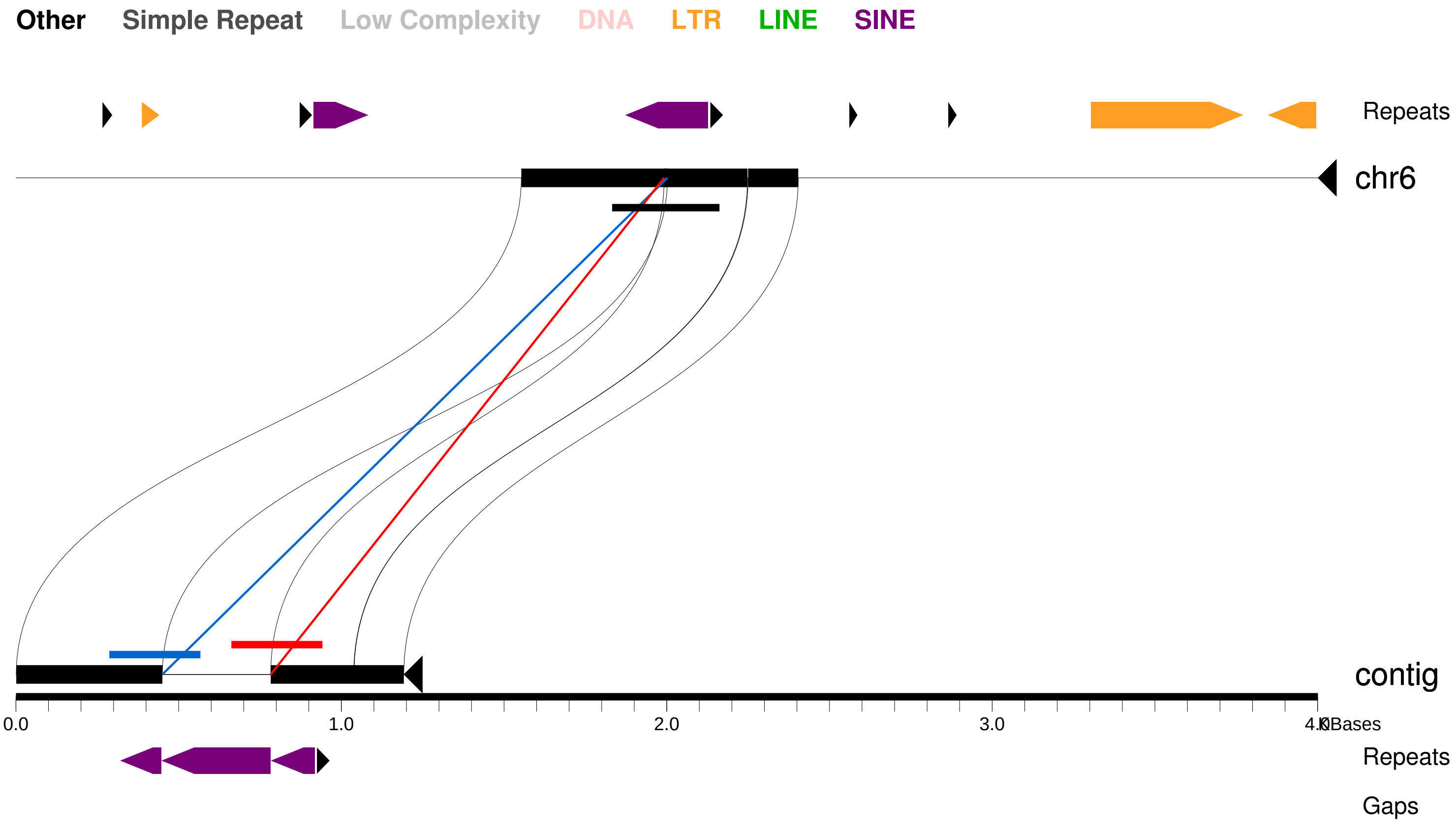

siteID: chr6_72580478 Scafftig3 - Breakpoint: chr6:72580483
chr6:72580437-72580542
Overlap Length: 14 INS size: 304 Genotyped: Yes

```
left     -CTCCAAAATT-----CATGTTGAAACCTAATCACAAAGGTGATGGTATTAG-------AAGATGGTTGA
chrom    -CTCCAAAATT-----CATGTTGAAACCTAATCACAAAGGTGATGGTATTAG-------AAGATGGTTGA
right    ACTCCAGCCTGGGCGACAGAGCGAGACTCCATCTCAAAAAAAAAAAAAAAAAAAAAAAAAAGATGGTTGA
         1*****111*111111**1111**1**111***1****1111*1111*11*11111111***********

left     GTCGCCGGGCGCGGTGGCTCACGCCTGTAATCCCAGCACTTTGGGAGGCCGAGGCGGGCGGA
chrom    GTCATGAGG----GAACCTCCCTCATGAATTG-------ATTA--AGGCCTTATAAAAAGGG
right    GTCATGAGG----GAACCTCCCTCATGAATTG-------ATTA--AGGCCTTATAAAAAGGG
         ***2222**2222*222***2*2*2**2*2*222222222**222*****222222222**2
```

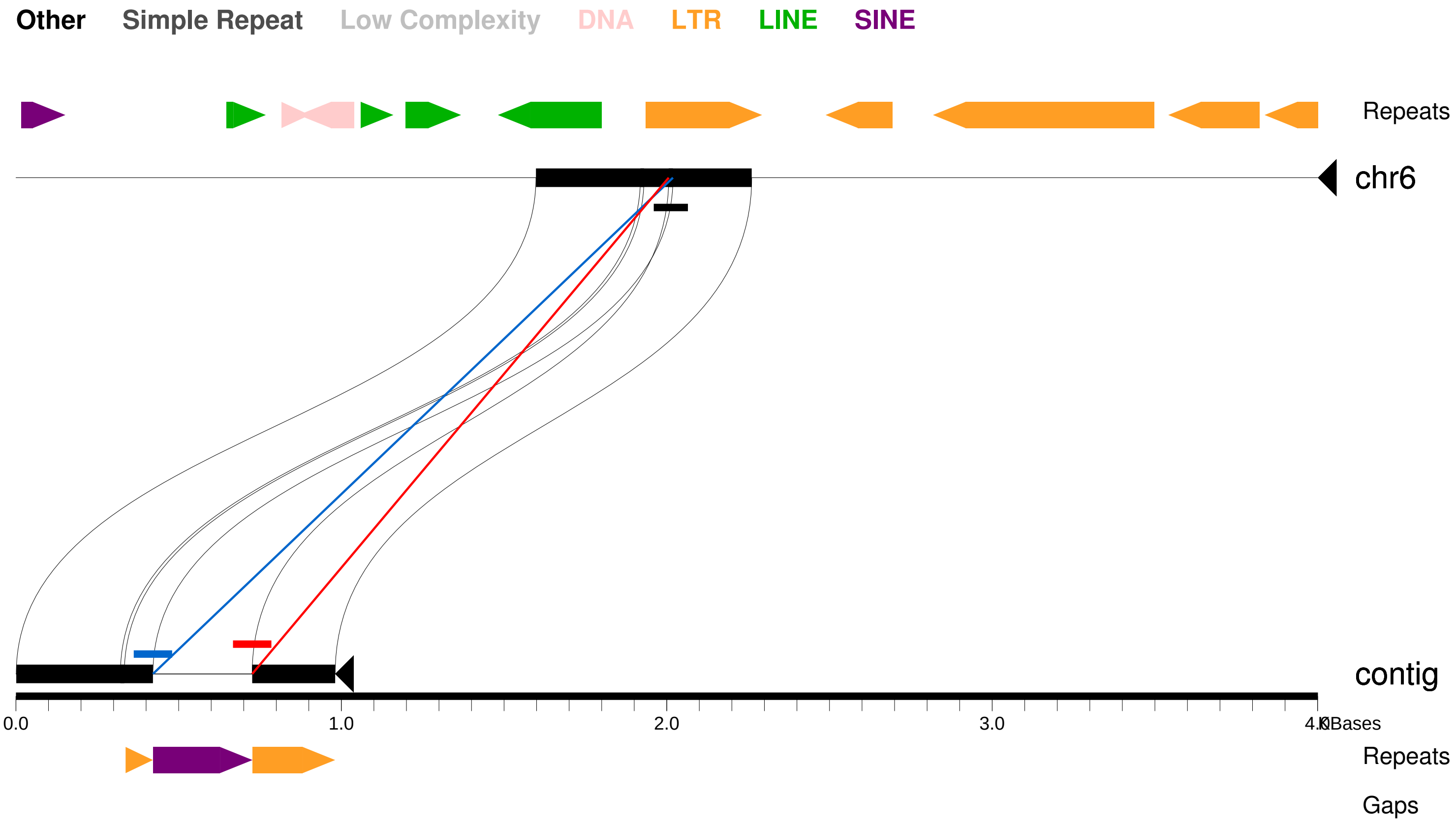

siteID: chr6_74504862 Scafftig11 + Breakpoint: chr6:74504838
chr6:74504678-74505015
Overlap Length: 18 INS size: 299 Genotyped: Yes

```
left     T-----------------GGTATATTGTGTGCCATGCTAAACACAAATGAGACCTTTACACATTGGCATAA
chrom    TACCAAGTGGTCTCTGTGGTATATTGTGTGCCATGCTAAACACAAATGAGATCTTTACACATTGGCATAA
right    GA------GG---CGGGAGAATGGCGTGAACCCGGG--------AGGCGGAGCTT---------------
         12      22   2N2N1*1**111***11**11*111111111*1111**N***111111111111111

left     TGGCTGTAATTAAGGAGATAAGTAAAAATTATTTGGGATTGTCATGAATGAACGTTGTACCTTTTGAAAA
chrom    TGGCTGTAATTAAGGAGATAAGTAAAAATTATTTGGGATTGTCATGAATGAACGTTGTACCTTTTGAAAA
right    --GCAGTGAGC--GGAGATCCCGCCACTGCACTCCAGCCTGGGC-GACAGAGCGAGACTCCGTCTCAAAA
         11**1**1*1111******111111*1111*1*111*11**1111**11**1**11111**1*1*1****

left     CAAGATTCCAATTTGTATTCAAGATTTAGTTTTTCTGGCCGGGCGTGGTGGCT-CAC--------GCCTG
chrom    CAAGATTCCAATTTGTATTCAAGATTTAGTTTTTCTGGTTTGAAGTAGAAACTACATAGGAATTAGACTG
right    AAAAAAAAAAAA----AAAAAAGATTTAGTTTTTCTGGTTTGAAGTAGAAACTACATAGGAATTAGACTG
         1**1*1111**11111*111*****************222*22**2*222**2**222222222*2***

left     --------TAATC---CCAGCA--CTTTGGGA--GGCCGAGGCGGGCGGATCACG-AGGTCAGGAGAT-C
chrom    GTGCTTTATAATTTGGCATACATTCTTTGGGATAGTTTAATACAATAAGGTAAAATATTTCAGAAAATGC
right    GTGCTTTATAATTTGGCATACATTCTTTGGGATAGTTTAATACAATAAGGTAAAATATTTCAGAAAATGC
         22222222****2222*222**22********22*2222*22*22222*2*2*222*22****2*2**2*

left     GAGA-------CCATCCCGGCTAAA-----ATGG--TGAAACCCCGTCTCTACTAC-A
chrom    CACATATAACTCCATATTTACATATGCTTTATGAACTGTAACTCTGGCTAATCAAATA
right    CACATA--ACTCCATATTTACATATGCTTTATGAACTGTAACTC--------------
         2*2*22  222****22222*22*222222***222**2***2*N1N11NNN1N1N 1
```

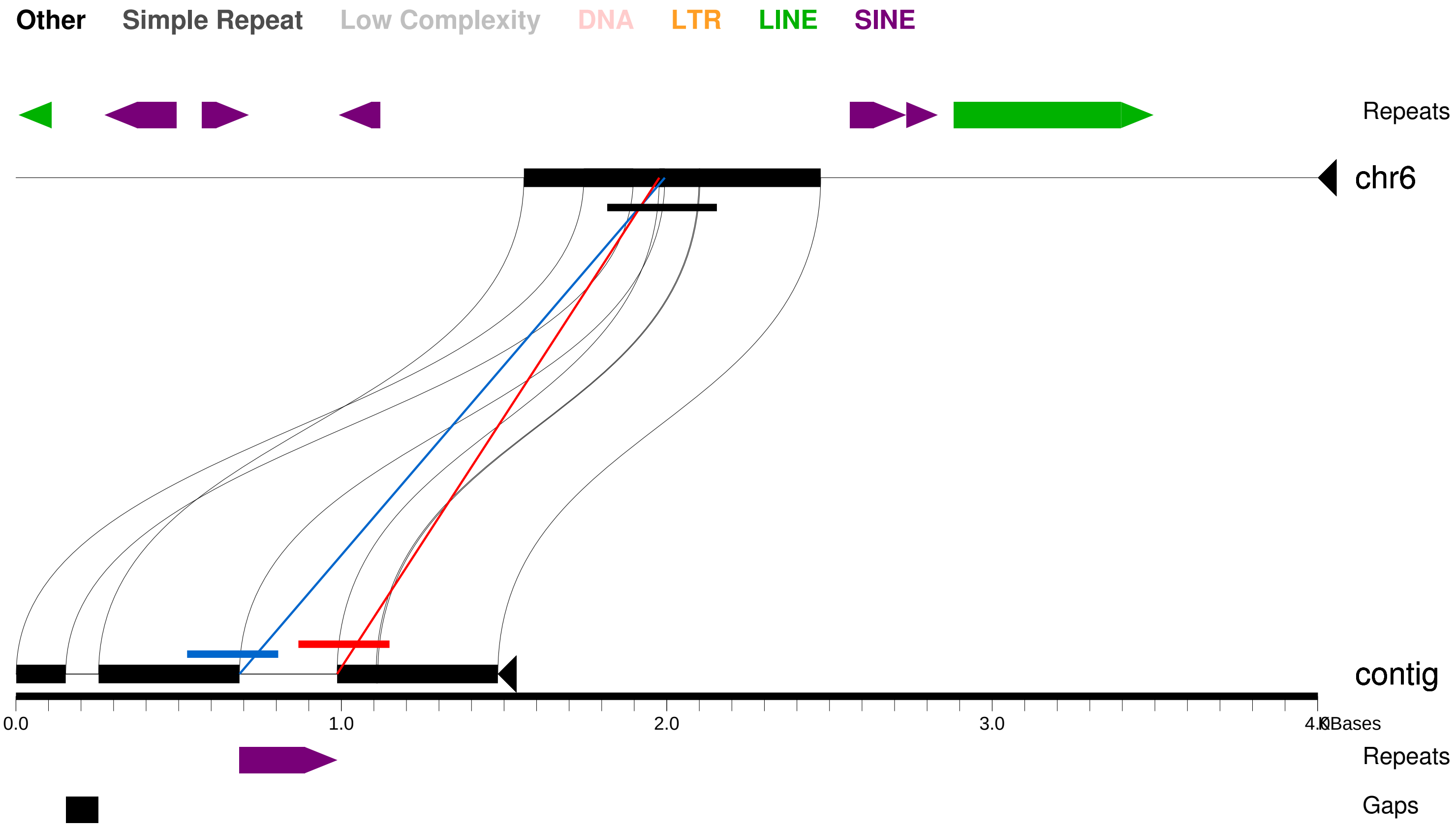

siteID: chr6_75338250 Scafftig1 + Breakpoint: chr6:75338221
chr6:75338061-75338396
Overlap Length: 16 INS size: 427 Genotyped: No

```
left    CA---------------CACCAAAATATAAAGACAGATAACACTATGAAGAAACTGCATCAAATAATGTG
chrom   CAGCTACTGCAAAAACACACCAAAATATAAAGACAGATAACACTATGAAGAAACTGCATCAAATAATGTG
right   NNNNNNNNNNNAAAAAA-----------------------------------------------------
        11NNNNNNNNN2222N211111111111111111111111111111111111111111111111111111

left    CAAAATAACCAGCTAGCATCATGACGACAGGATCAAATTCACACATAGCAATACTAACCTTAAATGTAAA
chrom   CAAAATAACCAGCTAGCATCATGACGACAGGATCAAATTCACACATAGCAATACTAACCTTAAATGTAAA
right   ----------------------------------------------------------------------
        1111111111111111111111111111111111111111111111111111111111111111111111

left    TGGGCTAAGTGCCCACAATTAAAAGACACAGACTGG---------------GCCGGGCGC----GGTG-G
chrom   TGGGCTAAGTGCCCACAATTAAAAGACACAGACTGGCAAATTGGATAAAGAGTCAACAACCATTGGTGTG
right   --------------------AAAAGACACAGACTGGCAAATTGGATAAAGAGTCAACAACCATTGGTGTG
        11111111111111111111****************222222222222222*2*22222*2222****2*

left    CTCACGCCTGTAATCCCAGCACTT-TGGGAGGCCG-----AGGC-----------GGGCGGATCACGAGG
chrom   CTGTATTCAGGAGACCCATCTCATGTGCAAAGACACACATAGGCTCAAAAAAAAGGGATGGAGGAAAATT
right   TTGTATTCAGGAGACCCATCTCATGTGCAAAGACACACATAGGCTCAAAAAAAAGGGATGGAGGAAAAT-
        1*22222*2*2*22****2*2*2*2**22*2*2*222222****22222222222**22***22*22*2N

left    TC---AGGAGATCGAGACCATCCTGGCTAACACGG--TGAAACCCTGTCTCTACTAAA
chrom   TACCAAGCAAATGGAAAGCA--CAAAATAGCAGGGGTTGCAATACTAGTTCTGACAAA
right   ----------------------------------------------------------
        1N   11N1N11N11N1N11221NNNN11N11N11  11N11NN11NNN111NNN111
```

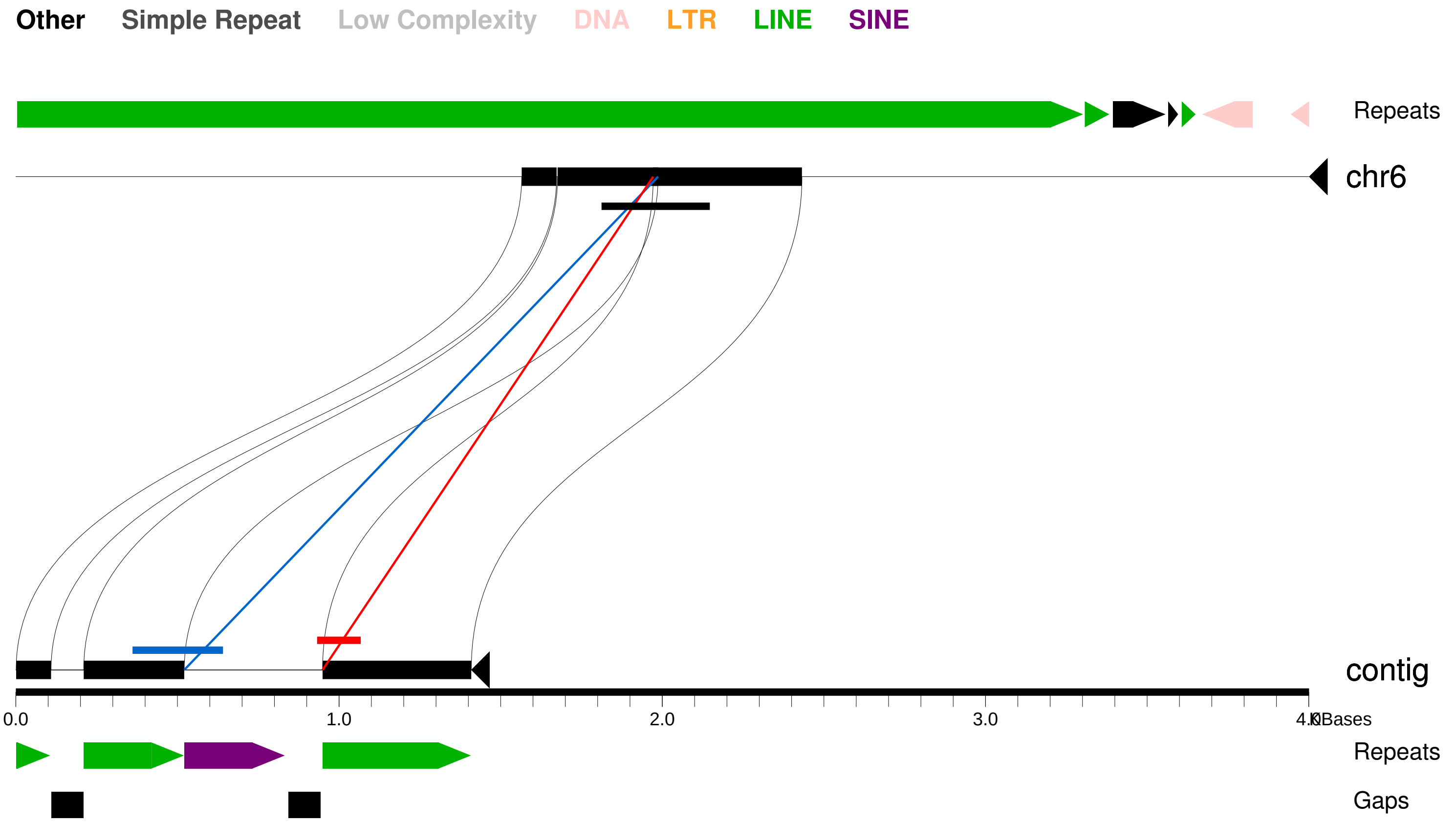

siteID: chr6_77548623 Scafftig12 + Breakpoint: chr6:77548608
chr6:77548448-77548781
Overlap Length: 14 INS size: 452 Genotyped: No

```
left   A--------------GCTAATGTTCTGCATAATAATGTGAAAAGAATTGAAATGCAGAAATTTTGCAGAG
chrom  AACTGGGTAGCAGGAGCTAATGTTCTGCATAATAATGTGAAAAGAATTGAAATGCAGAAATTTTGCAGAG
right  NNNNNNNNNNNA------------------AAAAA----AAAAAAA-------------------------
       1NNNNNNNNNN2   111111111111111**1**1111****1**1111111111111111111111111

left   GAAAGATTCCTGCACAGCTTGGCAGAAAATGAGCACCGTCTTTTTAAAGAAAGATGGTTAAGTCTTAAAG
chrom  GAAAGATTCCTGCACAGCTTGGCAGAAAATGAGCACCGTCTTTTTAAAGAAAGATGGTTAAGTCTTAAAG
right  ----------------------------------------------------------------------
       1111111111111111111111111111111111111111111111111111111111111111111111

left   CCTCATATTTTAGTAACTTTAAAGCTTATCTTTTGCCGGGCGCGGTGGCTCACGCCTG-----TAATCCC
chrom  CCTCATATTTTAGTAACTTTAAAGCTTATCTTTTACTTCAATACTTAA-TGAAGGCTGCTACATATTCAA
right  --------------------AAAGCTTATCTTTTACTTCAATACTTAA-TGAAGGCTGCTACATATTCAA
       11111111111111111111*************2*222222222*222*2*2*2***22222**2**22

left   AGC-----ACTTTGGGAGGCTGAGGCGGGTG----GATC-----ATG--------AGGTCAGGAGATCGA
chrom  AGATAAATATTAATGAAGTCATCTTGGGGTGCTATGATCTTAACATGTCCCCCAAAATTCATGTGTTGGA
right  AGATAAATATTAATGAAGTCATCTTGGGGTGCTATGATCTTAACATGTCC--------------------
       **222222*2*222*2**2*222222*****2222****22222***222     1NN111N1N1N1N11

left   GAC-----CATCCTGGCTAACA--------AGGTGAAACCCCGTCTCTACTAAAA
chrom  AACTTAATCACCAATGCCAACGGTGTTGGGAGGTGGTGGGGACTTTCAAGAGATG
right  -------------------------------------------------------
       N11     11N1NNN11N111N        11111NNNNNNNNN1N11N1NNN1NN
```

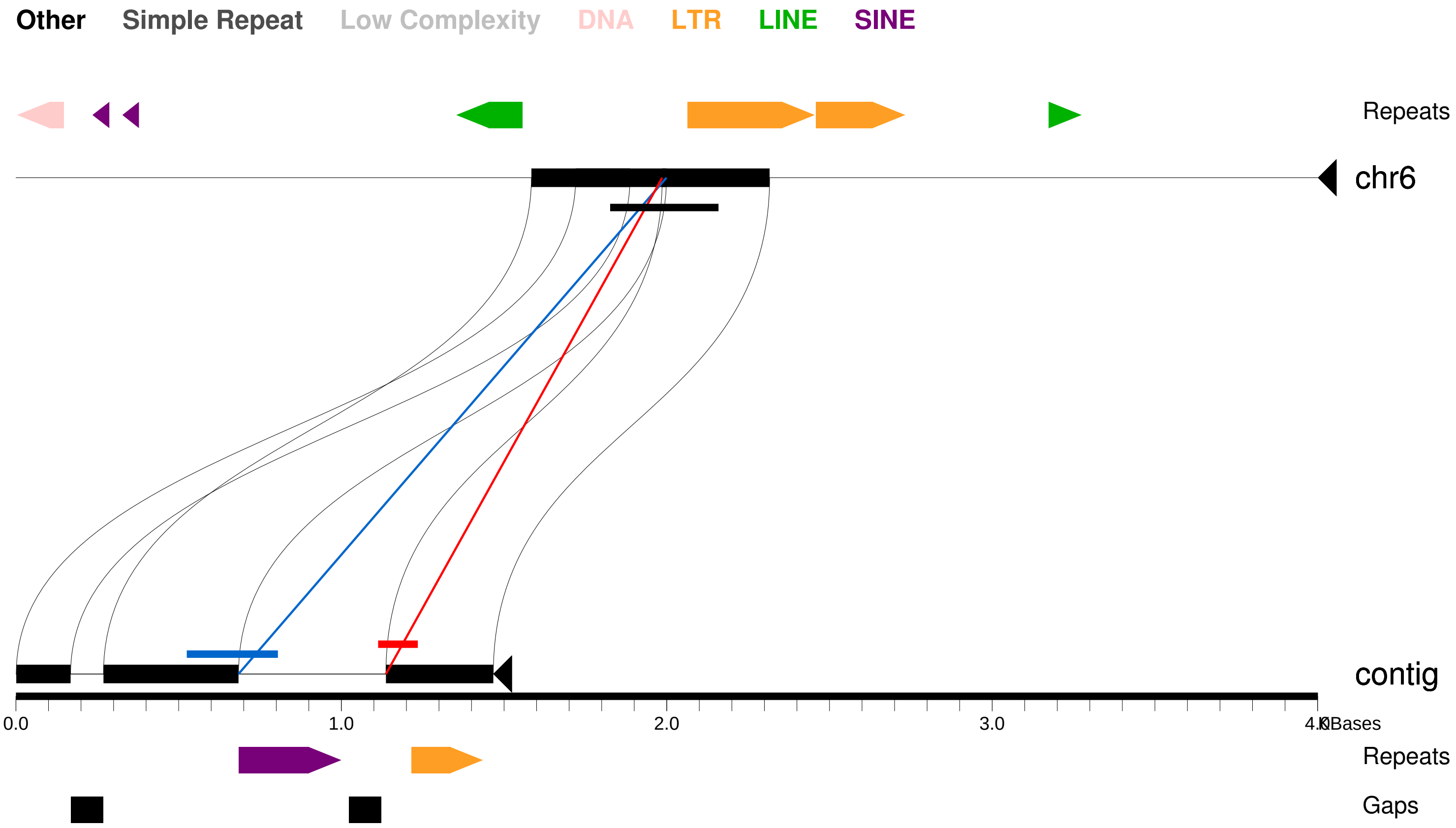

siteID: chr6_78364264 Scafftig5 - Breakpoint: chr6:78364255
chr6:78364212-78364314
Overlap Length: 17 INS size: 459 Genotyped: No

```
left     TCTTTATCCATAG----------------ATTCAAACCAACCACAGATTGAATATGTTTAAAAAATTGAA
chrom    TCTTTATCCATAG----------------ATTCAAACCAACCACAGATTGAATATGTTTAAAAAATTGAA
right    NNNNNNNNNNNNNNNNNNNNNNNNNNNNNNNNNNNNAAAAAAAAAAAAAAAAAAAAAAAAAAAAAATTGAA
         111111111111111111111111111111111111*11**11*1*1*111**1*11111**********

left     AACAATGGGCGCGGTGGCTCACGCCTGTAATCCCAGCACTTTGGGAGGCCGAGGCGGGCGGATCA
chrom    AACAATAAT-----------ACAACACTAAAAAAAAAAT-----GAATAAAAGTACTACTACTGT
right    AACAATAAT-----------ACAACACTAAAAAAAAAAT-----GAATAAAAGTACTACTACTGT
         ******22222222222222**22*22***2222*22*222222**22222**22222*222*22
```

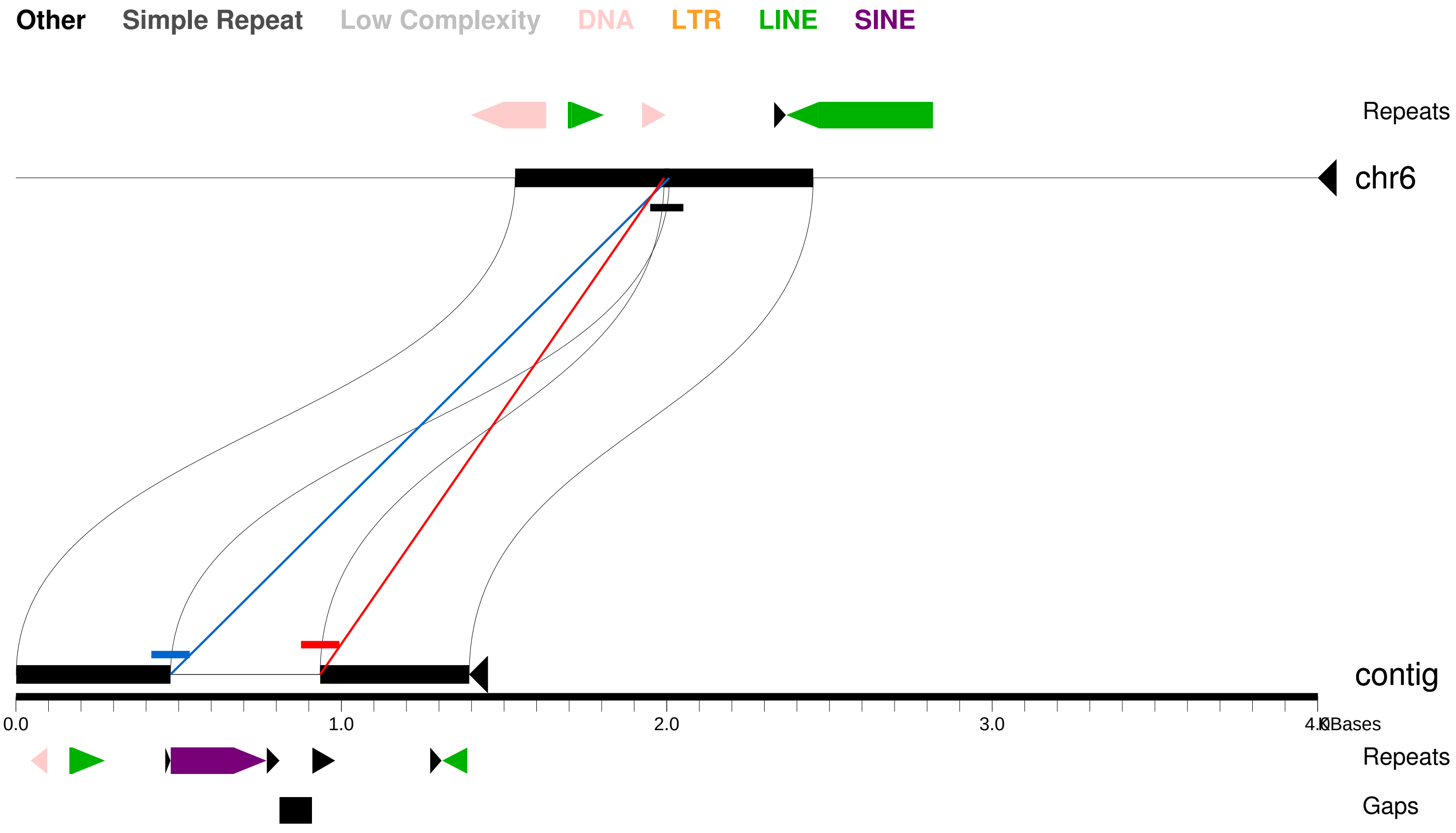

siteID: chr6_78416747 Scafftig2 - Breakpoint: chr6:78416754
chr6:78416710-78416813
Overlap Length: 16 INS size: 305 Genotyped: Yes

```
left     TGT---GACGTTGCTATAAAG--ATAC-CTG---------AAAATGTGGATGTGGTTTTGAAAGTGGGCA
chrom    TGT---GACGTTGCTATAAAG--ATAC-CTG---------AAAATGTGGATGTGGTTTTGAAAGTGGGCA
right    ACTCCAGCCTGGGCGACAGAGCGAGACTCCGTCTCAAAAAAAAAAAAAAAAAAAAAAAAGAAAGTGGGCA
         11*111*1*111**1*1*1**11*1**1*1*111111111****11111*111111111***********

left     ATGGGGCCGGGCGCGGTGGCTCACGCCTGTAATCCCAGCACTTTGGGAGGCCGAGGCGGGCGGA
chrom    ATGGG-CAGAGTTTGGGAGA----GTTTGGAG----AGC---TAAGAAG---AAGACAGGAAGC
right    ATGGG-CAGAGTTTGGGAGA----GTTTGGAG----AGC---TAAGAAG---AAGACAGGAAGC
         *****2*2*2*222**22*22222*22**2*22222***222*22*2**2222**2*2**22*2
```

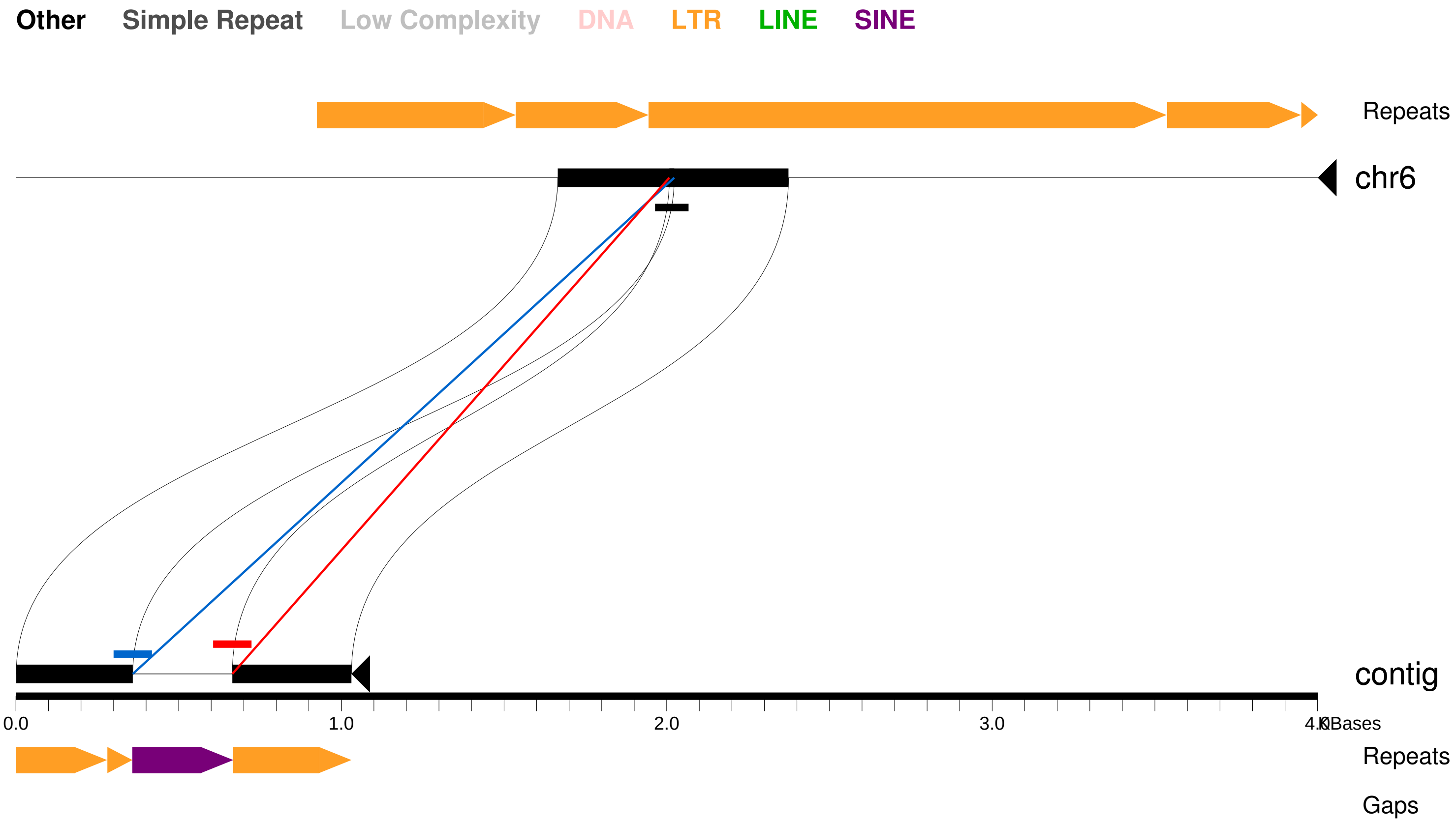

siteID: chr6_78775161 Scafftig2 - Breakpoint: chr6:78775170
chr6:78775164-78775229
Overlap Length: 54 INS size: 439 Genotyped: No

```
left    GAGCTC-----------------------------------------------------TTTCAATGAGC
chrom   GAGCTC-----------------------------------------------------TTTCAATGAGC
right   NNNNNNNNNNNNNNNNNNNNNNNNNNNNNNNNNNNNNNNNNNNNNNNNNNNNNNNNNNNTTTCAATGAGC
        11111111111111111111111111111111111111111111111111111111111***********

left    TCCTTTGTTTCTTTGACAAACTCCTATTAAAATGGTGTTTTTGGCCGGGCGCGGTGGCTCACGCCTGTAA
chrom   TCCTTTGTTTCTTTGACAAACTCCTATTAAAATGGTGTTTTTG----------------------TTT--
right   TCCTTTGTTTCTTTGACAAACTCCTATTAAAATGGTGTTTTTG----------------------TTT--
        *******************************************2222222222222222222222*2*22

left    TCCCAGCACCTTGGGAGGCCGAGGCGGGCGGA
chrom   ----------TTG-------------------
right   ----------TTG-------------------
        2222222222***2222222222222222222
```

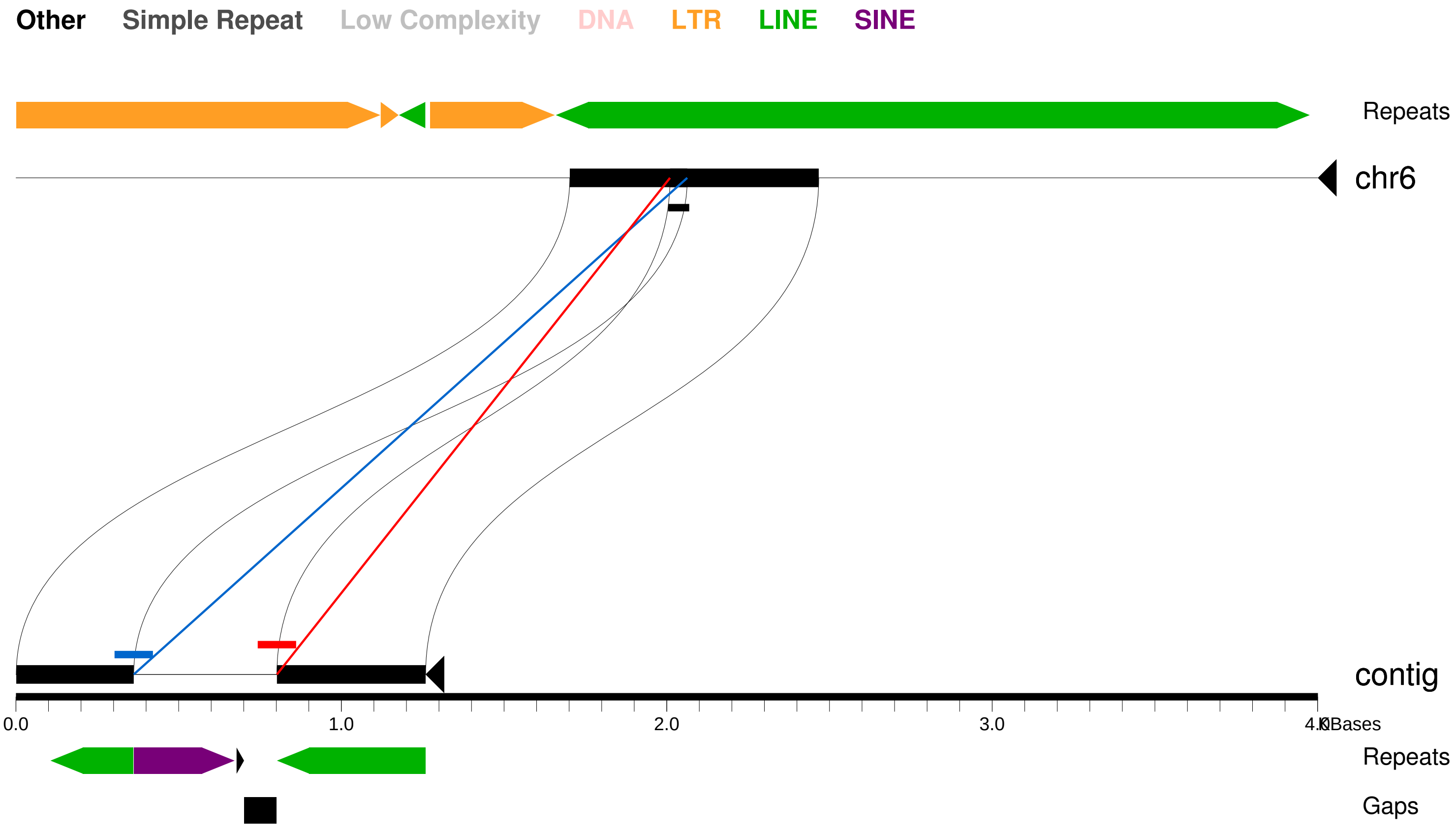

siteID: chr6_78843181 Scafftig9 + Breakpoint: chr6:78843172
chr6:78843012-78843344
Overlap Length: 13 INS size: 281 Genotyped: Yes

```
left    ----------CAGAAATGGAACATGGGAGGGGACAAATAAGGAAATAAAAGCTGGCCACCCCAGCTAGCA
chrom   TCTGATAGGACAGAAATGGAACATGGGAGGGGACAAATAAGGAAATAAAAGCTGGCCACCCCAGCTAGCA
right   TAATTTT---------TGTATTTTTAGTAGAGAC------GGGGTTTCACCGTGTT------AGCCAGGA
        2NNNN2N   111111**1*111*11*11*1***111111**111*11*111**11111111***1**1*

left    GTGGCAACCCACTTGGGTCCCCTTCCATGCAGTGAAAGCTTTGTTTTTTTGTTCTTCACAATAAACCTTG
chrom   GTGGCAACCCACTTGGGTCCCCTTCCATGCAGTGAAAGCTTTGTTTTTTTGTTCTTCACAATAAACCTTG
right   TGGTC--------TCGATCTCCTGACCTC--GTGA----TCCGCCCGTCTCGGCCTCCCAAAGTGC--TG
        11*1*11111111*1*1**1***11*1*111****1111*11*1111*1*111*1**1***1111*11**

left    CTACTGCCCACTCTTTGGGTCCCTGCCATCTTTTTTTTTTTTTTTTTTTTGAG-ACGG------AGTTTC
chrom   CTACTGCCCACTCTTTGGGTCCCTGCCATCTTTAAGAGCTGTAACACTCACCGCACAGGTCCACAGCTTC
right   GGATTACAGGC-----GTGACCCTGCCATCTTTAAGAGCTGTAACACTCACCGCACAGGTCCACAGCTTC
        11*1*1*111*11111*1*1*************222222*2*22222*2222*2**2*222222**2***

left    GTTCT----GTCGCCCAGGCTGGAGTGC-AGTGGCGTGA------TCTTGACTCACT----GCAAGCTCC
chrom   ATTCTTGAAGTCAATGAGACCACAAACCCACTGGAAGGAACCAATTCTGGACACAATTGGTGCCCACTCA
right   ATTCTTGAAGTCAATGAGACCACAAACCCACTGGAAGGAACCAATTCTGGACACAATTGGTGCCCACTCA
        2****2222***2222**2*222*222*2*2***222**222222***2***2**2*2222**222***2

left    G-----CC----TCCCGGG-----TTCACGCCA----TTC-TCCTGCCTCAG-
chrom   GGATTTCCAAGTTCGAGGGAATTGTTCAGGCTACGGTTTCATCATGGGACAAT
right   GGATTTCCAAGTTCGAGGGAATTGTTCAGGCTACGGTTTCATC----------
        *22222**2222**22***22222****2**2*2222***2**N11NNN11N
```

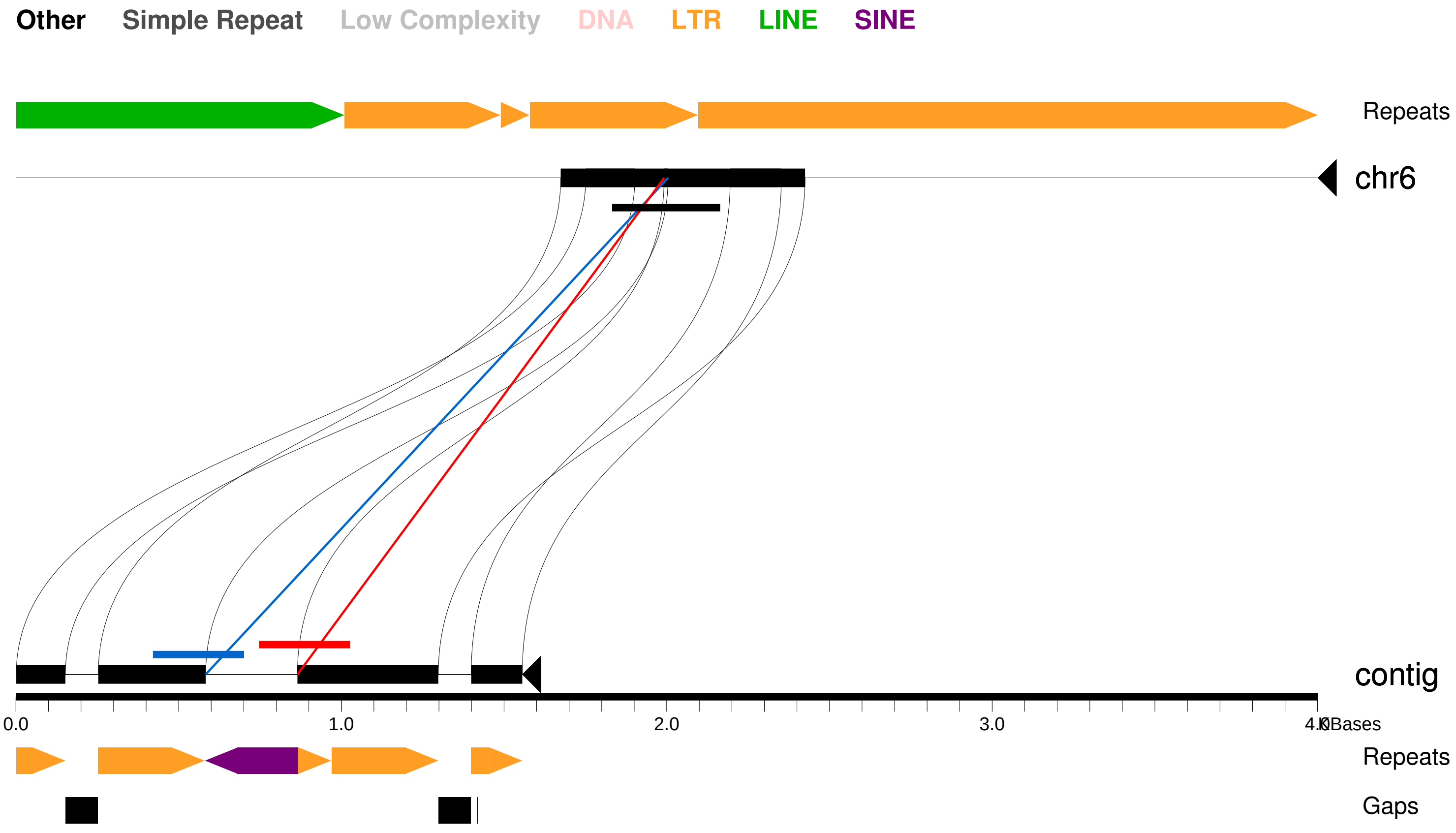

siteID: chr6_79723803 Scafftig1 - Breakpoint: chr6:79723785
chr6:79723740-79723844
Overlap Length: 15 INS size: 379 Genotyped: No

```
left   GTGCACACCAAA---------TAATTAACACTGTTTTCTAGAAATAGAGGGTTT-----TACAAACCTTA
chrom  GTGCACACCAAA---------TAATTAACACTGTTTTCTAGAAATAGAGGGTTT-----TACAAACCTTA
right  GCGCCCGCCACTACGCCCGGCTAATTTTTTGTATTTTTAGTAGAGACGGGGTTTCACCGTACAAACCTTA
       *1**1*1***11111111111*****11111*1****1111*1*1*11******11111***********

left   TTTCNNNNNNNNNNNNNNNNNNNNNNNNNNNNNNNNNNNNNNNNNNNNNNNNNNNNNNNNNNN
chrom  TTTCTTACCTAATGTATTCTAAGGCACAGCCTTAAAGATAGCTAAAGCT--------------
right  TTTCTTACCTAATGTATTCTAAGGCACAGCCTTAAAGATAGCTAAAGCT--------------
       ****22222222222222222222222222222222222222222222222222222222222
```

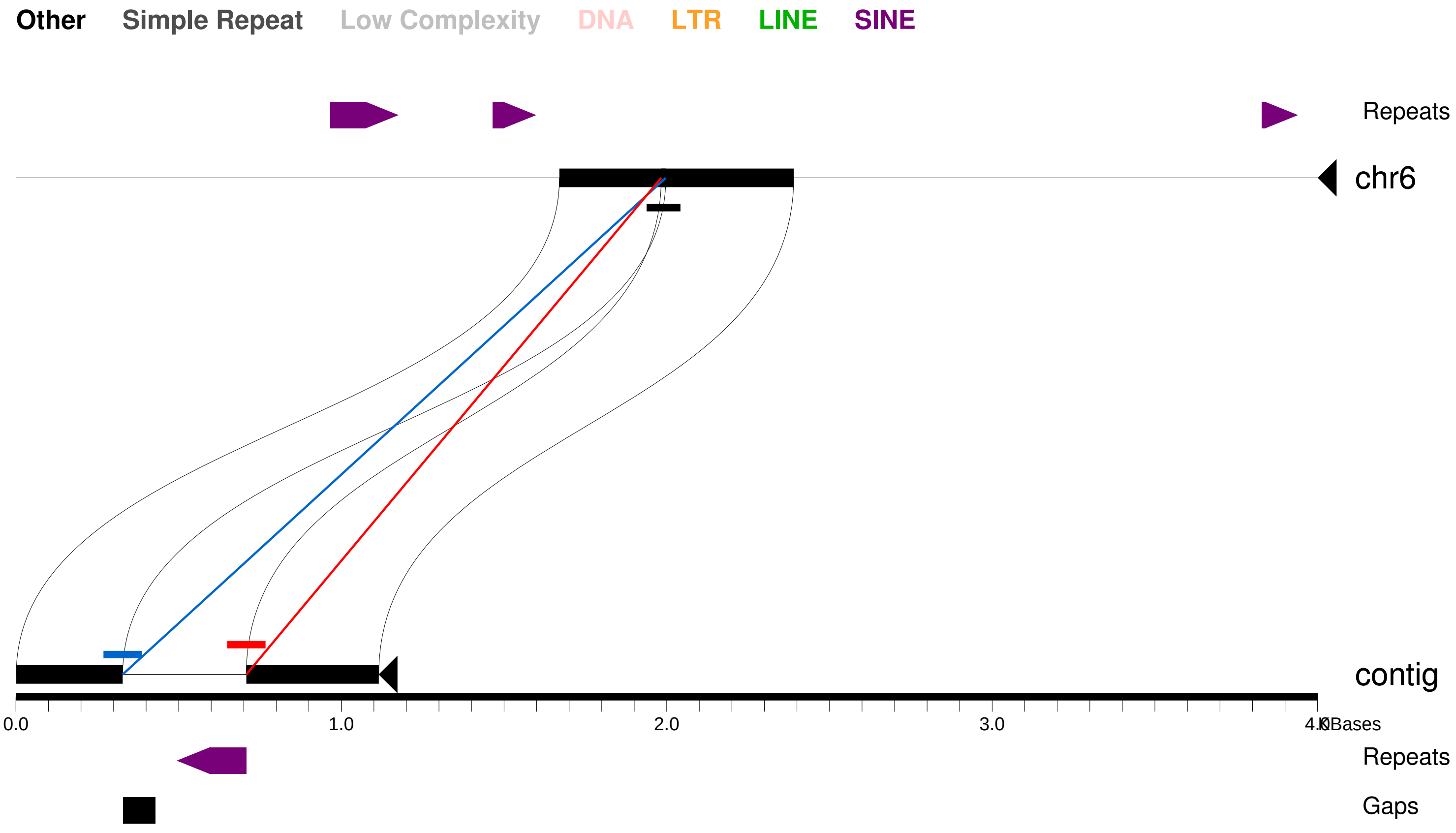

siteID: chr6_80086786 Scafftig1 + Breakpoint: chr6:80086746
chr6:80086695-80086805
Overlap Length: 9 INS size: 422 Genotyped: No

```
left     AAA----TATTAGACTTAGAATGTTGTTTGCATTAC-----TAATCATCAGCGCTCTCATAAAGTCAAAN
chrom    AAA----TATTAGACTTAGAATGTTGTTTGCATTAC-----TAATCATCAGCGCTCTCATAAAGTCAAAG
right    CCACCCGCCTCGGCCTCCCAAAGT-GCTGGGATTACAGGCGTGAGCCACCGCGCCCGGCCAAAGTCAAAG
         11*111111*11*1**111**1**1*1*1*1*****11111*1*1*11*1****1*1111*********2

left     NNNNNNNNNNNNNNNNNNNNNNNNNNNNNNNNNNNNNNNNNNNNNNNNNNNNNNNNNN
chrom    TTTTATTCTTTCTTACTATTCAGATGTTTTACTCTCCAGTCATGCTTCAC--------
right    TTTTATTCTTTCTTACTATTCAGATGTTTTACTCTCCAGTCATGCTTCAC--------
         2222222222222222222222222222222222222222222222222222222222
```

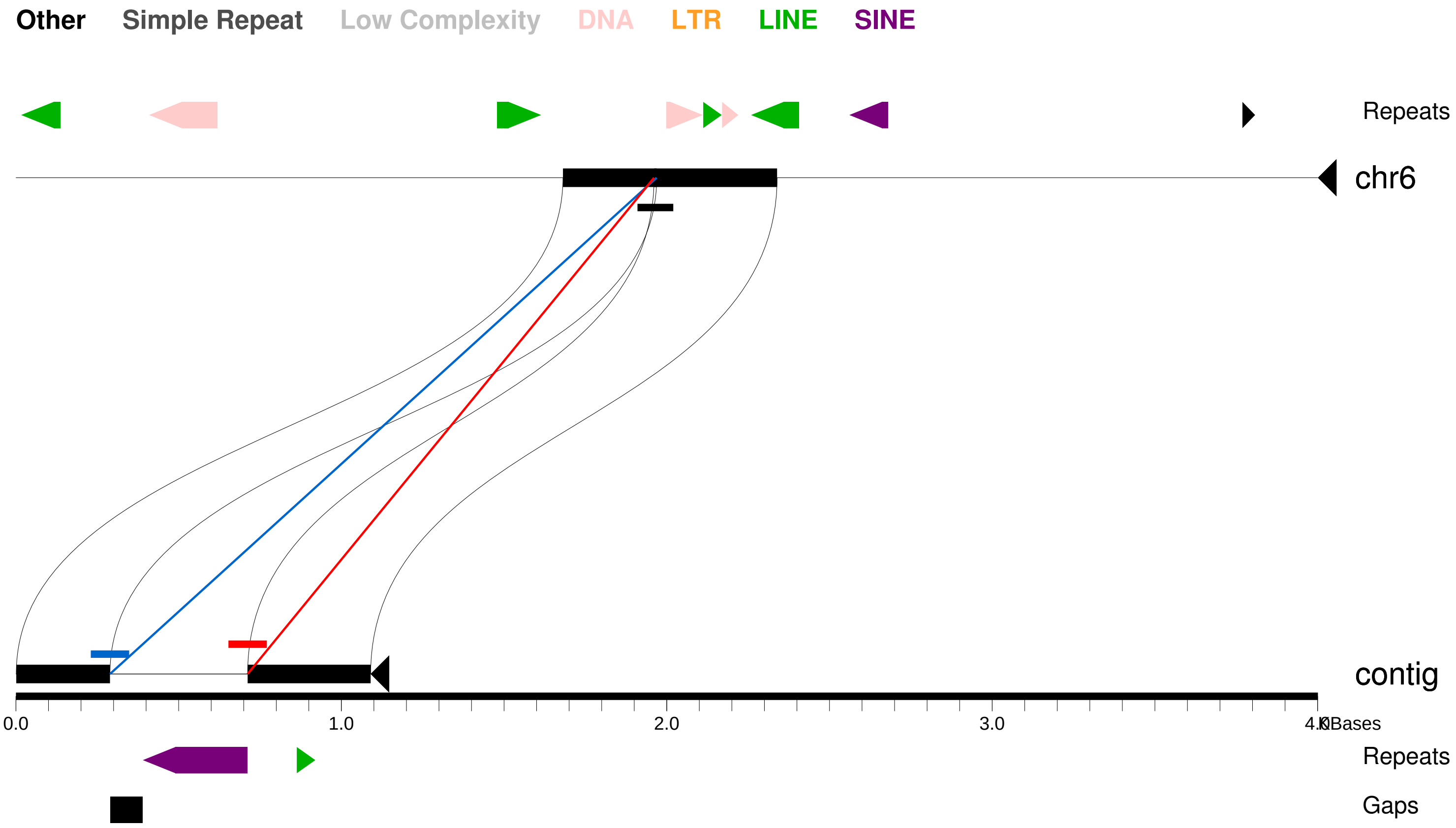

siteID: chr6_81381045 Scafftig5 - Breakpoint: chr6:81381047
chr6:81381002-81381089
Overlap Length: 15 INS size: 299 Genotyped: Yes

```
left    ---GCTCATATCT-----------TAAATAATGGAATACA-----------------ATAAAAGAGTAAA
chrom   ---GCTCATATCT-----------TAAATAATGGAATACA-----------------ATAAAAGAGTAAA
right   TGAGCCGAGATCGCGCCACTGCACTCCAGCCTGGGCGACAGAGCGAGACTCCGTCTCAAAAAAAAAAAAA
        111**11*1***111111111111*11*111***111***11111111111111111*1****1*11***

left    ATAGTTAAAAACATAGCTTAGGCCGGGCGCGGTGGCTCACGCCTGTAATCCCAGCACTTTGGGAGGCCGA
chrom   ATAGTTAAAAACATAGCTTAG-----------TGA------AATGTAA----ATTAATATATGAAGACAA
right   AAAAAAAAAAACATAGCTTAG-----------TGA------AATGTAA----ATTAATATATGAAGACAA
        *1*111**************22222222222**222222222*****2222*22*2*2*22**2*2*2*

left    GGCGGGCGGA
chrom   ----------
right   ----------
        2222222222
```

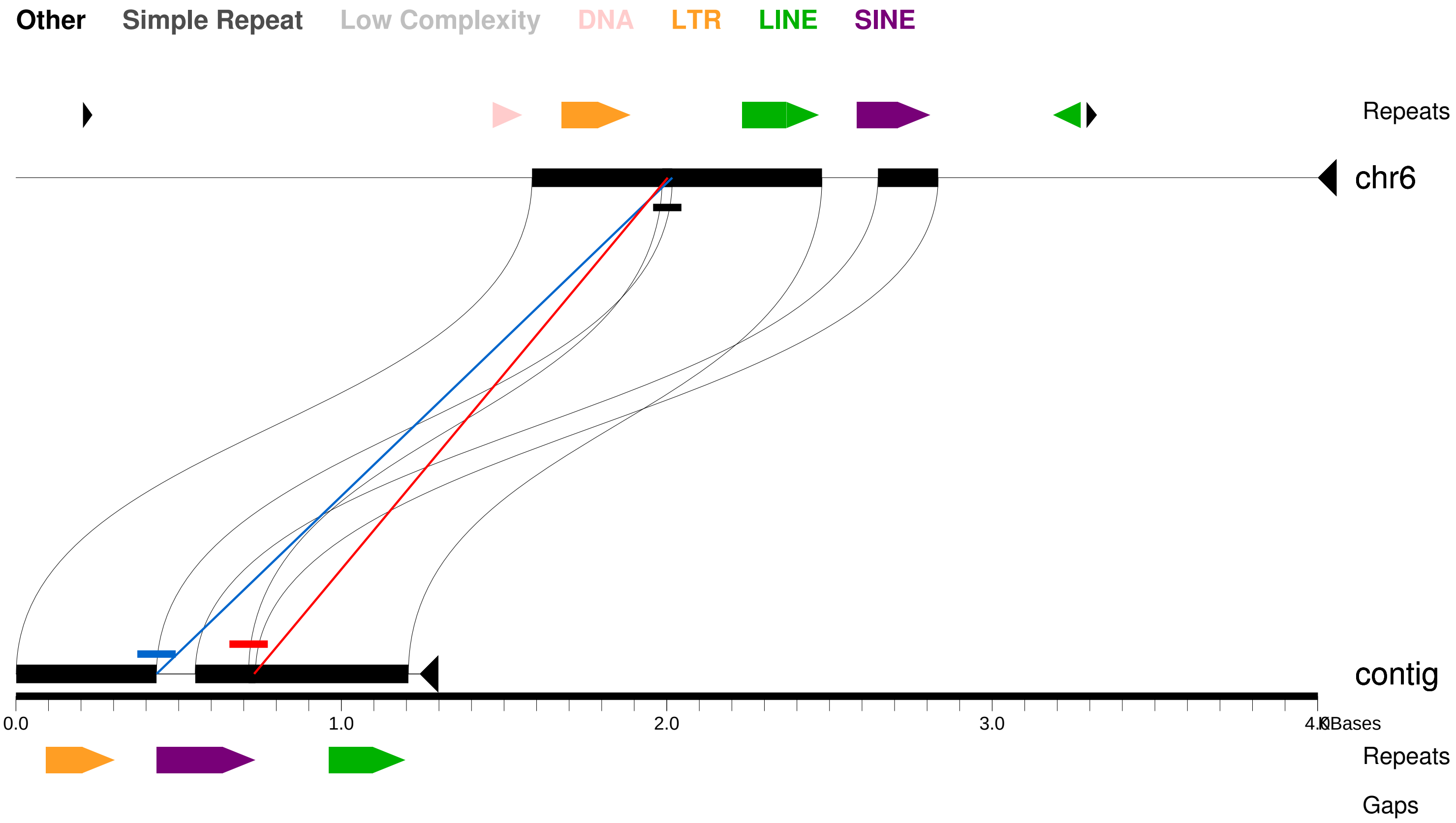

siteID: chr6_82297440 Scafftig2 - Breakpoint: chr6:82297423
chr6:82297263-82297598
Overlap Length: 16 INS size: 429 Genotyped: No

```
left    TC-------------------------------------------GTTGCATAGTATTCTATGGTGTATA
chrom   TCATCCATGTCCCTTCAAAG-GACATGAACTCATCCTTCTTTATCGTTGCATAGTATTCTATGGTGTATA
right   T------------TTAGTAGAGACGGGGTTTCACCGTG--TTAGC----CAGA-------ATGGTCTCGA
        *1           22NNN221222NN2NNN222N2N2N  222N21111**1*1111111*****1*11*

left    TGTGCCATATTTTCTTAATCCAGTCTGTCATTGATGGGCATTTGGGTTGG-TTCCAAGTCTTTGCTATTG
chrom   TGTGCCATATTTTCTTAATCCAGTCTGTCATTGATGGGCATTTGGGTTGG-TTCCAAGTCTTTGCTATTG
right   TCTCC--TGACCTCGTGATCC-GCCCGCCTCGGCCTCCCAAAGTGCTGGGATTACAGGCGTGAGCCACCG
        *1*1*11*1111**1*1****1*1*1*1*111*11111**1111*1*1**1**1**1*11*11**1*11*

left    TGAATAGTGCTGCAATAAACATGTGTGCATGTATCTTTTNNNNNNNNNNN--------------------
chrom   TGAATAGTGCTGCAATAAACATGTGTGCATGTATCTTTATAGTAGAATGATTTATAATCCTTTGGGTATA
right   CGCC-----CGGCC--------GTGTGCATGTATCTTTATAGTAGAATGATTTATAATCCTTTGGGTATA
        1*1111111*1**111111111***************22222222222222222222222222222222

left    ----------------------------------------------------------------------
chrom   TACCCAGTAATGGGATTGCTGGGTCAAACGATATTTCTGGTTCTAGATCCTTGAGAAATCACCACACTGT
right   TACCCAGTAATGGGATTGCTGGGTCAAACGATATTTCTGGTTCTAGATCCTTGAGAAATCACCACACTGT
        2222222222222222222222222222222222222222222222222222222222222222222222

left    ----------------------------------------------------------
chrom   CTTCCACAATGGTTGAACTAATTTACACTCCCAACAGTGTAAAAGCGTTCCTATCGCT
right   CTTCCACAATGGTTGAACTAATTTACACTCCCAACAGTGTAA----------------
        222222222222222222222222222222222222222222
```

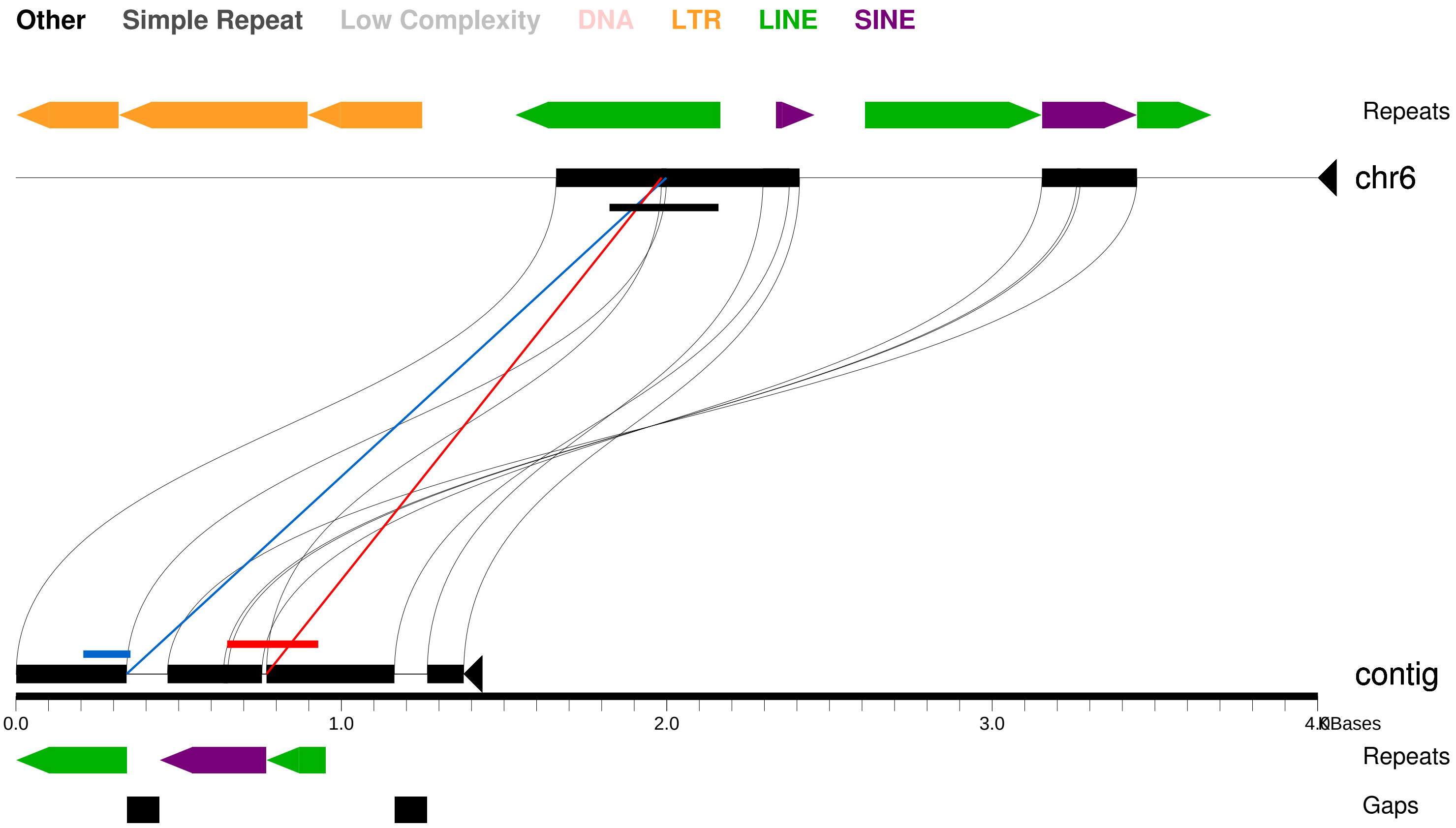

siteID: chr6_88698059 Scafftig2 + Breakpoint: chr6:88698068
chr6:88698018-88698124
Overlap Length: 10 INS size: 296 Genotyped: Yes

```
left     AAATG-GTGC----TGGGAT---AACTGGCTAGC--CATATGCAGAAG--ATTGAAACTGGGCCCCTTTC
chrom    AAATG-GTGC----TGGGAT---AACTGGCTAGC--CATATGCAGAAG--ATTGAAACTGGGCCCCTTTC
right    TGATCCGTCCGCCTCGGCCTCCCAAAGTGCTGGGATTACAGGCGTGAGCCACCGCGCCCGGCCCCCTTTC
         11**11**1*11111**11*111**111***1*1111*1*1**111**11*11*111*1**1********

left     TTTTTTTTTTTTTTTTTTGAGACGGAGTCTCGCTCTGTCGCCCAGGCTGGAGTGCAGTGGC
chrom    TTAAACTATATA----CAAAAATTAACTCAAGATTGATTA--AAGGCTTAAATAT------
right    TTAAACTATATA----CAAAAATTAACTCAAGATTGATTA--AAGGCTTAAATAT------
         **2222*2*2*22222222*2*222*2**22*2*222*22222*****22*2*22222222
```

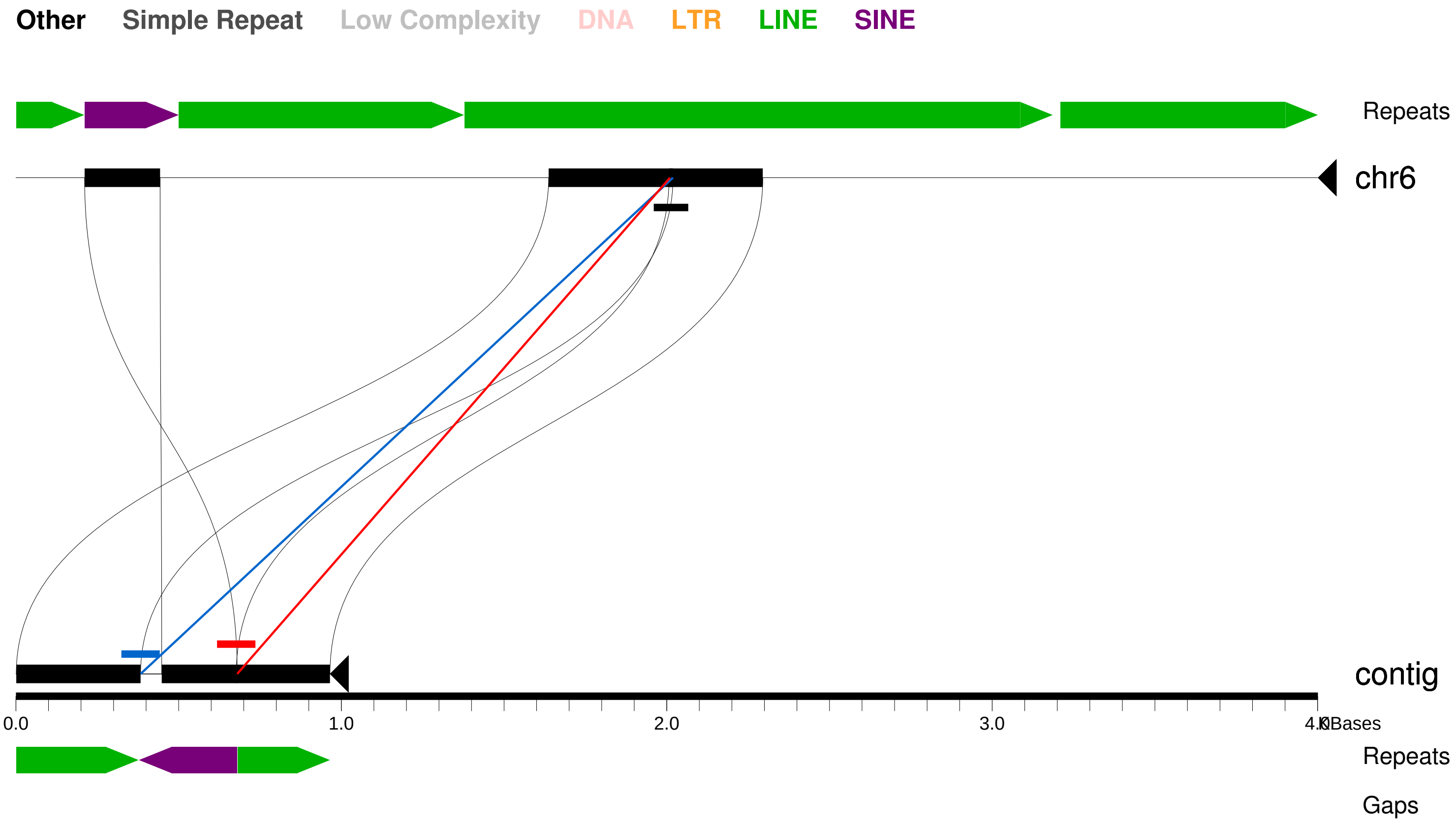

siteID: chr6_93032717 Scafftig1 + Breakpoint: chr6:93032745
chr6:93032699-93032804
Overlap Length: 14 INS size: 316 Genotyped: Yes

```
left    A----------GGT-----AAAATACAGG--TTACAG---TAAGTCAAAGGGCTTTCAAGTTTGGGTATA
chrom   A----------GGT-----AAAATACAGG--TTACAG---TAAGTCAAAGGGCTTTCAAGTTTGGGTATA
right   CCACCCGCCTCGGCCTCCCAAAGTGCTGGGATTACAGGCGTGAGCCACCGCGCCCG-------GCCTATA
        11111111111**111111***1*1*1**11******111*1**1**11*1**1111111111*11****

left    TATGCTTTTTTTTTTTTTTTTTTTTTTTTTTTTTTTTTTTGAGACGGAGTCTCGCTCTGTCGCCCAGGCCG
chrom   TATGCTTTTTTAAGAAACTTTAATGGAAGAATTATAT---GGCTTGAGT-----TCTAATGCCAA-----
right   TATGCTTTTTTAAGAAACTTTAATGGAAGAATTATAT---GGCTTGAGT-----TCTAATGCCAA-----
        **********2222222***22*2222222**2*2*2222*222****22222***222***2*22222
```

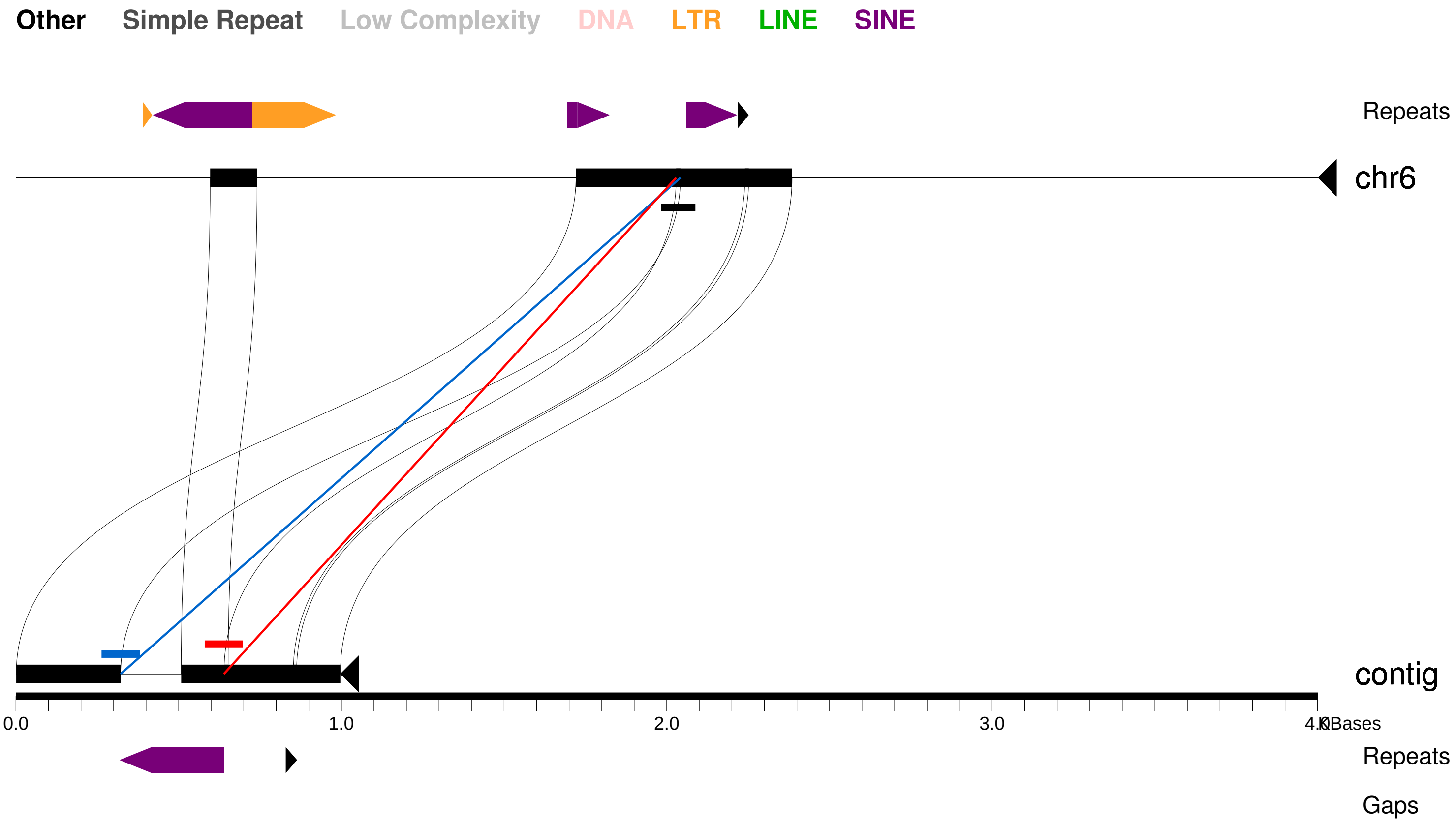

siteID: chr6_93576141 Scafftig1 - Breakpoint: chr6:93576135
chr6:93575975-93576308
Overlap Length: 14 INS size: 301 Genotyped: Yes

```
left   --------------AGTTTTCATGATATAAATTAAGGATATGCCAGACAATAAATTACTAATTTGATCAA
chrom  GCCTCCTTTTAACCAGTTTTCATGATATAAATTAAGGATATGCCAGACAATAAATTACTAATTTGATCAA
right  GAGGC------AGGAGA----ATGGCGTGAACCCGGGA---GGCGGAGC-------------TTG--CAG
       2NNN2      2NN**11111***111*1**1111***111*1*1**111111111111111***11**1

left   TTAATCCATACCAGATAATTTGATTAGCCAAATGAATACTTGTTGAGTTCCTGCAATATCCTAGGTTCTG
chrom  TTAATCCATACCAGATAATTTGATTAGCCAAACGAATACTTGTTGAGTTCCTGCAATATCCTAGGTTCTG
right  TGAGCCGAGATCCC------------GCCACTGCACTCCAGCCTGGGCGACAGAGCGAGACTCCGT-CT-
       *1*11*1*1*1*111111111111111****11N1*1*1*1111**1*111*1*1111*11**11**1**1

left   TGCATGCTAAACACCAGCATAAAAAGACTGTATGGGCCGGGCGCGGT--GGCTCACGCCTGTA-ATCCCA
chrom  TGCATGCTAAACACCAGCATAAAAAGACTGTATGACACAATCCTTGTCTGACTCTTGCTCCAAGATTCAT
right  --CAAAAAAAAAAAAAAAAAAAAAAGACTGTATGACACAATCCTTGTCTGACTCTTGCTCCAAGATTCAT
       11**1111***1*11*11*1**************222*222*222**22*2***22**2222*2**2*22

left   GCACTTTGGGAGGCCGAGGCGGGCGGA--TCACGAG------GTCAGGAGATTGAGACCATCCCGGCT--
chrom  AAACACTGTTAGACCAGATAGATCTGTCTTCCCTATAGATCTGTCTTCCCTGTTACACCAGCTGGATTTG
right  AAACACTGTTAGACCAGATAGATCTGTCTTCCCTATAGATCTGTCTTCCCTGTTACACCAGCTGGATTTG
       22**22**22**2**22222*22*2*222**2*2*2222222***2222222*2*2****2*22*22*22

left   -AAAACG---------GTGAAACCCCGTCT------------CTACTAAA----
chrom  GACAACATGAGTTGCTGTGAGACCTAGACTTAGAGTAGAGGCCTAAGGAAGAAT
right  GACAACATGAGTTGCTGTGAGACCTAGACTTAGAGTAGAGGCCTA---------
       2*2***2222222222****2***22*2**222222222222***NNN11
```

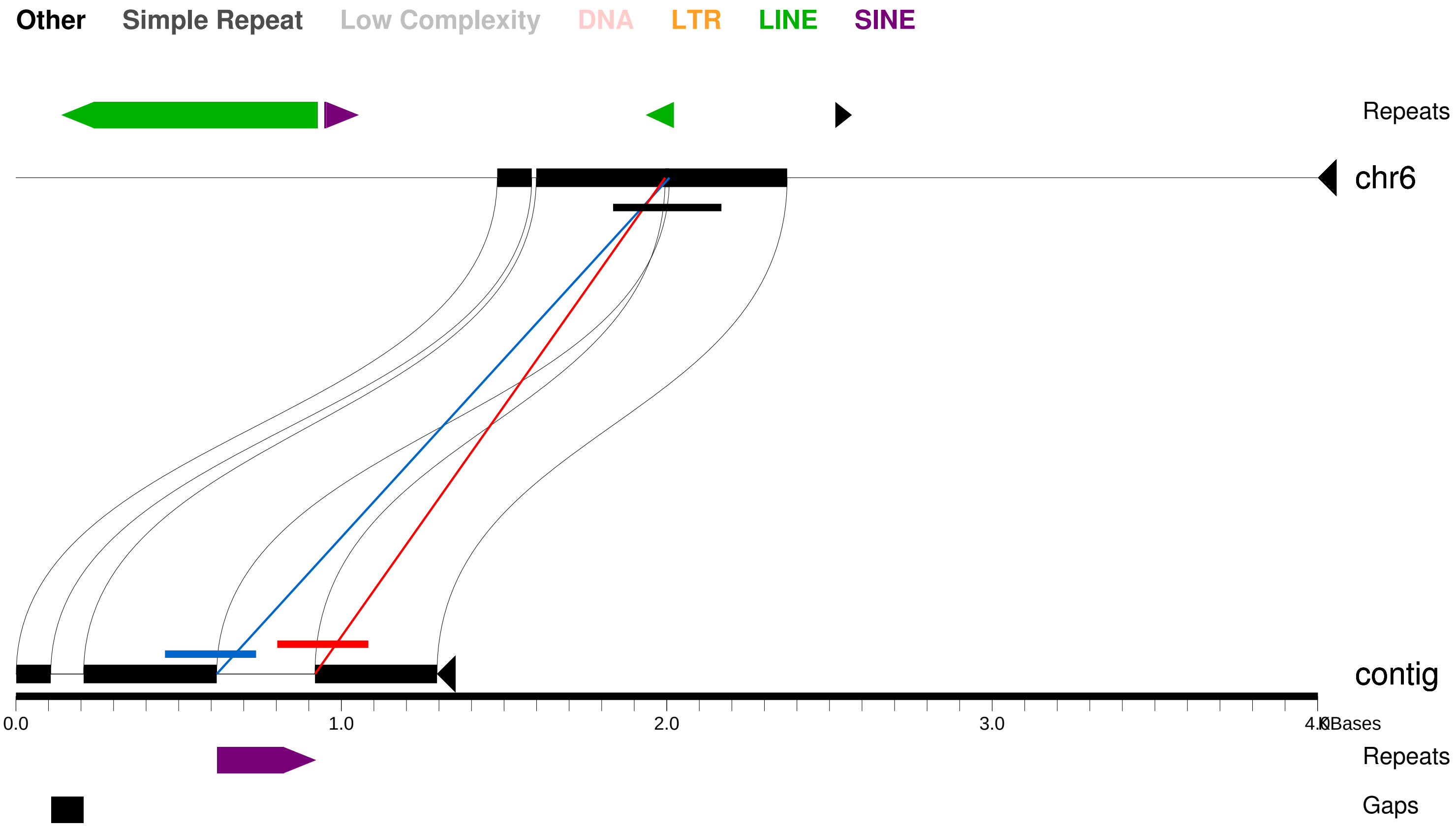

siteID: chr6_9419656 Scafftig1 - Breakpoint: chr6:9419569
chr6:9419526-9419628
Overlap Length: 17 INS size: 171 Genotyped: Yes

```
left    --------------AGAGAATTTGGCTTATATT--AATTATAATTACAGATGTAGTTATAAGATATGCAA
chrom   --------------AGAGAATTTGGCTTATATT--AATTATAATTACAGATGTAGTTATAAGATATGCAA
right   CTCCAGCCTGGGCGACAGAGCGAGACTCCGTCTCAAAAAAAAAAAAAAAAAAAAAAAAAAAGATATGCAA
        11111111111111*1***1111*1**11111*11**11*1**11*1*1*111*111*1***********

left    ACTTTAGCCGGGCGTGGTAGCGGGTGCCTGTAGTCCCAGCTACTCGGGAGGCTGAGGCAGGAGAA
chrom   ACTTTATT---------TAAACCATCC---TATTAGCACCAAAGCAGTAC----AGATTGAACAC
right   ACTTTATT---------TAAACCATCC---TATTAGCACCAAAGCAGTAC----AGATTGAACAC
        ******22222222222**22222*2*222**2*22**2*2*22*2*2*22222**222*2*2*2
```

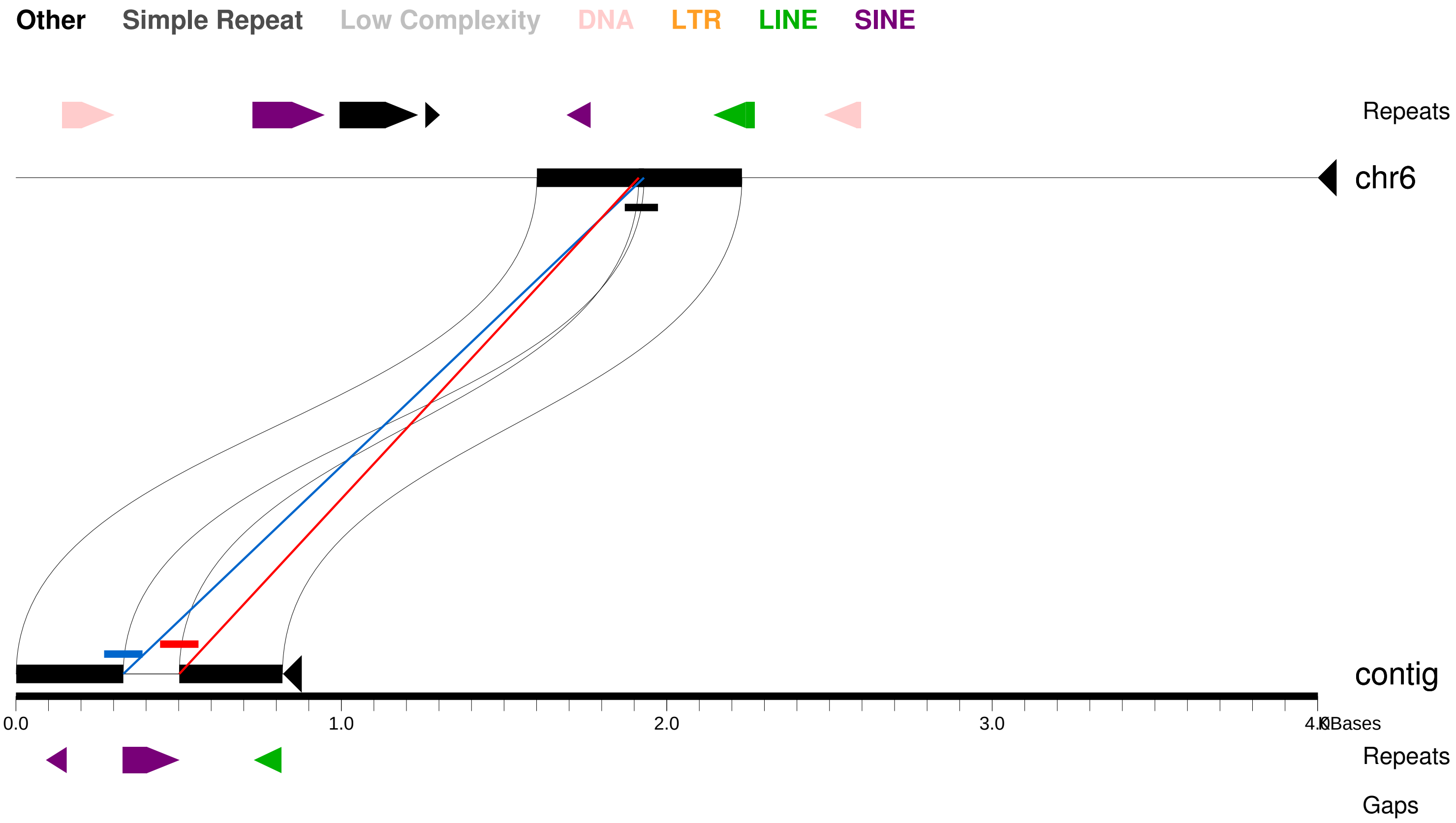

siteID: chr6_94930780 Scafftig4 + Breakpoint: chr6:94930771
chr6:94930669-94930829
Overlap Length: 8 INS size: 426 Genotyped: No

```
left    GACCATTTCCTTTCTTGTTTCTATTTATAATTAATAATTCTGTAGCTCTGTAAAATAACTCAGTCTCAAG
chrom   GACCATTTCCTTTCTTGTTTCTATTTATAATTAATAATTCTGTAGCTCTGTAAAATAACT--GTCTCAAG
right   --CCA---CCCGCCTCGGCC-----------------TCCCAAAGTGCTGGGA--TTACA--GGCGTGAG
        11***111**111**1*11111111111111111111*1*111**11***11*11*1**122*1*111**

left    TTTCAAATCTATAAAATTCTTGCTTTATCTATTTCCAACTCTNNNNNNN---------------------
chrom   TTTCAAATCTATAAAATTCTTGCTTTATCTATTTCCAACTCTCTTTTGAATATAAAAAAATTACATGTAA
right   CCACCGCGC---------CCGGCCT---------CCAACTCTCTTTTGAATATAAAAAAATTACATGTAA
        111*1111*111111111*11**1*111111111********2222222222222222222222222222

left    -----------------------
chrom   ACTTACCTTTGTAATTTAAAAAT
right   ACTTACCTTTGTAATTTAAAAAT
        22222222222222222222222
```

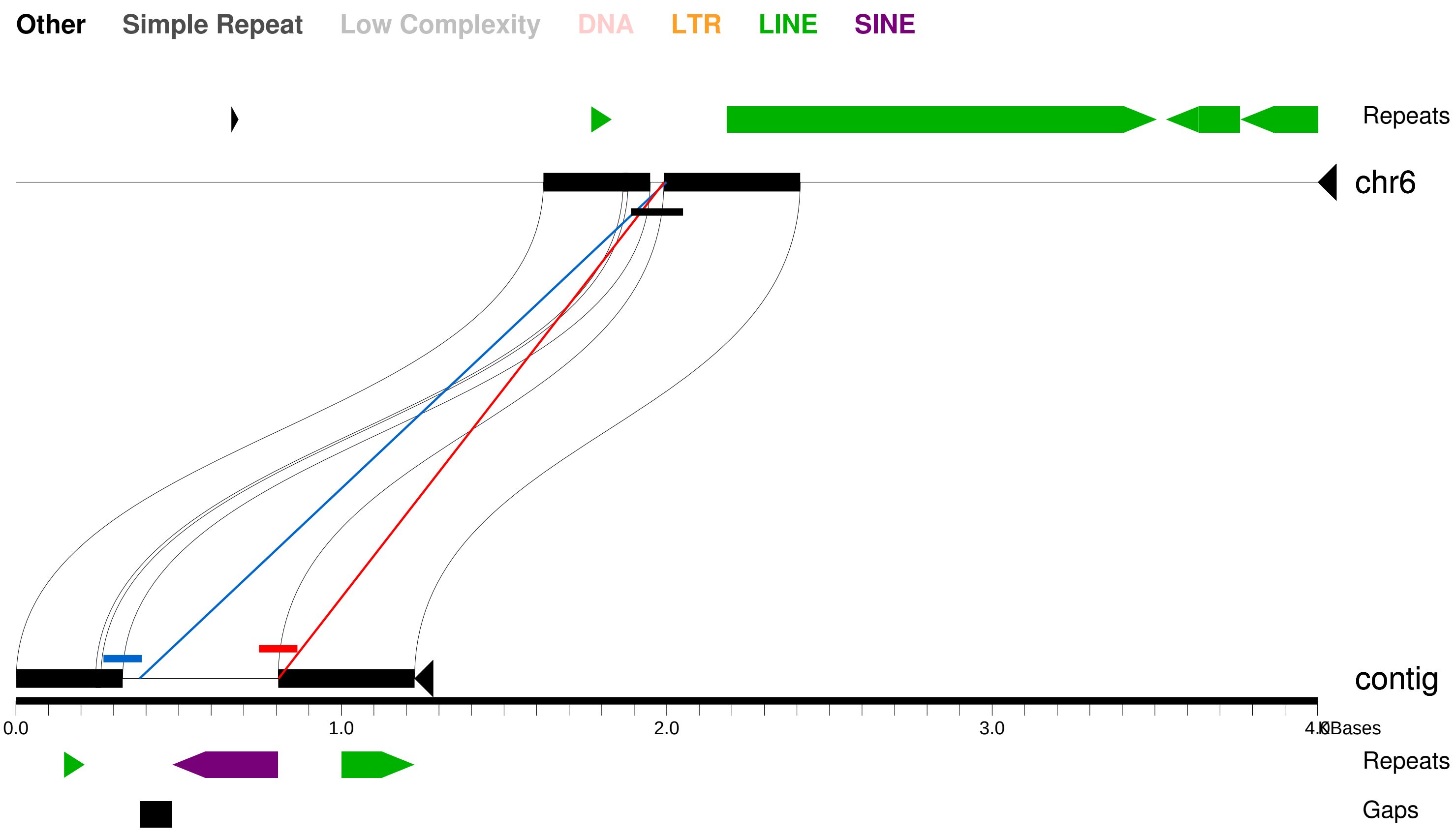

siteID: chr6_95368336 Scafftig4 + Breakpoint: chr6:95368360
chr6:95368314-95368419
Overlap Length: 14 INS size: 317 Genotyped: Yes

```
left    TCA--TGGTTTTACTTTCAGATAATCTT---TTAAATTCCTG-GCTACTTTT-------TTCATTCACTA
chrom   TCA--TGGTTTTACTTTCAGATAATCTT---TTAAATTCCTG-GCTACTTTT-------TTCATTCACTA
right   CCACCCGCCTCGGCCTCCCAAAGTGCTGGGATTACAGGCGTGAGCCACCGCGCCCGGCCTTCATTCACTA
        1**111*11*111*1*1*11*1111**1111***1*11*1**1**1**11111111111***********

left    TCTTTTTTTTTTTTTTTTTTTTTTTTTTTTTTTGGGACGGAGTCTCGCTCTGTCGCCCAGGCC-
chrom   TCTAGTGAACTTAATATATAGGTT--------------GATTCAATTTCTTTCCATTATTTTT
right   TCTAGTGAACTTAATATATAGGTT--------------GATTCAATTTCTTTCCATTATTTTT
        ***22*2222**22*2*2*222**22222222222222**2**2222***2**2222*22222
```

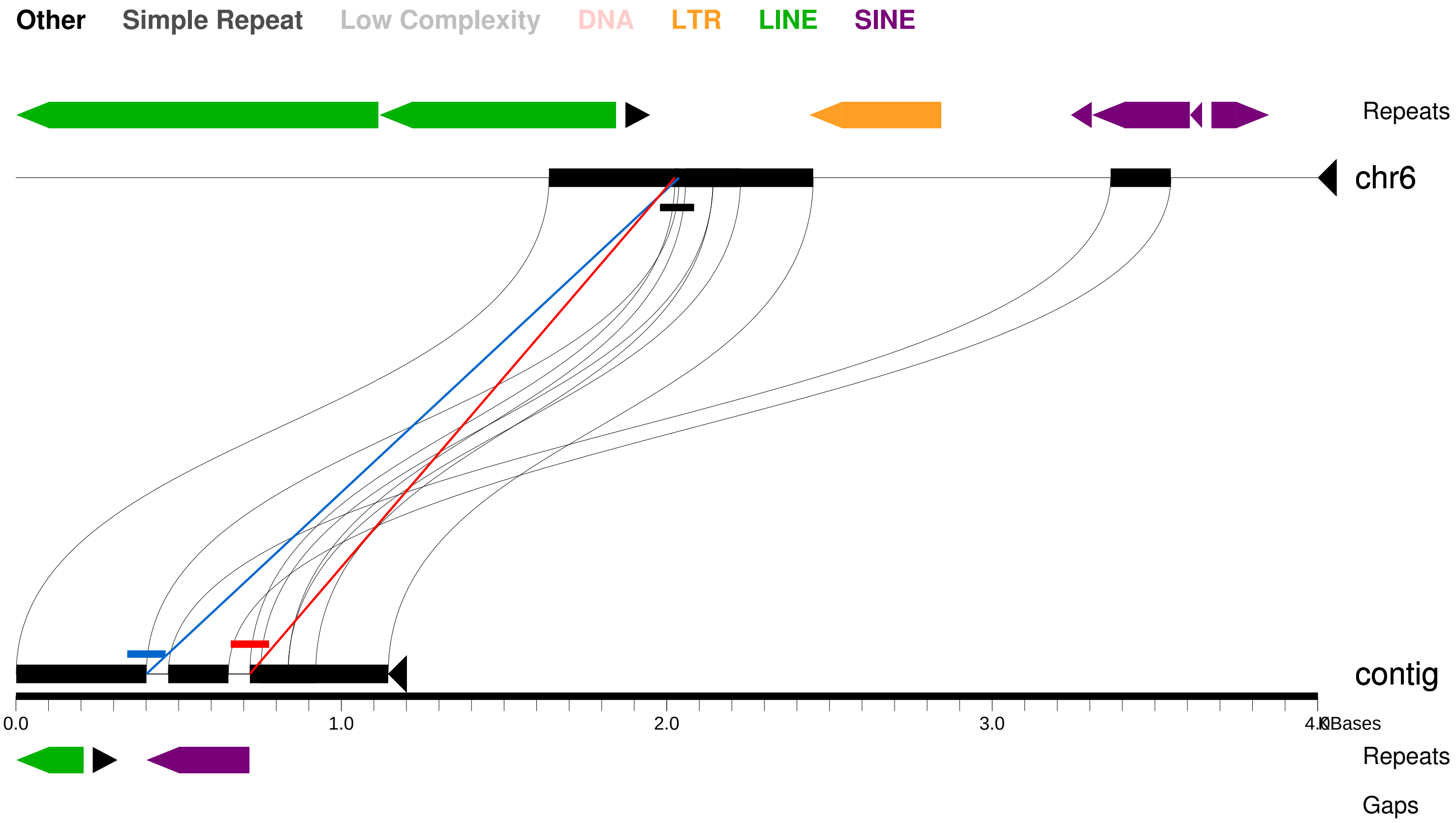

siteID: chr6_96009444 Scafftig1 - Breakpoint: chr6:96009406
chr6:96009246-96009578
Overlap Length: 13 INS size: 387 Genotyped: No

```
left   --------------------------------------ATTAAACCTGGAGTGAACACAGCACATGTCTC
chrom  CTTTTTTAACGAAGCACATTCTGCATAGCCCTAAATCCATTAAACCTGGAGTGAACACAGCACATGTCTC
right  -TTTAGTAGAGACGGGTTTCACCGT-------------TTTAGCCGGGA-TGGTCTCG----ATCTC-C
        222NN22NN22N2NNNN222NN2N2            1**1*1**1***1**11*1*11111**1**1*

left   TGCAAGCACAGGGTTGGGGCTAGGGTTACAGATTAACAGCATCTCAAGGCAGAAGAATTTTTCTTAGTAC
chrom  TGCAAGCACAGGGTTGGGGCTAGGGTTACAGATTAACAGCATCTCAAGGCAGAAGAATTTTTCTTAGTAC
right  TG--ACCTCGTGATCCGCCC--GCCT----------CGGCCTCCCAAAGTGCTGGGATTAC----AGGCG
       **11*1*1*11*1*11*11*11*11*1111111111*1**1**1***1*11111*1***111111**111

left   AGAACAAAATGGAGTCTCTTATGTCTACTTCTTNNNNNNNNNNN--------------------------
chrom  AGAACAAAATGGAGTCTCTTATGTCTACTTCTTTCTACACAGACACAGTAACAGTCTGATCTCTCTTTCT
right  TGAGCCACCGCGCC-CGCCCATGTCTACTTCTTTCTACACAGACACAGTAACAGTCTGATCTCTCTTTCT
       1**1*1*1111*111*1*11*************2222222222222222222222222222222222222

left   ----------------------------------------------------------------------
chrom  TTTCTCCTTATCTTTACTTCTACCTTTCTACGTGTAGGTCTTTATTTACGGATTTTGGTCTAAACAATTG
right  TTTCTCCTTATCTTTACTTCTACCTTTCTACGTGTAGGTCTTCATTTACGGATTTTGGTCTAAACAATTT
       2222222222222222222222222222222222222222222N22222222222222222222222222N

left   -----------------------------------------------------
chrom  TGGTGCCCTCTTATATCCAAGTTTTACATGCCCCTTTATCTTATAAAAACCCC
right  TGGTGCCCTCTTATATCCAAGTTTTACATGCCCCTTTATC-------------
       2222222222222222222222222222222222222222
```

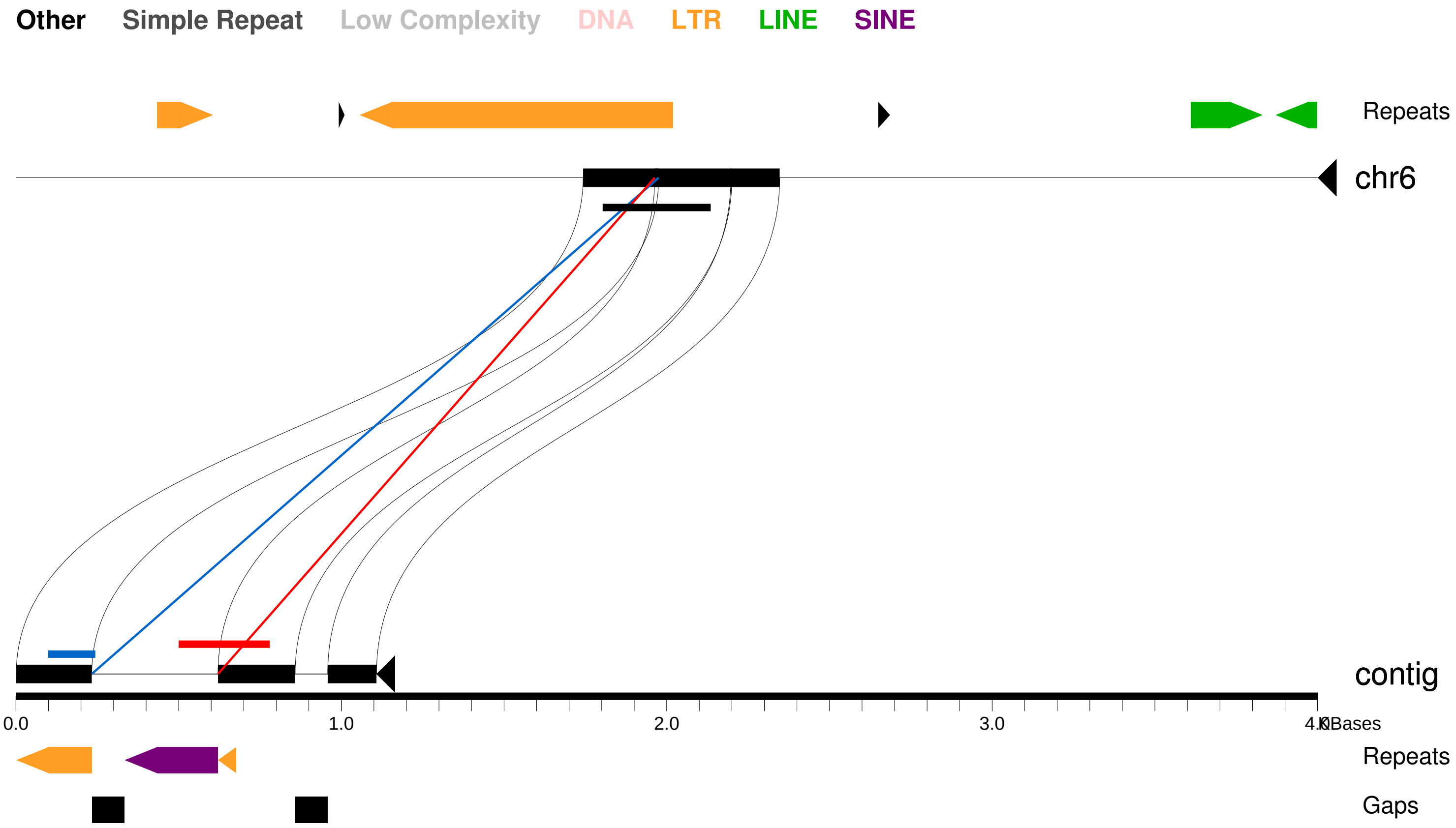

siteID: chr6_97017682 Scafftig1 + Breakpoint: chr6:97017670
chr6:97017611-97017730
Overlap Length: 0 INS size: 416 Genotyped: No

```
left    --ATTTGTATGGATTTTTTTTCCATCCCTTATTTTT---ATTCTATATGTGTCTTTTTAGG---TGAANN
chrom   --ATTTGTATGGATTTTTTTTCCATCCCTTATTTTT---ATTCTATATGTGTCTTTTTAGG---TGAAGT
right   CCACCCGCCTCGG---------CCTCCCAAAGTGCTGGGATTACAGGCGTGAGCCACCGCGCCCGGCCGT
        11*111*11*1*1111111111*1****11*1*11*111***11*111***111111111*1111*1122

left    NNNNNNNNNNNNNNNNNNNNNNNNNNNNNNNNNNNNNNNNNNNNNNNNNNNNNNNNNNNNN-
chrom   GAGTTTCTTTTAGGCAGCATATACTTAGGTCTTTTTTTTTTTTTAATTCATTCAGCTAG
right   GAGTTTCTTTTAGGCAGCATATACTTAGGTCTTTTTTTTTTTTTAATTCATTCAGCTAG
        2222222222222222222222222222222222222222222222222222222222222
```

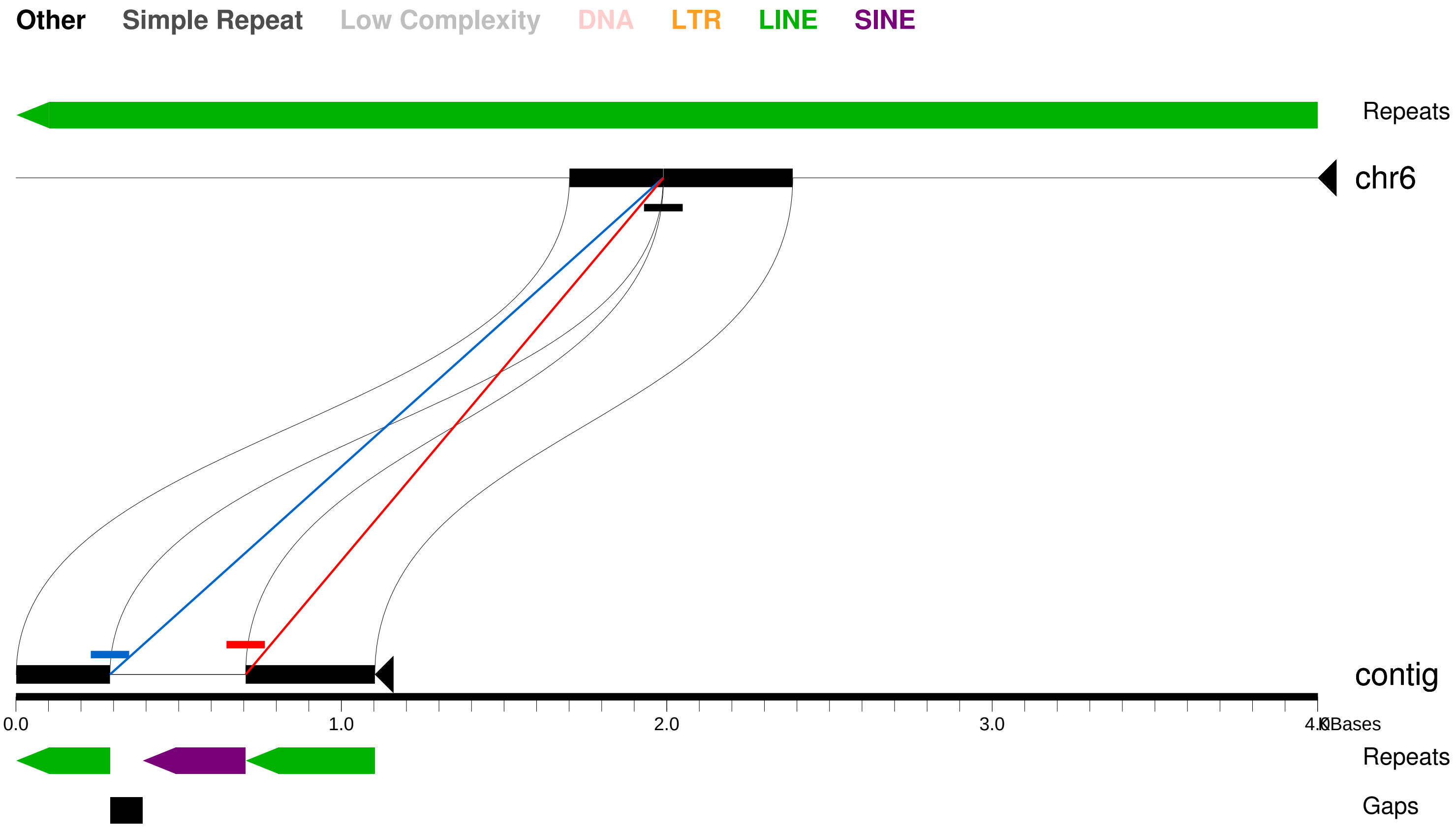

siteID: chr6_9702239 Scafftig2 - Breakpoint: chr6:9702239
chr6:9702079-9702410
Overlap Length: 12 INS size: 310 Genotyped: Yes

```
left    ------------GGAGATCTCAGAGGACCA--TAGGGACAGCTGTTCATCCTCCCCAAATGTCTTCACAT
chrom   ACATCCTTCTAAGGAGATCTCAGAGGACCA--TAGGGACAGCTGTTCATCCTCCCCAAATGTCTTCACAT
right   -------------GAGAATGGCGTGAACCCGGGAGGCGGAGCT-TGCAGTGAGCCGAGATCCCGCCAC-T
                    1****11111*1*1***1111***111****1*1**11111**1*1**11*11***1*

left    GTGATGTGGCTGTAGGCCCAGCCCCAGCACCCTGTCTACTCAATATGCACATCAGGCATCTGGGAGAAAT
chrom   GTGATGTGGCTGTAGGCCCAGCCCCAGCACCCTGTCTACTCAATATGCACATCAGGCATCTGGGAGAAAT
right   GCACTCCAGCCTGGGCGACAGAGCGAG-ACTCCGTCT---CAAAAAAAAAAAAA----------AAAAAA
        *111*111**1111*111***11*1**1**1*1****111***1*111*1*11*1111111111*1***1

left    TGCAAGGTGGTCCACTCAGAATGCAGACAGCATTGG-----------CCGGG---------CGCGGTGGC
chrom   TGCAAGGTGGTCCACTCAGAATGCAGACAGCATTTCTCTCTACATCTCCTGCTCATCAATCCTCGATGCC
right   AAAAA------------AAAAAGCAGACAGCATTTCTCTCTACATCTCCTGCTCATCAATCCTCGATGCC
        111**111111111111*1**1************2222222222222**2*2222222222*2**2**2*

left    TCACGCCTGTAATC--CCAGCACTTTGGGAGGCCGAGGCGG-GCGGATCACGAGGT--CAGGAGATC---
chrom   ACAGTCACCTTATTATCCTGGAGAATGAGAGGTAGGAACGTAGATGTTATCCAGCTGGCTGCAGCTCAGT
right   ACAGTCACCTTATTATCCTGGAGAATGAGAGGTAGGAACGTAGATGTTATCCAGCTGGCTGCAGCTCAGT
        2**22*222*2**222**2*2*222**2****22*222**22*22*2*22*2**2*22*2*2**2**222

left    -----GAGAC-CATCCCGG--CTAAA----ATGGTGAAACCCCGTCTCTACTAAA
chrom   TCACTGAGAATCAGCAGGGTGCTAATCGGCAGGGTGAAAAAAA-TTTAGAGATGG
right   TCACTGAGAATCAGCAGGGTGCTAATCGGCAGGGTGAAAAAA-------------
        22222****22**2*22**22****22222*2*******222N21N1NN1NNNNN
```

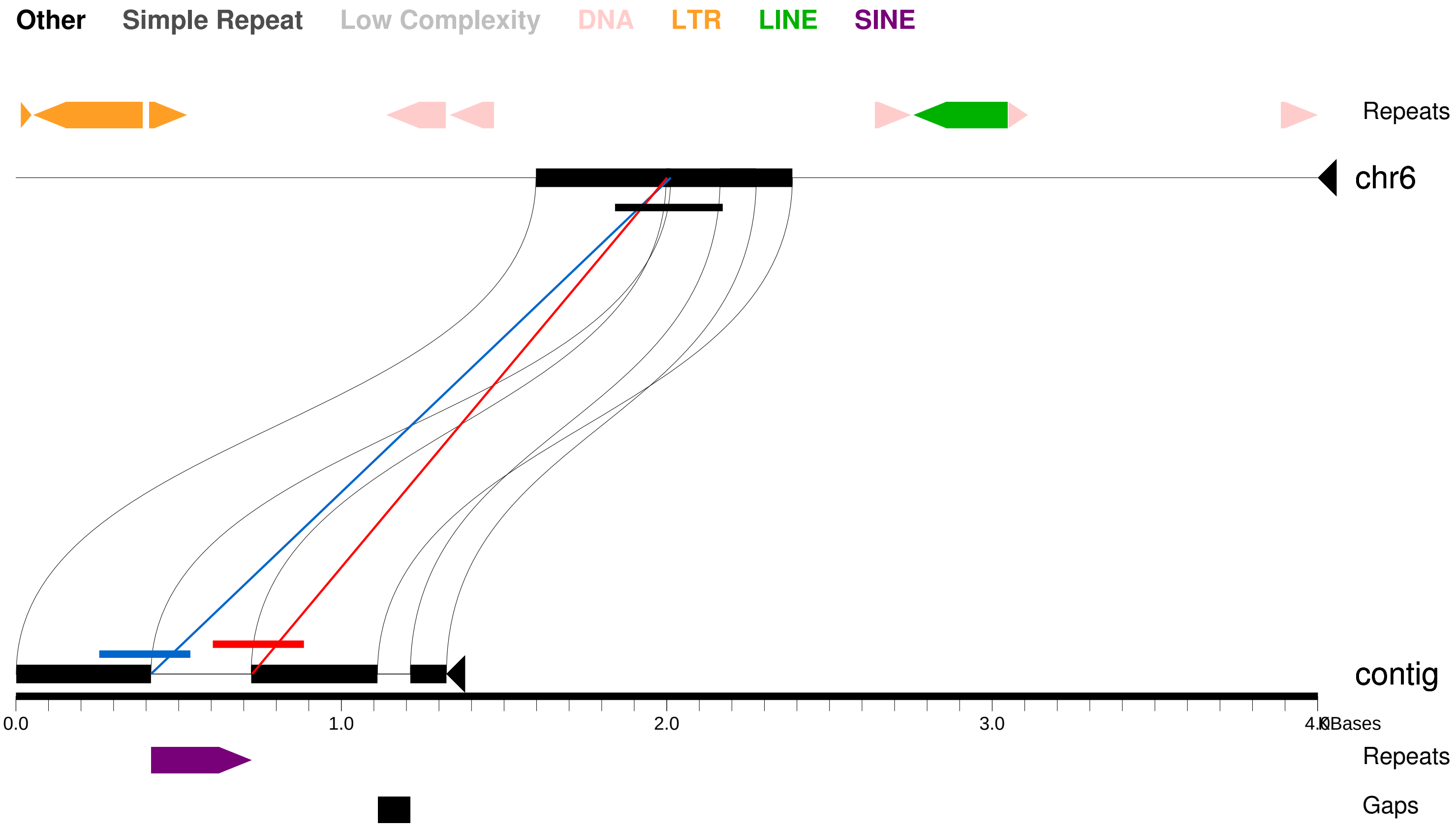

siteID: chr6_98251348 Scafftig1 - Breakpoint: chr6:98251329
chr6:98251281-98251388
Overlap Length: 12 INS size: 295 Genotyped: Yes

```
left   T------TTCGTTATCAGTCAGAGT--GATGACATTATTACATGTTTTACGGGCTC---TAGACCATTTC
chrom  T------TTCGTTATCAGTCAGAGT--GATGACATTATTACATGTTTTACGGGCTC---TAGACCATTTC
right  TCCTGACCTCGTGATCCGCCCGCCTCGGCCTCCCAAAGTGCTGGGATTACAGGCGTGAGTAGACCATTTC
       *1111111****1***1*1*1*11*11*1111*111*1*1*11*11****1***11111***********

left   TTTTTTTTTTTTTTTTTTTTTTTTTTTTTTGAGACGGAGTCTCGCTCTGTCGCCCAGGCT
chrom  TCTGTACATCTGTGAGACAATGAAGGT---GAAAGGGAAAATGACATTTTAG--------
right  TCTGTACATCTGTGAGACAATGAAGGT---GAAAGGGAAAATGACATTTTAG--------
       *2*2*222*2*2*2222222*22222*222**2*2***222*22*22*2*2*22222222
```

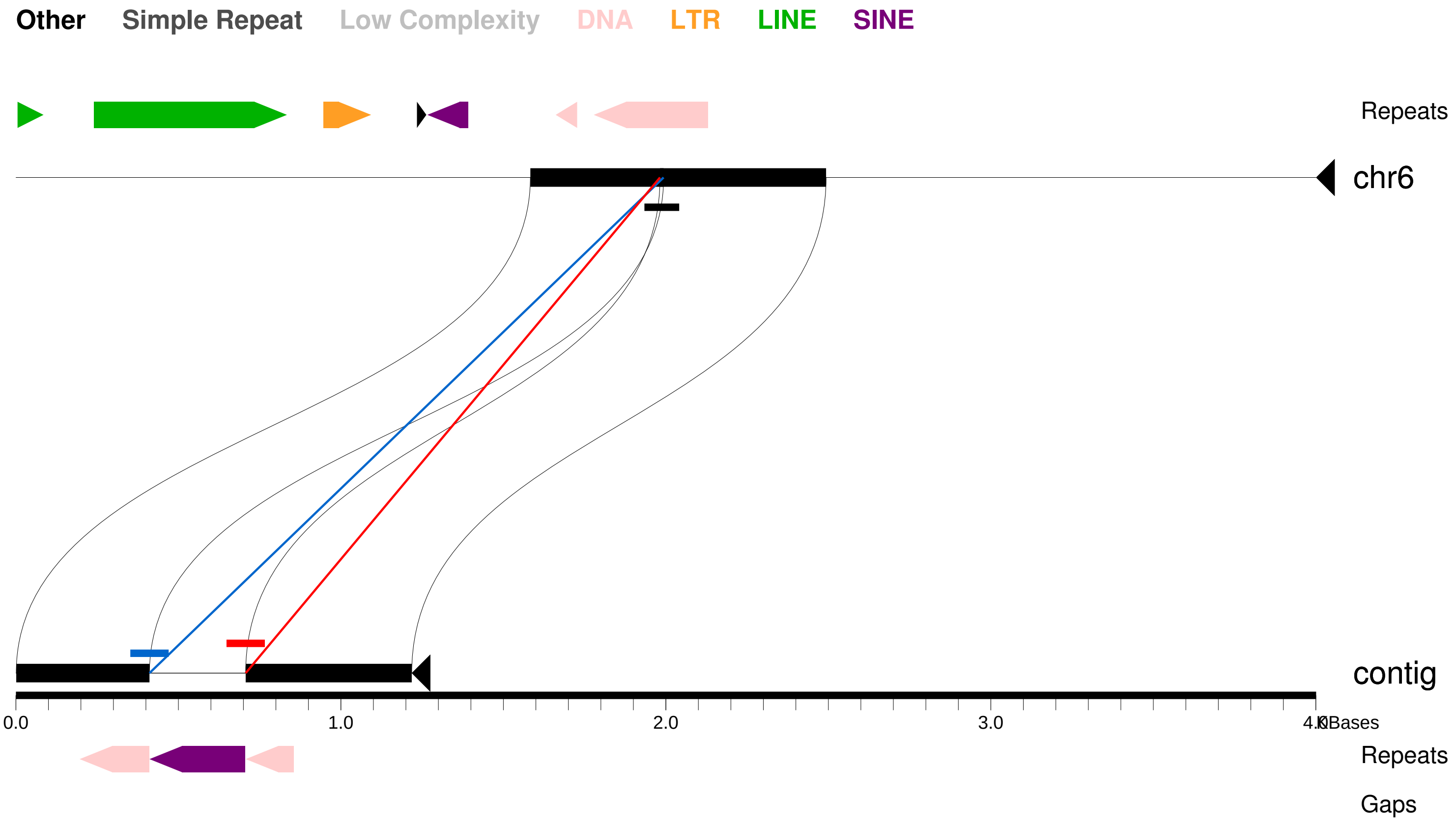

siteID: chr6_98320406 Scafftig2 + Breakpoint: chr6:98320284
chr6:98320232-98320343
Overlap Length: 8 INS size: 315 Genotyped: No

```
left    GGGTGGATAACTTAGA-----AATGTG-TGACCTTAGATACTT--ACCTTCTCTAAGCCTCAGATTGTNN
chrom   GGGTGGATAACTTAGA-----AATGTG-TGACCTTAGATACTT--ACCTTCTCTAAGCCTCAGATTGTGT
right   TCC-GCCCACCTCGGCCTCCCAAAGTGCTGGGATTACAGGCGTGAGCCACCGCGCCCGGCCAGATTGTGT
        1111*111*1**11*111111**1***1**111***1*11*1*111**11*1*1111111********22

left    NNNNNNNNNNNNNNNNNNNNNNNNNNNNNNNNNNNNNNNNNNNNNNNNNNNNNNNNN
chrom   TTTCTTAATGGGCGATAATAATGGAACCAATCTCAGAGGGTAATTTTGTG-------
right   TTTCTTAATGGGCGATAATAATGGAACCAATCTCAGAGGGTAATTTTGTG-------
        222222222222222222222222222222222222222222222222222222222
```

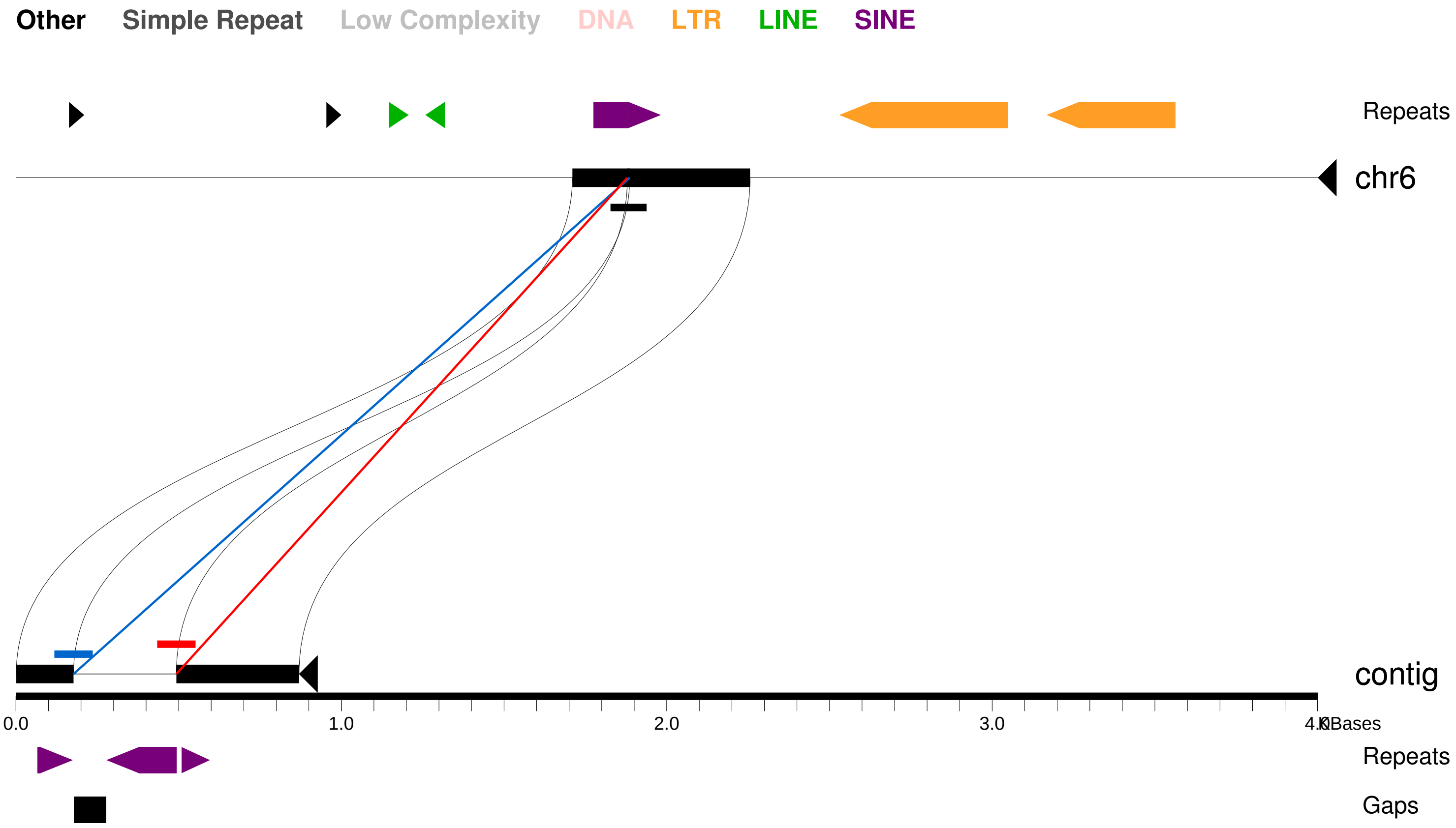

siteID: chr6_9876901 Scafftig3 - Breakpoint: chr6:9876874
chr6:9876870-9876933
Overlap Length: 56 INS size: 379 Genotyped: No

```
left    AAGG-------------------------------------------------------AAAGGAGGTCA
chrom   AAGG-------------------------------------------------------AAAGGAGGTCA
right   NNNNNNNNNNNNNNNNNNNNNNNNNNNNNNNNNNNNNNNNNNNNNNNNNNNNNNNNNNNAAAGGAGGTCA
        11111111111111111111111111111111111111111111111111111111111***********

left    GTCAAAATTTTCTAATGGTCTGTATTTTTGAAAATAAAGGAATGGGCCGGGCGCGGTGGCTCACGCCTGT
chrom   GTCAAAATTTTCTAATGGTCTGTATTTTTGAAAATAAAGGAATGG-------------------------
right   GTCAAAATTTTCTAATGGTCTGTATTTTTGAAAATAAAGGAATGG-------------------------
        *********************************************2222222222222222222222222

left    AATCCCAGCACTTTGGGAGGCCGAGACGGGCGGA
chrom   -----------------------AGA-------A
right   -----------------------AGA-------A
        22222222222222222222222***2222222*
```

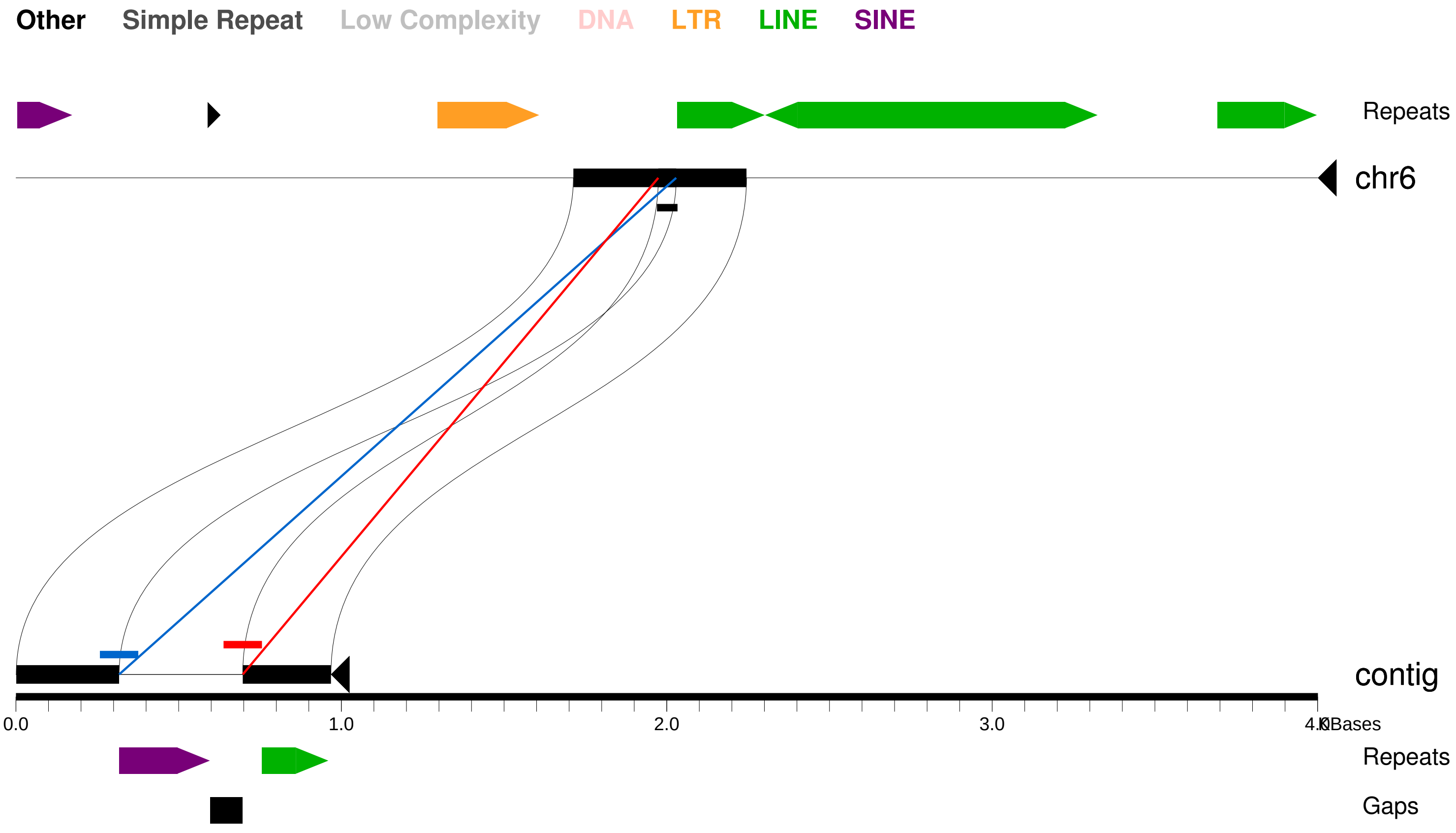

siteID: chr7_10014476 Scafftig1 - Breakpoint: chr7:10014478
chr7:10014477-10014566
Overlap Length: 59 INS size: 452 Genotyped: No

```
left    A------------------------------ATAAATACATGTTGTCTCCATCAGTAGATTTTTTTTAAA
chrom   A------------------------------ATAAATACATGTTGTCTCCATCAGTAGATTTTTTTTAAA
right   ACCGCGCCCGGCCAAAGTTTTAATTACTACAATAAATACATGTTGTCTCCATCAGTAGATTTTTTTTAAA
        *111111111111111111111111111111***************************************

left    AAAAATAGTAAATATTTTGANNNNNNNNNNNNNNNNNNNNNNNNNNNNNNNNNNNNNNNNNNNNNNNNNN
chrom   AAAAATAGTAAATATTTTGATACAGTAGGCAACTGAAAATATAGGTACAT--------------------
right   AAAA-TAGTAAATATTTTGATACAGTAGGCAACTGAAAATATAGGTACAT--------------------
        ****1***************22222222222222222222222222222222222222222222222222

left    NNNNNNNNN
chrom   ---------
right   ---------
        222222222
```

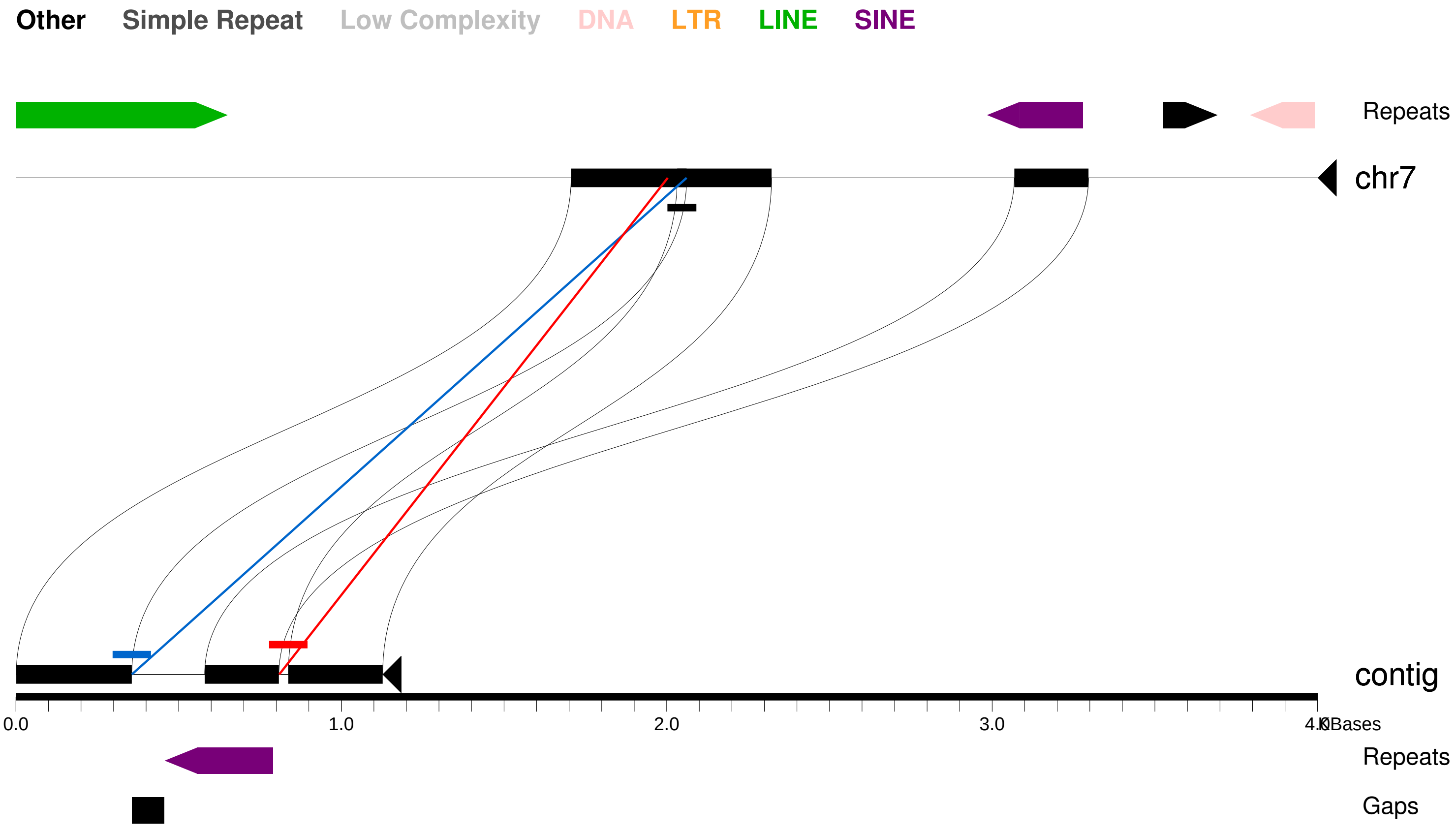

siteID: chr7_104052599 Scafftig1 - Breakpoint: chr7:104052601
chr7:104052558-104052660
Overlap Length: 17 INS size: 296 Genotyped: Yes

```
left    -------ATAAGATCTTCCTT-----TAGTATTAA---CAAGAGCTAT-GATTTGGAAAGTATTTAGTAT
chrom   -------ATAAGATCTTCCTT-----TAGTATTAA---CAAGAGCTAT-GATTTGGAAAGTATTTAGTAT
right   CCGCCCGCCTCGGCCTCCCAAAGTGCTGGGATTACAGGCGTGAGCCACCGCGCCCGGCCGTATTTAGTAT
        11111111111*11**1**1111111*1*1****1111*11****1*11*11111*111***********

left    ACTTTTTTTTTTTTTTTTTTTTGAGACGGAGTCTCGCTCTGTCGCCCAGGCTGGAGTGCAGTGGCG
chrom   ACTTTTAAGTGCTGTATTAT---------GTC-CTTTAAGTATACCG---TATTGTTCATTT---
right   ACTTTTAAGTGCTGTATTAT---------GTC-CTTTAAGTATACCG---TATTGTTCATTT---
        ******222*22*2*2**2*222222222***2*22*22**222**2222*222**2**2*2222
```

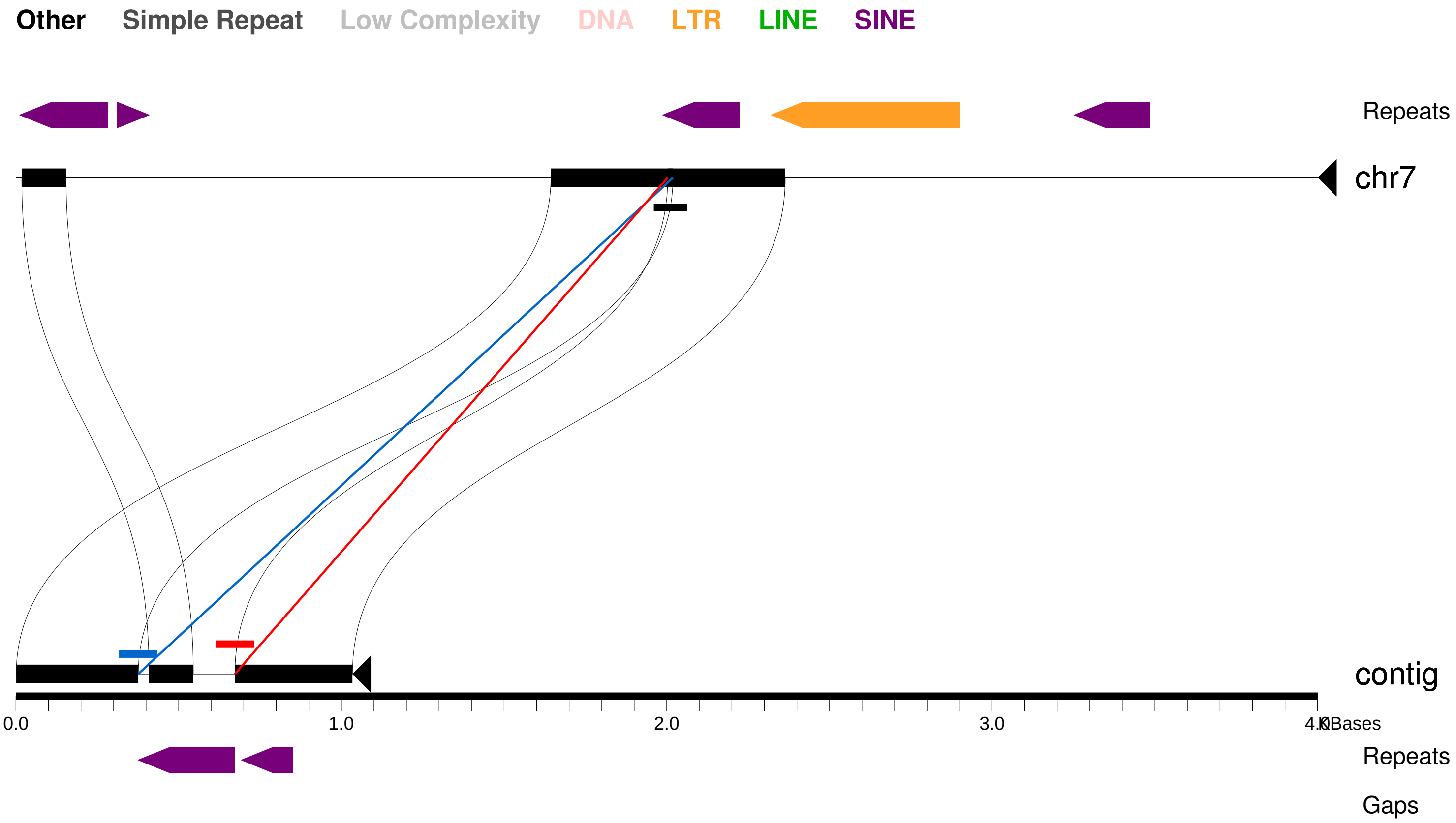

siteID: chr7_105466012 Scafftig1 - Breakpoint: chr7:105466061
chr7:105466010-105466120
Overlap Length: 9 INS size: 333 Genotyped: No

```
left    --AGTGGAATT-GCCAATATT---GCCTAATATTG-GTAGACAAAG-ACAGACTCAATAAGCATGTTTNN
chrom   --AGTGGAATT-GCCAATATT---GCCTAATATTG-GTAGACAAAG-ACAGACTCAATAAGCATGTTTTT
right   CTACAGGCGCCCGCCACCACGCCCGGCTAATTTTTTGTATTTTTAGTAGAGACGGGGTTAGCATGTTTTT
        11*11**11111****11*11111*1*****1**11***11111**1*1****1111*1*********22

left    NNNNNNNNNNNNNNNNNNNNNNNNNNNNNNNNNNNNNNNNNNNNNNNNNNNNNNNNNNNN
chrom   AAGACACAAGGAAATAAATACTCCTTGGCTCTGTAAGTGAACAGTATAG--------
right   AAGACACAAGGAAATAAATACTCCTTGGCTCTGTAAGTGAACAGTATAG--------
        22222222222222222222222222222222222222222222222222222222222
```

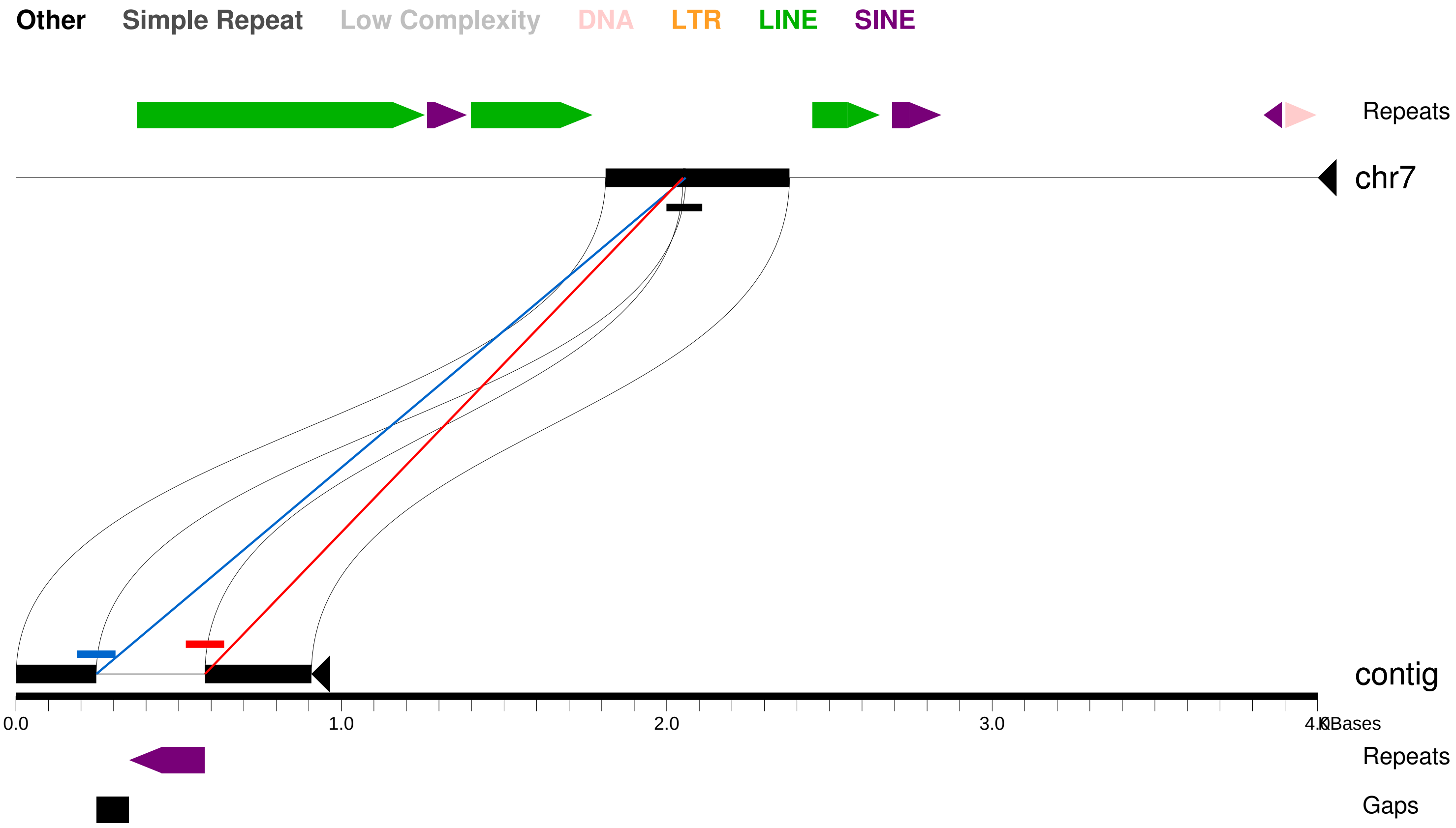

siteID: chr7_105564470 Scafftig5 - Breakpoint: chr7:105564456
chr7:105564413-105564515
Overlap Length: 10 INS size: 305 Genotyped: Yes

```
left   -TAGAATAAGGTTGGCA-AGA---ACTGGGTG-----------AGCAGGGATAGGAATTAAGACTGAGGG
chrom  -TAGAATAAGGTTGGCA-AGA---ACTGGGTG-----------AGCAGGGATAGGAATTAAGACTGAGGA
right  CTCCAGCCTGGGCGACAGAGCGAGACTCCGTCTCAAAAAAAAAAAAAAAAAAAAAAAAAAGACTGAGGA
       1*11*1111**11*1**1**1111***11**111111111111*11*111*1*11**11**********2

left   CCGGGCGC--GGTGGCTCACGCCTGTAATCCCAGCACTTTGGGAGGCCGAGGCGGGCGGATCACGAG
chrom  CTGGGCTGAGGGTAGGTCAGGGGT-------------TCTGGCAG--CAACACAGCTGGA--ATGA-
right  CTGGGCTGAGGGTAGGTCAGGGGT-------------TCTGGCAG--CAACACAGCTGGA--ATGA-
       *2****2222***2*2***2*22*2222222222222*2***2**22*2*22*2*22***22*2**2
```

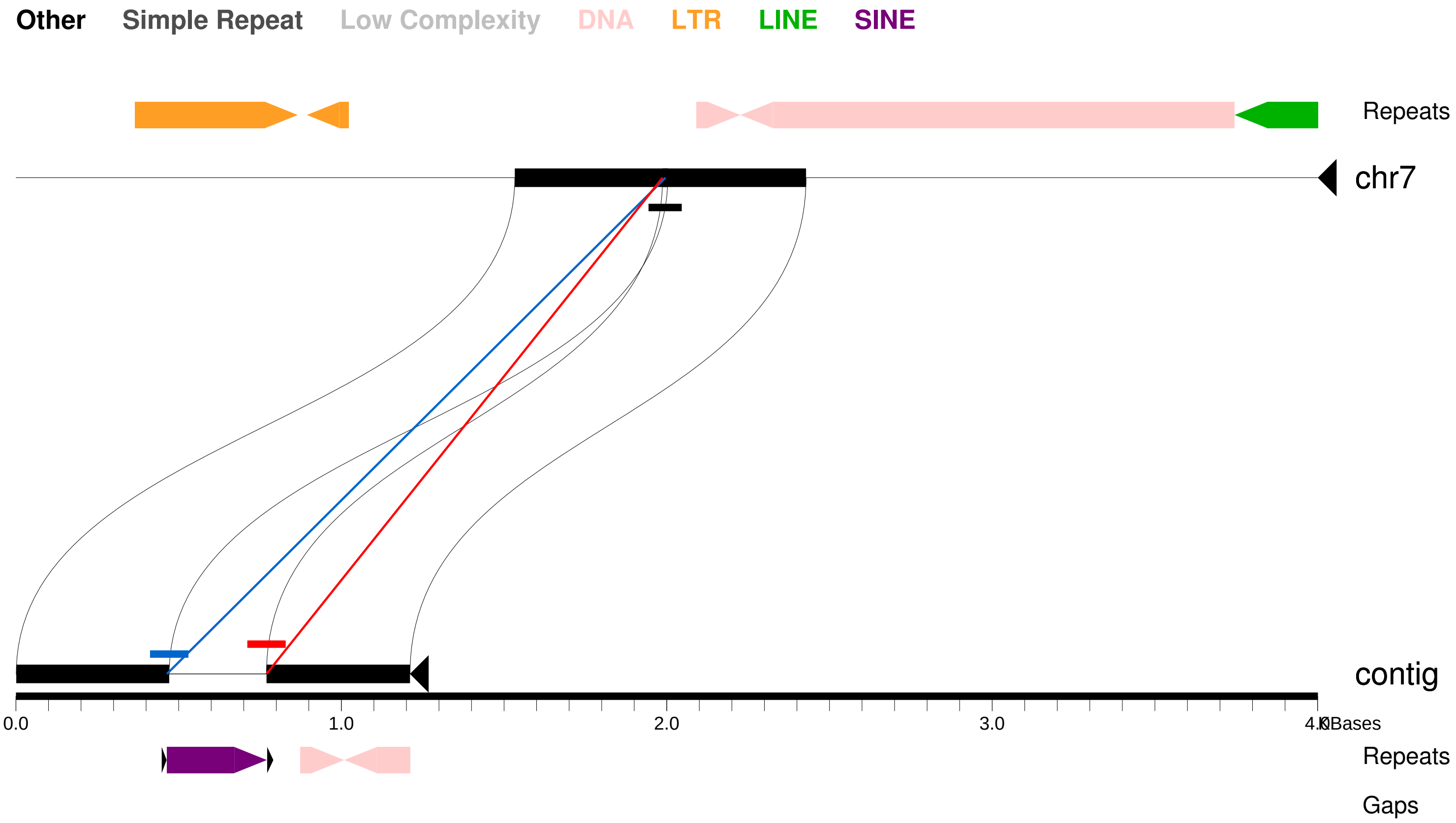

siteID: chr7_108392032 Scafftig1 + Breakpoint: chr7:108392005
chr7:108391961-108392064
Overlap Length: 16 INS size: 265 Genotyped: Yes

```
left     TAGTAACTAAAAAGTAAA----GTTTCTTAGGGTAA---AAGATAGGTTAG---------CAAGAGGGAC
chrom    TAGTAACTAAAAAGTAAA----GTTTCTTAGGGTAA---AAGATAGGTTAG---------CAAGAGGGAC
right    T-GTATTTTTAGTAGAGACGGGGTTTCACCTTGTTAGCCAGGATGGTCTCGATCTCCTGACAAGAGGGAC
         *1***11*11*1111*1*1111*****11111**1*111*1***1*11*1*111111111**********

left     ATTTTTTGTTTTTTTGTTTTTTGTTTTTTTTTTTTTTTTGTTTTTGTTTTTTTTGAG-ACGGAGTCTCG
chrom    ATTTTTAAGAC---------AGAATAAATGTATTC-------TGTTAATAACAGCACATCAAAATA
right    ATTTTTAAGAC---------AGAATAAATGTATTC-------TGTTAATAACAGCACATCAAAATA
         ******222222222222222*22*222*2*2**22222222****22*222**2**222222222
```

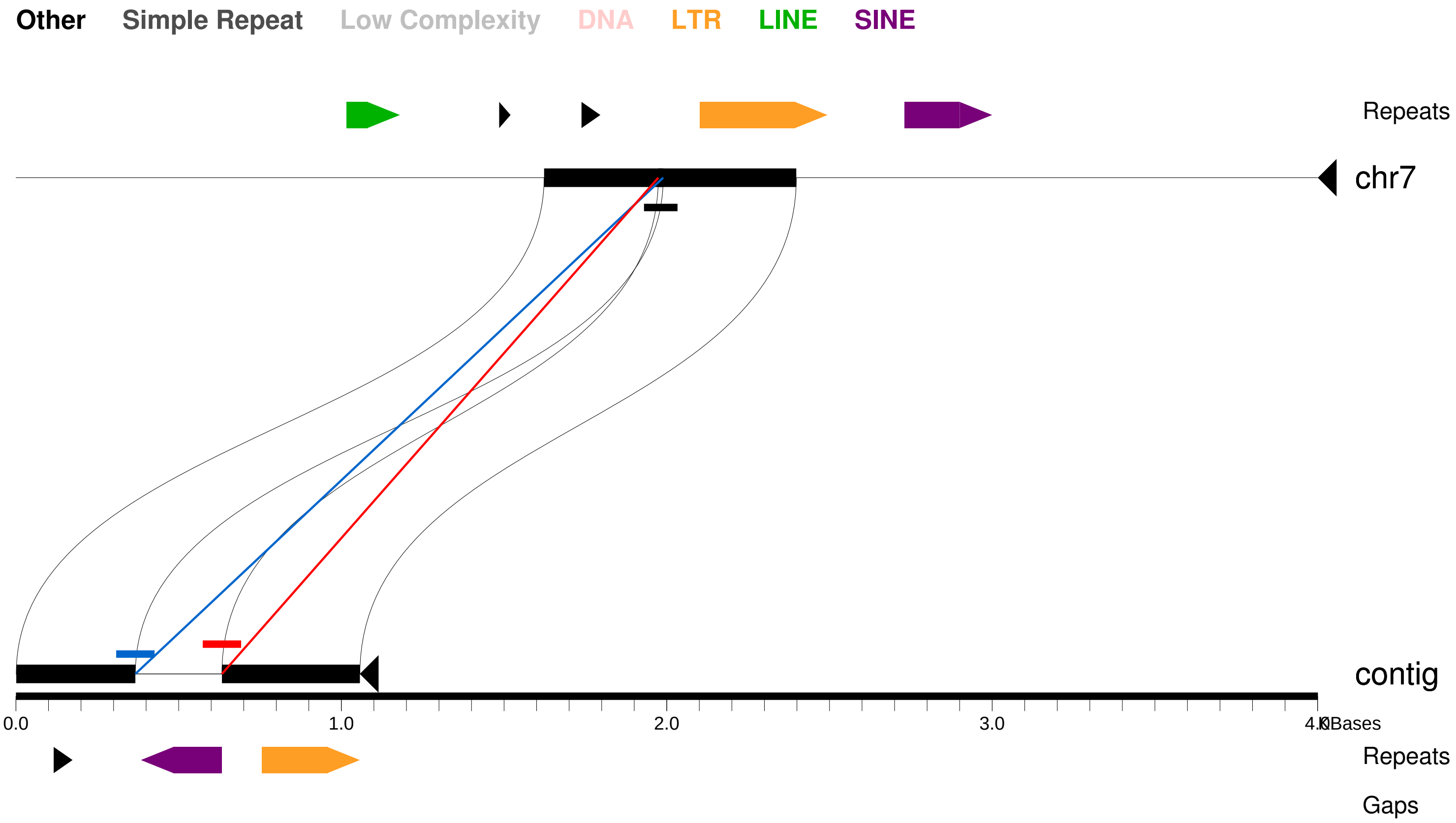

siteID: chr7_108542970 Scafftig1 + Breakpoint: chr7:108542975
chr7:108542929-108543034
Overlap Length: 14 INS size: 419 Genotyped: No

```
left     GGATCAT--TCAGGCTAC--AAGGTCTACC-TTATGGAAG---GATATAGAAGT-----CAGGCTTCCTT
chrom    GGATCAT--TCAGGCTAC--AAGGTCTACC-TTATGGAAG---GATATAGAAGT-----CAGGCTTCCTT
right    CCACCCGCCTCGGCCTCCCAAAGTGCTGGGATTACAGGCGTGAGCCACCGCGCCCGGCCCAGGCTTCCTT
         11*1*1111**1*1**1*11***11**1111***11*11*111*11*11*111111111***********

left     TTTTNNNNNNNNNNNNNNNNNNNNNNNNNNNNNNNNNNNNNNNNNNNNNNNNNNNNNNNNNN
chrom    TTTT-------------ATAGAACCATCATCTTCATCCTCATCTCGATTACTTTCTTTTTCTA
right    TTT-------------ATAGAACCATCATCTTCATCTTCATCTCGATTACTTTCTTTTTCTA
         ***222222222222222222222222222222222N2222222222222222222222222
```

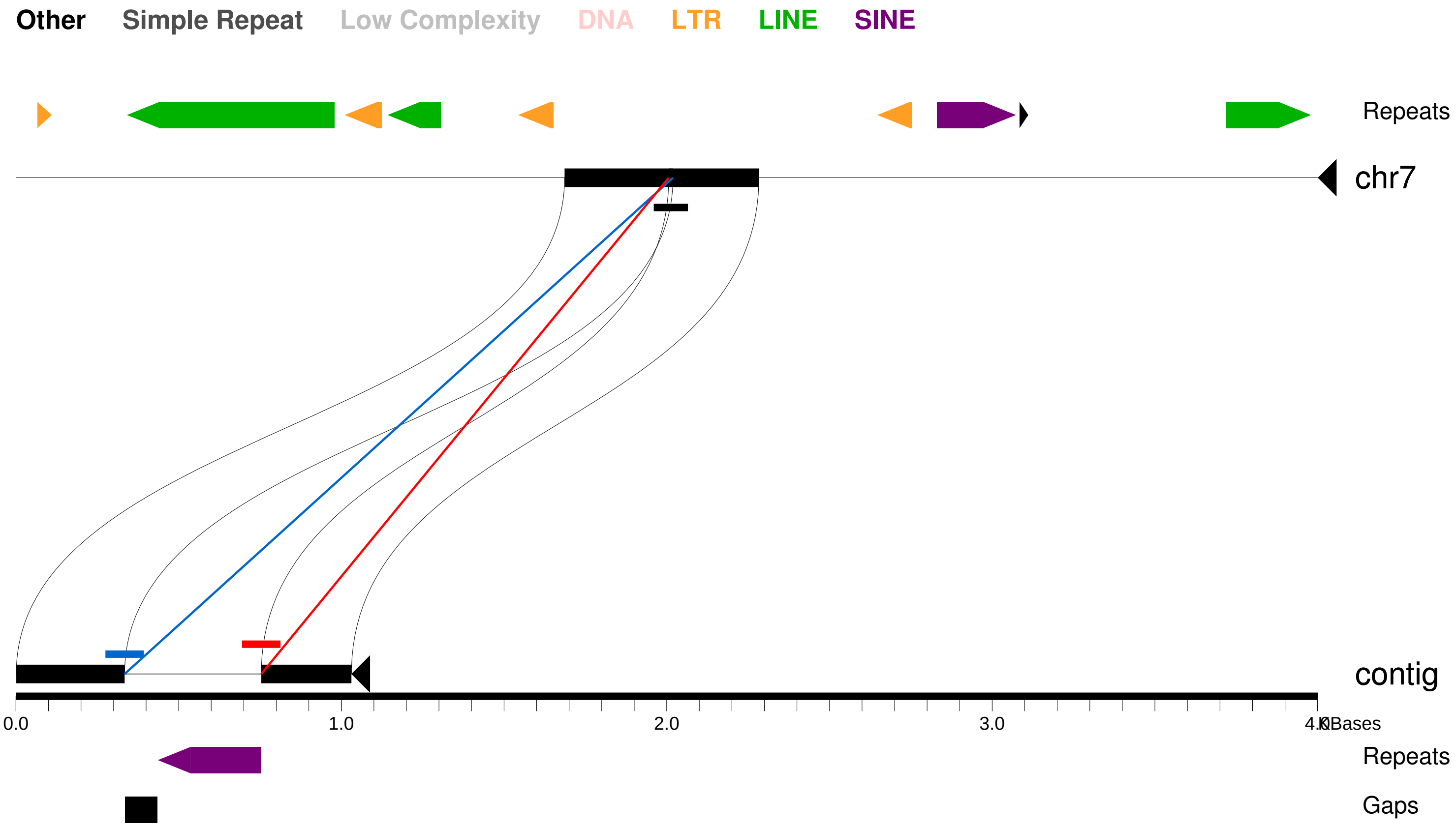

siteID: chr7_109283337 Scafftig1 + Breakpoint: chr7:109283339
chr7:109283293-109283398
Overlap Length: 14 INS size: 487 Genotyped: No

```
left    CCAGTCCTTTTTAAGTTGGAGGCTGAGCTTGGTGAGGTGTGTTTTT-------------AAAAGACCATT
chrom   CCAGTCCTTTTTAAGTTGGAGGCTGAGCTTGGTGAGGTGTGTTTTT-------------AAAAGACCATT
right   NNNNNNNNNNNNNNNNNNNNNNNNNNNNNNNNNNNNNNNNNNNNNNNNNNNNNNNNAAAAAAAGACCATT
        11111111111111111111111111111111111111111111111111111111111***********

left    AGTGGCCGGGCGCGGTGGCTCACGCCTGTAATCCCAGCACTTTGGGAGGCCG-AGGCGGGTGG
chrom   AGTCT--------GTTTTACCTTTCCTGAAGACTGAGGA---TGGTAAGTGGTATGAGGG---
right   AGTCT--------GTTTTACCTTTCCTGAAGACTGAGGA---TGGTAAGTGGTATGAGGG---
        ***2222222222*2*2222*222****2*22*22**2*222***2*2*22*2*2*2***222
```

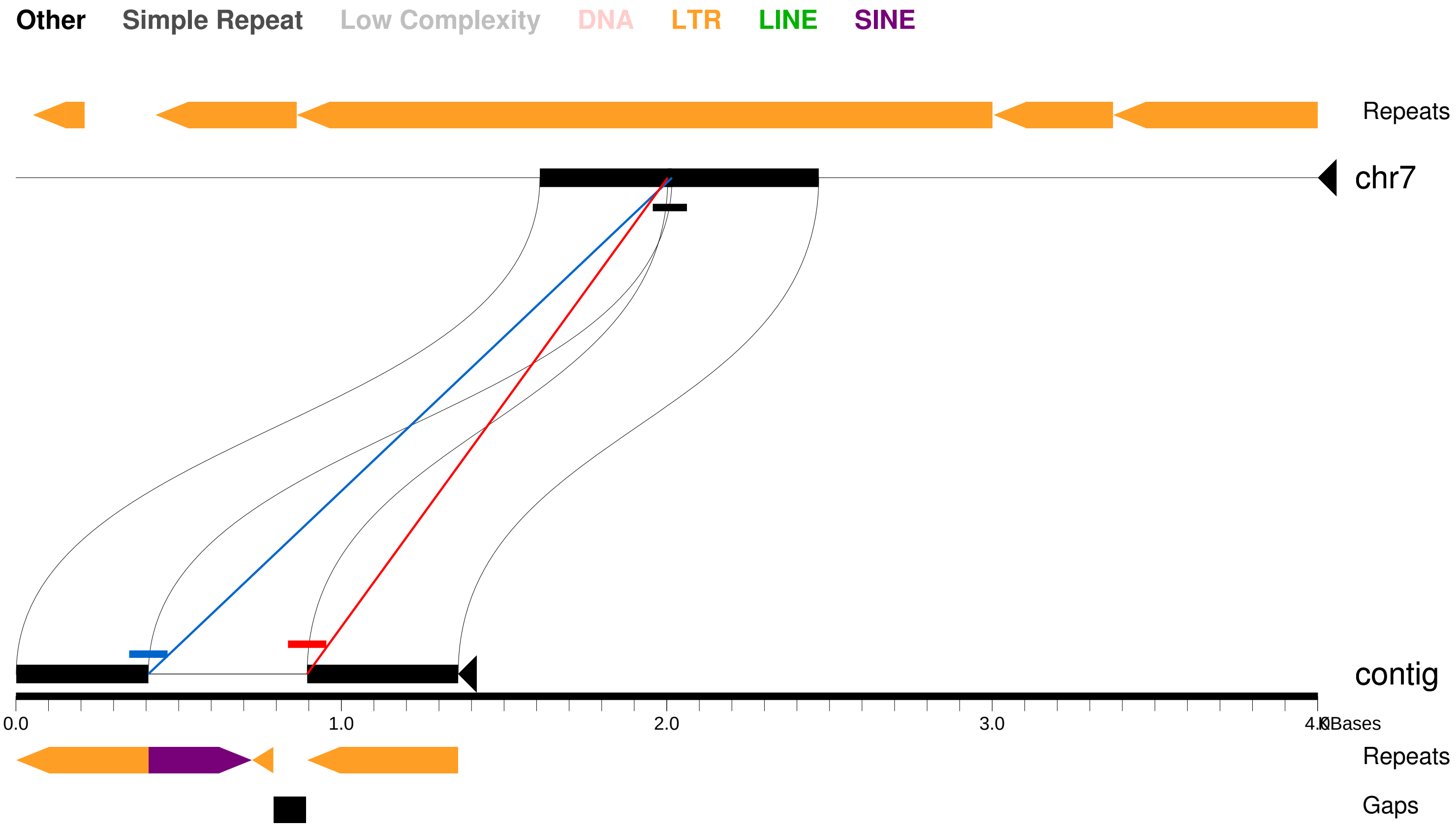

siteID: chr7_113868905 Scafftig2 + Breakpoint: chr7:113868868
chr7:113868826-113868927
Overlap Length: 18 INS size: 270 Genotyped: No

```
left    AAGGAAACAAAGTATAGAACTACAAAAAAATCAATGAAAC-----------------ACAAAGAGAGCAA
chrom   AAGGAAACAAAGTATAGAACTACAAAAAAATCAATGAAAC-----------------ACAAAGAGAGCAA
right   NNNNNNNNNNNNNNNNNNNNNNNNNNNNNNNNNNNNNNNNNNNNNNNNNNNNNNNNNAAAAAGAGAGCAA
        111111111111111111111111111111111111111111111111111111111*1***********

left    GACAGGGCCGGGCGCGGTGGCTCACGCCTGTAATCCCAGCACTTTGGGAGGCCGAGGCGGGCGGAT
chrom   GACAGGG------AAAAAGGGACAAAAGAGCTA---CAAAAC------AGAAAGAAACAACT--AA
right   GACAGGG------AAAAAGGGACAAAAGAGCTA---CAAAAC------AGAAAGAAACAACT--AA
        *******22222222222**22**22222*22*222**22**222222**222**22*222222*2
```

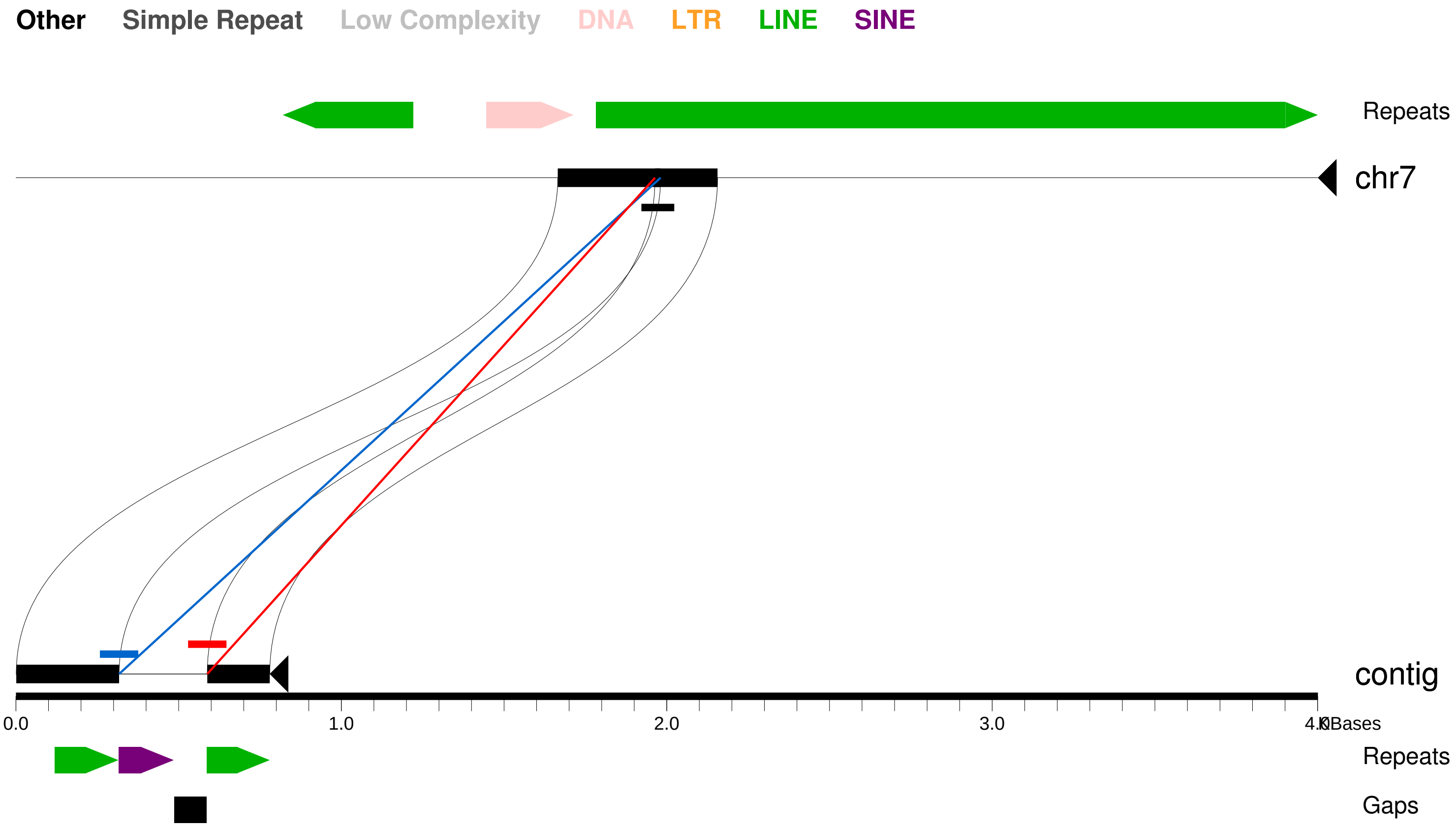

siteID: chr7_115865166 Scafftig2 - Breakpoint: chr7:115865227
chr7:115865176-115865286
Overlap Length: 9 INS size: 311 Genotyped: Yes

```
left   CTAACACTTCCACCATCACAACCCTCAGTGCTCATTTTTTAAAGTATAGTT------------AAAAACA
chrom  CTAACACTTCCACCATCACAACCCTCAGTGCTCATTTTTTAAAGTATAGTT------------AAAAACA
right  G---CCTGGGCGACAGAGCGAGACTCCGT-CTCAAAAAAAAAAAAAAAAAAAAACAAAAACAAAAAAACA
       1111*11111*11**111*1*11***1**1****111111***11*1*111111111111111*******

left   CTGGCCGGGCGCGGTGGCTCACGCCTGTAATCCCAGCACTTTGGGAGGCCGAGGCGGGCGG
chrom  CTT--------CAACATATCCTGCCAAAACTAAAATTTATTATTGTTTCCTAGAATGTAGC
right  CTT--------CAACATATCCTGCCAAAACTAAAATTTATTATTGTTTCCTAGAATGTAGC
       **222222222*222222**22***222*2*222*2222**222*222**2**222*22*2
```

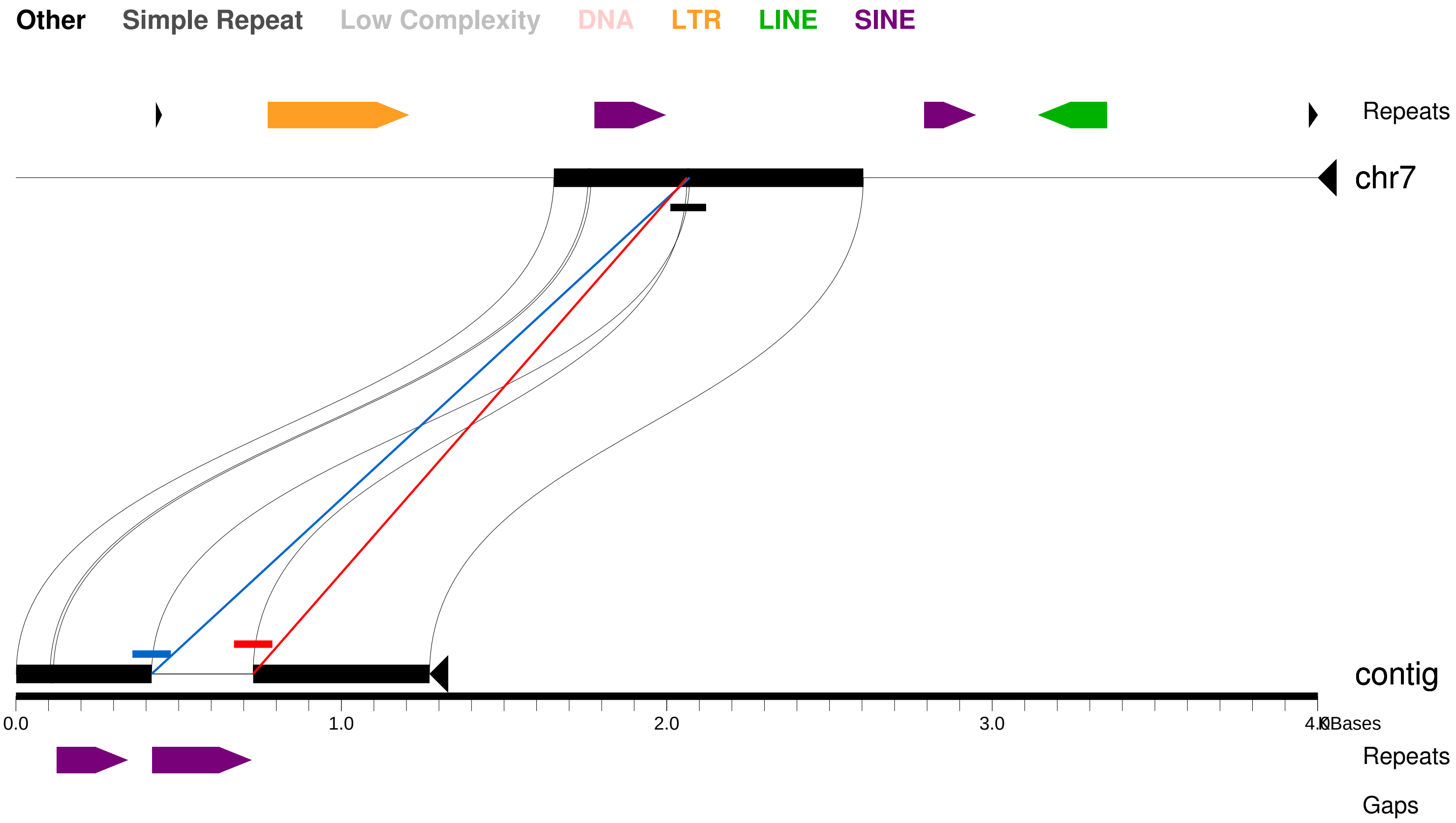

siteID: chr7_117356236 Scafftig1 + Breakpoint: chr7:117356239
chr7:117356192-117356299
Overlap Length: 13 INS size: 306 Genotyped: Yes

```
left    TTTTTTTTCTTTGTATAAGAGCCCAGATAGTATA----------AAAGGACTCGCTTTAAAAAATAACT-
chrom   TTTTTTTTCTTTGTATAAGAGCCCAGATAGTATA----------AAAGGACTCGCTTTAAAAAATATCT-
right   CCCGCCT-CGGCCTCCCAAAGTGCTGGGATTACAGGCGTGAGCCACCGCGCCCGGCCTAAAAAATAACTT
        111111*1*1111*111*1**11*1*11*1**1*1111111111*11*11*1**111*********N**1

left    -TTTTTTTTTTTTTTTTTTTGAGACGGAGTCTCGCTCTGTCGCCCAGGCCGGACTGCGGAC
chrom   -TAAACAA--------AGCTGCCCAGAACTTTGACTTTCAGTACTA---CTTTTTTTTTGT
right   TTAAACAA--------AGCTGCCCAGAACTTTGACTTTCAGTACTA---CTTTTTTTTTGT
        1*22222222222222222**2222*2*2*2*22**2*22222*2*222*2222*222222
```

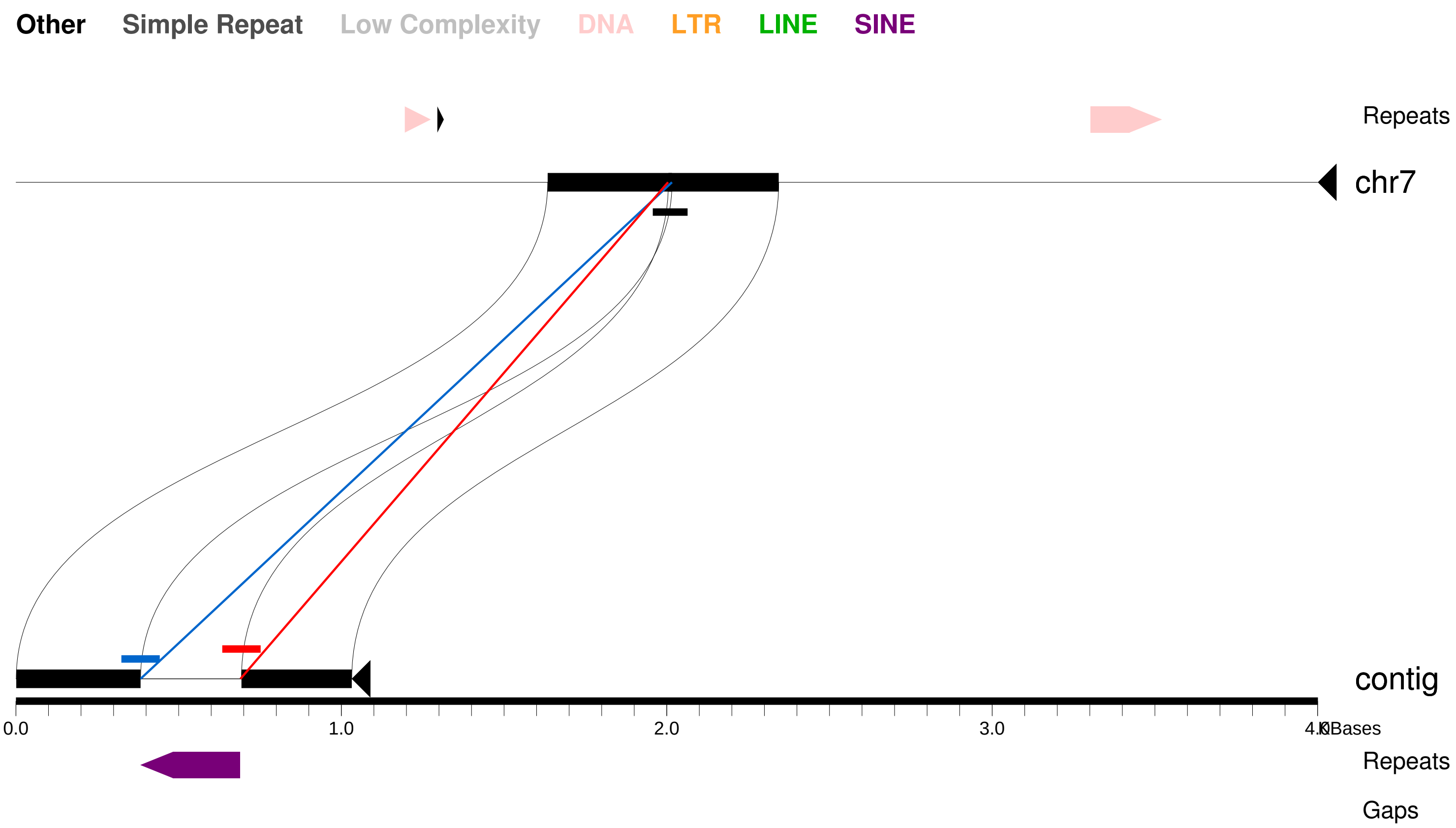

siteID: chr7_118083316 Scafftig6 - Breakpoint: chr7:118083302
chr7:118083255-118083361
Overlap Length: 13 INS size: 296 Genotyped: Yes

```
left   TT-CCTCTATAATTTTATTTT----TTAAGTAAATTCTTTTT-------ATGTGAGCTTAAAAATAATTC
chrom  TT-CCTCTATAATTTTATTTT----TTAAGTAAATTCTTTTT-------ATGTGAGCTTAAAAATAATTC
right  GCGCCACTGCACTCCAGCCTGGGCGACAGCGAGACTCCGTCTCAAAAAAAAAAAAAAAAAAAAAATAATTC
       111**1**11*1*111111*1111111*111*1*1**11*1*1111111*1111*1111***********

left   CTGGCCGGGCGCGGTGGCTCACGCCTGTA----ATCCCAGCACTTTGGGAGGCCGAGGCGGGCGG
chrom  CTTATTGTTTACCA-GGATTATGACTGAAGATTATATATAAACTTTTAAA---------------
right  CTTATTGTTTACCA-GGATTATGACTGAAGATTATATATAAACTTTTAAA---------------
       **2222*2222*222**2*2*2*2***2*2222**222222*****222*222222222222222
```

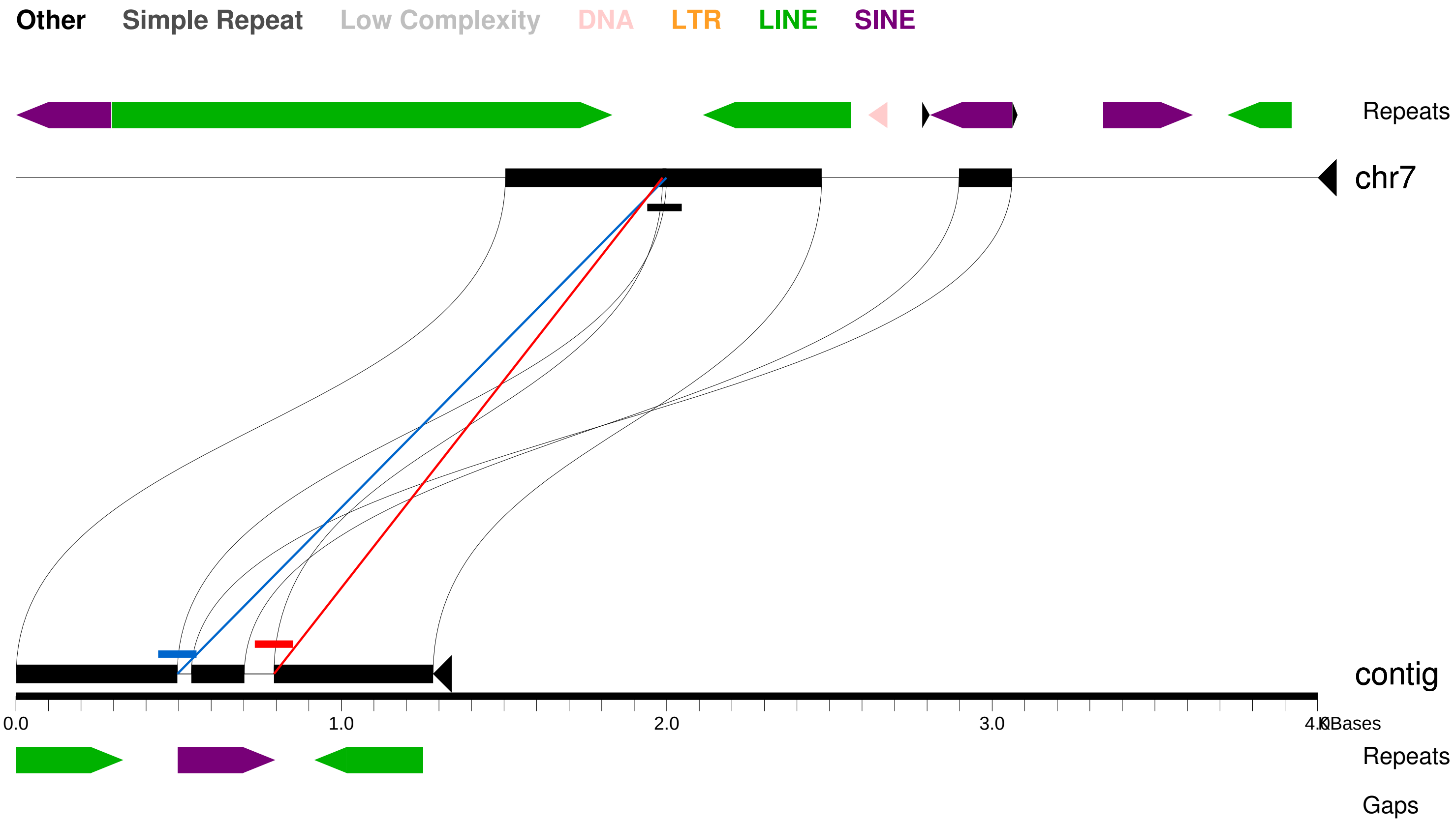

siteID: chr7_119259827 Scafftig1 + Breakpoint: chr7:119259809
chr7:119259649-119259975
Overlap Length: 7 INS size: 304 Genotyped: Yes

```
left     -----TTTTGATGATTTCTTACAGT-CTAAACACTGTCATTGTAAGACTGTTCTGTACACAACCTTAAGT
chrom    CCTCATTTTGATGATTTCTTACAGTTCTAAACACTGTCATTGTAAGACTGTTCTGTACACAACCTTAAGT
right    TTTAGTAGAGACGGGGTTTCACCGTTTTAGCC---GGGATGGTCTCGATCTCCTG------ACCTCGTGA
         NN2NN*111**1*111*1*1**1**21**11*111*11**1**11111*1*1***111111****111*1

left     TCCAAAATGAGGAAGGACATTTTCTAGGCTTCATACTGTGTTTGGAGAGAATGAGGTTAATATTTTCCCT
chrom    TCCAAAATGAGGAAGGACATTTTCTAGGCTTCATACTGTGTTTGGAGAGAATGAGGTTAATATTTTCCCT
right    TCC-------GCCCGC-------CTCGGCCTCCCAAAGTGCTGGGA----------TTA-----------
         ***1111111*111*11111111**1***1**11*11***1*1***1111111111***11111111111

left     CAGCAGTGAGGATCTGC----AGTCCAGCATTTTTTTTTT---------------------------TTT
chrom    CAGCAGTGAGGATCTGC----AGTCCAGCATCTTTAATATAGAGACTGCCAGGGGAACAGGAAGCAATGA
right    CAGGCGTGAGCCACCGCGCCCGGCCCAGCATCTTTAATATAGAGACTGCCAGGGGAACAGGAAGCAATGA
         ***11*****111*1**11111*1*******2***22*2*222222222222222222222222222*22

left     TTTTTTTTTTTTGAGACGGAGTCTCGCTCTGTCGCCCAGGCTGGA---GTGCAGTGGCGCAATCTCGGCT
chrom    TGGGAATTACTTTTTCCCTTTTCCCTCTGTGAAAACTATGAGGAATATGTCTAAAAGGGTAAAATGATTT
right    TGGGAATTACTTTTTCCCTTTTCCCTCTGTGAAAACTATGAGGAATATGTCTAAAAGGGTAAAATGATTT
         *22222**22**2222*2222**2*2**2**2222*2*2*22*2*222**22*222*2*2**22*2222*

left     CACTGCAAGCTCCGCCTCCCGGGTTCACGCCATTCTC-----CTGC-----
chrom    AACTTAGAGATTGGGTAGAAGATTACATTTCTTACTCTTTTTCTTCTCATT
right    AACTTAGAGATTGGGTAGAAGATTACATTTCTTACTCTTTTTCTT------
         2***222**2*22*222222*22*2**222*2*2***22222**21
```

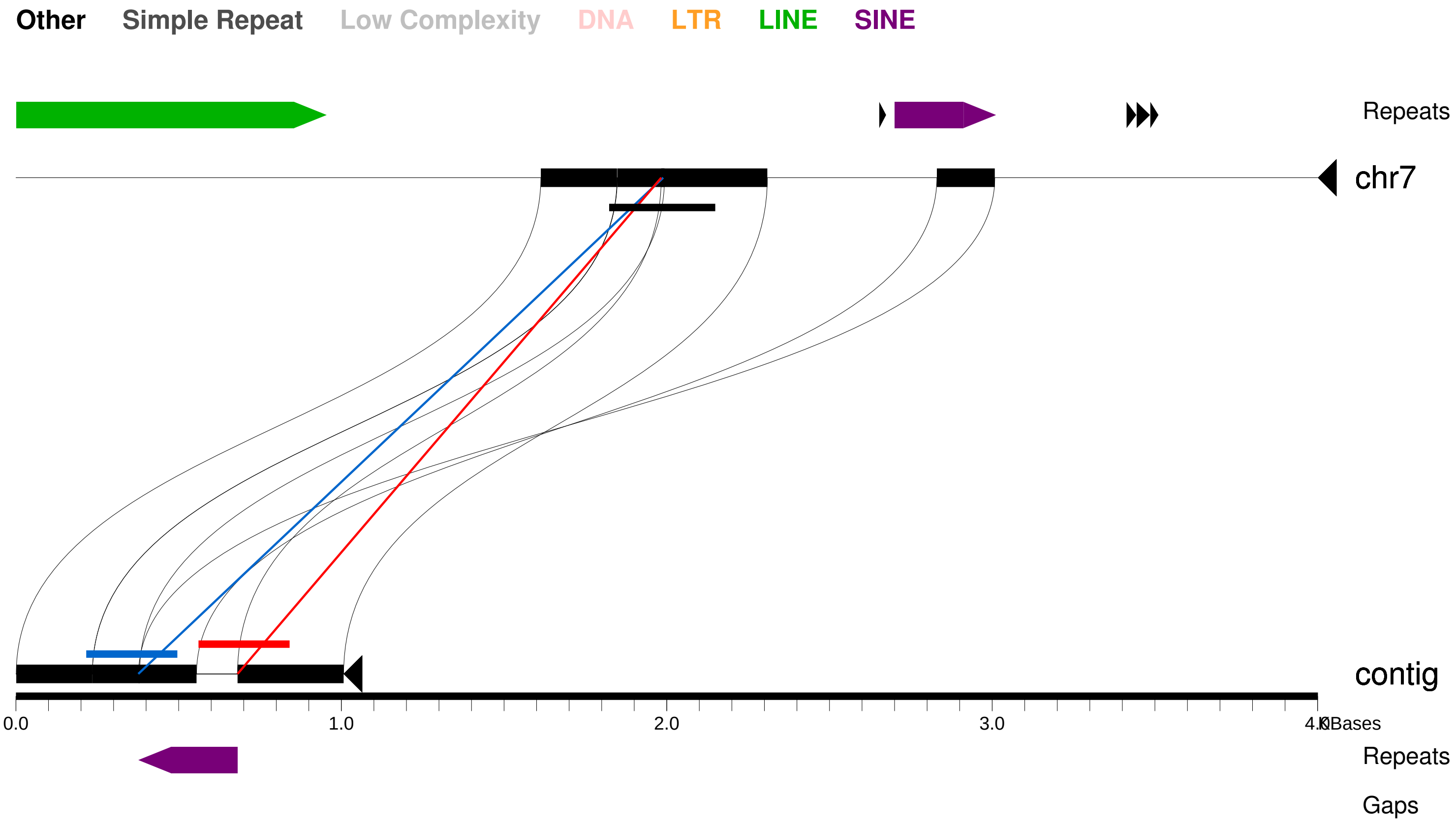

siteID: chr7_119558030 Scafftig1 - Breakpoint: chr7:119558036
chr7:119557993-119558095
Overlap Length: 12 INS size: 421 Genotyped: No

```
left    TTTTCTG--TGATACATTTAATTTACTTGAAA-ACAA-CCTGATCAT------------TTCTATGTATA
chrom   TTTTCTG--TGATACATTTAATTTACTTGAAA-ACAA-CCTGATCAT------------TTCTATGTATA
right   CCGCCCGCCTCGGCCTCCCAAAGTGCTGGGATTACAGGCGTGAGCCACCGCGCCCGGCCTTCTATGTATA
        1111*1*11*1111*1111**11*1**1*1*11***11*1***1*111111111111111***********

left    TATATATATTTTTTTTTTTTTTNNNNNNNNNNNNNNNNNNNNNNNNNNNNNNNNNNNNNNNNNNNN
chrom   TTTTTAACCACTGTTATGTTT-----------------AAAATCCCAGGGACATTTGGATTGGGCA
right   TTTTTAACCACTGTTATGTTT-----------------AAAATCCCAGGGACATTTGGATTGGGCA
        *2*2**22222*2**2*2***222222222222222222222222222222222222222222222
```

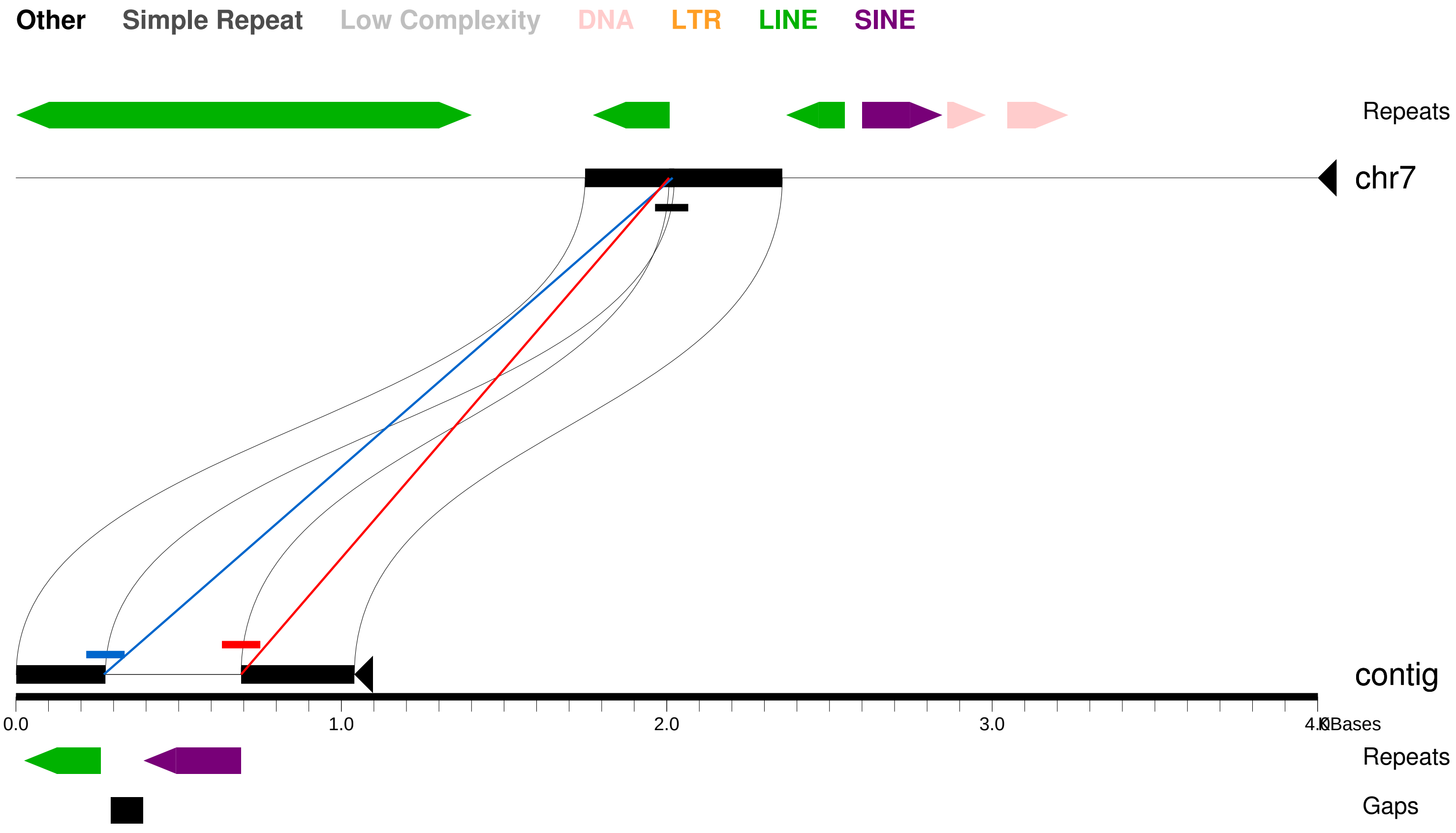

siteID: chr7_120103478 Scafftig1 + Breakpoint: chr7:120103450
chr7:120103413-120103509
Overlap Length: 9 INS size: 306 Genotyped: Yes

```
left    TCTCT--------------AAAGT--------TAGATGCATAGGTATATAAGCAAGCATTAAATTTTTTT
chrom   TCTCT--------------AAAGT--------TAGATGCATAGGTATATAAGCAAGCATTAAATTTTTAT
right   CCGCCCGCCTCGGCCTCCCAAAGTGCTGGGATTACAGGCGTGAGCCACCGCGCCCGGCCTAAATTTTTAT
        1*1*11111111111111*****11111111**1*1**1*11*1111111**11*111*********2*

left    TTTTTTTTTTTTTTTTTTTTTTTGAGACGGAGTCTCGCTCTGTCGCCCAGGCTGGAGTGCAGTGGCGGGA
chrom   TCATTAACTTTTGCAAACTAATTG-------GTAT-----------------TGAAAAGTATTGCCAGTA
right   TCATTAACTTTTGCAAACTAATTG-------GTAT-----------------TGAAAAGTATTGCCAGTA
        *22**222****222222*22***2222222**2*22222222222222222**2*22*2*2**2*2*2*

left    T--
chrom   ATA
right   ATA
        222
```

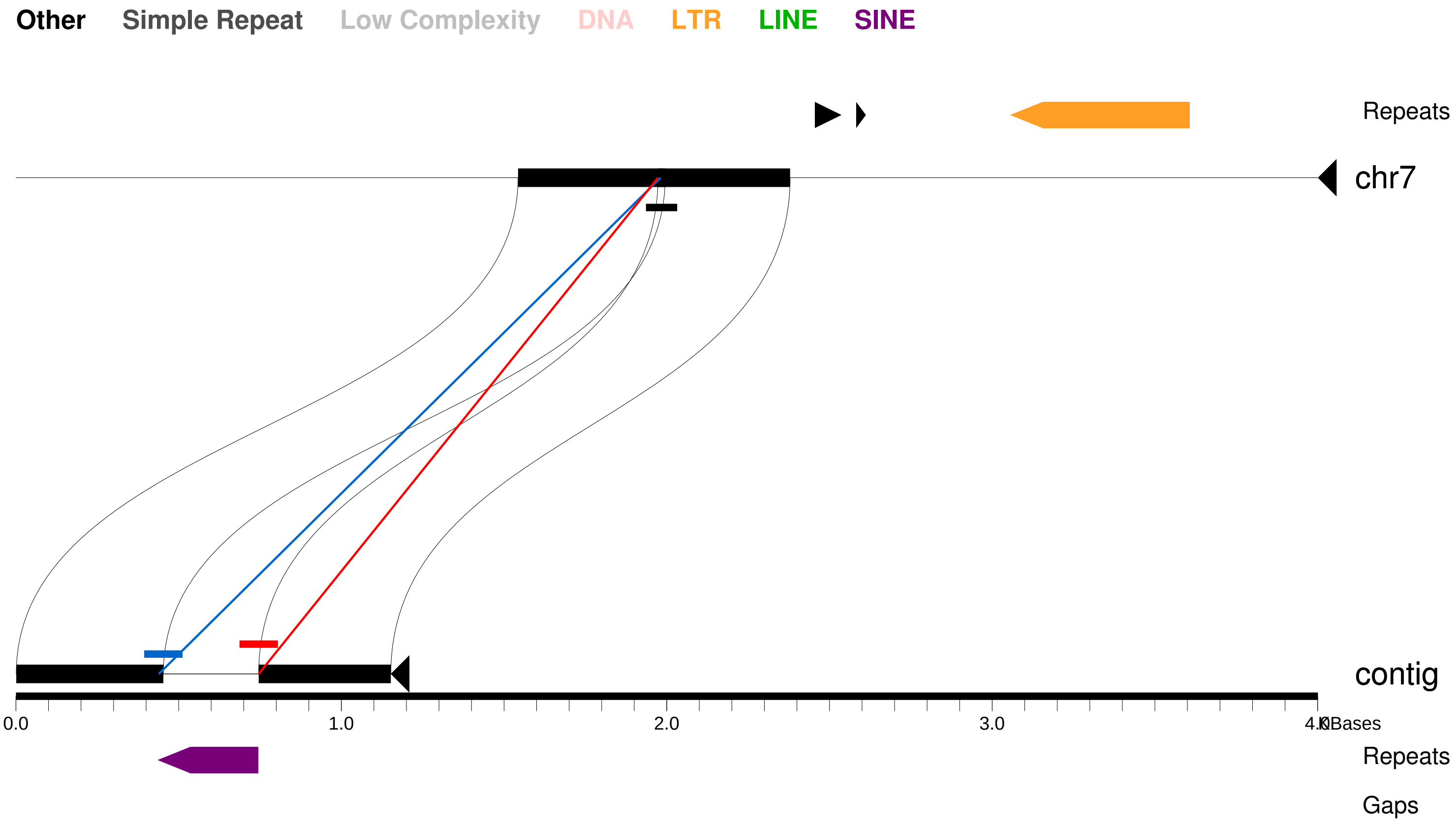

siteID: chr7_120537974 Scafftig1 + Breakpoint: chr7:120538075
chr7:120537915-120538246
Overlap Length: 12 INS size: 302 Genotyped: Yes

```
left    AA--------AAATAAGTTCTAAACAAAAAATGGAAAACATTGCTATGATAATGATCCAATTAAAAAAAT
chrom   AATTTATTAAAAATAAGTTCTAAACAAAAAATGGAAAACATTGCTATGATAATGATCCAATTAAAAAAAT
right   --TTTAGTAGAGACAGGGTTTCACCG-----TTTTAGCC---GGGATGGTCTCGATCTCCTGACCTC---
        112222N22N*1*1*1*1*1*1*1*111111*111*11*111*11***1*111****111*1*1111111

left    AGGTACTTTTCAAGATTTGTCAAAGAAAAATGCAATCATGTAAATACTAAAAGTAAATAATTTGGGGTTT
chrom   AGGTACTTTTCAAGATTTGTCAAAGGAAAATGCAATCATGTAAATACTAAAAGTAAATAATTTGGGGTTT
right   --GTGATCCGCCCGCCTCGGCCTCCCAAAGTGC-----TGGGATTAC-AGGCGTGAGCCACCGCGCCC--
        11**11*111*11*11*1*1*1111N***1***11111**11*1***1*111**1*111*1111*11111

left    TTAAAAAATTGGTTTTTTTTCAATAAACTTTTT--------TTTTTTTTTTTTTTTTTTCAGACG--GAG
chrom   TTAAAAAATGGGTTTTTTTTCAATAAACTTTTAACACAGACTATAAAATTATACTGCTACAAAGGAGGAG
right   ---------GGCCTT-----CAATAAACTTTTAACACAGACTATAAAATTATACTGCTACAAAGGAGGAG
        1111111112*11**11111************222222222*2*2222**2*22*22*2**2*2*22***

left    T-----------------CTCGCTCTGTCGC----CCAGGCTGGA------------GTGCAGTGGCGGG
chrom   TAAGAATTATTTTACATACTATCTGTATCCCTAAACCAGGGTCAGCTTTCCAAACAGGTGAAATGAATGG
right   TAAGAATTATTTTACATACTATCTGTATCCCTAAACCAGGGTCAGCTTTCCAAACAGGTGAAATGAATGG
        *22222222222222222**22**2*2**2*2222*****2*222222222222222***2*2**222**

left    ATCTCGGTT-CACTGCAAGCTCCGCCTCCCGGGTTCACGCCATTCTCCTGCTT
chrom   CAGTGGGAGGCAGTTGGTGCACACTATCCCACCTACTTCTGACCCTCCA-CGT
right   CAGTGGGAGGCAGTTGGTGCACACTATCCCACCTACTT---------------
        222*2**222**2*2222**2*2222****222*2*22NNN1NN1111N21N1
```

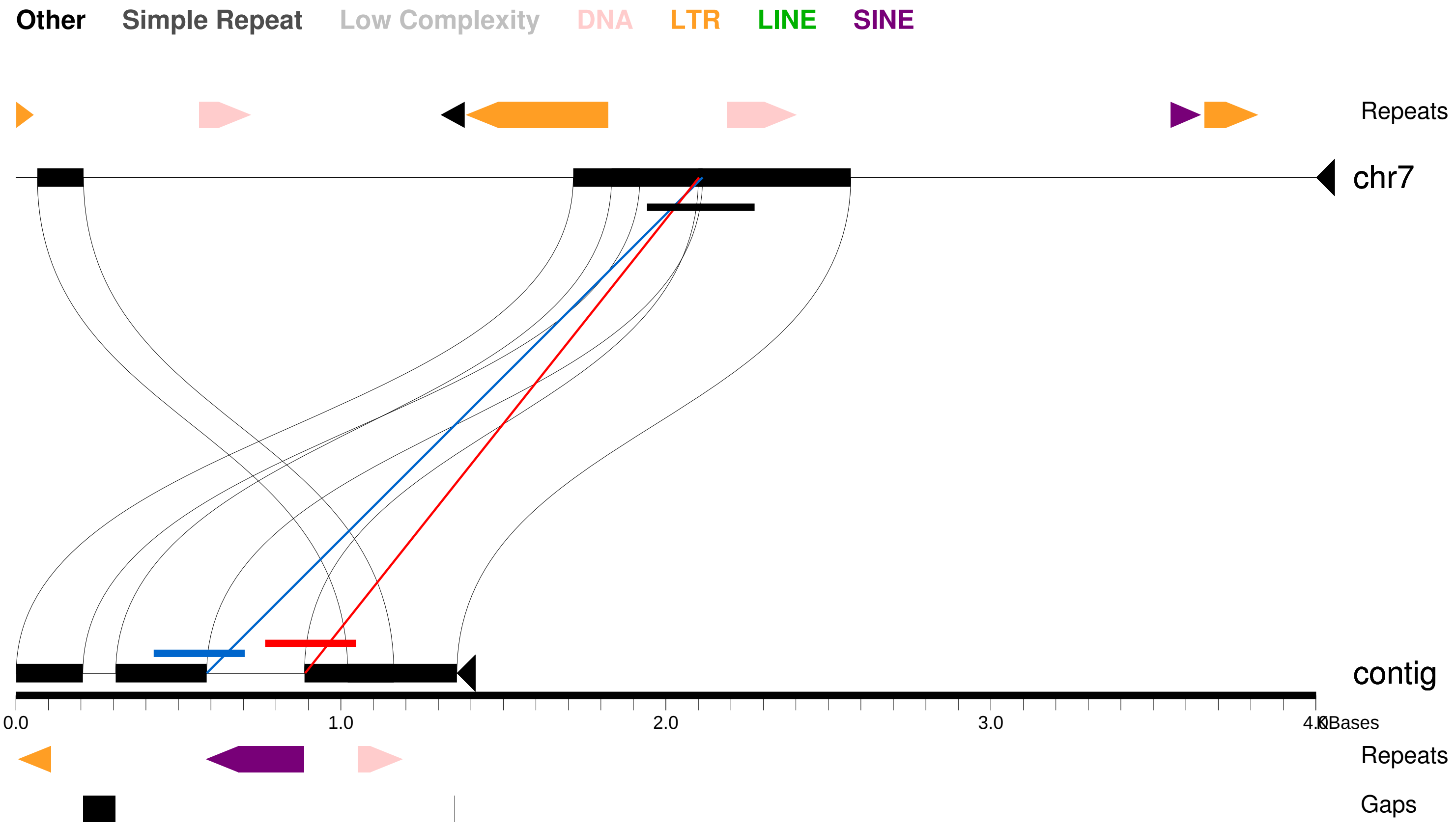

siteID: chr7_124593023 Scafftig8 - Breakpoint: chr7:124593011
chr7:124592965-124593059
Overlap Length: 14 INS size: 307 Genotyped: Yes

```
left    A----------TAATTAGTAT--------CTTCAGAGTGAGCCAA------AATATTACAAAAATATTAG
chrom   A----------TAATTAGTAT--------CTTCAGAGTGAGCCAA------AATATTACAAAAATATTAG
right   CGCCACTGCAGTACGCAGTCCGGCCTGGGCGACAGAGCGAGACTCCGTCTCAAAAAAAAAAAAAAAAAAA
        11111111111**111***1111111111*11*****1***1*11111111**1*11*1*****1*11*1

left    AAATAAAGGGACTTGGCCGGGCGCGGTGGCTCACGCCTGTAATCCCAGCACTTTGGG-AGGCCGAGGCGG
chrom   AAATAAAGGGACTTA---------------TTAC------AAATATAGAATGTTAAACAAGTATAAACTA
right   AAAAAAAGGGACTTA---------------TTAC------AAATATAGAATGTTAAACAAGTATAAACTA
        ***1**********2222222222222222*2**222222**2222**2*22**2222*2*222*22*22

left    GTGG
chrom   ----
right   ----
        2222
```

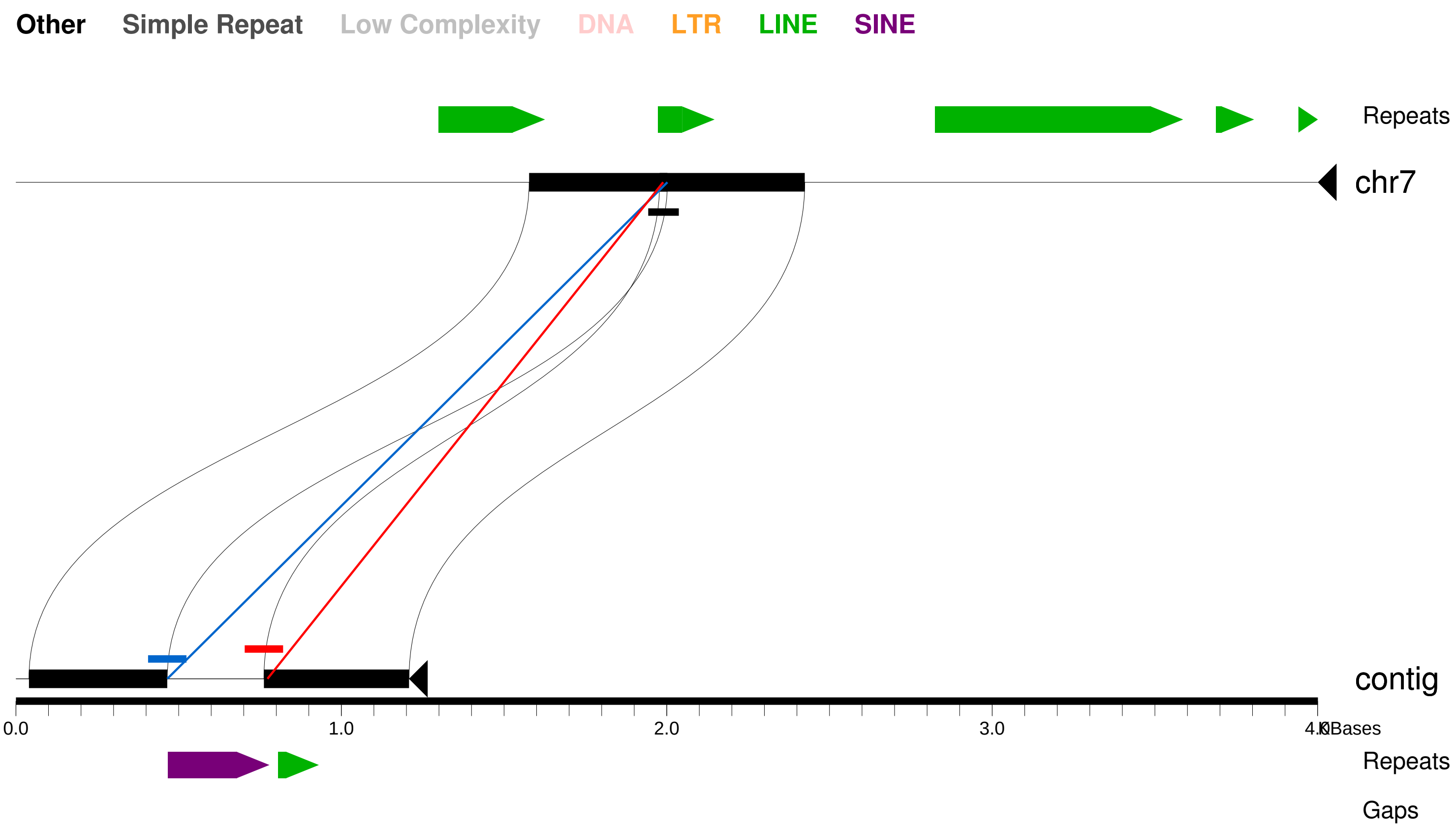

siteID: chr7_124814720 Scafftig1 - Breakpoint: chr7:124814691
chr7:124814531-124814865
Overlap Length: 15 INS size: 387 Genotyped: No

```
left   NNNNNNNN----------AATAGTAATGGCAAACATGACTAAAACCTTAAAATTTTTAGACTGTTAAATG
chrom  ACTTCTTTCTACTCACTGAATAGTAATGGCAAACATGACTAAAACCTTAAAATTTTTAGACTGTTAAATG
right  NNNNNNNNNNNNTACA------------GGCG----TGA-------------------------------G
       NNNNNNNNNNNNNN22  111111111***11111***1111111111111111111111111111111*

left   CCTTACAATGAAAAATGCATGTAAAAATCCTACCTTCTCCCTTGGAAGGCATTTTATTTGGTTAAAAAAA
chrom  CCTTACAATGAAAAATGCATGTAAAAATCCTACCTTCTCCCTTGGAAGGCATTTTATTTGGTTAAAAAAA
right  CC-----------------------------ACC--------------GCG-------------------
       **11111111111111111111111111111***11111111111111**1111111111111111111

left   ATCCCAACTGATTATGTGCCTGGGCAATGATTTTTTTTTTTTTTTTTTTTGAGACGGAGTCTC--------
chrom  ATCCCAACTGATTATGTGCCTGGGCAATGATTTTTATAGATTAGCTTTA---ACAGAGTATCTGCAATAT
right  --CCCA--------------TGGGCAATGATTTTTATAGATTAGCTTTA---ACAGAGTATCTGCAATAT
       11****11111111111111**************2*222**222***2222**2****2**22222222

left   -----GCTCTGTCGCCCAGGTCGGACTGCGGA----CTGCAGTGGC-------GCAATCTCGGCTC----
chrom  TAGATGTTATTACCACCATGCCAAAGTATTTAATAGCTTCTTTGCCTAGCTATGGAATCTGTCCTATAAT
right  TAGATGTTATTACCACCATGCCAAAGTATTTAATAGCTTCTTTGCCTAGCTATGGAATCTGTCC------
       22222*2*2*22*22***2*2*22*2*2222*2222**2*22**2*2222222*2*****222*1N

left   ------------ACTGCAAGCTCCGCTTCCCGGGTTCACGCCAT-TCTCCTG------
chrom  GTGGGGAGATAGAGTGGGTGCTTTCCTCCTAAGATCCTCCTAAGATCTTAGGAGGTGC
right  ----------------------------------------------------------
                   1N11NNN111NNN11N1NNN1N1N1N1NNN1N 111NNN1
```

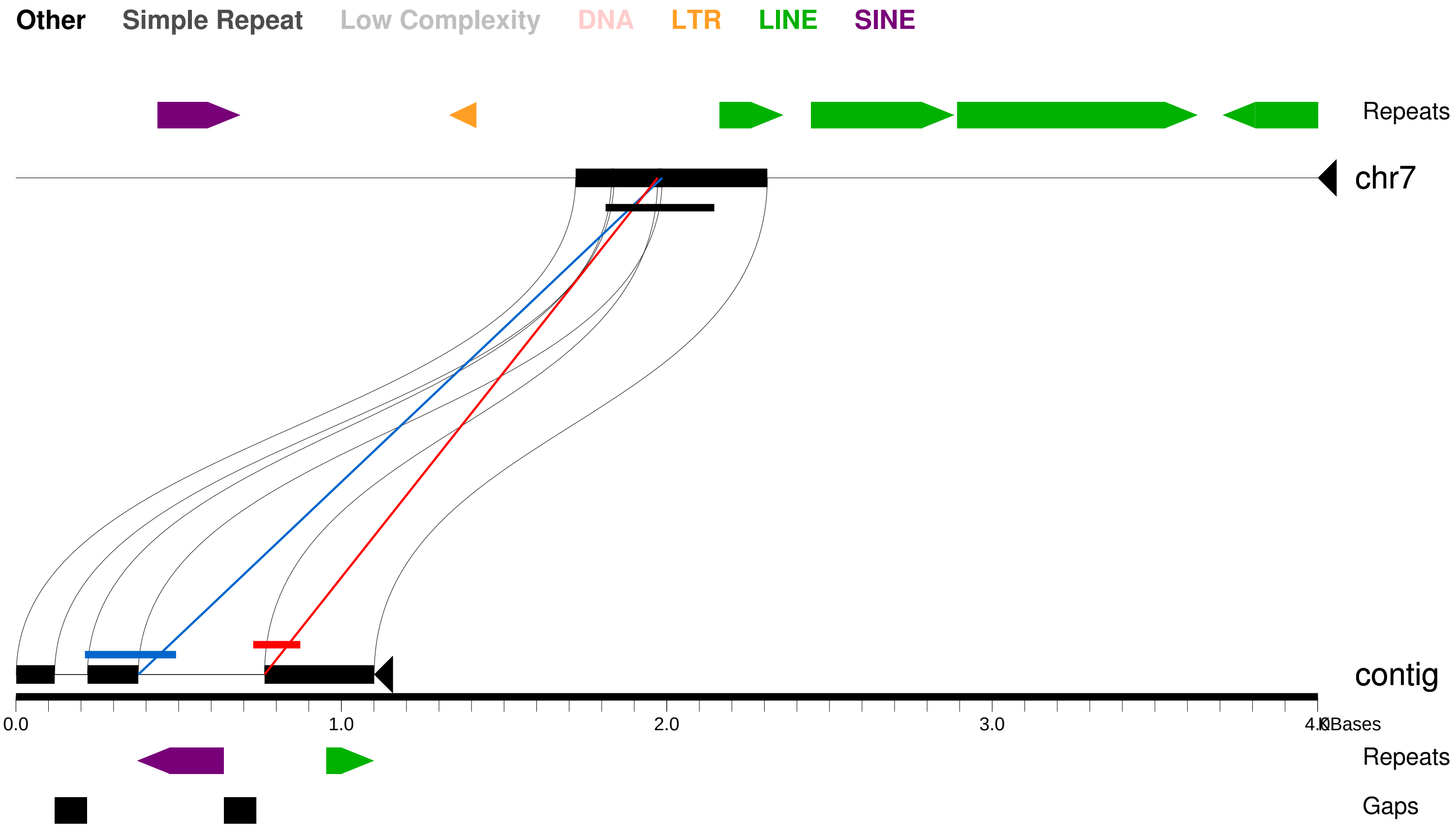

siteID: chr7_125861728 Scafftig1 - Breakpoint: chr7:125861734
chr7:125861685-125861790
Overlap Length: 11 INS size: 303 Genotyped: Yes

```
left     AAAATAAT------TTATCCCT---CCATTCAC-GAAGCTCAGTTTCACTGGGTACAAA---ATTTTTGG
chrom    AAAATAAT------TTATCCCT---CCATTCAT-GAAGCTCAGTTTCACTGGGTACAAA---ATTTTTGG
right    ACTGCAGTCCGCAGTCCGGCCTGGGCGACAGAGCGAGACTCCGTCTCAAAAAAAAAAAAAAAATTCTTGG
         *1111*1*111111*1111***111*1*111*N1**11***1**1***111111*1***111***1****

left     CTGGGCTGGGCGCGGTGGCTCACGCCTGTAATCCCAGCACTTTGGGAGGCCGAGGCGGGTGG
chrom    CTG--ATAATCATTTTGTTTAAGGAGGCTAAAAA--------TGGGA--CCCAATCCCTTC-
right    CTG--ATAATCATTTTGTTTAAGGAGGCTAAAAA--------TGGGA--CCCAATCCCTTC-
         ***222*222*2222**22*2*2*2222***2222222222*****22**2*22*222*22
```

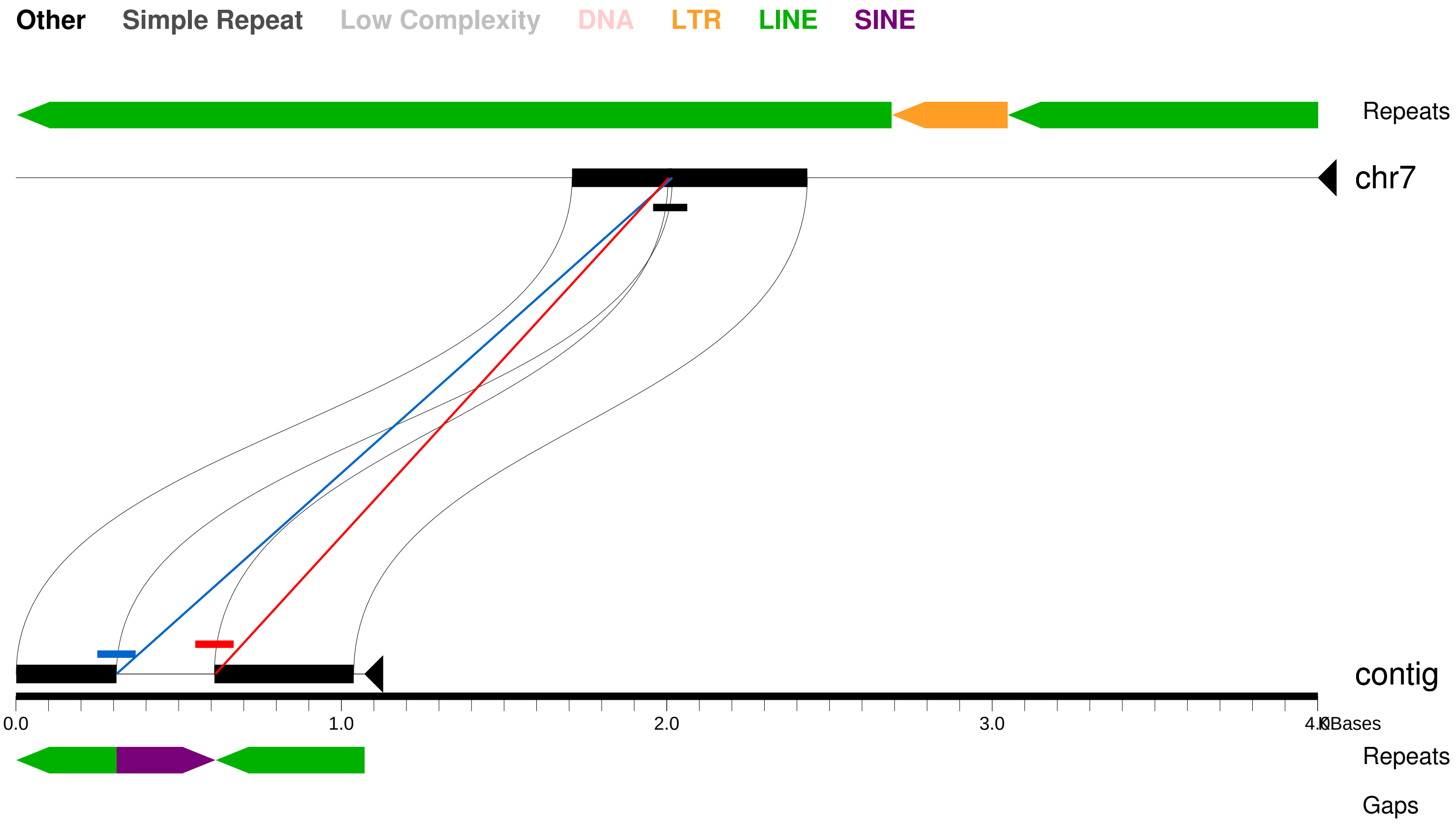

siteID: chr7_126661396 Scafftig1 + Breakpoint: chr7:126661392
chr7:126661345-126661451
Overlap Length: 13 INS size: 296 Genotyped: Yes

```
left    CCCAGCTGCTT---CCTTT-AAGGAACTG--AAGCCAGAA---AGGTACTGC---AGAGGGAATTTTTTT
chrom   CCCAGCTGCTT---CCTTT-AAGGAACTG--AAGCCAGAA---AGGTACTGC---AGAGGGAAATTTTTT
right   TCCGCCCGCCTCGGCCTCCCAAAGTGCTGGGATTACAGGCGTGAGCCACCGCGCCCGGCGGAAATTTTTT
        1**11*1**1*111***111**1*11***11*111***11111**11**1**1111*11****2******

left    TTTTTTTTTTTTTTTTTTGAGACGGAGTCTCGCTCTGTCGCCCAGGCTGGAGTGCAGTGGC
chrom   TTAAAGAAATCAACTTCTCAGAAATATAATA-------AGCCCAAGCC----TAAACTGT-
right   TTAAAGAAATCAACTTCTCAGAAATATAATA-------AGCCCAAGCC----TAAACTGT-
        **2222222*2222**2*2***222*222*222222222*****2**22222*22*2**22
```

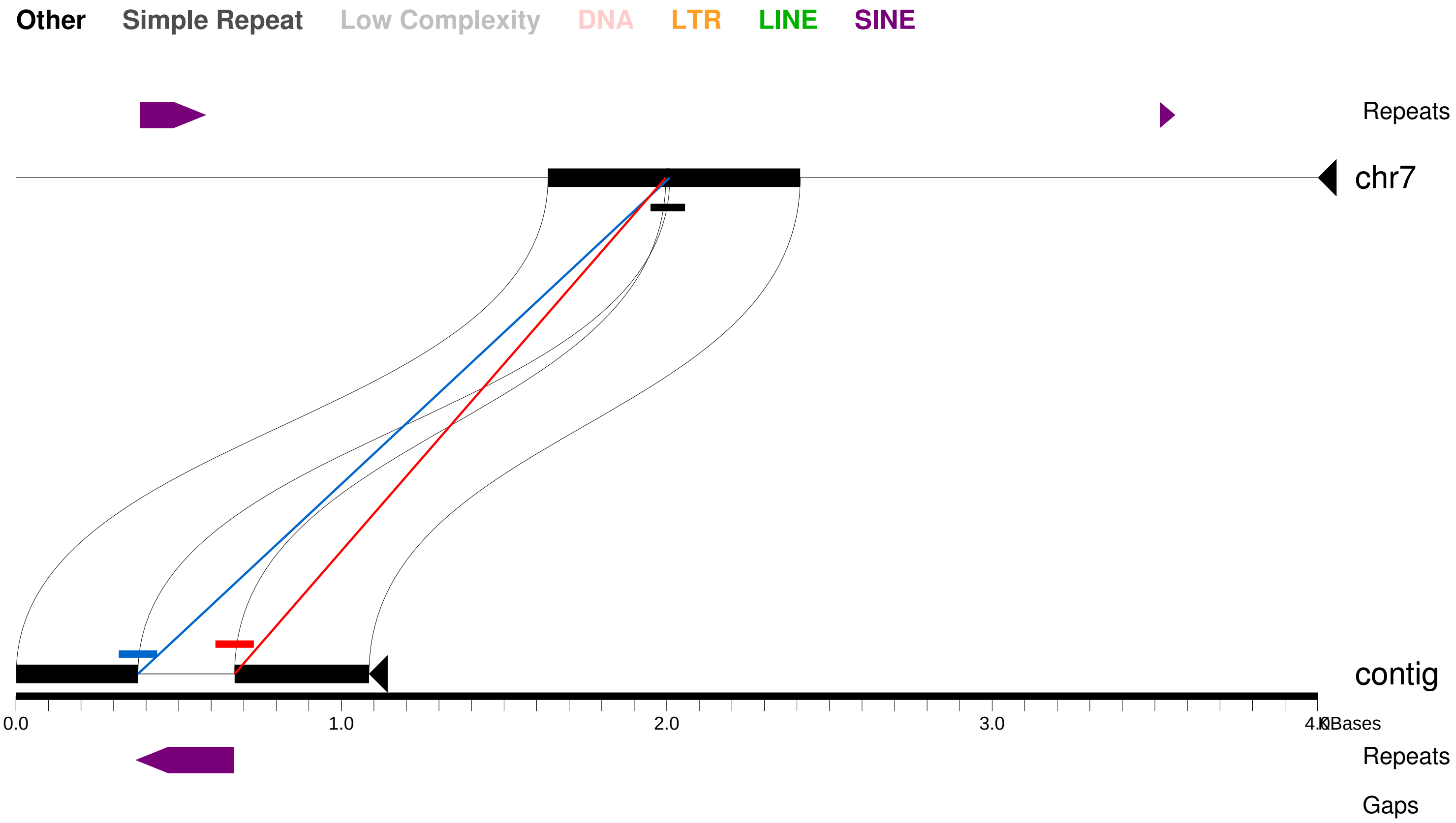

siteID: chr7_129726838 Scafftig1 + Breakpoint: chr7:129726863
chr7:129726811-129726922
Overlap Length: 8 INS size: 452 Genotyped: No

```
left    TTTATATAAA-CATTTACTTATAACCCAG----TA-AGGGAACAGATACTATTGCTGTCCCC-TTTTNNN
chrom   TTTATATAAA-CATTTACTTATAACCCAG----TA-AGGGAACAGATACTATTGCTGTCCCC-TTTTTTA
right   TCCGCCCGCCTCGGCCTCCCAAAGTGCTGGGATTACAGGCGTGAGCCACCGCGCCCGGCCCCCTTTTTTA
        *1111111111*11111*11*1*111*1*1111**1***1111**11**11111*1*1****1*****222

left    NNNNNNNNNNNNNNNNNNNNNNNNNNNNNNNNNNNNNNNNNNNNNNNNNNNNNNN
chrom   TAGTTAAGGAAGCTGGGGTACCCAGGACAAAACCTTGTCCAAGGACATA-------
right   TAGTTAAGGAAGCTGGGGTACCCAGGACAAAACCTTGTCCAAGGACATA-------
        2222222222222222222222222222222222222222222222222222222
```

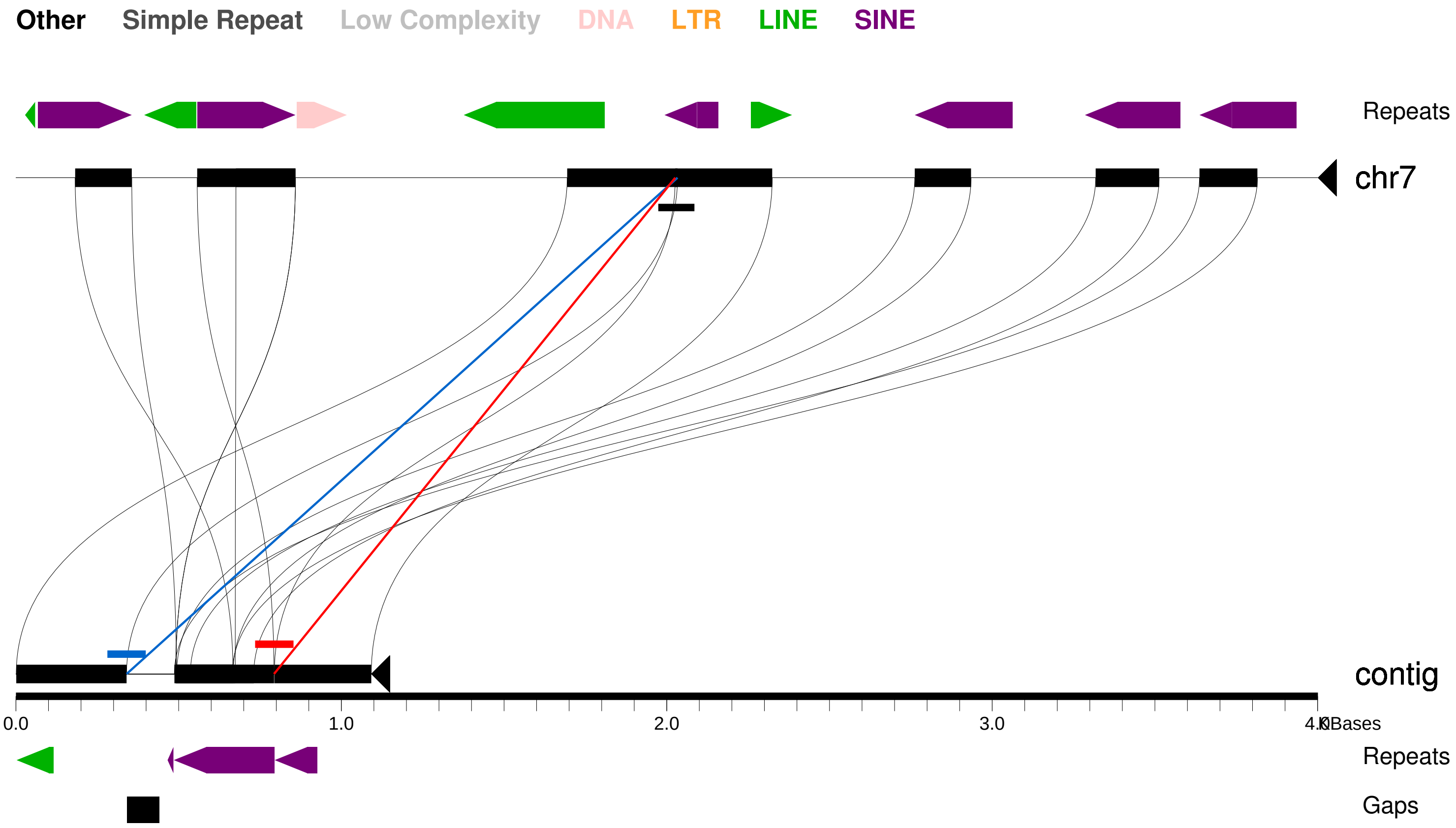

siteID: chr7_13250247 Scafftig2 + Breakpoint: chr7:13250252
chr7:13250208-13250311
Overlap Length: 16 INS size: 314 Genotyped: Yes

```
left     TTTCCTGCA-CATTCTCACCAA------------CACTTGTTATCTTTTGT--CTTTTTGATAATACTTA
chrom    TTTCCTGCA-CATTCTCACCAA------------CACTTGTTATCTTTTGT--CTTTTTGATAATACTTA
right    CCACCCGCCTCGGCCTCCCAAAGTGCTGGGATTACAGGCGTGAGCTACCGCGCCCGGCCGATAATACTTA
         111**1**11*111***1*1**111111111111**111**1*1**111*111*11111***********

left     TTCTTTTTTTTTTTTTTTTTTTTTTTGAGACGGAGTCTCGCTCTGTCGCCCAGGCCGGACTGCG
chrom    TTCTTA-------------AAGGTGTCAGATG--ATATGTCATTGTGGTTTTAACTTGCATTCC
right    TTCTTA-------------AAGGTGTCAGATG--ATATGTCATTGTGGTTTTAACTTGCATTCC
         *****22222222222222222*2*2***2*222*2*22*22***2*222222*22*22*2*2
```

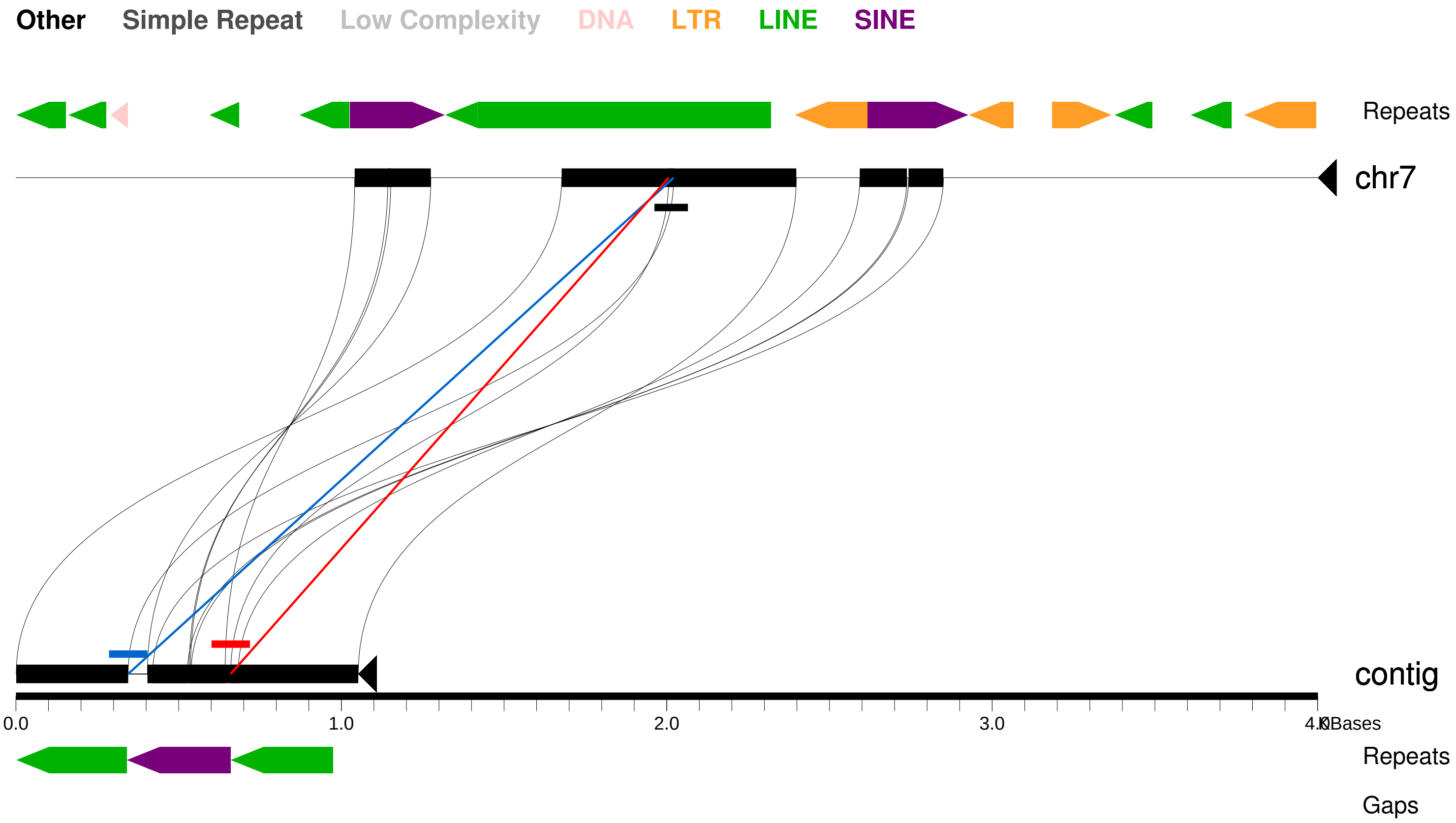

siteID: chr7_134424662 Scafftig1 - Breakpoint: chr7:134424642
chr7:134424597-134424701
Overlap Length: 15 INS size: 482 Genotyped: No

```
left    ----CAAACTAAAGCCT--CAAATT----GGAGTCCAAGAGTAA-CAATCAA---ATGGCATAATTTTAT
chrom   ----CAAACTAAAGCCT--CAAATT----GGAGTCCAAGAGTAA-CAATCAA---ATGGCATAATTTTAT
right   TCCGCCCGCCTCGGCCTCCCAAAGTGCTGGGATTACAGGCGTGAGCCACCGCGCCCGGCCATAATTTTAT
        1111*111*1111****11****1*1111***1*1**1*1**1*1*1*1*1111111*1***********

left    TCTTTTTTTTNNNNNNNNNNNNNNNNNNNNNNNNNNNNNNNNNNNNNNNNNNNNNNNNNNNNN
chrom   TCTTAAGT--------------CACCTATTAAATGATGTCCACTACATTCTTCACTGATAATC
right   TCTTAAGT--------------CACCTATTAAATGATGTCCACTACATTCTTCACTGATAATC
        ****222*2222222222222222222222222222222222222222222222222222222
```

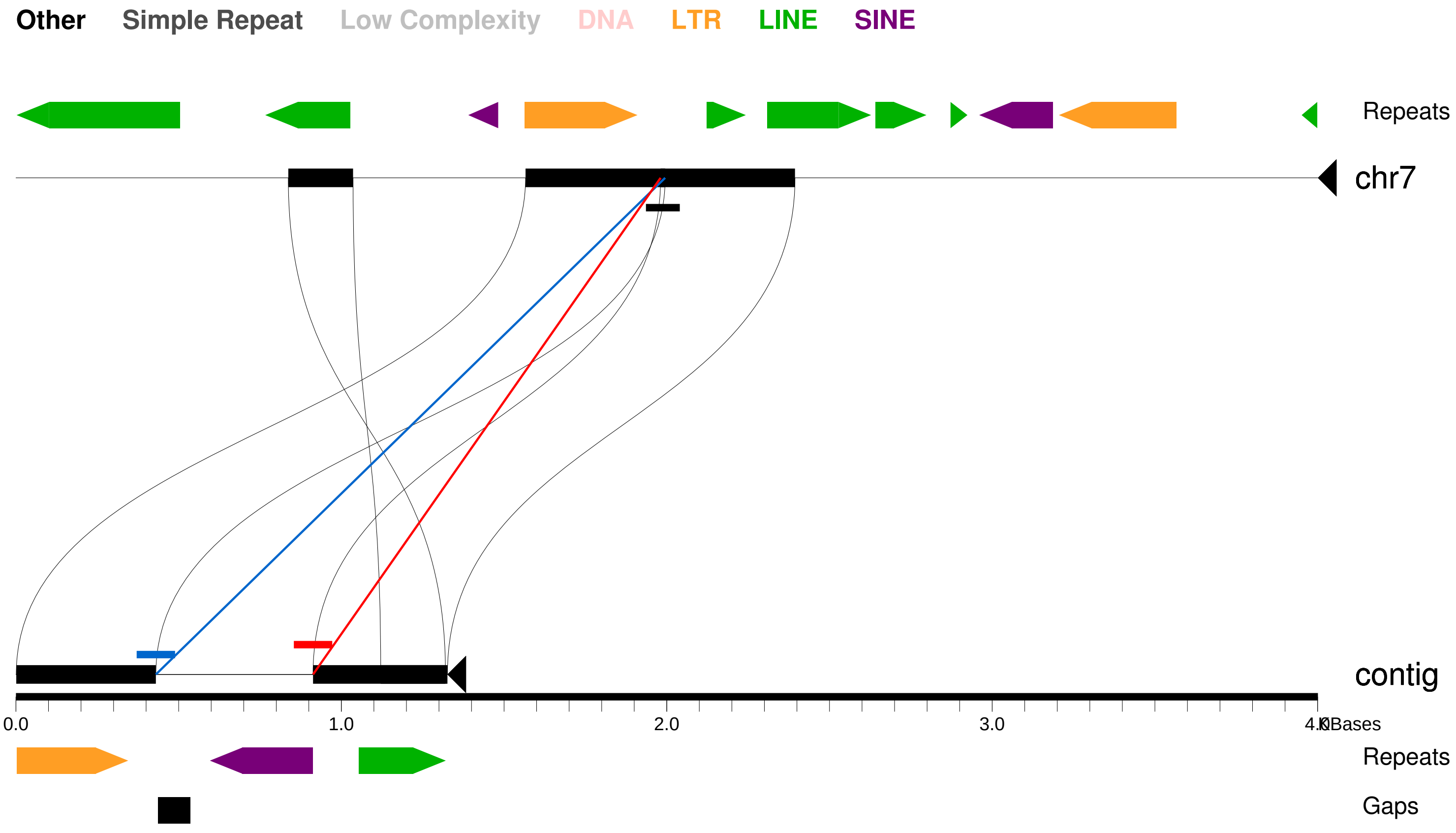

siteID: chr7_136178789 Scafftig2 - Breakpoint: chr7:136178795
chr7:136178750-136178854
Overlap Length: 15 INS size: 299 Genotyped: Yes

```
left    TT-CAGTAGTCCAGAC-------CACCTGAAATAGG------ATGGCAACTGAGGTGACAGAAAACAGGG
chrom   TT-CAGTAGTCCAGAC-------CACCTGAAATAGG------ATGGCAACTGAGGTGACAGAAAACAGGG
right   CCACTGCACTCCAGCCTGGGCGACAGCGAGACTCCGTCTCAAAAAAAAAAAAAAAAAAAAGAAAACAGGG
        111*1*1*1*****1*1111111**1*111*1*11*111111*1111**111*1111*1***********

left    AATAGGCCGGGCGCGGTGGCTCACGCCTGTAATCCCAGC-ACTTTGGGAGGCCGAGGCGGGCGG
chrom   AATA--CCTGAAAGAATGAA-----AATGGAAAGACAGTGACTATGGGATGCAAAA--------
right   AATA--CCTGAAAGAATGAA-----AATGGAAAGACAGTGACTATGGGATGCAAAA--------
        ****22**2*222222**222222222**2**222***22***2*****2**22*222222222
```

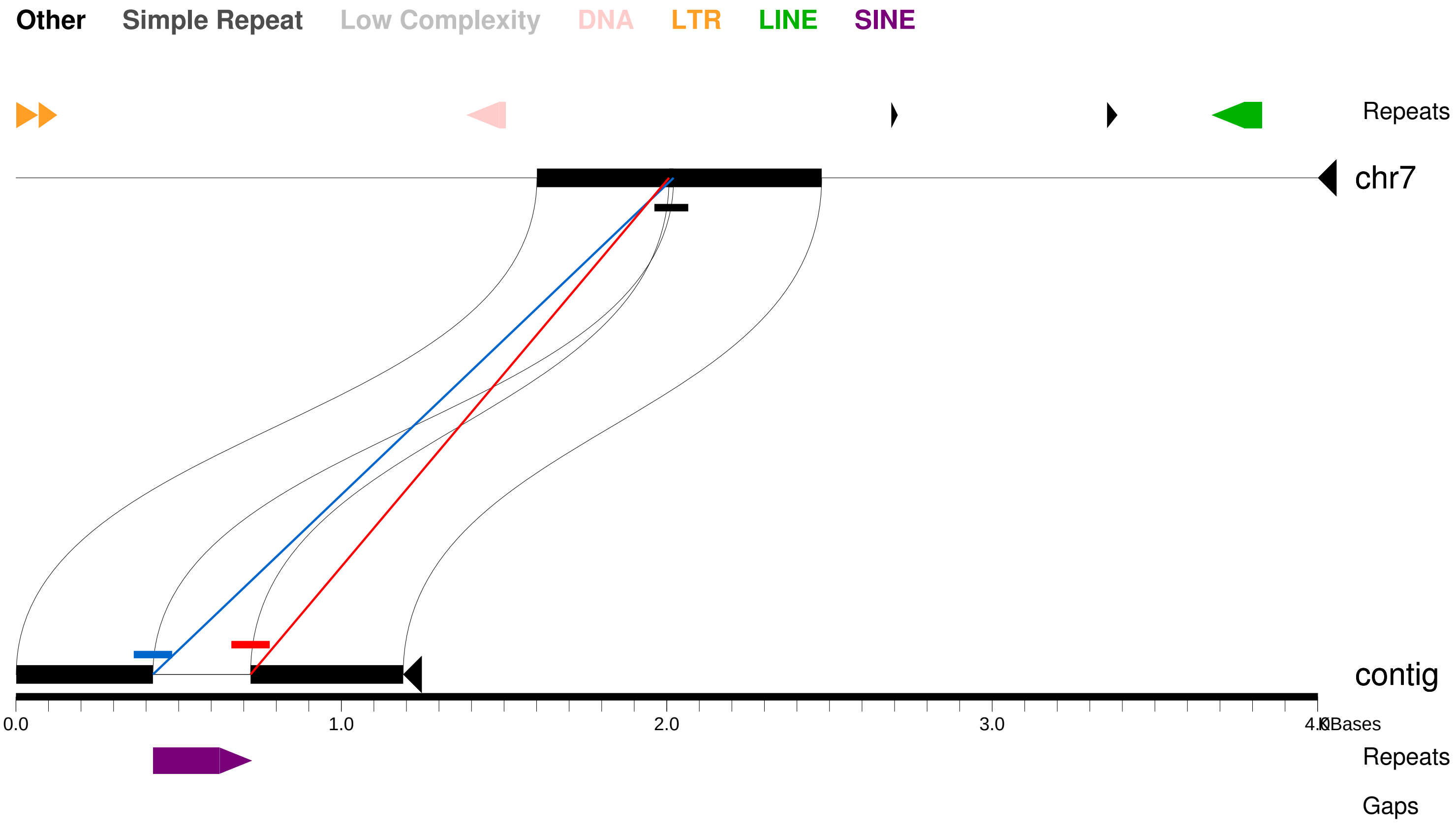

siteID: chr7_141013580 Scafftig1 - Breakpoint: chr7:141013582
chr7:141013531-141013639
Overlap Length: 9 INS size: 295 Genotyped: No

```
left    AATGGAGATGTGGCTTCCCGTGGGCAAACTGGACATATTTTTATGTTTA----------AAAAAATTCTG
chrom   AATGGAGATGTGGCTTCCCGTGGGCAAACTGGACATATTTTTATGTTTA----------AAAAAATTCTG
right   NNNNNNNNNNNNNNNNNNNNNNNNNNNNNNNNNNNNNNNNNNNNNNNNNNNNNNNNNNNNNAAAATTCTG
        1111111111111111111111111111111111111111111111111111111111111*********

left    GGCCGGG-CGCGGTGGCTCACGCCTGTAATCCCAGCACTTTGGGAGGCCGAGGCGGGCGG-
chrom   CATTTGCACTTTGTGCATTATTGATGT-------GCGTCTTTGGA-----AGGAGGGCATT
right   CATTTGCACTTTGTGCATTATTGATGT-------GCGTCTTTGGA-----AGGAGGGCATT
        22222*22*222***22*2*2222***2222222**222**2***22222***2****222
```

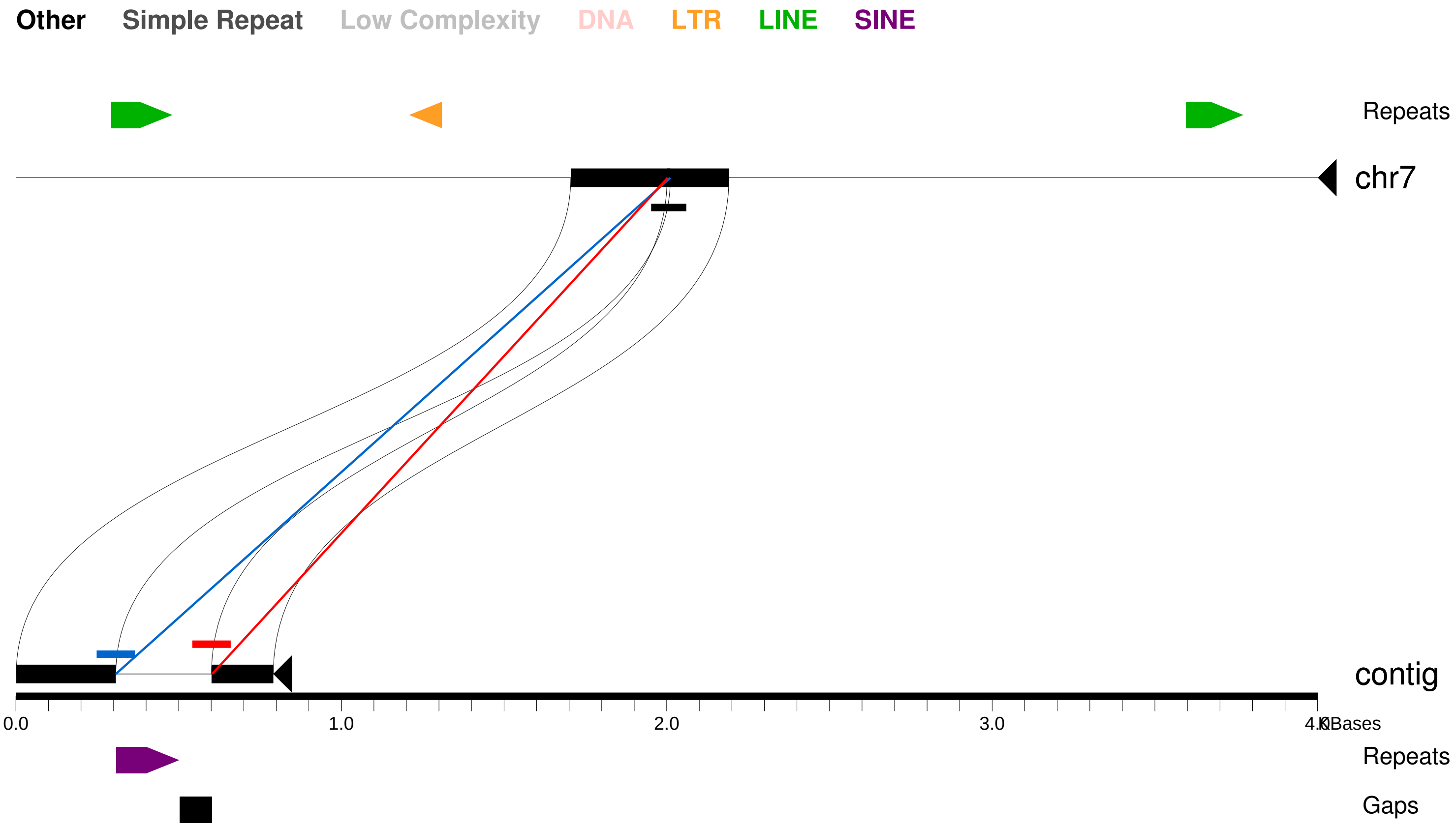

siteID: chr7_147585792 Scafftig3 + Breakpoint: chr7:147585776
chr7:147585731-147585835
Overlap Length: 15 INS size: 302 Genotyped: Yes

```
left     TAGACAG------------AAATTACA--GATTATTTACAAAAGAATTGAAATTAAACTGACCATTGACT
chrom    TAGACAG------------AAATTACA--GATTATTTACAAAAGAATTGAAATTAAACTGACCATTGACT
right    CCGCCCGCCTCGGCCTCCCAAAGTGCTGGGATTACAGGCGTGAGCCACCGCGCCCGGCCGACCATTGACT
         11*1*1*111111111111***1*1*111*****1111*111**1111111111111*1***********

left     TCTTTTTTTTTTTTTTTTTTTTTTTTGAGACGGAGTCTCGCTCTGTCGCCCAGGCTGGAGTGCA
chrom    TCTTGACAATGGAACCCTGAA----GAAACAAACTAT---TGTG------AGAAAATAAC-CA
right    TCTTGACAATGGAACCCTGAA----GAAACAAACTAC---TGTG------AGAAAATAAC-CA
         ****22222*2222222*2222222**2**22*2*21222*2**222222**22222*222**
```

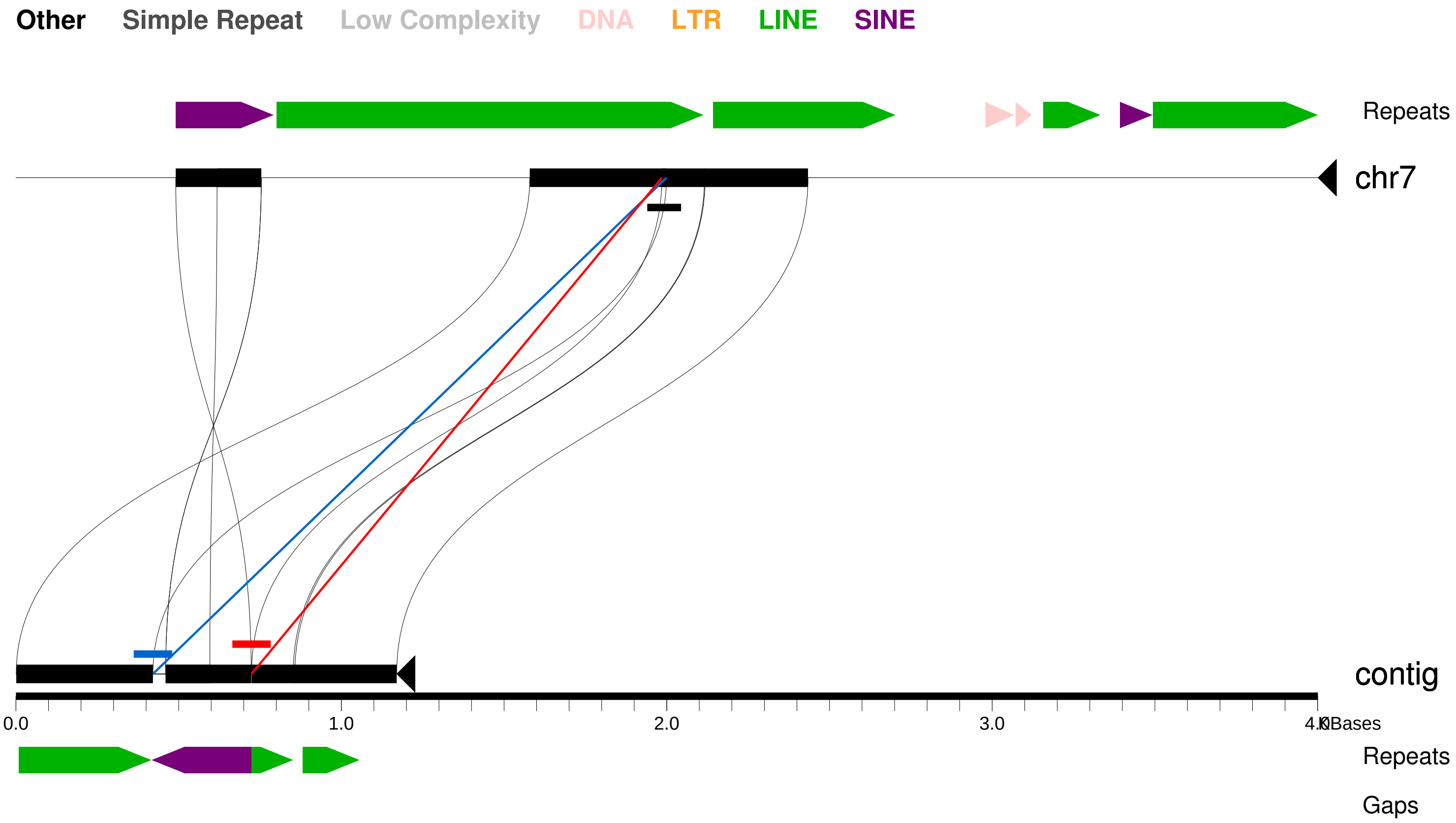

siteID: chr7_147704410 Scafftig1 + Breakpoint: chr7:147704390
chr7:147704342-147704446
Overlap Length: 12 INS size: 307 Genotyped: Yes

```
left     AAATGTAGGACTTCGTTGTACTTGGGGGATTACTTC----------------AATTGCCTTAATAGTCTA
chrom    AAATGTAGGACTTCGTTGTACTTGGGGGATTACTTC----------------AATTGCCTTAATAGTCTA
right    CACTCCAG--CCTGGGCGACAGAGCGAGACTCCGTCTCAAAAAAAAAAAAAAAAAAAAAAAAAAGTCTA
         1*1*11**11*1*1*11*11111*1*1**1*1*1**1111111111111111**1111111**1******

left     CCAGAGGCCGGGCGCGGTGGCTCACGCCTGTAATCCCAGCACTTTGGGAGGCCGAGGCGGGCGGA
chrom    CCAGAGT-----AGAAATATTACATTCTTTAAAGTTCAGATAAATA--------ATTTGTGCTG-
right    CCAGAGA-----AAAAATATTACATTCTTTAAAGTTCAGATAAATA--------ATTTGTGCTG-
         ******N2222221222*2222**22*2*22**222***22222*222222222*222*2**2*2
```

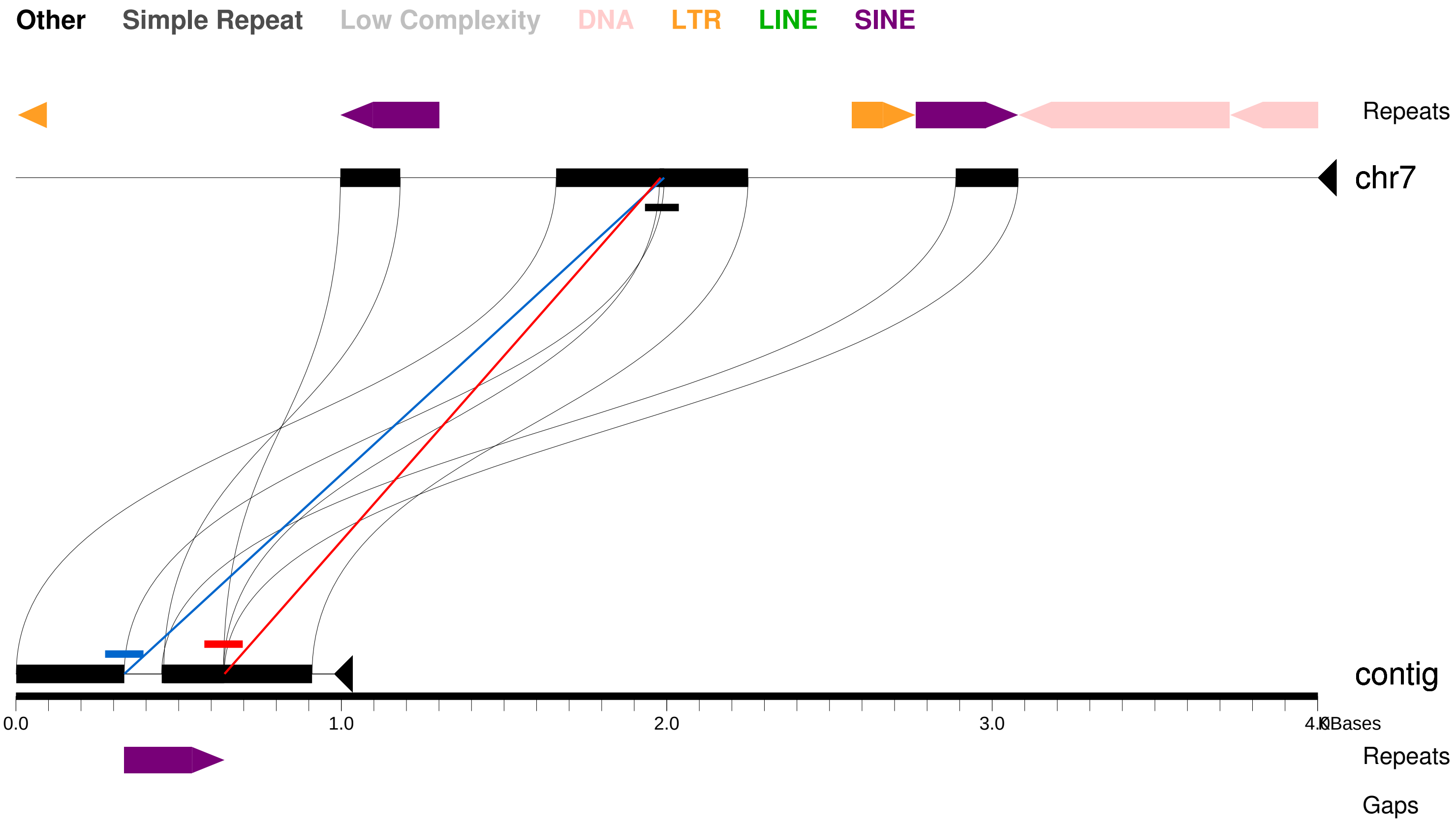

siteID: chr7_147919503 Scafftig2 - Breakpoint: chr7:147919509
chr7:147919349-147919725
Overlap Length: 57 INS size: 338 Genotyped: No

```
left   G---------------------------------------------------------------------
chrom  GTAATTTA-TAAAGAAAAGGAATTTATTTCTTATAGTTATGGAGGCTAAGAAGTCTGAGGCCAAAGGGCC
right  ----TTTAGTAGAGACGGGG------TTTCACCTTGTTAGCCAGGATG----GTCTCGATCT------CC
       1   2222122N222NNN22      2222NNN2N2222NNN222N2N    2222NNNN2N      22

left   --------------CCTTATTACTGGTGGGAACTCTCTGCAGTTCTGAGACAGTGCAGGGCATGACATGG
chrom  AAACCTGGTGAGGGCCTTATTACTGGTGGGAACTCTCTGCAGTTCTGAGACAGTGCAGGGCATGACATGG
right  TGACCTCATGAT--CC-----ACCCGC-------CTCGGC----CTCCCAAAGTGCTGGG-ATTACA-GG
       NN2222NN222N  **11111**11*11111111***1**1111**111*1*****1***1**1***1**

left   CGAGGTTAAGCTGCCAGCTCAGGCCTCTCTTCCTCTTCTTTTAGACAGTCCCACTCCCATGAGAACCTGT
chrom  CGAGGTTAAGCTGCCAGCTCAGGCCTCTCTTCCTCTTCTTATAGACAGTCCCACTCCCATGAGAACCTGT
right  CGTG----AGCCACCGCGCCCGGCCTCT--TCCTCTTCTTATAGACAGTCCCACTCCCATGAGAACCTGT
       **1*1111***11**1111*1*******11**********2*****************************

left   TAATCCATNNNNNNNNNNN---------------------------------------------------
chrom  TAATCCATTAACCCATTAATCCATGAATCTATGAATGGATCATTCCATGAATCGATGAATGGATCATTCC
right  TAATCCATTAACCCATTAATCCATGAATCTATGAATGGATCATTCCATGAATCGATGAATGGATCATTCC
       *********2222222222222222222222222222222222222222222222222222222222222

left   ----------------------------------------------------------------------
chrom  ATGAATCGATGAATGGATCATTCCATGAATCTATGAATGGATCATTCCATTCATGAGGACAGAGTCCTCA
right  ATGAATCTATGAATGGATCATTCCATGAATCTATGAATGGATCATTC-----------------------
       2222222N222222222222222222222222222222222222222

left   ----------------------------
chrom  TGATCCAATCACCTCTTAAAGGGCTCAC
right  ----------------------------
```

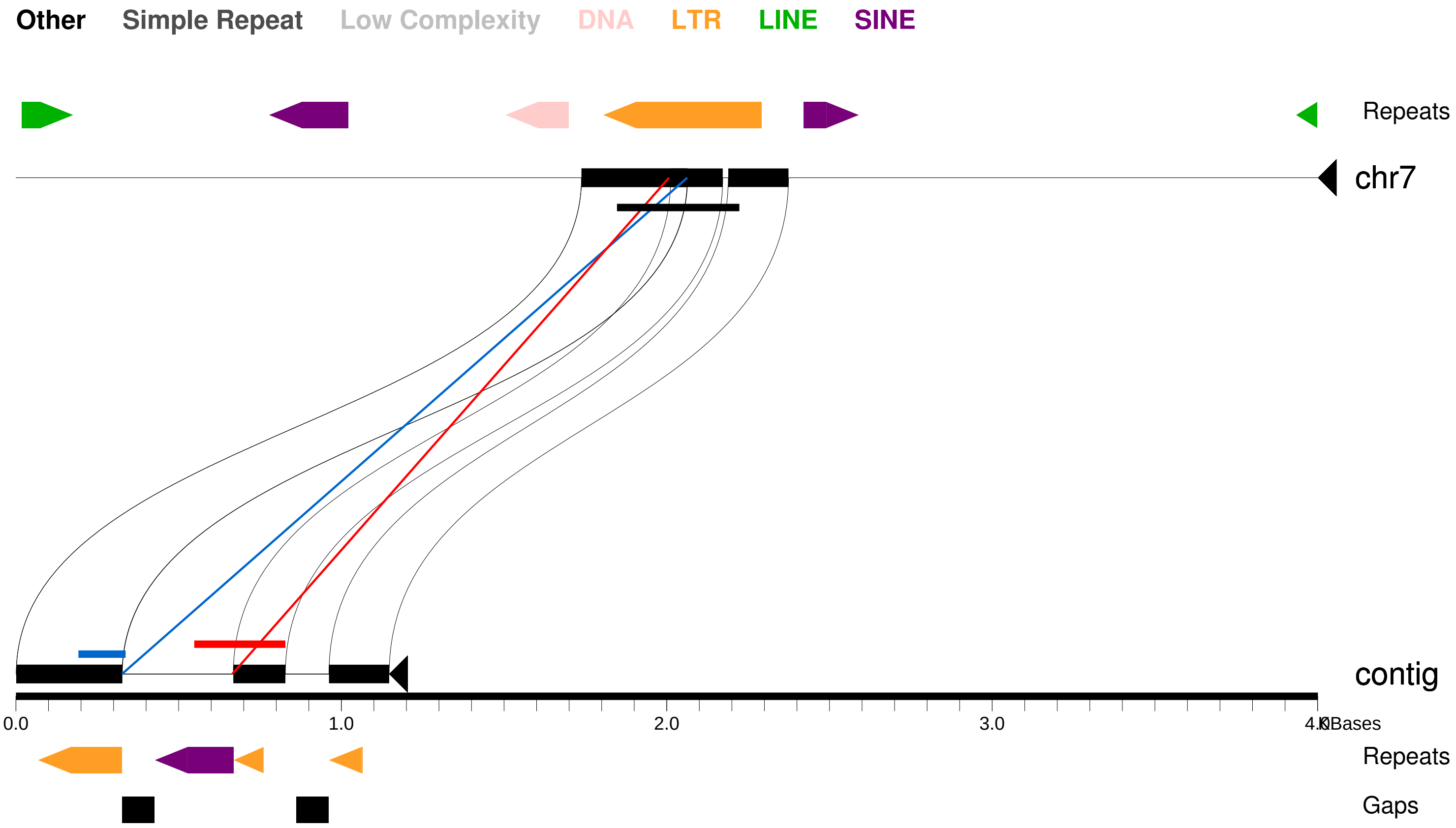

siteID: chr7_15139417 Scafftig1 - Breakpoint: chr7:15139425
chr7:15139377-15139481
Overlap Length: 12 INS size: 383 Genotyped: No

```
left    TATGTTCCAGTTATAGGAAGTAGGAGAAAAGACAAAGATGGAGGT--------------AATGAAAAAAG
chrom   TATGTTCCAGTTATAGGAAGTAGGAGAAAAGACAAAGATGGAGGT--------------AATGAAAAAAG
right   NNNNNNNNNNNNNNNNNNNNNNNNNNNNNNNNNNNNNNNNNNNNNNNNNNNNNNNNNAAAAAGAAAAAAG
        11111111111111111111111111111111111111111111111111111111111**1********

left    TCTGTTGGGAGGCCGAGGCGGGCGGATCACGAGGTCAGGAGATCGAGACCATCCTGGCTAAAA
chrom   TCTGA----AGACCCTGTAA-----ATAAGTAGACCTGCATTCTCTTTTCATAC-----ACAC
right   TCTGA----AGACCCTGTAA-----ATAAGTAGACCTGCATTCTCTTTTCATAC-----ACAC
        ****22222**2**22*22222222**2*22**22*2*2*222222222***2*22222*2*2
```

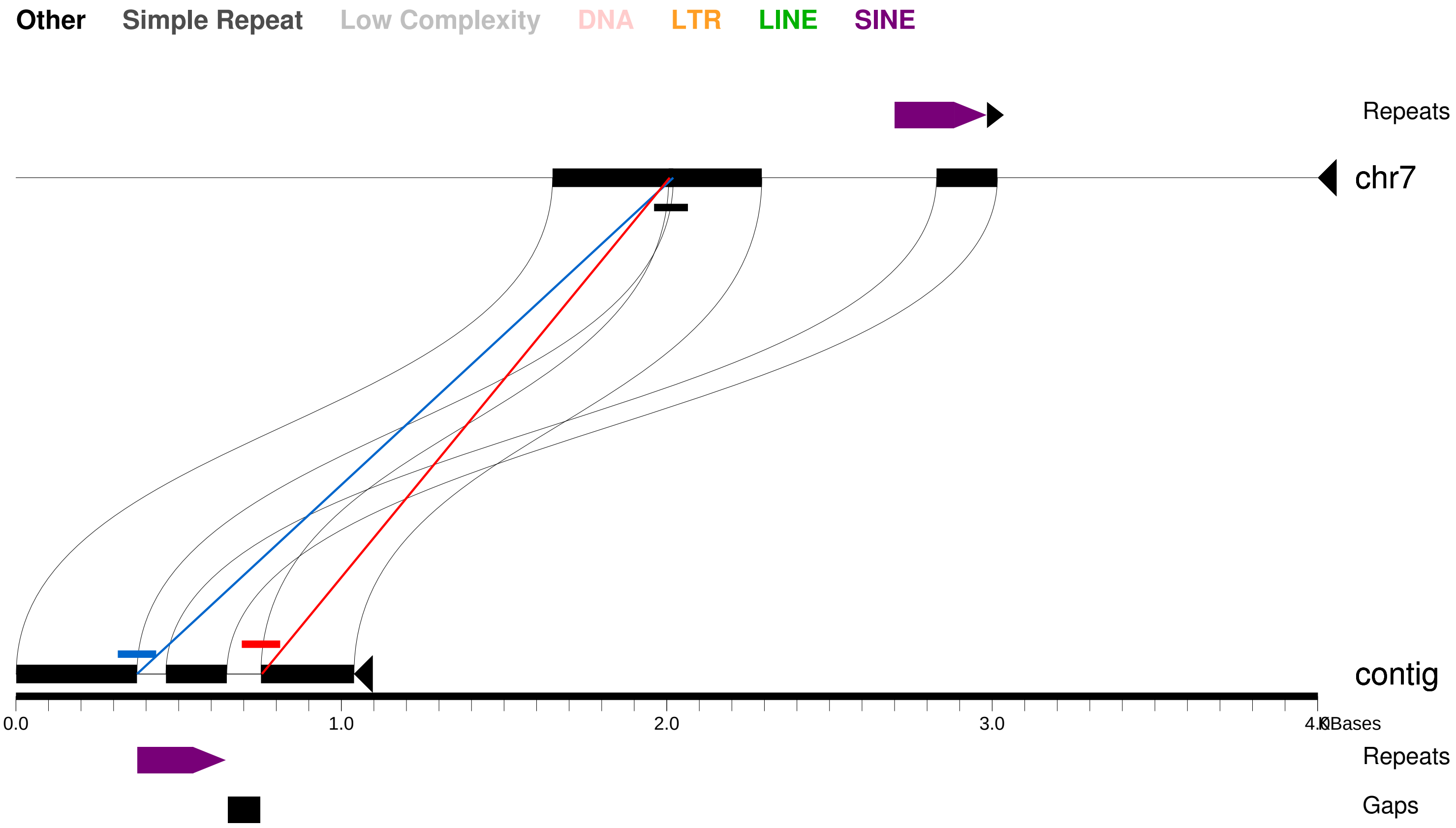

siteID: chr7_153206578 Scafftig3 - Breakpoint: chr7:153206602
chr7:153206442-153206771
Overlap Length: 10 INS size: 307 Genotyped: Yes

```
left   NNNNNNNNNNNNNNNNNNNNNNNNNNN-------TTTTAAAATTATAATAACCCTTCTAACGCCAAGTAA
chrom  TAAAAATTAAGCACAATTAGGAATAAGAAAACATTTTTAAAAGTATAATAACCCTTCTAACGCCAAGTAA
right  T---------------TTAGTA----GAGACGGGGTTTCA------------CCTTGTTA-GCCAGGATG
       2NNNNNNNNNNNNNNN2222N2NNNN22N2NNNN1***1*11N111111111****1*1*1****1*111

left   AAACCTATTGCTTGACTTCA-GCTTATTGATTTTCTTCTTCTTAGCTTCCTTG--TTACCAAGGCAAAAG
chrom  AAACCTATTGCTTGACTTCA-GCTTATTGATTTTCTTCTTCTTAGCTTCCTTG--TTACCAAGGCAAAAG
right  GTCTCGATCTCCTGACCTCATGATCCACCCGCCTCGGCCTCCCAAAGTGCTGGGATTAC--AGGCGTGAG
       1111*1**11*1****1***1*1*111111111**11*1**11*111*1**1*11****11****111**

left   AGAAAAAGCATTAACTTACATTTCTGCTACTTTTT----------TTTTTTTTTTTTTTTTGAGACGGAG
chrom  AGAAAAAGCATTAACTTACATTTCTGCTACTTTCTGAAGACAAACTTTCTGCTTTATTTATCAGCTTTAT
right  CCACCGCGCCCGGCCT-------CTGCTACTTTCTGAAGACAAACTTTCTGCTTTATTTATCAGCTTTAT
       11*1111**11111**1111111*********2*2222222222***2*22***2***2*2**2222*2

left   TCTCGCTCTGTCGCC-----------CAGGCCGGACTGC-------------GGAC--TGCAGTGG----
chrom  CCTGTTACTGAATTCAAATGAGAATGCAGTCAGGAAAACCAGGTAACATAATGGACAATGCATTTAAATA
right  CCTGTTACTGAATTCAAATGAGAATGCAGTCAGGAAAACCAGGTAACATAATGGACAATGCATTTAAATA
       2**2222***2222*22222222222***2*2***222*2222222222222****22****2*222222

left   CGCAATCTCAGCTCACTGCAAGCTCCGCTTCCCG--GGTTCACGCCATTC-TCC
chrom  CACATTTTTAAAACA-TGTAGACATGTTGTCCAAATGGATGAGTGTATACCTCT
right  CACATTTTTAAAACA-TGTAGACATGTTGTCCAAATGGATGAG-----------
       *2**2*2*2*222**2**2*22*222222***2222**2*2*2NNN11N1 11N
```

Other **Simple Repeat** **Low Complexity** **DNA** **LTR** **LINE** **SINE**

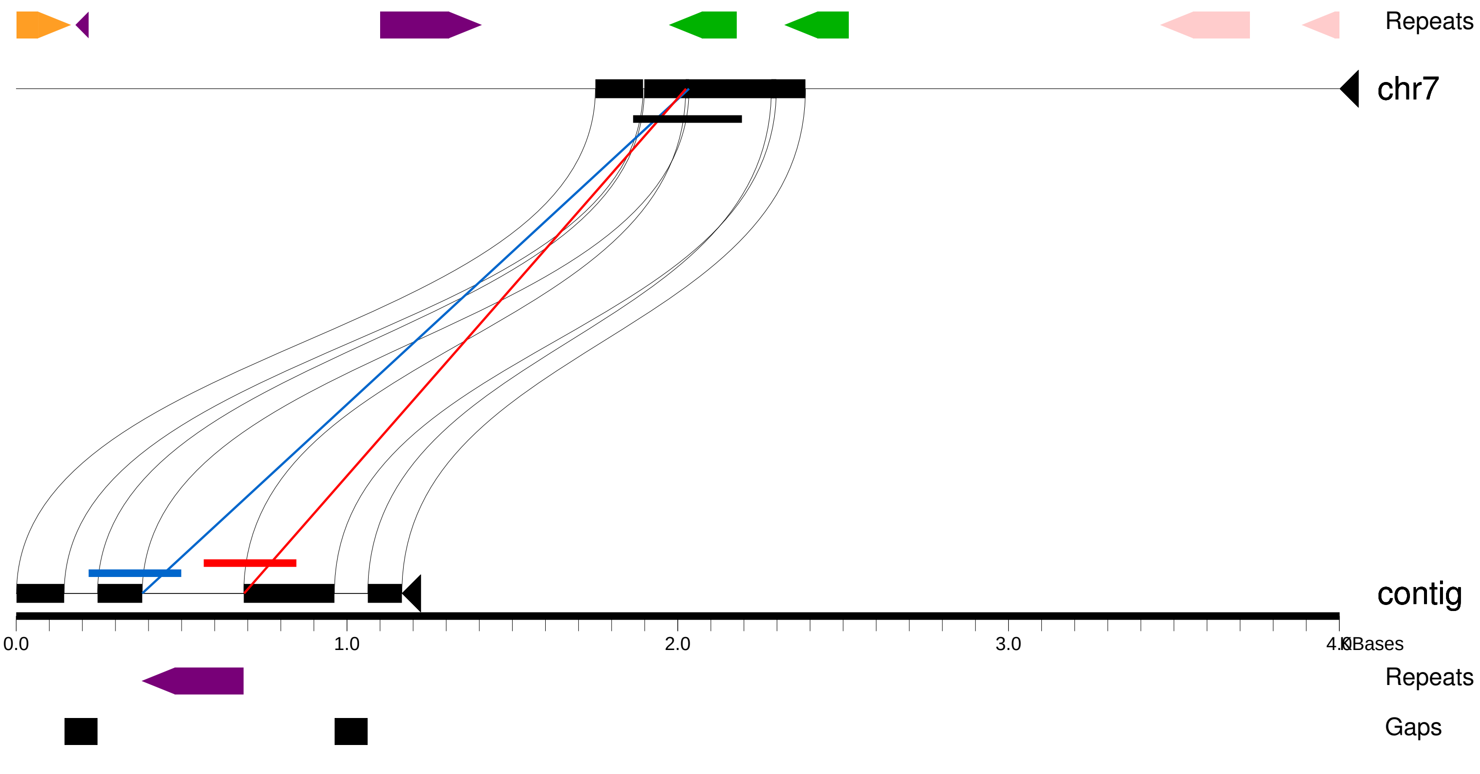

siteID: chr7_153966048 Scafftig18 - Breakpoint: chr7:153966037
chr7:153965991-153966096
Overlap Length: 14 INS size: 299 Genotyped: Yes

```
left    TAA--CACAAGATCCTGT-CATCTCA-CTAGACTATGT---------AAATTTGCAGATAAAAATGCCCA
chrom   TAA--CACAAGATCCTGT-CATCTCA-CTAGACTATGT---------AAATTTGCAGATAAAAATGCCCA
right   CACTGCACTCCAGCCTGGGCGACAGAGCGAGACTCCGTCTCAAAAAAAAAAAAAAAAAAAAAAAATGCCCA
        1*111***111*1****11*11*11*1*1*****11**111111111***11111*1*1***********

left    TCT--GGCCGGGCG--CGGTGGCTCACGCCTGTAATCCCAGCACTTTGGGAGGCCGAGGCGGGCGG
chrom   TCTTAGATTGGTCAGCCTGGGGGTGAAAA-TGGACTCA-ACCACTT--------CTATC-------
right   TCTTAGATTGGTCAGCCTGGAGGTGAAAA-TGGACTCA-ACCACTT--------CTATC-------
        ***22*222**2*222*2*21*2*2*2222**2*2**22*2*****22222222*2*222222222
```

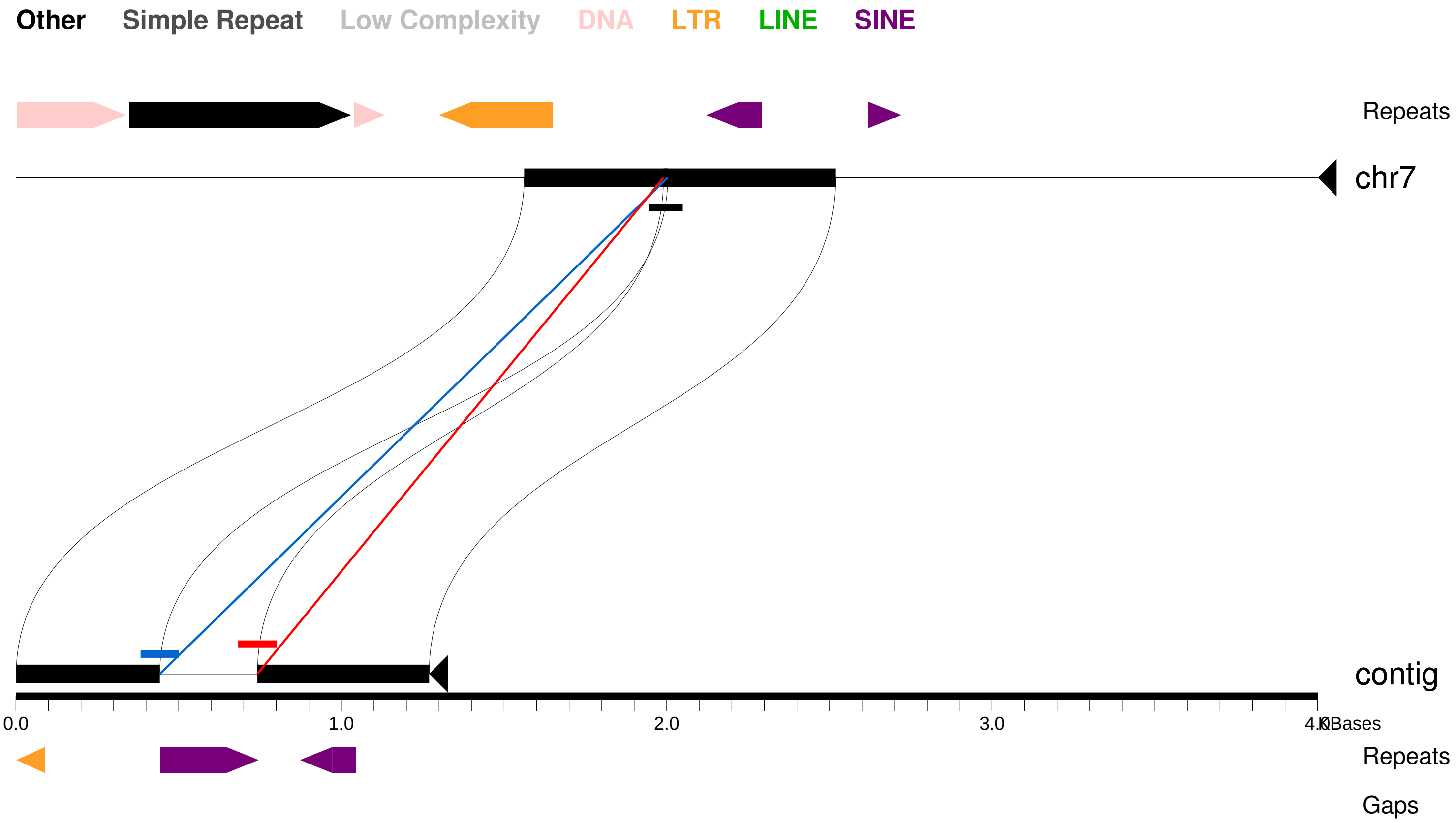

siteID: chr7_154858211 Scafftig9 + Breakpoint: chr7:154858207
chr7:154858161-154858266
Overlap Length: 14 INS size: 298 Genotyped: Yes

```
left     TATCATAAAA--TCCTCATGGAGTTTTTTCCCATTACAGGT-----ACTCCTA--------GTAACATAA
chrom    TATCATAAAA--TCCTCATGGAGTTTTTTCCCATTACAGGT-----ACTCCTA--------GTAACATAA
right    CCGCCCGCCTCGGCCTCCCAAAGTGCTGGG--ATTACAGGCGTGAGCCACCGCGCCCGGCCGTAACATAA
         111*111111111****1111***11*11111********1111111*1**1111111111*********

left     GCTTTTTTTTTTTTTTTTTTTTGAGACGGAGTCTCGCTCTGTCGCCCAGGCTGGAGTGCAGTG-G
chrom    GCTTTGCAAAT-------CATAAAGCCCAAG-------CAGTTGGGCTGCTTTCACTGCAGCCAG
right    GCTTTGCAAAT-------CATAAAGCCCAAG-------CAGTTGGGCTGCTTTCACTGCAGCCAG
         *****22222*222222222*22**2*22**2222222*2**2*22*2*22*22*2*****222*
```

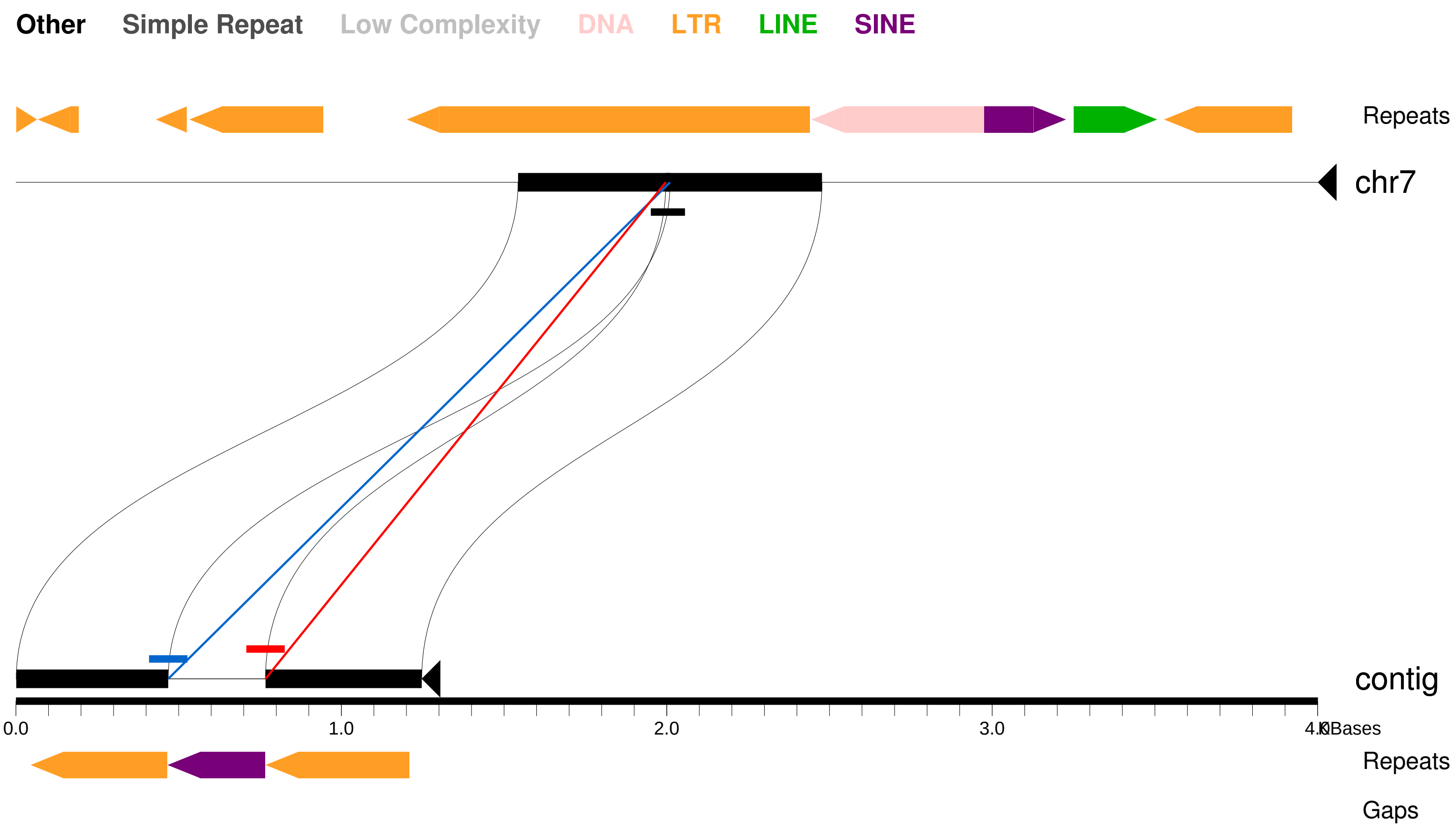

siteID: chr7_157350779 Scafftig1 + Breakpoint: chr7:157350779
chr7:157350619-157350954
Overlap Length: 16 INS size: 324 Genotyped: Yes

```
left   CCA--------------GGAGGACAGGGAATTGTGCAAAATATCCTGGTAAGTTTCTATCGACTGCTGGA
chrom  CCATCCCCAATACTCCAGGAGGACAGGGAATTGTGCAAAATATCCTGGTAAGTTTCTATCGACTGCTGGA
right  -------------TTTAGTAGAGACGGGGTTTCACCTTGTTAGCCAGGATGGTCTCGATCTCCTG-----
       111          2NN2*1**1111***11**111*1111**1**1**111**1**1***11***11111

left   CCATCCCATAATACTGTTGAGGTGCTGGCAGCATCAACGCGGAGACACCGGACCTCTGCACCCCTGTGCA
chrom  CCATCCCATAATACTGTTGAGGTGCTGGCAGCATCAACGCGGAGACACCGGACCTCTGCACCCCTGTGCA
right  --ACCTCATGATCCA---------CCCGCCTCGGCCTCCCAAAGTGCTGGGAT---TACAGGCGTGAGCC
       11*1*1***1**1*1111111111*11**11*11*11*1*11**11111***1111*1**11*1**1**1

left   GGCGAGCACAGCTTCACGATTCAAGGGGGGATTGTT----------------TTTTTTTTTTTTTTTTTT
chrom  GGCGAGCACAGCTTCACAATTCAAGGGGGGATTGTTAAAAGGGAAAGACGAATACGATGGCTCCACAATT
right  ACCGCGCCCGGCCT------TCAAGGGGGGATTGTTAAAAGGGAAAGACGAATACGATGGCTCCACAATT
       11**1**1*1**1*111N11****************2222222222222222*2222*222*222222**

left   TTTTTTTTTTTTTTTTTGAGACGGAGTCTCGCTCTGTCGCCCAGGCCGGACTGCGGACTGCAGT--GGCG
chrom  CCGAGTCCTATCTGTGTGTGA---ACACGTGCACATTGGTGC--GACGCGGTGCCCACCGCAGTTTGCTG
right  CCGAGTCCTATCTGTGTGTGA---ACACGTGCACATTGGTGC--GACGCGGTGCCCACCGCAGTTTGCTG
       22222*22*2*2*2*2**2**222*22*22**2*22*2*22*22*2**222***22**2*****22*22*

left   CAATCTCGGCTCACTGC-------AA-----------GCTCCGCTTC----CCGG------
chrom  CCATCCCACCTACCTGCCCTGCACAACCAGTGCCAGGGCACCGGGGCAGGACCGAGATGCA
right  CCATCCCACCTACCTGCCCTGCACAACCAGTGCCAGGGCACCGG-----------------
       *2***2*22**22****2222222**22222222222**2***2NN1    111N
```

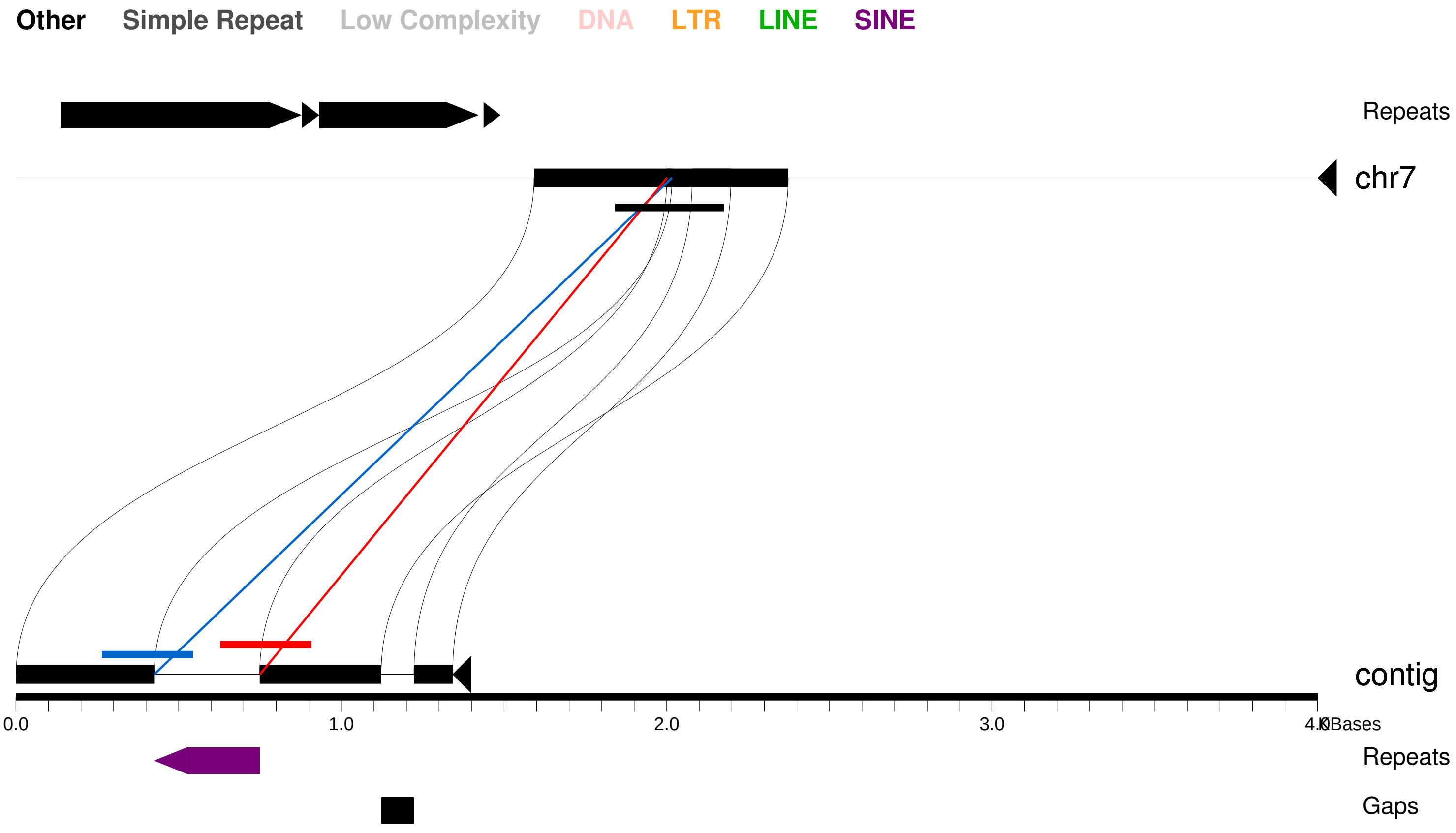

siteID: chr7_157448783 Scafftig4 - Breakpoint: chr7:157448773
chr7:157448613-157448947
Overlap Length: 15 INS size: 302 Genotyped: Yes

```
left   -------------TGTCACAGCAGTGACATTTATTTACCCAAATGACGCTTGCTGGCCTTGAGTGCCAAG
chrom  CCTCCTGCCTTGCTGTCACAGCAGTGACATTTATTTACCCAAATGACGCTTGCTGGCCTTGAGTGCCAAG
right  TTT----------------AGTAGAGACGGGGTTTCACC--------GTTT--TAGCCGGGATGGTCTCG
       NN2          111111**1**1***11111**1***11111111*1**11*1***11**11*1*11*

left   GAGACAGCAGCTGCTTCCGAGTCAGAGGCCCAAAACGGCAGCGCACATGGTAGGCACTAACAGACGTGTA
chrom  GAGACAGCAGCTGCTTCCGAGTCAGAGGCCCAAAACGGCAGCGCACATGGTAGGCACTAACAGACGTGTA
right  -----ATCTTCTGA--CCTCGTGATCCGCCCGCCTCGGCCTCCCAAAGTGCTGGGATTA-CAGGCGTGAG
       11111*1*11***111**11**1*111****1111****11*1**1*11*11**1*1**1***1****11

left   TTCCACTGAAAACTAAATAGCGGGTGCTCACTCTT-------------TTTTTTTTTTTTTTTTTTTTTT
chrom  TTCCACTGAAAACTAAATAGCGGGTGCTCACTCTTACACACCAGGGACTGTGTGTGGGGGCTGCACGTTC
right  --CCACCGCGCCCGGC----CGGGTGCTCACTCTTACACACCAGGGACTGTGTGTGGGGGCTGCACGTTC
       11****1*1111*1111111***************2222222222222*2*2*2*222222*22222**2

left   GAGACGGAGTCTCGCTCTGTCGCCCAGGCTGGAG----TGCAGTGGCGCGATCTC-----GGCTCACTGC
chrom  CAGGCAGAAGCACGGCAAAGATCTCAGGAAGTAAATATTCTTGTTTATCTAAATTAAGAAGACTCCCTGC
right  CAGGCAGAAGCACGGCAAAGATCTCAGGAAGTAAATATTCTTGTTTATCTAAATTAAGAAGACTCCCTGC
       2**2*2**22*2**22222222*2****22*2*22222*222**2222*2*22*222222*2***2****

left   A-----AGC--------TCCGCCTC---------CCGGGTTCACGCCATTCTCCTGCC
chrom  AGAAAGAGCCCTGCCTGTCAGCCTCTGCAGCGGCAGGGTGCA-GCCATCATC--GCC
right  AGAAAGAGCCCTGCCTGTCAGCCTCTGCAGCGGCAGGGTG-----------------
       *22222***22222222**2*****222222222*2****211211111NN1122111
```

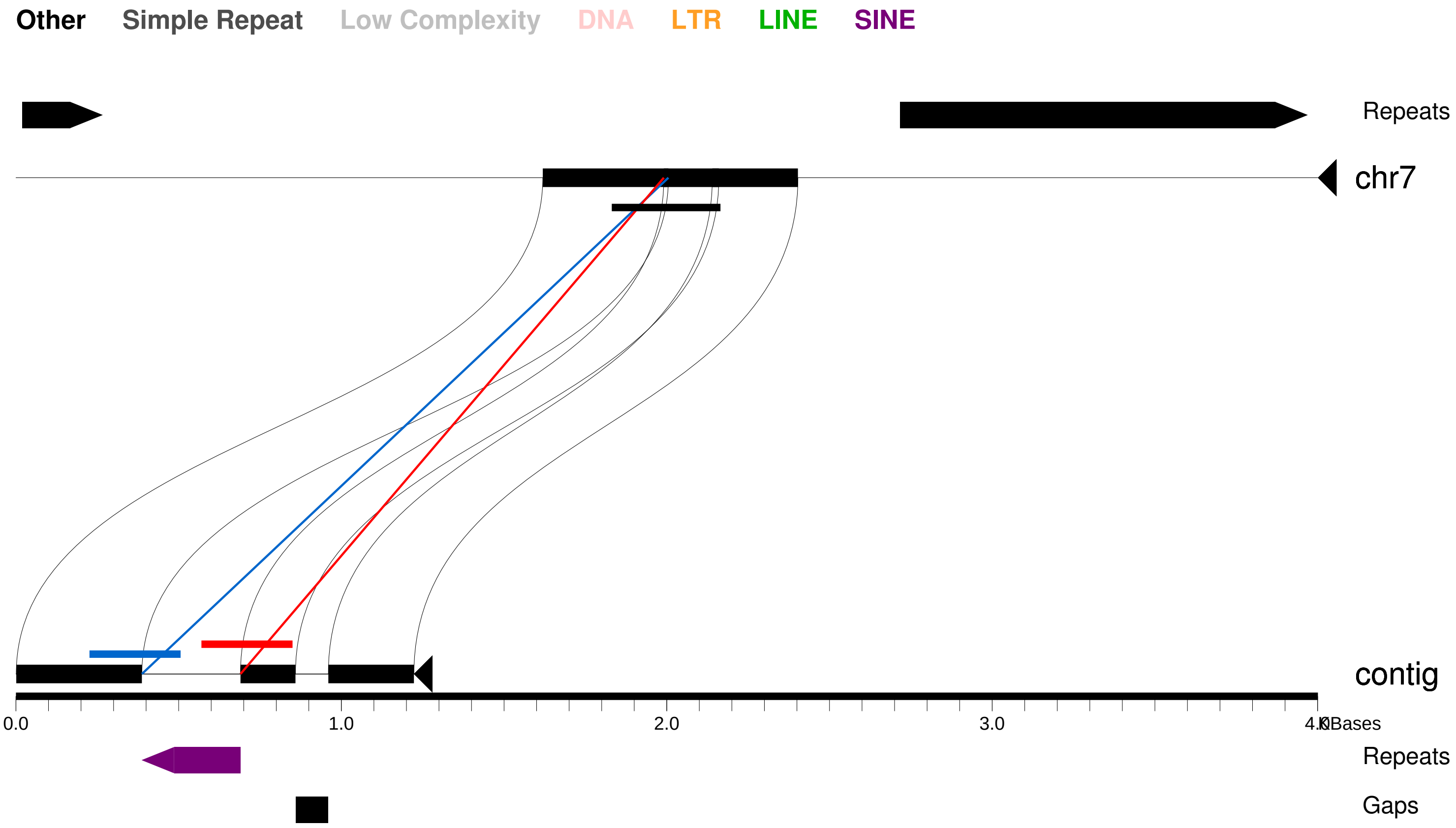

siteID: chr7_18168364 Scafftig1 + Breakpoint: chr7:18168358
chr7:18168304-18168417
Overlap Length: 6 INS size: 422 Genotyped: No

```
left    ACAGATGTAT-----TCAAAAAATT---GAATGGCATGTTTTACCTAAATGGTAGGGGTTAGGGAAGANN
chrom   ACAGATGTAT-----TCAAAAAATT---GAATGGCATGTTTTACCTAAATGGTAGGGGTTAGGGAAGATA
right   CCACCCGCCTCGGCCTCCCAAAGTGCTGGGATTACAGGCGTGAGCCACCGCGCCCGGCC---GGAAGATA
        1**111*11*11111**11***1*1111*1**11**1*11*1*1*1*1111*111**11111******22

left    NNNNNNNNNNNNNNNNNNNNNNNNNNNNNNNNNNNNNNNNNNNNNNNNNNNNNNNNN
chrom   GCTTCTTAATGGGTCTTTGTGCCATGTCTCTTTATTCCTTATGATTAGTACA-----
right   GCTTCTTAATGGGTCTTTGTGCCATGTCTCTTTATTCCTTATGATTAGTACA-----
        222222222222222222222222222222222222222222222222222222222
```

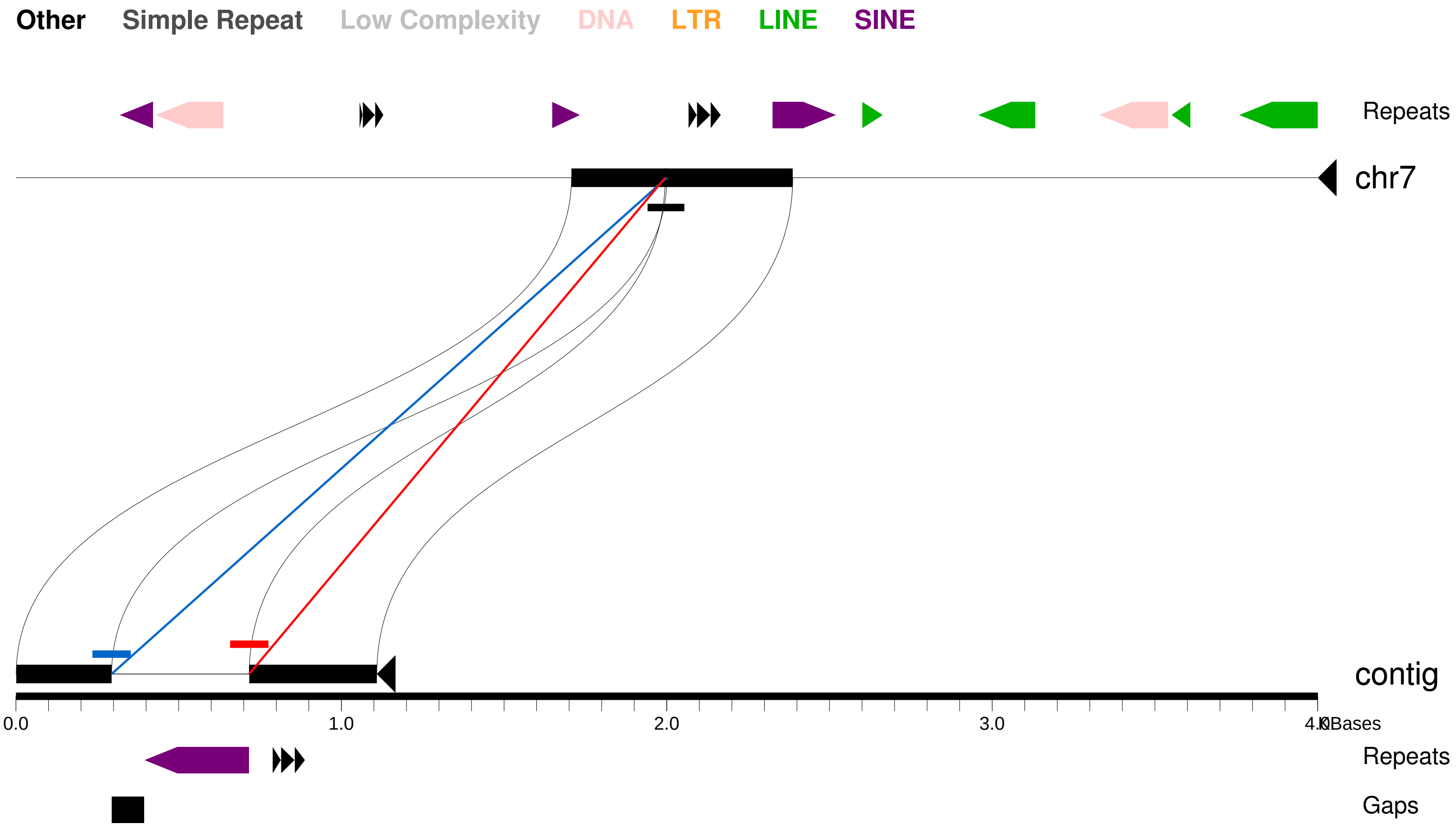

siteID: chr7_18322587 Scafftig3 - Breakpoint: chr7:18322589
chr7:18322541-18322648
Overlap Length: 12 INS size: 376 Genotyped: No

```
left    -----GTAATAAATAGGGTA-TGTGAAACTCTTTGACCT--TTTTCTGGT--TCTCAG--TGCCTTGAAA
chrom   -----GTAATAAATAGGGTA-TGTGAAACTCTTTGACCT--TTTTCTGGT--TCTCAG--TGCCTTGAAA
right   TCACCGTGTTAGCCAGGATGGTCTCGATCTCCT-GACCTCGTGATCCGCCCGCCTCGGCCTGCCTTGAAA
        11111**11**111***1*11*1*11*1***1*1*****11*11**1*11111***1*11**********

left    TTNNNNNNNNNNNNNNNNNNNNNNNNNNNNNNNNNNNNNNNNNNNNNNNNNNNNNNNNNNN
chrom   TTTCTATCTCAATTTTGTAGATACTCAACACTCTAACCCAGCAAGGGGAG-----------
right   TTTCTATCTCAATTTTGTAGATACTCAACACTCTAACCCAGCAAGGGGAG-----------
        **22222222222222222222222222222222222222222222222222222222222
```

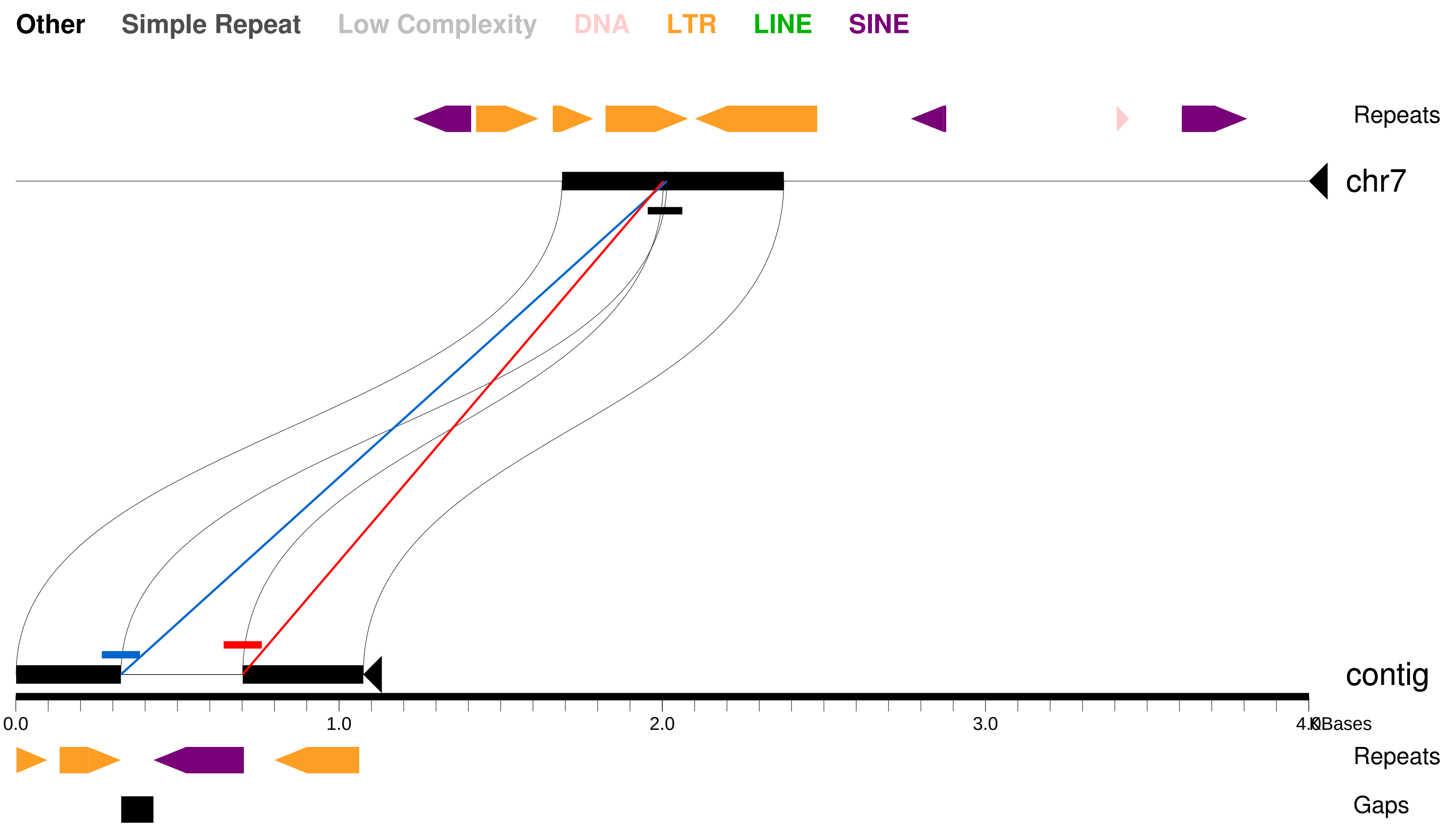

siteID: chr7_18345295 Scafftig1 - Breakpoint: chr7:18345307
chr7:18345265-18345366
Overlap Length: 18 INS size: 297 Genotyped: Yes

```
left     T-------------TTGAATGACACATAG---TTCTGTTGTACCCTTGTAGAGCACACAAA-GAAACAAA
chrom    T-------------TTGAATGACACATAG---TTCTGTTGTACCCTTGTAGAGCACACAAA-GAAACAAA
right    CACTGCACTCCAGCCTGGGCGACAGAGCGAGACTCCGTCTCAAAAAAAAAGAAAAAAAAAAAGAAACAAA
         111111111111111**111****1*11*1111**1**111*1111111***11*1*1***1********

left     GTTTCTGGCCGGGCGCGGTGGCTCACGCCTGTAATCCCAGCACTTTGGGAGGCCGAGGCGGGCGGA
chrom    GTTTCTGAT----CTCTGGG---CAGGGGTCTAAT---AG----ATGAGAAGACAAGACATAT---
right    GTTTCTGAT----CTCTGGG---CAGGGGTCTAAT---AG----ATGAGAAGACAAGACATAT---
         *******222222*2*2*2*222**2*22*2****222**22222**2**2*2*2**2*2222222
```

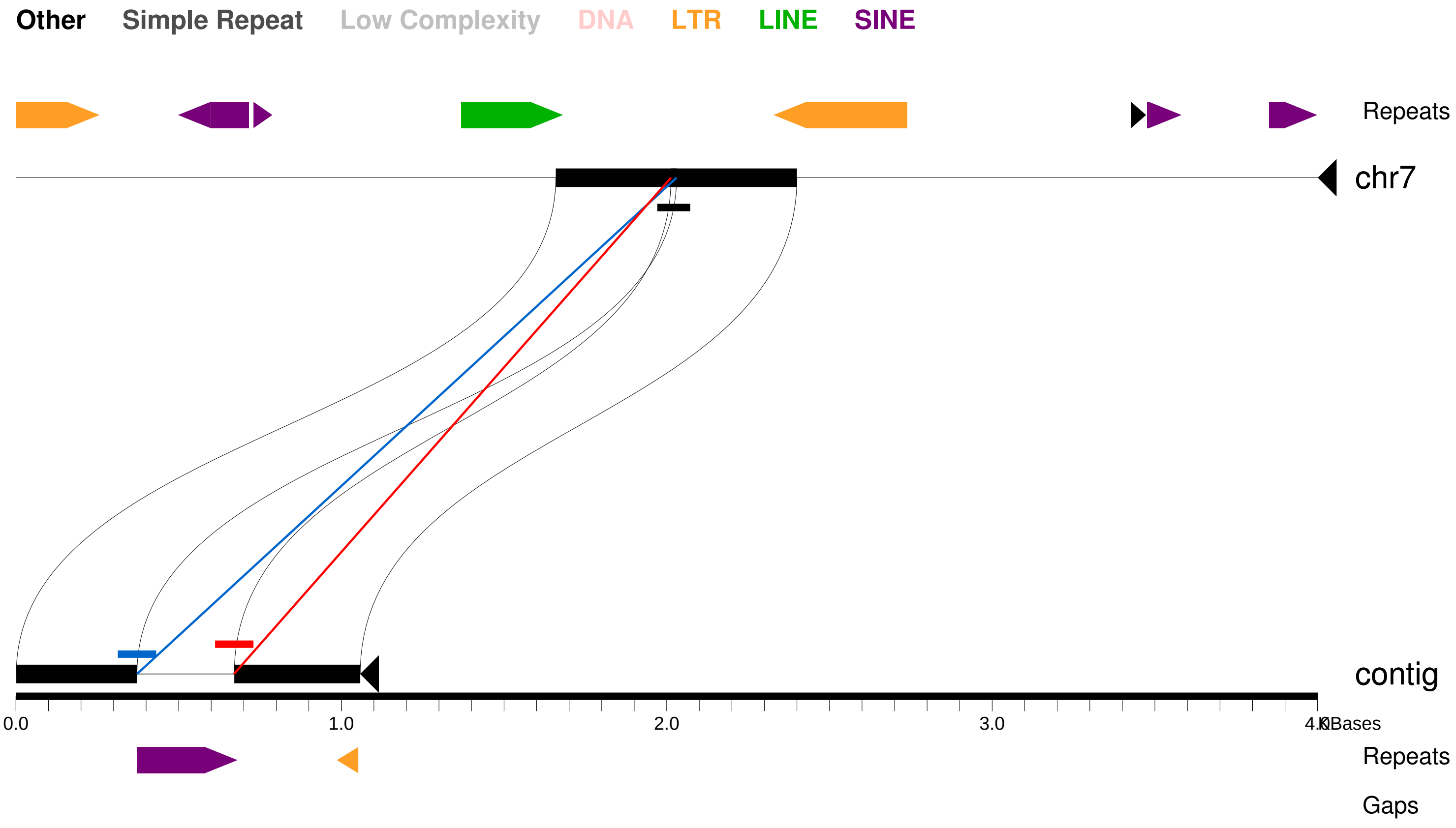

siteID: chr7_22799821 Scafftig4 + Breakpoint: chr7:22799808
chr7:22799756-22799867
Overlap Length: 8 INS size: 431 Genotyped: No

```
left   TC-----AATAATCAAACATAATT--TAACAACTCAAGAAAAGGAAAGTCTTGTTTTTACTGATTGTNNN
chrom  TC-----AATAATCAAACATAATT--TAACAACTCAAGAAAAGGAAAGTCTTGTTTTTACTGATTGTTTT
right  CCGCCCGCCTCGGCCTCCCAAAGTGCTGGGATTACAGGCGTGAGCCACCGCGCCCGGCCCTGATTGTTTT
       1*1111111*111*111*11**1*11*111*111**1*11111*11*111111111111********222

left   NNNNNNNNNNNNNNNNNNNNNNNNNNNNNNNNNNNNNNNNNNNNNNNNNNNNNNNN
chrom  ACTCATCCCATGTTCTTTCTTCCTTCCTACTGTTCCAAGACTTTTTATT-------
right  ACTCATCCCATGTTCTTTCTTCCTTCCTACTGTTCCAAGACTTTTTATT-------
       22222222222222222222222222222222222222222222222222222222
```

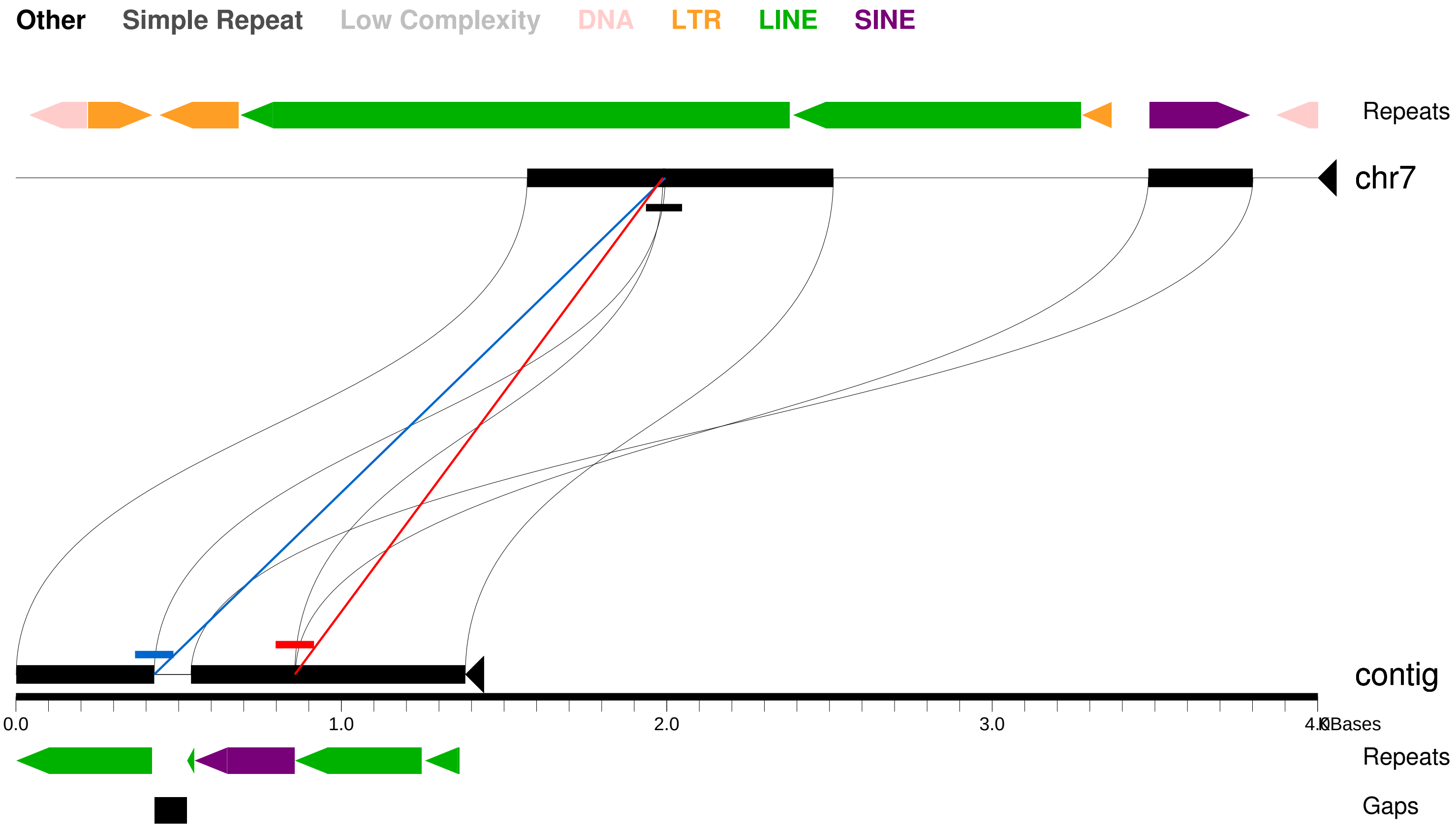

siteID: chr7_24476603 Scafftig1 + Breakpoint: chr7:24476614
chr7:24476455-24476784
Overlap Length: 11 INS size: 433 Genotyped: No

```
left   ----------ACATTATATTACACTGCATTGCCCAAGGTCATTATCAATCCAAACCCCAGTGGAACTAGG
chrom  GCTTCTTTCAACATTATATTACACTGCATTGCCCAAGGTCATTATCAATCCAAACCCCAGTGGAACTAGG
right  NNNNNNNNNNNNAA--------------------------------------------------------
       NNNNNNNNNN11*111111111111111111111111111111111111111111111111111111111

left   CCAAGCTGCTCATAAACTTCTCTTTGCGATCTACCACAGGAATGTCCTCCATCTCTCAAAAAGGGAGTTC
chrom  CCAAGCTGCTCATAAACTTCTCTTTGCGATCTACCACAGGAATGTCCTCCATCTCTCAAAAAGGGAGTTC
right  ----------------------------------------------------------------------
       1111111111111111111111111111111111111111111111111111111111111111111111

left   ATTCTGTACCAATGATGTTAAGACCTTTACGGCCGGG------CGCGG--------------TGGCTCAC
chrom  ATTCTGTACCAATGATGTTAAGACCTTTACAATGTAGATTTAACTTGAAAGAATATAGAATATAGGAAAC
right  -------------------AAGACCTTTACAATGTAGATTTAACTTGAAAGAATATAGAATATAGGAAAC
       1111111111111111111***********222222*222222*22*222222222222222*2*222**

left   GCCTGTAATCCCA-GCACTTTGGGAGGCCGAGGCGGGTGGATCA-TGAGGTCAGGAGATCGA-----GAC
chrom  TCATATAATCTTATGGTAACAGGGTCTAAAATTTATATTCATCACTCAGGTATGTAAAACAAACATAGTT
right  TCATATAATCTTATGGTAACAGGGTCTAAAATTTATATTCATCACTCAGGTATGTAAAACAAACATAGT-
       2*2*2*****22*2*222222***222222*222222*22****2*2****22*2*2*2*2*22222*2N

left   CATCC--TGGCTA---ACAAGGTGAAACCC-------CGTCTCTACTAAA
chrom  TATCTATTCCCTATAAATAATCTGACATTCTTCAGGCCTACCTTATTGAC
right  --------------------------------------------------
       N111N  1NN111   1N11NN111N1NN1       1NN1NN11N1N1N
```

Other Simple Repeat Low Complexity DNA LTR LINE SINE

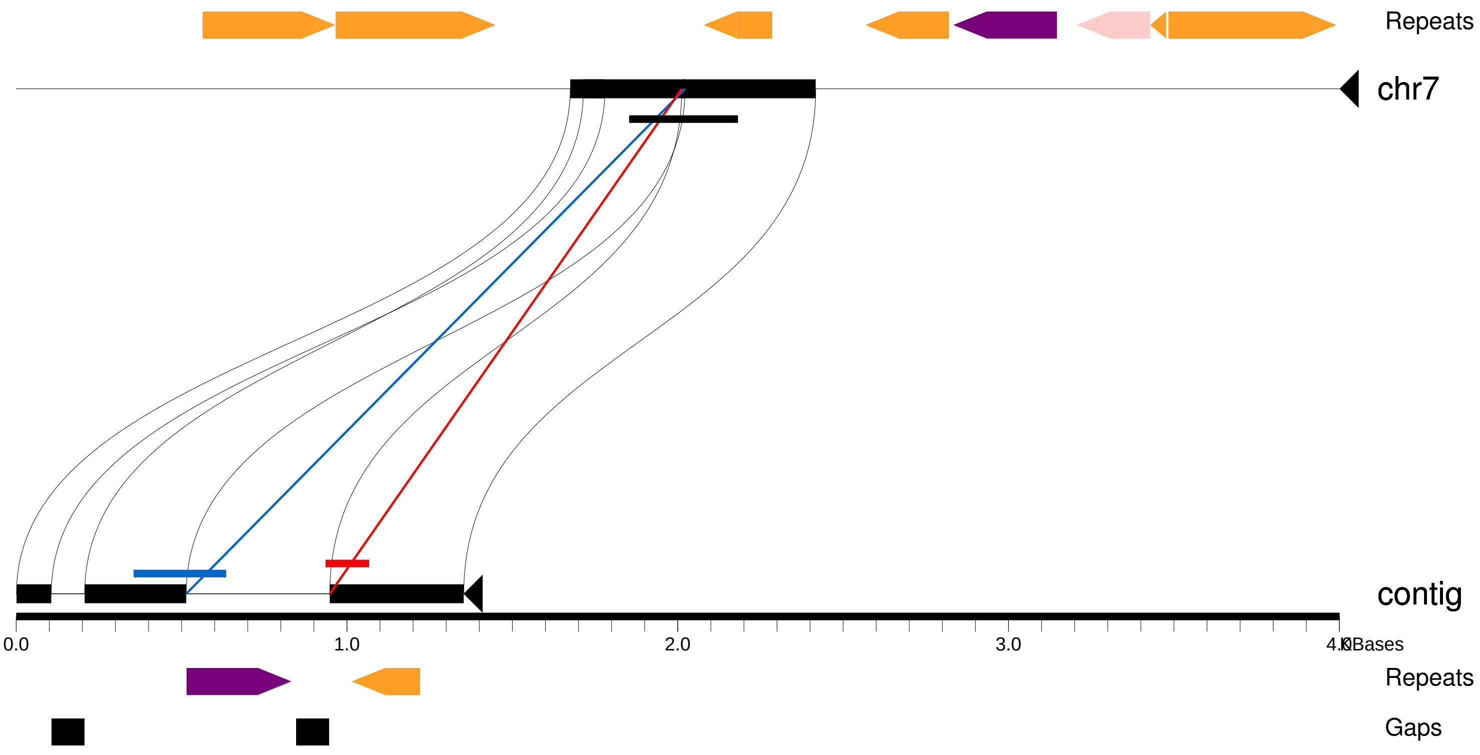

siteID: chr7_3090713 Scafftig4 - Breakpoint: chr7:3090694
chr7:3090659-3090753
Overlap Length: 25 INS size: 430 Genotyped: No

```
left    TATGAAACAGGCTGCCTGAATAAAGGAACAGGTCA------------------------TGGAAATTAAA
chrom   TATGAAACAGGCTGCCTGAATAAAGGAACAGGTCA------------------------TGGAAATTAAA
right   NNNNNNNNNNNNNNNNNNNNNNNNNNNNNNNNNNNNNNNNNNNNNNNNNNNNNNNNNNNTGGAAAAAAAA
        11111111111111111111111111111111111111111111111111111111111******11***

left    AATAGGGTTGTCCGGCCGGGCGCGGTGGCTCACGCCTGTAATCCCAGCACTTTGGGAGGCCG-AGGCGGG
chrom   AATAGGGTTGTTCG-----------------AATAATAAAATAAAA----TTTAACATACAGTAGAAGGG
right   AATAGGGTTGTCCG-----------------AATAATAAAATAAAA----TTTAACAGACAGTAGAAGGG
        ***********N**22222222222222222*2222*22***222*2222***222*N2*2*2**22***

left    CGGA
chrom   ----
right   ----
        2222
```

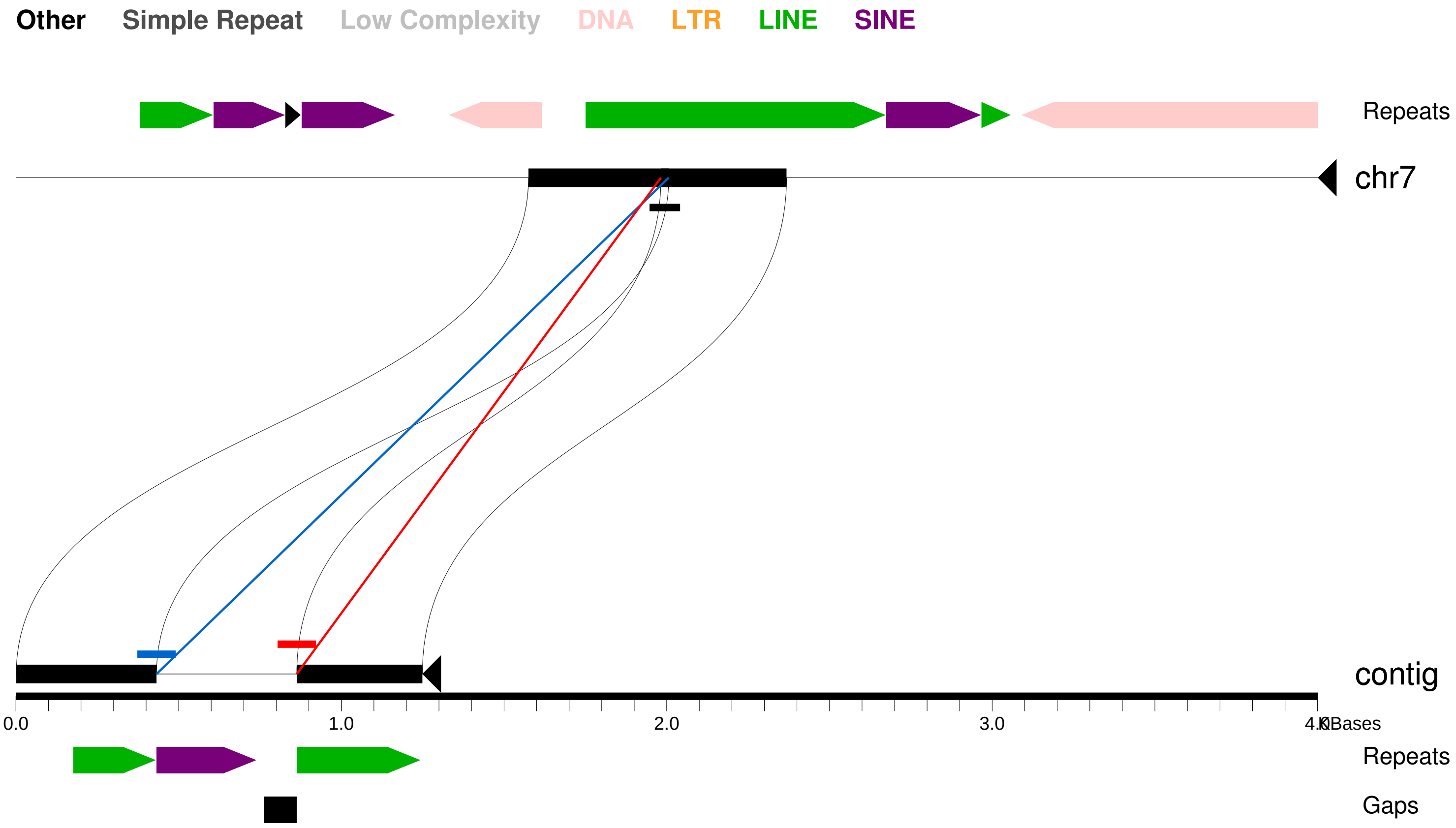

siteID: chr7_33195310 Scafftig1 - Breakpoint: chr7:33195318
chr7:33195270-33195377
Overlap Length: 12 INS size: 343 Genotyped: Yes

```
left   T-----GGCT--GTGTTACA---TTCTAGAAAACTTTGTGTCTACT-CTGTCTCAGGTAAGAAATATTTT
chrom  T-----GGCT--GTGTTACA---TTCTAGAAAACTTTGTGTCTACT-CTGTCTCAGGTAAGAAATATTTT
right  CCACCCGCCTCGGCCTCCCAAAGTGCTGGGATTACAGGCGTGAGCCACCGCGCCCGGCCAGAAATATTTT
       111111*1**11*11*11**111*1**1*1*111111*1**111*11*1*111*1**11***********

left   TTTTTTCTTTTCTTTTTTTTTTTTTTTTTTTTTTTTTTTTTTTTTTTTTTTTTTTTTGAGACGG
chrom  TA---------CCAATGTAGAATTTATATTACAAATTGGGCTTAATGTTATATGAGAA--
right  TA---------CCAATGTAGAATTTATATTACAAATTGGGCTTAATGTTATATGAGAA--
       *2222222222*222*2*2222***2*2**22222**2222**22*2**2*2*****222
```

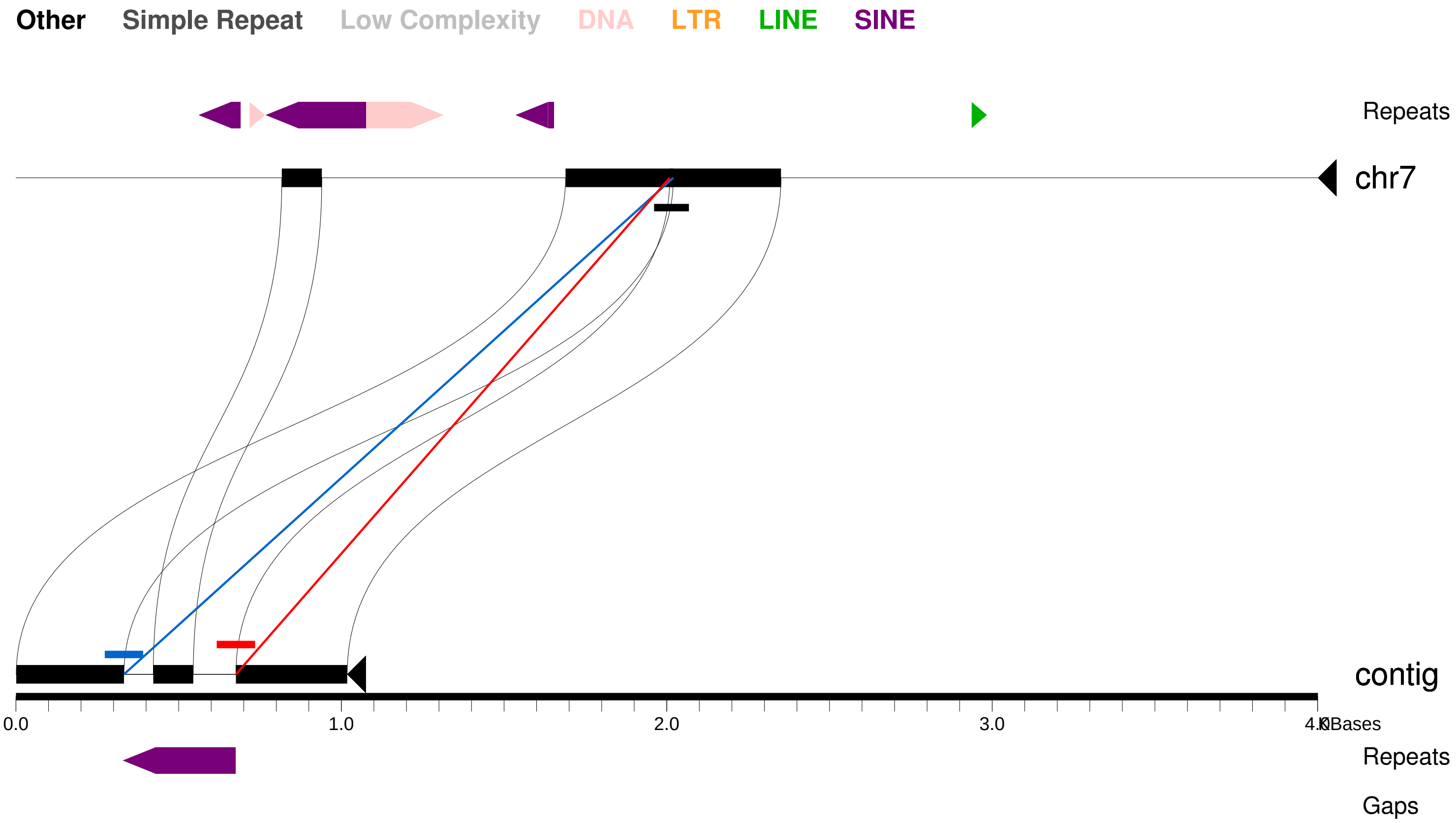

siteID: chr7_33529736 Scafftig1 + Breakpoint: chr7:33529714
chr7:33529666-33529773
Overlap Length: 12 INS size: 424 Genotyped: No

```
left    GTGACACCTGTTTAATTTGTTAAGTGAACTAGAAAGTGAGTTATTTTT-----------AAAACTAGTTG
chrom   GTGACACCTGTTTAATTTGTTAAGTGAACTAGAAAGTGAGTTATTTTT-----------AAAACTAGTTG
right   NNNNNNNNNNNNNNNNNNNNNNNNNNNNNNNNNNNNNNNNNNNNNNNNNNNNNNNNNNNAAAAACTAGTTG
        11111111111111111111111111111111111111111111111111111111111***********

left    TGGCCGGGCGCGGTGGCTCACGCTTGTAATCCCAGCACTTTGGGAGGCCGAGGCGGGCGG
chrom   TAATCCATACCTCTCT-TAACAGATG--CTCTCATC-CTCATATAGTCTATTT-------
right   TAATCCATACCTCTCT-TAACAGATG--CTCTCATC-CTCATATAGTCTATTT-------
        *222*22222*22*222*2**222**222**2**2*2**22222**2*222222222222
```

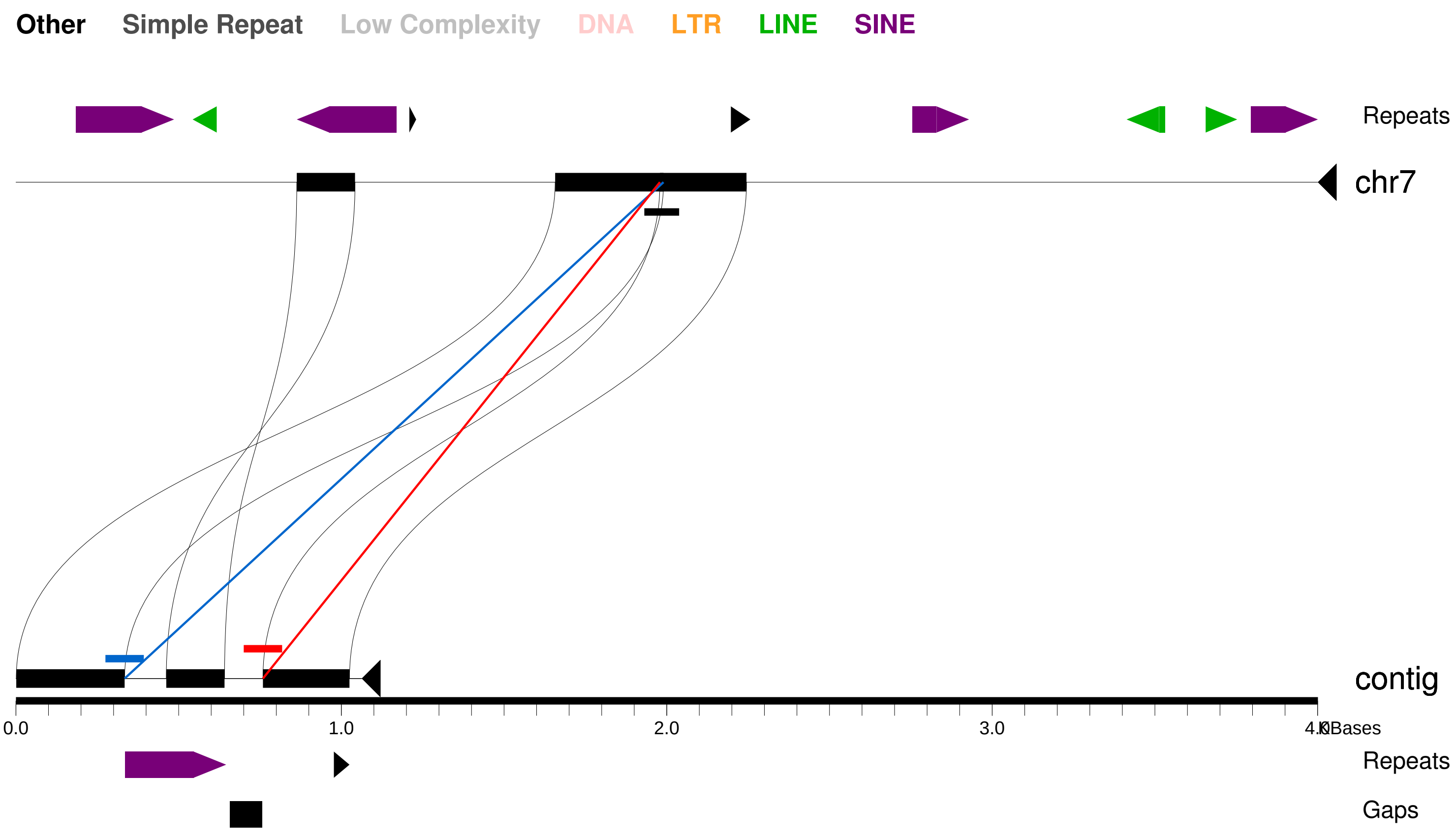

siteID: chr7_34492793 Scafftig2 + Breakpoint: chr7:34492788
chr7:34492742-34492847
Overlap Length: 14 INS size: 305 Genotyped: Yes

```
left   -ATTATAATCTCATTTATCATGTGATTCCTGG------------AGGTTTTTTGGAAGGAGAATTCTAGG
chrom  -ATTATAATCTCATTTATCATGTGATTCCTGG------------AGGTTTTTTGGAAGGAGAATTCTAGG
right  CACTCCAGCCTGGGCGACAGAGCGAGACTCCGTCTCAAAAAAAAAAAAATAATAAAATAAGAATTCTAGG
       1*1*11*11**11111*1111*1**11*111*111111111111*1111*11*11**11***********

left   GCTGGGCCTGGCGCGGTGGCTCACGCCTGTAATCCCAGCACTTCGGGAGGCCGAGGCGGGCG
chrom  GCTAAATATAA-----TATAACCTGCGAATGAT--GATAATTTTG-----CCTATTTGT-CA
right  GCTAAATATAA-----TATAACCTGCGAATGAT--GATAATTTTG-----CCTATTTGT-CA
       ***22222*2222222*2222*22**222*2**222*22*2**2*22222**2*222*22*2
```

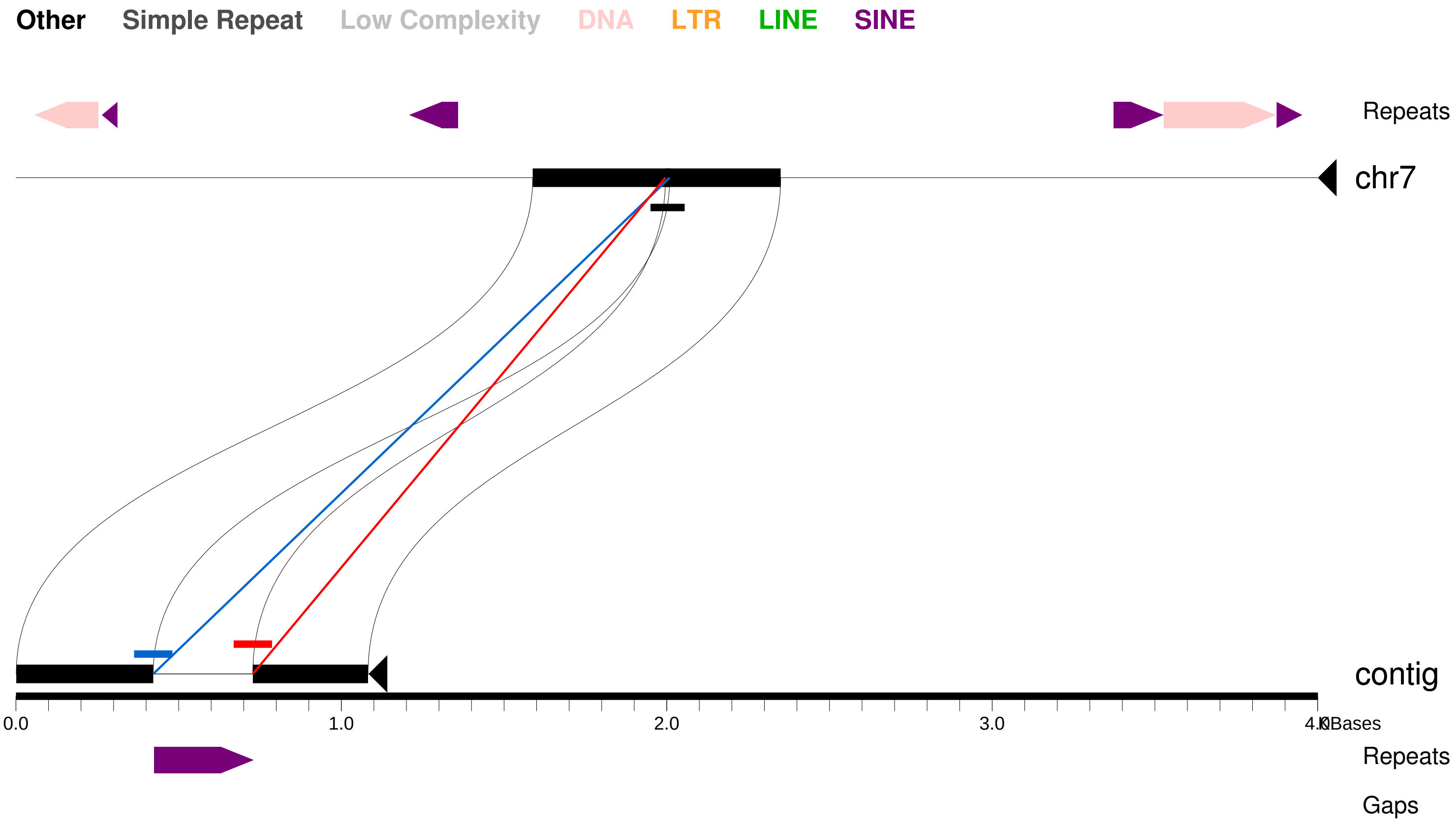

siteID: chr7_36164928 Scafftig1 + Breakpoint: chr7:36164880
chr7:36164275-36165041
Overlap Length: 58 INS size: 413 Genotyped: No

```
left   ----------------------------------------------------------------
chrom  AGAAAGCTGGAGGCATCACGCTACCTGACTTCAAACTATACTACAAGGCTACAGTAATCAAAACAGCATG
right  NNNNNNNNNNN-----------------------------------------------------
       NNNNNNNNNNN

left   ----------------------------------------------------------------
chrom  GTACTGGTACCAAAACAGATATATAGACCAATGGAACAGAACAGAGGCCTCAGAAATAACACCAGACATC
right  ----------------------------------------------------------------

left   ----------------------------------------------------------------
chrom  TACAGCCATCTGATCTTTGACAAACCTGACAAAAACAAGAAATGGGGAAAGGATTCCCTACTTATAAATA
right  ----------------------------------------------------------------

left   ----------------------------------------------------------------
chrom  GTGCTGGGAAAACTGGCTAGCCATATGTAGAAAGCTGAAACTGGATCCCTTCCTTATACCTTATACAAAA
right  ----------------------------------------------------------------

left   ----------------------------------------------------------------
chrom  ATTAAGTCAAGATGGATTAAAGACTTAAATGTAAGACCTAAAACCATAAAAACCTTAGAAGAAAACCTAG
right  ----------------------------------------------------------------

left   ----------------------------------------------------------------
chrom  GCAATACCATTCAGGACATAGGCATGGGCAAAGACTTCATGACTAAAACACCAAAAGCAAAGGCAACAAA
right  ----------------------------------------------------------------

left   ----------------------------------------------------------------
chrom  AGCCAAAATTGACAAATGGGATCTAATTAAACTAAAGAGCTTCTGCACAGCAAAATAAATTATCAGCAGA
right  ----------------------------------------------------------------

left   --------------CTACAGAATGGGAGAAAATTTTTGCAATCTATCAATCTGACAAAGAGCTAATACCCA
chrom  GTGAACAGGCAACCTACAGAATGGGAGAAAATTTTTGCAATCTATCAATCTGACAAAGAGCTAATACCCA
right  ----------------------------------------------------------------
                     111111111111111111111111111111111111111111111111111111111

left   GAATCTACAAAGAACTTAAACAAATTTACAAGAAAGAAAGAAACAACCCCATCAAAATGTGGGCAAAGGA
chrom  GAATCTACAAAGAACTTAAACAAATTTACAAGAAAGAAAGAAACAACCCCATCAAAATGTGGGCAAAGGA
right  ---------------------------------------------ACCCCATCAAAATGTGGGCAAAGGA
       111111111111111111111111111111111111111111111*************************

left   TATGAACAGACACTTCTCAAAAGAAGACATTTACACTTTGCCAGGCCGAGGCGGGTGGATCATGAGGTCA
chrom  TATGAACAGACACTTCTCAAAAGAAGACATTTA------TGCAGCCAACAGACATATGAAAAAATGCTCA
right  TATGAACAGACACTTCTCAAAAGAAGACATTTA------TGCAGCCAACAGACATATGAAAAAATGCTCA
       *********************************2222222*2**2*2222*222222**22*222*2***

left   [illegible]ATACAAAAAAA-TTAGCCGG
chrom  TCATTACTGG-CCATCAGAGAAATGCAAATGAAAACCACAATGAGATACCATCTCACACCAGTTAGAATG
right  TCATTACTGG-CCATCAG-------[illegible]-----------------------------
       22*222*22*2*****22N1NN1NNN1NN111[illegible]N11NNNN1NNNN11NN1NNN11N1NN1 1111NNN1

left   GCGCGGTGGCGGGCG
chrom  GCAA-----------
right  ---------------
       11NN22222222222
```

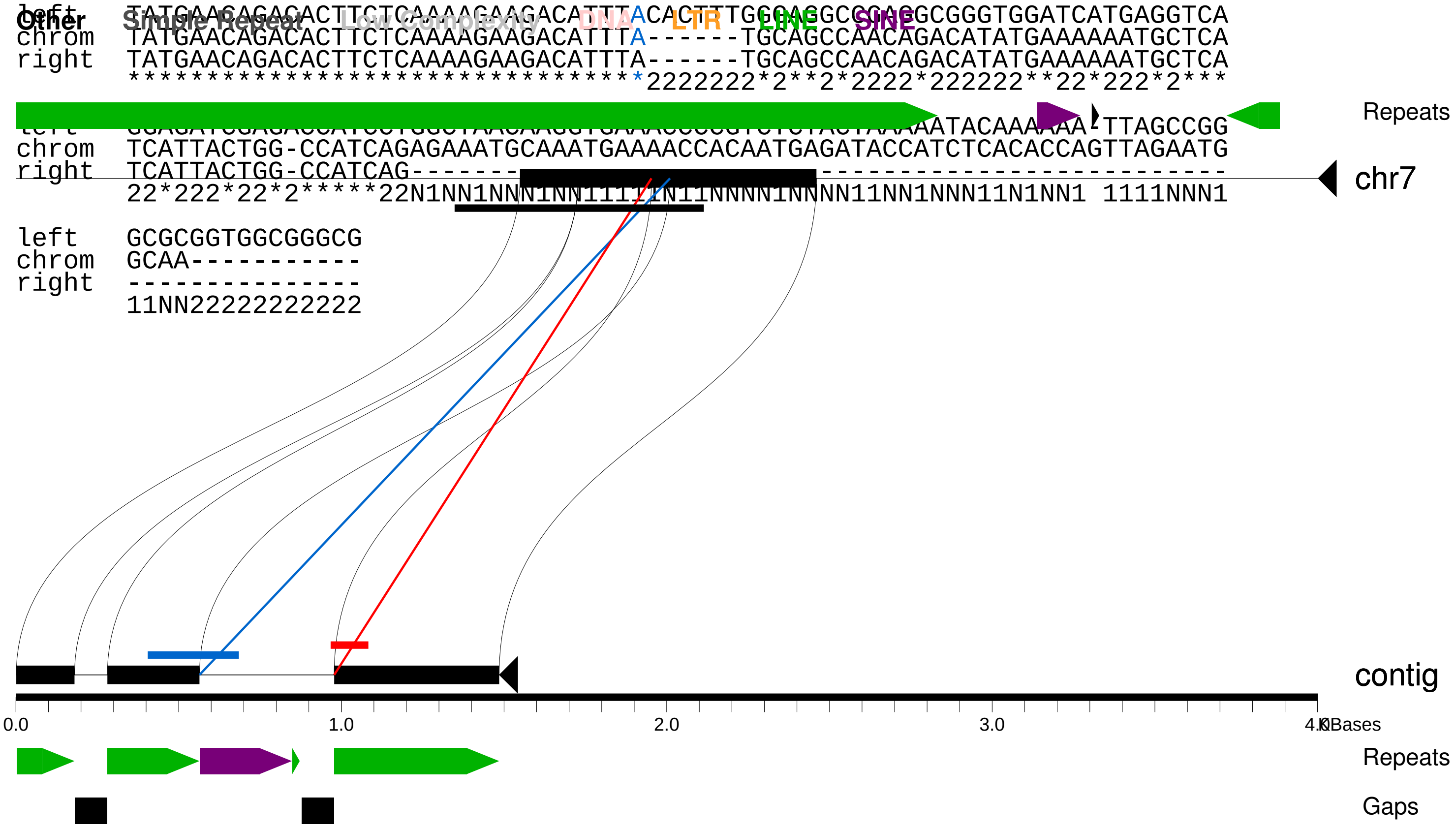

siteID: chr7_37836654 Scafftig8 + Breakpoint: chr7:37836637
chr7:37836592-37836696
Overlap Length: 15 INS size: 294 Genotyped: Yes

```
left     A-----TCATCAG-------AATTGTTTTTTTTCCCAAAA--AGGTAATGAGGATTGATTGCTCCCCTCT
chrom    A-----TCATCAG-------AATTGTTTTTTTTCCCAAAA--AGGTAATGAGGATTGATTGCTCCCCTCT
right    CCGCCCGCCTCGGCCTCCCAAAGTGCTGGGATTACAGGCGTGAGCCACCGCGCCCGGCCTGCTCCCCTCT
         1111111*1**1*1111111**1**1*1111**1*1111111**11*11*1*1111*11***********

left     CTTTTTTTTTTTTTTTTTGAGACGGAGTCTCGCTGTCGCCCAGGCTGGA-GTGCAGTGGCGCAA
chrom    CTTTAGAGAACCATT------ACC-AGT-TCTATATTTACCATACAATATGTTCAAT-------
right    CTTTAGAGAACCATT------ACC-AGT-TCTATATTTACCATACAATATGTTCAAT-------
         ****222222222**222222**22***2**22*2*222***22*222*2**2**2*2222222
```

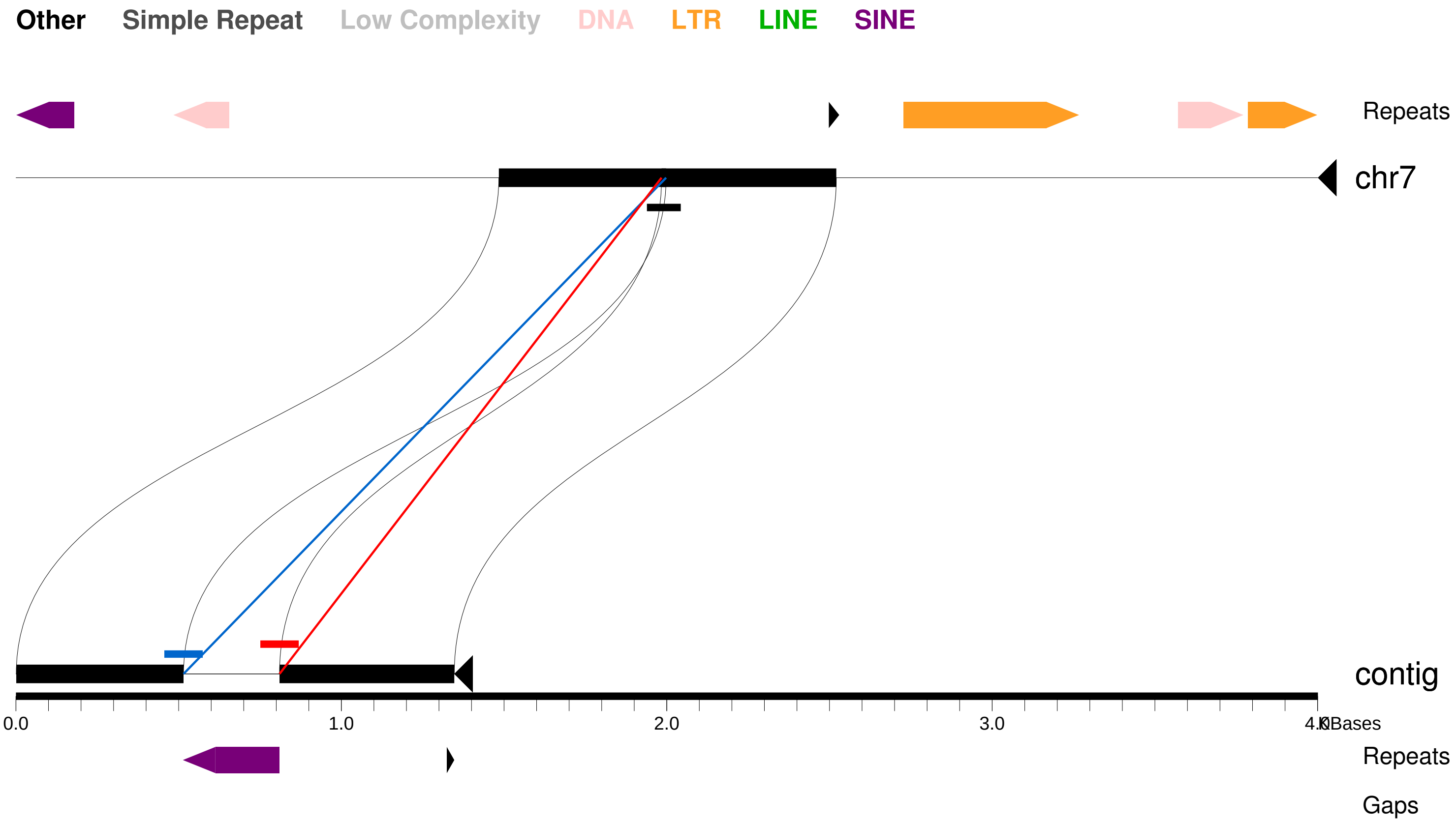

siteID: chr7_38116214 Scafftig1 + Breakpoint: chr7:38116190
chr7:38116142-38116246
Overlap Length: 12 INS size: 426 Genotyped: No

```
left    -----------GGTCTTC-----TTGTTGAATTTAACCCTTTA-CCATTATGTAATGCTCTTCTTTGTCT
chrom   -----------GGTCTTC-----TTGTTGAATTTAACCCTTTA-CCATTATGTAATGCTCTTCTTTGTCT
right   CCACCCGCCTCGGCCTCCCAAAGTGCTGGGATTACAGGCGTGAGCCACCGCGCCCGGCCCTT---TGTCT
        11111111111**1**1*11111*11*1*1***11*11*1*1*1***1111*1111**1***111*****

left    TATTTTTTTTTNNNNNNNNNNNNNNNNNNNNNNNNNNNNNNNNNNNNNNNNNNNNNNNNNNNNNN
chrom   TATTTTTTAAT--------------CTTTGTTGGTTTATAGTCTGTTTTGTGTGAAATTAGGATTGG
right   TATTTTTAAT--------------CTTTGTTGGTTTATAGTCTGTTTTGTGTGAAATTAGGATTGG
        *******22*2222222222222222222222222222222222222222222222222222222
```

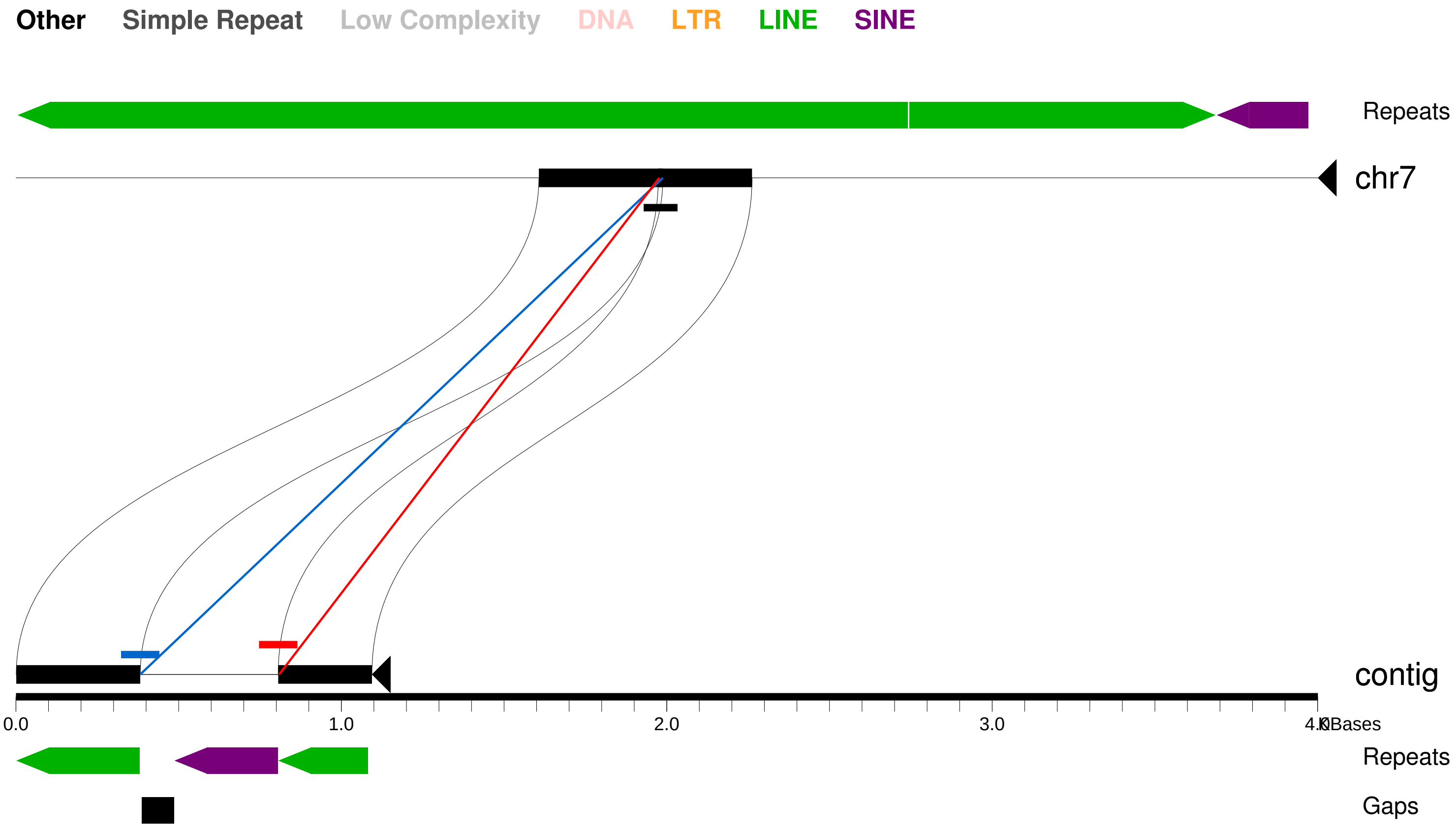

siteID: chr7_38209207 Scafftig3 - Breakpoint: chr7:38209199
chr7:38209039-38209360
Overlap Length: 0 INS size: 415 Genotyped: No

```
left   ------------------------------ACCTCAATAACTCTATCAGTTTCTGATTATAGGAAGT-GAG
chrom  CTAATTTTTAAAACAAACAAACAAAAAAGTACCTCAATAACTCTATCAGTTTCTGATTATAGGAAGT-GAG
right  ------TTTAGTAGAGACGGGGTTTCA---CCGTTTTAGCCGGGATGGTCTC-GATCTCCTGACCTCGTG
             2222NN2N2N22NNNNNNNN2   1**1111**1*1111111**1**1***11111**11*1*1*

left   TTCACCACAAATGAATATTGATTATTATAATGTTGTCCGTTTGTATTATTTTTCCTTTTCAAAAAAGCAA
chrom  TTCACCACAAATGAATATTGATTATTATATTGTTGTCCGTTTGTATTATTTTTCCTTTTCAAAAAAGCAA
right  ATCCGCCCGCCTCG-----GCCTCCCAAAGTGCTG-------GGATTACAGG-CGTG--------AGCCA
       1**11*1*111*1111111*11*111*1*N**1**1111111*1****11111*1*111111111***1*

left   CTTTTATGGATAGCATAATTTTNNNNNNNNNNN------------------------------------
chrom  CTTTTATGGATAGCATAATTTTACGGAGAAACTTTTATATCAATATCTTGCTGGCAAAAGTTAAGAATTA
right  CCGCGCCCGGCCG---------ACGGAGAAACTTTTATATCAATATCTTGCTGGCAAAAGTTAAGAATTA
       *1111111*111*111111111222222222222222222222222222222222222222222222222

left   ----------------------------------------------------------------------
chrom  CTAGCAATGGTTTCATTTGAATTCTTTTTTAAACACAGAATCAGCATCAGGTTTCACATGCTTACAAAAA
right  CTAGCAATGGTTTCATTTGAATTCTTTTTTAAACACAGAATCAGCATCAGGTTTCACATGCTTACAAAAA
       2222222222222222222222222222222222222222222222222222222222222222222222

left   -------------------------------------------
chrom  GACTAAGAAAACCCCCAATAACTCCAAAAGGCAATGTGGATAA
right  GACTAAGAAAACCCCCAATAACTCCAAAAGGCAATGTGGAT--
       22222222222222222222222222222222222222222
```

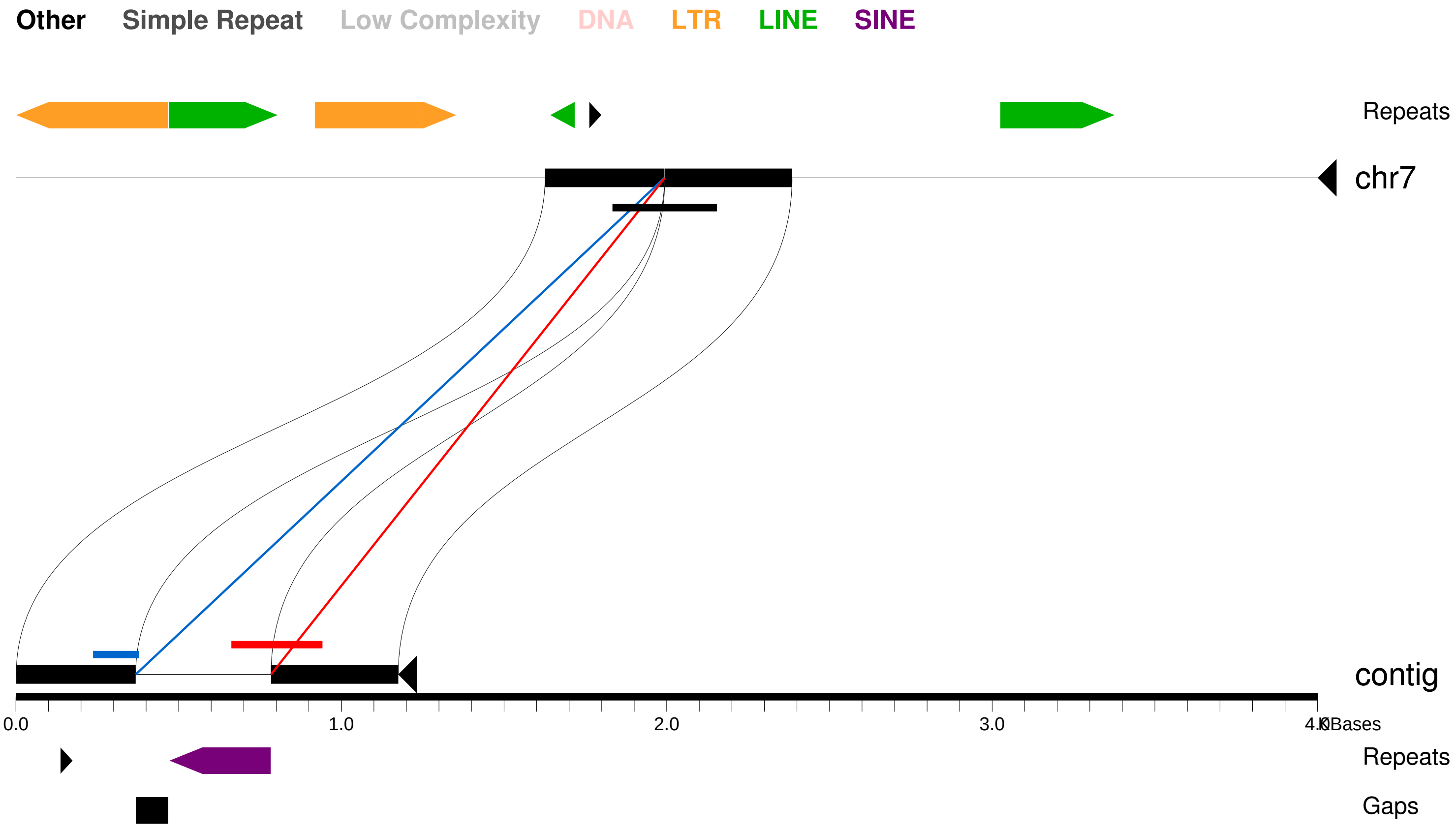

siteID: chr7_38759709 Scafftig5 - Breakpoint: chr7:38759700
chr7:38759656-38759759
Overlap Length: 16 INS size: 306 Genotyped: Yes

```
left     TGACTCTTTCC--------CTAAA-----ACCCTCACCTCTCAATTCTCCTCACTTT---AGAAAGCTTC
chrom    TGACTCTTTCC--------CTAAA-----ACCCTCACCTCTCAATTCTCCTCACTTT---AGAAAGCTTC
right    GTCCGCAGTCCGGCCTGGGCGACAGAGCGAGACTC-CGTCTCAAAAAAAAAAAAAAAAAAAGAAAGCTTC
         111*1*11***11111111*1*1*11111*11***1*1******11111111*1111111**********

left     ACTCTTGGCCGGGCGCGGTGGCTCACGCCTGTAATCCCAGCACTTTGGGAGGCCGAGGCGGGTGG
chrom    ACTCTTAATC---CTCTATGGAACTCTAGGCTAATTCCTGC-CTATCTAAAGAT-----------
right    ACTCTTAATC---CTCTATGGAACTCTAGGCTAATTCCTGC-CTATCTAAAGAT-----------
         ******222*222*2*22***22*2*22222****2**2**2**2*222*2*2222222222222
```

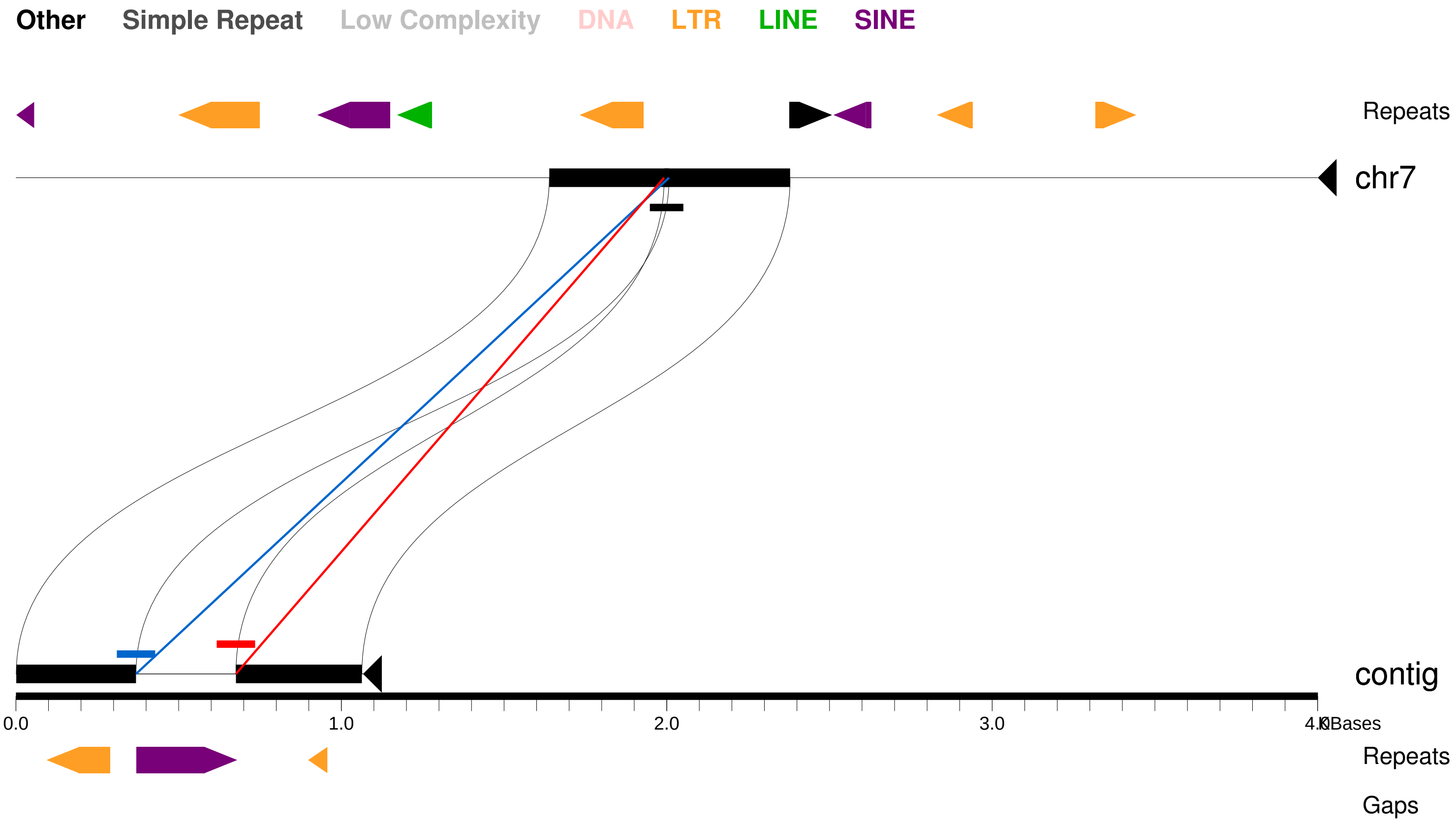

siteID: chr7_39749700 Scafftig2 - Breakpoint: chr7:39749694
chr7:39749650-39749753
Overlap Length: 16 INS size: 430 Genotyped: No

```
left    AA----CATCAGT-----AAAGAGGCAGCATTCCTTTGGC-AGCC-CAGAGCTTT----CCTCCCATATT
chrom   AA----CATCAGT-----AAAGAGGCAGCATTCCTTTGGC-AGCC-CAGAGCTTT----CCTCCCATATT
right   CACCCGCCTCGGCCTCCCAAAGTGCTGGGATTACAGGCGTGAGCCACCGCGCCCGGCCACCTCCCATATT
        1*1111*1**1*111111****1*111*1***1*1111*11****1*1*1**1111111***********

left    TTTTTTTTNNNNNNNNNNNNNNNNNNNNNNNNNNNNNNNNNNNNNNNNNNNNNNNNNNNNNNNNN
chrom   TTTTTT---------------AACTGCAAAATCATTTCAGTCCTCTACTGTAGCAGTTCTGCATG
right   TTTTT----------------AACTGCAAAATCATTTCAGTCCTCTACTGTAGCAGTTCTGCATG
        ******222222222222222222222222222222222222222222222222222222222222
```

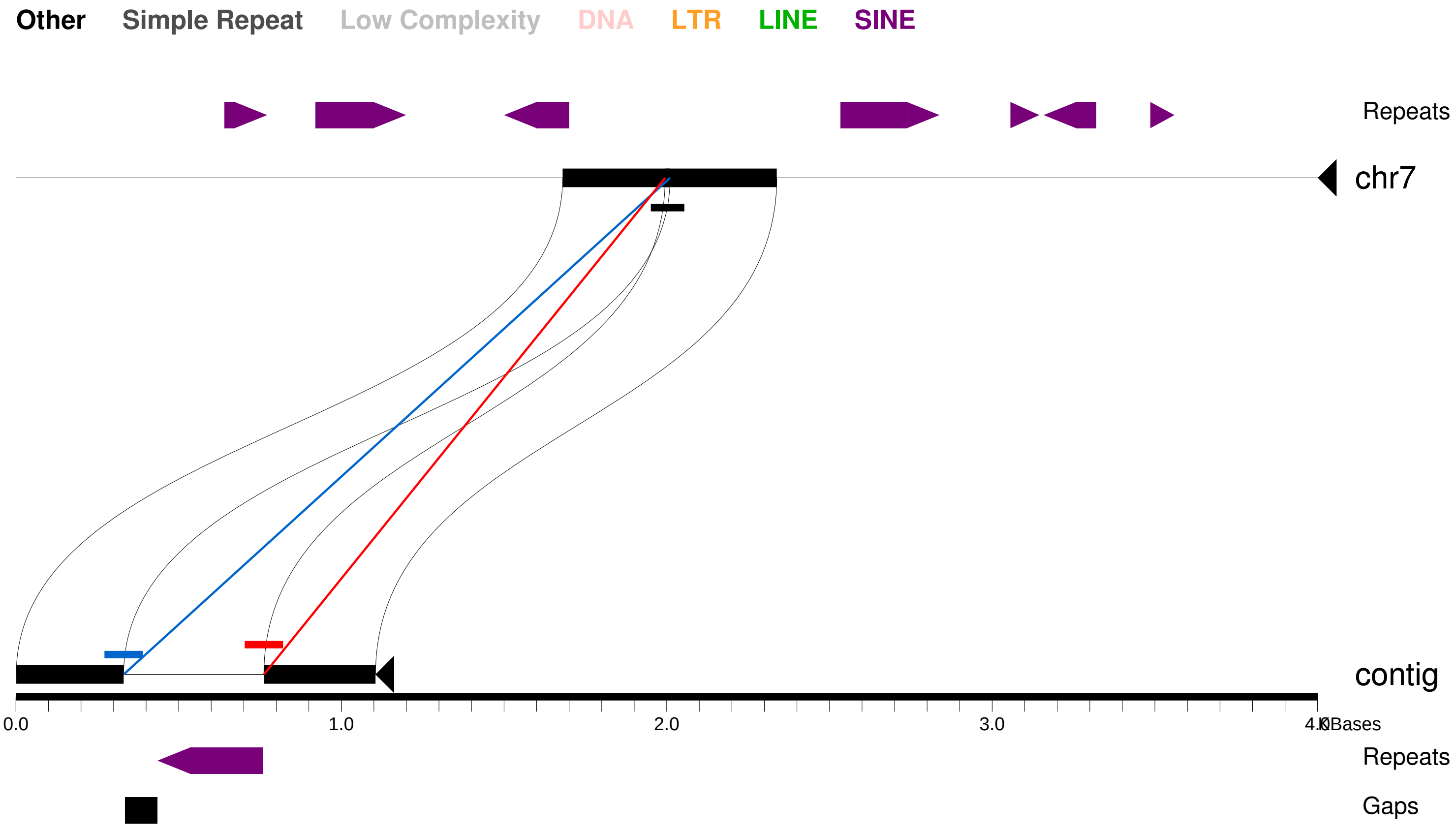

siteID: chr7_42647659 Scafftig1 + Breakpoint: chr7:42647644
chr7:42647601-42647703
Overlap Length: 17 INS size: 306 Genotyped: Yes

```
left     T--GACAGTGTCTAACAGGAGACCTCCCTGGATAA--------AGCTGCAACA------CCAAAAGTCTT
chrom    T--GACAGTGTCTAACAGGAGACCTCCCTGGATAA--------AGCTGCAACA------CCAAAAGTCTT
right    TCCGCCCGCCTCGGCCTCCCAAAGTGCTGGGATTACAGGCGTGAGCCACCGCGCCCGGCCCAAAAGTCTT
         *11*1*1*11**111*11111*11*1*11****1*11111111***11*11*1111111***********

left     TATCTTTTTTTTTTTTTTTTTTTTTTTTTTTTTTGAGACGGAGTCTCGCTCTGTCGCCCAGGCTGGA
chrom    TATCTTAAGCATAAGTATGAACT----------AGAAATAAACTCTCCCCATCCTCCT------A
right    TATCTTAAGCATAAGTATGAACT----------AGAAATAAACTCTCCCCATCCTCCT------A
         ******22222*222*2*2222*2222222222***222*22***2*2*22**22**2222222*
```

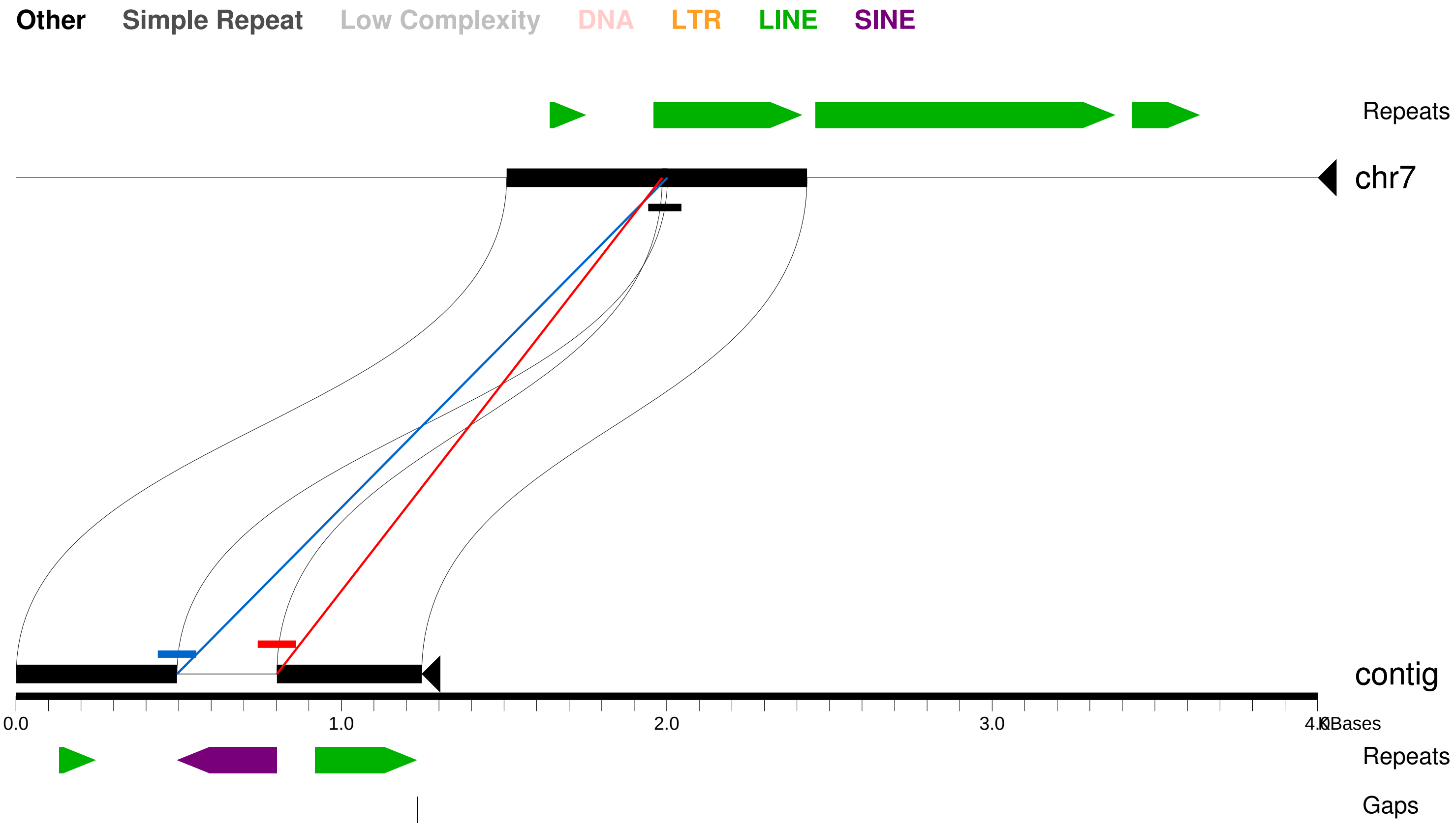

siteID: chr7_45329421 Scafftig2 - Breakpoint: chr7:45329396
chr7:45329250-45329605
Overlap Length: 19 INS size: 159 Genotyped: Yes

```
left   ----------------------------------------------------------------------
chrom  -TAAGCCAAGTATAATAAAGCAAACCAATCCTACCATGATTTGTCTTTAGCAAAAATGGAAAACTGGAGA
right  CTCGGCTCACT--------GCAAGCTCCGCCTCCCGGG--------------------------------
       12NN22NN2N2        2222N2NNNN222N22NN2

left   -------------------------------ACTCTAGTCTTGTCTAATGTTTTTCAGTTTTTATTATTT
chrom  GAGAAAAATTATGTTTCAAAAACTATAGTACACTCTAGTCTTGTCTAATGTTTTTCAGTTTTTATTATTT
right  --------------TTCA------------CGCCATTCTCCTGCCT---------CAGC-----------
                     2222            21*11*11**1**1**111111111***111111111111

left   CTACAGTTCTGGCAGAATTTTTTCTT--CT-----TTTTCT--------TTTTCTTTTTTTTTTTTTTTT
chrom  CTACAGTTCTGGCAGAATTTTTTCTTGGCTACAAGTCTTCAAAATAATGTTTTCATTATTTTTTCTTTTT
right  CTCCC--TCTGGCAGAATTTTTTCTTGGCTACAAGTCTTCAAAATAATGTTTTCATTATTTTTTCTTTTT
       **1*111*******************22**22222*2***222222222*****2**2******2*****

left   TT-----TTTTTT----TTTTTTTTGAGACGGAGTCTC----------GCTCTG---TCGCCCAGGCCGG
chrom  TTCCTGCTTTTTTCCCATTTTTCCTAATTTGGAGTCACTGAAAACTAAGCTGTGCTTTCTTAAAACCCTG
right  TTCCTGCTTTTTTCCCATTTTTCCTAATTTGGAGTCACTGAAAACTA-----------------------
       **22222******2222*****22*2*222******2*222222222 111N11   11NNNN1NN11N1

left   ---ACTGCGG-------ACTG-------------------CAGTGGCGCAAT---------------CT-
chrom  CCAACTGAAGCCAAACAACTTAAATTAAAAAAAAAAAAAACAGCAACCCATTTACATACATAAGCCACTT
right  ----------------------------------------------------------------------
          1111NN1       111N                   111NNN1N11N1               11

left   -----CG
chrom  TCATACC
right  -------
            1N
```

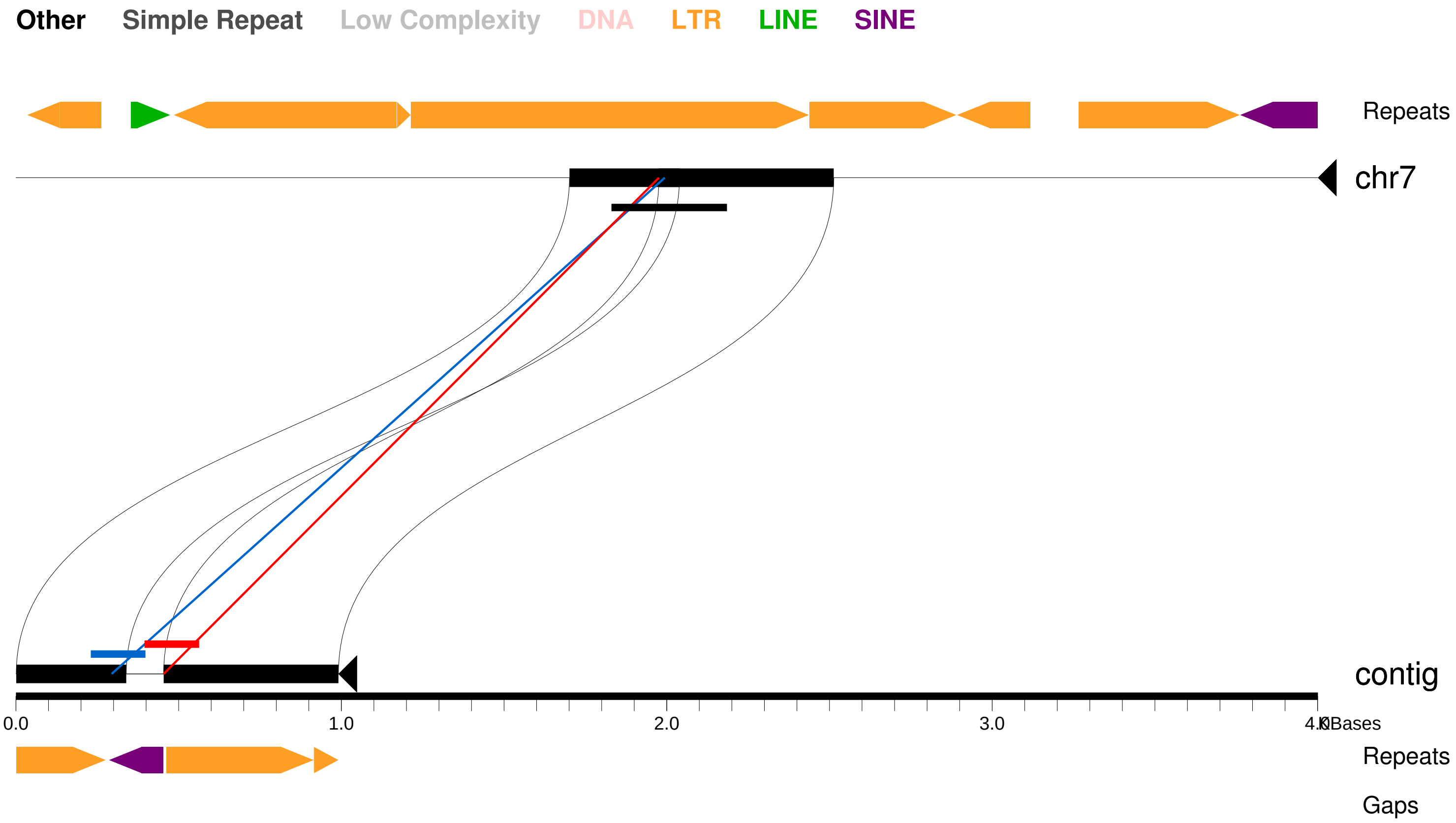

siteID: chr7_46102169 Scafftig3 + Breakpoint: chr7:46102164
chr7:46102135-46102223
Overlap Length: 14 INS size: 297 Genotyped: Yes

```
left    G-----------------AGAGAGCAAGAAAGACA------ACCCAAAACTG-------AAGTCTGTTTT
chrom   G-----------------AGAGAGCAAGAAAGACA------ACCCAAAACTG-------AAGTCTGTTTT
right   CGCCCGCCTCGGCCTCCCAAAGTGCTGGGATTACAGGCGTGAGCCACCGCGCCCGGCCCAAGTCTGTTTT
        111111111111111111*1**1**11*1*11***111111*1***111*111111111***********

left    TTTTTTTTTTTTTTTTGAGACGGAGTCTCGCTCTGTCGCCCAGGCTGGAGTGCAGTGGCGGGATCTCGGC
chrom   TTTATAACCTCTTTTTG----GGAGT-----------GACCACTCATCA---------------CTTGGG
right   TTTATAACCTCTTTTTG----GGAGT-----------GACCACTCATCA---------------CATGGG
        ***2*2222*2******2222*****22222222222*2***22*222*222222222222222*12**2

left    TCACTGCAA
chrom   CCATTGGTT
right   CCATTGGTT
        2**2**222
```

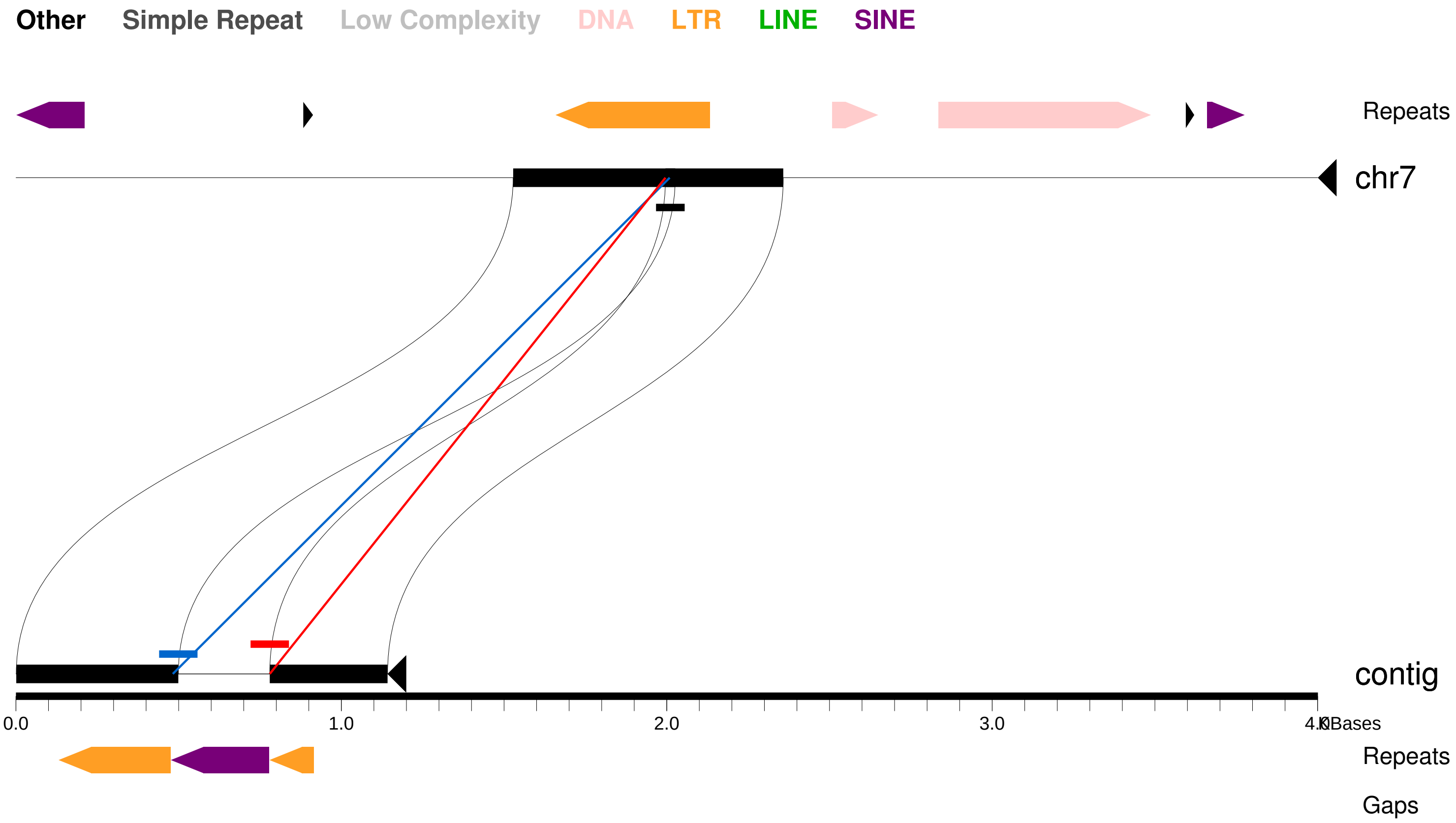

siteID: chr7_47551084 Scafftig2 + Breakpoint: chr7:47551077
chr7:47550917-47551249
Overlap Length: 13 INS size: 324 Genotyped: Yes

```
left   ----------------------------------------ACAGAATCACAAAAAAAAATGCACCTCCTC
chrom  TGCATGCAAGGGTAACCAGCCTCTTGATTAAGGCTGCATAACAGAATCACAAAAAAAAATGCACCTCCTC
right  AATTTTTT---GTA-------TTTTTAGTAGAG-------ACGGGGTTTCACCGTGGTCTCGATCTCCTG
       NNNN2NNN   222       2N22N2N22NN2       **1*11*11**11111111*11*1*****1

left   TCATTTTGTTGTTTTGAAACATAGTTATTTTTTATTAAGCTTATGTTCATTTTAACATGTAATTGGTTCA
chrom  TCATTTTGTTGTTTTGAAACATAGTTATTTTTTATTAAGCTTATGTTCATTTTAACATGTAATTGGTTCA
right  ACCTCGTGATCCGCCCG--CCTCGGCCTCCCAAAGT--GCTGGGATT-------ACAGG---------CG
       1*1*11**1*111111111*1*1*111*11111*1*11***1111**1111111***1*111111111*1

left   TTA-CCATTGCTTTTAACTGACTTAAATATTTTATTTTATTTTTTTTTTGTTTGTTTGTTTTTTGTTTTT
chrom  TTA-CCATTGCTTTTAACTGACTTAAATATTTTAAAT-ACTCATCAGTTTCCCATTTCTAATAGGGTAAA
right  TGAGCCACCGCGC----CCGGCTTAAATATTTTAAAT-ACTCATCAGTTTCCCATTTCTAATAGGGTAAA
       *1*1***11**111111*1*1*************22*2*2*22*222**22222***2*22*22*2*222

left   TTTTTTTTGTTTTTTTTTTTTTGAGACG-GAGTCTCGTTCTGTCGCCCAGG-CGGGAGTGCTGTG-GCGCG
chrom  TCGTGATGAATACAAACCACACTGACAAGAGCCCTTTAGGGTCTTCTAGGACCCTAGCAGTGGGTGCCCG
right  TCGTGATGAATACAAACCACACTGACAGGAGCCCTTTAGGGTCTTCTAGGACCCTAGCAGTGGGTGCCCG
       *22*22*222*222222222222***2N***2*222*222***22*2***2*222**222**2*2**2**

left   ATCTCCGCTC----ACTGCAAGCTCCG--CCTTCTGGGTTCACGCCATTCT-----
chrom  AGACCCGCAGCGCGAGGGCAA-CCCCGTTCATTCTCCTTGCCTGAGGCTCTCAGAA
right  AGACCCGCAGCGCGAGGGCAA-CCCCGTTCATTCTCCTTGCCTG------------
       *222****222222*22****2*2***22*2****222*2*22*NNNN111
```

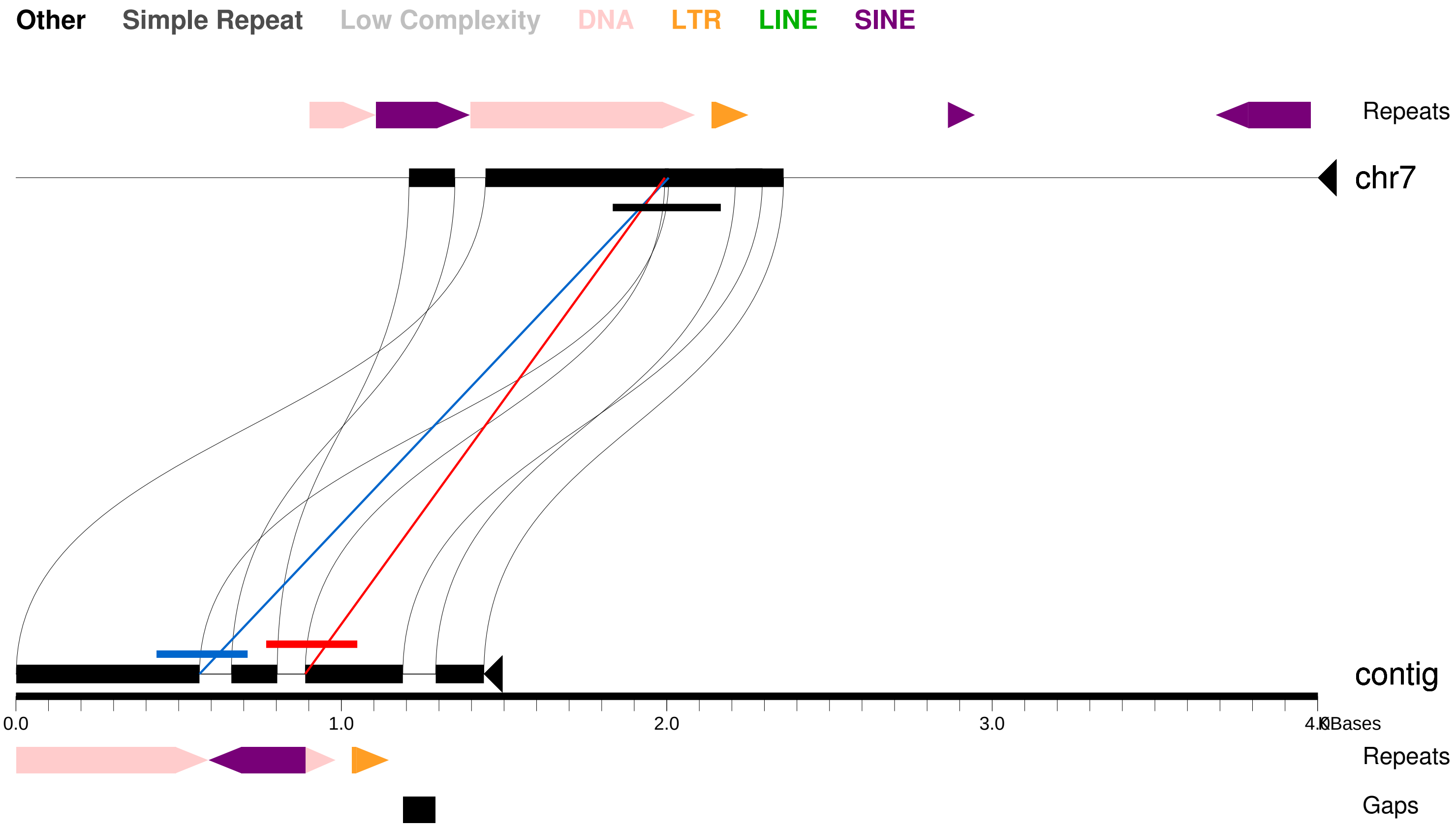

siteID: chr7_47832640 Scafftig1 - Breakpoint: chr7:47832646
chr7:47832594-47832702
Overlap Length: 8 INS size: 520 Genotyped: No

```
left    AATGTACGTATACAGATATACATGTAAATGCTACAGTTAATAATGCCTA----------CATAAAAAAAA
chrom   AATGTACGTATACAGATATACATGTAAATGCTACAGTTAATAATGCCTA----------CATAAAAAAAA
right   NNNNNNNNNNNNNNNNNNNNNNNNNNNNNNNNNNNNNNNNNNNNNNNNNNNNNNNNNNNCAAAAAAAAAA
        11111111111111111111111111111111111111111111111111111111111**1********

left    AACTATTGGCCGGGCGCGGTGGCTCACGCCTGTAATCCCAGCACTTTGGGAGGCCGAGG
chrom   --CTATTGAATGGA-------TTTAAAGGCAGTAATGC-ATCATGCTGCTATGGGAAAA
right   --CTATTGAATGGA-------TTTAAAGGCAGTAATGC-ATCATGCTGCTATGGGAAAA
        22******222**2222222222*2*2*2*2*****2*2*2**222**22*2*222*22
```

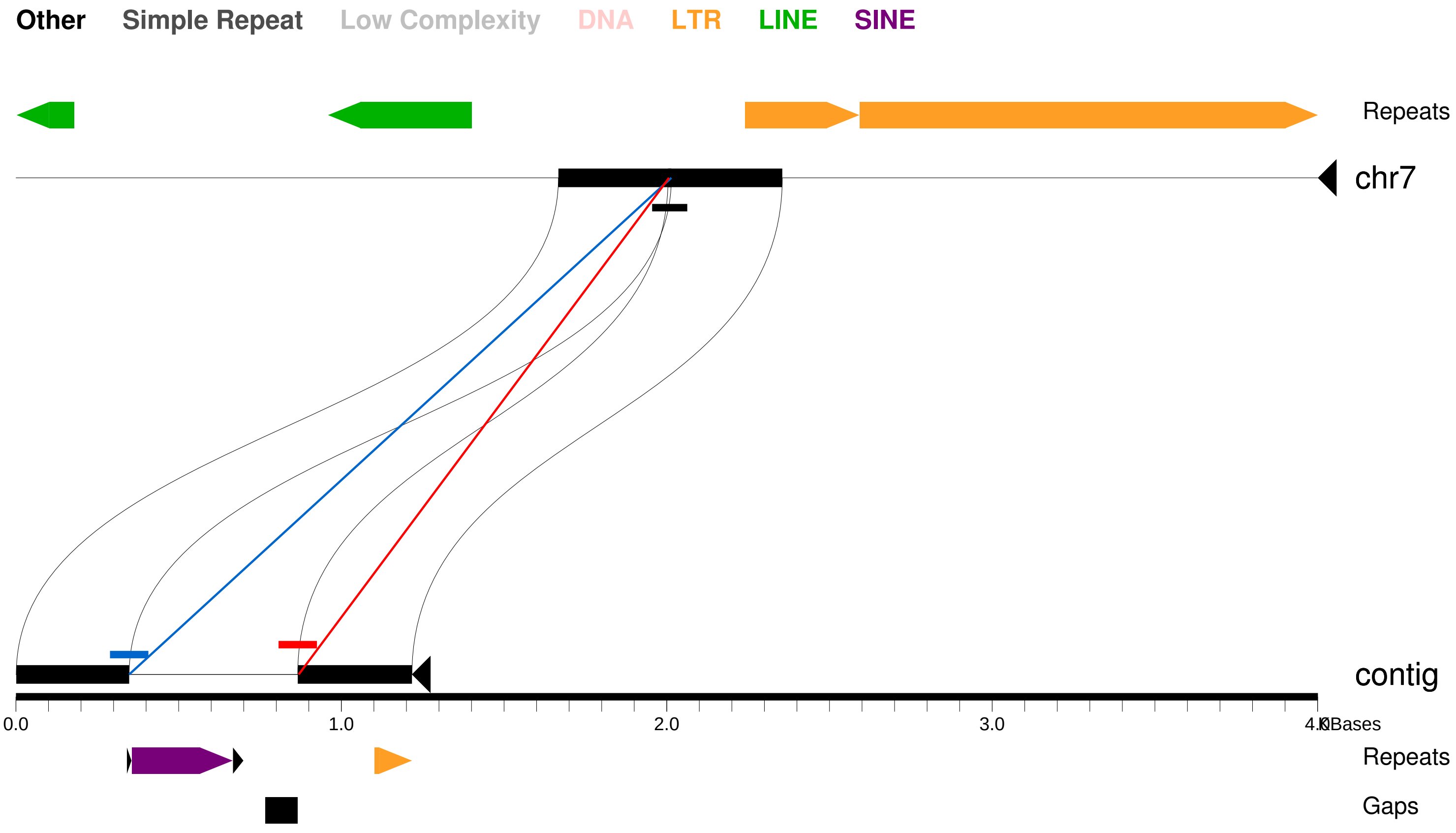

siteID: chr7_49057615 Scafftig5 + Breakpoint: chr7:49057608
chr7:49057565-49057667
Overlap Length: 17 INS size: 295 Genotyped: Yes

```
left     T-----------------TCCCAAATTGGTGT--ATACATTCAGTGCAGTCCCATTCAAAATGCCTGTAG
chrom    T-----------------TCCCAAATTGGTGT--ATACATTCAGTGCAGTCCCATTCAAAATGCCTGTAG
right    GATCCGCCCGCCTCGGCCTCCCAAAGTGCTGGGATTACAGGC-GTG-AGCCACCG-CGCCCGGCCTGTAG
         111111111111111111*******1**1**1111****11*1***1**1*1*111*11111********

left     CTTGGTTCTTTTTTTTTTTTTTTTTTGAGACGGAGTCTCGCTCTGTCGCCCAGGCTGGAGTGCAGTGG
chrom    CTTGGTTCTAACAAATGGCATTCT--------GATTTTACCA-TGT-----ATATAGAAATGTAAA--
right    CTTGGTTCTAACAAATGGCATTCT--------GATTTTACCA-TGT-----ATATAGAAATGTAAA--
         *********222222*2222**2*22222222**2*2*22*22***22222*2222*2*2**2*2222
```

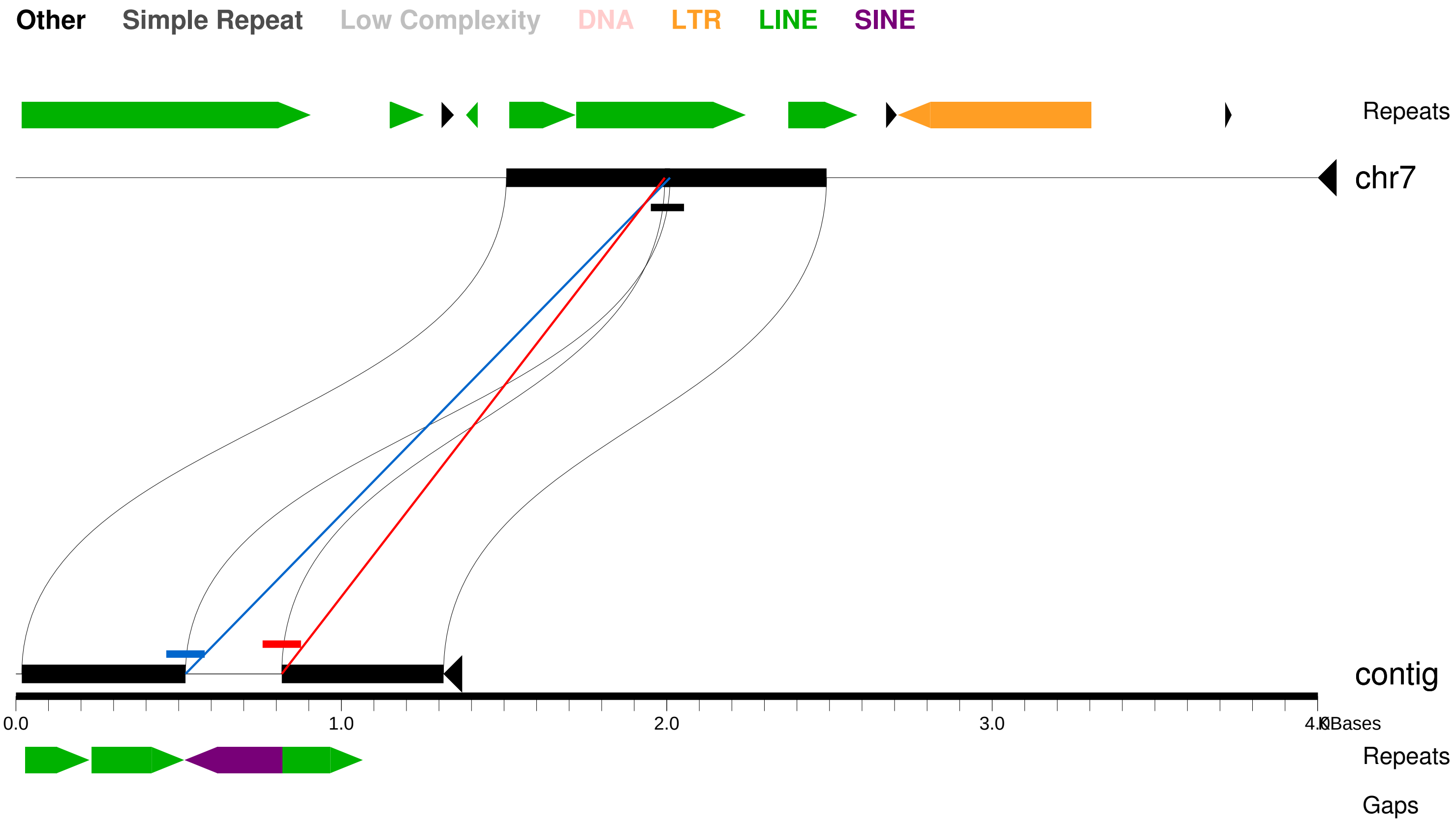

siteID: chr7_52403908 Scafftig6 + Breakpoint: chr7:52403910
chr7:52403750-52404076
Overlap Length: 7 INS size: 261 Genotyped: Yes

```
left    -------GTCAGTCCAAGTGAAAGTGAAGAGAGGCTGGGATGAAGGGTGCAAAGGATAGTAAAGAAAGCA
chrom   TGTCAGAGTCAGTCCAAGTGAAAGTGAAGAGAGGCTGGGATGAAGGGTGCAAAGGATAGTAAAGAAAGCA
right   ---CAGGCGCGCGCCACC-------------ATGCCCGGCTAATTTTTGTATTTT-TAGTAGAGACGGGG
           222N11*111***111111111111111*1**11**1*1*1111**1*11111*****1***11*11

left    TCTTTGAGATCTAGAACAGAATAATGGGTTGTGGAGGGAGGTATTGAGGATAGGAAAGTATATGGGTTTG
chrom   TCTTTGAGATCTAGAACAGAATAATGGGTTGTGGAGGGAGGTATTGAGGATAGGAAAGTATATGGGTTTG
right   TTTC------------------ACCGTGTTAGCCAGGATGGTCTCGATCTCCTGACCTCGT---GATCCG
        *1*1111111111111111111*11*1***1111***11***1*1**111111**11111*111*1*11*

left    GCACCATGGGGTGGATAGGCAAAACAATTTTTTTTTTTTTTTTTTGAGGCGGAGTTTCGCT---------
chrom   GCACCATGGGGTGGATAGGCAAAACAACTTTATTGATAAGGTGCAGATCCTGAAATAACCTGTAAGACTT
right   CCCGCCTCGGCCTCCCAAA-AAAACAACTTTATTGATAAGGTGCAGATCCTGAAATAACCTGTAAGACTT
        1*11*1*1**111111*111*******2***2**22*2222*222**22*2**22*222**222222222

left    --CTGTTGCCCAGGCTGG------AGTGCAGTGGC---GCGA-TCTCGACTCACTGCAAGCTCC------
chrom   GTCTGGTTTTTAGACAGGTAAAATAGGGGAATTGTAAGGAGAGTTTATAGGCTTTAGAAGCCCATGTTGT
right   GTCTGGTTTTTAGACAGGTAAAATAGGGGAATTGTAAGGAGAGTTTATAGGCTTTAGAAGCCCATGTTGT
        22***2*2222**2*2**222222**2*2*2*2*2222*2**2*2*22*22*22*22****2*2222222

left    -GCC---------TCCCGGGTTTACGCCATTCTCCTGCCT-CAGCC-
chrom   AGCAGGTGAGTGATAATGGGCTTTAACCTTTTTAAAGCCTGCTGTGG
right   AGCAGGTGAGTGATAATGGGCTTTAACCTTTTTAAAGCCT-------
        2**2222222222*222***2**222**2**2*222**** 1N1NN
```

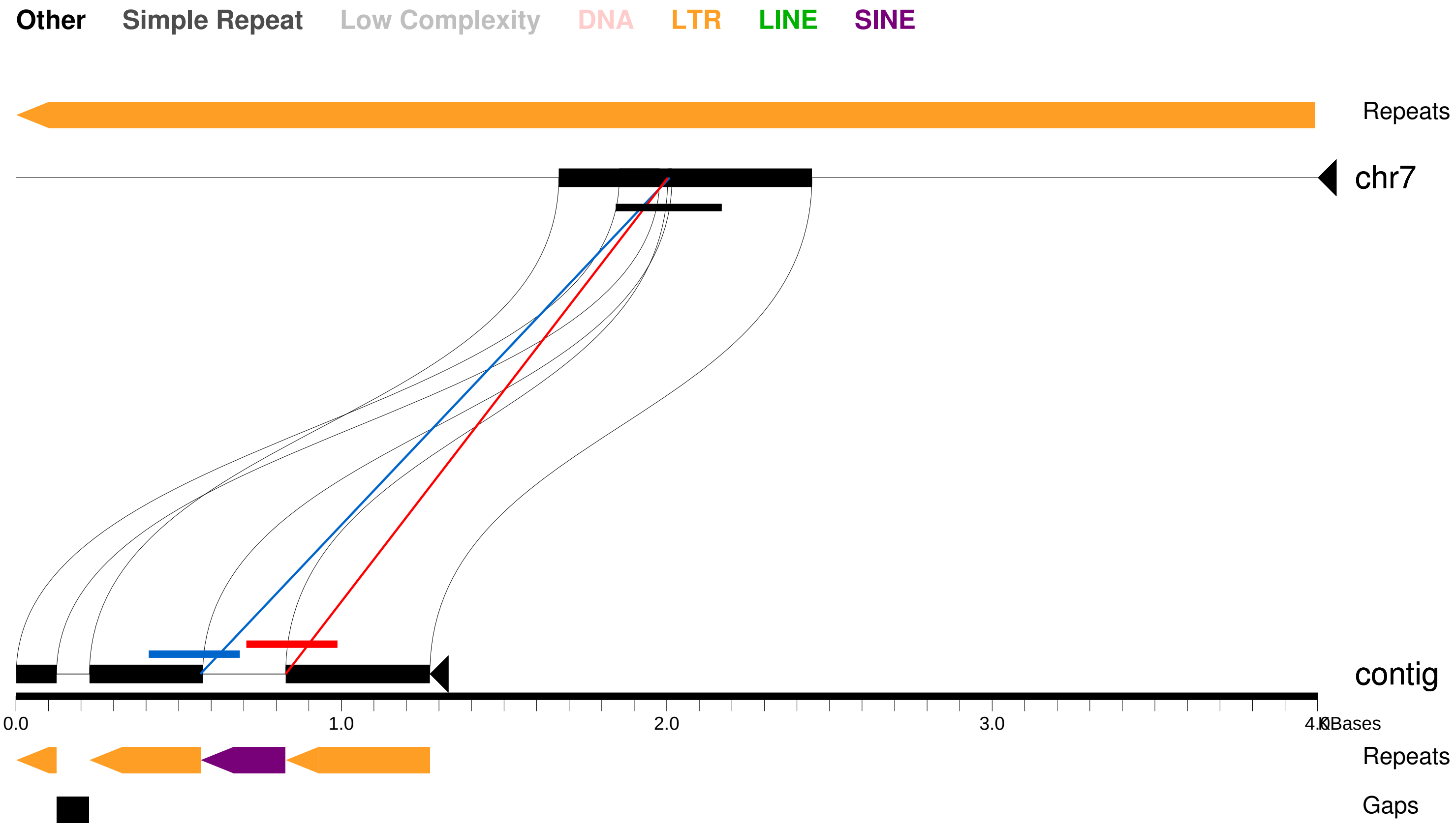

siteID: chr7_52808315 Scafftig1 + Breakpoint: chr7:52808304
chr7:52808257-52808360
Overlap Length: 13 INS size: 414 Genotyped: No

```
left    TCAAAGCATGTTATGTCTGCTGTTACACCATGGATTACTCC---------------ATTAAGAAATGACA
chrom   TCAAAGCATGTTATGTCTGCTGTTACACCATGGATTACTCC---------------ATTAAGAAATGATA
right   NNNNNNNNNNNNNNNNNNNNNNNNNNNNNNNNNNNNNNNNNNNNNNNNNNNNNNAAAAAAAAAAATGATA
        11111111111111111111111111111111111111111111111111111111*11**1******2*

left    ATAAGGCCGGGCGCGGTGGCTCACGCCTGTAATCCCAGCACTTTGGGAGGCCGAGGC---GGGCGGA
chrom   ATAAG-----------------AAGCATAATATTCATATCCTTGGCTTGACCTTGACACAGAGCTG-
right   ATAAG-----------------AAGCATAATATTCATATCCTTGGCTTGACCTTGACACAGAGCTG-
        *****22222222222222222*2**2*222**2*22222***2*222*2**22*2*222*2**2*2
```

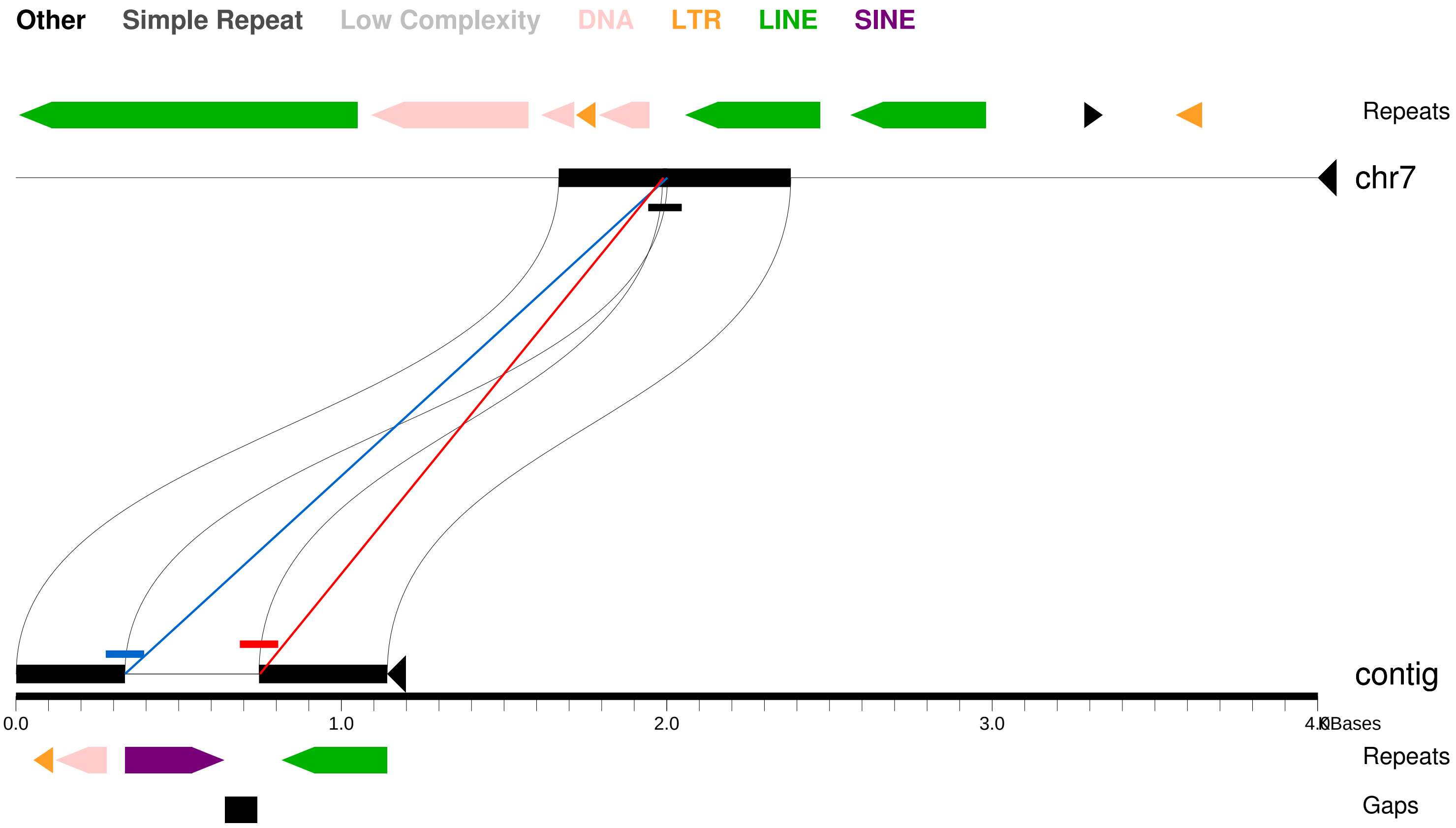

siteID: chr7_53358278 Scafftig4 - Breakpoint: chr7:53358285
chr7:53358236-53358344
Overlap Length: 11 INS size: 292 Genotyped: Yes

```
left    TACACATAGGTGAGT--ATTTTTGCCTGCTTTAAAAATTATTTTTGTTTTT-----------AAAAAATT
chrom   TACACATAGGTGAGT--ATTTTTGCCTGCTTTAAAAATTATTTTTGTTTTT-----------AAAAAATT
right   GAT---TGCGCCACTGCACTCCAGCCTGGGCGACAGCGAGACTCTGTCTCAAAAAAAAAAAAAAAAAAATT
        1*1111*11*11*1*11*1*111*****1111*1*1111111*1***1*1111111111111********

left    GACGGCCGGGCGCGGTGGCTCACGCCTGTAATCCCAGCACTTTGGGAGGCCGAGGCGGGCGG
chrom   GACAATAAAATTGTATG---TACTTATGTACAACATGATGTTTTCAA-------GTGTATAT
right   GACAATAAAATTGTATG---TACTTATGTACAACATGATGTTTTCAA-------GTGTATAT
        ***222222222222**2222**222****222*22*222***222*2222222*2*22222
```

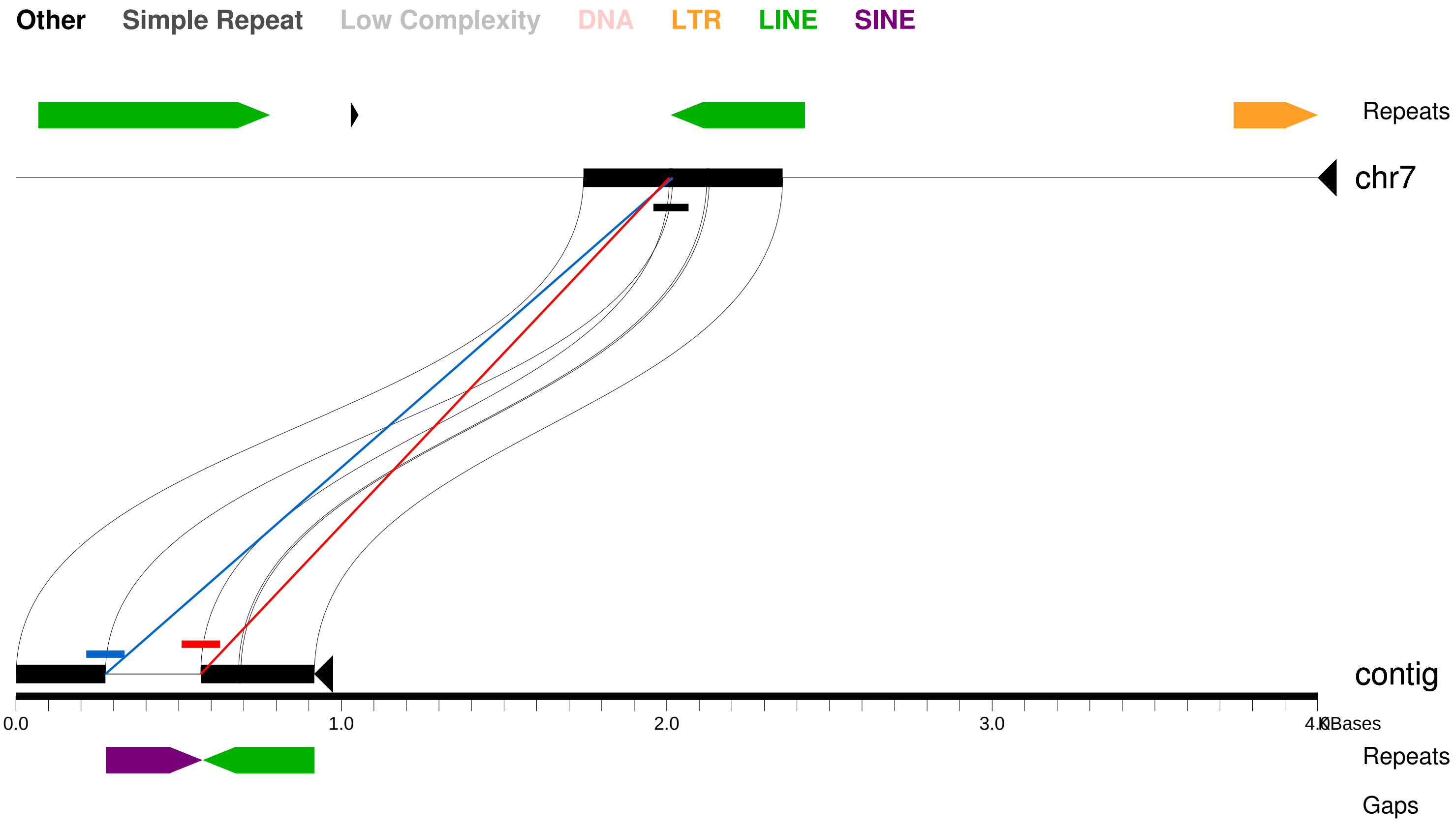

siteID: chr7_56244912 Scafftig13 - Breakpoint: chr7:56244904
chr7:56244744-56245078
Overlap Length: 15 INS size: 307 Genotyped: Yes

```
left    ----------------CTCCCACATTGGTGAGGGCCAGCCTTCTTTACTCAGTCTACTGATTCAAATGCT
chrom   G-GAATGGATGGTGTCCTCCCACATTGGTGAGGGCCAGCCTTCTTTACTCAGTCTACTGATTCAAATGCT
right   GAGAATGGCGTGAATCC----------GGGAAGCGGAGCTTGC---AGTGAGCCGA--GATTGC---GCC
        21222222NNN2NN22*1111111111*1**1*111***1*1*111*1*1**1*1*11****11111**1

left    AATCTCTTCCAGAAACATCTTCACAGATGCACCTGGAAATAATGTTTTACCAGCCATCTGGGCATCCCTT
chrom   AATCTCTTCCAGAAACATCTTCACAGATGCACCTGGAAATAATGTTTTACCAGCCATCTGGGCATCCCTT
right   ACTG-----CAG-------TCCGCAGTCCGACCTGGGC--------------GACAGAGCGAGACTCCGT
        *1*111111***1111111*1*1***1111******1111111111111111*1**1111*11*11**1*

left    ACCCCAGTCAAGTTGACACATAAAAGTAATCATCACGGCCG-----GGCGCGG--TGGCTCACGCC----
chrom   ACCCCAGTCAAGTTGACACATAAAAGTAATCATCACATTGGTATATGAAGAGGATTAAGTCACTCAGTTT
right   CTCAAAAAAAAAAAAAAAAAAAAAAAGTAATCATCACATTGGTATATGAAGAGGATTAAGTCACTCAGTTT
        11*11*111**1111*1*1*1***************2222*22222*22*2**22*222****2*22222

left    ----------TGTAATCCCAGCACTTTGGG-----AGGCCGAGGCGGGTGGATCATGAGGTCAGGAGATC
chrom   TGAATAGGTTTGTGAGTGCTGAACTTTCTTTGTCAAGGCTGATTCGAGTTCATAATGA--TCTGCTCACT
right   TGAATAGGTTTGTGAGTGCTGAACTTTCTTTGTCAAGGCTGATTCGAGTTCATAATGA--TCTGCTCACT
        2222222222***2*222*2*2*****22222222****2**22**2**22**2****22**2*222*22

left    GAGACCATCCTGGC----TAACAAGG----TGAAACCCCGTCTCTGCTAA-------A
chrom   GTGTCCCTGGAGAAGAAGTAACAGGGGAAATGATGCTCATGGACTGTCATGGTCGTTA
right   GTGTCCCTGGAGAAGAAGTAACAGGGGAAATGATGCTCATGGACTGT-----------
        *2*2**2*222*222222*****2**2222***22*2*22222***2N1N       1
```

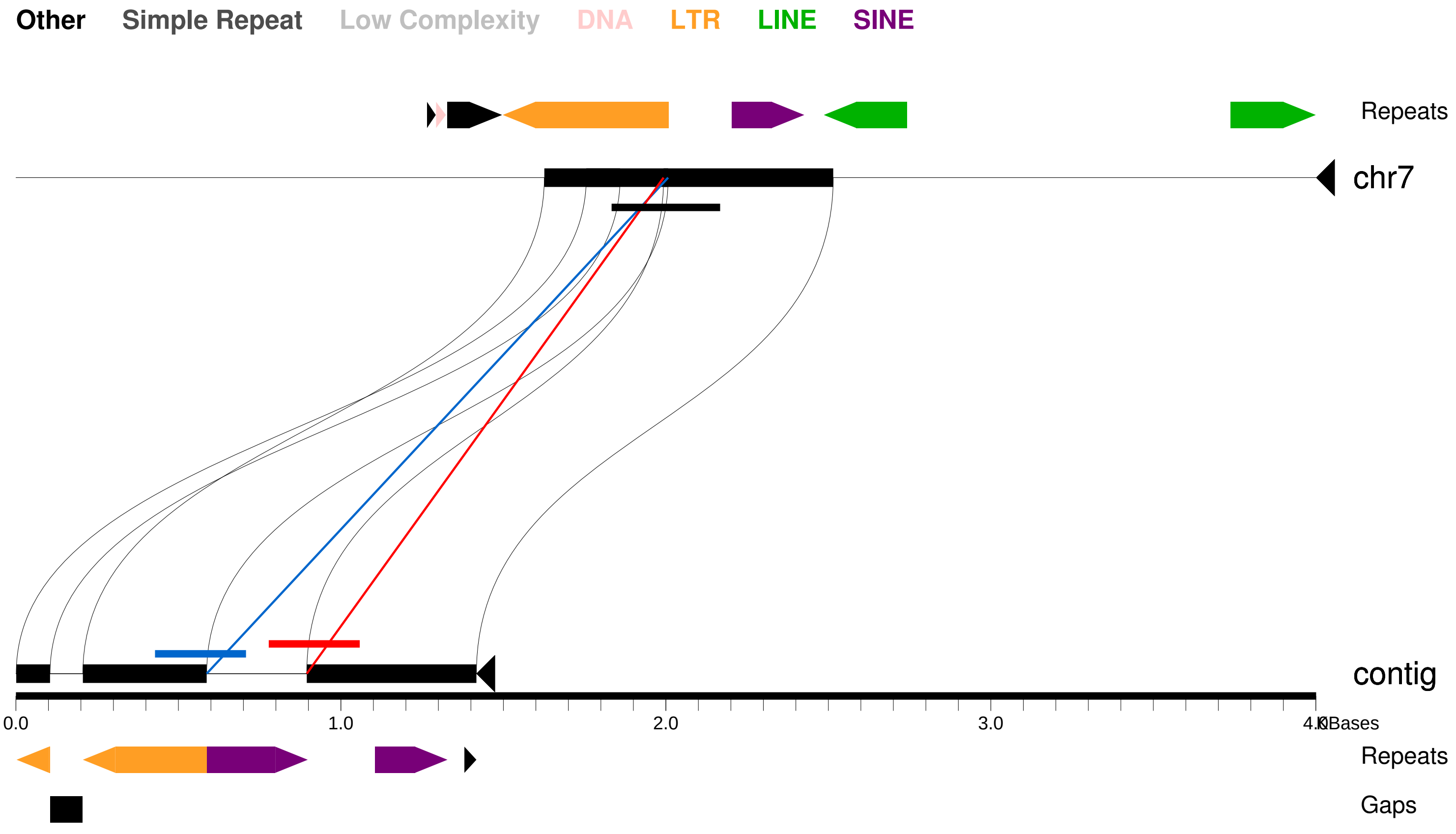

siteID: chr7_61674434 Scafftig2 + Breakpoint: chr7:61674460
chr7:61674417-61674519
Overlap Length: 17 INS size: 376 Genotyped: No

```
left    TTGGAGCAAGATATAAAATTTAGACTTGATTTTTTAAAGATTT----------------TTCAAGATGTA
chrom   TTGGAGCAAGATATAAAATTTAGACTTGATTTTTTAAAGATTT----------------TTCAAGATGTA
right   NNNNNNNNNNNNNNNNNNNNNNNNNNNNNNNNNNNNNNNNNNNNNNNNNNNNNNNNNNNTTCAAGATGTA
        11111111111111111111111111111111111111111111111111111111111***********

left    CTTCTTGGCCGGGCGCGGTGGCTCACGCCTGTAATCCCAGCACTTTGGGAGGCCGAGGCGGGCGG
chrom   CTTCTTA-----------TTTCTTTTTGTTCTATTTAACATTCTCTGAGTTTCCTATATT-----
right   CTTCTTA-----------TTTCTTTTTGTTCTATTTGACATTCTCTGAGTTTCCTATATA-----
        ******222222222222*22**222222*2**2*2N22222**2**2*222**2*222N22222
```

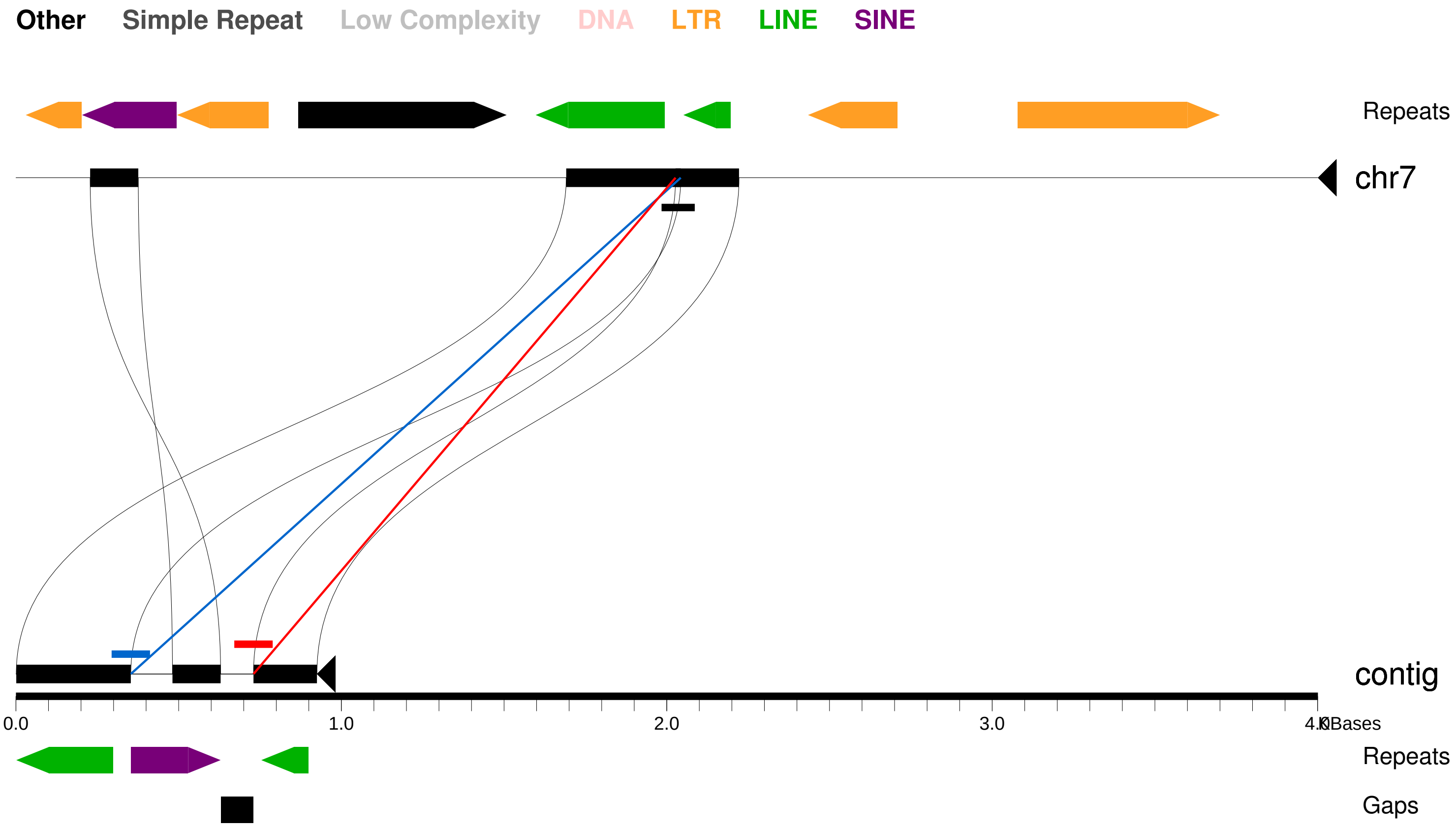

siteID: chr7_62502693 Scafftig1 - Breakpoint: chr7:62502699
chr7:62502652-62502758
Overlap Length: 13 INS size: 428 Genotyped: No

```
left   GCAAAGATATGGAATCAACTTAGGTGCCCATTAGCAGTGGACTGA------------ATAAAGAAAATGT
chrom  GCAAAGATATGGAATCAACTTAGGTGCCCATTAGCAGTGGACTGA------------ATAAAGAAAATGT
right  NNNNNNNNNNNNNNNNNNNNNNNNNNNNNNNNNNNNNNNNNNNNNNNNNNNNNNNNNAAAAAGAAAATGT
       111111111111111111111111111111111111111111111111111111111*1***********

left   GGGCTGGGCGCGGTGGCTCACGCCTGTAATCCCAGCACTTTGGGAGGCCGA----GGCGGGCGGA
chrom  GGTATAT--------GTACAC-CATGGAAT------ACTATGG-AGCCATAAAAAGGAAGGAAAT
right  GGTATAT--------GTACAC-CATGGAAT------ACTATGG-AGCCATAAAAAGGAAGGAAAT
       **22*2222222222*22***2*2**2***222222***2***2**2*22*2222**22**2222
```

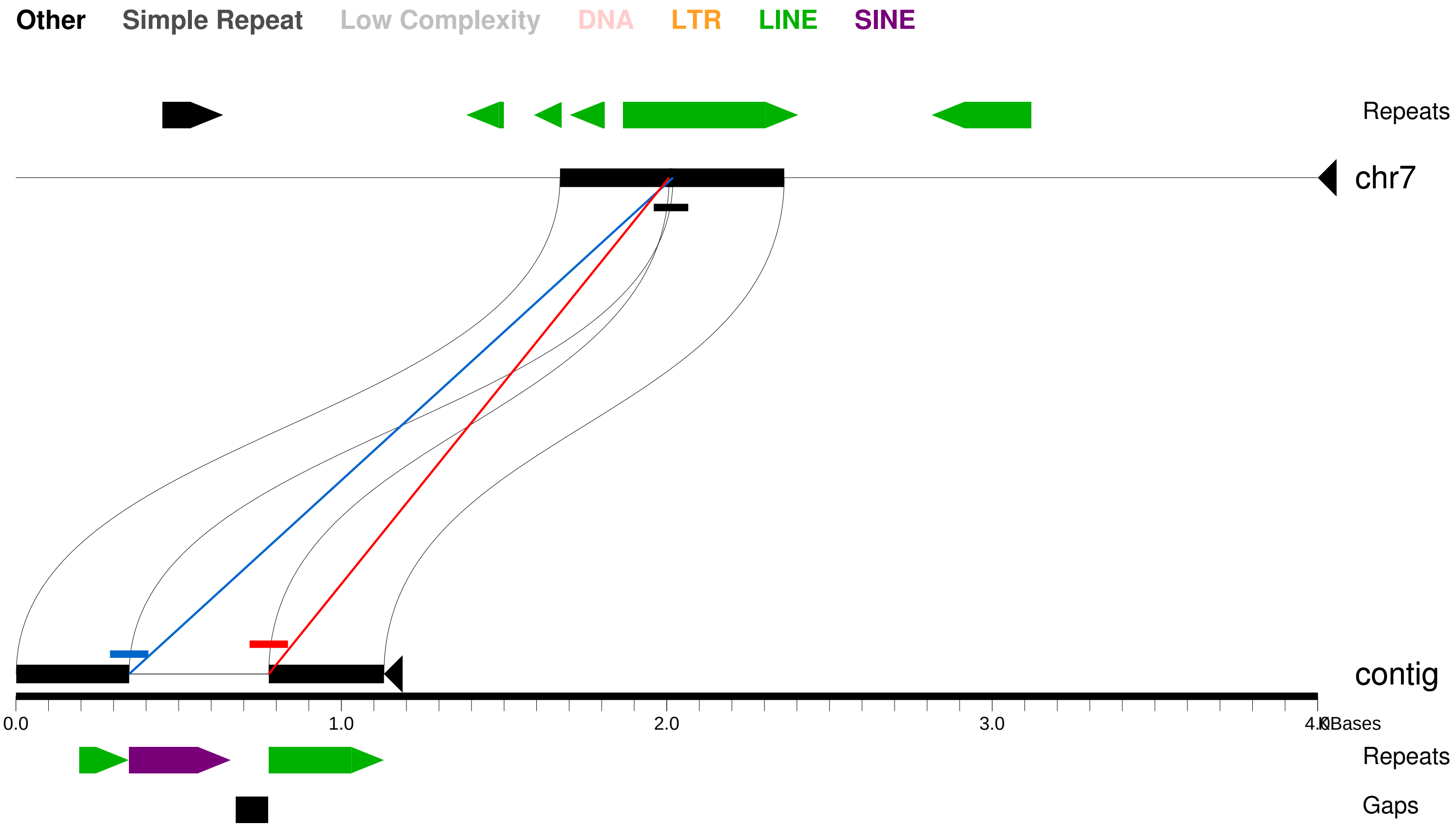

siteID: chr7_65932852 Scafftig1 + Breakpoint: chr7:65932851
chr7:65932691-65933027
Overlap Length: 17 INS size: 411 Genotyped: No

```
left    ----------------CTAAAGAAAATAAAACACTGTTACTGCTGAGAAATTAACTTTAAAATGGCTAAC
chrom   TGTATATCTGGAAGGCCTAAAGAAAATAAAACACTGTTACTGCTGAGAAATTAACTTTAAAATGGCTAAC
right   NNNNNNNNNNNNAA---------AAAA-------------------------------------------
        NNNNNNNNNNN22   111111****11111111111111111111111111111111111111111111

left    TTTCATAAAAGAATTCATATTTATGTGAAATAAGCTAAATAGGTACACGAAATCTGTGGAACTCTTGTAA
chrom   TTTCATAAAAGAATTCATATTTATGTGAAATAAGCTAAATAGGTACACGAAATCTGTGGAACTCTTGTAA
right   ----------------------------------------------------------------------
        1111111111111111111111111111111111111111111111111111111111111111111111

left    CAGACAAACAATAACAAGTTAGAAAGTATAAAAAGAGGC--CGGGCGCGGTGGC---------TCACGCC
chrom   CAGACAAACAATAACAAGTTAGAAAGTATAAAAAGAGATATCAGTCAAAATAGCATGAATATGTAAAGAC
right   --------------------AGAAAGTATAAAAAGAGATATCAGTCAAAATAGCATGAATATGTAAAGAC
        11111111111111111111****************2222*2*2*2222*2**222222222*2*2*2*

left    TG-------------TAATCCCAG----------CACTTTGGGAGGCCGAGGCGGGCGGATCACGAGGTC
chrom   TTATAGAAAATATTTTAATGAGAAAATTATAATCCACATGGAAACACTGAGATATACTTAACGCAATGTG
right   TTATGGAAAATATTTTAATGAGAAAATTATAATCCACATGGAAACACTGAGATATACTTAACGCAATGTG
        *222N2222222222****222*22222222222***2*2*22*22*2***22222*22*2*2*2*2**2

left    AGGAGATCGAGACCATTC----TGGCTAACACGGTGAAACCCCGTC--TCTACTA-AA
chrom   CGAAAATAAAATCTTATAACCATGTCAAACATTCC-AAAATGTGTAAGTTTACTACAA
right   CGAAAATAA-------------------------------------------------
        2*2*2**221NN1NNN1N    11N1N1111NNNN2111NNNN11N  1N11111 11
```

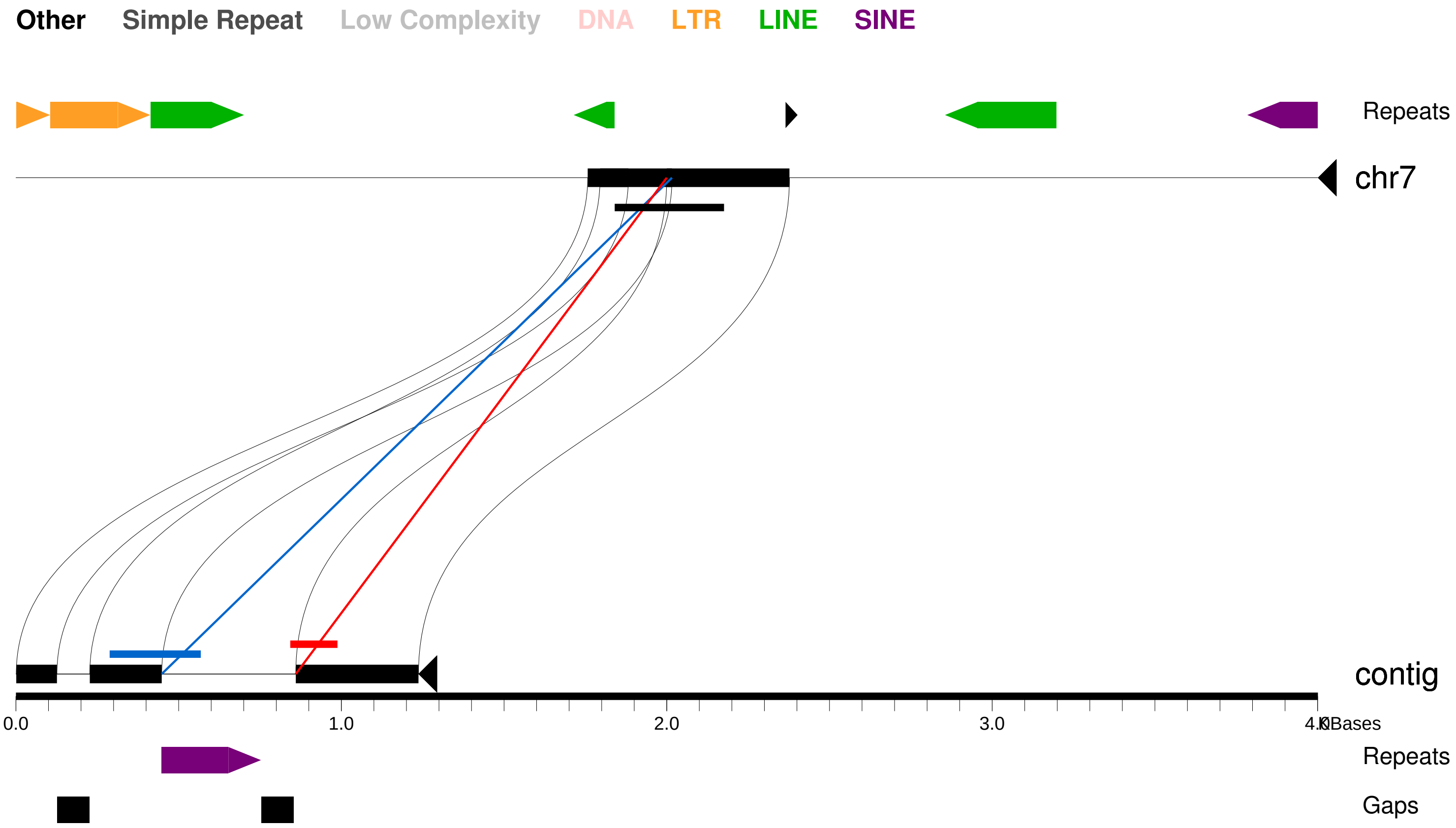

siteID: chr7_77737216 Scafftig6 + Breakpoint: chr7:77737193
chr7:77737170-77737249
Overlap Length: 14 INS size: 314 Genotyped: Yes

```
left    T-----------------------------CTCTGATTTTATTTATTTG-----------GGTCTTCCCTC
chrom   T-----------------------------CTCTGATTTTATTTATTTG-----------GGTCTTCCCTC
right   TGATCCGCCCGCCTCGGCCTCCCAAAGTGCTGGGATTACAGGCGTGAGCCACCGCGCCCGGCCTTCCCTC
        *11111111111111111111111111111**11****11*1111*11*11111111111**1********

left    TTTCTTTTTTTTTTTTTTTTTTTTTTTTTTTTTTTTTTTTTTTGAGACGGAGTCTTGCTCTGTCGCCCAGGCT
chrom   TTTCTTAGTATTGATTTTGTTTATCTTTT---------------------------CAAAAAACCAAGCT
right   TTTCTTAGTATTGATTTTGTTTATCTTTT---------------------------CAAAAAACCAAGCT
        ******22*2**22****2***2*2****222222222222222222222222222*222222***2***

left    GGAGTGCAGTGGCGGGAT
chrom   ----TTCATT--------
right   ----TTCATT--------
        2222*2**2*22222222
```

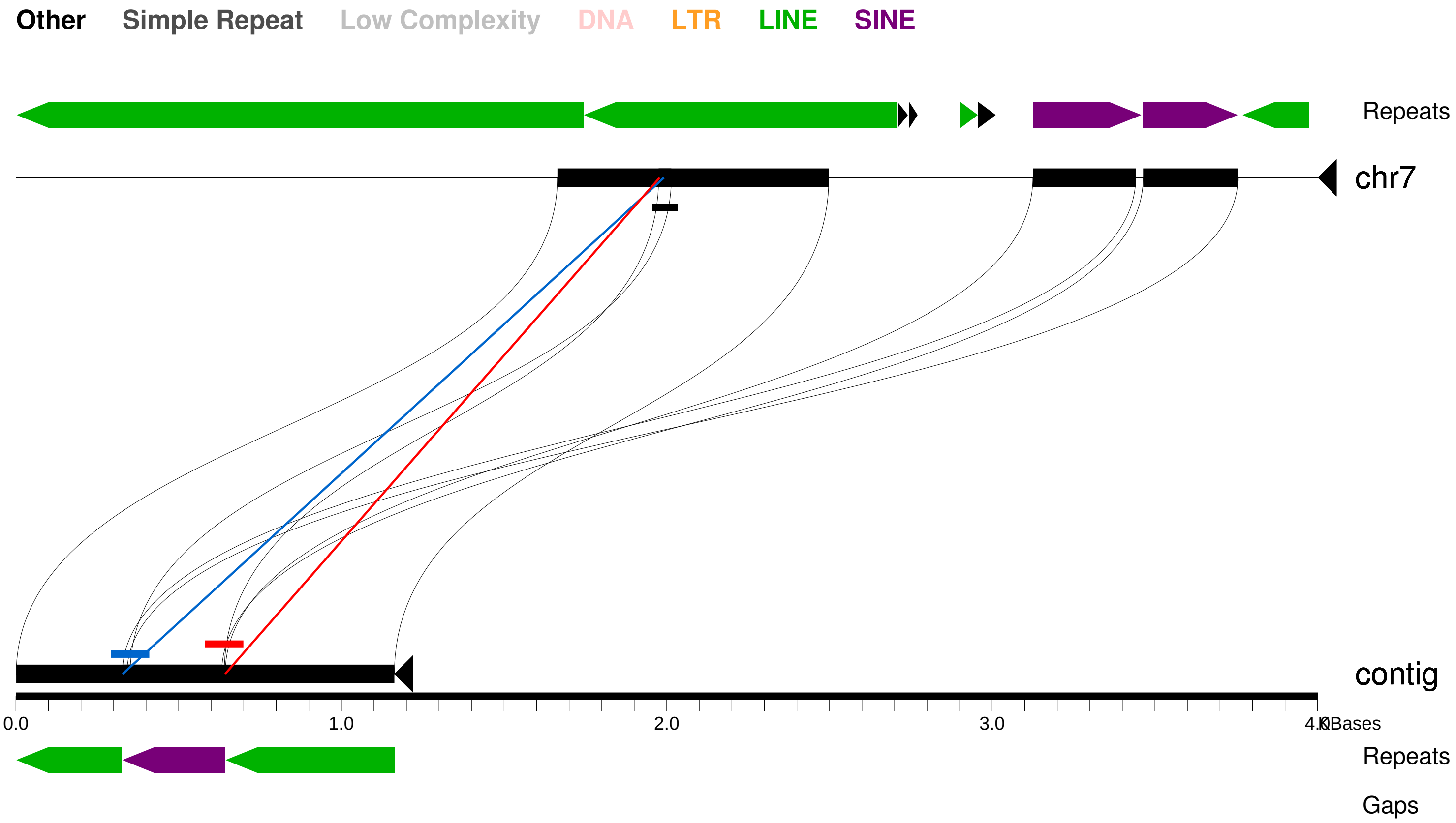

siteID: chr7_82178798 Scafftig1 - Breakpoint: chr7:82178789
chr7:82178744-82178845
Overlap Length: 15 INS size: 464 Genotyped: No

```
left     ATTATATATCTGTATCTCTCTG-----------------ACACTACAACCCTACTGTGTAAGAAATAGCT
chrom    ATTATATATCTGTATCTCTCTG-----------------ACACTACAACCCTACTGTGTAAGAAATAGCT
right    NNNNNNNNNNNNNNNNNNNNNNNNNNNNNNNNNNNNNNAAAAAAAAAAAAAAAAAAAAAAAAAAAAAAGCT
         111111111111111111111111111111111111111*1*11*1**1111*111111**1***1****

left     GGCACACGGGCCGGGCGCGGTGGCTCACGCCTGTAATCCCAGCACTTTGGGAGGCCGAGGCGGGTG
chrom    GGCACACTTTATAA-------GGAAAACATCT-TATTGTTAACTCAAT---------AAGCACATA
right    GGCACACTTTATAA-------GGAAAACATCT-TATTGTTAACTCAAT---------AAGCACATA
         *******22222222222222**222**22**2**2*222*2*2*22*222222222*2**222*2
```

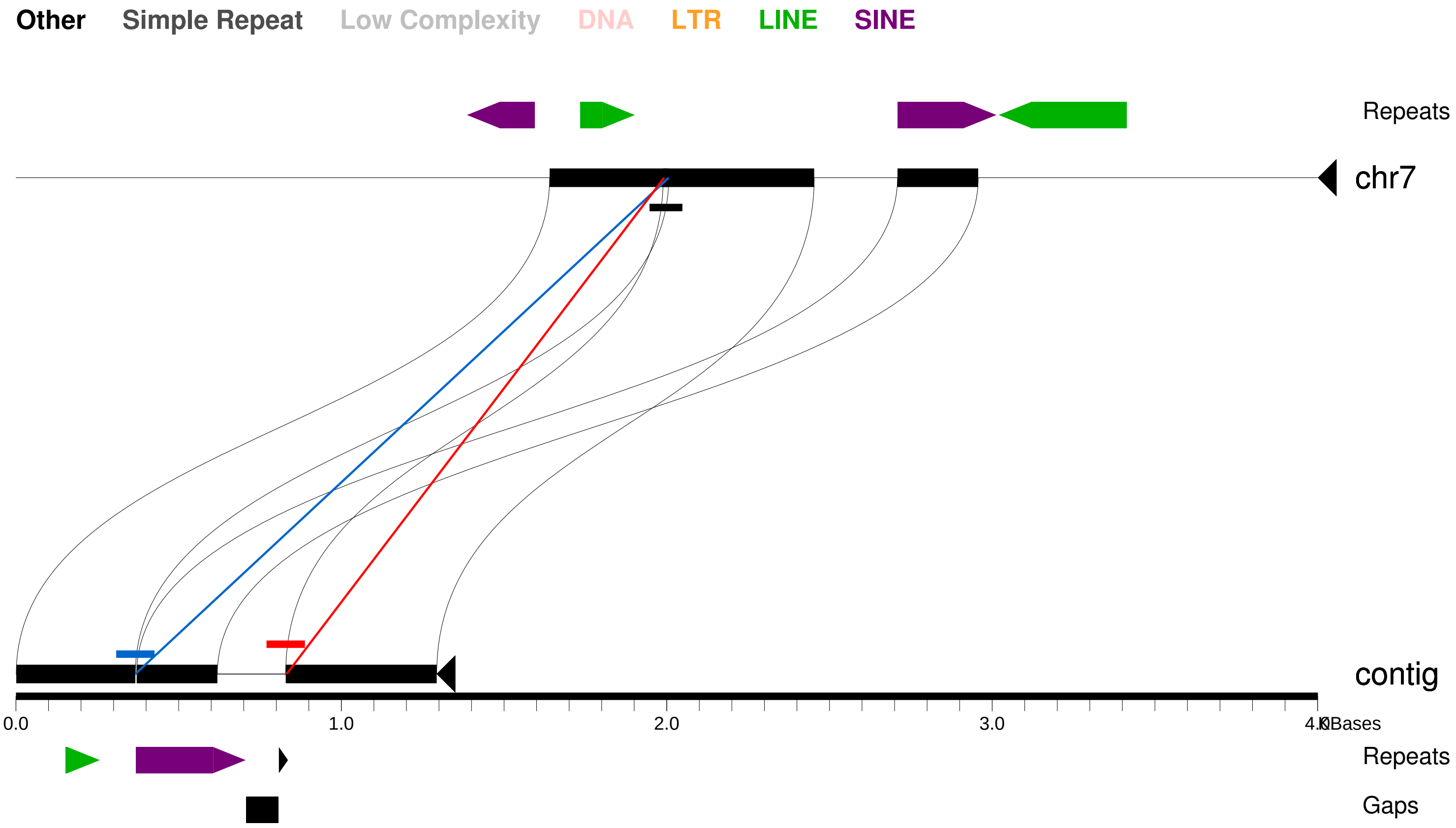

siteID: chr7_82851736 Scafftig1 + Breakpoint: chr7:82851743
chr7:82851626-82851802
Overlap Length: 14 INS size: 300 Genotyped: Yes

```
left     TCTAATCTTGTGTGTTTGTTTTTCAGTACTTT--TTACATGGATGTATTCAGTGACAAAAAGAAGTTCTC
chrom    TCTAATCTTGTGTGTTTGTTTTTCAGTACTTT--TTACATGGATGTATTCAGTGACAAAAAG---TTCTC
right    TCCACCCGCCTCGGCCT--TCCAAAGTGCTGGGATTACAGGCGTG-----AGCCAC-----------CGC
         **1*11*111*11*11*11*1111***1**1111*****1*11**11111**11**11111122211*1*

left     ACACTGGTAGGCGAAAACTGATTCTTTTTTATTTGTGGTAATAAGTAGTCATCAGAACCAGTTTTTTTTTT
chrom    ACACTGGTAGGCGAAAACTGATTCTTTTTTATTTGTGGTAATAAGTAGTCATCAGAACCAGTTTTTTAAAC
right    GCCC-----GGC----------------------------------------CAGAACCAGTTTTTAAAC
         1*1*11111***1111111111111111111111111111111111111111*************2222

left     TTTTTTT-TTGAG-----ACGGAG-TCTCGCTCTGTTG----
chrom    TTGTTCCATTGATCAAAAACTCACATCACATTTTCTTAACCC
right    TTGTTCCATTGATCAAAAACTCACATCACATTTTCTTAACCC
         **2**222****222222**22*22**2*22*2*2**22222
```

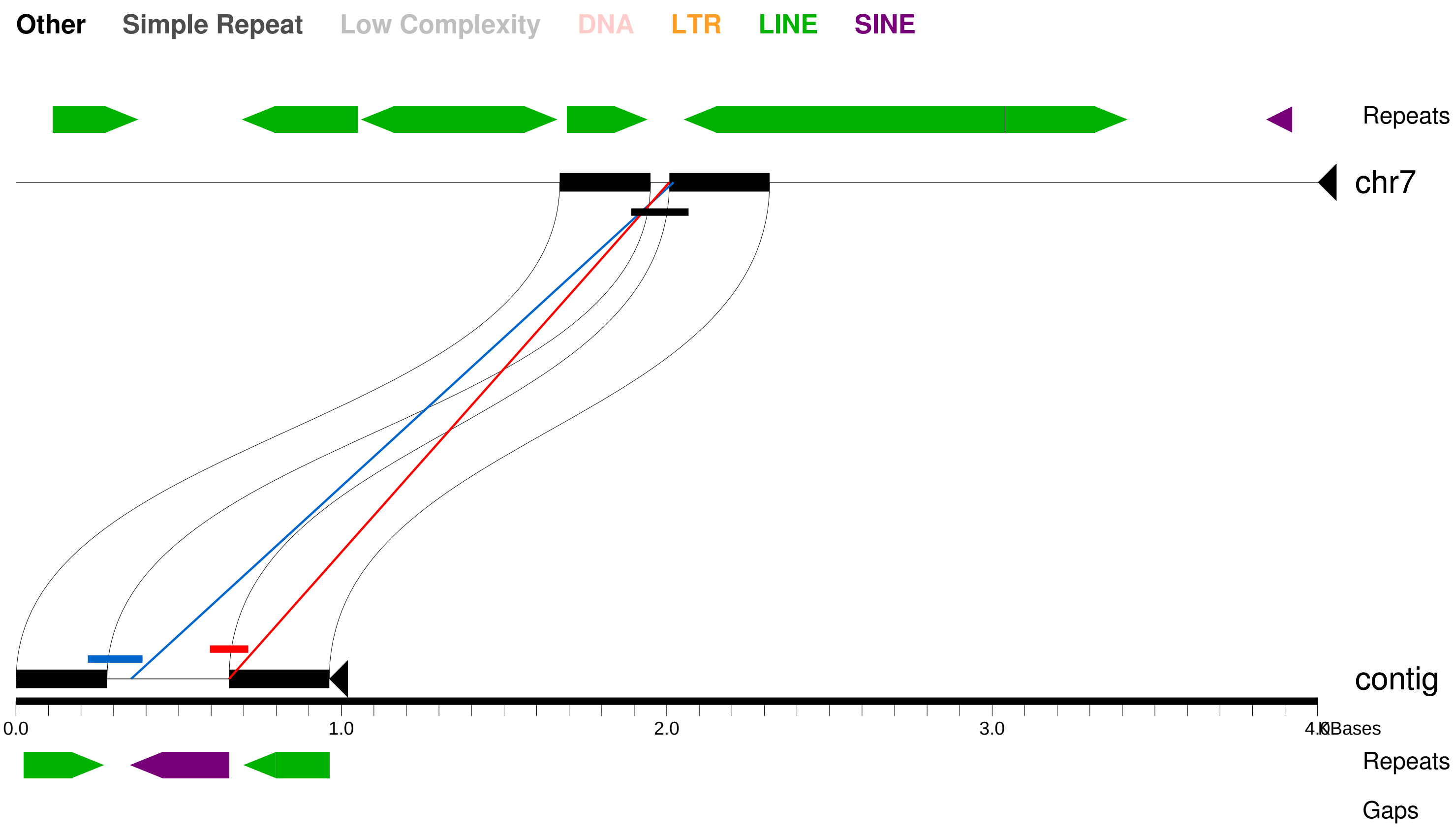

siteID: chr7_85013804 Scafftig3 - Breakpoint: chr7:85013789
chr7:85013629-85013962
Overlap Length: 14 INS size: 296 Genotyped: Yes

```
left   --------------CTTTTCGATAATAGAATAAGTCTAAGACAGTGAGTCCCATAAGATATTGGATTACA
chrom  TTCGGATTTTCATACTTTTCGATAATAGAATAAGTCTAAGACAGTGAGTCCCATAAGATATTGGATTACA
right  G-CTGA--------------GGCAGGAGAATG-GCGTGAACCCGGGAGGCG-----GAGCTTGCAGTG--
       N 2N22        111111*11*11*****11*11*1*11*1*1***1*111111**11***1*1*111

left   AATCTTAGAATCACATTCCATGAGTACATTTTCCTCCCTCAATAAAATTCCATCAATCTATATTAAACCA
chrom  AATCTTAGAATCACATTCCATGAGTACATTTTCCTCCCTCAATAAAATTCCATCAATCTATATTAAACCA
right  -AGCCGAGA------TTGCGCCACTGCACT--CCAGCCTGGGCGACAGAGCGAGACTCCGTCTCAAAAAA
       1*1*11***111111**1*111*1*1**1*11**11***11111*1*111*111*1**11*1*1***11*

left   TTAAGACTCAAATGGTTTTAAAAATTGGCAAGTAGGCCG----GGCGCGGTGGCTCAC--GCCTGTAATC
chrom  TTAAGACTCAAATGGTTTTAAAAATTGGCAAGTAAATAAAAGAGTCATAGGGTTTTATTGGACTTTAAGT
right  AAAAAA---AAA--------AAAATTGGCAAGTAAATAAAAGAGTCATAGGGTTTTATTGGACTTTAAGT
       11**1*111***11111111*************222222222*2*222*2*22*2*222*2**2***22

left   CCA-GCACTTTGGGAGGCCGAGGCGGGCGGATCACGAGGTCAGGAGAT------CGAGACCAT-CCCGGC
chrom  CCACGCATTCCTTAAATTATACCATGGGCAAACATAAATTTACAATGTATCTGTCTTAACTTTGCCCCTA
right  CCACGCATTCCTTAAATTATACCATGGGCAAACATAAATTTACAATGTATGTGTCTTAACTTTGCCCCTA
       ***2***2*22222*22222*2222**222*2**22*22*2*22*22*22N222*222**22*2***222

left   TAACACG----GTGAAACCCCGTCTCTA-------CTAAA--------------
chrom  TATTTTGTGAAGTGAAAACATTTCAATTTCAGGAGCTAAGCAATTACAGTGTCC
right  TATTTTGTGAAGTGAAAACATTTCAATTTCAGGAGCTAAGCAAT----------
       **2222*2222******2*222**22*22222222****22222
```

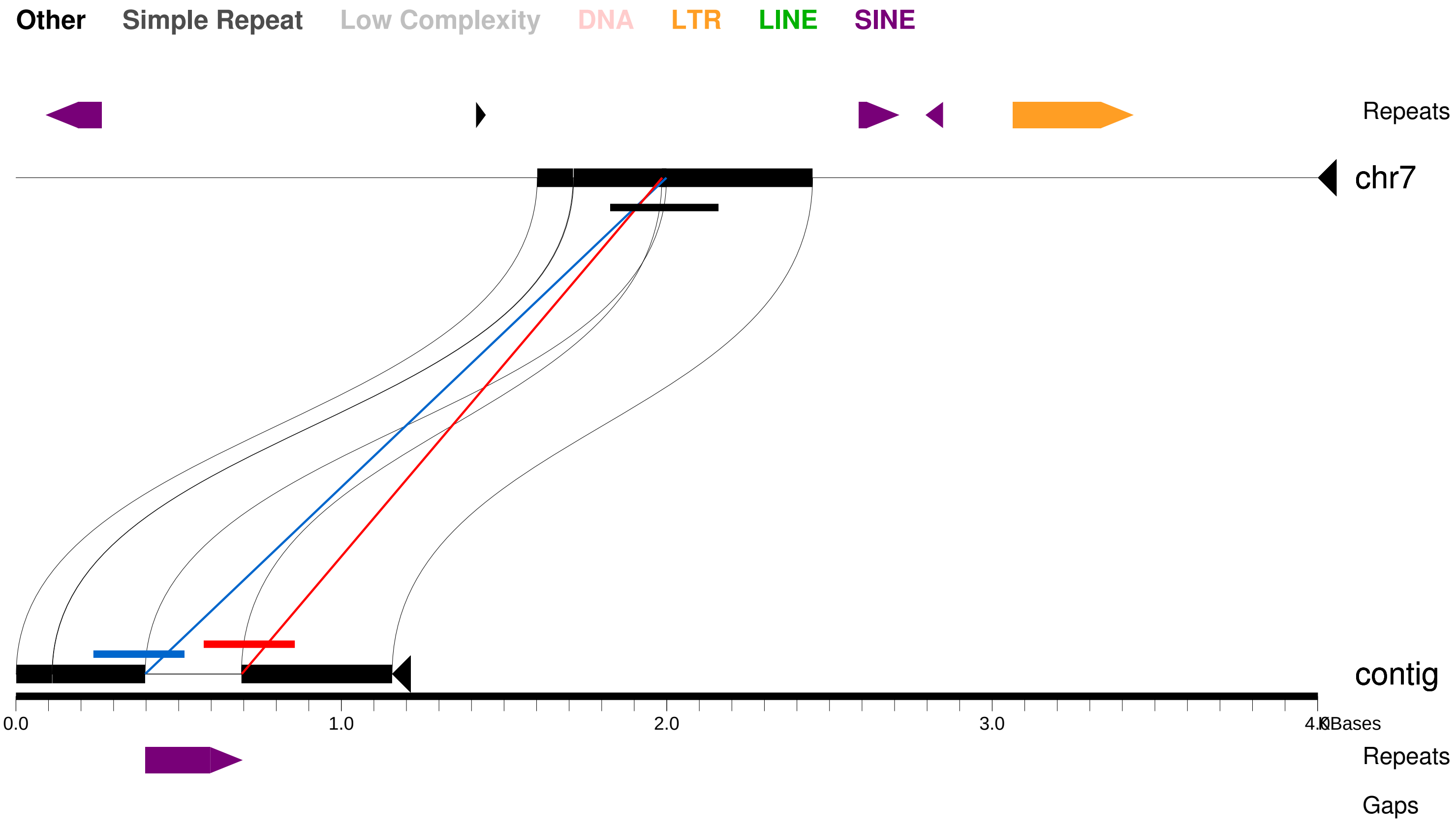

siteID: chr7_8517303 Scafftig1 - Breakpoint: chr7:8517275
chr7:8517231-8517334
Overlap Length: 13 INS size: 314 Genotyped: Yes

```
left    CT-----------CTTAATGTAATTCTAGATTTAATGGCA----TTTTGGTGCTTGGGTCAGACCAGTGA
chrom   CT-----------CTTAACGTAATTCTAGATTTAATGGCA----CTTTGGTGCTTGGGTCAGACCAGTGA
right   CCGCCCGCCTCGGCCTCCCAAAGTGCTGGGATTACAGGCGTGAGCCACAGCGCCCGGCCCAGACCAGTGA
        *111111111111*1*11211*1*1**1*11***11***1111121111*1**11**11***********

left    TTTTTTTTTTTTTTTTTTTTTTTTTTTTTTTTTTTGAGACGGAGTCTCGCTCTGTCGCCCAGGC-
chrom   TTCTTAATAGGAATCACCTCCTGCTGC-------AGGGATGGTG-------CTGGGGG--AGGAT
right   TTCTTAATAGGAATCACCTCCTGCTGC-------AGGGATGGTG-------CTGGGGG--AGGAT
        **2**22*22222*2222*22*22*2222222222*2**2**2*2222222***22*222***22
```

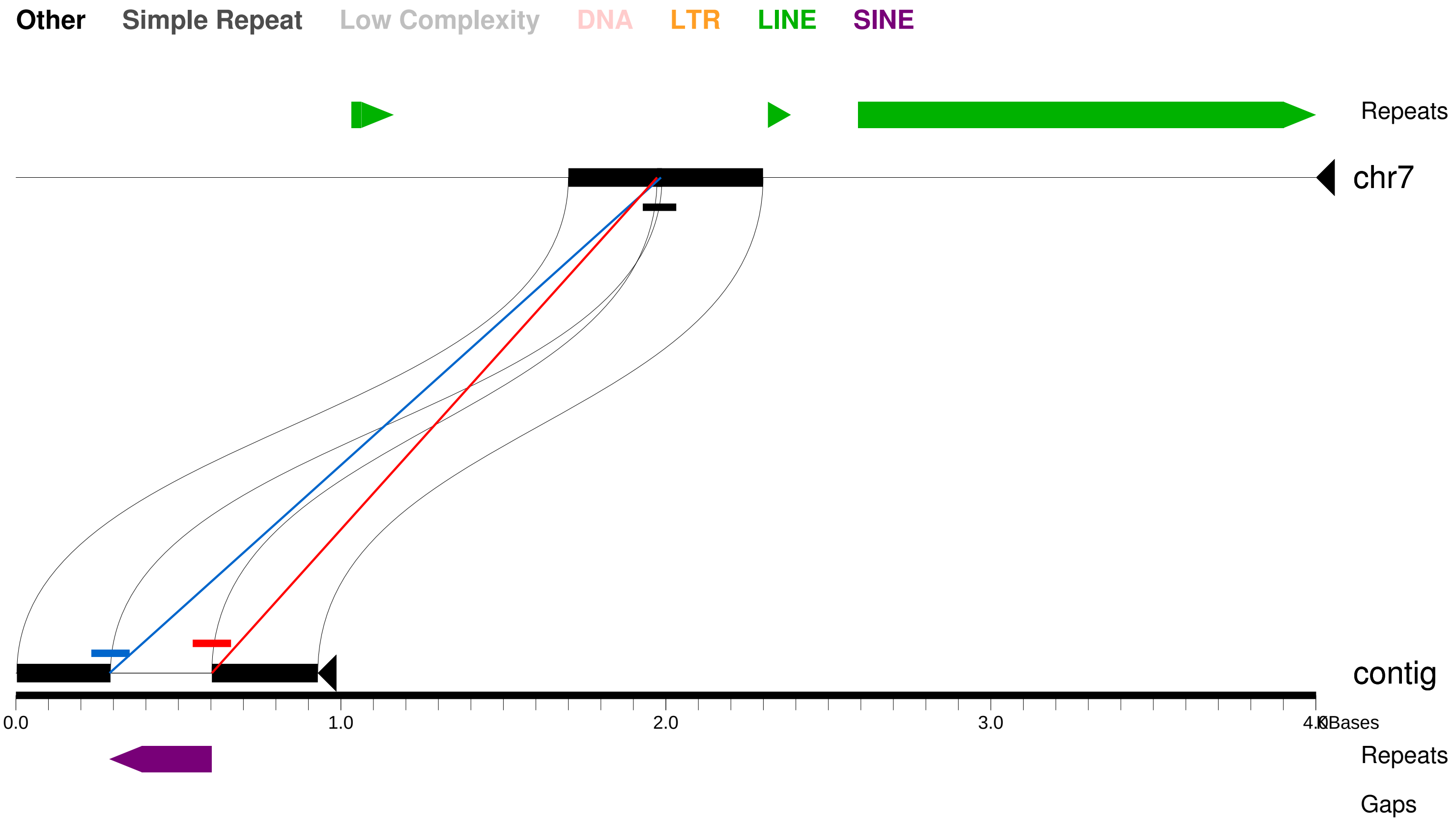

siteID: chr7_8534969 Scafftig1 + Breakpoint: chr7:8534953
chr7:8534907-8535012
Overlap Length: 14 INS size: 303 Genotyped: Yes

```
left     T-----CTTAAGGCCTTC--AACTGATTTGATGA-------GACCCACCCACATTATGGAGAGTAATCAG
chrom    T-----CTTAAGGCCTTC--AACTGATTTGATGA-------GACCCACCCACATTATGGAGAGTAATCAG
right    CACCCGCCTC-GGCCTCCCAAAGTGCTGGGATTACAGGCGTGAGCCACCGCGCCCGGCCTGAGTAATCAG
         111111*1*11*****1*11**1**1*11***1*1111111**1*****11111111111**********

left     CTTTTTTTTTTTTTTTTGAGACGGAGTCTCGCTCTGTCGCCCAGGCTGGACTGCGGACTGCAG
chrom    CTTTAATCTAAGTCT-----ACTGA-------TTTAAAGATTAATCACATCTAAAAAATATT-
right    CTTTAATCTAAGTCT-----ACTGA-------TTTAAAGATTAATCACATCTAAAAAATATT-
         ****22*2*222*2*22222**2**2222222*2*222*222*22*2222**2222*2*2222
```

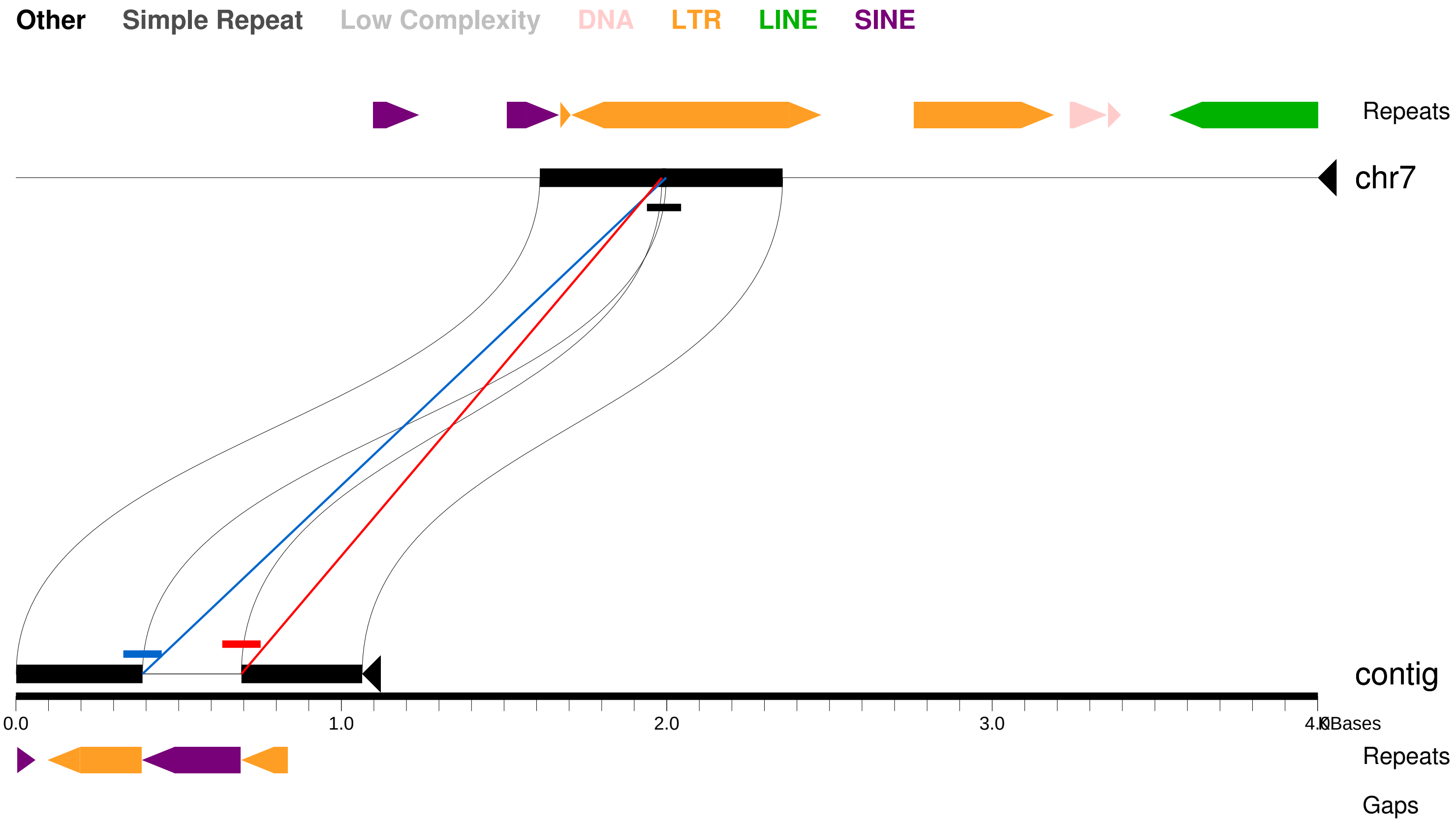

siteID: chr7_88380340 Scafftig2 - Breakpoint: chr7:88380340
chr7:88380285-88380399
Overlap Length: 5 INS size: 409 Genotyped: No

```
left    CTTGAATTGTATGTAAATGAAATGACAGTTTGTAGCCATTTGTGTCTCAATTCTT----TTGCTNNNNNN
chrom   CTTGAATTGTATGTAAATGAAATGACAGTTTGTAGCCATTTGTGTCTCAATTCTT----TTGCTTATTTT
right   CCGCCCGCCTCGGCCTCCCAAAGTGCTGGGATTACAGGCGTGAGCCACCGCGCCCGGCCTTGCTTATTTT
        *11111111*11*111111***111*1*1111**111111**1*1*1*1111*111111*****222222

left    NNNNNNNNNNNNNNNNNNNNNNNNNNNNNNNNNNNNNNNNNNNNNNNNNNNNNN
chrom   TATGTCTAGGATATTAATATGTGTTAAGATATGAACATTGCAAGCAAGT----
right   TATGTCTAGGATATTAATATGTGTTAAGATATGAACATTGCAAGCAAGT----
        222222222222222222222222222222222222222222222222222222
```

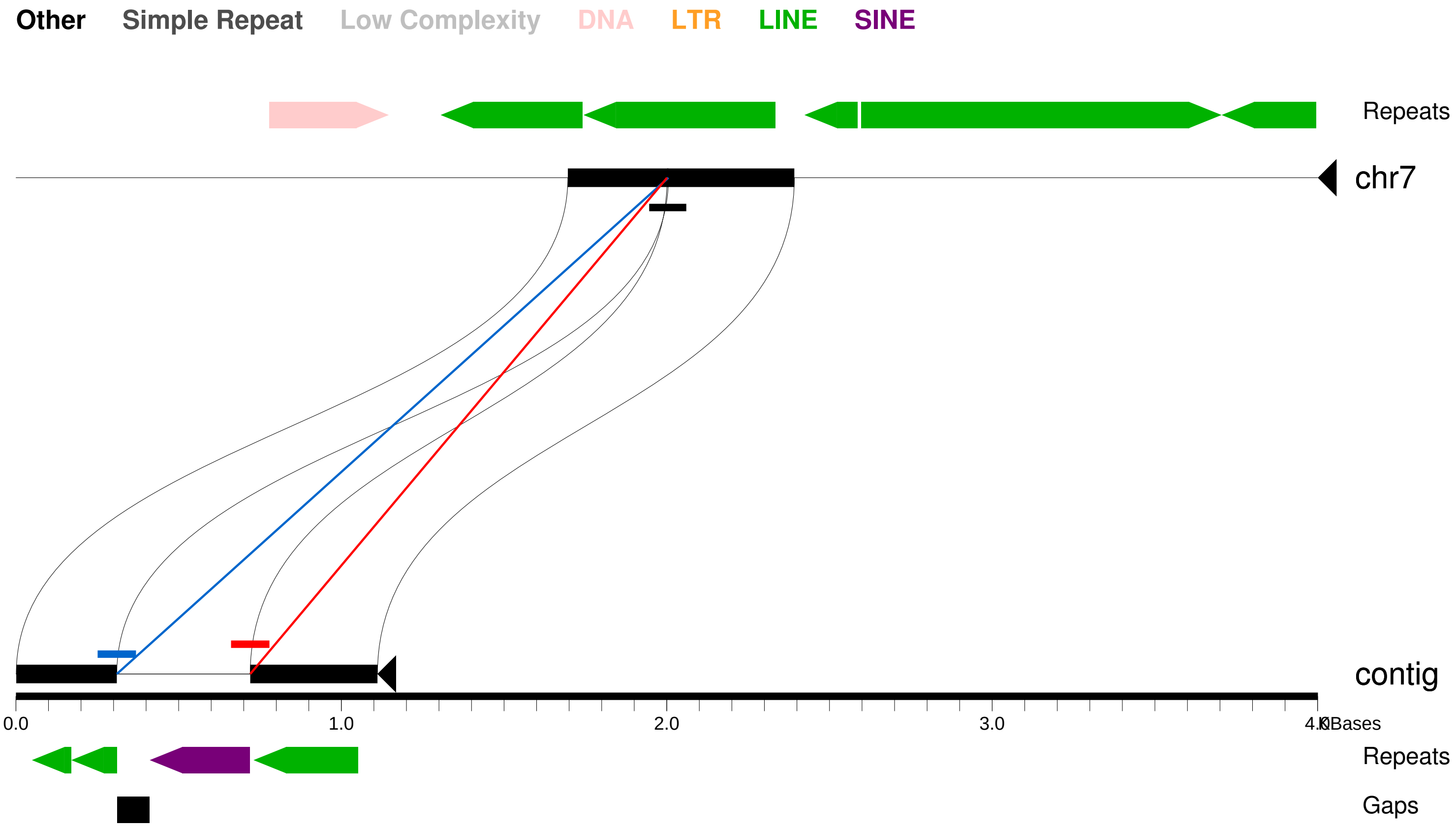

siteID: chr7_91751560 Scafftig3 - Breakpoint: chr7:91751535
chr7:91751493-91751594
Overlap Length: 18 INS size: 297 Genotyped: Yes

```
left     C----CTGTA--ACAA--------ATAAATTGAATTAGT---AAGTAAAGTCTTCTCAGAAAGAAAAGCA
chrom    C----CTGTA--ACAA--------ATAAATTGAATTAGT---AAGTAAAGTCTTCTCAGAAAGAAAAGCA
right    CGCCACTGCACTCCAGCCTGGGCGACAGCGAGACTCCGTCTCAAAAAAAAAAAAAAAAAAAAGAAAAGCA
         *1111***1*111**111111111*1*1111**1*11**111**11***11111111*1***********

left     CAGATCTGGCCGGGCGCGGTGGCTCACGCCTGTAATCCCAGCACTTTGGGAGGCCGAGGCGGGCGG
chrom    CAGATCTAGATGA--------TTTCACTGCTG-AATTTTTGCAAATATGTAAAAAA--------GG
right    CAGATCTAGATGA--------TTTCACTGCTG-AATTTTTGCAAATATGTAAAAAA--------GG
         *******2*22*22222222222****22***2***2222***22*22*2*2222222222222**
```

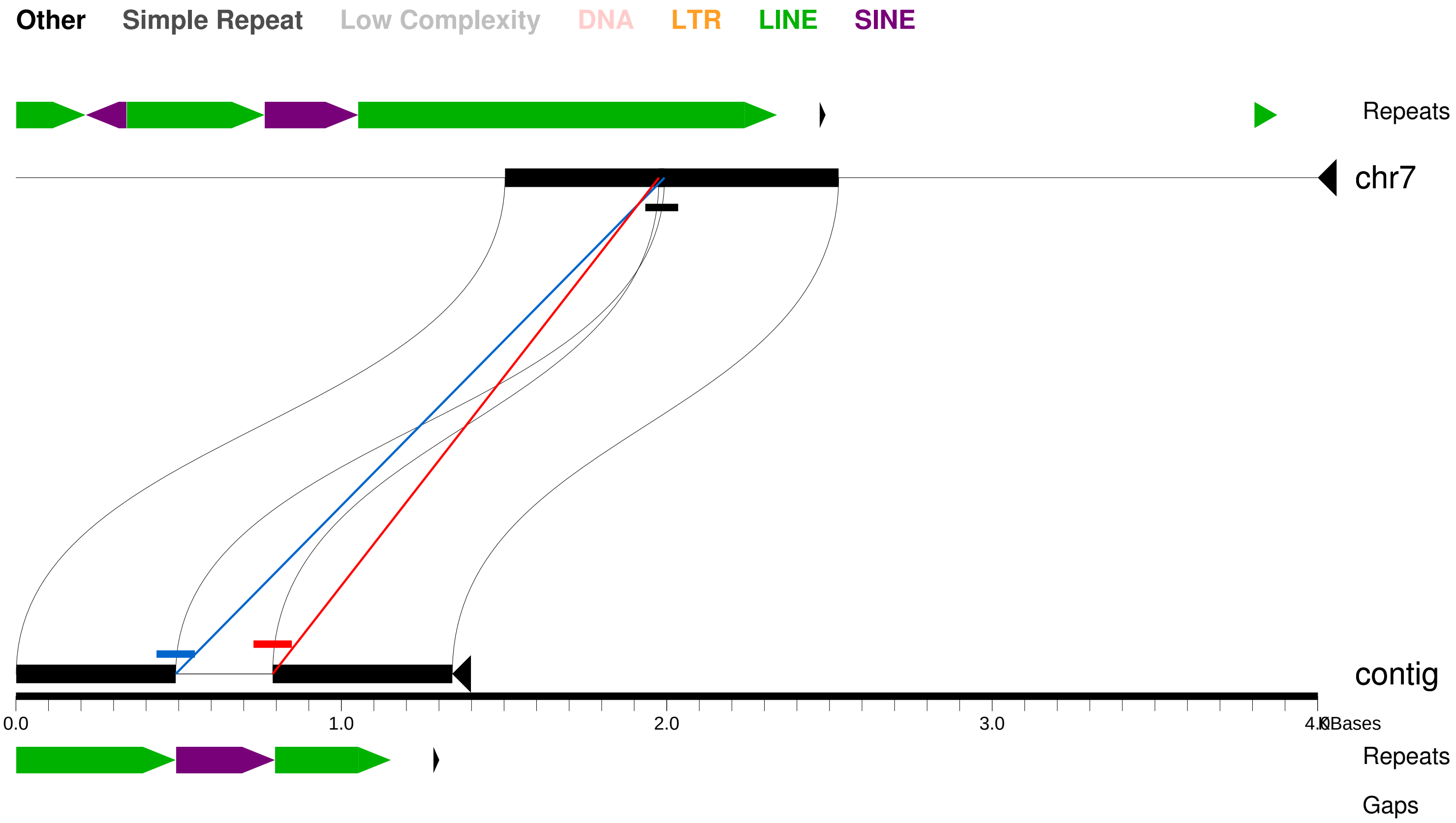

siteID: chr7_92205693 Scafftig1 - Breakpoint: chr7:92205693
chr7:92205648-92205752
Overlap Length: 15 INS size: 217 Genotyped: No

```
left     AATAAATGTTAGCTATCATTATCATGAGACTAGCTCTTCTGCTTT---------------AAAAGACAAAG
chrom    AATAAATGTTAGCTATCATTATCATGAGACTAGCTCTTCTGCTTT---------------AAAAGACAAAG
right    NNNNNNNNNNNNNNNNNNNNNNNNNNNNNNNNNNNNNNNNNNNNNNNNNNNNNNNNNNAAAAAAGACAAAG
         111111111111111111111111111111111111111111111111111111111111***********

left     AGAAGGCCGGGCGCGGTGGCTCACGCCTGTAATCCCAGCACTTTGGGAGGCCGAGGCGGGCGG
chrom    AGAAAACCAA---CATTTACTGAGTACT----TTCTAGATGCTAGGTA-------TTTTGCCA
right    AGAAAACCAA---CATTTACTGAGTACT----TTCTAGATGCTAGGTA-------TTTTGCCA
         ****22**22222*22*22**2*222**2222*2*2**2222*2**2*22222222222**22
```

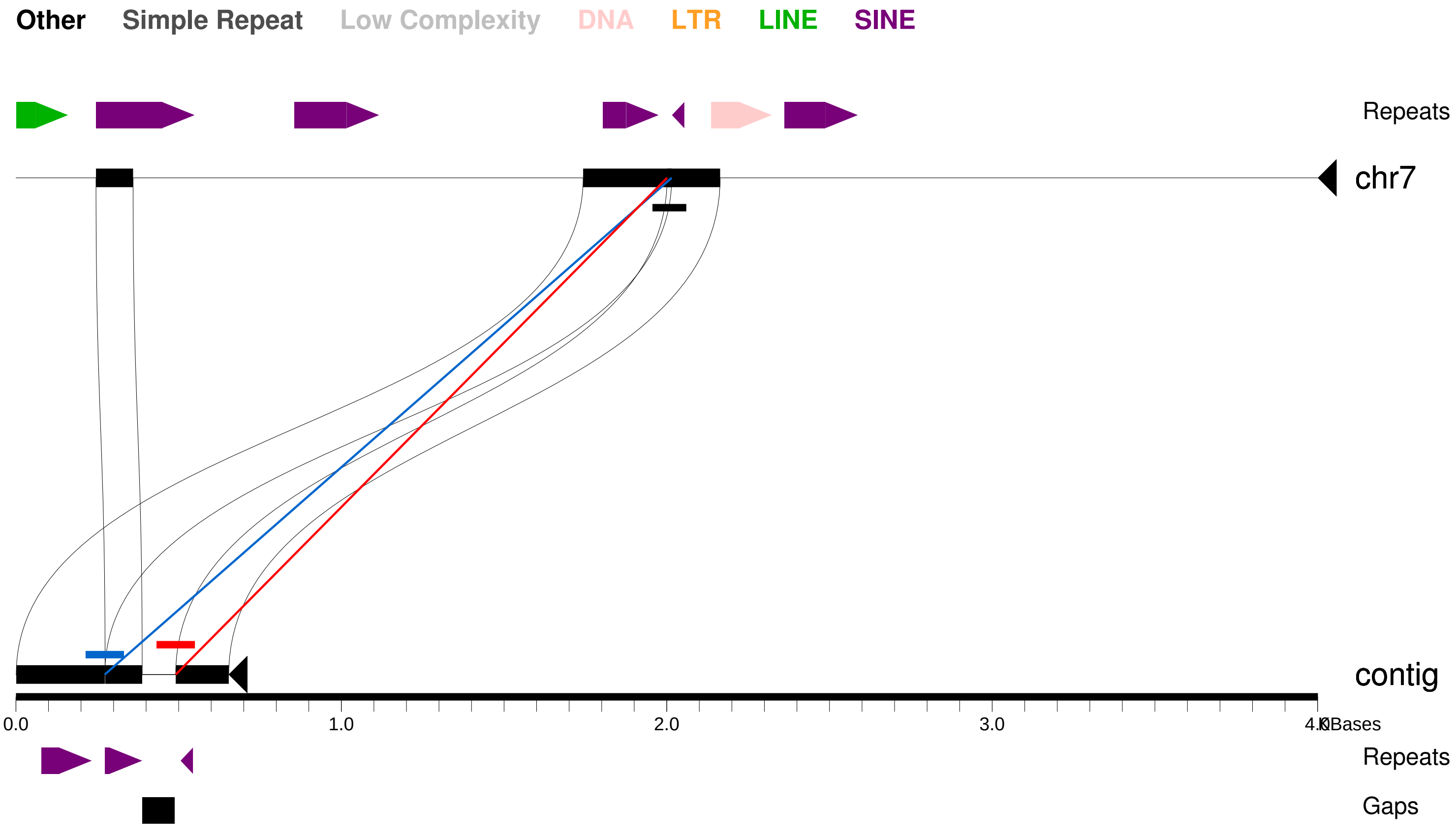

siteID: chr7_93357403 Scafftig1 - Breakpoint: chr7:93357378
chr7:93357218-93357572
Overlap Length: 35 INS size: 433 Genotyped: No

```
left    ------------------------------------------------------------TGTCTACTTT
chrom   CTTTAAAAGATTTGATTCCCTTGACTTCATTACACCCTCATAGTTTTGTTTCCATCTCCCTGTCTACTTT
right   GTAGAGACGGGGT--TTCACCT---TTTTTTTTAGCCG----GGATGGTCTCGATCTCC-TGAC--CTTG
        N2NN2N2N2NNN2  222N2N2   22NN22NN2N22N    2NN2N22N22N222222 **1*11***1

left    ACCTCTGTTAGGCTTTTAACTGTAGGAGTCCTGATGGTTTCACACTTGTCTCCCTTTTCTTCTCTCTTTA
chrom   ACCTCTGTTAGGCTTTTAACTGTAGGAGTCCTGATGGTTTCACACTTGTCTCCCTTTTCTTCTCTCTTTA
right   TGATCCGCCCGCCTCGGC-CTCCCAAAGTGCTGG-GATTACAGGCGTGAGCCAC----------------
        111**1*111*1**11111**11111***1***11*1**1**11*1**111*1*1111111111111111

left    CTTGTTCTGCCTGATTTTTACCATTTCTATGTCTTTTTTTTCATCTATATAGTGGNNNNNNNNNNN----
chrom   CTTGTTCTGCCTGATTTTTACCATTTCTATGTCTTTAATTCCATCTATATAGTGGTTATTCCCAACTTTA
right   CGCGCCCGGCC---------CCATTTCTATGTCTTTAATTCCATCTATATAGTGGTTATTCCCAACTTTA
        *11*11*1***111111111***************22**2**************222222222222222

left    ----------------------------------------------------------------------
chrom   TATCTCTAGCTCAAATTTATCTTCCAGGCTCTAGAAAAAAATATCAAGCATTCTATTATAAATCTCCACC
right   TATCTCTAGCTCAAATTTATCTTCCAGGCTCTAGAAAAAAATATCAAGCATTCTATTATAAATCTCCACC
        2222222222222222222222222222222222222222222222222222222222222222222222

left    ----------------------------------------------------------------------
chrom   ATACATTTCATGGGTACTTCAAAATTACCATGCCCCAAACAGAAGTTATGATCCTCCCATCACACGAGCA
right   ATACATTTCATGGGTACTTCAAAATTACCATGCCCCAAAC------------------------------
        ----------------------------------------
        2222222222222222222222222222222222222222

left    -----
chrom   ACCCC
right   -----
```

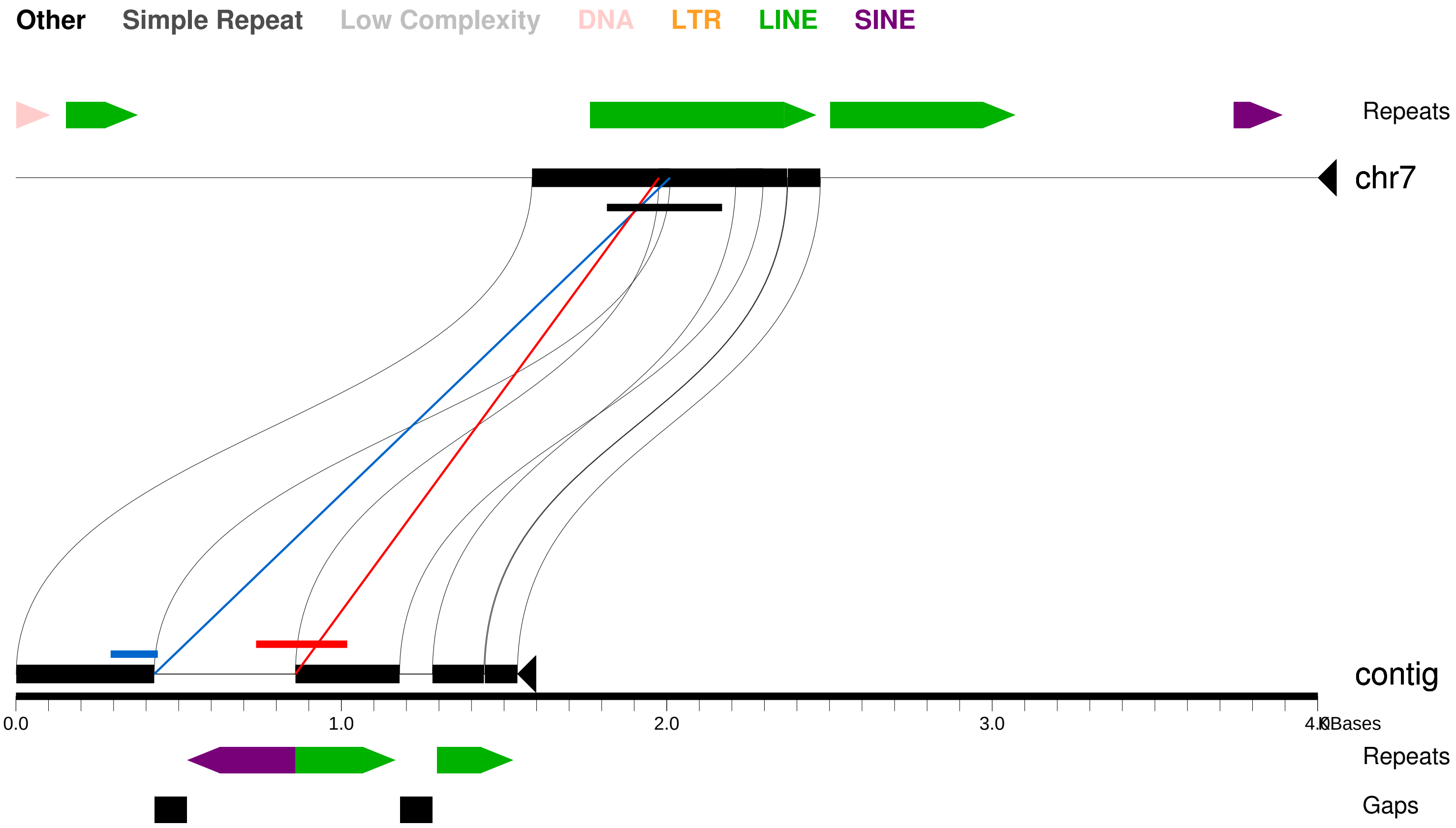

siteID: chr7_94779038 Scafftig3 - Breakpoint: chr7:94779052
chr7:94779006-94779091
Overlap Length: 14 INS size: 314 Genotyped: Yes

```
left    ------ACTACA---------------------AAACTGTGAT-CAAAGTTTTGAAATAATTAATAAATG
chrom   ------ACTACA---------------------AAACTGTGAT-CAAAGTTTTGAAATAATTAATAAATG
right   TGCGCCACTGCAGTCCGCAGTCCGGCCTGGGCGACAGAGCGAGACTCCGTCTCAAAAAAAAAAAAAAAAA
        111111***1**1111111111111111111111*1*11*1**11*111**1*11***1**11**1***11

left    GTAA-----GATATTCTACATTCGGCCGGGCGCGGTGGCTCACGCCTGTA-ATCCCAGCACTTTGGGAGG
chrom   GTAA-----GATATTCTACATTCA-----------TGGATAA-----GAAGATGTCAGTTCTT-------
right   AAAAAAAAAGATATTCTACATTCA-----------TGGATAA-----GAAGATGTCAGTTCTT-------
        11**11111*************222222222222***2*2*22222*2*2**22***22***2222222

left    CCGAGGCGGGTGG
chrom   CC-----------
right   CC-----------
        **22222222222
```

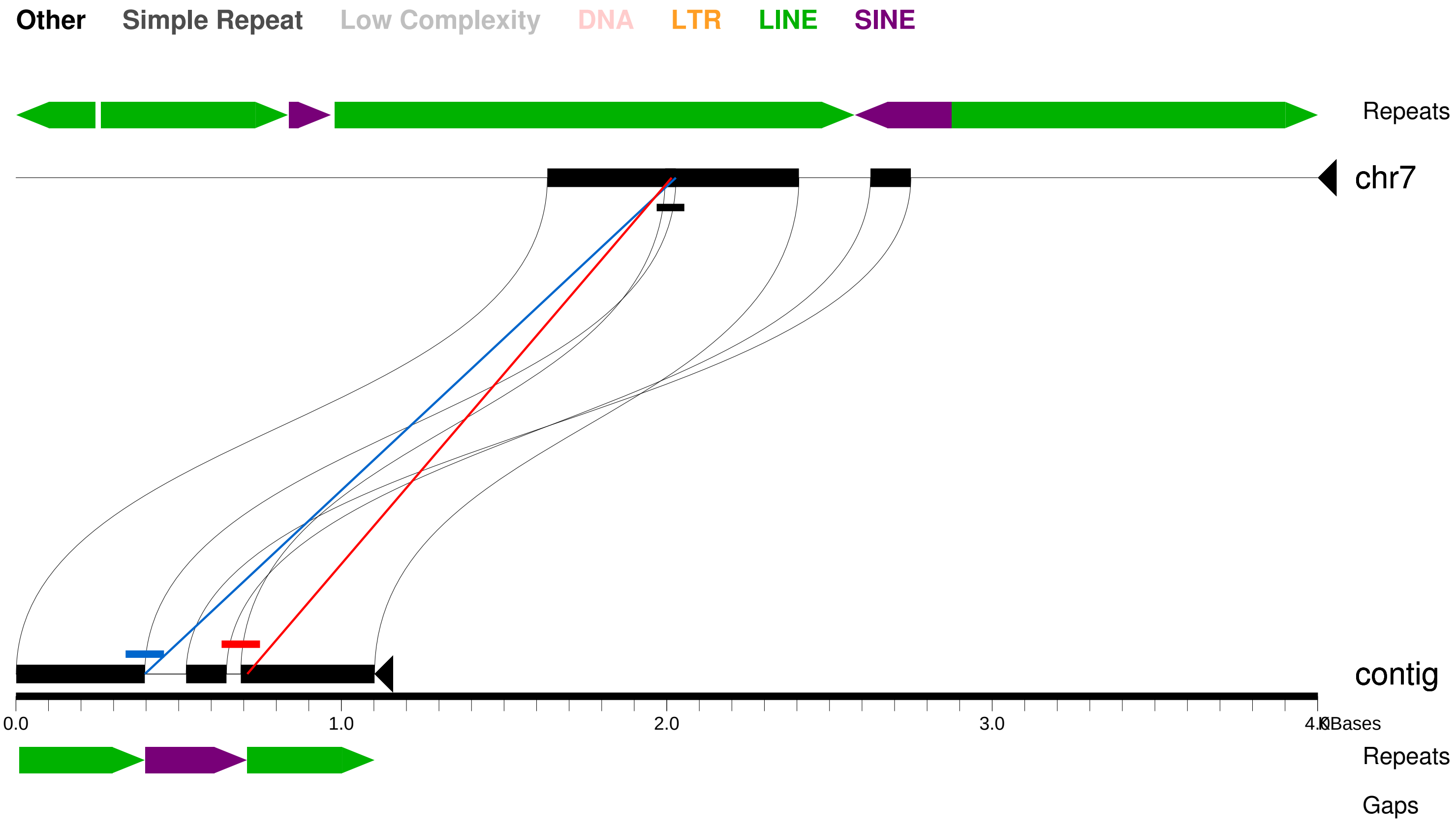

siteID: chr7_94877494 Scafftig1 - Breakpoint: chr7:94877499
chr7:94877464-94877558
Overlap Length: 10 INS size: 273 Genotyped: Yes

```
left   TGTT---TAGTTTTATCAA-ATATACAATTTCAAAGATT--------------------TAAAAATATAT
chrom  TGTT---TAGTTTTATCAA-ATATACAATTTCAAAGATT--------------------TAAAAATATA-
right  TGTTAGCCAGGATGGTCTCGATCTCCTGACCTCATGATCCGCCCGCCTCGGCCTCCCAATAAAAATATA-
       ****1111**11*11**111**1*1*1111111*1***111111111111111111111**********2

left   ATTTTTTTTTTTTTTTTTTTTTTTTTTTTTGAGACGGAGTCTCGCTCTGTCGCCCAGGCTGGAGTGCAGTG
chrom  -------------TTTTAAATTACTTTTAAAGCAATATTCTTTAT-TGT---------TTCAATGGTCTT
right  -------------TTTTAAATTACTTTTAAAGCAATATTCTTTAT-TGT---------TTCAATGGTCTT
       2222222222222****222**22****22**2222*2***222*2***222222222*22*2**222*2

left   GCG
chrom  TTA
right  TTA
       222
```

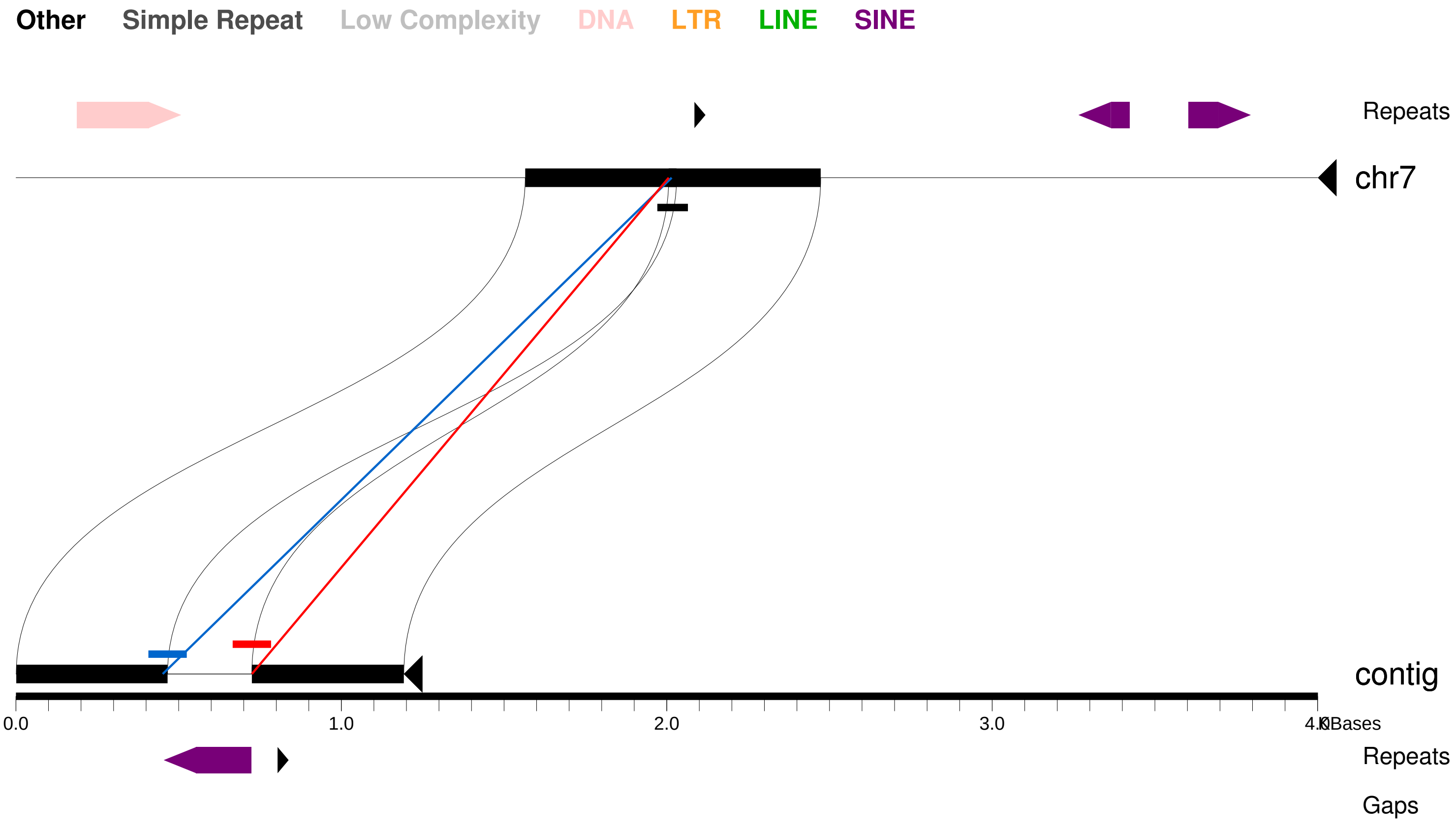

siteID: chr7_95101200 Scafftig1 + Breakpoint: chr7:95101183
chr7:95101133-95101242
Overlap Length: 10 INS size: 286 Genotyped: Yes

```
left    --------AT--TTTATGTGTGCTAACACTATGCTTTCTTAGGCAGGTACTATTATTATCTGCTTTTTTTT
chrom   --------AT--TTTATGTGTGCTAACACTATGCTTTCTTAGGCAGGTACTATTATTATCTGCTTTTTTTT
right   TAGCCAGGATGGTCTCGATCTCCTGACCTCATGATCCACCCGCCTCGGCCTCCCAAAGT-TGCTTTTTTTT
        11111111**11*1*111*1*1**1**111***1*111111*1*11*11**111*111*1**********

left    TTTTTTTTTTTTTTTTTTTTTTTTTTTTTTTTTTGAGACGGAGTCTCGCTCTGTCGCCCA----
chrom   AACAGATGAAGAAACC--------------GAGCCACACAGAGGTTAAATAACTTGCCCAAGGG
right   AACAGATGAAGAAACC--------------GAGCCACACAGAGGTTAAATAACTTGCCCAAGGG
        222222*2222222222222222222222222222*2**2***22*222*222*2*****2222
```

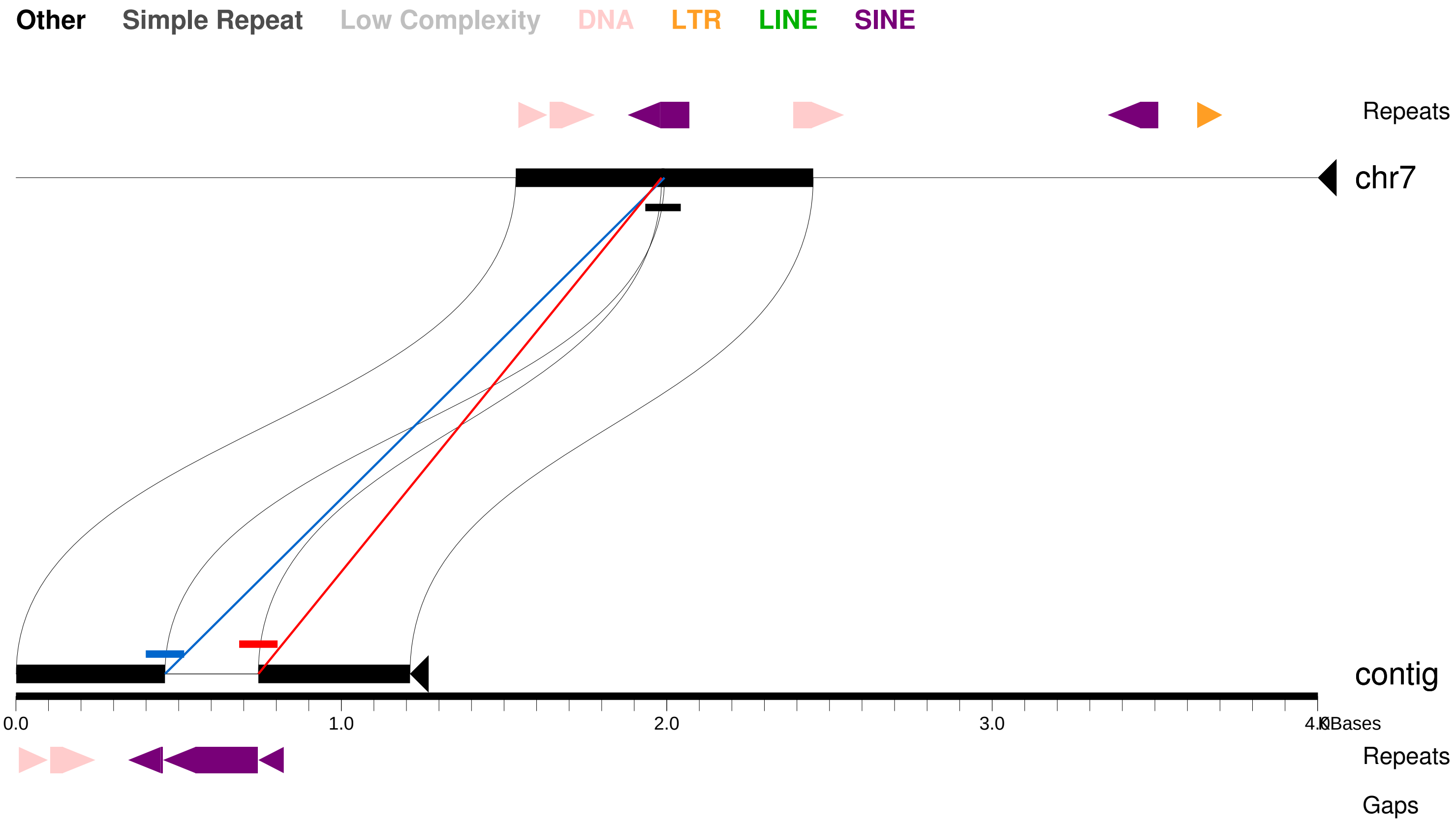

siteID: chr7_95264771 Scafftig1 + Breakpoint: chr7:95264780
chr7:95264739-95264839
Overlap Length: 5 INS size: 311 Genotyped: Yes

```
left    TT-----CAAAAGCCCATCACCTTAAT--AAATAAATGTTTGGATTTT-----------TGCATTTTTTTT
chrom   TT-----CAAAAGCCCATCACCTTAAT--AAATAAATGTTTGGATTTT-----------TGCAT-----T
right   CCGCCCGCCTCGGCCTCCCAAAGTGCTGGGATTACAGGCGTGAGCCACCGCGCCCGGCCTGCAT-----T
        1111111*1111***111**111*11*111*1**1*1*11**111111111111111111*****22222*

left    TATTTTTTTTTTTTTTTTTTTTTGAGACGGAGTCTCGCTCTGTCGCCCAGGCTGGAGTGCAGTGGCG
chrom   TATTCTTTATTTTAATCT------AGATGAATGCTTAATTCCTCTTACA---TTTAAT-CATAA---
right   TATTCTTTATTTTAATCT------AGATGAATGCTTAATTCCTCTTACA---TTTAAT-CATAA---
        ****2***2****22*2*222222***2*2*22**222*222**222**222*22*2*2**222222
```

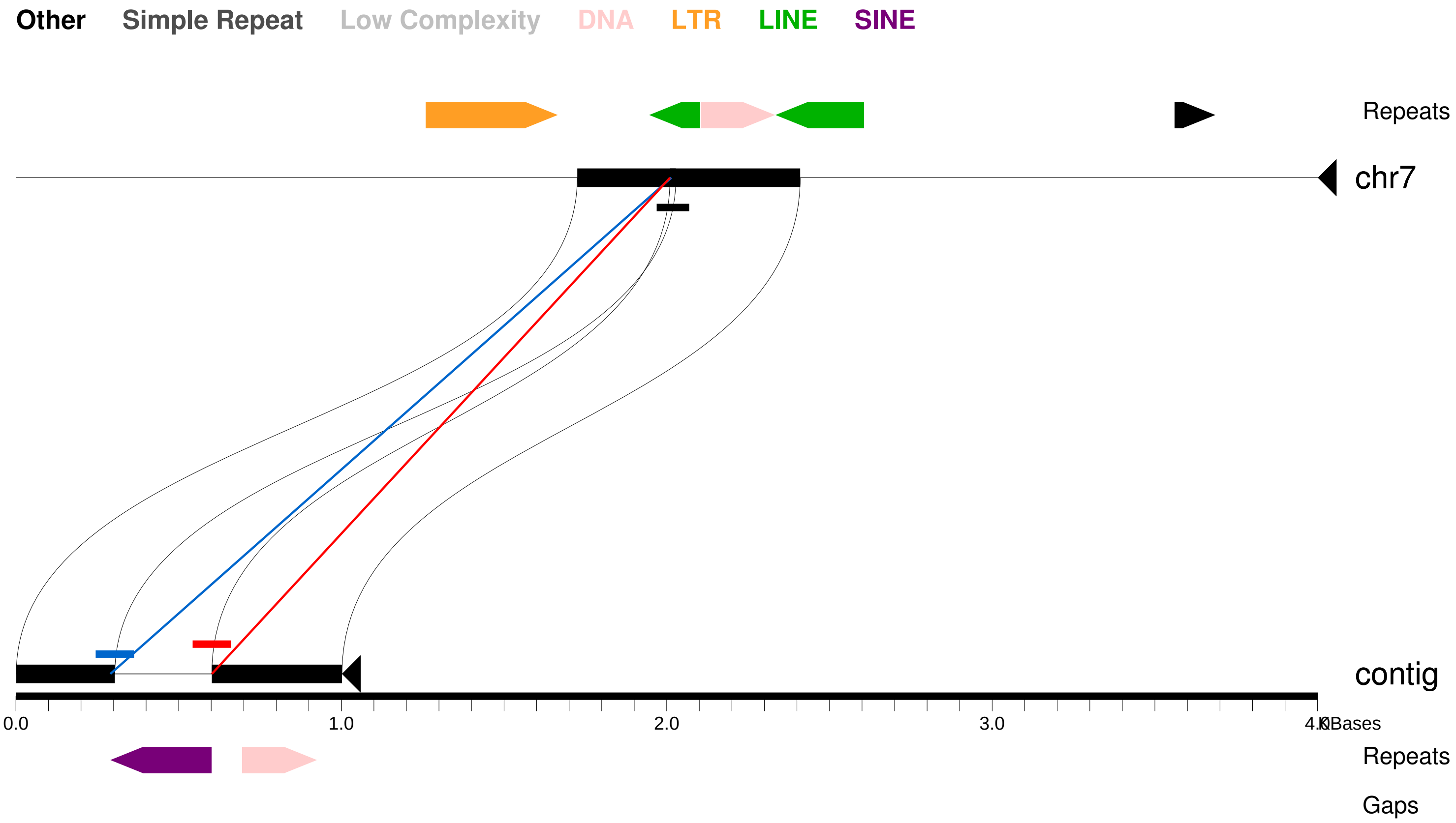

siteID: chr7_97067530 Scafftig10 + Breakpoint: chr7:97067522
chr7:97067479-97067581
Overlap Length: 17 INS size: 301 Genotyped: Yes

```
left     AT-GACATCTGTATACATGCAAAATGAAACAAT--CTGGCAATATA--------------CATTTATTACT
chrom    AT-GACATCTGTATACATGCAAAATGAAACAAT--CTGGCAATATA--------------CATTTATTACT
right    TCCGCCCGCCTCGGCCTCCCAAAGTGCTGGGATTACAGGCGTGAGCCACCGCGCCCGGCCATTTATTACT
         111*1*11*111111*111****1**11111**11*1***111*111111111111111***********

left     ATATTTTTTTTT----TTTTTTTTTTTTGAGACGGAGTCTCGCTCTGTCGCCCAGGCTGGAGTGCAGTG
chrom    ATATTTGACAAAGAAATTCTTAAATTATAATGTGAAACCTCATTTTATA--------------------
right    ATATTTGACAAAGAAATTCTTAAATTATAATGTGAAACCTCATTTTATA--------------------
         ******2222222222**2**222**2*2*222*2*22***22*2*2*222222222222222222222
```

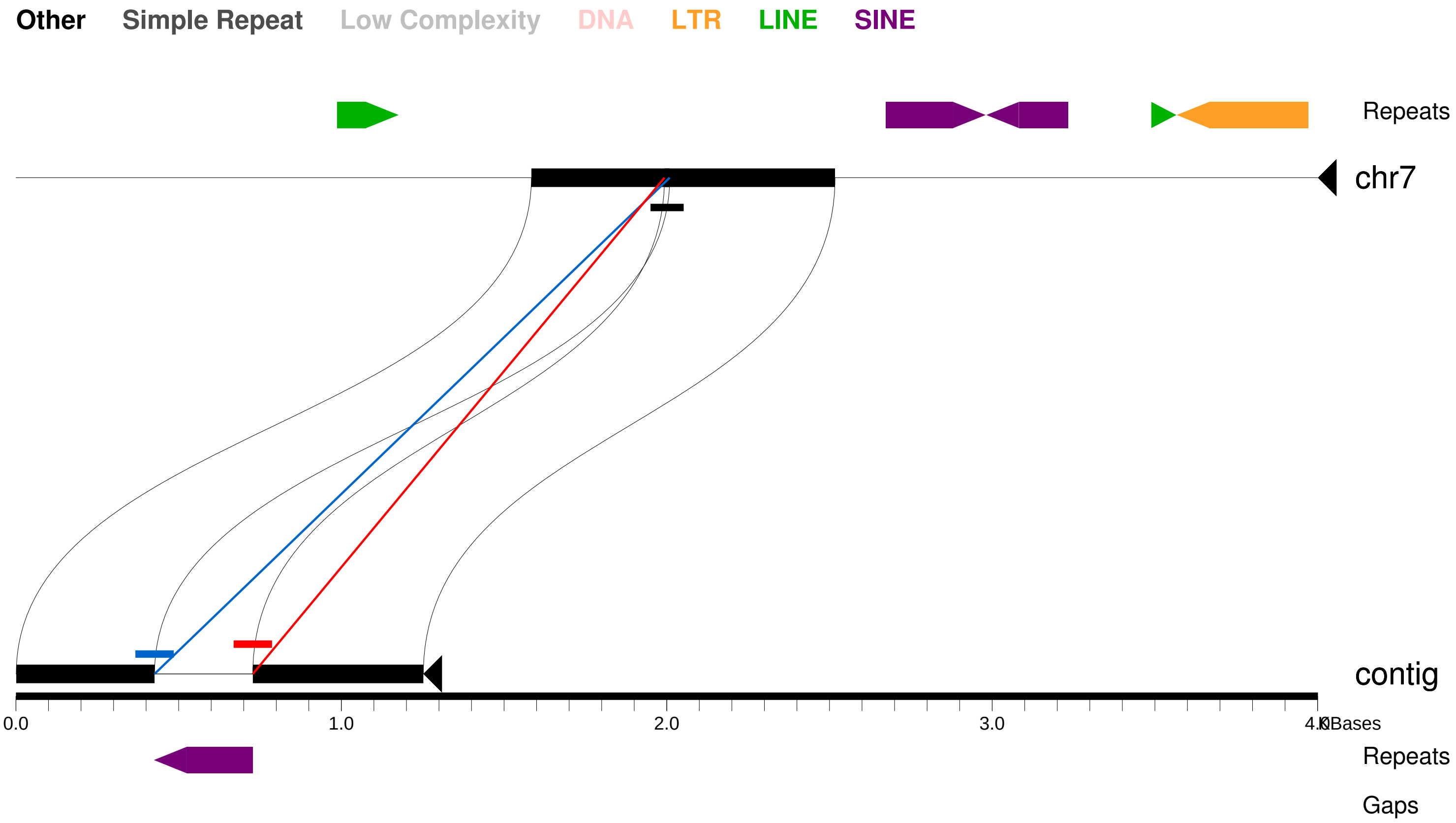

siteID: chr7_gl000195_random_56203 Scafftig5 - Breakpoint: chr7_gl000195_random:56179
chr7_gl000195_random:56019-56355
Overlap Length: 17 INS size: 313 Genotyped: No

```
left   ------------------------------------------TATTGAGTGACTCAGTTTTTTATTGAAT
chrom  CT-ATGACAGAGGAAGTCTTCACAAAGTCTCCCAGTACTAGGTATTGAGTGACTCAGTTTTTTATTGAAT
right  TTTAGTAGAGACGGGGT-TTCACCTTGTTAGCCAGGAT--GGTCTCGATCTCCTGACCTCATGATCCACC
       N212NN2N222N2NN22 22222NNN22NNN2222N2N  22*1*1**1111**1*11*11*1**11*11

left   AAAATGGAATACTTCCTGATGCCAGTACTATGGCCCTTCGGTTTTGAGGAAAATATCATCTTGTATGTTG
chrom  AAAATGGAATACTTCCTGATGCCAGTACTATGGCCCTTCGGTTTTGAGGAAAATATCATCTTGTATGTTG
right  CGCCTCGG---CCTCCCAA----AGTGCT---------CGGATTACAGGC----------------GTGA
       1111*1*1111*1***11*1111***1**111111111***1**11***1111111111111111**11

left   GCTAACAAGGAGATAGGAGTTCAAATCAAATTTGTTTTTTTGTTTT---TTTGTTTTTTTTTTTTGAGAC
chrom  GCTAACAAGGAGATAGGAGTTCAAATCAAATTTGTTTTGTCATACTGGCTTTAAGGCAGTGATTAGAAAA
right  GCCACC---GCGCCTGGCCT-CAAATCAAATTTGTTTTGTCATACTGGCTTTAAGGCAGTGATTAGAAAA
       **1*1*111*1*111**11*1****************2*22*22*222***2222222*22**2**2*2

left   GG------AGTCTCGCTCTGTCGCCCAG-GCTGGACTGCGGACTGCAGTGGCGCAATCTCTGCT-CAC-T
chrom  GGCCTAATAGGTGGGTTCTGTAGGGGATTGCTGGAAGGAAAGTAGGAATATGGAAAGTCATGAGACATAT
right  GGCCTAATAGGTGGGTTCTGTAGGGGATTGCTGGAAGGAAAGTAGGAATATGGAAAATCATGAGACATAT
       **222222**2222*2*****2*222*22******22*222222*2*2*222*2**N222**222**22*

left   GCAAGCTCCGCTTCCCGGGTTCACGCCATTCTCCTGCC---TCAGCCTCCCA-AGTAGC
chrom  ACAGTCATCTCTTCTTGTTTCCTCACAGGTCACATACAAATTCAGGGAGAGTTAGTAT-
right  ACAGTCATCTCTTCTTGTTTCCTCACAGGTCACATACAAA-------------------
       2**22*22*2****22*22*2*2*2*222**2*2*2*222 1111NNNNNNN 1111N2
```

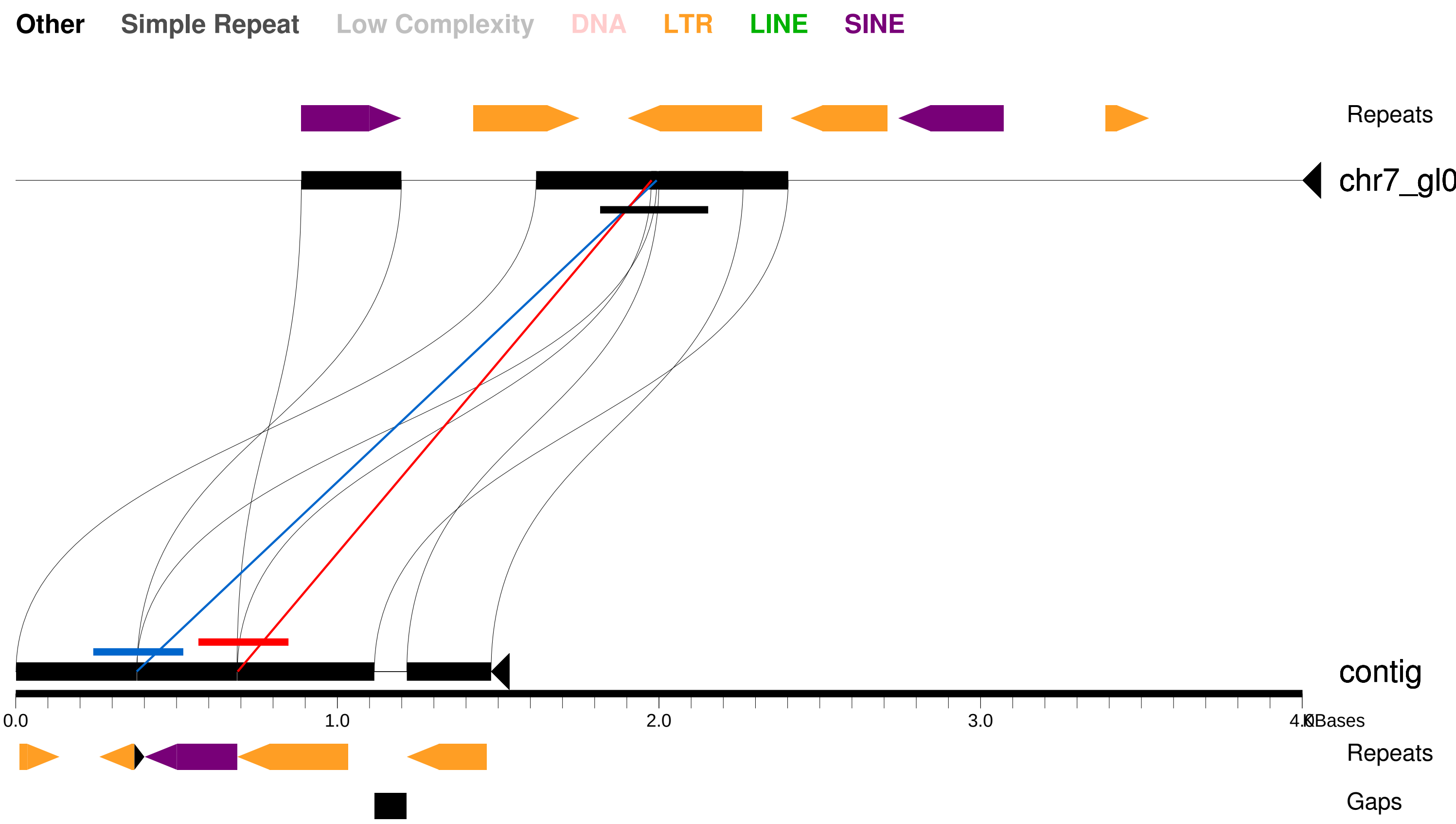

siteID: chr8_10132606 Scafftig1 + Breakpoint: chr8:10132592
chr8:10132549-10132651
Overlap Length: 13 INS size: 315 Genotyped: Yes

```
left    AGGACAT-----GACGTGCAAG-TGCATTATTTTCATCAGTTTAGATAT-----------GAGAATAATT
chrom   AGGACAT-----GACGTGCAAA-TGCATTATTTTCATCAGTTTAGATAT-----------GAGAATAATT
right   CCGCCCGCCTCGGCCTCCCAAAGTGCTGGGATTACAGGCGTG-AGCCACCGCGCCCGGCCGAGAATAATT
        11*1*1111111*1*111***21***11111**1**111**11**11*111111111111**********

left    CTTTTTTTTTTTTTTTTTTTTTTTTTTTTTTTTTTTTGAGACGGAGTCTCGCTCTGTCGCCCAGGC
chrom   CTTATTTGCTGCCTTTCATATTCCAGGTGTCCTTTTT---------TCTGAAAATTTCT-------
right   CTTATTTGCTGCCTTTCATATTCCAGGTGTCCTTTTT---------TCTGAAAATTTCT-------
        ***2***22*222***22*2**22222*2*22*****222222222***22222*2**22222222
```

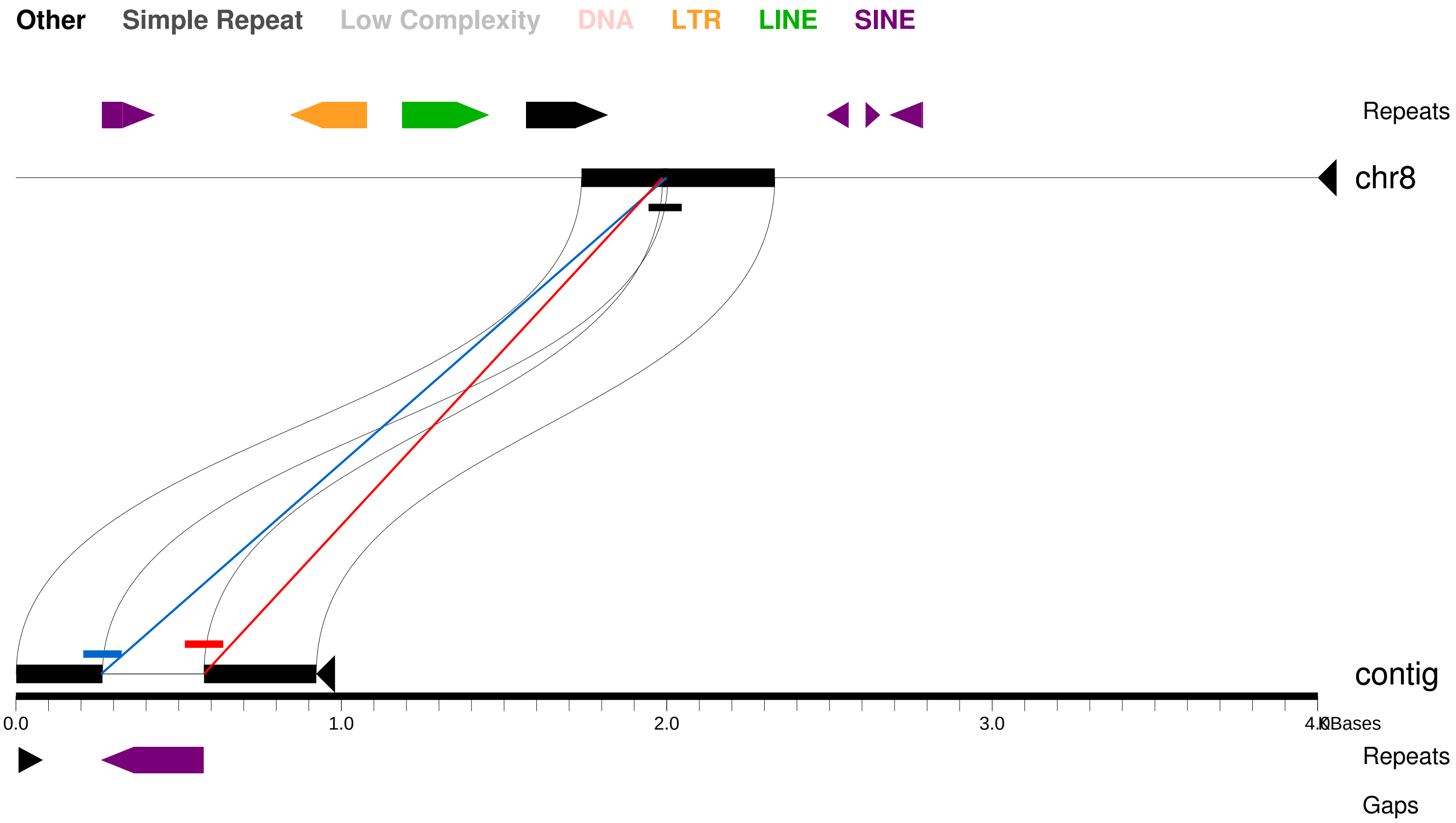

siteID: chr8_106664528 Scafftig4 - Breakpoint: chr8:106664512
chr8:106664352-106664682
Overlap Length: 11 INS size: 319 Genotyped: Yes

```
left   TT------ACTGGGTGGTTCTTTTTGGACCCACTTTACTGGTAAATAGTAACAGTGTCTGTTTC-AGCTC
chrom  TTGCTATTACTGGGTGGTTCTTTTTGGACCCACTTTACTGGTAAATAGTAACAGTGTCTGTTTC-AGCTC
right  TT--TAGTAGAGACGGGGTTT-------CACCGTTT------------TAGCCGGGATGGTCTCGATCCC
       **  22N2*11*111**1*1*1111111*1*11***111111111111**1*1*1*111**1**1*1*1*

left   TTCACTAGGGGAAGGTCAGTCAGAAACCTCTACTCTTTGCTCTAATACCTGAAATTGGAGGCTTTTCATT
chrom  TTCACTAGGGGAAGGTCAGTCAGAAACCTCTACTCTTTGCTCTAATACCTGAAATTGGAGGCTTTTCATT
right  CTGACCTCGTGA---TCCGCCCG---CCTCGGC-CT---CCCAAAGTGCTGGGATTACAGGCGTGAG---
       1*1**111*1**111**1*1*1*111****11*1**111*1*1**111***11***11****1*111111

left   TTACCACTGATGTAGAAGTCAAGCCCCTTTTT------TTTTTTGAGACGGA----GTCTCGCT------
chrom  TTACCACTGATGTAGAAGTCAAGCCCATTTTTGAAGCCTATTTTTAAAATTAAAACGTCACCTTATATGC
right  ---CCACCGCGCCCGG---CCAGCCCATTTTTGAAGCCTATTTTTAAAATTAAAACGTCACCTTATATGC
       111****1*11111*1111*1*****2*****222222*2****2*2*222*2222***2*22*222222

left   CTGTCGCCCAGG----CTGGAGTGCAGTGGCAGGATCTCG--GCTCACTGC--------AAGC---TCCG
chrom  CTGACATACAGTAGTACTTCAGAGAAGTTGTATTCTCATGAGGCTGTCTGAGGCCTGTGAAGACTGTCAA
right  CTGACATACAGTAGTACTTCAGAGAAGTTGTATTCTCATGAGGCTGTCTGAGGCCTGTGAAGACTGTCAA
       ***2*222***22222**22**2*2***2*2*222**22*22***22***222222222***2222**22

left   CCTCCCG--GGTTCACGCCATTCTCCTGCCTCAGCCTCCCAAG---------
chrom  ACAACAGTAGGGTCAACCAAATAGTATACCATGGTGTTGCAAGCAAGAGTAT
right  ACAACAGTAGGGTCAACCAAATAGTATACCATGGTGTTGCA-----------
       2*22*2*22**2***22*2*2*2222*2**222*22*22**11
```

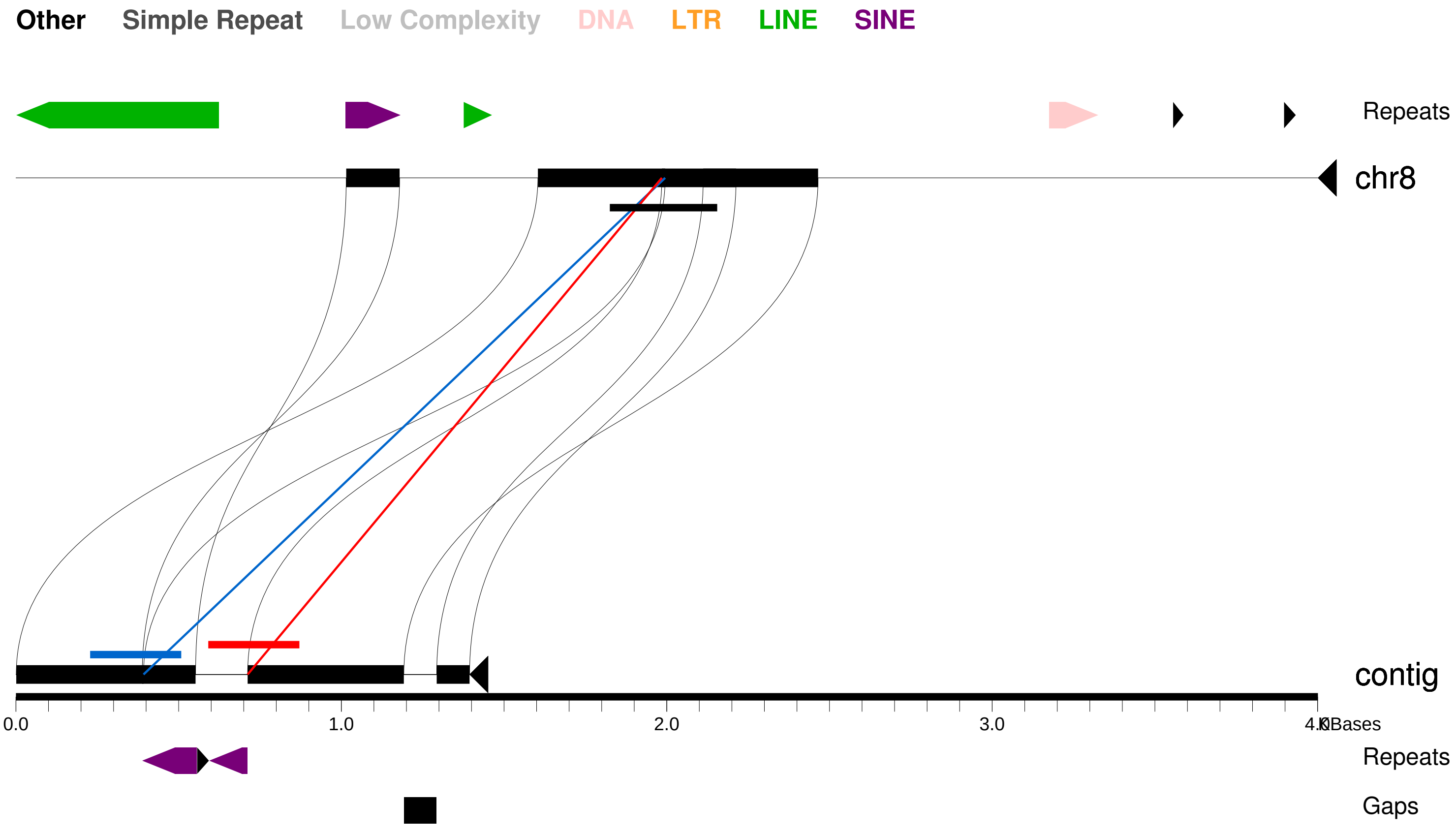

siteID: chr8_106920752 Scafftig1 + Breakpoint: chr8:106920743
chr8:106920694-106920802
Overlap Length: 2 INS size: 419 Genotyped: No

```
left     TTCTGTGGGTTTGCCGCTCCTATGATAATTACTCCTATAAGATGTCACT----------CTTGGCCGGGC
chrom    TTCTGTGGGTTTGCCGCTCCTATGATAATTACTCCTATAAGATGTCACT----------CTAG--CAGGG
right    NNNNNNNNNNNNNNNNNNNNNNNNNNNNNNNNNNNNNNNNNNNNNNNNNNNNNNNNNNNNCTAG--CAGGG
         111111111111111111111111111111111111111111111111111111111111**2*22*2**2

left     GCGGTGGCTCACGCCTGTAATCCCAGCACTTGGGGAGGCCGAG-GCGGGCGGATCACGAG
chrom    GCAGTTGCTT---CTTGTAG---CAGCACCTGGGCA--CCGTTTGTAGTTTGCTCAAT-G
right    GCAGTTGCTT---CTTGTAG---CAGCACCTGGGCA--CCGTTTGTAGTTTGCTCAAT-G
         **2**2***2222*2****2222******2****2*22***222*22*222*2***222*
```

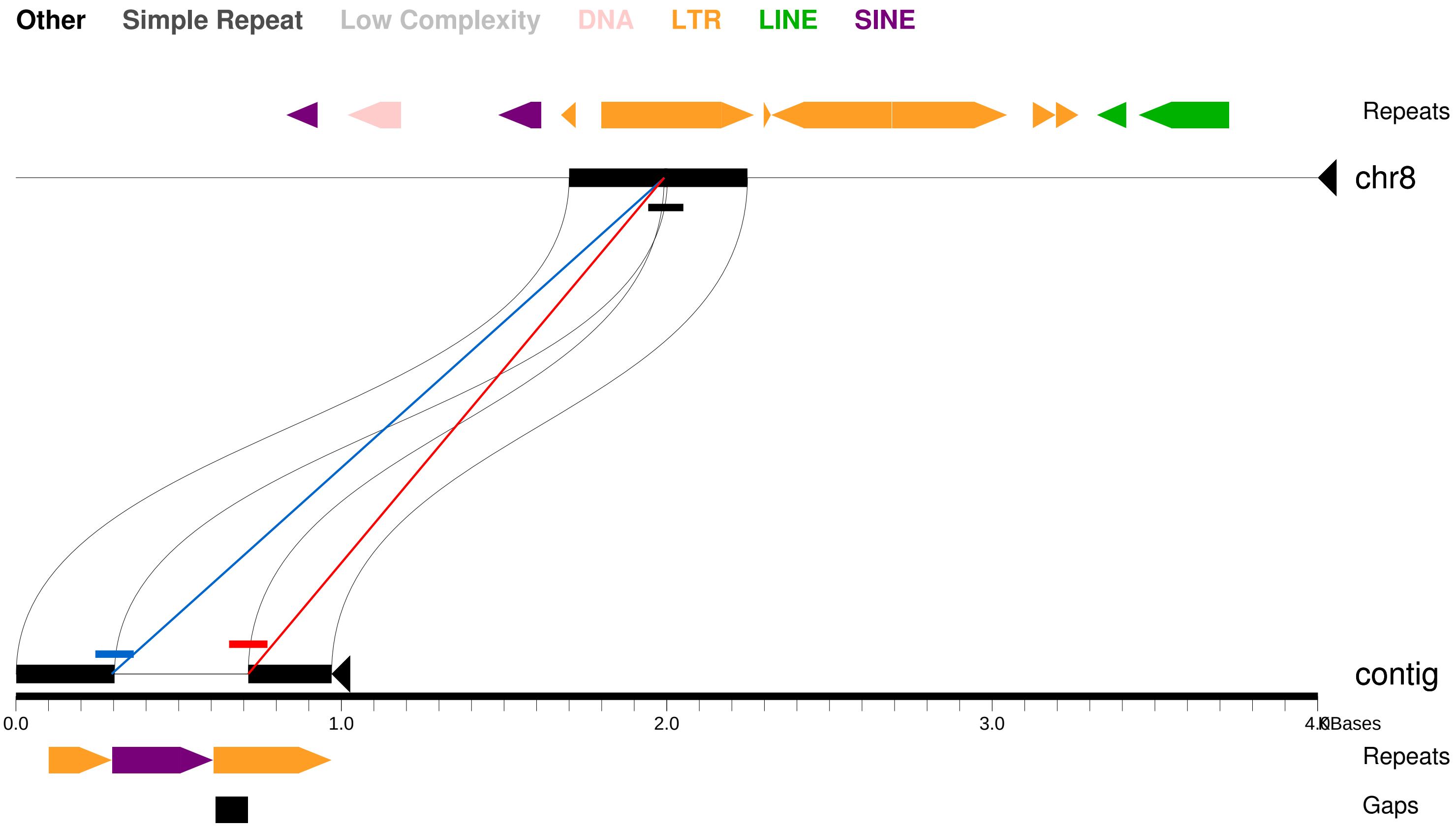

siteID: chr8_107207895 Scafftig8 + Breakpoint: chr8:107207879
chr8:107207829-107207938
Overlap Length: 10 INS size: 302 Genotyped: Yes

```
left   GTCTTCTT--TCTGAATTC--ATTTTTTG----TCCA--CTTGAATTTTC-CTCCTGTTAAGACACTTTT
chrom  GTCTTCTT--TCTGAATTC--ATTTTTTG----TCCA--CTTGAATTTTC-CTCCTGTTAAGACACTTTT
right  TCCACCCGCCTCGGCCTCCCAAAGTGCTGGGATTACAGGCGTGAGCCACCGCGCCCGGC--GACACTTTT
       11*11*1111**1*11*1*11*11*11**1111*1**11*1***11111*1*1**1*1111*********

left   TTTTTTTTTTTTTTTTGAGACGGAGTCTCGCTCTGTCGCCCAGGCCGGACTGCGGACTGC
chrom  TAACTTAATACAGAT-GAGCCATTG-CTTTCTCTGTCTAGT-GTCC------CTAATTTA
right  TAACTTAATACAGAT-GAGCCATTG-CTTTCTCTGTCTAGT-GTCC------CTAATTTA
       *222**22*22222*2***2*222*2**22******22222*2**222222*22*2*22
```

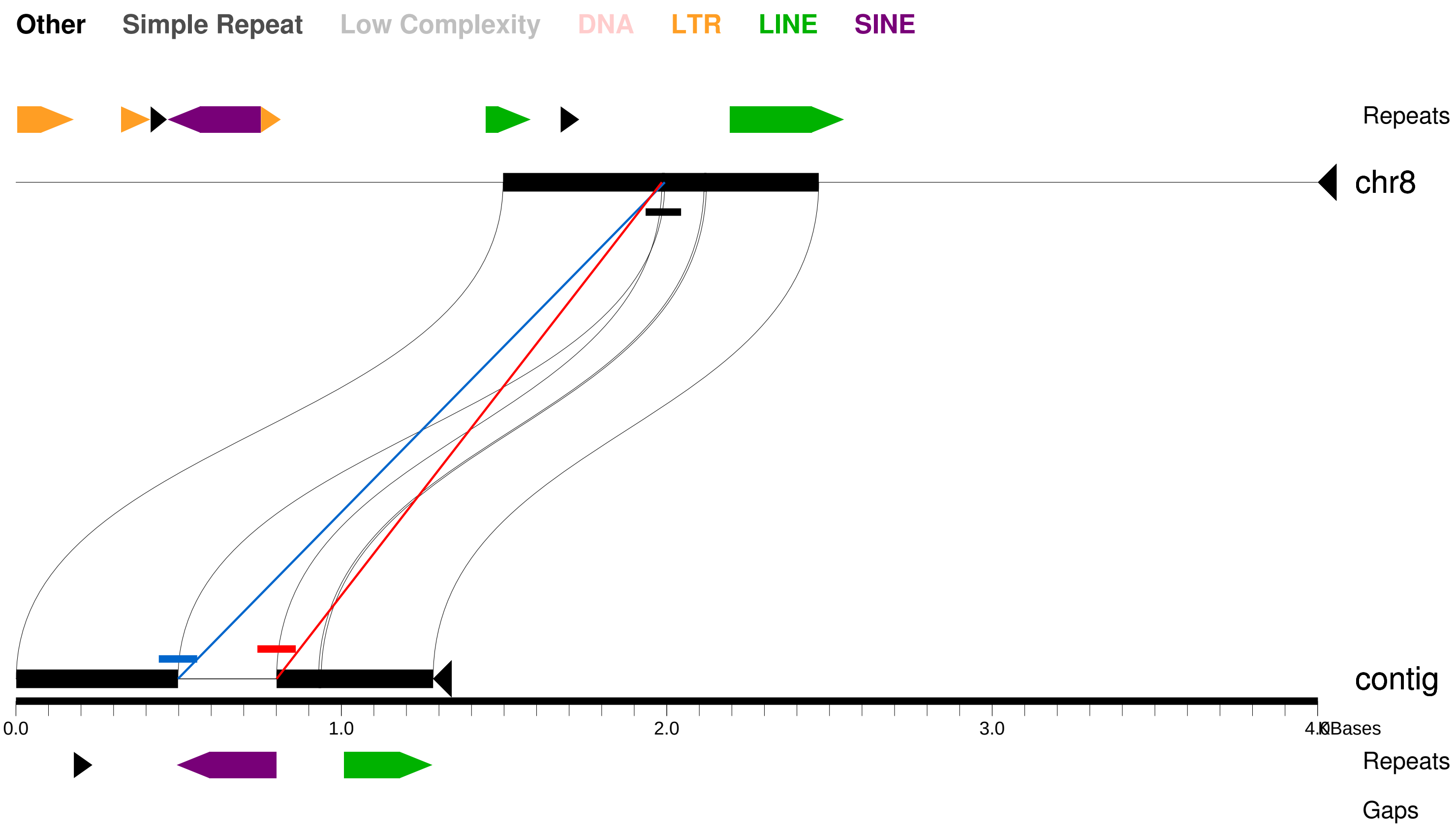

siteID: chr8_107465198 Scafftig1 + Breakpoint: chr8:107465111
chr8:107464951-107465287
Overlap Length: 17 INS size: 259 Genotyped: No

```
left   C-------------------------------------------------CCAGGATTCTTTCTATGTGG
chrom  CTTGTCCTTTGCTTTTTGTTGTTTGTTTTGAAACTTGTTGAAAATTCTTCCCAGGATTCTTTCTATGTGG
right  -------------TTTAGTAGA-------GACGGGGTTTCACCGTTTTAGCCAGGATGGTCTCGATCTCC
       1            222N22N2N       22NNNNNN22N2NNN22N2NN*******11*1**1**1*11

left   AATCCAATTTA-CAGTACATTTTAAGGGCCTTTTTAAAAAACATAAAATGAAACATAGGCAATCCTTTAG
chrom  AATCCAATTTA-CAGTACATTTTAAGGGCCTTTTTAAAAAACATAAAATGAAACATAGGCAATCCTTTAG
right  TGACCTCGTGATCCGCCCGCCTC---GGCCT----------CCCAAAGTGCT-----GGGATTACAGGCG
       111**111*1*1*1*11*111*1111*****1111111111*11***1**1111111**1*1*1*1111*

left   TGGATCTGTCCTGTTTTAGGACTAAATTGATATCTTTT--------------------------------
chrom  TGGATCTGTCCTGTTTTAGGACTAAATTGATATCTTTTAATAGATGTAAGGCCTTTTGGATTATCTGCCC
right  TGAGCCA---CCGCGCCCGGCCTAAATTGATATCTTTTAATAGATGTAAGGCCTTTTGGATTATCTGCCC
       **111*1111*1*11111**1*****************22222222222222222222222222222222

left   ----------------------------------------------------------------------
chrom  CGAACTTCTCCCACTATTGATAGGCATCTTGTACTAAGCCATAGCCATATATTGAAAATTGCCCCAAATG
right  CGAACTTCTCCCACTATTGATAGGCATCTTGTACTAAGCCATAGCCATATATTGAAAATTGCCCNNNNNN
       2222222222222222222222222222222222222222222222222222222222222222NNNNNN

left   ---------------------TTTTTTTNNNNNNNNNNN-------------------
chrom  TGCTCTGCTCTTTCTCACCTCTTTGTTTTTGCACAAGCTTACCTCTGCCCAGGATGCC
right  NNNNNNNNNNNNNNNNNNNNNNNNNNNNNNNNNNNNNNNNNN----------------
       NNNNNNNNNNNNNNNNNNNNN111N111NNNNNNNNNNNNNN
```

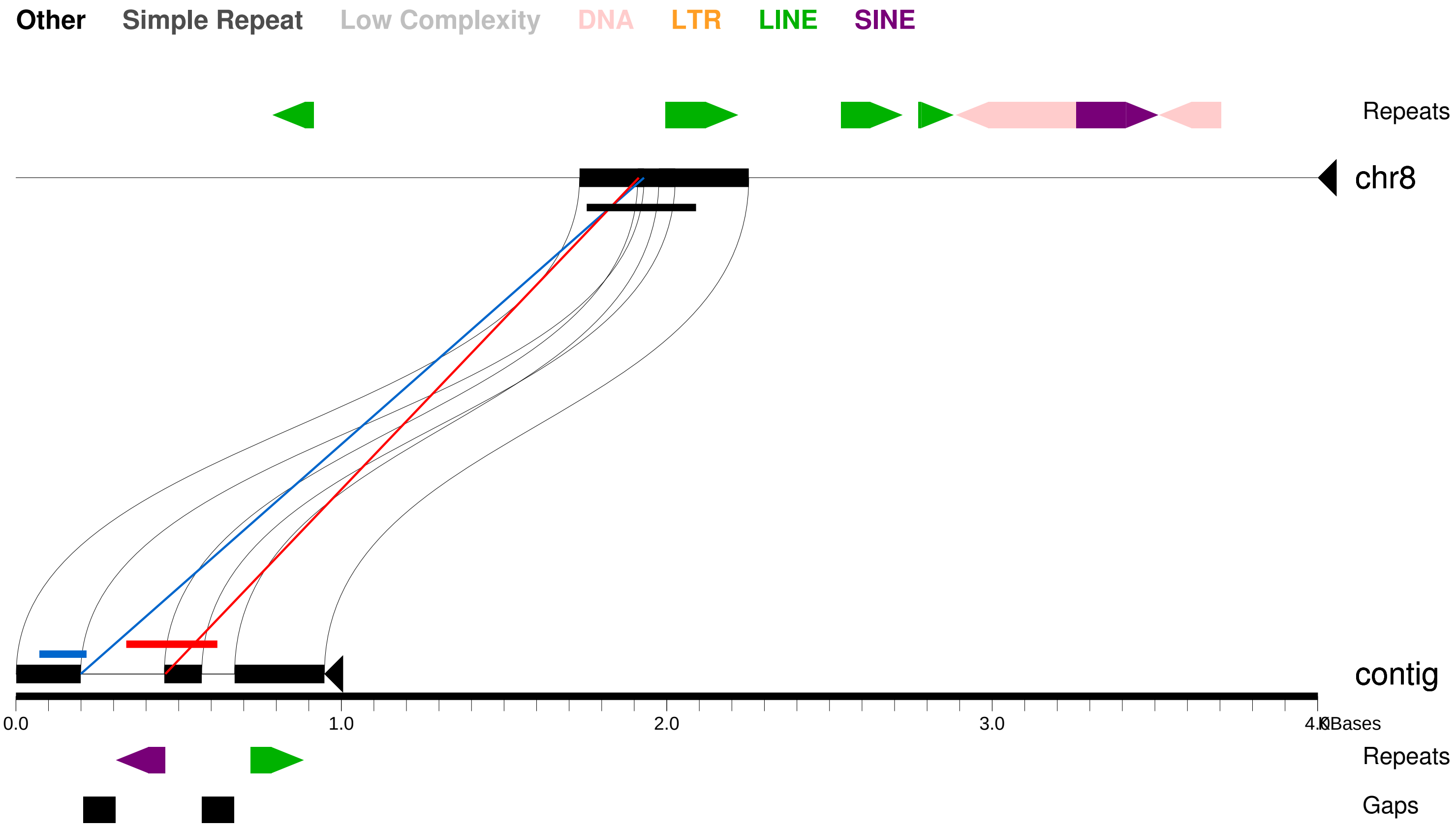

siteID: chr8_108277409 Scafftig1 - Breakpoint: chr8:108277360
chr8:108277310-108277419
Overlap Length: 10 INS size: 444 Genotyped: No

```
left   CCCATCTGTTTAATATGGCA---TTCTTCGAAGAACCTCTCTTACTATCTAGA------AACATTTCTTT
chrom  CCCATCTGTTTAATATGGCA---TTCTTCGAAGAACCTCTCTTACTATCTAGA------AACATTTCTTT-
right  CCACCCGCCTCGGCCTCCCAAAGTGCTGGGACTACAGGCGTGAGCCACCGCGCCCGGCCAACATTTCTT-
       **111*111*11111*11**111*1**11**11*1111*11111*1*1*11*1111111**********2

left   NNNNNNNNNNNNNNNNNNNNNNNNNNNNNNNNNNNNNNNNNNNNNNNNNNNNNNNNNNNNN
chrom  --------ATATAAAACTTGCATTAACCTAGATCAGTGGCCCTCTAATGGATGTGCAT
right  --------ATATAAAACTTGCATTAACCTAGATCAGTGGCTCTCTAATGGATGTGCAT
       2222222222222222222222222222222222222N2222222222222222
```

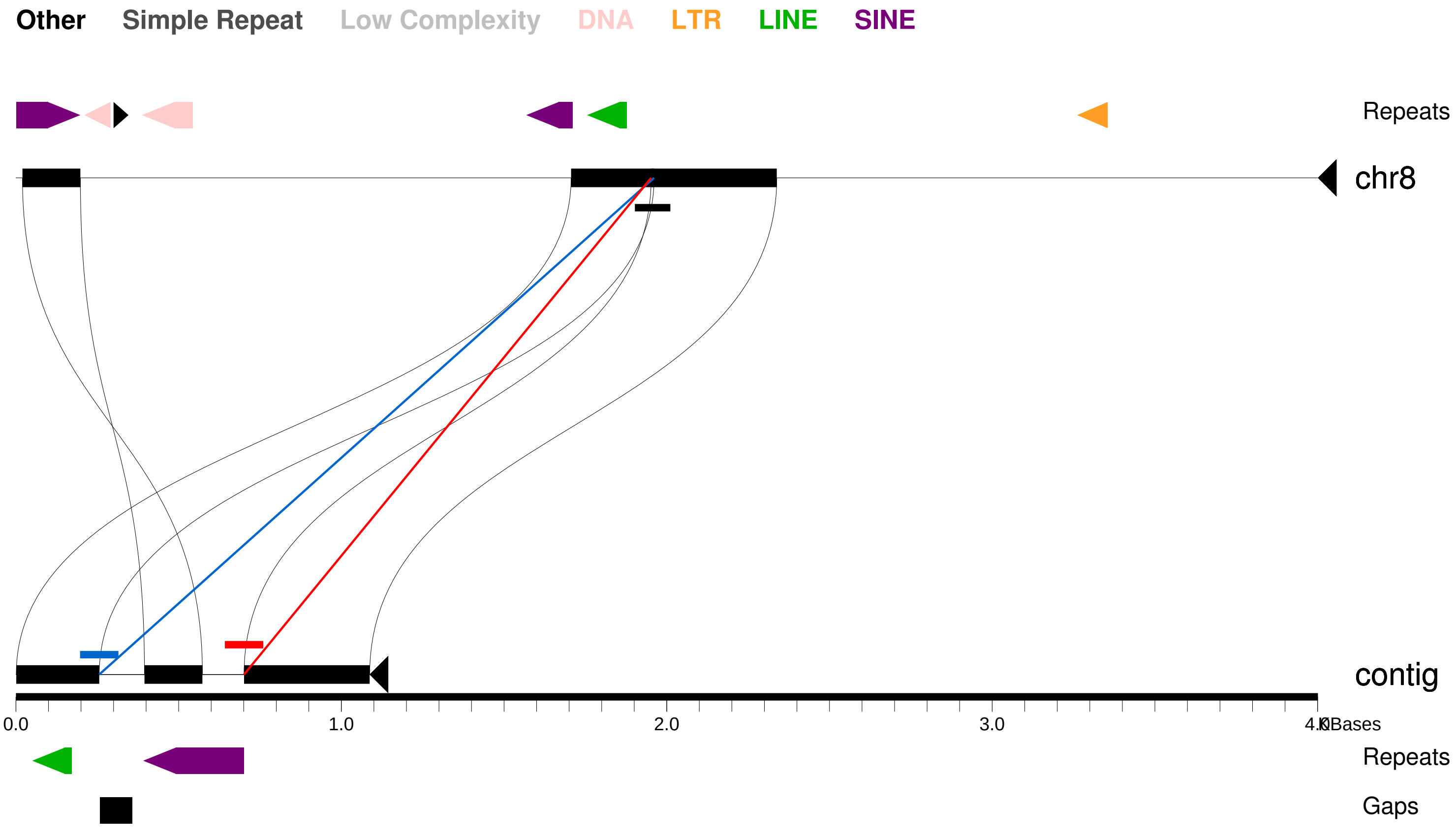

siteID: chr8_110079109 Scafftig2 + Breakpoint: chr8:110079085
chr8:110079048-110079144
Overlap Length: 16 INS size: 139 Genotyped: Yes

```
left    TTTTATTGGGT---TGTTTGTGTTCTTATTGTTTAGTTTT-------------------AAGAATTCTTT
chrom   TTTTATTGGGT---TGTTTGTGTTCTTATTGTTTAGTTTT-------------------AAGAATTCTTT
right   AGCCTGGGCGACAGAGCGAGACTCCGTCTCAAAAAAAAAAAAAAAAAAAAAAAAAAAAAAAAGAATTCTTT
        1111111*1*11111*111*11*1*1*1*11111*111111111111111111111111***********

left    GTATACTTGGGAGGCTGAGGCAGGAGAATGGCGTGAACCCGGGAGGCGGAGCTTGCAGTGAGCCGAGATC
chrom   GTATATTATGGAC-------------AATTTTTT-------TGTTGTTATTTTTTTGATTTTTTTAAAT-
right   GTATATTATGGAC-------------AATTTTTT-------TGTTGTTATTTTTTTGATTTTTTTAAAT-
        *****2*22***22222222222222***2222*22222222*22*222222**2222*222222*2**2

left    C
chrom   -
right   -
        2
```

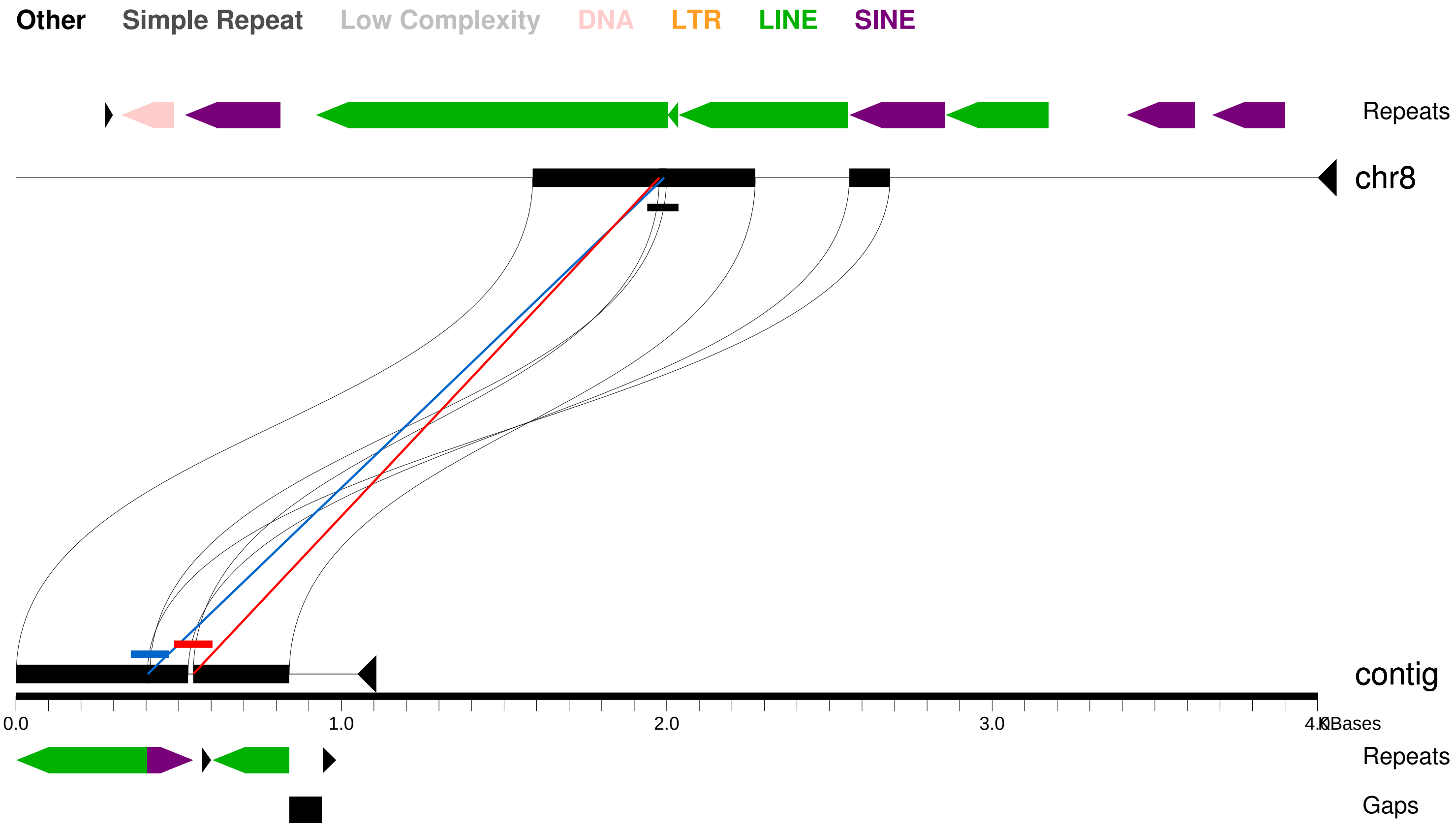

siteID: chr8_110101597 Scafftig1 + Breakpoint: chr8:110101593
chr8:110101546-110101652
Overlap Length: 13 INS size: 314 Genotyped: Yes

```
left   TCATATTTCTTG-------AAAG------GAATATAGTTCTGTGTACATAGCTAGAAACTGAGTGTACTT
chrom  TCATATTTCTTG-------AAAG------GAATATAGTTCTGTGTACATAGCTAGAAACTGAGTGTACTT
right  CCGCCCGCCTCGGCCTCCCAAAGTGCTGGGATTACAGGCGTGAGC-CACCGCGCCCGGCCGAGTGTACTT
       1*111111**1*1111111****111111**1**1**111**1*11**11**111111*1**********

left   TGTTTTTTTTTTTTTTTTTTTTTTTTTTTTTTTTTTTGAGACGGAGTCTCGCTGTCGCCCAG
chrom  TGTAAAAAAGTATACATATAA-----------TCATTCACACTGTTACTTCATAAACTCCA-
right  TGTAAAAAAGTATACATATAA-----------TCATTCACACTGTTACTTCATAAACTCCA-
       ***2222222*2*222*2*2222222222222*22**2*2**2*222**222*22222***2
```

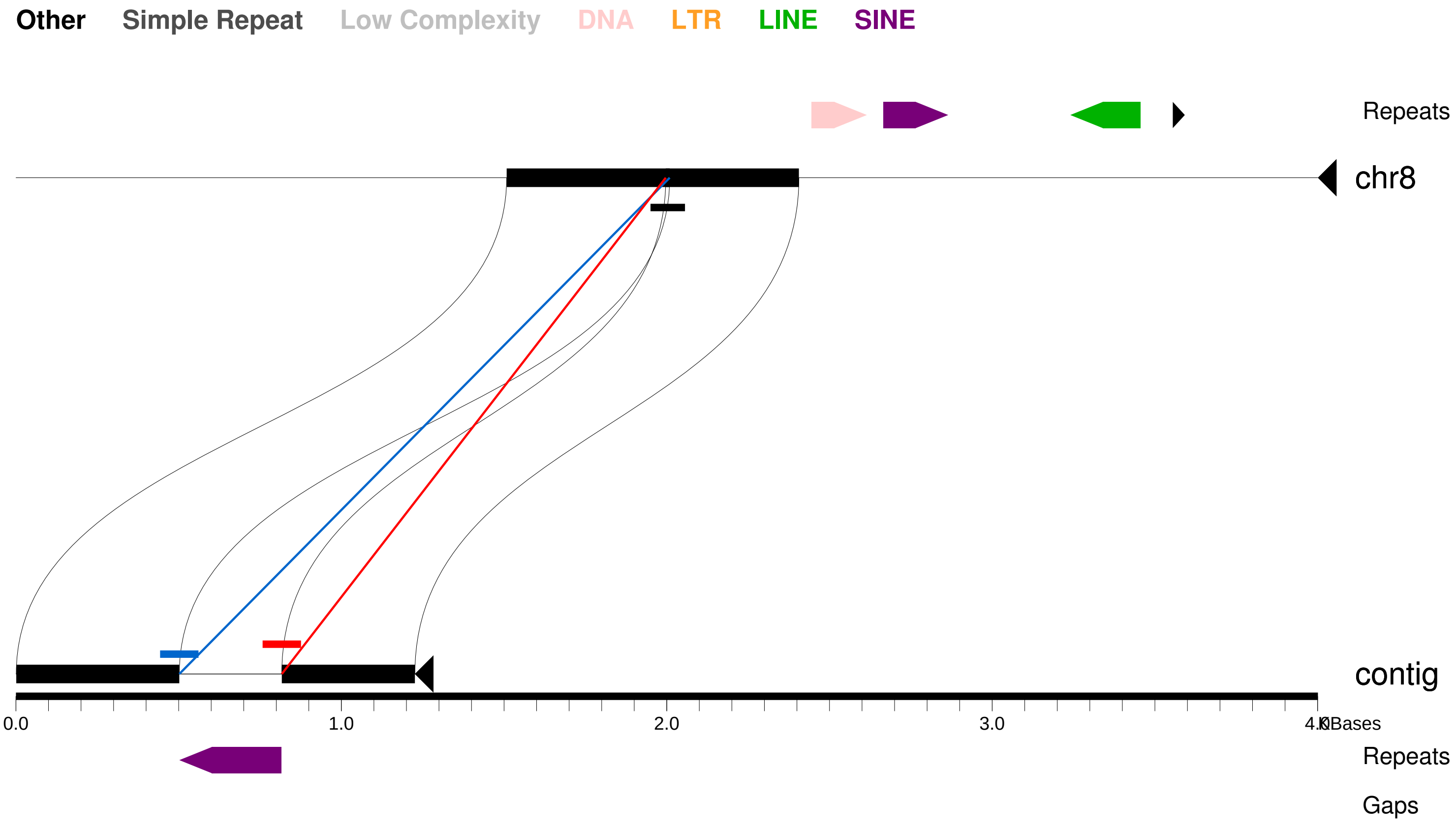

siteID: chr8_110349290 Scafftig2 - Breakpoint: chr8:110349298
chr8:110349239-110349357
Overlap Length: 1 INS size: 399 Genotyped: No

```
left    TCACACAATAAGAAAAAGAGGTGC--ATCTGTTGCTGACTTTATTTTTGGATG---ATGGGAGTCNNNNN
chrom   TCACACAATAAGAAAAAGAGGTGC--ATCTGTTGCTGACTTTATTTTTGGATG---ATGGGAGTCATTTA
right   -----CAGGATGGTCTCGATCTCCTGACCTCGTGATCCGCCCGCCTCGGCCTCCCAAAGTGCTGCATTTA
        11111**11*1*11111**11*1*11*1**11**1*111111111*11*11*1111*1*1*111*22222

left    NNNNNNNNNNNNNNNNNNNNNNNNNNNNNNNNNNNNNNNNNNNNNNNNNNNN
chrom   AAGTTTTAAGAAAGGAAATGACTAGATCACATTTACACTTTAGGAATCTTACTC
right   AAGTTTTAAGAAAGGAAATGACTAGATCACATTTACACTTTAGGAATCTTACTC
        222222222222222222222222222222222222222222222222222222
```

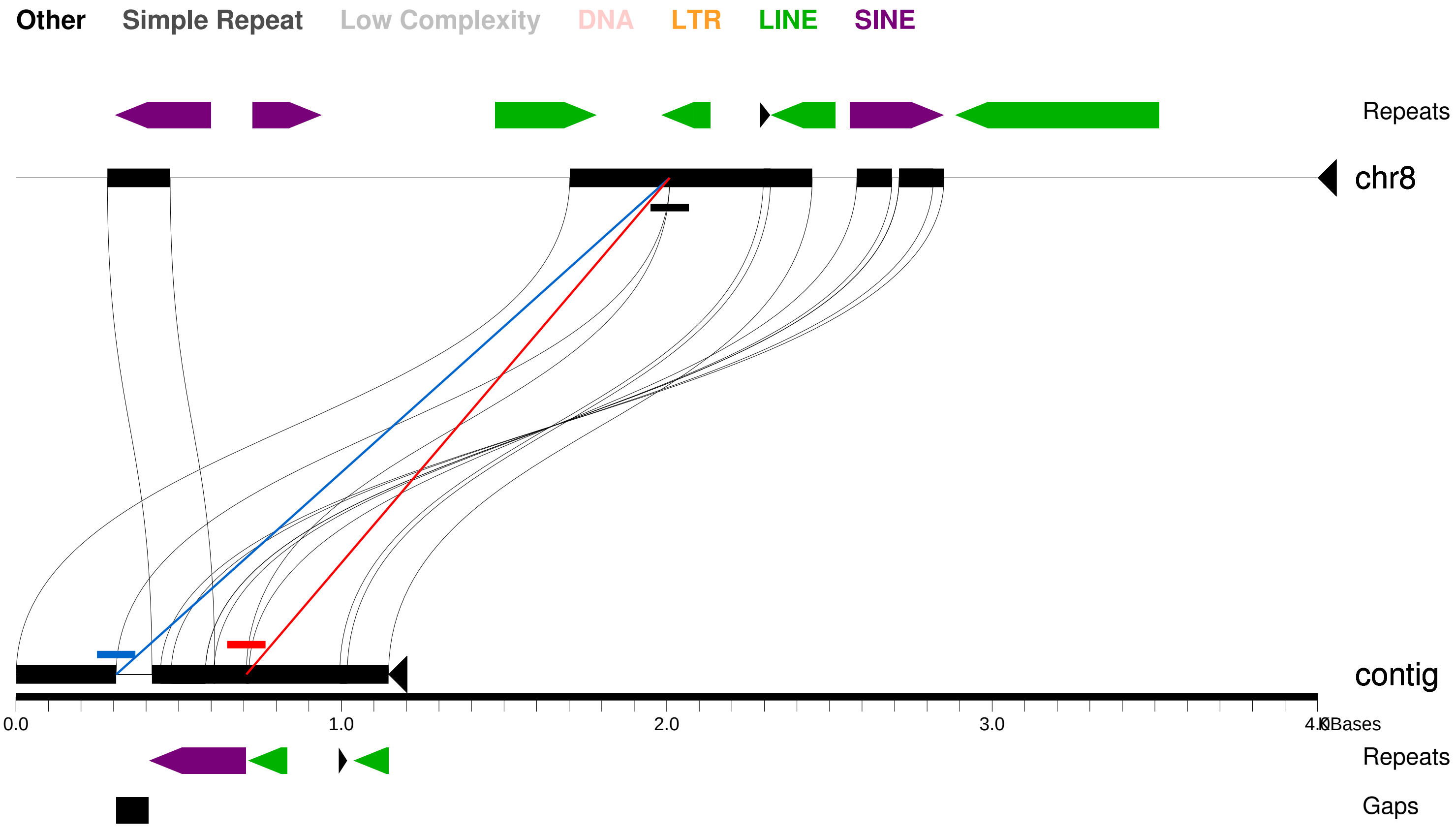

siteID: chr8_110370871 Scafftig2 + Breakpoint: chr8:110370873
chr8:110370713-110371046
Overlap Length: 14 INS size: 272 Genotyped: Yes

```
left    -------------ATCAATCCCTTATCATTTTCTTATTGCTTTTAG--GTCAAGGGACCAATAAGTTTAA
chrom   TGCAAGAATTTCTATCAATCCCTTATCATTTTCTTATTGCTTTTAG--GTCAAGGGACCAATAAGTTTAA
right   -----GAATGGCG-TGAACCCGGGAAGCGGAGCTT---GCAGTGAGCCGAGATTGCGCCACTG-------
             2222NN2N1*1**1**111*1111111***111**11*1**11*11*11*11***1*11111111

left    AAACAGTCTCTTTTTTCACCATATGGGCCAGTAGAAC-AGACTACATAATTTGTGAGGTCTCATGCAAAA
chrom   AAACAGTCTCTTTTTTCACCATATGGGCCAGTAGAAC-AGACTACATAATTTGTGAGGTCTCATGCAAAA
right   ---CAGTCCGCAGTCTGGCC---TGGGCGAC-AGAGCGAGACTCC------------GTCTCAAAAAAAA
        111*****11111*1*11**111*****1*11***1*1*****1*111111111111******111****

left    TGAAAAGGGGAAGTCCCTTGCTCAAAAAATATTAAGG----CACTTTGGGAGGCCGAG---------GCG
chrom   TGAAAAGGGGAAGTCCCTTGCTCAAAAAATATTAAGGATCCCAAGATGACAG-CAGAGTATTAAACCGAG
right   AAAAAAAA--AA-----------AAAAAAATATTAAGGATCCCAAGATGACAG-CAGAGTATTAAACCGAG
        11****1111**11111111111*************2222**222**22**2*2***22222222*2*

left    GGTGGATC--ATGAGGT----CAGGAGATCGAGACCATCCTGGC----TAACAAGGGG------AAACCC
chrom   TATGGGCCCTATGAGATTGCACAGGTCACACTCACTATCCTGGCCCTTTAGGCAGGGTTTTGTTAGAGCT
right   TATGGGCCCTATGAGATTGCACAGGTCACACTCACTATCCTGGCCCTTTAGGCAGGGTTTTGTTAGAGCT
        22***22*22*****2*2222****22*22222**2********2222**222****2222222*2*2*2

left    CGTCTCTACTAAAAATACAAAAAATTAGCCGGGCACGGTGG-CGGGC-----------
chrom   AGATTTTTGAAAACCTGCAAAAGAAACGCGGGCTTTAATGATCAGGATTCTACTTTCA
right   AGATTTTTGAAAACCTGCAAAAGAAACGCGGGCTTTAATGATCAGGATTC--------
        2*22*2*222***22*2*****2*222**2**222222**22*2**2222
```

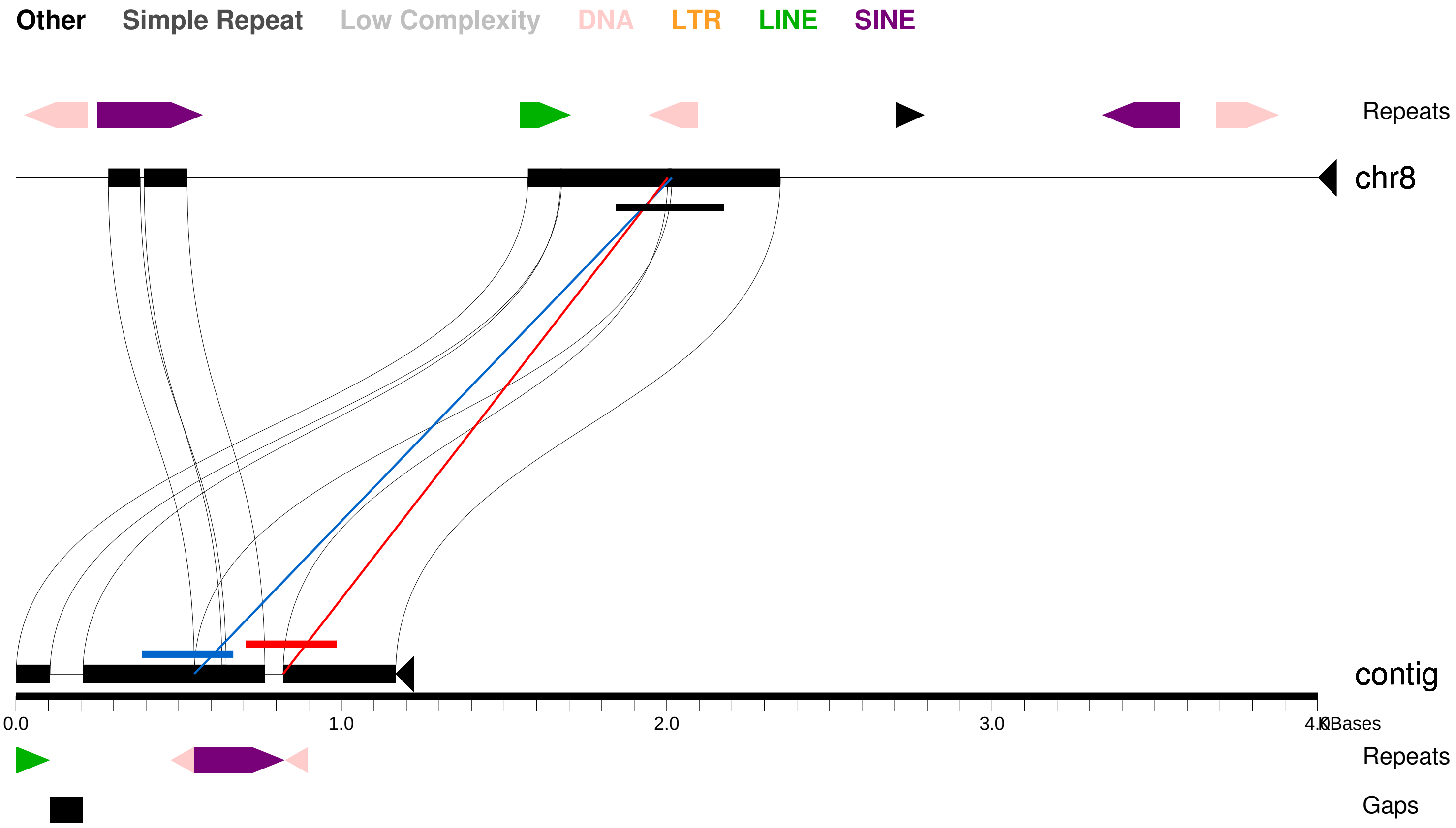

siteID: chr8_114741551 Scafftig2 - Breakpoint: chr8:114741566
chr8:114741418-114741968
Overlap Length: 13 INS size: 291 Genotyped: Yes

```
left   TTATTATGCTAAATTTAACAGCCACTCTTTCTTGAAGTTTTATTATTTTCAGAAAATTAAATGAAGGAAG
chrom  T------GCTAAATTTAACAGCCACTCTTTCTTGAAGTTTTATTATTTTCAGAAAATTAAATGAAGGAAG
right  T------TTTGTATTT---------TTTTAGTAGAGACGGGGTT----TCACCGTGTTA------GCCAG
       *22222211*11****111111111*1**11*1**1111111**1111***11111***111111*11**

left   AAGTGACTCAGTGTGTAATATTTTAAAATTGTCATGTTATATGGAACTCATCTGACTCGTGCAAAAAACT
chrom  AAGTGACTCAGTGTGTAATATTTTAAAATTGTCATGTTATATGGAACTCATCTGACTCGTGCAAAAAACT
right  GATGGTCTCG-------ATCTCCTGACCTCGTGATCC------GCCCGCCTCGGCCTCC--CAAAGTGCT
       1*11*1***11111111**1*11*1*11*1**1**11111111*11*1*1**1*1***111****111**

left   GCCTTT-------TGAAACTAAAGTTGTTTTCTT----------TTT-----------------------
chrom  GCCTTT-------TGAAACTAAAGTTGTTTTCTTAAAAATTCGATTTAAAAATAAGATTGATTTCAGTTG
right  GGGATTACAGGCGTGAGCCACAAGTTGTTTTCTTAAAAATTCGATTTAAAAATAAGATTGATTTCAGTTG
       *111**1111111***11*11*************2222222222***22222222222222222222222

left   -------------------------------TTTTTTTTTTT---TTTT--------TGAGACGGAGTC-
chrom  ATCCTAAAAATTGGTCAATGTGATAAACACATTTTTTGTCTTCAATTTTCAGTAAACTGATATGTAGTCA
right  ATCCTAAAAATTGGTCAATGTGATAAACACATTTTTTGTCTTCAATTTTCAGTAAACTGATATGTAGTCA
       2222222222222222222222222222222******2*2**222****22222222***2*2*2****2

left   ----------------------TC-------------------------GCTCTGTCGCCC-AGGCTG--
chrom  ACATTTTATACATAATTTACCTTCTTTTGGGGGATTTAGATAATATAGTGCACTTTTGGCATAGTCTGAT
right  ACATTTTATACATAATTTACCTTCTTTTGGGGGATTTAGAT-----------------------------
       2222222222222222222222**22222222222222222        11N11N1N1N1N 11N111

left   ---GAG-------TGCAGTGGC------------------GCGATC--TCGGCTCACTGCAAGCTCCGCC
chrom  CTTGAGCATGAAATACAGAGAAATCAAACTGATTTCCACAGCCATCATTCAG-TAAGTGCAATATCTACC
right  ----------------------------------------------------------------------
          111       1N111N1NN                  11N111  11N121N1N11111NN11NN11

left   -------TCCCGGGTTC-------------------------------ACGCCA----------------
chrom  ATCTATATCACTGATTCTGACATCTTGAGAAACGGAGACTGCTCGGAAACACCAATGAACTAATTTACTT
right  ----------------------------------------------------------------------
              11N1N1N111                               11N111

left   ---------------------------------------------TTCCC--------------------
chrom  ACAGCTAAATGTATATATAATTAAAAATTTTTTTTTGAAAATCTATTGCCTCATAACTTGCTTTATAATA
right  ----------------------------------------------------------------------
                                                    11N11

left   --CTG
chrom  GTCTT
right  -----
         11N
```

Other **Simple Repeat** **Low Complexity** **DNA** **LTR** **LINE** **SINE**

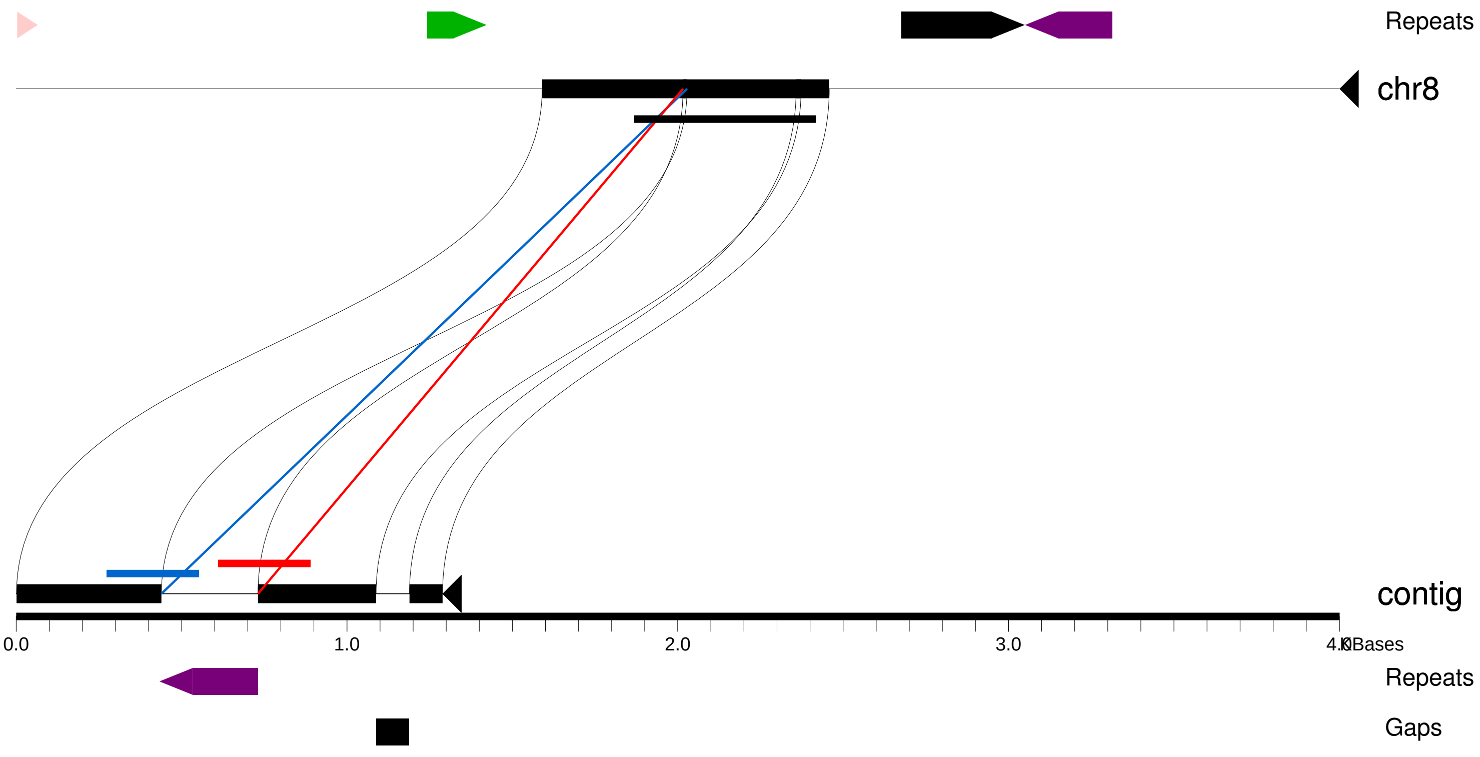

siteID: chr8_114792908 Scafftig1 + Breakpoint: chr8:114792909
chr8:114792870-114792968
Overlap Length: 12 INS size: 416 Genotyped: No

```
left    ---TCCTCCTAAAAGTCAAAAAGTGATC----TATAAACATAATAT-------------CCCAAGGTATA
chrom   ---TCCTCCTAAAAGTCAAAAAGTGATC----TATAAACATAATAT-------------CCCAAGGTATA
right   CCGCCCGTCTCGGCCTCCCAAAGTGCTGGGATTACAGGCGTGAGCCACCGCGCCCGGCCCCCAAGGTATA
        1111**11**11111**11******1*11111**1*11*1*1*1111111111111111***********

left    TATATATATTTNNNNNNNNNNNNNNNNNNNNNNNNNNNNNNNNNNNNNNNNNNNNNNNNNNNNNNNNNN
chrom   TTTTTATCTT--------------------CCATGTACACTGACTGGTTACTTAGTAGCAAGTCTGTCA
right   TTTTTATCTT--------------------CCATGTACACTGACTGGTTACTTAGTAGCAAGTCTGTCA
        *2*2***2**22222222222222222222222222222222222222222222222222222222222
```

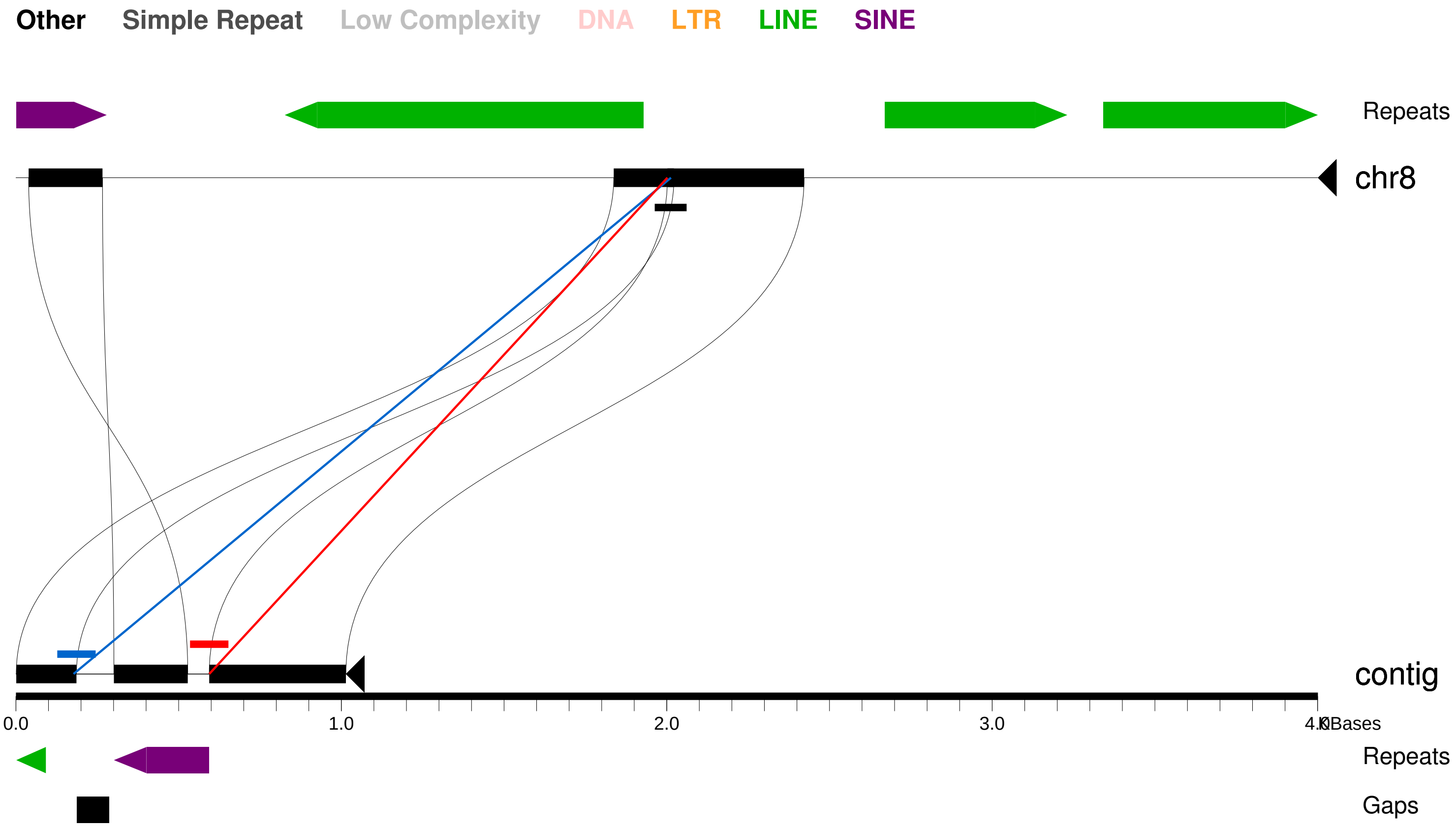

siteID: chr8_117046755 Scafftig8 - Breakpoint: chr8:117047006
chr8:117046960-117047062
Overlap Length: 14 INS size: 263 Genotyped: Yes

```
left     GAAAACTATGGATACATTCTGTATTAGCTGTTCTCATGCT----------------ACTAATAAAGACAT
chrom    GAAAACTATGGATACATTCTGTATTAGCTGTTCTCATGCT----------------ACTAATAAAGACAT
right    CACTGCACTCCAGCCTGGGCGACAGAGCGAAACTCCGTCTCAAAAAAAAAAAAAAAAAAAAAAAAGACAT
         1*111*11*11*11*11111*1111***1111***111**1111111111111111*11**1********

left     AACCAATTGGGAGGCCGAGACGGGCGGATCACGAGGTCAGGAGATCGAGACCATCCTGGCTAACA-
chrom    AACCAA-------GCC----TGGGTAATTTATAAAGGAAAGAGGTTTA----AT--TGACTCATAG
right    AACCAA-------GCC----TGGGTAATTTATAAAGGAAAGAGGTTTA----AT--TGACTCATAG
         ******2222222***22222***2222*2*22*2*22*2***2*22*2222**22**2**2*2*2
```

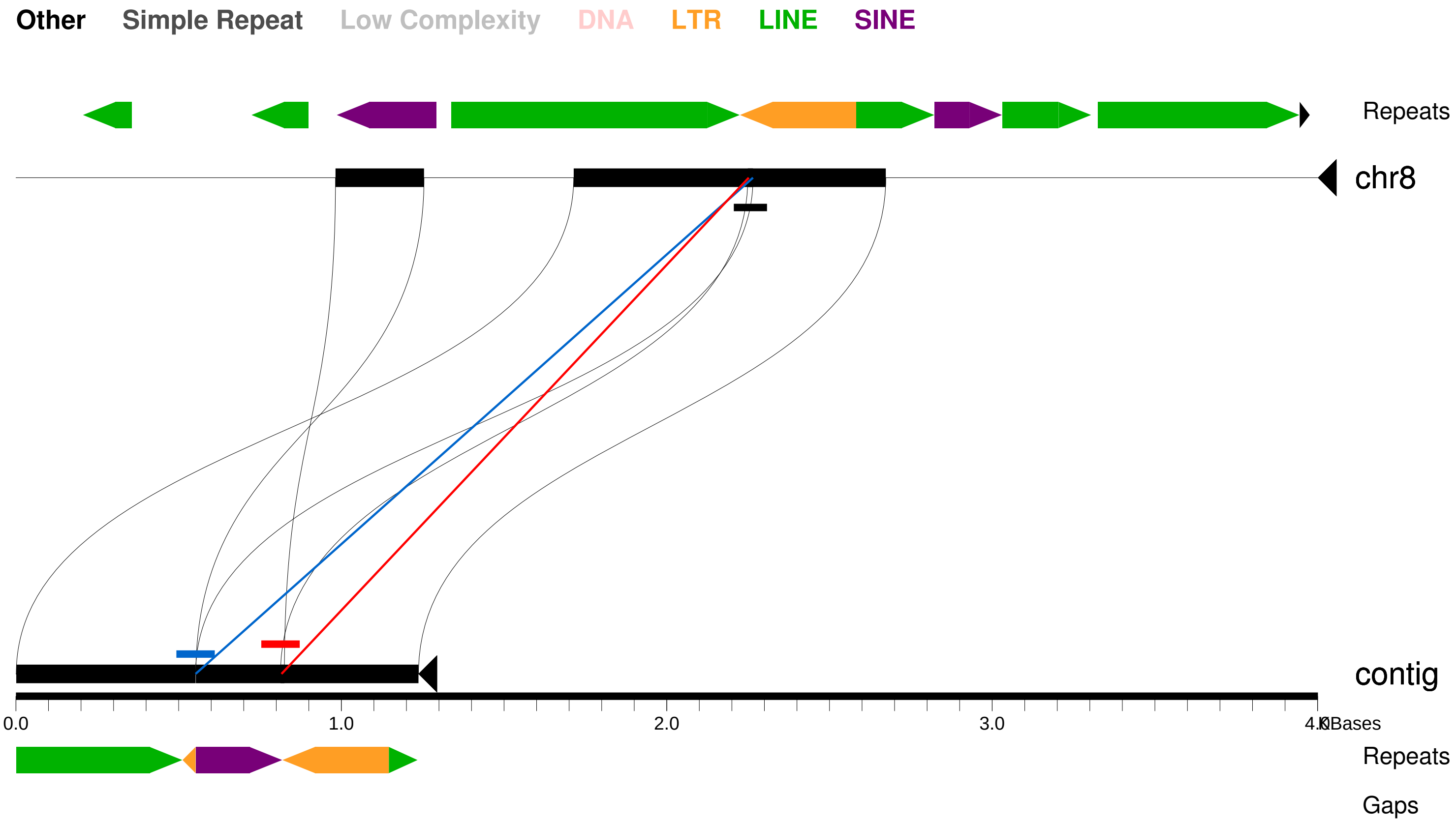

siteID: chr8_119491413 Scafftig1 - Breakpoint: chr8:119491312
chr8:119491271-119491371
Overlap Length: 16 INS size: 268 Genotyped: No

```
left     ATTCTTTTGCACAAAACTCGGCATTGGCTCCCCATTTCTCT------------------CAAAATAAAAG
chrom    ATTCTTTTGCACAAAACTCGGCATTGGCTCCCCATTTCTCT------------------CAAAATAAAAG
right    NNNNNNNNNNNNNNNNNNNNNNNNNNNNNNNNNNNNNNNNNNNNNNNNNNNNNNNNNNNCAAAATAAAAG
         11111111111111111111111111111111111111111111111111111111111***********

left     TCAAGGCCGGGCGCGGTGGCTCACGCCTGTAATCCCAGCACTTTGGGAGGCCAAGGCGGGCGGATCA
chrom    TCAAGTCC------------TTACAGCAA-ACTACAATGTCTTTC-----CCAATCTGTGTCTCAGT
right    TCAAGTCC------------TTACAGCAA-ACTACAATGTCTTTC-----CCAATCTGTGTCTCAGT
         *****2**222222222222*2**22*222*2*2*2*222****222222****222*2*2222222
```

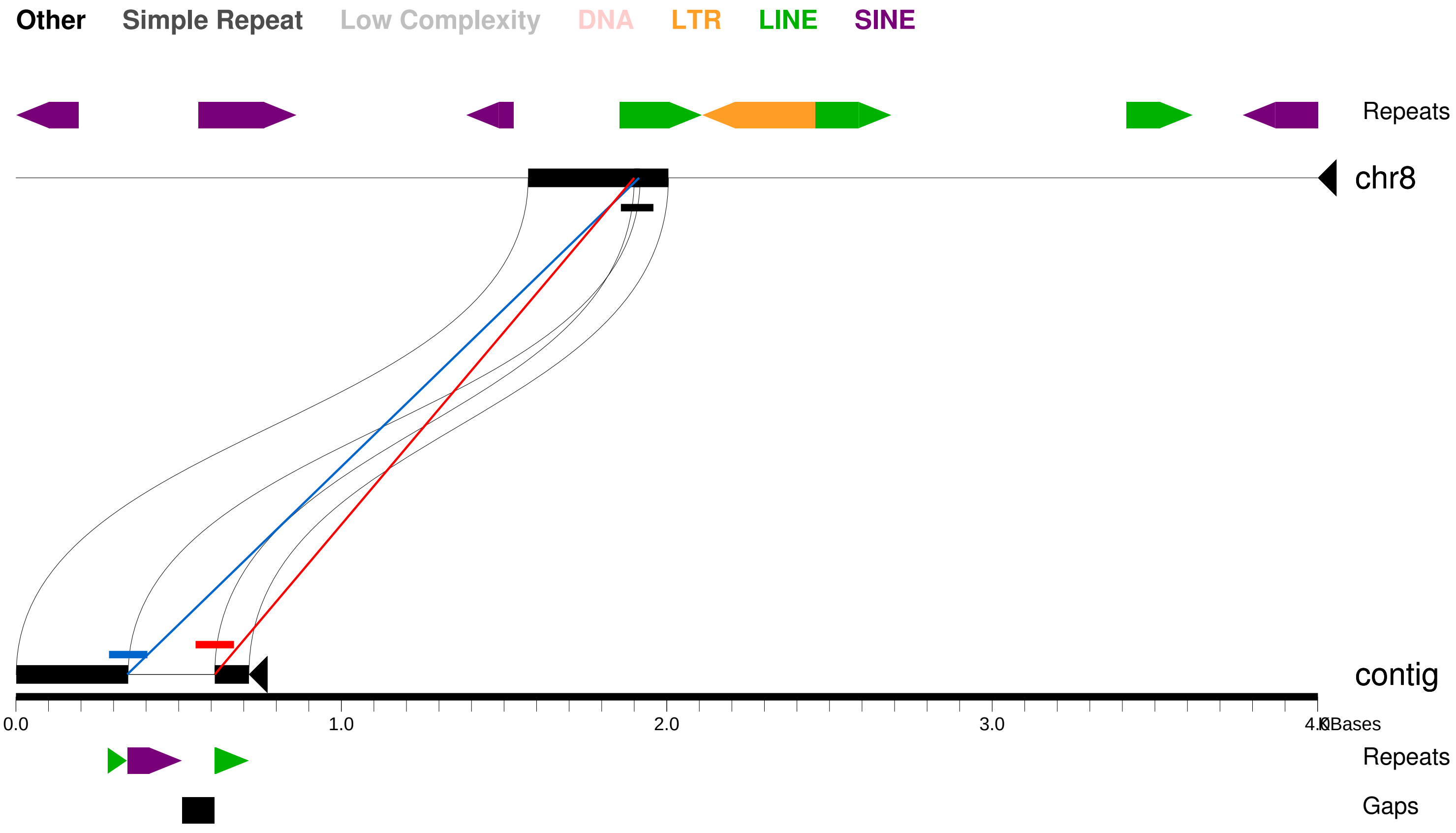

siteID: chr8_120800789 Scafftig7 - Breakpoint: chr8:120800779
chr8:120800733-120800820
Overlap Length: 14 INS size: 221 Genotyped: Yes

```
left    A--CCAATAT-----------------------------GTATGCTTATGTGTACAGAGAAATATAAATC
chrom   A--CCAATAT-----------------------------GTATGCTTATGTGTACAGAGAAATATAAATC
right   GAGCCGAGATTGCGCCACTGCACTCCAGCCTGGGCGACAGCGAGACTCCGTCTCAAAAAAAAAAAAAAAA
        111**1*1**11111111111111111111111111111*111*11*11**1*11*1*1***1*1***11

left    AAGATATAAAATATTACACAACCATCCTGGCTAACACGGTGAAACCCCGTCTCTACTAAAAATACAAAAA
chrom   AAGATATAAAATATTACACAA-----------AA-----TGA------GTATCTGG-----AGACCTAGA
right   AAAAAAAAAAATATTACACAA-----------AA-----TGA------GTATCTGG-----AGACCTAGA
        **1*1*1*************22222222222**22222***222222**2***2222222*2**22*2*

left    ATTAGCCGGG
chrom   --TAGGCA--
right   --TAGGCA--
        22***2*222
```

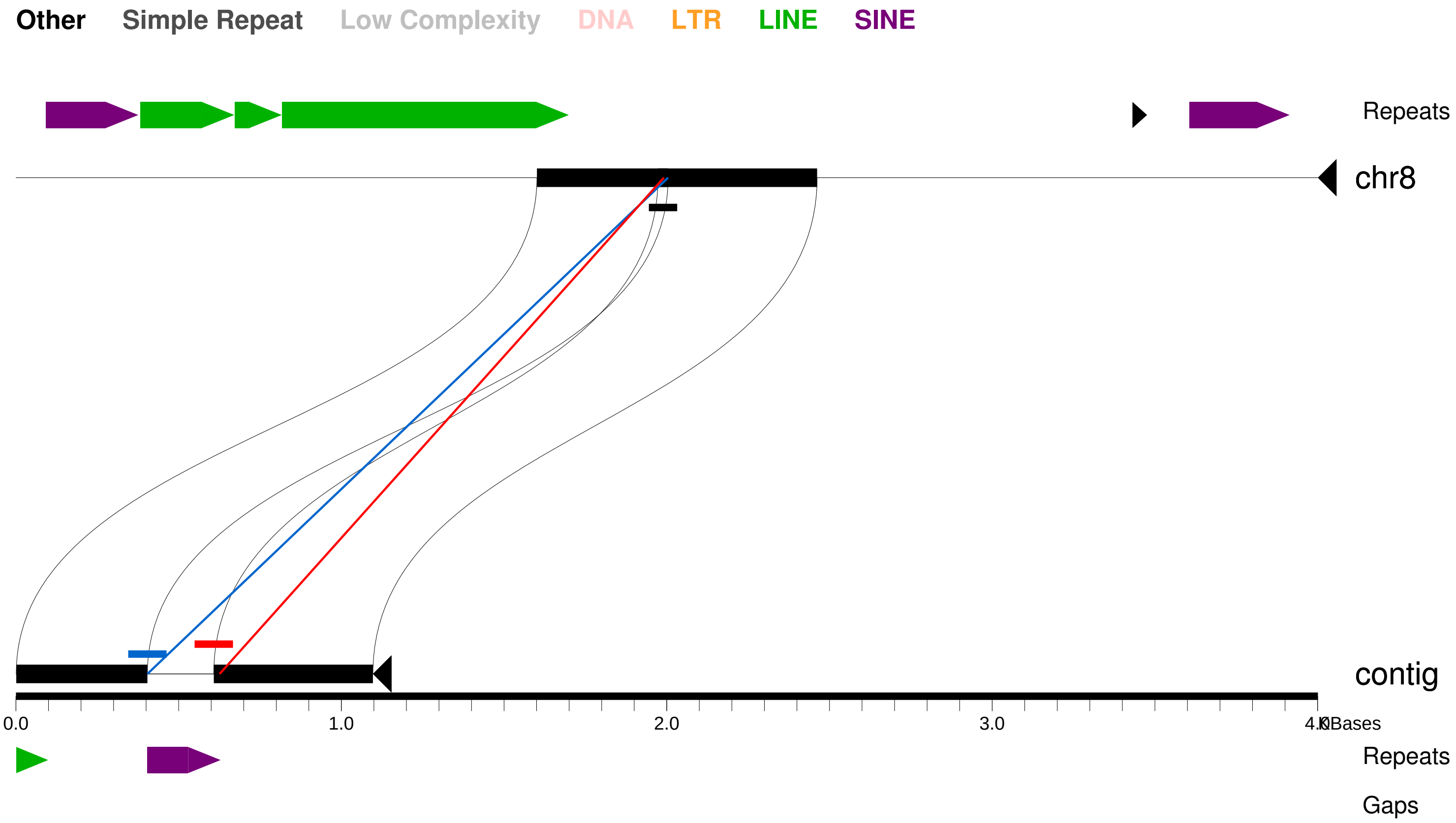

siteID: chr8_122155288 Scafftig8 - Breakpoint: chr8:122155278
chr8:122155228-122155337
Overlap Length: 10 INS size: 306 Genotyped: Yes

```
left    G-CTAAAGATTAAACTCAATTATAAAATT----TCCGG----AAAGGTTTTCACTTGTTAAAAAAAAATTG
chrom   G-CTAAAGATTAAACTCAATTATAAAATT----TCCGG----AAAGGTTTTCACTTGTTAAAAAAAAATTA
right   GTCCGCAGTCCGACCTGGGCGACAGAGCGAGACTCCGTCTCAAAAAAAAAAAAAAAAAAAAAAAAAAATTA
        *1*111**1111*1**11111*1*1*1111111****11111***1111111*111111**********2

left    GCCGGGCGCAGTGGCTCACGCCTGTAATCCCAGCACTTTGGGAGGCCGAGGCGGGTGG
chrom   TTTATGA-CACTTGTTAAAGGTGGTAAGGC-AG-ATTTT---ATTCAAAGG---GTGT
right   TTTATGA-CACTTGTTAAAGGTGGTAAGGC-AG-ATTTT---ATTCAAAGG---GTGT
        22222*22**2*2*2*2*2*222****22*2**2*2***222*22*22***222***2
```

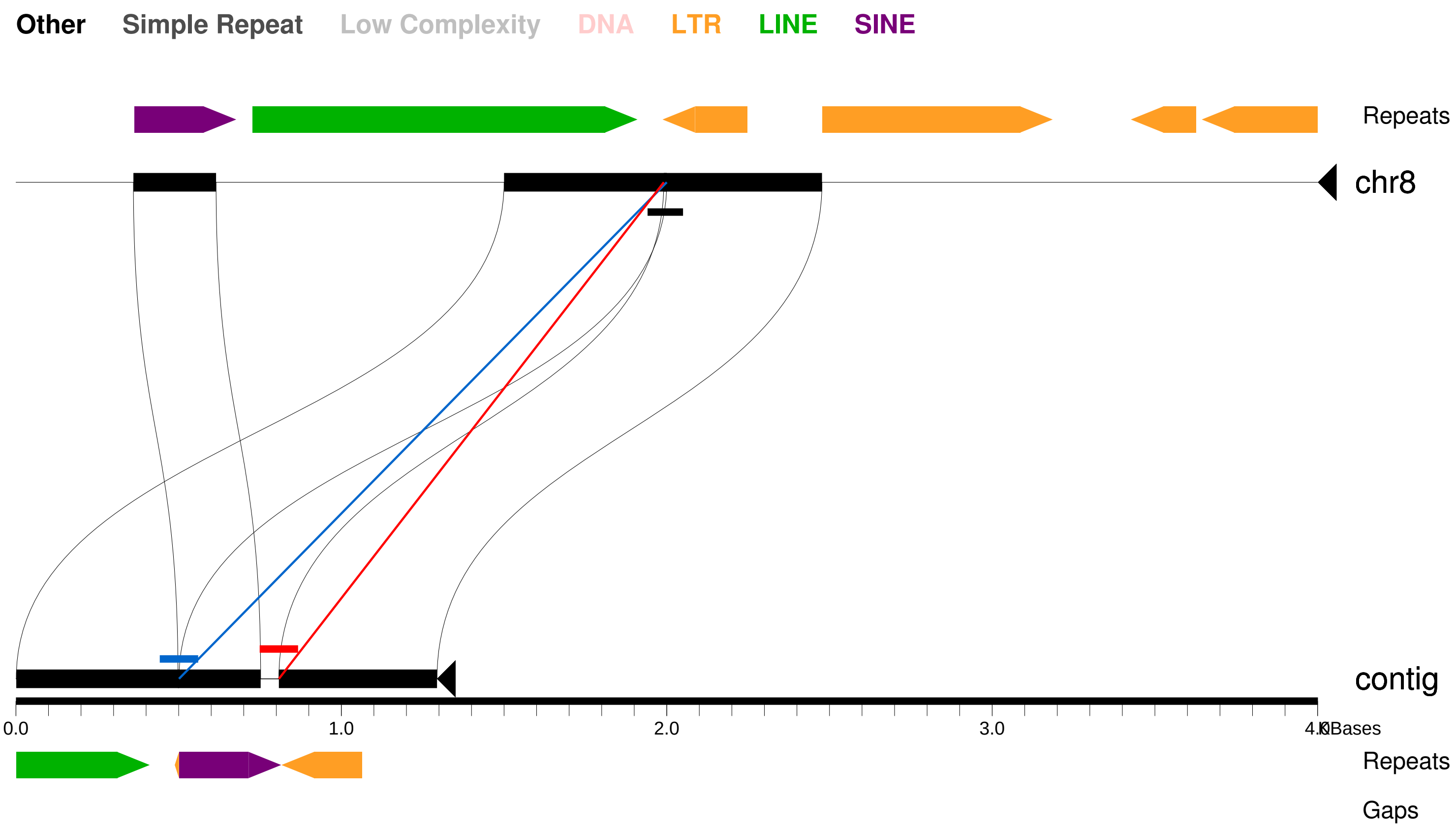

siteID: chr8_122541986 Scafftig1 + Breakpoint: chr8:122541998
chr8:122541955-122542057
Overlap Length: 17 INS size: 459 Genotyped: No

```
left    G--GACTCCCTCAG-------AATTTCTG--ATCA-----GTGCGTCTTTTGTGGAGCTCAAGAATTTGC
chrom   G--GACTCCCTCAG-------AATTTCTG--ATCA-----GTGCGTCTTTTGTGGAGCTCAAGAATTTGC
right   TCCGCCCGCCTCGGCCTCCCAAAGTGCTGGGATTACAGGCGTGAGCCACCGCGCCCGGCCAAGAATTTGC
        111*1*11****1*1111111**1*1***11**1*11111***1*1*111111111*11***********

left    ATTTCTTTTNNNNNNNNNNNNNNNNNNNNNNNNNNNNNNNNNNNNNNNNNNNNNNNNNNNNNNNN
chrom   ATTTCTAATACATTTCCAAGTAATTCTGCTAGTCCAGCATTGCCCTGAG----------------
right   ATTTCTAATACATTTCCAAGTAATTCTGCTAGTCCAGCATTGCTCTGAG----------------
        ******22*2222222222222222222222222222222222N222222222222222222222
```

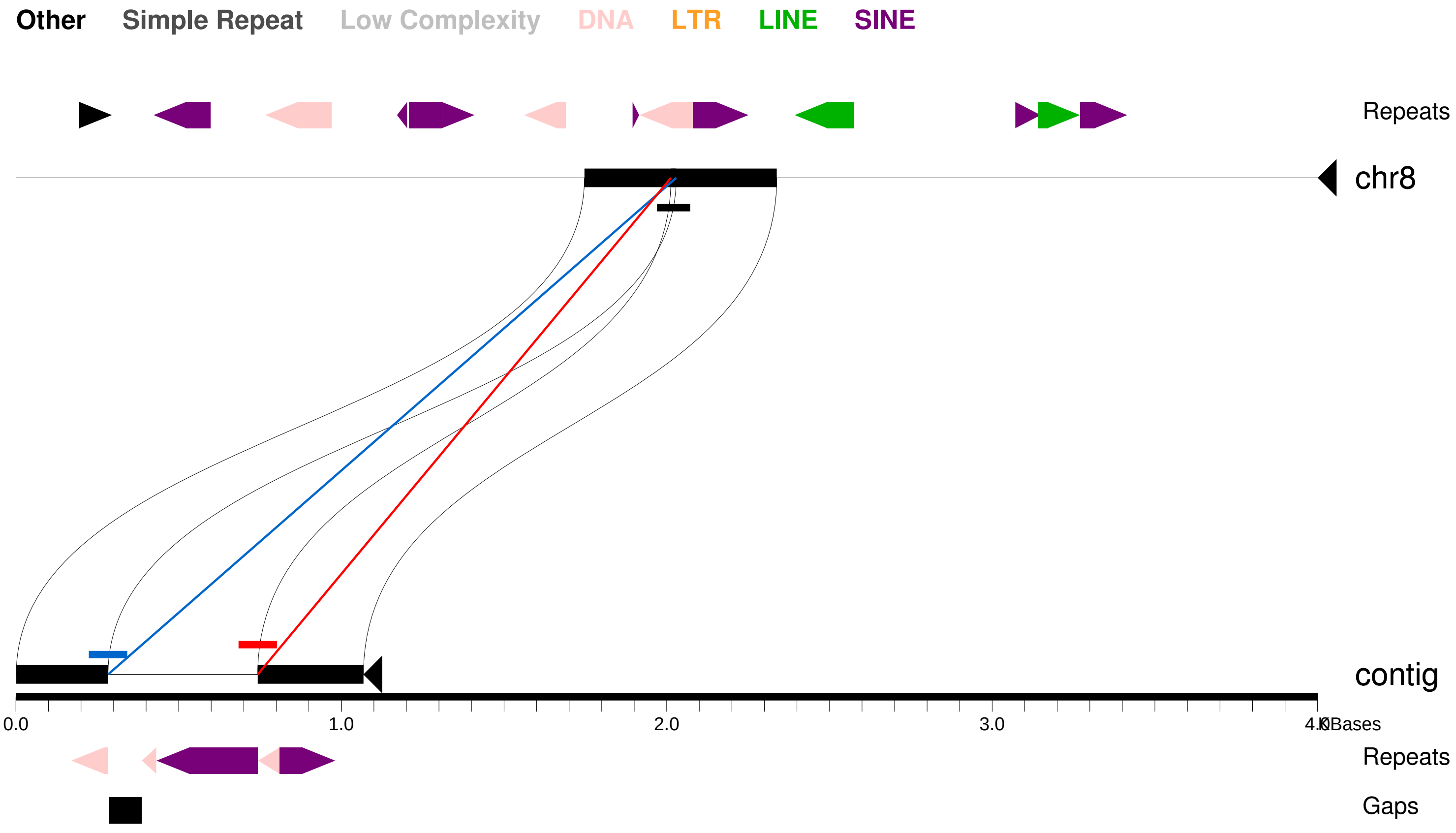

siteID: chr8_122731019 Scafftig1 - Breakpoint: chr8:122731008
chr8:122730848-122731180
Overlap Length: 13 INS size: 312 Genotyped: Yes

```
left   -----------TGATGCAATACTGGGCAAGTCACGTCACTCTCAGATTCATTCTCTCCTCTCTTCTCTGA
chrom  CAT-GTGGCCATGATGCAATACTGGGCAAGTCACGTCACTCTCAGATTCATTCTCTCCTCTCTTCTCTGA
right  TTTAGTAGAGACGGGGTTTCACCTTGTTAGCCAGGATGGTCTC-GAT-------CTCCTGACCTC-----
       NN2122N2NN21*11*1111**111*11**1**1*1111****1***1111111*****11*1**11111

left   AAACTGATGAGGGCAGACTAAATAATCCTTT-CCTTTCAAGGTTTAAGATCTTTTTATCCCCTGAATGGG
chrom  AAACTGATGAGGGCAGACTAAATAATCCTTT-CCTTTCAAGGTTTAAGATCTTTTTATCCCCTGAATGGG
right  ------ATGA--------TCCACCCGCCTCGGCCTCCCAAAGT----GCTGGGATTA---CAGGCGTGAG
       111111****11111111*11*1111***111***11***1**1111*1*1111***111*11*11**1*

left   ATGTTTTGTCAAAGAGCTTCTTCCATCTTGTCTTTTTTTTTTTTTTTTTTTTTTTTTTT----------T
chrom  ATGTTTTGTCAAAGAGCTTCTTCCATCTTGTCTTTAAGAGTAACTTTTTATAAGCTATGCCCTGGCAAGT
right  C--------CACCGCGCCCGGCCCATCTTGTCTTTAAGAGTAACTTTTTATAAGCTATGCCCTGGCAAGT
       111111111**11*1**11111*************22222*222*****2*2222*2*22222222222*

left   GAGA--CGGA--GTCTCGCTCTGTCGCCC--AGGCCGGACTGC-----GGACTGCA---------GTGGC
chrom  GAGAAACTGAAAGACAGGATTAGCAGTACTAAGACCAAAGTGGTTTATGGGCTTTAAAAAATACTGTTGC
right  GAGAAACTGAAAGACAGGATTAGCAGTACTAAGACCAAAGTGGTTTATGGGCTTTAAAAAATACTGTTGC
       ****22*2**22*2*22*2*22*22*22*22**2**22*2**222222**2**22*222222222**2**

left   GCA--AT-CTCGGCTCACTG--CAAGCTCCGCT-------TCCCGGGTTCACGCC
chrom  TCAGGATGCTTTATTCATAGAGCAAAATCTAATGATGAACTCAGCATTTCCATTG
right  TCAGGATGCTTTATTCATAGAGCAAAATCTAATGATGAACTCA------------
       2**22**2**2222***22*22***22**222*2222222**2NNNN111NNNNN
```

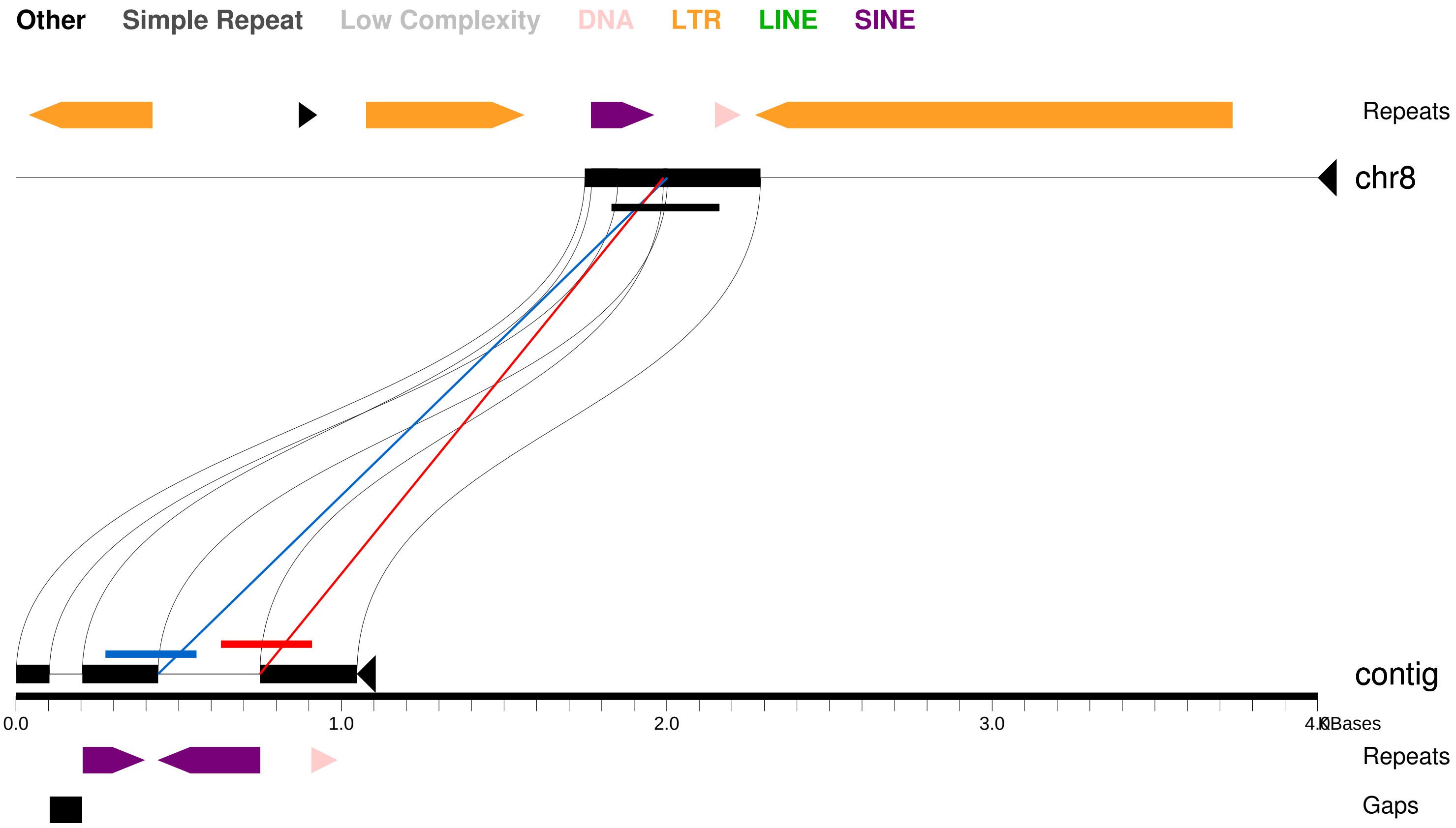

siteID: chr8_124263774 Scafftig1 - Breakpoint: chr8:124263723
chr8:124263679-124263777
Overlap Length: 16 INS size: 451 Genotyped: No

```
left     TTCTGACCAGAATTAGTATCCATTACAGTGTTAATAT--------------------ATAACATAAGAAT
chrom    TTCTGACCAGAATTAGTATCCATTACAGTGTTAATAT--------------------ATAACATAAGAAT
right    NNNNNNNNNNNNNNNNNNNNNNNNNNNNNNNNNNNNNNNNNNNNNNNNNNNNNNNNAAAAAAAAAAGAAT
         111111111111111111111111111111111111111111111111111111111*1**1*1******

left     TATCTATACAGGGCCGGGCGCGGTGGCTCACGCCTGTAATCCCAGCACTTTGGGAGGCCGAGGCGGGTG
chrom    TATCTATACAAAATTA---AAGGTCAAACTAAACTGTA----CAGAAAA-----ATTCACA--------
right    TATCTATACAAAATTA---AAGGTCAAACTAAACTGTA----CAGAAAA-----ATTCACA--------
         **********22222222222***2222*2222*****2222***2*2222222*22*22*22222222
```

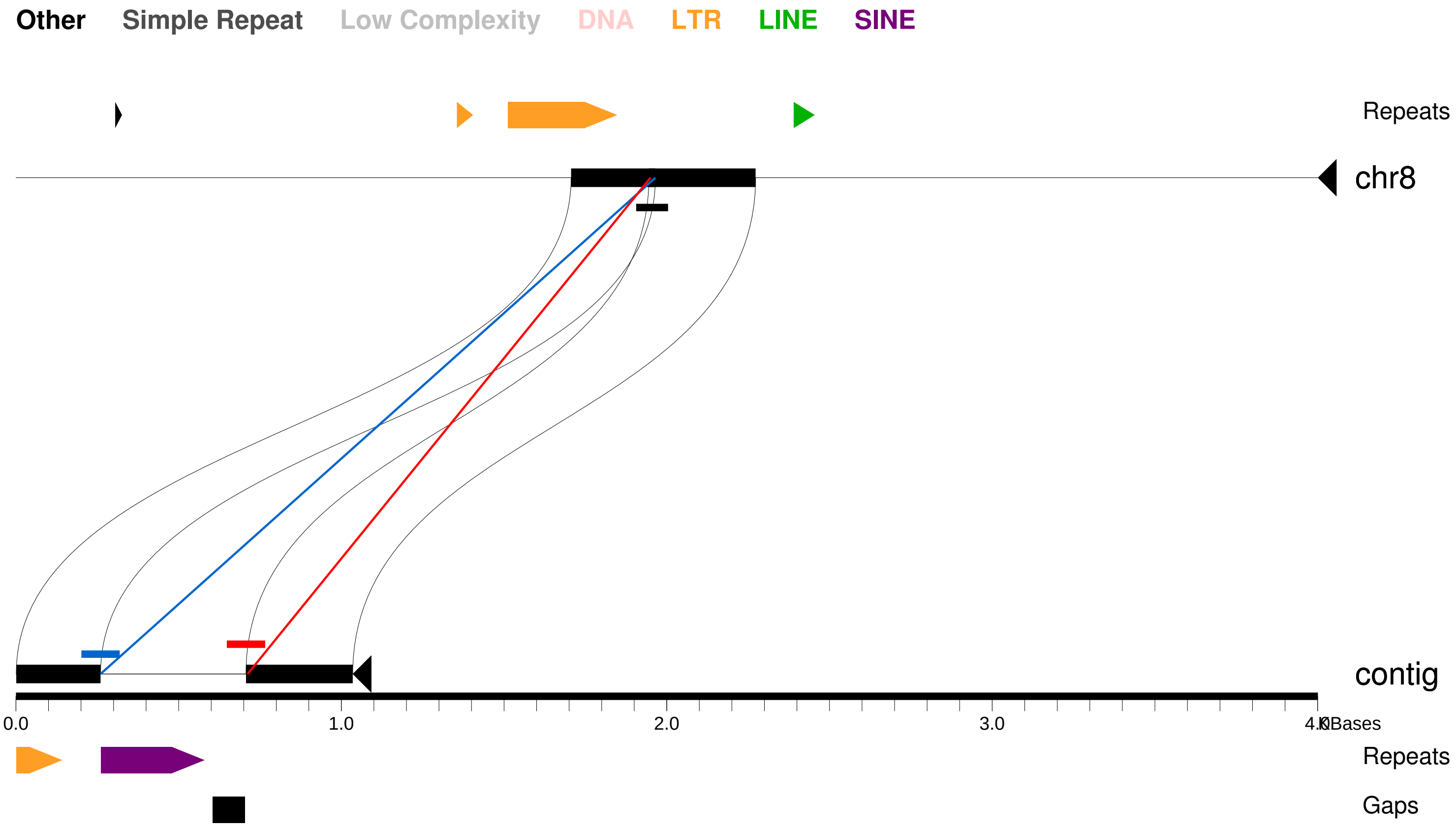

siteID: chr8_124680190 Scafftig4 + Breakpoint: chr8:124680170
chr8:124680124-124680229
Overlap Length: 14 INS size: 474 Genotyped: No

```
left   TCCTCGATATTATCTTGGGAGAGGGGGTGATGACTGGGTTCCTG-------------AGAAAAGAACTCA
chrom  TCCTCGATATTATCTTGGGAGAGGGGGTGATGACTGGGTTCCTG-------------AGAAAAGAACTCA
right  NNNNNNNNNNNNNNNNNNNNNNNNNNNNNNNNNNNNNNNNNNNNNNNNNNNNNNAAAAAAAAAGAACTCA
       111111111111111111111111111111111111111111111111111111111*1***********

left   GGTGGCCGGGCGCGGTGGCTCACGCCTGTAATCCCAGCACTTTGGGAGGCCGAGGCGGGCGG
chrom  GGTAA--GACAACTGTAACTT---TCTCAAATTTTAAGACTTCGGGAT--CAATTT------
right  GGTAA--GACAACTGTAACTT---TCTCAAATTTTAAGACTTCGGGAT--CAATTT------
       ***2222*2222*2**22**22222**22***222*22****2****222*2*222222222
```

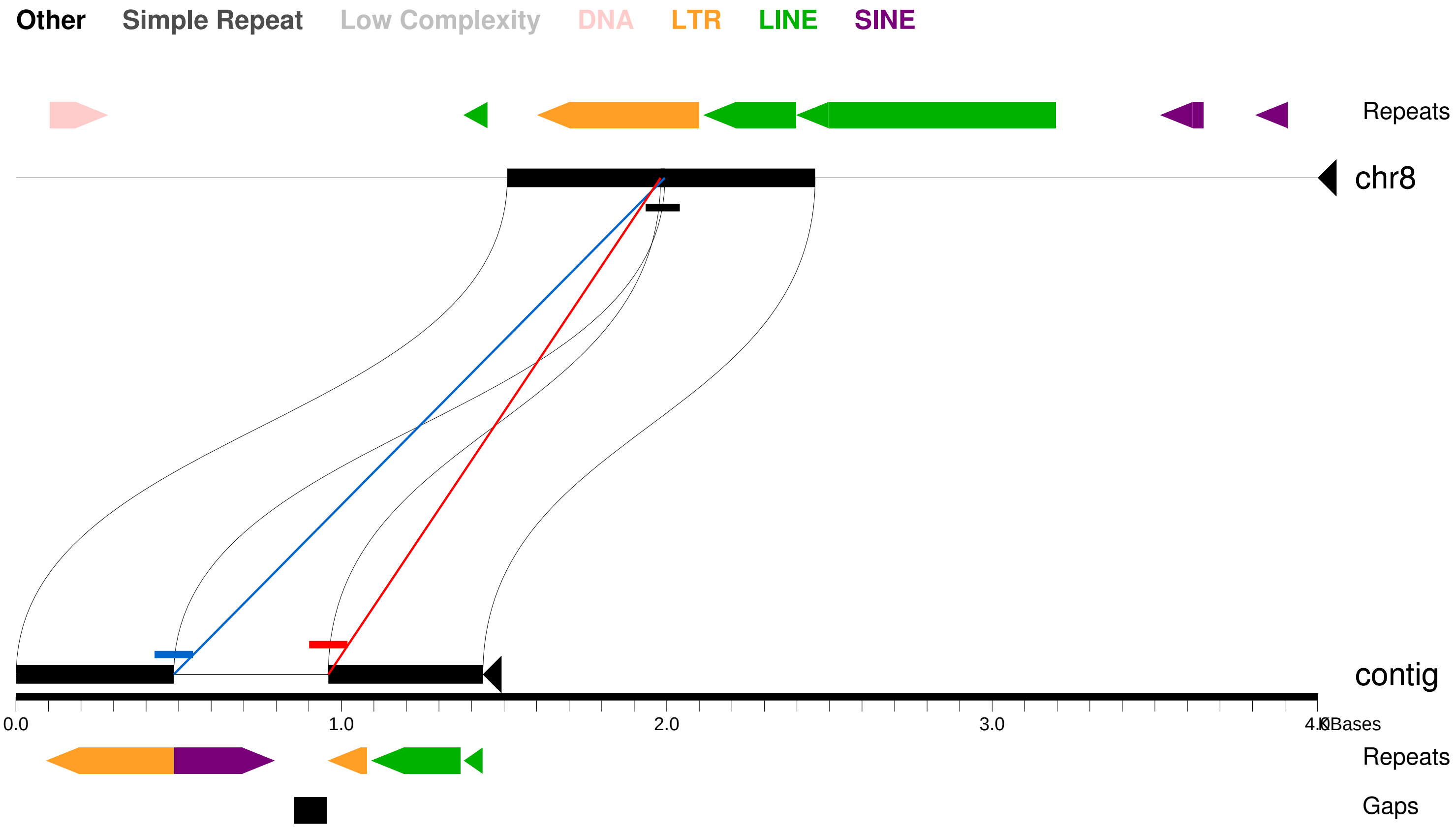

siteID: chr8_125889977 Scafftig13 - Breakpoint: chr8:125889958
chr8:125889911-125890017
Overlap Length: 10 INS size: 274 Genotyped: Yes

```
left    ---AATTATCTTCTTTTGAGGAT-TATTCAGCCTCTTGCTTCT-CCTTCCAT-------TTCTTCTGATT
chrom   ---AATTATCTTCTTTTGAGGAT-TATTCAGCCTCTTGCTTCT-CCTTCCAT-------TTCTTCTGATC
right   CGGGATGGTCTCGATCTCCTGACCTCGTGATCCGCCTGCCTCGGCCTCCCAAAGTGCTGTTCTTCTGATC
        1111**11***111*1*111**11*11*1*1**1*1***1**11***1***11111111**********2

left    TTTTTTTTTTTTTTTTTTTTTCTGAGATGGAGTCTCGCTCTGTCGCCCAGGCTGGAGTGCA
chrom   TTATAGACATTTCATAGCTT---GAGA--GAATCA-------TTTAAGAGGAAAGGAAGCA
right   TTATAGACATTTCATAGCTT---GAGA--GAATCA-------TTTAAGAGGAAAGGAAGCA
        **2*22222***22*222**222****22**2**22222222*22222***222*222***
```

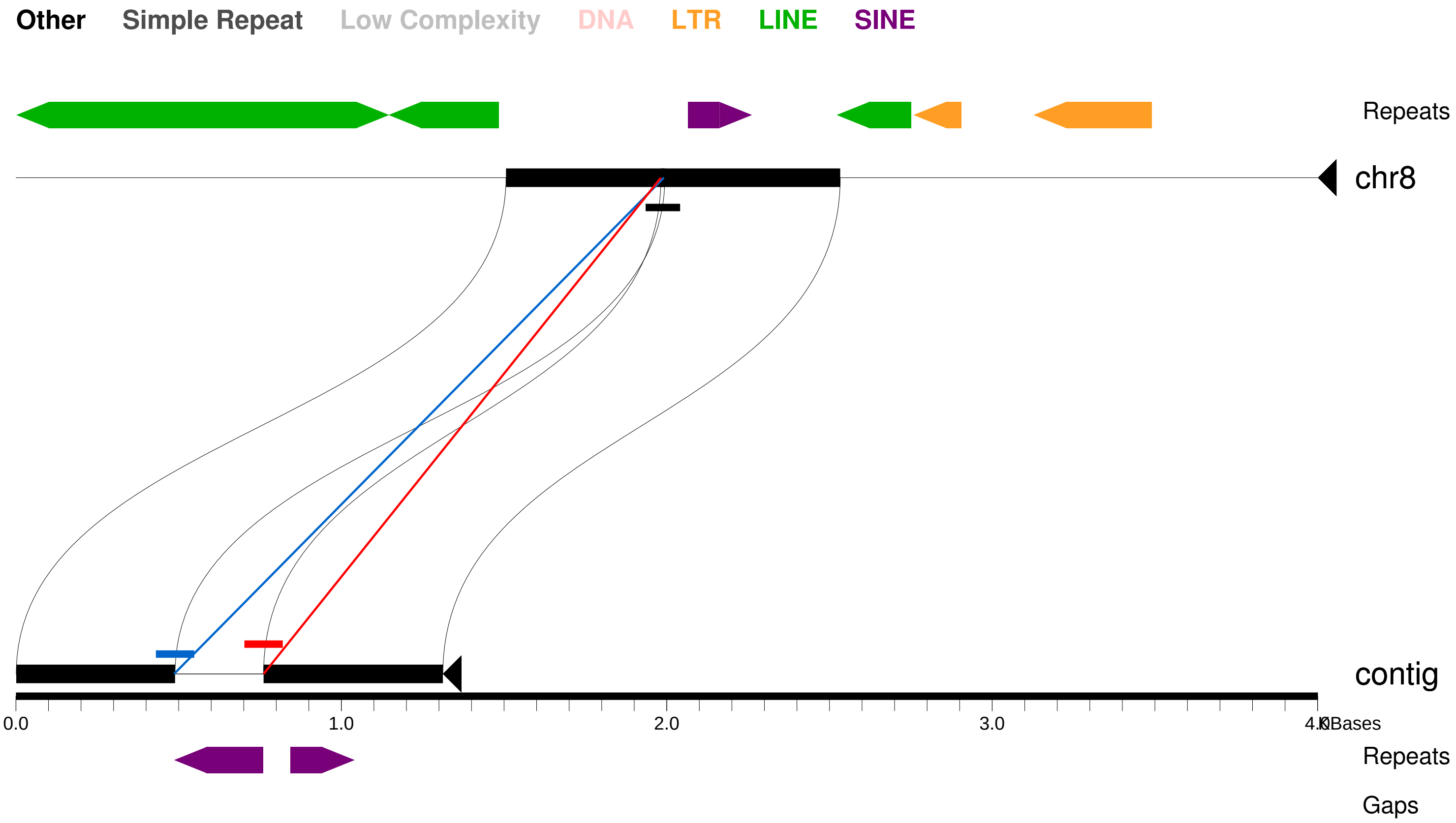

siteID: chr8_126024122 Scafftig8 + Breakpoint: chr8:126024104
chr8:126024060-126024163
Overlap Length: 8 INS size: 334 Genotyped: Yes

```
left    ----------GTGCT-----AGT-CAGGGAAACAACAGGTAAAAAACTGACCTTGACCTAGAGAAGGCCT
chrom   ----------GTGCT-----AGT-CAGGGAAACAACAGGTAAAAAACTGACCTTGACCTAGAGAAGGCAA
right   CGCCCGCCTCGGCCTCCCAAAGTGCTGGGATTACAGGCGTGAGCCACCGCGCCCGGCCC-GAGAAGGCAA
        1111111111*11**11111***1*1****1111*111**1*111**1*11*11*1**11********22

left    TTTTCTTTTTTTCTTTTTTTTTTTTTTTTTTTTTTTTTTTTTTTTTTTTTTTGAGACGGAGTCTCGCT
chrom   TTTTCTACTTAGGAATGTAGACAGATAATTTTACA---------------ACACTGTTGATAAAT
right   TTTTCTACTTAGGAATGTAGACAGATAATTTTACA---------------ACACTGTTGATAAAT
        ******22**22222*2*2222222*22****222222222222222222*2**2*2222*222*
```

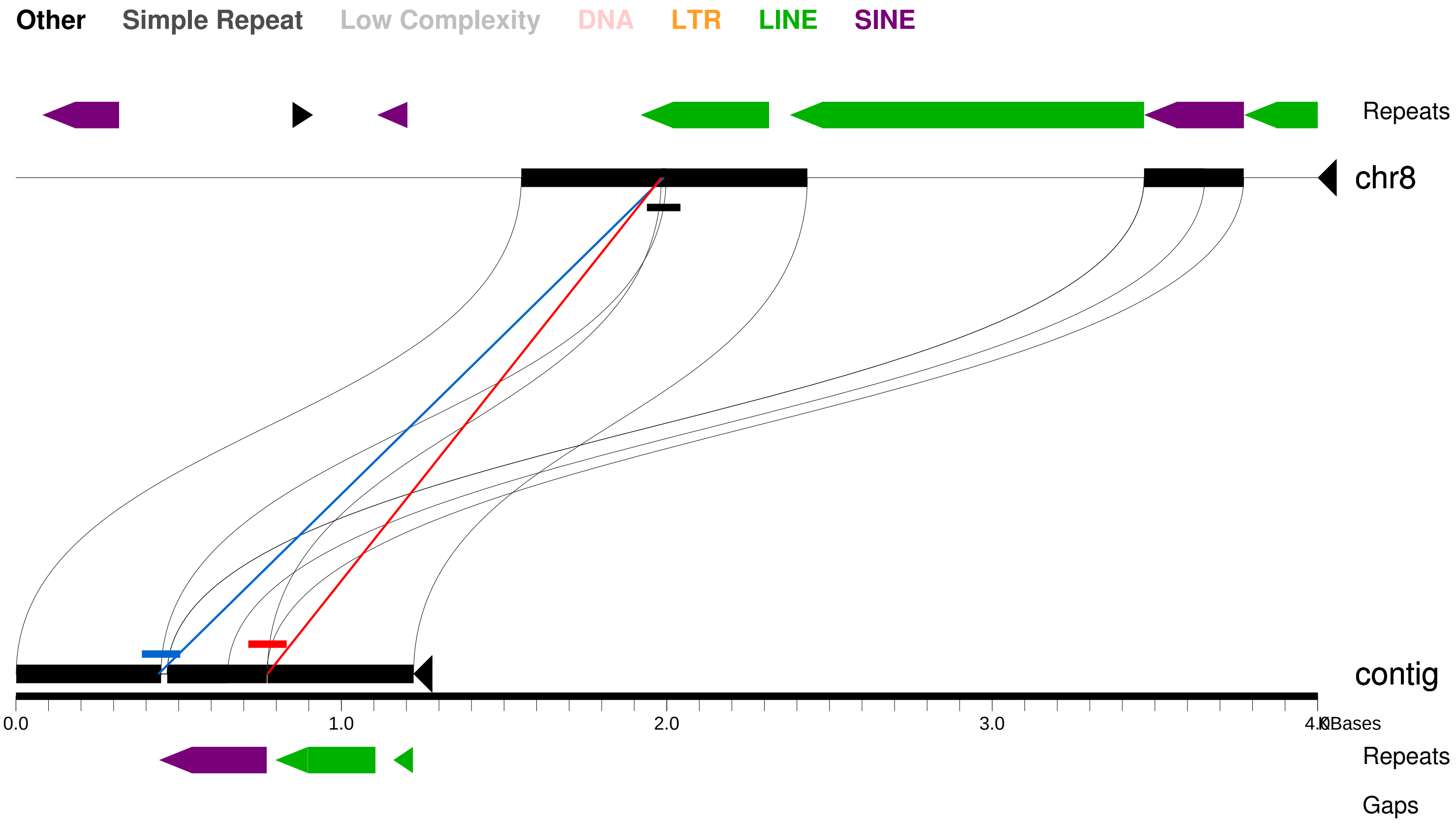

siteID: chr8_127865480 Scafftig4 - Breakpoint: chr8:127865467
chr8:127865420-127865521
Overlap Length: 13 INS size: 279 Genotyped: Yes

```
left     CAACTTTAAATTGGAAGAAAGGATCAGA----GTCTCATTTACACT--------------AACATAAAAA
chrom    CAACTTTAAATTGGAAGAAAGGATCAGA----GTCTCATTTACACT--------------AACATAAAAA
right    CA-CTCCAGCCTGGGCGACAGAGCGAGACTCCGTCTCAAAAAAAAAAAAAAAAAAAAAAAAAAAAAAAAA
         **1**11*111***11**1**1111***1111******111*1*1111111111111111**1*1*****

left     ACAATGTATTGGGAGGCCGAGGCGGGCGGATCACGAGGTCAGGAGATCGAGACCATCCCGGCTAAAA
chrom    ACAATGTAAT---ATGTCATGTCTCC---AACA-GACGTTTGTTCATTAA-------CTGGTTC---
right    ACAATGTAAT---ATGTCATGTCTCC---AACA-GACGTTTGTTCATTAA-------CTGGTTC---
         ********2*222*2*2*22*2*222222*2**2**2**22*222**22*2222222*2**2*2222
```

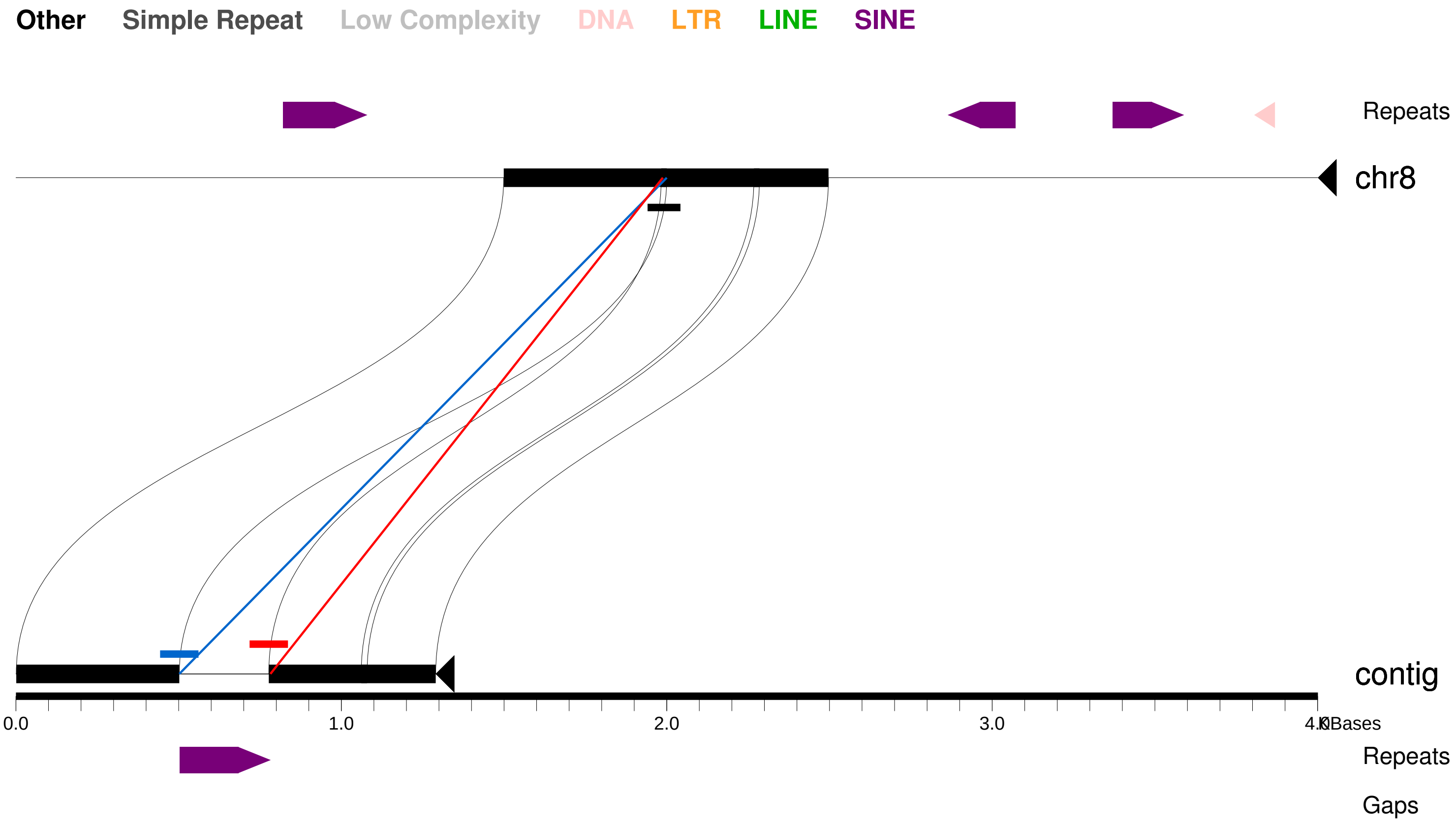

siteID: chr8_128038109 Scafftig1 - Breakpoint: chr8:128038124
chr8:128037964-128038284
Overlap Length: 1 INS size: 324 Genotyped: Yes

```
left   T-GCTTTTTGTTATAACAGCATTAGAAACCTAGTACAGATTCTGATAGCTAGAAGTGGGGTGCTGCAGTA
chrom  TTGCTTTTTGTTATAACAGCATTAGAAACCTAGTACAGATTCTGATAGCTAGAAGTGGGGTGCTGCAGTA
right  C--------GTGAACCCGG-----GAAGCGGAGCT----TGCAGTGAGCCGAGATTGCGCCACTGCAGTC
       1 1111111**1*111*1*11111***1*11**111111*1*1*11***1111*1**1*111*******1

left   ACAAATAACTAAAAATGTGAATGTGTCTTTGGAATTGGCCAAGGGGCAGGGGCTGGAAGAATTTTGAGTA
chrom  ACAAATAACTAAAAATGTGAATGTGTCTTTGGAATTGGCCAAGGGGCAGGGGCTGGAAGAATTTTGAGTA
right  CGCAGTCCG---------------GCCTGGGCGACAGAGCGAGACTCCGTCTCAAAAAAAAAAAAAAAAA
       111*1*1111111111111111111*1**11*11*11*11*1**111*1*111*111**1**11111*11*

left   GCATGTTAGAAAAAGCTTAGAGGC-CGGGCGCGG-----TGGCTCACGCCTGTAATCC------CAGCAC
chrom  GCATGTTAGAAAAAGCTTAGATTCTCTTGAACAGACTGTTAGTAGAAATATGGAGTTAACGATTCTGCTA
right  AAAAAAAAAAAAAAAAAAAA-ATTTTCTTGAAAAGACTGTTAGTAGAAATATGGAGTTAACGATTCTGCTA
       11*1111*1*****1111*1*2212*22*2212*22222*2*222*2222**2*2*22222222*2**22

left   TTTGGGAGGCCGAGGCGGGTGGATCATGAGGTCAGGAG-ATCGAGACCATCCT---GGCTAACAAG---G
chrom  ATGAGGACTCAGAAGAAAGTGAAGGACACAGTAAAAAATATATGTGTCATTTTATAGACTACCTAAATTG
right  ATGAGGACTCAGAAGAAAGTGAAGGACACAGTAAAAAATATATGTGTCATTTTATAGACTACCTAAATTG
       2*22***22*2**2*222***2*22*2222**2*22*22**222222***22*222*2***2*2*2222*

left   TGAAACCCCGTCTCT----------------ACTAAA----
chrom  TCATATGTAGACTGTTGGTAGAAATATGAATATTAAAGGCA
right  TCATATGTAGACTGTTGGTAGAAATATGAATATT-------
       *2*2*2222*2**2*2222222222222222*2*111
```

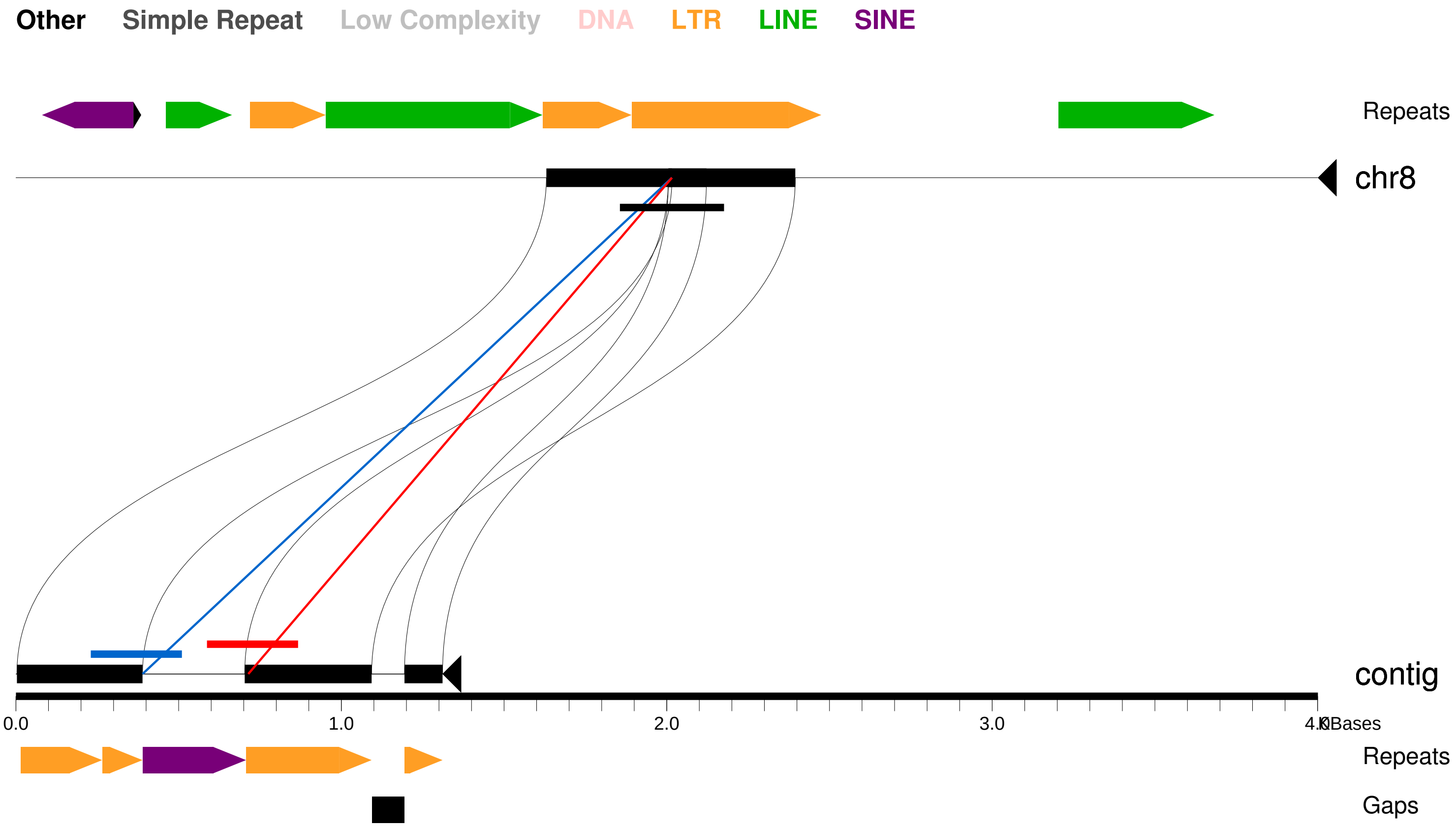

siteID: chr8_128356671 Scafftig2 - Breakpoint: chr8:128356653
chr8:128356608-128356712
Overlap Length: 15 INS size: 161 Genotyped: Yes

```
left    TGACT---TTTCCATCTTTACAATCTTCTTTCACTTTGTAGATGCATT-----------AAAAATTGCCT
chrom   TGACT---TTTCCATCTTTACAATCTTCTTTCACTTTGTAGATGCATT-----------AAAAATTGCCT
right   AGACTCCGTCTCAAAAAAAAAAAAAAAAAAAAAAAAAAAAAAAAAAAAAAAAAAAAAAAAAAAATTGCCT
        1****111*1**1*11111*1**111111111*111111*1*111*1111111111111**********

left    GGCTGGGAGGCTGAGGC-AGGAGAATGGCGT-GAACCCGGGAAGCGGAGCTTGCAGTGAGCCGAG
chrom   GGCTAA----CTGATGTGAGTAATATGTCATTGAGCCC---AAG--------GCCATGGGGA-AT
right   GGCTAA----TTGATGTGAGTAATATGTCATTGAGCCC---AAG--------GCCATGGGGA-AT
        *****2222221***2*22**2*22***2*2*2**2***222***22222222**22**2*222*2
```

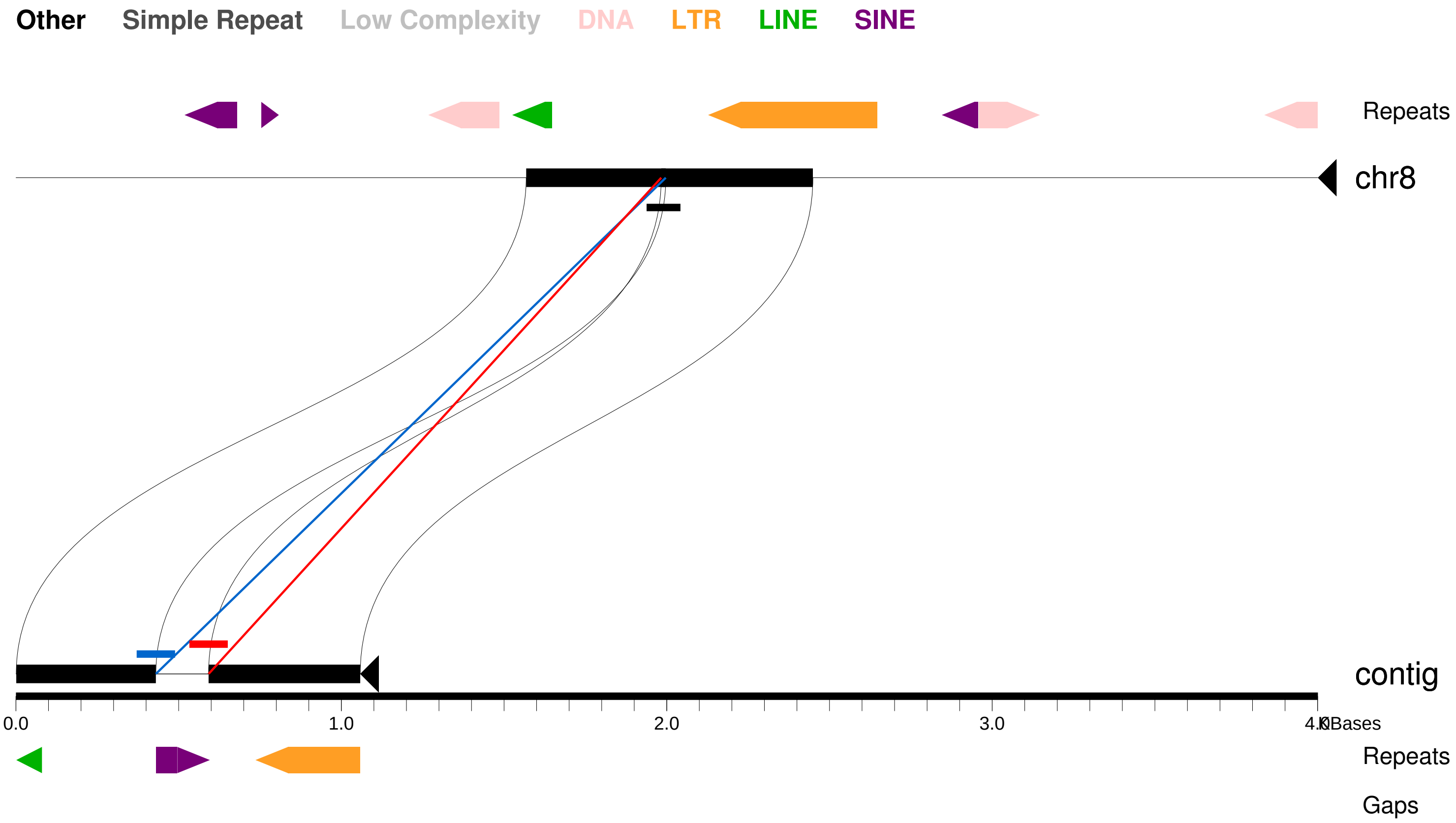

siteID: chr8_129708025 Scafftig4 + Breakpoint: chr8:129708031
chr8:129707873-129708196
Overlap Length: 6 INS size: 377 Genotyped: No

```
left    NNNNNNNNNNNNNTTGGAATAAGGCTAGGTCATCTGCAGTATTGTGCTTGAGTAGGGTTGTTATTGTCTA
chrom   TTGCTTGCATGACTTGGAATAAGGCTAGGTCATCTGCAGTATTGTGCTTGAGTAGGGTTGTTATTGTCTA
right   NNNNNNNNNNN---------------------------------------------------------------
        NNNNNNNNNNNNN1111111111111111111111111111111111111111111111111111111111111

left    AAAGTTTTCTGTCTCACTAACTGGCCCCTTTCCTCATCCTTGGCTAGAGAGATCAGGCTTTTATTGAAGC
chrom   AAAGTTTTCTGTCTCACTAACTGGCCCCTTTCCTCATCCTTGGCTAGAGAGATCAGGCTTTTATTGAAGC
right   ----------------------------------------------------------------------
        1111111111111111111111111111111111111111111111111111111111111111111111

left    TCTTGTAAAAAAAAA-TCTGTGCCG---GG-----CGCGGTGGCTCACGCCTGTAATCCCA-----GCAC
chrom   TCTTGTAAAAAAAAAATCTGTGCCCATTGGTATTTCTAGTTTGATGGCTTCTCTAGTCCTAAGTGTGGTA
right   ------------------TGTGCCCATTGGTATTTCTAGTTTGATGGCTTCTCTAGTCCTAAGTGTGGTA
        111111111111111 11******2222**22222*22*2*2*2*22*22**2**2***2*22222*222

left    TTTGGGAGGCCGAG----------GCGGGCGGATCACGAGGTCAGGA-----GATC-GAGA---CCATCC
chrom   TATATTAGGCAAAAACAAAACCTAGCTAACTTACCACCTTGTCATTCCTCAGGATCCGAGGTTTCTAGCT
right   TATATTAGGCAAAAACAAAACCTAGCTAACTTACCACCTTGTCATTCCTCAGGATCTGAGGTTTCTAGCT
        *2*222****22*22222222222**222*22*2***222****22222222****N***2222*2*2*2

left    CGGCT----AAAACGGTGAAACCCCGTCTCTAC-TAAAA-----
chrom   GGTCTGCTCAGAGTCTTCTTATGCTTGCTTTATGTACAATATCC
right   GGTCTGCTCAGAG-------------------------------
        2*2**2222*2*2NNN1NNN1NN1NNN11N11N 11N11
```

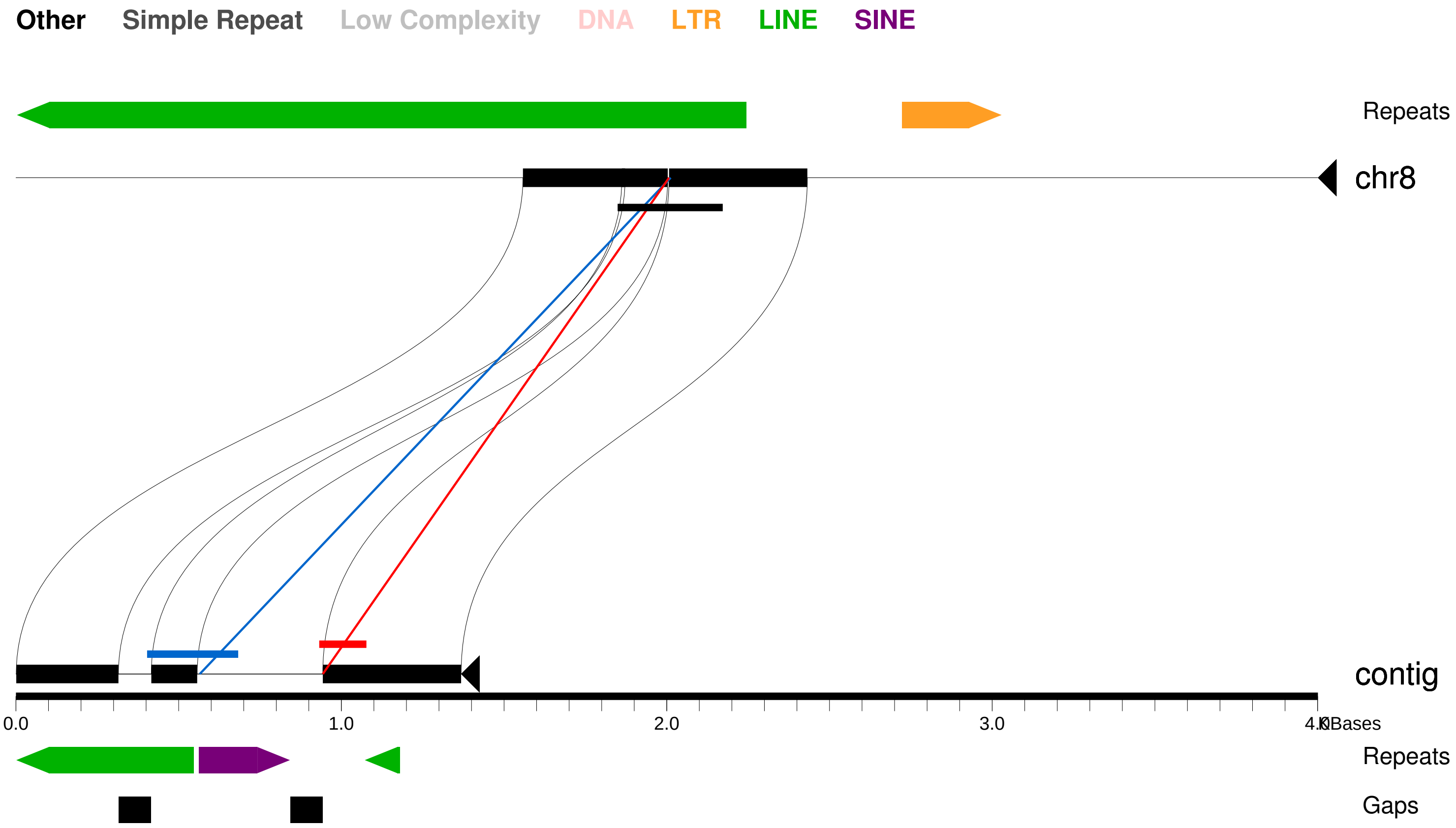

siteID: chr8_129955730 Scafftig1 - Breakpoint: chr8:129955753
chr8:129955708-129955812
Overlap Length: 15 INS size: 443 Genotyped: No

```
left     CATTAGTCAATCTACACTTAAGGTATAATTCCAACCATTGCT--------------ATCAAGAAAGAGAT
chrom    CATTAGTCAATCTACACTTAAGGTATAATTCCAACCATTGCT--------------ATCAAGAAAGAGAT
right    NNNNNNNNNNNNNNNNNNNNNNNNNNNNNNNNNNNNNNNNNNNNNNNNNNNNNNAAAAAAAGAAAGAGAT
         11111111111111111111111111111111111111111111111111111111*11***********

left     GGTGGCCGGGCGCGGTGGCTCACGCCTGTAATCCCAGCACTTTGGGAGGCCGAGGCGGGCGGA
chrom    GGTGAA-------GTTAGCTA----CTGGCAGCAGAGAAAGTTCAATA---TTGGCTTACACC
right    GGTGAA-------GTTAGCTA----CTGGCAGCAGAGAAAGTTCAATA---TTGGCTTACACC
         ****222222222*2*2***22222***22*2*22**2*22**2222222222***222*222
```

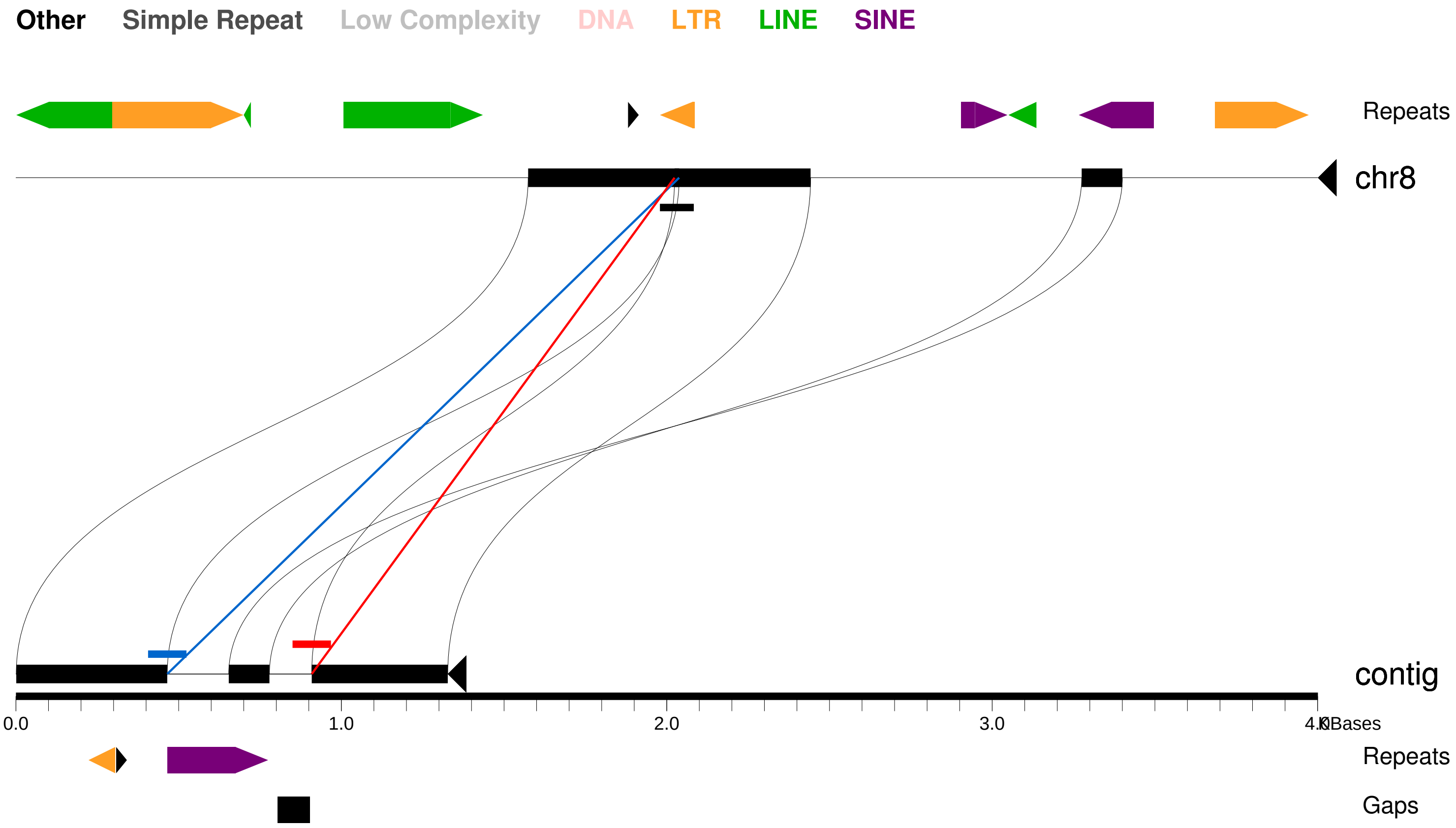

siteID: chr8_130270664 Scafftig1 + Breakpoint: chr8:130270490
chr8:130270447-130270549
Overlap Length: 17 INS size: 213 Genotyped: No

```
left    AGGTTCAATCTCAACAACTCAACAAGCGAGAAACAACCCC----------------ATTAAAAAGTAGGC
chrom   AGGTTCAATCTCAACAACTCAACAAGCGAGAAACAACCCC----------------ATTAAAAAGTAGGC
right   NNNNNNNNNNNNNNNNNNNNNNNNNNNNNNNNNNNNNNNNNNNNNNNNNNNNNNNNAAAAAAAAGTAGGC
        1111111111111111111111111111111111111111111111111111111*11***********

left    AAAGGAGGCCGAGGCGGGCGGATCACGAGGTCAGGAGATCGAGACCATCCTGGCTAACACGGTGA
chrom   AAAGGACACAAATA--GACATTTCTCAA---AAGATGAT--ATACAAGCA--GCCAAC-------
right   AAAGGACACAAATA--GACATTTCTCAA---AAGAGGAT--ATACAAGCA--GCCAAC-------
        ******22*22*2222*2*222**2*2*2222**2N***22*2**2*2*222**2***2222222
```

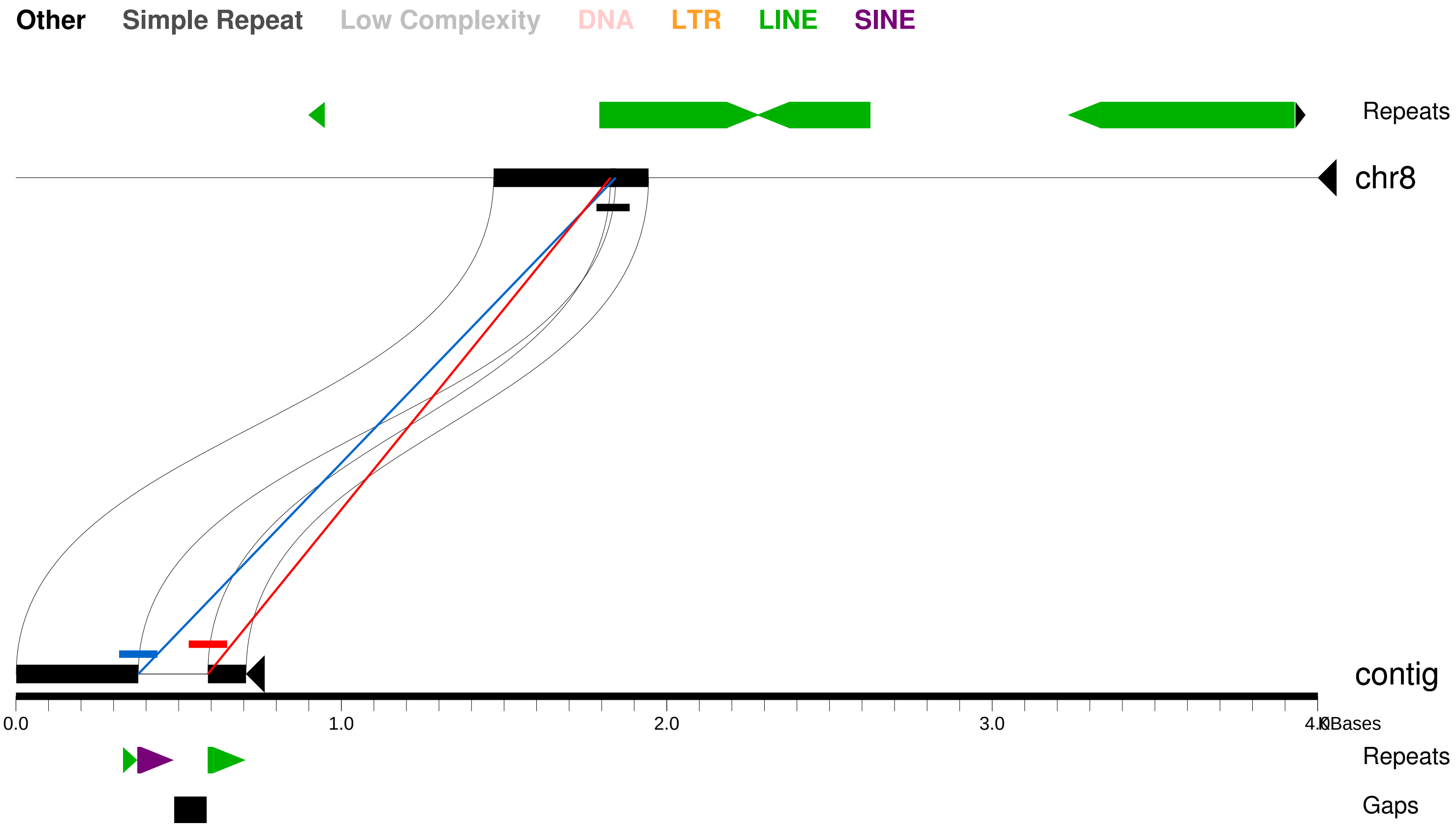

siteID: chr8_132139147 Scafftig1 - Breakpoint: chr8:132139145
chr8:132138985-132139312
Overlap Length: 8 INS size: 312 Genotyped: Yes

```
left   C----------ACCTCCTTATTCC-ACCTTAGTGTGTGCTAAGCTTTTGCTTTCTATGAACATGACTATC
chrom  CCAATGAC--CACCTCCTTATTCC-ACCTTAGTGTGTGCTAAGCTTTTGCTTTCTATGAACATGACTATC
right  AGAATGGCGTGAACCCGGAAGGCGGAGCTTGCAGTGAGCGGAGATCGTGCC--------ACAGCACTCTC
       1N2222N211N*1*1*111*11*11*1***111***1**11**1*11***11111111***11***1**

left   CTCCTTTCGAACTTTATACTCCTGTAGTTCCAAGCTTCTTCTTGTTTCCCAACACTCTTTGTTCTTTTAT
chrom  CTCCTTTCGAACTTTATACTCCTGTAGTTCCAAGCTTCTTCTTGTTTCCCAACACTCTTTGTTCTTTTAT
right  GCCTGGGCGAC--------------AGAGCGAGACTCCGTCT-------CAAAAAAAAAATAAAAAATAA
       11*1111***111111111111111**11*1*11**1*1***1111111***1*1111111111111**1

left   GTGTCAGGGAAATAAGAGTCATTAAGAAGTTGGCCGGGC-GCGGTGGC----TCACGCCTGTA-------
chrom  GTGTCAGGGAAATAAGAGTCATTAAGAAGTTTTAATCAATGAGGTGATATAGTCAGATTTGCATTTTTGA
right  AAAAAAAAAAAATAAAAAAAAAAAAGAAGTTTTAATCAATGAGGTGATATAGTCAGATTTGCATTTTTGA
       11111*111******1*111*11********222222222*2****222222***2222**2*2222222

left   ---ATCCCAG--CACTTTGGGAGGCC--GAGGCGGGCGG---ATCAC---GAGGTC-----------AGG
chrom  AAGATGGCAGGCCATTTTGGAAGAGCAGGAGACCAGCTGGAAACCACAGAGAGGTCTGAGCACAAATAGC
right  AAGATGGCAGGCCATTTTGGAAGAGCAGGAGACCAGCTGGAAACCACAGAGAGGTCTGAGCACAAATAGC
       222**22***22**2*****2**22*22***2*22**2*222*2***222******22222222222**2

left   AGATCGAGACCACGGTGA---AACCCCGTCTCTACTAAAAATACAAAAAAT
chrom  AGATGTAGGATGGAGAGACAGAAACCTTTTTAGCGGCAGAAATTGATAGTT
right  AGATGTAGGATGGAGAGACAGAAACCTTTT---------------------
       ****22**222222*2**222**2**22*21NNNNNN1N11NNNN1N1NN1
```

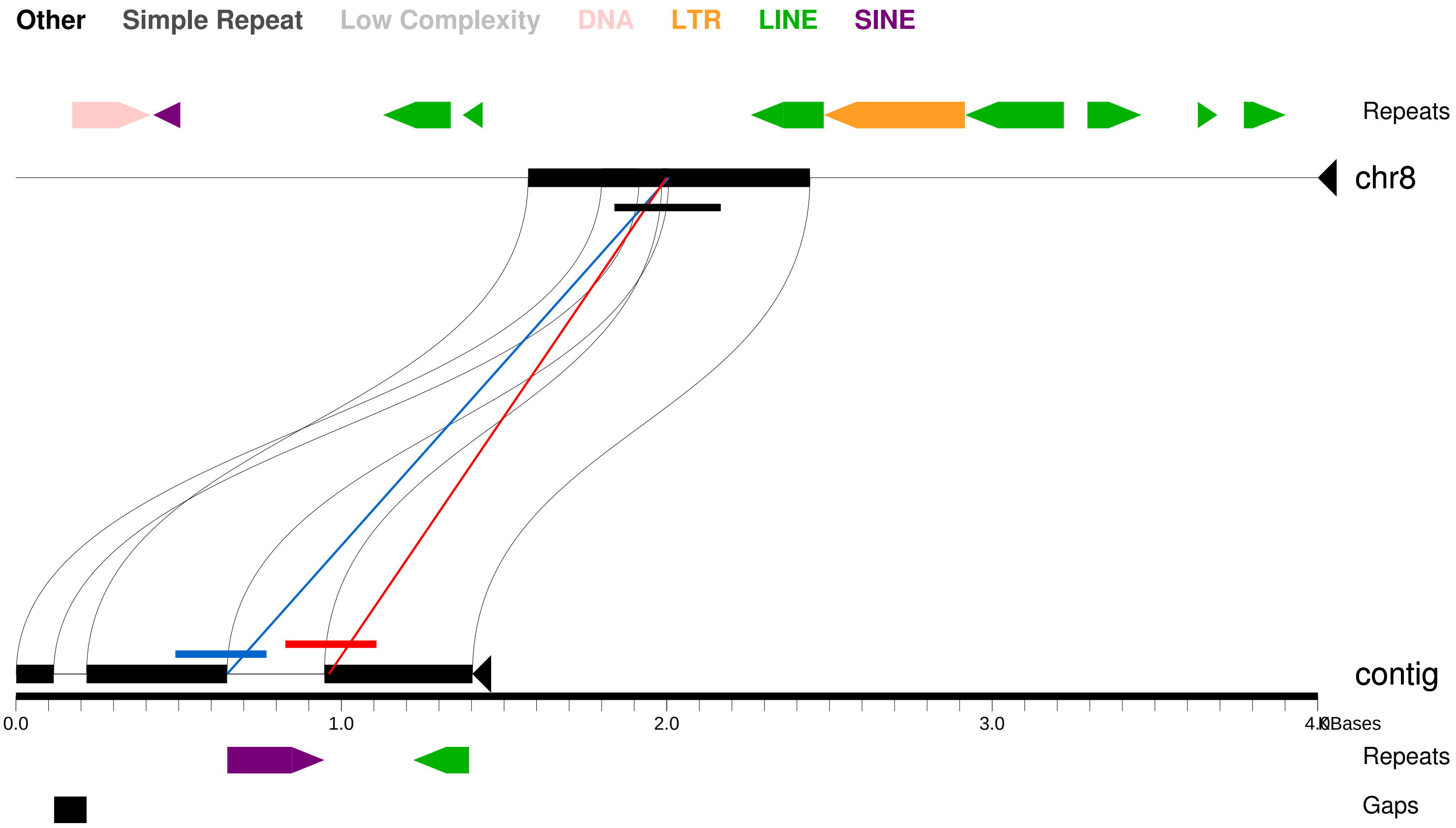

siteID: chr8_13650909 Scafftig1 - Breakpoint: chr8:13650884
chr8:13650841-13650943
Overlap Length: 14 INS size: 421 Genotyped: No

```
left    -CATCACCTCAATGTTTATCTCAGTTTTGTTGAATATTTATGTA----------------CGCCATCTTT
chrom   -CATCACCTCAATGTTTATCTCAGTTTTGTTGAATATTTATGTA----------------CGCCATCTTT
right   TCCGCCCGCCTCGGCCTCCCAAAGTGCTGG-GATTACAGGCGTGAGCCACCGCGCCCGGCCGCCATCTTT
        1*11*1*11*111*11*11*11***11**11**1**11111**1111111111111111**********

left    TTTTNNNNNNNNNNNNNNNNNNNNNNNNNNNNNNNNNNNNNNNNNNNNNNNNNNNNNNNNNNNNNN
chrom   TTTTCCTAGATCTATGATACAGCTGAATTACTTAATGCACATTAATTTTG----------------
right   TTTTCCTAGATCTATGATACAGCTGAATTACTTAATGCACATTAATTTTG----------------
        ****22222222222222222222222222222222222222222222222222222222222222
```

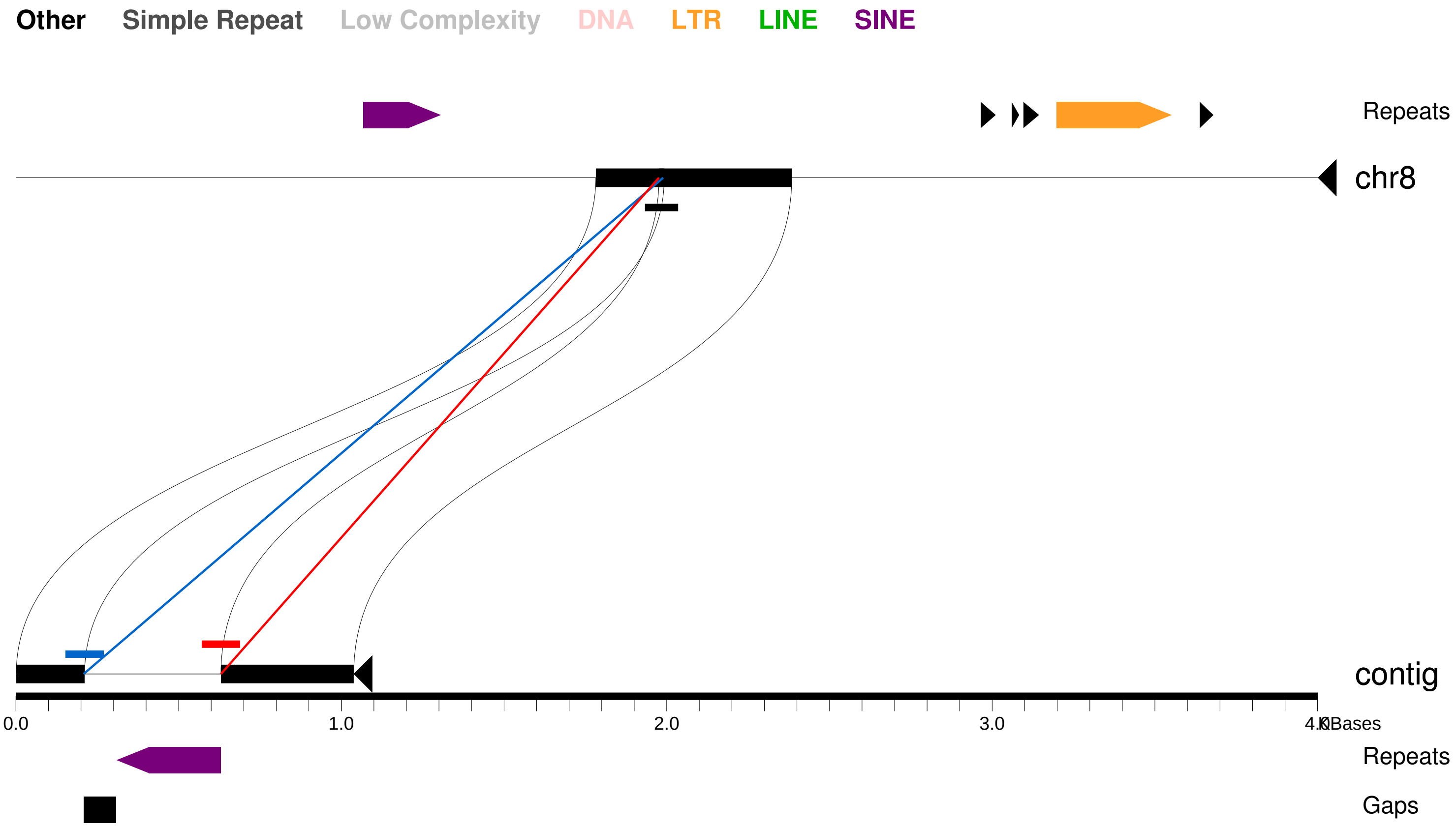

siteID: chr8_137062186 Scafftig1 - Breakpoint: chr8:137062192
chr8:137062146-137062251
Overlap Length: 14 INS size: 304 Genotyped: Yes

```
left   G--GTGCTTCCTTCCC------------CTTTGTCTTCCACCATGATTGTAAGCTTCCTTAAACCTCCCC
chrom  G--GTGCTTCCTTCCC------------CTTTGTCTTCCACCATGATTGTAAGCTTCCTTAAACCTCCCC
right  GCACTCCAGCCTGGGCAAAAGAGCGAGACTCTGTCTCAAAAAAAAAAAAAAAAAAAAAAAAAACCCCCCC
       *111*1*11***111*111111111111**1*****111*11*11*1111**11111111*****1****

left   AGCCGGCCGGGCGCGGTGGCTCACGCCTGTAATCCCAGCACTTTGGGAGGCCGAGGCAGGCGG
chrom  AGCCCTGCAGAA-CTGTGAGCCAA---TTAAACCTCTTTCCTTTATAA---------ATTACC
right  -GCCCTGCAGAA-CTGTGAGCCAA---TTAAACCTCTTTCCTTTATAA---------ATTACC
       1***222*2*222*2***222**2222*22**2*2*2222****222*222222222*22222
```

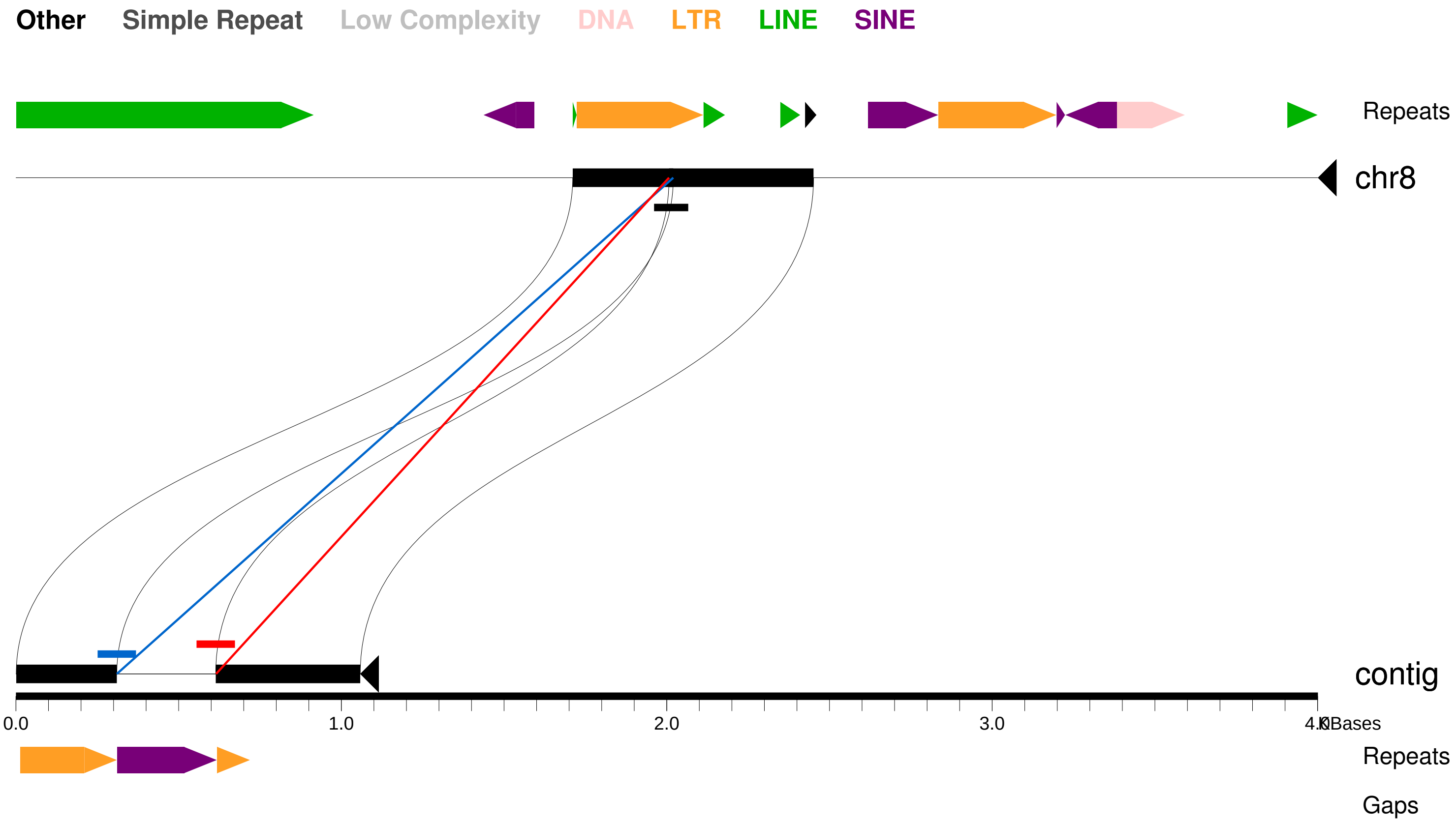

siteID: chr8_138295140 Scafftig6 - Breakpoint: chr8:138295184
chr8:138295072-138295243
Overlap Length: 12 INS size: 305 Genotyped: Yes

```
left     AGGAACCACTTCCAGCTTCTTCTTTCTCTACCGCCTATACTCGCTGAGGCACCATCCTTGGCCCATGCCC
chrom    AGGAACCATTTCCAGCTTCTTCTTTCACTACCGCCTATACTCGCTCAGGCACCATCGTTG----------
right    ------------CCGC--------------CCGCCTCGGC---CTC-----CCAAAGTG-----------
         11111111N111*1**1111111111N111******111*111**211111***112*112222222222

left     TACTCTTACTTCAGTGCTGGTGCTAGAGTGCTCCCTTCAGGAAGTAGCAGGAACTGAAGGTATTGTT---
chrom    -ACTCTTACTTCAGTGCTGGTGCTAGAGTGCTCCCTTCAGGAAGTAGCAGAAACTGAAGGTATTGATAGA
right    --CTGGGATTACAG-GCGTGAGCCACCGCGCCC----------------GGCCCTGAAGGTATTGTTAGA
         21**111*1*1***1**11*1**1*11*1**1*1111111111111111*N11************N*222

left     -TTTTTTTTTTTTTTT-------TGAGA---CGGAGTTTCGCT
chrom    GTTCATGTTCCCTTCTCCCTCACTGATCTCTCTCAAATTAACA
right    GTTCATGTTCCCTTCTCCCTCACTGATCTCTCTCAAATTAACA
         2**22*2**222**2*2222222***22222*22*22**22*2
```

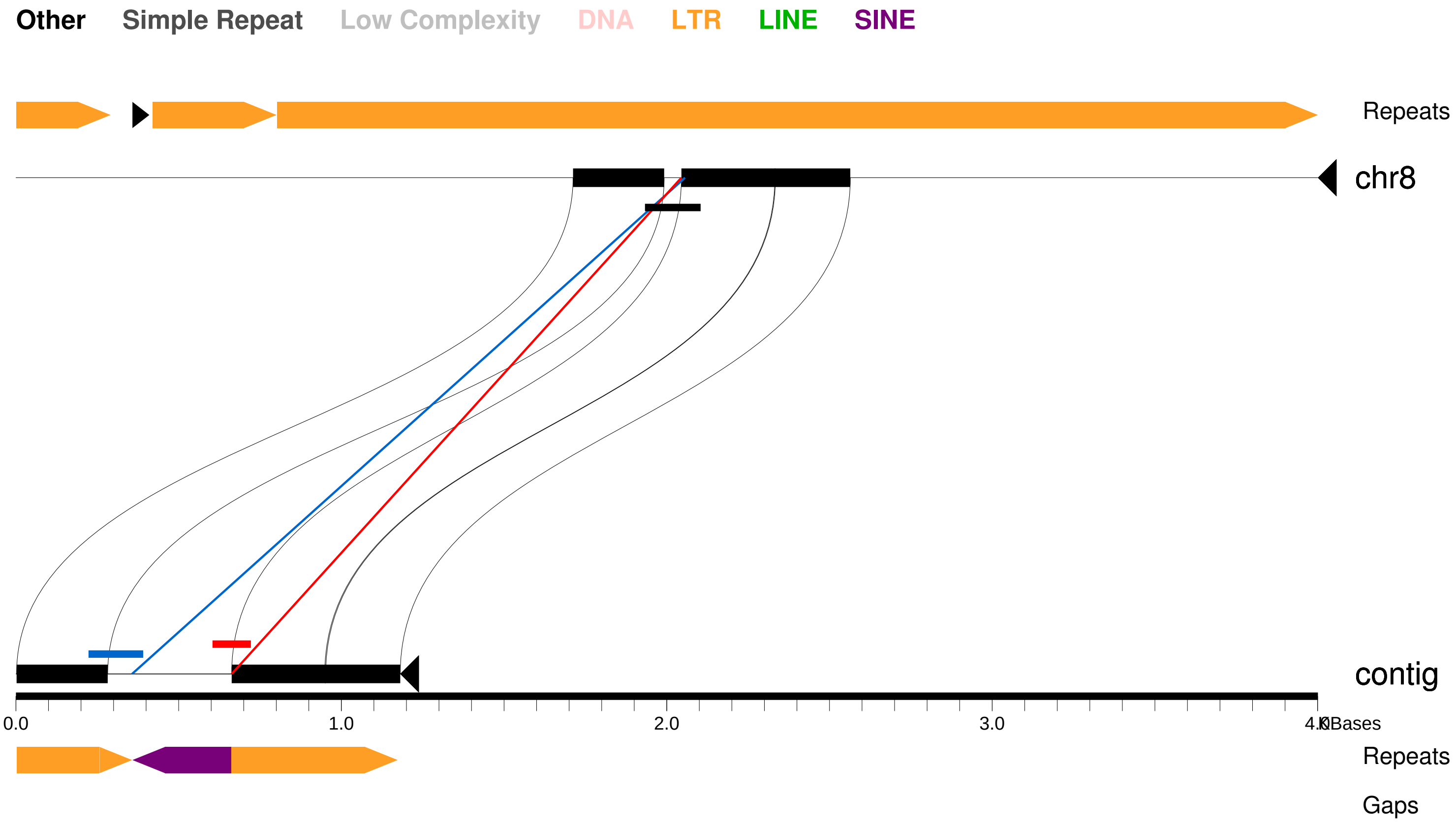

siteID: chr8_139117399 Scafftig1 - Breakpoint: chr8:139117405
chr8:139117358-139117499
Overlap Length: 13 INS size: 328 Genotyped: Yes

```
left    CTTAGAGGGGTTAAGTCACATGTCTGAGGTCTCACAGATACATTTTTAAAAGAGTCTGCAGGCCGGGCGC
chrom   CTTAGAGGGGTTAAGTCACATGTCTGAGGTCTCACAGATACATTTTTAAAAGAGTCTGCATACAAAGCAC
right   AAAAAA------AAAAAAAA-------------AAAAAAAAA-----AAAAGAGTCTGCATACAAAGCAC
        111*1*111111**111*1*1111111111111*1*1*1*1*11111************22*222**2*

left    ----GGTGGCTCA-CGCCTGTA-------ATCCCAGCACTT-TGGGAGGCCGAGGCGGGC----------
chrom   TTGAGGTGAATGA-TAAATGAAGGTATGAATGAGTGAATAAATGAAAGTATGAATAAGTAAATTAATGAA
right   TTGAGGTGAATGAATAAATGAAGGTATGAATGAGTGAATAAATGAAAGTATGAATAAGTAAATTAATGAA
        2222****22*2*12222**2*2222222**2222*2*2222**22**222**2222*222222222222

left    -GG
chrom   TGA
right   TGA
        2*2
```

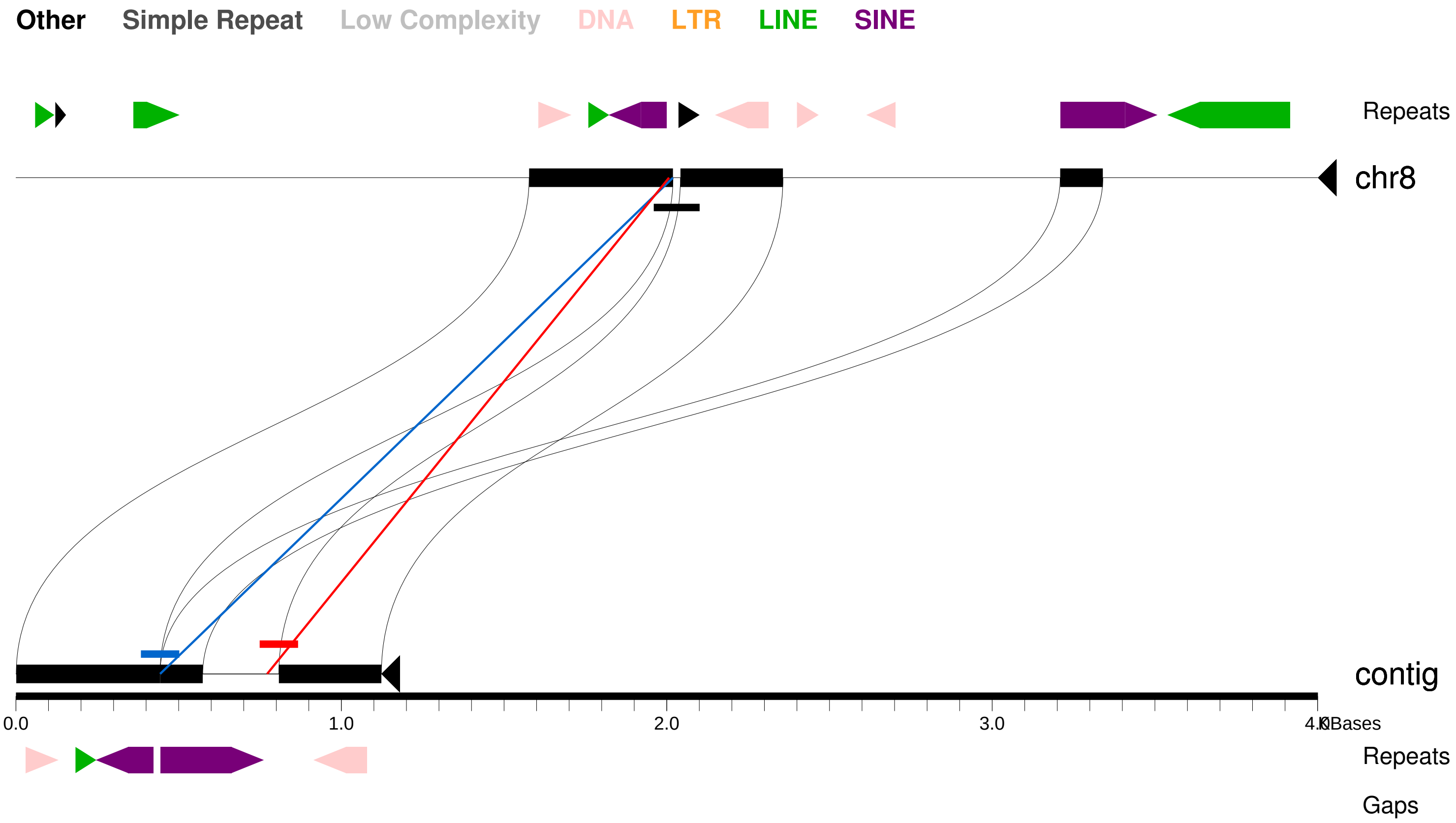

siteID: chr8_13975432 Scafftig1 - Breakpoint: chr8:13975419
chr8:13975374-13975478
Overlap Length: 15 INS size: 309 Genotyped: Yes

```
left    G-----------------AGAATGATGTTAT--CTAGCTTCATCCATGTCCCTGCAAAGGACATGAACTC
chrom   G-----------------AGAATGATGTTAT--CTAGCTTCATCCATGTCCCTGCAAAGGACATGAACTC
right   CGCCCGCCTCGGCCTCCCAAAGTGCTGGGATTACAGGCGTGAGCCA-----CCGCGCCCGGCCCGAACTC
        111111111111111111*1*1**1**11**11*11**1*1*1***11111*1**1111*1*11******

left    ATCCTTTTTTTTTTTTTTTTTTTTTTTTTTTTTTTTGAGACGGAGTCTCGCTCTGTCGCCCAGGCTGG
chrom   ATCCTTTTTATGGTTGCTTAGTATTCCATGGTTTATATG--------------TGCCACATTTTCTTT
right   ATCCTTTTTATGGTTGCTTAGTATTCCATGGTTTATATG--------------TGCCACATTTTCTTT
        *********2*22**22**22*2**222*22***2*22*22222222222222**2*2*22222**22
```

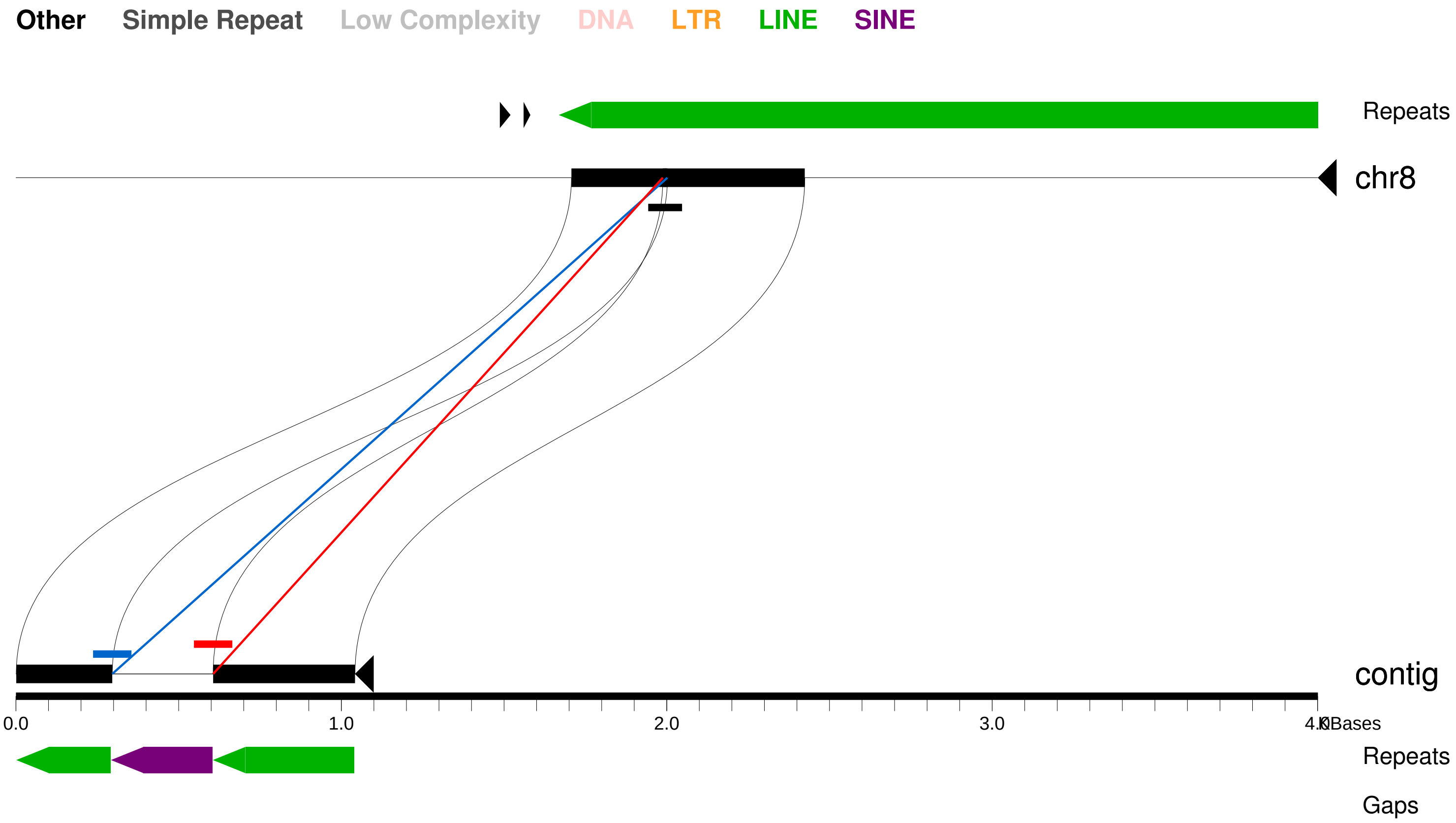

siteID: chr8_140081486 Scafftig1 - Breakpoint: chr8:140081519
chr8:140081359-140081748
Overlap Length: 70 INS size: 419 Genotyped: No

```
left   C---------------------------------------------------------------------
chrom  CAGCCTACACCATGACAGACACTGTGCTGGACTTTGGCACTGGACTTTGGCACTGTGCTGGATGAGAAGG
right  TTTAGTAGAGACGGGGTTTCACCGT-------TTTAGC-CGGGATGGTCTCGATCTCCTG----ACCTCG
       1NNNN22N2NNNN2NNNNN222N22       222N22 2N222NNN2NN2NN2N2N222    2NNNN2

left   --------------------------CCACACTGAACTGACAATGTAGTTGGGGTGGCAGTAAACAGAAG
chrom  TGAGAAAAAATAGGCATGATGTCTGCCCACACTGAACTGACAATGTAGTTGGGGTGGCAGTAAACAGAAG
right  TGA------------------TCCGCCCGCCTCGGCCTCCCAAAGT-GCTGGGACTACAGGCGTGAGC--
       222                  22N22**1*111*11**11***1**1*1****1111***11111**111

left   GTCACAAAATCAGCATAAAATTTCACCTATTCTAAGTGTTAGAAAGCAGAATATAGGAGGCGATGAGAAT
chrom  GTCACAAAATCAGCATAAAATTTCACCTATTCTAAGTGTTAGAAAGCAGAATATAGGAGGCGATGAGAAT
right  --CACCGCGCCCGGCCAA--TTTCACCTATTCTAAGTGTTAGAAAGCAGAATATAGGAGGCGATGAGAAT
       11***11111*1*111**11**************************************************

left   GGATGGTGGTGAATAAGGGANNNNNNNNNNN---------------------------------------
chrom  GGATGGTGGTGAATAAGGGAAGAAGCCATTACAGGCTTTGGGGATCAGAGGCCCTGTGATGAGGAGAGCC
right  GGATGGTGGTGAATAAGGGAAAAAGCCATTACAGGCTTTGGGGATCAGAGGCCCTGTGATGAGGAGAGCC
       ********************2N222222222222222222222222222222222222222222222222

left   ----------------------------------------------------------------------
chrom  TAGCAAGAGTACGGAACTGAGGTCCAGGGACCCTGGTGCTGAGCAGGGGAGGCATTGGATGAGGCTGGAG
right  TAGCAAGAGTACGGAACTGAGGTCCAGGGACCCTGGTG--------------------------------
       22222222222222222222222222222222222222

left   ----------------------------------------
chrom  AAACGGAGAGCACCGGATCACTCAGGACCTGGTAGGCTAT
right  ----------------------------------------
```

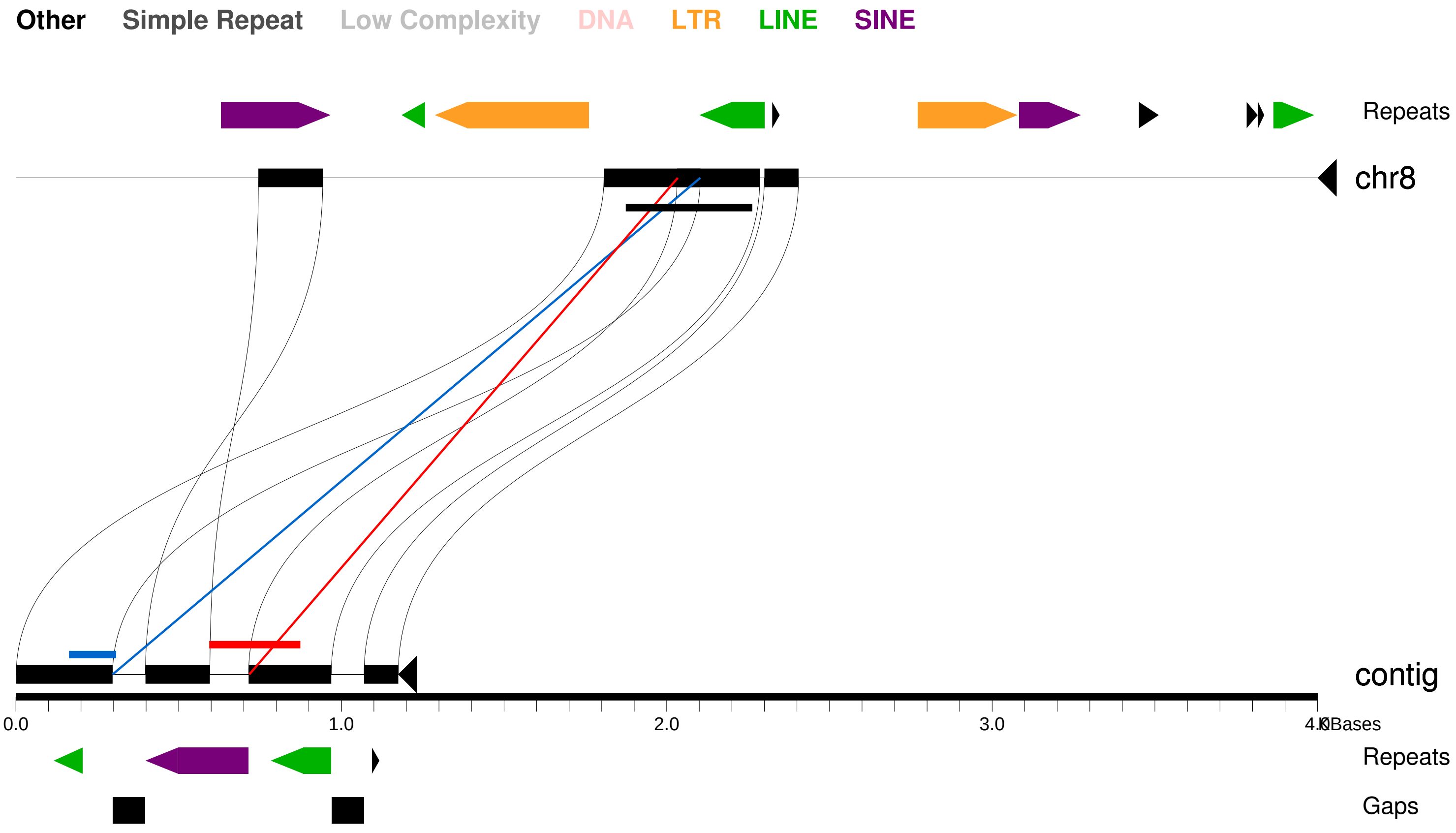

siteID: chr8_140593328 Scafftig1 + Breakpoint: chr8:140593322
chr8:140593222-140593378
Overlap Length: 11 INS size: 306 Genotyped: Yes

```
left   GCTTGAAACAGGGTTAAAATAGCAAATACCATGAGAAGAGAAACTTCAGAAGGAAAAAAAAGCCTTCAGT
chrom  GCTTGAAACAGGGTTAAAATAGCAAATACCATGAGAAGAGAAACTTCAGAAGGAAAAAAA-GCCTTCAGT
right  CC----ACCCGCCTCG-----GCC--TCCCA----AAGTG---CT------GGGATTACA-GGCGTGAGC
       1*1111*1*1*11*1111111**111*1***1111***1*111**111111**1*11*1*2*1*1*1**1

left   ATCAATCATAGAGGTGAAACATGTAATAATGGTTAAATTTTT-----------------------------
chrom  ATCAATCATAGAGGTGAAACATGTAATAATGGTTAAATTTTTAAAGCTCGCTAGCACTTGACTACAAAAA
right  ------CACCGCGCCCGGCCATG--------GTTAAATTTTTAAAGCTTGCTAGCACTTGACTACAAAAA
       111111**11*1*111111****11111111**********222222N22222222222222222222222

left   ----TTTTT-------TT
chrom  CAAATGTTTACCCTAATC
right  CAAATGTTTACCCTAATC
       2222*2***2222222*2
```

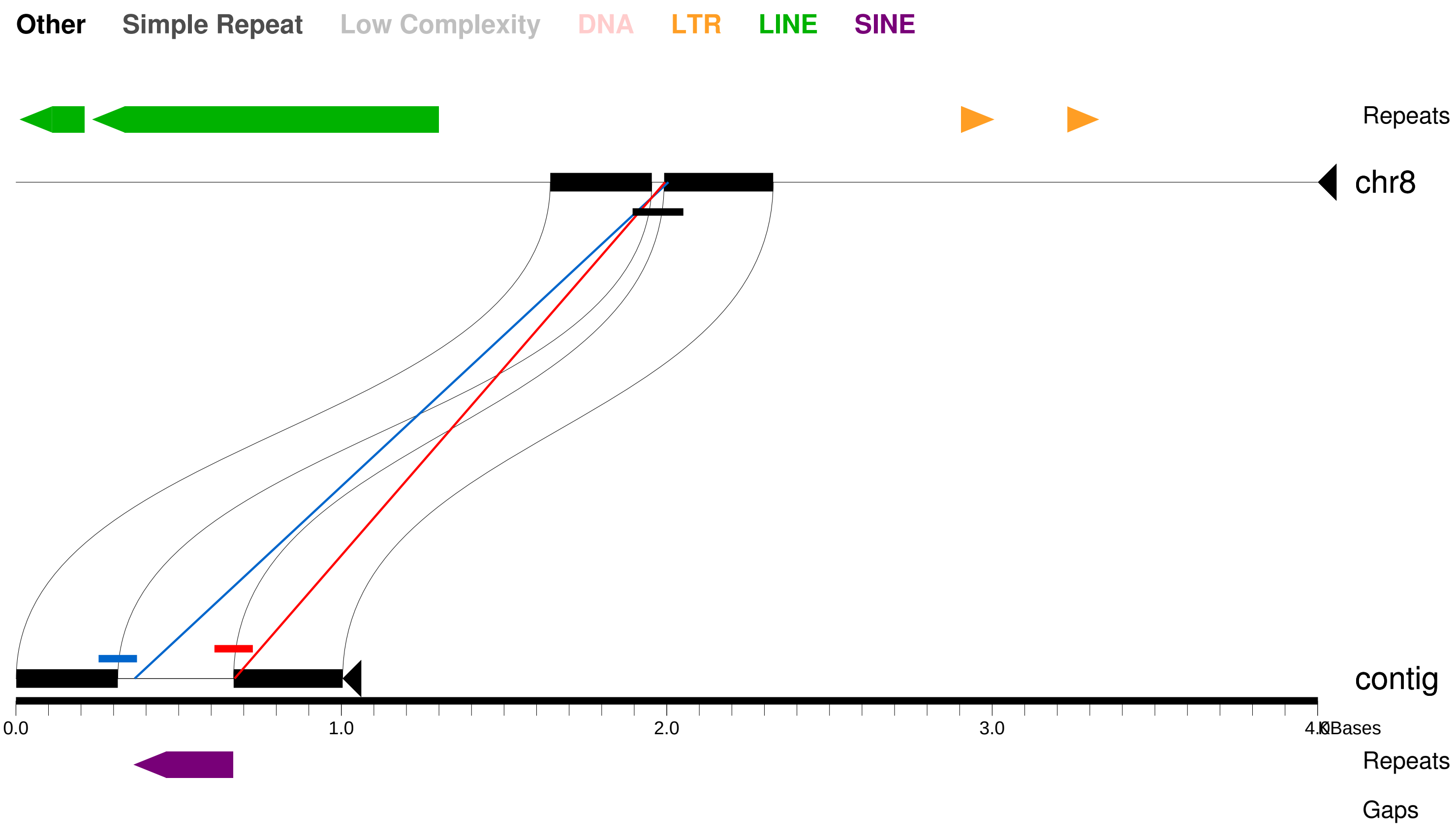

siteID: chr8_141048050 Scafftig3 - Breakpoint: chr8:141048052
chr8:141048003-141048108
Overlap Length: 11 INS size: 306 Genotyped: Yes

```
left     ACACACCC----------CAGAGCTTCCCTCGTGCTGCATGGTAGGAAAGTTGCTGAAA---GCTGTATT
chrom    ACACACCC----------CAGAGCTTCCCTCGTGCTGCATGGTAGGAAAGTTGCTGAAA---GCTGTATT
right    GCACTCCAGCCTGGGCGACAGAGCGAGACTCCGTCTCAAAAAAAAAAAAAAAAAAAAAAAAAGCTGTATT
         1***1**11111111111******1111***111**11*1111*11***1111111***111********

left     GTTGGCCGGGCGCGGTGGCTCACGCCTGTAATCCCAGCACTTTGGGAGGCCGAGGCGGGCGG-
chrom    GTTTCACAC---CTGTGT-----GCCTGAAATGAATGTCCTTA------CCCACTCTCTCCAA
right    GTTTCACAC---CTGTGT-----GCCTGAAATGAATGTCCTTA------CCCACTCTCTCCAA
         ***222*22222*2***222222*****2***2222*22***2222222**2*22*222*222
```

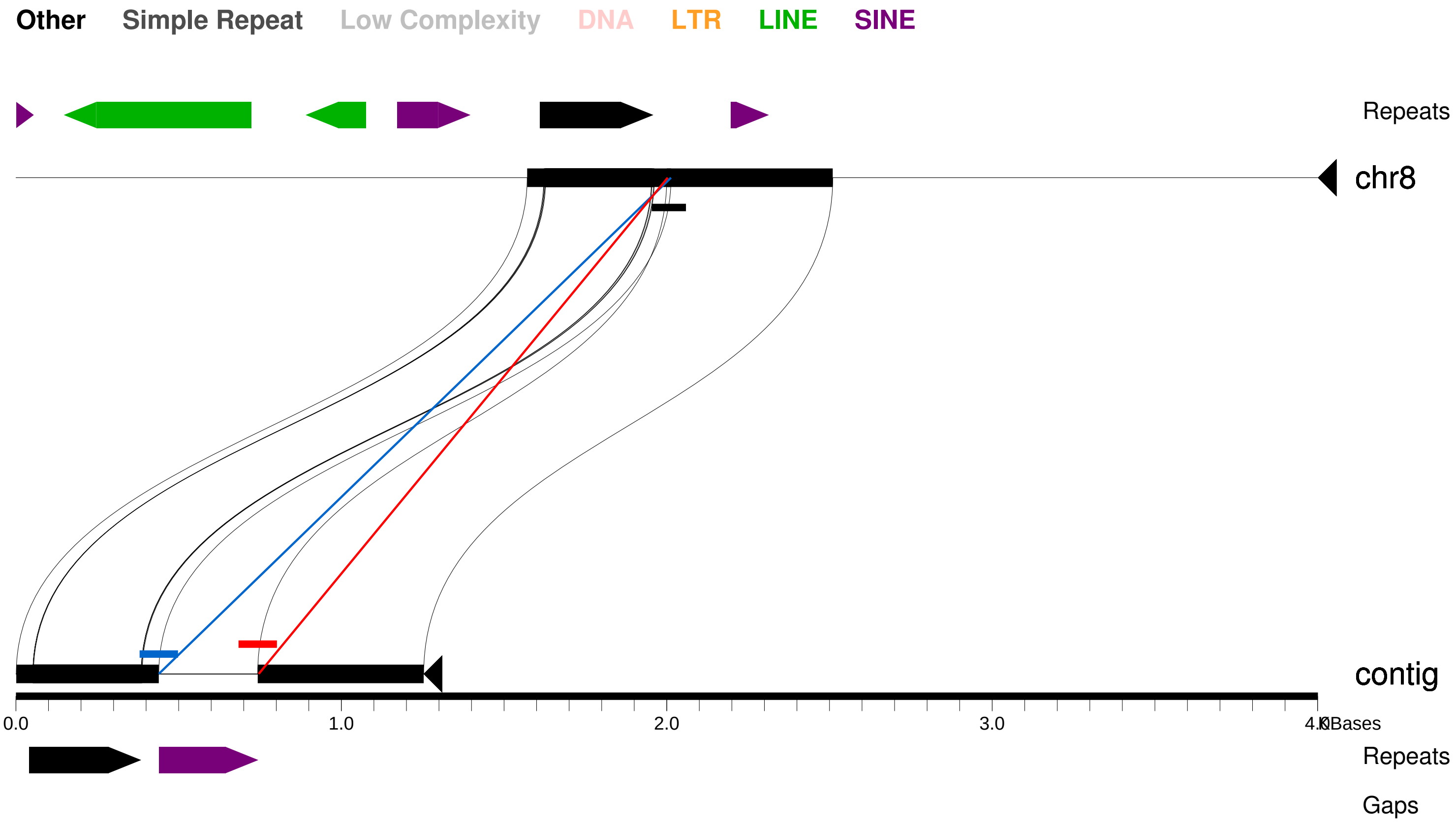

siteID: chr8_142940041 Scafftig5 + Breakpoint: chr8:142940044
chr8:142939996-142940103
Overlap Length: 12 INS size: 388 Genotyped: No

```
left     GATACAACTTCTAGATCATCATAACCATCATGATTACCAC-------CATCACCA-----------TCAC
chrom    GATACAACTTCTAGATCATCATAACCATCATGATTACCAC-------CATCACCA-----------TCAC
right    -----ATGGTCTCGATC-TCCTGACC-TCGTGATCCGCCCGCCTCGGCCTCCCAAAGTGCTGGGATTCAC
         11111*111***1****1**1*1***1**1****111*1*1111111*1**1*1*11111111111****

left     TACTTCTTTTTTTNNNNNNNNNNNNNNNNNNNNNNNNNNNNNNNNNNNNNNNNNNNNNNNNNNNNN
chrom    TACTTCTTACTATTATTGTTGCTGTTGTTTTTTTTTAAGACTGGGAGTTAGAAGAT-----------
right    TACTTCTTACTATTATTGTTGCTGTTGTTTTTTTTTAAGACTGGGAGTTAGAAGAT-----------
         ********22*2*22222222222222222222222222222222222222222222222222222
```

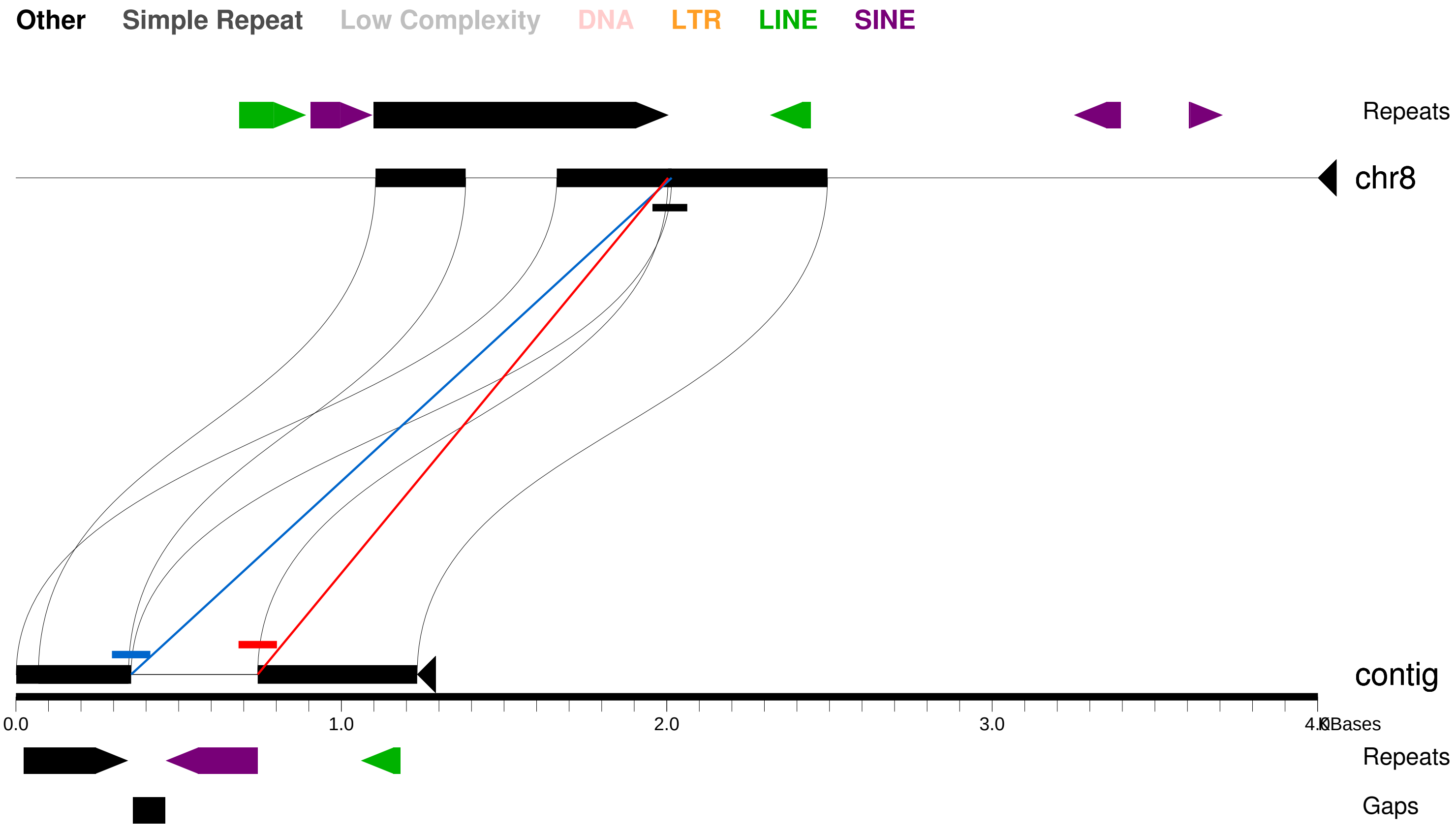

siteID: chr8_15149716 Scafftig1 - Breakpoint: chr8:15149768
chr8:15149608-15149942
Overlap Length: 15 INS size: 422 Genotyped: No

```
left   ------------------------------------------CTCAACTTAACTGTATATTAATTTGCAA
chrom  ATGTATTACCTCTTTCATGTATACCAAATTGAGATGTCTTATCTCAACTTAACTGTATATTAATTTGCAA
right  -------------TTCA-GTA---------GAGACGGGGTTTCACCGTTTTAGCCGGGATGGTCTCG--A
                    2222 222         2222N2NNN2N2*1*111**1*111111**1111*1*11*

left   TTAAATGTACTCTAAACCATGGCTCTTTGACATTTTGAAAAGTGCCAGTTTTATCAATTAAAGTTGAGAC
chrom  TTAAATGTACTCTAAACCATGGCTCTTTGACATTTTGAAAAGTGCCAGTTTTATCAATTAAAGTTGAGAC
right  TCTCCTGACCTCGTGATCCGCCCGCCTCGGCCTCCC--AAAGTGCTGGGATTAC-----AGGCGTGAGCC
       *1111**11***111*1*1111*1*1*1*1*1*11111*******11*11***111111*1111****1*

left   TTTGAGGTTACCAAATGGTTTGATTACTTATTCTTTTNNNNNNNNNNN----------------------
chrom  TTTGAGGTTACCAAATGGTTTGATTACTTATTCTTGTACTTGTGTATTCCACCATTATTTATTGGACACT
right  ACCGCGC---CC----GGCCTGATTACTTATTCTTGTACTTGTGTATTCCACCATTATTTATTGGACACT
       111*1*1111**1111**11***************2*222222222222222222222222222222222

left   ----------------------------------------------------------------------
chrom  GTACTAAGTGCTGGGGATACAAAAGTAAACAAGACAGAATTTTATGAGTCAATAAATGATGAAATGCAAA
right  GTACTAAGTGCTGGGGATACAAAAGTAAACAAGACAGAATTTTATGAGTCAATAAATGATGAAATGCAAA
       2222222222222222222222222222222222222222222222222222222222222222222222

left   -------------------------------------------------------
chrom  ATTTATTATTTCATAATGATTGTAATTAAATGCTCTCAACAAAGAAGTATAGGCA
right  ATTTATTATTTCATAATGATTGTAATTAAATGCTCTCAAC---------------
       2222222222222222222222222222222222222222
```

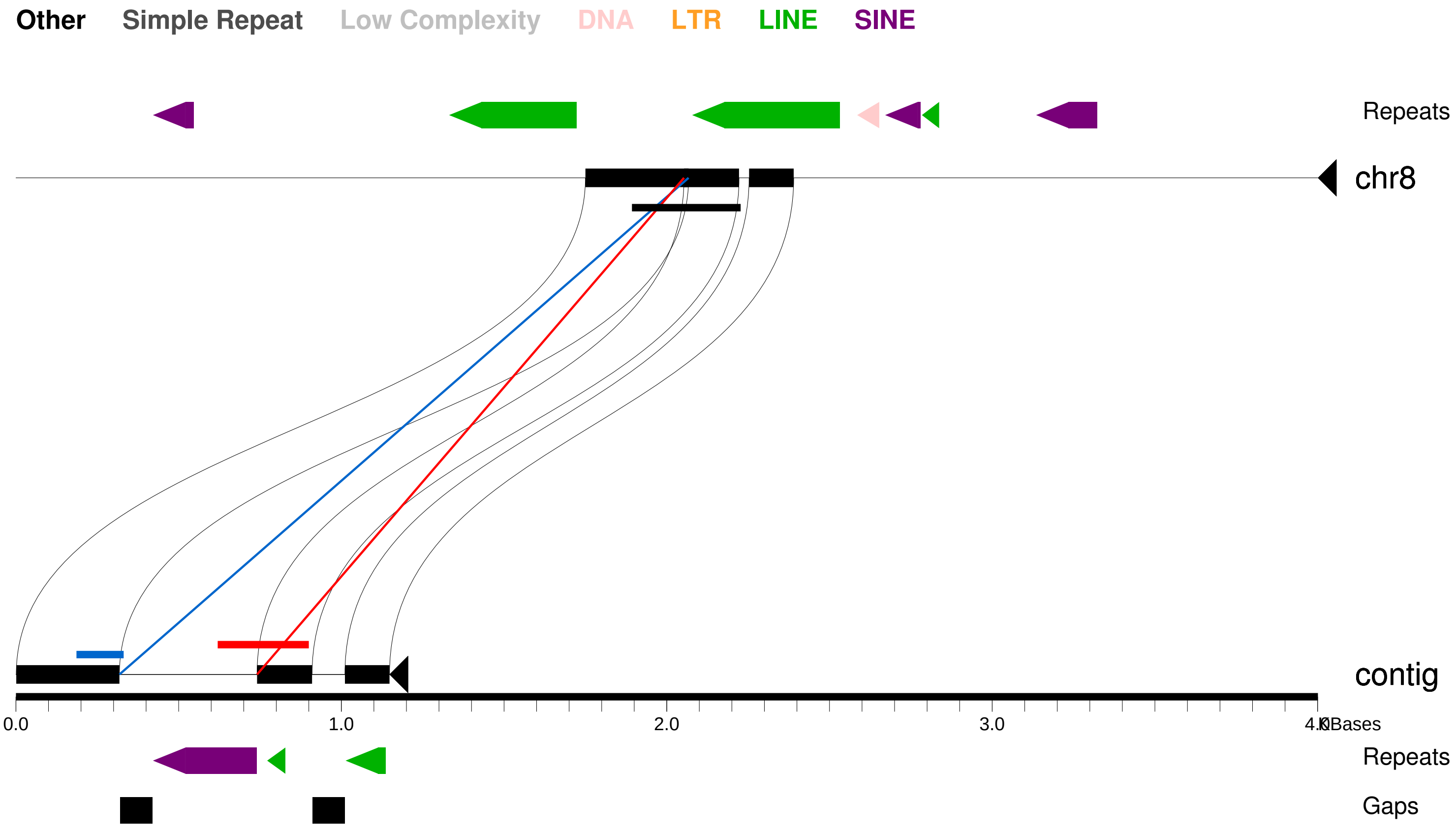

siteID: chr8_15399319 Scafftig1 + Breakpoint: chr8:15399339
chr8:15399289-15399397
Overlap Length: 10 INS size: 418 Genotyped: No

```
left    ATTCCAAGAGTTTTAGATTTAGGAGTTCACGAG-----------AAAGGTAGAAGAGTGAAGAAAAAGTA
chrom   ATTCCAAGAGTTTTAGATTTAGGAGTTCACGAG-----------AAAGGTAGAAGAGTGAAGAAAAAGTA
right   NNNNNNNNNNNNNNNNNNNNNNNNNNNNNNNNNNNNNNNNNNNNNNNNNNNNAAAAAAGA-GAAAAAGTA
        1111111111111111111111111111111111111111111111111111**1*11**1*********

left    GGCCGGGCGCGGTGGCTCACGCCTGTAATCCCAGCACTTTGGGAGGCCGAGGCGGGCGGA
chrom   GACAGTTTTTTTTG---------TGGATTACCAAAGCAAAGGGCAATAAAAGATGATG-A
right   GACAGTTTTTTTTG---------TGGATTACCAAAGCAAAGGGCAATAAAAGATGATG-A
        *2*2*2222222**222222222**2*2*2***222*222***222222*2*22*22*2*
```

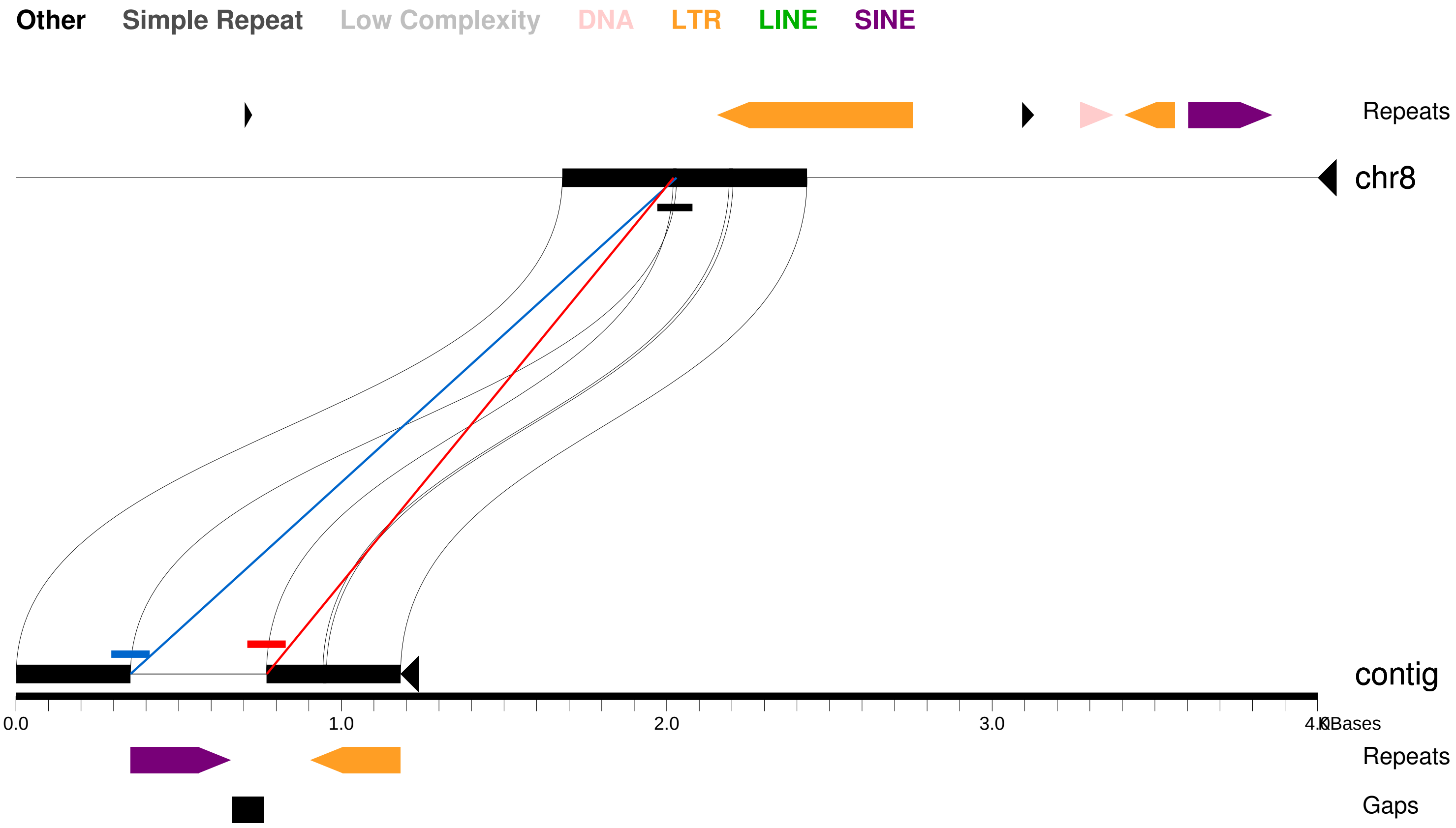

siteID: chr8_15548529 Scafftig2 - Breakpoint: chr8:15548507
chr8:15548347-15548680
Overlap Length: 14 INS size: 311 Genotyped: Yes

```
left    ----------CATTGGTCTGTGTGTCTGTATTTGTACCGGTACAATGGTGTTGTGGTTACTGTAGCATTG
chrom   TATTCTGTTCCATTGGTCTGTGTGTCTATATTTGTACCGGTACAATGGTGTTGTGGTTACTGTAGCATTG
right   TTTAGTAGA------GACGGGGTTTC-ACCTTGTTAGCC--AGGATGGTCTCG-----------------
        2N2NN2NNN 11111*1*1*1**1**1211**11**1*111*11*****1*1*11111111111111111

left    CAGTATAGTTTGAAGTCTGGTAATGCCAAATGCCTCCAGCTTTGCCCATTTTGCTTAGGATTGCCTTGGC
chrom   CAGTATAGTTTGAAGTCTGGTAATGCCAAATGCCTCCAGCTTTGCCCATTTTGCTTAGGATTGCCTTGGC
right   ----ATCTCCTGACCTCATG--AT-CCACCCGCCTCGGCCT---CCCAAAGTGCTG-GGATTACAGGCGT
        1111**1111***11**11*11**1***111*****111**111****111****11*****1*1111*1

left    TATTCAGGCTATTTTTTGGTTCCATATGAATTTTTTTTTTTTTTTTTTTTTTTTTTTTGAGACGGA-GTC-
chrom   TATTCAGGCTATTTTTTGGTTCCATATGAATTTTTAAAATAGTTTTTTTCCTAATTCTGTGAAGAATGTCC
right   GAGCCA--CCGCGCCCGGCCTCCATATGAATTTTTAAAATAGTTTTTTTCCTAATTCTGTGAAGAATGTCC
        1*11**11*11111111*11**************2222*22*******22*22**2**2**2*2*2***2

left    ------------TCGCTCTGTCGCCCAGGTCG--GACTGCGGACTGCAGTGGCGCAATCTCGGCTC-ACT
chrom   TTGGTAGTTTGATAGAAATATCACTGAATTTGTGGATTGCTTTGGGCAGTGTGGCCATTTTAGCTATAGT
right   TTGGTAGTTTGATAGAAATATCACTGAATTTGTGGATTGCTTTGGGCAGTGTGGCCATTTTAGCTATAGT
        222222222222*2*222*2**2*22*22*2*22**2***22222******22**2**2*22***22*2*

left    GC--------AAGC------------------TCCGCTTCCCGGGTTCACGCCA
chrom   GATTCTTCCTAATCATGAACATGAAATGTTTTTCCATTTGTTTGTATCATCTCT
right   GATTCTTCCTAATCATGAACATGAAATGTTTTTCCATTTG--------------
        *222222222**2*222222222222222222***22**2NNN1NN111NNN1N
```

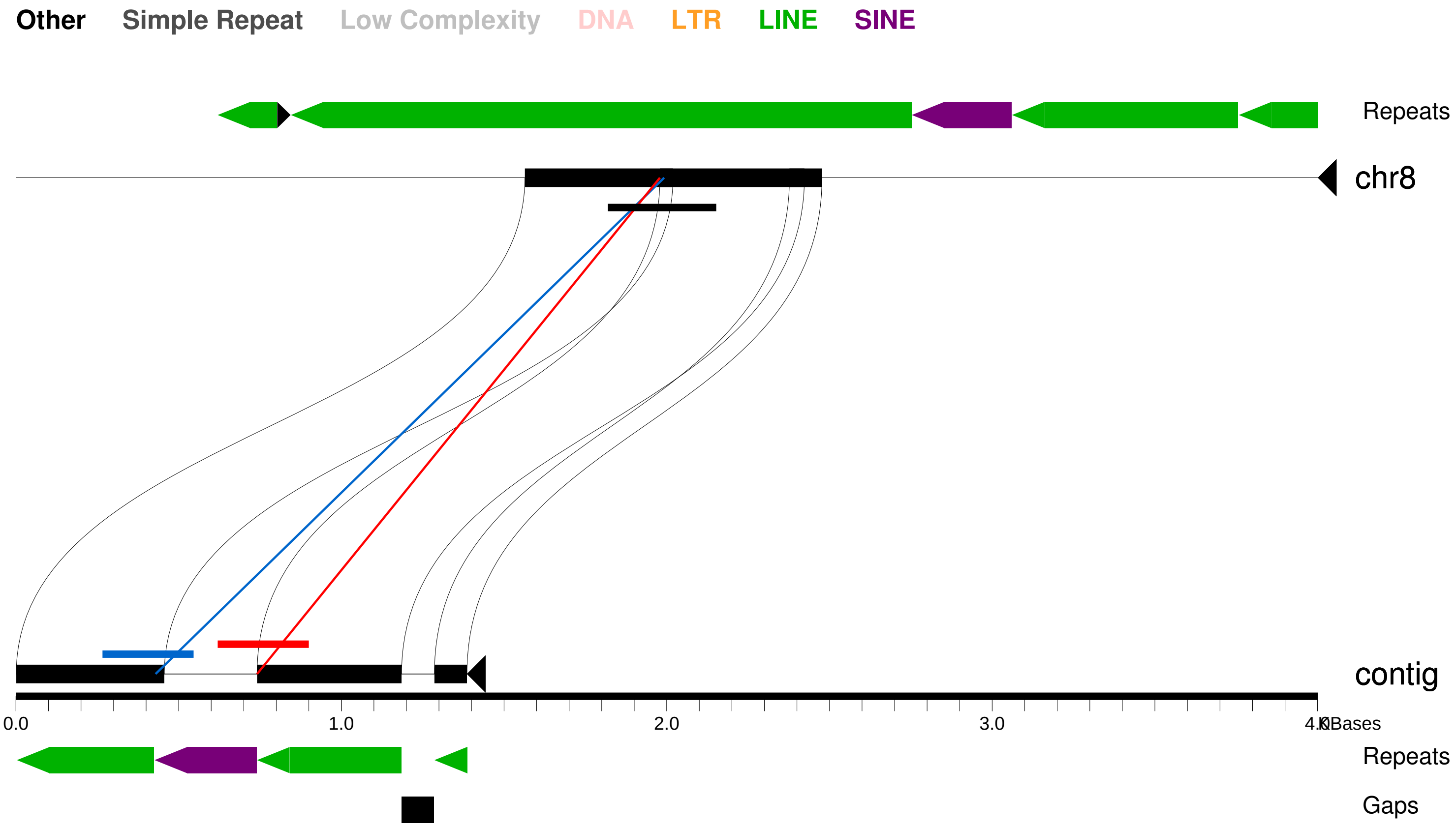

siteID: chr8_16102805 Scafftig3 - Breakpoint: chr8:16102807
chr8:16102763-16102866
Overlap Length: 16 INS size: 260 Genotyped: Yes

```
left     T------ATG-TCTCTGTGTTCATATAT----ATCCTTACATATATGTGTGTATA----CATATTATGGA
chrom    T------ATG-TCTCTGTGTTCATATAT----ATCCTTACATATATGTGTGTATA----CATATTATGGA
right    AGCCGGGATGGTCTCGATCTCCTGACCTCGTGATCCGCCCGCCTCGGCCTCCCAAAGTGCATATTATGGA
         1111111***1****11*1*1*11*11*1111****111*111*11*11*1111*1111***********

left     TTCTTTTTTTTTTTTTTTGAGACGGAGTCTCGCTCTGTCGCCCAGGCTGGAGTGCAGTGGCGGG
chrom    TTCTTAAAAATATATAAGCATATGTACA-----TGTGTTGT--ATGTATGCGTACA--------
right    TTCTTAAAAATATATAAGCATATGTACA-----TGTGTTGT--ATGTATGCGTACA--------
         *****22222*2*2*2222*2*2*2*2222222*2***2*222*2*222*2**2**22222222
```

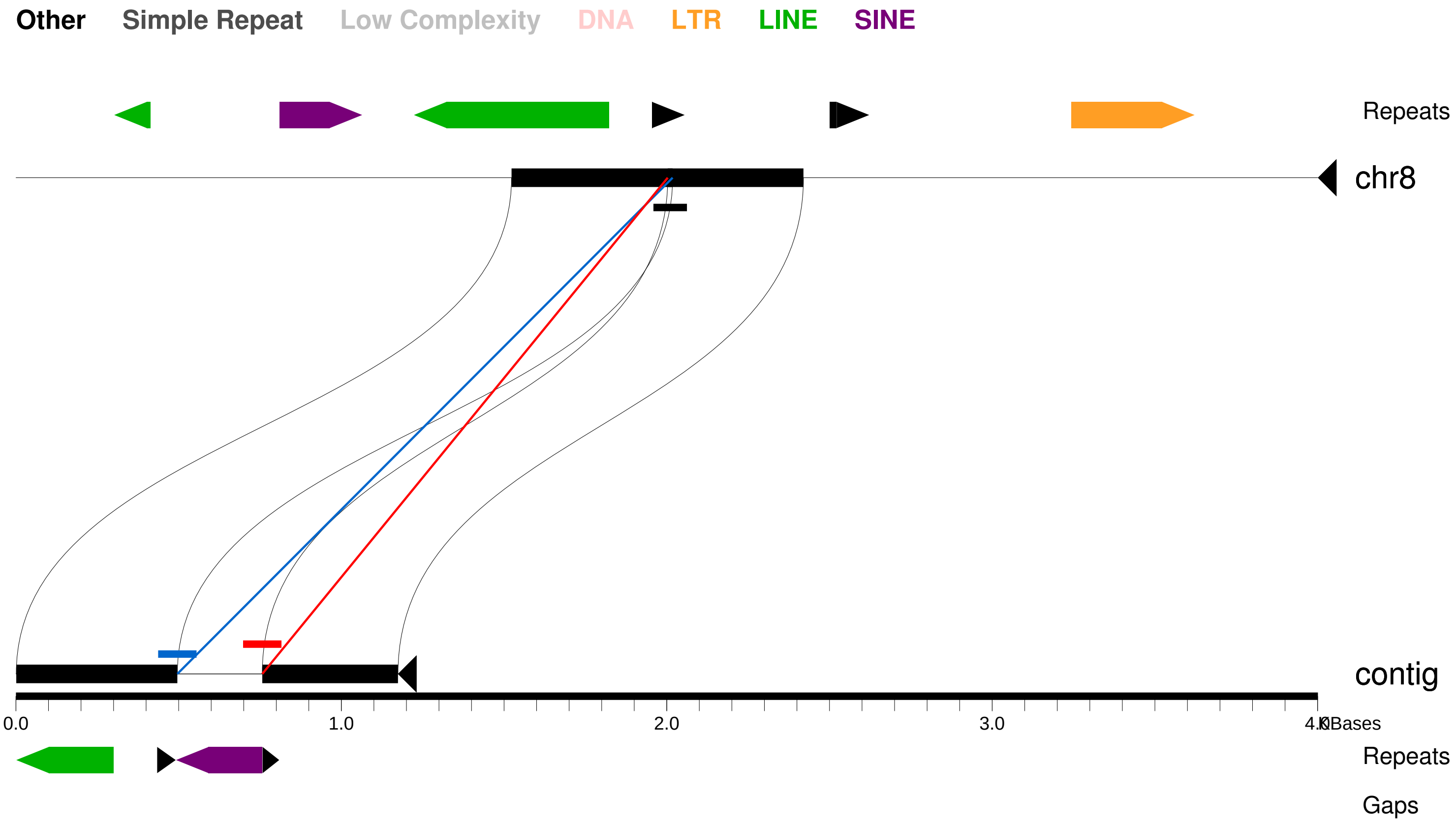

siteID: chr8_17336025 Scafftig2 - Breakpoint: chr8:17336010
chr8:17335956-17336069
Overlap Length: 3 INS size: 411 Genotyped: No

```
left   GGGACCTCCTAGTC-TGCCATCTTGCTTACGTG-CAGTACTTTGAATCTTCATATG---AAACATNNNNN
chrom  GGGACCTCCTAGTC-TGCCATCTTGCTTACGTG-CAGTACTTTGAATCTTCATATG---AAATATTCTAT
right  CCGCCCGCCTCGGCCTCCCAAAGTGCTGGGATTACAGGCGTGAGCCACCGCGCCCGGCCAAATATTCTAT
       11*1**1***1*1*1*1***111****1111*11***111*11*111*11*1111*111***2**22222

left   NNNNNNNNNNNNNNNNNNNNNNNNNNNNNNNNNNNNNNNNNNNNNNNNNNNNNN
chrom  TACAGCTAAATTGACAAATTTATTGGCATATGGTTGCTCATAATAGTCC-----
right  TACAGCTAAATTGACAAATTTATTGGCATATGGTTGCTCATAATAGTCC-----
       222222222222222222222222222222222222222222222222222222
```

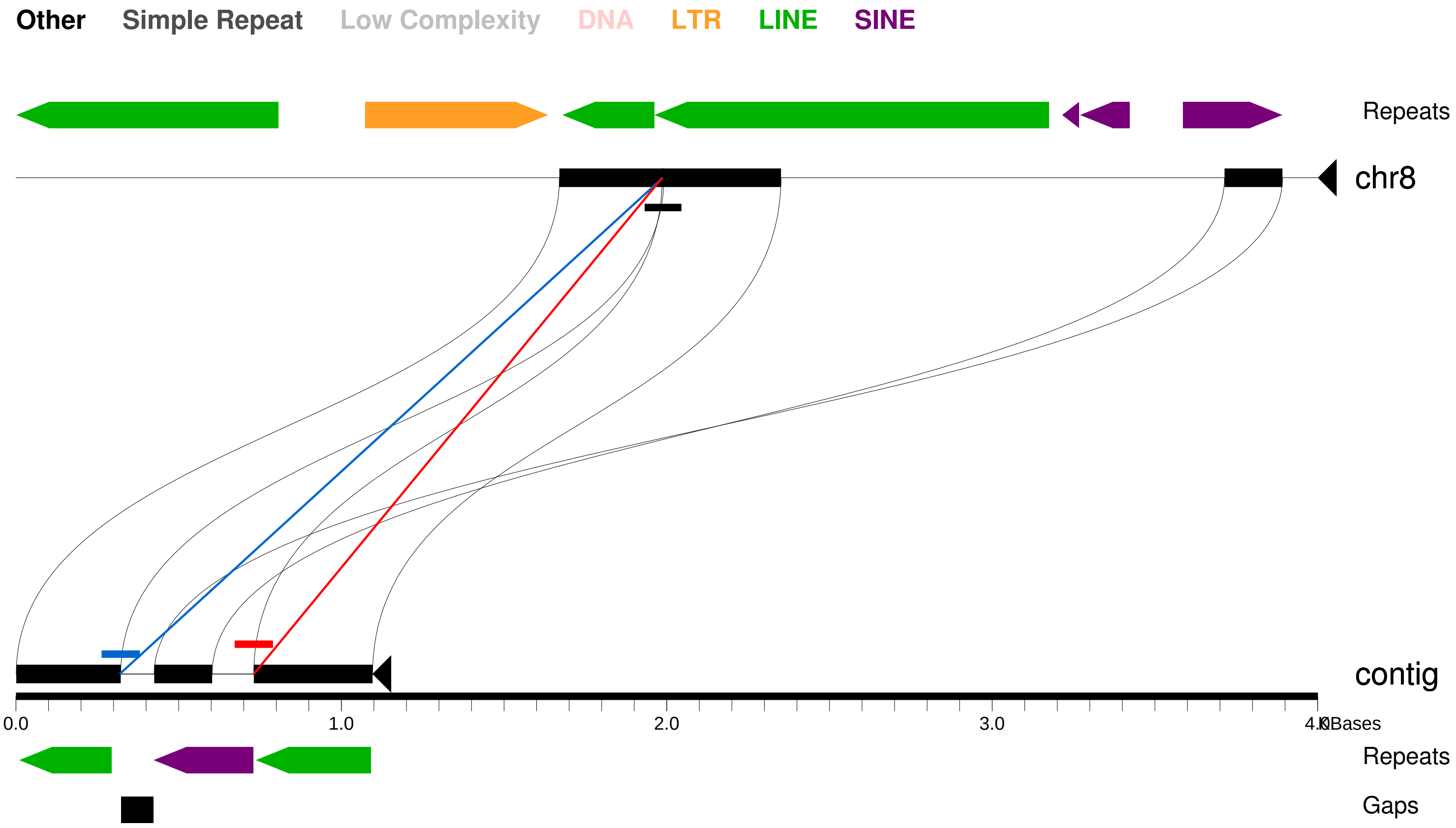

siteID: chr8_18617470 Scafftig3 + Breakpoint: chr8:18617463
chr8:18617418-18617522
Overlap Length: 15 INS size: 501 Genotyped: No

```
left    TTTCTGTATATAATGAGAAATGGAGAATTATCAACCACC--------------AGAGTTAGAAATGCAGA
chrom   TTTCTGTATATAATGAGAAATGGACAATTATCAACCACC--------------AGAGTTAGAAATGCAGA
right   NNNNNNNNNNNNNNNNNNNNNNNNNNNNNNNNNNNNNNNNNNNNNNNNNNNNNAAAAAAAGAAATGCAGA
        111111111111111111111111N1111111111111111111111111111*1*111***********

left    GCAGGGCCGGGCGCGGTGGCTCACGCCTGTAATCCCAGCACTTTGGGAGGCCGAGGCGGGTGG
chrom   GCAGATTCGATAGAAGA----------TAGAAACGCAGCT-TTTCTGTTACAAACAAGAA---
right   GCAGATTCGATAGAAGA----------TAGAAACGCAGCT-TTTCTGTTACAAACAAGAA---
        ****222**222*22*22222222222*22**2*2****22***22*222*22*222*22222
```

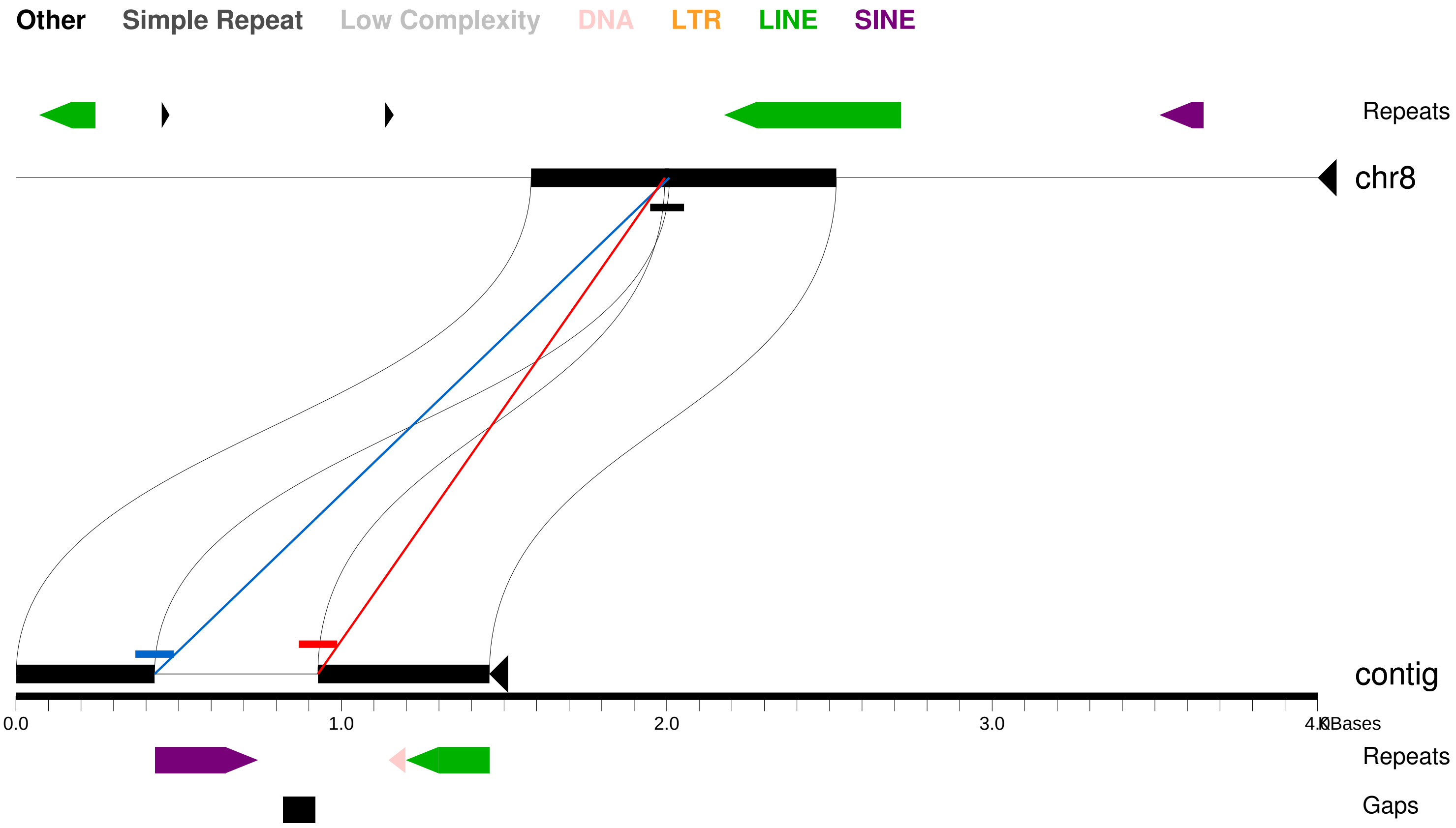

siteID: chr8_2275581 Scafftig2 + Breakpoint: chr8:2275558
chr8:2275544-2275617
Overlap Length: 46 INS size: 482 Genotyped: No

```
left   -----------------------------GAGAACA---------CACTCTG-------TATAATTTCTA
chrom  -----------------------------GAGAACA---------CACTCTG-------TATAATTTCTA
right  CCGCCCGCCTCGGCCTCCCAAAGTGCTGGGATTACAGGCGTGAGCCACCGCGCCCAGCCTATAATTTCTA
       11111111111111111111111111111**11***111111111***111*1111111***********

left   TTTTTTAAATTTGTTTCATACGTGGTCTATCTTGGNNNNNNNNNNNNNNNNNNNNNNNNNNNNNNNNNNN
chrom  TTTTTAAAATTTGTTTCATACGTGGTCTATCTTGGTGAATACTCCATTC---------------------
right  TTTTTAAAATTTGTTTCATACGTGGTCTATCTTGGTGAATACTCCATTC---------------------
       *****2*****************************22222222222222222222222222222222222

left   NNNNNNNNNNNNNNNNNNNNNNNN
chrom  ------------------------
right  ------------------------
       222222222222222222222222
```

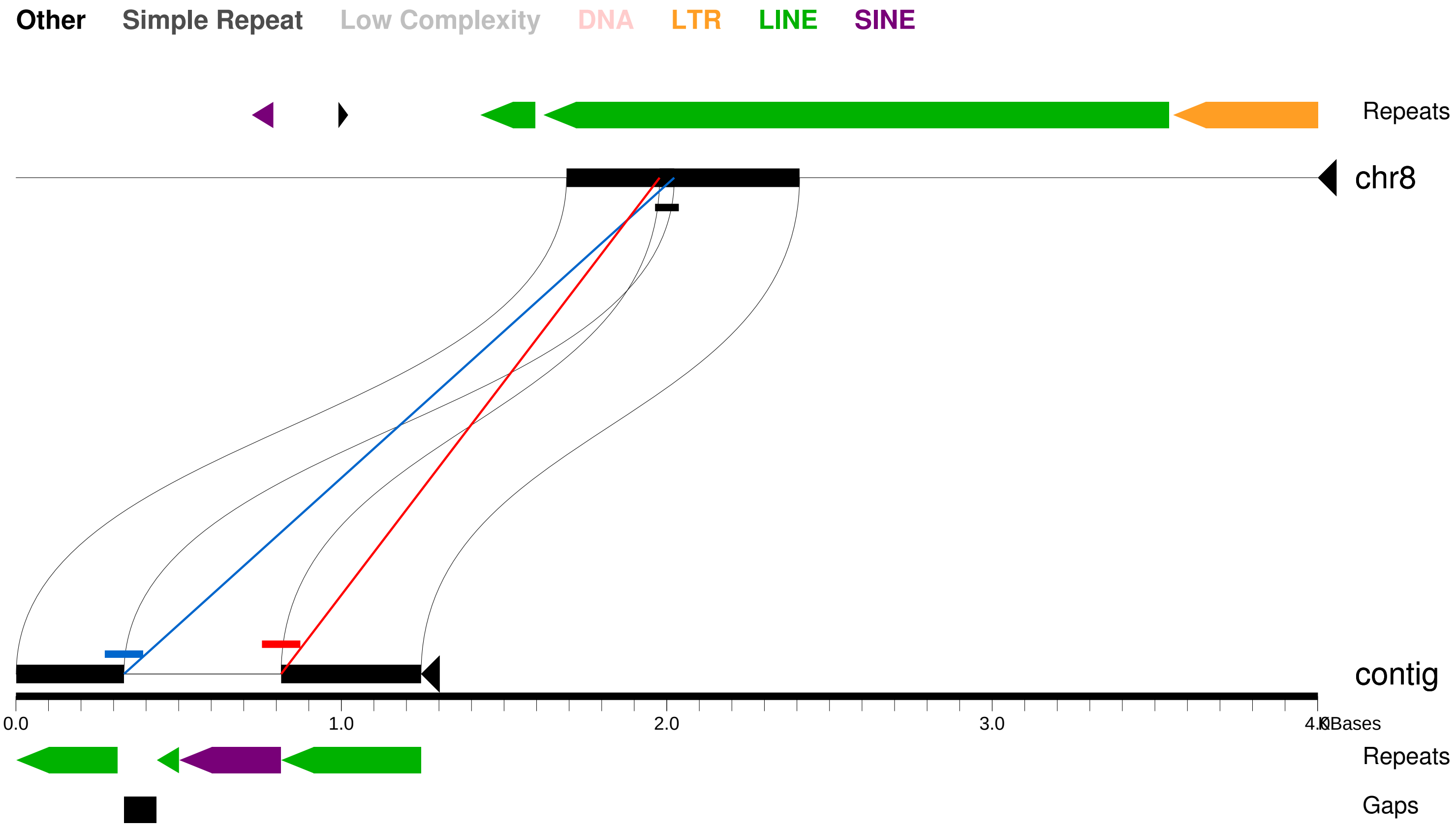

siteID: chr8_23042389 Scafftig4 + Breakpoint: chr8:23042409
chr8:23042360-23042468
Overlap Length: 11 INS size: 430 Genotyped: No

```
left   CCACGAGCAATGTCAATTAGCCAAACCTTTCAAT----------TTTCTATCAGATTTTAAAGAATATTT
chrom  CCACGAGCAATGTCAATTAGCCAAACTTTTCAAT----------TTTCTATCAGATTTTAAAGAATATTT
right  NNNNNNNNNNNNNNNNNNNNNNNNNNNNNNNNNNNNNNNNNNNNNNNNNNNNAAAAAAAAAAGAATATTT
       11111111111111111111111111N1111111111111111111111111*1*1111**********

left   GGCCGGGC----GCAGTGGCTCACGCCTGTAATCCCAGCACTTTGGGAGGCCGAGGCGGGCGG
chrom  TACAATGTACTTGCA-TGGATCATTGATGACATACTAGGACTTT-------CTATTT------
right  TACAATGTACTTGCA-TGGATCATTGATGACATACTAGGACTTT-------CTATTT------
       22*222*22222***2***2***2222**22**2*2**2*****2222222*2*222222222
```

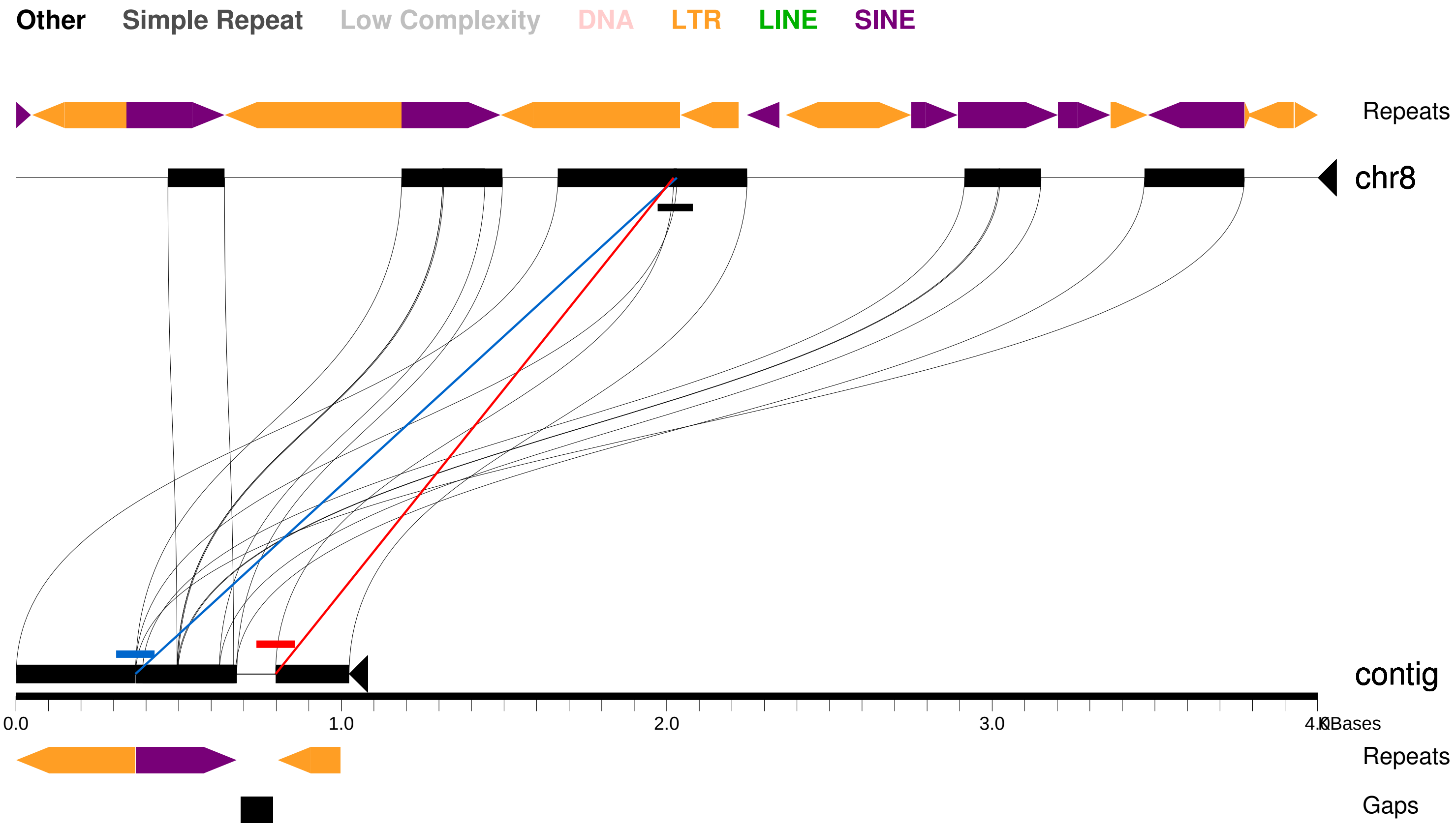

siteID: chr8_24989905 Scafftig1 + Breakpoint: chr8:24989993
chr8:24989941-24990052
Overlap Length: 8 INS size: 147 Genotyped: Yes

```
left    ---AGG---AAGAAACT-TCATGAAAAATCTA--TAAGTAAATGTTTCTCAAAATCAGAATGGTTTTTCT
chrom   ---AGG---AAGAAACT-TCATGAAAAATCTA--TAAGTAAATGTTTCTCAAAATCAGAATGGTTTTTCA
right   CCCGGGTTCACGCCATTCTCCTGCCTCAGCCTCCCAAGTAGCTGGGACT--ACAGGCGCCCGGTTTTTCA
        1111**111*1*11*1*1**1**1111*1*11111*****11**111**11*1*111*111********2

left    TTTTTTTTTTTTTTTGAGACGGAGTCTCGCTCTGTCGCCCAGGCTGGAGTGCAGTGGC
chrom   TGACAGAAATC-------AAGAAGTCTTTCAAAGATAAAAATGACAAAACTAAAAGAT
right   TGACAGAAATC-------AAGAAGTCTTTCAAAGATAAAAATGACAAAACTAAAAGAT
        *22222222*22222222*2*2*****22*222*222222*2*2222*2222*22*22
```

Other Simple Repeat Low Complexity DNA LTR LINE SINE

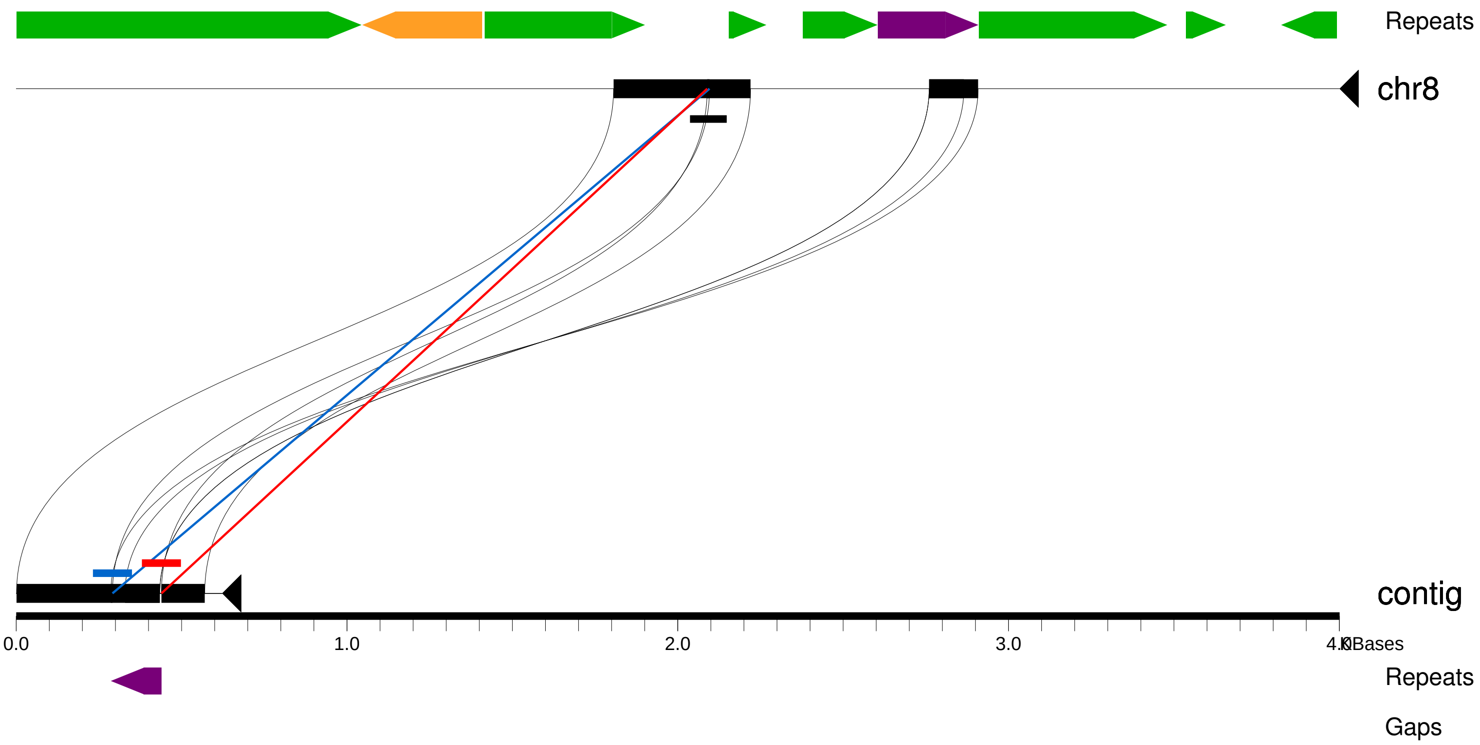

siteID: chr8_25172668 Scafftig11 + Breakpoint: chr8:25172647
chr8:25172606-25172706
Overlap Length: 14 INS size: 288 Genotyped: Yes

```
left     GCTGAAAAC---ATCTTACTGACT-----TTCATTTATGTTG-------AGG-GTAA---ATATAAAAAT
chrom    GCTGAAAAC---ATCTTACTGACT-----TTCATTTATGTTG-------AGG-GTAA---ATATTAAAAT
right    -CTGACCTCGTGATCCGCCCGCCTCGGCCTCCCAAAGTGCTGGGATTACAGGCGTGAGCCATATAAAAAT
         1****111*111***111*1*1**11111*1*11111**1**1111111***1**1*111****N*****

left     TCTTTTTTTTTTTTTTTTTTTTTGAGACAGAGTCTCGCTCTGTTGCCCAGGCTGGAGTGCAGTGGCGG
chrom    TCTTAATTTG-------------CCCACCG-GCCTCAG---GGGGATCTGACTT-ATATCTGTCTCTG
right    TCTTAATTTG-------------CCCACCG-GCCTCAG---GGGGATCTGACTT-ATATCTGTCTCTA
         ****22***22222222222222222**2*2*2***22222*22*22*2*2**22*222*2**22*21
```

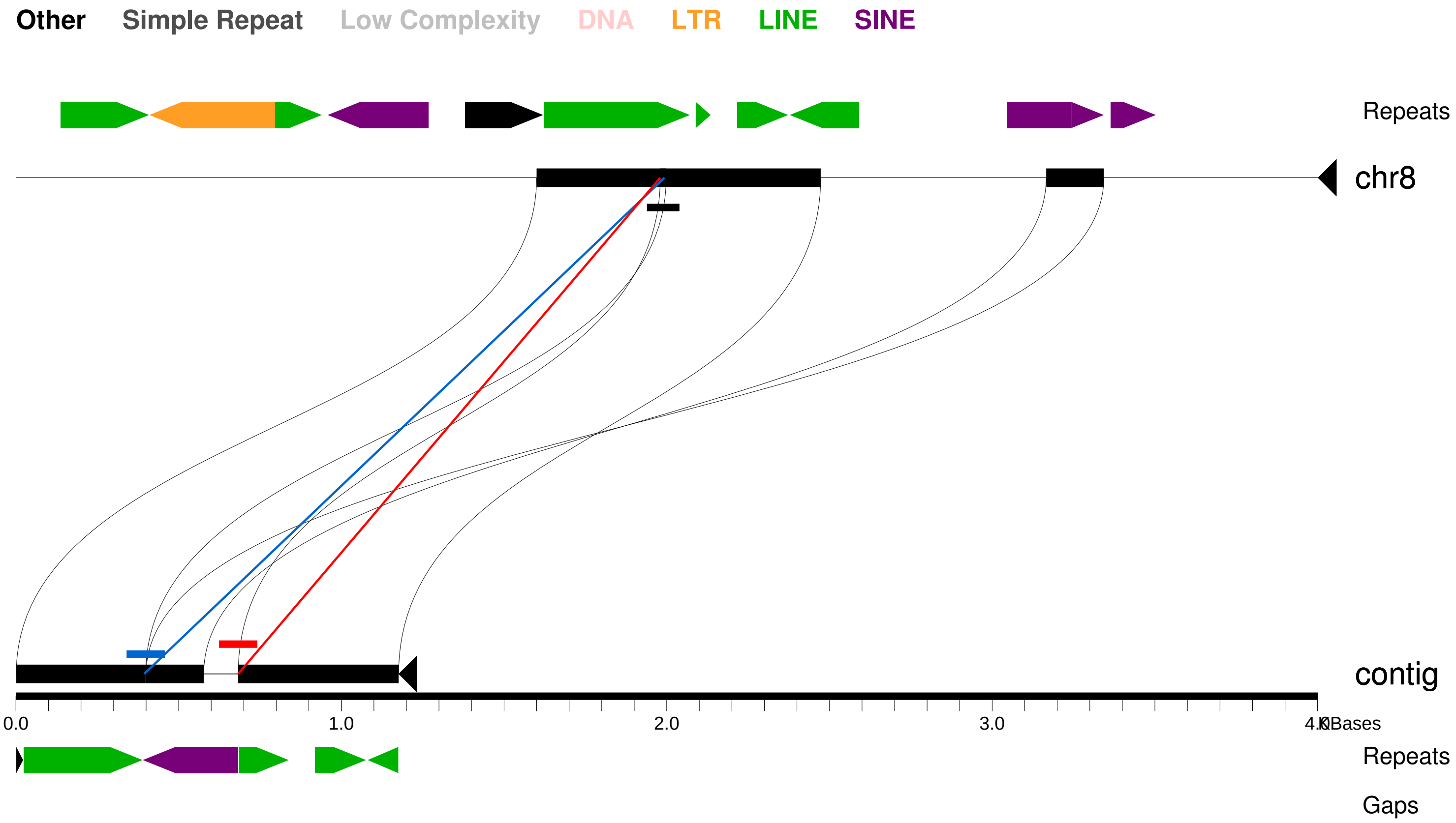

siteID: chr8_32359993 Scafftig13 + Breakpoint: chr8:32359972
chr8:32359927-32360031
Overlap Length: 15 INS size: 298 Genotyped: Yes

```
left   -----CAACATGTG-------ATTTGGTGGGGACACACATTTAACCCATATCAACTA--CTAAGCTCATT
chrom  -----CAACATGTG-------ATTTGGTGGGGACACACATTTAACCCATATCAACTA--CTAAGCTCATT
right  TCCGCCCGCCTCGGCCTCCCAAAGTGCTGGGATTACAGGCGTGAGCCACCGCACCCGGCCTAAGCTCATT
       11111*11*1*11*1111111*11**1****111***1111*1*1***111**1*1111***********

left   CTTTTTTTTTTTTTTTTTTTTTTGAGACGGAGTCTCGCTCTGTCGCCCAGGCTGGAGTGCAGTG
chrom  CTTTAAAACCTTATAAAT-----ACACCCATTCA---------GTCCTAGCCAGCGTGGCCAC
right  CTTTAAAACCTTATAAAT-----ACACCCATTCA---------GTCCTAGCCAGCGTGGCCAC
       ****222222**2*222*22222*2**22*2**2222222222*2**22**22*2***22222
```

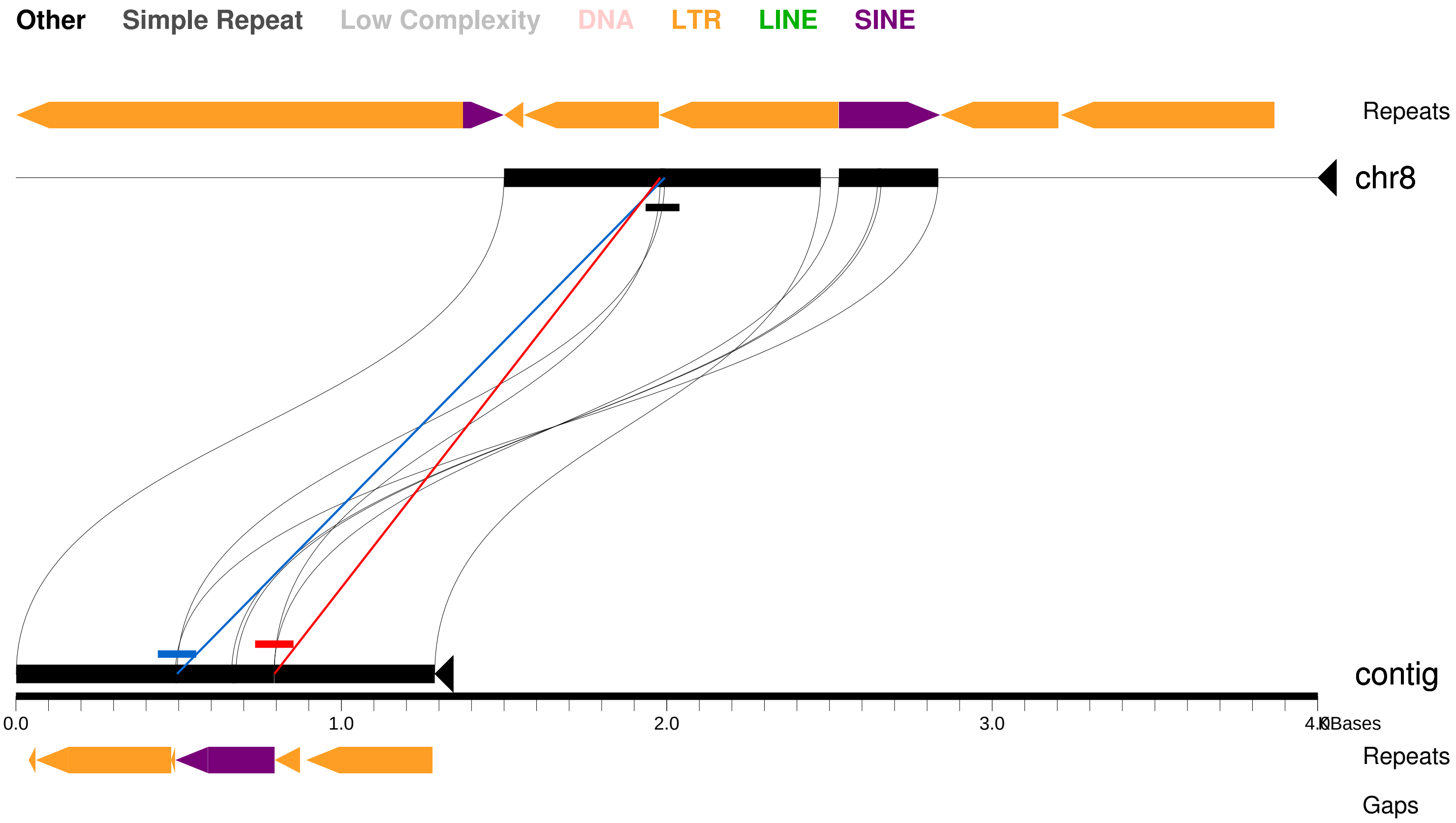

siteID: chr8_33210000 Scafftig2 - Breakpoint: chr8:33210034
chr8:33209989-33210093
Overlap Length: 15 INS size: 289 Genotyped: Yes

```
left     AAGTTAT-------TTAACAATGT------ATCA-AGCCAAGTATCCCATTTTTATTTTTGATTCTTTTT
chrom    AAGTTAT-------TTAACAATGT------ATCA-AGCCAAGTATCCCATTTTTATTTTTGATTCTTTTT
right    CCGCCCGCCTCGGCCTCCCAAAGTGCTGGGATTACAGGCGTGAGCCACCGCGCCCGGCCTGATTCTTTTT
         11*111111111111*11***1**111111**1*1**1*11*111*1*11111111111***********

left     TTTTTTTTTTTTGAGACGGAGTCTCGCTCTGTCGCCCAGGCTGGAGTGCAGTGGCACGATCTC
chrom    TTTTAAATGA---ACCCACATAAGTGTATTTTCACTTATAATG-AG---AAAGACA-------
right    TTTTAAATGA---ACCCACATAAGTGTATTTTCACTTATAATG-AG---AAAGACA-------
         *****222*22222*22*22*22222*222*2**2*22*222**2**222*22*2**2222222
```

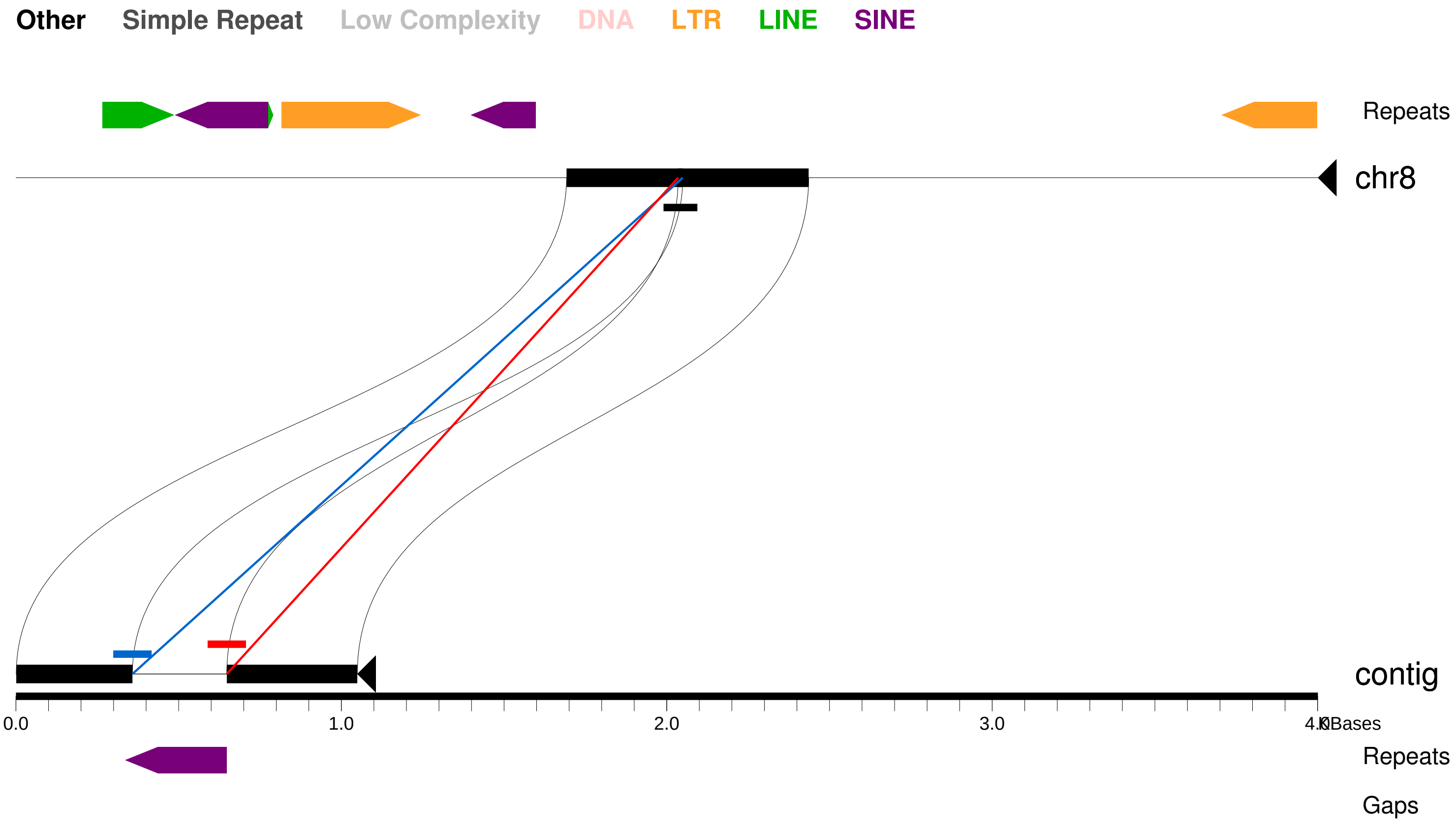

siteID: chr8_34065325 Scafftig1 - Breakpoint: chr8:34065286
chr8:34065230-34065364
Overlap Length: 4 INS size: 493 Genotyped: No

```
left     TGCCTACATTCAAGAATCTGTTGCTTTT-CTTCTGTGATAGGCTTTTCTTTTACATTAATCNNNNNNNNN
chrom    TGCCTACATTCAAGAATCTGTTGCTTTT-CTTCTGTGATAGGCTTTTCTTTTACATTAATCTTAACACTA
right    AG------TGCTGGATTACAGGCGTGAGCCACCGCGCCCGGCCT-------------AATCTTAACACTA
         1*111111*1*11*1**11111**1*111*11*1*1*111***1*111111111111****222222222

left     NNNNNN-NNNNNNNNNNNNNNNNNNNNNNNNNNNNNNNNNNNNNNNNNNNN----------------
chrom    ACAAGA-AGGCCTGATATATCCTAAACATTTTCTTGTTCTTTAGCATTTTTTTAAAATACTCTATTG
right    ACAAGACAGGCCTGATATATCCTAAACATTTTCTTGTTCTTTAGCATTTTTTTAAAATACTCTATTG
         2222221222222222222222222222222222222222222222222222222222222222222
```

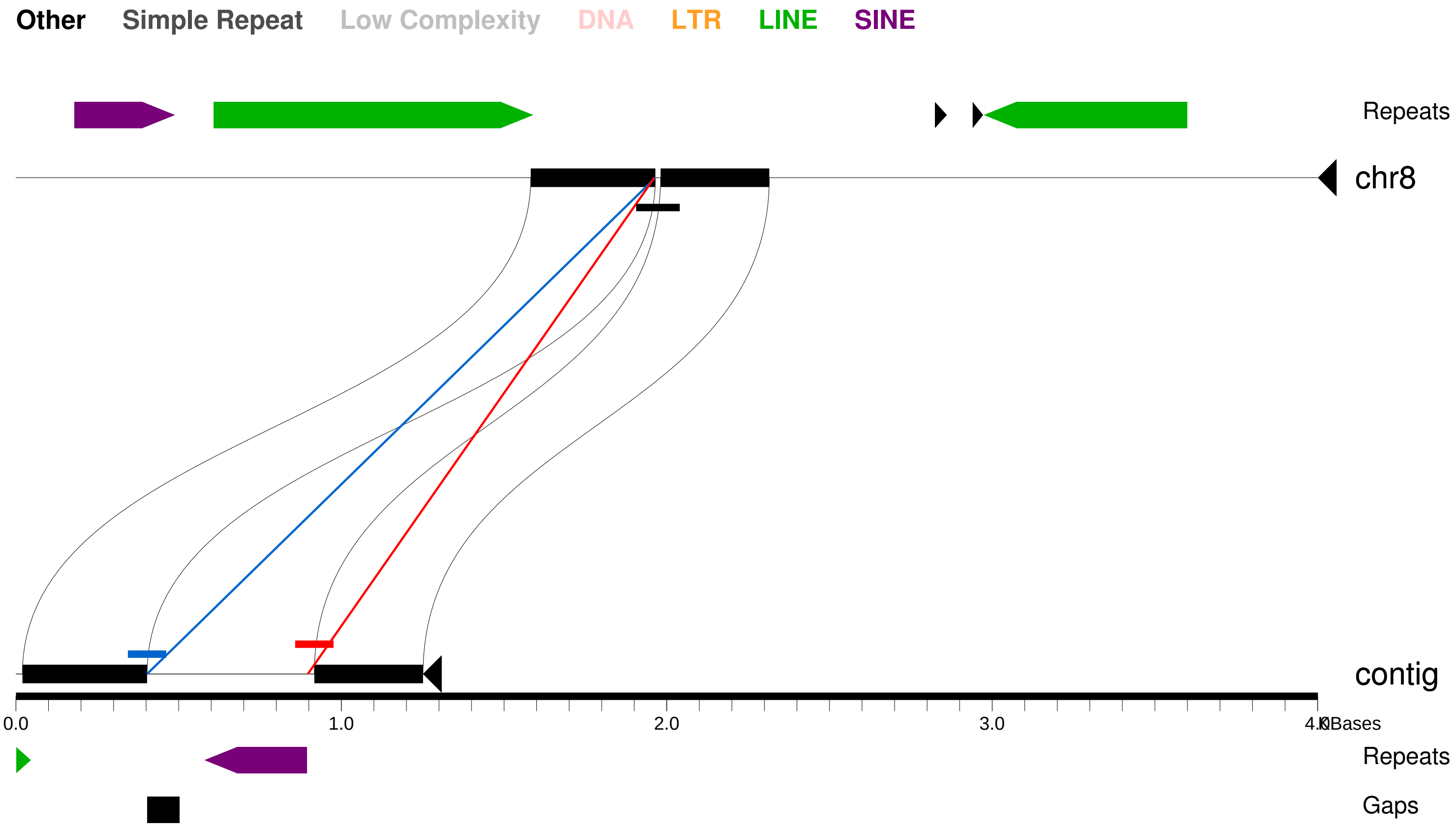

siteID: chr8_34258124 Scafftig1 + Breakpoint: chr8:34258114
chr8:34258070-34258173
Overlap Length: 13 INS size: 305 Genotyped: No

```
left    TTCCTGAGTT----TACTACA---CTGAGGTTGCTTATGATAGTTATT--------CATGACAACATTCT
chrom   TTCCTGAGTT----TACTACA---CTGAGGTTGCTTATGATAGTTATT--------CATGACAACATTCT
right   CGCCCGCCTCGGCCTCCCAAAGTGCTGGGATTACAGGCGTGAGCCACCGCGCCCGGCCCGACAACATTCT
        11**1*11*11111*1*1*1*111***1*1**1*1111*11**11*1111111111*11***********

left    TTTTTTTTNNNNNNNNNNNNNNNNNNNNNNNNNNNNNNNNNNNNNNNNNNNNNNNNNNNNNNNN
chrom   TTATTCATGACAATTATGTGACCATATGTAAATAATAAAAATACCATGA---------------
right   TTATTCATGACAATTATGTGACCATATGTAAATAATAAAAATACCATGA---------------
        **2**22*********************************************************
```

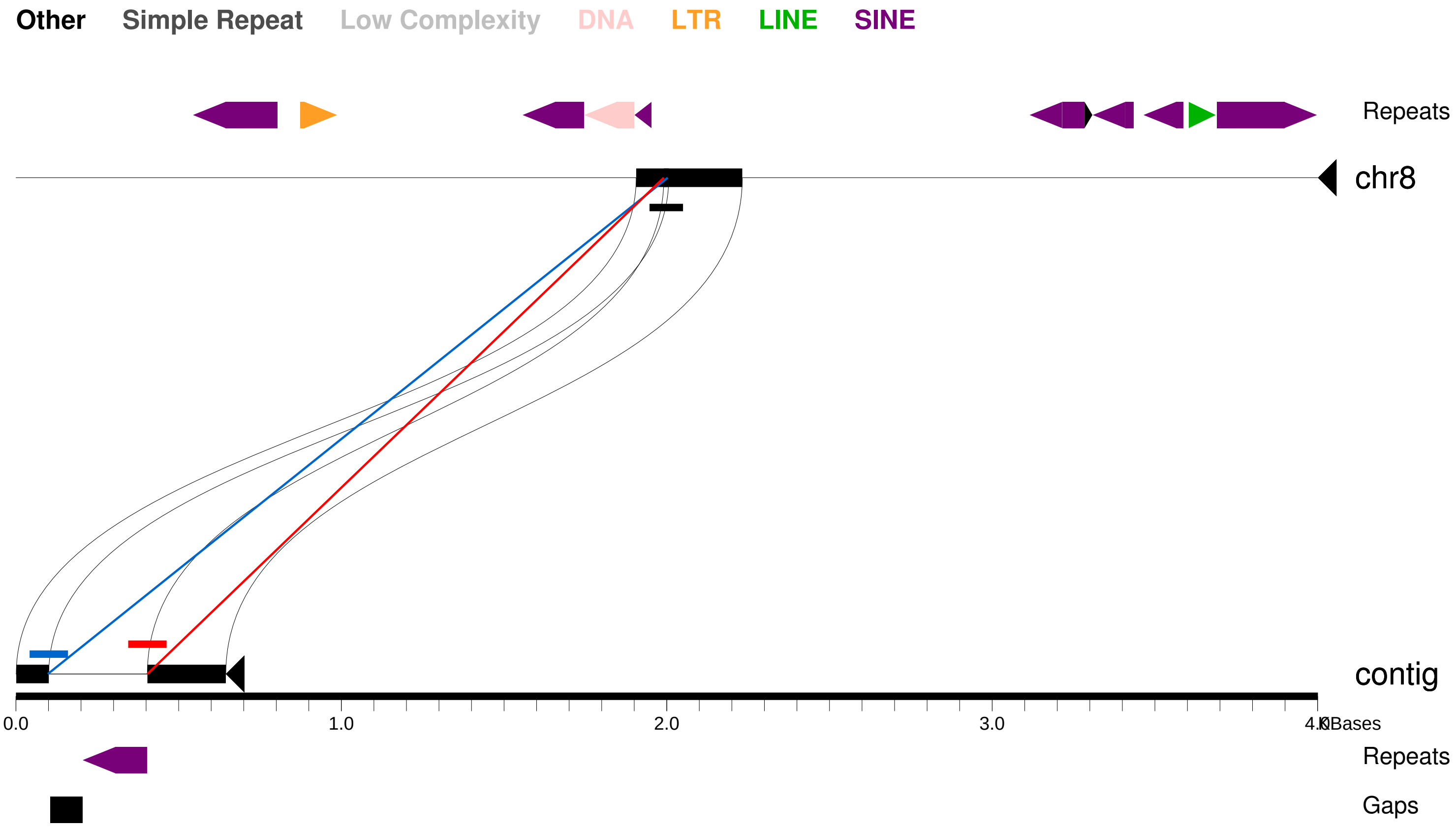

siteID: chr8_34469663 Scafftig1 + Breakpoint: chr8:34469648
chr8:34469488-34469819
Overlap Length: 12 INS size: 404 Genotyped: No

```
left   G-------------CTGGAAAACTCAATGAGGCAAGAGGAGAGAAACTTCACCCCATTGGGAAAGGCTCA
chrom  GATTTAGCCTGTTGCTGGAAAACTCAATGAGGCAAGAGGAGAGAAACTTCACCCCATTGGGAAAGGCTCA
right  NNNNNNNNNNN-----------------------------------------------------------
       1NNNNNNNNNN   11111111111111111111111111111111111111111111111111111111

left   AATACTTAAGAACATACAGAAATCTCTACATATGACTAGATTTCTTTAGGTCATCCTGTTCAAGATTTTA
chrom  AATACTTAAGAACATACAGAAATCTCTACATATGACTAGATTTCTTTAGGTCATCCTGTTCAAGATTTTA
right  ----------------------------------------------------------------------
       1111111111111111111111111111111111111111111111111111111111111111111111

left   AAAATGTGTCAGAAATGTTAAAAAATAAACATTGGCC----GGGCGCG-GTGGCTCAC-GCCTGTAA-TC
chrom  AAAATGTGTCAGAAATGTTAAAAA-TAAACATTTTTTTTTGTGTGTGTGTGTGTGTGAGTGTAACTG
right  --------------------AAAA-TAAACATTTTGTGTGTGTGTGTGTGTGTGTGTGAGTGTAACTG
       11111111111111111111****2********22N2N2N2*2*2*2*2***22*2222*22*****2*2

left   CCAGCACTTTGGGAGG---CCGAGGCGGGTGG-ATCATGAGGT--------CAGGAGATCGAGACCATCC
chrom  AAATAAGTTTGATATGTGTCCCACCCAAATCTCATCTTGAATTGTAATCCCCAGTATAG-GAGGTGGGGC
right  AAATAAGTTTGATATGTGTCCCACCCAAATCTCATCTTGAATTGTAATCCCCAGTATAG-GAGGTGGGGC
       22*22*2****22*2*222**2*22*222*222***2***22*22222222***2*2*22***222222*

left   TGGCTAACAAGG---TGAAACCCCG------TCTCTACTAA------------A
chrom  CCAGTAAGAGGGAATTGAATCATGGGGTGGATTTCTCATGAGTGGGGCCCAGTA
right  CCAGTAAGAGGGAATTG-------------------------------------
       2222***2*2**222**11N1NNN1      1N111NN1N1            1
```

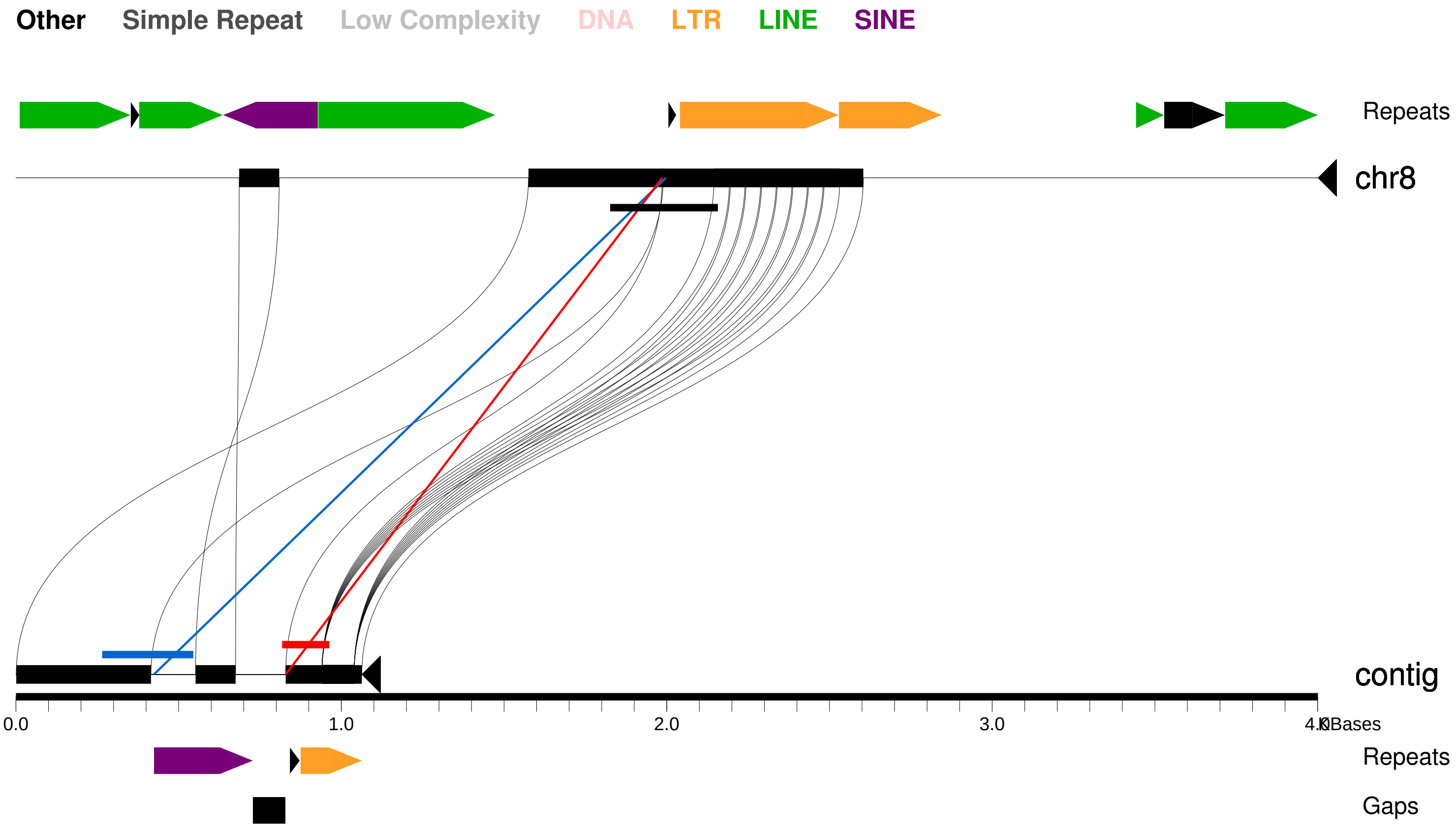

siteID: chr8_35069017 Scafftig6 - Breakpoint: chr8:35069023
chr8:35068978-35069082
Overlap Length: 15 INS size: 292 Genotyped: Yes

```
left    TACAACCATCTCATGATCACACAGGAAAAGAGAG-GACAT---GTCTGC----------AAAAGAGTAAC
chrom   TACAACCATCTCATGATCACACAGGAAAAGAGAG-GACAT---GTCTGC----------AAAAGAGTAAC
right   ATCCCGCCACTGCACTCCAGCCTGGGCGACAGAGCGAGACTCCGTCTCAAAAAAAAAAAAAAAGAGTAAC
        11*111*11**111111**11*1**111*1****1**1*1111****111111111111***********

left    TCCCGGCCGGGCGCGGTGGCTCACGCCTGTAATCCCAGCACTTTGGGAGGCCGAGGCGGGCGG
chrom   TCCCTTTACAGCAATG-GAATCAGGACACTTATCACTAAA-------------AGAATGACCA
right   TCCCTTTACAGCAATG-GAATCAGGACACTTATCACTAAA-------------AGAATGACCA
        ****222222**222*2*22***2*2*22*2***2*222*2222222222222**222*2*22
```

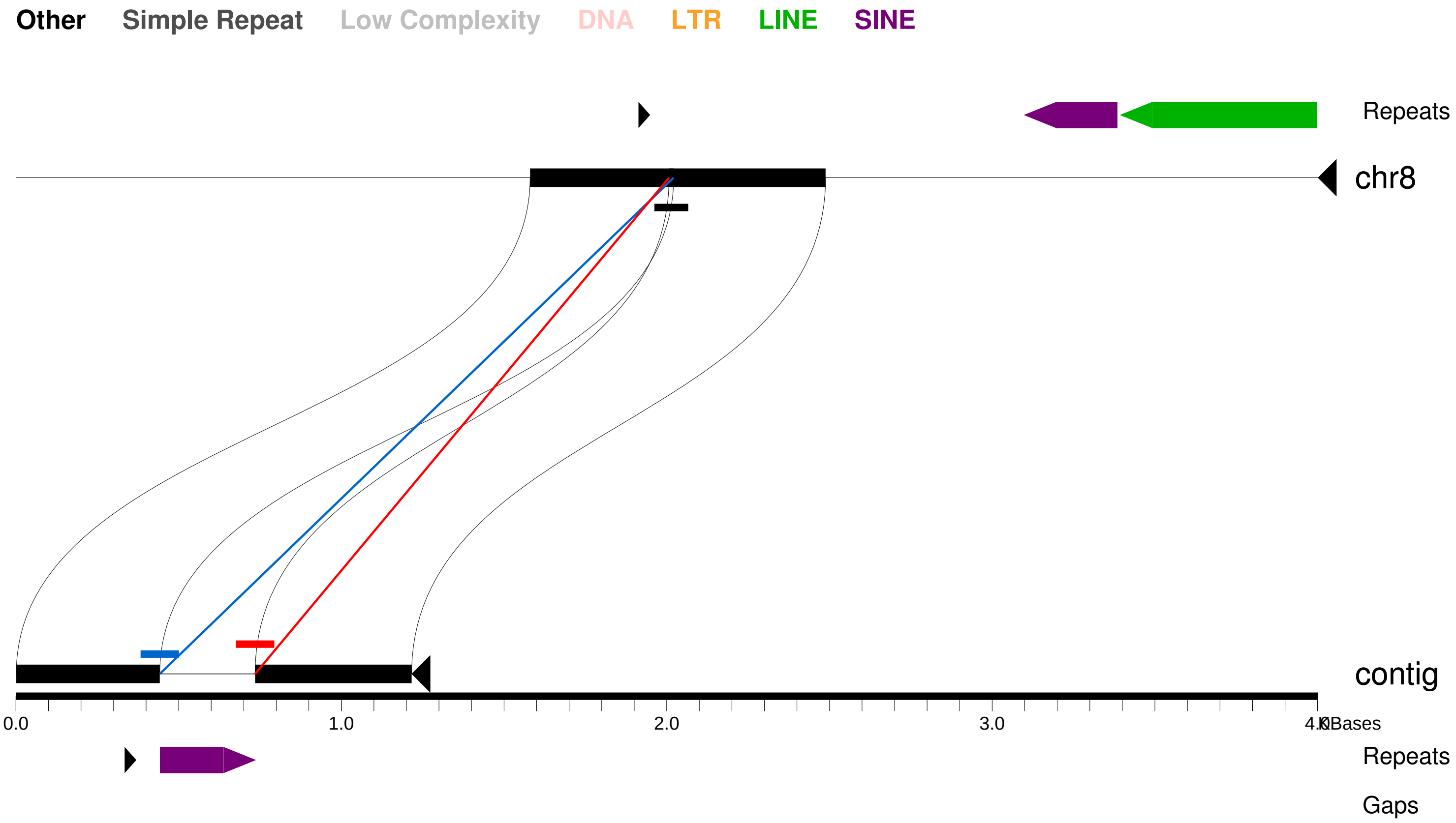

siteID: chr8_38864072 Scafftig12 - Breakpoint: chr8:38864061
chr8:38864018-38864120
Overlap Length: 17 INS size: 294 Genotyped: Yes

```
left    G-----ATTGGA-TCAAA---GGAGGAGAGTATGAGAGTAGGG-------AGACCAGGTAAAAAGGTATC
chrom   G-----ATTGGA-TCAAA---GGAGGAGAGTATGAGAGTAGGG-------AGACCAGGTAAAAAGGTATC
right   CGCGCCACTGCACTCCAGCGTGGGCGACAGAGCGAGACTCCGTCTCAAAAAAAAAAAAAAAAAAGGTATC
        111111*1**1*1**1*1111**11**1**111****1*11*11111111*1*11*111***********

left    ATAGTCGGCCGG--GCGCGGTGGCTCACGCCTGTAATCCCAGCACTTTGGGAGGCCGAGGCGGGCGG
chrom   ATAGTCATCTAGAGGTGAGGTTAGTAAGG---GTTAAGCCA-----TTGGTAATAAT----------
right   ATAGTCATCTAGAGGTGAGGTTAGTAAGG---GTTAAGCCA-----TTGGTAATAAT----------
        ******22*22*22*2*2***222*2*2*222**2*22***22222****2*222222222222222
```

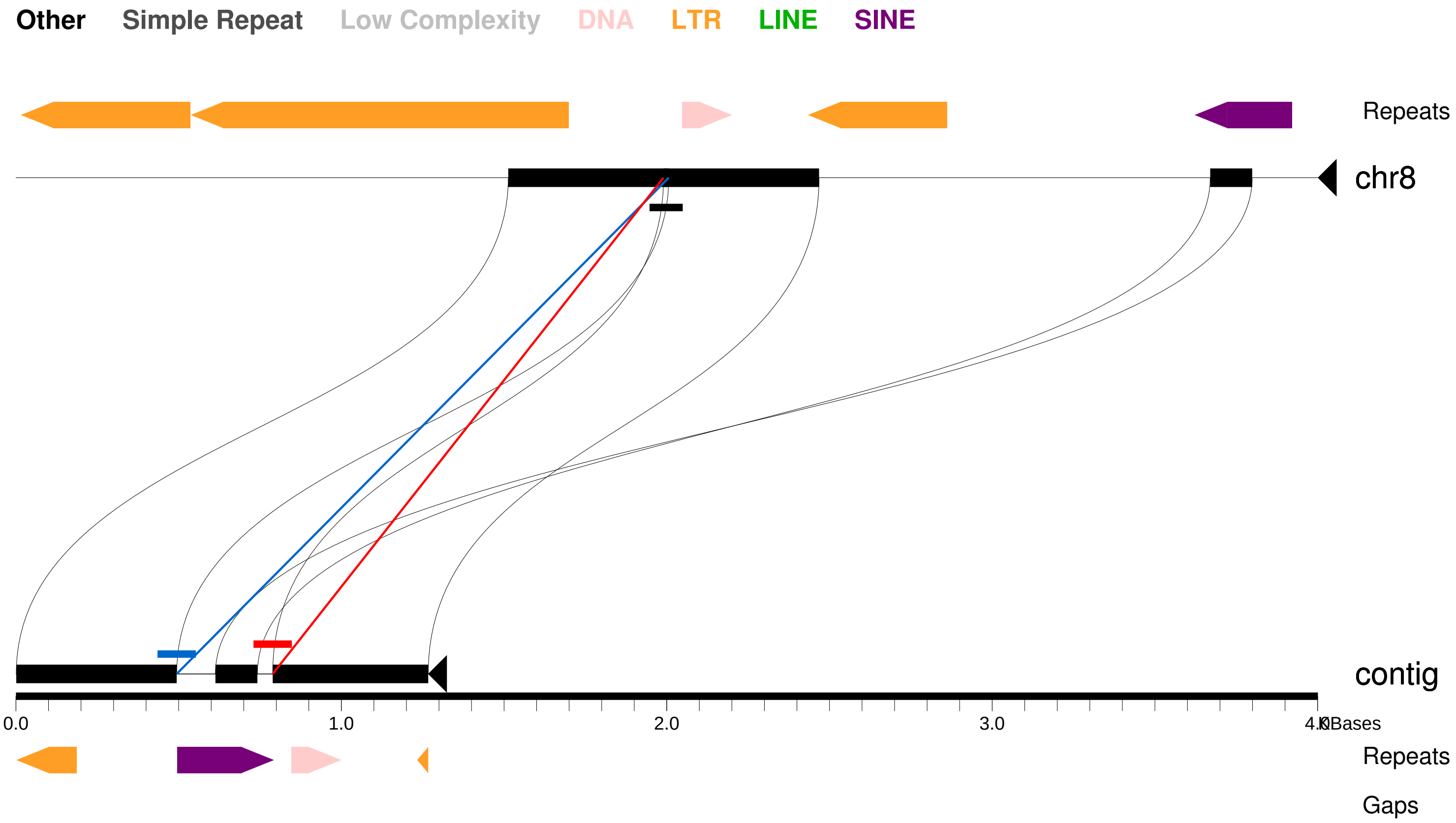

siteID: chr8_39700448 Scafftig2 + Breakpoint: chr8:39700461
chr8:39700416-39700520
Overlap Length: 15 INS size: 440 Genotyped: No

```
left    AATCCAAACAATACTAAATCAAGACTTATTATAATCAAACTGTCA--------------AAAATTAAAGA
chrom   AATCCAAACAATACTAAATCAAGACTTATTATAATCAAACTGTCA--------------AAAATTAAAGA
right   NNNNNNNNNNNNNNNNNNNNNNNNNNNNNNNNNNNNNNNNNNNNNNNNNNNNNNNNNNNAAAATTAAAGA
        11111111111111111111111111111111111111111111111111111111111***********

left    GAAGCCGGGCGCGGTGGCTCACGCCTGTAATCCCAGCACTTTGGGAGGCCGAGGCGGGCGGAT
chrom   GAAGATCCAAAAAGTAGCAAGAGAAAGGAAGCACATCAC------ACACAAAAGA--------
right   GAAGATCCAAAAAGTAGCAAGAGAAAGGAAGCACATCAC------ACACAAAAGA--------
        ****222222222**2**2222*222*2**2*2**2***222222*22*22*2*222222222
```

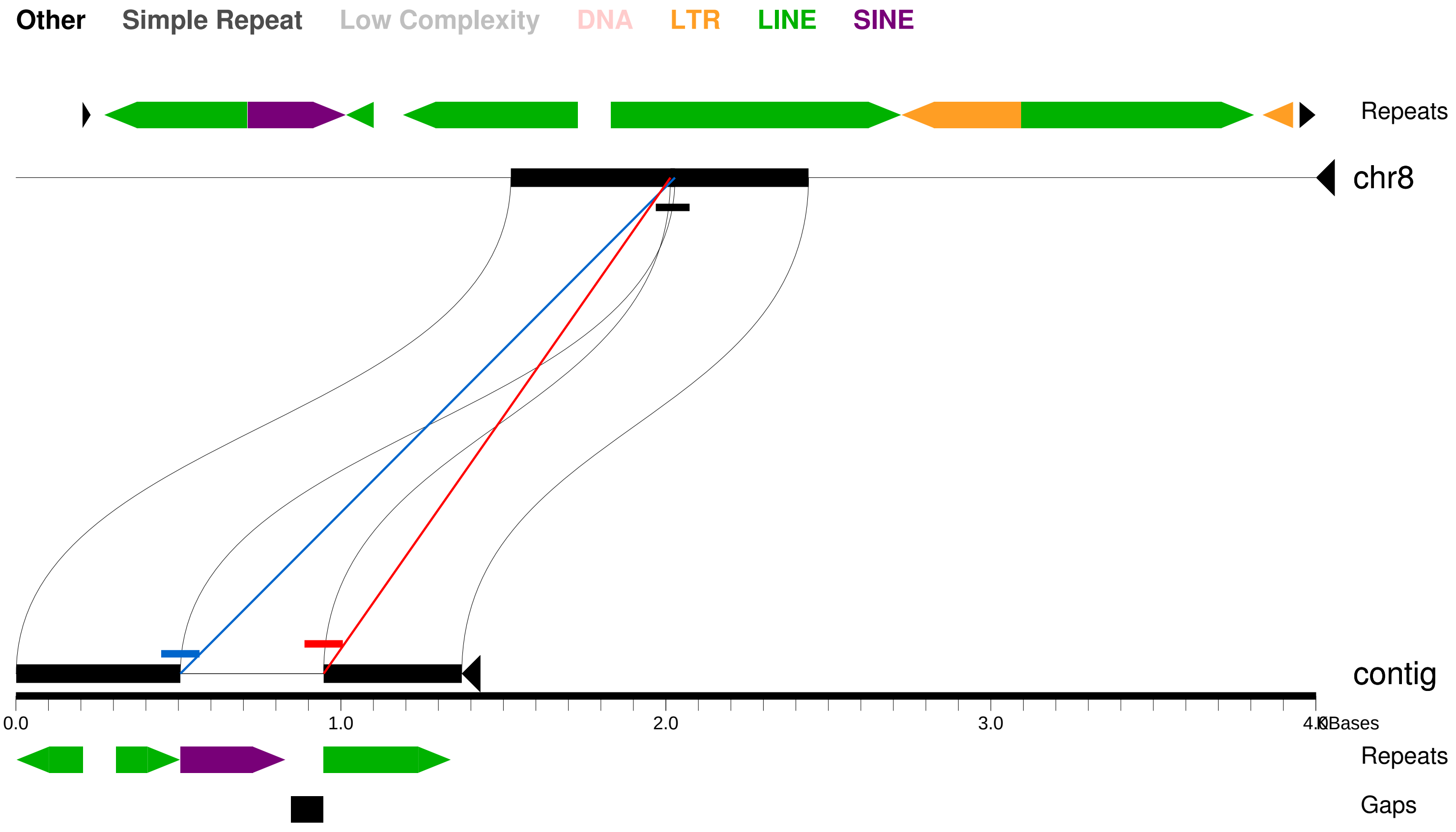

siteID: chr8_41270463 Scafftig3 - Breakpoint: chr8:41270459
chr8:41270299-41270628
Overlap Length: 10 INS size: 449 Genotyped: No

```
left    ----------------------------------------------------AGCCTGGCAAGAGACAGG
chrom   GCCTTCTCCCTCTTAGAAGCCTAGTAGAGATTTGACTCCAATGGACTGAGCCAGCCTGGCAAGAGACAGG
right   T-----------TTAGTAG---AGACGGGGTTTCACCT---TG---TTAGCCAGGATGGTCTC-GATCTC
        N           2222N22   22NN2N2N222N22NN   22   2N2222**11***11111**1111

left    CAGTTGTCATCATTCCCAGCAAGCAAAGCTGTTCCCAGGGCCCTCCCCATGCAGACTGCTAGCACTGATC
chrom   CAGTTGTCATCATTCCCAGCAAGCAAAGCTGTTCCCAGGGCCCTCCCCATGCAGACTGCTAGCACTGATC
right   CTGACCTCATGAT--CCACCCGCCTCGGCC--TCCCAAAGTGCTGGGATTACAGGCGTGAGCCACCGCGC
        *1*111****1**11***1*111*111**111*****11*11**11111*1***1*111111***1*11*

left    TTGGACCTGGAAGATCTCAAGACACATTTTTTTT------------------------------------
chrom   TTGGACCTGGAAGATCTCAAGACACATTTTAGTTACTCAGAAAAACCAGGTTCCCTAGCCTCTGTTGGAC
right   CCGGCC--------------GACACATTTTAGTTACTCAGAAAAACCAGGTTCCCTAGCCTCTGTTGGAC
        11**1*11111111111111**********22**222222222222222222222222222222222222

left    ----------------------------------------------------------------------
chrom   TTACCCCATAAATATGTAATGTGATGGGCAGTGGGGCTTGTTCTCTGTGGAGTGCCCTGGGTGGAACATT
right   TTACCCCATAAATATGTAATGTGATGGGCAGTGGGGCTTGTTCTCTGTGGAGTGCCCTGGGTGGAACATT
        2222222222222222222222222222222222222222222222222222222222222222222222

left    -----------------------------TTTNNNNNNNNNNNN-------
chrom   AGTCCTCTGGCCATGCTGCACCTAGGATGTTTAGAACCAGACTATTCTGT
right   AGTCCTCTGGCCATGCTGCACCTAGGATGTTTAGAACCAG----------
        22222222222222222222222222222***22222222NNN
```

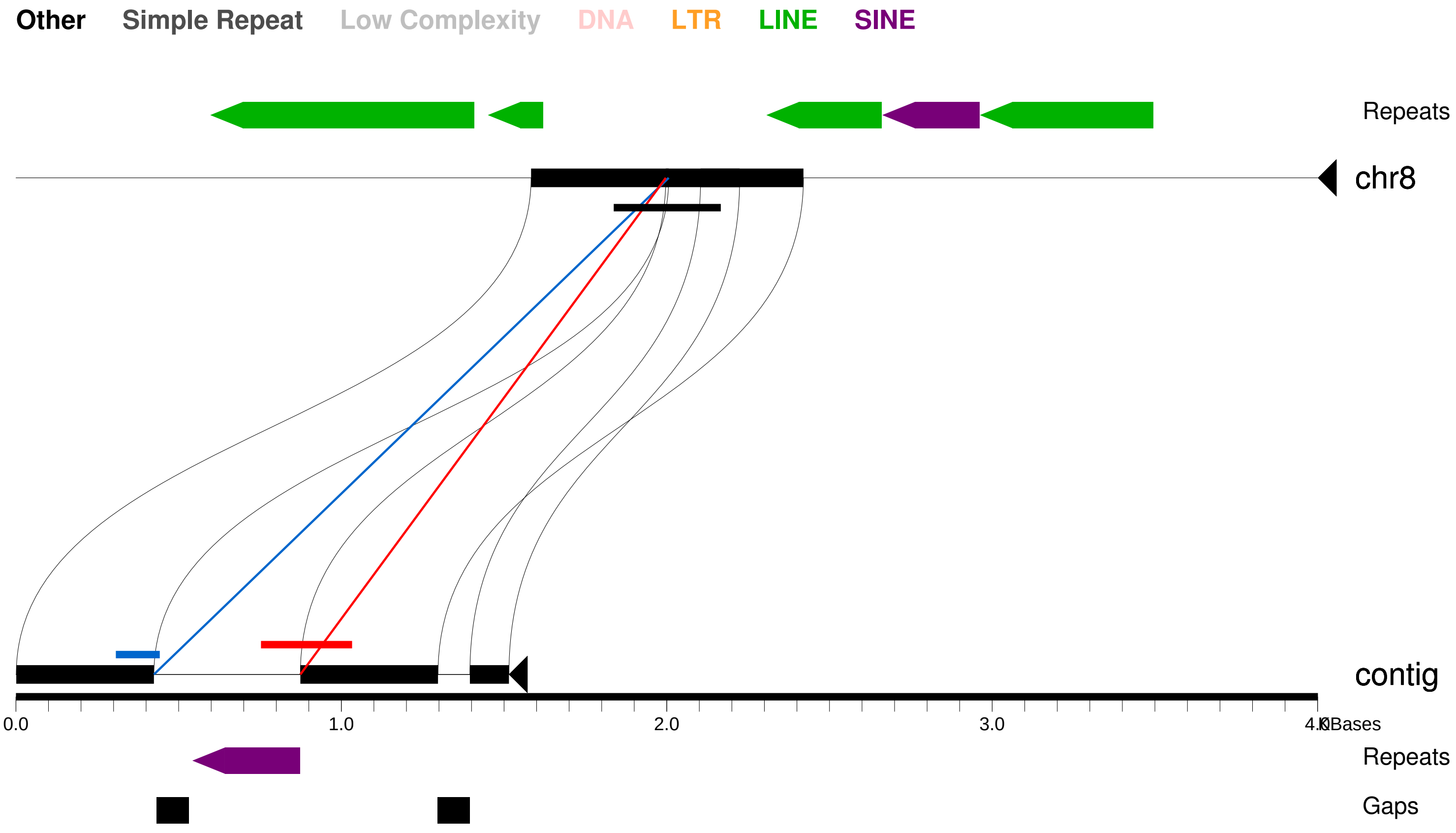

siteID: chr8_49045483 Scafftig2 + Breakpoint: chr8:49045481
chr8:49045443-49045540
Overlap Length: 14 INS size: 458 Genotyped: No

```
left    ATG---------GCTT-----ATTTCTCTGTTTAAATTCTTTAGAAAGATAT-------TTTAATAAAGT
chrom   ATG---------GCTT-----ATTTCTCTGTTTAAATTCTTTAGAAAGATAT-------TTTAATAAAGT
right   CCGCCCGCCTCGGCCTCCCAAAGTGCTGGGATTACAAGCGTGAGCCACCGCGCCCGGCCTTTAATAAAGT
        11*111111111**1*11111*1*1**11*1***1*11*1*1**11*111111111111***********

left    TTTTTTTTTTTTTNNNNNNNNNNNNNNNNNNNNNNNNNNNNNNNNNNNNNNNNNNNNNNNNNNNNNNNN
chrom   TTTATAATTTT---------------------CCTCACAAAAATCTTATTTATCTTTTGTTAGATTTAGT
right   TTTATAATTTT---------------------CCTCACAAAAATCTTATTTATCTTTTGTTAGATTTAGT
        ***2*22****22222222222222222222222222222222222222222222222222222222222
```

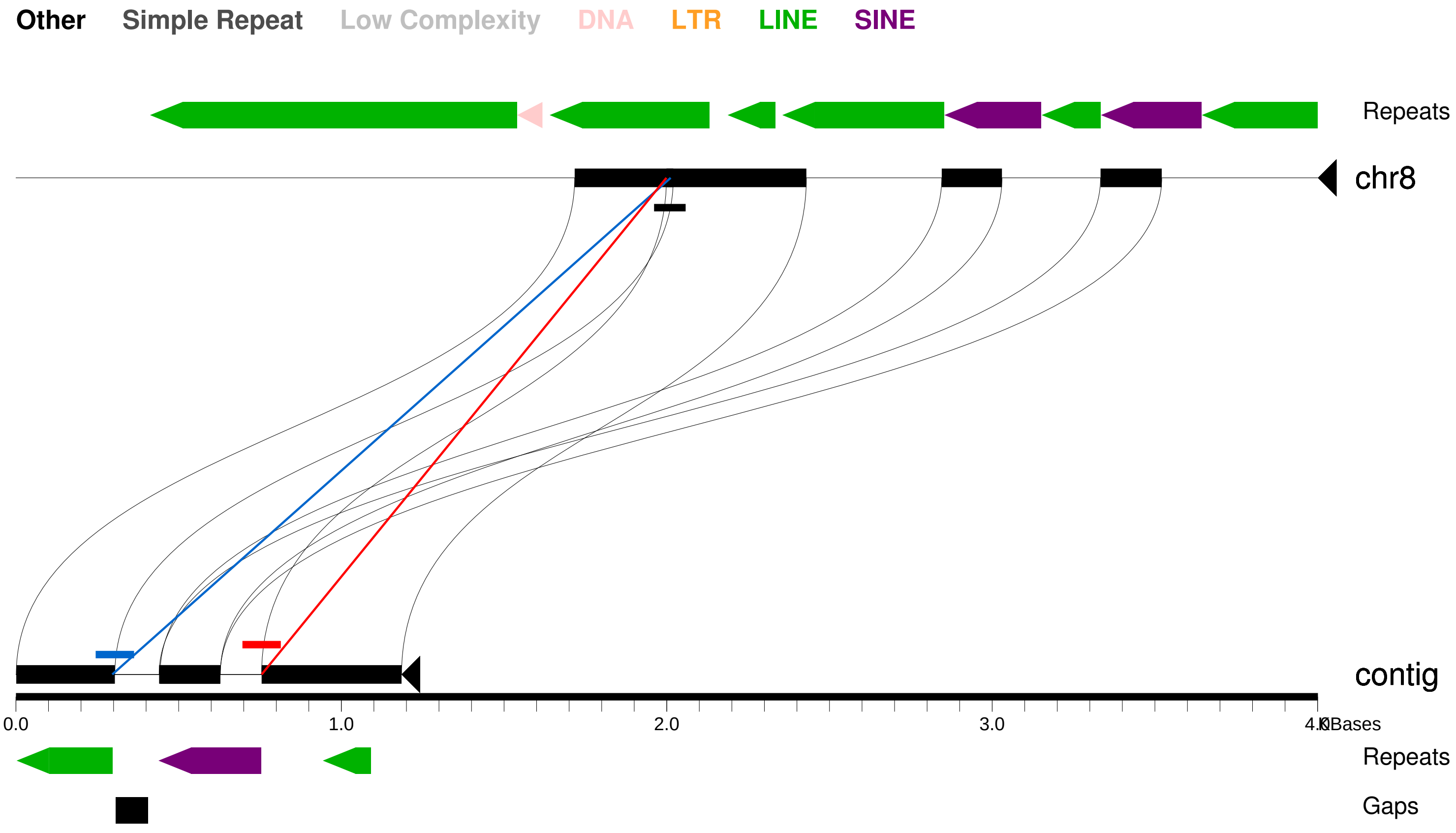

siteID: chr8_50550385 Scafftig1 + Breakpoint: chr8:50550364
chr8:50550313-50550422
Overlap Length: 9 INS size: 361 Genotyped: No

```
left    TCAAAATGTT----TTTACATTTTTCTT---TTA---GCATTTGCAACTACATACTATTTTGTGCTGAAT
chrom   TCAAAATGTT----TTTACATTTTTCTT---TTA---GCATTTGCAACTACATACTATTTTGTGCTGAAT
right   CCGCCCGCCTCGGCCTCCCAAAGTGCTGGGATTACAGGCGTGAGCCACCGCGCCCGGCCT-GTGCTGAAT
        1*1111111*11111*11**111*1**1111***111**1*11**1**11*111*1111*1*********

left    NNNNNNNNNNNNNNNNNNNNNNNNNNNNNNNNNNNNNNNNNNNNNNNNNNNNNNNNNNN
chrom   GCTTTTTAAAAGTATAGTTGTAAATTTATTTTGCTGTATTATTTATGATT---------
right   GCTTTTTAAAAGTATAGTTGTAAATTTATTTTGCTGTATTATTTATGATT---------
        22222222222222222222222222222222222222222222222222222222222
```

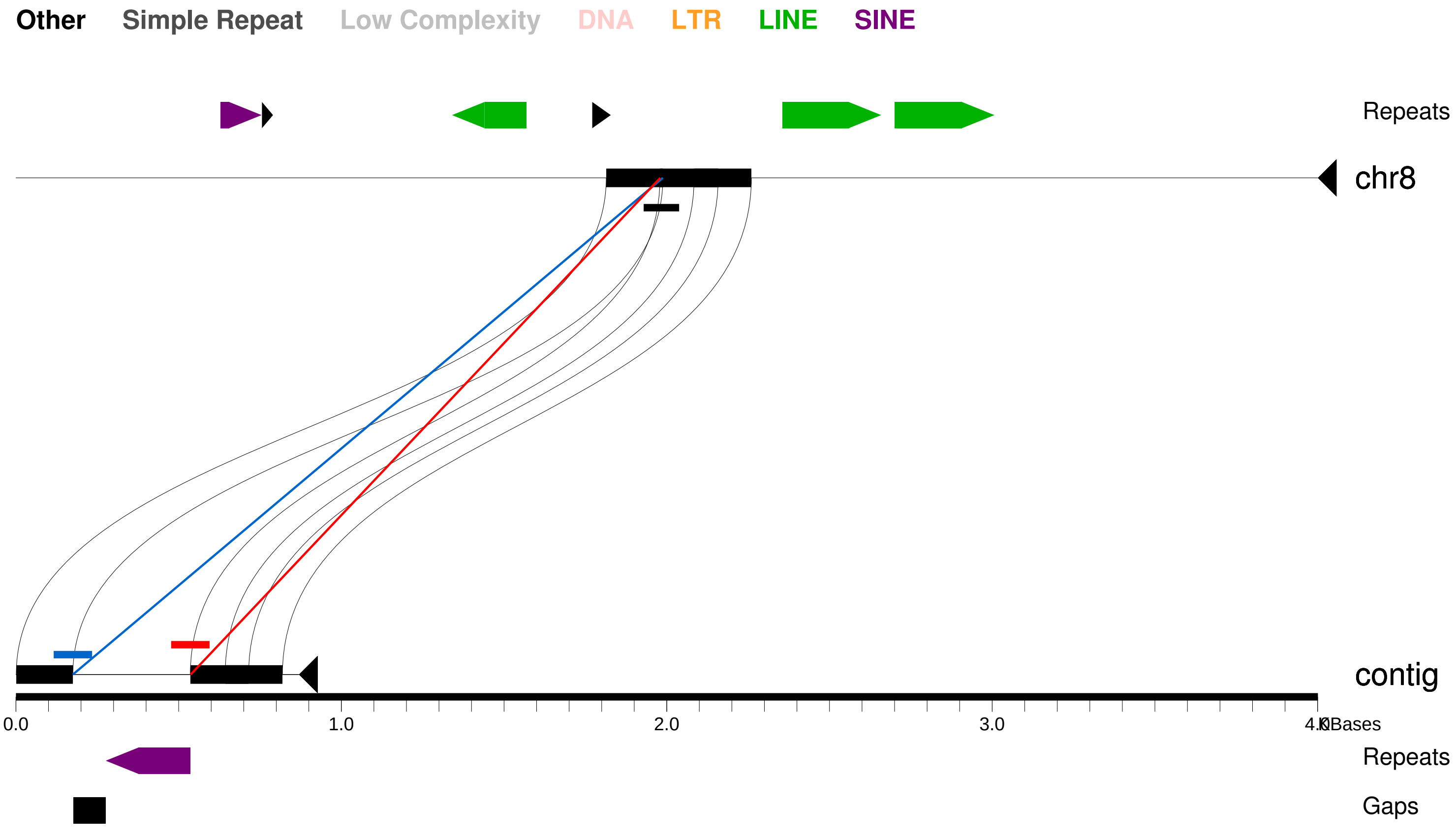

siteID: chr8_51442451 Scafftig2 - Breakpoint: chr8:51442457
chr8:51442405-51442516
Overlap Length: 8 INS size: 415 Genotyped: No

```
left     TT----GTATTATTTT----AAGTA--TGGTTTCTAAGAGCAGGCCACTTTAGATAATTAAACAAAATAA
chrom    TT----GTATTATTTT----AAGTA--TGGTTTCTAAGAGCAGGCCACTTTAGATAATTAAACAAAATAA
right    CCACCCGCCTCGGCCTCCCAAAGTGCTGGGATTACAGGCGTGAGCCACC---GCGCCCGGCCCAAAATAA
         111111*11*11111*1111****1111**1**11*1*1*111*****1111*111111111********

left     NNNNNNNNNNNNNNNNNNNNNNNNNNNNNNNNNNNNNNNNNNNNNNNNNNNNNNNNNNNNN
chrom    TACTTTTTATAAGGCTGTACATTTTCCTTTATAAAGTCTTAATGTTTCTGAA-------
right    TACTTTTTATAAGGCTGTACATTTTCCTTTATAAAGTCTTAATGTTTCTGAA-------
         2222222222222222222222222222222222222222222222222222222222222
```

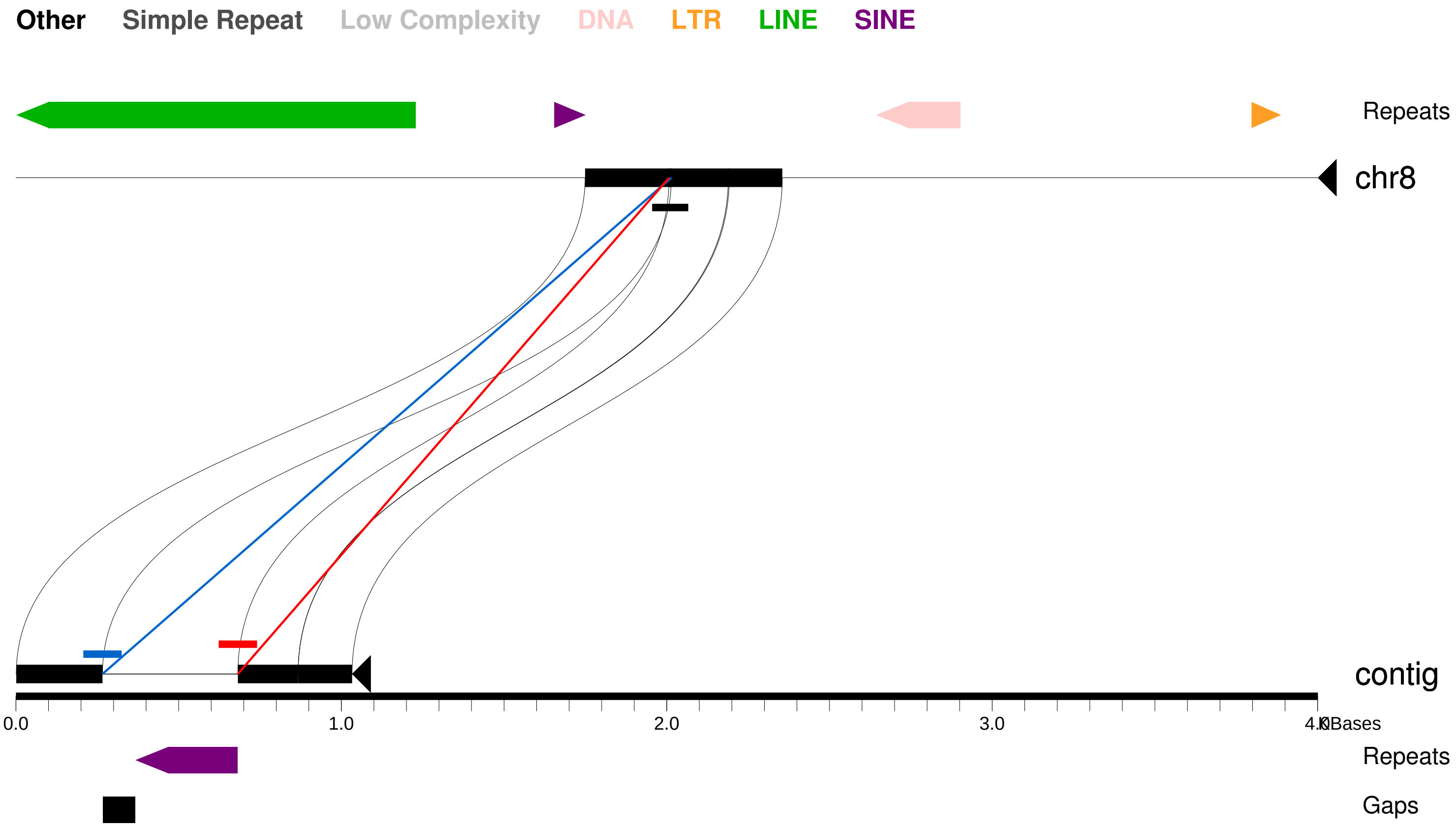

siteID: chr8_53173024 Scafftig3 - Breakpoint: chr8:53173018
chr8:53172971-53173077
Overlap Length: 13 INS size: 202 Genotyped: Yes

```
left    GTCTTGTTCTTATTTATCATGTGACAT-GTGGCATCACCAGCTGCATG-----------AAATCTGGCTG
chrom   GTCTTGTTCTTATTTATCATGTGACAT-GTGGCATCACCAGCTGCATG-----------AAATCTGGCTG
right   TCCAGCCTGGGCGACAGAGCGAGACTCCGTCTCAAAAAAAAAAAAAAAAAAAAAAAAAAAAATCTGGCTG
        11*1111*1111111*1111*1***111**11**11*11*11111*1111111111111***********

left    AACCCCGTCTCTACTAAAAATACAAAAAATTAGCCGGGCGTAGTGGCGGGCGCCTGTAG-TC
chrom   AA----GATTC--CTTTGCACACTTTATAT--GCCTGATGAAGTGAAA-----CTTTAGATC
right   AA----GATTC--CTTTGCACACTTTATAT--GCCTGATGAAGTGAAA-----CTTTAGATC
        **2222*22**22**2222*2**222*2**22***2*22*2****22222222**2***2**
```

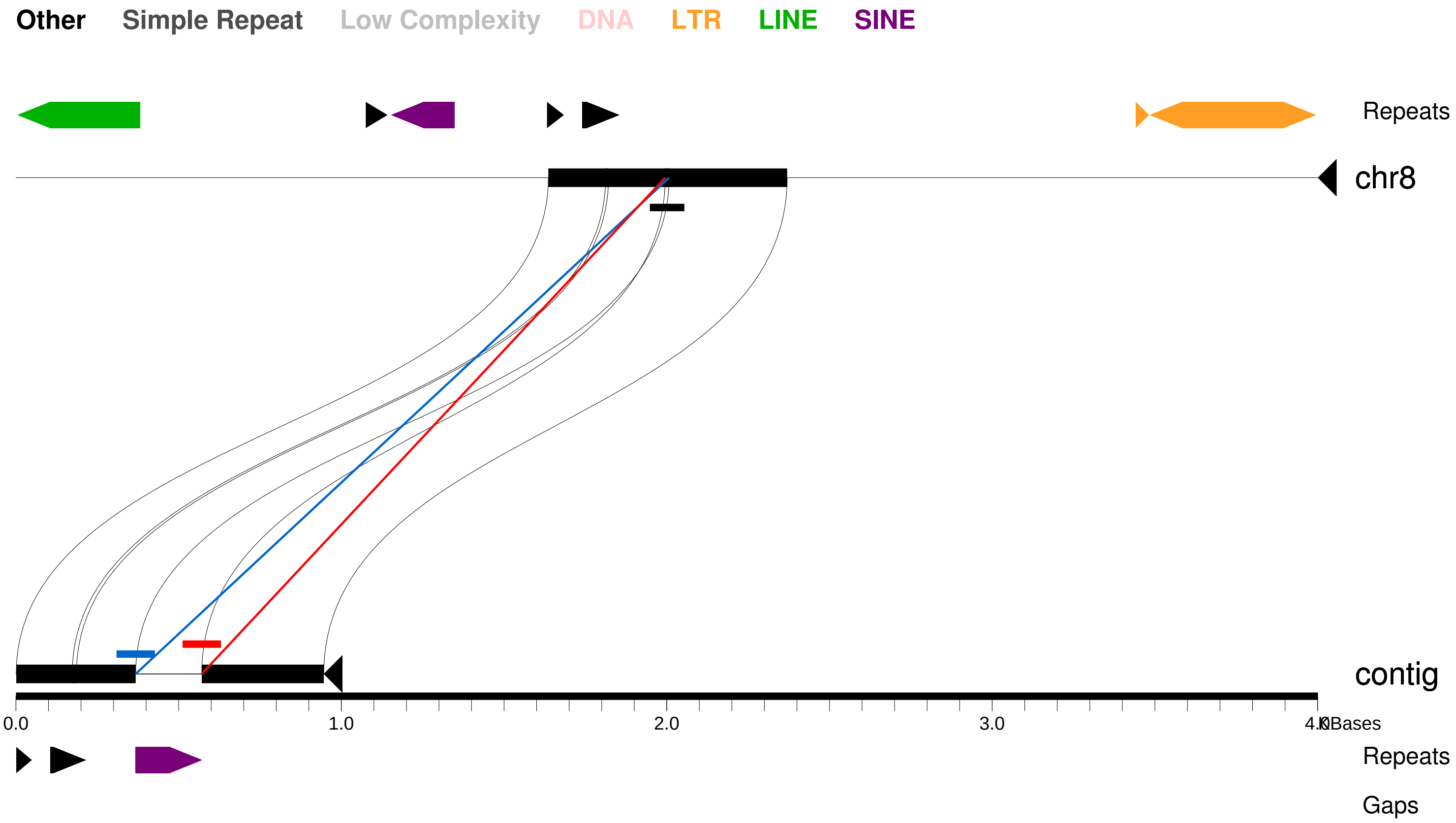

siteID: chr8_53791044 Scafftig2 - Breakpoint: chr8:53791041
chr8:53791001-53791087
Overlap Length: 13 INS size: 241 Genotyped: Yes

```
left   -----------ATTTTCATCATCATCATCAGTA----------TT----GTT--------GAAGGGCTAT
chrom  -----------ATTTTCATCATCATCATCAGTA----------TT----GTT--------GAAGGGCTAT
right  TACGCCCGGCTAATTTTTTTGT-ATTTTTAGTAGAGACGGGGTTTCACCGTTTTAGCCGGGATGGTCTCG
       11111111111*1***11*11*1**11*1****1111111111**1111***11111111**1**1**11

left   TTTTAGCTGAATTTTTTTTTTTTTTTTTTTTTTTTTTTTTTTTTTTTTTGAGACAGAGTCTCGCTCTGTT
chrom  TTTTAGCTGAATTTTTAATTTTTGTGAATCTGCT----------------AGGTAAAATCACGTTT----
right  ATCTAGCTGAATTTTTAATTTTTGTGAATCTGCT----------------AGGTAAAATCACGTTT----
       1*1*************22*****2*222*2*22*2222222222222222**22*2*2**2**2*22222

left   GCCCAGGCTGGA
chrom  ------------
right  ------------
       222222222222
```

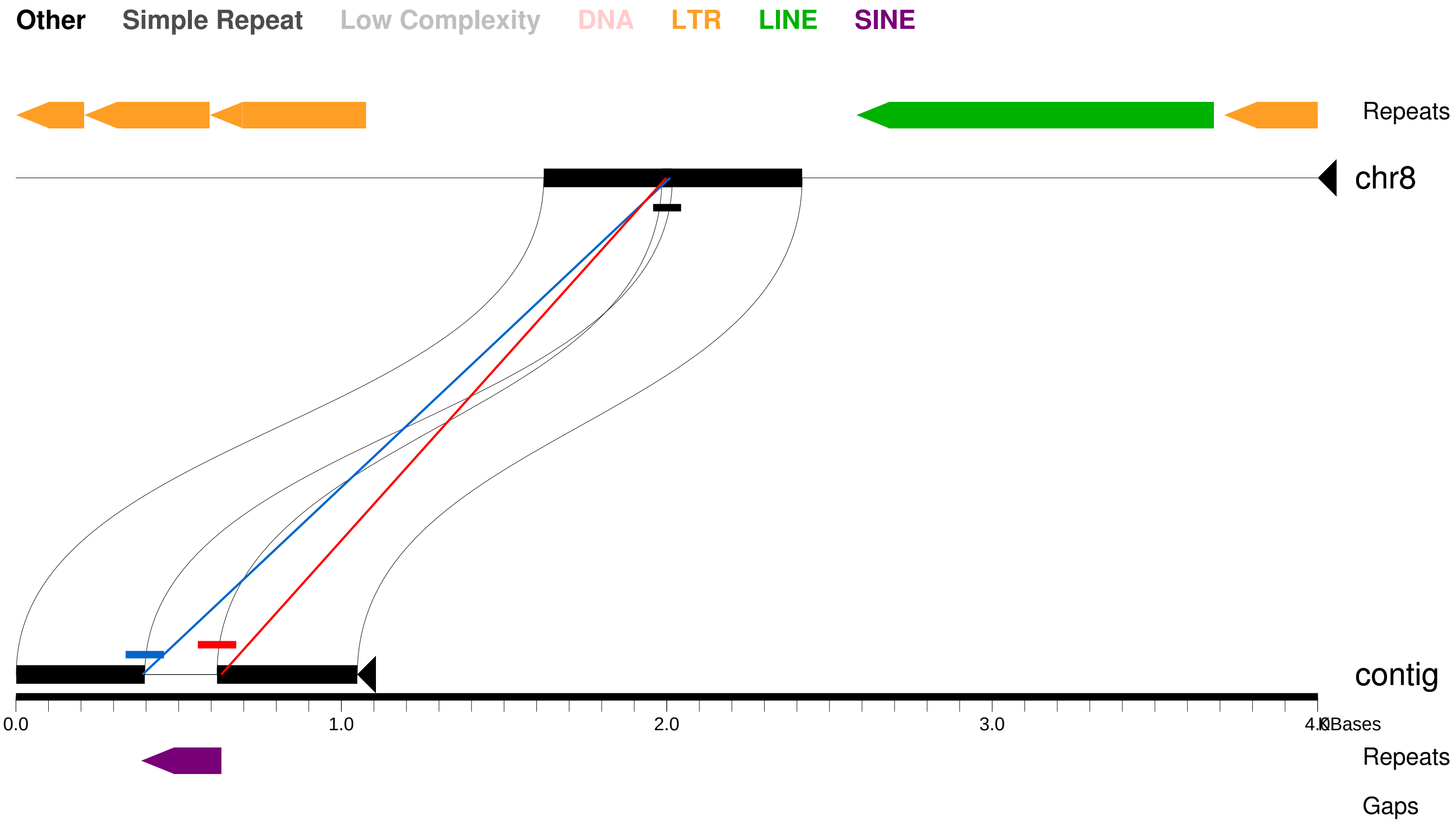

siteID: chr8_5506280 Scafftig1 - Breakpoint: chr8:5506268
chr8:5506215-5506327
Overlap Length: 7 INS size: 439 Genotyped: No

```
left    AGGCACACCTGTT--------AGAGCAAGCTATATATGCTCTAAGCCTCTTTGTTGCTTTTGAGAAATTC
chrom   AGGCACACCTGTT--------AGAGCAAGCTATATATGCTCTAAGCCTCTTTGTTGCTTTTGAGAAATAT
right   CCGCCCGCCTCGGCCTCCCAAAGTGCTGGGATTACAGGCG-TGAGCCACCGCGCC-CGGCCGAGAAATAT
        11**1*1***11111111111**1**11*111**1*1**11*1****1*111*111*1111*******22

left    CTGCTGAACCGCTCTTCCGATCNNNNNNNNNNNNNNNNNNNNNNNNNNNNNNNNNNNN
chrom   TTTT------GTTTTTCCTCCTAAAATGAAAATCAGCCCAGAAAAGGAAGTCAAGGA
right   TTTT------GTTTTTCCTCCTAAAATGAAAATCAGCCCAGAAAAGGAAGTCAAGGA
        2*22222222*2*2****222222222222222222222222222222222222222
```

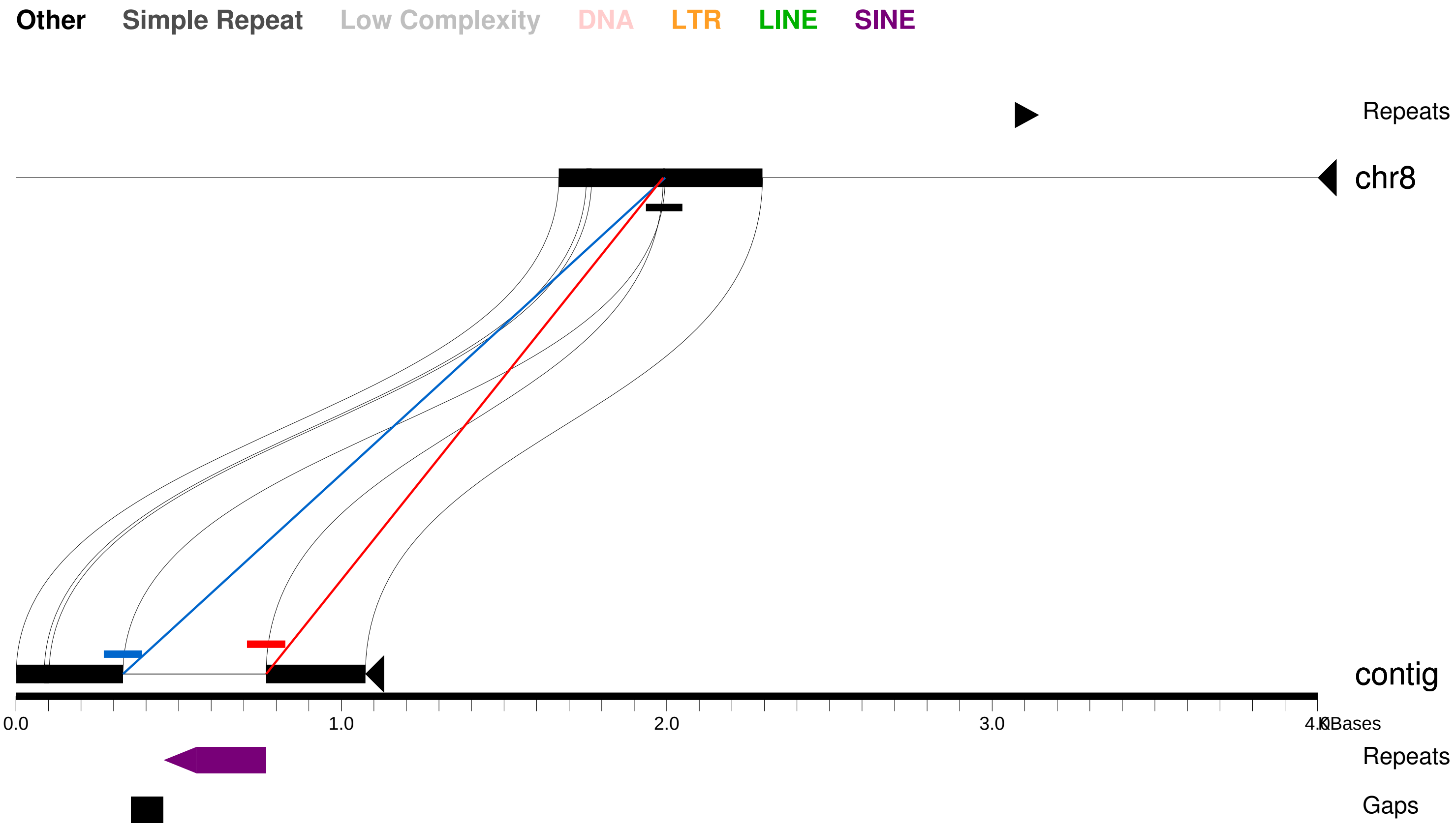

siteID: chr8_57847925 Scafftig1 - Breakpoint: chr8:57847920
chr8:57847877-57847979
Overlap Length: 17 INS size: 418 Genotyped: No

```
left    GGACAGCCTTCCAGAGTCCCTTGCACACCAGTAAGGTGAGG----------------AGAAAGATGCTAG
chrom   GGACAGCCTTCCAGAGTCCCTTGCACACCAGTAAGGTGAGG----------------AGAAAGATGCTAG
right   NNNNNNNNNNNNNNNNNNNNNNNNNNNNNNNNNNNNNNNNNNNNNNNNNNNNNNNNNNNAAAAAGATGCTAG
        1111111111111111111111111111111111111111111111111111111111*1***********

left    GAGTCCTGGCCGGGCGCGGTGGCTCACGCCTGTAATCCCAGCACTTTGGGAGGCCGAGGCGGGCG
chrom   GAGTCCCA-CAAGCCTCTATGGGGCACAGGAGTA---------CTTAGAG-GGCTGGTG-----G
right   GACCCCCA-CAAGCCTCTATGGGGCACAGGAGTA---------CTTAGAG-GGCTGGTG-----G
        **11**222*22*2*2*22***22***2222***222222222***2*2*2***2*22*22222*
```

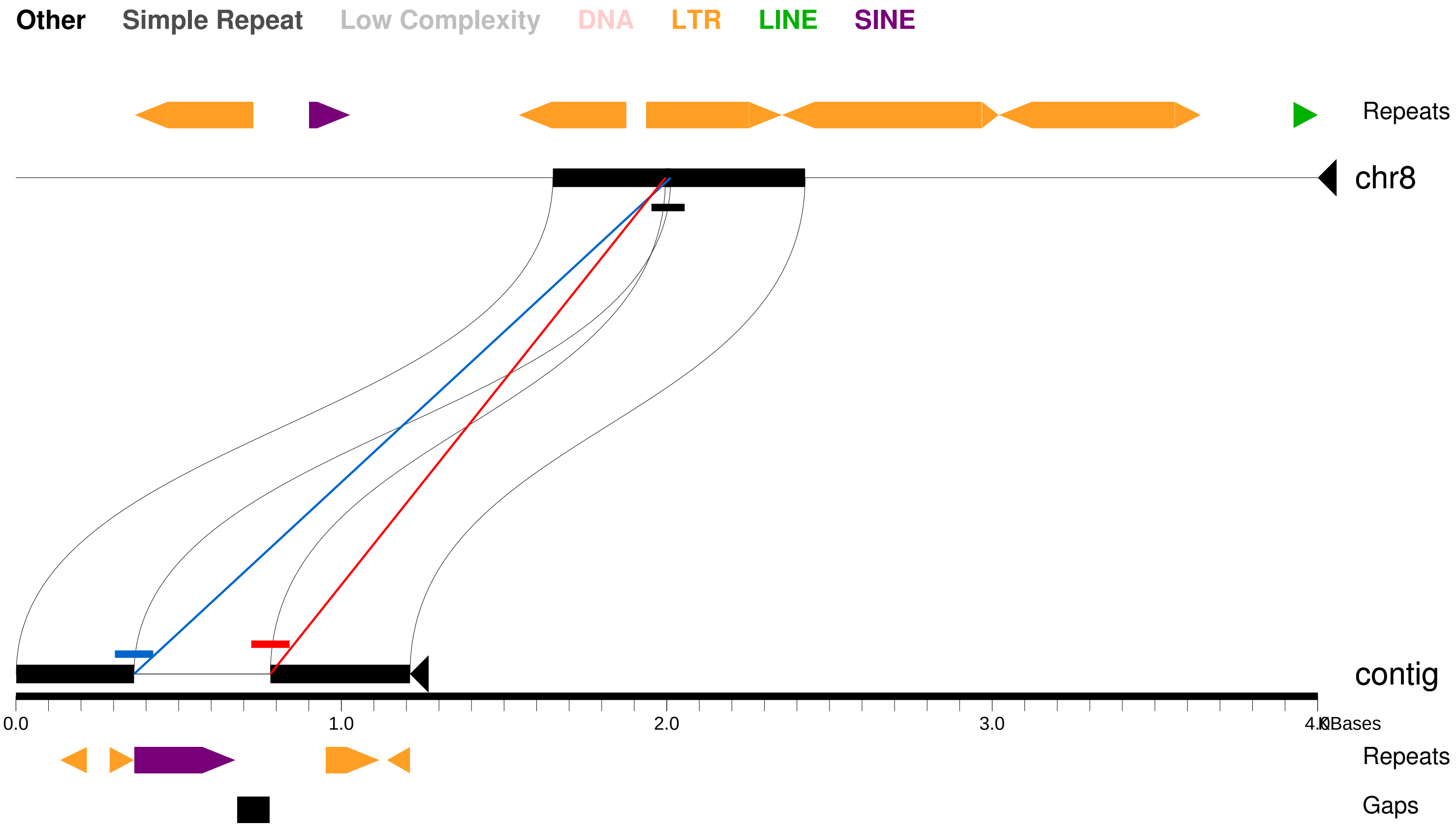

siteID: chr8_58704426 Scafftig1 - Breakpoint: chr8:58704391
chr8:58704231-58704568
Overlap Length: 18 INS size: 423 Genotyped: No

```
left   G-----------------------------------------------------GGTTATAGGGTACAAACAT
chrom  GAGACTTGGAAGGGTGTGTGA--TGGGGAGGGAGAAAGATGAAGAGAAGTGGGTTATAGGGTACAAACAT
right  TTTAGTAGAGACGGGGTTTCACCTTGTTAGCCAGGATGGTCTCGATCTCCTGACCTCATGATCCACCCGC
       1NN2N2N2NN2N22N22N2N2112N2NN22NN22N2N2N2NNN22NNNNNN*11111*1*1*1**11*11

left   ACGATAAGATAGAAGAAATAAATACAATGTTTGATAGTAGAGCAGGGTGACTACACTTAACAAAAGTGTA
chrom  ACGATAAGATAGAAGAAATAAATACAATGTTTGATAGTAGAGCAGGGTGACTACACTTAACAAAAGTGTA
right  C--------TCGGCCTCCCAAAG----TGCTGGG-ATTACAG--GCGTGAGC-CACCGCGC---------
       111111111*1*1111111***11111**1*1*11*1**1**11*1****111***1111*111111111

left   TTGTGCTGGACAATAGTTAATGGACACCCTAAATATTCTT------------------------------
chrom  TTGTGCTGGACAATAGTTAATGGACACCCTAAATATTCTTATTTGATTACTTGGCACTATAGACATTTAA
right  -----CCGGCC-----------GACACCCTAAATATTCTTATTTGATTACTTGGCACTATAGACATTTAA
       11111*1**1*11111111111*****************222222222222222222222222222222

left   -------------------------TTTTTNNNNNNNNNNN-----------------------------
chrom  CAAAATTTCACAAAGATCCCATTAATTTTTAAATTTATAAAATAATAAAATAAAGGACAATAAATCAATA
right  CAAAATTTCACAAAGATCCCATTAATTTTTAAATTTATAAAATAATAAAATAAAGGACAATAAATCAATA
       2222222222222222222222222*****2222222222222222222222222222222222222222

left   ------------------------------------------------------------
chrom  ATAAATAATGTAATACATTTAAAACATTAAGTTAAATATATTTTTAAAATATGGATTTTC
right  ATAAATAATGTAATACATTNNNNNNNNNNNNNNNNNNNNNNN------------------
       2222222222222222222NNNNNNNNNNNNNNNNNNNNNNN
```

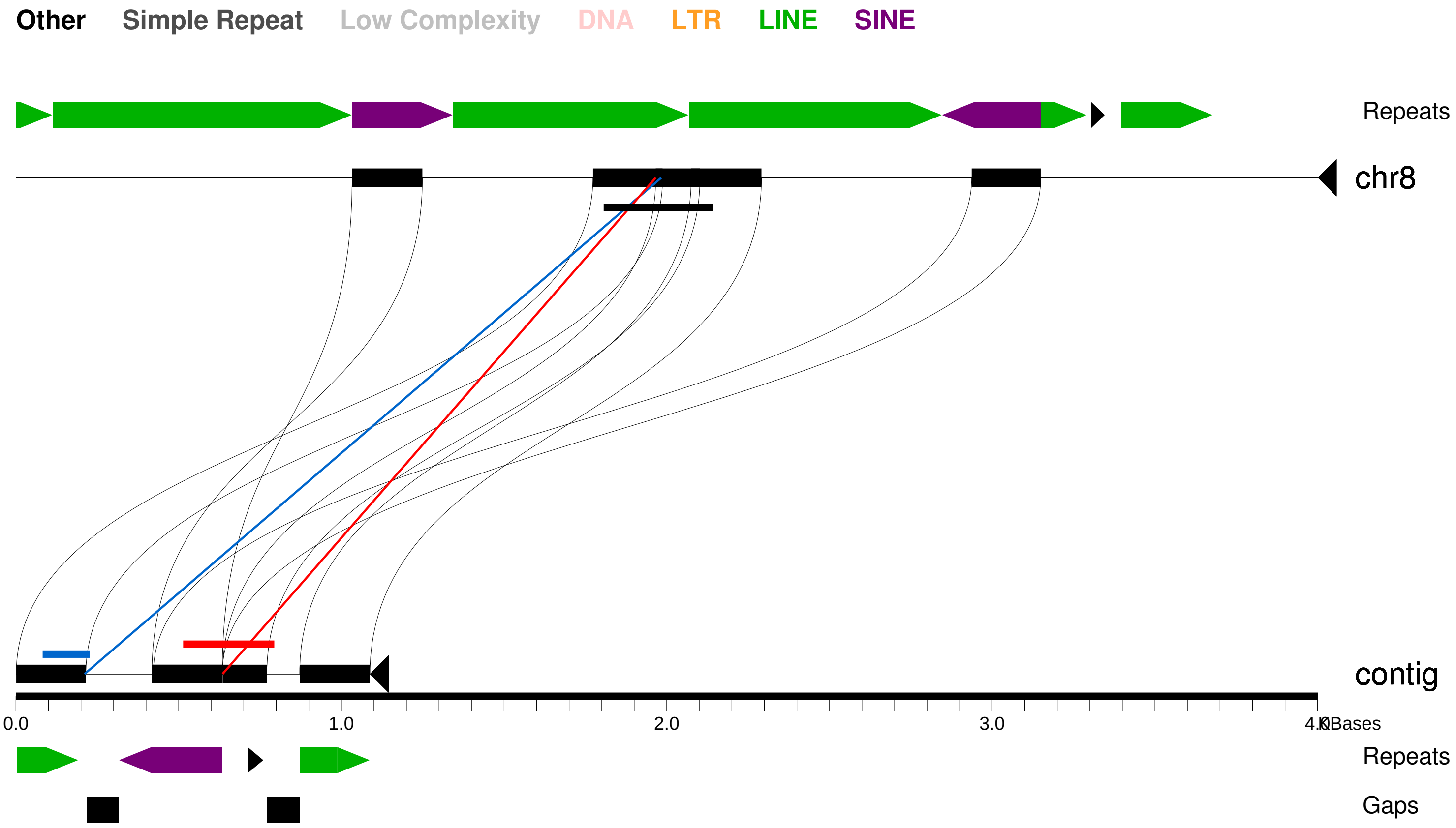

siteID: chr8_59615993 Scafftig4 - Breakpoint: chr8:59615978
chr8:59615931-59616037
Overlap Length: 13 INS size: 309 Genotyped: Yes

```
left    TAGCAACA--------------ACAAAATTAGACAT-GTATCAAAATGAGTGTTTTCCTTTTCAAAGTCA
chrom   TAGCAACA--------------ACAAAATTAGACAT-GTATCAAAATGAGTGTTTTCCTTTTCAAAGTCA
right   AGTCCGCAGTCCGGCCTGGGCCACAGAGCGAGACTCCGTCTCAAAAAAAAAAAAAAAAAA---CAAAGTCA
        111*11**11111111111111***1*111****111**1******11*1111111111111********

left    CAATA--GGCAGGGCGCGGTGGCTCACGCCTGTAATCCCAGCACTTTGGGAGGCCGAGGCGGGTGG
chrom   CAATATTGGCAAG------TTGAACACT----TACTAGAAAGATTTT----ATCTAAAACAGTGAC
right   CAATATTGGCAAG------TTGAACACT----TACTAGAAAGATTTT----ATCTAAAACAGTGAC
        *****22****2*222222*2*22***22222**2*222*22*2***222222*22*22*2*2222
```

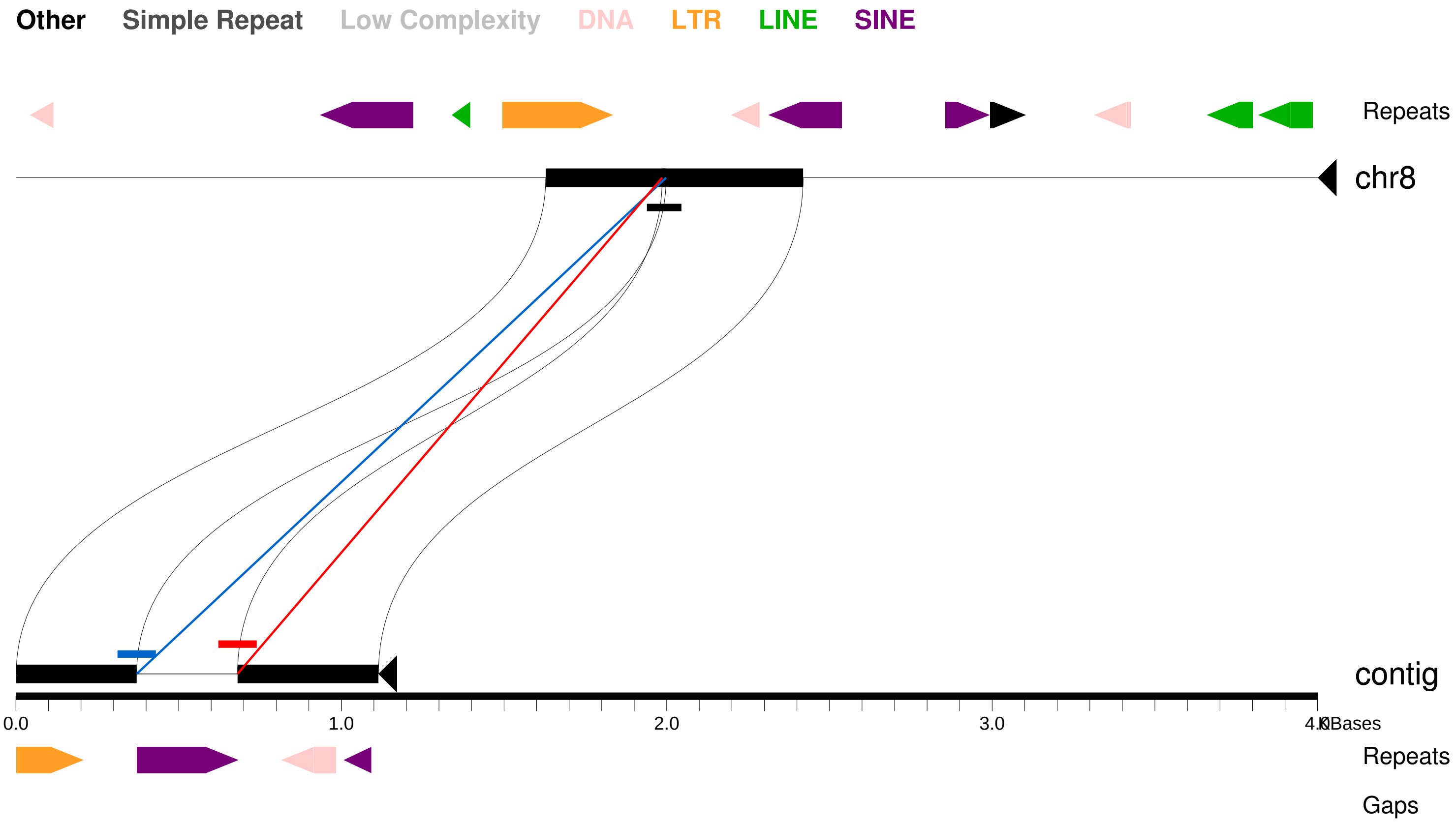

siteID: chr8_59645630 Scafftig3 + Breakpoint: chr8:59645627
chr8:59644978-59645787
Overlap Length: 5 INS size: 311 Genotyped: Yes

```
left   T----------------------------------------------------------------------
chrom  TGAGGTGGGCCACCTCCCACAGCTCAGGAGGATGTGATGGTTGTGATTGAGGGGGTGTAGCTGATGCCAG
right  T-----------------------TTAGTAGA-----------------GACGGGGTTT------------
       *                       2N22N22N                 22N22222N2

left   -----------------------------------------------------------------------
chrom  GGGTTACCTACTCCTCTCTCCTTCTTTCTTGCTGGAAGAGCCACCTTGATTCAGTGTTGGTATTCACTCT
right  -----------------------------------------CACCTTGTT--AG----------------
                                                2222222N2  22

left   -----------------------------------------------------------------------
chrom  TCTCTTCCAGGGCCAGAAAATGAATGTTCACAAGTCTAAGCCAATCGAAGTGCTTCCCTTCTCCTGGTCA
right  ------CCAGG------------ATGGTC---------------TCGA-----------TCTCCTG----
             22222            222N22               2222           2222222

left   -----------------------------------------------------------------------
chrom  CTGATTGGTTTAGGTGTGGGCCTGTAACACTGTCCTGGGAAATGAGACTTAAAAGTCATTCTTTAGACAG
right  -----------------------------------------------------------------------

left   -----------------------------------------------------------------------
chrom  AACTCAGTAAAGGTTTGTCTCTGGATAGAAGGAGACAAGCAAGGAGACACCTCCTTTCCTGGCTTTGACT
right  ------------------------------------------------ACCTC-----------------
                                                       22222

left   -----------------------------------------------------------------------
chrom  ACAGTTGTGTGAAAACATGTATAGTATCCACGCTGTGATCATGAAAGGACATGCCTGAGGATGAACCCTA
right  ----------------ATG------ATCCACCC-------------------GCCTCGG-------CCTC
                       222      222222N2                   2222NN2       222N

left   -----------------------------------------------------------------------
chrom  ACAGAGTGAGGATGGGAGAGCAGAAATAGGGCAAGCTGTTGGGTCCTTGTTGATATCATAGCATTCCTGA
right  CCAAAGTGC---TGGGAT-----------------------------------------------------
       N22N2222N   22222N

left   ---ACAAACCTGGGCCCTTCTTTTCTGTCTCTGTATGTTTTGCTGTTTATTGTTATATAAGATAACACAC
chrom  AGTACAAACCTGGGCCCTTCTTTTCTGTCTCTGTATGTTTTGCTGTTTATTGTTATATAAGATAACACAC
right  --TACAGGCGTGAGCC---------------------------------------------ACCGCGC
         2***11*1**1***1111111111111111111111111111111111111111111111*1*1*1*

left   CTTTCTTGTTTAAGCCACTTTCCATTATTCTGTTACATGGACTTAAAAGATTCTAACGGATACAGAAGGT
chrom  CTTTCTTGTTTAAGCCACTTTCCATTATTCTGTTACATGGACTTAAAAGATTCTAACGGATACAGAAGGT
right  C--------------------------------------------------------CGG-----------
       *11111111111111111111111111111111111111111111111111111111***11111111111

left   TTCCCAAAGTAGTGAAGATTAGTTTTTTTTTTT[illegible]TTTTTTTTTTTTTGAGACGGAGTCT
chrom  TTCCCAAAGTAGTGAAGATTAGTTCGTGCTTTAAAGGGCCAAAGATATTCACCTTTGGGAGTTGATGGGT
right  --CC----------------TAGTTCGTGCTTTAAAGGGCCAAAGATATTCACCTTTGGGAGTTGATGGGT
       11**11111111111111*****22*22***2222222222222*2**2222***22***22*22*22*

left   CGCTCTGTCGCC-CAGGC[illegible]-----TGCAG[illegible]ATCTCGGCTCACT[illegible]GCAA
chrom  GGAAGAATGGGAGCAGGCCACTCCAGGGAAGAGCTTGTGCAAAAGCTTAGAAACACAGAGCAATATGGAA
right  GGAAGA[illegible]GGAGCAGGCCACTCCA[illegible]AAAAGCTTAGAAACACAGAGCAA[illegible]AA
       2*22222*2*222*****222*222***2222*222****222**22*22*2*2*2*22**2*22*2**

left   GCTC--------------CGCTTCCCGGGTTCACGCCATT
chrom  GGTTTGAGATAGGACAAGCGTTTCTCACATGGGTAGGATG
right  GGTTTGAGATAGGACAAGCGTTTCTCACATGGGTAGGAT-
       *2*2222222222222**2***2*222*2222222**N
```

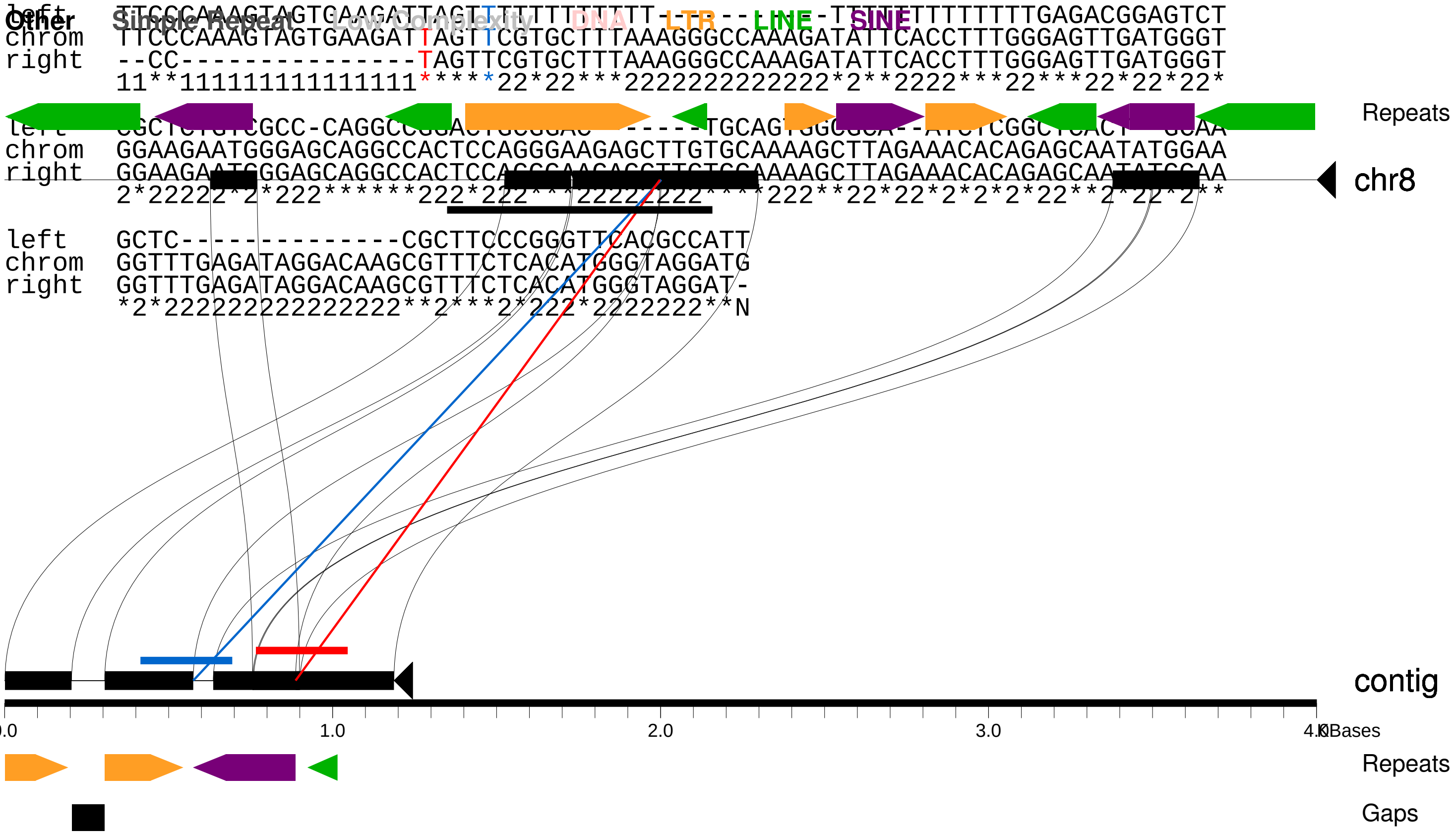

siteID: chr8_59906439 Scafftig2 + Breakpoint: chr8:59906451
chr8:59906291-59906613
Overlap Length: 3 INS size: 409 Genotyped: No

```
left    ---AAATTAAGATGACAGACCTTTACTCTTGAGTCATTAGTTTTATCCTTTTATATGGATTGCATTCTCA
chrom   TTAAAATTAAGATGACAGACCTTTACTCTTGAGTCATTAGTTTTATCCTTTTATATGGATTGCATTCTCA
right   NNNNNNNNNNN-----------------------------------------------------------
        NNN1111111111111111111111111111111111111111111111111111111111111111111

left    GTTTACTGTAGAGGTTCTGGCAATTCCATCATCTTCCTAAGGGGACCTAAGGGGAAAAGGCATCTTGCCA
chrom   GTTTACTGTAGAGGTTCTGGCAATTCCATCATCTTCCTAAGGGGACCTAAGGGGAAAAGGCATCTTGCCA
right   ----------------------------------------------------------------------
        1111111111111111111111111111111111111111111111111111111111111111111111

left    TACCTTGTTCAGAATACAGAGAAGGCCGGGCGCGG--TGGCTCAC--GCCTGTA---ATCCCAGCA----
chrom   TACCTTGTTCAGAATACAGAGAAAAGCATTCCCCGAATGGCTGAGTGGCCAGTTGCCATGGCAGCATAAA
right   --------------------GAAAAGCATTCCCCGAATGGCTGGGTGGCCAGTTGCCATGGCAGCATAAA
        11111111111111111111***222*222*2*2*22*****21222***2**2222**22*****2222

left    --CTTTGGGA---GGCCGAGGCGGGCGGATCAC-----------GAGGTCAGG------------AGATC
chrom   GACGTTGTGAATTGGTCTACCTGTGGGAAAAATTTCCAAAAATAGAACACAGTCTATTTTCACCTAGATC
right   GACGTTGTGAATTGGTCTACCTGTGGGAAAAATTTCCAAAAATAGAACACAGTCTATTTTCACCTAGATC
        22*2***2**222**2*2*222*2*2*2*22*222222222222**222***2222222222222*****

left    GA-GACC-ATCCTGGCTAACACGGTGAAACCCCGTCTCTACTAAA
chrom   CACGACATATTCTTGCCAGTAT--TCATCAAGAGTTTCTAGAGAC
right   CACGACATATTCTTG------------------------------
        2*2***22**2**2*1N1NN1N221N1NNNNNN11N1111NNN1N
```

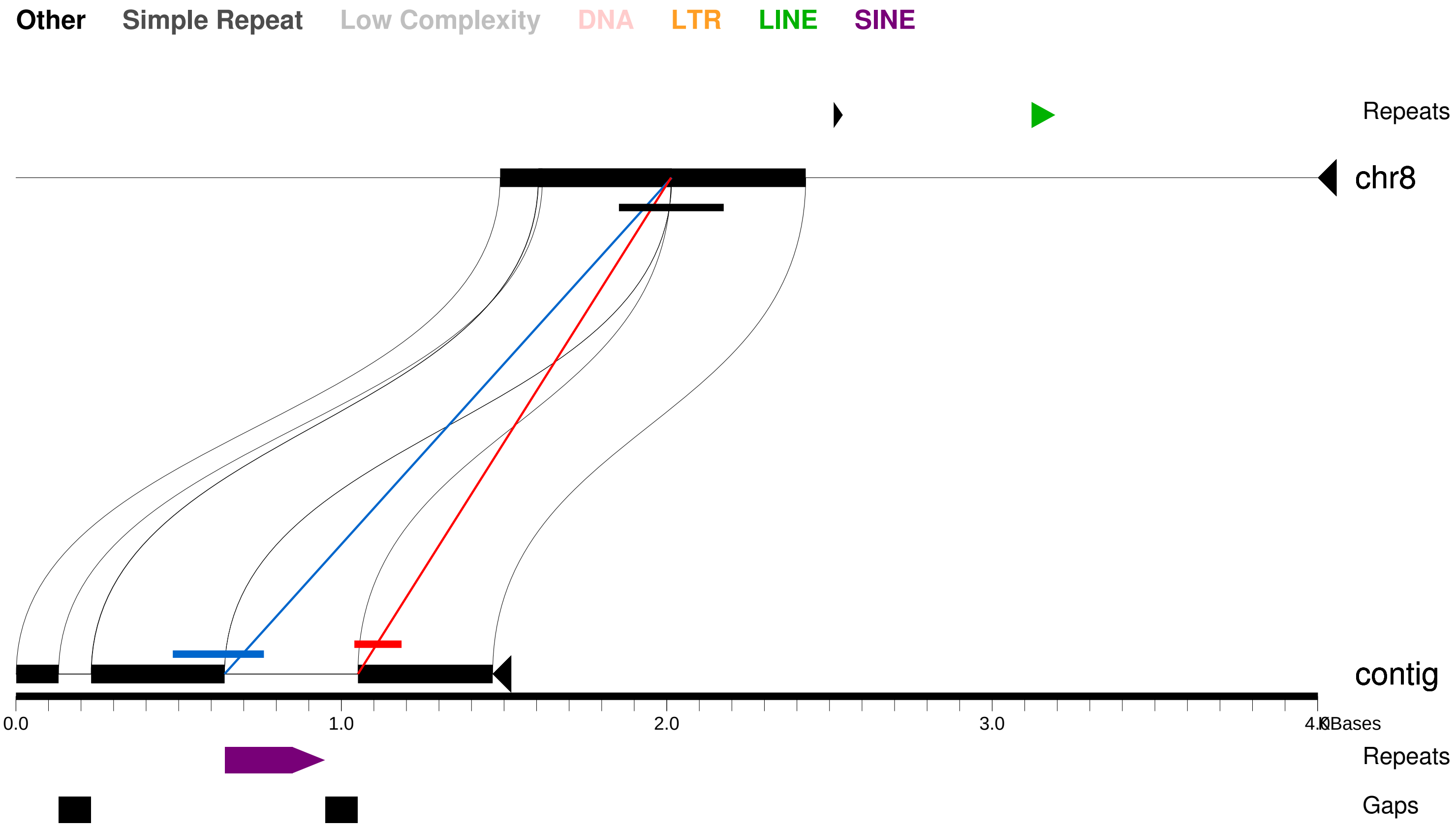

siteID: chr8_6044976 Scafftig6 - Breakpoint: chr8:6044960
chr8:6044918-6045019
Overlap Length: 18 INS size: 300 Genotyped: Yes

```
left     TCCACTCACCAGCC---------GTCCATTAGTTCTTC----------AGAAGTATTGATCAATATGCAA
chrom    TCCACTCACCAGCC---------GTCCATTAGTTCTTC----------AGAAGTATTGATCAATATGCAA
right    TGCACTC--CAGCCTGGGCGACAGAGCGAGACTCCGTCTCAAAAAAAAAAAAAAAAAAAAAAATATGCAA
         *1*****11*****111111111*11*111*1*1*1**1111111111*1**11*111*11*********

left     CACTTAAGGCCGGGCGCGGTGGCTCACGCCTGTAATCCCAGCACTTTGGGAGGCCGAGGCGGGCGGAT
chrom    CACTTAAGGTT--------TCAAACAACACTTAAAT----GCAATTTTTGA-----AGGTATCTCAAG
right    CACTTAAGGTT--------TCAAACAACACTTAAAT----GCAATTTTTGA-----AGGTATCTCAAG
         *********2222222222*2222**222**22***2222***2***22**22222***2222222*2
```

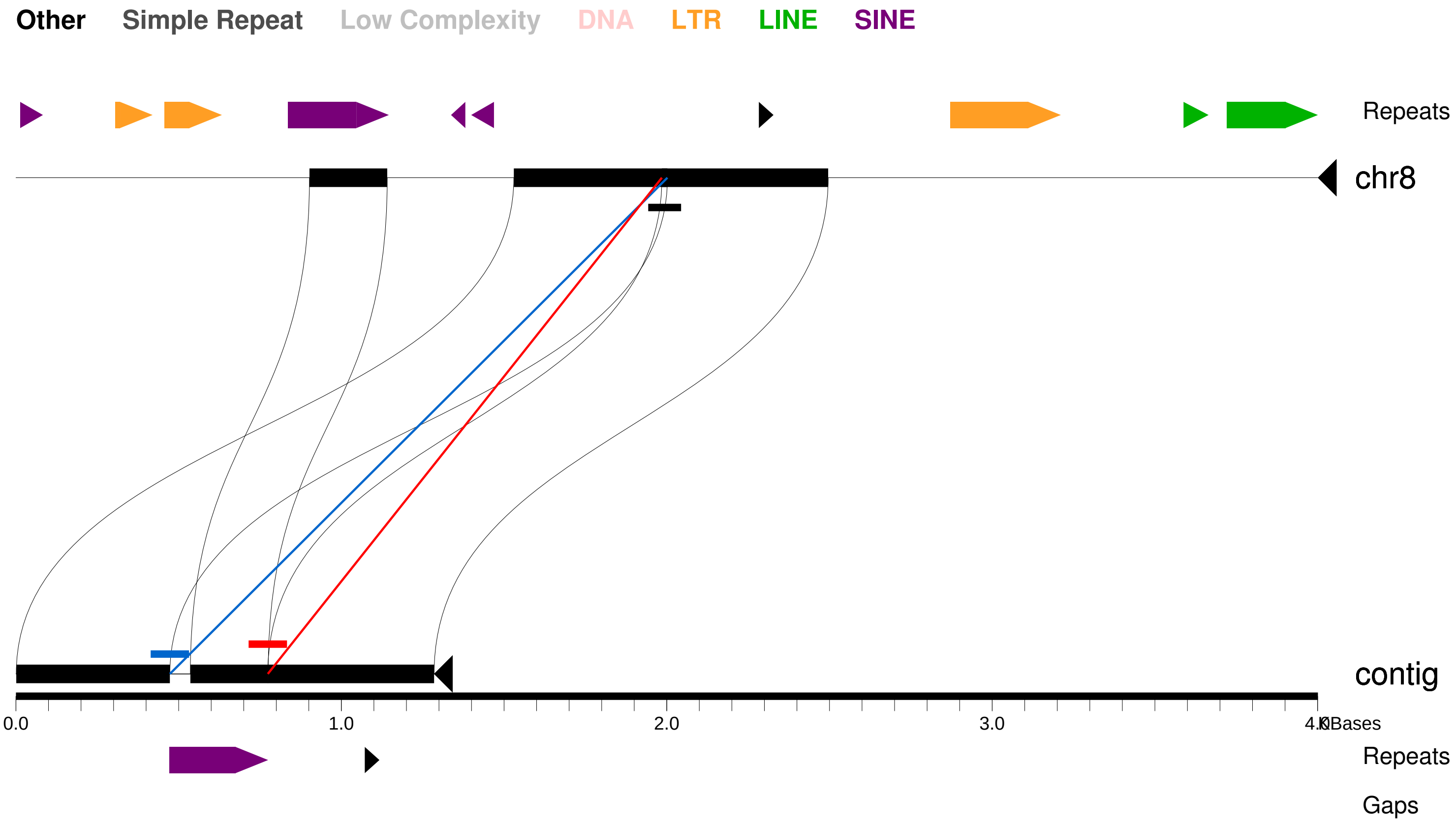

siteID: chr8_61194851 Scafftig2 - Breakpoint: chr8:61194841
chr8:61194794-61194900
Overlap Length: 13 INS size: 298 Genotyped: Yes

```
left     CCTTAAACGT--GCTTCTC--AGT-CTAGTGTTAACGACTTCAGCC------CTCTGT-TCCTGTGCCTT
chrom    CCTTAAACGT--GCTTCTC--AGT-CTAGTGTTAACGACTTCAGCC------CTCTGT-TCCTGTGCCTT
right    CCGCCCGCCTCGGCCTCCCAAAGTGCTGGGATTACAGGCGTGAGCCACCGCGCCCGGCCTCCTGTGCCTT
         **11111*1*11**1**1*11***1**1*11***11*1*1*1****111111*1*1*11***********

left     TTTTTTTTTTTTTTTTTTTGAGACGGAGTCTCGCTCTGTCGCCC--AGGCTGG---AGTGCAG----TGG
chrom    TT------------------AAA---AGCCCTGGTCAGTAACCCCTAAGTAGGTTAACTCCACATTTTTA
right    TT------------------AAA---AGCCCTGGTCAGTAACCCCTAAGTAGGTTAACTCCACATTTTTA
         **222222222222222222*2*222**2*22*2**2**22***22*2*22**222*2*2**22222*22
```

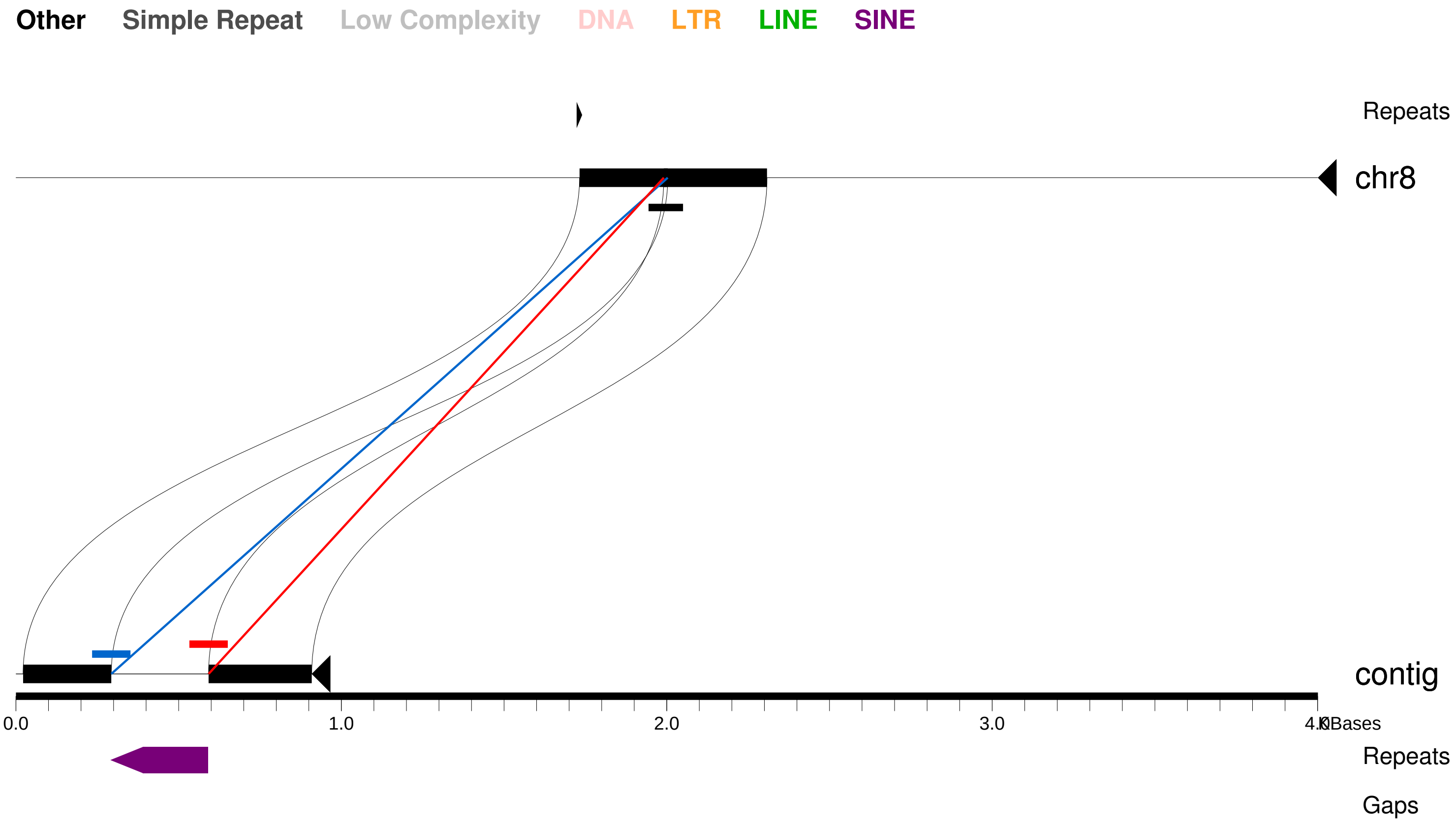

siteID: chr8_61526780 Scafftig2 - Breakpoint: chr8:61526780
chr8:61526738-61526839
Overlap Length: 18 INS size: 308 Genotyped: Yes

```
left    TAAAGACCC---------------TGTTAGCACAATATCCGTGAG--ACCTGCCTTAATCCAGCCCTGCA
chrom   TAAAGACCC---------------TGTTAGCACAATATCCGTGAG--ACCTGCCTTAATCCAGCCCTGCA
right   TCCGCCCGCCTCGGCCTCCCAAAGTGCTGGGATTACAGGCGTGAGCCACCGCGCCCGGCCCAGCCCTGCA
        *11111*1*111111111111111**1*1*1*11*1*11******11***111*11111***********

left    TTCTTCTTTTTTTTTTTTTTTTTTTTTTTTTTTTTGAGACGGAGTCTCGCTCTGTCGCCCAGGCTG
chrom   TTCTTCTATCGGTTAGTGCAGAAAGTTTTTTTCTT------------CCATCTTT-----ATGGAG
right   TTCTTCTATCGGTTAGTGCAGAAAGTTTTTTTCTT------------CCATCTTT-----ATGGAG
        *******2*222**22*22222222******2**222222222222*22***2*22222*2*22*
```

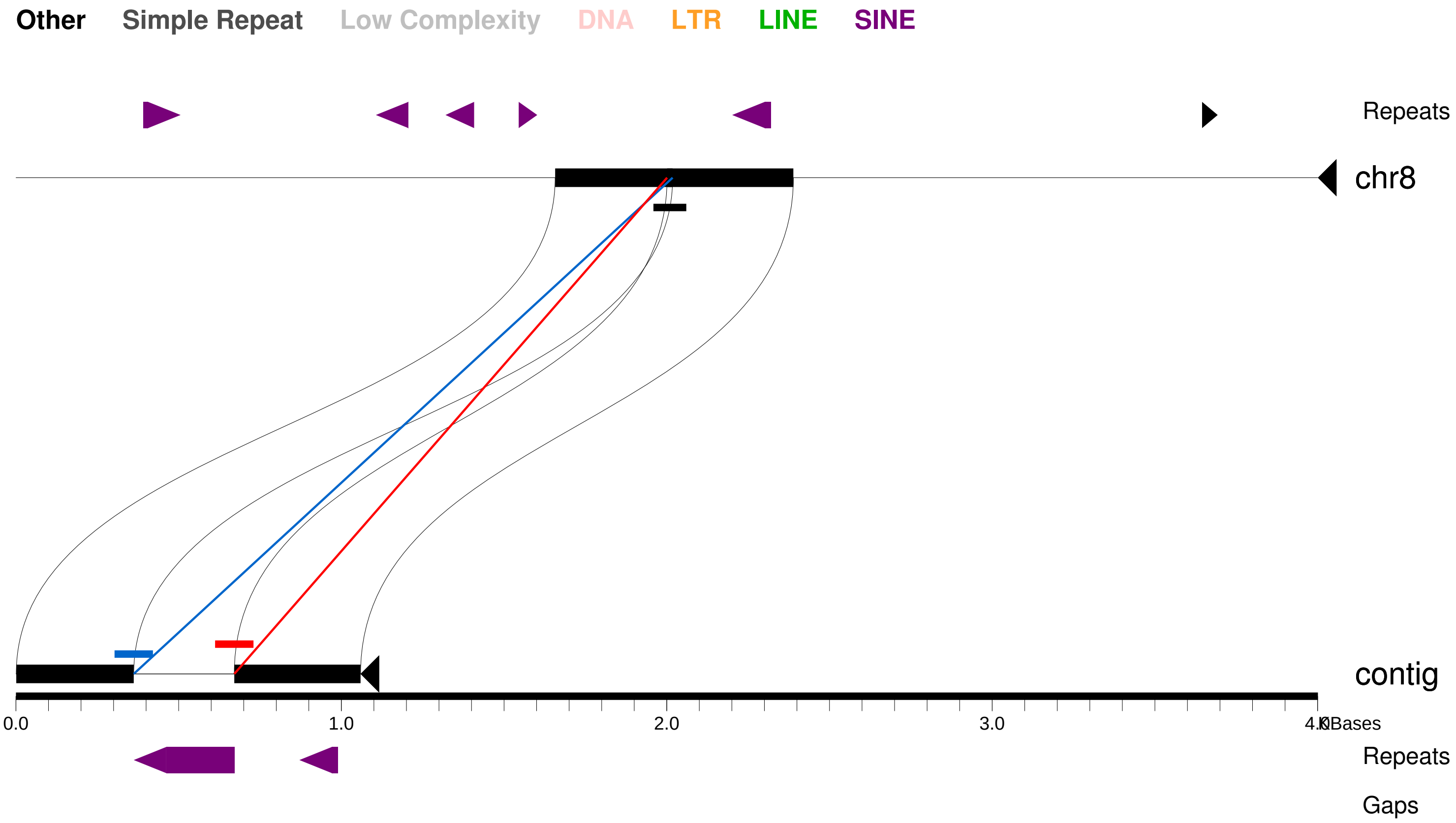

siteID: chr8_63344453 Scafftig2 + Breakpoint: chr8:63344470
chr8:63344386-63344529
Overlap Length: 12 INS size: 426 Genotyped: No

```
left   ATGCTCTCAGGAGAACTGAAGAGAAGCTTATAAAAAGGAAAAATAAAAAAAAA-GCTTTTTCTAAGAACT
chrom  ATGCTCTCAGGAGAACTGAAGAGAAGCTTATAAAAAGGAAAAAAAAAAAAAAAAGCTTTTTCTAAGAACT
right  NNNNNNNNNNNNNNNNNNNNNNNNNNNNNNNNNNNNNNNNNNNNNNNNNNNNNNNNNNNA-----------
       1111111111111111111111111111111111111111111N111111111N1111111111111111

left   GTTTTCACCCAATTAAACTATAACTG----GGGCCGGGCGCGGTGGCTCACGCC----------------
chrom  GTTTTCACCCAATTAAACTATAACTGATTTGTGGTTAGCCCA-TGTCATAAGCCTTTAGGTTTTGTAGGA
right  --------------AAACTATAACTGATTTGTGGTTAGCCCA-TGTCATAAGCCTTTAGGTTTTGTAGGA
       11111111111111************2222*2*2222**2*22**2*22*2***2222222222222222

left   -----
chrom  AATAA
right  AATAA
       22222
```

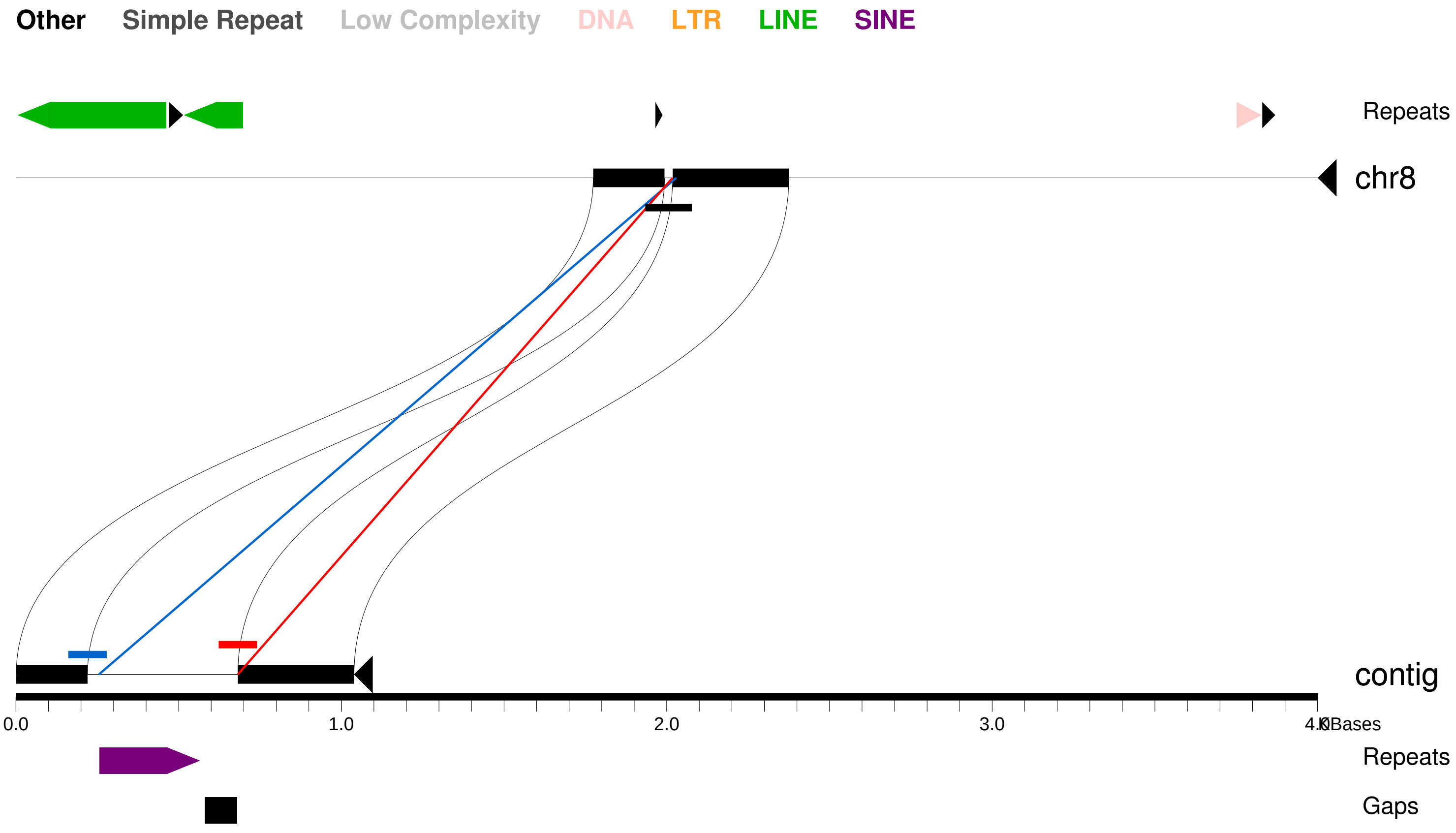

siteID: chr8_63856004 Scafftig1 + Breakpoint: chr8:63855938
chr8:63855928-63855997
Overlap Length: 50 INS size: 407 Genotyped: Yes

```
left    --------AAT-----------TTAAAAA------------------------------TCAAAAAAGAA
chrom   --------AAT-----------TTAAAAA------------------------------TCAAAAAAGAA
right   CGGAAAAAAATGTGAAGATTCCTTAAAGAAGCAAAAATAGATCTGCCATTTGATCCAGCTCAAAAAAGAA
        11111111***11111111111*****1*111111111111111111111111111111***********

left    TAGATGTTGGCATGGATGTGGTGAAAAGGGAACACTTTTTTTTTTTTTTTTTTTTGAGACGGAGTCTCGCT
chrom   TAGATGTTGGCATGGATGTGGTGAAAAGGGAACACTTTTTATACT--------------------------
right   TAGATGTTGGCATGGATGTGGTGAAAAGGGAACACTTTTTATACT--------------------------
        ***************************************2*22*22222222222222222222222222

left    CTGTCGCCCAGGCTGGAGTGCAGTGGCG
chrom   -----------GCTGG------------
right   -----------GCTGG------------
        22222222222*****222222222222
```

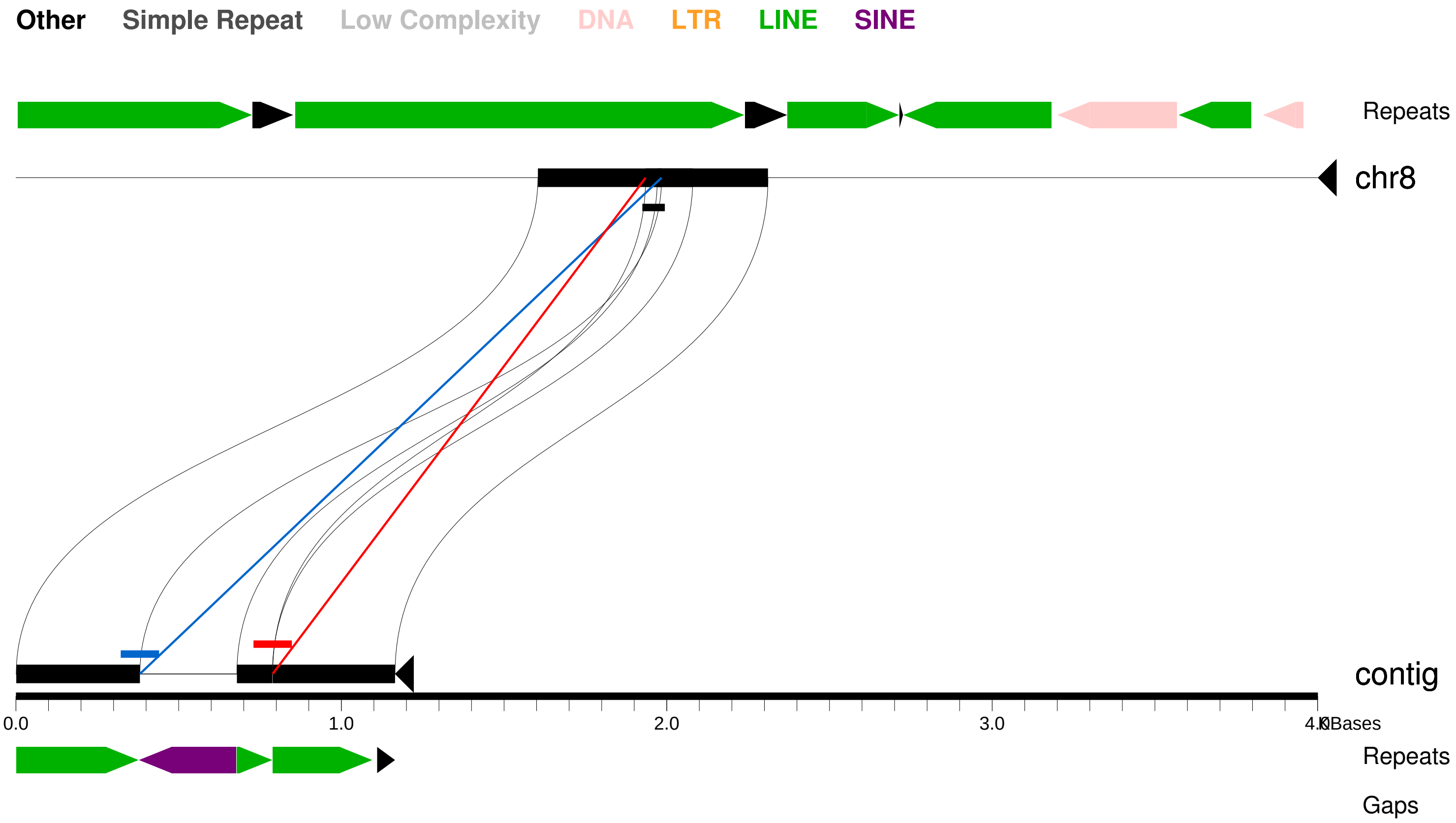

siteID: chr8_68981806 Scafftig5 - Breakpoint: chr8:68981793
chr8:68981749-68981852
Overlap Length: 16 INS size: 315 Genotyped: Yes

```
left    TATAGATGTATTACTGTATTGGTATATTTTGTTT------------------AATTTACGTTAGAAATTT
chrom   TATAGATGTATTACTGTATTGGTATATTTTGTTT------------------AATTTACGTTAGAAATTT
right   CCGGCCTGGGCGACAGA---GCGAGACTCCGTCTCAAAAAAAAAAAAAAAAAAAAAAAAAAAAGAAATTT
        111111**1111**1*1111*11*1*1*11**1*111111111111111111**111*1111********

left    TATATTTTGGCCGGGCGCGGTGGCTCACGCCTGTAATCCCAGCACTTTGGGAGGCCGAGGCGGGTGG
chrom   TATATTTTAA----GAAACATAATTTAGAA---AAATCTTTGTATGTGTGTATGTCATA--------
right   TATATTTTAA----GAAACATAATTTAGAA---AAATCTTTGTATGTGTGTATGTCATA--------
        ********222222*22222*222*2*2222222****222*2*22*22*2*2*2*222222222222
```

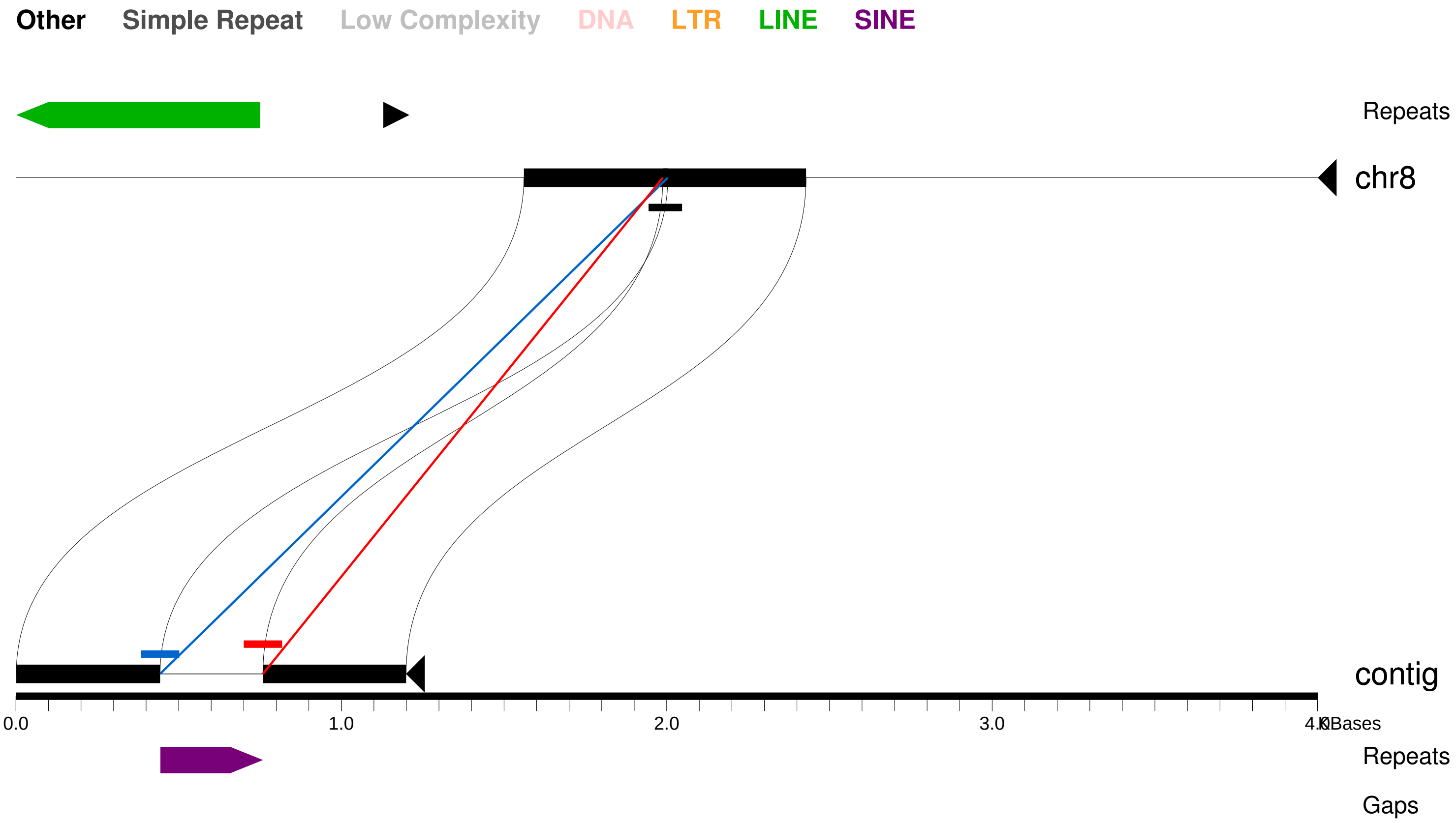

siteID: chr8_71774804 Scafftig1 + Breakpoint: chr8:71774791
chr8:71774748-71774850
Overlap Length: 14 INS size: 302 Genotyped: Yes

```
left     TTTGAATGTAATTGTTCTTTAAAA---CACATATTATA--CTTAA--CAT----------CTGTTCATAT
chrom    TTTGAATGTAATTGTTCTTTAAAA---CACATATTATA--CTTAA--CAT----------CTGTTCATAT
right    TCCGCCCGCC-TCGGCCTCCCAAAGTGCTGGGATTACAGGCGTGAGCCACCGCGCCCGGCCTGTTCATAT
         *11*111*111*1*11**111***111*1111****1*11*1*1*11**11111111111**********

left     TTTTTTTTTTTTTTTTTTTTTTTTTTGAGACGGAGTCTCGCTCTGTCGCCCAGGCTGGAGTGCAGTG---
chrom    TTTTCTT-------------------AGAGGCAGTATGGTTTAATAAAAAGAACTTTAATTCATTAAAC
right    TTTTCTT-------------------AGAGGCAGTATGGTTTAATAAAAAGAACTTTAATTCATTAAAC
         ****2**2222222222222222222***2*2***2*2*2*222*22222222**22*2*2**2*2222
```

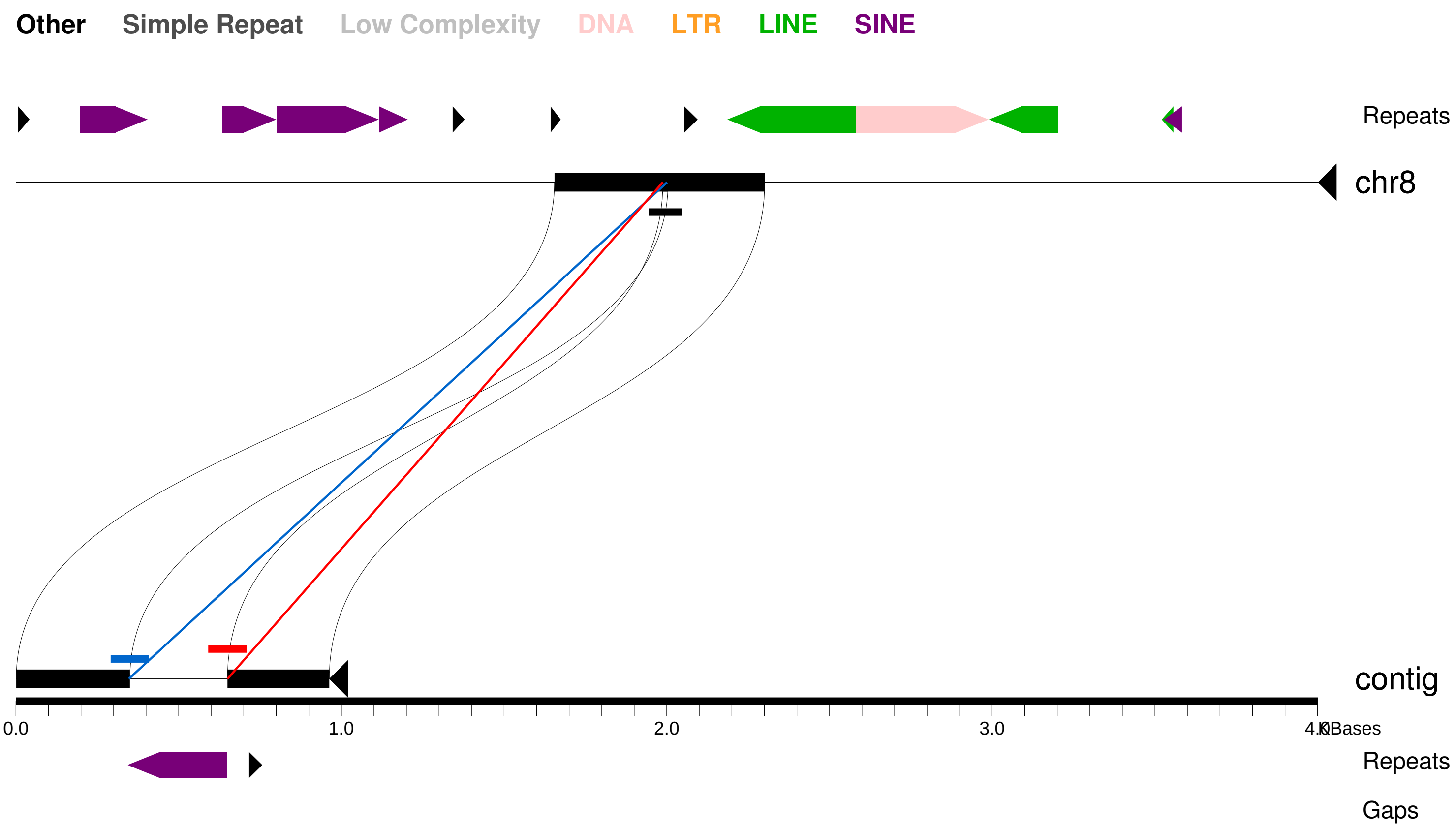

siteID: chr8_71914580 Scafftig1 - Breakpoint: chr8:71914579
chr8:71914419-71914751
Overlap Length: 13 INS size: 321 Genotyped: Yes

```
left    -------------GTTAATTCTATCATGTCCTAACAGGCTGGGTCAGTGGTCAAATTGTTGGAGAGATTG
chrom   TTTAGTAAATGTAGTTAATTCTATCATGTCCTAATAGGCTGGGTCAGTGGTCAAATTGTTGGAGAGATTG
right   C---GTGAACCCGGG-AAGCGGAGCTTG----------------CAGTGAGCCGA--GATTGCGCCACTG
        N   22N22NNNN*11**1111*1*1**111111N111111111*****11*11*11*1*1*1*11*1**

left    GGGTTGACGATAGTCTATAAACTACGAGGAAGGGCTCACCATATGTGGTAAGAGTTTTGTTTCAAATAAT
chrom   GGGTTGACGATAGTCTATAAACTACGAGGAAGGGCTCACCATATGTGGTAAGAGTTTTGTTTCAAATAAT
right   CAGTCCGC---AGTCCC--ACCTGGGCGACAGAGCGAGAC-TCCGTCTCAAAAAAAAA-----AAATAAA
        11**111*111****1111*1**11*1*11**1**1111*1*11**111**1*1111111111******1

left    TTTAATCTAATTTAATGTTTAAAATAAAAGCTAG-GCCGGGCGCGGT----GGCTCACGCCTG---TAAT
chrom   TTTAATCTAATTTAATGTTTAAAATAAAAGCTAAAGCCGTGGTAGGTTTTTGTTTTTTTTCCGAAATAAT
right   TA-AATAAAATAAAAA----AAAATAAAAGCTAAAGCCGTGGTAGGTTTTTGTTTTTTTTCCGAAATAAT
        *11***11***11**11111*************22****2*222***2222*22*22222*2*222****

left    CCCAGCACT----------------------TTGGGAGGCCGAGGCGGGTGGATCATGAGGTCAGGAGAT
chrom   TCCAGTTGTGTTTATCTATATTATTTTTCTTTTAGTATGTTCTGTCTC-TCAATTAACAGCATAGTATAT
right   TCCAGTTGTGTTTATCTATATTATTTTTCTTTTAGTATGTTCTGTCTC-TCAATTAACAGCATAGTATAT
        2****222*2222222222222222222222**2*2*2*2222*2*222*22**2*22**222**2*2**

left    ---CGAGACCATCCTGGCTAACA-------AGGTGAAACCCCGTCTCTACTAAA-
chrom   TAGCTTTCCAATTCTGTTTATTGTATCTTTAAGT-AAATTTAATTTATATAAAAT
right   TAGCTTTCTAATTCTGTTTATTGTATCTTTAAGT-AAACTT--------------
        222*222212**2***22**2222222222*2**2***N22NN1N1N11NN111
```

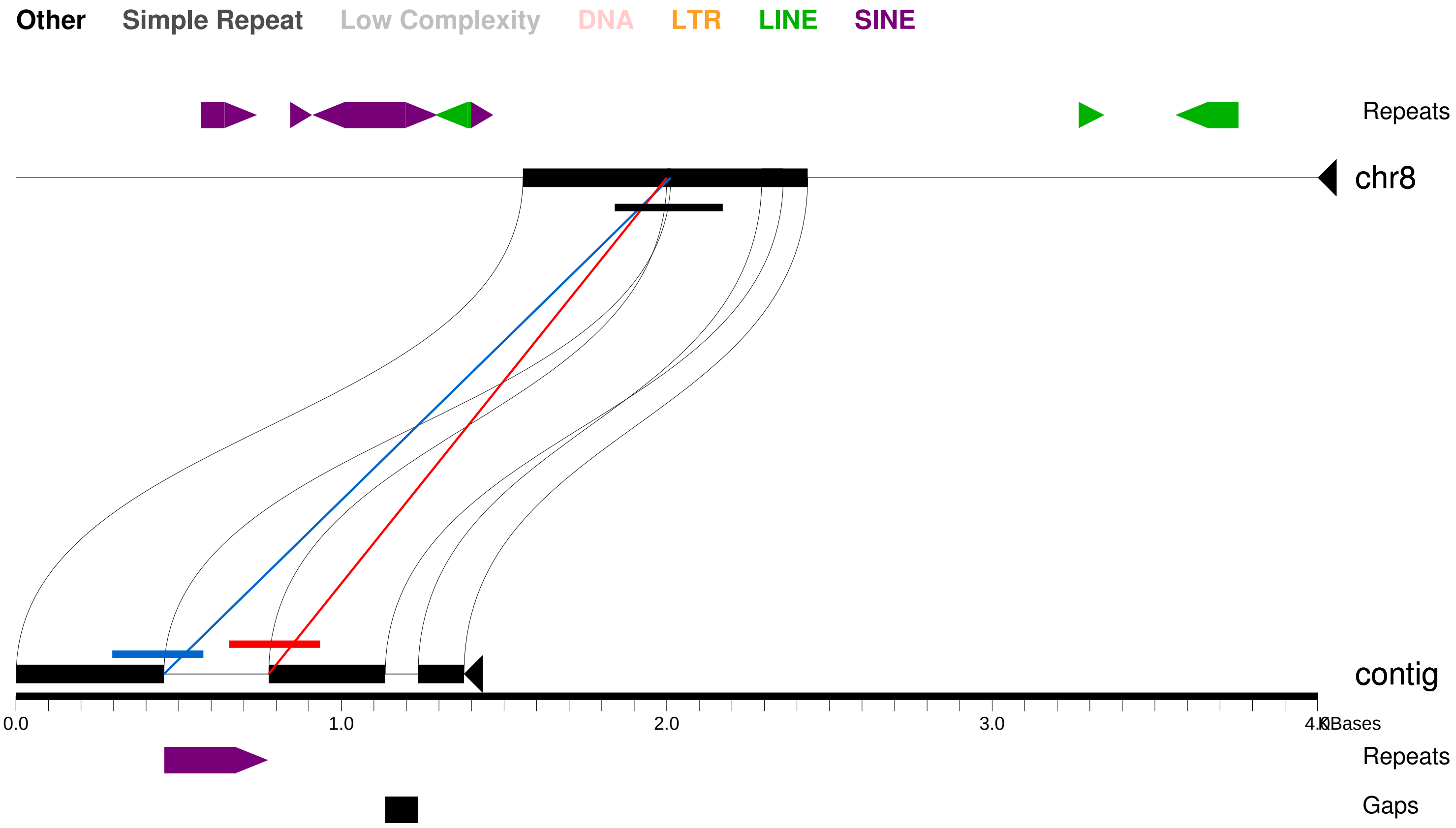

siteID: chr8_72510295 Scafftig2 - Breakpoint: chr8:72510303
chr8:72510254-72510359
Overlap Length: 10 INS size: 307 Genotyped: Yes

```
left     AGCATGTCTGGATGCTATGGGAAAG---GTGATGTCAGCTCAGAGGGTC--------------AAGAATG
chrom    AGCATGTCTGGATGCTATGGGAAAG---GTGATGTCAGCTCAGAGGGTC--------------AAGAATG
right    -GCA---CTCCAGCCTGGGCGACAGAGCGAGACTCCGTCTCAAAAAAAAAAAAAAAAAAAAAAAAAAATG
         1***111**11*11**11*1**1**111*1**111*11****1*1111111111111111111**1****

left     GGCTCTCGGCCGGGCGCGGTGGCTCACG-CCTGTAATCCCAGCACTTTGGGAGGCCGAGGCGGGCGG
chrom    GGCTCT---CCGAG-GAGGTGACATTTGACCTGAGAT----GTACATGATGAGAGAGAGAC------
right    GGCTCT---CCGAG-GAGGTGACATTTGACCTGAGAT----GTACATGATGAGAGAGAGAC------
         ******222***2*2*2****2*2222*2****22**2222*2**2*222***222***2*222222
```

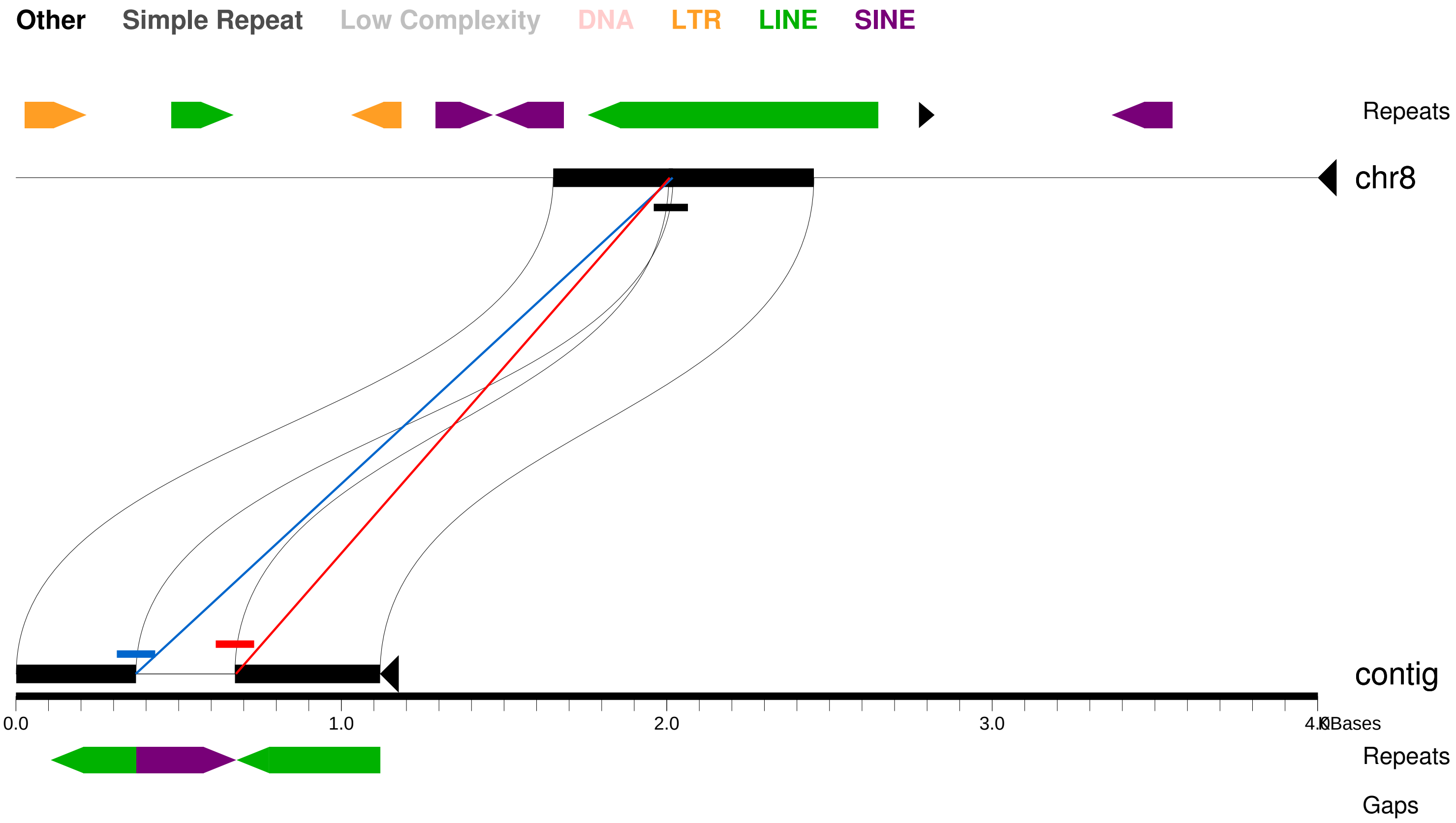

siteID: chr8_73092956 Scafftig2 - Breakpoint: chr8:73092947
chr8:73092815-73093156
Overlap Length: 78 INS size: 470 Genotyped: No

```
left   ----------------------------------------------------------------------
chrom  AAACTAGTATAGTGCTAACAGTTCTAGTGGTGATCAATGAAAAGCAATCCATAATCCAGAAACTAGTAAT
right  CCACCCGCCTCGGCCTCCCAA----AGTGCTGGG--ATTACAGGCG------------------------
       NN22NN2NN2N2NN22NN22N    2222N22NN  22N2N2N22N

left   ------------------------------CAGCTACAAAGTAAAGTGTTCTCATGCTGAGATACGTTTA
chrom  ATGAAACACTTAATTCAGTACTTAAATATTCAGCTACAAAGTAAAGTGTTCTCATGCTGAGATACGTTTA
right  -----------------------------TGAGCCACCGCGC--------------CCGGCCTACGTTTA
                                    21***1**111*111111111111111*1*111********

left   TCTTCAATATAATATTTGCTTATTTAACTGGAAACTGTTGTCTTCATGGCATGTTGTGTGTTAACACTGT
chrom  TCTTCAATATAATATTTGCTTATTTAACTGGAAACTGTTGTCTTCATGGCATGTTGTGTGTTAACACTGT
right  TCTTCAATATAATATTTGCTTATTTAACTGGAAACTGTTGTCTTCATGGCATGTTGTGTGTTAACACTGT
       **********************************************************************

left   NNNNNNNNNNNNNNNNNNNNNNNNNNNNNNNNNNNNNNNNNNNNNNNNNNNNNNNNNNN-----------
chrom  GTTTGTAGATATTTTCAAATCCTGGGTTTTTTTTCCTTTTTCTATTCTATGGTTTTTAATATGCTTCTAA
right  GTTTGTAGATATTTTCAAATCCTGGGTTTTTT--------------------------------------
       22222222222222222222222222222222NNNNNNNNNNNNNNNNNNNNNNNNNNN

left   --------------------------------------------------------------
chrom  TGTGTTTAAATAAATAACGAAGGAAGTTGAGAATGAAAATATTGATGCAGAGATGCTTATTA
right  --------------------------------------------------------------
```

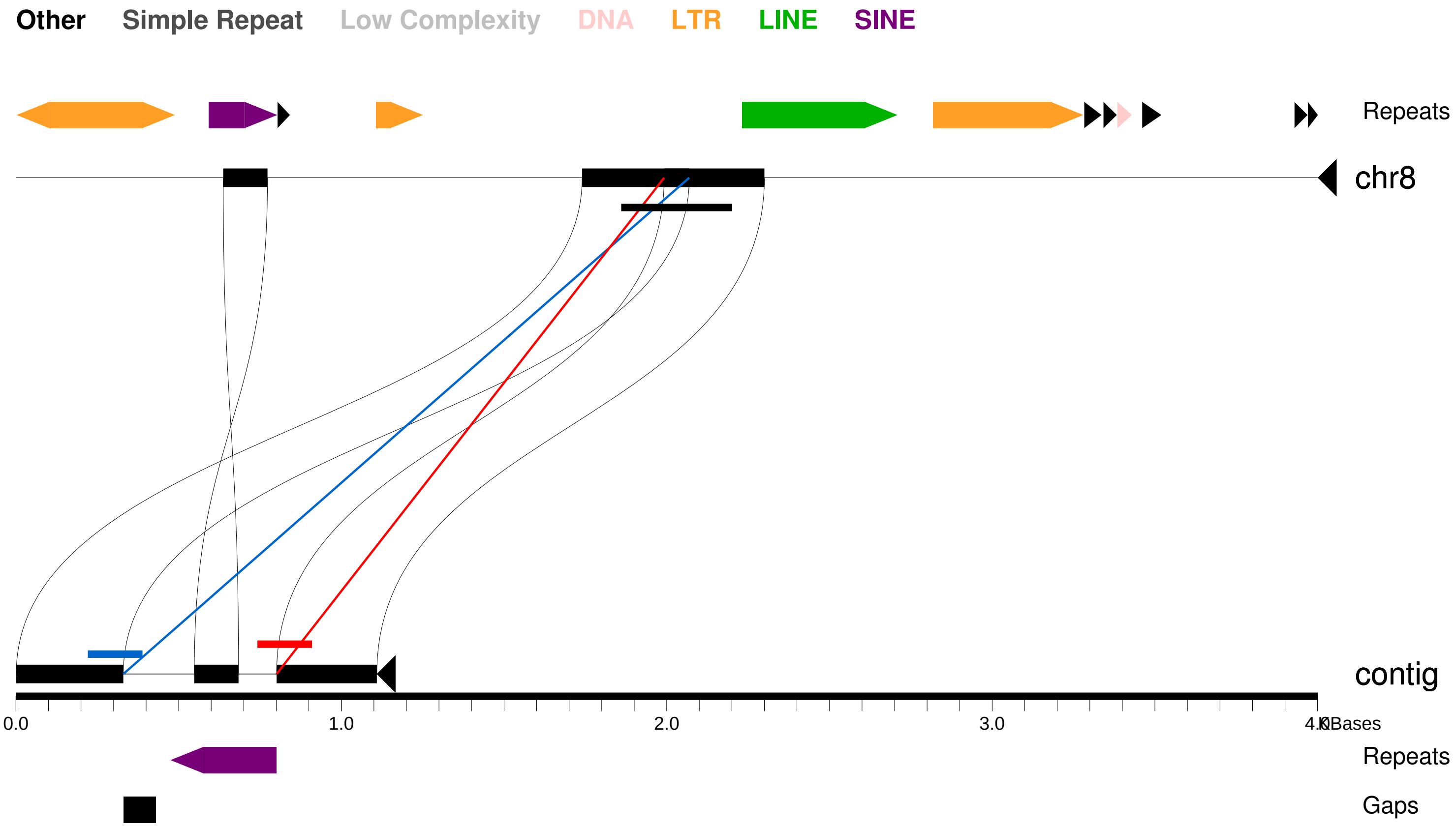

siteID: chr8_73936918 Scafftig2 + Breakpoint: chr8:73936920
chr8:73936760-73937094
Overlap Length: 15 INS size: 302 Genotyped: Yes

```
left    ---------------TTGATTATGTTTTATTTCTGAAACTGGGTAATGGATACTCTGGTTTATAATACAT
chrom   GGGATTTTAACCATATTGATTATGTTTTATTTCTGAAACTGGGTAATGGATACTCTGGTTTATAATACAT
right   G----------CAG---GAGAATGGCGT------GAACCCGGGAAGCGGA-------GCTTGCAGTG---
        2          22N 11**11***111*111111***1*1***1*11***1111111*1**11*1*1111

left    ATCTTTTTGAATCTGTCTAAAATGTAAAATATTTTTTAAAATGAG-GAGAG-GCCAGAAGAAGTCAGAAA
chrom   ATCTTTTTGAATCTGTCTAAAATGTAAAATATTTTTTAAAATGAG-GAGAG-GCCAGAAGAAGTCAGAAA
right   AGCCGA--GATTGCGCC---ACTGCAGTCCGCAGTCCGACCTGGGCGACAGAGCGAGACTCCGTCTCAAA
        *1*11111**1*11*1*111*1**1*11111111*111*11**1*1**1**1**1***1111***11***

left    AAGGTACATTGGCATGCCATTTAAAACTTAATTTTAAGGCCGGGCGCGGTGGCTCACGCCTGTAA-----
chrom   AAGGTACATTGGCATGCCATTTAAAACTTAATTTTAAAAATATCTGTG-TCTCTTGAGTTTGTAAAGTGA
right   AAAAAAAAAAAA-----------AAAACTTAATTTTAAAAATATCTGTG-TCTCTTGAGTTTGTAAAGTGA
        **111*1*11111111111111**************22222222*2*2*22**222*22*****22222

left    --TCCCAGCACTT------TGGGAGGCCGAGGCGGGTGGATCA-----------TGAGGTCAGGAGATCG
chrom   TTTCTTTTCAATTAAATAATATGTGACAAAAATCGTTGTATCTGTAACTTTGTGTGATTTATGTTAATAT
right   TTTCTTTTCAATTAAATAATATGTGACAAAAATCGTTGTATCTGTAACTTTGTGTGATTTATGTTAATAT
        22**2222**2**222222*22*2*2*22*2222*2**2***222222222222***22*22*222**22

left    AGACCATC------CT---GGCTAACAAGGTGAAACCCCGT--------CTCTACTAAA
chrom   ATTTCTTCTGTTTTCTTTAGCCTTTCAAAAGCAAATTGCTTATGATAATCTCT-CAGAA
right   ATTTCTTCTGTTTTCTTTAGCCTTTCAAAAGCAAATTGCTTATGATA-----------
        *222*2**222222**222*2**22***2222***222*2*222222  111121NN11
```

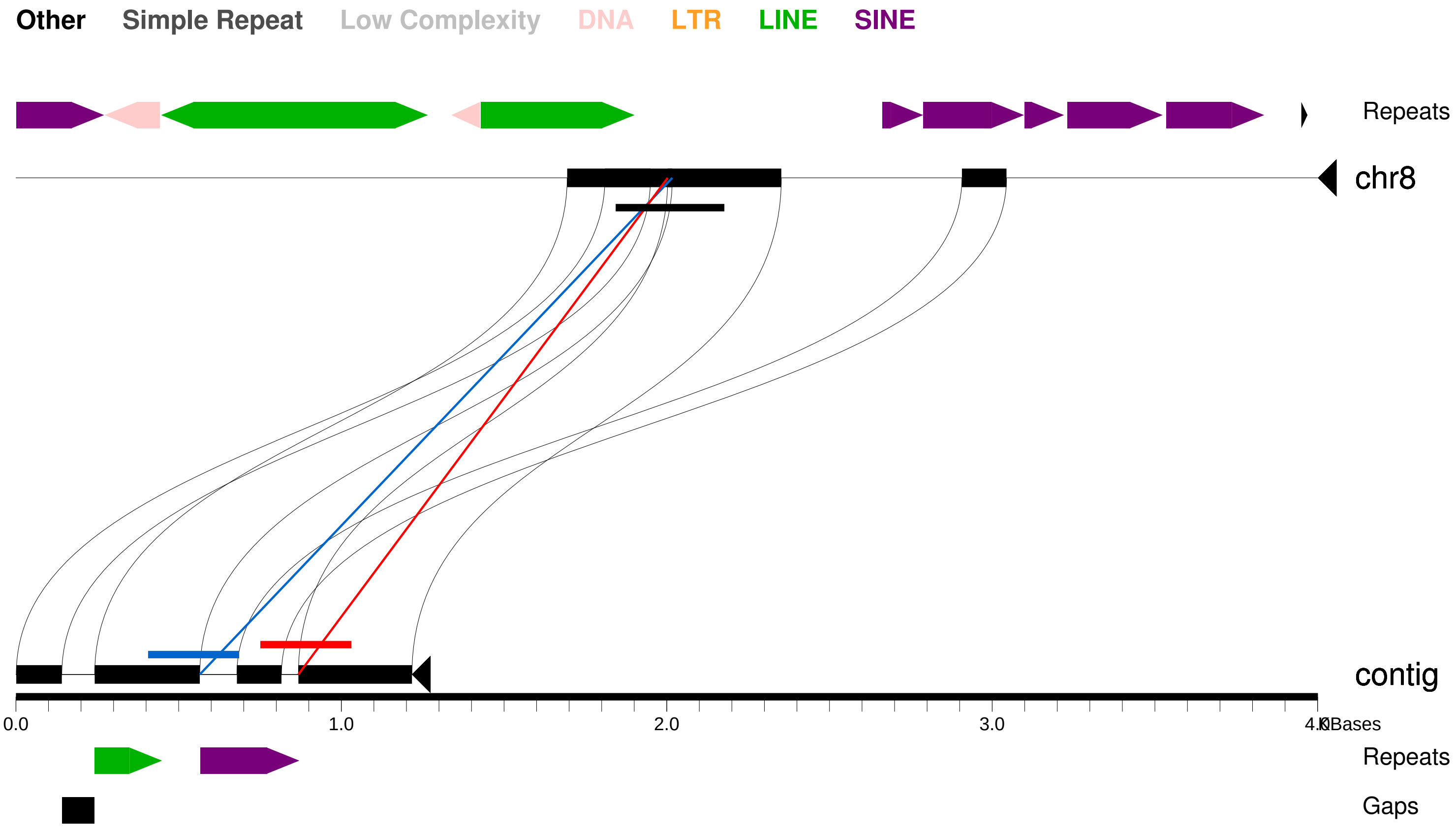

siteID: chr8_75062575 Scafftig6 - Breakpoint: chr8:75062560
chr8:75062514-75062616
Overlap Length: 14 INS size: 306 Genotyped: Yes

```
left     GAATGAAA---TGTACC-CAGAA-GTGACAC-GTGGCACTTCTAGGTAG----------AAGAATTTAAG
chrom    GAATGAAA---TGTACC-CAGAA-GTGACAC-GTGGCACTTCTAGGTAG----------AAGAATTTAAG
right    GCACTCCAGCCTGGGCGACAGAGCGAGACTCCGTCTCAAAAAAAAAAAAAAAAAAAAAAAAAAATTTAAG
         *1*1111*111**11*11****11*1***1*1**11**11111*111*11111111111**1********

left     AGTAGG--CCGGGCGCGGTGGCTCACGCCTGTAATC-CCAGCACTTTGGGAGGCCGAGGCGGGCGGAT
chrom    AGTAGGTGCATAATTTTCTTTTTCCCTCATAGATGCACCTGCACTATGG-------------------
right    AGTAGGTGCATAATTTTCTTTTTCCCTCATAGATGCCCCTGCACTATGG-------------------
         ******22*222222222*222**2*2*2*22*22*N**2*****2***2222222222222222222
```

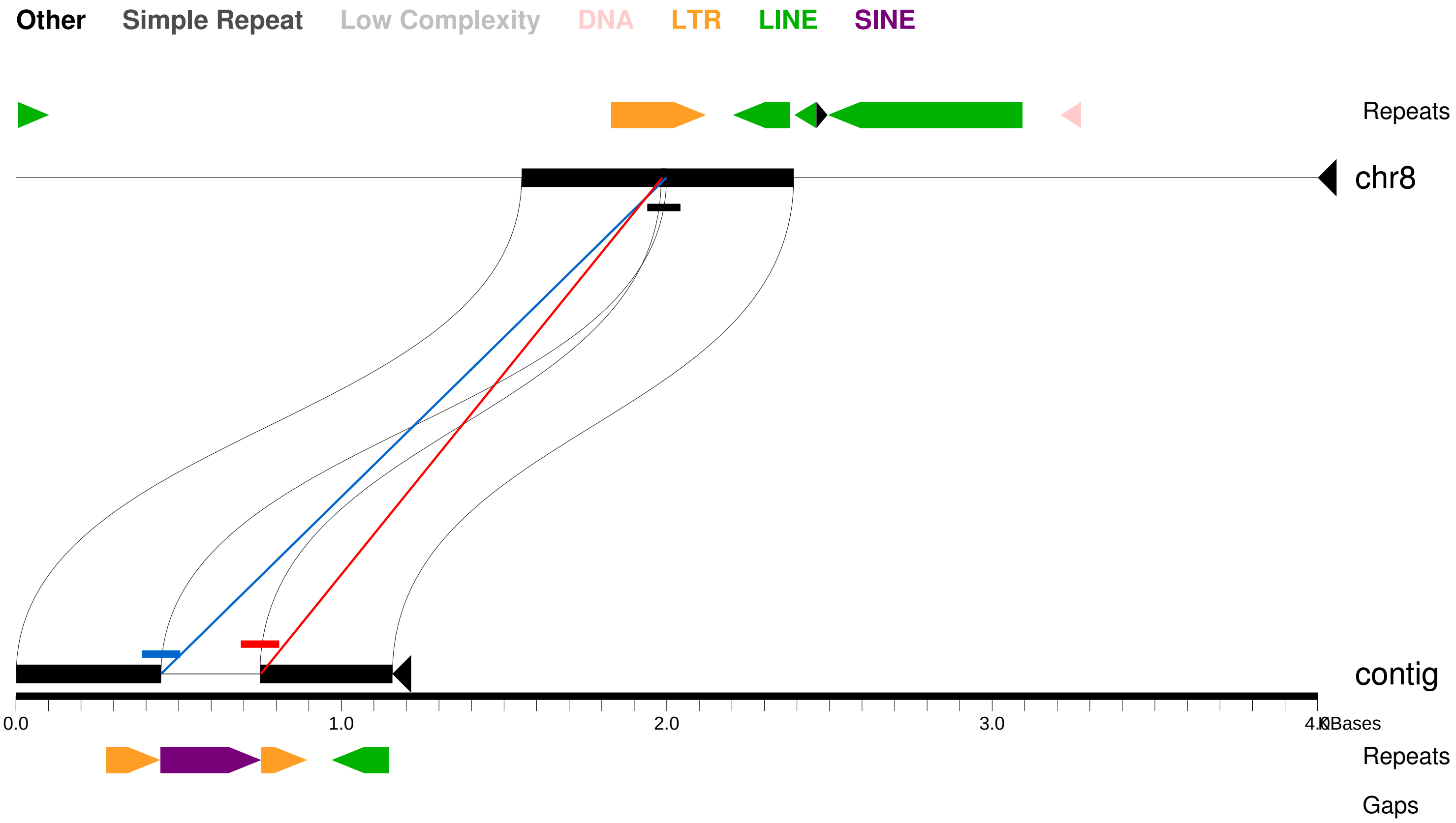

siteID: chr8_76317951 Scafftig1 + Breakpoint: chr8:76317965
chr8:76317915-76318024
Overlap Length: 10 INS size: 235 Genotyped: No

```
left   TTT-CTTGCTCCTGGCACAGG----AGGATT---GGCAGTGT--TACCTAAACTCTTCTCCGGGGTTTTT
chrom  TTT-CTTGCTCCTGGCACAGG----AGGATT---GGCAGTGT--TACCTAAACTCTTCTCAGGGGTTTTT
right  TCGGCTCACTGCAAGCTCCGCTTCCCGGGTTCACGCCATTCTCCTGCCTCAGC-CTCCCGAGGGGTTTTT
       *111**11**1*11**1*1*111111**1**111*1**1*1*11*1***1*1*1**1*112*********

left   TTTTTTTTTTTTTTNNNNNNNNNNNNNNNNNNNNNNNNNNNNNNNNNNNNNNNNNNNNNN
chrom  AAAAATT---------GAACAAATGCAATGTTTTTCTTAGATAATAACTAAGACTTCAC
right  AAAAATT---------GAACAAATGCAATGTTTTTCTTAGATAATAACTAAGACTTCAC
       22222**2222222222222222222222222222222222222222222222222222
```

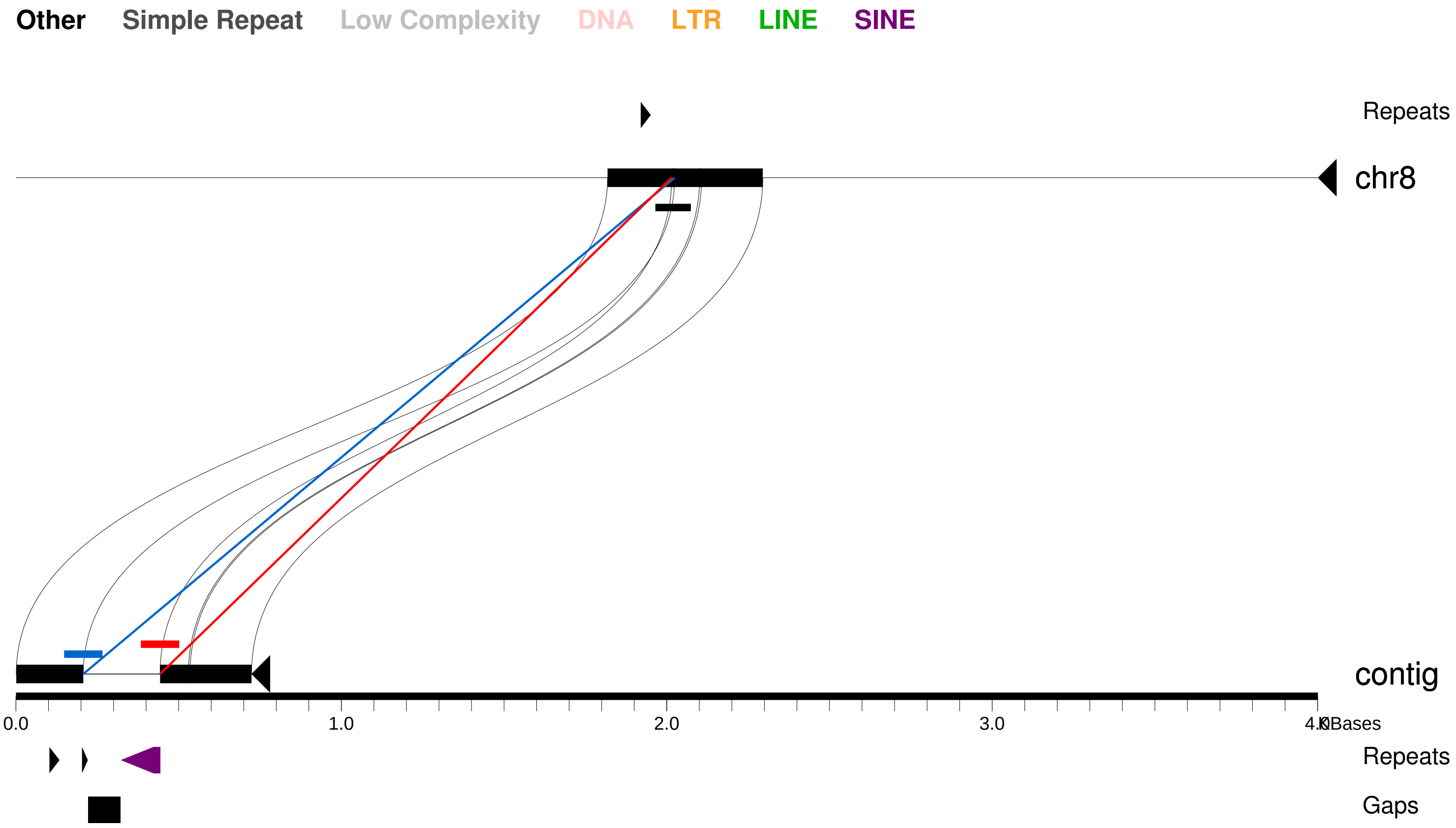

siteID: chr8_77867092 Scafftig3 + Breakpoint: chr8:77867080
chr8:77866920-77867251
Overlap Length: 12 INS size: 303 Genotyped: Yes

```
left   A------------TTTCAGAAATGTGTTAAAATAGAAATGATGTAATATGAAAAATATGAAACATCAGTT
chrom  AATCATCCATACATTTCAGAAATGTGTTAAAATAGAAATGATGTAATATGAAAAATATGAAACATCAGTT
right  A-----------------GAATGGCGTGAACCCGGGA--GGCGGAGCTTGCAG----TGAGCCGAGATCC
       *            11111***11*1**1**1111*1*11*11*1*111**1*11111***11*111*111

left   TGACAATT-AGTCCTTAAAATTTTATAAACTAAAATTAACACTGTATTTTTATATTAATAAAATTCCACA
chrom  TGACAATT-AGTCCTTAAAATTTTATAAACTAAAATTAACACTGTATTTTTATATTAATAAAATTCCACA
right  CGCCACTGCACTCC----AGCCTGGGCGAC--AGAGCGAGACTCCGTCTCAAAAAAAAAAAAA----AAA
       1*1**1*11*1***1111*111*11111**11*1*111*1***111*1*11*1*11**1****1111*1*

left   AACCCATTGCTTTTTGTAATTAAAAAAAATTTTAGGCCGGGCGCGGTGGCTCACGCCTGTAATCCCA--GC
chrom  AACCCATTGCTTTTTGTAATTAAAAAAAATTTTAAAAATAACAAAATGAAGTTTATTTTTATTGCCATAGG
right  AA---------------AAAAAAAAAAAAATTTAAAAATAACAAAATGAAGTTTATTTTTATTGCCATAGG
       **111111111111111**11********1****2222222*2222**222222222*2**2*2***22*2

left   ACTTTGGGAGGCCGA---------------GGCGGGCGGATCACGAGGTCAGG--AGA-----TCGAGAC
chrom  AGATTGACAAAATAAAAATGATTAACTTTTGGCTTCCAGTCTACTTTTTCAATCTAGAAATATTCTAGAA
right  AGATTGACAAAATAAAAATGATTAACTTTTGGCTTCCAGTCTACTTTTTCAATCTAGAAATATTCTAGAA
       *22***22*22222*222222222222222***222*2*222**2222***2222***22222**2***2

left   CATCCT--GGCTAA----CAAGGTGAAACCCCGT---------CTCTACTAAA
chrom  ATGACTTAGTCTTAGCCTCAGGGTAGAGCCCGGCTCATATGACCTCTTGAAAA
right  ATGACTTAGTCTTAGCCTCAGGGTAGAGCCCGGCTCATATGACCTCTTG----
       2222**22*2**2*2222**2***22*2***2*2222222222****22N111
```

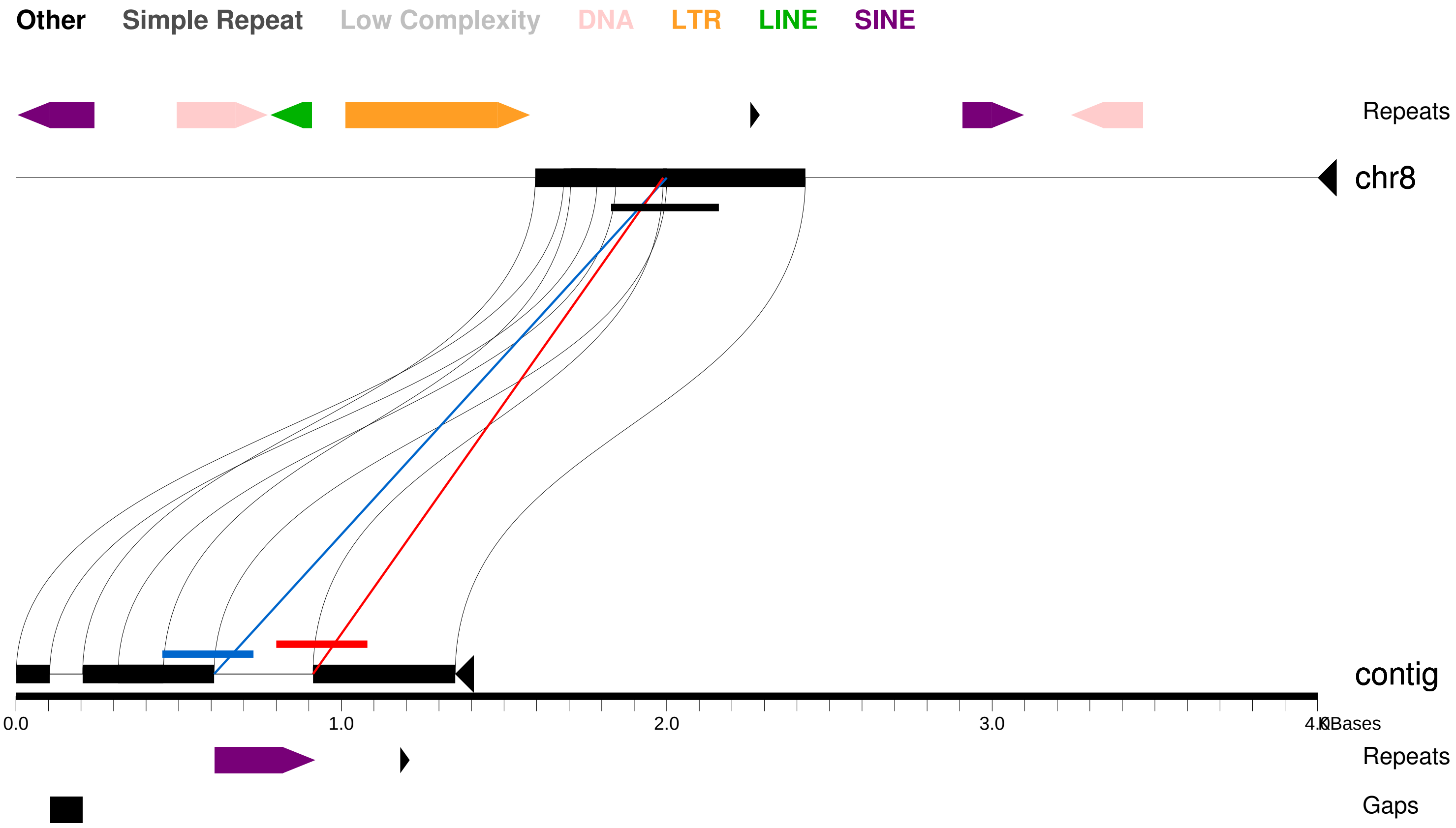

siteID: chr8_80466333 Scafftig3 - Breakpoint: chr8:80466334
chr8:80466174-80466539
Overlap Length: 46 INS size: 432 Genotyped: No

```
left   ----------------------------------------------------------------------
chrom  TT--GTCGTTACC---TTTTGACAACTTAATAATAATGTGTCTCAGTTGATGTTCTTTGTACCTTTGTAT
right  TTTAGTAGAGACGGGGTTTCACCGTTTTAGCCAGGATG-GTCTC----GATCTCCTG---ACCTC-----
       221122N2NN22N111222NNN2NNN222NNN2NN222 22222    222N2N22N   2222N

left   ------CTGCAAATCTGAAGAAATGGAGCCACCTCTACCAATCTTTATGGACTGGTTTGGCAGGGAAAGA
chrom  TGGTGGCTGCAAATCTGAAGAAATGGAGCCACCTCTACCAATCTTTATGGACTGGTTTGGCAGGGAAAGA
right  --GTG-------ATCCGC----------CCGCCTCGGCC--TCCCAAAGTGCTGGGATTACAGGCGT-GA
         222 111111***1*11111111111**1****11**11**111*1*11****11*11****1111**

left   TCTTCCTAGCTAGAAATTCTGGAAGCAGCTTAAACCTTTTCTATAACTGTGTCTTCTCTTTATTTGTGCA
chrom  TCTTCCTAGCTAGAAATTCTGGAAGCAGCTTAAACCTTTTCTATAACTGTGTCTTCTCTTTATTTGTGCA
right  GCCACCGCGC--------CCGGC--CAGCTTAAACCTTTTCTATAACTGTGTCTTCTCTTTATTTGTGCA
       1*11**11**11111111*1**111*********************************************

left   TNNNNNNNNNNN----------------------------------------------------------
chrom  TTTAAATTCTTAAATAAAATGTTTTACCAATTTCTTTTTTTTTTAAGAGCCCATTGTATCTTGCTTCCTC
right  TTTAAATTCTTAAATAAAATGTTTTACCAATTTCTTTTTTTTTTAAGAGCCCATTGTATCTTGCTTCCTC
       *222222222222222222222222222222222222222222222222222222222222222222222

left   ----------------------------------------------------------------------
chrom  TGGTATCTGACTGCTATATCAGGGTCAGGACTTACAGGCACACTTCACTTCTCTCCCTCTTTCCCTGTGG
right  TGGTATCTGACTGCTATATCAGGGTCAGGACTTACAGGCACACTTC------------------------
       2222222222222222222222222222222222222222222222

left   ---------------------
chrom  AGAAGCCATGAGTTCTATGCT
right  ---------------------
```

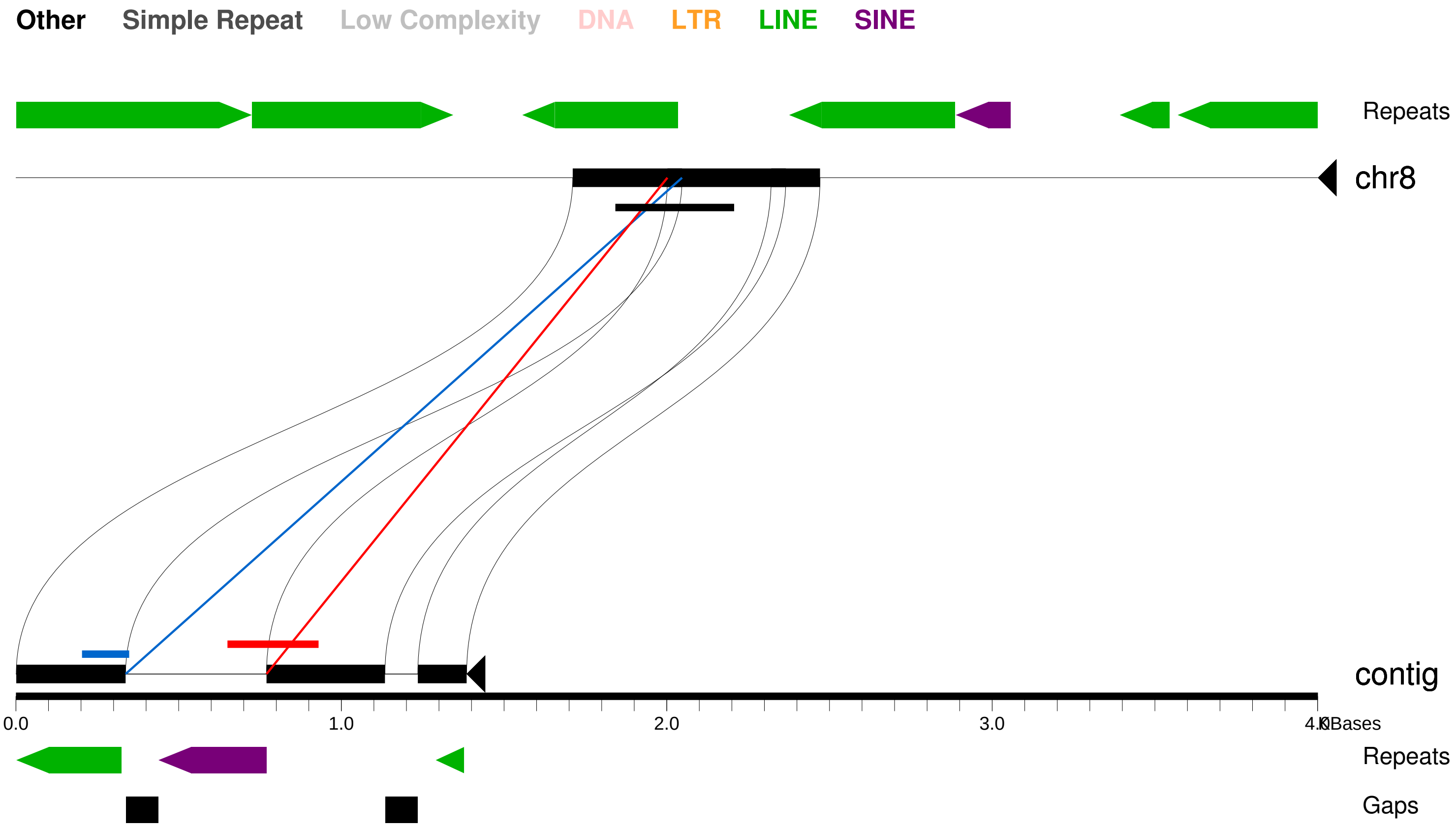

siteID: chr8_81767572 Scafftig2 - Breakpoint: chr8:81767581
chr8:81767536-81767640
Overlap Length: 15 INS size: 301 Genotyped: Yes

```
left    ATCAATTTCTTTTCATTTAGAAATATTGTTA-TCCGGTTCTTGTCT--------------AAAAAATCTT
chrom   ATCAATTTCTTTTCATTTAGAAATATTGTTA-TCCGGTTCTTGTCT--------------AAAAAATCTT
right   CTGCACTCCAGC-CTGGGCGACAGAGCGAGACTCCGTCTCAAAAAAAAAAAAAAAAAAAAAAAAAATCTT
        1*11*1*1*1111*11111**1*1*11*11*1****11**11111111111111111111**********

left    TGCTTGGCCAGGCACGGTGGCTCACGCCTGTAATCCCAGCACTTTGGGAGGCCGAGGCGGGCGG
chrom   TGCTTA---AGATATGGAA--TCAA-CCTAAATGCCCATCCATGAATGA--------CTGGATA
right   TGCTTA---AGATATGGAA--TCAA-CCTAAATGCCCATCCATGAATGA--------CTGGATA
        *****2222**22*2**2222***22***22*22****2*22*2222**22222222*2**222
```

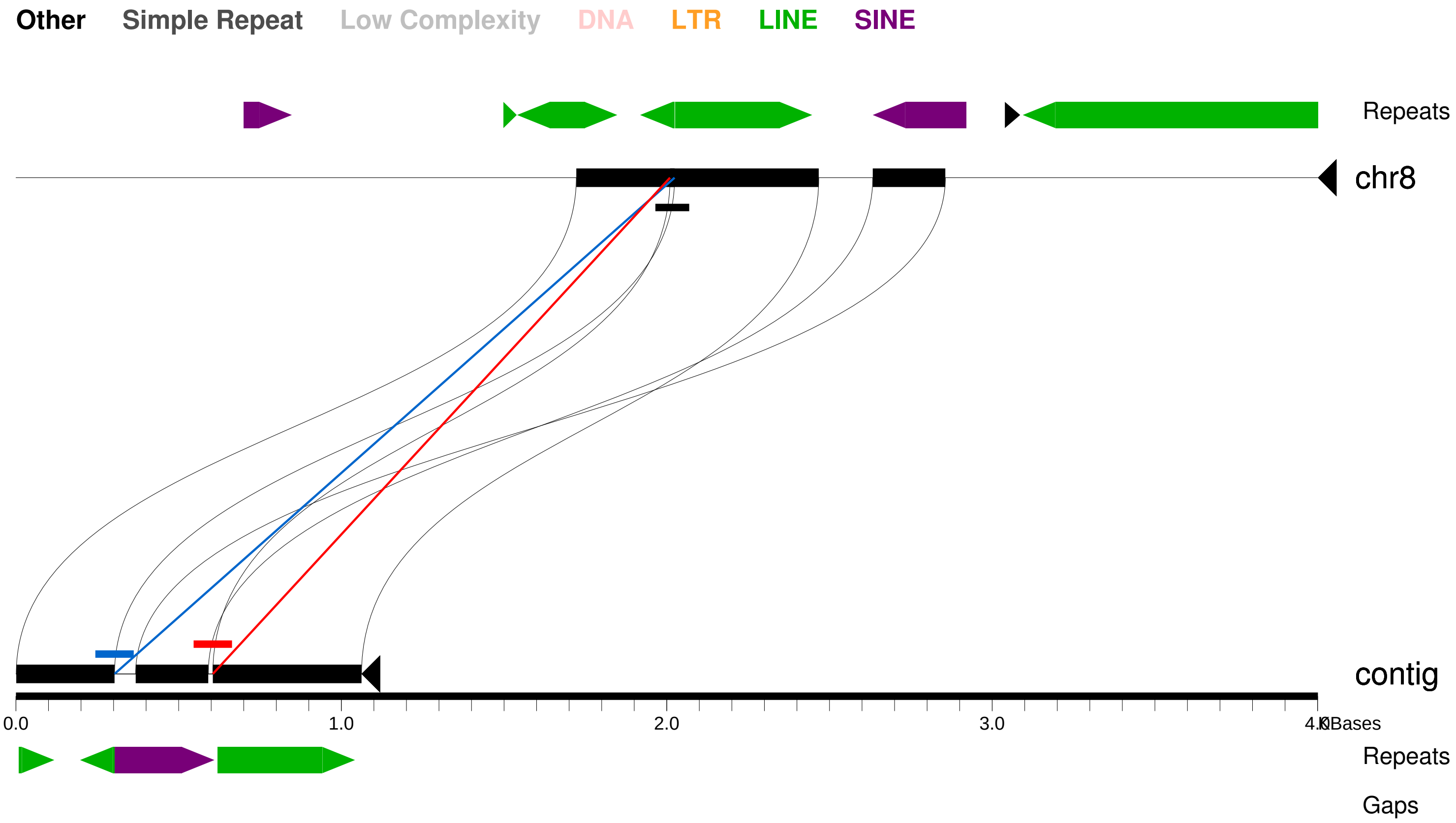

siteID: chr8_82262941 Scafftig2 + Breakpoint: chr8:82262946
chr8:82262899-82263005
Overlap Length: 13 INS size: 390 Genotyped: No

```
left    TGCTCG------TACATTAGATATGA-GAACTAAC-ATCAAACCA-----GCTTAAAAACATATTTATTT
chrom   TGCTCG------TACATTAGATATGA-GAACTAAC-ATCAAACCA-----GCTTAAAAACATATTTATTT
right   TTTTAGCCGGGATGGTCTCGATCTCCTGACCTCGTGATCCGCCCGCCTCGGCCTCCCAA-ATATTTATTT
        *11*1*111111*1111*1***1*111**1**1111***111**111111**1*111**1**********

left    CTTTNNNNNNNNNNNNNNNNNNNNNNNNNNNNNNNNNNNNNNNNNNNNNNNNNNNNNNNNNN
chrom   CTTATATTCTCAGCTTTTCACAGCTTGGTGGGCAGGAACTAAAATTATTT------------
right   CTTATATTCTCAGCTTTTCACAGCTTGGTGGGCAGGAACTAAAATTATTT------------
        ***2*222222222222222222222222222222222222222222222222222222222
```

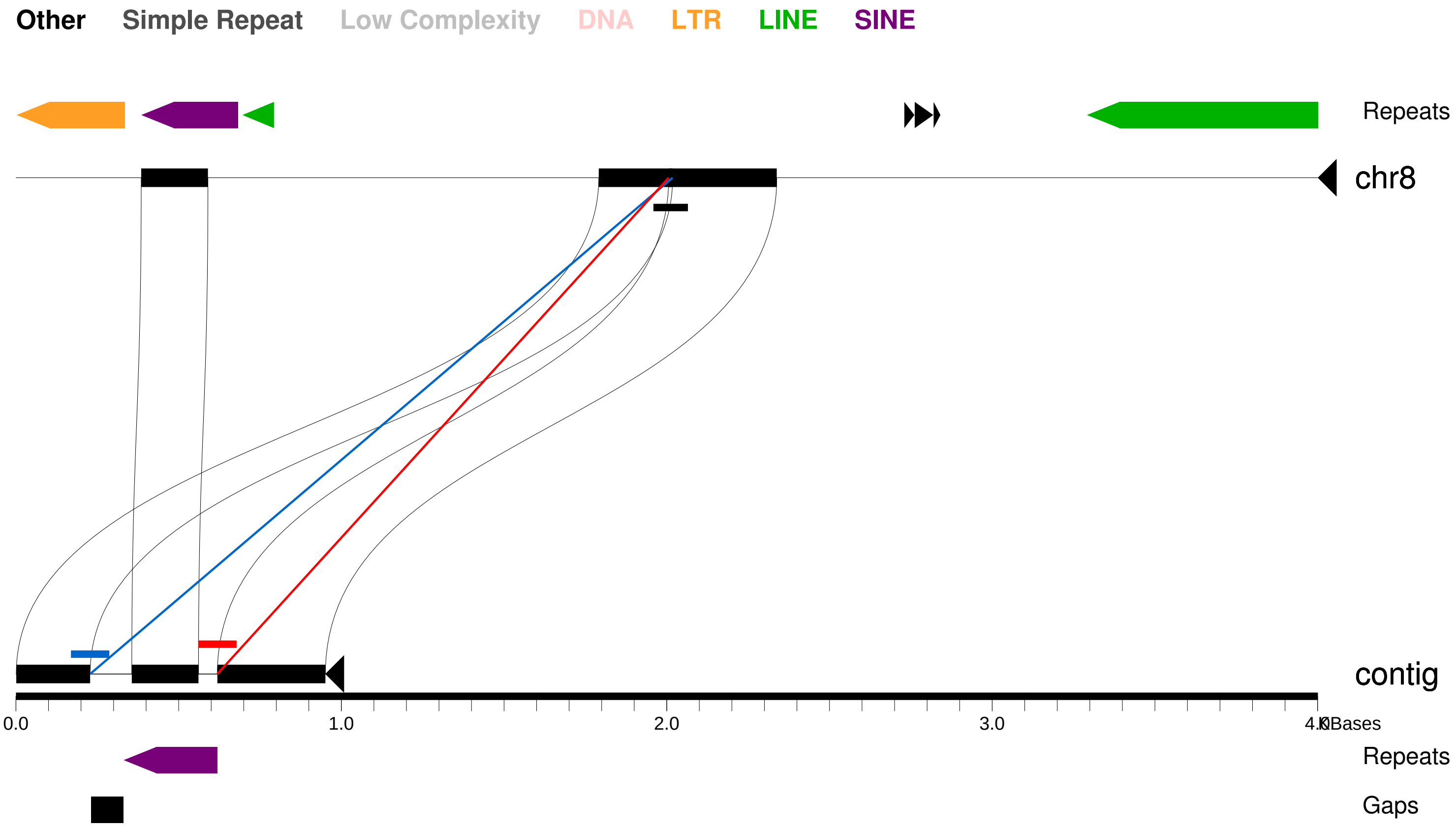

siteID: chr8_82424964 Scafftig2 + Breakpoint: chr8:82424998
chr8:82424988-82425057
Overlap Length: 50 INS size: 258 Genotyped: No

```
left    ATGAAAGCAA-------------------------------------------------CAACCAAAAAT
chrom   ATGAAAGCAA-------------------------------------------------CAACCAAAAAT
right   NNNNNNNNNNNNNNNNNNNNNNNNNNNNNNNNNNNNNNNNNNNNNNNNNNNNNNNNNNNCAACCAAAAAT
        11111111111111111111111111111111111111111111111111111111111***********

left    CCCTATCCTCTTAGAGCTTGTCAAGAAAAGCAGCTTTGTGGCCGGGCGCGGTGGCTCACGCCTGTAATCC
chrom   CCCTATCCTCTTAGAGCTTGTCAAGAAAAGCAGCTTTGTT----------------------TTTAA---
right   CCCTATCCTCTTAGAGCTTGTCAAGAAAAGCAGCTTTGTT----------------------TTTAA---
        ***************************************22222222222222222222222*2***222

left    CAGCACTTTGGGAGGCCGAGGCGGGCGG
chrom   -ATAA-----------------------
right   -ATAA-----------------------
        2*22*22222222222222222222222
```

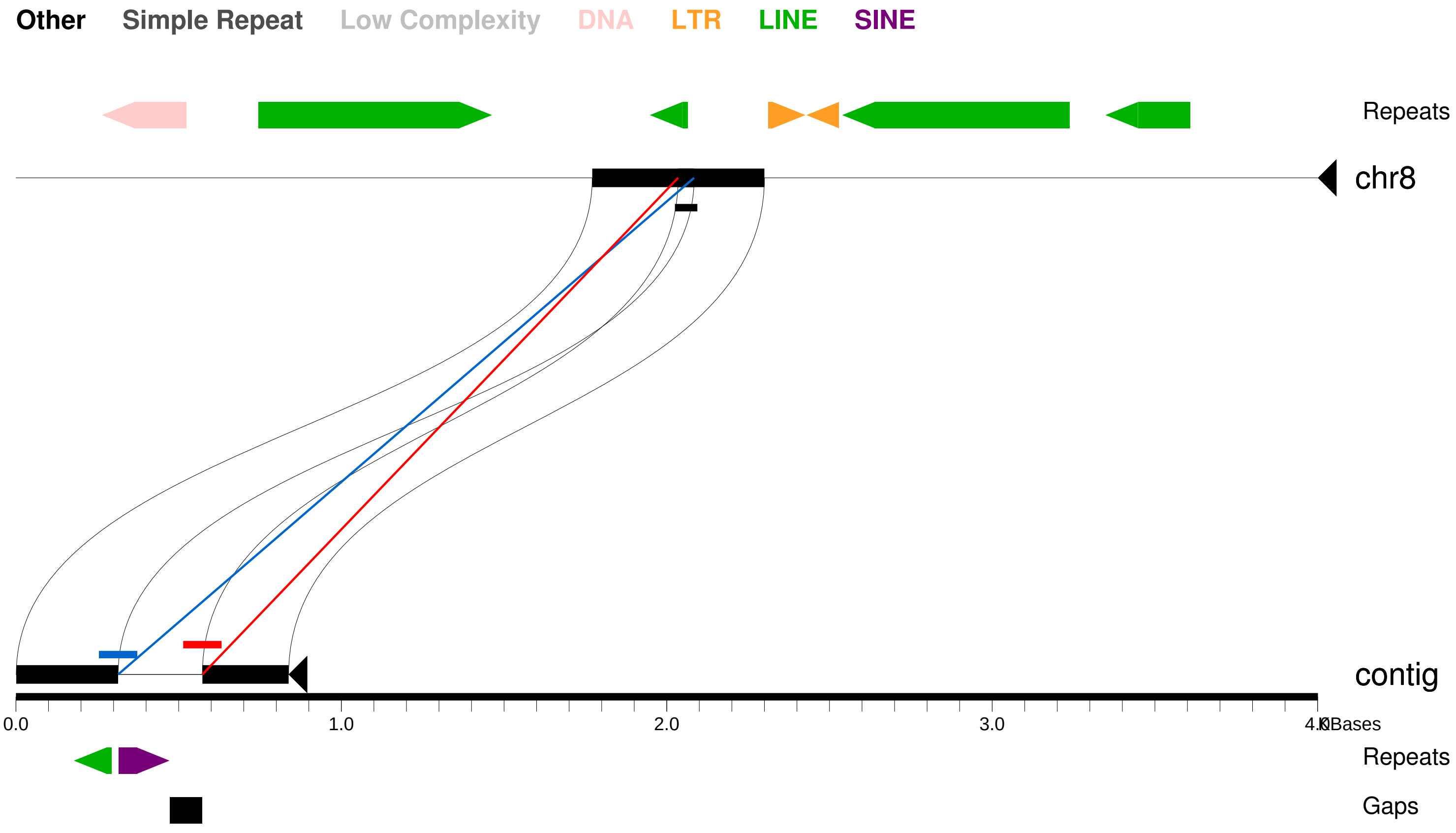

siteID: chr8_83161613 Scafftig2 - Breakpoint: chr8:83161625
chr8:83161566-83161684
Overlap Length: 1 INS size: 400 Genotyped: No

```
left    AATTACACAAAAGTTCCCTGAATTATAGAGTGAATGGTGTGATTAAGAACTGAGATGGGGTGGCCGGGCG
chrom   AATTACACAAAAGTTCCCTGAATTATAGAGTGAATGGTGTGATTAAGAATTGAGATGGGGAAGA--GGAG
right   NNNNNNNNNNNNNNNNNNNNNNNNNNNNNNNNNNNNNNNNNNNNNNNNNNNNNNNNNNNGAAGA--GGAG
        1111111111111111111111111111111111111111111111111N111111111*22*222**2*

left    CAGTGGCTCACGCCTGTAATCCCAGCACTTTGGGAGGCCGAGGCGGGC---------G
chrom   GAGAAACAGAG----TTAATT---GGAGTTTAGGAAGAAAAATCAAAAACTCATTTTG
right   GAGAAACAGAG----TTAATT---GGAGTTTAGGAAGAAAAATCAAAAACTCATTTTG
        2**222*22*222222****2222*2*2***2***2*222*22*2222222222222*
```

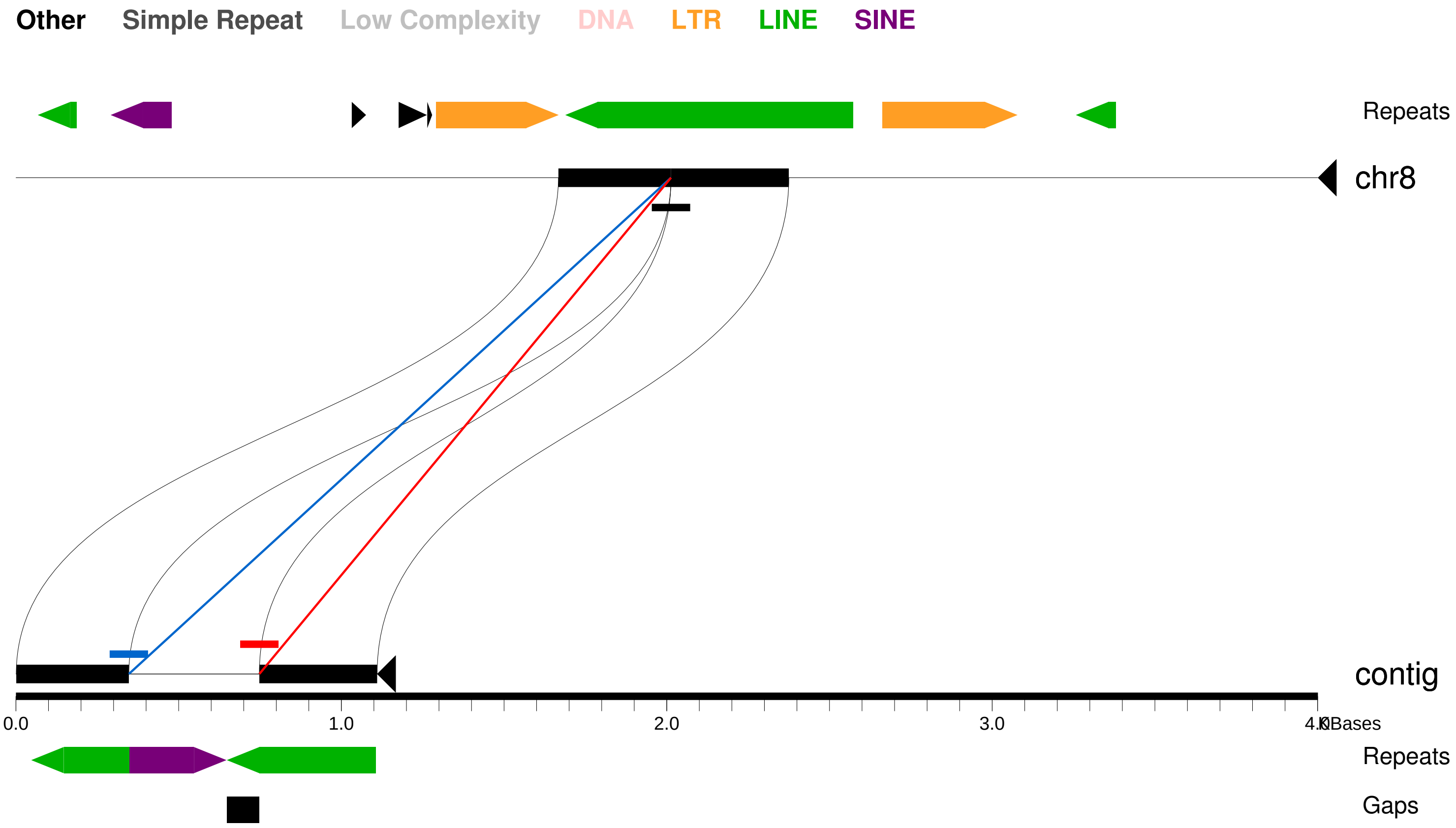

siteID: chr8_83173547 Scafftig1 - Breakpoint: chr8:83173532
chr8:83173481-83173591
Overlap Length: 9 INS size: 403 Genotyped: No

```
left    AA---CATGTAAA---GTCCATCTAGGATTCAAGCATAAATATGAAGGTTTGTGTAT---AAAAATTTTN
chrom   AA---CATGTAAA---GTCCATCTAGGATTCAAGCATAAATATGAAGGTTTGTGTAT---AAAAATTTTT
right   AGGCGCCCGCCACCGCGCCCGGCTAATTTTTT-GTATTTTTAGTAGAGACGGGGTTTCACAAAAATTTTT
        *1111*11*11*1111*1**11***111**111*1**111**11*11*111*1**1*111*********2

left    NNNNNNNNNNNNNNNNNNNNNNNNNNNNNNNNNNNNNNNNNNNNNNNNNNNNNNNNNN
chrom   AGCTTTATTGAATACTTACTATGTACGGGGTAGGCGTAAGTACTTTCATA--------
right   AGCTTTACTGAATACTTACTATGTACGGGGTAGGCGTAAGTACTTTCATA--------
        2222222N22222222222222222222222222222222222222222222222222
```

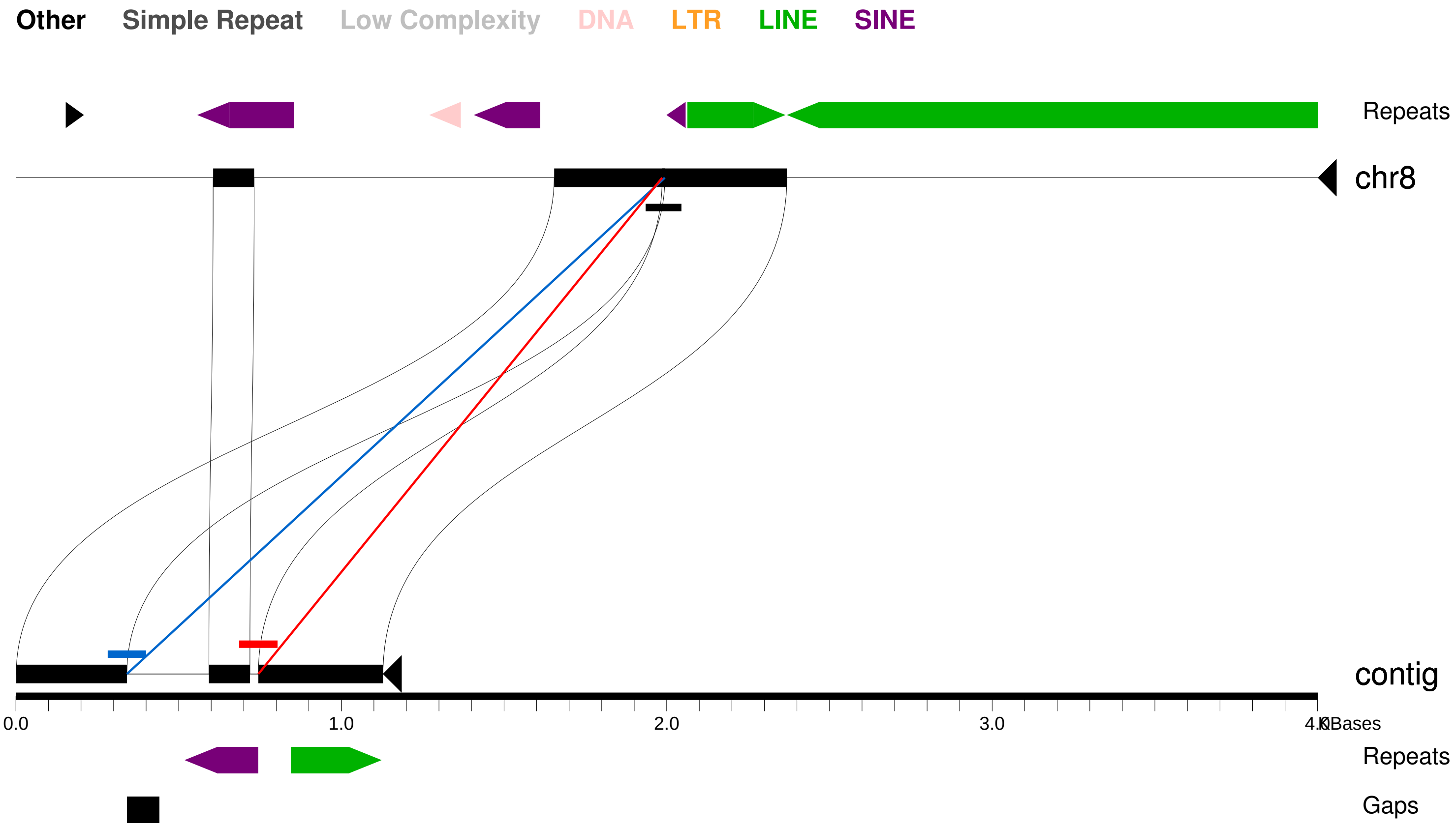

siteID: chr8_83272926 Scafftig2 - Breakpoint: chr8:83272971
chr8:83272811-83273144
Overlap Length: 14 INS size: 114 Genotyped: No

```
left   NNNNNNNNNNNNNNNNNNNNNNNNNNNNNNNNNNNNNNNNNNNNNNNNNNNNNNNNNNNNNNNNNNNNNNN
chrom  CTGAGAAATTAAATGTCTTTCTTTGCACTGAAAAACAAATGCAGAACAGCTCATTTTCCTTTGCTGAGAG
right  G---GCTCTTTTTTTTTTTTTTTTT--------------------------TTTTTTTTTTTTTTTGAGAC
       NNNN2NNN22NNN2N2N222N222NNNNNNNNNNNNNNNNNNNNNNNNNN2NN2222NN222NN22222N

left   NNNNNNNNNNNA------------AAGAATTATTCTTAATATAGATGAAAAATATTTCCCCAAAGCATTG
chrom  GCAGAGCTAGAACATAGGTATGCAAAGAATTATTCTTAATATAGATGAAAAATATTTCCCCAAAGCATTG
right  GGAGT-CTCGCTCT---GTC-GCCCAGGCTGG-----AGTGCAGTGGCGGGATCTCGGCTCACTGCA---
       2N22NN22N2N12N    22N 22N1**11*1111111*1*11**11*1111**1*111*1**11***111

left   AAAATTC-CCTTTTAGTCTAA---GCATAGTGGCTCTTTTTTTTTTTTTTTTT----TTTTT--------T
chrom  AAAATTC-CCTTTTAGTCTAA---GCATAGTGGCTCTTAAATAGAGACTTGCACAATCGTTAACACCAAT
right  -AGCTCCGCCTCCCGGGTTCACGCGCATAGTGGCTCTTAAATAGAGACTTGCACAATCGTTAACACCAAT
       1*11*1*1***1111*11*1*111*************222*222222**222222*22**22222222*

left   TTTTTT---------TTTGAGACGGAGTCTCG-----CTCTGTCGCCCAGGCTGGAGTGCAGTGGCGG--
chrom  TTATGTAACAGGCAATTTGAAAAGACTTTTAGAAATCCTGGGAAGTTACGACTACAACACATTTGTTCAT
right  TTATGTAACAGGCAATTTGAAAAGACTTTTAGAAATCCTGGGAAGTTACGACTACAACACATTTGTTCAT
       **2*2*222222222*****2*2*222*2*2*22222**22*22*2222*2**22*222**2*2*22222

left   --GATCTCGG-CTCACTGC----AAGC---TCCGCCTC-----CCGGGTTCACGCGCATA
chrom  TAGCTCTCAGTCTAATTACCACAAAGCCATTCACCCTCACCTTCCTGAATCAATG--TTC
right  GAGCTCTCAGTCTAATTACCACAAAGCCATTCACCCTCACCTTC----------------
       N2*2****2*2**2*2*2*2222****222**22****22222*1N1NN111NNN22N1N
```

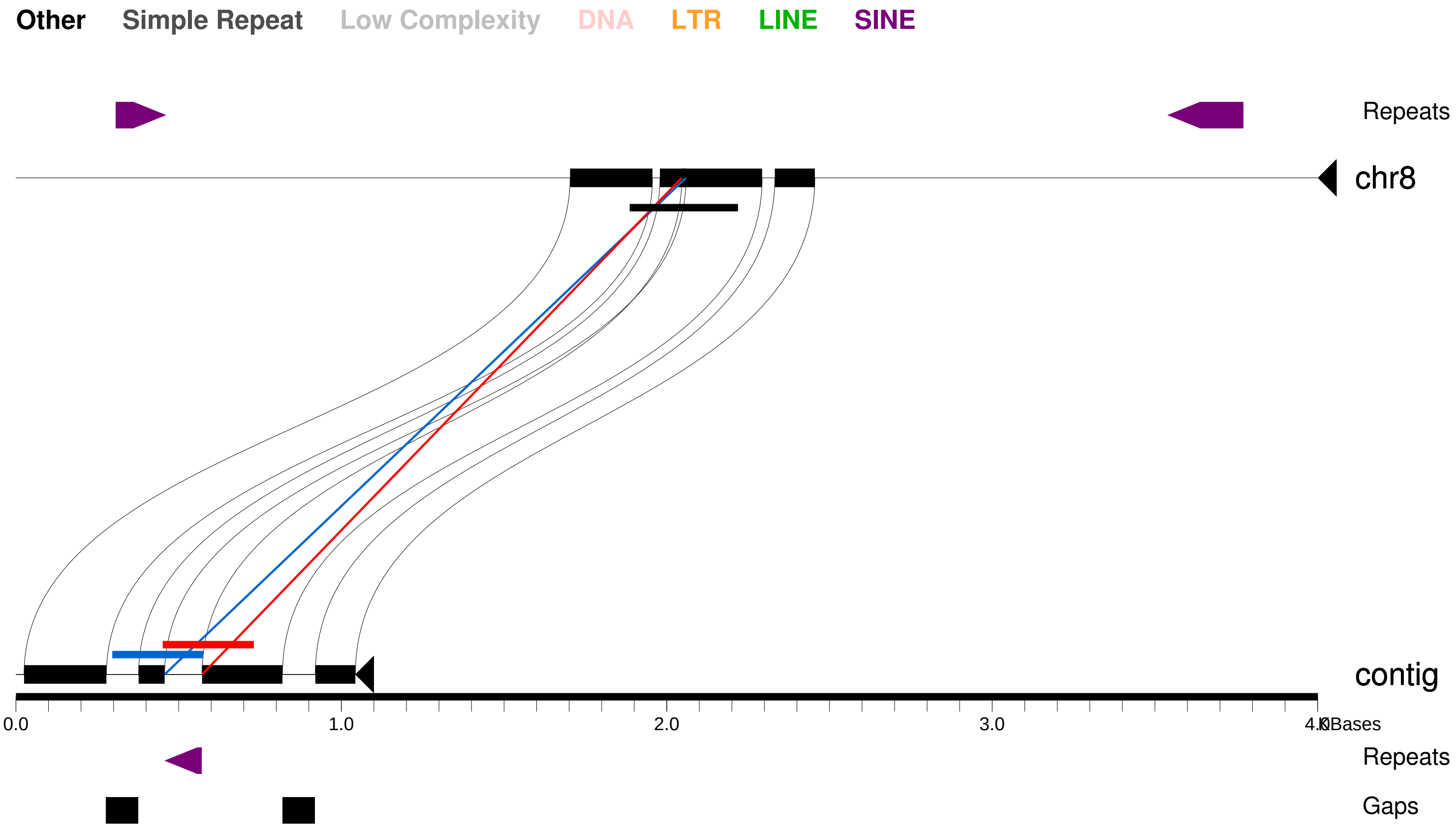

siteID: chr8_83351482 Scafftig5 - Breakpoint: chr8:83351463
chr8:83351419-83351522
Overlap Length: 16 INS size: 306 Genotyped: Yes

```
left     TCTACATTCTACGTTTATGAGAGCAACTATTGTAGCTCTCACAT----------------AAGAGTAAAA
chrom    TCTACATTCTACGTTTATGAGAGCAACTATTGTAGTTCTCACAT----------------AAGAGTAAAA
right    CCGCAGTCCGGCCTGGGCGACAG-AGCGAGACTCCGTCTCAAAAAAAAAAAAAAAAAAAAAAGAGTAAAA
         1*1111*1*11*1*1111**1**1*1*1*111*11N*****1*1111111111111111**********

left     AGGTGGCCGGGCGCGGTGGCTCACGCCTGTAATCCCAGCACTTTGGGAGGCCGAGGCGGGTGGAT
chrom    AGGTGG------GATTTTTTTCTT-CCTGTG---CCTGGG--TTGTTTCACCT---CACATAATG
right    AGGTGG------GATTTTTTTCTT-CCTGTG---CCTGGG--TTGTTTCACCT---CACATAATG
         ******222222*222*222**222*****2222**2*2222***22222**2222*222*2222
```

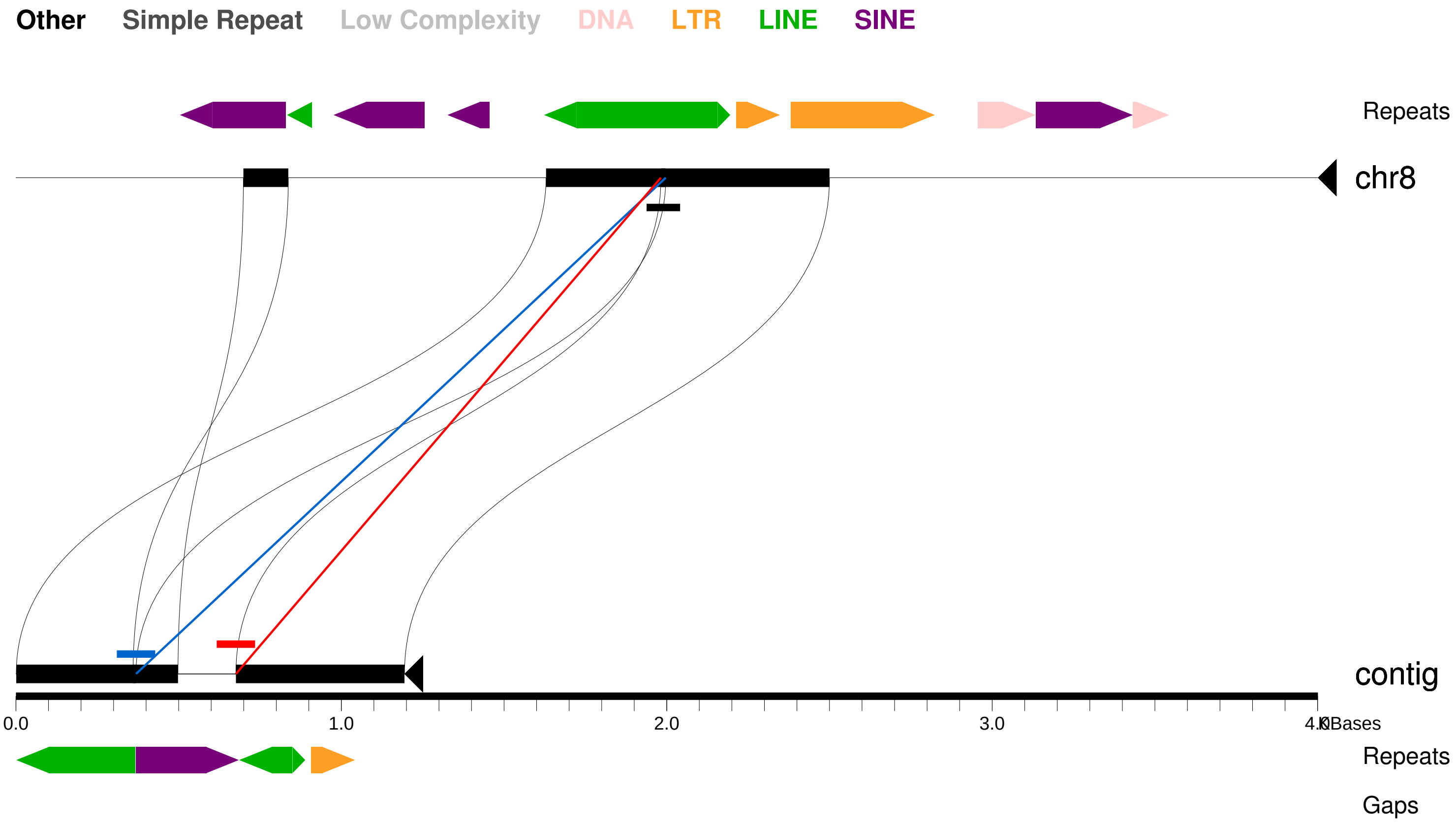

siteID: chr8_85437281 Scafftig3 + Breakpoint: chr8:85437265
chr8:85437215-85437321
Overlap Length: 10 INS size: 297 Genotyped: Yes

```
left   GGCAAGATCCCTG-TTAGCCTTTCGGGGTAGAGCAATT-CA---TTAAAATA-------CGGCCTCAATT
chrom  GGCAAGATCCCTG-TTAGCCTTTCGGGGTAGAGCAATT-CA---TTAAAATA-------CGGCCTCAATT
right  GTGATCCGCCCGCCTCGGCCTCCCAAAGTGCTGGGATTACAGGCGTGAGCCACCGCGCCCGACCTCAATT
       *11*1111***111*11****11*111**111*11***1**1111*1*111*1111111**1********

left   TTTTTTTTTTTTTTTTTTGAG-ACGGAGTCTCGCTCTGTCGCCCAGGCTGGAGTGCAGTGGC
chrom  TTAACTAAGTAAAATTAAAAATACAGTGTCTTAATC--------ATACTAG---CCAGAT--
right  TTAACTAAGTAAAATTAAAAATACAGTGTCTTAATC--------ATACTAG---CCAGAT--
       **222*222*2222**222*22**2*2****222**22222222*22**2*2222***2222
```

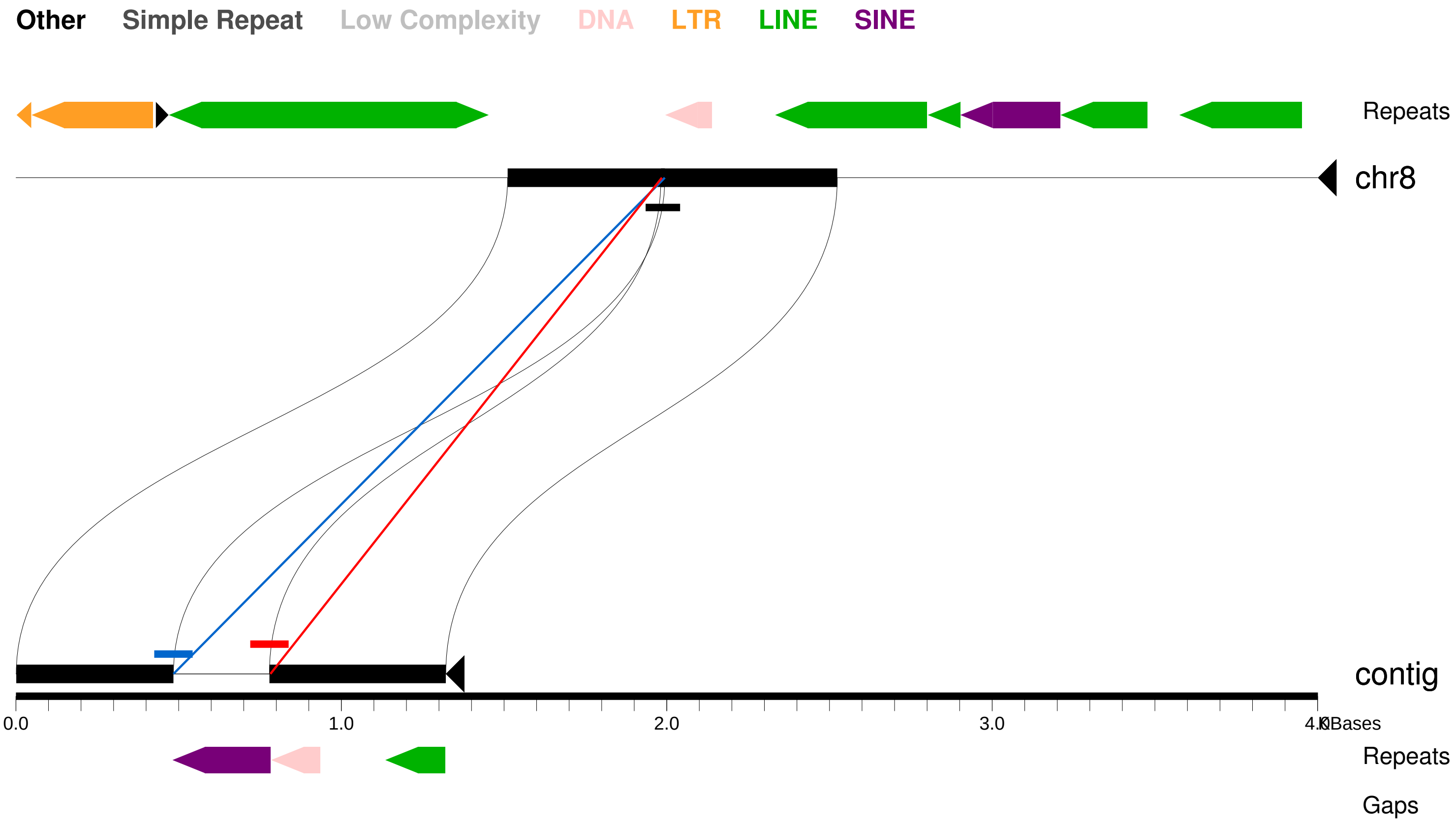

siteID: chr8_87079625 Scafftig1 - Breakpoint: chr8:87079621
chr8:87079578-87079666
Overlap Length: 17 INS size: 311 Genotyped: Yes

```
left    TTGT------------AAAGTGC----CT--------------AGCACAGAGGAGTAGCAATAAACACTG
chrom   TTGT------------AAAGTGC----CT--------------AGCACAGAGGAGTAGCAATAAACACTG
right   CAGTCCGGCCTGGGCGACAGAGCGAGACTCCGTCTCAAAAAAAAAAAAAAAAAAAAAAAAAAAAAAAAAA
        11**111111111111*1**1**1111**11111111111111*11*1*1*11*11*11**1***1*111

left    AACAAAAGAATCATTCACTGGCTCACGCCTGTAATCCCAGCACTTTGGGAGGCCGAGGCGGGTGGATCAT
chrom   AACAAAAGAATCATTCACTGAAT---ACATATTATTCC------------------------TGAATCAT
right   AAAAAAAGAATCATTCACTGAAT---ACATATTATTCC------------------------TGAATCAT
        **1****************22*2222*2*2*2**2**222222222222222222222222**2*****

left    GAGGTCAGG
chrom   CATATA---
right   CATATA---
        2*22*2222
```

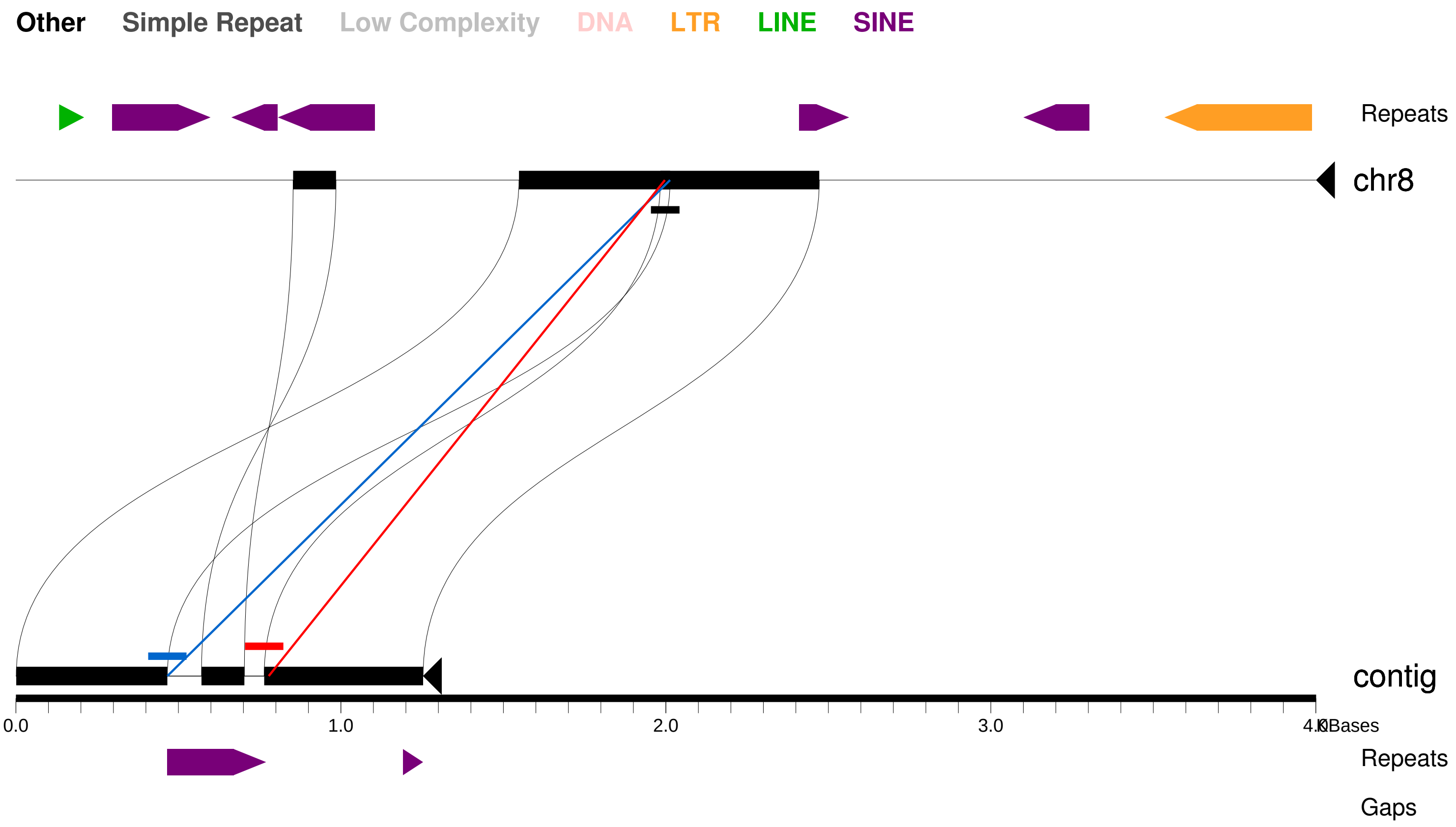

siteID: chr8_88410428 Scafftig3 + Breakpoint: chr8:88410425
chr8:88410406-88410484
Overlap Length: 18 INS size: 266 Genotyped: Yes

```
left    -------ATGTTAT--AT------------TAAT-------------------AGATTTGAAATATTTTC
chrom   -------ATGTTAT--AT------------TAAT-------------------AGATTTGAAATATTTTC
right   AGCCGGGATGGTCTCGATCTCCTGACCTCGTGATCCGCCCGCCTCGGCCTCCCAAAGTGGAAATATTTTC
        1111111***1*1*11**111111111111*1**1111111111111111111*1*1*1***********

left    TTTTTTTTTTTTTTTTTTTTTTTTTTGAGACGGAGTCTCGCTCTGTCGCCCAGGCTGGAGTGCAGTGGCG
chrom   TTATTTTCTTATAAATCTATTTTCTGGAGA-------------TATC---TAAGC---------------
right   TTATTTTCTTATAAATCTATTTTCTGGAGA-------------TATC---TAAGC---------------
        **2****2**2*222*2*2****2*2****2222222222222*2**2222*2**222222222222222

left    GGATCTCGGCTCACTGCAA
chrom   --AT------TCA-TGTAT
right   --AT------TCA-TGTAT
        22**222222***2**2*2
```

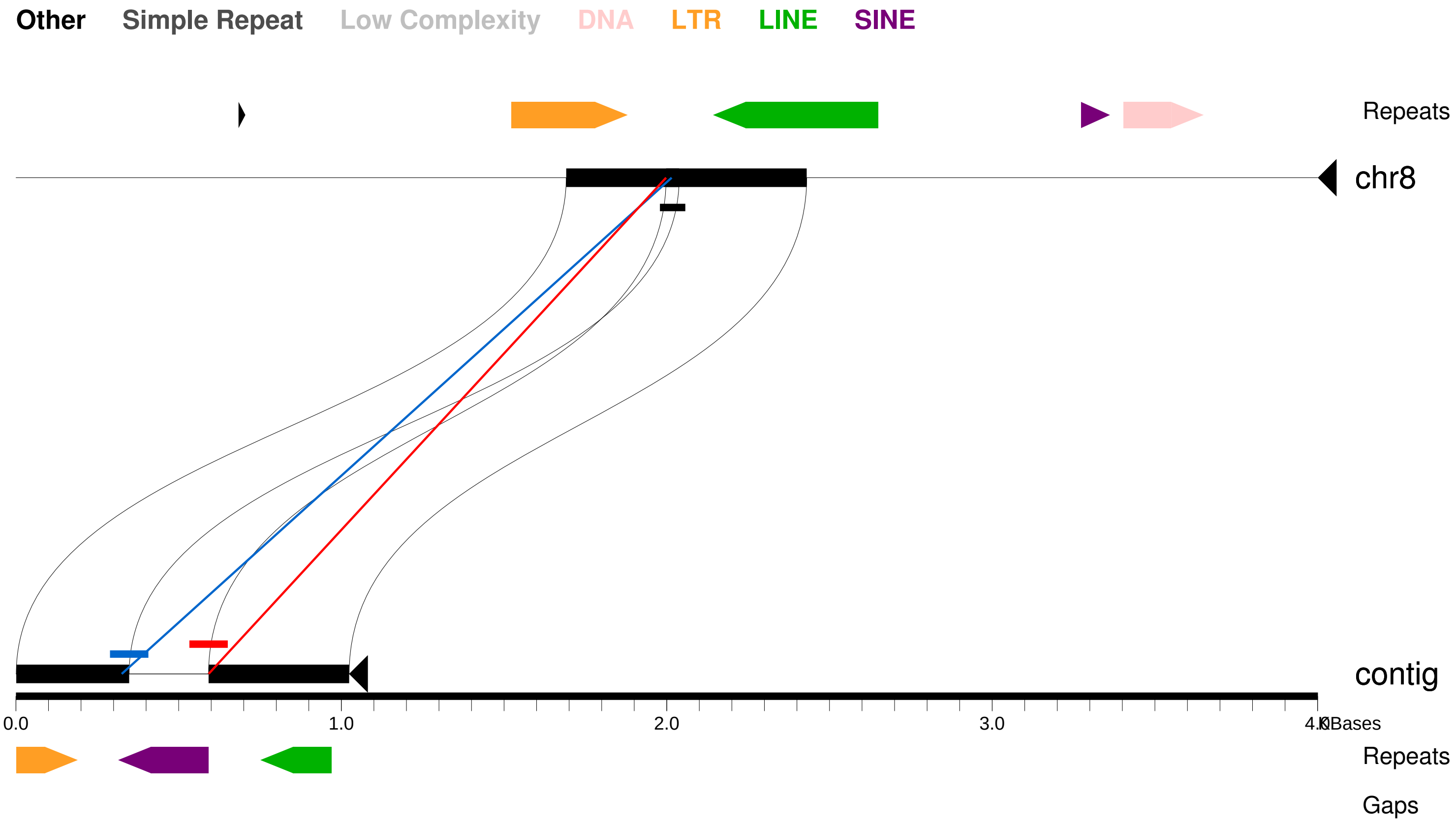

siteID: chr8_88756994 Scafftig1 - Breakpoint: chr8:88757009
chr8:88756954-88757068
Overlap Length: 5 INS size: 488 Genotyped: No

```
left    CTGTCAAAACTATGCTAGGTCTAGTCGTAGACATATGATTGGTTTAAAAAATTGT----ATTGGGCCGGG
chrom   CTGTCAAAACTATGCTAGGTCTAGTCGTAGACATATGATTGGTTTAAAAAATTGT----ATTGGCAATTT
right   NNNNNNNNNNNNNNNNNNNNNNNNNNNNNNNNNNNNNNNNNNNNNNNNNNNNNNNNNNNATTGGCAATTT
        11111111111111111111111111111111111111111111111111111111111*****222222

left    CGCGGTGGCTCACGCCTGTAATCCCAGCACTTTGGGA---GGCCGAGGCGGGCGGA-
chrom   -----TGTTTTAATACT---ATTCCATTCCTTCAGAATCAGACATAATTGTTAAGTT
right   -----TGTTTTAATACT---ATTCCATTCCTTCAGAATCAGACATAATTGTTAAGTT
        22222**22*2*222**222**2***222***22*2*222*2*22*222*2222*22
```

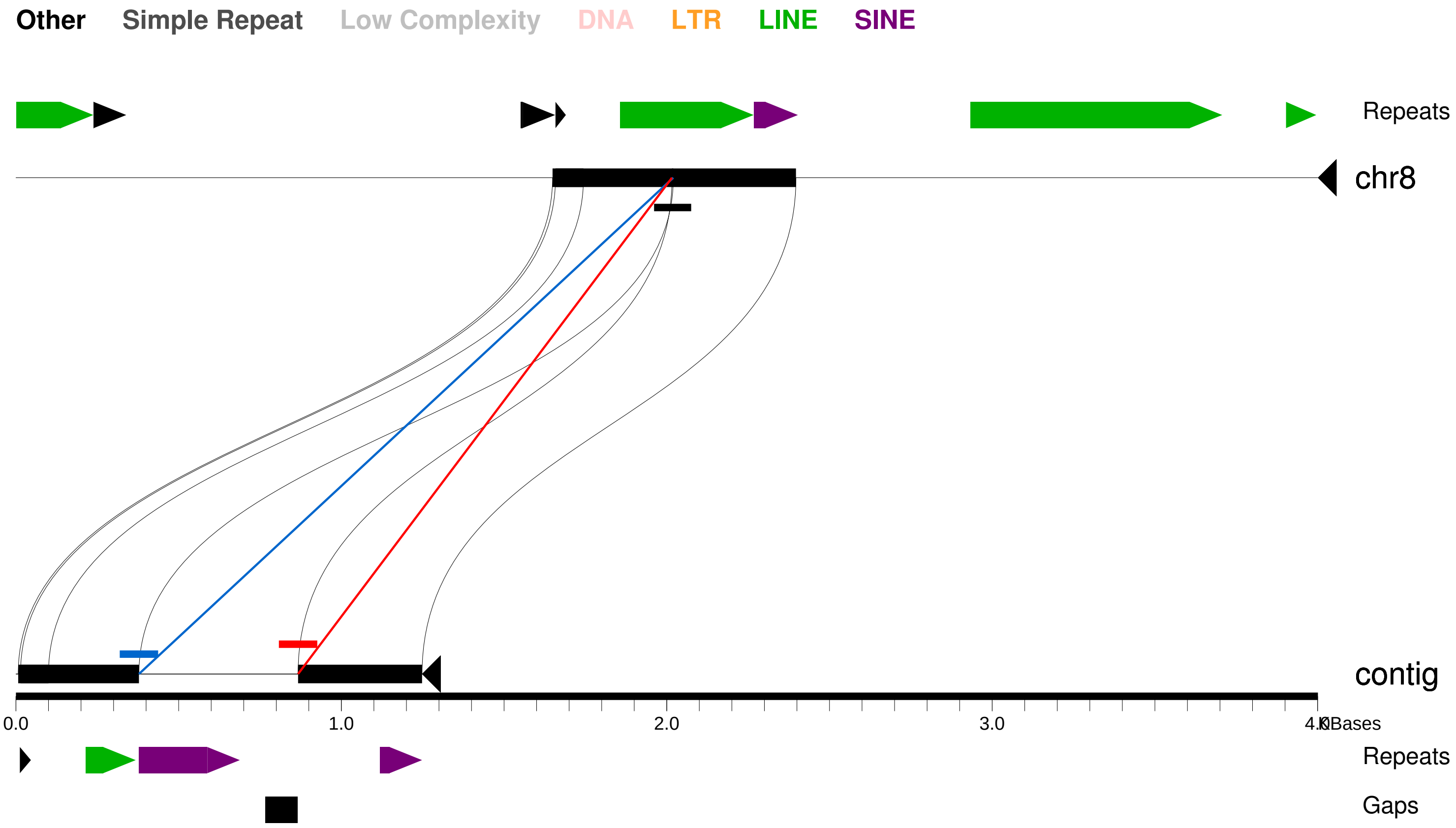

siteID: chr8_8920098 Scafftig8 - Breakpoint: chr8:8920115
chr8:8919955-8920287
Overlap Length: 13 INS size: 308 Genotyped: Yes

```
left   AAA-------------GAATGTTTTCTAAGTTGGCACCAGAGTTGGGGAGTTTTAAGAGGTTTAGAAGCC
chrom  AAAAGAGTGCAAAAAAGAATGTTTTCTAAGTTGGCACCAGAGTTGGGGAGTTTTAAGAGGTTTAGAAGCC
right  AATGGCGTGAACCCGGGAAG-----CGGAGCTTGCA---------GTGAGCC----GAGATTGCGCCACT
       **1N2N222N2NNNNN***111111*11**1*1***111111111*1***111111***1**11*111*1

left   TGGCTGTCAATACTTACAACAGTTATGGAGGCA-AGGGAAACAGGCCCTTGAAAAGAAGGTAATGTGGAG
chrom  TGGCTGTCAATACTTACAACAGTTATGGAGGCA-AGGGAAACAGGCCCTTGAAAAGAAGGTAATGTGGAG
right  --GCAGTCCGCAGTCCGACCTG----GGCGACAGAGCGAGACTCCGTCTCAAAAAAAAAAAAA-------
       11**1***111*1*111*1*1*1111**1*1**1**1**1**11111**11****1**111**1111111

left   TGGGTAGCCTCCGTATTGATTAAGAAGGGGACGGGG----CCGGGCGTG-GTGGCTCACGC-------CT
chrom  TGGGTAGCCTTCGTATTGATTAAGAAGGGGACGGACTTAACCTCCACTGTGAGAGTTAATCAGAGTATCT
right  --------------AAAAAAAAAGAAGGGGACGGACTTAACCTCCACTGTGAGAGTTAATCAGAGTATCT
       1111111111N111*111*11*************222222**22222**2*2*22*2*22*2222222**

left   GTAATCCCAGCACTTTGGGAGGCCGAGGC-----GGGTGGATCA------------TGAGGTCAGGAGAT
chrom  GTGAT---GGTCCTGTAGGCTTCCGAGGCAATCCGGCAGTGTCAGTCTACAGCCGCTAAGCTGAGAAGAT
right  GTGAT---GGTCCTGTAGGCTTCCGAGGCAATCCGGCAGTGTCAGTCTACAGCCGCTAAGCTGAGAAGAT
       **2**2222*22**2*2**222*******22222**22*22***222222222222*2**2*2**2****

left   C-------GAGACCATCCTGGCTAACAAGGTGAAACCCC----GTCTCTACTAAA--
chrom  CTGGGAAGGAGTCAGTCAGAGAGCCTTGGGCCAGAGTTCCAGGGGCTCTGGGAGTGG
right  CTGGGAAGGAGTCAGTCAGAGAGCCTTGGGCCAGAGTTGCAGGGGCTCT--------
       *2222222***2*22**222*2222222**22*2*22212222*2****NNN1NN
```

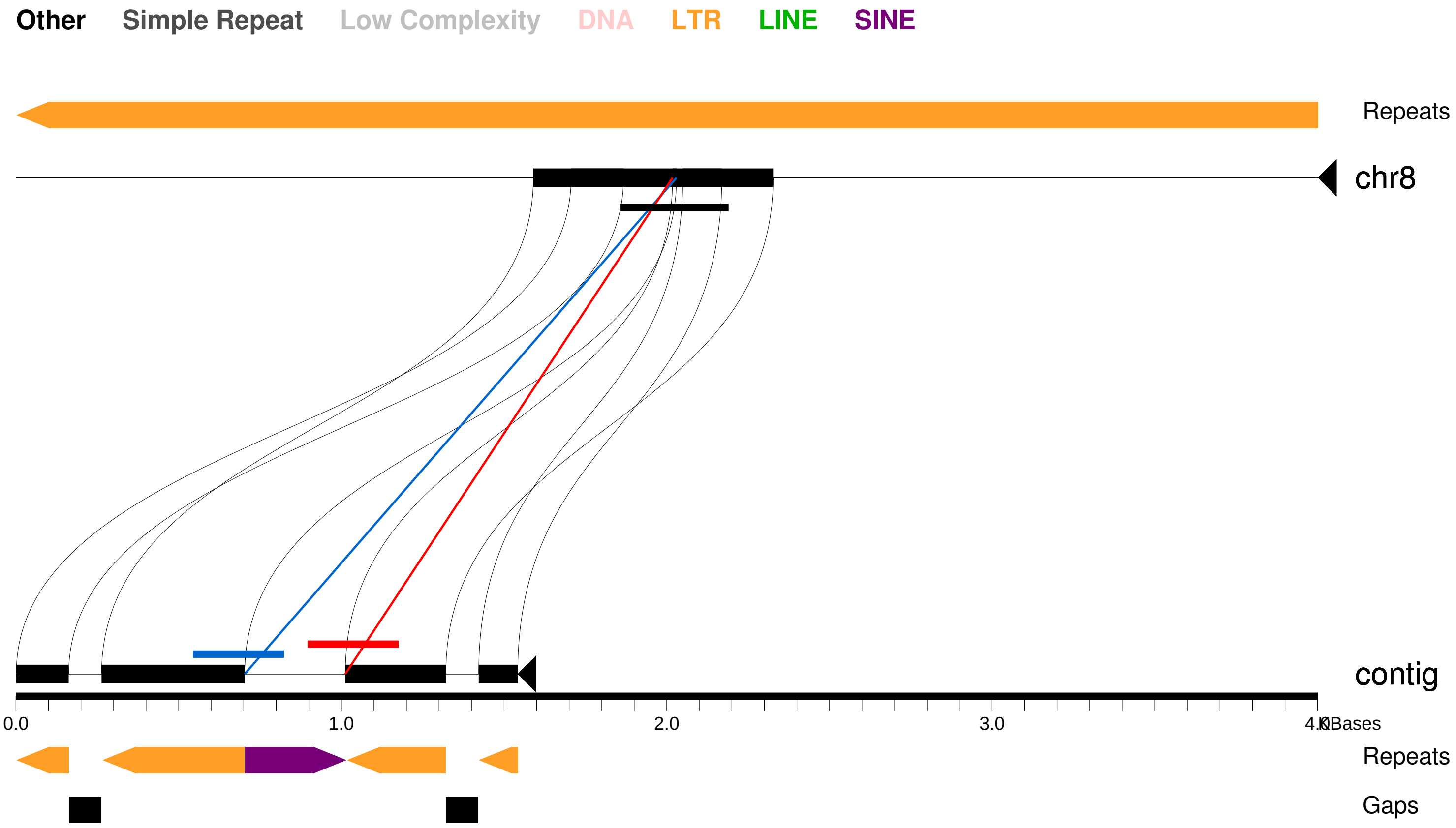

siteID: chr8_9012978 Scafftig1 - Breakpoint: chr8:9012956
chr8:9012907-9013014
Overlap Length: 11 INS size: 428 Genotyped: No

```
left   ---------------TTAAACAGTGTTCCTTTATACATGTGTTCACATCAGCTTTCTGCTCATCCAGACA
chrom  ---------------TTAAACAGTGTTCCTTTATACATGTGTTCACATCAGCTTTCTGCTCATCCAGAAA
right  CCGCCCGCCTCGGCCTCCCAAAGTGCTGGGAT-TACAGGCGTGAGCCACCGCGCCCGGCCC---CAGAAA
       111111111111111*111*1****1*1111*1****1*1**111*11*1**111*1**1*111****2*

left   CTTTTTNNNNNNNNNNNNNNNNNNNNNNNNNNNNNNNNNNNNNNNNNNNNNNNNNNNNNNNNNNNNN
chrom  CTTTTT-----------AAAAAGGATTGCAAATGTTTTACCTCCTTCTCAACTTAATGCATTTCA
right  CTTTT-----------AAAAAGGATTGCAAATGTTTTACCTCCTTCTCAACTTAATGCATTTCA
       *****2222222222222222222222222222222222222222222222222222222222222
```

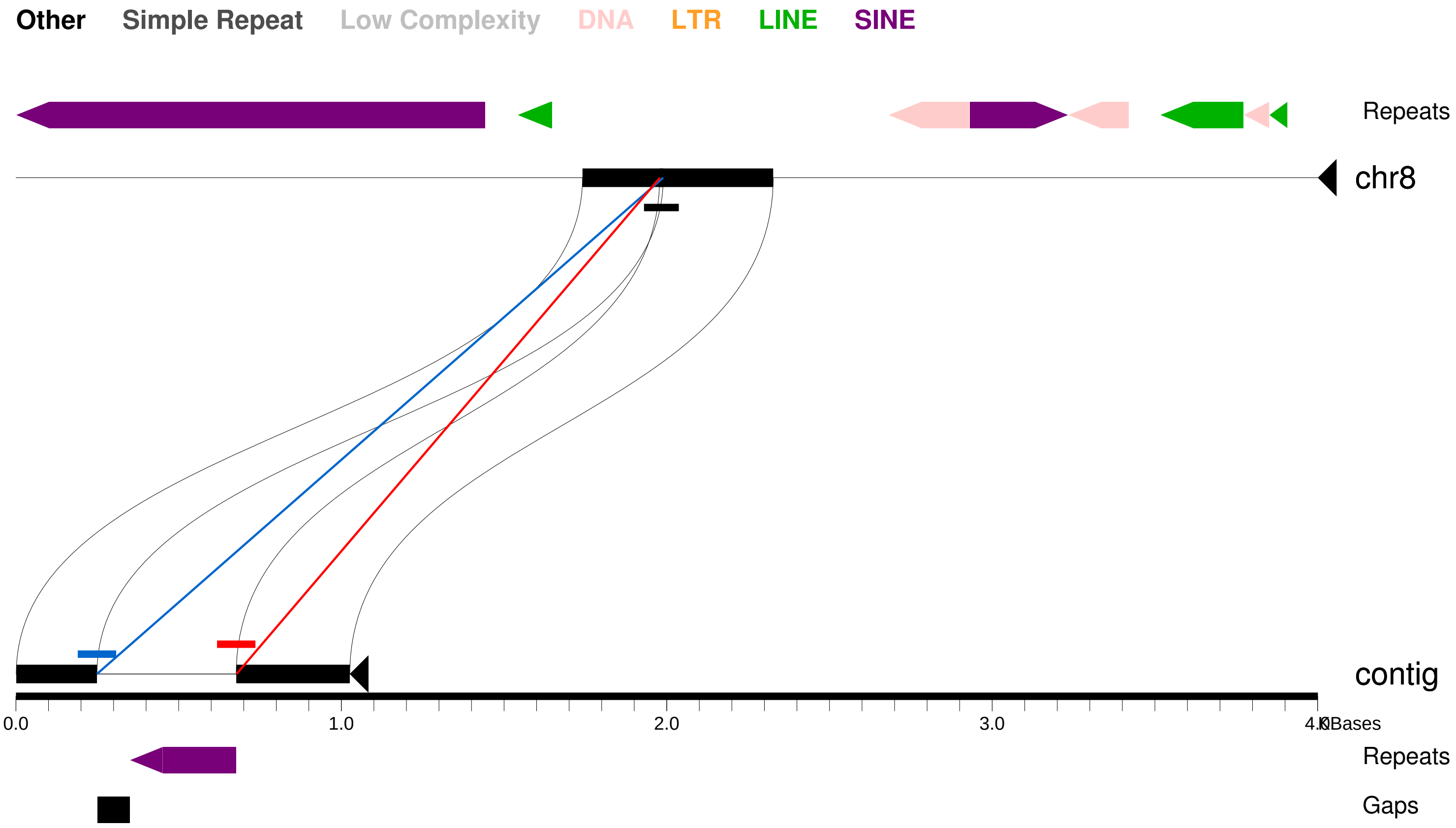

siteID: chr8_93373079 Scafftig1 + Breakpoint: chr8:93373100
chr8:93373050-93373159
Overlap Length: 10 INS size: 167 Genotyped: No

```
left    GACCTCAAGAGACTCACAGTCCACTGGAGTTCAGCAACATGTTAAAAAAT---------GTCTATAATAG
chrom   GTCCTCAAGAGACTCACAGTCCACTGGAGTTCAGCAACATGTTAAAAAAT---------GTCTATAATAT
right   NNNNNNNNNNNNNNNNNNNNNNNNNNNNNNNNNNNNNNNNNNNNNNNNNNNNNNNNNNNGTCTATAATAT
        1N111111111111111111111111111111111111111111111111111111111**********2

left    GCCGGGCGCGGTGGC-TCACGCCTGTAATCCCAGCACTTTGGGAGGCCGAGGCGGGGGG
chrom   ACCATAAAAAGTGCAATCATAATGGTAGTATC--CACTT----ATTGCCATACAG----
right   ACCATAAAAAGTGCAATCATAATGGTAGTATC--CACTT----ATTGCCATACAG----
        2**2222222***222***22222***2*22*22*****2222*222*2*22*2*2222
```

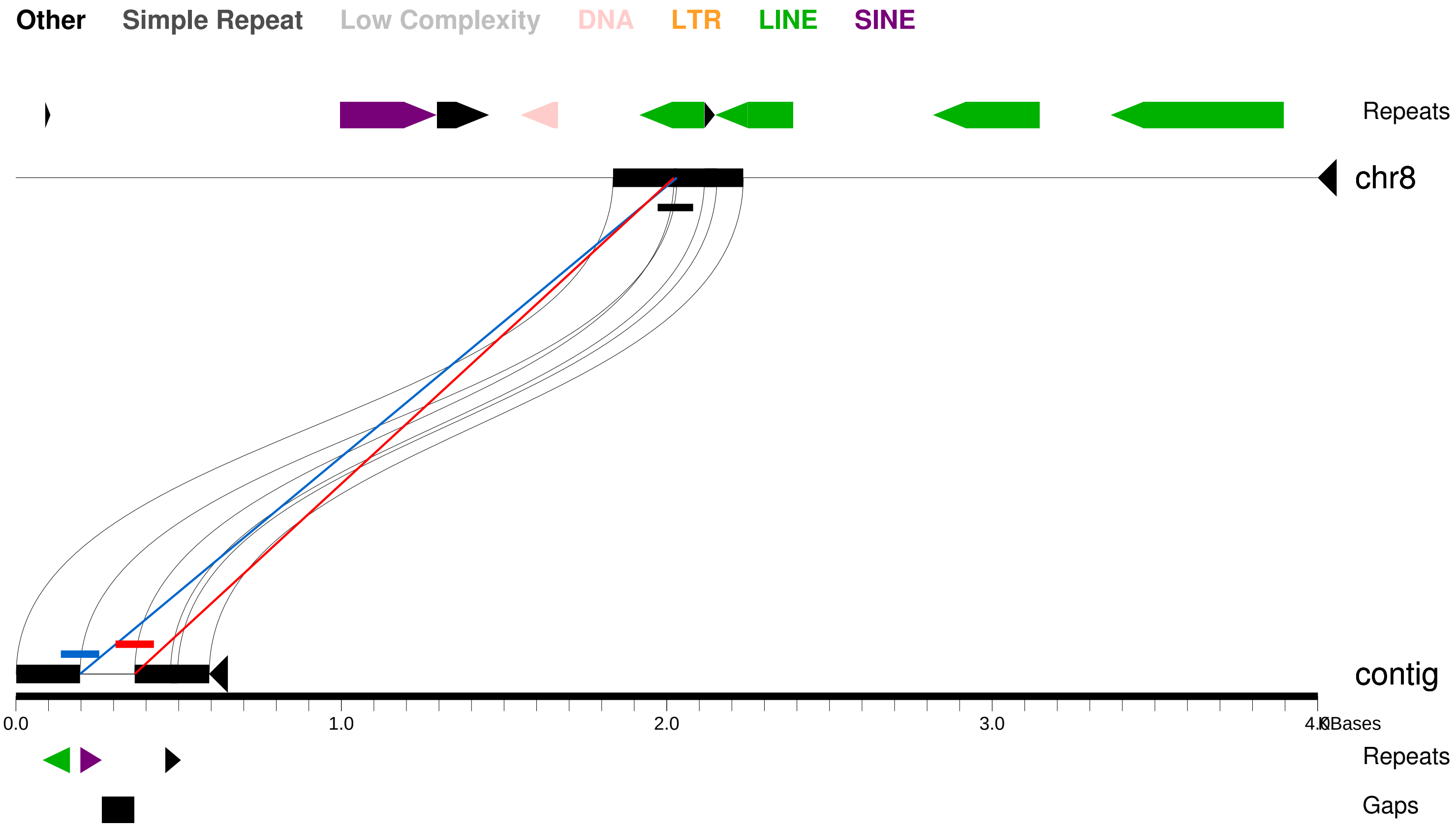

siteID: chr8_94162476 Scafftig6 - Breakpoint: chr8:94162472
chr8:94162429-94162531
Overlap Length: 17 INS size: 299 Genotyped: Yes

```
left    --CATATATGTAGGT-----ATGTTTCTGGACTCTCTATTCTGATATATT---------GGTTTATTTAT
chrom   --CATATATGTAGGT-----ATGTTTCTGGACTCTCTATTCTGATATATT---------GGTTTATTTAT
right   TCCGCCCGTCTCGGCCTCCCAAAGTGCTGGGATTACAGGCGTGAGCCACCGCGCCCGGCGGTTTATTTAT
        11*11111*1*1**111111*111*1****11*11*11111***111*11111111111***********

left    TTTTCTTTTTTTTTTTTTTTTTTTTTGAGGCGGAGTTTTGCTCTGTCGCCCAGGCTGGAGTGCAG
chrom   TTTTCTGTACCAATATCTCACTGTT------GTAATTAT----TGTAGC------TTTATAACCA
right   TTTTCTGTACCAATATCTCACTGTT------GTAATTAT----TGTAGC------TTTATAACCA
        ******2*22222*2*2*222*2**222222*2*2**2*2222***2**222222*22*222*22
```

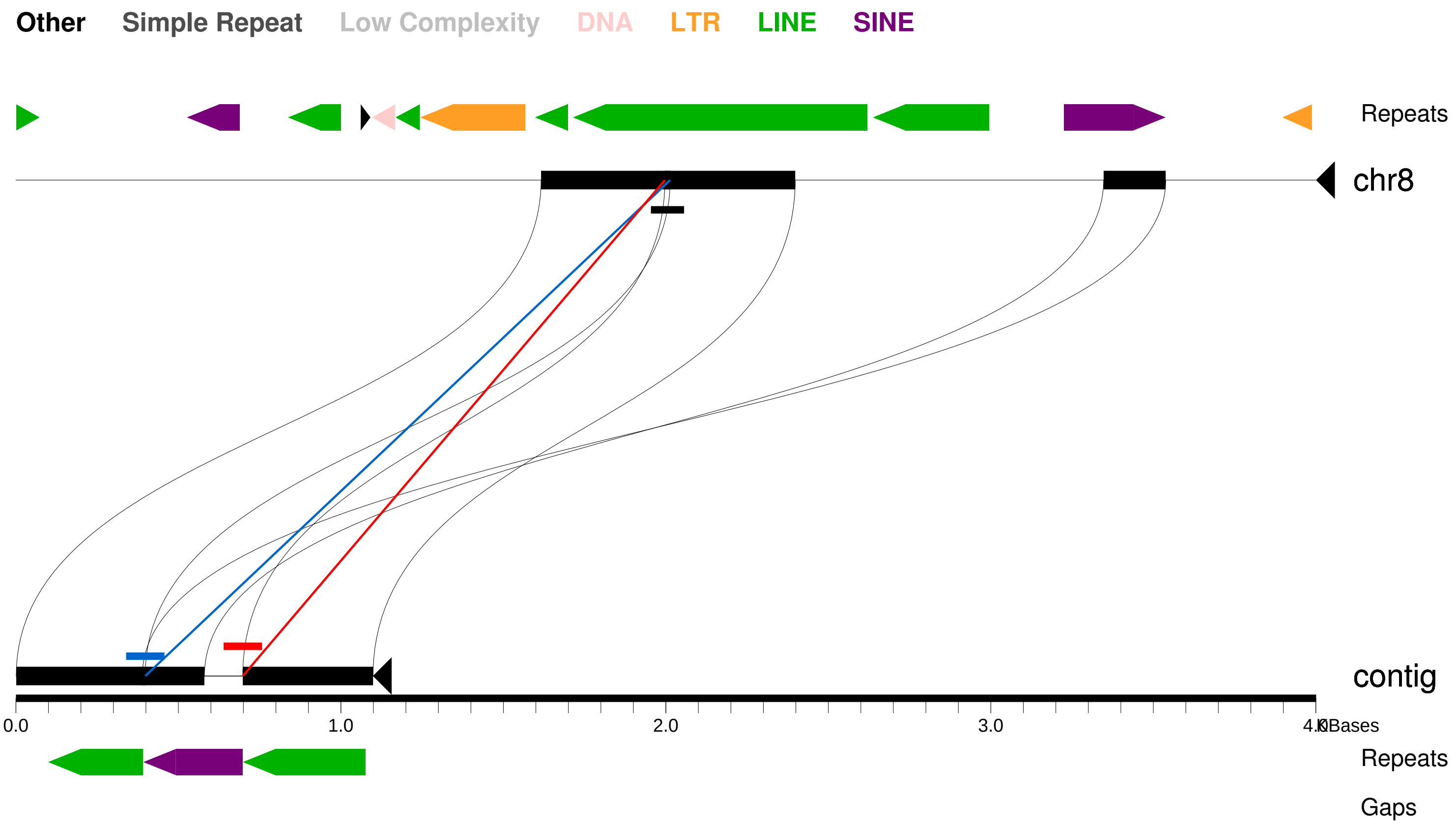

siteID: chr8_9463657 Scafftig7 - Breakpoint: chr8:9463650
chr8:9463620-9463717
Overlap Length: 12 INS size: 300 Genotyped: Yes

```
left    TTGAGTT--AATGTGTTTGTGT----GGTGTG------------CGGT-AAGAGTTATTTT-----TTTT
chrom   TTGAGTT--AATGTGTTTGTGT----GGTGTG------------CGGT-AAGAGTTATTTTGTG--TTTT
right   CGGCCTCCCAAAGTGCTGGGATTACAGGCGTGAGCCACCGCGCCCGGCCAAGAGTTATTTTGTGTGTTTT
        11*11*111**1***1*1*11*1111**1***111111111111***11************22211****

left    TATTTTTATTTTTTGAGACGGAGTCTCGCTCTGTCGCCCAGGCTGGAGTGCAGTGGCGGGATCTCGGCTC
chrom   TTTTTTTTTCTTTTCTATATAGAGAATCCGT-TGT--------------TTCAG---CATGATTTG-----
right   TTTTTTTTTCTTTTCTATATAGAGAATCCGT-TGT--------------TTCAG---CATGATTTG-----
        *2*****22****22*2*22***22**22*2***22222222222222*2***222*22***2*222222

left    ATT
chrom   -TT
right   -TT
        2**
```

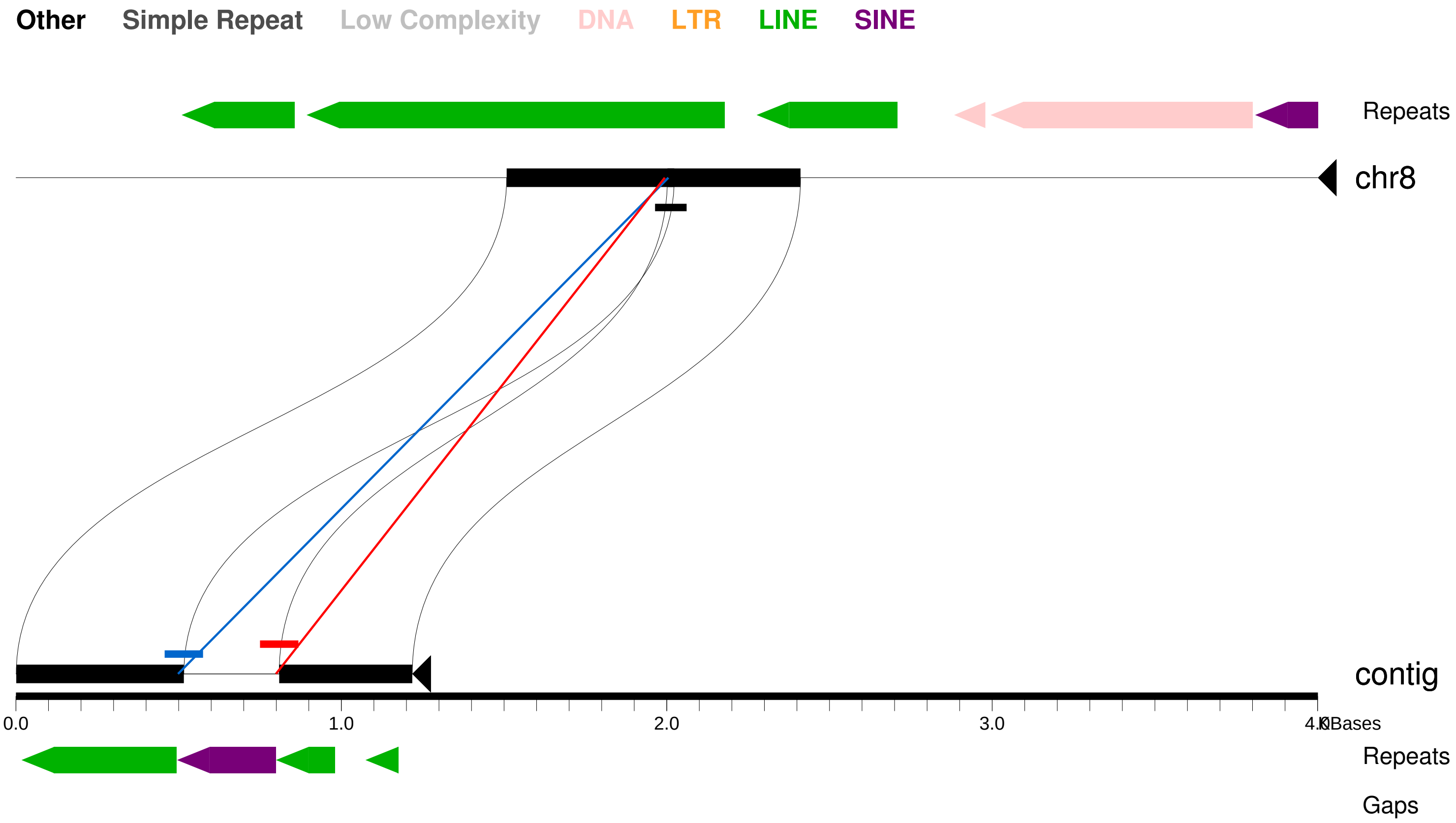

siteID: chr8_95794736 Scafftig3 + Breakpoint: chr8:95794721
chr8:95794677-95794780
Overlap Length: 16 INS size: 295 Genotyped: Yes

```
left     AATACTAGCATTCCCTCCAAGA---CCATGACCATAGG-GAGAGGAAT-----------TACTGTGGCCA
chrom    AATACTAGCATTCCCTCCAAGA---CCATGACCATAGG-GAGAGGAAT-----------TACTGTGGCCA
right    CCACCCGCCTCGGCCTCCCAAAGTGCTGGGATTACAGGCGTGAGCCACCGCGCCCGGCCTACTGTGGCCA
         1111*111*1111*****1*1*111*111**11*1***1*1***11*111111111111***********

left     TTTTTTTTTTTTTTTTTTTTGA--GACGGAGTCTCGCTGTCGCCCAGGCTGGAGTGCAGTGGCGCA
chrom    TTTTTTGTAACCAGTCTATTCCATGGATGGAAACTAT----------GAAATAACTACAT-------
right    TTTTTGTAACCAGTCTATTCCATGGATGGAGACTAT----------GAAATAACTACAT-------
         *****2*222222*2*2**22*22**2***N2**222222222222*22222*2*2**22222222
```

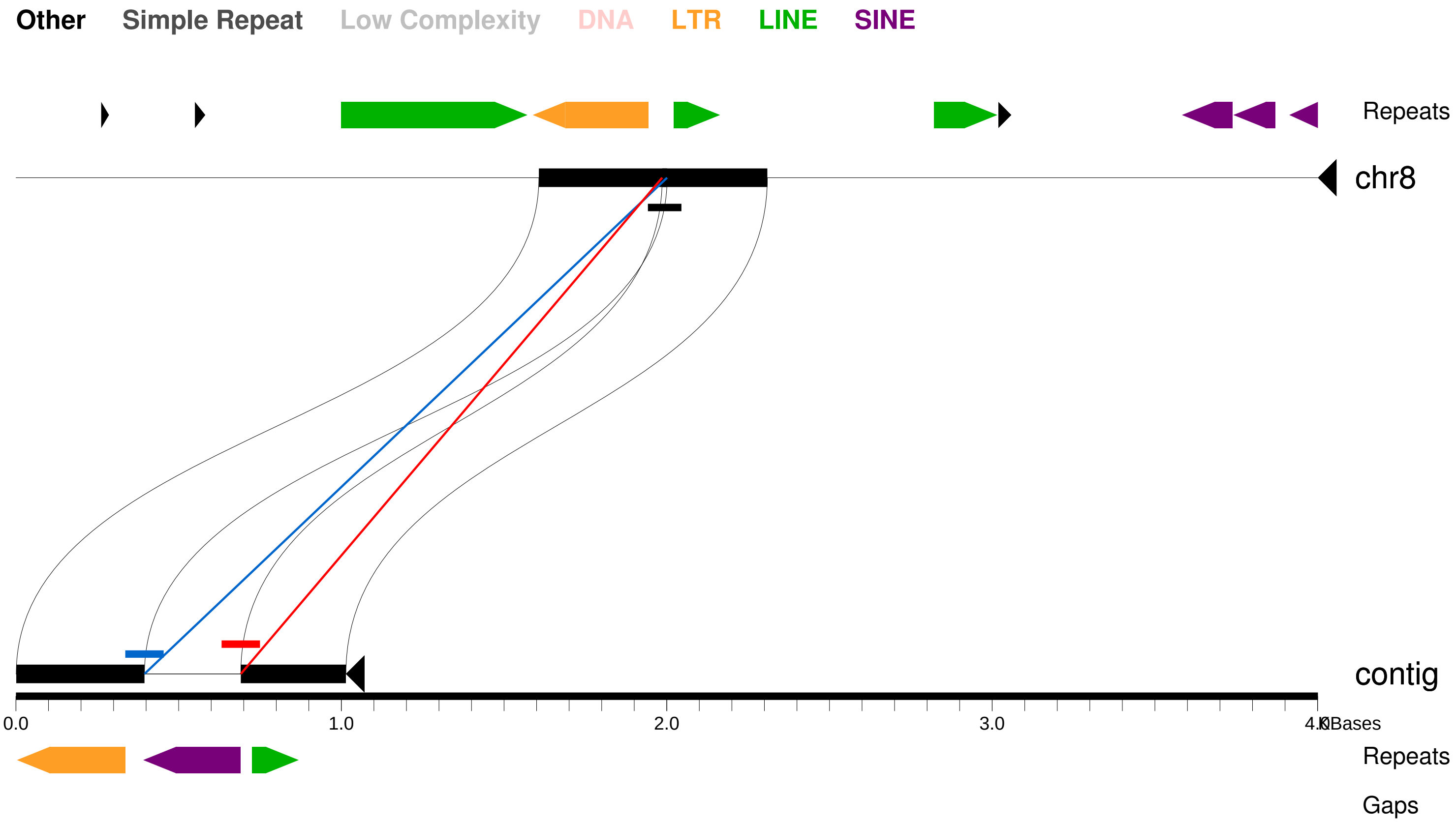

siteID: chr8_96467775 Scafftig1 - Breakpoint: chr8:96467779
chr8:96467735-96467838
Overlap Length: 9 INS size: 307 Genotyped: Yes

```
left    CCTT---TCTCTGGTT-------TGCTGGGACCTC-----TTAGATGGACTGTTAGTTTTCCCTGTCCCC
chrom   CCTT---TCTCTGGTT-------TGCTGGGACCTC-----TTAGATGGACTGTTAGTTTTCCCTGTCCAC
right   CCGCCCGCCTCGGCCTCCCAAAGTGCTGGGATTACAGGCGTGAGCCACCGTGCCCGGCCTCCCTGTCCAC
        **111111***1*11*1111111********111*11111*1**111111**111*111*********2*

left    TTTTTTTTTTTTTTTTTTTTTTTTGAGACGGAGTCTCGCTCTGTCGCCCAGGCTGGAGTGCAGT
chrom   TCTTTAA------------GACTCAAGTCTGGTTGTTGAAGTGTCCTTAATGATGG---GAATC
right   TCTTTAA------------GACTCAAGTCTGGTTGTTGAAGTGTCCTTAATGATGG---GAATC
        *2***2222222222222222*22**2*2*22*2*2*222****2222*2*2***222*2*22
```

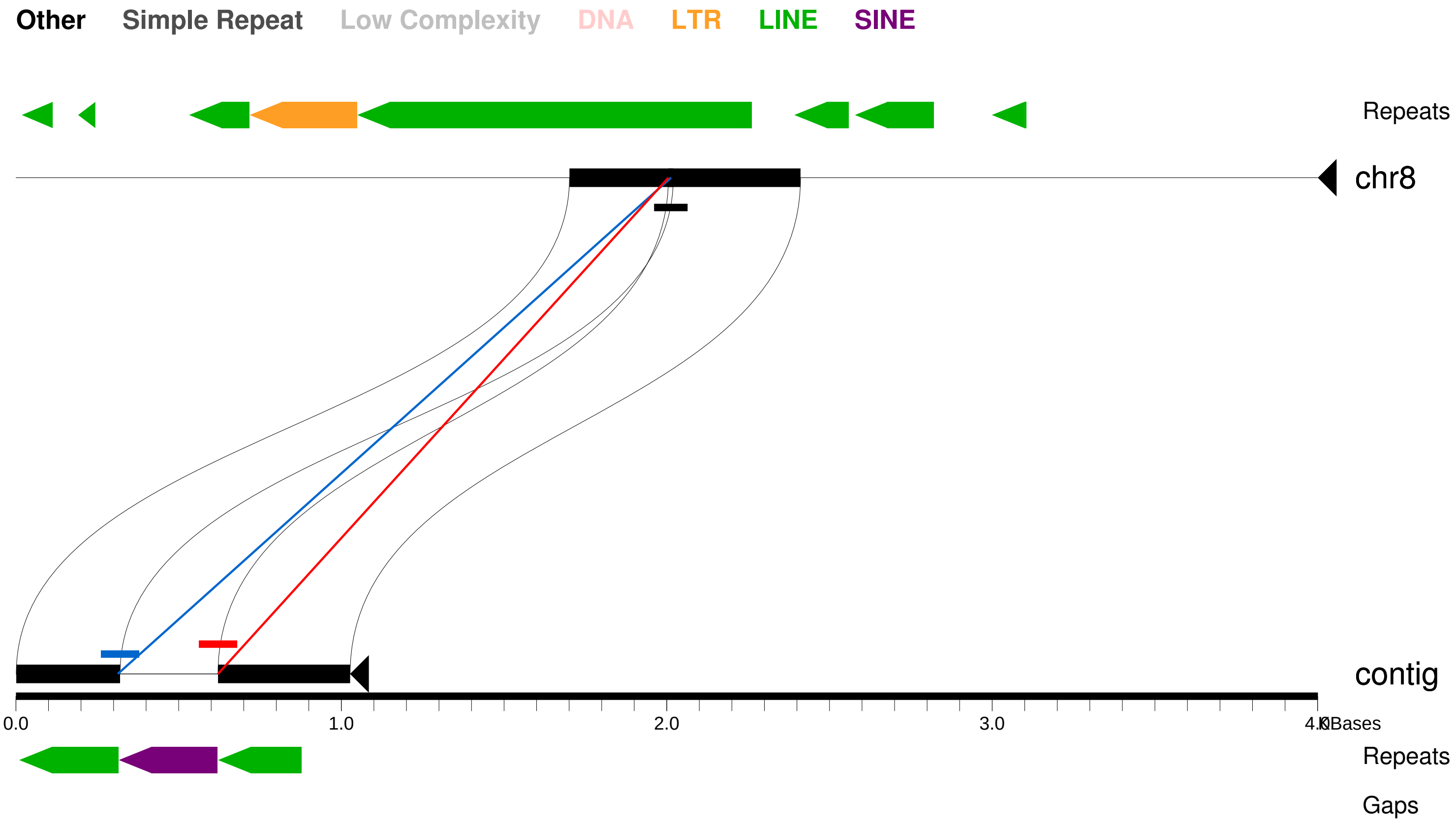

siteID: chr8_9665640 Scafftig2 - Breakpoint: chr8:9665657
chr8:9665614-9665716
Overlap Length: 17 INS size: 493 Genotyped: No

```
left    TTTTACCTCAAACCACCACCCTTAGGTCTCTCTTGAAGTGG----------------ATAGAAGATCTTC
chrom   TTTTACCTCAAACCACCACCCTTAGGTCTCTCTTGAAGTGG----------------ATAGAAGATCTTC
right   NNNNNNNNNNNNNNNNNNNNNNNNNNNNNNNNNNNNNNNNNNNNNNNNNNNNNNNAAAAAGAAGATCTTC
        111111111111111111111111111111111111111111111111111111111*1***********

left    ACTGGCGGCCGGGCGCGGTGGCTCACGCCTGTAATCCCAGCACTTTGGGAGGCCGAGGCGGGTGG
chrom   ACTGGCAATCT-----------TCAC--CAATACTCCAAAAAGTGT---ACTCCAATACTTTCAC
right   ACTGGCAATCT-----------TCAC--CAATACTCCAAAAAGTGT---ACTCCAATACTTTCAC
        ******222*222222222222****22*22**2***2*22*2*2*222*22**2*22*222222
```

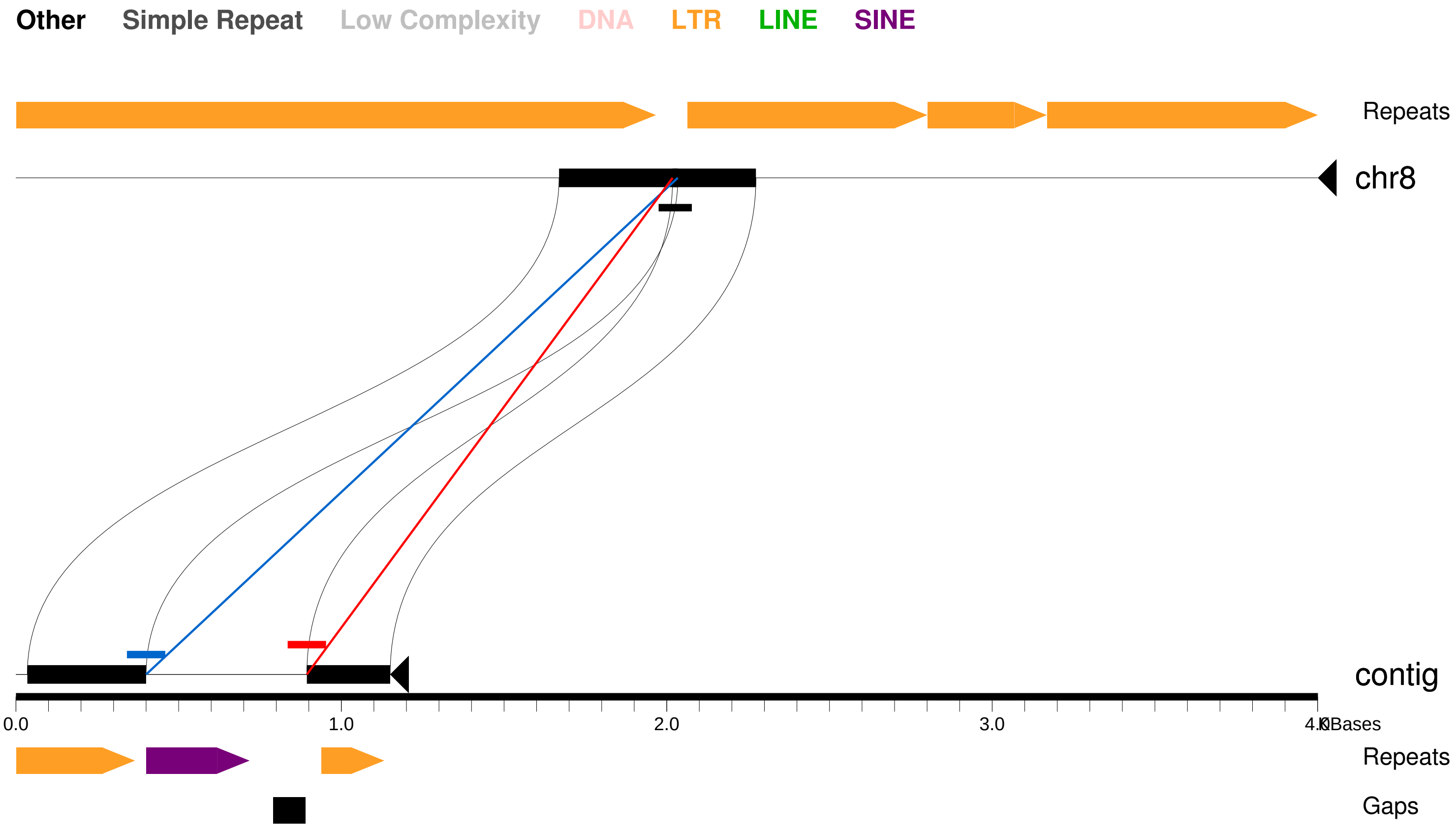

siteID: chr8_96657277 Scafftig3 + Breakpoint: chr8:96657267
chr8:96657221-96657326
Overlap Length: 14 INS size: 316 Genotyped: Yes

```
left     AC-CTTGAATAGCGAGGTAGCCTTGTACTCAATCTTCAGATGAGAAG--------------TAACAGTTC
chrom    AC-CTTGAATAGCGAGGTAGCCTTGTACTCAATCTTCAGATGAGAAG--------------TAACAGTTC
right    CCACCCGCCT--CGGCCTCCCAAAGTGCTGGGATTACAGGCGTGAGCCACCGCGCCCGGCCTAACAGTTC
         1*1*11*11*11**111*11*111**1**11111*1***11*1**1111111111111111*********

left     TCATTTTTTTTTTTTTTTTTTTTTTTTTTTTTTTTTGAGACGGAGTCTCGCTCTGTCGCCCAGGTCG
chrom    TCATTCATGGACACTTTTGTATAATT-------------GGGTGTATCTGTTTACACTATCCTC
right    TCATTCATGGACACTTTTGTATAATT-------------GGGTGTATCTGTTTACACTATCCTC
         *****22*222222****2*2*22**2222222222222*2**2*22**2*2*222*2*22222
```

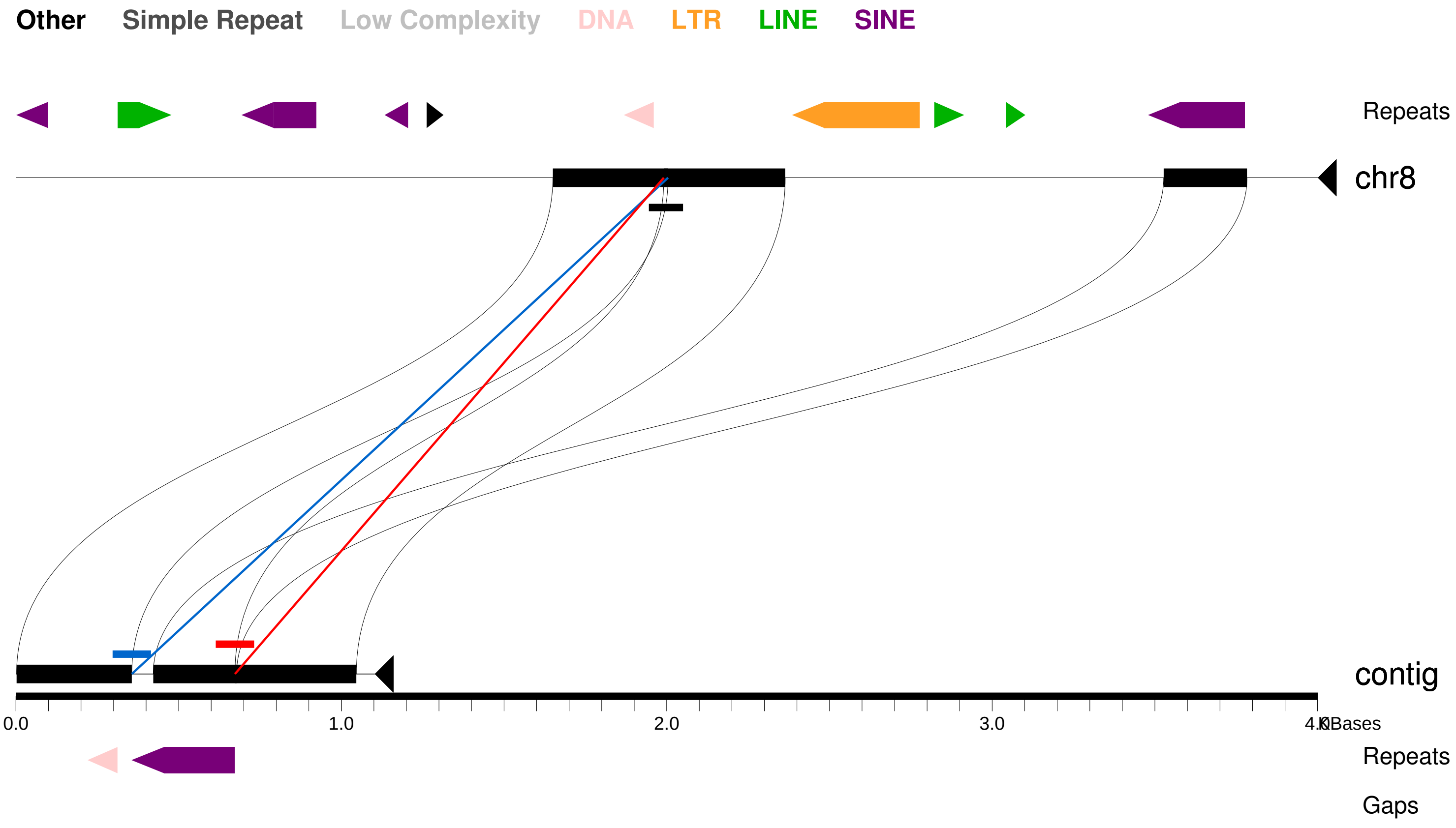

siteID: chr8_96783528 Scafftig2 - Breakpoint: chr8:96783534
chr8:96783460-96783592
Overlap Length: 15 INS size: 469 Genotyped: No

```
left   GAGGATAGTTCTATTGCTTGCAATAGGTTTTCAAATGCCATGTTAACTCAAAAAAAAAAAAAAAATCACAT
chrom  GAGGATAGTTCTATTGCTTGCAATAGGTTTTCAAATGCCATGTTAATTCAAAAAAAAAAAA----TCACAT
right  NNNNNNNNNNNNNNNNNNNNNNNNNNNNNNNNNNNNNNNNNNNNNNNNNNNNNNNNNNNNNNA----------
       11111111111111111111111111111111111111111111N111111111111*2222111111

left   ACATTGTAAAATAGAATAACAGAGGCCGGGC--GCGGTGGCT--------CACGCCTGT--------
chrom  ACATTGTAAAATAGAATAACAGAATCTAAATATGCAGAAACTTTAATTACCAAGAATTTTTGGTCTT
right  --------AAATAGAATAACAGAATCTAAATATGCAGAAACTTTAATTACCAAGAATTTTTGGTCTT
       11111111**************22*2222222**2*222**22222222**2*22*2*22222222
```

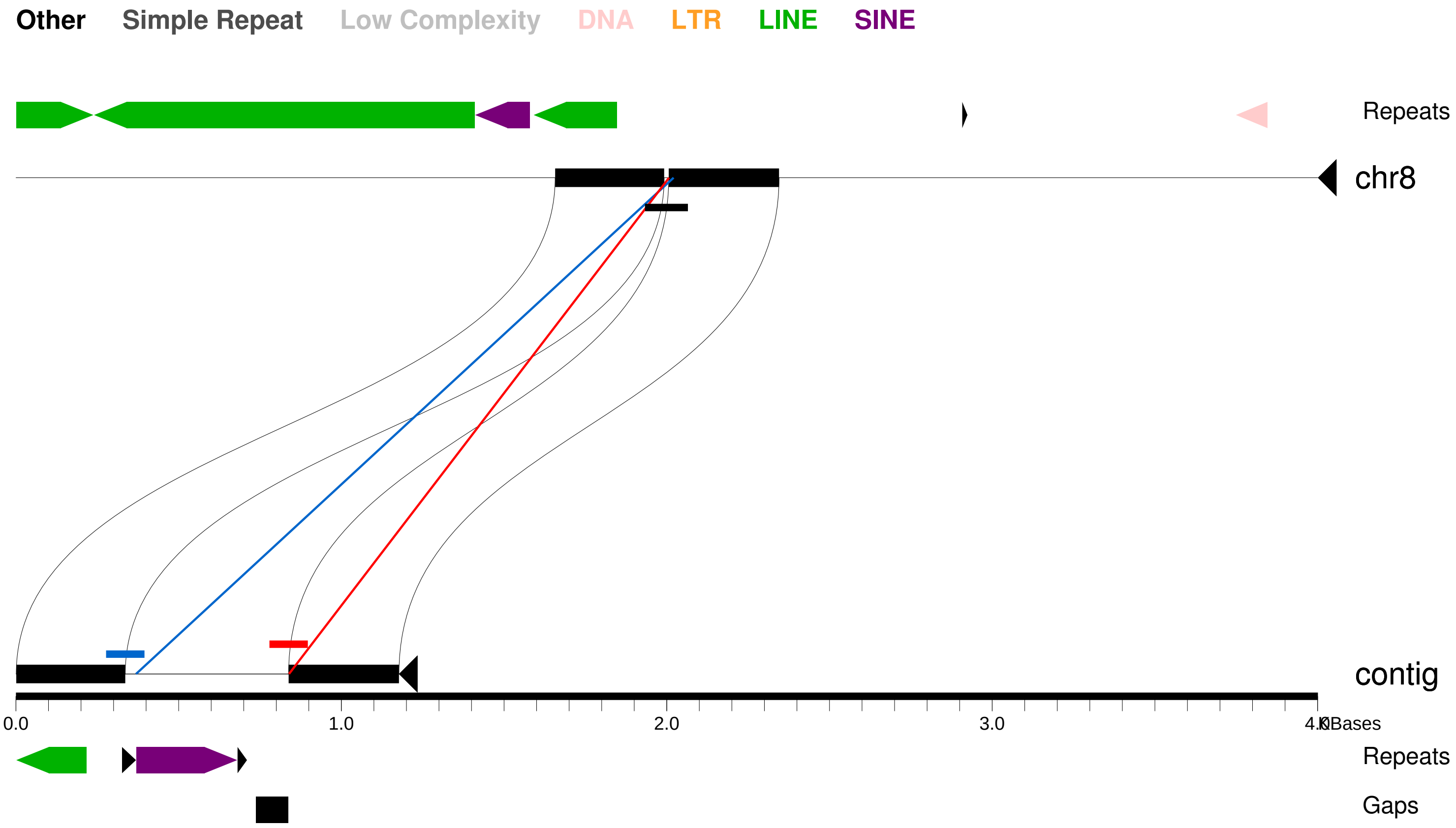

siteID: chr8_9807884 Scafftig6 + Breakpoint: chr8:9807891
chr8:9807731-9808061
Overlap Length: 11 INS size: 309 Genotyped: Yes

```
left    ------------GCAAGTAATGTGGCTATTTCTATCATTCCAGTGAAAAGTCATCCCTGACGATTGGGAG
chrom   TGTGTGTAATTTGCAAGTAATGTGGCTATTTCTATCATTCCAGTGAAAAGTCATCCCTGACGATTGGGAG
right   -GAATGGCGTGAACCCGGGAAGCGG--------AGC-TTGCAGTGAGCCGAGATTGC-GCCACT--GCAG
         2NN22NNN2NN1*11*11*1*1**11111111*1*1**1******111*11**11*1*1*11*11*1**

left    AGCTCATAGGCTATGGCCTTT-CAAAACATTCTTCACTAAGGGGAGAGCTGAGTCTGGTGAGGATGGAGG
chrom   AGCTCATAGGCTATGGCCTTT-CAAAACATTCTTCACTAAGGGGAGAGCTGAGTCTGGTGAGGATGGAGG
right   TCCGCAG----TCTGGCCTGGGCGACAGA------------GCGAGA-CTCCGTCTCAAAAAAAAA----
        11*1**11111*1******111*1*1*1*111111111111*1****1**11****1111*11*111111

left    GCTGCGGTTCAAGTCAGCTATAAAAGAGTCTG--------------------GGGCC-GGGCGCGG---T
chrom   GCTGCGGTTCAAGTCAGCTATAAAAGAGTCTGAACTCTCTCCTAGTCTCTTTGGTCCAGGGCGATGACTT
right   ----------AAAAAAAAAAAAAAAGAGTCTGAACTCTCTCCTAGTCTCTTTGGTCCAGGGCGATGACTT
        1111111111**111*111*1***********22222222222222222222**2**2*****22*222*

left    GGCTCACGCCTGT--AATCCCA----GCACTTTGGG-AG-------GCCGAGGCGGGTG--GATCATGAG
chrom   GTCTGGGAAATGTGGAATACAACAAAGGAGTCTGTGTAGACAACTTGCCTAGTTTGGTGCTGATTTTTAG
right   GTCTGGGAAATGTGGAATACAACAAAGGAGTCTGTGTAGACAACTTGCCTAGTTTGGTGCTGATTTTTAG
        *2**222222***22***2*2*2222*2*2*2**2*2**2222222***2**222****22***22*2**

left    GTCAG-GAGATCGAGACCATCCTGG--CTAACAAGGTGAAACCCCGTCTCTACTAAA
chrom   GTAAGTGAGAAGTAAAACCTGCAAGTGCTTCTTTCCTGAAGCA---TCTCAAGGA--
right   GTAAGTGAGAAGTAAAACCTGCAAGTGCTTCTTTCCTGAAGCA---TC---------
        **2**2****222*2*2*2*2*22*22**2222222****2*2222**11N1NN122
```

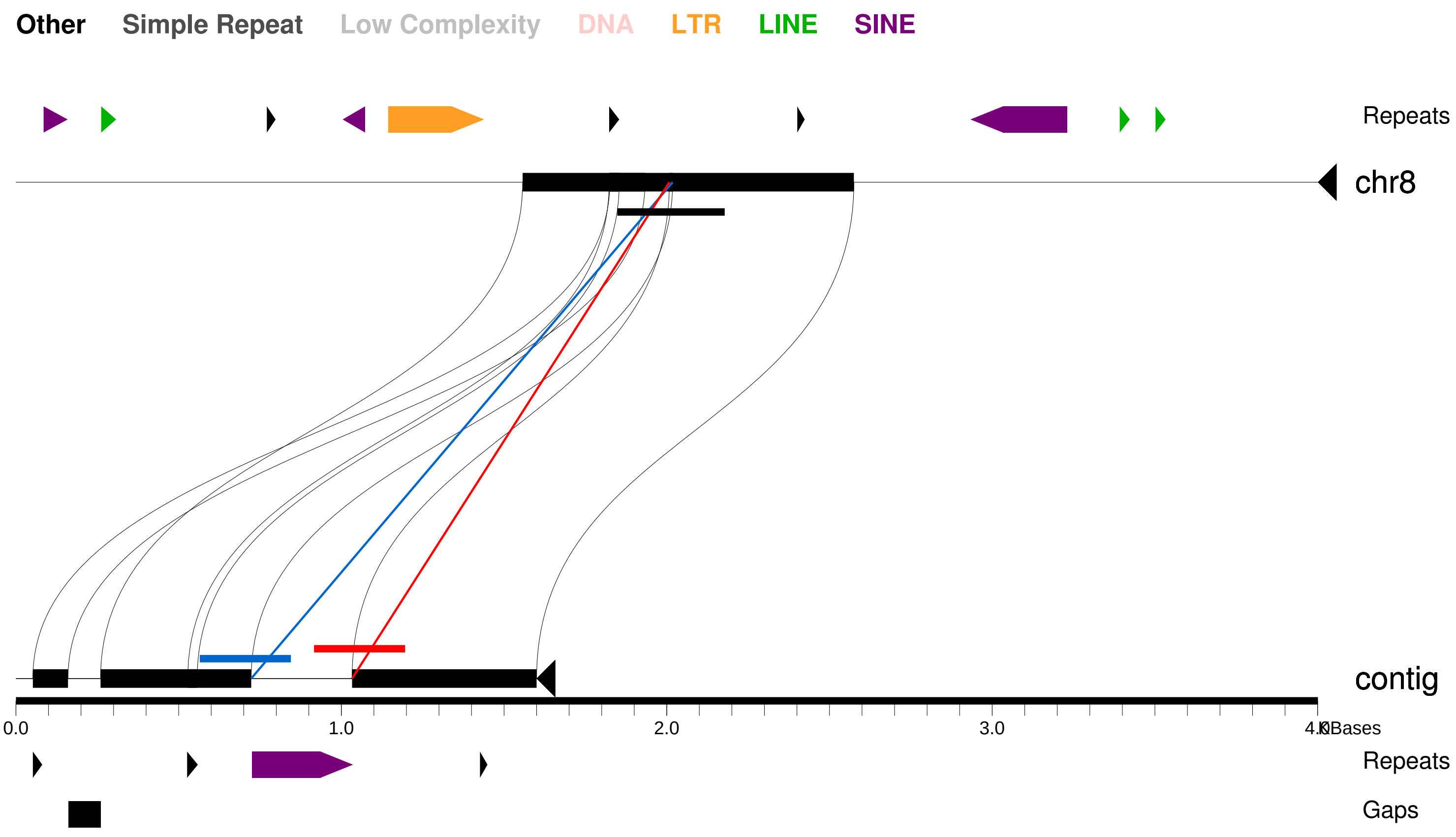

siteID: chr9_100124946 Scafftig2 + Breakpoint: chr9:100124918
chr9:100124758-100125123
Overlap Length: 46 INS size: 470 Genotyped: No

```
left   ---------------------------------------------CAGGCTTTTCCTCAGAAGGCACCAC
chrom  AGTCCATCCCCCCACACAAAGGCAGGAGATGACCTATGGCTTTCCCAGGCTTTTCCTCAGAAGGCACCAC
right  NNNNNNNNNNN-----------------------------------------------------------
       NNNNNNNNNNN                                  1111111111111111111111111

left   AATGCTCATGGCTCTGGGACTCCACGGGGCGCAGTGAGGGGTGAGCGCCATCCTTAGTTTGCTGGTTGTC
chrom  AATGCTCATGGCTCTGGGACTCCACGGGGCGCAGTGAGGGGTGAGCGCCATCCTTAGTTTGCTGGTTGTC
right  ----------------------------------------------------------------------
       1111111111111111111111111111111111111111111111111111111111111111111111

left   CATGGTGCTGATGGATCTCAGCCGTGCCACCTTCCGCACACGGTCATGGTTAGAAAGACGGGGCAG--GC
chrom  CATGGTGCTGATGGATCTCAGCCGTGCCACCTTCCACACACGGTCATGGTTAGAAAGACGGGGCAGATGG
right  --------------------GCCGGGCCCCCTGCCACACACGGTCATGGTTAGAAAGACGGGGCAGATGG
       11111111111111111111****1***1***1**2*****************************22*2

left   CGGGCGCGGTGGCTCACGCCTGTAATCCCAGCACT--TTGGGAGGCCGAGGCGGGCGGATCACGAGG--T
chrom  CTTTCCTGGAGAGACATCACAG-AGCCACAGAAAGAGTTCTG-GGCCTTGGCGCCCTGCCCACCAAGCCT
right  CTTTCCTGGAGAGACATCACAG-AGC--------------------------------------------
       *222*22**2*222**222*2*2*221N111N1NN  11NN121111NN1111NN1N1NN111N1N1  1

left   CAG-GAGATCG--AGAC------------CATCCTGGCTAAC------AAGGTG--------AAACCCCG
chrom  CAGTGGGGTTGTTAGACTCAAAGTTTACCCCACATGCATATTTTATGGAAGGTGCATATCAGAAGCCCCA
right  ----------------------------------------------------------------------
       111 1N1N1N1  1111            1NN1N11NN11NN      111111        11N1111N

left   TCTCT-ACTAAA------
chrom  TGGCTGAGTAGACTCACT
right  ------------------
       1NN11 1N11N1
```

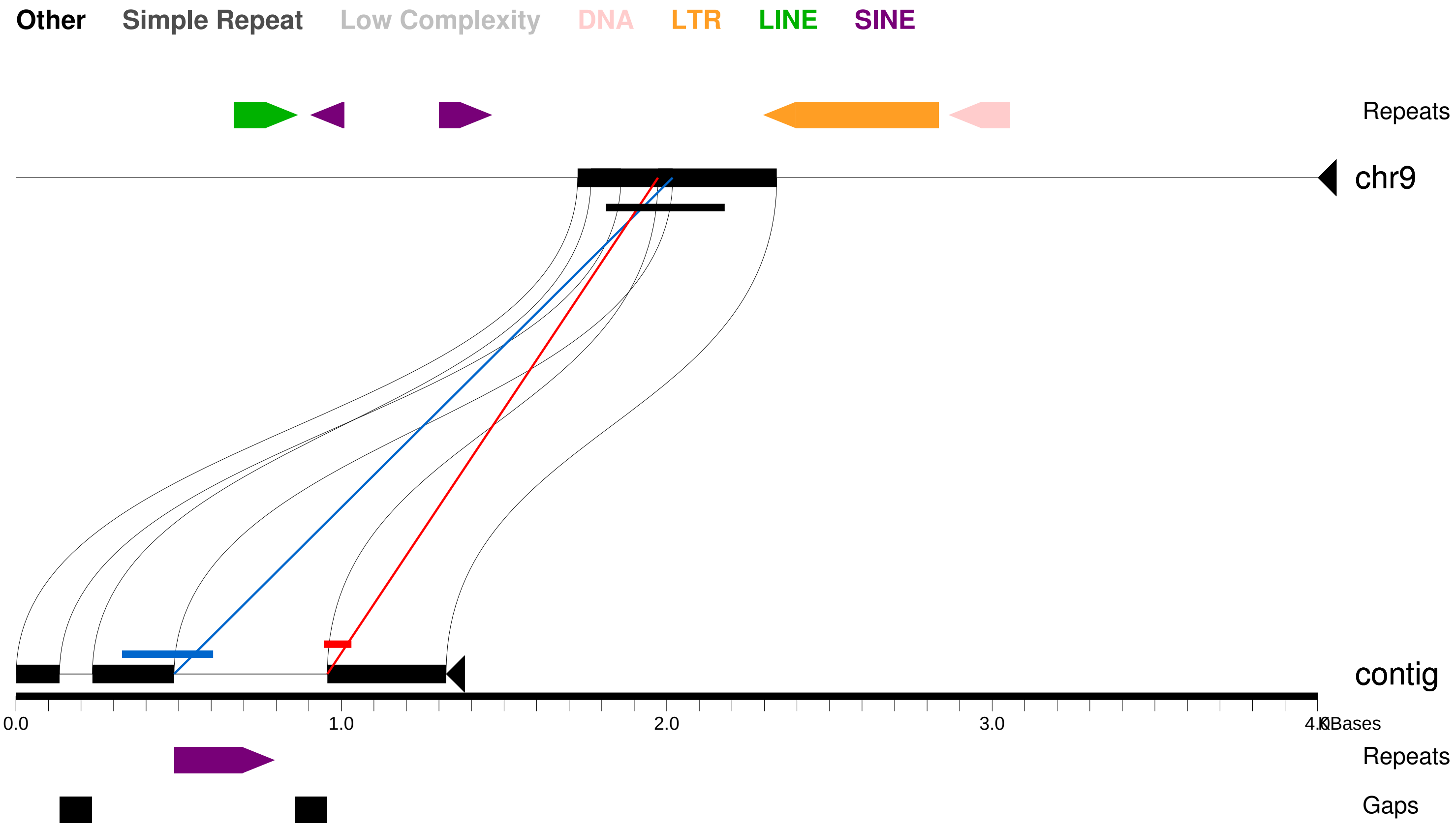

siteID: chr9_101737247 Scafftig1 - Breakpoint: chr9:101737272
chr9:101737112-101737446
Overlap Length: 15 INS size: 467 Genotyped: No

```
left   ---------------------------------------------------------------------------
chrom  GTGTGTGGGAATGGTAGAAACCCAGGAGGGGCACCTTCAGCCTTTCCCAGCACTCGGTGCAGAGAAACAG
right  TT---------TAGTAGAGAC---GGGGTTTCACCGTG----TTAGCCAGGAT--GGTCTCGAT---CTC
       N2         2N22222N22   22N2NNN2222N2N    22NN2222N2N  222NNN22N    2NN

left   -----------------------------CTAGGCCTGGCTTGGCCAAACTCACTGCATGGCCT-CAGAC
chrom  CAGAGGAGGGAAAGGAGGCGTTATGGGTTCTAGGCCTGGCTTGGCCAAACTCACTGCATGGCCT-CAGAC
right  CTGACCT-----------CGTGATCCG-------CCCGTCTCGGCCTCCCAAAGTGCTGGGATTACAGGC
       2N22NNN           222N22NN2  11111**1*1**1****111*11*1***11**11*1***1*

left   CTGCTCTCTTGGGTCCCTTAACAGACCTTTCGCTTT-----TTTTTTTNNNNNNNNNNN-----------
chrom  CTGCTCTCTTGGGTCCCTTAACAGACCTTTCGCTTTGAGTCTGTTGTTGGGATCCCGGTTCACAGCGCAG
right  GTGAGCCACCGCG-CCCGGCCCAGACCTTTCGCTTTGAGTCTGTTGTTGGGATCCCGGTTCACAGCGCAG
       1**11*1111*1*1***1111***************22222*2**2**222222222222222222222

left   ---------------------------------------------------------------------------
chrom  GTGGAAAGCCTTCCTTGCAGTTGGTGGAAGCGCAGTTCCCGTAGAGACCGCCAGGAGCCGCTGCCGCGCA
right  GTGGAAAGCCTTCCTTGCAGTTGGTGGAAGCGCAGTTCCCGTAGAGCCCGCCAGGAGCCGCTGCCGCGCA
       2222222222222222222222222222222222222222222222N22222222222222222222222

left   -----------------------------------------------------------
chrom  CGGTCCCGGGGCGTCCTCAGCCTCCCTCGCGCCTCCGTCCGGACATATCCGCAGCG
right  CGGTCCCGGGGCGTCCTCAGCNNNNNNNNNNNNNNNNNNNN---------------
       222222222222222222222NNNNNNNNNNNNNNNNNNNN
```

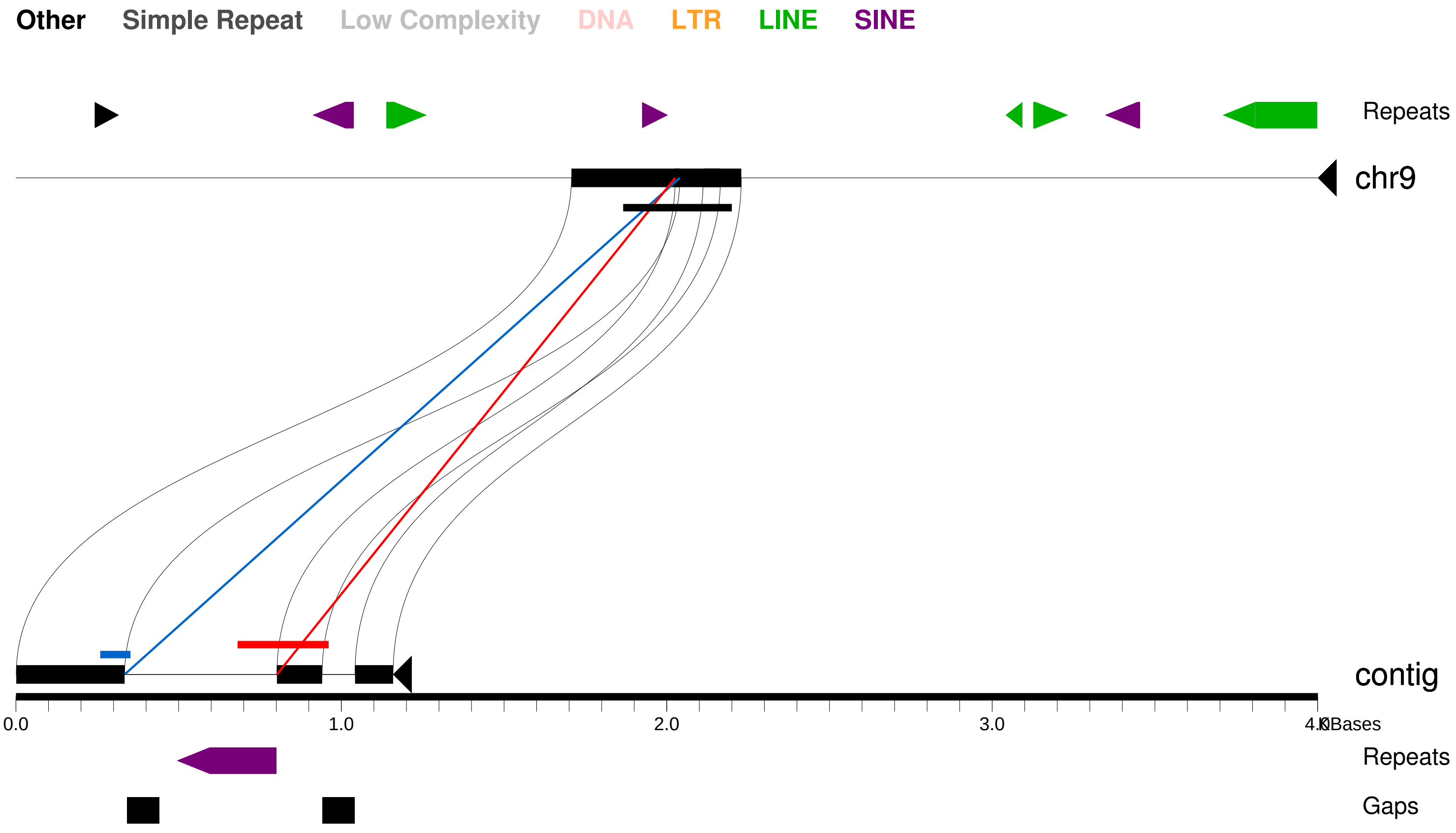

siteID: chr9_102849294 Scafftig3 + Breakpoint: chr9:102849297
chr9:102849251-102849356
Overlap Length: 14 INS size: 282 Genotyped: No

```
left    CTCTGAACGTGAAGTAGGAATTACATTATCCCTATTTTATAAATAA-------------AAAAAAAAACT
chrom   CTCTGAACGTGAAGTAGGAATTACATTATCCCTATTTTATAAATAA-------------AAAAAAAAACT
right   NNNNNNNNNNNNNNNNNNNNNNNNNNNNNNNNNNNNNNNNNNNNNNNNNNNNNNNNNNNAAAAAAAAACT
        11111111111111111111111111111111111111111111111111111111111***********

left    TTGGCCGGGCGCGGTGGCTCACGCCTGTAATCCC-AGCACTTTGGGAGGCCGAGGCGGGCGGA
chrom   TTG--CAGTCTC--TGGCATA---CAGTAAAAGGGAGAATATTTATTAAATGAAT-------A
right   TTG--CAGTCTC--TGGCATA---CAGTAAAAGGGAGAATATTTATTAAATGAAT-------A
        ***22*2*2*2*22****22*222*2****22222**2*22**22222222**222222222*
```

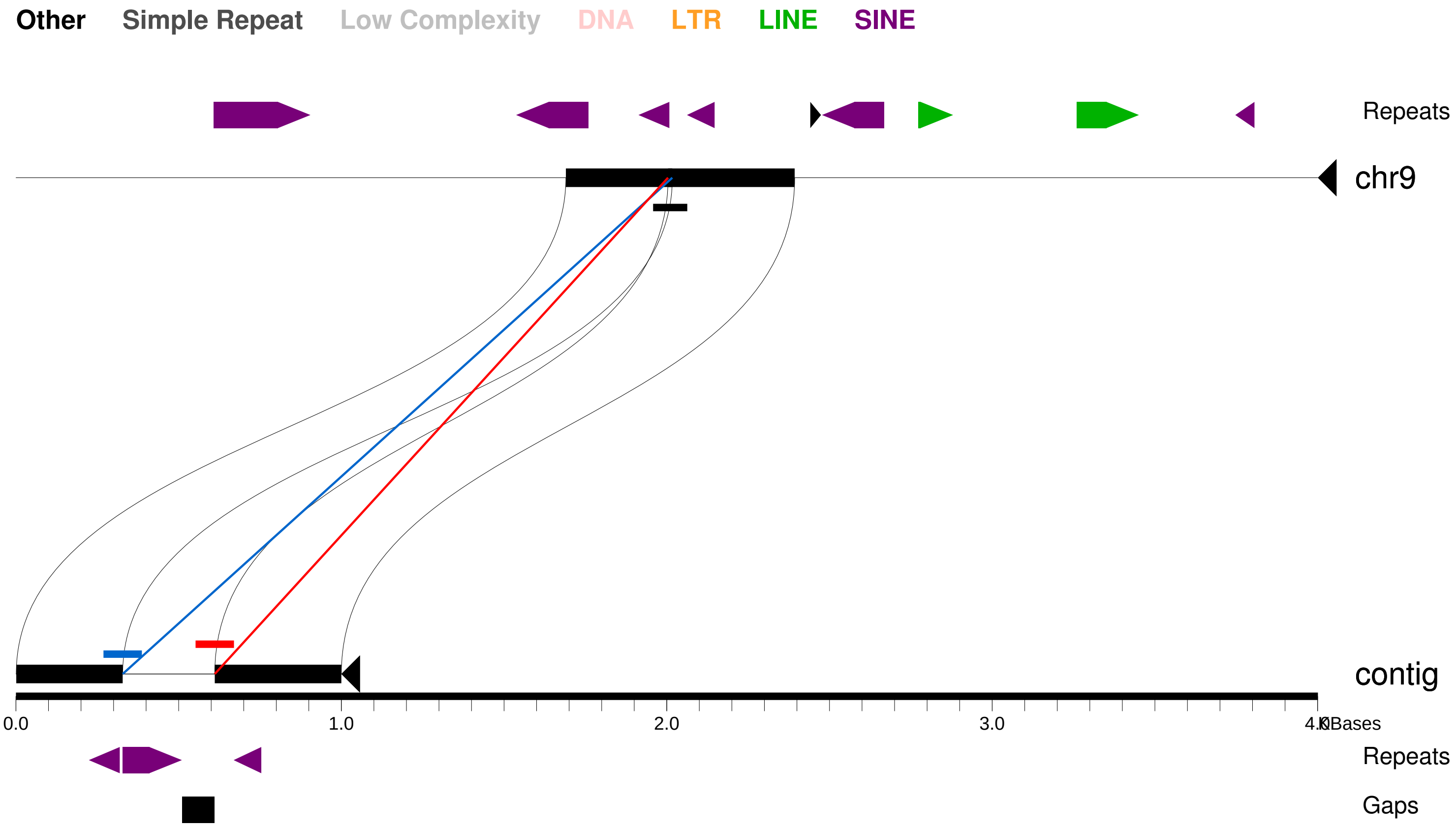

siteID: chr9_103223386 Scafftig1 - Breakpoint: chr9:103223389
chr9:103223349-103223448
Overlap Length: 17 INS size: 402 Genotyped: No

```
left    ATCTCTAGTCTTA---------AGTGCTAG-ACTCAAAGGGTTTCTGACT---------CCCAGGAAAGC
chrom   ATCTCTAGTCTTA---------AGTGCTAG-ACTCAAAGGGTTTCTGACT---------CCCAGGAAAGC
right   TCCACCCGCCTCGGCCTCCCAGAGTGCTGGGATTACAAGCGTGAGCCACCGCGCCCGGCCCCAGGAAAGC
        11*1*11*1**11111111111******1*1*1*11***1**11111**1111111111***********

left    ATTCTTTTTNNNNNNNNNNNNNNNNNNNNNNNNNNNNNNNNNNNNNNNNNNNNNNNNNNNNNNNNNNNN
chrom   ATTCTTATTATTTTGTGGCTTGCTTTACCACTTTACCATATCTTTACTT-------------------
right   ATTCTTATTATTTTGTGGCTTGCTTTACCACTTTACCATATCTTTACTT-------------------
        ******2**222222222222222222222222222222222222222222222222222222222222
```

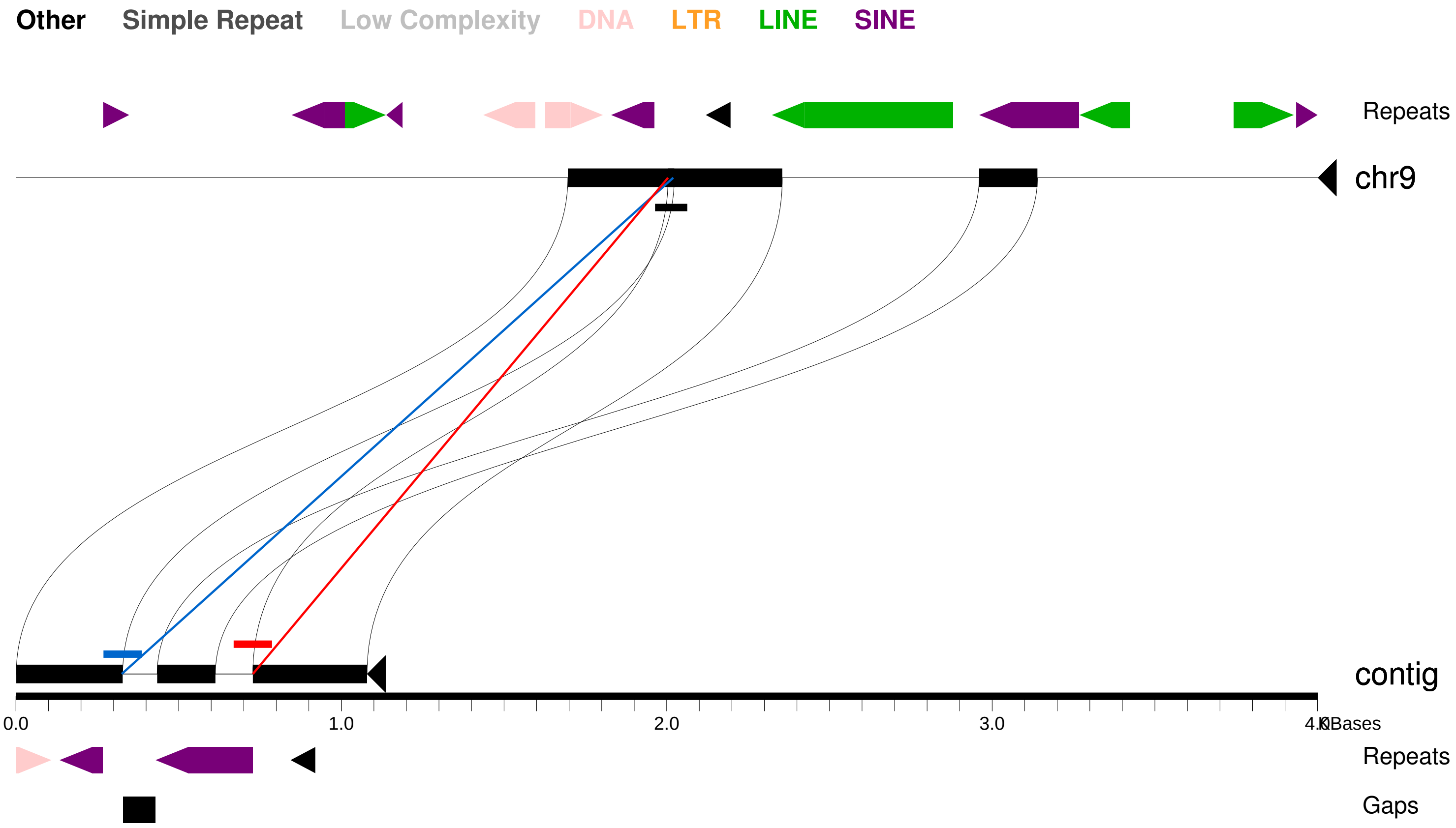

siteID: chr9_104565396 Scafftig1 + Breakpoint: chr9:104565397
chr9:104565237-104565570
Overlap Length: 14 INS size: 257 Genotyped: No

```
left   NNNNNNNNNNNNNNNNNNNNNNNNNNNNNNNNNNNNNNNNNNNNNNNNNNNNNNNNNNNNNNNNNNNNNNNN
chrom  GCTGACCCCATCTGATGGCACTGGCACTGGTTCCTGCCCTCAAGATGCAGTCTTCTGCCTGCTGCCTGTA
right  -CTGA-----------GGCAGGAGAA-TGG--CGTGAACCCGGGAGGCGGAGCT-TGCAGTGAGCCGAGA
       N2222NNNNNNNNNNN2222NNN2N2N222NN2N22NN2N2NN22N22N2NNN2N222NNNNN222NNN2

left   NNNNNNNNNN--------------GTGCCCTACTGCTGCAAGAGCTGTGGCAAAAGCAGCTGGCTAGGCA
chrom  TTGCCCTTCACTGCAAGCAGGACTGTGCCCTACTGCTGCAAGAGCTGTGGCAAAAGCAGCTGGCTAGGCA
right  T--CCCGCCACTGCACTCCAGCCTGGGC---------GACAGAGCG--------AGACTCCGTCTCAAAA
       2NN222NN2222222NN2NN2N22*1**111111111*11*****111111111**111*1*1**1111*

left   GATGCAAAAATAGTGGGGTTAAAATATGTCATTAGGGAGGCCGAGGC-----------------GGGCGG
chrom  GATGCAAAAATAGTGGGGTTAAAATATGTCATTAAAATGGTCAACACCAAAATAAGAAACTTCTGGGTAA
right  AAAAAAAAAAAA---------AAAATATGTCATTAAAATGGTCAACACCAAAATAAGAAACTTCTGGGTAA
       1*111*****1*11111111**************2222**2*2*22*222222222222222222***222

left   A-TCACGAGGTCAGGAGATCG---AG-ACCATCCCGGCTAAAACGGTGA-AACCCCGTCTCTAC-TAAAA
chrom  AGTCAGACCCTGAGGAAATGGGTCAGCATGATCCAAAGAGATAGGTCGACAACTCAGAGTTTGCCTTGGA
right  AGTCAGACCCTGAGGAAATGGGTCAGCATGATCCAAAGAGATAGGTCGACAACTCAGAGTTTGCCTTGGA
       *2***22222*2****2**2*222**2*22****222222*2*2*22**2***2*2*22*2*2*2*222*

left   ATACAAAA---------AATTAGCCGGGCGTAGTGGCGGGCGCCTGTA------
chrom  ATAGAAGAGGATTTTGGAGGTGGACAGGTGAAAAGGAGAGGGAAGAAAGAGAGA
right  ATAGAAGAGGATTTTGGAGGTGGACAGGTGAAAAGGAGAGGGAA----------
       ***2**2*222222222*22*2*2*2**2*2*22**2*2*2*22NNN1
```

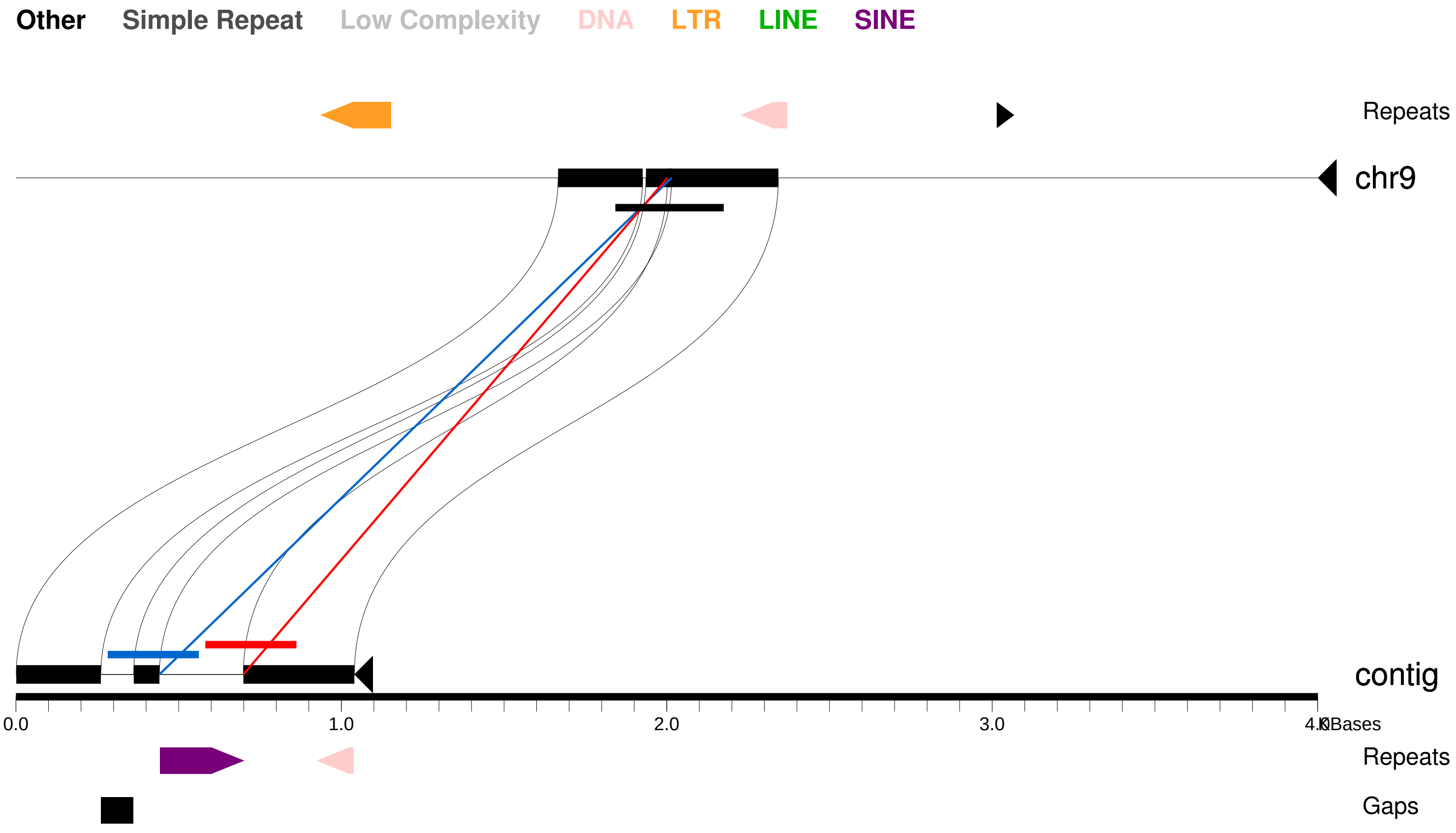

siteID: chr9_105450614 Scafftig7 + Breakpoint: chr9:105450605
chr9:105450445-105450779
Overlap Length: 15 INS size: 295 Genotyped: Yes

```
left   --------------AAAACAGTATGGACACATACATGCGCATGCACACACACACACACTCTCTCTCTC
chrom  TATGGTTAATGACAAAAACAGTATGGACACATACATGCGCATGCACACACACACACACTCTCTCTCTCTC
right  -------AGGCAGGAGAATGGCGTGAACCCGGG-AGGCGGA-GCTTGCAGTGAGCCGAGATCCCGC-CAC
              2NNN2NN*1**11*11**1**1*1111*1***1*1**111**111*11*11N1**1*1*1*1*

left   TCTCTCTCCCCCCAAATGTCTTTTGGATTTAAAAGAAAGTATCTGAATATTGAACATTCAGCCATTATAT
chrom  TCTCTCTCCCCCCAAATGTCTTTTGGATTTAAAAGAAAGTATCTGAATATTGAACATTCAGCCATTATAT
right  TG-CACTCCAGCC----------TGGGCGACAGAGCGAGACTCCG--TCTCAAAAAAAAA----------
       *11*1****11**1111111111***11111*1**11**11**1*11*1*11**1*111*1111111111

left   ACAGTCATAAAACCTCTTTTAAAAAAAAACAAGCAGGCCGGGCGCGGTGGCTCACGCCTGTAATCCCAGCA
chrom  ACAGTCATAAAACCTCTTTTAAAAAAAAACAAGCAGAATGACTCTAAAGGCTGAATCTCTTCTTAGTAGAC
right  ------AAAAAA--------AAAAAAAAACAAGCAGAATGACTCTAAAGGCTGAATCTCTTCTTAGTAGAC
       111111*1****11111111****************222*22222222****2*22*222*22*222**22

left   CTTTGGGAGGCCG-----------AGGCGGGCGGATCACGAGGT--CAGGAGATCGAGACCATCCCGGCT
chrom  TTTTAAAAAGTAATATACATATAAAAAGATGCAAAGCATAAAGTTTCAGCTCATCAAATTTATACCTAGT
right  TTTTAAAAAGTAATATACATATAAAAAGATGCAAAGCATAAAGTTTCAGCTCATCAAATTTATACCTAGT
       2***222*2*22222222222222*22222**22*2**22*2**22***222***2*2222**2**222*

left   AAAA----CGGTGAAAC-CCCGTCTCTACTAA----------------------A
chrom  AAACAAACCTATGTAACTCCCATCCACATGAAGAAAGAGAATATGATAGTCACCA
right  AAACAAACCTATGTAACTCCCATCCACATGAAGAAAGAGAATATGATA-------
       ***22222*22**2***2***2**222*22**2222222222222222      1
```

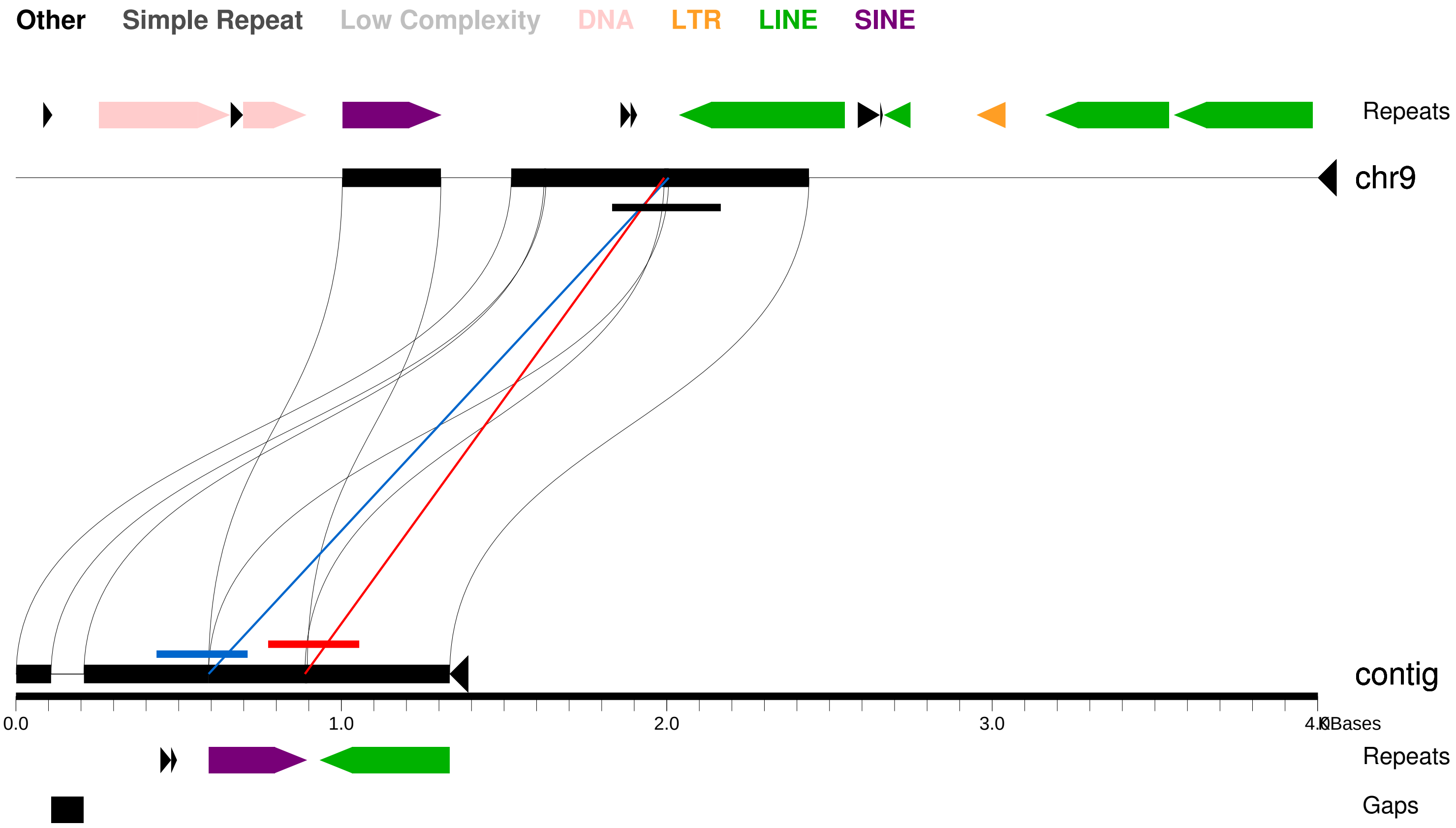

siteID: chr9_106244189 Scafftig4 - Breakpoint: chr9:106244169
chr9:106244009-106244342
Overlap Length: 14 INS size: 438 Genotyped: No

```
left   --------------------------------------------CTAGAGACCTAGGTTTACTCATCTTT
chrom  AAATCTAAGAGTAAGAATAAGAGATATCACTGAGATATATGAACCTAGAGACCTAGGCTTACTCATCTTT
right  ---TTTAGTAG-----AGACGGGGTTTCACCTTGTTAG-----CCAGGA----TGGTCTCGATC-TCCTG
          2N22NN22     2N2N2N2N2N2222NNN2N22N     2*11**1111*1*12*111**1**1*1

left   ACATCTCAATACCTAACATAATTCTAGACACATAGAAAGTGCTCAAAAAAGATCCACTGAATGAATGAAT
chrom  ACATCTCAATACCTAACATAATTCTAGACACATAGAAAGTGCTCAAAAAAGATCCACTGAATGAATGAAT
right  AC--CTCATGATCCACCCGC---CTCGGC-CTCCCAAAGTGCTGG-----GATT-ACAGGCGTAAGCCAC
       **11****11*1*1*1*111111**1*1*1*1111********1111111***11**1*1111**111*1

left   CGGGAATGTTTTATAGCATACTGGATGCACTTCT-----------------------TTTTTNNNNNNNN
chrom  CGGGAATGTTTTATAGCATACTGGATGCACTTCTAACATGTTAGTATAATGATGTTATTTTTATTTTTGG
right  CGCGCCCG-------GCC--CTGGATGCACTTCTAACATGTTAGTATAATGATGTTATTTTTATTTTTGG
       **1*111*1111111**111**************22222222222222222222222*****22222222

left   NNN-------------------------------------------------------------------
chrom  GTGGGAGATAGGGAGAGTAGGTGCTTCATGTTAAAAAAAAAAA--TCACATTTGTCCAGCCCTGAGTGAT
right  GTGGGAGATAGGGAGAGTAGGTGCTTCATGTTAAAAAAAAAAAAATCACATTTGTCCAGCCCTGAGTGAT
       2222222222222222222222222222222222222222222112222222222222222222222222

left   --------------------------------------------------------
chrom  ATAGTTTTAGTGTTTAGTTTTATTTTGTTTTGCCATATTTCACATAAGTTTAAAAT
right  ATAGTTTTAGTGTTTAGTTTTATTTTGTTTTGCCATATTT----------------
       2222222222222222222222222222222222222222
```

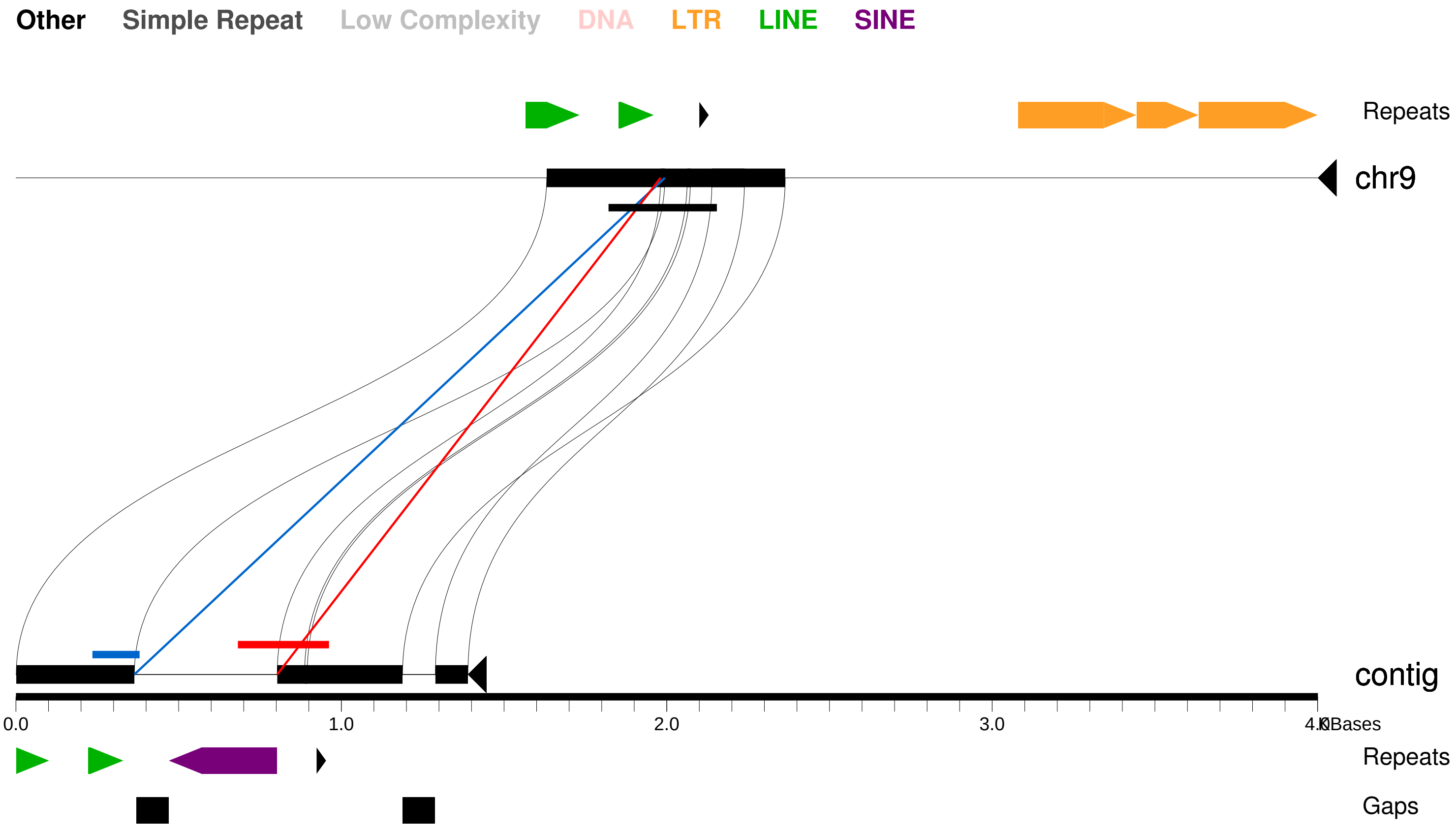

siteID: chr9_111949736 Scafftig1 + Breakpoint: chr9:111949724
chr9:111949687-111949783
Overlap Length: 8 INS size: 310 Genotyped: Yes

```
left    T-----GCATTTGC-----AAAGTCTTAG---TCCATGGTTCATTTT---------GTTGACTGTTTTTT
chrom   T-----GCATTTGC-----AAAGTCTTAG---TCCATGGTTCATTTT---------GTTGACTGTTTCTA
right   CCACCCGCCTCGGCCTCCCAAAGTGCTGGGATTACAGGCGTGAGCCACCGCGCCCGGCCGACTGTTTCTA
        111111**1*11**11111*****11*1*111*1**1*11*1*1111111111111*11********2*2

left    TTTTTTTTTTTTTTTTTTTGAGACGGAGTCTCGCTCTGTCGCCCAGGCCGGACTGCGGACTGCAGTGGCG
chrom   AATCTAGTTTTTAGTCA-----AGTGAATCCATCTGAGTCACC-----------------TGCAGTTAAC
right   AATCTAGTTTTTAGTCA-----AGTGAATCCATCTGAGTCACC-----------------TGCAGTTAAC
        22*2*22*****22*2222222*22**2**222**22***2**22222222222222222******2222

left    C
chrom   T
right   T
        2
```

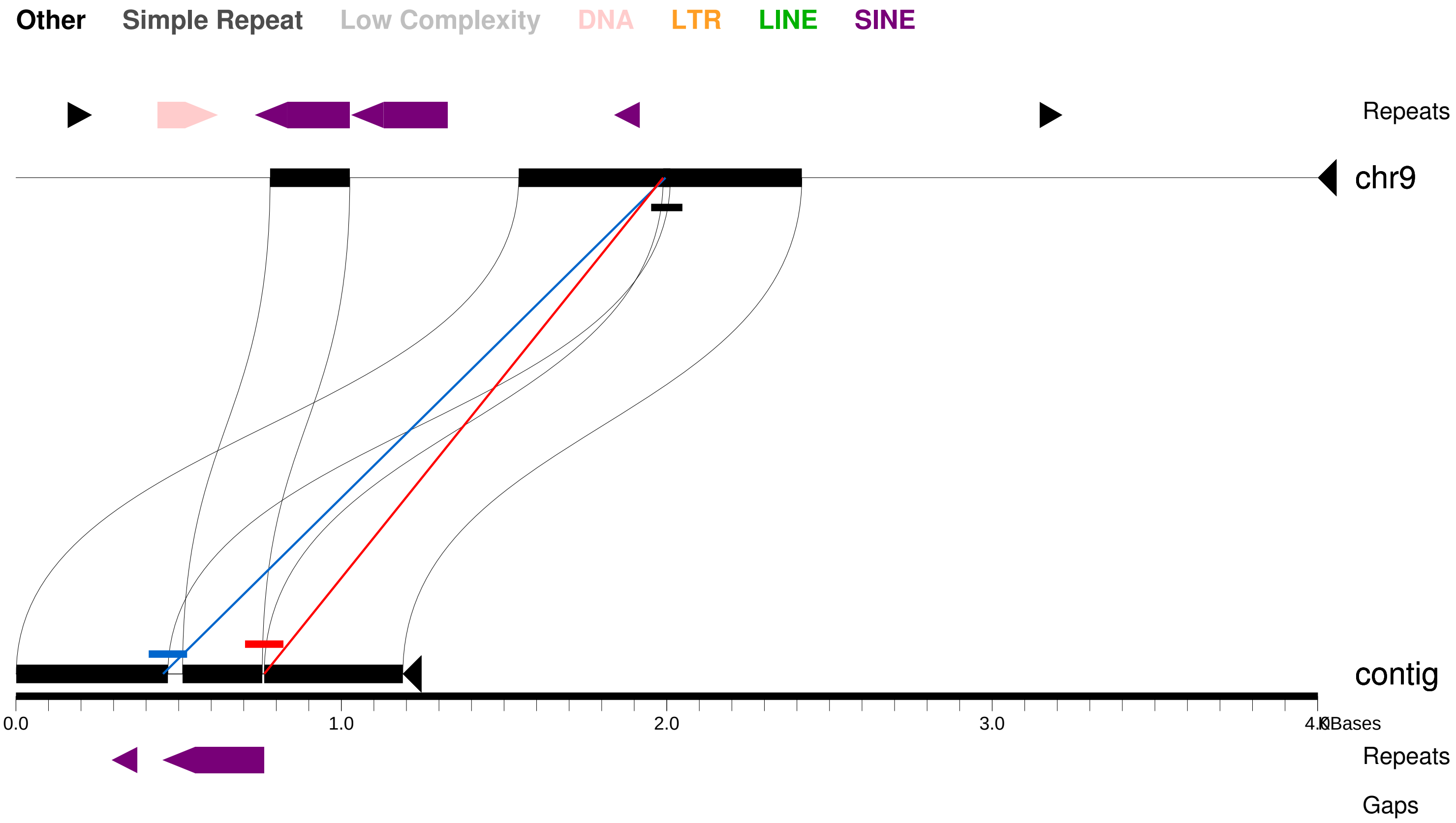

siteID: chr9_112261398 Scafftig7 - Breakpoint: chr9:112261384
chr9:112261334-112261432
Overlap Length: 10 INS size: 305 Genotyped: Yes

```
left   TATTT-ATTGAGCACCTATTCTGGTTTTTCTCGCGTGGGG--------------------AACAAATAGA
chrom  TATTT-ATTGAGCACCTATTCTGGTTTTTCTCGCGTGGGG--------------------AACAAATAGA
right  CCCGCCACTGCACTCC-AGCCTGGGCGACAGAGCGAGACTCCGTCTCAAAAAAAAAAAAAAAAAAAAAAA
       111111*1**11*1**1*11****11111111***1*11111111111111111111111**1***1*1*

left   CAAAAATTCTGGGCCGGGCGCGGTGGCTCACGCCTGTAATCCCAGCACTTTGGGAGGCCGAGGCGGG---
chrom  CAAAAATTCTGTCCCC----CAGTGG-----------AGT--------TTGGGGAAAGAGGGACAGGTAA
right  AAAAAATTCTGTCCCC----CAGTGG-----------AGT--------TTGGGGAAAGAGGGACAGGTAA
       1**********22**22222*2****22222222222*2*22222222**2****2222*2*2*2**222

left   CGG
chrom  CAG
right  CAG
       *2*
```

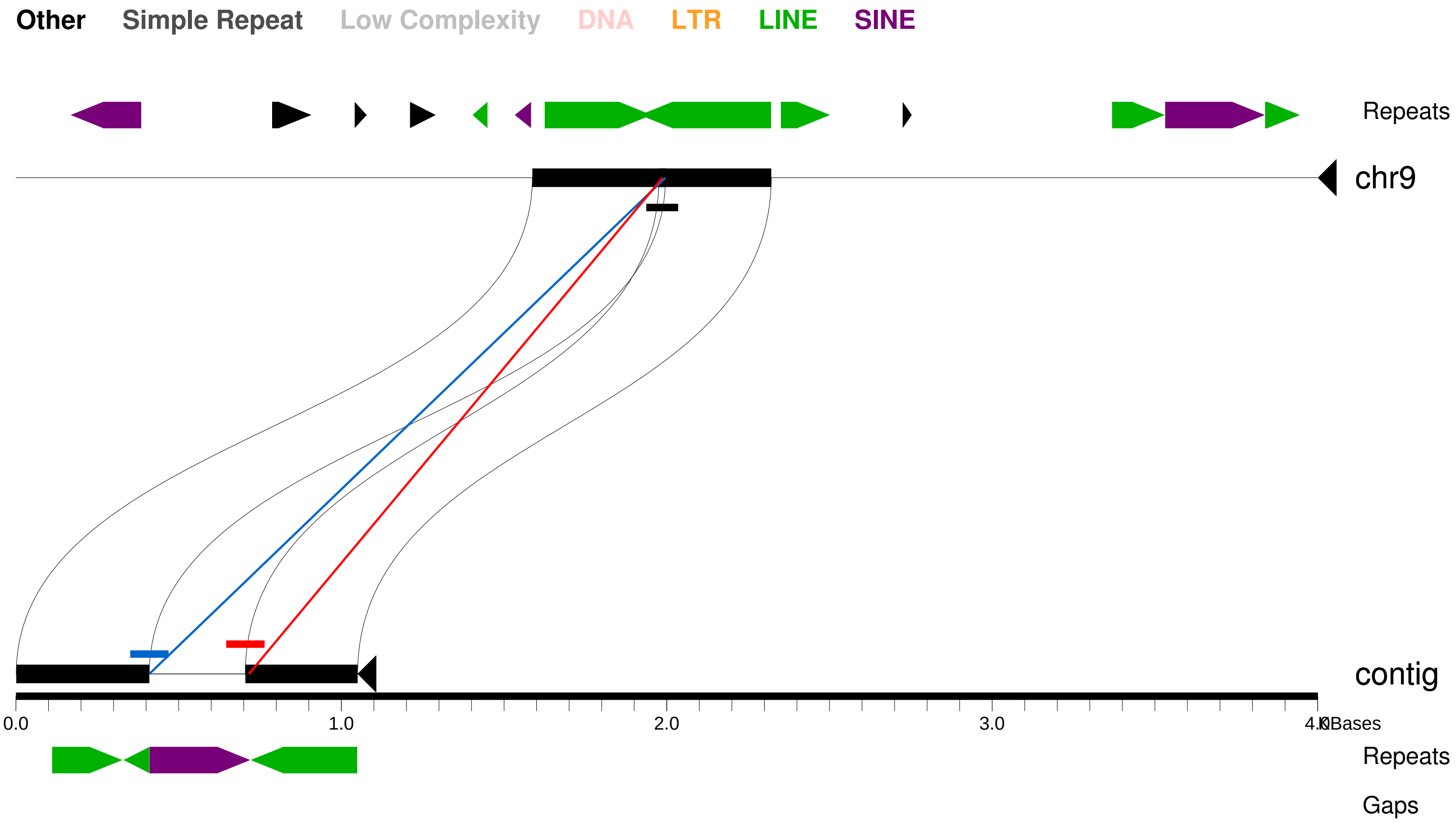

siteID: chr9_11329333 Scafftig1 - Breakpoint: chr9:11329314
chr9:11329154-11329486
Overlap Length: 13 INS size: 316 Genotyped: Yes

```
left   ----------AAAAATATTTTAGACTTCTTTTGCTATGCTTAGTGAATTTTTGAGGCTATCTAAATGACT
chrom  CATACCTGCAAAAAAATATTTTAGACTTCTTTTGCTATGCTTAGTGAATTTTTGAGGCTATCTAAATGACT
right  TTTA---GTAGAGACGGGGTTTCACCTTGTTAGCCAGGATG-----------GTCTCGATCTCCTGACCT
       NN22   2N21*1*11111**11**1*11**1**1*1*1*111111111111*111*1****111111**

left   CATGATATTTAGATCTTTGCTATGGATTATATTTTTGTCACTATTATGTGTCTTTTTGTTTTAATTATTT
chrom  CATGATATTTAGATCTTTGCTATGGATTATATTTTTGTCACTATTATGTGTCTTTTTGTTTTAATTATTT
right  CATGATCCACCCGCCTCGGCC-------------TCCCAAAGTGCTGGGATTACAGGCGTGAGCCACCG
       ******11111111**11**111111111111111*11**111*11**1*11*1111*11*1*111*111

left   CACTTTTAATTCAATGTTGTCAGATATTAATTTTTT---------TTTTTTTTTT---------TTTTT
chrom  CACTTTTAATTCAATGTTGTCAGATATTAATTTCTTAATCACTGCTTTCTCATTTTGCATTTGCCTTTCT
right  CGCCCGGCC-----------CAGATATTAATTTCTTAATCACTGCTTTCTCATTTTGCATTTGCCTTTCT
       *1*111111111111111111************2**222222222***2*22****222222222***2*

left   TT--------TTTTTTTGAGACGGAGTCTCGCTC-TGTCGCCC---AGGTCG---GACTGC---GGACTG
chrom  CTCTTCTCCATTTTCTTGTTAGAGTAAATTCCTCATGAAGCCATAAATGTAGCTTGAAAGAAAGGGAGTG
right  CTCTTCTCCATTTTCTTGTTAGAGTAAATTCCTCATGAAGCCATAAATGTAGCTTGAAAGAAAGGGAGTG
       2*22222222****2***22*22*2222*22***2**22***2222*2**2*222**22*2222***2**

left   CAGT-GGCGCAAT--CTCGGCTCACTGCAAGCTCC-GCTTCCCGGGTTCAC---
chrom  AAGTAGGAGAAATGACTAGGAAG-CTTCAGCCAACTGCTTATGTGCTTCGCTTC
right  AAGTAGGAGAAATGACTAGGAAG-CTTCAGCCAACTGCTTA-------------
       2***2**2*2***22**2**2222**2**22*22*2****2NNN1N111N1
```

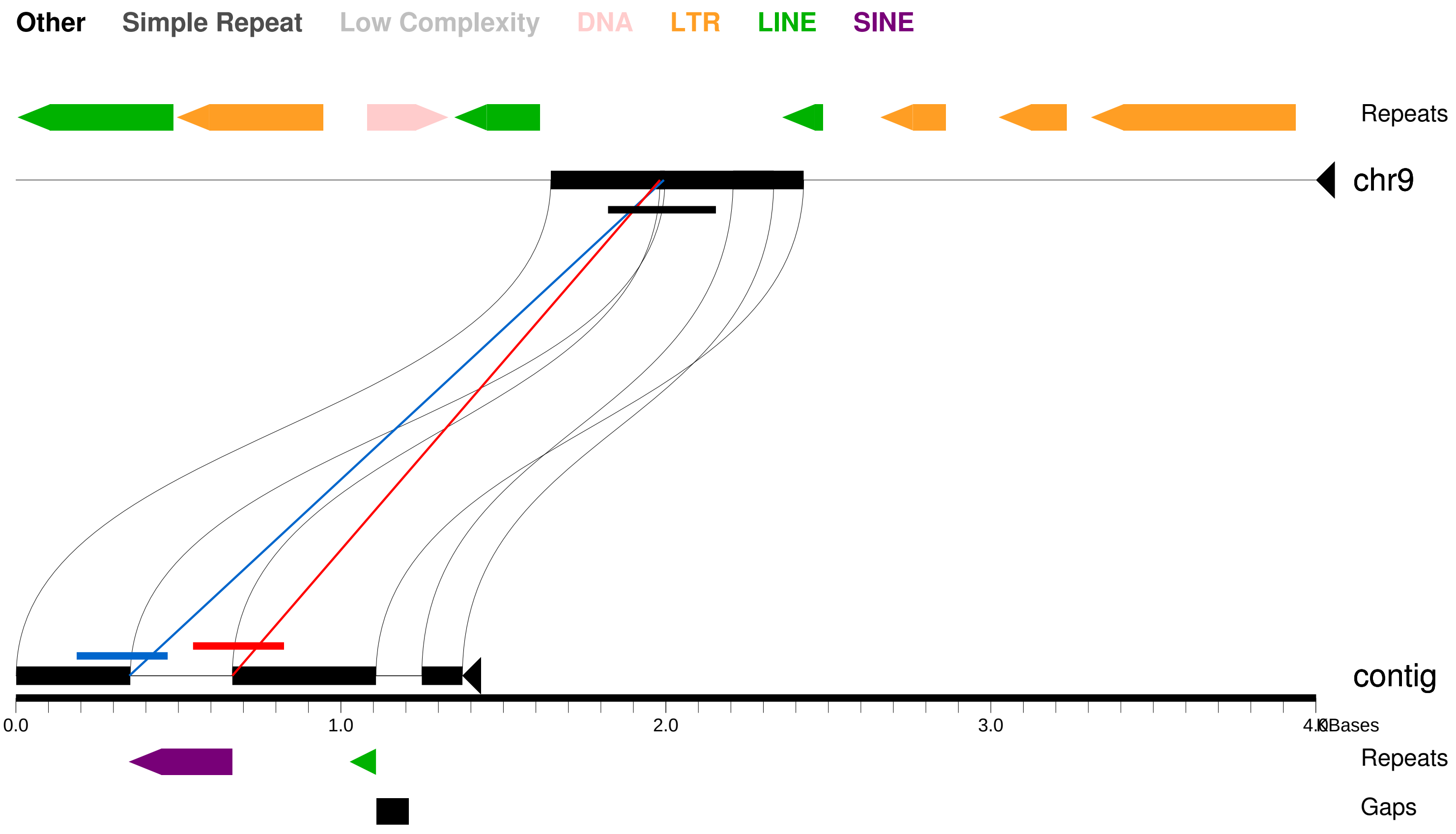

siteID: chr9_113571232 Scafftig4 + Breakpoint: chr9:113571236
chr9:113571191-113571394
Overlap Length: 15 INS size: 295 Genotyped: Yes

```
left    TTAGAGCTGTGATTAAAATTAGAAGTGTTGGCATGACACTGCTTTCTGACTTTCTTTTTTT-----TTTT-
chrom   TTAGAGCTGTGATTAAAATTAGAAGTGTTGGCATGACACTGCTTTCTGACTTTCTTTTTTTAGCCATGTTG
right   AAAGTGCTGGGATTACA----GGCGTGA-GCCACCGCGCCCGGCCCTGACTTTCTTTTTTTAGCCATGTTG
        11**1****1*****1*1111*11***11*1**111*1*111111****************22222*2**2

left    ---------------------------TTTTTTTTT---TTGAGACGG------AGTCTCGCTG------
chrom   ACACCGTGGTGGTTAGTGTGATGCTCCTTCTTATTTCAATTGATGCTTTACTCAAGTATGGATGAAACAT
right   ACACCGTGGTGGTTAGTGTGATGCTCCTTCTTATTTCAATTGATGCTTTACTCAAGTATGGATGAAACAT
        222222222222222222222222222**2**2***222****22*22222222***2*2*2**222222

left    ---------TCGCC----------CAGGCTGGAGT-----------------GCAGTGGCGC-A
chrom   AATTTAAAGTCACCTCTTCTTCTGCTATCTTGATTTTTAAAATTATTTGGATGCATTATATTTA
right   AA-----AGTCACCTCTTCTTCTGCTATCTTGATTTTTAAAATTATTTGGATGCATTATATTTA
        22     22**2**2222222222*222**2**2*22222222222222222***2*222222*
```

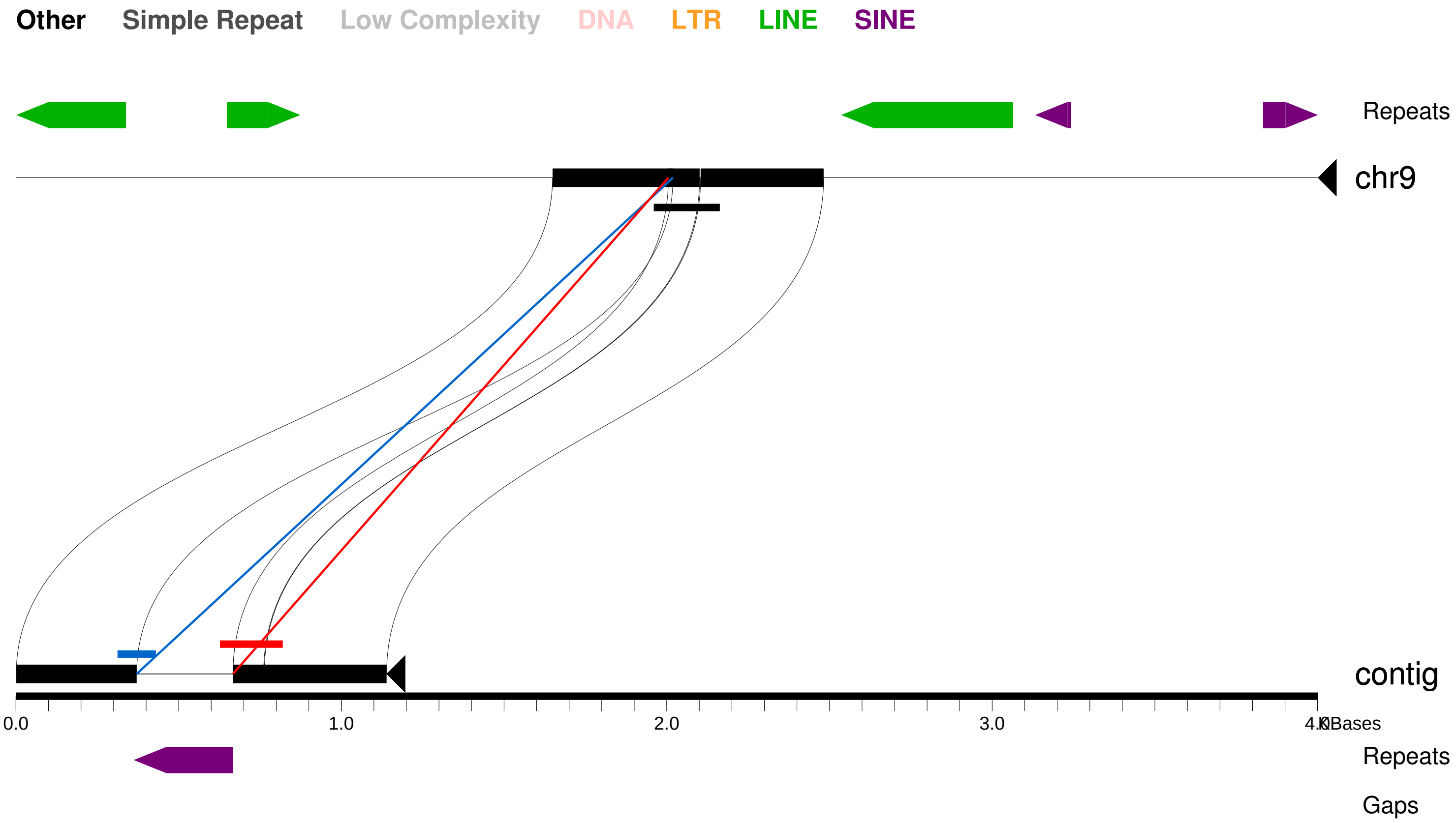

siteID: chr9_115663996 Scafftig1 + Breakpoint: chr9:115664011
chr9:115663852-115664184
Overlap Length: 14 INS size: 354 Genotyped: No

```
left   A-------------GCAGTTCTCTGGTTATCTTCTCTATTTGGGGCTTTTATGCCTTTTCATCATGTTTA
chrom  AAATCAAGCAATTAGCAGTTCTCTGGTTATCTTCTCTATTTGGGGCTTTTATGCCTTTTCATCATGTTTA
right  NNNNNNNNNNN-----------------------------------------------------------
       1NNNNNNNNNN   11111111111111111111111111111111111111111111111111111111

left   TTTTTTCTTTCTAAGTTTAAAGATAATTCTCCTTTCCCTTGAACACAGTGTAGTGTGGTGGCTAAGAGTG
chrom  TTTTTTCTTTCTAAGTTTAAAGATAATTCTCCTTTCCCTTGAACACAGTGTAGTGTGGTGGCTAAGAGTG
right  ----------------------------------------------------------------------
       1111111111111111111111111111111111111111111111111111111111111111111111

left   CAGGCTCTGAAATCAGACTAGAACTTGAGTCCTG-CCGG--GC------GCGGTG---------GCTCAC
chrom  CAGGCTCTGAAATCAGACTAGAACTTGAGTCCTATCCTGATGCTTACTAGCTGTGTGACCTTAAGGTCTC
right  -------------------AGAACTTGAGTCCTATCCTGATGCTTACTAGCTGTGTGACCTTAAGGTCTC
       1111111111111111111**************22**2*22**222222**2***222222222*2**2*

left   GCCTGTAAT-----CCCAGCACTTT---GGGAGGCCGAGGCGGGTGGATCATGAGGTCAG-GAGATCGAG
chrom  TCGTATCATATTTTCTCATCTGTAAAACGGGAATCATATTAGGACTTACCTTGAGAATTGCGAAAACTAA
right  TCGTATCATATTTTCTCATCTGTAAAACGGGAATCATATTAGGACTTACCTTGAGAATTGCGAAAACTAA
       2*2*2*2**22222*2**2*22*22222****22*22*222**2222*2*2****2222*2**2*2*2*2

left   ACCA-TCCTGGCTAACAAG-GTGAAACCCCGTC-TCTACTAAAA---------
chrom  ATGAGTTAATGCATGCAAGAGTTAATGCATGTAATATGTTTAAAATAGTCTCA
right  ATGAGTTAAT-------------------------------------------
       *22*2*222211NNN1111 11N11NN1NN11N 1N1NN1N111
```

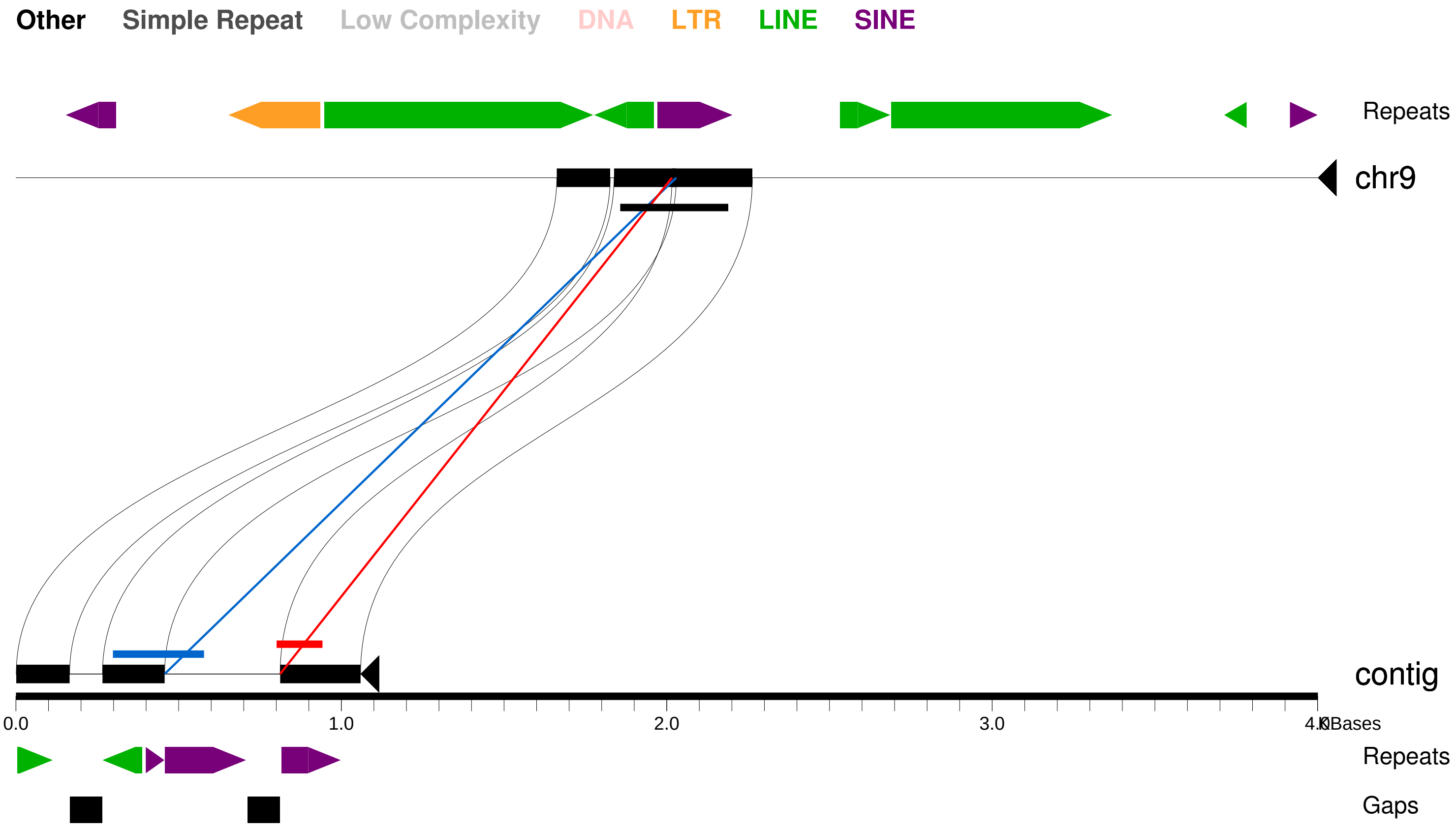

siteID: chr9_117928285 Scafftig1 - Breakpoint: chr9:117928267
chr9:117928222-117928322
Overlap Length: 15 INS size: 278 Genotyped: Yes

```
left     TGGGTTAATAGA-------------GA-AGAACTCATAGCATTCTTAAGAGGACTAAAA----GAAAGAG
chrom    TGGGTTAATAGA-------------GA-AGAACTCATAGCATTCTTAAGAGGACTAAAA----GAAAGAG
right    CGCGCCACTGCACTCCAGCCTGGGCGACAGAGCGAGACTCCGTCTCAAAAAAAAAAAAAAAAAGAAAGAG
         1*1*11*1*11*1111111111111**1***1*111111*11***1**1*11*11****1111*******

left     CATAGTACGTGTAATCCCAGCACTTTGGGAGGCCGAGGCGGGCGGATCACGAGGTCAGGAGATCGAG
chrom    CATAGTACAT---ACACATGTAGTTTTCAA---CTTGCTGCCTTTATCT-----TCAGTA-------
right    CATAGTACAT---ACACATGTAGTTTTCAA---CTTGCTGCCTTTATCT-----TCAGTA-------
         ********2*222*22*22*2*2***222*222*22*22*22222***222222****2*2222222
```

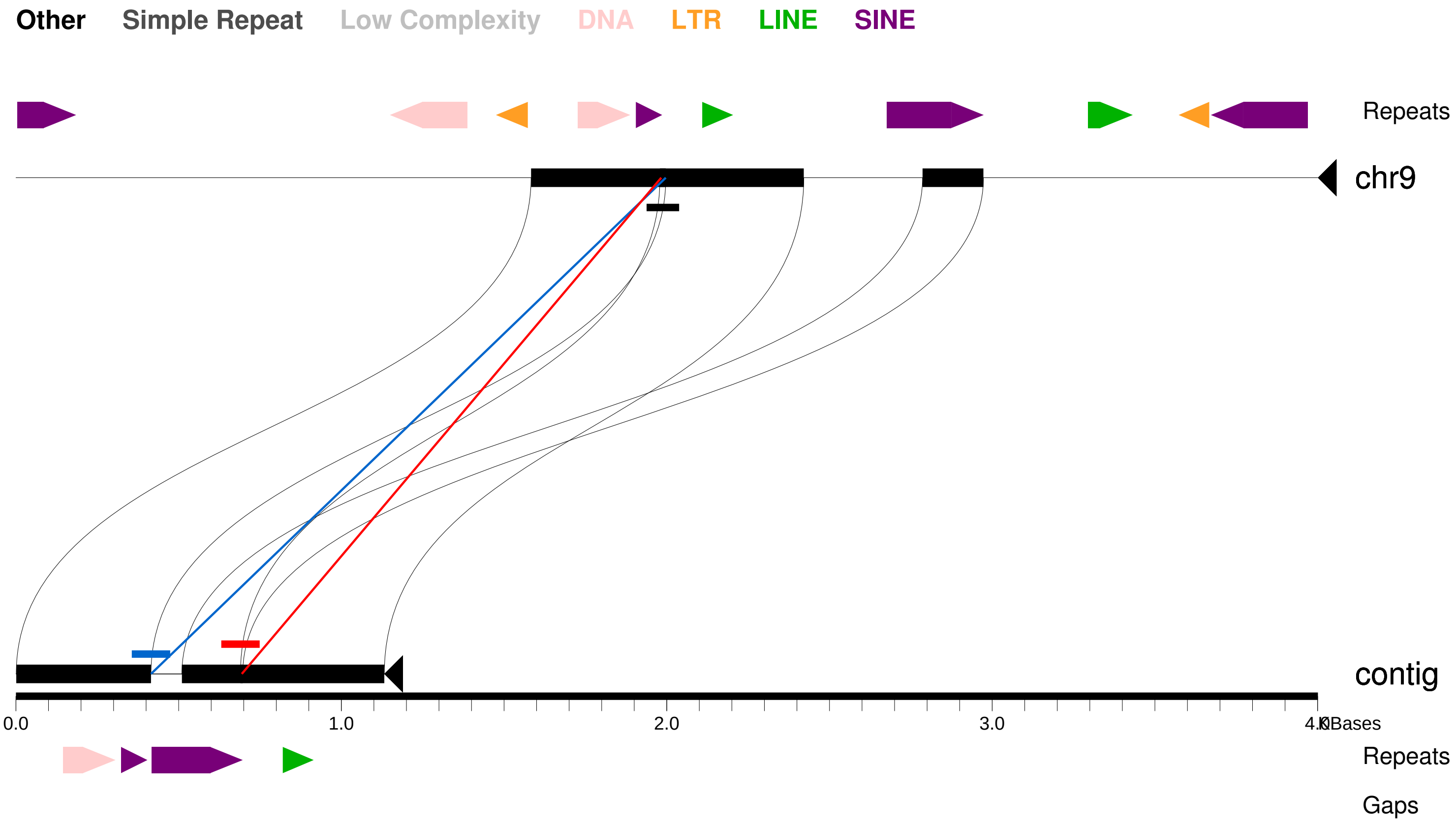

siteID: chr9_119496802 Scafftig1 + Breakpoint: chr9:119496823
chr9:119496776-119496882
Overlap Length: 13 INS size: 344 Genotyped: No

```
left    TAAATATGACTTATGAGGTCCCTTTATATTCAGAAATTCCCAGC------------ATTAAAGACATGAA
chrom   TAAATATGACTTATGAGGTCCCTTTATATTCAGAAATTCCCAGC------------ATTAAAGACATGAA
right   NNNNNNNNNNNNNNNNNNNNNNNNNNNNNNNNNNNNNNNNNNNNNNNNNNNNNNNAAAAAAAGACATGAA
        1111111111111111111111111111111111111111111111111111111*11***********

left    TTG--GCCGGGCGCGGTGGCTCACGCCTGTAATCCCAGCACTTTGGGAGGCCGAGGCGGGCGG
chrom   TTATAGTCTGGAGAGGAGG---AGGAGAGAAG----AGAA-----GGGGGAGGAGAAGAAC--
right   TTATAGTCTGGAGAGGAGG---AGGAGAGAAG----AGAA-----GGGGGAGGAGAAGAAC--
        **222*2*2**2*2**2**222*2*222*2*22222**2*22222**2**22***22*22*22
```

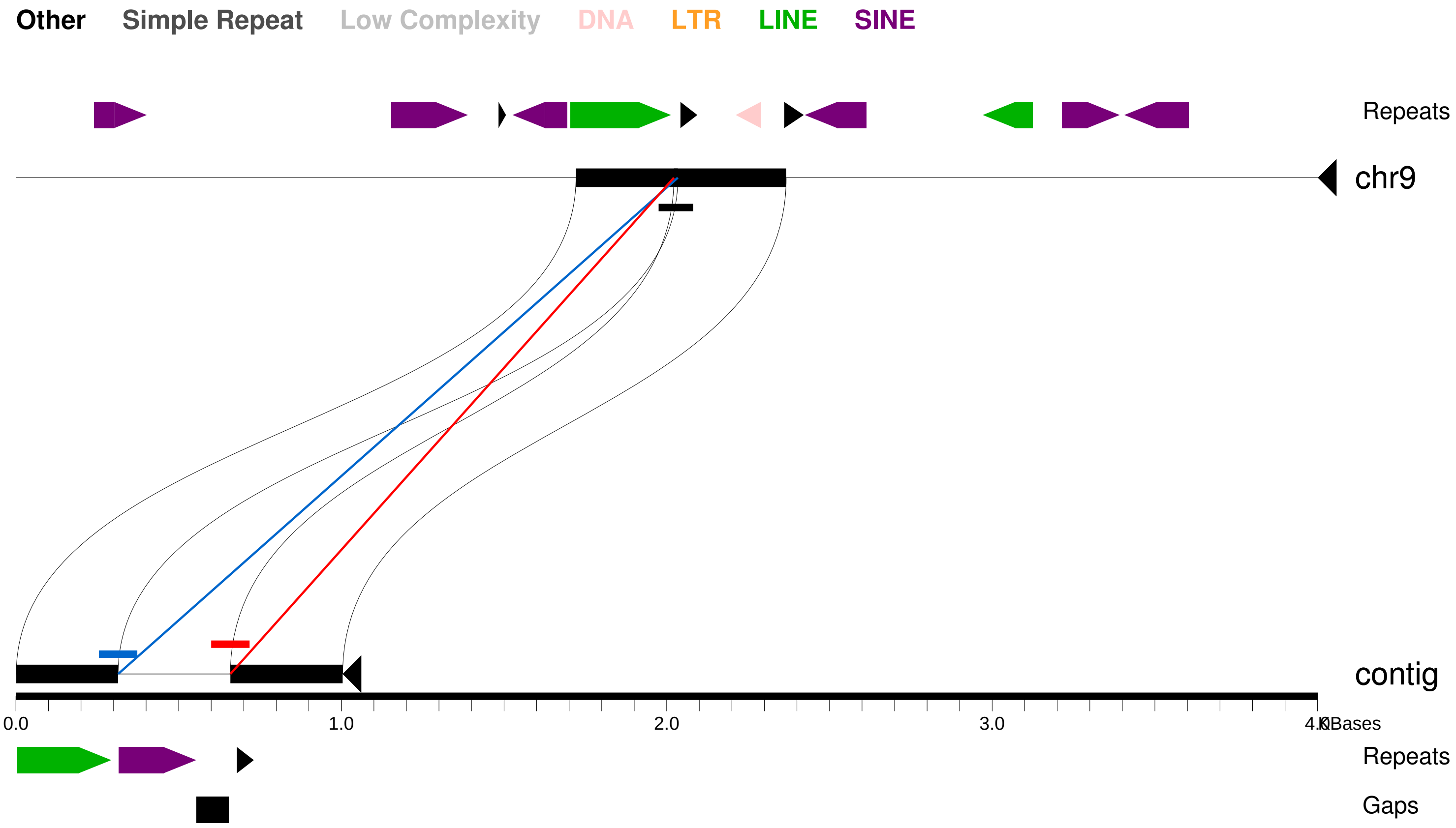

siteID: chr9_120390725 Scafftig1 - Breakpoint: chr9:120390727
chr9:120390674-120390786
Overlap Length: 7 INS size: 436 Genotyped: No

```
left    AAACCCTTCCTTGGAAACCATAGCATGTGCCATAGTGAGCTTACTTGAAAGTA------GTCTCCTGGCC
chrom   AAACCCTTCCTTGGAAACCATAGCATGTGCCATAGTGAGCTTACTTGAAAGTA------GTCTCCTAAGA
right   NNNNNNNNNNNNNNNNNNNNNNNNNNNNNNNNNNNNNNNNNNNNNNNNNNNNNNNNNNNGTCTCCTAAGA
        11111111111111111111111111111111111111111111111111111111111*******2222

left    GGGCCTGGTG-GCTCACGCCTGTAATCCCAGCACTTTGGGAGGCCGAGGCGGGTGG
chrom   GAGTCAAAACAGCTGCATCCTGAAGAAATAGCCTTTTGAGAAGGCAAAG-------
right   GAGTCAAAACAGCTGCATCCTGAAGAAATAGCCTTTTGAGAAGGCAAAG-------
        *2*2*222222***2222****2*22222***22****2**2*2*2*2*2222222
```

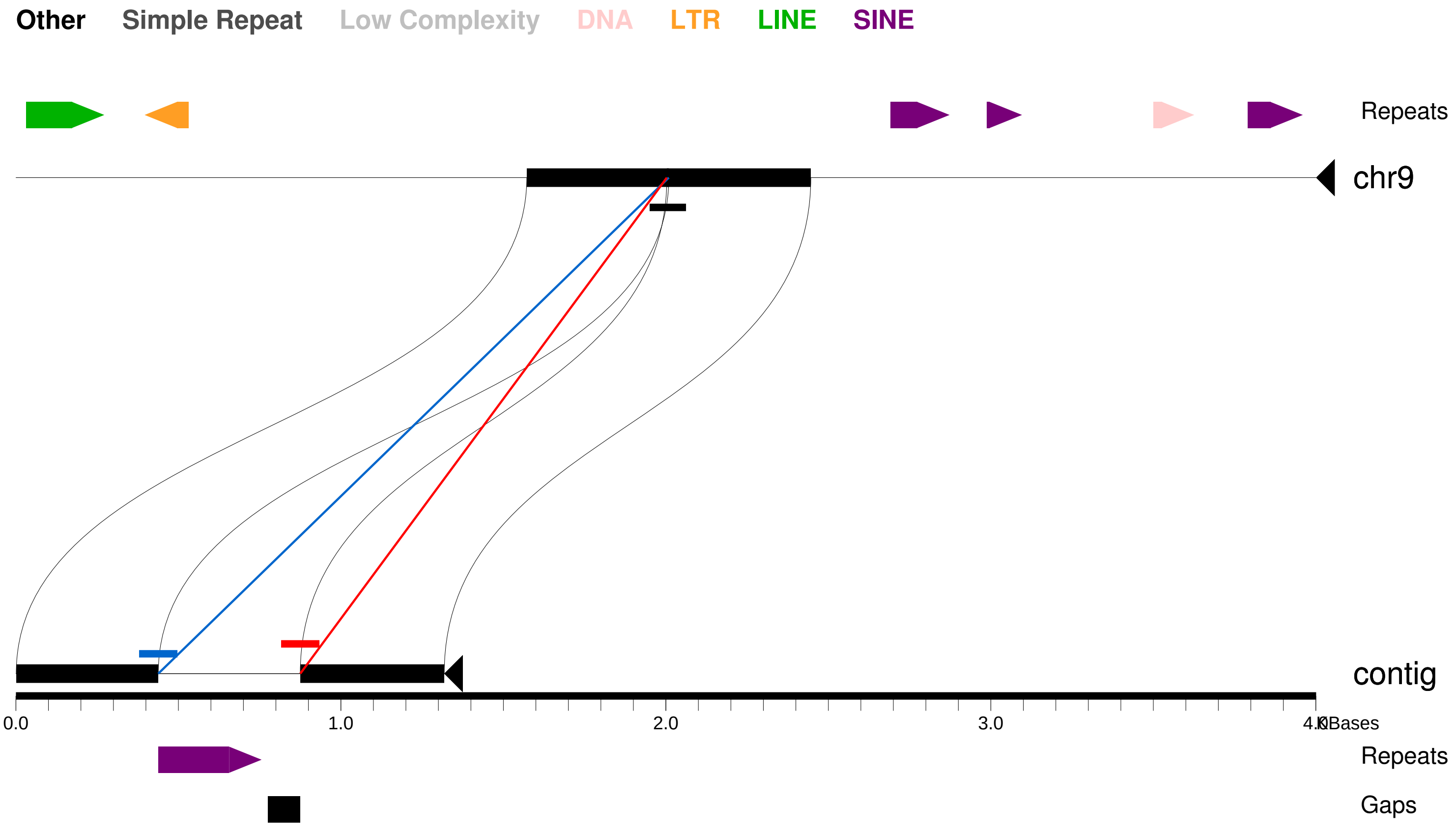

siteID: chr9_124039925 Scafftig3 + Breakpoint: chr9:124039933
chr9:124039776-124040107
Overlap Length: 15 INS size: 466 Genotyped: No

```
left   ------------AAGTCCCTTGTTCAGGGCTAAAGACCCAGTGACAGTAATAGGGTCTAGCCTGGAGCCC
chrom  GACTTAAGAGGCAAGTCCCTTGTTCAGGGCTAAAGACCCAGTGACAGTAATAGGGTCTAGCCTGGAGCCC
right  NNNNNNNNNNNNAAA-------------------------------------------------------
       NNNNNNNNNNNN**11111111111111111111111111111111111111111111111111111111

left   AGATCTTTTAATGTCTTGTCTTTGACATCTGACTTATCTGTCTATTTTATCTGCAGCTATGAGTTTCTAG
chrom  AGATCTTTTAATGTCTTGTCTTTGACATCTGACTTATCTGTCTATTTTATCTGCAGCTATGAGTTTCTAG
right  ----------------------------------------------------------------------
       1111111111111111111111111111111111111111111111111111111111111111111111

left   AATCTGAACAGCCCTTAAAATACTTTGCTGCTGGCCGG----GCGCGGTGGCTCACGCCT--GTAATCCC
chrom  AATCTGAACAGCCCTTAAAATACTTTGCTGCTTAATTATGTAGCAAAGTAGGTTCTTCTTTAGGAAAGAG
right  -----------------AAATACTTTGCTGCTTAATTATGTAGCAAAGTAGGTTCTTCTTTAGGAAAGAG
       11111111111111111**************2222222222**222**2*2*2222*2*22*2**2222

left   AG---CACTTTGGGAGGCC----GAGGCGGGCGGATCACGAGGT--CAGGAGATCGAGACCATC-CCGGC
chrom  AGTGACTCAGTAGAAAGCCATTGGAAGGGGGAGAAATAC-AGCTAGCATTTGATCAACCCTTTCACCAGA
right  AGTGACTCAGTAGAAAGCCATTGGAAG-------------------------------------------
       **222*2*22*2*2*2***2222**2*N111N1N1NN11211N1  11NNN1111N1NN1NN11 11N1N

left   ------TAAAACGGTGA----AACC---CCGTCTCTACT---------AAA--
chrom  AGTTTTTATACTTGAGATTATAAACATTCTTTCTGTATTTATGGATGCAAACT
right  -----------------------------------------------------
             11N1NNN1N11    11N1   1NN111N11N1         111
```

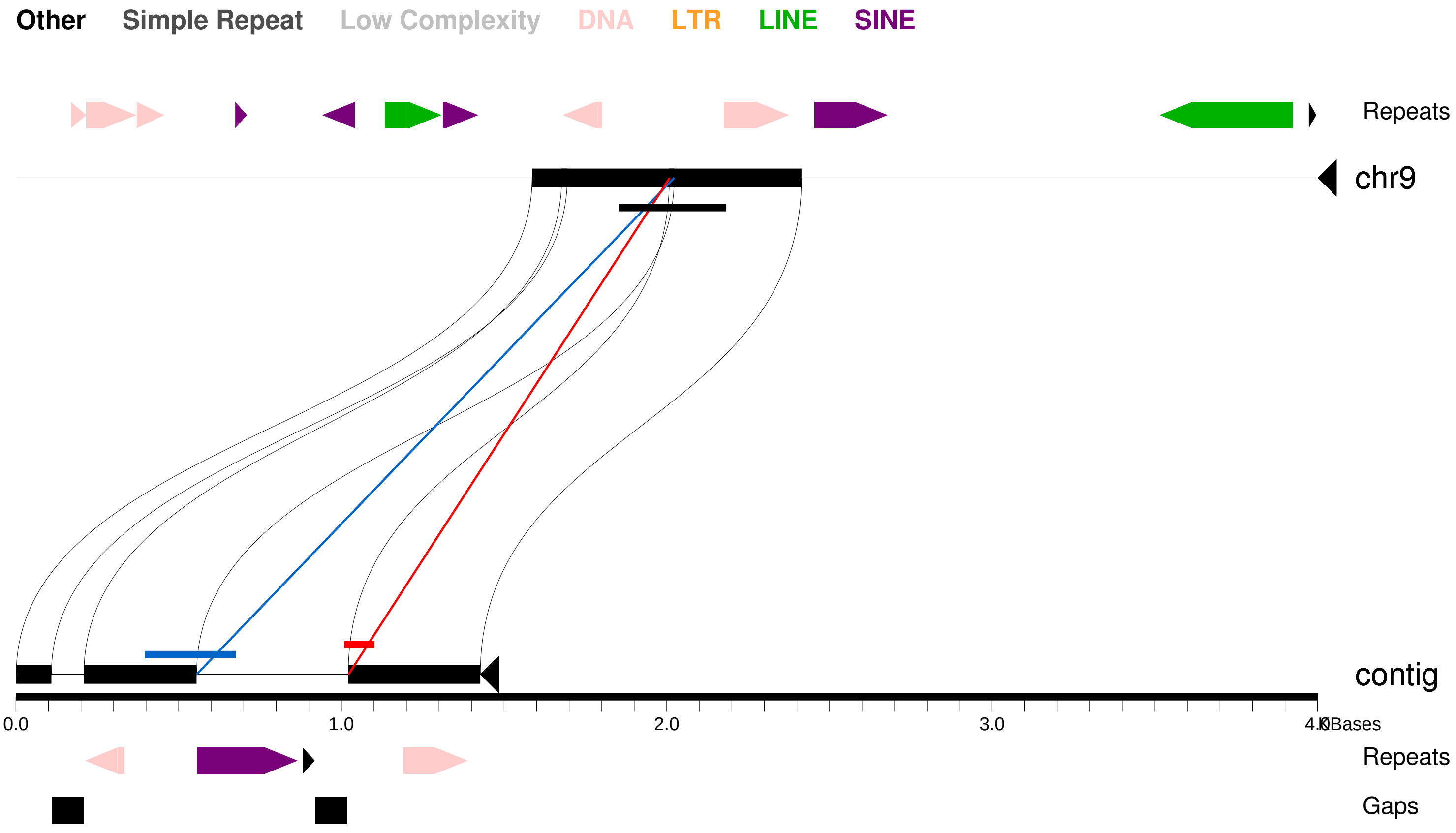

siteID: chr9_12601807 Scafftig5 + Breakpoint: chr9:12601788
chr9:12601741-12601847
Overlap Length: 13 INS size: 436 Genotyped: No

```
left   TGTATGGAATAAATGAATAAATAATTACATGAATAATTGAATGGTTT------------AAAAAGTAAAT
chrom  TGTATGGAATAAATGAATAAATAATTACATGAATAATTGAATGGTTT------------AAAAAGTAAAT
right  NNNNNNNNNNNNNNNNNNNNNNNNNNNNNNNNNNNNNNNNNNNNNNNNNNNNNNNNNNNNAAAAAAAGAGTAAAT
       1111111111111111111111111111111111111111111111111111111111***1*******

left   ATGGCCGGGCGCGGTGGCTCACGCCTGTAATCCCAGCACTTTGGGAGGCCGAGGCGGGCGG
chrom  ATTAAAAA------TGGAAAAAGTCT-TAATGGCAGC---TTGTGTGTAAAATAT--TCTG
right  ATTAAAAA------TGGAAAAAGTCT-TAATGGCAGC---TTGTGTGTAAAATAT--TCTG
       **222222222222***222*2*2**2****22****222***2*2*2222*222222*2*
```

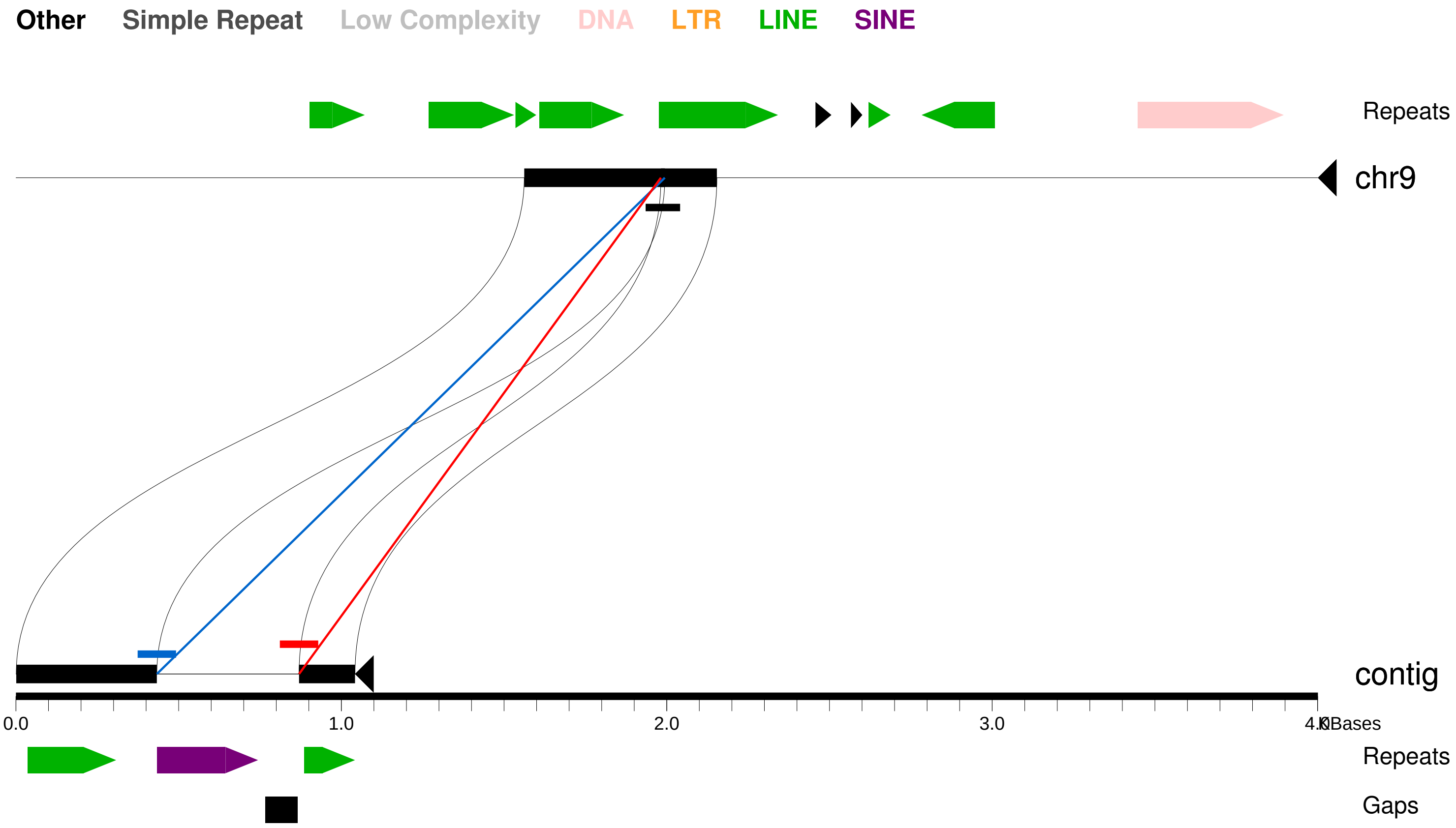

siteID: chr9_128980173 Scafftig4 - Breakpoint: chr9:128980166
chr9:128980117-128980220
Overlap Length: 11 INS size: 167 Genotyped: Yes

```
left    CCAAAAAG-----GCCAC-----CACCTCCTTTGCCCTATTGACAGCCTTGCTTAAGAA-----TCTCTC
chrom   CCAAAAAG-----GCCAC-----CACCTCCTTTGCCCTATTGACAGCCTTGCTTAAGAA-----TCTCTC
right   CACTCCAGCCTGGGCGACAGAGCCAGACGCTGTCTCAAAAAAAAAAAAAAAAAAAAAAAAAAAATCTCTC
        *11111**11111**1**11111**1111**1*11*11*111*1*111111111**1**11111******

left    ACCTTGCGGTGGCGGGCTCCTGTAGTCCCAGCTACTTGAGAGGCTGAGGCAGGAGAATGGCGTG
chrom   ACCTTAA---------CCCGTGCATTTTTACAAACT------GCTGCAGCCAGATCAGTTTAGG
right   ACCTTAA---------CCCGTGCATTTTTACAAACT------GCTGCAGCCAGATCAGTTTAGG
        *****22222222222*2*2**2*2*222*222***222222****22**22**22*222222*
```

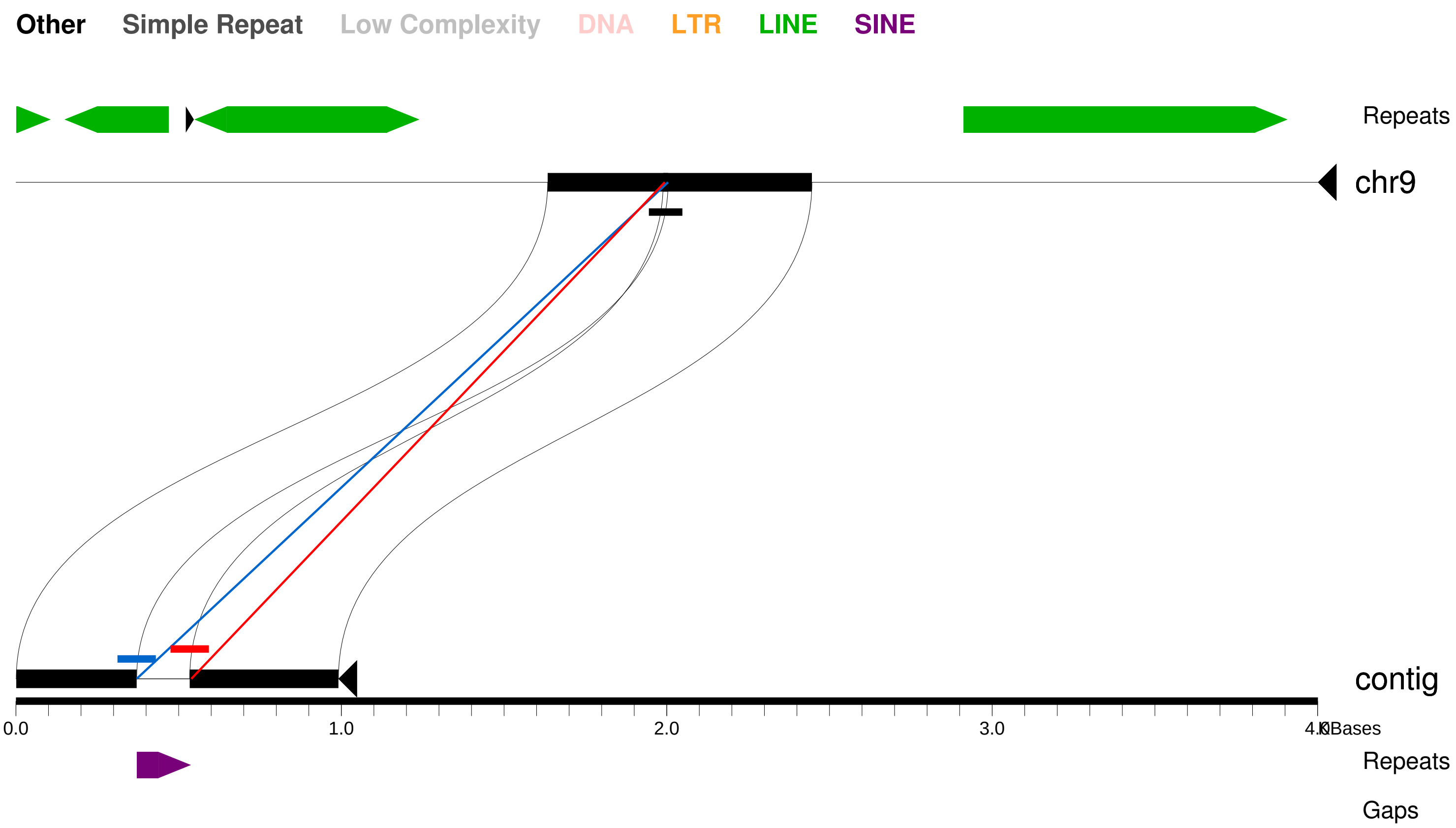

siteID: chr9_14877433 Scafftig2 + Breakpoint: chr9:14877434
chr9:14877274-14877605
Overlap Length: 12 INS size: 297 Genotyped: Yes

```
left   C-----TGGGTGGTGTATAAAAGACATCTCACACTTTATGAAGCCAAAATTGAGCTCTCAATCTTCCCTC
chrom  CATCTCTGGGTGGTGTATAAAAGACATCTCACACTTTATGAAGCCAAAATTGAGCTCTCAATCTTCCCTC
right  T---TGTATTTTTAGTAGAGACGGGGTTTCAC-CTTGTT--AGCCAGGATGG---TCTCGATCT-CCTGA
       1   2N*111*111***1*1*1*111*1****1***11*11*****11**1*111****1****1**111

left   CCTCAGTCTTCCTTACCTTAATTAACAGCAACTCCAACTTTTCAGTTGTTCCTTTCTTCATCACACATTC
chrom  CCTCAGTCTTCCTTACCTTAATTAACAGCAACTCCAACTTTTCAGTTGTTCCTTTCTTCATCACACATTC
right  CCTCA-TGATCC--ACCCGCCT---CGGC--CTCCCAAAGTGCTGGGATTACAGGCGTGAGC-CAC----
       *****1*11***11***1111*111*1**11****1*111*1*1*111**1*111*1*1*1*1***1111

left   TAAATCTAGACAAAAAATCTTGTTGTTTCTTTTTTTTTTTTTTTTTTTTTTGAGACGG-------------
chrom  TAAATCTAGACAAAAAATCTTGTTGTTTCTTTATGGTAGACAGATCTTAGGTGACCCCCCAAATAATTCC
right  ------------------CGTGTTGTTTCTTTATGGTAGACAGATCTTAGGTGACCCCCCAAATAATTCC
       111111111111111111*1************2*22*2222222*2**22*2***222222222222222

left   AGTCTCGCTCTGTCGCCCAGGCCGGAC----TGCG-GACTGCAG----TGGCGCAATCTCGGCTCACTGC
chrom  AGTCTCCTAGTATCCAAAAGTTCCTTCCCCTTGAGTGCCAGCAGAATCTGTAACACTTCCAACCAACAGA
right  AGTCTCCTAGTATCCAAAAGTTCCTTCCCCTTGAGTGCCAGCAGAATCTGTAACACTTCCAACCAACAGA
       ******2222*2**2222**22*222*2222**2*2*2*2****2222**222**2*22*22*22**2*2

left   A---AGCTCCGCTTCCCGGGTTCACGCC----ATT-----------------
chrom  ATTGAACAAAGGTGATGGGTGTCACTCCTATGATTACATTTTGTTATACAGG
right  ATTGAACAAAGGTGATGGGTGTCACTCCTATGATTACATTTT----------
       *222*2*222*2*2222**22****2**2222***2222222
```

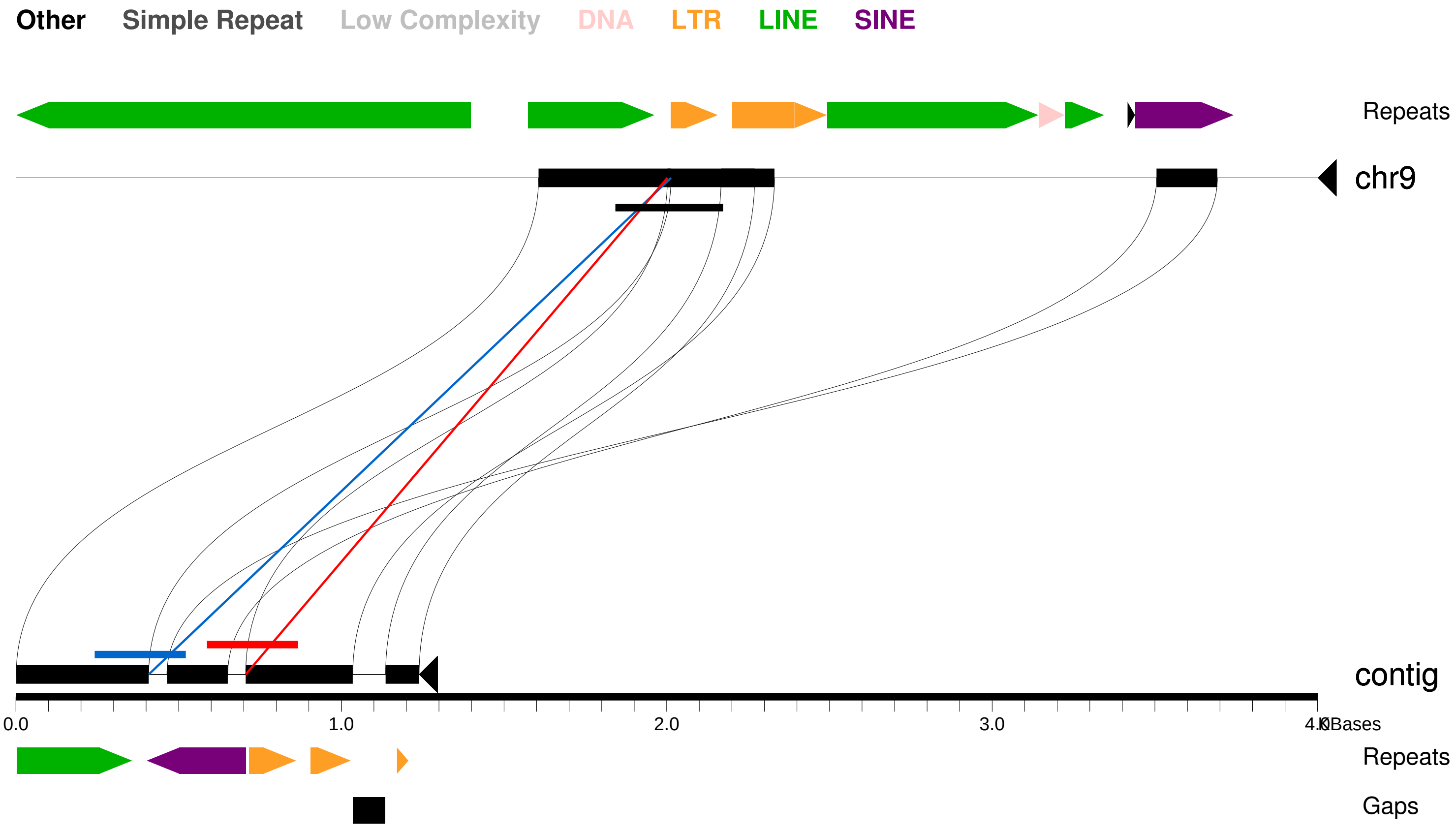

siteID: chr9_15831802 Scafftig5 + Breakpoint: chr9:15831808
chr9:15831762-15831867
Overlap Length: 14 INS size: 291 Genotyped: Yes

```
left    AGTAACTAC-TTTACCTTGAAATGTGCA------AAAGGAATCA---ATAGG---CTTACAAAACTGCCT
chrom   AGTAACTAC-TTTACCTTGAAATGTGCA------AAAGGAATCA---ATAGG---CTTACAAAACTGCCT
right   TCCGCCCGCCTCGGCCTCCCAAAGTGCTGGGATTACAGGCGTGAGCCACCGCGCCCGGCCAAAACTGCCT
        11111*11*1*111***111**1****1111111*1***11*1*111*11*1111*111***********

left    TTTTTTTTTTTTTTGAGACGGAGTCTCGCTCTGTCGCCCAGGCTGGAGTGCAGTGGCGGGAT
chrom   TTTAAAAAATGTTTGTAA-----TCATATTTTTCCTGGTAGCCTGAATATCA--------AT
right   TTTAAAAAATGTTTGTAA-----TCATATTTTTCCTGGTAGCTTGAATATCA--------AT
        ***222222*2****22*22222**2222*2*22*2222**21**2*222**22222222**
```

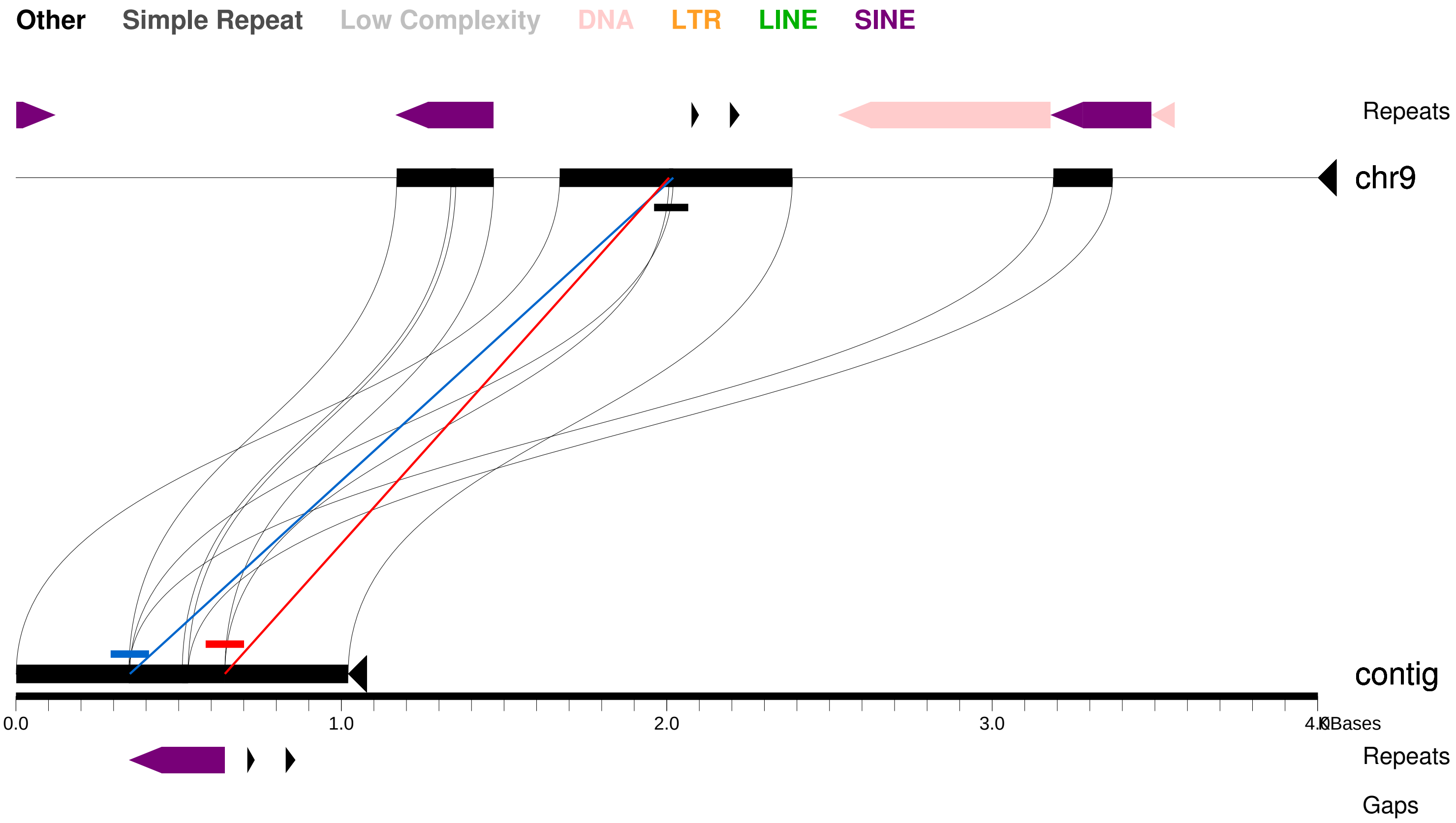

siteID: chr9_1631825 Scafftig4 + Breakpoint: chr9:1631814
chr9:1631779-1631873
Overlap Length: 13 INS size: 331 Genotyped: Yes

```
left    CCCCCAACTCCTCCTCCTAAA-----------ACAG--------CAAGGTTTTC-----ATATGTTTTCT
chrom   CCCCCAACTCCTCCTCCTAAA-----------ACAG--------CAAGGTTTTC-----ATATGTTTTCT
right   CACCCGCCTCGGCCTCCCAAAGTGCTGGGATTACAGGCGTGAGCCACCGCGCCCGGCCTATATGTTTTCT
        *1***11***11*****1***11111111111****11111111**11*1111*11111***********

left    TTTTTTTTTTTTTTTTTTTTTTTTTTTTTTTTTTTTTTTTTTTTTGAGACGGAGTCTCGCTCTGTCGCCCA
chrom   TTGTTGCCTTCTTTGGAAGATTAGATTTAGAAATAATTTCT-----GAAAGAGT----------------
right   TTGTTGCCTTCTTTGGAAGATTAGATTTAGAAATAATTTCT-----GAAAGAGT----------------
        **2**222**2***222222**222***22222*22***2*22222**22****2222222222222222

left    GGC
chrom   ---
right   ---
        222
```

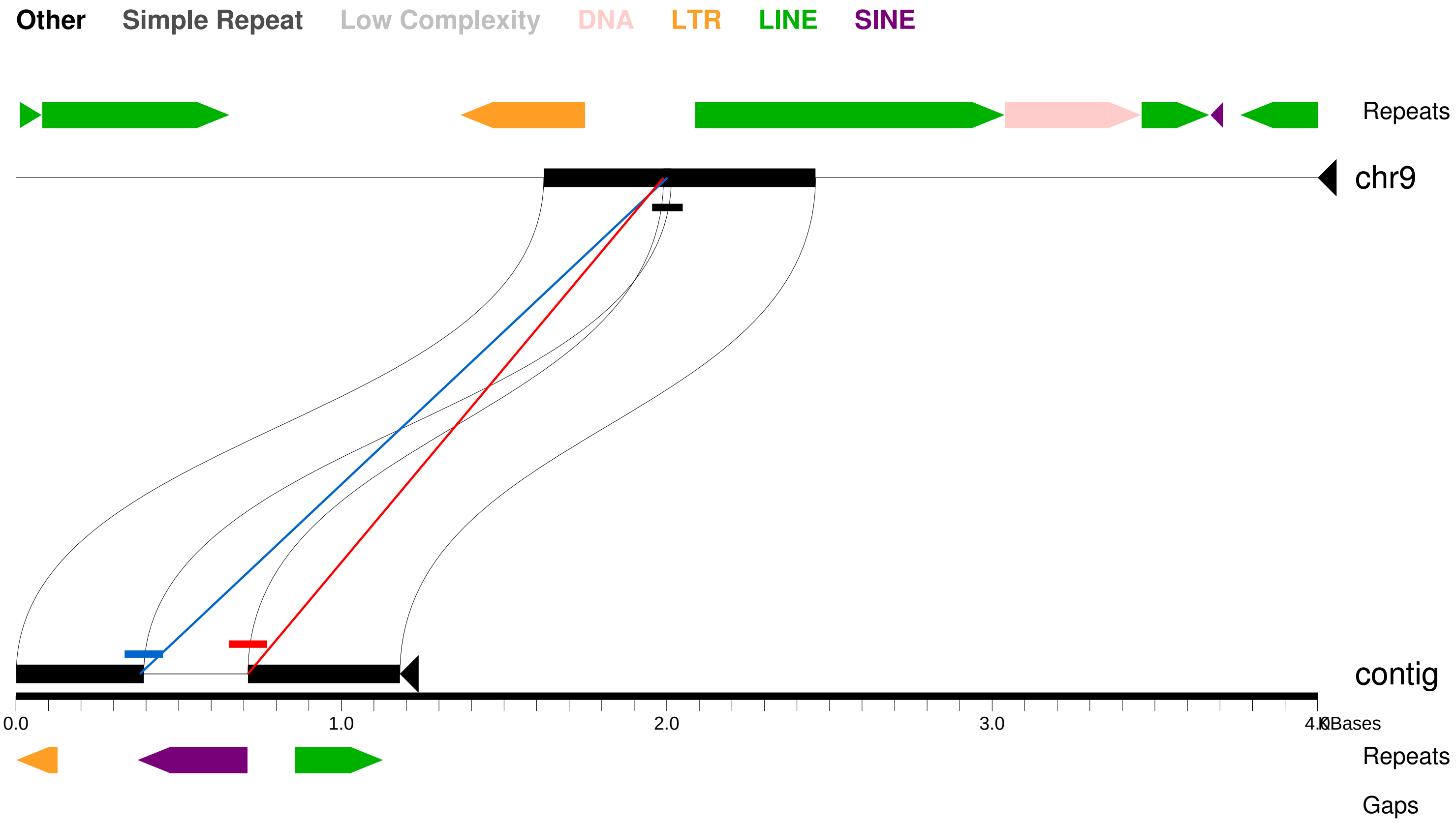

siteID: chr9_1665594 Scafftig1 - Breakpoint: chr9:1665610
chr9:1665450-1665824
Overlap Length: 55 INS size: 389 Genotyped: No

```
left   ----------------------------------------------------------------------
chrom  CATGTAACATACAATTCATGCACTTAAAGTGTATAATTAAATGGCTTTTGGTACATTCATAGGGCTGTGC
right  TTTAGTAGAGACGGGGTTT-CACCT----TGTTAGCCAGGATGG-TCTCGATC---TCCT-GACCTCATG
       NN2NNN2N2N22NNNNNN2 222N2    222NNNNNNNN2222 2N2N2N2N   22N2 2NN22NNNN

left   ----------ATAATCAAGAAAATATGTAAATTTTGAAAGACAAAAGGCCAAGGCCATCTATTTGCCACT
chrom  AACCACCATCATAATCAAGAAAATATGTAAATTTTGAAAGACAAAAGGCCAAGGCCATCTATTTGCCACT
right  ATCCACCCGCCTCGGCCTCCCAAAGTGC-----TGGGATTACA---GGCG-----------TGAGCCAC-
       2N22222NN21*111*11111**11**111111*1*1*11***111***111111111111*11*****1

left   TCTGATTGTCACTGCTGGTAAGCACTGGGTTTTCTTGGCACCCCTTTTGTAGTATCCAGTCACCAGCTTC
chrom  TCTGATTGTCACTGCTGGTAAGCACTGGGTTTTCTTGGCACCACTTTTGTAGTATCCAGTCACCAGCTTC
right  ---------CGCGCCCGGCCAGCACTGGGTTTTCTTGGCACCACTTTTGTAGTATCCAGTCACCAGCTTC
       111111111*1*11*1**11**********************2***************************

left   TTATANNNNNNNNNNN------------------------------------------------------
chrom  TTATATGGCAACAGGCATTTGCAGCAGCAGTGGTGGCTTCCTGGTGACTTGATTGAGCCTCCTAATTATT
right  TTATATGGCAACAGGCATTTGCAGCAGCAGTGGTGGCTTCCTGGTGACTTGATTGAGCCTCCTAATTATT
       *****22222222222222222222222222222222222222222222222222222222222222222

left   ----------------------------------------------------------------------
chrom  ATGGCAATTGTAGCTTTCCTGGCTTATTATTTCTGCAGCCATTTTCTAGGCATTATTACCGAAAATTCAA
right  ATGGCAATTGTAGCTTTCCTGGCTTATTATTTCTGCAGCC------------------------------
       2222222222222222222222222222222222222222

left   -------------------------
chrom  CTTAGAACCTGCGCCTTCGGCCTTT
right  -------------------------
```

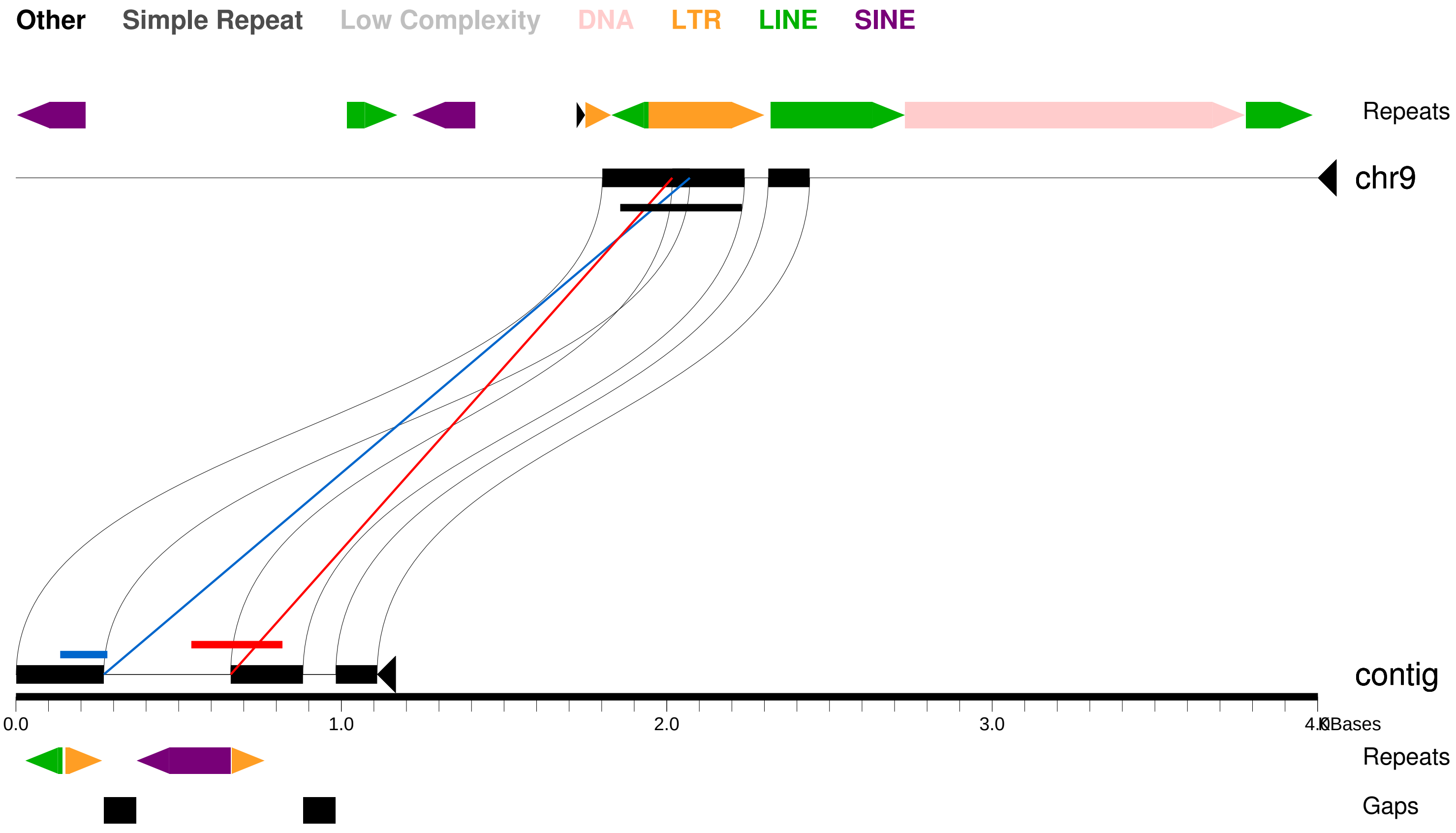

siteID: chr9_16682312 Scafftig5 - Breakpoint: chr9:16682300
chr9:16682140-16682473
Overlap Length: 14 INS size: 289 Genotyped: Yes

```
left   -----------TTTTGTTTAGTTCATACTGTTGTTAAAAAATATAGAGAATTTGCTGGCTTCATTGTGAG
chrom  AGAACTTCAGATTTTGTTTAGTTCATACTGTTGTTAAAAAATATAGAGAATTTGCTGGCTTCATTGTGAG
right  TGTATTT----------TTAGTAGAGACGGGGTTTCACCGTGTTAGCCAGGATGGTCTCGATCTCCTGAC
       N2N2N22    111111*****11*1**1*111**1*111111***11*111**1*11*1111*11***1

left   TTCATAAACATGACCAGCTTTGCCCCAGTAGGTTTATCATGTAATAAAAAGTTTCAAGACACCCTATACA
chrom  TTCATAAACATGACCAGCTTTGCCCCAGTAGGTTTATCATGTAATAAAAAGTTTCAAGACACCCTATACA
right  CTCGTGATCC-GCCCGCCTCGGCCTCCCAAAGTGC----TGGGATTACAAGCGTGA-GCCACCGCG----
       1**1*1*1*11*1**11**11***1*111*1**111111**11**1*1***11*1*1*1****1111111

left   AAATTAATTAACTTCTCCAACATCATAGGAATTTTTTTTTTTTTTT--TGA--GACGGAGTCTCGCT---
chrom  AAATTAATTAACTTCTCCAACATCATAGGAATTTAAATCCGTTCATAGTGATCGACAGACT-TCGATGAG
right  --------------------CATCATAGGAATTTAAATCCGTTCATAGTGATCGACAGACT-TCGATGAG
       11111111111111111111*************222*222**22*22***22***2**2*2***2*222

left   ---CTGTGG-----CCC--AGGCAGGAGTGCAGTGGC-GCA-------ATCTCGGCTCACTGC------A
chrom  CAGCTTTTGGTTCACCCTTATGCTGCATTTAACTAGCAGCAGCAGCCGAGCTCATCTCCGTGATTCCAGA
right  CAGCTTTTGGTTCACCCTTATGCTGCATTTAACTAGCAGCAGCAGCCGAGCTCATCTCAGTGATTCCAGA
       222**2*2*22222***22*2**2*2*2*22*2*2**2***2222222*2***22***N2**2222222*

left   AGCTCCGCCTCCAGGGT------TCAC--GCCA--TTCTCCT--GCCTCAGCCTC
chrom  AGTGAATGCTGCAGGTTGTTTAATAACCTGGCAAATTCACAGTAGCTTGTCAAAT
right  AGTGAATGCTGCAGGTTGTTTAATAACCTGGCAAATTCACAG-------------
       **222222**2****2*222222*2**22*2**22***2*22  11N1NNNNNNN
```

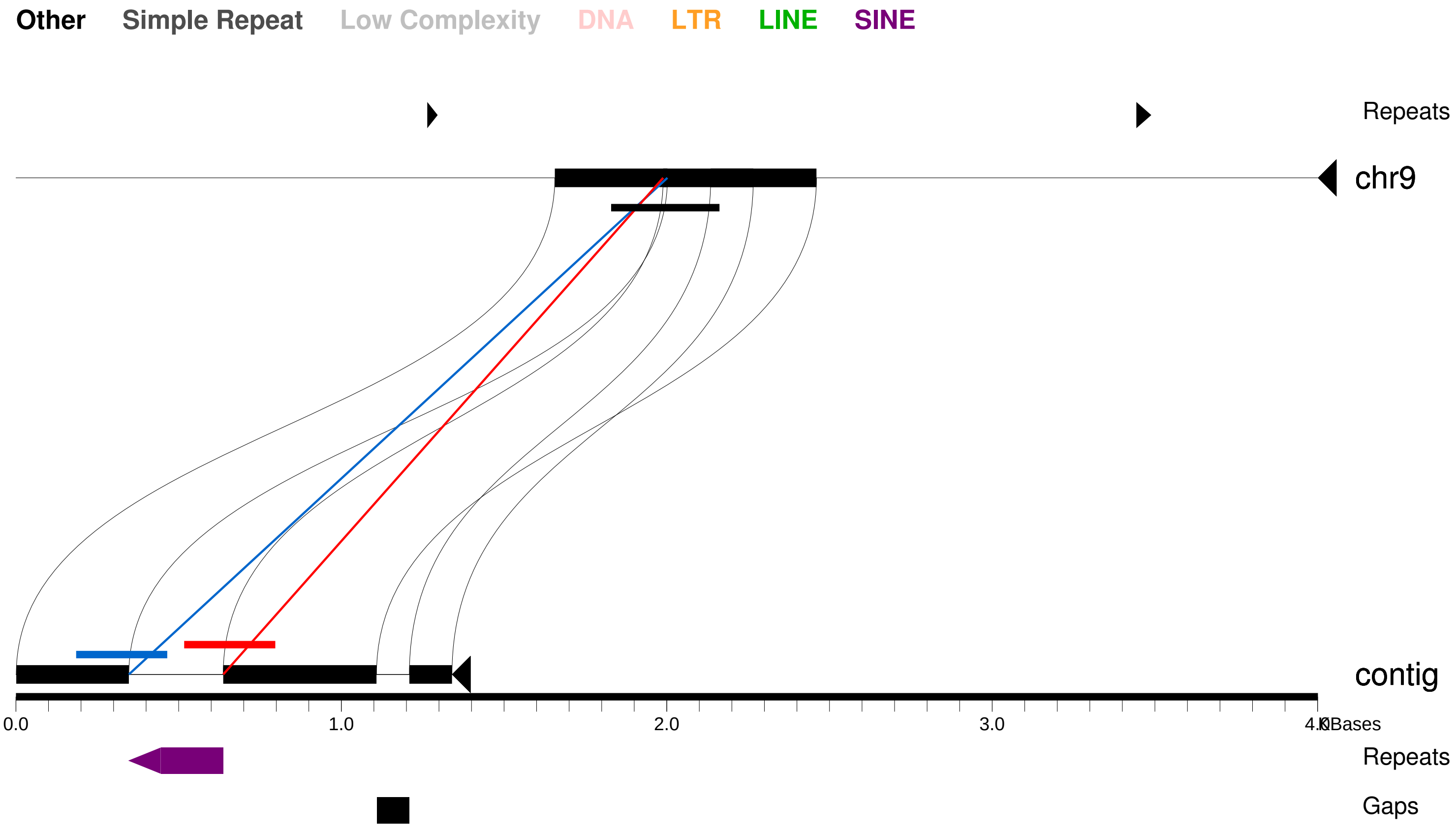

siteID: chr9_17874422 Scafftig1 + Breakpoint: chr9:17874426
chr9:17874294-17874635
Overlap Length: 78 INS size: 439 Genotyped: No

```
left   ----------------------------------------------------------------------
chrom  AGAAGGAATGCAGCTTTGCCAACACCATGATTTTGCTGTTGTAAGGCACATTTCAGACTTCTGCTGTGAG
right  CCAC------CCGCCTCGGCCTCCCAAAG----TGCTG------GG---ATTACAGGC-------GTGAG
       NN2N      2N22N2N2N2NN2N2N2N2    22222      22   222N222N2       22222

left   ------------------------------TTTGTGACTATTTATTACAGCAGCAATAGGAAACTAATAC
chrom  AAAATAGATTCGTGTTGCTTTAAACCACTGTTTGTGACTATTTATTACAGCAGCAATAGGAAACTAATAC
right  ------------------------CCACCG-----------------------CGCCCGGCCACTAATAC
                               2222N211111111111111111111111*1111**11********

left   AGTTTTTAAGAAAACATTGTTCCTGTATTAGGCTGTTCTTGCATTGAAATGCCTGAGACTGGGTAATTTA
chrom  AGTTTTTAAGAAAACATTGTTCCTGTATTAGGCTGTTCTTGCATTGAAATGCCTGAGACTGGGTAATTTA
right  AGTTTTTAAGAAAACATTGTTCCTGTATTAGGCTGTTCTTGCATTGAAATGCCTGAGACTGGGTAATTTA
       **********************************************************************

left   NNNNNNNNNNNNNNNNNNNNNNNNNNNNNNNNNNNNNNNNNNNNNNNNNNNNNNNNNNN-----------
chrom  TAAAGAAAAGAGGTTTAATTGGCTTGTGGTTCTGTAGGCTTTACAGGAAGCATGATGCTGGCATCTGCTC
right  TAAAGAAAAGAGGTTTAATTGGCTTGTGGTTC--------------------------------------
       22222222222222222222222222222222NNNNNNNNNNNNNNNNNNNNNNNNNNN

left   --------------------------------------------------------------
chrom  ACCTTCTGAGGAGGCCTCAGGAAACTTACAATCATGGCAGAAGGAGAAGGGGGAGCAGGTAC
right  --------------------------------------------------------------
```

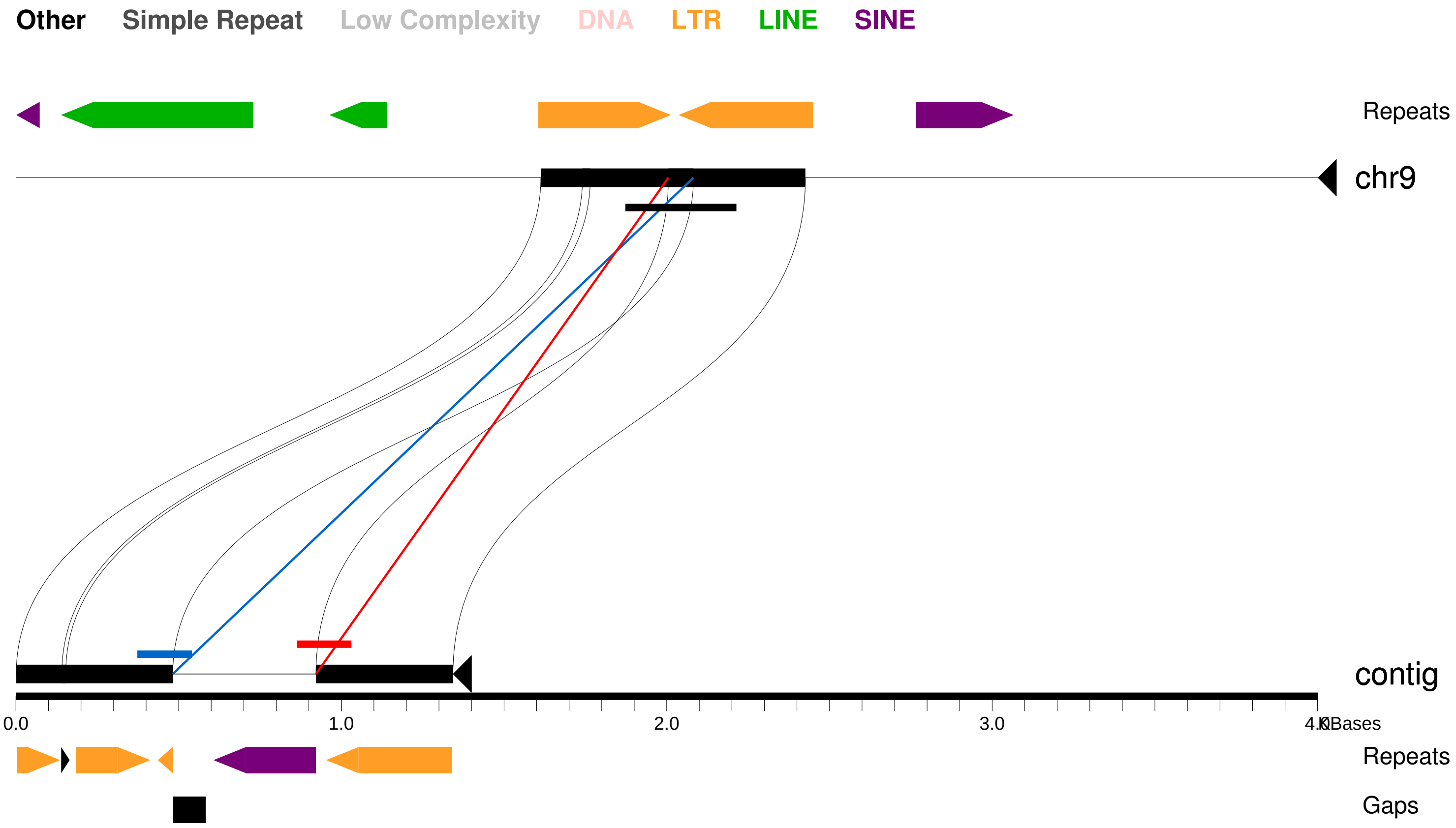

siteID: chr9_21261929 Scafftig1 + Breakpoint: chr9:21261940
chr9:21261890-21261999
Overlap Length: 10 INS size: 465 Genotyped: No

```
left   ATTAGTTCCATAATGCTGTGAATGCAGTTAATACTACTGAACTGTATACC---------AAAAAATGGTG
chrom  ATTAGTTCCATAATGCTGTGAATGCAGTTAATACTACTGAACTGTATACC---------AAAAAATGGTT
right  NNNNNNNNNNNNNNNNNNNNNNNNNNNNNNNNNNNNNNNNNNNNNNNNNNNNNNNNNNNAAAAAAAATGGTT
       11111111111111111111111111111111111111111111111111111111111*********2

left   GCCGGGCGCGGTGGCTCACGCCTGTAATCCCAGCACTTTGGGAGGCCGAGGCGGGCGG
chrom  AA-------GGTGG-TAAATTTTCTGCTAACTGTACTTTATCTTAATTTTAAAAATA-
right  AA-------GGTGG-TAAATTTTCTGCTAACTGTACTTTATCTTAATTTTAAAAATA-
       222222222*****2*2*2222*2*22*22*2*2*****222222222222222222
```

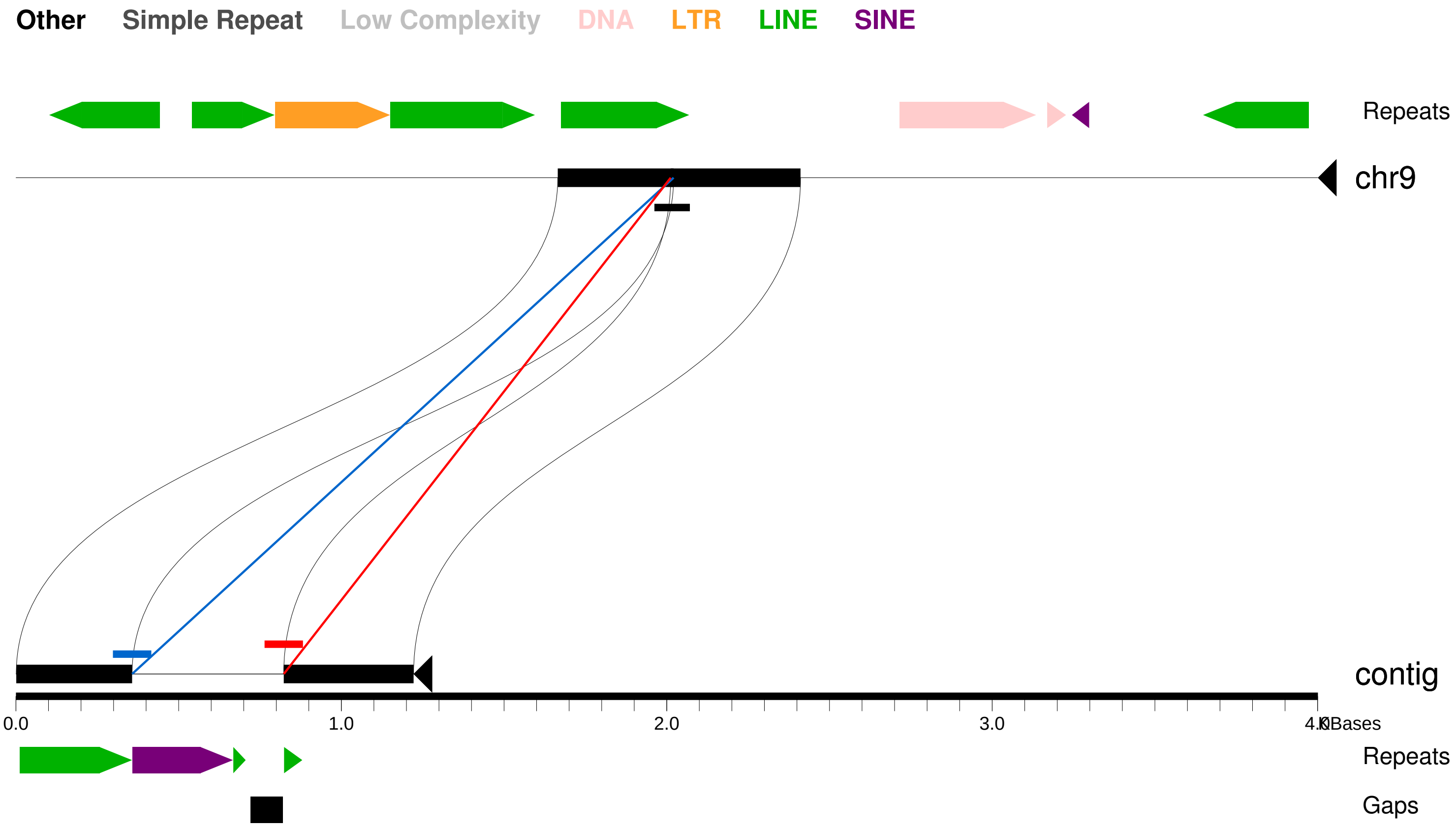

siteID: chr9_22288723 Scafftig3 + Breakpoint: chr9:22288708
chr9:22288665-22288767
Overlap Length: 17 INS size: 314 Genotyped: Yes

```
left    ATGAGATGGTGTGC------AAGATTTGATCTTC----------CCCCCACCACAGATTGCCTGTGTTTC
chrom   ATGAGATGGTGTGC------AAGATTTGATCTTC----------CCCCCACCACAGATTGCCTGTGTTTC
right   CCGCCCGCCTCGGCCTCCCAAAGTGCTGGGATTACAGGCGTGAGCCACCGCGCCCGGCCGCCTGTGTTTC
        11*111111*11**111111***111**111**11111111111**1**1*11*1*111***********

left    CTTTCTTTTTTTTTTTTTTTTTTTTTTTTTTTTTTTTTTTTGAGACGGAGTCTCGCTCTGTCGCCCA
chrom   CTTTCTACTCAAATGTCAGTGAATGCTTTCAACTGTTTTCTAAGGGAAC-----------------
right   CTTTCTACTCAAATGTCAGTGAATGCTTTCAACTGTTTTCTAAGGGAAC-----------------
        ******22*2222*2*222*222*22***2222*2****222*2***2222222222222222222
```

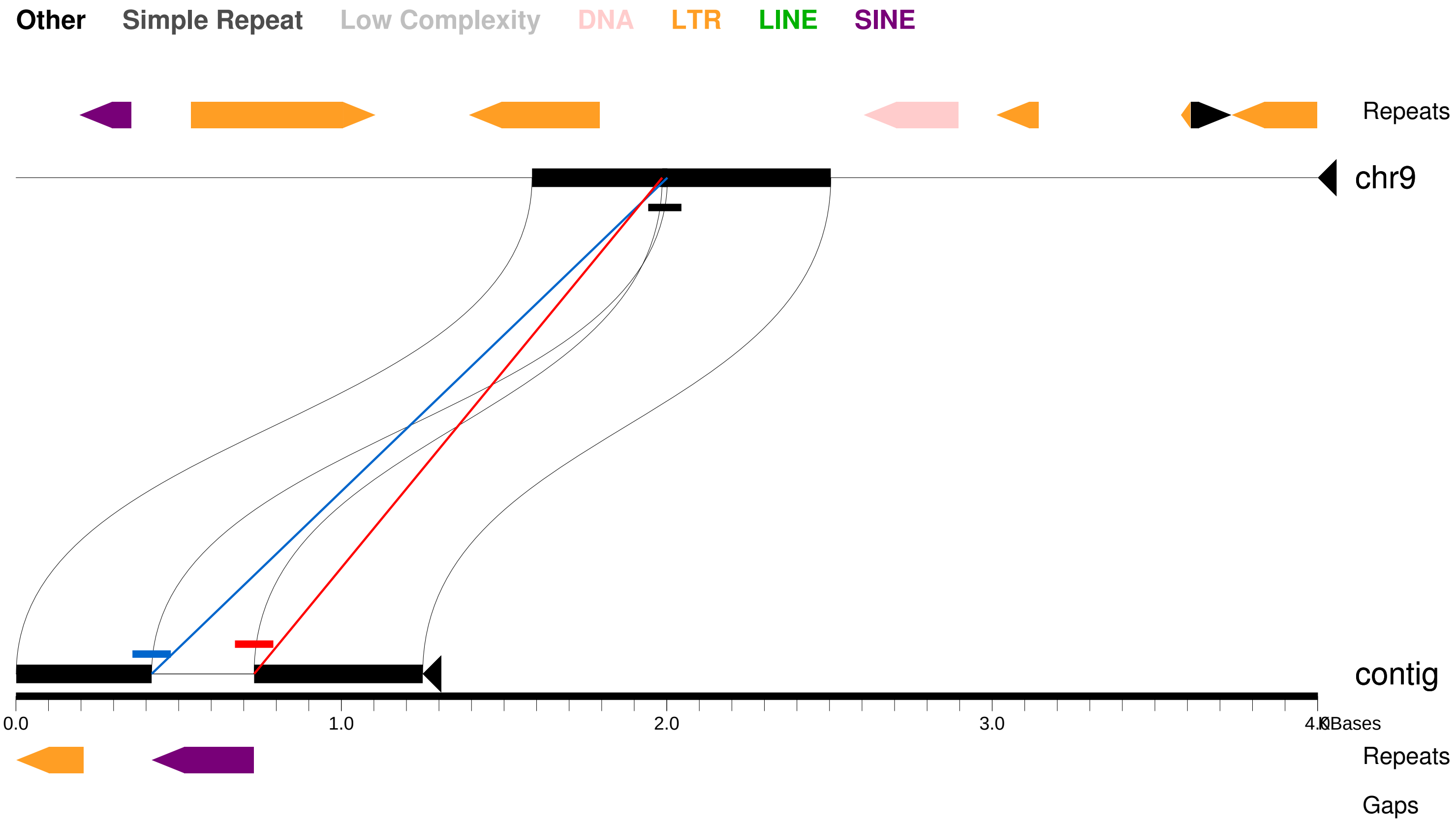

siteID: chr9_2351279 Scafftig4 - Breakpoint: chr9:2351288
chr9:2351244-2351347
Overlap Length: 16 INS size: 299 Genotyped: Yes

```
left   -ATAGTATTTTTTCCTTTC--TCTTTTCTA-ACTCTTT-TCATAACTCT----------AAGAATTATGA
chrom  -ATAGTATTTTTTCCTTTC--TCTTTTCTA-ACTCTTT-TCATAACTCT----------AAGAATTATGA
right  CACTGCACTCCAGCCTGGGCGACAGAGCGAGACTCCGTCTCAAAAAAAAAAAAAAAAAAAGAATTATGA
       1*11*1*1*1111***111111*1111*1*1****11*1***1**111111111111111**********

left   TTTCTGGCCGGGCGCGGTGGCTCACGCCTGTAA-TCCCAGCACTTTGGGAGGCCGAGGCGGGCGG
chrom  TTTCTTTCTCTTCCCCAATTTTGTTGGCTATGACTTCTAGTACAT----------------GTCA
right  TTTCTTTCTCTTCCCCAATTTTGTTGGCTATGACTTCTAGTACAT----------------GTCA
       *****22*2222*2*222222*222*2**2*2*2*2*2**2**2*2222222222222222*222
```

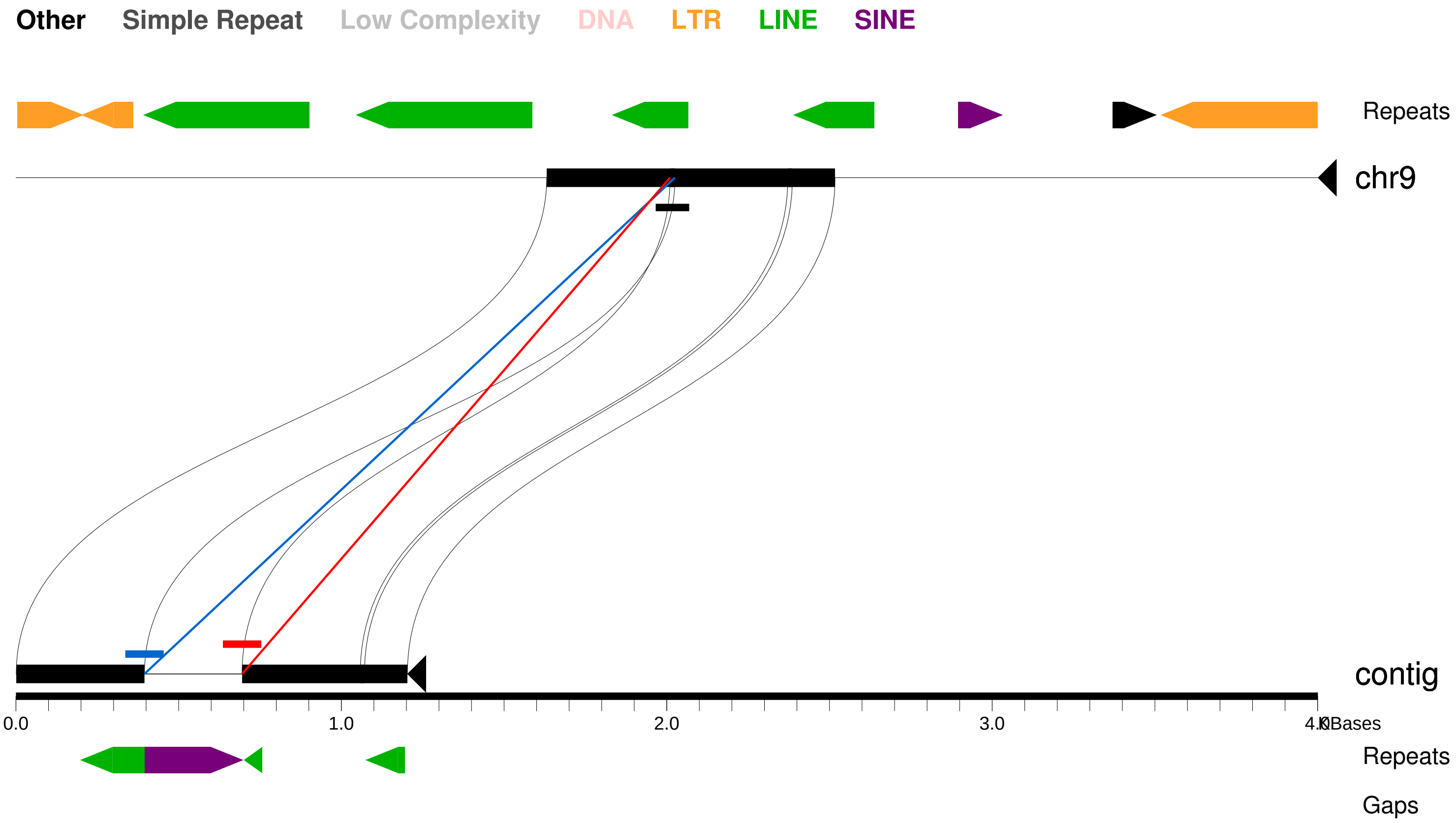

siteID: chr9_25600975 Scafftig1 - Breakpoint: chr9:25600953
chr9:25600909-25601012
Overlap Length: 16 INS size: 161 Genotyped: Yes

```
left     TTTTATTT--TTCACTTTTTGTTTCTATAATGTATGC--TTTTCCTAA-----------TTGAAGAGAAC
chrom    TTTTATTT--TTCACTTTTTGTTTCTATAATGTATGC--TTTTCCTAA-----------TTGAAGAGAAC
right    CTCGGCTCACTGCAAGCTCCGCCTCCCGGGTTCACGCCATTCTCCTGCCTCAGCCTCCCTTGAAGAGAAC
         1*1111*111*1**111*11*11**11111*11*1**11**1****1111111111111***********

left     TTTCTTTTTTTTTTTTTTTTTTTTTTTTTTTTTTTTTTTTTTTTTTTTTTTTTTTTTTTTTGAGAC
chrom    TTTCTGAGACTACATCTAATTTATAGATGTGTTC---------------TCCTCTCTCCCAACA
right    TTTCTGAGACTACATCTAATTTATAGATGTGTTC---------------TCCTCTCTCCCAACA
         *****22222*222*2*22***2*222*2*2**2222222222222222*22*2*2*222*222
```

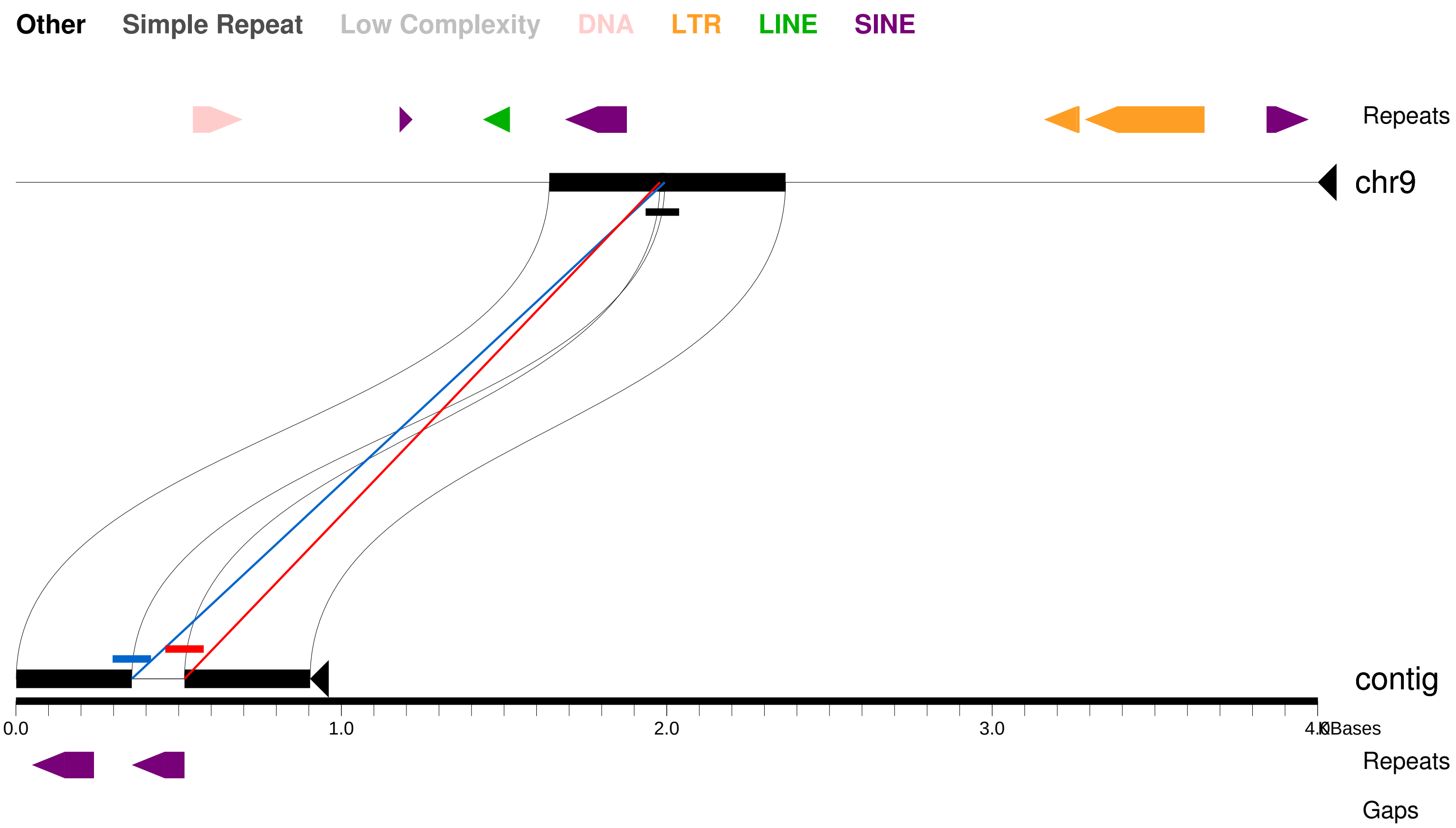

siteID: chr9_26476445 Scafftig1 + Breakpoint: chr9:26476356
chr9:26476314-26476415
Overlap Length: 18 INS size: 104 Genotyped: Yes

```
left     AAGAGTAA------TGGGAATA---AATTAGGAAGAAAAATTTAGGCTGAA--------TATAAGGAACA
chrom    AAGAGTAA------TGGGAATA---AATTAGGAAGAAAAATTTAGGCTGAA--------TATAAGGAACA
right    CTCTGTCGCCCAGGCGGGACTGCGGACTGCAGTGGCGCAATCTCGGCTCACTGCAAGCTTATAAGGAACA
         1111**111111111****1*1111*1*111*11*111***1*1****1*111111111***********

left     TTTTCTTTTTTTTTTTTTTTTTTTTTTTTTTTTTTTTTTTGAGACGGAGTCTCGCTCTGTCGCCCAGG
chrom    TTTTCTTAATCATTAAATCAATTAGACAATGGAATTTA-AAATTGGGTAT---------------
right    TTTTCTTAATCATTAAATCAATTAGACAATGGAATTTA-AAATTGGGTAT---------------
         *******22*22**222*222**222222*2222***22*2*22*2**2*2222222222222222
```

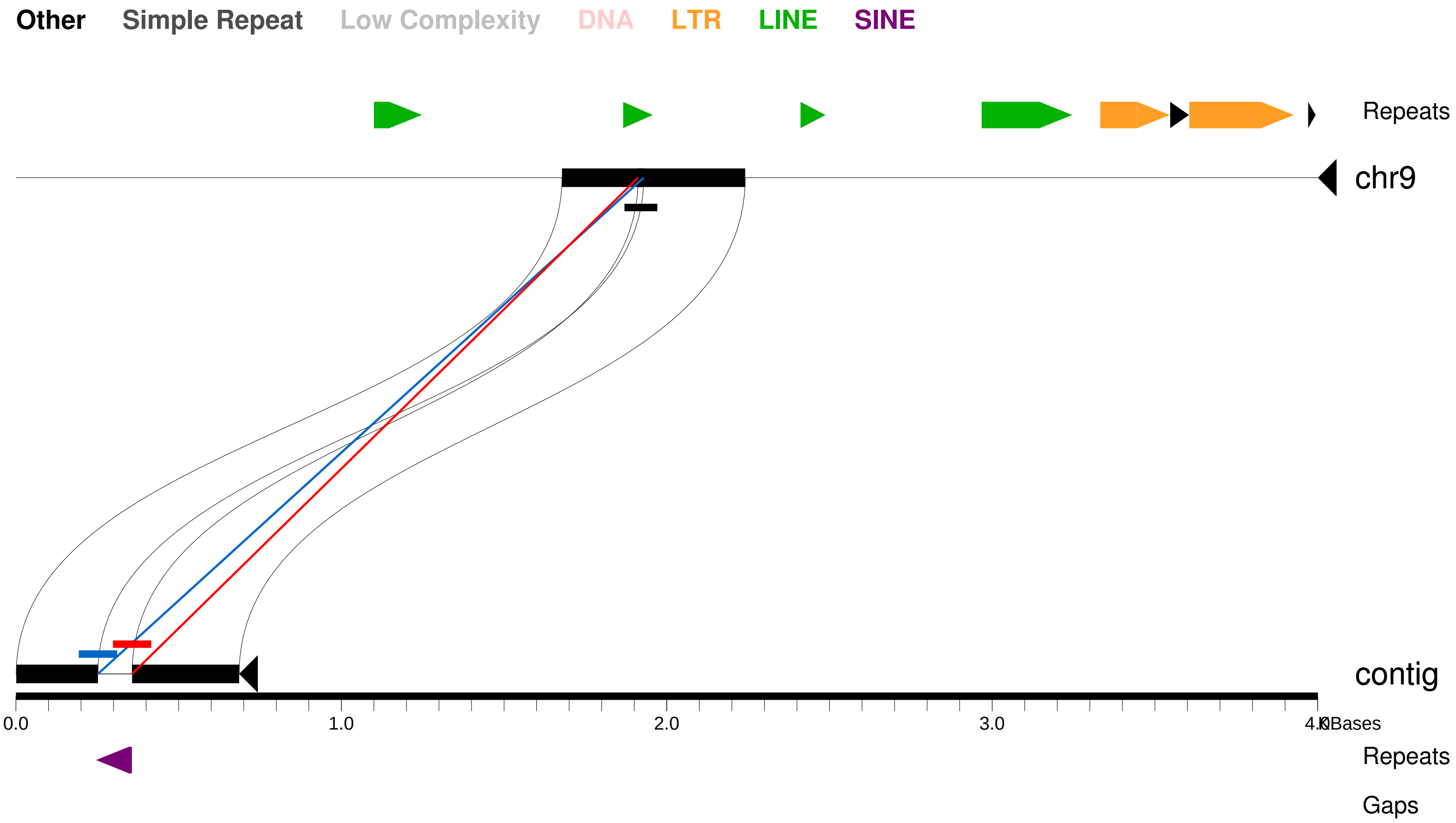

siteID: chr9_29103301 Scafftig2 - Breakpoint: chr9:29103305
chr9:29103250-29103364
Overlap Length: 5 INS size: 410 Genotyped: No

```
left   TGTATCAAGTTACAAAGAACTTCTACAGCAGCTAGAAAACTTTATAAAGAATGCA----CTTGTGGCCGG
chrom  TGTATCAAGTTACAAAGAACTTCTACAGCAGCTAGAAAACTTTATAAAGAATGCA----CTTGTTA--GT
right  NNNNNNNNNNNNNNNNNNNNNNNNNNNNNNNNNNNNNNNNNNNNNNNNNNNNNNNNNNNCTTGTTA--GT
       11111111111111111111111111111111111111111111111111111111111*****2222*2

left   GCGCGGTGGC-TCAC--GCCTGTAATCCCAGCACTTTGGGAGGCCGAGGCGGGCGG
chrom  CTACAGTGACATCAATGGACTTTAAGACT--GATTTTTGAAGA---AATGATGAAA
right  CTACAGTGACATCAATGGACTTTAAGACT--GATTTTTGAAGA---AATGATGAAA
       222*2***2*2***222*2**2***22*2222*2***2*2**2222*22222*222
```

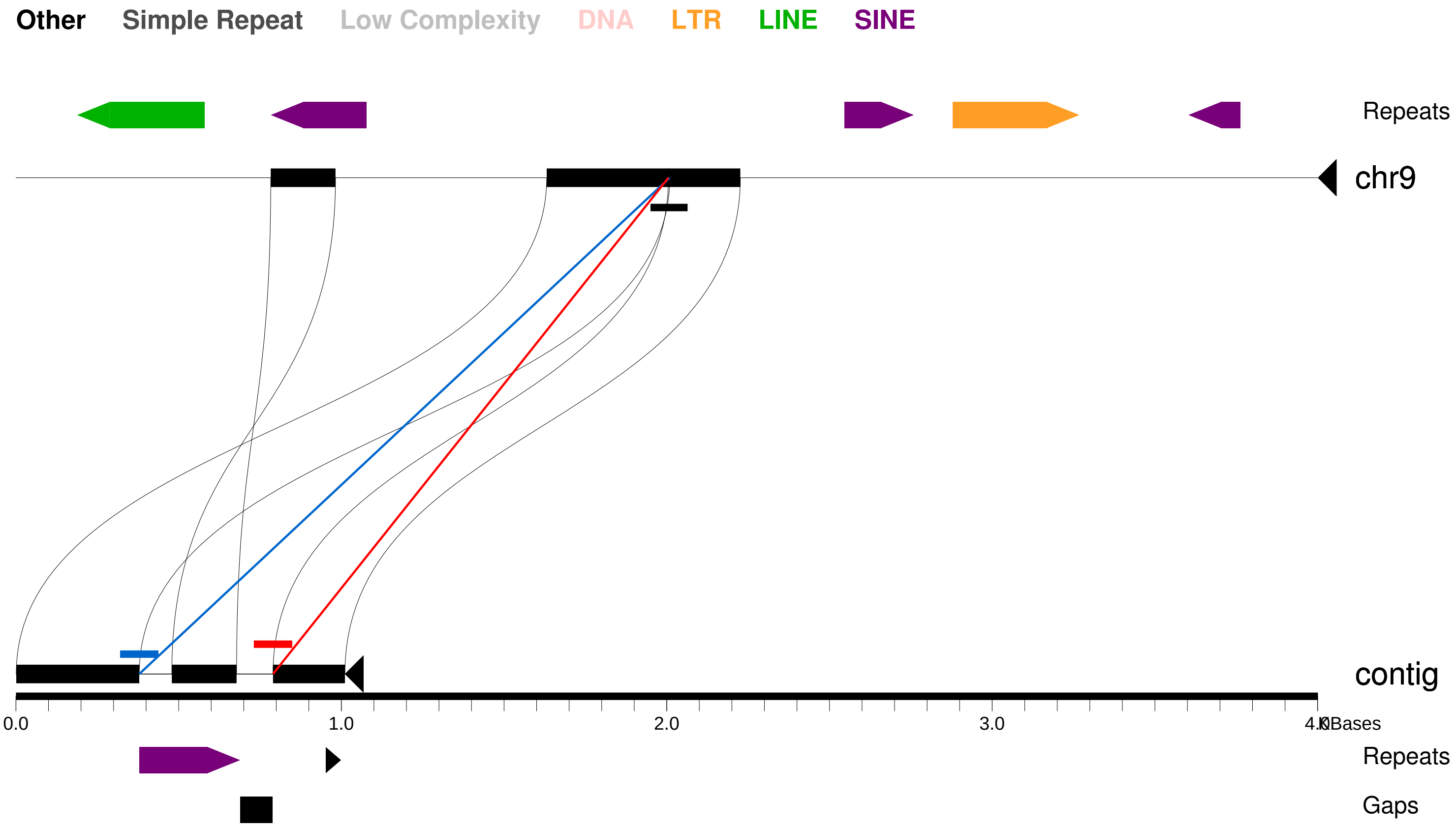

siteID: chr9_30734685 Scafftig3 - Breakpoint: chr9:30734676
chr9:30734625-30734735
Overlap Length: 9 INS size: 300 Genotyped: Yes

```
left    ATGGTATAC-----TTAACAACTGACCT---CATAAACTCTAACTTTAAAAACTTGTATAAACATGTTGG
chrom   ATGGTATAC-----TTAACAACTGACCT---CATAAACTCTAACTTTAAAAACTTGTATAAACATGTT--
right   ACTGCACTCCAGCCTGGGCGACAGAGCGAGACTCCGTCTCAAAAAAAAAAAAAAAAAAAAAACATGTT--
        *11*1*11*11111*111*1**1**1*1111*11111***1**1111*****11111*1*********22

left    CCGGGCGCGGTGGCTCACGCCTGTAATCCCAGCACTTTGGGAGGCCGAGGCGGGCGG
chrom   ACAGACCAGTTTTATTATGTTCTCAA--CATGTACTTT--GAAGCTAATAAAACT--
right   ACAGACCAGTTTTATTATGTTCTCAA--CATGTACTTT--GAAGCTAATAAAACT--
        2*2*2*22*2*222*2*2*22222**22*22*2*****22**2**22*222222222
```

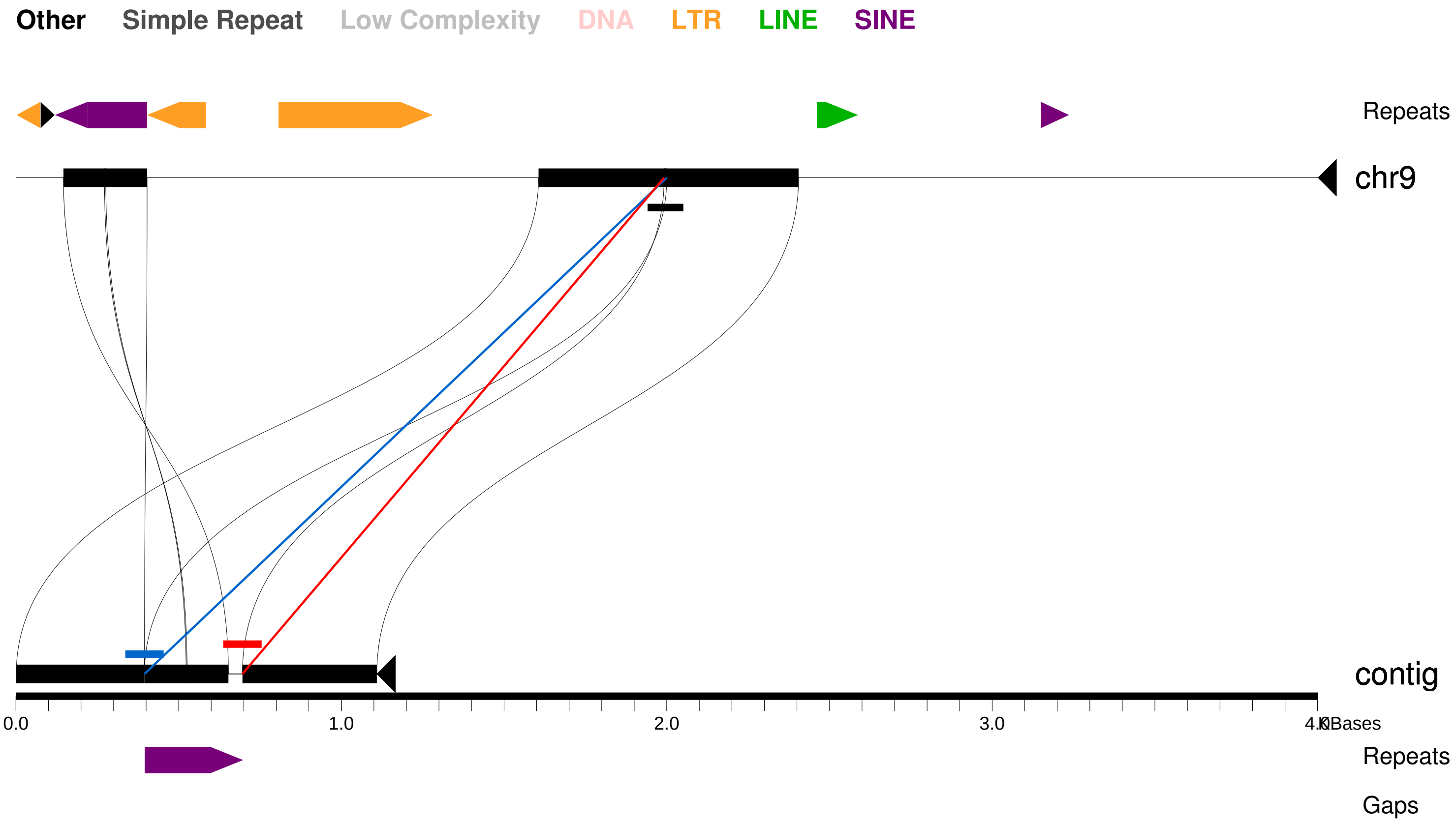

siteID: chr9_32080555 Scafftig2 + Breakpoint: chr9:32080536
chr9:32080506-32080595
Overlap Length: 12 INS size: 313 Genotyped: Yes

```
left    TC-----ATTCTTCCTCCAAAACAAAT--AATTATAT----------------------AAGAAGGTTTT
chrom   TC-----ATTCTTCCTCCAAAACAAAT--AATTATAT----------------------AAGAAGGTTTT
right   CCGCCCGCCTCGGCCTCCCAAAGTGCTGGGATTACAGGCGTGAGCCACCGCGCCCGGCCAAGAAGGTTTT
        1*1111111**11*****1***1111*111****1*1111111111111111111111***********

left    TTGTTTTTTTTTTTTTTTTTTTTTTTTTTTTGAGACGGAGTCTCGCTCTGTCGCCCAGGCTGGAGTGCAGT
chrom   TAAATAGTGTTTTGCTTTT-------------CACAGAGA-----------GCAAAAGTATTTATACAAT
right   TAAATAGTGTTTTGCTTTT-------------CACAGAGA-----------GCAAAAGTATTTATACAAT
        *222*22*2****22****22222222222222**2***222222222222**22*2*222222*2**2*

left    GGCGGGAC
chrom   A-----AG
right   A-----AG
        222222*2
```

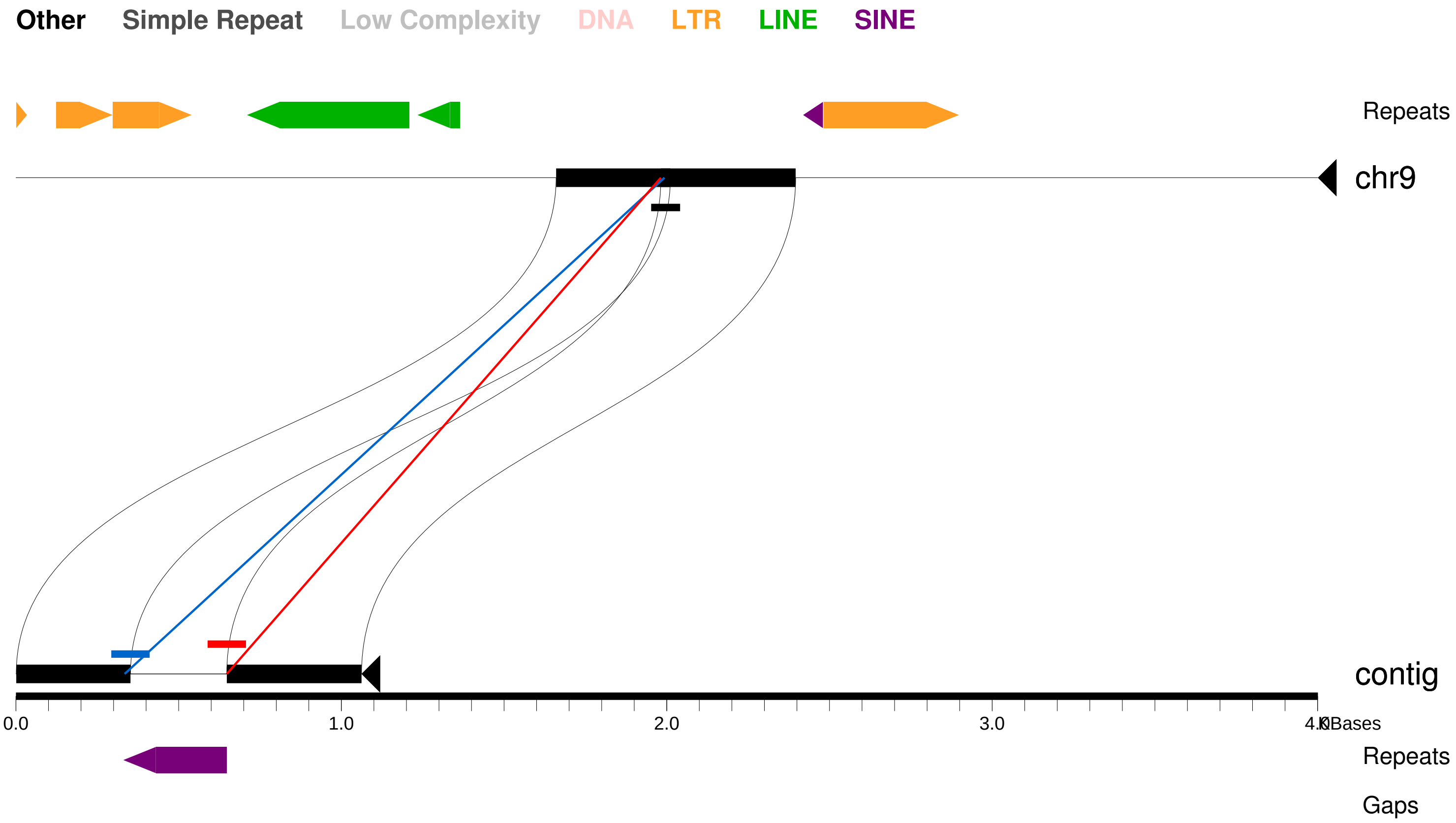

siteID: chr9_33700126 Scafftig1 + Breakpoint: chr9:33700119
chr9:33700075-33700178
Overlap Length: 16 INS size: 301 Genotyped: Yes

```
left     TAAT---GTCAAGTTCACACTAGAGTCTACGAT----GGTGT----CAAAGAGCT----CTATATTTTTC
chrom    TAAT---GTCAAGTTCACACTAGAGTCTACGAT----GGTGT----CAAAGAGCT----CTATATTTTTC
right    TCCACCCGCCTCGGCCTCCCAAAGTGCTGGGATTACAGGCGTGAGCCACCGCGCCCGGCCTATATTTTTC
         *111111*1*11*11*1*1*1*1111**11***1111**1**1111**11*1**11111***********

left     TTCTTCTTTTTTTTTTTTTGAGACGGAGTCTCGCTCTGTTGCCCAGGCCGGACTGCGGACTGCA
chrom    TTCTTGAGTCACTTTGAT--------ATCCTCCCACAGTA----AGACC--ATTACAGT-TGGT
right    TTCTTGAGTCACTTTGAT--------ATCCTCCCACAGTA----AGACC--ATTACAGT-TGGT
         *****222*222***22*22222222*22***2*2*2**22222**2**22*2*2*2*22**22
```

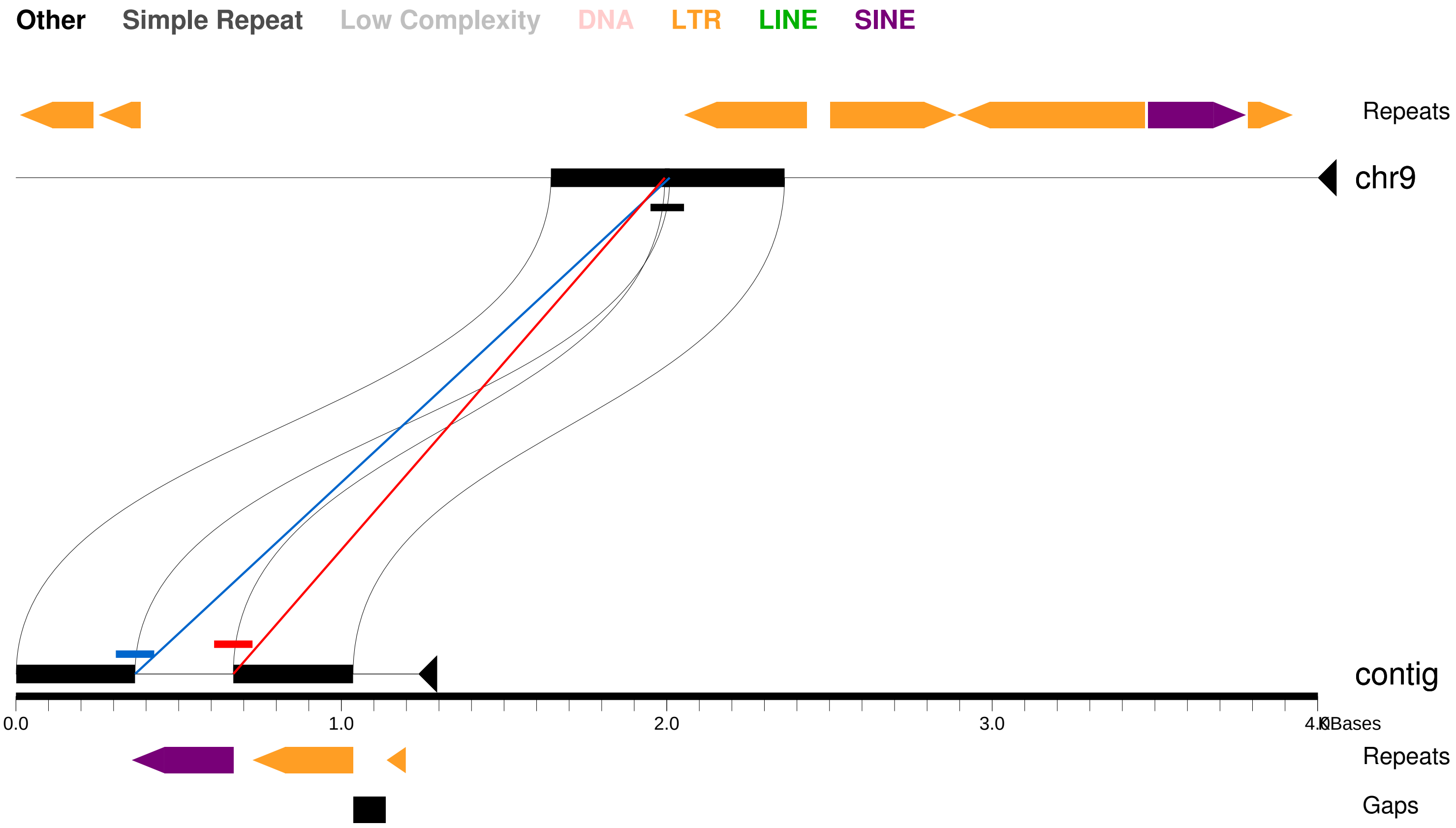

siteID: chr9_37596340 Scafftig4 + Breakpoint: chr9:37596332
chr9:37596172-37596508
Overlap Length: 17 INS size: 311 Genotyped: Yes

```
left    T------------------ATTGTCCAATGACAGTGGGTTTAGGTGAGGC-AAATTGAGAAAATATGCTT
chrom   TTGGCATGTAGGTGGTGGTATTGTCCAATGACAGTGGGTTTAGGTGAGGC-AAATTGAGAAAATATGCTT
right   G--------AGAATGGCGTGAACCCCAGGGGGCGGAGCCTGCAGTGAGCCGAGATTGCGCCACTGCACTC
        1        22NNN2NN2211111***11*111*11*11*111*****1*1*1****1*11*1*111**1

left    CAGGAAGTCAACATTGGATGGAGCTATTTCATATCTTGGACCCCTCATTCTTCCTCAATGATGGGATGAT
chrom   CAGGAAGTCAACATTGGATGGAGCTATTTCATATCTTGGACCCCTCATTCTTCCTCAATGATGGGATGAT
right   CAG--------CCTGGGCGACAGCGA------------GACTCCG---TCTCAAAAAAAAAAAAAAAAAA
        ***11111111*1*1**1111***1*111111111111***1**1111***11111**11*1111*11*1

left    TAGAGGTTGGGTCTAATCTTTAAAGTTGCCCACATTTC-----AGGCCGGG---CGCAGTGGCTCACGCC
chrom   TAGAGGTTGGGTCTAATCTTTAAAGTTGCCCACATTTCTAAAAATGCTGTAATTCTGTGTTCATCTTTCC
right   AAAAA---------AAAAAAAAAAGTTGCCCACATTTCTAAAAATGCTGTAATTCTGTGTTCATCTTTCC
        1*1*1111111111**11111****************22222*2**2*22222*222**222**222**

left    TGTAATCCCAGCA---CTTTGGG----AGGCCGAGGCGGGCGGAT-CAC-GAGGTC---------AGG--
chrom   TGTTCTTCCTACATAACCTTGAACTAAAAGCTAAGAGCAAAGGATTCACTGTGATTTGCCGTGGAAGGCA
right   TGTTCTTCCAACATAACCTTGAACTAAAAGCTAAGAGCAAAGGATTCACTGTGATTTGCCGTGGAAGGCA
        ***22*2**N2**222*2***222222*2**22**222222****2***2*2*2*2222222222***22

left    -AGATCGAGACCATCCTGGC------TAACACGGTGAAACCCTGTCTCTACT---AAA
chrom   AAAATAAAAAGTATGCGCGCATAATTTAATATTGATATTTGTATTATTTAGTCTGATC
right   AAAATAAAAAGTATGCGCGCATAATTTAATATTGATATTTG------------------
        2*2**22*2*22**2*22**222222***2*22*22*2222NNN1N1N11N1   1NN
```

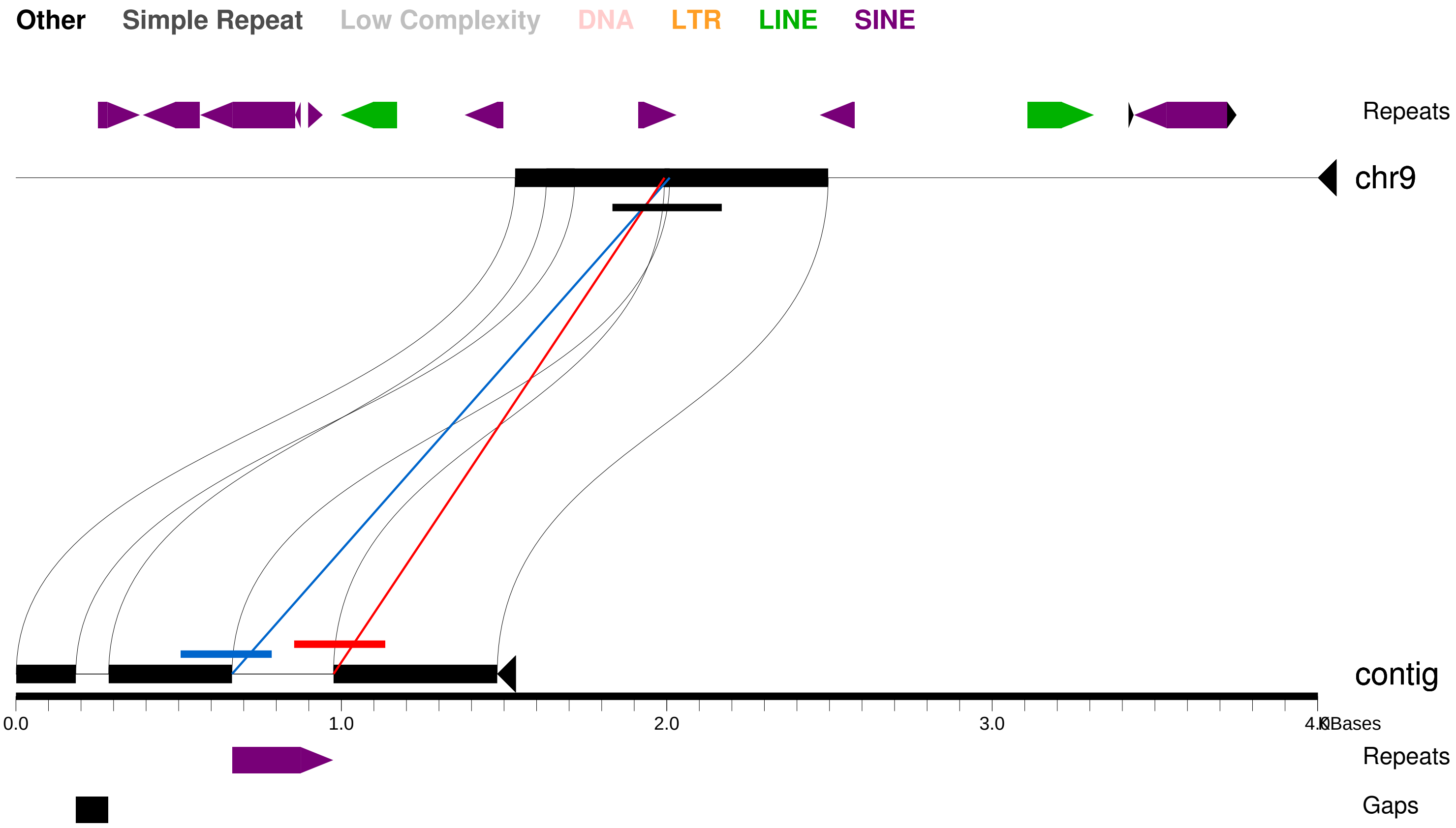

siteID: chr9_38767754 Scafftig6 + Breakpoint: chr9:38767761
chr9:38767601-38767934
Overlap Length: 14 INS size: 300 Genotyped: Yes

```
left   --------------CCCTCTTGTGCTTTCAGTGCTCCCTTGATTTCTGACATAACAAGGTAACTGTTCAC
chrom  TTGCTCTGGCGCAGCCCTCTTGTGCTTTCAGTGCTCCCTTGATTTCTGACATAACAAGGTAACTGTTCAC
right  ----------GCAG-------GAGAATGGCGTGAACCCCAGGGGGCGGAGCCTGCA--------GTGAGC
                 22221111111*1*11*111***11***11*1111*1**11111**11111111**111*

left   CGACTACTTCTATTGCCCAGACCCGGAATTAACCATTTCCTCAAGGACTGGCATTATTATTATTAATGCG
chrom  CGACTACTTCTATTGCCCAGACCCGGAATTAACCATTTCCTCAAGGACTGGCATTATTATTATTAATGCG
right  CGAG-------ATTGCGC---CACTGCACT--CCAG--CCTGGGCGACAGCGAGACTCCGTCTCAA----
       ***11111111*****1*111*1*1*1*1*11***111***1111***1*11*111*111*1*1**1111

left   GTATAACTTACACAGAGTATAAGAAAGTACACTAGGC---CGGGCGCGGTGG------CT----CACGCC
chrom  GTATAACTTACACAGAGTATAAGAAAGTACACTAATCTTACATGTACAATTCAATGAACTTTTACATACT
right  --AAAAAAAAAAAAAAAAAAAAGAAAGTACACTAATCTTACATGTACAATTCAATGAACTTTTACATACT
       11*1**111*1*1*1*11*1*************22*222*22*22*22*22222222**2222**22*2

left   TGTA----ATCCCAGCACTT-TGGGAGGCCGAGG----------CGGGCAGATCACGAGGTC---AGGAG
chrom  TATACAGCATACAACCACTTCTCATATAAAGATATACAACATTTCTAGCACCTAGAAAGTTCCCCAGGGG
right  TCTACAGCATACAACCACTTCTCATATAAAGATATACAACATTTCTAGCACCTAGAAAGTTCCCCAGGGG
       *N**2222**2*2*2*****2*222*2222**222222222222*22***22*2222**2**222***2*

left   --------ATCGAGACCATCCTGGCTAACACGGTGAAACCCCGTCTCTACTAAA
chrom  CCCATTGCATGTATTACCTCCTCCCCCATGCTGTATGCAGTGGTAATTACAATA
right  CCCATTGCATGTATTACCTCCTCCCCCATGCTGTATGCAGTGGTAA--------
       22222222**22*222*2****22*22*22*2**22222222**22N111N1N1
```

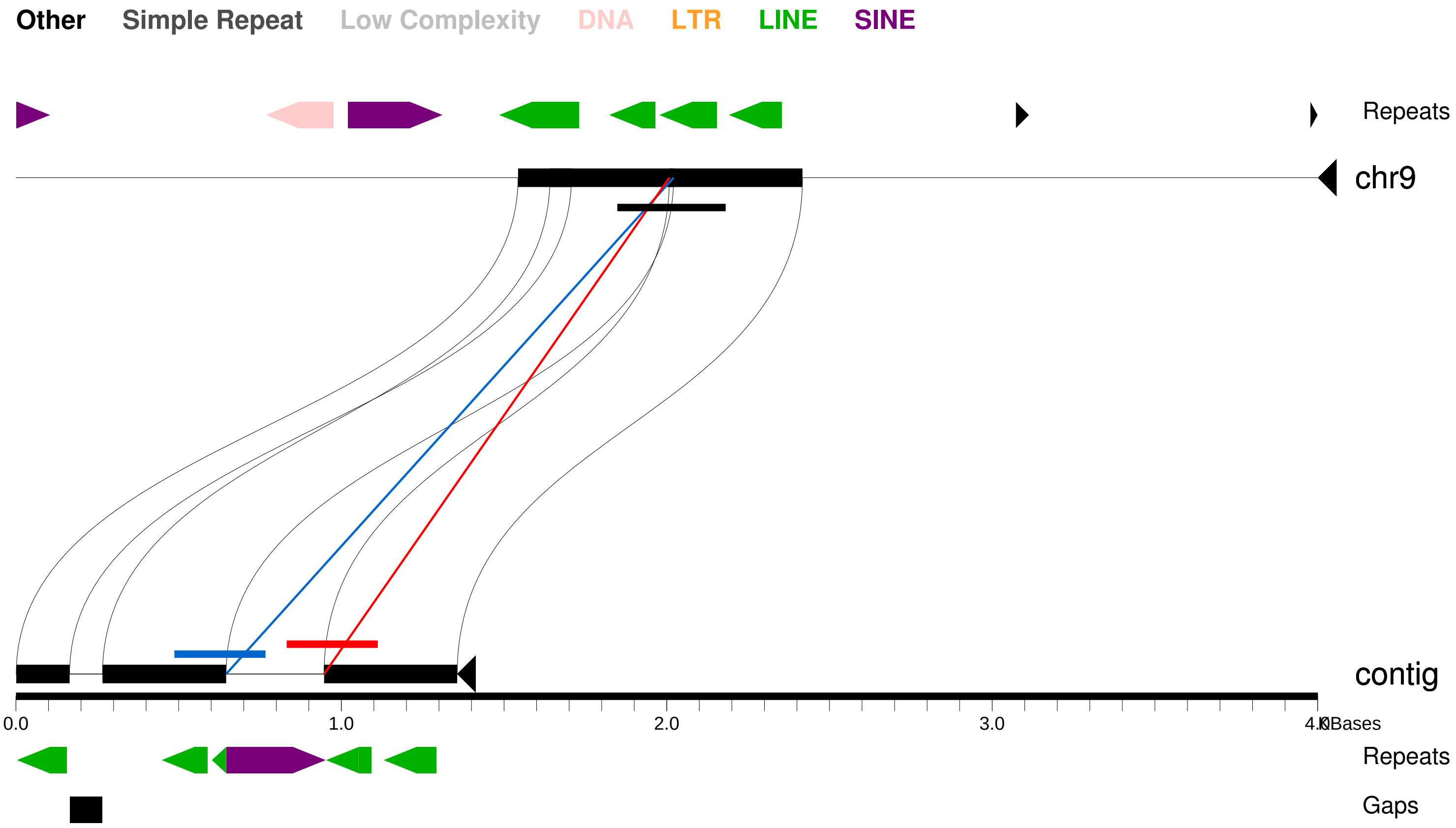

siteID: chr9_4237120 Scafftig3 + Breakpoint: chr9:4237130
chr9:4237082-4237189
Overlap Length: 12 INS size: 281 Genotyped: Yes

```
left   AAGTA-TAAAGAATCACCAACATGTTCACT-TCCTTCACTGATT---------TATTTCAAAAATAATCC
chrom  AAGTA-TAAAGAATCACCAACATGTTCACT-TCCTTCACTGATT---------TATTTCAAAAATAATCC
right  CTGCACTCCAGCCTGGGCGACAGAGCGAGACTCTGTCTCAAAAAAAAAAAAAATAAAATAAAAATAATCC
       11*1*1*11**11*111*1***11111*111**11**1*11*11111111111**1111***********

left   T--GCCTGTAATCCCAGCACTTTGGGAGGCCGAGGCGGGCGGATCACGAGGTCAGGAGATC-G
chrom  TAAGGAAATAATCCAAA-ACAAGGGAAAACTGT------------ATTATGT-ATTATAGCAG
right  TAAGGAAATAATCCAAA-ACAAGGGAAAACTGT------------ATTATGT-ATTATAGCAG
       *22*2222******2*22**222**2*22*2*2222222222222*22*2**2*22*2*2*2*
```

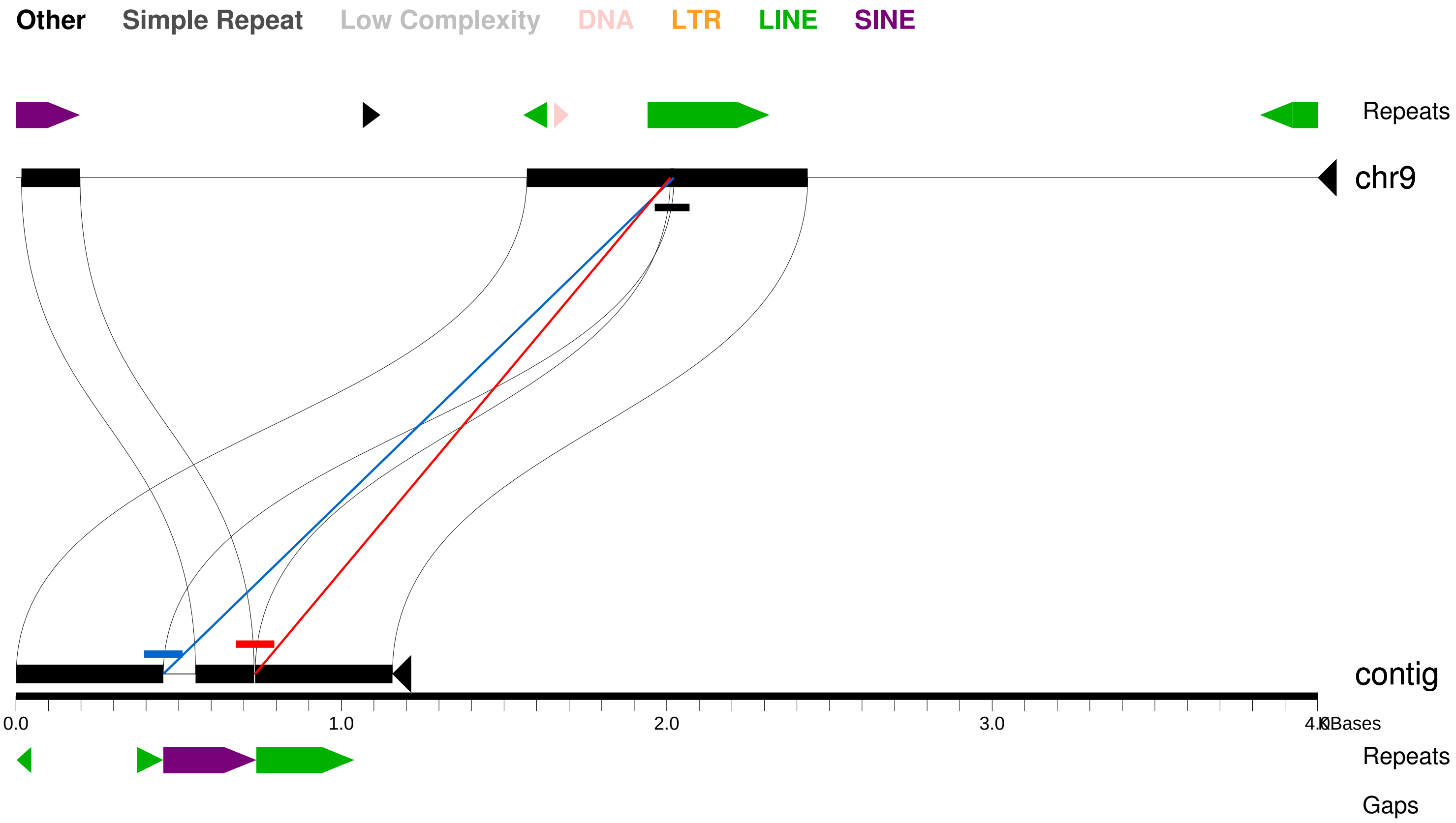

siteID: chr9_68434021 Scafftig7 + Breakpoint: chr9:68434050
chr9:68433890-68434223
Overlap Length: 14 INS size: 295 Genotyped: Yes

```
left   --------------CATAATTATGAATGCATACTTTATTCAAATATTAGCATTTTAAGTAAAATTTATTT
chrom  TTTAAAGACAAGAACATAATTATGAATGCATACTTTATTCAAATATTAGCATTTTAAGTAAAATTTATTT
right  TG---AGGCAAGAGAATG-----GCATGAACCAGGGAGGCAGAGCTT-GCAGTGAGCCGAGA--------
       2N   22N22222N1**111111*1***1*111111*11**1*11**1***1*111111*1*11111111

left   TCTTCACAATTAGAAAACATGAAAAGGCATATACAATGCCTTTGGTGTTTTGAATTTAAGAATCAATGTC
chrom  TCTTCACAATTAGAAAACATGAAAAGGCATATACAATGCCTTTGGTGTTTTGAATTTAAGAATCAATGTC
right  TCGTGCCACT--GCACTCCAGCCTGGGCG---ACAGAGCCA-----GACTCTGCTTAAAAAATAAATAAA
       **1*11**1*11*1*11*11*1111***1111***11***111111*11*1111**1**1***1***111

left   TGAGGGACTTTTGTGTGTGAAAATAAATATTCATGGCCGG---------------GCGCGGTGGCTCACG
chrom  TGAGGGACTTTTGTGTGTGAAAATAAATATTCATATACATTTTTAGTTGTTTAATGTTTGATGTATTACA
right  T-------------------AAATAAATATTCATACACATTTTTCGTTGTTTAATGTTTGATGTATTACA
       *1111111111111111111*************2N2*222222N2222222222*222*2**22*2**2

left   C------CTGTAATCCTAGCACTTTGGGAGGCCGAGG--CGGGTGGATCACGA----GGTCAGGAGATCG
chrom  CTGCTTTCTATTAAAC-AAAACTTTAAAAA-CTGATTTTCTTGTGTATCTAAATCTGGGTTATAAATTTG
right  CTGCTTTCTATTAAAC-AAAACTTTAAAAA-CTGATTTTCTTGTGTATCTAAATCTGGGTTATAAATTTG
       *222222**2*2*22*2*22*****222*22*2**2222*22***2***222*2222***2*22*22*2*

left   ---AGACCA--TCCTGG--CTAACATGGTG-------AAACCCCGTCTCTACTAAA
chrom  GTTAGCTTAACTCCTGTAACAAATATAATGTTTATTTATAACTTGTATTTGGTTGA
right  GTTAGCTTAACTCCCGTAACAAATATAGTGTTTATTTATAACTTGTATT-------
       222**222*22***1*222*2**2**2N**2222222*2*2*22**2*21NN1NN1
```

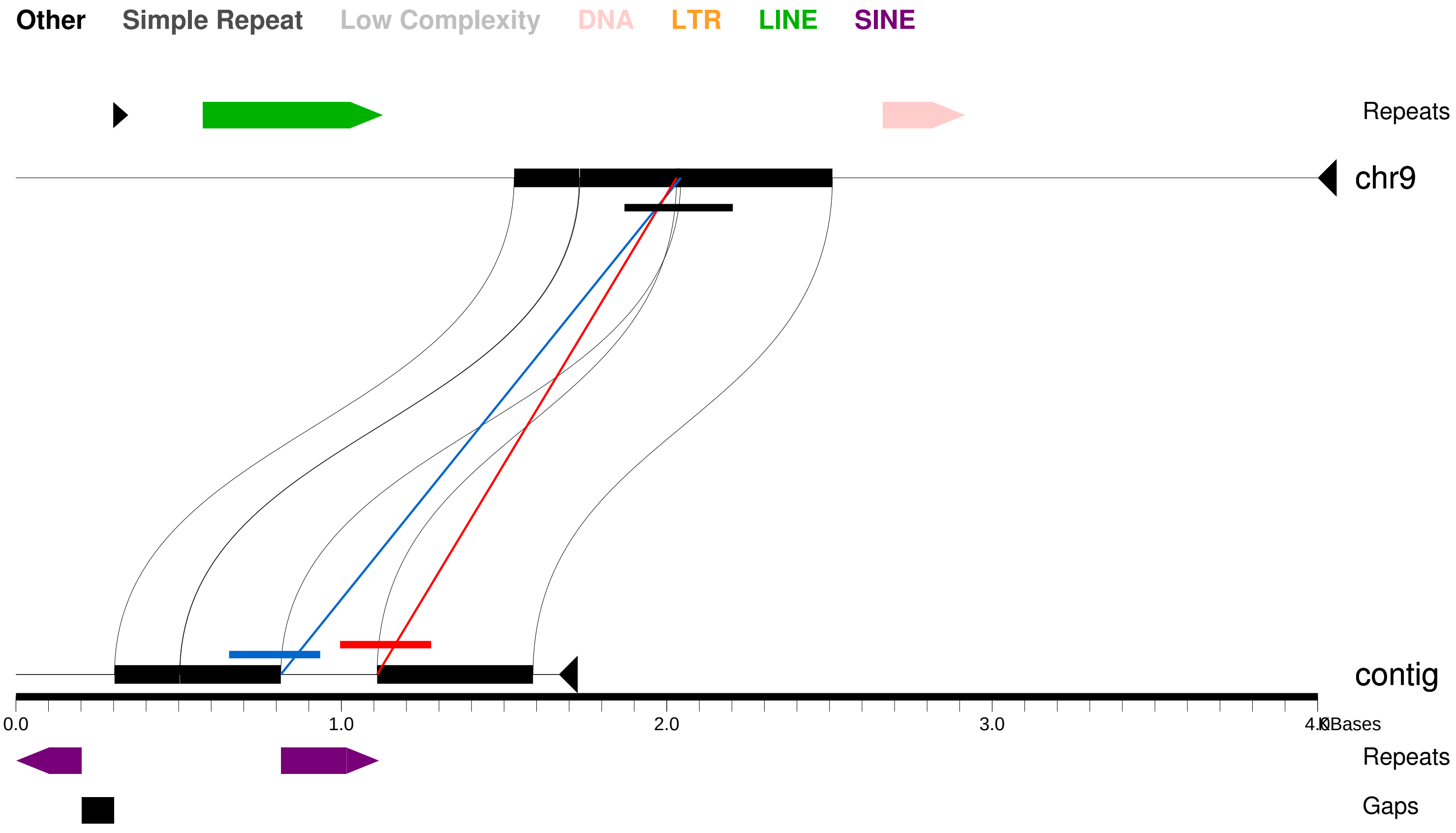

siteID: chr9_74544794 Scafftig6 - Breakpoint: chr9:74544780
chr9:74544733-74544839
Overlap Length: 13 INS size: 428 Genotyped: No

```
left   GCTATAGGAACACATGTTCCACTTGTAACTTAATAGCATGCCTTAAA------------CTTAAAGATAT
chrom  GCTATAGGAACACATGTTCCACTTGTAACTTAATAGCATGCCTTAAA------------CTTAAAGATAT
right  NNNNNNNNNNNNNNNNNNNNNNNNNNNNNNNNNNNNNNNNNNNNNNNNNNNNNNNNNNNCTTAAAGATAT
       11111111111111111111111111111111111111111111111111111111111***********

left   CTGGCCGGGCGCGGTGGCTCAC--GCCTGTAATCCCAGCACTTTGGGAGGCCGAGGCGGGTGG
chrom  CTTTTTATGGATTCTGCCTCTGCTGCTTCTGATTA----ATTTTGTGA--CC--------TCA
right  CTTTTTATGGATTCTGCCTCTGCTGCTTCTGATTA----ATTTTGTGA--CC--------TCA
       **222222*22222**2***2222**2*2*2**222222*2****2**22**22222222*22
```

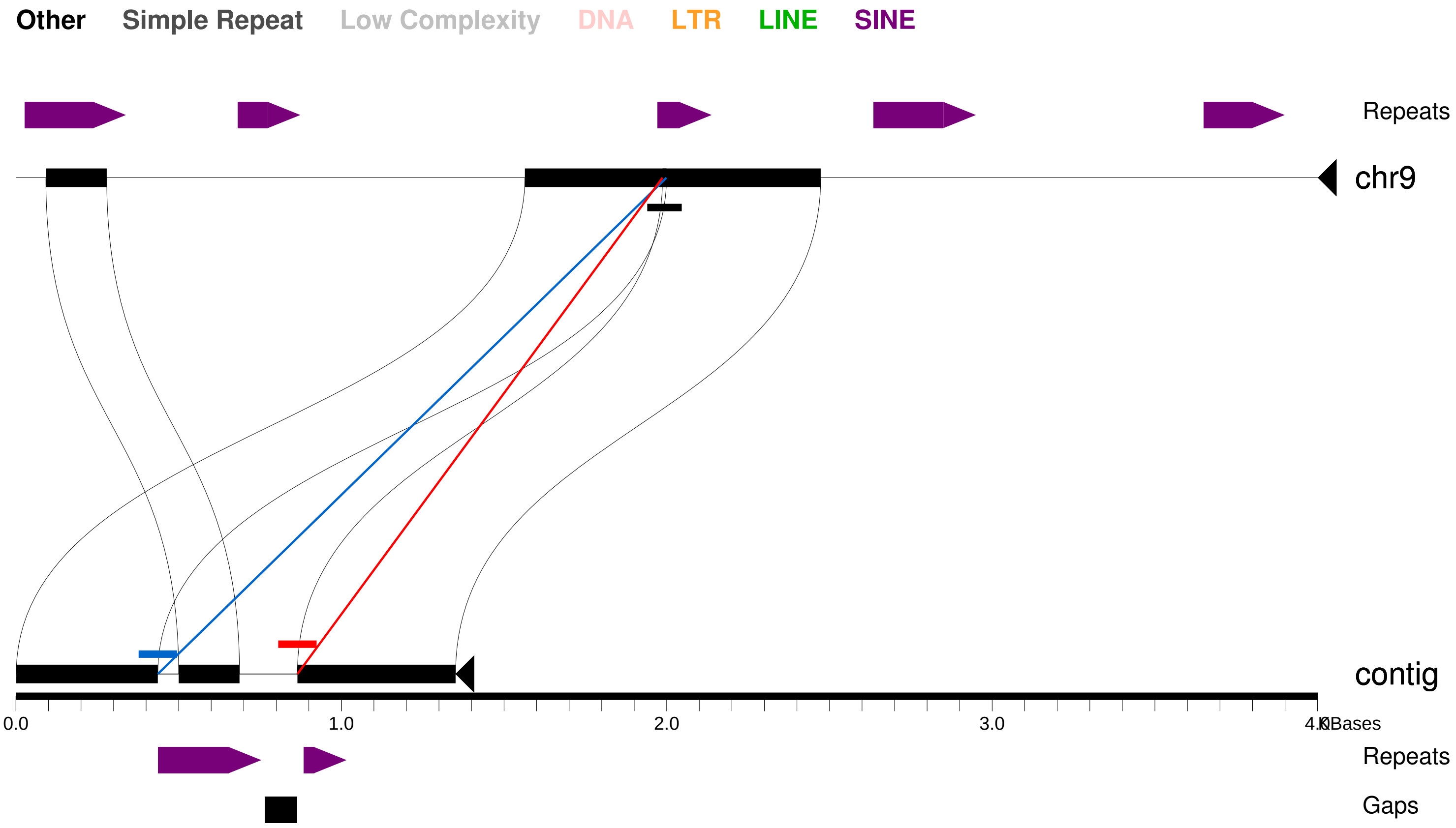

siteID: chr9_75231549 Scafftig1 - Breakpoint: chr9:75231534
chr9:75231487-75231590
Overlap Length: 13 INS size: 303 Genotyped: Yes

```
left     GGTTGTGTTAGT-------ACTGAGCAGTATATCTTTT--------ATTGTTGAAGTCCAATAAGAACAG
chrom    GGTTGTGTTAGT-------ACTGAGCAGTATATCTTTT--------ATTGTTGAAGTCCAATAAGAACAG
right    CTGCACTCCAGCCTGGGCGACAGAGCGAGACTCCGTCTCAAAAAAAAAAAAAAAAAAAAAAAAGAACAG
         111111111**11111111**1****111*111*1*1*11111111*111111**1111**1********

left     AGCAGGCCGGGCGTGGTGGCTCACGCCTGTAATCCCAGCACTTTGGGAGGCCGAGGCGGGCGGA
chrom    AGCAGATA--------TCAACCTAATTTGTAAGTGATGCATTTT------CCTAAACTAAATG-
right    AGCAGATA--------TCAACCTAATTTGTAAGTGATGCATTTT------CCTAAACTAAATG-
         *****22222222222*2222*22222*****22222***2***222222**2*22*22222*2
```

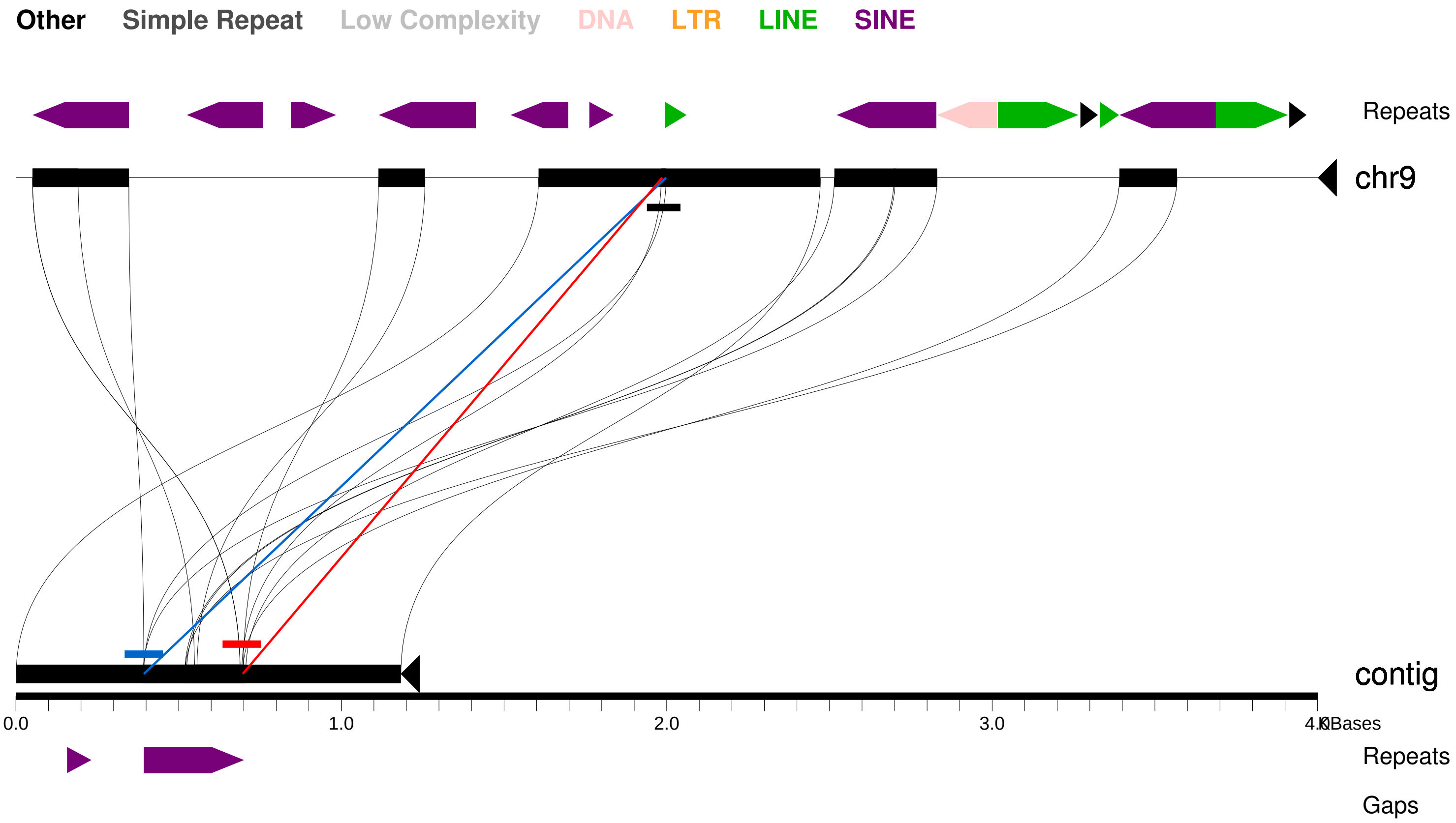

siteID: chr9_75542977 Scafftig2 - Breakpoint: chr9:75542970
chr9:75542926-75543029
Overlap Length: 16 INS size: 333 Genotyped: Yes

```
left    AA-GTGACT---TCTCCCATTTTGTCCCGGAGTAAAGTAGGACAGTTC-----------TAAAGTAGCCA
chrom   AA-GTGACT---TCTCCCATTTTGTCCCGGAGTAAAGTAGGACAGTTC-----------TAAAGTAGCCA
right   AGCGAGACTCCGTCTCAAAAAAAAAACAAAAAAAAAAAAACAAAAAAAAAAAAAAAAATAAAGTAGCCA
        *11*1****111****11*1111111*111*11***11*11*1*111111111111111***********

left    CATTTTGGCCGGGCGCGGTGG-CTCACGCCTGTAATCCCAGCACTTTGGGAGGCCGAGGCGGGTG
chrom   CATTTCTTAGTACCACCGAGGTCTTAC---TCTCCTCTCA--ACTTTCTG----CTAT-------
right   CATTTCTTAGTACCACCGAGGTCTTAC---TCTCCTCTCA--ACTTTCTG----CTAT-------
        *****22222222*2*2*2**2**2**222*2*22**2**22*****22*2222*2*22222222
```

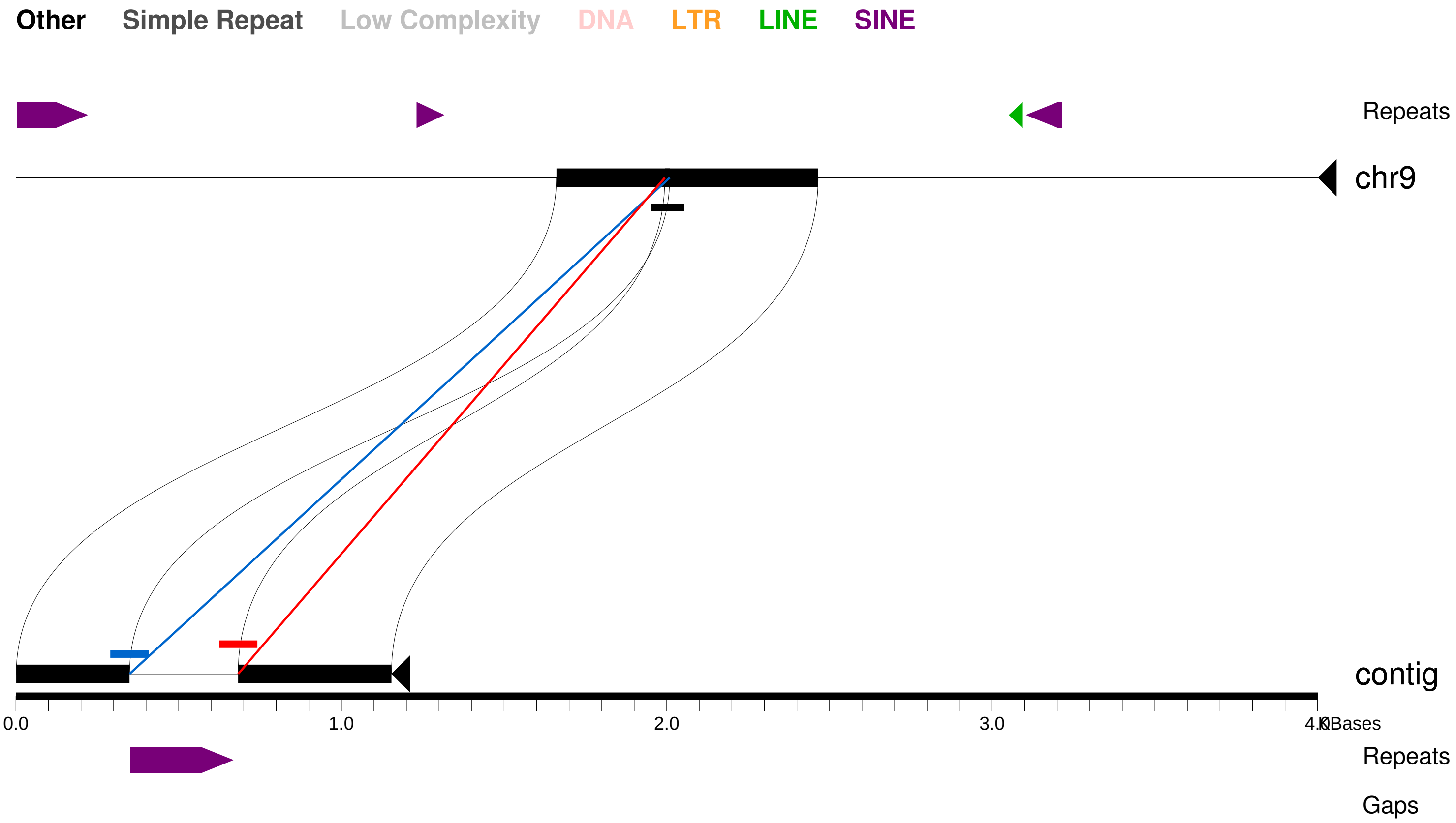

siteID: chr9_75996057 Scafftig1 - Breakpoint: chr9:75996031
chr9:75996004-75996090
Overlap Length: 33 INS size: 473 Genotyped: No

```
left    CTCTAGGGCAGGGGCAAAATGCCACAA--------------------------------GTCTCTTTGCT
chrom   CTCTAGGGCAGGGGCAAAATGCCACAA--------------------------------GTCTCTTTGCT
right   NNNNNNNNNNNNNNNNNNNNNNNNNNNNNNNNNNNNNNNNNNNNNNNNNNNNNNNNNNNGTCTCTTTGCT
        11111111111111111111111111111111111111111111111111111111111***********

left    AAAGCACAGTTAAGAGTGACCTGGCCGGGCGCGGTGGCTCACGCCTGTAATCCCAGCACTTTGGGAGGCC
chrom   AAAGCACAGTTAAGAGTGACCT----------------TTACTCCAGT--TCCCAATAAGTTC-----CT
right   AAAGCACAGTTAAGAGTGACCT----------------TTACTCCAGT--TCCCAATAAGTTC-----CT
        **********************2222222222222222*2**2**2**22*****22*22**222222*2

left    GAGGCGGGCGG
chrom   CA---------
right   CA---------
        2*222222222
```

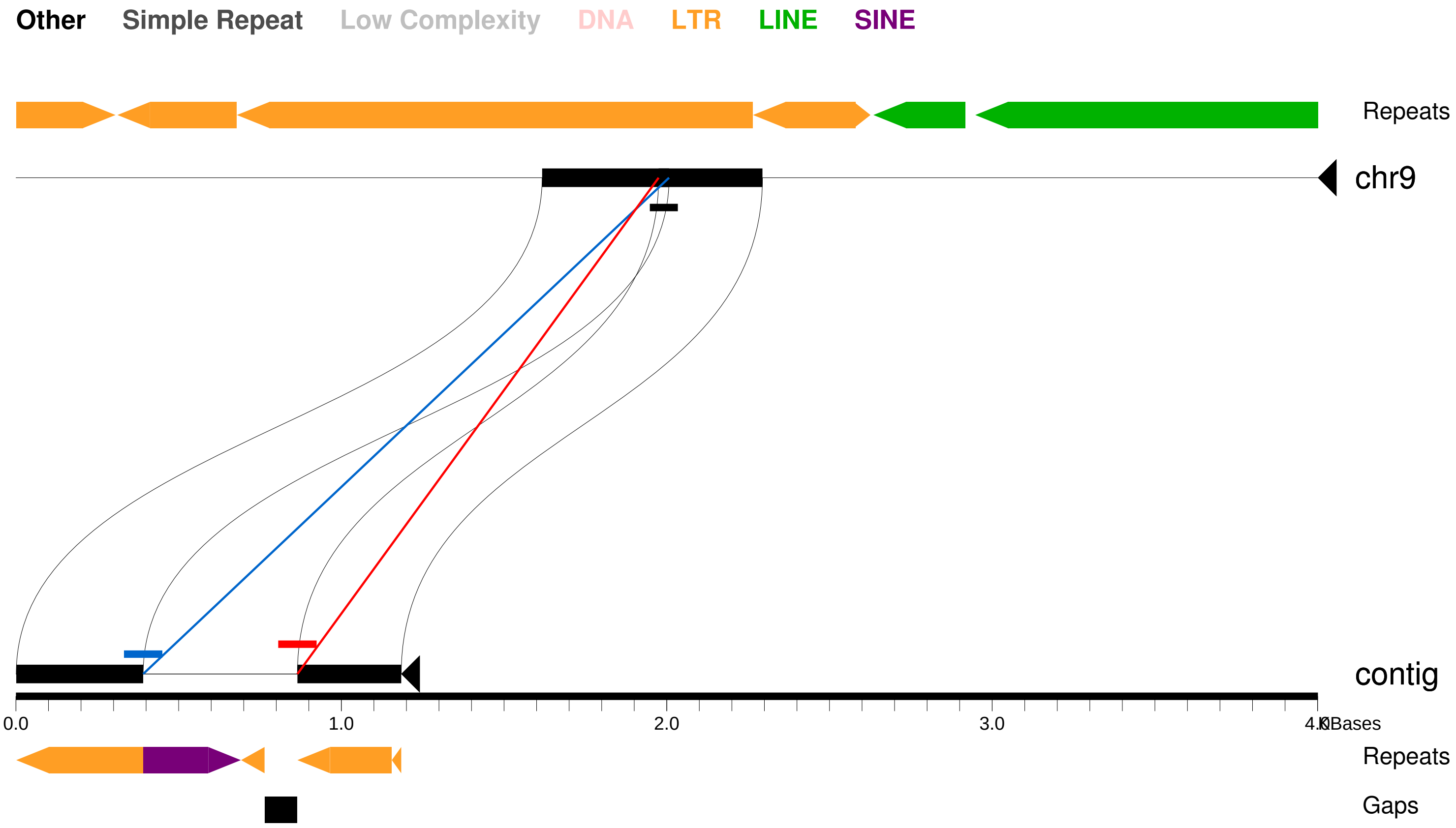

siteID: chr9_76197797 Scafftig4 + Breakpoint: chr9:76197791
chr9:76197745-76197850
Overlap Length: 10 INS size: 311 Genotyped: Yes

```
left    AGA-----------ATCTCAAAACTATGTGAGTTCATTTTTTTTT--ATTGATTTTCAGTGAGCAGTTATC
chrom   AGA-----------ATCTCAAAACTATGTGAGTTCATTTTTTTTT--ATTGATTTTCAGTGAGCAGTTATT
right   CCACCCGCCTCGGCCTCCCAAAGTGCTGGGATTACAGGCGTGAGCCACCGCGCCCGGCCGAGCAGTTATT
        11*111111111111**1****1111**1**1*1**1111*11111*11*111111111**********2

left    CTTTTTTTTTTTTTTTTTTTTTTGAGACGGAGTCTCGCTCTGTCGCCCAGGCCGGACTGCGGA
chrom   CTTAACAGAA-------GATTTCAAACTAACCATTACT-TATCCCTTAAGTCAAGTT-----
right   CTTAACAGAA-------GATTTCAAACTAACCATTACT-TATCCCTTAAGTCAAGTT-----
        ***2222222222222222***2*2**22*222*22**2*2**2*22*2*2*2222*22222
```

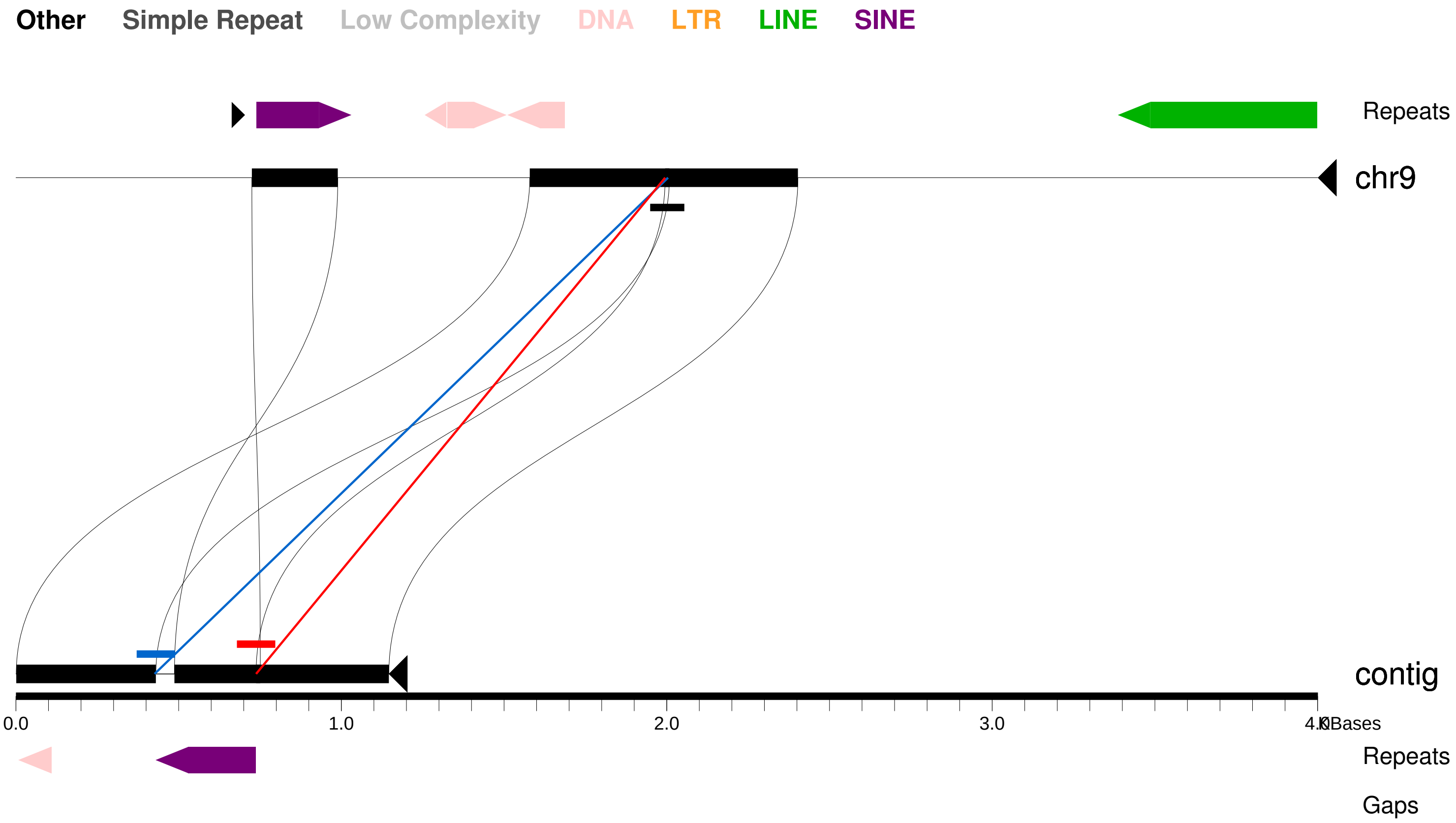

siteID: chr9_78401676 Scafftig1 + Breakpoint: chr9:78401651
chr9:78401599-78401710
Overlap Length: 8 INS size: 347 Genotyped: No

```
left   C--------CTA---TGGGTGCCATTGCATGTGAGATGGGATTCTTGAAGACAGGATACAGTTGGGTCTT
chrom  C--------CTA---TGGGTGCCATTGCATGTGAGATGGGATTCTTGAAGACAGGATACAGTTGGGTCTT
right  CACGCCCGGCTAATTTTTTTGTATTTTTAGTAGAGACGGGGTT--TCACCGTGTTAGCCAG--GGGTCTT
       *11111111***111*111**111**11*111****1***1**11*1*1111111*11***11*******

left   GGTNNNNNNNNNNNNNNNNNNNNNNNNNNNNNNNNNNNNNNNNNNNNNNNNNNNNNNNNNNNN
chrom  GATTCTTTATCCAACTTGCCACTCTGTGCCTTTTAAGTGGAGCATTTAGCCCG-------
right  GATTCTTTATCCAACTTGCCACTCTGTGCCTTTTAAGTGGAGCATTTAGCCCG-------
       *2*2222222222222222222222222222222222222222222222222222222222222
```

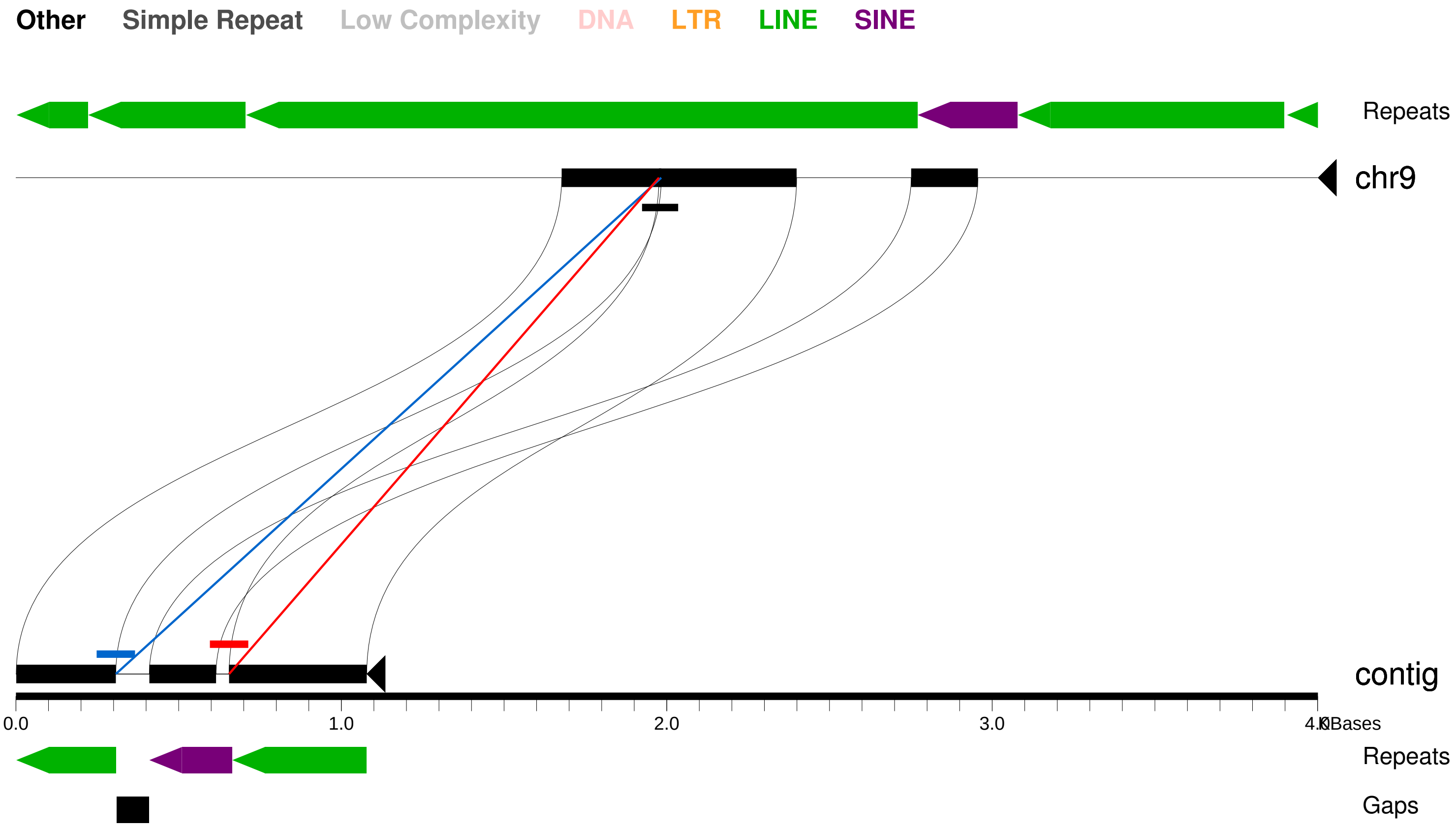

siteID: chr9_7852545 Scafftig9 - Breakpoint: chr9:7852524
chr9:7852479-7852583
Overlap Length: 15 INS size: 314 Genotyped: Yes

```
left    GATAG--TGATAGGAACATCAATGACTACGGTTTTGGTCAATCTTTC-------------AAAATTGAGA
chrom   GATAG--TGATAGGAACATCAATGACTACGGTTTTGGTCAATCTTTC-------------AAAATTGAGA
right   TCCGGCCTGGGCGACAGAGCGA-GACTCCGTCTCAAAAAAAAAAAAAAAAAAAAAAAAAAAAAAATTGAGA
        1111*11**111*11*1*1*1****1**11*111111**1111111111111111111111**********

left    AAATGGGCCGGGCGCGGTGGCTCACGCCTGTAGTCCCAGCACTTTGGGAGGCCGAGGCGGGTGG
chrom   AAATGA-CCAAAAGGGGGGAAT------TGTTAAAGCAA-ACTAAATATGGCCT------GAGA
right   AAATGA-CCAAAAGGGGGGAAT------TGTTAAAGCAA-ACTAAATATGGCCT------GAGA
        *****22**2222*2**2*22*222222***22222**22***222222****2222222*2*2
```

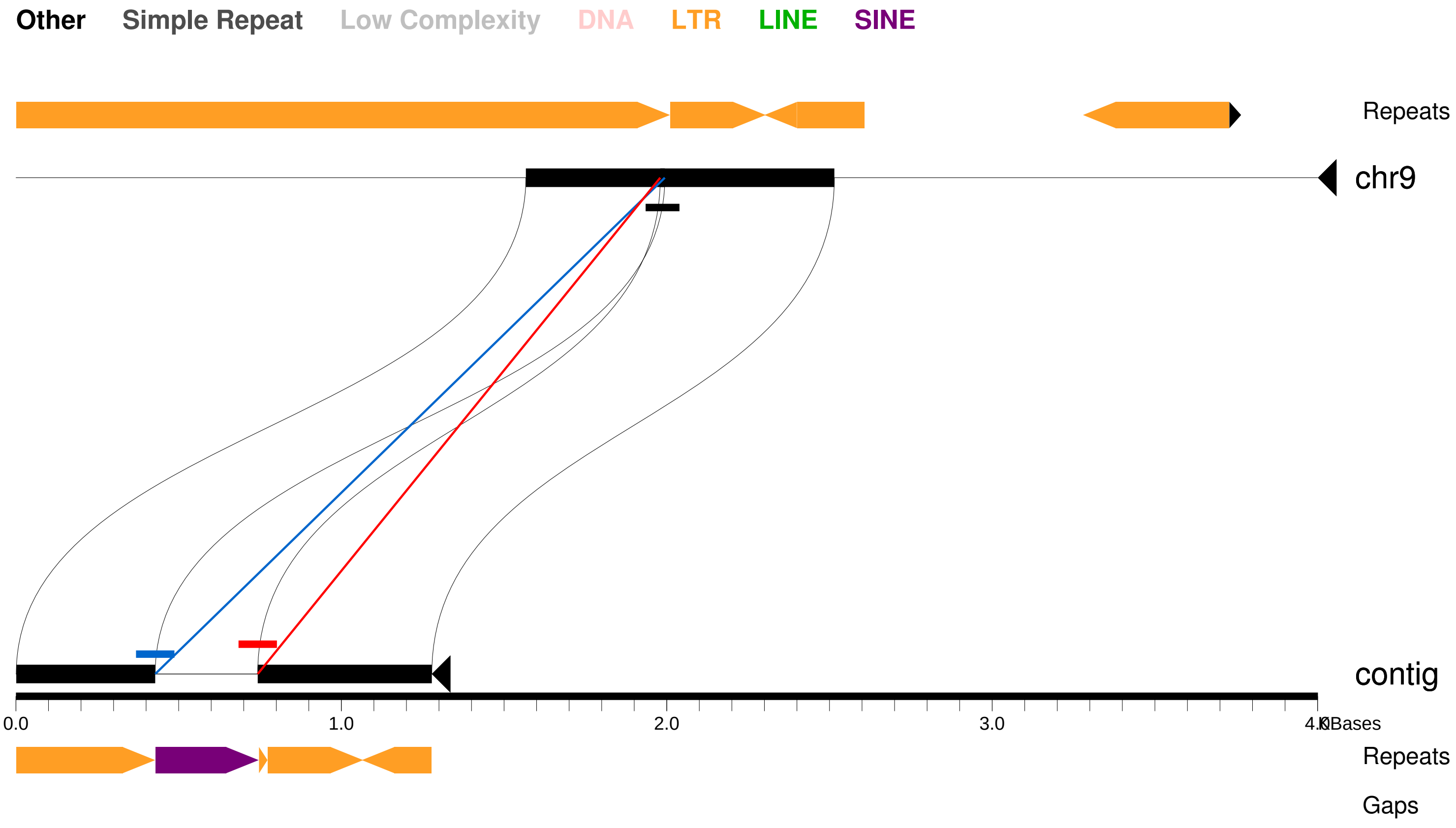

siteID: chr9_79330938 Scafftig1 - Breakpoint: chr9:79330944
chr9:79330902-79331003
Overlap Length: 18 INS size: 381 Genotyped: No

```
left     TTATGTAT--ATAAAGGCCTCCAAAT----ACAT--TTATAAACCC---------TTGAAAGAAGACACA
chrom    TTATGTAT--ATAAAGGCCTCCAAAT----ACAT--TTATAAACCC---------TTGAAAGAAGACACA
right    CGTGTTAGCCAGGATGGTCTCGATCTCCTGACCTCGTGATCCGCCCGCCTCGGCCTCCCAAGAAGACACA
         11111**111*11*1**1***1*11*1111**1*11*1**111***111111111*111***********

left     CATTTCTNNNNNNNNNNNNNNNNNNNNNNNNNNNNNNNNNNNNNNNNNNNNNNNNNNNNNNNNNNNNNN
chrom    CATTTCTAAGAGATTTTTACATATAACAAAATTCTCACTACACTCCTGG-----------------
right    CATTTCTAAGAGATTTTTACATATAACAAAATTCTCACTACACTCCTGG-----------------
         ********2222222222222222222222222222222222222222222222222222222222
```

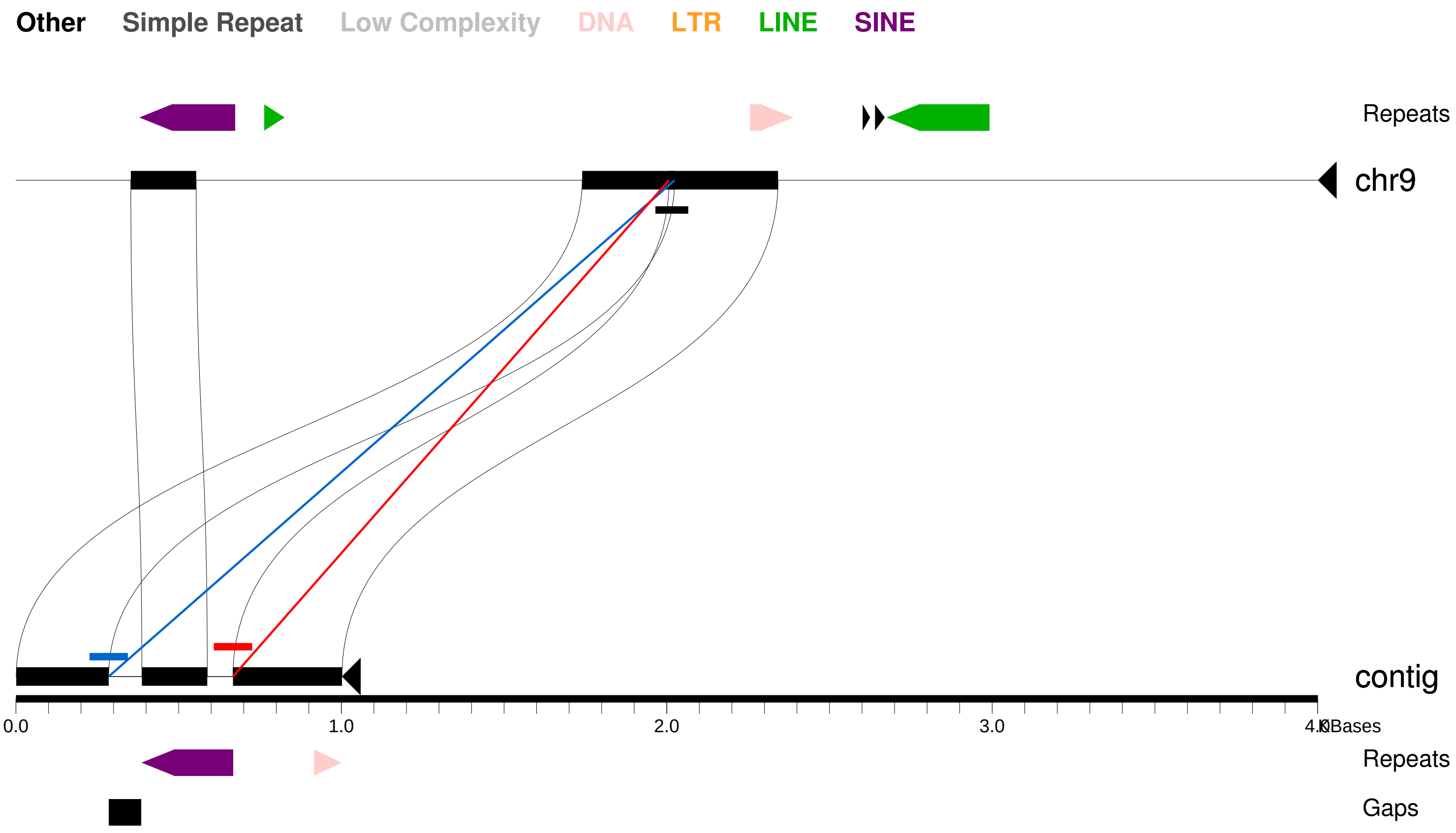

siteID: chr9_79583153 Scafftig3 - Breakpoint: chr9:79583156
chr9:79583113-79583215
Overlap Length: 17 INS size: 298 Genotyped: Yes

```
left    GCCGGTCCCTCCTGCCAATCCAAAATG--GACATGTAGTGTGAGG--------------AAAAAAAAAAG
chrom   GCCGGTCCCTCCTGCCAATCCAAAATG--GACATGTAGTGTGAGG--------------AAAAAAAAAAG
right   CCACTGCACTCCAGCCTGGGCGACAGAGCGAGACTCCGTCTCAAAAAAAAAAAAAAAAAAAAAAAAAAAG
        1*1111*1****1***1111*1*1*1111**1*1111**1*1*1111111111111111***********

left    CTTTGAGGCCGGGCGCGGTGGCTCACGCCTGTAATCCCAGCACTTTGGGAGGCCGAGGCGGGCGG
chrom   CTTTGATGTTT-------TATTCCAAGGAGATCATACG-GCTATTTG---TTCCTACA-----GC
right   CTTTGATGTTT-------TATTCCAAGGAGATCATACG-GCTATTTG---TTCCTACA-----GC
        ******2*2222222222*2222**2*2222*2**2*22**22****22222**2*2222222*2
```

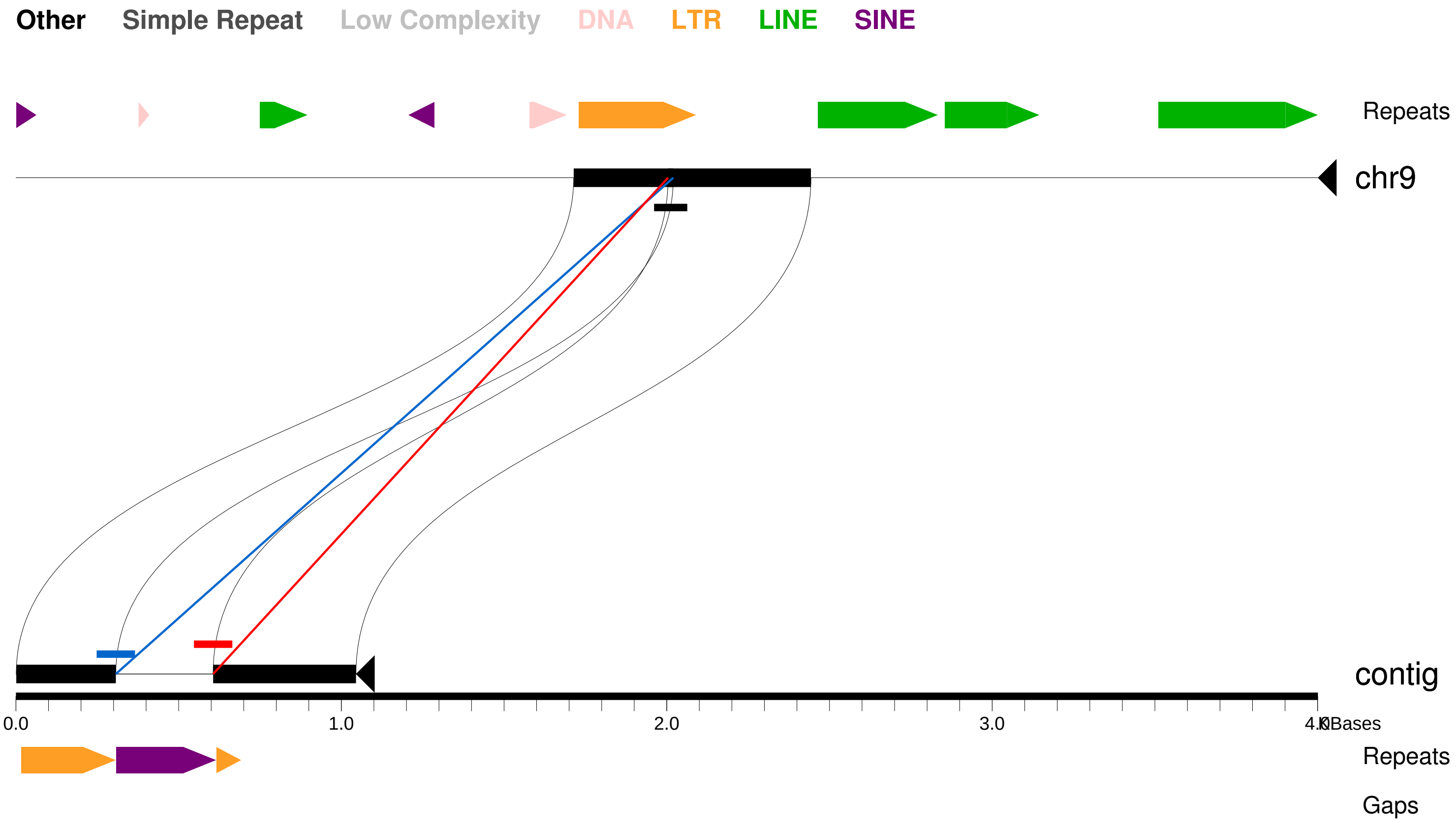

siteID: chr9_80089995 Scafftig1 + Breakpoint: chr9:80089945
chr9:80089899-80090004
Overlap Length: 2 INS size: 306 Genotyped: Yes

```
left     TAG---GACTGGG-------AGGTCATGGAAATAGGAGCATGATG----GCTTAATGGGTCCTTTTTTTT
chrom    TAG---GACTGGG-------AGGTCATGGAAATAGGAGCATGATG----GCTTAATGGGTCCAGTGTTTC
right    CCGCCTGCCTCGGCCTCCCAAAGTGCTGGGATTACAGGCGTGAGCCACCGCGCCC-GGCCCCAGTGTTTC
         11*111*1**1**1111111*1**11***1*1**111**1***111111**11111**11**22*2***2

left     TTTTTTTTTTTTTTTTGAGACGGAGTCTCGCTCTGTCGCCCAGGCTGGAGTGCAGTGGTGGGA
chrom    TTTTATGG--------GGGACATAAAAT---TTTGTGATCCACACCCAAGTTGGCTGGCAG--
right    TTTTATGG--------GGGACATAAAAT---TTTGTGATCCACACCCAAGTTGGCTGGCAG--
         ****2*2222222222*2***22*222*222*2***222***22*222***2222***22*22
```

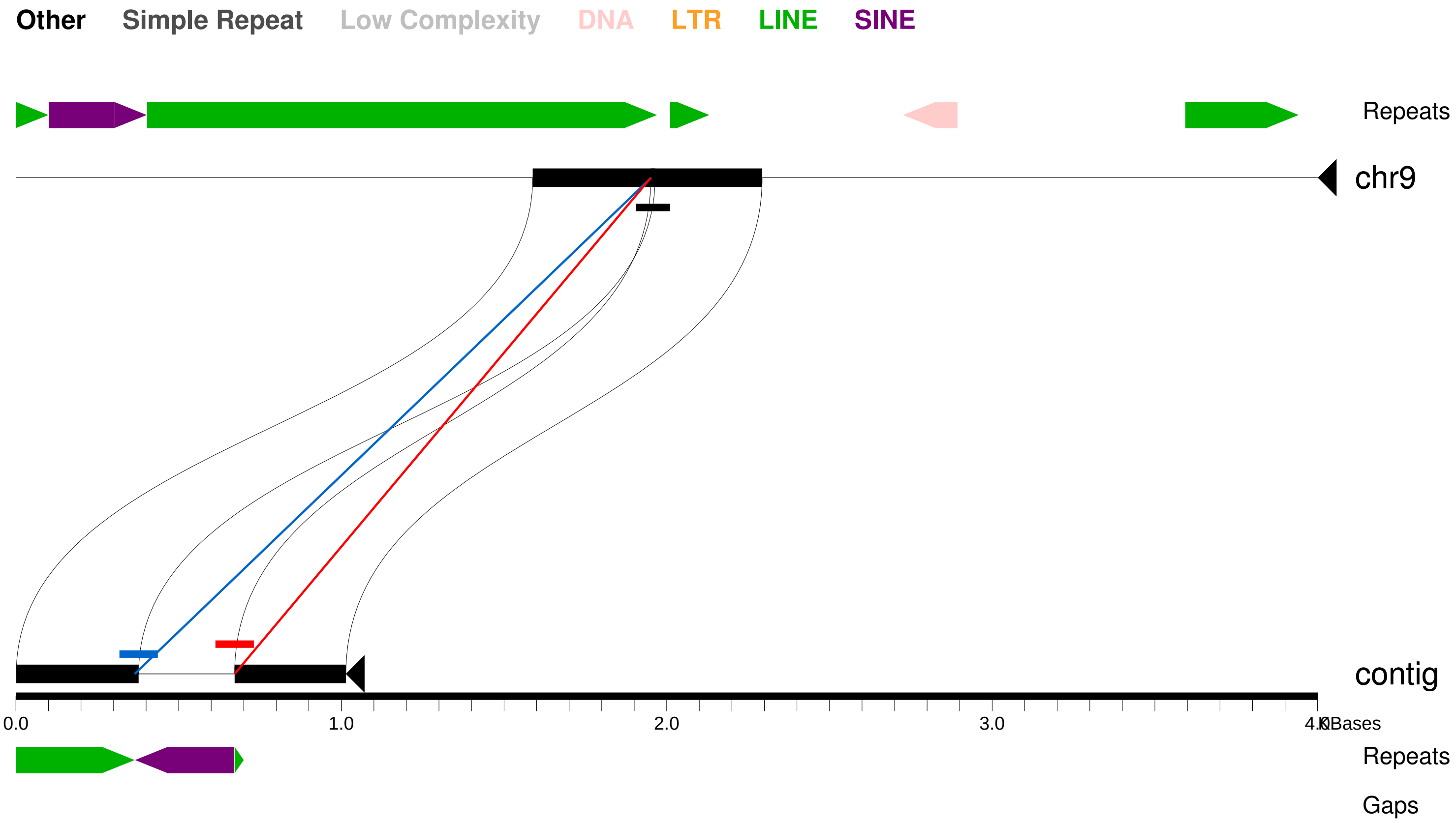

siteID: chr9_81596110 Scafftig1 - Breakpoint: chr9:81596119
chr9:81596075-81596162
Overlap Length: 16 INS size: 303 Genotyped: Yes

```
left   T------TTTCCTTTGAAAAATTTTCTGAGAGGC-------------------------AAAATAGTACA
chrom  T------TTTCCTTTGAAAAATTTTCTGAGAGGC-------------------------AAAATAGTACA
right  CGAGATCCCGCCACTGCACTCCAGCCTGGGCGACAGAGCGAGACTCCGTCTCAAAAAAAAAAAAAAAAAA
       1111111111**11**1*1111111***1*1*1*1111111111111111111111111****1*11*1*

left   ACATTAAGAAATACAATGATGGCCGGGCGCGGTGGCTCACGCCTGTAATCCCAGCACTTTGGGAGGCCGA
chrom  ACATTAAGAAATACAATGATG-----------TGGCAAAC-------AGCCCTTC--TTTGGG-------
right  AAAAAAAGAAATACAATGATG-----------TGGCAAAC-------AGCCCTTC--TTTGGG-------
       *1*11***************22222222222****22**2222222*2***22*22******2222222

left   GGCGGGCGGA
chrom  -GCTTTC---
right  -GCTTTT---
       2**2221222
```

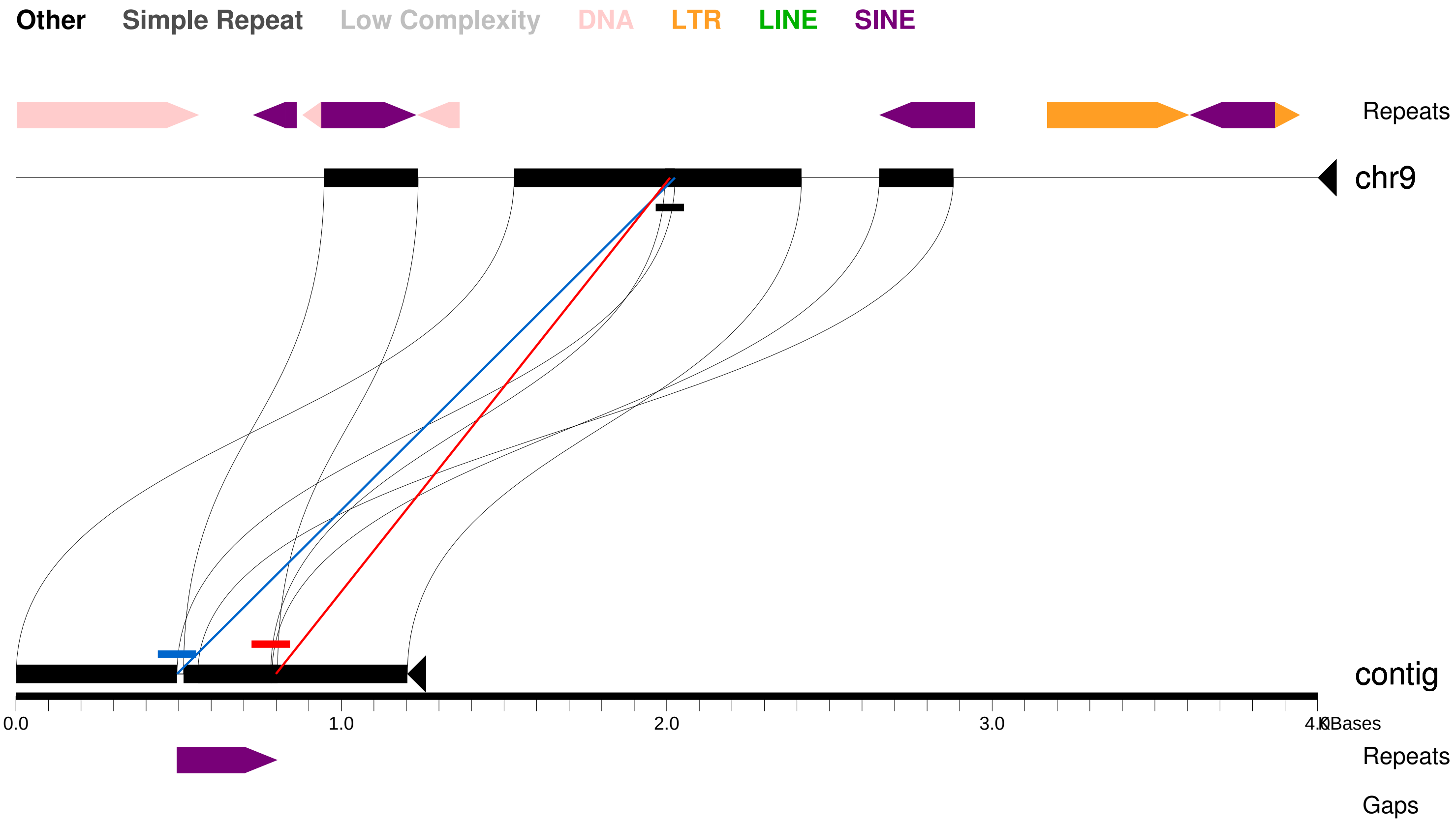

siteID: chr9_86164569 Scafftig1 + Breakpoint: chr9:86164536
chr9:86164484-86164595
Overlap Length: 8 INS size: 298 Genotyped: Yes

```
left   GCG----TATAAGAGTTTCCTTTT-CT----TTGCAACCTTGCCAGCATCAGTTATTTTTTGACTTTTTT
chrom  GCG----TATAAGAGTTTCCTTTT-CT----TTGCAACCTTGCCAGCATCAGTTATTTTTTGACTTTTTA
right  CCGCCCGCCTCGGCCTCCCAAAGTGCTGGGATTACAGGCGTG--AGCCACCGCGCCCGGCCGACTTTTTA
       1**111111*11*11*11*1111*1**1111**1**11*1**11***11*1*111111111********2

left   TTTTTTTTTTTTTTGAGACGGAGTCTCGCTCTGTCGCCCAGGCTGGAGTGCAGTGGCG
chrom  ATAATAGCCATTTTGACT-GGTGTGAGATGCTATCTC--ATTGTGCTTTTGATT----
right  ATAATAGCCATTTTGACT-GGTGTGAGATGCTATCTC--ATTGTGCTTTTGATT----
       2*22*22222*****222**2**222222**2**2*22*222**222*22*2*2222
```

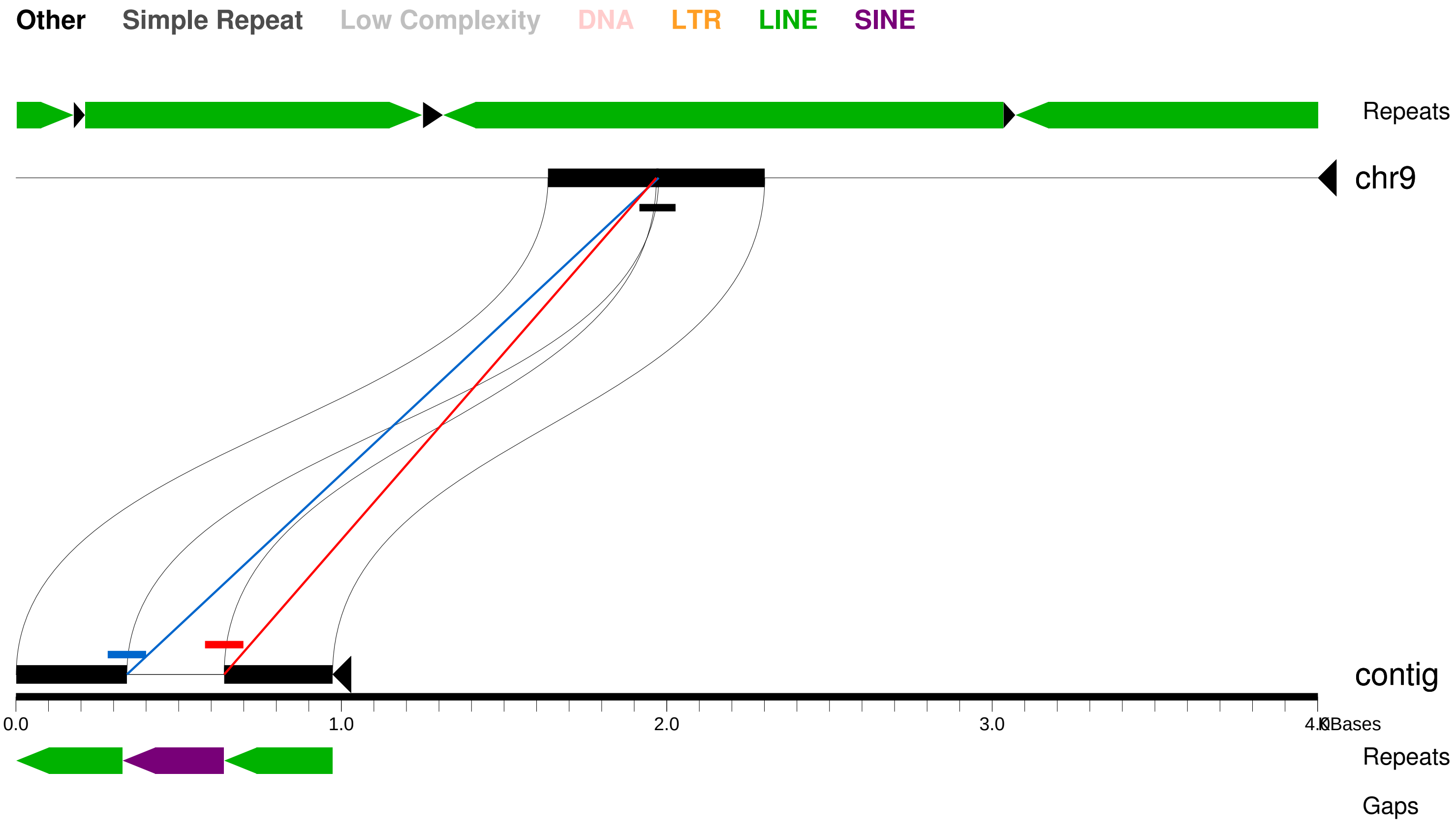

siteID: chr9_8645763 Scafftig3 + Breakpoint: chr9:8645764
chr9:8645719-8645823
Overlap Length: 15 INS size: 259 Genotyped: Yes

```
left    --CACTTGCACTTTCTA-TCAATGTTAG---GTTATTATTTCT---------AACTGCTTGAAAGATTAC
chrom   --CACTTGCACTTTCTA-TCAATGTTAG---GTTATTATTTCT---------AACTGCTTGAAAGATTAC
right   GCCACT-GCACTCCAGCCTGGACGACAGAGCGAGACTCCGTCTCAAAAAAAAAAAAAAAAGAAAGATTAC
        11****1*****111111*11*1*11**111*11*1*111***111111111**111111**********

left    CTTCCTTGGGAGGCCGAGGCGGGCGGATCACGAGGTCAGGAGATCGAGACCATCCCGGCTAAAA
chrom   CTTCC--AGCATCTCAAA--------AT--TGTTGTGTTGAAAAC-ATACCATGTCATTTGCC-
right   CTTCC--AGCATCTCAAA--------AT--TGTTGTGTTGAAAAC-ATACCATGTCATTTGCC-
        *****222*2*222*2*222222222**222*22**222**2*2*2*2*****22*222*2222
```

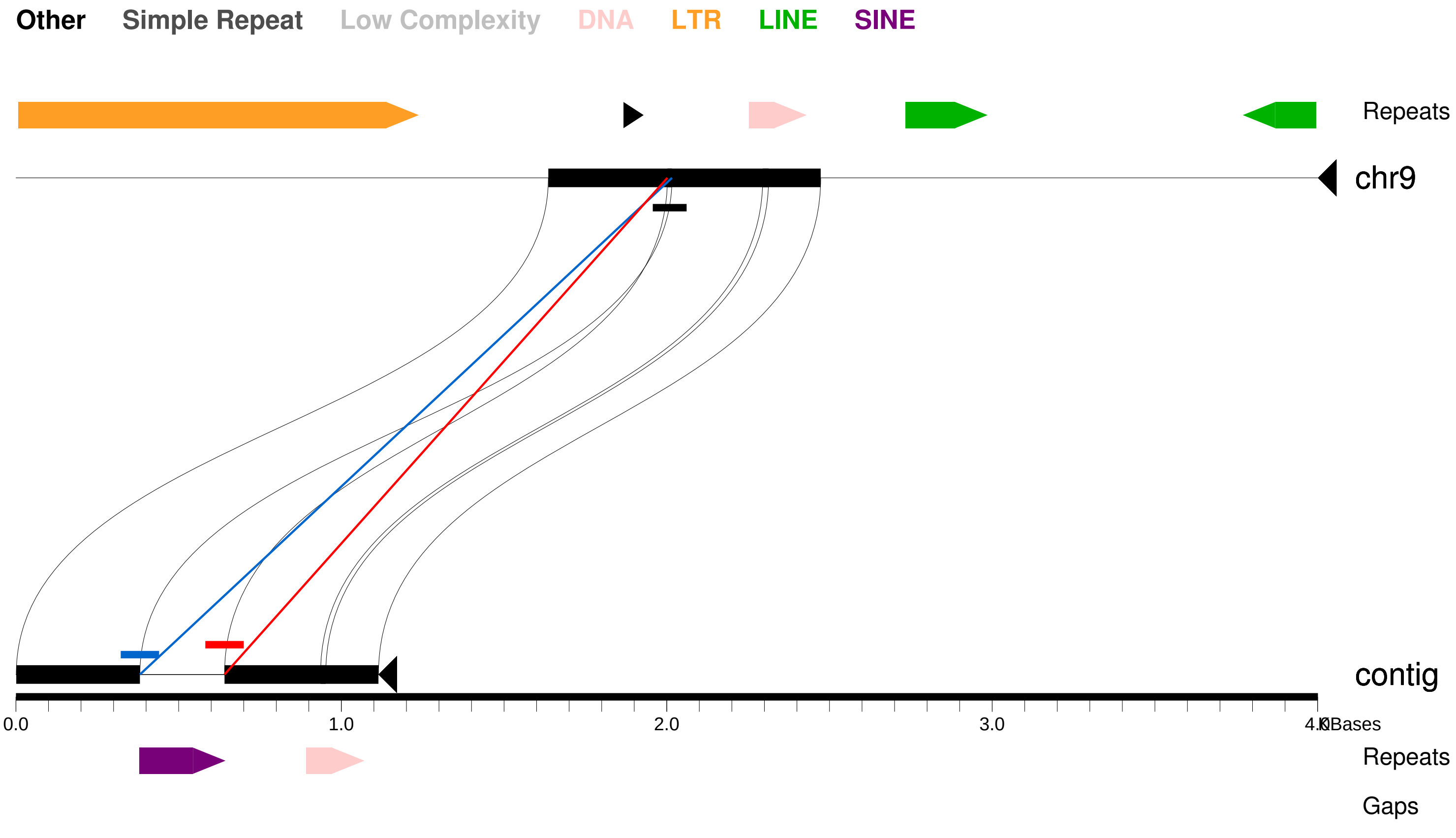

siteID: chr9_89946026 Scafftig2 - Breakpoint: chr9:89946013
chr9:89945968-89946072
Overlap Length: 15 INS size: 310 Genotyped: Yes

```
left    ---------GTCAGA--GAAAAT--GT-TCAAATCAAAAGTGCAGGCGTAAGACCTTAGAAAGATTTGAG
chrom   ---------GTCAGA--GAAAAT--GT-TCAAATCAAAAGTGCAGGCGTAAGACCTTAGAAAGATTTGAG
right   AGCCTGGCGACAGAGCGAGACTCCGTCTCAAAAAAAAAAAAAAAAAAAAAAAAAAAAAAAAAGATTTGAG
        111111111*1****11**1*1*11**1*****11****1111*11111**1*1111*1***********

left    ACAGGCCGGGCGCGGTGGCTCACGCCTGTAATCCCAGCACTTTGGGAGGCCGA--GGCGGGCGGA
chrom   ACAG-----------TGCCTCAA-----TAACCTTCTTCATTTCCAAGACAGAAGGGCTTCCAAG
right   ACAG-----------TGCCTCAA-----TAACCTTCTTCATTTCCAAGACAGAAGGGCTTCCAAG
        ****22222222222**2****222222***2*2222222***222**2*2**22***222*222
```

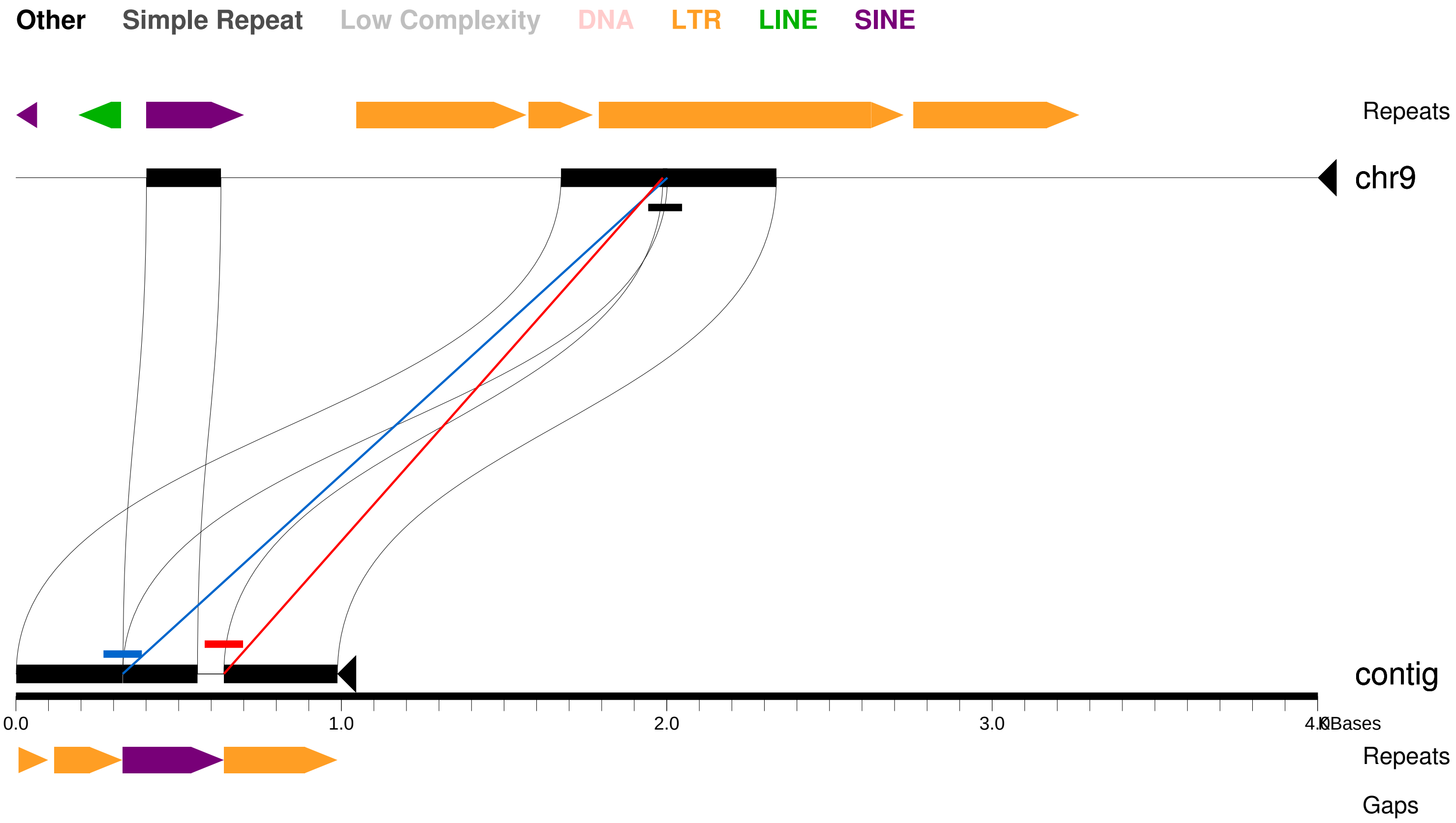

siteID: chr9_90845815 Scafftig2 + Breakpoint: chr9:90845791
chr9:90845753-90845850
Overlap Length: 22 INS size: 176 Genotyped: Yes

```
left    GAA----AATACAGA---------ACAGC-AGA---TGCCCCATTGTAAGCACTC----AATTATATTGT
chrom   GAA----AATACAGA---------ACAGC-AGA---TGCCCCATTGTAAGCACTC----AATTATATTGT
right   CCACTGCACTCCAGCCTGGGCGACAGAGCGAGACTCTGTCTCAAAAAAAAAAAAAAAAAATTATATTGT
        11*1111*1*1***1111111111*1***1***111**1*1**1111**11*1111111***********

left    CTTTTTTTTTCATACAAAAAATTAGCCGGGCGCGGTGGCGGGCGCCTGTAGTCCCAGCTACTCGGGAGGC
chrom   CCTTTTTTTTTCTT--------TTTTCTTT-----------TACTTTTTTCATTCCTGAAACATTATAG--
right   CTTTTTTTTTTCTT--------TTTTCTTT-----------TACTTTTTTCATTCCTGAAACATTATAG--
        *N********2*22222222**22*2222222222222222*222*2*22*2**2*22**22222**22
```

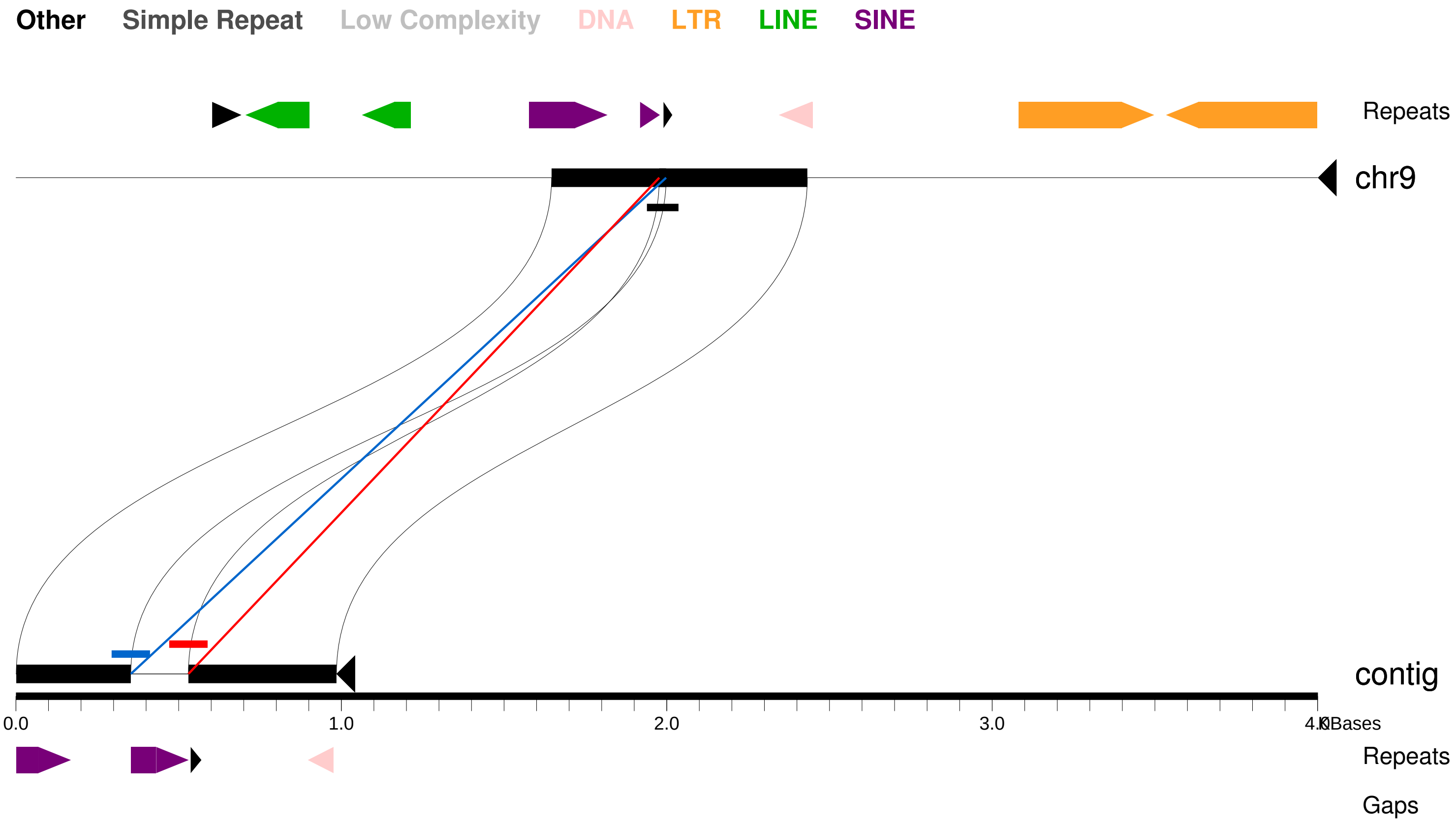

siteID: chr9_91947707 Scafftig3 + Breakpoint: chr9:91947692
chr9:91947648-91947751
Overlap Length: 9 INS size: 323 Genotyped: No

```
left    ------TGTAAGGAATT--GCTAAACTATTAAAT-ATCTCCCAAAGTGGCTGG------AACGTTTTTTC
chrom   ------TGTAAGGAATT--GCTAAACTATTAAAT-ATCTCCCAAAGTGGCTGT------AACGTTTTTCA
right   CTCCGCTTCCCGGGTTCACGCCATTCTCCTGCCTCAGCCTCCCCAGTAGCTGGGACTACAACGTTTTTCA
        111111*1111**11*111**1*11**11*111*1*1*11**11***1****N111111*********22

left    TTCTTTTTTTTTTTTTTTNNNNNNNNNNNNNNNNNNNNNNNNNNNNNNNNNNNNNNNNNNNNNN
chrom   TTCTT---------------ACCAGCAATGGATAAGGATTTCTGTTGTCAAAGTGTTCTGGATT
right   TTCTT---------------ACCAGCAATGGATAAGGATTTCTGTTGTCAAAGTGTTCTGGATT
        *****22222222222222222222222222222222222222222222222222222222222
```

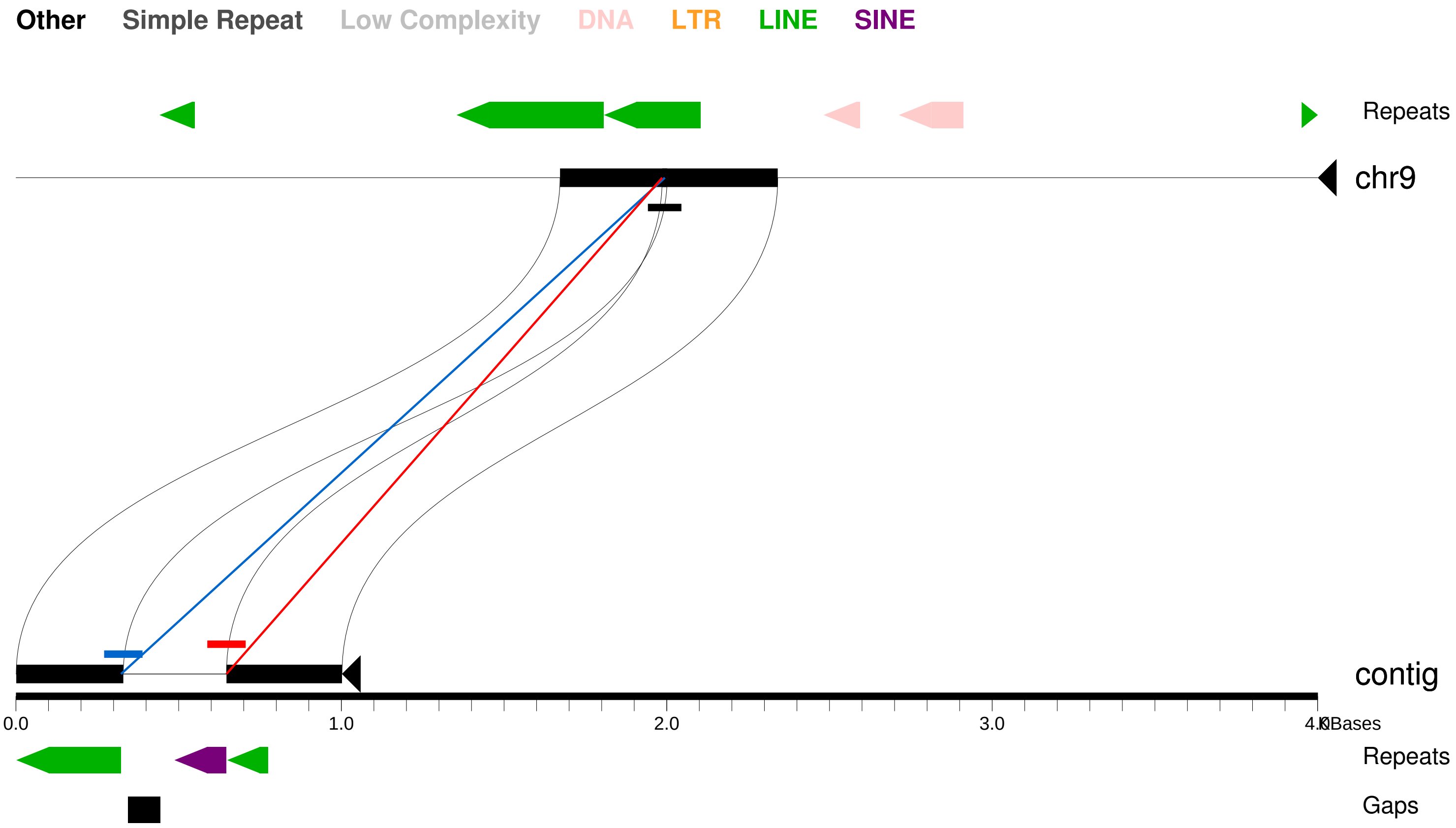

siteID: chr9_93057526 Scafftig1 - Breakpoint: chr9:93057512
chr9:93057352-93057682
Overlap Length: 11 INS size: 301 Genotyped: Yes

```
left   A---------CAAGATTATCCACGTCTTAGTTCATAAAACAAGTCTTGAAAAATTTACAAAGATTAAAAT
chrom  AATAAGTCTACAAGATTATCCACGTCTTAGTTCATAAAACAAGTCTTGAAAAATTTACAAAGATTAAAAT
right  TTTA-GTAGAGACGGGGTTTCAC--CTT-GTTAGCCAGGATGGTCTCGA----TCTCCTGACCTCGTGAT
       1N22 22NN21*1*1111*1***11***1***1111*11111****1**1111*1*1*11*11*1111**

left   CATACTAAGTATCTTTTCTGACCATAAGGGAATAAAACTAGAAATCAATAGCAAAAGGAGAGCTGAAAAA
chrom  CATACTAAGTATCTTTTCTGACCATAAGGGAATAAAACTAGAAATCAATAGCAAAAGGAGAGCTGAAAAA
right  CCGCCCGC----CTCGGCCTCCCA-AAGTG-------CTGGGATT--------ACAGGCGTG-------A
       *111*1111111**111*111***1***1*1111111**1*1*1*11111111*1***1*1*1111111*

left   AACACTAATATGTGGAAAGT-AACACACTGTTTTTTTTTTTTTT----------------------TT--
chrom  AACACTAATATGTGGAAAGT-AACACACTGTTAAATAATGGGTTAAGGAAGAGGTCACAAGGAAAATTAG
right  GCCACC-----GCGCCCGGCCAACACACTGTTAAATAATGGGTTAAGGAAGAGGTCACAAGGAAAATTAG
       11***111111*1*1111*11**********222*22*222**2222222222222222222222**22

left   ----TTTTTTGAGACGGAGTCTCGCTC---TGTCGCCCAGGCTGGAG----TGCAGTGGCGCGATCTCGG
chrom  AAAATATCTCGAACCAAAGGAACACACGTTTGTTTAACATACTAATTCTGCTTCAGTGTCACATTGAAGG
right  AAAATATCTCGAACCAAAGGAACACACGTTTGTTTAACATACTAATTCTGCTTCAGTGTCACATTGAAGG
       2222*2*2*2**22*22**222*2*2*222***2222**22**22222222*2*****2*2*22*222**

left   CTCACTGCAAGC---TCCGCCTCCCGGGTTCACGCCATTCTCCTGCCTC---
chrom  GAAATGTTTACCCTTTCAGATTTTTTTTTCCAGAAAATAGGTGGGCATTCAA
right  GAAATGTTTACCCTTTCAGATTTTTTTTTCCAGAAAATAGG-----------
       222*22222*2*222**2*22*222222*2**2222**222NNN11N1N
```

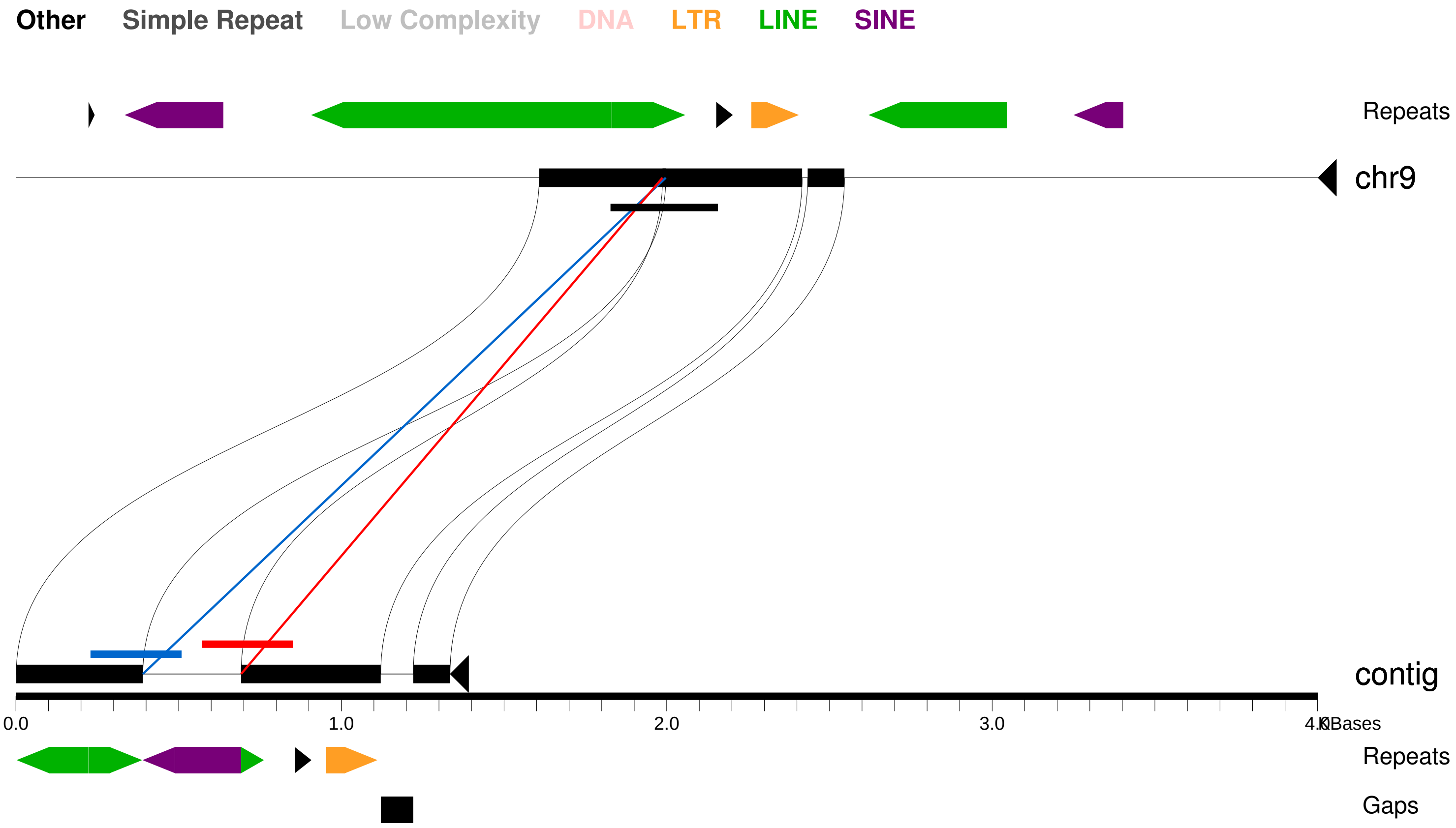

siteID: chr9_93194551 Scafftig4 + Breakpoint: chr9:93194532
chr9:93194485-93194590
Overlap Length: 13 INS size: 293 Genotyped: Yes

```
left    --TTAA-TTGTTAG---------TAGGCTA--AAAAGAGAAAG--TTAAATGTTATAGCTTTTAATTTTA
chrom   --TTAA-TAGTTAT---------TAGGCTA--AAAAGAGAAAG--TTAAATGTTATAGCTTTTAATTTTA
right   CCTGACCTCGTGATCCGCCCGCCTCGGCCTCCCAAAGTGCTGGGATTACAGGCGTGAGCT---AATTTTA
        11*1*11*N**1*2111111111*1***11111****1*111*11***1*1*1111****111*******

left    AGTTTTTTTTTTTGTTGTTGTTGTTTTTTTTTGAGACGGAGTCTCGCTCTGTCACCCAGGCTGGAG
chrom   AGTTTTAACTTA--------AGCATTTTATTTAGTC--ATTCTTAACCTGGCA---ATGAATAAA
right   AGTTTTAACTTA--------AGCATTTTATTTAGTC--ATTCTTAACCTGGCA---ATGAATAAA
        ******222**2222222222*22****2**2**2*22*2***2222***2**222*2*2222*2
```

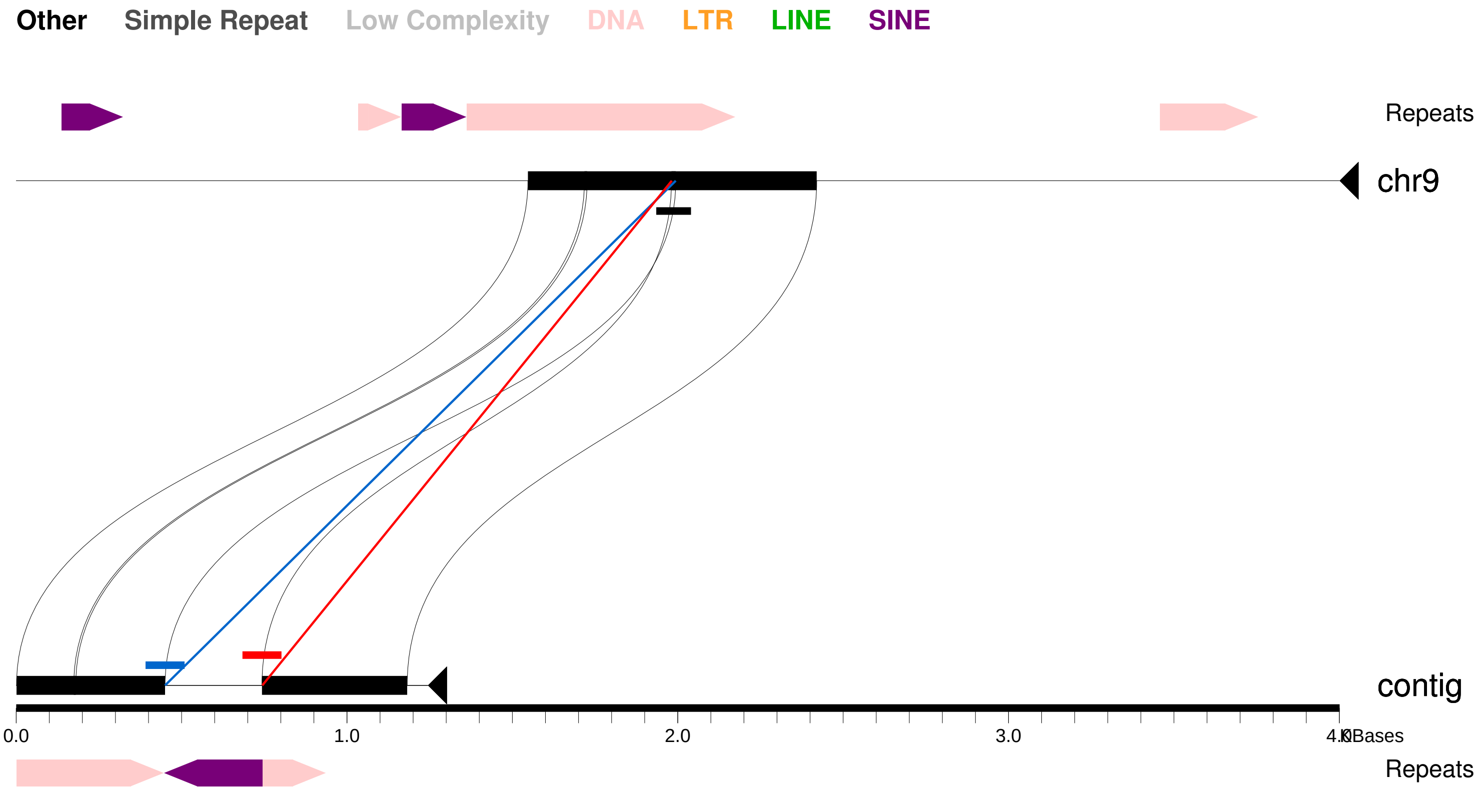

siteID: chr9_96371393 Scafftig5 + Breakpoint: chr9:96371373
chr9:96371327-96371432
Overlap Length: 14 INS size: 304 Genotyped: Yes

```
left   TTTCTCCTCTCAGCATATCAATAACGCTTTTTTAAAAAACTTTTTT--------------TTAGTGGTTT
chrom  TTTCTCCTCTCAGCATATCAATAACGCTTTTTTAAAAAACTTTTTT--------------TTAGTGGTTG
right  CCGCCCGCCTCGGCCTCCCAA-AGTGCTGGGATTACAGGCGTGAGCCACCGCGCCCGGCCTTAGTGGTTG
       111*1*11***1**1*11***1*11***1111*1*1*11*1*111111111111111111*********2

left   TTTTTTTTTTTTTTTTTTTTTTTGA-GACGGAGTCTCGCTCTGTCGCCCAGGCTGGAGTGCAGT
chrom  TTTTAAAATGTGCGATATACGTTTATGACTAATCCAAGTCCTCT--------------TTCAAA
right  TTTTAAAATGTGCGATATACGTTTATGACTAATCCAAGTCCTCT--------------TTCAAA
       ****2222*2*2222*2*222**2*2***22*22*22*22**2*22222222222222*2**22
```

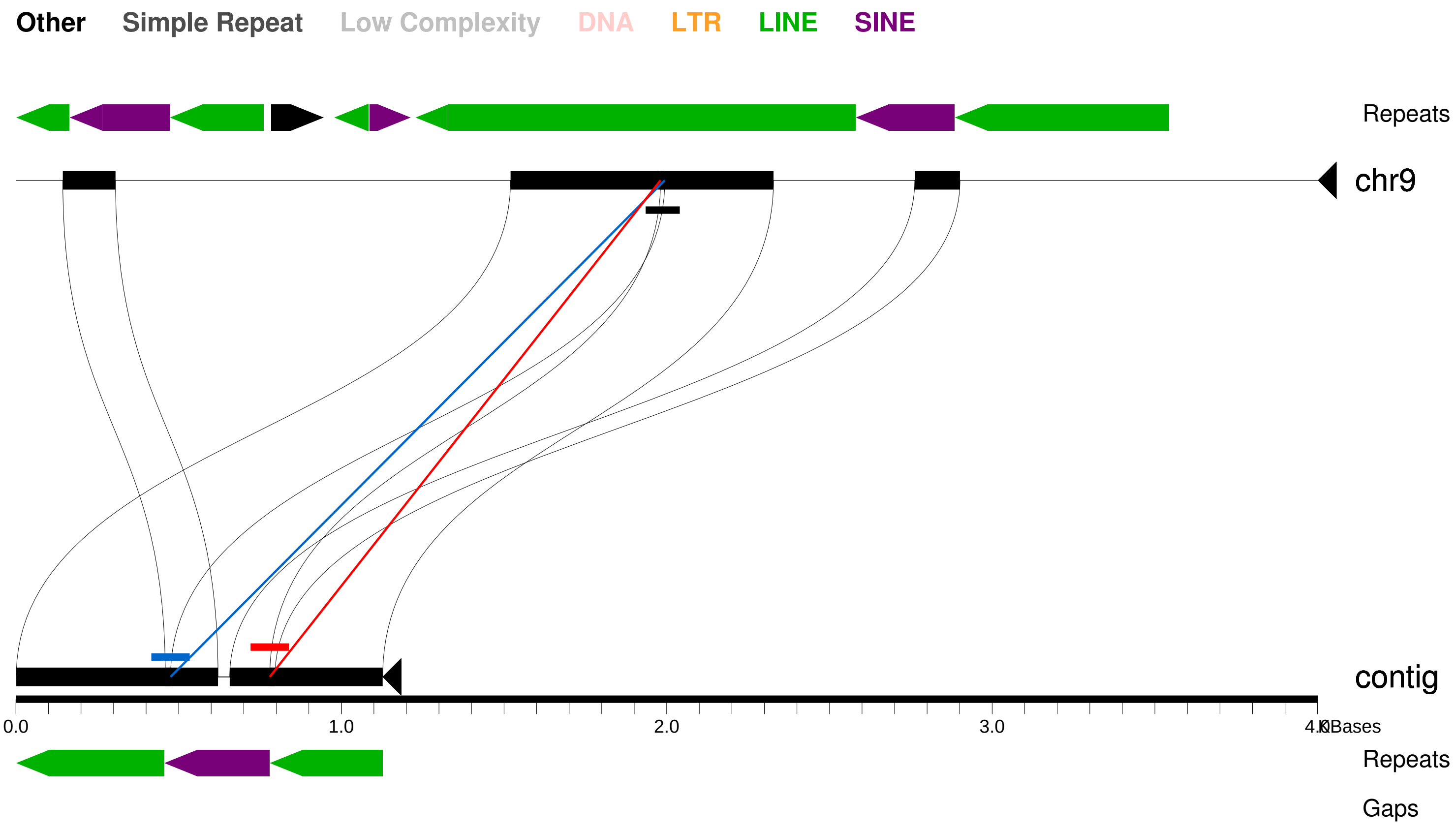

siteID: chr9_97314263 Scafftig9 - Breakpoint: chr9:97314242
chr9:97314197-97314301
Overlap Length: 15 INS size: 302 Genotyped: Yes

```
left     TTAA--------------AAAAAAAAAGAGTTCATACCAAAGGTAGTAATACTACGATTAAAAGAGAGCT
chrom    TTAA--------------AAAAAAAAAGAGTTCATACCAAAGGTAGTAATACTACGATTAAAAGAGAGCT
right    TGCACTCCAGCCTGGGCGACAGAGCGAGACTCCGTCTCAAAAAAAAAAAAAAAAAAAAAAAAAGAGAGCT
         *11*11111111111111*1*1*111***1*1*1*11****111*11**1*11*11*11***********

left     CAACGGCCGGGCGCGGTGGCTCACGCCTGTAATCCCAGCACTTTGGGAGGCCGAGGCGGGCGG
chrom    CAACAC----------TG---CAGGTTCTTAAAGGCAAAAGTTAATAAAGCCTTGTTTTT-GG
right    CAACAC----------TG---CAGGTTCTTAAAGGCAAAAGTTAATAAAGCCTTGTTTTT-GG
         ****222222222222**222**2*2222***222**22*2**2222*2***22*222222**
```

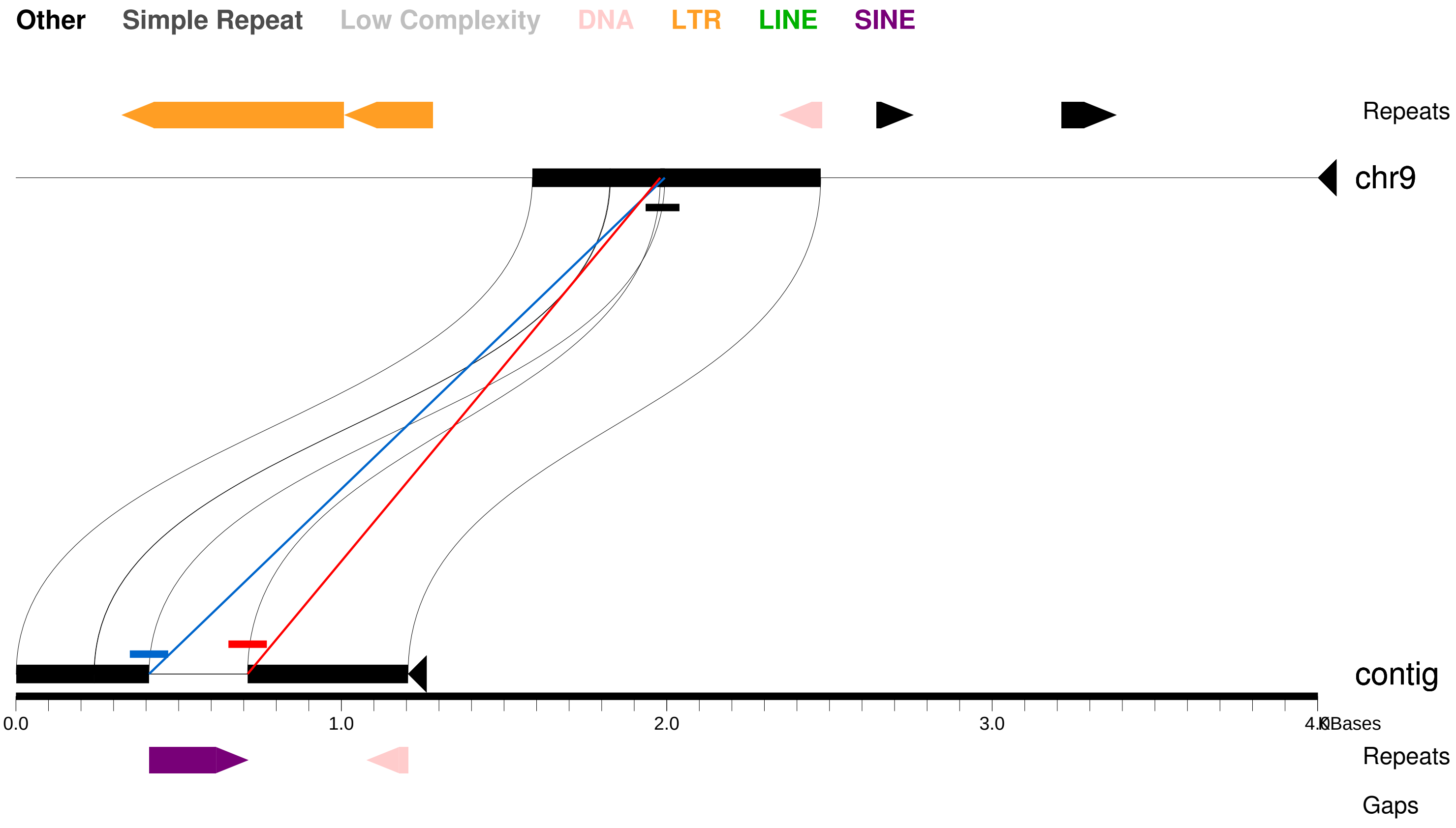

siteID: chr9_97710728 Scafftig1 - Breakpoint: chr9:97710699
chr9:97710654-97710758
Overlap Length: 15 INS size: 430 Genotyped: No

```
left    GAA--GTCAAGATGCCCCA-----CTGA-ATTTCTGGAATTGGCC------CTCAGGATCCCATTCTAAC
chrom   GAA--GTCAAGATGCCCCA-----CTGA-ATTTCTGGAATTGGCC------CTCAGGATCCCATTCTAAC
right   CGCCCGCCTCGGCCTCCCAAAGTGCTGGGATTACAGGCGTGAGCCACCGCGCCCGGCCCCCCATTCTAAC
        11111*1*11*1111****11111***11***1*1**11*11***111111*1*1*111***********

left    TCTTTTTTTTTNNNNNNNNNNNNNNNNNNNNNNNNNNNNNNNNNNNNNNNNNNNNNNNNNNNNNN
chrom   TCTTGTAGTT--------------CATCATTTTCTCTTTTAAGTGCTCACAAACTGTTCAATA
right   TCTTGTAGTT--------------CATCATTTTCTCTTTTAAGTGCTCACAAACTGTTCAATA
        ****2*22**22222222222222222222222222222222222222222222222222222
```

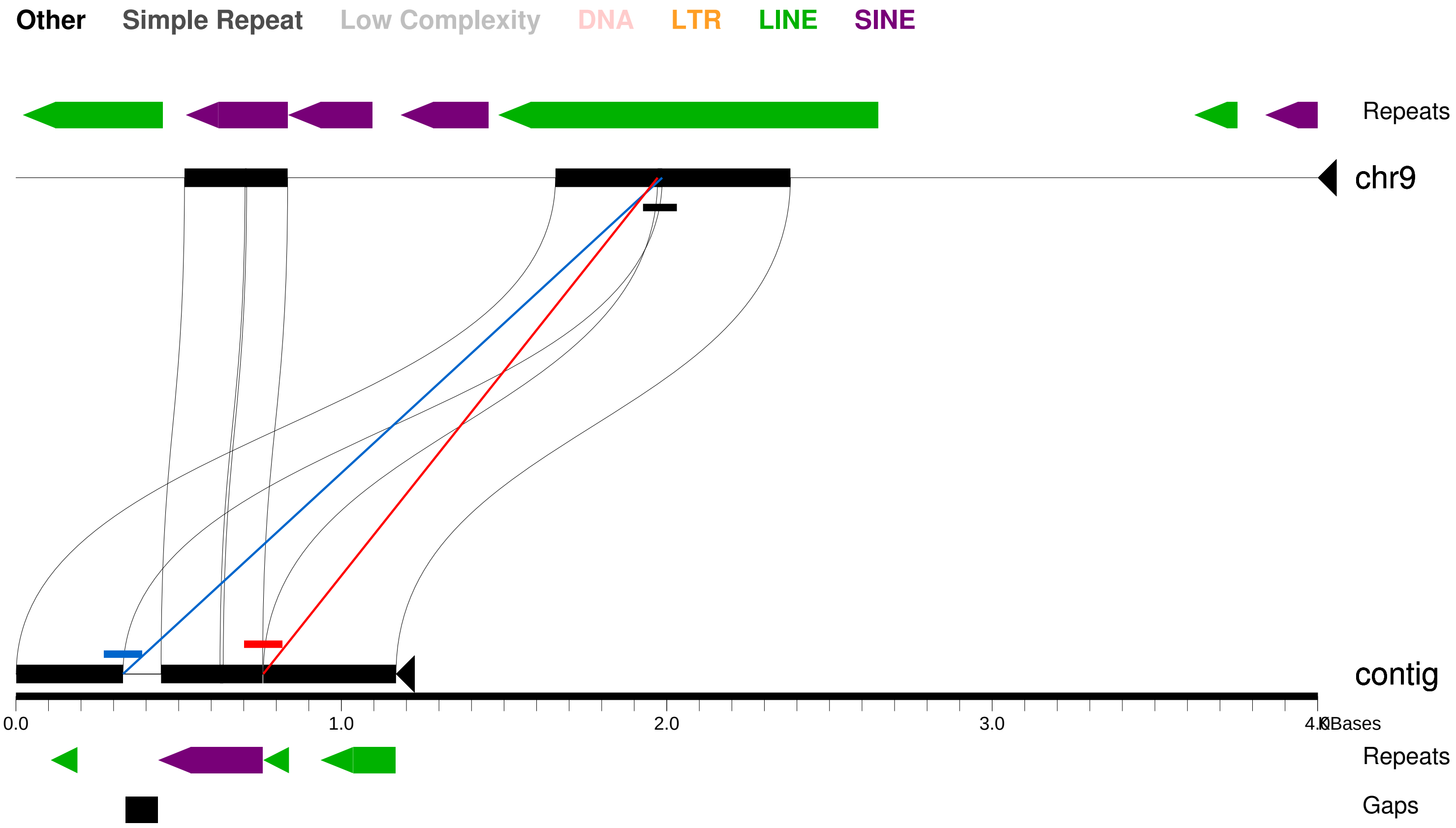

siteID: chr9_98696731 Scafftig4 + Breakpoint: chr9:98696737
chr9:98696687-98696796
Overlap Length: 10 INS size: 303 Genotyped: Yes

```
left   AGAAATAAGTAGTA---------TGCTTATAGAAAAAAATGAATCTAGCAAAACCAATATGTGATTTTTT
chrom  AGAAATAAGTAGTA---------TGCTGATAGAAAAAAATGAATCTAGCAAAACCAATATGTGATTTTTA
right  CCGCCCGCCTCGGCCTCCCAAAGTGCTGGGATTACAAGCGTGAGCCACCGCGCCCGGCCTGTGATTTTTA
       111111111*1*11111111111****211*11*1**11111*1*1*1*1111**1111*********2

left   TTTTTTTTTTTGAGACGGAGTCTCGCTCTGTGGCCCAGGTGGGAGTGCAGTGGCGCAA
chrom  TACTAATAATAAAAACTGCCAAAAAAATATGT----CTTTAGTGACTTCTCTTC-----
right  TACTAATAATAAAAACTGCCAAAAAAATATGT----CTTTAGTGACTTCTCTTC-----
       *22*22*22*22*2**2*22222222*2***2222*2222*2**2*2*22*2222222
```

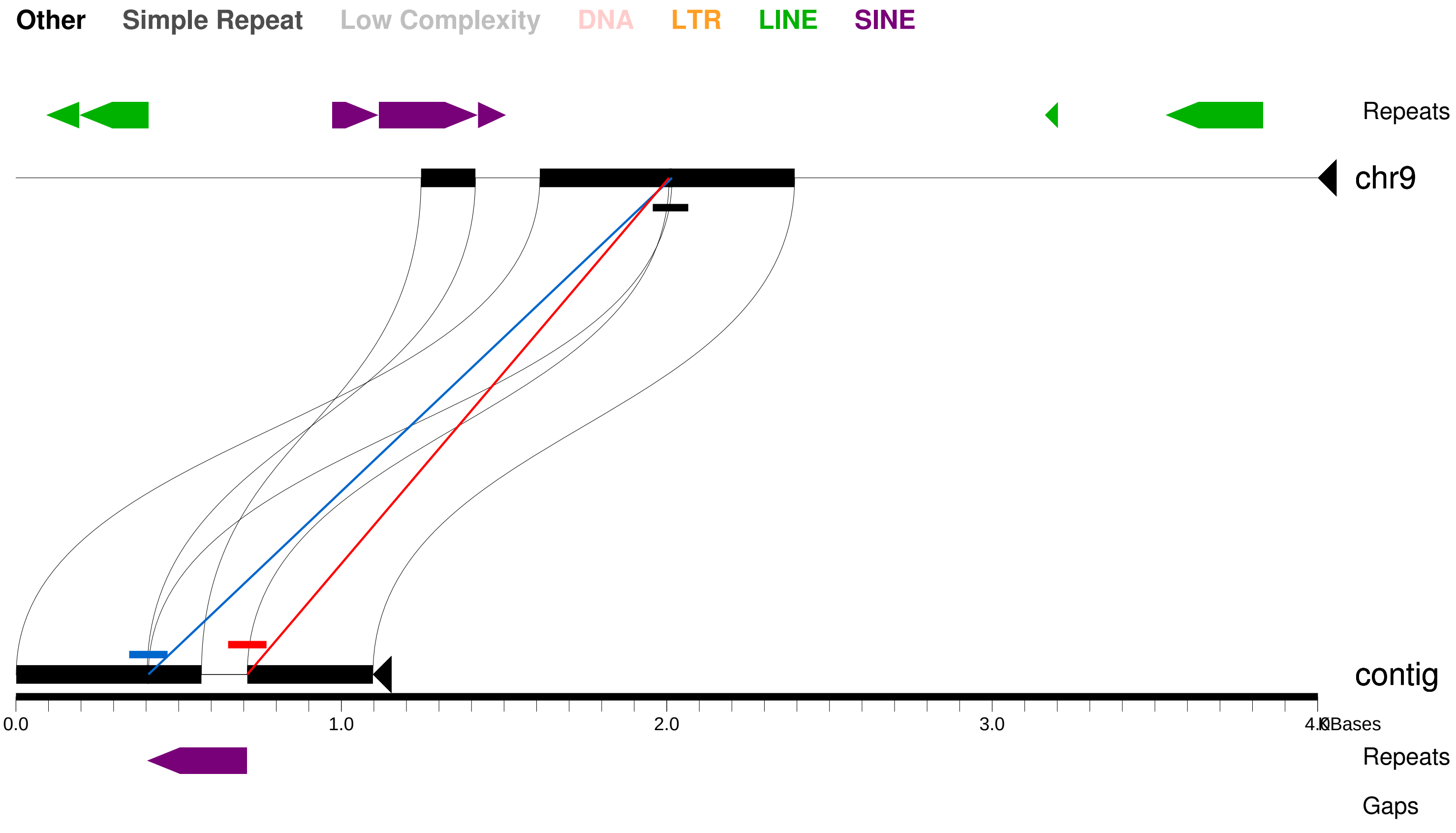

siteID: chr9_99554806 Scafftig2 - Breakpoint: chr9:99554775
chr9:99554738-99554834
Overlap Length: 15 INS size: 456 Genotyped: No

```
left    GGTAATA-------TCCCCTT-TGCTG---TTTC-----TGATT------GTTTTTATTTGGATCTTCTC
chrom   GGTAATA-------TCCCCTT-TGCTG---TTTC-----TGATT------GTTTTTATTTGGATCTTCTC
right   CGCCCGCCTCGGCCTCCCAAAGTGCTGGGATTACAGGCGTGAGCCACCGCGCCCGGCCATGGATCTTCTC
        1*111111111111****1111*****111**1*11111***11111111*11111111***********

left    TCTTCTTTTTTTNNNNNNNNNNNNNNNNNNNNNNNNNNNNNNNNNNNNNNNNNNNNNNNNNNNNNNNNNN
chrom   TCTTTTCTTATTAGTCTAGCTAGTGGTCTAATTAATTTAAAAAAATAAG---------------------
right   TCTTTTCTTATTAGTCTAGCTAGTGGTCTAATTAATTTAAAAAAATAAG---------------------
        ****2*2**2**2222222222222222222222222222222222222222222222222222222222

left    N
chrom   -
right   -
        2
```

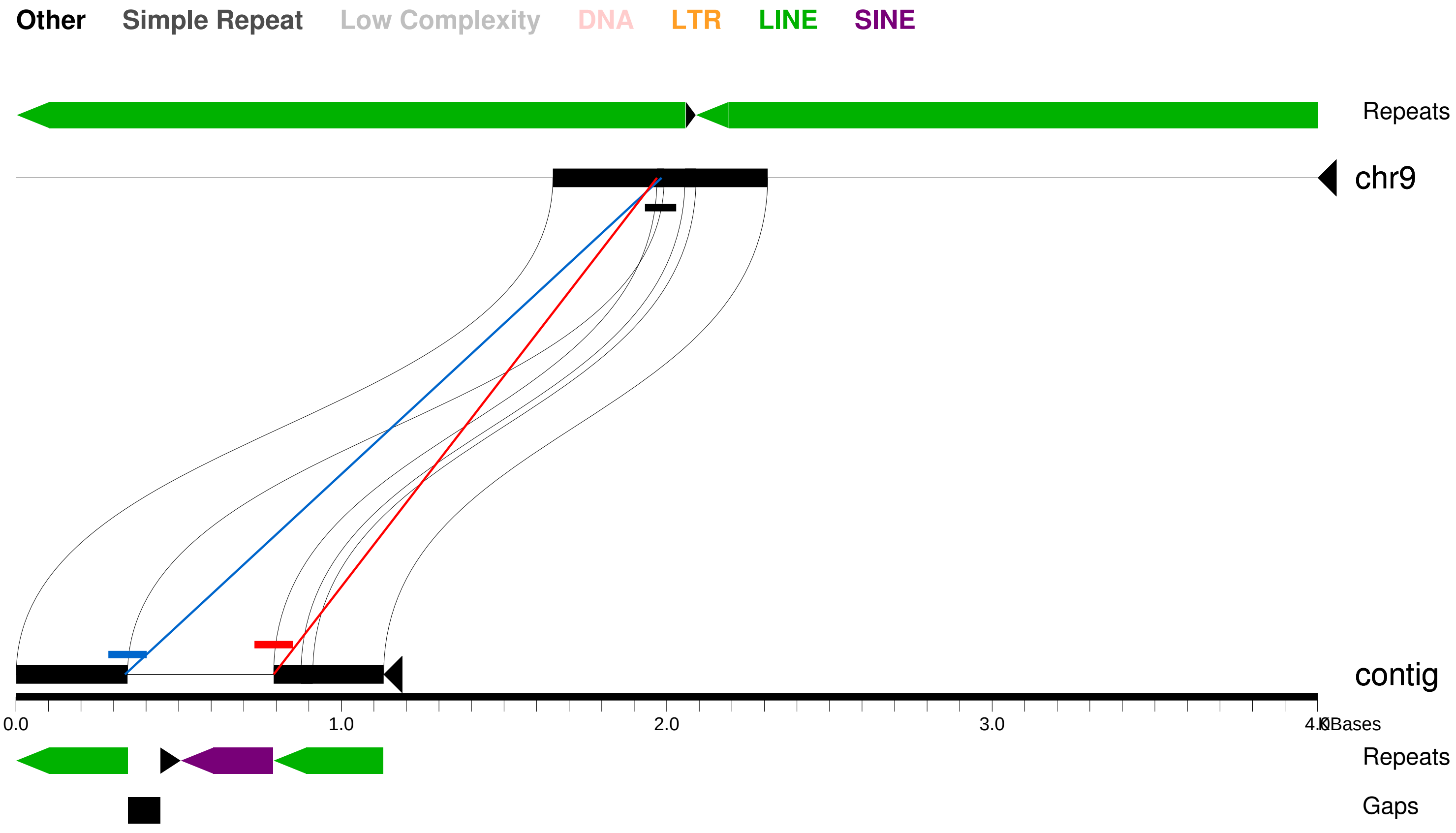

siteID: chrUn_gl000212_25718 Scafftig2 - Breakpoint: chrUn_gl000212:25693
chrUn_gl000212:25664-25752
Overlap Length: 16 INS size: 327 Genotyped: No

```
left    TCT-----------TTATC-------AAAGTCAGTTG--ACTGTGTCCA----------TATTTGTGTTG
chrom   TCT-----------TTATC-------AAAGTCAGTTG--ACTGTGTCCA----------TATTTGTGTTG
right   CCTCAGCCTCCCGAGTAGCTGGGACTACAGGCGCCCGCCACCGCGCCCGGCTAATTTTTTATTTGTGTTG
        1**111111111111**1*1111111*1**1*1111*11**1*1*1**11111111111***********

left    TTTTTTTTTTTTTTTTTTTTTTTTTTTNNNNNNNNNNNNNNNNNNNNNNNNNNNNNNNNNNNNNNNNNNN
chrom   TTTTTGTCCTCCCTGTTTTTGTCCAT------------------------------AGTTCTAGGTATTT
right   TTTTTGTCCTCCCTGTTTTTGTCCAT------------------------------AGTTCTAGGTATTT
        *****2*22*222*2*****2*222*222222222222222222222222222222222222222222222

left    NNNNNNNNN
chrom   CTTTTTATG
right   CTTTTTATG
        222222222
```

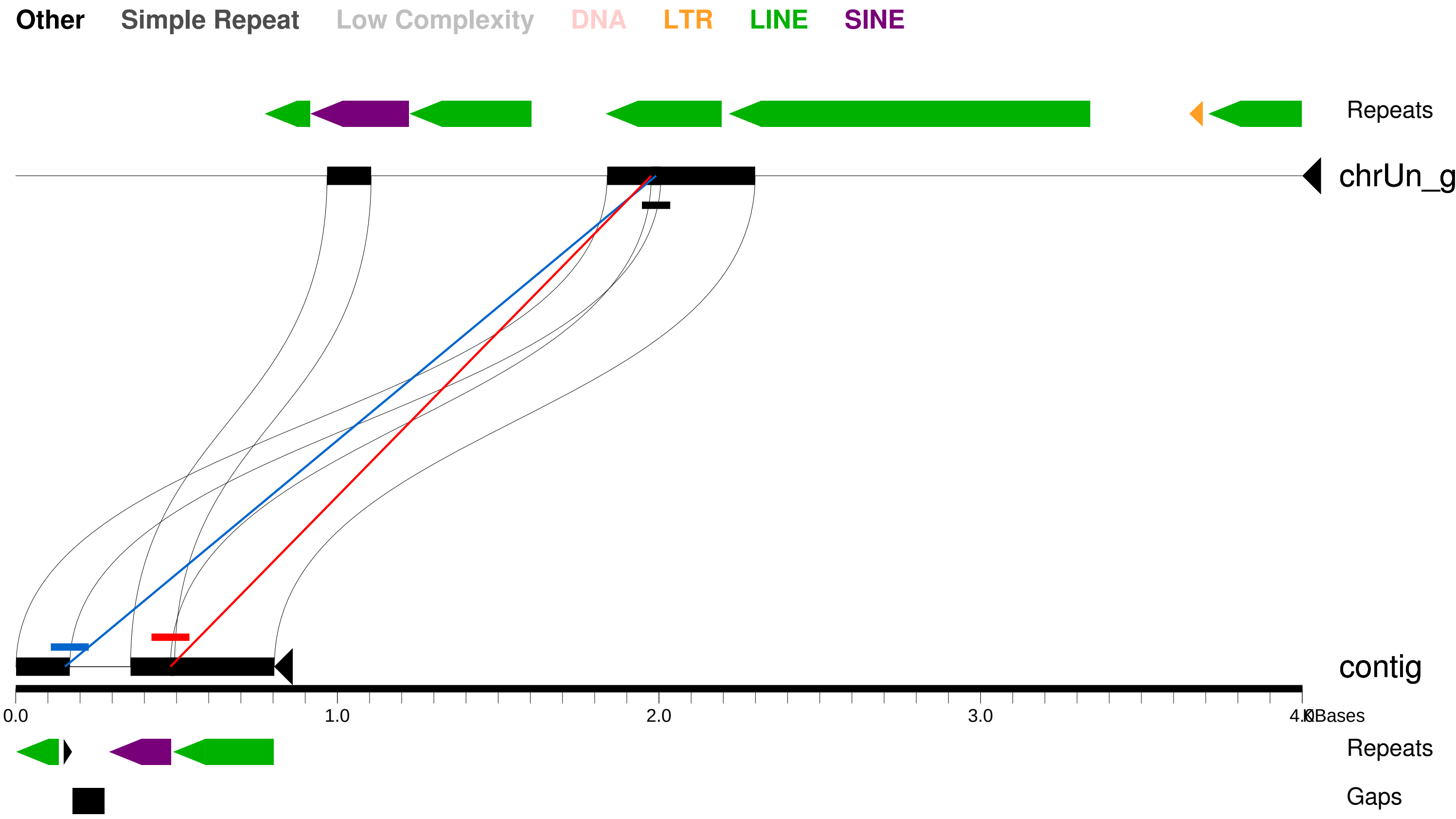

siteID: chrX_106661472 Scafftig2 + Breakpoint: chrX:106661455
chrX:106661415-106661520
Overlap Length: 20 INS size: 414 Genotyped: No

```
left     CATGGAAGCCCCTTGAGGT--AAGTGCTGTTCTT-CA---CTGAT------TGCCCTGC-AACACTGCTT
chrom    CATGGAAGCCCCTTGAGGT--AAGTGCTGTTCTT-CA---CTGAT------TGCCCTGC-AACACTGCTT
right    TCCGCCCGCCTCGGCCTCCCAAAGTGCTGGGATTACAGGCGTGAGCCACCGCGCCCGGCCAACACTGCTT
         111*111***1*111111111********111**1**1111***11111111****1**1**********

left     TCTTNNNNNNNNNNNNNNNNNNNNNNNNNNNNNNNNNNNNNNNNNNNNNNNNNNNNNNNNNN
chrom    TCT-------------GAACATCTACACTTCACCCCTTGCTGCCTTTATCCACAATTCATTA
right    TCT-------------GAACATCTACACTTCACCCCTTGCTGCCTTTATCCACAATTCATTA
         ***22222222222222222222222222222222222222222222222222222222222
```

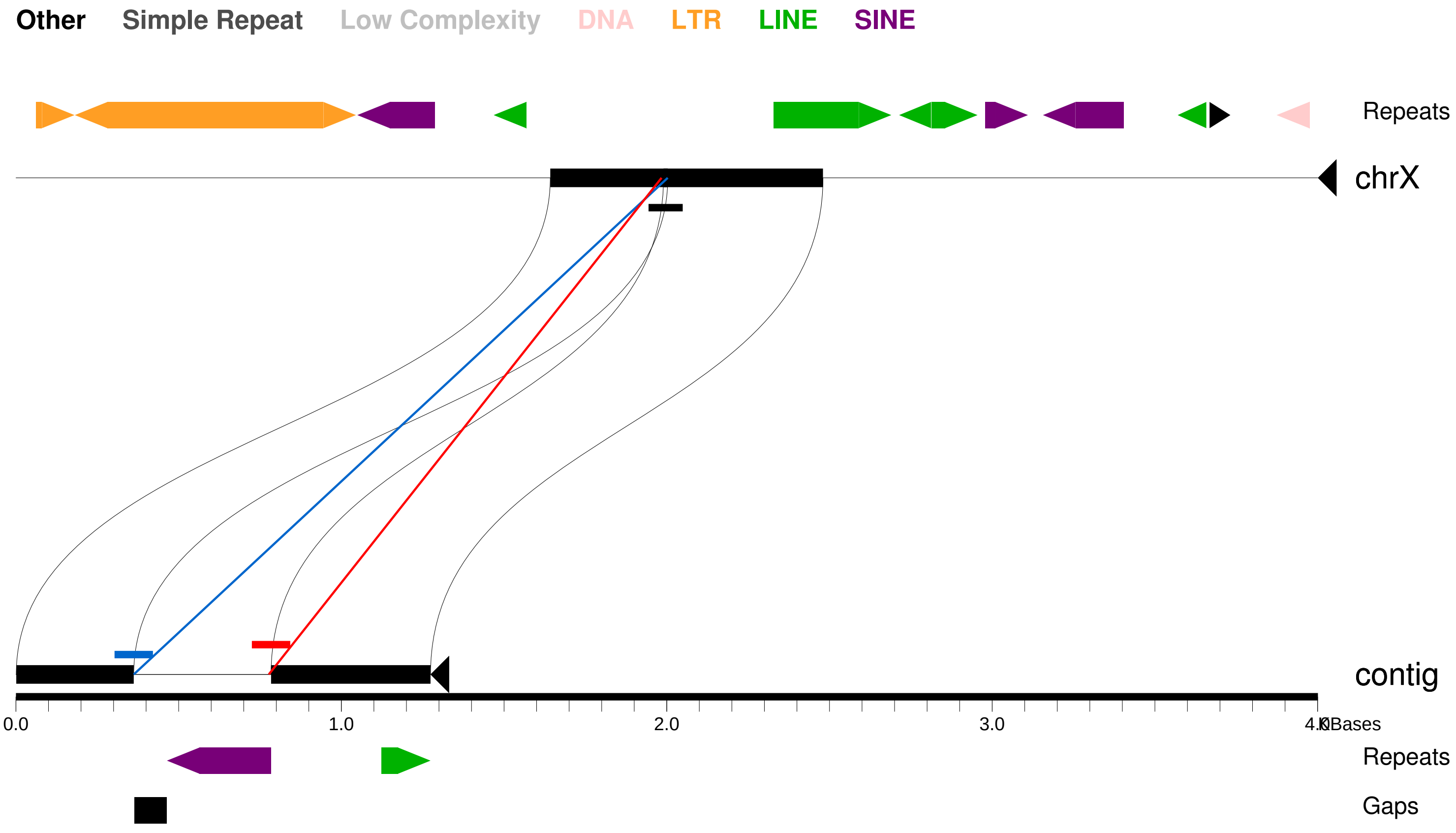

siteID: chrX_116438542 Scafftig1 - Breakpoint: chrX:116438551
chrX:116438391-116438715
Overlap Length: 5 INS size: 381 Genotyped: No

```
left    T-----GGTACATAGGTTTTGAAGCCTAGCAAAATTAGTCTTAAAACCCAGTTATGGCTTCAAGATGGCT
chrom   TACAGTGGTACATAGGTTTTGAAGCCTAGCAAAATTAGTCTTAAAACCCAGTTATGGCTTCAAGATGGCT
right   NNNNNNNNNNN-----------------------------------------------------------
        1NNNNN1111111111111111111111111111111111111111111111111111111111111111

left    GACTAGATGCATGTTGTAACTGCCCCCTCCAAAAAGTATCAAAAATAGCTAATAGATAATCATATTTCAA
chrom   GACTAGATGCATGTTGTAACTGCCCCCTCCAAAAAGTATCAAAAATAGCTAATAGATAATCATATTTCAA
right   ----------------------------------------------------------------------
        1111111111111111111111111111111111111111111111111111111111111111111111

left    ATTGATCATGTAAGAGTGAATGCTG----TTGGGAGGCCGAGG-CGGG---CGGATCACG-AGGTCAGGA
chrom   ATTGATCATGTAAGAGTGAATGCTGGAATTTCACAGACAAATGACAGGAAACACCTAAGGCAAGAAAGGA
right   --------------------TGCTGGAATTTCACAGACAAATGACAGGAAACACCTAAGGCAAGAAAGGA
        11111111111111111111*****2222**222**2*22*2*2*2**222*222*2*2*2*2*22****

left    GATCGAG--------ACCATCCCGGCTAAAACGG--TGAAACCCC----GTCTCTACTAAAAATACAAA-
chrom   GAGGGAAGTGAGGCAACCTGCTCAGCTGAGATTGGCTGGAGCCTGAAGAGGCTCCCCAATTCAGGGAAAG
right   GAGGGAAGTGAGGCAACCTGCTCAGCTGAGATTGGCTGGAGCCTGAAGAGGCTCCCCAATTCAGGGAAAG
        **22**222222222***22*2*2***2*2*22*22**2*2**222222*2***22*2*222*222***2

left    ---AAATTAGCCGGGCGTGGTGGCGGG------CGCC------TG
chrom   AGTAAATGAGAGATTTCCAGTGGTCTGTATTCCCACCATGAAGTT
right   AGTAAAT--------------------------------------
        222****N11NNNNNNNNN1111NNN1      1N11      1N
```

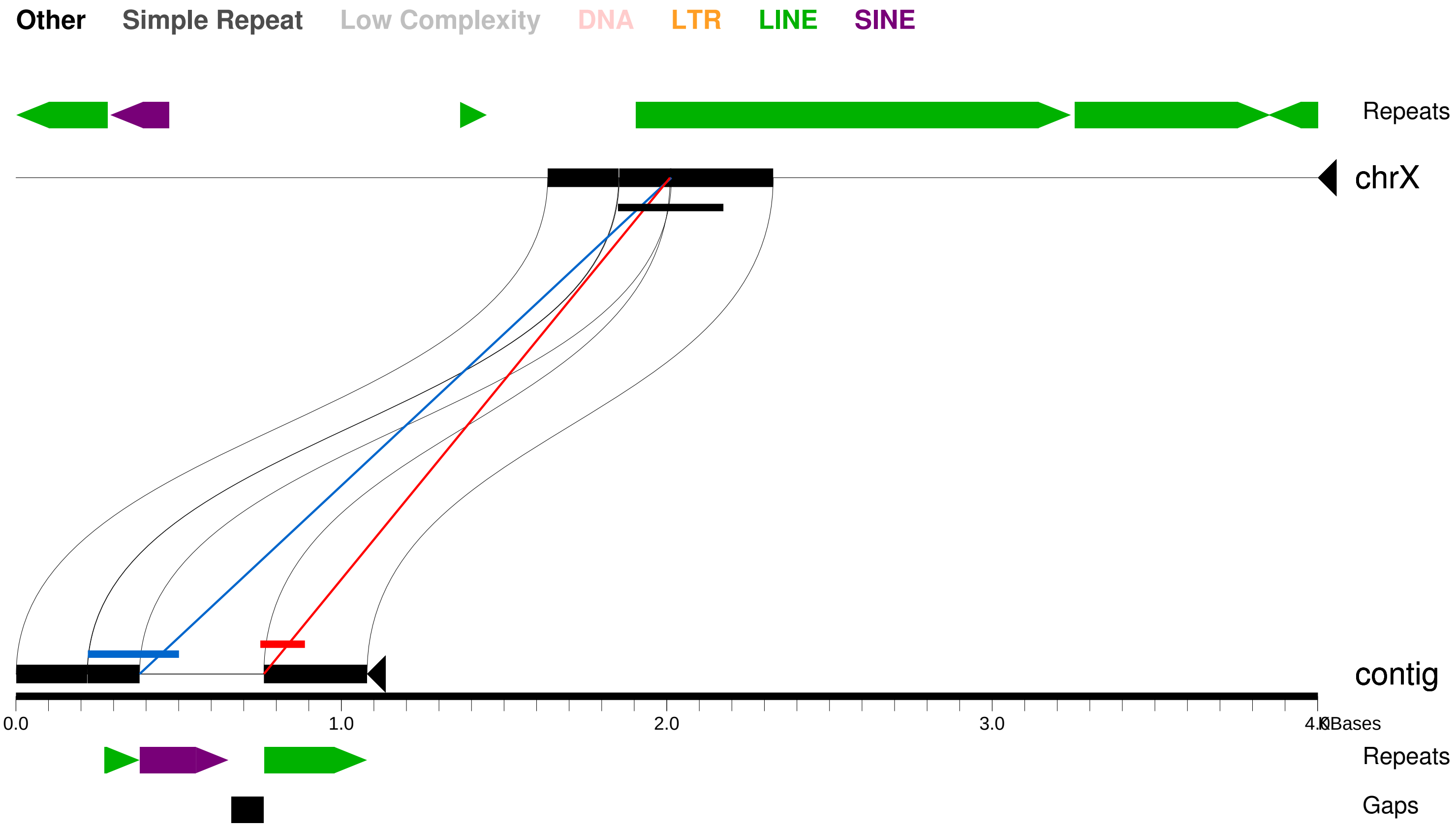

siteID: chrX_116627629 Scafftig4 + Breakpoint: chrX:116627631
chrX:116627587-116627690
Overlap Length: 13 INS size: 309 Genotyped: Yes

```
left   AAATATCCACATTAAAGCAAA--------AGT-------TGAAAAAGAAAGGAAAATTTAAGTAAAGTAC
chrom  AAATATCCACATTAAAGCAAA--------AGT-------TGAAAAAGAAAGGAAAATTTAAGTAAAGTAC
right  CACCCGCCTCGGCCTCCCAAAGTGCTGGGATTACAGGCGTGAGCCACCGCGCCCGGCCCAAGTAAAGTAC
       1*1111**1*1111111****11111111*1*1111111***111*1111*11111111***********

left   TTTTTTTTTTTTTTTTTTTTGAGACGGAGTCTCGCTCTGTCGCCCAGGCCGGACTGCGGACTGC
chrom  TTATTACCACTTAATGT----------AATTCCCATCT-----CCTAAAATGTTGGCTTATTTT
right  TTATTACCACTTAATGT----------AATTCCCATCT-----CCTAAAATGTTGGCTTATTTT
       **2**22222**22*2*2222222222*2*22*22***22222**222222*222**22*2*22
```

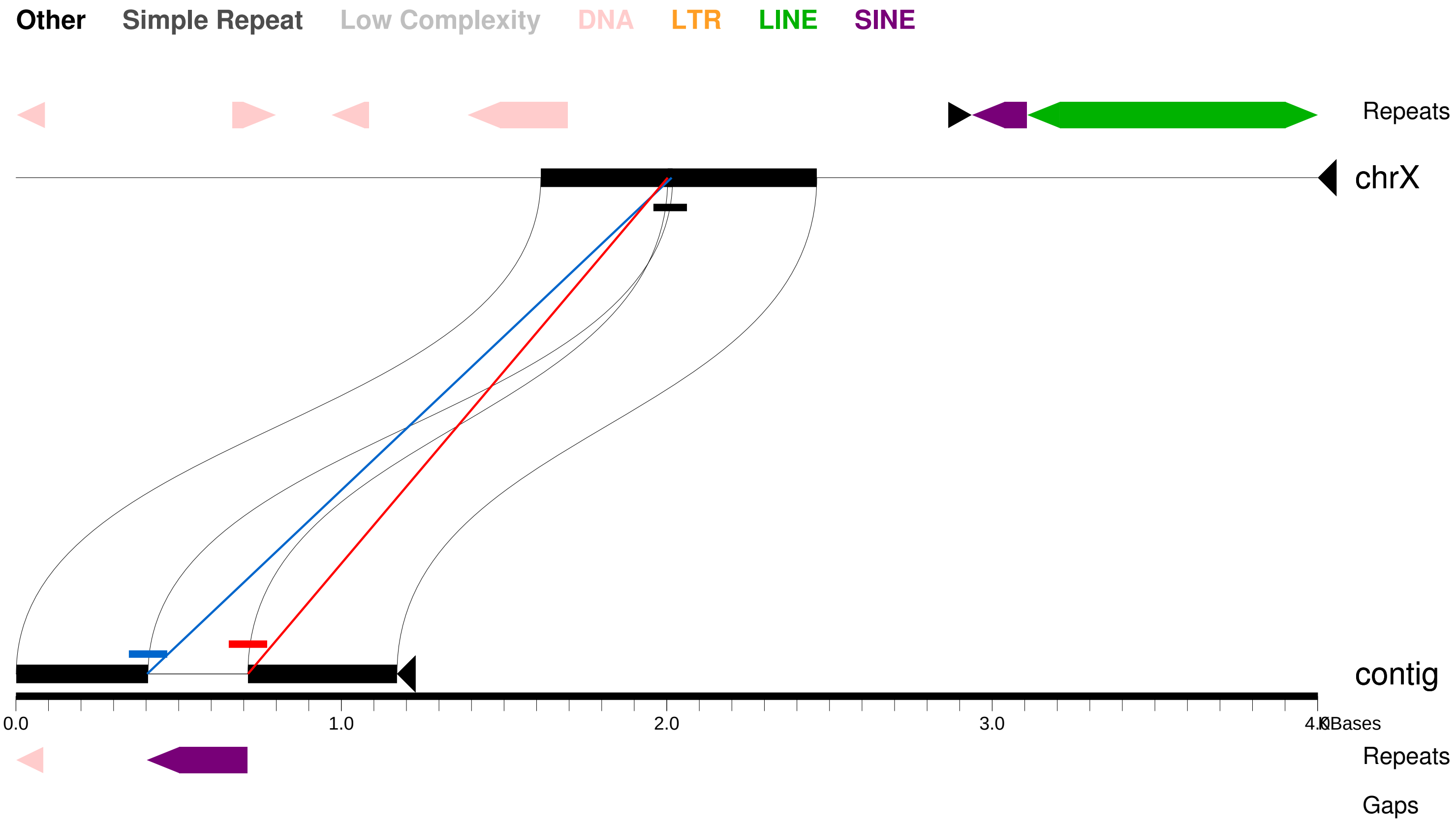

siteID: chrX_116759204 Scafftig1 - Breakpoint: chrX:116759187
chrX:116759130-116759246
Overlap Length: 3 INS size: 418 Genotyped: No

```
left    AACCCTAAGCAGCAGAATTTCAGAAATACTCATTTCCTAATCATTA---ATCCTGACTGG-AAANNNNNN
chrom   AACCCTAAGCAGCAGAATTTCAGAAATACTCATTTCCTAATCATTA---ATCCTGACTGG-AAAACAATC
right   TCCGCCC-GCCTCGGCCTCCCA-AAGTGCTGGGATTACAGGCGTGAGCCACCGCGCCCGGCAAAACAATC
        11*1*111**11*1*11*11**1**1*1**1111*111*11*1*1*111*1*11*1*1**1***222222

left    NNNNNNNNNNNNNNNNNNNNNNNNNNNNNNNNNNNNNNNNNNNNNNNNNNNNN
chrom   TTACCCAGCTATGATATTAAAAAGATATCAAACTGATACAAAGGAGCAACT--
right   TTACCCAGCTCTGATATTAAAAAGATATCAAACTGATACAAAAGAGCAACT--
        2222222222N2222222222222222222222222222222N2222222222
```

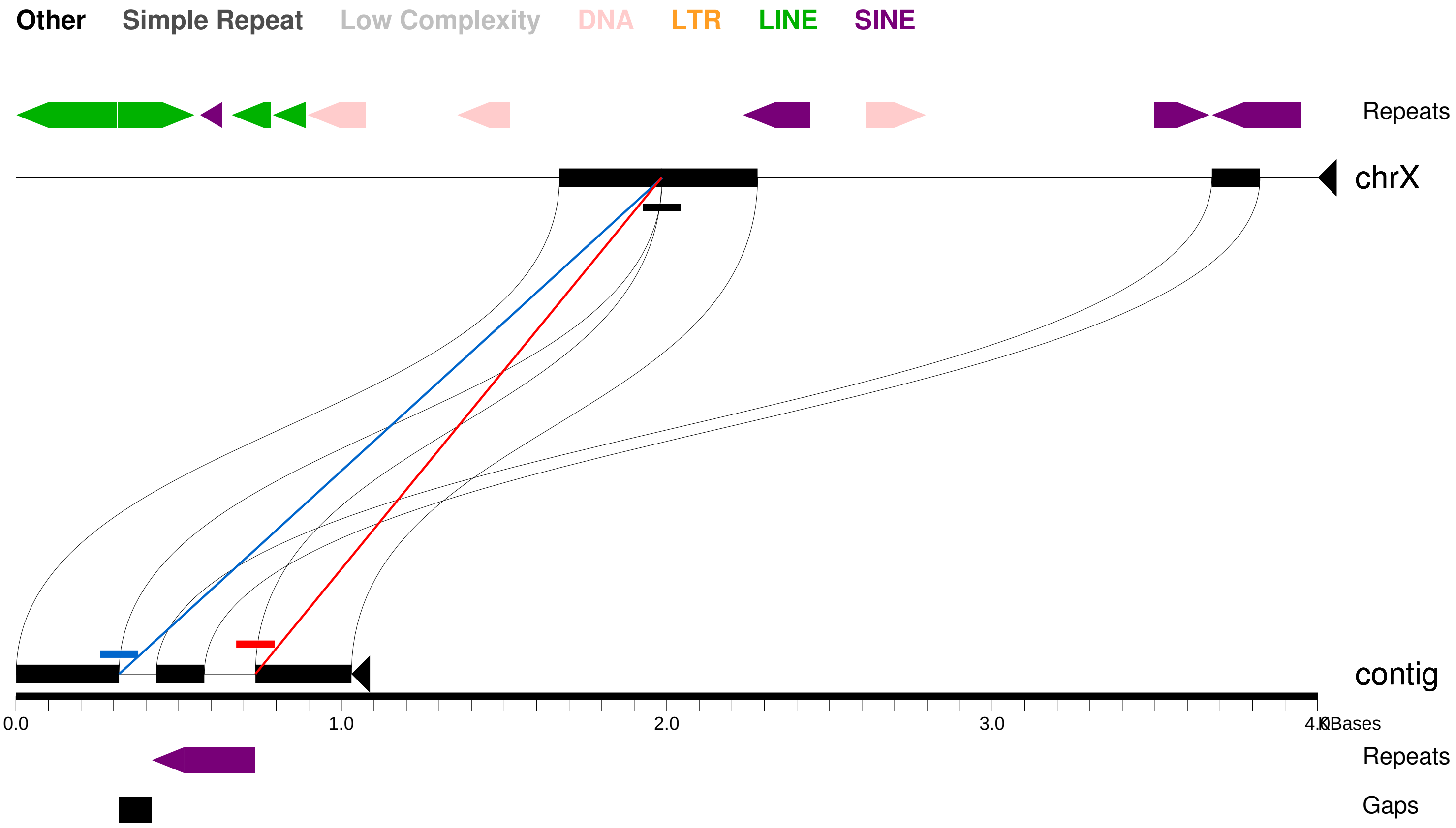

siteID: chrX_116838875 Scafftig2 + Breakpoint: chrX:116838851
chrX:116838805-116838910
Overlap Length: 14 INS size: 317 Genotyped: Yes

```
left     TCTT----------TGCATTTCCAACCAATATTTCTATAATTAACATGG--AATGCTT-TAATAATAAAT
chrom    TCTT----------TGCATTTCCAACCAATATTTCTATAATTAACATGG--AATGCTT-TAATAATAAAT
right    CGACAGAGCGAGACTCCGTCTCAAAAAAAAAGAAAAAAAAATAATAATAATAATAATAATAATAATAAAT
         11111111111111*1*1*1**1**11**1*11111*1**1***1*11111***11*11***********

left     CAAGGCCGGGCGCGGTGGCTCACGCCTGTAATCCCAGCACTTTGGGAGGCCGAGGCGGGCGG
chrom    CAAAAACAAAAAAGGAAAA--------GGAAAAAAAGAAAATTGT-----CTTGGGAATTGT
right    CAAAAACAAAAAAGGAAAA--------GGAAAAAAAGAAAATTGT-----CTTGGGAATTGT
         ***222*222222**222222222222*2**2222**2*22***222222*22**22222*2
```

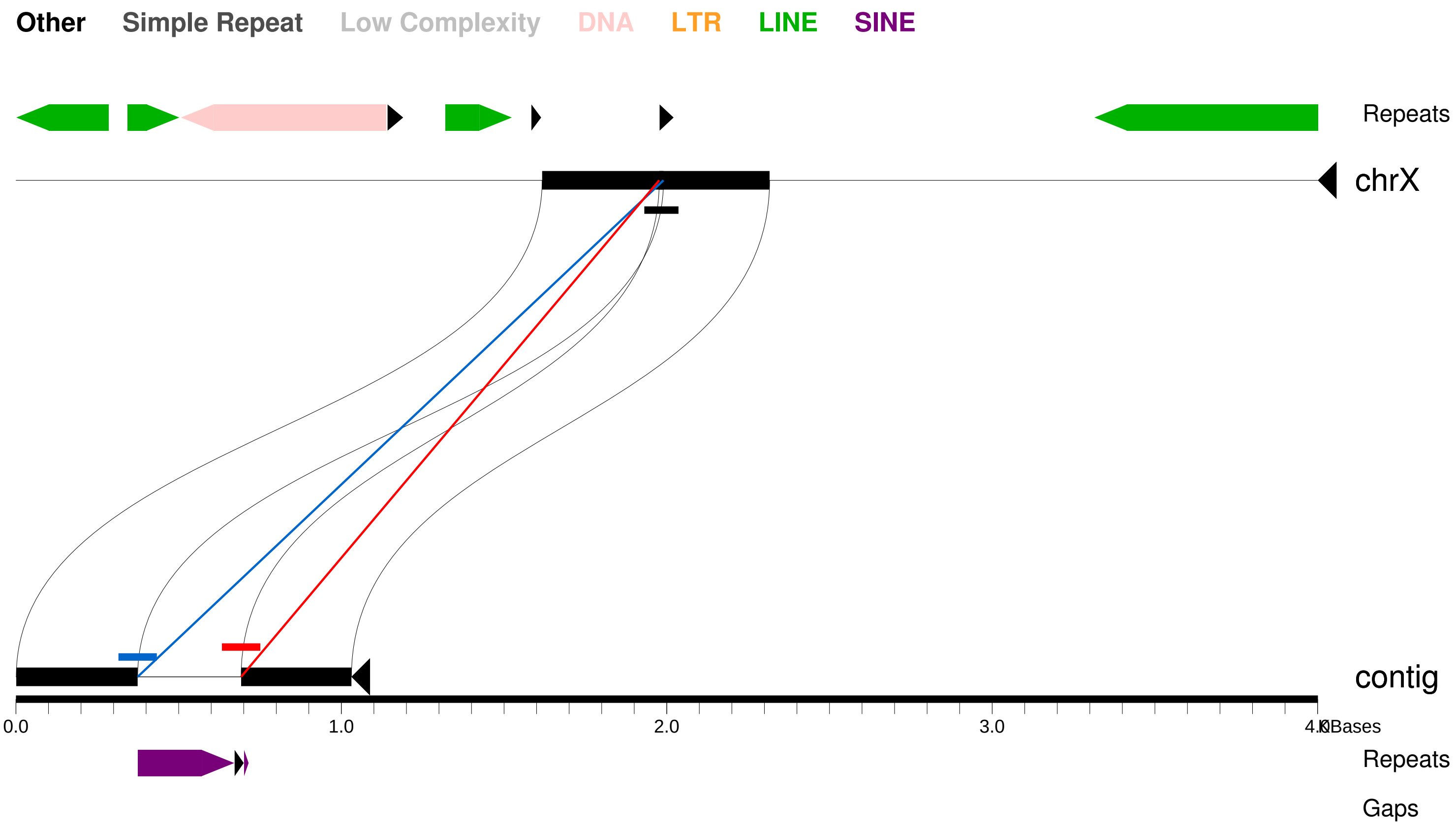

siteID: chrX_119245648 Scafftig1 + Breakpoint: chrX:119245645
chrX:119245604-119245704
Overlap Length: 19 INS size: 416 Genotyped: No

```
left    AACAAATTCCAGAAAACCCACCTCTTCAAAAAAGTACCACC------------------AAAAAGAAATA
chrom   AACAAATTCCAGAAAACCCACCTCTTCAAAAAAGTACCACC------------------AAAAAGAAATA
right   NNNNNNNNNNNNNNNNNNNNNNNNNNNNNNNNNNNNNNNNNNNNNNNNNNNNNNNNNAAAAAAAGAAATA
        11111111111111111111111111111111111111111111111111111111111***********

left    TAAATCCTGGCCGGGCGCGG--TGGCTCACGCCTGTAATCCCAG-CACTTTGGGAGGCCGAGGCGGGCGG
chrom   TAAATCCTTAGATGGATAGAAATTCCTCAAG------AGAACAGTCACTTAAACA---------------
right   TAAATCCTTAGATGGATAGAAATTCCTCAAG------AGAACAGTCACTTAAACA---------------
        ********22222**222*222*22****2*222222*222***2*****2222*222222222222222
```

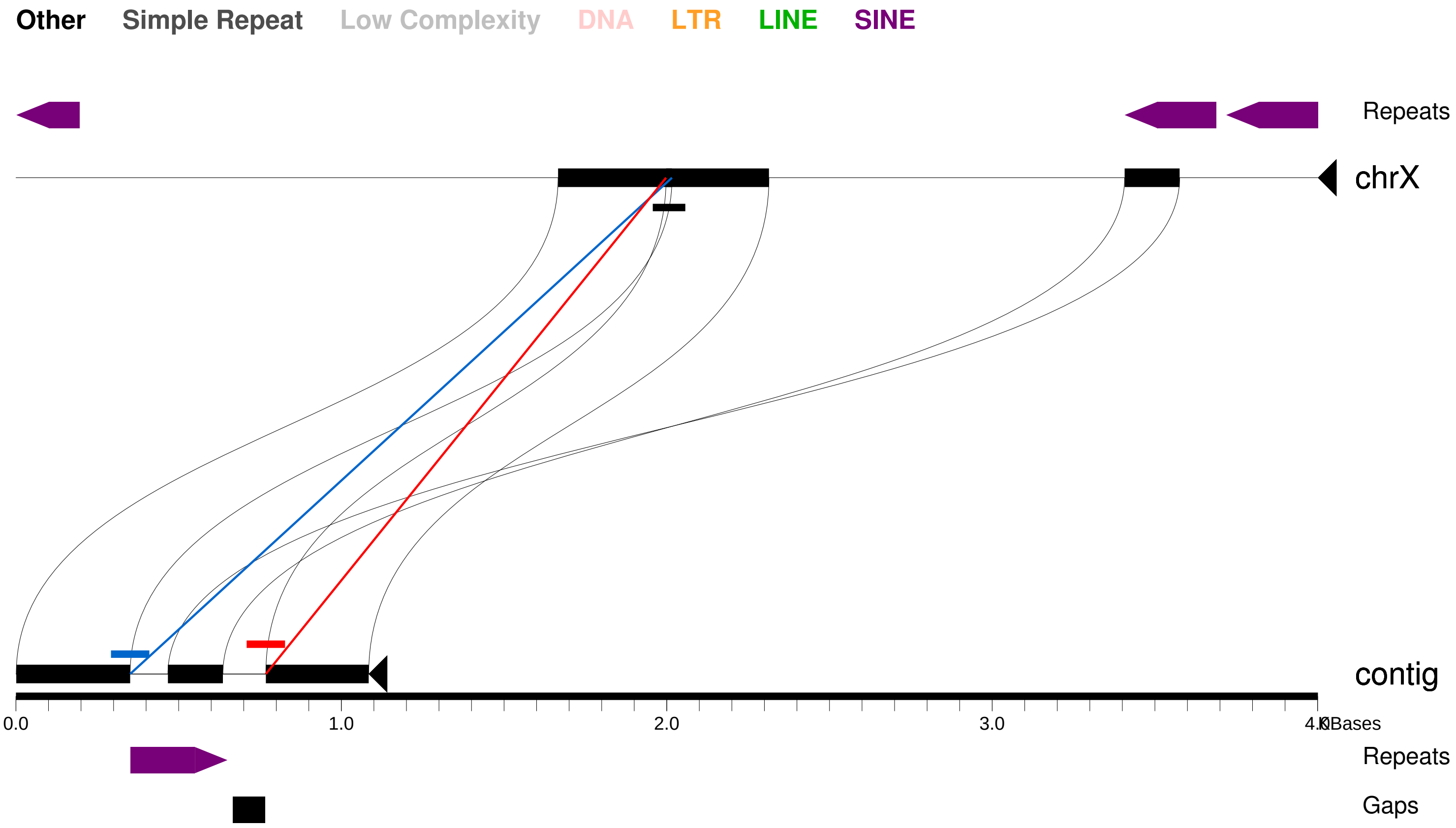

siteID: chrX_119527856 Scafftig3 + Breakpoint: chrX:119527843
chrX:119527797-119527902
Overlap Length: 14 INS size: 294 Genotyped: Yes

```
left     CTGCA-GCTTTGTTCTT----ACTGCTCAAAAT---TGTTTTAGTTATGT-----GGATTCTGTTGTGGT
chrom    CTGCA-GCTTTGTTCTT----ACTGCTCAAAAT---TGTTTTAGTTATGT-----GGATTCTGTTGTGGT
right    CCGCCCGCCTCGGCCTCCCAAAGTGCTGGGATTACAGGCGTGAGCCACCGCGCCCGGCCTCTGTTGTGGT
         *1**11**1*1*11**11111*1****111*1*1111*11*1**11*11111111**11***********

left     TTTTTTTTTTTTTTTTGAGACGGAGTCTCGCTCTGTCGCCCAGGCTGGAGTGCAGTGGCGGG
chrom    TTTATAAGCATTTTA-------GATTTTTTTTCTATTAAAAA------AGTCAAAAAATAAC
right    TTTATAAGCATTTTA-------GATTTTTTTTCTATTAAAAA------AGTCAAAAAATAAC
         ***2*22222****22222222**2*2*222***2*22222*222222***22*22222222
```

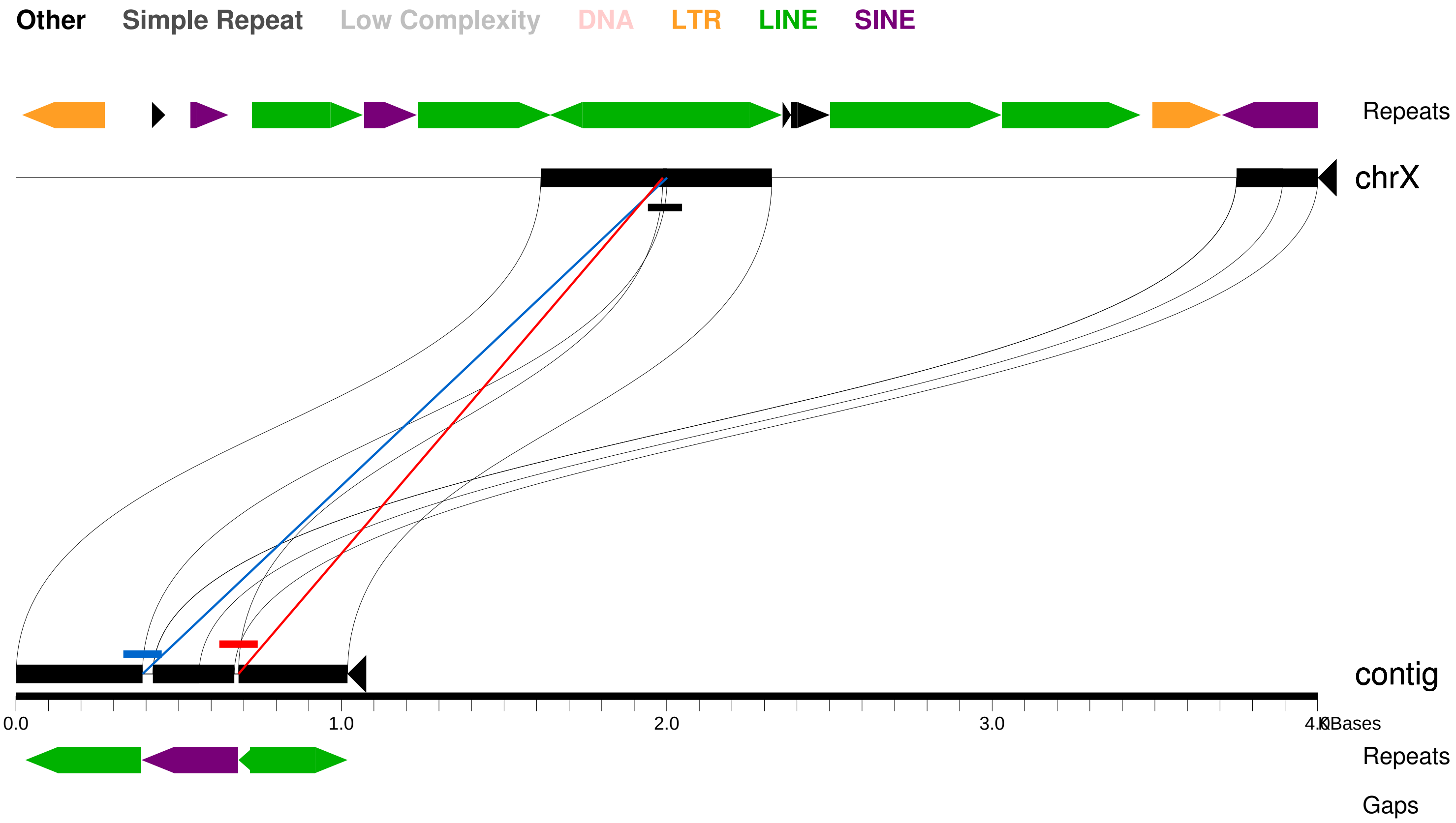

siteID: chrX_121569310 Scafftig1 + Breakpoint: chrX:121569312
chrX:121569290-121569371
Overlap Length: 14 INS size: 315 Genotyped: Yes

```
left    GAA-----------------------TGTTTTTCCA--CTTG------------CTTTTGTCATCTACAA
chrom   GAA-----------------------TGTTTTTCCA--CTTG------------CTTTTGTCATCTACAA
right   CCACCCGCCTCGGCCTCCCAAAGTGCTGGGATTACAGGCGTGAGCCACCGCGCCCGGCCGTCATCTACAA
        11*11111111111111111111111**111**1**11*1**111111111111*1111***********

left    TTTTTTTTTTTTTTTTTTTTTTTTTTTTGAGACGGAGTCTCGCTCTGTCGCCCAGGCGGGACTGCGGACT
chrom   TTTCTTTCTTCAGTGTTTTGTACTTTT-----CATTGTTGACATCTTTC-----------ACT-------
right   TTTCTTTCTTCAGTGTTTTGTACTTTT-----CATTGTTGACATCTTTC-----------ACT-------
        ***2***2**222*2****2*22****22222*222**22222***2**22222222222***2222222

left    GCAGCGGCGCAATCTC
chrom   --------------TC
right   --------------TC
        22222222222222**
```

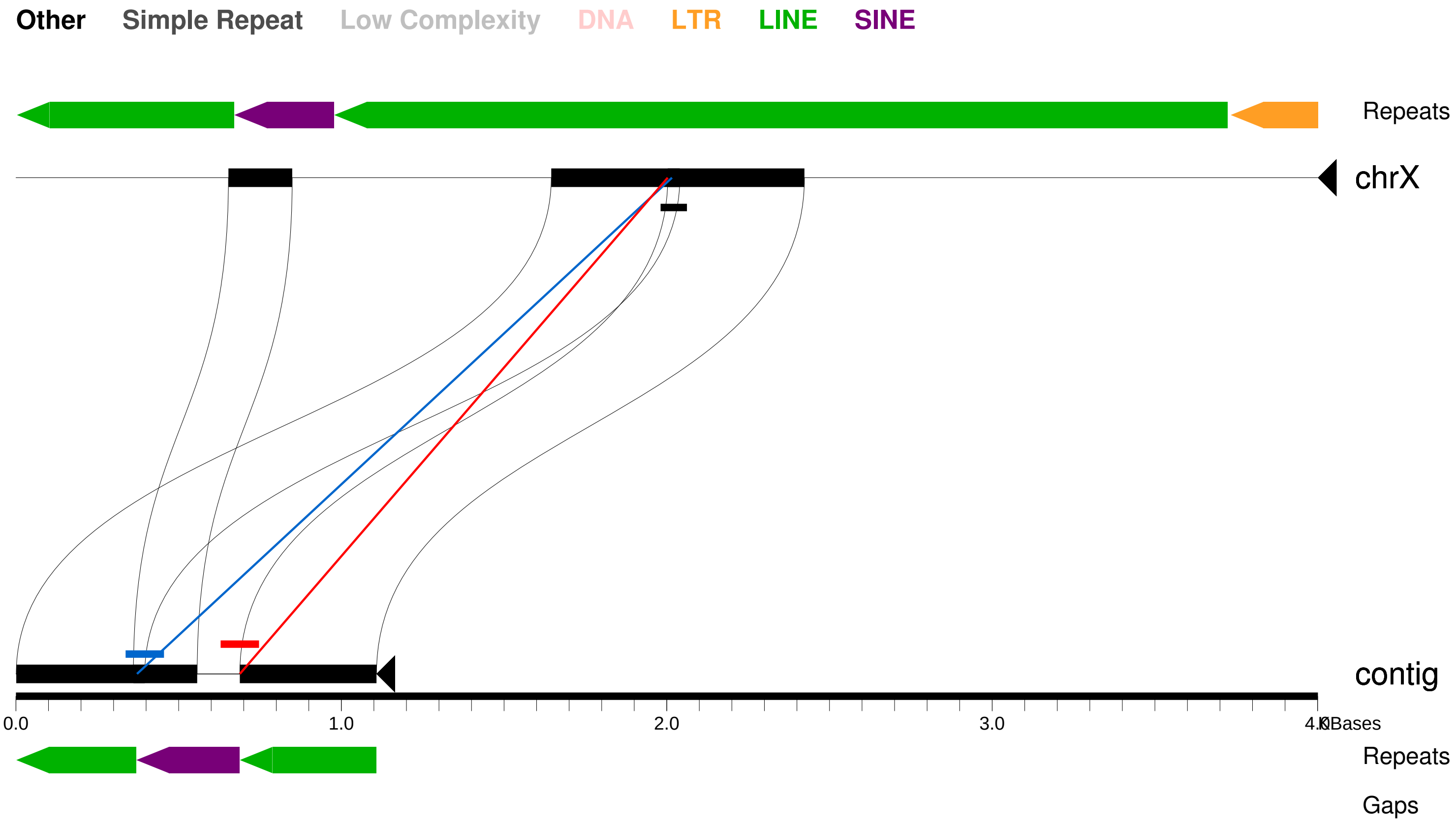

siteID: chrX_122672390 Scafftig1 + Breakpoint: chrX:122672364
chrX:122672319-122672423
Overlap Length: 15 INS size: 320 Genotyped: Yes

```
left     C------TTCTTAAAAACAATTATTTTGAATTCTTT--GTGAGGTCACTTGTA------GATCTCCATTT
chrom    C------TTCTTAAAAACAATTATTTTGAATTCTTT--GTGAGGTCACTTGTA------GATCTCCATTT
right    CACCCGCCTCGGCCTCCCAAAGTGCTGGGATTACAGGCGTGAGCCACCGCGCCCGGCCCGATCTCCATTT
         *1111111**1111111***11111*1*1***111111*****1111*11*11111111***********

left     CTTTTTTTTTTTTTTTTTTTTTTTTTTTTTTTTTTGAGACGGAGTCTCGCTCTGTCGCCCAGG
chrom    CTTTAGAGTTGGTCCCTGGTGCTTTATTTTGTTCC---------TTTGGTGGTGTCAT-----
right    CTTTAGAGTTGGTCCCTGGTGCTTTATTTTGTTCC---------TTTGGTGGTGTCAT-----
         ****2222**22*222*22*22***2****2**22222222222*2*2*222****2222222
```

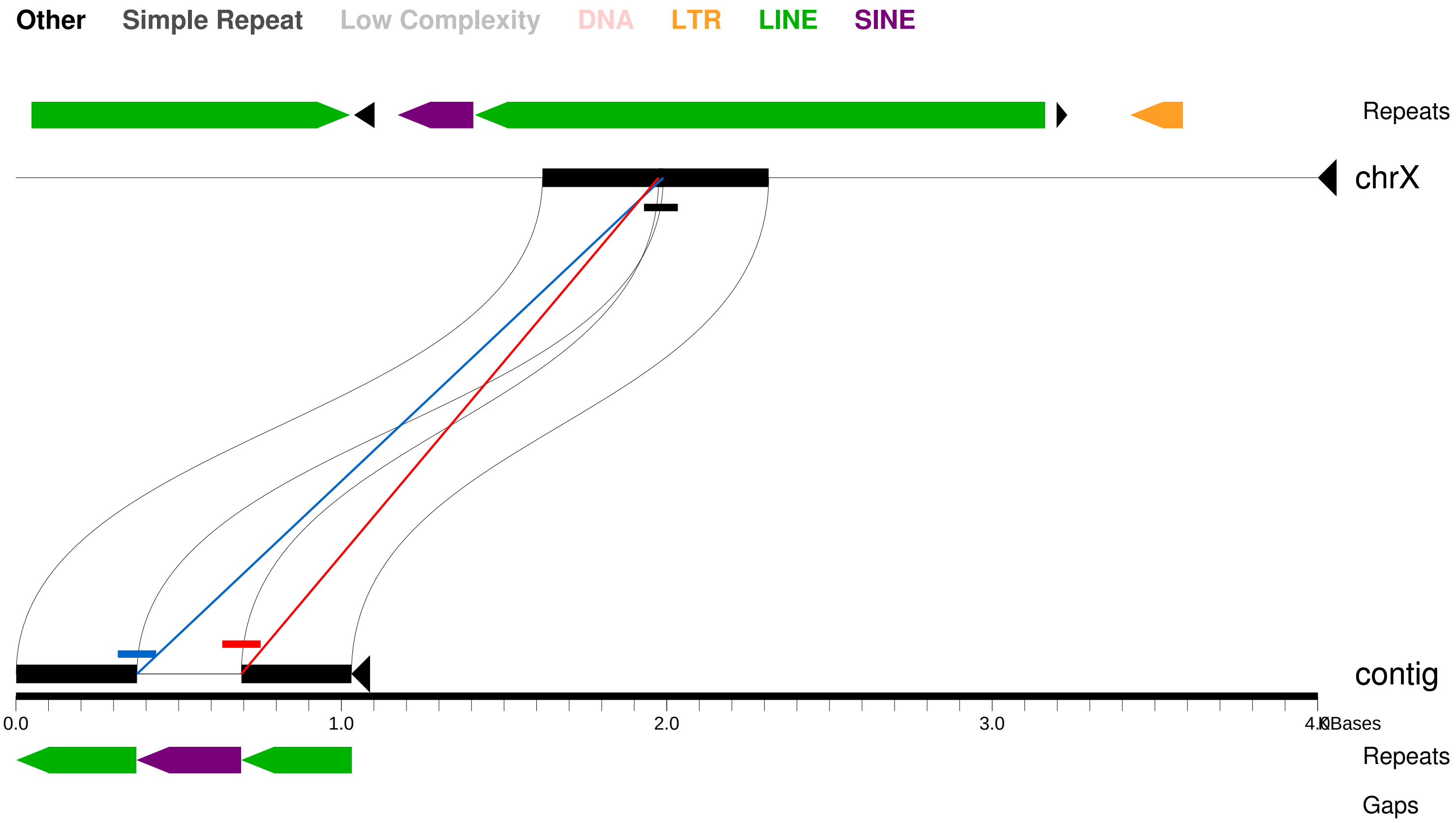

siteID: chrX_125560574 Scafftig1 - Breakpoint: chrX:125560557
chrX:125560397-125560728
Overlap Length: 12 INS size: 416 Genotyped: No

```
left   -------------------------------------GCTTCATTTTATCCCAGATAATTTTCCATTTCA
chrom  TTGCTTTTGCTATATCTTATGATTTTTGGTAGGTTGTGCTTCATTTTATCCCAGATAATTTTCCATTTCA
right  TT-----TAGTAGA------GAC----GG--GGTTTCAC--CGTGTTAGCCAGGATGGTCT-CGATCTCC
       22     2NN22N2      22N    22  2222NN1*11*1*1***1**11***11*1*1*1**1**1

left   CTTGTGATGTCAAGGATGTATTGTTTGTTTGGGATATATTGTACTGTTTAATTTTCACATACTGTGTATT
chrom  CTTGTGATGTCAAGGATGTATTGTTTGTTTGGGATATATTGTACTGTTTAATTTTCACATACTGTGTATT
right  ----TGACCTCG----TGATCCGCCCGCCTCGGCC-TCCCAAAGTGCTGGGATT--ACAGGC-GTGAGCC
       1111***11**11111**1111*111*11*1**111*11111*1**1*1111**11***11*1***1111

left   ATCAGAATTCCTTCGGTTATTGATTTCTAATTNNNNNNNNNNN---------------------------
chrom  ATCAGAATTCCTTCGGTTATTGATTTCTAATTTCATTCCATGTAATCAGAGTGTCTTAAACTGTTTGGGC
right  ACCG-----CGCCCGGCC--TGATTTCTAATTTCATTCCATGTAATCAGAGTGTCTTAAACTGTTTGGGC
       *1*111111*111***1111************22222222222222222222222222222222222222

left   ----------------------------------------------------------------------
chrom  TGCTATAATAAAATATCATAGACAAGTAGCTTATAAACAACAGAAATTTATTTTTCGGTTTTGGAGTTTG
right  TGCTATAATAAAATATCATAGACAAGTAGCTTATAAACAACAGAAATTTATTTTTCGGTTTTGGAGTTTG
       2222222222222222222222222222222222222222222222222222222222222222222222

left   ----------------------------------------------------
chrom  AGAAGTCCAAGGTCAGGACACCAGCAGATTTGATGTCTGATGAAGGCCTGCT
right  AGAAGTCCAAGGTCAGGACACCAGCAGATTTGATGTCTGA------------
       2222222222222222222222222222222222222222
```

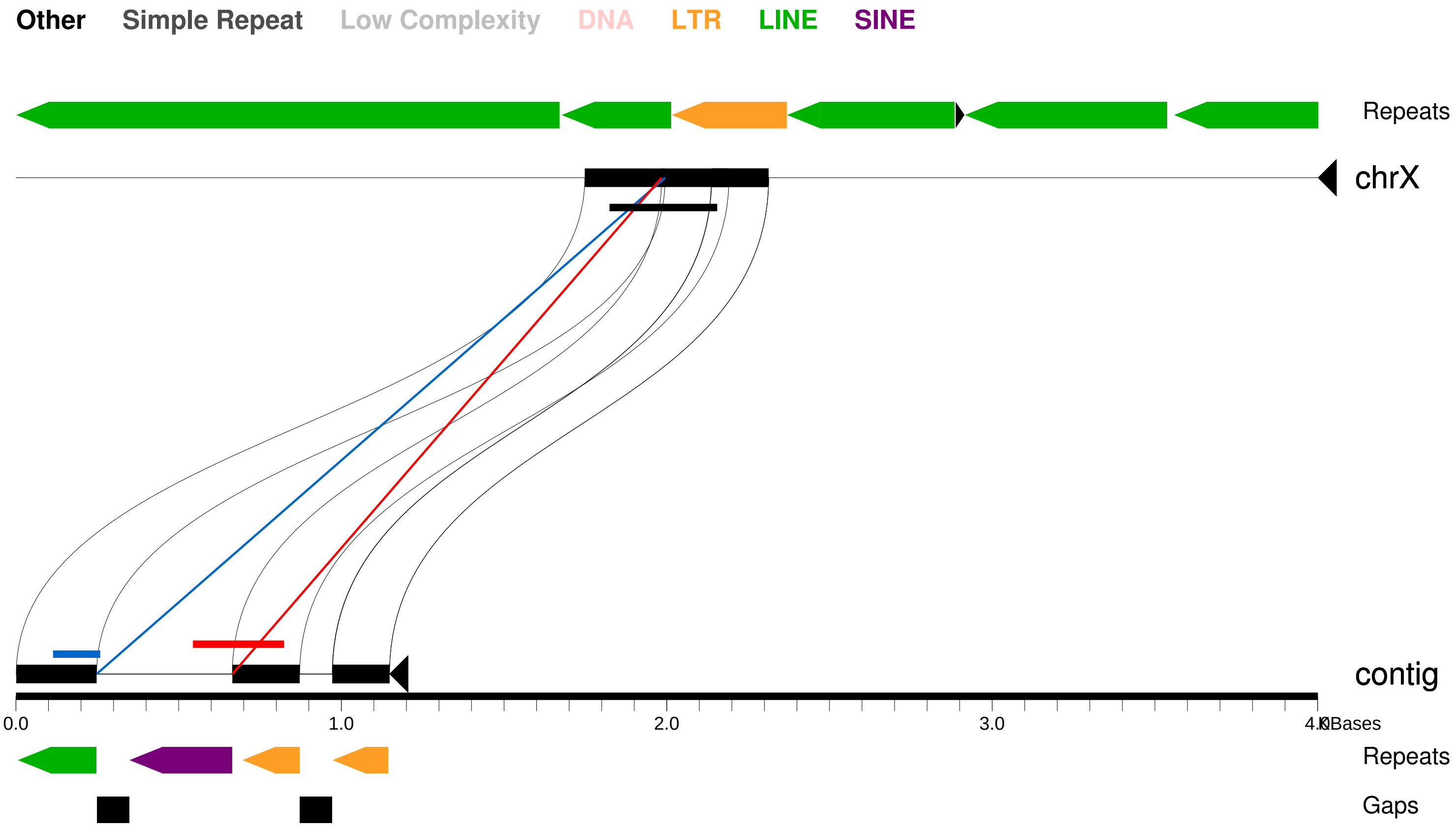

siteID: chrX_126290670 Scafftig4 + Breakpoint: chrX:126290677
chrX:126290634-126290736
Overlap Length: 17 INS size: 268 Genotyped: Yes

```
left    --ATT-------CAGGTTTTCCCTCTTCCTCAGGTT-------TTATTTATTTATTTATTTATTTTTACA
chrom   --ATT-------CAGGTTTTCCCTCTTCCTCAGGTT-------TTATTTATTTATTTATTTATTTTTACA
right   CGATCTCCTGACCTCGTGATCCGCCCGCCTCGGCCTCCCAAAGTGCTGGGATTACAGGCTTATTTTTACA
        11**11111111*11**11***11*11****1*11*1111111*11*1111***11111***********

left    ATTCTTTTTTTTTTTTTTTTTTTTTTTTTTTTTTTTGAGACGGAGTCTCGCTCTGTCGCCCAGGC
chrom   ATTCTTGTAGGCTATATAATTCTGAGGTGTAAACTTCAG----------GCCTTTTC----AG--
right   ATTCTTGTAGGCTATATAATTCTGAGGTGTAAACTTCAG----------GCCTTTTC----AG--
        ******2*2222*2*2*22**2*2222*2*2222**2**2222222222**22*2**2222**22
```

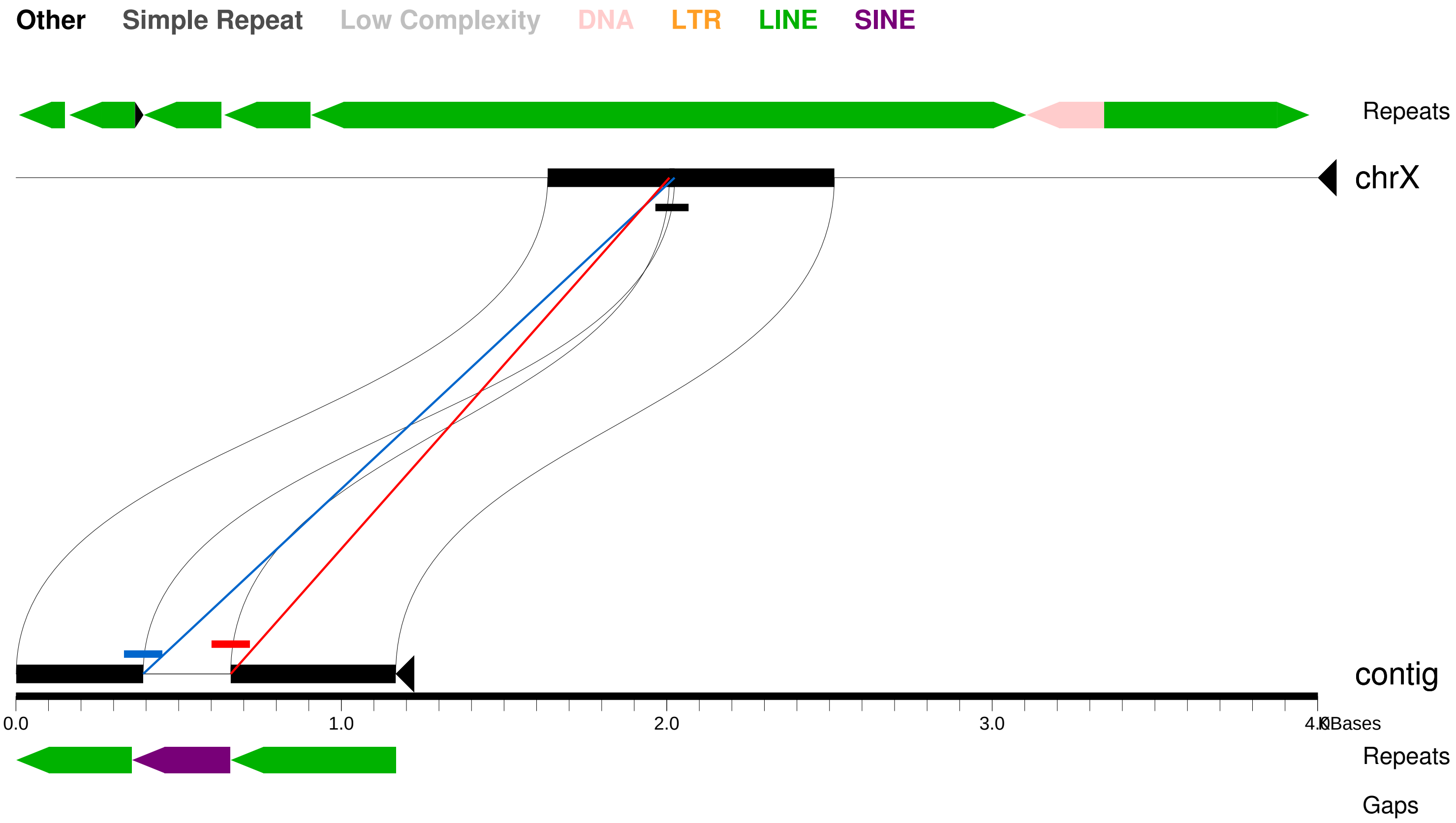

siteID: chrX_130921946 Scafftig2 + Breakpoint: chrX:130921943
chrX:130921896-130922002
Overlap Length: 10 INS size: 300 Genotyped: Yes

```
left    -------GATTTTATTCCTTT--TTCTGGCTCAATAATCTT---CCTTTTTATATGTATGTCACATTTTT
chrom   -------GATTTTATTCCTTT--TTCTGGCTCAATAATCTT---CCTTTTTATATGTATGTCACATTTTC
right   CCGCCCGCCTCGGCCTCCCAAAGTGCTGGGATTACAGGCGTGAGCCACCGCGCCCGGCCGTCACATTTTC
        111111111*11111***11111*1****1111*1*11*1*111**111111111*111**********2

left    TTTTTTTTTTTTTTTTTTGAGACGGAGTCTCGCTTTGTCGCCCAGGCTGGAGTGCAGTGGCG
chrom   TTAATCCATTCATCCGTCAGT-GAACACTTAGGTTG------ATTCT--ATATCTTTG---
right   TTAATCCATTCATCCGTCAGT-GAACACTTAGGTTG------ATTCT--ATATCTTTG---
        **22*222**22*222*2**22*2*22**2222***222222*22**22*222*22**222
```

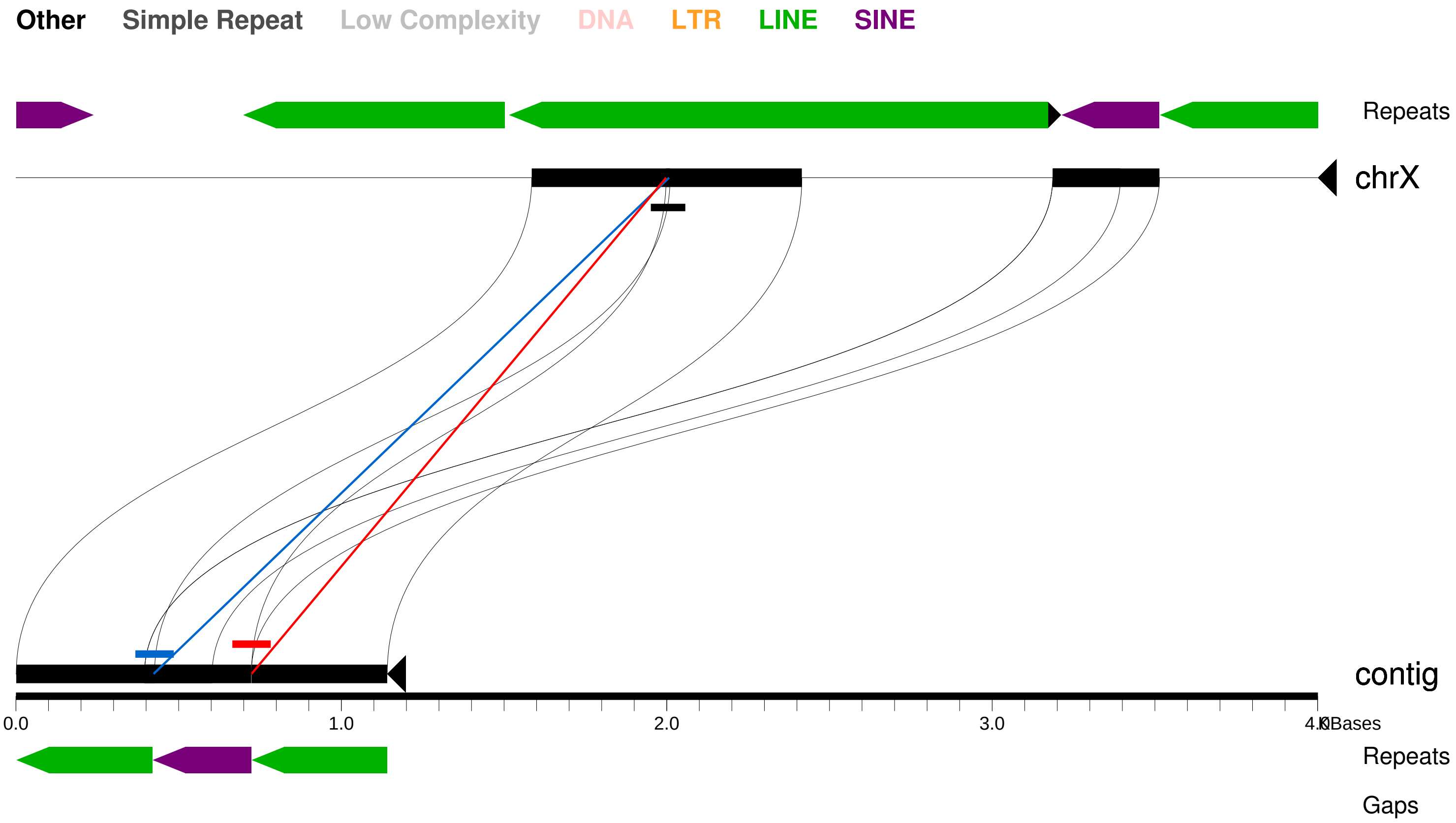

siteID: chrX_136349834 Scafftig2 + Breakpoint: chrX:136349822
chrX:136349115-136349982
Overlap Length: 19 INS size: 308 Genotyped: Yes

```
left   A--------------------------------------------------------------------
chrom  AGATATCCTCTTCCATTTTAATAGACGGATGGGCAGAATTACATACAGGTCAGTCTGTAGATTTGGTAGT
right  GGA--------------------------------GAAT----------------------------------
       122                                2222

left   ----------------------------------------------------------------------
chrom  AGGAAGGTCAGGGAATCTGAAGTTTTCCAATTTCTAATGTGAATTTGAAAGTGAGTTCATTTGCTTGGAG
right  ------------------------------------------------------------------GGCG
                                                                         22N2

left   ----------------------------------------------------------------------
chrom  TGAAAGTTGGGGTGGCTGAAAGATAATGGAGTATGAATAGGATAAACATGTGAACTAACTTCTTTGGAAA
right  TGAA------------------------------------------------------------------
       2222

left   ----------------------------------------------------------------------
chrom  GTAGGTGAGAGCATTGGTGTGGTTCTCAAACGTAAGCATGCATTAGAATCCCCTGGAGGGCTTGCTAAAA
right  --------------------------------------------------CCCGGGAGG-----------
                                                         222N22222

left   ----------------------------------------------------------------------
chrom  TAGAGTGCTGGGGCCCACTCACAGGGGCTCGGATTCTGTAGGTCTAGGATGAGCTGAGAATTTGCATTTC
right  -----------------------------CGGAGCTTGCAG--------TGAGCCGAGA-----------
                                    2222NNN22N22        22222N2222

left   ----------------------------------------------------------------------
chrom  TGACAAGCTCCCAGCTGATGCTGATGCTGCTGGTCCAGAGAACACACTTCAACAACCACTGGACTTGCCA
right  --------TCCC-------------GCCACTG------------CACTCCAGC-----CTGGGC------
               2222              22NN222             2222N22N2     2222N2

left   ----------------------------------------------------------------------
chrom  AGTAGCACTGACACTGGAAATTATAAATATTGACAGTGATGGTAGGGCAAGGTATGAAAGGTATGAAGAT
right  -------------------------------GACAG----------------------------------
                                      22222

left   ----------------------------------------------------------------------
chrom  GGTCAAGAAAGTTTGCAATTGTGGAGAATGTGAGAGAATTGACTAGAGAAACAATATAAATAGTGTCAGT
right  -------------------------------------------AGCGAGAC-------------TCCGT
                                                  22N22N22               22N22

left   -------AAGTTAAAGTTTTGACTGAGGCATCAAGATACCATAGATATTGAAGCAATTAGTGAGACAGGC
chrom  GTAACAAAAGTTAAAGTTTTGACTGAGGCATCAAGATACCATAGATATTGAAGCAATTAGTGAGACAGGC
right  CTCAAAAAAAAAAAA-------------------------------------------------------
       N2N2N22**111***1111111111111111111111111111111111111111111111111111111

left   CACCCTGTATGAATTTCAAAGTAGCGAACCAATAACTGATTTCAAAACTCTTAAGCCCTTCACTCAGTAT
chrom  CACCCTGTATGAATTTCAAAGTAGCGAACCAATAACTGATTTCAAAACTCTTAAGCCCTTCACTCAGTAT
right  -----------------AAA-----------AAAAA--------AAAA-------------------------
       11111111111111111***1111111111**1**11111111****1111111111111111111111111

left   GGGTTT[illegible]AATCCCAGC[illegible]TGGGA
chrom  GGGTTTTAAAAGCTCTGGTTGGTGGATATTAGGGGTACTTG-TAAGGAA--TATTTCTAGTAAGAGGGCT
right  -------AAAAGCTCTGGTTGGTG[illegible]AGGAA--TATTTCTAGTAAGAGGGCT
       1111111*****************[illegible]222*2*2*2*2*2222**2*2*2**2*2222**22

left   GGCC---GAGGCGGGCGGATCACGAGGTCAGGAGATCGAGACCATC-CCGGCTAAAAC------GGTGAA
chrom  GTTCTGTGAGCCCTCCTTCCCAAGATGGAAGGGTACAGAGTGCTTCTCCCTCTACATCAAGTCAGGAGAG
right  GTTCTGTGAGCCCTCCTTCCCAAGATGGAAGGGTACAGAGTGCTTCTCCCTCTACATCAAGTCAGGAGAG
       *22*222***2*222*2222**2**2*22***22*22***22*2**2**22***2*2*222222**2**2

left   A-------------CCCCGTCTCTAC-TAAA---
chrom  AGGAATTTTAAGGACCACTTAGCTTGATATG---
right  AGGAATTTTAAGGACCACTTAGCTTGATATGTGA
       *2222222222222**2*2*22**222**22111
```

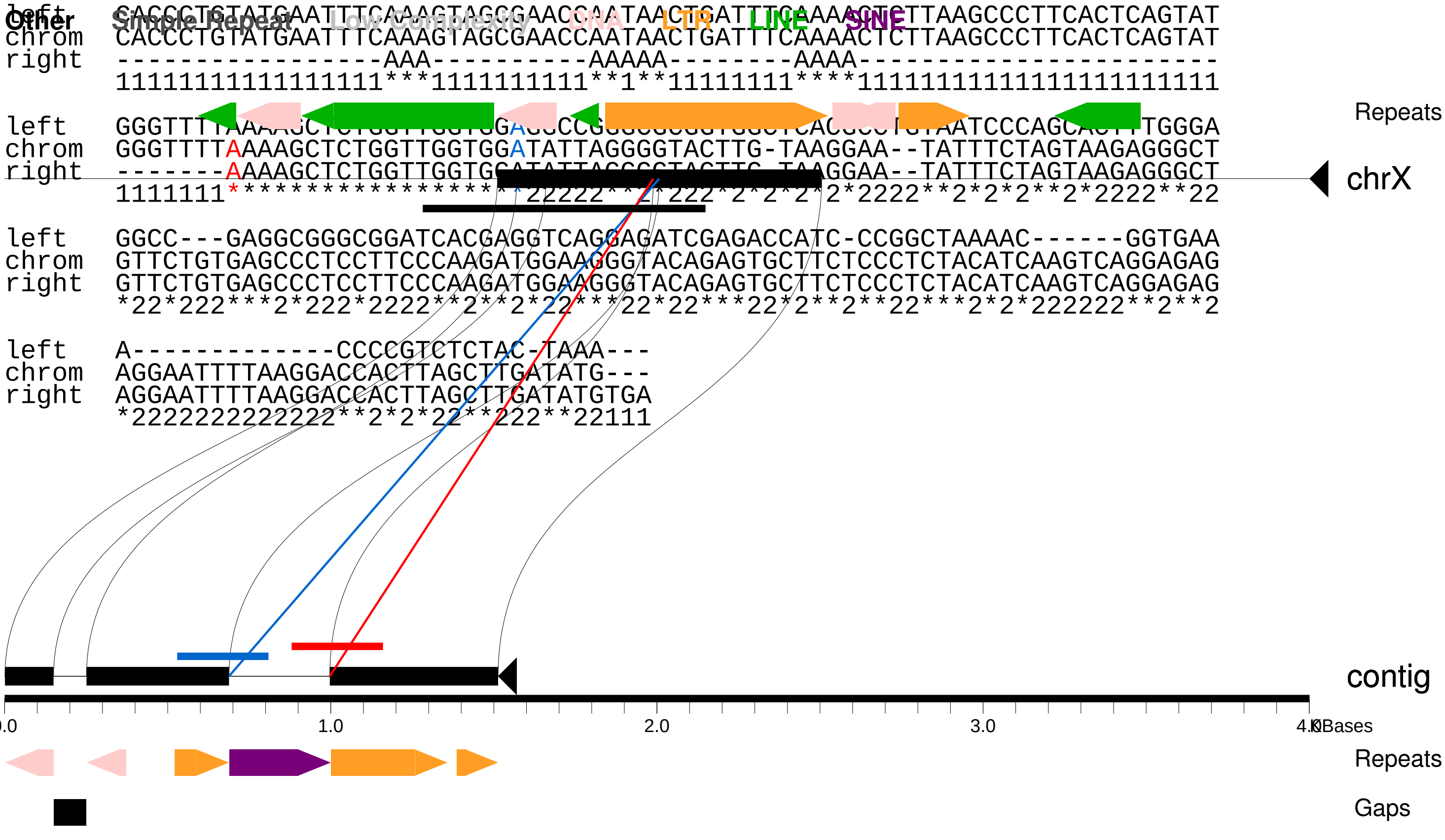

siteID: chrX_136957311 Scafftig3 - Breakpoint: chrX:136957286
chrX:136957238-136957342
Overlap Length: 12 INS size: 395 Genotyped: No

```
left     ATGTGTCAAAACAATAATGT-----TCTATTCAACTCAGAGCAC----ATTA-----GGGATGAAGTGTG
chrom    ATGTGTCAAAACAATAATGT-----TCTATTCAACTCAGAGCAC----ATTA-----GGGATGAAGTGTG
right    ATCTCCTGACCTCGTGATCCGCCCGTCTCGGCCTCCCAAAGTGCTGGGATTACAGGCGTGAAGAAGTGTG
         **1*1111*11111*1**1111111***111*11*1**1**11*1111****11111*1**1********

left     ATTTTTTTNNNNNNNNNNNNNNNNNNNNNNNNNNNNNNNNNNNNNNNNNNNNNNNNNNNNNNN
chrom    ATTT--------------AAAATCAGGAAATAAGCCCATTTTGTACTTCTCTGATTACACTGG
right    ATTT--------------AAAATCAGGAAATAAGCCCATTTTGTACTTCTCTGATTACACTGG
         ****22222222222222222222222222222222222222222222222222222222222
```

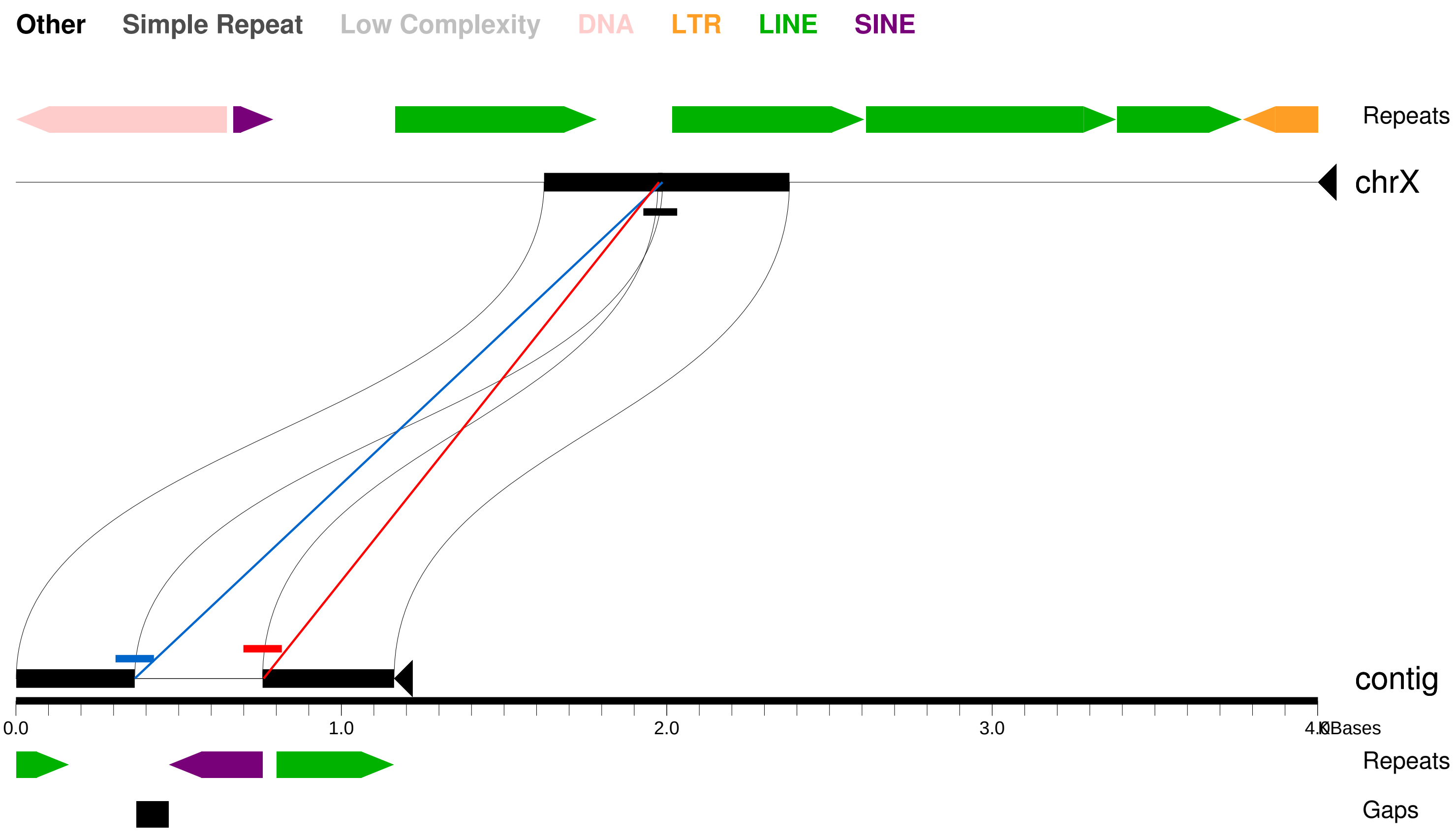

siteID: chrX_138744440 Scafftig1 + Breakpoint: chrX:138744467
chrX:138744439-138744526
Overlap Length: 16 INS size: 296 Genotyped: Yes

```
left    -----------------CCTAAATTGATA----TACAGAT-TGAAAA---------GAACCCCTATAAAG
chrom   -----------------CCTAAATTGATA----TACAGAT-TGAAAA---------GAACCCCTATAAAG
right   TCCGCCCGCCTCGGCCTCCCAAAGTGCTGGGATTACAGGCGTGAGCCACCGCGCCCGGCCCCCTATAAAG
        11111111111111111**1***1**1*11111*****111***111111111111*11***********

left    ATTTTTTTTTTTTTTTTTTTTTGAGGCGGAGTTTCGCTCTGTCGCCCAGGCTGGAGTGCAGTGGCGCGAT
chrom   ATTTTAGCTCGCTTTTTTCTTACA---GAAATTAA-----------CAGG-TGGA-------------AC
right   ATTTTAGCTCGCTTTTTTCTTACA---GAAATTAA-----------CAGG-TGGA-------------AC
        *****222*222******2**22*222*2*2**2222222222222****2****2222222222222*2

left    CTCGACTCAC
chrom   CTAAA---AC
right   CTAAA---AC
        **22*222**
```

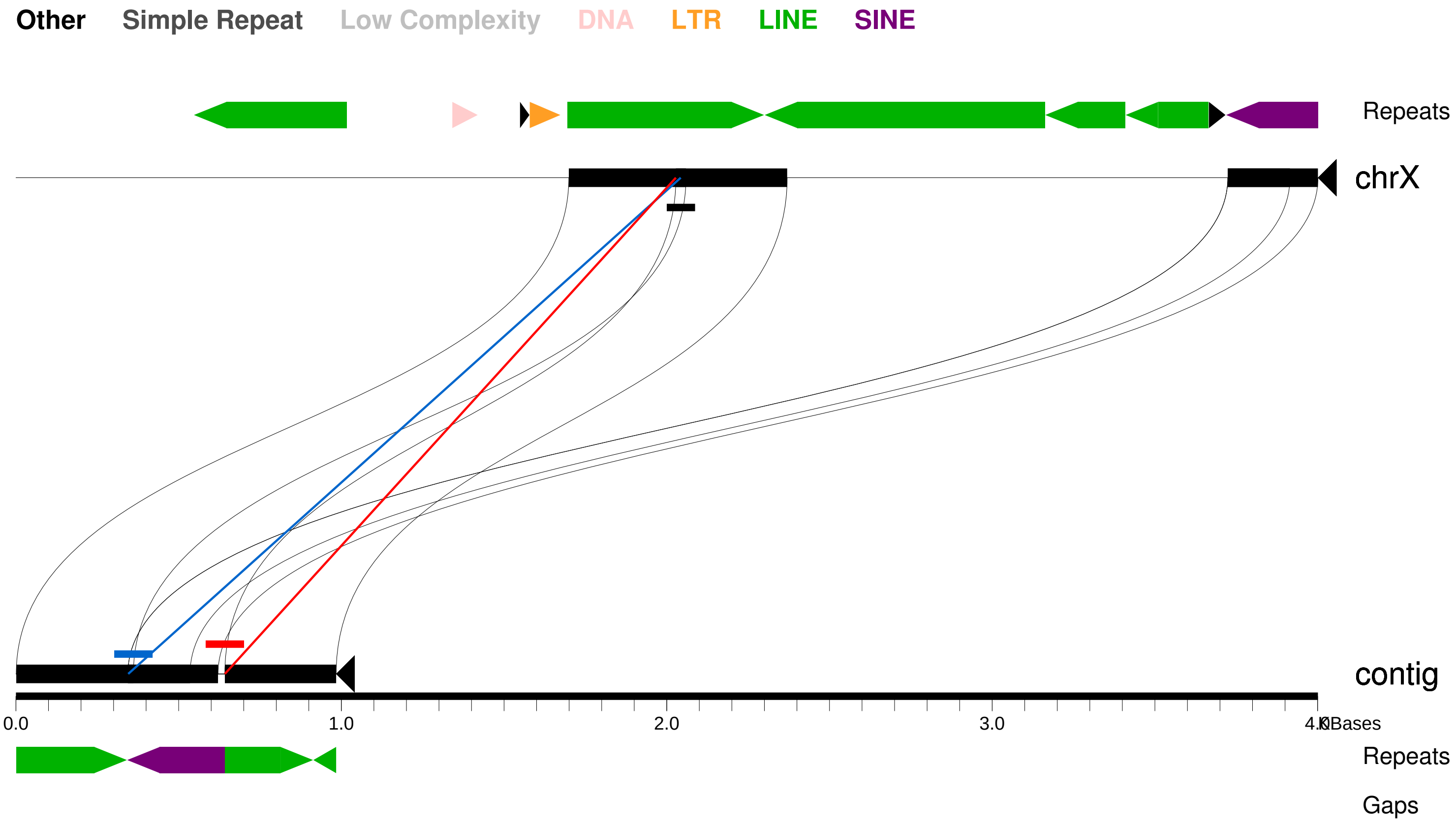

siteID: chrX_141138864 Scafftig1 - Breakpoint: chrX:141138866
chrX:141138814-141138923
Overlap Length: 8 INS size: 412 Genotyped: No

```
left    GGAACCCCTAAGTATATCAAAAATCATATCCAATATAAATGTTCTAAACA---------GTCCAGTTGAG
chrom   GGAACCCCTAAGTATATCAAAAATCATATCCAATATAAATGTTCTAAACA---------GTCCAGTTGAA
right   NNNNNNNNNNNNNNNNNNNNNNNNNNNNNNNNNNNNNNNNNNNNNNNNNNNNNNNNNNNNNCCAGTTGAA
        1111111111111111111111111111111111111111111111111111111111111********2

left    GCCGGGCGCGGTGGCTCAC-GCCTGTAATCCCAGCACTTTGGGAGGCCGAGGCGGGTGG
chrom   AAGCAGAGAC-TATCAGACTGGATGTAAAAGCAAGACTTGACTAT----ATGCT-----
right   AAGCAGAGAC-TATCAGACTGGATGTAAAAGCAAGACTTGACTAT----ATGCT-----
        22222*2*222*22*22**2*22*****222**22****2222*22222*2**222222
```

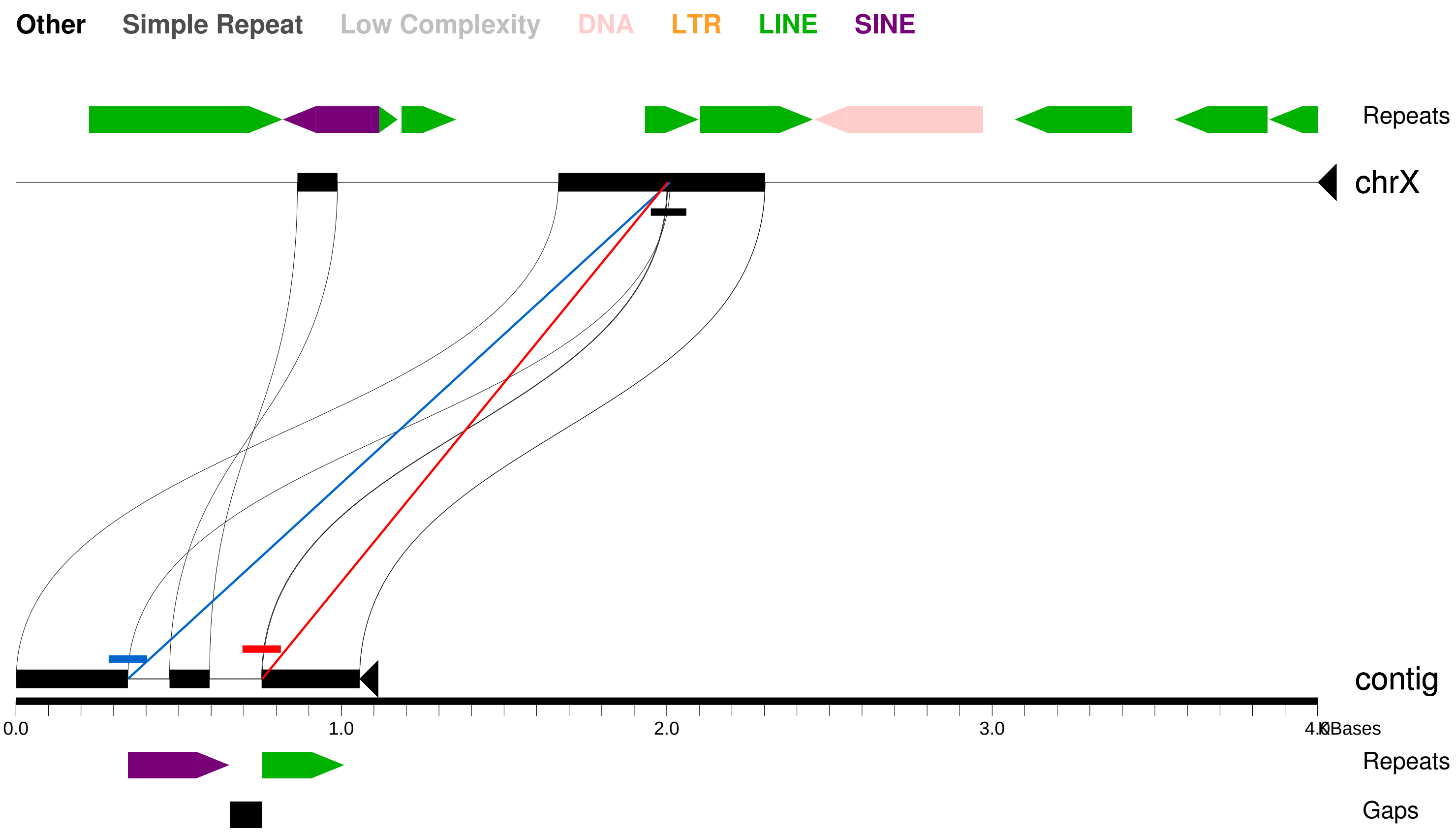

siteID: chrX_142926117 Scafftig1 - Breakpoint: chrX:142926114
chrX:142926067-142926172
Overlap Length: 10 INS size: 419 Genotyped: No

```
left   ------------GCTTTGTAATATATTTTGAAGTCAGGTAGTGTG---ATGCCTCCAGCTTTGCTCTTTT
chrom  ------------GCTTTGTAATATATTTTGAAGTCAGGTAGTGTG---ATGCCTCCAGCTTTGCTCTTTT
right  CCGCCCGCCTCGGCCTCCCAAAGTGCTGGGATTACAGGC-GTGAGCCACTGCGCCCGGCCT-GCTCTTTT
       111111111111**1*111**11*11*11**111****11***1*1111***11**1**1*1********

left   TTTTTNNNNNNNNNNNNNNNNNNNNNNNNNNNNNNNNNNNNNNNNNNNNNNNNNNNNNNNNNNNN
chrom  TTGTTCAGTATTTCTTTGGCATTTCAGGGTCTTTACGGTTTAATATAAAAT-------------
right  TTGTTCAGTATTTCTTTGGCATTTCAGGGTCTTTACGGTTTAATATAAAAT-------------
       **2**222222222222222222222222222222222222222222222222222222222222
```

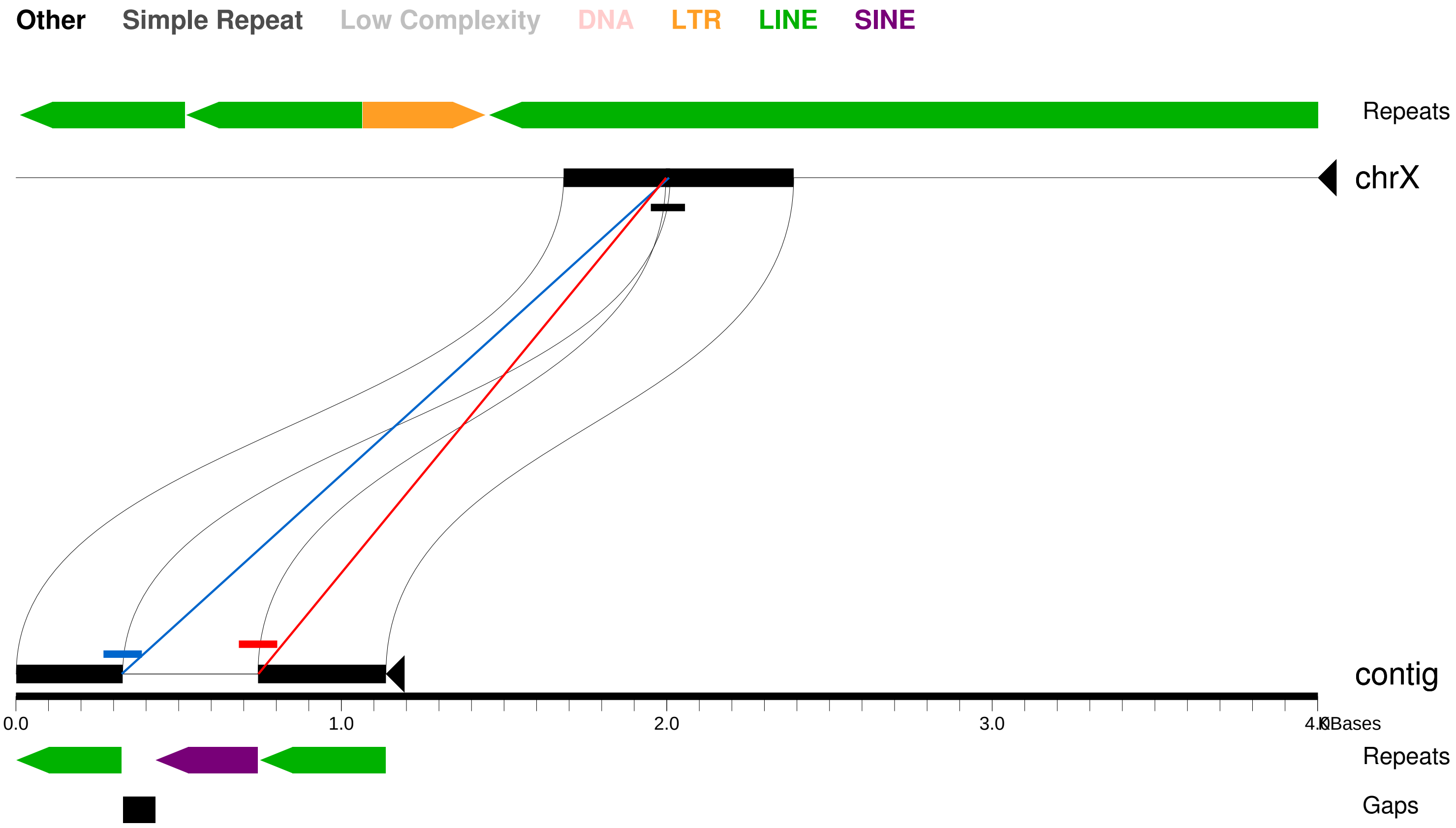

siteID: chrX_143723308 Scafftig1 - Breakpoint: chrX:143723330
chrX:143723292-143723389
Overlap Length: 22 INS size: 425 Genotyped: No

```
left    TGATGATGTTTTAAATAAAAGAAAGAAATATTTTAAAG--------------------AAAGTATAAAA
chrom   TGATGATGTTTTAAATAAAAGAAAGAAATATTTTAAAG--------------------AAAGTATAAAA
right   NNNNNNNNNNNNNNNNNNNNNNNNNNNNNNNNNNNNNNNNNNNNNNNNNNNNNNNNNNAAAGTATAAAA
        1111111111111111111111111111111111111111111111111111111111***********

left    GCATGTGTTAGGCCGGGCGCGGTGGCTCACGCCTGTAATCCCAGCACTTTGGGAGGCCGAGGCGGCGGA
chrom   GCATGTGTTAG------AGAAATATAACA-GAAAATAAATATAATATTAAATAAT-------------A
right   GCATGTGTTAG------AGAAATATAACA-GAAAATAAATATAATATTAAATAAT-------------A
        ***********2222222*222*2222**2*2222***2222*22*2*22222*22222222222222*
```

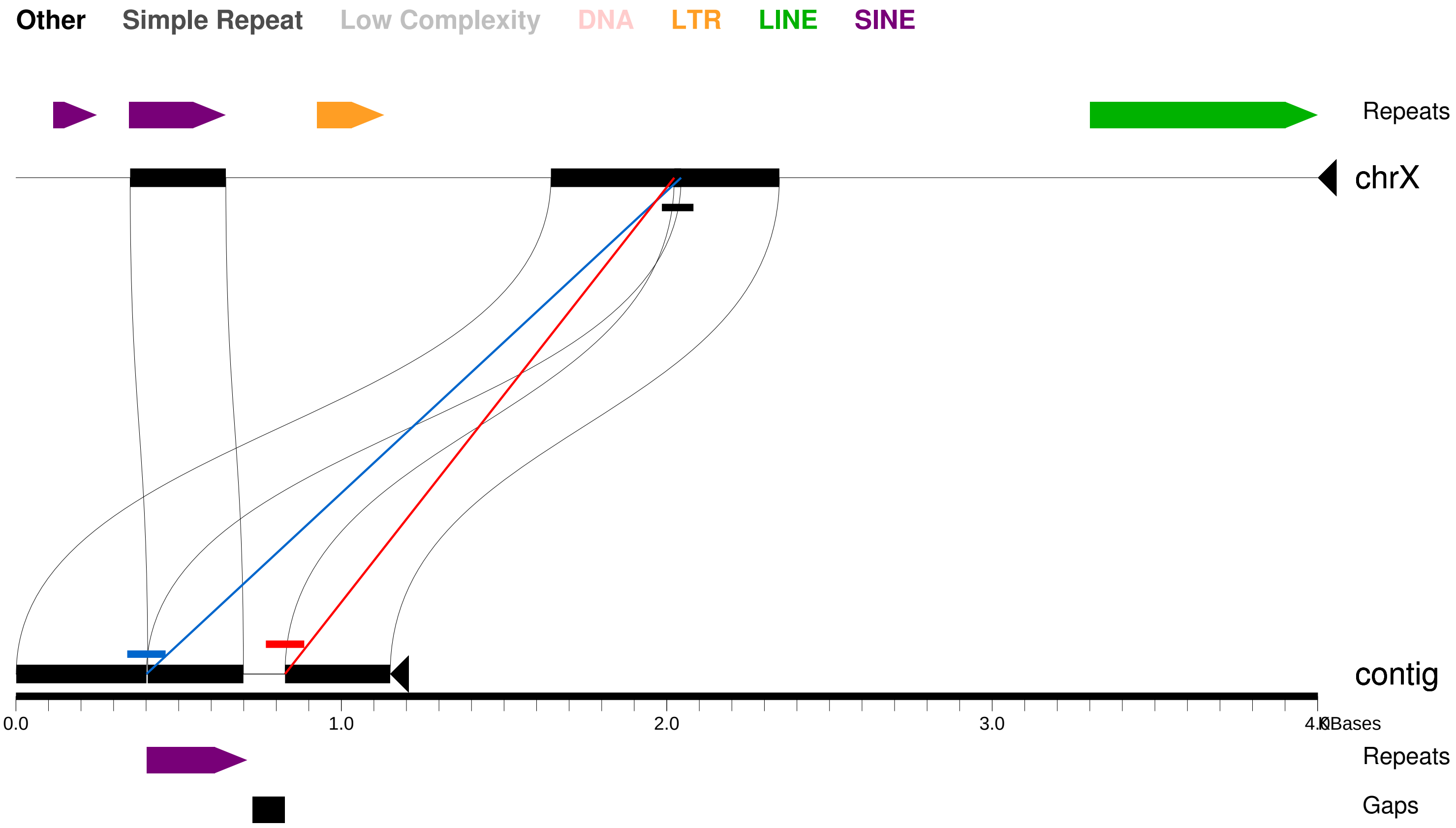

siteID: chrX_144443234 Scafftig1 + Breakpoint: chrX:144443219
chrX:144443171-144443278
Overlap Length: 12 INS size: 299 Genotyped: Yes

```
left    TACCTCATTAAAGA---TAAAAGAG---GAAATACAACCTAG---CACTGTGGTTTA--TCAATGACTTT
chrom   TACCTCATTAAAGA---TAAAAGAG---GAAATACAACCTAG---CACTGTGGTTTA--TCAATGACTTT
right   CCGCCCGCCTCGGCCTCCCAAAGTGCTGGGATTACAGGCGTGAGCCACCGCGCCCGGCCTCAATGACTTT
        111*1*111111*111111****1*111*1*1****11*11*111***1*1*1111111***********

left    TTTTTTTTTTTTTTTTTTTGAGACGGAGTCTCGCTCTGTCGCCCAGGCTGGAGTGCAGTG
chrom   TAAAGAG--------ATCTGACAACTGGAAAATATATTTTTTAAAGTATATATATTTAT---
right   TAAAGAG--------ATCTGACAACTGGAAAATATATTTTTTAAAGTATATATATTTAT---
        *222222222222222*2***2*2222*222222*2*2*22222**22*22*2*222222
```

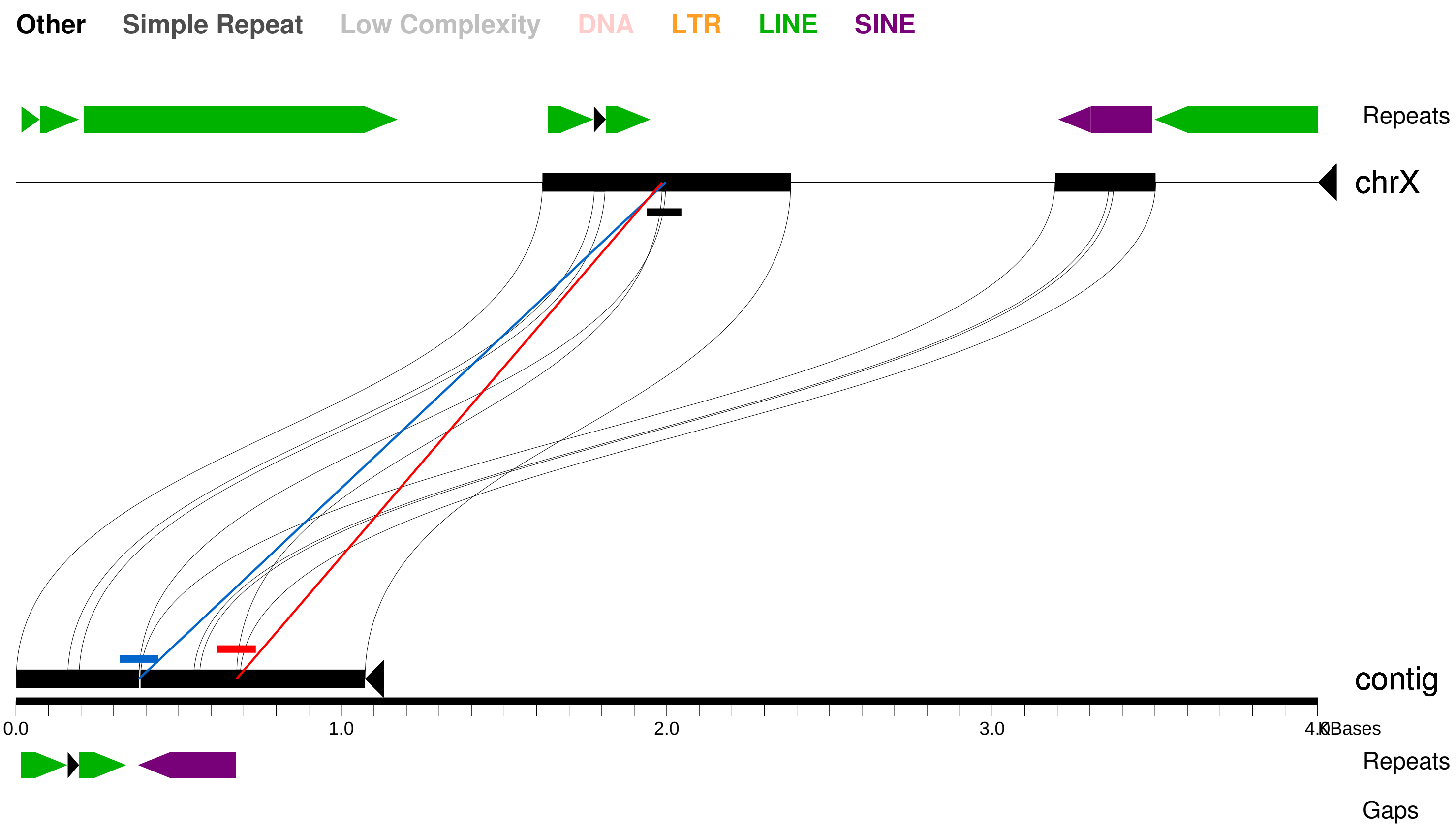

siteID: chrX_145539109 Scafftig7 - Breakpoint: chrX:145539108
chrX:145539060-145539164
Overlap Length: 12 INS size: 313 Genotyped: Yes

```
left     AATAGAAACTACAAAGCAA----CCAGCT--------AACAACTTCACGATAGTATCAAA--ACTTCACC
chrom    AATAGAAACTACAAAGCAA----CCAGCT--------AACAACTTCACGATAGTATCAAA--ACTTCACC
right    AGCCTGGGCGACAGAGCGAGACTCCGTCTCAAAAAAAAAAAAAAAAAAAAAAAAAACAAAAAACTTCACC
         *1111111*1***1***1*1111**11**11111111**1**1111*11*1*11*1****11********

left     TATCGGCCGGGCGCGGTGGC-TCATGCCTGTAATCCCAGCACTTTGGGAGGCTGAGGCGGGCGG
chrom    TATCAATATAAACCTTAAGTGTTAATCGTCTAAACGCACCACTTAAAA---------------G
right    TATCAATATAAACCTTAAGTGTTAATCGTCTAAACGCACCACTTAAAA---------------G
         ****222222222*2222*22*2*22*2*2***2*2**2*****2222222222222222222*
```

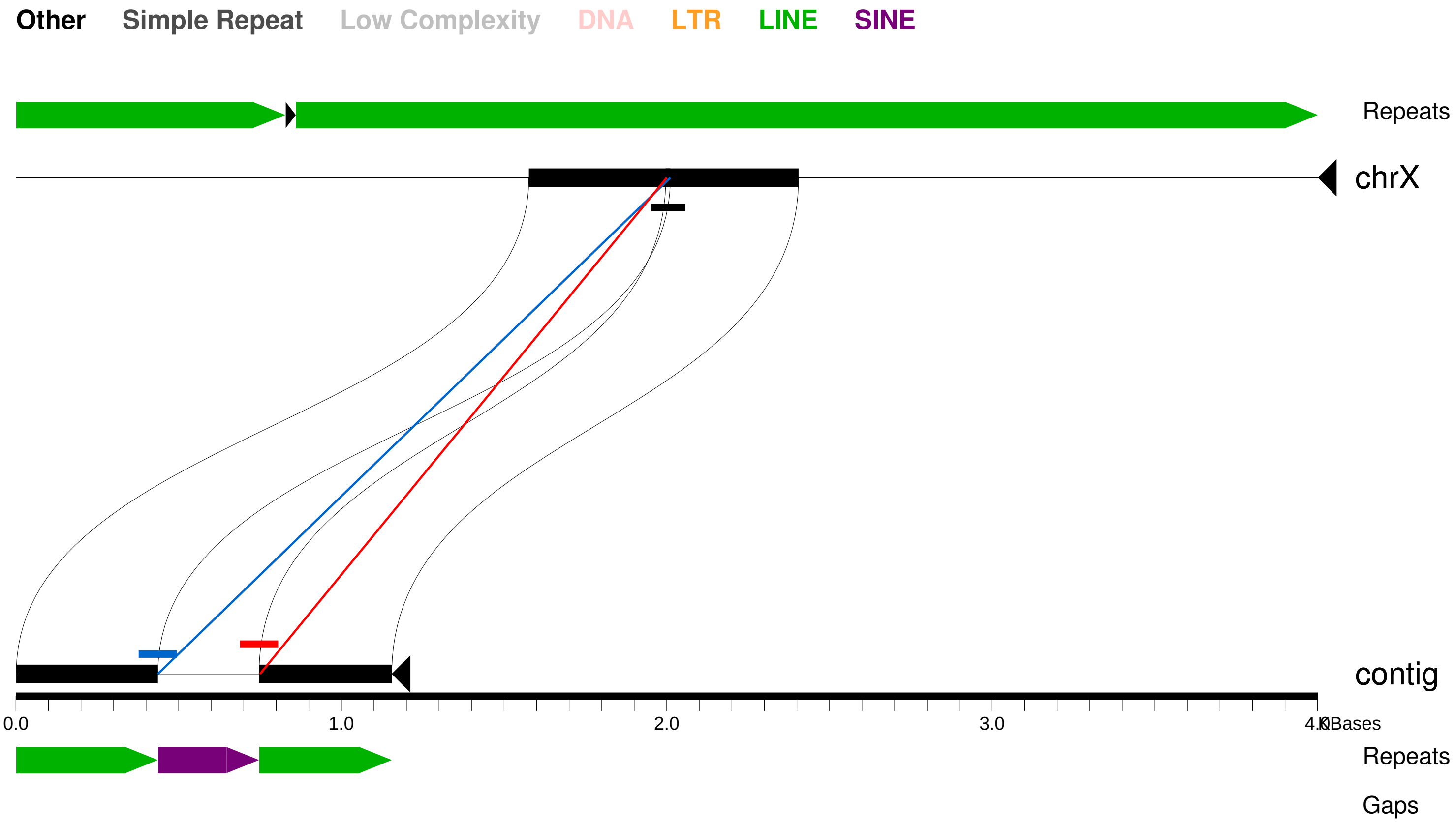

siteID: chrX_148539099 Scafftig1 - Breakpoint: chrX:148539112
chrX:148538952-148539275
Overlap Length: 4 INS size: 318 Genotyped: Yes

```
left   T-----CACTGGGTTGAATCCACCTCAGTCTGGTTGTTACCAAACCGCATTACTAAGGTTTAACTTCTAA
chrom  TGAGGTCACTGGGTTGAATCCACCTCAGTCTGGTTGTTACCAAACCGCATTACTAAGGTTTAACTTCTAA
right  -GCGTGAACCCGG--GAAGCGG----AG-CTTGCAGTGAGC----CG--------AGATTGCGCCACTGC
       12N2NN1**11**11***1*111111**1**1*11**1*1*1111**11111111**1**111*11**11

left   ATACCAAAGTTTCAGGGCATCGAGAGAAGAGGGGAGAAAGTGGTACTGAGGCACTGAATGGAGACAGAGG
chrom  ATACCAAAGTTTCAGGGCATCGAGAGAAGAGGGGAGAAAGTGGTACTGAGGCACTGAATGGAGACAGAGG
right  AGTCCGCAGTCT---GGCCTGG---------GCGACAGAGCGAGACTCCGTCTCAAAAAAAAAAAAAAAA
       *11**11***1*111***1*1*111111111*1**1*1**1*11***11*1*1*11**111*1*1*1*11

left   GGAGATGGGTTGACCAGAACTCTCGG--GCCGGGCGCGGTGGCT-CACGCCTGTAATCCCAG----CACT
chrom  GGAGATGGGTTGACCAGAACTCTCAGCAGCTAGGCCAATTGGGTTCTAGATTCCAAAGAAAGAGGTCCAT
right  AAAAA-------AAAAAAAATCTCAGCAGCTAGGCCAATTGGGTTCTAGATTCCAAAGAAAGAGGTCCAT
       11*1*1111111*11*1**1****2*22**22***2222***2*2*22*22*22**2222**2222*22*

left   T-----TGGGAGGCCGAGGCGGGTGG------ATC-ATGAGG----TCAGGAGATCGAGACCAT-----C
chrom  TATTGCTGGGATGCCCAGAATTATTGTCCCAAATCCACTAGAAGCTTGTGGGAATTAACACTCTTTATTC
right  TATTGCTGGGATGCCCAGAATTATTGTCCCAAATCCACTAGAAGCTTGTGGGAATTAACACTCTTTATTC
       *22222*****2***2**22222*2*222222***2*22**22222*22**22**22*2**22*22222*

left   CTGGCTAACAAGGTGAAA------CCCCGTCT-CTAC---TAAA
chrom  TTTTTTTAAGCTGTGAAATATCTGCAGCGTGTTCTTGGGATAAA
right  TTTTTTTAAGCTGTGAAATATCTGCAGCGTGTTCTTGGGA----
       2*222*2*2222******222222*22***2*2**222221111
```

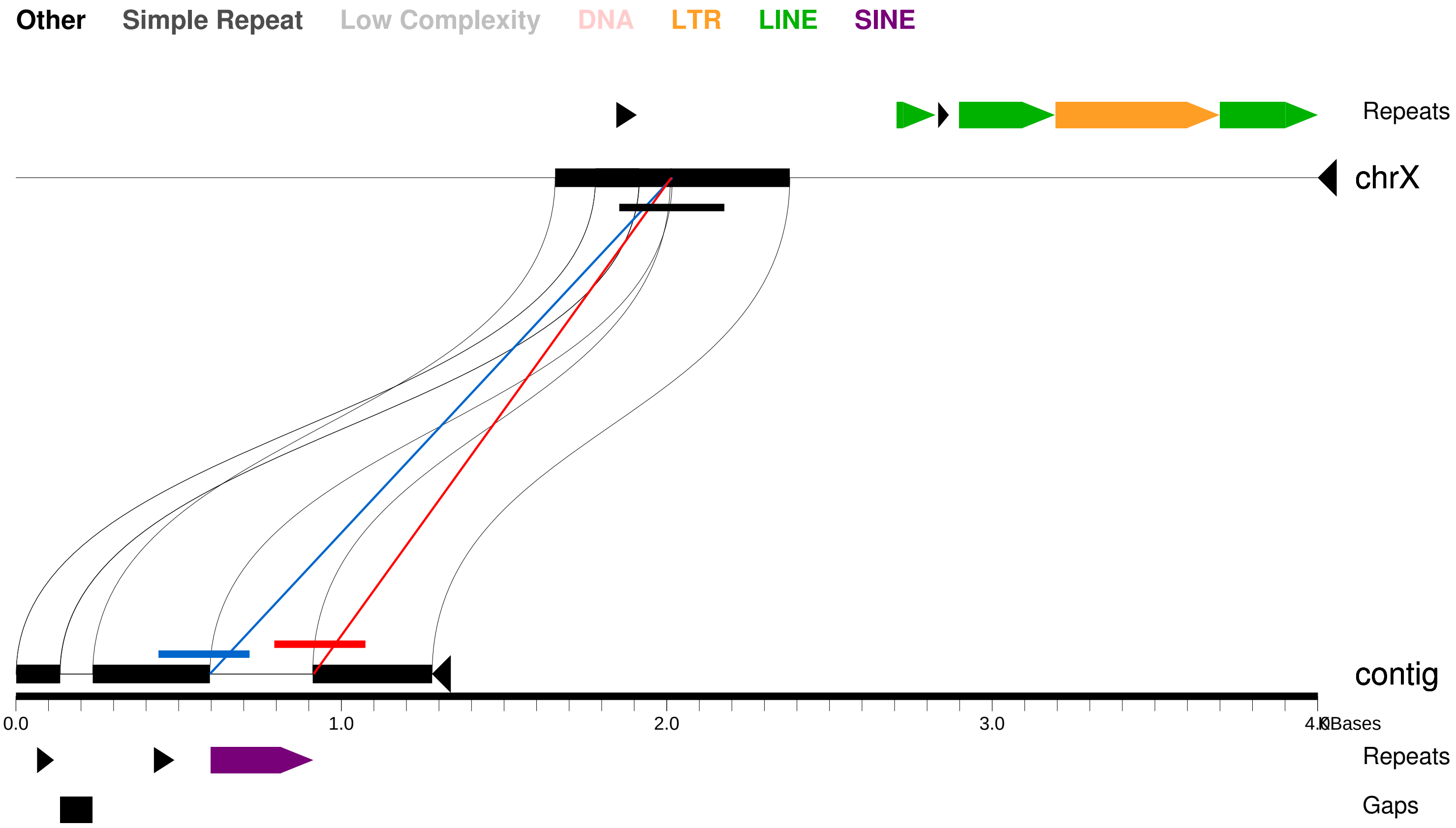

siteID: chrX_152001788 Scafftig1 - Breakpoint: chrX:152001791
chrX:152001746-152001850
Overlap Length: 15 INS size: 436 Genotyped: No

```
left    CTTCCAGCCAACAGTAAGCTATATTAGTGAAGTTTTAGGTG--------------AGTCAAAAGTTATAT
chrom   CTTCCAGCCAACAGTAAGCTATATTAGTGAAGTTTTAGGTG--------------AGTCAAAAGTTATAT
right   NNNNNNNNNNNNNNNNNNNNNNNNNNNNNNNNNNNNNNNNNNNNNNNNNNNNAAAAAAAAAAGTTATAT
        1111111111111111111111111111111111111111111111111111111*111***********

left    GTGGGCCG--GGCGCGGTGGCTCAC-GCTTGTAATCCCAGCACTTTGGGAGGCCGAGGCGGGCGGA
chrom   GTGGATTTTTGACTTCGGGGCTCCGTGCCCCTAACCCC--CACATTGTTC---------------A
right   GTGGATTTTTGACTTCGGGGCTCCGTGCCCCTAACCCC--CACATTGTTC---------------A
        *****222222*2*222*2*****222**222***2***22***2***222222222222222222*
```

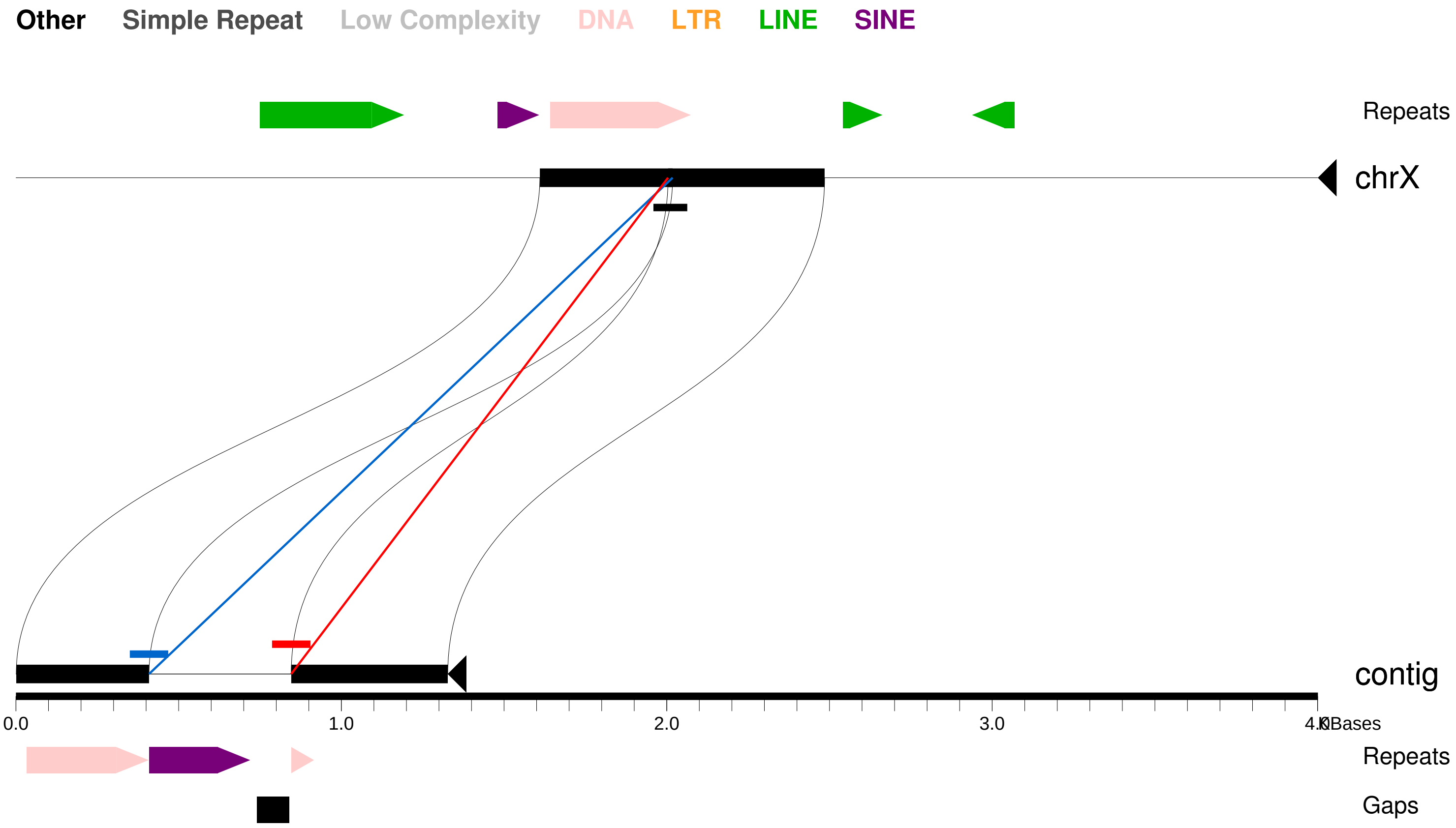

siteID: chrX_1683497 Scafftig1 + Breakpoint: chrX:1683504
chrX:1683344-1683674
Overlap Length: 11 INS size: 326 Genotyped: Yes

```
left   -----GGCGTTGGGAGAGGGGGGAGGGAGAGCATTAGGAGATATACCTAGTGTAAATGACGAGTTAATGG
chrom  CGTGGGGCGTTGGGAGAGGGGGGAGGGAGAGCATTAGGAGATATACCTAGTGTAAATGACGAGTTAATGG
right  TTT-------TAGTAGAGACGGG--GTTTCACCTTGTTAGCCAGGA-TGGTCTCGATCTCCTGACCTCGT
       NN2  11111*1*1****11***11*11111*1**111**11*1111*1**1*11**11*11*11111*1

left   GTGCAGCACACCAACATGGCACATGTATACGTATGTAACAAACCTGCACGTTGTGCACACGTACCCTAGA
chrom  GTGCAGCAAACCACCATGGCACATGTATACGTATGTAACAAACCTGCACGTTGTGCACACGTACCCTAGA
right  GATCCGC---CCGCCTCGGCC-------------TCCCAAAG-TGCTGGGATTACAGGCGTGAGCCACC
       *11*1**1N1**12*11***111111111111111*11****11***11*111*1**11***111*1*11

left   ACTTAAAGGATAAAAATTTAAAAAAATTTTTTTTTTTTTTTTTTTTTTTTTTTTTTTT--------TTTT--
chrom  ACTTAAAGGATAAAAATTTAAAAAAATTTTTAAAAACTGGGGCTGAGGGTGTGGGTGGGGAAGCTCATGA
right  GCGCCCGGCA----------AAAAAATTTTTAAAAACTGGGGCTGAGGGTGTGGGTGGGGAAGCTCATGA
       1*11111*1*1111111111*****2*****222222*22222*22222*2*222*22222222*22*22

left   ------TTTTTTTTTGAGACGGAGTCTCGCTCTGTCGCCCAGGTC---GGACTGCG-GACTGC----AGT
chrom  ACAGCCTCAGCTCTTCCAGTGGAGCTTTGCTGTGGGCAGAAGGTCAATGTCCTCCCCGAATTCCCACATT
right  ACAGCCTCAGCTCTTCCAGTGGAGCTTTGCTGTGGTCAGAAGGTCAATGTCCTCCCCCAATTCCCACATT
       222222*2222*2**22222****22*2***2**2N2222*****222*22**2*221*2*2*2222*2*

left   GGCGCAATCTCG-----GCTCACTGCAAGCTCC----------------GC
chrom  GATGTAACCACCAACAAGATGATGTTAAGAGGCTTTGGGAGGTGACGAGGT
right  GATGTAACCACCAACAAGATGATGTTAAGAGGCTTTGGG------------
       *22*2**2*2*222222*2*2*2222***222*222222          1N
```

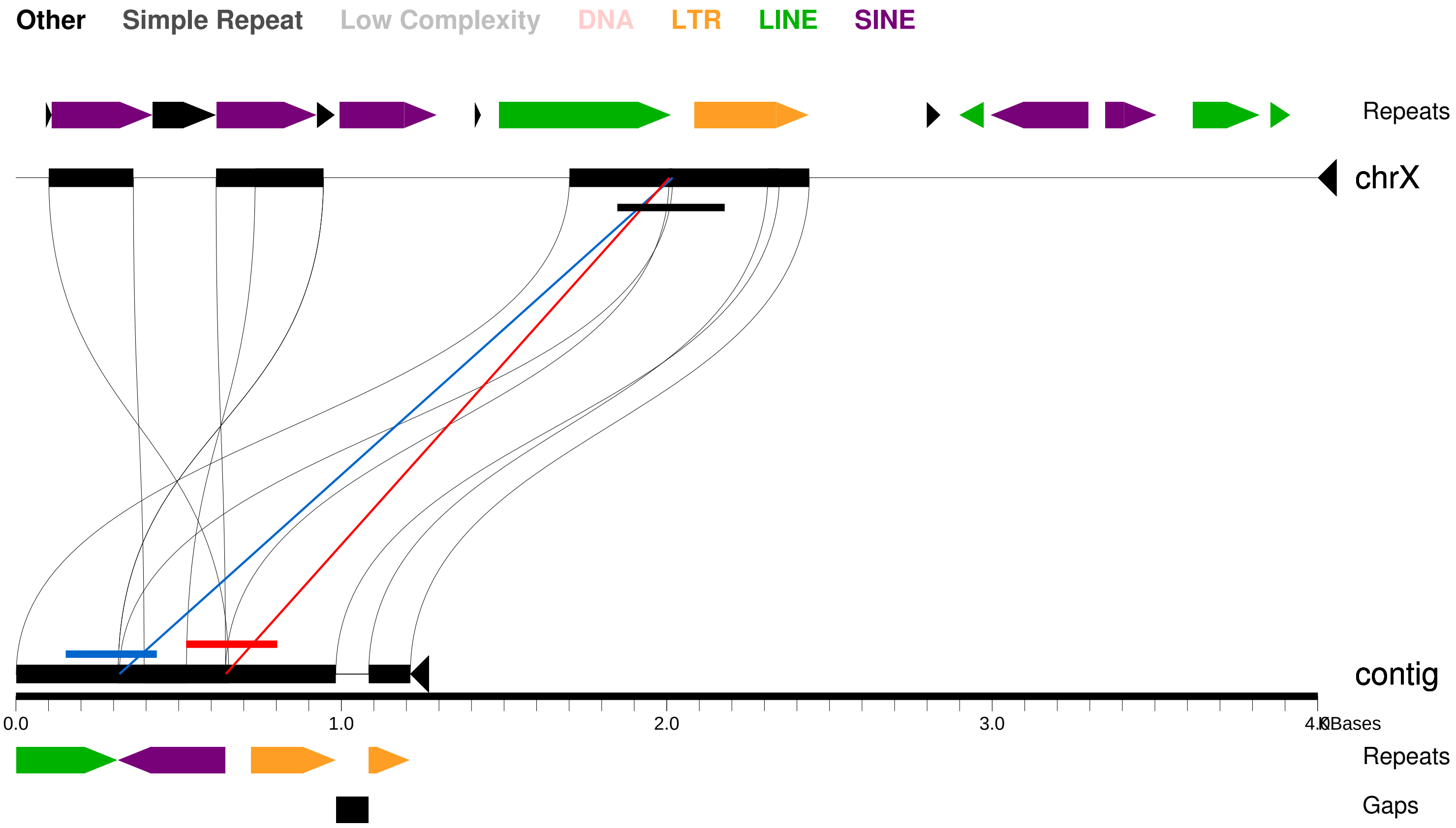

siteID: chrX_22365095 Scafftig1 - Breakpoint: chrX:22365072
chrX:22365015-22365128
Overlap Length: 3 INS size: 421 Genotyped: No

```
left    CTCTCATTAAGCATAATATTCTCCACGTTC----ATCTACAT---TGTAGTATGGGCCAATGCTTCCNNN
chrom   CTCTCATTAAGCATAATGTTCTCCACGTTC----ATCTACAT---TGTAGTATGGGCCAATGCTTCCTTT
right   GA-TCCGCCCGCCTCGGCCTCCCAAAGTGCTGGGAT-TACAGGCGTGAGCCACCGCGCCCGGCCTCCTTT
        111**11111**1*111N1**1*1*1**1*1111**1****1111**1111*11*11*111**1***222

left    NNNNNNNNNNNNNNNNNNNNNNNNNNNNNNNNNNNNNNNNNNNNNNNNNNNNNNNN
chrom   CTTTTTATGGATGAATAATATTCCATTTTACGGATACACAACATTTTGTTT-----
right   CTTTTTATGGATGAATAATATTCCATTTTACGGATACACAACATTTTGTTT-----
        22222222222222222222222222222222222222222222222222222222
```

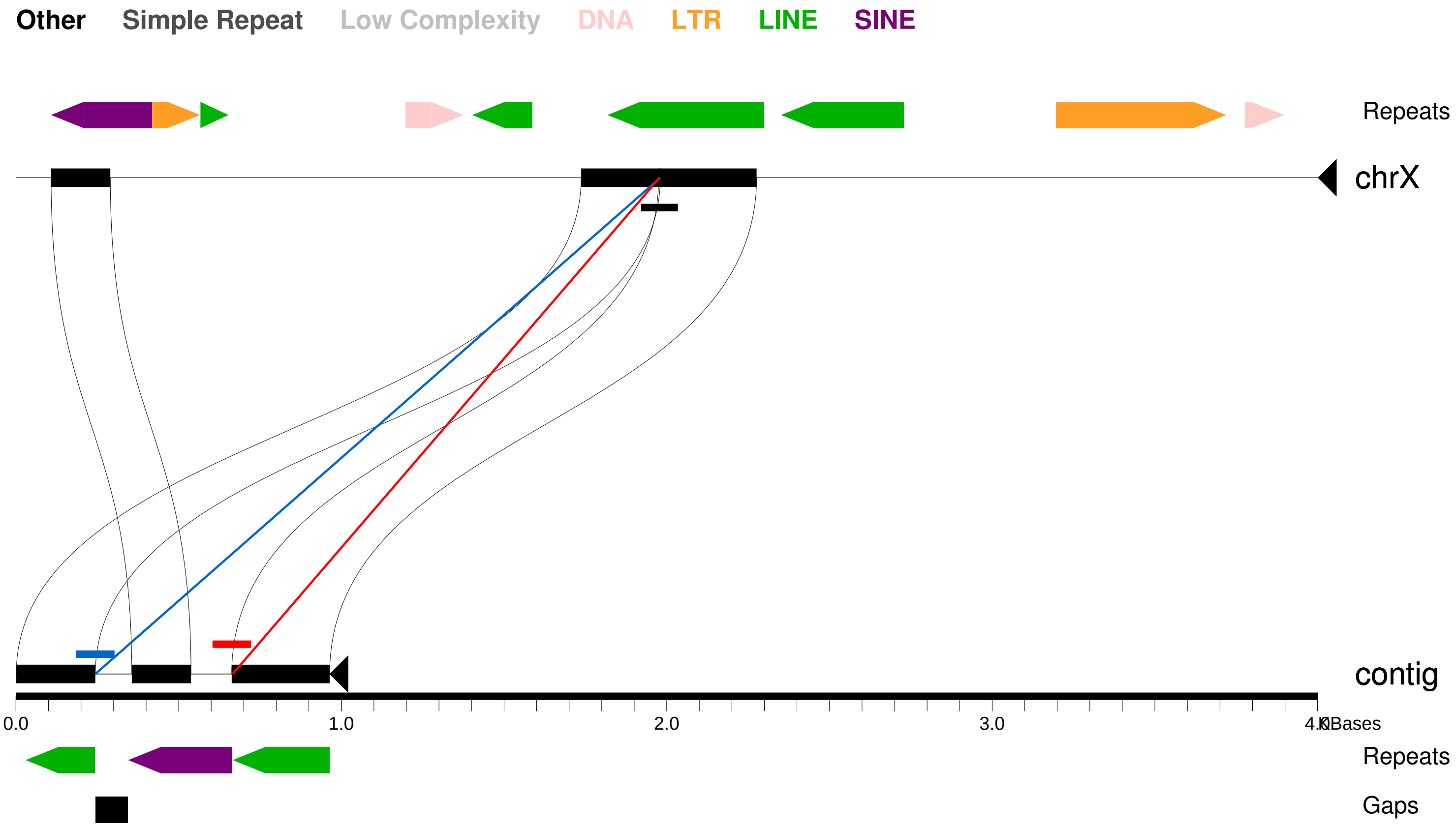

siteID: chrX_24451510 Scafftig1 + Breakpoint: chrX:24451542
chrX:24451498-24451601
Overlap Length: 16 INS size: 94 Genotyped: Yes

```
left    GCCTAGGAA-----------------AGGAGG-AGAGATAAGGCTCACTAGCCACAGAAAAACAGGCAGT
chrom   GCCTAGGAA-----------------AGGAGG-AGAGATAAGGCTCACTAGCCACAGAAAAACAGGCAGT
right   TGAGACGGAGTCTCGCTCTGTGGCCCAGGCGGGAGTGCAGTGGCGCAAT---CTCGGCTCACCAGGCAGT
        1111*1*1*11111111111111111***1**1**1*1111***1**1*111*1*1*111*1********

left    TAATTTTTTTTTTTTTTTTTTTTTTTTTTTTTTTTTTTTTTTTTGAGACGGAGTCTCGCTCTGTGGC
chrom   TAATTTTTAAAGGACTCCAGCTCTTTC----------TCTTTCTCAGGGGGAAT-----TGGGTTTT
right   TAATTTTTAAAGGACTCCAGCTCTTTC----------TCTTTCTCAGGGGGAAT-----TGGGTTTT
        ********2222222*22222*2***22222222222*2***2*2**22***2*22222*22**222
```

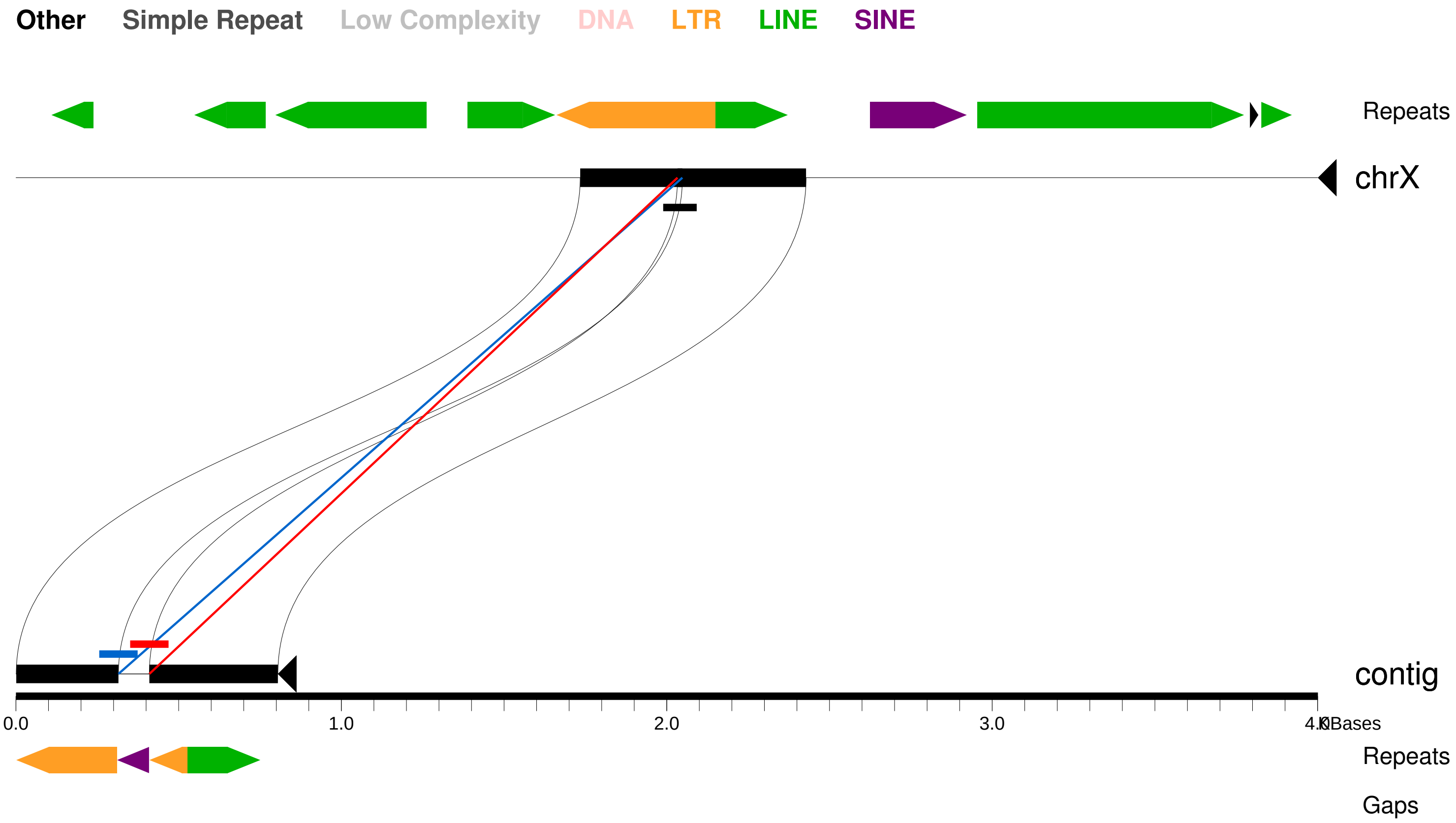

siteID: chrX_32001073 Scafftig1 + Breakpoint: chrX:32001073
chrX:32001027-32001132
Overlap Length: 11 INS size: 477 Genotyped: No

```
left    T------TTTATTCTTTCTCAAGTA--------ACATTATTCAGCTCCTCTAATATAATTTCAGAGCTTT
chrom   T------TTTATTCTTTCTCAAGTA--------ACATTATTCAGCTCCTCTAATATAATTTCAGAGCTTT
right   CCGCCCGCCTCGGCCTCCCAAAGTGCTGGGATTACAGGCGTGAGCCAC-CGCACCTGGCCTCAGAGCTTT
        111111111*111*1*1*11****111111111***1111*1***11*1*11*11*1111**********

left    TTTTTTTTTNNNNNNNNNNNNNNNNNNNNNNNNNNNNNNNNNNNNNNNNNNNNNNNNNNNNNN
chrom   TGTTAAT-------------GGTATTTTTCTACATACTAGTTTTGAATAAATTCATGTCTACC
right   TGTTAAT-------------GGTATTTTTCTACATACTAGTTTTGAATAAATTCATGTCTACC
        *2**22*22222222222222222222222222222222222222222222222222222222
```

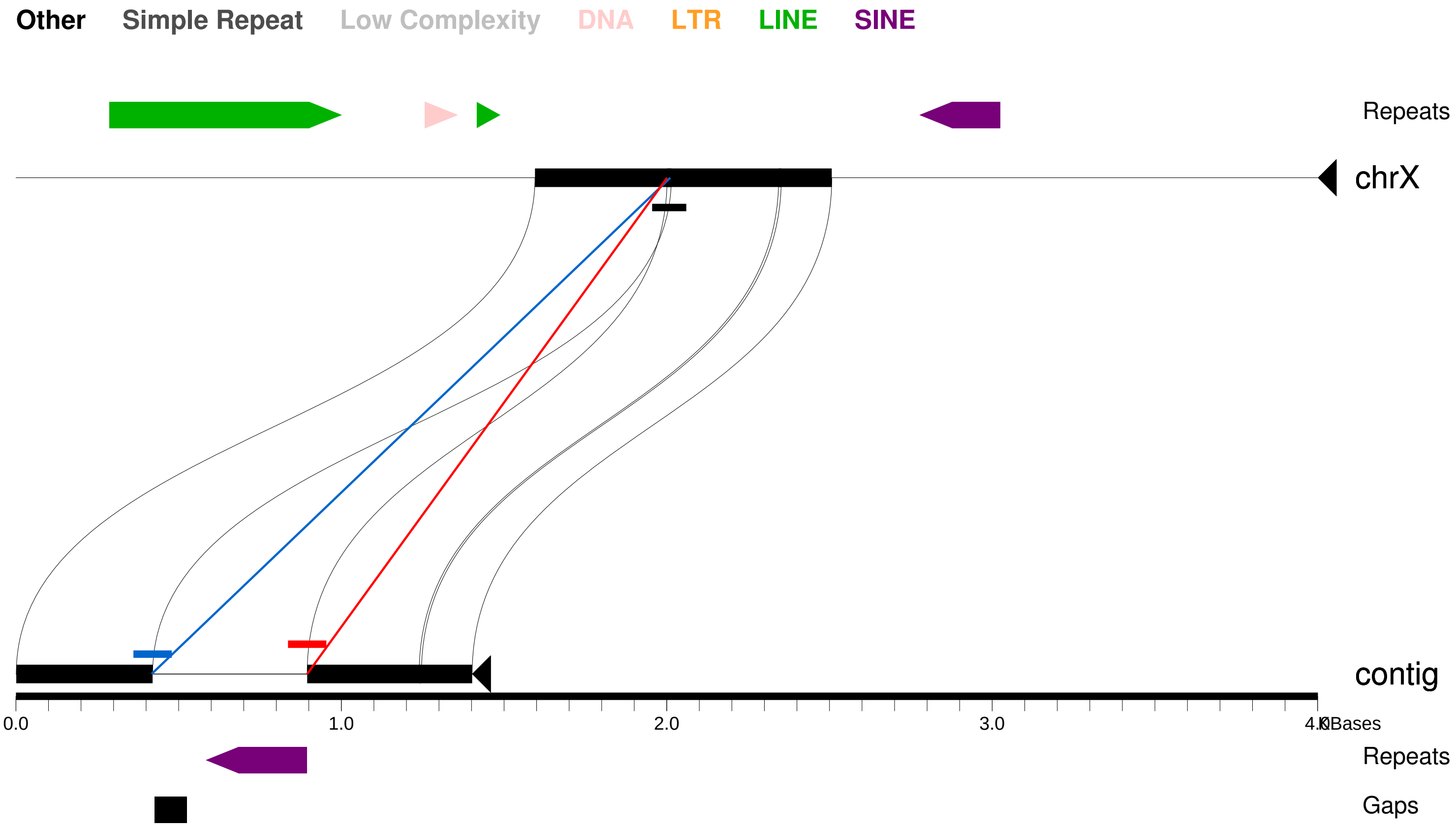

siteID: chrX_33302846 Scafftig1 + Breakpoint: chrX:33302847
chrX:33302687-33303010
Overlap Length: 4 INS size: 420 Genotyped: No

```
left   ----AATATATTTACTGACTATCAATGCTCTCTTCAAATTTGGTATAGGTATATTTTTCAGCCTTTCCCC
chrom  CTGCAATATATTTACTGACTATCAATGCTCTCTTCAAATTTGGTATAGGTATATTTTTCAGCCTTTCCCC
right  NNNNNNNNNNN-----------------------------------------------------------
       NNNN111111111111111111111111111111111111111111111111111111111111111111

left   CTTCTTGTCTCCAGTTACAGGCCCTTTTATGGGCCTATAACGTTGGTTTTCTATCTCAATTCAAAATTCA
chrom  CTTCTTGTCTCCAGTTACAGGCCCTTTTATGGGCCTATAACGTTGGTTTTCTATCTCAATTCAAAATTCA
right  ----------------------------------------------------------------------
       1111111111111111111111111111111111111111111111111111111111111111111111

left   AAAGAAAAGACTTTAAAAATCATCCGCGGTGGCTCACGCC--TG--TAAT------CCCAGCACTTTGGG
chrom  AAAGAAAAGACTTTAAAAATCATCTTTTTTTTTTTGCCCTAATGCTTAATTTAGAGCGTAGCACATAGAT
right  --------------------CATCTTTTGTCTTT-GCCCTAATGCTTAATTTAGAGCGTAGCACATAGAT
       11111111111111111111****2222N*N22*N2*2*222**22****222222*22*****2*2*22

left   AGGCCGAGGCGGGTG--------------GATCAT-GAGGTC----AGGAGATCGAGACCATCCTGGCTA
chrom  ATTCCCAATATGTTCTCATTAAAGGTATTGATCTTTGATTTTTAAAAGTAAGTAACAACAAAACTG--TG
right  ATTCCCAATATGTTCTCATTAAAGGTATTGATCTTTGATTTTTAAAAGTAAGTAACAACAAAACTG--TG
       *22**2*2222*2*22222222222222222****2*2**22*22222**2*22*2222**2*22***22*2

left   ACA-AGGTGAAACC----CCGT------CTCTACTAAAAATACAA-
chrom  AGAGAGGAGCAGCTTTTTCCTTTGAGAACTGTGGTTTAAATGCAGT
right  AGAGAGGAGC------------------------------------
       *2*2***2*21N1N    11N1      11N1NN1NN1111N11N
```

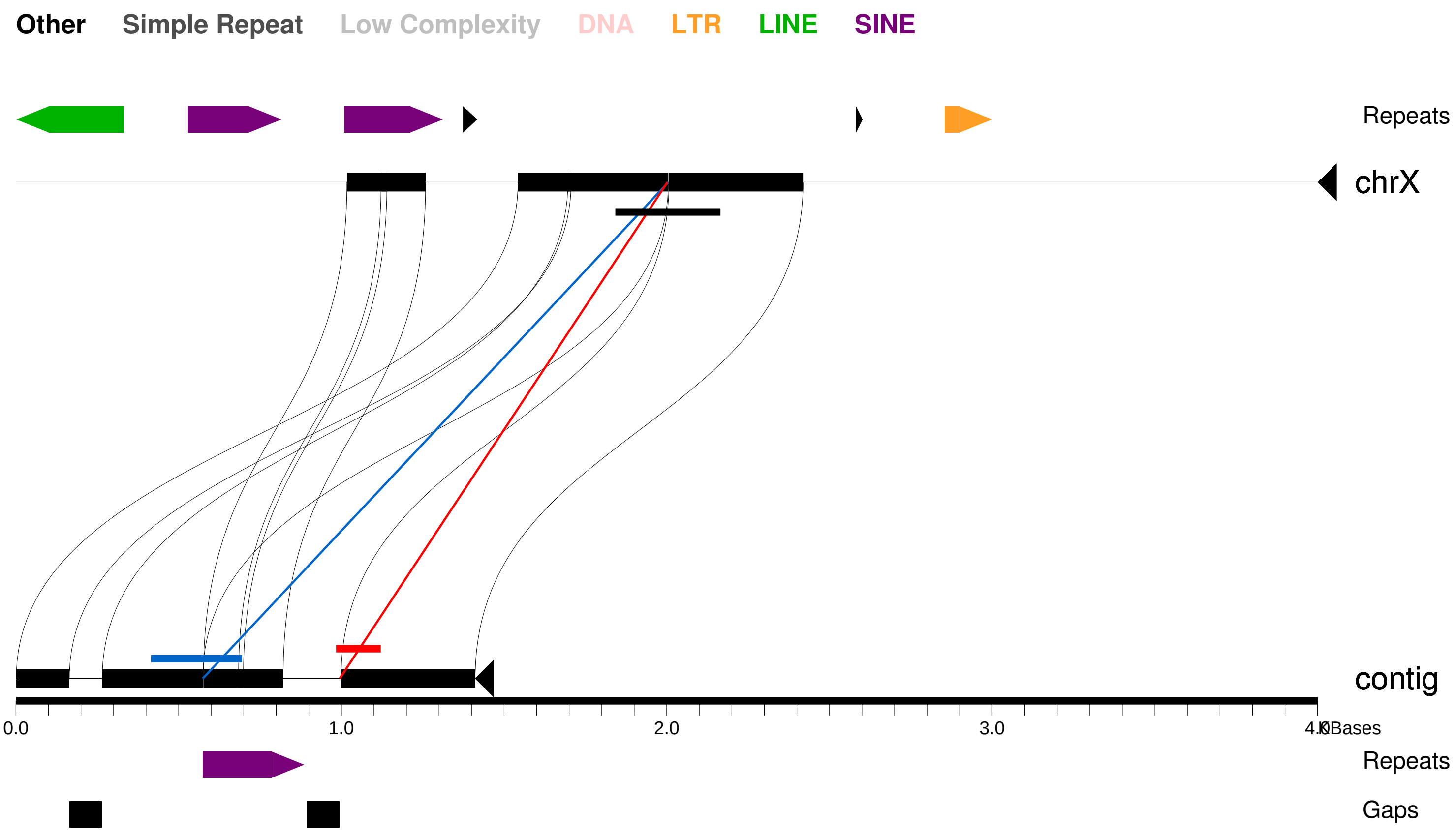

siteID: chrX_34242456 Scafftig1 - Breakpoint: chrX:34242444
chrX:34242395-34242503
Overlap Length: 11 INS size: 451 Genotyped: No

```
left    GGCAAGTAATTTCATGGCTATTTGAAACACAAGAAATAACTCT----------AGAAGTAAAGAAATATG
chrom   GGCAAGTAATTTCATGGCTATTTGAAACACAAGAAATAACTCT----------AGAAGTAAAGAAATATG
right   NNNNNNNNNNNNNNNNNNNNNNNNNNNNNNNNNNNNNNNNNNNNNNNNNNNNNAAAAAAAAAAAGAAATATG
        1111111111111111111111111111111111111111111111111111*1**11***********

left    GCCGGGCGCGGTGGCTCACGCTTGTAATCCCAGC-ACTTTGGGAGGCCGAGGCGGGCGGA
chrom   ------AGTGATGG---AGAGTTGTA--CCTAGAGAAGCTCTCAGCCTGGAATAATACAT
right   ------AGTGATGG---AGAGTTGTA--CCTAGAGAAGCTCTCAGCCTGGAATAATACAT
        2222222*2*2***222*222*****22**2**22*222*222**2*2*22222222222
```

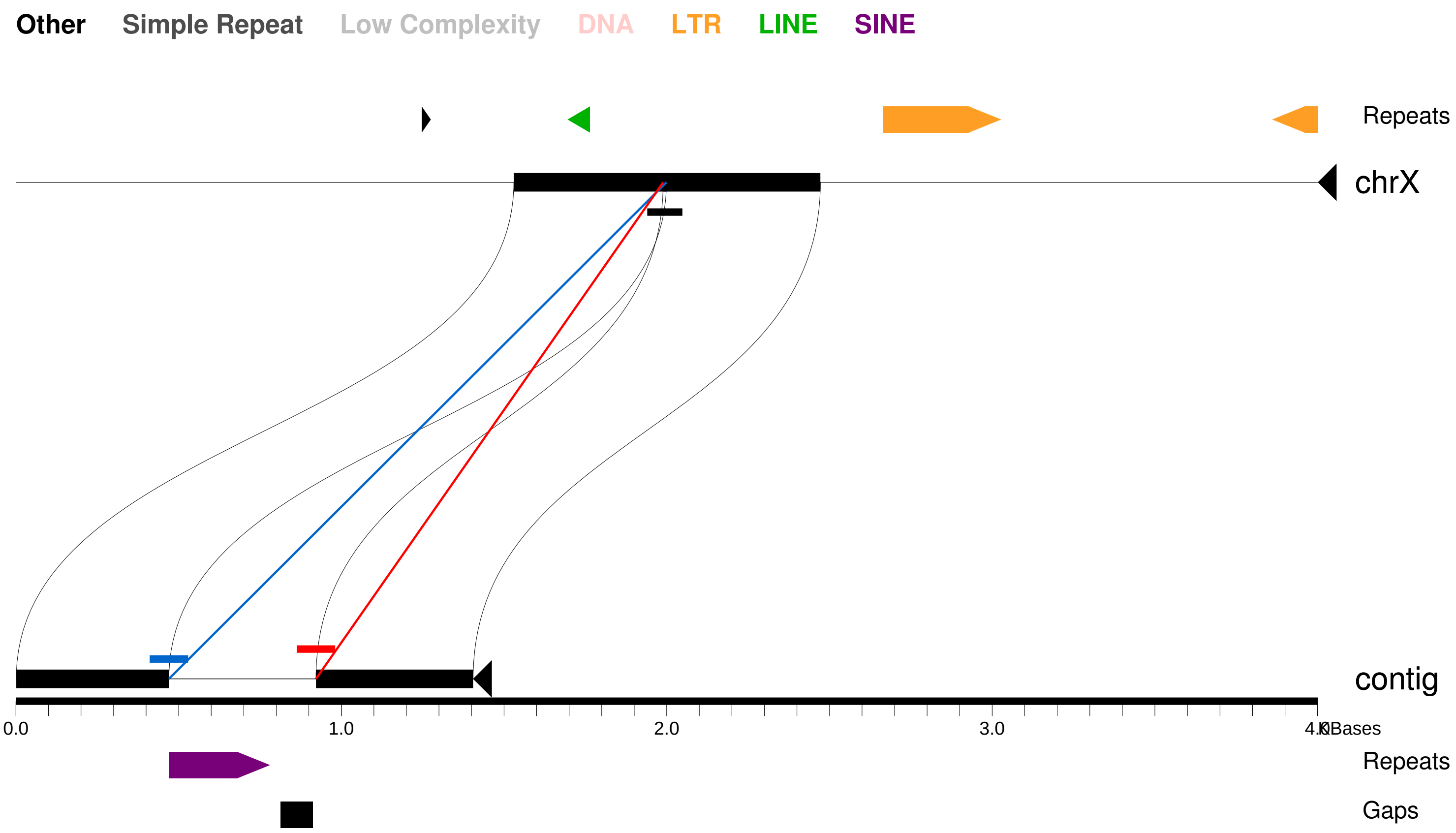

siteID: chrX_35028081 Scafftig6 + Breakpoint: chrX:35028089
chrX:35027930-35028263
Overlap Length: 15 INS size: 435 Genotyped: No

```
left   -------------ATCTGCTTAAGAAGAAATGGGACTTCACTTCCTGTGTCCTCTTGATTCCTAGAAAGC
chrom  TGTAATAGTTAAAATCTGCTTAAGAAGAAATGGGACTTCACTTCCTGTGTCCTCTTGATTCCTAGAAAGC
right  NNNNNNNNNNNAAA-------AA-----------------------------------------------
       NNNNNNNNNNN22*1111111**11111111111111111111111111111111111111111111111

left   AAGATTTTACTTTCCTCACTTTGATATAAGATAGACCATTTATTTTCTGGTGAGATTGAGTCAGAGAAAA
chrom  AAGATTTTACTTTCCTCACTTTGATATAAGATAGACCATTTATTTTCTGGTGAGATTGAGTCAGAGAAAA
right  ----------------------------------------------------------------------
       1111111111111111111111111111111111111111111111111111111111111111111111

left   TCAGTACTTGGGTATACTTAAAAACGGCAATGAGGCCGGGCGCGGTGGCTCACGCTTGTA------ATC-
chrom  TCAGTACTTGGGTATACTTAAAAACGGCAATGAGCTCATACATAAAAACTAGTACATGAAGTAAATATCT
right  -------------------AAAAACGGCAATGAGCTCATACATAAAAACTAGTACATGAAGTAAATATCT
       1111111111111111111**************22*222*2222222**2222*2**2*222222***2

left   --CCAGCACTTTGGGAGGCCGAGGCGGGCGGATCACGAGG---------TCAGGAGATCGAGACCATCCT
chrom  ATCCAGAATGGTGACATTCCTAG---TGCCTATCTCCAAAAGACCCCACTCAGCTGTCCCAAACCACACA
right  ATCCAGAATGGTGACATTCCTAG---TGCCTATCTCCAAAAGACCCCACTCAGCTGTCCCAAA-------
       22****2*222**22*22**2**2222**22***2*2*22222222222****22*22*2*2*111NN1N

left   GGCTA----ACAC-----------------GGTGAAACCCCGTCT----CTACTAAA
chrom  GGCTGTTTTATACTACCTAGCCAAAATATAGGAGGATTCCATTTTGCGACAATTGAT
right  ---------------------------------------------------------
       1111N    1N11                 11N1N1NN11NN1N1    1N1N1N1N
```

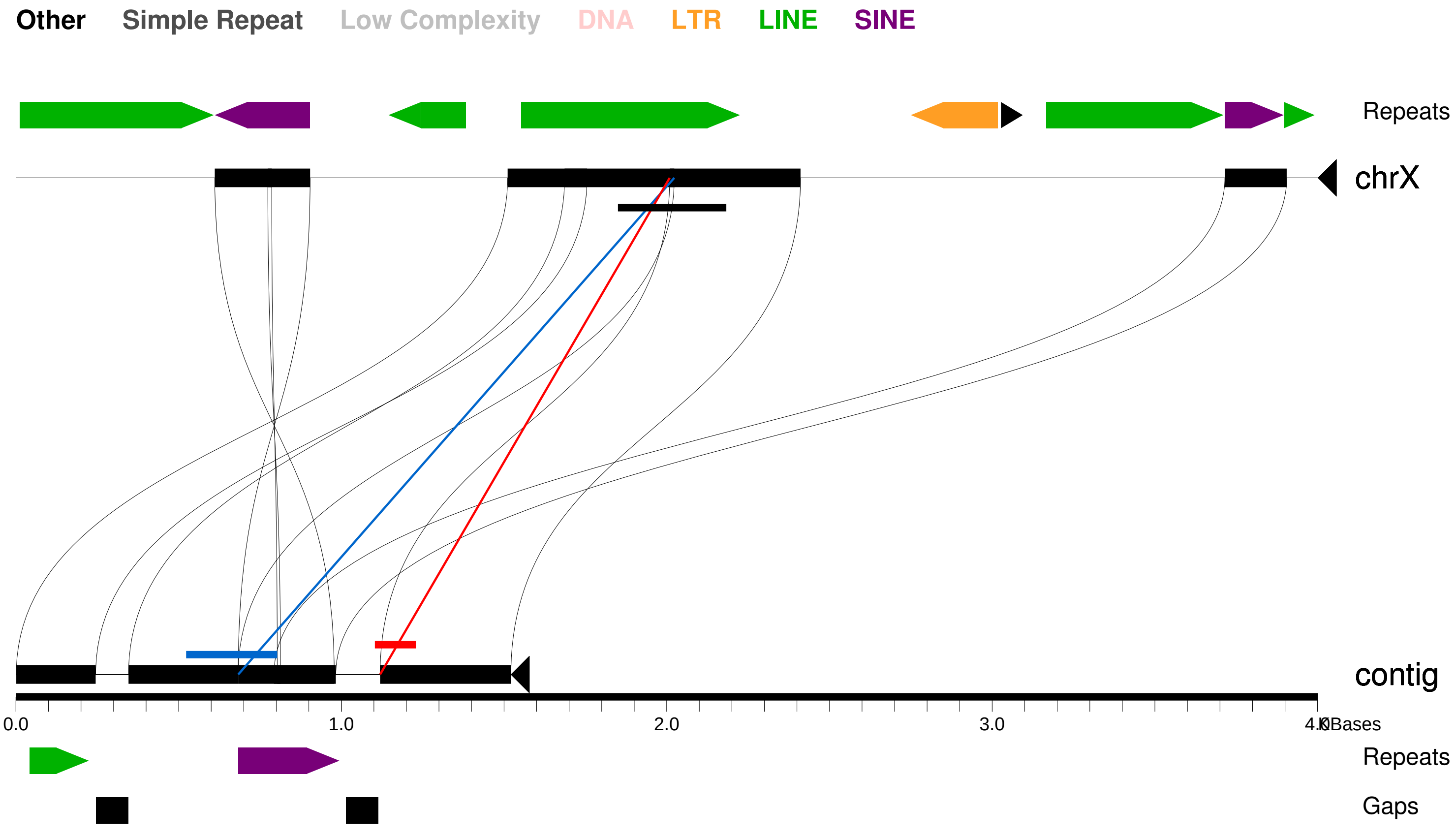

siteID: chrX_37324643 Scafftig1 + Breakpoint: chrX:37324634
chrX:37324579-37324693
Overlap Length: 5 INS size: 325 Genotyped: No

```
left    GACCCCTGGAGCTACCCTCAGAATCGATGAACTTGTTTTTCTTTTAAAGAACAGT----GATCC------
chrom   GACCCCTGGAGCTACCCTCAGAATCGATGAACTTGTTTTTCTTTTAAAGAACAGT----GATCCATAGGT
right   NNNNNNNNNNNNNNNNNNNNNNNNNNNNNNNNNNNNNNNNNNNNNNNNNNNNNNNNNNNGATCCATAGGT
        11111111111111111111111111111111111111111111111111111111111*****222222

left    CATCCTGGCTAACACGGTGAAACCCCGTCTCTACTAAAAATACAAAAAATTAGCCGGGC
chrom   CATGCAGACCTCCTTGATGACATCC----ACAAGTTTGATTGGAACTGATGAG------
right   CATGCAGACCTCCTTGATGACATCC----ACAAGTTTGATTGGAACTGATGAG------
        ***2*2*2*222*22*2***2*2**22222*2*2*222*2*22**222**2**222222
```

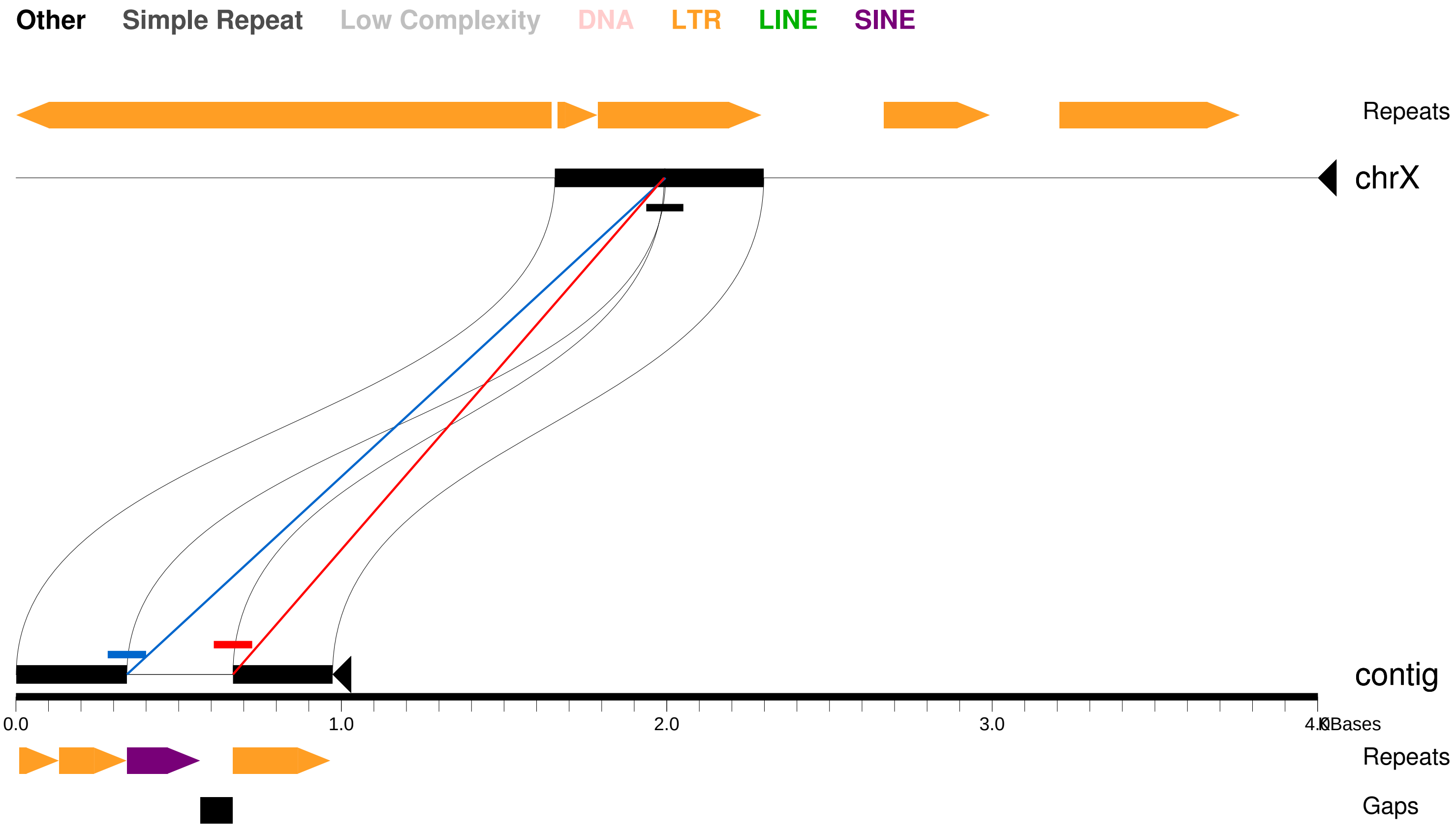

siteID: chrX_50640924 Scafftig1 - Breakpoint: chrX:50640900
chrX:50640740-50641070
Overlap Length: 11 INS size: 387 Genotyped: No

```
left   G------------------------------------GGACACCCAGAACACCCAAAAGTTTGGCCTCAG
chrom  GTGCTCATGCCAAACTTGCCTTCTTTCAATTCCTTTGGGACACCCAGAACACCCAAAAGTTTGGCCTCAG
right  TTTAGTAG---AGACGGGG----TTTCA---CCGT---GTTAGCCAGGA------------TGGTCTCGA
       12NNNN2N   2N22NN2N    22222   22N2  1*11*1****1*111111111111***1***11

left   AGTCTTTATATTGGTGTATCCTCTTCCTGGAATATTCTTCCTCCAAATCTTTACCTGCTGAGTTCCTACT
chrom  AGTCTTTATATTGGTGTATCCTCTTCCTGGAATATTCTTCCTCCAAATCTTTACCTGCTGAGTTCCTACT
right  TCTCCTGACCTCG-TG-ATCCGCCCGCC-------TCGGCCTCCCAAAGTG---CTGG-GATTACAGGCG
       11**1*1*11*1*1**1****1*111*11111111**11*****1**11*1111***11**1*1*111*1

left   TAACCTTTATATACCAGCTTAAATATTTCTTNNNNNNNNNNN----------------------------
chrom  TAACCTTTATATACCAGCTTAAATATTTCTTCTTTGAGTGAATCTTCCTGGACCCATCTGACTAAATTCG
right  TGAGCCACCGCGCCCGGCC-AAATATTTCTTCTTTGAGTGAATCTTCCTGGACCCATCTGACTAAATTCG
       *1*1*11111111**1**11***********222222222222222222222222222222222222222

left   ----------------------------------------------------------------------
chrom  GTCTTCCATCCCACCTTCATATCCTGCTGGTTTATTTTCCCAGGGGAGCCCATAATTTCCCTTCATAGCA
right  GTCTTCCATCCCACCTTCATATCCTGCTGGTTTATTTTCCCAGGGGAGCCCATAATTTCCCTTCATAGCA
       2222222222222222222222222222222222222222222222222222222222222222222222

left   ---------------------------------------------------
chrom  CTCGACAACTGAGATGAGTGACTGAGGTATAAGTCTCAATCACTGCGGTTT
right  CTGGACAACTGAGATGAGTGACTGAGGTATAAGTCTCAAT-----------
       22N2222222222222222222222222222222222222
```

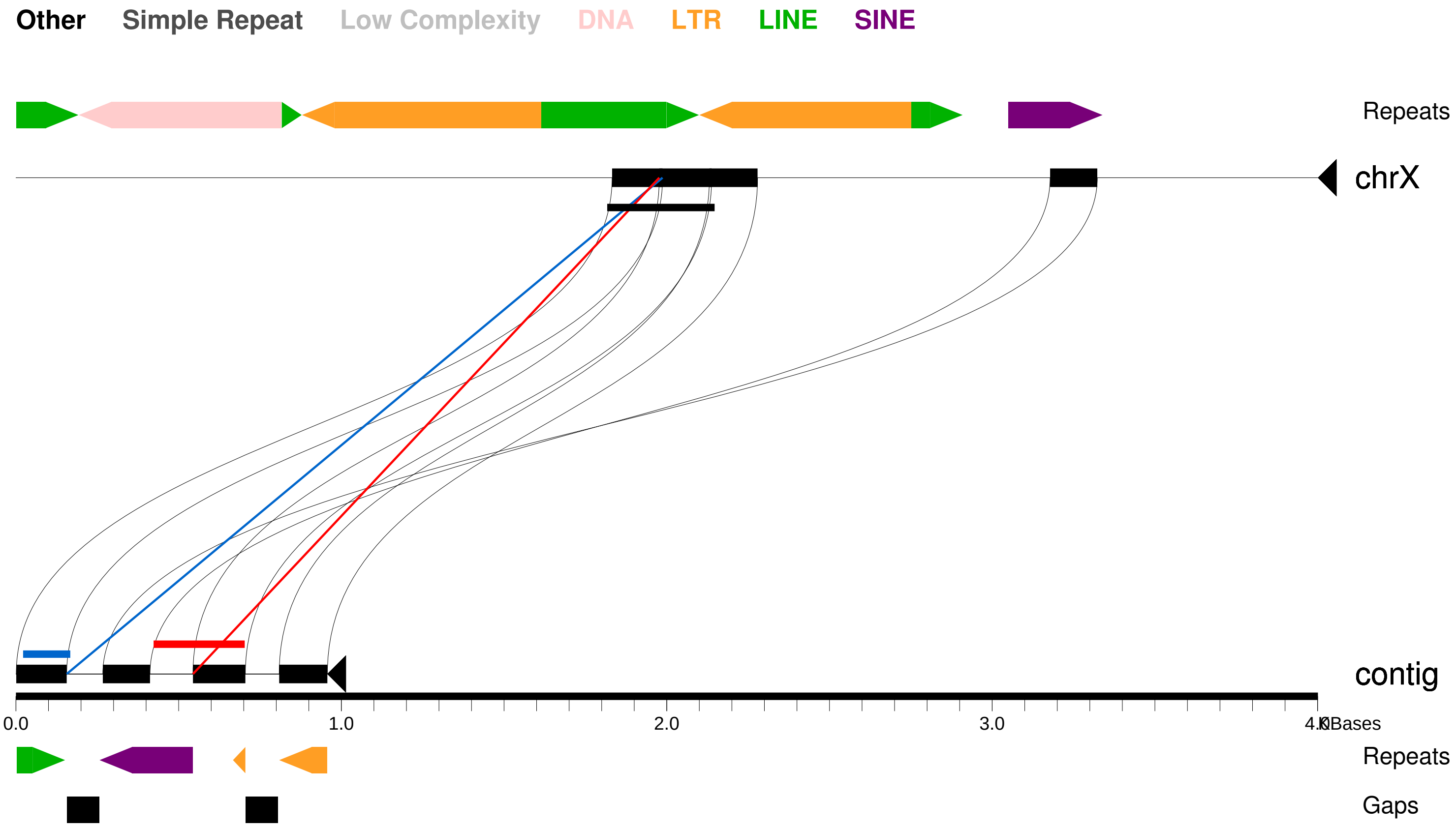

siteID: chrX_5781750 Scafftig4 + Breakpoint: chrX:5781742
chrX:5781695-5781801
Overlap Length: 1 INS size: 320 Genotyped: Yes

```
left   AGCCAATGTCATTTTGTTTTATAAGGACTAGAGTT---GGAATGAG-----------GGATTTTTTTTTT
chrom  AGCCAATGTCATTTTGTTTTATAAGGACTAGAGTT---GGAATGAG-----------GGATTGCTGTATT
right  --CCGCCCGCCTCGGCCTCCCAAAGTGCTGGGATTACAGGCGTGAGCCACCGCGCCCGGCCTGCTGTATT
       11**11111*1*11111*1111***11**1*11**111**11****11111111111**11*22*2*2**

left   TTTTTTTTTTTTTTTTTTTTTTTTTTTTTTTGAG-ACGGAGTCTCGCTCTGTCGCCCAGGCTGG
chrom  GTTTAGAAGGGGTAGGCACTTAAGCCCAGTTGACCACAAAGA-------TGAGTGCAA------
right  GTTTAGAAGGGGTAGGCACTTAAGCCCAGTTGACCACAAAGA-------TGAGTGCAA------
       2***22222222*222222**22222222****22**22**22222222**2222*2*222222
```

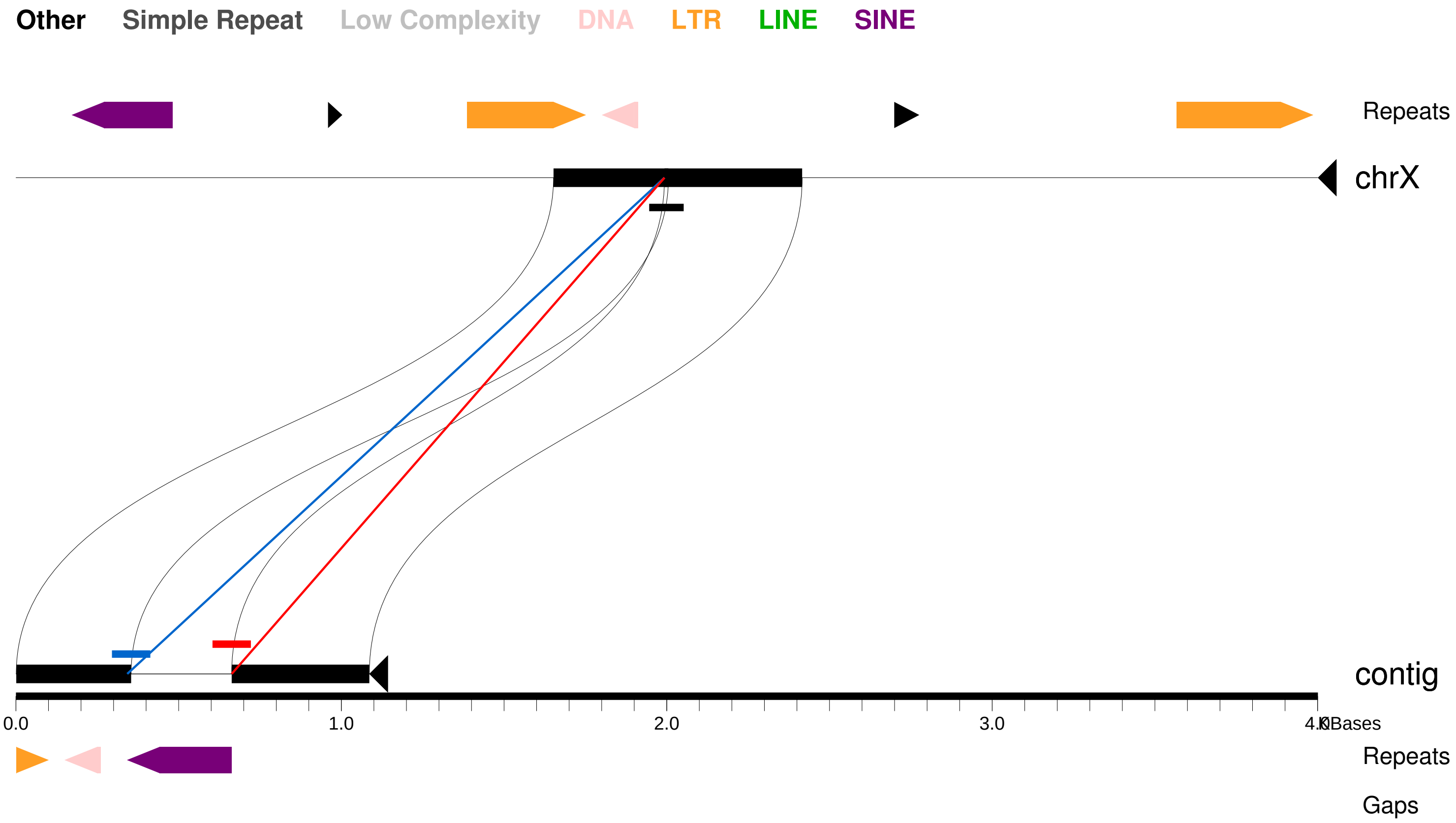

siteID: chrX_63440818 Scafftig1 + Breakpoint: chrX:63440806
chrX:63440757-63440865
Overlap Length: 11 INS size: 408 Genotyped: No

```
left    GTGAGTATAAGTGTCCCATTCTAAGAACTGAAACCTTCAAGTCTAAAAA----------ACCAAGGTATT
chrom   GTGAGTATAAGTGTCCCATTCTAAGAACTGAAACCTTCAAGTCTAAAAA----------ACCAAGGTATT
right   NNNNNNNNNNNNNNNNNNNNNNNNNNNNNNNNNNNNNNNNNNNNNNNNNNNNNNNNNNNACCAAGGTATT
        11111111111111111111111111111111111111111111111111111111111***********

left    GGGAGGCCGAGGCGGGCGGATCACGAG-GTCAGGAGATCGAGACCATCCCGGCTAAAATG--
chrom   -------CATGGCTGTC--TTCAGTTTAGTCTGCA-ACCTAG---CTCCTGTTCAAATTCAC
right   -------CATGGCTGTC--TTCAGTTTAGTCTGCA-ACCTAG---CTCCTGTTCAAATTCAC
        2222222*22***2*2*222***22222***2*2*2*2*2**2222***2*222***2*222
```

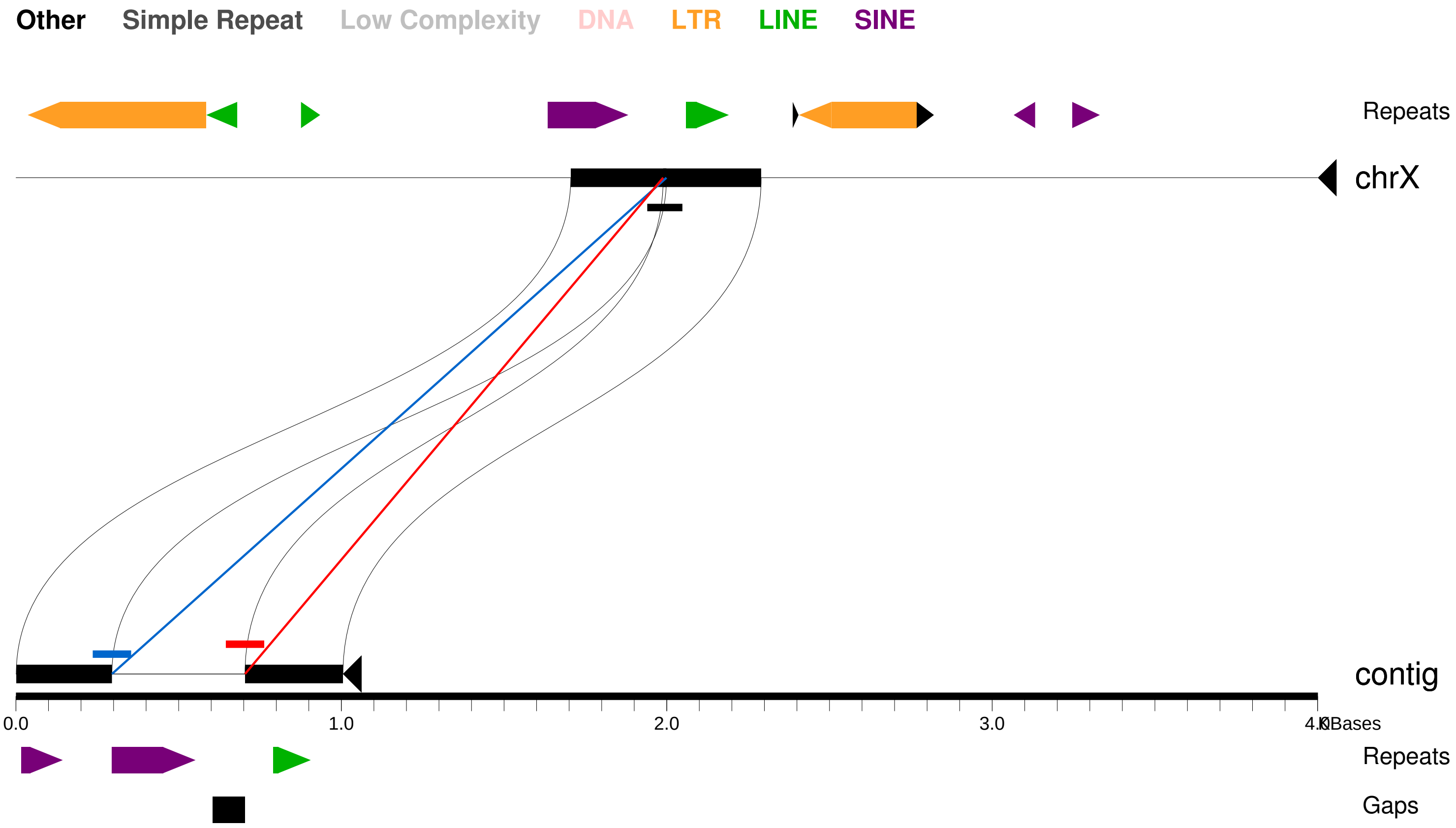

siteID: chrX_71609439 Scafftig1 - Breakpoint: chrX:71609522
chrX:71609477-71609580
Overlap Length: 15 INS size: 471 Genotyped: No

```
left    CACTTCTAGTTAAAGGATCAGAAC----------------CCCAAATTGTTTGGCTCAAAAGCTCATGTT
chrom   CACTTCTAGTTAAAGGATCAGAAC----------------CCCAAATTGTTTGGCTCAAAAGCTCATGTT
right   NNNNNNNNNNNNNNNNNNNNNNNNNNNNNNNNNNNNNCCTCCCAAAGTGCTGGGATTACA-GCTCATGTT
        1111111111111111111111111111111111111111******1**1*1**1*1*1*1*********

left    CTTTTTNNNNNNNNNNNNNNNNNNNNNNNNNNNNNNNNNNNNNNNNNNNNNNNNNNNNNNNNNNNNN
chrom   CTTTTTACCCAGCCACAGGCTCCAAAAGAAAACAAAGCTTAACCTCTAGG---------------
right   CTTTTTACCCAGCCACAGGCTCCAAAAGAAAACAAAGCTTAACCTCTAGG---------------
        ******22222222222222222222222222222222222222222222222222222222222
```

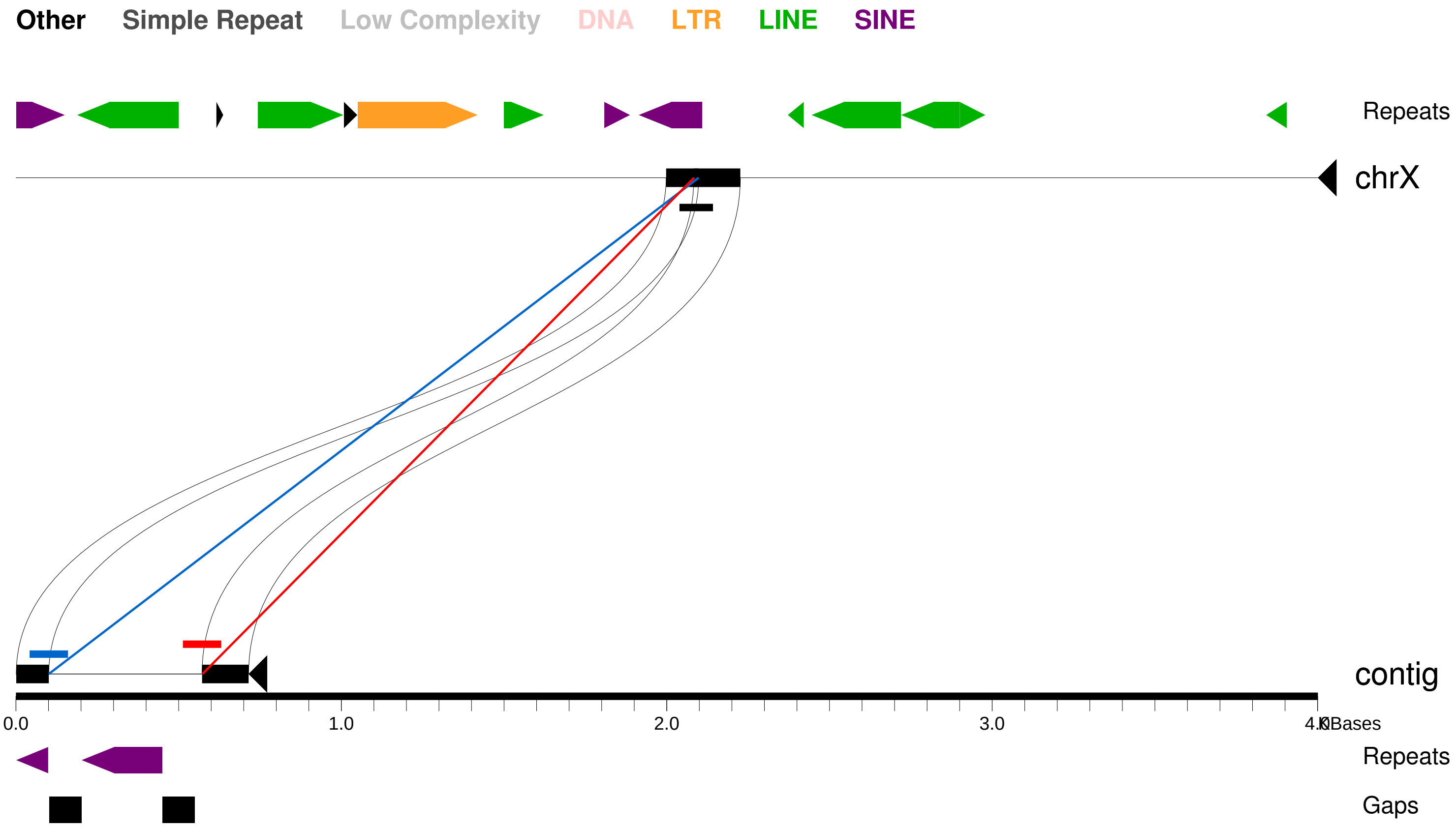

siteID: chrX_7548184 Scafftig1 + Breakpoint: chrX:7548160
chrX:7548112-7548219
Overlap Length: 12 INS size: 419 Genotyped: No

```
left    TGCTGAGATTACACAACCAAATGGTGCCTTCTCTGATGTCTTTAAAAA-----------TAATCACATTT
chrom   TGCTGAGATTACACAACCAAATGGTGCCTTCTCTGATGTCTTTAAAAA-----------TAATCACATTT
right   NNNNNNNNNNNNNNNNNNNNNNNNNNNNNNNNNNNNNNNNNNNNNNNNNNNNNNNNNNNTAATCACATTT
        11111111111111111111111111111111111111111111111111111111111***********

left    GGCCGGGCGCGGTGGCTCACGCCTGT-AATCCCAGCACTTTGGGAGGCCGAGGCGGGCGGA
chrom   GCTCTTATAAGGAATATTTTCTGTTTCAATCTAAGAACATTTCTA------------CAGT
right   GCTCTTATAAGGAATATTTTCTGTTTCAATCTAAGAACATTTCTA------------CAGT
        *22*222222**2222*222222*2*2****22**2**2**222*222222222222*2*2
```

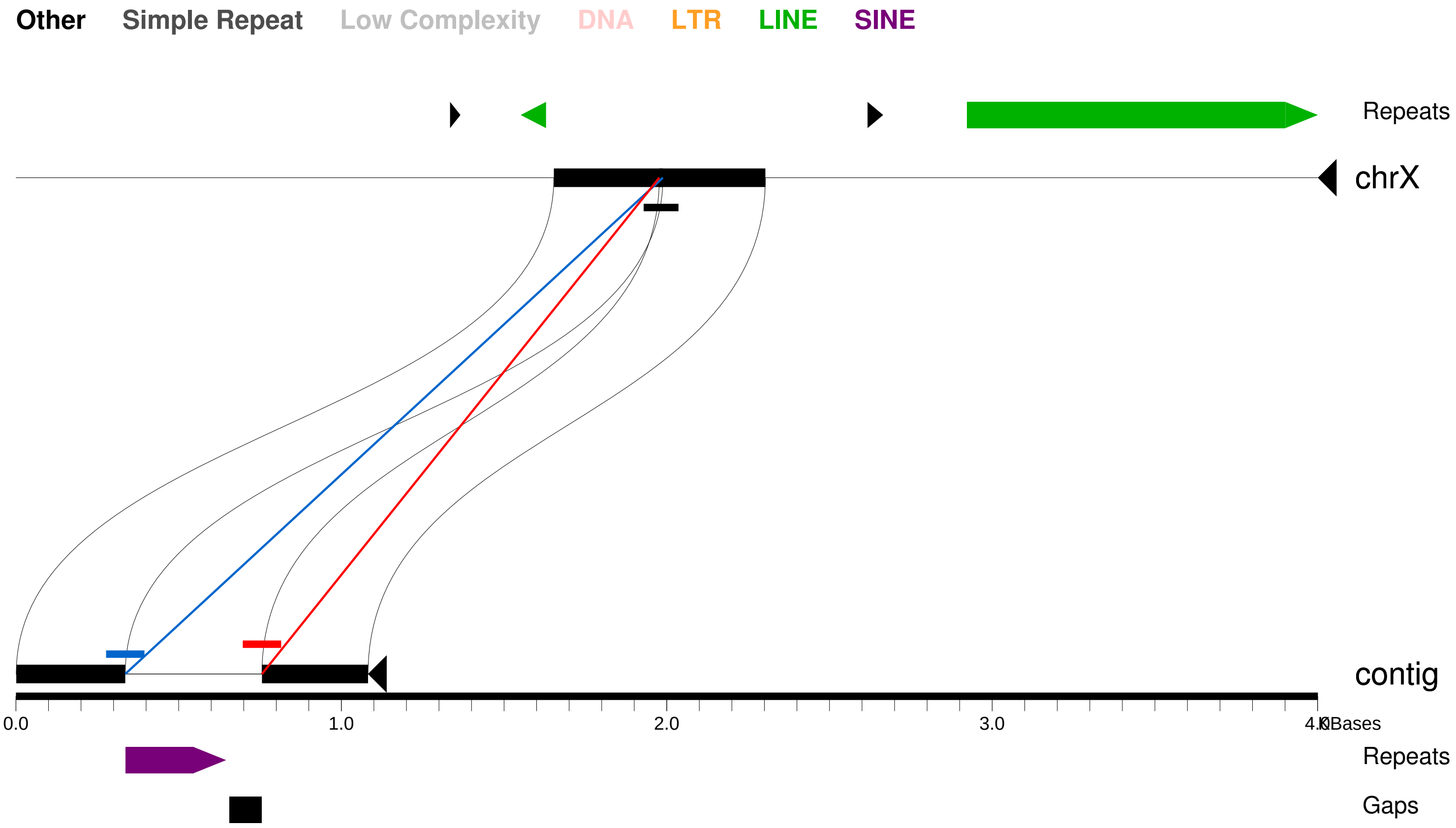

siteID: chrX_87112828 Scafftig1 - Breakpoint: chrX:87112817
chrX:87112762-87112876
Overlap Length: 5 INS size: 414 Genotyped: No

```
left     TTTCTTTTAAAGTCATAATTGTAAAA-GGTGTGTTTA-AG--GTAAAACACAAATATATGCTCANNNNNN
chrom    TTTCTTTTAAAGTCATAATTGTAAAA-GGTGTGTTTA-AG--GTAAAACACAAATATATGCTCATTCTGT
right    GATCCGCCCGCCTCGGCCTCCCAAAGTGCTGGGATTACAGGCGTGAGCCACCGCGCCCGGCTCATTCTGT
         11**11111111**1111*111***11*1**1*1***1**11**1*11***11111111*****222222

left     NNNNNNNNNNNNNNNNNNNNNNNNNNNNNNNNNNNNNNNNNNNNNNNNNNNN
chrom    TTTTGATAAAGCTCGAGATACAAGCTCATGTGAAATATTTAGTCTATAT----
right    TTTTGATAAAGCTCGAGATACAAGCTCATGTGAAATATTTAGTCTATAT----
         2222222222222222222222222222222222222222222222222222
```

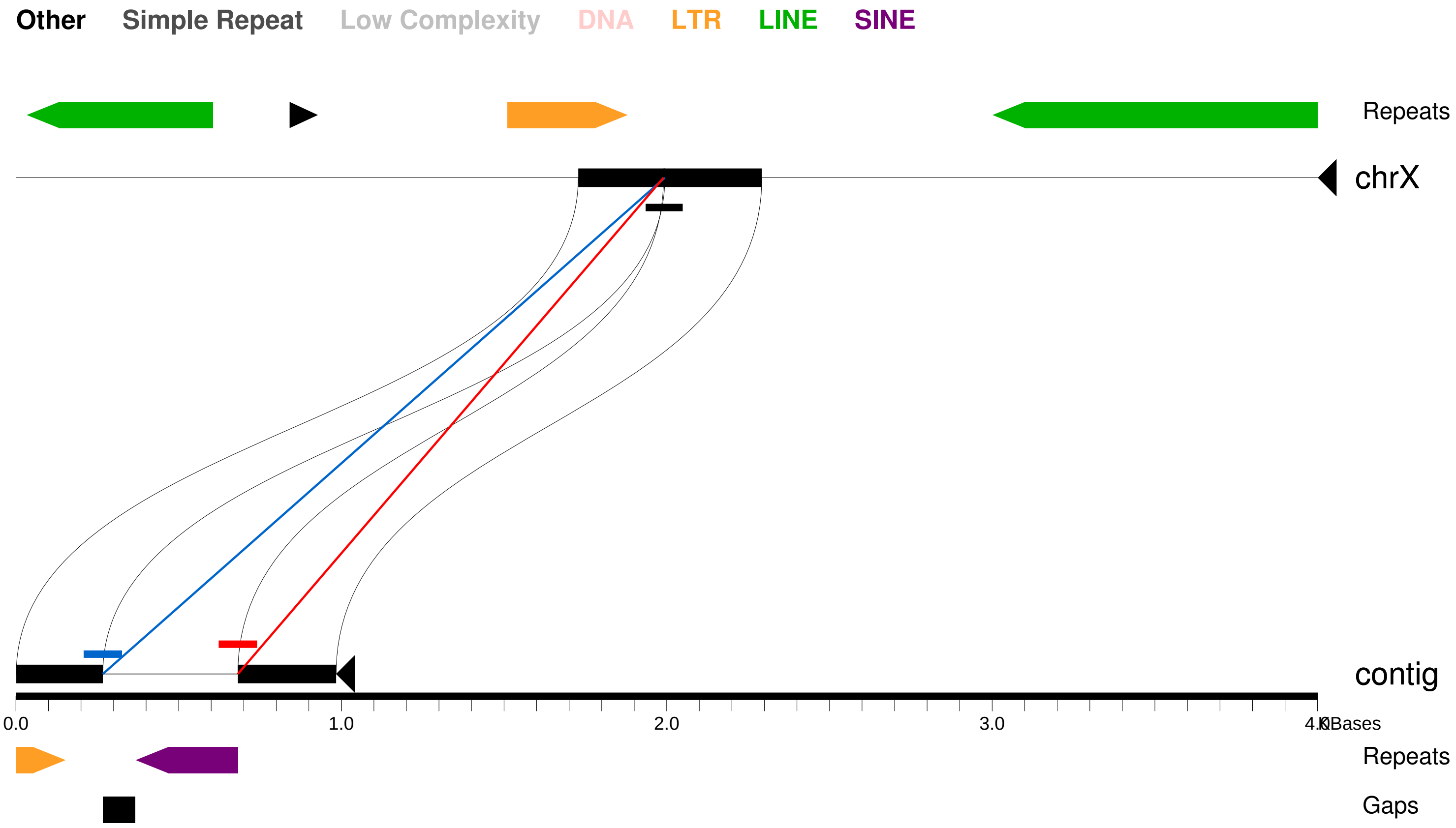

siteID: chrX_90534142 Scafftig2 - Breakpoint: chrX:90534150
chrX:90534098-90534209
Overlap Length: 3 INS size: 307 Genotyped: No

```
left   TGTTCTCAGAAGTTATCTTAGGGCCTCTCATGTCTGCATTAAGAGTGGCAAG-------ACAGATCG---
chrom  TGTTCTCAGAAGTTATCTTAGGGCCTCTCATGTCTGCATTAAGAGTGGCAAG-------ACAAAACGGAT
right  NNNNNNNNNNNNNNNNNNNNNNNNNNNNNNNNNNNNNNNNNNNNNNNNNNNNNNNNNNNACAAAACGGAT
       11111111111111111111111111111111111111111111111111111111111***2*2**222

left   --AGACCATCCTGGCTAACAAGGTGAAACCCCGTCTCTACTAAAAATACAAAAAATTAGCC
chrom  AAAAATAATTCAGTCGACTGAGAAAAAATCT----TTTTCCAGAAAAACAAGA--------
right  AAAAATAATTCAGTCGACTGAGAAAAAATCT----TTTTCCAGAAAAACAAGA--------
       22*2*22**2*2*2*2*222**222***2*22222*2*2*2*2***2****2*22222222
```

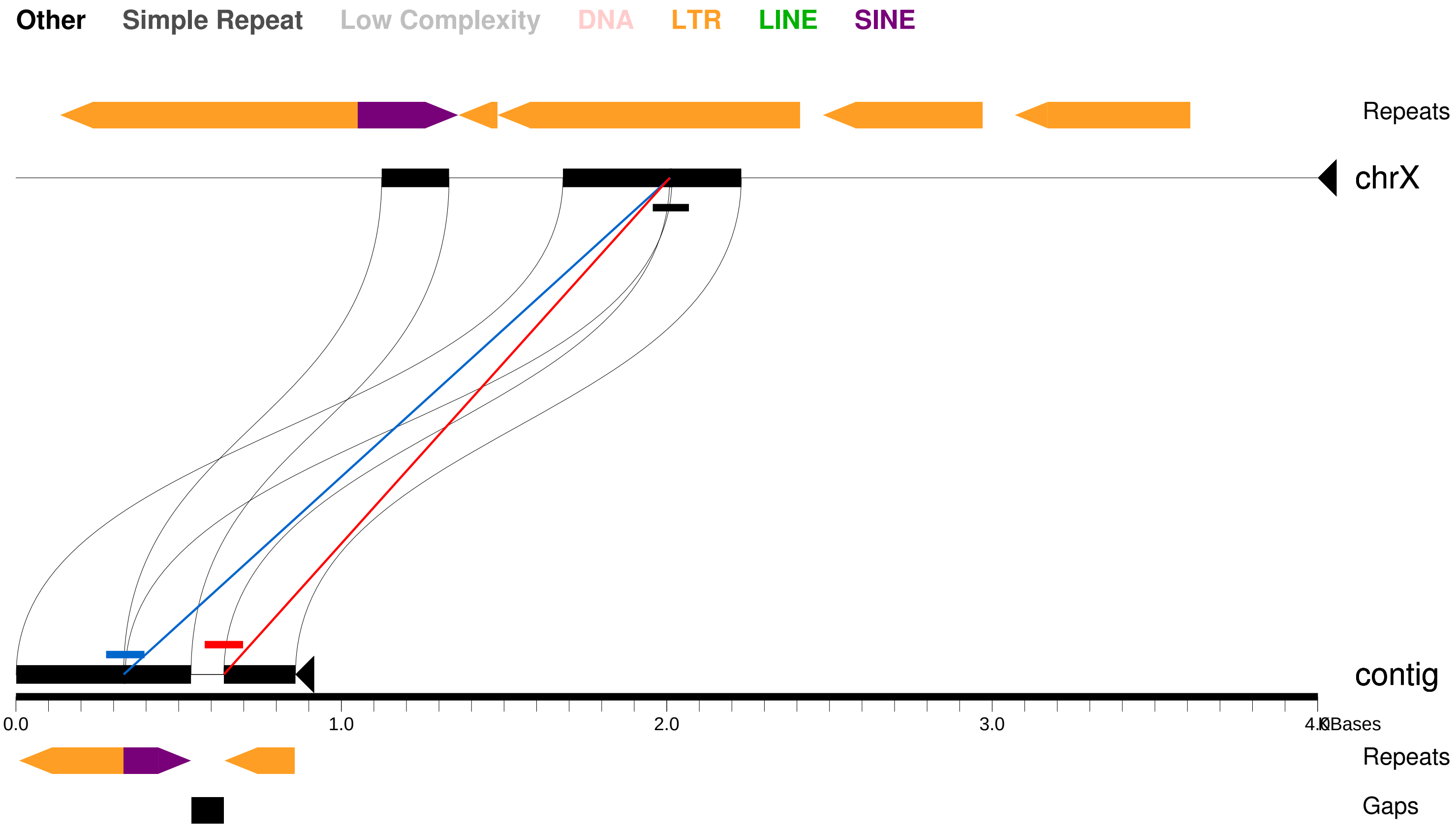

siteID: chrX_92744103 Scafftig1 - Breakpoint: chrX:92744076
chrX:92744041-92744135
Overlap Length: 9 INS size: 311 Genotyped: Yes

```
left    AAC-------------------AATGA--GAAAAATTGTCGTAA-CCATTTTTTAAG--CCCAATTTTTT
chrom   AAC-------------------AATGA--GAAAAATTGTCGTAA-CCATTTTTTAAG--CCCAATTTTCT
right   TCCGCCCGCCTCGGCCTCCCAAAGTGCTGGGATTACAGGCGTGAGCCACCGCGCCCGGCCCCAATTTTCT
        11*1111111111111111111*1**111*1*11*11*1***1*1***11111111*11*********2*

left    TTTTTTTTTTTTTTTTTTTTTTTTTTTTGAGACGGAGTCTCGCTCTGTCGCCCAGGCTGGAGTGCAGTGG
chrom   TAATCCTTGTATTTCGGGTAT----------ACAGAGT-TCAACCTT-------------AATCAACTCT
right   TAATCCTTGTATTTCGGGTAT----------ACAGAGT-TCAACCTT-------------AATCAACTCT
        *22*22**2*2***2222*2*2222222222**2****2**222**22222222222222*2*22*2*22

left    CGG
chrom   CTG
right   CTG
        *2*
```

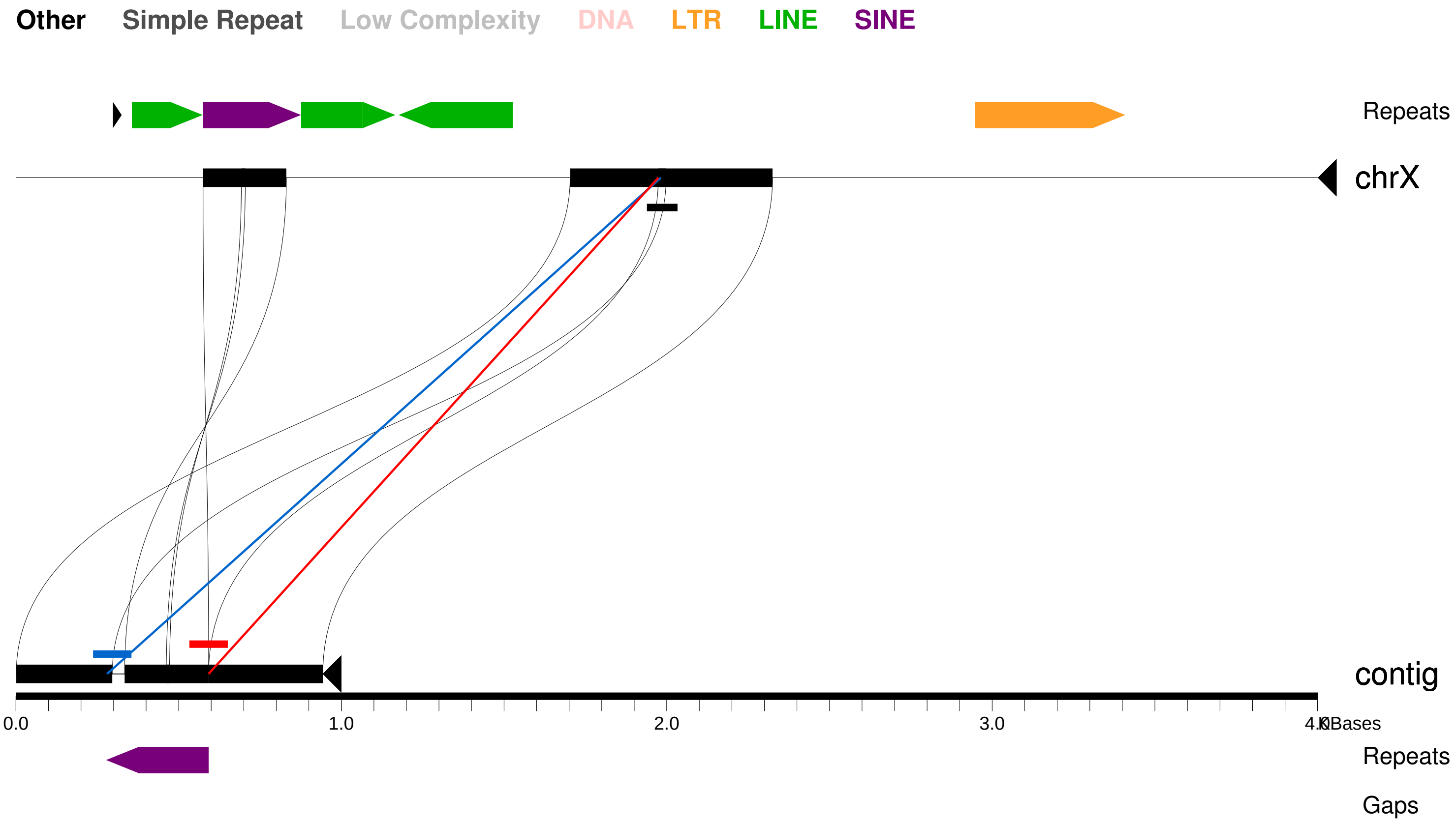

siteID: chrY_21611998 Scafftig2 + Breakpoint: chrY:21611993
chrY:21611938-21612052
Overlap Length: 3 INS size: 423 Genotyped: No

```
left   TTCCAGG-GCCAACTCCAACCAAGTTAAAGTTAAAACAGGTTTGATCTTCTTATGT-----TATNNNNNN
chrom  TTCCAGG-GCCAACTCCAACCAAGTTAAAGTTAAAACAGGTTTGATCTTCTTATGT-----TATATTTCT
right  --CCACCCGCCTCGGCCTCCCAAAGTGCTGGGATTACAGGCGTGAGCCACCGCGCCCGGCCTATATTTCT
       11***111***1111**11****11*111*11*11*****11***1*11*11111111111***222222

left   NNNNNNNNNNNNNNNNNNNNNNNNNNNNNNNNNNNNNNNNNNNNNNNNNNNNNNN
chrom  TTATCTTCCCCTGCTATTGCTAGTCCTCTCCTTATTAACGTAACCAGGTCA----
right  TTATCTTCCCCTGCTATTGCTAGTCCTCTCCTTATTAACGTAACCAGGTCA----
       2222222222222222222222222222222222222222222222222222222
```

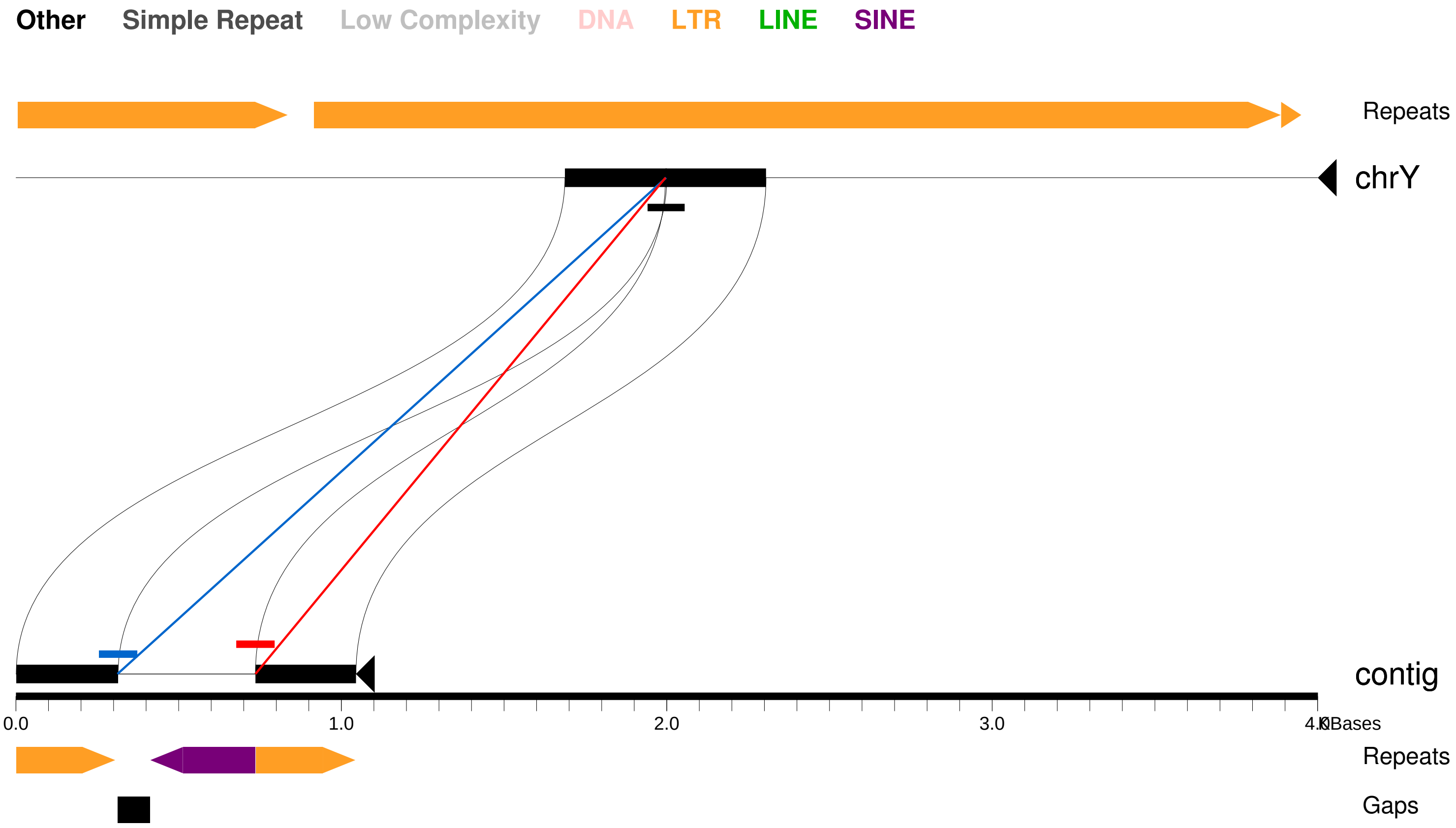

Supplement: SUPPLEMENTARY DATA [file supp_gkv1089_nar-01397-h-2015-File006.pdf]
