# Supplementary material for: Discovery and characterization of Alu repeat sequences via precise local read assembly
Source: Nucleic Acids Res. 2015 Oct 25;43(21):10292–307. doi: 10.1093/nar/gkv1089 (PMC4666360; doi:10.1093/nar/gkv1089)
Supplement: SUPPLEMENTARY DATA [file supp_gkv1089_nar-01397-h-2015-File010.pdf]

A.

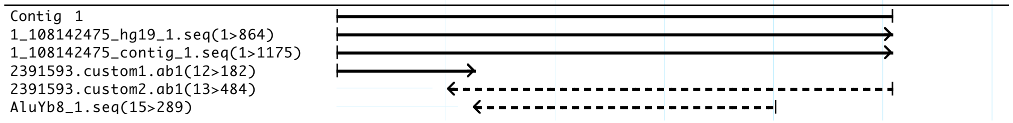

C.

genotyped

siteID: chr1_108142475
sample sequenced: HGDP00713
Alu subfamily: AluYb8

B.

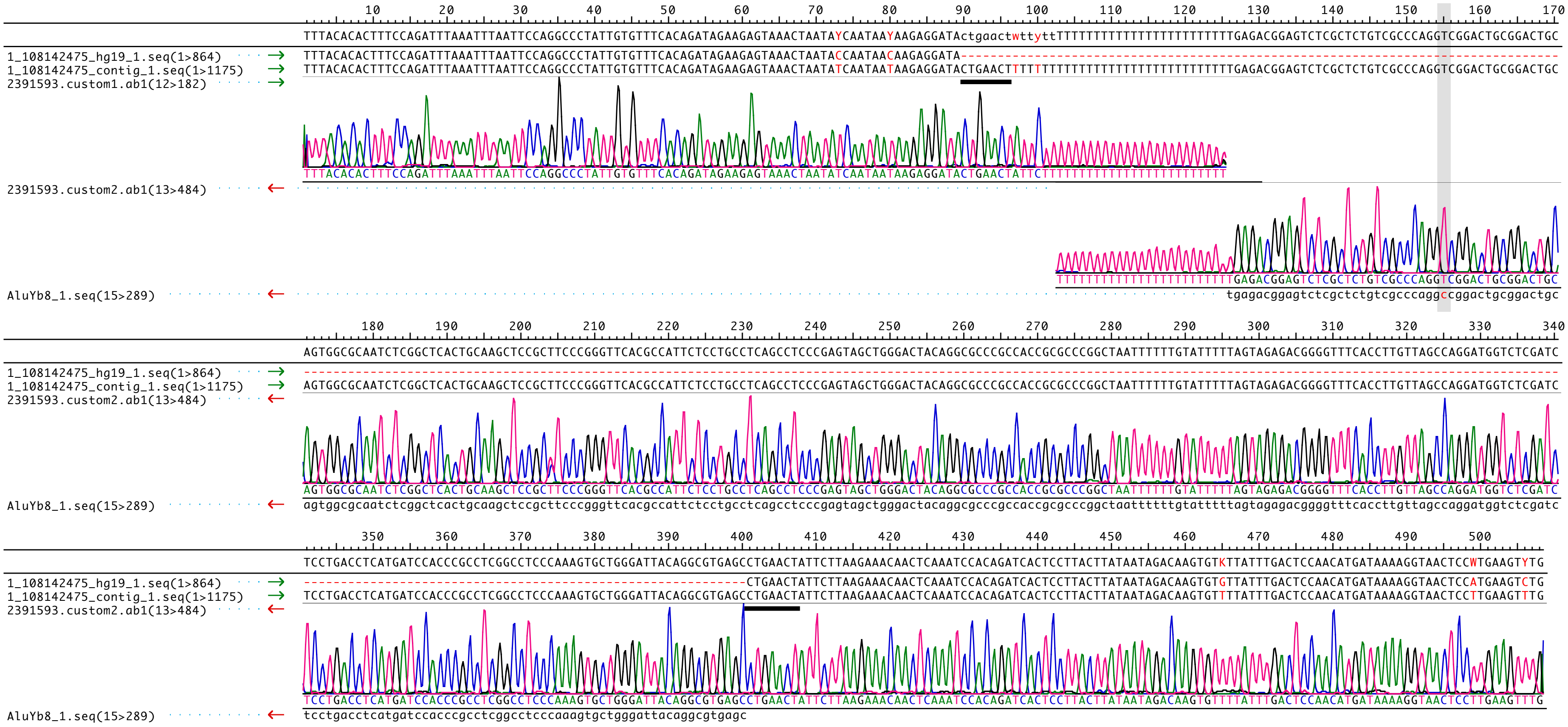

Figure S5.1

A.

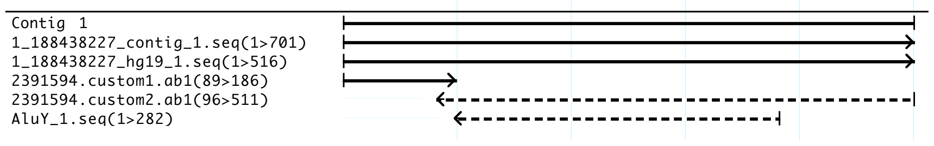

B.

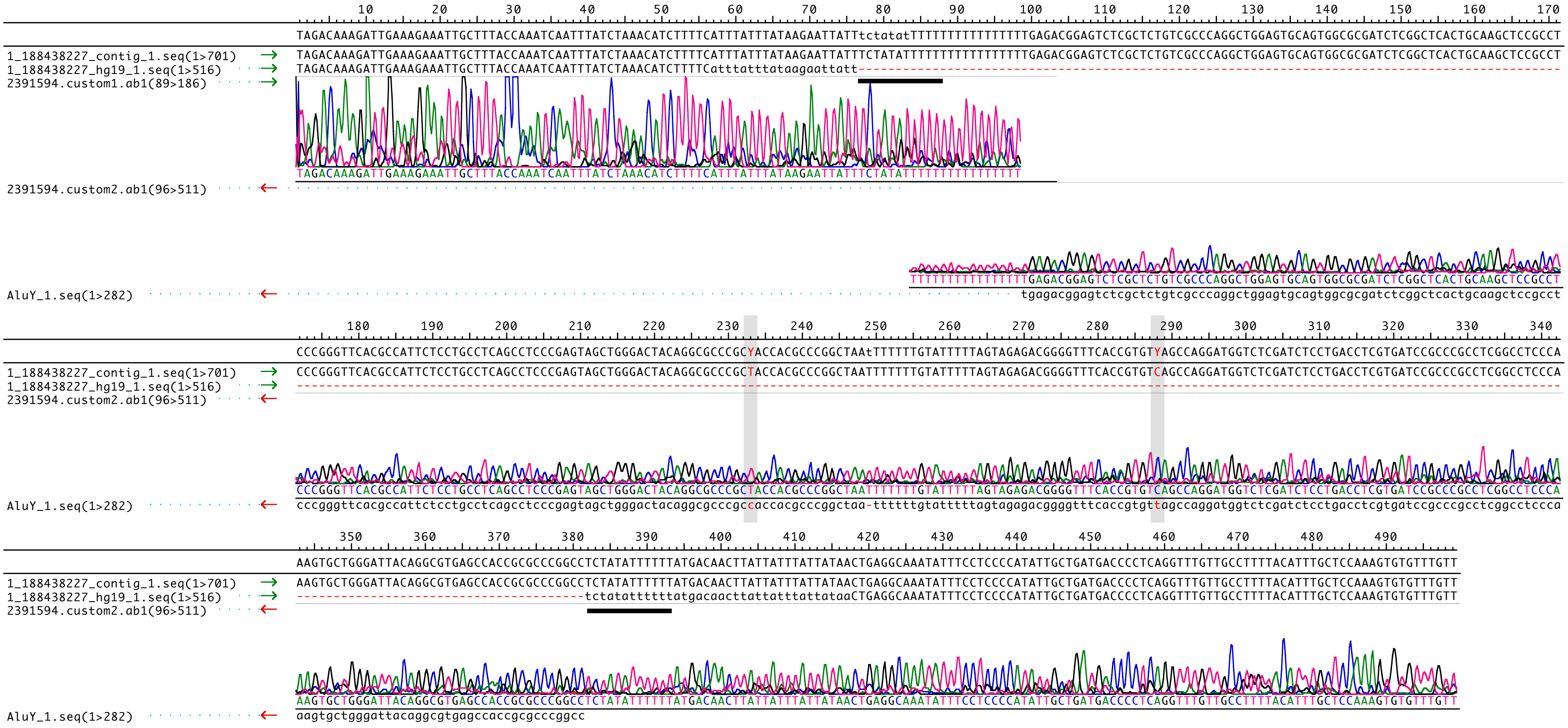

C.

genotyped

siteID: chr1_188438227
sample sequenced: HGDP00474
Alu subfamily: AluYc1

Figure S5.2

A.

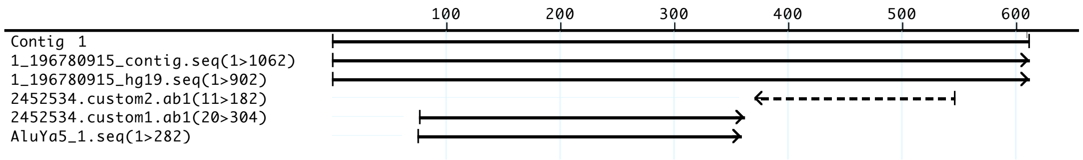

C.

genotyped

siteID: chr1_196780915
sample sequenced: HGDP00222
Alu subfamily: AluYa5
5’ truncation: 166

B.

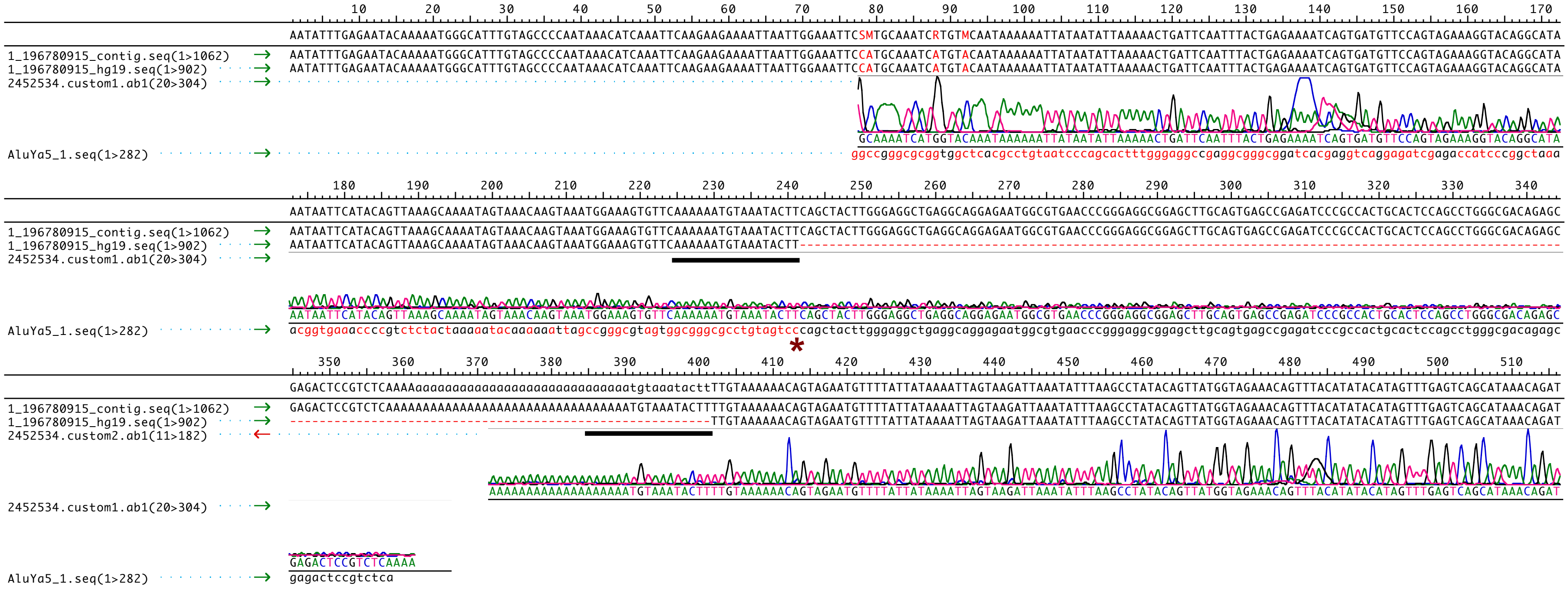

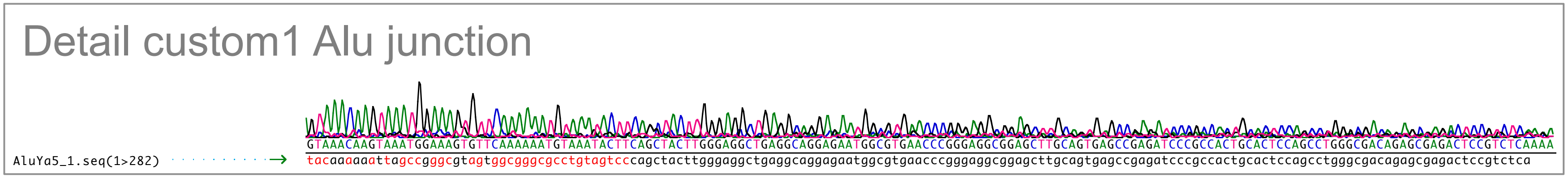

Figure S5.3

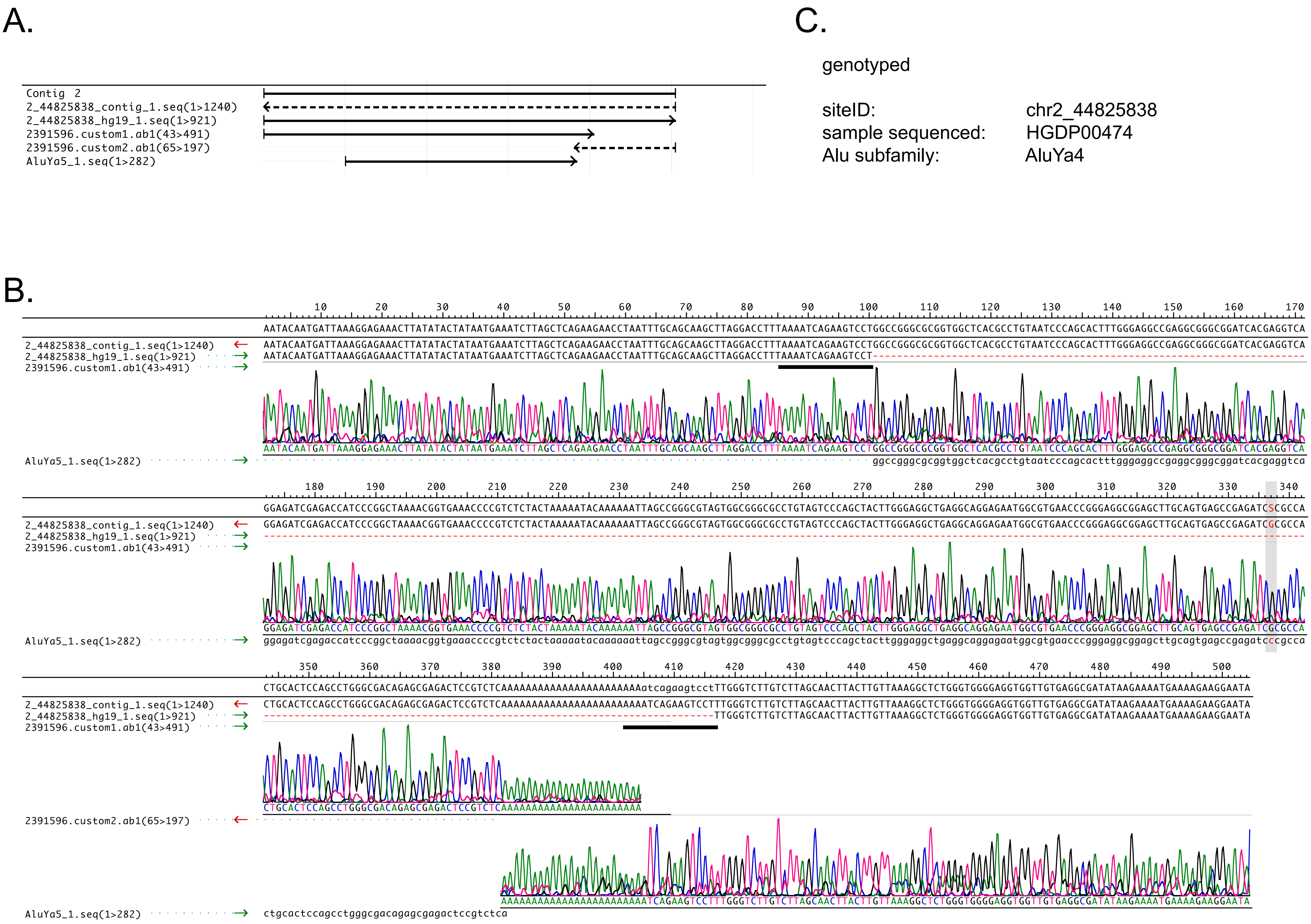
A.
Contig 2
2_44825838_contig_1.seq(1>1240)
2_44825838_hg19_1.seq(1>921)
2391596.custom1.ab1(43>491)
2391596.custom2.ab1(65>197)
AluYa5_1.seq(1>282)
B.
2_44825838_contig_1.seq(1>1240)
2_44825838_hg19_1.seq(1>921)
2391596.custom1.ab1(43>491)
AluYa5_1.seq(1>282)
2391596.custom2.ab1(65>197)
C.
genotyped
siteID: chr2_44825838
sample sequenced: HGDP00474
Alu subfamily: AluYa4

Figure S5.4

A.

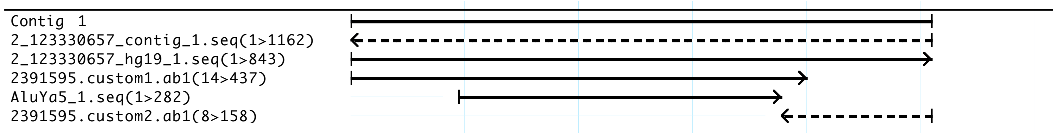

C.

genotyped

siteID: chr2_123330657
sample sequenced: HGDP00474
Alu subfamily: AluYa5

B.

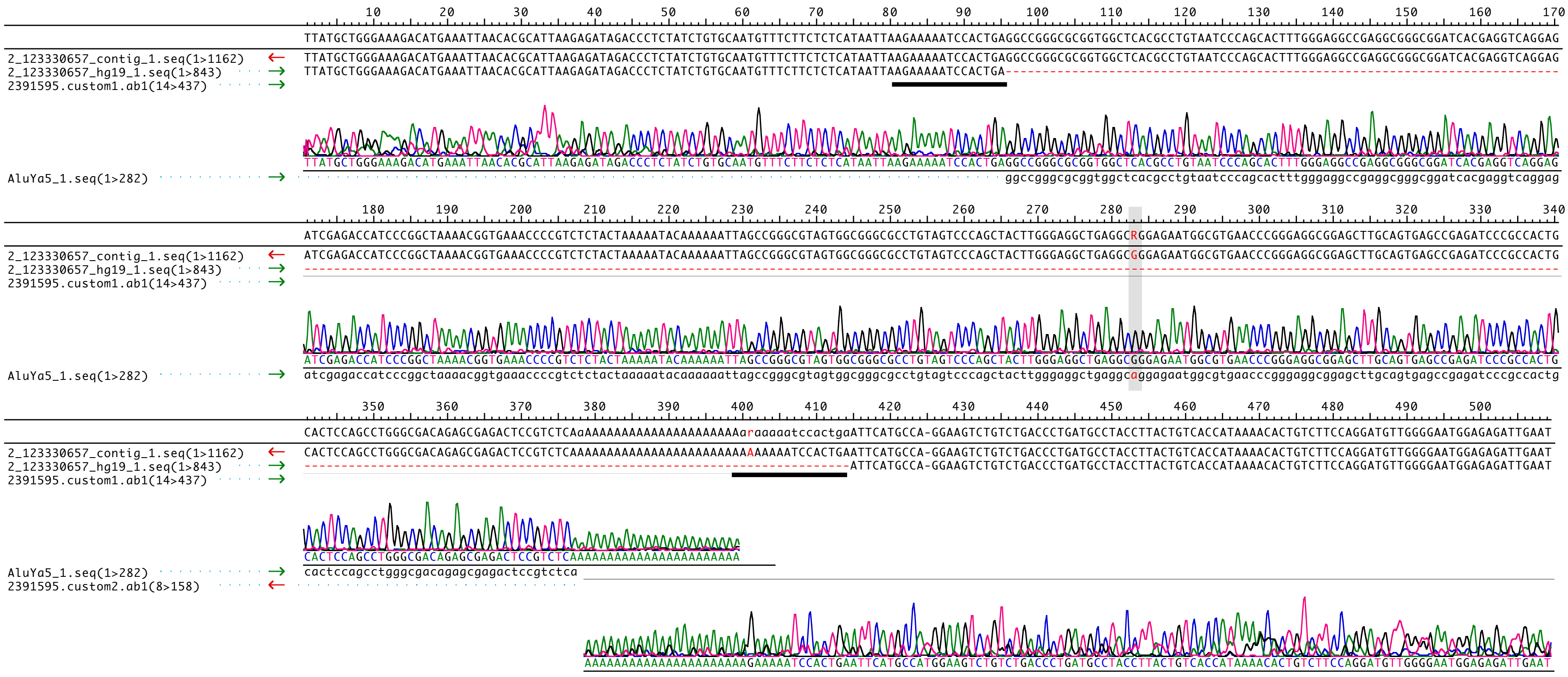

Figure S5.5

A.

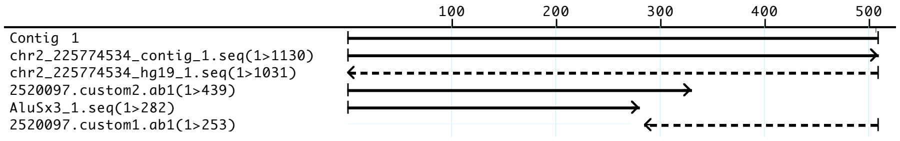

C.

genotyped

siteID:	chr2_225774534
sample sequenced:	HGDP00877
Alu subfamily:	unclassified

B.

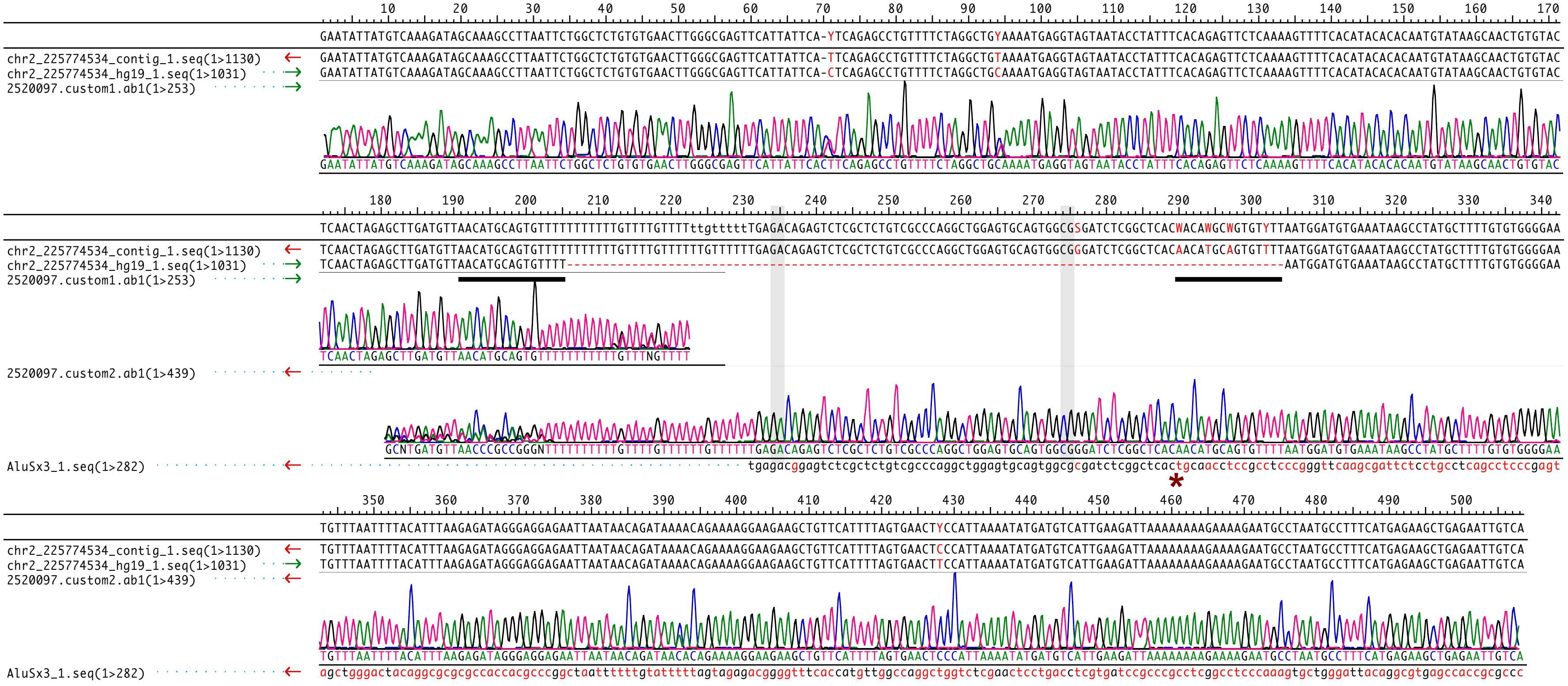

Figure S5.6

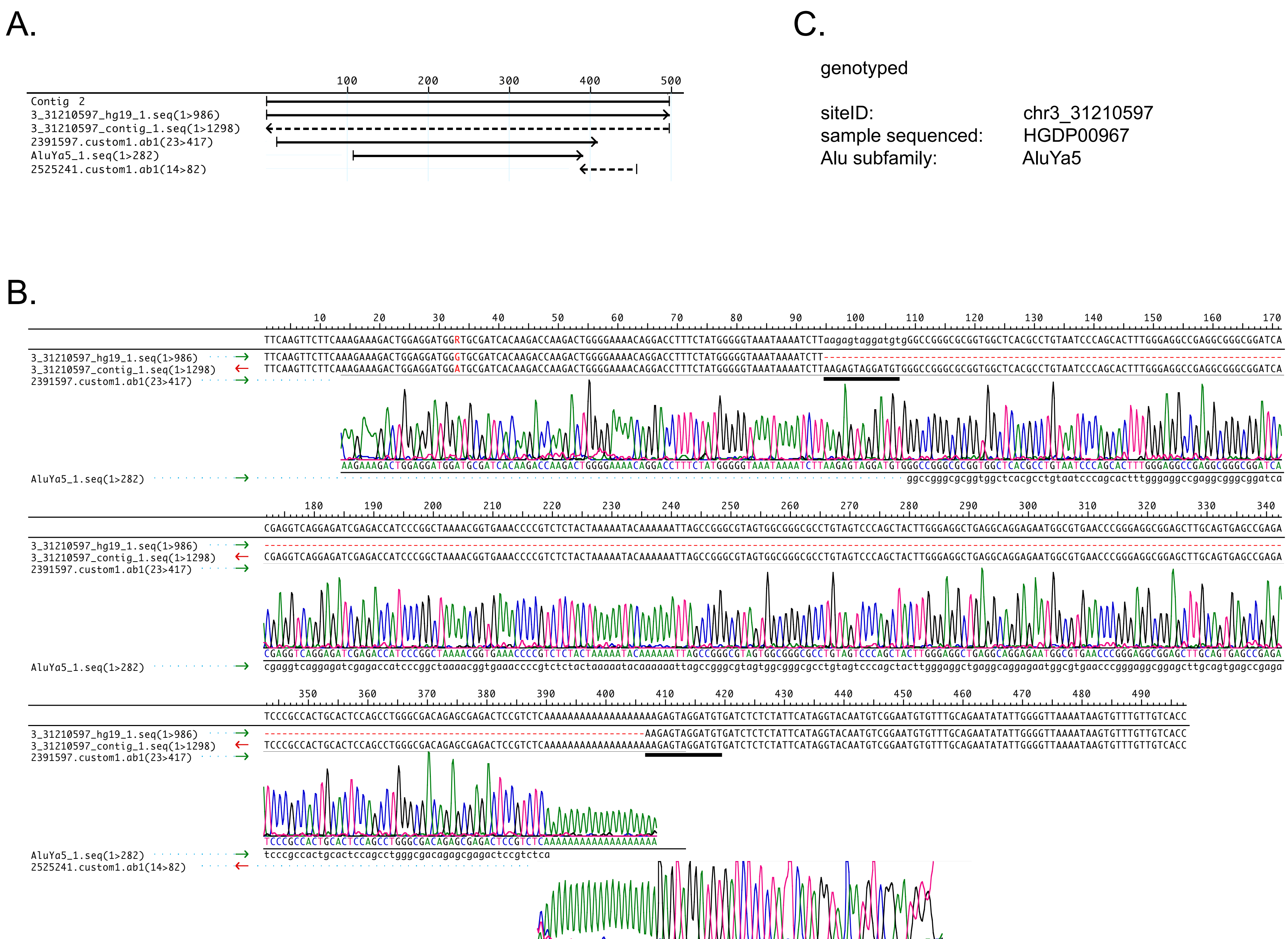
A.
Contig 2
3_31210597_hg19_1.seq(1>986)
3_31210597_contig_1.seq(1>1298)
2391597.custom1.ab1(23>417)
AluYa5_1.seq(1>282)
2525241.custom1.ab1(14>82)
B.
C.
genotyped
siteID: chr3_31210597
sample sequenced: HGDP00967
Alu subfamily: AluYa5

Figure S5.7

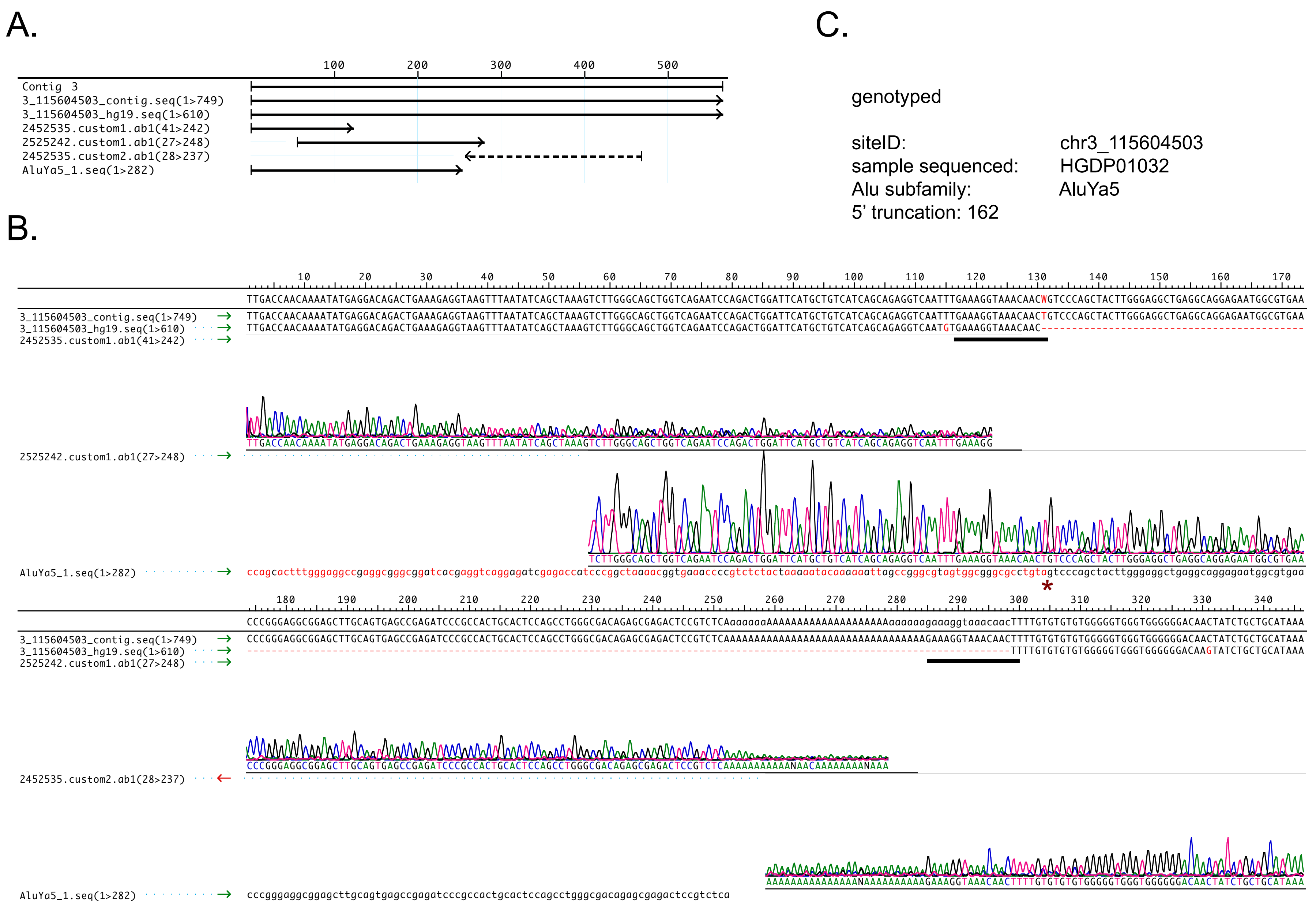
A.
Contig 3
3_115604503_contig.seq(1>749)
3_115604503_hg19.seq(1>610)
2452535.custom1.ab1(41>242)
2525242.custom1.ab1(27>248)
2452535.custom2.ab1(28>237)
AluYa5_1.seq(1>282)
B.
C.
genotyped
siteID: chr3_115604503
sample sequenced: HGDP01032
Alu subfamily: AluYa5
5' truncation: 162

Figure S5.8

A.

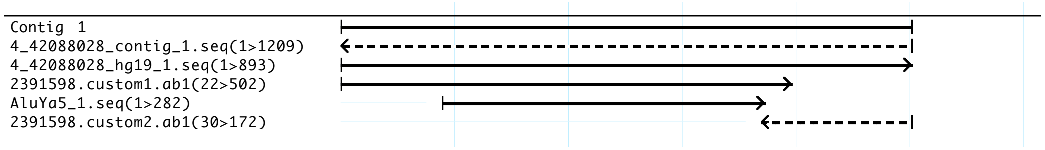

C.

genotyped

siteID:	chr4_42088028
sample sequenced:	HGDP00713
Alu subfamily:	AluYa4

B.

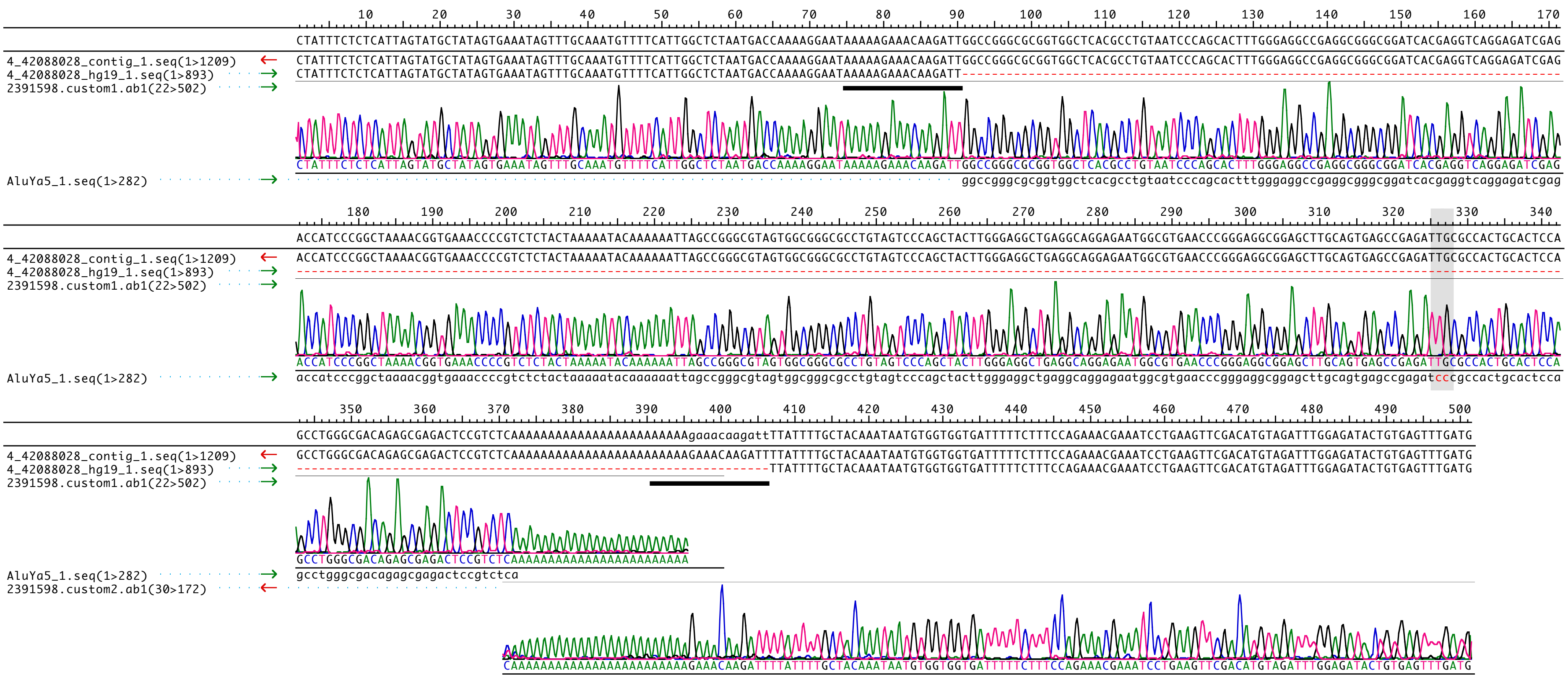

Figure S5.9

A.

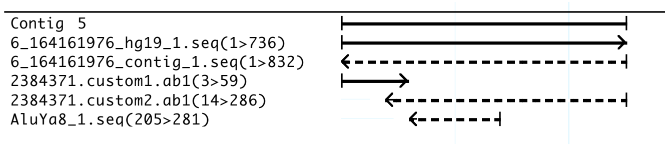

C.

not genotyped

siteID: chr6_164161976
sample sequenced: HGDP00959
Alu subfamily: unclassified

B.

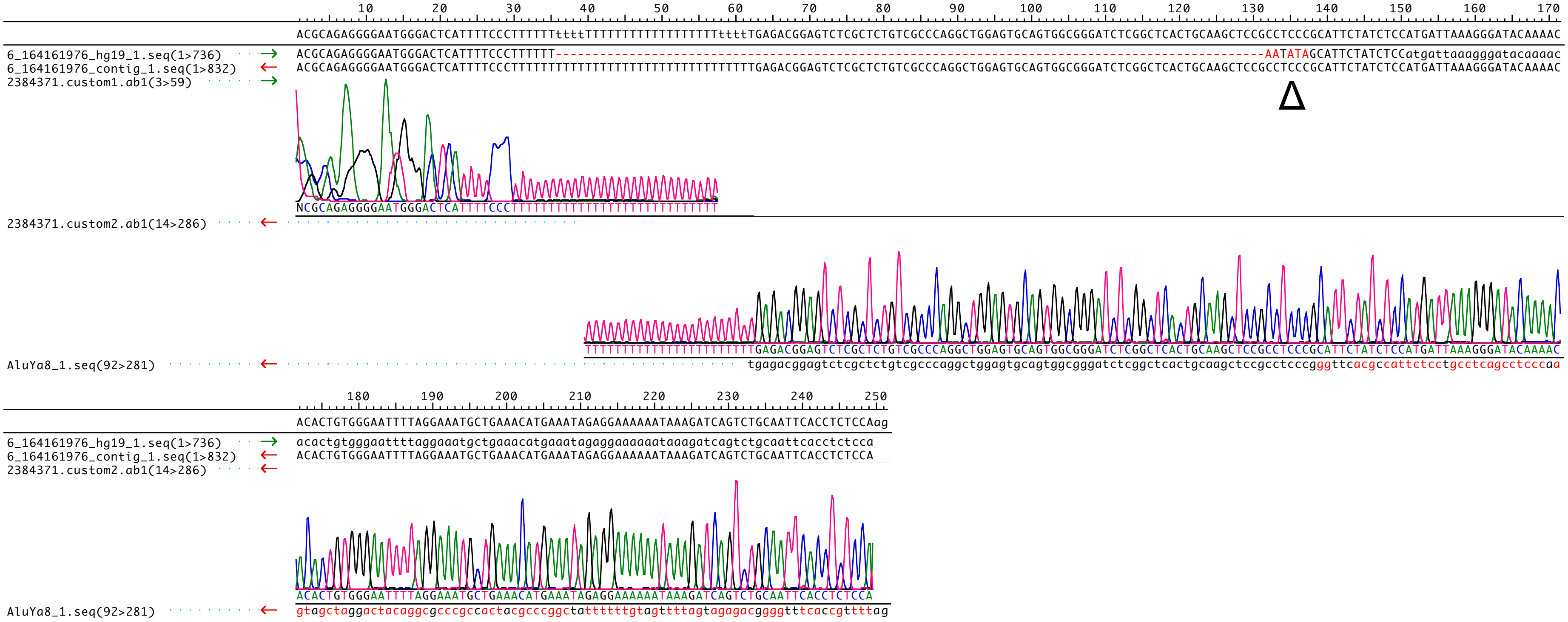

Figure S5.10

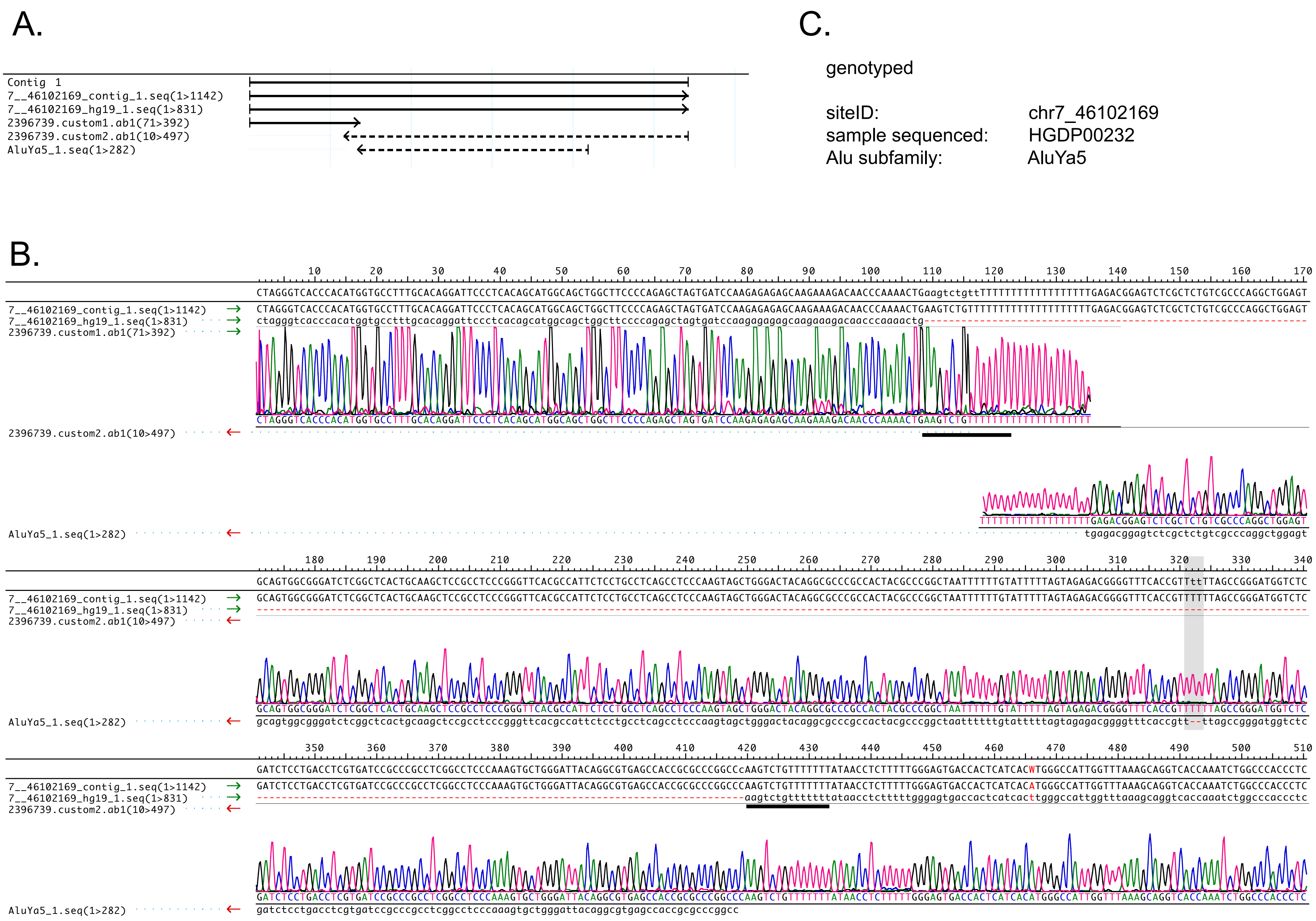
A.
Contig 1
7__46102169_contig_1.seq(1>1142)
7__46102169_hg19_1.seq(1>831)
2396739.custom1.ab1(71>392)
2396739.custom2.ab1(10>497)
AluYa5_1.seq(1>282)
B.
C.
genotyped
siteID: chr7_46102169
sample sequenced: HGDP00232
Alu subfamily: AluYa5

Figure S5.11

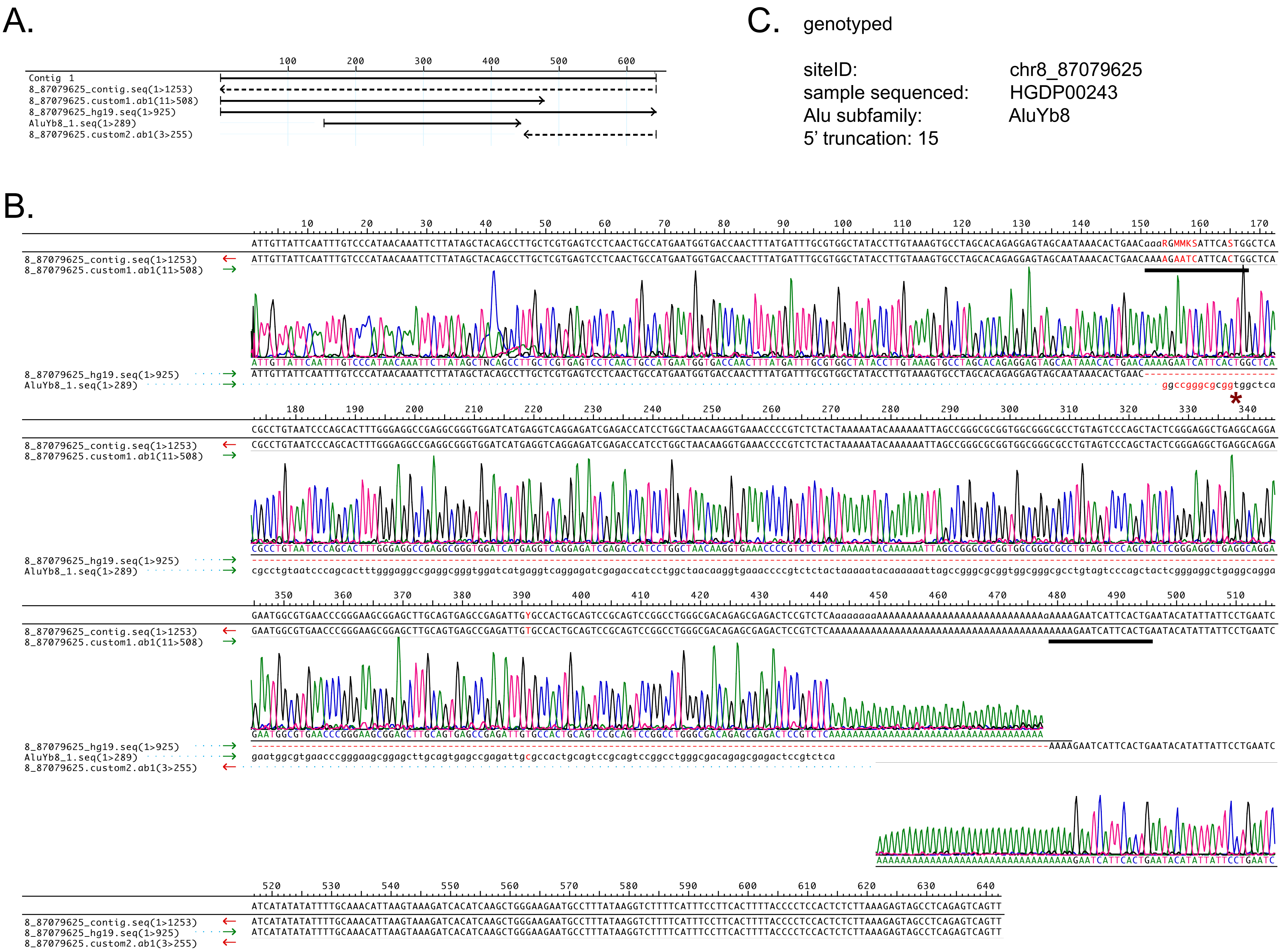
A.
Contig 1
8_87079625_contig.seq(1>1253)
8_87079625.custom1.ab1(11>508)
8_87079625_hg19.seq(1>925)
AluYb8_1.seq(1>289)
8_87079625.custom2.ab1(3>255)
C.
genotyped
siteID: chr8_87079625
sample sequenced: HGDP00243
Alu subfamily: AluYb8
5' truncation: 15
B.

Figure S5.12

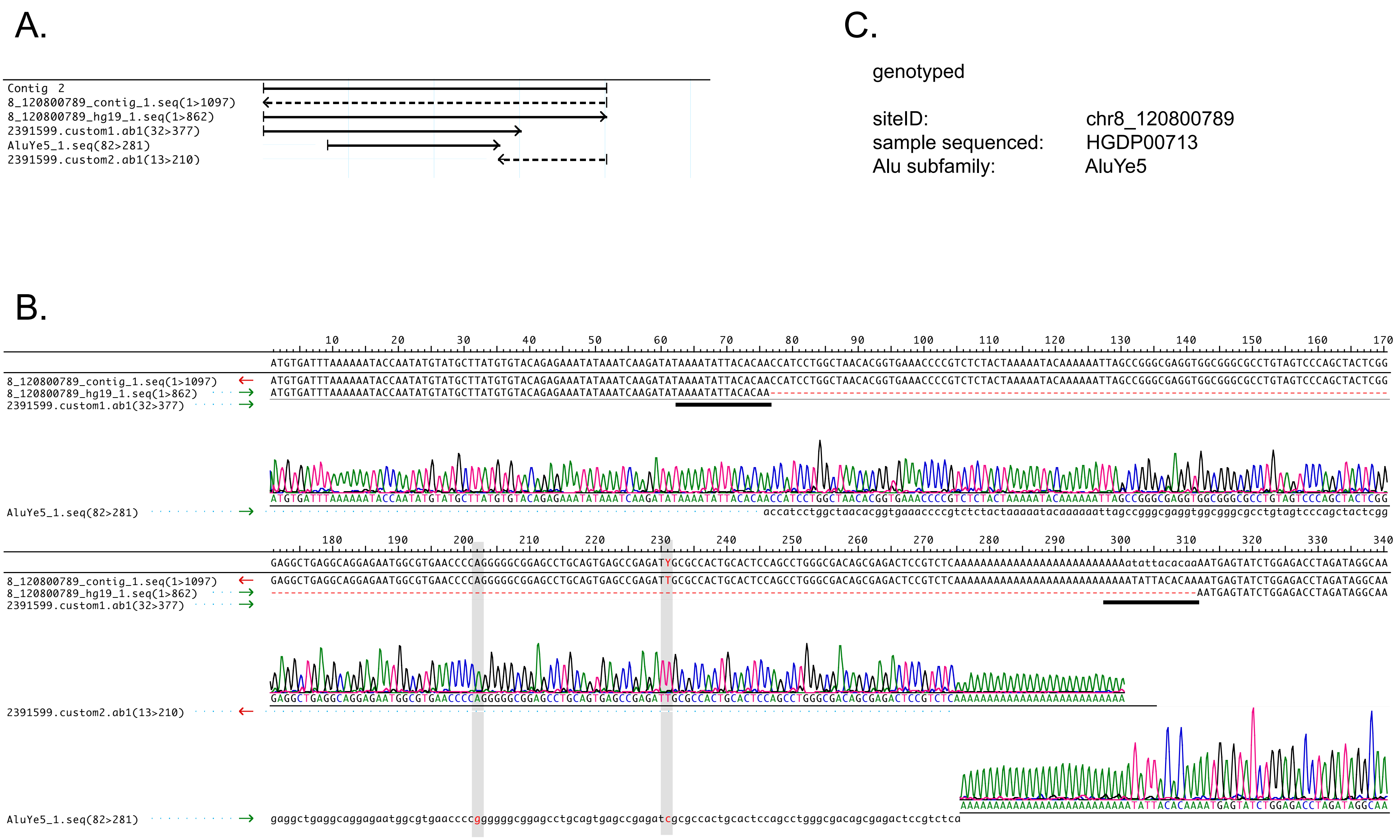
A.
Contig 2
8_120800789_contig_1.seq(1>1097)
8_120800789_hg19_1.seq(1>862)
2391599.custom1.ab1(32>377)
AluYe5_1.seq(82>281)
2391599.custom2.ab1(13>210)
C.
genotyped
siteID: chr8_120800789
sample sequenced: HGDP00713
Alu subfamily: AluYe5
B.
8_120800789_contig_1.seq(1>1097)
8_120800789_hg19_1.seq(1>862)
2391599.custom1.ab1(32>377)
AluYe5_1.seq(82>281)
2391599.custom2.ab1(13>210)

Figure S5.13

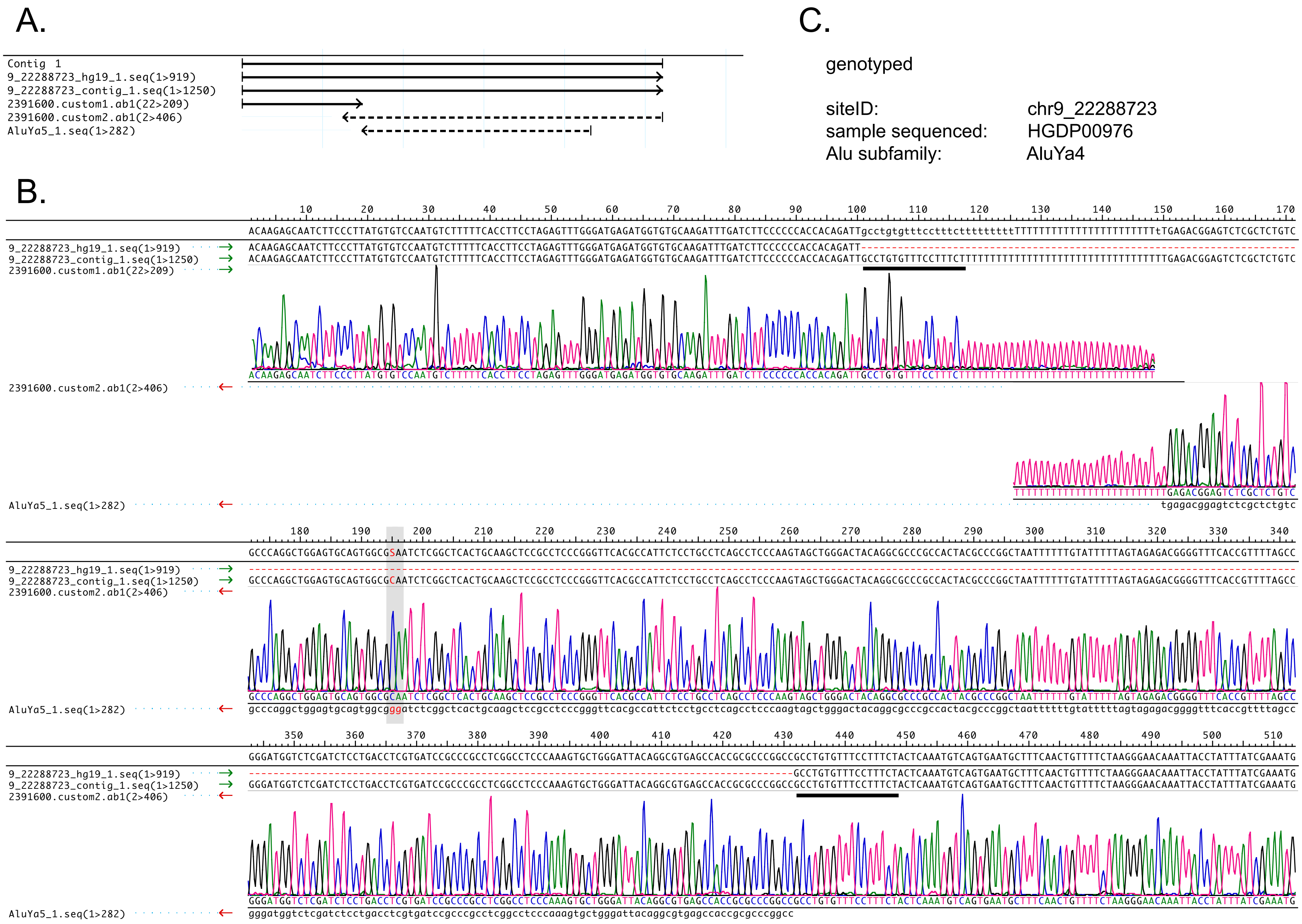
A.
Contig 1
9_22288723_hg19_1.seq(1>919)
9_22288723_contig_1.seq(1>1250)
2391600.custom1.ab1(22>209)
2391600.custom2.ab1(2>406)
AluYa5_1.seq(1>282)
B.
C.
genotyped
siteID: chr9_22288723
sample sequenced: HGDP00976
Alu subfamily: AluYa4

Figure S5.14

A.

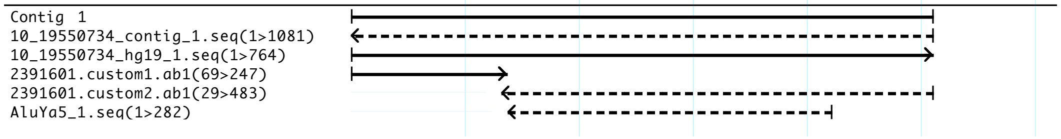

C.

genotyped

siteID: chr10_19550734
sample sequenced: HGDP00474
Alu subfamily: AluYa5

B.

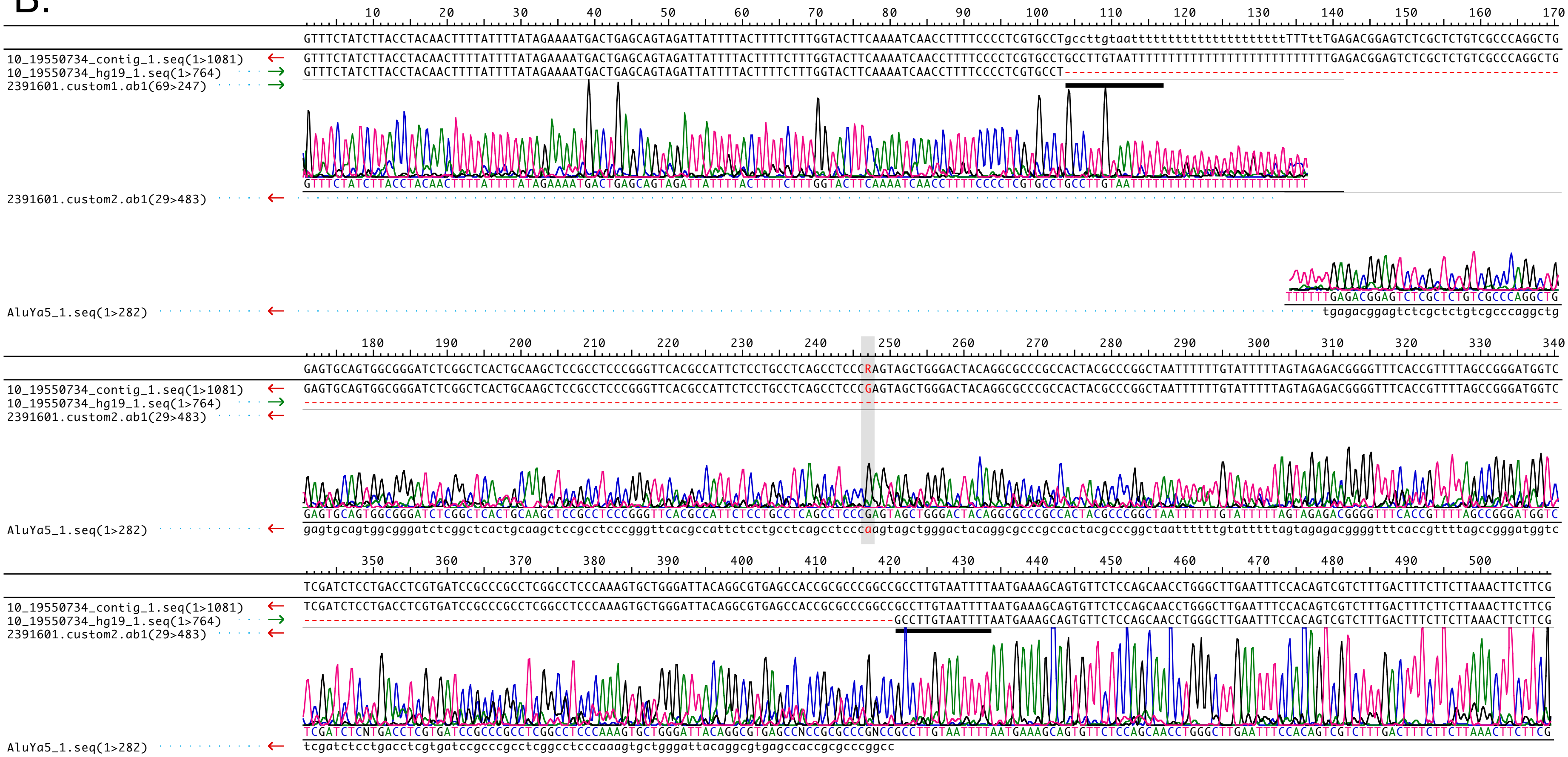

Figure S5.15

A.

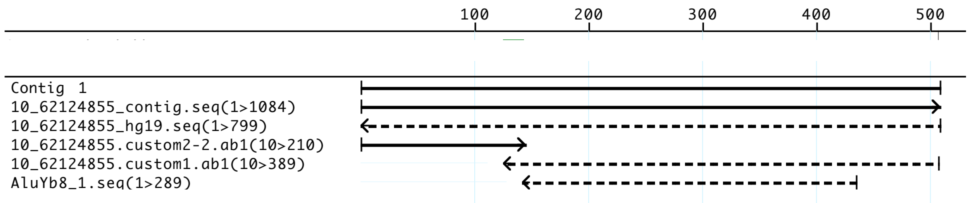

C.

genotyped

siteID: chr10_62124855
sample sequenced: HGDP01029
Alu subfamily: AluYb8
5’ truncation: 30

B.

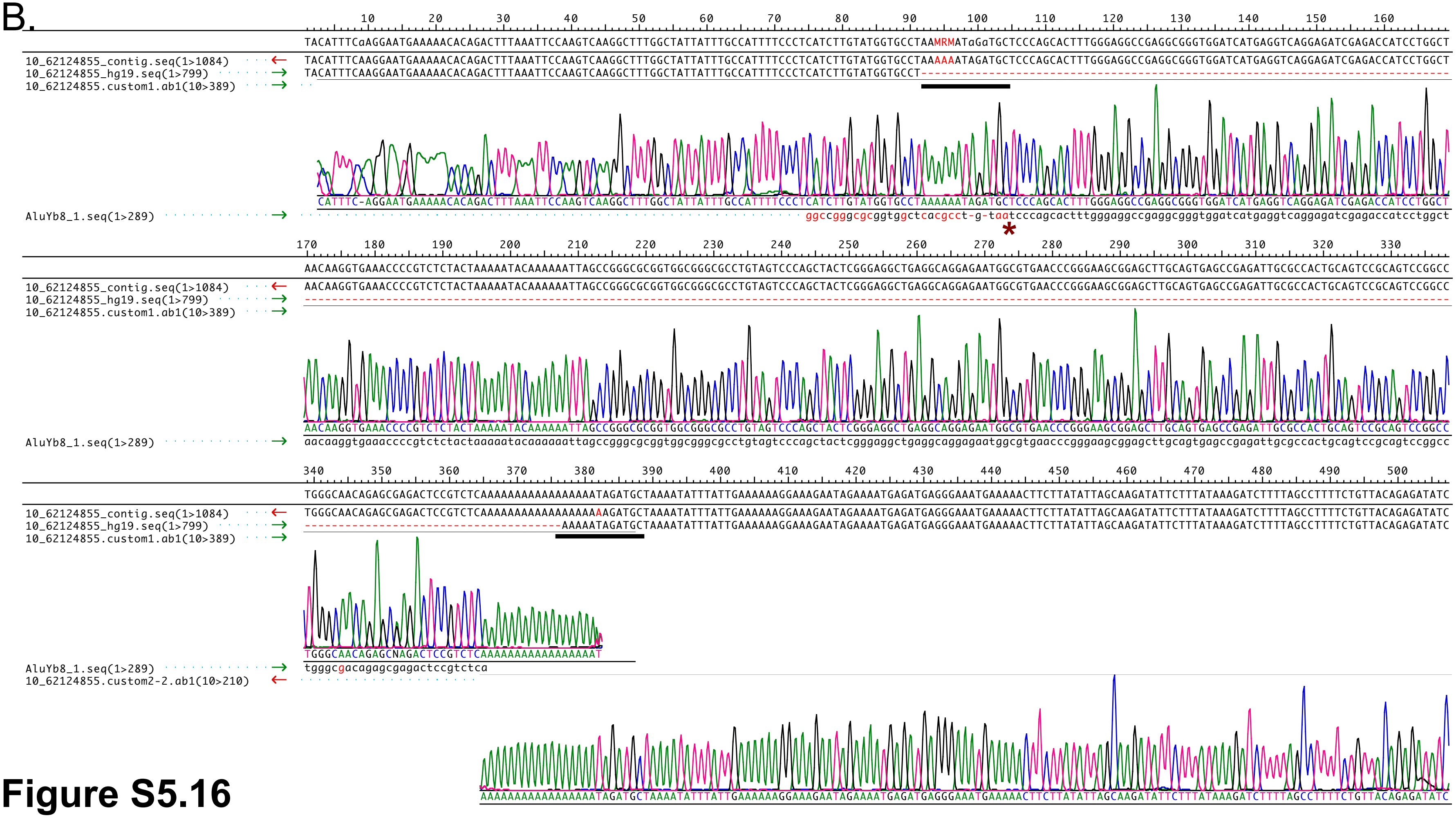

Figure S5.16

A.

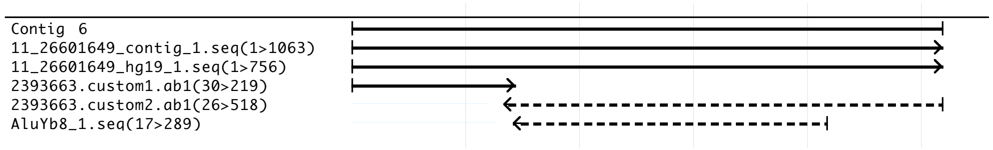

C.

genotyped

siteID: chr11_26601649
sample sequenced: HGDP00713
Alu subfamily: AluYb8

B.

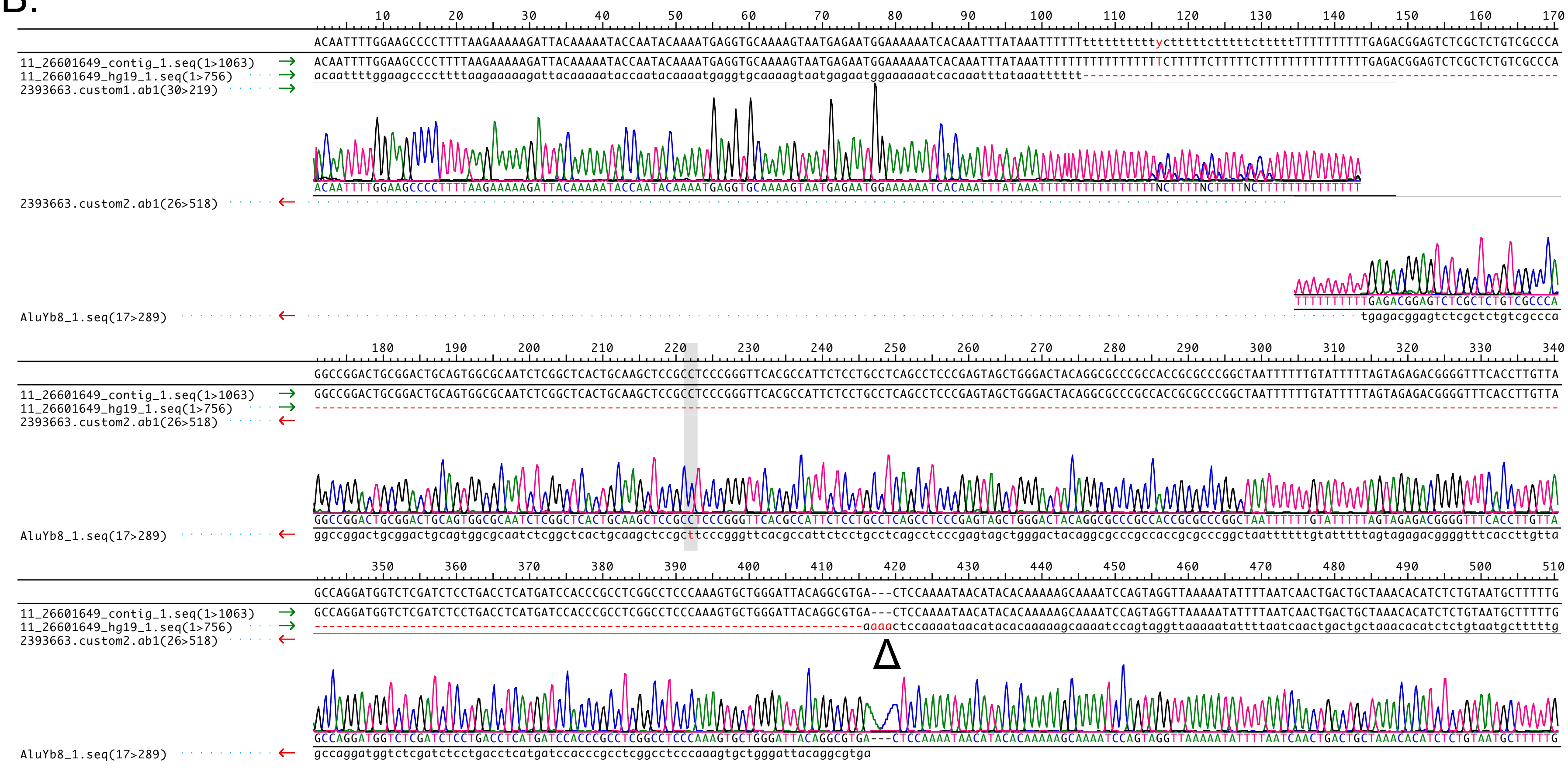

Figure S5.17

A.

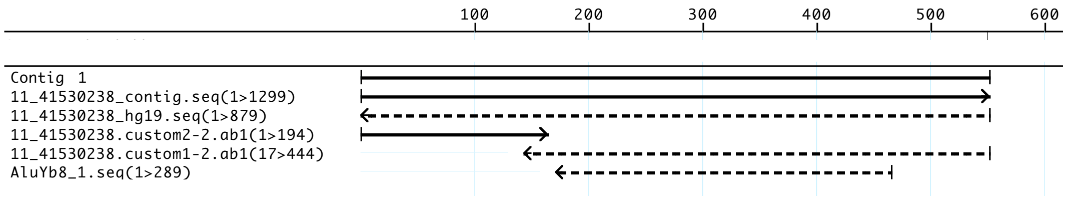

C.

genotyped

siteID: chr11_41530238
sample sequenced: HGDP0721
Alu subfamily: AluYb8
5’ truncation: 35

B.

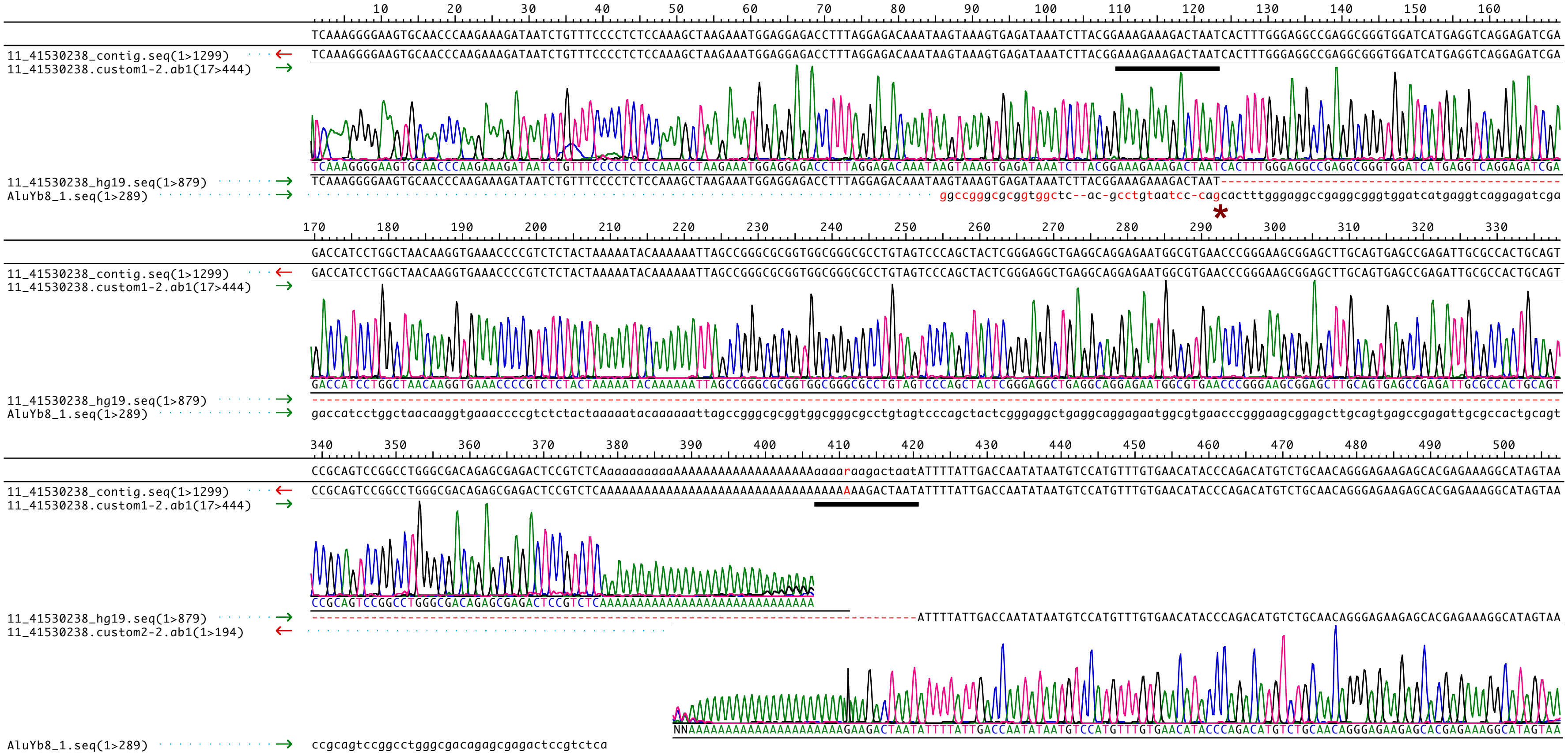

Figure S5.18

A.

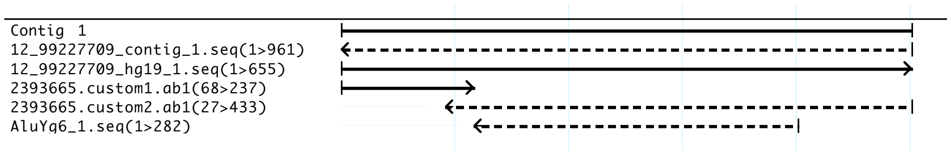

C.

genotyped

siteID: chr12_99227709
sample sequenced: HGDP00476
Alu subfamily: AluYg6

B.

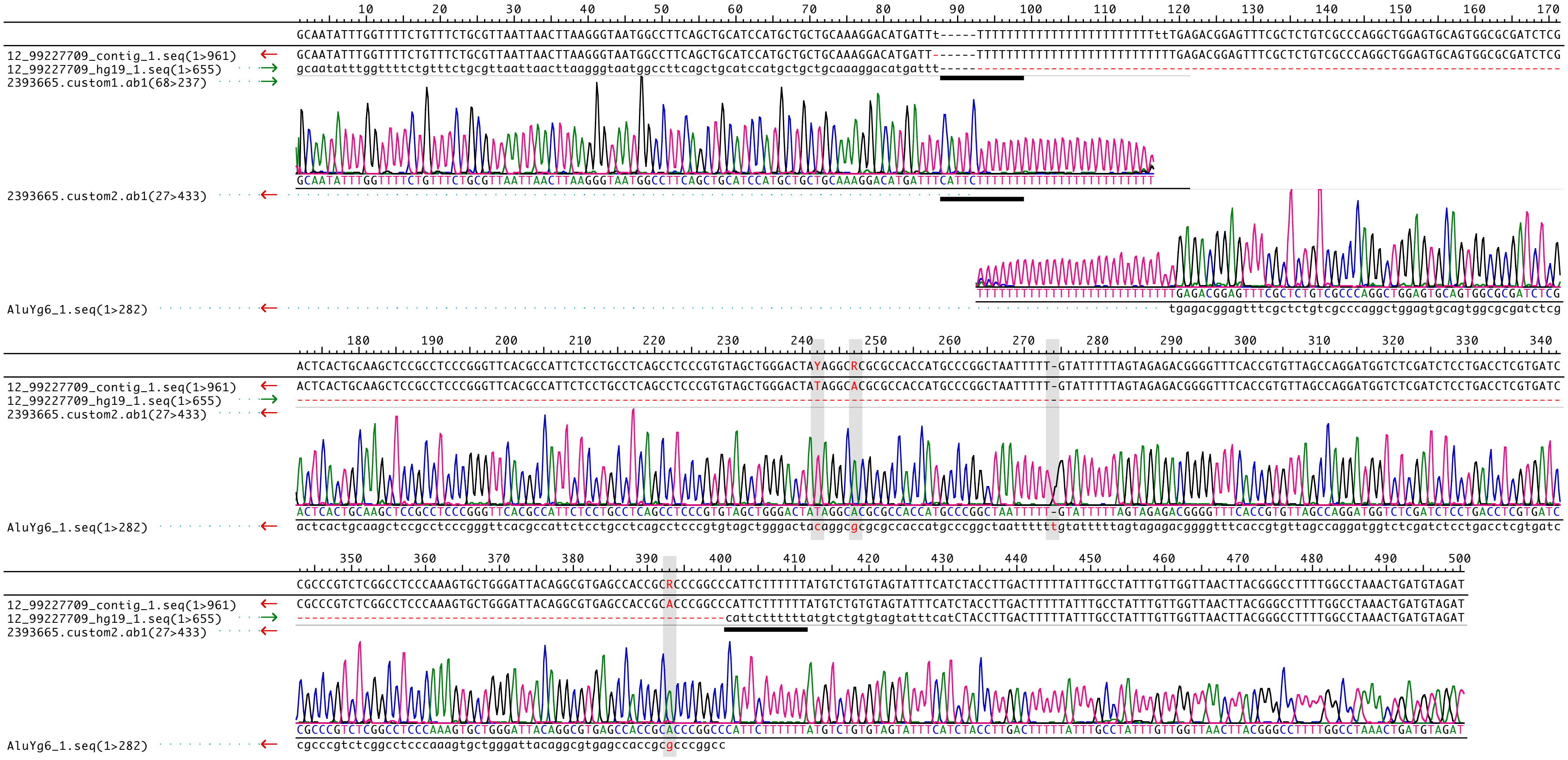

Figure S5.19

A.

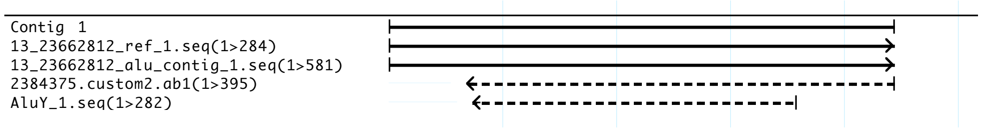

C.

genotyped

siteID: chr13_23662812
sample sequenced: HGDP00721
Alu subfamily: AluY

B.

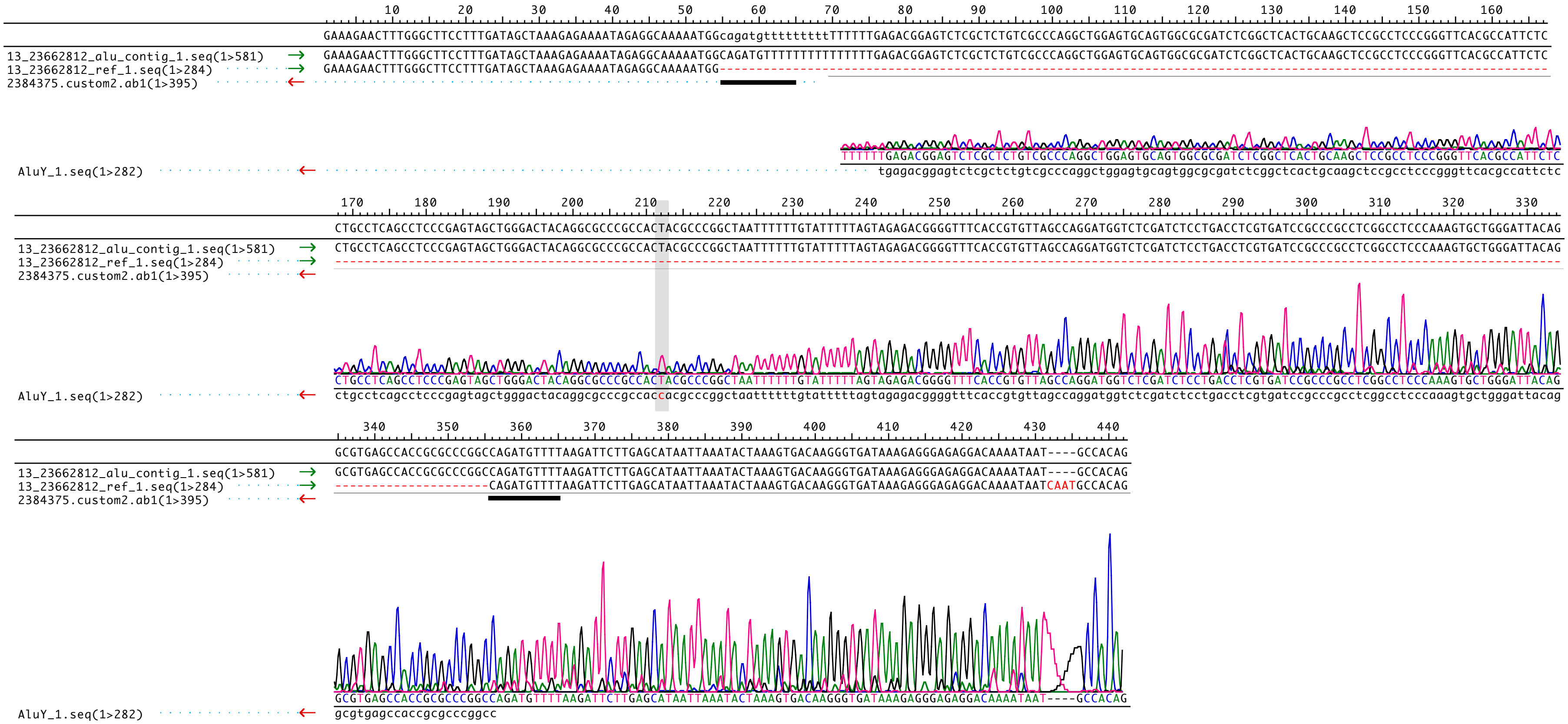

Figure S5.20

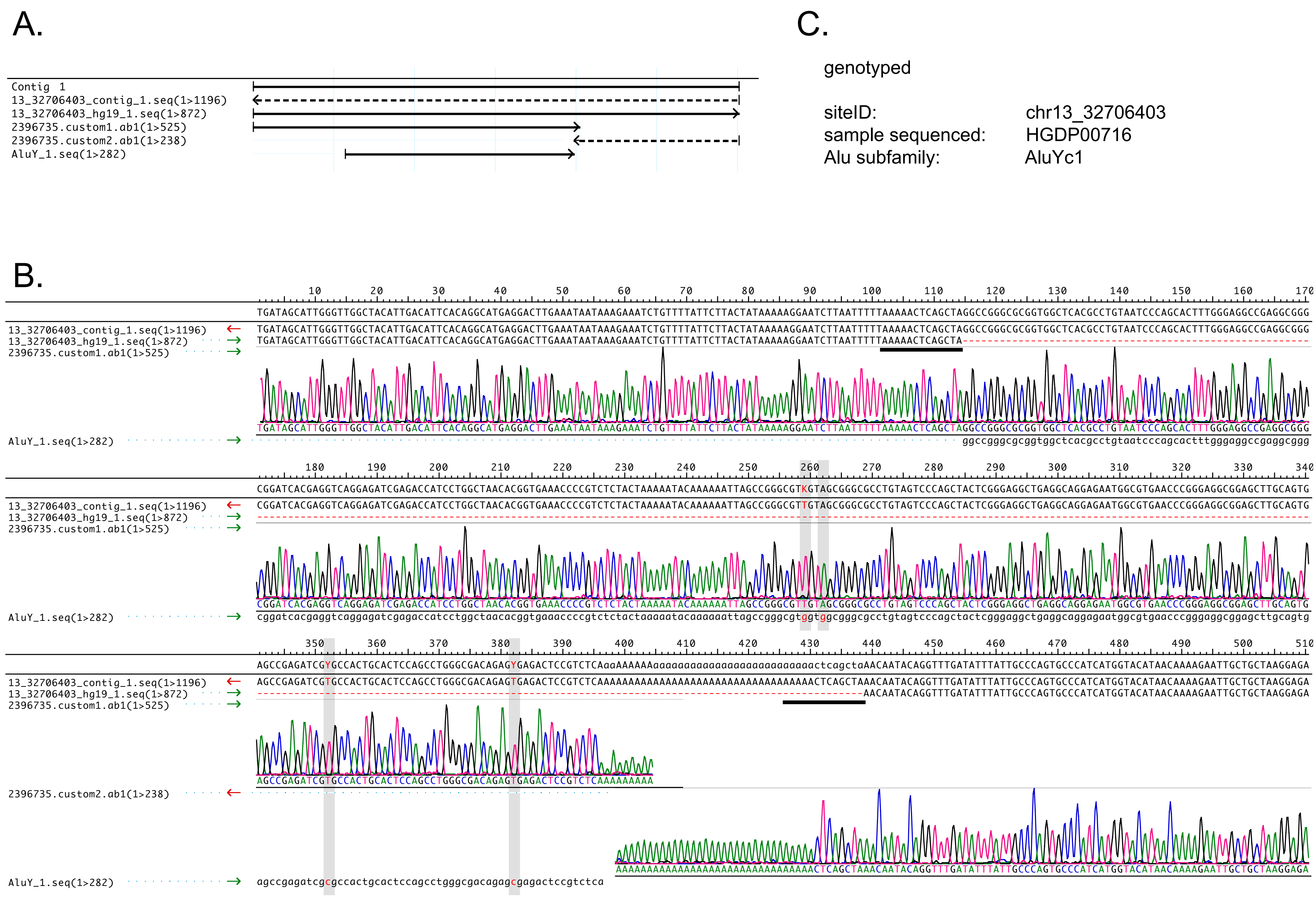
A.
Contig 1
13_32706403_contig_1.seq(1>1196)
13_32706403_hg19_1.seq(1>872)
2396735.custom1.ab1(1>525)
2396735.custom2.ab1(1>238)
AluY_1.seq(1>282)
C.
genotyped
siteID: chr13_32706403
sample sequenced: HGDP00716
Alu subfamily: AluYc1
B.
13_32706403_contig_1.seq(1>1196)
13_32706403_hg19_1.seq(1>872)
2396735.custom1.ab1(1>525)
2396735.custom2.ab1(1>238)
AluY_1.seq(1>282)

Figure S5.21

A.

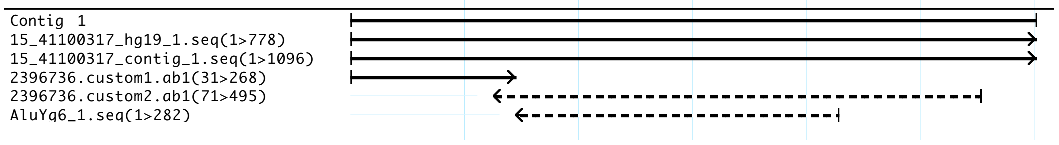

C.

genotyped

siteID: chr15_41100317
sample sequenced: HGDP01264
Alu subfamily: AluYg6

B.

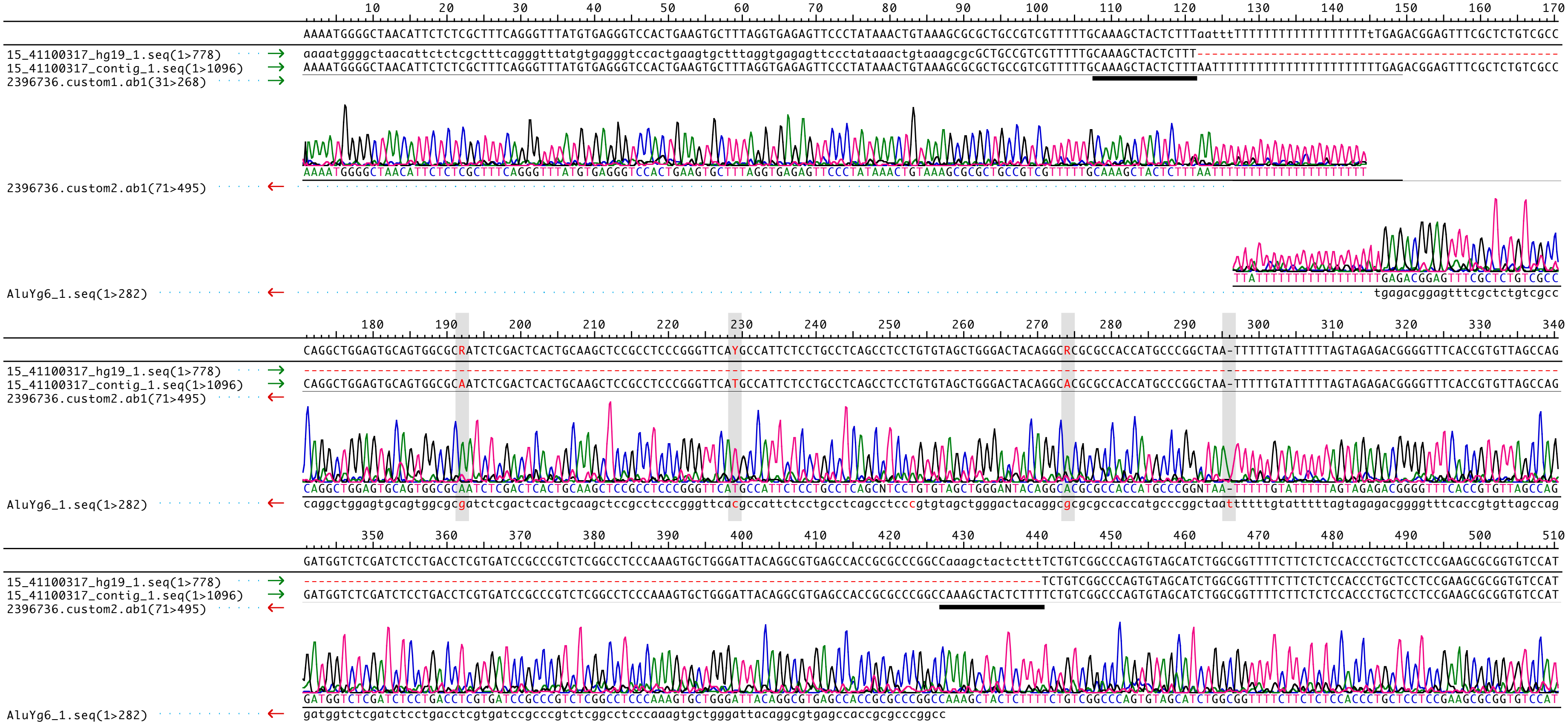

Figure S5.22

A.

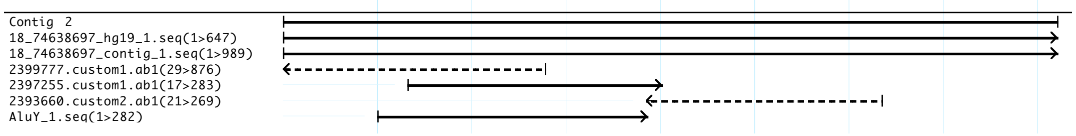

C.

genotyped

siteID: chr18_74638697
sample sequenced: HGDP00222
Alu subfamily: AluY

B.

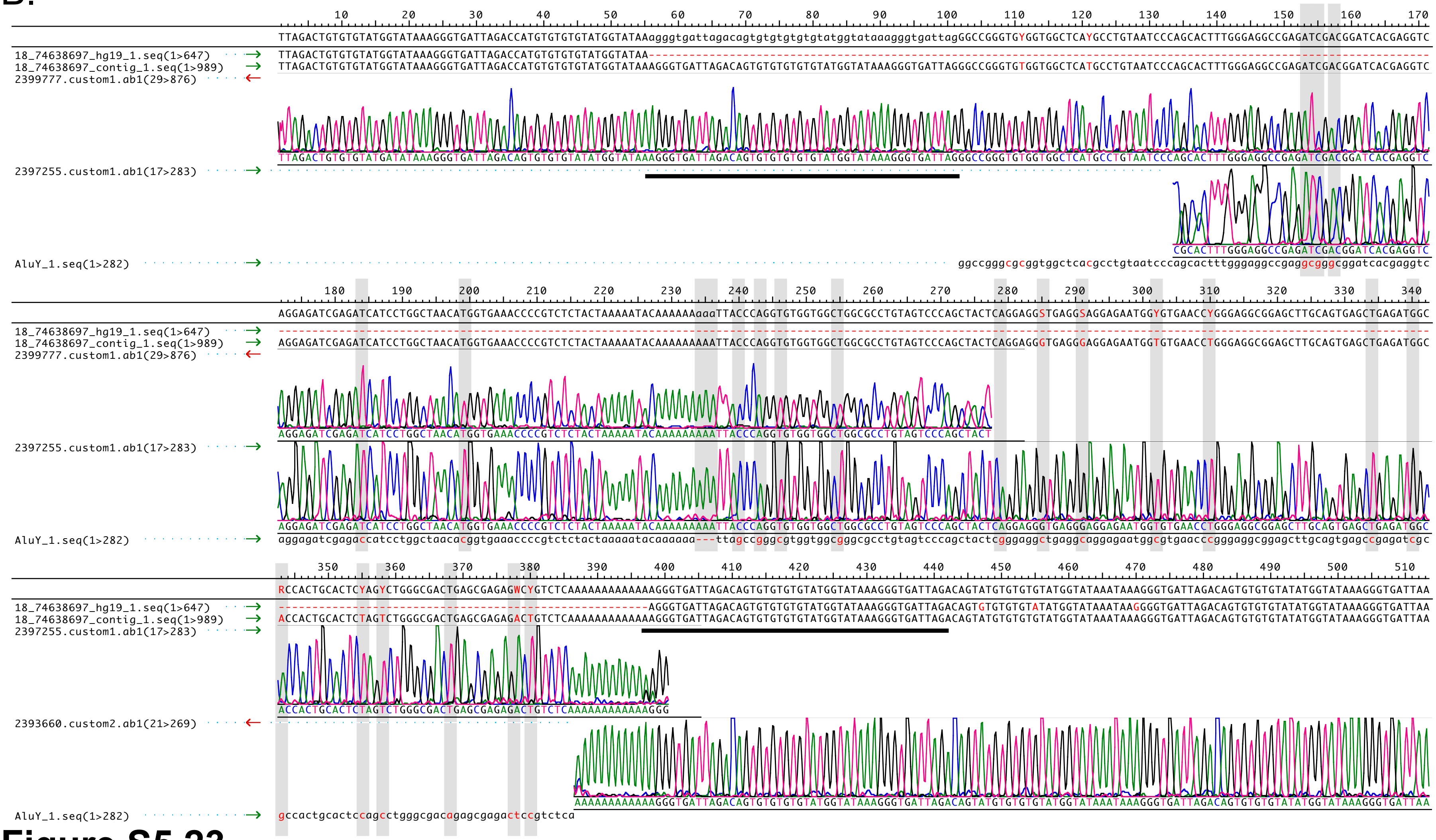

Figure S5.23

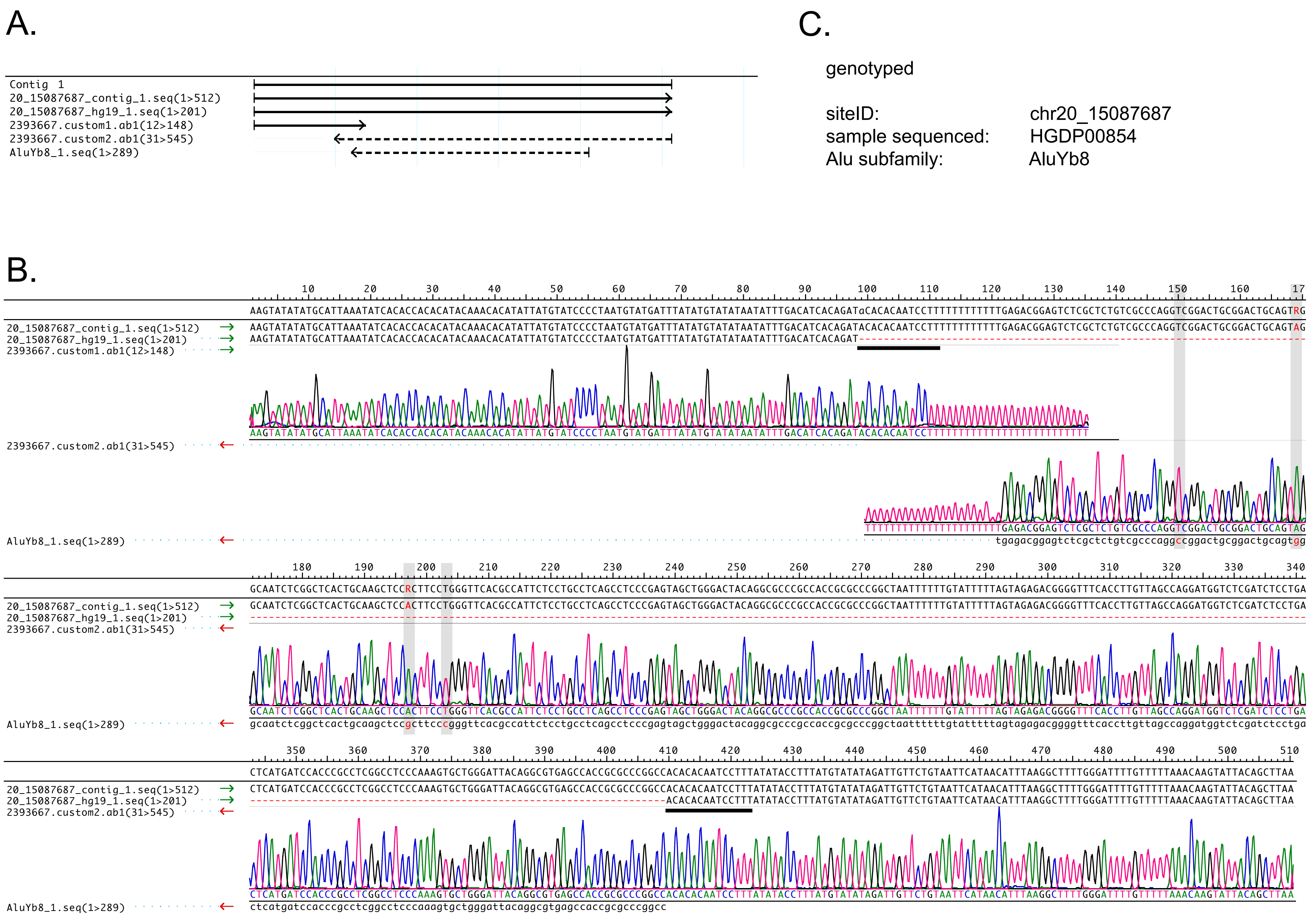
A.
Contig 1
20_15087687_contig_1.seq(1>512)
20_15087687_hg19_1.seq(1>201)
2393667.custom1.ab1(12>148)
2393667.custom2.ab1(31>545)
AluYb8_1.seq(1>289)
B.
C.
genotyped
siteID: chr20_15087687
sample sequenced: HGDP00854
Alu subfamily: AluYb8

Figure S5.24

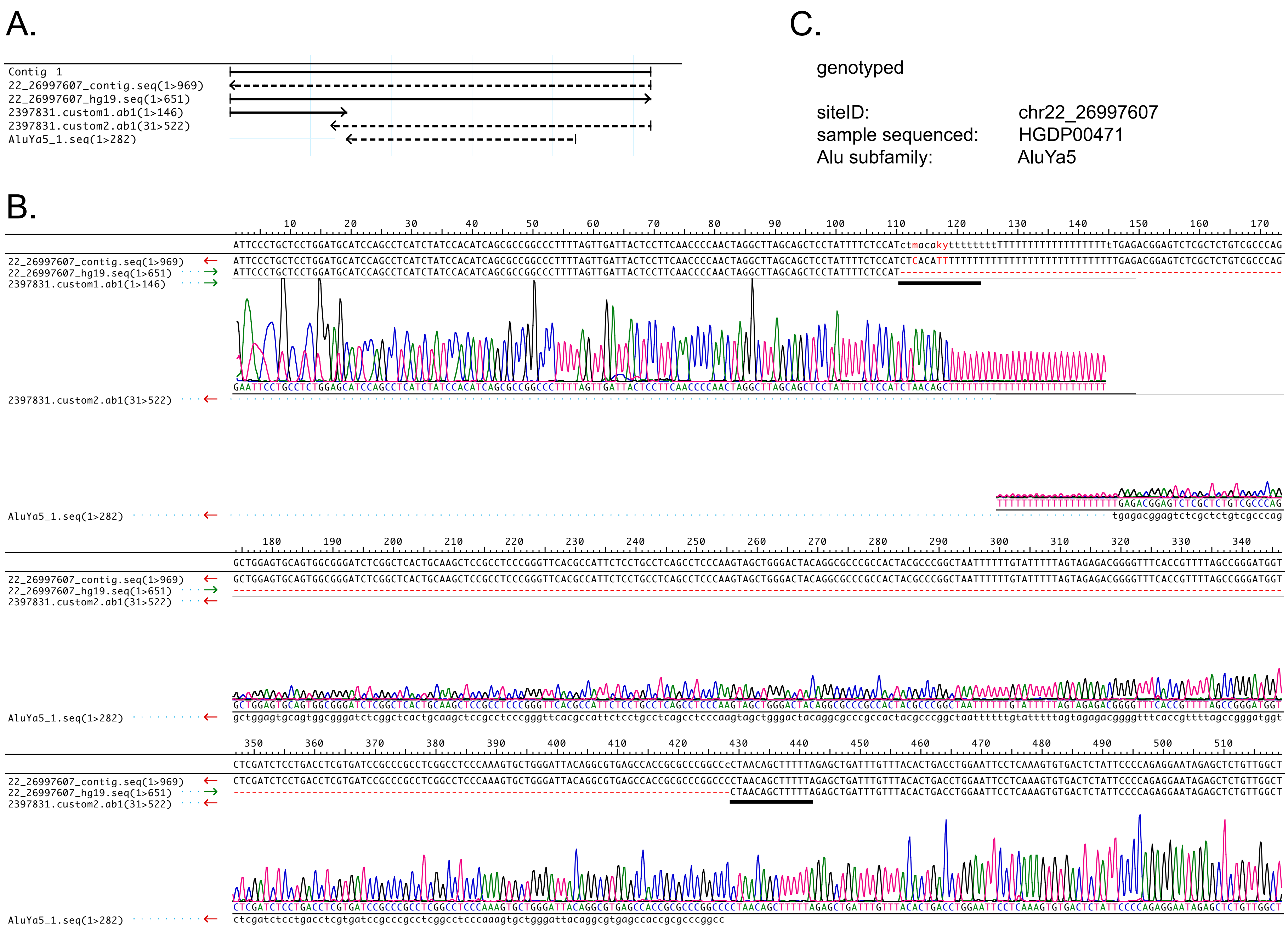
A.
B.
C.
genotyped
siteID: chr22_26997607
sample sequenced: HGDP00471
Alu subfamily: AluYa5

Figure S5.25

A.

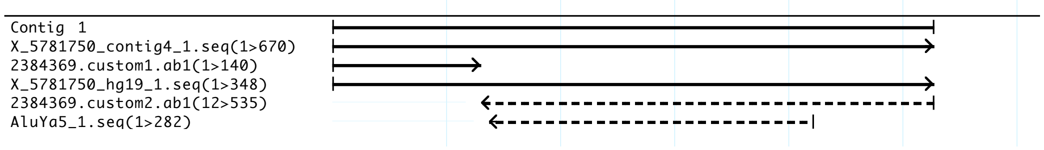

C.

genotyped

siteID: chrX_5781750
sample sequenced: HGDP00222
Alu subfamily: AluYa5

B.

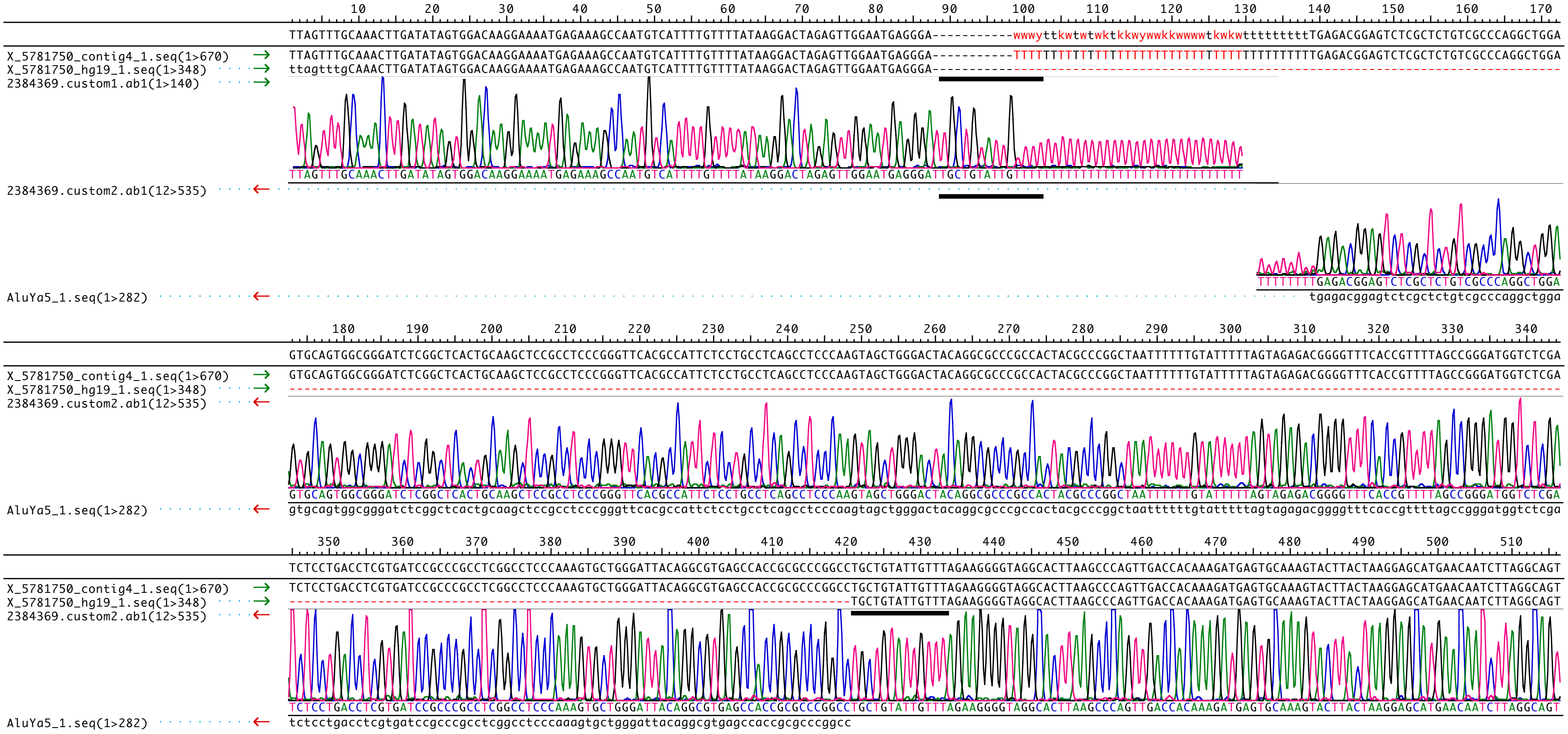

Figure S5.26

A.

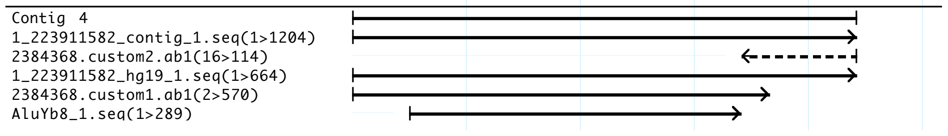

C.

not genotyped

siteID: chr1_223911582
sample sequenced: HGDP00967
Alu subfamily: AluYb8

B.

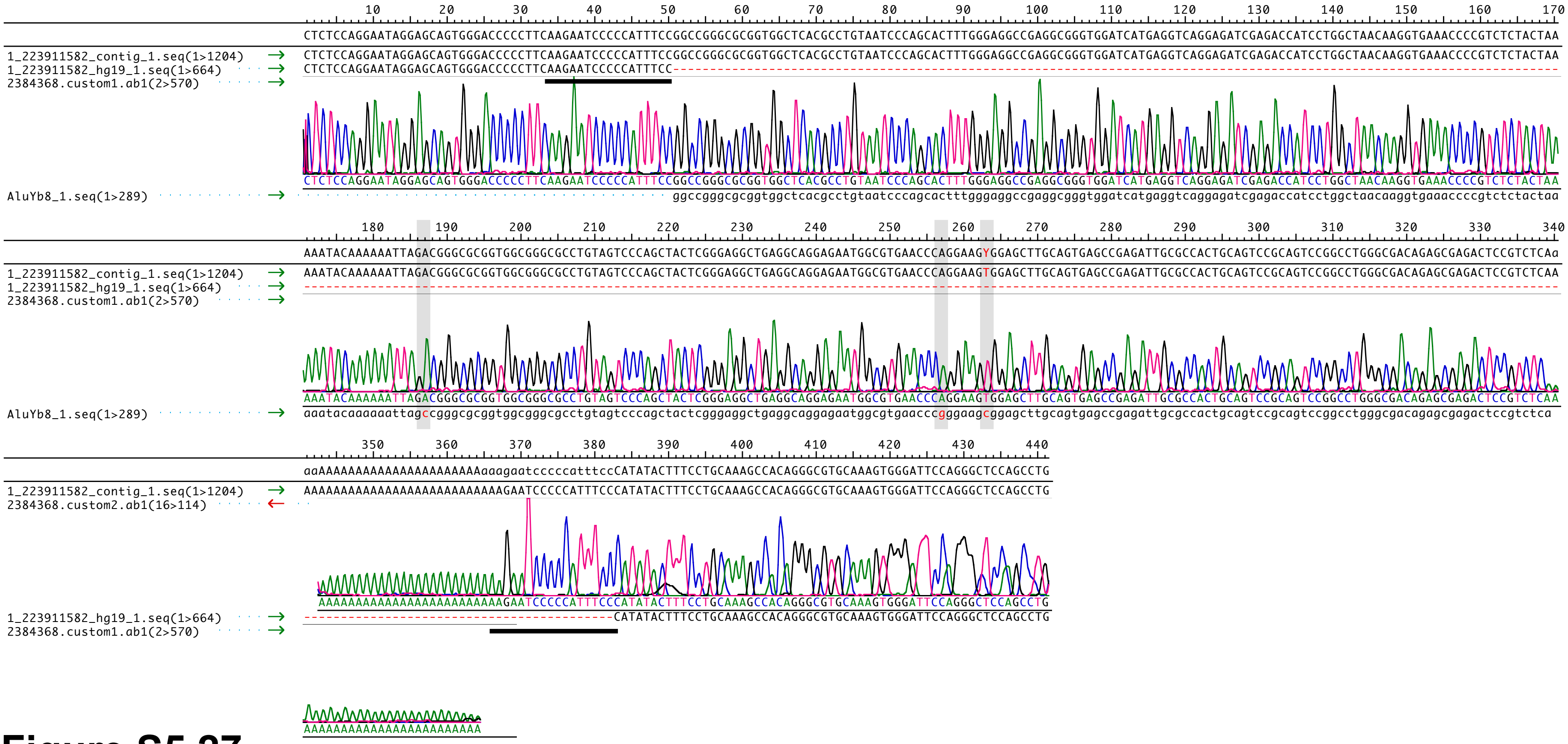

Figure S5.27

A.

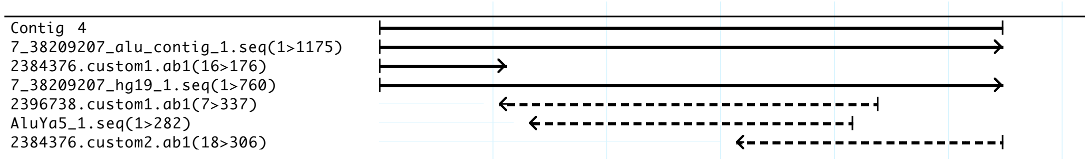

C.

not genotyped

siteID: chr7_38209207
sample sequenced: HGDP00232
Alu subfamily: AluYa4

B.

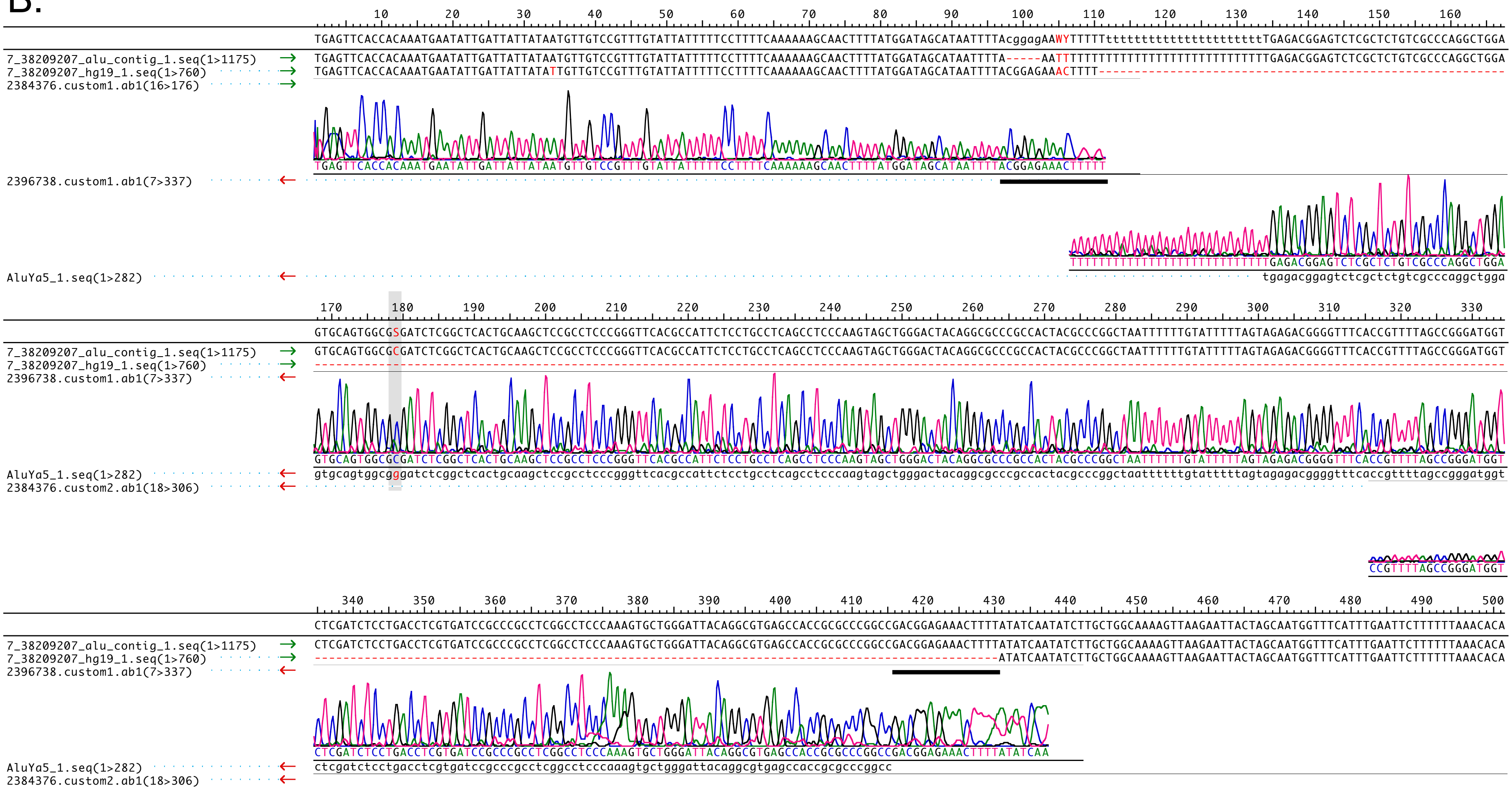

Figure S5.28

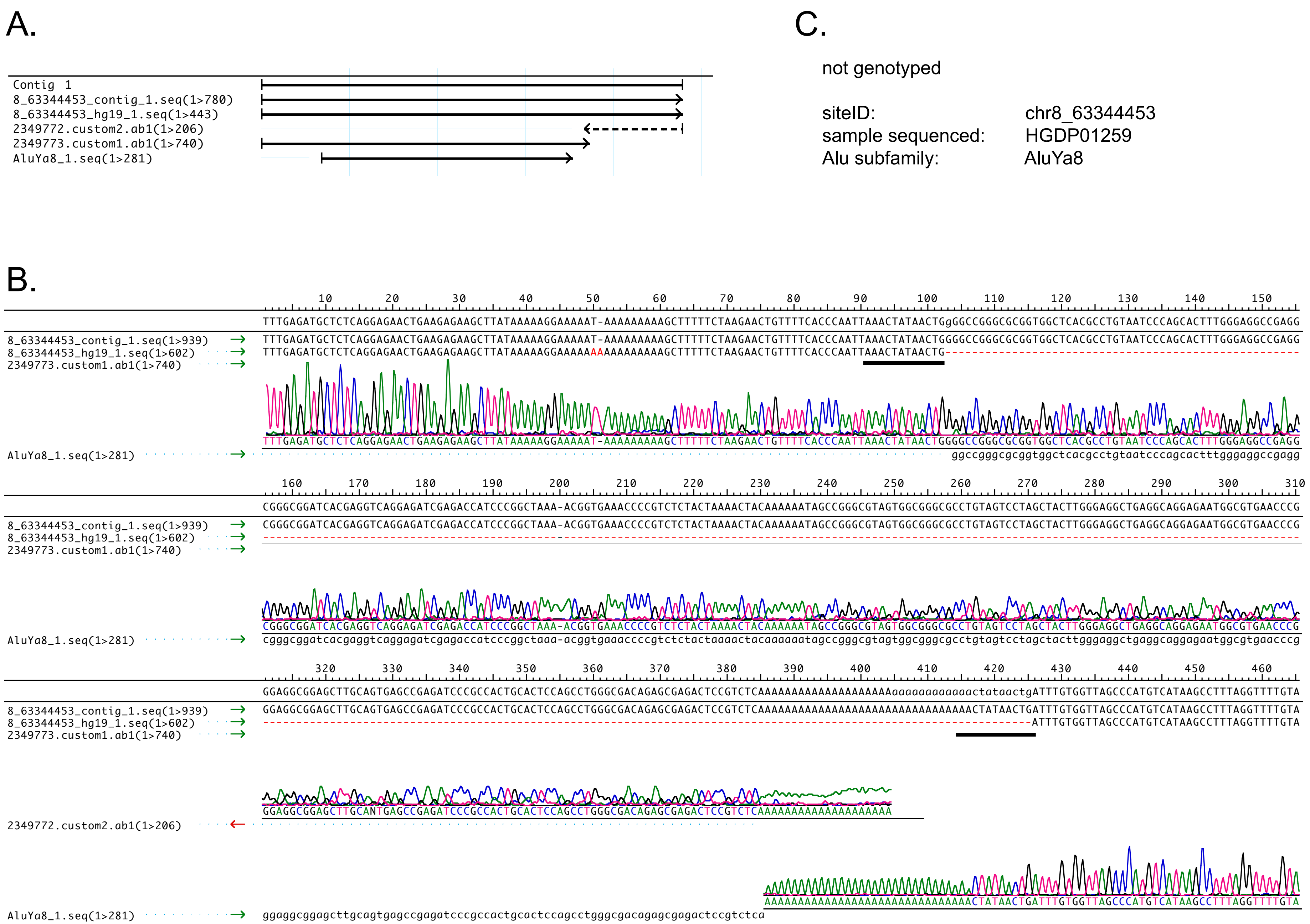

C.

not genotyped

siteID: chr8_63344453
sample sequenced: HGDP01259
Alu subfamily: AluYa8

Figure S5.29

A.

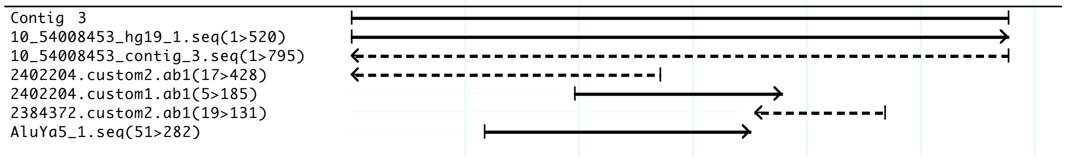

C.

not genotyped

siteID: chr10_54008453
sample sequenced: HGDP00992
Alu subfamily: AluYa5

B.

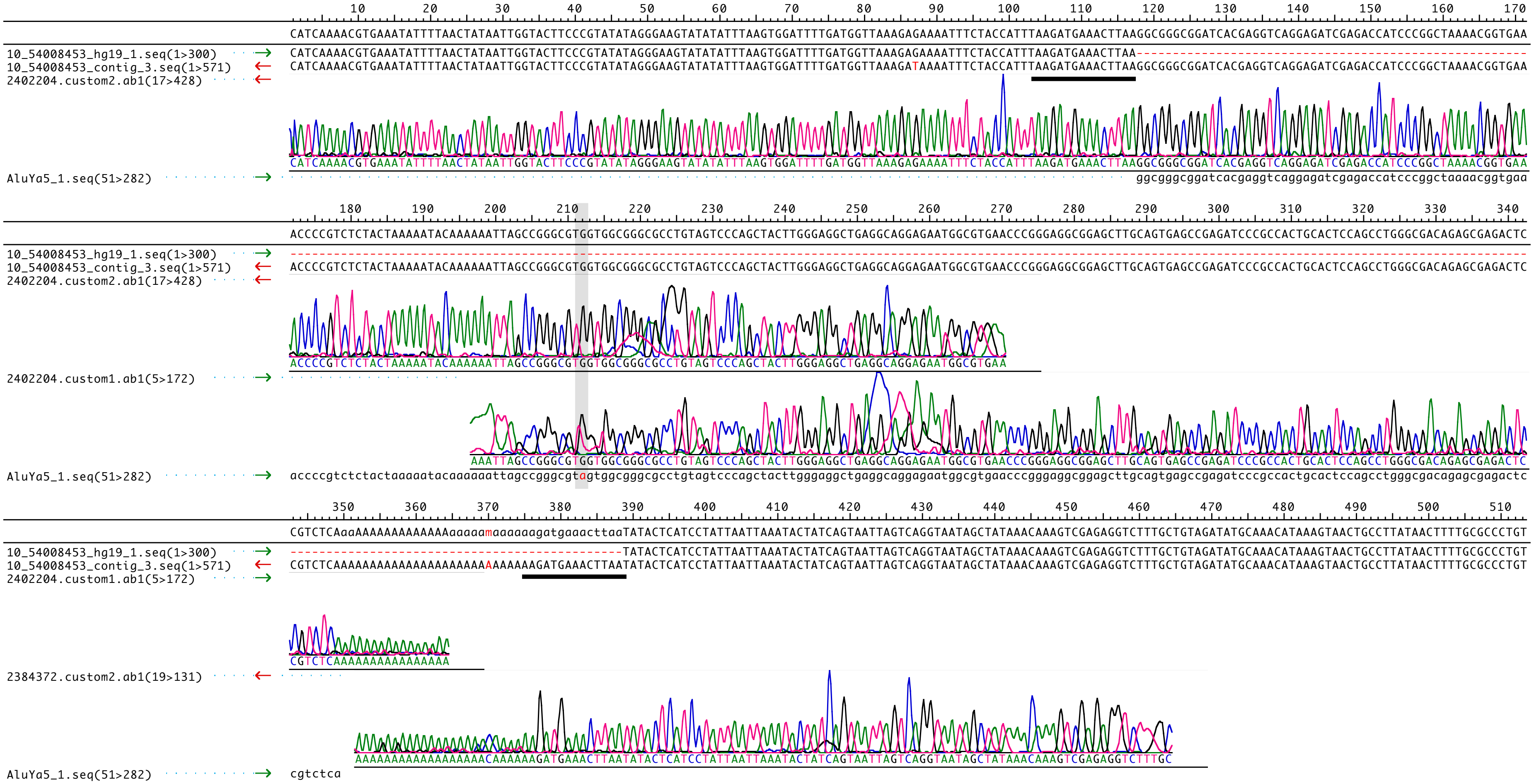

Figure S5.30

A.

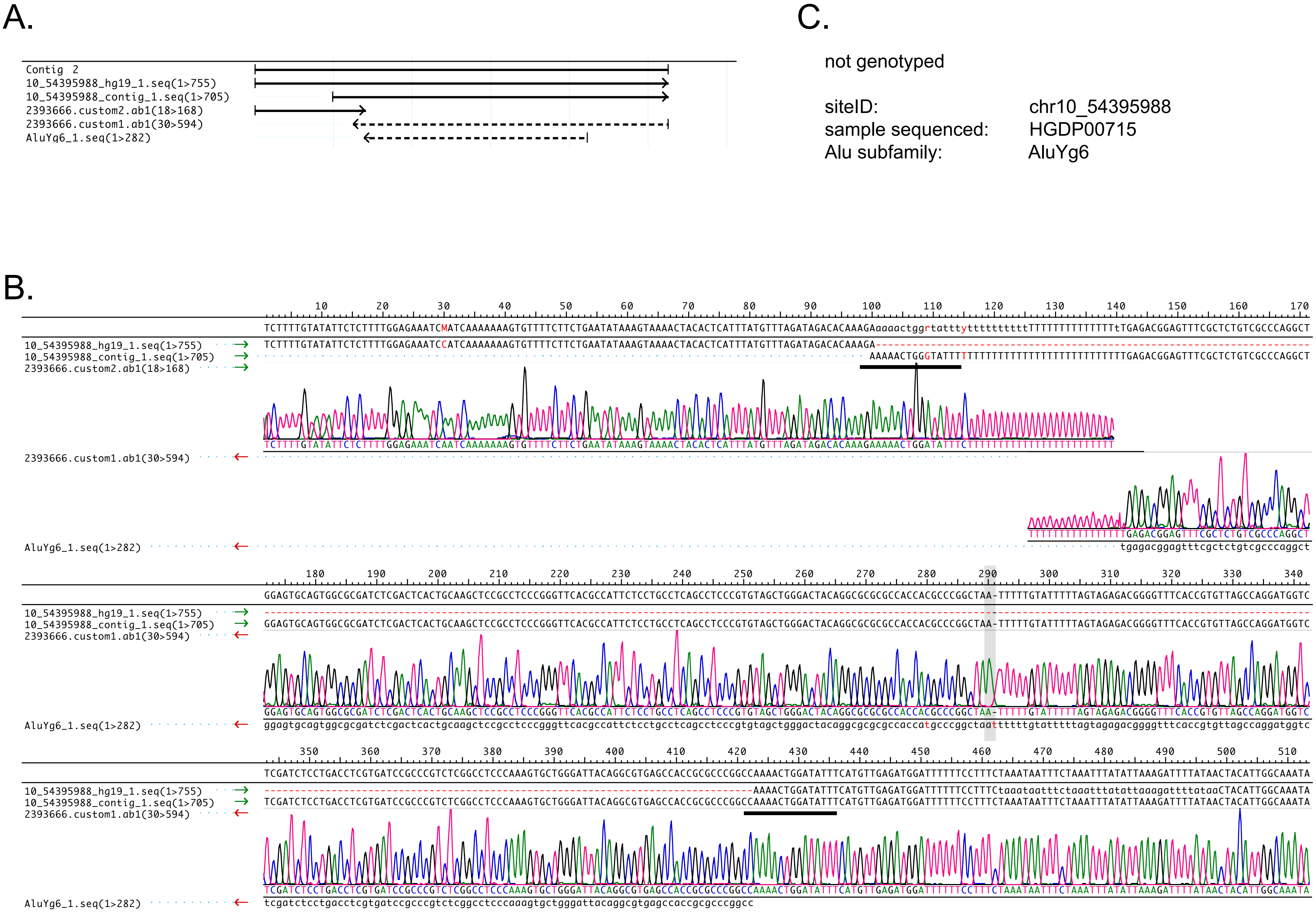

C.

not genotyped

siteID: chr10_54395988
sample sequenced: HGDP00715
Alu subfamily: AluYg6

Figure S5.31

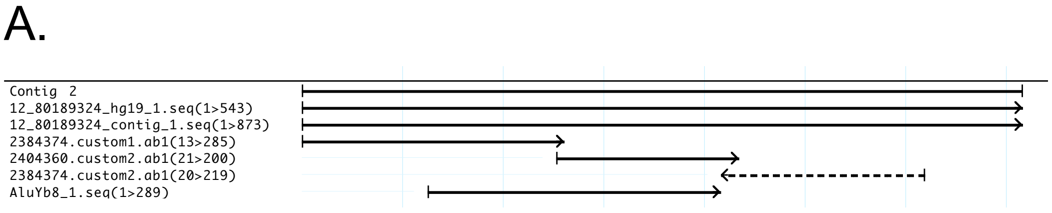

C.

not genotyped

siteID: chr12_80189324

sample sequenced: HGDP001029

Alu subfamily: AluYb8

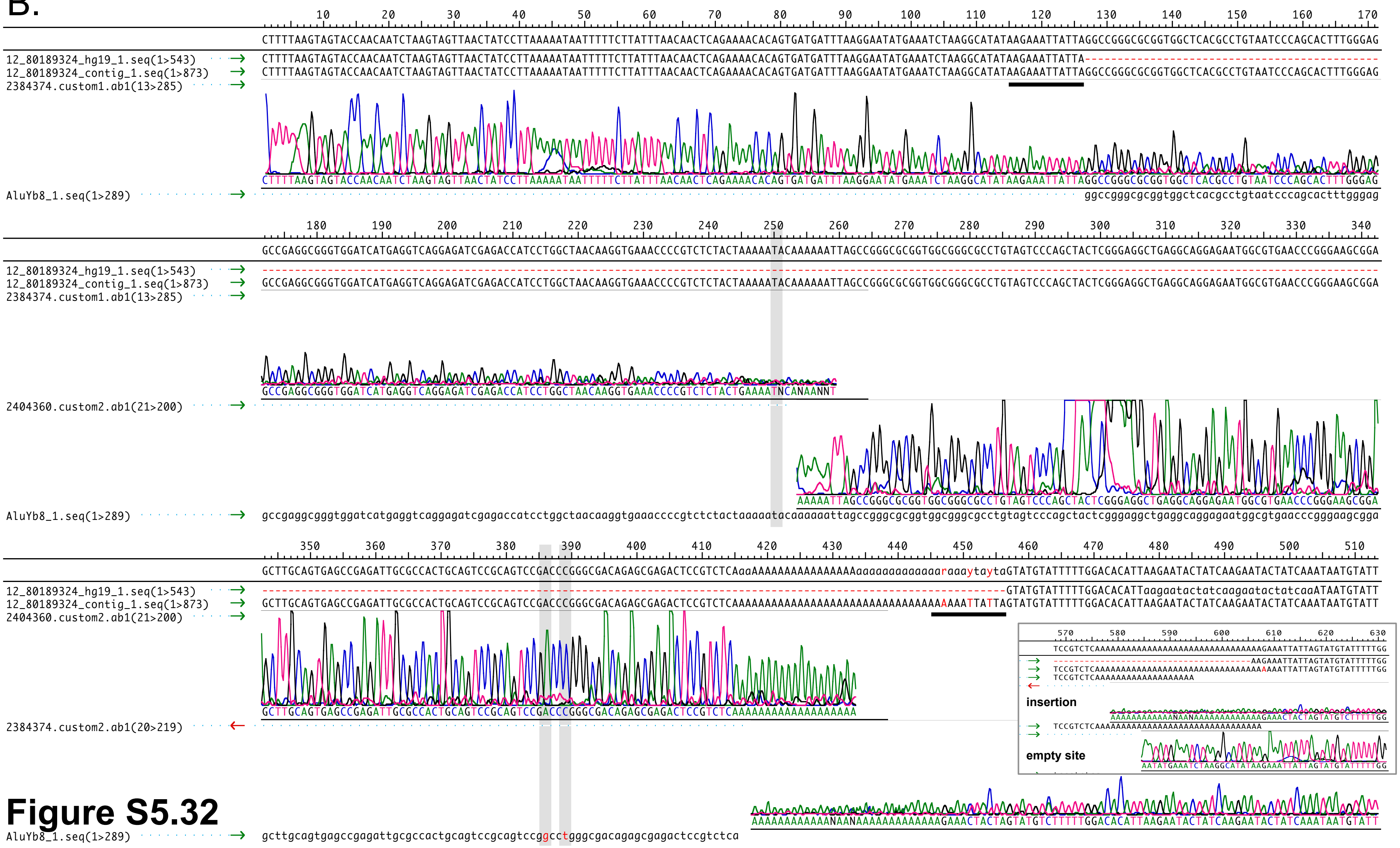

Figure S5.32

A.

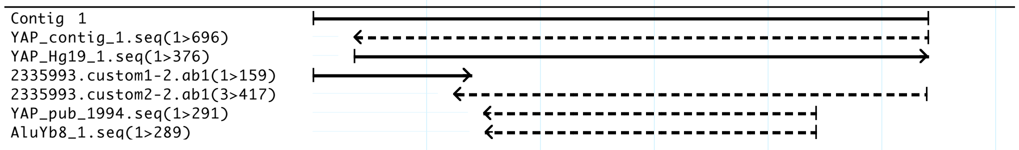

C.

not genotyped

siteID: chrY_21611998
sample sequenced: HGDP00213
Alu subfamily: AluYb8

B.

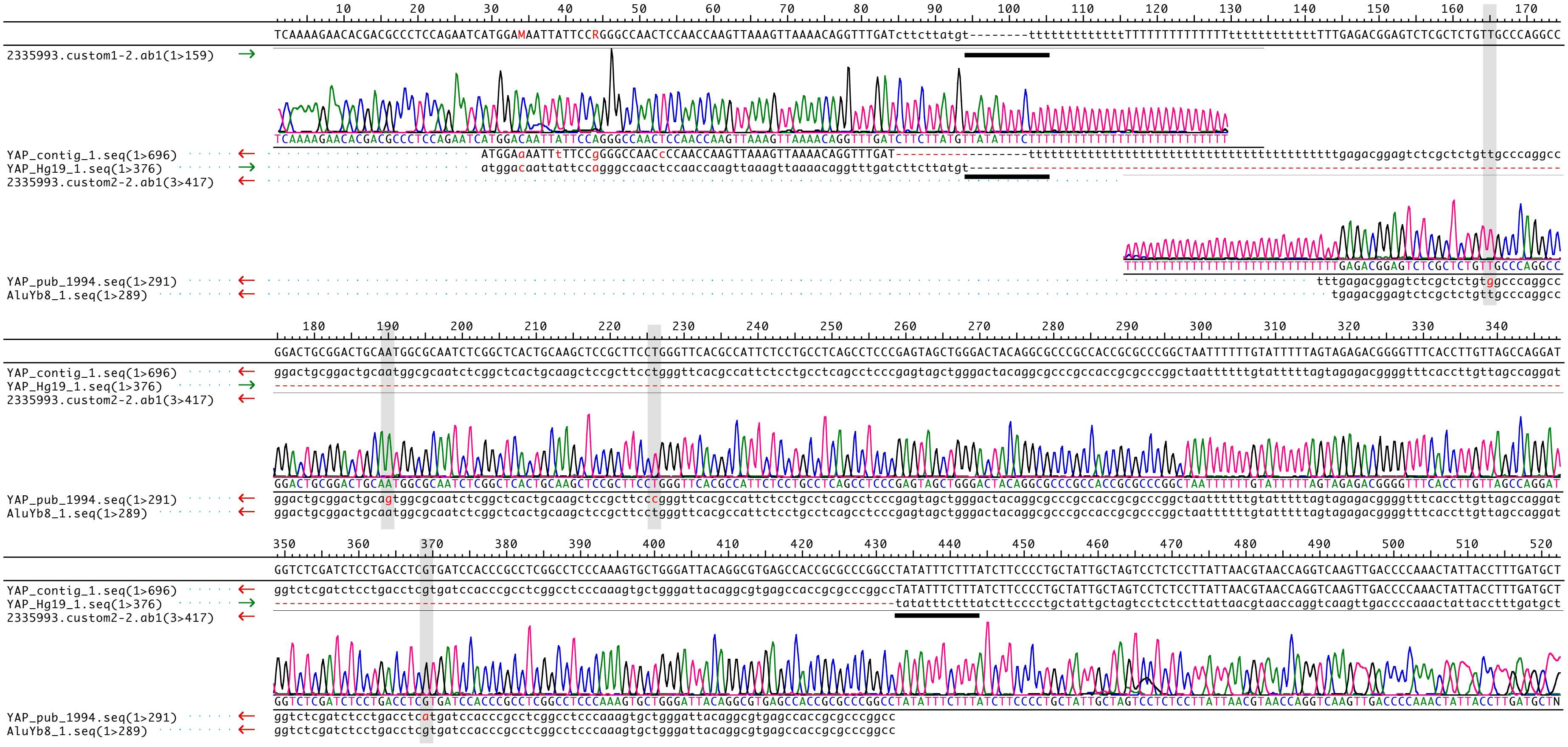

Figure S5.33
