# Supplementary material for: Discovery and characterization of Alu repeat sequences via precise local read assembly
Source: Nucleic Acids Res. 2015 Oct 25;43(21):10292–307. doi: 10.1093/nar/gkv1089 (PMC4666360; doi:10.1093/nar/gkv1089)
Supplement: SUPPLEMENTARY DATA [file supp_gkv1089_nar-01397-h-2015-File011.pdf]

A.

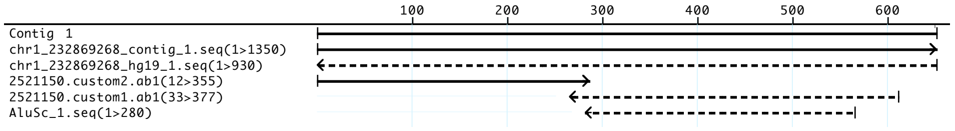

C.

genotyped

siteID:	chr1_232869268
sample sequenced:	HGDP00476
Alu subfamily:	AluSc
percent divergence	11.0%

B.

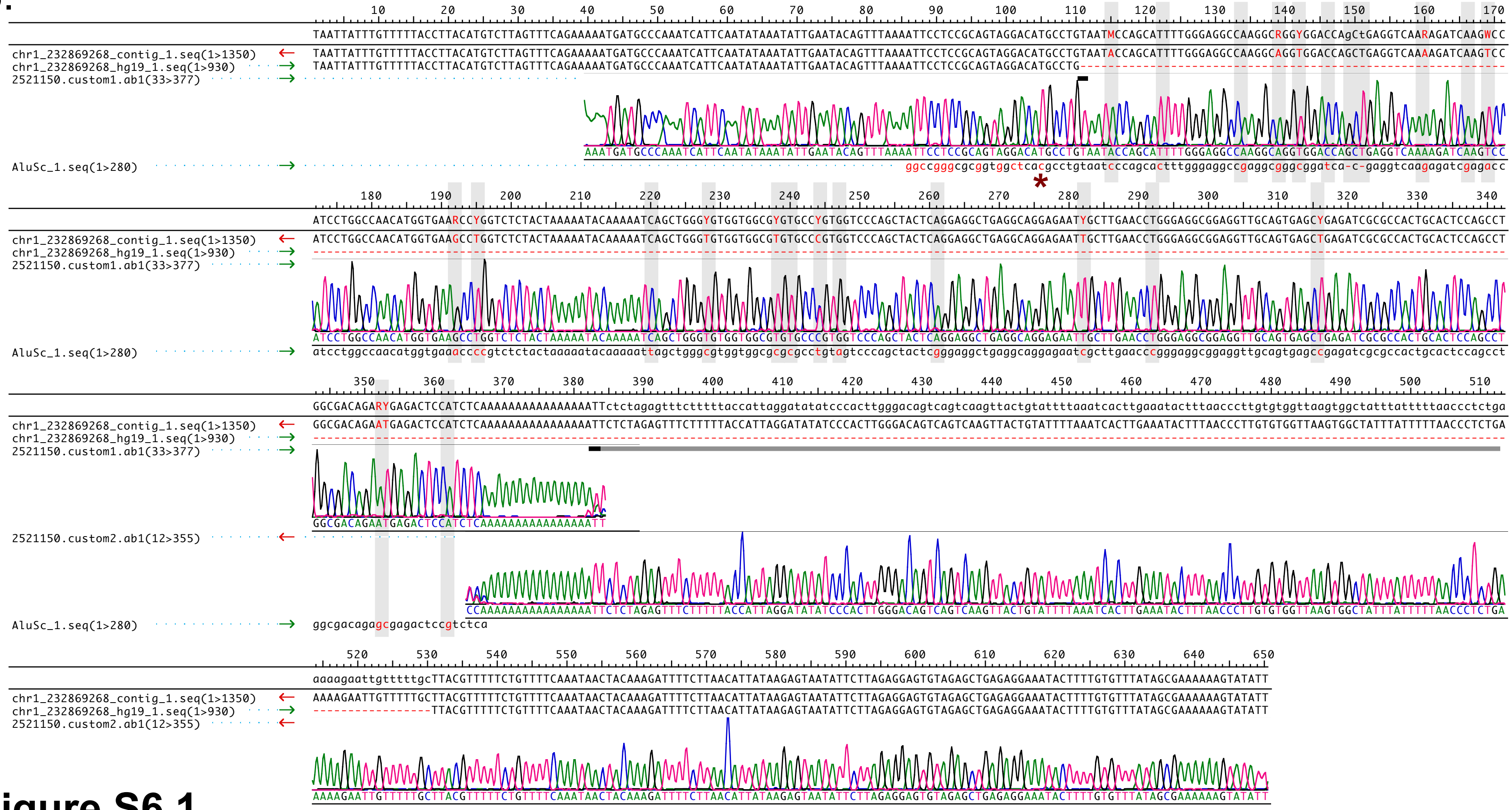

Figure S6.1

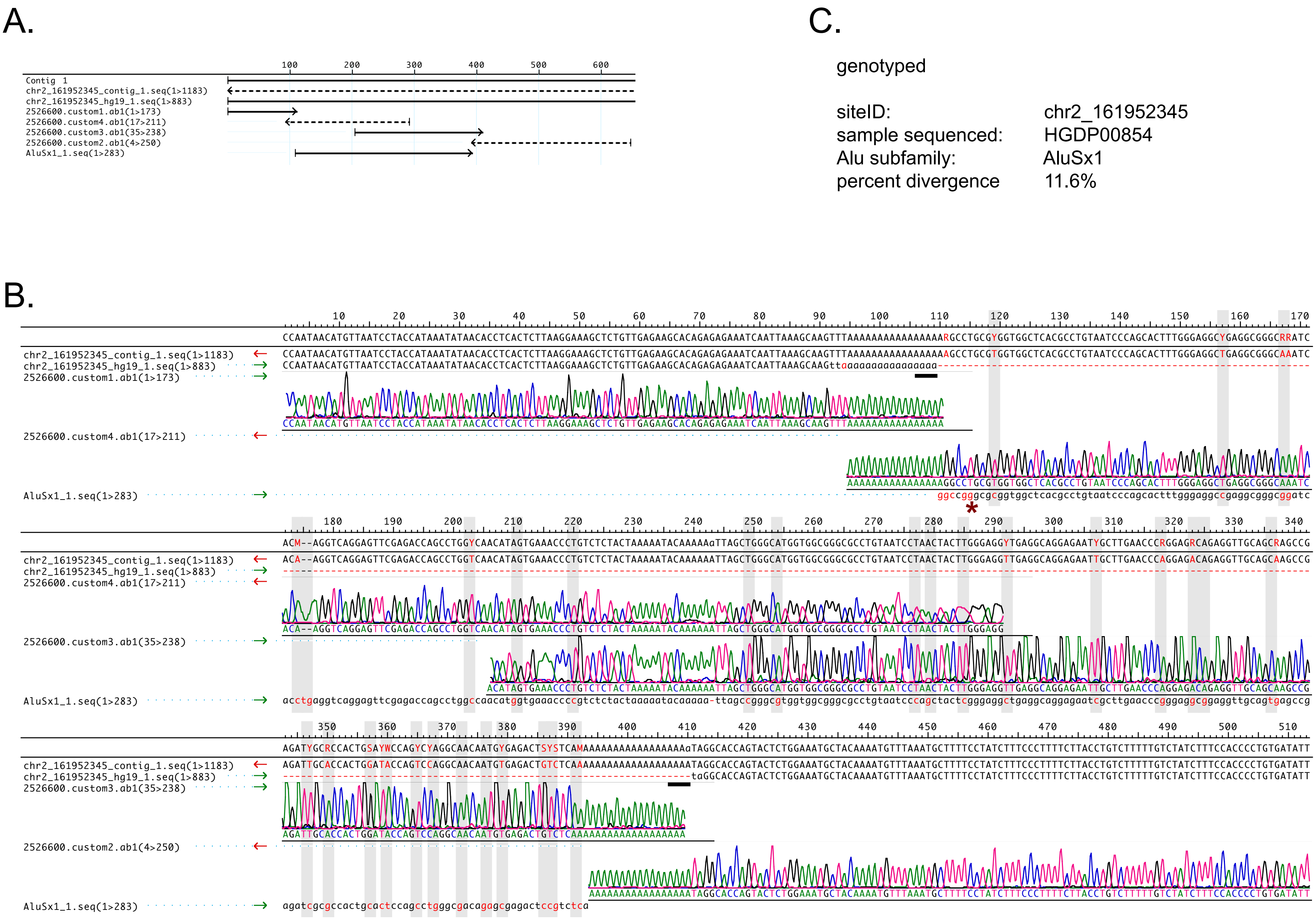

C.

genotyped

siteID: chr2_161952345
sample sequenced: HGDP00854
Alu subfamily: AluSx1
percent divergence 11.6%

Figure S6.2

A.

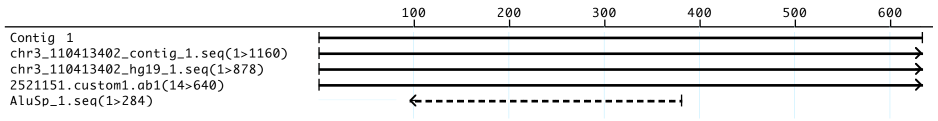

C.

genotyped

siteID:	chr3_110413402
sample sequenced:	HGDP00711
Alu subfamily:	AluSp
percent divergence	11.2%

B.

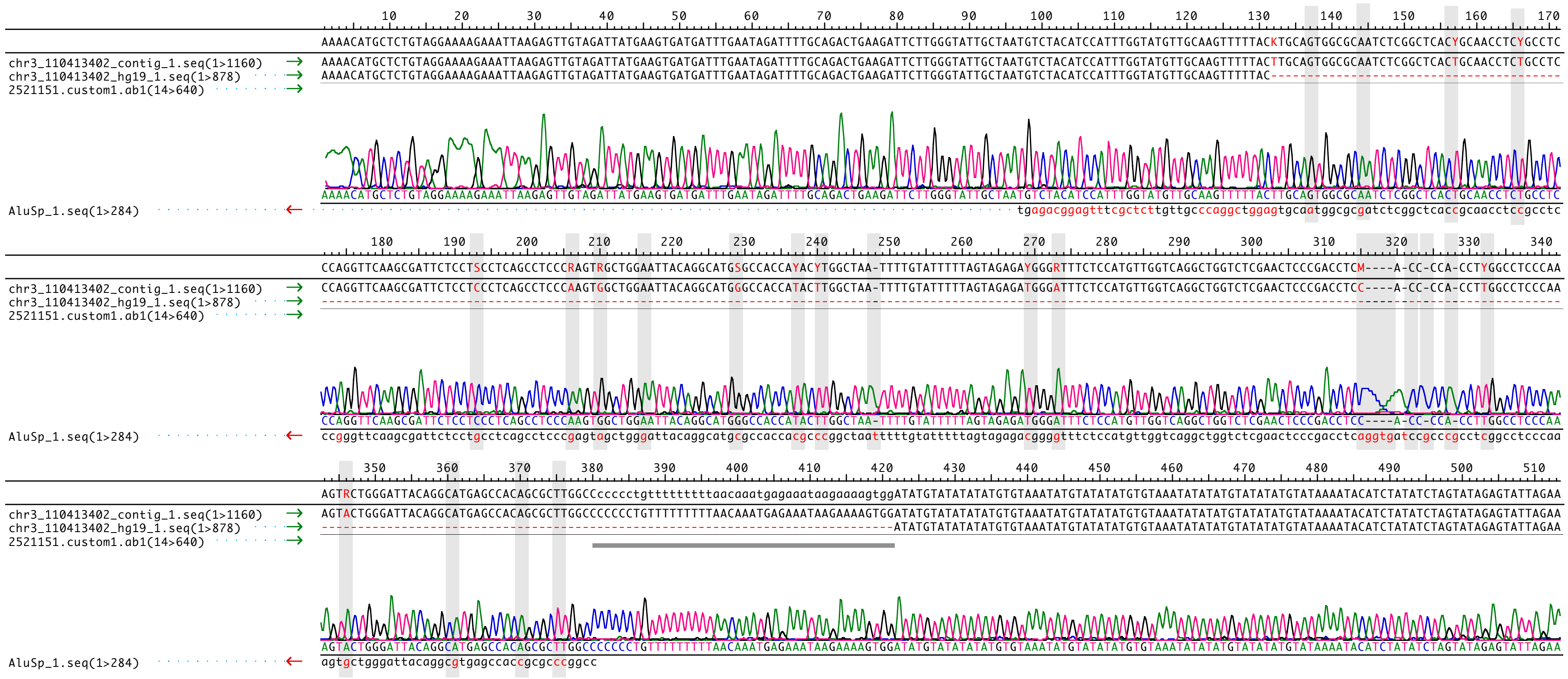

Figure S6.3

A.

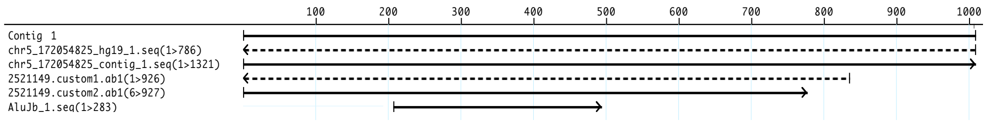

C.

genotyped

siteID:	chr5_172054825
sample sequenced:	HGDP00948
Alu subfamily:	AluJb
percent divergence	13.8%

B.

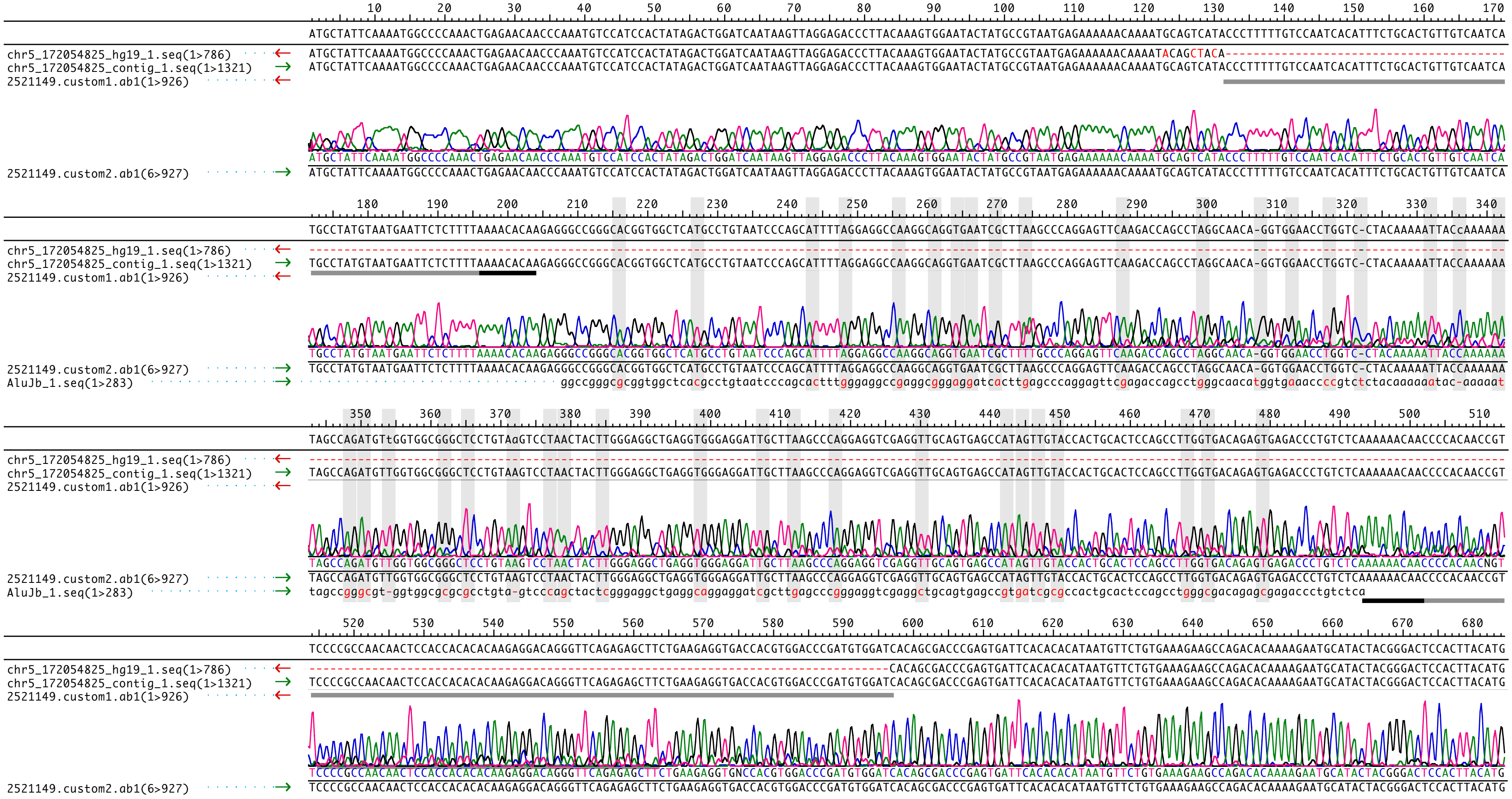

Figure S6.4

A.

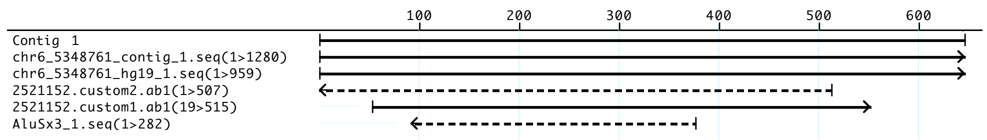

C.

genotyped

siteID:	chr6_5348761
sample sequenced:	HGDP00456
Alu subfamily:	AluSx3
percent divergence	13.9%

B.

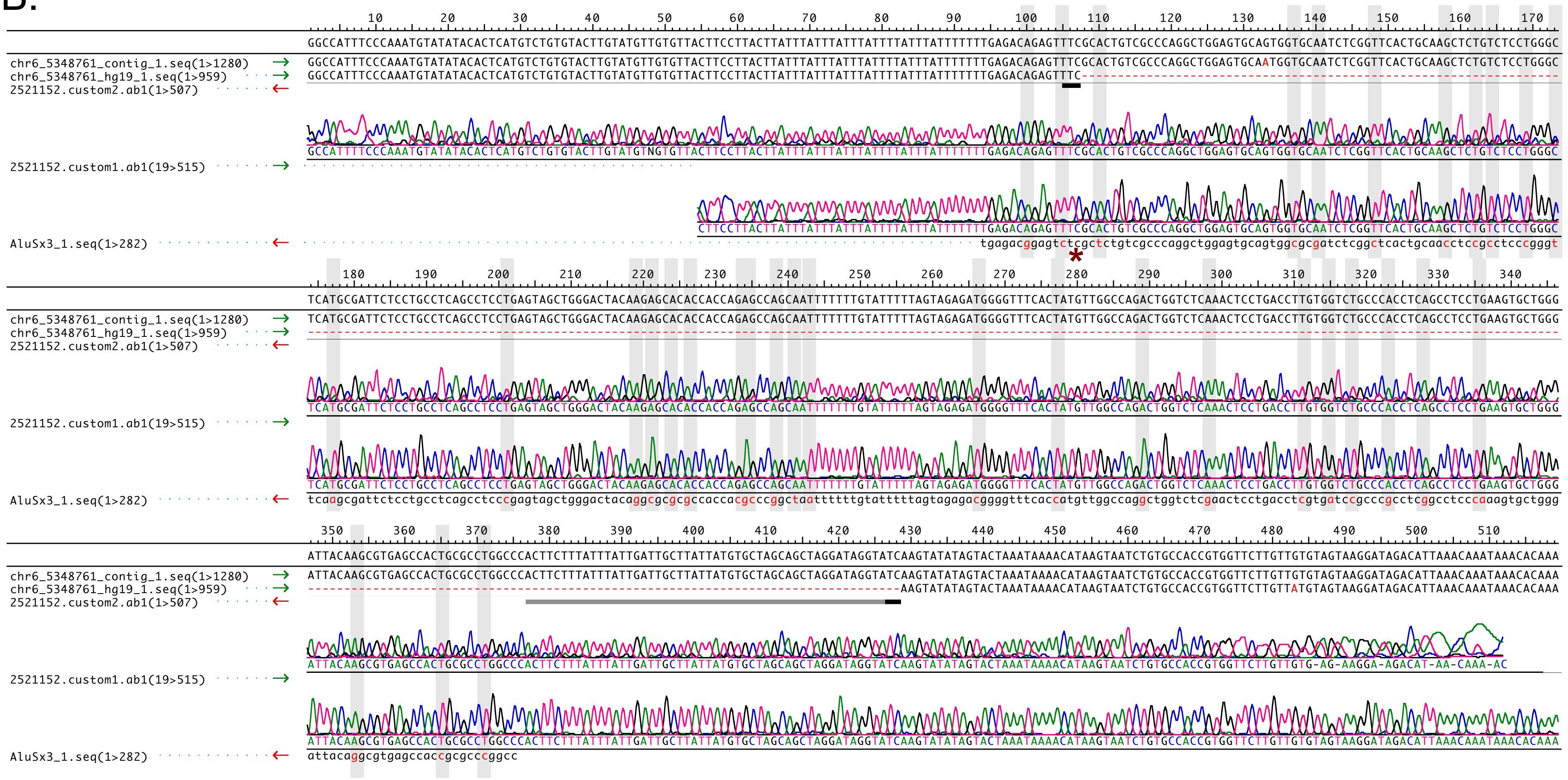

Figure S6.5

A.

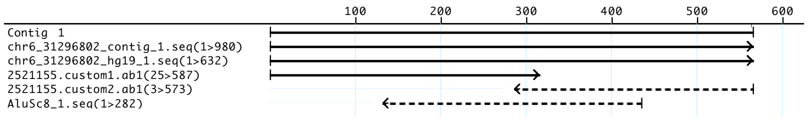

B.

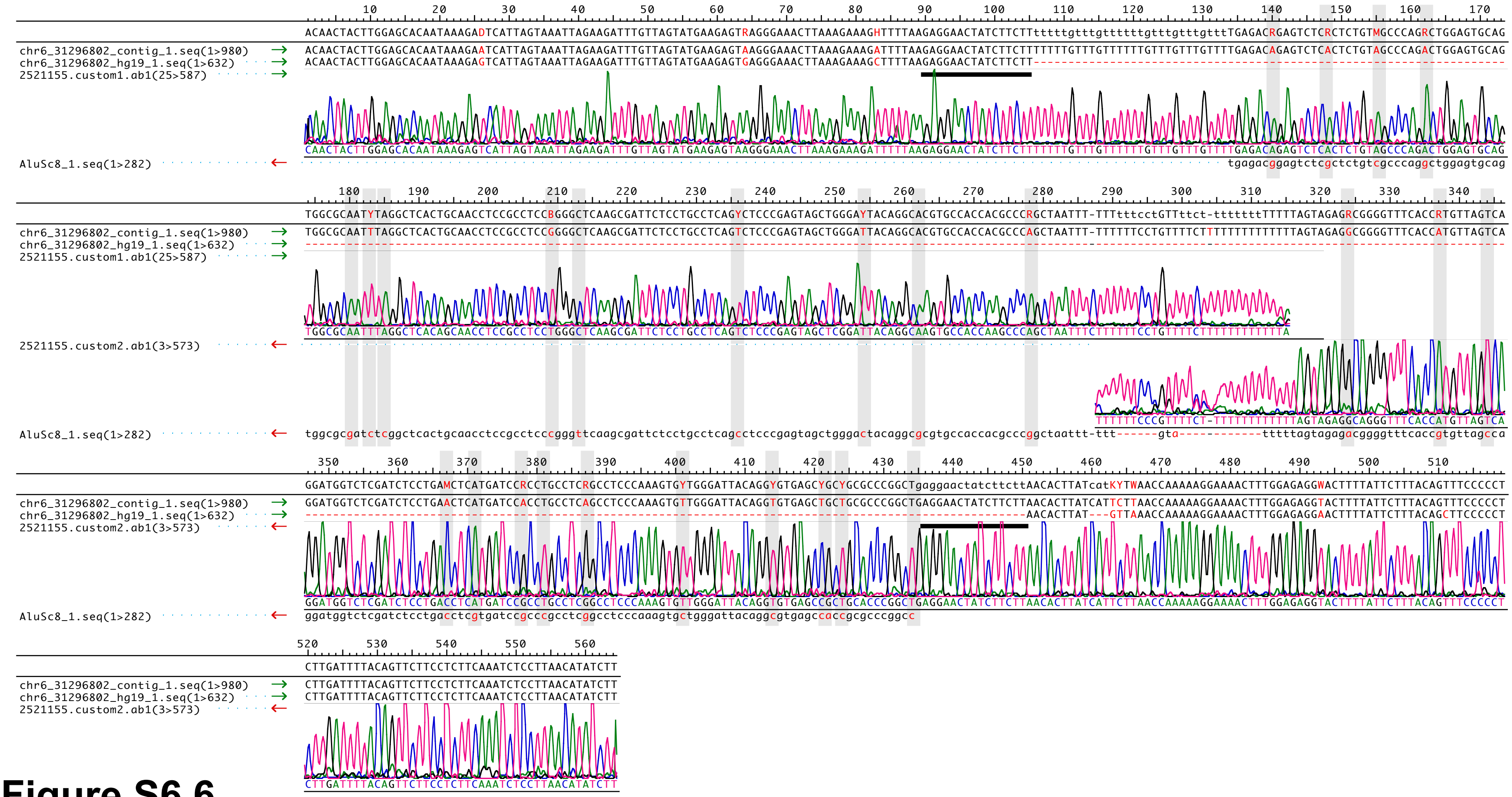

C.

genotyped

siteID:	chr6_31296802
sample sequenced:	HGDP00232
Alu subfamily:	AluSc8
percent divergence	9.8%

Figure S6.6

A.

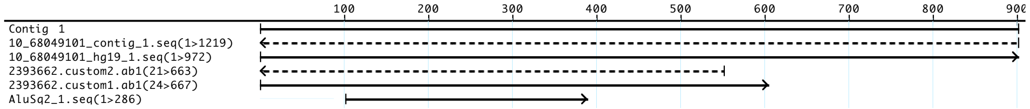

B.

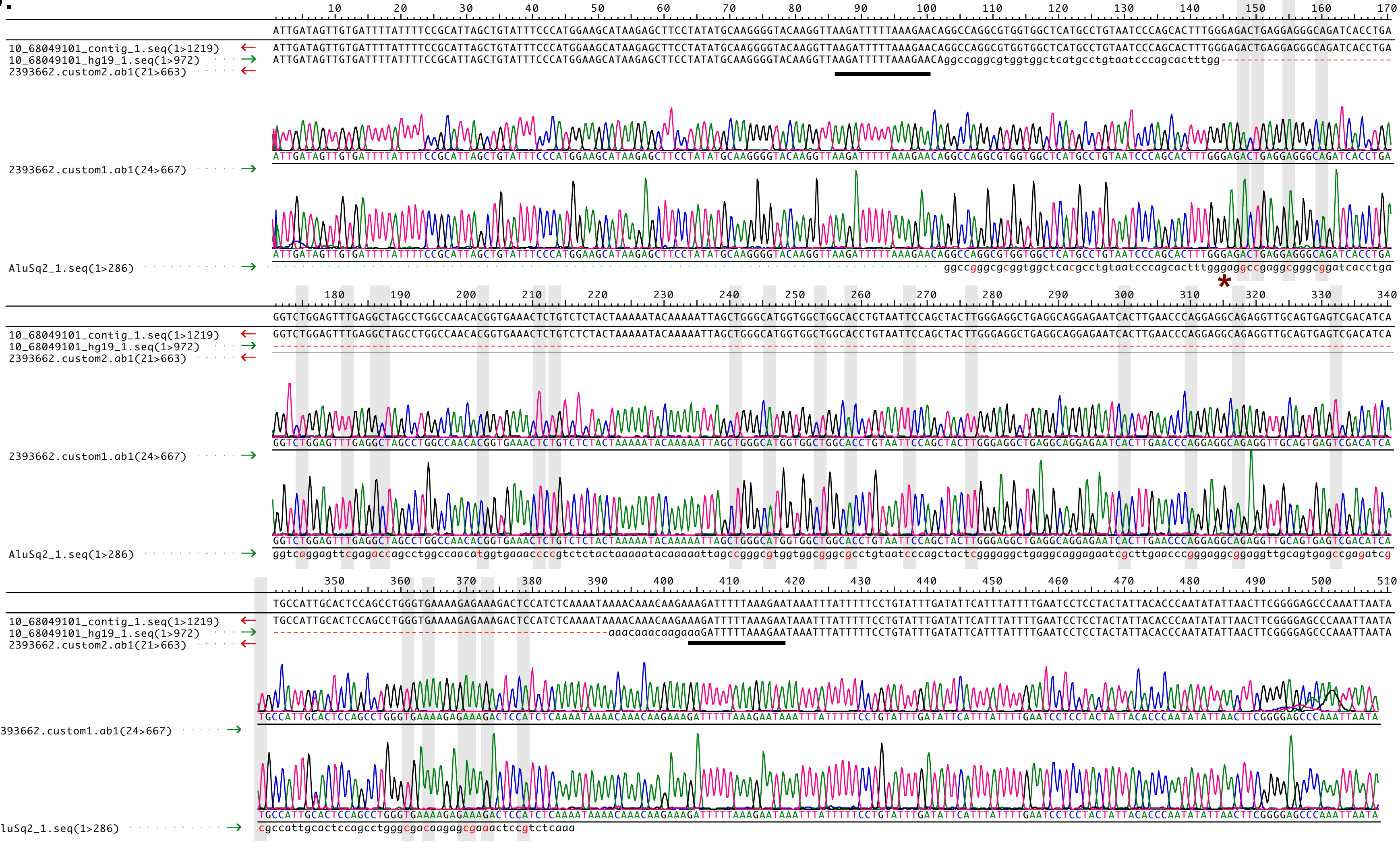

C.

genotyped

siteID:	chr10_68049101
sample sequenced:	HGDP00474
Alu subfamily:	AluSq2
percent divergence	12.2%

Figure S6.7

A.

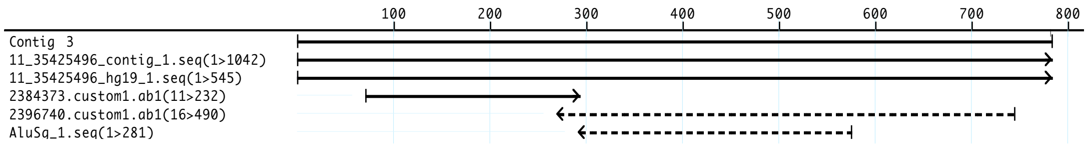

C.

genotyped

siteID:	chr11_35425496
sample sequenced:	HGDP00857
Alu subfamily:	AluSx3
percent divergence	13.0%

B.

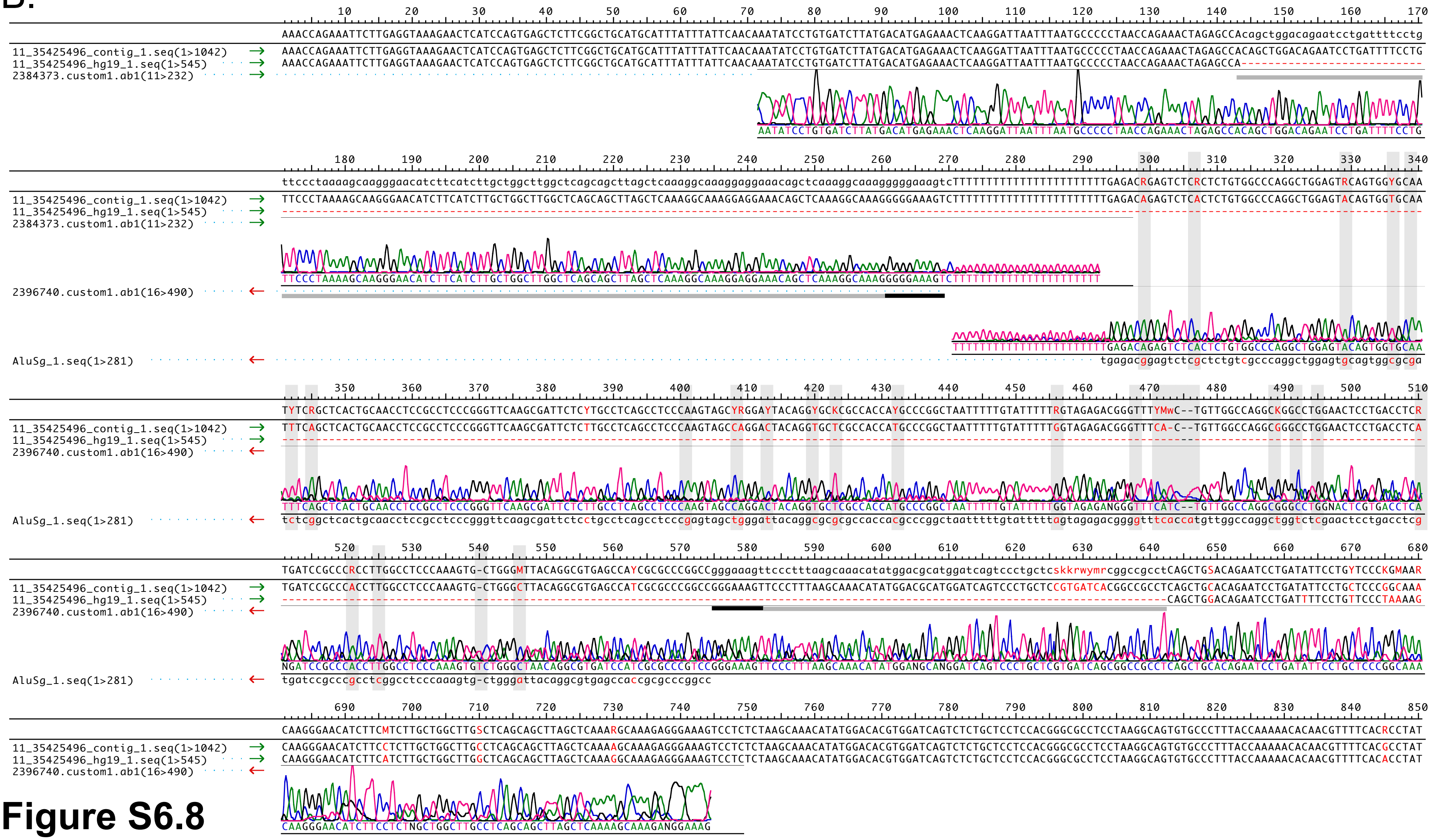

Figure S6.8

A.

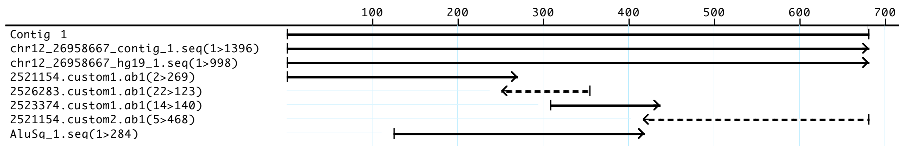

B.

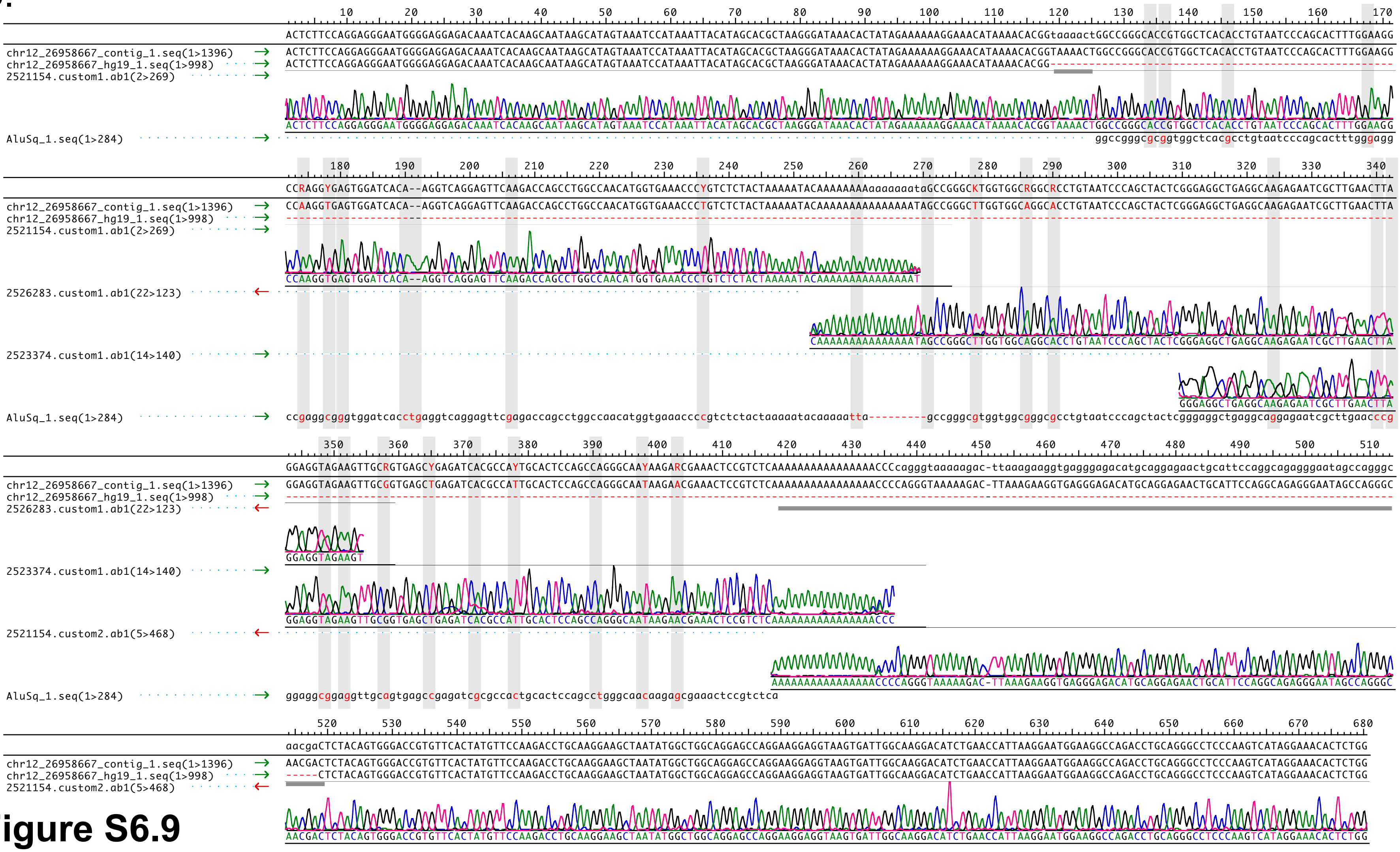

C.

genotyped

siteID:	chr12_26958667
sample sequenced:	HGDP00471
Alu subfamily:	AluSq
percent divergence	9.4%

Figure S6.9

A.

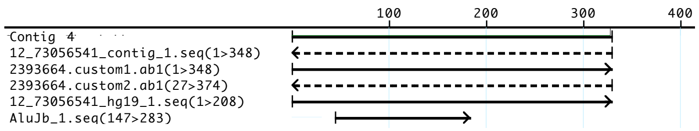

B.

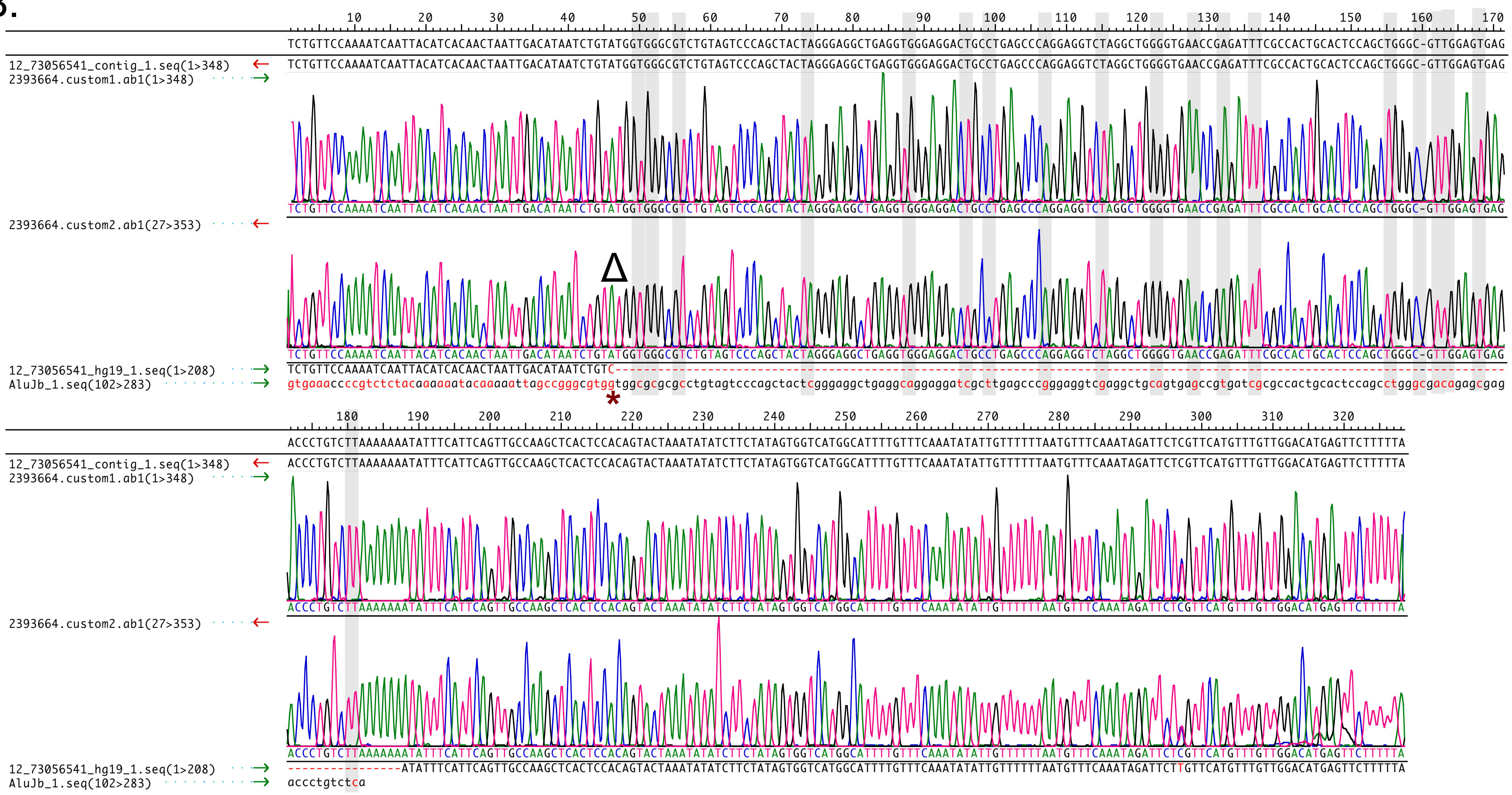

C.

not genotyped

siteID:	chr12_73056541
sample sequenced:	HGDP00987
Alu subfamily:	AluJb
percent divergence	14.2%

Figure S6.10

A.

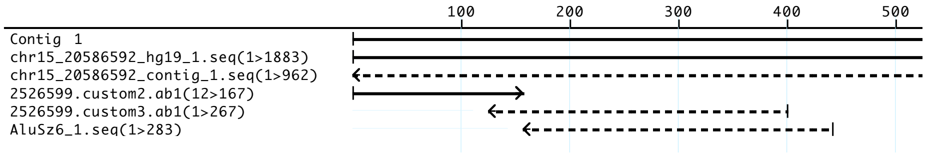

C.

genotyped

siteID: chr15_20586592
sample sequenced: HGDP00854
Alu subfamily: AluSz6
percent divergence 3.1%

B.

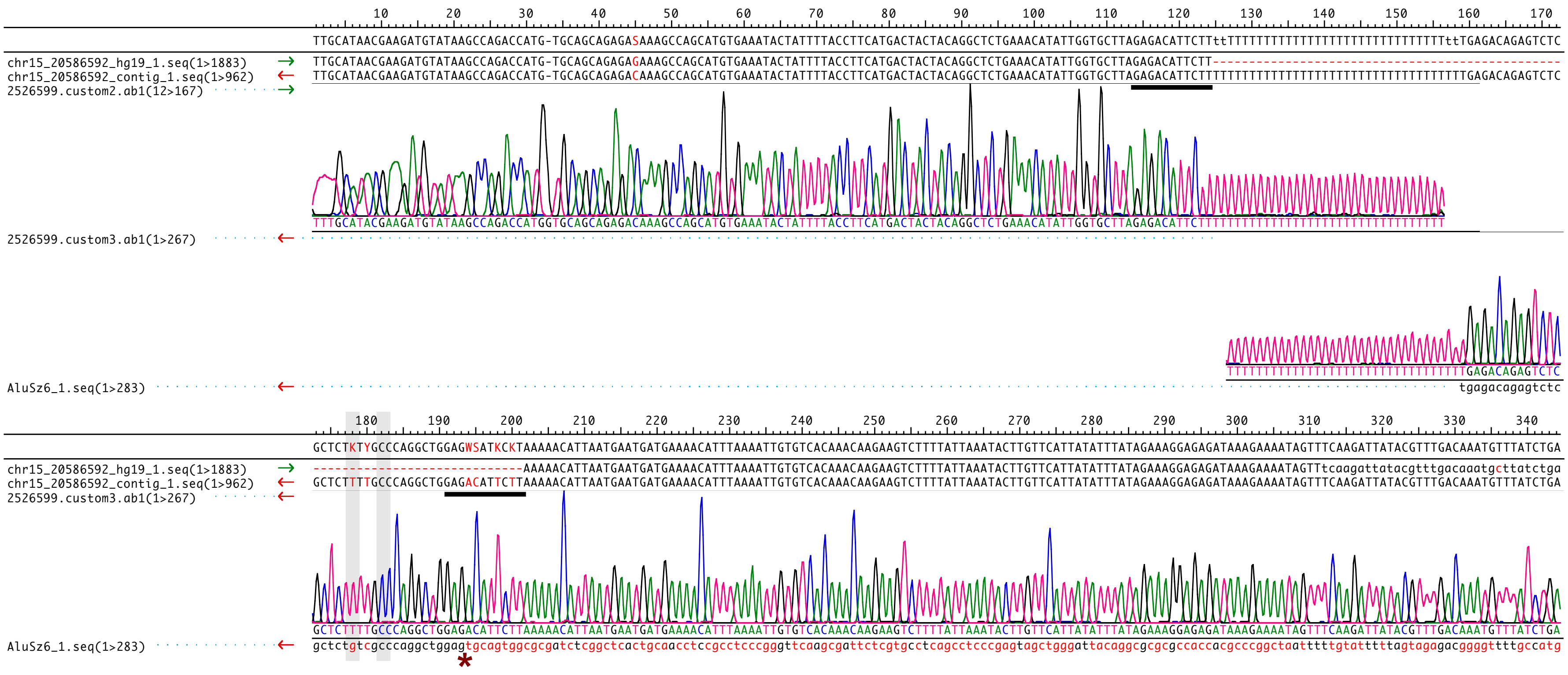

Figure S6.11

A.

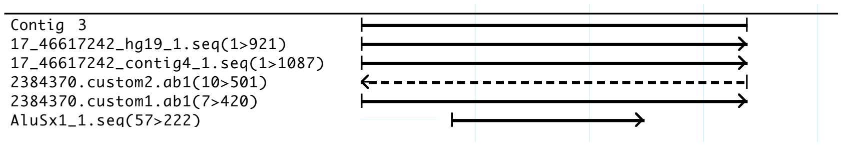

C.

genotyped

siteID:	chr17_46617242
sample sequenced:	HGDP00858
Alu subfamily:	AluSx3
percent divergence	12.2%

B.

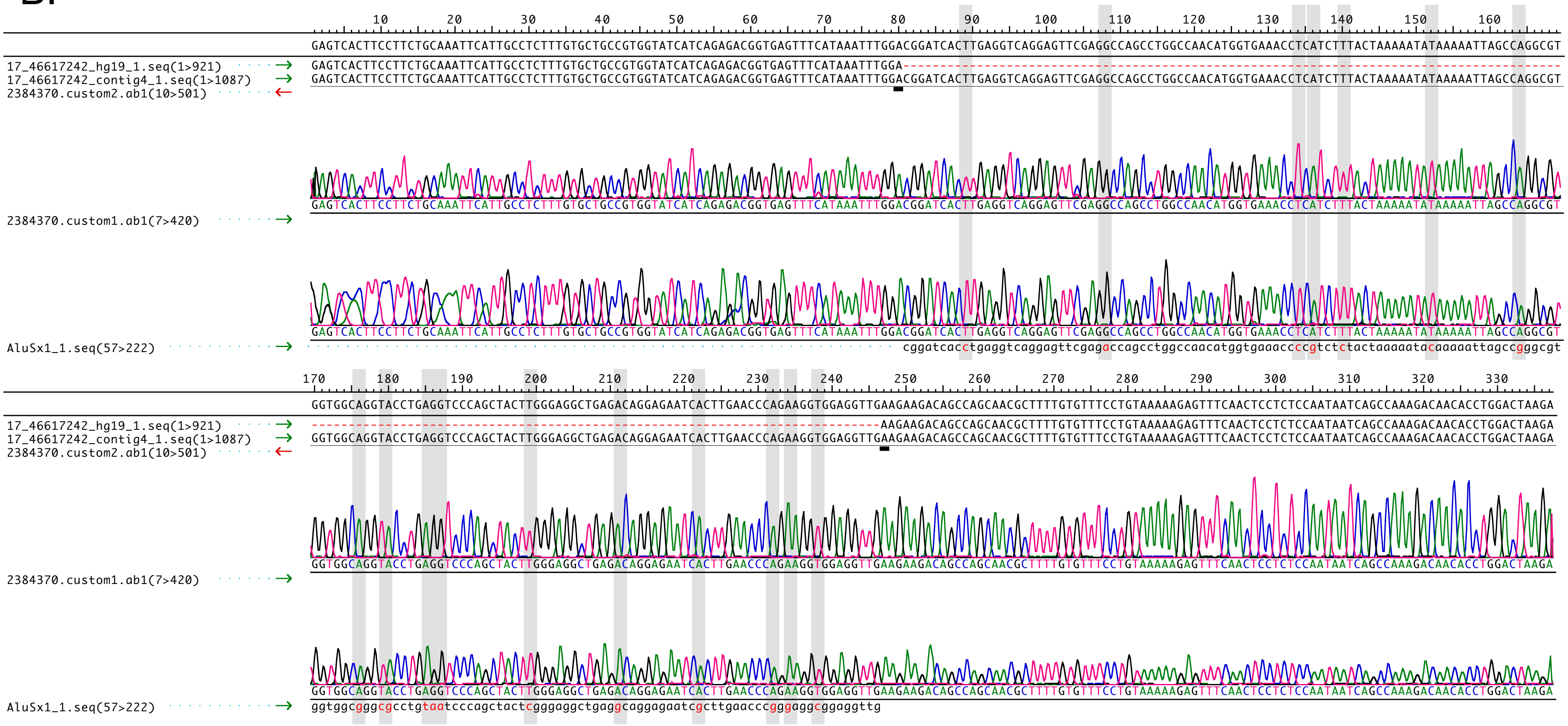

Figure S6.12

A.

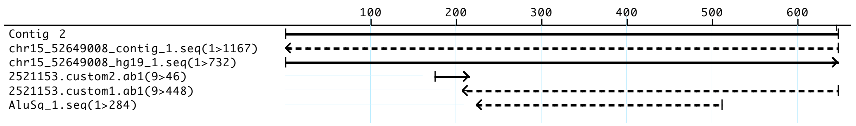

C.

not genotyped

siteID:	chr15_52649008
sample sequenced:	HGDP01036
Alu subfamily:	AluSq
percent divergence	9.0%

B.

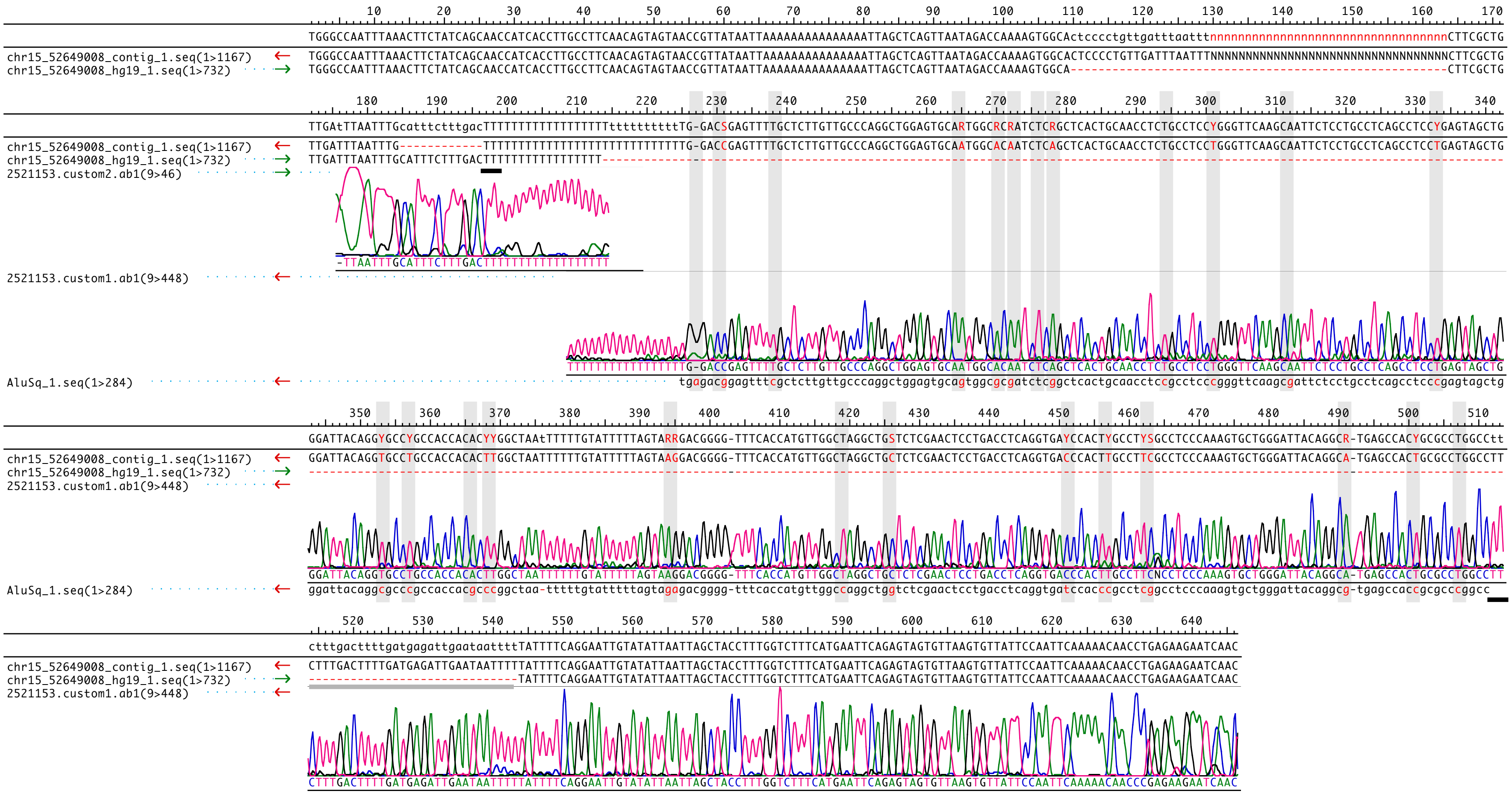

Figure S6.13
