# Supplementary material for: Discovery and characterization of Alu repeat sequences via precise local read assembly
Source: Nucleic Acids Res. 2015 Oct 25;43(21):10292–307. doi: 10.1093/nar/gkv1089 (PMC4666360; doi:10.1093/nar/gkv1089)
Supplement: SUPPLEMENTARY DATA [file supp_gkv1089_nar-01397-h-2015-File014.pdf]

siteID: chr11_35425496
Yellow : matches to reference hg19 based on blat
Light blue: indel rs371417146, A → GAGGAAACAGCTCAAAGGCAAAGG
Purple: AluSx
Red: Additional sequence
Bold Green: Region of similarity
Bold Underline: Candidate Target Site Duplication
!

```
>hg19_dna range=chr11:35425003-35425797
CTGGTAAGAGGATTTCATGCAAGCCTGCACATGTGTGGCTGCCCTGTAGGACTGGCTACATAATGT
GTGGGTCTGCACGTAATTTGGGCAAAATGAAATCTTGAGACCCCTTGTTCAAATTAAACATTTCAA
GATGATAAGAGCAGAGCATTAAACCGTGGACTGCCTAAGTCGCATATCCATAAAGCAGGTCCTGCT
TCCCAGGGTTCCTATGTTTATGCCCAAATAATCTAAGATTGTCTTGAAGGAGAAACCAGAAATTCT
TGAGGTAAAGAACTCATCCAGTGAGCTCTTCGGCTGCATGCATTTATTTATTCAACAAATATCCTG
TGATCTTATGACATGAGAAACTCAAGGATTAATTTAATGCCCCCTAACCAGAAACTAGAGCCACAG
CTGGACAGAATCCTGATTTTCCTGTTCCCTAAAAGCAAGGGAACATCTTCATCTTGCTGGCTTGGC
TCAGCAGCTTAGCTCAAAGGCAAAGAGGGAAAGTCCTCTCTAAGCAAACATATGGACACGTGGATC
AGTCTCTGCTCCTCCACGGGCGCCTCCTAAGGCAGTGTGCCCTTTACCAAAAACACAACGTTTTCA
CACCTATTTTAATTTTGTTGCTTGCTTCCCATTGGGTTAAAAGCTAGACTTTGGGGGTGGCTGGTA
AGTCATTTTGAAAAGAAATACCTGTTTTCATGCCTCCATTCTTGTGTTGTTGATTTTGGGGCCTTT
CCAACCCTTTCCAAATGTTTTTTCAATCCCTTGAGTGTCTAGGCTTCCTGCTTTTAAGGCCTCCCT
TCT

>contig
CTGGTAAGAGGATTTCATGCAAGCCTGCACATGTGTGGCTGCCCTGTAGGACTGGCTACATAATGT
GTGGGTCTGCACGTAATTTGGGCAAAATGAAATCTTGAGACCCCTTGTTCAAATTAAACATTTCAA
GATGATAAGAGCAGAGCATTAAACCGTGGACTGCCTAAGTCGCATATCCATAAAGCAGGTCCTGCT
TCCCAGGGTTCCTATGTTTATGCCCAAATAATCTAAGATTGTCTTGAAGGAGAAACCAGAAATTCT
TGAGGTAAAGAACTCATCCAGTGAGCTCTTCGGCTGCATGCATTTATTTATTCAACAAATATCCTG
TGATCTTATGACATGAGAAACTCAAGGATTAATTTAATGCCCCCTAACCAGAAACTAGAGCCACAG
CTGGACAGAATCCTGATTTTCCTGTTCCCTAAAAGCAAGGGAACATCTTCATCTTGCTGGCTTGGC
TCAGCAGCTTAGCTCAAAGGCAAAGGAGGAAACAGCTCAAAGGCAAAGGGGGAAAGTCTTTTTTTT
TTTTTTTTTTTTTTTGAGACAGAGTCTCACTCTGTGGCCCAGGCTGGAGTACAGTGGTGCAATTTC
AGCTCACTGCAACCTCCGCCTCCCGGGTTCAAGCGATTCTCTTGCCTCAGCCTCCCAAGTAGCCAG
GACTACAGGTGCTCGCCACCATGCCCGGCTAATTTTTGTATTTTTGGTAGAGACGGGTTTCACTGT
TGGCCAGGCGGGCCTGGAACTCCTGACCTCATGATCCGCCCACCTTGGCCTCCCAAAGTGCTGGGC
TTACAGGCGTGAGCCATCGCGCCCGGCCGGGAAAGTTCCCTTTAAGCAAACATATGGACGCATGGA
TCAGTCCCTGCTCCGTGATCACGGCCGCCTCAGCTGCACAGAATCCTGATATTCCTGCTCCCGGCA
AACAAGGGAACATCTTCCTCTTGCTGGCTTGCCTCAGCAGCTTAGCTCAAAAGCAAAGAGGGAAAG
TCCTCTCTAAGCAAACATATGGACACGTGGATCAGTCTCTGCTCCTCCACGGGCGCCTCCTAAGGC
AGTGTGCCCTTTACCAAAAACACAACGTTTTCACGCCTATTTTAATTTTGTTGCTTGCTTCCCATT
GGGTTAAAAGCTAGACTTTGCGGGTGGCTGGTAAGTCATTTTGAAAAGACAAATACCTGTTTTCAT
GCCTCCATTCTTGTGTTGTTGATTTTGGGGCCTTTCCAACCCTTTCCAAATGTATTTTCAATCCCT
TGAGTGTCTAGGCTTCCTGCTTTTAAGGCCTCCCTTCT
```

Alignment (89.5% identity)

```
CAGCTGGACAGAATCCTGATTTTCCTGTTCCCTAAAAGCAAGGGAACATC
|||||| ||||||||||||| |||||| ||||   || ||||||||||||
CAGCTGCACAGAATCCTGATATTCCTGCTCCCGGCAAACAAGGGAACATC

TTCATCTTGCTGGCTTGGCTCAGCAGCTTAGCTCAAAGGCAAAGA
||| ||||||||||||| ||||||||||||||||||| |||||||
TTCCTCTTGCTGGCTTGCCTCAGCAGCTTAGCTCAAAAGCAAAGA
```
