# Supplementary material for: Discovery and characterization of Alu repeat sequences via precise local read assembly
Source: Nucleic Acids Res. 2015 Oct 25;43(21):10292–307. doi: 10.1093/nar/gkv1089 (PMC4666360; doi:10.1093/nar/gkv1089)
Supplement: SUPPLEMENTARY DATA [file supp_gkv1089_nar-01397-h-2015-File009.pdf]

A.

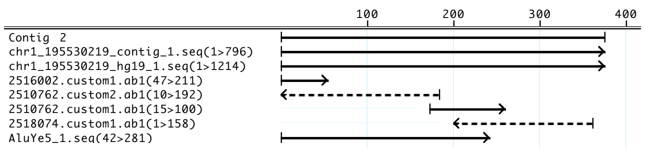

C.

genotyped

siteID: chr1_195530219
sample sequenced: HGDP00213
Alu subfamily: AluYe5

B.

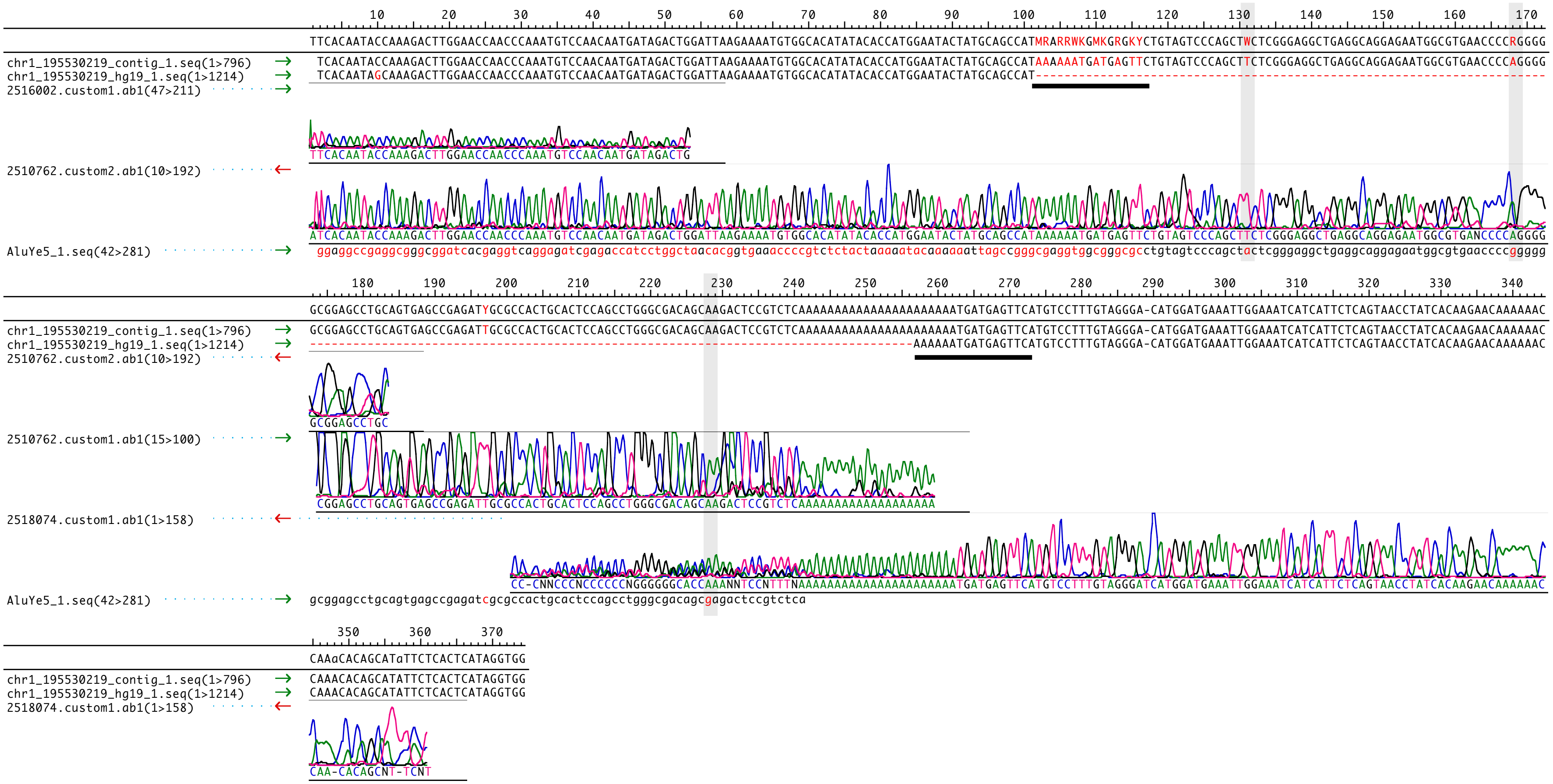

Figure S4.1

A.

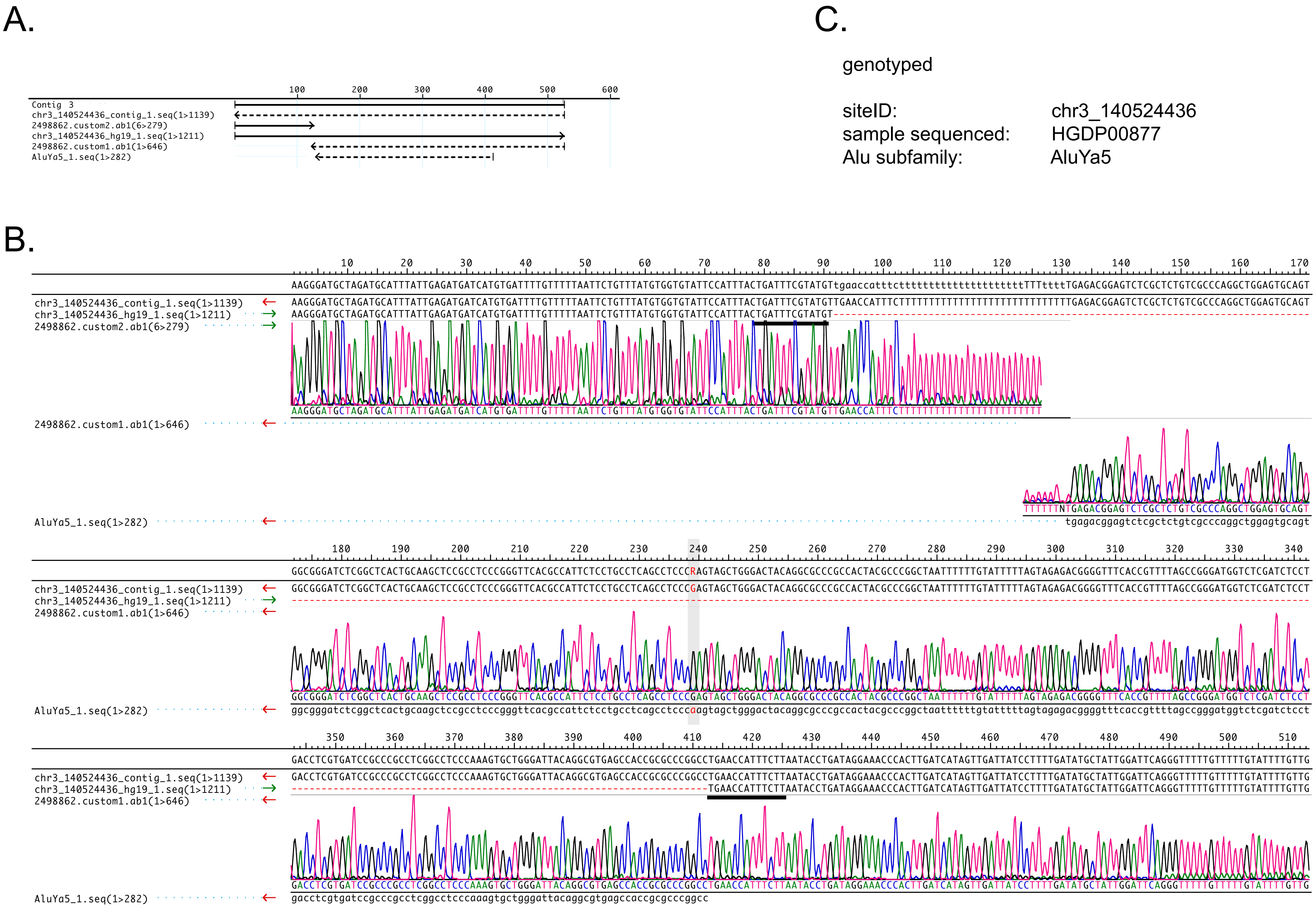

C.

genotyped

siteID: chr3_140524436
sample sequenced: HGDP00877
Alu subfamily: AluYa5

Figure S4.2

A.

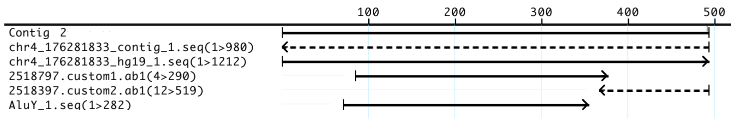

C.

genotyped

siteID:	chr4_176281833
sample sequenced:	HGDP00711
Alu subfamily:	AluY

B.

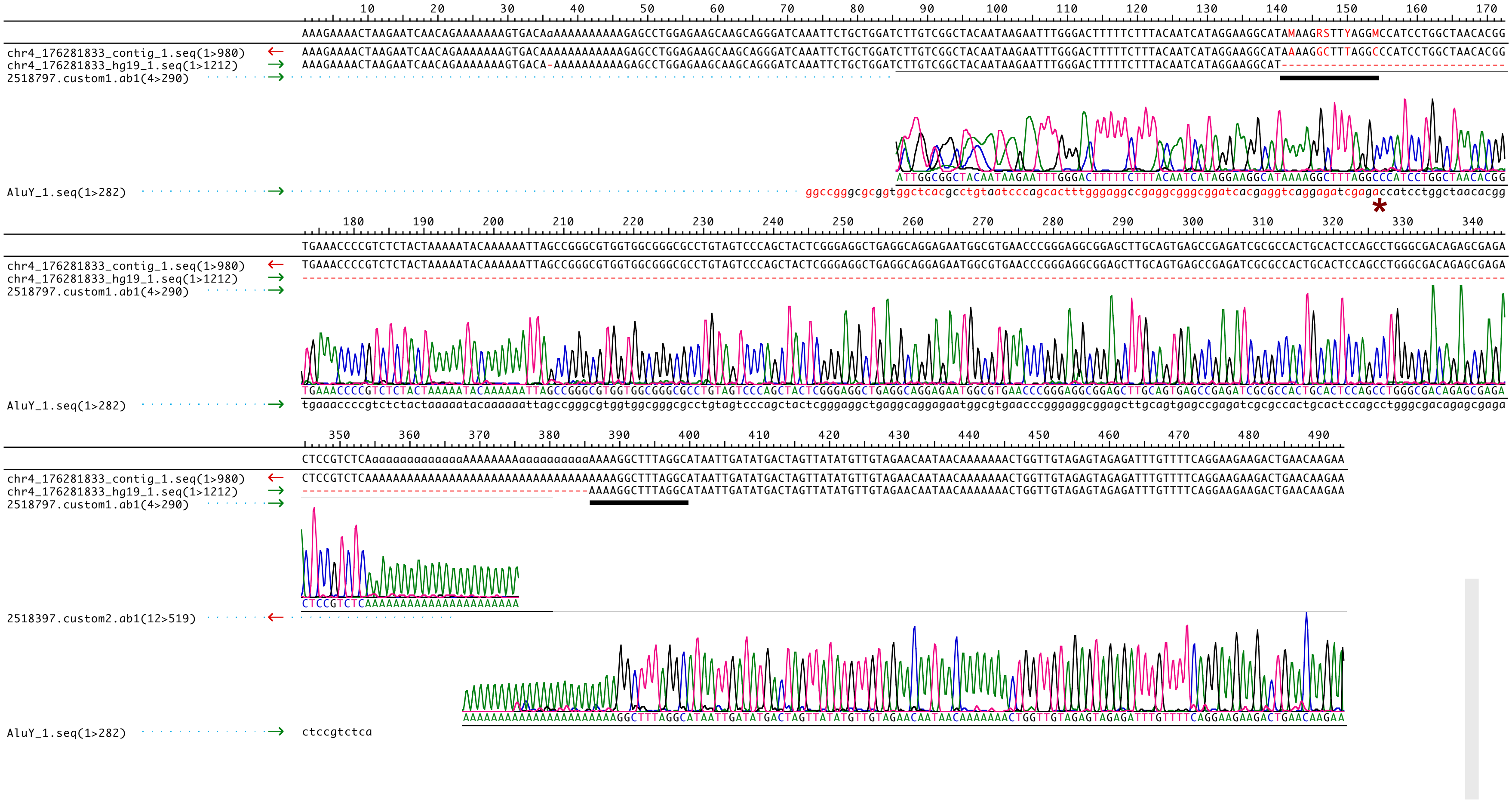

Figure S4.3

A.

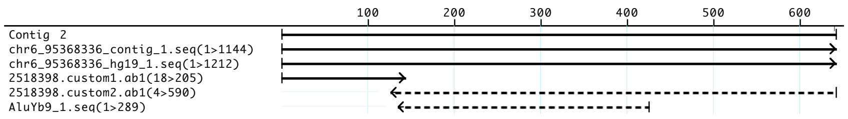

C.

genotyped

siteID:	chr6_95368336
sample sequenced:	HGDP00474
Alu subfamily:	AluYb9

B.

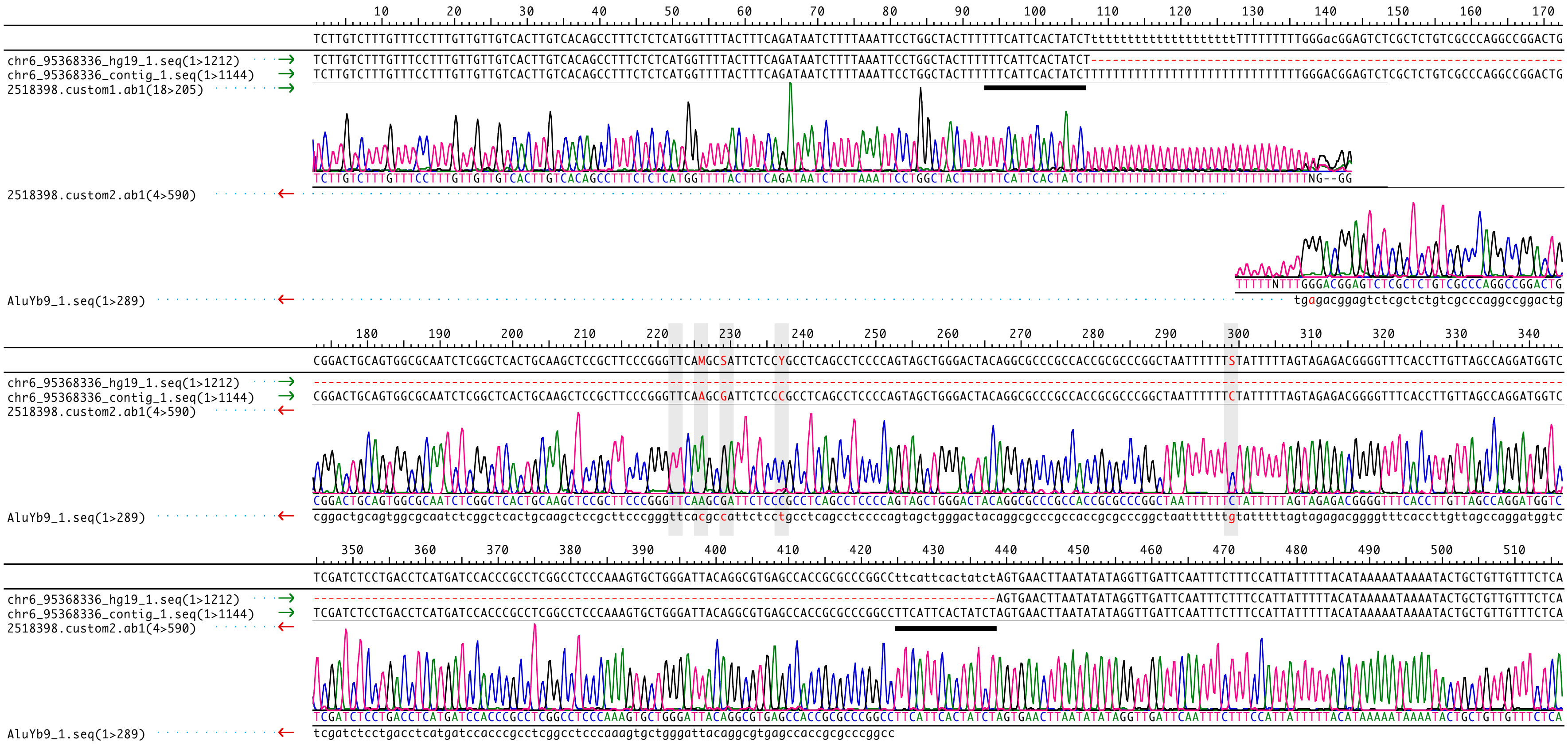

Figure S4.4

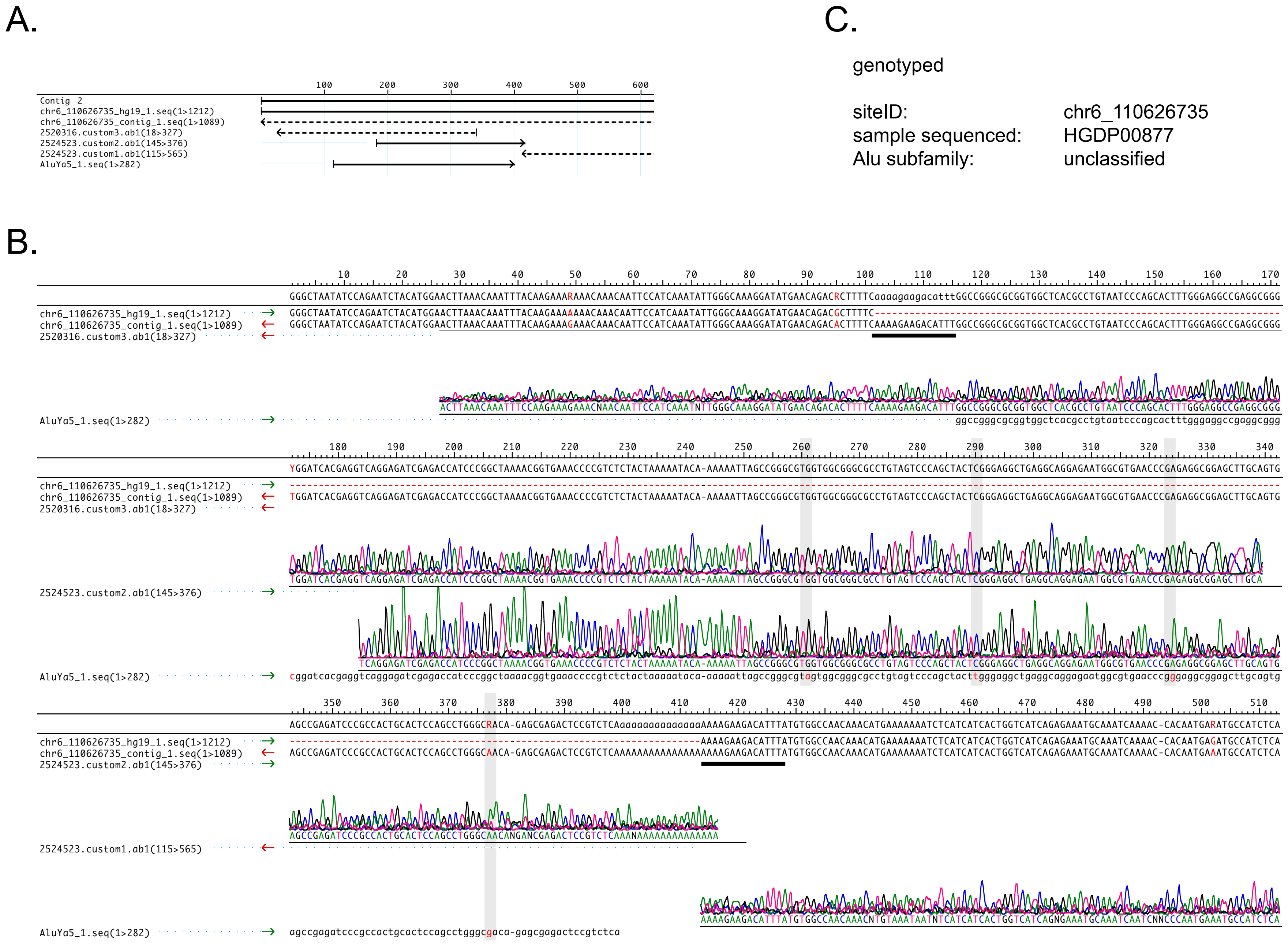
A.
B.
C.
genotyped
siteID: chr6_110626735
sample sequenced: HGDP00877
Alu subfamily: unclassified

Figure S4.5

A.

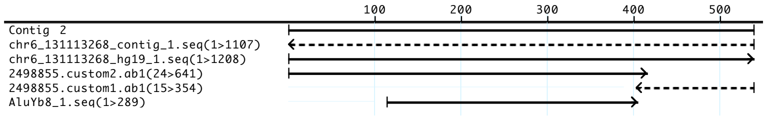

C.

genotyped

siteID:	chr6_131113268
sample sequenced:	HGDP00232
Alu subfamily:	AluYb8

B.

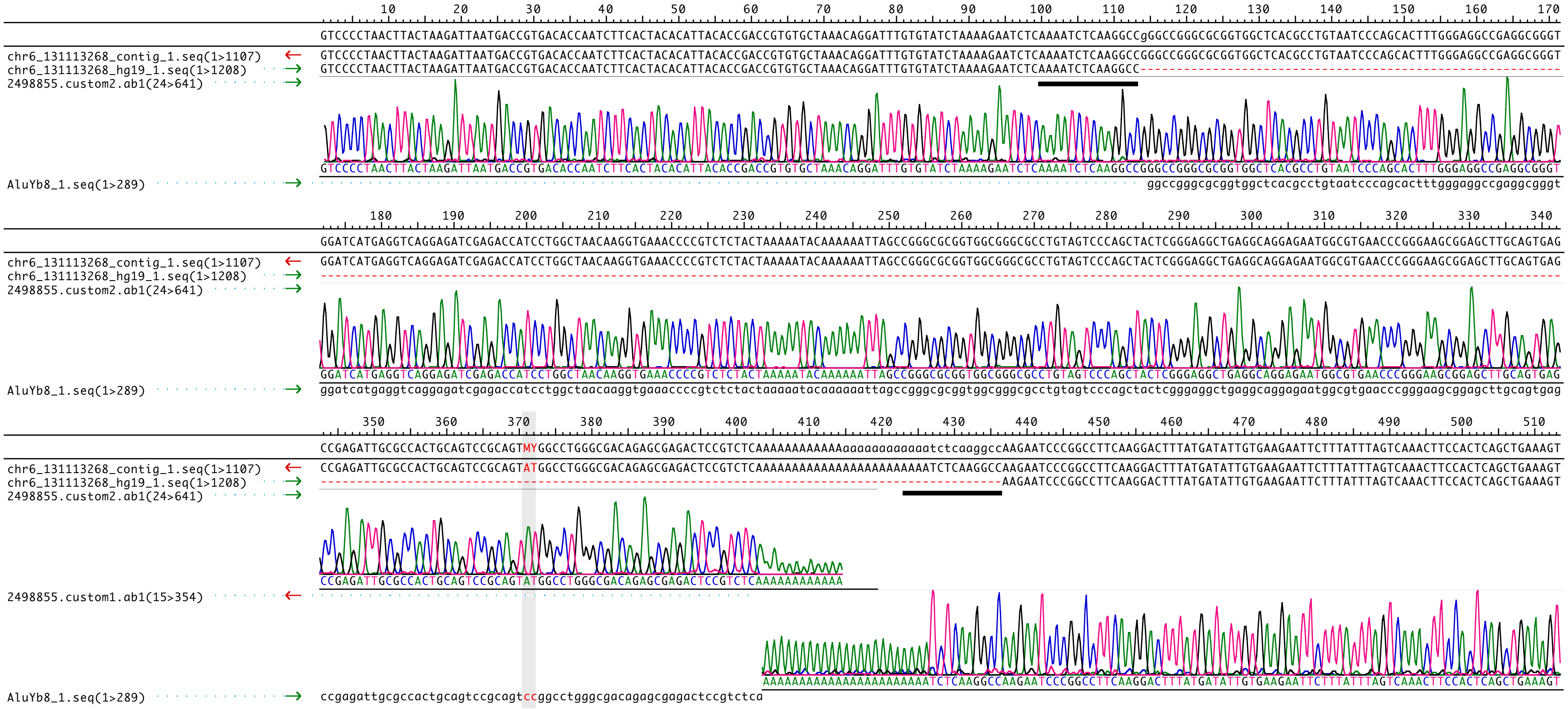

Figure S4.6

A.

C.

genotyped

siteID:	chr7_49057615
sample sequenced:	HGDP00711
Alu subfamily:	AluYc1

B.

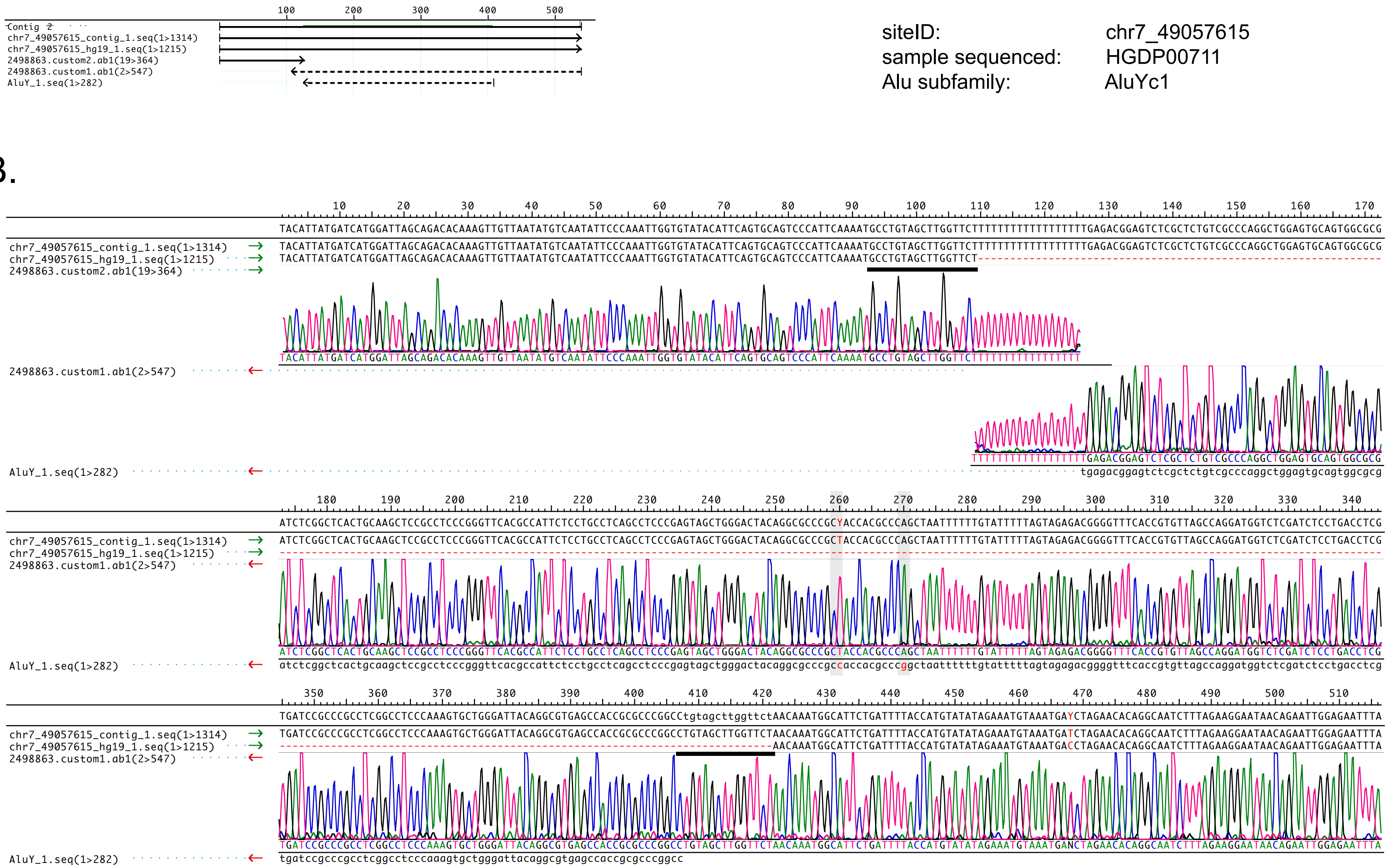

Figure S4.7

A.

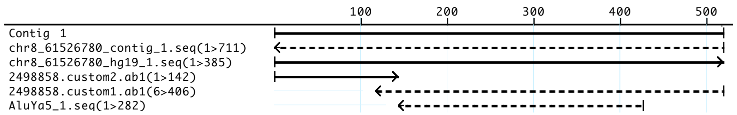

C.

genotyped

siteID:	chr8_61526780
sample sequenced:	HGDP00716
Alu subfamily:	AluYa4

B.

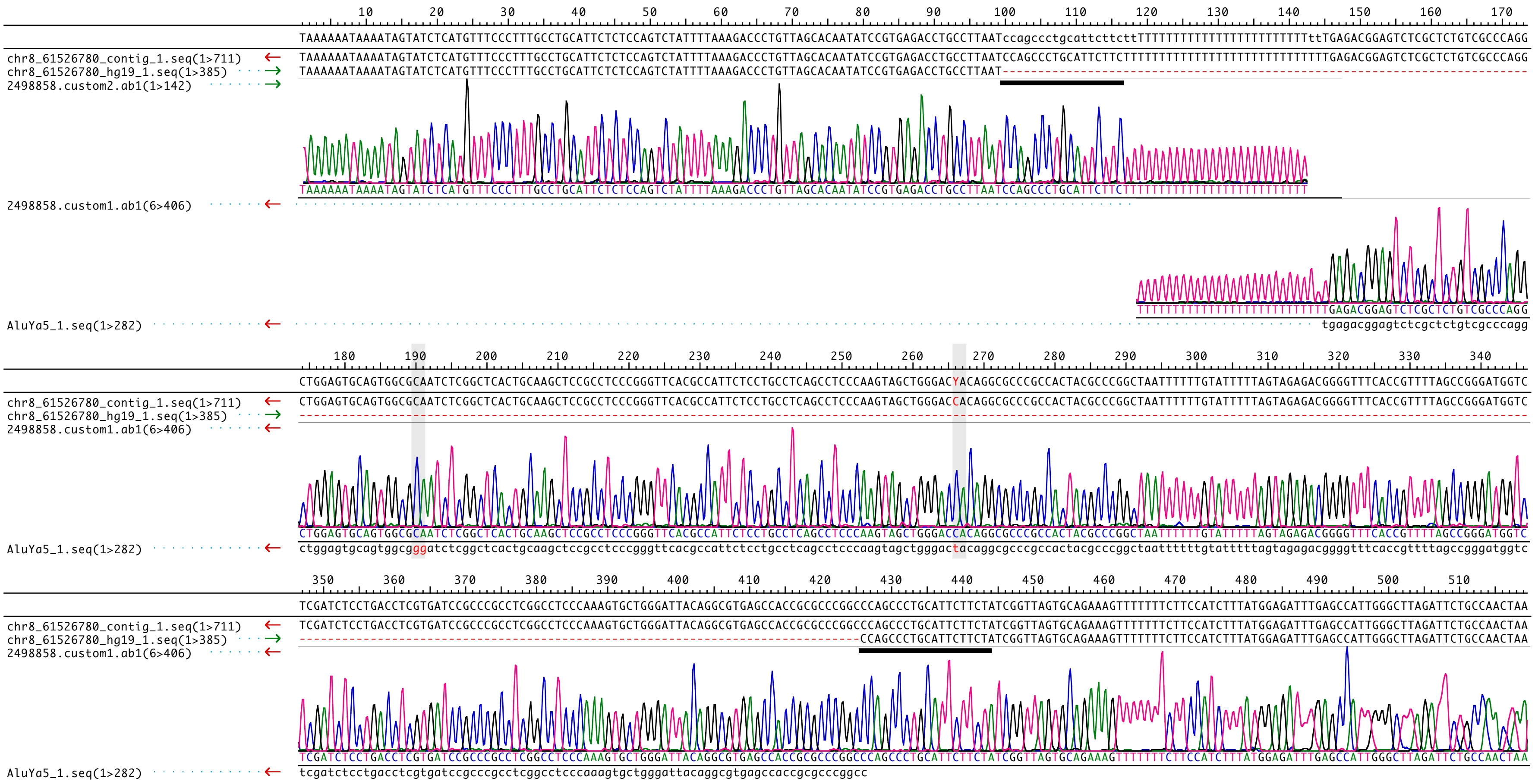

Figure S4.8

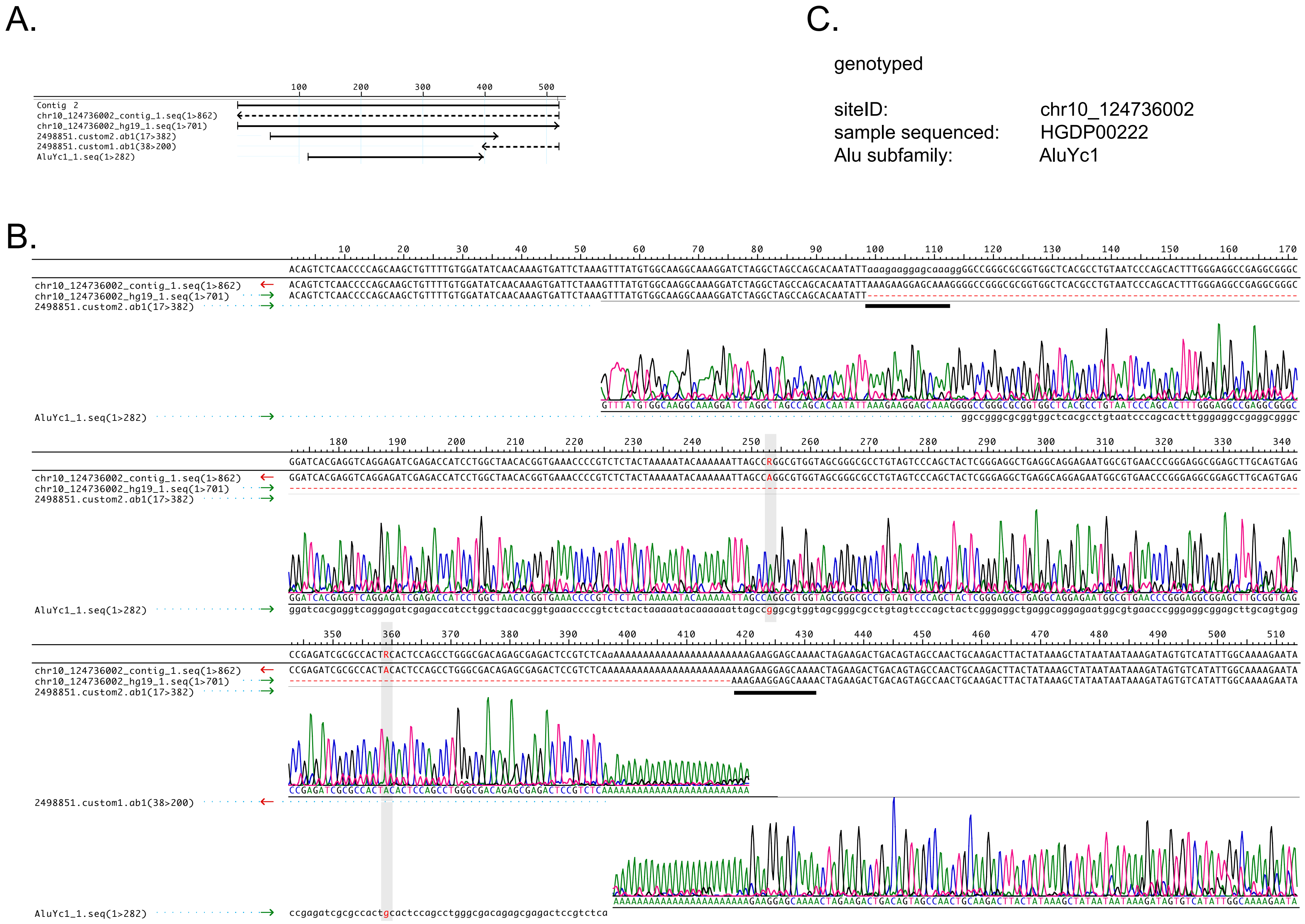

A.
Contig 2
chr10_124736002_contig_1.seq(1>862)
chr10_124736002_hg19_1.seq(1>701)
2498851.custom2.ab1(17>382)
2498851.custom1.ab1(38>200)
AluYc1_1.seq(1>282)
C.
genotyped
siteID: chr10_124736002
sample sequenced: HGDP00222
Alu subfamily: AluYc1
B.

Figure S4.9

A.

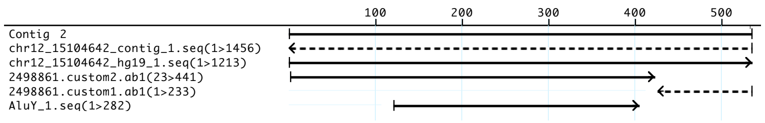

C.

genotyped

siteID: chr12_15104642
sample sequenced: HGDP00471
Alu subfamily: AluYc1

B.

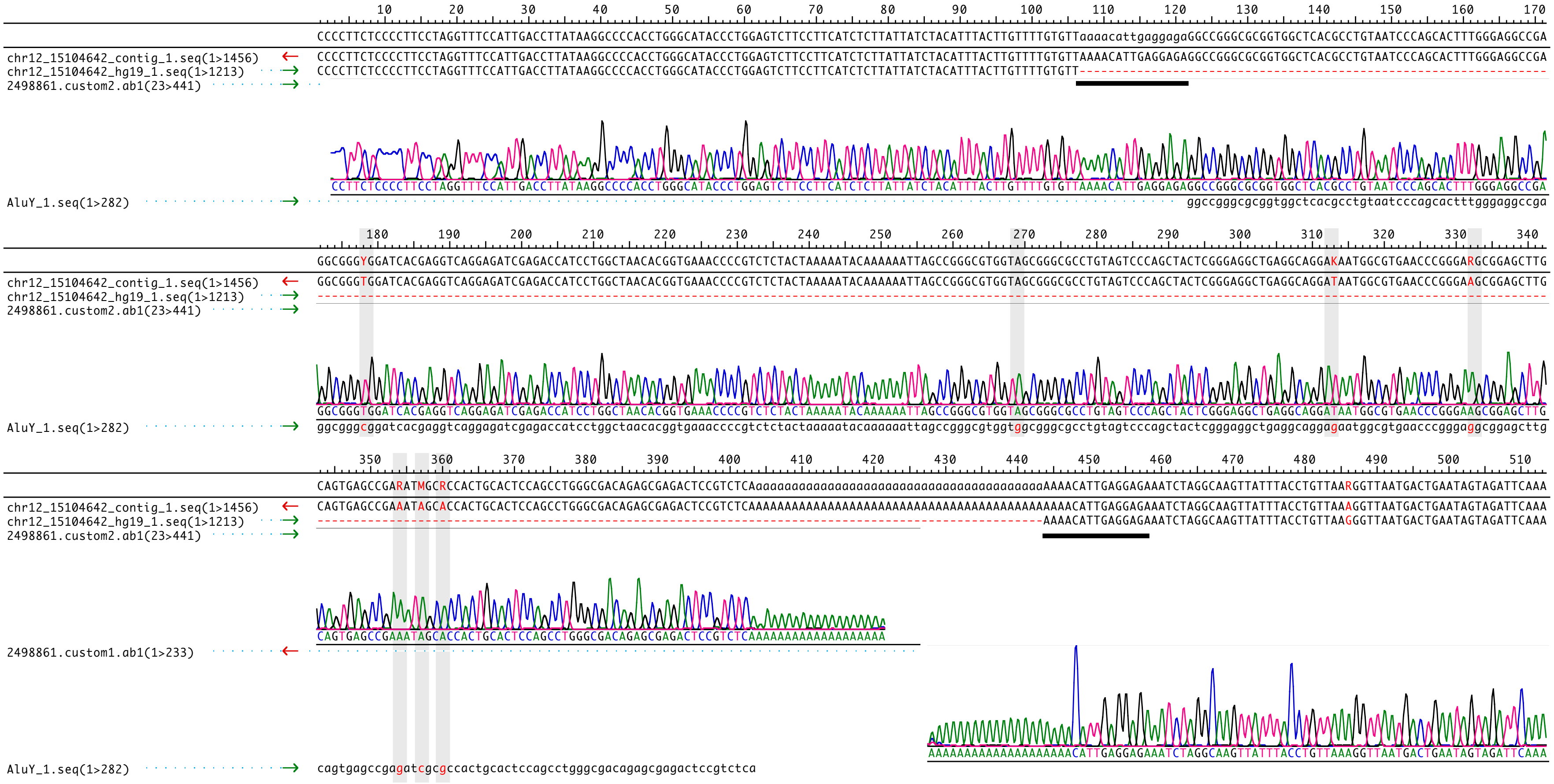

Figure S4.10

A.

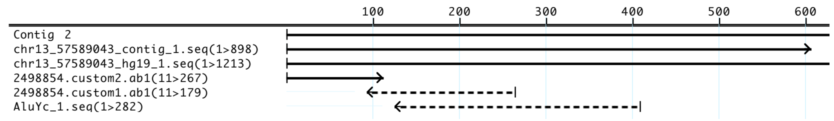

C.

genotyped

siteID:	chr13_57589043
sample sequenced:	HGDP00877
Alu subfamily:	unclassified

B.

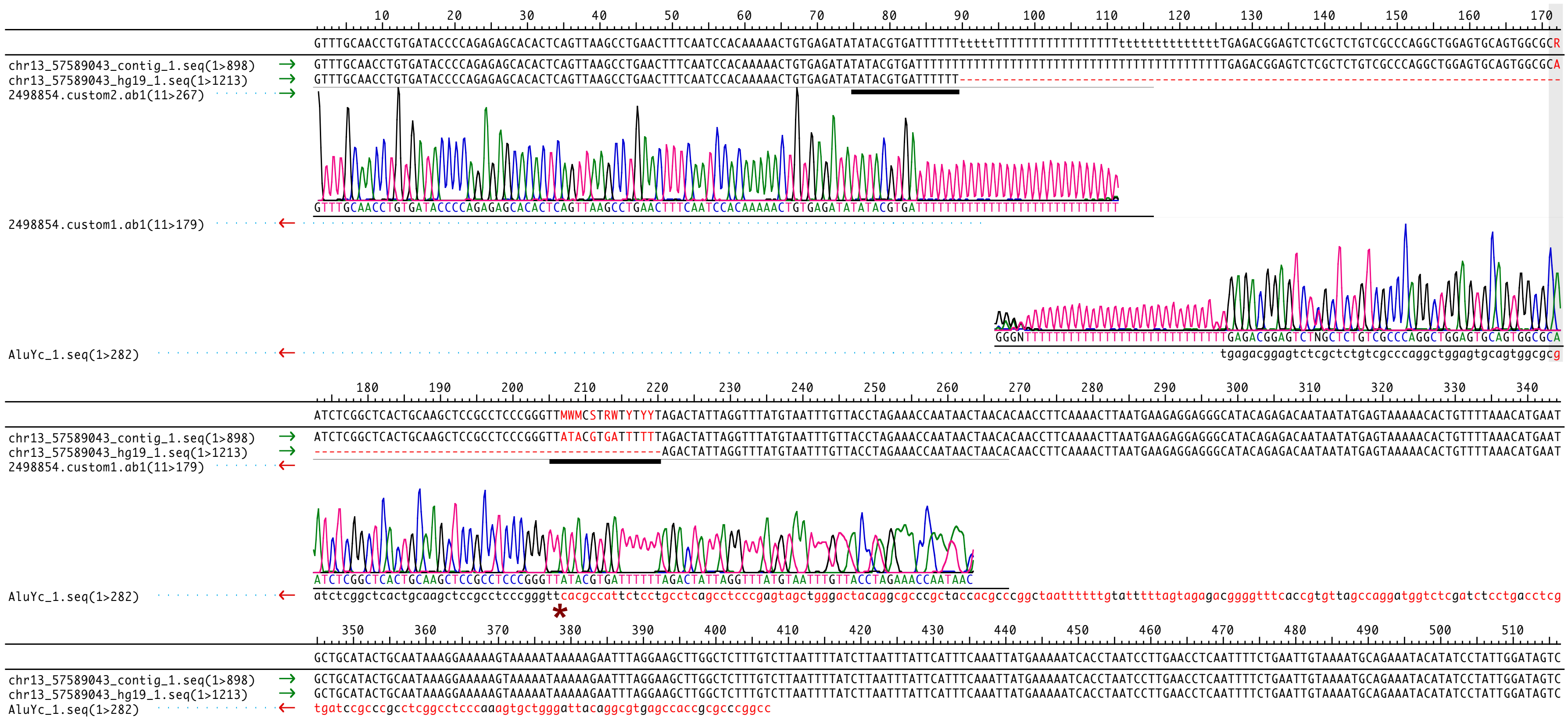

Figure S4.11

A.

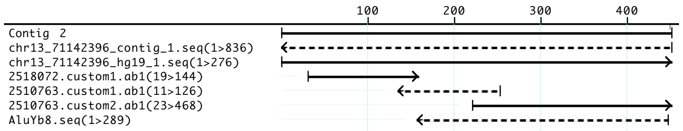

B.

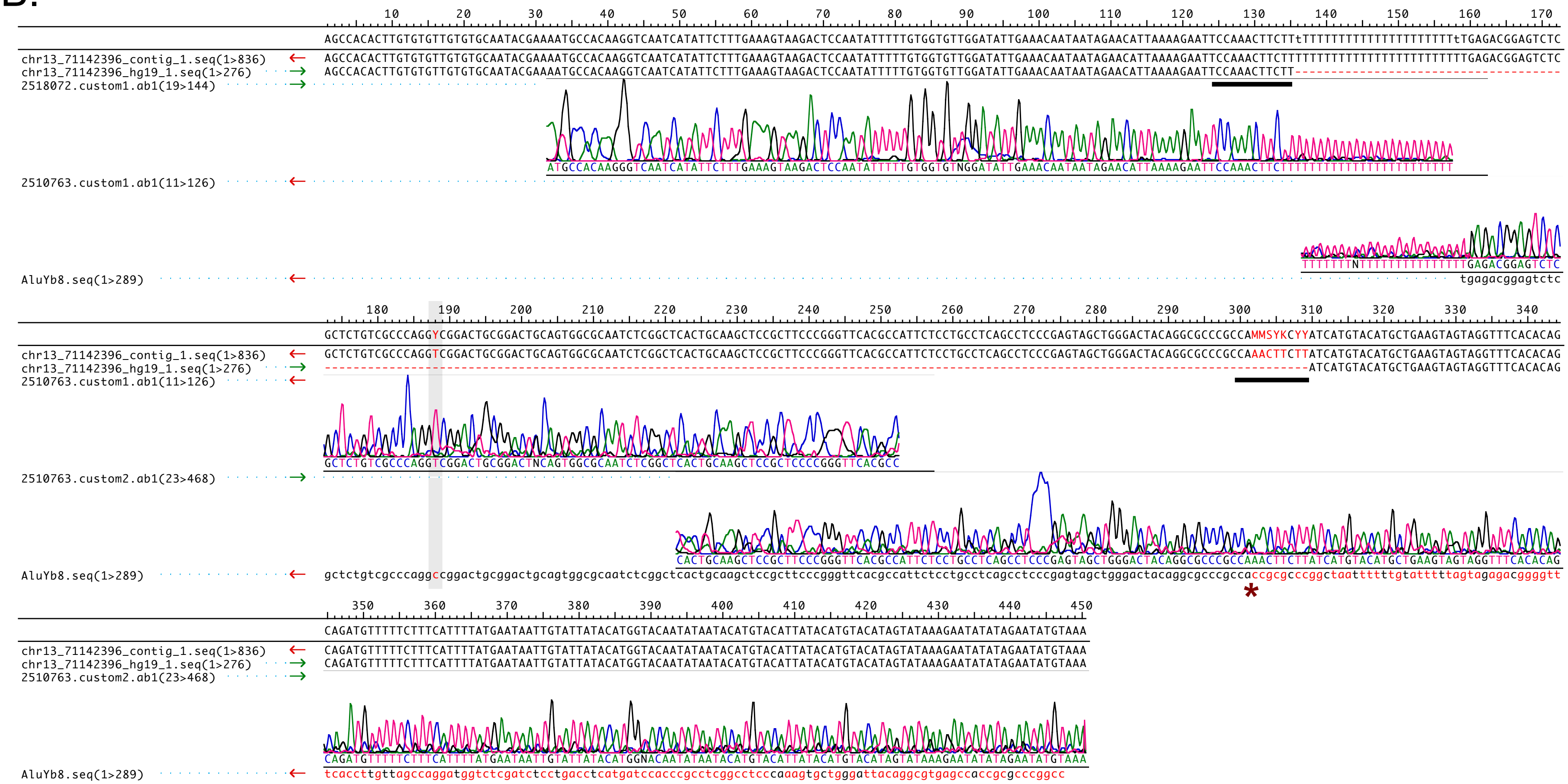

C.

genotyped

siteID:	chr13_71142396
sample sequenced:	HGDP00213
Alu subfamily:	AluYb8

Figure S4.12

A.

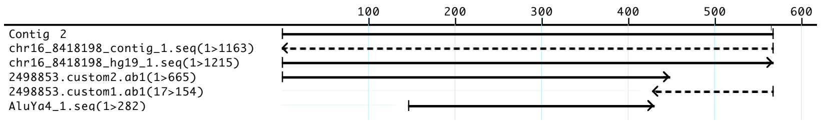

C.

genotyped

siteID: chr16_8418198
sample sequenced: HGDP00721
Alu subfamily: AluYa4

B.

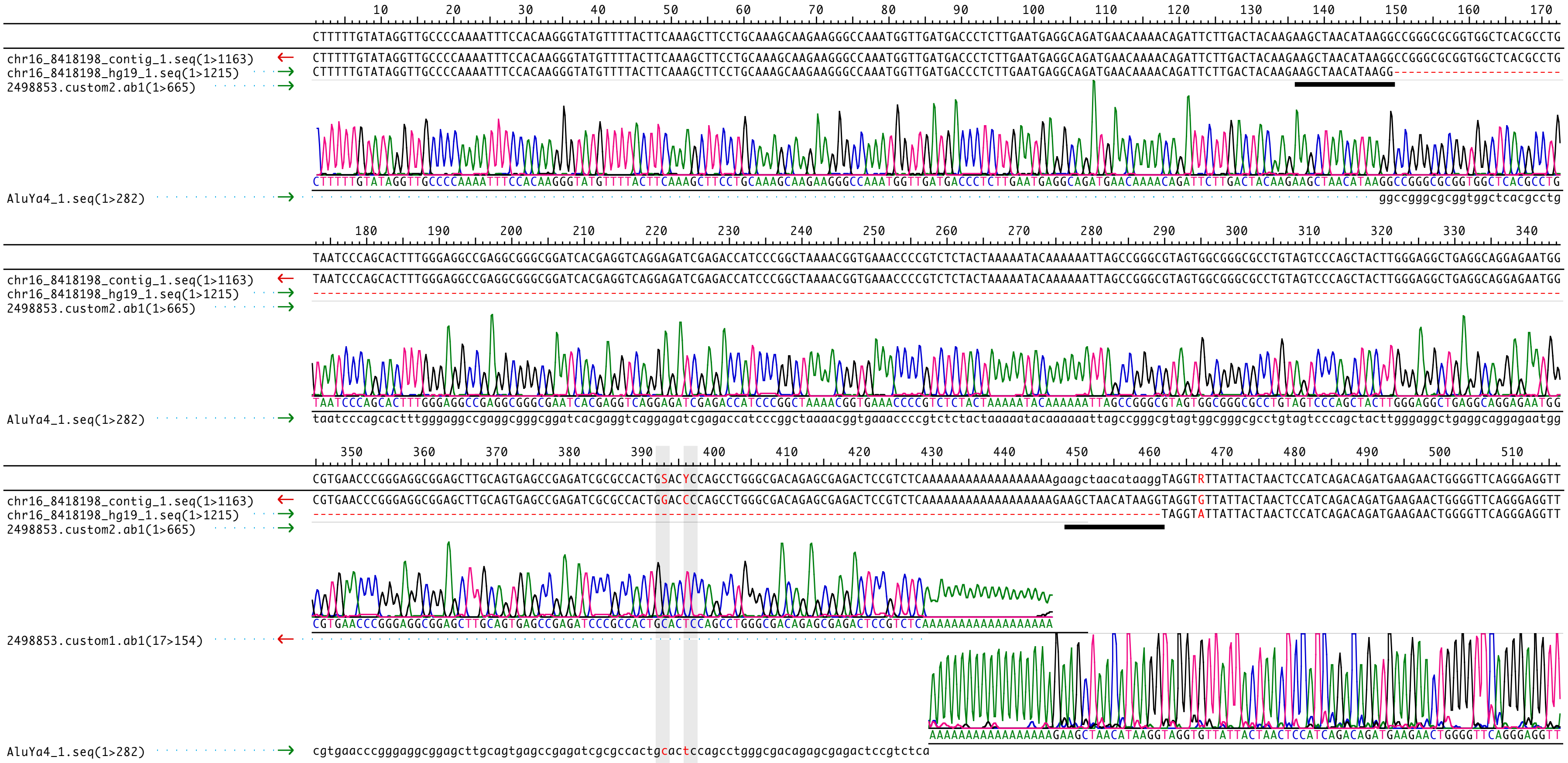

Figure S4.13

A.

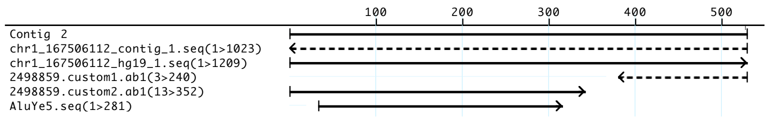

C.

not genotyped

siteID: chr1_167506112
sample sequenced: HGDP00959
Alu subfamily: AluYe5

B.

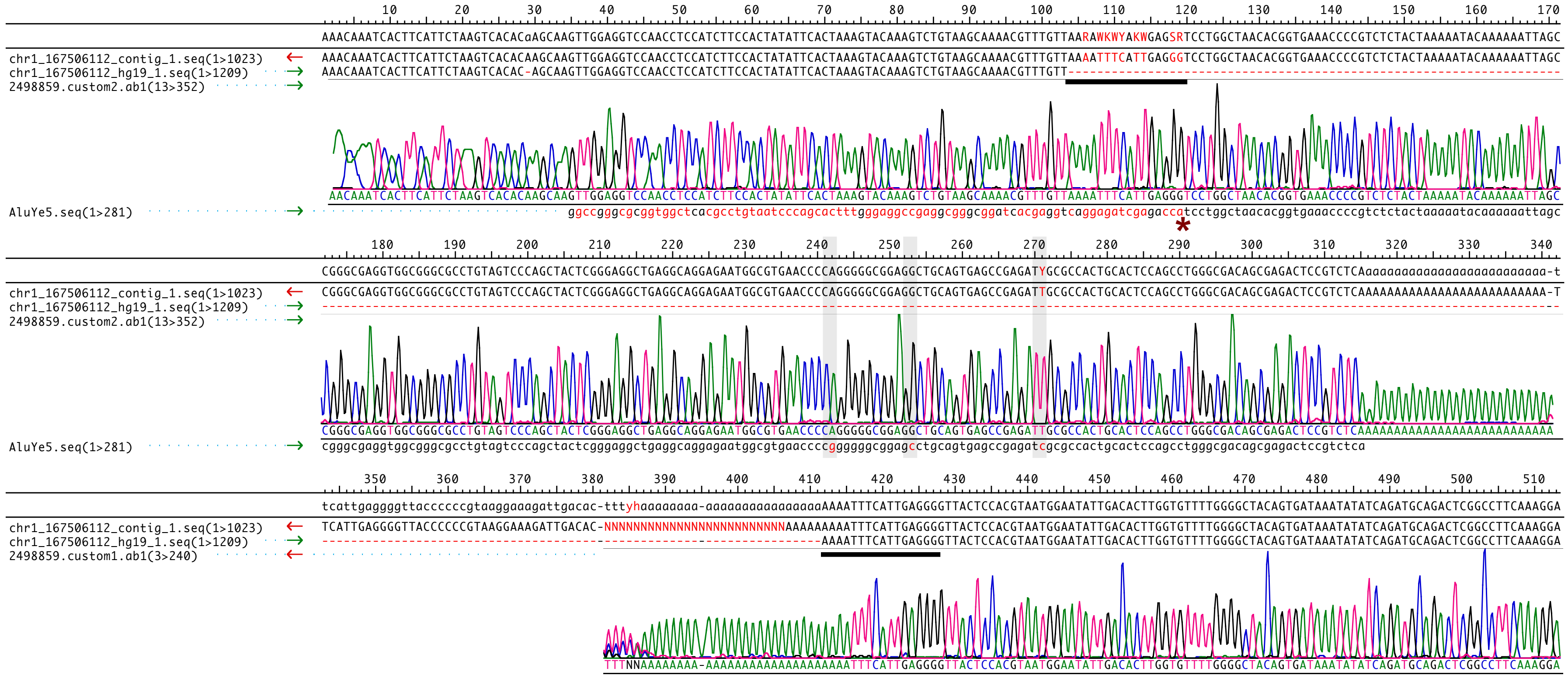

Figure S4.14

A.

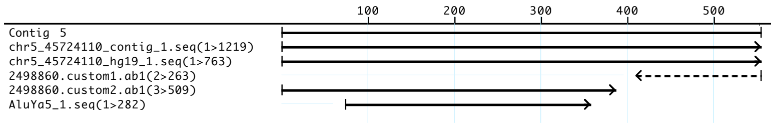

C.

not genotyped

siteID: chr5_45724110
sample sequenced: HGDP00856
Alu subfamily: AluYa5

B.

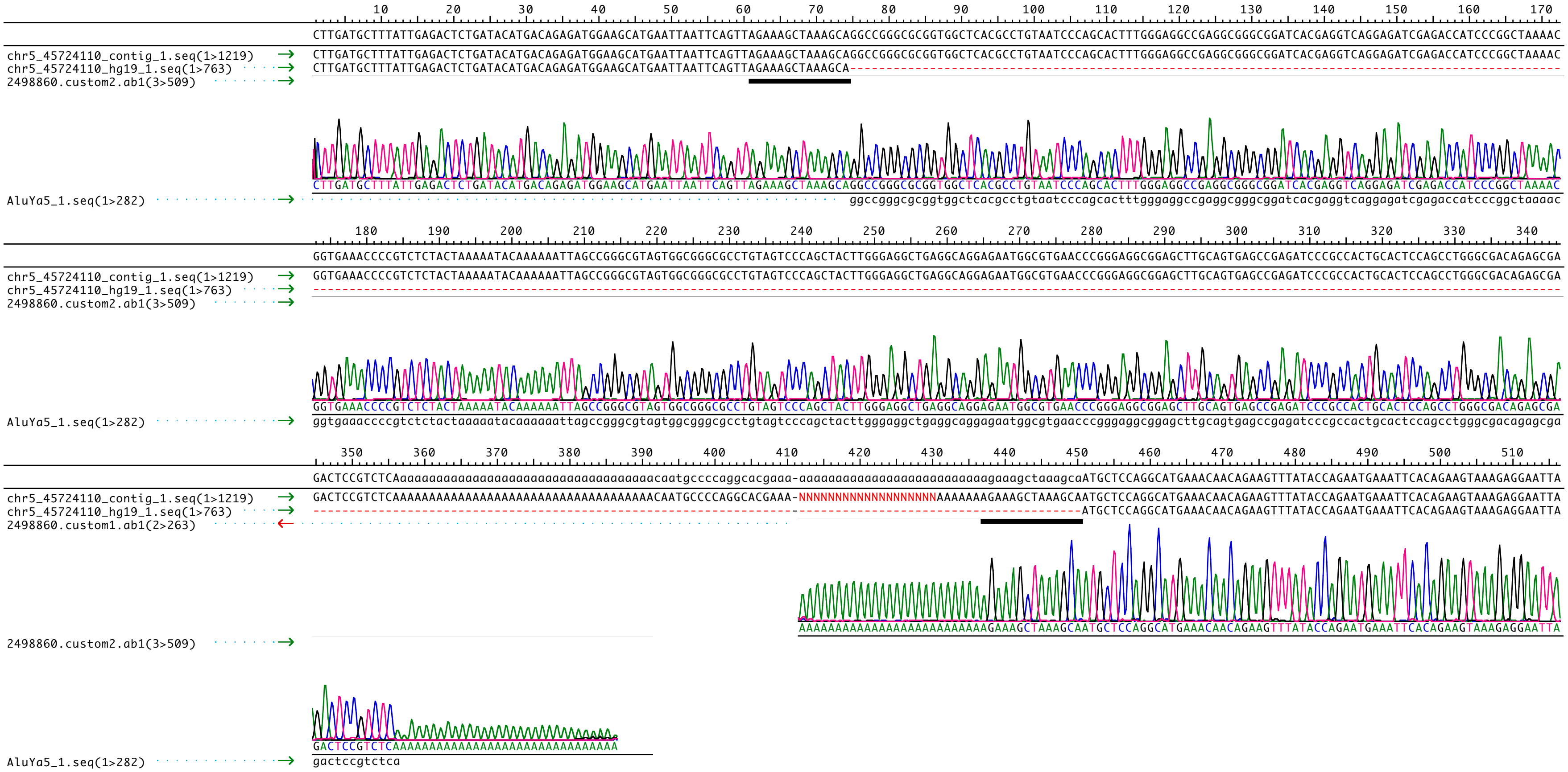

Figure S4.15

A.

C.

not genotyped

siteID: chr5_119485627
sample sequenced: HGDP00987
Alu subfamily: unclassified

B.

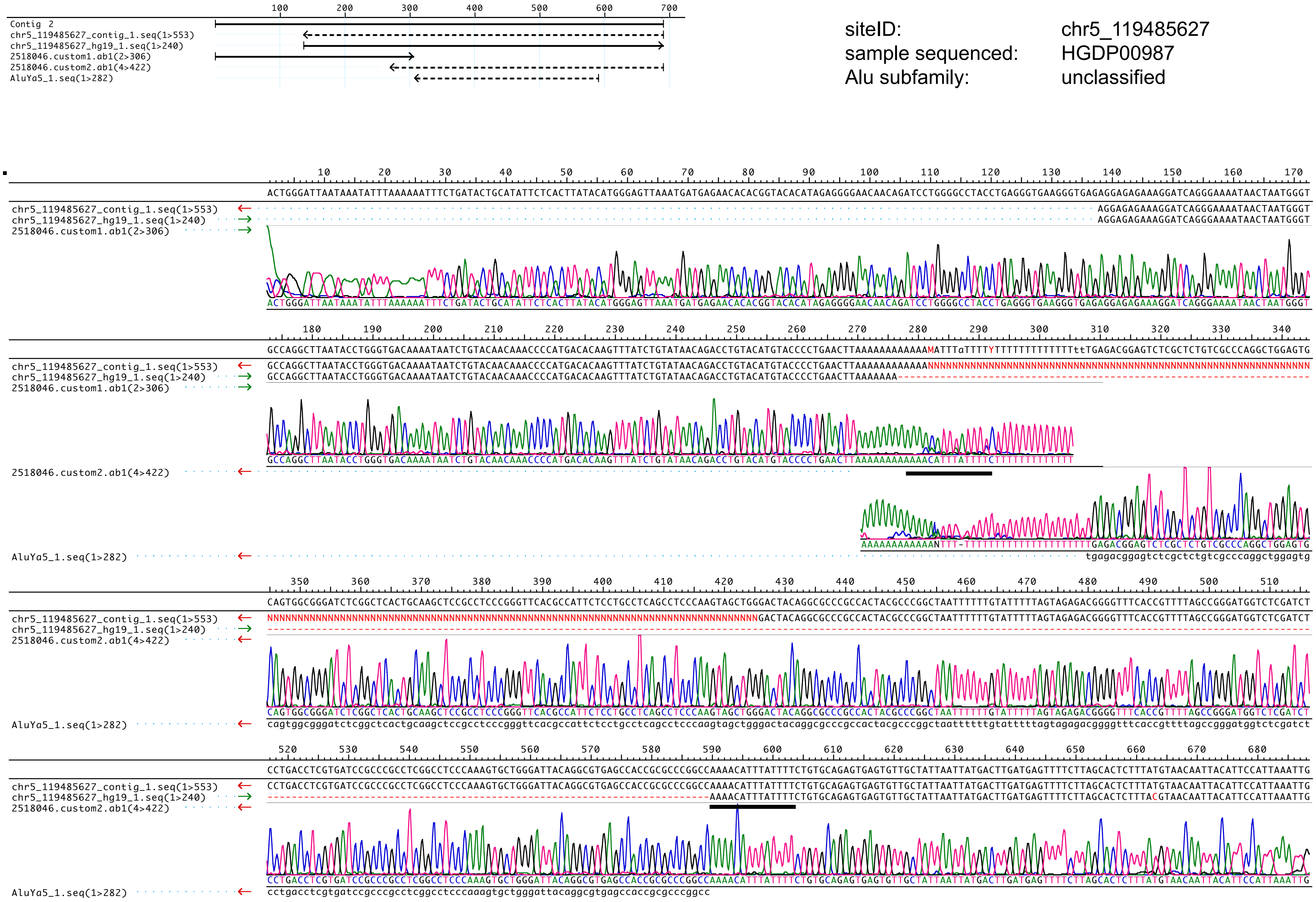

Figure S4.16

A.

C.

not genotyped

siteID:	chr6_156541106
sample sequenced:	HGDP00711
Alu subfamily:	AluYb8

B.

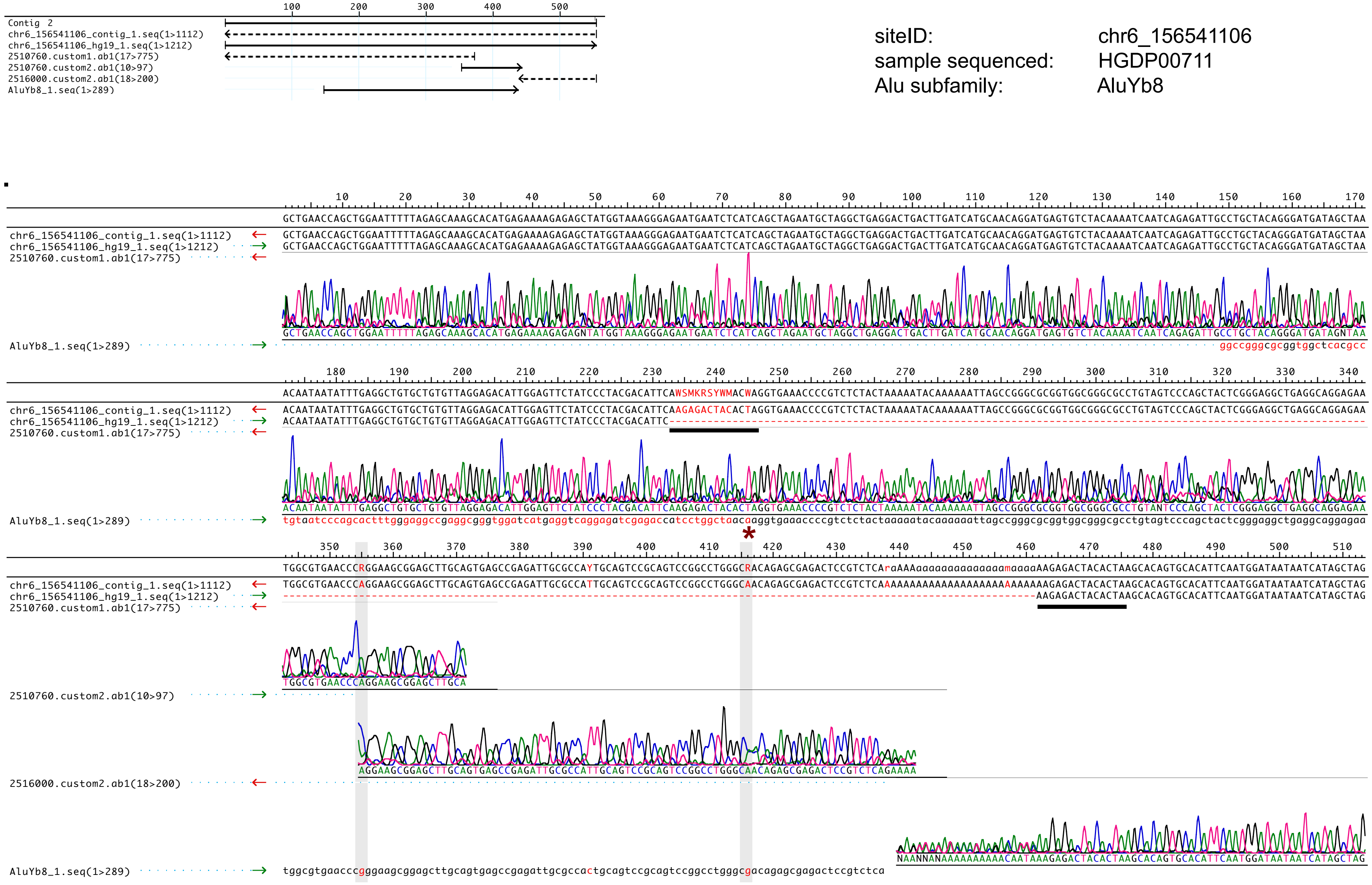

Figure S4.17

A.

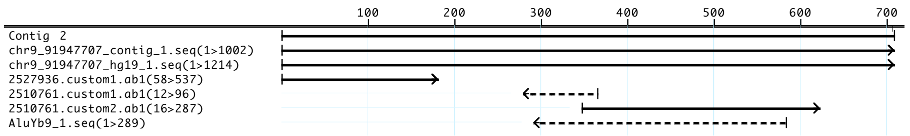

C.

not genotyped

siteID: chr9_91947707
sample sequenced: HGDP00992
Alu subfamily: AluYb9

B.

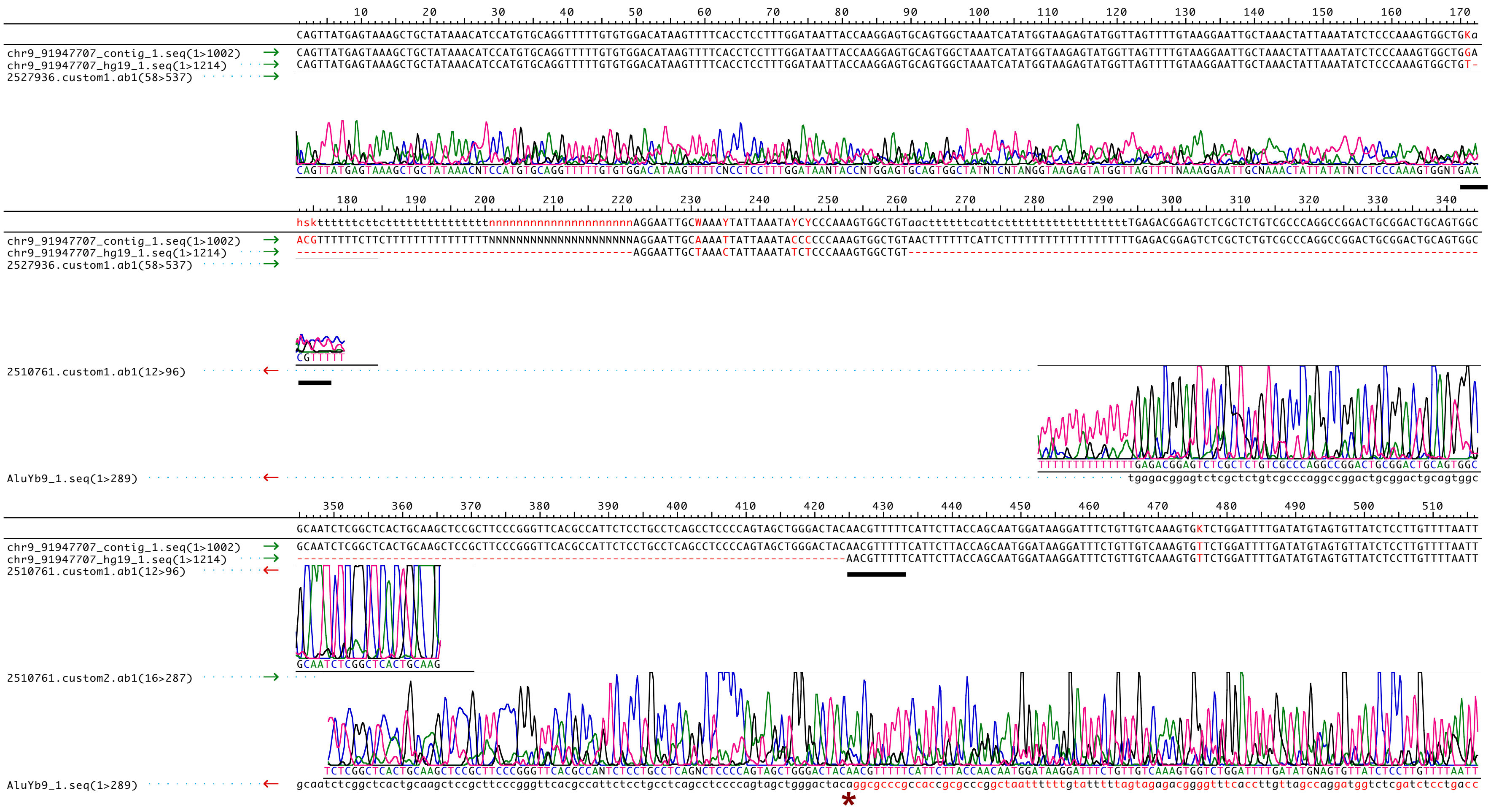

Figure S4.18

A.

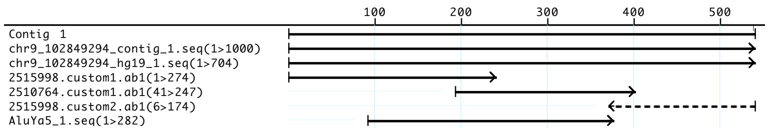

C.

not genotyped

siteID:	chr9_102849294
sample sequenced:	HGDP00456
Alu subfamily:	unclassified

B.

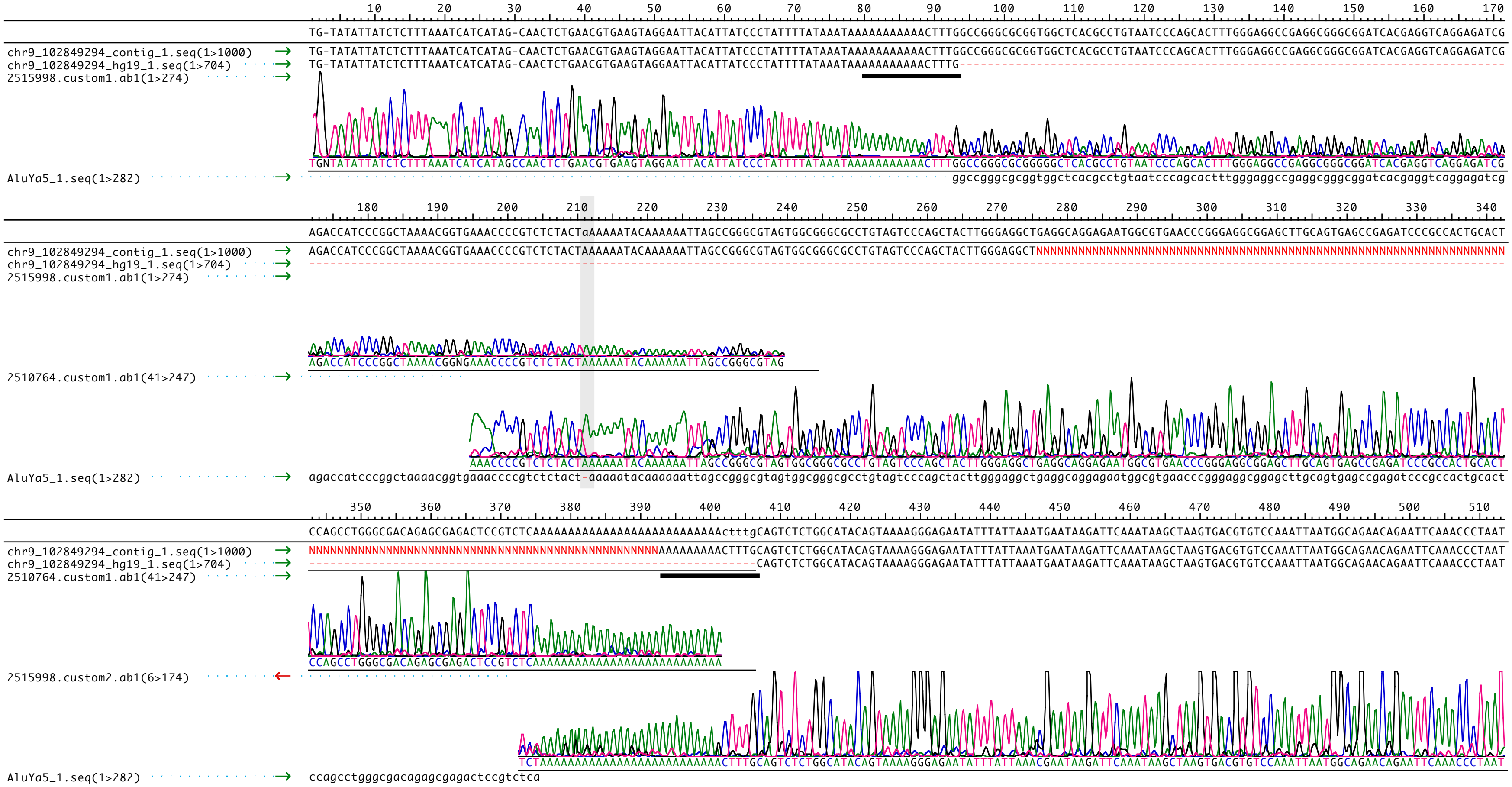

Figure S4.19

A.

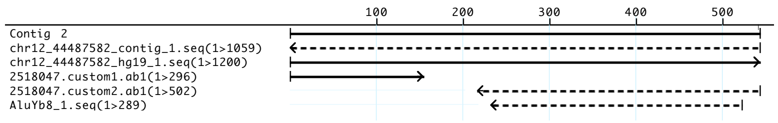

C.

not genotyped

siteID:	chr12_44487582
sample sequenced:	HGDP00471
Alu subfamily:	AluYb8

B.

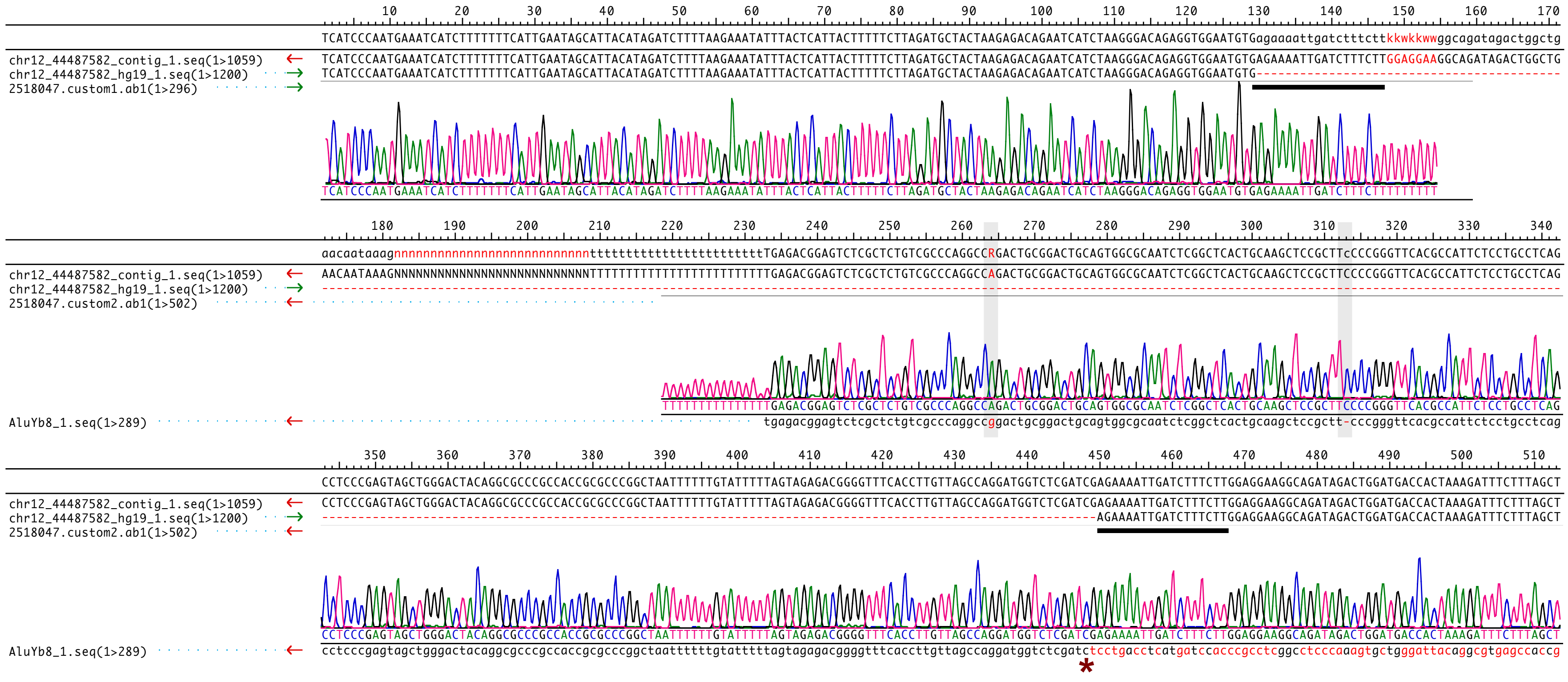

Figure S4.20
